# Mental health status and related factors influencing healthcare workers during the COVID-19 pandemic: A systematic review and meta-analysis

**DOI:** 10.1371/journal.pone.0289454

**Published:** 2024-01-19

**Authors:** Jia Huang, Zhu-Tang Huang, Xin-Ce Sun, Ting-Ting Chen, Xiao-Tian Wu

**Affiliations:** 1 The Second Clinical Medical College, Lanzhou University, Lanzhou, Gansu, China; 2 The First Clinical Medical College, Lanzhou University, Lanzhou, Gansu, China; Universidad Complutense de Madrid Facultad de Psicologia, SPAIN

## Abstract

**Background:**

The mental health of healthcare workers during the coronavirus-2019 pandemic was seriously affected, and the risk of mental health problems was high. The present study sought to systematically evaluate the mental health problems of healthcare workers worldwide during the pandemic and to determine the latest global frequency of COVID-19 associated mental health problems.

**Methods:**

Data in the Cumulative Index to Nursing and Allied Health Literature (CINAHL), EMBASE, Elsevier, MEDLINE, PubMed, PsycINFO and the Web of Science before November 11, 2022, were systematically searched. Cohort, case-control and cross-sectional studies were included. The meta-analysis used a random effects model to synthesize the comprehensive prevalence rate of mental health problems. Subgroup analyses were performed based on time of data collection; whether the country was or was not developed; continent; doctors and nurses; doctors/nurses vs. other healthcare workers; and psychological evaluation scale.

**Results:**

A total of 161 studies were included, including 341,014 healthcare workers worldwide, with women accounting for 82.8%. Occupationally, 16.2% of the healthcare workers were doctors, 63.6% were nurses and 13.3% were other medical staff. During the pandemic, 47% (95% confidence interval [CI], 35–60%) of healthcare workers reported job burnout, 38% (95% CI, 35–41%) experienced anxiety, 34% (95% CI 30–38%) reported depression, 30% (95% CI, 29–31%) had acute stress disorder, and 26% (95% CI, 21–31%) had post-traumatic stress disorder.

**Conclusions:**

The study found that there were common mental health problems among health care workers during the COVID-19 pandemic. The most common was job burnout, followed by anxiety, depression, acute stress and post-traumatic stress disorder. Although the global pandemic has been brought under control, its long-term impact on the mental health of healthcare workers cannot be ignored. Additional research is required to develop measures to prevent, monitor and treat psychological disorders among healthcare workers.

## 1. Introduction

On January 9, 2020, a kind of novel coronavirus 2019-NCoV was officially identified as the pathogen of a viral pneumonia outbreak in Wuhan, China [1]. As of January 19, 2023, the cumulative number of confirmed cases of COVID-19 worldwide has reached 663,001,898, including 6,707,959 deaths, and these numbers are still increasing [2].

The pandemic situation of COVID-19, like other pandemics, has had a significant impact on the mental health of ordinary people, but because of the particularity of the working conditions of medical staff, they suffered a greater impact on their mental health [3]. Lack of treatment experience and protective equipment, understaffing and work overload, and ethical dilemmas in allocating scarce resources to other patients who also needed help had deleterious effects on the mental health of healthcare workers. Other factors, such as the potential risk of infecting family members and the and the emergency deployment of many non-respiratory and infectious disease professionals to front-line intensive care units with high risk in the face of a sharp increase in the number of patients, not only put healthcare workers worldwide at higher risk of infection, but caused great psychological stress [4–7]. Studies of the SARS pandemic and MERS epidemic showed that these psychological stresses increased the risk of mental health diseases among healthcare workers, including anxiety, depression, post-traumatic stress symptoms and job burnout [8, 9]. These conditions not only had adverse effects on healthcare workers, but had serious adverse consequences for their co-workers, families, and society in general [10].

Many articles on this subject have been published recently, most of which showed an increase in the prevalence of mental diseases among health workers, but most of these studies were either aimed at all social people or people in non-medical fields or in specific medical personnel, such as front-line personnel, psychiatric professionals, rehabilitation areas, and some studies were carried out in specific countries and regions [11–15]. At present, the literature search deadline of the published research is mostly in 2020 or 2021 [16–19], and there were few studies included health care worker prevalence published in 2022 [20]. Because of the ongoing nature of the COVID-19 pandemic and the continuous change of the global pandemic situation, the mental health status of global healthcare workers is in a state of dynamic change. Our analysis, which included studies published through November 2022, evaluated the existing scientific evidence on the mental health status of medical staff during the COVID-19 pandemic. These findings can therefore provide the latest estimates on the rates of depression, anxiety, burnout, acute stress and post-traumatic stress disorder among healthcare workers.

This study conducted an innovative subgroup analysis according to the time of data collection included in the article. Compared with the previous subgroup analysis based on the publication time of the included articles, our findings can more accurately determine the mental health status of health care workers around the world at different times.

## 2. Materials and methods

This systematic review and meta-analysis adhered to the Preferred Reporting Items for Systematic Reviews and Meta-Analyses (PRISMA) guidelines [21], and detailed information is provided in **S1 Table in S2 File**. The study protocol has been registered in the International Prospective Register of Systematic Reviews (PROSPERO) as No. CRD42023392547.

### 2.1. Search strategy and selection criteria

To find all relevant studies, two independent researchers (J. Huang and T. T. Chen) searched the Cumulative Index to Nursing and Allied Health Literature (CINAHL), EMBASE, Elsevier, MEDLINE, PubMed, PsycINFO and Web of Science databases for all studies published through November 11, 2022, using medical subject word (MeSH) terms and specific keywords. The specific keywords used to search the databases included COVID-19 OR COVID 19 OR COVID 19 OR Infection SARS-CoV-2 OR SARS CoV 2 Infection OR SARS-CoV-2 Infections OR 2019 Novel Coronavirus Disease OR 2019 Novel Coronavirus Infection OR 2019-nCoV Disease OR 2019 nCoV Disease OR 2019-nCoV Diseases OR Disease, 2019-nCoV OR COVID-19 Virus Infection OR COVID-19 Virus Infection OR COVID-19 Virus Infections OR Infection, COVID-19 Virus OR Virus Infection, COVID-19 OR Coronavirus Disease 2019 OR Disease 2019, Coronavirus OR Coronavirus Disease-19 OR Coronavirus Disease 19 OR Severe Acute Respiratory Syndrome Coronavirus 2 Infection OR SARS Coronavirus 2 Infection OR COVID-19 Virus Disease OR COVID 19 Virus Disease OR COVID-19 Virus Diseases OR Disease, COVID-19 Virus OR Virus Disease, COVID-19 OR 2019-nCoV Infection OR 2019 nCoV Infection OR 2019-nCoV Infections OR Infection, 2019-nCoV OR COVID19 OR COVID-19 Pandemic OR COVID 19 Pandemic OR Pandemic, COVID-19 OR COVID-19 Pandemics AND Mental Health OR Health, Mental OR Mental Hygiene OR Hygiene, Mental AND Personnel, Health OR Healthcare Providers OR Healthcare Provider OR Provider, Healthcare OR Healthcare Workers OR Healthcare Worker OR Healthcare Professionals OR Healthcare Professional OR Professional, Healthcare OR "Health Personnel". The retrieval strategy is described in detail in the supplementary document (**S1 File**).

### 2.2. Eligibility criteria

All the retrieved articles were screened independently by two researchers (J. Huang and T.T. Chen). Title and abstract were screened initially, with all articles that did not meet the inclusion criteria excluded. The full texts of the remaining articles were screened subsequently.

Criteria for inclusion in the systematic review and meta-analysis were: (a) studies of professionals who worked as healthcare workers during the COVID-19 pandemic; (b) cohort, cross-sectional and case-control studies; (c) studies that used a validated structured assessment scale; (d) studies of sample size ≥100 persons; and (e) studies with original and independent data. In addition, studies that reported mental health outcomes during the pandemic had to include at least one of the following categories: anxiety, depression, job burnout, post-traumatic stress disorder and acute stress disorder, with the prevalence rate being reported or able to be calculated. Studies were excluded if they reporting results from people other than healthcare workers, such as the general public, students, or police or were written in a language other than English.

### 2.3. Data extraction

Two researchers (X.C. Sun and Z.T. Huang) comprehensively and independently extracted the basic data included in the study, including: first author, data collection time, national and economic level, continent, study design, sample size, age, gender (percentage of males), occupation; numbers of individuals with depression, anxiety, job burnout, post-traumatic stress disorder, and acute stress disorder; and assessment tools used. After the data was extracted, the two reviewers resolved any differences by consensus; if necessary, a third reviewer (J. Huang) was consulted.

### 2.4.Quality assessment

The quality of each study included study in this analysis was evaluated using the American Institute for Healthcare Quality and Research (AHRQ) cross-sectional study evaluation criteria [22] and the Newcastle-Ottawa observational study scale (NOS) [23]. The AHRQ consists of 11 items, each with a score of 0 or 1; with total scores of ≤ 3, 4–7, and ≥ 8 indicating low, medium quality, and high quality, respectively. The NOS consists of eight items that are used to evaluate cohort and case-control studies; scores range from 0–9, with scores <6 regarded as low quality and those ≥6 as high quality. Three reviewers (J. Huang, Z.T. Huang and X.C. Sun) evaluate the quality of each study independently, with any differences resolved by discussions.

### 2.5. Statistical analysis

The comprehensive prevalence rate of mental health problems among health workers during the COVID-19 pandemic was calculated as the main effect, with the metaprop command in STATA used meta-analysis [24]. The random effect model was used to calculate the estimated comprehensive prevalence of mental health disorders to take into account the expected heterogeneity caused by changes in the characteristics of the study. Variability was assessed using the I^2^ index, with indices of 25%, 50% and 75% indicating low, medium and high heterogeneity, respectively, in the random effects model [25]. Subgroup analyses were performed based on six criteria: time of data collection, whether the country was or was not developed, continent, doctors and nurses, doctors/nurses vs. other healthcare workers, and assessment tools. The data collection time was divided into three groups based on the year of data collection, rather than the year of publication: 2020, 2021, and 2022. Countries around the world were divided into developed and developing countries according to the human development index(HDI) proposed by the United Nations Development Programme [26]. The study of other healthcare workers was abandoned when studying doctors and nurses, followed by the scale used by the study. Except for the three subgroups of data collection time, continent and evaluation tool, the other subgroups were defined as binary. The six models were evaluated independently, with each factor examined separately. All statistical analyses were performed using STATA16.0 software, with a two-side p-value <0.05 defined as statistically significant.

## 3. Results

### 3.1. Search results

**Fig 1:**
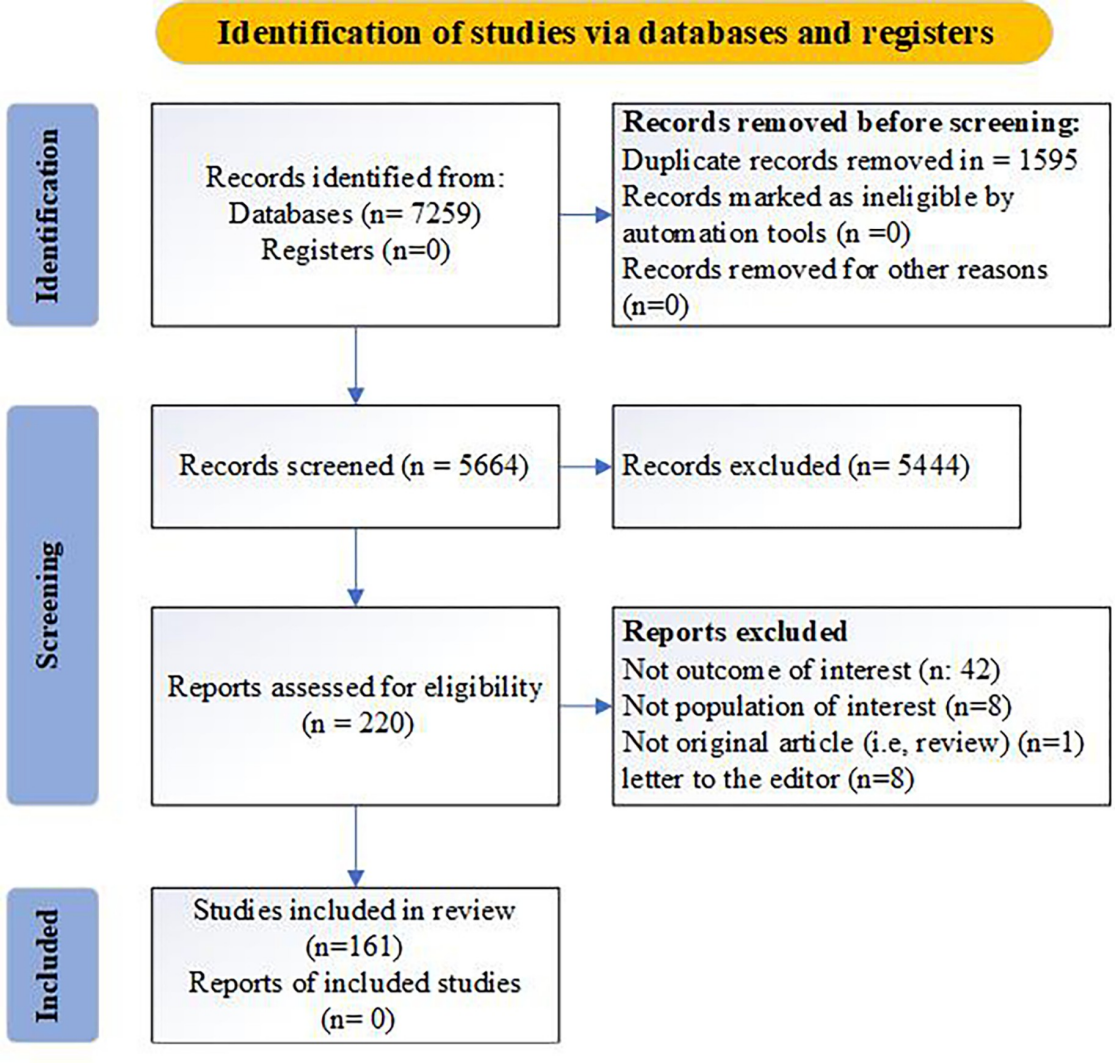
Prisma flow-diagram. *From*: Page MJ, McKenzie JE, Bossuyt PM, Boutron I, Hoffmann TC, Mulrow CD, et al. The PRISMA 2020 statement: an updated guideline for reporting systematic reviews. BMJ 2021;372:n71. doi: 10.1136/bmj.n71. For more information, visit: http://www.prisma-statement.org/.

The literature search identified 7,259 citations, of which 1,595 were deleted due to repetition, and 5,444 were excluded after reading their titles and abstracts. A total of 220 articles were evaluated and screened,59 of these studies were excluded. specifically, 8 studies were excluded due to topic mismatch, 42 studies were excluded due to inconsistent results, 1 study was excluded as it was not an original research, and 8 studies were excluded as they were letters to journal editors. Detailed reasons for the exclusions can be found in **S2 Table in S3 File**. Hence, a total of 161 studies were incorporated into this systematic review and meta-analysis, and all of them are appropriately cited in the reference section [27–187]. **Fig 1** shows the flow diagram of the search process and study selection, following the PRISMA protocol.

### 3.2. Study characteristics

All 161 included studies focused on the mental health of health workers during the pandemic; of these, 136 studies (84.5%) evaluated anxiety, 134 (83.2%) evaluated depression, 18 (11.2%) evaluated job burnout, 45 (27.9%) evaluated post-traumatic stress disorder, and two (1.2%) evaluated acute stress disorder. Of the 161 included studies, 158 (98.1%) were cross-sectional and three (1.9%) were cohort studies. The studies evaluated healthcare workers from 49 countries on five continents, of which 94 (58.4%), 37 (23.0%), 11 (6.8%), 10 (6.2%), 5 (3.1%) and 4 (2.5%) studies were conducted in Asia, Europe, Africa, North America, South America and Oceania, respectively. The highest number of studies, totaling 34 (21.1%), originated from China, followed by 10 studies (6.2%) from Italy. The United States and Iran tied for third place, each with 8 studies (5.0%). Additionally, three studies (1.9%) were conducted across multiple countries. The 161 studies included 341,014 participants, 82.8% of whom were women. Although not all studies reported age, those that reported age found that participants ranged in age from 18 to ≥60 years. Evaluation of their occupations showed that 16.2% of the participants were doctors, 63.6% were nurses, and 13.3% were other medical personnel. Data for 138 studies (85.7%) were collected in 2020, whereas data for 15 (9.3%) and two (1.2%) studies were collected in 2021 and 2022, respectively, with data collection time not reported for the remaining six (3.7%) studies. Of the 155 studies reporting data collection time, 138 (89%) were collected in 2020, 15 (9.7%) were collected in 2021, and 2 (1.3%) were collected in 2022. **S3 Table in S4 File and S4, S5 Tables in S5 File** present detailed information and quality assessment scores of all included studies.

### 3.3. Meta-analysis of mental health problems among healthcare workers during the COVID-19 pandemic

#### 3.3.1. Burnout

**Fig 2:**
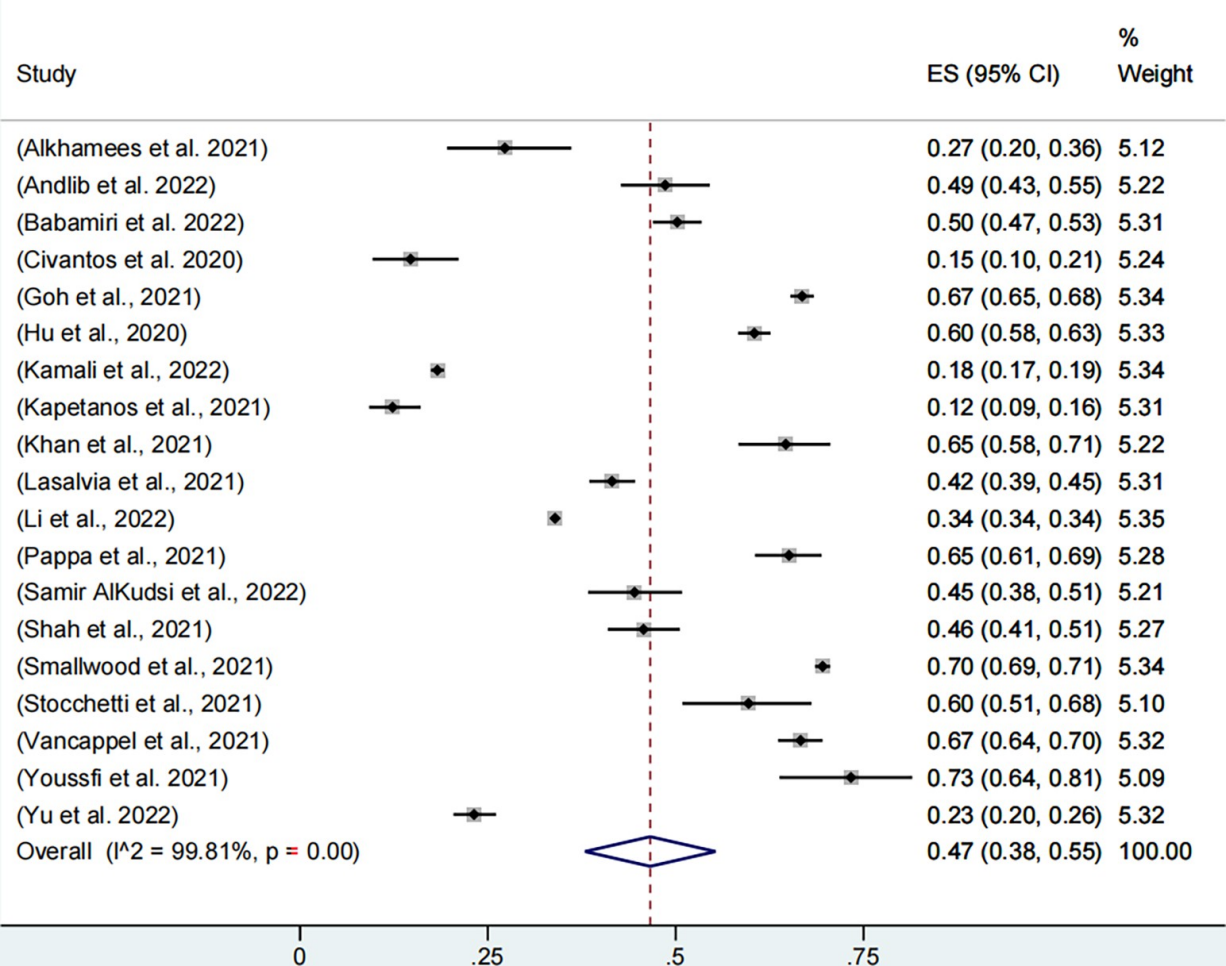
Meta-analysis and pooled estimate of burnout in health care workers during the COVID-19 pandemic.

Nineteen studies, including 165,770 participants, reported the prevalence of job burnout, with the combined prevalence rate of job burnout in health workers being 47% (95% CI, 38%-55%, **Fig 2**). Individual study estimates ranged from 12% to 73% and there was evidence of high between-study heterogeneity (I2 = 99.81%, p<0.001).

*Subgroup analysis*: *Burnout*. As shown in **Table 1**, the combined estimate for 2020 was 47% (95% CI, 33%-61%), and the combined estimate for 2021 was 44% (95% CI, 35%-53%). There was no significant difference in prevalence among different years (p = 0.731).

**Table 1:** Subgroup analyses for studies on burnout.

Subgroup analysis		No. of studies	Prevalence % (95% CI)	I^2^	Between-group difference
Data collection time	2020	15	47 (33–61)	99.82%	
	2021	3	44 (35–53)	-	
					p = 0.731
Continent	Oceania	1	70 (69–71)	-	
	Europe	5	60 (50–70)	-	
	Africa	2	52 (48–56)	-	
	Asia	8	37 (28–46)	99.67%	
	North America	2	31 (28–33)	-	
	South America	1	15 (10–21)	-	
					P<0.001
Assessment tools	OLBI	1	67 (65–68)	-	
	MBI	14	47 (37–57)	99.83%	
	SPFI	1	46 (41–51)	-	
	ProQoL	2	28 (25–31)	-	
	Mini-Z	1	15 (10–21)	-	
					p<0.001

Abbreviation: MBI = Maslach Burnout Inventory; Mini-Z = a non-proprietary, single-item burnout measure; OLBI = Oldenburg Burnout Inventory; ProQoL = Professional Quality of Life scale; SPFI = Stanford Professional Fulfillment Index Questionnaire

It was estimated that the prevalence rate of job burnout was significantly different in different regions (p<0.001). Studies from Oceania had the highest estimated combined prevalence rate (70%; 95% CI, 69%-71%) and the lowest in South America (15%; 95% CI, 10%-21%).

Fourteen studies used the Maslach Job Burnout scale (MBI) [188], and the combined prevalence of all these studies was estimated at 47% (95% CI, 37%-57%). The highest combined prevalence rate was calculated from the study using the Oldenburg Burnout Inventory (OLBI) [189] (67%; 95% CI, 65%-68%), and the study using the single-item Mini-Z burnout assessment [190] obtained the lowest comprehensive estimate (15%; 95% CI, 10%-21%). The combined estimated values of these subgroups were significantly different (p<0.001).

There was no evidence of differential prevalence estimates across other subgroups: the degree of national development(p = 0.515); doctors and nurses (p = 0.417); and doctors and nurses vs other healthcare workers (p = 0.079).

The burnout variable was chosen as the main independent variables in the Burnout variable and regression analyses were performed. Next, sensitivity analyses and bias tests were conducted. The results of the sensitivity analyses showed that the burnout variable had a small effect on the results at different values. For example, when the burnout variable was increased by 1 unit, the results changed within ±9.79% (S1A Fig). The robustness of the results was verified. The results of the bias test showed that we eliminated the potential bias of the burnout variable through the propensity score matching method. The test results showed that the association between the burnout variable and the results was significant (p<0.05) in the matched sample, indicating that the results of the study were affected by significant bias (S1B and S1C Fig).

**S1-S6 Figs in S6 File** display the specific results of subgroup analyses.

#### 3.3.2. Anxiety

**Fig 3:**
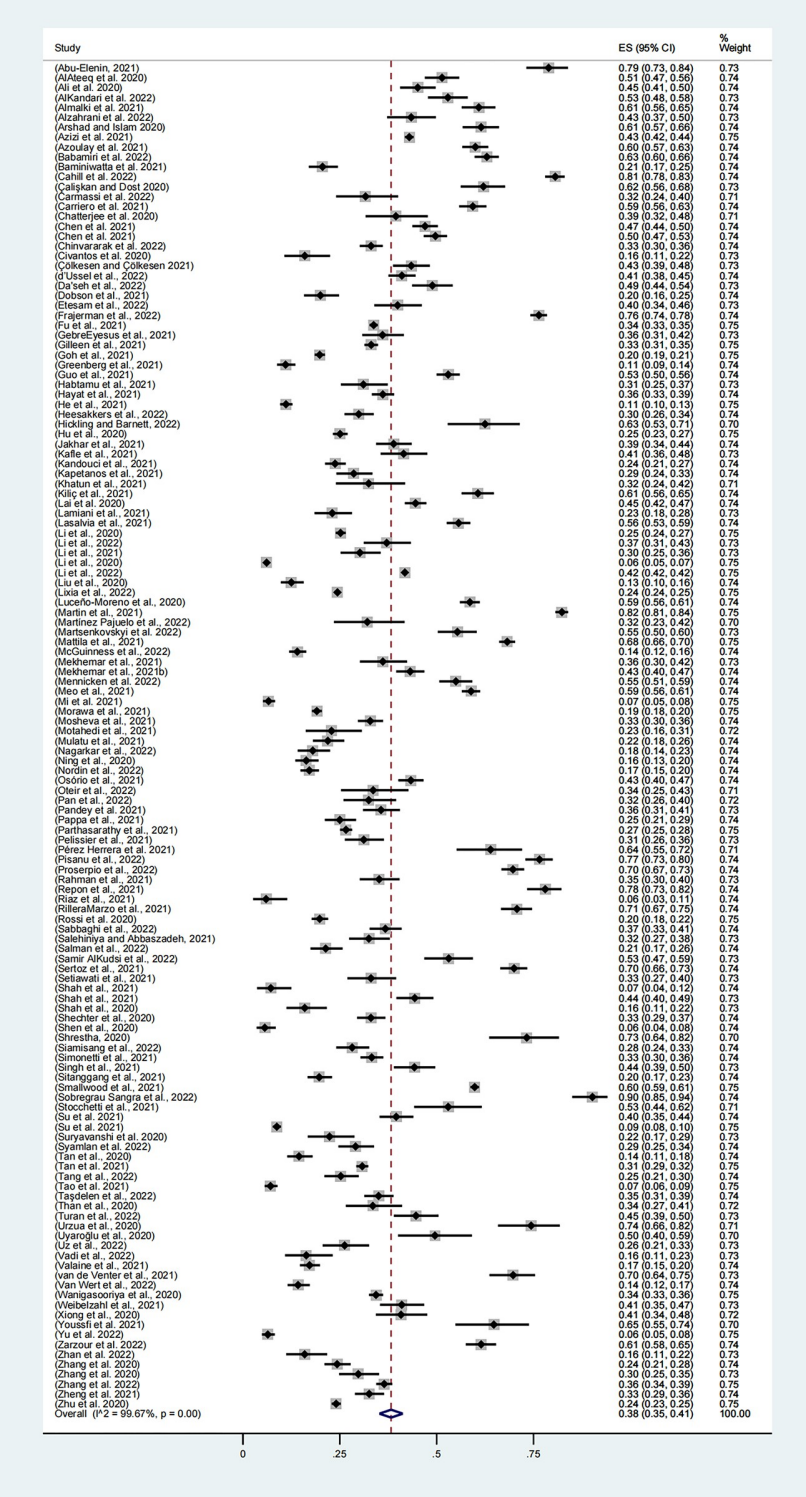
Meta-analysis and pooled estimate of anxiety in health care workers during the COVID-19 pandemic.

A total of 136 studies, involving 112,805 participants, reported the prevalence of anxiety disorders, with the combined prevalence rate of anxiety among health workers being 38% (95% CI, 35–41%, **Fig 3**). Individual study estimates ranged from 6% to 90% and there was evidence of high heterogeneity between studies (I2 = 99.67%, p<0.001).

Subgroup analysis: anxiety. As shown in **Table 2**, the combined estimate for 2020 was 38% (95% CI, 35%-42%), the combined estimate for 2021 was 41% (95% CI, 34%-47%), and the combined estimate for 2022 was 29% (95% CI, 26%-33%). The prevalence rate of 2022 was lower than that of 2021, and there were significant differences in different years (p<0.001).

**Table 2:** Subgroup analyses for studies on anxiety.

Subgroup analysis		No. of studies	Prevalence % (95% CI)	I^2^	Between-group difference
Data collection time	2020	117	38 (35–42)	99.60%	
	2021	14	41 (34–47)	98.80%	
	2022	2	29 (26–33)	-	
					p<0.001
Assessment tools	STAI	6	56 (39–73)	99.08%	
	GHQ-28	2	55 (52–58)	-	
	PROMIS	2	53 (51–55)	-	
	GAD-7/BAI	1	50 (40–59)	-	
	HADS	19	43 (32–55)	99.67%	
	ISR	1	41 (35–47)	-	
	GAD-7	54	39 (34–45)	99.73%	
	DASS-42	4	38 (28–47)	92.05%	
	DASS-21	27	36 (30–41)	99.04%	
	GAD-2	4	29 (22–36)	97.45%	
	SAS	9	25 (14–37)	99.69%	
	PHQ-4	2	18 (17–19)	-	
	PHQ-2	1	16 (11–22)	-	
	CAS	2	11 (10–13)	-	
	SCL-90	2	10 (8–12)	-	
					p<0.001

Abbreviation: BAI = Beck Anxiety Inventory; CAS = Coronavirus Anxiety Scale; DASS-21 = The Depression Anxiety Stress Scale-21; DASS-42 = The Depression Anxiety Stress Scale-42; GAD-2 = The 2-item Generalized Anxiety Disorder Scale; GAD-7 = General Anxiety Disorder-7; GHQ-28 = General Health Questionnaire; HADS = The Hospital Anxiety and Depression Scale; ISR = The self-report questionnaire ICD-10 Symptom Rating; PROMIS = National Institution of Mental Health (NIMH) Patient‐Reported Outcomes Measurement Information System; SAS = Self-Rating Anxiety Scale; SCL-90 = Symptom Checklist 90; STAI = State Trait Anxiety Inventory Scale; PHQ-2 = The 2-item Generalized Anxiety Disorder Scale; PHQ-4 = Patient Health Questionnaire-4

A total of 54 studies used the Generalized Anxiety Disorder 7 (GAD-7) questionnaire [191], and the combined prevalence rate of all these studies was estimated at 39% (95% CI, 34%-45%). The highest combined prevalence rate was calculated from the study using the State-Trait Anxiety Inventory (STAI) [192] (56%; CI, 39%-73%), and the study using Symptom Checklist 90 (SCL-90) [193] produced the lowest comprehensive estimate (10%; 95% CI, 8%-12%). The combined estimated values of these subgroups were significantly different (p<0.001).

There was no evidence of differential prevalence estimates across other subgroups: the degree of national development(p = 0.435); continents (p = 0.298); doctor and nurse (p = 0.796); and doctors and nurses vs other healthcare workers (p = 0.378). **S7-S12 Figs in S6 File** display the specific results of subgroup analyses.

The anxiety variable was chosen as the main independent variable and regression analyses were performed. Next, sensitivity analyses and bias tests were conducted. The results of the sensitivity analysis showed that the anxiety variable had a small effect on the results at different values, with a combined effect size of 0.26 (0.25,0.27, CI95%) (S2A Fig). The robustness of the results was verified. The bias test showed that we eliminated the potential bias of the anxiety variable by the propensity score matching method. The test results showed a significant association between the anxiety variable and the outcome in the matched sample (p<0.05), indicating that the findings were affected by significant bias (S2B and S2C Fig).

#### 3.3.3. Depression

**Fig 4:**
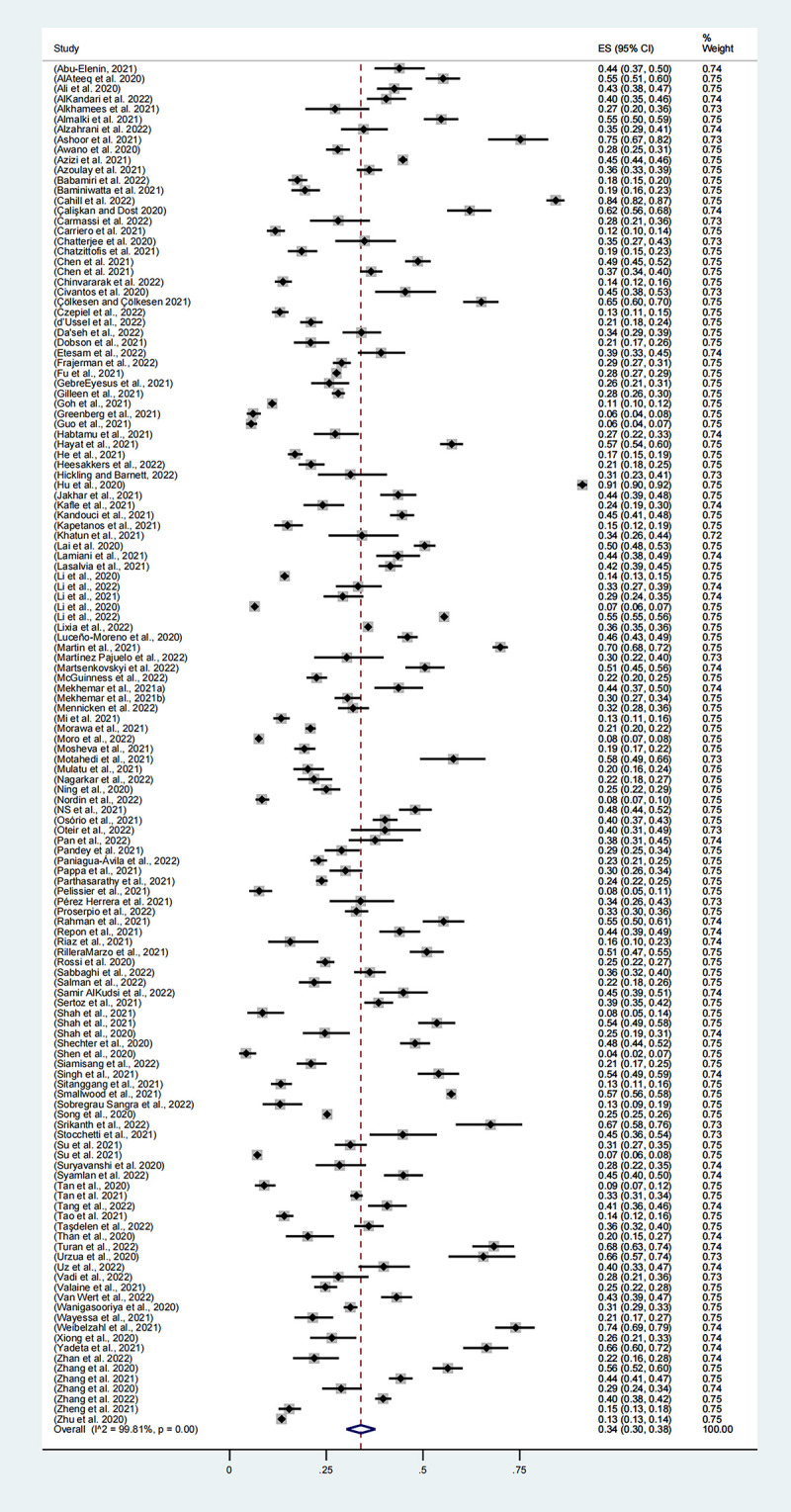
Meta-analysis and pooled estimate of depression in health care workers during the COVID-19 pandemic.

A total of 134 studies, involving 316,891 participants, reported the prevalence of depression, with the combined prevalence rate of depression among healthcare workers being 34% (95% CI, 30–38%, **Fig 4**). Estimates for a single study ranged from 4% to 91%, and there was evidence of high heterogeneity between studies (I2 = 99.81%, p<0.001).

Subgroup analysis: depression. As shown in **Table 3**, the combined estimate for 2020 was 33% (95% CI, 30%-37%), the combined estimate for 2021 was 38% (95% CI, 27%-48%), and the combined estimate for 2022 was 38% (95% CI, 34%-41%). The prevalence rate in 2021 and 2022 was higher than that in 2020, although statistical analysis showed that there was no significant difference in prevalence among different years (p = 0.255).

**Table 3:** Subgroup analyses for studies on depression.

Subgroup analysis		No. of studies	Prevalence % (95% CI)	I^2^	Between-group difference
Data collection time	2020	113	33 (30–37)	99.68%	
	2021	16	38 (27–48)	99.64%	
	2022	2	38 (34–41)	-	
					p = 0.255
Assessment tools	GAD-7	1	28(25–31)	-	
	GHQ-28	1	18(15–20)	-	
	HAM-D	1	44(38–49)	-	
	GAD-2	1	25(19–31)	-	
	IRS	1	74(69–79)	-	
	SAS	1	44(41–47)	-	
	PHQ-9	54	33(29–38)	99.69%	
	DASS-21	27	32(27–38)	98.94%	
	HADS	19	35(27–43)	99.42%	
	PHQ-8	2	68(66–70)	-	
	BDI	3	24(8–40)	-	
	PHQ-2	7	39(27–52)	98.58%	
	SDS	5	42(19–65)	99.92%	
	CES-D	3	42(25–59)	-	
	DASS-42	3	29(20–38)	-	
	SCL-90	2	8(6–10)	-	
	PHQ-4	3	23(14–32)	-	
					p<0.001

Abbreviation: BDI = Beck Depression Inventory; CES-D = The Center for Epidemiologic Studies Depression Scale

DASS-21 = The Depression Anxiety Stress Scale-21; DASS-42 = The Depression Anxiety Stress Scale-42; GAD-2 = The 2-item Generalized Anxiety Disorder Scale; GAD-7 = General Anxiety Disorder-7; GHQ-28 = General Health Questionnaire; HADS = The Hospital Anxiety and Depression Scale; HAM-D = Hamilton Depression Scale; ISR = The self-report questionnaire ICD-10 Symptom Rating; PHQ-4 = Patient Health Questionnaire-4; PHQ-8 = The 8-item Patient Health Questionnaire; PHQ-9 = The Patient Health Questionnaire, 9-item version; SAS = Self-Rating Anxiety Scale; SCL-90 = Symptom Checklist 90; SCL-90 = Symptom Checklist 90; SDS = Self-Rating Depression Scale

Fifty-four studies used the patient Health questionnaire (PHQ-9) [194], and the combined prevalence rate of all these studies was estimated at 33% (95% CI, 29%-38%). The highest combined prevalence rate was calculated from the study using the ICD-10 symptom Checklist 90 (ISR) [195, 196] (74%; 95% CI, 69%-79%), while the study using the symptom list 90 (SCL-90) [193] yielded the lowest comprehensive estimate (8%; 95% CI, 6%-10%). The combined estimated values of these subgroups were significantly different (p<0.001).

There was no evidence of differential prevalence estimates across other subgroups: the degree of national development(p = 0.321); continents (p = 0.352); doctor and nurse (p = 0.746); and doctors and nurses vs other healthcare workers (p = 0.196). **S13-S18 Figs in S6 File** display the specific results of subgroup analyses.

The depression variable was chosen as the main independent variable and regression analyses were performed. Next, sensitivity analyses and bias tests were conducted. The results of the sensitivity analysis showed that the depression variable had a small effect on the results at different values, with a combined effect size of 0.25 (0.24,0.26, CI95%) (S3A Fig). The robustness of the results was verified. The results of the bias test showed that we eliminated the potential bias of the depression variable by the propensity score matching method. The test results showed that the association between the depression variable and the results was not significant (p>0.05) in the matched sample, indicating that the results of the study were not affected by significant bias (S3B and S3C Fig).

#### 3.3.4. Acute stress disorder

**Fig 5:**
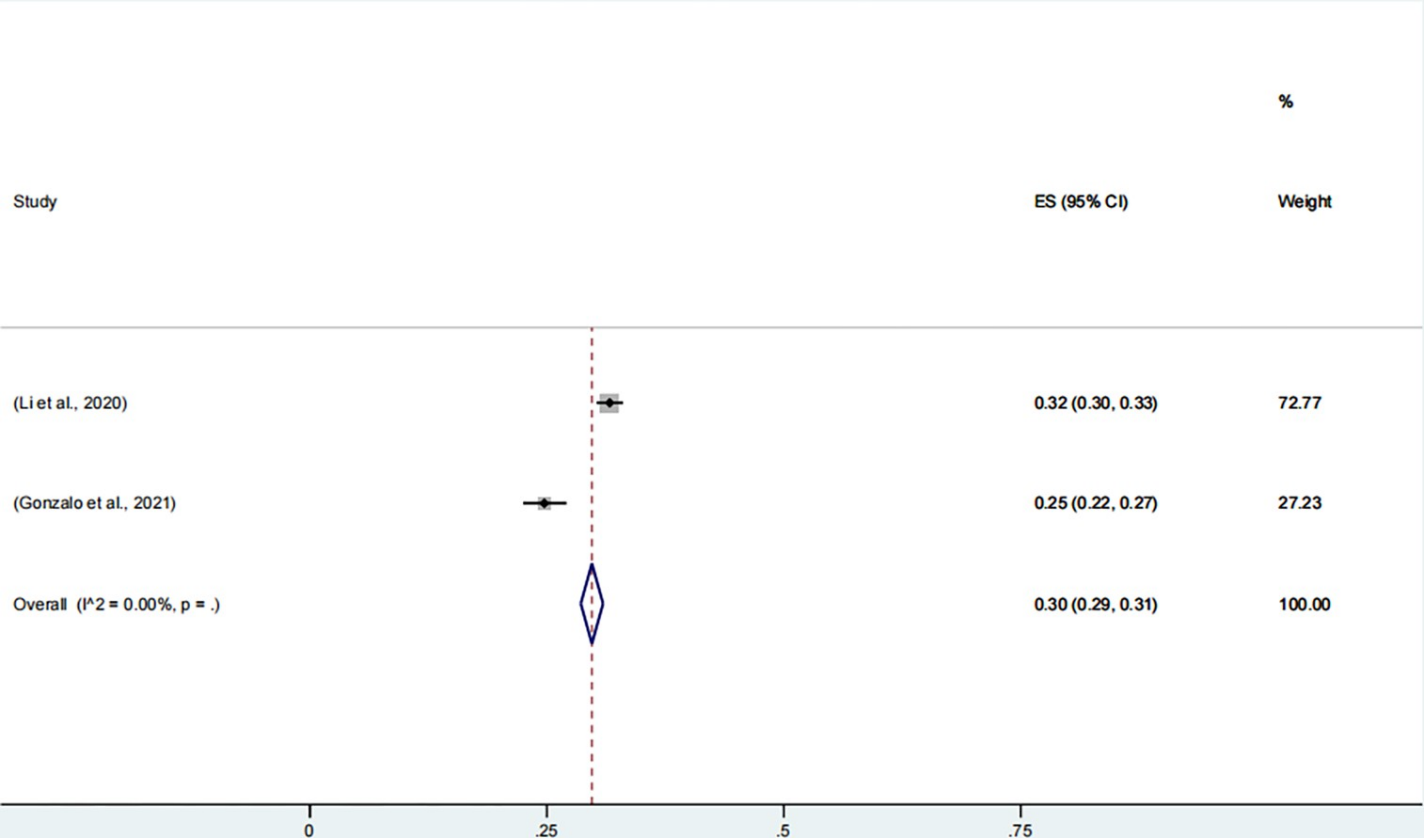
Meta-analysis and pooled estimate of acute stress disorder in health care workers during the COVID-19 pandemic.

Two studies, involving 5,776 participants, reported the prevalence of acute stress disorder, with the combined prevalence rate of acute stress disorder among healthcare workers being 30% (95% CI, 29–31%, **Fig 5**). The data were collected in 2020. Two evaluation scales were used: Standford acute Stress Reaction Questionnaire (SARS-Q) [197] and Impact of Event Scale-Revised Questionnaires (IES-R) [198].

No subgroup analysis was performed because few studies reported the prevalence of acute stress disorder (<3).

The Acute stress disorder variable was selected as the main independent variable and regression analyses were performed. Next, sensitivity analyses and bias tests were conducted. The results of the sensitivity analysis showed that the Acute stress disorder variable had a small effect on the results at different values, with a combined effect size of 0.14 (0.13,0.16, CI95%) (S4A Fig). The robustness of the results was verified. The results of the bias test showed that we eliminated the potential bias of the Acute stress disorder variable by the propensity score matching method. The test results showed that the association between Acute stress disorder variables and the outcomes was not significant (p>0.05) in the matched sample, indicating that the findings were not affected by significant bias (S4B and S4C Fig).

#### 3.3.5. Post-traumatic stress disorder

**Fig 6:**
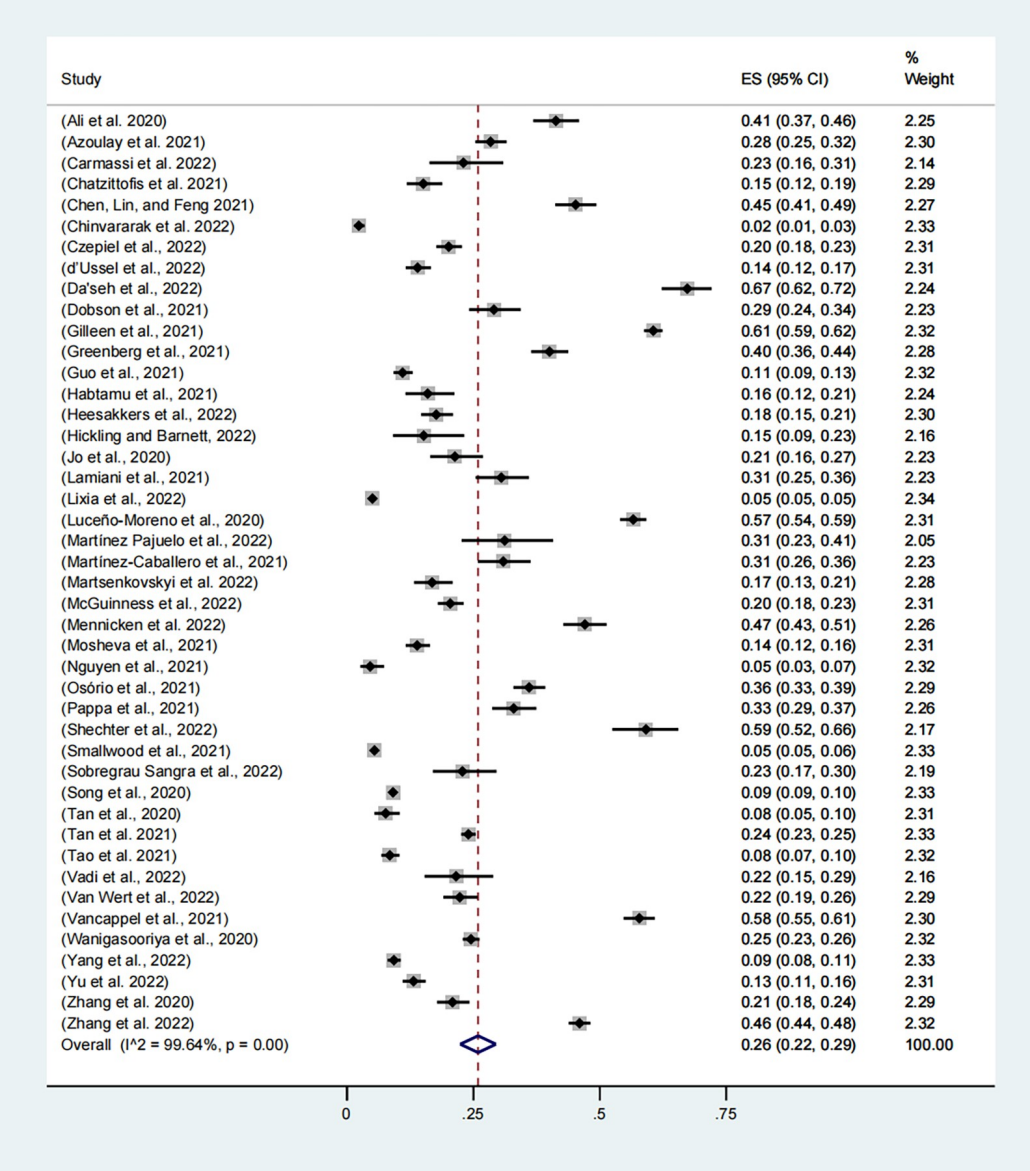
Meta-analysis and pooled estimate of post-traumatic stress disorder in health care workers during the COVID-19 pandemic.

Forty-four studies, including 89,977 participants, reported the prevalence of post-traumatic stress disorder (PTSD), with the combined prevalence rate of PTSD among healthcare workers being 26% (95% CI, 22–29%, **Fig 6**). Individual study estimates ranged from 2% to 67% and there was evidence of high between-study heterogeneity (I2 = 99.64%, p<0.001).

Subgroup analysis: post-traumatic stress disorder. As shown in **Table 4**, the combined estimate for 2020 was 25% (95% CI, 21%-29%) and for 2021 was 28% (95% CI, 13%-42%). The prevalence rate was similar in different years and there was no significant difference (p = 0.733).

**Table 4:** Subgroup analyses for studies on post-traumatic stress disorder.

Subgroup analysis		No. of studies	Prevalence % (95% CI)	I^2^	Between-group difference
Data collection time	2020	36	25(21–29)	99.66%	
	2021	7	28(13–42)	99.51%	
					p = 0.733
Continent	Oceania	3	18(4–32)	-	
	Europe	17	33(25–42)	99.25%	
	Africa	1	16(12–21)	-	
	Asia	17	19(15–23)	99.54%	
	North America	4	27(12–43)	98.38%	
	South America	2	35(33–38)	-	
					p<0.001
Assessment tools	IES-R	22	32(23–41)	99.74%	
	PCL	19	20(17–22)	99.02%	
	DTS-8	1	31(26–36)	-	
	ASDS	2	9(8–11)	-	
					p<0.001

Abbreviation: ASDS = Acute Stress Disorder Scale; DTS-8 = The Davidson Trauma Scale; IES-R = The 22-item Impact of Event Scale-Revised; PCL = Post Traumatic Stress Disorder Checklist

It was estimated that the prevalence rate of post-traumatic stress disorder was significantly different in different regions (p<0.001). The study from South America produced the highest estimated combined prevalence rate (35%; 95% CI, 33%-38%) and the lowest in Africa (16%; 95% CI, 12%-21%).

Twenty-two studies used the Impact of Event Scale-Revised (IES-R) [198], and the combined prevalence rate of all these studies was estimated at 32% (95% CI, 23%-41%), which was the highest. The study using the Acute Stress Disorder Scale (ASDS) [199] yielded the lowest comprehensive estimate (9%; 95% CI, 8%-11%). The combined estimated values of these subgroups were significantly different (p<0.001).

There was no evidence of differential prevalence estimates across other subgroups: the degree of national development(p = 0.107); doctors and nurses (p = 0.336); and doctors and nurses vs other healthcare workers (p = 0.473). **S19-S24 Figs in S6 File** display the specific results of subgroup analyses.

### 3.4. Quality assessment

The quality of the 158 cross-sectional studies was evaluated using the AHRQ [22], which found that the average score was 6.03 ±1.12. Of these 158 studies, 17 (10.8%) had scores ≥ 8, indicating high-quality, and the remaining 141 (89.2%) had scores of 4 to 7, indicating medium-quality; none had scores <4, indicating low quality. Evaluation of the three cohort studies with the NOS scale [23] showed that their average score was 6.67 ±0.47, with all three studies having scores ≥ 6, indicating high quality. **S4 and S5 Tables in S5 File** provides details of the study quality scores.

## 4. Discussion

This meta-analysis of 161 studies involving 341,014 healthcare workers was performed to provide an update on the prevalence of multiple mental health symptoms among healthcare workers worldwide. During the COVID-19 pandemic, the pooled prevalence rates of burnout, anxiety, depression, acute stress disorder and post-traumatic stress disorder among healthcare workers were 47%, 38%, 34%, 30% and 26%, respectively. These rates were higher than in the general population [200–202], suggesting that mental health risks were greater for healthcare workers during the pandemic.

Evaluation of 136 studies showed that the prevalence rate of anxiety among medical workers was 38%, higher than the 26.9% [200] and 20% [201] rates reported in the general population. The 134 studies that assessed depression reported a prevalence among healthcare workers of 34%, higher than that of 28% [200] and 30% [201] rates in the general population. Anxiety and depression among medical staff during COVID-19 were related to many factors, such as the shortage of personal protective equipment, the lack of other medical resources and the sacrifice of colleagues [5]. Most healthcare workers believed that, despite having adequate personal protective equipment, they felt unsafe when contacting and treating patients. Moreover, they were also worried about their families becoming infected, especially if they take care of these infected patients, with the possibility that infection may spread to their families [4, 203]. This pandemic resulted in an unprecedented restructuring of the healthcare system, with a large number of medical staff, especially nurses, being transferred to intensive care units [204]. These workers had to absorb a lot of information and master new technical skills associated with unfamiliar areas of expertise in a short period of time, which could also lead to mental health disorders [205].

Few studies in a previous meta-analysis focused on job burnout of healthcare workers,our analysis showed that the combined prevalence rate of burnout among medical workers in 18 studies was 47%, higher than that of 37% in previous studies [18]. Job burnout among healthcare workers was high even before the pandemic [206]. During the pandemic, burnout was further exacerbated by long-term intense and stressful work, emotional exhaustion, deteriorating mental health, fear of infection and lack of support [207].The increasing death toll of critically ill patients not only led to depression and pain among healthcare workers, but reduced their sense of professional achievement [208]. This not only had a negative impact on the physical and mental health and interpersonal relationships of medical personnel, but also affected their attitudes toward work and their efficiency. In addition, the present meta-analysis showed that the prevalence of acute stress disorder among health care workers was 30%, and the prevalence of PTSD was 26%, rates significantly higher than earlier in the pandemic [209].

When interpreting the pooled prevalence estimates calculated in this review and meta-analysis, it is need to note that the percentage of variability (I^2^) in the prevalence estimates due to heterogeneity was very high. When a large number of studies are included in meta-analyses, the I^2^ is extremely sensitive, while a high I^2^ is often inevitable [210]. The I^2^ may therefore detect only a small amount of heterogeneity, which is not clinically important. Despite this, we discussed the heterogeneity through subgroup analysis. Although the subgroup analyses suggested evidence of between-group differences in prevalence estimates across a number of variables (e.g. data collection time, continent, evaluation tool), intra-group heterogeneity is still high and does not fully explain the existence of heterogeneity.

Our subgroup analysis found that the prevalence of anxiety remained at a high level in 2020 and 2021, and showed a significant downward trend in 2022. The prevalence rates of job burnout, depression, and post-traumatic stress disorder (PTSD) had consistently remained high, and there was an increasing trend in depression and PTSD, which was consistent with previous research conducted on SARS [211]. PTSD has been evaluated infrequently in previous studies, as it usually occurs weeks or months after the acute stress disorder; that is, it appears later in the "repair phase" after the virus has been roughly controlled. In addition to having adverse psychological consequences, PTSD can lead to changes in a variety of physiological functions and have deleterious effects on multiple organ systems, especially increasing the risk of cardiovascular disease. Moreover, the presence of post-traumatic stress disorder (PTSD) among healthcare workers could potentially have detrimental effects on the healthcare system. Therefore, we suggest implementing screening measures and interventions for healthcare personnel [212, 213]. The decline in the prevalence of anxiety may be due to the rapid and positive response of all countries and regions around the world after the pandemic, including expanding the production of medical supplies and calling on citizens to wear masks [214].The major breakthrough in novel coronavirus’s research has led to the successful research and development of vaccines [215] and the advent of drugs aimed at novel coronavirus [216], as well as the accumulated experience of medical staff and broad social support, which quickly stabilized the tense situation and reduced the negative impact of the pandemic.

However, it should be noted that the pandemic is not really over [217], and the continuous emergence of new virulent strains such as AY.4.2 [218] and XBB.1.5 [219], as well as changes in governments’ pandemic prevention and control policies, may cause a new round of outbreaks at any time. Repeated psychological damage to health care workers will not only increase the risk of illness and lead to the deterioration of psychological disorders, but also lead to an increase in the prevalence and death of serious diseases [213]. Mental health disorders of medical staff are not as easy to detect as physical injuries, but they can also cause great pain to medical staff and damage the health care system and public safety[211]. Therefore, the prevention and control of the pandemic should not be taken lightly, and global leaders and all stakeholders should devote resources to the mental health of health care workers to screen for mental health problems and take effective interventions to help them solve their mental health disorders.

In addition, this study also has some limitations. Although seven major databases were searched, some of the other unsearched databases were still omitted and the search was mainly focused on English literature, and there were no grey literature. Secondly, during the pandemic, in order to clarify the biological characteristics of COVID-19 virus and develop better diagnosis and treatment strategies, a large number of studies related to novel coronavirus were carried out and published [220], but the quality of some of them was limited [221]. And most of the studies on the prevalence of mental disorders are cross-sectional studies, and the lower level of evidence in evidence-based medicine affects the credibility of the study [222].Because different pandemic stages of each study lead to higher heterogeneity in the estimated prevalence rate, and different pandemic stages in different countries, there is no very ideal indicator to define different periods of the global epidemic. These also affect the accuracy of the survey results. Higher heterogeneity in Meta-analysis may be due to differences in 1. participant characteristics, participants in a study may come from different populations, such as different ages, genders, ethnic groups, geographic locations, and so on. Differences in the characteristics of these populations may lead to increased heterogeneity in the results of the study. 2. differences in study design. There may be differences in the design of different studies with respect to sample size, random allocation, control group selection, measurement tools and indicator definitions, etc. These differences may also lead to increased heterogeneity. For example, some studies may be more rigorously designed, while others may have some potential bias or methodological problems. A similar issue arose in Zhou et al study of the causes of the prevalence of post-traumatic stress symptoms under the influence of public health emergencies over the last 20 years, where a high degree of heterogeneity between studies and within subgroups, which he attributed to differences in study sampling, was observed [223]. Therefore, improvements need to be made to the study in these areas mentioned above.

## 5. Conclusions

Following the outbreak of the COVID-19 pandemic, the prevalence rates of mental health disorder among healthcare workers worldwide increased significantly, resulting in serious damage to their physical and mental health. Although the pandemic has been brought under control, it has not really disappeared. Thus, future outbreaks may further aggravate mental health disorders in healthcare workers. In addition, later-occurring mental health disorders such as PTSD can have serious adverse consequences. Governments and relevant organizations should pay close attention to the mental health status of healthcare workers and implement intervention measures. Moreover, additional research is needed to better prevent and reduce mental health disorders among healthcare workers.

## Supporting information

S1 File Retrieval strategy. (DOCX)

S2 File PRISMA statement and checklist (S1 Table). (DOCX)

S3 File Reasons for exclusion during full- text screening (S2 Table). (DOCX)

S4 File Main characteristics of the included studies (S3 Table). (DOCX)

S5 File Quality assessment (S4 and S5 Tables). (DOCX)

S6 File Other subgroup analysis results (S1-S24 Figs). (DOCX)

S1 Fig (JPG)

S2 Fig (JPG)

S3 Fig (JPG)

S4 Fig (JPG)

S1 Data (ZIP)
