# Supplementary figures and images for: Mental health status and related factors influencing healthcare workers during the COVID-19 pandemic: A systematic review and meta-analysis
Source: PLoS One. 2024 Jan 19;19(1):e0289454. doi: 10.1371/journal.pone.0289454 (PMC10798549; doi:10.1371/journal.pone.0289454)

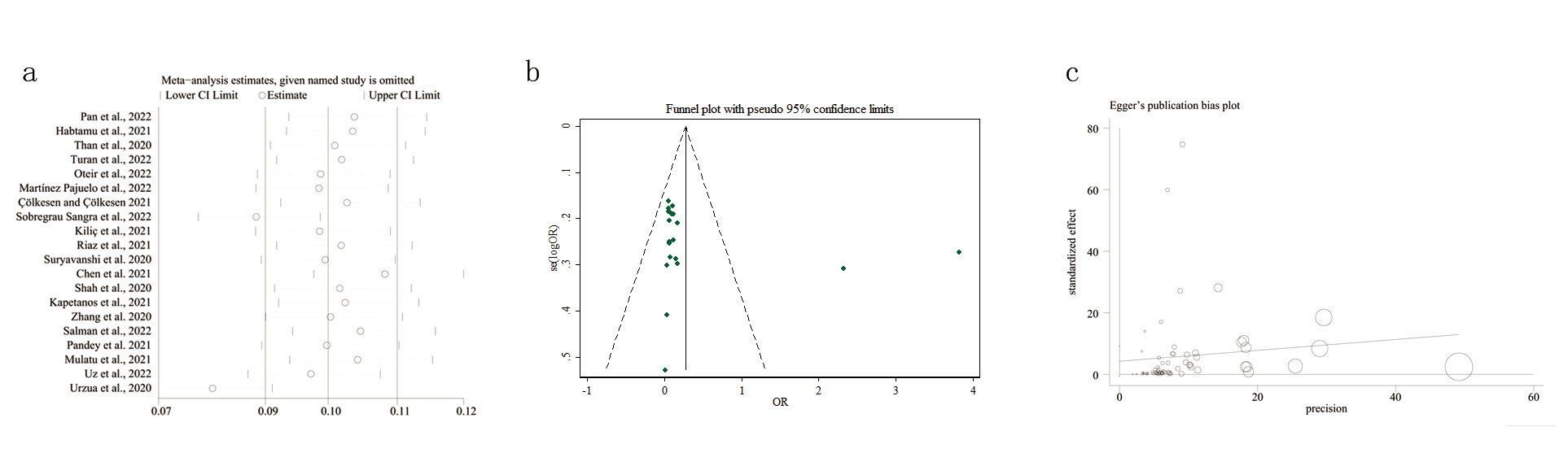

Supplement: S1 Fig — (JPG) [file pone.0289454.s007.jpg]

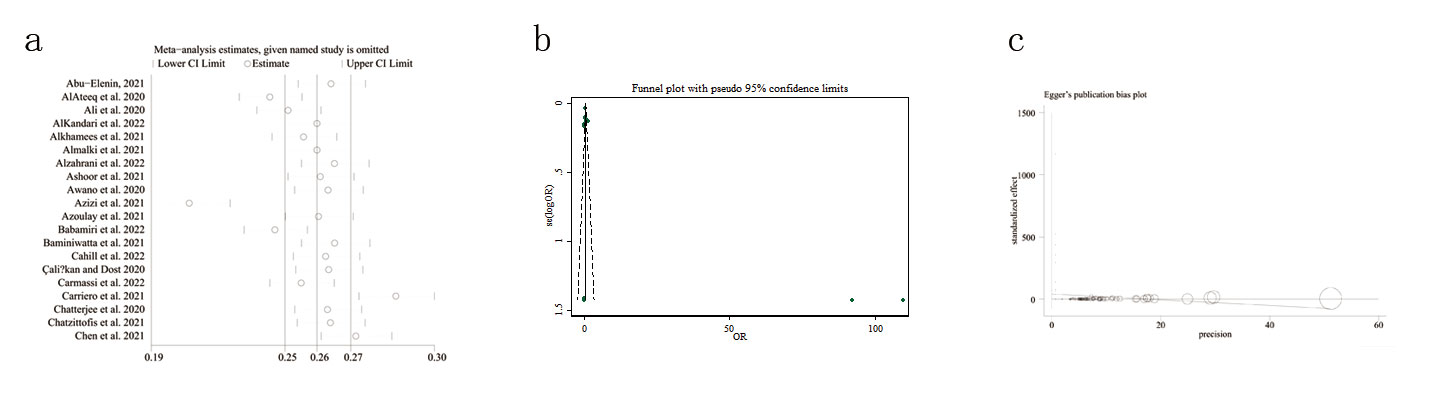

Supplement: S2 Fig — (JPG) [file pone.0289454.s008.jpg]

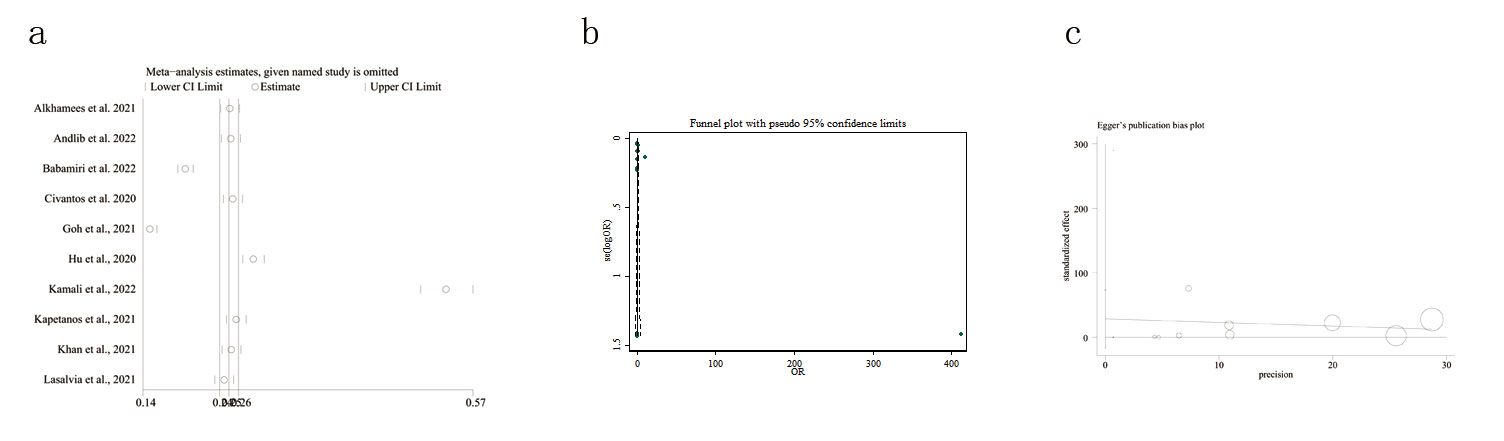

Supplement: S3 Fig — (JPG) [file pone.0289454.s009.jpg]

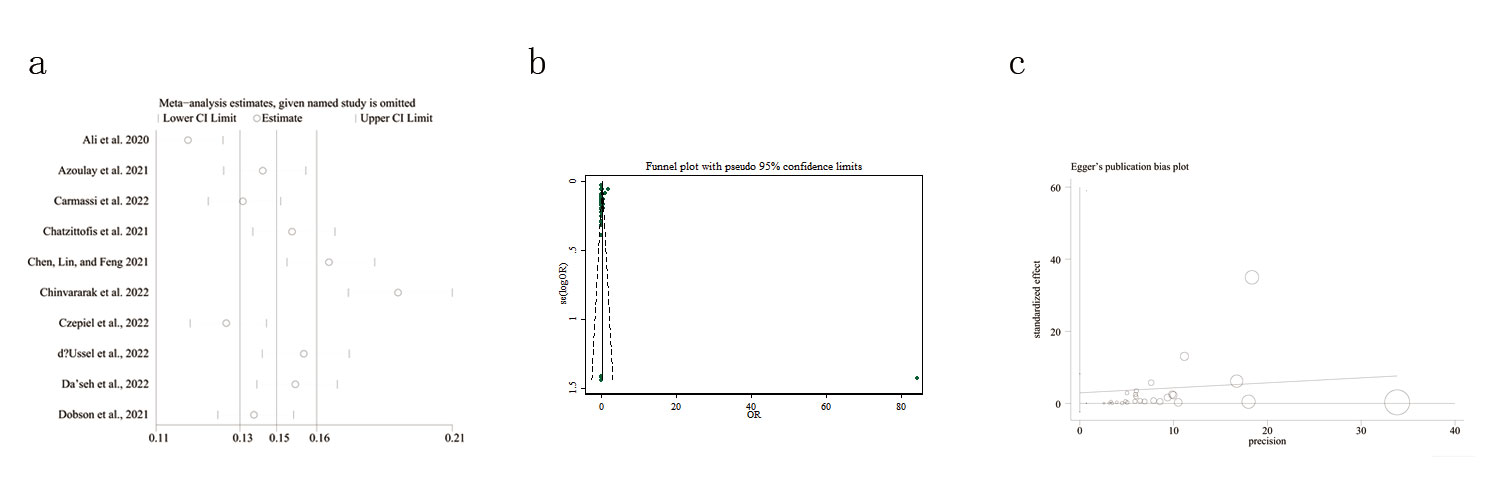

Supplement: S4 Fig — (JPG) [file pone.0289454.s010.jpg]
