# Supplementary material for: Mental health status and related factors influencing healthcare workers during the COVID-19 pandemic: A systematic review and meta-analysis
Source: PLoS One. 2024 Jan 19;19(1):e0289454. doi: 10.1371/journal.pone.0289454 (PMC10798549; doi:10.1371/journal.pone.0289454)
Supplement: S1 Data — (ZIP) [file pone.0289454.s011.zip › literatures/10.pdf]

Received: 15 May 2021 | Accepted: 15 January 2022

DOI: 10.1111/inr.12750

International Nursing Review | WILEY

ORIGINAL ARTICLE

Burnout and psychological distress among Pakistani nurses providing care to COVID-19 patients: A cross-sectional study

Sonia Andlib RN, RM, BSN[1] | **Shahzad Inayat RN, BSN, MHR**[2] | **Kehkishan Azhar MBBS**[3] | **Faisal Aziz BSN, MPH**[4]

[1] Benazir Bhutto Hospital, Rawalpindi, Pakistan

[2] Isra College of Nursing, Isra University, Islamabad, Pakistan

[3] Rawal Surgical Hospital, Wah Cantt, Pakistan

[4] Interdisciplinary Metabolic Medicine Trials Unit, Department of Endocrinology and Diabetology, Medical University of Graz, Graz, Austria

Correspondence
Faisal Aziz, Interdisciplinary Metabolic Medicine Trials Unit, Department of Endocrinology and Diabetology, Medical University of Graz, Auenbruggerplatz 15, 8036, Graz, Austria.
Email: faisal.aziz@stud.medunigraz.at

Abstract

Aim: The aim of this study is to assess the burden of burnout and psychological distress and its association among Pakistani nurses providing care to patients with COVID-19.

Background: Nurses may experience an elevated risk of burnout and psychological distress during epidemics.

Methods: A cross-sectional study was conducted using a convenience sample of 288 nurses. Maslach Burnout Inventory was administered to measure burnout and its domains of emotional exhaustion, depersonalization, and personal accomplishment. Screening Tool for Psychological Distress was administered to measure depression, anxiety, stress, anger, and low social support.

Results: Burnout was present in 48.6% of nurses, severe emotional exhaustion in 37.2%, severe depersonalization in 36.8%, and low personal accomplishment in 46.9% of nurses. Psychological distress was present in 45% of nurses. Burnout and psychological distress were significantly higher in nurses who: were working in public hospitals, did not receive training for COVID-19 prevention, and were dealing with increased patient load. Burnout and its domains were significantly associated with depression, anxiety, stress, anger, and low social support.

Conclusion: Nurses are experiencing high levels of burnout and psychological distress during the COVID-19 pandemic with a significant moderate-to-strong association between these conditions. These findings accentuate the need for institution-based interventions to mitigate burnout and preserve the mental health of nurses.

Implications for nursing and health policy: Hospitals must screen nurses frequently for the presence of significant burnout and psychological distress and offer supportive interventions to protect their mental health and well-being.

KEYWORDS
burnout, COVID-19, mental health, nurses, psychological distress

INTRODUCTION

During any pandemic or emergency, nurses being the frontline healthcare workers provide direct care to infected patients, participate in infection containment strategies, deal with a higher number of patients, and work extended hours (Mehta et al., 2021; Ross, 2020). These situations elevate their risk of contracting the infection, put an enormous strain on their physical, emotional, and mental well-being, and thereby exacerbate their susceptibility to psychological distress and burnout (Ross, 2020). During earlier breakouts of severe acute respiratory syndrome (SARS) and Middle East respiratory syndrome (MERS), high levels of fear, anxiety, depression, posttraumatic stress disorder, and burnout were reported among healthcare workers including nurses (Brier et al., 2020; Kim & Choi, 2016).

Coronavirus disease 2019 (COVID-19) is an infectious disease, which erupted in December 2019 in Wuhan, China, before becoming a pandemic in a short time (WHO, 2020). Evidence suggests a high risk of SARS-CoV-2 infection (10.1%) in healthcare workers (Sahu et al., 2020) and a higher seroprevalence than the general population worldwide (17.1% vs. 8.0%) (Chen et al., 2021). Research also shows that the prevalence of psychological distress, burnout, insom-

 © 2022 International Council of Nurses

nia, and other mental health problems has increased among healthcare workers dealing with COVID-19 patients (Çelmeçe & Menekay, 2020; Lai et al., 2020).

In Pakistan, the first case of COVID-19 was reported on February 26, 2020. As of January 31, 2021, a total of 544 214 cases of COVID-19 and 11 623 deaths have been reported across the country (Government of Pakistan, 2021). This pandemic has put the healthcare system of Pakistan in crisis as hospitals are facing a severe shortage of healthcare workers, personal protective equipment, and resources to implement infection-safety measures (Rana et al., 2020). In addition, a study carried out in a hospital in Pakistan, before this pandemic, reported a high burden of burnout (79%) due to increased workload (Naz et al., 2016). Given the previously reported high burden of burnout and the current crisis caused by COVID-19, nurses in Pakistan face an excessive risk of experiencing psychological distress and burnout. This may compromise the well-being and performance of nurses and the quality of patient care. This study assessed the burden of burnout and psychological distress among nurses providing care to COVID-19 patients in Pakistan. The relationship of burnout with psychological distress was also examined.

METHODS

Study design, study site, and study population

A cross-sectional study was conducted at three public and five private hospitals located in Rawalpindi and Islamabad, Pakistan. Both public and private hospitals were selected to recruit a representative sample of nurses. Public hospitals are much larger than private hospitals in terms of bed capacity and human resource; therefore, more private hospitals were included in the study. The study population comprised of nurses working in general wards, emergency department, and critical care units of participating hospitals.

Inclusion and exclusion criteria

Nurses providing direct care to COVID-19 patients for at least two weeks and agreeing to participate in the study were included in this study. Nurse managers were excluded because they are not involved in direct patient care. Nursing students were also excluded from the study.

Data collection

A sample of 288 nurses was required for this study. This was estimated based on the prevalence of burnout (79%) reported by an earlier study (Naz et al., 2016) and assuming the confidence interval of 95%, the margin of error of 0.05, and the nonresponse rate of 10%.

After obtaining approval from each participating hospital, two researchers approached nurse managers of departments designated for COVID-19 patients at each participating hospital. Nurses were then invited to assess their eligibility and willingness to participate. A written informed consent was obtained from each participant. Questionnaires with unique identification numbers were distributed to all eligible nurses who filled the questionnaires at their convenient time and handed them over to their nurse managers. The researchers collected all filled questionnaires from nurse managers and stored them in a secure place to maintain the confidentiality and privacy of data.

Study variables and data collection tools

Burnout was assessed with Maslach Burnout Inventory–Human Services Survey (MBI-HSS) (Maslach & Jackson, 1981). The MBI is a globally validated 22-item tool that measures burnout in terms of emotional exhaustion, depersonalization, and diminished personal accomplishment (Maslach & Jackson, 1981). Each question measures an aspect of work-related burnout using a seven-point Likert scale with responses ranging from never (0) to every day (6). Emotional exhaustion comprises nine items with a score range of 0–54, which is further categorized as low (0–16), moderate (17–26), and high (≥ 27) burnout. Depersonalization comprises five items with a score range of 0–30, which is further categorized as low (0–6), moderate (7–12), and high (≥ 13) burnout. Personal accomplishment comprises eight items with a score range of 0–48, which is further categorized as low (0–31), moderate (32–38), and high (≥ 39) burnout. Overall burnout was defined as high scores in emotional exhaustion or depersonalization domains.

Psychological distress was assessed with the Screening Tool of Psychological Distress (STOP-D) (Young et al., 2007). It is a validated single-item tool that assesses depression, anxiety, stress, anger, and lack of social support. Each dimension of psychological distress is assessed with a question comprising a nine-point Likert scale that ranges from 0 (not at all) to 9 (severely). The presence of significant psychological distress is defined as a score of ≥4 for depression and anxiety each, while the score of ≥5 for stress, anger, and social support each.

Other variables measured in this study included age, sex, marital status, education, type of employment contract, type of hospital, years of work experience, nursing specialty, number of working hours per week, number of COVID-19 patients attended per shift, and whether nurses received infection prevention training while dealing with COVID-19.

Ethical considerations

Ethical approval was obtained from the Institutional Review Board and Ethics Committee (IRB and EC) of the hospital (Ethics approval #IRBEC/BIH/012/2020). In addition, permissions for data collection were obtained from the administration of each study hospital. Written informed consents were obtained from all study participants. The identities of participating hospitals and study participants were anonymized by

assigning unique identification numbers to maintain the confidentiality of the data.

Statistical analysis

Data were analyzed in Stata version 16.1. Categorical variables were tabulated as frequencies with corresponding percentages (%). Quantitative variables including scores of MBI domains and STOP-D items were summarized as mean and standard deviation (±SD). The prevalence of burnout and psychological distress was estimated as per recommended cutoff values.

The chi-square test was applied to compare binary variables of burnout and psychological distress with categorical variables, and the one-way analysis of variance test was applied to compare the mean scores of burnout domains with categorical variables. The Pearson correlation test was applied to assess the degree of correlations between burnout and psychological distress scores. Simple and multiple logistic regressions were applied to assess the associations of overall burnout with binary items of psychological distress. Simple and multiple linear regressions were applied to assess the associations of each burnout dimension score (emotional exhaustion, depersonalization, personal accomplishment) with binary items of psychological distress. Both multiple logistic and linear regressions were adjusted for age, sex, marital status, education level, type of employment, work experience, working hours, number of patients, training on COVID-19 prevention, and types of the hospital by simultaneously adding these variables in the respective models. The results of logistic regression were reported as unadjusted and adjusted odds ratios (OR) with corresponding 95% confidence intervals (CI) and p values. The results of linear regression were reported as unadjusted and adjusted beta coefficients (β) with corresponding 95% CI and p values. The p value of < 0.05 was chosen to decide statistical significance.

RESULTS

Characteristics of participants

A total of 288 nurses participated in the study. The mean age was 27.7 ± 4.4 years, and the majority (74.6%) were females. More than 40% of nurses worked in critical care units and 69.3% received training regarding COVID-19. The nurses had an average work experience of 4.8 ± 4.3 years, worked for the median number of 48 hours per week, and provided care to the median number of 10 patients during each shift (Table 1).

Levels of burnout and psychological distress

The prevalence of overall burnout was 48.6%. The mean MBI scores indicate that the nurses reported moderate levels of emotional exhaustion (22.6 ± 11.5) and depersonalization (10.7 ± 6.3) each, while low levels of personal accomplishment (30.4 ± 11.9). Concurrently, most nurses reported high

TABLE 1 Characteristics and levels of burnout and psychological distress among nurses ($N = 288$)

Variables	Statistics
Age, mean ± SD	27.7 ± 4.4
Sex, n (%)	
Male	67 (23.3)
Female	221 (76.7)
Marital status, n (%)	
Single	165 (57.3)
Married	116 (40.3)
Separated/divorced	7 (2.4)
Education, n (%)	
Diploma in nursing	182 (63.2)
Post-RN BSN	56 (19.4)
BSN	49 (17.0)
MSN	1 (0.4)
Type of employment, n (%)	
Contractual	70 (24.3)
Regular	218 (75.7)
Type of hospital, n (%)	
Public	136 (47.2)
Private	152 (52.8)
Department, n (%)	
Emergency	54 (18.7)
Critical care	119 (41.3)
General ward	86 (29.9)
Other	29 (10.1)
Received training on COVID-19 prevention, n (%)	199 (69.3)
Work experience—years, mean ± SD	4.8 ± 4.3
Working hours—per week, median (IQR)	48 (6)
Number of COVID-19 patients attended per shift, median (IQR)	10 (23)
**MBI**	
Overall Burnout[†], n (%)	140 (48.6)
Emotional exhaustion score, mean ± SD	22.6 ± 11.5
Low, n (%)	89 (30.9)
Moderate, n (%)	92 (31.9)
High, n (%)	107 (37.2)
Depersonalization score, mean ± SD	10.7 ± 6.3
Low, n (%)	89 (30.9)
Moderate, n (%)	93 (32.3)
High, n (%)	106 (36.8)
Personal accomplishment score, mean ± SD	30.4 ± 11.9
Low, n (%)	135 (46.9)
Moderate, n (%)	69 (23.9)
High, n (%)	84 (29.2)

(Continues)

TABLE 1 (Continued)

Variables	Statistics
**Screening Tool for Psychological Distress (STOP-D)**	
Depression score, mean ± SD	3.5 ± 2.3
Depression score ≥ 4, n (%)	131 (45.5)
Anxiety score, mean ± SD	3.5 ± 2.2
Anxiety score ≥ 4, n (%)	140 (48.6)
Stress score, mean ± SD	4.2 ± 2.4
Stress score ≥ 5, n (%)	136 (47.2)
Anger score, mean ± SD	3.9 ± 2.4
Anger score ≥ 5, n (%)	126 (43.7)
Low social support score, mean ± SD	4.1 ± 2.7
Low social support score ≥ 5, n (%)	132 (45.8)

Abbreviations: BSN, Bachelor of nursing; MSN, Master of nursing; RN, Registered Nurse; IQR, Interquartile Range; SD, Standard Deviation.
†High score in emotional exhaustion and/or depersonalization domains of the MBI.

levels of emotional exhaustion (37.2%) and depersonalization (36.8%) and low levels of personal accomplishment (46.9%) (Table 1).

The mean scores of STOP-D items show that nurses had little to moderate levels of depression (3.5 ± 2.3), anxiety (3.5 ± 2.2), stress (4.2 ± 2.4), anger (3.9 ± 2.4), and lack of social support (4.1 ± 2.7) each. The prevalence of significant psychological distress was approximately 45% in each item (Table 1).

Comparison of burnout with variables

Burnout was significantly higher among nurses who: were employed in public hospitals (< 0.001), had no training on COVID-19 prevention ($p = 0.005$), worked for < 48 hours per week ($p = 0.021$), and provided care to an increased number of COVID-19 patients per shift ($p < 0.001$). The mean emotional exhaustion score was significantly different by education level ($p = 0.004$), types of the hospital ($p < 0.001$), training status on COVID-19 ($p < 0.001$), the number of working hours per week ($p = 0.004$), and the number of COVID-19 patients attended per shift ($p = 0.002$). The mean depersonalization score differed significantly by education level ($p = 0.020$), type of employment ($p = 0.049$), type of hospital ($p < 0.001$), training status on COVID-19 ($p < 0.001$), the number of working hours per week ($p < 0.001$), and the number of COVID-19 patients attended per shift ($p < 0.001$). The mean personal accomplishment score was significantly different by education level ($p = 0.040$) and the type of hospital ($p = 0.006$) (Table 2).

Comparison of psychological distress with variables

Depression was significantly higher among nurses aged above 30 years ($p = 0.017$) and those who did not receive any training on COVID-19 prevention ($p = 0.005$). Anxiety was significantly higher among nurses having a postgraduate degree ($p = 0.023$), contractual job ($p = 0.028$), and no training on COVID-19 ($p = 0.008$). Stress was significantly higher ($p = 0.007$) in nurses who had no training on COVID-19 prevention compared with those with training. Anger was significantly higher among nurses with a postgraduate degree ($p = 0.002$), employed at public hospitals ($p = 0.043$), and no training on COVID-19 ($p = 0.031$) (Table 2).

Association between psychological distress and burnout

The simple and multiple logistic and linear regression analyses showed significant positive associations of psychological distress items with burnout and its domains. The adjusted odds of burnout were 3.81 ($p < 0.001$) for depression, 4.00 ($p < 0.001$) for anxiety, 4.21 ($p < 0.001$) for stress, 3.20 ($p < 0.001$) for anger, and 4.48 ($p < 0.001$) for low social support. Among burnout domains, emotional exhaustion had the strongest association with psychological distress followed by depersonalization. While personal accomplishment was significantly associated with stress, anger, and social support only (Table 3).

DISCUSSION

This is the first study that assessed burnout and psychological distress and their relationship among nurses working with COVID-19 patients at public and private hospitals of Pakistan. Our analysis showed a high burden of burnout and its domains and psychological distress such as depression, anxiety, stress, anger, and low social support among nurses. Each psychological distress item was positively associated with burnout and its domains. Moreover, nurses employed at public hospitals, having no training on COVID-19 prevention, and dealing with a large number of COVID-19 patients reported a higher burden of burnout and psychological distress.

This study found burnout in 48.6%, high emotional exhaustion in 37.2%, depersonalization in 36.8%, and low personal accomplishment in 46.9% of nurses working with COVID-19 patients. This estimate is 4 times the global prevalence of 11.2% (Woo et al., 2020) before this pandemic, thereby suggesting the drastic impact of the COVID-19 on elevating burnout among nurses. Other countries tackling with COVID-19 pandemic have also assessed its burden among healthcare workers including nurses. For instance, a recent meta-analysis has reported a similar pooled prevalence of emotional exhaustion (34.1%), whereas a lower prevalence of depersonalization (12.6%) and personal accomplishment (15.2%) compared with our study (Galanis et al., 2021). Another large cross-sectional study from China has reported emotional exhaustion in 41.5% of nurses, depersonalization in 27.6%, and personal accomplishment in 38.3% of nurses (Hu et al., 2020). Likewise, the prevalence of burnout was 55.3%, high emotional exhaustion

International Nursing Review

TABLE 2 Comparison of burnout and psychological distress with variables among nurses (*N* = 288)

	Burnout				Psychological Distress				
	Overall Burnout[†]	Emotional Exhaustion	Depersonal-ization	Personal Accomplish-ment	Depression[1]	Anxiety[2]	Stress[3]	Anger[4]	No Social Support[5]
Variables	n (%)	Mean ± SD	Mean ± SD	Mean ± SD	n (%)	n (%)	n (%)	n (%)	n (%)
Age									
< 30 years	100 (37.4)	22.1 ± 11.7	10.7 ± 6.6	31.1 ± 11.9	86 (40.8)	95 (45.0)	93 (44.1)	86 (40.8)	94 (44.5)
30+ years	37 (51.4)	24.1 ± 10.8	10.7 ± 5.5	28.4 ± 11.9	41 (56.9)	42 (58.3)	39 (54.2)	37 (51.4)	35 (48.6)
P value	*0.558*	*0.200*	*0.971*	*0.091*	*0.017*	*0.051*	*0.138*	*0.116*	*0.550*
Sex									
Male	27 (40.3)	20.8 ± 12.7	10.6 ± 7.2	28.3 ± 12.2	27 (40.3)	28 (41.8)	30 (44.8)	25 (37.3)	30 (44.8)
Female	113 (51.1)	23.1 ± 11.1	10.8 ± 6.1	31.0 ± 11.8	104 (47.1)	112 (50.7)	106 (48.0)	101 (45.7)	102 (46.1)
P value	*0.120*	*0.144*	*0.817*	*0.098*	*0.330*	*0.202*	*0.647*	*0.225*	*0.843*
Marital status									
Single	75 (45.5)	22.1 ± 11.7	10.5 ± 6.7	31.1 ± 12.4	70 (42.4)	75 (45.5)	71 (43.0)	63 (38.2)	73 (44.2)
Married	63 (54.3)	23.6 ± 11.3	11.1 ± 5.8	29.7 ± 11.0	58 (50.0)	62 (53.5)	63 (54.3)	60 (51.7)	57 (49.1)
Separated/ divorced	2 (28.6)	17.3 ± 9.7	9.3 ± 5.4	23.7 ± 14.3	3 (42.9)	3 (42.9)	2 (28.6)	3 (42.9)	2 (28.6)
P value	*0.193*	*0.277*	*0.583*	*0.201*	*0.450*	*0.399*	*0.107*	*0.079*	*0.468*
Education									
Diploma in nursing	85 (46.7)	21.0 ± 11.1	10.1 ± 6.1	29.3 ± 12.4	76 (41.8)	78 (42.9)	77 (42.3)	68 (37.4)	80 (44.0)
Post-RN BSN	34 (60.7)	26.3 ± 11.0	12.8 ± 12.8	31.5 ± 10.7	31 (55.4)	35 (62.5)	32 (57.1)	36 (64.3)	28 (50.0)
BSN	21 (42.9)	24.7 ± 12.4	10.9 ± 10.9	33.9 ± 10.1	24 (49.0)	27 (55.1)	27 (55.1)	22 (44.9)	24 (49.0)
P value	*0.123*	*0.004*	*0.020*	*0.040*	*0.178*	*0.023*	*0.075*	*0.002*	*0.656*
Type of employment									
Contractual	32 (45.7)	22.1 ± 9.2	9.4 ± 5.9	30.3 ± 11.9	37 (52.9)	42 (60.0)	33 (47.1)	31 (44.3)	32 (45.7)
Regular	108 (49.5)	22.7 ± 12.2	11.1 ± 6.4	30.4 ± 11.9	94 (43.1)	98 (44.9)	103 (47.3)	95 (43.6)	100 (45.9)
P value	*0.577*	*0.690*	*0.049*	*0.966*	*0.155*	*0.028*	*0.988*	*0.917*	*0.982*
Type of hospital									
Private	51 (37.5)	19.9 ± 11.8	9.4 ± 6.4	28.4 ± 13.1	56 (41.2)	65 (47.8)	59 (43.4)	51 (37.5)	55 (40.4)
Public	89 (58.5)	25.0 ± 10.8	12.0 ± 6.1	32.2 ± 10.4	75 (49.3)	75 (49.3)	77 (50.7)	75 (49.3)	77 (50.7)
P value	*< 0.001*	*< 0.001*	*< 0.001*	*0.006*	*0.165*	*0.793*	*0.217*	*0.043*	*0.082*
Department									
Emergency	25 (46.3)	21.4 ± 12.2	12.3 ± 6.4	30.2 ± 10.9	22 (40.7)	22 (40.7)	24 (44.4)	20 (37.0)	29 (53.7)
Critical care	61 (51.3)	23.0 ± 10.4	10.5 ± 5.8	30.8 ± 12.3	57 (47.9)	55 (46.2)	55 (46.2)	54 (45.4)	53 (44.5)
General ward	44 (51.2)	23.9 ± 11.9	10.8 ± 6.8	30.2 ± 11.5	44 (51.2)	48 (55.8)	43 (50.0)	38 (44.2)	41 (47.7)
Other	10 (34.5)	19.1 ± 13.1	8.7 ± 6.7	30.0 ± 13.9	8 (27.6)	15 (51.7)	14 (48.3)	14 (48.3)	9 (31.0)
P value	*0.393*	*0.198*	*0.090*	*0.978*	*0.131*	*0.319*	*0.920*	*0.714*	*0.231*
Received training on COVID-19 prevention									
Yes	86 (43.2)	21.1 ± 10.8	9.8 ± 6.0	30.3 ± 12.6	80 (40.2)	86 (43.2)	83 (41.7)	79 (39.7)	78 (39.2)
No	54 (61.4)	26.0 ± 12.5	12.9 ± 6.5	30.8 ± 10.3	51 (57.9)	53 (60.2)	52 (59.1)	47 (53.4)	54 (61.4)
P value	*0.005*	*< 0.001*	*< 0.001*	*0.720*	*0.005*	*0.008*	*0.007*	*0.031*	*0.001*
Work experience									
< 1 year	30 (50.0)	22.1 ± 12.4	11.4 ± 6.8	31.7 ± 12.1	25 (41.7)	31 (51.7)	29 (48.3)	25 (41.7)	32 (53.3)
1–5 years	60 (42.5)	21.9 ± 10.9	9.9 ± 6.0	30.0 ± 11.9	67 (47.5)	65 (46.1)	60 (42.5)	55 (39.0)	57 (40.4)

(Continues)

TABLE 2 (Continued)

	Burnout				Psychological Distress				
	Overall Burnout[†]	Emotional Exhaustion	Depersonal-ization	Personal Accomplish-ment	Depression[1]	Anxiety[2]	Stress[3]	Anger[4]	No Social Support[5]
Variables	n (%)	Mean ± SD	Mean ± SD	Mean ± SD	n (%)	n (%)	n (%)	n (%)	n (%)
5+ years	48 (58.5)	24.8 ± 11.8	11.7 ± 6.4	29.9 ± 12.1	38 (46.3)	42 (51.2)	46 (56.1)	44 (53.7)	42 (51.2)
P value	*0.069*	*0.163*	*0.101*	*0.625*	*0.745*	*0.670*	*0.148*	*0.097*	*0.139*
Working hours—per week									
< 48 hours	63 (56.8)	25.0 ± 11.9	12.3 ± 6.3	31.0 ± 11.1	55 (49.5)	56 (50.5)	54 (48.7)	50 (45.1)	54 (48.7)
48+ hours	73 (42.7)	21.0 ± 11.1	9.7 ± 6.2	30.4 ± 12.4	74 (43.3)	82 (47.9)	78 (45.6)	75 (43.9)	76 (44.4)
P value	*0.021*	*0.004*	*< 0.001*	*0.681*	*0.301*	*0.682*	*0.618*	*0.845*	*0.489*
Number of COVID-19 patients attended per shift									
< 5	42 (35.0)	20.2 ± 10.9	8.7 ± 5.8	30.4 ± 13.3	52 (43.3)	58 (48.3)	52 (43.3)	46 (38.3)	48 (40.0)
5–10	21 (58.3)	24.3 ± 12.4	12.5 ± 6.8	30.6 ± 9.8	22 (61.1)	20 (55.6)	19 (52.8)	20 (55.6)	17 (47.2)
11–20	21 (53.9)	23.7 ± 10.4	12.1 ± 6.2	29.6 ± 10.4	20 (51.3)	21 (53.9)	21 (53.9)	20 (51.3)	22 (56.4)
20+	51 (67.1)	26.3 ± 11.4	12.7 ± 5.9	31.9 ± 10.7	32 (42.1)	34 (44.7)	38 (50.0)	36 (47.4)	39 (51.3)
P value	*< 0.001*	*0.002*	*< 0.001*	*0.741*	*0.210*	*0.669*	*0.566*	*0.206*	*0.231*

Abbreviations: BSN, Bachelor of nursing; MSN, Master of nursing; RN, Registered Nurse; IQR, Interquartile Range; SD, Standard Deviation.
[†]High score in emotional exhaustion and/or depersonalization domains of the MBI.
[1]Cutoff score: ≥ 4.
[2]Cutoff score: ≥ 4.
[3]Cutoff score: ≥ 5.
[4]Cutoff score: ≥ 5.
[5]Cutoff score: ≥ 5.

was 53.0%, and depersonalization was 13.3% among nurses in Iran (Jalili et al., 2020). These findings indicate that nurses working during the COVID-19 pandemic are experiencing extremely high burnout that needs to be addressed immediately for preserving their physical and mental well-being.

In this study, psychological distress measured as depression, anxiety, stress, anger, and low social support was nearly 45% among nurses dealing with COVID-19 patients. Although earlier studies from Pakistan and other countries have also assessed the impact of COVID-19 on the mental health of nurses, it is difficult to compare psychological distress across studies due to vast variations in screening tools, defining criteria, nursing specialties, and study settings. For instance, a study found a significant impact of fear from COVID-19 on the psychological distress among Pakistani nurses (Khattak et al., 2020). Similarly, a study showed that 24.0%, 44.0%, and 16.0% of nurses in Pakistan experienced symptoms of depression, anxiety, and stress, respectively, during the COVID-19 pandemic (Arshad et al., 2020). Comparison with other countries revealed the prevalence of 40% and 45% for depression and anxiety among frontline nurses in China, respectively (Hu et al., 2020). Despite the differences in measurement tools of psychological distress, the findings of studies conducted in previous and current pandemics confirm that a vast majority of nurses and other frontline healthcare workers experience psychological distress and thus require adequate psychological support during such disasters.

Both burnout and psychological distress are considered important indicators of mental well-being, work performance, and quality of care. However, these conditions represent different facets of mental well-being. Burnout represents a person's response to chronic work-related stressors (Maslach & Jackson, 1981), whereas psychological distress comprises various psychological risk factors and mental health problems that may encompass some facets of burnout and share some risk factors of burnout as well. These underlying similarities might explain why these two conditions coexist and are strongly associated. Our study and many other studies carried out before and during the COVID-19 pandemic have confirmed that burnout dimensions such as emotional exhaustion and depersonalization have a moderate-to-strong relationship with various facets of psychological distress (Dobson et al., 2021; Hu et al., 2020; Koutsimani et al., 2019; Zou et al., 2016). In addition, some studies have also suggested that burnout leads to the development of psychological distress. However, the nature and direction of this relationship is complex due to the presence of mediating factors and methodological shortcomings in existing studies (Hamama

TABLE 3 Association of burnout and its domains with psychological distress among nurses, unadjusted and adjusted logistic and linear regression analyses ($N = 288$)

STOP-D tool	Burnout OR (95% CI)	EE β (95% CI)	DP β (95% CI)	PA β (95% CI)
Depression				
Unadjusted	3.43 (2.11–5.57)**	8.31 (5.79–10.82)**	3.70 (2.28–5.11)**	3.27 (0.51–6.02)*
Adjusted	3.81 (2.14–6.79)**	7.01 (4.40–9.63)**	3.13 (1.70–4.56)**	2.35 (−0.51–5.20)
Anxiety				
Unadjusted	3.32 (2.05–5.38) **	9.16 (6.70–11.62)**	3.56 (2.15–4.98)**	2.41 (−0.34–5.17)
Adjusted	4.00 (2.24–7.14) **	7.99 (5.42–10.57)**	3.34 (1.91–4.76)**	0.71 (−2.17–3.58)
Stress				
Unadjusted	4.53 (2.76–7.44) **	9.53 (7.09–11.98)**	4.51 (3.13–5.89)**	3.74 (1.00–6.48)*
Adjusted	4.21 (2.39–7.43) **	7.56 (4.99–10.12)**	3.60 (2.20–5.00)**	2.52 (−0.31–5.35)
Anger				
Unadjusted	3.56 (2.18–5.80) **	8.67 (6.16–11.17)**	4.60 (3.21–5.98)**	3.55 (1.81–7.29)*
Adjusted	3.20 (1.83–5.61) **	6.82 (4.18–9.46)**	3.71 (2.30–5.13)**	3.32 (0.47–6.18)*
Low social support				
Unadjusted	4.85 (2.94–7.99) **	9.49 (7.04–11.94)**	4.59 (3.21–5.97)**	3.90 (1.16–6.64)*
Adjusted	4.48 (2.54–7.89) **	7.98 (5.43–10.53)**	3.28 (1.86–4.69)*	3.53 (0.72–6.35)*

Abbreviations: STOP-D: Screening Tool for Psychological Distress; EE: Emotional Exhaustion; DP: Depersonalization; PA: Personal Accomplishment; OR: Odds Ratio, CI: Confidence Interval.
†Burnout: High score in emotional exhaustion and/or depersonalization domain of the MBI.
Cutoff scores for psychological distress: depression ≥ 4, anxiety ≥ 4, stress ≥ 5, anger ≥ 5, and social support ≥ 5.
Model was adjusted for age, sex, marital status, education level, type of employment, work experience, working hours, number of patients, training on COVID-19 prevention, and type of hospital.
*Association is significant at the 0.05 level.
**Association is significant at the 0.001 level.

et al., 2021; Zou et al., 2016). Therefore, we recommend more prospective studies to better understand this relationship to develop effective preventive and mitigating strategies.

Studies have extensively investigated factors associated with burnout and psychological distress of nurses during the COVID-19 pandemic. We also compared these conditions with factors and found that burnout and psychological distress were higher among nurses working at public hospitals, those who did not receive any training on COVID-19 prevention, and those providing care to a large number of COVID-19 patients during each shift. These findings are not surprising as public hospitals in Pakistan are designated to manage COVID-19 patients and therefore receive more COVID-19 patients than private hospitals. As workload is a well-recognized risk factor for compromised mental health and burnout among nurses (Galanis et al., 2021; Matsuo et al., 2020), our subgroup analysis also confirms that nurses working at public hospitals provided care to a substantially higher median number of patients (22 vs. 3 patients, $p < 0.001$) than those working at private hospitals. While similar to our findings, a study from Pakistan and recent systematic reviews and meta-analyses have reported the beneficial impact of COVID-19 prevention training on burnout and psychological distress of healthcare professionals (Brier et al., 2020; Salman et al., 2020; Galanis et al., 2021). These findings accentuate the need for organizing such training, managing workload, and implementing supportive psychological interventions during pandemics to protect and preserve the mental health of healthcare workers including nurses.

Previous research before and during the COVID-19 pandemic suggests that demographic factors (age, gender, marital status, education level), department, and work experience significantly influence burnout in nurses (Galanis et al., 2021; Molina-Praena et al., 2018). Except for education level, we did not find any of these factors to be significantly related to burnout and therefore need to be studied further. Despite the insignificant relationship of age and experience with burnout and psychological distress, nurses were relatively younger and less experienced in our study. We carried out this study after approximately 10 months of the COVID-19 outbreak in Pakistan, which indicates a chronic exposure of nurses to the high-risk working environment. We speculate that these reasons are responsible for the high burden of burnout and psychological distress in this study. Two recent meta-analyses support our speculations that younger and less experienced nurses and those working for a longer time in high-risk conditions are more likely to experience burnout (Galanis et al., 2021). Regarding the education level, nurses with post-RN BSN degree had higher levels of burnout and psychological distress than BSN and diploma holders. This difference might exist because the majority of nurses (~70%) were employed at public hospitals.

Limitations

This study has several limitations. First, this study was conducted in hospitals that are located in two cities of Pakistan, and therefore its findings can not be generalized to the entire nursing population of the country. Second, this study adopted a convenience sampling approach and collected data via a self-administered questionnaire. Hence, some degree of reporting bias is expected in the responses of nurses. We minimized this bias by using validated tools, designing questions in a neutral and simple language, and providing necessary instructions in the questionnaire. Third, psychological distress was not diagnosed as per the criteria of diagnostic and statistical manual of mental disorders, which may have resulted in the misclassification of these disorders among nurses. Fourth, the cross-sectional nature of our study did not allow us to ascertain whether psychological distress leads to burnout or vice versa. Therefore, we recommend more prospective studies to understand the nature of relationship between these conditions and the impact of mitigating burnout on psychological distress. Last, despite using validated tools for screening burnout and psychological distress in nurses, comparison across studies may not be feasible due to drastic variations in defining criteria, study settings, study population, and healthcare infrastructure.

Implications for nursing and health policy

This study demonstrated that a high proportion of Pakistani nurses providing care to COVID-19 patients are experiencing psychological distress and burnout. These alarming findings suggest that hospitals must screen nurses frequently for the presence of significant burnout and mental health problems and offer supportive interventions to protect their mental well-being and improve resilience. Moreover, hospitals must organize frequent training on COVID-19, manage the workload of nurses, and establish psychological support services for nurses working with COVID-19 patients.

CONCLUSIONS

Nurses working with COVID-19 patients in Pakistan are experiencing high levels of burnout and psychological distress with a significant moderate-to-strong association between these conditions. Moreover, nurses who were employed at public hospitals, had no training about COVID-19 prevention, and dealing with an increased workload of patients reported higher levels of burnout and psychological distress. These findings indicate the urgent need for conducting frequent COVID-19 prevention training and implementing psychological and institution-based supportive interventions to mitigate burnout and preserve the mental well-being of nurses dealing with COVID-19 patients.

DATA AVAILABILITY STATEMENT

Data can be provided upon a reasonable request from the corresponding author.

CONFLICT OF INTEREST

All authors declare no competing interests related to this study.

FUNDING

This study did not receive any funding.

AUTHOR CONTRIBUTIONS

Conceived idea: FA and SI; design, data collection, data curation, and writing of the manuscript: SA, SI, and KA; analyzed the data, interpreted the results, and wrote the first draft of the manuscript: KA, FA, and SA; guaranteers of the data integrity: FA and SI; critical review and final draft of the manuscript: SI and FA.

ETHICS STATEMENT

The study was approved by the Ethics committee of Benazir Bhutto hospital, Rawalpindi, Pakistan.

ORCID

Sonia Andlib RN, RM, BSN https://orcid.org/0000-0003-3692-0776

Faisal Aziz BSN, MPH https://orcid.org/0000-0002-0109-2969

REFERENCES

Alshawish, E. & Nairat, E. (2020) Burnout and psychological distress among nurses working in primary health care clinics in West Bank-Palestine. *International Journal of Mental Health*, 49(4), 321–335.

Arshad, M.S., Hussain, I., Nafees, M., Majeed, A., Imran, I., Saeed, H. et al. (2020). Assessing the impact of COVID-19 on the mental health of healthcare workers in three metropolitan cities of Pakistan. *Psychology Research and Behavior Management*, 13, 1047–1055. https://doi.org/10.2147/PRBM.S282069

Brier, N.D., Stroobants, S., Vandekerckhove, P. & Buck, E.D. (2020). Factors affecting mental health of health care workers during coronavirus disease outbreaks (SARS, MERS & COVID-19): a rapid systematic review. *PloS One*, 15(12), e0244052.

Çelmeçe, N. & Menekay, M. (2020). The effect of stress, anxiety and burnout levels of healthcare professionals caring for COVID-19 patients on their quality of life. *Frontiers in Psychology*, 11, 597624. https://doi.org/10.3389/fpsyg.2020.597624

Chen, X., Chen, Z., Azman, A.S., Deng, X., Chen, X., Lu, W. et al . (2021). Serological evidence of human infection with SARS-CoV-2: a systematic review and meta-analysis. *The Lancet Global Health*, 9(5), E598–E609. https://doi.org/10.1016/S2214-109X(21)00026-7

Dobson, H., Malpas, C.B., Burrell, A. Jc, Gurvich, C., Chen, L., Kulkarni, J. & Winton-Brown, T. (2021) Burnout and psychological distress amongst Australian healthcare workers during the COVID-19 pandemic. *Australasian Psychiatry*, 29(1), 26–30.

Galanis, P., Vraka, I., Fragkou, D., Bilali, A. & Kaitelidou, D. (2021). Nurses' burnout and associated risk factors during the COVID-19 pandemic: a systematic review and meta-analysis. *Journal of Advanced Nursing*, 77(8), 3286–3302. https://doi.org/10.1111/jan.14839.

Government of Pakistan (2021) COVID-19 Live Dashboard - Pakistan. [Online]. Available from: http://datastudio.google.com/reporting/1PLVi5amcc_R5Gh928gTE8-8r8-fLXJQF/page/R24IB?feature=opengraph [Accessed 16th February 2021].

Hamama, L., Marey-Sarwan, I., Hamama-Raz, Y., Nakad, B. & Asadi, A. (2021). Psychological distress and perceived job stressors among hospital nurses and physicians during the COVID-19 outbreak. *Journal of Advanced Nursing*. https://doi.org/10.1111/jan.15041

Hu, D., Kong, Y., Li, W., Han, Q., Zhang, X., Zhu, L.X. et al. (2020) Frontline nurses' burnout, anxiety, depression, and fear statuses and their associated factors during the COVID-19 outbreak in Wuhan, China: a large-scale cross-sectional study. *EClinicalMedicine*, 24, 100424. https://doi.org/10.1016/j.eclinm.2020.100424

Jalili, M., Niroomand, M., Hadavand, F., Zeinali, K. & Fotouhi, A. (2020). Burnout among healthcare professionals during COVID-19 pandemic: a cross-sectional study. *International Archives of Occupational and Environmental Health*, 94(6), 1345–1352. https://doi.org/10.1007/s00420-021-01695-x

Khattak, S.R., Saeed, I., Rehman, S.U. & Fayaz, M. (2020) Impact of fear of COVID-19 pandemic on the mental health of nurses in Pakistan. *Journal of Loss and Trauma*, 26(5), 421–435. https://doi.org/10.1080/15325024.2020.1814580

Kim, J.S. & Choi, J.S. (2016) Factors influencing emergency nurses' burnout during an outbreak of Middle East respiratory syndrome coronavirus in Korea. *Asian Nursing Research*, 10(4), 295–299.

Koutsimani, P., Montgomery, A. & Georganta, K. (2019) The Relationship between burnout, depression, and anxiety: a systematic review and meta-analysis. *Frontiers in Psychology*, 10, 284. https://doi.org/10.3389/fpsyg.2019.00284

Lai, J., Ma, S., Wang, Y., Cai, Z., Hu, J., Wei, N. et al. (2020) Factors associated with mental health outcomes among health care workers exposed to coronavirus disease 2019. *JAMA Network Open*, 3(3), e203976. https://doi.org/10.1001/jamanetworkopen.2020.3976

Maslach, C. & Jackson, S.E. (1981) The measurement of experienced burnout. *Journal of Organizational Behavior*, 2(2), 99–113. https://doi.org/10.1002/job.4030020205

Matsuo, T., Kobayashi, D., Taki, F., Sakamoto, F., Uehara, Y., Mori, N. & Fukui, T. (2020) Prevalence of health care worker burnout during the coronavirus disease 2019 (COVID-19) pandemic in Japan. *JAMA Network Open*, 3(8), e2017271. https://doi.org/10.1001/jamanetworkopen.2020.17271

Mehta, S., Machado, F., Kwizera, A., Papazian, L., et al. (2021) COVID-19: a heavy toll on health-care workers. *The Lancet Respiratory Medicine*, 9(3), 226–228. https://doi.org/10.1016/S2213-2600(21)00068-0

Molina-Praena, J., Ramirez-Baena, L., Gómez-Urquiza, J.L. & Cañadas, G. R. (2018) Levels of burnout and risk factors in medical area nurses: a meta-analytic study. *International Journal of Environmental Research and Public Health*, 15(12), 2800. https://doi.org/10.3390/ijerph15122800

Naz, S., Hashmi, A.M. & Asif, A. (2016) Burnout and quality of life in nurses of a tertiary care hospital in Pakistan. *JPMA. The Journal of the Pakistan Medical Association*, 66(5), 532–536.

Rana, W., Mukhtar, S. & Mukhtar, S. (2020) Mental health of medical workers in Pakistan during the pandemic COVID-19 outbreak. *Asian Journal of Psychiatry*, 51, 51102080. https://doi.org/10.1016/j.ajp.2020.102080

Ross, J. (2020) The exacerbation of burnout during COVID-19: a major concern for nurse safety. *Journal of Perianesthesia Nursing*, 35(4), 439–440.

Sahu, A.K., Amrithanand, V.T., Mathew, R., Aggarwal, P., Nayer, J. & Bhoi, S. (2020) COVID-19 in health care workers – a systematic review and meta-analysis. *The American Journal of Emergency Medicine*, 38(9), 1727–1731. https://doi.org/10.1016/j.ajem.2020.05.113

Salman, M., Raza, M.H., Mustafa, Z.U., Khan, T. M., Asif, N., Tahir, H. et al. (2020) The psychological effects of COVID-19 on frontline healthcare workers and how they are coping: a web-based, cross-sectional study from Pakistan. *medRxiv*, https://doi.org/10.1101/2020.06.03.20119867

WHO (2020) Weekly operational update on COVID-19. [online]. Available at: https://www.who.int/publications/m/item/weekly-epidemiological-update—14-december-2020 [Accessed 15th February 2021].

Woo, T., Ho, R., Tang, A. & Tam, W. (2020). Global prevalence of burnout symptoms among nurses: a systematic review and meta-analysis. *Journal of Psychiatric Research*, 123, 9–20. https://doi.org/10.1016/j.jpsychires.2019.12.015

Young, Q.-R., Ignaszewski, A., Fofonoff, D. & Kaan, A. (2007). Brief screen to identify 5 of the most common forms of psychosocial distress in cardiac patients: validation of the screening tool for psychological distress. *The Journal of Cardiovascular Nursing*, 22(6), 525–534. https://doi.org/10.1097/01.JCN.0000297383.29250.14

Zou, G., Shen, X., Tian, X., Liu, C., Li, G., Kong, L. & Li, P. (2016) Correlates of psychological distress, burnout, and resilience among Chinese female nurses. *Industrial Health*, 54(5), 389–395. https://doi.org/10.2486/indhealth.2015-0103

SUPPORTING INFORMATION

Additional supporting information may be found in the online version of the article at the publisher's website.

How to cite this article: Andlib, S., Inayat, S., Azhar, K. & Aziz, F. (2022) Burnout syndrome and psychological distress among Pakistani nurses providing care to COVID-19 patients: A cross-sectional study. *International Nursing Review*, 1–9. https://doi.org/10.1111/inr.12750
