# Supplementary material for: Mental health status and related factors influencing healthcare workers during the COVID-19 pandemic: A systematic review and meta-analysis
Source: PLoS One. 2024 Jan 19;19(1):e0289454. doi: 10.1371/journal.pone.0289454 (PMC10798549; doi:10.1371/journal.pone.0289454)
Supplement: S1 Data — (ZIP) [file pone.0289454.s011.zip › literatures/100.pdf]

International Journal of
Environmental Research and Public Health

Article

Comparison of Generalized Anxiety and Sleep Disturbance among Frontline and Second-Line Healthcare Workers during the COVID-19 Pandemic

Sultan Ayoub Meo *, Joud Mohammed Alkhalifah, Nouf Faisal Alshammari and Wejdan Saud Alnufaie

Department of Physiology, College of Medicine, King Saud University, Riyadh 11461, Saudi Arabia; Jude.alkhalifah@gmail.com (J.M.A.); nouf6alshammari@gmail.com (N.F.A.); wejdan.alnufaie@gmail.com (W.S.A.)
* Correspondence: sultanmeo@hotmail.com or smeo@ksu.edu.sa

Abstract: Severe Acute Respiratory Syndrome Coronavirus-2 (SARS-CoV-2) infection, also known as COVID-19, has developed into an alarming situation around the world. Healthcare workers are playing the role of frontline defense to safeguard the lives of everyone during the COVID-19 pandemic. The present study aimed to investigate the anxiety levels and sleep quality among frontline and second-line healthcare workers during the COVID-19 pandemic. In this cross-sectional study, a validated, self-administered, electronic questionnaire was distributed through email to healthcare workers. The selection of 1678 healthcare workers was based on a convenience sampling technique. The General Anxiety Disorder-7 (GAD-7) and Pittsburgh Sleep Quality Index (PSQI) instrument scales were used to assess healthcare workers' anxiety levels and sleep quality during the COVID-19 pandemic. Out of 1678 respondents, 1200 (71.5 %) were frontline healthcare workers, while 478 (28.5%) were second-line healthcare workers. Among all the healthcare workers, 435 (25.92%) were experiencing moderate to severe anxiety. Among them, 713 (59.4%) frontline healthcare workers were experiencing anxiety in comparison with 277 (57.9%) second-line healthcare workers. Severe anxiety symptoms were seen in 137 (11.41%) frontline healthcare workers compared to 44 (9.20%) second-line healthcare workers. In total, 1376 (82.0%) healthcare workers were found to have poor sleep quality; 975 (58.10%) were frontline, and 407 (23.89%) were second-line healthcare workers. The highest poor sleep quality levels were found among 642 (84.6%) of the healthcare workers who work in frontline areas (emergency departments, intensive care units, and wards) compared to 734 (79.9%) of the healthcare workers who work in second-line areas. These findings provide a substantial contribution to the consolidation of evidence concerning the negative impact of the pandemic on the mental health of healthcare workers (HCWs). These results have established an association that the COVID-19 pandemic causes larger negative psychological symptoms in frontline healthcare workers, such as severe anxiety and poor sleep quality. Preventive measures to minimize anxiety levels and maintain sleep quality, addressing this issue nationally and globally, are essential to support the healthcare workers who are sacrificing their mental health for the future of our nations.

Keywords: anxiety; sleep disturbance; frontline; healthcare workers; COVID-19 pandemic

check for updates

Citation: Meo, S.A.; Alkhalifah, J.M.; Alshammari, N.F.; Alnufaie, W.S. Comparison of Generalized Anxiety and Sleep Disturbance among Frontline and Second-Line Healthcare Workers during the COVID-19 Pandemic. *Int. J. Environ. Res. Public Health* **2021**, *18*, 5727. https://doi.org/10.3390/ijerph18115727

Academic Editor: Nicola Magnavita

Received: 10 April 2021
Accepted: 16 May 2021
Published: 26 May 2021

Publisher's Note: MDPI stays neutral with regard to jurisdictional claims in published maps and institutional affiliations.

Copyright: © 2021 by the authors. Licensee MDPI, Basel, Switzerland. This article is an open access article distributed under the terms and conditions of the Creative Commons Attribution (CC BY) license (https://creativecommons.org/licenses/by/4.0/).

1. Introduction

Severe Acute Respiratory Syndrome-2 (SARS-CoV-2) infection, also known as COVID-19, has developed into a global challenge and threatening situation with long-lasting health and financial effects [1,2]. On 30 April 2021, worldwide, the total number of SARS-CoV-2 cases was 150,110,310, with a mortality rate of 3,158,792 (2.10%). The incidence and prevalence of SARS-CoV-2 cases are continuously increasing [3].

Multiple factors are involved in the rising incidence and mortality of SARS-CoV-2 cases and deaths. Pre-existing health conditions may exacerbate the severity of SARS-CoV-2 disease. These conditions include old age [4], hypertension, coronary artery disease, and diabetes mellitus [5–7]. Moreover, weather conditions, such as a cold climate, humidity [8,9],

and environmental pollution are leading causative factors in increasing SARS-CoV-2 cases and deaths [10,11].

The COVID-19 pandemic has caused tremendous health problems in different sub-populations, both in developed and developing countries. The literature demonstrates that it has raised numerous challenges for the global healthcare system to safely manage patients [12]. The public has identified that the COVID-19 pandemic has caused emotional detachment from their families, fellows, and friends [13]. The present pandemic has caused a dramatic loss of human lives worldwide and developed into an unprecedented situation for public health.

Healthcare workers are fighting against the COVID-19 pandemic to protect global communities from this outbreak. Frontline healthcare workers have experienced high work volume, personal risk, and societal pressure to meet extraordinary demands for healthcare [14]. Moreover, the working environment of frontline and second-line healthcare workers during the COVID-19 pandemic has been stressful. The adverse effects of stress can lead to mental health problems, such as anxiety and sleep disturbances, which may affect work performance, family, and social relationships. Healthcare providers are frequently in contact with SARS-CoV-2 patients, and therefore more prone to fear, stress, insomnia [15], and depression [16].

While mental health changes among the general population during the pandemic have been widely noted, data about frontline and second-line healthcare workers is limited, despite the fact that they are sacrificing their mental health for the future of our nations. The present study is the first of its kind to investigate and compare generalized anxiety and sleep disturbance among frontline and second-line healthcare workers during the COVID-19 pandemic.

2. Subjects and Methods

2.1. Study Design and Settings

The present questionnaire-based observational–analytical study was conducted in the Department of Physiology, College of Medicine, King Saud University, Riyadh, Saudi Arabia, from July–December 2020.

2.2. Selection of Healthcare Workers and Data Collection

The targeted population of the current study was all healthcare workers (HCWs) employed in various healthcare hospitals in Saudi Arabia. The HCWs were on duty across all departments and sub-specialties during the data collection period. A structured, self-administered survey of HCWs was conducted via email, using a scale to assess their generalized anxiety levels and sleep disturbance during the COVID-19 pandemic. The demographical data of the HCWs were obtained through an online survey. The validated, self-administered electronic questionnaire was distributed through email to healthcare workers. The selection of healthcare workers was made using the convenience sampling technique. Data collectors were assigned to ensure that the data was inclusive of all healthcare workers in Saudi Arabia. The power formula was employed to calculate the sample size; as per an earlier published study [17], the required sample size for both groups was 1458; however, in this study, the sample size of 1678 was more than the size of the sample needed to detect the effect size at 80% power.

Based on the job titles and duties in the various units in hospitals, healthcare workers were classified as either frontline or second-line healthcare workers. Healthcare workers who were directly involved in the management of patients, such as consultants, physicians, nurses, and clinical pharmacists, were considered frontline healthcare workers. However, healthcare workers who were not directly involved in the management of patients but were working in hospitals, such as lab technicians in biochemistry, hematology, or molecular labs, were considered second-line healthcare workers. Participants who were working in the hospital as receptionists or clerical and administrative staff were considered "others".

2.3. Inclusion and Exclusion Criteria

All healthcare workers on duty during the COVID-19 pandemic were included in the study. Participants with any previous frequent complaints of headaches, vertigo, anxiety, depression, and/or sleep disturbances before the beginning of the COVID-19 pandemic were excluded from the study. Participants with a known history of chronic debilitating diseases, such as neurological or psychological disorders, or malignancy were excluded. These debilitating conditions may cause stress and impair sleep quality; hence, we excluded these participants. Moreover, healthcare workers who were not on duty during the pandemic period were also excluded from the study. The information about health status was gathered from the participants in a questionnaire.

2.4. Questionnaire

The survey questionnaire consisted of a total of 25 items; 17 items were focused on the assessment of sleep quality using the Pittsburgh Sleep Quality Index (PSQI) scale [18], and the other eight items were focused on the anxiety assessment using the General Anxiety Disorder-7 (GAD-7) scale [19]. For the Pittsburgh Sleep Quality Index (PSQI) scale, individual items were scored out of 3, with the minimum score being 0 and the maximum score being 3, where lower values indicated better sleep quality [18]. Scores of 5, 10, and 15 were taken as the cut-off points for mild, moderate, and severe anxiety, respectively [19]. The survey was comprised of an initial page for informed consent, and all contributors were given the option to participate or not. The healthcare workers who opted to participate were led through a series of questions. No reward of any kind was given to the participants, and the information was kept entirely confidential. We used validated and reliable instruments to investigate sleep [18] and anxiety [19].

2.5. General Anxiety Disorder-7 (GAD-7) and Pittsburgh Sleep Quality Index (PSQI) Scales

The psychometric characteristics of the GAD-7 are reliable to measure anxiety allied with clinical features [20] with strong consistency (Cronbach's alpha = 0.92) and good test–retest reliability (r = 0.88). The PSQI has [16] internal uniformity and a reliability coefficient (Cronbach's alpha) of 0.83 for its seven components. The overall PSQI global score correlation coefficient for test–retest reliability was r = 0.87. The survey was distributed through email, healthcare facilities' Human Resources departments, and data collectors to 1806 healthcare workers; 128 responses were excluded for not matching the inclusion/exclusion criteria, or missing information. Finally, 1678 responses were received, with a response rate of 92.91%.

2.6. Statistical Analysis

The findings were analyzed using SPSS software version 26.0 for Mac [20]. All demographical variables, including age, gender, and occupation status, were summarized and reported using frequency and percentage. The total response score was summarized and reported using mean and standard deviation. The comparisons between the variables with demographic and clinical factors were analyzed using independent samples t-tests, ANOVA, and Chi-square tests with a degree of freedom (df). A p-value <0.05 was considered as significant.

2.7. Ethics Statement

This study was executed in harmony with the Declaration of Helsinki, and the Ethics Committee Institutional Review Board (IRB), College of Medicine Research Centre, King Saud University, Riyadh, Saudi Arabia, approved the protocol (ref: E-20-4939).

3. Results

The total number of HCWs included in this study was 1678 (83.9%); 859 (51.2%) were females, and 819 (48.8%) were males. The mean age of the participants was 34.1 years.

Of the included respondents, 1200 (71.5%) were frontline healthcare workers, while 478 (28.5%) were second-line healthcare workers (Table 1).

Table 1. The demographics of healthcare workers (n = 1678).

Parameters	Number (N) and Percentage (%)
**Age (years)**	
20 to 30	805 (48.0)
31 to 40	475 (28.3)
41 to 50	238 (14.2)
51 to 60	117 (7.0)
61 or above	43 (2.6)
**Gender**	
Male	819 (48.8)
Female	859 (51.2)
**Profession**	
**Frontline healthcare workers**	**1200 (71.5)**
Consultants	289 (17.2)
Physicians	447 (26.6)
Nurses	259 (15.4)
Clinical pharmacists	205 (12.2)
**Second-line healthcare workers**	**478 (28.5)**
Lab technicians	64 (3.8)
Others	414 (24.7)
**Area of workplace**	
Emergency department	219 (13.1)
Ward	413 (24.6)
Intensive care unit (ICU)	127 (7.6)
Labs	87 (5.2)
Other	832 (49.6)
**HCWs' involvement with COVID-19 patients**	
No involvement	534 (31.8)
Diagnosis	229 (13.6)
Treatment	303 (18.1)
Nursing care	151 (9.0)
Other	461 (27.5)

3.1. Generalized Anxiety Disorder-7 (GAD-7)

The results show that among the participants, 33.0% had mild anxiety, 15.1% had moderate anxiety, and 10.8% had severe anxiety. In total, 25.9% of the healthcare workers had moderate to severe anxiety as assessed by the GAD-7 scale (Figure 1). This finding further indicates that 273 (31.7%) females had moderate to severe anxiety compared to 162 (15.38%) males (Table 2). Healthcare workers with 1–5 years of experience suffer from severe anxiety, 12.6% higher than healthcare workers with more years of experience.

3.2. Anxiety Levels between Frontline and Second-Line Healthcare Workers

As demonstrated in Table 2, out of 1678 respondents, 1200 (71.5%) were frontline healthcare workers, while 478 (28.5%) were second-line healthcare workers. Of the total frontline and second-line healthcare workers, 435 (25.92%) had moderate to severe anxiety. There were 713 (59.4%) frontline healthcare workers suffering from anxiety overall, compared to 277 (57.9%) second-line healthcare workers. The results further indicate that there were 137 (11.41%) frontline healthcare workers suffering from severe anxiety, compared to 44 (9.20%) second-line healthcare workers. It has also been identified that moderate to severe anxiety was higher in physicians and nurses (184 (15.33%)) compared to consultants (66 (5.55%)) (Table 2, Figure 2).

Table 2. Association between anxiety and study variables.

Survey Statement	Minimal Anxiety N (%)	Mild Anxiety N (%)	Moderate Anxiety N (%)	Severe Anxiety N (%)	Total N (%)	Chi (*df*)	*p*-Value
**Age (years)**							
20 to 30	301 (37.4)	266 (33.0)	142 (17.6)	96 (11.9)	805 (100.0)	30.007 (12)	0.003
31 to 40	199 (41.9)	161 (33.9)	62 (13.0)	53 (11.1)	475 (100.0)		
41 to 50	97 (40.8)	87 (36.5)	31 (13.0)	23 (9.6)	238 (100.0)		
51 to 60	65 (55.5)	28 (23.9)	16 (13.6)	8 (6.8)	117 (100.0)		
61 above	26 (60.5)	13 (30.2)	3 (6.9)	1 (2.3)	43 (100.0)		
**Gender**							
Male	390 (47.6)	267 (32.6)	99 (12.0)	63 (7.7)	819 (100.0)	41.226 (3)	0.000
Female	298 (34.7)	288 (33.5)	155 (18.0)	118 (13.7)	859 (100.0)		
**Professional title**							
Consultant	121 (41.8)	102 (35.3)	33 (11.4)	33 (11.4)	289 (100.0)	27.460 (15)	0.025
Physician	187 (41.8)	146 (32.6)	61 (13.6)	53 (11.8)	447 (100.0)		
Nurses	104 (40.1)	87 (33.6)	47 (18.1)	21 (8.1)	259 (100.0)		
Clinical pharmacist	75 (36.6)	73 (35.6)	27 (13.1)	30 (14.6)	205 (100.0)		
Lab technician	38 (59.4)	15 (23.4)	6 (9.3)	5 (7.8)	64 (100.0)		
Others	163 (39.3)	132 (31.9)	80 (19.3)	39 (9.4)	414 (100.0)		
**HCWs' involvement with COVID-19 patients**							
No involvement	239 (44.8)	173 (32.4)	79 (14.8)	43 (8.1)	534 (100.0)	34.536 (12)	0.001
Diagnosis	94 (41.0)	73 (31.9)	30 (13.1)	32 (14.0)	229 (100.0)		
Treatment	99 (32.7)	97 (32.0)	55 (18.2)	52 (17.2)	303 (100.0)		
Nursing care	63 (41.7)	47 (31.1)	30 (19.9)	11 (7.3)	151 (100.0)		
SOther	193 (41.9)	165 (35.8)	60 (13.0)	43 (9.3)	461 (100.0)		

Degree of freedom (df).

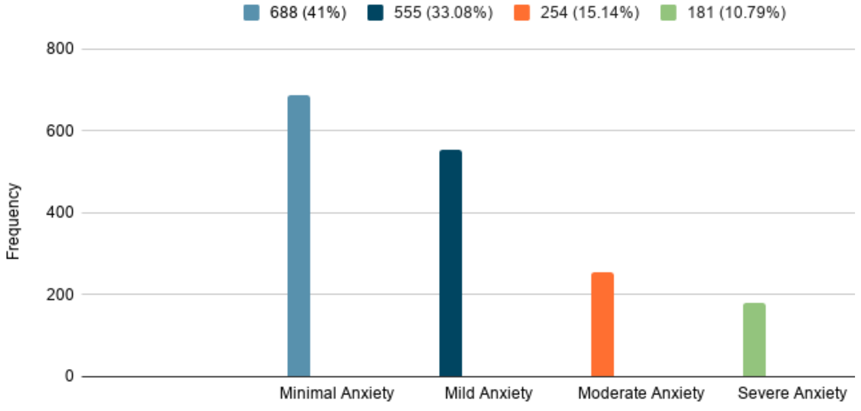

Figure 1. General Anxiety Disorder-7 (GAD-7) among healthcare workers.

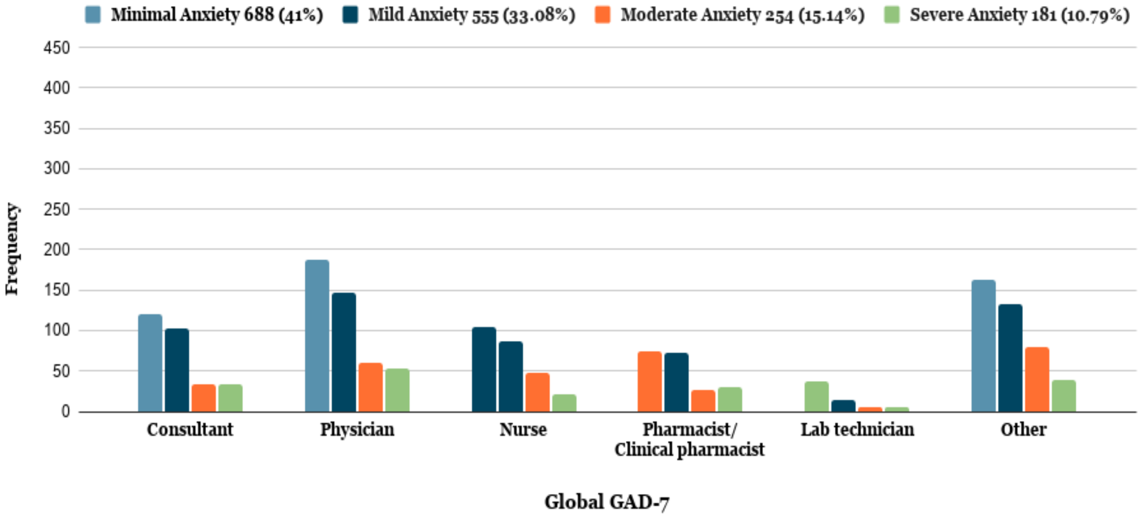

Figure 2. General Anxiety Disorder-7 (GAD-7) among healthcare workers concerning their profession.

3.3. Anxiety Levels and COVID-19 Involvement

As demonstrated in Table 2, out of 1678 respondents, 683 (40.7%) were directly involved with confirmed COVID-19 cases, while 995 (59.3%) were not directly involved. Involvement with confirmed COVID-19 cases was defined as either diagnosing, treating, or providing nursing care, and among those, 210 (30.75%) were suffering from moderate to severe anxiety in comparison with 225 (22.6%) non-directly involved healthcare workers. The percentage of moderate to severe anxiety among directly involved healthcare workers was as following: 29.5% among healthcare workers who were involved in diagnosing COVID-19 patients, 35.3% among healthcare workers who were involved in treating COVID-19 patients, and 27.2% among healthcare workers who were providing nursing care to COVID-19 patients.

3.4. Pittsburgh Sleep Quality Index (PSQI)

Table 3 demonstrates that 1376 (82.0%) of the healthcare worker respondents had poor sleep quality (≥5 global PSQI score). The results show that 975 (58.10%) were frontline healthcare workers, and 407 (23.89%) were second-line healthcare workers who suffered from poor sleep quality. The highest poor sleep quality levels were identified among healthcare workers who work in frontline areas (emergency departments, intensive care units, and wards) (642 (84.6%)), in comparison with others who work in second-line areas (734 (79.9%)) (Table 3, Figure 3). The results further indicate some important relationships with demographic factors, such as the type of healthcare facility; the results show that healthcare workers who work in secondary or tertiary care hospitals and specialized hospitals have the highest poor sleep quality (781 (46.54%)). The highest poor sleep quality was identified among those directly involved in treating COVID-19 patients (Table 3, Figure 3).

Table 3. Association between sleep quality and study variables.

Survey Statement	Good Sleep Quality <5 N (%)	Poor Sleep Quality ≥5 N (%)	Total N (%)	Chi (Degree of Freedom-*df*)	*p*-Value
**Healthcare facility**					
Primary healthcare center	76 (21.9)	270 (78.0)	346 (100.0)		
Secondary or tertiary hospital	88 (16.9)	432 (83.0)	520 (100.0)		
Specialized hospital	61 (14.8)	349 (85.1)	410 (100.0)		0.026
Polyclinic	9 (11.2)	71 (88.75)	80 (100.0)	12.775 (5)	
Laboratories	8 (15.3)	44 (84.6)	52 (100.0)		
Others	60 (22.2)	210 (77.7)	270 (100.0)		
**Work area**					
Emergency department	49 (22.3)	170 (77.6)	219 (100.0)		
Ward	51 (12.3)	362 (87.6)	413 (100.0)		
Intensive care unit (ICU)	17 (13.4)	110 (86.6)	127 (100.0)	16.979 (4)	0.002
Labs	15 (17.2)	72 (82.7)	87 (100.0)		
Other	170 (20.4)	662 (79.5)	832 (100.0)		
**HCWs' involvement with COVID-19**					
No involvement	117 (21.9)	417 (78.0)	534 (100.0)		
Diagnosis	32 (13.9)	197 (86.0)	229 (100.0)		
Treatment	32 (10.5)	271 (89.4)	303 (100.0)	21.733 (4)	0.00
Nursing care	34 (22.5)	117 (77.4)	151 (100.0)		
Others	87 (18.8)	374 (81.1)	461 (100.0)		
**Profession**					
Consultant	65 (22.5)	224 (77.5)	289 (100.0)	7.658 (5)	
Physician	74 (16.6)	373 (83.4)	447 (100.0)		
Nurse	53 (20.5)	206 (79.5)	259 (100.0)		
Clinical pharmacist	33 (16.1)	172 (83.9)	205 (100.0)		0.176
Lab technician	12 (18.8)	52 (81.2)	64 (100.0)		
Other	65 (15.7)	349 (84.3)	414 (100.0)		
**Global PSQI Score** **Mean ± SD** (8.944 ± 3.79)	302 (18.0)	1376 (82.0)	1678 (100.0)		

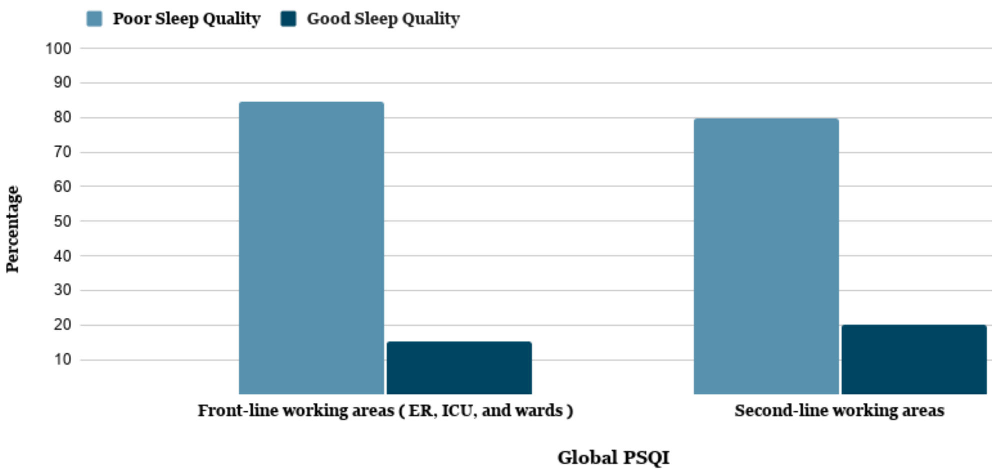

Figure 3. Pittsburgh Sleep Quality Index (PSQI) among frontline and second-line healthcare workers in their working areas.

3.5. Sleep Quality and COVID-19 Involvement

As demonstrated in Table 3, out of 1678 respondents, 683 (40.7%) were directly involved with confirmed COVID-19 cases, while 995 (59.3%) were not directly involved. Involvement with confirmed COVID-19 cases was defined as either diagnosing, treating, or providing nursing care, and among the HCWs that met these criteria, 585 (85.7%) were suffering from poor sleep quality in comparison with 791 (79.5%) non-directly involved healthcare workers. The percentage of poor sleep quality among directly involved healthcare workers was 86% among healthcare workers who were involved in diagnosing COVID-19 patients, 89.4% among healthcare workers who were involved in treating COVID-19 patients, and 77.4% among healthcare workers who were providing nursing care to COVID-19 patients.

4. Discussion

The COVID-19 pandemic has developed into a threatening situation around the world. Healthcare workers are fighting against the pandemic and building a safe environment for global communities. Healthcare providers are highly prone to SARS-CoV-2 infection [7]. The present study findings during this critical time in the COVID-19 pandemic highlights the sleep health and anxiety levels of healthcare workers. Here, we have identified that frontline healthcare workers have more anxiety and sleep disturbance than second-line healthcare workers. Moreover, the highest poor sleep quality levels were detected among healthcare workers who work in frontline areas, including emergency departments, intensive care units, and wards.

A study conducted in China demonstrated that frontline workers who were exposed to COVID-19 experienced severe insomnia, and the female gender predicted a greater risk of psychological stress among the HCWs [21]. A systematic study found that the prevalence of anxiety among healthcare workers was 23.2%, and the prevalence of insomnia was 38.9% [22]. Healthcare workers had anxiety- and sleep-allied health symptoms during the pandemic; these symptoms may be due to the nature of the job, their responsibilities while managing infected patients, and fear of the risk of infection during the pandemic. Moreover, their duties in various units, particularly high-risk medical units, were associated with poor mental health outcomes [23,24].

The literature demonstrates that frontline workers who performed their duties in high-risk units had poorer outcomes than workers who performed their duties in lower-risk environments [25]. Frontline healthcare workers are at greater risk of getting infected and spreading the infection to their families, in addition to their increased workload, which can be psychologically debilitating.

Liang et al. [26] compared HCWs in COVID-19-associated departments to other HCWs. The authors found that HCWs experienced clinically depressive symptoms, but no significant differences between frontline HCWs and non-frontline HCWs were found. The most probable reason for their insignificant findings was the small sample size of 23 doctors and 36 nurses. Based on such a small study, it is not possible to reach an appropriate conclusion. Another study conducted by Cai et al. [27] investigated the psychological relationship of COVID-19 and anxiety on 534 frontline medical-staff members; they found that HCWs experienced more anxiety. It has been reported that approximately one-third of nurses working during the COVID-19 pandemic were suffering from psychological symptoms [28]. Early studies demonstrate that while providing care to COVID-19 patients, healthcare workers are more susceptible and are at high risk of acquiring the infection, with physical and mental consequences [29,30].

Similarly, Chew et al. [31] assessed stress and anxiety in healthcare workers in Singapore and India. It was identified that 48 (5.3%) workers faced moderate to severe depression, and 79 (8.7%) had moderate to extremely severe depression. Moreover, 54 (6%) healthcare workers experienced moderate to extremely severe stress. Salari et al. [24] reported that the prevalence of stress, anxiety, and depression within frontline healthcare workers who were involved in the management of COVID-19 patients was high. In another study, Rossi et al. [32] found that the rate of depression was 24.73%, the rate of anxiety was 19.80%, and the rate of insomnia was 8.27% among healthcare workers. The present study findings are in line with earlier reports confirming a substantial proportion of mental health problems, particularly among frontline HCWs.

Alamri et al. [33] conducted a study on the prevalence of anxiety among the general population in Saudi Arabia during the COVID-19 Pandemic. The authors found that 10% of people had moderate to severe anxiety symptoms. However, in the present study, it was identified that 25.92% of healthcare workers have moderate to severe anxiety. The rate of frontline healthcare workers suffering from anxiety was 59.4%, compared to 57.9% of second-line healthcare workers. There were significantly higher anxiety levels in healthcare workers compared to the general population in Saudi Arabia.

Alharbi et al. 2021 [34] conducted a study during lockdown and reported that 55% of the general Saudi population suffered from poor sleep quality. However, in the present study, the data indicate that 82% of healthcare workers in Saudi Arabia suffered from poor sleep quality during the same period. The study findings show that the healthcare workers in Saudi Arabia had poorer sleep quality than the general public.

Magnavita et al. [35] performed a study on clinical symptoms in a cohort of HCWs during the COVID-19 pandemic. In this study, 11 participants were physicians, 58 were nurses, and 7 were technicians. The authors demonstrated that the rate of anxiety and depression in HCWs was not higher than generally recorded in the population working at the same organization. However, in COVID-19 cases, there was a significant risk of anxiety, mainly in those who had low sleep quality. Similarly, Magnavita et al. [36] assessed the health situations of 90 anesthesiologists out of 155 workers in a COVID-19 hospital in Italy; 71.1% reported high work-related stress among workers, with an imbalance between high effort and low reward. The workers also reported insomnia (36.7%), anxiety (27.8%), and depression (51.1%). In another study, Magnavita et al. [37] investigated 152 out of 205 workers; 105 were physicians, and 47 were nurses. The authors found that a high workload, isolation at work, uncertainty about safety procedures, and a decrease in time devoted to meditation and relaxation have led to a significant increase in stress. In three studies [35–37], the findings support the hypothesis of increased stress, anxiety, and depression among HCWs. However, the main limitation of these three studies [35–37] was a small sample size and a minimal number of frontline HCWs.

During this pandemic, worldwide, people have faced multiple challenges related to lockdown, quarantine, travel restrictions, and emotional detachment from family, fellows, and friends [13]. However, healthcare workers are also facing other issues, including increased job responsibilities, higher chances of infection, and carrying the infection from

hospital to their family members. These factors may further enhance their psychological burden.

Healthcare workers are at the heart of the unprecedented crisis of COVID-19, facing many challenges while managing SARS-CoV-2 patients, minimizing the spread of infection, and being involved in hospital strategy. Moreover, HCWs are still engaged in treating non-COVID-19 patients and maintaining their personal responsibilities, including taking care of their families and themselves. The literature shows high rates of burnout and psychological stress in HCWs [38]. The results presented here show that HCWs who experienced anxiety and/or sleep disturbance, mainly due to direct or indirect contact with SARS-CoV-2 patients, can develop infection and anxiety about the safety of themselves and their family members.

5. Study Strengths and Limitations

To our knowledge, this is the first large sample size study conducted on the comparison of the anxiety levels and sleep disturbance among frontline and second-line healthcare workers during the COVID-19 pandemic. The sample size was appropriate and represents healthcare workers from various primary, secondary, and tertiary healthcare workers in Saudi Arabia. This study has some limitations. First, due to the convenience sampling procedure, representativeness was difficult. Second, a few psychosocial work conditions were not surveyed. Third, the cross-sectional design limited the interpretation of the causal relationship that COVID-19 is associated with elevated anxiety and sleep disturbance in HCWs. The fourth limitation of this study was that the data were collected from one country only; it would be more appropriate to additionally collect data from other neighboring countries.

6. Conclusions

The present study results have established that the COVID-19 pandemic has more extensive negative psychological features on the mental health of frontline healthcare workers, associated with severe anxiety and poor sleep quality, than second-line healthcare workers. The study findings confer great opportunities for healthcare officials and policymakers to account for workplace context when planning for mental health prevention programs for healthcare workers during the pandemic period. The protection of mental health in HCWs is of paramount importance. Medical and paramedical staff must take appropriate rest, have a healthy diet, and engage in regular physical exercise to reduce anxiety levels and maintain sleep quality. Health officials must support the healthcare workers who are sacrificing their mental health for the future of our nations. This study highlights the importance of providing comprehensive support strategies to reduce the psychological allied symptoms of the COVID-19 outbreak among healthcare workers under pandemic conditions.

Author Contributions: S.A.M.: research conceptualization, supervision, manuscript writing and editing. J.M.A., literature review, data collection and manuscript writing. N.F.A., W.S.A.: data collection, analysis, and literature review. All authors have read and agreed to the published version of the manuscript.

Funding: Deanship of Scientific Research, King Saud University, Riyadh, Saudi Arabia (RGP-181).

Institutional Review Board Statement: The study was conducted according to the guidelines of the "Declaration of Helsinki, and approved by the Institutional Review Board of College of Medicine, King Saud University, Riyadh, KSA" (E-20-4939).

Informed Consent Statement: Informed consent was obtained from all the participants.

Data Availability Statement: Data may be provided on reasonable request to corresponding author.

Acknowledgments: We thank the "Deanship of Scientific Research, King Saud University, Riyadh, Saudi Arabia, for supporting this work through a research group project (RGP-181)". We also thank to May Ahmed Babaeer for her support in data collection and analysis.

Conflicts of Interest: The authors declare no competing financial interests.

References

1. Meo, S.A.; Bukhari, I.A.; Akram, J.; Meo, A.S.; Klonoff, D.C. COVID-19 vaccines: Comparison of biological, pharmacological characteristics and adverse effects of Pfizer/BioNTech and Moderna Vaccines. *Eur. Rev. Med. Pharmacol. Sci.* **2021**, *25*, 1663–1669. [CrossRef] [PubMed]
2. Meo, S.A.; Zaidi, S.Z.A.; Shang, T.; Zhang, J.Y.; Al-Khlaiwi, T.; Bukhari, I.A.; Akram, J.; Klonoff, D.C. Biological, molecular and pharmacological characteristics of chloroquine, hydroxychloroquine, convalescent plasma, and remdesivir for COVID-19 pandemic: A comparative analysis. *J. King Saud Univ. Sci.* **2020**, *32*, 3159–3166. [CrossRef]
3. World Health Organization. Coronavirus (COVID-19) Dashboard. Available online: https://covid19.who.int/ (accessed on 30 April 2021).
4. Ge, Y.; Sun, S.; Shen, Y. Estimation of case-fatality rate in COVID-19 patients with hypertension and diabetes mellitus in the New York state: A preliminary report. *Epidemiol. Infect.* **2021**, *149*, e14. [CrossRef] [PubMed]
5. Gupta, A.; Nayan, N.; Nair, R.; Kumar, K.; Joshi, A.; Sharma, S.; Singh, J.; Kapoor, R. Diabetes Mellitus and Hypertension Increase Risk of Death in Novel Corona Virus Patients Irrespective of Age: A Prospective Observational Study of Co-morbidities and COVID-19 from India. *SN Compr. Clin. Med.* **2021**, *3*, 937–944. [CrossRef] [PubMed]
6. Lima-Martínez, M.M.; Carrera, B.C.; Madera-Silva, M.D.; Marín, W.; Contreras, M. COVID-19 and diabetes: A bidirectional relationship. *Clin. Investig. Arterioscler.* **2020**. [CrossRef]
7. Guan, W.J.; Ni, Z.Y.; Hu, Y.; Liang, W.H.; Ou, C.Q. Clinical Characteristics of Coronavirus Disease 2019 in China. *N. Engl. J. Med.* **2020**, *382*, 1708–1720. [CrossRef]
8. Meo, S.A.; Abukhalaf, A.A.; Alomar, A.A.; Sumaya, O.Y.; Sami, W.; Shafi, K.M.; Meo, A.S.; Usmani, A.M.; Akram, J. Effect of heat and humidity on the incidence and mortality due to COVID-19 pandemic in European countries. *Eur. Rev. Med. Pharmacol. Sci.* **2020**, *24*, 9216–9225. [CrossRef] [PubMed]
9. Meo, S.A.; Abukhalaf, A.A.; Alomar, A.A.; Al-Beeshi, I.Z.; Alhowikan, A.; Shafi, K.M.; Meo, A.S.; Usmani, A.M.; Akram, J. Climate and COVID-19 pandemic: Effect of heat and humidity on the incidence and mortality in world's top ten hottest and top ten coldest countries. *Eur. Rev. Med. Pharmacol. Sci.* **2020**, *24*, 8232–8238. [CrossRef] [PubMed]
10. Meo, S.A.; Abukhalaf, A.A.; Alomar, A.A.; Alessa, O.M.; Sami, W.; Klonoff, D.C. Effect of environmental pollutants PM-2.5, carbon monoxide, and ozone on the incidence and mortality of SARS-COV-2 infection in ten wildfire affected counties in California. *Sci. Total. Environ.* **2021**, *757*, 143948. [CrossRef]
11. Meo, S.A.; Abukhalaf, A.A.; Sami, W.; Hoang, T.D. Effect of environmental pollution PM2.5, carbon monoxide, and ozone on the incidence and mortality due to SARS-CoV-2 infection in London, United Kingdom. *J. King Saud Univ. Sci.* **2021**, *33*, 101373. [CrossRef]
12. Li, W.; Yang, Y.; Liu, Z.H. Progression of Mental Health Services during the COVID-19 Outbreak in China. *Int. J. Biol. Sci.* **2020**, *16*, 1732–1738. [CrossRef]
13. Meo, S.A.; Abukhalaf, A.A.; Alomar, A.A.; Sattar, K.; Klonoff, D.C. COVID-19 Pandemic: Impact of Quarantine on Medical Students' Mental Wellbeing and Learning Behaviors. *Pak. J. Med. Sci.* **2020**, *36*, S43–S48. [CrossRef] [PubMed]
14. Johnson, S.B.; Butcher, F. Doctors during the COVID-19 pandemic: What are their duties and what is owed to them? *J. Med. Ethics* **2021**, *47*, 12–15. [CrossRef] [PubMed]
15. Xiang, Y.T.; Zhao, Y.J.; Liu, Z.H.; Li, X.H.; Zhao, N.; Cheung, T.; Ng, C.H. The COVID-19 outbreak and psychiatric hospitals in China: Managing challenges through mental health service reform. *Int. J. Biol. Sci.* **2020**, *16*, 1741–1744. [CrossRef]
16. Zhang, H.H.; Zhao, Y.J.; Wang, C.; Zhang, Q.; Yu, H.Y.; Cheung, T.; Hall, B.J.; An, F.R.; Xiang, Y.T. Depression and its relationship with quality of life in frontline psychiatric clinicians during the COVID-19 pandemic in China: A national survey. *Int. J. Biol. Sci.* **2021**, *17*, 683–688. [CrossRef] [PubMed]
17. Mohd Noor, N.; Che Yusof, R.; Yacob, M.A. Anxiety in Frontline and Non-Frontline Healthcare Providers in Kelantan, Malaysia. *Int. J. Environ. Res. Public Health* **2021**, *18*, 861. [CrossRef] [PubMed]
18. Buysse, D.J.; Reynolds, C.F.; Monk, T.H.; Berman, S.R.; Kupfer, D.J. The Pittsburgh Sleep Quality Index: A new instrument for psychiatric practice and research. *Psychiatry Res.* **1989**, *28*, 193–213. [CrossRef]
19. Spitzer, R.L.; Kroenke, K.; Williams, J.B.; Löwe, B. A brief measure for assessing generalized anxiety disorder: The GAD-7. *Arch. Intern. Med.* **2006**, *166*, 1092–1099. [CrossRef]
20. IBM Support. Using (SPSS) Software Version 26.0 for Mac. Available online: https://www.ibm.com/support/pages/downloading-ibm-spss-statistics-26 (accessed on 12 July 2020).
21. Lai, J.; Ma, S.; Wang, Y.; Cai, Z.; Hu, J.; Wei, N.; Wu, J.; Du, H.; Chen, T.; Li, R.; et al. Factors Associated With Mental Health Outcomes Among Health Care Workers Exposed to Coronavirus Disease 2019. *JAMA Netw. Open* **2020**, *3*, e203976. [CrossRef]
22. Pappa, S.; Ntella, V.; Giannakas, T.; Giannakoulis, V.G.; Papoutsi, E.; Katsaounou, P. Prevalence of depression, anxiety, and insomnia among healthcare workers during the COVID-19 pandemic: A systematic review and meta-analysis. *Brain Behav. Immun.* **2020**, *88*, 901–907. [CrossRef]
23. Alsulimani, L.K.; Farhat, A.M.; Borah, R.A.; AlKhalifah, J.A.; Alyaseen, S.M.; Alghamdi, S.M.; Bajnaid, M.J. Health care worker burnout during the COVID-19 pandemic: A cross-sectional survey study in Saudi Arabia. *Saudi Med. J.* **2021**, *42*, 306–314. [CrossRef] [PubMed]

24. Salari, N.; Khazaie, H.; Hosseinian-Far, A.; Khaledi-Paveh, B.; Kazeminia, M.; Mohammadi, M.; Shohaimi, S.; Daneshkhah, A.; Eskandari, S. The prevalence of stress, anxiety, and depression within frontline healthcare workers caring for COVID-19 patients: A systematic review and meta-regression. *Hum. Resour. Health* **2020**, *18*, 100. [CrossRef] [PubMed]
25. Huang, Y.; Zhao, N. Generalized anxiety disorder, depressive symptoms and sleep quality during COVID-19 outbreak in China: A web-based cross-sectional survey. *Psychiatry Res.* **2020**, *288*, 112954. [CrossRef]
26. Liang, Y.; Chen, M.; Zheng, X.; Liu, J. Screening for Chinese medical staff mental health by S.D.S. and S.A.S. during the outbreak of COVID-19. *J. Psychosom. Res.* **2020**, *133*, 110102. [CrossRef] [PubMed]
27. Cai, H.; Tu, B.; Ma, J.; Chen, L.; Fu, L.; Jiang, Y.; Zhuang, Q. Psychological Impact and Coping Strategies of Frontline Medical Staff in Hunan Between January and March 2020 During the Outbreak of Coronavirus Disease 2019 (COVID-19) in Hubei, China. *Med. Sci. Monit.* **2020**, *26*, e924171.
28. Al-Maqbali, M.; Al-Sinani, M.; Al-Lenjawi, B. Prevalence of stress, depression, anxiety, and sleep disturbance among nurses during COVID-19 pandemic. A systematic review and meta-analysis. *J. Psychosom. Res.* **2021**, *141*, 110343. [CrossRef] [PubMed]
29. Lin, M.; Beliavsky, A.; Katz, K.; Powis, J.E.; Ng, W.; Williamsm, V. What can early Canadian experience screening for COVID-19 teach us about how to prepare for a pandemic? *Can. Med. Assoc. J.* **2020**, *192*, E314–E318. [CrossRef]
30. Koh, D. Occupational risks for COVID-19 infection. *Occup. Med.* **2020**, *70*, 3–5. [CrossRef]
31. Chew, N.W.S.; Lee, G.K.H.; Tan, B.Y.Q.; Jing, M.; Goh, Y.; Ngiam, N.J.H.; Yeo, L.L.L.; Ahmad, A. A multinational, multicentre study on the psychological outcomes and associated physical symptoms amongst healthcare workers during the COVID-19 outbreak. *Brain Behav. Immun.* **2020**, *88*, 559–565. [CrossRef]
32. Rossi, R.; Socci, V.; Pacitti, F.; Di Lorenzo, G.; Di Marco, A.; Siracusano, A.; Rossi, A. Mental Health Outcomes Among Frontline and Second-Line Health Care Workers During the Coronavirus Disease 2019 (COVID-19) Pandemic in Italy. *JAMA Netw. Open* **2020**, *3*, e2010185. [CrossRef]
33. Alamri, H.S.; Algarni, A.; Shehata, S.F.; Al Bshabshe, A.; Alshehri, N.N.; ALAsiri, A.M.; Hussain, A.H.; Alalmay, A.Y.; Alshehri, E.A.; Alqarni, Y.; et al. Prevalence of Depression, Anxiety, and Stress among the General Population in Saudi Arabia during the Covid-19 Pandemic. *Int. J. Environ. Res. Public Health* **2020**, *17*, 9183. [CrossRef]
34. Alharbi, A.S.; Alshahrani, S.M.; Alsaadi, M.M.; Al-Jahdali, H.H.; Wali, S.O.; BaHammam, A.S. Sleep quality and insomnia during the COVID-19 lockdown among the Saudi public. *Saudi Med. J.* **2021**, *42*, 384–390. [CrossRef] [PubMed]
35. Magnavita, N.; Tripepi, G.; Di Prinzio, R.R. Symptoms in Health Care Workers during the COVID-19 Epidemic. A Cross-Sectional Survey. *Int. J. Environ. Res. Public Health* **2020**, *17*, 5218. [CrossRef] [PubMed]
36. Magnavita, N.; Soave, P.M.; Ricciardi, W.; Antonelli, M. Occupational Stress and Mental Health among Anesthetists during the COVID-19 Pandemic. *Int. J. Environ. Res. Public Health* **2020**, *17*, 8245. [CrossRef]
37. Magnavita, N.; Soave, P.M.; Antonelli, M. Prolonged stress causes depression in frontline workers facing the COVID-19 pandemic. A repeated cross-sectional study. *Int. J. Environ. Res. Public Health* **2021**, 2021050129. [CrossRef]
38. Santarone, K.; McKenney, M.; Elkbulim, A. Preserving mental health and resilience in frontline healthcare workers during COVID-19. *Am. J. Emerg. Med.* **2020**, *38*, 1530–1531. [CrossRef] [PubMed]
