# Supplementary material for: Mental health status and related factors influencing healthcare workers during the COVID-19 pandemic: A systematic review and meta-analysis
Source: PLoS One. 2024 Jan 19;19(1):e0289454. doi: 10.1371/journal.pone.0289454 (PMC10798549; doi:10.1371/journal.pone.0289454)
Supplement: S1 Data — (ZIP) [file pone.0289454.s011.zip › literatures/101.pdf]

AIDS and Behavior (2021) 25:18–27
https://doi.org/10.1007/s10461-020-03073-z

ORIGINAL PAPER

Mental Health Problems of HIV Healthcare Providers During the COVID-19 Pandemic: The Interactive Effects of Stressors and Coping

Tianyue Mi[1] · Xueying Yang[1,4] · Shufang Sun[2] · Xiaoming Li[1] · Cheuk Chi Tam[1] · Yuejiao Zhou[3] · Zhiyong Shen[3]

Accepted: 21 October 2020 / Published online: 30 October 2020
© Springer Science+Business Media, LLC, part of Springer Nature 2020

Abstract
HIV healthcare providers might be vulnerable to mental health problems during the COVID-19 pandemic. Guided by the stress and coping paradigm, the current study aimed at examining the interactive effects of COVID-19-related stressors and coping on mental health problems. A cross-sectional online survey was conducted among 1029 HIV healthcare providers in Guangxi, China. The prevalence of depression and anxiety in the current study was 13.31% and 6.61%, respectively. Results from path analyses revealed that the main effects of COVID-19-related stressors and coping were significant on both depression and anxiety. The interaction of coping and COVID-19-related stressors had significant effects on depression and anxiety. Simple slope tests revealed that more coping behaviors buffered against the negative effect of COVID-19-related stressors on mental health problems. Coping acted as a protective factor that alleviated the harm of COVID-19-related stressors on mental health. Intervention targeting coping management might benefit the mental health of HIV healthcare providers.

Keywords COVID-19 · HIV healthcare providers · Mental health · Coping · China

Introduction

The coronavirus disease 2019 (COVID-19) has become a global public health crisis, causing enormous pressure on residents and governments [1]. The COVID-19 pandemic has triggered massive human casualties and severe economic loss [2], as well as substantial adverse psychological impacts [3]. What is worse, the number of cases has continued to escalate exponentially in some countries [4]. The unprecedentedly epidemic and substantial lockdown in China has caused great distress and burden, disproportionately among healthcare providers [5]. Healthcare providers are among those at high risk of infection due to occupational exposure when treating the patients. A study conducted in Wuhan reported that healthcare providers accounted for 29% of all COVID-19 cases [6]. Recent studies have consistently shown the elevated prevalence of mental health problems among healthcare providers, such as frontline nurses and physicians [7]. However, mental health problems among HIV healthcare providers have rarely been investigated.

Despite the strict COVID-19 control measures implemented in China, the healthcare system was still largely overwhelmed by such a public health emergency [8]. Many hospitals still operate near or over capacity and face a severe shortage of personnel due to increasing daily COVID-19 cases and the insufficient preparedness of the healthcare system [8]. To adapt the personnel shortage during the COVID-19 pandemic, many hospitals in China reassigned HIV healthcare providers to fight against the COVID-19 outbreak. As a result, HIV healthcare providers might put aside the ongoing needs of people living with HIV (PLWH) [9] while lacking alternative strategies to maintain core HIV services (e.g., pick-up points for refills, virtual support platforms) [10–13]. As a consequence, HIV care services have been significantly interrupted by the COVID-19 pandemic

✉ Xueying Yang
xueyyang@mailbox.sc.edu

1 Department of Health Promotion Education and Behavior, SC SmartState Center for Healthcare Quality, University of South Carolina, Columbia, USA

2 Department of Behavioral and Social Sciences, Brown University School of Public Health, Providence, RI, USA

3 Guangxi Center for Diseases Control and Prevention, Nanning, China

4 University of South Carolina, 915 Greene St, Room 529, Columbia, SC 29208, USA

due to strict quarantine enforcement and transportation lockdown in various cities across China [9]. A national anonymous survey in China reported 32.6% of PLWH were at risk of antiretroviral therapy discontinuation during the pandemic [14]. The intersection of the COVID-19 and HIV pandemics also render HIV healthcare providers more vulnerable to mental health problems.

Healthcare providers working on the frontlines and in general wards were all exposed to various COVID-19-related stressors [15]. These unprecedented COVID-19-related stressors were associated with adverse psychological consequences among healthcare providers [16, 17]. Quantitative studies have shown that frontline healthcare providers treating COVID-19 patients were vulnerable to higher risks of mental health problems, such as anxiety, depression, and insomnia [18]. Another cross-sectional study conducted among Chinese healthcare workers reported high rates of depressive (50.4%) and anxiety symptoms (44.6%) during the COVID-19 pandemic [19]. Despite the vulnerability, limited studies have explored protective factors confronting the stress influences on psychological outcomes in HIV healthcare providers.

Coping behaviors may be robust factors protecting the psychological health of HIV healthcare providers. The stress and coping paradigm, an important theory for psychological intervention, suggests that coping as a dynamic reaction process can buffer the negative influences of adversities on psychological outcomes [20, 21]. When a life stressor is cognitively appraised as threatening, harmful or challenging, appropriate coping strategies will be elicited to handle the stress and protect the individual from emotional distress and maladjustment [22]. Existing COVID-19 studies have documented various coping behaviors in response to the COVID-19 pandemic among healthcare providers. The results from a US study showed that 80% of healthcare providers adopted at least one type of coping behavior to manage COVID-19-related stress, with physical activity/exercise being the most commonly endorsed behavior [17]. In addition, self-care as one type of coping behavior was extremely vital to minimize potential adverse long-term effects from COVID-19. The strategies of self-care included taking breaks and being aware of local resources [23]. Although several coping behaviors have been identified, the role of coping behaviors in the association of COVID-19-related stressors and mental health problems in HIV healthcare providers is understudied.

HIV healthcare providers have faced stressors due to the need to manage the dual pandemics of HIV and COVID-19, but few studies have examined the interactive effects between COVID-19-related stressors and coping on mental health problems among such a population. The current study aimed to address these gaps by evaluating mental health problems and the role of coping among HIV healthcare providers under such a stressful situation during the COVID-19 outbreak. The two specific aims were: (a) investigate the prevalence of depression and anxiety symptoms among HIV healthcare providers; and (b) examine the main and interactive effects of COVID-19-related stressors and coping on mental health problems. The results from this research can serve as a piece of evidence to inform future intervention efforts aiming to improve the mental health of HIV healthcare providers during the COVID-19 pandemic.

Methods

Study Site, Participants, and Procedure

Data were collected from a web-based anonymous online survey among a convenience sample of HIV healthcare providers in Guangxi Zhuang Autonomous Region (Guangxi), China. Guangxi has been one of the HIV/AIDS epicenters across China for decades, with the fast growing and high accumulated number of HIV/AIDS cases. In 2018, over 86,000 confirmed HIV/AIDS cases were reported in Guangxi [24], and Guangxi ranked the third among 31 provinces across China in terms of the documented HIV positive cases [25].

The detailed procedure of this study was reported elsewhere [26]. In brief, the online survey was initiated in April 2020 and lasted for a month. In collaboration with Guangxi Center for Disease Control and Prevention (CDC), our research team reached out to healthcare providers who provide HIV care in Guangxi and invited them to participate in the online survey. The inclusion criteria included: (1) currently providing HIV-related care and services; (2) being 18 years of age or older; (3) being residents in Guangxi. A total of 1,280 HIV healthcare providers gave consent and participated in the online survey. Data from 251 participants were excluded due to their responses were identified as random or careless ($n=76$), answers from out of Guangxi ($n=63$), and outliers ($n=112$). A final sample size of 1029 participants were included in the current study.

The online survey was presented through SO JUMP system technology, a widely used Chinese online survey platform. The survey was anonymous and voluntary, taking about 15 min. Upon the completion of the survey, we provided a guideline developed by counseling psychologists, on psychological distress management under COVID-19 pandemic. No financial incentive was offered for participation. The research protocol was approved by the Institutional Review Boards at both the University of South Carolina in the United States and the Guangxi CDC in China.

Measures

Demographics

Participants were asked to provide their individual and occupational demographics, including age, gender (male *vs.* female), marital status (single *vs.* married/remarried), and educational attainment (high school or below *vs.* college *vs.* bachelor or above). Occupational information included years of engaging in medical care services, years of engaging in HIV care services, professional position (physician *vs.* nurse *vs.* CDC staff *vs.* other), professional ranking (no ranking *vs.* entry level *vs.* middle level *vs.* senior/advanced level), administrative ranking (no ranking *vs.* department leader *vs.* hospital/CDC director *vs.* other), and level of institutional affiliation (province/city *vs.* country *vs.* community).

COVID-19-Related Stressors

A 20-item checklist was developed in the current study to evaluate the COVID-19-related stressors among HIV healthcare providers. Participants were asked to report whether they have experienced any of the 20 potential stressful events during the COVID-19 outbreak (0 = no, 1 = yes). Stressful events included daily life disturbance (e.g., "Disturbance of daily life, study or work plan due to the COVID-19 outbreak"), care of COVID-19 patients (e.g., "Treated/provided care for HIV seropositive patients affected by COVID-19"), occupational overload (e.g., "Hospital was overloaded due to the COVID-19 outbreak"), difficulties in the delivery of healthcare (e.g., "Difficulties in advising HIV patients during the COVID-19"), and self/family infection with COVID-19 (e.g., "I was diagnosed with COVID-19 or a suspect case"). A sum score was calculated with a higher score indicating more exposure to stressors during the COVID-19 outbreak. The Cronbach's alpha of this scale was 0.78 in the current study.

Coping with COVID-19

Coping with COVID-19 was assessed with a coping scale developed in the current study, measuring the frequency of adopting numerous coping strategies to handle the influences of the COVID-19 pandemic. This scale included six items describing commonly used personal coping approaches such as "called psychological counseling hotline provided my clinics to relieve stress" and "tried to find an appropriate time and place to express my negative feelings." Participants responded to the items on a five-point scale ranging from 1 (never) to 5 (always). A sum score was generated with a higher score representing a greater level of coping strategy usage in response to the outbreak. The Cronbach's alpha of this scale was 0.72 in the current study.

Depression and Anxiety

Depression and anxiety were assessed by the 4-item Patient Health Questionnaire-4 (PHQ-4), which has been used among Chinese healthcare providers [27]. PHQ-4 is an efficient ultra-brief tool for identifying common mental disorders, with two items measuring depression and two items measuring anxiety. Participants were asked about the frequency of experiencing depression and anxiety symptoms during the COVID-19 outbreak. A sample item of depression was "little interest or pleasure in doing things," and a sample item of anxiety was "feeling nervous, anxious or on edge." Items were rated on a four-point scale ranging from 0 (not at all) to 3 (nearly everyday). Two sum scores were generated with a higher score representing more severe depression or anxiety. The Cronbach's alpha was 0.67 for depression and 0.77 for anxiety in the current study.

Data Analysis

Data were analyzed with SPSS 26.0 and Amos 26. Descriptive statistics were performed, and correlations (Pearson correlation for continuous variables, Point-biserial correlation for dichotomic variables) were calculated on key variables (i.e., COVID-19-related stressors, depression, anxiety, coping) and demographics. To better control the covariances between two endogenous factors (i.e., depression and anxiety) and account for the family-wise error, path analysis was employed to examine the hypothesized interaction. COVID-19-related stressors and coping were centered before calculating the interaction term (i.e., multiplying centered stressor and coping). Based on existing literature [19, 28, 29], demographic variables and occupational information (i.e., age, gender, marital status, educational attainment, years of engaging in medical care services, years of engaging in HIV care services, professional position, professional ranking, administrative ranking and level of institutional affiliation) were considered as potential confounders and being controlled in the following path analysis. Path analysis using maximum likelihood estimation was employed to examine the interactive effects of stressors and coping on depression and anxiety, providing estimates of path coefficients (β) and bootstrapped 95% confidence interval (95% CI) based on 5000 resamples. Model's goodness of fit was determined by several indices, including the comparative fit index (CFI), the Tucker-Lewis index (TLI), root mean square of approximation (RMSEA), and the standardized root mean squared residual (SMSR). If path analysis yielded significant interactive effects, simple slope analyses would be employed to provide details of the interaction. A p-value of less than 0.05 was applied to indicate statistical significance.

Results

Descriptive Results

Among the 1029 participants, the average age was 38.39 years old (SD = 9.20). The majority of the participants were female (61.6%), married or remarried (81.6%), and had a degree of college or above (81.9%). Regarding the professional position, 37.6% were CDC staff, and 21.3% were physicians. In terms of professional ranking, 31.8% has no ranking, 37.5% were entry-level, and 30.8% were middle level or above. Regarding affiliated institute level, 16.6% affiliated to province/city level, 31.7% affiliated to the county level, and 51.8% affiliated to the community level. For administrative ranking, 26.7% were department leaders or hospital/CDC leaders. The average length of engaging in medical care services and HIV care services was 15.23 years (SD = 9.53; range 0–52), and 6.24 years (SD = 5.21; range 0–39), respectively (Table 1). The mean scores of COVID-19-related stressors and coping with COVID-19 were 6.24 (SD = 3.35; range 0–18) and 18.48 (SD = 4.99; range 6–30), respectively. The most common coping strategy was "positive attitude" (52.09%), followed by "physical exercise" (25.85%) and "expression feeling/emotion" (22.16%). With a cut-point of 3 for each subscale to identify participants with depressive and anxiety symptoms [30, 31], 13.31% and 6.61% of participants had symptoms of depression and anxiety, respectively.

Associations Among Main Study Variables

As displayed in Table 2, COVID-19-related stressors was positively associated with depression ($r = 0.27$, $p < 0.01$), anxiety ($r = 0.34$, $p < 0.01$) and working in urban level institutes ($r = 0.08$, $p < 0.05$). Coping was negatively related to depression ($r = -0.07$, $p < 0.05$), anxiety ($r = -0.07$, $p < 0.05$) and working in urban level institutes ($r = -0.08$, $p < 0.01$). Depression was positively associated with anxiety ($r = 0.71$, $p < 0.01$), working in the urban level institute ($r = 0.13$, $p < 0.01$), and higher educational level ($r = 0.10$, $p < 0.01$); while negatively associated with older age ($r = -0.11$, $p < 0.01$), being married ($r = -0.17$, $p < 0.01$), and longer years of engaging in medical care services ($r = -0.09$, $p < 0.01$). Likewise, anxiety was positively associated with being female ($r = 0.11$, $p < 0.01$), working in the urban level institute ($r = 0.10$, $p < 0.01$), and higher educational level ($r = 0.06$, $p < 0.05$); while negatively associated with older age ($r = -0.09$, $p < 0.05$) and being married ($r = -0.12$, $p < 0.01$) (Table 2).

Table 1 The descriptions of socio-demographics and key variables

Variable	Range	Mean (SD)/frequency (column %)
COVID-19-related stressors	0–18	6.24 (3.35)
Coping with COVID-19	6–30	18.48 (4.99)
Depression	0–5	1.12 (1.18)
Anxiety	0–4	.96 (1.09)
Age (years)	19–78	38.39 (9.20)
Years of engaging in medical care services	0–52	15.23 (9.53)
Years of engaging in HIV care services	0–39	6.24 (5.21)
Gender		
Female		634 (61.61%)
Male		395 (38.39%)
Marital status		
Single		189 (18.37%)
Married/remarried		840 (81.63%)
Educational attainment		
High school or below		186 (18.08%)
College		491 (47.72%)
Bachelor's degree or above		352 (34.21%)
Institute level		
Province/city level		170 (16.52%)
County level		326 (31.68%)
Community level		533 (51.80%)
Professional position		
Physician		268 (26.04%)
Nurse		219 (21.28%)
CDC staff		155 (15.06%)
Other		387 (37.61%)
Professional ranking		
No ranking		327 (31.78%)
Entry level		386 (37.51%)
Middle level		229 (22.25%)
Senior/advanced level		87 (8.45%)
Administrative ranking		
No ranking		610 (59.28%)
Department leader		229 (22.25%)
Hospital/CDC director		45 (4.37%)
Other		145 (14.09%)

Distributions of continuous variables are reported as mean (standardized deviation). Distributions of categorical variables are reported as frequency/(column percent)

Path Analysis

As shown in Fig. 1, we delineated the interactive effects of stressors and coping on depression and anxiety after controlling all the potential confounders. The specified model resulted in the following good indices of fit: $\chi^2(56) = 155.99$, $\chi^2/df = 2.79$, $p < 0.01$, CFI = 0.99,

Table 2 Correlation matrix among socio-demographics and key variables

		1	2	3	4	5	6	7	8	9	10
1	COVID-19-related stressors										
2	Coping	.004									
3	Depression	.27**	−.07*								
4	Anxiety	.34**	−.07*	.71**							
5	Age	−.04	−.03	−.11**	−.09*						
6	Gender	−.01	−.03	.06	.11**	−.12**					
7	Marital status	−.02	−.003	−.17**	−.12**	.42**	.03				
8	Institute level	.08*	−.08**	.13**	.10**	.09**	.11**	−.02			
9	Education attainment	.03	−.001	.10**	.06*	−.18**	.13**	−.03	.29**		
10	Years of engaging in medical care services	−.02	−.04	−.09**	−.06	.88**	−.05	.39**	.13**	−.11**	
11	Years of engaging in HIV care services	.02	−.02	−.06	−.06	.48**	−.13**	.26**	.12**	−.03	.52**

Gender: 1 = male, 2 = female; marital status: 0 = other, 1 = married/remarried; institute level: 0 = community, 1 = others; education attainment: 0 = high school or below, 1 = college or above

*$p < .05$; **$p < .01$

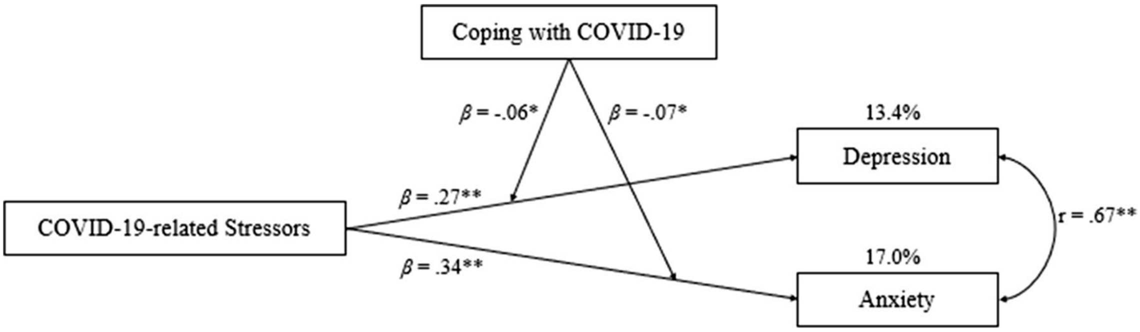

Fig. 1 The interactive effects of stressors and coping on depression and anxiety. *$p < .05$, **$p < .01$. Model fit: $\chi^2(56) = 155.99$, $p < .01$, $\chi^2/df = 2.79$, CFI = .99, TLI = .95, RMSEA = .04, SRMR = .03. Demographic variables and occupational information were controlled in the model as covariates, including age, gender, marital status, educational attainment, institute level, professional position, professional ranking and administrative ranking. To assure simplicity, covariates for dependent variables, as well as correlations between covariates and independent variable have been omitted. The standardized coefficient, 95% CI and p-value of COVID-19-related stressors and covariates on depression/anxiety are reported in Table 3

TLI = 0.95, RMSEA = 0.04, SRMR = 0.03. The correlation between depression and anxiety was significant ($r = 0.67$, $p < 0.01$). The model explained 13.4% variance of depression and 17.0% variance of anxiety.

As shown in Table 3, the main effect of COVID-19-related stressors on depression ($\beta = 0.27$, 95% CI [0.03, 0.21], $p < 0.01$) and anxiety ($\beta = 0.34$, 95% CI [0.28, 0.40], $p < 0.01$) was significantly positive. The main effect of coping on depression ($\beta = -0.07$, 95% CI [−0.13, −0.01], $p < 0.05$) and anxiety ($\beta = -0.08$, 95% CI [−0.13, −0.02], $p < 0.01$) was significantly negative.

The interactive effects of stressors and coping on depression ($\beta = -0.06$, 95% CI [−0.11, −0.00], $p < 0.05$) or anxiety ($\beta = -0.07$, 95% CI [−0.13, −0.02], $p < 0.01$) were both significant after adjusting for all the covariates. Regarding the covariates in the path analysis, being married ($\beta = -0.13$, 95% CI [−0.20, −0.06], $p < 0.01$) and affiliated with the county level ($\beta = -0.10$, 95% CI [−0.20, −0.01], $p < 0.05$) and community level ($\beta = -0.13$, 95% CI [−0.24, −0.02], $p < 0.05$) were all negatively associated with depression; while being female ($\beta = 0.11$, 95% CI [0.04, 0.17], $p < 0.01$), and no administrative ranking ($\beta = 0.10$, 95% CI [0.01, 0.18], $p < 0.05$) were positively associated with anxiety; and being married ($\beta = -0.09$, 95% CI [−0.16, −0.03], $p < 0.01$) and working as a nurse ($\beta = -0.10$, 95% CI [−0.17, −0.03], $p < 0.01$) were positively associated with anxiety.

Simple slope analysis showed an attenuated association between COVID-19-related stressors and depression, with a high level of coping having a greater protective effect (see Fig. 2). At 1 SD below the mean level of coping, the association between stressors and depression was stronger, $b = 0.11$,

Table 3 Standardized regression weights in path analysis on depression

	Depression					Anxiety				
	β	SE	95% CI		p-value	β	SE	95% CI		p-value
			Lower bound	Upper bound				Lower bound	Upper bound	
COVID-19-related stressors	.271	.030	**.213**	**.331**	**.000**	.341	.029	**.284**	**.396**	**.000**
Coping	−.071	.029	**−.126**	**−.013**	**.015**	−.075	.028	**−.130**	**−.021**	**.007**
Stressor × coping	−.058	.028	**−.113**	**−.002**	**.043**	−.073	.027	**−.126**	**−.019**	**.006**
Age (years)	−.028	.077	−.182	.118	.718	−.250	.079	.060	.238	.238
Years of engaging in medical care services	.038	.079	−.113	.199	.607	.086	.084	−.073	.262	.289
Years of engaging in HIV care services	−.040	.035	−.110	.028	.242	−.064	.035	−.133	.005	.067
Gender										
Male	Reference group									
Female	.049	.034	−.016	.116	.143	.108	.033	**.043**	**.172**	**.001**
Marital status										
Single	Reference group									
Married/remarried	−.132	.036	**−.202**	**−.063**	**.000**	−.092	.033	**−.158**	**−.029**	**.003**
Educational attainment										
High school or below	Reference group									
College	.085	.046	−.007	.176	.071	.019	.046	−.070	.111	.671
Bachelor's degree or above	.082	.050	−.015	.182	.096	.028	.050	−.069	.129	.565
Institute level										
Province/city level	Reference group									
County level	−.103	.047	**−.197**	**−.012**	**.026**	−.068	.046	−.158	.023	.144
Community level	−.133	.056	**−.244**	**−.023**	**.015**	−.064	.053	−.166	.040	.231
Professional position										
Other	Reference group									
Physician	−.022	.036	−.093	.051	.546	−.048	.036	−.120	.024	.190
Nurse	−.052	.037	−.125	.022	.166	−.100	.036	**−.170**	**−.029**	**.005**
CDC staff	.008	.046	−.081	.099	.865	−.038	.044	−.124	.047	.374
Professional ranking										
No ranking	Reference group									
Entry level	−.060	.039	−.137	.015	.119	−.062	.038	−.136	.015	.104
Middle level	−.003	.045	−.088	.088	.944	.050	.046	−.041	.141	.272
Senior/advanced level	−.042	.042	−.123	.039	.308	.022	.043	−.061	.105	.628
Administrative ranking										
Other	Reference group									
No ranking	.085	.045	−.007	.171	.068	.098	.044	**.011**	**.183**	**.027**

Table 3 (continued)

	Depression					Anxiety				
	β	SE	95% CI		p-value	β	SE	95% CI		p-value
			Lower bound	Upper bound				Lower bound	Upper bound	
Department leader	.018	.045	− .072	.106	.672	.050	.044	− .038	.138	.256
Hospital/CDC director	− .013	.033	− .076	.051	.678	−.030	.034	.103	.320	.320

Bold means variables with statistical significance $p < 0.05$

95% CI = [0.08, 0.14]; while at 1 SD above the mean level of coping, the association between stressors and depression was significant yet weaker, $b = 0.07$, 95% CI = [0.04, 0.10]. A similar simple slope analysis pattern was showed in associations among stressors, coping, and anxiety (see Fig. 2). At 1 SD below the mean level of coping, the association between stressors and anxiety was stronger, $b = 0.13$, 95% CI = [0.11, 0.16]; while at 1 SD above the mean level of coping, the association between stressors and anxiety was still significant though weaker, $b = 0.08$, 95% CI = [0.06, 0.11].

Discussion

This study is one of the first efforts to characterize the mental health problems and the protective role of coping in the relationship between COVID-19-related stressors and mental health problems among HIV healthcare providers in the context of COVID-19 pandemic. The results showed that 13.31% of healthcare providers screened positive for depression and 6.61% for anxiety. The prevalence of mental health issues in the current study was lower than those reported in the previous surveys among frontline healthcare providers treating patients with COVID-19 [32]. A systematic review examined the effects of COVID-19 outbreak on the mental health of frontline healthcare providers, revealing the prevalence of anxiety and depression of 23.2% and 22.8%, respectively [32]. Such discrepancy can possibly be explained by the difference in the target population and survey timing. First, not all HIV healthcare providers were assigned to the frontline to treat COVID-19 patients. They might, therefore, experience fewer stressors such as less fear of exposure to the virus due to indirect and less frequent contact with the patients [8, 33]. Second, rather than having been conducted in the early stage of the outbreak (late December 2019 and early January 2020) as Pappa et al. reviewed, the current study was conducted in April 2020, when the pandemic was generally controlled in China.

The associations between stressors and depression/anxiety were significantly positive, which was consistent with previous research and suggested that healthcare providers were facing a critical situation with a higher risk of developing mental health symptoms [19]. This finding is in line with studies conducted in other public health crisis contexts. For example, during the 2003 Severe Acute Respiratory Syndrome (SARS) outbreak, healthcare providers who experienced a high level of stress reported adverse psychological reactions including depressive and anxiety symptoms [34–36].

Coping behaviors were found to protect healthcare providers from the negative effect of stressors on mental health. Participants who were able to adopt various coping behaviors during the pandemic experienced less mental health

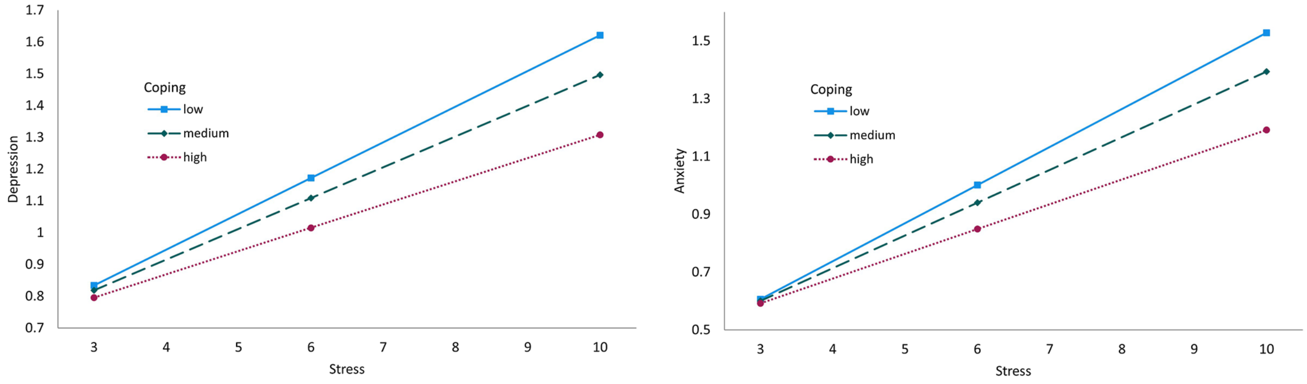

Fig. 2 Significant interactive effects of stressors, coping strategies and mental health problems (i.e., depression, anxiety) plotted using mean values ± 1 SD from coping scale. At 1 SD below the mean level of coping, the association between stressors and depression was stronger, $b = .11$, 95% CI = [.08, .14]; while at 1 SD above the mean level of coping, the association between stressors and depression was significant yet weaker, $b = .07$, 95% CI = [.04, .10]. At 1 SD below the mean level of coping, the association between stressors and anxiety was stronger, $b = .13$, 95% CI = [.11, .16]; while at 1 SD above the mean level of coping, the association between stressors and anxiety was still significant though weaker, $b = .08$, 95% CI = [.06, .11]

issues. Such interactive effects of stressors and coping could be explained by the transactional theory of stress and coping [22]. This theory posits that individuals who are equipped with coping resources and abilities would initiate effective coping responses and, as a result, experience less psychological distress [37]. Although limited research has directly examined the interactive effects of stressors and coping on mental health issues among HIV healthcare providers, various coping strategies were adopted in physicians or nurses during the COVID-19 outbreak [38]. In line with previous studies, the current study found that "a positive attitude and physical exercise" were more frequently used; while coping strategies such as "accepting the reality" were not as common [38].

This result highlighted the importance of coping management among stressful HIV healthcare providers. The mental health of healthcare providers can benefit from better coping resources and increased coping abilities. To provide adequate coping resources, hospitals could set counseling for physicians seeking help. For example, a longitudinal study indicated that counseling for physicians effectively reduced job stress and emotional exhaustion [39]. To facilitate coping abilities, interventions to accelerate adaptive coping may be conducted for healthcare providers during times of the COVID-19 crisis. For example, a coping-enhancing program called Effective Coping and Communication Skills for Physicians showed significant efficacy in improving healthcare providers' stress management skills and decreasing the level of emotional exhaustion [40]. Future intervention may benefit from tailoring and adapting this program for HIV healthcare providers.

This study has several limitations. First, the study was cross-sectional with a lack of longitudinal follow up. Long term psychological monitoring and intervention of this population worth further investigation. Moreover, the nature of the cross-sectional study hindered the causal inference. Second, the participants were recruited via a convenience sampling approach, and only participants who are familiar with Internet-based platforms were reached. This sampling method may be subject to selection bias. Third, we asked participants to recall their experienced stressors and self-report the frequency of coping behaviors, which was subjective to recall bias and social desirability. Fourth, some scales used in the current study were self-developed with reliability and validity left unknown among HIV healthcare providers in China. For example, to keep the survey brief and clean, we listed only six items to assess the number of coping strategies, without distinguishing different types of coping (e.g., adaptive vs. maladaptive). Findings can be strengthened if validated and structured measures are available in the future. Fifth, the screening tool for depression and anxiety used in the current study (PHQ–4) consisted of only two items for each mental disorder and the Cronbach's alphas were low. Although it has been proved to be efficient and useful for busy clinicians, it did not evaluate other important conditions (e.g., suicidal thoughts) of depression and anxiety [41]. Future studies may use a longer and clinician interviewed assessment of depression and anxiety. Finally, some health providers might be recruited from the same hospital, but we did not collect data to identify such information. Future studies may benefit from collecting such information and examining clustering effects.

Despite these limitations, this study still presented the mental health problems among HIV healthcare providers in the context of the COVID-19 outbreak and the rarely reported moderating role of coping in attenuating the adverse effects of COVID-19-related stressors on mental health problems. Given the low utilization of psychological

counseling services, HIV healthcare providers are encouraged to receive emergency psychological crisis interventions in a timely manner from mental health practitioners, and to seek help from psychological assistance hotlines or user-friendly online mental health education systems [39]. Reducing COVID-19-related stressors would also be beneficial to the improvement of psychological well-being. In the context of COVID-19, recommendations for the HIV response, such as adapting differentiated service delivery for HIV by increasing out-of-facility and decentralized service points, would possibly alleviate the provider's workload without compromising the continuity of testing and prevention activities. Coping strategies served as an intrapersonal protective factor to moderate the likelihood of mental health problems following the COVID-19-related stressors. Reinforcement of such coping strategies would be beneficial for strengthening the psychological well-being of HIV healthcare providers.

Acknowledgements Research reported in this publication was supported by the National Institute of Allergy and Infectious Diseases of the National Institutes of Health under Award Number R01AI127203-4S1 and the University of South Carolina Office of Vice President for Research COVID-19 Grant (USCIP 80003673). Work by Shufang Sun was also supported by National Institute of Health (T32MH078788 and K23AT011173). The content is solely the responsibility of the authors and does not necessarily represent the official views of the National Institutes of Health and University of South Carolina.

References

1. Horton R. Offline: don't let COVID-19 divert us completely. Lancet (London, England). 2020;395(10236):1534.
2. Baldwin R, di Mauro BW. Economics in the time of COVID-19. London: CEPR Press; 2020.
3. Pfefferbaum B, North CS. Mental health and the Covid-19 pandemic. N Engl J Med. 2020;383:510–2.
4. World Health Organization. Coronavirus disease 2019 (COVID-19) situation report 163. 2020. https://www.who.int/docs/default-source/coronaviruse/situation-reports/20200701-covid-19-sitrep-163.pdf?sfvrsn=c202f05b_2.
5. Moazzami B, Razavi-Khorasani N, Moghadam AD, Farokhi E, Rezaei N. COVID-19 and telemedicine: Immediate action required for maintaining healthcare providers well-being. J Clin Virol. 2020. https://doi.org/10.1016/j.jcv.2020.104345.
6. Wang D, Hu B, Hu C, Zhu F, Liu X, Zhang J, et al. Clinical characteristics of 138 hospitalized patients with 2019 novel coronavirus–infected pneumonia in Wuhan, China. JAMA. 2020;323(11):1061–9.
7. Santarone K, McKenney M, Elkbuli A. Preserving mental health and resilience in frontline healthcare workers during COVID-19. Am J Emerg Med. 2020. https://doi.org/10.1016/j.ajem.2020.04.030.
8. Liu Q, Luo D, Haase JE, Guo Q, Wang XQ, Liu S, et al. The experiences of health-care providers during the COVID-19 crisis in China: a qualitative study. Lancet Glob Health. 2020. https://doi.org/10.1016/S2214-109X(20)30204-7.
9. Sun S, Hou J, Chen Y, Lu Y, Brown L, Operario D. Challenges to HIV care and psychological health during the COVID-19 Pandemic among people living with HIV in China. AIDS Behav. 2020. https://doi.org/10.1007/s10461-020-02903-4.
10. Flowers T. ART services in the time of COVID-19: adaptations to differentiated service delivery (DSD) models with a focus on those struggling with ART'. Médecins Sans Frontières, South Africa, (IAS webinar). https://www.iasociety.org/Web/WebContent/File/EduFund/COVID-webinars/Session_1_Presentation_TracyFlowers_SA.pdf. Accessed 21 Apr 2020.
11. Wilcher R, Akolo C. Five strategies for preserving key population-focused HIV programmes in the era of COVID-19. 2020. https://www.iasociety.org/HIV-Programmes/Cross-cutting-issues/COVID-19-and-HIV/Five-strategies-for-preserving-key-population-focused-HIV-programmes-in-the-era-of-COVID-19. Accessed 10 Aug 2020.
12. Songo I. Perspectives from recipients of care during the COVID-19 Pandemic'. CQUIN DSD and COVID-19 Webinar series. https://www.differentiatedcare.org/Portals/0/adam/Content/lZkUqjU6BkO4zqmcniS1tA/File/CQUIN%20ITPC%20RoC%20Perspectives_MASTER%20FINAL21April-compressed.pdf. Accessed 21 Apr 2020.
13. Zvandiri A. Adaptation of the CATS model during COVID-19', in 'COVID-19 and HIV: continuing services with and for marginalised people. https://frontlineaids.org/wp-content/uploads/2020/05/COVID-19-and-HIV-Webinar-slides-FINAL1.pdf. Accessed 15 May 2020.
14. Guo W, Weng H, Bai H, Liu J, Wei X, Zhou K, et al. Quick community survey on the impact of COVID-19 outbreak for the healthcare of people living with HIV. Zhonghua liu Xing Bing xue za zhi= Zhonghua Liuxingbingxue Zazhi. 2020;41(5):663–7.
15. Wu Y, Wang J, Luo C, Hu S, Lin X, Anderson AE, et al. A comparison of burnout frequency among oncology physicians and nurses working on the front lines and usual wards during the COVID-19 epidemic in Wuhan, China. J Pain Symptom Manage. 2020. https://doi.org/10.1016/j.jpainsymman.2020.04.008.
16. Shanafelt T, Ripp J, Trockel M. Understanding and addressing sources of anxiety among health care professionals during the COVID-19 pandemic. JAMA. 2020;323(21):2133–4.
17. Shechter A, Diaz F, Moise N, Anstey DE, Ye S, Agarwal S, et al. Psychological distress, coping behaviors, and preferences for support among New York healthcare workers during the COVID-19 pandemic. Gen Hosp Psychiatry. 2020;66:1–8.
18. Liu S, Yang L, Zhang C, Xiang Y-T, Liu Z, Hu S, et al. Online mental health services in China during the COVID-19 outbreak. Lancet Psychiatry. 2020;7(4):e17–8.
19. Lai J, Ma S, Wang Y, Cai Z, Hu J, Wei N, et al. Factors associated with mental health outcomes among health care workers exposed to coronavirus disease 2019. JAMA Netw Open. 2020;3(3):e203976.
20. Lazarus RS. The stress and coping paradigm. Fifty years of the research and theory of RS Lazarus: an analysis of historical and perennial issues. Hove: Psychology Press; 1998. p. 182–220.
21. Billings AG, Moos RH. Coping, stress, and social resources among adults with unipolar depression. J Personal Soc Psychol. 1984;46(4):877.
22. Folkman S, Lazarus RS. Stress, appraisal, and coping. New York: Springer; 1984.
23. Wallace CL, Wladkowski SP, Gibson A, White P. Grief during the COVID-19 pandemic: considerations for palliative care providers. J Pain Symptom Manage. 2020. https://doi.org/10.1016/j.jpainsymman.2020.04.012.
24. Guangxi Center for Disease Control and Prevention. Know your status. Embrace Health. 2018. https://www.gxcdc.com/zxdt/2018/1204/9594.html. Accessed 15 Aug 2020.
25. Wu Z, Chen J, Scott SR, McGoogan JM. History of the HIV Epidemic in China. Curr HIV/AIDS Rep. 2019;16(6):458–66.

26. Tam CC, Sun S, Yang X, Li X, Zhou Y, Shen Z. Psychological distress among HIV healthcare providers during the COVID-19 pandemic in China: mediating roles of institutional support and resilience. AIDS Behav. 2020. https://doi.org/10.1007/s10461-020-03068-w.
27. Zhang W-R, Wang K, Yin L, Zhao W-F, Xue Q, Peng M, et al. Mental health and psychosocial problems of medical health workers during the COVID-19 epidemic in China. Psychother Psychosom. 2020;89(4):242–50.
28. Du J, Dong L, Wang T, Yuan C, Fu R, Zhang L, et al. Psychological symptoms among frontline healthcare workers during COVID-19 outbreak in Wuhan. Gen Hosp Psychiatry. 2020. https://doi.org/10.1016/j.genhosppsych.2020.03.011.
29. Guo J, Liao L, Wang B, Li X, Guo L, Tong Z, et al. Psychological effects of COVID-19 on hospital staff: a national cross-sectional survey of China Mainland. SSRN. 2020;3550050:2020. https://doi.org/10.2139/ssrn.3550050.
30. Kroenke K, Spitzer RL, Williams JB. The Patient Health Questionnaire-2: validity of a two-item depression screener. Med Care. 2003;41:1284–92.
31. Kroenke K, Spitzer RL, Williams JB, Monahan PO, Löwe B. Anxiety disorders in primary care: prevalence, impairment, comorbidity, and detection. Ann Intern Med. 2007;146(5):317–25.
32. Pappa S, Ntella V, Giannakas T, Giannakoulis VG, Papoutsi E, Katsaounou P. Prevalence of depression, anxiety, and insomnia among healthcare workers during the COVID-19 pandemic: a systematic review and meta-analysis. Brain Behav Immun. 2020. https://doi.org/10.1016/j.bbi.2020.05.026.
33. Lee SM, Kang WS, Cho A-R, Kim T, Park JK. Psychological impact of the 2015 MERS outbreak on hospital workers and quarantined hemodialysis patients. Compr Psychiatry. 2018;87:123–7.
34. Maunder R, Hunter J, Vincent L, Bennett J, Peladeau N, Leszcz M, et al. The immediate psychological and occupational impact of the 2003 SARS outbreak in a teaching hospital. CMAJ. 2003;168(10):1245–51.
35. Bai Y, Lin C-C, Lin C-Y, Chen J-Y, Chue C-M, Chou P. Survey of stress reactions among health care workers involved with the SARS outbreak. Psychiatr Serv. 2004;55(9):1055–7.
36. Chua SE, Cheung V, Cheung C, McAlonan GM, Wong JW, Cheung EP, et al. Psychological effects of the SARS outbreak in Hong Kong on high-risk health care workers. Can J Psychiatry. 2004;49(6):391–3.
37. Lazarus RS, Folkman S. Stress, appraisal, and coping. New York: Springer; 1984.
38. Cai H, Tu B, Ma J, Chen L, Fu L, Jiang Y, et al. Psychological impact and coping strategies of Frontline Medical Staff in Hunan between January and March 2020 during the outbreak of coronavirus disease 2019 (COVID-19) in Hubei, China. Med Sci Monit. 2020;26:e924171–81.
39. Ro KEI, Tyssen R, Hoffart A, Sexton H, Aasland OG, Gude T. A three-year cohort study of the relationships between coping, job stress and burnout after a counselling intervention for help-seeking physicians. BMC Public Health. 2010;10(1):213.
40. Penberthy JK, Chhabra D, Ducar DM, Avitabile N, Lynch M, Khanna S, et al. Impact of coping and communication skills program on physician burnout, quality of life, and emotional flooding. Saf Health Work. 2018;9(4):381–7.
41. Kroenke K, Spitzer RL, Williams JB, Löwe B. An ultra-brief screening scale for anxiety and depression: the PHQ–4. Psychosomatics. 2009;50(6):613–21.

Publisher's Note Springer Nature remains neutral with regard to jurisdictional claims in published maps and institutional affiliations.
