# Supplementary material for: Mental health status and related factors influencing healthcare workers during the COVID-19 pandemic: A systematic review and meta-analysis
Source: PLoS One. 2024 Jan 19;19(1):e0289454. doi: 10.1371/journal.pone.0289454 (PMC10798549; doi:10.1371/journal.pone.0289454)
Supplement: S1 Data — (ZIP) [file pone.0289454.s011.zip › literatures/102.pdf]

Check for updates

Received: 18 March 2020 | Revised: 7 September 2020 | Accepted: 18 October 2020

DOI: 10.1111/jocn.15549

ORIGINAL ARTICLE

Journal of Clinical Nursing WILEY

Anxiety of Nurses to support Wuhan in fighting against COVID-19 Epidemic and its Correlation With Work Stress and Self-efficacy

Yuanyuan Mo Master of Medicine, Clinical Nurse[1] | Lan Deng Master of Medicine, Head Nurse[1] | Liyan Zhang Bachelor of Medicine, Head Nurse[1] | Qiuyan Lang Bachelor of Medicine, Head Nurse[2] | He Pang Bachelor of Medicine, Clinical Nurse[1] | Chunyan Liao Bachelor of Medicine, Head Nurse[3] | Nannan Wang Bachelor of Medicine, Head Nurse[4] | Pinyue Tao Master of Medicine, Head Nurse[1] | Huiqiao Huang Master of Medicine, Head of the Nursing Department[1]

[1]The Second Affiliated Hospital of Guangxi Medical University, Nanning, China

[2]The First Affiliated Hospital of Guangxi Medical University, Nanning, China

[3]Affiliated hospital of Guilin Medical University, Guilin, China

[4]Liuzhou Worker's Hospital, Liuzhou, China

Correspondence
Pinyue Tao and Huiqiao Huang, Department of Nursing, the Second Affiliated Hospital of Guangxi Medical University, Daxue east road 166, Nanning, Guangxi, China.
Emails: 15878198319@163.com (PT); huanghuiqiaonn@163.com (HH)

Funding information
the Key Research and Development Project of Guangxi, Grant/Award Number: 2020AB39028; Scientific Research Project of the Second Affiliated Hospital of Guangxi Medical University, Grant/Award Number: EFYKY2020013; Self-funded Scientific Research Project of Guangxi Health Commission, Grant/Award Number: Z20201402

Abstract

Aims and objectives: We aimed to investigate the anxiety of nurses who are supporting Wuhan in fighting against coronavirus disease 2019 (COVID-19) infection and explore relevant influencing factors.

Background: The COVID-19 outbreak poses a major threat to public health worldwide. Nurses play an important role in this epidemic. However, available data on the mental health among these nurses are limited.

Design: A descriptive, cross-sectional survey was performed.

Methods: An online questionnaire was completed by 200 nurses who went to Wuhan to help to fight against COVID-19 from another province. Data collection tools include the Chinese version of the Stress Overload Scale (SOS), the Self-Rating Anxiety Scale (SAS) and General Self-Efficacy Scale (GSES). Descriptive, single-factor correlation and multiple regression analyses were used in exploring related influencing factors. Reporting followed the STROBE guidelines.

Results: The scores of SAS, SOS and GSES range from 20 to 80, 22 to 110 and 10 to 40, respectively, and the SAS (31.79 ± 7.32) and SOS (40.19 ± 12.92) and GSES scores (24.83 ± 6.60) were obtained. Anxiety was positively correlated with stress ($r = .679$, $p < .001$) but negatively correlated with self-efficacy ($r = -.326$, $p < .001$). Multiple regression analysis showed that professional qualification, sleep, stress and self-efficacy were the main factors affecting nurse anxiety ($p = .006$, <.001, <.001, .039, respectively).

Conclusions: Nurses who are supporting Wuhan in fighting against COVID-19 were under a low level of anxiety.

Yuanyuan Mo and Lan Deng contributed equally to this work.

 © 2020 John Wiley & Sons Ltd

13652702, 2021, 3-4, Downloaded from https://onlinelibrary.wiley.com/doi/10.1111/jocn.15549 by CochraneChina, Wiley Online Library on [19/12/2022]. See the Terms and Conditions (https://onlinelibrary.wiley.com/terms-and-conditions) on Wiley Online Library for rules of use; OA articles are governed by the applicable Creative Commons License

Relevance to clinical practice: The current study suggests work stress reduction might be a key factor in reducing anxiety and maintaining mental health to support nurses who are fighting against COVID-19 infection.

KEYWORDS
anxiety, COVID-2019, mental health, nurses, social support, work stress, Wuhan

1 | INTRODUCTION

In China, the unfolding emergency caused by the novel coronavirus or corona virus disease 2019 (COVID-19) in Wuhan City is putting nursing services under intense pressure as thousands of people became infected with an uncertain but potentially fatal respiratory infection; the infection spread across China and other countries (Catton, 2020). In order to restrain the spread of the epidemic, from 27 January 2020, all the provinces in China that not only Hubei initiated an emergency response to major public health emergencies (The Beijing News, 2020; WHO, 2020a). On 30 January 2020, the World Health Organization (WHO) declared the COVID-19 outbreak as a global health emergency (WHO, 2020b). As the epidemic spreads around the world, on 11 March 2020, WHO officially classified COVID-19 as a pandemic (WHO, 2020c). As of 10:00 on 27 April 2020, a total of 2,883,603 confirmed cases and 198,842 deaths have been reported globally. The worst-hit European region has confirmed 1,359,380 cases (29,659 new cases) and 124,525 deaths (2,307 new cases). COVID-19 has been reported in 212 countries and regions (WHO, 2020d). In the absence of the speedy breakthrough of an successful treatment or the discovery of an effective vaccine that can be mass produced and widely distributed, this pandemic may cause close to half a billion deaths, that is 6% of the global population (Grech, 2020). There is no doubt that COVID-19 presents a vast public health challenge around the world.

Keypoints

A cross-sectional survey was completed by 200 nurses who are supporting Wuhan in fighting against COVID-19. These nurses were under a low level of anxiety. Anxiety was positively correlated with stress but negatively correlated with selfefficacy. Nurse leaders should pay attention to the mental health of the nurses who are fighting against COVID-19 infection, and offer solutions to retain mental health among these nurses.

2 | BACKGROUND

Nurses have always played an important role in infection prevention, infection control, isolation, containment and public health (Smith et al., 2020). Dr. Tedros said that 'Nurses are the backbone of any health system. Today, many nurses find themselves on the front line in the battle against COVID-19' (WHO, 2020e). Many countries, such as China (Health News, 2020), the United States (Global Times, 2020) and Peru (CCTV News, 2020a), called on nurses to join the fight against the epidemic and support the worst-affected areas.

Anti-epidemic nurses show a strong sense of responsibility and professionalism, but the truth is they are putting their lives at risk in the course of their duties (Catton, 2020). Twenty per cent of the number of confirmed cases during the 2003 SARS outbreak in China (around 1,100) were medical workers (Hung, 2003). This outbreak of COVID-19 even more devastating and more than 3,000 medical workers in China were infected (National Health Commission of the People's Republic of China, 2020a), over 8,000 in Italy (CCTV News, 2020b), over 19,000 in Spain (CCTV News, 2020c). The unfolding emergency caused by the COVID-19 is putting nursing services under intense pressure. Actually, those nurses who fighting against COVID-19 infection not only vulnerable to a higher risk of infection but also mental health problems (Smith et al., 2020). Maintaining the mental health of nursing staff is essential to control infectious diseases (Kang et al., 2020; Xiang et al., 2020).

Research on the COVID-2019 epidemic has mostly focused on epidemiological investigation, prevention, control, diagnosis and treatment. Fewer studies have investigated the mental health issues of nurses. The present study aimed to investigate anxiety of Chinese nurses fighting against COVID-19 epidemic and its correlation with work stress and social support.

3 | METHODS

3.1 | Design

A descriptive, cross-sectional survey was performed. The study followed the reporting guideline-Strengthening the Reporting of Observational Studies in Epidemiology (STROBE), for completed checklist see (Appendix S1).

3.2 | Participants

By means of convenient sampling, nurses from Guangxi supporting Wuhan were selected to carry out the survey. The inclusion criteria were as follows: nurses who from Guangxi who are

13652702, 2021, 3-4, Downloaded from https://onlinelibrary.wiley.com/doi/10.1111/jocn.15549 by CochraneChina, Wiley Online Library on [19/12/2022]. See the Terms and Conditions (https://onlinelibrary.wiley.com/terms-and-conditions) on Wiley Online Library for rules of use; OA articles are governed by the applicable Creative Commons License

involved in fighting against COVID-19, those who have entered the clinical front line to participate in the rescue work and those who volunteered to participate in this study. As of 22 February 2020, all the nurses (around 250) were invited to participate in this study. The response rate was 80.00%, which resulted in a sample of 200 nurses.

3.3 | Measurements

3.3.1 | Demographic and COVID-19-specific survey form

According to literature review and expert consultation, self-made general information and demographic questionnaires were prepared. The following data were obtained: gender, age, nursing experience, education, professional qualification, marital status, got kids, only-child, get the support of their extended family, have been trained in SARS-CoV-2 prevention and control knowledge, self-assessment of the mastery of COVID-19 prevention and control knowledge, participation in protective skills training, self-assessment of the mastery of the protection skills, experience in related departments such as fever clinic infectious diseases, respiratory, critical medicine, and emergency, volunteered to participate in the support work, whether they regret participating in the support work, whether they have completed the support work with confidence, severity of patients, working hours per week, support duration, whether adapt to the current diet and self-evaluation sleep.

3.3.2 | Self-Rating Anxiety Scale

The Self-Rating Anxiety Scale (SAS), which was developed by Professor Zung (1971), is a simple clinical tool used to analyse subjective anxiety. The questionnaire consists of 20 items and a 4-point scale for evaluating the frequency of symptoms. The standard scoring of SAS is as follows: '1' indicates no or limited time, and '4' indicates most or all the time. Cronbach's α value is 0.777, and the half-coefficient is 0.757, which has good reliability and validity (Tian et al., 2019). The total score of SAS is the cumulative score of each item. The higher the score is, the more severe the anxiety will be.

3.3.3 | Chinese version of the Stress Overload Scale

Amirkhan (2012) developed the Stress Overload Scale (SOS), and Su and Guo (2014) introduced it and conducted cultural commissioning. The Chinese version of the SOS has good reliability and validity. Cronbach's α value is 0.936, and the content validity is 0.860. The scale consists of two dimensions: event load (12 entries) and individual vulnerability (10 entries), with 22 entries. A 5-point scoring method is used ('never before' scores 1 point and 'always there' scores 5 points), and the total score is 22–110 points. The higher the score is, the greater the pressure load will be.

3.3.4 | General Self-Efficacy Scale

The General Self-Efficacy Scale (GSES) was developed by German psychologist Zhang and Schwarzer (1995), and the Chinese version was revised by Wang et al. (2001). This tool has a total of 10 items and is used to measure individuals' self-confidence when facing challenges in various environments. The Chinese version has internal consistency coefficient Cronbach's α value of 0.87, retest reliability of $r = .83$ ($p < .001$), and half-reliability of $r = .82$. Moreover, this version has high reliability and validity. The scale uses a Likert 4-point scale. Each question is scored as 1 to 4 points, which represent completely incorrect, somewhat correct, most correct and completely correct, respectively, with a total score of 40 points. The scale is divided into three levels: low (10–20 points), medium (21–30 points) and high (31–40 points). The higher the score is, the stronger the sense of self-efficacy will be.

3.4 | Survey methods and data collection

Online survey (via a questionnaire website platform) was send to the heads of each batch of nurses from Guangxi who are supporting in Wuhan, whom were asked to send on to nurses. The participants could complete the questionnaire via computer or smart phone that can open a web link or scan a Quick Response code. The online survey was sent to the potential participants with an invitation letter containing information regarding purpose, anonymity and confidentiality. Consent was implied if participants connected to the website link and completed the questionnaire.

3.5 | Data analysis

Counting data were expressed by frequency and percentage, and measurement data were expressed by $\bar{x} \pm s$. Two groups were compared using two independent sample t tests, and multiple groups were compared using single-factor ANOVA. Correlation among anxiety, stress and self-efficacy was analysed using Pearson correlation, and multiple linear regression was used for multivariate analysis. All statistical analyses were performed with SPSS for Windows 22.0, with two-tailed $p < .05$ considered as statistically significant.

3.6 | Ethics

The procedures of this study were reviewed and approved by the Review Board of the investigator's institution (No. 2020--KY0005).

13652702, 2021, 3-4, Downloaded from https://onlinelibrary.wiley.com/doi/10.1111/jocn.15549 by CochraneChina, Wiley Online Library on [19/12/2022]. See the Terms and Conditions (https://onlinelibrary.wiley.com/terms-and-conditions) on Wiley Online Library for rules of use; OA articles are governed by the applicable Creative Commons License

Participation was voluntary; informed consent, including permission to publish the results of the research, was obtained.

4 | RESULTS

4.1 | Demographic and COVID-19-specific survey information of aid nurses

Of the 200 nurses who participated in the survey, 22 were male (11.0%), and 178 were female (89.0%). Nurses were aged 21 years to 48 years (32.12 ± 7.65) and had 2–32 years of working (7.89 ± 5.68). Most have an undergraduate education (80.00%, n = 160). The majority professional title were senior nurses (58.50%, n = 117) and supervisors (30.50%, n = 61). Respondents' demographic and COVID-19-specific survey information is outlined in Table 1.

4.2 | Anxiety, stress and self-efficacy of nurses

The total SAS score of 200 nurses who assisted in combating COVID-19 was 31.79 ± 7.32. The total SOS score was 40.19 ± 12.92 points, of which the event load score was 21.22 ± 6.81 points and the individual vulnerability score was 18.97 ± 7.03 points. The total GSES score was 24.83 ± 6.60 points.

4.3 | Single-factor analysis of anxiety of nurses

The results showed that different professional qualifications, disease severity, working hours per week, support hours, diet and sleep status influenced the anxiety scores of the nurses ($p < .05$, Table 1).

4.4 | Multiple-factor analysis of anxiety on nurses

When the total SAS score was used as a dependent variable, the single-factor analysis of the SAS had statistically significant titles: professional qualification (i.e., junior = 1, middle = 2, sub-senior = 3, senior = 4), disease severity (i.e., suspected = 1, mild = 2, common = 3, severe = 4 and critically ill = 5), working hours per week (i.e., <20 h = 1, 20–25 h = 2, 26–30 h = 3, 31–35 h = 4 and >35 h = 5), support duration (i.e., <7 days = 1, 7–15 days = 2, 16–30 days = 3 and >30 days = 4), whether they adapted to the current diet (i.e., yes = 1 and no = 2), self-evaluation sleep (i.e., very good = 1, good = 2, average = 3, not very good = 4 and very bad = 5), and SOS and GSES total score (substituting the actual value) as independent variables for multiple linear regression analysis. Professional qualification, sleep status, stress and self-efficacy explained 54.4% of the total variation and were the main factors influencing the anxiety of nurses assisting in the fight against COVID-19 (Table 2).

4.5 | Correlation among anxiety, stress and self-efficacy of nurses

According to Pearson correlation analysis, the SAS score was positively correlated with the total SOS score and each dimension (r = .679, .600, .667); that is, the greater the stress is, the more obvious the anxiety is. Moreover, the SAS score was negatively correlated with the GSES score (r = −.326); that is, the higher the self-efficacy is, the lower the anxiety is (Table 3).

5 | DISCUSSION

The total SAS score of this study was 31.79 ± 7.32 points, which was higher than the national standard points (29.78 + 0.46), and the difference was statistically significant (t = 3.893, $p < .001$). When we compare with previous studies, we find that our study is lower than the research of Wu et al. (2020), Zhang et al. (2020) and other studies (Kane, 2009; Lin et al., 2014), similar with Chew NWS's research (2020). That may attributed to the high level of conscientiousness and preparation for nurses in this study. Conscientiousness is the strongest driving force of the work role performance (Ellershaw et al., 2016). Nurses on the front line exude a sense of responsibility and collective action (Kupferschmidt & Cohen, 2020). All the nurses in this study volunteered to support Wuhan and have been trained in COVID-19 knowledge and occupational protection.

Multiple regression analysis showed that professional qualification, sleep status, stress and self-efficacy were the main factors influencing the anxiety of nurses assisting in the fight against COVID-19. This new type of SARS-CoV-2 infection is mainly transmitted by respiratory droplets and close contact and is highly contagious (Huang et al., 2019). Medical workers have close contact with infected patients (Li et al., 2020). The limited knowledge of the COVID-19 and the overwhelming news may lead to anxiety and fear (Bao et al., 2020; Shigemura et al., 2020). In the early stage of the epidemic, nurses with lower professional titles were more likely to worry that they would be incompetent for work because of the inadequate grasp of the admission process and personal protective measures for patients with COVID-19. Strengthening specialist training and preparation is the only effective measure to alleviate the psychological pressure of the medical staff (Wong et al., 2007). Thus, preservice training for such nursing staff is important. Strengthening the training of nursing team's business capabilities, formulating a detailed training plan for the outbreak of new coronary pneumonia, and using multimedia network platforms are recommended to promote the orderly participation of all nurses in training and learning.

In this study, 10.50% of the participants reported sleep disturbance, lower than that former research (36.1%) (Lin et al., 2014). A systematic review by Becker et al. (2017) revealed that sleep disorders and anxiety promote and influence each other. The main reason is that the occurrence of anxiety is related to the abnormalities of neurotransmitters, such as serotonin (5-HT) and norepinephrine (NE). Moreover, sleep disorders are associated with synthesis of neurotransmitters, such

13652702, 2021, 3-4, Downloaded from https://onlinelibrary.wiley.com/doi/10.1111/jocn.15549 by CochraneChina, Wiley Online Library on [19/12/2022]. See the Terms and Conditions (https://onlinelibrary.wiley.com/terms-and-conditions) on Wiley Online Library for rules of use; OA articles are governed by the applicable Creative Commons License

TABLE 1 Univariate associations of demographic and COVID-19-specific variables with SAS (n = 200).

Variables	Number (%)	SAS Scores (M ± SD)	t/F	p-Value	SOS Scores (M ± SD)	GSES Scores (M ± SD)
Gender						
Male	21 (10.50)	31.55 ± 7.15	0.026	.871	30.38 ± 13.35	24.52 ± 6.71
Female	179 (89.50)	31.82 ± 7.35			40.40 ± 12.89	24.87 ± 6.60
Education						
Junior college	37 (18.50)	32.30 ± 7.91	0.286	.752	38.86 ± 13.80	24.16 ± 7.11
Undergraduate	160 (80.00)	31.73 ± 7.23			40.41 ± 12.74	24.92 ± 6.45
Graduate	3 (1.50)	29.17 ± 4.39			44.33 ± 14.74	28.00 ± 9.16
Professional qualification						
Junior	8 (4.00)	26.72 ± 3.89	2.888	.037	19.63 ± 7.36	19.63 ± 7.36
Middle	117 (58.50)	32.66 ± 7.67			24.93 ± 6.42	24.93 ± 6.42
Sub-senior	61 (30.50)	31.56 ± 7.17			24.93 ± 6.54	24.93 ± 6.54
Senior	14 (7.00)	28.48 ± 3.77			26.50 ± 7.21	26.50 ± 7.21
Marital status						
Unmarried	94 (47.00)	32.23 ± 8.02	0.563	.570	40.49 ± 12.41	25.69 ± 6.65
Married	100 (50.00)	31.53 ± 6.76			39.53 ± 13.34	23.91 ± 6.55
Divorced or widowed	6 (3.00)	29.38 ± 3.68			46.33 ± 14.15	26.67 ± 4.96
Got kids						
Yes	85 (42.50)	31.60 ± 7.81	0.100	.751	40.59 ± 12.23	26.12 ± 6.53
No	115 (57.50)	31.93 ± 6.95			39.89 ± 13.45	23.88 ± 6.51
Only-child						
Yes	18 (9.00)	32.85 ± 7. 84	0.409	.523	49.33 ± 10.40	22.89 ± 6.42
No	182 (91.00)	31.69 ± 7.27			39.28 ± 12.81	25.02 ± 6.60
Get the support of their extended family						
Yes	200 (100.00)	31.79 ± 7.32			40.19 ± 12.92	24.83 ± 6.60
Have been trained in SARS-CoV-2 prevention and control knowledge						
Yes	200 (100.00)	31.79 ± 7.32			40.19 ± 12.92	24.83 ± 6.60
Self-assessment of the mastery of COVID-19 prevention and control knowledge						
Very good	41 (21.50)	37.76 ± 13.18	0.413	.662	37.76 ± 13.18	26.27 ± 6.78
Good	131 (65.50)	39.92 ± 12.02			39.92 ± 12.02	24.64 ± 6.67
General	28 (14.00)	45.00 ± 15.60			45.00 ± 15.60	23.61 ± 5.80
Poor	0 (0.00)					
Participated in protective skills training						
Yes	200 (100.00)	31.79 ± 7.32			40.19 ± 12.92	24.83 ± 6.60
Self-assessment of the mastery of the protection skills						
Very good	60 (30.00)	38.37 ± 13.01	5.197	.024	38.37 ± 13.01	26.15 ± 6.73
Good	120 (60.00)	40.35 ± 12.34			40.35 ± 12.34	24.73 ± 6.47
General	18 (9.00)	45.28 ± 16.11			45.28 ± 16.11	20.72 ± 5.81
Poor	2 (1.00)	39.00 ± 0.00			39.00 ± 0.00	28.00 ± 1.41
Volunteered to participate in the support work						
Yes	200 (100.00)	31.79 ± 7.32			40.19 ± 12.92	24.83 ± 6.60
Regret participated in the support work						
No	200 (100.00)	31.79 ± 7.32			40.19 ± 12.92	24.83 ± 6.60
Whether they have completed the support work with confidence						
Yes	200 (100.00)	31.79 ± 7.32			40.19 ± 12.92	24.83 ± 6.60

(Continues)

13652702, 2021, 3-4, Downloaded from https://onlinelibrary.wiley.com/doi/10.1111/jocn.15549 by CochraneChina, Wiley Online Library on [19/12/2022]. See the Terms and Conditions (https://onlinelibrary.wiley.com/terms-and-conditions) on Wiley Online Library for rules of use; OA articles are governed by the applicable Creative Commons License

TABLE 1 (Continued)

Variables	Number (%)	SAS Scores (*M* ± SD)	*t*/*F*	*p*-Value	SOS Scores (*M* ± SD)	GSES Scores (*M* ± SD)
Severity of patients						
Suspected	12 (6.00)	28.65 ± 3.67	6.069	<.01	35.67 ± 7.35	28.17 ± 6.92
Mild	92 (46.00)	33.04 ± 7.89			41.84 ± 13.65	23.97 ± 6.45
Common	51 (25.50)	28.73 ± 4.35			36.43 ± 10.70	25.49 ± 6.97
Severe	34 (17.00)	32.10 ± 6.70			40.68 ± 12.31	25.50 ± 6.40
Critically ill	11 (5.50)	38.07 ± 10.49			47.18 ± 17.82	23.27 ± 5.46
Working hours per week						
<20 h	74 (37.00)	30.69 ± 6.50	3.194	.014	36.28 ± 11.86	25.81 ± 6.22
20–25 h	54 (27.00)	30.12 ± 5.51			40.65 ± 15.33	23.82 ± 5.72
26–30 h	55 (27.50)	34.20 ± 8.77			47.57 ± 12.22	26.14 ± 8.74
31–35 h	7 (3.50)	33.75 ± 7.64			50.80 ± 10.94	21.20 ± 6.76
>35 h	10 (5.00)	34.38 ± 9.43			40.55 ± 12.92	25.23 ± 6.60
Support duration						
<7 days	87 (43.50)	30.17 ± 5.75	5.307	.002	38.90 ± 11.86	24.76 ± 6.41
7–15 days	55 (27.50)	32.77 ± 8.56			41.36 ± 15.33	25.11 ± 7.27
16–30 days	47 (23.50)	34.55 ± 8.04			41.57 ± 12.22	24.17 ± 6.28
>30 days	11 (5.50)	27.95 ± 2.64			38.55 ± 12.22	26.82 ± 6.25
Whether adapt to the current diet						
Adapt	154 (77.00)	30.82 ± 6.58	12.56	<.01	38.86 ± 12.14	24.98 ± 6.42
Inadaptation	46 (23.00)	35.05 ± 8.68			44.61 ± 14.52	24.33 ± 7.15
Self-evaluation sleep						
Great	20 (10.00)	26.50 ± 1.44	13.13	<.01	31.45 ± 11.70	27.40 ± 6.84
Good	64 (32.00)	28.98 ± 5.05			36.25 ± 9.93	24.73 ± 6.71
General	95 (47.50)	33.29 ± 7.25			43.07 ± 12.36	24.28 ± 6.52
Not good	12 (6.00)	38.65 ± 8.86			48.67 ± 13.58	25.00 ± 5.34
Bad	9 (4.50)	38.61 ± 10.83			45.78 ± 21.04	25.33 ± 7.56

TABLE 2 Multiple-factor analysis of SAS of nurses

Dependent variable	Regression coefficient	SE	Standardised regression coefficient	*t*-Value	*p*-Value
Constant	16.766	3.009		5.571	<.001
Professional qualification	−1.467	0.531	−0.136	−2.761	.006
Sleep status	1.804	0.434	0.224	4.153	<.001
Total SOS points	0.312	0.032	0.551	9.678	<.001
Total GSES points	−0.118	0.057	−0.107	−2.074	.039

R^2 = .565, adjust R^2 = .544, F = 27.406, p < .001. Only statistically significant results are listed.
Abbreviations: GSES, General Self-Efficacy Scale; SAS, the Self-Rating Anxiety Scale; SOS, the Chinese version of the Stress Overload Scale.

as 5-HT and NE (Zeng & Tan, 2019). Good sleep quality can promote recovery of body function rapidly, relieve work fatigue, and maintain sufficient energy, physical strength, and healthy mental state (Azevedo Da Silva et al., 2014). The government of Wuhan arranged hotel for medical workers. In order to ensure nurses obtain enough sleep, medical institutions should reasonably manage the human resources.

This study found that work stress and self-efficacy were closely correlated with anxiety. The correlation analysis indicated that work stress was moderately positively correlated with anxiety. The more evident stress is, the more intense the anxiety that the nurses will feel. Many previous studies have also shown that nurses with high stress lead to anxiety, frustration, depression and other psychological disorders and emotions (Malinauskiene et al., 2011; Teles et al., 2014). Social support plays an important role in alleviating mental stress (Su & Guo, 2015). Strengthening social support among nurses could mitigate the effect of job strain on

13652702, 2021, 3-4, Downloaded from https://onlinelibrary.wiley.com/doi/10.1111/jocn.15549 by CochraneChina, Wiley Online Library on [19/12/2022]. See the Terms and Conditions (https://onlinelibrary.wiley.com/terms-and-conditions) on Wiley Online Library for rules of use; OA articles are governed by the applicable Creative Commons License

TABLE 3 Correlation analysis of SAS with SOS and GESE (n = 200).

	SAS	SOS	Event load	Individual vulnerability	GESE
SAS		0.679[a]	0.600[a]	0.667[a]	−0.326[a]
SOS	0.679[a]		0.931[a]	0.936[a]	−0.332[a]
Event load	0.600[a]	0.931[a]		0.744[a]	−0.275[a]
Individual vulnerability	0.667[a]	0.936[a]	0.744[a]		−0.343[a]
GESE	−0.326[a]	−0.332[a]	−0.275[a]	−0.343[a]	

Abbreviations: GSES, General Self-Efficacy Scale; SAS, the Self-Rating Anxiety Scale; SOS, the Chinese version of the Stress Overload Scale.
[a]p < .01.

health, as has been mentioned in the literature (Garcia-Rojas et al., 2015; Schmidt, 2013). Different from the past, due to the strong transmission power of virus, in order to reduce cross-infection, nurses had to stay alone in the room when they finished the work (National Health Commission of the People's Republic of China, 2020b). They may feel lonely, anxiety. Thus, actively mobilising the nurse's social support system is necessary. Managers should encourage and guide nurses at the right time and create a harmonious, safe, and encouraging work environment simultaneously. Leisure activities and training on how to relax should be properly arranged to help staff reduce stress.

Self-efficacy is a subjective judgement of nurses on their competence in nursing work. In this study, 163 (84.00%) nurses had a self-efficacy score of >20, which is high. The correlation analysis also indicated that self-efficacy was negatively correlated with anxiety. The more evident self-efficacy is, the less intense the anxiety that the nurses will feel. In a longitudinal study conducted with an adult population in China, general self-efficacy was also negatively correlated with stress and anxiety (Chang et al., 2016). Work experience, social support, and physical and mental status can affect self-efficacy (Conner, 2015). Self-efficacy is important to improve work efficiency, enhance work motivation and improve work attitude (Holland et al., 2017; Molero Jurado et al., 2019; Papadopoulou et al., 2017). People with a strong sense of self-efficacy have the courage to overcome difficulties, and exhibit good behavioural and emotional states. Thus, nursing managers should pay attention to cultivate nurses' self-efficacy, to enhance their self-confidence in the face of stress, reducing their anxiety, and improving their ability to treat epidemics.

6 | LIMITATIONS

Several limitations should be considered. First, this study only investigated the nurses from Guangxi who are supporting in Wuhan to fight against COVID-19 without additional data from nurses who came from other provinces. Thus, the results cannot be generalised to all Chinese nurses. Second, as a cross-sectional design, this study could only evaluate the mental health at the time without the longitudinal observation of the subjects. Finally, we just conducted a questionnaire survey and did not perform an intervention because of time constraints. Future research should increase the sample size.

7 | CONCLUSIONS

The COVID-19 brings remarkable challenges to social prevention and control and front line treatment. In public health emergencies, the mental health of nurses should be given focus. Nurses who are supporting Wuhan in fighting against COVID-19 were under a low level of anxiety. Professional qualification, sleep status, stress and self-efficacy were the main factors that affected nurses' mental health. Nurse managers should strengthen nurses' operational ability training, properly allocate human resources, mobilise social support systems and attach importance to the cultivation of self-efficacy for nurses to promote the mental health and improve their ability to fight COVID-19.

8 | RELEVANCE TO CLINICAL PRACTICE

The current study suggests that work stress reduction should be the key factor in reducing anxiety and maintaining mental health to support nurses who are fighting against COVID-19 infection. Our patients and nurses should be cared for, and only nurses who are physically and mentally healthy can provide improved care for patients. Nurse leaders should pay attention to the mental health of the nurses who are fighting against COVID-19 infection, and offer solutions to retain mental health among these nurses.

ACKNOWLEDGMENTS

This work was supported by funds from the Key Research and Development Project of Guangxi (grant no. 2020AB39028), Self-funded Scientific Research Project of Guangxi Health Commission (grant no. Z20201402) and Scientific Research Project of the Second Affiliated Hospital of Guangxi Medical University (grant no. EFYKY2020013). The authors thank the research participants for their participation in the study and the heads of each batch of nurses who distributed the questionnaires to the nurses.

CONFLICT OF INTEREST

We declare that we have no competing interests.

AUTHORS' CONTRIBUTIONS

All authors have contributed extensively to this work. Huiqiao Huang and Pinyue Tao conceived the study. Yuanyuan Mo created

13652702, 2021, 3-4, Downloaded from https://onlinelibrary.wiley.com/doi/10.1111/jocn.15549 by CochraneChina, Wiley Online Library on [19/12/2022]. See the Terms and Conditions (https://onlinelibrary.wiley.com/terms-and-conditions) on Wiley Online Library for rules of use; OA articles are governed by the applicable Creative Commons License

and performed the literature search, Lan Deng and Qiuyan Lang built the data extraction file, Yuanyuan Mo and Liyan Zhang performed the data extraction, and Huiqiao Huang and MQ supervised the process. Yuanyuan Mo obtained the results. All authors interpreted the data and contributed substantially to the writing and revision of the manuscript. All authors have read and approved the final version of the manuscript.

ETHICAL APPROVAL

The procedures of this study were reviewed and approved by the Institutional Review Board of The second affiliated hospital of Guangxi Medical University (No. 2020--KY0005). Written informed consent was obtained from each participant. All data collected from the subjects were kept anonymous and confidential to protect the privacy of the study subjects.

ORCID

Yuanyuan Mo https://orcid.org/0000-0001-7031-0870

REFERENCES

Amirkhan, J. H. (2012). Stress overload: A new approach to the assessment of stress. *American Journal of Community Psychology*, *49*(1–2), 55–71. https://doi.org/10.1007/s10464-011-9438-x

Azevedo Da Silva, M., Singh-Manoux, A., Shipley, M. J., Vahtera, J., Brunner, E. J., Ferrie, J. E., Kivimäki, M., & Nabi, H. (2014). Sleep duration and sleep disturbances partly explain the association between depressive symptoms and cardiovascular mortality: the Whitehall II cohort study. *Journal of Sleep Research*, *23*(1), 94–97. https://doi.org/10.1111/jsr.12077

Bao, Y., Sun, Y., Meng, S., Shi, J., & Lu, L. (2020). 2019-nCoV epidemic: address mental health care to empower society. *The Lancet*, *395*(10224), e37–e38. https://doi.org/10.1016/S0140-6736(20)30309-3

Becker, N. B., Jesus, S. N., João, K., Viseu, J. N., & Martins, R. (2017). Depression and sleep quality in older adults: a meta-analysis. *Psychology, Health & Medicine*, *22*(8), 889–895. https://doi.org/10.1080/13548506.2016.1274042

Catton, H. (2020). Global challenges in health and health care for nurses and midwives everywhere. *International Nursing Review*, *67*(1), 4–6. https://doi.org/10.1111/inr.12578

CCTV News (2020a). *More than 19,000 health workers in Spain were infecte*. http://news.cctv.com/2020/04/07/ARTI31KBThRCTIAhKvbTeO6L200407.shtml

CCTV News (2020b). *A total of 28,699 cases have been confirmed in Peru*. http://m.news.cctv.com/2020/04/28/ARTIcuvd4YBRrclZA4sQjzms200428.shtml

CCTV News (2020c). *More than 8,000 medical workers in Italy were infected with COVID 19, resulting in 63 deaths among doctor*. http://news.cctv.com/2020/03/31/ARTIGTy4tRs5xuQTEb1RDpfT200331.shtml

Chang, L.-C., Yu, P., & Chang, S.-Y. (2016). Longitudinal relationships between two self-efficacy types and stress in active older adults in taichung city, taiwan. *International Journal of Mental Health Promotion*, *18*(2), 95–105.

Chew, N., Lee, G., Tan, B., Jing, M., Goh, Y., Ngiam, N., Yeo, L., Ahmad, A., Ahmed Khan, F., Napolean Shanmugam, G., Sharma, A. K., Komalkumar, R. N., Meenakshi, P. V., Shah, K., Patel, B., Chan, B., Sunny, S., Chandra, B., Ong, J., … Sharma, V. K. (2020). A multinational, multicentre study on the psychological outcomes and associated physical symptoms amongst healthcare workers during COVID-19 outbreak. *Brain, Behavior, and Immunity*, *88*, 559–565. https://doi.org/10.1016/j.bbi.2020.04.049

Conner, M. (2015). Self-efficacy, stress, and social support in retention of student registered nurse anesthetists. *AANA Journal*, *83*(2), 133–138.

Ellershaw, J., Fullarton, C., Rodwell, J., & Mcwilliams, J. (2016). Conscientiousness, openness to experience and extraversion as predictors of nursing work performance: A facet-level analysis. *Journal of Nursing Management*, *24*(2), 244–252. https://doi.org/10.1111/jonm.12306

Garcia-Rojas, I. J., Choi, B. K., & Krause, N. (2015). Psychosocial job factors and biological cardiovascular risk factors in Mexican workers. *American Journal of Industrial Medicine*, *58*(3), 331–351. https://doi.org/10.1002/ajim.22410

Global Times (2020). *The governor of New York has asked for help from health workers across the United States*. https://weibo.com/1686546714/IBc17a6fm?type=comment

Grech, V. (2020). Unknown unknowns - COVID-19 and potential global mortality. *Early Human Development*, *144*, 105026. https://doi.org/10.1016/j.earlhumdev.2020.105026

Health News (2020). *The white coat went out to battle*. http://szb.jkb.com.cn/jkbpaper/html/2020-03/12/node_3.htm

Holland, B., Gosselin, K., & Mulcahy, A. (2017). The effect of autogenic training on self-efficacy, anxiety, and performance on nursing student simulation. *Nursing Education Perspectives*, *38*(2), 87–89. https://doi.org/10.1097/01.NEP.0000000000000110

Huang, H., Liu, L., Yang, S., Cui, X., Zhang, J., & Wu, H. (2019). Effects of job conditions, occupational stress, and emotional intelligence on chronic fatigue among Chinese nurses: A cross-sectional study. *Psychology Research and Behavior Management*, *12*, 351–360. https://doi.org/10.2147/PRBM.S207283

Hung, L. S. (2003). The SARS epidemic in Hong Kong: what lessons have we learned? *Journal of the Royal Society of Medicine*, *96*(8), 374–378. https://doi.org/10.1258/jrsm.96.8.374

Kane, P. P. (2009). Stress causing psychosomatic illness among nurses. *Indian Journal of Occupational and Environmental Medicine*, *13*(1), 28–32. https://doi.org/10.4103/0019-5278.50721

Kang, L., Li, Y., Hu, S., Chen, M., Yang, C., Yang, B. X., Wang, Y., Hu, J., Lai, J., Ma, X., Chen, J., Guan, L., Wang, G., Ma, H., & Liu, Z. (2020). The mental health of medical workers in Wuhan, China dealing with the 2019 novel coronavirus. *The Lancet. Psychiatry*, *7*(3), e14. https://doi.org/10.1016/S2215-0366(20)30047-X

Kupferschmidt, K., & Cohen, J. (2020). Can China's COVID-19 strategy work elsewhere? *Science*, *367*(6482), 1061–1062. https://doi.org/10.1126/science.367.6482.1061

Li, W., Yang, Y., Liu, Z. H., Zhao, Y. J., Zhang, Q., Zhang, L., Cheung, T., & Xiang, Y. T. (2020). Progression of Mental Health Services during the COVID-19 Outbreak in China. *International Journal of Biological Sciences*, *16*(10), 1732–1738. https://doi.org/10.7150/ijbs.45120

Lin, S. H., Liao, W. C., Chen, M. Y., & Fan, J. Y. (2014). The impact of shift work on nurses' job stress, sleep quality and self-perceived health status. *Journal of Nursing Management*, *22*(5), 604–612. https://doi.org/10.1111/jonm.12020

Malinauskiene, V., Leisyte, P., Romualdas, M., & Kirtiklyte, K. (2011). Associations between self-rated health and psychosocial conditions, lifestyle factors and health resources among hospital nurses in Lithuania. *Journal of Advanced Nursing*, *67*(11), 2383–2393. https://doi.org/10.1111/j.1365-2648.2011.05685.x

Molero Jurado, M., Pérez-Fuentes, M., Oropesa Ruiz, N. F., Simón Márquez, M., & Gázquez Linares, J. J. (2019). Self-efficacy and emotional intelligence as predictors of perceived stress in nursing professionals. *Medicina*, *55*(6), 237. https://doi.org/10.3390/medicina55060237

National Health Commission of the People's Republic of China (2020a). *Transcript of the press conference on March 20, 2020*. http://www.

13652702, 2021, 3-4, Downloaded from https://onlinelibrary.wiley.com/doi/10.1111/jocn.15549 by CochraneChina, Wiley Online Library on [19/12/2022]. See the Terms and Conditions (https://onlinelibrary.wiley.com/terms-and-conditions) on Wiley Online Library for rules of use; OA articles are governed by the applicable Creative Commons License

nhc.gov.cn/xcs/s3574/202003/4ad24ab68e2441668b569757b147c100.shtml

National Health Commission of the People's Republic of China (2020b). *Notice on issuing a new coronary virus pneumonia diagnosis and treatment plan*. http://www.nhc.gov.cn/yzygj/s7653p/202002/8334a8326dd94d329df351d7da8aefc2.shtml

Papadopoulou, C., Kotronoulas, G., Schneider, A., Miller, M. I., McBride, J., Polly, Z., Bettles, S., Whitehouse, A., McCann, L., Kearney, N., & Maguire, R. (2017). Patient-reported self-efficacy, anxiety, and health-related quality of life during chemotherapy: Results from a longitudinal study. *Oncology Nursing Forum*, *44*(1), 127–136. https://doi.org/10.1188/17.ONF.127-136

Schmidt, D. R. C. (2013). Demand-Control model and occupational stress among nursing professionals: Integrative review. *Revista Brasileira de Enfermagem*, *66*(5), 779–788. https://doi.org/10.1590/s0034-71672013000500020

Shigemura, J., Ursano, R. J., Morganstein, J. C., Kurosawa, M., & Benedek, D. M. (2020). Public responses to the novel 2019 coronavirus (2019-nCoV) in Japan: Mental health consequences and target populations. *Psychiatry and Clinical Neurosciences*, *74*(4), 281–282.

Smith, G. D., Ng, F., & Ho Cheung Li, W. (2020). COVID-19: Emerging compassion, courage and resilience in the face of misinformation and adversity. *Journal of Clinical Nursing*, *29*(9–10), 1425–1428. https://doi.org/10.1111/jocn.15231

Su, Q., & Guo, L. (2014). Reliability and validity test of pressure load scale in Chinese nurses. *Chinese Journal of Nursing*, *49*(10), 1264–1268.

Su, Q., & Guo, L. (2015). Relationship between psychological elasticity, workstress and social support of clinical female nurses. *Chinese Occupational Medicine*, *42*(1), 55–58.

Teles, M. A., Barbosa, M. R., Vargas, A. M., Gomes, V. E., Ferreira, E. F., Martins, A. M., & Ferreira, R. C. (2014). Psychosocial work conditions and quality of life among primary health care employees: a cross sectional study. *Health and Quality of Life Outcomes*, *12*, 72. https://doi.org/10.1186/1477-7525-12-72

The Beijing News (2020). *Emergency response was activated in 31 provinces* (in Chinese). http://wwwbjnewscomcn/feature/2020/01/28/680524html

Tian, Y., Wang, Y., Li, J., Wang, M., & Dang, S. (2019). Reliability and validity evaluation of anxiety and depression scale in clinical application of patients with liver cirrhosis. *Journal of Practical Liver Disease*, *22*(1), 105–108.

Wang, C., Hu, Z., & Liu, Y. (2001). Reliability and validity of general self-efficacy scale. *Applied Psychology*, *7*(1), 37–40.

Wong, W. C., Wong, S. Y., Lee, A., & Goggins, W. B. (2007). How to provide an effective primary health care in fighting against severe acute respiratory syndrome: The experiences of two cities. *American Journal of Infection Control*, *35*(1), 50–55. https://doi.org/10.1016/j.ajic.2006.06.009

World Health Organization (2020). *Novel coronavirus-China*. https://wwwwhoint/csr/don/12-january-2020-novel-coronavirus-china/en/

World Health Organization (2020b). *Statement on the second meeting of the International Health Regulations (2005) Emergency Committee regarding the outbreak of novel coronavirus (2019-nCoV)*. https://wwwwhoint/news-room/detail/30-01-2020-statement-on-the-second-meeting-of-the-international-health-regulations-(2005)-emergency-committee-regarding-the-outbreak-of-novel-coronavirus-(2019-ncov)

World Health Organization (2020c). *WHO and partners call for urgent investment in nurses 7 April 2020*. https://www.who.int/news-room/detail/07-04-2020-who-and-partners-call-for-urgent-investment-in-nurses

World Health Organization (2020d). *The new crown pneumonia outbreak has acquired pandemic characteristics*. http://news.cctv.com/2020/03/12/ARTI9mWo9pDI29dMKMh5nPzP200312.shtml

World Health Organization (2020e). *Coronavirus disease 2019 (COVID-19) situation Report-98*. https://www.who.int/docs/default-source/coronaviruse/situation-reports/20200427-sitrep-98-covid-19.pdf?sfvrsn=90323472_4

Wu, J., Wu, X., Wu, F., Dia, Y., Dechun, C., & Gong, X. (2020). Survey of sleep quality of clini-cal front-line nurses and its influencing factors in the fight against new coronavirus pneumonia. *Nursing Research*, *34*(4), 558–562. http://kns.cnki.net/kcms/detail/14.1272.r.20200214.1136.004.html

Xiang, Y.-T., Yang, Y., Li, W., Zhang, L., Zhang, Q., Cheung, T., & Ng, C. H. (2020). Timely mental health care for the 2019 nov-el coronavirus outbreak is urgently needed. *The Lancet Psychiatry*, *7*(3), 228–229. https://doi.org/10.1016/S2215-0366(20)30046-8

Zeng, T., & Tan, H. (2019). Asymptomatic cerebral infarction sleep disorders and cognitive executive function impairment and coke Correlation of anxiety depression. *Journal of Applied Medicine*, *35*(2), 3522–3526.

Zhang, J. X., & Schwarzer, R. (1995). Measuring optimistic self-beliefs: A Chinese adaptation of the general self-efficacy scale. *Psychologia: An International Journal of Psychology in the Orient*, *38*(3), 174–181.

Zhang, W., Li, E. P., Zheng, L., & Zhang, Y. (2020). Investigation and counter-measures of nurse anxiety in a hospital with a new coronavirus pneumonia in Hangzhou. *Health Research*, *2020*(02), 1–4. http://kns.cnki.net/kcms/detail/33.1359.R.20200205.1810.004.html

Zung, W. W. (1971). A rating instrument for anxiety disorders. *Psychosomatics*, *12*(6), 371–379. https://doi.org/10.1016/S0033-3182(71)71479-0

SUPPORTING INFORMATION

Additional supporting information may be found online in the Supporting Information section.

How to cite this article: Mo Y, Deng L, Zhang L, et al. Anxiety of Nurses to support Wuhan in fighting against COVID-19 Epidemic and its Correlation With Work Stress and Self-efficacy. *J Clin Nurs*. 2021;30:397–405. https://doi.org/10.1111/jocn.15549

13652702, 2021, 3-4, Downloaded from https://onlinelibrary.wiley.com/doi/10.1111/jocn.15549 by CochraneChina, Wiley Online Library on [19/12/2022]. See the Terms and Conditions (https://onlinelibrary.wiley.com/terms-and-conditions) on Wiley Online Library for rules of use; OA articles are governed by the applicable Creative Commons License
