# Supplementary material for: Mental health status and related factors influencing healthcare workers during the COVID-19 pandemic: A systematic review and meta-analysis
Source: PLoS One. 2024 Jan 19;19(1):e0289454. doi: 10.1371/journal.pone.0289454 (PMC10798549; doi:10.1371/journal.pone.0289454)
Supplement: S1 Data — (ZIP) [file pone.0289454.s011.zip › literatures/103.pdf]

INQUIRY: The Journal of Health Care Organization, Provision, and Financing
Volume 58: 1–8
© The Author(s) 2021
Article reuse guidelines: sagepub.com/journals-permissions
DOI: 10.1177/00469580211025225
journals.sagepub.com/home/inq

COVID-19: Fear and Anxiety among Healthcare Workers in Saudi Arabia. A Cross-Sectional Study

Syed Fareed Mohsin[1], Muhammad Atif Agwan[1], Sameer Shaikh[2], Ziyad Ahmad Alsuwaydani[1], and Saleh Ahmad AlSuwaydani[3]

Abstract

The novel coronavirus (COVID-19) is an extremely contagious respiratory disease. It poses a serious threat to healthcare workers which may cause substantial degree of psychological distress. This study aimed to evaluate the level of fear and anxiety among healthcare workers. A cross sectional study was designed to collect data from healthcare workers in Saudi Arabia using an online questionnaire. A non-probability convenient sampling technique was used to collect the data from 1st August to 10th August 2020 with the exclusion of non-healthcare worker and general public. A 5-point Likert scale was used to evaluate the level of fear and anxiety. The data was analyzed by using Spss version 23 .The descriptive data were represented as frequencies and percentage. Multinomial logistic regression analysis was applied to control the confounding factors and assess the relationship between variables. Data analyzed 737 participants revealed that 10.7%, 73.5%, and 15.7% of HCWs had a mild, moderate, and severe degree of fear and anxiety respectively. The multinomial logistic regression analysis revealed that gender and specialty of the participants were significantly associated with moderate and severe anxiety level. This study demonstrates that two-third of the healthcare workers had a moderate risk of fear and anxiety. However, females participants found to have elevated fear and anxiety level. Social distancing has a major impact on social well-being therefore its important to address social support through a policy. Further studies are needed to explore the mental health condition of healthcare workers in order to develop evidence based strategies.

Keywords

Fear, Anxiety, COVID-19, Healthcare Workers, Saudi Arabia

What do we know about this topic?
Due to COVID-19 pandemic frontline healthcare workers face a greater risk of disease contraction and mortality.

How does your research contribute to the field?
This study was able to Identify the level of fear and anxiety among healthcare workers.

What are your research implications toward theory, practice, or policy?
Our study revealed that two-third of the healthcare workers had a moderate risk of fear and anxiety.

Introduction

Coronavirus Disease 2019 (COVID-19) is a respiratory infection that has affected individuals, causing unsurpassed deaths, and extreme psychological trauma across the world.[1] The World Health Organization (WHO) declared the spread of coronavirus disease of 2019 (COVID-19) as a pandemic in March 2020.[2] As of August 31 2020, COVID-19 has spread to 213 countries, infecting 26 million people and leading to 850 000 deaths globally.[3]

Any natural or human-made catastrophe will increase the risk of (or worsen) existing psychiatric and emotional issues in

[1]College of Dentistry Ar Rass, Qassim University, Ar Rass, Qassim, Saudi Arabia
[2]College of Dentistry, University of Hail, Hail, Saudi Arabia
[3]Unaizah College of Medicine, Qassim University, Unaizah, Qassim, Saudi Arabia

Received 25 November 2020; revised 23 May 2021; revised manuscript accepted 24 May 2021

Corresponding Author:
Syed Fareed Mohsin, College of Dentistry Ar Rass, Qassim University, Alshunanah street, Ar Rass, 52719, Saudi Arabia
Email: sfareedmohsin@gmail.com

Creative Commons Non Commercial CC BY-NC: This article is distributed under the terms of the Creative Commons Attribution-NonCommercial 4.0 License (https://creativecommons.org/licenses/by-nc/4.0/) which permits non-commercial use, reproduction and distribution of the work without further permission provided the original work is attributed as specified on the SAGE and Open Access pages (https://us.sagepub.com/en-us/nam/open-access-at-sage).

those who are vulnerable or affected. In the current COVID-19 pandemic, the danger of becoming infected with the virus and the relative inadequacy of global healthcare systems in preventing and treating the virus at a national level can cause considerable stress and severe mental health problems, particularly among vulnerable individuals. Fear of being ill or dying, feelings of helplessness, depression, anxiety, loneliness, and stigma are among the most prominent psychiatric symptoms.[4] During disease outbreaks, frontline healthcare workers are vulnerable to temporary and long-term psychological issues due to the fears of disease contraction and being quarantined, the possibility of their family and friends being infected, caring for fellow workers as a patient, feelings of stigmatization, and rejection by others in their locality.[5,6]

Predominantly, the psychological issues confronting the HCWs include high levels of depression, fear, anxiety, insomnia, and distress.[7]

Suicide has been stated as a severe psychological consequence of COVID-19. In extreme cases, COVID-19-related suicides have been reported in various countries.[4] According to the studies on COVID-19, both the general public and healthcare professionals are at risk for increased psychiatric distress, including suicidality.[8] The HCWs faced significant psychological issues during the previous coronavirus disease outbreaks of SARS and MERS.[9,10]

During the Severe Acute Respiratory Syndrome (SARS) outbreak in 2003, mental stress and psychological distress were confronted by 68% and 57% HCWs, respectively.[11]

Similarly, during the Middle East Respiratory Syndrome (MERS) outbreak in 2015, high levels of mental stress were exhibited by HCWs dealing with the patients infected with the MERS.[12]

Fear and anxiety are closely associated with essential preventive activities like spatial distancing and hand hygiene behaviors. Unfortunately, fear and anxiety may exacerbate the disease's effects.[13,14] The psychological problems due to traumatic situations were found to adversely affect the physical health and quality of work in HCWs.[15]

Among the Gulf Cooperation Council (GCC) countries, Saudi Arabia has the largest number of reported cases of COVID-19, raising the probability of pressures on the healthcare system and the risk of contamination. This aspect is assumed to be of significance in the causation of psychological issues among HCWs in Saudi Arabia.[16]

The current study aims to estimate the psychological impacts of COVID-19 among the HCWs in various cities of Saudi Arabia. The findings from this study will help to elaborate on the importance of mental health interventions in HCWs for tackling physiological issues during the public health crisis.

Material and Methods

Study Design and Participants

A cross-sectional study was designed to gather information from healthcare workers employed in major cities of Saudi Arabia. The data was collected by using an online survey from 1st August to 10th August 2020. By using non-probability convenient sampling, a questionnaire was distributed among participants using WhatsApp, Twitter, Telegram, and email. All healthcare workers, including doctors, dentists, pharmacist, nutritionist, and nurses were approached to fill the questionnaire whereas non healthcare workers and the general public were excluded in this survey.

Sample Size

The sample size was calculated by using Epi info software version 5.4.1, assuming the prevalence of 50% with a 4% margin of error and a 95% confidence level. A minimal sample size of 567 was required for study.[17]

Ethical Consideration

The study was approved by Qassim University Ethical Review Board (ERB) number DRC/0016FA/20. An online informed consent was obtained prior to the data collection.

Data Collection Procedure

This survey used the COVID-19 fear and anxiety self-reported questionnaire that was initially in English. The authors translated the study in Arabic and converted it back into English to ensure that translation retained the meaning of the original English version. A proforma was prepared to collect the participant's information. The first part of the proforma was related to sociodemographic characteristics like age, gender, marital status, and place of work. In the second part, a 5-point Likert scale was used to evaluate the level of fear and anxiety among healthcare workers. Each question was scored 1 (Strongly disagree), 2 (disagreed), 3 (Neutral), 4 (Agreed), and 5 (Strongly agreed) a high score suggest a more significant stressful impact and vice versa. A cut-off value of $\geq$50 was used to demonstrate moderate-to-severe impact.[18]

The Cronbach's alpha coefficient of the fear and anxiety questionnaire was .88 in our sample, reflecting satisfactory internal consistency.[19]

Besides, an open-ended question was used to assess the individual perspective about the widespread impact of COVID-19.

Statistical Analyses

Data was analyzed by using SPSS version 23. The qualitative data presented as frequency and percentage. Logistic regression analysis was used to control the confounding factors and assess the relationship between variables A significant difference was recorded as $P < .05$.

Results

Amongst the 737 health care workers, 570 (77%) were between 18 and 30 years of age, 146 (20%) between 31 and

Table 1. Baseline Demographics of Healthcare Workers.

		Anxiety level		
		Mild	Moderate	Severe
Characteristics	n (%)	n (%)	n (%)	n (%)
Age				
18-30 year	570 (77.3)	47 (8.2)	436 (76.5)	87 (15.3)
31-40 year	146 (19.8)	25 (17.1)	92 (63.0)	21 (19.9)
41-50 year	21 (2.8)	7 (33.3)	14 (66.7)	0 (0)
Gender				
Male	450 (61.1)	69 (15.3)	325 (72.2)	56 (12.4)
Female	287 (38.9)	10 (3.5)	217 (75.6)	60 (20.9)
Marital status				
Married	261 (35.4)	34 (13.0)	178 (68.2)	49 (18.8)
Single	476 (64.6)	45 (9.5)	364 (76.5)	67 (14.1)
Speciality				
Applied health science	180 (24.4)	8 (4.4)	119 (66.1)	53 (29.4)
Dentistry	243 (33.0)	36 (14.8)	169 (69.5)	38 (15.6)
Medicine	242 (32.8)	33 (13.6)	191 (78.9)	18 (7.4)
Pharmacy	72 (9.8)	2 (2.8)	63 (87.5)	7 (9.7)
Years of experience				
1-5 years	570 (77.3)	47 (8.2)	436 (76.5)	87 (15.3)
6-10 years	146 (19.8)	25 (17.1)	92 (63.0)	22 (19.9)
More than 15 years	21 (2.8)	7 (33.3)	14 (66.7)	0 (0)
Anxiety score		79 (10.7)	542 (73.5)	116 (15.7)

40 years, and 21 (3%) between 41 and 50 years of age. Four hundred fifty (61%) were males, and 287 (39%) were females. Four hundred seventy-six (65%) of workers were single while 261 (35%) were married. Two hundred forty-three (33%) and 242 (33%) of workers belonged to Dentistry and Medicine specialties while 180 (24%) belonged to College of Applied Health Sciences and 72 (10%) to Pharmacy. Five hundred seventy (77%) of workers had a working experience between 1 and 5 years, 146 (20%) between 6 and 10 years while 21 (3%) above 15 years. According to the scoring category, severe scores were observed in 116 (16%) of workers, moderate in 542 (74%) of workers while mild in 79 (11%) of healthcare workers (Table 1).

Five hundred and fourteen (70%) of healthcare workers agreed that they were infected with COVID-19. Four hundred seventy-seven (65%) agreed that someone in their immediate family was infected with COVID-19. Two hundred eighty-eight (39%) disagreed that thinking about COVID-19 made them uncomfortable. Three hundred thirty-seven (46%) of workers agreed that they feared losing their life to COVID-19. Two hundred eleven (29%) and 106 (14%) disagreed and strongly disagreed to becoming nervous or frightened when watching the news and stories regarding COVID-19. Two hundred ninety (39%) of healthcare workers disagreed of being fearful about the fear and anxiety caused by COVID-19 among the society. Four hundred fifty-six (62%) disagreed and 166 (22.5%) strongly disagreed to the fact the negative impact of COVID-19 has or will make the economy suffer, making them anxious. Two hundred eighty-eight (39%) workers disagreed of getting infected with COVID-19 by others. Two hundred fifty-four (35%) disagreed that they feared quarantine duration (14 days) due to COVID-19. Two hundred thirty-six (32%) strongly disagreed, and 207 (28%) disagreed that uncertainty of when COVID-19 will be eradicated made them nervous. Three hundred sixty-seven (50%) of workers strongly agreed to the fact that getting infected with COVID-19 even after taking all precautionary measures in patient treatment's made them upset. Two hundred twelve (29%) of workers disagreed of having disturbing thought of getting infected with COVID-19. Two hundred thirty-four (32%) of workers disagreed of having any disturbing thought that some people that they might come in contact with be having COVID-19. Five hundred forty-nine (75%) strongly agreed on feeling dizzy, lightheaded, or faint when listening to or reading about COVID-19 news. Six hundred nine (83%) of workers strongly agreed of being unable to sleep due to fear of COVID-19. Four hundred three (55%) of works strongly agreed to lose interest in eating when thinking about or being exposed to COVID-19. Four hundred fifty-five (62%) of workers strongly agreed of feeling nausea and other stomach problem when thinking about COVID-19 (Table 2).

Table 2. Assessment of Fear and Anxiety among Healthcare Workers in the Study.

Variable	n (%)				
	Strongly agree	Agree	Neutral	Disagree	Strongly disagree
Have you been infected with COVID-19?	196 (26.6)	514 (69.7)	27 (3.7)	0 (0.0)	0 (0.0)
Anyone in your immediate family that had been infected with the COVID 19?	246 (33.4)	477 (64.7)	14 (1.9)	0 (0.0)	0 (0.0)
It makes me uncomfortable to think about COVID-19	97 (13.2)	95 (12.9)	186 (25.2)	288 (39.1)	71 (9.6)
I am fearful of losing my life because of COVID-19	123 (16.7)	337 (45.7)	162 (22.0)	115 (15.6)	0 (0.0)
When watching news and stories about COVID-19 on social media, I become nervous or frightened	124 (16.8)	117 (15.9)	179 (24.3)	211 (28.6)	106 (14.4)
I am fearful about the fear and anxiety caused by COVID 19 in the society	102 (13.8)	81 (11.0)	160 (21.7)	290 (39.3)	104 (14.1)
The negative impact of the disease on the current and future economy makes me anxious	78 (10.6)	12 (1.6)	25 (3.4)	456 (61.9)	166 (22.5)
I fear of getting infected by others due to COVID-19	56 (7.6)	38 (5.2)	172 (23.3)	288 (39.1)	183 (24.8)
I fear of the duration of quarantine (14 days) due to COVID-19	73 (9.9)	182 (24.7)	177 (24.0)	254 (34.5)	51 (6.9)
The uncertainty of when COVID-19 will be eradicated makes me nervous	51 (6.9)	83 (11.3)	160 (21.7)	207 (28.1)	236 (32.0)
Getting infected by COVID-19 despite taking all precautionary measures during the patient's treatment makes me upset	367 (49.8)	202 (27.4)	166 (22.5)	2 (0.3)	0 (0.0)
Having disturbing thoughts that I may have caught the COVID-19	187 (25.4)	129 (17.5)	135 (18.3)	212 (28.8)	74 (10.0)
I had disturbing thoughts that certain people I came in contact with may have the COVID-19	131 (17.8)	89 (12.1)	188 (25.5)	234 (31.8)	95 (12.9)
I felt dizzy, lightheaded, or faint when I read or listened to news about the COVID-19	549 (74.5)	141 (19.1)	47(6.4)	0 (0.0)	0 (0.0)
I am unable to sleep due to fear of COVID-19	609 (82.6)	93 (12.6)	0 (0.0)	35 (4.7)	0 (0.0)
I lost interest in eating when I thought about or was exposed to information about the COVID-19	403 (54.7)	213 (28.9)	87 (11.8)	34 (4.6)	0 (0.0)
I feel nausea and have stomach problems on thinking about COVID-19	455 (61.7)	156 (21.2)	70 (9.5)	56 (7.6)	0 (0.0)

The multinomial logistic regression analysis showed that gender and specialty of the participants were significantly associated with moderate anxiety where participants who were female had significantly higher odds of having moderate anxiety than those who were male (OR = 3.78; 95% CI 1.78-8.05; P = .001) and participants who belonged to the faculty of dentistry and medicine had significantly lower odds of having moderate anxiety than those who belonged to the applied health sciences (OR = 0.07; 95% CI 0.01-0.36; P = .001 and OR = 0.14; 95% CI 0.03-0.70; P = .016 respectively) (Table 3).

The multinomial logistic regression analysis further showed that gender and specialty of the participants were significantly associated with severe anxiety where participants who were female had significantly higher odds of having severe anxiety than those who were male (OR = 4.21; 95% CI 1.78-9.92; P = .001) and participants who belonged to the faculty of dentistry and medicine had significantly lower odds of having severe anxiety than those who belonged to the applied health sciences (OR = 0.13; 95% CI 0.02-0.77; P = .025 and OR = 0.10; 95% CI 0.01-0.63; P = .014 respectively) (Table 4).

Discussion

This cross-sectional study was designed to evaluate the fear and anxiety level among HCWs during COVID-19 pandemic in Saudi Arabia. For this purpose, a questionnaire-based study was formulated to collect information about HCWs fear and anxiety. Survey-based studies are proven for

Table 3. Logistic Regression Analysis for Associations of Socio-Demographic Characteristics with Moderate Anxiety.

	Moderate anxiety			
		95% CI		
Variable (n = 542)	OR	Lower	Upper	*P*
Gender				
Female	3.78	1.78	8.05	<.001
Marital status				
Married	1.5	0.675	3.34	.318
Specialty				
Applied health sciences	0.32	0.06	1.74	.189
Dentistry	0.07	0.01	0.36	<.001
Medicine	0.14	0.03	0.70	.016

Table 4. Logistic Regression Analysis for Associations of Socio-Demographic Characteristics with Severe Anxiety.

	Severe anxiety			
		95% CI		
Variable (n = 116)	OR	Lower	Upper	*P*
Gender				
Female	4.21	1.78	9.92	<.001
Marital status				
Married	1.58	0.62	4.01	.33
Specialty				
Applied health sciences	1.03	0.15	6.79	.975
Dentistry	0.13	0.02	0.77	.025
Medicine	0.10	0.01	0.63	.014

collecting information on participant behaviors, opinions, and experiences; however, careful data collection and analysis are required.[20] The questionnaire used in this study collected information systematically. Any incomplete responses have been discarded.

In pandemics, psychological impacts such as fear and anxiety are natural, especially if the number of infected individuals and death rates is sharply increasing. In this study, data revealed that 10.7%, 73.5%, and 15.7% of HCWs had a mild, moderate, and severe degree of fear and anxiety, respectively. Several factors are related to moderate to high level of fear and anxiety; these includes personal, social aspects, and society. Personal factors are linked with an elevated level of anxiety is being a smoker or living with someone with chronic illness. Social factors are associated with increased exposure to media news and the economic impact of this disease. Society is another aspect of high level of anxiety associated to living with older people having respiratory and immune-mediated disease. Our findings are consistent with other studies conducted on the impact of COVID-19 on HCWs.[21,22]

During COVID-19 outbreak, our study reported a fear and anxiety prevalence of 42.72% among 737 healthcare workers. A study conducted in Saudi Arabia found an anxiety prevalence of 51% among health care providers.[23]

A similar study in Saudi Arabia found that 68.5% of study participants had moderate to severe anxiety level in healthcare workers.[21] Other recent findings in general Saudi population observed a lower 29.9% prevalence of anxiety compared to our study.[24]

Moreover, similar findings were found in other studies showed a prevalence of severe anxiety of 15.3% and 16% compared to our study. Our results are consistent with the severe anxiety level found among healthcare workers in Saudi Arabia.[24,25] Also, a Chinese study reported a prevalence of 44.6% of anxiety among 1257 health-care providers.[26]

The prevalence of anxiety, depression, and insomnia during COVID-19 was evaluated in a systematic review and meta-analysis conducted by Pappa et al[27] they assessed 12 studies and reported a prevalence of 23.2%. The findings of Pappa et al[27] favors the result of present study as anxiety and depression was experienced by healthcare workers during COVID-19. Moreover, Labrague and De Los Santos[28] found that 37.8% of nurses had dysfunctional anxiety level. These findings support the present study results by demonstrating

that frontline workers are affected by anxiety during COVID-19 pandemic.

Studies on the previous outbreak in 2017, a study reported a 52% prevalence of anxiety among HCWs working in level I trauma center in Saudi Arabia. Work-related pressures and high job responsibilities can explain anxiety in emergency situations. Other research conducted among health care professionals during the SARS outbreak indicated some causes of distress: social stigma; family members; social isolation; loss of control; self-health, family, and others; changes in work; and spread of the virus.[29,30] There is still no vaccine or approved therapy, which further raises fear about the thought of being infected. Healthcare staff who deal with patients continuously are at greater risk of contracting infectious diseases, creating an immense psychological toll.[11] Another genuine concern about HCWs is that this disease is carried to their families from their practices. As coronavirus last several hours to days on various surfaces this, along with its extended incubation time before the development of symptoms, are factors that make it especially difficult to restrict its transmission.[31]

In our study, multinomial logistic regression analysis showed that gender and specialty of the participants were significantly associated with moderate and severe anxiety. A similar finding was revealed in general population where female had a higher level of fear and anxiety as compared to males.[24] Similar results have been reported in the recent Jordanian study, which described women as a higher risk category for anxiety, 70.8% among healthcare providers.[32] With regard to specialty of participants similar findings were observed amongst physicians and dentist in other studies around the world.[33-36]

The COVID-19 has effected the people in many ways in our study majority of participants responded that this disease had caused a substantial impact on the economy, psychological life, and social life. The economical impact showed that multiple businesses has been shut down with an increase in the unemployment rate. Social life is also severely affected. People are bound to stay at home. All these factors have a psychological impact on individuals mental status that leads to an increase in anxiety and fear. Early diagnosis of anxiety is of prime importance; however, studies showed that failure to identify signs of anxiety could lead to severe psychological consequences.[37]

We agreed that our study has highlighted the higher levels of anxiety in HCWs that could assist decision-makers and clinicians in recognizing and providing assistance to professionals with elevated levels of anxiety. This study has some limitations. First, data was collected in a brief period of time. Second, our responses are mainly from major cities of Saudi Arabia as the response from the rural health centers still not known. Third, this study did not evaluate the mental health condition of participants which might be existed before COVID-19 pandemic. Fourth, our study did not use Arabic version of COVID-19 fear scale (FCV-19S) to assess the psychological impact of this disease in Saudi population. Moreover, convenience sampling and cross-sectional design of study act as a barrier in making a casual conclusion. In addition, an online survey makes it difficult to generate random sampling as respondents are only those who use the internet as survey participants do not have the opportunity to ask clarifying questions. Therefore the findings of this study should be carefully interpreted and not to be generalized.

We suggest that further research on fear and anxiety must include the variables we were unable to address in this study, like socioeconomic status and mental health status of HCWs. Furthermore, SAVE-9[38] rating scale should be used in future research to validate the systematic evaluation of stress and anxiety level in HCWs during COVID-19 pandemic.

Conclusion

In conclusion, despite having prior knowledge, healthcare workers are in a state of fear and anxiety. Our study demonstrated that two-third of the healthcare workers had moderate risk of fear and anxiety. However, females participants found to have elevated fear and anxiety level. In future, larger population studies are needed to confirm our findings. Our research was able to capture the impact of COVID-19 pandemic on mental health. The healthcare agencies must pay attention to their employees' well-being and encourage early detection and evaluation of psychological distress. Due to social distancing, there are less opportunities for social interaction and attendance at events, it's often important to address social support through a policy. Social support systems are crucial in safeguarding health-care workers and lowering the prevalence of psychological distress.

Author Contributions

SFM conceived and designed the study. SFM, ASA, and SS did manuscript writing, drafted the article, and revised it critically for important content. ZAS and SAS data collection and curation. SFM, ASA, and SS analyzed the data and editing of manuscript. All Authors revised and approved the final version.

Declaration of Conflicting Interests

The author(s) declared no potential conflicts of interest with respect to the research, authorship, and/or publication of this article.

Funding

The author(s) received no financial support for the research, authorship, and/or publication of this article.

ORCID iD

Syed Fareed Mohsin https://orcid.org/0000-0002-5357-3967

Supplemental Material

Supplemental material for this article is available online.

References

1. Zhu N, Zhang D, Wang W, et al. A novel coronavirus from patients with pneumonia in China, 2019. *N Engl J Med*. 2020;382(8):727-733. doi:10.1056/NEJMoa2001017
2. Gupta KV, Murthy PR, Kumar AA. Is anxiety a rising concern during COVID-19 pandemic among healthcare workers? *Indian J Crit Care Med*. 2020;24(5):369-370. doi:10.5005/jp-journals-10071-23434
3. WHO. WHO coronavirus disease (COVID-19) dashboard. Published 2020. Accessed August 31, 2020. https://covid19.who.int/
4. Mamun MA, Sakib N, Gozal D, et al. The COVID-19 pandemic and serious psychological consequences in Bangladesh: a population-based nationwide study. *J Affect Disord*. 2021; 279:462-472. doi:10.1016/j.jad.2020.10.036
5. Temsah M, Al-Sohime F, Alamro N, et al. The psychological impact of COVID-19 pandemic on health care workers in a MERS-CoV endemic country. *J Infect Public Health*. 2020;13(6):877-882. doi:10.1016/j.jiph.2020.05.021
6. Sakib N, Akter T, Zohra F, Bhuiyan AKMI, Mamun MA, Griffiths MD. Fear of COVID-19 and depression: a comparative study among the general population and healthcare professionals during COVID-19 pandemic crisis in Bangladesh. *Int J Ment Health Addict*. 2021;19:1-17. doi:10.1007/s11469-020-00477-9
7. Khan YH, Mallhi TH, Alotaibi NH, Alzarea AI. Work related stress factors among healthcare professionals during COVID-19 pandemic; a call for immediate action. *Hosp Pract*. 2020; 48(5):244-245. doi:10.1080/21548331.2020.1793519
8. Mamun MA, Akter T, Zohra F, et al. Prevalence and risk factors of COVID-19 suicidal behavior in Bangladeshi population: are healthcare professionals at greater risk? *Heliyon*. 2020;6(10):e05259.
9. Shaukat N, Ali DM, Razzak J. Physical and mental health impacts of COVID-19 on healthcare workers: a scoping review. *Int J Emerg Med*. 2020;13(1):40. doi:10.1016/j.heliyon.2020.e05259
10. De Brier N, Stroobants S, Vandekerckhove P, De Buck E. Factors affecting mental health of health care workers during coronavirus disease outbreaks (SARS, MERS & COVID-19): a rapid systematic review. *PLoS One*. 2020;15(12):e0244052. doi:10.1371/journal.pone.0244052
11. Tam CWC, Pang EPF, Lam LCW, Chiu HFK. Severe acute respiratory syndrome (SARS) in Hong Kong in 2003: stress and psychological impact among frontline healthcare workers. *Psychol Med*. 2004;34(7):1197-1204. doi:10.1017/s0033291704002247
12. Oh N, Hong N, Ryu DH, Bae SG, Kam S, Kim K. Exploring nursing intention, stress, and professionalism in response to infectious disease emergencies: the experience of local public hospital nurses during the 2015 MERS outbreak in South Korea. *Asian Nurs Res*. 2017;11(3):230-236. doi:10.1016/j.anr.2017.08.005
13. Ahorsu DK, Lin CY, Imani V, Saffari M, Griffiths MD, Pakpour AH. The fear of COVID-19 scale: development and initial validation. *Int J Ment Health Addict*. 2020;27:1-9. doi:10.1007%2Fs11469-020-00270-8
14. Pakpour AH, Griffiths MD, Lin CY. Assessing psychological response to the COVID-19: the fear of COVID-19 scale and the COVID stress scales. *Int J Ment Health Addict*. 2020;29:1-4. doi:10.1007/s11469-020-00334-9
15. Kang X, Fang Y, Li S, et al.The benefits of indirect exposure to trauma: the relationships among vicarious posttraumatic growth, social support, and resilience in ambulance personnel in China. *Psychiatry Investig*. 2018;15(5):452-459. doi:10.30773/pi.2017.11.08.1
16. Al-Hanawi MK, Mwale ML, Alshareef N, et al. psychological distress amongst health workers and the general public during the COVID-19 pandemic in Saudi Arabia. *Risk Manag Healthc Policy*. 2020;13:733-742. doi:10.2147/RMHP.S264037
17. CDC. Epiinfo. sample size calculator software version 5.4.1. https://www.cdc.gov/epiinfo/
18. Adelson JL, McCoach DB. Measuring the mathematical attitudes of elementary students: the effects of a 4-Point or 5-Point Likert-Type Scale. *Educ Psychol Meas*. 2010;70(5):796-807. doi:10.1177/0013164410366694
19. Taber KS. The use of Cronbach's Alpha when developing and reporting research instruments in science education. *Res Sci Educ*. 2018;48(6):1273-1296. doi:10.1007/s11165-016-9602-2
20. Lydeard S. The questionnaire as a research tool. *Fam Pract*. 1991;8(1):84-91. doi:10.1093/fampra/8.1.84
21. Alenazi TH, BinDhim NF, Alenazi MH, et al. Prevalence and predictors of anxiety among healthcare workers in Saudi Arabia during the COVID-19 pandemic. *J Infect Public Health*. 2020;13(11):1645-1651. doi:10.1016/j.jiph.2020.09.001
22. Shanafelt T, Ripp J, Trockel M. Understanding and addressing sources of anxiety among health care professionals during the COVID-19 pandemic. *JAMA*. 2020;323(21):2133-2134. doi:10.1001/jama.2020.5893
23. AlAteeq DA, Aljhani S, Althiyabi I, Majzoub S. Mental health among healthcare providers during coronavirus disease (COVID-19) outbreak in Saudi Arabia. *J Infect Public Health*. 2020;13(10):1432-1437. doi:10.1016/j.jiph.2020.08.013
24. Alkhamees AA, Alrashed SA, Alzunaydi AA, Almohimeed AS, Aljohani MS. The psychological impact of COVID-19 pandemic on the general population of Saudi Arabia. *Compr Psychiatry*. 2020;102:152192. doi:10.1016/j.comppsych.2020.152192
25. Alharthy N, Alrajeh OA, Almutairi M, Alhajri A. Assessment of anxiety level of emergency health-care workers by generalized anxiety disorder-7 tool. *Int J Appl Basic Med Res*. 2017;7(3):150-154. doi:10.4103/2229-516X.212963
26. Lai J, Ma S, Wang Y, et al. Factors associated with mental health outcomes among health care workers exposed to coronavirus disease 2019. *JAMA Netw Open*. 2020;3(3):e203976. doi:10.1001/jamanetworkopen.2020.3976
27. Pappa S, Ntella V, Giannakas T, Giannakoulis VG, Papoutsi E, Katsaounou P. Prevalence of depression, anxiety, and insomnia among healthcare workers during the COVID-19 pandemic: a systemic review and meta-analysis. *Brain Behav Immun*. 2020;88:901-907. doi:10.1016/j.bbi.2020. 05.026
28. Labrague LJ, De Los Santos J. COVID-19 anxiety among frontline nurses: predictive role of organizational support, personal resilience and social support. *J Nurs Manag*. 2020;22(7):1653–1661. doi:10.1111/jonm.13121

29. Koh D, Lim MK, Chia SE, et al. Risk perception and impact of Severe Acute Respiratory Syndrome (SARS) on work and personal lives of healthcare workers in Singapore: what can we learn? *Med Care*. 2005;43(7):676-682. doi:10.1097/01.mlr.0000167181.36730.cc
30. Wong TW, Yau JK, Chan CL, et al. The psychological impact of severe acute respiratory syndrome outbreak on healthcare workers in emergency departments and how they cope. *Eur J Emerg Med*. 2005;12(1):13-18. doi;10.1097/00063110-200502000-00005
31. van Doremalen N, Bushmaker T, Morris DH, et al. Aerosol and surface stability of SARS-CoV-2 as compared with SARS-CoV-1. *N Engl J Med*. 2020;382(16):1564-1567. doi:10.1056/NEJMc2004973
32. Naser AY, Dahmash EZ, Al-Rousan R, et al. Mental health status of the general population, healthcare professionals, and university students during 2019 coronavirus disease outbreak in Jordan: a cross-sectional study. *Brain Behav*. 2020;10(8):e01730. doi:10.1002/brb3.1730
33. Mrklas K, Shalaby R, Hrabok M, et al. Prevalence of perceived stress, anxiety, depression, and obsessive-compulsive symptoms in health care workers and other workers in Alberta during the COVID-19 pandemic: cross-sectional survey. *JMIR Mental Health*. 2020;7(9):e22408. doi:10.2196/22408
34. Elhadi M, Msherghi A, Elgzairi M, et al. Psychological status of healthcare workers during the civil war and COVID-19 pandemic: a cross-sectional study. *J Psychosom Res*. 2020;137:110221. doi:10.1016/j.jpsychores.2020.110221
35. Amin F, Sharif S, Saeed R, Durrani N, Jilani D. COVID-19 pandemic-knowledge, perception, anxiety and depression among frontline doctors of Pakistan. *BMC Psychiatry*. 2020;20:459. doi:10.1186/s12888-020-02864-x
36. Kinariwala N, Samaranayake LP, Perera I, Patel Z. Concerns and fears of Indian dentists on professional practice during the coronavirus disease 2019 (COVID-19) pandemic. *Oral Dis*. 2021;27(suppl 3):730-732. doi:10.1111/odi.13459
37. Tanios CY, Abou-Saleh MT, Karam AN, Salamoun MM, Mneimneh ZN, Karam EG. The epidemiology of anxiety disorders in the Arab world: a review. *J Anxiety Disord*. 2008;23(4):409-419. doi:10.1016/j.janxdis.2008.10.009
38. Tavormina G, Tavormina MGM, Franza F, et al. A new rating scale (SAVE-9) to demonstrate the stress and anxiety in the healthcare workers during the COVID-19 viral epidemic. *Psychiatr Danub*. 2020;32:5-9.
