# Supplementary material for: Mental health status and related factors influencing healthcare workers during the COVID-19 pandemic: A systematic review and meta-analysis
Source: PLoS One. 2024 Jan 19;19(1):e0289454. doi: 10.1371/journal.pone.0289454 (PMC10798549; doi:10.1371/journal.pone.0289454)
Supplement: S1 Data — (ZIP) [file pone.0289454.s011.zip › literatures/104.pdf]

NNJ NEPHROLOGY NURSING JOURNAL

Mental Health and Health-Related Quality of Life Among Nephrology Nurses: A Survey-Based Cross-Sectional Study

Vicki Montoya, PhD, APRN, FNP-BC, Katie Donnini, BA, Marjolaine Gauthier-Loiselle, PhD, Myrlene Sanon, MPH, Martin Cloutier, MSc, Jessica Maitland, MScPH, Annie Guérin, MSc, Paula Dutka, MSN, RN, CNN, Lillian Pryor, MSN, RN, CNN, Charlotte Thomas-Hawkins, PhD, RN, FAAN, Arthur Voegel, MA, Mark Hoffmann, PhD, Samuel Savin, BA, Alissa Kurzman, MPH, and Tamara Kear, PhD, RN, CNN, FAAN

Copyright 2021 American Nephrology Nurses Association.

Montoya, V., Donnini, K., Gauthier-Loiselle, M., Sanon, M., Cloutier, M., Maitland, J., Guérin, A., Dutka, P., Pryor, L., Thomas-Hawkins, C., Voegel, A., Hoffman, M., Savin, S., Kurzman, A., & Kear, T. (2021). Mental health and health-related quality of life among nephrology nurses: A survey-based cross-sectional study. *Nephrology Nursing Journal, 48*(5), 447-461. https://doi.org/10.37526/1526-744X.2021.48.5.447

Nephrology nurses face health and wellness challenges due to significant work-related stressors. This survey, conducted online between July 24 and August 17, 2020, assessed the psychological well-being of nephrology nurses in the United States during the COVID-19 pandemic (*n* = 393). Respondents reported feeling burned out from work (62%), symptoms of anxiety (47% with Generalized Anxiety Disorder-7 [GAD-7] scores ≥ 5), and major depressive episodes (16% with Patient Health Questionnaire-2 [PHQ-2] scores ≥ 3). Fifty-six percent (56%) of survey respondents reported caring for COVID-19 patients, and 62% were somewhat or very worried about COVID-19. Factors, including high workload, age, race, and the COVID-19 pandemic, may partially explain the high proportion of nephrology nurses who reported symptoms of burnout, anxiety, and depression.

Key Words:
Nephrology nurses, mental health outcomes, health-related quality of life, mental health survey.

Nephrology nurses provide complex technological care to large numbers of patients who have a substantial clinical burden (Li et al., 2018). This care can also involve providing long-term support to motivate and comfort patients and their families in the face of declining health and mortality due to the progressive nature of chronic kidney disease (CKD) (Bednar & Latham, 2014; Hayes & Bonner, 2010; Meguid El Nahas & Bello, 2005; Neild, 2017; Thompson et al., 2015). The close relationships nephrology nurses have with their patients, combined with the pressures of a demanding workload, can often result in emotional and physical work-related stress (Ashker et al., 2012; Flynn et at., 2009; Hayes & Bonner, 2010; Kapucu et al., 2009). This stress can have an adverse effect on the well-being of nephrology nurses (Ashker et al., 2012). Factors that influence the lack of well-being of nephrology nurses include poor interpersonal relationships with colleagues, the inability to deliver quality patient care, and long shifts (Ashker et al., 2012; Hayes & Bonner, 2010). Planful problem solving, seeking social support, self-control, positive reappraisal, and positive interpersonal relationships with colleagues are associated with increased well-being and decreased job stress levels (Ashker et al., 2012; Hayes & Bonner, 2010). However, Hayes and Bonner (2010), in a review of the literature, also found that interpersonal relationships with physicians were identified as having both a positive and negative influence on job stress and burnout.

Employers experience major challenges in recruiting, training, and retaining nephrology nurses (Gaietto & Brooks, 2019; Gardner et al., 2007; Mehrotra et al., 2011; Wolfe, 2014). Gardner and colleagues (2007) found evidence of high attrition among this essential workforce. In a study by Ulrich and Kear (2018), 40% of the nephrology nurse respondents reported that they planned to leave their current positions in the next three years, with most planning to retire or take another position in clinical/patient care nursing. The attrition rate for nephrology nurses is anticipated to continue rising as factors that contribute to work-related stress persist (Gaietto & Brooks, 2019; Mehrotra et al., 2011). This is particularly concerning given the time it takes to train nephrology nurses and the increased need for skilled nephrology nurses to address the growing clinical burden of chronic kidney disease (CKD) and its associated morbidities (e.g., diabetes, hypertension) (Gaietto & Brooks, 2019; Sharif et al., 2016).

The current pandemic due to the novel coronavirus disease of 2019 (COVID-19) has amplified the health and wellness challenges faced by nurses (Lai et al., 2020; Neto et al., 2020). In a survey of frontline health care providers

Note: Author biographical statements appear on next page.

treating patients with COVID-19, 72% experienced some form of distress, 45% reported symptoms of anxiety, 50% reported symptoms of depression, and 34% reported insomnia (Lai et al., 2020). Despite recognition of the broad challenges faced by health care providers during the pandemic and the demanding workplace environments of nephrology nurses, there is limited research exploring the well-being of nephrology nurses. For this reason, a web-based, cross-sectional survey was conducted, with a primary objective of assessing the mental health and well-being of nephrology nurses in the workplace and a secondary objective of assessing the additional psychological burden related to COVID-19.

Methods

Study Design

This cross-sectional online survey was designed to collect information from eligible nurses who were members of the American Nephrology Nurses Association (ANNA) at the time of the survey and who agreed to participate. The survey was conducted between July 24 and August 17, 2020. The survey had a targeted completion time of 15 to 20 minutes and consisted of two sections: 1) a short list of screening questions to confirm respondents' eligibility to participate (4 questions), and 2) a series of questions designed to collect information on demographic characteristics (5 questions), general workplace background (11 questions), work-related impact of COVID-19 (3 questions), health-related quality of life (2 questions), and psychological well-being (5 questions).

The survey was approved by an Institutional Review Board/Independent Ethics Committee and conducted in accordance with an established protocol. Respondent consent was obtained at the time of participation, and respondents could opt out of participating by terminating the survey at any time, if they desired. The survey was anonymous, and confidentiality was assured through de-identification of participants and their responses. No compensation was provided as part of the survey.

Respondents

At the time of the survey, eligible respondents were licensed and employed as nephrology nurses in the United States, members of ANNA, and comfortable reading and understanding English. The target sample size was determined by the nurses' willingness to participate in the study and was not pre-specified.

Measures, Outcomes, and Statistical Analysis

The survey focused on psychological well-being with symptoms indicative of anxiety, depression, and burnout, as well as other work-related factors, such as absenteeism, effects of health on work performance, compassion fatigue, and worry about COVID-19. Health-related quality of life was assessed by self-reported perceptions of overall health and physical conditions.

Psychological well-being was assessed using a series of questions that included the Generalized Anxiety Disorder-7 (GAD-7) (range 0 to 21) (Spitzer et al., 2006) and the Patient Health Questionnaire-2 (PHQ-2) (range 0 to 6) (Kroenke et al., 2001). The total scores of these measurement tools are interpreted as follows: GAD-7, no anxiety (0

Vicki Montoya, PhD, APRN, FNP-BC, is an Assistant Professor, Nursing Practice Department, College of Nursing, University of Central Florida, Orlando, FL; the Chair of ANNA's Research Committee (2020-2021), and a member of ANNA's Sunshine State Chapter.

Katie Donnini, BA, is Director of Patient Advocacy & Stakeholder Management, Otsuka America Pharmaceutical Inc., Princeton, NJ.

Marjolaine Gauthier-Loiselle, PhD, is a Manager, Analysis Group, Inc., Montreal, Quebec, Canada.

Myrlene Sanon, MPH, was the Director of Health Economics and Outcomes Research, Otsuka Pharmaceutical Development & Commercialization, Inc., Princeton, NJ, when this study was completed.

Martin Cloutier, MSc, is Vice President, Analysis Group, Inc., Montreal, Quebec, Canada.

Jessica Maitland, MScPH, is a Senior Research Professional, Analysis Group, Inc., Montreal, Quebec, Canada.

Annie Guérin, **MSc,** is a Managing Principal, Analysis Group, Inc., Montreal, Quebec, Canada.

Paula Dutka, **MSN, RN, CNN**, is Director, Education and Research, Nephrology Network, NYU Langone Hospital – Long Island, Long Island, NY; a member of the *Nephrology Nursing Journal* Editorial Board, and a member of ANNA's Long Island Chapter.

Lillian Pryor, MSN, RN, CNN, is a Renal Clinic Nurse, Atlanta VAMC, Decatur, GA; a Past President of the American Nephrology Nurses Association, and a member of ANNA's Dogwood Chapter.

Charlotte Thomas-Hawkins, **PhD, RN, FAAN,** is Associate Dean and Associate Professor, Rutgers University School of Nursing, Newark, NJ, and a member of ANNA's Garden State Chapter.

Arthur Voegel, **MA,** is a Senior Research Professional, Analysis Group, Inc., Montreal, Quebec, Canada.

Mark Hoffmann, PhD, is a Managing Director, High Lantern Group, Philadelphia, PA.

Samuel Savin, BA, is a Director, High Lantern Group, Philadelphia, PA

Alissa Kurzman, MPH, is an Associate, High Lantern Group, Philadelphia, PA

Tamara Kear, PhD, RN, CNN, FAAN, is the Executive Director, American Nephrology Nurses Association, Pitman, NJ; an Associate Adjunct Professor, M. Louise Fitzpatrick College of Nursing, Villanova University, Villanova, PA; a Past President of ANNA, and a member of ANNA's Keystone Chapter.

Acknowledgments: This study was funded by Otsuka Pharmaceutical Development & Commercialization. Clint Earnheart, PhD, of BioScience Communications assisted in drafting the manuscript, activity that was also funded by Otsuka.

Figure 1
IWPS-R Workload Scale Components

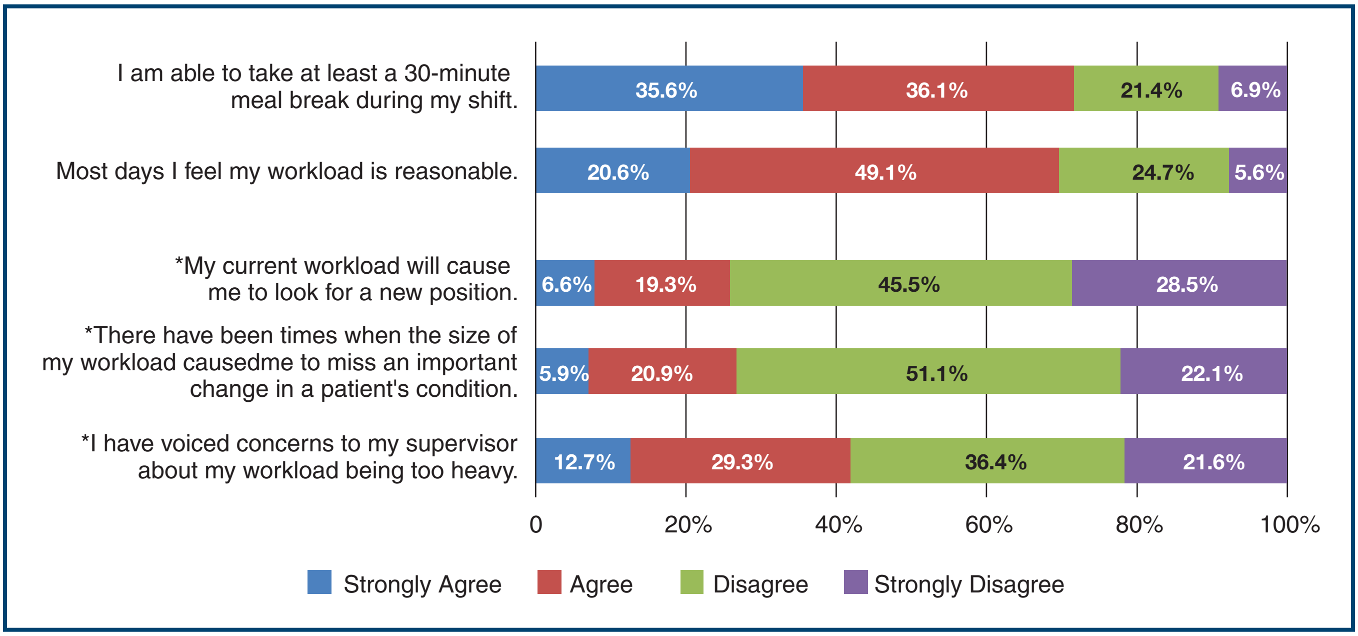

*Negatively worded question.
Note: IWPS-R = Individual Workload Perception Scale-Revised.

to 4), mild anxiety (5 to 9), moderate anxiety (10 to 14), and severe anxiety (15 to 21) (Spitzer et al., 2006); for PHQ-2, a score of 3 or higher is suggestive of a major depressive episode (Kroenke et al., 2001). Burnout from work was assessed based on two validated questions: 1) "During the past month, have you felt burned out from your work?" and 2) "During the past month, have you worried that your work is hardening you emotionally?" (West et al., 2012). The survey also assessed if respondents had recently (in the past 2 weeks) felt that problems with their physical or mental health affected their work performance.

Demographic characteristics reported include age, gender identity, and race/ethnicity. Respondents also provided information about their nursing education level (highest nursing degree) and nursing experience (number of years practicing as a nephrology nurse). Data collected on work-related characteristics included work schedule, position, facility setting, and geographic location. The workload subscale instrument from the revised Individual Workload Perception Scale (IWPS-R) (Cox et al., 2003) and a rating of patient safety in unit/work area (Sorra et al., 2016) were also included. Absenteeism was assessed in the two weeks prior to data collection and was calculated as weekly hours of work missed due to problems with physical or mental health)/weekly hours of work missed due to problems with physical or mental health + weekly hours worked. Respondents were asked whether they were responsible for the care of patients with diagnosed or suspected COVID-19 or patients who died with diagnosed or suspected COVID-19. They were also asked about their level of worry regarding COVID-19.

Descriptive statistics were calculated for all variables and outcomes. Continuous variables were summarized using means, medians, and standard deviations (SDs), while categorical variables were summarized using frequency counts and percentages. Stratified analyses were replicated separately for the outcomes described above based on age, race, and United States census region. Logistic regression models were estimated to identify factors associated with anxiety symptoms (defined as a GAD score of greater than 5) and feeling burned out from work.

Results

Demographic Characteristics

A total of 393 respondents were included in the analysis. An overview of respondent demographics is presented in Table 1. The majority of respondents self-identified as female (92%), of non-Hispanic White race (79%), and had a mean age (± SD) of 52.9 years (± 10.5 years). Respondents had a mean of 25.5 years (± 12.9 years) of experience in nursing, including a mean of 18.2 ± 12.0 years in nephrology nursing (see Table 2). Of the nurses who responded, 49% worked in chronic outpatient hemodialysis settings; 32% worked in acute inpatient dialysis care; 48% worked as a clinical/staff

Table 1
Demographics

	N = 393
**Age, mean ± SD**	52.9 ± 10.5
**Gender identity, *n* (%)**	
Female	362 (92.1%)
Male	28 (7.1%)
Prefer not to answer	3 (0.8%)
**Race, *n* (%)**	
Caucasian or White	310 (78.9%)
Asian or Pacific Islander	31 (7.9%)
African American or Black	28 (7.1%)
Hispanic or Latino	18 (4.6%)
Native American or Alaskan Native	3 (0.8%)
Other	2 (0.5%)
Prefer not to answer	8 (2.0%)
**Highest nursing degree, *n* (%)**	
Diploma	27 (6.9%)
Associate	111 (28.2%)
Baccalaureate of Science in nursing	173 (44.0%)
Master of Science in nursing	70 (17.8%)
Doctorate	6 (1.5%)
Other	6 (1.5%)
**Number of years in the field of nursing, mean ± SD**	25.5 ± 12.9
**Number of years in the field of nephrology nursing, mean ± SD**	18.2 ± 12.0

Notes: SD = standard deviation.

nurse. Forty-seven percent (47%) of respondents practiced in urban settings and 36% in suburban settings. Respondents were located in the South (35%), Midwest (25%), Northeast (20%), West (19%), or Other (1%) regions of the United States, with the most respondents located Texas (8%), New York (7%), and California (6%).

Work Environment and Workload

As a whole, the nephrology nurses surveyed worked an average (± SD) of 35.5 ± 13.2 hours per week in the two weeks prior to data collection. Of respondents, the mean number of hours per week worked by clinical/staff nurses and was 34.1; the mean number of hours per week worked by non-clinical/staff nurses was 36.9. Slightly more than half of the respondents reported working 40 or more hours per week (51%; $n = 202$); 23% reported working 45 hours or more per week ($n = 89$) (see Table 3).

The average score (± SD) on the IWPS-R workload scale was 10.6 ± 3.3, with 28% of respondents reporting a workload score of 13 or higher, reflecting a moderate to high workload. Among respondents, 30% disagreed or strongly disagreed that their workload was reasonable. Twenty-seven percent (27%) reported missing an important change in a patient's condition because of workload, and 42% of respondents reported voicing concerns to a supervisor about the workload being too heavy. Twenty-six percent (26%) indicated that their workload will cause them to look for a new position. Regarding patient safety, 23% of respondents rated patient safety as excellent, 39% of respondents rated it as very good, and 24% rated it as good.

Health-Related Quality of Life and Psychological Well-Being

Responding nephrology nurses reported having very good or excellent health (45%) (see Table 4). The most frequent self-reported conditions included hypertension/high blood pressure (29%), anxiety (27%), sleep disturbances or insomnia (27%), and depression (25%). More respondents

Table 2
Workplace Characteristics

	N = 393
**Position, *n* (%)**	
Clinical/staff nurse	190 (48.3%)
Nurse manager/supervisor	86 (21.9%)
Educator	31 (7.9%)
Nurse practitioner	23 (5.9%)
Other	63 (16.0%)
**Primary Area of Practice[1], *n* (%)**	
Chronic outpatient hemodialysis	194 (49.4%)
Acute inpatient dialysis care	124 (31.6%)
Peritoneal dialysis	83 (21.1%)
Home hemodialysis	53 (13.5%)
Nursing education	22 (5.6%)
Pediatric nephrology	15 (3.8%)
Other	91 (23.2%)
**Region of Practice, *n* (%)**	
South	137 (34.9%)
Midwest	100 (25.4%)
Northeast	78 (19.8%)
West	76 (19.3%)
Other	2 (0.5%)
**Geographic Setting of Practice, n (%)**	
Urban	184 (46.8%)
Suburban	140 (35.6%)
Rural	63 (16.0%)
Prefer not to answer	6 (1.5%)

[1]More than one option could have been selected (i.e., responses were not mutually exclusive).

aged 65 years and older reported very good or excellent health (56%, $n = 25$) as compared to nurses less than 65 years of age (43%, $n = 151$).

Forty-seven percent (47%) of responding nephrology nurses reported symptoms of mild, moderate, or severe anxiety, and the mean (± SD) GAD-7 score was 5.4 ± 5.3 (considered mild anxiety). Symptoms associated with severe anxiety were reported by 8% of respondents (see Figure 2A). Twenty percent (20%) of survey respondents reported "feeling nervous, anxious, or on edge;" 21% reported "trouble relaxing;" and 22% reported "becoming easily annoyed/irritable" more than half the days or nearly every day in the two weeks prior to completing the survey. Thirty-eight percent (38%) of responding nephrology nurses reported "feeling afraid, as if something awful might happen" several days a week to nearly every day in the two weeks prior to completing the survey. Nearly one-third of respondents (32%) reported "being so restless it was hard to sit still" several days a week to nearly every day. Respondents less than 65 years of age were approximately twice as likely to have mild to severe anxiety compared to nurses aged 65 and over (50% vs. 27%). Non-White/Hispanic respondents were less likely to have at least mild anxiety based on the GAD-7 scale than non-Hispanic White respondents (39% vs. 50%).

The mean (± SD) PHQ-2 score was 1.2 ± 1.7, and 16% of respondents scored 3 or higher (i.e., likelihood of a major depressive episode) (see Figure 2B). Nearly one-fifth of non-Hispanic White nurses (19%) who responded reported symptoms compatible with a major depressive episode,

Table 3
Work Environment and Workload

	N = 393
**Current work schedule, *n* (%)**	
Days (majority of hours are before 6/7pm)	359 (91.3%)
Nights (majority of hours are after 6/7pm)	12 (3.1%)
Rotating schedule between days and nights	20 (5.1%)
Prefer not to answer	2 (0.5%)
**Number of hours worked per week[1], mean ± SD**	35.5 ± 13.2
< 20.0 hours, *n* (%)	39 (9.9%)
20.0 to 34.9 hours, *n* (%)	107 (27.2%)
35.0 to 39.9 hours, *n* (%)	42 (10.7%)
40.0 to 44.9 hours, *n* (%)	113 (28.8%)
≥ 45.0 hours, *n* (%)	89 (22.6%)
Prefer not to answer, *n* (%)	3 (0.8%)
**Absenteeism due to problems with physical or mental health[1,2], %, mean ± SD**	1.8% ± 10.5%
**Score on the IWPS Workload Scale[3], mean ± SD**	10.6 ± 3.3
**Rating of patient safety in unit/work area[4], mean ± SD**	3.7 ± 1.0
1 (poor), *n* (%)	7 (1.8%)
2 (fair), *n* (%)	45 (11.5%)
3 (good), *n* (%)	95 (24.2%)
4 (very good), *n* (%)	155 (39.4%)
5 (excellent), *n* (%)	91 (23.2%)

Notes: IWPS-R = Individual Workload Perception Scale – Revised, SD = standard deviation.

[1]Workplace characteristics were measured based on recollection of events from the previous 2 weeks at the time of data collection.

[2]Absenteeism was calculated as (weekly hours of work missed due to problems with physical or mental health)/[(weekly hours of work missed due to problems with physical or mental health) + (weekly hours worked)] and was evaluated for respondents who provided a value for both outcomes (*n* = 384).

[3]Scores range from 5 to 20 with higher scores indicating higher workloads. Source: Cox et al., 2003.

[4]Sorra et al., 2004.

while 4% of non-White/Hispanic nurses who responded reported the same.

Sixty-two percent (62%) of nephrology nurses who responded to the survey reported feeling burned out from work (see Table 4). Approximately one-quarter of respondents (23%) reported problems with their physical/mental health affecting work performance, and almost half (47%) reported worrying that work is hardening them emotionally. Two out of three respondents less than 65 years old reported feeling burned out from work, twice as high as the percentage of respondents 65 years and older (31%) who felt burned out.

Experiences During the COVID-19 Pandemic

Eighty-three percent (83%) of the survey respondents had patients with COVID-19 at their work facility, and 56% were responsible for the care of patients with COVID-19 at some time during the pandemic. Among those nurses responsible for the care of patients with COVID-19, 59% responded that the patient(s) they cared for had died following diagnosed or suspected COVID-19. Few respondents (4%) reported having been diagnosed with COVID-19 or having a suspected case of COVID-19. Of survey respondents, 62% were somewhat worried to very worried about COVID-19 (38% and 24%, respectively). Non-White/Hispanic nephrology nurses who responded more often (count) reported having been responsible for patients with COVID-19 than non-Hispanic White respondents (64% and 53%, respectively) and were almost twice as likely to report being very worried about COVID-19

Table 4
Health-Related Quality of Life and Psychological Well-Being by Age

	Overall *N* = 393	< 65 Years Old[1] *N* = 348	≥ 65 Years Old[1] *N* = 45
**Self-rated overall health, *n* (%)**			
Poor	4 (1.0%)	4 (1.1%)	0 (0.0%)
Fair	39 (9.9%)	38 (10.9%)	1 (2.2%)
Good	174 (44.3%)	155 (44.5%)	19 (42.2%)
Very good	138 (35.1%)	119 (34.2%)	19 (42.2%)
Excellent	38 (9.7%)	32 (9.2%)	6 (13.3%)
**Physical conditions[2], *n* (%)**			
Hypertension/high blood pressure	112 (28.5%)	96 (27.6%)	16 (35.6%)
Anxiety	107 (27.2%)	100 (28.7%)	7 (15.6%)
Sleep disturbances or insomnia	107 (27.2%)	100 (28.7%)	7 (15.6%)
Depression	100 (25.4%)	92 (26.4%)	8 (17.8%)
Headaches or migraines	80 (20.4%)	75 (21.6%)	5 (11.1%)
Substantial weight loss or gain (e.g., ≥ 10 pounds)	68 (17.3%)	66 (19.0%)	2 (4.4%)
Chronic respiratory disease (e.g., asthma, chronic lung disease)	42 (10.7%)	38 (10.9%)	4 (8.9%)
Diabetes	35 (8.9%)	29 (8.3%)	6 (13.3%)
Gastrointestinal symptoms (e.g., nausea, vomiting, diarrhea)	36 (9.2%)	35 (10.1%)	1 (2.2%)
Chronic fatigue syndrome	24 (6.1%)	24 (6.9%)	0 (0.0%)
Immunosuppressed condition (e.g., cancer)	14 (3.6%)	13 (3.7%)	1 (2.2%)
Other	64 (16.3%)	60 (17.2%)	4 (8.9%)
None	66 (16.8%)	53 (15.2%)	13 (28.9%)
**Score on the GAD-7 scale[3], mean ± SD**	5.4 ± 5.3	5.66 ± 5.4	3.20 ± 4.1
< 5 (no anxiety)	208 (52.9%)	175 (50.3%)	33 (73.3%)
5 to 9 (mild anxiety)	114 (29.0%)	107 (30.7%)	7 (15.6%)
10 to 14 (moderate anxiety)	40 (10.2%)	36 (10.3%)	4 (8.9%)
15 to 21 (severe anxiety)	31 (7.9%)	30 (8.6%)	1 (2.2%)

continued on next page

Table 4 (continued)
Health-Related Quality of Life and Psychological Well-Being by Age

	Overall *N* = 393	< 65 Years Old[1] *N* = 348	≥ 65 Years Old[1] *N* = 45
**Score on the PHQ-2 scale[4], mean ± SD [median]**	1.2 ± 1.7 [0.0]	1.2 ± 1.7 [0.0]	0.9 ± 1.5 [0.0]
≥ 3 (likelihood of major depressive episode), *n* (%)	63 (16.0%)	57 (16.4%)	6 (13.3%)
**Problems with physical/mental health affecting work performance[5], *n* (%)**			
Yes	90 (22.9%)	82 (23.6%)	8 (17.8%)
No	265 (67.4%)	232 (66.7%)	33 (73.3%)
Unsure	35 (8.9%)	31 (8.9%)	4 (8.9%)
Prefer not to answer	3 (0.8%)	3 (0.9%)	0 (0.0%)
**Feeling burned out from work[6], *n* (%)**			
Yes	245 (62.3%)	231 (66.4%)	14 (31.1%)
No	145 (36.9%)	114 (32.8%)	31 (68.9%)
Prefer not to answer	3 (0.8%)	3 (0.9%)	0 (0.0%)
**Worrying that work is hardening emotions[6], *n* (%)**			
Yes	184 (46.8%)	175 (50.3%)	9 (20.0%)
No	204 (51.9%)	168 (48.3%)	36 (80.0%)
Prefer not to answer	5 (1.3%)	5 (1.4%)	0 (0.0%)

Notes: GAD-7 = Generalized Anxiety Disorder-7, PHQ-2 = Patient Health Questionnaire-2, SD = standard deviation.
[1]Results were stratified based on respondents age at the time of data collection. No respondents were excluded from the stratification.
[2]More than one option could have been selected (i.e., responses were not mutually exclusive).
[3]GAD-7 items were measured based on recollection of events from the previous two weeks at the time of data collection. Source: Spitzer et al., 2006.
[4]PHQ-2 items were measured based on recollection of events from the previous two weeks at the time of data collection. Source: Kroenke et al., 2003.
[5]Problems with physical/mental health were measured based on recollection of events from the previous two weeks at the time of data collection.
[6]Items were measured based on recollection of events from the previous month at the time of data collection. Source: West et al., 2012.

Figure 2A
GAD-7 Scale Components

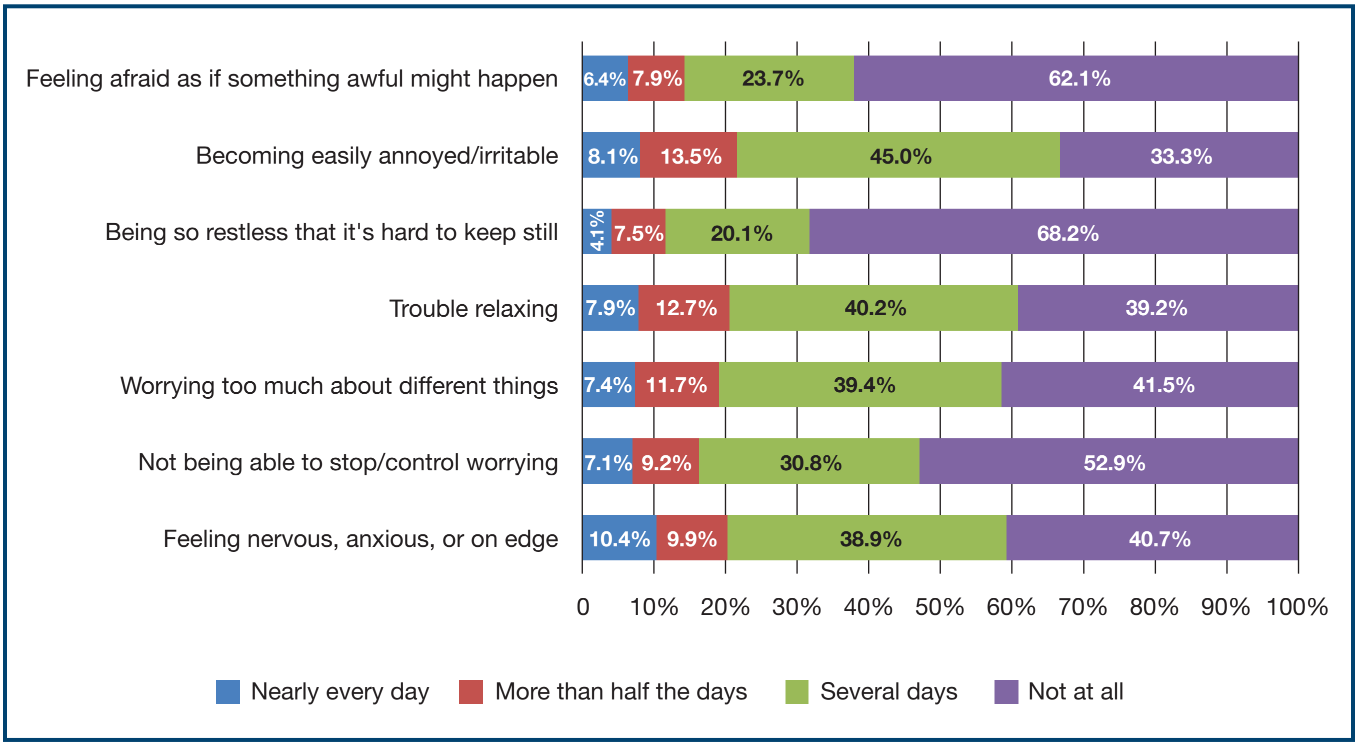

Notes: GAD-7 scale (Generalized Anxiety Disorder-7) items were measured based on recollection of events from the previous 2 weeks at the time of data collection.

Figure 2B
PHQ-2 Scale Components

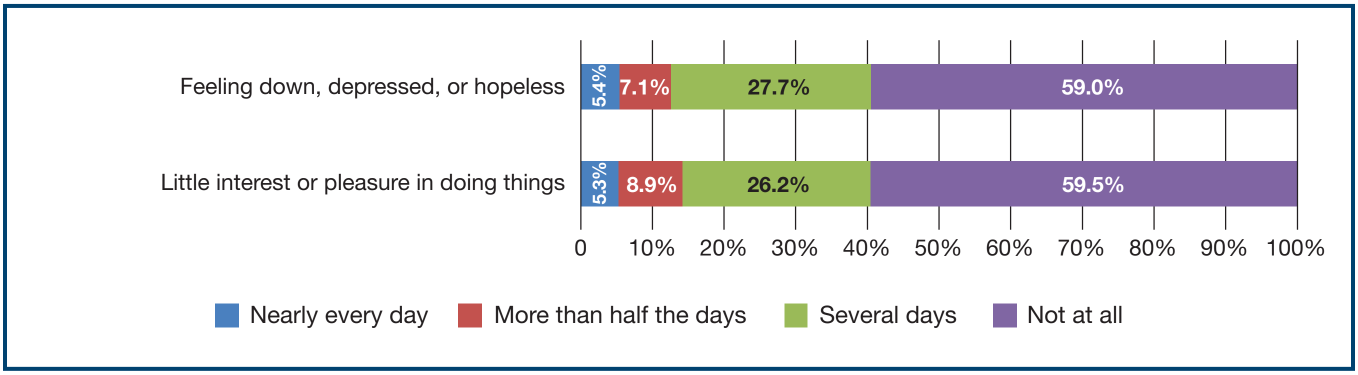

Notes: PHQ-2 scale (Patient Health Questionnaire-2) items were measured based on recollection of events from the previous 2 weeks at the time of data collection.

(39% and 20%, respectively). Respondents who practiced in the Northeast were responsible for the care of patients with COVID-19 more often than nurses in other regions (Northeast: 68%; South: 55%; Midwest: 56%; and West: 46%). Respondents in the Northeast were also more likely to report being very worried about COVID-19 (Northeast: 37%; South: 25%; Midwest: 13%; and West: 22%).

Factors Associated with Anxiety and Burnout from Work

In the regression analysis, workload was the main driver of anxiety among respondents (IWPS-R workload scale score 9 to 12: odds ratio [OR] = 2.73; $p = 0.001$; score ≥ 13: OR = 6.30; $p < 0.001$, relative to score 5 to 8) (see Table 5). Odds of anxiety were also higher among respondents who indicated that their position was educator (OR = 3.29; $p = 0.011$) or nurse manager/supervisor (OR = 1.96; $p = 0.035$) relative to those who identified as a clinical/staff nurse. Older age was associated with decreased odds of anxiety (OR = 0.92; $p < 0.001$). Odds of anxiety were higher among respondents who indicated being somewhat/very worried about COVID-19 (OR = 2.39; $p = 0.001$) or having at least one comorbidity associated with an increased risk for COVID-19 complications (OR = 1.78; $p = 0.024$).

Workload was also the main driver of feeling burned out from work among respondents (IWPS-R workload scale score 9 to 12: OR = 4.99; score ≥ 13: OR = 20.54; all $p < 0.001$, relative to score 5 to 8), while a very good or excellent rating of patient safety was associated with decreased odds of feeling burned out from work (OR = 0.53; $p = 0.033$) (see Table 5). The position of respondents predicted feeling burnout from work, with nurse managers and supervisors having increased odds of feeling burned out (OR = 2.35; $p = 0.020$) relative to clinical/staff nurse. Older age was associated with decreased odds of feeling burned out from work (OR = 0.95; $p = 0.002$). Odds of feeling burned out from work were increased for nurses responsible for the care of patients with COVID-19 (OR = 2.39; $p = 0.001$).

Discussion

Findings from this cross-sectional online survey underscore the high psychological burden experienced by nephrology nurses; nearly two-thirds (62%) of respondents reported feeling burned out from work, and about one of five (18%) reported moderate to severe anxiety. In addition, 51% of the nephrology nurses who responded reported working 40 or more hours per week; 23% reported working 45 hours or more per week. While this survey did not determine why respondents worked extra hours, it may be that employers required extra hours for some and that others chose to take on extra hours due to the COVID-19 pandemic. Factors related to the COVID-19 pandemic, along with other factors, such as age and race, may explain the high proportion of nurses who experienced anxiety, depression, and burnout.

Consistent with nurses' experiences globally, poor psychological well-being demonstrated in the current study is likely exacerbated by the position of nephrology nurses at the frontline of the COVID-19 pandemic (Luo et al., 2020; Marshall 2020; Pappa et al., 2020). Global prevalence of anxiety since the start of the COVID-19 pandemic among all health care providers has been estimated at about 26%, with substantial heterogeneity between studies (Luo et al., 2020; Pappa et al., 2020). Likewise, rates of depression have also been elevated to about 25%, with substantial heterogeneity between studies (Luo et al., 2020; Pappa et al., 2020). Nurses have been particularly affected by increased psychological stress during the pandemic, with a 30% to 63% prevalence of anxiety (Lai et al., 2020; Luo et al., 2020; Pappa et al., 2020; Thiete et al., 2021). More than half of the nephrology nurses participating in this study reported caring for COVID-19 patients (56%), and 59% of these nurses reported caring for COVID-19 patients who died from this illness. In a survey administered by the American Nurses Foundation (ANF) and the American Nurse Association (ANA) between March 20 to April 10, 2020 (N = 32,174 nurses), 87% of respondents indicated that they were somewhat or very concerned or afraid to come to work because of the COVID-19 pandemic (ANF & ANA, 2020). Concerns commonly centered around adequate training, appropriate facilities, available personal protective equipment, and abilities to provide care to vulnerable patients. In this study, nearly two-thirds of nephrology nurses responded that they were somewhat or very worried about COVID-19. Worry about COVID-19 as well as conditions associated with an increased risk for COVID-19 complications were both associated with increased anxiety in this study. Challenges related to the COVID-19 pandemic changed rapidly throughout 2020. Differences in responses between the current survey and the ANF and the ANA survey may be due to the relationship between the timing of each survey and changing perceptions of COVID-19 risk, as well as the populations that were assessed. These experiences, coupled with the routine psychological burden of caring for a complex and medically fragile population of patients (Marshall, 2020), have contributed to the reported symptoms of anxiety (47% with GAD-7 scores of 5 or higher), major depressive episodes (16% with PHQ-2 scores of 3 or higher), and feelings of burnout from work (62%) seen in this study.

Minority populations and minority health care workers are disproportionately affected by COVID-19 (Nguyen et al., 2020; Rentsch et al., 2020). Non-White/Hispanic nurses in the United States may have an increased awareness of COVID-19 incidence and mortality, which is likely reflected in the racial differences in COVID-19 concern seen in this study. Not only were non-White/Hispanic nephrology nurses almost twice as likely to report being very worried about COVID-19 than non-Hispanic White nurses, but they were also more commonly responsible for the care of patients with COVID-19. Similar results have been seen in another study recently completed (C. Thomas-Hawkins, personal

Table 5
Factors Associated with Anxiety and Feeling Burned Out from Work

	Anxiety[1]			Feeling Burned Out from Work[2]		
**Number of respondents[3]**	***N* = 384**			***N* = 384**		
**Number of respondents with the outcome, *n* (%)**	**177 (46.1%)**			**233 (60.7%)**		
**Covariates[4]**	**OR[4]**	**[95% CI]**	***p*-Value**	**OR[5]**	**[95% CI]**	***p*-Value**
**Demographics and workplace characteristics**						
Age	0.92	[0.90, 0.94]	<0.001*	0.95	[0.92, 0.98]	0.002*
Male (ref.: female)	0.72	[0.30, 1.75]	0.471	1.70	[0.61, 4.70]	0.307
Non-White/Hispanic (ref.: non-Hispanic White)	0.41	[0.22, 0.75]	0.004*	0.80	[0.41, 1.56]	0.516
Technical position (ref.: clinical/staff nurse)						
Nurse manager/supervisor	1.96	[1.05, 3.67]	0.035*	2.35	[1.14, 4.84]	0.020*
Educator	3.29	[1.32, 8.24]	0.011*	1.37	[0.61, 3.07]	0.439
Nurse practitioner	1.94	[0.73, 5.17]	0.185	1.74	[0.65, 4.61]	0.269
Other[6]	2.03	[0.99, 4.18]	0.053	1.64	[0.77, 3.49]	0.203
Score on the IWPS-R workload scale (ref.: 5–8)[7]						
9 to 12	2.73	[1.47, 5.07]	0.001*	4.99	[2.71, 9.21]	<0.001*
≥ 13	6.30	[3.05, 12.99]	<0.001*	20.54	[8.60, 49.06]	<0.001*
Very good/excellent rating of patient safety in unit/work area (ref.: poor/fair/good)[8]	0.71	[0.42, 1.22]	0.216	0.53	[0.30, 0.95]	0.033*
**Experiences with COVID-19**						
At least one comorbidity associated with an increased risk for COVID-19 complications[9]	1.78	[1.08, 2.93]	0.024*	1.61	[0.95, 2.72]	0.079
Somewhat/very worried about COVID-19 (ref.: not at all/a little worried)[10]	2.39	[1.45, 3.95]	0.001*	1.62	[0.94, 2.78]	0.081
Responsible for the care of patients with COVID-19 at any time	1.16	[0.70, 1.91]	0.557	2.39	[1.41, 4.08]	0.001*

Notes appear on next page.

Table 5 (continued)
Factors Associated with Anxiety and Feeling Burned Out from Work

Notes: CI = confidence interval, COVID-19 = coronavirus disease 2019, GAD-7 = Generalized Anxiety Disorder-7, IWPS-R = Individual Workload Perception Scale – Revised, OR = odds ratio, ref = reference.
*Significant at the 5% level.

[1]Respondents were considered to have anxiety symptoms if they had a score ≥5 on the GAD-7. Source: Kroenke et al., 2006.

[2]Feeling burned out from work was self-reported by the respondent based on recollection of events from the previous month at the time of data collection. Source: West et al., 2012.

[3]Respondents who did not provide their race or gender were excluded for the regression analyses (n = 9).

[4]The following covariates were assessed but were not included in the final regression models as they were not statistically significantly associated with either outcome: region, geographical setting (urban/suburban/rural), household composition (children, adults ≥ 65 years old), work schedule (i.e., day/night/rotating), number of hours worked per week, and being responsible for patients who died from COVID-19. The following interactions were also found to be insignificant: level of worry about COVID-19 and household composition, and being responsible for the care of patients with COVID-19 and household composition.

[5]ORs estimated from a logistic regression. An OR greater than 1 indicates a higher odds of having the outcome.

[6]This category includes "Administrator," "Case manager," "Critical nurse specialist," "Researcher," and "Other."

[7]Scores range from 5 to 20, with a higher score indicating a higher workload. Source: Cox et al., 2003.

[8]Source: Sorra et al., 2004.

[9]Comorbidities that were considered as associated with an increased risk for COVID-19 complications included chronic respiratory disease (e.g., asthma, chronic lung disease), hypertension/high blood pressure, diabetes, immunosuppressed condition (e.g., cancer).

[10]The reference category also includes respondents who responded "Prefer not to answer" (n = 2).

communication, 2020). This higher level of worry among non-White/Hispanic nurses may also reflect personal worry for themselves and their families.

In this study, nephrology nurses who practiced in the Northeast were more commonly responsible for the care of patients with COVID-19 and more likely to report being very worried about COVID-19 compared with nurses in other regions. Although many factors may influence the impact of COVID-19 on nephrology nurses, Northeastern states, particularly New York, were experiencing a high burden of COVID-19 mortality around the time of this survey. The increased concern among nephrology nurses may not be surprising given that these nurses were positioned on the frontlines of a pandemic hotspot and were likely concerned that their exposure to COVID-19 increased the risk of transmission to themselves and family members at home. Best practices for COVID-19 treatment evolved as the pandemic affected different regions of the United States. Respondents working in regions initially affected by COVID-19, such as Washington state or New York, may have had fewer treatment options for rapidly deteriorating patients, further contributing to the level of worry associated with COVID-19. In addition, variations in state-level COVID-19 responses in terms of stay-at-home orders and closing of non-essential services, as well as local regulations concerning wearing masks in public places, restrictions on indoor activities (e.g., indoor dining), and social distancing, may have resulted in regional discrepancies that affected the degree of worry.

In a Belgian study of health care workers conducted during the COVID-19 pandemic, nurses were significantly more likely to have burnout, insomnia, and anxiety than physicians, as determined by a multivariate analysis (Thiete et al., 2021). However, whether the facility was a center dedicated to treating COVID-19 was not associated with an increased risk of burnout, insomnia, and anxiety, suggesting that being a nurse had a greater impact on these outcomes than working at a COVID-19 center. While this has not been demonstrated in other studies (Lai et al., 2020; Lu et al., 2020), data from Belgium highlight the vulnerability of nurses to decreased psychological well-being and health-related quality of life.

In the present study, workload was the main driver for feeling burned out from work and experiencing anxiety, while position and younger age were also strong predictors. By contrast, a very good or excellent rating of patient safety decreased the odds of feeling burned out. Similarly, Flynn and colleagues (2009) have reported that nurses with the highest workloads were five times as likely to be burned out compared to nurses with the lowest workloads. In addition, nurses who reported leaving three or more necessary patient care activities undone at the end of a shift were more than twice as likely to be burned out than their colleagues who reported completing all necessary patient care activities (Flynn et at., 2009). Using system dynamics modeling to quantify and understand the effects of nursing workload on nurse burnout, absenteeism, and quality of

patient care, Farid and colleagues (2020) also demonstrated that medical errors increase with nurses' feelings of burnout, longer work shifts and workweeks, and mandatory overtime. This, in turn, can lead to health problems for nurses and safety risks for patients (Farid et al., 2020). Twelve-hour shifts doubled the burnout rate of nurses and increased medical errors by 50% compared to 8-hour shifts (Farid et al., 2020). In a study of 104 staff nurses in hemodialysis units, 40% of respondents reported a high workload (registered nurse [RN] workload score of 13 or higher), and 34% reported three or more care activities left undone (Thomas-Hawkins et al., 2020). Low RN staffing, high RN workloads, and RN nursing care left undone were key contributors to unsafe patient shift change periods and lower overall safety ratings in hemodialysis facilities (Thomas-Hawkins et al., 2020). While shift length was not assessed in the current survey, data that link long shifts to feelings of burnout and medical errors are particularly concerning given that shifts for nephrology nurses often last up to 12 hours in both in-hospital and outpatient clinics and sometimes up to 24 hours with additional mandatory on-call hours. Factors that have also been previously associated with job stress and burnout among nephrology nurses include difficult interpersonal relationships with physicians, difficult facets of patient care, and lack of access to ongoing education (Flynn et at., 2009; Hayes & Bonner, 2010).

Multiple strategies at the personal and organizational level have been proposed to help address high levels of anxiety and burnout among health care providers (National Academies of Sciences, Engineering, and Medicine, 2018; National Academy of Medicine, 2021). Increased levels of personal resilience, organizational support, and social support are associated with decreased levels of burnout, compassion fatigue, anxiety, depression, and psychological distress (Labrague et al., 2020; Mealer et al., 2017). Our findings point to the need for nephrology nurses to consider the long-term benefits of protecting their mental health through intentional strategies, such as self-care, group resiliency rounds, and counselling. It has previously been shown that nephrology nurses successfully employ personal coping techniques, such as planful problem solving, self-controlling, positive reappraisal, and social support, to address difficult situations (Ashker et al., 2012). Self-care among nurses has such important links to optimal care for patients, that the ANA Code of Ethics for Nurses explicitly states that nurses must adopt self-care as an ethical obligation to self in addition to their duty to provide care to patients (Linton & Koonmen, 2020). Sampson and colleagues (2020) have demonstrated that training newly licensed RNs to use mindfulness techniques resulted in an immediate significant improvement in depressive symptoms and job satisfaction, as well as sustained positive effects on stress, anxiety, depressive symptoms, job satisfaction, and healthy lifestyle behaviors. Mindfulness strategies/techniques to reduce burnout and protect the mental health of health care workers may be helpful for nephrology nurses, and organizations may want to assess whether inclusion of these programs would be helpful (Penque, 2019). Healthy work environments have been associated with positive patient and nurse outcomes (Ulrich et al., 2019), and increased organizational support for these techniques may help provide a healthy work environment, which can decrease factors related to burnout and improve psychological health. In addition, increasing staff within mental health management systems, revising policies (i.e., rules and regulations around health management, and a new employee support system), implementation of screening tools, expanding counselling opportunities, setting up collaborative meetings with staff and faculty, and starting a mental health awareness campaigns (National Academies of Sciences, Engineering, and Medicine, 2018).

Limitations

The study included a relatively small sample size of members of ANNA who agreed to complete an online survey. As such, it may not be representative of all the members of ANNA or the entire population of nephrology nurses in the United States. Additionally, situations during the COVID-19 pandemic have been rapidly evolving throughout 2020 and 2021. Survey results may have been impacted by the timing of the study (July 24, 2020 to August 17, 2020) in relation to the COVID-19 pandemic in the United States and may not be generalizable to the current situation or to the situation in other countries. Another limitation common among survey questionnaires that may also apply to the current study is the reliance on respondents' recollection of past events. Recall bias, or errors in the accuracy or completeness of respondents' recalled experiences, can be an issue, particularly if past memories are influenced by more recent events. The survey aimed to minimize recall bias by asking respondents to recall events that occurred in the recent past, when possible. Finally, this study was observational in nature; therefore, no causal inference can be drawn.

Conclusion

This study describes clinically relevant insights about the effect of demanding workplace environments on the physical and psychological well-being of nephrology nurses who are members of ANNA. It also provides a timely update about how the COVID-19 pandemic has contributed to and even worsened the symptoms of burnout, anxiety, and depression experienced by nephrology nurses.

Nephrology nurses face major challenges related to their psychological well-being and health-related quality of life. Workplace issues that existed prior to the pandemic may have been exacerbated by COVID-19, including demanding workloads, as well as physical and emotional stress. The present survey also revealed that anxiety and burnout stand out as two key issues nephrology nurses commonly face. The nephrology community must come together to develop additional tools and resources to support the mental health of nephrology nurses in the work-

place. A multidimensional approach is needed to provide evidence-based strategies aimed at improving the mental well-being of nephrology nurses. Individual clinicians, health care administrators, and those influencing policy change have an obligation to systematically address the key factors that lead to increased rates of nephrology nurse burnout, depression, anxiety, and stress.

References

American Nurses Foundation (ANF) & American Nurses Association (ANA). (2020). *COVID-19 survey results*. https://www.nursingworld.org/practice-policy/work-environment/health-safety/disaster-preparedness/coronavirus/what-you-need-to-know/covid-19-survey-results/

Ashker, V.E., Penprase, B., & Salman, A. (2012). Work-related emotional stressors and coping strategies that affect the well-being of nurses working in hemodialysis units. *Nephrology Nursing Journal, 39*(3), 231-236.

Bednar, B., & Latham, C. (2014). The changing landscape of the nephrology nursing care environment in the United States over the last 45 years. *Nephrology Nursing Journal, 41*(2), 183-191.

Cox, K. (2003). *Individual Workload Perception Scale user's manual.* Children's Mercy Hospitals and Clinics.

Farid, M., Purdy, N., & Neumann, W.P. (2020). Using system dynamics modelling to show the effect of nurse workload on nurses' health and quality of care. *Ergonomics, 63*(8), 952-964. https://doi.org/10.1080/00140139.2019.1690674

Flynn, L., Thomas-Hawkins, C., & Clarke, S.P. (2009). Organizational traits, care processes, and burnout among chronic hemodialysis nurses. *Western Journal of Nursing Research, 31*(5), 569-582. https://doi.org/10.1177/0193945909331430

Gaietto, K.J., & Brooks, M.V. (2019). The shortage of expert nephrology nurses in hemodialysis: A literature review. *Nephrology Nursing Journal, 46*(6), 577-585.

Gardner, J.K., Thomas-Hawkins, C., Fogg, L., & Latham, C.E. (2007). The relationships between nurses' perceptions of the hemodialysis unit work environment and nurse turnover, patient satisfaction, and hospitalizations. *Nephrology Nursing Journal, 34*(3), 271-282.

Hayes, B., & Bonner, A. (2010). Job satisfaction, stress and burnout associated with haemodialysis nursing: a review of literature. *Journal of Renal Care, 36*(4), 174-179. https://doi.org/10.1111/j.1755-6686.2010.00194.x

Kapucu, S.S., Akku , Y., Akdemir, N., & Karacan, Y. (2009). The burnout and exhaustion levels of nurses working in haemodialysis units. *Journal of Renal Care, 35*(3), 134-140. https://doi.org/10.1111/j.1755-6686.2009.00108.x

Kroenke, K., Spitzer, R.L., & Williams, J.B. (2003). The Patient Health Questionnaire-2: Validity of a two-item depression screener. *Medical Care, 41*(11), 1284-1292. https://doi.org/10.1097/01.MLR.0000093487.78664.3C

Labrague, L.J., & De Los Santos, J. (2020). COVID-19 anxiety among front-line nurses: Predictive role of organisational support, personal resilience and social support. *Journal of Nursing Management, 28*(7), 1653-1661. https://doi.org/10.1111/jonm.13121

Lai, J., Ma, S., Wang, Y., Cai, Z., Hu, J., Wei, N., Wu, J., Du, H., Chen, T., Li, R., Tan, H., Kang, L., Yao, L., Huang, M., Wang, H., Wang, G., Liu, Z., & Hu, S. (2020). Factors associated with mental health outcomes among health care workers exposed to coronavirus disease 2019. *JAMA Network Open, 3*(3), e203976. https://doi.org/10.1001/jamanetworkopen.2020.3976

Li, H., Xie, L., Yang, J., & Pang, X. (2018). Symptom burden amongst patients suffering from end-stage renal disease and receiving dialysis: A literature review. *International Journal of Nursing Sciences, 5*(4), 427-431. https://doi.org/10.1016/j.ijnss.2018.09.010

Linton, M., & Koonmen, J. (2020). Self-care as an ethical obligation for nurses. Nursing ethics, 969733020940371. *Nursing Ethics, 27*(8), 1684-1702. https://doi.org/10.1177/0969733020940371

Lu, W., Wang, H., Lin, Y., & Li, L. (2020). Psychological status of medical workforce during the COVID-19 pandemic: A cross-sectional study. *Psychiatry Research, 288*, 112936.

Luo, M., Guo, L., Yu, M., Jiang, W., & Wang, H. (2020). The psychological and mental impact of coronavirus disease 2019 (COVID-19) on medical staff and general public – A systematic review and meta-analysis. *Psychiatry Research, 291*, 113190. https://doi.org/10.1016/j.psychres.2020.113190

Marshall, B. (2020). Impact of COVID-19 on nurses' mental health. *Issues in Mental Health Nursing, 41*(10), 853-854. https://doi.org/10.1080/01612840.2020.1819083

Mealer, M., Jones, J., & Meek, P. (2017). Factors affecting resilience and development of posttraumatic stress disorder in critical care nurses. *American Journal of Critical Care, 26*(3), 184-192. https://doi.org/10.4037/ajcc2017798

Meguid El Nahas, A., & Bello, A.K. (2005). Chronic kidney disease: the global challenge. *Lancet (London, England), 365*(9456), 331-340. https://doi.org/10.1016/S0140-6736(05)17789-7

Mehrotra, R., Shaffer, R.N., & Molitoris, B.A. (2011). Implications of a nephrology workforce shortage for dialysis patient care. *Seminars in Dialysis, 24*(3), 275-277. https://doi.org/10.1111/j.1525-139X.2011.00933.x

National Academies of Sciences, Engineering, and Medicine; Health and Medicine Division; Board on Global Health; Global Forum on Innovation in Health Professional Education. (2018). Appendix B, The importance of well-being in the health care workforce. In E.H. Forstag, & P.A. Cuff (Eds.), *A design thinking, systems approach to well-being within education and practice: Proceedings of a workshop* (pp. 61-74). National Academies Press (US). https://www.ncbi.nlm.nih.gov/books/NBK540859/

National Academy of Medicine. (2021). *Clinician well-being knowledge hub*. https://nam.edu/clinicianwellbeing/causes/

Neild, G.H. (2017). Life expectancy with chronic kidney disease: An educational review. *Pediatric Nephrology (Berlin, Germany), 32*(2), 243-248. https://doi.org/10.1007/s00467-016-3383-8

Neto, M., Almeida, H.G., Esmeraldo, J.D., Nobre, C.B., Pinheiro, W.R., de Oliveira, C., Sousa, I., Lima, O., Lima, N., Moreira, M.M., Lima, C., Júnior, J.G., & da Silva, C. (2020). When health professionals look death in the eye: The mental health of professionals who deal daily with the 2019 coronavirus outbreak. *Psychiatry Research, 288*, 112972. https://doi.org/10.1016/j.psychres.2020.112972

Nguyen, L.H., Drew, D.A., Graham, M.S., Joshi, A.D., Guo, C.G., Ma, W., Mehta, R.S., Warner, E.T., Sikavi, D.R., Lo, C.H., Kwon, S., Song, M., Mucci, L.A., Stampfer, M.J., Willett, W.C., Eliassen, A.H., Hart, J.E., Chavarro, J.E., Rich-Edwards, J.W., Davies, R., … Coronavirus Pandemic

Epidemiology Consortium (2020). Risk of COVID-19 among front-line health-care workers and the general community: a prospective cohort study. *The Lancet. Public Health, 5*(9), e475-e483. https://doi.org/10.1016/S2468-2667(20)30164-X

Pappa, S., Ntella, V., Giannakas, T., Giannakoulis, V.G., Papoutsi, E., & Katsaounou, P. (2020). Prevalence of depression, anxiety, and insomnia among healthcare workers during the COVID-19 pandemic: A systematic review and meta-analysis. *Brain, Behavior, and Immunity, 88*, 901-907. https://doi.org/10.1016/j.bbi.2020.05.026

Penque S. (2019). Mindfulness to promote nurses' well-being. *Nursing Management, 50*(5), 38-44. https://doi.org/10.1097/01.NUMA.0000557621.42684.c4

Rentsch, C.T., Kidwai-Khan, F., Tate, J.P., Park, L.S., King, J.T., Jr., Skanderson, M., Hauser, R.G., Schultze, A., Jarvis, C.I., Holodniy, M., Re, V.L., 3rd, Akgün, K.M., Crothers, K., Taddei, T.H., Freiberg, M.S., & Justice, A.C. (2020). Covid-19 by race and ethnicity: A national cohort study of 6 million United States veterans. *medRxiv,* 2020.05.12.20099135. https://doi.org/10.1101/2020.05.12.20099135

Sampson, M., Melnyk, B.M., & Hoying, J. (2020). The MIND-BODYSTRONG Intervention for new nurse residents: 6-month effects on mental health outcomes, healthy lifestyle behaviors, and job satisfaction. *Worldviews on Evidence-Based Nursing, 17*(1), 16-23. https://doi.org/10.1111/wvn.12411

Sharif, M.U., Elsayed, M.E., & Stack, A.G. (2016). The global nephrology workforce: emerging threats and potential solutions! *Clinical Kidney Journal, 9*(1), 11-22. https://doi.org/10.1093/ckj/sfv111

Sorra, J., Gray, L., Streagle, S., Famolaro, T., Yount, N., & Behm, J. (2016). *AHRQ hospital survey on patient safety culture: User's guide.* Agency for Healthcare Research and Quality.

Spitzer, R.L., Kroenke, K., Williams, J.B., & Löwe, B. (2006). A brief measure for assessing generalized anxiety disorder: the GAD-7. *Archives of Internal Medicine, 166*(10), 1092-1097.

Tiete, J., Guatteri, M., Lachaux, A., Matossian, A., Hougardy, J.M., Loas, G., & Rotsaert, M. (2021). Mental health outcomes in healthcare workers in COVID-19 and non-COVID-19 care units: A cross-sectional survey in Belgium. *Frontiers in Psychology, 11*, 612241. https://doi.org/10.3389/fpsyg.2020.612241

Thomas-Hawkins, C., Flynn, L., & Dillon, J. (2020). Registered nurse staffing, workload, and nursing care left undone, and their relationships to patient safety in hemodialysis units. *Nephrology Nursing Journal, 47*(2), 133-142. https://doi.org/10.37526/1526-744X.2020.47.2.133

Thompson, S., James, M., Wiebe, N., Hemmelgarn, B., Manns, B., Klarenbach, S., Tonelli, M., & Alberta Kidney Disease Network (2015). Cause of death in patients with reduced kidney function. *Journal of the American Society of Nephrology, 26*(10), 2504-2511. https://doi.org/10.1681/ASN.2014070714

Ulrich, B., Barden, C., Cassidy, L., & Varn-Davis, N. (2019). Critical care nurse work environments 2018: Findings and implications. *Critical Care Nurse, 39*(2), 67-84. https://doi.org/10.4037/ccn2019605

Ulrich B.T., & Kear T.M. (2018). The health and safety of nephrology nurses and the environments in which they work: Important for nurses, patients, and organizations. *Nephrology Nursing Journal, 45*(2), 117-139, 168.

West, C.P., Dyrbye, L.N., Satele, D.V., Sloan, J.A., & Shanafelt, T.D. (2012). Concurrent validity of single-item measures of emotional exhaustion and depersonalization in burnout assessment. *Journal of General Internal Medicine*, 27(11), 1445-1452. https://doi.org/10.1007/s11606-012-2015-7

Wolfe, WA. (2014). Are word-of-mouth communications contributing to a shortage of nephrology nurses? *Nephrology Nursing Journal, 41*(4), 371-376, quiz 377.

Katie Donnini disclosed that she is an employee of Otsuka.

Myrlene Sanon disclosed that she was an employee of Otsuka at the time of the study.

Marjolaine Gauthier-Loiselle, Arthur Voegel, Martin Cloutier, and Jessica Maitland disclosed that they are employees of Analysis Group, Inc., a consulting company that provided paid consulting services to Otsuka.

Mark Hoffmann, Samuel Savin, Alissa Kurzman disclosed that they are employees of High Lantern Group, a consulting company that provided paid consulting services to Otsuka.
