# Supplementary material for: Mental health status and related factors influencing healthcare workers during the COVID-19 pandemic: A systematic review and meta-analysis
Source: PLoS One. 2024 Jan 19;19(1):e0289454. doi: 10.1371/journal.pone.0289454 (PMC10798549; doi:10.1371/journal.pone.0289454)
Supplement: S1 Data — (ZIP) [file pone.0289454.s011.zip › literatures/105.pdf]

ELSEVIER

Since January 2020 Elsevier has created a COVID-19 resource centre with free information in English and Mandarin on the novel coronavirus COVID-19. The COVID-19 resource centre is hosted on Elsevier Connect, the company's public news and information website.

Elsevier hereby grants permission to make all its COVID-19-related research that is available on the COVID-19 resource centre - including this research content - immediately available in PubMed Central and other publicly funded repositories, such as the WHO COVID database with rights for unrestricted research re-use and analyses in any form or by any means with acknowledgement of the original source. These permissions are granted for free by Elsevier for as long as the COVID-19 resource centre remains active.

Journal of Psychosomatic Research 144 (2021) 110415

Contents lists available at ScienceDirect

Journal of Psychosomatic Research

journal homepage: www.elsevier.com/locate/jpsychores

Psychosocial burden and working conditions during the COVID-19 pandemic in Germany: The VOICE survey among 3678 health care workers in hospitals

Eva Morawa [a,*], Caterina Schug [a], Franziska Geiser [b], Petra Beschoner [c], Lucia Jerg-Bretzke [c], Christian Albus [d], Kerstin Weidner [e], Nina Hiebel [b], Andrea Borho [a], Yesim Erim [a]

[a] Department of Psychosomatic Medicine and Psychotherapy, University Hospital of Erlangen, Friedrich-Alexander University Erlangen-Nürnberg (FAU), Erlangen, Germany
[b] Department of Psychosomatic Medicine and Psychotherapy, University Clinic of Bonn, Bonn, Germany
[c] Department of Psychosomatic Medicine and Psychotherapy, Ulm University Medical Center, Ulm, Germany
[d] Department of Psychosomatics and Psychotherapy, University Hospital of Cologne, Cologne, Germany
[e] Department of Psychotherapy and Psychosomatic Medicine, Faculty of Medicine, Technische Universität Dresden, Dresden, Germany

A R T I C L E I N F O

Keywords:
Anxiety
COVID-19
Depression
Health care
Mental health
Working conditions

A B S T R A C T

Objective: The aim of this cross-sectional web-based study was to examine self-reported mental distress, psychosocial burdens, working conditions and potential risk and protective factors for depressive and anxiety symptoms during the COVID-19 pandemic in health care workers (HCW).

Methods: In the largest survey on mental health of HCW conducted during the first wave of COVID-19 in Europe (N = 8071 HCW), we investigated depressive (Patient Health Questionnaire-2, PHQ-2), and anxiety symptoms (Generalized Anxiety Disorder-2, GAD-2), working conditions, and psychosocial burden of 3678 HCW of three health care professions in hospitals: physicians (n = 1061), nurses (n = 1275), and medical technical assistants (MTA, n = 1342).

Results: The prevalence of clinically significant levels of depressive and anxiety symptoms was 17.4% and 17.8% for physicians, 21.6% and 19.0% for nurses, and 23.0% and 20.1% for MTA, respectively. All three professions demonstrated significantly elevated PHQ-2 and GAD-2 scores, when compared with general German population before the pandemic, but lower scores in relation to that during the pandemic. Multiple linear regression analyses revealed that higher levels of depressive symptoms were associated with insufficient recovery during leisure time, increased alcohol consumption, and less trust in colleagues in difficult situations at work. In addition, elevated anxiety scores were related to increased fear of becoming infected with COVID-19.

Conclusion: During the pandemic HCW demonstrated a lower burden of mental distress compared to the general population. Nevertheless, a high percentage of HCW demonstrates psychosocial distress, so that the establishment of regular mental health screening and prevention programmes for HCW is indicated.

1. Introduction

The outbreak of the coronavirus SARS-CoV-2 has had a significant impact on the social lives and wellbeing of the population [1,2], and especially the health care workers (HCW) who are directly confronted with issues of potential infection. It is known from previous epidemics of SARS and Ebola-virus and the novel coronavirus SARS-CoV-2 that these conditions can lead to detrimental mental health outcomes such as post-traumatic, depressive and anxiety symptoms in HCW [3,4]. In a systematic review about the prevalence of mental symptoms among HCW in Asia during the COVID-19 pandemic, a pooled prevalence of 23.2% for anxiety, 22.8% for depression, and 38.9% for insomnia were

* Corresponding author at: Department of Psychosomatic Medicine and Psychotherapy, University Hospital Erlangen, Schwabachanlage 6, 91054 Erlangen, Germany.

E-mail addresses: eva.morawa@uk-erlangen.de (E. Morawa), caterina.schug@uk-erlangen.de (C. Schug), franziska.geiser@ukbonn.de (F. Geiser), petra.beschoner@uniklinik-ulm.de (P. Beschoner), lucia.bretzke@uni-ulm.de (L. Jerg-Bretzke), christian.albus@uk-koeln.de (C. Albus), kerstin.weidner@uniklinikum-dresden.de (K. Weidner), nina.hiebel@ukbonn.de (N. Hiebel), andrea.borho@uk-erlangen.de (A. Borho), yesim.erim@uk-erlangen.de (Y. Erim).

https://doi.org/10.1016/j.jpsychores.2021.110415
Received 17 November 2020; Received in revised form 25 February 2021; Accepted 8 March 2021
Available online 10 March 2021
0022-3999/© 2021 Elsevier Inc. All rights reserved.

reported [5]. A meta-analysis that included 62 studies from 17 countries (10 from Europe) found only slightly higher prevalence of anxiety (26%) and depression (25%) among HCW during the COVID-19 pandemic [6].

Only few studies reported about the European circumstances, in Spain [7], Turkey [8,9], Norway [10], Serbia [11] and France [12], respectively. From Germany Paffenholz et al. [13] stated that 78.9% of HCW felt at least moderately threatened by COVID-19. Only two studies from Germany have employed validated anxiety or depression questionnaires in HCW. In a study conducted between 10 and 31 march 2020, Skoda et al. [14] observed at least moderate generalized anxiety symptoms (GAD-7 $\geq$ 10) in 5.89% of physicians (n = 492) and 11.41% of nursing staff (n = 1511).

Two of the most commonly and consistently reported risk factors of increased mental burden are female gender [5,6,15,16] and being a nurse (in comparison with physicians) [5,6,15]. Working on the frontline with direct contact with patients infected with COVID-19 was shown to be a correlate for adverse mental health outcomes among HCW [16]; however, the opposite finding has also been demonstrated, e.g., Li et al. [17]. Previous research also suggests that worry about becoming infected or about infecting others is a common mental health problem [15,16].

To sum up, previous meta-analyses on the mental health outcomes of HCW during the pandemic included only [5], or mainly, Asian studies, and there have been few European studies so far [4,6]. Although several smaller surveys have been conducted in HCW, published data from large samples and validated symptom questionnaires are still missing. Thus, the central objective of this study was to explore the working conditions, psychosocial burden, and mental health of HCW in a large German sample taking into account profession-related differences. The analyses of our survey have been conducted in the scope of the newly set-up cooperation network of university medicine by the German Ministry of Education and Research (BMBF). The objective of this national network is to develop scalable and evidence based interventions in university hospitals and hospitals of maximum care. Therefore this article focuses on the sample of HCW in university hospitals and hospitals of maximum care. Since the available scientific literature has so far focused on hospital settings, the choice of this sample also allows for extensive comparability.

The aims of the present study were:

1. To explore the symptom severity and frequency of clinically significant levels of depressive and anxiety symptoms among physicians, nurses, and medical technical assistants (MTA) and to analyze the differences between the three professions (also in comparison with the German general population before and during the pandemic).
2. To examine the frequency of COVID-19-related psychosocial problems and the assessment of the working conditions by the three professions.
3. To detect risk and protective factors for depressive and anxiety symptoms (for the total sample and stratified for profession).

2. Method

2.1. Statement of Ethics

The present study was approved by the Ethics Committee of the Medical Faculty of the Friedrich-Alexander University Erlangen-Nürnberg (FAU) and registered on ClinicalTrials (DRKS-ID: DRKS00021268). All respondents provided their online informed consent.

2.2. Data collection

The online survey was conducted between April 20 and July 5, 2020 by the psychosomatic departments of the university hospitals of Erlangen, Bonn, Ulm, Cologne, and Dresden and was shared via online platforms or mailing lists. Further hospitals and various professional associations and online platforms promoted participation in the survey. In Fig. 1 the data collection period is presented in the context of the development of the COVID-19 pandemic situation in Germany in terms of the total number of infected, dead and recovered persons and regarding the difference of cases of infection in comparison to the previous week, respectively (based on the data of the Robert Koch Institut [18]).

The 15 min survey (77 items) was programmed with two academic online survey tools, Unipark (www.unipark.com) and SoSci Survey (www.soscisurvey.de). Inclusion criteria were a minimum age of 18 years, working in the health care sector, residence/working place in Germany, and sufficient German language skills.

2.3. Measures

2.3.1. Sociodemographic, occupational, and COVID-19-related variables

The online questionnaire consisted of the following sociodemographic data: age, gender, living alone (or not), having children (in the household or not in the household or not having children), and migration background. Occupational characteristics were the working setting, profession, years of professional experience, and employment status. The following COVID-19-related variables were assessed: presently working in home office, displacement of the department due to the pandemic, degree of occupancy of the wards, having direct contact at work with COVID-19 infected patients proved by a test, contact with contaminated material during work, belonging to an at-risk group because of age or a chronic illness.

2.3.2. Depressive and anxiety symptoms

Depressive and general anxiety symptoms were measured with the PHQ-4 (Patient Health Questionnaire). This ultrashort form (4 items) of the Patient Health Questionnaire (PHQ—D) can be used as a total score or can be divided into a depression (PHQ-2) and a generalized anxiety module (GAD-2). The total PHQ-2 and GAD-2 sum scores range from 0 to 6. A cut-off-value of $\geq$3 has been suggested to detect probable cases of clinically significant levels of depressive and anxiety symptoms. The psychometric characteristics of the PHQ-4 are well documented [19]. In the present sample, the validated German version obtained Cronbach's Alpha scores of 0.755 for the PHQ-2 and 0.778 for the GAD-2.

2.3.3. Working conditions during the COVID-19 pandemic

Working conditions during the COVID-19 pandemic were assessed with 5 items on a scale from 0 "strongly disagree" to 4 "strongly agree" (with regard to the last two weeks): “There is sufficient personnel protection equipment for the staff (including mouth protection).”; “I work more than before the COVID-19 pandemic.”; “There is sufficient staff for the current work load.”; “I can recover sufficiently during my free time.”; and “I can trust in my colleagues, when it gets difficult during work.”

2.3.4. Potential problems in the COVID-19 pandemic

Potential problems in the COVID-19 pandemic were measured with 9 items on a scale from 0 "strongly disagree" to 4 "strongly agree" (with regard to the last two weeks): “I was afraid to become infected.”; “I was afraid to infect relatives or my family.”; “I felt protected by measures taken by national and local authorities.”; “I felt protected by measures taken by the hospital.”; “I had trouble sleeping.”; “I felt physically or mentally exhausted.”; “I smoked more.”; “I drank more alcohol.”; and “I took more antidepressants/tranquilizers.” These items were derived from a former study [20].

The survey also included questionnaires measuring post-traumatic symptoms, psychosocial resources, work family conflict, and effort and reward imbalance at work. The results for these questionnaires will be analyzed in other publications.

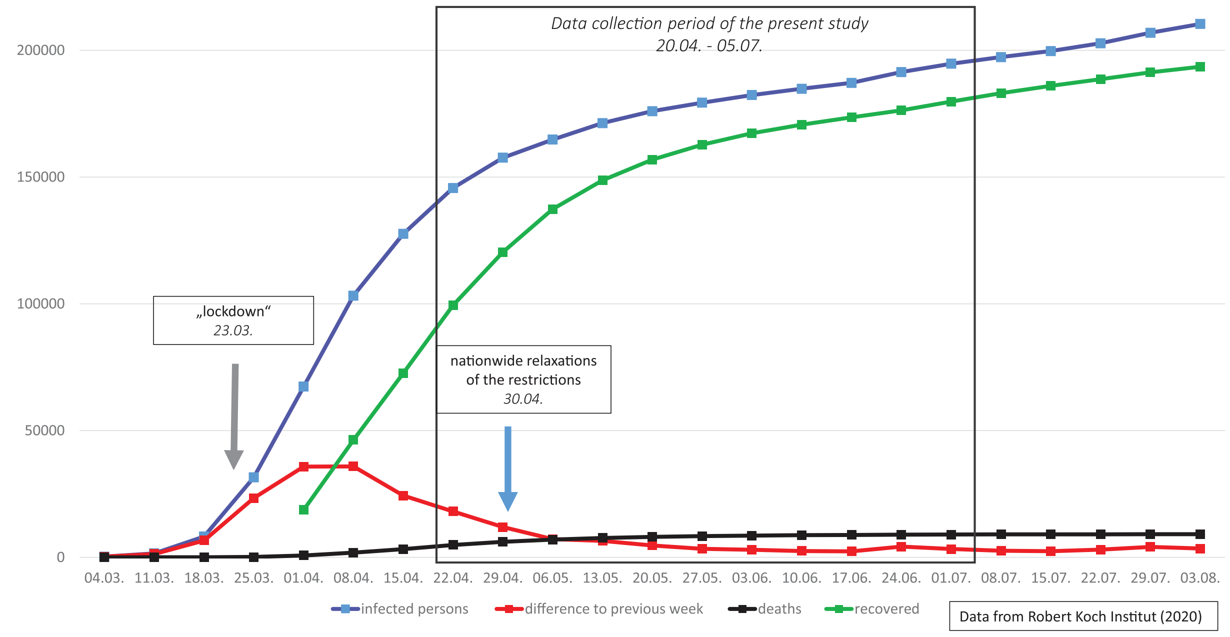

Fig. 1. Data collection period in the context of the course of COVID-19 in Germany.

2.4. *Statistical analysis*

Data analyses were performed with SPSS V. 24. Missing values were found in 7.6% for each of the PHQ-4 items, between 3.1 and 3.2% for the COVID-19-related psychosocial problems, and between 21.4 and 21.7% for the items concerning working conditions during the COVID-19 pandemic. Missing data were imputed using the expectation-maximization algorithm. Descriptive statistics (absolute and relative frequencies) were computed to describe the sociodemographic, occupational, and COVID-19-related characteristics of the total sample and the three professions. Group differences were tested with the χ^2-test for categorical variables, the two sample *t*-test or the univariate analysis of variance (ANOVA, with Bonferroni adjusted post hoc analysis) for continuous variables, where appropriate. Comparisons with norm/reference values were performed with the one sample *t*-test for continuous variables. The effect sizes (Cohen's d, η^2 and Cramer's V) are also reported ($d \geq 0.2$ = small, $d \geq 0.5$ = medium and $d \geq 0.8$ = large effect size; $\eta^2 \geq 0.01$ = small, $\eta^2 \geq 0.06$ = medium and $\eta^2 \geq 0.14$ = large effect size; $V \geq 0.1$ = small, $V \geq 0.3$ = medium and $V \geq 0.5$ = large effect size) [21]. Multiple linear regression analyses were performed to investigate the associations among sociodemographic, occupational, and COVID-related variables, working conditions during the COVID-19 pandemic, and psychosocial problems due to the COVID-19 pandemic with the severity of depressive and anxiety symptoms reported for the total sample and stratified for profession. A level of significance of $p < .05$ (two-tailed) was predetermined in all analyses except for the case of alpha error correction (then explicitly reported in the text).

3. Results

A total of 8071 health care professionals participated in the online survey. In this paper, we present data from health care professionals working in the hospital setting, namely in university hospitals (n = 2290, 62.3%) and further hospitals of maximum care (n = 1388, 37.7%). 28.8% of the total sample analyzed in this paper (N = 3678) were classified as physicians (n = 1061), 34.7% as nurses (n = 1275), and 36.5% as medical technical assistants (MTA; n = 1342). The term MTA refers to allied medical staff such as laboratory or radiology or pharmaceutical-technical assistants. Ninety percent of 3678 HCW analyzed in this paper participated in our survey between 20 April and 28 May.

3.1. *Response rate and gender proportion*

Due to the heterogeneous recruitment strategies, the response rate for the total sample could not be measured. However, we report the response rates for the four university hospitals with the largest proportions of respondents (Table 1). The highest average response rate was found for the group of MTA (24.5%), followed by physicians (10.0%), and nurses (8.9%). Among physicians the gender proportion (females to

Table 1
Response rates and gender proportion of the physicians, nurses and MTA in the four participating university hospitals (UH) with the largest proportion of respondents.

	Response rate			Gender proportion (women : men)	
	Number of study participants from the four UH	Number of employees in the four UH	Response rate, % (range of the four UH)	Study sample (4 UH)	Four UH
Physicians	520	5181	10.0 (8.1–13.3)	60.2 : 39.8	49.0 : 51.0
Nurses	941	10.624	8.9 (5.9–10.1)	75.4 : 24.6	77.9 : 22.1
MTA*	465	1896	24.5 (13.8–37.4)	82.5 : 17.5	92.7 : 7.3

* The group consisted of the following professional subgroups: Medical assistants, Medical-technical laboratory assistants, Medical-technical radiology assistants, Pharmaceutical-technical assistants.

Table 2
Response rates and gender proportion of the physicians, nurses and MTA in one participating university hospital.

	Response rate (women/men/total)	Gender proportion (women : men)	
		Study sample	University hospital
Physicians	9.9/6.5/8.1	58.1 : 41.9	47.6 : 52.4
Nurses	8.7/11.2/9.3	76.4 : 23.6	80.6 : 19.4
MTA*	11.7/47.7/13.8	81.1 : 18.9	94.6 : 5.4

* The group consisted of the following professional subgroups: Medical assistants, Medical-technical laboratory assistants, Medical-technical radiology assistants, Pharmaceutical-technical assistants.

males) was 60.2% : 39.8% for study participants and the mean gender proportion for physicians of the four university hospitals was 49.0% : 51.0%. In the group of nurses the gender ratio among participants (75.4% : 24.6%) was similar to that found in the hospitals (77.9% : 22.1%). Among the MTA the gender proportion was 82.5% : 17.5% for respondents and 92.7% : 7.3% in hospitals. In Table 2 the response rates and gender proportions for the three professional groups of one of the four university hospitals are presented.

3.2. Sociodemographic, occupational and COVID-19-related variables

Three quarters of the participants (74.8%) were female, almost half of the sample (48.6%) was younger than 41 years (Table 3). More than half of the sample (54.8%) reported having direct contact with COVID-19 infected patients (confirmed by a test) during work hours (Table 4). Only approximately 1% of the participants in each of the three investigated professions respectively stated that they had been infected with COVID-19. A quarter (25.9%) of the HCW assessed the degree of the occupancy of the wards as strongly/slightly above average. Almost 14% of the total sample had to change their departments due to the pandemic.

3.3. Prevalence of clinically significant levels of depressive and anxiety symptoms

The prevalence of clinically significant levels of depressive symptoms (cut-off-value of ≥3) was 17.4% for physicians, 21.6% for nurses, and 23.0% for MTA (Table 5). The rate of clinically significant levels of anxiety symptoms (cut-off-value of ≥3) was 17.8% for physicians, 19.0% for nurses and 20.1% for MTA (Table 5). In terms of the GAD-2 the difference between professions was not significant ($p = 0.359$, Cramer-V = 0.024); regarding the PHQ-2 the difference was significant, but of negligible effect size ($p = 0.003$, Cramer-V = 0.056).

3.4. Severity of depressive and generalized anxiety symptoms

The ANOVA showed a significant main effect of profession on the PHQ-2 score ($F(2, 3675) = 21.911$, $p < 0.001$, $\eta^2 = 0.012$) and the GAD-2 score ($F(2, 3675) = 7.468$, $p = 0.001$, $\eta^2 = 0.004$). In the direct comparison, MTA revealed significantly higher levels of depressive and anxiety symptoms than physicians and nurses (Table 6); nurses showed significantly higher scores than physicians. When compared with physicians and nurses after the COVID-19 outbreak in Germany [14], both physicians and nurses in our study demonstrated significantly increased levels of depressive symptoms of large effect size (Table 6).

In comparison with a representative sample of the general German population before the pandemic [19], all three professions demonstrated significantly elevated PHQ-2 and GAD-2 scores of medium to large effect sizes (Table 6). In relation to the general population in

Table 3
Socio-demographic and occupational characteristics of the study sample.

	Total sample N = 3678	Physicians n = 1061	Nurses n = 1275	MTA* n = 1342
**Gender, n (%)**				
Women	2751 (74.8)	634 (59.8)	960 (75.3)	1157 (86.2)
Men	912 (24.8)	422 (39.8)	307 (24.1)	183 (13.6)
Divers	15 (0.4)	5 (0.5)	8 (0.6)	2 (0.1)
**Age, years, n (%)**				
18–30	812 (22.1)	152 (14.3)	369 (28.9)	291 (21.7)
31–40	974 (26.5)	365 (34.4)	316 (24.8)	293 (21.8)
41–50	820 (22.3)	250 (23.6)	265 (20.8)	305 (22.7)
51–60	899 (24.4)	234 (22.1)	277 (21.7)	388 (28.9)
61–70	167 (4.5)	56 (5.3)	46 (3.6)	65 (4.8)
>70	6 (0.2)	4 (0.4)	2 (0.2)	0 (0.0)
**Living alone, n (%)**				
Yes	869 (23.6)	189 (17.8)	338 (26.5)	342 (25.5)
No	2809 (76.4)	872 (82.2)	937 (73.5)	1000 (74.5)
**Children, n (%)**				
Yes, in the household	1385 (37.7)	497 (46.8)	442 (34.7)	446 (33.2)
Yes, but not in the household	472 (12.8)	115 (10.8)	157 (12.3)	200 (14.9)
No	1821 (49.5)	449 (42.3)	676 (53.0)	696 (51.9)
**Migration background, n (%)**				
Yes	429 (11.7)	161 (15.2)	152 (11.9)	116 (8.6)
No	3246 (88.3)	899 (84.7)	1121 (87.9)	1226 (91.4)
Missing	3 (0.1)	1 (0.1)	2 (0.2)	0 (0.0)
**Professional experience in patient care**				
<3 years	317 (8.6)	132 (12.4)	94 (7.4)	91 (6.8)
3–6 years	441 (12.0)	142 (13.4)	184 (14.4)	115 (8.6)
>6 years	2498 (67.9)	749 (70.6)	968 (75.9)	781 (58.2)
Not in patient care	418 (11.4)	37 (3.5)	29 (2.3)	352 (26.2)
Missing	4 (0.1)	1 (0.1)	0 (0.0)	3 (0.2)
**Employment**				
Full-time	2456 (66.8)	772 (72.8)	790 (62.0)	894 (66.6)
Part-time	1217 (33.1)	288 (27.1)	485 (38.0)	444 (33.1)
Missing	5 (0.1)	1 (0.1)	0 (0.0)	4 (0.3)

* The group consisted of the following professional subgroups: Medical assistants, Medical-technical laboratory assistants, Medical-technical radiology assistants, Pharmaceutical-technical assistants.

Table 4
COVID-19-related characteristics of the study sample.

	Total sample N = 3678	Physicians n = 1061	Nurses n = 1275	MTA* n = 1342
**Contact with infected patients, n (%)**				
Yes	2017 (54.8)	599 (56.5)	712 (55.8)	706 (52.6)
No	1637 (44.5)	454 (42.8)	556 (43.6)	627 (46.7)
Missing	24 (0.7)	8 (0.8)	7 (0.5)	9 (0.7)
**Contact with contaminated material, n (%)**				
Yes	2035 (55.3)	514 (48.4)	687 (53.9)	834 (62.1)
No	1614 (43.9)	538 (50.7)	581 (45.6)	495 (36.9)
Missing	29 (0.8)	9 (0.8)	7 (0.5)	13 (1.0)
**Risk group because of age, n (%)**				
Yes	474 (12.9)	106 (10.0)	157 (12.3)	211 (15.7)
No	3176 (86.4)	947 (89.3)	1111 (87.1)	1118 (83.3)
Missing	28 (0.8)	8 (0.8)	7 (0.5)	13 (1.0)
**Risk group because of previous illness, n (%)**				
Yes	685 (18.6)	130 (12.3)	259 (20.3)	296 (22.1)
No	2965 (80.6)	923 (87.0)	1009 (79.1)	1033 (77.0)
Missing	28 (0.8)	8 (0.8)	7 (0.5)	13 (1.0)
**Risk group, n (%)**				
Yes	991 (26.9)	209 (19.7)	359 (28.2)	423 (31.5)
No	2659 (72.3)	844 (79.5)	909 (71.3)	906 (67.5)
Missing	28 (0.8)	8 (0.8)	7 (0.5)	13 (1.0)
**Infection with SARS-CoV-2 virus, n (%)**				
Yes	46 (1.3)	15 (1.4)	19 (1.5)	12 (0.9)
No	1949 (53.0)	635 (59.8)	636 (49.9)	678 (50.5)
I don't know	1654 (45.0)	402 (37.9)	613 (48.1)	639 (47.6)
Missing	29 (0.8)	9 (0.8)	7 (0.5)	13 (1.0)
**Occupancy rate of the wards, n (%)**				
Strongly below average	514 (14.0)	223 (21.0)	131 (10.3)	160 (11.9)
Slightly below average	1090 (29.6)	367 (34.6)	308 (24.2)	415 (30.9)
Average	1092 (29.7)	251 (23.7)	449 (35.2)	392 (29.2)
Slightly above average	604 (16.4)	129 (12.2)	253 (19.8)	222 (16.5)
Strongly above average	349 (9.5)	82 (7.7)	127 (10.0)	140 (10.4)
Missing	29 (0.8)	9 (0.8)	7 (0.5)	13 (1.0)
**Displacement of the department due to the pandemic, n (%)**				
Yes	514 (14.0)	178 (16.8)	236 (18.5)	100 (7.5)
No	3140 (85.4)	875 (82.5)	1032 (80.9)	1233 (91.9)
Missing	24 (0.7)	8 (0.8)	7 (0.5)	9 (0.7)
**Presently working in home office, n (%)**				
Yes, exclusively	30 (0.8)	12 (1.1)	6 (0.5)	12 (0.9)
Yes, partly	330 (9.0)	165 (15.6)	50 (3.9)	115 (8.6)
No	3314 (90.1)	883 (83.2)	1219 (95.6)	1212 (90.3)
Missing	4 (0.1)	1 (0.1)	0 (0.0)	3 (0.2)

* The group consisted of the following professional subgroups: Medical assistants, Medical-technical laboratory assistants, Medical-technical radiology assistants, Pharmaceutical-technical assistants.

Table 5
Prevalence of clinically significant levels of depressive and anxiety symptoms of the study sample.

	Total sample N = 3678	Physicians n = 1061	Nurses n = 1275	MTA* n = 1342
Depressive symptoms (PHQ-2 ≥ 3), %	20.9	17.4	21.6	23.0
Anxiety symptoms (GAD-2 ≥ 3), %	19.1	17.8	19.0	20.1

* The group consisted of the following professional subgroups: Medical assistants, Medical-technical laboratory assistants, Medical-technical radiology assistants, Pharmaceutical-technical assistants.

Germany in the pandemic [2] the physicians and nurses showed significantly lower scores in the PHQ-2 of small and the MTA of very small effect size; regarding the GAD-2 all three groups reported significantly lower scores of small effect sizes (Table 6).

3.5. Working conditions during the COVID-19 pandemic

Strongest agreement was observed regarding the question about trust in colleagues when it gets difficult during work: 69.5% of the participants strongly or quite strongly agreed with this statement (Fig. 2). Only 38.6% strongly or quite strongly agreed that they were able to recover sufficiently during their leisure time and 23.2% stated that they were working more than before the COVID-19 pandemic. In terms of each single item concerning the working conditions, the differences between the three professional groups were of a negligible effect size, except for having sufficient staff. Nurses agreed with this item less frequently than physicians and MTA. The difference was significant and showed a small effect size ($p < 0.001$, Cramer-V = 0.107).

3.6. Potential problems in the COVID-19 pandemic

More than a quarter (27.8%) of the sample agreed strongly or quite strongly to be afraid of becoming infected with COVID-19 (Fig. 3). The fear of infecting relatives was reported almost twice as frequently (54.6%). A considerable proportion of the HCW reported physical or emotional exhaustion (42.3%) and sleep problems (28.8%).

3.7. Variables associated with mental health

To examine the associations of various factors of interest with the severity of depressive and anxiety symptoms, multiple linear regression analyses were performed for the total sample (Table 7: Model 1: depressive symptoms, adjusted $R^2 = 20.9\%$; Model 2: anxiety symptoms, adjusted $R^2 = 22.7\%$) and for the three professions individually

Table 6
Severity of depressive (PHQ-2) and generalized anxiety symptoms (GAD-2) for physicians, nurses and MTA, the differences between the three investigated occupational groups and in comparison with general population and reference groups before and during the COVID-19 pandemic.

Depressive symptoms						
	M (SD)	Pairwise comparisons p# (effect size Cohen's d)	Comparison with German normal population before the pandemic (N = 5036)[a]	Comparison with German general population in the early period of the pandemic (N = 6509)[b]	Comparison with physicians after the outbreak of the pandemic (N = 492)[c]	Comparison with nurses after the outbreak of the pandemic (N = 1511)[c]
Physicians (A)	1.48 (1.35)	A vs. B: 0.001 (0.157)	<0.001 (0.438)	<0.001 (0.382)	<0.001 (0.829)	–
Nurses (B)	1.70 (1.44)	B vs. C: 0.008 (0.115)	<0.001 (0.605)	<0.001 (0.249)	–	<0.001 (0.876)
MTA* (C)	1.86 (1.45)	C vs. A: <0.001 (0.274)	<0.001 (0.735)	<0.001 (0.149)	–	–
**Generalized anxiety symptoms**						
	M (SD)	Pairwise comparisons p# (effect size Cohen's d)	Comparison with German normal population before the pandemic	Comparison with German general population in the early period of the pandemic	Comparison with physicians after the outbreak of the pandemic	Comparison with physicians after the outbreak of the pandemic
Physicians (A)	1.45 (1.41)	A vs. B: 1.0 (0.023)	<0.001 (0.543)	<0.001 (0.338)	–	–
Nurses (B)	1.48 (1.48)	B vs. C: 0.006 (0.119)	<0.001 (0.559)	<0.001 (0.318)	–	–
MTA* (C)	1.66 (1.50)	C vs. A: 0.001 (0.144)	<0.001 (0.703)	<0.001 (0.215)	–	–

*The group consisted of the following professional subgroups: Medical assistants, Medical-technical laboratory assistants, Medical-technical radiology assistants, Pharmaceutical-technical assistants.
[#] p-values from Bonferroni adjusted post hoc analysis of the ANOVA.
[a] Löwe et al. (2010).
[b] Petzold et al. (2020).
[c] Skoda et al. (2020).

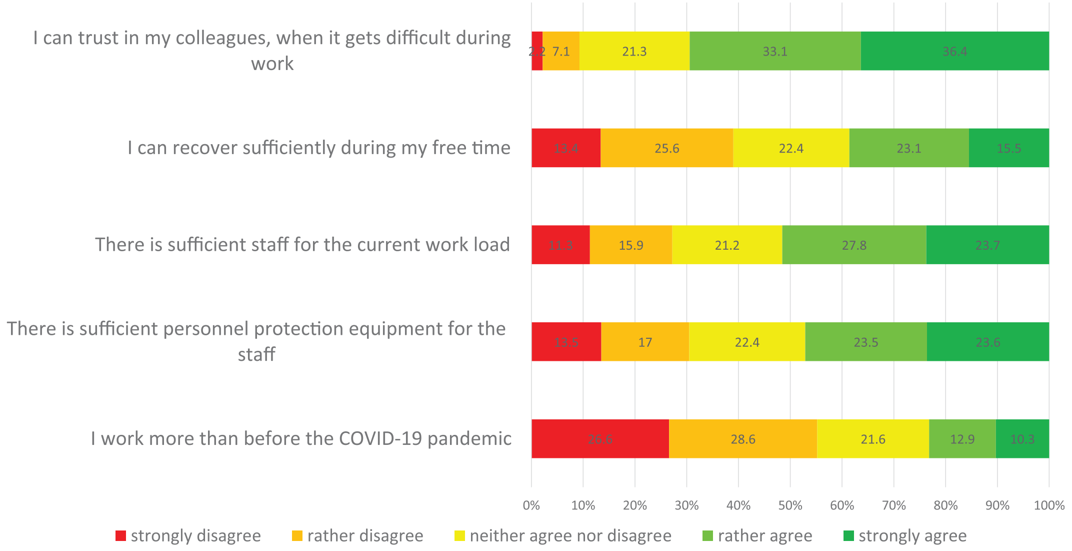

Fig. 2. Assessment of working conditions in the COVID-19 pandemic by health care workers.

(Tables A.1–3).

Significantly and clinically relevant variables (standardized $\beta > 0.1$) that were associated with higher levels of depressive symptoms in the total sample were: having insufficient recovery during leisure time, increased alcohol consumption, and less trust in colleagues when it becomes difficult at work. Elevated scores of anxiety were related to increased fear of becoming infected with COVID-19, insufficient recovery during leisure time, and increased alcohol consumption.

In the regression analyses performed separately for the three professions, the most important variables associated with increased severity of depressive symptoms were insufficient recovery during leisure time and increased alcohol consumption. The three most important variables related to elevated anxiety scores in all three groups were the fear of getting infected with COVID-19, insufficient recovery during leisure time, and increased alcohol consumption.

4. Discussion

To the best of our knowledge, the present study of mental health during the COVID-19 pandemic and its associations with working conditions and COVID-19-related problems is the largest of this type conducted to date among health care professionals in Europe.

4.1. Depressive and anxiety symptoms

When comparing the prevalences of clinically significant levels of

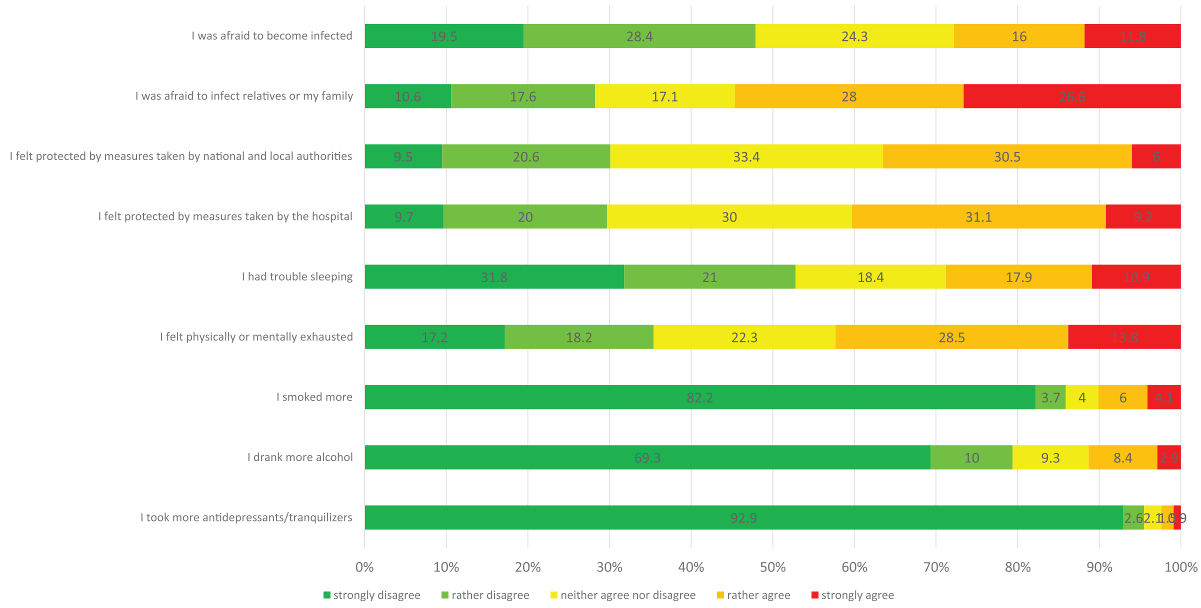

Fig. 3. Assessment of potential problems in the COVID-19 pandemic by health care workers.

depressive and anxiety symptoms with those of other studies on medical staff using the same questionnaire (PHQ-4), the rates shown in the present study are lower than those reported for New York HCW [22] less than one month since the start of the COVID-19 pandemic but higher than those detected in China less than two months after the pandemic outbreak [23]. A possible explanation for these differences may be the different timing of the studies, as this was associated with different degrees of distress and unsafety. The difference could also be due to socio-cultural and political characteristics of societies, health care structures and the course of the pandemic.

In comparison with normative values of the general German population (established pre-COVID because appropriate comparative values during the COVID-19 pandemic do not exist yet), respondents from all three professions revealed considerably increased PHQ-2 and GAD-2 scores. However, in a recently published population-based study from the early period of the pandemic in Germany [2], the average scores for depressive and anxiety symptoms were higher than those reported by HCW in our study. Despite the high level of work-related distress, it can be postulated that HCW have special knowledge and competencies that allow them to better deal with the pandemic situation than the general population. In another study conducted in Germany with HCW in the beginning of the pandemic [14], lower depression scores were observed than those reported in the current study. This may be related to the later timing of our study, at that time the full impact of the pandemic was visible and had putatively higher detrimental effects.

Different professions are not affected equally in the pandemic. In our study, the MTA group reported increased levels of depressive symptoms as compared to physicians. MTA were under immense pressure in the early phase of the pandemic to establish innovative tests and then to increase the testing capacity. Both factors may contribute to an enhanced risk of distress. The elevated symptom burden of MTA may also be explained by their lower hierarchical position at work or/and by their lower socioeconomic status in comparison with physicians. There is strong empirical evidence demonstrating that depression is associated with socioeconomic position [24,25]. Lower occupational position has also been shown to be related to a higher risk of depression as compared to higher occupational position [26]. The increased symptom severity among MTA in relation to physicians found in our study underlines the importance of investigating and supporting not only the mental health of HCW in the frontline but also indispensable professionals in the background.

4.2. Risk and protective factors

Insufficient recovery was an important risk factor for both depression and anxiety in our study. The opportunity to recreate between shifts naturally contributes to mental stability. The participants did not indicate a general increase in tasks, and most staff felt that there was enough manpower. For these reasons, it is necessary to investigate why the failure to recover occurred. One possible factor would be uncertainty due to the virus, whose routes of spread were not sufficiently known at that time. So, possibly, ruminative thinking and anxiety in the employees and/or additional commitments such as homeschooling led to a lack of recreation.

Increased alcohol consumption was related with increased depression and anxiety levels. Prior research indicates that substance abuse is a common risk factor among HCW [27–29]. Alcohol is putatively consumed to regulate stress. However, due to the cross-sectional study design of the present survey the opposite explanation could also be true, namely that alcohol is presumed to contribute to elevated anxiety levels. The results of our survey highlight the importance of measures that detect individual stress levels among professionals in the health sector and supporting them to cope adequately with psychological distress.

We have found out that lack of trust in one's own working team is a risk factor for generalized anxiety and depression. A central feature of activity on the ward occurs through cooperation in information exchange and actions. Furthermore, belonging to a team conveys feelings of security and self-esteem. In the pandemic situation, the demands on the functioning of the team have increased. Thus, a lack of trust in one's own working group is accompanied by high psychological stress.

Generalized anxiety was associated with the fear of being infected with the virus. In the first weeks and months of the pandemic, because the pathways of infection and hygiene measures were not clear, and, the hospitals were confronted with supply bottlenecks regarding protective clothing, fear of becoming infected increased [13]. Therefore this correlation is in line with our assumptions.

Lack of opportunity for recovery and increased alcohol consumption were confirmed as the most important predictors in the three different occupational groups indicating the robustness of these indicators. Together with trust in the team, these three variables provide a good

Table 7
Linear regression analyses for severity of depressive (PHQ-2) and generalized anxiety symptoms (GAD-2) for the total sample of healthcare workers.

Independent variables	Model 1: PHQ-2 (R^2_{adj} = 20.9%)					Model 2: GAD-2 (R^2_{adj} = 22.7%)				
	Regression coefficient (95% CI)	SE	Beta	T	P	Regression coefficient (95% CI)	SE	Beta	T	P
Constant	2.439 (1.927, 2.952)	0.261	-	9.333	<0.001	1.729 (1.206, 2.252)	0.267	-	6.481	<0.001
Gender (men vs. women)	0.020 (−0.083, 0.123)	0.053	0.006	0.385	0.700	0.173 (0.068, 0.278)	0.054	0.051	3.225	**0.001**
Age (<41 vs. ≥41 years)	−0.011 (−0.109, 0.088)	0.050	−0.004	−0.213	0.831	0.122 (0.021, 0.222)	0.051	0.041	2.367	**0.018**
Living alone (no vs. yes)	0.034 (−0.072, 0.140)	0.054	0.010	0.626	0.532	0.018 (−0.091, 0.126)	0.055	0.005	0.318	0.750
Children (no vs. yes)	−0.180 (−0.284, −0.076)	0.053	−0.063	−3.390	**0.001**	−0.083 (−0.190, 0.023)	0.054	−0.028	−1.536	0.125
Ethnic Germans (no vs. yes)	−0.060 (−0.190, 0.071)	0.067	−0.013	−0.894	0.371	−0.010 (−0.143, 0.123)	0.068	−0.002	−0.148	0.882
Nurses (Ref. = Physicians)	0.087 (−0.025, 0.198)	0.057	0.029	1.525	0.127	−0.150 (−0.264, −0.036)	0.058	−0.049	−2.584	**0.010**
MTA (Ref. = Physicians)	0.227 (0.111, 0.343)	0.059	0.077	3.835	**<0.001**	−0.089 (−0.207, 0.030)	0.060	−0.029	−1.468	0.142
Patient care (no vs. yes)	0.168 (0.021, 0.315)	0.075	0.037	2.241	**0.025**	0.188 (0.038, 0.338)	0.076	0.041	2.461	**0.014**
Fulltime (full- vs. part-time)	0.060 (−0.040, 0.159)	0.051	0.020	1.171	0.242	0.142 (0.040, 0.244)	0.052	0.046	2.736	**0.006**
Home office (no vs. yes)	−0.039 (−0.182, 0.104)	0.073	−0.008	−0.532	0.595	0.026 (−0.120, 0.172)	0.074	0.005	0.352	0.725
Deplacement of the department (no vs. yes)	0.181 (0.059, 0.303)	0.062	0.044	2.914	**0.004**	0.126 (0.001, 0.250)	0.063	0.030	1.980	**0.048**
Contact with infected patients (no vs. yes)	−0.073 (−0.185, 0.038)	0.057	−0.026	−1.291	0.197	−0.084 (−0.198, 0.029)	0.058	−0.029	−1.453	0.146
Contact with contaminated material (no vs. yes)	−0.104 (−0.213, 0.004)	0.055	−0.036	−1.888	0.059	−0.010 (−0.121, 0.101)	0.056	−0.003	−0.176	0.860
Risk group (yes vs. no)	−0.019 (−0.118, 0.081)	0.051	−0.006	−0.365	0.715	−0.121 (−0.222, −0.019)	0.052	−0.037	−2.332	**0.020**
Occupancy of the wards+	0.028 (−0.013, 0.070)	0.021	0.023	1.332	0.183	0.030 (−0.013, 0.072)	0.022	0.024	1.376	0.169
Protection equipment*	0.008 (−0.031, 0.048)	0.020	0.007	0.413	0.680	0.012 (−0.028, 0.052)	0.021	0.010	0.594	0.552
Higher work load*	0.011 (−0.032, 0.053)	0.022	0.008	0.486	0.627	0.027 (−0.016, 0.070)	0.022	0.021	1.218	0.223
Sufficiant staff*	0.016 (−0.028, 0.060)	0.022	0.013	0.728	0.467	0.012 (−0.033, 0.057)	0.023	0.010	0.536	0.592
Sufficient recovery*	−0.281 (−0.323, −0.238)	0.022	−0.224	−12.937	**<0.001**	−0.253 (−0.296, −0.210)	0.022	−0.196	−11.423	**<0.001**
Trust in colleages*	−0.157 (−0.206, −0.108)	0.025	−0.101	−6.294	**<0.001**	−0.144 (−0.194, −0.094)	0.026	−0.089	−5.648	**<0.001**
Fear of becoming infected*	0.078 (0.035, 0.122)	0.022	0.069	3.538	**<0.001**	0.233 (0.189, 0.277)	0.023	0.199	10.304	**<0.001**
Fear to infect family*	0.070 (0.028, 0.111)	0.021	0.064	3.304	**0.001**	0.077 (0.035, 0.119)	0.022	0.068	3.561	**<0.001**
Measures (national)*	−0.065 (−0.111, −0.018)	0.024	−0.048	−2.713	**0.007**	−0.052 (−0.099, −0.004)	0.024	−0.037	−2.129	**0.033**
Measures (hospital)*	−0.121 (−0.169–0.073)	0.025	−0.094	−4.908	**<0.001**	−0.068 (−0.118, −0.019)	0.025	−0.051	−2.720	**0.007**
Smoking*	0.033 (−0.008, 0.075)	0.021	0.025	1.574	0.116	0.055 (0.013, 0.098)	0.022	0.040	2.558	**0.011**
Alcohol*	0.184 (0.144, 0.224)	0.020	0.143	9.096	**<0.001**	0.168 (0.127, 0.208)	0.021	0.126	8.121	**<0.001**
Pre-Covid-19 burdon#	0.105 (0.060, 0.149)	0.023	0.069	4.587	**<0.001**	0.115 (0.070, 0.161)	0.023	0.074	4.960	**<0.001**
Infected cases in Germany†	−6.407E-7 (<0.001, <0.001)	<0.001	−0.007	−0.467	0.640	−1.708E-6 (<0.001, <0.001)	<0.001	−0.019	−1.220	0.223

R^2_{adj} = adjusted R^2; SE = standard error; +1 = strongly below average, 2 = slightly below average, 3 = average, 4 = slightly above average, 5 = strongly above average; *0 = strongly disagree, 1 = rather disagree, 2 = neither agree nor disagree, 3 = rather agree, 4 = strongly agree; #0 = not at all, 1 = little, 2 = middle, 3 = strong, 4 = very strong; †data from Robert Koch Institut (2020); significant predictors are marked in bold.

Table 8
Recommendations for optimizing the mental health of health care workers.

Most influential variables	Recommendations
Alcohol consumption	Activate addiction prevention
Insufficient recovery	Screening for distress and recovery
Trust in colleagues	Team building activities
Fear of becoming infected	Transparent hygiene measures

basis for recommendations for prevention of mental health disorders (Table 8).

4.3. Strengths and limitations

To the best of our knowledge, the present study is the largest survey on mental health of healthcare professionals during the first wave of the COVID-19 pandemic in Europe. The study sample can be regarded as partially representative of HCW from three professions (physicians, nurses and MTA) in university hospitals in Germany due to the representative gender proportion and the relatively good average response rate for some of these subgroups. Nevertheless, our study has also some limitations. The cross-sectional design does not allow causal conclusions concerning the measured variables to be drawn. The response rate for most of the subgroups mentioned above was low. Thus, the results of our study cannot be generalized to the group of all HCW of university hospitals. In addition, we did not recruit a control group from the general population. Future research should examine the mental health of medical staff using representative samples and control groups from the general population and prospective studies to protect the health and wellbeing of workers and, as a result, to sustain the work ability of these key members of the public health response during epidemics.

5. Conclusion

Due to the limitations of the study the conclusions should be treated with caution. HCW demonstrated increased levels of depression and anxiety in comparison with the normal population in Germany before the pandemic. During the pandemic HCW revealed a lower burden of mental distress compared to the general population. Nevertheless, a high percentage of HCW demonstrated psychosocial distress, so that the establishment of regular mental health screening and prevention programmes for HCW is indicated. Our study suggested that MTA seem to be the most vulnerable group. This result underlines the importance of investigating and supporting not only the mental health of HCW in the frontline but also indispensable professionals in the background. The risk factors revealed in the present study need to be investigated and replicated in further studies. Nevertheless, on the basis of the results uncovered here, the following recommendations can be formulated (Table 8): special screening and programs are required to prevent substance abuse in medical staff. Targeted training to perceive and regulate distress levels should be offered to HCW. Concerning insufficient recovery implementation of regular screening of recovery and distress (e.g. via distress thermometer) as well as psychoeducation are recommended. A well-functioning team in which trust prevails can more sufficiently master the challenge in the pandemic situation. Thus, interventions that strengthen the team spirit should be established in daily work. The fear of becoming infected can best be diminished by transparent hygiene measures. The challenges encountered by HCW can also have productive results, leading to meaningful changes in the health care system.

Acknowledgment

This research is embedded in the EgePan Unimed study and was funded by the German Federal Ministry of Education and Research (BMBF, funding number: 01KX2021) headed by Prof. Dr. Jochen Schmitt and Dr. Michael von Wagner. The funders had no role in the study design; in the collection, analysis, and interpretation of data; in the writing of the manuscript; and in the decision to submit the paper for publication. The corresponding author had full access to all the data in the study and had final responsibility for the decision to submit for publication.

We thank Dipl.-Psych. Marietta Lieb for administrative and technical support, Dr. Tobias Hepp for statistical support, and Frederik Wuchenauer for data visualisation.

Disclosure statement

The authors declare that no conflict of interest exists.

Appendix A. Supplementary data

Supplementary data to this article can be found online at https://doi.org/10.1016/j.jpsychores.2021.110415.

References

[1] A. Bäuerle, M. Teufel, V. Musche, B. Weismüller, H. Kohler, M. Hetkamp, et al., Increased generalized anxiety, depression and distress during the COVID-19 pandemic: a cross-sectional study in Germany, J. Public Health (2020), https://doi.org/10.1093/pubmed/fdaa106.

[2] M.B. Petzold, A. Bendau, J. Plag, L. Pyrkosch, L. Mascarell Maricic, F. Betzler, et al., Risk, resilience, psychological distress, and anxiety at the beginning of the COVID-19 pandemic in Germany, Brain Behav. (2020), e01745, https://doi.org/10.1002/brb3.1745.

[3] S.K. Brooks, R. Dunn, R. Amlot, G.J. Rubin, N. Greenberg, A systematic, thematic review of social and occupational factors associated with psychological outcomes in healthcare employees during an infectious disease outbreak, J. Occup. Environ. Med. 60 (3) (2018) 248–257.

[4] G.S. de Pablo, J.V. Serrano, A. Catalan, C. Arango, C. Moreno, F. Ferre, et al., Impact of coronavirus syndromes on physical and mental health of health care workers: systematic review and meta-analysis, J. Affect. Disord. 275 (2020) 48–57.

[5] S. Pappa, V. Ntella, T. Giannakas, V.G. Giannakoulis, E. Papoutsi, P. Katsaounou, Prevalence of depression, anxiety, and insomnia among healthcare workers during the COVID-19 pandemic: a systematic review and meta-analysis, Brain Behav. Immun. 88 (2020) 901–907.

[6] M. Luo, L. Guo, M. Yu, W. Jiang, H. Wang, The psychological and mental impact of coronavirus disease 2019 (COVID-19) on medical staff and general public - a systematic review and meta-analysis, Psychiatry Res. 291 (2020) 113190.

[7] C.S. Romero, C. Delgado, J. Catala, C. Ferrer, C. Errando, A. Iftimi, et al., COVID-19 psychological impact in 3109 healthcare workers in Spain: the PSIMCOV group, Psychol Med. (2020) 1–7, https://doi.org/10.1017/S0033291720001671.

[8] Y. Hacimusalar, A.C. Kahve, A.B. Yasar, M.S. Aydin, Anxiety and hopelessness levels in COVID-19 pandemic: a comparative study of healthcare professionals and other community sample in Turkey, J. Psychiatr. Res. 129 (2020) 181–188.

[9] M.K. Sahin, S. Aker, G. Sahin, A. Karabekiroglu, Prevalence of depression, anxiety, distress and insomnia and related factors in healthcare workers during COVID-19 pandemic in Turkey, J. Community Health 45 (6) (2020) 1168–1177.

[10] S.U. Johnson, O.V. Ebrahimi, A. Hoffart, PTSD symptoms among health workers and public service providers during the COVID-19 outbreak, PLoS One 15 (10) (2020), e0241032.

[11] J. Antonijevic, I. Binic, O. Zikic, S. Manojlovic, S. Tosic-Golubovic, N. Popovic, Mental health of medical personnel during the COVID-19 pandemic, Brain Behav. (2020), e01881, https://doi.org/10.1002/brb3.1881.

[12] F. Georger, E. Dos Santos, L. Gazagne, P. Berdague, A. Saib, S. Nahon, et al., COV IMPACT: Stress exposure analysis among hospital staff in 2 hospitals in France during the COVID-19 pandemic, Ann. Cardiol. Angeiol. (Paris) 69 (5) (2020) 227–232.

[13] P. Paffenholz, A. Peine, M. Hellmich, S.V. Paffenholz, L. Martin, M. Luedde, et al., Perception of the 2020 SARS-CoV-2 pandemic among medical professionals in Germany: results from a nationwide online survey, Emerg. Microb. Infect. 9 (1) (2020) 1590–1599.

[14] E.M. Skoda, M. Teufel, A. Stang, K.H. Jöckel, F. Junne, B. Weismüller, et al., Psychological burden of healthcare professionals in Germany during the acute phase of the COVID-19 pandemic: differences and similarities in the international context, J. Public Health (Oxf). (2020), https://doi.org/10.1093/pubmed/fdaa124.

[15] S. Cabarkapa, S.E. Nadjidai, J. Murgier, C.H. Ng, The psychological impact of COVID-19 and other viral epidemics on frontline healthcare workers and ways to address it: a rapid systematic review, Brain Behav. Immun. Health 8 (2020) 100144.

[16] A.E. Muller, E.V. Hafstad, J.P.W. Himmels, G. Smedslund, S. Flottorp, S. O. Stensland, et al., The mental health impact of the covid-19 pandemic on healthcare workers, and interventions to help them: a rapid systematic review, Psychiatry Res. 293 (2020) 113441.

[17] Z. Li, J. Ge, M. Yang, J. Feng, M. Qiao, R. Jiang, et al., Vicarious traumatization in the general public, members, and non-members of medical teams aiding in COVID-19 control, Brain Behav. Immun. 88 (2020) 916–919.
[18] Robert Koch Institut, COVID-19: Case numbers in Germany and worldwide, 2020. Available from: https://www.rki.de/DE/Content/InfAZ/N/Neuartiges_Coronavirus/Fallzahlen.html.
[19] B. Löwe, I. Wahl, M. Rose, C. Spitzer, H. Glaesmer, K. Wingenfeld, et al., A 4-item measure of depression and anxiety: validation and standardization of the Patient Health Questionnaire-4 (PHQ-4) in the general population, J. Affect. Disord. 122 (1–2) (2010) 86–95.
[20] K. Matsuishi, A. Kawazoe, H. Imai, A. Ito, K. Mouri, N. Kitamura, et al., Psychological impact of the pandemic (H1N1) 2009 on general hospital workers in Kobe, Psychiatry Clin. Neurosci. 66 (4) (2012) 353–360.
[21] Lenhard W., Lenhard A. [Calculation of effect sizes]. Available from: https://www.psychometrica.de/effektstaerke.html.
[22] A. Shechter, F. Diaz, N. Moise, D.E. Anstey, S. Ye, S. Agarwal, et al., Psychological distress, coping behaviors, and preferences for support among New York healthcare workers during the COVID-19 pandemic, Gen. Hosp. Psychiatry 66 (2020) 1–8.
[23] W.R. Zhang, K. Wang, L. Yin, W.F. Zhao, Q. Xue, M. Peng, et al., Mental health and psychosocial problems of medical health workers during the COVID-19 epidemic in China, Psychother. Psychosom. 89 (4) (2020) 242–250.
[24] S.A. Everson, S.C. Maty, J.W. Lynch, G.A. Kaplan, Epidemiologic evidence for the relation between socioeconomic status and depression, obesity, and diabetes, J. Psychosom. Res. 53 (4) (2002) 891–895.
[25] M. Marmot, M. Shipley, E. Brunner, H. Hemingway, Relative contribution of early life and adult socioeconomic factors to adult morbidity in the Whitehall II study, J. Epidemiol. Community Health 55 (5) (2001) 301–307.
[26] A.C. Volkers, G.P. Westert, F.G. Schellevis, Health disparities by occupation, modified by education: a cross-sectional population study, BMC Public Health 7 (2007) 196.
[27] K. Nilan, T.M. McKeever, A. McNeill, M. Raw, R.L. Murray, Prevalence of tobacco use in healthcare workers: a systematic review and meta-analysis, PLoS One 14 (7) (2019), e0220168.
[28] A. Medisauskaite, C. Kamau, Prevalence of oncologists in distress: systematic review and meta-analysis, Psychooncology 26 (11) (2017) 1732–1740.
[29] F.M. Wurst, H.J. Rumpf, G.E. Skipper, J.P. Allen, I. Kunz, P. Beschoner, et al., Estimating the prevalence of drinking problems among physicians, Gen. Hosp. Psychiatry 35 (5) (2013) 561–564.
