# Supplementary material for: Mental health status and related factors influencing healthcare workers during the COVID-19 pandemic: A systematic review and meta-analysis
Source: PLoS One. 2024 Jan 19;19(1):e0289454. doi: 10.1371/journal.pone.0289454 (PMC10798549; doi:10.1371/journal.pone.0289454)
Supplement: S1 Data — (ZIP) [file pone.0289454.s011.zip › literatures/107.pdf]

Received: 18 October 2020 | Revised: 11 January 2021 | Accepted: 17 January 2021

DOI: 10.1002/da.23140

RESEARCH ARTICLE

ANXIETY AND DEPRESSION ASSOCIATION OF AMERICA WILEY

The association between witnessing patient death and mental health outcomes in frontline COVID-19 healthcare workers

Mariela Mosheva[1,2] | Raz Gross[1,2] | Nimrod Hertz-Palmor[1,3] | Ilanit Hasson-Ohayon[4] | Rachel Kaplan[1] | Rony Cleper[2] | Yitshak Kreiss[1,2] | Doron Gothelf[1,2] | Itai M. Pessach MD, PhD, MPH[1,2]

[1]The Chaim Sheba Medical Center, Ramat Gan, Israel

[2]Sackler Faculty of Medicine, Tel Aviv University, Tel Aviv, Israel

[3]School of Psychological Sciences, Tel Aviv University, Tel Aviv, Israel

[4]Bar-Ilan University, Ramat Gan, Israel

Correspondence

Itai M. Pessach, MD, PhD, MPH, The Edmond and Lily Safra Children's Hospital, Sheba Medical Center, Tel Hashomer 52621, Israel.
Email: Itai.Pessach@Sheba.gov.il

Funding information

Foundation Dora and the Binational Science Foundation, Grant/Award Number: 2017369

Abstract

Background: Healthcare workers (HCW) treating coronavirus disease 2019 (COVID-19) patients face high levels of psychological stress. We aimed to compare mental health outcomes, risk and protective factors for posttraumatic stress symptoms (PTSS), probable depression, and anxiety between HCW working in COVID-19 and non-COVID-19 wards.

Methods: A self-report survey, administered in a large tertiary hospital in Israel during the peak of the COVID-19 outbreak was completed by 828 HCW (42.2% physicians, 57.8% nurses. Patient-Reported Outcomes Measurement Information System; the Patient Health Questionnaire-9; the Primary Care-Post Traumatic Stress Disorder Screen for DSM-5 (PC-PTSD-5) were used for assessing anxiety, depression, and PTSS, respectively. Pandemic-related stress factors, negative experiences, and potential protective factors were also assessed.

Results: Median PC-PTSD scores differed significantly between study teams (χ^2 [5] = 17.24; *p* = .004). Prevalence of probable depression and anxiety were similar in both groups. Risk factors for mental health outcomes included mental exhaustion, anxiety about being infected and infecting family. Overall, higher proportion of the COVID-19 team witnessed patient deaths as compared to the non-COVID-19 team (50.2% vs. 24.7%). Witnessing patient death at the COVID-19 wards was associated with a four-fold increased likelihood of PTSS (odds ratio [OR] = 3.97; 95% confidence interval [CI], 1.58–9.99; *p* = .0007), compared with the non-COVID-19 wards (OR 0.91; 95% CI, 0.51–1.61; *p* = .43).

Conclusions: Witnessing patient death appears to be a risk factor for PTSS unique to HCW directly engaged in treating patients with COVID-19. Our findings suggest that helping HCW cope with COVID-19 related deaths might reduce their risk of posttraumatic stress.

KEYWORDS

anxiety, COVID-19, depression, mental health, PTSD, stress

Mariela Mosheva and Raz Gross equal contribution as first author.

© 2021 Wiley Periodicals LLC

1 | INTRODUCTION

The coronavirus disease 2019 (COVID-19) pandemic is an unprecedented healthcare crisis that is challenging and overwhelming both for institutions and individuals. Media coverage indicates that the pandemic has created widespread fear, anxiety, depression, and stress in the public in general (Ettman et al., 2020; Gao et al., 2020; Huang & Zhao, 2020; Lu et al., 2020; Wang et al., 2020) as well as among frontline responders, most noticeably physicians and nurses (Chen et al., 2020; Dewey et al., 2020; Kisely et al., 2020; Lai et al., 2020; Mosheva et al., 2020; Ning et al., 2020; Pappa et al., 2020; Tan et al., 2020; Taylor & Blackford, 2020; Williamson et al., 2020). Protecting the mental health of healthcare workers (HCW) is a crucial task in the struggle to maintain the availability of healthcare services during pandemics and medical crises. This can only be achieved by understanding the characteristics and mental effects of a unique crisis.

Hence, in the present study we aim to focus on the mental health of HCW assigned to work at the 'epicenter' of the pandemic, that is, at acute COVID-19 containment wards.

Studies conducted during previous emerging viral outbreaks and the current ongoing COVID-19 pandemic have shown that level of exposure (Carmassi et al., 2020), fear of infecting family members and friends (Goulia et al., 2010; Maunder et al., 2003), uncertainty about the health consequences of the disease (Goulia et al., 2010), perception of risk to oneself (Nickell et al., 2004; Styra et al., 2008), living with children (Nickell et al., 2004), being quarantined (Bai et al., 2004; Carmassi et al., 2020), and stigmatization and social isolation (Bai et al., 2004; Can et al., 2005; Maunder et al., 2003; Nickell et al., 2004) were all related to psychological distress. A large national survey of physicians in Israel found that mental exhaustion, anxiety about being infected and about infecting family, and sleep difficulties were associated with higher anxiety scores (Mosheva et al., 2020). Posttraumatic stress is common within disaster-exposed occupations (Brooks et al., 2019; Naushad et al., 2019), is associated with risk of developing other psychiatric conditions, such as depression and substance abuse (Ursano et al., 1999), and its prevalence has been shown to increase over time among disaster rescue and recovery workers (Lowell et al., 2018). Negative experiences, such as witnessing patients' suffer and death, have been linked to stress among medical personnel (Laposa & Alden, 2003). However, to our knowledge, they have not been studied during the current COVID-19 pandemic.

Data on the frequency of traumatic stress and its presentation among HCW during the COVID-19 pandemic are thus far scarce and limited by lack of direct comparison with non-COVID-19 HCW (Liu et al., 2020), loosely defined sample, unquantifiable response rate, self-reported classification of exposure level (frontline or second-line; Kang et al., 2020; Rossi et al., 2020), and limited data on stress factors and pertinent experiences (Li et al., 2020; Sun et al., 2020).

In the present study, we set to assess the prevalence of posttraumatic stress symptoms (PTSS), probable anxiety, and depression in frontline (high exposure group) compared with second-line (low exposure group) HCW. We further aimed to explore the association between the study's mental health outcomes and pandemic-related stress factors (PRSF) and experiences. Based on prior studies, we hypothesized that PRSF, mainly mental exhaustion, anxiety about being infected and infecting family would be associated with mental health outcomes in both groups, whereas witnessing patients' death and other negative experiences would be more strongly associated with mental health outcomes in the COVID-19 team.

2 | METHODS

2.1 | Participants

We conducted a cross-sectional study of physicians and nurses, working at a large tertiary medical center in central Israel. Participants were recruited by means of text messages sent to their mobile phone numbers as registered in the hospital listing, and to their hospital email address. The study protocol was approved by the Institutional Review Board of the Sheba Medical Center, and the standards and ethics of the American Association for Public Opinion Research reporting guidelines were followed (The American Association for Public Opinion Research, 2016). This study followed the Strengthening the Reporting of Observational Studies in Epidemiology (STROBE) reporting guidelines (von Elm et al., 2007). As part of the preparation for the surge of COVID-19-patients expected to be treated at the hospital, specialized COVID-19 care wards were set up and separated from the non-COVID-19 care areas. These included two intensive care units, a separate emergency department, five inpatient wards, and a psychiatry ward, totaling with almost 400 COVID-19-specialized beds. Organic teams composed of hospital personnel were nonvoluntarily allocated to the COVID-19 wards, with minimal relocations of medical stuff between teams (Leshem et al., 2020).

The analytic sample included responders who consented to participate in the study between April 19 and April 23, 2020. During that time, the number of confirmed COVID-19 cases in Israel peaked from 13,319 (1.5 confirmed cases per each 1000 people) to 14,611 (1.64 cases per each 1000 people).

2.2 | Assessments

A self-administered anonymous questionnaire was completed by the participants online through a secured digital platform (Qualtrics). Participants indicated whether they were assigned to the COVID-19 teams, provided sociodemographic data, completed questions about their physical and mental health, and a yes/no item about being quarantined.

2.3 | Work-related positive and negative experiences

Questions aimed at eliciting positive experiences included: feeling mission driven to work, feeling protected by government and hospital, altruistic acceptance of risk ("Because I wanted to help the COVID-19 patients, I was willing to accept the risks involved"). Questions concerning negative experiences included: exposure to

patients' physical and mental suffering, the number of patient deaths witnessed over the past month (none/one/more than one; Laor-Maayany et al., 2020), and a self-perceived physical health question from the Short-Form Health Status Survey (SF-12; poor/fair/good/very good/excellent; Amir et al., 2002).

2.4 | Outcome measures

Anxiety was assessed with the 8-item Hebrew version of the National Institution of Mental Health (NIMH) Patient-Reported Outcomes Measurement Information System (PROMIS) anxiety module (Bevans et al., 2014; Health, 2017; Mosheva et al., 2020; Yardeni et al., 2020). PROMIS has an established coding system validated by the NIMH, with standardized "T" scores ranging between 36.3 and 82.7 (PROMIS® Scoring Manuals). The cutoff point for probable anxiety was set at T ≥ 62.3, considered as equivalent to the GAD-7 standard cutoff score for moderate anxiety (= 10; Spitzer et al., 2006). Depression was assessed by means of the Hebrew version of the Patient Health Questionnaire-9 (PHQ-9; Geuolayov et al., 2009). The PHQ-9 has a score range of 0–27. A score of ≥ 10, established as the standard cutoff for moderate depression (Levis et al., 2019), was used to identify probable depression. Posttraumatic stress symptoms were screened with the validated Hebrew version of the Primary Care PTSD Screen for DSM-5 (PC-PTSD-5; Spoont et al., 2015), which has a score range of 0–5. The standard cutoff score of ≥ 3, was used to classify as probable PTSD.

The PRSF were measured with an inventory compiled from questions that proved to be pertinent in research carried out during the SARS and N1H1 pandemics (Imai et al., 2010; Mosheva et al., 2020; Tam et al., 2004), and in the current COVID-19 pandemic (Mosheva et al., 2020). A 4-point Likert-type scale was used for scoring the items (from 0 = never to 3 = always). Resilience was assessed using the well-validated 10-item Connor–Davidson Resilience Scale (CD-RISC) in which items are rated from 0 (not true at all) to 4 (true nearly all the time), and final score ranges 0–40 (Campbell-Sills & Stein, 2007).

2.5 | Data analysis

Differences in baseline characteristics, PRSF, negative and positive experiences, resilience, and mental health outcomes between the COVID-19 and non-COVID-19 teams were determined using Z test for two independent proportions for binary variables. The χ^2 tests were used for categorical variables with more than two categories. Student's *t*-tests were used for continuous variables. Missing values in the PROMIS Anxiety module, PHQ-9, and CD-RISC questionnaires were imputed by the participant's mean score in other items (Altman & Bland, 2007).

Group differences in outcome continuous measures (PHQ-9 and PROMIS Anxiety) were tested by Analysis of Covariance (ANCOVA) with sociodemographics (age, sex, and physician\nurse) as covariates. Group differences in ordinal measures (PC-PTSD-5, PRSF, negative, and positive experiences) were tested with Mann–Whitney U test for independent samples. Within-person comparisons of ordinal measures (PRSF) were tested by Wilcoxon test for related samples.

We conducted two-step hierarchical logistic regression (Stoltzfus, 2011) for each mental health outcome (PC-PTSD-5; PHQ-9; and PROMIS Anxiety) separately. In Step 1, we computed crude odds ratios (ORs) for differences between COVID-19 and non-COVID-19 teams, with the latter set as reference group (OR = 1.00). The following variables were included in Step 2: (1) baseline characteristics (age, sex, physician \nurse, past psychological problems); (2) PRSF that were previously shown to be associated with physicians' anxiety, that is, anxiety about being infected, anxiety about infecting family, and mental exhaustion (Mosheva et al., 2020); (3) negative experiences: exposure to physical and/or mental suffering of patients, witnessing patient death (none/one or more), negative self-perception of physical health, and feeling unprotected by the hospital; (4) resilience.

To explore more explicitly how the effect of witnessing patient death on risk of PTSS was modified by work at the COVID-19 ward, we stratified the data to compute the OR and 95% CI for this association, for each study group. To explore interaction between witnessing patient death and work in COVID-19 ward, we tested for heterogeneity of effect by computing ORs and 95% CIs for the effect of witnessing patient death on the risk of each mental health outcome, using stratified by study team analysis. To confirm this interaction, we also used a multivariate logistic regression model with PTSS as dependent variable and an interaction term that includes COVOD-19 × witnessing patient death as covariate. An ancillary analysis was conducted for assessing effect modification by sex and occupation on the association between witnessing patient death and PTSS, by stratifying the data on each of those variables separately.

Alpha was set at .05, and all tests were two-tailed. Statistical analyses were conducted with IBM SPSS v25 software.

3 | RESULTS

A total of 828 HCW (42.1% physicians, 57.9% nurses, 189 from the COVID-19 team (42.3% of total COVID-19 team members) and 639 from non-COVID-19 team (20.1% of total non-COVID-19 team members) participated in the study. The characteristics of the study sample are presented in Table 1. Sample flow across COVID-19 and non-COVID-19 physicians and nurses is presented in Supplementary 1.

Compared with the non-COVID-19 teams, the COVID-19 team members had more male workers (41.3% vs. 30.7%), were younger (36.91 ± 8.81 vs. 43.14 ± 11.35 years), had fewer years of professional experience (9.91 ± 9.40 vs. 15.45 ± 12.32), and were more likely to live alone (23.7% vs. 12.2%) and be single (31.2% vs. 15.3%). Significantly more HCW from the COVID-19 team had to enter a 14-day quarantine (21.7% vs. 12.2%, Table 1).

Overall, members of both teams were significantly more anxious about infecting their families (48.7% reported feeling this way "often" or "always") than about contracting infection themselves (21.7%; $Z = 17.09$; $p < .001$). The COVID-19 team reported significantly more anxiety about infecting their families (54.3% vs. 47.0%; $Z = 2.46$; $p = .014$), had higher

TABLE 1 Sociodemographic and clinical characteristics of the study sample

Characteristics	Total sample (*n* = 828) No. (%)	COVID-19 team (*n* = 189) No. (%)	Non-COVID-19 team (*n* = 639) No. (%)	*p* value
Age, mean (*SD*), year	41.7 (11.1)	36.9 (8.8)	43.1 (11.3)	<.001
Sex, female	557 (67.2)	111 (58.7)	443 (69.3)	.006
Physician/nurse	349/479 (42.1/57.9)	73/116 (38.6/61.4)	276/363 (43.2/56.8)	.262
Quarantined	139 (16.7)	41 (21.7)	78 (12.2)	.001
History of physical illness	225 (26.7)	47 (25.0)	178 (27.9)	.417
Past psychological problems	297 (35.2)	82 (43.4)	215 (33.6)	.014
Living alone	122 (14.8)	44 (23.7)	78 (12.2)	<.001
Professional experience, mean (*SD*), year	14.2 (11.9)	9.9 (9.4)	15.4 (12.3)	<.001
Marital status				
Single	157 (18.9)	59 (31.2)	98 (15.3)	<.001
Married	584 (70.6)	114 (60.3)	470 (73.5)	
Divorced	61 (7.3)	11 (5.8)	50 (7.8)	
Other	26 (3.1)	5 (2.6)	21 (3.2)	
Religion				
Jewish	687 (82.7)	131 (69.7)	556 (86.5)	<.001
Muslim	69 (8.3)	36 (19.1)	33 (5.1)	
Christian	6 (0.7)	2 (1.1)	4 (0.6)	
Atheist	56 (6.7)	17 (9.0)	39 (6.1)	
Other	13 (1.6)	2 (1.1)	11 (1.7)	

Abbreviation: COVID-19, coronavirus disease 2019.

rates of mental exhaustion (36.4% vs. 25.6%; $Z = 3.39$; $p = .001$), felt more protected by the hospital (49.7% vs 28.3%; $Z = 5.11$; $p < .001$), and reported more altruistic acceptance of the risk working with COVID-19 patients (68.6% vs. 36.4%; $Z = 9.22$; $p < .001$, Table 2).

A significantly higher proportion of the COVID-19 team witnessed one or more patient deaths as compared with the non-COVID-19 team (50.2% vs. 24.7%, respectively). During March–April 2020 there have been 33 deaths (11 female, 32%) in the COVID-19 wards. Mean age at death was younger in the COVID-19 wards (71.23 ± 10.12 vs. 78.30 ± 13.72 in the non-COVID-19 wards). The number of deaths during March–April 2020, as compared with the parallel period in 2019, is presented in Figure 1. The mean number of hospital days between admission and death in the COVID-19 wards (16.69 ± 14.92) was significantly higher than in the non-COVID-19 wards (8.71 ± 9.37; $p < .001$).

3.1 | Posttraumatic stress symptoms, depression, and anxiety

The median PC-PTSD-5 scores differed significantly between study teams (χ^2 [5] = 17.24; $p = .004$). More than half (52.4%) of the COVID-19 team reported at least one PTSS compared with 41.8% in the non-COVID-19 team ($Z = 2.57$; $p = .009$, Table 2). The most commonly reported symptoms among the COVID-19 HCW were dissociation (30.5% vs. 19.5% in the non-COVID-19 team, $Z = 3.20$; $p = .001$) and intrusiveness (31.0% vs. 23.5% in the non-COVID-19 team; $p < .05$). The prevalence of PTSS score ≥ 3 in the total sample was 13.9%, with somewhat higher prevalence in the COVID-19 team: 16.9% versus 12.9% ($Z = 1.44$; $p = .150$). Depression was reported by 19.3% of the participants (COVID-19 teams: 25.0%; non-COVID-19 teams: 17.7%; $p = .025$). Anxiety was reported by 32.9% of the participants (COVID-19 teams: 37.0%; non-COVID-19 teams: 32.2%; $p = .23$). Differences were attenuated after controlling for age, sex and physician/nurse (Table 2).

The ORs and 95% CIs of the association between work in the COVID-19 wards, sociodemographic characteristics, PRSF, and negative experiences, and the mental health outcomes are presented in Table 3. Anxiety about being infected (OR, 1.58; 95% CI, 1.15–2.19; $p < .001$), infecting family (OR, 2.02; 95% CI, 1.43–2.85; $p < .0001$), and mental exhaustion (OR, 2.25; 95% CI, 1.68–3.01; $p < .0001$) were all found to be strongly associated with above cutoff PTSS. Depression and anxiety were associated with anxiety about infecting family (PHQ-9: OR, 1.81; 95% CI, 1.32–2.49; $p < .001$; PROMIS: OR, 1.72, 95% CI, 1.31–2.26; $p < .001$), and mental exhaustion (PHQ-9: OR, 3.75, 95% CI, 2.76–5.09; $p < .001$; PROMIS: OR, 2.69, 95% CI, 2.06–3.51; $p < .001$). Anxiety about being infected was associated with anxiety only (OR, 3.71; 95% CI, 2.64–5.22; $p < .001$, Table 3).

TABLE 2 Posttraumatic stress symptoms, depression, anxiety, resilience, stress factors, and subjective experiences by study team

Measure	COVID-19 team (*n* = 189) No. (%)	Non-COVID-19 team (*n* = 639) No. (%)	*p* value
PC-PTSD-5, No. of symptoms, median (IQR)	1 (0–2)	0 (0–1)	.004
0 symptoms	88 (46.6)	371 (57.7)	
1–2 symptoms	67 (35.5)	186 (28.9)	
1–2 symptoms	32 (16.9)	83 (12.9)	
PHQ-9, mean (*SD*)	6.6 (4.9)	5.4 (5.0)	.079
PHQ-9 ≥ 10	47 (25.0)	113 (17.7)	.025
PROMIS Anxiety, mean (*SD*)	58.2 (7.8)	57.9 (7.8)	.427
PROMIS ≥ 62.3	70 (37.0)	207 (32.2)	.234
CD-RISC, mean (*SD*)	30.3 (6.6)	29.7 (6.5)	.074
Pandemic-related stress factors (endorsing "often" or "always")			
Anxiety about being infected	34 (18.1)	131 (20.4)	.350
Anxiety about infecting family	102 (53.9)	301 (47.1)	.014
Lack of knowledge about infectiveness and virulence	37 (19.6)	112 (17.5)	.700
Lack of knowledge about prevention and protection	28 (14.8)	92 (14.4)	.126
Financial concerns	59 (31.7)	208 (32.6)	.387
Mental exhaustion	68 (36.0)	163 (25.5)	.001
Negative experiences			
High exposure to physical suffering of patients (often\always)	154 (81.5)	483 (75.6)	.004
High exposure to mental suffering of patients (often\always)	140 (74.9)	510 (80.0)	.442
Negative self-perceived health (fair\poor)	17 (9.0)	62 (9.8)	.191
Witnessing patient death	95 (50.2)	179 (24.7)	<.001
None	92 (48.7)	480 (74.7)	<.001
One	34 (18.0)	89 (13.8)	
More than one	61 (32.2)	70 (10.9)	
Positive experiences (endorsing "often" or "always")			
Altruistic acceptance of risk	127 (68.6)	225 (36.4)	<.001
Feeling mission driven to work	131 (70.0)	411 (64.4)	.001
Feeling protected by the hospital	93 (49.7)	181 (28.3)	<.001
Feeling protected by the government	29 (15.9)	102 (16.0)	.529

Abbreviations: CD-RISC, Connor–Davidson Resilience Scale; PC-PTSD-5, Primary Care-Post Traumatic Stress Disorder Screen for DSM-5; PHQ-9, Patient Health Questionnaire-9; PROMIS, Patient-Reported Outcomes Measurement Information System.

*PC-PTSD-5, PRSF, negative and positive experiences were tested with Mann–Whitney U test for independent samples. PHQ-9, PROMIS Anxiety, and CD-RISC were tested with ANCOVA adjusted for age, sex, and physician/nurse. Above/below cutoff proportions of PHQ-9, PROMIS Anxiety, and CD-RISC were tested with Z-test for two-population proportions.

3.2 | Witnessing patient death and posttraumatic stress symptoms

Witnessing patient death in the COVID-19 team was associated with a two-fold increased likelihood of PTSS as compared with the non-COVID 19 team (26.3% vs. 13.2%). This association was observed only in the COVID-19 team (OR, 3.97; 95% CI, 1.58–9.99; $p = .0007$), but not in the non-COVID-19 team (OR, 0.91; 95% CI, 0.51–1.61; $p = .92$, Table 4). The effect of witnessing patient death on PTSS in the COVID-19 team was stronger in females as compared with males (aOR 4.97 vs. aOR 2.91, respectively) and in physicians as compared with nurses (aOR 8.32 vs. 3.15, respectively, Supplementary 2). To further confirm this association, multivariate logistic regression model was used with PTSS as dependent variable and an interaction term of COVOD-19 x witnessing

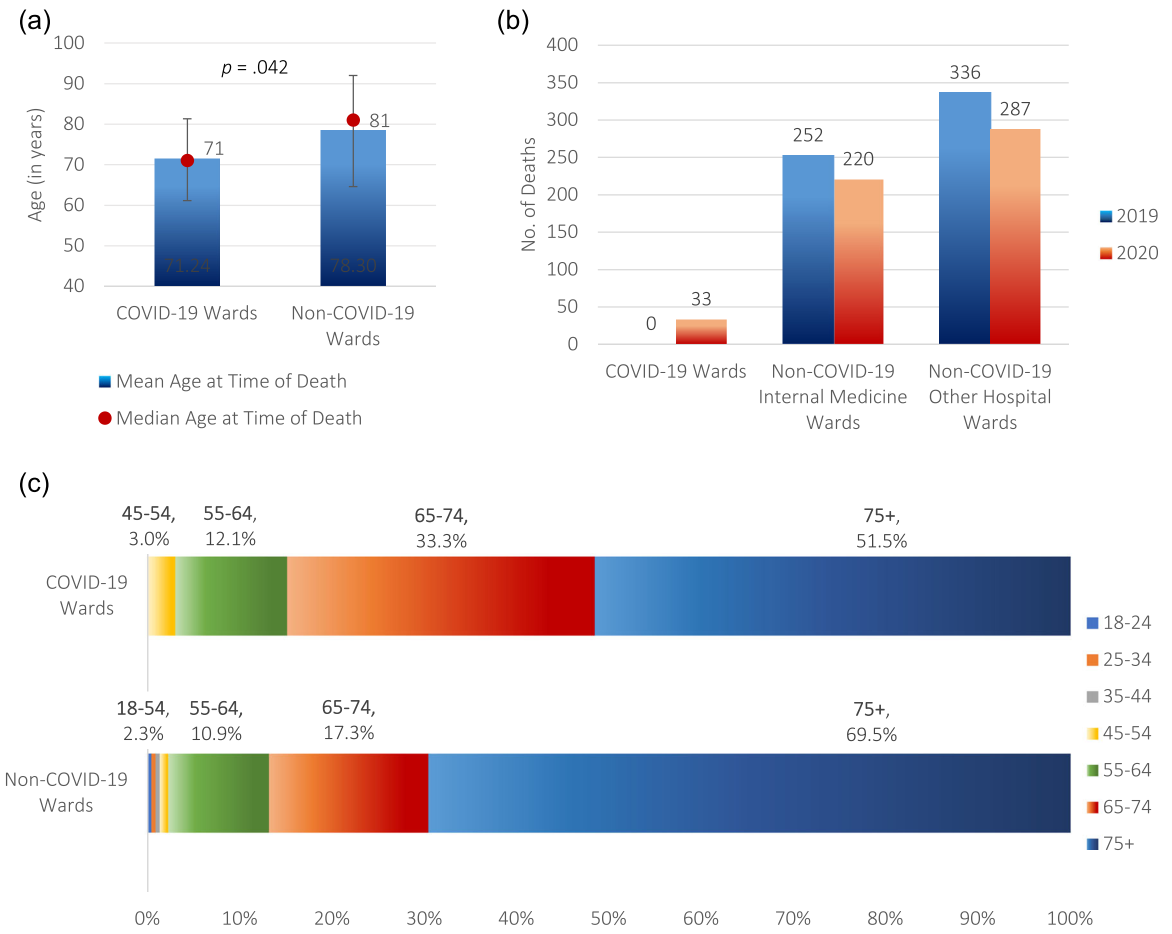

FIGURE 1 Total deaths and age at death in the coronavirus disease 2019 (COVID-19) versus non-COVID-19 wards. (a) Mean and median age at time of death in the COVID-19 versus non-COVID-19 wards during March–April 2020. (b) Number of deaths in COVID-19 versus non-COVID-19 wards during March–April in 2019 and 2020. (c) Age at death, by age categories in COVID-19 and non-COVID-19 wards

patient death as a covariate. The adjusted OR for this interaction term was 3.97 (95% CI, 1.34–11.77; $p = .013$).

4 | DISCUSSION

In this study of a sample involving more than 800 HCW from a large, tertiary medical center, conducted during a peak of inpatient admissions for COVID-19 in Israel, three independent risk factors for PTSS, probable depression and anxiety were identified: anxiety about infecting family, anxiety about being infected, and mental exhaustion. A fourth risk factor, unique to risk of PTSS that emerged from our data was witnessing patient death.

Witnessing death in different settings was shown to be related to PTSD symptoms among professionals (Alden et al., 2008; Lee et al., 2017). In a recently published study 50% of HCW working in a medical center in New Work City rated patient deaths as highly distressing (Shechter et al., 2020). Our study, however, is the first to report an independent, robust and statistically significant association between witnessing patient death and PTSS in COVID-19 frontline HCW. This finding was stronger among physicians as compared with nurses and among females as compared males. The latter strengthens the face validity of our findings, as sex differences in PTSD are well documented (Olff et al., 2007). The finding that physician are more likely to report PTSS might be explained by more susceptibility to feelings of uncertainty about effective lifesaving treatment protocols for COVID-19 which might lead to sense of despair and anguish.

Our finding of an association between witnessing patient death and PTSS might be attributed to several additional factors, unique to the COVID -19 setting. First, the COVID-19 team witnessed higher number of deaths, while at the same time there has been a decline in non-COVID-19 hospital admissions and subsequently a decline in non COVID-19 related in-hospital mortality (Figure 1). Second, unpredictable deterioration of young and previously healthy patients, in contrast to the typical non-COVID-19 deaths, might be experienced as traumatic. Third, we found that the mean age, at time of patient death in the COVID-19 wards, was significantly younger compared with the non-COVID-19 wards, and it has been shown that HCW are more distressed by death of patients that are closer to them in age (Alhazzani et al., 2020; Laposa & Alden, 2003). Lastly, the longer duration of hospital stay of patients in the COVID-19 ward, coupled with the fact that families were not allowed to be with their

TABLE 3 The association between mental health outcomes, study team, pandemic related stress factors, and subjective experiences

	PC-PTSD ≥ 3		PHQ-9 ≥ 10		PROMIS anxiety ≥ 62.3	
	STEP I	STEP II	STEP I	STEP II	STEP I	STEP II
	OR (95% CI)	OR (95% CI)	OR (95% CI)	OR (95% CI)	OR (95% CI)	OR (95% CI)
STEP I						
Working in a specialized COVID-19 ward	1.26 (0.79–2.00)	0.95 (0.53– 1.69)	1.48 (1.00– 2.21)	1.11 (0.65– 1.90)	1.22 (0.86– 1.73)	1.15 (0.70– 1.89)
STEP II- PRSF, negative experiences, and resilience						
Age		0.99 (0.96–1.01)		0.98 (0.96–1.01)		1.01 (0.99– 1.03)
Female		1.14 (0.63–2.06)		0.86 (0.50–1.49)		1.88 (1.15–3.06)*
Physician (ref = nurse)		1.18 (0.67–2.09)		0.91 (0.54–1.55)		1.02 (0.66–1.58)
Anxiety about being infected		1.58 (1.15–2.19)**		1.28 (0.94–1.75)		3.59 (2.57–5.01)***
Anxiety about infecting family		2.02 (1.43–2.85)***		1.82 (1.33–2.48)***		1.74 (1.32–2.28)***
Feeling protected by the hospital		0.82 (0.60–1.13)		0.93 (0.69–1.25)		1.04 (0.80–1.35)
Mental exhaustion		2.25 (1.68–3.01)***		3.72 (2.75–5.03)***		2.60 (2.01–3.37)***
Poor self-perceived health		0.98 (0.76–1.26)		0.81 (0.64–1.03)		0.81 (0.68–1.04)
Past psychological problems		1.62 (0.99– 2.65)		1.61 (1.01–2.56)*		0.99 (0.63–1.56)
High exposure to physical suffering of patients		0.92 (0.64–1.31)		1.35 (0.96–1.92)		0.89 (0.67–1.18)
High exposure to mental suffering of patients		1.21 (0.83–1.76)		0.93 (0.65–1.22)		1.12 (0.83–1.53)
Witnessing patient death		1.28 (0.75–2.19)		0.71 (0.43–1.20)		0.96 (0.93–0.99)*
Resilience		0.97 (0.94–1.01)		0.97 (0.94–1.01)		1.01 (0.99–1.03)

Note: Covariates in the model included: Perceived physical health; exposure to patients' physical suffering; exposure to patients' mental suffering.
Abbreviations: CI, confidence interval; OR, odds ratio; PC-PTSD-5, primary care-posttraumatic stress disorder screen for DSM-5; PHQ-9, Patient Health Questionnaire-9; PROMIS, Patient-Reported Outcomes Measurement Information System; PRSF, pandemic related stress factors.

*$p < .05$.

**$p < .01$.

***$p < .001$.

dying relative during their last moments, leaving the HCW to be those separating from the deceased, and having to communicate with and comfort the surviving family members, might have further contributed to the psychological burden on HCW (Lichtenthal et al., 2020; Selman et al., 2020).

This study extends our previously reported findings from another cohort of Israeli physicians on risk factors for COVID-19 related anxiety (Mosheva et al., 2020). In the present study we show that the factors that we reported to be associated with anxiety (mental exhaustion, anxiety about being infected, and infecting family) are also associated with depression and PTSS.

Curiously, resilience, shown previously as mitigating for anxiety of HCW (Mosheva et al., 2020), was not found to be associated with the outcome measures in our current investigation. That might be explained by the overwhelming and insoluble effects of the stressors and negative experiences, mainly witnessing death of patients, at the time of the pandemic.

A main strength of our study is that the study group (COVID-19 team) and the comparison group (non-COVID-19 team) were sampled from the same underlying cohort of physicians and nurses who share occupational, organizational, and hospital leadership features. Additional strength is that data were collected in "real-time," at the height of the pandemic in Israel, and not retrospectively, thus reducing likelihood of recall bias. We also had objective information on participants' hospital allocation, used well-validated instruments, and had a very low proportion of missing data.

Our study has several limitations. First, its cross-sectional design limits conclusions about directionality. However, the time sequence of our main finding, that is, that witnessing patient death in the COVID-19 ward is associated with PTSS, is unequivocal. In addition, we were able to establish temporality by anchoring of the traumatic stress questions specifically to the COVID-19 pandemic. Second, the relatively low response rate, among the non-COVID-19 team, although might be viewed as reasonable considering the circumstances under which recruitment carried out, might have introduced selection bias. However, responders were overall similar to non-responders with regard to their baseline characteristics. Thus, it is likely that nonresponse does not threaten considerably the internal validity of our findings (Johnson & Wislar, 2012). Third, information bias might have occurred if HCW tend to underreport mental health symptoms due to fear of labeling and stigma, and if responses on certain items were influenced by social desirability bias (Latkin et al., 2017; Tourangeau & Yan, 2007). However, the fact that questionnaires were anonymous makes this bias less likely. Fourth, we

TABLE 4 The association between witnessing patient death and mental health outcomes, stratified by study group (COVID-19 and non-COVID-19 teams)

	Full sample ($n = 829$)				COVID-19 team ($n = 189$)				Non-COVID-19 team ($n = 639$)			
	Witnessing patient death No. (%)	Not witnessing patient death No. (%)	Crude OR (95% CI)	Adjusted OR[a] (95% CI)	Witnessing patient death No. (%)	Not witnessing patient death No. (%)	Crude OR (95% CI)	Adjusted OR[a] (95% CI)	Witnessing patient death No. (%)	Not witnessing patient death No. (%)	Crude OR (95% CI)	Adjusted OR[a] (95% CI)
PC-PTSD ≥ 3	46 (18.1)	69 (12.0)	1.46 (0.96–2.23)	1.36 (0.88–2.10)	25 (26.3)	7 (7.6)	3.83 (1.54–9.49)*	3.97 (1.58–9.99)**,b	21 (13.2)	62 (12.9)**	0.96 (0.55–1.66)	0.91 (0.51–1.61)
PC-PTSD < 3	208 (81.9)	504 (88.0)	1.00	1.00	70 (73.7)	85 (92.4)	1.00	1.00	138 (86.8)	419 (87.1)	1.00	1.00
PHQ-9≥10	57 (22.4)	103 (17.9)	1.16 (0.79–1.69)	1.06 (0.72–1.57)	27 (28.4)	20 (21.5)	1.29 (0.65–2.54)	1.32 (0.64–2.71)	30 (18.9)	83 (17.3)	0.97 (0.60–1.58)	0.91 (0.55–1.50)
PHQ-9<10	197 (77.6)	471 (82.1)	1.00	1.00	68 (71.6)	73 (78.5)	1.00	1.00	129 (81.8)	398 (82.7)	1.00	1.00
PROMIS Anxiety ≥ 62.3	92 (36.2)	185 (32.0)	1.13 (0.82–1.56)	1.12 (0.80–1.56)	37 (38.9)	33 (35.1)	1.14 (0.62–2.07)	1.14 (0.62–2.10)	55 (34.6)	152 (31.5)	1.06 (0.71–1.57)	1.05 (0.69–1.59)
PROMIS Anxiety > 62.5	162 (63.8)	392 (68.0)	1.00	1.00	58 (61.1)	61 (64.9)	1.00	1.00	104 (65.4)	331 (68.5)	1.00	1.00

Abbreviations: CI, confidence interval; COVID-19, coronavirus disease 2019; OR, odds ratio; PC-PTSD-5, primary care-posttraumatic stress disorder screen for DSM-5; PHQ-9, Patient Health Questionnaire-9; PROMIS, Patient-Reported Outcomes Measurement Information System.

*$p < .01$.

**$p < .001$.

[a]Adjusted OR and 95% CI for age, sex, physician/nurse.

[b]Adjusted OR and 95% CI for COVID-19*witnessing patient death interaction term: 3.97 (1.34–11.77), $p = .013$.

used conventional thresholds of validated screening instruments, rather than gold standard diagnostic interviews, to identify probable PTSD, depression and anxiety. Fifth, although the odds ratio is a valid measure of association, its use as an approximation to the relative risk tends to exaggerate the magnitude of the association, more notably when the prevalence of the outcome is common, as in our study (Rigby, 1999). Finally, although higher number of deaths in the COVID-19 vs non-COVID-19 wards is universally reported (More people dying at home during Covid-19 pandemic – UK analysis), our study is based on data from a single medical center in Israel, and thus generalizability of its findings might be limited.

Our main finding that witnessing patient death in the past month was associated with PTSS, among the COVID-19 HCW only, have several potentially important implications for healthcare organizations and leaders in times of grave public health crisis. Those include actions aimed at prevention, detection, and management of traumatic stress in frontline HCW, particularly among those who have witnessed patient death. Suggested actions include educating frontline HCW about PTSS, providing support by their hospital and team leaders to better cope with traumatic events and bereavement, facilitate effective team cohesion, and implement strategies to support teams' day-to-day work, including informal debriefing and peer support (Selman et al., 2020).

Further research should focus on longitudinal studies of long-term mental health consequences among HCW who care for patients with COVID-19, with special attention to timely detection of post-traumatic stress and the role of potential risk and protective factors. Furthermore, interventional trials of potential strategies to reduce mental health burden among HCW, such as Balint groups (Van Roy et al., 2015) and end of life education, promote health programs that address bereavement issues (Kokou-Kpolou et al., 2020), and improving attitude to dying patients are needed (Lichtenthal et al., 2020). With growing awareness of the needs of HCW in COVID-19 teams and the recognition of the risk for PTSD, it is important that health organizations will take a proactive role in screening and intervening to address those needs. Those needs were recently reported in a Spanish study, where the majority of HCW working in COVID-19 units argued that psychological assistance should be provided at their centers, and almost half of the sample estimated that they will need mental health care in the future due to their COVID-19 related experiences (Martínez-López et al., 2020; Nguyen et al., 2020). As the COVID-19 pandemic is still ongoing, it is clear that maintaining the mental agility of the teams standing at the frontline is key for the endurance of the health care community facing this immense challenge (Fraher et al., 2020).

ACKNOWLEDGMENTS

Supported by grants from Foundation Dora and the Binational Science Foundation (Grant No. 2017369). The funding source had no role in the study design, collection, analysis, or interpretation of data, the writing of the article, or decision to submit the article for publication.

CONFLICT OF INTERESTS

The authors declare no potential conflicts of interest with respect to the research, authorship, and/or publication of this article.

AUTHOR CONTRIBUTIONS

All authors contributed to, reviewed and approved the final manuscript. Conceiving and designing the study: Mariela Mosheva, Raz Gross, Nimrod Hertz-Palmor, Doron Gothelf, and Itai M. Pessach. Data collection: Mariela Mosheva, Nimrod Hertz-Palmor, and Itai M. Pessach. Statistical analyses: Nimrod Hertz-Palmor. Data interpretation: Mariela Mosheva, Raz Gross, Nimrod Hertz-Palmor, Ilanit Hasson-Ohayon, Rachel Kaplan, Rony Cleper, Yitshak Kreiss, Doron Gothelf, and Itai M. Pessach. Writing the final manuscript: Mariela Mosheva, Raz Gross, Nimrod Hertz-Palmor, and Doron Gothelf.

PEER REVIEW

The peer review history for this article is available at https://publons.com/publon/10.1002/da.23140.

DATA AVAILABILITY STATEMENT

The data that support the findings of this study are available on request from the corresponding author. The data are not publicly available due to privacy or ethical restrictions.

ETHICS STATEMENT

The work described has been carried out in accordance with The Code of Ethics of the World Medical Association (Declaration of Helsinki) for experiments involving humans. Informed consent was obtained from all the subjects included in the study.

ORCID

Mariela Mosheva http://orcid.org/0000-0001-6404-0376
Nimrod Hertz-Palmor http://orcid.org/0000-0001-6837-9553

REFERENCES

Alden, L. E., Regambal, M. J., & Laposa, J. M. (2008). The effects of direct versus witnessed threat on emergency department healthcare workers: Implications for PTSD Criterion A. *Journal of Anxiety Disorders*, *22*, 1337–1346. https://doi.org/10.1016/j.janxdis.2008.01.013

Alhazzani, W., Møller, M. H., Arabi, Y. M., Loeb, M., Gong, M. N., Fan, E., Oczkowski, S., Levy, M. M., Derde, L., Dzierba, A., Du, B., Aboodi, M., Wunsch, H., Cecconi, M., Koh, Y., Chertow, D. S., Maitland, K., Alshamsi, F., Belley-Cote, E., … Rhodes, A. (2020). Surviving sepsis campaign: guidelines on the management of critically ill adults with coronavirus disease 2019 (COVID-19). *In Intensive Care Medicine*, *46*, 854–887. https://doi.org/10.1007/s00134-020-06022-5

Altman, D. G., & Bland, J. M. (2007). Missing data. *British Medical Journal*, *334*(7590), 424. https://doi.org/10.1136/bmj.38977.682025.2C

Amir, M., Lewin-Epstein, N., Becker, G., & Buskila, D. (2002). Psychometric properties of the SF-12 (Hebrew version) in a primary care population in Israel. *Medical Care*, *40*(10), 918–928. https://doi.org/10.1097/00005650-200210000-00009

Bai, Y. M., Lin, C. C., Lin, C. Y., Chen, J. Y., Chue, C. M., & Chou, P. (2004). Survey of stress reactions among health care workers involved with the SARS outbreak. *Psychiatric Services*, *55*(9), 1055–1057. https://doi.org/10.1176/appi.ps.55.9.1055

Bevans, M., Ross, A., & Cella, D. (2014). Patient-Reported Outcomes Measurement Information System (PROMIS): Efficient, standardized tools to measure self-reported health and quality of life. *Nursing Outlook*, *62*(5), 339–345. https://doi.org/10.1016/j.outlook.2014.05.009

Brooks, S. K., Rubin, G. J., & Greenberg, N. (2019). Traumatic stress within disaster-exposed occupations: Overview of the literature and suggestions for the management of traumatic stress in the workplace. *British Medical Bulletin*, *129*(1), 25–34. https://doi.org/10.1093/bmb/ldy040

Campbell-Sills, L., & Stein, M. B. (2007). Psychometric analysis and refinement of the Connor-Davidson Resilience Scale (CD-RISC): Validation of a 10-item measure of resilience. *Journal of Traumatic Stress*, *20*, 1019–1028. https://doi.org/10.1002/jts.20271

Can, W., Learn, W., Koh, D., Lim, M. K., Chia, S. E., & Ko, S. M. (2005). Risk perception and impact of Severe Acute Respiratory Syndrome (SARS) on work and personal lives of. *Medical Care*, *43*(7), 676–682.

Carmassi, C., Foghi, C., Dell'Oste, V., Cordone, A., Bertelloni, C. A., Bui, E., & Dell'Osso, L. (2020). PTSD symptoms in healthcare workers facing the three coronavirus outbreaks: What can we expect after the COVID-19 pandemic. *Psychiatry Research*, *292*(May), 113312. https://doi.org/10.1016/j.psychres.2020.113312

Chen, Q., Liang, M., Li, Y., Guo, J., Fei, D., Wang, L., He, L., Sheng, C., Cai, Y., Li, X., Wang, J., & Zhang, Z. (2020). Mental health care for medical staff in China during the COVID-19 outbreak. *The Lancet Psychiatry*, *7*(4), e15–e16. https://doi.org/10.1016/S2215-0366(20)30078-X

Dewey, C., Hingle, S., Goelz, E., & Linzer, M. (2020). Supporting clinicians during the COVID-19 pandemic. *Annals of Internal Medicine*, *172*(11), 752–753. https://doi.org/10.7326/M20-1033

von Elm, E., Altman, D. G., Egger, M., Pocock, S. J., Gøtzsche, P. C., & Vandenbroucke, J. P. (2007). Strengthening the Reporting of Observational Studies in Epidemiology (STROBE) statement: Guidelines for reporting observational studies. *BMJ*, *335*(7624), 806–808. https://doi.org/10.1136/bmj.39335.541782.AD

Ettman, C. K., Abdalla, S. M., Cohen, G. H., Sampson, L., Vivier, P. M., & Galea, S. (2020). Prevalence of depression symptoms in U.S. adults before and during the COVID-19 pandemic. *JAMA Network Open*, *9*. https://doi.org/10.1001/jamanetworkopen.2020.19686

Fraher, E. P., Pittman, P., Frogner, B. K., Spetz, J., Moore, J., Beck, A. J., Armstrong, D., & Buerhaus, P. I. (2020). Ensuring and sustaining a pandemic workforce. *New England Journal of Medicine*, *382*(23), 2181–2183. https://doi.org/10.1056/NEJMp2006376

Gao, J., Zheng, P., Jia, Y., Chen, H., Mao, Y., Chen, S., Wang, Y., Fu, H., & Dai, J. (2020). Mental health problems and social media exposure during COVID-19 outbreak. *PLoS One*, *15*(4), 1–10. https://doi.org/10.1371/journal.pone.0231924

Geuolayov, G., Jungerman, T., Moses, S., Friedman, N., Miron, R., & Gross, R. (2009). Validation of the Hebrew version of the PHQ-9, a screening instrument for depression in primary care. *Israel Journal of Psychiatry*, *46*(1), 36.

Goulia, P., Mantas, C., Dimitroula, D., Mantis, D., & Hyphantis, T. (2010). General hospital staff worries, perceived sufficiency of information and associated psychological distress during the A/H1N1 influenza pandemic. *BMC Infectious Diseases*, *10*(1), 322. https://doi.org/10.1186/1471-2334-10-322

National Institutes of Health. (2017). PROMIS: Dynamic tools to measure health outcomes from the patient perspective.

Huang, Y., & Zhao, N. (2020). Generalized anxiety disorder, depressive symptoms and sleep quality during COVID-19 outbreak in China: A web-based cross-sectional survey. *Psychiatry Research*, *299*(112954). https://doi.org/10.1016/j.psychres.2020.112954

Imai, H., Matsuishi, K., Ito, A., Mouri, K., Kitamura, N., Akimoto, K., & Mita, T. (2010). Factors associated with motivation and hesitation to work among health professionals during a public crisis: A cross sectional study of hospital workers in Japan during the pandemic (H1N1) 2009. *BMC Public Health*, 10. https://doi.org/10.1186/1471-2458-10-672

Johnson, T. P., & Wislar, J. S. (2012). Response rates and nonresponse errors in surveys. *Journal of the American Medical Association*, *307*(17), 1805–1806. https://doi.org/10.1001/jama.2012.3532

Kang, L., Ma, S., Chen, M., Yang, J., Wang, Y., Li, R., Yao, L., Bai, H., Cai, Z., Xiang Yang, B., Hu, S., Zhang, K., Wang, G., Ma, C., & Liu, Z. (2020). Impact on mental health and perceptions of psychological care among medical and nursing staff in Wuhan during the 2019 novel coronavirus disease outbreak: A cross-sectional study. *Brain, Behavior, and Immunity*, *87*(March), 11–17. https://doi.org/10.1016/j.bbi.2020.03.028

Kisely, S., Warren, N., McMahon, L., Dalais, C., Henry, I., & Siskind, D. (2020). Occurrence, prevention, and management of the psychological effects of emerging virus outbreaks on healthcare workers: rapid review and meta-analysis. *BMJ (Clinical Research Ed.)*, *369*, m1642. https://doi.org/10.1136/bmj.m1642

Kokou-Kpolou, C. K., Fernández-Alcántara, M., & Cénat, J. M. (2020). Prolonged grief related to COVID-19 seaths: Do we have to fear a steep rise in traumatic and disenfranchised griefs? *Psychological Trauma: Theory, Research, Practice, and Policy*, *12*(c), 94–95. https://doi.org/10.1037/tra0000798

Lai, J., Ma, S., Wang, Y., Cai, Z., Hu, J., Wei, N., Wu, J., Du, H., Chen, T., Li, R., Tan, H., Kang, L., Yao, L., Huang, M., Wang, H., Wang, G., Liu, Z., & Hu, S. (2020). Factors associated With mental health outcomes among health care workers exposed to Coronavirus Disease 2019. *JAMA Network Open*, *3*(3), e203976. https://doi.org/10.1001/jamanetworkopen.2020.3976

Laor-Maayany, R., Goldzweig, G., Hasson-Ohayon, I., Bar-Sela, G., Engler-Gross, A., & Braun, M. (2020). Compassion fatigue among oncologists: The role of grief, sense of failure, and exposure to suffering and death. *Supportive Care in Cancer*, *28*(4), 2025–2031. https://doi.org/10.1007/s00520-019-05009-3

Laposa, J. M., & Alden, L. E. (2003). Posttraumatic stress disorder in the emergency room: Exploration of a cognitive model. *Behaviour Research and Therapy*, *41*(1), 49–65. https://doi.org/10.1016/s0005-7967(01)00123-1

Latkin, C. A., Edwards, C., Davey-Rothwell, M. A., & Tobin, K. E. (2017). The relationship between social desirability bias and self-reports of health, substance use, and social network factors among urban substance users in Baltimore, Maryland. *Addictive Behaviors*, *73*, 133–136. https://doi.org/10.1016/j.addbeh.2017.05.005

Lee, J. H., Lee, D., Kim, J., Jeon, K., & Sim, M. (2017). Duty-related trauma exposure and posttraumatic stress symptoms in professional Firefighters. *Journal of Traumatic Stress*, *30*, 133–141. https://doi.org/10.1002/jts.22180

Leshem, E., Klein, Y., Haviv, Y., Berkenstadt, H., & Pessach, I. M. (2020). Enhancing intensive care capacity: COVID-19 experience from a Tertiary Center in Israel. *Intensive Care Medicine*, 1–2. https://doi.org/10.1007/s00134-020-06097-0

Levis, B., Benedetti, A., & Thombs, B. D. (2019). Accuracy of Patient Health Questionnaire-9 (PHQ-9) for screening to detect major depression: Individual participant data meta-analysis. *BMJ*, *365*, l1476. https://doi.org/10.1136/bmj.l1476

Li, Z., Ge, J., Yang, M., Feng, J., Qiao, M., Jiang, R., Bi, J., Zhan, G., Xu, X., Wang, L., Zhou, Q., Zhou, C., Pan, Y., Liu, S., Zhang, H., Yang, J., Zhu, B., Hu, Y., Hashimoto, K., … Yang, C. (2020). Vicarious traumatization in the general public, members, and non-members of medical teams aiding in COVID-19 control. *Brain, Behavior, and Immunity*, *88*, 916–919. https://doi.org/10.1016/j.bbi.2020.03.007

Lichtenthal, W. G., Roberts, K. E., & Prigerson, H. G. (2020). Bereavement Care in the Wake of COVID-19: Offering condolences and referrals. *Annals of Internal Medicine*, 1–4. https://doi.org/10.7326/m20-2526

Liu, S., Yang, L., Zhang, C., Xiang, Y. T., Liu, Z., Hu, S., & Zhang, B. (2020). Online mental health services in China during the COVID-19

outbreak. *The Lancet Psychiatry*, *7*(4), e17–e18. https://doi.org/10.1016/S2215-0366(20)30077-8

Lowell, A., Suarez-Jimenez, B., Helpman, L., Zhu, X., Durosky, A., Hilburn, A., Schneier, F., Gross, R., & Neria, Y. (2018). 9/11-related PTSD among highly exposed populations: A systematic review 15 years after the attack. *Psychological Medicine*, *484*, 537–553. https://doi.org/10.1017/S0033291717002033

Lu, W., Wang, H., Lin, Y., & Li, L. (2020). Psychological status of medical workforce during the COVID-19 pandemic: A cross-sectional study. *Psychiatry Research*, *288*, 1–5. https://doi.org/10.1016/j.psychres.2020.112936

Martínez-López, J. Á., Lázaro-Pérez, C., Gómez-Galán, J., Fernández-Martínez, M., & del, M. (2020). Psychological impact of COVID-19 emergency on health professionals: Burnout incidence at the most critical period in Spain. *Journal of Clinical Medicine*, *9*, 3029. https://doi.org/10.3390/jcm9093029

Maunder, R., Hunter, J., Vincent, L., Bennett, J., Peladeau, N., Leszcz, M., & Mazzulli, T. (2003). The immediate psychological and occupational impact of the 2003 SARS outbreak in a teaching hospital. *Canadian Medical Association Journal/Journal de l'Association Medicale Canadienne*, *168*(10), 1245–1251.

More people dying at home during Covid-19 pandemic – UK analysis. Retrieved from The Guardian website: https://www.theguardian.com/society/2020/may/08/more-people-dying-at-home-during-covid-19-pandemic-uk-analysis

Mosheva, M., Hertz-Palmor, N., Dorman Ilan, S., Matalon, N., Pessach, I. M., Afek, A., & Gothelf, D. (2020). Anxiety, pandemic-related stress and resilience among physicians during the COVID-19 pandemic. *Depression and Anxiety*, 1–7. https://doi.org/10.1002/da.23085

Naushad, V. A., Bierens, J. J., Nishan, K. P., Firjeeth, C. P., Mohammad, O. H., Maliyakkal, A. M., ChaliHadan, S., & Schreiber, M. D. (2019). A systematic review of the impact of disaster on the mental health of medical responders. *Prehospital and Disaster Medicine*, *34*(6), 632–643. https://doi.org/10.1017/S1049023X19004874

Nguyen, L. H., Drew, D. A., Graham, M. S., Joshi, A. D., Guo, C.-G., Ma, W., Mehta, R. S., Warner, E. T., Sikavi, D. R., Lo, C. H., Kwon, S., Song, M., Mucci, L. A., Stampfer, M. J., Willett, W. C., Eliassen, A. H., Hart, J. E., Chavarro, J. E., Rich-Edwards, J. W., … Zhang, F. (2020). Risk of COVID-19 among front-line health-care workers and the general community: A prospective cohort study. *The Lancet Public Health*, *5*, e475–e483. https://doi.org/10.1016/s2468-2667(20)30164-x

Nickell, L. A. (2004). Psychosocial effects of SARS on hospital staff: Survey of a large tertiary care institution. *Canadian Medical Association Journal/Journal de l'Association Medicale Canadienne*, *170*(5), 793–798 . https://doi.org/10.1503/cmaj.1031077

Ning, X., Yu, F., Huang, Q., Li, X., Luo, Y., Huang, Q., & Chen, C. (2020). The mental health of neurological doctors and nurses in Hunan Province, China during the initial stages of the COVID-19 outbreak. *BMC Psychiatry*, *20*(1), 436. https://doi.org/10.1186/s12888-020-02838-z

Olff, M., Langeland, W., Draijer, N., & Gersons, B. P. R. (2007). Gender differences in posttraumatic stress disorder. *Psychological Bulletin*, *133*, 183–204. https://doi.org/10.1037/0033-2909.133.2.183

Pappa, S., Ntella, V., Giannakas, T., Giannakoulis, V. G., Papoutsi, E., & Katsaounou, P. (2020). Prevalence of depression, anxiety, and insomnia among healthcare workers during the COVID-19 pandemic: A systematic review and meta-analysis. *Brain, Behavior, and Immunity*. https://doi.org/10.1016/j.bbi.2020.05.026

PROMIS® Scoring Manuals. Retrieved from https://www.healthmeasures.net/promis-scoring-manuals

Rigby, A. S. (1999). Statistical methods in epidemiology. III. The odds ratio as an approximation to the relative risk. *Disability and Rehabilitation*, *21*(4), 145–151. https://doi.org/10.1080/096382899297756

Rossi, R., Socci, V., Pacitti, F., Di Lorenzo, G., Di Marco, A., Siracusano, A., & Rossi, A. (2020). Mental health outcomes among frontline and second-line health care workers during the Coronavirus disease 2019 (COVID-19) pandemic in italy. *JAMA Network Open*, *3*(5), e2010185. https://doi.org/10.1001/jamanetworkopen.2020.10185

Van Roy, K., Vanheule, S., & Inslegers, R. (2015). Research on Balint groups: A literature review. *Patient Education and Counseling*, *98*(6), 685–694. https://doi.org/10.1016/j.pec.2015.01.014

Selman, L. E., Chao, D., Sowden, R., Marshall, S., Chamberlain, C., & Koffman, J. (2020). Bereavement Support on the Frontline of COVID-19: Recommendations for Hospital Clinicians. *Journal of Pain and Symptom Management*, *60*(2), e81–e86. https://doi.org/10.1016/j.jpainsymman.2020.04.024

Shechter, A., Diaz, F., Moise, N., Anstey, D. E., Ye, S., Agarwal, S., Birk, J. L., Brodie, D., Cannone, D. E., Chang, B., Claassen, J., Cornelius, T., Derby, L., Dong, M., Givens, R. C., Hochman, B., Homma, S., Kronish, I. M., Lee, S. A. J., … Abdalla, M. (2020). Psychological distress, coping behaviors, and preferences for support among New York healthcare workers during the COVID-19 pandemic. *General Hospital Psychiatry*, *66*(May), 1–8. https://doi.org/10.1016/j.genhosppsych.2020.06.007

Spitzer, R. L., Kroenke, K., Williams, J. B. W., & Löwe B. (2006). A brief measure for assessing generalized anxiety disorder the GAD-7. *Archives of Internal Medicine*, *166*(10), 1092–1097. https://doi.org/10.1001/archinte.166.10.1092

Spoont, M. R., Williams, J. W. J., Kehle-Forbes, S., Nieuwsma, J. A., Mann-Wrobel, M. C., & Gross, R. (2015). Does this patient have posttraumatic stress disorder?: Rational clinical examination systematic review. *Journal of the American Medical Association*, *314*(5), 501–510. https://doi.org/10.1001/jama.2015.7877

Stoltzfus, J. C. (2011). Logistic regression: A brief primer. *Academic Emergency Medicine*, *18*(10), 1099–1104. https://doi.org/10.1111/j.1553-2712.2011.01185.x

Styra, R., Hawryluck, L., Robinson, S., Kasapinovic, S., Fones, C., & Gold, W. L. (2008). Impact on health care workers employed in high-risk areas during the Toronto SARS outbreak. *Journal of Psychosomatic Research*, *64*(2), 177–183. https://doi.org/10.1016/j.jpsychores.2007.07.015

Sun, D., Yang, D., Li, Y., Zhou, J., Wang, W., Wang, Q., Lin, N., Cao, A., Wang, H., & Zhang, Q. (2020). Psychological impact of 2019 novel coronavirus (2019-nCoV) outbreak in health workers in China. *Epidemiology and Infection*, *148*, e96. https://doi.org/10.1017/S0950268820001090

Tam, C. W. C., Pang, E. P. F., Lam, L. C. W., & Chiu, H. F. K. (2004). Severe acute respiratory syndrome (SARS) in Hongkong in 2003: Stress and psychological impact among frontline healthcare workers. *Psychological Medicine*, *34*(7), 1197–1204. https://doi.org/10.1017/S0033291704002247

Tan, B. Y. Q., Chew, N. W. S., Lee, G. K. H., Jing, M., Goh, Y., Yeo, L. L. L., Zhang, K., Chin, H. K., Ahmad, A., Khan, F. A., Shanmugam, G. N., Chan, B. P. L., Sunny, S., Chandra, B., Ong, J. J. Y., Paliwal, P. R., Wong, L. Y. H., Sagayanathan, R., Chen, J. T., … Sharma, V. K. (2020). Psychological impact of the COVID-19 pandemic on health care workers in Singapore. *Annals of Internal Medicine*, *173*, 317–320. https://doi.org/10.7326/m20-1083

Taylor, W. D., & Blackford, J. U. (2020). Mental health treatment for front-line clinicians during and after the Coronavirus disease 2019 (COVID-19) pandemic: A plea to the medical community. *Annals of Internal Medicine*, *173*, 574–575. https://doi.org/10.7326/M20-2440

The American Association for Public Opinion Research. (2016). *Standard definitions: Final dispositions of case codes and outcome rates for surveys* (9th ed.). AAPOR.

Tourangeau, R., & Yan, T. (2007). Sensitive questions in surveys. *Psychological Bulletin*, *133*(5), 859–883. https://doi.org/10.1037/0033-2909.133.5.859

Ursano, R. J., Fullerton, C. S., Vance, K., & Kao, T. C. (1999). Posttraumatic stress disorder and identification in disaster workers. *American Journal of Psychiatry*, *156*(3), 353–359. https://doi.org/10.1176/ajp.156.3.353

Wang, C., Pan, R., Wan, X., Tan, Y., Xu, L., Ho, C. S., & Ho, R. C. (2020). Immediate psychological responses and associated factors during the initial stage of the 2019 coronavirus disease (COVID-19) epidemic among the general population in China. *International Journal of Environmental Research and Public Health*, *17*(5), 1729. https://doi.org/10.3390/ijerph17051729

Williamson, V., Murphy, D., & Greenberg, N. (2020). COVID-19 and experiences of moral injury in front-line key workers. *Occupational Medicine*. https://doi.org/10.1093/occmed/kqaa052

Yardeni, M., Abebe-Campino, G., Bursztyn, S., Shamir, A., Toren, A., Mekori-Domachevsky, E., & Gothelf, D. (2020). A three-tier process for screening depression and anxiety among children and adolescents with cancer. *Psycho-Oncology*, *29*, 2019–2027.

SUPPORTING INFORMATION

Additional Supporting Information may be found online in the supporting information tab for this article.

How to cite this article: Mosheva M, Gross R, Hertz-Palmor N, et al. The association between witnessing patient death and mental health outcomes in frontline COVID-19 healthcare workers. *Depress Anxiety*. 2021;38:468–479. https://doi.org/10.1002/da.23140
