# Supplementary material for: Mental health status and related factors influencing healthcare workers during the COVID-19 pandemic: A systematic review and meta-analysis
Source: PLoS One. 2024 Jan 19;19(1):e0289454. doi: 10.1371/journal.pone.0289454 (PMC10798549; doi:10.1371/journal.pone.0289454)
Supplement: S1 Data — (ZIP) [file pone.0289454.s011.zip › literatures/109.pdf]

ELSEVIER

Since January 2020 Elsevier has created a COVID-19 resource centre with free information in English and Mandarin on the novel coronavirus COVID-19. The COVID-19 resource centre is hosted on Elsevier Connect, the company's public news and information website.

Elsevier hereby grants permission to make all its COVID-19-related research that is available on the COVID-19 resource centre - including this research content - immediately available in PubMed Central and other publicly funded repositories, such as the WHO COVID database with rights for unrestricted research re-use and analyses in any form or by any means with acknowledgement of the original source. These permissions are granted for free by Elsevier for as long as the COVID-19 resource centre remains active.

Journal of Affective Disorders Reports 6 (2021) 100246

Contents lists available at ScienceDirect

Journal of Affective Disorders Reports

journal homepage: www.sciencedirect.com/journal/journal-of-affective-disorders-reports

Research Paper

The prevalence of common mental disorders among healthcare professionals during the COVID-19 pandemic at a tertiary Hospital in Addis Ababa, Ethiopia

Hailu Abera Mulatu [a,*], Muluken Tesfaye [b], Esubalew Woldeyes [a], Tola Bayisa [a], Henok Fisseha [a], Rodas Asrat Kassu [c]

[a] *Department of Internal Medicine, St. Paul's Hospital Millennium Medical College, P.O.Box 1784 Code 1250 Addis Ababa, Ethiopia*
[b] *Department of psychiatry, St. Paul's Hospital Millennium Medical College, Addis Ababa, Ethiopia*
[c] *Department of Neurosurgery, St. Paul's Hospital Millennium Medical College, Addis Ababa, Ethiopia*

ARTICLE INFO

Keywords:
Addis Ababa
COVID-19
Ethiopia
Health professional
Mental disorder
Prevalence

ABSTRACT

Background: Coronavirus disease 2019 (COVID-19) has resulted in unprecedented morbidity, mortality, and health system crisis leading to a significant psychological destress on healthcare workers (HCWs). The study aimed to determine the prevalence of symptoms of common mental disorders among HCWs during the COVID-19 pandemic at St. Paul's Hospital, Ethiopia.
Methods: A self-administered cross-sectional study was conducted to collect socio-demographic information and symptoms of mental disorders using validated measurement tools. Accordingly, PHQ-9, GAD-7, ISI, and IES-R were used to assess the presence of symptoms of depression, anxiety, insomnia, and distress, respectively. Chi-square test, non-parametric, and logistic regression analysis were used to detect risk factors for common mental disorders.
Results: A total of 420 healthcare workers participated in the survey. The prevalence of symptoms of depression, anxiety, insomnia, and psychological distress was 20.2%, 21.9%, 12.4%, and 15.5% respectively. Frontline HCWs had higher scores of mental health symptoms than non-frontline healthcare workers. Binary logistic regression analysis showed that being married was associated with a high level of depression. Furthermore, working in a frontline position was an independent risk factor associated with a high-level of symptoms of depression, anxiety, and psychological distress.
Limitations: It is a single-center cross-sectional study and the findings may not be nationally representative or reveal causality.
Conclusions: A significant proportion of healthcare workers are suffering from symptoms of mental disorders. Frontline HCWs were at a greater risk of severe symptoms. Therefore, psychological interventions should be implemented to support health professionals, especially frontline workers.

1. Introduction

Coronavirus disease 2019 (COVID-19) is a mild to severe respiratory illness that is caused by a coronavirus named severe acute respiratory syndrome coronavirus 2 (SARS COV2). It was first recognized in China in late December 2019 as an unknown respiratory disease outbreak. Due to its high risk of contiguity and human-to-human transmission, it has reached a global pandemic level in a very short period of time (Guan et al., 2020; Lai et al., 2020a).

Healthcare workers (HCWs) dealing with COVID-19 are under increased psychological pressure, and experience high rates of psychiatric morbidity, resembling the situation during the previous severe acute respiratory syndrome (SARS) and influenza epidemics (Bohlken et al., 2020; Li et al., 2020). Depression, anxiety, insomnia, and psychological distress are the common mental health disorders that occur during such a dramatic global health crisis (Liu et al., 2020; Pappa et al., 2020). A study among 1257 healthcare professionals in a tertiary hospital in China, revealed a high prevalence of mental health symptoms

* Corresponding author.
E-mail address: dhailu2001@gmail.com (H.A. Mulatu).

https://doi.org/10.1016/j.jadr.2021.100246
Received 20 May 2021; Received in revised form 19 September 2021; Accepted 1 October 2021
Available online 8 October 2021
2666-9153/© 2021 The Author(s). Published by Elsevier B.V. This is an open access article under the CC BY-NC-ND license (http://creativecommons.org/licenses/by-nc-nd/4.0/).

among HCWs during the early period of the pandemic. Overall, 50.4%, 44.6%, 34.0%, and 71.5% of health workers reported symptoms of depression, anxiety, insomnia, and distress, respectively. Nurses, females, and frontline HCWs were affected more than others with these mental disorders (Lai et al., 2020b). Likewise, the prevalence of symptoms of depression, anxiety, insomnia and distress was observed in 77.6%, 60.2%, 50.4%, and 76.4% respectively among healthcare workers in Turkey (Şahin et al., 2020). More recently, symptoms of depression and anxiety were reported in 15.7% and 41.2% frontline healthcare workers respectively in Bangladish (Tasnim et al., 2021). In a systematic review of 74 studies from 10 areas in Asia, Europe, North America and South America, the pooled prevalence of depression, anxiety, mental distress, and post-traumatic stress symptoms was 23.9%, 23.2%, 11.6%, and 28% respectively among health professionals during the COVID-19 epidemic (Zhao et al., 2021). In a recent study from Ethiopia, anxiety was observed among 26.8% of health professionals during the early period of COVID-19 pandemic (Dagne et al., 2021). In another study, psychological distress was reported in 40.2% of HCWs in South West Ethiopia (Hajure et al., 2021).

To date, studies on the psychological impact of COVID-19 among HCWs in Ethiopia are limited. Therefore, the present study tries to fill the gap of information and identifies the prevalence of depression, anxiety, insomnia, and psychological distress and associated factors among HCWs in Ethiopia.

2. Methods and materials

2.1. Participants

The study was conducted at St. Paul's Tertiary Hospital, the second-largest public hospital in Addis Ababa, Ethiopia. A total of 2308 frontline and non-frontline HCWs were involved in clinical care of patients suffering from COVID-19 in the hospital during the month of August 2020. Here, frontline HCWs are those participants who were directly engaged in clinical activities of diagnosing, treating, or providing nursing care to patients with confirmed COVID-19. Non-frontline HCWs are those participants who were not directly engaged in clinical activities of diagnosing, treating, or providing nursing care to patients with confirmed COVID-19 but could be indirectly exposed while involved in the care of other patients who might be in a pre-symptomatic stage of COVID-19.

A cross-sectional survey was conducted to assess the prevalence of depression, anxiety, insomnia, and mental distress among the HCWs at St. Paul's Hospital from August 1st, 2020 up to August 30, 2020. Sample size was calculated based on a single population formula for a finite population of 2308 HCWs with a 95% confidence interval, 5% margin of error, and taking the highest of the prevalence's of depression, anxiety, insomnia, and distress of 71.5% from similar studies (C. Lai et al., 2020; Zhu et al., 2020). By considering a 10% non-response rate and applying a sample correction formula, the total sample size was 272 HCWs. However, to allow for subgroup analysis, we amplified the sample size by 50% to reach 430 HCWs. HCWs 18 years or older and currently involved in patient care were included in the study. Those with previous mental illnesses or did not give consent were excluded from the study.

2.2. Procedure

Participants were recruited using a simple random sampling method from their alphabetical list prepared by the human resource development directorate after first stratifying them into four groups (doctors, nurses/ midwives, laboratory professionals, and pharmacy professionals) using probability proportional to size method. Three-fourth of the sample size was recruited from the COVID-19 treatment unit where participants were directly involved with the care of COVID-19 patients (frontline). The rest was from other units where participants might be exposed indirectly (non-frontline).

2.3. Measures

Data were collected using a self-administered questionnaire to assess for symptoms of depression, anxiety, insomnia, and distress using validated measurement tools in the country's official language (Amharic).

Patient Health Questionnaire-9 (PHQ-9) is a nine-item self-report scale designed to screen for depression. It examines how frequently participants were bothered by problems in the previous two weeks. Items are rated on a 4-point Likert-type scale, ranging from 0 (not at all) to 3 (nearly every day). The total scores range from 0 to 27, and was interpreted as: normal (0–4), mild (5–9), moderate (10–14), and severe (15–21) depression. The cut-off score for diagnosis of depression was 10 (Gelaye et al., 2014; Hanlon et al., 2015; Johnson et al., 2019). In the present study, the overall Cronbach's alpha of the PHQ-9 for depression subscale was 0.86, indicating good internal consistency.

General Anxiety Disorder-7 (GAD-7) is a seven-item self-report questionnaire designed to screen for anxiety. GAD-7 examines how frequently participants have been disturbed by problems in the preceding two weeks. Items are rated on a 4-point Likert-type scale, ranging from 0 (not at all) to 3 (nearly every day). Total scores range from 0 to 21. It was used to assess the severity of symptoms of anxiety, and was interpreted as: normal (0–4), mild (5–9), moderate (10–14), and severe (15–21) anxiety. The cut-off score for diagnosis of anxiety was 9 (Ong, and Suh, 2013; Manzar et al., 2021). In this study, the overall Cronbach's alpha of the GAD-7 questionnaire was 0.88, showing good reliability.

The Insomnia Severity Index (ISI) is a seven-item self-report questionnaire evaluating the nature, severity, and impact of insomnia. The ISI assesses participants' difficulty in falling asleep, difficulty in remaining asleep, very early waking, the satisfaction derived from the sleep pattern, impairments emerging in day to day functioning, awareness of sleep-related impairments, and stress levels caused by sleep problems in the previous two weeks. Items are rated on a 5-point Likert-type scale, ranging from 0 (no problem) to 4 (very severe problem). Total scores range from 0 to 28, and was interpreted as: normal (0–7), sub-threshold (8–14), moderate (15–21), and severe (22–28) insomnia. The cut-off score for diagnosis of insomnia was 15 (Ong, J. C., and Suh, 2013; Johnson et al., 2019; Manzar et al., 2020). In our study, the overall Cronbach's alpha of the ISI questionnaire for insomnia subscale was 0.81, indicating good internal consistency.

The Impact of Event Scale-Revised (IES-R) is a 22-item self-report questionnaire evaluating psychological distress. The IES-R investigates how frequently participants have been troubled by problems in the previous two weeks. Items are rated on a 5-point Likert-type scale, ranging from 0 (not at all) to 4 (extremely). Total scores range from 0 to 88, and was interpreted as: normal (0–8), mild (9–25), moderate (26–43), and severe (44–88) distress. A cut-off score for diagnosis of distress was 33 (Creamer et al., 2003). In the present study, the overall Cronbach's alpha of the IES-R for distress subscale was 0.83, indicating good reliability.

Demographic data were self-reported by the participants, including educational level (graduate, post-graduate), profession (doctor, nurse/ midwife, laboratory professional or pharmacy professional), sex (male or female), age (18–25, 26–30, 31–40, or >40 years), marital status, monthly net salary, work experience (<5, 5-10, or >10 years), and technical title (junior or senior). The different technical titles of respondents refer to the professional titles certificated by the Ethiopian Food, Medicine and Health Care Administration, and Control Authority (EFMHACA). Participants were asked whether they were directly engaged in clinical activities of diagnosing, treating, or providing nursing care to patients with confirmed COVID-19. Those who responded yes were defined as frontline workers, and those who answered no were defined as non-frontline workers.

2.4. Statistical analysis

Data were first entered and cleaned using EPI data version 7 and then exported into SPSS version 23 for statistical analysis. All categorical variables were presented as numbers and percentages. The original scores of the 4 measurement tools were continuous, not normally distributed, and thus presented as a median and interquartile range (IQR). A nonparametric Mann-Whitney U test and Kruskal-Wallis H test were applied to compare the severity of each symptom between two or more groups. The ranked data, which were derived from the counts of each level for symptoms of depression, anxiety, insomnia, and distress, were presented as numbers and percentages. To determine for factors associated with symptoms of depression, anxiety, insomnia, and distress, first a univariate analysis was performed for all variables and those with $p \leq 0.2$ on univariate analysis were entered into a multivariable logistic regression analysis. The associations between risk factors and outcomes were presented as odds ratios (ORs) and 95% CIs, after adjustment for confounders, including age, sex, marital status, profession, educational level, technical rank, monthly salary, work experience, and working position (frontline or non-frontline). Hosmer-Lemeshow was used for the evaluation of the calibration of the regression model. Finally, a p-value <0.05 in the multivariable model was considered as significant.

2.5. Ethical approval

This study was approved by the Institutional Review Board of St. Paul's Hospital Millennium Medical College (Reference No. PM 23/10). Written informed consent was obtained from all participants. Each staff's information was collected by residents and nurses using an anonymous pre-coded structured questionnaire that was assigned to each selected HCW. The code was blinded to both data collectors and data entry clerks. The information was kept confidential among the investigators. A staff that had severe symptoms of mental disorder was traced by a psychiatrist (one of the investigators) and advised on further evaluation and treatment. Those who volunteered were linked to our psychiatric clinic.

3. Results

3.1. Socio-demographic characteristics of study participants

A total of 420 participants completed the questionnaire making a response rate of 97.7%. Of these, 115 (27.4%) participants were doctors, 237 (56.4%) were nurses and midwives, 40 (9.5%) were laboratory professionals and 28 (6.7%) were pharmacy professionals. The response rates for doctors, nurses or midwives, laboratory professionals, and pharmacy professionals were 92%, 100%, 100%, and 100% respectively. The mean age of the participants was 28±5.4 [range: 20–57] years. Among the participants, 212 (50.5%) were in the age range of 26–30 years. Two-hundred-forty-six (58.6%) participants were men and 296 (70.5%) were unmarried. The qualification of participants indicated that 295 (70.2%) had undergraduate level and 125 (29.8%) had a postgraduate level of education. Regarding their technical title, 237 (56.4%) were junior and 183 (43.6%) were senior staffs in their professional career. The mean monthly salary of respondents was 167± 84.7 United States Dollar (USD) and 213 (50.7%) respondents earn between 111 and 222 USD. The majority (70.5%) of respondents had a work experience of fewer than five years and 296 (70.5%) were frontline healthcare professionals. A significant proportion of respondents working as frontline were aged between 26 and 30 years, unmarried, and nurses or midwives. They had an undergraduate level of education and work experience of fewer than five years (Table 1).

3.2. The prevalence of symptoms of mental disorders

The prevalence of symptoms of the mental disorders was computed from the pre-specified cut-off values of PHQ-9 score ≥10 for depression, GAD-7 score ≥9 for anxiety, ISI score ≥15 for insomnia, and IES-R score ≥ 33 for psychological distress diagnosis. Based on the above cut-off scores, the prevalence of depression, anxiety, insomnia, and psychological distress was found to be 20.2%, 21.9%, 12.4%, and 15.5% respectively. The median (IQR) scores on the PHQ-9, GAD-7, ISI, and IES-R for all respondents were 4.0(1.0–9.0), 3.0(0–8.0), 4.0(1.0–9.0), and 11.0(4.0–25.0), respectively. Symptoms of depression, anxiety,

Table 1
Socio-demographic characteristics of participants, St. Paul's Hospital, 2020.

Characteristic		Number (%)			*p*-value
		Overall N=420	Frontline N=296	Non-frontline N=124	
Age, year	18-25	121 (28.8)	84 (28.4)	37 (29.8)	<0.001
	26-30	212 (50.5)	165 (55.7)	47 (37.9)	
	31-40	75 (17.9)	44 (14.9)	31 (25.0)	
	>40	12 (2.9)	3 (1.0)	9 (7.3)	
Sex	Male	246 (58.6)	176 (59.5)	70 (56.5)	0.56
	Female	174 (41.4)	120 (40.5)	54 (43.5)	
Marital status	Unmarried	296 (70.5)	223 (75.3)	73 (58.9)	0.001
	Ever married*	124 (29.5)	73 (24.7)	51 (41.1)	
Profession	Doctor	115 (27.4)	73 (24.7)	42 (33.9)	<0.001
	Nurse/Midwife	237 (56.4)	177 (59.8)	60 (48.4)	
	Laboratory professional	40 (9.5)	34 (11.5)	6 (4.8)	
	Pharmacy professional	28 (6.7)	12 (4.0)	16 (12.9)	
Level of Education	undergraduate	295 (70.2)	218 (73.6)	77 (62.1)	0.02
	Postgraduate	125 (29.8)	78 (26.4)	47 (37.9)	
Technical title	Junior	237 (56.4)	171 (57.8)	66 (53.2)	0.45
	Senior	183 (43.6)	125 (42.2)	58 (46.8)	
Monthly salary, USD	<111	110 (26.2)	87 (29.4)	23 (18.5)	0.01
	111-222	213 (50.7)	151 (51.0)	62 (50.0)	
	>222	97 (23.1)	58 (19.6)	39 (31.5)	
Work experience, y	<5	301 (71.7)	217 (73.3)	84 (67.7)	0.02
	5-10	93 (22.1)	67 (22.6)	26 (21.0)	
	>10	26 (6.2)	12 (4.1)	14 (11.3)	

USD, United States Dollar; SD, standard deviation;
* Ever married included widowed and divorced participants; y, year

Table 2
Number of cases and scores of depression, anxiety, insomnia, and psychological distress measurements in total cohort and subgroups among HCWs at St. Paul's Hospital, 2020.

Scale	Total cases (%)†	Total Score, median (IQR)	Working position Score, median (IQR) Frontline	Non-frontline	P -value
PHQ-9, depression symptoms	85 (20.2)	4.0 (1.0–9.0)	5.0 (2.0–9.0)	2.0 (0–6.0)	<0.001
GAD-7, anxiety symptoms	92 (21.9)	3.0 (0–8.0)	4.0 (0–9.0)	2.5 (0–5.0)	0.003
ISI, insomnia symptoms	52 (12.4)	4.0 (1.0–9.0)	5.0 (1.0–10.0)	4.0 (1.0–7.0)	0.003
IES-R, distress symptoms	65(15.5)	11.0 (4.0–25.0)	13.0 (4.25–29.0)	8.0 (3.0–16.0)	<0.001

Abbreviations: GAD-7, 7-item Generalized Anxiety Disorder; IES-R, 22-item Impact of Event Scale–Revised; ISI, 7-item Insomnia Severity Index; PHQ-9, 9-item Patient Health Questionnaire, IQR, interquartile range, †number of participants who were diagnosed with the specific disorder (number of cases/ total number of respondents).

Table 3
Severity categories of depression, anxiety, stress, insomnia, and psychological distress in total cohort and subgroups, St. Paul's Hospital, 2020.

Severity Category	Total, N (%) $N = 420$	Working position, n (%) Frontline $N = 296$	Non-frontline $N = 124$	P-value
PHQ-9, depression symptoms				
Normal	216 (51.4)	132 (44.6)	84 (67.7)	<0.001
Mild	119 (28.3)	94 (31.8)	25 (20.2)	
Moderate	57 (13.6)	51 (17.2)	6 (4.8)	
Severe	28 (6.7)	19 (6.4)	9 (7.3)	
GAD-7, anxiety symptoms				
Normal	248 (59.0)	161 (54.4)	87 (70.2)	0.02
Mild	99 (23.6)	75 (25.3)	24 (19.4)	
Moderate	49 (11.7)	40 (13.5)	9 (7.3)	
Severe	24 (5.7)	20 (6.8)	4 (3.2)	
ISI, insomnia symptoms				
Absence	290 (69.0)	194 (65.5)	96 (77.4)	0.04
Sub-threshold	78(18.6)	59 (19.9)	19 (15.3)	
Moderate	43 (10.2)	34 (11.5)	9 (7.3)	
Severe	9 (2.1)	9 (3.0)	–	
IES-R, distress symptoms				
Normal	177 (42.2)	114 (38.5)	63 (50.8)	<0.001
Mild	142 (33.8)	94 (31.8)	48 (38.7)	
Moderate	71(16.9)	58 (19.6)	13(10.5)	
Severe	30 (7.1)	30 (10.1)	–	

Abbreviations: GAD-7, 7-item Generalized Anxiety Disorder; IES-R, 22-item Impact of Event Scale–Revised; ISI, 7-item Insomnia Severity Index; PHQ-9, 9-item Patient Health Questionnaire

insomnia, and distress were reported in higher proportion of frontline healthcare workers (i.e. depression among frontline vs non-frontline: 70 [23.6%] vs 15 [17.6%]; $P<0.001$; anxiety among frontline vs non-frontline: 75[25.3%] vs 17 [13.7%]; $P = 0.003$; insomnia among frontline vs non-frontline: 43[14.5%] vs 9 [7.3%]; $P = 0.003$; and distress among frontline vs non-frontline: 60 [20.3%] vs 5 [4.0%]; $P < 0.001$) (Table 2).

3.3. Severity of symptoms of mental disorders and associated factors

Moderate symptoms of depression, anxiety insomnia, and distress was reported in 57(13.6%), 49(11.7%), 43(10.2%), and 71(16.9%) respondents respectively. Severe symptoms of depression, anxiety insomnia, and distress was also reported in 28(6.7%), 24(5.7%), 9 (2.1%), and 30(7.1%) respondents respectively. The proportion of frontline HCWs who experienced moderate to severe symptoms of anxiety, insomnia, and distress was greater than non-frontline HCWs (Table 3).

3.4. Perception of threat of COVID-19 and effects of psychological protective measures

The following five common risk factors of perception of threat of COVID-19 were assessed: (i) feel of exposure to COVID-19; (ii) resigning thoughts; (iii) worry of life-threatening situation; (iv) feel of family members and friends' avoidance; and (v) feel of infection of self or family members by COVID-19. Feel of exposure to COVID-19 was reported in 53 (62.4%), 49 (53.3%), 35 (67.3%), and 48 (73.8%) HCWs with symptoms of depression, anxiety, insomnia, and psychological distress respectively. Thought of resigning from work due to COVID-19 was reported in significantly higher proportion of respondents with symptoms of depression, anxiety, insomnia, and psychological distress (p-values: 0.005, 0.007, <0.001, and <0.001 respectively) (Table 4).

The study also assessed whether or not the respondents were satisfied with the psychological protective measures implemented at St. Paul's Hospital. The common protective measures assessed were as follows: (i) care provided by hospital administrators; (ii) work-shift arrangement; (iii) coverage of the department with personal protective equipment; and (iv) logistic and accommodation support. Sixty-three (74.1%) respondents with symptoms of depression were not satisfied with the care provided by the hospital administrator. Similarly, 66 (71.7%), 35 (67.3), and 48 (73.8%) HCWs who showed symptoms of anxiety, insomnia, and psychological distress respectively were not satisfied with the care provided by the hospital administrator. Respondents who were satisfied with the work-shift arrangement suffered less from insomnia and psychological distress than those who were not satisfied with this protective measure (p-values: 0.004 and 0.008 respectively) (Table 5).

3.5. Factors associated with symptoms of mental health disorders

A binary logistic regression analysis was conducted to determine the demographic and relevant contextual factors that were associated with mental health illnesses. In the adjusted logistic regression analysis, several factors were independently associated with depression (PHQ-9 score ≥ 10), anxiety (GAD-7 score ≥ 9), insomnia (ISI score ≥ 15), and psychological distress (IES-R score ≥ 33). Factors that were independently associated with a higher risk of depression included being married (OR, 2.5; 95% CI, 1.4–4.6; $p = 0.03$) and working in frontline position (OR, 2.4; 95% CI, 1.2–4.5; $p = 0.009$). Working in frontline position is also associated with more severe symptoms of anxiety (OR, 2.1; 95% CI, 1.1–3.9; $p = 0.02$) and psychological distress (OR, 5.9; 95% CI, 2.2–15.5; $p<0.001$). (Table 6).

4. Discussion

Our study revealed a high prevalence of mental health symptoms among HCWs at St. Paul's Hospital, with close to one-fifth of respondents screened positive on at least one of the four established measuring tools. The prevalence of depression, anxiety, insomnia, and psychological distress in the study among health professionals was 20.2%, 21.9%, 12.4%, and 15.5% respectively. We also found out that the proportion of frontline HCWs experiencing these adverse mental outcomes was significantly higher than that of non-frontline. Finally, we discovered that being married, having undergraduate level of education, and earning monthly salary below 222 USD were associated with development of severe symptoms of mental health disorders. As a result,

Table 4
Perception of threat of the COVID-19 in binary logistic regression analysis among HCWs dealing with the COVID-19 at St. Paul's Hospital, 2020.

Variable		Diagnosis		OR (95% CI)	P value
		Depression (%) (N=85)	Non-depression (N=335)		
Feel of exposure to COVID-19	Yes	53 (62.4)	199 (59.4)	1.2 (0.7-2.1)	0.44
	No	32 (37.6)	136 (40.6)	Ref.	
Thought of resigning from work due to COVID-19	Yes	29 (34.1)	51 (15.2)	2.3 (1.3-4.0)	0.005
	No	56 (65.9)	284 (84.8)	Ref.	
Worried about a life-threatening situation once infected	Yes	54 (63.5)	143 (42.7)	1.8 (1.0-3.0)	0.03
	No	31(36.5)	192 (57.3)	Ref.	
Feel of family members or friends avoidance	Yes	67 (78.8)	216 (64.5)	0.7 (0.4-1.2)	0.20
	No	18 (21.2)	119 (35.5)	Ref.	
Worried of infection of self or family members	Yes	80 (94.1)	282 (84.2)	0.5 (0.2-1.5)	0.23
	No	5 (5.9)	53 (15.8)	Ref.	
		Anxiety (N=92)	Non-anxiety N=328)		
Feel of exposure to COVID-19	Yes	49 (53.3)	203 (61.9)	1.8 (1.1-3.0)	0.02
	No	43 (46.7)	125 (38.1)	Ref.	
Thought of resigning from work due to COVID-19	Yes	28 (30.4)	52 (15.9)	2.2 (1.2-3.8)	0.007
	No	64 (69.6)	276 (84.1)	Ref.	
Worried about a life-threatening situation once infected	Yes	52 (56.5)	145 (44.2)	0.7 (0.4-1.1)	0.15
	No	40 (43.5)	183 (55.8)	Ref.	
Feel of family members or friends avoidance	Yes	68 ((73.9)	215 (65.5)	0.8 (0.4-1.4)	0.39
	No	24 (26.1)	113 (34.5)	Ref.	
Worried of infection of self or family members	Yes	82 (89.1)	280 (85.4)	0.9 (0.4-1.9)	0.71
	No	10 (10.9)	48 (14.6)	Ref.	
		Insomnia (N=52)	Non-insomnia (N=368)		
Feel of exposure to COVID-19	Yes	35 (67.3)	217 (59.0)	1.1 (0.6-2.1)	0.77
	No	17 (32.7)	151 (41.0)	Ref.	
Thought of resigning from work due to COVID-19	Yes	26 (50.0)	54 (14.7)	5.0 (2.6-9.6)	<0.001
	No	26 (50.0)	314 (85.3)	Ref.	
Worried about a life-threatening situation once infected	Yes	30 ((57.7)	167 (45.4)	1.1 (0.6-2.2)	0.7
	No	22 (42.3)	201 (54.6)	Ref.	
Feel of family members or friends avoidance	Yes	44 ((84.6)	239 (64.9)	0.5 (0.2-1.2)	0.2
	No	8 (15.4)	129 (35.1)	Ref.	
Worried of infection of self or family members	Yes	51 (98.1)	311 (84.5)	0.2 (0.02-1.3)	0.1
	No	1 (1.9)	57 (15.5)	Ref.	
		Distress (N=65)	Non-distress (N=355)		
Feel of exposure to COVID-19	Yes	48 (73.8)	204 (57.5)	0.8 (0.4-1.4)	0.4
	No	17 (26.2)	151 (42.5)	Ref.	
Thought of resigning from work due to COVID-19	Yes	35 (53.8)	45 (12.7)	6.5 (3.5-12.0)	<0.001
	No	30 (46.2)	310 (87.3)	Ref.	
Worried about a life-threatening situation once infected	Yes	39 (60.0)	158 (44.5)	1.1 (0.6-2.0)	0.75
	No	26 (40.0)	197 (55.5)	Ref.	
Feel of family members or friends avoidance	Yes	56 (86.2)	227 (63.7)	0.5 (0.2-1.1)	0.08
	No	9 (13.8)	128 (36.1)	Ref.	
Worried of infection of self or family members	Yes	64 (98.5)	298 (83.9)	0.2 (0.02-1.2)	0.08
	No	1 (1.5)	57 (16.1)	Ref.	

Abbreviation: Ref, reference category

our findings raise a concern about the psychological well-being of HCWs involved in the care and treatment of COVID-19.

The findings of our survey is consistent with several other studies (Huang and Zhao, 2020; Que et al., 2020; Zhu et al., 2020) conducted during the COVID-19 pandemic. A report by Zhu et al. among 5062 HCWs in China found a significant percentage of mental health symptoms; 13.5% for depression, 24.1% for anxiety, and 29.8% for distress among healthcare workers. Another study by Que et al. from China among 2285 HCWs also identified moderate to severe symptoms of depression, anxiety, and insomnia in 12.8%, 11.6%, and 6.8% of respondents respectively. Similarly, in a recent meta-analysis of thirteen studies, the pooled prevalence of depression was 22.8% and that of anxiety was 23.2% among the HCWs involved in COVID-19 care (Pappa et al., 2020). In the contrary, a report by Lai et al. from China reported depression, anxiety, insomnia, and psychological distress among 50.4%, 44.6%, 34%, and 71.5% of HCWs respectively. This discrepancy is probably due to the differences in the methodology, where by Lai et al. used lower threshold values (scores) for the diagnosis of symptoms of depression, anxiety, insomnia, and distress that might have overrated the prevalence. Had Lai et al. utilized a similar cut-off values to our current study, the prevalence of depression, anxiety, insomnia, and distress would have been 14.8%, 12.3%, 7.7%, and 35% respectively. Therefore, the use of different measuring tools and methodologies, and the use of different classifications even if the same scale was used, leads to very different results being reported for the prevalence of mental disorders (Pappa et al., 2020).

The current study revealed that working in a frontline position was independently associated with higher adverse mental health outcomes, compared to working in a non-frontline environment. Our finding is in line with other recent studies (Alkhamees et al., 2020; Huang and Zhao, 2020; Lai et al., 2020; Que et al., 2020; Zhu et al., 2020), which also reported a significantly higher proportion of severe symptoms of mental disorders among frontline than non-frontline HCWs. Some of the reasons could be; frontline HCWs encounter and witness the suffering of seriously ill and dying-alone patients. They might also be under enormous workload abiding with the strict protective measures, which could make them uncomfortable (Cai et al., 2020; Xiang et al., 2020). However, we did not observe a significantly higher rate of seeking help or receiving treatment for mental problems among these subjects. The phenomenon that HCWs have difficulty accepting and disclosing mental health issues is not unique to the COVID-19 outbreak (Tysser et al., 2004; Fridner et al., 2012). Several studies reported high prevalence of psychological

Table 5
Psychological protective measures in binary logistic regression analysis among HCWs dealing with the COVID-19 at St. Paul's Hospital, 2020.

Variable		Diagnosis, N (%)		OR (95% CI)	P value
		Depression (N=85)	Non-depression (N=335)		
Satisfied with care provided by hospital administrator	Yes	22 (25.9)	115 (34.3)	Ref.	0.45
	No	63 (74.1)	220 (65.7)	1.3 (0.7-2.2)	
Satisfied with coverage of department with personal protective equipment	Yes	17 (20.0)	81 (24.2)	Ref.	0.95
	No	63 (80.0)	254 (75.8)	1.0 (0.5-1.8)	
Satisfied with work-shift arrangement	Yes	32 (37.6)	174 (51.9)	Ref.	0.054
	No	53 (62.4)	161 (48.1)	1.6 (0.99-2.7)	
Satisfied with logistic support and comfortable accommodation	Yes	16 (18.8)	93 (27.8)	Ref.	0.31
	No	69 (81.2)	242 (72.2)	1.4 (0.7-2.6)	
		Anxiety (N=92)	Non-anxiety (N=328)		
Satisfied with care provided by department and hospital administrator	Yes	26 (28.3)	111 (33.8)	Ref.	0.55
	No	66 (71.7)	217 (66.2)	1.2 (0.7-2.1)	
Satisfied with coverage of department with personal protective equipment	Yes	19 (20.7)	79 (24.1)	Ref.	0.91
	No	73 (79.3)	249 (75.9)	1.0 (0.6-1.9)	
Satisfied with work-shift arrangement	Yes	37 (40.2)	169 (51.5)	Ref.	0.09
	No	55 (59.8)	159 (48.5)	1.5 (0.9-2.5)	
Satisfied with logistic support and comfortable accommodation	Yes	22 (23.9)	87 (26.5)	Ref.	0.94
	No	70 (76.1)	241 (73.5)	1.0 (0.6-1.7)	
		Insomnia (N=52)	Non-insomnia (N=368)		
Satisfied with care provided by hospital administrator	Yes	17 (32.7)	120 (32.6)	Ref.	0.93
	No	35 (67.3)	248 (67.4)	0.5 (0.3-1.0)	
Satisfied with coverage of department with personal protective equipment	Yes	5 (9.6)	93 (25.3)	Ref.	0.07
	No	47 (90.4)	275 (74.7)	2.5 (0.9-6.8)	
Satisfied with work-shift arrangement	Yes	13 (25.0)	193 (52.4)	Ref.	0.004
	No	39 (75.0)	175 (47.6)	2.7 (1.4-5.5)	
Satisfied with logistic support and accommodation	Yes	2 (3.8)	107 (29.1)	Ref.	0.002
	No	50 (96.2)	261 (70.9)	9.6 (2.2-41.5)	
		Distress (N=65)	Non-distress (N=355)		
Satisfied with care provided by hospital administrator	Yes	17 (26.2)	120 (33.8)	Ref.	0.61
	No	48 (73.8)	235 (66.2)	0.8 (0.4-1.6)	
Satisfied with coverage of department with personal protective equipment	Yes	10 (15.4)	88 (24.8)	Ref.	0.56
	No	55 (84.6)	267 (75.2)	1.3 (0.6-2.7)	
Satisfied with work-shift arrangement	Yes	19 (29.2)	187 (52.7)	Ref.	0.008
	No	46 (70.8)	168 (47.3)	2.2 (1.2-4.0)	
Satisfied with logistic support and comfortable accommodation	Yes	4 (6.2)	105 (29.6)	Ref.	0.002
	No	61 (93.8)	250 (70.4)	5.5 (1.9-16.1)	

Abbreviation: Ref, reference category

distress among HCWs (Cai et al., 2020; Lai et al., 2020; Que et al., 2020; Şahin et al., 2020). However, many of whom do not seek psychological support from their colleagues, because they either think they did not need or are embarrassed to seek help and worried about confidentiality (Fridner et al., 2012; Greenberg et al., 2020). These findings remind that psychologists or psychotherapists should pay more attention to HCWs with mental health problems.

In our study, thought of resigning from work due to COVID-19 was

Table 6
Factors associated with mental health symptoms in binary logistic regression analysis among HCWs dealing with the COVID-19 at St. Paul's Hospital, 2020.

Variable		No. of cases[†] / No. of total cases (%)	Adjusted OR (95% CI)[a]	P value
Depression				
Marital status	Unmarried	53/296 (17.9)	2.5 (1.4-4.5)	0.003
	Ever married	32/124 (25.8)	1	
Education level	Undergraduate	73/295 (24.7)	2.1 (1.4-6.2)	0.04
	Post-graduate	12/125 (19.8)	1	
Working position	Frontline	70/296 (23.6)	2.4 (1.2-4.5)	0.009
	Non-frontline	15/124 (12.1)	1	
Anxiety				
Monthly salary, USD	≤ 222	88/323 (27.2)	8.1 (2.5-30.4)	0.001
	> 222	4/97 (4.1)	1	
Working position	Frontline	75/296 (25.3)	2.1 (1.1-3.9)	0.02
	Non-frontline	17/124 (13.7)	1	
Insomnia				
Monthly salary, USD	≤ 222	49/323 (15.2)	4.2 (1.5-25.3)	0.03
	> 222	3/97 (3.1)	1	
Psychological distress				
Working position	Frontline	60/296 (20.3)	5.9 (2.2-15.5)	<0.001
	Non-frontline	5/124 (4.0)	1	

Abbreviation: 1, reference; GAD-7, 7-item Generalized Anxiety Disorder; IES-R, 22-item Impact of Event Scale–Revised; ISI, 7-item Insomnia Severity Index; OR, odds ratio; PHQ-9, 9-item Patient Health Questionnaire; USD, United States Dollar;

[†] Number of respondents with diagnosis of specific adverse mental health outcome based on the symptom scores;

[a] Adjusted for age, sex, marital status, profession, educational level, technical title, salary, work experience, and working position, when appropriate.

associated with higher risk of severe symptoms of depression, anxiety, insomnia, and distress. On the other hand, being satisfied with the work-shift arrangements and logistic support implemented by the hospital administration were the protective factors associated with lower risk of severe symptoms of insomnia and psychological distress. A similar finding was reported from a study conducted during the earlier stage of COVID-19 pandemic in China (Zhu et al., 2020).

Similar to a study from China (Zhu et al., 2020), our finding further indicated that HCWs who were married reported more severe symptoms of depression. This might be due to increased occupational exhaustion and family responsibilities among married than unmarried HCWs. Furthermore, depression was observed in HCWs who have undergraduate as compared with post-graduate level of education. Similar findings were reported by Zhang et al. and Lai et al. This might be attributed to excessive workloads, frequent night shifts, and frequent contact with patients by the undergraduate HCWs who were predominantly nurses and general practitioners than HCWs with postgraduate qualification who were specialist doctors. High levels of anxiety and insomnia were reported in HCWs earning a monthly income of less than 222 USD. This might be due to the fact that a low income is known to be associated with more frequent mental health problems. People of the low income are estimated to be two to three times more likely to have a mental disorder than are those with the highest income (Fang et al., 2021; Kim and Cho, 2020).

The strength of the study was the use of random sampling method with adequate number of participants. However, our study has several limitations which should be addressed in future studies. First, it was a single-center study and the findings may not be nationally representative. Second, it was a cross-sectional study and was not the best method of determining correlation and causation of factors to mental disorders. Third, all the data collected were self-reported by the respondents and could be exposed to social desirability bias. Therefore, a multi-centric and longitudinal study is warranted to address these issues.

5. Conclusion and recommendation

In conclusion, the study demonstrates that a significant proportion of healthcare workers are suffering from symptoms of mental health disorders during the COVID-19 pandemic at St. Paul's Hospital. Being married, low monthly income, undergraduate level of education, and working at frontline position were independently associated with a greater risk of experiencing severe symptoms of mental disorders.

Strategies to provide psychological support to HCWs should be implemented by the hospital administration for the mental health of HCWs in order to control the impact of the Pandemic. Long-term surveillance should be implemented to monitor the mental health of HCWs during the COVID-19 pandemic.

Funding

The work was funded by St. Paul's Hospital Millennium Medical College Research Directorate under a protocol number; PM 23/10. The funder had no role in the design, data collection, analysis, interpretation, and in writing the manuscript.

Authors' contributions

HAM and MT conceived and developed the study protocol and drafted the manuscript. All authors involved in data collection. Interpretation and final review were done by HAM, MT, EW, and TB. All authors approved the final manuscript.

Declaration of Competing Interest

None.

Acknowledgments

We would like to thank St. Paul's Hospital Millennium Medical College Research Directorate for funding the study. We would like to thank all healthcare workers who, despite the increased workload during the COVID-19 crisis, have dedicated their time to answer the survey.

Supplementary materials

Supplementary material associated with this article can be found, in the online version, at doi:10.1016/j.jadr.2021.100246.

References

Alkhamees, A.A., Alrashid, S.A., Alzunaydi, A.A., Almohimeed, A.S., Aljohani, M.S., 2020. The psychological impact of COVID-19 pandemic on the general population of Saudi Arabia. Compr. Psychiatry 102, 152192. https://doi.org/10.1016/j.comppsych.2020.152192.

Fridner, A., Belkić, K., Marini, M., Sendén, M.G., Schenck-Gustafsson, K., 2012. Why don't academic physicians seek needed professional help for psychological distress ? Swiss Med. Wkly. 142, w13626. https://doi.org/10.4414/smw.2012.13626.

Bohlken, J., Schömig, F., Lemke, M.R., Pumberger, M., Riedel-Heller, S.G., 2020. COVID-19 Pandemic : Stress Experience of Healthcare Workers, 47. Psychiat Prax, pp. 190–197.

Cai, Q., Feng, H., Huang, J., Wang, M., Wang, Q., et al., 2020. The mental health of frontline and non-frontline medical workers during the coronavirus disease 2019 (COVID-19) outbreak in China: a case-control study. J. Affect. Disord. 275, 210–215.

Creamer, M., Bell, R., Failla, S., 2003. Psychometric properties of the Impact of Event Scale- Revised. Behav. Res. Ther. 41, 1489–1496. https://doi.org/10.1016/j.brat.2003.07.010.

Dagne, H., Atnafu, A., Alemu, K., Azale, T., Yitayih, S., et al., 2021. Anxiety and associated factors among Ethiopian health professionals at early stage of COVID-19 pandemic in Ethiopia. PLoS ONE 16 (6), e0252664. https://doi.org/10.1371/journal.pone.0252664 doi.org/.

Fang, D., Thomsen, M.R., Nayga, R.M, 2021. The association between food insecurity and mental health during the COVID-19 pandemic. BMC Public Health 21, 607. https://doi.org/10.1186/s12889-021-10631-0 doi.org/.

Gelaye, B., Williams, M.A., Lemma, S., Deyessa, N., Bahretibeb, Y., et al., 2014. Validity of the Patient Health Questionnaire-9 for depression screening and diagnosis in East Africa. Psychiatry Res. 210 (2) https://doi.org/10.1016/j.psychres.2013.07.015.

Greenberg, N., Docherty, M., Gnanapragasam, S., Wessely, S., 2020. Managing mental health challenges faced by healthcare workers during covid-19 pandemic Early support. BMJ 368, m1211. https://doi.org/10.1136/bmj.m1211.

Guan, W., Ni, Z., Hu, Y., Liang, W., Ou, C., et al., 2020. Clinical characteristics of coronavirus disease 2019 in China. N. Engl. J. Med. 382 (18), 1708–1720. https://doi.org/10.1056/NEJMoa2002032.

Hajure, M., Dibaba, B., Shemsu, S., Desalegn, D., Reshad, M., et al., 2021. Psychological Distress among Health Care Workers in Health Facilities of Mettu Town during COVID-19 Outbreak, South West Ethiopia. Front. Psychiatry 12, 574671. https://doi.org/10.3389/fpsyt.2021.574671 doi.org/.

Hanlon, C., Medhin, G., Selamu, M., Breuer, E., Worku, B., et al., 2015. Validity of brief screening questionnaires to detect depression in primary care in Ethiopia. J. Affect. Disord. 186, 32–39. https://doi.org/10.1016/j.jad.2015.07.015.

Huang, Y., Zhao, N., 2020. Generalized anxiety disorder, depressive symptoms and sleep quality during COVID-19 outbreak in China: a web-based cross-sectional survey. Psychiatry Res. J. 288, 112954 https://doi.org/10.1016/j.psychres.2020.112954.

Kim, Y.-.M., Cho, S.-ii., 2020. Socioeconomic status, work-life conflict, and mental health. Am. J. Ind. Med. 63, p703–p712. https://doi.org/10.1002/ajim.23118.

Que, J., Shi, L., Deng, J., Lui, J., Zhang, L., et al., 2020. Psychological impact of the COVID- 19 pandemic on healthcare workers: a cross-sectional study in China. General Psychiatry 33, e100259.

Johnson, S.U., Ulvenes, P.G., Øktedalen, T., Hoffart, A., 2019. Psychometric Properties of the General Anxiety Disorder 7-Item (GAD-7) Scale in a Heterogeneous Psychiatric Sample. Front. Psychol. 10, 1713. https://doi.org/10.3389/fpsyg.2019.01713.

Lai, C., Shih, T., Ko, W., Tang, H., Hsueh, P., 2020a. Severe acute respiratory syndrome coronavirus 2 (SARS-CoV-2) and coronavirus disease-2019 (COVID-19): the epidemic and the challenges. Int. J. Antimicrobial Agents. Elsevier B.V. 55 (3), 105924 https://doi.org/10.1016/j.ijantimicag.2020.105924.

Lai, J., Ma, S., Wang, Y., Cai, Z., Hu, J., et al., 2020b. Factors Associated With Mental Health Outcomes Among Health Care Workers Exposed to Coronavirus Disease 2019. JAMA Netw. Open 3 (3), e203976. https://doi.org/10.1001/jamanetworkopen.2020.3976.

Li, S., Wang, Y., Xue, J., Zhao, N., Zhu, T., 2020. The Impact of COVID-19 Epidemic Declaration on Psychological Consequences : a Study on Active Weibo Users. Int. J. Environ. Res. Public Health 17, 2032.

Liu, X., Kakade, M., Fuller, C.J., Fan, B., Fang, Y., Kong, J., Guan, Z., Wu, P., 2020. Depression after exposure to stressful events: lessons learned from the severe acute respiratory syndrome epidemic. Compr. Psychiatry 53, 15–23. https://doi.org/10.1016/j.comppsych.2011.02.003.

Manzar, M.D., Alghadir, A.H., Anwer, S., Alqahtani, M., Salahuddin, M., et al., 2021. Psychometric properties of the general anxiety disorders-7 scale using categorical

data methods: a study in a sample of university attending Ethiopian young adults. Neuropsychiatr. Dis. Treat. 17, p893–p903. doi.org/10.2147/NDT.S295912.

Manzar, M.D., Noohu, M.M., Salahuddin, M., Nureye, D., Albougami, A., et al., 2020. Insomnia Symptoms and Their Association with Anxiety and Poor Sleep Hygiene Practices among Ethiopian University Students. Nat. Sci. Sleep 12, p575–p582. doi.org/10.2147/NSS.S246994.

Ong, J.C., Suh, S., 2013. Diagnostic Tools for Insomnia. Encyclopedia of Sleep. Elsevier Inc., pp. 268–273

Pappa, S., Ntella, V., Giannakas, T., Giannakoulis, V.G., Papoutsi, E., 2020. Prevalence of depression, anxiety, and insomnia among healthcare workers during the COVID-19 pandemic: a systematic review and meta-analysis. Brain Behav. Immun. 88, 901–907 doi: https://doi.org/10.1016/j.bbi.2020.05.026.

Şahin, M., Aker, S., Şahin, G., et al., 2020. Prevalence of Depression, Anxiety, Distress and Insomnia and Related Factors in Healthcare Workers During COVID-19 Pandemic in Turkey. J. Community Health 45 (6), 1168–1177. https://doi.org/10.1007/s10900-020-00921-w doi.org/.

Tasnim, Rafia, Sujan, M.S.H, Islam, M.S., et al., 2021. Prevalence and correlates of anxiety and depression in frontline healthcare workers treating people with COVID-19 in Bangladesh. BMC Psychiatry 21, 271. https://doi.org/10.1186/s12888-021-03243-w. In this issue.

Tysser, R., Røvik, J.O., Vaglum, P., Gronvold, N.T., Ekeberg, O., 2004. Help-seeking for mental health problems among young physicians: is it the most ill that seeks help ? A longitudinal and nationwide study. Soc. Psychiatry Psychiatr. Epidemiol. 39, 989–993. https://doi.org/10.1007/s00127-004-0831-8.

Xiang, Y., Zhao, Y., Liu, Z., Li, X., Zhao, N., Cheun, T., Ng, C., 2020. The COVID-19 outbreak and psychiatric hospitals in China: managing challenges through mental health service reform. Int. J. Biol. Sci. 16, 1741–1744. https://doi.org/10.7150/ijbs.45072.

Zhao, Y.J., Jin, Y., Rao, W.W., Li, W., Zhao, N., et al., 2021. The prevalence of psychiatric comorbidities during the SARS and COVID-19 epidemics: a systematic review and meta-analysis of observational studies. J. Affect. Disord. 287, 145–157. doi.org/10.1016/j.jad.2021.03.016.

Zhu, Z., Xu, S., Wang, H., Wu, J., Li, G., et al., 2020. COVID-19 in Wuhan: Immediate Psychological Impact on 5062 Health Workers. medRxiv, p. 1095. https://doi.org/10.1101/2020.02.20.20025338.
