# Supplementary material for: Mental health status and related factors influencing healthcare workers during the COVID-19 pandemic: A systematic review and meta-analysis
Source: PLoS One. 2024 Jan 19;19(1):e0289454. doi: 10.1371/journal.pone.0289454 (PMC10798549; doi:10.1371/journal.pone.0289454)
Supplement: S1 Data — (ZIP) [file pone.0289454.s011.zip › literatures/110.pdf]

frontiers | Frontiers in Psychiatry

ORIGINAL RESEARCH
published: 26 July 2022
doi: 10.3389/fpsyt.2022.895788

The Impact of COVID-19 on Psychological and Social Stigma for Indonesian Nurses: A Cross-Sectional Survey

Mustikasari Mustikasari[1]*, Harif Fadhillah[2,3], Apri Sunadi[4], Nursalam Nursalam[3,5], Ati Surya Mediawati[3] and Muhammad Adam[1]

[1] Faculty of Nursing, Universitas Indonesia, Jakarta, Indonesia, [2] Faculty of Health Science, Muhammadiyah University of Jakarta, Jakarta, Indonesia, [3] Indonesian National Nursing Association, Jakarta, Indonesia, [4] Universitas Respati Indonesia, Yogyakarta, Indonesia, [5] Faculty of Nursing, Universitas Airlangga, Surabaya, Indonesia

OPEN ACCESS

Edited by:
Kevin A. Pelphrey,
University of Virginia, United States

Reviewed by:
Fitriana Kurniasari Solikhah,
Poltekkes Kemenkes Malang, Indonesia
Riza Fikriana,
Sekolah Tinggi Ilmu Kesehatan Kepanjen, Indonesia
Dluha Mafula,
Gadjah Mada University, Indonesia
Dongmei Diao,
Xi'an Jiaotong University, China

****Correspondence:***
Mustikasari Mustikasari
ns.mustikasari@gmail.com

Specialty section:
This article was submitted to Social Neuroscience, a section of the journal Frontiers in Psychiatry

Received: *14 March 2022*
Accepted: *21 June 2022*
Published: *26 July 2022*

Citation:
Mustikasari M, Fadhillah H, Sunadi A, Nursalam N, Mediawati AS and Adam M (2022) The Impact of COVID-19 on Psychological and Social Stigma for Indonesian Nurses: A Cross-Sectional Survey. Front. Psychiatry 13:895788. doi: 10.3389/fpsyt.2022.895788

Introduction: Nurses are on the front line and are at high risk of experiencing a mental health crisis during the pandemic due to the psychological impact and stigma. The aim of this study was to identify the role of psychological status and social stigma in anxiety, fear, depression, and mental health crises during the pandemic.

Materials and Methods: A cross-sectional design during December 2020–August 2021. A total of 2,156 nurses who work in health facilities, either hospitals, or communities based on the criteria of nurses who interact directly with COVID-19 patients, work at least 3 months, age 20–54 years, are literate, have internet access, and have the ability to access the electronic form. The eligible participants filled in online questionnaires that were sent to them via WhatsApp. Data were analyzed using Spearman rho correlation test with statistically significant p value < 0.05.

Results: A total of 2,156 respondents responded to the questionnaire, and the response rate was 100%. The psychological status of nurses was 78.4% moderate, 18.5% experienced social stigma, 44.0% showed an anxiety response, 53.5% fear, 64.5% depression in the very severe category, and 63.5% fell into a mental health crisis. The results of the inferential analysis showed that all $P < 0.05$ which indicated that psychological status and social stigma had a significant relationship with anxiety, fear, depression, and mental health crisis in nurses.

Conclusion: The psychological status and social stigma experienced by nurses during the COVID-19 pandemic indicate a bad situation and lead to a mental health emergency crisis.

Keywords: COVID-19, social stigma, mental health, anxiety, nursing

INTRODUCTION

Indonesia often faces infectious disease problems because Indonesia is a tropical country with the highest number of infectious diseases in the world (1, 2). However, at the end of 2019, Indonesia was still surprised by the emergence of a new infectious disease caused by the SARS-CoV-2, virus which was later known as coronavirus disease 2019 (COVID-19) (3, 4). Facing a pandemic that is also taking place in countries around the world, Indonesia is also experiencing the same crisis, both in the health sector as well as in all sectors of the country's structure. The impact that is felt the most is the health impact felt by the community, especially health workers (5, 6).

The existence of the COVID-19 pandemic at the beginning of the emergence of cases made Indonesia really keep tight so as not to cause transmission of the number of COVID-19 cases, so detailed analysis and in-depth tracing were applied to trace cases of close contact with COVID-19 spreaders, but public awareness which is still low and the information circulating about COVID-19 makes the tightness decrease (7). The result of not complying with the health protocol is the spike in COVID-19 cases in Indonesia, especially in the May–August 2020 period. The surge in COVID-19 cases had slowed down in Indonesia around February–May 2021, but the repatriation of Indonesian Health workers from abroad caused variants. The new COVID-19, namely the delta variant, spread to Indonesia and a second wave of COVID-19 emerged. The second wave caused a lot of health crises in Indonesia. Health facilities were full of patients, health workers were overwhelmed, and many died and fell ill with COVID-19 (8).

Health workers who have the highest mortality rate are nurses, nurses as staff who provide care for 24 h make them have to have close contact with patients (9, 10). Nurses who have used level 3 personal protective equipment in Indonesia still do not guarantee that they are not infected, because there are several other factors, such as decreased immunity, interaction with other health workers without a protocol when eating, and interaction with the community, that they are infected with COVID-19 are also very important allows. Based on the number of nurse deaths in Indonesia during January–July 2021, Indonesia has lost more than 300 nurses due to COVID-19 (11).

The pandemic atmosphere in Indonesia shows very tense conditions, especially during the spike period of the first wave and second wave of COVID-19. Feelings of fear of being infected, fear of sudden death, and fear of people who have had COVID-19 make people wary of each other (12, 13). This also creates conditions of anxiety, panic, fear, and even depression and mental health crises, due to stigma in society and psychological conditions (9, 10). It is very important to evaluate the psychological condition and social stigma felt by nurses, because nurses who are directly involved in care certainly feel a greater impact than just the physical impact, but also psychologically and socially. There are so many nurses who experience injustice in society, are ostracized, stigmatized, to experience discrimination and are expelled (7). Conditions like this in society make it easy for nurses to experience feelings of anxiety, fear, and mental health crises. Therefore, the purpose of this study was to determine the role of psychological conditions and social stigma on the incidence of anxiety, fear, depression and mental health crises in Indonesian nurses during the COVID-19 pandemic.

MATERIALS AND METHODS

This research has been conducted in Indonesia on nurses who provide direct care for COVID-19. The research was carried out from December 2020 to August 2021.

Study Design

We chose to use a quantitative study with a cross sectional approach to nurses in health facilities in Indonesia. The main reason for choosing the research design was to conduct a comprehensive survey by distributing questionnaires to find out the nurses' reactions to the COVID-19 pandemic. Ethical approval was obtained from the local Ethics Committee

TABLE 1 | Sociodemographic characteristics of respondents.

Characteristics of respondent	n (%)
**Ages**	
20–29 years	961 (44.6)
30–39 years	542 (25.1)
40–49 years	417 (19.3)
50 years and above	236 (10.9)
**Gender**	
Male	700 (32.5)
Female	1456 (67.5)
**Marital status**	
Married	677 (31.4)
Single	1455 (67.5)
Widow	24 (1.1)
**Ethnic**	
Javanese	1454 (67.5)
Sundanese	400 (18.6)
Cirebon	22 (1.0)
Betawi	185 (8.6)
Madura	73 (3.4)
Boyam	22 (1.0)
**Religion**	
Moeslem	2004 (92.9)
Buddhist	35 (1.6)
Hindu	31 (1.4)
Cristian	86 (4.0)
**Educational background**	
Diploma	662 (30.8)
Bachelor	1252 (58.1)
Master	242 (11.2)
**Income**	
< Minimum regional income	900 (41.7)
> Minimum regional income	1256 (58.3)

of the University of Indonesia with the number Ket-198/UN2.F12.D1.2.1/PPM.00.02/2021. Researchers in collecting data ensure that respondents voluntarily and without coercion are willing to fill out research questionnaires.

Participants and Settings

The sample included 2,156 nurses who worked in health facilities, either hospitals or communities, and were recruited from all nursing staff in Indonesia. Based on the respondent selection criteria, the main criteria in this study were nurses who interacted directly with COVID-19 patients, worked in health services, had worked for at least 3 months, aged 20–54 years, were willing to take part in the study, were literate, had internet access, and had the ability to access the electronic form. The following exclusion criteria were applied: immigrant nursing, being unwilling or unable to continue contributing to the study.

Recruitment

Patient recruitment was conducted between December 2020 and August 2021. The researcher, via online, informed the nurses about the purpose of the study and asked them whether they were willing to be contacted by the research and willing to fill out the questionnaire. The study coordinator contacted the interested nurses by online form and screened the eligible patients until the target number of 710 patients was reached. The eligible participants were informed about the voluntary nature of their participation, and online informed consent was obtained from them. After that, online questionnaires were sent to them via WhatsApp, and they were asked to fill out the questionnaires. The nurse who did not return the questionnaire was given the opportunity for 2 weeks. The researcher confirmed that if she was not willing to continue, the researcher did not force it. The research data was collected by giving initials without writing names, and the researchers also strictly maintained the confidentiality of the respondents.

Measures

Data were collected using an online questionnaire form. A socio-demographic form developed by the researchers was used to assess the patient's sociodemographic characteristics (age, gender, religion, marital status, and educational level). The questionnaire was developed by the researchers from the COVID-19 Social Psychological Survey to assess psychological condition and social stigma. This questionnaire consists of 27 items with a rating using a 4-point Likert scale starting from 1 (strongly disagree), 2 (disagree), 3 (agree), and 4 (strongly disagree), on unfavorable questions, the assessment is reversed. The result of score interpretation shows that < 56 has a low psychological condition,

TABLE 2 | Psychological condition, stigma, and crisis mental health during pandemic situation.

Psychological condition	n (%)
**Psychological status**	
Bad	271 (12.6)
Moderate	1690 (78.4)
Good	195 (9.0)
**Social stigma**	
Yes	399 (18.5)
No	1757 (81.5)
**Anxiety**	
Normal	505 (23.4)
Mild	256 (11.9)
Moderate	241 (11.2)
Severe	206 (9.6)
Very severe	948 (44.0)
**Fear**	
Normal	254 (11.8)
Mild	220 (10.2)
Moderate	340 (15.8)
Severe	188 (8.7)
Very severe	1154 (53.5)
**Depression**	
Normal	118 (5.5)
Mild	192 (8.9)
Moderate	215 (10.0)
Severe	236 (10.9)
Very severe	1395 (64.7)
**Crisis mental health**	
Mild	182 (8.4)
Moderate	604 (28.0)
Severe	1370 (63.5)

TABLE 3 | Predictors psychological condition caused anxiety, fear, depression, and crisis mental health during pandemic.

Variable	Psychological Status			*P*-value
	Bad	Moderate	Good	
Anxiety				0.000
Normal	51 (10.1)	416 (82.4)	38 (7.4)	
Mild	48 (18.8)	198 (77.3)	10 (3.9)	
Moderate	10 (4.1)	219 (90.9)	12 (5.0)	
Severe	47 (22.8)	139 (67.5)	20 (9.7)	
Very severe	115 (12.1)	718 (75.7)	115 (12.1)	
Fear				0.000
Normal	37 (14.6)	185 (72.8)	32 (12.6)	
Mild	12 (5.5)	202 (91.8)	6 (2.7)	
Moderate	56 (16.5)	270 (79.4)	14 (4.1)	
Severe	4 (2.1)	176 (93.6)	8 (4.3)	
Very severe	162 (14.0)	857 (74.3)	135 (11.7)	
Depression				0.000
Normal	16 (13.6)	76 (64.4)	26 (22.0)	
Mild	27 (14.1)	159 (82.8)	6 (3.1)	
Moderate	10 (4.7)	195 (90.7)	10 (4.7)	
Severe	46 (19.5)	184 (78.0)	6 (2.5)	
Very severe	172 (12.3)	1076 (77.1)	147 910.5)	
Crisis mental health				0.008
Normal	18 (9.9)	136 (74.7)	28 (15.4)	
Mild	64 (10.6)	516 (85.4)	24 (4.0)	
Moderate	189 (13.8)	1038 (75.8)	143 (10.4)	
Severe	271 (12.6)	1690 (78.4)	195 (9.0)	

56–65 has a moderate psychological condition and > 65 indicates a high psychological condition. While the social stigma is said to experience social stigma if the score obtained is < 24. Anxiety, fear, and depression were measured using a modified depression, anxiety, stress scale questionnaire which consisted of 21 questions using a 0–3 scale, namely a score of zero indicates never, a value of 1 is sometimes, a value of 2 is quite often and a value of 3 is very often. Interpretation score tNormal = 0–7, mild anxiety level = 8–9, moderate = 10–14, severe = 15–19 and very severe = 20, while the assessment for depression normal = 0–14, mild depression = 15–18, moderate = 19–25, severe = 26–33 and very severe 34. Measurement of the mental health crisis in Indonesian nurses during the COVID-19 pandemic was measured using a mental health questionnaire that had been developed by researchers. Question items consist of 9 questions with a Likert scale of 4 points; 0 points never, 1 point every day (1–7 days), 2 points more often (7–12 days), and 4 points almost every day (13–14 days). The result of the interpretation assessment is a score of 0–4: No referral is required at this time. 5–9: Clients may benefit from natural support or mental health services. 10–19: Clients should seek professional mental health services. 20–27: Clients should seek health crisis services as soon as possible. All questionnaires have been tested for validity first with 100 respondents, all questions show valid results, the calculated r value is between 0.772 and 0.985, so that it is greater than the r table value of 0.1638. While the questionnaire shows reliable with Cronbach's Alpha value between 0.875 and 0.995.

TABLE 4 | Predictors social stigma caused anxiety, fear, depression, and crisis mental health during pandemic.

Variable	Social Stigma		*p*-value
	Yes	No	
Anxiety			0.000
Normal	68 (13.5)	437 (86.5)	
Mild	32 (12.5)	224 (87.5)	
Moderate	38 (15.8)	203 (85.2)	
Severe	39 (18.9)	167 (81.1)	
Very severe	222 (23.4)	726 (76.6)	
Fear			0.000
Normal	37 (14.6)	217 (85.4)	
Mild	25 (11.4)	195 (88.6)	
Moderate	46 (13.5)	294 (86.5)	
Severe	30 (16.0)	158 (84.0)	
Very severe	261 (22.6)	893 (77.4)	
Depression			0.000
Normal	16 (13.6)	102 (86.4)	
Mild	28 (14.6)	164 (85.4)	
Moderate	26 (12.1)	189 (87.9)	
Severe	30 (12.7)	206 (87.3)	
Very severe	299 (21.4)	1096 (78.6)	
Crisis mental health			0.000
Normal	25 (13.7)	157 (86.3)	
Mild	94 (15.6)	510 (84.4)	
Moderate	280 (20.4)	1090 (79.6)	
Severe	399 (18.5)	1757 (81.5)	

Statistical Analysis

This study analyzed using descriptive and inferential analysis. A compliance test for normal distribution was applied using the Kolmogorov–Smirnov test. A descriptive value such as means, SDs, frequencies, and percentages was analyzed with frequent distribution. A Chi-square test was performed to compare the groups concerning demographic, independent, and dependent variables. The relationship between psychological condition and social stigma with anxiety, fear, depression, and crisis mental health was analyzed using Spearman rho correlation test with statistically significant p value < 0.05. The analyzes were conducted with SPSS® for Windows® version 22.0.

RESULTS

The total of 2,156 respondents responded to the questionnaire and the response rate was 100%. The results showed that the average nurse was 20–29 years old and the majority were women (67.5%). Most of the nurses were single (67.5%) and came from Java (67.5%). The religion of the majority of nurses is Islam (92.9%), the most educated level is at the undergraduate level (58.1%), and the income is above the minimum regional income (58.3%) (**Table 1**).

The psychological condition of nurses in a pandemic situation reflects the actual condition of nurses while providing care to patients, especially in the second wave of COVID-19. The psychological status of nurses based on the survey results showed moderate results (78.4%). Nurses who experienced social stigma were 399 (18.5%). The nurse showed a very severe anxiety response of 44.0%, as well as fear, which showed very severe (53.5%), and depression which showed very severe (64.5%). This condition allows nurses to experience a severe mental health emergency crisis of 63.5% (**Table 2**).

A bad psychological condition can lead to the emergence of accompanying psychological symptoms that worsen the nurse's condition and cause her to fall into a state of mental health crisis. Nurses with poor psychological status experienced severe anxiety (22.8%), very severe fear (14.0%), very bad depression (12.3%), and 12.6% of nurses who had fallen into a very bad mental health crisis (**Table 3**).

It is almost the same as psychological status conditions that can cause problems, including anxiety, fear, depression, and mental health crisis. This condition can also be exacerbated by the situation of social stigma shown by the community, the emergence of this stigma makes conditions facing a pandemic even more difficult. The results of a survey of Indonesian nurses showed that 23.4% of nurses who experienced social stigma showed a very poor anxiety response. Likewise, the fear experienced by nurses was also very high, namely, 22.6% who experienced stigma, they definitely showed very severe fear, and 21.4% experienced depression. Nurses who experienced social stigma also fell into a mental health crisis as much as 15.6% in mild conditions, 20.4% moderate, and 18.5% severe (**Table 4**).

DISCUSSION

Psychological status and social stigma have a high contribution to the incidence of anxiety, fear, depression, and mental health emergency crises in nurses who provide care in COVID-19 isolation rooms. Nurses revealed that carrying out their duties in isolation rooms is a challenge and vigilance that they must exercise caution, because it can cause various problems if they contract COVID-19 and the bad impact is on the health condition of each individual (11, 14). Nurses as the front line must not be afraid and retreat from the pandemic situation, nurses cannot choose to give up and must keep fighting because of the professionalism and professional oath that have been made (15, 16). Nurses in Indonesia and all countries are facing the same burden of problems, namely dealing with COVID-19, especially the psychological impact caused by the crisis during the pandemic (8).

The results showed that the average nurse was 20–29 years old and most were women, in line with several studies which stated that most of the nurses were women and the nurses who served in the isolation room were nurses who were young and did not have comorbidities (17, 18). Nurses in Indonesia who carry out their duties in isolation rooms are mostly single nurses, because the nurses on duty are volunteer nurses and fresh graduates who have been given the necessary training in the isolation room (8, 19, 20). Nurses who work in isolation rooms must be carefully considered and ensured that they still have excellent health to prevent transmission and worsening of the disease if infected with COVID-19 (9, 21).

The psychological condition of nurses in a pandemic situation reflects the actual condition of nurses while providing care to patients, especially in the second wave of COVID-19 (17, 19). The psychological status of nurses based on the survey results showed moderate results, also in line with several studies conducted in Indonesia. Indeed the pandemic situation caused an unfavorable psychological impact for nurses, especially in wave 2 of COVID-19 (5, 22). Nurses who experience social stigma also show high results, nurses are stigmatized because they are considered to be spreaders of the virus because they work in hospitals, there are lots of rejections given by the community, especially in the early stages of the emergence of COVID-19 (23, 24). As a result, this condition gave rise to symptoms in nurses such as responses to anxiety, fear, and depression which were in the very severe category (7, 20). Nurses who are on the front line in providing patient care, screening, tracing and becoming vaccination officers certainly create situations of excessive anxiety and fear, because COVID-19 can trigger a rapid worsening of the condition and death. Many nurses also fall into depression and need counseling with other health workers and psychiatrists (14, 25). If this condition makes it possible for nurses to experience a severe mental health emergency crisis, if left unchecked, health workers will become victims in a pandemic situation that continues.

It is almost the same as psychological status conditions that can cause problems including, anxiety, fear, depression, and mental health crisis (26, 27). This condition can also be exacerbated by the situation of social stigma shown by the community, the emergence of this stigma makes conditions facing a pandemic even more difficult. The results of a survey on Indonesian nurses who experienced social stigma showed a very bad response to anxiety, fear, and depression. Nurses who experience social stigma are also very many who fall into a mental health crisis, as a result, many nurses are traumatized and remember the extraordinary events of the pandemic (28, 29). The conditions of anxiety, fear, depression, and mental health crises not only occur due to the pandemic situation, nurses who have experienced the loss of their closest family members and sources of life support so far have also shown a very bad response, the deep trauma experienced by nurses is also felt to become a memorable memory. bad for him. Many nurses died as a result of battling the COVID-19 pandemic (7, 11).

The results of this study have research limitations, namely they have not measured the level of depth of the psychological feelings of nurses qualitatively, so that the results obtained are nurses expressing their hearts. So it is very important to pay attention to digging deeper into the psychological state of nurses, especially those who provide treatment in isolation rooms, nurses who have trauma from the pandemic and nurses who still have symptoms of long haul COVID-19. It is hoped that further researchers can continue and make innovations in resolving the psychological impact of the pandemic among nurses and other health workers.

CONCLUSION

The psychological status and social stigma experienced by nurses during the COVID-19 pandemic indicate a bad situation, many nurses are stigmatized in society, resulting in very bad anxiety, fear, depression, and causing a mental health emergency crisis.

DATA AVAILABILITY STATEMENT

The original contributions presented in the study are included in the article/supplementary material, further inquiries can be directed to the corresponding author.

ETHICS STATEMENT

The studies involving human participants were reviewed and approved by the Local Ethics Committee University of Indonesia with the number Ket-198/UN2.F12.D1.2.1/PPM.00.02/2021. Written informed consent to participate in this study was provided by the participants' legal guardian/next of kin.

AUTHOR CONTRIBUTIONS

MM, HF, AS, NN: study conception and design. NN, MA, AM: data collection, analysis, and interpretation of results. NN: manuscript preparation. All authors contributed to the article and approved the submitted version.

REFERENCES

1. Liberati A, Altman DG, Tetzlaff J, Mulrow C, Gøtzsche PC, Ioannidis JPA, et al. The PRISMA statement for reporting systematic reviews and meta-analyses of studies that evaluate health care interventions: explanation and elaboration. *PLoS Med.* (2009) 6:e1000100. doi: 10.1371/journal.pmed.1000100
2. Yao H, Chen JH, Xu YF. Rethinking online mental health services in China during the COVID-19 epidemic. *Asian J Psychiatr.* (2020) 50:102015. doi: 10.1016/j.ajp.2020.102015
3. Zulkipli ZN, Idris SH, Rahman NA. COVID-19 ANXIETY: MALAYSIAN REGULATORY MECHANISMS ON MENTAL HEALTH. *Int J Adv Sci Technol.* (2020) 29:2472–7.
4. Liu S, Yang L, Zhang C, Xiang YT, Liu Z, Hu S, et al. Online mental health services in China during the COVID-19 outbreak. *Lancet Psychiatry.* (2020) 7:e17–e18. doi: 10.1016/S2215-0366(20)30077-8
5. Peitl V, Zatezalo VG, Karlović D. Mental health issues and psychological crisis interventions during the COVID-19 pandemic and earthquakes in Croatia. *Arch Psychiatry Res.* (2020) 56:193— 8. doi: 10.20471/dec.2020.56.02.07
6. Zhu Z, Ph D, Xu S, Ph D, Wang H, Med M, et al. COVID-19 in Wuhan: immediate psychological impact on 5062 health workers. *medRxiv.* (2020) [Preprint]. doi: 10.1101/2020.02.20.20025338
7. Nursalam N, Sukartini T, Priyantini D, Mafula D, Efendi F. Risk factors for psychological impact and social stigma among people facing covid-19: a systematic review'. *Syst Rev Pharm.* (2020) 11:1022–8.
8. Priyantini D, Nursalam N, Sukartini T. Analysis of factors affecting the mental health crisis of coronavirus disease infection in Java Island. *J Ners.* (2021) 16:60–6. doi: 10.20473/jn.v16i1.23321
9. Gao Y, Li T, Han M, Li X, Wu D, Xu Y, et al. Diagnostic utility of clinical laboratory data determinations for patients with the severe COVID-19. *J Med Virol.* (2020) 92:791–6. doi: 10.1002/jmv.25770
10. Hu, B, Wang D. Clinical features of critically ill patients with COVID-19 infection in China. *Res Sq.* (2020) 8:1–21. doi: 10.21203/rs.3.rs-16250/v1
11. Priyantini D, Nursalam N, Sukartini T. The first psychological management intervention towards community resilience facing covid-19 infection. *Int J Pharm Res.* (2021) 13:4485–92. doi: 10.31838/ijpr/2021.13.01.654
12. Li Y, Xia L. Coronavirus disease 2019 (COVID-19): role of chest ct in diagnosis and management. *AJR Am J Roentgenol.* (2020) 214:1280–6. doi: 10.2214/AJR.20.22954
13. Liang H, Acharya G. Novel corona virus disease (COVID-19) in pregnancy: what clinical recommendations to follow? *Acta Obstet Gynecol Scand.* (2020) 99:439–42. doi: 10.1111/aogs.13836
14. Sohrabi C, Alsafi Z, O'Neill N, Khan M, Kerwan A, Al-Jabir A, et al. World health organization declares global emergency: a review of the 2019 novel coronavirus (COVID-19). *Int J Surg [Internet].* (2020) 76:71–6. doi: 10.1016/j.ijsu.2020.02.034
15. Metlay JP, Waterer GW, Long AC, Anzueto A, Brozek J, Crothers K, et al. Diagnosis and treatment of adults with community-acquired pneumonia. *Am J Respir Crit Care Med.* (2019) 200:E45— E67. doi: 10.1164/rccm.201908-1581ST
16. Zhou F, Yu T, Du R, Fan G, Liu Y, Liu Z, et al. Clinical course and risk factors for mortality of adult inpatients with COVID-19 in Wuhan, China: a retrospective cohort study. *Lancet.* (2020) 395:1054–62. doi: 10.1016/S0140-6736(20)30566-3
17. Percudani M, Corradin M, Moreno M, Indelicato A, Vita A. Mental health services in lombardy during COVID-19 outbreak. *Psychiatry Res.* (2020) 288:1–3. doi: 10.1016/j.psychres.2020.112980
18. Ping NPT, Shoesmith WD, James S, Nor Hadi NM, Yau EKB, Lin LJ. Ultra brief psychological interventions for covid-19 pandemic: introduction of a locally-adapted brief intervention for mental health and psychosocial support service. *Malaysian J Med Sci.* (2020) 27:51–6. doi: 10.21315/mjms2020.27.2.6
19. Li S, Wang Y, Xue J, Zhao N, Zhu T. The impact of covid-19 epidemic declaration on psychological consequences: a study on active weibo users. *Int J Environ Res Public Health.* (2020) 17:2032. doi: 10.3390/ijerph17062032
20. Mafula D, Nursalam N, Tintin S. Quality of nursing worklife based on caring model for improving nurse performance in hospitals. *Indones Nurs J Educ Clin.* (2020) 5:172–84. doi: 10.24990/injec.v5i2.339
21. Wang S, Guo L, Chen L, Liu W, Cao Y, Zhang J, et al. A case report of neonatal COVID-19 infection in China. *Clin Infect Dis.* (2020) 71:853–7. doi: 10.1093/cid/ciaa225
22. Lupe SE, Keefer L, Szigethy E. Gaining resilience and reducing stress in the age of COVID-19. *Curr Opin Gastroenterol.* (2020) 36:295–303. doi: 10.1097/MOG.0000000000000646
23. Mukhtar S. Mental health of medical workers in Pakistan during the pandemic COVID- 19 outbreak. *Asian J Psychiatr.* (2020) 51:19–21. doi: 10.1016/j.ajp.2020.102080
24. Stankovska G, Memedi I, Dimitrovski D. Coronavirus Covid-19 disease, mental health and psychosocial support. *Soc Regist.* (2020) 4:33–48. doi: 10.14746/sr.2020.4.2.03
25. Covid-19 National Emergency Response Center, Epidemiology and Case Management Team, Korea Centers for Disease Control and Prevention. Early epidemiological and clinical characteristics of 28 cases of coronavirus disease in South Korea. *Osong Public Heal Res Perspect.* (2020) 11:8–14. doi: 10.24171/j.phrp.2020.11.1.03
26. Oikarainen A, Mikkonen K, Kenny A, Tomietto M, Tuomikoski A, Meriläinen M. Educational interventions designed to develop nurses ' cultural competence: a systematic review. *Int J Nurs Stud.* (2019) 98:75–86. doi: 10.1016/j.ijnurstu.2019.06.005
27. Guo Y-R, Cao Q-D, Hong Z-S, Tan Y-Y, Chen S-D, Jin H-J, et al. The origin, transmission and clinical therapies on coronavirus disease 2019 (COVID-19) outbreak – an update on the status. *Mil Med Res.* (2020) 7:11. doi: 10.1186/s40779-020-00240-0
28. Li W, Yang Y, Liu ZH, Zhao YJ, Zhang Q, Zhang L, et al. Progression of mental health services during the COVID-19 outbreak in China. *Int J Biol Sci.* (2020) 16:1732–8. doi: 10.7150/ijbs.45120
29. De Sousa A, Mohandas E, Javed A. Psychological interventions during COVID-19: challenges for low and middle income countries. *Asian J Psychiatr.* (2020) 51:102128. doi: 10.1016/j.ajp.2020.102128

Conflict of Interest: The authors declare that the research was conducted in the absence of any commercial or financial relationships that could be construed as a potential conflict of interest.

Publisher's Note: All claims expressed in this article are solely those of the authors and do not necessarily represent those of their affiliated organizations, or those of the publisher, the editors and the reviewers. Any product that may be evaluated in this article, or claim that may be made by its manufacturer, is not guaranteed or endorsed by the publisher.

Copyright © 2022 Mustikasari, Fadhillah, Sunadi, Nursalam, Mediawati and Adam. This is an open-access article distributed under the terms of the Creative Commons Attribution License (CC BY). The use, distribution or reproduction in other forums is permitted, provided the original author(s) and the copyright owner(s) are credited and that the original publication in this journal is cited, in accordance with accepted academic practice. No use, distribution or reproduction is permitted which does not comply with these terms.
