# Supplementary material for: Mental health status and related factors influencing healthcare workers during the COVID-19 pandemic: A systematic review and meta-analysis
Source: PLoS One. 2024 Jan 19;19(1):e0289454. doi: 10.1371/journal.pone.0289454 (PMC10798549; doi:10.1371/journal.pone.0289454)
Supplement: S1 Data — (ZIP) [file pone.0289454.s011.zip › literatures/112.pdf]

Journal of Multidisciplinary Healthcare

Dovepress
open access to scientific and medical research

Open Access Full Text Article

ORIGINAL RESEARCH

Depression, Anxiety, and Stress Among Nurses During the COVID-19 Wave III: Results of a Cross-Sectional Assessment

Fahad Nadeem[1]
Abdul Sadiq[2]
Abdul Raziq[3]
Qaiser Iqbal[1]
Sajjad Haider[1]
Fahad Saleem[1]
Mohammad Bashaar[4]

[1]Faculty of Pharmacy & Health Sciences, University of Balochistan, Quetta, Pakistan; [2]Department of Biochemistry, Jhalawan Medical College Khuzdar, Balochistan, Pakistan; [3]Department of Statistics, University of Balochistan, Quetta, Pakistan; [4]SMART Afghan International Trainings and Consultancy, Kabul, Afghanistan

Purpose: The current study was aimed to evaluate depression, anxiety, and stress among nurses during the COVID-19 (wave III) at a public hospital in Quetta city, Pakistan.

Participants and Methods: By using the validated Depression, Anxiety and Stress Scale - 21, three hundred and twenty-five nurses practicing at the Sandeman Provincial Hospital, Quetta were approached for data collection. Through SPSS, both descriptive and inferential statistics were used during data analysis. Chi-square test was used to assess the goodness-of-fit between observed values and demographic variables. The factors that were significantly associated with depression, anxiety, and stress were further assessed by binary logistic regression analysis. The statistical significance was set at 0.05.

Results: Out of the 325 nurses, 297 participated in the study with a response rate of 91.3%. Mild depression and stress were reported in the current cohort of nurses (6.00 ± 5.30 and 8.01 ± 4.47), respectively. The mean anxiety score was 6.68 ± 4.86 indicating moderate anxiety among the study respondents. Marital status, working department, and job experience were significantly associated with depression, anxiety, and stress with moderate effect size (>0.3). The logistic regression reported department in which nurses were stationed (adjusted OR = 2.54, 95% CI = 1.118–1.415, p < 0.001) and job experience (adjusted OR = 1.004, 95% CI = 1.001–1.215, p = 0.002) as significant predictors of depression, anxiety and stress. Nurses working in the corona ward were more likely to develop depression, anxiety and stress by a factor of 2.54 and married nurses with a factor of 1.004 provided controlling other confounding factors.

Conclusion: Moderate depression, anxiety, and stress were observed among nurses and it calls for hospital management to consider interventions for all nurses irrespective of their present working departments. Facilitating continuous and comprehensive support mechanisms aimed at protecting nurses' mental health is of great importance during pandemics. The findings of the study have some clear suggestions for mental health advocacy among nurses that are essential for improving the quality of services and patients' safety.

Keywords: depression, anxiety, stress, cross-sectional analysis, COVID wave III

Introduction

Among public health emergencies, an infectious disease outbreak is one of the most imminent threats. Infectious diseases are a leading cause of mortality and the biggest disablers of human functions.[1] Pathogenic microorganisms spread diseases resulting in minor ailments to the pandemic crises.[2] In this context, zoonotic diseases or zoonoses are the infectious diseases of animals that cause infections when transmitted to humans. Around the globe, approximately 60% of all infectious

Correspondence: Mohammad Bashaar
SMART Afghan International Trainings and Consultancy, Shahri Naw, Hospital Street No. 1, Kabul, Afghanistan
Tel +93 788233865
Email dr.mbashaar@gmail.com

Received: 13 September 2021
Accepted: 28 October 2021
Published: 6 November 2021

© 2021 Nadeem et al. This work is published and licensed by Dove Medical Press Limited. The full terms of this license are available at https://www.dovepress.com/terms.php and incorporate the Creative Commons Attribution – Non Commercial (unported, v3.0) License (http://creativecommons.org/licenses/by-nc/3.0/). By accessing the work you hereby accept the Terms. Non-commercial uses of the work are permitted without any further permission from Dove Medical Press Limited, provided the work is properly attributed. For permission for commercial use of this work, please see paragraphs 4.2 and 5 of our Terms (https://www.dovepress.com/terms.php).

diseases are zoonotic and have resulted in millions of deaths in recent years.[3] Furthermore, 30 new human pathogens were identified in the past three decades, 75% of which were originated from animals.[4] An increase in the emergence and transmission of zoonotic diseases is a major health catastrophe, today, and needs both short- and long-term strategies to minimize disease-related complications, injuries, and sufferings.

While discussing zoonotic diseases, the coronavirus disease (COVID-19) could be mentioned that was first detected in China, where it spread to 90 international locations.[5] Recognized as a global crisis, efforts around the globe are focused concomitantly on limiting the transmission and reducing the impact of the virus.[6] The outbreak of COVID-19 is a public health emergency that has generated an environment of uncertainty and chaos in all societies.[7] It is important to highlight that when the prognosis and treatment of the disease are known, it is relatively easier for health-care professionals to manage the disease. However, with a least known disease, like the COVID-19, health-care professionals face multiple challenges because of the limited evidence-based information, circulating myths, and the stigma developed by society.[8,9] Therefore, healthcare's capacity to respond to a pandemic plays a key role in disease management. At the same time, the mental health and approach of the health-care professionals are also essential in tackling disease-related complications and transmission. Correlating COVID-19 and the mental health of health-care professionals, Fernandez et al conducted a review of the global evidence in 2021. The authors included 10 systematic reviews involving 100 studies from 35 countries and concluded that there was a wide variation evident in the presence of depression, anxiety, and stress among the health-care professionals.[10] Another systematic review and meta-regression conducted in 2020 reported that 25.8% of the health-care professionals had anxiety while 45% of them had stress while caring for the COVID-19 patients. The increase in the sample size was inversely related to the prevalence of anxiety and stress with a statistically significant relationship.[11] Likewise, an umbrella review of meta-analyses carried out by Sahebi et al showed the prevalence of depression, anxiety, and stress was relatively high among health-care professionals during the COVID-19 pandemic. The authors also suggested that health-care professionals should be provided with resources to minimize the risk of developing depression, anxiety, and stress.[12] Summarizing, it is well acknowledged that health-care professionals are faced with increased depression, anxiety, and stress while managing patients with COVID-19 at different health-care settings and is supported by the results of the global prevalence of depression, anxiety and stress.[13]

In line with what is being discussed, among all the health-care professionals, nurses play a pivotal role as they are at the frontline for patient care.[14,15] Although the literature report about the mental health of nurses while dealing with the COVID-19[16–18] mixed results are reported from literature. Within this context, Alnazly et al in their study with the Jordanian nurses concluded that their participants were extremely depressed and had severe anxiety and depression while managing patients with COVID-19. Additionally, being male, married, aged >40 years, and having more clinical experience were the factors related to mental health.[19] Additionally, a systematic review and meta-analysis focusing prevalence of depression, anxiety and stress during the COVID-19 concluded that almost one-third of nurses had depression, anxiety, and stress during the COVID-19 pandemic.[18] Anxiety and depression were again reported among nurses in China whereas the three most important variables that accounted for depression, anxiety, and stress were resilience, coping styles, and sleep quality.[20] Similarly, a study from Portuguese demonstrated high depression and anxiety among nurses where physical activity, rest between shifts, and spending less time searching for information about COVID-19 were related to improved mental health.[21] Correspondingly, high depression, anxiety, and stress were also reported in nurses from Brazil and the authors highlighted the units in which nurses were working and less experience as important factors in developing depression, anxiety and stress.[22]

Shifting our concerns to the evidence reported from Pakistan, there is a scarcity of published literature that is too reported from the metropolitan cities. A study from three major cities of Pakistan reported that depression, anxiety, and stress were frequent among nurses. In terms of predictors, age and residence were significantly associated with depression, anxiety, and stress.[23] Abid et al identified a predictive link between fear of COVID-19 and depression, anxiety, and stress. Furthermore, a significant mediating effect of certain demographic features was also discovered by the authors.[24] Continuing with our arguments, Khan et al reported moderate to severe levels of generalized anxiety and depressive disorder among nurses. By using the regression model, the authors managed to

identify female gender, less job experience, and no training on how to handle COVID patients as predicting variables influencing depression, anxiety, and stress.[25] High and frequent depression, anxiety, and stress were also reported among nurses from other parts of Pakistan with a variety of demographics having a significant relationship with the development of depression, anxiety, and stress.[26,27] As the COVID-19 is a recent pandemic, it is imperative to explore the mental health of nurses who work in a risky and chaotic environment, while dealing with it. Moreover, as there are more myths than evidence-based information around the globe, evaluation of nurses' mental health becomes indispensable. For that reason, we evaluated the mental health of nurses involved in the management of COVID-19 at a public hospital in Quetta, Pakistan. The paucity of related evidence from this particular area of Pakistan was a predisposition for this study. Furthermore, we were interested to assess the burden of depression, anxiety, and stress as well to investigate if socio-demographic factors have an effect on these mental distress variables among the nurses working in the COVID-19 pandemic, in Pakistan.

Materials and Methods

Study Design and Setting

A cross-sectional study was conducted from 1st May to 15th June 2021 among all nurses working in Sandeman Provincial Hospital, Quetta (SPHQ). Established in 1939 and located in the center of the city, SPHQ is 800-bedded tertiary care, teaching institute. The hospital provides both inpatients as well as outpatient services. Patients typically prefer to approach SPHQ because of affordability and hence SPHQ is the institute of choice for the majority of the residents.[28] The hospital has a separate unit with facilities, supplies, and modern equipment required to manage patients with COVID-19.

Sampling, Study Participants, and Criteria

Nurses in SPHQ are either on contract (employed by the hospital for six months) or permanent (employed by the Ministry of Health). By using a universal sampling approach, all nurses (n = 325; official data from the Directorate General of Health office) providing care to patients in the hospital were approached for data collection. Nurses not willing to participate in the study were excluded.

Data Instrument

In addition to the demographic variables, we used the validated Urdu (National language of Pakistan) version of Depression, Anxiety and Stress Scale - 21 Items (DASS-21) for data collection.[29] Originally developed by Lovibond et al, the DASS-21 is a set of three self-report scales designed to measure the emotional states of depression, anxiety, and stress.[30] Each of the three DASS-21 scales contains 7 items, divided into subscales with similar content. The recommended cut-off scores for conventional severity labels are presented in Table 1.

The DASS-21 is available in public domains and hence permission is not needed from the authors. However, as we used the Urdu version of DASS-21, permission was taken from the authors who were involved in the validation of the questionnaire in Urdu. The DASS-21 was scored by using the recommended cut-off scores for conventional severity labels [normal, moderate, severe] as proposed by Lovibond et al.[30]

To ensure the reliability of DASS-21, we conducted a pilot study. The tool was rated reliable with an alpha value of 0.85. For the main study, the first author (FN) contacted the respondents for their consent of participation in the present study. By using the face-to-face approach, the first author offered the research instrument (paper and pencil based) to the nurses which was completed in his presence.

Data Coding and Statistical Analysis

The data were coded and entered into SPSS v 25.0 for formal analysis. The Kolmogorov–Smirnov test was used for normality assessment and non-parametric tests were used accordingly. Descriptive statistics (frequency, percentage) were used for socio-demographic variables. The demographic variables were compared with the depression, anxiety, and stress scores by using Pearson's Chi-square test of independence. Later, the binary logistic regression was applied to identify the predictors of depression, anxiety, and stress. For this purpose, all three variables (depression, anxiety, and

Table 1 Cut-off Scores for DASS-21

Labels	Depression	Anxiety	Stress
Normal	0–4	0–3	0–7
Mild	5–6	4–5	8–9
Moderate	7–10	6–7	10–12
Severe	11–13	8–9	13–16
Extremely severe	≥14	≥10	≥17

stress) were converted into dichotomous forms. For all analyses, a p-value of <0.05 was of statistical significance.

Results

Demographic Characteristics

Out of the 325 nurses, 297 participated in the study with a response rate of 91.3% (Table 2). Thirty (10.1%) worked in COVID-19 wards while the rest were stationed at the regular wards. Most participating nurses were female 91.5% (n = 272), and most were married 62.2% (n = 185). As many as 51.5% (n = 152) had been working in the hospital for 10 years or less and 48.8% (n = 145) had a nursing diploma as the highest level of education in nursing.

Frequency of Depression, Anxiety, and Stress Among Study Respondents

The depression profile and scores are described in Table 3. Forty-seven (15.8%) nurses felt that life was meaningless while 34 (11.4%) felt that they are worthless as a person. The mean depression score in the current cohort was 6.00±5.30 indicating mild depression among the study respondents.

Table 4 presents the anxiety levels among the nurses enrolled in the current study. Thirty-nine (13.1%) of the nurses felt scared without a good reason while 36 (12.1%) were aware of the action of their heart in the absence of physical exertion. Overall, the mean anxiety score in the current cohort was 6.68±4.86 indicating moderate anxiety among the study respondents.

Stress scores are calculated and provided in Table 5. Forty (13.5%) of our respondents felt that they were rather touchy while 32 (10.8%) found themselves getting agitated. The mean stress score in the current cohort was 8.01±4.47 indicating mild stress among the study respondents.

Relationship Between Depression, Anxiety and Stress Scores and Nurses' Demographic Characteristics

The associations between the demographic variables and depression, anxiety, and stress are presented in Table 6. Where significant associations were reported, the effect size was calculated by using the Phi and Cramer V coefficients. Marital status, department, and job experience were significantly associated ($p < 0.05$) with all three variables (depression, anxiety, and stress) having moderate correlations ($r = 0.3–0.5$) as shown in Table 6.

A binary logistic regression analysis of the model was performed after entering the considered independent variables and comparing them with depression, anxiety, and stress. In the logistic analysis, age, gender, marital status, current position, education, department, and job experience were included as study parameters. The created model showed significant goodness-of-fit as the Omnibus

Table 2 Demographic Characteristics of the Study Respondents

Characteristics	Frequency	Percentage
Age		
28–37	226	76.0
38–47	59	19.9
>47	12	4.1
Gender		
Male	25	8.5
Female	272	91.5
Marital status		
Single	112	37.7
Married	185	62.2
Current position		
Matron	2	0.7
Head nurse	20	6.7
Staff nurse	275	92.6
Education		
BS Nursing	95	31.9
Nursing diploma	145	48.8
Post BS (BSC-N)	57	19.1
Department		
Medicine	53	17.8
Surgery	55	18.5
Paediatrics	40	13.4
Gynaecology/Obstetrics	30	10.1
Cardiac	34	11.4
Urology	15	5.05
ENT	20	6.73
Neurology	20	6.73
Corona ward	30	10.1
Job experience		
<5 years	152	51.1
5–10 years	102	34.4
>10 years	43	14.5
Have you attended training/workshops/seminars on infectious diseases management?		
Yes	120	40.4
No	177	59.6

Table 3 Assessment of Depression Among Study Respondents

Questions	Responses			
	N* N (%)	S* N (%)	O* N (%)	AA* N (%)
I could not seem to experience any positive feeling at all	144 (48.5)	88 (29.6)	41 (13.8)	24 (8.1)
I found it difficult to work up the initiative to do things	133 (44.8)	93 (31.3)	42 (14.1)	29 (9.8)
I felt that I had nothing to look forward to	154 (51.9)	71 (23.9)	46 (15.5)	26 (8.8)
I felt down-hearted and blue	129 (43.4)	91 (30.6)	52 (17.5)	25 (8.4)
I was unable to become enthusiastic about anything	117 (39.4)	104 (35.0)	44 (14.8)	32 (10.8)
I felt I was not worth much as a person	171 (57.6)	49 (16.5)	43 (14.5)	34 (11.4)
I felt that life was meaningless	176 (59.3)	47 (15.8)	27 (9.1)	47 (15.8)

Notes: The depression scale ranged from a minimum 0 to a maximum 21. Depression was measured as (0–4 normal, 5–6 mild, 7–10 moderate, 11–13 severe, and ≥ 14 extremely severe). The mean depression score in the current cohort was 6.00±5.30 indicating mild depression among the study respondents. *Did not apply to me at all = Never (coded as 0), applied to me to some degree, or some of the time = Sometimes (coded as 1), applied to me to a considerable degree, or a good part of time = Often (coded as 2), and applied to me very much, or most of the time = Almost always (coded as 3).

Test of Model Coefficient was highly significant (Chi-square = 13.224, p = 0.004, df = 3), indicating that the model was advisable. Department in which nurses were stationed (adjusted OR = 2.54, 95% CI = 1.118–1.415, p < 0.001) and job experience (adjusted OR = 1.004, 95% CI = 1.001–1.215, p = 0.002) emerged as significant predictors of depression, anxiety and stress. Nurses working in the corona ward were more likely to develop depression, anxiety, and stress by a factor of 2.54, and less experienced nurses with a factor of 1.004 provided controlling other confounding factors.

Discussion

The third wave of COVID-19 in Pakistan started in mid-March 2021. Most of the positive cases were reported from the provinces of Punjab and Khyber Pakhtunkhwa. Increased frequency was reported till May 2021, and since then, there is a gradual decline in the number of positive cases and new death numbers. Till 19th September 2021, 1,265,650 confirmed cases of COVID-19 were reported with 28,300 deaths and 1,211,710 recoveries. Pakistan still has 25,640 active cases of COVID-19 that require pharmaceutical care at health-care institutes.[31]

Table 4 Assessment of Anxiety Among Study Respondents

Questions	Responses			
	N* N (%)	*S* N (%)	O N (%)	AA* N (%)
I was aware of the dryness of my mouth	110 (37.0)	130 (43.8)	25 (8.4)	32 (10.8)
I experienced breathing difficulty (eg, excessively rapid breathing, breathlessness in the absence of physical exertion)	113 (38.0)	113 (38.0)	45 (15.2)	16 (8.8)
I experienced trembling (eg, in the hands)	107 (36.0)	129 (43.4)	40 (13.5)	21 (7.1)
I was worried about situations in which I might panic and make a fool of myself	101 (34.0)	111 (37.4)	54 (18.2)	31 (10.4)
I felt I was close to panic	181 (60.9)	45 (15.2)	38 (12.8)	33 (11.1)
I was aware of the action of my heart in the absence of physical exertion (eg, sense of heart rate increase, heart missing a beat)	103 (34.7)	115 (38.7)	43 (14.5)	36 (12.1)
I felt scared without any good reason	102 (34.3)	113 (38.0)	43 (14.5)	39 (13.1)

Notes: The anxiety scale ranged from a minimum 0 to a maximum 21. Anxiety was measured as (0–3 normal, 4–5 mild, 6–7 moderate, 8–9 severe, and ≥ 10 extremely severe). The mean anxiety score in the current cohort was 6.68±486 indicating moderate anxiety among the study respondents. *Did not apply to me at all = never (coded as 0), applied to me to some degree, or some of the time = sometimes (coded as 1), applied to me to a considerable degree, or a good part of time = often (coded as 2), and applied to me very much, or most of the time = almost always (coded as 3).

Table 5 Assessment of Stress Among Study Respondents

Questions	Responses			
	N* N (%)	S* N (%)	O* N (%)	AA* N (%)
I found it hard to wind down	119 (40.1)	135 (45.5)	28 (9.4)	15 (5.1)
I tended to over-react to situations	91 (30.6)	134 (45.1)	50 (16.8)	22 (7.4)
I felt that I was using a lot of nervous energy	100 (33.7)	108 (36.4)	60 (20.2)	29 (9.8)
I found myself getting agitated	75 (25.3)	138 (46.5)	52 (17.5)	32 (10.8)
I found it difficult to relax	93 (31.3)	125 (42.1)	50 (16.8)	29 (9.8)
I was intolerant of anything that kept me from getting on with what I was doing	139 (46.8)	86 (29.0)	44 (14.8)	28 (9.4)
I felt that I was rather touchy	106 (35.7)	98 (33.0)	53 (17.8)	40 (13.5)

Notes: The stress scale ranged from a minimum of 0 to a maximum of 21. Stress was measured as (0–7 normal, 8–9 mild, 10–12 moderate, 13–16 severe, and ≥ 17 extremely severe). The mean stress score in the current cohort was 8.01±4.47 indicating mild stress among the study respondents. *Did Not Apply To Me At All = Never (coded as 0), Applied to me to some degree, or some of the time = Sometimes (coded as 1), Applied to me to a considerable degree, or a good part of time = Often (coded as 2), and applied to me very much, or most of the time = Almost always (coded as 3).

While managing patients with communicable diseases, nursing is one of the most stressful professions in both developed and developing countries.[32,33] In this context, Jamali et al, in their latent class regression analysis, concluded that mental health problems are frequently reported among nurses when compared to other health-care practitioners.[34] Under the umbrella of mental health issues, depression, anxiety, and stress are time and again reported among nurses and hence are of keen interest for both clinical and social scientists.[32,35] Based on what is being discussed here, we hypothesized that depression, anxiety, and stress among nurses during a pandemic must be severe, as fear of getting sick during the provision of health-care services makes the condition worse. Specifically, concerning COVID-19, the development of disease-related complications as well as increased casualties has led to reservations among nurses, hence resulting in emotional confusion, out-of-control behaviors, and increased depression, anxiety and stress. Therefore, it is crucial to assess nurses' mental health during the COVID-19 pandemic and for that reason, the current study was undertaken.

Nurses, in the current study, reported mild stress and depression, and moderate anxiety during the COVID-19. On the contrary, Zheng et al reported a high prevalence of depression, anxiety, and stress among nurses during the outbreak of COVID-19.[36] Increased depression, anxiety and stress were also reported from studies of the same nature from other parts of the world.[37,38] Maqbali et al, in their systematic review and meta-analysis, found that approximately one-third of nurses working during the COVID-19 pandemic were suffering from psychological symptoms.[18] A possible explanation of these conflicting results is attributed to the episodes of COVID-19. In March 2021, the Government of Pakistan launched the COVID-19 vaccination drive, and priority was given to frontline health workers. Understandably, vaccination must have provided confidence to health-care

Table 6 Relationship Between Depression, Anxiety, and Stress Scores and Nurses' Demographic Characteristics

Demographics	P-value*		
	Depression	Anxiety	Stress
Age	0.554	0.848	0.523
Gender	0.61	0.582	0.996
Marital status	0.002 (ϕ = 0.318)	0.003 (ϕ = 0.300)	0.007 (ϕ = 0.388)
Current position	0.457	0.249	0.973
Education	0.557	0.299	0.410
Department	0.005 (φc = 0.415)	0.001 (φc = 0.399)	0.004 (φc = 0.382)
Job experience	0.008 (φc = 0.4.01)	0.015 (φc = 0.408)	0.006 (φc = 0.381)

Notes: *Chi-square test; Phi = ϕ, Cramer V = φc. (Values in brackets reveal effect size which are interpreted as >0.5 = high association, 0.3–0.5 = moderate association, 0.1–0.3 = low association and 0–0.1 = little if any association.

professionals while managing patients with COVID-19. Our discussion with the nurses during the data collection revealed that vaccination did provide them a sense of protection and hence has improved their mental health (informal communication). Furthermore, compared with the early days of the pandemic, the assured and guaranteed availability of COVID-19 related essentials (personal protective equipment, mask, etc.), medicines, and supplies in the hospitals also provided an additional sense of protection to the nurses. Finally, the decreasing frequency of COVID-19 in Pakistan, in general, and Quetta, in particular, is another contributing factor for mild or moderate depression, anxiety, and stress among nurses in the current cohort.[31] Additionally, the difference in the study settings and tools used for data collection also clarify the variation of mental distress between the reported studies.[39]

The Chi-square test revealed that marital status was significantly associated with depression, anxiety and stress. Mixed results are reported in the literature when marital status is compared against depression, anxiety, and stress. At some instances, married nurses were more likely to suffer from depression, anxiety and stress.[40–42] On the contrary, Lasebikan & Oyetunde reported that single nurses were depressed and had high anxiety than the married participants.[43] The explanation for this, in our study, is straightforward and the inference is understandable. A family is considered one of the most important units of society. It provides the support that is significant for the wellbeing of the members of society and the continuance of social order. In Pakistan also, family is the core of social life; where parents, their sons, and the sons' families often live together in the same household. Therefore, the fear of being infected with COVID-19 or becoming a carrier might increase the chances that the infection may get transmitted to the family members including elders and children. Continuously living with this fear may have increased DA&S among nurses practicing in the hospitals during the pandemic.

The logistic regression analysis reported job experience as a significant predictor where nurses with less experience were more likely to develop depression, anxiety, and stress. Our observations are in line with what was reported by Humpel & Caputi. The authors concluded that nurses with a shorter experience had personal self-doubt and faced depression, anxiety, and stress.[44] A study conducted in the Punjab province of Pakistan also reconfirmed our findings whereby the authors concluded that young nurses (≤3 years) had an increased likelihood of developing depression and anxiety.[25] Parallel to our finding, Roberts et al in their regression model also reported less experienced nurses with higher levels of anxiety and depression.[45] The relationship of job experience with the development of depression, anxiety, and stress is understandable. When job experience increases, nurses also expand their skills and knowledge while practicing safe patient care. Experience also plays a primary role in developing best practices that differentiate it from mere technical interventions.[46] Hence, it is concluded that as the experience increases, the skills and expertise in handling emergencies and crises are also enhanced. Additionally, experienced nurses are often more prepared mentally and are equipped with proficiency in dealing with crises.[47] Understandably, since all such skills and capabilities take time to be learned and adopted, less experienced nurses do feel depression, anxiety, and stress during their practice. As expected, they feel burnt-out and pressure as compared to their experienced counterparts.[48]

In addition to job experience, nurses working in the corona ward were also likely to develop depression, anxiety, and stress. This relationship is also explainable as nurses working in the corona ward are in continuous contact with the patients and chances of being infected with the COVID-19 are higher as compared to other wards of the hospital. Besides, COVID-19 being exceedingly communicable has a greater risk of transmission through close contact, particularly in the corona ward. Our assumption is supported by a study reported from Jordan. The regression model highlighted that nurses dealing with corona-positive patients were more depressed and had high anxiety.[49] Likewise with increased cases of COVID-19 in China, nurses who were involved in direct patient care developed a wide range of psychological disturbances, ranging from posttraumatic stress disorder to depression and anxiety.[50] Another study from China again reported that nurses involved in providing direct patient care to COVID-19 patients experienced rapid changes in their psychological status with elevated depressive symptoms.[51] Increased depression, anxiety, and stress were also evident in nurses involved in managing COVID-19 patients from other parts of the world.[21,39,52]

Limitations

The study was conducted in a single health-care institute and the generalizability is always questioned. We do recommend a nationwide study to get a clear picture of the nurses' mental health during the recent pandemic.

Conclusion

COVID-19 is a global emergency that has developed depression, anxiety, and stress among health-care professionals including nurses. Although mild to moderate depression, anxiety and stress were observed among nurses, yet it calls for hospital management to consider interventions (mental health protective measures on COVID-19) for all nurses irrespective of their present working departments. However, we also recommend targeted interventions especially for those nurses who are directly involved with the corona patients. The findings of the study have some clear implications for mental health advocacy among nurses that are essential for improving the quality of services and safety of patients and staff in high-pressure conditions such as COVID-19.

Ethics Approval and Consent to Participate

This study was carried out in accordance with the Declaration of Helsinki. This study was approved by the Institutional Ethical Committee at Faculty of Pharmacy & Health Sciences, Quetta (GSO/UOB:578/18). The informed written consent was also obtained from all nurses.

Acknowledgments

We acknowledge the nurses and the hospital administration for their support during the study.

Funding

This study was not funded.

Disclosure

The authors declare that they have no competing interests.

References

1. Lam SK, Kwong EW, Hung MS, Pang SM, Chiang VC. Nurses' preparedness for infectious disease outbreaks: a literature review and narrative synthesis of qualitative evidence. *J Clin Nurs*. 2018;27(7–8): e1244–e1255. doi:10.1111/jocn.14210
2. World Health Organization. Infectious diseases. Available from: http://www.emro.who.int/health-topics/infectious-diseases/index.html. Accessed September 5, 2021.
3. Petersen E, Petrosillo N, Koopmans M, et al. Emerging infections—an increasingly important topic: review by the emerging infections task force. *Clin Microbiol Infect*. 2018;24(4):369–375. doi:10.1016/j.cmi.2017.10.035
4. Jones KE, Patel NG, Levy MA, et al. Global trends in emerging infectious diseases. *Nature*. 2008;451(7181):990–993. doi:10.1038/nature06536
5. Centers for Disease Control and Prevention. Coronavirus disease 2019 (COVID-19): situation summary. Available from: https://www.cdc.gov/coronavirus/2019-ncov/index.html. Accessed September 4, 2021.
6. World Health Organization. Report of the WHO-China joint mission on coronavirus disease 2019 (COVID-19). Available from: https://www.who.int/docs/default-source/coronaviruse/who-china-joint-mission-on-covid-19-final-report.pdf. Accessed August 5, 2021.
7. Nyblade L, Stockton MA, Giger K, et al. Stigma in health facilities: why it matters and how we can change it. *BMC Med*. 2019;17(1):25. doi:10.1186/s12916-019-1256-2
8. Zarei N, Joulaei H, Darabi E, Fararouei M. Stigmatized attitude of healthcare providers: a barrier for delivering health services to HIV positive patients. *Int J Community Based Nurs Midwifery*. 2015;3 (4):292.
9. Kohrt BA, Jordans MJ, Turner EL, et al. Reducing stigma among healthcare providers to improve mental health services (RESHAPE): protocol for a pilot cluster randomized controlled trial of a stigma reduction intervention for training primary healthcare workers in Nepal. *Pilot Feasibility Stud*. 2018;4(1):36. doi:10.1186/s40814-018-0234-3
10. Fernandez R, Sikhosana N, Green H, et al. Anxiety and depression among healthcare workers during the COVID-19 pandemic: a systematic umbrella review of the global evidence. *BMJ Open*. 2021;11(9):e054528. doi:10.1136/bmjopen-2021-054528
11. Salari N, Khazaie H, Hosseinian-Far A, et al. The prevalence of stress, anxiety and depression within front-line healthcare workers caring for COVID-19 patients: a systematic review and meta-regression. *Hum Resour Health*. 2020;18(1):1–14. doi:10.1186/s12960-020-00544-1
12. Sahebi A, Nejati B, Moayedi S, Yousefi K, Torres M, Golitaleb M. The prevalence of anxiety and depression among healthcare workers during the COVID-19 pandemic: an umbrella review of meta-analyses. *Prog Neuropsychopharmacol Biol Psychiatry*. 2021;107:110247. doi:10.1016/j.pnpbp.2021.110247
13. Mahmud S, Hossain S, Muyeed A, Islam MM, Mohsin M. The global prevalence of depression, anxiety, stress, and, insomnia and its' changes among health professionals during COVID-19 pandemic: a rapid systematic review and meta-analysis. *Heliyon*. 2021;7: e07393. doi:10.1016/j.heliyon.2021.e07393
14. Kieft RA, de Brouwer BB, Francke AL, Delnoij DM. How nurses and their work environment affect patient experiences of the quality of care: a qualitative study. *BMC Health Serv Res*. 2014;14(1):249. doi:10.1186/1472-6963-14-249
15. Hughes R. *Patient Safety and Quality: An Evidence-Based Handbook for Nurses*. Vol. 3. MD: Agency for Healthcare Research and Quality Rockville; 2008.
16. Fathiariani L, Nassimbwa J. Investigating the burden of mental distress among nurses at a provincial COVID-19 referral hospital in Indonesia: a cross-sectional study. *BMC Nurs*. 2021;20(1):1–8.
17. Talevi D, Socci V, Carai M, et al. Mental health outcomes of the CoViD-19 pandemic. *Riv Psichiatr*. 2020;55(3):137–144.
18. Al Maqbali M, Al Sinani M, Al-Lenjawi B. Prevalence of stress, depression, anxiety and sleep disturbance among nurses during the COVID-19 pandemic: a systematic review and meta-analysis. *J Psychosom Res*. 2020;141:110343.
19. Alnazly E, Khraisat OM, Al-Bashaireh AM, Bryant CL. Anxiety, depression, stress, fear and social support during COVID-19 pandemic among Jordanian healthcare workers. *PLoS One*. 2021;16(3): e0247679. doi:10.1371/journal.pone.0247679
20. Pang Y, Fang H, Li L, Chen M, Chen Y, Chen M. Predictive factors of anxiety and depression among nurses fighting coronavirus disease 2019 in China. *Int J Ment Health Nurs*. 2021;30(2):524–532. doi:10.1111/inm.12817

21. de Pinho LG, Sampaio F, Sequeira C, Teixeira L, Fonseca C, Lopes MJ. Portuguese nurses' stress, anxiety, and depression reduction strategies during the covid-19 outbreak. *Int J Environ Res Public Health*. 2021;18(7):3490. doi:10.3390/ijerph18073490
22. Appel AP, Carvalho A, Santos R. Prevalence and factors associated with anxiety, depression and stress in a COVID-19 nursing team. *Revista Gaúcha de Enfermagem*. 2021;42(spe):e20200403. doi:10.1590/1983-1447.2021.20200403
23. Arshad MS, Hussain I, Nafees M, et al. Assessing the impact of COVID-19 on the mental health of healthcare workers in three metropolitan cities of Pakistan. *Psychol Res Behav Manag*. 2020;13:1047. doi:10.2147/PRBM.S282069
24. Abid M, Riaz M, Bano Z, Parveen T, Fayyaz MU, Qureshi SH. Association between fear of COVID-19 and emotional distress in nurses with mediating role of socio-demographic features. *Front Psychol*. 2021;12. doi:10.3389/fpsyg.2021.734623
25. Khan TM, Tahir H, Salman M, et al. General anxiety predictors among frontline warriors of COVID: cross-sectional study among nursing staff in Punjab, Pakistan. *Arch Pharm Pract*. 2021;1:40. doi:10.51847/A0j3y5qmHp
26. Fatima A, Ali S, Ghani N, Shah BA. LEVEL of stress, anxiety and depression among nurses working at hayatabad medical complex Peshawar Pakistan. *Khyber Med Univ J*. 2020;12(4):321–325.
27. Mehdi M, Waseem M, Rehm MH, Aziz N, Anjum S, Javid MA. Depression and anxiety in health care workers during COVID-19. *Biomedica.* 2020;36:247–252. doi:10.51441/BioMedica//BioMedica/5-414
28. Shahzad F, Saleem F, Iqbal Q, et al. A cross-sectional assessment of health literacy among hypertensive community of Quetta City, Pakistan. *Biomed J*. 2018;11(4):8685–8693.
29. Aslam N, Kamal A. Translation, validation and effectiveness of depression, anxiety and stress (DASS-21) in assessing the psychological distress among flood affected individuals. *J Pak Psychiatr Soc*. 2017;14(4):16–20.
30. Lovibond SH, Lovibond PF. *Manual for the Depression Anxiety & Stress Scales*. 2nd ed. Psychology Foundation; 1995.
31. Government of Pakistan. Pakistan cases details. Available from: https://covid.gov.pk/stats/pakistan. Accessed October 19, 2021.
32. Tran TTT, Nguyen NB, Luong MA, et al. Stress, anxiety and depression in clinical nurses in Vietnam: a cross-sectional survey and cluster analysis. *Int J Ment Health Syst*. 2019;13(1):1–11. doi:10.1186/s13033-018-0257-4
33. Eurofound. Sixth European working conditions survey - overview report; 2016.
34. Jamali J, Roustaei N, Ayatollahi SMT, Sadeghi E. Factors affecting minor psychiatric disorder in Southern Iranian nurses: a latent class regression analysis. *Nurs Midwifery Stud*. 2015;4(2):e28017. doi:10.17795/nmsjournal28017
35. Letvak S, Ruhm CJ, McCoy T. Depression in hospital-employed nurses. *Clin Nurse Specialist*. 2012;26(3):177–182. doi:10.1097/NUR.0b013e3182503ef0
36. Zheng R, Zhou Y, Fu Y, et al. Prevalence and associated factors of depression and anxiety among nurses during the outbreak of COVID-19 in China: a cross-sectional study. *Int J Nurs Stud*. 2021;114:103809. doi:10.1016/j.ijnurstu.2020.103809
37. Chowdhury SR, Sunna TC, Das DC, et al. Mental health symptoms among the nurses of Bangladesh during the COVID-19 pandemic. *Middle East Current Psychiatry.* 2021;28(1):1–8. doi:10.1186/s43045-021-00103-x
38. Basit M, Peni MR. Psychological stress of nurses during the Covid-19 pandemic. *KnE Life Sci*. 2021;43:256–267.
39. Heidarijamebozorgi M, Jafari H, Sadeghi R, Sheikhbardsiri H, Kargar M, Amiri Gharaghani M. The prevalence of depression, anxiety, and stress among nurses during the coronavirus disease 2019: a comparison between nurses in the frontline and the second line of care delivery. *Nurs Midwifery Stud*. 2021;10(3):188–193.
40. Yao Y, Yao W, Wang W, Li H, Lan Y. Investigation of risk factors of psychological acceptance and burnout syndrome among nurses in C hina. *Int J Nurs Pract*. 2013;19(5):530–538.
41. Xie Z, Wang A, Chen B. Nurse burnout and its association with occupational stress in a cross-sectional study in Shanghai. *J Adv Nurs*. 2011;67(7):1537–1546. doi:10.1111/j.1365-2648.2010.05576.x
42. Al-Turki HA, Al-Turki RA, Al-Dardas HA, et al. Burnout syndrome among multinational nurses working in Saudi Arabia. *Ann Afr Med*. 2010;9(4):226–229. doi:10.4103/1596-3519.70960
43. Lasebikan VO, Oyetunde MO. Burnout among nurses in a Nigerian general hospital: prevalence and associated factors. *Int Scholarly Res Not*. 2012;5:402157.
44. Humpel N, Caputi P. Exploring the relationship between work stress, years of experience and emotional competency using a sample of Australian mental health nurses. *J Psychiatr Ment Health Nurs*. 2001;8(5):399–403. doi:10.1046/j.1365-2850.2001.00409.x
45. Roberts N, McAloney-Kocaman K, Lippiett K, Ray E, Welch L, Kelly C. Levels of resilience, anxiety and depression in nurses working in respiratory clinical areas during the COVID pandemic. *Respir Med*. 2021;176:106219. doi:10.1016/j.rmed.2020.106219
46. Arbon P. Understanding experience in nursing. *J Clin Nurs*. 2004;13(2):150–157. doi:10.1046/j.1365-2702.2003.00861.x
47. Vasli P, Dehghan-Nayeri N. Emergency nurses' experience of crisis: a qualitative study. *Jpn J Nurs Sci*. 2016;13(1):55–64. doi:10.1111/jjns.12086
48. Shen X, Zou X, Zhong X, Yan J, Li L. Psychological stress of ICU nurses in the time of COVID-19. Springer; 2020.
49. Al-Amer RM, Malak MZ, Aburumman G, et al. Prevalence and predictors of depression, anxiety, and stress among Jordanian nurses during the coronavirus disease 2019 pandemic. *Int J Ment Health*. 2021:1–12. doi:10.1080/00207411.2021.1916701
50. Huang Y, Zhao N. Generalized anxiety disorder, depressive symptoms and sleep quality during COVID-19 outbreak in China: a web-based cross-sectional survey. *Psychiatry Res*. 2020;288:112954. doi:10.1016/j.psychres.2020.112954
51. An Y, Yang Y, Wang A, et al. Prevalence of depression and its impact on quality of life among frontline nurses in emergency departments during the COVID-19 outbreak. *J Affect Disord*. 2020;276:312–315. doi:10.1016/j.jad.2020.06.047
52. Spoorthy MS, Pratapa SK, Mahant S. Mental health problems faced by healthcare workers due to the COVID-19 pandemic – a review. *Asian J Psychiatr*. 2020;51:102119. doi:10.1016/j.ajp.2020.102119

Journal of Multidisciplinary Healthcare Dovepress

Publish your work in this journal

The Journal of Multidisciplinary Healthcare is an international, peer-reviewed open-access journal that aims to represent and publish research in healthcare areas delivered by practitioners of different disciplines. This includes studies and reviews conducted by multidisciplinary teams as well as research which evaluates the results or conduct of such teams or healthcare processes in general. The journal covers a very wide range of areas and welcomes submissions from practitioners at all levels, from all over the world. The manuscript management system is completely online and includes a very quick and fair peer-review system. Visit http://www.dovepress.com/testimonials.php to read real quotes from published authors.

Submit your manuscript here: https://www.dovepress.com/journal-of-inflammation-research-journal
