# Supplementary material for: Mental health status and related factors influencing healthcare workers during the COVID-19 pandemic: A systematic review and meta-analysis
Source: PLoS One. 2024 Jan 19;19(1):e0289454. doi: 10.1371/journal.pone.0289454 (PMC10798549; doi:10.1371/journal.pone.0289454)
Supplement: S1 Data — (ZIP) [file pone.0289454.s011.zip › literatures/113.pdf]

Psychiatria Danubina, 2022; Vol. 34, No. 1, pp 164-170 https://doi.org/10.24869/psyd.2022.164 *Original paper*
© Medicinska naklada - Zagreb, Croatia

PSYCHOLOGICAL AND MENTAL HEALTH BURDEN ON HEALTH CARE PROVIDERS IN A CANCER CENTRE DURING COVID-19 PANDEMIC OUTBREAK IN INDIA

Raj Nagarkar[1], Roshankumar Patil[2], Kavita Gadade[3], Nishtha Paleja[4] & Yasam Venkata Ramesh[5]

[1]Department of Surgical Oncology, HCG Manavata Cancer Centre, Nashik, Maharashtra, India
[2]Department of Radiation Oncology, HCG Manavata Cancer Centre, Nashik, Maharashtra, India
[3]Department of Psycho-Oncology, HCG Manavata Cancer Centre, Nashik, Maharashtra, India
[4]Department of Clinical Research, HCG Manavata Cancer Centre, Nashik, Maharashtra, India
[5]Department of Academics, HCG Manavata Cancer Centre, Nashik, Maharashtra, India

received: 7.6.2021; revised: 30.9.2021; accepted: 30.10.2021

SUMMARY

Background: *The outbreak of novel coronavirus (COVID-19) is severely affecting the public health and posing a challenge to health care providers, especially working as front-line medical staff. This study was aimed to understand the psychological impact and mental burden of the present outbreak on Indian health care providers who are working at cancer care centre.*

Subjects and methods: *A self-reporting online questionnaire was given to the multidisciplinary staff (n=344) and their mental health was assessed using various scales via GAD-7 scale for anxiety, PHQ-9 scale for depression, ISI for insomnia, K-10 for distress, and STAI for stress along with five self-made Pandemic specific questions.*

Results: *Response rate was 91% (n=344) among 190 (55%) were male and 154 (45%) were female. The frontline and second-line workers were 178 (52%) and 166 (48%), respectively. Symptoms of anxiety, depression, insomnia and distress was observed in 62 (18%), 75 (22%), 42 (12%), and 60 (17%) of the participants, respectively. They were predominantly influenced by variables such as gender (female), education (≥graduation), co-morbidities, and level of work (frontline). Followed by other less dominant variables such as contact with patients (frequent), and working in hospital (<3 years), respectively.*

Conclusion: *A mild to moderate level of psychological burden was observed in the health care providers. Overall, there is a need to address the mental health issues by providing, timely training, counselling, rotation in shifts, lowering workload and intensify the awareness programmes of the staff during this COVID-19 pandemic for better outcomes and promoting resilience in the staff.*

Key words: *COVID-19 pandemic - psychological burden - healthcare providers - cancer care - India*

* * * * *

INTRODUCTION

Cancer has become the second most common cause of death globally in 2018, accounting for almost 9.6 million deaths (Bray et al. 2018). Because of the multidimensional influencing factors associated with cancer and its treatment, survival rate among patients was observed to be low, causing emotional burden to both the patient and the staff involved. Many studies have proved that medical practitioners, nurses and other health care providers working in oncology centres have significantly greater emotional distress than their peers working in other specialisations (Jones et al. 2013, Beresford et al. 2018). This might be due to the frequent dealings with end-of-life scenarios, tackling difficult-to-treat diseases, and lack of training in handling emotional aspects of medical care (Daruvala et al. 2019).

The recent outbreak of global pandemic novel coronavirus (COVID-19) has ameliorating the existing psychological situation. The COVID-19 was first reported in Wuhan, China on 08th December 2019 (Chen & Yu 2020). Within no time, the novel virus has become a pandemic and the outbreak has spread around the world causing a major public health emergency. To curb the situation, many countries have imposed strict travel bans and lockdowns over a prolonged period of time. According to World Health Organization (WHO) assessment, these situations have largely threatened mental health and psychological condition of the affected populations (World Health Organization 2020). Along with the public, the situation has created a panic in cancer patients, which in turn multiplying its effects and pressure on the treating and handling staff working at cancer care centres.

It was also observed and proved during the previous outbreaks (Roy et al. 2020) such as SARS, EBOLA, HIN1 etc. Many studies have reported adverse psychological reactions related to the outbreaks among health care workers. This COVID-19 outbreak is currently causing a severe mental health burden in worst-hit countries such as India with 124981 active cases and 7200 deaths by 8 June 2020 (MoHFW 2020). With the Government of India extending the nationwide lockdown throughout the country, the districts across the states are classified as red, orange and green zones with varying levels of restrictions aimed to curb the further spread of COVID-19. Present classification was done depending on the incidence of cases, extent of testing, severity of the outbreak, and surveillance

feedback in the respective places. Present study was conducted in a classified red zone by Government of India (MoHFW 2020). It was designed and aimed to understand and assess the psychological impact and mental health burden of the present pandemic COVID-19 outbreak on Indian health care providers who are working at cancer care centre.

SUBJECTS AND METHODS

Participants

All the staff working at a specialized cancer care centre, India, had participated in the present study.

Study design and recruitment

This is a cross-sectional study, performed via an online survey conducted from 24th May to 30th May 2020. All the hospital staff was enquired about the previous 60 days mental health and psychological impact of COVID-19, following WHO announcement of the COVID-19 as a pandemic outbreak on 11th March 2020 and declaration of first lockdown (24th March 2020) by Indian Government. All the necessary ethical approvals for the present study were obtained from the Institutional Ethical Committee. Written informed consent was obtained from participants. Participants were allowed to terminate the survey at any time they desired. The identity of participants was blinded in the survey, and information confidentiality was assured.

Data collection tool

A self-administered, internet-based questionnaire was developed that included demographic characteristics, personal clinical history, job-related characteristics, work-related and other stressors, ideations, and stress management questions of the selected instruments.

Instruments / questionnaires

The initial measurements in the online questionnaire survey included demographic data, marital status, living with family, co-morbidities, role in the hospital, and exposure with patients. Depending on the responsibilities and technical titles attributed by the hospital, the staff engaged in clinical activities were further defined as front-line and second-line workers. In this survey, various internationally recognized validated scales were used such as Generalized Anxiety Disorder (GAD-7 scale, range 0-21) for anxiety (Huang & Zhao 2020, Li et al. 2020), Patient Health Questionnaire (PHQ-9 scale, range 0-27) for depression (Li et al. 2020), Insomnia Severity Index (ISI, range 0-28) for insomnia (Bastien et al. 2001), Kessler Psychological Distress Scale (K-10, range 0-50) for distress (Moccia et al. 2020), and the State-Trait Anxiety Inventory (STAI, range 0-80 for each form) for stress (Julian 2011) along with five self-made Pandemic specific questions.

GAD-7 and PHQ-9 scales consists of 7 and 9 questions, respectively. Each question carries a score of 0-3. These scales are used as screening tools to measure the severity of anxiety and depression symptoms based on the scores received, respectively. Recorded score was interpreted based on the individual classification as follows: GAD-7, minimal (0-4), mild (5-9), moderate (10-14), and severe (15-21); PHQ-9, minimal (1-4), mild (5-9), moderate (10-14), moderately severe (15-19), and severe (20-27) (Huang & Zhao 2020, Li et al. 2020).

Insomnia was assessed using the ISI scale, where the respondent’s sleep, sleep patterns, frequent sleep breaks and its impact on daily routine, and the overall level of distress created by the sleep problem were measured. It is a seven-item questionnaire categorized as no clinically significant insomnia (0-7), subthreshold insomnia (8-14), moderate clinical insomnia (15-21), and severe clinical insomnia (22-28) depending on the score received (Bastien 2001).

K-10 scale was used to clinically measure the psychological distress and negative symptoms in the past 60 days, including characteristics like fatigue, worthlessness, sadness, hopelessness, and nervousness. It is a five-level response scale with each question score ranging from 1 to 5, with the total scores ranging from 10 (Likely to be well) to 50 (likely to have severe disorder).

Another scale, STAI is 4-point Likert scale and consists of 40 questions divided into two parts as Y-1 (S-Anxiety subscale) and Y-2 (T-Anxiety subscale). The incidence and severity of current symptoms of anxiety and a generalized propensity to be anxious were measured. The range of subtest total scores for each subtest varies from 20-80, where the lower score indicating lower anxiety and higher score indicating higher anxiety (Julian 2011, Moccia et al. 2020).

All the questions of various scales used in this self-reporting survey were shuffled to avoid a particular pattern, during answering the questions, which may influence the answer of the next questions.

Statistical analysis

A p-value less than 0.05 (typically ≤ 0.05) was considered as statistically significant. The score of all the 5 scales were non-normally distributed and presented as number (n) and percentages (%). Actual psychological burden within our staff were noted in the form of score and stratified according to each manual. Multivariate logistic regression analysis was performed to determine the independent risk factors for symptoms of anxiety, depression, insomnia, and distress of all the participants involved in this study and the outcomes were presented as odd ratios and 95% confidence interval (95% CI). All statistical data analyses were performed using SPSS (IBM SPSS Statistics version 22.0, IBM Corp., USA).

Table 1. Demographical Parameters of the staff

Parameters<br>Variable	Number<br>(n)	Percentage<br>(%)
Age		
<25	115	33%
25-35	164	48%
36-45	49	14%
>45	16	5%
Gender		
Male	190	55%
Female	154	45%
Income		
Low	262	76%
Medium	44	13%
High	38	11%
Dependent Number of family members		
<4	136	40%
≥4	208	60%
Education		
<Graduate	188	54%
≥Graduate	156	46%
Marital status		
Single / Divorcee	148	43%
Married	196	57%
Living		
Alone	62	18%
With family	282	82%
Comorbidities		
HT	10	3%
DM	5	1.5%
Thyroid	7	2%
Mental disorder	2	0.5%
Others	4	1%
No comorbidities	316	92%
Working in the hospital as		
Physicians / Nurses	153	45%
Allied Health care professionals	30	9%
Clerical staff	89	26%
Maintenance workers	72	20%
Contact with patients		
Almost always	232	67%
Sometimes	63	19%
Almost never	49	14%
Health Worker		
Front line	178	52%
Second line	166	48%
Working in the hospital		
<3 years	186	54%
>3 years	158	46%

RESULTS

A total of 344 health care workers participated in the survey. The demographic data of the participants were presented in table 1. Of which, 190 (55%) were male and 154 (45%) were female, with median age of 29 years. Over 188 participants (54%) finished their graduation,

Table 2. Psychological outcomes of health care providers

Instrument<br>Severity		Number<br>(n)	Percentage<br>(%)
GAD7			
No		282	82%
Mild Anxiety		44	13%
Moderate Anxiety		11	3%
Severe Anxiety		7	2%
PHQ9			
Minimal or none		269	78%
Mild		56	17%
Moderate		13	3.5%
Moderately severe		2	0.5%
Severe		4	1%
ISI			
No		302	88%
Subthreshold		35	10%
Moderate		3	0.8%
Severe		4	1.2%
K10			
No / Minimal		284	83%
Mild		40	11%
Moderate		13	4%
Severe		7	2%
STAI			
S – Anxiety	<40	216	63%
	≥40	128	37%
T - Anxiety	<40	218	64%
	≥40	126	36%

178 (52%) are working as frontline workers in various roles such as physicians, nurses, and allied health care professionals and 232 (67%) participants are in direct contact with patients almost always. Among 344, 158 (46%) participants are working with the hospital more than 3 years.

Table 2 and table 3 shows the psychological outcomes and multivariate logistic regression analysis data of scales used, respectively. From GAD-7, 282 (82%) participants have no signs of anxiety, 44 (13%) with mild anxiety, and 18 (5%) with moderate to severe anxiety. From multivariate analysis, gender [Female vs Male (OR, 3.03; 95% CI, 1.53-5.99; p=0.001)], education [≥Graduate vs <Graduate (OR, 2.85; 95% CI, 1.43-5.07; p=0.002)], and working experience with hospital [<3 years vs >3 years (OR, 2.02; 95% CI, 1.07-3.81; p=0.03)] were found to influence anxiety. From PHQ-9, 56 (17%) participants were observed to have depression symptoms, among them 19 (5%) were with moderate to severe depression mostly influenced by gender [Female vs Male (OR, 2.96; 95% CI, 1.57-5.56; p=0.01)], education [≥Graduate vs <Graduate (OR, 2.10; 95% CI, 1.13-3.92; p=0.01)], comorbidities [Yes vs No (OR, 2.40; 95% CI, 1.60-5.99; p=0.04)], and contact with patients [Frequent vs Never (OR, 2.91; 95% CI, 1.41-5.98; p=0.004)]. Subthreshold levels of insomnia (ISI) and mild distress (K10) was witnessed in 35 (10%) and 40 (11%) participants, respectively. Insomnia was mostly

Table 3. Independent Risk factors for psychological manifestations identified by multivariate analysis

Variable		Odds Ratio (95% Confidence Interval)	P-Value
GAD 7			
Age	< 35 VS > 35	1.6 (0.64-4.04)	0.29
Gender	Female vs Male	3.03 (1.53-5.99)	0.001
Education	≥Graduate vs < Graduate	2.85 (1.43-5.07)	0.002
Comorbidities	Yes vs No	2.12 (0.91-5.72)	0.07
Contacts with patients	Frequent vs Never	1.12 (0.23-1.10)	0.08
Level of work	Frontline vs Second line	1.40 (0.40-1.59)	0.54
Working in hospital since	< 3 years vs > 3 years	2.02 (1.07-3.81)	0.03
PHQ 9			
Age	< 35 VS > 35	1.63 (0.68-3.95)	0.27
Gender	Female vs Male	2.96 (1.57-5.56)	0.01
Education	≥Graduate vs < Graduate	2.10 (1.13-3.92)	0.01
Comorbidities	Yes vs No	2.40 (1.60-5.99)	0.040
Contacts with patients	Frequent vs Never	2.91 (1.41-5.98)	0.004
Level of work	Frontline vs Second line	1.21 (0.65-2.25)	0.63
Working in hospital since	< 3 years vs > 3 years	1.04 (0.58-1.86)	0.69
ISI			
Age	< 35 VS > 35	1.80 (0.57-5.70)	0.31
Gender	Female vs Male	1.56 (0.70-3.44)	0.15
Education	≥Graduate vs < Graduate	3.73 (1.64-8.48)	0.002
Comorbidities	Yes vs No	2.97 (1.12-7.83)	0.028
Contacts with patients	Frequent vs Never	3.52 (1.46-8.30)	0.005
Level of work	Frontline vs Second line	1.25 (0.72-3.82)	0.13
Working in hospital since	< 3 years vs > 3 years	2.41 (1.10-5.30)	0.028
K10			
Age	< 35 VS > 35	0.92(0.37-2.29)	0.86
Gender	Female vs Male	3.69(1.83-7.46)	0.01
Education	≥Graduate vs < Graduate	2.74 (1.44-5.21)	0.002
Comorbidities	Yes vs No	2.57 (1.01-6.53)	0.04
Contacts with patients	Frequent vs Never	1.36 (0.59-3.10)	0.46
Level of work	Frontline vs Second line	2.60 (1.31-5.18)	0.006
Working in hospital since	< 3 years vs > 3 years	1.53 (0.80-2.92)	0.18
STAI-S			
Age	< 35 VS > 35	1.83 (0.80-2.0)	0.99
Gender	Female vs Male	1.55 (0.94-2.55)	0.07
Education	≥Graduate vs < Graduate	1.47 (1.20-2.30)	0.02
Comorbidities	Yes vs No	1.30 (0.58-2.89)	0.51
Contacts with patients	Frequent vs Never	1.07 (0.56-2.03)	0.82
Level of work	Frontline vs Second line	1.79 (1.09-2.96)	0.03
Working in hospital since	< 3 years vs > 3 years	1.40 (0.90-2.17)	0.13
STAI-T			
Age	< 35 VS > 35	1.12 (0.90-1.32)	0.77
Gender	Female vs Male	1.50 (0.94-2.64)	0.84
Education	≥Graduate vs < Graduate	1.52 (0.99-2.52)	0.06
Comorbidities	Yes vs No	2.01 (0.89-4.54)	0.09
Contacts with patients	Frequent vs Never	1.03 (0.53-1.99)	0.92
Level of work	Frontline vs Second line	1.94 (1.15-3.26)	0.013
Working in hospital since	< 3 years vs > 3 years	1.88 (1.18-2.99)	0.007

influenced by education [≥Graduate vs <Graduate (OR, 3.73; 95% CI, 1.64-8.48; p=0.002), comorbidities [Yes vs No (OR, 2.97; 95% CI, 1.12-7.83; p=0.02)], contact with patients [Frequent vs Never (OR, 3.52; 95% CI, 1.46-8.30; p=0.005), and working with hospital [<3 years vs >3 years (OR, 2.41; 95% CI, 1.10-5.30; p=0.02)].

Multivariate analysis showed influence of gender [Female vs Male (OR, 3.69; 95% CI, 1.83-7.46; p=0.01), education [≥Graduate vs <Graduate (OR, 2.74; 95% CI, 1.44-5.21; p=0.002), comorbidities [Yes vs No (OR, 2.57; 95% CI, 1.01-6.53; p=0.04), and level of work [Frontline vs Second line (OR, 2.60; 95% CI, 1.31-5.18; p=0.006)]

Table 4. Pandemic specific questions

Pandemic specific questions	Yes (n)	Percentage (%)	No (n)	Percentage (%)
Updated about COVID-19	323	(94%)	19	6%
Fear of infection	170	49%	174	51%
Fear of family members getting infection	180	52%	164	48%
Disconnected from loved ones	47	14%	297	86%
Proud of their Job as a health worker in COVID-19 pandemic	309	89%	35	11%

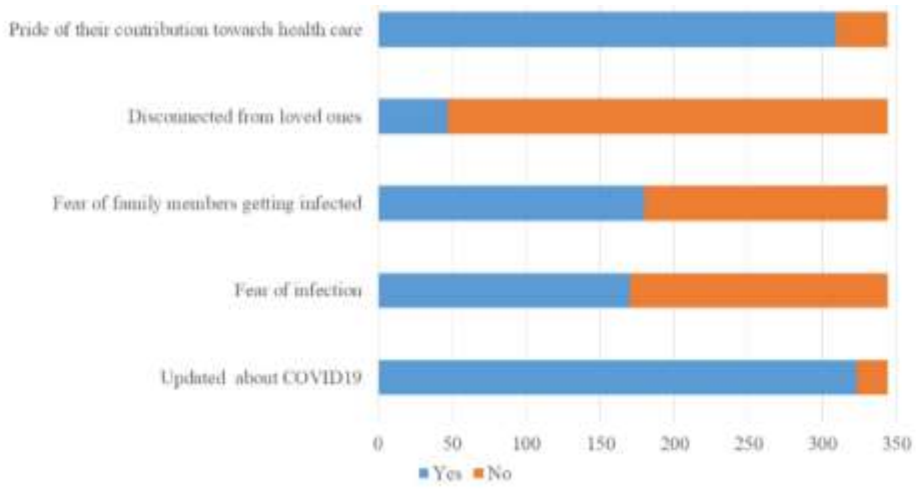

Figure 1. Pandemic specific questions for health care providers

on distress. From STAI, the psychophysiological state and personality characteristics were derived by keeping the cut-off score as ≥40 (mild anxiety symptoms), where 128 (37%) participants have shown mild to severe state anxiety and 126 (36%) participants showed trait anxiety rarely to almost always. STAI-S was found to be influenced by education [≥Graduate vs <Graduate (OR, 1.47; 95% CI, 1.20-2.30; p=0.02)], and level of work [Frontline vs Second line (OR, 1.79; 95% CI, 1.09-2.96; p=0.03)], whereas STAI-T by level of work [Frontline vs Second line (OR, 1.94; 95% CI, 1.15-3.26; p=0.001)] and working with hospital [<3 years vs >3 years (OR, 1.88; 95% CI, 1.18-2.99; p=0.007)].

Overall, in all the participants, anxiety, depression, insomnia, and distress symptoms were predominantly influenced by variables such as gender, education, comorbidities, and level of work, followed by other less dominant variables such as contact with patients, and working in hospital with a significance value of p<0.05 (Table 3). To determine the other influencing factors for psychological burden, pandemic specific questions were included in the survey questionnaire as shown in table 4. From self-made pandemic specific questionnaire of figure 1, all the participants have shown positive hope towards the COVID-19 situation by updating themselves with the latest information and coping mechanism (n=323), and by feeling pride of their contribution in health care (n=309) in these critical times. However, they had a fear of infection [170 (49%)], fear of family members getting infected [180 (52%)], and disconnected from loved ones [47 (14%)].

DISCUSSION

The viral epidemics and their impact on psychological distress dates back to more than 100 years (Menniger 1919, Moccia et al. 2020). Such distress in medical professionals especially working in hospital settings were proven over time. Possible reasons for such related distress might be due to insufficient understanding of the virus, control knowledge, infodemic (O'Sullivan 2020, Zarocostas 2020), limitations of freedom (Barbisch et al. 2015), feeling separation from loved ones (Brooks et al. 2020), fear of infecting family members, lack of sufficient rest, high risk of exposure to patients with COVID-19, fear of getting infected, long-term workload, feelings of extreme fear and uncertainty (CDC 2020), limited access to mental health services for managing psychological distress, anxiety, and depression (Zhang et al. 2020). Additionally, an increase in the number of new cases on a daily basis with time, panic buying, hoarding, misuse, and shortage of medical protective equipment were also observed to be contributing factors influencing the health care workers psychologically (Chaib 2020).

Compared to other disease treating centres, COVID-19 pandemic has shown a large negative impact and disruption on the full spectrum of medical cancer care services and cancer care centres, which will undoubtedly have a large effect on cancer-related mortality. A 5–10% decrease in survival of patients from high-income countries has been predicted due to the treatment delays caused by COVID-19. Its impact is expected to be more in low-income and middle income coun-

tries like India (The Lancet Oncology 2020). Medical health workers working in cancer centres play a very important role in continuing cancer patients care. However, such services are coming at a cost of healthcare workers psychological distress at many centres. To find out such influencing and potential risk factors, all the working staff at our centre were assessed for their anxiety, depression, insomnia, and distress conditions (Khan 2020, Wang et al. 2020).

In our study, we found more anxiety and depressive symptoms in female staff which was consistent with previously published reports, where females are at higher risk of anxiety compared to males in developing GAD and depression (Alharthy et al. 2017, Salk et al. 2017). We also found, strong association of comorbidities such as hypertension, diabetes mellitus, CHD on anxiety and developing depressive symptoms, which are in consistent with the literature (Player & Peterson 2011, Zhang et al. 2018). It was also observed from the recent studies that health care providers with associated comorbidities are more prone to COVID-19 and its related deaths, which was causing them much stress than normal, while working in the hospital. Distress and insomnia related problems were observed to be more in frontline workers, less than 3 years of experience in the hospital and workers in frequent contacts with patients. Reports suggest a strong correlation between medical health profession and the prevalence of insomnia (Huang et al. 2018). However, on the contrary, our insomnia results are not in consistency with previously reported studies conducted during past epidemics (Brooks et al. 2018, Zhang et al. 2020). COVID-19 has caused a huge panic in the initial stages, especially in the medical staff working <3 years with the institute. Later on with brief training sessions and safety measures maintained at the centre has worked really well in giving a positive hope to the staff, which ultimately might have helped in decreesing the distress and insomnia related symptoms.

Majority of the staff in our study sample displayed no clinically intervening level of anxiety, depression, insomnia, and distress. This might be due to the still relatively short exposure to the pandemic, previous experiences from past epidemics, continuous updating of guidelines, stable policies, training, counselling and psycho-education, rotation in shifts, lowering workload and job demands, individual interventions, timely support, experience sharing and conducting recovery programmes which were all worked really well for promoting resilience in the staff.

Individuals experiencing clinically prominent symptoms and psychological stress were identified at an early stage and helped them to undergo psychological and cognitive-behavior based therapies. Few of them had been referred to a psychiatrist for further counselling and pharmacological therapies.

Strength of this study is it has been carried out within staff working in a single cancer centre, having the same working environment. Almost 91% of our staff have participated in this study indicating reliable information. To the best of our knowledge, this is first cross-sectional study, where the psychological burden of healthcare professionals working in a cancer care centre had been assessed especially during the peak phase of COVID-19 pandemic in India.

The study has its own limitations. First, it's a seven days' cross-sectional study without a longitudinal follow-up. Second, the impact of pandemic COVID-19 may worsen over time in India and it may bring increasingly arduous situations for health care professionals. Third, the reliability of self-administered internet-based questionnaire may be partially biased. Fourth, assessment through clinical interviews should be carried out to get a more comprehensive assessment.

CONCLUSION

Our results are a reminder of effectiveness of the positive approach, impact of special interventions, reassurance, psychological counselling, continuous support and its consequences on the prevalence of psychological symptoms in health care professionals working at cancer care centres especially during this peak pandemic phases of COVID-19. So, it is also very important to provide proper healthcare protection, protective equipment and timely psychological support to health care workers to boost their overall well-being, which will in-turn help in improving the fighting spirit and resilient behavior during these tough times like pandemic COVID-19.

Acknowledgements: None.

Ethical approval:

Ethics committee approval was received for this study from the Ethics Committee of Manavata Clinical Research Institute, India (HEPSICO19/2020).

Informed Consent: Written informed consent was obtained from the participants.

Conflict of interest: None to declare.

Contribution of individual authors:

Raj Nagarkar: design, supervision, material, critical review, approval of the final version.

Roshankumar Patil: concept, design, supervision, material, data collection and/or processing, analysis and/or interpretation, critical review, approval of the final version.

Kavita Gadade & Nishtha Paleja: design, material, data collection and/or processing.

Yasam Venkata Ramesh: concept, design, supervision, material, data collection and/or processing, analysis and/or interpretation, critical review, literature search, writing manuscript, approval of the final version.

References

1. Alharthy N, Alrajeh O, Almutairi M, Alhajri A: Assessment of anxiety level of emergency health-care workers by generalized anxiety disorder-7 tool. Int J Appl Basic Med Res 2017; 7:150-54
2. Barbisch D, Koenig KL, Shih FY: Is There a Case for Quarantine? Perspectives from SARS to Ebola. Disaster Med Public Health Prep 2015; 9:547-53
3. Bastien CH, Vallières A, Morin CM: Validation of the insomnia severity index as an outcome measure for insomnia research 2001; 2:297-307
4. Beresford B, Gibson F, Bayliss J, Mukherjee S: Preventing work-related stress among staff working in children's cancer Principal Treatment Centres in the UK: a brief survey of staff support systems and practices. Eur J Cancer Care (Engl) 2018; 27:e12535
5. Bray F, Ferlay J, Soerjomataram I, Siegel RL, Torre LA, Jemal A: Global cancer statistics 2018: GLOBOCAN estimates of incidence and mortality worldwide for 36 cancers in 185 countries. CA Cancer J Clin 2018; 68:394-424
6. Brooks SK, Dunn R, Amlôt R, Rubin GJ, Greenberg N: A Systematic, Thematic Review of Social and Occupational Factors Associated with Psychological Outcomes in Healthcare Employees during an Infectious Disease Outbreak. J Occup Environ Med 2018; 60:248-57
7. Brooks SK, Webster RK, Smith LE, Woodland L, Wessely S, Greenberg N, et al: The psychological impact of quarantine and how to reduce it: rapid review of the evidence. Lancet 2020. https://doi.org/10.1016/S0140-6736(20)30460-8
8. CDC: Mental health and Coping during COVID-19. Centers Dis Control Prev 2020. https://doi.org/10.1001/jama.2020.2633
9. Chaib F: Shortage of personal protective equipment endangering health workers worldwide. Who 2020
10. Chen X, Yu B: First two months of the 2019 Coronavirus Disease (COVID-19) epidemic in China: real-time surveillance and evaluation with a second derivative model. Glob Heal Res Policy 2020. https://doi.org/10.1186/s41256-020-00137-4
11. Daruvala R, Ghosh M, Fratazzi F, Norzan S, Laha A, Ahmed R, et al: Emotional exhaustion in cancer clinicians: A mixed methods exploration. Indian J Med Paediatr Oncol 2019; 40:111-20
12. Huang CLC, Wu MP, Ho CH, Wang JJ: Risks of treated anxiety, depression, and insomnia among nurses: A nationwide longitudinal cohort study. PLoS One 2018; 13:e0204224
13. Huang Y, Zhao N: Generalized anxiety disorder, depressive symptoms and sleep quality during COVID-19 outbreak in China: a web-based cross-sectional survey. Psychiatry Res 2020. https://doi.org/10.1016/j.psychres.2020.112954
14. Jones MC, Wells M, Gao C, Cassidy B, Davie J: Work stress and well-being in oncology settings: A multidisciplinary study of health care professionals. Psychooncology 2013; 22:46-53
15. Julian LJ: Measures of Anxiety. Arthritis Care 2011; 63: S467-72
16. Khan S, Siddique R, Li H, Ali A, Shereen MA, Bashir N, et al: Impact of coronavirus outbreak on psychological health. J Glob Health 2020. https://doi.org/10.7189/JOGH.10.010331
17. Li J, Yang Z, Qiu H, Wang Y, Jian L, Ji J, et al: Anxiety and depression among general population in China at the peak of the COVID-19 epidemic. World Psychiatry 2020; 19:249–50. https://doi.org/10.1002/wps.20758
18. Menninger KA: Psychoses associated with influenza. JAMA 1919; 72:235-41
19. MoHFW | Home n.d. https://www.mohfw.gov.in/ (accessed August 17, 2020)
20. Moccia L, Janiri D, Pepe M, Dattoli L, Molinaro M, De Martin V, et al: Affective temperament, attachment style, and the psychological impact of the COVID-19 outbreak: an early report on the Italian general population. Brain Behav Immun 2020. https://doi.org/10.1016/j.bbi.2020.04.048
21. O'Sullivan D: Meet the woman fighting coronavirus misinformation online. CNN Bus 2020
22. Player MS, Peterson EL: Anxiety disorders, hypertension, and cardiovascular risk: A review. Int J Psychiatry Med 2011; 41:365-77
23. Roy D, Tripathy S, Kar SK, Sharma N, Verma SK, Kaushal: Study of knowledge, attitude, anxiety & perceived mental healthcare need in Indian population during COVID-19 pandemic. Asian J Psychiatr 2020. https://doi.org/10.1016/j.ajp.2020.102083
24. Salk RH, Hyde JS, Abramson LY: Gender differences in depression in representative national samples: Meta-analyses of diagnoses and symptoms. Psychol Bull 2017; 143:783-822
25. The Lancet Oncology. Safeguarding cancer care in a post-COVID-19 world. Lancet Oncol 2020. https://doi.org/10.1016/S1470-2045(20)30243-6
26. Zhang C, Yang L, Liu S, Ma S, Wang Y, Cai Z, et al: Survey of Insomnia and Related Social Psychological Factors Among Medical Staff Involved in the 2019 Novel Coronavirus Disease Outbreak. Front Psychiatry 2020. https://doi.org/10.3389/fpsyt.2020.00306
27. Zhang Y, Chen Y, Ma L: Depression and cardiovascular disease in elderly: Current understanding. J Clin Neurosci 2018; 47: 1-5
28. Zhang WR, Wang K, Yin L, Zhao WF, Xue Q, Peng M, et al: Mental Health and Psychosocial Problems of Medical Health Workers during the COVID-19 Epidemic in China. Psychother Psychosom 2020. https://doi.org/10.1159/000507639
29. Zarocostas J: How to fight an infodemic. Lancet (London, England) 2020. https://doi.org/10.1016/S0140-6736(20)30461-X
30. Wang C, Pan R, Wan X, Tan Y, Xu L, Ho CS, et al: Immediate psychological responses and associated factors during the initial stage of the 2019 coronavirus disease (COVID-19) epidemic among the general population in China. Int J Environ Res Public Health 2020. https://doi.org/10.3390/ijerph17051729
31. World Health Organization: Mental Health and Psychosocial Considerations During COVID-19 Outbreak. World Heal Organ 2020: (accessed August 17, 2020)

Correspondence:
Raj Nagarkar, MS
Department of Surgical Oncology, HCG Manavata Cancer Centre
Nashik, 422011, Maharashtra, India
E-mail: academics@manavatacancercentre.com
