# Supplementary material for: Mental health status and related factors influencing healthcare workers during the COVID-19 pandemic: A systematic review and meta-analysis
Source: PLoS One. 2024 Jan 19;19(1):e0289454. doi: 10.1371/journal.pone.0289454 (PMC10798549; doi:10.1371/journal.pone.0289454)
Supplement: S1 Data — (ZIP) [file pone.0289454.s011.zip › literatures/115.pdf]

JYX JYVÄSKYLÄN YLIOPISTO UNIVERSITY OF JYVÄSKYLÄ

This is a self-archived version of an original article. This version may differ from the original in pagination and typographic details.

Author(s): Nicolaou, Christiana; Menikou, Joanna; Lamnisos, Demetris; Lubenko, Jelena; Presti, Giovambattista; Squatrito, Valeria; Constantinou, Marios; Papacostas, Savvas; Aydın, Gokcen; Chong, Yuen Yu; Chien, Wai Tong; Cheng, Ho Yu; Ruiz, Francisco J.; Segura-Vargas, Miguel A.; Garcia-Martin, Maria B.; Obando-Posada, Diana P.; Vasiliou, Vasilis S.; McHugh, Louise; Höfer, Stefan; Baban, Adriana; Dias [illegible]

Title: Mental Health Status of Healthcare Workers During the COVID-19 Outbreak : An International Study

Year: 2021

Version: Published version

Copyright: © 2021The Author(s)

Rights: CC BY 4.0

Rights url: https://creativecommons.org/licenses/by/4.0/

Please cite the original version:

Nicolaou, C., Menikou, J., Lamnisos, D., Lubenko, J., Presti, G., Squatrito, V., Constantinou, M., Papacostas, S., Aydın, G., Chong, Y. Y., Chien, W. T., Cheng, H. Y., Ruiz, F. J., Segura-Vargas, M. A., Garcia-Martin, M. B., Obando-Posada, D. P., Vasiliou, V. S., McHugh, L., Höfer, S., . . . Gloster, A. T. (2021). Mental Health Status of Healthcare Workers During the COVID-19 Outbreak : An International Study. European Journal of Psychology Open, 80(1-2), 62-76. https://doi.org/10.1024/2673-8627/a000010

https://econtent.hogrefe.com/doi/pdf/10.1024/2673-8627/a000010 - Wednesday, October 06, 2021 11:44:11 PM - University of Jyvaskyla IP Address:130.234.90.20

Original Article

Mental Health Status of Healthcare Workers During the COVID-19 Outbreak

An International Study

Christiana Nicolaou[1], Joanna Menikou[2], Demetris Lamnisos[2], Jelena Lubenko[3], Giovambattista Presti[4], Valeria Squatrito[4], Marios Constantinou[5], Savvas Papacostas[6], Gokcen Aydın[7], Yuen Yu Chong[8], Wai Tong Chien[8], Ho Yu Cheng[8], Francisco J. Ruiz[9], Miguel A. Segura-Vargas[9], Maria B. Garcia-Martin[10], Diana P. Obando-Posada[10], Vasilis S. Vasiliou[11], Louise McHugh[12], Stefan Höfer[13], Adriana Baban[14], David Dias Neto[15], Ana Nunes da Silva[16], Jean-Louis Monestès[17], Javier Alvarez-Galvez[18], Marisa Paez-Blarrina[19], Francisco Montesinos[20], Sonsoles Valdivia-Salas[21], Dorottya Ori[22], Bartosz Kleszcz[23], Raimo Lappalainen[24], Iva Ivanović[25], David Gosar[26], Frederick Dionne[27], Rhonda M. Merwin[28], Angelos P. Kassianos[29,30], Maria Karekla[30], and Andrew T. Gloster[31]

[1]Department of Nursing, School of Health Sciences, Cyprus University of Technology, Limassol, Cyprus
[2]Department of Health Sciences, European University Cyprus, Nicosia, Cyprus
[3]Psychological Laboratory, Faculty of Public Health and Social Welfare, Riga Stradiņš University, Riga, Latvia
[4]Kore University Behavioral Lab (KUBeLab), Faculty of Human and Social Sciences, Kore University of Enna, Italy
[5]Department of Social Sciences, School of Humanities and Social Sciences, University of Nicosia, Cyprus
[6]Cyprus Institute of Neurology and Genetics, Nicosia, Cyprus
[7]Department of Psychological Counseling and Guidance, Faculty of Education, Hasan Kalyoncu University, Gaziantep, Turkey
[8]The Nethersole School of Nursing, Faculty of Medicine, The Chinese University of Hong Kong, Hong Kong
[9]Faculty of Psychology, Fundación Universitaria Konrad Lorenz, Bogotá, Colombia
[10]Faculty of Psychology, University de La Sabana, Chía, Colombia
[11]School of Applied Psychology, University College Cork, Ireland
[12]School of Psychology, University College Dublin, Ireland
[13]Department of Medical Psychology, Medical University Innsbruck, Austria
[14]Department of Psychology, Babeş-Bolyai University (UBB), Cluj-Napoca, Romania
[15]ISPA – Instituto Universitário; APPsyCI – Applied Psychology Research Center Capabilities & Inclusion, Lisboa, Portugal
[16]Faculdade de Psicologia, Universidade de Lisboa, Alameda da Universidade, Lisboa, Portugal
[17]LIP/PC2S, Univ. Grenoble Alpes, Grenoble, France
[18]Department of Biomedicine, Biotechnology and Public Health, University of Cadiz, Spain
[19]Instituto ACT, Madrid, Spain
[20]Department of Psychology, European University of Madrid, Spain
[21]Department of Psychology and Sociology, University of Zaragoza, Spain
[22]Department of Mental Health, Heim Pal National Pediatric Institute, Budapest, Hungary
[23]Private Practice, Sosnowiec, Poland
[24]Department of Psychology, University of Jyväskylä, Finland
[25]Clinical Centre of Montenegro, Institute for Children's diseases, Department for Child Psychiatry, Podgorica, Montenegro
[26]Ljubljana University Medical Centre, Ljubljana, Slovenia

European Journal of Psychology Open (2021), 80(1–2), 62–76
https://doi.org/10.1024/2673-8627/a000010
© 2021 The Author(s) Distributed as a Hogrefe OpenMind article
under the license CC BY 4.0 (https://creativecommons.org/licenses/by/4.0)

[27]Department of Psychology, Université du Québec à Trois-Rivières, Canada
[28]Department of Psychiatry and Behavioral Science, Duke University, Durham, NC, USA
[29]Department of Applied Health Research, University College London, United Kingdom
[30]Department of Psychology, University of Cyprus, Nicosia, Cyprus
[31]Division of Clinical Psychology & Intervention Science, Department of Psychology, University of Basel, Switzerland

https://econtent.hogrefe.com/doi/pdf/10.1024/2673-8627/a000010 - Wednesday, October 06, 2021 11:44:11 PM - University of Jyvaskyla IP Address:130.234.90.20

Abstract: *Background:* The COVID-19 pandemic is a massive health crisis that has exerted enormous physical and psychological pressure. Mental healthcare for healthcare workers (HCWs) should receive serious consideration. This study served to determine the mental-health outcomes of 1,556 HCWs from 45 countries who participated in the COVID-19 IMPACT project, and to examine the predictors of the outcomes during the first pandemic wave. *Methods:* Outcomes assessed were self-reported perceived stress, depression symptom, and sleep changes. The predictors examined included sociodemographic factors and perceived social support. *Results:* The results demonstrated that half of the HCWs had moderate levels of perceived stress and symptoms of depression. Half of the HCWs (n = 800, 51.4%) had similar sleeping patterns since the pandemic started, and one in four slept more or slept less. HCWs reported less perceived stress and depression symptoms and higher levels of perceived social support than the general population who participated in the same project. Predictors associated with higher perceived stress and symptoms of depression among HCWs included female sex, not having children, living with parents, lower educational level, and lower social support. *Discussion:* The need for establishing ways to mitigate mental-health risks and adjusting psychological interventions and support for HCWs seems to be significant as the pandemic continues.

Keywords: healthcare workers, COVID-19, pandemic, mental health, psychological problems

In the 18 months since COVID-19 was named as a pandemic (WHO, 2020a), more than 216,303,376 confirmed cases of COVID-19 have occurred, including almost 4,498,451 deaths in more than 160 countries globally (WHO, 2020b). The number of confirmed cases and deaths related to COVID-19 continues to escalate, with a sharp increase in the number of cumulative cases worldwide (WHO, 2020c). Infectious disease outbreaks are known to impact the mental health of the general population (Brooks et al., 2020; Cabarkapa et al., 2020; Chong et al., 2021; Chou et al., 2020; Dias Neto et al., 2021; Gloster, Lamnisos et al., 2020; Huang & Zhao, 2020; Ji et al., 2017; Kamara et al., 2017; Tian et al., 2020). COVID-19 is highly contagious and has a high mortality rate in some populations, seriously threatening human physical and mental health (Gao et al., 2020; Guan et al., 2020; P. Zhou et al., 2020). The mandated lockdowns unexpectedly and dramatically impacted the daily routines, work, travel, and leisure activities of humans around the world to a degree unprecedented by most people living outside of war zones (Gloster, Lamnisos et al., 2020). Reducing activities, stimuli, and routines in the population's daily life potentially negatively affects their well-being and increases their psychological distress (Morina et al., 2021). The mental-health outcomes commonly reported among the general population were psychological stress, depression, boredom (Dias Neto et al., 2021; Gloster, Lamnisos et al., 2020), poor sleep quality (Lin et al., 2021), obsessive compulsion, interpersonal sensitivity, phobic anxiety, and psychoticism (Tian et al., 2020).

The pandemic crisis of COVID-19 severely impacted healthcare systems as well, including healthcare workers (HCWs), through the increased number of patients, the construction of COVID-19 wards, the increased number of hospital intensive unit beds and ventilators, and of course the lack of knowledge regarding the best treatment of this specific disease. Moreover, the lack of inpatient beds and protection supplies in clinical settings was a major problem for many countries worldwide during the first phase of the COVID-19 outbreak (Giang et al., 2020). Indeed, HCWs are critically important in mitigating an infectious disease outbreak anytime, facing immense pressures. Hence, they are one of the most vulnerable groups at risk of mental and physical health problems during a pandemic (Pfefferbaum & North, 2020; Yamamoto et al., 2020). Not only do HCWs have to deal with drained resources, they also are often forced to make difficult decisions to prioritize and triage patients while working extended hours (Giang et al., 2020). HCWs are also particularly vulnerable to emotional distress in the current pandemic, given that, to do their job, they must risk exposure to the virus (Gloster, Zacharia et al., 2020). This stressor is compounded by concerns about infecting and caring for their loved ones and involvement in emotionally and ethically fraught resource-allocation decisions (Pfefferbaum & North, 2020; Yamamoto et al., 2020).

The mental-health outcomes and related risk factors of HCWs have been one focus of interest, especially among Chinese scientists since the onset of the current pandemic. A high prevalence of psychological stress, depression, anxiety, and sleep disturbances were among the most frequently reported mental-health outcomes (Brooks et al., 2020; Kang, Li et al., 2020; Kang, Ma et al., 2020; S. Liu et al., 2020; Z. R. Liu et al., 2020; Moderato et al., 2021; B. Wang et al., 2020; Xiang et al., 2020; W. R. Zhang et al., 2020). Similar psychological reactions were reported by HCWs in previous infection outbreaks, i.e., SARS-CoV-1, the Middle East respiratory syndrome (MERS)-CoV,

© 2021 The Author(s) Distributed as a Hogrefe OpenMind article
under the license CC BY 4.0 (https://creativecommons.org/licenses/by/4.0)

https://econtent.hogrefe.com/doi/pdf/10.1024/2673-8627/a000010 - Wednesday, October 06, 2021 11:44:11 PM - University of Jyvaskyla IP Address:130.234.90.20

(Chua et al., 2004; Grace et al., 2005; Lee et al., 2018; Maunder et al., 2003; Tam et al., 2004; Wong et al., 2005; Wu et al., 2009).

In several studies, HCWs were found to demonstrate worse psychological outcomes than the general population (Simione & Gnagnarella, 2020; Tian et al., 2020; Y. Zhou et al., 2020). In the current study, we hypothesized that HCWs report lower levels of mental-health outcomes than the general population. Their role is generally recognized by governments and the public as crucial to the provision of healthcare services. Currently, in every country, HCWs are being praised for their efforts and hardships in carrying out their jobs during the pandemic, and countless encouraging messages are being relayed to HCWs through the mass media (Kim et al., 2020). We followed hypotheses derived from the cognitive-behavioral literature, thus assuming that the specific social support has had a protective effect on the mental health of HCWs, potentially influencing job-related stress reduction and even enhancing self-esteem (Shirey, 2004). It can also make them feel more positive and capable of justifying the emotional stress of the work (Whealin et al., 2008). The appraisal of positive meaning is associated with resilience and with the ability to cope effectively with potentially traumatic events (Bonanno & Mancini, 2008; Connor & Davidson, 2003; Whealin et al., 2008). Obviously, HCWs who appraise pandemic events positively and find their engagement meaningful are not expected to develop trauma-related psychopathology to the same degree as those who are excessively negative and fail to find meaning in these events (Ehlers & Clark, 2000; Resick & Schnicke, 1993).

Given this unprecedented crisis, it is important to identify what factors trigger potential mental burdens and psychological barriers among HCWs. This could direct public-health officials and organizations to identify high-risk HCWs' subgroups and provide them with the necessary support early on, before problems develop. Sociodemographic characteristics such as being female, having a lower educational background level, younger age, being unmarried or not in a relationship, and access to social support were identified as predicting factors associated with the psychological symptoms among HCWs during the current pandemic (Alnazly et al., 2021; Gupta et al., 2021; Kim et al., 2020; Labrague & De Los Santos, 2020; Lai et al., 2020; Li et al., 2021; Pouralizadeh et al., 2020). However, generalization of these findings seems limited as most of the studies were conducted nationally and investigated mainly nurses in Asian countries, where, in fact, nursing is mostly a female profession. Based on the previous literature, we hypothesized that a higher prevalence of stress and depression symptoms is found among females, older age, those who have no children, and those who live with their parents (Alnazly et al., 2021; Pouralizadeh et al., 2020; Simione & Gnagnarella, 2020; Tella et al., 2020). We also followed hypotheses derived from the social support literature (Berkman & Kawachi, 2000; Cohen & McKay, 1984; Dunkel-Schetter, 1984; Helgeson, 2003; House, 1981). Thus, we expect that HCWs demonstrate higher levels of perceived social support than the general population. We also assumed that the support derived either formally i.e., from the different strategies implemented by governments to reduce the psychological burden of HCWs (Kang, Li et al., 2020; Rana et al., 2020), or informally, i.e., from the public (Kim et al., 2020) or their family and broader social context (Muller et al., 2020), positively influences the perception of HCWs of their social support. Interestingly, perceived social support among Chinese nurses was found to be significantly higher than among the patients with COVID-19, which was positively related to the psychological health and quality of life of their nurses (Li et al., 2021).

Notably, lack of perceived social support was among the predictors of mental-health problems examined in several studies. For example, one review showed that perceived social support was the most frequently reported protective factor of the mental-health problems of HCWs (Muller et al., 2020), which is consistent with the stress-buffering hypothesis model that posits that support "buffers" (i.e., protects) persons from the potentially pathogenic influence of stressful events (Cohen, 1992; Cohen & Wills, 1985; House, 1981). Indeed, support may intervene between the stressful event and a stress reaction by attenuating or preventing a stress appraisal response. Adequate support may intervene between the experience of stress and the onset of the pathological outcome by reducing or even eliminating the stress reaction, or by directly influencing physiological processes (Cohen & Wills, 1985). Increasingly, evidence is indicating that social support is positively related to psychological health and quality of life among different groups of the population, i.e., children with malignancies and their families (Fayed et al., 2010; Klassen et al., 2011; Nicolaou et al., 2015), adult patients with malignancies (Grunfeld et al., 2004), patients with heart problems (Lee et al., 2005), and women with postpartum depression (Surkan et al. 2006; Vaezi et al., 2019).

Social support, both formal and informal, was also crucial to confronting potentially negative psychological effects of previous pandemics and lockdowns among HCWs and the general population (Chua et al., 2004; Ji et al., 2017; Nasser & Overholser, 2005). Thus, it is important to study social support, especially given that this is a malleable parameter that can be targeted with psychosocial interventions. Though we assumed that individuals with higher levels of perceived social support have fewer psychological problems, existing knowledge about predicting factors of mental-health problems related to COVID-19 and the

© 2021 The Author(s) Distributed as a Hogrefe OpenMind article under the license CC BY 4.0 (https://creativecommons.org/licenses/by/4.0)

https://econtent.hogrefe.com/doi/pdf/10.1024/2673-8627/a000010 - Wednesday, October 06, 2021 11:44:11 PM - University of Jyvaskyla IP Address:130.234.90.20

role of perceived social support among HCWs is limited. The existing studies were mainly conducted nationally in Asian countries, surveying either the general population or subgroups of HCWs (Alnazly et al., 2021; Grey et al., 2020; Kim et al., 2020; Labrague & De Los Santos, 2020; Li et al., 2021; Pouralizadeh et al., 2020; Yu et al., 2020). Therefore, further international surveys are required to explore knowledge and understanding of these issues, using also more diverse samples.

Given the scope of the COVID-19 pandemic, it is important to understand to what degree the pandemic crisis affects the mental health of HCWs. To our knowledge, this is the first international survey examining the mental-health outcomes of HCWs related to the COVID-19 pandemic and the predicting factors in comparison to the general population, based on a sample from 78 countries/regions. Because pandemic refers to "an epidemic occurring worldwide, crossing the boundaries of several countries and usually affecting a large number of people" (Porta, 2008), international studies seem very important as the national ones are limited to national data and are valuable during epidemic outbreaks. We expect that this international survey will add precious information to better understand the psychological impact of the pandemic on HCWs. Thus, the purpose of this study was to determine the mental-health outcomes of HCWs and potential predictors, such as sociodemographic characteristics and perceived social support.

Methods

Design and Sample

The present study is part of the larger research project, the "COVID-19 IMPACT Study." In brief, it employed an international online survey, aiming to explore the behavioral and psychological impacts of the COVID-19 pandemic. The overall sample of the COVID-19 IMPACT project consisted of 9,565 participants from 78 countries. The inclusion criteria were being $\geq$ 18 years of age and being able to understand one of the 18 languages of the online survey (English, Greek, German, French, Spanish, Turkish, Dutch, Latvian, Italian, Portuguese, Finnish, Slovenian, Polish, Romanian, Hong Kong Chinese, Hungarian, Montenegrin, and Persian). Concerning the research questions of the current study, the sample included 1,556 HCWs from 45 countries. The remaining 7,819 participants of the survey who reported not being an HCW were used only for comparisons between HCWs and the general population. The countries with the largest samples of HCWs were Italy (n = 236), Austria (n = 210), Latvia (n = 122), Cyprus (n = 121), Switzerland (n = 120), France (n = 89), Portugal (n = 80), Hungary (n = 63), Hong Kong (n = 60), and Germany (n = 54). Most of HCWs came from European countries (n = 1,325, 85%). Further information on the HCWs sample can be found at https://doi.org/10.23668/psycharchives.5071

Procedure

A random sampling approach followed by snowball sampling was carried out to collect data over a 2-month period, between 7 April 2020 and 7 June 2020. Data were collected through an online survey using a variety of methods, such as local press (e.g., newspapers, newsletters, and radio stations), social media platforms (e.g., Facebook, Instagram, Twitter, and WeChat), professional networks, local hospitals, and health centers, email lists of HCW professional groups, and social institutions, as well as the mass emailing at the participating universities. Ethics approval was obtained by the Cyprus National Bioethics Committee (ref.: EEBK EΠ 2020.01.60) followed by site approvals from different research teams involved in data collection. Participants who self-selected and enrolled in the study were invited to provide informed consent and completed a 20-min online survey via a secured Google platform.

Measures

Predictors

Sociodemographic Status

A structured, closed-questioned tool was used to obtain sociodemographic information, which was defined as predictors and included age, sex, marital status, having children, living situation, employment status, and educational background.

Perceived Social Support

Perceived social support was measured using the Oslo Social Support Scale (OSS) (Dalgard, 1996), which consists of three items assessing the number of people an individual can count on for important personal problems, the interest others take in that person, and the difficulty a person getting help from neighbors. The scale is scored on one 4-point Likert type scale and two 5-point Likert type scales, which are summed and provide three levels of social support: *low* (scored 3–8), *moderate* (scored 9–11), and *high* (scored 12–14). The coefficient alpha value for the OSS scale was 0.54.

Outcomes

Perceived Stress

Perceived stress was measured using the Perceived Stress Scale (PSS) (Cohen et al., 1983), the most widely used tool for measuring perceived stress and assessing the feelings

© 2021 The Author(s) Distributed as a Hogrefe OpenMind article under the license CC BY 4.0 (https://creativecommons.org/licenses/by/4.0)

https://econtent.hogrefe.com/doi/pdf/10.1024/2673-8627/a000010 - Wednesday, October 06, 2021 11:44:11 PM - University of Jyvaskyla IP Address:130.234.90.20

and thoughts in different situations. The PSS includes 10 items that measure an individual's appraisal of how stressful situations in their life are. The items focus on assessing people's feelings and thoughts during the last month and are ranked on a Likert scale ranging from 0 = *never* to 4 = *very often*. Total scores are obtained by reversing the scores on the four positively worded items (items 4, 5, 7, 8) and then summing across all 10 items. The total scores range from 0 to 40: 0–13 indicates *low perceived stress*, 14–26 *moderate stress*, and 27–40 a *high level of stress*. The coefficient alpha value for PSS was 0.89.

Depressive Symptoms

Depressive symptoms were assessed using two items from the disengagement subscale of the Multidimensional State Boredom Scale (MSBS; Fahlman et al., 2011). We chose the subscale we believed would be relevant to the unique situation of a lockdown. At the time of the first lockdown, people were not allowed to leave their homes often and only as necessary, which represents a potential depletion of reinforcements and has been associated with depression.

These items measured (a) whether a person is willing to do pleasurable things but not finding anything appealing (i.e., boredom) and (b) the person's wasting time. The items are scored on a 4-point Likert scale, from 1 = *very little* to 4 = *extremely*, with higher scores indicating higher depressive symptomatology. The MSBS disengagement subscale score was significantly correlated with measures of trait boredom, the Boredom Proneness Scale (BPS), (r = 0.61), and depression, the Center for Epidemiologic Studies Depression Scale (CESD), (r = 0.56) (Fahlman et al., 2011). There was also an additional item based on reinforcement deprivation concepts which measures the pleasure people experience during activities; it was also scored on a 4-point Likert scale. The coefficient alpha value for the three items used to measure depressive symptomatology was 0.67.

Sleep Changes

One structured, closed question was used to assess responders' sleep change, who were asked to rate how much their sleep had changed since lockdown, the options being *I sleep more*, *I sleep less*, and *I sleep about the same*.

Statistical Analysis

All statistical analyses were performed using SPSS version 26. The current data followed the normal distribution. Descriptive statistics, including frequency (n), percentage (%), mean (M), and standard deviation (SD), were used to describe the sociodemographic data, perceived stress, depressive symptoms, perceived social support, and sleep change. All predictors of sociodemographic data and social support were treated as categorical variables, except for age, which was treated as a continuous variable. The outcome variables of perceived stress and depressive symptoms were treated as continuous variables and the outcome of sleep changes as a categorical variable. To examine the bivariable associations between the mental-health outcomes of HCWs and predictors, we conducted independent-sampled t-tests and one-way analyses of variance (ANOVAs) for the categorical predictors, and linear regression was used for the numerical predictor of age. Games-Howell post-hoc analysis or Tukey post-hoc analysis was conducted to investigate whether the differences between groups were statistically significant. Independent-sample t-tests for numerical variables and chi-square tests for categorical variables were used to examine the differences between the HCWs and the general population in the scores of perceived stress, depressive symptoms, perceived social support, and sleep change. Multivariable associations between each outcome variable of HCWs and all predictors were investigated with multiple linear regression analysis for the variables of perceived stress and depressive symptoms, and ordinal logistic regression analysis in the case of the sleep changes variable. Finally, we applied hierarchical multiple regression to determine whether the addition of social support improved the prediction of perceived stress and depression symptoms over and above the sociodemographic characteristics alone.

We assessed the strength of the associations between predictor variables and dependent variables by computing standardized regression coefficients and Cramer's V as effect sizes. To evaluate the effect sizes, we used the following values in the case of standardized regression coefficients: tiny $\leq$ .05, very small from .05 to .10, small from .10 to .20, medium from .20 to .30, large from .30 to .40, and very large $\geq$ .40 (Funder & Ozer, 2019); and in the case of Cramer's V: weak from .10 to .30, medium from .4 to .5, and strong > .5. Statistical significance was defined as p < .05.

Results

Descriptive

Predictors

Sociodemographic Data

The mean age of HCWs was 40.52 (SD = 11.57) years. The majority were female (n = 1,307, 84%), working fulltime (n = 1,040, 66.8%), with a postgraduate education (n = 775, 49.8%), married (n = 728, 46.8%), living with their own family (n = 1,092, 70.2%), and having children

© 2021 The Author(s) Distributed as a Hogrefe OpenMind article under the license CC BY 4.0 (https://creativecommons.org/licenses/by/4.0)

Table 1. Comparison of PSS, OSS, and MSBS scores among HCWs and the general population

	Bivariate analysis			
	HCWs	General population	Mean difference (95% CI)	Effect size
PSS score (*M, SD*)	16.10 (6.89)	17.28 (7.57)	−1.18 (−1.58, −0.77)	0.06†
MSBS (*M, SD*)	6.14 (2.12)	6.68 (2.27)	−0.53 (−0.65, −0.41)	0.09†
OSS level (*n*, %)			0.1†††	
Low	265 (17.0%)	2,027 (25.9%)		
Moderate	813 (52.2%)	4,090 (52.3%)		
High	478 (30.7%)	1,702 (21.8%)		
Sleep change (*n*, %)††			0.06†††	
Sleep less	373 (24.0%)	1,671 (21.4%)		
Sleep more	383 (24.6%)	2,554 (32.7%)		
Sleep about the same	800 (51.4%)	3,594 (46.0%)		

Note. Effect size interpretation with Cramer's *V*: weak (0.1–0.3), medium (0.4–0.5), strong (> 0.5). Effect size interpretation with standardized regression coefficients: tiny (0–0.05), very small (0.05–0.10), small (0.10–0.20), medium (0.20–0.30), large (0.30–0.40), very large (> 0.40). †Standardized regression coefficients; ††Results as *f*(%) from chi-square analysis; †††Cramer's *V* value.

https://econtent.hogrefe.com/doi/pdf/10.1024/2673-8627/a000010 - Wednesday, October 06, 2021 11:44:11 PM - University of Jyvaskyla IP Address:130.234.90.20

(*n* = 818, 52.6%). The sociodemographic characteristics of the HCWs and general population were similar with no significant differences apart from sex, where the percentage of women was higher among HCWs than in the general population (ES = 0.67). In particular, the mean age of general population was 36.12 (*SD* = 13.44). Most were female (*n* = 5,980, 76.5%), working fulltime (*n* = 3,973, 50.8%), with a postgraduate education (*n* = 2,453, 31.4%), married (*n* = 2,641, 33.8%), living with their own family (*n* = 3,966, 50.7%), and not having children (*n* = 4,820, 61.6%). More detailed information about sociodemographic characteristics can be found at https://doi.org/10.23668/psycharchives.5071

Perceived Social Support

The mean score of the perceived social support among HCWs was 10.39 (*SD* = 2.05), indicating that the HCWs had a moderate level of perceived social support. Approximately half of the HCWs (*n* = 813, 52.2%) had a moderate level of perceived social support, one-third (*n* = 478, 30.7%) had a high level, and less than one-fifth (*n* = 265, 17%) had a low level of perceived social support. More participants from the general population sample than among the HCWs demonstrated low levels of perceived social support (25.9%), and also fewer participants from the general population perceived high levels of social support (21.8%) (*ES* = 0.1) than among the HCWs (Table 1).

Outcomes

Perceived Stress

Most HCWs (*n* = 887, 57%) demonstrated moderate levels of perceived stress, some HCWs (*n* = 562, 36.1%) showed low levels of perceived stress, and the remaining ones (*n* = 107, 6.9%) showed high levels of perceived stress. The general population reported higher levels of perceived stress than the HCWs, with a statistically significant mean difference (95% CI [−1.58, −0.77]) (Table 1).

Depressive Symptoms

The HCWs had moderate symptoms of depression (*M* = 6.14, *SD* = 2.12). Their average depressive symptoms score was lower than that of the general population, with a statistically significant mean difference (95% CI [−0.65, −0.41]) (Table 1).

Sleep Changes

During the lockdown or self-isolation, half of the HCWs (*n* = 800, 51.4%) continued to sleep about the same, whereas 383 (24.6%) slept more, and 373 (24.0%) slept less. Regarding the quality of their sleep, 925 HCWs (59.4%) reported having fairly good or very good sleep, 340 (21.9%) neither good nor bad sleep, and 291 (18.7%) fairly poor or very poor sleep. Similar patterns were observed with reported sleep changes of the general population (ES = 0.1). Almost one-half of them continued to sleep about the same (*n* = 3,594, 46.0%), one-third slept more (*n* = 2,554, 32.7%), and one-fifth slept less (*n* = 1,671, 21.4%). Concerning their sleep quality, most of them reported their sleep quality to be fairly good or very good (*n* = 4,298, 54.9%), 26.3% (*n* = 2,057) indicated neither poor nor good sleep quality, and a minority indicated that their sleep quality was fairly poor or very poor (18.7%, *n* = 1,464) (Table 1).

Bivariate Analyses

Perceived Stress

The results of bivariate analyses with perceived stress as an outcome are available at https://doi.org/10.23668/psycharchives.5071 Predictors associated with higher

© 2021 The Author(s) Distributed as a Hogrefe OpenMind article under the license CC BY 4.0 (https://creativecommons.org/licenses/by/4.0)

https://econtent.hogrefe.com/doi/pdf/10.1024/2673-8627/a000010 - Wednesday, October 06, 2021 11:44:11 PM - University of Jyvaskyla IP Address:130.234.90.20

perceived stress among HCWs included female sex (95% CI [−2.67, −0.80]), not having children (95% CI [−1.83, −0.46]), and living with parents (95% CI [0.63, 3.06]). Another predictor of higher perceived stress was lower levels of perceived social support. In particular, the perceived stress among HCWs was significantly different for different levels of perceived social support group, Welch's $F(2, 661.639) = 42.404$, $p < .00$, see https://doi.org/10.23668/psycharchives.5071 There was no other statistically significant association between PSS score and sociodemographic characteristics.

Depressive Symptoms

The results of bivariate analyses with depressive symptoms as outcome are available at https://doi.org/10.23668/psycharchives.5071 A lower educational level $F(5, 1{,}543) = 4.411$, $p = .001$, living with their parents $F(4, 1{,}548) = 10.836$, $p < .01$, not having children −0.53 (95% CI [−0.74, −0.32]), $p < .01$, and lower level of perceived social support Welch's $F(2, 661.639) = 42.404$, $p < .01$ proved to be predictors of higher levels of depressive symptoms in HCWs. No other sociodemographic data appeared to have a statistically significant association with the prevalence of depressive symptoms.

Sleep Changes

The results of bivariate analyses with sleep changes as outcome are available at https://doi.org/10.23668/psycharchives.5071 There was a statistically significant association between sleep changes and age ($p < .001$), employment status ($\chi^2(4) = 11.347$, $p = .023$), marital status ($\chi^2(10) = 20.123$, $p = .028$), having or not children ($\chi^2(2) = 27.555$, $p < .001$), and living situation ($\chi^2(8) = 16.721$, $p = .033$). In particular, across the marital status group, widowed HCWs (33.3%) appear to sleep fewer hours. In addition, older HCWs ($M = 42.24$, $SD = 11.90$), not working at that time (29.3%), having children (25.1%), and living with parents (28.7%) were sleeping fewer hours as well. Sex and educational status did not demonstrate a statistically significant association with sleep changes.

Multivariate Analyses

Perceived Stress

The results of the multivariate analyses for the outcome of perceived stress can be seen in Table 2. The strongest predictor of perceived stress was perceived social support. Both high (−4.65, 95% CI [−5.65, −3.65]) and moderate levels (−2.92, 95% CI [−3.84, −2.00]) of perceived social support proved protective against perceived stress. Other protective factors of perceived stress were male sex (−1.81, 95% CI [−2.73, −0.90]), younger age (−0.12, 95% CI [−0.16, −0.09]), living with their own family (−1.68, 95% CI [−2.60, −0.16]), and having children (−1.04, 95% CI [−1.95, −0.12]). Positive predictors of perceived stress were being in a relationship/engaged (1.55, 95% CI [0.37, 2.73]), and being married (1.10, 95% CI [0.12, 2.73]). No statistically significant differences were found in the results when countries with more than 50 participants ($n = 10$) were added as a covariate in the analysis (see https://doi.org/10.23668/psycharchives.5071). The hierarchical multiple regression analysis indicated that the full model of sociodemographic characteristics and social support was statistically significant in predicting perceived stress $R^2 = 0.10$, $F(22, 1{,}532) = 7.867$, $p < .001$, adjusted $R^2 = 0.089$. The addition of social support to the prediction of perceived stress led to a statistically significant increase in R^2 of 0.039, $F(2, 1{,}532) = 33.393$, $p < .001$ (see https://doi.org/10.23668/psycharchives.5071).

Depressive Symptoms

The results of multivariate analyses for the outcome of depressive symptomatology can be seen in Table 2. Predictors associated with less depressive symptoms were older age (−0.03, 95% CI [−0.04, 0.23]) and living with friends or roommates (−0.04, 95% CI [−0.62, 0.55]). Perceived social support was also protective against depressive symptoms for both moderate (−0.96, 95% CI [−1.24, −0.68]) and high levels (−1.68, 95% CI [−1.99, −1.38]) of perceived social support. No statistically significant differences were observed in the results when countries with more than 50 participants ($n = 10$) were added to the analysis as a covariate (see https://doi.org/10.23668/psycharchives.5071). According to the hierarchical multiple regression analysis, the full model of sociodemographic characteristics and social support was statistically significant in predicting depression symptoms $R^2 = 0.12$, $F(22, 1{,}532) = 9.992$, $p < .001$, adjusted $R^2 = 0.113$. The addition of social support to the prediction of depression symptoms led to a statistically significant increase in R^2 of 0.067, $F(2, 1{,}532) = 58.443$, $p < .001$ (see https://doi.org/10.23668/psycharchives.5071).

Sleep Changes

Results of multivariate analyses for the outcome of sleep changes can be seen at https://doi.org/10.23668/psycharchives.5071 The positive predictor of sleep changes was working part-time (0.46, 95% CI [0.07, 0.85]), and the negative predictor was having children (−0.43, 95% CI [−0.70, −0.17]). In addition, the effect of depression was controlled in the analysis, and no significant changes were observed.

Discussion

The COVID-19 pandemic is a public-health emergency of international concern and poses a big challenge to both

© 2021 The Author(s) Distributed as a Hogrefe OpenMind article under the license CC BY 4.0 (https://creativecommons.org/licenses/by/4.0)

https://econtent.hogrefe.com/doi/pdf/10.1024/2673-8627/a000010 - Wednesday, October 06, 2021 11:44:11 PM - University of Jyvaskyla IP Address:130.234.90.20

Table 2. Multivariate analysis of predictors of perceived stress and depression symptoms

	Perceived Stress Scale		MSBS Depression Symptoms	
Variable	Mean difference (95% CI) for categorical variable and regression coefficient (95% CI) for continuous variable	Effect size†	Mean difference (95% CI) for categorical variable and regression coefficient (95% CI) for continuous variable	Effect size†
Sex				
Female	Ref	Ref	Ref	Ref
Male	−1.81 (−2.73, −0.90)	−0.10	0.06 (−0.22, 0.33)	0.01
Age (years)	−0.12 (−0.16, −0.09)	−0.21	−0.03 (−0.04, 0.23)	−0.15
Employment status				
Not working at that moment	Ref	Ref	Ref	Ref
Working (full-time)	−0.89 (−2.14, 0.35)	−0.06	0.13 (−0.25, 0.51)	0.03
Working (part-time)	−0.69 (−2.03, 0.65)	−0.04	0.07 (−0.34, 0.48)	0.01
Educational background				
Higher education	−0.36 (−2.26, 1.55)	−0.01	0.31 (−0.27, 0.90)	0.03
Some college/university	0.27 (−1.48, 2.01)	0.01	0.22 (−0.31, 0.76)	0.02
Graduate college/university	−0.13 (−1.25, 0.99)	−0.01	0.13 (−0.21, 0.47)	0.03
Master/postgraduate studies	−0.42 (−1.38, 0.54)	−0.03	−0.05 (−0.34, 0.25)	−0.01
Doctoral studies	Ref	Ref	Ref	Ref
Marital status				
Single	Ref	Ref	Ref	Ref
In a relationship/engaged	1.55 (0.37, 2.73)	0.10	−0.15 (−0.51, 0.21)	−0.03
Married	1.10 (0.12, 2.73)	0.08	−0.18 (−0.59, 0.22)	−0.04
Divorced	−0.11 (−1.28, 2.03)	−0.00	0.06 (−0.46, 0.58)	0.01
Living situation				
Live alone	Ref	Ref	Ref	Ref
Live with parents	−0.57 (−1.98, 0.93)	−0.02	0.30 (−0.15, 0.74)	0.04
Live with one parent	0.13 (−2.46, 2.64)	0.00	−0.16 (−0.94, 0.61)	−0.01
Live with own family (partner and/or children)	−1.68 (−2.60, −0.16)	−0.11	−0.19 (−0.57, 0.20)	−0.04
Live with friends/roommates	−0.50 (−2.40, 1.41)	−0.01	−0.04 (−0.62, 0.55)	0.14
Having children				
Yes	Ref	Ref	Ref	Ref
No	−1.04 (−1.95, −0.12)	−0.07	−0.05 (−0.32, 0.23)	−0.00
Perceived social support				
Low	Ref	Ref	Ref	Ref
Moderate	−2.92 (−3.84, −2.00)	−0.21	−0.96 (−1.24, −0.68)	−0.23
High	−4.65 (−5.65, −3.65)	−0.31	−1.68 (−1.99, −1.38)	−0.36
R^2	0.11		0.12	

Note. Effect size interpretation: *tiny* (0–0.05), *very small* (0.05–0.10), *small* (0.10–0.20), *medium* (0.20–0.30), *large* (0.30–0.40), *very large* (>0.40). †Standardize regression coefficients.

HCWs and the general population (Rajkumar, 2020; Ren et al., 2020; Vindegaard & Benros, 2020). In our international survey, we examined the mental health and the predictive factors reported by 1,556 HCWs across 45 countries/regions fighting against the COVID-19 pandemic, compared to a sample of 7,819 participants from the general population who reported not being HCW. We found that, at the height of the first lockdown, almost half of the HCWs demonstrated moderate levels of perceived stress, and 7% reported the highest levels of perceived stress. Participants reported also moderate depressive symptoms with most continuing to sleep about the same, having fairly good or very good sleep. Perceived social support was found to be the strongest protective factor against perceived stress and depression symptoms in HCWs. Female sex, not having children, and living with parents predicted higher levels of perceived stress; further, having a lower educational level predicted higher depressive symptoms. HCWs who were

© 2021 The Author(s) Distributed as a Hogrefe OpenMind article under the license CC BY 4.0 (https://creativecommons.org/licenses/by/4.0)

https://econtent.hogrefe.com/doi/pdf/10.1024/2673-8627/a000010 - Wednesday, October 06, 2021 11:44:11 PM - University of Jyvaskyla IP Address:130.234.90.20

widowed, older, not presently working, with children, and living with parents reported sleeping fewer hours.

These results suggest that there are subgroups among HCWs who are suffering psychologically and may be at risk for developing mental health difficulties in the future. The current findings corroborate the findings of other studies, demonstrating the need to improve mental healthcare of HCWs. However, they do not reveal more serious mental-health problems than those observed especially in China where the outbreak of COVID-19 first emerged (Chew et al., 2020; Kang, Li et al. 2020; Que et al., 2020; L .Q. Wang et al., 2020; Zhu et al., 2020a). It seems that Chinese HCWs reported worse mental health being the first to suddenly be faced with an unknown and highly contagious virus. Healthcare systems and HCWs had to struggle with the rapid spread of the Coronavirus and the unprecedented number of deaths, also within healthcare systems that appear not to have been prepared for this pandemic (Doumas et al., 2020). The rapid increase of infected patients and the uncertainty of transmission in the early stage of the outbreak increased the enormous workload and psychological burden of medical workers (Q. Cai et al., 2020). Some studies that examined frontline HCWs who were directly working with infected COVID-19 patients found higher symptoms of depression, anxiety, somatization, and insomnia than nonfrontline workers or the general population (H. Cai et al., 2020; Q. Cai et al., 2020; Lai et al., 2020; Que et al., 2020; B. Wang et al., 2020; W. R. Zhang et al., 2020; Y. Zhou et al., 2020; Zhu, Sun, et al., 2020). Such differences in reported psychological outcomes further suggest variations in outcomes among different subgroups of HCWs and may warrant further examination in association with individual differences and other coping factors.

HCWs, and especially those at the frontline, are susceptible to mental and physical exhaustion and risk of sleep disturbances (Du et al., 2020; Lai et al., 2020; Lin et al., 2021; Shaukat et al. 2020; C. Zhang et al., 2020). However, the current study found that half of the responders continued to sleep about the same, and only one in four reported sleep disturbances. HCWs presenting with sleep problems were those who also reported higher levels of perceived stress and depressive symptoms. Sociodemographic characteristics such as being widowed, older, not working at that time, having children, and living with parents predicted fewer sleeping hours.

In line with our hypothesis and like other studies (Q. Cai et al., 2020; Tan et al., 2020; C. Wang et al., 2020; D. Wang et al., 2020), HCWs presented with lower perceived stress and depressive symptoms than the general population. This is not entirely surprising, since HCWs tend to work under stressful situations that probably makes them generally more resilient to stress, so they may have been more adept at better handling the stress caused by the COVID-19 pandemic. Resilience to stress potentially increases their self-efficacy, which has been correlated with positive emotions (Folkman & Moskowitz, 2000) and increased feelings of controllability in situations that appear uncontrollable (Whealin et al., 2008). HCWs may have had an advantage over all others especially during the first lockdown period when this study was conducted: They were engaged in their normal working patterns and had greater contact with others – albeit via their work – compared to many individuals who were confined to their homes and had to adjust to new working and life conditions in addition to the stress associated with the pandemic. For instance, teleworking and digital workers reported higher levels of anxiety, stress, fatigue, and feelings of social isolation and loneliness than office-based workers (Eurofound, 2020; Gifford, 2020; Samek Lodovici et al., 2021). The pandemic may have provided HCWs with structure, and they may also have felt extremely useful during this crisis, which in turn may have provided some balance against the stress experienced. It seems that returning to work and participating in minimizing the spread of the pandemic could improve their self-esteem leading to a better quality of life, less depression and stress, and better immunity (Evans & Repper, 2000; Modini et al., 2016). It is also possible that HCWs experienced less perceived stress because of their professional knowledge and skills, which potentially made them more adept at effectively handling stressful situations in their daily practice. HCWs as a group tend to also have higher education levels, a possible protective factor against both stress and depression (Gao et al., 2020; Mazza et al., 2020).

Perceived social support emerged as a strong mental-health protective factor in this study. HCWs reported significantly greater perceived social support than the general population. This might be another means of protection against mental-health problems, where HCWs may fair better than the general population. HCWs potentially received support from their social environment since those who lived with friends or roommates reported less depression symptomatology and those who lived with their family and had children exhibited less perceived stress. A previous study (Brooks et al., 2018) noted that, as a group, HCWs rely greatly on social support and contact with family and friends as a way of dealing with stressors. Perceived social support was found to contribute to reductions in anxiety and stress levels and increased self-efficacy among HCWs (Brooks et al., 2018; Muller et al., 2020; Xiao et al., 2020).

Female HCWs who live with their parents and are childless were found to present with higher levels of perceived stress and depressive symptoms. As a group female HCWs are reported in the literature to be more vulnerable to stress, anxiety, and depression than male HCWs (Gupta et al., 2021; Lai et al., 2020; Naser et al., 2020;

© 2021 The Author(s) Distributed as a Hogrefe OpenMind article under the license CC BY 4.0 (https://creativecommons.org/licenses/by/4.0)

https://econtent.hogrefe.com/doi/pdf/10.1024/2673-8627/a000010 - Wednesday, October 06, 2021 11:44:11 PM - University of Jyvaskyla IP Address:130.234.90.20

Simione & Gnagnarella, 2020; Tella et al., 2020; W. R. Zhang et al., 2020; Y. Zhou et al., 2020; Zhu, Sun, et al., 2020; Zhu et al., 2020b). However, the view that depression rates are universally higher in women is challenged, as biological determinants, sex-role changes, and social factors might also contribute to this difference (Parker & Brotchie, 2010). We assumed that older HCWs experience higher levels of perceived stress and depression symptoms because of their potential fear of being at high risk of severe respiratory distress as a result of COVID-19, which increases with age (Centers for Disease Control and Prevention, 2020). However, in fact, older HCWs demonstrated better mental health than younger ones, than those who were living with their parents and had no children. This may be an artifact of the type of work carried out by younger HCWs or because those who have no children who are generally employed as frontline workers and are thus more prone to being in contact with infected patients and are exposed to more stressful situations (Chua et al., 2004). Also, younger HCWs may worry more about contracting the virus and spreading it to their family members, friends, and colleagues (Johal, 2009).

Much evidence demonstrates the dramatic psychological impact of the current pandemic on HCWs and the importance of devising dedicated interventions to deal with mental-health problems, such as stress, anxiety, depressive symptoms (Brooks et al., 2020; Kang, Li et al., 2020; Kang, Ma et al., 2020; S. Liu et al., 2020; Xiang et al., 2020). Support programs should be designed and implemented according to workers' individual needs, respecting their desire concerning the type, timing, and content of such interventions (Shechter et al., 2020). Moreover, the stressor support specificity model of the buffering process posits that the type of support provided is effective when the type of support matches the coping requirements elicited by a particular stressor or stress experience (Cohen and McKay, 1984). Mechanisms should be developed to identify those HCWs who also possess demographic characteristics associated with more mental-health difficulties and may be more at risk of developing mental-health problems. Also, provisions should be made for early psychological aid for these identified HCWs, and this should constitute a priority for healthcare systems.

Our study had several limitations. First, sample composition is an important limitation of the current study. Although we obtained an international sample, we did not distinguish the subgroups of HCWs, in terms of types of workers (physicians, nurses, healthcare assistants, and others), types of medical or nursing specialties, years of clinical experiences, etc. Therefore, we were unable to assess how mental-health outcomes differed between different the various HCWs subgroups. Furthermore, in many countries, the sample was very small at less than five participants. This could bias the findings of the study. Second, the results are based on cross-sectional analysis and correlations, so that causation cannot be inferred and any delayed impact of the pandemic and lockdown on the mental health of HCWs was not captured. Third, we used online self-report questionnaires, which can be subject to retrospective response bias. Further research is required, applying a qualitative approach to collect data from different HCWs subgroups, in longitudinal studies with multiple time points to confirm or refute the present findings. The current survey was conducted during the first wave of the pandemic and before pandemic fatigue may have set in (Zou, et al., 2021). Longitudinal studies would thus be beneficial, especially if they examine long-term fluctuations in stress and depressive symptoms among HCWs. Finally, the country-specific incidence rates and lockdown measures differed across countries. All these factors may bias the results of the study and should be considered in future research for a better understanding of the impact of the COVID-19 pandemic on the health status of HCWs.

Conclusion

In conclusion, our international survey documents the mental-health outcomes and sleep quality among a broad sample of HCWs during the COVID-19 global pandemic crisis. It is noticeable that being female, having lower levels of perceived social support, lower educational background, not having children, and living with parents were predictors of higher levels of perceived stress and depressive symptoms in HCWs. Current findings highlight the need for implementing appropriate supportive measures and timely treatment for COVID-19-related mental-health problems of HCWs. As the pandemic is ongoing, healthcare systems need to continue to perfect the psychological support system, providing early psychological interventions targeting vulnerable groups both in HCWs and the general population.

References

Alnazly, E., Khraisat, O. M., Al-Bashaireh, A. M., & Bryant, C. L. (2021). Anxiety, depression, stress, fear and social support during COVID-19 pandemic among Jordanian healthcare workers. *PLoS One, 16*(3), Article e0247679. https://doi.org/10.1371/journal.pone.0247679

Berkman, L. F., & Kawachi, I. (2000). *Social epidemiology*. Oxford University Press.

Bonanno, G. A., & Mancini, A. D. (2008). The human capacity to thrive in the face of potential trauma. *Pediatrics, 121*(2), 369–375. https://doi.org/10.1542/peds.2007-1648

© 2021 The Author(s) Distributed as a Hogrefe OpenMind article under the license CC BY 4.0 (https://creativecommons.org/licenses/by/4.0)

https://econtent.hogrefe.com/doi/pdf/10.1024/2673-8627/a000010 - Wednesday, October 06, 2021 11:44:11 PM - University of Jyvaskyla IP Address:130.234.90.20

Brooks, S. K., Dunn, R., Amlôt, R., Rubin, G. J., & Greenberg, N. (2018). A systematic, thematic review of social and occupational factors associated with psychological outcomes in healthcare employees during an infectious disease outbreak. *Journal of Occupational & Environmental Medicine, 60*(3), 248–257. https://doi.org/10.1097/jom.0000000000001235

Brooks, S. K., Webster, R. K., Smith, L. E., Woodland, L., Wessely, S., Greenberg, N., & Rubin, G. J. (2020). The psychological impact of quarantine and how to reduce it: Rapid review of the evidence. *Lancet, 395*(10227), 912–920. https://doi.org/10.2139/ssrn.3532534

Cabarkapa, S., Nadjidai, S. E., Murgier, J., & Ng, C. H. (2020). The psychological impact of COVID-19 and other viral epidemics on frontline healthcare workers and ways to address it: A rapid systematic review. *Brain, Behavior, & Immunity – Health, 8*, Article 100144. https://doi.org/10.1016/j.bbih.2020.100144

Cai, H., Tu, B., Ma, J., Chen, L., Fu, L., Jiang, Y., & Zhuang, Q. (2020). Psychological impact and coping strategies of frontline medical staff in Hunan between January and March 2020 during the outbreak of coronavirus disease 2019 (COVID19) in Hubei, China. *Medical Science Monitor, 26*, 1–16. https://doi.org/10.12659/msm.924171

Cai, Q., Feng, H., Huang, J., Wang, M., Wang, Q., Lu, X., Xie, Y., Wang, X., Liu, Z., Hou, B., Ouyang, K., Pan, J., Li, Q., Fu, B., Deng, Y., & Liu, Y. (2020). The mental health of frontline and non-frontline medical workers during the coronavirus disease 2019 (COVID-19) outbreak in China: A case-control study. *Journal of Affective Disorders, 275*, 210–215. https://doi.org/10.1016/j.jad.2020.06.031

Centers for Disease Control and Prevention. (2020). *Older adult at greater risk of requiring hospitalization or dying if diagnosed with COVID-19*. US Department of Health and Human Services, National Institutes of Health. https://www.cdc.gov/coronavirus/2019-ncov/needextra-precautions/older-adults

Chew, N. W. S., Lee, G. K., Tan, B. Y., Jing, M., Goh, Y., Ngiam, N. J., Yeo, L. L., Ahmad, A., Ahmed Khan, F., Napolean Shanmugam, G., Sharma, A. K., Komalkumar, R., Meenakshi, P., Shah, K., Patel, B., Chan, B. P., Sunny, S., Chandra, B., Ong, J. J., … Sharma, V. K. (2020). A multinational, multicentre study on the psychological outcomes and associated physical symptoms amongst healthcare workers during COVID-19 outbreak. *Brain, Behavior, and Immunity, 88*, 559–565. https://doi.org/10.1016/j.bbi.2020.04.049

Chong, Y. Y., Chien, W. T., Cheng, H. Y., Lamnisos, D., Ļubenko, J., Presti, G., Squatrito, V., Constantinou, M., Nicolaou, C., Papacostas, S., Aydin, G., Ruiz, F. J., Garcia-Martin, M. B., Obando-Posada, D. P., Segura-Vargas, M. A., Vasiliou, V. S., McHugh, L., Höfer, S., Baban, A., … Kassianos, A. P. (2021). Patterns of psychological responses among the public during the early phase of COVID-19: A cross-regional analysis. *International Journal of Environmental Research and Public Health, 18*(8), 1–19. https://doi.org/10.3390/ijerph18084143

Chou, R., Dana, T., Buckley, D. I., Selph, S., Fu, R., & Totten, A. M. (2020). Epidemiology and risk factors for coronavirus infection in health care workers: A living rapid review. *Annals of Internal Medicine, 173*(2), 120–136. https://doi.org/10.7326/m20-1632

Chua, S. E., Cheung, V., Cheung, C., McAlonan, G. M., Wong, J. W., Cheung, E. P., Chan, M. T., Wong, M. M., Tang, S. W., Choy, K. M., Wong, M. K., Chu, C. M., & Tsang, K. W. (2004). Psychological effects of the SARS outbreak in Hong Kong on high-risk healthcare workers. *The Canadian Journal of Psychiatry, 49*(6), 391–393. https://doi.org/10.1177/070674370404900609

Cohen, S. (1992). Stress, social support, and disorder. In H. O. F. Veiel & U. Baumann (Eds.), *The meaning and measurement of social support* (pp. 109–124). Hemisphere.

Cohen, S., Kamarck, T., & Mermelstein, R. (1983). A global measure of perceived stress. *Journal of Health and Social Behavior, 24*(4), 385–396. https://doi.org/10.2307/2136404

Cohen, S., & McKay, G. (1984). Social support, stress and the buffering hypothesis: A theoretical analysis. In A. Baum, J. E. Singer, & S. E. Taylor (Eds.), *Handbook of psychology and health 4* (pp. 253–267). Erlbaum.

Cohen, S., & Wills, T. A. (1985). Stress, social support, and the buffering hypothesis. *Psychological Bulletin, 98*(2), 310–357.

Connor, K. M., & Davidson, J. R. (2003). Development of a new resilience scale: The Connor-Davidson Resilience Scale (CD-RISC). *Depression and Anxiety, 18*(2), 76–82. https://doi.org/10.1002/da.10113

Dalgard, O. (1996). Community health profile as tool for psychiatric prevention. *Promote Mental Health, 5*, 681–695.

Dias Neto, D., Nunes da Silva, A., Roberto, M. S., Lubenko, J., Constantinou, M., Nicolaou, C., Lamnisos, D., Papacostas, S., Höfer, S., Presti, G., Squatrito, V., Vasiliou, V. S., McHugh, L., Monestès, J., Baban, A., Alvarez-Galvez, J., Paez-Blarrina, M., Montesinos, F., Valdivia-Salas, S., … Kassianos, A. P. (2021). Illness perceptions of COVID-19 in Europe: Predictors, impacts and temporal evolution. *Frontiers in Psychology, 12*, 1–11. https://doi.org/10.3389/fpsyg.2021.640955

Doumas, M., Imprialos, K. P., Patoulias, D., Katsimardou, A., & Stavropoulos, K. (2020). COVID-19: The Waterloo of governments, healthcare systems, and large health organizations. *European Journal of Internal Medicine, 77*, 153–155. https://doi.org/10.1016/j.ejim.2020.05.043

Du, J., Dong, L., Wang, T., Yuan, C., Fu, R., Zhang, L., Liu, B., Zhang, M., Yin, Y., Qin, J., Bouey, J., Zhao, M., & Li, X. (2020). Psychological symptoms among frontline healthcare workers during COVID-19 outbreak in Wuhan. *General Hospital Psychiatry, 67*, 144–145. https://doi.org/10.1016/j.genhosppsych.2020.03.011

Dunkel-Schetter, C. (1984). Social support and cancer: Findings based on patient interviews and their implications. *Journal of Social Issues, 40*(4), 77–98. https://doi.org/10.1111/j.1540-4560.1984.tb01108.x

Ehlers, A., & Clark, D. M. (2000). A cognitive model of posttraumatic stress disorder. *Behaviour Research and Therapy, 38*(4), 319–345. https://doi.org/10.1016/s0005-7967(99)00123-0

Eurofound. (2020). *Telework and ICT-based mobile work: Flexible working in the digital age. New forms of employment series*. Publications Office of the European Union. https://www.eurofound.europa.eu/sites/default/files/ef_publication/field_ef_document/ef19032en.pdf

Evans, J., & Repper, J. (2000). Employment, social inclusion and mental health. *Journal of Psychiatric and Mental Health Nursing, 7*(1), 15–24. https://doi.org/10.1046/j.1365-2850.2000.00260.x

Fahlman, S. A., Mercer-Lynn, K. B., Flora, D. B., & Eastwood, J. D. (2011). Development and validation of the multidimensional state boredom scale. *Assessment, 20*(1), 68–85. https://doi.org/10.1177/1073191111421303

Fayed, N., Klassen, A. F., Dix, D., Klaassen, R., & Sung, L. (2010). Exploring predictors of optimism among parents of children with cancer. *Psycho-Oncology, 20*(4), 411–418. https://doi.org/10.1002/pon.1743

Folkman, S., & Moskowitz, J. T. (2000). Positive affect and the other side of coping. *American Psychologist, 55*(6), 647–654. https://doi.org/10.1037/0003-066x.55.6.647

Funder, D. C., & Ozer, D. J. (2019). Evaluating effect size in psychological research: Sense and nonsense. *Advances in Methods and Practices in Psychological Science, 2*(2), 156–168. https://doi.org/10.1177/2515245919847202

© 2021 The Author(s) Distributed as a Hogrefe OpenMind article under the license CC BY 4.0 (https://creativecommons.org/licenses/by/4.0)

https://econtent.hogrefe.com/doi/pdf/10.1024/2673-8627/a000010 - Wednesday, October 06, 2021 11:44:11 PM - University of Jyvaskyla IP Address:130.234.90.20

Gao, J., Zheng, P., Jia, Y., Chen, H., Mao, Y., Chen, S., Wang, Y., Fu, H., & Dai, J. (2020). Mental-health problems and social media exposure during COVID-19 outbreak. *PLoS One, 15*(4), Article e0231924. https://doi.org/10.1371/journal.pone.0231924

Giang, T. L., Vo, D. T., & Vuong, Q. H. (2020). COVID-19: A Relook at healthcare systems and aged populations. *Sustainability, 12*(10), 1–10. https://doi.org/10.3390/su12104200

Gifford, C. (2020). *COVID-19 raises questions about employee surveillance technology*. European CEO. https://www.europeanceo.com/home/featured/COVID-19-raisesquestions-about-employee-surveillance-technology/

Gloster, A. T., Lamnisos, D., Lubenko, J., Presti, G., Squatrito, V., Constantinou, M., Nicolaou, C., Papacostas, S., Aydın, G., Chong, Y. Y., Chien, W. T., Cheng, H. Y., Ruiz, F. J., Garcia-Martin, M. B., Obando-Posada, D. P., Segura-Vargas, M. A., Vasiliou, V. S., McHugh, L., Höfer, S., . . . Karekla, M. (2020). Impact of COVID-19 pandemic on mental health: An international study. *PLoS One, 15*(12), Article e0244809. https://doi.org/10.1371/journal.pone.0244809

Gloster, A. T., Zacharia, M., & Karekla, M. (2020). Psychological aid for frontline healthcare workers. *Clinical Neuropsychiatry, 17*(4), 253–254. https://doi.org/10.36131/cnfioritieditore20200406

Grace, S. L., Hershenfield, K., Robertson, E., & Stewart, D. E. (2005). The occupational and psychosocial impact of SARS on academic physicians in three affected hospitals. *Psychosomatics, 46*(5), 385–391. https://doi.org/10.1176/appi.psy.46.5.385

Grey, I., Arora, T., Thomas, J., Saneh, A., Tohme, P., & Abi-Habib, R. (2020). The role of perceived social support on depression and sleep during the COVID-19 pandemic. *Psychiatry Research, 293*, Article 113452. https://doi.org/10.1016/j.psychres.2020.113452

Grunfeld, E., Coyle, D., Whelan, T., Clinch, J., Reyno, L., Earle, C. C., Willan, A., Viola, R., Coristine, M., Janz, T., & Glossop, R. (2004). Family caregiver burden: Results of a longitudinal study of breast cancer patients and their principal caregivers. *Canadian Medical Association Journal, 170*(12), 1795–1801. https://doi.org/10.1503/cmaj.1031205

Guan, W., Ni, Z., Hu, Y., Liang, W., Ou, C., He, J., Liu, L., Shan, H., Lei, C., Hui, D. S., Du, B., Li, L., Zeng, G., Yuen, K., Chen, R., Tang, C., Wang, T., Chen, P., Xiang, J., . . . Zhong, N. (2020). Clinical characteristics of coronavirus disease 2019 in China. *New England Journal of Medicine, 382*(18), 1708–1720. https://doi.org/10.1056/nejmoa2002032

Gupta, S., Prasad, A. S., Dixit, P. K., Padmakumari, P., Gupta, S., & Abhisheka, K. (2021). Survey of prevalence of anxiety and depressive symptoms among 1124 healthcare workers during the coronavirus disease 2019 pandemic across India. *Medical Journal Armed Forces India, 77*, S404–S412. https://doi.org/10.1016/j.mjafi.2020.07.006

Helgeson, V. S. (2003). Social support and quality of life. *Quality of Life Research, 12*(suppl), 25–31. https://doi.org/10.1023/a:1023509117524

House, J. S. (1981). *Work stress and social support*. Addison-Wesley.

Huang, Y., & Zhao, N. (2020). Generalized anxiety disorder, depressive symptoms and sleep quality during COVID-19 outbreak in China: A web-based cross-sectional survey. *Psychiatry Research, 288*, Article 112954. https://doi.org/10.1016/j.psychres.2020.112954

Ji, D., Ji, Y., Duan, X., Li, W., Sun, Z., Song, X., Meng, Y., Tang, H., Chu, F., Niu, X., Chen, G., Li, J., & Duan, H. (2017). Prevalence of psychological symptoms among Ebola survivors and healthcare workers during the 2014–2015 Ebola outbreak in Sierra Leone: A cross-sectional study. *Oncotarget, 8*(8), 12784–12791. https://doi.org/10.18632/oncotarget.14498

Johal, S. S. (2009). Psychosocial impacts of quarantine during disease outbreaks and interventions that may help to relieve strain. *New Zealand Medical Journal, 122*(1296), 47–52.

Kamara, S., Walder, A., Duncan, J., Kabbedijk, A., Hughes, P., & Muana, A. (2017). Mental healthcare during the Ebola virus disease outbreak in Sierra Leone. *Bulletin of the World Health Organization, 95*(12), 842–847. https://doi.org/10.2471/blt.16.190470

Kang, L. J., Li, Y., Hu, S., Chen, M., Yang, C., Yang, B. X., Wang, Y., Hu, J., Lai, J., Ma, X., Chen, J., Guan, L., Wang, G., Ma, H., & Liu, Z. (2020). The mental health of medical workers in Wuhan, China, dealing with the 2019 novel coronavirus. *The Lancet Psychiatry, 7*(3), Article e14. https://doi.org/10.1016/s2215-0366(20)30047-x

Kang, L. J., Ma, S., Chen, M., Yang, J., Wang, Y., Li, R., Yao, L., Bai, H., Cai, Z., Xiang Yang, B., Hu, S., Zhang, K., Wang, G., Ma, C., & Liu, Z. (2020). Impact on mental health and perceptions of psychological care among medical and nursing staff in Wuhan during the 2019 novel coronavirus disease outbreak: A cross-sectional study. *Brain, Behavior, and Immunity, 87*, 11–17. https://doi.org/10.1016/j.bbi.2020.03.028

Kim, Y., Lee, S., & Cho, J. (2020). A study on the job retention intention of nurses based on social support in the COVID-19 situation. *Sustainability, 12*(18), 1–9. https://doi.org/10.3390/su12187276

Klassen, A. F., Raina, P., McIntosh, C., Sung, L., Klaassen, R. J., O'Donnell, M., Yanofsky, R., & Dix, D. (2011). Parents of children with cancer: Which factors explain differences in health-related quality of life. *International Journal of Cancer, 129*(5), 1190–1198. https://doi.org/10.1002/ijc.25737

Labrague, L. J., & De Los Santos, J. A. (2020). COVID-19 anxiety among front-line nurses: Predictive role of organisational support, personal resilience and social support. *Journal of Nursing Management, 28*(7), 1653–1661. https://doi.org/10.1111/jonm.13121

Lai, J., Ma, S., Wang, Y., Cai, Z., Hu, J., Wei, N., Wu, J., Du, H., Chen, T., Li, R., Tan, H., Kang, L., Yao, L., Huang, M., Wang, H., Wang, G., Liu, Z., & Hu, S. (2020). Factors associated with mental-health outcomes among healthcare workers exposed to coronavirus disease 2019. *JAMA Network Open, 3*(3), Article e203976. https://doi.org/10.1001/jamanetworkopen.2020.3976

Lee, D. T., Yu, D. S., Woo, J., & Thompson, D. R. (2005). Health-related quality of life in patients with congestive heart failure. *European Journal of Heart Failure, 7*(3), 419–422. https://doi.org/10.1016/j.ejheart.2004.08.004

Lee, S. M., Kang, W. S., Cho, A., Kim, T., & Park, J. K. (2018). Psychological impact of the 2015 MERS outbreak on hospital workers and quarantined hemodialysis patients. *Comprehensive Psychiatry, 87*, 123–127. https://doi.org/10.1016/j.comppsych.2018.10.003

Li, Z., Ge, J., Feng, J., Jiang, R., Zhou, Q., Xu, X., Pan, Y., Liu, S., Gui, B., Wang, Z., Zhu, B., Hu, Y., Yang, J., Wang, R., Su, D., Hashimoto, K., Yang, M., Yang, C., & Liu, C. (2021). Less social support for patients with COVID-19: Comparison with the experience of nurses. *Frontiers in Psychiatry*, *12*. https://doi.org/10.3389/fpsyt.2021.554435

Lin, L. Y., Wang, J., Ou-Yang, X., Miao, Q., Chen, R., Liang, F., Zhang, Y., Tang, Q., & Wang, T. (2021). The immediate impact of the 2019 novel coronavirus (COVID-19) outbreak on subjective sleep status. *Sleep Medicine, 77*, 348–354. https://doi.org/10.1016/j.sleep.2020.05.018

Liu, S., Yang, L., Zhang, C., Xiang, Y., Liu, Z., Hu, S., & Zhang, B. (2020). Online mental health services in China during the COVID-19 outbreak. *The Lancet Psychiatry, 7*(4), e17–e18. https://doi.org/10.1016/s2215-0366(20)30077-8

© 2021 The Author(s) Distributed as a Hogrefe OpenMind article under the license CC BY 4.0 (https://creativecommons.org/licenses/by/4.0)

https://econtent.hogrefe.com/doi/pdf/10.1024/2673-8627/a000010 - Wednesday, October 06, 2021 11:44:11 PM - University of Jyvaskyla IP Address:130.234.90.20

Liu, Z. R., Han, B., Jiang, R., Huang, Y., Ma, C., Wen, J., Zhang, T., Wang, Y., Chen, H., & Ma, Y. (2020). *Mental health status of doctors and nurses during COVID-19 epidemic in China*. Preprint. https://doi.org/10.2139/ssrn.3551329

Maunder, R., Hunter, J., Vincent, L., Bennett, J., Peladeau, N., Leszcz, M., Sadavoy, J., Verhaeghe, L. M., Steinberg, R., & Mazzulli, T. (2003). The immediate psychological and occupational impact of the 2003 SARS outbreak in a teaching hospital. *Canadian Medical Association Journal, 168*(10), 1245–1251.

Mazza, C., Ricci, E., Biondi, S., Colasanti, M., Ferracuti, S., Napoli, C., & Roma, P. (2020). A nationwide survey of psychological distress among Italian people during the COVID-19 pandemic: Immediate psychological responses and associated factors. *International Journal of Environmental Research and Public Health, 17*(9), 1–14. https://doi.org/10.3390/ijerph17093165

Moderato, L., Lazzeroni, D., Oppo, A., Dell'Orco, F., Moderato, P., & Presti, G. (2021). Acute stress response profiles in health workers facing SARS-Cov-2. *Frontiers in Psychology*, *12*. https://doi.org/10.3389/fpsyg.2021.660156

Modini, M., Joyce, S., Mykletun, A., Christensen, H., Bryant, R. A., Mitchell, P. B., & Harvey, S. B. (2016). The mental health benefits of employment: Results of a systematic meta-review. *Australasian Psychiatry, 24*(4), 331–336. https://doi.org/10.1177/1039856215618523

Morina, N., Kip, A., Hoppen, T. H., Priebe, S., & Meyer, T. (2021). A potential impact of physical distancing on physical and mental health. A rapid narrative umbrella review of meta-analyses on the link between social isolation and health. *BMJ Open, 11*, Article e042335. https://doi.org/10.1101/2020.10.06.20207571

Muller, A. E., Hafstad, E. V., Himmels, J. P., Smedslund, G., Flottorp, S., Stensland, S. Ø., Stroobants, S., Van de Velde, S., & Vist, G. E. (2020). The mental health impact of the COVID-19 pandemic on healthcare workers, and interventions to help them: A rapid systematic review. *Psychiatry Research, 293*, 1–11. https://doi.org/10.1016/j.psychres.2020.113441

Naser, A. Y., Dahmash, E. Z., Al-Rousan, R., Alwafi, H., Alrawashdeh, H. M., Ghoul, I., Abidine, A., Bokhary, M. A., Al-Hadithi, H. T., Ali, D., Abuthawabeh, R., Abdelwahab, G. M., Alhartani, Y. J., Muhaisen, H. A., Dagash, A., & Alyami, H. S. (2020). Mental health status of the general population, healthcare professionals, and university students during 2019 coronavirus disease outbreak in Jordan: A cross-sectional study. *Brain and Behavior, 10*(8), 1–13. https://doi.org/10.1002/brb3.1730/v2/response1

Nasser, E. H., & Overholser, J. C. (2005). Recovery from major depression: The role of support from family, friends, and spiritual beliefs. *Acta Psychiatrica Scandinavica, 111*(2), 125–132. https://doi.org/10.1111/j.1600-0447.2004.00423.x

Nicolaou, C., Kouta, C., Papathanassoglou, E. D. E., & Middleton, N. (2015). Perceived social support among Greek-Cypriot mothers of children with cancer and mothers of healthy children. *International Journal of Caring Sciences, 8*(2), 241–255.

Parker, G., & Brotchie, H. (2010). Gender differences in depression. *International Review of Psychiatry, 22*(5), 429–436. https://doi.org/10.3109/09540261.2010.492391

Pfefferbaum, B., & North, C. S. (2020). Mental health and the COVID-19 pandemic. *New England Journal of Medicine, 383*(6), 510–512. https://doi.org/10.1056/nejmp2008017

Porta, M. A. (2008). *Dictionary of epidemiology*. Oxford University Press.

Pouralizadeh, M., Bostani, Z., Maroufizadeh, S., Ghanbari, A., Khoshbakht, M., Alavi, S. A., & Ashrafi, S. (2020). Anxiety and depression and the related factors in nurses of Guilan University of Medical Sciences hospitals during COVID-19: A web-based cross-sectional study. *International Journal of Africa Nursing Sciences, 13*, 1–6. https://doi.org/10.1016/j.ijans.2020.100233

Que, J., Shi, L., Deng, J., Liu, J., Zhang, L., Wu, S., Gong, Y., Huang, W., Yuan, K., Yan, W., Sun, Y., Ran, M., Bao, Y., & Lu, L. (2020). Psychological impact of the COVID-19 pandemic on healthcare workers: A cross-sectional study in China. *General Psychiatry, 33*(3), Article e100259. https://doi.org/10.1136/gpsych-2020-100259

Rajkumar, R. P. (2020). COVID-19 and mental health: A review of the existing literature. *Asian Journal of Psychiatry, 52*, 1–5. https://doi.org/10.1016/j.ajp.2020.102066

Rana, W., Mukhtar, S., & Mukhtar, S. (2020). Mental health of medical workers in Pakistan during the pandemic COVID-19 outbreak. *Asian Journal of Psychiatry, 51*, 1–2. https://doi.org/10.1016/j.ajp.2020.102080

Ren, X., Huang, W., Pan, H., Huang, T., Wang, X., & Ma, Y. (2020). Mental health during the COVID-19 outbreak in China: A meta-analysis. *Psychiatric Quarterly, 91*(4), 1033–1045. https://doi.org/10.1007/s11126-020-09796-5

Resick, P. A., & Schnicke, M. K. (1993). *Cognitive processing therapy for rape victims: A treatment manual*. Sage.

Samek Lodovici, M., Ferrari, E., Paladino, E., Pesce, F., Frecassetti, P., Aram, E., & Hadjivassiliou, K. (2021). *The impact of teleworking and digital work on workers and society*. European Parliament. https://www.europarl.europa.eu/Reg Data/etudes/STUD/2021/662904/IPOL_STU(2021)662904_EN.pdf

Shaukat, N., Ali, D. M., & Razzak, J. (2020). Physical and mental health impacts of COVID-19 on healthcare workers: A scoping review. *International Journal of Emergency Medicine, 13*(1), 1–8. https://doi.org/10.1186/s12245-020-00299-5

Shechter, A., Diaz, F., Moise, N., Anstey, D. E., Ye, S., Agarwal, S., Birk, J. L., Brodie, D., Cannone, D. E., Chang, B., Claassen, J., Cornelius, T., Derby, L., Dong, M., Givens, R. C., Hochman, B., Homma, S., Kronish, I. M., Lee, S. A., ... Abdalla, M. (2020). Psychological distress, coping behaviors, and preferences for support among New York healthcare workers during the COVID-19 pandemic. *General Hospital Psychiatry, 66*, 1–8. https://doi.org/10.1016/j.genhosppsych.2020.06.007

Shirey, M. R. (2004). 2004 AONE award winner profiles. *Nurse Leader, 2*(6), 14–23. https://doi.org/10.1016/j.mnl.2004.09.008

Simione, L., & Gnagnarella, C. (2020). Differences between health workers and general population in risk perception, behaviors, and psychological distress related to COVID-19 spread in Italy. *Frontiers in Psychology, 11*, 1–17. https://doi.org/10.3389/fpsyg.2020.02166

Surkan, P. J., Peterson, K. E., Hughes, M. D., & Gottlieb, B. R. (2006). The role of social networks and support in postpartum women's depression: A multiethnic urban sample. *Maternal and Child Health Journal, 10*(4), 375–383. https://doi.org/10.1007/s10995-005-0056-9

Tam, C. W., Pang, E. P., Lam, L. C., & Chiu, H. F. (2004). Severe acute respiratory syndrome (SARS) in Hong Kong in 2003: Stress and psychological impact among frontline healthcare workers. *Psychological Medicine, 34*(7), 1197–1204. https://doi.org/10.1017/s0033291704002247

Tan, B. Y., Chew, N. W., Lee, G. K., Jing, M., Goh, Y., Yeo, L. L., Zhang, K., Chin, H., Ahmad, A., Khan, F. A., Shanmugam, G. N., Chan, B. P., Sunny, S., Chandra, B., Ong, J. J., Paliwal, P. R., Wong, L. Y., Sagayanathan, R., Chen, J. T., ... Sharma, V. K. (2020). Psychological impact of the COVID-19 pandemic on healthcare workers in Singapore. *Annals of Internal Medicine, 173*(4), 317–320. https://doi.org/10.7326/m20-1083

Tella, M. D., Romeo, A. A., Benfante, A., & Castelli, L. (2020). Mental health of healthcare workers during the COVID-19 pandemic in Italy. *Journal of Evaluation in Clinical Practice, 26*, 1583–1587. https://doi.org/10.22541/au.158878917.77777713

© 2021 The Author(s) Distributed as a Hogrefe OpenMind article under the license CC BY 4.0 (https://creativecommons.org/licenses/by/4.0)

https://econtent.hogrefe.com/doi/pdf/10.1024/2673-8627/a000010 - Wednesday, October 06, 2021 11:44:11 PM - University of Jyvaskyla IP Address:130.234.90.20

Tian, F., Li, H., Tian, S., Yang, J., Shao, J., & Tian, C. (2020). Psychological symptoms of ordinary Chinese citizens based on SCL-90 during the level I emergency response to COVID-19. *Psychiatry Research, 288*, 1–9. https://doi.org/10.1016/j.psychres.2020.112992

Vaezi, A., Soojoodi, F., Banihashemi, A. T., & Nojomi, M. (2019). The association between social support and postpartum depression in women: A cross sectional study. *Women and Birth, 32*(2), e238–e242. https://doi.org/10.1016/j.wombi.2018.07.014

Vindegaard, N., & Benros, M. E. (2020). COVID-19 pandemic and mental health consequences: Systematic review of the current evidence. *Brain, Behavior, and Immunity, 89*, 531–542. https://doi.org/10.1016/j.bbi.2020.05.048

Wang, B., Sun, J., Gao, F., Chen, J., Shi, L., Li, L., Tang, Y., Wang, K., & Lu, H. (2020). *A study on mental health status among the staff in a designated hospital for COVID-19*. Preprint. https://doi.org/10.21203/rs.3.rs-23224/v1

Wang, C., Pan, R., Wan, X., Tan, Y., Xu, L., Ho, C. S., & Ho, R. C. (2020). Immediate psychological responses and associated factors during the initial stage of the 2019 coronavirus disease (COVID-19) epidemic among the general population in China. *International Journal of Environmental Research and Public Health, 17*(5), 1–25. https://doi.org/10.3390/ijerph17051729

Wang, D., Hu, B., Hu, C., Zhu, F., Liu, X., Zhang, J., Wang, B., Xiang, H., Cheng, Z., Xiong, Y., Zhao, Y., Li, Y., Wang, X., & Peng, Z. (2020). Clinical characteristics of 138 hospitalized patients with 2019 novel coronavirus–infected pneumonia in Wuhan, China. *Journal of the American Medical Association, 323*(11), 1061–1069. https://doi.org/10.1001/jama.2020.1585

Wang, L. Q., Zhang, M., Liu, G., Nan, S., Li, T., Xu, L., Xue, Y., Zhang, M., Wang, L., Qu, Y., & Liu, F. (2020). Psychological impact of coronavirus disease (2019) (COVID-19) epidemic on medical staff in different posts in China: A multicenter study. *Journal of Psychiatric Research, 129*, 198–205. https://doi.org/10.1016/j.jpsychires.2020.07.008

Whealin, J. M., Ruzek, J. I., & Southwick, S. (2008). Cognitive-behavioral theory and preparation for professionals at risk for trauma exposure. *Trauma, Violence, & Abuse, 9*(2), 100–113. https://doi.org/10.1177/1524838008315869

Wong, T. W., Yau, J. K., Chan, C. L., Kwong, R. S., Ho, S. M., Lau, C. C., Lau, F. L., & Lit, C. H. (2005). The psychological impact of severe acute respiratory syndrome outbreak on healthcare workers in emergency departments and how they cope. *European Journal of Emergency Medicine, 12*(1), 13–18. https://doi.org/10.1097/00063110-200502000-00005

World Health Organization (WHO). (2020a). *WHO Director-General's opening remarks at the media briefing on COVID-19–11 March 2020*. https://www.who.int/director-general/speeches/detail/who-director-general-s-opening-remarks-at-the-media-briefing-on-covid-19—11-march-2020

World Health Organization (WHO). (2020b). *WHO coronavirus disease (COVID-19) dashboard*. https://covid19.who.int/

World Health Organization (WHO). (2020c). *WHO coronavirus disease (COVID-19) dashboard*. https://covid19.who.int/table

Wu, P., Fang, Y., Guan, Z., Fan, B., Kong, J., Yao, Z., Liu, X., Fuller, C. J., Susser, E., Lu, J., & Hoven, C. W. (2009). The psychological impact of the SARS epidemic on hospital employees in China: Exposure, risk perception, and altruistic acceptance of risk. *The Canadian Journal of Psychiatry, 54*(5), 302–311. https://doi.org/10.1177/070674370905400504

Xiang, Y., Yang, Y., Li, W., Zhang, L., Zhang, Q., Cheung, T., & Ng, C. H. (2020). Timely mental healthcare for the 2019 novel coronavirus outbreak is urgently needed. *The Lancet Psychiatry, 7*(3), 228–229. https://doi.org/10.1016/s2215-0366(20)30046-8

Xiao, H., Zhang, Y., Kong, D., Li, S., & Yang, N. (2020). The effects of social support on sleep quality of medical staff treating patients with coronavirus disease 2019 (COVID-19) in January and February 2020 in China. *Medical Science Monitor, 26*, 1–8. https://doi.org/10.12659/msm.923549

Yamamoto, K., Kimoto, K., Watanabe, N., Hoshiyama, S., Kamiyama, S., Onishi, Y., & Mikami, K. (2020). Taking social support into account regarding the mental health of healthcare practitioners involved in treating patients with COVID-19. *Psychosomatics, 61*(5), 575–577. https://doi.org/10.1016/j.psym.2020.06.001

Yu, H., Li, M., Li, Z., Xiang, W., Yuan, Y., Liu, Y., Li, Z., & Xiong, Z. (2020). Coping style, social support and psychological distress in the general Chinese population in the early stages of the COVID-19 epidemic. *BMC Psychiatry, 20*(1), 1–11. https://doi.org/10.1186/s12888-020-02826-3

Zhang, C., Yang, L., Liu, S., Ma, S., Wang, Y., Cai, Z., Du, H., Li, R., Kang, L., Su, M., Zhang, J., Liu, Z., & Zhang, B. (2020). Survey of insomnia and related social psychological factors among medical staff involved with the 2019 novel coronavirus disease outbreak. *Frontiers in Psychiatry, 11*, 1–9. https://doi.org/10.2139/ssrn.3542175

Zhang, W. R., Wang, K., Yin, L., Zhao, W., Xue, Q., Peng, M., Min, B., Tian, Q., Leng, H., Du, J., Chang, H., Yang, Y., Li, W., Shangguan, F., Yan, T., Dong, H., Han, Y., Wang, Y., Cosci, F., . . . Wang, H. (2020). Mental health and psychosocial problems of medical health workers during the COVID-19 epidemic in China. *Psychotherapy and Psychosomatics, 89*(4), 242–250. https://doi.org/10.1159/000507639

Zhou, P., Yang, X., Wang, X., Hu, B., Zhang, L., Zhang, W., Si, H., Zhu, Y., Li, B., Huang, C., Chen, H., Chen, J., Luo, Y., Guo, H., Jiang, R., Liu, M., Chen, Y., Shen, X., Wang, X., . . . Shi, Z. (2020). A pneumonia outbreak associated with a new coronavirus of probable bat origin. *Nature, 579*(7798), 270–273. https://doi.org/10.1038/s41586-020-2012-7

Zhou, Y., Wang, W., Sun, Y., Qian, W., Liu, Z., Wang, R., Qi, L., Yang, J., Song, X., Zhou, X., Zeng, L., Liu, T., Li, Z., & Zhang, X. (2020). The prevalence and risk factors of psychological disturbances of frontline medical staff in China under the COVID-19 epidemic: Workload should be concerned. *Journal of Affective Disorders, 277*, 510–514. https://doi.org/10.1016/j.jad.2020.08.059

Zhu, J., Sun, L., Zhang, L., Wang, H., Fan, A., Yang, B., Li, W., & Xiao, S. (2020). Prevalence and influencing factors of anxiety and depression symptoms in the first-line medical staff fighting against COVID-19 in Gansu. *Frontiers in Psychiatry, 11*, 1–6. https://doi.org/10.3389/fpsyt.2020.00386

Zhu, Z., Xu, S., Wang, H., Liu, Z., Wu, J., Li, G., Miao, J., Zhang, C., Yang, Y., Sun, W., Zhu, S., Fan, Y., Hu, J., Liu, J., & Wang, W. (2020a). COVID-19 in Wuhan: Immediate psychological impact on 5062 health workers. *E. Clinical Medicine, 24*, 1–24. https://doi.org/10.1101/2020.02.20.20025338

Zhu, Z., Xu, S., Wang, H., Liu, Z., Wu, J., Li, G., Miao, J., Zhang, C., Yang, Y., Sun, W., Zhu, S., Fan, Y., Hu, J., Liu, J., & Wang, W. (2020b). COVID-19 in Wuhan: Sociodemographic characteristics and hospital support measures associated with the immediate psychological impact on healthcare workers. *Clinical Medicine, 24*, 1–11. https://doi.org/10.2139/ssrn.3578747

Zou, X., Liu, S., Li, J., Chen, W., Ye, J., Yang, Y., Zhou, F., & Ling, L. (2021). Factors associated with healthcare workers' insomnia symptoms and fatigue in the fight against COVID-19, and the role of organizational support. *Frontiers in Psychiatry, 12*, 1–12. https://doi.org/10.3389/fpsyt.2021.652717

© 2021 The Author(s) Distributed as a Hogrefe OpenMind article under the license CC BY 4.0 (https://creativecommons.org/licenses/by/4.0)

https://econtent.hogrefe.com/doi/pdf/10.1024/2673-8627/a000010 - Wednesday, October 06, 2021 11:44:11 PM - University of Jyvaskyla IP Address:130.234.90.20

History
Received June 1, 2021
Accepted September 1, 2021
Published online September 21, 2021

Author Notes
ATG, MK, and APK designed and implemented the study together. CN, ATG, APK, and MK should be considered joint senior authors.

Open Science
The open material can be found at https://doi.org/10.23668/psycharchives.5071.

Christiana Nicolaou
Department of Nursing
School of Health Sciences
Cyprus University of Technology
15, Vragadinou St
3041, Limassol
Cyprus
c.nicolaou@cut.ac.cy

© 2021 The Author(s) Distributed as a Hogrefe OpenMind article under the license CC BY 4.0 (https://creativecommons.org/licenses/by/4.0)
