# Supplementary material for: Mental health status and related factors influencing healthcare workers during the COVID-19 pandemic: A systematic review and meta-analysis
Source: PLoS One. 2024 Jan 19;19(1):e0289454. doi: 10.1371/journal.pone.0289454 (PMC10798549; doi:10.1371/journal.pone.0289454)
Supplement: S1 Data — (ZIP) [file pone.0289454.s011.zip › literatures/117.pdf]

International Journal of
Environmental Research and Public Health

MDPI

Article

The Mental Health of Malaysia's Northwest Healthcare Workers during the Relaxation of COVID-19 Restrictions and Its Associated Factors

Syazwan Nordin [1], Nor Azwany Yaacob [1,*], Johny Kelak [2], Ahmad Hazri Ilyas [2] and Aziah Daud [1]

[1] Department of Community Medicine, School of Medical Sciences, Health Campus, Universiti Sains Malaysia, Kubang Kerian 16150, Kelantan, Malaysia; drahmadsyazwan@student.usm.my (S.N.); aziahkb@usm.my (A.D.)
[2] Ministry of Health State Department, Alor Setar 05400, Kedah, Malaysia; johnyclark@moh.gov.my (J.K.); drahmadhazri@gmail.com (A.H.I.)
* Correspondence: azwany@usm.my; Tel.: +60-767-3000

Abstract: The COVID-19 pandemic has affected people in many ways, including mental health status. Depression, anxiety, and stress (DAS) are terms often used to describe mental health status worldwide. The present study describes the prevalence of DAS and its associated factors among healthcare workers (HCWs) in the northwest region of Malaysia, during the early phase of recovery of movement control order (RMCO), where some restrictions were lifted, and cases are reducing in number. This cross-sectional study used HCW's mental health surveillance data using the DASS-21 questionnaire. A total of 981 data collected between 1 July and 31 August 2020 were randomly sampled. Socio-demographic factors, occupational characteristics, and health backgrounds were extracted and analyzed using multiple logistic regression. The prevalences of DAS are 8.4% (6.7, 10.3), 17.1% (14.8, 19.6), and 6.4% (5.0, 8.1), respectively. Age is significantly associated with depression (Adjusted Odd Ratio (Adj.OR) 0.96 (0.93, 0.99)) and stress (Adj.OR 0.96 (0.93, 0.997)). Working at the hospital is associated with depression (Adj.OR 1.88 (1.19, 2.97)) as well as anxiety (Adj.OR 1.91 (1.36, 2.68). HCWs with a degree or postgraduate education level are more stressed compared to those with lower educational levels (Adj.OR 8.43 (1.95, 36.37)). Mental health surveillance helps to identify those at risk. Those younger in age, working in hospitals, and with more responsibility in management are the most affected. With the easing of COVID-19 pandemic restrictions, which lead to the release of certain movement control, the mental health status of HCWs was less affected. Those working directly with COVID-19 patients and with more responsibility in management are the most affected.

Keywords: mental health; depression; anxiety; stress; healthcare workers; COVID-19 pandemic; Malaysia

check for updates

Citation: Nordin, S.; Yaacob, N.A.; Kelak, J.; Ilyas, A.H.; Daud, A. The Mental Health of Malaysia's Northwest Healthcare Workers during the Relaxation of COVID-19 Restrictions and Its Associated Factors. *Int. J. Environ. Res. Public Health* **2022**, *19*, 7794. https://doi.org/10.3390/ijerph19137794

Academic Editor: Paul B. Tchounwou

Received: 27 April 2022
Accepted: 20 June 2022
Published: 25 June 2022

Publisher's Note: MDPI stays neutral with regard to jurisdictional claims in published maps and institutional affiliations.

Copyright: © 2022 by the authors. Licensee MDPI, Basel, Switzerland. This article is an open access article distributed under the terms and conditions of the Creative Commons Attribution (CC BY) license (https://creativecommons.org/licenses/by/4.0/).

1. Introduction

The novel coronavirus disease 2019 (COVID-19) pandemic has attracted concerns globally after its emergence from Wuhan City, Hubei Province, China, and is primarily transmitted through person-to-person contact and respiratory droplets [1]. The World Health Organization (WHO) declared the COVID-19 outbreak in the Public Health Emergencies of International Concern (PHEIC) on 30 January 2020 following the recommendations by the Emergency Committee [2]. More than 140 million individuals had been infected as of 18 April 2021, with around 3 million deaths globally [3].

Malaysia reported its first imported case in Johor Bharu on 25 January 2020; the first locally transmitted COVID-19 case was in Kedah on 6 February 2020 [4,5]. In response to the increase in cases of COVID-19 in Malaysia, the National Security Council imposed a Movement Control Order (MCO) or lockdown, which prohibited mass assemblies and movements, and imposed quarantine upon overseas arrivals and the closure of non-essential service sectors from 18 March to 4 May 2020 [6–8]. Public health services

in Malaysia are administered by the Ministry of Health through its central, state, and district offices. The tax-funded healthcare services run primary healthcare centers and hospitals. These provide a comprehensive low-cost healthcare system [9]. However, the high numbers of suspected and confirmed COVID-19 cases forced the health services to adapt to the situation and demanded healthcare workers (HCWs) to perform multi-tasking. The usual duties of HCWs were either replaced or added on with tasks in managing the pandemic, either direct or indirect patient care, and contact tracing. In an earlier phase of the pandemic, there were many uncertainties concerning the disease, such as the mode of transmission, best preventive measures, treatment, and future situations. These uncertainties led to the rearrangement of place and interruption of regular work processes among HCWs. The pandemic increased HCWs' work demands and limited their opportunity to rest and recover adequately, subsequently exposing them to a significant risk of adverse mental health implications [10].

COVID-19 as a novel infectious disease has the potential to affect the mental health of HCWs. Mental health problems affects physical health and well-being, and might lead to psychosomatic problems, burn-out, and subsequently affect the productivity and quality of health services. Mental health problems among HCWs also might be overlooked or ignored. Thus, the mental health psychosocial support services (MHPSSs) [11] were activated to curb the effect of the pandemic on HCWs in March 2020. A mental health screening assessment using a DASS21 questionnaire was conducted in regular intervals on various types of HCWs. Those who were affected were immediately referred for psychological support and occupational intervention.

Social demographic factors, such as being female, single, of a younger age, and living alone, were found to be associated with higher incidences of depression, anxiety, and stress (DAS) [12–16]. Studies that were conducted before the COVID-19 pandemic identified a high workload, uncooperative colleagues, and fear of committing mistakes as contributing factors to stress among HCWs in Malaysia [13,17]. Subsequently, a study conducted during the early stages of the COVID-19 pandemic in Malaysia reported an increase in the workload of healthcare personnel while limiting their ability to relax and recover sufficiently, placing them in danger of adverse mental health effects [10]. HCWs who worked at the frontline and high-risk department for COVID-19 together with those experienced with treatment for infectious disease, were associated with psychological distress [15,18,19].

The previous studies were conducted in hospitals and did not include other HCWs working in peripheral health-care services, which were also affected by the pandemic [10,20]. Moreover, these were conducted during different phases of the pandemic, which might be translated into different restrictions, workloads, and disease trends. With the easing of the pandemic restrictions, whereby cases were reducing, more restrictions (recovery of movement control order (RMCO)) were eased in June 2020. It is important to know how the easing of restrictions during the pandemic affects the mental health status of HCWs, both in the community and hospital service, and those who are the most affected among them. Thus, this study examines how the easing of the pandemic affects the mental health status of HCWs, both at the community and hospital service levels in terms of the prevalence of DAS, and its associated factors among HCWs in the northwest region of Malaysia during the early phase of the RMCO.

2. Materials and Methods

2.1. Study Setting and Design

This cross-sectional study used secondary data from the mental health surveillance data of the northwest region of Malaysia for HCWs from July 2020 until August 2020. The surveillance is a part of the Ministry of Health's activity of monitoring the mental health of healthcare workers. This region's healthcare system is under the Ministry of Health overseeing all government health facilities, which consist of 9 hospitals, 12 health district offices, and 80 health clinics [21]. It is an online, self-administered web-based

screening in the Google form format, using a Malay version of Depression, Anxiety, and Stress Scale 21 (DASS-21) questionnaire. The Google form link was provided by the MHPSS of Kedah Health State Department to HCWs through their Head of Department via WhatsApp messaging. All responses of mental health screening during 1 July–31 August 2020 were extracted.

Sociodemographic variables (age, gender, marital status, ethnicity, religion, living arrangements, education level, and household income), occupational characteristics (type of workplace, working station, and working position), and health characteristics (smoking status, comorbidity, and overweight or obesity) were also extracted.

2.2. Study Population and Sample Size

The HCWs worked at the Ministry of Health of Malaysia, consisting of hospitals, district health office, and health clinics, were included in this study. They were from all categories, ranging from health professionals to support personnel. Respondents' data with self-declared pre-existing mental illness and with one or more missing variables were excluded.

A sample size of 981 was estimated by a dichotomous two-proportion formula based on the factors associated with DAS in a study [15] at 80% power with an additional 10% to cover incomplete data. Simple random sampling was applied to the total of 1100 data. All cases were given anonymous codes. This study was approved by the Medical Research and Ethics Committee (NMRR-20-2543-57241), Human Research Ethics Committee (JePEM) of Universiti Sains Malaysia (USM/JEPeM/20110582), and permission from the Ministry of Health was obtained.

2.3. Study Instrument and Data Collection

DASS-21 is a globally used screening tool and a quantitative measure of distress along the axes of DAS [22]. The tool contains a 21-item Depression, Anxiety and Stress Questionnaire (DASS-21), each factor measured using a 4-point Likert scale (from 0 to 3) to indicate the severity of the individuals' symptoms over the previous week. A higher score denotes a higher level of the respective subscale's symptoms. This study used the Malay version of the DASS-21. The validated Malay version, the Cronbach's alphas were 0.84, 0.74, and 0.79 for DAS, respectively [23]. Scores of more than 5 were interpreted as "depressed", more than 4 as "anxiety", and more than 7 as "stressed", respectively, based on the items of each subdomain [11].

2.4. Operational Definition

HCWs were workers who were involved in the screening process, handling contaminated medical supplies, clinical samples and waste, contact tracing, logistics, administrative, data management, treatment process, or any other activities related to managing COVID-19 cases [24], and working at any healthcare premise under the Ministry of Health Malaysia.

Living arrangement is the number of people living together at a residence, including the HCWs. Type of workplace was classified into hospital and peripheral healthcare service, which included both clinical and public health sections.

Working station was categorized to reflect HCWs' exposure and their involvement in managing the COVID-19 pandemic. Direct clinical contact was defined as individuals who had direct contact with a COVID-19 patient or people under investigation while performing clinical work, such as taking swabs and patient care, whereas direct nonclinical contact was defined as individuals performing non-clinical work, such contact tracing and customer service. Non-direct contact was defined as individuals who did not have any direct contact with a COVID-19 patient or people under investigation, for example, those coordinating logistics for screening, quarantine, and treatment. Laboratory was defined as individuals who worked in the laboratory who handled the clinical samples.

The healthcare worker category was based on the International Classification of Health Workers (ICHW) [25]. Health professionals are individuals who analyze, consult on, or

offer healthcare services based on theoretical and factual knowledge in diagnosing and treating illness. Health Associate Professionals assist in the diagnosis and treatment process, typically planned by health professionals. Personal Care Workers in Health Service provide direct personal care service in healthcare, help with procedures, and conduct various easy and routine services. Health Management and Support Personnel are those who manage the health service or provide a service that supports the healthcare system [25]. Overweight/obesity is based on the Asian classification of which BMI $\geq$ 23 kg/m^2 is considered overweight/obesity [26].

2.5. Data Analysis

Data were analyzed using IBM SPSS Statistic for Windows, version 26 software (IBM Corp., Armonk, NY, USA). Factors with p-value < 0.25 on simple logistic regression were analyzed using forward methods of multiple logistic regression. The level of significance was set at a p-value of less than 0.05.

3. Results

3.1. Characterictics of Respondent

The majority of the respondents were female, Malay, Muslim, married, health professionals, and working in a peripheral healthcare service (Table 1).

Table 1. Sociodemographic, occupational, and health characteristics of respondents (n = 981).

Variables	n (%)
Age (Year) [1]	36.43 (7.28)
Gender	
Male	250 (25.5)
Female	731 (74.5)
Ethnicity	
Malay	909 (92.7)
Non-Malay	72 (7.3)
Religion	
Islam	916 (93.4)
Non-Islam	65 (6.6)
Education Level	
Primary/secondary	155 (15.8)
Diploma	604 (61.6)
Degree/postgraduate	222 (22.6)
Marital Status	
Married	812 (82.8)
Single/divorced/widowed	169 (17.2)
Living Arrangement	
Alone	50 (5.0)
Living with others	932 (95.0)
Household Income	
<MYR 3000	163 (16.6)
MYR 3000–7000	574 (58.5)
MYR 7001–14,000	207 (21.2)
>MYR 14,001	37 (3.8)
Type of Workplace	
Peripheral healthcare service	642 (65.4)
Hospital	339 (34.6)

Table 1. *Cont.*

Variables	n (%)
Working Station	
Direct contact clinical	634 (64.6)
Direct contact non-clinical	86 (8.8)
Non-direct contact	226 (23.0)
Laboratory	35 (3.6)
Health Worker Category	
Health professionals	561 (57.2)
Health associate professionals	220 (22.4)
Personal care workers in health services	72 (7.3)
Health management and support personnel	128 (13.0)
Medical Comorbidity	
No	884 (90.1)
Yes	102 (9.9)
Smoking	
No	949 (96.7)
Yes	32 (3.3)
Overweight/Obesity	
No	603 (61.5)
Yes	378 (38.5)

[1]: Mean (SD).

3.2. Prevalence of DAS

The majority of the HCWs scored a normal result on the DASS-21 (Table 2). There were only 82 (8.4% (6.7, 10.3)) individuals who had depression, 168 (17.1% (14.8, 19.6)) anxiety, and 63 (6.4% (5.0, 8.1)) stress.

Table 2. Percentage of DAS by DASS-21 among respondents (n = 981).

Variables	Classification	
	Normal n (%)	Above Normal n (%)
Depression	899 (91.6)	82 (8.4)
Anxiety	813 (82.9)	168 (17.1)
Stress	918 (93.6)	63 (6.4)

3.3. Factors Associated with DAS

Age was found to be significantly associated with depression and stress; being older significantly decreased the odds of depression (Adj.OR 0.96, 95% CI: 0.93 to 0.99)) and stress (Adj.OR 0.96, 95% CI: 0.92 to 0.997).

HCWs' type of workplace was found to be significantly associated with depression and anxiety; working in the hospital increased the odds of depression (Adj.OR 1.88, 95% CI: 1.19 to 2.97) and anxiety (Adj.OR 1.91, 95% CI: 1.36 to 2.68) compared to other HCWs in peripheral healthcare service.

Education level was found to be significantly associated with stress; having a degree/postgraduate education significantly increased the odds of having stress (Adj.OR 8.43, 95% CI: 1.95 to 36.37) compared to HCWs with primary/secondary education levels (Table 3).

Table 3. Factors associated with depression, anxiety, and stress (n = 981).

Variables	Adjusted OR (95% CI)			p-Value [1]
	Depression	Anxiety	Stress	
Age (Years)	0.96 (0.93, 0.99)		0.96 (0.92, 0.997)	0.015, 0.035
Type of Workplace				
Peripheral healthcare service	1	1		
Hospital	1.88 (1.19, 2.97)	1.91 (1.36, 2.68)		0.007, <0.001
Education Level				
Primary/secondary			1	
Diploma			3.97 (0.94, 16.83)	0.062
Degree/postgraduate			8.43 (1.95, 36.37)	0.004

[1]: Multiple logistic regression, forward method.

4. Discussion

4.1. Prevalence of DAS

This study observed the prevalence of DAS among various types of HCWs, including those with non-direct contact, which might explain the findings that are lower compared to other similar studies [19,27,28]. This was reflected by the significance of workplace as one of the associated factors for depression and anxiety. The prevalence may also vary according to the time of the study in relation to the pandemic phase and incidence of the area of study. This surveillance was conducted during the relaxation of the movement control order where the restriction at that time was reduced, compared to earlier MCOs. The interstate travel ban was lifted, social and economic activities became permissible, non-essential sectors, such as tourism, were allowed to re-open. The relaxation of restrictions was based on the reduced number of cases; thus, the workload of managing the pandemic was also reduced. As the DASS-21 detected a subclinical (mild) category of DAS, it might be the combination of reducing workload and the personal soothing feeling of being free from restrictions, especially interstate travel, which reduced stress levels, as well as depression and anxiety. However, the lifting of such restrictions may also create anxiety for some individuals who realized that this would also increase the risk of disease transmission, as reflected by the relatively higher prevalence of anxiety compared to depression n stress. On the other hand, a small number of individuals with severe depression and anxiety should not be put aside and indicate the importance of mental health surveillance in crisis situations, and the relaxation of the restrictions may not result in the immediate relief for some individuals.

4.2. Factors Associated with DAS

4.2.1. Age

The age of HCWs is a significant protective factor against depression and stress. Other studies reported a similar protective effect of age against depression and stress during the various phases of the pandemic [29,30]. This was supported by the report that depression does not occur naturally as part of the aging process [31]. Age has also been viewed as a factor in the provision of family care [32]. Older-aged individuals are more likely to settle down and establish a more structured support system away from families and friends, where they can focus more on their jobs without worrying about their children's routine and well-being. Age also has a strong correlation with the duration of the service [33]. A longer service period may be associated with more job experience, such as participating in disaster management and handling outbreaks [34,35]. A longer service period may be associated with better familiarization, communication, and recognition among colleagues. Positive workplace relationships among workers also improve mental health outcomes [36]. A longer duration of service may also be associated with a higher job position in an organization, which translates into a higher salary and better discussion that drives employee decisions. Seniority is also associated with better working conditions and

more favorable duty rosters, including time off. Junior colleagues may be prioritized in outstation deployment to localities with clusters or higher risk areas.

4.2.2. Workplace

Individuals working in a hospital have a significantly higher chance of attaining depression and anxiety than those working in the peripheral healthcare service. Working in a hospital in Malaysia is associated with working seven-to-nine hour shifts or being actively on call for 24 h, in comparison to individuals working in peripheral healthcare service facilities who work during office hours or perform passive on-call duties. Even if individuals are required to stay for extra hours after work, they do not need to stay overnight at their workplace. COVID-19 also infected HCWs or HCWs that are in close contact with a COVID-19-positive case, and this forces the staff to be quarantined. These work absences, even in small numbers, may lead to double shifts and increased days of active on-call duties that might disrupt the sleep patterns and family-time arrangements of workers, thus exacerbating mental health problems. This situation would also affect individuals required and obligated to be absent from the workplace, as they might feel guilty and irresponsible [37].

All COVID-19 patients in Malaysia were admitted to hospitals, regardless of their clinical stage during this phase of the pandemic, thus increasing the workloads and possibly increasing the risk of HCWs becoming infected due to greater exposure to and contact with patients. The HCWs need to use full-suit PPE to prevent exposure to patients. This is known to be very uncomfortable and tiring, especially in the hot climate of Malaysia.

A study conducted in Finland found that hospital employees faced a series of work-related stress and anxiety problems due to their unfamiliarity to the new situation that changed the usual protocols and work routines, and not because of a realistically high chance of becoming infected at work [38]. Work procedures, such as doffing and donning, designated the zoning facilities, especially for COVID-19 cases, diagnostic criteria, and treatment guidelines in the hospital in Malaysia, which rapidly changed due to new findings and discoveries, compared to the work process in peripheral healthcare service facilities. Working in a hospital may impose greater risks for HCWs to become infected, as they are in direct contact with infected patients during the diagnostic and treatment processes [39]. These uncertainties and risks of infections may explain the results for this study, in which anxiety prevalence is much higher than depression and stress.

4.2.3. Education Level

Having a degree or postgraduate education is associated with a higher risk of stress among HCWs. This category of workers mainly represents medical officers and specialists, including public health practitioners, dentists, pharmacists, and science officers, who are more likely to be officers in managerial and decision-making positions in the health system. Health professionals with managerial positions have higher stress symptoms, job dissatisfaction, and emotional demands [40]. Increased work demands may prolong the working hours for this group without monetary compensation or adequate recovery times, compared to other work categories, which may benefit from overtime payments. The uncertainty of the management of cases and close contacts with frequent changes in standard operating procedures may also contribute to stress among administrators at the middle and top levels, and clinical managers. However, the lower precision of the finding indicated by the wide CI was contributed to by a smaller number (222 (22.6%)) of HCWs with a degree/postgraduate education, compared to 759 (77.4%) who had no degree/postgraduate education.

4.3. Implications and Recommendations

The findings of the study suggest the need for administrative management, considerations of work-task rotations for employees, and the provision of flexible hours or time off.

Annual leave for employees to rest and recover is a good way of releasing the workload pressure, especially with the easing of restrictions.

Mental health and psychological support service (MHPSS) and peer-support activities should be prioritized, especially for junior staff, those working in the hospital, and the managerial and professional groups. Continuing medical education (CME) should be performed on a regular basis, updating workers during the pandemic.

4.4. Strength and Limitation

The sample of this study is comprehensively represented by HCWs from all levels of worker categories and all departments of health facilities. This study can be easily replicated by using other sampling frames or different work settings. A two-month sampling frame can ensure an extensive portrayal of the mental health of HCWs during the relaxation of COVID-19 restrictions.

This study was based on secondary data obtained from healthcare worker surveillance during the RMCO only; thus, a comparison of mental health status between different phases of the pandemic MCO could not be performed. Nevertheless, in the last two years, the understanding and management of the coronavirus have significantly changed. A self-reported DASS-21 questionnaire was used, which is do not provide a clinical diagnosis as it is only detects subclinical factors and reflects the underlying continuity of the severity of symptoms, so this DAS may not reflect the actual occurrence among HCWs. This study was unable to verify health status with medical records, as secondary data were anonymous as part of ensuring the privacy and confidentiality of the responders. The actual effect of the pandemic on the mental health of HCWs could not be proved as the cross-sectional study design simultaneously assessed the exposure and outcomes, for which previous mental health screenings before the pandemic were not available for comparison.

The HCWs may have also already undergone screening before the sampling period, and those who experienced symptoms would have undergo clinical intervention. Healthy worker bias was also possible as those who were affected were released from work as part of mental health intervention, and were thus not available for this investigation. Other events that can trigger depression, anxiety, or stress unrelated to the job and its environment, such as financial and family issues, as well as information on the duration of exposure to the confirmed and probable COVID-19 cases during work, were also not captured in this study.

5. Conclusions

The systematic, ongoing mental health surveillance helps to identify individuals at risk. The relaxation of the restriction movement control order as COVID-19 cases reduced showed a lower prevalence of DAS among HCWs. Individuals of a younger age, working in hospitals, or possessing degree/postgraduate qualifications were found to be more vulnerable, which translated to those working directly with COVID-19 patients, and individuals with more responsibility in the management sector were the most affected. A more comprehensive study is needed to ascertain the effect of the pandemic and to explore the root cause and how to effectively manage the psychological implications of the pandemic in enhancing mental resilience among HCWs.

Author Contributions: Conceptualization, S.N. and N.A.Y.; Methodology, S.N. and N.A.Y.; Project administration, J.K. and A.H.I.; Resources, J.K. and A.H.I.; Software, S.N.; Supervision, N.A.Y.; Visualization, A.D.; Writing—original draft, S.N.; Writing—review and editing, S.N., N.A.Y., J.K., A.H.I. and A.D. All authors have read and agreed to the published version of the manuscript.

Funding: This research received no external funding.

Institutional Review Board Statement: The study was conducted in accordance with the Declaration of Helsinki, and approved by the Ethics Committee of Universiti Sains Malaysia (protocol code USM/JEPeM/20110582 and date of approval on 28 January 2021) and Ethics Committee of Ministry of Health Malaysia (protocol code NNMR-20-2543-57241 and date of approval on 14 December 2020).

Informed Consent Statement: Informed consent was obtained from all subjects involved in the study.

Data Availability Statement: Not applicable.

Acknowledgments: We would like to thank the Director General of Health Malaysia for his permission to publish this article. This study was conducted in collaboration with the Department of Community Medicine, School of Medical Sciences Universiti Sains Malaysia, Non-communicable Disease Unit, Kedah State Health Department. We would like to thank them for their support and assistance in this research.

Conflicts of Interest: The authors declare no conflict of interest.

References

1. Guo, Y.-R.; Cao, Q.-D.; Hong, Z.-S.; Tan, Y.-Y.; Chen, S.-D.; Jin, H.-J.; Tan, K.-S.; Wang, D.-Y.; Yan, Y. The origin, transmission and clinical therapies on coronavirus disease 2019 (COVID-19) outbreak—An update on the status. *Mil. Med Res.* **2020**, *7*, 11. [CrossRef] [PubMed]
2. WHO. Statement on the Second Meeting of the International Health Regulations. Emergency Committee Regarding the Outbreak of Novel Coronavirus (2019-nCoV). 2005. Available online: https://www.who.int/news/item/30-01-2020-statement-on-the-second-meeting-of-the-international-health-regulations-(2005)-emergency-committee-regarding-the-outbreak-of-novel-coronavirus-(2019-ncov) (accessed on 30 November 2020).
3. WHO. WHO Coronavirus (COVID-19) Dashboard. Available online: https://covid19.who.int/ (accessed on 14 April 2021).
4. WHO. COVID-19 Situation Overview in Malaysia. Available online: https://www.who.int/docs/default-source/wpro---documents/countries/malaysia/coronavirus-disease-(covid-19)-situation-reports-in-malaysia/situation-report-malaysia-23-april-2020-final.pdf?sfvrsn=22ad02ca_6 (accessed on 15 December 2020).
5. Ahmad, N.A.; Chong, Z.L.; Abd Rahman, S.; Ghazali, M.H.; Nadzari, E.E.; Zakiman, Z.; Redzuan, S.; Md Taib, S.; Kassim, M.S.A.; Wan Mohamed Noor, W.N. First Local Transmission Cluster of COVID-19 in Malaysia: Public Health Response. *Int. J. Travel Med. Glob. Health* **2020**, *8*, 124–130. [CrossRef]
6. Bunyan, J. PM: Malaysia under movement control order from Wed until March 31, all shops closed except for essential services. *The Malay Mail*, 16 March 2020.
7. The Associated Press. Malaysia Closes Borders, Shuts Most Businesses in Lockdown. Available online: https://abcnews.go.com/Health/wireStory/malaysia-bans-mass-gatherings-shuts-businesses-69620354 (accessed on 10 October 2020).
8. Tang, A. Malaysia Announces Movement Control Order after Spike in COVID-19 Cases. Available online: https://www.thestar.com.my/news/nation/2020/03/16/malaysia-announces-restricted-movement-measure-after-spike-in-covid-19-cases (accessed on 29 November 2020).
9. Safurah, J.; Kamaliah, M.H.; Khairiyah, A.M.; Nour, H.O.; Healy, J. Malaysia Health System Review. *Malays. Health Syst. Rev.* **2013**, *3*, 103.
10. Mohd Fauzi, M.F.; Mohd Yusoff, H.; Muhamad Robat, R.; Mat Saruan, N.A.; Ismail, K.I.; Mohd Haris, A.F. Doctors' Mental Health in the Midst of COVID-19 Pandemic: The Roles of Work Demands and Recovery Experiences. *Int. J. Environ. Res. Public Health* **2020**, *17*, 7340. [CrossRef]
11. Ibrahim, N.b.; Hashim, N.A.b.M.; Ramasamy, S.; Kaur, K. Mental Health and Psychosocial Support in COVID-19. Guidelines COVID-19 Management. No. 5/2020. Available online: http://covid-19.moh.gov.my/garis-panduan/garis-panduan-kkm/Annex_33_Mental_health_and_Psychosocial_support_23032020.pdf (accessed on 15 December 2020).
12. Shahruddin, S.A.; Saseedaran, P.; Salleh, A.D.; Azmi, C.A.A.; Alfaisal, N.H.I.M.; Abdullah, M.R. Prevalence and Risk Factors of Stress, Anxiety and Depression among House Officers in Kota. *Educ. Med. J.* **2016**, *8*, 31–40. [CrossRef]
13. Ghawadra, S.F.; Abdullah, K.L.; Choo, W.Y.; Phang, C.K. Psychological distress and its association with job satisfaction among nurses in a teaching hospital. *J. Clin. Nurs.* **2019**, *28*, 4087–4097. [CrossRef]
14. Ismail, M.; Lee, K.Y.; Sutrisno Tanjung, A.; Ahmad Jelani, I.A.; Abdul Latiff, R.; Abdul Razak, H.; Ahmad Shauki, N.I. The prevalence of psychological distress and its association with coping strategies among medical interns in Malaysia: A national-level cross-sectional study. *Asia-Pac. Psychiatry* **2020**, *13*, e12417. [CrossRef]
15. Liu, Z.; Han, B.; Jiang, R.; Huang, Y.; Ma, C.; Wen, J.; Zhang, T.; Wang, Y.; Chen, H.; Ma, Y. Mental Health Status of Doctors and Nurses During COVID-19 Epidemic in China. *SSRN Electron. J.* **2020**, *1*, 1–10. [CrossRef]
16. Chew, Q.; Chia, F.L.-A.; Ng, W.K.; Lee, W.C.I.; Tan, P.L.L.; Wong, C.S.; Puah, S.H.; Shelat, V.G.; Seah, E.-J.D.; Huey, C.W.T.; et al. Perceived Stress, Stigma, Traumatic Stress Levels and Coping Responses amongst Residents in Training across Multiple Specialties during COVID-19 Pandemic—A Longitudinal Study. *Int. J. Environ. Res. Public Health* **2020**, *17*, 6572. [CrossRef]
17. Yusoff, M.S.B.; Ying Jie, T.; Esa, A.R. Stress, Stressors And Coping Strategies Among House Officers In A Malaysian Hospital. *ASEAN J. Psychiatry* **2011**, *12*, 85–94.
18. Lebares, C.C.; Guvva, E.V.; Ascher, N.L.; O'Sullivan, P.S.; Harris, H.W.; Epel, E.S. Burnout and Stress Among US Surgery Residents: Psychological Distress and Resilience. *J. Am. Coll. Surg.* **2018**, *226*, 80–90. [CrossRef]

19. Alshekaili, M.; Hassan, W.; Al Said, N.; Al Sulaimani, F.; Jayapal, S.K.; Al-Mawali, A.; Chan, M.F.; Mahadevan, S.; Al-Adawi, S. Factors associated with mental health outcomes across healthcare settings in Oman during COVID-19: Frontline versus non-frontline healthcare workers. *BMJ Open* **2020**, *10*, e042030. [CrossRef]
20. Razali, S.; Abdullah, M.N.; Rahman, N.A.; Azhar, N.A.; Yaacob, S.S. Psychological Distress of Health Care Workers in the Main Admitting Hospital and Its Related Health Care Facilities in Malaysia during the COVID-19 Pandemic. *Asia-Pac. J. Public Health* **2021**, *33*, 667–669. [CrossRef]
21. MOH. Senarai Klinik Kesihatan Negeri Kedah. Available online: https://fh.moh.gov.my/v3/index.php/direktori/direktori-klinik/kedah/klinik-kesihatan/ (accessed on 11 January 2021).
22. Lovibond, S.H.; Lovibond, P.F. Manual for the Depression Anxiety Stress Scales. Available online: www.psy.unsw.edu.au/dass/ (accessed on 10 November 2020).
23. Musa, R.; Fadzil, M.A.; Zain, Z. Translation, validation and psychometric properties of Bahasa Malaysia version of the Depression Anxiety and Stress Scales (DASS). *ASEAN J. Psychiatry* **2007**, *8*, 82–89.
24. WHO. *The World Health Report 2006: Working Together for Health*; WHO: Geneva, Switzerland, 2006.
25. WHO. Classifying Health Workers: Mapping Occupations to the International Standard Classification. Available online: http://www.who.int/hrh/statistics/workforce_statistics/en/ (accessed on 30 November 2020).
26. WHO. Public Health Appropriate Body-Mass Index for Asian Populations and Its Implications for Policy and Intervention Strategies. Available online: https://www.who.int/nutrition/publications/bmi_asia_strategies.pdf (accessed on 10 January 2021).
27. Chew, N.; Lee, G.K.H.; Tan, B.Y.Q.; Jing, M.; Goh, Y.; Ngiam, N.J.H.; Yeo, L.L.L.; Ahmad, A.; Ahmed Khan, F.; Napolean Shanmugam, G.; et al. A multinational, multicentre study on the psychological outcomes and associated physical symptoms amongst healthcare workers during COVID-19 outbreak. *Brain Behav. Immun.* **2020**, *88*, 559–565. [CrossRef]
28. Kiat, S.S.; Liang, L.B.; Ramli, S.R.Z.; Matrikulasi, S.; Abdul Muti, N.H.; Unimas, S.A.S.H.; Abdul Rahman, N.M.A.; Balachandran, J.P.; Hamzah, A.S. Psychological symptoms among healthcare workers handling COVID-19 patients. *Med. J. Malays.* **2021**, *76*, 138–144.
29. Mazza, M.G.; De Lorenzo, R.; Conte, C.; Poletti, S.; Vai, B.; Bollettini, I.; Melloni, E.M.T.; Furlan, R.; Ciceri, F.; Rovere-Querini, P.; et al. Anxiety and depression in COVID-19 survivors: Role of inflammatory and clinical predictors. *Brain Behav. Immun.* **2020**, *89*, 594–600. [CrossRef]
30. Liang, Y.; Chen, M.; Zheng, X.; Liu, J. Screening for Chinese medical staff mental health by SDS and SAS during the outbreak of COVID-19. *J. Psychosom. Res.* **2020**, *133*, 1101–1102. [CrossRef]
31. Casey, D.A. Depression in Older Adults: A Treatable Medical Condition. *Prim. Care Clin. Off. Pract.* **2017**, *44*, 499–510. [CrossRef]
32. AlHaidar, A.M.; AlShehri, N.A.; AlHussaini, M.A. Family Support and Its Association with Glycemic Control in Adolescents with Type 1 Diabetes Mellitus in Riyadh, Saudi Arabia. *J. Diabetes Res.* **2020**, *2020*, 5151604. [CrossRef]
33. Chung, J.; Park, J.; Cho, M.; Park, Y.; Kim, D.; Yang, D.; Yang, Y. A study on the relationships between age, work experience, cognition, and work ability in older employees working in heavy industry. *J. Phys. Ther. Sci.* **2015**, *27*, 155–157. [CrossRef]
34. Chua, K.B. Nipah virus outbreak in Malaysia. *J. Clin. Virol. Off. Publ. Pan Am. Soc. Clin. Virol.* **2003**, *26*, 265–275. [CrossRef]
35. Peeri, N.C.; Shrestha, N.; Siddikur Rahman, M.; Zaki, R.; Tan, Z.; Bibi, S.; Baghbanzadeh, M.; Aghamohammadi, N.; Zhang, W.; Haque, U. The SARS, MERS and novel coronavirus (COVID-19) epidemics, the newest and biggest global health threats: What lessons have we learned? *Int. J. Epidemiol.* **2021**, *49*, 717–726. [CrossRef]
36. Mackenzie, C.R.; Keuskamp, D.; Ziersch, A.M.; Baum, F.E.; Popay, J. A qualitative study of the interactions among the psychosocial work environment and family, community and services for workers with low mental health. *BMC Public Health* **2013**, *13*, 796. [CrossRef]
37. Cabarkapa, S.; King, J.A.; Ng, C.H. The psychiatric impact of COVID-19 on healthcare workers. *Aust. J. Gen. Pract.* **2020**, *49*, 791–795. [CrossRef]
38. Mattila, E.; Peltokoski, J.; Neva, M.H.; Kaunonen, M.; Helminen, M.; Parkkila, A.-K. COVID-19: Anxiety among hospital staff and associated factors. *Ann. Med.* **2021**, *53*, 237–246. [CrossRef]
39. Shih, F.-J.; Gau, M.-L.; Kao, C.-C.; Yang, C.-Y.; Lin, Y.-S.; Liao, Y.-C.; Sheu, S.-J. Dying and caring on the edge: Taiwan's surviving nurses' reflections on taking care of patients with severe acute respiratory syndrome. *Appl. Nurs. Res.* **2007**, *20*, 171–180. [CrossRef]
40. Peter, K.A.; Schols, J.M.G.A.; Halfens, R.J.G.; Hahn, S. Investigating work-related stress among health professionals at different hierarchical levels: A cross-sectional study. *Nurs. Open* **2020**, *7*, 969–979. [CrossRef]
