# Supplementary material for: Mental health status and related factors influencing healthcare workers during the COVID-19 pandemic: A systematic review and meta-analysis
Source: PLoS One. 2024 Jan 19;19(1):e0289454. doi: 10.1371/journal.pone.0289454 (PMC10798549; doi:10.1371/journal.pone.0289454)
Supplement: S1 Data — (ZIP) [file pone.0289454.s011.zip › literatures/12.pdf]

COVID-19 and Anxiety amongst Doctors: A Pakistani Perspective

Abdul Rehman Arshad[1] and Farrukh Islam[2]

[1]*Department of Nephrology, Combined Military Hospital Peshawar, Peshawar Cantt, Pakistan*
[2]*Department of Nephrology, Combined Military Hospital Malir, Karachi, Pakistan*

ABSTRACT

Objective: To assess anxiety in Pakistani doctors in context of COVID-19 pandemic and evaluate possible causes.
Study Design: Cross-sectional study.
Place and Duration of Study: Departments of Nephrology, Combined Military Hospital Peshawar and Combined Military Hospital Malir, Karachi, during March 2020.
Methodology: Doctors working in different parts of Pakistan were approached online through snowball sampling technique. Those with history of psychiatric disorders were excluded. They were administered a questionnaire including Seven-item Generalised Anxiety Disorder Scale (GAD-7). Reasons why they felt anxious were also explored.
Results: Responses from 431 doctors, including 238 (55.2%) males, were evaluated. Most of them were younger than 30 years (286; 66.4%), in training (335; 77.7%), and working in public hospitals (347; 80.5%). Mild, moderate and severe anxiety was seen in 120 (27.8%), 103 (23.9%) and 42 (9.7%) doctors, respectively. Median score on GAD-7 was 6 (interquartile range = 3 - 11). Lady doctors had higher scores than males (7 *vs.* 5; p=0.024). No significant differences in scores were found amongst doctors from different workplaces or of different professional status. A greater proportion of females had anxiety as compared to males (67.9% *vs.* 56.3%; p=0.014). Frequency of anxiety was not different amongst doctors of different professional status, types of workplace and amongst different age groups. Commonest reasons for anxiety were lack of personal protective equipment (83.8% doctors); and the fear that they could spread infection to family members (79.8% doctors).
Conclusion: COVID-19 pandemic has had a major impact on the psychological well-being of doctors. Greater attention needs to be paid towards lady doctors to ensure their mental well-being.

Key Words: *Anxiety disorders, Pandemic, Personal protective equipment, Workforce.*

How to cite this article: Arshad AR, Islam F. COVID-19 and Anxiety amongst Doctors: A Pakistani Perspective. *J Coll Physicians Surg Pak* 2020; **30(Supp)**:S106-S109.

INTRODUCTION

Coronavirus Disease 2019 (COVID-19) has grabbed the whole world over the last several months. What initially appeared to be a mild flu-like illness, affecting just a city, has spread like wildfire, affecting each and every sphere of life globally. As of now, there are more than 13 million people affected. With 579,319 confirmed deaths, the pandemic is at nowhere near end.[1] This unprecedented situation has never been faced by mankind over generations. And, hence, human response to this catastrophe has also never been assessed or gauged in the past.

Correspondence to: Dr. Abdul Rehman Arshad, Department of Nephrology, Combined Military Hospital Peshawar, Peshawar Cantt, Pakistan
E-mail: maj.abdulrehman@gmail.com

....
Received: April 10, 2020; Revised: July 02, 2020;
Accepted: August 13, 2020
DOI: https://doi.org/10.29271/jcpsp.2020.Supp.S106

Healthcare workers (HCW) are at the frontline to fight the pandemic. Various concerns including fear of contact, overwhelming workload, scarcity of personnel protective equipment (PPE), lack of specific treatment, social stigma and uncertainty regarding the disease are factors adversely affecting the medical staff. They are at high risk to develop psychological problems, including stress, anxiety and depression. In 2003, severe acute respiratory syndrome (SARS) outbreak, psychological problems in HCW were identified.[2-4] High levels of stress, anxiety and depressive symptoms were seen then, although the scale of damage due to SARS and its fear was much less as compared to current situation.[5] Similar concerns related to physical and mental health problems of HCW are arising all over the world; and our country is not different. Medical profession in itself predisposes individuals to anxiety related symptoms; and in one of a local study, it was found that mild to moderate anxiety was found in 34% and severe anxiety in 7.2% among doctors working in a tertiary care hospital in Lahore.[6]

Healthcare authorities are so much intensely involved in limiting the spread of COVID-19 that some of the related issues remain overlooked. Protecting and caring for the frontline force in this life-threatening situation needs to be highlighted and addressed

at an earliest so as to keep the integrity of the health system intact. While there is an increasing focus on protection from contacting the infection among doctors and nurses who care for patients with COVID-19, there has been limited research to examine the role of anxiety in contributing to these occupational stressors.

Therefore, this study was planned to get an estimate of the problem scientifically, so that appropriate interventions could be made timely, and address the issue effectively. From the policy--making perspective, the results of this study would increase awareness amongst administrators and help identify areas for improvement to mitigate the psychological impact of COVID-19 infection.

The aim of this study was to evaluate prevalence and severity of anxiety among HCW treating patients with COVID-19 by quantifying the magnitude of symptoms of anxiety, insomnia, and distress; and by analysing potential risk factors associated with these symptoms.

METHODOLOGY

This cross-sectional study was carried out at the Departments of Nephrology, Combined Military Hospital Peshawar and Combined Military Hospital Malir, Karachi during the last week of March 2020. The study protocol was approved by Ethics Review Board of Combined Military Hospital Peshawar (Approval No. 13, dated 20 March 2020). Doctors of any professional status and gender, working in Pakistan, who were willing to participate, were selected through snowball sampling technique. Invitations for participation in this study were sent *via* WhatsApp and email messages to doctors amongst close contacts of the authors of this study. Those doctors were also asked to extend invitations for participation to other doctors in their contact lists. Doctors working outside Pakistan and those with known history of psychiatric disorders (based on disclosure by the respondents) were excluded.

The questionnaire was administered in English language online using Google forms. It consisted of three parts. The first one was concerning the basic demographic data of the participants. In the second step, questions included in the 7-item Generalized Anxiety Disorder Scale (GAD-7) were asked. Lastly, we explored reasons why the doctors felt anxious during this time period. The respondents were asked to choose from eight common/possible themes as they thought relevant in their case. Details of these questions are provided in the Results section. Apart from e-mail addresses, no personal information that could help identify the respondents was recorded. All subjects filled out the questionnaires at their own convenience. All the responses were finally downloaded in the form of a single MS Excel file from the Google Drive at the expiry of the data collection window.

Data, particularly demographic data of the respondents, was reviewed before analysis. In case of multiple responses from any doctor, the first one was retained and the rest were deleted. Data was analysed using IBM SPSS Statistics for Windows, Version 20.0 (IBM Corp, Armonk, NY). Categorical variables were expressed as frequencies and percentages. Since the GAD-7 scores had a non-parametric distribution, they were described as median and interquartile range. Independent samples Mann-Whitney U-test was used to compare the difference in median - GAD-7 scores amongst different groups. Chi-square test was done to determine the significance of different demographic factors associated with anxiety (assessed as a categorical variable). *P-values* <0.05 were considered significant.

RESULTS

A total of 464 doctors responded by filling the questionnaire. Out of these, 33 responses were deleted, 23 being duplicate and another 10 from doctors working outside Pakistan. Baseline characteristics of the remaining 431 doctors are shown in Table I. Median score on GAD-7 scale was 6 (interquartile range = 3 - 11). Mild, moderate and severe grades of anxiety were seen in 120 (27.84%), 103 (23.90%) and 42 (9.74%) patients, respectively; whereas, 166 (38.52%) patients did not have anxiety.

Table I: Baseline characteristics.

Parameter		Number of doctors
Age	Up to 30 years	286 (66.36%)
	31- 40 years	121 (28.07%)
	41- 50 years	21 (4.87%)
	>50 years	3 (0.70%)
Gender	Male	238 (55.22%)
	Female	193 (44.78%)
Professional status	House officer / resident	335 (77.73%)
	Consultant	96 (22.27%)
Type of practice	Public	347 (80.51%)
	Private	84 (19.49%)
Province of practice	Punjab	179 (41.53%)
	Sindh	103 (23.90%)
	KPK	90 (20.88%)
	Balochistan	22 (5.10%)
	Islamabad (Federal area)	21 (4.87%)
	Azad Kashmir & Northern Areas	8 (1.86%)
	Not declared	8 (1.86%)

Amongst males, 134 (56.30%) had anxiety, in contrast to 131 (67.88%) out of 193 females. This difference was statistically significant (p=0.014). The reported frequency of anxiety was not different amongst doctors of different professional status, type of workplace and amongst different age groups. Details are shown in Figure 1.

Median GAD-7 score was 5 (interquartile range 2- 11 years) amongst male doctors and 7 (interquartile range 4-11 years) amongst female doctors. This difference was statistically significant (p=0.024). However, no significant differences in median GAD-7 scores were found amongst doctors from different workplaces (6 (interquartile range 3- 11 years) amongst doctors in public sectors and 6 (interquartile range 3-11 years) amongst doctors in private sector; p= 0.771) or of different professional status (6 (interquartile range 3- 11 years) amongst house officers/ residents and 5 (interquartile range 1-12 years) amongst consultants; p=0.247).

Different predisposing factors for anxiety are shown in Figure 2. Majority (n: 241; 55.92%) found that the problems posed by COVID-19 had made it somewhat difficult for them to take care of things at home, or getting along with other people. Sixty (13.92%) found it very difficult, and 12 (2.78%) found it extremely difficult. Rest of the patients (n: 118; 27.38%) did not perceive any impact of the disease on relationships.

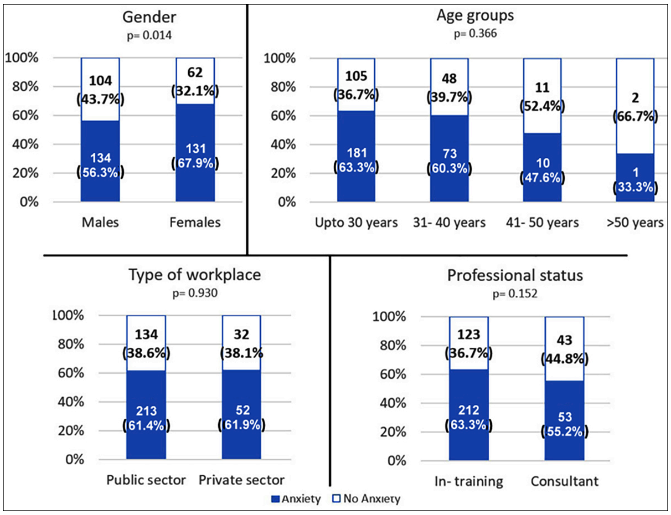

Figure 1: Determinants of anxiety amongst doctors. Data labels represent number of respondents and percentages within group.

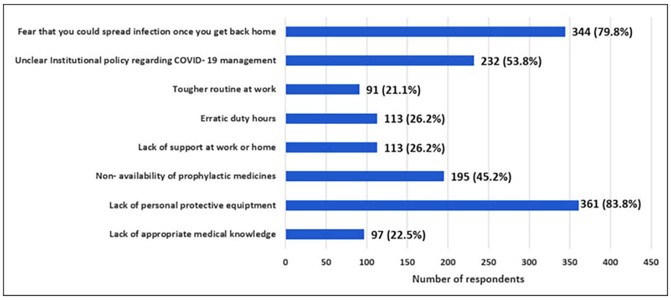

Figure 2: Possible reasons for anxiety amongst doctors.

DISCUSSION

Anxiety is generally described as a feeling of helplessness or fear, often related to a sense of losing control that frequently accompanies life with illness, as well as being generated by death-related factors. It is a common response to adverse life events and if excessively manifested can lead to long term psychological implications. From a mental health perspective, COVID-19 has been a major concern for doctors and the general population alike. More than 0.1 million people have already been infected with COVD-19 globally. This includes more than 3300 HCW from China.[7] The situation is far worse in Italy, with at least 61 deaths reported amongst HCW so far. Protecting HCW from this disease is not only important for their own selves, but also to ensure that effective medical care continues to be provided to all patients.

GAD-7 questionnaire was used in this study. This questionnaire was developed in 2006 primarily to screen patients for anxiety in primary care settings. Numerous studies have since been done to assess its reliability and validity, with encouraging results. Johnson, *et al.* have demonstrated good internal consistency (Cronbach's alpha 0.83- 0.93), convergent validity (r=0.76) as well as discriminant validity (Cohen's d=0.5).[8] Similar results were reported by Kertz *et al*. as well.[9] We used this questionnaire because of its strong psychometric properties and ease of administration in an online medium.

During this current pandemic, a study done on 1257 HCW in China found 45% respondents to have anxiety, based on GAD-7.[10] This was more marked in women, consistent with our results. Amongst the respondents of this study, there was an increased trend towards higher GAD-7 scores amongst junior doctors. They are generally the first ones to establish contact with suspected/-confirmed cases and this predisposition could simply push up their anxiety levels. Difference from GAD-7 scores amongst consultants was statistically insignificant.

Two major reasons were found for anxiety amongst the respondents: lack of PPE and the risk of transmitting infection to their family members. Lack of PPE is not just an issue for developing countries like Pakistan only. Shortage of face masks, for instance, has arisen as the epidemic led to seized production in China, the country that contributes the most to production.[11] British Medical Association has already recognised considerable risk to doctors because of shortage of PPE.[12] In the current crisis situation, Centers for Disease Control and Prevention has recommended limited reuse of respirators and masks that are actually meant for one-time use only.[13] Pakistan has already instituted measures to conserve PPEs as suggested elsewhere; and an increase in local production of face masks and hand sanitizers is being contemplated.[14] There is a dire need for the regulatory bodies to ensure equitable distribution of resources.

Concerns raised by doctors about the risk of transmission to family members are truly valid. There have been numerous reports of transmission to household contacts.[15] Healthcare facilities in Pakistan have already downsized the services, with all outdoor clinics, elective admissions and procedures having been cancelled. Duties for the hospital staff have also been reduced and staggered to reduce the possibility of cumulative exposure to the virus. The junior colleagues need to be educated about possible preventive measures at home, including removing shoes, changing clothes as well as taking a shower immediately after returning home. They could also be told about separate living spaces at home. It is important to realize that the evidence to support all this is weak. Nevertheless, it is important for doctors to monitor themselves for signs of illness and immediately disengage from patient care, should they have problems themselves.

The major strength of this study has been the inclusion of a large diverse population of doctors, covering all geographical regions of the country, different professional status and wide range of age as well as targeting both public and private healthcare setups. This would make the results applicable to almost the entire country. However, it is important to recognise as the pandemic is still continuing to evolve in Pakistan. The incidence of anxiety amongst doctors might actually increase proportionate to the rise in number of COVID-19 confirmed cases.

We had asked doctors with known psychiatric disorders not to fill this form. Still, we cannot completely negate the fact that some of the respondents might actually be having anxiety prior to this pandemic, thus magnifying the figures reported above. It is very much possible for the general population to be anxious because of misinformation as well as the effects of lockdown. The lack of a control group makes it difficult to say whether the degree of anxiety documented in this study is only related to workplace-based stressors only. This observational study is limited by our ability to establish a cause-effect relationship because of its design. The short duration as well as the lack of follow-up are also important considerations.

Nevertheless, being one of the very first studies to be reported from Pakistan, the results would serve as a curtain-raiser for concerned authorities to implement measures to help mitigate the problems faced by the brave soldiers of this country. It is important that the medical administrators at all levels make necessary efforts to ensure availability of PPE for healthcare workers, provide training opportunities to doctors at all levels and arrange for treatment guidelines specific to the needs and the available resources at different hospitals. This would be beneficial in alleviating anxiety.

CONCLUSION

COVID-19 pandemic has had a major impact on the psychological well-being of doctors in Pakistan. There is a need to keep them safe during this difficult time so that they could continue to fight with full force. A deliberate effort needs to be made to ensure adequate strategic planning and distribution of resources to give the doctors a peace of mind. More attention needs to be paid towards female doctors to ensure their mental well-being.

ETHICAL APPROVAL:

The study protocol was approved by Ethics Review Board of Combined Military Hospital Peshawar prior to initiation of data collection (Approval No. 13 dated 20 March 2020).

PATIENTS' CONSENT:

Informed consents were obtained from all respondents to publish the data.

CONFLICT OF INTEREST:

The authors declared no conflict of interest.

AUTHORS' CONTRIBUTION:

ARA: Designed the study, acquired data, critically revised the manuscript, approved the final version, agreed to be accountable for all aspects.

FI: Conceived the study, analysed data, drafted the manuscript, approved the final version, agreed to be accountable for all aspects.

REFERENCES

1. Coronavirus disease (COVID-19). World Health Organization. http://www.who.int/emergencies/diseases/novel-coronavirus-2019. (Accessed on 16/7/2020)
2. Maunder R, Hunter J, Vincent L, Bennett J, Peladeau N, Leszcz M, *et al*. The immediate psychological and occupational impact of the 2003 SARS outbreak in a teaching hospital. *CMAJ* 2003; **168(10)**:1245-51.
3. Bai Y, Lin CC, Lin CY, Chen JY, Chue CM, Chou P. Survey of stress reactions among health care workers involved with the SARS outbreak. *Psychiatr Serv* 2004; **55(9)**:1055-7. doi: 10.1176/appi.ps.55.9.1055.
4. Chua SE, Cheung V, Cheung C, McAlonan GM, Wong JWS, Cheung EPT, *et al*. Psychological effects of the SARS outbreak in Hong Kong on high-risk health care workers. *Can J Psychiatry* 2004; **49(3)**:391-3. doi: 10.1177/ 070674370404900609.
5. Lee AM, Wong JG, McAlonan GM, Cheung V, Cheung C, Sham PC, *et al*. Stress and psychological distress among SARS survivors 1 year after the outbreak. *Can J Psychiatry* 2007; **52(4)**:233-40. doi: 10.1177/070674370705200405.
6. Atif K, Khan HU, Ullah MZ, Shah FS, Latif A. Prevalence of anxiety and depression among doctors; the unscreened and undiagnosed clientele in Lahore, Pakistan. *Pak J Med Sci* 2016; **32(2)**:294-8. doi: 10.12669/pjms.322.8731.
7. The Lancet. COVID-19: Protecting health-care workers. *Lancet* 2020; **395(10228)**:922. doi: 10.1016/ S0140-6736(20)30644-9.
8. Johnson SU, Ulvenes PG, Øktedalen T, Hoffart A. Psychometric properties of the general anxiety disorder 7-Item (GAD-7) scale in a heterogeneous psychiatric sample. *Front Psychol* 2019; 10:1713. doi: 10.3389/fpsyg.2019.01713.
9. Kertz S, Bigda-Peyton J, Bjorgvinsson T. Validity of the generalized anxiety disorder-7 scale in an acute psychiatric sample. *Clin Psychol Psychother* 2013; **20(5)**:456-64. doi: 10.1002/cpp.1802.
10. Lai J, Ma S, Wang Y, Zhongxiang Cai, Jianbo Hu, Ning Wei, *et al*. Factors associated with mental health outcomes among health care workers exposed to coronavirus disease 2019. *JAMA Netw Open* 2020; **3(3)**:e203976. doi: 10.1001/jamanetworkopen.2020.3976.
11. Ranney ML, Griffeth V, Jha AK. Critical supply shortages: The need for ventilators and personal protective equipment during the Covid-19 pandemic. *N Engl J Med* 2020; **382(18)**:e41. doi: 10.1056/NEJMp2006141.
12. Iacobucci G. Covid-19: Doctors still at "considerable risk" from lack of PPE, BMA warns. *BMJ* 2020; **368**:m1316. doi: 10.1136/bmj.m1316.
13. Recommended guidance for extended use and limited reuse of N95 filtering facepiece respirators in healthcare settings. Centers for Disease Control and Prevention, The National Institute for Occupational Safety and Health http://www.cdc.gov/niosh/topics/hcwcontrols/recommendeduidanceextuse.html. (Accessed on 4/2/2020).
14. Livingston E, Desai A, Berkwits M. Sourcing personal protective equipment during the COVID-19 pandemic [published online ahead of print, 2020 Mar 28]. *JAMA* 2020; doi:10.1001/jama.2020.5317.
15. Bai Y, Yao L, Wei T, Tian F, Jin D, Chen L, *et al*. Presumed asymptomatic carrier transmission of COVID-19. *JAMA* 2020; **323(14)**:1406-7. doi: 10.1001/jama.2020.2565.
