# Supplementary material for: Mental health status and related factors influencing healthcare workers during the COVID-19 pandemic: A systematic review and meta-analysis
Source: PLoS One. 2024 Jan 19;19(1):e0289454. doi: 10.1371/journal.pone.0289454 (PMC10798549; doi:10.1371/journal.pone.0289454)
Supplement: S1 Data — (ZIP) [file pone.0289454.s011.zip › literatures/120.pdf]

**Odikpo *et al*., Afr., J. Infect. Dis. (2021) 15 (2): 16-23**
https://doi.org/10.21010/ajid v15i2. 4

# KNOWLEDGE AND REASONS FOR ANXIETY AMONG NURSES TOWARDS COVID -19 IN NIGERIA

**Linda Chihurumnanya Odikpo[1*]; Helen Ogechi Abazie[2]; Duke Emon[3]; Mary Oluwafunmilola Mobolaji-Olajide[2]; Dorothy Dooshima Gbahabo[2]; Aisha Musa-Malikki[4]**

[1]Department of Nursing Science, Nnamdi Azikiwe University Nnewi Campus, Awka Nigeria. [2]Department of Nursing Science, Faculty of Clinical Sciences, College of Medicine, University of Lagos, Nigeria. [3]Department of Nursing Science, Faculty of Allied Medical Sciences, University of Calabar, Calabar, Nigeria. [4]Department of Nursing Science ABU Zaria.

**[*]Corresponding Author's E-mail:** lc.odikpo@unizik.edu.ng

***Article History***
*Received:* 14th Sept. 2020
*Revised Received:* 28th Jan. 2021
*Accepted:* 29th Jan 2021
*Published Online:* 18th March 2021

## Abstract

**Background:** Nurses in Nigeria and the whole world are facing an unprecedented severe level of anxiety in their professional and individual lives, compounded by not knowing what the future holds especially with regards to the present COVID-19 pandemic. This research is to evaluate the knowledge and reasons for anxiety toward COVID 19 among nurses in Nigeria.
**Materials and Methods:** An online cross-sectional quantitative survey that utilized a multistage sampling technique and data was collected with questionnaire instrument from 418 nurses using Google form for a period of eight weeks. Analysis of the result was with the aid of the Statistical Package for Social Science (SPSS) version 20 software. Descriptive data of participants was presented in tables while the test of the inferential data was with Chi-square at 95% level of significance ($p = 0.05$).
**Results:** The result revealed that 81.3% of the respondents are female, with a mean age of $37.81 \pm 8.21$ years and mean years of experience of $13.1 \pm 8.44$ years. One hundred and eighty (56.9%) of the respondents have good knowledge of COVID -19, with mean of $10.67 \pm 1.19$. 88.5% were anxious because they are front line workers and having direct contact with COVID-19 patients. The relationship between identified reasons for the anxiety experienced among Nigerian nurses and level of knowledge of COVID-19 were not significant ($p > 0.05$).
**Conclusion:** Although nurses in Nigeria are knowledgeable about the COVID-19, they have reasons for being anxious. Addressing the reasons for their anxiety will promote their physical and psychological well-being.

**Keywords:** Knowledge, Reasons, Anxiety, Nurses, COVID-19

**List of Abbreviations:** COVID-19**:** *C*oronavirus disease 2019, MERS- CoV: Middle East Respiratory Syndrome Coronavirus, SARS: Severe Acute Respiratory Syndrome.

## Introduction

Globally, there is a record of over 7.2 million cases of Coronavirus Disease-2019 (COVID -19) with over 408,794 deaths as of the time of this study (John Hopkins University Coronavirus Resource Center, 2020). In Africa, a record of an estimate of 190,767 cases and 5,344 deaths equally exist, and the number is still increasing. Nigeria reported its first case of COVID-19 on 27th February 2020 and have recorded 12,486 cases with over 300 deaths at the time of this study (Nigeria Center for Disease Control, 2020). COVID-19 is spread by human-to-human transmission through the droplet, faecal-oral, and direct contact and has an incubation period of 2-14 days (Bao-Liang *et al*., 2020).

Coronavirus is a zoonotic pathogen transmitted via animal-to-human and human-to-human interactions (Li *et al*., 2020). Multiple epidemic outbreaks of Severe Acute Respiratory Syndrome (SARS) occurred in 2002, with the death of approximately 800 patients, and in 2012, Middle East Respiratory Syndrome Coronavirus (MERS-CoV), with 860 deaths, also occurred (Shehab, 2019; Li *et al*., 2020). About eight years after the MERS-CoV epidemic, the current outbreak of COVID-19 in Wuhan City, Hubei Province, China, emerged as a global outbreak and significant public health issue (Lai *et al*., 2020).

COVID-19 has infected many healthcare providers, and some of them died while caring for patients with the disease. Nurses are among the first front line medical personnel caring for patients with COVID-19 all over the world, and Nigerian nurses are not left out. As posited by the International Council of Nurses (2020), information from 30 countries' National Nursing Associations puts

government figures, and media reports at over 90,000 of health workers believed to have been infected with COVID 19 and possibly twice that amid reports of a continuing shortage of personal protective equipment. Also, more than 260 nurses have lost their lives to the disease. Nurses are always in contact with patients, and this exposes them to infected cases in health care settings. Thus, nurses are at high risk of contracting COVID-19. Because of this risk, most nurses have an unprecedented level of anxiety at this moment that a common enemy, COVID-19, is being fought world over. Brittany *et al*. (2020) stated that nurses are facing unprecedented stressors in their professional and personal lives, compounded by uncertainty about the future especially those whose works require direct interface with COVID-19 patients, and fears including being put in the position to care for patients with an inadequate workforce or without life-saving personal protective equipment, which could lead to contracting the disease. The stressors extend to nurses working in other specialty areas, such as outpatient clinics, mental health/psychiatric facilities, and home care settings, who may worry about contracting COVID-19 from an asymptomatic patient.

Few studies have emerged on the knowledge and reasons for anxiety of nurses towards COVID-19. Most of the studies discussed the knowledge, the attitude of nurses and also psychological and mental effects of COVID-19 stressors for healthcare workers especially nurses but none said anything about the reasons for anxiety experienced by nurses, especially in Nigeria. Ogolodom *et al*. (2020) on Knowledge, Attitudes and Fears of Healthcare Workers Toward the Corona Virus Disease (COVID-19) Pandemic in South-South, Nigeria reported that majority of health workers were highly knowledgeable of the pandemic. Similarly, Huynh *et al*. (2020) stated that health workers in China showed good knowledge of COVID -19. Nemati *et al*. (2020) also assessed the knowledge and anxiety of Iranian nurses toward COVID 19 and discovered that nurses' level of anxiety is high, especially with regards to infection of their family members. Lai *et al*. (2020) found that a considerable portion of healthcare workers in direct contact with patients with COVID-19 in China reported symptoms of anxiety. It was reported by Li Z *et al*. (2020) that nurses working with patients who have COVID-19 exposure experienced much higher vicarious traumatization scores than those working in other facilities.

Anxiety and Psychological distress rates were significantly higher for nurses caring for COVID-19 patients, specifically in all of the studies (Brittany *et al*., 2020). Nurses are inquisitive about their welfare and that of their family members. They equally bothered about the possibility of getting infected and infecting others, including family members and colleagues, especially as there are many cases of deaths of their colleagues in many settings (Steve, 2020). Nurses with children seem to be more anxious regarding the care and safety of their children in the present period of the pandemic. Therefore, there is a need to support nurses who may be experiencing anxiety due to reasons peculiar to the situations around them. The support can come through offering verbal support, assuring them (nurses) that their feelings are normal while remaining nonjudgmental and not taking their negative feelings to heart, encouraging professional help, supporting a safe work environment and sharing information that could help to allay the anxiety of nurses (Brittany *et al*., 2020). Thus, the need for a study of this kind cannot be overemphasized especially in Nigeria where many health workers in private and public hospitals have been infected with patients disguising their symptoms and covering their travel history in order to receive medical attention and many have also lost their lives as a result of the disease. Therefore, this study assessed knowledge of COVID-19 and reasons for anxiety experienced towards disease among nurses in Nigeria. The objectives of the study were to determine the knowledge of nurses towards COVID-19 in Nigeria and to identify reasons for anxiety experienced by nurses in Nigeria towards COVID-19.

Hypothesis was tested to determine if significant relationship exist between the knowledge of nurses towards COVID-19 and the identified reasons for anxiety.

## Materials and Methods

### Research design

This study is an online cross-sectional quantitative survey that assessed the knowledge and reason for anxiety toward COVID 19 among Nurses in Nigeria.

### Area of study

Nigeria comprises six geopolitical zones. From the six geopolitical zones, three isolation centres/hospitals from each of the states were selected for the study. The zones and states included were North central, Benue and Nassarawa; South-west, Lagos and Ogun; North–west, Kaduna and Kano; South-south, Rivers and Akwa-Ibom; North-East, Bauchi and Taraba; South-East, Abia and Anambara states.

### Population/sample for the study

Using Naing formula for sample size calculation (Naing *et al*., 2006) the total population of nurses working in Nigeria are 250,000 (Nursing and Midwifery Council of Nigeria, 2020). With confidence level of 95%, margin of error 5% = 384. At a 70% response rate, we adjusted the sample size to 550.

### Sampling technique

We utilized a multistage sampling technique to assess the knowledge and reason for anxiety toward COVID 19 among Nurses in Nigeria. Simple purposive sampling technique guided the selection of two states (with high cases of COVID-19) from the six geopolitical zones and simple random sampling guided the selection of three isolation centres/hospitals from each of the states.

The zones and states included were North central, Benue and Nassarawa; Southwest, Lagos and Ogun; Northwest, Kaduna and Kano; Southsouth, Rivers and Akwa-Ibom; Northeast, Bauchi and Taraba; Southeast, Abia and Anambara states respectively. Consecutive recruitment method guided the selection of the emails of the nurses that participated in the study. The decision to use the above recruitment method was because of the lockdown.

**Instrument for data collection**

The research instrument used to generate the data was a structured questionnaire. The questionnaire consists of three sections. Section A consists of 8 items which were used to elicit information on the demographic characteristics of the nurses, section B consists of 16 items which were used to elicit information on the knowledge of COVID-19 among nurses and section C contains 6 items which were used to elicit information on the reasons for anxiety towards COVID-19 among nurses.

**Method of data collection**

Data was collected using Google form via email, WhatsApp and Instagram. Data collection was for a period of eight weeks and we stopped as a result of no influx of data after a period of additional one week

**Ethical consideration**

Research was conducted in line with the ethical standard guiding the conduct of research involving humans. Ethical approval was obtained from the Health and Research Ethical Committee of Lagos University Teaching Hospital with approval number-LUTHHREC/EREV/0620/49. The researchers presented the informed consent form in addition to the questionnaire before the main study questions to the nurses that agreed to participate in the study. We adhered to confidentiality and other ethical principles in the course of data collection and analysis.

**Data analysis**

We recorded a return rate of 76% (418) and analyzed the data with the help of the Statistical Package for Social Science (SPSS) version 20 software. Participants' descriptive data were presented in tables, charts, percentages, mean and standard deviation while the inferential data were tested with Chi-square, at a significance level of 0.05.

## Results

**Table 1: Socio-Demographic variables of Nurses in Nigeria**

| Variable | Frequency (n=418) | Percentage (%) |
|---|---|---|
| **Gender** | | |
| Female | 340 | 81.3 |
| Male | 78 | 18.7 |
| **Age (year)** | | |
| 16 – 29 | 96 | 23.0 |
| 30 – 49 | 210 | 50.2 |
| 50+ | 112 | 26.8 |
| Mean age =37.81±8.21years | | |
| **Marital status** | | |
| Divorced | 6 | 1.4 |
| Married | 278 | 66.5 |
| Separated | 4 | 1.0 |
| Single | 108 | 25.8 |
| Widow/Widower | 22 | 5.3 |
| **Highest Educational Level** | | |
| B.Sc/B.N.Sc | 214 | 51.2 |
| Double Qualified | 84 | 20.1 |
| M.Sc | 68 | 16.3 |
| Ph. D | 8 | 1.9 |
| RN | 44 | 10.5 |
| **Position in the institution** | | |
| Assistant Director of nursing service | 36 | 8.6 |
| Chief nursing officer | 90 | 21.5 |
| Deputy director of nursing | 20 | 4.8 |
| Director of nursing | 8 | 1.9 |
| Nursing officer 1 | 118 | 28.2 |
| Nursing officer 11 | 58 | 13.9 |
| Principal nursing officer | 64 | 7.7 |
| Senior Nursing Officer | 56 | 13.4 |
| **Years of experience** | | |
| 1-5 | 126 | 30.1 |
| 6-10 | 88 | 21.1 |

| | | |
|---|---|---|
| 11-15 | 46 | 11.0 |
| 16 – 20 | 22 | 5.3 |
| 21 – 25 | 48 | 11.5 |
| 26 – 30 | 48 | 11.5 |
| Above 30 | 40 | 9.6 |
| Mean years of experience =13.1±8.44years | | |
| **Have vehicle to aid mobility to work** | | |
| No | 128 | 30.6 |
| Yes | 290 | 69.4 |
| **Living with others in a family** | | |
| No | 46 | 11.0 |
| Yes | 372 | 89.0 |

Table 1 shows that the majority (340 [81.3%]) of the respondents are females. Their mean age is 37.81±8.21 years, with 278 (66.5%) married and 214 (51.2%) having B.Sc/B.N.Sc qualification. 118 (28.2%) are Nursing officer 1 (Nursing officer 1) at the time of the study with mean years of experience of 13.1±8.44 years. Most of the nurses (290 [69.4%]) have vehicle to aid their mobility to work, and 372 (89%) are living with others in a family.

**Table 2a: Knowledge of coronavirus among nurses in Nigeria.**

| **Variable** | Frequency (n=418) | Percentage (%) |
|---|---|---|
| **Have you heard about COVID19?** | | |
| No | 0 | 0.0 |
| Yes | 418 | 100.0 |
| **If yes, where did you hear of that?** | | |
| All of the above | 2 | .5 |
| Both | 2 | .5 |
| Friends/family/colleagues | 16 | 3.8 |
| Mass media | 276 | 66.0 |
| Online training | 2 | .5 |
| Read about it | 2 | .5 |
| Social media | 116 | 27.8 |
| Working place | 2 | .5 |
| **Are you trained to manage COVID-19 patients?** | | |
| No | 230 | 55.0 |
| Yes | 188 | 45.0 |

All the participants (100%) have heard of COVID-19, 276 (66%) heard from mass media, only 188 (45%) got training on how to manage COVID-19 patients (Table 2a).

**Table 2b: Knowledge of coronavirus among nurses in Nigeria**

| Variables n=418 | True | False | Don't Know |
|---|---|---|---|
| The major clinical presentations of COVID-19 are fever, fatigue, dry cough, and myalgia. | 370(88.5) | 48(11.5) | |
| Common cold, congested nose, runny nose, and sneezing are rare in persons infected with COVID-19 | 210(50.2) | 196(46.9) | 12(2.9) |
| There is no effective cure for COVID-19, currently, but early symptomatic and supportive treatment can help most patients recover. | 410(98.1) | 6(1.4) | 2(0.5) |
| Not all persons with COVID-19 will develop severe cases. | 408(97.6) | 8(1.9) | 2(0.5) |
| Severe cases are seen only in the elderly with chronic illnesses and obesity | 262(62.7) | 146(34.9) | 10(2.4) |
| Consuming or contacting wild animals would result in COVID-19. | 98(23.4) | 272(65.1) | 48(11.5) |
| Persons with COVID-19 cannot infect another person with the virus when a fever is not present. | 28(6.7) | 374(89.5) | 16(3.8) |
| COVID-19 virus spreads through respiratory droplets of infected individuals. | 406(97.1) | 12(2.9) | 0(0.0) |
| Residents can put on general medical masks to prevent COVID-19 infection. | 374(89.5) | 38(9.1) | 6(1.4) |
| It is not essential for children and young adults to adopt measures to prevent COVID-19 infection. | 30(7.2) | 384(91.9) | 4(1.0) |
| To prevent COVID-19 infection, individuals should avoid going to crowded places such as train stations and avoid taking public transportations. | 388(92.8) | 26(6.2) | 4(1.0) |
| Isolation and management of people diagnosed with COVID-19 are effective ways to reduce the spread of the virus. | 410(98.1) | 8(1.9) | 0(0.0) |
| Contact persons with someone infected with COVID-19 should be immediately isolated and observed for 14 days. | 410(98.1) | 8(1.9) | 0(0.0) |

A majority (370 [88.5%]) identified the central clinical systems of COVID-19, while 410 (98.1%) indicated that there is no cure for COVID-19. Only 28 (6.7%) indicated that persons with COVID-19 cannot infect another person with the virus when a fever is not present. 406 (97.1%) of them know that COVID-19 virus spreads via respiratory droplets of infected individuals while 374 (89.5%) know that residents can wear general medical masks to prevent the infection by the COVID-19. Also, 410 (98.1%) know that isolation and treatment of people with COVID-19 are useful means of minimizing the escalation of the virus and 410 (98.1%) indicated that contact persons with someone infected with COVID-19 should be immediately isolated and observed for 14 days (Table 2b).

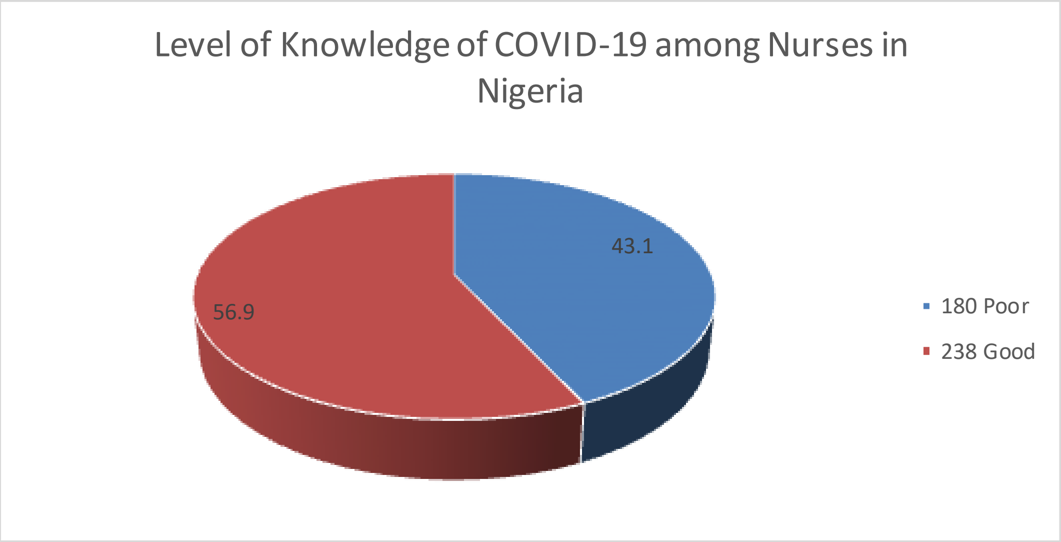


Most (238 [56.9%]) of the participants have good knowledge of COVID-19 with mean of 10.67±1.19. The mean of the correct options on knowledge of Coronavirus questions was determined, scores below the mean were rated poor, while above the mean were rated good knowledge.

**Table 3: The Reasons for the Anxiety Experienced by Nurses in Nigeria during COVID-19**

| Items n = 418 | SA | A | D | SD | Total |
|---|---|---|---|---|---|
| I am anxious because I know that COVID-19 is highly contagious | 240(57.4) | 134(32.1) | 40(9.6) | 4(1.0) | 418(100.0) |
| I am anxious because I am a frontline worker and having direct contact with patients | 252(61.7) | 112(26.8) | 44(10.5) | 4(1.0) | 418(100.0) |
| Many people including my colleagues have died from Covid-19 | 158(37.8) | 162(38.8) | 76(18.2) | 22(5.3) | 418(100.0) |
| Fear of infecting my family/patients/colleagues and losing them | 256(61.2) | 112(26.8) | 44(10.5) | 6(1.4) | 418(100.0) |
| Due to inadequate personal protective equipment in my institution, I feel I can get the disease | 210(50.2) | 136(32.5) | 60(14.4) | 12(2.9) | 418(100.0) |
| Due to scary news from media with regards to the disease, I am anxious I might get infected | 158(37.8) | 144(34.4) | 98(23.4) | 18(4.3) | 418(100.0) |
| Afraid my children might be vulnerable as I care for them | 182(43.5) | 152(36.4) | 64(15.3) | 20(4.8) | 418(100.0) |

SD- strongly disagreed, D-Disagreed, A-Agreed, SA-Strongly Agreed

Most (374 [89.5%]) of the participants agreed that they are anxious because they know that COVID-19 is highly contagious. 364 (88.5%) were anxious because they are front line workers and having direct contact with COVID-19 patients, while 320 (76.6%) agreed that many people, including their colleagues, have died from Covid-19. However, 368 (88%) are afraid of infecting their family/patients/colleagues and losing them while 346 (82.7%) feel they can get the disease due to inadequate personal protective equipment in their institution. 302 (72.2%) are anxious they might get infected due to scary news from media with regards to the COVID-19, and 334 (79.9%) are afraid that their children might be vulnerable as they care for them (Table 3).

**Table 4: Relationship between identified reasons for the anxiety experienced among Nigerian nurses and level of knowledge of COVID-19**

| n=418 | **Level of Knowledge** | | | | |
|---|---|---|---|---|---|
| **Variables** | **Poor** | **Good** | **Total** | **$X^2$** | **p-value** |
| Anxious because I know that COVID-19 is highly contagious | | | | | |
| A | 62(46.3%) | 72(53.7%) | 134(100%) | 2.429 | 0.488 |
| D | 20(50%) | 20(50%) | 40(100%) | | |
| SA | 98(40.8%) | 142(59.2%) | 240(100%) | | |
| SD | 0 | 4 | 4(100%) | | |
| Anxious because I am a frontline worker and having direct contact with patients | | | | | |
| A | 46(41.1%) | 66(58.9%) | 112(100%) | 3.984 | 0.263 |

| | | | | | |
|---|---|---|---|---|---|
| D | 26(59.1%) | 18(40.9%) | 44(100%) | | |
| SA | 108(41.9%) | 150(58.1%) | 258(100%) | | |
| SD | 0 | 2(100%) | 2(100%) | | |
| Many people, including my colleagues, have died from Covid-19. | | | | | |
| A | 70(43.2%) | 92(56.8%) | 162(100%) | 0.604 | 0.895 |
| D | 30(39.5%) | 46(60.5%) | 76(100%) | | |
| SA | 73(45.6%) | 86(54.4%) | 158(100%) | | |
| SD | 8(36.4%) | 14(63.6%) | 22(100%) | | |
| Fear of infecting my family Patients colleagues and losing them | | | | | |
| A | 52(46%) | 60(53.6%) | 112(100%) | 0.778 | 0.855 |
| D | 16(36.4%) | 28(63.6%) | 44(100%) | | |
| SA | 110(43%) | 146(57%) | 256(100%) | | |
| SD | 2(33.3%) | 4(66.7%) | 6(100%) | | |
| Due to inadequate personal protective equipment in my institution, I feel I can get the disease. | | | | | |
| A | 58(42.6%) | 78(57.4%) | 136(100%) | 2.767 | 0.429 |
| D | 22(36.7%) | 38(63.3%) | 60(100%) | | |
| SA | 98(46.7%) | 112(53.3%) | 210(100%) | | |
| SD | 2(16.7%) | 10(83.3%) | 12(100%) | | |
| Due to scary news from media with regards to the disease, l am anxious I might get infected. | | | | | |
| A | 66(45.8%) | 78(54.2%) | 144(100%) | 2.390 | 0.496 |
| D | 38(38.8%) | 60(61.2%) | 98(100%) | | |
| SA | 72(45.6%) | 86(54.4%) | 158(100%) | | |
| SD | 4(22.2%) | 14(77.8%) | 18(100%) | | |
| I am afraid my children might be vulnerable as I care for them. | | | | | |
| A | 64(42.1%) | 88(57.9%) | 152(100%) | 0.825 | 0.843 |
| D | 24(37.5%) | 40(62.5%) | 64(100%) | | |
| SA | 84(46.2%) | 98(53.8%) | 182(100%) | | |
| SD | 8(40%) | 12(60%) | 20(100%) | | |

SD- strongly disagreed, D-Disagreed, A-Agreed, SA-Strongly Agreed

The result from Table 4 above shows significant relationship did not exist between identified reasons for the anxiety experienced among Nigerian nurses and their level of knowledge of COVID-19 as all the p-values were > 0.05.

## Discussion

On the knowledge of COVID-19 among nurses in Nigeria, the result shows that nurses in Nigeria (56.9%) have good knowledge of the COVID-19 disease with mean of 10.67±1.19. Almost all the nurses had heard of COVID-19 mainly through the mass media even when 55% received no training on how to manage COVID-19 patients. Majority of the nurses have good knowledge of the main clinical symptoms of COVID-19. They were also aware that COVID-19 has no known cure. The nurses are also knowledgeable on the means of transmission, the preventive measures and also the effective means to prevent the escalation of COVID-19 disease. This finding is in agreement with the study by Dorcas *et al*. (2020) who reported that regarding COVID-19, 62.7% had "good" knowledge about the outbreak and their major source of information was the internet. This study equally revealed that the major source of the nurses' information was the mass media, including social media which invariably is from the internet. Ogolodom *et al*. (2020) reported a similar finding in their study titled "Knowledge, Attitudes and Fears of Healthcare Workers Towards the COVID-19 Pandemic in South-South, Nigeria". The result shows that majority of health workers were highly aware of the pandemic, although their most common source of information was their colleagues. However, the above finding is not in total agreement with the present study's finding where the participants' principal source of information was mass media. Huynh *et al*. (2020) reported a similar finding in their study carried out in a District 2 Hospital, Ho Chi Minh City. They discovered the health workers showed good knowledge of what COVID-19 is, knew the mode of transmission, the isolation period and the treatment. Against this backdrop, Benedict *et al*. (2020) in their study reported limited knowledge about the COVID-19 disease in Nigeria among health workers as about 1 in 4 respondents could outline significant symptoms of COVID-19 while few could outline at least four primary preventive measures and the treatment options available at the moment. The similarity discovered in this study with the other ones may be as a result of similarity in participants' professional characteristics while the difference may be as a result of environmental characteristics.

On the reasons for anxiety experienced by nurses during this COVID-19 pandemic, most of the study participants accepted that their anxiety was because: they know that COVID-19 is highly contagious; they are front line workers and are having direct contact with

COVID-19 patients; many people including their colleagues have died from Covid-19; they are afraid of infecting their family/patients/colleagues and losing them. Many of the nurses also feel anxious because they can get the disease due to inadequate personal protective equipment in their institution, scary news from media with regards to the COVID-19 and that their children might be vulnerable as they care for them. These findings on reasons for anxiety experienced by nurses agree with the findings by Brittany *et al.* (2020) that nurses are facing unprecedented stressors in their professional and personal lives, compounded by uncertainty about the future especially those working directly with patients diagnosed of COVID-19. Their fears include being made to care for patients with an inadequate number of the workforce or without life-saving personal protective equipment. The result agrees with the study by Nemati *et al*. (2020), that the nurses' level of anxiety is high, especially with regards to the infection of their family members.

Similarly, the result was also supported by the studies by Lai *et al*. (2020) that nurses caring for patients with COVID-19 exposure experienced a high level of vicarious traumatisation scores than those working in other places. Similarly, nurses in this study reported that they are anxious about their welfare and that of their families. They are equally anxious about the possibility of getting infected and infecting others, including family members and colleagues. The rampant deaths of their colleagues, also reported by Steve (2020), equally heighten their level of anxiety. From the above discussion, there are pieces of evidence that nurses in Nigeria are anxious about the COVID-19 disease, although they have been given the professional mandate to care for others no matter the situation. There is a need, however, to support nurses, especially those who may be experiencing anxiety by offering verbal support and making sure they work in a secure and supportive environment. The similarity observed in this study with other studies reviewed might be as a result of similarity in participant's characteristics.

The hypothesis tested using a non-parametric tool (Chi-square) shows that significant relationship did not exist between identified reasons for the anxiety experienced among Nigerian nurses and their level of knowledge of COVID-19. This revelation shows that knowledge of nurses with regards to the COVID-19 disease had nothing to do with the anxiety they experience and the reasons for the anxiety because although the nurses in this study were knowledgeable about the disease, its signs and symptoms, transmission mode, preventive measures and treatment, they were anxious about the disease and strongly identified the reasons for the anxiety they experience over this pandemic. This discovery is in line with a result from a study that anxiety and psychological distress rates were significantly higher for nurses caring for COVID-19 patients specifically, which had nothing to do with their knowledge of the disease as found in this study (Brittany *et al*., 2020).

The strength of the study lies on the uniqueness of this study in Nigeria as literature reviewed shows there are few studies completed outside Nigeria with regards to this topic but none has been completed in Nigeria at the moment the study was carried out hence it will add to the body of knowledge in this regards. The weakness is majorly on the methods of data collection although the method was chosen as a result of the lockdown from COVID-19 pandemic which may have effect on the result. The study cannot be completed without limitation as the major limitation lies on the incomplete sample. The required sample for the study could not be attained as the researchers could not get any further response from the respondents which prompted the researchers to stop data collection and thereby having incomplete sample which may also have affected the statistical power

## Conclusion

Although Nurses in Nigeria are knowledgeable about the COVID-19 disease, they still give reasons for being anxious which in any way had nothing to do with their knowledge of the disease. There is, therefore, the need to support the nurses physically and psychologically in their day to day task to allay their anxiety. Also, hospital administrators should employ all the necessary strategies to prevent them from being infected, and those seen to be experiencing chronic anxiety should receive good counsel and support.

**Disclosure of Conflict of Interest:** The authors declare no conflict of interest in connection with this study.

## References


1. Bao-Liang, Z., Wei, l., Hai-Mei, L., Qian-Qian, Z., Xiao-Ge, L., Wen-Tian, L., and Yi, L. (2020). Knowledge, attitude and practices towards COVID-19 among Chinese residents during the rapid rise period of the COVID-19 outbreak: a quick online cross-sectional survey. *International Journal of Biological Sciences*, 16(10): 1745–1752.
2. Benedict, E. A., Chukwu, O. A., Abubakar, D. and Onwukwe, E. V. (2020). COVID-19 Outbreak: Awareness and Knowledge among Healthcare Professionals in Nigeria. https://www.researchgate.net/publication/341216680_COVID-19_Outbreak_Awareness_and_Knowledge_among_Healthcare_Professionals_in_Nigeria. Accessed 12th July 2020.
3. Brittany, H., Mathew, S., and Marci, Z. (2020). Easing the psychological impact of COVID-19 for nurses. American Nurse at: www.HealthComMedia.com.
4. Dorcas, S., Emmanuel, L., Anthony, B. A., Ephraim, K.S. and Jude K.A. (2020). Knowledge, risk perception and preparedness towards COVID-19 outbreak among Ghanaians: A quick online cross-sectional survey. *Pan African Medical Journal*, 35(2); 44.
5. Huynh, G.,Nguyen, T.N., Van, K.T., Kim, N.V., Van, T.V. and Le, A.P. (2020). Knowledge and attitude toward COVID-19 among healthcare workers at District 2 Hospital, Ho Chi Minh. *Asian Pacific Journal of Tropical Medicine* 13 · DOI: 10.4103/1995-7645.280396.
6. International Council of Nurses (2020). Immediate and serious threat: ICN calls on WHO member states to collect and share data on health workers COVID-19 infection rates and deaths. www.icn.ch.
7. John Hopkins university coronavirus resource centre (2020). COVID-19 dashboard by the centre for systems science and engineering (CSSE) at John Hopkins University.
8. Lai, C., Shih, T, Ko W., Tang, H., and Hsueh, P. (2020). Severe acute respiratory syndrome coronavirus-2 (SARS-CoV-2) and coronavirus disease-2019 (COVID-19): The epidemic and the challenges. *International Journal* of *Antimicrobial Agents*, 55(3):105924 [doi: 10.1016/j.ijantimicag.2020.105924].

9. Lai, J., Ma, S., Wang, Y., Zhongxiang C., Jianbo H., Ning W., Jiang W., Hui D., Tingting C., Ruiting L., Huawei T., Lijun K., Lihua Y., Manli H., Huafen W., Gaohua W., Zhongchun L., and Shaohua H. (2020). Factors associated with mental health outcomes among health care workers exposed to coronavirus disease in 2019. *JAMA Netw Open.* ;3(3):e203976.
10. Li,Q., Guan, X., Wu, P., Wang, X., Zhou, L., Tong, Y., Ruiqi, R., Kathy, S. M., Eric, H.Y., Jessica, Y.W., Xuesen, X., Nijuan, X., Yang, W., Chao, L., Qi, C., Dan, L., Tian, L., Jing, Z.L., Wenxiao T., Chuding, C., Lianmei J., Rui Y., Qi W., SZhou, Rui W., Hui L., Yinbo L.,, Yuan L., Ge S.,, Huan L., Zhongfa T., Yang Y., Zhiqiang D., Boxi L., Zhitao M.,, Yanping Z., Guoqing S., Tommy T. Y., Joseph T.W., George F.G., Benjamin J. C., Bo Y., Gabriel.M. L., and Zijian F. (2020). Early Transmission Dynamics in Wuhan, China, of Novel Coronavirus-Infected Pneumonia. *New England Journal of Medicine,* 26;382 (13):1199-1207.doi: 10.1056/NEJMoa2001316].
11. Li, Z., Ge, J., Yang, M., Jianping, F., Mei, Q., Riyue, J., Jiangjiang, B., Gaofeng Z., Xiaolin, X., Long, W., Qin, Z., Chenliang Z., Yinbing P., Shijiang L., Haiwei Z., Jianjun, Y., Bin Z., Yimin Hu., Kenji, H., Yan J., Haofei W., Rong W., Cunming L., and Chun Y. (2020). Vicarious traumatisation in the general public, members, and non-members of medical teams aiding in COVID-19 control. *Brain*, *Behavior*, and *Immunity*, S0889-1591(20):30309-3.
12. Nigeria Center for Disease Control (2020). Nigeria report on COVID-19. https://covid19.ncdc.gov.ng/contact. Accessed 24th March 2020.
13. Naing, L., Winn, T., and Rusli, B. (2006). Practical issues in calculating the sample size for prevalence studies. *Archives of Orofacial Sciences, 1*: 9-14.
14. Nemati, M., Ebrahimi, B., and Nemati, F. (2020). Assessment of Iranian Nurses' Knowledge and Anxiety Toward COVID-19 During the Current Outbreak in Iran. *Arch Clin Infect Disease,* Online ahead of Print; 15(COVID-19): e102848. DOI: 10.5812/archcid.102848.
15. Nursing and Midwifery Council of Nigeria (2020). https://www.nmcn.gov.ng. Accessed 27th March 2020.
16. Ogolodom, M. P., Mbaba, A. N., Alazigha, N., Erondu O.F., Egbe N.O., Golden I., Ugwuanyi D.C., Achi G.I., and Eke C.M**.** (2020.) Knowledge, Attitudes and Fears of Healthcare Workers towards the Corona Virus Disease (COVID-19) Pandemic in South-South, *Nigeria. Health Science Journal*, Iss 1: 002. DOI: 10.36648/1791-809X.S1.002.
17. Shehab, A. (2019). The Story of Mysterious Pneumonia and the Response to Deadly Novel Coronavirus (2019-nCoV): So Far!. *NEMJ,* 21;1(1):7-10. DOI: 10.2174/0250688202001010007.
18. Steve, F. (2020). Global nursing body issues warning on nurse mental health during COVID-19 crisis. UK Nursing Times. Available at: https://www.nursingtimes.net/news/mental-health/global-nursing-body-issues-warning-on-nurse-mental-health-during-covid-19-crisis-30-04-2020/.
