# Supplementary material for: Mental health status and related factors influencing healthcare workers during the COVID-19 pandemic: A systematic review and meta-analysis
Source: PLoS One. 2024 Jan 19;19(1):e0289454. doi: 10.1371/journal.pone.0289454 (PMC10798549; doi:10.1371/journal.pone.0289454)
Supplement: S1 Data — (ZIP) [file pone.0289454.s011.zip › literatures/122.pdf]

South African Journal of Psychiatry
ISSN: (Online) 2078-6786, (Print) 1608-9685

Post-traumatic stress disorder among the staff of a mental health hospital: Prevalence and risk factors

Authors:
Anthony A. Olashore[1]
Oluyemi O. Akanni[2]
Keneilwe Molebatsi[1]
John A. Ogunjumo[3]

Affiliations:
[1]Department of Psychiatry, University of Botswana, Botswana

[2]Clinical Services, Federal Neuropsychiatric Hospital, Benin, Nigeria

[3]Department of Family Medicine, University of Botswana, Botswana

Corresponding author:
Anthony Olashore,
olawaleanthonya@gmail.com

Dates:
Received: 28 Mar. 2018
Accepted: 12 July 2018
Published: 30 Aug. 2018

How to cite this article:
Olashore AA, Akanni OO, Molebatsi K, Ogunjumo JA. Post-traumatic stress disorder among the staff of a mental health hospital: Prevalence and risk factors. S Afr J Psychiat. 2018;24(0), a1222. https://doi.org/10.4102/sajpsychiatry.v24i0.1222

Copyright:
© 2018. The Authors. Licensee: AOSIS. This work is licensed under the Creative Commons Attribution License.

Read online:
Scan this QR code with your smart phone or mobile device to read online.

Background: Mental health service providers are frequently exposed to stress and violence in the line of duty. There is a dearth of data concerning the psychological sequelae of the frequent exposure to stress and violence, especially among those who work in resource-limited countries such as Botswana.

Aim: To determine the prevalence and predictors of post-traumatic stress disorder (PTSD) among mental health workers in a tertiary mental health institute in Botswana.

Setting: The study was conducted in Sbrana Psychiatric Hospital, which is the only referral psychiatric hospital in Botswana.

Methods: The study used a descriptive cross-sectional design. A total of 201 mental health workers completed a researcher-designed psycho-socio-demographic questionnaire, which included one neuroticism item of the Big Five Inventory, and a PTSD Checklist-Civilian Version (PCL-C), which was used to assess symptoms of PTSD.

Results: Majority of the study participants were general nurses (n = 121, 60.5%) and females (n = 122, 60.7%). Thirty-seven (18.4%) of the participants met the criteria for PTSD. Exposure to violence in the past 12 months (AOR = 3.26; 95% CI: 1.49–7.16) and high neuroticism score (AOR = 2.72; 95% CI: 1.19–6.24) were significantly associated with the diagnosis of PTSD among the participants.

Conclusion: Post-traumatic stress disorder could result from stressful events encountered in the course of managing patients in mental health institutes and departments. Pre-placement personality evaluation of health workers to be assigned to work in psychiatric units and post-incident trauma counselling of those exposed to violence may be beneficial in reducing the occurrence of PTSD in mental hospital health care workers.

Introduction

Post-traumatic stress disorder (PTSD) is a consequence of exposure to death, threatened death, actual or threatened serious injury, or actual or threatened sexual violence, either by direct exposure, by being a witness to the trauma or by learning that a close friend or family member was exposed to trauma. It is characterised by re-experiencing the traumatic event, for example, having nightmares and flashbacks; hyper-arousal; avoidance of stimuli-related to the traumatic event; negative thoughts or feelings evidenced by excessive blame of self or others for causing the trauma; isolating self; and inability to remember important details about the trauma.[1] Persistence of these symptoms and other associated symptoms beyond a 30-day period from the time of exposure to the stressful or triggering events is required for the diagnosis to be made according to the Diagnostic and Statistical Manual of Mental Disorders (DSM-5).[1]

Although overwhelming stress or trauma is the principal causal factor in the development of PTSD,[1,2] not everyone experiences PTSD after a traumatic event. For example, in a study where participants were exposed to a significantly high level of stress, only 3.5% developed PTSD symptoms,[3] thus suggesting the role of other factors in the aetiology of PTSD. Evidence from prior studies suggests that there are some factors which increase vulnerability to developing PTSD. These include female gender, neuroticism, inadequate social support, substance use, personality disorder, genetic predisposition to mental illness and pre-existing mental illness, especially the internalising type.[2]

The prevalence of PTSD is estimated to be about 3% – 4% in the general population, even though rates range from 5% to 80% in high-risk populations.[2,3,4,5] In addition to victims of war, disasters or domestic violence, high rates have been reported among workers who are regularly exposed to

stress and violence in the line of duty. Prevalence among health care providers ranges between 0% and 30%.[4,5] For example, a rate of 24% has been reported among nurses who deal with trauma and critically ill patients daily, whereas about 30% has been reported among paediatric staff.[5] Health workers in mental institutions are also at risk of developing PTSD from frequent exposure to threats and violence caused by their patients.[6] Rates ranging from 0% to 17% have been found among mental health care providers.[6,7,8]

Mental health care workers (MHCWs) in resource-limited countries are at an elevated risk of exposure to violence or threats, which often results from inadequate facilities or staffing, long waiting time, inadequate attention to health needs of the patients and the use of inexperienced or unqualified staff.[9] Moreover, inadequate resources or facilities have been associated with an increase in frustration, irritability, anger, possible violence by relatives and/or mentally ill patients against MHCWs.[9,10] All these increase the risk of MHCWs developing psychiatric disorders such as mood and anxiety disorders, and PTSD. Mental health professionals, who are working in Botswana, are not exempted from this, mainly because of the current shortage of mental health personnel. When not identified and treated, PTSD may have a severe consequence on social and occupational functioning, most importantly, low productivity in health care delivery.[4]

There is currently no data on the psychological effects of trauma experienced by mental health workers in Botswana. Therefore, this study aimed to determine the prevalence and predictors of PTSD among mental health workers in a tertiary mental health institute in Botswana.

Methods

Study design and setting

The study was a cross-sectional descriptive design which was conducted at Sbrana Psychiatric Hospital (SPH), the only referral mental hospital in Botswana. The hospital is located in the south-western part of the country and has a capacity of 300 beds. The hospital resources are mainly for adult mental health care.

Study procedure

All members of the hospital staff who could read and write were included in the study, except the support staff (security guards and the cleaners as they were privately employed) and all the administrative staff, because they have little or no contact with the patients. Staff who did not consent and those who had worked at the hospital for less than 1 month were also excluded. Two sets of questionnaires, socio-demographic questionnaire and PTSD Checklist-Civilian Version (PCL-C), were administered to every consenting participant by the principal investigator (PI) (A.A.O.) and a research assistant. The participants were instructed not to disclose or share their responses with their colleagues. The completed questionnaires were collected only by the PI in a pool and locked in a cabinet to maintain confidentiality. The period of the study lasted for 4 months, between 1 August and 30 November, 2016, to allow those on shift duty and those on leave to be part of the study.

Measures

The definition of physical violence according to Dhumad et al.[11] was adopted in this study. Thus, we captured physical violence as any act of physical aggression during which the patient deliberately and forcefully hits a caregiver with any part of his or her body or with an object, thereby inflicting pain or injury to the victim. Non-work-related traumatic events such as domestic violence and rape, which may increase the risk to develop PTSD, were excluded from the questionnaire based on the agreement with the Independent Review Board.

The psycho-socio-demographic questionnaire was designed by the authors to inquire about the socio-demographic characteristics of the participants. In addition to age, gender and other demographic characteristics, variables that have been shown to be associated with PTSD such as a history of physical violence, previous history of a mood or anxiety disorder, level of neuroticism, and social support were included. Neuroticism was assessed out of convenience by using one of the neuroticism items of the Big Five Inventory (Do you get nervous easily?) and rated on a five-point Likert scale. It was rated from 'strongly agree', which was scored 4, to 'strongly disagree', scored 0. A higher score indicated a higher level of neuroticism. Neuroticism is one of the five-factor model of personality that measures negative emotionality such as feeling anxious, nervous, sad and tense. There are well-established scales recommended for the measurement of neuroticism with other traits such as the Revised Neuroticism Extraversion Openness Personality Inventory (NEO-PI-R); however, researchers over the years have derived short scales such as the 10-item Big Five Inventory (2 items of the scale have been standardised to measure neuroticism). Social support was assessed subjectively by asking the participants a single question to rate the support received from a combination of family, friends and colleagues following a physical attack at work. It was rated 'poor', 'moderate' and 'strong' and scored, respectively, as 0, 1 and 2. A higher score indicated a higher level of social support.

PTSD Checklist-Civilian Version[12] is a 17-item PTSD self-report checklist which assesses trauma in response to stressful experiences. It is a widely used PTSD-research instrument with the items consistent with the Diagnostic and Statistical Manual of Mental Disorders, 4th edition (DSM-IV) criteria for the diagnosis of PTSD.[13] It was modified to reflect response or reaction following work-related violence or assault and has been used in Africa with a test-retest consistency of $\alpha = 0.89$.[14] The scale has a Likert score system ranging from 1 to 5. The instrument is divided into three parts: the first part consists of items relating to intrusive thoughts and feelings, the second part consists of those relating to avoidance and the

last part relates to hyper-arousal. The scores were converted to PTSD diagnosis according to Weathers et al.[12] An individual is given a diagnosis of PTSD if he has positive responses to at least 1 out of 5 questions in the first part of the questionnaire, 3 out of 7 questions in the second part and 2 out of 5 questions in the last part. Only the symptoms of PTSD which occurred during the period of employment and related to experiences of violence by mentally ill patients were considered as caseness, that is, previous history or experience of PTSD symptoms was excluded.

Data analysis

The statistical analysis was performed using Statistical Package for Social Sciences (SPSS) for Windows version 16. Descriptive statistics were performed to determine the characteristics of the sample. Participants' ages were dichotomised using the median score as the cut-off point. Chi-square test was used to test the association of PTSD with variables such as gender, age, profession and history of violence. The association of PTSD with continuous variables such as neuroticism and social support was tested using independent *t*-test. Neuroticism was furthermore dichotomised into low and high, based on the mean score of 1.67, to carry out a binary logistic regression. A regression analysis was performed to identify predictors of PTSD caseness. Basic demographics such as gender and age were included into the regression analysis. The level of significance was set at $p < 0.05$.

Ethical considerations

The authors obtained approval from the University of Botswana Independent Review Board (IRB). Permission to undertake the study was obtained from the Ministry of Health and the management of Sbrana Psychiatric Hospital. The purpose of the study was explained to every eligible participant, and a written informed consent was obtained from everyone who agreed to participate.

Results

A total of 201 mental (95.7%) MHCWs out of the 210 who were eligible to participate in the study during the study period consented to participate. Table 1 depicts the socio-demographic and clinical variables of the study population. The median age of the respondents was 32 years. Majority were females ($n = 122$, 60.7%) and nurses ($n = 121$, 60.5%). The median duration of practice in a psychiatric hospital for the respondents was 4 years.

One hundred and forty-seven (73.1%) reported being physically assaulted by patients at least once in their period of employment in the psychiatric hospital, and 39.3% ($n = 79$) had been assaulted within the past year. According to the PCL-C score, 37 (18.4%) of the respondents met the criteria for PTSD diagnosis.

Chi-square test revealed a significant association between the experience of physical assault over the preceding 12 months and PTSD ($\chi^2 = 5.79$; $p = 0.02$) (Table 2). The mean score for neuroticism was found to be significantly ($t = -3.07$; $p = 0.022$) higher in those who met the criteria for PTSD than those who did not, but no significant relationship was found between social support and a PTSD diagnosis (Table 3).

The two variables that were significantly associated with PTSD on bivariate analyses, namely neuroticism and previous year exposure to violence, were then further investigated using binary logistic regression to determine the predictors of PTSD caseness. Those with an experience of violence in the last 12 months were three times more likely to have PTSD compared with those who did not experience any violence. This was statistically significant (95% CI [1.49, 7.16], $p = 0.03$). Also, those with a high level of neuroticism were about two times more likely to have PTSD compared with those with a low level of neuroticism (95% CI [1.19, 6.24], $p = 0.02$) (Table 4).

TABLE 1: Socio-demographic characteristics of the participants.

Characteristics	Number of participants	Percentage
**Gender**	**201‡**	**100.0**
Male	79	39.3
Female	122	60.7
**Marital status**	**200‡**	**100.0**
Single	122	61.0
Married	76	38.0
Divorced	1	0.5
Widowed	1	0.5
**Religion**	**197‡**	**100.0**
Christianity	188	95.4
Islam	1	0.5
African traditional religion	4	2.0
Others	4	2.0
**Profession**	**200‡**	**100.0**
Psychiatrist	4	2.0
Medical officer	6	3.0
Psychiatric nurse	34	17.0
General nurse	121	60.5
Student nurse	3	1.5
Psychologist	4	2.0
Others	28	14.0
**Income per month**	**198‡**	**100.0**
≤ 5,000	15	7.6
6–10,000	59	29.8
11–12,000	109	55.1
> 20,000	15	7.5
**PTSD caseness**	**201‡**	**100.0**
No	164	81.6
Yes	37	18.4
**Lifetime violence attack†**	**201‡**	**100.0**
No	54	26.9
Yes	147	73.1
**Past 12 months violence attack†**	**201‡**	**100.0**
No	122	60.7
Yes	79	39.3

PTSD, post-traumatic stress disorder.
The median age is 32 years.
The median duration of practice is 4 years.
Others include social worker, occupational therapist, pharmacist, record officer and lab technician.
†, Violence by patients; ‡, Figure does not add up to 206 because of missing data.

TABLE 2: Association of post-traumatic stress disorder caseness with risk variables.

Variables	Post-traumatic stress disorder						χ^2	*p*
	No		Yes		Total			
	Frequency (*n*)	Percentage	Frequency (*n*)	Percentage	Frequency (*n*)	Percentage		
**Gender**								
Male	65	39.6	14	37.8	79	37.8	0.04	0.84
Female	99	60.4	23	62.2	122	60.7	-	-
**Age (years)**								
≤ 32	81	51.3	21	60.0	102	52.8	0.88	0.35
> 32	77	48.7	14	40.0	91	47.2	-	-
**Lifetime violence†**								
No	47	28.7	7	18.9	54	26.9	1.46	0.23
Yes	117	71.3	30	81.1	147	73.1	-	-
**12 months violence†**								
No	106	64.6	16	43.2	122	60.7	5.79	**0.02**
Yes	58	35.4	21	56.8	79	39.3	-	-
**History of mood disorder**								
No	155	95.1	34	91.9	189	94.5	0.59	0.44
Yes	8	4.9	3	8.1	11	5.5	-	-
**Duration of practice**								
≤ 4	80	49.4	14	40.0	94	47.7	1.02	0.31
> 4	82	50.6	21	60.0	103	52.3	-	-
**Profession‡**								
Doctors	34	21.4	6	16.2	40	20.4	7.00	0.07
Nurses	98	61.6	30	81.1	128	65.3	-	-
Other clinical	20	12.6	-	-	20	10.2	-	-
Non-clinical	7	4.4	1	2.7	8	4.1	-	-

Significant association in bold.
Degree of freedom (*df*) = 1.
†, Violence by patients; ‡, *df* = 3.

TABLE 3: Neuroticism and social support comparison between post-traumatic stress disorder caseness and non-caseness.

Variables	PTSD	Mean	s.d.	*t*	*p*
**Neuroticism**				-3.07	**0.022**
	No	1.52	1.30		
	Yes	2.03	1.17		
**Social support**				-1.82	0.068
	No	1.62	0.56		
	Yes	1.43	0.50		

PTSD, post-traumatic stress disorder; s.d., standard deviation.
Significant association in bold.

TABLE 4: Predictors of post-traumatic stress disorder caseness.

Variables	B	s.e.	Wald	Sig.	AOR	95% CI	
						Lower	Upper
**Gender**							
Male (ref)	1.00	-	-	-	-	-	-
Female	0.28	0.41	0.48	0.49	1.33	0.60	2.95
**Age**							
≤ 32 (ref)	1.00	-	-	-	-	-	-
> 32	-0.12	0.41	0.08	0.77	0.89	0.40	1.98
**Neuroticism**							
Low (ref)	1.00	-	-	-	-	-	-
High	1.00	0.42	5.59	**0.02**	2.72	1.19	6.24
**12 months violence†**							
No (ref)	1.00	-	-	-	-	-	-
Yes	1.18	0.40	8.68	**0.03**	3.26	1.49	7.16

AOR, adjusted odds ratio; CI, confidence interval; s.e., standard error.
Significant test of association in bold.
Degree of freedom (*df*) = 1.
†, Violence by patients.

Discussion

The prevalence of PTSD in our sample was 18% and is comparable with reports from previous hospital studies.[5,6] A similar study conducted by Richter and Berger using PCL-C found a prevalence rate of 17% among psychiatric hospital staff in Germany.[6] Another study conducted among nurses using a different instrument, the Mississippi Scale for PTSD, reported a prevalence of 18%.[5] In contrast, a lower rate (10%) was reported by Jacobowitz,[8] while no case was found in a study among nursing staff of a forensic psychiatric security unit in Sweden.[7] Variation in organisational structures across settings, sincerity in response to questions, methodological differences, cultural influence on reporting and attrition from work have been suggested to account for disparities in rates.[7] For example, in the study among forensic unit nurses[7], avoidance of emotion-triggering questions, the protective effect of highly organised environment and high nurse–patient ratio were among the reasons suggested for no participant meeting the criteria of PTSD. Nevertheless, the rate found in this study which is several times higher than what has been reported in the general population (2.3%)[3], emphasises the need for more attention to the health and safety of MHCWs. Addressing this problem may also have both direct and indirect positive influence on the patients' care and mental health care system at large.

A significantly high proportion of those who experienced physical assault over the preceding 12 months met the criteria for PTSD in this study compared to those who had not experienced assault. This study also revealed that the odds of developing PTSD are more than thrice as high following

exposure to assault in the past 12 months which emphasises the role of physical violence in the development of PTSD. An essential factor in the development of PTSD is exposure to overwhelming stress,[2,15] especially work-related repeated exposure, as highlighted in DSM-5.[1] In the health sector, this may include caring for patients with terminal diseases, verbal assault from superior officers and patient's relatives, and even physical assault by the patients, particularly those who are mentally ill.[5,7,8]

The root of the core symptoms of PTSD has been associated with threat or exposure to physical violence.[1,15] For example, the Cognitive theory has postulated that the frequent intrusion of unwanted memories of past traumatic events and impaired processing of the emotionally charged information (about the events) which is then stored in an unprocessed form are essential in PTSD development.[16] Furthermore, the Pavlovian theory posited that stimuli experienced at the time of trauma such as violence in a mental hospital setting might become associated with fear and avoidance.[17] MHCWs are at higher risk of being assaulted frequently compared to other health workers and the general population,[9,10] and this may have severe consequences on the victims, especially if there are no structures in place to minimise the effects of violence on the workers.[18] Thus, this highlights the need for frequent training in the handling of violent patients, partaking in post-incident trauma counselling, and careful use of critical incident debriefing (CID). CID has been reported to play a significant role in facilitating the development of resilience to PTSD in exposed health care workers;[18] although its role has been found to be mixed; some showed null or adverse effects.[19] Two Cochrane reviews concluded that debriefing might increase the risk of development of PTSD. They also established that findings from randomised clinical trials are not adequate to inform the use of CID in clinical practice.[20,21] Conversely, some authors have suggested that CID may be beneficial in reducing the risk of developing PTSD and associated psychological complications in persons exposed to trauma.[18,20,23] Periodic rotation of staff to facilities with less potential for violence will likewise assist in reducing exposure to emotion-triggering activities.

In addition to physical violence, those with high neuroticism score were two times more likely to develop PTSD than those with a low score. This finding again emphasises the role of personality or individual differences in the development of PTSD. Individuals with high neuroticism score have been shown to have a higher risk of developing internalising disorders such as PTSD compared to the general population. Engelhard et al.[15] demonstrated a strong relationship between pre-trauma neuroticism and PTSD symptoms, particularly the arousal symptoms. A more recent study also revealed that neuroticism score at baseline significantly increased the relative risk of PTSD after a traumatic exposure.[24] Our finding underscores the importance of pre-job placement personality testing, in which persons with high-risk traits are placed in the departments with low risk of violence.

Although the mean score of social support was found to be higher among those without PTSD when compared with those diagnosed with PTSD, this association was not statistically significant. Good social support is expected to be protective against the development of PTSD as reported in a previous study.[2] The relationship between social support and the development of PTSD is a composite one and is dependent on various factors. For example, there are three different dimensions of social support, namely the subjective, which is felt by oneself; the objective, which is evident to people; and support utilisation, which is merely the degree of use.[25] Of the three, the subjective support and utilisation were reported to be more related to prevention and recovery from psychological disorders, because an individual's psychological perception of reality influences his behaviour and growth.[25] Another study suggested that there is no relationship between PTSD and social support in the presence of negative attitudes, beliefs and expectations towards support networks utilisation.[26] These suggest that the relationship between PTSD and social support is dependent on so many other factors not explored in this study, hence the lack of association in this study and the need for further studies.

Conclusion

Post-traumatic stress disorder is a psychological disorder that could result from stressful events encountered in the course of managing violent patients in mental health institutes and departments. Pre-placement personality evaluation of health workers to be assigned to work in psychiatric units and, regular, frequent training on the management of violent patients should help to reduce the occurrence of stressful events that could result in PTSD. Post-incident trauma counselling and emotional support should be made accessible to all health workers who experience stressful events.

Limitations

Over-reporting or under-reporting cannot be entirely ruled out as a result of the use of self-report questionnaires. Secondly, the participants may have recall biases because they were required to report about past incidents. Also, avoidance is a feature of PTSD which may limit disclosure of information regarding events relating to the stressor. Thirdly, other traumatic life events, indirect exposure to violence and stressors outside work should have been controlled for as potential risk factor of PTSD. Another limitation is that causality cannot be inferred with the cross-sectional nature of the study. It is possible that the trauma predated the construct of 'getting nervous easily'; however, the supposed stable nature of personality trait which is posited to have begun from adolescent counters this argument. Finally, the use of a single-item statement to measure neuroticism and social support is not a gold standard; applying a standardised scale might be more utile.

Acknowledgements

The authors would like to thank the staff of Sbrana Psychiatric Hospital for their participation in this study.

Competing interests

The authors declare that they have no financial or personal relationships that may have inappropriately influenced them in writing this article.

Authors' contributions

A.A.O. and O.O.A. developed the concept and designed the study. A.A.O. and O.O.A. drafted the initial manuscript. K.M. and J.A.O. also made substantial intellectual contributions to the final manuscript. All the authors read and approved the final manuscript for publication.

References

1. American Psychiatric Association. Diagnostic manual of mental disorders. 5th ed. Arlington, TX: American Psychiatric Association; 2013.
2. Ozer EJ, Best SR, Lipsey TL, Weiss DS. Predictors of posttraumatic stress disorder and symptoms in adults: A meta-analysis. Psychol Bull. 2003;129(1):52. https://doi.org/10.1037/0033-2909.129.1.52
3. Atwoli L, Stein DJ, Williams DR, et al. Trauma and posttraumatic stress disorder in South Africa: Analysis from the South African stress and health study. BMC Psychiatr [serial online]. 2013 [cited 2018 Mar 20];13(1):182. Available from: http://bmcpsychiatry.biomedcentral.com/articles/10.1186/1471-244X-13-182
4. Sendler DJ, Rutkowska A, Makara-Studzinska M. How the exposure to trauma has hindered physicians' capacity to heal: Prevalence of PTSD among healthcare workers. Eur J Psychiatr. 2016;30(4):321–334.
5. Mealer ML, Shelton A, Berg B, Rothbaum B, Moss M. Increased prevalence of post-traumatic stress disorder symptoms in critical care nurses. Am J Respir Crit Care Med [serial online]. 2007 [cited 2018 Mar 20];175(7):693–697. Available from: http://www.atsjournals.org/doi/abs/10.1164/rccm.200606-735OC
6. Richter D, Berger K. Post-traumatic stress disorder following patient assaults among staff members of mental health hospitals: A prospective longitudinal study. BMC Psychiatr [serial online]. 2006 [cited 2018 Mar 20];6(1):15. Available from: http://bmcpsychiatry.biomedcentral.com/articles/10.1186/1471-244X-6-15
7. Lauvrud C, Nonstad K, Palmstierna T. Occurrence of post-traumatic stress symptoms and their relationship to professional quality of life (ProQoL) in nursing staff at a forensic psychiatric security unit: A cross-sectional study. Health Qual Life Outcomes [serial online]. 2009 [cited 2018 Mar 20];7(1):31. Available from: http://hqlo.biomedcentral.com/articles/10.1186/1477-7525-7-31
8. Jacobowitz W. The rate of PTSD in hospital-based psychiatric healthcare workers: A descriptive cross-sectional design study. Ann Psychiatr Ment Health. 2015; 3(5):1039.
9. Phillips JP. Workplace violence against health care workers in the United States. In Longo DL, editor. N Engl J Med [serial online]. 2016 [cited 2018 Mar 20];374(17):1661–1669. Available from: http://www.nejm.org/doi/10.1056/NEJMra1501998
10. Davison SE. The management of violence in general psychiatry. Adv Psychiatr Treat [serial online]. 2005 [cited 2018 Mar 20];11(5):362–370. Available from: https://www.cambridge.org/core/product/identifier/S1355514600002509/type/journal_article
11. Dhumad S, Wijeratne A, Treasaden I. Violence against psychiatrists by patients: Survey in a London mental health trust. Psychiatrist. 2007;31(10):371–374. https://doi.org/10.1192/pb.bp.106.012815
12. Weathers FW, Huska JA, Keane TM. PCL-C for DSM-IV. Boston, MA: National Center for PTSD-Behavioral Science Division. Google Scholar; 1991.
13. American Psychiatric Association (APA). Diagnostic and statistical manual of mental disorders: DSM-IV-TR®. American Psychiatric Association; 2000. Washington, DC.
14. Okulate GT, Jones OB. Post-traumatic stress disorder, survivor guilt and substance use-a study of hospitalised Nigerian army veterans. S Afr Med J. 2006;12(1):37–40. https://doi.org/10.4102/sajpsychiatry.v12i1.53
15. Engelhard IM, van den Hout MA, Kindt M. The relationship between neuroticism, pre-traumatic stress, and post-traumatic stress: A prospective study. Personality and individual differences, 2003;35(2):381–388. https://doi.org/10.1016/S0191-8869(02)00200-3
16. Ehlers A, Clark DM. Post-traumatic stress disorder: The development of effective psychological treatments. Nord J Psychiatry. 2008;62(47):11–18.
17. VanElzakker MB. Dahlgren MK. Davis FC. Dubois S. Shin LM. From Pavlov to PTSD: The extinction of conditioned fear in rodents, humans, and anxiety disorders. Neurobiol Learn Mem. 2014;113:3–18. https://doi.org/10.1016/j.nlm.2013.11.014
18. Rothbaum BO, Kearns MC, Price M, et al. Early intervention may prevent the development of posttraumatic stress disorder: A randomised pilot civilian study with modified prolonged exposure. Biol Psychiatr [serial online]. 2012 [cited 2018 Mar 20];72(11):957–963. Available from: http://www.ncbi.nlm.nih.gov/pubmed/22766415
19. McNally RJ, Bryant RA, Ehlers A. Does early psychological intervention promote recovery from posttraumatic stress? Psychol Sci Public Interest. 2003;4(2):49–72. https://doi.org/10.1111/1529-1006.01421
20. Rose SC, Bisson J, Churchill R, Wessely S. Psychological debriefing for preventing post-traumatic stress disorder (PTSD). Cochrane Database Syst Rev. 2002;(2): CD000560. https://doi.org/10.1002/14651858.CD000560
21. Bastos MH, Bick D, Rowan CJ, Small R, McKenzie-McHarg K. Debriefing for the prevention of psychological trauma in women following childbirth. Cochrane Database Syst Rev. 2008;2. https://doi.org/10.1002/14651858.CD000560
22. Campfield KM, Hills AM. Effect of timing of critical incident stress debriefing (CISD) on posttraumatic symptoms. J Trauma Stress. 2001;14(2):327–340. https://doi.org/10.1023/A:1011117018705
23. Deahl MP, Srinivasan M, Jones N, Neblett C, Jolly A. Evaluating psychological debriefing: Are we measuring the right outcomes? J Trauma Stress. 2001;14(3): 527–529. https://doi.org/10.1023/A:1011160606866
24. Breslau N, Schultz L. Neuroticism and post-traumatic stress disorder: A prospective investigation. Psychol Med. 2013;43(8):1697–702. https://doi.org/10.1017/S0033291712002632
25. Dai W, Chen L, Tan H, et al. Association between social support and recovery from post-traumatic stress disorder after flood: A 13–14 year follow-up study in Hunan, China. BMC Public Health [serial online]. 2016 [cited 2018 Mar 20];16(1):194. Available from: http://www.biomedcentral.com/1471-2458/16/194
26. Clapp JD, Gayle Beck J. Understanding the relationship between PTSD and social support: The role of negative network orientation. Behav Res Ther [serial online]. 2009 [cited];47(3):237–244. Available from: https://doi.org/10.1016/j.brat.2008.12.006
