# Supplementary material for: Mental health status and related factors influencing healthcare workers during the COVID-19 pandemic: A systematic review and meta-analysis
Source: PLoS One. 2024 Jan 19;19(1):e0289454. doi: 10.1371/journal.pone.0289454 (PMC10798549; doi:10.1371/journal.pone.0289454)
Supplement: S1 Data — (ZIP) [file pone.0289454.s011.zip › literatures/123.pdf]

Impact of COVID-19 pandemic outbreak on mental health of the hospital front-line healthcare workers in Chile: a difference-in-differences approach

Pedro Olivares-Tirado[1,2], Rosendo Zanga-Pizarro[1,3]

[1]Research and Development Department, Superintendency of Health of Chile, Santiago, Chile.
[2]Health Service Development Research Center. University of Tsukuba, 7921501 Tsukuba, Japan.
[3]School of Public Health, Faculty of Medicine, University of Chile, 7921501 Santiago, Chile
Address correspondence to Rosendo Zanga-Pizarro, E-mail: rzanga@uchile.cl

ABSTRACT

Background Most of the evidence about impact of COVID-19 pandemic on the mental health of healthcare workers (HCWs) comes from symptom questionnaires. It is important to evaluate main mental health diagnoses in hospital front-line HCW's during the early acute phase of the COVID-19 pandemic in Chile.

Methods An individual-level cross-sectional study using administrative data was conducted. A Difference-in-Difference (DiD) approach was used to estimate the impact of the COVID-19 pandemic on sick leave rates of depression, anxiety and acute stress reaction among hospital front-line HCW's in comparison with other private insured workers.

Results DiD estimates showed a significant reduction of depression (17%), anxiety (8%) and acute stress reaction (8%) sick leave rate, in the front-line HCWs during the COVID-19 pandemic. Reduction of the three mental disorders sick leave rates was higher in men than women. Except for depression, front-line HCW's from the Santiago region's sick leave rates of anxiety and acute stress reaction decreased more than other regions'.

Conclusion Opposite to our hypothesis the results suggests a remarkable resilience level and compromise of front-line HCW's. To address threats to the mental health of HCW's is key to promotes programs for their psychological well-being and safety.

Keywords COVID-19, healthcare workers, mental disorders, sick leave, resilience, Chile

Introduction

The COVID-19 outbreak originated from Wuhan, China, at the end of 2019 and has now rapidly spread over the world, was declared a pandemic on 11 March 2020, by the World Health Organization (WHO).[1] The COVID-19 pandemic continues to spread across most of the world's populations with significantly high mortality and morbidity, devastating economic and social impact and unprecedented health consequences.[2,3]

Reports of the mental health impacts of previous severe health epidemics have focused primarily on those individuals most directly affected by disease, e.g. disease survivors, friends or family members, healthcare workers (HCWs) more than others from affected communities.[4] Female, age under 40 years old, people with chronic diseases and history of medical/psychiatric illness and student status were associated with higher levels of mental disorders during pandemic outbreak.[5]

Previous literature showed that HCWs are at particular risk of negative psychological impact during pandemics, especially if they were front-line workers.[1] In the SARS epidemic, hospital HCWs in China were found to be particularly vulnerable to psychological disorders during and after the acute pandemic wave.[6]

To date, several researches on the psychological symptoms of HCWs during the COVID-19 pandemic have been published. Most were carried out in China using self-reported questionnaires such as Patient Health Questionnaire-9 (PHQ-9), General Health Questionnaire version 12 questions (GHQ-12) and General Anxiety Disorder-7 (GAD-7) to explore the mental health outcomes among HCWs exposed to COVID-19.[1,7] However, these self-reported scales measure

Pedro Olivares-Tirado
Rosendo Zanga-Pizarro

© The Author(s) 2022. Published by Oxford University Press on behalf of Faculty of Public Health. All rights reserved. For permissions, please e-mail: journals.permissions@oup.com

symptoms rather than actual diagnoses. Although both symptoms and diagnoses are important, the difference between them is relevant in the pandemic context. A diagnosis of a psychological disorder, for example, anxiety requires that individuals recognize their emotional distress as excessive or unreasonable, but GAD-7 does not capture this aspect.[8]

Findings showed that front-line HCWs were associated with a higher risk of psychological symptoms and reported more severe degrees of mental health symptoms than other HCWs.[1,9–14] Results from a recent systematic review about mental health among HCWs, including 19 studies, predominantly in hospital settings conducted in a wide range of countries, showed range estimates of 20–51% for depression, 12–45% for anxiety, 34–37% for sleep problems and 31% for acute stress disorder.[7]

Few studies in Latin American countries are available. In Mexico, a self-reported questionnaire for screening applied to a sample of 5938 participants, to compare mental health problems among the front-line with other HCWs. Insomnia (52%), depression (38%) and post-traumatic stress disorder (PTSD) (38%) were the most frequent mental health problems all of which were more frequent in front-line HCWs. A lack of rest time, mourning the death of friends or loved ones due to pandemic and personal COVID-19 status was the main risk factor for insomnia, depression and for PTSD, respectively.[3]

In Chile, results from 'COVID-19 Health Care Workers Study', a 30 countries collaboration project, based on an online questionnaire including PHQ-9 and GHQ-12, applied to 954 Chilean HCWs, showed that the most frequent symptoms were eating disorders (38.6%), sleep disorders (32.7%), moderate to severe depressive symptoms (31.4%), asthenia and fatigue (37.3%), loss of concentration (19.0%) and apathy and anhedonia (16.3%). Furthermore, results showed that HCWs have more than three times the prevalence of mental disorders found in previous population studies in Chile.[15,16]

It is worth mentioning that once the first cases of COVID-19 were detected in Chile, government authorities implemented an integrated public–private hospital care system, to guarantee the availability of critical beds and mechanic ventilators, for the treatment of severe cases of the COVID-19. Additionally, the Chilean government launched an early vaccination program for the entire population prioritizing vulnerable groups, including health workers.

We hypothesized that front-line HCWs may have a higher risk of adverse mental health outcomes associated with higher psychological distress due to the nature of their works, particularly sustained working stressing conditions (e.g. directly involved in care and death of COVID-19 patients, burnout, staff shortages, shift work, unplanned shifts, etc.) during this unprecedented pandemic. We aim to evaluate the prevalence and burden of main mental health disorders in hospital front-line HCWs insured by private health insurances, during the early acute phase of the COVID-19 pandemic in Chile.

Methods

Administrative data recording sick leaves form from the Chilean Private Health Insurance (ISAPRE) was used to investigate depressive episode, anxiety and acute stress reaction sick leave rates, in front-line HCWs. This database offers superior reliability to survey data since the records are directly registered by physicians.

Participants in this study comprised Chilean public hospital front-line HCWs insured by the ISAPREs as of 1 January 2019 to 30 June 2021. As front-line HCWs, we include nurses, physicians, kinesiologists and paramedical staff, assuming they were directly involved in hospital COVID-19 patients' care. As a comparison group, we use the same database to identify other ISAPREs insured workers different from HCWs, with the same diagnoses codes.

The dependent variables in our analysis were the sick leave rate and the total rest days' rate of selected mental disorders. The dependent variables were accounted as the rate (× 1000 insured) of the total number of sick leave and total rest days by the patient in both pre-pandemic and pandemic period, to the front-line HCWs and other ISAPREs insured workers.

To evaluate the impact of pandemic COVID-19 on the mental health of front-line HCWs, we use the Difference-in-Difference (DiD) approach. This method compares the change in outcomes in a group before and after an exposition or intervention (treatment group) to the change in outcomes in another group not affected by the exposition or intervention (comparison group).[17] The DiD estimates were derived from regression models on sick leave rates and total rest day rates for the three selected mental disorders. Models were adjusted by age, sex and location variables. Statistically, the COVID-19 outbreak impact on three selected mental disorders—DiD estimates—were estimated by examining the interaction between time and groups dummies. If the association exists, this interaction term will be significantly different from zero. All analyses were performed with Stata 14.1 and defined statistical significance as P-value < 0.05.

Results

In March 2021, total hospital HCWs amount to 116 667 representing 79% of total public health workers in the country. Front-line HCWs represented 60% of total hospital HCWs, and during the pandemic period, they increased by 15%.

Additionally, 41% of total front-line HCWs are insured by ISAPREs.

The three selected mental disorders represent 95% of total sick leaves by affective and neurotic, stress-related disorders in front-line HCWs, during pre-pandemic and pandemic periods. Adult psychiatrists issued 60% of total sick leaves and the remaining cases were diagnosed by other medical specialties.

The prevalence rates of depression episodes, anxiety and acute stress reaction in front-line HCWs during the pre-pandemic period were 4.6%, 3.8% and 5.9%, respectively. During the pandemic period, the prevalence rates increased to 5.2% for depression episodes to 5.4% for anxiety and to 8.2% for the acute stress reaction. The prevalence rates in the comparison group during the pre-pandemic period were 2.5%, 2.4% and 2.5% for depression episodes, anxiety and acute stress reaction, respectively. These figures decreased to 2.4% in depression and increased to 2.9% and 3.0% in anxiety and acute stress reaction, respectively. In both the groups, the prevalence rates were twice or more in women than in men. The differences in prevalence rates between both periods in both groups were statistically significant. Tables 1–3 show demographic, geographic, professional characteristics and variables related to the samples of the three mental disorders analyzed.

The crude depression sick leave rate decreased 2% in the treatment group, whereas the comparison group increased 6%, during the pandemic period. Front-line HCWs are younger with a higher proportion of women and less concentrated in the Santiago region, than other insured workers. During the pandemic period, the relative proportion of nurses with depression increased in front-line HCWs and administrative and teachers decreased in the comparison group. Both treatment and comparison groups, similarly increased the average rest days by sick leave in the pandemic period.

During the pandemic period, the crude anxiety sick leaves rate increased by 32% and 45% in front-line HCWs and comparison groups, respectively. No significant age differences were observed in both groups. Front-line HCWs show a higher proportion of women and they are less concentrated in the Santiago region than other insured workers. During the pandemic period, the relative proportion of nurses with anxiety increased, meanwhile administrative and teachers decreased and technicians and sellers increased, in the comparison group.

In the acute stress reaction sample, during the pandemic period, the crude sick leave rate increased by 27% and 36% in front-line HCWs and comparison groups, respectively. No significant age differences were observed in both groups. Front-line HCWs show a higher proportion of women and they are less concentrated in the Santiago region, as the other insured workers. During the pandemic period, the relative proportion of nurses with acute stress reaction increased; meanwhile, administrative and teachers decreased and technicians and sellers increased in the comparison group.

According to the DiD approach, the estimates represent the difference in means of sick leave rates between front-line HCWs and other ISAPREs-insured workers during previous and pandemic periods. To interpret these DiD estimates, we must assume that the COVID-19 pandemic in the other private insured workers reflects how the COVID-19 pandemic would have changed in front-line HCWs had they not suffered particular psychological distress by the COVID-19 outbreak—the common trends assumption. In Table 4, we present DiD estimates of the effect of the COVID-19 pandemic outbreak on the front-line HCW's sick leave rate and rest days' sick leave rate of the three mental disorders analyzed. According to R^2s, models fit the data well.

The estimates of depression sick leave rate in front-line HCWs between pandemic and pre-pandemic periods, dropped by about −0.035; meanwhile, other insured workers increased marginally about 0.00045. Then the difference between these two changes is the DiD estimate (−0.036), actually a significant negative effect of the COVID-19 pandemic outbreak on the depression sick leave rate of front-line HCWs, which amounts to a 17% reduction of the baseline rate. On the other hand, the effect of the COVID-19 pandemic is associated with 0.36 significant reductions in depression rest days of sick leave rate, which amounts to a 9.0% reduction of the baseline rate (Table 4).

Also, the effect of COVID-19 pandemic outbreak is associated with a significant −0.011 reduction in sick leave rate for anxiety and acute stress reaction in front-line HCWs, which amounts to an 8.0% reduction of the baseline rate of both mental disorders. Moreover, the COVID-19 pandemic outbreak effect corresponds to a significant 16% and 10% increase in the total rest days' rate for anxiety and acute stress reaction in front-line HCWs, respectively.

As there are important differences in sex and location distributions on mental disorders samples, we run separates DiD models by sex and location to explore the impact of the COVID-19 pandemic outbreak in front-line HCWs of both sex and metropolitan region and other regions of the country.

DiD models estimate that the effect of the COVID-19 pandemic outbreak, on women front-line HCWs were associated with a significant reduction in sick leave rate for depression, anxiety and acute stress reaction, which amounts to 16%, 7% and 6% decreases of the baseline rate of the three mental disorders, respectively. In men front-line HCWs, with

Table 1 Characteristics of depression episode sample of the hospital front-line HCWs and other private insured workers, Chile

	Sick leave by Depression episode (ICD-10 code: F32)			
	Before COVID-19 outbreak		*After COVID-19 outbreak*	
	Front-line HCWs (n: 1554)	*Other workers (n: 57 212)*	*Front-line HCWs (n: 1802)*	*Other workers (n: 51 871)*
Age (years), mean (SD)	37.4 (8.95)	40.3 (10.93)	37.6 (8.55)	41.0 (10.95)
Women (%)	77.5%	60.8%	77.1%	58.5%
Geographic location (%)				
Santiago Metropolitan region	36.5%	64.5%	41.2%	67.0%
Occupation (%,women)				
nurses	54.2% (86%)	—	57.4% (86%)	—
Physicians	33.7% (65%)	—	31.7% (64%)	—
paramedics	6.1% (78%)	—	5.5% (75%)	—
kinesiologists	6.0% (73%)	—	5.4% (65%)	—
administratives	—	28.2% (67%)	—	24.0% (65%)
other professionals	—	25.0% (66%)	—	27.1% (65%)
technicians	—	8.9% (32%)	—	11.5% (26%)
teachers	—	10.6% (77%)	—	8.1% (82%)
sellers	—	8.5% (69%)	—	8.9% (65%)
others	—	18.7% (54%)	—	20.4% (48%)
Number of sick leave by person/year (% women)				
1	45% (77%)	46% (60%)	47% (77%)	42% (59%)
2	24% (75%)	21% (63%)	25% (75%)	21% (61%)
3	12% (79%)	11% (64%)	12% (82%)	12% (62%)
4 and more	19% (82%)	23% (65%)	17% (82%)	25% (62%)
Sick leave rate (x 100 insured)	17.1	8.3	16.7	8.8
women	25.6	14.4	24.7	8.4
men	7.6	4.9	7.4	9.5
Average rest days by sick leave (SD)	17.9 (6.82)	18.0 (6.98)	19.9 (6.89)	20.3 (6.86)

Other professionals: includes executives and others professionals. Technicians: also includes operators, craftsman and manual workers. Others: includes cleaner, unclassified and other insured workers.

exception of depression, the sick leave rates of anxiety and acute stress reaction compared with women, shows a significantly higher decrease regarding the baseline rates. Opposite to the depression, the anxiety and acute stress reaction rest days' sick leave rates, increased in both sex front-line HCWs during the pandemic period (data no shown).

DiD models estimate that the effect of the COVID-19 pandemic outbreak, on women front-line HCWs were associated with a significant reduction in sick leave rate for depression, anxiety and acute stress reaction, which amounts to 16%, 7% and 6% decreases of the baseline rate of the three mental disorders, respectively. In men front-line HCWs, with exception of depression, the sick leave rates of anxiety and acute stress reaction compared with women shows a significantly higher decrease regarding the baseline rates. Opposite to the depression, the anxiety and acute stress reaction rest days' sick leave rates, increased in both sex front-line HCWs during the pandemic period (data no shown).

During the pandemic period, the sick leave rate in front-line HCWs residents in Santiago metropolitan region showed a significant decrease of 8%, 10% and 10% with respect to the baseline rate of depression, anxiety and acute stress reaction, respectively. Furthermore, rest days' sick leave rate in front-line HCWs residents in Santiago metropolitan region decreased in depression and increased in anxiety and acute stress reaction, respectively. In the other regions, the front-line HCWs sick leave rate during the pandemic period showed a significant decrease of 13%, 7% and 7% with respect to the baseline rate of depression, anxiety and acute stress reaction, respectively. In turn, the rest days' sick leave rate of the front-line HCWs residents in other regions, decreased in depression, and increased in anxiety and acute stress reaction, respectively.

Table 2 Characteristics of anxiety sample of the hospital front-line HCWs and other private insured workers, Chile

	Sick leave by anxiety (ICD-10 code: F41)			
	Before Covid-19 outbreak		*After COVID-19 outbreak*	
	Front-line HCWs (n: 1,309)	*Other workers (n: 57,647)*	*Front-line HCWs (n: 1,844)*	*Other workers (n: 64,493)*
Age (years), mean (SD)	37.3 (8.94)	38.8 (10.57)	37.5 (8.77)	39.5 (10.66)
Women (%)	73.4%	54.1%	75.7%	49.4%
Geographic location (%)				
Santiago Metropolitan region	37.6%	67.3%	40.6%	67.6%
Occupation (%,women)				
Nurses	54.7% (81%)	—	58.7% (86%)	—
Physicians	30.8% (60%)	—	26.6% (59%)	—
Paramedics	7.5% (78%)	—	8.5% (69%)	—
Kinesiologists	7.0% (69%)	—	6.3% (56%)	—
Administratives	—	26.1% (58%)	—	22.3% (57%)
Other professionals	—	25.3% (61%)	—	24.8% (59%)
Technicians	—	11.6% (25%)	—	15.5% (24%)
Teachers	—	9.9% (75%)	—	6.7% (80%)
Sellers	—	7.7% (58%)	—	8.5% (57%)
Others	—	19.5% (45%)	—	22.3% (40%)
Number of sick leave by person/year (% women)				
1	69% (73%)	65% (56%)	65% (74%)	56% (52%)
2	19% (70%)	20% (56%)	22% (78%)	21% (50%)
3	6% (81%)	7% (56%)	7% (84%)	9% (50%)
4 and more	6% (86%)	8% (57%)	6% (80%)	13% (52%)
Sick leave rate (×100 insured)	8.9	5.4	11.8	7.8
Women	12.6	8.1	17.1	10.3
Men	4.9	3.8	5.6	6.3
Average rest days by sick leave (SD)	14.0 (7.07)	14.7 (6.62)	15.6 (6.93)	17.0 (6.80)

Other professionals: includes executives and others professionals. Technicians: also includes operators, craftsman and manual workers. Others: includes cleaner, unclassified and other insured workers.

Discussion

Main finding of this study

We hypothesized that front-line HCWs due to the nature of their works, particularly sustained and stressful working conditions during the current COVID-19 pandemic may have a higher risk of adverse mental health outcomes.

Despite an increase in the prevalence rate of three mental disorders in front-line HCWs during pandemic period, DiD estimates showed a significant reduction of depression, anxiety and acute stress reaction sick leave rates in front-line HCWs during the COVID-19 pandemic period. These paradoxical outcomes in sick leave rates could be explained by a relatively high representation of women, particularly nurses in our study.

Nurses have been identified as a risk group for psychological distress, but also some of their work behaviors could be considered a protective factor.[7] Nurses personal and professional characteristics (i.e. mutual support among colleagues, sense of control, coping ability, sense of duty and altruism, acceptance of the risk) and safe and supportive work environment are important protective factors for mental health, then these unobservable effects could be affecting our results.

Then, our findings seem to be more related to the resilience of HCWs against the psychological stress imposed by the pandemic. Then, evaluating resilience in HCWs during the COVID-19 pandemic must be a priority to discuss.

What is already known on this topic

The COVID-19 pandemic has strain on the whole of society. Particularly affected have been HCWs. Most of the studies highlighted that HCWs during the COVID-19 pandemic have experienced elevated psychological distress and increased risk

Table 3 Characteristics of acute stress reaction sample of the hospital front-line HCWs and other private insured workers, Chile

	Sick leave by acute stress reaction (ICD-10 code: F43)			
	Before COVID-19 outbreak		*After COVID-19 outbreak*	
	Front-line HCWs (n: 2027)	*Other workers (n: 59 493)*	*Front-line HCWs (n: 2770)*	*Other workers (n: 66 043)*
Age (years), mean (SD)	37.0 (8.85)	39.0 (10.64)	36.7 (8.29)	39.1 (1.50)
Women (%)	69.5%	53.5%	70.5%	47.5%
Geographic location (%)				
Santiago Metropolitan region	32.2%	62.7%	37.0%	64.7%
Occupation (%,women)				
Nurses	49.6% (82%)	—	50.6% (84%)	—
Physicians	39.3% (54%)	—	39.0% (55%)	—
Paramedics	5.8% (78%)	—	5.2% (74%)	—
Kinesiologists	5.3% (56%)	—	5.2% (54%)	—
Administratives	—	24.0% (58%)	—	20.8% (55%)
Other professionals	—	27.0% (61%)	—	26.2% (59%)
Technicians	—	11.7% (24%)	—	15.7% (17%)
Teachers	—	10.6% (75%)	—	6.9% (78%)
Sellers	—	6.7% (56%)	—	7.4% (54%)
Others	—	20.0% (43%)	—	23.0% (37%)
Number of sick leave by person/year (% women)				
1	69% (69%)	67% (56%)	65% (71%)	59% (50%)
2	20% (71%)	20% (56%)	22% (71%)	22% (50%)
3	6% (78%)	7% (56%)	7% (77%)	9% (49%)
4 and more	5% (72%)	6% (59%)	7% (75%)	10% (49%)
Sick leave rate (×100 insured)	13.6	5.1	17.3	6.9
women	17.7	7.7	23.3	8.8
men	9.0	3.7	10.5	5.9
Average rest days by sick leave (SD)	12.9 (6.68)	14.3 (6.72)	15.0 (6.90)	17.1 (6.87)

Other professionals: includes executives and others professionals. Technicians: also includes operators, craftsman and manual workers. Others: includes cleaner, unclassified and other insured workers.

Table 4 COVID-19 pandemic impact on sick leave rate of mental disorders on front-line HCWs: DiD estimation

Outcome variable	*n obs*	*Unadjusted models*		*Adjusted models*	
		DiD (SE)	*Adj-R^2*	*DiD (SE)*	*Adj-R^2*
Sick leave rate					
Depression	112.439	−0.036*** (0.0013)	0.4520	−0.036*** (0.0013)	0.4523
Anxiety	125.293	−0.011*** (0.0006)	0.5770	−0.011*** (0.0006)	0.5776
Acute reaction to stress	130.333	−0.011*** (0.0006)	0.5610	−0.011*** (0.0006)	0.5613
Rest days sick leave rate					
Depression	112.439	−0.359*** (0.0297)	0.3715	−0.359*** (0.0297)	0.3719
Anxiety	125.293	0.191*** (0.0129)	0.4274	0.189*** (0.0129)	0.4280
Acute reaction to stress	130.333	0.169*** (0.0137)	0.3694	0.169*** (0.0137)	0.3700

The DiD estimator was estimates using linear regression models adjusting for covariates including age, sex and Santiago metropolitan location. In parentheses, standard errors (SE).

The three asterisks indicate significance at the 0.000 level.

of mental health disorders, particularly if they are young, women, nurses and front-line workers.[10]

A systematic review across 19 studies focused on HCWs predominantly in a hospital setting—most conducted in China—has identified a wide range of the prevalence of depression (20–51%), anxiety (12–45%), acute stress (31%) and sleep problems (34–37%) among HCWs during the COVID-19 pandemic.[9] A study in Japan, suggested that COVID-19 outbreaks had a greater psychological impact on HCWs than non-HCWs and demonstrated that psychological distress among HCWs was sustained at a similar level during all four waves.[13]

Regarding to HCWs resilience during the COVID-19 pandemic, Robles *et al*.[3] found that nurses and psychologists had lower frequencies of almost all mental health problems than the other HCWs and Alvarado *et al*.[18] who reported that the vast majority of hospital HCW's reported feeling support from their coworkers (83%) and loved ones (94%). Furthermore, only 51% say they need psychological support, being higher in women (56%) than in men (38%). This finding suggests important factors contributing to a resilience level in front-line HCWs.

What this study adds

Most of the results of the COVID-19 epidemic impact on the mental health of the HCWs proceed from self-reported surveys, collected in short periods and based on scales measure symptoms. Then, to have data about current diagnoses from medical leave registers—not only symptom—extended by specialists, certainly it is a more precise approach about the real impact of the COVID-19 pandemic on the mental health of the front-line HCWs.

The use of the regression models to estimate DiD estimates allows us to adjust these estimates by pertinent variables and also offer a way to estimate the statistical significance of the differences between the DiD estimates, which certainly must be considered as a methodological contribution.

Limitations of this study

Limitation of this study includes a sample representing only a tier of the total hospital HCWs in Chile, which may impact generalizability. Professional heterogeneity of the front-line group could be leveled out the effect of pandemic COVID-19 on mental disorder sick leave rates. The effect of the government measures to control the pandemic, such as quarantines, threatening disruption of work or job loss in some occupations, induced the use/abuse of sick leave as a legal way to maintain work contracts and incomes, then an overrepresentation of mental disorders in the comparison group it was possible.

Conclusions

In conclusion, the emergence of the COVID-19 pandemic has exacerbated conditions and determinants of poor mental health in the whole population but particularly in HCW's. However, the impact this global crisis will have on HCW's mental health, in the long term is unknown. In dealing with COVID-19 epidemic outbreaks, the ability to adapt and overcome existing difficulties—resilience—is needed. The WHO has indicated that governments and leaders of health organizations must address threats to the health and safety of its workers and patients, promoting policies and programs for their safety, as well as mental health and psychological wellbeing is key to ensuring the functioning of the health system and society in a pandemic scenario.[12,19]

Supplementary data

Supplementary data are available at the *Journal of Public Health* online.

References

1. Talevi D, Socci V, Carai M *et al.* Mental health outcomes of the covid-19 pandemic. *Riv Psichiatr 1 de mayo de* 2020;**55**(**3**):137–44.
2. Santomauro DF, Mantilla Herrera AM, Shadid J *et al.* Global prevalence and burden of depressive and anxiety disorders in 204 countries and territories in 2020 due to the COVID-19 pandemic. *Lancet [Internet]*. 6 de noviembre de 2021 [citado 22 de noviembre de 2021];**398**(**10312**):1700–12.
3. Robles R, Rodríguez E, Vega-Ramírez H *et al.* Mental health problems among healthcare workers involved with the COVID-19 outbreak. *Rev Bras Psiquiatr [Internet]*. octubre de 2021 [citado 22 de noviembre de 2021];**43**(**5**):494–503.
4. Dawel A, Shou Y, Smithson M *et al.* The effect of COVID-19 on mental health and wellbeing in a representative sample of Australian adults. *Front Psych*6 de octubre de 2020;**11**:1026.
5. Xiong J, Lipsitz O, Nasri F, Lui LMW, Gill H, Phan L, *et al.* Impact of COVID-19 pandemic on mental health in the general population: a systematic review. *J Affect Disord [Internet]*. 1 de diciembre de 2020 [citado 22 de noviembre de 2021];**277**:**55**. Disponible en: /pmc/articles/PMC7413844/
6. O'Connor K, Wrigley M, Jennings R *et al.* Mental health impacts of COVID-19 in Ireland and the need for a secondary care mental health service response. *Ir J Psychol Med [Internet]*1 de junio de 2021[citado 22 de noviembre de 2021];**38**(**2**):1 Disponible en: /pmc/articles/PMC7471570/.
7. Uphoff EP, Lombardo C, Johnston G *et al.* Mental health among healthcare workers and other vulnerable groups during the COVID-19 pandemic and other coronavirus outbreaks: a rapid systematic review. *PLoS One [Internet]*. 1 de agosto de 2021 [citado 22 de noviembre de 2021];**16**(**8**):e0254821.
8. Taquet M, Holmes EA, Harrison PJ. Depression and anxiety disorders during the COVID-19 pandemic: knowns and unknowns. *Lancet*

*(London, England) [Internet]*noviembre de 2021[citado 22 de noviembre de 2021];**398**(**10312**):1665–6.

9. Lai J, Ma S, Wang Y *et al.* Factors associated with mental health outcomes among health care workers exposed to coronavirus disease 2019. *JAMA Netw Open [Internet]*. 2 de marzo de 2020 [citado 22 de noviembre de 2021];**3**(**3**):e203976–6.
10. Søvold LE, Naslund JA, Kousoulis AA *et al.* Prioritizing the mental health and well-being of healthcare workers: an urgent global public health priority. *Front public Heal [Internet]*. 7 de mayo de 2021 [citado 22 de noviembre de 2021];**9**. Disponible en. https://pubmed.ncbi.nlm.nih.gov/34026720/.
11. Khanal P, Devkota N, Dahal M *et al.* Mental health impacts among health workers during COVID-19 in a low resource setting: a cross-sectional survey from Nepal. *Global Health [Internet]*. 25 de septiembre de 2020 [citado 22 de noviembre de 2021];**16**(**1**) Disponible en. https://pubmed.ncbi.nlm.nih.gov/32977818/.
12. Pappa S, Ntella V, Giannakas T *et al.* Prevalence of depression, anxiety, and insomnia among healthcare workers during the COVID-19 pandemic: a systematic review and meta-analysis. *Brain Behav Immun [Internet]*. 1 de agosto de 2020 [citado 22 de noviembre de 2021];**88**:901–7.
13. Sasaki N, Asaoka H, Kuroda R *et al.* Sustained poor mental health among healthcare workers in COVID-19 pandemic: a longitudinal analysis of the four-wave panel survey over 8 months in Japan. *J Occup Health [Internet]*. 1 de enero de 2021 [citado 22 de noviembre de 2021];**63**(**1**):e12227.
14. Urzúa A, Samaniego A, Caqueo-Urízar A *et al.* Salud mental en trabajadores de la salud durante la pandemia por COVID-19 en Chile. *Rev Med Chil [Internet]*. 1 de agosto de 2020 [citado 22 de noviembre de 2021];**148**(**8**):1121–7.
15. *Primer informe The COVID-19 Health Care Workers Study - Escuela de Salud Pública - Universidad de Chile [Internet]*. [citado 22 de noviembre de 2021]. Disponible en: http://www.saludpublica.uchile.cl/noticias/167313/primer-informe-the-covid-19-health-care-workers-study.
16. *INFORME PRELIMINAR N° 4 CHILE Condiciones Laborales - Buscar con Google [Internet]*. [citado 22 de noviembre de 2021]. Disponible en: https://www.google.com/search?q=INFORME+PRELIMINAR+No+4+CHILE+Condiciones+Laborales&rlz=1C1ALOY_esCL956CL956&oq=INFORME+PRELIMINAR+N0+4+CHILE+Condiciones+Laborales&aqs=chrome..69i57.332j0j9&sourceid=chrome&ie=UTF-8.
17. Fredriksson A, de Oliveira GM. Impact evaluation using difference-in-differences. *RAUSP Manag J* 9 de diciembre de 2019;**54**(**4**):519–32.
18. Alvarado R, Ramirez J, Cortes M *et al.* El impacto de la pandemia de COViD-19 en la salud mental de los trabajadores de la salud en Chile. *Rev Med Chil* 2021;**149**:1205–14.
19. Baskin RG, Bartlett R. Healthcare worker resilience during the COVID-19 pandemic: an integrative review. *J Nurs Manag [Internet]*. 1 de noviembre de 2021 [citado 22 de noviembre de 2021];**29**(**8**):2329–42.
