# Supplementary material for: Mental health status and related factors influencing healthcare workers during the COVID-19 pandemic: A systematic review and meta-analysis
Source: PLoS One. 2024 Jan 19;19(1):e0289454. doi: 10.1371/journal.pone.0289454 (PMC10798549; doi:10.1371/journal.pone.0289454)
Supplement: S1 Data — (ZIP) [file pone.0289454.s011.zip › literatures/125.pdf]

CSP CADERNOS DE SAÚDE PÚBLICA
REPORTS IN PUBLIC HEALTH

The impact of the COVID-19 pandemic on the mental health of healthcare professionals

O impacto da pandemia de COVID-19 na saúde mental dos profissionais de saúde

El impacto de la pandemia de COVID-19 en la salud mental de los profesionales de la salud

Felipe Ornell [1,2,3]
Silvia Chwartzmann Halpern [1,2]
Felix Henrique Paim Kessler [1,2]
Joana Corrêa de Magalhães Narvaez [4]

doi: 10.1590/0102-311X00063520

The 2019 novel coronavirus pandemic (COVID-19) is an international public health emergency unprecedented in modern history [1]. Besides the biological context, and due to the wide and long-lasting changes in daily life it may cause, coping with it represents a challenge to psychological resilience. Previous studies have shown that epidemics and contamination outbreaks of diseases have been followed by drastic individual and social psychosocial impacts, which eventually become more pervasive than the epidemic itself [2,3]. Currently, due to this pandemic, high levels of anxiety, stress and depression have already been observed in the general population [4,5].

Considering this scenario, it is essential that health authorities identify groups with high risk of developing emotional issues – in addition to the biological peril, already well established and publicized – to monitor their mental health and carry out early psychological and psychiatric interventions [3,4]. Amongst these are the healthcare workers assisting patients with known or suspected COVID-19. Primary care workers, such as nurses, nursing technicians and medical doctors who are in direct contact with patients and their body fluids, are those most vulnerable to infection [3,6].

During pandemics, as the world faces a shutdown or slowdown in daily activities and individuals are encouraged to implement social distancing so as to reduce interactions between people, consequently reducing the possibility of new infections [7], health professionals usually go in the opposite direction. Due to the exponential increase in the demand for healthcare, they face long work shifts, often with few resources and precarious infrastructure [8], and with the need of wearing Personal Protective Equipment (PPE) that may cause physical discomfort and difficulty breathing [6]. In addition, many professionals may feel unprepared to carry out the clinical intervention of patients infected with a new virus, about which little is known, and for which there are no well-established clinical protocols or treatments [6]. Also, there is the fear of autoinoculation, as well as the concern about the possibility of spreading the virus to their families, friends or colleagues [9,10]. This can lead them to isolate themselves from their family nuclear or extended, change their routine and narrow down their social support network [6].

These factors can result in different levels of psychological pressure, which may trigger feelings of loneliness and helplessness, or a series of dysphoric emotional states, such as stress, irritability, physical and mental fatigue, and despair [6]. The work overload and the symptoms related to stress make health professionals especially vulnerable to psychological suffering [8,9,10], which increases the chance

[1] Hospital de Clínicas de Porto Alegre, Universidade Federal do Rio Grande do Sul, Porto Alegre, Brasil.
[2] Programa de Pós-graduação em Psiquiatria e Ciências do Comportamento, Universidade Federal do Rio Grande do Sul, Porto Alegre, Brasil.
[3] Faculdade lBGEN – Grupo Uniftec, Centro Universitário e Faculdades, Porto Alegre, Brasil.
[4] Universidade Federal de Ciências da Saúde de Porto Alegre, Porto Alegre, Brasil.

Correspondence
F. Ornell
Centro de Pesquisas em Álcool e Drogas, Hospital das Clínicas de Porto Alegre, Universidade Federal do Rio Grande do Sul.
Rua Prof. Álvaro Alvim 400, Porto Alegre, RS
90420-020, Brasil.
felipeornell@yahoo.com.br

This article is published in Open Access under the Creative Commons Attribution license, which allows use, distribution, and reproduction in any medium, without restrictions, as long as the original work is correctly cited.

of developing psychiatric disorders [11]. If, on the one hand, healthcare teams – mainly in emergency services – may be used to feeling physical fatigue and mental weariness, on the other hand, due to the fear, insecurity and uncertainty caused by a pandemic, these well-known factors could now impact human relationships. Historically, catastrophes can mobilize teams due to commotion, but they are usually exempt from fear of the transmissibility of the infection, as despite the threat being invisible, possible negative outcomes are an inconvenient and frightening reality. Therefore, the recognition of risks and planning of interventions aimed at reducing the damage to the psychological health of professionals involved in the care of patients infected by COVID-19 (Brazilian Intensive Care Association – AMIB) should be a priority, and actions need to be established and implemented.

During the severe acute respiratory syndrome (SARS) outbreak in 2003, 18 to 57% of health professionals experienced serious emotional problems and psychiatric symptoms during and after the event [12]. In 2015, during the Middle East respiratory syndrome (MERS) outbreak, also caused by coronavirus, dysphoria and stress were observed among health professionals. These conditions were a predictor of misconduct, delays in treatment due to communication failures and absenteeism, among others. In these situations, it is common for feelings that are not verbally expressed by the teams to end up being expressed in the work environment through absences and omissions. Frontline professionals were also shown to be at higher risk of developing post-traumatic stress disorder (PTSD), which persisted even after a period of absence from work [12]. There are also studies reporting that the mental health implications for health workers involved in healthcare during epidemics can be persistent. High levels of stress, depression, anxiety and PTSD were observed after some time had transpired since the end of the emergency [2,3].

The Burnout syndrome was also reported by health professionals involved in assisting patients during an epidemic caused by another type of coronavirus that occurred in Korea in 2016 [13]. Although this condition is usually established longitudinally and is related to organizational factors (such as institutional climate, moral harassment, excessive workload, low wages, among others), the severity of the pandemic can trigger emotional exhaustion [14].

Vicarious trauma or secondary traumatic stress, a phenomenon in which health professionals experience symptoms similar to the patients' due to continued exposure, is also common during catastrophes. The main symptoms of indirect trauma are appetite loss, fatigue, physical decline, sleep and attention disorders, irritability, numbness, fear and despair [2]. In addition, professionals involved directly in the care of a disease with high potential of contagion may suffer stigma. At the other end of the spectrum, a trend that in COVID-19 is more triggered is to give health professionals a status of super heroes, and if on the one hand it adds value, on the other hand, it has additional pressure, because superheroes don't fail, don't give up or get sick. This can be reinforced by the media due to the sensational character of an event with worldwide proportions, demarcating the need for emotional support, encouragement and appreciation [15]. Moral suffering also can lead to situations such as the collapse of the health system, preventing health professionals from making adequate decisions due to internal (fear, inability to face suffering, lack of knowledge) or external pressures (hierarchical pressure, communication and organizational problems, lack of resources and support from other services).

Currently, there are few scientific studies addressing epidemiological data and intervention models focused on the mental health of health professionals involved in assisting patients with COVID-19. The majority of these studies available have been conducted in China. Therefore, besides the language barrier, these data may not be generalized to other locations with different socio-cultural characteristics, especially in developing countries such as Brazil, in which specificities associated with the structure of its health system need to be addressed.

Recently, a study with nurses and physicians involved in the treatment of COVID-19 found a high incidence of stress, anxiety and PTSD, with higher levels of anxiety in women and nurses compared to men and physicians, respectively. This can be explained by the fact that nurses have longer work shifts and closer contact with patients, which can easily lead to fatigue and tension. Another study with a similar sample found that the physicians' level of social support was significantly associated with efficacy and quality of sleep, and negatively associated with anxiety and stress [16].

Health professionals who are in direct contact with infected patients need to have their mental health regularly screened and monitored, especially in relation to depression, anxiety and suicidal

ideation. In the same way, it is essential to identify professionals with a history of exposure to psychosocial risk factors. Therefore, psychiatric treatments should be provided to those with more serious mental health problems. Specifically regarding the mental health of healthcare professionals in the context of COVID-19, it is important to identify secondary psychosocial factors that may potentially generate stress, e.g., professionals with chronic diseases, living with young children or older family members, among others [15].

It is suggested that somatic symptoms such as insomnia, anxiety, anger, rumination, decreased concentration, depression and loss of energy are evaluated and managed at the institution by the mental health professionals. It is also recommended that psychological/psychiatric care is provided to professionals in hospitals or other healthcare settings. In addition, strict measures must be implemented to prevent infection and ensure a safe environment for consultations, as well as practical training on how to use PPE properly [12] (Box 1).

Box 1

Mental health care models and interventions strategies for healthcare professionals.

**1 Psychosocial response team**<br>*Marketing and press*<br>• Disseminate mental health care actions and strategies<br>• Disseminate informative and psychoeducational materials towards the identification of physical and emotional symptoms<br>• Disseminate self-applied instruments for monitoring mental health and early identification of symptoms<br>• Disseminate information about psychosocial support services	**2 Technical support team for psychological intervention**<br>*Psychologists and psychiatrists*<br>• Implement mental health care actions<br>• Define tools for monitoring mental health<br>• Develop psychoeducational and informative folders promoting mental health care and to the recognition of signs and symptoms linked to mental health<br>• Define theoretical framework and format of psychological interventions<br>• Provide training, guidance and technical supervision to intervention teams<br>• Develop actions focused on mental health care after the end of the epidemic<br>• Establish a preventive contingency plan and strategies to deal with more serious psychiatric symptoms
**3 Psychological/Social/Psychiatric intervention team**<br>*Psychologists, psychiatrists, social workers*<br>• Carry out clinical psychological interventions with health professionals<br>• Provide psychological support for health professionals to facilitate emotional expression<br>• Provide emergency care and psychological first aid<br>• Identify professionals with previous psychiatric conditions (depression, burnout, anxiety disorders, bipolar disorder, trauma...)<br>• Identify professionals with secondary psychosocial risks to work (chronic diseases, young children or elderly family members, difficulty in transportation)<br>• Identify professionals who need special attention, provide initial care and refer for longitudinal follow-up, if necessary<br>• Provide psychological counseling<br>• Develop strategies for managing anxiety<br>• Develop strategies that promote empathy and support among the team through therapeutic groups, for example<br>• Identify the occurrence of burnout and provide care<br>• Establish team communication	**4 Psychological front line teams**<br>*Volunteers (health professionals or students) who received training and oversight*<br>• Provide remote psychological assistance (phone, mobile applications) 24 hours a day, 7 days a week<br>• Provide face-to-face or remote guidance to assist in crisis management<br>• Provide psychological counseling<br>• Provide emotional psychoeducation strategies, guide the identification of PTSD symptoms, anxiety and depression, so they can identify themselves and their peers early on<br>• Provide remotely psychological support for professionals involved in patient care, aiming to facilitate emotional expression<br>• Identify the occurrence of burnout (physical and mental exhaustion resulting from work), provide help in the identification of needs and concerns, and in strategy development<br>• Provide psychological intervention in the context of crisis – psychological first aid<br>• Assist in self-monitoring of somatic symptoms: insomnia, sadness, fatigue, anhedonia, anguish, fear, anxiety, anger, suicidal thoughts

(continues)

Box 1 (continued)

Screening instruments	Possible interventions
• Burnout: *Maslach Burnout Inventory – Human Services Survey* (MBI-HSS)<br>• Stress at work: *Job Stress Scale* (JSS)<br>• Tracking non-psychotic mental disorders: S*elf-Reporting Questionnaire* (SRQ-20)<br>• Tracking Mental Disorders: *DSM-5 Cross-Level Symptom Scale 1*<br>• Alcohol and drugs: *Alcohol, Smoking and Substance Involvement Screening Test* (ASSIST)/WHO<br>• Quality of life: *World Health Organization Quality of Life –* Short version (WHOQOL-bref)<br>• Operation: *Brief Scale of Operation* (FAST)<br>• Depression: *Hamilton scale*<br>• Anxiety: *Hamilton scale*<br>• Resilience: *Resilience Pillar Scale* (EPR)	• Mindfulness techniques<br>• Guided meditation<br>• Psychoeducation interventions<br>• Burnout prevention protocols<br>• Therapeutic groups<br>• Stress management strategies<br>• Anxiety and depression brief interventions<br>• Post-traumatic stress interventions<br>• Suicide protocols<br>• Psychological first aid "kits"<br>• Mindfulness and meditation techniques<br>• Positive psychology and wellbeing strategies for individuals and environmental setting<br>• Individual counseling<br>• Small group therapies and supervision<br>• Telephone and virtual assistance<br>• Mental health applications<br>• Nutrition and exercise reinforcement<br>• Psychiatric and pharmacological treatment for severe cases

PTSD: post-traumatic stress disorder; WHO: World Health Organization.

It is essential that psychological support measures take into consideration the professionals' emotional expression. Psychological intervention (PI) in the context of a crisis also aims to offer coping strategies to deal with intrusive thoughts and anticipatory or situational anxiety. In addition, emotional interventions aim to facilitate intra-team support, empathy, and compassion towards more fragile colleagues [15]. In healthcare settings without enough safety and prevention facilities, informative folders promoting mental health and online psychological counseling and psychotherapy [5] are recommended. Digital communication can also be a viable complementary strategy [15] and should be available 24 hours a day [5].

PI teams and healthcare models were implemented at the RenMin Hospital of Wuhan University and at the Wuhan Mental Health Center. They were responsible for preparing materials and providing guidance and technical supervision. The PI medical team, made up mainly of psychiatrists, participated in the interventions. There were also teams from the psychological assistance hotline (volunteers trained in PI) providing guidance by phone or virtual chat to help the health professionals deal with their mental health problems [9].

It is also important to highlight the need to develop communication within healthcare teams, in order to establish a climate of reciprocity and empathic cooperation, allowing the expression of feelings and symptoms such as burnout and emotional exhaustion. In addition, psychoeducation and guidance regarding symptoms of post-traumatic stress, anxiety and depression must be provided to the teams [15] so that they are able to identify these symptoms in themselves and in their peers in their early stages. Furthermore, the health professionals must be capable of identifying the emotional aspects arising from the patients and their families to map them out and indicate adequate psychological support resources and interventions available in the healthcare system. Promoting the mental health of healthcare teams is essential, and has clinical, political and social effects [11].

Finally, there are strategies for dealing with the emotional helplessness and mental suffering expressed by some professionals after the end of the epidemic. In this sense, it is suggested to create proper spaces for listening and exchanging feelings in order to prevent burnout [15].

In Brazil, where the contagion curve is currently escalating, healthcare strategies for frontline professionals also need to be intensified. If they are not prioritized, in addition to the possible collapse of the health system, healthcare professionals will be at risk of experiencing an emotional breakdown. It should be noted that Brazil is characterized by strong socioeconomic disparities. However, the country offers a universal health system that aims to provide equal access to all its citizens. Therefore, the access to different levels of healthcare dramatically exposes the country's socioeconomic structure and weaknesses in this moment of crisis.

Thus, strategies for combating COVID-19 require primary attention to the general health of the population, including economic measures that support the necessary guidelines during this period of contingency. It is important to highlight the complexity of the Brazilian healthcare system, in which healthcare professionals who operate on different complexity levels may require different information, support or intervention strategies. Moreover, historically, there are few mental health programs available to healthcare professionals.

Two issues need to be considered: (1) the availability of human and other resources, due to the current precariousness of the health system; (2) the availability of healthcare in distant areas. It is essential that governmental guidelines are developed to support training programs and implement municipal strategies. Regional interventions are necessary,considering that the local health determinants may differ between regions. As complementary strategies, it is possible to establish partnerships with civil society institutions and implement remote assistance systems. Currently, different initiatives are being developed by universities and other private mental health clinics to provide online and telephone support to healthcare professionals.

In summary, therefore, government and healthcare agencies have the responsibility to protect the psychological well-being of the healthcare community worldwide [4]. It must be emphasized that physical distance is not synonymous with emotional distance [15], and that the fear of interaction should be restricted to caring for the virus, since isolation must be distinguished from loneliness. In addition, it is imperative that resources are invested in order to significantly promote the mental health of these frontline professionals, both in terms of research, prevention and treatment.

Contributors

F. Ornell contributed to the article's design, writing and final review, and to the literature review. S. C. Halpern contributed to the literature review, and to the article's writing and proofreading. F. H. P. Kessler contributed to the article's writing and proofreading. J. C. M. Narvaez contributed to the article's design and orientation, writing, and final review.

Additional informations

ORCID: Felipe Ornell (0000-0002-3881-4283); Silvia Chwartzmann Halpern (0000-0002-3882-7762); Felix Henrique Paim Kessler (0000-0001-7059-2564); Joana Corrêa de Magalhães Narvaez (0000-0001-5972-4722).

Acknowledgments

To the Brazilian National Drug Policy Secretariat (SENAD).

References

1. World Health Organization. Novel coronavirus (2019-nCoV) situation reports. Geneva: World Health Organization; 2020.
2. Li Z, Ge J, Yang M, Feng J, Qiao M, Jiang R, et al. Vicarious traumatization in the general public, members, and non-members of medical teams aiding in COVID-19 control. Brain Behav Immun 2020; [Epub ahead of print].
3. Ornell F, Schuch JB, Sordi AO, Kessler FHP. "Pandemic fear" and COVID-19: mental health burden and strategies. Braz J Psychiatry 2020; [Epub ahead of print].
4. Wang C, Pan R, Wan X, Tan Y, Xu L, Ho CS, et al. Immediate psychological responses and associated factors during the initial stage of the 2019 Coronavirus disease (COVID-19) epidemic among the general population in China. Int J Environ Res Public Health 2020; 17:E1729.
5. Zhu Y, Chen L, Ji H, Xi M, Fang Y, Li Y. The risk and prevention of novel coronavirus pneumonia infections among inpatients in psychiatric hospitals. Neurosci Bull 2020; 36:299-302.

6. Huang JZ, Han MF, Luo TD, Ren AK, Zhou XP. Mental health survey of 230 medical staff in a tertiary infectious disease hospital for COVID-19. Zhonghua Lao Dong Wei Sheng Zhi Ye Bing Za Zhi 2020; 38:E001.
7. Wilder-Smith A, Freedman DO. Isolation, quarantine, social distancing and community containment: pivotal role for old-style public health measures in the novel coronavirus (2019-nCoV) outbreak. J Travel Med 2020; 27:taaa020.
8. Shigemura J, Ursano RJ, Morganstein JC, Kurosawa M, Benedek DM. Public responses to the novel 2019 coronavirus (2019-nCoV) in Japan: mental health consequences and target populations. Psychiatry Clin Neurosci 2020; 74:281-2.
9. Kang L, Li Y, Hu S, Chen M, Yang C, Yang BX, et al. The mental health of medical workers in Wuhan, China dealing with the 2019 novel coronavirus. Lancet Psychiatry 2020; 7:e14.
10. Xiang Y-T, Yang Y, Li W, Zhang L, Zhang Q, Cheung T, et al. Timely mental health care for the 2019 novel coronavirus outbreak is urgently needed. Lancet Psychiatry 2020; 7:228-9.
11. Malta M, Rimoin AW, Strathdee SA. The coronavirus 2019-nCoV epidemic: is hindsight 20/20? EClinicalMedicine 2020; 20:100289.
12. Lee SM, Kang WS, Cho AR, Kim T, Park JK. Psychological impact of the 2015 MERS outbreak on hospital workers and quarantined hemodialysis patients. Compr Psychiatry 2018; 87:123-7.
13. Kim JS, Choi JS. Factors influencing emergency nurses' Burnout during an outbreak of Middle East respiratory syndrome coronavirus in Korea. Asian Nurs Res (Korean Soc Nurs Sci) 2016; 10:295-9.
14. Herrer MG. Coronar la cumbre. Riesgos emocionales y cuidado del personal sanitario ante el COVID-19. https://humanizandoloscuidadosintensivos.com/es/coronar-la-cumbre-riesgos-emocionales-y-cuidado-del-personal-sanitario-ante-el-covid-19/ (accessed on Mar/2020).
15. Associação de Medicina Intensiva Brasileira. Recomendações para o bem-estar emocional da equipe multidisciplinar durante a pandemia pelo Sars-Cov-2. https://www.amib.org.br/fileadmin/user_upload/amib/2020/marco/18/corona_psico_amib_15h56_18032020.pdf (accessed on Mar/2020).
16. Xiao H, Zhang Y, Kong D, Li S, Yang N. The effects of social support on sleep quality of medical staff treating patients with Coronavirus disease 2019 (COVID-19) in January and February 2020 in China. Med Sci Monit 2020; 26:e923549.

Submitted on 31/Mar/2020
Final version resubmitted on 06/Apr/2020
Approved on 06/Apr/2020
