# Supplementary material for: Mental health status and related factors influencing healthcare workers during the COVID-19 pandemic: A systematic review and meta-analysis
Source: PLoS One. 2024 Jan 19;19(1):e0289454. doi: 10.1371/journal.pone.0289454 (PMC10798549; doi:10.1371/journal.pone.0289454)
Supplement: S1 Data — (ZIP) [file pone.0289454.s011.zip › literatures/126.pdf]

frontiers in Psychiatry

ORIGINAL RESEARCH
published: 28 July 2021
doi: 10.3389/fpsyt.2021.662742

Risk and Protective Factors for the Mental Health of Brazilian Healthcare Workers in the Frontline of COVID-19 Pandemic

Flávia L. Osório[1,2], Isabella Lara Machado Silveira[1], Karina Pereira-Lima[3], José Alexandre de Souza Crippa[1,2], Jaime Eduardo Cecílio Hallak[1,2], Antônio Waldo Zuardi[1,2] and Sonia Regina Loureiro[1,2]*

[1] Medical School of Ribeirão Preto, São Paulo University, São Paulo, Brazil, [2] National Institute for Science and Techonology, Translational Medicine, National Council for Scientific and Technological Development, Brasília, Brazil, [3] Department of Psychiatry and Medical Psychology, Federal University of São Paulo (UNIFESP), São Paulo, Brazil

OPEN ACCESS

Edited by:
Joao Mauricio Castaldelli-Maia, Faculdade de Medicina do ABC, Brazil

Reviewed by:
Lise Haddouk, Université de Rouen, France
Marcos C. Baptista, Universidade de São Paulo, Brazil

***Correspondence:**
Flávia L. Osório
flaliosorio@gmail.com

Specialty section:
This article was submitted to Public Mental Health, a section of the journal Frontiers in Psychiatry

Received: *01 February 2021*
Accepted: *07 July 2021*
Published: *28 July 2021*

Citation:
Osório FL, Silveira ILM, Pereira-Lima K, Crippa JAdS, Hallak JEC, Zuardi AW and Loureiro SR (2021) Risk and Protective Factors for the Mental Health of Brazilian Healthcare Workers in the Frontline of COVID-19 Pandemic. Front. Psychiatry 12:662742. doi: 10.3389/fpsyt.2021.662742

The objective was to compare the mental health indicators of health workers providing care to individuals with COVID-19 in Brazil, considering sociodemographic and occupational variables and the risk perception of contamination by the Sars-CoV-2 of workers from different professions, identifying risk and protective factors. A sample of 916 health workers was assessed: physicians, nursing workers, and workers from other professions (psychologists, physical therapists, nutritionists, speech therapists, occupational therapists, dentists, pharmacists, and social workers). REDCAP software was used to collect data online, using standardized instruments to assess anxiety, depression, posttraumatic stress, and insomnia, and one questionnaire addressed risk and protective variables. Statistical techniques for comparing groups were used along with logistic regression analysis. The results revealed that all the groups presented indicators of significant mental health problems (>36%), especially the nursing group. A larger percentage of participants, regardless of the profession, presented a high rate of insomnia disorders, while posttraumatic stress was the least expressive. Occupational variables stand out as risk factors for mental health, with specificities among the different groups. A protective factor for all the groups was having positive professional prospects. The protective factors for the physicians group included support provided by co-workers, being older and a man, while being satisfied with physical protective measures implemented by the employing institution was a protective factor for the groups composed of nursing workers and other professionals. These findings are relevant for devising mental health care strategies.

Keywords: mental health, risk, COVID-19, health workers, Brazil

INTRODUCTION

The impact of the Sars-CoV-2 and COVID-19 on health workers has been reported worldwide. The increased demand for healthcare services is a challenge for health workers because the virus is highly transmissible so that interactions represent a risk for contamination while the disease may have a severe presentation in 19% of cases (1, 2). Moreover, the effectiveness of clinical management protocols is uncertain (3).

Studies conducted in various countries reveal that healthcare providers working in the frontline against COVID-19 have experienced the highest rates of mental disorders (4). The meta-analysis conducted by Garcia-Iglesias et al. reports high levels of anxiety (26 to 45%), depression (8 to 25%), preoccupation/insomnia (24 to 38%), and stress (3.8 to 68.3%) (5). Other meta-analyses report that 63% of workers have a general concern with their health, 44% fear contagion, and 38% experience insomnia (6); women and nursing workers more frequently experience these problems (7).

Heeding the mental health of healthcare providers ensures the quality of their immediate professional skills, which are translated into effective care provided to patients (8) and minimizes the long-term effects of the pandemic. Various mental disorders are likely to develop in this context, such as major depressive disorder, generalized anxiety, and posttraumatic stress, among others, the impact of which on the health and well-being of these workers have not been fully established (9).

Thus, numerous specificities and occupation-related conditions of the different professionals in the health field, such as the hierarchy of technical and social relationships established at work, gender differences, and unequal recognition, need to be taken into account to implement appropriate actions and strategies intended to promote, protect, and assist these workers (10).

Thus far, few studies have considered the workers' profession a relevant variable, specifically focusing on medical and nursing workers (11). Some studies, mostly conducted in developed countries, included other professionals in their samples such as radiology and laboratory technicians, psychologists, physical therapists, dentists, pharmacists, public health workers, and health sciences students, who integrate multidisciplinary teams providing care to patients with COVID-19 (8, 12–17). These studies report that nursing workers are more intensively affected by the pandemic, presenting higher levels of anxiety (12–15, 17), somatization (8, 17), dissociation (14), depression (16, 17), hopelessness (13), acute stress (17), and compassion fatigue (12).

To our knowledge, no study has analyzed sociodemographic and occupational variables together with these workers' risk perception. These variables can either work as risk or protective factors, influencing the mental health of workers from different professions (18–20). Such information might be relevant when planning interventions at a personal or institutional level.

Studies conducted in low-and-middle-income countries (21), such as Brazil, are opportune because specific sociocultural aspects can be identified and assessed, shedding light on risk and protective factors.

Hence, this study's aim is to compare mental health indicators among the different health workers providing care to individuals with COVID-19 in Brazil, considering sociodemographic and occupational variables along with their perception of risk contamination by Sars-CoV-2 to identify potential risk and protective factors.

METHOD

Design

observational, cross-sectional study with group comparison. This study integrates a larger longitudinal study, the objective of which is to assess and monitor mental health indicators and levels of emotional overload among Brazilian health workers during the COVID-19 pandemic. The sample, estimated at 1,500 participants, was based on the Chinese pioneer study (11).

Sample

Non-probabilistic sample, composed of Brazilian health workers from different professions providing care to patients with COVID-19 during the pandemic, namely: physicians (regardless of the specialty), nursing workers (nurses, nursing technicians/aids, and radiology technicians), and other professionals with a college degree (psychologists, physical therapists, nutritionists, speech therapists, occupational therapists, dentists, pharmacists, and social workers). The participants were recruited through social media (Facebook, Instagram, WhatsApp), traditional media (TV and radio), and contacting professional practice councils and relevant health institutions located in different Brazilian regions. Participation in the study was voluntary, and the participants confirmed their agreement by signing online free and informed consent forms. The study was submitted to and approved by the Institutional Review Board (Process No. 4.032.190).

Assessment Instruments

Sample Characterization

- Questionnaire addressing sociodemographic, occupational, and perception of contamination risk: self-report questionnaire with close-ended questions developed for this study. The guidelines provided by the Brazilian Society of Intensive Care (22) and the study by Pfefferbaum & North (4) guided the selection of questions.

Assessment of Outcomes

The outcomes of interest were chosen based on previous studies conducted in different countries reporting the mental health conditions of healthcare providers (8, 9, 11, 12, 23) and studies addressing the mental health conditions of health providers working in the pandemic context (24–26).

- Generalized Anxiety Disorder-7 (GAD-7): 7-item self-report instrument that screens anxiety-associated symptoms rated on a three-point scale ranging from 0 (never) to 3 (almost every day). It was proposed by Spitzer et al. (27) and validated in Brazil by Moreno et al. (28); its cut-off score $\geq$ 10 presents 89% of sensitivity and 82% of specificity;

- Patient Health Questionnaire-9 (PHQ-9): 9-item self-report instrument intended to assess depression indicators. It was proposed by Kroenke et al. (29) and validated in Brazil by Osório et al. (30). Its items are rated from 0 ("never") to 3 ("almost every day"), and the cut-off score $\geq$ 10 presents 100% sensitivity and 98% of specificity;

- Posttraumatic Stress Disorder Checklist for DSM-5 (PCL-5): self-report instrument used to assess posttraumatic stress disorder symptoms using the criteria established by the DSM-5. Its short version (8 items), which was translated, adapted, and psychometrically assessed Osório et al. (31) and Pereira-Lima et al. (32), was used. Its cutoff point $\geq$ 21 presents sensitivity equal to 0.79 and specificity equal to 0.76.

- Insomnia Severity Index (ISI): 7-item self-report instrument rated on a 5-point Likert scale intended to assess the severity of insomnia in the last 2 weeks. It was adapted and validated in Brazil by Castro (33), with a cutoff point $\geq$ 8 and sensitivity of 73% and specificity of 80% to detect positive and negative cases of chronic insomnia.

Procedures

Data were collected and managed through REDCap software (Research Electronic Data Capture), in which the SURVEY application generated an electronic link through which the participants could access the instruments. Data were collected during 3 months, from May 19th to August 23rd, 2020. When the study began, the first COVID-19 case had been officially diagnosed in Brazil 82 days before, while the number of confirmed cases had reached 271,628 and deaths totaled 17,971, with different peaks in the various Brazilian regions. At the end of data collection, the total number of accumulated cases was 3,605,783, with 114,744 deaths (34).

Data Analysis

Only the responses of the participants who thoroughly answered all the instruments were analyzed. SPSS was used for the statistical analysis. Sociodemographic and occupational data were descriptively analyzed and compared between groups (Chi-square and ANOVA) together with the participants' perceptions of risk contamination and mental health indicators. Due to multiple comparisons, the Bonferroni correction was applied to the level of significance. The final level of significance obtained in three bivariate comparisons was 0.017. Later, a multivariate logistic regression analysis was independently performed using the backward stepwise method (35) for each group of professionals. The model included the continuous variables "age and years of work experience" and nominal variables "age, marital status, live with (no one/with a spouse and/or children), have children (yes, no), workplace (public/private/mixed), type of facility (secondary or tertiary), COVID-19 referral facility (yes, no), work in the COVID-19 frontline (yes, no), increased working hours due to the pandemic (yes, no), desire to quit the job (never-rarely/often-always), positive professional prospects (yes, no), satisfied with the physical and mental protective measures implemented by the employing institution (never-rarely/often-always), receive social/emotional support from co-workers (yes, no), Sar-CoV-2 infection (yes, no), concern with being infected or the possibility of infecting family members (yes/no), a perception that people avoid social contact due to your job (yes, no). The model was considered significant when p-value < 0.05.

RESULTS

The initial sample was composed of 1,522 participants; 606 did not fully answer all the instruments and were excluded. The final sample was composed of 916 participants: 41% were nursing workers ($N = 376$), 30% physicians ($N = 275$), and 29% ($N = 265$) other professions (11.4% were physical therapists, 6.2% psychologists, 3.1% nutritionists, 2.8% pharmacists, 2.0% speech therapists, 1.7% social workers, 1.1% dentists, and 0.7% were occupational therapists). All the Brazilian states were represented, but most participants were from the southeast (62%). **Table 1** presents the sample characterization.

Table 1 shows a predominance of women in the three groups, with a partner and/or children, 9 years of work experience on average, with a position in a COVID-19 referral center, working in the frontline, and reporting a concern with being infected or with the risk of infecting a family member. Regarding the physicians' group, a smaller share of women composes this group along with a larger number of people living alone. This group also differs from the remaining because it gathers older and more experienced participants. The percentage of nursing professionals working in public hospitals is larger than that of other professionals, while physicians predominate in tertiary hospitals. More nursing professionals are working in the frontline against the COVID-19 and are also the least satisfied with their employers' physical and mental protective measures. Note that all the groups reported low levels of satisfaction with protective measures. The group composed of nursing workers and workers from other professions presented the highest percentage of individuals concerned with being infected by the Sars-CoV-2 and individuals receiving social and emotional support from co-workers. No differences were found between the groups in relation the variables extra workload (most answered yes), desire to quit the job (most answered no), and positive professional prospects (most answered yes). The initial differences regarding a concern with the possibility of infecting family members and a perception that people avoided contact due to their work did not remain in the *post hoc* analyses.

Table 2 presents the groups' mental health indicators and shows that all the groups presented considerably high indicators of mental health problems (>36%), mainly nursing workers, who present significantly higher indicators of anxiety, depression, and insomnia than the remaining groups. The percentage of problems linked to insomnia was the most expressive among all workers. Comparatively, the physicians presented the lowest percentage of problems linked to insomnia. No differences were found between the groups with regard posttraumatic stress, which presented the lowest percentages.

Table 3 presents the final logistic regression models for each of the outcomes and groups. This table shows that occupational

TABLE 1 | Sociodemographic and occupational characterization and risk perception of contamination by the Sars-CoV-2, considering three groups of workers ($n = 916$).

Variables	Nursing *N* (%)	Physicians *N* (%)	Other professionals *N* (%)	Total *N* (%)	Statistics (*)
**Sociodemographic**					
Female	314 (83.5)$^{\#}$	186 (67.6)$^{\#\circ}$	230 (86.8)$^{\circ}$	730 (79.7)	<0.001
Age X (SD)	33.7 (±8.4)$^{\#}$	38.5 (±10.5)$^{\#\circ}$	33.9 (±7.9)$^{\circ}$	35.2 (±9.2)	<0.001
Married/stable union	178 (47.3)	141 (51.3)	115 (43.4)	434 (47.4)	0.19
Live with spouse and/or children	328 (87.2)$^{\#}$	205 (74.5)$^{\#\circ}$	224 (84.5)$^{\circ}$	757 (82.6)	<0.001
No children	188 (50.0)$^{\propto}$	160 (58.2)$^{\circ}$	181(68.3)$^{\circ\propto}$	529 (57.8)	<0.0001
**Occupational**					
Years of professional experience	9.0 (7.4)$^{\#}$	12.4 (10.7) $^{\#\circ}$	9.5 (7.7)$^{\circ}$	10.2 (8.7)	<0.001
Public hospital	246 (65.4)$^{\#}$	144 (52.4)$^{\#\circ}$	182 (68.7)$^{\circ}$	572 (62.4)	<0.001
Tertiary facility	152 (40.4)$^{\#}$	172 (62.5)$^{\#\circ}$	121 (45.7)$^{\circ}$	445 (48.6)	<0.001
COVID-19 referral center	251 (66.8)	175 (63.6)	175 (66.0)	601 (65.6)	0.70
Work in the frontline of the COVID-19	329 (87.5)$^{\#\propto}$	190 (69.1)$^{\#}$	193 (72.8)$^{\propto}$	712 (77.7)	<0.001
Increased working hours due to the pandemic	199 (52.9)	124 (45.1)	137 (51.7)	460 (50.2)	0.12
Desire to quit job	72 (19.1)	40 (14.5)	37 (14.0)	149 (6.3)	0.14
**COVID-19 perceptions**					
Satisfied with physical protective measures	140 (37.2)$^{\propto}$	125 (45.5)	135 (50.9)$^{\propto}$	400 (43.7)	0.002
Satisfied with mental protective measures	51 (13.6)$^{\propto}$	47 (17.1)	62 (23.4)$^{\propto}$	160 (17.5)	0.005
Social and emotional support provided by co-workers	238 (63.3)$^{\propto}$	179 (65.1)$^{\circ}$	205 (77.4)$^{\propto\circ}$	622 (67.9)	<0.001
Positive professional prospects	284 (75.5)	209 (76.0)	214 (80.8)	707 (77.2)	0.26
Infected with Sars-CoV-2 (self-report)	71 (18.9)$^{\#}$	28 (10.8)$^{\#}$	32 (12.1)	131 (14.3)	0.003
Concern with being infected	317 (84.3)$^{\#}$	196 (71.3)$^{\#\circ}$	215 (81.1)$^{\circ}$	728 (79.5)	<0.001
Concern with infect a family member	364 (96.8)	257 (93.5)	258 (97.4)	879 (96.0)	0.04
People avoid social contact due to the job	235 (62.5)	148 (53.8)	144 (54.3)	527 (57.5)	0.04

**post-hoc analysis ($p < 0.017$); $^{\#}$difference between nursing and physicians; $^{\circ}$significant difference between physicians and other professions; $^{\propto}$significant difference between nursing and other professions.*

TABLE 2 | Indicators of mental health problems, considering the cut-off point of each instrument for the three groups of workers.

Variable/ instrument	Nursing *N* (%)	Physicians *N* (%)	Other professions *N* (%)	Total *N* (%)	Statistics*
Anxiety/ GAD-7	189 (50.3)$^{\#\propto}$	102 (37.1)$^{\#}$	106 (40.0)$^{\propto}$	397 (43.3)	0.002
Depression/ PHQ-9	170 (45.2)$^{\#}$	97 (35.3)$^{\#}$	101 (38.1)	368 (40.2)	0.03
TEPT/ PCL-5	146 (38.9)	89 (32.4)	95 (35.8)	330 (36.0)	0.24
Insomnia/ ISI	242 (64.4)$^{\#}$	148 (53.8)$^{\#\circ}$	173 (65.3)$^{\circ}$	563 (61.5)	0.008

*%, percentage of participants with scores above the instrument's cut-off point; *post-hoc analysis ($p < 0.017$); $^{\#}$difference between nursing and physicians; $^{\circ}$significant difference between physicians and other professions; $^{\propto}$ significant difference between nursing and other professions.*

factors stood out compared to sociodemographic factors in terms of potential risk and occupational protective measures.

Among the protective factors, having positive professional prospects stood out in all the groups for all psychopathological outcomes. The support provided by co-workers also emerged as an important protective variable, especially among the physicians. Being satisfied with the physical protective measures adopted by the employing institution was the most important protective factor for the groups composed of nursing workers and workers from other professions. Being older was the only protective factor linked to sociodemographic variables that remained in the predictive model for the outcomes anxiety/stress in the three groups; the likelihood of not experiencing pathological levels of anxiety/stress increased from 3 to 5% with every additional year of experience. Being a man and having more years of experience in the profession were protective factors for the physicians' group.

Regarding risk factors, greater specificity was found between the groups. In the nursing group, a concern with being infected by the Sars-CoV-2 was the variable most frequently associated

TABLE 3 | Predicting variables that compose the final regression model for anxiety, depression, posttraumatic stress, and insomnia according to group.

**Anxiety**		**β**	**IC (95%)**
Nursing	Concern with being infected	3.64	1.88–6.99
	Increased working hours due to the pandemic	1.91	1.24–2.93
	Positive professional prospects	0.51	0.31–0.85
	Satisfaction with mental health protective measures	0.51	0.27–0.98
Physicians	Work in the COVID-19 frontline	4.65	2.17–9.98
	Desire to quit job—yes	3.04	1.29–7.15
	No children	2.41	1.03–5.61
	Age—increase (per year)	0.95	0.91–0.99
	Being a man	0.51	0.26–0.96
	Positive professional prospects—yes	0.40	0.19–0.83
	Social/emotional support provided by co-workers—yes	0.28	0.14–0.56
Others	Concern with being infected	4.66	2.03–10.68
	Being older (per year)	0.94	0.91–0.98
	Positive professional prospects	0.19	0.09–0.40
**Depression**			
Nursing	Concern with being infected	4.51	2.19–9.26
	Longer professional experience (per year)	0.96	0.93–0.99
	Social/emotional support provided by co-workers—yes	0.47	0.29–0.76
	Positive professional prospects	0.41	0.24–0.70
	Satisfaction with mental health protective measures	0.18	0.08–0.44
Physicians	No children	2.37	1.14–4.91
	Work in the COVID-19 frontline	2.24	1.12–4.52
	Single/no partner	2.18	1.09–4.37
	Perception that people avoid social contact due to the work	1.88	1.03–3.42
	Social/emotional support provided by co-workers	0.37	0.19–0.70
	Positive professional prospects	0.24	0.12–0.47
	Being a man	0.21	0.10–0.43
Others	Desire to quit job	2.20	1.02–4.74
	Satisfaction with mental health protective measures	0.40	0.20–0.80
	Positive professional prospects	0.37	0.19–0.73
**Post traumatic stress**			
Nursing	Concern with being infected	2.85	1.44–5.68
	Age—increase (per year)	0.97	0.95–0.99
	Positive professional prospects	0.48	0.29–0.80
	Satisfaction with mental health protective measures	0.35	0.17–0.74
Physicians	Desire to quit job	3.11	1.38–7.02
	Perception that people avoid social contact due to the work	2.55	1.37–4.76
	Single/no partner	2.54	1.30–4.93
	Longer professional experience (per year)	0.96	0.93–0.99
	Social/emotional support provided by co-workers	0.49	0.26–0.94
**Post traumatic stress**			
	Being a man	0.37	0.19–0.74
	Positive professional prospects	0.30	0.15–0.62
Others	Perception that people avoid social contact due to the work	2.65	1.50–4.71
	Desire to quit job	2.61	1.19–5.73
	Positive professional prospects	0.26	0.13–0.52
**Insomnia**			
Nursing	Increased working hours due to the pandemic	1.66	1.08–2.57
	Social/emotional support provided by co-workers	0.56	0.35–0.90
	Positive professional prospects	0.43	0.24–0.75
Physicians	Single/no partner	2.84	1.69–4.78
	COVID-19 referral center	2.21	1.18–4.12
	Extra workload	1.91	1.14–3.20
	Tertiary hospital	0.51	0.28–0.95
	Positive professional prospects	0.41	0.22–0.76
Others	Increased working hours due to the pandemic	1.92	1.14–3.24
	Positive professional prospects	0.33	0.15–0.73

with the risk of experiencing mental health problems (OR > 2.85) such as anxiety, depression, and stress followed by extra workload (OR>1.66) for anxiety and insomnia. In the physicians' group, working in the COVID-19 frontline was a variable of significant risk for outcomes such as anxiety and depression (OR > 2.24), along with a desire to quit the job for anxiety and posttraumatic stress (OR > 3.11). A perception that people avoided social contact due to the professionals' job also emerged as a risk variable for physicians and other workers, especially in relation posttraumatic stress (OR > 2.55). In the group of other professionals, concern with being infected by the Sar-CoV-2 was a risk variable for anxiety (OR = 4.66), and a desire to quit the job was a risk variable for depression and posttraumatic stress (OR > 2.20). Sociodemographic variables (not having children or a spouse) were associated with risk factors for all the outcomes in the physicians' group only. The facility's characteristics, such as being a secondary hospital or a referral COVID-19 center, emerged as risk factors for insomnia in the physicians' group.

Few specificities were found when analyzing the psychopathological outcomes in isolation, with different variables standing out among the different professions.

DISCUSSION

There were many new cases and deaths during the data collection (34), revealing the pandemic's impact in Brazil. Consequently, an intense demand was imposed on healthcare providers. This demand became explicit in the results presented as most workers (regardless of their professions) were in the frontline against the COVID-19. Approximately half of the workers reported

extra workload due to the pandemic, regardless of the facility's level of complexity or whether it was a referral COVID-19 center. Nonetheless, most did not report a desire to quit their jobs and manifested positive expectations about their professional prospects.

The rates of mental health disorders were high (above 30%) among all the workers regardless of the profession, with higher rates for insomnia, revealing the healthcare providers' high personal and emotional involvement in this challenging context (7). Higher rates of insomnia are a relevant warning sign, considering that sleep deprivation is one of the leading causes of impaired neurobehavioral performance, possibly compromising patient safety when affecting healthcare providers (14). It is worth noting that insomnia rates are much higher (61.5%) than those reported by previous studies conducted in China (20 to 36%) (36, 37), Nepal (33.5%) (16), and Paraguay (27.8%) (13). Thus, it requires attention and a search for measures intended to decrease the risk factors associated with this condition, such as excessive workload.

Nursing workers presented the highest rates of anxiety, depression, and stress. These indicators align with the results reported by studies addressing health workers from different countries (4–6), with nursing workers, especially those in the frontline (7), experiencing greater vulnerability (11, 38).

In contrast, physicians reported the lowest rates of problems for all the outcomes addressed here. This finding aligns with other studies conducted during the pandemic regarding depressive symptoms (14–16, 39) and insomnia (40, 41).

Most studies compare nursing workers and physicians, and some of these studies considered specificities such as these workers' specialties and qualifications (e.g., medical residents, nursing students). However, most studies addressing the group Other Professions did not provide details about the professions included, qualifications, or whether the participants were healthcare providers or healthcare managers. Having more information about the context of these workers is relevant, especially in the context of low-and-middle-income countries, and this is one of this study's contributions (21).

Overall, current indicators found among Brazilian health workers are high compared to the parameters reported before the pandemic, suggesting that the rate of common mental disorders increased among healthcare workers during the COVID-19 pandemic. For instance, studies addressing primary health care workers before the pandemic report that 16% experienced minor mental disorders (42); 23.39% of the physicians working in ICUs, emergency rooms, and wards (43) and 25.2% of mental health workers on average presented such disorders (44). Another study reports that the percentage of depression indicators was 28.4% among ICUs nurses (45).

Comparisons of the respondents' sociodemographic profiles revealed characteristics shared by all the professions, such as a predominance of women living with a partner and/or children. Various studies conducted in the pandemic context report that more significant emotional distress, anxiety, stress, depression, insomnia, somatic symptoms, and worse overall health status are associated with being a woman (12, 16, 46–48). Note that emotional problems are more prevalent and severe among women, as reported by a study conducted with the general population (49) in an occupational context. Female workers' vulnerability is an issue regardless of the current context and may be associated with a lack of appreciation/recognition and having to reconcile a paid job with domestic chores and the care provided to the family (8). Extra working hours during the pandemic, combined with direct contact with infected patients and social isolation measures, which often involve distancing themselves from their loved ones to avoid contagion, impose additional pressure on these women, especially when they have children and/or elderly relatives at home (16). Another aspect to be highlighted is that women, more frequently than men, tend to pay greater attention to their inner experiences and others' emotional conditions (8).

The physicians presented the lowest percentage of women and were mainly composed of older and more experienced workers. These appear as protective factors in this group and possibly favored a drop in emotional vulnerability indicators. These characteristics, also identified in other studies addressing the pandemic context, favored fewer mental health problems among these health workers (4, 50). Additionally, being a man was associated with a lower prevalence of anxiety, depression, and dissociation symptoms (14); being older was associated with less frequent anxiety disorders (47), depression (12), and improved mental health, though not improved physical health (51). Living alone was associated with less frequent anxiety disorders among these workers (47). In this study, living alone possibly explains why these workers are less afraid of contaminating family members. Professional experience also contributed to less frequent mental health problems, as reported by an Australian study in which older medical workers reported significantly lower levels of depression than workers from other professions (39).

In relation to the occupational characteristics, most of the nursing group reported working in a public hospital in the frontline against the COVID-19, elements that emerged as risk factors for mental health problems and may have contributed to this groups' higher rates of problems. The reason is that the context of a public hospital in Brazil is very different from that of a private hospital in terms of human and material resources. Moreover, few public hospitals are considered centers of excellence, providing low-quality services and dealing with a high demand of patients. These factors, coupled with low salaries and few benefits, directly impact working conditions and occupational safety against many risks (52, 53). Previous studies report that healthcare providers from private institutions enjoy improved mental health (51) while working in the frontline against COVID-19 was associated with higher levels of anxiety, depression, insomnia, and distress (11). Additionally, excess workload, longer hours delivering care (15), and mainly fear of being infected by Sars-CoV-2 represented risk factors for mood and stress problems in the nursing group. This finding is relevant, considering that this group reported a higher rate of contamination, which seems to reflect peculiarities of the profession that involves close and constant contact with patients (13); which is an important contamination risk in the COVID-19 context (1). Magnavita et al. (54) report that nurses are

the workers most frequently infected, a factor associated with physical symptoms, anxiety, and depression.

Even though physicians present the lowest rate of mental distress, the risk factors linked to their occupational characteristics were quite prominent, such as being a frontline worker, working in a referral center and/or secondary health care service, working extra hours, having a desire to quit their jobs, and a perception that people avoid social contact due to their jobs. Personal conditions, such as not having a partner or children, compounded the risks. Excessive workload and burnouts caused by the medical profession have been long reported (55). One Brazilian study reports that 46% of the participant physicians routinely worked more than 50 h/week, a workload that seems to have become even heavier during the pandemic (55). Job dissatisfaction is recurrent and linked to other factors such as low remuneration, difficulties in making a living as a private practice physician, lack of professional autonomy, and poor working conditions, which favor uncertainty and pessimistic professional prospects (55). It is noteworthy that there are few secondary care facilities in Brazil linked to universities of excellence providing health care programs (most are tertiary), which would provide more work opportunities and facilitate discussions with the health staff and supervisors, previously reported as an indicator of quality in healthcare services (53).

The group composed of workers from other professions presented fewer risk indicators; the main one was fear of being infected by the Sars-CoV-2. Fear is associated with fatigue, discomfort, helplessness, and an inability to adopt self-care strategies (14). Approximately 73% of these professionals were in the frontline of the COVID-19 fight, though in many services, only occasionally mental care was provided to individuals infected with COVID-19.

The indicators suggest a high level of psychological distress among all the groups during the pandemic, revealing that health workers are dealing with high work demands, which may have an immediate impact on their long-term mental health (8). In addition to the effects caused to the health of these workers, individuals experiencing psychological distress tend to less frequently engage in relationships with patients and more frequently make mistakes, with the potential to compromise the patients' clinical outcomes (8).

All the participants reported a low level of satisfaction (<50%) with the protective measures implemented in their workplaces, for example, insufficient personal protective equipment (PPEs). Risk perception (3) possibly reflects on a large number of workers afraid of being infected (79%) and of contaminating their families and loved ones (96%), also influencing their perception that people avoid social contact due to their jobs (57%); the media widely disseminate health workers' poor working conditions. Not having access to protective equipment was associated with a greater risk of experiencing acute stress (46), while previous studies report that having protection and access to PPEs predicted improved physical health and lower levels of distress (51). Likewise, fear of being infected was associated with a high prevalence of anxiety, depression, and dissociation (14), higher risk of experiencing acute stress, and concern with becoming sick (46), and concern with the possibility of infecting a family member and with one's own life, was associated with higher stress levels (48). Additionally, experiencing discrimination and stigma may affect one's work performance and the ability to concentrate (15).

Dissatisfaction with the care provided by the work institutions to the employees' mental health is even more emphasized (82%). This information is worth noting, considering the restrictions imposed by the mental health policies implemented in Brazil. In theory, these policies should include primary health care to high complex care actions, though, in practice, the focus is restricted to care provided to severe and acute cases. No system is structured to provide expanded care, not in the employing institutions or the Brazilian Unified Health System (SUS). Being a public system, the SUS, which is regulated by the constitution, is supposed to provide all Brazilian citizens integral, universal, and free of charge access to healthcare services (56).

Receiving mental care in the current context emerged as a protective factor, especially for the nursing group, decreasing up to 50% the risk of anxiety problems. Therefore, the importance of implementing care measures should not be neglected, especially after the pandemic, when such an impact will likely affect the lives of many workers due to posttraumatic stress. Hence, health managers in the different governmental and institutional spheres will likely face this challenge. Interestingly, receiving social and emotional support from co-workers was a protective factor for the nursing and medical groups, though they less frequently reported support from co-workers. Social support perception has been reported to have mediating effects on protecting health providers' mental health (57). In the current social stigma context, having a more cohesive workgroup is crucial, and different strategies should be implemented to promote team cohesiveness.

Analysis of this set of sociodemographic and occupational variables and the workers' perceptions reveals considerable impact on the mental health conditions of workers from different professions, especially occupational variables. Having positive professional prospects was relevant to decrease risks among all the groups, suggesting that it is essential to continually show professional appreciation and recognition to support workers coping with challenging situations, such as a pandemic. It represents a challenge for managers worldwide but especially for those in low-and-middle-income countries. Healthcare professionals are vital to mitigate the pandemic effects, and the media and society have widely recognized their work. As these workers have long sought to improve working conditions, the current moment seems opportune for demands of this nature to be considered and met, improve labor conditions.

Limitations and contributions: to our knowledge, this is the first study addressing Brazilian health workers' mental health conditions and comparing risk and protective factors among different professions in the health field. Such knowledge is relevant for devising preventive measures and care actions at an occupational and institutional level, considering the importance of the current context. The most relevant limitations include the online collection of data and associated bias; the cross-sectional methodological design, which does not allow establishing a cause and effect relationship; the differentiated

incidence of the pandemic in the different Brazilian regions, as a more significant number of participants were from the southeast, a region with the highest number of cases. Additionally, the participants' previous mental health conditions were not assessed, so that rates may have been underestimated, considering that those with important symptoms may not have adhered to the study or provided incomplete answers, possibly not reporting symptoms such as apathy or lack of concentration, among others. Future studies with a longitudinal design are needed to monitor these different profiles at different points in time.

DATA AVAILABILITY STATEMENT

The raw data supporting the conclusions of this article will be made available by the authors, without undue reservation.

ETHICS STATEMENT

The studies involving human participants were reviewed and approved by Comitê de Ética em Pesquisa do Hospital das Clínicas da Faculdade de Medicina de Ribeirão Preto–USP. The patients/participants provided their written informed consent to participate in this study.

AUTHOR CONTRIBUTIONS

FO, IS, KP-L, JC, JH, AZ, and SL: conception and design or analysis and interpretation of data and final approval of the version to be published. FO and SL: substantial contributions to drafting the article or revising it critically for important intellectual content. All authors contributed to the article and approved the submitted version.

FUNDING

National Council for Scientific and Technological Development (CNPq–Process No. 401058/2020-4; 465458/2014-9; Productivity Research Fellows: no. 302601/2019-8 (FO); 307945/2018-9 (SL); The São Paulo Research Foundation no. 2014/50891-1). The funders had no role in the design, analysis, interpretation, or publication of this study.

REFERENCES

1. Kang L, Li Y, Hu S, Chen M, Yang C, Yang BX, et al. The mental health of medical workers in Wuhan, China dealing with the 2019 novel coronavirus. *Lancet Psychiatry.* (2020) 7:3. doi: 10.1016/S2215-0366(20)30047-X
2. Hu B, Guo H, Zhou P, Shi ZL. Characteristics of SARS-CoV-2 and COVID-19. *Nat Rev Microbiol.* (2021) 19:141–54. doi: 10.1038/s41579-020-00459-7
3. Pfefferbaum B, North CS. Mental health and the covid-19 pandemic. *N Engl J Med.* (2020) 383:6. doi: 10.1056/NEJMp2008017
4. Maciaszek J, Ciulkowicz M, Misiak B, Szczesniak D, Luc D, Wieczorek T, et al. Mental health of medical and non-medical professionals during the peak of the covid-19 pandemic: a cross-sectional nationwide study. *J Clin Med.* (2020) 9:8. doi: 10.3390/jcm9082527
5. García-Iglesias JJ, Gómez-Salgado J, Martín-Pereira J, Fagundo-Rivera J, Ayuso-Murillo D, Martínez-Riera JR, Ruiz-Frutos C. Impact of SARS-CoV-2 (Covid-19) on the mental health of healthcare professionals: a systematic review. *Rev Esp Salud Publica.* (2020) 94:e202007088.
6. Pablo GS, Vaquerizo-Serrano J, Catalan A, Arango C, Moreno C, Ferre F, et al. Impact of coronavirus syndromes on physical and mental health of health care workers: systematic review and meta-analysis. *J Affect Disord.* (2020) 275:48–57. doi: 10.1016/j.jad.2020.06.022
7. Pappa S, Ntella V, Giannakas T, Giannakoulis VG, Papoutsi E, Katsaounou P. Prevalence of depression, anxiety, and insomnia among healthcare workers during the COVID-19 pandemic: a systematic review and meta-analysis. *Brain Behav Immun.* (2020) 88:901–7. doi: 10.2139/ssrn.3594632
8. Conti C, Fontanesi L, Lanzara R, Rosa I, Porcelli P. Fragile heroes. The psychological impact of the COVID-19 pandemic on health-care workers in Italy. *PLoS ONE.* (2020) 15:11. doi: 10.1371/journal.pone.0242538
9. Rana W, Mukhtar S, Mukhtar S. Mental health of medical workers in Pakistan during the pandemic COVID-19 outbreak. *Asian J Psychiatr.* (2020) 51:102080. doi: 10.1016/j.ajp.2020.102080
10. Teixeira CFS, Soares CM, Souza EA, Lisboa ES, Pinto ICM, Andrade LR, et al. The health of healthcare professionals coping with the Covid-19 pandemic. *Ciênc Saúde Coletiva.* (2020) 25:9. doi: 10.1590/1413-81232020259.19562020
11. Lai, J, Ma S, Wang Y, Cai Z, Hu J, Wei N, et al. Factors associated with mental health outcomes among health care workers exposed to coronavirus disease. *JAMA Netw Open.* (2020) 3:3. doi: 10.1001/jamanetworkopen.2020.3976
12. Samaniego, A, Urzúa, A, Buenahora, M, Vera-Villarroel, P. Symptomatology associated with mental health disorders in health workers in Paraguay: COVID-19 effect. *Interam J Psychol.* (2020) 54:1. doi: 10.30849/ripijp.v54i1.1298
13. Hacimusalar Y, Kahve AC, Yasar AB, Aydin MS. Anxiety and hopelessness levels in COVID-19 pandemic: a comparative study of healthcare professionals and other community sample in Turkey. *J Psychiatr Res.* (2020) 129:181–8. doi: 10.1016/j.jpsychires.2020.07.024
14. Azoulay, E, Cariou, A, Bruneel, F, Demoule, A, Kouatchet, A, Reuter, D, et al. Symptoms of anxiety, depression, and peritraumatic dissociation in critical care clinicians managing patients with COVID-19. A cross-sectional study. *Am J Respir Crit Care Med.* (2020) 202:10. doi: 10.1164/rccm.202006-2568OC
15. Khanal P, Devkota N, Dahal M, Paudel K, Joshi D. Mental health impacts among health workers during COVID-19 in a low resource setting: a cross-sectional survey from Nepal. *Global Health.* (2020) 16:1. doi: 10.1186/s12992-020-00621-z
16. Cunill, M, Aymerich, M, Serdà, B, Patiño-Masó, J. The impact of COVID-19 on spanish health professionals: a description of physical and psychological effects. *Int J Ment Health Promot.* (2020) 22:3. doi: 10.32604/IJMHP.2020.011615
17. Wang, Y, Duan, Z, Peng, K, Li, D, Ou, J, Wilson, A, et al. Acute stress disorder among frontline health professionals during the COVID-19 outbreak: a structural equation modelling investigation. *Psychosom Med.* (2020) 83:373–9. doi: 10.1097/PSY.0000000000000851
18. Simms A, Fear NT, Greenberg N. The impact of having inadequate safety equipment on mental health. *Occup Med.* (2020) 20:70:278–81. doi: 10.1093/occmed/kqaa101
19. Evanoff BA, Strickland JR, Dale AM, Hayibor L, Page E, Duncan JG, Kannampallil T, Gray DL. Work-related and personal factors associated with mental well-being during the covid-19 response: survey of health care and other workers. *J Med Internet Res.* (2020) 22:e21366. doi: 10.2196/21366
20. Bekele F, Hajure M. Magnitude and determinants of the psychological impact of COVID-19 among health care workers: a systematic review. *SAGE Open Med.* (2021) 9:20503121211012512. doi: 10.1177/20503121211012512
21. Ornell F, Schuch JB, Sordi AO, Kessler FHP. "Pandemic fear" and COVID-19: mental health burden and strategies. *Braz J Psychiatry.* (2020) 42:3. doi: 10.1590/1516-4446-2020-0008
22. Associação de Medicina Intensiva Brasileira. Recomendações para o bem-estar emocional da equipe multidisciplinar durante a pandemia pelo Sars-Cov-2. Available online at: https://www.amib.org.br/fileadmin/user_upload/amib/2020/marco/18/corona_psico_amib_15h56_18032020.pdf (accessed October 20, 2020).
23. Ornell F, Halpern SC, Kessler FHP, Narvaez JCM. The impact of the COVID-19 pandemic on the mental health of healthcare professionals. *Cad Saúde Pública.* (2020) 4:e00036520. doi: 10.1590/0102-311x00063520

24. Øyane NM, Pallesen S, Moen BE, Akerstedt T, Bjorvatn B. Associations between night work and anxiety, depression, insomnia, sleepiness and fatigue in a sample of Norwegian nurses. *PLoS One.* (2013) 8:e70228. doi: 10.1371/journal.pone.0070228
25. Huang CL, Wu MP, Ho CH, Wang JJ. Risks of treated anxiety, depression, and insomnia among nurses: A nationwide longitudinal cohort study. *PLoS ONE.* (2018) 13:e0204224. doi: 10.1371/journal.pone.0204224
26. Huang CL, Weng SF, Wang JJ, Hsu YW, Wu MP. Risks of treated insomnia, anxiety, and depression in health care-seeking physicians: a nationwide population-based study. *Medicine.* (2015) 94:e1323. doi: 10.1097/MD.0000000000001323
27. Spitzer RL, Kroenke K, Williams JB, Löwe B. A brief measure for assessing generalized anxiety disorder: the GAD-7. *Arch Intern Med.* (2006) 166:10. doi: 10.1001/archinte.166.10.1092
28. Moreno AL, DeSousa DA, Souza AMFLP, Manfro GG, Salum GA, Koller SH, et al. Factor structure, reliability, and item parameters of the brazilian-portuguese version of the GAD-7 questionnaire. *Temas Psicol.* (2016) 24:1. doi: 10.9788/TP2016.1-25
29. Kroenke K, Spitzer RL, Williams JB. The PHQ-9: validity of a brief depression severity measure. *J Gen Intern Med.* (2001) 16:9. doi: 10.1046/j.1525-1497.2001.016009606.x
30. Osório FL, Mendes AV, Crippa JA, Loureiro SR. Study of the discriminative validity of the PHQ-9 and PHQ-2 in a sample of Brazilian women in the context of primary health care. *Perspect Psychiatr Care.* (2009) 45:3. doi: 10.1111/j.1744-6163.2009.00224.x
31. Osório FL, Silva TDA, Santos RG, Chagas MHN, Chagas NMS, Sanches RF, et al. Posttraumatic Stress Disorder Checklist for DSM-5 (PCL-5): transcultural adaptation of the Brazilian version. *Arch Clin Psychiatry.* (2017) 44:1. doi: 10.1590/0101-60830000000107
32. Pereira-Lima, K, Loureiro SR, Bolsoni LM, Silva, TDA, Osório FL. Psychometric properties and diagnostic utility of a Brazilian version of the PCL-5 (complete and abbreviated versions). *Eur J Psychotraumatol.* (2019) 10:1. doi: 10.1080/20008198.2019.1581020
33. Castro, LS. Adaptation and validation of the Insomnia Severity Index (ISI): population characteristics, normative values and associated factors [dissertation]. Universidade Federal de São Paulo, São Paulo (2011).
34. Public Tableau. Painel COVID-19 Brasil (2020). Available online at: https://public.tableau.com/profile/covid19estadosbr#!/vizhome/PainelNOVO/Painel (accessed August 23, 2020).
35. Tabachnick, B, Fidell, LS. *Using Multivariate Statistics*. New York, NY: Harper Collins (1996). p. 880.
36. Zhang C, Yang L, Liu S, Ma S, Wang Y, Cai Z, et al. Survey of insomnia and related social psychological factors among medical staff involved in the 2019 novel coronavirus disease outbreak. *Front Psychiatry.* (2020)11:306. doi: 10.3389/fpsyt.2020.00306
37. Morin CM, Carrier J. The acute effects of the COVID-19 pandemic on insomnia and psychological symptoms. *Sleep Med.* (2021) 77:346–7. doi: 10.1016/j.sleep.2020.06.005
38. Duarte MLC, Silva DG, Bagatini MMC. Nursing and mental health: a reflection in the midst of the coronavirus pandemic. *Rev Gaúcha Enferm.* (2020) 42. doi: 10.1590/1983-1447.2021.20200140
39. Dobson, H, Malpas, CB, Burrell, AJ, Gurvich, C, Chen, L, Kulkarni, J, et al. Burnout and psychological distress amongst Australian healthcare workers during the COVID-19 pandemic. *Australas Psychiatry.* (2020) 29:26–30. doi: 10.1177/1039856220965045
40. Rossi, R, Socci, V, Pacitti, F, Di Lorenzo, G, Di Marco, A, Siracusano, A, et al. Mental health outcomes among frontline and second-line health care workers during the coronavirus disease 2019 (COVID-19) pandemic in Italy. *JAMA Netw Open.* (2020) 3:5. doi: 10.1001/jamanetworkopen.2020.10185
41. Que, J, Shi, L, Deng, J, Liu, J, Zhang, L, Wu, S, et al. Psychological impact of the COVID-19 pandemic on healthcare workers: a cross-sectional study in China. *Gen Psychiatr.* (2020) 33:3. doi: 10.1136/gpsych-2020-100259
42. Dilélio AS, Facchini LA, Tomasi E, Silva SM, Thumé E, Piccini RX, et al. Prevalence of minor psychiatric disorders among primary healthcare workers in the South and Northeast regions of Brazil. *Cad Saúde Pública.* (2012) 28:3. doi: 10.1590/s0102-311x2012000300011
43. Cabana MCFL, Ludermir AB, Silva ER, Ferreira MLL, Pinto MER. Common mental disorders among physicians and their everyday practice. *J Bras Psiquiatr.* (2007) 56:1. doi: 10.1590/S0047-20852007000100009
44. Knuth BS, Silva RA, Oses JP, Radtke VA, Cocco RA, Jansen K. Mental disorders among health workers in Brazil. *Ciênc Saúde Coletiva.* (2015) 20:8. doi: 10.1590/1413-81232015208.05062014
45. Vargas D, Dias APV. Depression prevalence in Intensive Care Unit nursing workers: a study at hospitals in a northwestern city of São Paulo State. *Rev Latino-Am Enfermagem.* (2011) 19:5. doi: 10.1590/S0104-11692011000500008
46. Gonzalo RM, Ana RG, Patricia CA, Laura AL, Nathalia GT, Luis C, et al. Short-term emotional impact of COVID-19 pandemic on Spaniard health workers. *J Affect Disord.* (2021) 278:390–4. doi: 10.1016/j.jad.2020.09.079
47. Zandifar A, Badrfam R, Mohammadian Khonsari N, Mohammadi MR, Asayesh H, Qorbani M. Prevalence and associated factors of posttraumatic stress symptoms and stigma among health care workers in contact with covid-19 patients. *Iran J Psychiatry.* (2020) 15:4. doi: 10.18502/ijps.v15i4.4303
48. Zhu Z, Xu S, Wang H, Liu Z, Wu J, Li G, et al. COVID-19 in Wuhan: sociodemographic characteristics and hospital support measures associated with the immediate psychological impact on healthcare workers. *EClinMed.* (2020) 24:100443. doi: 10.1016/j.eclinm.2020.100443
49. Höglund P, Hakelind, C, Nordin S. Severity and prevalence of various types of mental ill-health in a general adult population: age and sex differences. *BMC Psychiatry.* (2020) 20:1. doi: 10.1186/s12888-020-02557-5
50. Alzaid, EH, Alsaad, SS, Alshakhis, N, Albagshi, D, Albesher, R, Aloqaili, M, et al. Prevalence of COVID-19-related anxiety among healthcare workers: a cross-sectional study. *J Family Med Prim Care.* (2020) 9:9. doi: 10.4103/jfmpc.jfmpc_674_20
51. Zhang SX, Liu J, Afshar Jahanshahi A, Nawaser K, Yousefi A, Li J, et al. At the height of the storm: healthcare staff's health conditions and job satisfaction and their associated predictors during the epidemic peak of COVID-19. *Brain Behav Immun.* (2020) 87:144–6. doi: 10.1016/j.bbi.2020.05.010
52. Santos, AS, Monteiro, JK, Dilélio, AS, Sobrosa, GMR, Borowski, SBV. Public and private hospital context: impact on mental illness of health workers. *Trab Educ Saúde.* (2017) 15:2. doi: 10.1590/1981-7746-sol00054
53. Lima Júnior J, Alchieri JC, Maia EMC. Assessment of the work conditions in hospitals of Natal, Rio Grande do Norte State, Brazil. *Rev Esc Enferm USP.* (2009) 43:3. doi: 10.1590/S0080-62342009000300024
54. Magnavita N, Tripepi G, Di Prinzio RR. Symptoms in health care workers during the covid-19 epidemic. a cross-sectional survey. *Int J Environ Res Public Health.* (2020) 17:14. doi: 10.3390/ijerph17145218
55. Nogueira-Martins LA. Mental health of health care workers. *Rev Bras Med Trab.* (2003) 1:59-71.
56. Bernardo MH, Garbin AC. Work-related mental health care in the Brazilian national health system: challenges and possibilities. *Rev Bras Saúde Occup.* (2011) 36:123. doi: 10.1590/S0303-76572011000100010
57. Gomes, RK, Oliveira, VB. Depression, anxiety and social support in nursing professional. *Bol Psicol.* (2013) 63:23–33.

Conflict of Interest: The authors declare that the research was conducted in the absence of any commercial or financial relationships that could be construed as a potential conflict of interest.

Publisher's Note: All claims expressed in this article are solely those of the authors and do not necessarily represent those of their affiliated organizations, or those of the publisher, the editors and the reviewers. Any product that may be evaluated in this article, or claim that may be made by its manufacturer, is not guaranteed or endorsed by the publisher.

Copyright © 2021 Osório, Silveira, Pereira-Lima, Crippa, Hallak, Zuardi and Loureiro. This is an open-access article distributed under the terms of the Creative Commons Attribution License (CC BY). The use, distribution or reproduction in other forums is permitted, provided the original author(s) and the copyright owner(s) are credited and that the original publication in this journal is cited, in accordance with accepted academic practice. No use, distribution or reproduction is permitted which does not comply with these terms.
