# Supplementary material for: Mental health status and related factors influencing healthcare workers during the COVID-19 pandemic: A systematic review and meta-analysis
Source: PLoS One. 2024 Jan 19;19(1):e0289454. doi: 10.1371/journal.pone.0289454 (PMC10798549; doi:10.1371/journal.pone.0289454)
Supplement: S1 Data — (ZIP) [file pone.0289454.s011.zip › literatures/127.pdf]

BMJ Open

Depression, anxiety and insomnia among frontline healthcare workers amid the coronavirus pandemic (COVID-19) in Jordan: a cross-sectional study

Alaa O Oteir ,[1,2] Mohammad S Nazzal ,[3] Ala'a F Jaber,[3] Mahmoud T Alwidyan ,[1] Liqaa A Raffee [4]

To cite: Oteir AO, Nazzal MS, Jaber AF, *et al*. Depression, anxiety and insomnia among frontline healthcare workers amid the coronavirus pandemic (COVID-19) in Jordan: a cross-sectional study. *BMJ Open* 2022;**12**:e050078. doi:10.1136/bmjopen-2021-050078

► Prepublication history and additional supplemental material for this paper are available online. To view these files, please visit the journal online (http://dx.doi.org/10.1136/bmjopen-2021-050078).

Received 10 February 2021
Accepted 14 January 2022

Check for updates

© Author(s) (or their employer(s)) 2022. Re-use permitted under CC BY-NC. No commercial re-use. See rights and permissions. Published by BMJ.

[1]Department of Allied Medical Sciences, Jordan University of Science and Technology, Irbid, Jordan
[2]Department of Paramedicine, Monash University, Frankston, Victoria, Australia
[3]Department of Rehabilitation Sciences, Jordan University of Science and Technology, Irbid, Jordan
[4]Department of Accident and Emergency Medicine, Jordan University of Science and Technology, Irbid, Jordan

Correspondence to
Dr Alaa O Oteir;
aooteir@just.edu.jo

ABSTRACT

Objective This study aimed to explore the prevalence and factors associated with depression, anxiety and insomnia among frontline healthcare workers (HCWs) in Jordan.
Methods A cross-sectional design was conducted among 122 frontline HCWs who have dealt with suspected or confirmed cases of COVID-19. The study survey included standardised questionnaires of the 7-item Generalized Anxiety Disorder (GAD-7) Scale, 9-item Patient Health Questionnaire (PHQ-9) and the Insomnia Severity Index (ISI). Data were collected online during the active surge period of cases from 11 May 2020 to 13 June 2020. The statistical analysis included descriptive statistics, analysis of variance, bivariate correlation and multivariate linear regression analyses.
Results A total of 122 HCWs participated in the study (response rate=64.2%). Among the participants, 44.3% were physicians, 32.8% were nurses and 17.2% were paramedics. The mean age of participants was 32.1 (±5.8) years, and the majority were males (80.3%). The mean scores for GAD-7, PHQ-9 and ISI were 8.5 (±5.2), 9.5 (±5.7) and 11.2 (±6.4), respectively. Results showed that the participants reported severe symptoms of anxiety (29.5%), depression (34.5%) and insomnia (31.9%), with no observed differences based on gender, job title, marital status or educational level. Moreover, in the multivariate linear regression, none of the independent factors were associated with GAD-7, PHQ-9 or ISI scores, and the only exception was increased severity of insomnia among paramedics.
Conclusion The COVID-19 pandemic has exerted strenuous emotional, psychological and physical pressures on the health of frontline HCWs.

Strengths and limitations of this study

- To the extent of our knowledge, this is the first study to report mental health outcomes among frontline healthcare workers during the COVID-19 pandemic in Jordan using standardised questionnaires.
- The study provides evidence of high prevalence levels of anxiety, depression and insomnia among them which may urge the governing bodies to implement preventive measures and/or coping strategies.
- The associations between anxiety, depression and insomnia are complex bidirectional, intertwined and multifactorial.
- The cross-sectional design and self-reported questionnaires may have led to reporting bias.
- The study setting and interpretation of the results should be considered when generalising the current results.

INTRODUCTION

The global spread of COVID-19, which began in late 2019, has put an immediate halt to everyone's way of living and has affected our lives, psychosocial well-being and quality of life. Combating a new and unknown virus with no medication or vaccine, at the time, had a tremendous toll on the lives of medical workers worldwide. The overwhelming impact of handling patients with COVID-19 has been rising substantially since its initial spread, which led to the investigation of the psychological impacts of this pandemic on frontline healthcare workers (HCW).

The transactional theory of stress and coping, developed by Lazarus, viewed stress as a result of the individual's interaction with their environment.[1] Stress is defined as a threat to the individual's homeostasis caused by either a real or perceived event.[2] Lazarus exerted great emphasis on an individual's ability to appraise a situation in correlation to their own sense of well-being and how it affects their ability to effectively cope with available resources to meet the demands that stress is putting on them. Lazarus also stated that coping strategies are strongly related to the appraisal of stress, and encompasses 'cognitive and behavioural efforts made to master,

tolerate or reduce both external and internal demands and conflicts among them'. People from the same environment/event react and cope differently during stressful situations depending on their individual characteristics, which was noticed during the coronavirus pandemic.[3] Exposure to severe and chronic stressors among HCWs, such as dealing with patients with COVID-19, lockdowns and increased workload, has generated stress symptoms including sleep disorders and also a number of mental health problems, like depression and anxiety.[4] Depression is defined as a mood disorder that is characterised by feelings of constant sadness paired with physical and cognitive changes that significantly impair the individual's ability to participate in daily life activities.[5] Anxiety, on the other hand, is defined as an excessive anticipation of future threat that is often combined with cautious or avoidant behaviours.[5] Furthermore, insomnia is often defined as a complaint[6] of or difficulty with sleep.[7] The term insomnia, in sleep literature, is sometimes used to describe the existence of 'polysomnographic evidence of disturbed sleep'.[7] It is also known to be associated with daytime impairment or distress.[6 7] When anxiety and stress exist it can cause difficulty falling asleep or having a quality sleep. In fact, the literature shows complex bidirectional and intertwined associations between anxiety, depression and insomnia.[6 8]

Recent studies have shown a relationship between the immense pressure of handling patients with COVID-19 and the psychological well-being of HCWs.[9–12] Also, anxiety, depression and stress were found to be prevalent among HCWs who are at risk of exposure to patients confirmed or suspected to have COVID-19, due to the nature of their work.[9–11] Emerging evidence has divulged into the psychological and behavioural impacts of the pandemic on HCWs and investigated the possible related factors. For instance, a study by Lai *et al* showed that females were more prone to suffer from depression, anxiety and stress compared with their male colleagues.[10] Another study found that frontline responders suffered from clinically significant symptoms of anxiety, depression, insomnia and stress during the pandemic.[11] Zhang *et al* found that insomnia levels, in particular, were higher among those who had a high school education or less.[12] In addition, a study conducted in Italy, a country which was significantly affected by the high spreading rates of COVID-19, found that the death of a coworker was associated with an increased prevalence of post-traumatic stress symptoms among other coworkers.[13]

Recent reviews of COVID-19 impacts have reported high rates of mental health symptoms among HCWs. One systematic review revealed high prevalence rates of anxiety and depression among HCWs in 17 countries, where anxiety rates ranged from 7% to 57%, and depression ranged from 17% to 33%, with the rates being highest in studies conducted in Italy, China and Iran.[14] A second systematic review also reported high pooled prevalence rates of anxiety, depression and insomnia among HCWs, which were 23.2%, 22.8% and 38.9%, respectively.[15] The findings of these reviews also confirmed that females and nurses had a higher risk of developing anxiety and depression than males and other HCWs from various disciplines, such as paramedics and physicians.[14 15]

Physicians, nurses and paramedics in Jordan are the frontline workers when dealing with suspected and confirmed cases of COVID-19, which led to many reported cases of exposure and infection among HCWs and their families. Consequently, we hypothesised that the COVID-19 pandemic caused an increased prevalence and severity of depression, anxiety and insomnia among frontline HCWs. While few studies have assessed the psychological well-being of HCWs in Jordan, there is a lack of studies assessing it in the midst of response to a one-in-a-century pandemic. Therefore, this study aimed to explore the prevalence and associated factors of depression, anxiety and insomnia in the midst of COVID-19 response among frontline HCWs in Jordan. The current study contributes to the body of literature by highlighting the levels of depression, anxiety and insomnia symptoms and giving an insight into the importance of these symptoms. The results of this study may urge the governing bodies to implement preventive measures and/or coping strategies that will help reduce the impact of COVID-19, improve the quality of work and reduce burnout among frontline HCWs.

MATERIALS AND METHODS

Design, participants and settings

This cross-sectional study targeted a sample of Jordanian frontline HCWs who have dealt with suspected or confirmed COVID-19 cases, as part of their job, in the midst of COVID-19 response. The inclusion criteria encompassed HCWs who: (a) were adult responders (aged ≥18 years), (b) were healthcare professionals (ie, physicians, nurses, paramedics or of any other allied medical profession), (c) have dealt with confirmed or suspected COVID-19 cases at the time (performed testing, contact tracing and/or treating COVID-19 cases), and, finally (d) were able to complete the study questionnaire. Participants were excluded if they were diagnosed with a physical or psychological disease, refused to participate or had not dealt with suspected or confirmed COVID-19 cases.

The minimum sample of 92 participants was determined by G*power, to obtain a power of 0.8.[16] A total of 190 frontline HCWs were approached through professional emails, phone calls and their official supervisors and administrators. Of those, only 122 participants signed consents and were enrolled in the study.

Jordanian frontline HCWs in this study either worked in the Ministry of Health hospitals or provided prehospital care affiliated with the Jordanian civil defence. They were usually split into small groups and then dispersed into virus-infected areas for conduct testing and contact tracing. Each group encompassed a physician, nurse, paramedic, laboratory technician, a driver, along with a

police patrol, and were often equipped with full personal protective equipment.

Data collection and outcome measures

Data were collected during the active surge period of COVID-19 cases from 11 May 2020 to 13 June 2020, using an online survey created through Google Forms. The study survey had two sections: the first included demographic information, and the second included three standardised questionnaires. Standardised questionnaires consisted of the 9-item Patient Health Questionnaire (PHQ-9),[17] the 7-item Generalized Anxiety Disorder (GAD-7)[18] and the 7-item Insomnia Severity Index (ISI-7).[19] The data collection package consisted of multiple questionnaires that mainly required multiple-choice answers and yes/no questions to target several aspects, including:

A. Demographic information: included questions about the participants' demographics, health status, as well as work conditions such as working hours, type of activities during work, the practice of wearing personal protective equipment and standard precautions.

B. PHQ-9: a valid and reliable (Cronbach's α=0.89) 9-item questionnaire used to screen for depression and measure the severity of depressive symptoms.[17] The current study's Cronbach's α is 0.861 with 95% CI of 0.82 to 0.90. Participants can indicate the frequency of nine symptoms over the past 2weeks and how they affected their daily lives. Each of the nine items is ranked from 0 to 3 ('not at all' to 'nearly every day'). The PHQ-9 is scored on a scale of 0–27 in which scores of 0–4 indicate minimal depression severity, 5–9 mild, 10–14 moderate, 15–19 major (moderately severe) and 20 or higher a severe level of depression.[17 20]

C. GAD-7 Scale: a 7-item reliable (Cronbach's α=0.92) assessment tool used to evaluate anxiety, which is based on the Diagnostic and Statistical Manual of Mental Disorders, Fourth Edition diagnostic criteria.[18] The current study's Cronbach's α is 0.90 with 95% CI 0.88 to 0.93. Over the period of the past 2weeks, participants can indicate the frequency of symptoms and how it has affected their daily lives. Each item is given a score ranging from 0 to 3 ('not at all' to 'nearly every day'). GAD-7 is scored on a scale from 0 to 21, in which scores of 0–4 indicate a normal anxiety level, 5–9 mild anxiety symptoms, 10–14 moderate anxiety and 15–21 severe symptoms of anxiety.[18 20]

D. ISI-7: a 7-item scale that measures the level of insomnia (Cronbach's α=0.7).[19] The current study's Cronbach's α is 0.89 with 95% CI 0.87 to 0.92. Each item on ISI has five (0–4) potential scores. Items 1–3 range from 'none' to 'very severe', item 4 from 'very satisfied' to 'very dissatisfied' and items 5–7 from 'not at all interfering' to 'very much interfering'. The ISI is scored on a scale of 0–28, in which scores of 0–7 indicate no clinically significant insomnia, 8–14 subthreshold insomnia, 15–21 moderate severity of clinical insomnia and 22–28 severe clinical insomnia.[19]

Data analysis

The Statistical Package for Social Sciences (SPSS) V.23 was used to conduct all statistical analyses. The analysis plan included descriptive statistics of the sample demographics and main outcome measures. Descriptive analysis was also conducted and summarised into two groups: the continuous data as means and SDs, and the categorical data as frequencies and proportions. Groups were compared using an independent t-test, one-way analysis of variance or χ^2 tests as appropriate, and the internal consistency was calculated using Cronbach's α. Bivariate correlation tests were used to examine the associations between the outcome measures and to identify the demographic variables which are associated with the outcome measures (PHQ-9, GAD and ISI). Correlated variables with a p value of 0.2 or less were entered into a stepwise multivariate linear regression model to identify the factors associated with depressive symptoms, anxiety and insomnia among HCWs.[21 22] All reported p values were two sided, and a value of less than 0.05 was considered statistically significant.

Patient and public involvement

No patients or participants were involved.

RESULTS

A total sample of 122 participants was included in the study (response rate=64.2%). The mean age was 32.1 (SD±5.8) years and 80.3% (n=98) were males. The sample included HCWs which mainly comprised physicians (44.3%), nurses (32.8%) and paramedics (17.2%). More than two-thirds (67.2%) of the participants were married, and more than one-third (39.3%) had children. Participants reported marked changes in their work-related responsibilities during the COVID-19 pandemic, in which 81.1% reported increased workload and 50% reported an increase in working hours. Due to the nature of their work, the overwhelming majority of participants (94.3%) were afraid of infecting themselves or their family members with the virus. Table 1 summarises the demographic characteristics of the sample.

Summary of the outcome measures (GAD, PHQ-9 and ISI)

Severity of symptoms

The mean score for the GAD Scale was 8.5 (±5.2), and 29.5% of participants reported moderately severe and severe anxiety levels. Furthermore, the mean depression score was 9.5 (±5.7) with a proportion of 34.5% of participants which showed moderately severe and severe signs of depression. Finally, the ISI mean score was 11.2 (±6.4), with a percentage of 26.2% and 5.7% of participants who have reported moderately severe and severe clinical insomnia, respectively. The internal consistency reliability (Cronbach's α) of the GAD, PHQ-9 and ISI measures was α=0.905, 0.861 and 0.898, respectively, indicating acceptable internal consistency reliability.[23] Table 2 summarises the participants' scores on the main outcome measures.

Table 1 Participant demographics (n=122)

Demographic variables		Frequency (n=122)	Percentage (100%)
Age (years)	(Mean, SD)	32.1	5.8
Gender	Male	98	80.3
	Female	24	19.7
Education	Diploma	20	16.4
	Bachelor	76	62.3
	Postgraduate	26	21.3
Job title	Physicians	54	44.3
	Nurse	40	32.8
	Paramedic	21	17.2
	Others	7	5.7
Residence	Village	35	28.7
	City	87	71.3
Income (JOD)	<500	28	23.0
	501–1000	61	50.0
	1001–1501	5	4.1
	>2000	8	6.6
	Not disclosed	20	16.4
Marital status	Married	82	67.2
	Single	40	32.8
Having children	Yes	48	39.3
	No	74	60.7
Workload	Decreased	15	12.3
	Unchanged	8	6.6
	Increased	99	81.1
Work duration	Decreased	26	21.3
	Unchanged	35	28.7
	Increased	61	50.0
Fear of infection: self and family	Yes	115	94.3
	No	7	5.7

JOD, Jordanian dinar.

A correlation analysis between the different outcome measures was also conducted. GAD-7 was positively associated with both ISI (r=0.555, p<0.001) and PHQ-9 (r=0.739, p<0.001), and PHQ-9 was positively correlated with ISI (r=0.685, p<0.001).

Differences in outcomes

The comparative analysis revealed that paramedics had the highest mean score of anxiety on PHQ-9 (9.8±5.5), depression on GAD-7 (11.1±5.7) and insomnia on ISI (14±6.0), compared with other professions. Nonetheless, no statistically significant differences were observed in the different comparisons. Table 3 summarises the comparison of the participants' outcome measures based on their disciplines, gender, educational level, marital status, having children and fear of infection.

Factors associated with depression, anxiety and insomnia

Multivariate stepwise linear regression analysis of all outcome measures was conducted, and all items with a correlation p value of 0.2 or less were entered into the regression model. None of the entered demographics, such as age, gender, educational level and job title, were associated with the PHQ-9 or GAD-7. Being a paramedic was the only independent variable that had a statistically significant association with the ISI score (F=5.127, p=0.025, R^2=0.04). Online supplemental appendix A summarises the bivariate correlation and multivariate regression analyses.

DISCUSSION

The study aimed to explore the prevalence of anxiety, depression and insomnia symptoms using standardised instruments among frontline HCWs who have dealt with confirmed or suspected cases of patients with COVID-19, as part of their job. All outcome measures had an acceptable internal consistency and had moderate to strong correlations with each other.

Approximately one-third of our participants reported severe symptoms of anxiety (29.5%), depression (34.5%) and insomnia (31.9%). This was consistent with recent COVID-19-related studies from both China and Italy, which reported that frontline HCWs suffer from clinically significant symptoms of anxiety, depression, insomnia and stress.[10 13] They also reported that females and nurses were more prone to depression, anxiety and stress than

Table 2 Summary of outcome measures (n=122)

	**Mean (SD)**	**95% CI**	**Mild** (n, %)	**Moderate** (n, %)	**Moderately severe** (n, %)	**Severe** (n, %)
GAD-7	8.5 (5.2)	7.5 to 9.4	57 (46.7)	5 (4.1)	21 (17.2)	15 (12.3)
PHQ-9	9.5 (5.7)	8.5 to 10.5	47 (38.5)	7 (5.7)	17 (13.9)	25 (20.5)
	**Mean (SD)**	**95% CI**	**No clinically significant insomnia (n, %)**	**Subthreshold clinical insomnia (n, %)**	**Moderate severity clinical insomnia (n, %)**	**Severe clinical insomnia (n, %)**
ISI-7	11.2 (6.4)	10.0 to 12.4	38 (31.1)	45 (36.9)	32 (26.2)	7 (5.7)

GAD-7, 7-item Generalized Anxiety Disorder Scale; ISI-7, 7-item Insomnia Severity Index; PHQ-9, 9-item Patient Health Questionnaire.

Table 3 Differences in outcome measures based on participant characteristics (n=122)

			GAD-7		PHQ-9		ISI-7	
		Frequency (%)	Mean (SD)	P value	Mean (SD)	P value	Mean (SD)	P value
Gender	Male	98 (80.3)	8.6 (5.2)	0.60	9.5 (5.8)	0.97	11.2 (6.4)	0.96
	Female	24 (19.7)	8.0 (5.5)		9.5 (5.1)		11.3 (6.8)	
Education	Diploma	20 (16.4)	9.9 (5.3)	0.24	11.5 (5.3)	0.21	12.2 (6.1)	0.40
	Bachelor	76 (62.3)	8.5 (5.1)		9.0 (5.3)		10.6 (6.4)	
	Postgraduate	26 (21.3)	7.3 (5.3)		9.5 (6.7)		12.2 (6.8)	
Marital status	Married	82 (67.2)	8.2 (6.2)	0.73	9.2 (6.4)	0.69	11.3 (6.3)	0.82
	Single	40 (32.8)	8.6 (4.7)		9.6 (5.4)		11.0 (6.9)	
Have children	Yes	48 (39.3)	8.4 (4.8)	0.89	9.8 (5.6)	0.50	11.4 (6.5)	0.66
	No	74 (60.7)	8.4 (5.9)		9.1 (5.8)		10.9 (6.4)	
Profession	Paramedic	21 (17.2)	9.8 (5.5)	0.38	11.1 (5.7)	0.54	14.0 (6.0)	0.14
	Physicians	54 (44.2)	8.7 (5.7)		9.0 (6.2)		10.9 (6.7)	
	Nurse	40 (32.8)	7.7 (4.5)		9.4 (5.3)		10.1 (6.2)	
	Others	7 (5.7)	6.9 (3.2)		8.9 (2.5)		10.9 (5.4)	
Fear of infection	Yes	115 (94.3)	8.6 (5.3)	0.26	9.5 (5.8)	0.8	11.1 (6.5)	0.69
	No	7 (5.7)	6.3 (3.6)		9.0 (4.5)		12.1 (6.7)	

Others: refers to allied healthcare.
P value based on independent Student's t-test or one-way analysis of variance (ANOVA) test.
GAD-7, 7-item Generalized Anxiety Disorder Scale; ISI-7, 7-item Insomnia Severity Index; PHQ-9, 9-item Patient Health Questionnaire; SD, Standard Deviation.

male coworkers.[10 13] However, in our study, no differences between males and females were observed. This may be due to the low proportion of females, which only accounted for one-fifth of participants in our sample (19.7%, n=24), whereas they comprised approximately three-quarters of the sample in both studies by Lai *et al*[10] and Rossi *et al*.[13]

There were also no differences in this study based on profession. Outcome measures were compared between four groups (physicians, nurses, paramedics and other allied health professionals). Although paramedics had the highest mean scores of anxiety, depression and insomnia, no statistically significant differences were observed between the different professions. Also, there were no observed associations between the independent variables and anxiety, depression and insomnia. The outcome measures were compared based on gender, education, marital status and having children, with no differences detected. Moreover, although the majority of our sample reported concerns of COVID-19 infection to themselves and their families, this factor was not statistically associated with increased or decreased levels of anxiety, depression and insomnia.

The linear regression analysis revealed that being a paramedic was associated with increased ISI scores due to the increased workloads and extended shifts during the pandemic. Work-related pressures and the increased responsibilities made paramedics more vulnerable to exhibit fatigue, depression and persistent stress leading to sleep disturbances; however, this model only explained a very small percentage (4.0%) of the variance. On the contrary, other studies concluded that females and nurses reported more severe mental health issues when compared with their male counterparts in other health professions.[10 13]

A systematic review published in June 2020 reported mental health symptoms of patients within the general population and HCWs. Studies showed that China and Italy both have the highest prevalence of anxiety which ranged from 7% to 57%, depression from 17% to 33% and insomnia from 25% to 39% among their HCWs.[14] These findings were in congruence with another systematic review confirming that females and nurses had a higher prevalence of anxiety and depression compared with their counterparts.[14 15] Nevertheless, it can be explained that frontline HCWs in Jordan were found to have similar levels of anxiety, depression and insomnia because they are working in the same setting, dealing with the same cohort of patients and operating under the same circumstances including how the Jordanian Ministry of Health and other ministries dealt with this pandemic.

The interplay of the anxiety, depression and insomnia levels of the Jordanian frontline HCWs can still be explained through the multifactorial transactional Lazarus theory of stress. The pandemic and the governmental responses to the pandemic have created a magnitude of factors that prompted anxiety and stress responses. This prolonged anxiety of the pandemic, with no definite end of the pandemic on the horizon nor the existence of any knowledge on what outcomes this pandemic would bring to self, family or the world, may cause depressive symptoms. It is noted in the literature that depression is

more likely to happen when anxiety exists.[24] These levels also are dependent on how HCWs perceive this stress and how they cope with it. It is anticipated that HCWs may use a wide variety of coping strategies, whether positive or negative, to overcome their anxiety. Also, there are varying degrees of support systems that these HCWs have, whether in their family level, work institutions or society at large.

In this study, the coping of the HCWs during the pandemic was not investigated. By the end of the data collection phase in this study, there were a total of 953 confirmed COVID-19 cases in Jordan, 266 of whom were active cases.[25] Since August 2020, the number of COVID-19 cases started to increase drastically, and as of 24 January 2021, it reached a total of 31 232 cases.[25] Nowadays, with cumulative cases, reaching more than a million in Jordan,[25] frontline HCWs are expected to have fluctuating levels of mental health symptoms depending on the time of the data collection when there is an active surge or at the times of plateauing and reopening of the country. Therefore, with the dramatic increase in the number of patients with COVID-19, HCWs are expected to have higher levels of mental health symptoms if data collection for this study is replicated at the current time.

Jordan implemented one of the strictest preventive measures including complete and partial lockdowns, encouraging social distancing, universities and schools were forced to shut down and move to online teaching, as well as closing the shopping malls, mosques and churches. Jordan has also launched an application called 'AMAN' to help identify exposure to confirmed cases of COVID-19 and inform the application users to take appropriate measures in such cases. Other measures included conducting random tests and a 2-week quarantine was applied to anyone travelling to Jordan. All these strict measures may have also influenced the levels of anxiety, depression and insomnia. Furthermore, people are family oriented in Jordan and value close relationships with family members. These traits lead to a more cohesive and supporting environment to the loved ones, hence may influence the levels of anxiety, depression and insomnia.

Up to our knowledge, this is the first report on mental health outcomes among frontline HCWs during the COVID-19 pandemic in Jordan and Arab countries. However, the study setting as well as the interpretation of the results should be considered when generalising the current results. In this study, the percentages of anxiety, depression and insomnia reported by the HCWs during the COVID-19 pandemic are concerning. The Jordanian Ministry of Health and other governmental authorities should consider composing multidisciplinary mental health teams to provide psychological support services to all frontline HCWs. This may help preserve the quantity of working personnel in the field during the pandemic, preserve quality and working capacity while performing the job, as well as preventing any possible long-term mental health-related issues among the frontline HCWs.

Research implications

Due to the COVID-19 pandemic, frontline HCWs in Jordan have been confronted with overwhelming levels of psychological stress as evidenced by the high prevalence levels of anxiety, depression and insomnia among them. The pattern and sweeping prevalence of psychological stress across all subdomains, factors and job titles highlights the need for a national emergency plan to focus on their psychological aid to prevent eminent psychological burden. Multidisciplinary mental health teams are highly warranted to provide periodic psychological consultation and continuous support to frontline HCWs in Jordan. Moreover, healthcare administration as well as the national disaster management steering body should increase the staff of frontline HCWs, both quantity and quality; increasing quantity by hiring more HCWs to manage the surge of suspected cases. Hence, deployed HCWs on the field will work for lesser hours with fewer working shifts, which will help decrease their physical burden. Another way is to focus on increasing the quality by providing evidence-based protective equipment, providing crash courses and continuing education about the pandemic with ways to protect self and family. In fact, a study conducted in Jordan reported that 71.1% of 466 general HCW participants were willing to report to duty during a pandemic, if they were provided and equipped with adequate supplies of protective gear.[26] Another study reported that most participants (n=235, 96.4%) were willing to come to work during the pandemic.[27] Moreover, knowledge is power. A plan for constantly educating the HCWs about evidence-based practices geared towards the frontline personnel on handling the pandemic would increase preparedness and ease fears and anxiety. Finally, frontline HCWs working in Jordan need to be appreciated similarly to their counterparts around the world, and can be achieved by providing incentives, whether monetary or non-monetary. For example, public awareness and appreciation campaigns for frontline HCWs can boost the morale of HCWs and their productivity. Finally, a large-scale prospective study and/or qualitative study need to be conducted to understand the coping strategies and support system that Jordanian HCWs use to deal with a pandemic of this scale.

Limitations

The present study has some limitations. First, the cross-sectional design, self-reported questionnaires and the participant selecting method may have led to reporting bias and/or selection bias. However, this design was the most appropriate due to travel constraints, lockdowns and the availability of frontline HCWs during the active time of the pandemic. It is also important to note that the results should not be generalised without caution, as researchers must consider the study setting, participants' work settings as well as the interpretation of the results.

Second, the study did not compare the sample with non-frontline HCWs, although previous studies confirmed that frontline HCWs have higher levels of mental health

deficits compared with non-frontline HCWs. Furthermore, the study did not compare mental health symptoms before and after the pandemic, and whether there was an evolution over time.

CONCLUSION

This study explored the prevalence of anxiety, depression and insomnia and its associated factors among COVID-19 frontline HCWs. It was noted that a considerable proportion of the participants reported symptoms of anxiety, depression and insomnia. However, there were no associated factors with the outcome measures, with the exception of paramedics being the only factor associated with insomnia.

Twitter Alaa O Oteir @alaa_oteir

Contributors AOO collected the data, conducted the statistical analysis, wrote the manuscript and acts as the guarantor . AOO, MSN and AFJ conceived the study design and have made a considerable contribution to the study design. AOO, MSN, AFJ, MTA and LAR have made a considerable contribution to data collection, interpretation of results and writing of the manuscript. All authors have approved the final manuscript.

Funding The authors have not declared a specific grant for this research from any funding agency in the public, commercial or not-for-profit sectors.

Competing interests None declared.

Patient consent for publication Not required.

Ethics approval This study involves human participants and was approved by the Jordan University of Science and Technology Institutional Review Board (No: 20200365). Participants gave informed consent to participate in the study before taking part.

Provenance and peer review Not commissioned; externally peer reviewed.

Data availability statement Data are available upon reasonable request. The data generated and/or analysed during the current study are not publicly available because it contains information that could compromise research participant privacy/consent, but are available from the corresponding author on reasonable request.

Supplemental material This content has been supplied by the author(s). It has not been vetted by BMJ Publishing Group Limited (BMJ) and may not have been peer-reviewed. Any opinions or recommendations discussed are solely those of the author(s) and are not endorsed by BMJ. BMJ disclaims all liability and responsibility arising from any reliance placed on the content. Where the content includes any translated material, BMJ does not warrant the accuracy and reliability of the translations (including but not limited to local regulations, clinical guidelines, terminology, drug names and drug dosages), and is not responsible for any error and/or omissions arising from translation and adaptation or otherwise.

Open access This is an open access article distributed in accordance with the Creative Commons Attribution Non Commercial (CC BY-NC 4.0) license, which permits others to distribute, remix, adapt, build upon this work non-commercially, and license their derivative works on different terms, provided the original work is properly cited, appropriate credit is given, any changes made indicated, and the use is non-commercial. See: http://creativecommons.org/licenses/by-nc/4.0/.

ORCID iDs
Alaa O Oteir http://orcid.org/0000-0002-3156-2773
Mohammad S Nazzal http://orcid.org/0000-0003-1392-1477
Mahmoud T Alwidyan http://orcid.org/0000-0003-2428-3124
Liqaa A Raffee http://orcid.org/0000-0001-8020-8677

REFERENCES

1 Lazarus RS. *Psychological stress and the coping process*, 1966.
2 Motzer SA, Hertig V. Stress, stress response, and health. *Nurs Clin North Am* 2004;39:1–17.
3 Krohne HW. *Stress and coping theories. International encyclopedia of the social behavioral Sceinces*. , 2002: 22, 15163–70.
4 Secosan I, Bredicean C, Crainiceanu ZP, *et al*. Mental health in emergency medical clinicians: Burnout, STS, sleep disorders. A cross-sectional descriptive multicentric study. *Central Eur Ann Clin Res* 2019;1:1–16.
5 Edition F. Diagnostic and statistical manual of mental disorders. *Am Psychiatric Assoc* 2013;21.
6 Jansson-Fröjmark M, Lindblom K. A bidirectional relationship between anxiety and depression, and insomnia? A prospective study in the general population. *J Psychosom Res* 2008;64:443–9.
7 Roth T. Insomnia: definition, prevalence, etiology, and consequences. *J Clin Sleep Med* 2007;3:S7–10.
8 Morphy H, Dunn KM, Lewis M, *et al*. Epidemiology of insomnia: a longitudinal study in a UK population. *Sleep* 2007;30:274–80.
9 Anmella G, Fico G, Roca A, *et al*. Unravelling potential severe psychiatric repercussions on healthcare professionals during the COVID-19 crisis. *J Affect Disord* 2020;273:422–4.
10 Lai J, Ma S, Wang Y, *et al*. Factors associated with mental health outcomes among health care workers exposed to coronavirus disease 2019. *JAMA Netw Open* 2020;3:e203976.
11 Lin K, Yang BX, Luo D, *et al*. The mental health effects of COVID-19 on health care providers in China. *Am J Psychiatry* 2020;177:635–6.
12 Zhang C, Yang L, Liu S, *et al*. Survey of insomnia and related social psychological factors among medical staff involved in the 2019 novel coronavirus disease outbreak. *Front Psychiatry* 2020;11:306.
13 Rossi R, Socci V, Pacitti F, *et al*. Mental health outcomes among frontline and second-line health care workers during the coronavirus disease 2019 (COVID-19) pandemic in Italy. *JAMA Netw Open* 2020;3:e2010185.
14 Luo M, Guo L, Yu M, *et al*. The psychological and mental impact of coronavirus disease 2019 (COVID-19) on medical staff and general public - A systematic review and meta-analysis. *Psychiatry Res* 2020;291:113190.
15 Pappa S, Ntella V, Giannakas T, *et al*. Prevalence of depression, anxiety, and insomnia among healthcare workers during the COVID-19 pandemic: a systematic review and meta-analysis. *Brain Behav Immun* 2020;88:901–7.
16 Erdfelder E, Faul F, Buchner A. GPOWER: a general power analysis program. *Behav Res Methods Instruments Comp* 1996;28:1–11.
17 Kroenke K, Spitzer RL, Williams JB. The PHQ-9: validity of a brief depression severity measure. *J Gen Intern Med* 2001;16:606–13.
18 Spitzer RL, Kroenke K, Williams JBW, *et al*. A brief measure for assessing generalized anxiety disorder: the GAD-7. *Arch Intern Med* 2006;166:1092–7.
19 Bastien CH, Vallières A, Morin CM. Validation of the insomnia severity index as an outcome measure for insomnia research. *Sleep Med* 2001;2:297–307.
20 Jones S, Nagel C, McSweeney J, *et al*. Prevalence and correlates of psychiatric symptoms among first responders in a southern state. *Arch Psychiatr Nurs* 2018;32:828–35.
21 Stoltzfus JC. Logistic regression: a brief primer. *Acad Emerg Med* 2011;18:1099–104.
22 Bendel RB, AAJJotASa A. Comparison of stopping rules in forward "stepwise" regression 1977;72:46–53.
23 Darlow B, Perry M, Mathieson F, *et al*. The development and exploratory analysis of the back pain attitudes questionnaire (Back-PAQ). *BMJ Open* 2014;4:e005251.
24 Johnson EO, Roth T, Breslau N. The association of insomnia with anxiety disorders and depression: exploration of the direction of risk. *J Psychiatr Res* 2006;40:700–8.
25 Worldmeters. Jordan coronavirus cases 2021, 2020. Available: https://www.worldometers.info/coronavirus/ [Accessed 24 Jan 2021].
26 Alwidyan MT, Oteir AO, Trainor J. Working during pandemic disasters: views and predictors of EMS providers. *Disaster Med Public Health Prep* 2020:1–7.
27 Alwidyan M, Oteir A, Mohammad A, *et al*. Are healthcare professionals in Jordan willing to work and provide care for COVID-19 patients? *Australasia J Paramed* 2021;18.
