# Supplementary material for: Mental health status and related factors influencing healthcare workers during the COVID-19 pandemic: A systematic review and meta-analysis
Source: PLoS One. 2024 Jan 19;19(1):e0289454. doi: 10.1371/journal.pone.0289454 (PMC10798549; doi:10.1371/journal.pone.0289454)
Supplement: S1 Data — (ZIP) [file pone.0289454.s011.zip › literatures/128.pdf]

Received: 15 June 2020 | Accepted: 11 September 2020
DOI: 10.1111/appy.12427

ORIGINAL ARTICLE

ASIA-PACIFIC PSYCHIATRY
WILEY

Prevalence of mental health problems and associated risk factors among military healthcare workers in specialized COVID-19 hospitals in Wuhan, China: A cross-sectional survey

Xiao Pan PhD[1] | Ying Xiao Bachelor[2] | Ding Ren PhD[3] | Zheng-Mei Xu PhD[4] | Qian Zhang Bachelor[5] | Li-Ying Yang Bachelor[6] | Fen Liu Bachelor[7] | Yu-Shi Hao Bachelor[1] | Feng Zhao Bachelor[2] | Yong-Hai Bai PhD[1]

[1]Department of Medical Psychology, Changzheng Hospital, Second Military Medical University, Shanghai, China
[2]Hongkou District of Changhai Hospital, Second Military Medical University, Shanghai, China
[3]Department of Medical Psychology, PLA Navy No.905 Hospital, Second Military Medical University, Shanghai, China
[4]Changzheng Hospital, Second Military Medical University, Shanghai, China
[5]Department of Hyperbaric, Changhai Hospital, Second Military Medical University, Shanghai, China
[6]Department of Radiotherapy, Changhai Hospital, Second Military Medical University, Shanghai, China
[7]Department of Critical Medicine, Changhai Hospital, Second Military Medical University, Shanghai, China

Correspondence
Yong-Hai Bai, Department of Medical Psychology, Changzheng Hospital, Second Military Medical University, Shanghai 200005, China.
Email: baiyonghai179@126.com

Feng Zhao, Hongkou District of Changhai Hospital, Second Military Medical University, Shanghai 200081, China.
Email: fengziz@126.com

Funding information
Changzheng Hospital Pyramid Talent Project（2020）of Second Military Medical University; Research Project of Hubei Maternal and Child Health Hospital Guanggu District, Grant/Award Number: 2020-FYGG-017

Abstract

Introduction: China has been severely affected by coronavirus disease 2019 (COVID-19) since December 2019. Military healthcare workers in China have experienced many pressures when combating COVID-19. This study aimed to investigate the current psychological status and associated risk factors among military healthcare workers.

Methods: We collected data from 194 military healthcare workers from three inpatient wards in two specialized COVID-19 hospitals using a web-based cross-sectional survey. The survey covered demographic information, the patient health questionnaire-9, the Generalized Anxiety Disorder-7, and the patient health questionnaire-15. Hierarchical regression analysis was used to explore potential risk factors for mental health problems.

Results: The overall prevalence rates of depressive, generalized anxiety, and somatic symptoms were 37.6%, 32.5%, and 50%, respectively. Rates of severe depression, generalized anxiety, and somatic symptoms were 5.2%, 3.6%, and 15.5%, respectively. In 22.7% of cases, comorbidities existed between depression, generalized anxiety, and somatization. A junior-grade professional title was associated with depression, older age was associated with generalized anxiety and somatization, and short sleep duration and poor sleep quality were associated with all three symptoms.

Discussion: The prevalence of depression, generalized anxiety, and somatic symptoms among military healthcare workers in specialized COVID-19 hospitals is high

Abbreviations: COVID-19, coronavirus disease 2019; GAD-7, generalized anxiety disorder-7; LSD, least significance difference; PHQ-9, patient health questionnaire-9; PHQ-15, patient health questionnaire-15.

Xiao Pan, Ying Xiao, and Ding Ren contributed equally to this study.

 © 2020 John Wiley & Sons Australia, Ltd

during the current COVID-19 outbreak. A junior-grade professional title, older age, short sleep duration, and poor sleep quality significantly affect military healthcare workers' mental health. Continuous surveillance and monitoring of the psychological consequences of the COVID-19 outbreak should be routine to promote mental health among military healthcare workers.

KEYWORDS

coronavirus disease 2019, depressive symptoms, generalized anxiety, mental health, somatic symptoms

1 | INTRODUCTION

A novel pneumonia of unknown cause was reported in Wuhan, Hubei Province in December 2019. On February 11, 2020, the World Health Organization named this disease coronavirus disease 2019 (COVID-19) because it was caused by a novel coronavirus (Chinese National Healthcare Center, 2020b). As of February 22, 2020, there were 76 936 confirmed cases and 4148 suspected cases in mainland China. Health workers faced mounting psychological pressures because of the massive number of confirmed cases and deaths during the COVID-19 outbreak.

First-line health workers carry an inconceivable workload and are at huge risk for COVID-19 infection while working to save lives. According to Wang et al.'s (2020) research, infected healthcare workers accounted for 29% of all hospitalized COVID-19 patients in the early stage of COVID-19. In addition, Kang et al.'s (2020) research reported that these workers experienced psychological distress because of social isolation and possible discrimination. Mental health problems caused by such psychological distress tend to impair attention, cognitive function, and clinical decision-making (Panagioti et al., 2018), all of which increase the possibility of medical negligence. Consequently, it is necessary to pay more attention to the mental health of healthcare workers during the COVID-19 outbreak.

Healthcare workers from the military are a special group deployed by the central government to combat COVID-19. Military healthcare workers work in military hospitals. They are administered by the military and provide healthcare services for both military and civilian patients. They receive similar medical training to that of civilian healthcare workers, but receive additional training in the disposal, rescue, and treatment of war wounds. Compared with civilian healthcare workers, military healthcare workers participate in more emergency healthcare tasks in China, such as medical reinforcement for severe acute respiratory syndrome (SARS) in 2003, rescue in the Wenchuan earthquake in 2008, and other medical tasks for the Chinese army. On January 24, 2020, military healthcare workers arrived in Wuhan. They were the first group from other parts of China to carry out medical rescue in the context of inadequate equipment and little knowledge about COVID-19. Since military healthcare workers began to receive patients at COVID-19 specialized hospitals on February 4, 2020, they have experienced increasing pressures, including the concentration of patients, requirements of military discipline, and high-intensity work. Therefore, it is necessary to focus on the mental health of this special population.

Depression, generalized anxiety, and somatic symptoms are common symptoms among medical staff and patients under stress, and the comorbidity rate exceeds 50% (Löwe et al., 2008). Several studies have explored psychological effects during epidemics, such as SARS in 2003. Koh et al. (2005) reported 68% of first-line healthcare workers experienced severe work-related pressure and 57.0% experienced psychological disturbance during the SARS outbreak. Another study showed that 17.3% of healthcare workers experienced significant mental symptoms during the SARS epidemic (Lu, Shu, Chang, & Lung, 2006). A survey that was conducted among 255 Chinese patients with breast cancer reported the percentage of high somatic symptoms was 43.5% (Leonhart et al., 2017). Therefore, depression, general anxiety, and somatic symptoms should receive increased attention during the COVID-19 outbreak.

This study aimed to investigate the psychological status of first-line military healthcare workers and identify risk factors for developing depression, anxiety, and somatic symptoms to inform targeted mental health interventions for military healthcare workers during COVID-19 and future similar epidemic outbreaks.

2 | METHODS

A questionnaire survey was used for data collection. This cross-sectional survey was conducted from February 7, 2020 to February 21, 2020. We used cluster sampling to recruit 200 military healthcare workers who had worked for 3 weeks rescuing patients with COVID-19 from three inpatient wards in two specialized COVID-19 hospitals. These military healthcare workers worked in the designated COVID-19 specialized hospitals staffed only by military healthcare workers. The inclusion criteria were: (a) military healthcare workers, (b) working in COVID-19 specialized hospitals, and (c) no cognitive impairment. The exclusion criteria were: (a) an answer time for the 31 survey items of more than 30 minutes or less than 1 minute, and (b) having schizophrenia, severe mood disorders, or other mental disorders. The questionnaires were prepared using “Wen Juan Xing,” which is an online research tool that converts a paper scale into an electronic version.

This made the psychological assessment easily to access and conduct using cell phones or PC devices. Because of the high transmission risk associated with COVID-19, this tool has been widely applied in various studies(Zhang et al., 2020), including among our team.

The nine-item patient health questionnaire (PHQ-9) from the Diagnostic and Statistical Manual of Mental Disorders, Fifth Edition (DSM-5) was used to measure depression symptoms. The PHQ-9 evaluates the degree to which an individual has experienced depression symptoms over the past 2 weeks. Each item is rated from 0 (never) to 3 (nearly every day). The PHQ-9 total score ranges from 0 to 27, and a higher score indicates greater depression severity. Total scores of 5, 10, 15, and 20 represent cutoff points for low, medium, moderate-severe, and high depression symptom severity, respectively. Based on a study by Teymoori et al. (2020), a PHQ-9 score ≥5 was considered to represent the presence of depression symptoms. The Cronbach's alpha for the PHQ-9 was 0.815. Research using the Chinese version of the PHQ-9 showed satisfactory reliability and validity in the general population (Chen et al., 2010).

The generalized anxiety disorder-7 (GAD-7) scale from the DSM-5 was used to measure anxiety symptoms. These seven items assess the frequency of anxiety symptoms over the past 2 weeks on a 4-point Likert-scale ranging from 0 (never) to 3 (nearly every day). Higher scores indicate more severe functional impairment as a result of anxiety. Total scores of 5, 10, and 15 represent cutoff points for low, medium, and high anxiety symptom severity, respectively. Consistent with a study by Toussaint, a GAD-7 score ≥ 5 was considered to indicate the existence of anxiety symptoms (Toussaint et al., 2020). The Cronbach's alpha for the GAD-7 was 0.896. Research using the Chinese version of the GAD-7 showed satisfactory reliability and validity in the general population (He, Li, Qian, Chui, & Wu, 2010).

The patient health questionnaire-15 (PHQ-15) from the DSM-5 was used to measure the severity of somatic symptoms. The PHQ-15 measures 15 somatic symptoms, with each symptom scored from 0 (not bothered at all) to 2 (bothered a lot). Total scores of 5, 10, and 15 represent cutoff points for low, medium, and high somatic symptom severity, respectively. Cano-Garcia indicated that a PHQ-15 score ≥ 5 represented the presence of somatic symptoms (Cano-Garcia et al., 2020). The Cronbach's alpha for the PHQ-15 was 0.862. Research using the Chinese version of the PHQ-15 showed satisfactory reliability and validity in the general population (Qian, Ren, Yu, He, & Li, 2014).

Items in the questionnaire were modified by the present researchers after a pilot survey. Data were then collected through anonymous online surveys, which were disseminated via WeChat. This survey was approved by the ethics panel of the Medical Association Changzheng Hospital (No. 2020SL010). All participants in this study provided informed consent. Only one response to the questionnaire per IP address was permitted during the investigation. The questionnaire could only be submitted after all items had been completed. In addition, background monitoring was conducted in real time to ensure data reliability.

Statistical analyses were performed with IBM SPSS Statistics version 21.0. Descriptive analyses were conducted to describe participants' demographic characteristics and the overall prevalence rates of depression, generalized anxiety, and somatic symptoms. We used *t* tests, one-way variance (ANOVA), and nonparametric analyses to compare the prevalence of depression, generalized anxiety, and somatic symptoms between different subgroups. Hierarchical regression analysis was applied to identify independent variables associated with depression, generalized anxiety, and somatic symptoms among military healthcare workers. $P < .05$ was considered statistically significant.

3 | RESULTS

In total, 194 military healthcare workers were recruited for this study, among which 158 (81.4%) were females. Just over half (n = 102, 52.6%) were aged under 30 years, 87 (44.8%) were aged 30 to 50 years, and five (2.6%) were aged over 50 years. Forty-two (21.6%) participants were doctors, 148 (76.3%) were nurses, and four (2.1%) were command staff. A junior-grade professional title was held by 110 (56.7%) participants, 56 (28.9%) had a medium-grade professional title, and 28 (14.4%) had a senior-grade title. For doctors, nurses, and command staff, the terms “junior-grade,” “medium-grade,” and “senior-grade” reflect the grading system used in healthcare practice in China (also called professional titles). These designations comprehensively represent their professional years, performance, and academic level. Individuals must pass an examination and interview with the Professional Committee to achieve “medium” and “senior” professional grades.

In terms of the frequency of daily checking of COVID-19 news, 23 (11.9%) participants checked >10 times per day and 93 (47.9%) checked 3 to 10 times. Sixty-one (31.4%) participants worked ≥8 hours per day. The average daily sleep duration was <6 hours (n = 36, 18.6%), and 157 (80.9%) participants reported ordinary or bad sleep quality (Table 1).

Table 2 presents the average scores and overall prevalence rates of depression, generalized anxiety, and somatic symptoms as assessed by the PHQ-9, GAD-7, and PHQ-15, respectively. The average PHQ-9, GAD-7, and PHQ-15 scores were 3.79 ± 3.30, 3.09 ± 3.22, and 5.34 ± 4.44, respectively. With a cutoff score of 5, the overall prevalence of depression, generalized anxiety, and somatic symptoms was 37.6%, 32.5%, and 50%, respectively. Given that we defined a score ≥ 10 as severe symptoms, the prevalence of severe depression, severe generalized anxiety, and severe somatic symptoms was 5.2%, 3.6%, and 15.5%, respectively. In 22.7% of cases, comorbidities existed between depression, generalized anxiety, and somatization.

The scores for depression, generalized anxiety, and somatic symptoms by different subgroups are presented in Table 3. In terms of depression, participants with junior (P = .003) and medium (P = .012) grade professional titles had significantly higher scores than those with senior titles. Military healthcare workers that checked the daily news more than 10 times had significantly higher depression scores than those who checked 3 to 10 times (P = .009). Participants who

TABLE 1 Demographic information of participating military healthcare workers (*n*, %)

Characteristics	Total (*n* = 194)	Doctor (*n* = 42)	Nurse (*n* = 148)	Commanders (*n* = 4)
Age				
<30 years	102 (44.8%)	5 (2.6%)	96 (49.5%)	1 (0.5%)
30-50 years	87 (33.3%)	32 (16.5%)	52 (26.8%)	3 (1.5%)
>50 years	5 (21.9%)	5 (2.6%)	0	0
Gender				
Male	36 (18.6%)	33 (17.0%)	1 (0.5%)	2 (1.0%)
Female	158 (81.4%)	9 (4.6%)	147 (75.8%)	2 (1.0%)
Professional title				
Junior-grade	110 (56.7%)	0	108 (55.7%)	2 (1.0%)
Medium-grade	56 (28.9%)	15 (7.7%)	40 (20.6%)	1 (0.5%)
Senior-grade	28 (14.4%)	27 (13.9%)	0 (%)	1 (0.5%)
Daily checking news about COVID-19				
<3 times	78 (40.2%)	14 (7.2%)	63 (32.5%)	1 (0.5%)
3-10 times	93 (47.9%)	18 (9.3%)	72 (37.1%)	3 (1.5%)
>10 times	23 (11.9%)	10 (5.2%)	13 (6.7%)	0
Daily working hours				
<8 hours	133 (68.6%)	23 (11.9%)	108 (55.7%)	2 (1.0%)
≥8 hours	61 (31.4%)	19 (9.8%)	40 (20.6%)	2 (1.0%)
Daily sleep duration				
<6 hours	36 (18.6%)	6 (3.1%)	28 (14.4%)	2 (1.0%)
≥6 hours	158 (81.4%)	36 (18.6%)	120 (61.9%)	2 (1.0%)
Sleep quality				
Ordinary or bad	157 (80.9%)	26 (13.4%)	129 (66.5%)	2 (1.0%)
Good	37 (19.1%)	16 (8.2%)	19 (9.8%)	2 (1.0%)

TABLE 2 Average scores, prevalence, and comorbidity of depression, generalized anxiety, and somatic symptoms

	Average score ($\bar{x} \pm s$)	Prevalence (%)	Severe symptoms (score ≥ 10) (%)	Comorbidity (%)
PHQ-9	3.79 ± 3.30	37.6	5.2	22.7
GAD-7	3.09 ± 3.22	32.5	3.6	
PHQ-15	5.34 ± 4.44	50	15.5	

Abbreviations: GAD-7, generalized anxiety disorder-7; PHQ-9, patient health questionnaire-9; PHQ-15, patient health questionnaire-15.

had sleep duration per day of ≥6 hours ($P < .001$) and good sleep quality ($P < .001$) were less likely to report depression symptoms.

In terms of anxiety, participants with junior ($P = .049$) and medium ($P = .006$) grade professional titles had significantly higher scores than those with senior titles. Military healthcare workers that checked the news more than 10 times per day had significantly higher anxiety scores than those who checked 3-10 times ($P = .017$) and <3 times ($P = .014$) per day. There were significant differences in the daily working hours ($P = .019$), daily sleep duration ($P = .003$), and sleep quality ($P < .001$) among the subgroups.

In terms of somatic symptoms, the scores of females and doctors were significantly higher than those of males ($P = .008$) and nurses ($P = .011$). Participants with junior ($P = .004$) and medium ($P = .001$) grade professional titles had significantly higher somatic symptoms scores than those with senior titles. There were significant differences in the daily sleep duration ($P = .001$) and sleep quality ($P < .001$) among the subgroups.

Table 4 presents the results of the hierarchical logistic regression analysis. Professional title ($P = .010$), daily sleep duration ($P = .004$), and sleep quality ($P < .0001$) were significantly associated with PHQ-9 score. Age ($P = .013$) and sleep quality ($P = .001$) were associated with GAD-7 scores. Age ($P = .017$), daily sleep duration ($P = .018$), and sleep quality ($P < .0001$) were significantly associated with PHQ-15 scores.

4 | DISCUSSION

The COVID-19 outbreak was regarded as a public health emergency. It raised public health concerns and caused tremendous psychological distress. Although the increase of confirmed and suspected COVID-19

TABLE 3 Depression, generalized anxiety, and somatic symptoms scores in different subgroups

	PHQ-9 score		P	GAD-7 score		P	PHQ-15 score		P
Age	Mean	SD	.396	Mean	SD	.258	Mean	SD	.891
<30	3.93	2.98		2.72	3.01		5.10	4.07	
30-50	3.67	3.67		3.45	3.40		5.68	4.90	
>50	3.00	3.08		4.40	3.65		4.40	3.21	
Gender			.125			.385			.008**
Male	3.03	3.31		2.67	3.06		3.58	3.52	
Female	3.96	3.28		3.18	3.25		5.74	4.54	
Occupation			.160			.609			.036*
Doctor	2.93	3.22		2.67	3.02		3.78	3.26	LSD[a] .011*
Nurse	4.03	3.26		3.22	3.27		5.76	4.61	
Command group	3.75	4.79		2.75	3.59		6.00	5.94	
Professional title			.011*			.017*			.002**
Junior-grade	4.14	3.31	LSD[b] .003**	2.94	3.19	LSD[b] .049**	5.48	4.59	LSD[b] .004**
Medium-grade	3.96	3.28	LSD[c] .012*	3.96	3.35	LSD[c] .006*	6.32	4.46	LSD[c] .001**
Senior-grade	2.07	2.85		1.93	2.61		2.82	2.54	
Daily checking news about COVID-19			.027*			.037*			.704
<3 times	3.97	3.09		2.82	3.38	LSD[d] .014*	5.67	4.44	
3–10 times	3.27	3.28	LSD[e] .009**	2.91	3.02	LSD[e] .017*	5.13	4.55	
>10 times	5.26	3.70		4.70	3.11		5.09	4.07	
Daily working hours			.487			.019*			.896
<8 hours	3.68	3.17		2.72	2.97		5.37	4.64	
≥8 hours	4.03	3.58		3.89	3.60		5.28	4.00	
Daily sleep duration			<.001***			.003**			.001**
<6 hours	5.56	3.71		4.50	4.04		7.53	5.31	
≥6 hours	3.39	3.07		2.77	2.92		4.84	4.07	
Sleep quality			<.001***			<.001***			<.001***
Ordinary or bad	4.29	3.26		3.51	3.31		5.99	4.51	
Good	1.68	2.57		1.30	1.96		2.59	2.76	

Abbreviations: GAD-7, generalized anxiety disorder-7; LSD, least significance difference; PHQ-9, patient health questionnaire-9; PHQ-15, patient health questionnaire-15.
[a]Doctor vs Nurse.
[b]Junior-grade vs Senior-grade.
[c]Medium-grade vs Senior-grade.
[d]<3 times vs >10 times.
[e]3-10 times vs >10 times.
*$P < .05$.
**$P < .01$.
***$P < .001$.

cases has been constrained in China, first-line healthcare workers still need to keep working. Military healthcare workers, who were among the first group to engage in the epidemic prevention, experienced strict tests and more pressures because of the long work hours, meaning they became a group at high risk for mental health problems.

The prevalence of depression, generalized anxiety, and somatic symptoms in military healthcare workers in this study was high (37.6%, 32.5%, and 50%, respectively). These rates were higher than the prevalence of depression (18.1%) and anxiety (34.0%) reported for the public (Liu et al., 2020). There are three reasons that may explain this result. First, all patients in COVID-19-specialized hospitals were confirmed COVID-19 cases that had older ages and more underlying diseases, such as cognitive disorders and psychotic symptoms. Second, military healthcare workers were entrusted with this mission under critical circumstances. They needed to rescue patients in relatively unfamiliar places, with insufficient psychological and resource preparation. Finally, their living conditions and dwelling environments were poor. For example, they had to travel long distances from their residence to the hospitals, and often had irregular meals and insufficient sleep because of the heavy workload. However, it is worth

TABLE 4 Regression analysis with PHQ-9, GAD-7, and PHQ-15 scores as the dependent variables (*n* = 194)

Scale	Variables	*B*	β	*t*	*P* value
PHQ-9 score	Age	0.600	.528	1.137	.257
	Gender	−1.334	.936	−1.426	.156
	Occupation	−0.052	.762	−0.068	.946
	Professional title	−1.461	.564	−2.590	.010*
	Daily checking news about COVID-19	0.272	.346	0.786	.433
	Daily working hours	0.156	.510	0.305	.761
	Daily sleep duration	−1.732	.595	−2.912	.004**
	Sleep quality	−2.242	.600	−3.739	.000***
GAD-7 score	Age	1.301	.518	2.511	.013*
	Gender	−0.346	.919	−0.377	.707
	Occupation	0.210	.749	0.280	.780
	Professional title	−0.771	.554	−1.392	.166
	Daily checking news about COVID-19	0.548	.340	1.613	.108
	Daily working hours	0.770	.501	1.536	.126
	Daily sleep duration	−1.145	.584	−1.961	.051
	Sleep quality	−1.959	.589	−3.325	.001**
PHQ-15 score	Age	1.715	.714	2.403	.017*
	Gender	0.368	1.266	0.291	.771
	Occupation	0.820	1.031	0.795	.428
	Professional title	−0.710	.763	−0.931	.353
	Daily checking news about COVID-19	−0.322	.468	−0.689	.492
	Daily working hours	−0.499	.690	−0.723	.471
	Daily sleep duration	−1.923	.804	−2.391	.018*
	Sleep quality	−2.906	.811	−3.583	.000***

Abbreviations: B, unstandardized beta; β, standardized regression weight; GAD-7, generalized anxiety disorder-7; PHQ-9, patient health questionnaire-9; PHQ-15, patient health questionnaire-15.
*$P < .05$.
**$P < .01$.
***$P < .001$.

noting that the prevalence of mental health problems in this group was lower than that of healthcare workers in local hospitals during the COVID-19 outbreak. Huang and Zhao revealed that the prevalence of depression and anxiety among healthcare workers in local hospitals was as high as 50.7% and 44.7%, respectively (Huang & Zhao, 2020). Our study showed that 22.7% of military health workers with depression symptoms had comorbid anxiety and somatization, which was lower than that among primary care clinic patients in the United States (Löwe et al., 2008). However, military healthcare workers may have specific characteristics related to their military training that may act as mechanisms to protect their mental health to some extent.

Military healthcare workers with lower grade professional titles showed more depression symptoms, which was consistent with a previous study (Huang & Zhao, 2020), and may be related to the following reasons. People with junior-grade professional titles face more challenges, such as economic pressure and limited societal resources. In addition, to secure promotion, they need to consider other issues while treating patients with COVID-19 (eg, scientific research, publishing papers, and teaching tasks). Our study also showed that with the increase of age, more symptoms of anxiety and somatization appeared in military healthcare workers. This result indicated that older military healthcare workers may experience more occupational exhaustion and other pressures (eg, family responsibilities).

The present study found depression, generalized anxiety, and somatic symptoms were more prevalent among military health workers with short sleep duration and poor sleep quality. This was consistent with a previous study that showed poor sleep was strongly associated with symptoms of depression, anxiety, and posttraumatic stress disorder (Kobayashi, Boarts, & Delahanty, 2007). In our study, almost 81% of military healthcare workers had self-reported poor or ordinary sleep quality. The residence of the military healthcare workers was 21 km from the hospitals in which they worked. They traveled to and from the hospitals each day by auto-bus, which is a journey that takes about an hour each way. They did not live in barracks. Therefore, their sleep quality might have been negatively affected by factors such as living far from the hospital, time-consuming medical protection, generalized anxiety caused by severe epidemic situation, working in shifts leading to circadian rhythm disorder, and job burnout.

Many strong measures were implemented to maintain the mental health of the public in China during the pandemic. The National Health Commission issued guidelines for emergency psychological crisis intervention in COVID-19 (Chinese National Healthcare Center, 2020a). In these guidelines, intervention subjects are divided into four grades according to the degree of epidemic influence, with military healthcare workers regarded as the first grade. To improve the mental health of military healthcare workers in public health emergencies, we have put forward some practical suggestions based on the assessment of mental health conditions among these workers and the analysis of related risk factors. First, more attention should be paid to specific groups, such as nurses and those with junior- and medium-grade professional titles, with support provided via “physiological-psychological-social” interventions. Second, it is not advisable to check information about COVID-19 frequently. Finally, interventions to reduce stress are needed. For example, our psychiatrists have conducted various interventions for military healthcare workers, including: (a) virtual reality mindfulness stress reduction training and relaxation training; (b) psychological counseling; and (c) prescribing medications and other measures.

The present study had several limitations. First, because of the sudden appearance of COVID-19, this study could only use a cross-sectional design. Second, data for the baseline mental health of military healthcare workers before the COVID-19 outbreak was lacking. In addition, the relationship between mental health and other risk factors such as COVID-19 knowledge reserve and proportion of clinical classification of COVID-19 were not investigated. Finally, these results need to be explored in a further study with a larger sample size.

In conclusion, this study explored the prevalence of depression, generalized anxiety, and somatic symptoms in military healthcare workers during the COVID-19 outbreak. We also investigated related risk factors. Junior-grade professional title, older age, short sleep duration, and poor sleep quality have significant effects on the mental health of military healthcare workers. Some suggestions targeting military healthcare workers have been provided to help ensure their mental health. Such measures will promote military healthcare workers' combat effectiveness.

ACKNOWLEDGEMENTS

We acknowledge all project participants and the investigators for collecting data. This work was supported by Changzheng Hospital Pyramid Talent Project (2020) of the Second Military Medical University and Research Project of Hubei Maternal and Child Health Hospital Guanggu District (2020-FYGG-017).

CONFLICT OF INTEREST

The authors declare no conflicts of interest.

AUTHOR CONTRIBUTIONS

Xiao Pan, Yong-Hai Bai, Yu-Shi Hao, Feng Zhao, and Qian Zhang participated in the research design and drafted the manuscript. Xiao Pan, Feng Zhao, Ding Ren, Fen Liu, Yu-Shi Hao, and Ying Xiao performed the literature search. Ding Ren, Feng Zhao, and Li-Ying Yang designed the questionnaires. Qian Zhang, Li-Ying Yang, Fen Liu, and Ying Xiao participated in data analysis and data interpretation for this research. All authors participated in discussion of the outcomes and offered their unique contribution to this research.

ETHICS STATEMENT

This survey was approved by the ethics panel of the Medical Association Changzheng Hospital (No. 2020SL010). All participants in this study provided informed consent to participate.

ORCID

Yong-Hai Bai https://orcid.org/0000-0001-5818-7216

REFERENCES

Cano-Garcia, F. J., Munoz-Navarro, R., Sese Abad, A., Moretti, L. S., Medrano, L. A., Ruiz-Rodriguez, P., … Cano-Vindel, A. (2020). Latent structure and factor invariance of somatic symptoms in the patient health questionnaire (PHQ-15). *Journal of Affective Disorders*, *261*, 21–29. https://doi.org/10.1016/j.jad.2019.09.077

Chen, S., Chiu, H., Xu, B., Ma, Y., Jin, T., Wu, M., & Conwell, Y. (2010). Reliability and validity of the PHQ-9 for screening late-life depression in Chinese primary care. *International Journal of Geriatric Psychiatry*, *25*(11), 1127–1133. https://doi.org/10.1002/gps.2442

Chinese National Healthcare Center. (2020a). Circular on issuing guiding principles for emergency psychological crisis intervention for new coronavirus pneumonia. Retrieved from http://www.nhc.gov.cn/jkj/s3577/202001/6adc08b966594253b2b791be5c3b9467.shtml

Chinese National Healthcare Center. (2020b). Occupational injury insurance supports occupational injury “protection umbrella” for prevention and rescue personnel in the fight against new coronavirus pneumonia. Retrieved from http://www.nhc.gov.cn/xcs/fkdt/202001/0ffbb879ac1b4466b01de8b40a1372fc.shtml

He, X., Li, C., Qian, J., Chui, H., & Wu, W. (2010). Reliability and validity of a generalized anxiety scale in general hospital outpatients. *Shanghai Archives of Psychiatry*, *22*, 200–203. https://doi.org/10.3969/j.issn.1002-0829.2010.04.002

Huang, Y., & Zhao, N. (2020). Generalized anxiety disorder, depressive symptoms and sleep quality during COVID-19 outbreak in China: A web-based cross-sectional survey. *Psychiatry Research*, *288*, 112954. https://doi.org/10.1016/j.psychres.2020.112954

Kang, L., Li, Y., Hu, S., Chen, M., Yang, C., Yang, B., … Liu, Z. (2020). The mental health of medical workers in Wuhan, China dealing with the 2019 novel coronavirus. *The Lancet Psychiatry*, *7*, e14. https://doi.org/10.1016/S2215-0366(20)30047-X

Kobayashi, I., Boarts, J., & Delahanty, D. (2007). Polysomnographically measured sleep abnormalities in PTSD. *Psychophysiology*, *44*, 660–669. https://doi.org/10.1111/j.1469-8986.2007.537.x

Koh, D., Lim, M., Chia, S., Ko, S., Qian, F., Ng, V., … Fones, C. (2005). Risk perception and impact of severe acute respiratory syndrome (SARS) on work and personal lives of healthcare workers in Singapore. *Medical Care*, *43*, 676–682. https://doi.org/10.1097/01.mlr.0000167181.36730.cc

Leonhart, R., Tang, L., Pang, Y., Li, J., Song, L., Fischer, I., … Schaefert, R. (2017). Physical and psychological correlates of high somatic symptom severity in Chinese breast cancer patients. *Psychooncology*, *26*(5), 656–663. https://doi.org/10.1002/pon.4203

Liu, S., Yang, L., Zhang, C., Xiang, Y.-T., Liu, Z., Hu, S., & Zhang, B. (2020). Online mental health services in China during the COVID-19 outbreak. *The Lancet Psychiatry*, *7*, e17–e18. https://doi.org/10.1016/S2215-0366(20)30077-8

Löwe, B., Spitzer, R., Williams, J., Mussell, M., Schellberg, D., & Kroenke, K. (2008). Depression, anxiety and somatization in primary

care: Syndrome overlap and functional impairment. *General Hospital Psychiatry*, *30*, 191–199. https://doi.org/10.1016/j.genhosppsych.2008.01.001

Lu, Y. C., Shu, B. C., Chang, Y. Y., & Lung, F. W. (2006). The mental health of hospital workers dealing with severe acute respiratory syndrome. *Psychotherapy and Psychosomatics*, *75*(6), 370–375. https://doi.org/10.1159/000095443

Panagioti, M., Geraghty, K., Johnson, J., Zhou, A., Panagopoulou, E., Chew-Graham, C., … Esmail, A. (2018). Association between physician burnout and patient safety, professionalism, and patient satisfaction: A systematic review and meta-analysis. *JAMA Internal Medicine*, *178*, 1317. https://doi.org/10.1001/jamainternmed.2018.3713

Qian, J., Ren, Z. Q., Yu, D. H., He, X. Y., & Li, C. B. (2014). The value of the patient health questionnaire-15 (PHQ-15) for screening somatic symptoms in general hospital. *Chinese Mental Health Journal*, *28*, 173–178. https://doi.org/10.3969/j.issn.1000-6729.2014.03.003

Teymoori, A., Real, R., Gorbunova, A., Haghish, E. F., Andelic, N., Wilson, L., … von Steinbuchel, N. (2020). Measurement invariance of assessments of depression (PHQ-9) and anxiety (GAD-7) across sex, strata and linguistic backgrounds in a European-wide sample of patients after traumatic brain injury. *Journal of Affective Disorders*, *262*, 278–285. https://doi.org/10.1016/j.jad.2019.10.035

Toussaint, A., Husing, P., Gumz, A., Wingenfeld, K., Harter, M., Schramm, E., & Lowe, B. (2020). Sensitivity to change and minimal clinically important difference of the 7-item generalized anxiety disorder questionnaire (GAD-7). *Journal of Affective Disorders*, *265*, 395–401. https://doi.org/10.1016/j.jad.2020.01.032

Wang, D., Hu, B., Hu, C., Zhu, F., Liu, X., Zhang, J., … Peng, Z. (2020). Clinical characteristics of 138 hospitalized patients with 2019 novel coronavirus-infected pneumonia in Wuhan, China. *JAMA.*, *323*, 1061. https://doi.org/10.1001/jama.2020.1585

Zhang, W. R., Wang, K., Yin, L., Zhao, W. F., Xue, Q., Peng, M., … Wang, H. X. (2020). Mental health and psychosocial problems of medical health workers during the COVID-19 epidemic in China. *Psychotherapy and Psychosomatics*, *89*(4), 242–250. https://doi.org/10.1159/000507639

How to cite this article: Pan X, Xiao Y, Ren D, et al. Prevalence of mental health problems and associated risk factors among military healthcare workers in specialized COVID-19 hospitals in Wuhan, China: A cross-sectional survey. *Asia Pac Psychiatry*. 2022;14:e12427. https://doi.org/10.1111/appy.12427
