# Supplementary material for: Mental health status and related factors influencing healthcare workers during the COVID-19 pandemic: A systematic review and meta-analysis
Source: PLoS One. 2024 Jan 19;19(1):e0289454. doi: 10.1371/journal.pone.0289454 (PMC10798549; doi:10.1371/journal.pone.0289454)
Supplement: S1 Data — (ZIP) [file pone.0289454.s011.zip › literatures/129.pdf]

J Nepal Health Res Counc 2020 Oct-Dec;18(49): 655-60

DOI: https://doi.org/10.33314/jnhrc.v18i4.3190

Stress, Anxiety, Depression and Their Associated Factors among Health Care Workers During COVID -19 Pandemic in Nepal

Apsara Pandey,[1] Chandrakala Sharma,[1] Ram Hari Chapagain,[2] Narmada Devkota,[2] Kamal Ranabhat,[3] Suman Pant,[4] Kriti Adhikari[4]

[1]Maharajgunj Nursing Campus, Institute of Medicine, Tribhuwan University, Kathmandu, [2]Kanti Children Hospital, Maharajgunj, Kathmandu, [3]Central Department of Public Health, Institute of Medicine, Tribhuwan University, Kathmandu, Nepal, [4]Nepal Health Research Council, Ram Shah Path, Kathmandu, Nepal.

ABSTRACT

Background: Frontline Health Care Workers are at risk of developing mental and psychological distress during Corona virus disease 2019 pandemic. This study aimed to assess level of stress, anxiety, depression and their associated factors among health care workers during Corona virus disease 2019 pandemic in Nepal.

Methods: This is a web based cross-sectional survey conducted among 404 Health Care Workers during early phase of Corona virus disease 2019 pandemic. The participants were selected using convenience sampling technique and were invited to participate via various online networks. Depression, anxiety and stress were assessed using the Depression Anxiety Stress Scale-21 (DASS-21) and their associated factors were measured using structured questionnaires. Multivariable logistic regression was carried out to determine the associated factors.

Results: The symptoms of stress, anxiety and depression among health care workers were found to be 28.9%, 35.6% and 17.0% respectively. Females were found to be 2 fold more likely to have anxiety and depression than male. Nurses were found to be two times more likely to have anxiety than doctors. Laboratory personnel were almost three folds more likely to have anxiety than doctors. Health care workers with insufficient/ no PPE were almost three fold more likely to have depression. health care workers working in high-risk areas had almost 2 fold higher odds of having depression.

Conclusions: Health care workers experienced symptoms of stress, anxiety and depression in varied level of severity. This experience was higher among the nurses and laboratory workers than doctors. Pandemic preparedness assuring PPE and appropriate psychological interventions may be beneficial to promote mental health and well-being of health care workers.

Keywords: Anxiety; associated factors; depression; health workers; stress

INTRODUCTION

The dramatic spread of Corona Virus Disease 2019 (COVID-19) has caused a global public health crisis.[1] As of 13 July 2020, it had affected more than 216 countries and territories around the world and had infected more than 12768307people causing 566654 deaths (WHO).[2] With the increasing number of infection rates and death counts due to COVID-19, Health Care Workers (HCWs) are facing tremendous pressure and psychological distress.[3] Although few studies have investigated the psychological and mental health of general population, no study has been conducted among frontline HCWs during COVID-19 pandemic in seven provinces of Nepal. Therefore, the researchers aim to assess the level of, stress, anxiety, depression and their associated factors among frontline HCWs in Nepal.

METHODS

A web based cross-sectional analytical study was conducted among frontline HCWs working in Nepal during early phase of COVID-19 pandemic. Ethical approval was obtained from Nepal Health Research Council. Prior to starting the survey, informed consent was obtained from all the participants. Participants were informed about

Correspondence: Apsara Pandey, Department of Pediatric Nursing, Maharajgunj Nursing Campus, Institute of Medicine, Tribhuwan University, Kathmandu, Nepal. Email: pkapsara@gmail.com, Phone: +9779851186586.

the purpose of the study and no identifying information was asked from any of the participants. They were informed to withdraw from the study any time if they do not wish to participate. However, participants who lacked skills to complete an online survey, those who were severely ill and unable to answer the questions were excluded from the study. HCWs such as Doctors, Nurses and Laboratory personnel working at different health care facilities of seven provinces were considered as frontline HCWs and altogether 404 HCWs were invited to participate in this study using convenience sampling technique. Data was collected through electronic means. Self report, structured questionnaires were developed in the Google Forms and the link to the survey was posted on various social media platforms and was also shared via email, Face book, Viber, and other instant messaging applications inviting frontline HCWs to participate in the study. The system was set in such a way that one participant could submit only one form with one Google account. The online form was active from April 12 to May 12, 2020 and the data was collected until the desired sample size was achieved. In addition to information on demographic characteristics, work related factors, environmental factors and medical history the questionnaire included outcome variable of interest such as depression, anxiety and stress which was measured using validated Depression Anxiety Stress Scale-21 (DASS-21).[4] The DASS-21 has been validated and used in the Nepalese population to identify the symptoms of Depression, Anxiety, and Stress.[5-7]Cronbach's alpha coefficient for DASS-21 has been found to be ranging from 0.78-0.91.[6-9] Investigators discussed the study once and was explained by phone if any respondents had doubts on the questionnaire.

Scores for depression, anxiety, and stress were calculated by summing the scores for the relevant items.[10] Multivariate logistic regression was performed to measure the degree at which independent and dependent variables are related using SPSS version 22.0 .The significance level was fixed at 0.05 at 95.0% confidence interval for odds.

RESULTS

A total of 404 HCWs were enrolled in this study from seven provinces of Nepal. The socio demographic characteristics of the invited 404 HCWs those who participated in the study is shown in Table 1.

Table 1.Socio-demographic characteristics (n=404).

Characteristics	Number	Percent
**Age**		
<20	8	2
20-30	193	47.8
31-40	139	34.4
41-50	49	12.1
51-60	14	3.5
>60	1	0.2
Mean±SD	32.25 ± 8.23	
**Gender**		
Female	257	63.6
Male	147	36.4
Marital status		
Married	251	62.1
Unmarried	153	37.9
**Ethnic group**		
Dalit	3	0.7
Disadvantaged Janajati	23	5.7
Disadvantaged non-Dalit Terai	20	5.0
Relatively advantaged Janajati	71	17.6
Religious minorities	1	0.2
Upper-caste groups	286	70.8
**Socio-economic class**		
Upper	283	70.0
Upper Middle	119	29.5
Lower	2	0.5

Majority of the participants (70.0%) did not get any training or orientation on COVID-19 and among the participants who received training, only five percent got complete training (Donning, Doffing, IPC and Waste Management).The participant's duty hours ranged from six to ten hours per day for majority of participants (77.2%) with an mean of 8.25 hours(SD=2.48hours). Although 75.2% HCWs had the provision of Personal Protective Equipments (PPE) at their hospitals or workplace, they felt that the quantity was not adequate. Only 13.15% had provision of adequate PPE (Table 2).

Table 2. Work related information.

Variables	Number	Percent
**Profession (n=404)**		
Doctor	154	38.1
Nurses	188	46.5
Laboratory Personnel	62	15.3
NR**	3	0.7
**Training (n=404)**		
No	283	70.0
Yes	121	30.0
**Types of training (n= 121)**		

Complete training (Donning, Doffing, IPC* Waste Management)	20	5.0
Incomplete training	39	9.7
Online training	16	4.0
Orientation only	31	7.7
NR**	15	1.2
**Duty hours per day (n=404)**		
<5hours	16	4
6-10 hours	312	77.2
11-15 hours	70	17.3
>15 hours	6	1.5
Mean± SD	8.25±2.48	
**Provision of PPE (n=404)**		
No	47	11.6
Yes but not adequate	304	75.2
Yes, adequate	53	13.1

***Non response, * Infection prevention and control*

Analysis of three mental health conditions (stress, anxiety and depression) showed that symptoms of stress, anxiety and depression among health workers were found to be 17.1% 35.6% and 28.9%, respectively.

Analysis of severity of symptoms, it was found that 8.4%had mild stress, 4.7% had moderate stress whereas 2.5% and 1.5% had severe and extremely severe stress levels(Table 3).

Table 3. Level of stress (n=404).

Level (Score of stress subscale)	Number	Percent
Normal (0-14)	335	82.9
Mild (15-18)	34	8.4
Moderate (19-25)	19	4.7
Severe (26-33)	10	2.5
Extremely severe (+34)	6	1.5

Similarly, participants had symptoms of mild (8.2%) moderate (17.6%), severe (4.7%) and extremely severe (5.2%) level of anxiety. Likewise, participants had also symptoms of mild (13.1%), moderate (10.9%), severe and extremely severe (2.5%) level of depression (Table 4).

Table 4. Level of anxiety and depression (n=404).

Variables	Number	Percent
Anxiety level (Score of anxiety subscale)		
Normal (0-7)	260	64.4
Mild (8-9)	33	8.2
Moderate (10-14)	71	17.6
Severe (15-19)	19	4.7
Extremely severe (20+)	21	5.2
Depression level (score of depression subscale)		
Normal (0-9)	287	71.0
Mild (10-13)	53	13.1
Moderate (14-20)	44	10.9
Severe (21-27)	10	2.5
Extremely severe(28+)	10	2.5

Multivariable analysis revealed that female participants were more likely to have symptoms of anxiety (AOR=2.102, CI=1.030-4.290) and depression (AOR=2.137, CI=1.001-4.560) by two folds in comparison to males. Compared to doctors, nurses were found to be two times (AOR=2.373, CI=1.158-4.862) more likely to have anxiety symptoms. Similarly, Laboratory personnel were almost three folds (AOR=2.634, CI=1.241-5.587) more likely to have anxiety compared to doctors. HCWs living in the joint family were almost 2 folds (AOR=1.843, CI=1.117-3.042) more likely to have symptoms of anxiety compared to their counterparts living in nuclear family HCWs with insufficient or no PPE were almost three folds (AOR=2.990, CI=1.250-7.154) more likely to have symptoms of depression while the HCWs working in high-risk areas had almost 2 folds (AOR=1.902, CI=1.144-3.162) higher odds of having depressive symptoms. Surprisingly, those having some chronic conditions were found to have almost 68.0% lower risk (AOR=0.317, CI=0.110-0.914) of having depression (Table 5).

Table 5. Multivariate analysis of the factors associated with stress, anxiety and depression.

Characteristics	Stress			Anxiety			Depression		
	Odds ratio	95% CI		Odds ratio	95% CI		Odds ratio	95% CI	
		Lower	Upper		Lower	Upper		Lower	Upper
Female (Ref: Male)	2.163	0.873	5.359	2.102*	1.030	4.290	2.137*	1.001	4.560
Age (Ref: less than 25 years)									
26 to 35 years	1.038	0.436	2.473	1.329	0.647	2.733	1.404	0.647	3.047
More than 35 years	1.726	0.480	6.204	1.639	0.602	4.464	1.602	0.543	4.723
Married (Ref: Unmarried)	0.866	0.399	1.882	0.823	0.439	1.543	1.303	0.661	2.569
Training received (Ref: No)	1.168	0.617	2.211	0.702	0.416	1.186	1.203	0.694	2.086
Occupation (Ref: Doctors)									
Nurse	1.426	0.595	3.415	2.373*	1.158	4.862	1.543	0.725	3.283
Laboratory personnel	0.676	0.220	2.071	2.634*	1.241	5.587	1.274	0.552	2.942
Experience more than 5 years (Ref: 5 years or less)	0.438	0.183	1.050	0.682	0.346	1.346	0.666	0.326	1.362
Joint family (Ref: Nuclear)	1.437	0.763	2.706	1.843*	1.117	3.042	1.412	0.827	2.410
Insufficient or No PPE (Ref: sufficient PPE))	1.465	0.579	3.706	1.014	0.510	2.015	2.990*	1.250	7.154
More than 40 hours duty per week (Ref: 40 hours or less)	1.290	0.614	2.711	1.403	0.784	2.511	0.687	0.380	1.245
Staff quarantine facility (Ref: No)	1.301	0.707	2.396	1.553	0.959	2.514	1.551	0.927	2.598
Faced difficulty due to lockdown (Ref: No)	0.669	0.307	1.460	1.208	0.637	2.290	0.845	0.432	1.653
Living in high risk (Ref: No)	1.689	0.923	3.090	1.145	0.707	1.855	1.902*	1.144	3.162
Living distant from family (Ref: No)	1.601	0.892	2.873	1.200	0.743	1.938	1.216	0.730	2.026
having special condition (Ref: None)									
Chronic disease	0.869	0.294	2.568	1.285	0.574	2.877	0.317*	0.110	0.914
Pregnancy and lactation	0.686	0.209	2.252	1.018	0.377	2.745	0.735	0.261	2.071
Enough food supply (Ref: No)	0.737	0.368	1.478	0.576	0.323	1.028	0.636	0.350	1.156

** Significant at 0.05*

DISCUSSION

This cross sectional web based study describes the level of depression anxiety and stress using the DAS-21 questionnaire and identifies the factors associated with depression anxiety and stress among HCWs during COVID-19 pandemic in Nepal. Doctors, nurses and laboratory personnel were considered as HCWs for this study. The symptoms of depression, anxiety and stress among HCWs in this study were found to be 28.9%, 35.6% and 17.0% respectively, which is similar to a finding of a study conducted in China.[11] Considering the unexplored novel nature of the virus, rapid transmission, high mortality rate it is bound to create more mental health outcomes among HCWs specially those working in frontline. Mental health outcomes observed in this study among HCWs might due to stressful nature of their work and fear of getting infected and being isolated. In terms of severity, though a considerable number of participants had mild to moderate severity in our study, moderate to severe symptoms have been observed in various studies conducted in other parts of the world.[12] This difference in finding may be due to greater impact of COVID-19 in those parts of the world as compared to Nepal. Our study also found that among gender females and among HCWs, nurses and laboratory personnel were more likely to have symptoms of anxiety as compared to doctors. Similar results have been observed in a study conducted in Nepal and India among HCWs during COVID-19 pandemic where being a female and being a nurse were associated with greater mental health outcomes.[13,14] The fact that nurses spend a considerable

amount of time in patient care as compared to other HCWs might have resulted into this finding. Whereas, symptoms of anxiety in Laboratory personnel may be due to their heavy work load and roles and responsibilities in diagnosing COVID-19. Healthcare workers living in a joint family were found to be more likely have symptoms of anxiety than those living in a nuclear family , which may be due to fear of putting more family members at risk of infection. This is consistent with a qualitative study conducted in India which found that putting the family members at risk was one of the major fear among HCWs. [15]

One of the issues with containment of COVID -19 is training to the HCWs and adequacy as well as proper use of PPE and prolonged duty hours. Our study found about 55.6% had duty hours of 6-10 hours per day and the majority of the participants (70.0%) did not get any training/ orientation on COVID-19. The HCWs with insufficient or no PPE and those working in high risk areas were more likely to have depressive symptoms in this study. This type of scarcity is also observed in other places in the world [15-17] Long duty hours, less training and exposure, different departmental and home responsibility, working area, and working environment were some of the factors affecting stress anxiety and depression other studies.[18, 19] Though it is generally assumed that people having chronic disorders are more likely to have mental health outcomes as compared to others, our study found that, those having some chronic conditions had lower risk of having depression. Therefore, additional research with other chronic conditions is required for further insight into this counter intuitive finding.

In order to ensure mental and emotional well being of HCWs, the policy makers should consider the importance of psychological intervention to HCWs during the COVID-19 pandemic. It is imperative to focus on evidence based strategies to prevent people with early psychological distress from developing a psychiatric diagnosis Strategies either in the form of psychosocial support or more as a psycho-therapeutic intervention should also be developed .Online strategies such as self-care training package and psychological assistance hotline can be the first choice basic and essential supportive psychological intervention during the periods of social distancing rules to reduce the risk of infection. Government and health officials also need to ensure that an adequate amount of PPE is available to HCWs to protect themselves during the COVID-19 pandemic.

This study was conducted during the early phase of disease spread in Nepal with a cross-sectional study and lacks longitudinal follow-up. Since pre-existing mental health symptoms of the respondents has not been assessed in this study, it is difficult to distinguish between pre-existing mental health symptoms and new symptoms aroused due to this pandemic. Since the data was collected electronically using convenience sampling, it cannot be generalized to entire population of Nepal. The responses to the survey were self-administered and it may have resulted in to response bias affecting the results.

CONCLUSIONS

In a matter of few months COVID-19 pandemic has created an emergency state globally and has caused various psychological and mental distresses especially among HCWs. The evidence generated from this study suggests that healthcare workers exhibit a considerable amount of anxiety, stress and depressive symptoms due to various factors such as working in high risk areas, inadequate personal protective equipments, long duration of patient care and duty hours. Therefore, HCWs with symptoms of psychological distress and psychiatric morbidity should be identified and assessed further so that with appropriate strategies, techniques and intervention, mental health problems in HCWs can be improved.

ACKNOWLEDGMENTS

We would like to express our special thanks to all the laboratory personnel, nurses and doctors who participated in this study in spite of the busy duty schedule. We would like to express our heartfelt gratitude to the Nepal Health Research Council for providing financial support and supervision to this study.

REFERENCES

1. Singhal T. A Review of Coronavirus Disease-2019 (COVID-19). Indian J Pediatr. 2020;87(4):281-6.[Article]

2. WHO. Coronavirus Disease (COVID-19), Situation report-175 [Internet]. 2020 [cited 2020 Aug 1]. Available from: https://www.who.int/docs/default-source/coronaviruse/situation-reports/20200713-covid-19-sitrep-175.pdf?sfvrsn=d6acef25_2

3. Shechter A, Diaz F, Moise N, Anstey DE, Ye S, Agarwal S, et al. Psychological distress, coping behaviors, and preferences for support among New York healthcare workers during the COVID-19 pandemic. Gen Hosp Psychiatry. 2020;66:1-8.[Article]

4. Lovibond SH, Lovibond PF. Manual for the Depression Anxiety & Stress Scales. 1995(2nd Ed.) Sydney: Psychology Foundation

5. Kunwar D, Risal A, Koirala S. Study of depression, anxiety and stress among the medical students in two medical colleges of Nepal. Kathmandu Univ Med J. 2016;53(1):22–6.[Download PDF]

6. Tonsing KN. Psychometric properties and validation of Nepali version of the Depression Anxiety Stress Scales (DASS-21). Asian J Psychiatr. 2014;8:63–6.[Article]

7. Poudel OP, Thapa B, Bhandary S. Pre-departure psychological distress, depression, anxiety, stress and perception of risk factors at workplace among migrant workers of Nepal: A pilot study. J Gen Pract Emerg Med Nepal. 2019;6(8):20–5.[Download PDF]

8. Yusoff MSB. Psychometric properties of the depression anxiety stress scale in a sample of medical degree applicants. Int Med J. 2013;20(3):295–300.[Download PDF]

9. Coker AO, Coker OO, Sanni D. Psychometric properties of the 21-item Depression Anxiety Stress Scale (DASS-21). African Res Rev. 2018;12(2):135–42.[Download PDF]

10. Lovibond PF, Lovibond SH. The structure of negative emotional states: Comparison of the Depression Anxiety Stress Scales (DASS) with the Beck Depression and Anxiety Inventories. Behav Res Ther. 1995;33(3):335-43.[Article]

11. Du J, Dong L, Wang T, Yuan C, Fu R, Zhang L, et al. Psychological symptoms among frontline healthcare workers during COVID-19 outbreak in Wuhan. Gen Hosp Psychiatry. 2020;67:144-145. [PMC]

12. Chew NW, Lee GK, Tan BY, Jing M, Goh Y, Ngiam NJ, et al. A multinational, multicentre study on the psychological outcomes and associated physical symptoms amongst healthcare workers during COVID-19 outbreak. Brain Behav Immun. 2020.[Article]

13. Khanal P, Devkota N, Dahal M, Paudel K, Joshi D. Mental health impacts among health workers during COVID-19 in a low resource setting: a cross-sectional survey from Nepal. Globalization and Health. 2020;16(1):1-12.[BMC]

14. Wilson W, Raj JP, Rao S, Ghiya M, Nedungalaparambil NM, Mundra H, et al. Prevalence and predictors of stress, anxiety, and depression among healthcare workers managing COVID-19 pandemic in India: a nationwide observational study. Indian Journal of Psychological Medicine. 2020;42(4):353-8.[Article]

15. Mohindra R, Ravaki R, Suri V, Bhalla A, Singh SM. Issues relevant to mental health promotion in frontline health care providers managing quarantined/isolated COVID19 patients. Asian J Psychiatr. 2020;51:102084.[Article]

16. Kang L, Li Y, Hu S, Chen M, Yang C, Yang BX, et al. The mental health of medical workers in Wuhan, China dealing with the 2019 novel coronavirus. The Lancet Psychiatry. 2020;7(3):e14.[Article]

17. Liang Y, Chen M, Zheng X, Liu J. Screening for Chinese medical staff mental health by SDS and SAS during the outbreak of COVID-19. J Psychosom Res. 2020;133:110102.[PMC]

18. Spoorthy MS, Pratapa SK, Mahant S. Mental health problems faced by healthcare workers due to the COVID-19 pandemic–A review. Asian J Psychiatr. 2020;51:102119.[Article]

19. Shaukat N, Ali DM, Razzak J. Physical and mental health impacts of COVID-19 on healthcare workers: A scoping review. International Journal of Emergency Medicine. 2020;13(1):1-8.
