# Supplementary material for: Mental health status and related factors influencing healthcare workers during the COVID-19 pandemic: A systematic review and meta-analysis
Source: PLoS One. 2024 Jan 19;19(1):e0289454. doi: 10.1371/journal.pone.0289454 (PMC10798549; doi:10.1371/journal.pone.0289454)
Supplement: S1 Data — (ZIP) [file pone.0289454.s011.zip › literatures/13.pdf]

Received: 17 February 2021 | Accepted: 12 March 2021
DOI: 10.1002/lio2.556

ORIGINAL RESEARCH

Laryngoscope
Investigative Otolaryngology

# The psychological impact of the COVID-19 pandemic on otolaryngologists: Should we be concerned?

Mona Mohammedsaleh Ashoor MD | Noorah Jamal Almulhem MD | Zaid Abdulrahman AlMubarak MD | Ahmed Abdulrahman Alrahim MD | Saad Mohammed Alshammari MD | Fahad Saleh Alzahrani MD | Ali Radi Alhayek MD | Abdullah Alardhi MD

Department of Otolaryngology Head and Neck Surgery, King Fahad Hospital of the University, College of Medicine, Imam Abdulrahman Bin Faisal University, Dammam, Saudi Arabia

**Correspondence**
Mona Mohammedsaleh Ashoor, Department of Otolaryngology Head and Neck Surgery, King Fahad Hospital of the University, College of Medicine, Imam Abdulrahman Bin Faisal University, P.O. Box 2208 Alkhobar 31952, Saudi Arabia.
Email: mashoor@iau.edu.sa

## Abstract

**Objective:** Physicians on the frontline of the COVID-19 pandemic are at increased risk of contracting the disease. Otolaryngologists are amongst the high-risk practitioners, as they are in close proximity to patient's upper airway, which may induce their psychological stress.

**Methods:** A cross-sectional survey study, conducted among otolaryngologists in Saudi Arabia from June 11 to June 22. Survey consisted of sociodemographic questionnaire, Coronavirus Anxiety Scale (CAS), Obsession with COVID-19 Scale (OCS) and Patient Health Questionnaire-9 (PHQ-9). Available otolaryngologists with no history of mental health problems were included. A *P*-value lower than .05 was considered statistically significant.

**Results:** The study included 129 participants; 63.6% men and 36.4% women. 81.4% of participants were living with family, 57.4% living with either a child or an elderly family member. Nearly 7.8% of participants had dysfunctional COVID-19 anxiety and 75.2% had COVID-19-related depression symptoms ranging from minimal to severe. Obsession and dysfunctional thinking regarding COVID-19 were found in 26.4% of participants. Single otolaryngologists had significantly higher CAS ($P = .025$), OCS ($P = .048$), and PHQ-9 ($P < .001$) scores. Participants who lived with children or elderly individuals had significantly higher OCS scores ($P = .005$). When comparing job ranks, residents had significantly higher scores for the CAS ($P = .016$) and PHQ-9 ($P < .001$).

**Conclusion:** COVID-19 has a considerable psychological impact on otolaryngologists. Specifically, the young and single who have less social support. This indicates the importance of psychological support to this group. Additional thorough studies should explore the psychological impact of COVID-19 in this field as it may carry devastating long-term consequences if left unattended.

**Level of evidence:** Level 4.

This is an open access article under the terms of the Creative Commons Attribution-NonCommercial-NoDerivs License, which permits use and distribution in any medium, provided the original work is properly cited, the use is non-commercial and no modifications or adaptations are made.
© 2021 The Authors. *Laryngoscope Investigative Otolaryngology* published by Wiley Periodicals LLC on behalf of The Triological Society.

KEYWORDS
COVID-19, mental wellness, otolaryngologist, pandemic, psychiatric distress, SARS-CoV-2

# 1 | INTRODUCTION

In December 2019, the novel coronavirus disease, COVID-19, originated in Wuhan, China.[1] On March 2nd, 2020, the Ministry of Health of Saudi Arabia reported the first case in the Kingdom.[2] On March 11, 2020, with more than 118 000 cases in 114 countries, 4291 deaths, and thousands of cases in hospitals, the World Health Organization (WHO) declared a pandemic.[3]

Health care workers (HCWs) are on the frontline, managing patients. Therefore, HCWs are more at risk of contracting the disease than the general population. Many physicians and HCWs have been infected while caring for COVID-19 patients. In some countries, HCWs accounted for 29% of all infected patients and 12.3% of hospitalized COVID-19 patients.[4,5] Additionally, the infection rate among HCWs is as high as 10%.[6] This high infection rate can lead to psychological strain, which affects the cognitive functioning, and clinical decision making of HCWs.[7,8]

During the MERS-CoV outbreak in Saudi Arabia, 40% of cases were HCWs.[9] Consequently, medical institutions initiated interventions to foster proper prevention and control measures to provide a safe environment.

Due to high viral load in nasal and oropharyngeal mucosa, potential aerosol-generating procedures (eg, endoscopy, tracheostomy, and upper airway surgery) place otolaryngologists at higher risk of contracting the disease, particularly in procedures performed without proper personal protective equipment (PPE).[10] Consequently, otolaryngologists may experience significant anxiety, which may affect their ability to care for patients. Similar adverse effects were reported among HCWs exposed to the severe acute respiratory syndrome (SARS) during the outbreak of 2002-2003; they experienced significant psychological stress due to quarantine, and refused to provide medical care to their patients.[11] Our hypothesis is that COVID-19 pandemic has a psychological impact on Otolaryngologists in Saudi Arabia.

Therefore, the aim of our study is to assess the psychological impact of COVID-19 on otolaryngology practitioners in Saudi Arabia; this may help establish a support system for the affected physicians.

# 2 | METHODOLOGY

## 2.1 | Study setting

From June 11 to June 22, 2020, a cross-sectional study was conducted using an online 33-item survey, via Google Drive. The survey was distributed to otolaryngologists who are registered in Saudi commission of health specialties and are currently practicing otorhinolaryngology (ORL) in Saudi Arabia. Participants included trainee residents, specialists/fellows, and consultants practicing ORL during the pandemic. Other specialties were excluded, as well as individuals diagnosed with psychiatric disorders in the past and those who were taking psychiatric medications. Those who had incomplete survey were also excluded. Participation was voluntary and participants were allowed to terminate the survey at any time. Informed consent was obtained from all participants. The study was approved by the Institutional Review Board (IRB) of Imam Abdulrahman Bin Faisal University (IRB-2020-01-183).

## 2.2 | Study demographics

Fourteen baseline demographic characteristics were included in the study. Age, gender, and marital status were recorded. Additionally, we obtained the following data: living conditions (living alone or with family); having children or caring for the elderly; job title and type of hospital where they worked; change in workload during the pandemic; personal and family history of COVID-19 diagnosis; whether they were caring for suspected or confirmed COVID-19 cases at home; and quarantine history.

## 2.3 | Participants descriptions

1. ORL residents: Medical school graduates who are currently under training in ORL program. The residents are usually the first to encounter patients in the emergency department. Therefore, being the highest risk of COVID-19 exposure.
2. ORL specialists/fellow: physicians whom completed the ORL residency program and currently practicing ORL specialty, or being trained for sub-specialty in ORL. The specialists/fellows are usually the second inline to encounter the patients in the emergency department after or sometimes with the residents.
3. ORL consultants: Physicians who have been practicing the ORL specialty for a minimum of 3 years, or completed their fellowship. Consultants usually are the last who encounter the patients if their intervention is needed.

## 2.4 | Screening questionnaire

Three validated English questionnaires were used in this study: the Coronavirus Anxiety Scale (CAS),[12] the Obsession with COVID-19 Scale (OCS),[13] and the Patient Health Questionnaire-9 (PHQ-9).[14]

1. The *Coronavirus Anxiety Scale (CAS)* is a 5-item self-report mental health scale measuring dysfunctional anxiety associated with the

**TABLE 1** Descriptive statistics of the total score related to CAS, OCS and PHQ-9 questionnaires$^{(n = 129)}$

| Total score | N (%) | Mean ± SD | Mean (min-max) | 95% CI |
|---|---|---|---|---|
| CAS[a] | — | 1.58 ± 2.98 | 0.00 (0-14) | 1.116-2.124 |
| Level of CAS | | | | |
| • Dysfunctional COVID anxiety | 10 (07.8%) | — | — | — |
| • No COVID anxiety | 119 (92.2%) | — | — | — |
| OCS[b] | — | 4.28 ± 3.87 | 3.00 (1-15) | 3.636-4.992 |
| Level of OCS | | | | |
| • Dysfunctional thinking about COVID | 34 (26.4%) | — | — | — |
| • No dysfunctional thinking about COVID | 95 (73.6%) | — | — | — |
| PHQ-9[c] | — | 5.71 ± 6.19 | 4.00 (0-22) | 4.675-6.822 |
| Level of depression | | | | |
| • None | 22 (24.8%) | — | — | — |
| • Minimal | 43 (33.3%) | — | — | — |
| • Mild | 26 (20.2%) | — | — | — |
| • Moderate | 11 (08.5%) | — | — | — |
| • Moderately severe | 13 (10.1%) | — | — | — |
| • Severe | 04 (03.1%) | — | — | — |

[a]CAS—Coronavirus Anxiety Scale (total score: 14).
[b]OCS—Obsession with Coronavirus scale (total score: 15).
[c]PHQ-9—Patient Health Questionnaire (total score: 27).

coronavirus crisis. Each item of the CAS is rated on a 5-point scale. A total score ≥ 9 indicates probable dysfunctional coronavirus-related anxiety. Elevated scores on a particular item or a high total scale score (≥9) may indicate problematic symptoms for the individual that might warrant further assessment and/or treatment.

2. The *Obsession with COVID-19 Scale (OCS)* is a 4-item self-report mental health screening of persistent and disturbed thinking about COVID-19. Each item of the OCS is based on experiences over the past 2 weeks and is rated on a 5-point scale. This format is consistent with the DSM-5's crosscutting symptom measure. An OCS total score ≥ 7 indicates probable dysfunctional thinking about COVID-19. Elevated scores on a particular item or a high total scale score (≥7) may indicate problematic symptoms that might warrant further assessment and/or treatment.
3. The *Patient Health Questionnaire-9 (PHQ-9)* is a 9-item self-administered version of the PRIME-MD diagnostic instrument for common mental disorders. The PHQ-9 is the depression module, which is a valid tool for measuring the severity of depression. Each of the 9 DSM-IV (Diagnostic and Statistical Manual of Mental Disorders) items is rated in a 4-point scale. A PHQ-9 score ≥ 10 has a sensitivity of 88% and a specificity of 88% for major depression. PHQ-9 scores of 5, 10, 15, and 20 represent mild, moderate, moderately severe, and severe depression, respectively.

### 2.5 | Statistical analysis

Data were captured using numbers for all qualitative variables while the mean, SD, and median (min-max) were used to summarize all quantitative variables. Comparisons between CAS, OCS, and PHQ-9 scores and socio-demographic characteristics were calculated using the Mann-Whitney *U* test or the Kruskal-Wallis test. Normality, statistical interactions, and collinearity (ie, the variance inflation factor) were assessed using the Kolmogorov-Smirnov and Shapiro-Wilk tests. A *P*-value <.05 was considered statistically significant. Correlation procedures determined the linear agreement of the CAS, OCS, and PHQ-9. All data analyses were carried out using Statistical Packages for Software Sciences (SPSS) version 21 Armonk, New York, IBM Corporation.

## 3 | RESULTS

The study was distributed among 262 participants. Around 132 participants fully answered the questionnaire, with response rate of 50.3%. We excluded three candidates who had been diagnosed with depression in the past. We included 129 otolaryngologists in the study. The majority of participants were 25-35 years old (n = 66, 51.2%), followed by those 36-45 years old (n = 26, 20.2%), those 46-55 years old (n = 19, 14.7%), and those >55 years old (n = 18, 14%). Most participants were men (n = 82, 63.6%). Additionally, 65.1% (n = 84) were married. Furthermore, most participants lived with their families (n = 105, 81.4%) while the rest lived alone (n = 24, 18.6%). Of those who lived with their family, more than half (n = 74, 57.4%) had children younger than 12 years or elderly family members living with them. Regarding job rank, most participants were consultants (n = 50, 38.8%) followed by residents (n = 45, 34.9%), and specialists/fellows (n = 34, 26.4%). Among residents, over a fourth were in their third

**TABLE 2** Statistical association between CAS, OCS and PHQ-9 in relation to sociodemographic characteristics of otolaryngologists ($n = 129$)

| Factor | CAS Total score (14) Mean ± SD | OCS Total score (15) Mean ± SD | PHQ-9 Total score (27) Mean ± SD |
|---|---|---|---|
| Age group[a] | | | |
| • 25-35 | 1.98 ± 3.44 | 4.79 ± 4.12 | 7.53 ± 6.33 |
| • 36-45 | 1.81 ± 2.80 | 5.38 ± 4.13 | 6.58 ± 6.95 |
| • >45 | 0.70 ± 1.91 | 2.59 ± 2.53 | 1.84 ± 2.85 |
| *F*-test | 2.330 | 5.507 | 12.168 |
| *P*-value | 0.015* | 0.012* | <0.001* |
| Gender[b] | | | |
| • Men | 1.59 ± 2.80 | 3.87 ± 3.64 | 5.17 ± 6.14 |
| • Women | 1.57 ± 3.31 | 5.00 ± 4.18 | 6.64 ± 6.21 |
| *t*-test | 0.020 | −1.613; | −1.30 |
| *P*-value | 0.895 | 0.129 | 0.063 |
| Marital status[b] | | | |
| • Single | 1.84 ± 2.88 | 5.11 ± 3.95 | 8.22 ± 6.72 |
| • Married | 1.44 ± 3.04 | 3.83 ± 3.77 | 4.36 ± 5.46 |
| *t*-test | 0.732 | 1.804 | 3.530 |
| *P*-value | 0.025* | 0.048* | <0.001* |
| Living condition[b] | | | |
| • Living alone | 2.67 ± 3.94 | 4.33 ± 3.85 | 7.75 ± 7.20 |
| • Living with family | 1.33 ± 2.68 | 4.27 ± 3.89 | 5.24 ± 5.87 |
| *t*-test | 1.998 | 0.076 | 1.811 |
| *P*-value | 0.467 | 0.942 | 0.239 |
| Living with children <12 years or elderly at home | | | |
| • Yes | 1.59 ± 3.01 | 4.95 ± 4.16 | 5.57 ± 6.26 |
| • No | 0.71 ± 1.53 | 2.65 ± 2.54 | 4.45 ± 4.80 |
| *t*-test | 1.553 | 2.859 | 0.888 |
| *P*-value | 0.123 | 0.005* | 0.377 |
| Job title[a] | | | |
| • Resident | 2.38 ± 3.53 | 5.22 ± 4.39 | 8.76 ± 6.51 |
| • Specialist/Fellow | 1.24 ± 3.01 | 4.15 ± 3.26 | 4.65 ± 5.03 |
| • Consultant | 1.10 ± 2.25 | 3.52 ± 3.63 | 3.68 ± 5.59 |
| *F*-test | 2.543 | 2.371 | 9.841 |
| *P*-value | 0.016* | 0.132 | <0.001* |
| Resident level | | | |
| • PGY-1 | 1.20 ± 1.39 | 4.50 ± 4.55 | 7.40 ± 5.99 |
| • PGY-2 | 3.00 ± 3.61 | 5.33 ± 6.11 | 9.00 ± 9.00 |
| • PGY-3 | 3.42 ± 4.17 | 5.00 ± 3.38 | 10.3 ± 6.92 |
| • PGY-4 | 2.10 ± 3.69 | 3.30 ± 2.95 | 5.40 ± 5.04 |
| • PGY-5 | 2.40 ± 4.25 | 8.10 ± 5.36 | 11.5 ± 6.59 |
| *F*-test | 0.553 | 1.735 | 1.450 |
| *P*-value | 0.810 | 0.161 | 0.205 |
| Hospital level[b] | | | |
| • Primary/Secondary health center | 1.37 ± 3.03 | 4.41 ± 4.39 | 5.27 ± 6.15 |
| • Tertiary health center | 1.71 ± 2.97 | 4.20 ± 3.54 | 5.97 ± 6.23 |

(Continues)

**TABLE 2** (Continued)

| Factor | CAS Total score (14) Mean ± SD | OCS Total score (15) Mean ± SD | PHQ-9 Total score (27) Mean ± SD |
|---|---|---|---|
| *t*-test | −0.636 | 0.296 | −0.631 |
| *P*-value | 0.348 | 0.832 | 0.276 |
| Workload during COVID-19 pandemic[a] | | | |
| • Workload increased | 1.18 ± 2.32 | 4.47 ± 3.97 | 4.00 ± 4.32 |
| • Workload decreased | 1.77 ± 3.23 | 4.20 ± 3.77 | 6.11 ± 6.36 |
| • Workload remained the same | 0.69 ± 1.18 | 4.62 ± 4.72 | 4.85 ± 6.80 |
| *F*-test | 0.926 | 0.088 | 0.984 |
| *P*-value | 0.652 | 0.925 | 0.302 |
| Provided care to COVID-19 patients[b] | | | |
| • Yes | 2.30 ± 3.53 | 4.63 ± 4.09 | 6.56 ± 6.99 |
| • No | 0.71 ± 1.82 | 3.84 ± 3.55 | 4.66 ± 4.89 |
| *t*-test | 3.109 | 1.154 | 1.757 |
| *P*-value | 0.002 * | 0.470 | 0.326 |
| Have you been quarantined?[b] | | | |
| • Yes | 2.90 ± 4.17 | 5.13 ± 4.57 | 8.90 ± 6.67 |
| • No | 1.16 ± 2.37 | 4.01 ± 3.59 | 4.69 ± 5.69 |
| *t*-test | 2.912 | 1.410 | 3.439 |
| *P*-value | 0.022* | 0.340 | 0.001* |

*Significant at $P < .05$ level. [a]*P* value was calculated using the Kruskal Wallis test.
[b]*P*-value was calculated using the Mann Whitney *U* test.

post-graduate year (PGY-3) (n = 12, 26.7%), followed by PGY-1, PGY-4, and PGY-5 (each were n = 10, 22.2%). Most respondents worked at tertiary health centers (n = 80, 62%), followed by secondary health centers (n = 47, 36.4%) and primary health centers (n = 2, 1.6%). Regarding changes in workload during the COVID-19 pandemic, most participants (n = 99, 76.7%) stated that it decreased, while only 13.2% (n = 17) stated their workload increased. Around 55% of participants (n = 71) provided care to suspected or confirmed COVID-19 patients. Regarding positive testing for COVID-19, only 4.7% (n = 6) of participants had tested positive and 5.4% (n = 7) had family/household members who had tested positive, while 24% (n = 31) of participants had been quarantined. Descriptive statistics of total score related of CAS, OCS and PHQ-9 scales are presented in Table 1.

When measuring the association between the CAS, OCS, and PHQ-9 scores in relation to participants' sociodemographic characteristics, the higher age group (>45 years) had significantly lower CAS ($F = 2.330$; $P = .015$), OCS ($F = 5.507$; $P = .012$), and PHQ-9 scores ($F = 12.168$; $P < .001$). Furthermore, those who were single had significantly higher CAS ($t = 0.732$; $P = .025$), OCS ($t = 1.804$; $P = .048$), and PHQ-9 scores ($t = 3.531$; $P < .001$). Moreover, participants who lived with children under 12 years old or with elderly individuals had significantly higher OCS scores ($t = 2.859$; $P = .005$). When comparing between consultants, residents, and specialists/fellows, we found that residents had significantly higher CAS ($F = 2.543$; $P = .016$) and PHQ-9 scores ($F = 9.841$; $P < .001$). Additionally, participants who had been quarantined had significantly higher CAS ($t = 2.912$; $P = .022$) and PHQ-9 scores ($t = 3.439$; $P = .001$) (see Table 2).

The correlation between the CAS and OCS score was positively and highly statistically significant ($r = .538$; $P < .001$), suggesting that when the CAS increases the OCS will also likely increase (see Figure 1A).

The Correlation between the CAS and PHQ-9 was positively and highly statistically significant ($r = .648$; $P < .001$), indicating that as the CAS increases the PHQ-9 will also increase (see Figure 1B).

There was a positive and highly statistically significant correlation between OCS and PHQ-9 scores ($r = .568$; $P < .001$), which suggests that when the OCS score increases the PHQ-9 score will also likely increase (see Figure 1C).

While calculating post hoc analysis using Bonferroni test of CAS score, we found no statistical significant difference when comparing age group in years ($P > .05$) and similar findings were observed when calculating post hoc analysis of CAS score in job title ($P > .05$) (see Table 3).

However, while calculating post hoc analysis using Bonferroni test of OCS score, we found significant difference between age group 25-35 years and age group >45 years and vice versa ($P = .015$) and similar findings were observed between age group 36-45 years and age group >45 years and vice versa ($P = .013$) (see Table 4).

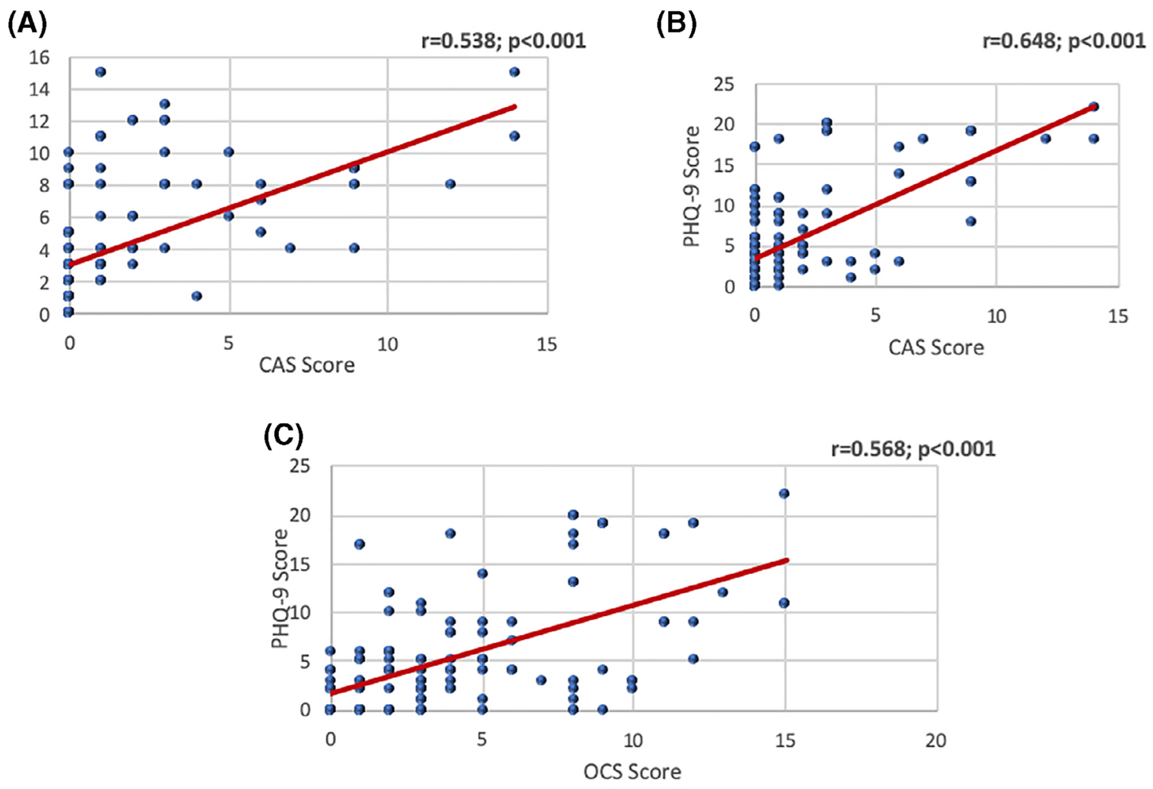


**FIGURE 1** A, Correlation between CAS and OCS scores. The correlation between the CAS and OCS score was positively and highly statistically significant ($r$ = .538; $P$ < .001) suggesting that when the CAS increases the OCS will also likely increase. B, Correlation between the CAS and PHQ-9, which was positively and highly statistically significant ($r$ = .648; $P$ < .001), indicating that as the CAS increases the PHQ-9 will also increase. C, There was a positive and highly statistically significant correlation between OCS and PHQ-9 scores ($r$ = .568; $P$ < .001), which suggests that when the OCS score increases the PHQ-9 score will also likely increase

**TABLE 3** Post hoc analysis for the CAS score

| | | Mean Diff. (I-J) | SE | *P*-value[a] |
|---|---|---|---|---|
| *Age group (I)* | *Age group (J)* | | | |
| Age 25-35 years | Age 36-45 years | −0.177 | 0.684 | 1.000 |
| | Age > 45 years | 1.282 | 0.606 | .109 |
| Age 36-45 years | Age 25-35 years | −0.177 | 0.684 | 1.000 |
| | Age > 45 years | 1.104 | 0.756 | .439 |
| Age > 45 years | Age 25-35 years | −1.282 | 0.606 | .109 |
| | Age 36-45 years | −1.105 | 0.756 | .439 |
| *Job Title (I)* | *Job Title (J)* | | | |
| Resident | Specialist/Fellow | 1.142 | 0.669 | .272 |
| | Consultant | 1.278 | 0.606 | .111 |
| Specialist/Fellow | Resident | −1.142 | 0.669 | .272 |
| | Consultant | 0.135 | 0.655 | 1.000 |
| Consultant | Resident | −1.278 | 0.606 | .111 |
| | Specialist/Fellow | −0.135 | 0.655 | 1.000 |

[a]*P*-value has been calculated using Bonferroni test.

Finally, while calculating post hoc analysis of PHQ-9, using Bonferroni test, there was a significant difference between age group 25-35 years and age group >45 years and vice versa ($P$ < .001). We also detected a significant difference between age group 36-45 years and age group >45 years and vice versa ($P$ = .005). Furthermore, there was a significant difference found between resident and specialist/

**TABLE 4** Post hoc analysis for the OCS score

| Age group (I) | Age group (J) | Mean Diff. (I-J) | SE | *P*-value[a] |
|---|---|---|---|---|
| Age 25-35 years | Age 36–45 years | −0.597 | 0.865 | 1.000 |
| | Age > 45 years | 2.193 | 0.768 | **.015*** |
| Age 36-45 years | Age 25-35 years | 0.597 | 0.865 | 1.000 |
| | Age > 45 years | 2.790 | 0.956 | **.013*** |
| Age > 45 years | Age 25-35 years | −2.193 | 0.768 | **.015*** |
| | Age 36-45 years | −2.790 | 0.956 | **.013*** |

*The mean difference is significant at the .05 level.
[a]*P*-value has been calculated using Bonferroni test.

**TABLE 5** Post hoc analysis for the PHQ-9 score

| | | Mean Diff. (I-J) | SE | *P*-value[a] |
|---|---|---|---|---|
| *Age group (I)* | *Age group (J)* | | | |
| Age 25-35 years | Age 36-45 years | 0.953 | 1.322 | 1.000 |
| | Age > 45 years | 5.692 | 1.172 | **<.001** * |
| Age 36-45 years | Age 25-35 years | −0.953 | 1.322 | 1.000 |
| | Age > 45 years | 5.692 | 1.172 | **.005** * |
| Age > 45 years | Age 25-35 years | −5.692 | 1.172 | **<.001** * |
| | Age 36-45 years | −4.739 | 1.461 | **.005** * |
| *Job Title (I)* | *Job Title (J)* | | | |
| Resident | Specialist/Fellow | 4.108 | 1.318 | **.007** * |
| | Consultant | 5.076 | 1.192 | **<.001** * |
| Specialist/Fellow | Resident | −4.108 | 1.318 | **.007** * |
| | Consultant | 0.967 | 1.289 | 1.000 |
| Consultant | Resident | −5.076 | 1.192 | **<.001** * |
| | Specialist/Fellow | −0.967 | 1.289 | 1.000 |

[a]*P*-value has been calculated using Bonferroni test.
*The mean difference is significant at the .05 level.

fellow and vice versa ($P$ = .007) while the comparison between resident and consultant and vice versa were also statistically significant ($P$ < .001) (see Table 5).

## 4 | DISCUSSION

Previous studies have documented the psychological impact of previous pandemic outbreaks on frontline HCWs.[15-17] Acute stress reactions have been reported among HCWs during the SARS and MERS-COV outbreaks.[16,18]

In China, COVID-19 had a high transmission rate amongst HCWs, as around 3000 reportedly contracted the infection and 22 of them died.[19] Hence, HCWs, particularly those in close proximity to patients' upper airway, are at a higher risk of contracting the virus, due to the high viral shedding from the nasal and oropharyngeal cavity tract.[20] For otolaryngologists, this adds to their psychological distress. Additionally, several procedures performed by otolaryngologists are aerosol-generating procedures (eg, tracheostomy, upper airway endoscopy, and endoscopic sinus surgery) which will add to their psychological stress during the epidemic.

Studies on the psychological impact of COVID-19 on HCWs' mental health confirmed they had significant anxiety and psychological stress.[1] However, when considering that the pandemic is ongoing, the psychological impact still needs further research, as it is expected to evolve over time.

In a systematic meta-analysis of 13 cross-sectional studies involving 33 062 HCWs, many reported significant anxiety and depression (23.2% and 22.8% respectively) during the COVID-19 pandemic.[21]

COVID-19 has severely affected the well-being of HCWs. In Hong Kong, medical and nursing staff were found vulnerable to burnout, anxiety, and mental exhaustion.[22] In Germany, doctors reported high levels of anxiety and depressive symptoms.[23]

Most studies on the psychological impact of COVID-19 on HCWs have addressed high-risk and low-risk areas of health care. Thus, it is difficult to standardize these results for all HCWs. As the present study focused on one specialty, its results are expected to have greater accuracy on the concerned group.

Our study was conducted from 11 to 22 June 2020, during the peak of COVID-19 pandemic in Saudi Arabia, with cases reaching up to 4700 per day and death cases during the same period reaching above 400. During this time, lockdown was still ongoing as well as strict precautions being taken.[24]

In our study, the psychological impact of the COVID-19 pandemic on otolaryngologists in Saudi Arabia included dysfunctional anxiety (7.8%) and moderate to severe depression (10.1%), which is comparable to a study conducted on Otolaryngologists in the United States where (7.4%) of participants displayed symptoms of anxiety in the sever range and (10.6%) where positive for depression.[25] Its worth mentioning that in this study they used the PHQ-2 depression scale while our study used a more accurate depression scale which is the PHQ_9. Similarly, another study reported that surgeons had significantly higher anxiety and depression levels during the pandemic in comparison the pre-pandemic period ($P < .001$).[26] In studies on HCWs of other specialties, anxiety and depression scores were lower than those of surgeons and otolaryngologists. In another Saudi study, high anxiety scores in response to COVID-19 were reported in 2% of HCWs.[5] Comparable findings were reported in HCWs in Singapore and India, where the prevalence of moderate to extremely-severe anxiety was 8.7% and moderate to very-severe depression was 5.3%.[27] Similarly, HCWs in China experienced mild psychological impact during the COVID-19 pandemic.[1] Otolaryngologists and surgeons are at higher risk of contracting the virus; hence, they are expected to have higher anxiety and depression than the entire population of HCWs.

Although anxiety and depression are relatively common among otolaryngologists and surgeons, they are less common than in the general population.[28,29] This can be explained by the fact that HCWs are accustomed to stressful environments such as public health emergencies. Similarly, the psychological impact of COVID-19 is higher in non-medically trained health care workers than in medically trained personnel.[18] Nevertheless, mild symptoms of depression and anxiety might be a sign that early intervention is needed before persisting psychological complications arise.[21]

In our study, there was no statistical gender difference among otolaryngologists regarding anxiety, depression, and obsession. In contrast, a meta-analysis of 13 cross-sectional studies demonstrated that the prevalence rate of anxiety and depression was higher in female HCWs. This may be because nurses (who are mostly women) were included in the population of HCWs during the meta-analysis, which might have biased the results. Nurses are at greater risk to contract COVID-19 because they spend more time on wards, provide direct care to patients, and are responsible for the collection of sputum for virus detection.[30] Moreover, they spend more time with infected patients and witness their suffering and passing.[21]

Our study showed that older otolaryngologists had significantly lower CAS, OCS, and PHQ scores (*P*-values of .015, .012, <.001, respectively). This may be because senior otolaryngologists are more experienced in dealing with stressful situations or because younger otolaryngologists are on the front line and have a higher chance of contracting the virus. The disparity between young and old otolaryngologists was further confirmed when assessing residents, who had significantly higher anxiety and depression scores (*P*-values of .016, <.001, respectively) than consultants, this was also documented in a study conducted on Otolaryngologists in the US, where burnout was experienced more in the residents in comparison to the attending physicians (14.7% vs 14.7%, *P*-value of .001).[25]

Contrary to what we expected, single otolaryngologists had significantly higher anxiety, obsession, and depression (*P*-values of .025, .048, <.001, respectively). This may be because of the comparatively higher social support that married otolaryngologists receive at home. A similar finding was reported by a study conducted on HCWs in China, in which social support reduced anxiety and stress and improved self-efficacy.[31] Social support can help medical staff reduce their anxiety, as friends and family provide social and emotional support.[32] Additionally, social interactions reduce negative emotions and can improve mood.[33] Thus, it is important to provide psychological support to HCWs during health crises. Our study suggests that health institutions must focus on having a good psychological support and intervention system for their single staff who have less social support at home.

Moreover, OCS scores of otolaryngologists living with young children or elderly family members were significantly higher than scores of those living alone (*P*-value .005). Although their anxiety and depression level may be lower due to the social support they receive at home, they are nevertheless concerned for their vulnerable family members and may fear infecting them more than infecting themselves.[5]

Furthermore, the statistical significance of the linear correlation between the three scoring systems applied in this study was high (CAS, OCS, and PHQ-9); the correlation demonstrates having a positive scale might predict involvement of other psychological aspects. Therefore, the otolaryngologist might need to be screened for other psychological disorders as well if one of these scales are positive.

Our study highlights the importance of providing otolaryngologists with psychological and emotional support and interventions by specialized personnel during health crises. Specifically, the most vulnerable otolaryngologists which include the single or unmarried, young, and those living with vulnerable family members at home. It has been found that evidence-based education and training of HCWs on readiness for a pandemic improves the experience, skills, and mental well-being of HCWs during the pandemic.[34]

This study has a few limitations. First, we used a cross-sectional electronic-based questionnaire; it would have been preferable to conduct face-to-face interviews rather than a self-reporting survey, but due to the health crisis, face-to-face interviews were not possible. Second, the self-reported levels of psychological impact in the form of anxiety, depression, and obsession may not be as accurate as those derived from an assessment performed by a mental health professional. Third, due to the ongoing changes in post-traumatic mental health, continuous long-term mental state follow-ups would be preferable. Forth, a larger sample size is needed to confirm our conclusions. Finally, our data is collected from a single country, therefore an international multicenter study would be suggested in the future to compare between otolaryngologists in different countries.

## 5 | CONCLUSIONS

COVID-19 has had a significant psychological impact on otolaryngologists. Particularly the single and younger group. Additional, thorough

mental health studies should be done in the field of otolaryngology, as the effects of the pandemic may carry devastating long-term consequences if left unattended.

## CONFLICT OF INTEREST

The authors have no financial conflicts of interest to disclose.

## ORCID

*Mona Mohammedsaleh Ashoor* https://orcid.org/0000-0003-1386-971X

## BIBLIOGRAPHY


1. Sun D, Yang D, Li Y, et al. Psychological impact of 2019 novel coronavirus (2019-nCoV) outbreak in health workers in China. *Epidemiol Infect*. 2020;148:1-6. https://doi.org/10.1017/S0950268820001090.
2. MOH News – MOH Reports First Case of Coronavirus Infection. https://www.moh.gov.sa/en/Ministry/MediaCenter/News/Pages/News-2020-03-02-002.aspx. Accessed June 26, 2020.
3. WHO Director-General's opening remarks at the media briefing on COVID-19 – 11 March 2020. https://www.who.int/dg/speeches/detail/who-director-general-s-opening-remarks-at-the-media-briefing-on-covid-19—11-march-2020. Accessed June 26, 2020.
4. Wang D, Hu B, Hu C, et al. Clinical characteristics of 138 hospitalized patients with 2019 novel coronavirus-infected pneumonia in Wuhan, China. *JAMA – J Am Med Assoc*. 2020;323(11):1061-1069. https://doi.org/10.1001/jama.2020.1585.
5. Temsah M, Al-sohime F, Alamro N, et al. Journal of infection and public health the psychological impact of COVID-19 pandemic on health care workers in a MERS-CoV endemic country. *J Infect Public Health*. 2020;13(6):877-882. https://doi.org/10.1016/j.jiph.2020.05.021.
6. Felice C, Di Tanna GL, Zanus G, Grossi U. Impact of COVID-19 outbreak on healthcare workers in Italy: results from a national E-survey. *J Community Health*. 2020;0123456789:675-683. https://doi.org/10.1007/s10900-020-00845-5.
7. Panagioti M, Geraghty K, Johnson J, et al. Association between physician burnout and patient safety, professionalism, and patient satisfaction: a systematic review and meta-analysis. *JAMA Intern Med*. 2018;178(10):1317-1330. https://doi.org/10.1001/jamainternmed.2018.3713.
8. Leblanc VR. The effects of acute stress on performance: implications for health professions education. *Acad Med*. 2009;84(10):25-33.
9. WHO | Middle East respiratory syndrome coronavirus (MERS-CoV) – The Kingdom of Saudi Arabia. *WHO*. Published online 2020. http://www.who.int/csr/don/24-february-2020-mers-saudi-arabia/en/. Accessed July 4, 2020.
10. American Academy of Otolaryngology–Head and Neck Surgery (AAO-HNS) (2020), Otolaryngologists and the COVID-19 Pandemic.
11. Can W, Learn W, Koh D, Lim MK, Chia SE, Ko SM. Risk perception and impact of severe acute respiratory syndrome (SARS ) on work and personal lives of. *Med Care*. 2005;43(7):676-682.
12. Lee SA. Coronavirus anxiety scale: a brief mental health screener for COVID-19 related anxiety. *Death Stud*. 2020;44(7):393-401. https://doi.org/10.1080/07481187.2020.1748481.
13. Lee SA. How much "thinking" about COVID-19 is clinically dysfunctional? *Brain Behav Immun*. 2020;87:97-98. https://doi.org/10.1016/j.bbi.2020.04.067.
14. Kroenke K, Spitzer RL, Williams JBW. The PHQ-9: validity of a brief depression severity measure. *J Gen Intern Med*. 2001;16(9):606-613. https://doi.org/10.1046/j.1525-1497.2001.016009606.x.
15. Wu P, Fang Y, Guan Z, et al. The psychological impact of the SARS epidemic on hospital employees in China: exposure, risk perception, and altruistic acceptance of risk. *Can J Psychiatry*. 2009;54(5):302-311. https://doi.org/10.1177/070674370905400504.
16. McAlonan GM, Lee AM, Cheung V, et al. Immediate and sustained psychological impact of an emerging infectious disease outbreak on health care workers. *Can J Psychiatry*. 2007;52(4):241-247. https://doi.org/10.1177/070674370705200406.
17. Li L, Wan C, Ding R, et al. Mental distress among Liberian medical staff working at the China Ebola Treatment Unit: a cross sectional study. *Heal Qual Life Outcomes*. 2015;13:1-7. https://doi.org/10.1186/s12955-015-0341-2.
18. Tan BYQ, Chew NWS, Lee GKH, et al. Psychological impact of the COVID-19 pandemic on Health Care Workers in Singapore. *Ann Intern Med*. 2020;16:5-7. https://doi.org/10.7326/m20-1083.
19. Adams JG, Walls RM. Supporting the health care workforce during the COVID-19 global epidemic. *JAMA – J Am Med Assoc*. 2020;323:1439-1440. https://doi.org/10.1001/jama.2020.3972.
20. Van Doremalen N, Bushmaker T, Morris DH, et al. Aerosol and surface stability of SARS-CoV-2 as compared with SARS-CoV-1. *N Engl J Med*. 2020;382:1564-1567. https://doi.org/10.1056/NEJMc2004973.
21. Pappa S, Ntella V, Giannakas T, Giannakoulis VG, Papoutsi E, Katsaounou P. Prevalence of depression, anxiety, and insomnia among healthcare workers during the COVID-19 pandemic: a systematic review and meta-analysis. *Brain Behav Immun*. 2020;88:901-907. https://doi.org/10.1016/j.bbi.2020.05.026.
22. Cheung T, Fong TKH, Bressington D. COVID-19 under the SARS cloud: mental health nursing during the pandemic in Hong Kong. *J Psychiatr Ment Health Nurs*. 2020;28:115-117. https://doi.org/10.1111/jpm.12639.
23. Bohlken J, Schömig F, Lemke MR, Pumberger M, Riedel-Heller SG. COVID-19 pandemic: stress experience of healthcare workers: a short current review. *Psychiatr Prax*. 2020;47:190-197. https://doi.org/10.1055/a-1159-5551.
24. Saudi Arabia: WHO Coronavirus Disease (COVID-19) Dashboard | WHO Coronavirus Disease (COVID-19) Dashboard. https://covid19.who.int/region/emro/country/sa. Accessed February 15, 2021.
25. Civantos AM, Byrnes Y, Chang C, et al. Mental health among otolaryngology resident and attending physicians during the COVID-19 pandemic: National study. *Head Neck*. 2020;42:1597-1609. https://doi.org/10.1002/hed.26292.
26. Xu J, hui XQ, ming WC, Wang J. Psychological status of surgical staff during the COVID-19 outbreak. *Psychiatry Res*. 2020;288:112955. https://doi.org/10.1016/j.psychres.2020.112955.
27. Chew NWS, Lee GKH, Tan BYQ, et al. A multinational, multicentre study on the psychological outcomes and associated physical symptoms amongst healthcare workers during COVID-19 outbreak. *Brain Behav Immun*. 2020;88:559-565. https://doi.org/10.1016/j.bbi.2020.04.049.
28. Wang C, Pan R, Wan X, et al. A longitudinal study on the mental health of general population during the COVID-19 epidemic in China. *Brain Behav Immun*. 2020;87:40-48. https://doi.org/10.1016/j.bbi.2020.04.028.
29. Wang C, Pan R, Wan X, et al. Immediate psychological responses and associated factors during the initial stage of the 2019 coronavirus disease (COVID-19) epidemic among the general population in China. *Int J Environ Res Public Health*. 2020;17(5):1-25. https://doi.org/10.3390/ijerph17051729.
30. Liu Z, Han B, Jiang R, et al. Mental health status of doctors and nurses during COVID-19 epidemic in China. *SSRN Electron J*. 2020. https://doi.org/10.2139/ssrn.3551329.
31. Xiao H, Zhang Y, Kong D, Li S, Yang N. The effects of social support on sleep quality of medical staff treating patients with coronavirus disease 2019 (COVID-19) in January and February 2020 in China. *Med Sci Monit*. 2020;26:e923549. https://doi.org/10.12659/MSM.923549.
32. Yang N, Xiao H, Wang W, Li S, Yan H, Wang Y. Effects of doctors' empathy abilities on the cellular immunity of patients with advanced prostate cancer treated by orchiectomy: the mediating role of patients' stigma, self-efficacy, and anxiety. *Patient Prefer Adher*. 2018;12:1305-1314. https://doi.org/10.2147/PPA.S166460.

33. Yang N, Xiao H, Cao Y, Li S, Yan H, Wang Y. Influence of oncology nurses' empathy on lung cancer patients' cellular immunity. *Psychol Res Behav Manag*. 2018;11:279-287. https://doi.org/10.2147/PRBM.S168649.
34. Aiello A, Young-Eun Khayeri M, Raja S, et al. Resilience training for hospital workers in anticipation of an influenza pandemic. *J Contin Educ Health Prof*. 2011;31(1):15-20. https://doi.org/10.1002/chp.20096.

**How to cite this article:** Ashoor MM, Almulhem NJ, AlMubarak ZA, et al. The psychological impact of the COVID-19 pandemic on otolaryngologists: Should we be concerned? *Laryngoscope Investigative Otolaryngology*. 2021;6:576–585. https://doi.org/10.1002/lio2.556
