# Supplementary material for: Mental health status and related factors influencing healthcare workers during the COVID-19 pandemic: A systematic review and meta-analysis
Source: PLoS One. 2024 Jan 19;19(1):e0289454. doi: 10.1371/journal.pone.0289454 (PMC10798549; doi:10.1371/journal.pone.0289454)
Supplement: S1 Data — (ZIP) [file pone.0289454.s011.zip › literatures/130.pdf]

Mental Health of Guatemalan Health Care Workers During the COVID-19 Pandemic: Baseline Findings From the HEROES Cohort Study

Alejandra Paniagua-Avila, MD, MPH, Dorian E. Ramírez, MD, MSCE, Aida Barrera-Pérez, MD, MSCE, Erwin Calgua, MD, MSCE, Claudia Castro, Ana Peralta-García, MD, Franco Mascayano, MPH, Ezra Susser, MD, DrPH, Rubén Alvarado, MD, PhD, and Victor Puac-Polanco, MD, DrPH, MSCE

Objectives. To assess the baseline prevalence of mental health conditions and associated exposures in a cohort of health care workers (HCWs) in Guatemala.

Methods. We analyzed baseline information from the 2020 Web-based COVID-19 Health Care Workers Study (HEROES)–Guatemala. Outcomes included mental distress and depressive symptoms. Exposures included COVID-19 experiences, sociodemographic characteristics, and job characteristics. We used crude and adjusted Poisson regression models in our analyses.

Results. Of the 1801 HCWs who accepted to participate, 1522 (84.5%) completed the questionnaire; 1014 (66.8%) were women. Among the participants, 59.1% (95% confidence interval [CI] = 56.6, 61.5) screened positive for mental distress and 23% (95% CI = 20.9, 25.2) for moderate to severe depressive symptoms. COVID-19 experiences, sociodemographic characteristics, and job characteristics were associated with the study outcomes. Participants who were worried about COVID-19 infection were at higher risk of mental distress (relative risk [RR] = 1.47; 95% CI = 1.30, 1.66) and depressive symptoms (RR = 1.51; 95% CI = 1.17, 1.96). Similarly, the youngest participants were at elevated risk of mental distress (RR = 1.80; 95% CI = 1.24, 2.63) and depressive symptoms (OR = 4.58; 95% CI = 1.51, 13.87).

Conclusions. Mental health conditions are highly prevalent among Guatemalan HCWs. (*Am J Public Health*. 2022;112(S6):S602–S614. https://doi.org/10.2105/AJPH.2021.306648)

COVID-19 was first detected in Guatemala on March 13, 2020, 2 days after the World Health Organization declared it a pandemic.[1,2] One year after the pandemic, evidence on the extent and severity of mental health conditions among health care workers (HCWs) coming from Latin American and low-and middle-income countries is scarce and limited by issues such as low response rates, nonprobabilistic samples, selection bias, and lack of prepandemic and pandemic comparisons.[3,4]

Guatemala's health system, with among the lowest public health investments (1% of gross domestic product) and HCW densities (12.5 per 100 000 population) in the Latin American region, had limited capacity to respond to the COVID-19 pandemic.[5–7] Guatemala's category as an upper-middle-income country masks marked inequalities in income distribution and human development across the population.[8,9] Multiple surges of COVID-19 cases have overburdened HCWs, and their opportunities to seek mental health services are reduced.[10] There is only 1 report to our knowledge regarding Guatemalan HCWs' mental health, a government-led cross-sectional survey showing that 25% of HCWs screened positive for depressive symptoms during a peak in COVID-19 cases.[11]

Similar to the general population, HCWs responding to COVID-19 are exposed to multiple stressors, including lockdowns, economic instability, and uncertainty.[5] Also, HCWs experience job-specific stressors such as fear of infecting themselves or their loved ones, isolation, increased workload stress, stigma, and harassment.[12] Recent country-specific studies have shown that COVID-19 is already affecting the mental health of HCWs.[13–16] According to a review of the literature, most HCWs had reported adverse psychological experiences during previous epidemics, and a significant subset exhibited mental health sequelae after the emergency.[12] Considering all of the characteristics related to the current pandemic, including generalized lockdowns and economic effects, an understanding of the frequency and severity of mental health issues among HCWs, as well as their long-term mental health, is essential.

More research is needed to close the gap in knowledge about the mental health status of HCWs during the COVID-19 pandemic in countries with vast health inequalities (e.g., countries of the Latin American region).[17] In this cross-sectional study, we analyzed baseline findings from the COVID-19 Health Care Workers Study (HEROES)–Guatemala, part of a larger investigation assessing the mental health of HCWs during the COVID-19 pandemic in 26 countries.[18]

METHODS

We used Guatemalan baseline data collected between July and September 2020 from a multicountry prospective cohort study assessing the mental health of HCWs at baseline, 6 months, and 12 months. Participants were recruited through health care institutions and union organizations with contact information (e-mail addresses or telephone numbers) databases of affiliates or employees working in health care settings across the country. The study team contacted each entity about the study objectives, design, and procedures. After authorization had been obtained, each entity sent out online invitations to potential participants via e-mail or social media. Invitations contained information about the study objectives and informed consent along with a self-administered Web-based survey. Approximately 2 to 3 weeks after the initial invitation to participants, reminders were sent to nonresponders in an attempt to achieve a higher participation rate.

Participants

Eligible individuals included adult HCWs (aged 18 years or older) affiliated with institutions serving patients suspected of having or diagnosed with COVID-19; these individuals were contacted through entities that agreed to participate in the study. All HCWs were eligible to participate, including health care professionals, technicians, support staff, and administrative personnel. Participants did not need to be deployed as frontline COVID-19 workers to be eligible to enroll, although we targeted entities involved in the COVID-19 response. Recruitment sites included public and private health services such as clinics, health posts, health centers, and hospitals (department, national, and specialized).

Sample Size

We used a nonprobabilistic purposive sampling approach to recruit participants. We calculated our target sample size with the formula $N = Z_{\alpha}{}^{2}P(1 - P)/d$.[2] Following the study conducted by Lai et al., we computed α as 0.05, $Z\alpha$ as 1.96, and a percentage of participants (P) with mental health conditions of 35% and calculated an estimated acceptable margin of error for proportion d ± 3%.[15] Accounting for 75% follow-up, we needed a total of at least 1423 completed questionnaires. Although this study was designed to be longitudinal, we report only on the first assessment here.

Measurements

The primary exposures were experiences with COVID-19 at work and outside work. Specifically, exposures included contact with patients with COVID-19 at work (yes, no, does not know), availability of personal protective equipment (PPE) at work (sufficient, insufficient), having a severe acute respiratory syndrome coronavirus 2 (SARS-CoV-2) test result (if available; negative, positive, unknown result), concerns about contracting COVID-19 (not worried, somewhat worried, very worried), and experiencing the death of a relative from COVID-19 (yes, no).

We also explored associations of our outcomes with sociodemographic and job characteristics and previous mental health disorders. With respect to sociodemographic and job characteristics, participants provided information about their age (continuous and 5 categories), sex (male or female), education (4 categories), and number of people living at home (total number, minors, adults older than 65 years, people with disabilities). The questionnaire also asked about the participant's job sector (private or public), job location, and

occupation. Questions on previous mental health disorders focused on prior mental disorder diagnoses (yes, no, prefers not to answer) and use of psychotropic medications (yes, no, prefers not to answer).

Outcomes

We assessed mental distress via the General Health Questionnaire (GHQ-12) and severity of depressive symptoms through the Patient Health Questionnaire (PHQ-9).

Developed by Goldberg in 1972, the GHQ is a widely used screening instrument for recognizing and measuring mental distress.[19] The GHQ-12 is a short version comprising 12 items (6 phrased positively and 6 phrased negatively), each scored from 0 to 3. We used the 0 to 12 scale and the bimodal scoring method whereby "less than usual" and "no more than usual" are scored as 0 and "rather more than usual" and "much more than usual" are scored as 1.[20] Items are summed to estimate a total score between 0 and 12. To our knowledge, there have not been previous validations of the GHQ-12 cut-off points in Guatemala. We used the standard two thirds cut-off point validated in multiple Spanish-speaking countries to classify individuals as having mental distress.[20–23] Our survey's 12 items had high internal consistency, as revealed by a Cronbach α value of 0.86 (95% confidence interval [CI] = 0.85, 0.87; Appendix A, available as a supplement to the online version of this article at http://www.ajph.org).

The PHQ-9 is a 9-item self-report instrument that screens for depressive symptoms and focuses on the preceding 2 weeks.[24] Items are rated on a Likert-type scale ranging from 0 (not at all) to 3 (nearly every day). Total scores range between 0 and 27. Total scores for depression are as follows: 0 to 4, minimal or none; 5 to 9, mild; 10 to 14, moderate; 15 to 19, moderately severe; and 20 to 27, severe. To our knowledge, no study has evaluated the optimal PHQ-9 cut-off points in Guatemala. As recommended in other Latin American surveys, we used a cut-off of 10 or more to classify individuals as having depressive symptoms.[24–26] The 9 items in our survey had high internal consistency, as shown by the Cronbach α value of 0.90 (95% CI = 0.89, 0.91; Appendix B, available as a supplement to the online version of this article at http://www.ajph.org).

Statistical Analysis

We collected data on participants' sociodemographic and job characteristics, COVID-19 experiences, and previous mental disorders. We examined the distributions between people who did and did not complete the survey. Item mean values for both outcome scales were examined for the overall sample and selected group categories. We performed unadjusted bivariate Poisson regression analyses with robust error variance to estimate relative risks (RRs) between predictors, COVID-19 exposures, mental distress, and depressive symptoms. Multivariable Poisson regression analyses with robust error variance were used to examine relative risks between each predictor and study outcomes adjusted for age, sex, education, and occupation. The sample of completed surveys was used in both unadjusted and adjusted models. (For details on the item–scale analyses, see Appendixes A and B. Appendixes C and D, available as supplements to the online version of this article at http://www.ajph.org, show the prevalence and distribution of the 2 study outcomes across the different sociodemographic and predictors.)

We assessed study outcomes with different cut-off thresholds and COVID–related exposures (Appendix E, available as a supplement to the online version of this article at http://www.ajph.org). We used a threshold wherein a score of 2 or above signifies risk for mental distress. This threshold has been recommended if the goal is to screen for psychiatric disorders in primary care settings. In contrast, the more stringent threshold of 3 points or above is preferred to discriminate between mood disorders and anxiety disorders.[27,28] We used Stata version 14 to conduct our statistical analyses.[29] Statistical significance was set at $P < .05$, and all tests were 2-tailed.

RESULTS

Of the 1801 individuals who agreed to participate, 1522 (84.5%) completed the online questionnaire. Table 1 shows distributions of sociodemographic characteristics, previous mental disorders, and COVID-19 experiences among the overall sample of participants, those who completed the survey, and those who agreed to participate but did not complete the survey. With the exception of job location, no differences were found between respondents who did and did not complete the survey.

Sociodemographic and Job Characteristics

Participants in the analytical sample (n = 1522) were mostly aged 49 years or younger (n = 1273; 87.2%), female

TABLE 1— Distribution of Exposure Variables Among the Total Sample of Participants, Those Who Completed the Survey, and Those Who Did Not Complete the Survey: HEROES–Guatemala Study, 2020

Characteristic	No./Total No. (%) or Median (IQR)			χ^2; *P*
	Total Sample (n = 1801)	Complete Surveys (n = 1522)	Incomplete Surveys (n = 279)	
Age, y				3.56; .31
18–34	898/1688 (53.2)	765/1461 (52.4)	133/227 (58.6)	
35–49	574/1688 (34.0)	508/1461 (34.8)	66/227 (29.1)	
50–59	162/1688 (9.6)	142/1461 (9.7)	20/227 (8.8)	
≥60	54/1688 (3.2)	46/1461 (3.1)	8/227 (3.5)	
Sex				2.36; .12
Female	1164/1762 (66.1)	1014/1519 (66.8)	150/243 (61.7)	
Male	598/1762 (33.9)	505/1519 (33.2)	93/243 (38.3)	
Education[a]				.65
Incomplete primary	3/1758 (0.2)	3/1522 (0.2)	0/236 (0.0)	
Primary	14/1758 (0.8)	14/1522 (0.9)	0/236 (0.0)	
High school	115/1758 (6.5)	103/1522 (6.8)	12/236 (5.1)	
Technical degree	293/1758 (16.7)	256/1522 (16.8)	37/236 (15.7)	
Professional degree	790/1758 (44.9)	680/1522 (44.7)	110/236 (46.6)	
Postgraduate degree	543/1758 (30.9)	466/1522 (30.6)	77/236 (32.6)	
No. of people living at home				
Total	4 (3–5)	4 (3–5)	4 (3–5)	
Minors	2 (1–2)	2 (1–2)	2 (1–2)	
Adults > 65 y	1 (1–2)	1 (1–2)	1 (1–2)	
People with disabilities	1 (1–1)	1 (1–1)	1 (1–1)	
Health care sector				0.32; .57
Public	1266/1735 (73.0)	1114/1522 (73.2)	152/213 (71.4)	
Private	469/1735 (27.0)	408/1522 (26.8)	61/213 (28.6)	
Job location by region				34.11; < .001
Metropolitan	876/1734 (50.5)	765/1522 (50.3)	111/212 (52.4)	
North	55/1734 (3.2)	49/1522 (3.2)	6/212 (2.8)	
Northeast	140/1734 (8.1)	127/1522 (8.3)	13/212 (6.1)	
Southeast	47/1734 (2.7)	38/1522 (2.5)	9/212 (4.2)	
Central	219/1734 (12.6)	190/1522 (12.5)	29/212 (13.7)	
Southwest	334/1734 (19.3)	305/1522 (20.0)	29/212 (13.7)	
Northwest	49/1734 (2.8)	42/1522 (2.8)	7/212 (3.3)	
Petén	14/1734 (0.8)	6/1522 (0.4)	8/212 (3.8)	
Occupation				15.10; .09
Physician	647/1708 (37.9)	566/1522 (37.2)	81/186 (43.6)	
Nurse	377/1708 (22.1)	326/1522 (21.4)	51/186 (27.4)	
Psychologist	28/1708 (1.6)	25/1522 (1.6)	3/186 (1.6)	
Social worker	24/1708 (1.4)	23/1522 (1.5)	1/186 (0.5)	
Hospital technician	99/1708 (5.8)	91/1522 (6.0)	8/186 (4.3)	
Nutritionist	31/1708 (1.8)	30/1522 (2.0)	1/186 (0.5)	
Dentist	125/1708 (7.3)	110/1522 (7.2)	15/186 (8.1)	
Administration	310/1708 (18.2)	289/1522 (19.0)	21/186 (11.3)	
Hospital staff	60/1708 (3.5)	55/1522 (3.6)	5/186 (2.7)	

Continued

TABLE 1— Continued

Characteristic	No./Total No. (%) or Median (IQR)			χ^2; *P*
	Total Sample (n = 1801)	Complete Surveys (n = 1522)	Incomplete Surveys (n = 279)	
Other	7/1708 (0.4)	7/1522 (0.5)	0/186(0.0)	
Contact with COVID-19 patients				0.81; .67
Yes	1038 (62.4)	954 (62.7)	84 (59.6)	
No	287 (17.3)	259 (17.0)	28 (19.9)	
Does not know	338 (20.3)	309 (20.3)	29 (20.6)	
SARS-CoV-2 test result[a]				.3
Negative	496/674 (73.6)	464/627 (74.0)	32/47 (68.1)	
Positive	161/674 (23.9)	146/627 (23.3)	15/47 (31.9)	
Unknown	17/674 (2.5)	17/627 (2.7)	0/47 (0.0)	
Personal protective equipment				0.11; .73
Insufficient	859/1621 (53.0)	792/1498 (52.9)	56/123 (45.5)	
Sufficient	762/1621 (47.0)	706/1498 (47.1)	67/123 (54.5)	
Worried about COVID-19 infection				0.31; .58
No or not a lot	400/1485 (26.9)	369/1379 (26.8)	31/106 (29.2)	
A lot or very worried	1085/1485 (73.1)	1010/1379 (73.2)	75/106 (70.8)	
Relative deceased because of COVID-19				0.27; .6
No	457/606 (75.4)	427/568 (75.2)	30/38 (79.0)	
Yes	149/606 (24.6)	141/568 (24.8)	8/38 (21.0)	
Prior mental disorder				
No	1235/1312 (94.1)	1234/1311 (94.1)		
Yes	59/1312 (4.5)	59/1312 (4.5)		
Prefer not to answer	18/1312 (1.4)	18/1312 (1.4)		
Taking medication for mental disorders				
No	1215/1312 (92.6)	1214/1311 (92.6)		
Yes	83/1312 (6.3)	83/1311 (6.3)		
Prefer not to answer	14/1312 (1.1)	14/1311 (1.1)		

Note. HEROES = COVID-19 Health Care Workers Study; IQR = interquartile range; SARS-CoV-2 = severe acute respiratory syndrome coronavirus 2.

[a] *P* value is from the Fisher exact test.

(n = 1014; 66.8%), and highly educated (professional or postgraduate degree; n = 1146; 75.3%); most worked in public health institutions (n = 1114; 73.2%), and half worked in Guatemala City (n = 765; 50.3%). The most frequent occupations were physician (n = 566; 37.2%) and nurse (n = 326; 21.4%). Only 4.5% of participants (n = 59) reported having a diagnosis of a previous mental disorder, and 6.3% (n = 83) reported having taken psychotropic medications.

COVID-19 Experiences

Regarding COVID-19 experiences, almost two thirds of participants reported having had contact with patients diagnosed with COVID-19 in the previous week (n = 954; 62.7%), and more than half reported insufficient PPE at work (n = 792; 53%). Approximately three quarters reported being very worried about contracting COVID-19 (n = 1010, 73.2%), and around a quarter reported having experienced the death of a relative from COVID-19 (n = 141; 24.8%). Of those who reported having been tested

for SARS-CoV-2 (n = 627; 41.2%), 23.3% (n = 146) had a positive result.

Outcomes

Table 2 shows mean scores for GHQ-12 items, overall and by gender, occupation, health care sector, and whether HCWs reported having had contact with patients diagnosed with COVID-19. Positive screens for mental distress were common, with 899 (59.1%; 95% CI = 56.6, 61.5) participants scoring 3 or more on the GHQ-12. Participants' mean GHQ-12 score was 3.88 (interquartile range [IQR] = 1–6), higher than the cut-off for positive mental distress screening. The most common negative mood symptom was feeling under stress (mean = 1.75). The most common positive mood symptom was being able to enjoy day-to-day activities (mean = 1.8). Mean tests for each GHQ-12 item revealed differences within all group categories. For example, physicians reported higher distress than nurses and those employed in other occupations (e.g., administrative staff, dentists, nutritionists) on all GHQ-12 items. HCWs who reported contact with patients diagnosed with COVID-19 had higher distress scores with the exception of the usefulness, making decisions, worthlessness, and happy feelings items.

Table 3 shows means for the PHQ-9 items, also by group characteristics. Depressive symptoms were prevalent among HCWs, with 22.9% (95% CI = 20.9, 25.2) reporting moderate, moderate to severe, or severe depression. The average PHQ-9 score for the sample overall was 6.11 (IQR = 1–9). The most common depressive symptom was feeling tired or having little energy (mean = 1.17). Mean tests also revealed differences for PHQ-9 items, with the prevalence of differences being highest for occupation and contact with patients diagnosed with COVID-19.

Appendix C shows the mean values and percentages of positive mental distress (GHQ-12) and moderate to severe depressive symptoms (PHQ-9) for the total sample of participants and by each exposure. Appendix D displays the distribution of depressive symptoms by severity category for each COVID-19–related exposure. Percentages of moderate to severe depressive symptoms were higher among those who had contact with COVID-19 patients, an unknown COVID-19 test result, and insufficient PPE and those who worried about being infected with COVID-19. In contrast, percentages of moderate to severe depressive symptoms were similar among those who had and had not experienced the death of a relative from COVID-19.

Associations Between Exposures and Outcomes

Table 4 shows crude and adjusted relative risks for the associations between exposures (sociodemographic characteristics, job characteristics, and COVID-19 experiences) and mental health conditions (mental distress and depressive symptoms).

Crude relative risks for mental distress and moderate to severe depressive symptoms were higher among participants 18 to 34 years of age (vs those aged 60 years or older), those with a postgraduate degree (vs those with a high school degree), physicians (vs administrative HCWs), and those working in the public sector (vs private sector workers). After adjustment, associations of mental distress and depressive symptoms with younger age (mental distress RR = 1.80; 95% CI = 1.24, 2.63; depressive symptoms RR = 4.58; 95% CI = 1.51, 13.87), holding a postgraduate degree (mental distress RR = 1.45; 95% CI = 1.16, 1.83; depressive symptoms RR = 2.31; 95% CI = 1.31, 4.07), being a physician (depressive symptoms RR = 1.58; 95% CI = 1.16, 2.16), and being a hospital technician (mental distress RR = 1.33; 95% CI = 1.10, 1.60) were attenuated but remained significant.

Associations between mental health conditions and being 35 to 49 years old, being a hospital technician, having an unknown SARS-CoV-2 test result, having a central region job location, experiencing the death of a relative from COVID-19, and taking medication for a mental disorder moved away from the null after adjustment. No associations were found with respect to sex, private versus public health care sector, number of people living at home, job location, or mental health conditions after adjustment.

In terms of COVID-19–related experiences, crude relative risks for mental distress and moderate to severe depression were higher among participants who reported contact with patients diagnosed with COVID-19 during the preceding week, those with insufficient PPE, and those who reported feeling somewhat or very worried about acquiring COVID-19 infection. After adjustment for age, sex, education, and occupation, relative risks for mental health conditions among those who reported contact with patients with COVID-19 (mental distress RR = 1.30; 95% CI = 1.13, 1.51; depressive symptoms RR = 1.96; 95% CI = 1.34, 2.87), insufficient PPE (mental distress RR = 1.25; 95% CI = 1.14, 1.36; depressive symptoms RR = 1.24; 95% CI = 1.02, 1.51), and feeling somewhat or very

AJPH Supplement 6, 2022, Vol 112, No. S6

TABLE 2— Mean Values for Each GHQ-12 Item, by Group Category: HEROES–Guatemala Study, 2020

GHQ-12 Item	Overall, Mean	Gender, Mean		Occupation, Mean			Health Care Sector, Mean		Contact With COVID-19 Patients, Mean	
		Female	Male	Physician	Nurse	Other	Public	Private	Yes	No
1. Able to concentrate	1.42	1.45	1.37	1.61	1.35	1.29*	1.46	1.32*	1.46	1.30*
2. Lost much sleep	1.41	1.46	1.32*	1.59	1.42	1.28*	1.46	1.29*	1.53	1.08*
3. Playing a useful part	0.70	0.69	0.72	0.88	0.48	0.62*	0.70	0.69	0.67	0.76
4. Capable of making decisions	0.86	0.87	0.85	0.99	0.65	0.83*	0.86	0.87	0.85	0.91
5. Under stress	1.75	1.80	1.64*	1.90	1.78	1.63*	1.79	1.65*	1.87	1.42*
6. Could not overcome difficulties	0.89	0.92	0.84	1.07	0.81	0.78*	0.91	0.85	0.94	0.76*
7. Enjoy your day-to-day activities	1.80	1.81	1.77	1.88	1.78	1.75*	1.83	1.71*	1.84	1.64*
8. Face up to problems	1.15	1.18	1.10	1.29	1.06	1.08*	1.18	1.08	1.20	1.04*
9. Feeling unhappy and depressed	1.14	1.18	1.07*	1.37	1.05	1.00*	1.18	1.05*	1.26	0.84*
10. Losing confidence	0.64	0.67	0.57	0.82	0.62	0.50*	0.66	0.56	0.70	0.48*
11. Thinking of self as worthless	0.35	0.33	0.37	0.50	0.32	0.24*	0.36	0.31	0.38	0.25
12. Feeling reasonably happy	1.10	1.11	1.10	1.25	1.09	1.00*	1.14	1.00*	1.13	1.03
Full sample statistics										
GHQ-12, mean (SD); median (IQR)	3.88 (3.14); 3 (1–6)									
GHQ-12, Cronbach α (95% CI)	0.86 (0.85, 0.87)									
Mental distress, no. (%; 95% CI)	899 (59.07; 56.57, 61.51)									

Note. CI = confidence interval; GHQ-12 = General Health Questionnaire; HEROES = COVID-19 Health Care Workers Study; IQR = interquartile range. The sample size was 1522. GHQ-12 scale items range from 0–3, with 0 representing nondistressed answers and 3 representing distressed answers. The distribution of responses to each of the items is shown in Appendix F (available as a supplement to the online version of this article at http://www.ajph.org).

*$P < .05$.

TABLE 3— Mean Values for Each PHQ-9 Item, by Group Category: HEROES–Guatemala Study, 2020

PHQ-9 Item	Overall, Mean	Gender, Mean		Occupation, Mean			Health Care Sector, Mean		Contact With COVID-19 Patients, Mean	
		Female	Male	Physician	Nurse	Other	Public	Private	Yes	No
1. Little interest or pleasure in doing things	0.72	0.73	0.70	0.95	0.58	0.59*	0.75	0.66	0.80	0.54*
2. Feeling down, depressed, or hopeless	0.68	0.71	0.63	0.88	0.58	0.56*	0.72	0.58*	0.78	0.47*
3. Trouble falling or staying asleep or sleeping too much	0.94	0.97	0.87	1.20	0.83	0.77*	0.96	0.88	1.05	0.65*
4. Feeling tired or having little energy	1.17	1.21	1.07*	1.45	1.10	0.97*	1.21	1.06*	1.30	0.76*
5. Poor appetite or overeating	0.87	0.91	0.81	1.16	0.77	0.68*	0.90	0.80	0.99	0.53*
6. Feeling bad about yourself	0.49	0.51	0.45	0.69	0.42	0.35*	0.51	0.43	0.57	0.32*
7. Trouble concentrating on things	0.65	0.68	0.61	0.88	0.62	0.49*	0.69	0.56*	0.75	0.44*
8. Moving or speaking slowly or fidgety or restless	0.45	0.45	0.46	0.52	0.48	0.39*	0.48	0.37	0.53	0.23*
9. Thoughts that you would be better off dead	0.13	0.12	0.16	0.20	0.09	0.10*	0.15	0.10	0.15	0.08
Full sample statistics										
PHQ-9, mean (SD); median (IQR)	6.11 (5.52); 5 (1–9)									
PHQ-9, Cronbach α (95% CI)	0.90 (0.89, 0.91)									
Depression moderate to severe, no. (%; 95% CI)	340 (22.97; 20.90, 25.19)									

Note. CI = confidence interval; HEROES = COVID-19 Health Care Workers Study; IQR = interquartile range; PHQ-9 = Patient Health Questionnaire. The sample size was 1480. PHQ-9 scale items range from 0–3, with 0 representing no days and 3 representing nearly every day. The distribution of responses to each of the items is shown in Appendix G (available as a supplement to the online version of this article at http://www.ajph.org).

*$P < .05$.

TABLE 4— Associations Between Sociodemographic and Job Characteristics, COVID-19–Related Exposures, and Mental Health Conditions Among Health Care Workers in Guatemala: HEROES–Guatemala Study, 2020

Characteristic	Positive for Mental Distress[a]		Positive for Moderate/Severe Depressive Symptoms[b]	
	Bivariate Model, RR (95% CI)	Multivariable Model,[c] RR (95% CI)	Bivariate Model, RR (95% CI)	Multivariable Model,[c] RR (95% CI)
Age, y				
18–34	1.69 (1.18, 2.44)	1.80 (1.24, 2.63)	4.51 (1.50, 13.54)	4.58 (1.51, 13.87)
35–49	1.43 (0.99, 2.07)	1.49 (1.02, 2.18)	2.94 (0.97, 8.90)	3.19 (1.05, 9.63)
50–59	1.04 (0.69, 1.57)	1.05 (0.69, 1.59)	1.06 (0.31, 3.70)	1.11 (0.32, 3.88)
≥ 60 (Ref)	1	1	1	1
Sex				
Male (Ref)	1	1	1	1
Female	1.06 (0.96, 1.16)	1.07 (0.98, 1.17)	1.08 (0.88, 1.32)	1.17 (0.96, 1.43)
Education				
High school or less (Ref)	1	1	1	1
Technical degree	0.99 (0.78, 1.26)	0.94 (0.73, 1.22)	1.22 (0.67, 2.23)	1.17 (0.62, 2.22)
Professional degree	1.40 (1.14, 1.71)	1.33 (1.07, 1.66)	2.22 (1.30, 3.76)	1.89 (1.09, 3.28)
Postgraduate degree	1.45 (1.18, 1.79)	1.45 (1.16, 1.83)	2.59 (1.52, 4.41)	2.31 (1.31, 4.07)
No. of people living at home				
Total	0.98 (0.96, 0.99)	0.99 (0.97, 1.01)	0.95 (0.90, 0.99)	0.98 (0.94, 1.03)
Minors	1.02 (0.96, 1.09)	1.03 (0.97, 1.09)	1.12 (0.98, 1.28)	1.13 (1.00, 1.26)
Adults aged > 65 y	1.05 (0.91, 1.22)	1.00 (0.86, 1.16)	0.94 (0.63, 1.41)	0.76 (0.52, 1.12)
People with disabilities	0.94 (0.66, 1.33)	0.90 (0.73, 1.12)	1.17 (0.61, 2.23)	0.96 (0.70, 1.32)
Health care sector				
Private (Ref)	1	1	1	1
Public	1.14 (1.03, 1.27)	1.10 (0.99, 1.23)	1.35 (1.07, 1.71)	1.21 (0.94, 1.54)
Job location by region				
Metropolitan (Ref)	1	1	1	1
North	0.83 (0.62, 1.11)	0.77 (0.56, 1.07)	0.64 (0.32, 1.28)	0.52 (0.25, 1.11)
Northeast	0.95 (0.81, 1.12)	1.07 (0.90, 1.26)	0.71 (0.48, 1.06)	0.87 (0.58, 1.31)
Southeast	1.21 (0.98, 1.49)	1.15 (0.93, 1.42)	1.53 (0.98, 2.40)	1.28 (0.87, 1.88)
Central	1.09 (0.97, 1.23)	1.15 (1.01, 1.30)	0.98 (0.74, 1.31)	1.02 (0.76, 1.36)
Southwest	0.98 (0.87, 1.09)	1.05 (0.94, 1.18)	0.87 (0.67, 1.12)	0.89 (0.68, 1.17)
Northwest	1.09 (0.87, 1.38)	1.06 (0.84, 1.34)	0.84 (0.45, 1.59)	0.86 (0.46, 1.59)
Petén	0.85 (0.38, 1.90)	0.82 (0.38, 1.76)	1.37 (0.44, 4.29)	1.35 (0.44, 4.15)
Occupation				
Administration (Ref)	1	1	1	1
Physician	1.36 (1.20, 1.54)	1.14 (1.00, 1.31)	2.24 (1.66, 3.02)	1.58 (1.16, 2.16)
Nurse	1.03 (0.88, 1.21)	1.05 (0.90, 1.24)	1.23 (0.86, 1.76)	1.14 (0.78, 1.67)
Psychologist	1.28 (0.93, 1.75)	1.07 (0.76, 1.51)	0.78 (0.26, 2.35)	0.42 (0.10, 1.72)
Social worker	1.3 (0.94, 1.79)	1.21 (0.86, 1.70)	0.31 (0.05, 2.15)	0.31 (0.05, 2.08)
Hospital technician	1.29 (1.07, 1.56)	1.33 (1.10, 1.60)	0.94 (0.53, 1.67)	0.93 (0.53, 1.63)
Nutritionist	1.33 (1.01, 1.75)	1.07 (0.81, 1.42)	2.10 (1.15, 3.84)	1.34 (0.75, 2.41)

Continued

TABLE 4— Continued

Characteristic	Positive for Mental Distress[a]		Positive for Moderate/Severe Depressive Symptoms[b]	
	Bivariate Model, RR (95% CI)	Multivariable Model,[c] RR (95% CI)	Bivariate Model, RR (95% CI)	Multivariable Model,[c] RR (95% CI)
Dentist	1.11 (0.90, 1.35)	1.05 (0.85, 1.30)	0.92 (0.53, 1.58)	0.89 (0.51, 1.53)
Hospital staff	0.98 (0.73, 1.31)	1.09 (0.81, 1.47)	0.83 (0.39, 1.75)	0.97 (0.46, 2.05)
Other	0.28 (0.05, 1.76)	0.28 (0.05, 1.66)		
Contact with COVID-19 patients during preceding week				
No (Ref)	1	1	1	1
Yes	1.38 (1.20, 1.58)	1.30 (1.13, 1.51)	2.65 (1.83, 3.84)	1.96 (1.34, 2.87)
Does not know	1.25 (1.06, 1.47)	1.24 (1.05, 1.46)	1.49 (0.96, 2.31)	1.34 (0.87, 2.07)
SARS-CoV-2 test result				
Negative (Ref)	1	1	1	1
Positive	0.78 (0.65, 0.93)	0.82 (0.69, 0.97)	0.90 (0.64, 1.26)	1.00 (0.72, 1.40)
Unknown	1.08 (0.79, 1.48)	1.07 (0.79, 1.46)	1.61 (0.89, 2.90)	1.72 (1.09, 2.74)
Personal protective equipment				
Sufficient (Ref)	1	1	1	1
Insufficient	1.35 (1.23, 1.47)	1.25 (1.14, 1.36)	1.52 (1.25, 1.85)	1.24 (1.02, 1.51)
Worried about COVID-19 infection				
No or not a lot (Ref)	1	1	1	1
Somewhat or very worried	1.53 (1.35, 1.74)	1.47 (1.30, 1.66)	1.65 (1.27, 2.15)	1.51 (1.17, 1.96)
Relative deceased because of COVID-19				
No (Ref)	1	1	1	1
Yes	1.10 (0.95, 1.27)	1.15 (1.00, 1.33)	1.15 (0.82, 1.60)	1.49 (1.09, 2.03)
Prior mental disorder				
No (Ref)	1	1	1	1
Yes	1.39 (1.20, 1.61)	1.26 (1.11, 1.44)	1.84 (1.32, 2.58)	1.44 (1.02, 2.03)
Prefer not to answer	1.39 (1.08, 1.78)	1.30 (0.96, 1.74)	0.53 (0.14, 1.95)	0.48 (0.13, 1.75)
Taking medication for mental disorders				
No (Ref)	1	1	1	1
Yes	1.51 (1.35, 1.68)	1.40 (1.24, 1.58)	2.21 (1.70, 2.88)	1.92 (1.45, 2.54)
Prefer not to answer	1.68 (1.44, 1.96)	1.75 (1.53, 2.01)	1.42 (0.61, 3.27)	1.51 (0.62, 3.70)

Note. CI = confidence interval; HEROES = COVID-19 Health Care Workers Study; RR = relative risk; SARS-CoV-2 = severe acute respiratory syndrome coronavirus 2. The sample size was 1522. Data are from bivariate and multivariable models.

[a]General Health Questionnaire score ≥ 3.
[b]Patient Health Questionnaire score ≥ 10.
[c]Multivariable models adjusted for age category, sex, education, and occupation.

worried about acquiring COVID-19 infection (mental distress RR = 1.47; 95% CI = 1.30, 1.66; depressive symptoms RR = 1.51; 95% CI = 1.17, 1.96) moved toward the null but remained significantly higher than risks among those who did not report COVID-19–related experiences.

The risk of mental distress, but not depression, was lower among those with a positive SARS-CoV-2 test result (RR = 0.82; 95% CI = 0.69, 0.97) than among those with a negative result. Depression was associated with experiencing the death of a relative from COVID-19 (RR = 1.49; 95% CI = 1.09, 2.03). Finally, adjusted models showed that mental distress was

associated with having a prior mental health diagnosis (RR = 1.26; 95% CI = 1.11, 1.44) and taking medication for a mental disorder (RR = 1.40; 95% CI = 1.24, 1.58). Depressive symptoms were also associated with a prior mental health diagnosis (RR = 1.44; 95% CI = 1.02, 2.03) and medication for a mental disorder (RR = 1.92; 95% CI = 1.45, 2.54).

Examinations of 2 cut-offs for classifying positive and negative cases revealed consistent associations between mental distress and COVID-19–related exposures (Appendix E). With respect to depression, mild and moderate cases were more consistent in being associated with COVID-19–related exposures.

DISCUSSION

We assessed the mental health of a cohort of Guatemalan HCWs during the height of the COVID-19 pandemic in Guatemala. Two important findings emerged from our baseline assessment. First, mental disorder symptoms were highly prevalent among Guatemalan HCWs, with close to 60% of the participants screening positive for mental distress and 23% for moderate to severe depressive symptoms. Second, mental distress and depressive symptoms were associated with sociodemographic and job characteristics such as younger age, higher education, and being a physician, as well as COVID-19 experiences such as potential exposure to COVID-19, concerns related to COVID-19 infection, and insufficient PPE. Having a history of a mental health disorder also was associated with mental distress and depression symptoms.

Most participants in this study were young, female, highly educated, and affiliated with a public health institution. Physician, nurse, and administrative staff were among the most common professions. Our sample comprised a more diverse health care workforce, including administrators, dentists, and hospital technicians, than most studies on this topic conducted in Asia, Europe, and the United States.[3,4,30–33] Moreover, whereas most studies have enrolled participants involved in the COVID-19 response at hospitals and emergency services, ours enrolled any HCW and included large and small health care facilities.

Our findings share similarities with those of systematic reviews and meta-analyses assessing the mental health of HCWs during COVID-19, despite methodological differences such as measurement scales, locations, and sampling strategies.[3,4] First, our prevalence estimates were somewhat similar to those found in pooled analyses, especially for depression (with a range of 24% to 30%).[3,4,31] Second, meta-analyses and systematic reviews have consistently shown that COVID-19–related exposures such as having contact with COVID-19 patients, having a COVID-19 infection, and having insufficient PPE seem to increase a broad spectrum of mental health conditions, including mental distress and depressive symptoms.[4,30,32] Our findings also revealed associations between mental health conditions and COVID-19–related exposures.

Third, regarding sociodemographic characteristics, Serrano-Ripoll and colleagues' meta-analysis revealed that younger HCWs seem especially vulnerable to depression and mental distress. We also found that younger populations were at higher risk of mental health conditions. Contrary to our results showing higher risks of mental health conditions among physicians and similar risks according to sex, other studies generally reveal that nurses and female HCWs fare worse than their counterparts.[4,30,32] This suggests that Guatemalan physicians may have other risk factors for mental health conditions in addition to COVID-19–related exposures. Contextual risk factors for mental health conditions such as low compensation or recognition for work during the pandemic, lack of support from government authorities, unequal allocation of resources, and nonexistence of mental health treatment options may play a role in these associations.

Studies from previous epidemics showed that HCWs with prior mental disorders were at increased risk of exhibiting severe and long-lasting mental health symptomatology during and after crises.[12] Reports of having prior mental diagnoses or taking psychotropic medications were associated with mental distress and depressive symptoms among Guatemalan HCWs. Although estimates were significant, less than 7% of participants reported having a history of a mental health disorder or taking medication, a result that warrants precaution when interpreting our findings.

To our knowledge, no prior study has documented the mental health of HCWs in Guatemala; thus, we compared our findings with those of previous Guatemalan studies focusing on other populations to shed light on the burden of mental health conditions. For example, the prevalence of depressive symptoms among HCWs during the COVID-19 pandemic was 12 times that of the general population.[34] Interestingly, our estimate of depressive symptoms was also 1.4 times higher than that shown among Guatemalan civil war refugees.[35] Although the

general and refugee populations of Guatemala do not represent an accurate comparison with HCWs working during the pandemic, they provide a reference to understand the potentially severe effects of COVID-19 on the health care workforce's mental well-being.

Limitations

Our results must be considered within the context of several limitations. First, we used a nonprobabilistic sampling technique for the enrollment of participants, meaning that our sample may not be representative of the universe of HCWs in Guatemala. However, given the pandemic's rapid evolution from week to week, the decision was made to sample HCWs via a nonrandom approach. A comparison of those who did and did not complete the survey revealed that only 1 variable differed between the 2 groups. While a generalization of our results to the universe of Guatemalan HCWs may be inaccurate, our findings shed light on the pandemic's potential mental health consequences.

Second, the cross-sectional design of this initial analysis limits our ability to assess time-variant associations between exposures and outcomes. However, 1500 participants will be followed at 6 and 12 months, and we will examine associations with longitudinal data methods.

Third, given that participants were recruited through academic institutions, union organizations, and associations, we did not have access to estimates of the numbers of HCWs who received the invitation to participate, preventing us from calculating a response rate. However, there was an 84% survey completion rate among those who received the invitation and agreed to participate in the study.

Fourth, the screening tools and cut-off points for the GHQ-12 and PHQ-9 have not been validated for Guatemala. However, both instruments and their cut-off points have been previously validated for many Latin American countries and Spain and have shown good psychometric properties.[20–26] According to our estimations, items in both scales had high internal consistency, as revealed by the Cronbach α values of 0.86 for the GHQ-12 and 0.90 for the PHQ-9 (Appendixes A and B).

Finally, despite our use of robust error variance, associations for depression models, especially the models for age categories, still showed wide 95% confidence intervals, which may indicate low precision and weak power. This limitation warrants caution when interpreting our depression results.

Public Health Implications

This report sheds light on mental health conditions and COVID-19–related factors among HCWs during the pandemic in Guatemala. Our estimates of the prevalence of mental health conditions among HCWs were higher than previous estimates among the Guatemalan general population and civil war refugees. Our descriptions of the characteristics of the most affected groups may guide surveillance efforts and direct psychological interventions to preserve HCWs' mental well-being. AJPH

ABOUT THE AUTHORS

Alejandra Paniagua-Avila, Franco Mascayano, and Ezra Susser are with the Department of Epidemiology, Columbia University Mailman School of Public Health, New York, NY. Dorian E. Ramírez, Aida Barrera-Pérez, and Erwin Calgua are with Facultad de Ciencias Médicas, Universidad de San Carlos de Guatemala, Guatemala City, Guatemala. Claudia Castro is an independent researcher in Guatemala City. Ana Peralta-García is with the School of Public Health & Tropical Medicine, Tulane University, New Orleans, LA. Rubén Alvarado is with the School of Public Health, Faculty of Medicine, Universidad de Chile, Santiago, Chile. Victor Puac-Polanco is with the Department of Health Care Policy, Harvard Medical School, Boston, MA.

CORRESPONDENCE

Correspondence should be sent to Victor Puac-Polanco, MD, DrPH, MSCE, Department of Health Care Policy, Harvard Medical School, Boston, MA, 180 Longwood Ave, Boston, MA 02115 (e-mail: puacpolanco@hcp.med.harvard.edu). Reprints can be ordered at http://www.ajph.org by clicking the "Reprints" link.

PUBLICATION INFORMATION

Full Citation: Paniagua-Avila A, Ramírez DE, Barrera-Pérez A, et al. Mental health of Guatemalan health care workers during the COVID-19 pandemic: baseline findings from the HEROES Cohort Study. *Am J Public Health*. 2022;112(S6): S602–S614.

Acceptance Date: November 29, 2021.

DOI: https://doi.org/10.2105/AJPH.2021.306648

CONTRIBUTORS

A. Paniagua-Avila, D. E. Ramírez, F. Mascayano, E. Susser, R. Alvarado, and V. Puac-Polanco substantially contributed to the work's conception. A. Paniagua-Avila, D. E. Ramírez, A. Barrera-Pérez, E. Calgua, C. Castro, A. Peralta-García, and V. Puac-Polanco worked on acquiring the data. A. Paniagua-Avila, D. E. Ramírez, A. Barrera-Pérez, E. Calgua, and V. Puac-Polanco worked on analyzing and interpreting the data. A. Paniagua-Avila, D. E. Ramírez, A. Barrera-Pérez, E. Calgua, A. Peralta-García, and V. Puac-Polanco drafted the text. All of the authors reviewed the final version.

ACKNOWLEDGMENTS

This research article is also being published in Spanish in the *Pan American Journal of Public Health* (doi: https://doi.org/10.26633/RPSP.2022.79).

We thank the COVID-19 Health Care Workers Study (HEROES) research group and all of the health care workers who took the time to complete the questionnaire for their valuable contribution to this work.

CONFLICTS OF INTEREST

The authors report no conflicts of interest.

HUMAN PARTICIPANT PROTECTION

This study was approved by the Health Research Bioethics Committee of the Facultad de Ciencias Médicas, Universidad de San Carlos de Guatemala, and the Pan American Health Organization's

Ethics Review Committee. All participants provided informed consent.

REFERENCES

1. Cucinotta D, Vanelli M. WHO declares COVID-19 a pandemic. *Acta Biomed.* 2020;91(1):157–160. https://doi.org/10.23750/abm.v91i1.9397
2. Ministerio de Salud Pública y Asistencia Social. Situación de COVID-19 en Guatemala. Available at: https://tablerocovid.mspas.gob.gt. Accessed November 25, 2020.
3. Saragih ID, Tonapa SI, Saragih IS, et al. Global prevalence of mental health problems among healthcare workers during the COVID-19 pandemic: a systematic review and meta-analysis. *Int J Nurs Stud.* 2021;121:104002. https://doi.org/10.1016/j.ijnurstu.2021.104002
4. Serrano-Ripoll MJ, Meneses-Echavez JF, Ricci-Cabello I, et al. Impact of viral epidemic outbreaks on mental health of healthcare workers: a rapid systematic review and meta-analysis. *J Affect Disord.* 2020;277:347–357. https://doi.org/10.1016/j.jad.2020.08.034
5. Avila C, Bright R, Gutierrez J, et al. *Guatemala Health System Assessment.* Bethesda, MD: Health Finance & Governance Project, Abt Associates Inc; 2015.
6. Economic Commission for Latin America and the Caribbean. Guatemala: national sociodemographic profile. Available at: https://estadisticas.cepal.org/cepalstat/Perfil_Nacional_Social.html?pais=GTM&idioma=english. Accessed January 4, 2021.
7. COVID-19 puts societies to the test. *Lancet Public Health.* 2020;5(5):e235. https://doi.org/10.1016/S2468-2667(20)30097-9
8. Class D, Rajkumar AS, Cavagnero E. Health financing profile: Guatemala. Available at: http://documents1.worldbank.org/curated/en/691171468283131455/pdf/893750BRI0P12300HFP0Guatemala0final.pdf. Accessed January 4, 2021.
9. United Nations Development Programme. The next frontier: human development and the Anthropocene. Available at: http://hdr.undp.org/sites/all/themes/hdr_theme/country-notes/GTM.pdf. Accessed April 28, 2021.
10. Kohn R, Ali AA, Puac-Polanco V, et al. Mental health in the Americas: an overview of the treatment gap. *Rev Panam Salud Publica.* 2018;42:e165. https://doi.org/10.26633/RPSP.2018.165
11. Ola AL. Lloraba por las noches, el impacto emocional en los médicos por la pandemia. Available at: https://www.prensalibre.com/pl-plus/guatemala/comunitario/lloraba-por-las-noches-el-impacto-emocional-en-los-medicos-por-la-pandemia. Accessed April 28, 2021.
12. Magill E, Siegel Z, Pike K. The mental health of frontline health care providers during pandemics: a rapid review of the literature. *Psychiatr Serv.* 2020;71(12):1260–1269. https://doi.org/10.1176/appi.ps.202000274
13. Awano N, Oyama N, Akiyama K, et al. Anxiety, depression, and resilience of healthcare workers in Japan during the coronavirus disease 2019 outbreak. *Intern Med.* 2020;59(21):2693–2699. https://doi.org/10.2169/internalmedicine.5694-20
14. Du J, Dong L, Wang T, et al. Psychological symptoms among frontline healthcare workers during COVID-19 outbreak in Wuhan. *Gen Hosp Psychiatry.* 2020;67:144–145. https://doi.org/10.1016/j.genhosppsych.2020.03.011
15. Lai J, Ma S, Wang Y, et al. Factors associated with mental health outcomes among health care workers exposed to coronavirus disease 2019. *JAMA Netw Open.* 2020;3(3):e203976. https://doi.org/10.1001/jamanetworkopen.2020.3976
16. Liang Y, Chen M, Zheng X, Liu J. Screening for Chinese medical staff mental health by SDS and SAS during the outbreak of COVID-19. *J Psychosom Res.* 2020;133:110102. https://doi.org/10.1016/j.jpsychores.2020.110102
17. Moreno C, Wykes T, Galderisi S, et al. How mental health care should change as a consequence of the COVID-19 pandemic. *Lancet Psychiatry.* 2020;7(9):813–824. https://doi.org/10.1016/S2215-0366(20)30307-2
18. Alvarado R. The COVID-19 Health Care Workers (HEROES) Study. Available at: https://mentalnet.cl/en/home. Accessed March 2, 2021.
19. Goldberg DP. *The Detection of Psychiatric Illness by Questionnaire: A Technique for the Identification and Assessment of Non-Psychotic Psychiatric Illness.* Oxford, England: Oxford University Press; 1972.
20. Goldberg DP, Gater R, Sartorius N, et al. The validity of two versions of the GHQ in the WHO study of mental illness in general health care. *Psychol Med.* 1997;27(1):191–197. https://doi.org/10.1017/S0033291796004242
21. Burrone MS, Abeldano A, Lucchese M, et al. [Psychometric properties and reliability of the General Health Questionnaire (GHQ-12) for adult patients in primary care centers in Cordoba, Argentina.] *Rev Fac Cien Med Univ Nac Cordoba.* 2015;72(4):236–242.
22. Gelaye B, Tadesse MG, Lohsoonthorn V, et al. Psychometric properties and factor structure of the General Health Questionnaire as a screening tool for anxiety and depressive symptoms in a multi-national study of young adults. *J Affect Disord.* 2015;187:197–202. https://doi.org/10.1016/j.jad.2015.08.045
23. Ruiz FJ, Garcia-Beltran DM, Suarez-Falcon JC. General Health Questionnaire-12 validity in Colombia and factorial equivalence between clinical and nonclinical participants. *Psychiatry Res.* 2017;256:53–58. https://doi.org/10.1016/j.psychres.2017.06.020
24. Kroenke K, Spitzer RL, Williams JB. The PHQ-9: validity of a brief depression severity measure. *J Gen Intern Med.* 2001;16(9):606–613. https://doi.org/10.1046/j.1525-1497.2001.016009606.x
25. Manea L, Gilbody S, McMillan D. Optimal cut-off score for diagnosing depression with the Patient Health Questionnaire (PHQ-9): a meta-analysis. *CMAJ.* 2012;184(3):E191–E196. https://doi.org/10.1503/cmaj.110829
26. Muñoz-Navarro R, Cano-Vindel A, Medrano LA, et al. Utility of the PHQ-9 to identify major depressive disorder in adult patients in Spanish primary care centres. *BMC Psychiatry.* 2017;17(1):291. https://doi.org/10.1186/s12888-017-1450-8
27. Anjara SG, Bonetto C, Van Bortel T, Brayne C. Using the GHQ-12 to screen for mental health problems among primary care patients: psychometrics and practical considerations. *Int J Ment Health Syst.* 2020;14(1):62. https://doi.org/10.1186/s13033-020-00397-0
28. Goldberg DP, Oldehinkel T, Ormel J. Why GHQ threshold varies from one place to another. *Psychol Med.* 1998;28(4):915–921. https://doi.org/10.1017/S0033291798006874
29. *Stata Statistical Software: Release 14* [computer program]. College Station, TX: StataCorp LP; 2015.
30. De Kock JH, Latham HA, Leslie SJ, et al. A rapid review of the impact of COVID-19 on the mental health of healthcare workers: implications for supporting psychological well-being. *BMC Public Health.* 2021;21(1):104. https://doi.org/10.1186/s12889-020-10070-3
31. Marvaldi M, Mallet J, Dubertret C, Moro MR, Guessoum SB. Anxiety, depression, trauma-related, and sleep disorders among healthcare workers during the COVID-19 pandemic: a systematic review and meta-analysis. *Neurosci Biobehav Rev.* 2021;126:252–264. https://doi.org/10.1016/j.neubiorev.2021.03.024
32. Muller AE, Hafstad EV, Himmels JPW, et al. The mental health impact of the Covid-19 pandemic on healthcare workers, and interventions to help them: a rapid systematic review. *Psychiatry Res.* 2020;293:113441. https://doi.org/10.1016/j.psychres.2020.113441
33. Sheraton M, Deo N, Dutt T, Surani S, Hall-Flavin D, Kashyap R. Psychological effects of the COVID 19 pandemic on healthcare workers globally: a systematic review. *Psychiatry Res.* 2020;292:113360. https://doi.org/10.1016/j.psychres.2020.113360
34. Puac-Polanco VD, Lopez-Soto VA, Kohn R, Xie D, Richmond TS, Branas CC. Previous violent events and mental health outcomes in Guatemala. *Am J Public Health.* 2015;105(4):764–771. https://doi.org/10.2105/AJPH.2014.302328
35. Sabin M, Lopes Cardozo B, Nackerud L, Kaiser R, Varese L. Factors associated with poor mental health among Guatemalan refugees living in Mexico 20 years after civil conflict. *JAMA.* 2003;290(5):635–642. https://doi.org/10.1001/jama.290.5.635
