# Supplementary material for: Mental health status and related factors influencing healthcare workers during the COVID-19 pandemic: A systematic review and meta-analysis
Source: PLoS One. 2024 Jan 19;19(1):e0289454. doi: 10.1371/journal.pone.0289454 (PMC10798549; doi:10.1371/journal.pone.0289454)
Supplement: S1 Data — (ZIP) [file pone.0289454.s011.zip › literatures/132.pdf]

ELSEVIER

Since January 2020 Elsevier has created a COVID-19 resource centre with free information in English and Mandarin on the novel coronavirus COVID-19. The COVID-19 resource centre is hosted on Elsevier Connect, the company's public news and information website.

Elsevier hereby grants permission to make all its COVID-19-related research that is available on the COVID-19 resource centre - including this research content - immediately available in PubMed Central and other publicly funded repositories, such as the WHO COVID database with rights for unrestricted research re-use and analyses in any form or by any means with acknowledgement of the original source. These permissions are granted for free by Elsevier for as long as the COVID-19 resource centre remains active.

Archives of Psychiatric Nursing 32 (2018) 2–6

Contents lists available at ScienceDirect

Archives of Psychiatric Nursing

journal homepage: www.elsevier.com/locate/apnu

Mental Health of Nurses Working at a Government-designated Hospital During a MERS-CoV Outbreak: A Cross-sectional Study

Ji-Seon Park[a], Eun-Hyun Lee[b,*], No-Rye Park[c], Young Hwa Choi[d]

[a] Seoul National University Bundang Hospital, Bundang, Republic of Korea
[b] Graduate School of Public Health, Ajou University, Suwon, Republic of Korea
[c] Graduate School of Public Health, Inje University, Seoul, Republic of Korea
[d] Department of Infectious Disease, School of Medicine, Ajou University, Suwon, Republic of Korea

ARTICLE INFO

KEYWORDS:
Middle East Respiratory Syndrome coronavirus
Mental health
Stigma
Hardiness
Stress
Nurse

ABSTRACT

BACKGROUND: During an epidemic of a novel infectious disease, many healthcare workers suffer from mental health problems.
OBJECTIVES: The aims of this study were to test the following hypotheses: stigma and hardiness exert both direct effects on mental health and also indirect (mediated) effects on mental health through stress in nurses working at a government-designated hospital during a Middle East Respiratory Syndrome coronavirus (MERS-CoV) epidemic.
METHODS: A total of 187 participants were recruited using a convenience sampling method. The direct and indirect effects related to the study hypotheses were computed using a series of ordinary least-squares regressions and 95% bootstrap confidence intervals with 10,000 bootstrap resamples from the data.
DISCUSSIONS: The influences of stigma and hardiness on mental health were partially mediated through stress in nurses working at a hospital during a MERS-CoV epidemic. Their mental health was influenced more by direct effects than by indirect effects.

INTRODUCTION

Middle East Respiratory Syndrome coronavirus (MERS-CoV) is a novel respiratory infection that was first reported in Saudi Arabia in 2012 (Zaki, van Bestebroer, & Osterhaus, 2012). The first case was discovered in Korea in May 2015, after which the rapid transmission of the MERS-CoV resulted in 186 patients being diagnosed within 6 weeks. On December 23, 2015, the Korean Ministry of Health and Welfare officially declared that the MERS-CoV outbreak had ended, with 186 laboratory-confirmed patients and 38 deaths. Among the MERS-confirmed Korean patients, 39 (21.0%) people were healthcare workers, 15 of whom (40%) were nurses (Korean Centers for Disease Control and Prevention, 2015). This was the largest MERS-CoV outbreak outside of the Arabian Peninsula (Cowling et al., 2015).

During an epidemic of an infectious disease, many healthcare workers including nurses on the front lines caring for patients with the disease suffer from mental health problems (Khee et al., 2004). At the peak of the 2003 Severe Acute Respiratory Syndrome (SARS) epidemic in Taiwan, nurses at a hospital caring for 27 suspected cases had trouble with psychological problems, such as anxiety, depression, and hostility (Chen, Cheng, Chung, & Lin, 2005). In Singapore, 27% of healthcare workers during the SARS outbreak were reported to have psychiatric symptoms (Chan & Huak, 2004). Fear and nervousness were the distressful experiences reported by healthcare workers taking care of patients with MERS-CoV in Saudi Arabia (Khalid, Khalid, Qabajap, Barnard, & Qushmaq, 2016). These findings indicate that mental health problems of nurses fighting such novel infectious diseases need to be considered.

Stigma is also a prominent issue to be considered among healthcare workers associated with infectious disease due to its transmission characteristics (Maunder et al., 2003). About 20% of healthcare workers involved with the SARS outbreak in Taiwan felt stigmatization and rejection from their neighborhood (Bai et al., 2004). In Singapore, 49% healthcare workers during the SARS outbreak experienced social stigmatization because of their jobs (Koh et al., 2005). In a similar vein, Korean nurses working at hospitals with MERS-CoV patients were distanced by from their significant others (e.g., family or friends) and prohibited from using elevators at their apartments, and even their children were not allowed to attend kindergartens and schools (Jung & Cho, 2015).

Stigma has been reported to adversely affect the mental health mainly of patients or their family caregivers (Cluver,

* Corresponding author at: Graduate School of Public Health, Ajou University, 164 Worldcup-ro, Yeongtong-Gu, Suwon-si, Gyeonggi-do 443-380, Republic of Korea.
E-mail addresses: sondreamer@snubh.org (J.-S. Park), ehlee@ajou.ac.kr (E.-H. Lee), Yhwa1805@aumc.ac.kr (Y.H. Choi).

http://dx.doi.org/10.1016/j.apnu.2017.09.006
Received 24 January 2017; Received in revised form 27 August 2017; Accepted 3 September 2017
0883-9417/ © 2017 Elsevier Inc. All rights reserved.

Gardner, & Operario, 2008; Demi, Bakeman, Moneyham, Sowell, & Seals, 1997; Furlotte & Schwartz, 2016; Tsutsumi et al., 2004). There is also rare empirical evidence of stigma among primary healthcare providers during the SARS outbreak in Singapore (Verma et al., 2004). Stigma was also considered as a source of perceived stress (Crocker, Major, & Steele, 1998; Rűsch et al., 2009). Consistent with this, previous studies have found stigma to be related to stress not only in people living with infectious disease (Charles et al., 2012) but also in the nurses caring for them (Hernandez, Morgan, & Parshall, 2016). Considering that perceived stress is well known to a predictor of mental health (Gomes, Faria, & Lopes, 2016; Lazarus & Folkman, 1984), stigma may indirectly influence mental health via stress. With a similar perspective, Hatzenbuehler, Phelan, and Link (2013) noticed that stress might mediate the linkage of stigma to health outcomes. Based on all of these findings, it was proposed in the present study that stigma exerts both direct effects on mental health and also indirect (mediated) effects on mental health via stress in nurses caring for patients with MERS-CoV.

Hardiness has been characterized by the following personality disposition: "persons high in hardiness involve themselves in whatever they are doing (commitment), believe and act as if they can influence the events forming their lives (control), and consider change to be not only normal but also a stimulus to development (challenge)" (Kobasa, Maddi, & Zola, 1983, p. 42). Previous studies have found, hardy nurses to have better mental health (Gito, Ihara, & Ogata, 2013; Woo & Suh, 2008), and that hardiness is inversely related to stress (McCalister, Dolbier, Webster, Mallon, & Steinhardt, 2006; Soderstrom, Dolbier, Leiferman, & Steinhardt, 2000). It might therefore also be conjectured that the personal characteristic of hardiness influences mental health directly and also indirectly via stress. To the best of our knowledge, hardiness has never been explored in nurses caring for patients with a novel communicable disease.

A simple partial-mediation model with two independent variables was hypothesized in this study: stigma and hardiness exert both direct effects on mental health and also indirect (mediated) effects on mental health via stress. The aims of this study were to identify the direct and indirect effects among the study variables in nurses working at a government-designated hospital during a MERS-CoV epidemic.

METHODS

DESIGN

A cross-sectional exploratory design was used.

PARTICIPANTS AND PROCEDURES

Participants were recruited from a government-designated hospital in Gyeonggi-do, South Korea from August 30 to September 21, 2015 using a convenience sampling method. The hospital has 1328 beds (1221 for general care and 107 for intensive care) and 28 operating rooms. The inclusion criterion was being a registered nurse working in high-risk areas for the MERS-CoV, such as isolation wards, intensive care units, emergency departments, infection-control office, respiratory words, and outpatient walk-in clinics. Those working in departments providing indirect support were excluded. A total of 187 nurses participated in this study. This sample size satisfied the required number for a multiple regression at a significance level of 0.05, an effect size of $f^2 = 0.15$, and a statistical power of 0.80, as calculated using G-Power 3.1.

After receiving approval from the institutional ethics committees (B-1508/312-306, AJIRB-SBR-SUR-15-277), one of the researchers met the chief director of a nursing service to explain the importance of this study. A list of potential participants was obtained from a nursing administration office. A researcher then met the potential participants and explained about the purpose of the study, its voluntary nature, and the maintenance of confidentiality. If they agreed to participate, they were asked to sign a written informed consent form and to complete a pack of questionnaires.

MEASURES

MENTAL COMPONENT SUMMARY

The Short Form-36 (SF-36) is a self-reported instrument that is widely used to measure the overall health status of general populations (Ware, 1994). It consists of 36 items in the following 8 subscales: physical function (PF), role physical (RP), bodily pain (BP), general health (GH), validity (VT), social functioning (SF), role emotional (RE), and mental health (MH). The first four subscales are categorized into the physical component summary, while the second four are categorized into the mental component summary (MCS). The MCS was used in this study to measure the mental health of nurses. The MCS score ranged from 0 to 100, with higher scores indicating a better mental health status. Cronbach's alpha of each subscale in the MCS ranged from 0.65 to 0.84 in this study: 0.77 for VT, 0.65 for SF, 0.84 for RE, and 0.80 for MH. The MCS was used with permission from QualityMetric Incorporated, and scored using Scoring Software (version 4.0).

PERCEIVED STRESS SCALE-10

The Perceived Stress Scale-10 (PSS-10) was developed to measure the degree to which situations in one's life are appraised as stressful (Cohen & Williamson, 1988). This scale comprises 10 items, each of which is scored on a 5-point Likert-type scale, with a higher score indicating higher perceived stress. In a systematic review of its measurement properties, Lee (2012) found that the PSS-10 was short, easy to use, and exhibited acceptable psychometric properties. The present study used the Korean version of the PSS-10 to measure the perceived stress of nurses. It exhibited excellent reliability and validity (Lee, Chung, Suh, & Jung, 2015), and its Cronbach's alpha was 0.73 in this study.

DISPOSITIONAL RESILIENCE SCALE-15

Hardiness was measured with the Dispositional Resilience Scale-15 (DRS-15) (Bartone, 2007). This 15-item scale comprises the 3 subscales of commitment, control, and challenge. The overall score ranges from 0 to 45, with higher scores indicating greater hardiness. In this study the DRS-15 was used with permission from KBmetric, which is the sole authorized distributor of this scale. The English version was translated into Korean using a translation and back-translation technique, with final acceptance received from the original developer (Paul T. Barton, PhD). In this study Cronbach's alpha of the Korean version of the DRS-15 was 0.78.

STIGMA

The scale for the perceived stigma of nurses regarding the MERS-CoV was newly developed to use in this study. The stigma scale comprises 13 items, each of which is scored on a 5-point Likert-type scale, with a higher score indicating that the nurses perceived greater stigma. The content validity of the scale was established by the content validity index exceeding 0.78 (Lynn, 1986). The stigma scale satisfied factorial construct validity using both exploratory factor analysis (explaining 71% of the variance) and confirmatory factor analysis (the ratio of the chi-square value to the degrees of freedom, comparative fit index, normed fit index, and standardized root mean square residual were 2.65, 0.95, 0.92, and 0.04, respectively), which indicates a good fit to the data (Browne & Cudeck, 1993) (see Supplementary File for the detailed results). Cronbach's alpha of the stigma questionnaire was 0.94.

DEMOGRAPHIC VARIABLES

Age, marital status, gender, marital status, work position, and the duration of clinical experience were examined.

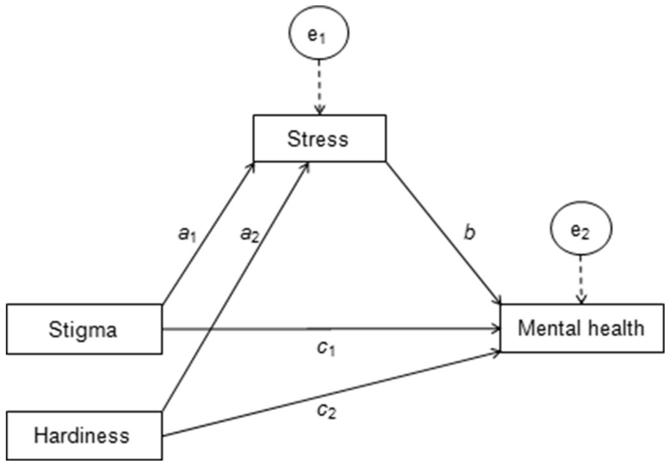

Fig. 1. Statistical diagram of a simple mediation model with two independent variables. a_1, a_2, b, c_1, and c_2: unstandardized regression coefficients; e_1 and e_2: unexplained parts of stress and mental health, respectively.
Two regression models: stress = constrant$_1$ + a_1·stigma + a_2·hardiness + e_1; mental health = constrant$_2$ + c_1·stigma + c_2·hardiness + b·stress + e_2
Indirect effect of stigma on mental health through stress was $a_1 \times b$, and the direct effect was c_1; indirect effect of hardiness on mental health through stress was $a_2 \times b$, and the direct effect was c_2.

ANALYSIS

Data were analyzed using SPSS Windows (version 22) and the PROCESS macro (version 2.16) for SPSS (Hays, 2013). Descriptive statistics was applied to the general characteristics and study variables. Correlations and mean differences between general characteristics and mental health were analyzed using Pearson's correlation, the *t*-test, and ANOVA. Pearson's coefficients were also computed for the bivariate correlations of study variables. Regarding the hypotheses of this study, the direct and indirect effects were computed using a series of ordinary least-squares regressions and 95% bootstrap confidence intervals (95% Boot CIs) with 10,000 bootstrap resamples from the data, based on Model Template for the SPSS PROCESS macro (Hays, 2013). As shown in the statistical diagram in Fig. 1, stigma and hardiness were entered as independent variables, mental health was entered as a dependent variable, and stress as a mediator. In addition, the effect size in Fig. 1 was calculated by the ratio of the indirect effect to direct effect of each sigma and hardiness on mental health (Sobel, 1982).

RESULTS

PARTICIPANTS

The participants were aged 31.15 ± 6.75 years (mean ± standard deviation), and all of them were female. Most of the participants were unmarried (n = 123, 65.8%) and worked as general nurses (n = 176, 94.1%). They had 8.4 ± 6.8 years of clinical experience.

The age and duration of clinical experience were not significantly correlated with mental health in Pearson's correlation analysis. The gender, marital status, and work position did not differ significantly with the mental health status in a *t*-test or ANOVA.

DESCRIPTIVE STATISTICS AND BIVARIATE CORRELATIONS OF STUDY VARIABLES

Descriptive statistics for the study variables are presented in Table 1. Bivariate correlations among the study variables were all significant at $p < 0.01$, with the exception of the relationship between stigma and hardiness.

Table 1
Descriptive statistics and bivariate correlations among study variables.

	Mean ± SD	1	2	3
1. Stigma	24.60 ± 11.94	–		
2. Stress	19.98 ± 4.25	0.229 (p = 0.002)	–	
3. Hardiness	24.59 ± 5.37	− 0.046 (p = 0.533)	− 0.401 (p < 0.001)	–
4. Mental health	40.89 ± 9.48	− 0.481 (p < 0.001)	− 0.562 (p < 0.001)	0.439 (p < 0.001)

HYPOTHESIS TESTING

The estimated regression coefficients of the simple partial-mediation model with two independent variables are summarized in Table 2. There was a significant direct effect of stigma on mental health when controlling for hardiness and stress (c_1 = − 0.306, t = − 7.2376, p < 0.001); that is, greater stigma was directly associated with worse mental health when hardiness and stress were constant. The 95% Boot CI for the indirect effect of stigma on mental health through stress ranged from − 0.107 to − 0.026, implying that stigma uniquely exerts an effect on mental health indirectly through stress (indirect effect = − 0.061, Boot SE = 0.020).

Hardiness exerted a significant direct effect on mental health when the other variables were kept constant (c_2 = 0.487, t = 4.8692, p < 0.001). The 95% Boot CI for the indirect effect of hardiness on mental health through stress was 0.146 to 0.401, and hence did not include 0, indicating a significant indirect effect of hardiness on mental health that was unique to hardiness (indirect effect = 0.251, Boot SE = 0.638). As hypothesized, the stigma and hardiness of nurses exerted direct effects on mental health and indirect effects on mental health via stress. In other words, the relationships of stigma and hardiness to mental health were partially mediated through stress.

The ratios of the indirect effects to the direct effects of stigma and hardiness on mental health were 0.199, and 0.516, respectively. In other words, the indirect effect of stigma on mental health was 19.9% of its direct effect on mental health, while the indirect effect of hardiness was 51.6% of its direct effect on the mental health of nurses working at a government-designated hospital during a MERS-CoV epidemic.

DISCUSSION

This study investigated the relationships of stigma, hardiness, and stress with mental health in nurses working at a government-designated hospital during a MERS-CoV epidemic in South Korea. The MERS-CoV was an unfamiliar communicable disease in South Korea before its outbreak in 2015. During the outbreak, 16 hospitals were designated to treat patients with the novel MERS-CoV. Nurses working at these hospitals were required to care for patients diagnosed with the disease or suspected of having the disease. Previous studies have found that staff nurses working at hospitals treating patients with infectious diseases (e.g., SARS and MERS-CoV) suffer from deteriorated mental health (Chen et al., 2005; Khalid et al., 2016). In a similar vein, the mental health score in the present study was worse than those of shift-work nurses (Kim, 2004) and scrub nurses (Jung, 2002) working at university hospitals not associated with a novel infectious disease in South Korea when their mental health was measured using the same instrument.

With the recent occurrences of novel contagious diseases (e.g., avian influenza, SARS, and MERS-CoV), the stigma and its consequences for front-line health providers have emerged as an important issue that needs to be addressed (Choi & Kim, 2016; Maunder et al., 2003). The present study has identified how stigma is linked to the mental health of nurses working at a hospital treating patients with the MERS-CoV—-stigma was related to mental health not only directly but also indirectly via stress. The direct linkage between stigma and mental health was

Table 2
Estimated regression coefficients from a simple mediation analysis with two independent variables.

		Consequent						
		Stress				Mental health		
		B	SE	p		B	SE	p
Stigma	a_1	0.075	0.023	0.002	c_1	− 0.306	0.042	< 0.001
Stress		–	–	–	b	− 0.810	0.130	< 0.001
Hardiness	a_2	− 0.310	0.051	< 0.001	c_2	0.487	0.100	< 0.001
Constant		25.751	1.455	< 0.001		52.649	4.202	< 0.001
		$R^2 = 0.205$				$R^2 = 0.510$		
		$F(2, 184) = 23.701, p < 0.001$				$F(3, 183) = 63.476, p < 0.001$		

B = unstandardized coefficient; SE = standard error.
a_1, a_2, b, c_1, and c_2: unstandardized regression coefficients; e_1 and e_2: unexplained parts of stress and mental health, respectively.
Two regression models: stress = constrant$_1$ + a_1·stigma + a_2·hardiness + e_1; mental health = constrant$_2$ + c_1·stigma + c_2·hardiness + b·stress + e_2.

congruent with the findings of an investigation of a SARS outbreak (Verma et al., 2004). The indirect linkage was similarly supported by stigma having a mediation effect on depression through stress in people with disabilities (Shin, Lee, Kim, & Lee, 2011). From these findings, it is suggested that hospital administrators and policymakers should make efforts to ensure that nurses do not suffer from infectious-disease-related stigma (e.g., social rejection, prejudice, or discrimination) during the early stages of an epidemic. This might result in the nurses perceiving less stress and maintaining better mental health, enabling them to concentrate on caring for their patients.

Hardiness has been studied in nurses at various workplaces, such as critical care units, emergency departments, and home care (Cho & Kim, 2014; Henderson, 2015; Judkins & Rind, 2005; Whitmer, Hurst, & Prins, 2009). The hardiness of nurses working in such settings was reported as an important direct predictor of their mental health (Harrison, Loiselle, Duquette, & Semenic, 2002; Lambert, Lambert, Petrini, Mei, & Zhang, 2007). This is consistent with the findings of the present study involving nurses working at hospitals treating patients with the MERS-CoV; this study is the first to investigate the hardiness of nurses working at hospitals treating patients with novel contagious diseases. In this study hardiness was also found to indirectly influence mental health via stress. Such a partial mediation effect of hardiness on a health outcome via stress was also found in a study of employees working in a corporate environment in the USA (Soderstrom et al., 2000). This personal trait of hardiness can be enhanced by ongoing education (Judkins, Massey, & Huff, 2006). It is therefore recommended to develop an educational program that strengthens the personal characteristics of commitment, control, and challenge among nurses.

Considering the effect sizes for how hardiness influenced mental health, the indirect effect through stress was about a half (51.6%) that for the direct effect. Combining a stress-reduction intervention with an educational program for enhancing hardiness might therefore be more effective in improving the mental health status of nurses during an infectious epidemic.

STRENGTHS AND LIMITATIONS

This study exhibited several strengths and limitations. A mediation effect refers to how an independent variable influences a dependent variable through a mediator (Field, 2013). The first strength of this study was therefore in identifying the mechanism underlying how stigma and hardiness influence the mental health of nurses working at hospitals treating patients with the MERS-CoV. The second strength was that a simple partial-mediation model of the influences of stigma and hardiness on mental health via stress was assessed using the powerful approach of bootstrapping. The most widely used approach is that suggested by Baron and Kenny (1986), but that approach has been criticized as having a low statistical power (Field, 2013; MacKinnon, Lockwood, Hoffman, West, & Sheets, 2002). An alternative is the Sobel test, which requires the assumption that the indirect effect is normally distributed (Hays, 2009). However, the distribution actually tends to exhibit skewness and kurtosis (Edwards & Lambert, 2007). Currently the most powerful approach for a mediating-effect model involves the application of bootstrapping, which provides the advantage of not needing to make any assumptions about the sampling distribution (Hays, 2009).

The participants were recruited from a single institute in this study. This limited the external validity for generalizing the findings. The mediation model in this study was tested with cross-sectional corrected data, which makes it difficult to identify the temporal sequence between exposure to the independent and mediating variables preceding the outcome variable of the mental health of the nurses.

Care is also needed when interpreting the effect sizes found in this study. The effect size was calculated as the ratio of an indirect effect to a direct effect. This approach is easy to estimate and is currently the most widely used measure; however, a very large sample is required to obtain stable estimations of this ratio measure (Tofighi, MacKinnon, & Yoon, 2009).

IMPLICATIONS FOR FURTHER RESEARCH

This study only involved nurses, whereas other kinds of healthcare workers were also involved in treating patients with the MRES-CoV. Studies of future outbreaks of MRES-CoV or other infectious diseases could include other health professionals such as physicians. In terms of the study design, future prospective longitudinal surveys are recommended for identifying the temporal sequence of the variables in the mediation model. An instrument is already available for measuring stigma related to chronic infectious diseases (e.g., HIV/AIDS) experienced by nurses providing healthcare services (Uys et al., 2009). However, there is no such instrument related to the MERS-CoV for nurses. The present study is the first to develop a scale for the stigma perceived by nurses related to the MERS-CoV, and the scale satisfies content validity, factorial-construct validity, and internal consistency reliability. Future studies should evaluate other psychometric properties of the scale, such as its convergent/discriminant validity, known-groups validity, and test–retest reliability. This instrument may also be suitable for application by infection control nurses or other healthcare providers at community health centers.

CONCLUSIONS

Stigma and hardiness influence mental health both directly and also indirectly via stress in nurses working at government-designated hospitals during a MERS-CoV epidemic. The mental health of nurses was more strongly determined by direct effects than by indirect effects. These findings suggest that the mental health of nurses caring for patients with infectious diseases could be greatly enhanced by the

application of an education/intervention program that considers all aspects of their stigma, hardiness, and stress.

Appendix A. SUPPLEMENTARY DATA

Supplementary data to this article can be found online at http://dx.doi.org/10.1016/j.apnu.2017.09.006.

REFERENCES

Bai, Y., Lin, C.-C., Lin, C.-Y., Chen, J.-Y., Chue, C.-M., & Chou, P. (2004). Survey of stress reactions among health care workers involved with the SARS outbreak. *Psychiatric Services, 55*, 1055–1057.

Baron, R. M., & Kenny, D. A. (1986). The moderator-mediator variable distinction in social psychological research: Conceptual, strategic, and statistical considerations. *Journal of Personality and Social Psychology, 52*, 1173–1182.

Bartone, P. T. (2007). Test-retest reliability of the Dispositional Resilience Scale-15, a brief hardiness scale. *Psychological Reports, 101*, 943–944.

Browne, M. W., & Cudeck, R. (1993). Alternatives ways of assessing model fit. In K. A. Bollen, & J. S. Long (Eds.). *Testing structural equation models* (pp. 136–162). Newbury Park, CA: Sage.

Chan, A. O. M., & Huak, C. Y. (2004). Psychological impact of the 2003 severe acute respiratory syndrome outbreak on health care workers in a medium size regional general hospital in Singapore. *Occupational Medicine, 54*, 190–196.

Charles, B., Jeyaseelan, L., Pandian, A. K., Sam, A. E., Thenmozhi, M., & Jayaseelan, V. (2012). Association between stigma, depression and quality of life of people living with HIV/AIDS (PLHA) in South India – A community based cross sectional study. *BMC Public Health, 12*, 463.

Chen, W.-K., Cheng, Y.-C., Chung, Y.-T., & Lin, C.-C. (2005). The impact of the SARS outbreak on an urban emergency department in Taiwan. *Medical Care, 43*, 168–172.

Cho, H. N., & Kim, S.-J. (2014). Relationship of job stress, hardness, and burnout among emergency room nurses. *Korean Journal of Occupational Health Nursing, 24*, 11–19.

Choi, J.-S., & Kim, J.-S. (2016). Factors influencing emergency nurses' ethical problems during the outbreak of MERS-CoV. *Nursing Ethics*. http://dx.doi.org/10.1177/0969733016648205.

Cluver, L., Gardner, F., & Operario, D. (2008). Effects of stigma on the mental health of adolescents orphaned by AIDS. *Journal of Adolescent Health, 42*, 410–417.

Cohen, S., & Williamson, G. (1988). Perceived stress in a probability sample of the United States. In S. Spacapan, & S. Oskamp (Eds.). *The social psychology of health: Claremont symposium on applied social psychology* (pp. 31–67). Newbury Park, CA: Sage.

Cowling, B. J., Park, M., Fang, V. J., Wu, P., Leung, G. M., & Wu, J. T. (2015). Preliminary epidemiology assessment of MERS-CoV outbreak in South Korea, May-June 2015. *Euro Surveillance, 20*, 7–13.

Crocker, J., Major, B., & Steele, C. (1998). Social stigma. In D. Gilbert, S. T. Fiske, & G. Lindzey (Eds.). *Handbook of social psychology* (pp. 504–553). (4th ed.). Boston: McGraw Hill.

Demi, A., Bakeman, R., Moneyham, L., Sowell, R., & Seals, B. (1997). Effects of resources and stressors on burden and depression of family members who provide care to an HIV-infected woman. *Journal of Family Psychology, 11*, 35–48.

Edwards, J. R., & Lambert, L. S. (2007). Methods for integrating moderation and mediation: A general analytical framework using moderated path analysis. *Psychological Methods, 12*, 1–22.

Field, A. (2013). *Discovering statistics using IBM SPSS statistics* (4th ed.). London: Sage.

Furlotte, C., & Schwartz, K. (2016). Mental health experiences of older adults living with HIV: Uncertainty, stigma, and approaches to resilience. *Canadian Journal on Aging, 36*(2), 125–140.

Gito, M., Ihara, H., & Ogata, H. (2013). The relationship of resilience, hardiness, depression and burnout among Japanese psychiatric hospital nurses. *Journal of Nursing Education and Practice, 3*(11), 12–18.

Gomes, A. R., Faria, S., & Lopes, H. (2016). Stress and psychological health: Testing the mediating role of cognitive appraisal. *Western Journal of Nursing Research, 38*, 1448–1468.

Harrison, M., Loiselle, C. G., Duquette, A., & Semenic, S. E. (2002). Hardiness, work support and psychological distress among nursing assistant and registered nurses in Quebec. *Journal of Advanced Nursing, 28*, 584–591.

Hatzenbuehler, M. L., Phelan, J. C., & Link, B. G. (2013). Stigma as a fundamental causes of population health inequalities. *American Journal of Public Health, 103*, 813–821.

Hays, A. F. (2009). Beyond Baron and Kenny: Statistical mediation analysis in the new millennium. *Communication Monographs, 76*, 408–420.

Hays, A. F. (2013). *Introduction to mediation, moderation, and conditional process analysis: A regression-based approach.* New York: Guilford Press.

Henderson, J. (2015). The effect of hardiness education on hardiness and burnout on registered nurses. *Nursing Economics, 34*(4), 204–209.

Hernandez, S. H. A., Morgan, B. J., & Parshall, M. B. (2016). Resilience, stress, stigma, and barriers to mental healthcare in U.S. Air Force nursing personnel. *Nursing Research, 65*(6), 481–486.

Judkins, S., Massey, C., & Huff, B. (2006). Hardiness, stress, and use of ill-time among nurse managers: Is there a connection? *Nursing Economics, 24*, 187–192.

Judkins, S., & Rind, R. (2005). Hardiness, job satisfaction, and stress among home health nurses. *Home Health Care Management and Practice, 17*, 113–118.

Jung, J. S. (2002). *Works of operation nurses and health status* (Unpublished thesis)Seoul, South Korea: Yonsei University.

Jung, W. S., & Cho, H. K. (2015). Punishment when refused to attend school of child with medical staff parents caring MERS-CoV infection patients. *The Kyunghyang Shinmun, 2, June 2015*http://news.khan.co.kr/kh_news/khan_art_view.html?artid=201506212253315&code=940100, Accessed date: 30 October 2015.

Khalid, I., Khalid, T. J., Qabajap, M. R., Barnard, A. G., & Qushmaq, I. A. (2016). Healthcare workers emotions, perceived stressors and coping strategies during a MERS-CoV outbreak. *Clinical Medicine & Research, 14*, 7–14.

Khee, K. S., Lee, L. B., Chai, O. T., Loong, C. K., Ming, C. W., & Kheng, T. H. (2004). The psychological impact of SARS on health care providers. *Critical Care and Shock, 7*, 99–106.

Kim, J. E. (2004). *Health evaluation of general hospital nurses by their working patterns* (Unpublished thesis)Seoul, South Korea: Yonsei University.

Kobasa, S. C., Maddi, S. R., & Zola, M. A. (1983). Type-A and hardiness. *Journal of Behavioral Medicine, 6*, 41–51.

Koh, D., Lim, M. K., Chia, S. E., Ko, S. M., Quin, F., Ng, V., et al. (2005). Risk perception and impact of severe acute respiratory syndrome (SARS) on work and personal lives of healthcare workers in Singapore: What can we learn? *Medical Care, 43*, 676–682.

Korean Centers for Disease Control and Prevention (2015). MERS data. http://www.mers.go.kr/mers/html/jsp/Menu_C/list_C1.jsp?fid=21, Accessed date: 29 June 2015.

Lambert, V. A., Lambert, C. E., Petrini, M., Mei, X., & Zhang, Y. J. (2007). Workplace and personal factors associated with physical and mental health in hospital nurses in China. *Nursing and Health Sciences, 9*, 120–126.

Lazarus, R. S., & Folkman, S. (1984). *Stress, appraisal and coping.* New York: Springer.

Lee, E.-H. (2012). Review of the psychometric evidence of the perceived stress scale. *Asian Nursing Research, 6*, 121–127.

Lee, E.-H., Chung, B. Y., Suh, C. H., & Jung, J. Y. (2015). Korean versions of the Perceived Stress Scale (PSS-14, -10 and -4): Psychometric evaluation in patients with chronic disease. *Scandinavian Journal of Caring Sciences, 29*, 183–192.

Lynn, M. R. (1986). Determination and quantification of content validity. *Nursing Research, 35*, 382–385.

MacKinnon, D. P., Lockwood, C. M., Hoffman, J. M., West, S. G., & Sheets, V. (2002). A comparison of methods to test mediation and other intervening variable effects. *Psychological Methods, 7*, 83–104.

Maunder, R., Hunter, J., Vincent, L., Bennett, J., Peladeau, N., Leszcz, M., et al. (2003). The immediate psychological and occupational impact of the 2003 SARS outbreak in a teaching hospital. *Canadian Medical Association Journal, 168*, 1245–1251.

McCalister, K. T., Dolbier, C. L., Webster, J. A., Mallon, M. W., & Steinhardt, M. A. (2006). Hardiness and support at work as predictors of work stress and job satisfaction. *American Journal of Health Promotion, 21*, 183–191.

Rűsch, N., Corrigan, P. W., Wassel, A., Michaels, P., Olschewski, M., Wilkniss, S., et al. (2009). A stress-coping model of mental illness stigma: I. Predictors of cognitive stress appraisal. *Schizophrenia Research, 110*, 59–64.

Shin, J.-S., Lee, K.-H., Kim, K.-S., & Lee, Y.-I. (2011). The impact of perceived social stigma on depression among people with disabilities living in Chungbuk. *Journal of Community Welfare, 36*, 361–385.

Sobel, M. E. (1982). Asymptotic confidence intervals for indirect effects in structural equation models. In S. Leinhardt (Ed.). *Sociological methodology* (pp. 290–312). Washington DC: American Sociological Association.

Soderstrom, M., Dolbier, C., Leiferman, J., & Steinhardt, M. (2000). The relationship of hardiness, coping strategies, and perceived stress to symptoms of illness. *Journal of Behavioral Medicine, 23*, 311–328.

Tofighi, D., MacKinnon, D. P., & Yoon, M. (2009). Covariances between regression coefficient estimate in a single mediator model. *British Journal of Mathematical and Statistical Psychology, 62*, 457–484.

Tsutsumi, A., Izutsu, T., Islam, A., Jalai, U. A., Nakahara, S., Takagi, F., et al. (2004). Depressive status of leprosy patients in Bangladesh: Association with self-perception of stigma. *Leprosy Review, 75*, 57–66.

Uys, L. R., Holzemer, W. L., Chirwa, M. L., Dlamini, P. S., Greeff, M., Kohi, T. W., et al. (2009). The development and validation of the HIV/AIDS Stigma Instrument-Nurse (HASI-N). *AIDS Care, 21*, 150–159.

Verma, S., Mythily, S., Chan, Y. H., Deslypere, J. P., Teo, E. K., & Chong, S. A. (2004). Post-SARS psychological morbidity and stigma among general practitioners and traditional Chinese medicine practitioners in Singapore. *Annals of the Academy of Medicine, Singapore, 33*, 743–748.

Ware, J. E. (1994). *SF-36 physical and mental health summary scales: A user's manual.* New England Medical Center, Boston: Health Assessment Lab.

Whitmer, M., Hurst, S., & Prins, M. (2009). Intergenerational views of hardiness in critical care nurses. *Dimensions of Critical Care Nursing, 28*, 214–220.

Woo, M. H., & Suh, K.-H. (2008). The influence of job stress and hardiness on physical and mental health among nursing home employees. *Korean Journal of Health Psychology, 13*, 91–109.

Zaki, A. M., van Bestebroer, B. S., & Osterhaus, A. D. (2012). Isolation of a novel coronavirus from a man with pneumonia in Saudi Arabia. *New England Journal of Medicine, 367*, 1814–1820.
