# Supplementary material for: Mental health status and related factors influencing healthcare workers during the COVID-19 pandemic: A systematic review and meta-analysis
Source: PLoS One. 2024 Jan 19;19(1):e0289454. doi: 10.1371/journal.pone.0289454 (PMC10798549; doi:10.1371/journal.pone.0289454)
Supplement: S1 Data — (ZIP) [file pone.0289454.s011.zip › literatures/133.pdf]

Asian Journal of Psychiatry 58 (2021) 102626

Contents lists available at ScienceDirect

Asian Journal of Psychiatry

journal homepage: www.elsevier.com/locate/ajp

Mental health issues among health care workers during the COVID-19 pandemic – A study from India

Rajani Parthasarathy [a], Jaisoorya TS [b,*], Thennarasu K [b], Pratima Murthy [b]

[a] Government of Karnataka, India
[b] NIMHANS, Bengaluru, India

ARTICLE INFO

Keywords:
Mental health issues
Correlates
Health care workers
COVID-19
India

ABSTRACT

Mental health issues among health care workers (HCWs) in treatment settings during COVID-19 remains understudied in India. This study examines its prevalence and correlates among HCWs in Karnataka State, India. HCWs who attended a workshop to improve mental health well-being during COVID-19 completed an anonymous online questionnaire. In addition to socio-demographics, domains assessed include occupational characteristics, COVID-19 related concerns, anxiety/depression, substance use, suicidality, lifestyle and family functioning. Of the 3083 HCWs who completed the survey (response rate-51.4 %), anxiety disorder and depression was highest among those with frontline COVID-19 responsibilities (anxiety disorder-26.6 %, depression-23.8 %). Prevalence was significantly higher among those with clinical responsibilities compared to those with supportive responsibilities (anxiety disorder: 23.9 % vs 15.5 %), (depression: 20.0 % vs 14.2 %). In the backward step-wise logistic regression analysis, HCWs with anxiety disorder were more likely to be doctors/nurses/hospital assistants, older, female, unmarried, without a leisure activity, report increased alcohol use and suicidal thoughts after pandemic onset, and having a history of receiving mental health interventions. Participants with depression additionally had family distress and hardly ever exercised. To conclude, mental health issues are common among HCWs in India. Interventions need to ensure that HCWs are protected from mental health consequences of working in COVID-19 treatment settings.

1. Introduction

India has witnessed a consistent spike in of COVID-19 cases since the beginning of July 2020. Currently it is among the most affected countries with close to 6.5 million infections and over 90,000 fatalities (WHO Coronavirus Dashboard, 2020). Reflecting this pattern, cases in the state of Karnataka, India have increased from around 15,000 in the beginning of July to 650,000 cases currently. The Government of Karnataka has been attempting to control this pandemic by initiating a series of robust public health measures to trace, track and test contacts using trained health workers. Much of the response is co-ordinated by the Government Health care system (COVID-19 pandemic, Karnataka, 2020).

In communities where the numbers of infected have hugely increased, the health care systems have become considerably stretched, with health care workers (HCWs) under tremendous stress (Remuzzi and Remuzzi, 2020). COVID-19 being highly infectious, an important reason for stress is the fear of getting infected and transmitting infection to their friends and family (Lai et al., 2020; Shechter et al., 2020; Spoorthy et al., 2020). Other workplace worries include adequacy of personal protection equipment, re-deployment outside their specialisation, inadequate training, higher patient load and longer working hours (Lai et al., 2020; Shechter et al., 2020; Spoorthy et al., 2020). Most experience difficulties with balancing work and household responsibilities (Spoorthy et al., 2020). Studies from countries initially affected by the pandemic like China, USA have reported rates of anxiety and depression to be in the excess of 30 % (Lai et al., 2020; Shechter et al., 2020). However, two studies from India examining HCWs at the beginning of the pandemic reported rates of anxiety to be of around 17 % and depression of 12 % (Chew et al., 2020; Wilson et al., 2020).

HCWs are a non-homogenous occupational category. In addition to doctors, most units require multi-disciplinary input from nurses, health assistants, lab-technicians, pharmacists, radiographers and administrative staff to ensure smooth functioning. However, the varying roles means that the duration of exposure varies and consequently the mental

* Corresponding author at: National Institute of Mental Health and Neurosciences, Hosur Road, Bangalore, Karnataka, 560029, India.
E-mail addresses: drrajanibalaji@gmail.com (R. Parthasarathy), tsjaisoorya@gmail.com (J. TS), kthenna@gmail.com (T. K), pratimamurthy@gmail.com (P. Murthy).

https://doi.org/10.1016/j.ajp.2021.102626
Received 1 November 2020; Received in revised form 19 February 2021; Accepted 7 March 2021
Available online 10 March 2021
1876-2018/© 2021 Elsevier B.V. All rights reserved.

health impact. Most studies have examined the psychological impact on doctors and nurses. Nurses, the largest part of the health force who work longer in high risk situations have reported higher rates of anxiety and depression (Hu et al., 2020; Said and Chiang, 2020). Mental health issues among health assistants and ancillary staff like pharmacists and laboratory technicians remain sparsely studied.

HCWs, compared to most other professions have comparably higher rates of psychiatric comorbidity, substance use and suicidality (Angres et al., 2003; Kalmoe et al., 2019). Those with pre-existing mental health vulnerabilities have a higher likelihood to worsen with the added psychological impact of the pandemic. Similarly, HCWs who have pre-existing chronic medical illness, like diabetes, chronic obstructive pulmonary disease or cardiac conditions, physical disorders known to have poor prognosis with COVID-19 may experience greater anxiety working in COVID-19 treatment settings (Nandy et al., 2020). Exercise, leisure activities, having friends to confide, supportive family have been reported to be protective among HCWs (Kisely et al., 2020; Mohindra et al., 2020).

Mental health issues among healthcare workers impact competency, motivation and increase risk of emotional exhaustion, hindering the health care response to COVID-19 (Kang et al., 2020). Occupational stress can also have a long-term impact on the psychological well-being of HCWs (Ruotsalainen et al., 2015). Recognising this, the Department of Health & Family Welfare, Government of Karnataka, in collaboration with NIMHANS, Bengaluru has initiated a program for mental health support of HCWs in COVID-19 treatment settings. The programme was designed on research reports from China, Europe and USA. As a part of the program, it was also felt necessary to evaluate the psychological issues among HCWs as the few existing studies from India have limited samples (<500) and restricted to few institutions (Chew et al., 2020; Mohindra et al., 2020; Wilson et al., 2020).

Hence this study examined mental health issues among a large sample of HCWs in COVID-19 treatment settings with the following objectives:

- To assess the prevalence of anxiety and depression among various occupational categories of HCWs in COVID-19 treatment settings
- To examine whether the nature of occupation, socio-demographic variables, life-style, family support, substance use and suicidality correlate with anxiety and depression among HCWs.

2. Methods

A cross-sectional survey of HCWs working under the Department of Health & Family Welfare, Government of Karnataka was conducted over a 2 month period between July 8, 2020 and September 7th 2020. HCWs across all the 30 districts of the state were invited to attend workshops to enhance their mental health and well-being. The workshops were conducted by specialist mental health personnel of the District Mental Health Program (DMHP) in all major hospitals of each district. The workshops were structured and based on a training manual prepared for the purpose (see supplementary material). Participants included all categories of HCWs including doctors, nurses, lab-technicians, radiographers, attenders/hospital assistants and hospital administrative staff. Prior to the workshop, participants were informed about the broad objectives of the survey and requested to complete an anonymous, self-rated online questionnaire. Decision to complete the questionnaire was voluntary and non-participation still entailed them to take part in the subsequent workshop. Informed consent was taken electronically. The study was approved by the ethical committee of NIMHANS, Bengaluru.

2.1. Survey instrument

The questionnaire, initially prepared in English was translated to Kannada (vernacular language) as per established guidelines (WHO, 2013). Participants could opt to answer either the English or Kannada version of the questionnaire.

The survey instrument assessed the following domains:

A check list was used to collect information regarding socio-demographics and occupational characteristics (job category/nature of COVID-19 treatment responsibilities). Participants were asked about their lifestyle including exercise, leisure activities and social relationships. The 3-item Brief assessment of family functioning scale was used to assess family functioning. The items were scored on a Likert scale with a collated score of >6 indicating family distress (Mansfield et al., 2019).

COVID-19 specific source of worries in clinical environment: Six concerns were selected by consensus among authors as common among HCWs working in COVID-19 settings in Karnataka. These include fear of getting infected, infecting family/loved ones, getting quarantined, inadequate personal protection equipment (PPE), inadequate training and work load. Participants were asked to rate on a 5-point Likert scale how often they were worried on any of these concerns in the prior 2 weeks, with the options ranging from 0 (none) to 4 (all of the time). HCWs who reported that they were either worried most of the time or all of the time were categorised as distressed.

Mental Health Screening:

Anxiety/Depression (PHQ-4)

The PHQ-4 was used to screen for anxiety and depression. The instrument has been validated as a screener for brief assessment of core symptoms of depression (PHQ-2) and uses a two item measure for anxiety (GAD-2). A positive screen for both depression and anxiety is indicated by a score ≥ 3 (range 0–6) (Löwe et al., 2010; Kroenke et al., 2009).

Substance Use: HCWs were asked whether there was a change in pattern of use of substances in the 3 months prior (signifying the period of the COVID-19 pandemic). The assessment was restricted to alcohol and tobacco (the commonest substances of abuse in India).

Suicidality: HCWs were asked about suicidal thoughts and attempts after the onset of the pandemic using single screening questions.

Finally, participants were asked whether they had ever sought consultation from specialist mental health professionals for mental health issues in their lifetime.

2.2. Statistical analysis

The data was analysed using STATA (version 14). The prevalence of anxiety and depression was reported across categories of HCWs (Doctor/Nurses/ /Hospital Assistants/Health care ancillary staff /Administrative staff) and further among the staff with clinical responsibilities (frontline COVID/general clinical). The prevalence rates were compared across various occupational categories using the chi-square test. The proportion of HCWs reporting distress about the common occupational worries related to COVID-19 was calculated.

Binary logistic regression analysis using backward step-wise method was done to identify the occupational, personal characteristics and mental health variables that correlated with anxiety and depression among HCWs. All tests were two tailed with $p < 0.05$ with results reported as odds ratio with 95 % confidence intervals.

3. Results

A total of 5995 HCWs who took part in the training program across all the 30 districts of the state of Karnataka were invited to take part in the survey. Of them, 3083 HCWs completed the survey, giving a response rate of 51.4 %.

The participants included doctors (n-826), nurses (n-504), hospital assistants/attenders (n-611), laboratory technicians/pharmacists/radiographers (n-508), and administrative staff (n-481). Both ancillary medical support staff and administrative staff were not involved in face to face patient care and for this study were collapsed to a single category of health care staff with supportive responsibilities. Among the

participants with clinical responsibilities, 531 (24.9 %) had frontline COVID-19 responsibilities while general clinical responsibilities were carried out by 1598 (75.1 %). The sample had a higher proportion of females (53.4 %) with a mean age of 36 years (standard deviation (SD)- 8.3 years). Other socio-demographic, personal, occupational characteristics have been described in Table 1.

Anxiety disorder and depressive disorder significantly varied between those with clinical responsibilities and supportive responsibilities (Anxiety: 23.9 % vs 15.4 % $p < 0.05$), (Depression: 20.0 % vs 14.2 % $p < 0.05$). HCWs with clinical responsibilities (doctors/nurses/hospital assistants) had significantly higher prevalence of anxiety disorder and depression compared to those with supportive responsibilities (Table 2). Among participants with clinical responsibilities, those with frontline COVID-19 responsibilities had significantly higher anxiety and depression compared to those with only general clinical duties (Anxiety: 26.6 % vs 20.6 % $p < 0.05$), (Depression: 23.8 % vs 17.6 % $p < 0.05$) (Table 2).

Participants with frontline COVID-19 responsibilities reported significantly higher COVID-19 occupational worries compared to those carrying out routine clinical responsibilities. The common worries in decreasing order of reporting were excessive work load (43.3 % vs 31 %); inadequate personal protection equipment (PPE) (38.2 % vs 26.5 %); infecting family/loved ones (29.4 vs 21.3 %); fear of getting infected (29.2 % vs 19.2 %); inadequate training (23.0 vs 17.3 %); and getting quarantined (21.7 vs 15.0 %) (Fig. 1).

In the backward step-wise method logistic regression analysis, participants with anxiety disorders were more likely to be doctors, nurses, hospital assistants/attenders compared to those only with health care supportive responsibilities. In addition, older age, being female, not married, not having a leisure activity, increased alcohol use and increased suicidal thoughts after pandemic onset, and lifetime history of receiving specialist input for mental health issues were correlated with anxiety disorders. Participants with depression over and above the correlates reported for anxiety disorder had family distress and did not exercise regularly. All other examined variables were not significant (Table 3).

4. Discussion

Our study is the largest to date from India to have examined the mental health impact of COVID-19 on HCWs. The survey was carried out during the period when COVID-19 cases in the state of Karnataka was consistently high. Our survey findings of 23.9 % of HCWs reporting anxiety disorder and 20 % depression, is approximately 50 % higher than two Indian studies among HCWs in the month of April which coincided with the beginning of the pandemic in the country (Chew et al., 2020; Wilson et al., 2020). Our prevalence rates also approximate the findings from a meta-analysis of anxiety and depression among HCWs during COVID-19 (Krishnamoorthy et al., 2020). A few large studies from China and USA have, however, reported higher rates (30–50 %) (Krishnamoorthy et al., 2020; Lai et al., 2020; Shechter et al., 2020; Spoorthy et al., 2020). Comparability with individual studies may be limited by the differences in the methodology, instruments used, cultural factors and occupational settings.

Table 1
Participant demographic and occupational characteristics (N-3083)©.

Characteristics	N (%)
**Occupation**	
Doctor	826 (26.8)
Nurses	504 (16.3)
Hospital Assistants/Attenders	611 (19.8)
Pharmacists/Laboratory Technician	508 (16.5)
Administrative staff	481 (15.6)
**Age (years) (mean ± SD)**	36.0 ± 8.3
**Gender**	
Female	1645 (53.4)
Male	1420 (47.6)
**Marital Status**	
Married	2488 (80.7)
Single	563 (18.3)
**Living arrangements**	
Living with family	2737 (88.8)
Living alone/hostel	346 (11.2)
**Having Children**	
Yes	2180 (71.5)
No	869 (28.5)

© Missing responses were excluded from analyses so samples sizes do not add to 3083 for all characteristics (average approximately 2%).

Table 2
Prevalence of screen positive for anxiety/depression among HCWs.

Occupational Category	Anxiety Disorder % (95 % CI)	Depression % (95 % CI)
**Overall (Clinical responsibilities)**	23.8 (21.9–25.8)	20.0 (18.2–21.9)
Doctors	23.5 (20.8–26.6)	21.0 (18.4–24.0)
Nurses	24.6 (20.9–28.7)	19.1 (15.8–22.8)
Hospital Assistants/Attenders	23.1 (19.9–26.6)	19.0 (16.0–22.4)
**Overall (Supportive responsibilities)**	15.4 (13.2–17.7)	14.2 (12.1–16.5)
Pharmacists/Laboratory Technicians	14.0 (11.0–17.2)	14.1 (11.1–17.4)
Administrative staff	17.1 (13.8–20.9)	14.3 (11.2–17.8)
**Predominant Clinical Responsibility**		
Frontline COVID-19 duties	26.6 (22.9–30.5)	23.8 (20.3–27.7)
General Clinical	20.6 (18.7–22.7)	17.6 (15.7–19.5)

CI – confidence interval.

In our study, the prevalence rates of anxiety and depression among doctors, nurses and hospital assistants were higher than among other HCWs with supportive responsibilities (pharmacists, laboratory technician, radiographers) or no clinical responsibilities (administrative staff). Previous studies comparing doctors and nurses have reported nurses to have higher rates of psychological issues as they spend more time in patient care (Lai et al., 2020; Shechter et al., 2020). However in our study there was no significant difference in prevalence amongst all categories of frontline staff. This could be owing to the fact that most hospitals in India have a higher patient load with doctors, nurses and hospital assistants being equally involved in direct patient care. Mental health issues among hospital assistants have been sparsely examined. Our finding that they experience rates of anxiety and depression comparable to doctors and nurses highlights the need to address their mental health needs. In COVID-19 treatment settings, hospital assistants have additional responsibilities involving disposing highly infectious medical waste, cleaning infective environments, shifting COVID-infected patients, and not uncommonly, dead bodies. They experience other occupational vulnerabilities like lower pay, poor job security (contractual employees) which also increases the likelihood of distress (Wiite, 1999). As a corollary, HCWs with frontline responsibilities in our study had significantly higher rates of both anxiety and depression compared to those carrying out non-COVID-19 clinical duties as reported in other studies (Cai et al., 2020; Shechter et al., 2020) Our frontline HCWs reported significantly higher fear of getting infected, infecting family/loved ones, getting quarantined, worries about PPE/training and work load. These worries are consistently reported among HCWs working in various infective settings (Hu et al., 2020; Kang et al., 2020; Mohindra et al., 2020; Shechter et al., 2020). In addition, many HCWs in the state of Karnataka have turned COVID-19 positive, adding to the stress of their colleagues. Other factors including speculations of mode of transmission, rapidity of spread and excessive media coverage may have fuelled mental health issues among our HCWs (Cai et al., 2020; Ho et al., 2020).

Female HCWs in our study had a higher risk of reporting both anxiety and depression. This finding of higher distress among females is consistent with findings both from COVID-19 treatment and other higher work load settings (Lai et al., 2020; Padkapayeva et al., 2018). Our HCWs who were older were at higher risk of reporting both anxiety

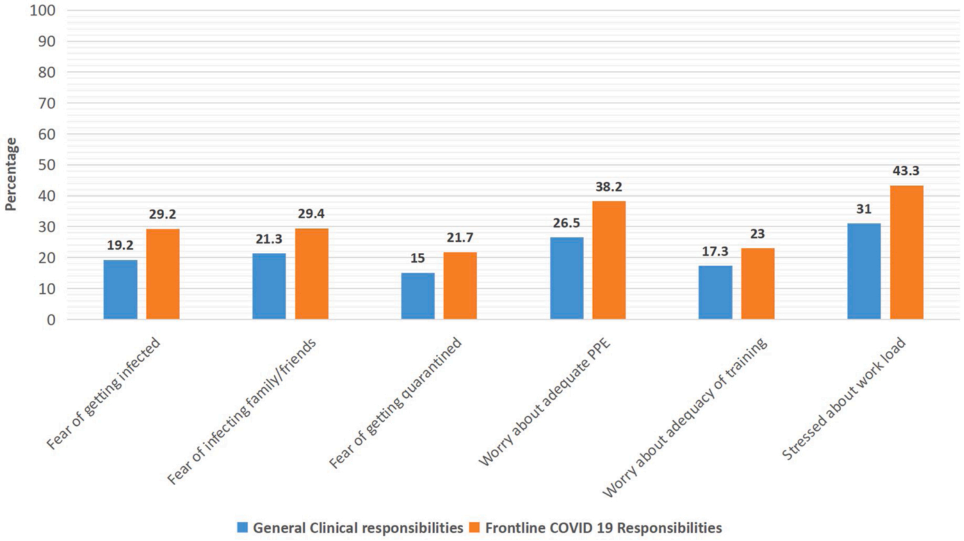

Fig. 1. COVID specific concerns among HCWs.

and depression. A previous study examining this aspect reported that older people reported increased stress due to exhaustion but the difference was not significant (Liang et al., 2020). HCWs in our study who were married had a lower risk of both anxiety and depression. Though living arrangements did not impact mental health issues, HCWs who reported poor quality of family relationships were more likely to report depression. Social and family support have been reported to be protective reducing anxiety and stress (Xiao et al., 2020). In our study, HCWs having a leisure time activity or exercised regularly had a lower risk of mental health issues. Positive lifestyle behaviours like exercise and relaxation have been reported to reduce occupational distress (Tsai and Liu, 2012). Co-occuring physical illness among our HCWs did not correlate with anxiety and depression. A previous study had reported that self-perceived poor physical health has been reported to increase mental health problems among HCWs (Kang et al., 2020). Participants in our study were young with a mean age below 40 years, making them less concerned about their physical health conditions, possibly explaining the lack of correlation with mental health issues. HCWs with a lifetime history of specialist mental health input in our study were significantly more likely to report both anxiety and depression. This is not unexpected, as pre-existing mental health vulnerability can predispose them to have worsening mental health during COVID-19 (Angres et al., 2003). Our HCWs who reported anxiety and depression experienced an increase in alcohol use (with tobacco use showing a trend towards significance) after the onset of the pandemic. Substance use is known share a bi-directional relationship with mental health issues. Some use substances as 'self-medication' to reduce distress and mental health issues for others can be outcome of increased use (Braquehais et al., 2014). Our HCWs with anxiety and depression have reported an increase in suicidal thoughts but not attempts after the onset of the pandemic. Occupationally HCWs have been consistently reported to have high suicide risk compared to most professions (Kõlves and De Leo, 2013). In addition, anxiety, depression and the workload in health care settings all known risk factors may have acted independently or synergistically to increase suicidality among our HCWs (Kõlves and De Leo, 2013).

Our findings have important public mental health implications. Longitudinal studies during the SARS pandemic have reported that mental health impacts on HCWs can be long term with distress persisting even after 2 years (Maunder et al., 2006). Sustained stress can increase long-term cardio-metabolic risk directly and indirectly through persistence of maladaptive behavioural patterns like substance use and disturbed sleep cycles (Theorell and Karasek, 1996). Even in the short term, sustaining the will, morale and capability of the HCWs is an important priority for the health care administrators. In countries like India, where specialist mental health input is sparse, the greater focus needs to be on mental health promotion strategies (Kakuma et al., 2011). Institutions need to foster a work-life balance, incentivize positive health behaviours and ensure mental health issues are discussed without stigma. More needs to be done to achieve this ideal but the current program which followed the assessment of mental health among HCWs attempts to ensure improved awareness of mental health issues, discuss self-care strategies and ensure a framework to screen and refer those with mental health difficulties to an appropriate level of services.

The study had its limitations. The study was cross-sectional, hence no inference can be made the direction of causality of examined correlates. The diagnosis of anxiety and depression was by using brief validated instruments. The data was self-reported and no diagnostic interview was conducted. The information collected was a part of the government program for its employees, increasing the likelihood of socially conforming responses. However, the large sample with participants belonging to all occupational categories, from various institutions, and the confidential nature of the responses makes our findings generalizable.

To conclude, anxiety and depression and COVID -19 related occupational worries are common among HCWs working in health care settings in India. Interventions needs to be optimized at both at the institutional and structural level to ensure that HCWs are supported and protected from the immediate and long-term mental health consequences of working in COVID-19 treatment settings.

Submission declaration

The article has not been published before, is not being considered for publication elsewhere and has been read and approved by all authors. The work if accepted for publication will not be published elsewhere either in English or in any other language.

We hereby transfer, assign, or otherwise convey all copyright ownership, including any and all rights incidental there to, exclusively to the journal, in the event that such work is published by the journal.

Contributors

Rajani TG: Conceptualisation, Data collection, Supervision, Program Implementation **Jaisoorya TS**: Conceptualization, Methodology Original draft preparation **Thennarasu K:** Methodology, Statistical analysis **Pratima Murthy**: Conceptualization, Methodology, Final draft

Table 3
Correlates of Anxiety/Depression among HCWs during COVID-19 (Total N – 3083)[α].

Variables	Total N	Anxiety Disorder N (%)	Odds Ratio (95 % CI) [$]	Depression	Odds Ratio (95 % CI) [$]
**Occupational category**					
Health care supportive staff[©]	988	150 (15.5)	Ref	136 (14.2)	Ref
Doctors	826	191 (23.6)	1.54 (1.15–2.05)	170 (21.0)	1.34 (0.99–1.81)
Nurses	504	121 (24.8)	1.79 (1.29–2.47)	96 (19.6)	1.47 (1.04–2.08)
Health assistants	611	139 (23.3)	1.77 (1.31–2.40)	112 (19.0)	1.53 (1.10–2.12)
**Age (Mean±SD) (years)**	36.0 ± 8.3	37.1 ± 8.0	1.02 (1.01–1.03)	38.9 ± 8.6	1.03 (1.01–1.04)
**Sex**					
Male	1645	246 (17.7)	Ref	226 (16.2)	Ref
Females	1420	369 (23.3)	1.74 (1.37–2.21)	304 (19.3)	1.68 (1.30–2.17)
**Marital Status**					
Single	563	127 (23.0)	Ref	110 (20.1)	Ref
Married	2488	488 (20.3)	0.70 (0.52–0.96)	419 (17.4)	0.63 (0.45 – 0.88)
**Children**					
No	869	175 (20.5)	–	157 (18.5)	–
Yes	2180	437 (20.7)		371 (17.6)	
**Living arrangement**					
Living with family	2737	530 (19.9)	–	462 (17.4)	–
Alone/Others	346	85 (27.2)		68 (21.8)	
**Family distress**					
Absent	1481	249 (17.2)	Ref	177 (12.2)	Ref
**Present**	1491	349 (24.0)	1.25 (0.98–1.53)	332 (22.9)	1.47 (1.14–1.88)
**Leisure time activity**					
No	496	166 (34.3)	Ref	163 (33.6)	Ref
Yes	2528	446 (18.1)	0.46 (0.35–0.60)	363 (14.8)	0.38 (0.29–0.51)
**Regular Exercise**					
Absent	1605	368 (23.9)	–	319 (20.8)	Ref
Present	1478	247 (17.3)		211 (14.7)	0.77 (0.61–0.98)
**Chronic Medical Illness**					
Absent	2665	470 (19.6)	–	406 (16.9)	–
Present	571	101 (23.9)		91 (21.6)	
**Increased Tobacco use after pandemic onset**					
No	253[β]	43 (17.0)	Ref	38 (15.0)	Ref
Yes	86[β]	29 (33.7)	1.78 (0.98–3.23)	29 (36.1)	1.82 (0.99–3.37)
**Increased Alcohol use after pandemic onset**					
No	525[β]	80 (15.2)	Ref	67 (12.7)	Ref
Yes	99[β]	37 (37.4)	1.76 (1.01–3.10)	36 (36.4)	1.78 (1.01–3.17)
**Suicidal thoughts after pandemic onset**					
No	2806	519 (18.8)	Ref	443 (16.1)	Ref
Yes	195	89 (45.9)	2.97 (2.05–4.30)	80 (41.5)	2.45 (1.66–3.61)
**Suicide attempts after pandemic onset**					
No	2909	569 (19.9)	–	491 (17.1)	–
Yes	86	41 (48.2)		35 (41.2)	
**Received specialist mental health input (Lifetime)**					
No	2764	495 (18.2)	Ref	425 (15.6)	Ref
Yes	245	114 (47.5)	3.41 (2.46–4.73)	100 (41.8)	3.42 (2.4–4.8)

"Ref" indicates reference category.
[$] Blanks indicate variables which were not correlated in the final model.
[α] Missing responses were excluded from analyses so samples sizes do not add to 3083 for all characteristics (average approximately 3%).
[©] Includes staff with administrative responsibilities and ancillary medical staff (pharmacists/laboratory technicians/radiographers).
[β] Total of lifetime users in sample.

preparation

All authors contributed to and have approved the final manuscript.

Funding

This research did not receive any specific grant from funding agencies in the public, commercial, or not-for-profit sectors.

Declaration of Competing Interest

The authors have no conflict of interest to declare.

Acknowledgements

We would like to acknowledge the contributions of all District Health Officers, District Mental Health Program Managers and all staff of the

District Mental Health Program who have collaborated with the project.

References

Angres, D.H., McGovern, M.P., Shaw, M.F., Rawal, P., 2003. Psychiatric comorbidity and physicians with substance use disorders: a comparison between the 1980s and 1990s. J. Addict. Dis. 22, 79–87. https://doi.org/10.1300/J069v22n03_0714621346.

Braquehais, M.D., Lusilla, P., Bel, M.J., Navarro, M.C., Nasillo, V., Díaz, A., et al., 2014. Dual diagnosis among physicians: a clinical perspective. J. Dual Diagn. 10 (3), 148–155, 3.

Cai, Q., Feng, H., Huang, J., Wang, M., Wang, Q., Lu, X., et al., 2020. The mental health of frontline and non-frontline medical workers during the coronavirus disease 2019 (COVID-19) outbreak in China: a case-control study. J. Affect. Disord. 275, 210–215.

Chew, N.W., Lee, G.K., Tan, B.Y., Jing, M., Goh, Y., Ngiam, N.J., et al., 2020. A multinational, multicentre study on the psychological outcomes and associated physical symptoms amongst healthcare workers during COVID-19 outbreak. Brain Behav. Immun. 88, 559–565.

COVID-19 pandemic in Karnataka. https://en.wikipedia.org/wiki/COVID-19_pandemic_in_Karnataka (accessed on 5th October 2020).

Ho, C.S., Chee, C.Y., Ho, R.C., 2020. Mental health strategies to combat the psychological impact of COVID-19 beyond paranoia and panic. Ann. Acad. Med. Singapore 49 (1), 1–3.

Hu, D., Kong, Y., Li, W., Han, Q., Zhang, X., Zhu, L.X., et al., 2020. Frontline nurses' burnout, anxiety, depression, and fear statuses and their associated factors during the COVID-19 outbreak in Wuhan, China: a Big-scale Cross-sectional study. Anxiety, Depression, and Fear Statuses and Their Associated Factors During the COVID-19 Outbreak in Wuhan, China: A Big-Scale Cross-Sectional Study. March, 27.

Kakuma, R., Minas, H., van Ginneken, N., Dal Poz, M.R., Desiraju, K., Morris, J.E., et al., 2011. Human resources for mental healthcare: current situation and strategies for action. Lancet 378 (9803), 1654–1663.

Kalmoe, M.C., Chapman, M.B., Gold, J.A., Giedinghagen, A.M., 2019. Physician suicide: a call to action. Mo Med. 116, 211–216, 31527944.

Kang, L., Li, Y., Hu, S., Chen, M., Yang, C., Yang, B.X., et al., 2020. The mental health of medical workers in Wuhan, China dealing with the 2019 novel coronavirus. Lancet Psychiatry 7 (3), e14, 1.

Kisely, S., Warren, N., McMahon, L., Dalais, C., Henry, I., Siskind, D., 2020. Occurrence, prevention, and management of the psychological effects of emerging virus outbreaks on healthcare workers: rapid review and meta-analysis. BMJ 369, m1642.

Kõlves, K., De Leo, D., 2013. Suicide in medical doctors and nurses: an analysis of the Queensland Suicide Register. J. Nerv. Ment. Dis. 201 (11), 987–990.

Krishnamoorthy, Y., Nagarajan, R., Saya, G.K., Menon, V., 2020. Prevalence of psychological morbidities among general population, healthcare workers and COVID-19 patients amidst the COVID-19 pandemic: A systematic review and meta-analysis. Psychiatry Res., 113382, 2020 August 11.

Kroenke, K., Spitzer, R.L., Williams, J.B., Löwe, B., 2009. An ultra-brief screening scale for anxiety and depression: the PHQ–4. Psychosomatics 50 (6), 613–621.

Lai, J., Ma, S., Wang, Y., Cai, Z., Hu, J., Wei, N., et al., 2020. Factors associated with mental health outcomes among health care workers exposed to coronavirus disease 2019. JAMA Netw Open. 3 (3), e203976.

Liang, Y., Chen, M., Zheng, X., Liu, J., 2020. Screening for Chinese medical staff mental health by SDS and SAS during the outbreak of COVID-19. J. Psychosom. Res. 133, 110102.

Löwe, B., Wahl, I., Rose, M., Spitzer, C., Glaesmer, H., Wingenfeld, K., et al., 2010. A 4-item measure of depression and anxiety: validation and standardization of the Patient Health Questionnaire-4 (PHQ-4) in the general population. J. Affect. Disord. 122 (1-2), 86–95.

Mansfield, A.K., Keitner, G.I., Sheeran, T., 2019. The Brief Assessment of Family Functioning Scale (BAFFS): a three-item version of the General Functioning Scale of the Family Assessment Device. Psychother. Res. 29 (6), 824–831.

Maunder, R.G., Lancee, W.J., Balderson, K.E., Bennett, J.P., Borgundvaag, B., et al., 2006. Long-term psychological and occupational effects of providing hospital healthcare during SARS outbreak. Emerg Infect Dis. 12 (12), 1924.

Mohindra, R., Ravaki, R., Suri, V., Bhalla, A., Singh, S.M., 2020. Issues relevant to mental health promotion in frontline health care providers managing quarantined/isolated COVID19 patients. Asian J. Psychiatr. 7 (51), 102084.

Nandy, K., Salunke, A., Pathak, S.K., Pandey, A., Doctor, C., Puj, K., et al., 2020. Coronavirus disease (COVID-19): a systematic review and meta-analysis to evaluate the impact of various comorbidities on serious events. Diabetes Metab. Syndr. 14 (5), 1017–1025.

Padkapayeva, K., Gilbert-Ouimet, M., Bielecky, A., Ibrahim, S., Mustard, C., Brisson, C., et al., 2018. Gender/sex differences in the relationship between psychosocial work exposures and work and life stress. Ann. Work Expo. Health 62 (4), 416–425.

Remuzzi, A., Remuzzi, G., 2020. COVID-19 and Italy: what next? Lancet (March), 13.

Ruotsalainen, J.H., Verbeek, J.H., Marine, A., Serra, C., 2015. Preventing occupational stress in healthcare workers. (Review). Cochrane Database Syst. Rev. 4 https://doi.org/10.1002/14651858.CD002892.pub5. CD002892.

Said, N.B., Chiang, V.C., 2020. The knowledge, skill competencies, and psychological preparedness of nurses for disasters: a systematic review. Int. Emerg. Nurs. 48, 100806.

Shechter, A., Diaz, F., Moise, N., Anstey, D.E., Ye, S., Agarwal, S., et al., 2020. Psychological distress, coping behaviors, and preferences for support among New York healthcare workers during the COVID-19 pandemic. Gen. Hosp. Psychiatry 66, 1–8.

Spoorthy, M.S., Pratapa, S.K., Mahant, S., 2020. Mental health problems faced by healthcare workers due to the COVID-19 pandemic–A review. Asian J. Psychiatr. 51, 102119.

Theorell, T., Karasek, R.A., 1996. Current issues relating to psychosocial job strain and cardiovascular disease research. J. Occup. Health Psychol. 1 (1), 9.

Tsai, Y.C., Liu, C.H., 2012. Factors and symptoms associated with work stress and health-promoting lifestyles among hospital staff: a pilot study in Taiwan. BMC Health Serv. Res. 12 (1), 199.

WHO, WHO Coronavirus Disease (COVID-19) Dashboard. https://covid19.who.int/region/searo/country/in (accessed on 5th October 2020).

Wilson, W., Raj, J.P., Rao, S., Ghiya, M., Nedungalaparambil, N.M., Mundra, H., et al., 2020. Prevalence and predictors of stress, anxiety, and depression among healthcare workers managing COVID-19 pandemic in India: a nationwide observational study. Indian J. Psychol. Med. 42 (4), 353–358.

World Health Organisation, 2013. Process of Translation and Adaptation of Instruments, 2013. https://www.who.int/substance_abuse/research_tools/translation/en/.

Xiao, H., Zhang, Y., Kong, D., Li, S., Yang, N., 2020. The effects of social support on sleep quality of medical staff treating patients with coronavirus disease 2019 (COVID-19) in January and February 2020 in China. Med. Sci. Monit. 26, e923549.
