# Supplementary material for: Mental health status and related factors influencing healthcare workers during the COVID-19 pandemic: A systematic review and meta-analysis
Source: PLoS One. 2024 Jan 19;19(1):e0289454. doi: 10.1371/journal.pone.0289454 (PMC10798549; doi:10.1371/journal.pone.0289454)
Supplement: S1 Data — (ZIP) [file pone.0289454.s011.zip › literatures/136.pdf]

Received: 20 January 2021 | Revised: 19 April 2021 | Accepted: 13 May 2021

DOI: 10.1002/alr.22831

IFAR: International Forum of Allergy & Rhinology

RESEARCH NOTE

Effect of the COVID-19 pandemic on the mental health, daily and occupational activities of otolaryngologists and allergists in Colombia: a national study

Lucia C. Pérez-Herrera MD[1,2] | **Sergio Moreno-López MSc[1,2]** | **Daniel Peñaranda MD[3]** | **Irene C. Pérez-García MD[4]** | **Elizabeth García MD[1,2,5]** | **Gloria Corredor-Rojas MD[6]** | **Edgardo Chapman MD[1,5]** | **Augusto Peñaranda MD, MSc[1,2,4]**

[1] Otolaryngology and Allergology Research Groups, Unidad Médico Quirúrgica de Otorrinolaringología (UNIMEQ-ORL), Bogotá, Colombia

[2] School of Medicine, Universidad de Los Andes, Bogotá, Colombia

[3] Otolaryngology Department, Fundación Universitaria de Ciencias de la Salud, Bogotá, Colombia

[4] Otolaryngology Department, Hospital Universitario Fundación Santa Fe de Bogotá, Bogotá, Colombia

[5] Pediatrics Department, and Allergy Section, Hospital Universitario Fundación Santa Fe de Bogotá, Bogotá, Colombia

[6] Otolaryngology Department, Fundación Universitaria Sanitas, Bogotá, Colombia

Correspondence
Augusto Peñaranda, MD, MSc, Hospital Universitario Fundación Santa Fe de Bogotá, Avenida 9 No. 116 -20, office 207. Postal code: 110111, Bogotá, Colombia.
Email: augpenar@gmail.com

Funding information
This work was supported by the Unidad Medico Quirúrgica de Otorrinolaringología (UNIMEQ-ORL) from Bogotá, Colombia.

KEYWORDS
allergists, anxiety, COVID-19, depression, otolaryngologists, prevalence, stress

INTRODUCTION

Healthcare specialists with higher rates of infection have higher occupational stress levels and risk of psychological symptoms.[1] Particularly, otolaryngologists who perform aerosol-generating procedures and allergists who are frequently exposed to respiratory diseases.[2,3] This study aimed to describe the prevalence and associated factors of depression, anxiety, and stress, as well as the changes in daily activities, among otolaryngologists and allergists during the coronavirus disease 2019 (COVID-19) pandemic in Colombia.

SUBJECTS AND METHODS

Study design

Observational, cross-sectional, survey-based study conducted between October-November 2020, approved by the ethics committee from the Fundación Santa Fe de Bogotá (CCEI-12489-2020). Anonymous online validated mental health questionnaires were applied to determine the frequency of anxiety, depression, and stress: the Generalized Anxiety Disorder−7 (GAD-7), the Patient Health Questionnaire-9 (PHQ-9), and the Perceived Stress

 © 2021 ARS-AAOA, LLC

Scale-10 (PSS-10), respectively. Sociodemographic and "Fear Score of COVID-19" questionnaires were also applied. The questionnaires were sent by the national otolaryngology (ACORL) and allergy societies from Colombia (ACAAI). Informed consent was obtained from all the participants. No incentives were offered for study participation.

Study population

Inclusion criteria were as follows: medical specialists who belonged to the ACORL or ACAAI, and conducted in-person consultations and/or telemedicine. Exclusion criteria were as follows: prior diagnosis of mental health disorders, and any acute/chronic condition limiting their ability to answer. Regarding the sample selection method, a non-probabilistic, consecutive sampling was conducted. A sample size of 146 participants was estimated considering a pooled prevalence of depression of 22.8%,[4] and the formula:

$$n \geq \frac{Z^2_{1-\frac{\alpha}{2}} * p * (1-p)}{d^2}$$

Besides, a precision of 7% and an adjustment for probable losses of 5% were considered.

Mental health, variables related to COVID-19, and daily activities questionnaires

Anxiety, depression, and stress were assessed using validated Spanish versions of the GAD-7, PHQ-9, and PSS-10. A cutoff of ≥10 points in the GAD-7 and PHQ-9 were used to determine the presence of general anxiety disorder[5] and major depression,[6] respectively. The score PSS-10 was classified as low-stress (0–13), moderate-stress (14–26), and high-stress (27–40).[7] A "Fear Score of COVID-19" was applied ranging from 1 to 5, as well as a questionnaire developed by "The New York Times"[8] on when the specialists would expect to resume daily/leisure activities.

Statistical analysis

Statistical analysis was performed using Stata 16MP software (StataCorp, College Station, TX, USA). Bivariate and multivariate exploratory analyses were used to explore the associations between the presence of anxiety and the covariates. These analyses were based on a penalized logistic regression analysis. Variables with clinical relevance, or those with a p value ≤0.2 in a Fisher test or a Mann-Whitney test, were included in the multivariate analysis. The goodness of fit of the model was assessed through a linearity test. Statistical significance for the multivariate models was established at $p < 0.05$.

RESULTS

A total of 133 individuals were included, of which 61.65% (n = 81) were otolaryngologists. The baseline demographics in both groups were similar (Table S1).

Prevalence of psychological outcomes and variables related to COVID-19

Table 1 shows the prevalence of psychological outcomes in the study population. Allergists were more likely to report symptoms of anxiety (76.47%), depression (43.14%), and stress (49.02%) than otolaryngologists (56.10%, 28.05%, and 28.05%, respectively). The "Fear Scores" of COVID-19 were also higher in the allergy specialists.

Factors associated with symptoms of anxiety, depression, and stress

Factors associated with anxiety, depression, and stress are shown in Table 2. Anxiety (odds ratio [OR] 0.34; 95% confidence interval [CI], 0.14–0.81) and stress (OR 0.38; 95% CI, 0.16–0.9) were less frequently found in otolaryngologists. A higher frequency of anxiety was found in participants that expressed a substantial reduction in consultations/surgeries during the pandemic (OR 1.03; 95% CI, 1.01–1.05), or a fear of the possibility of a negative outcome due to COVID-19 (OR 2.65; 95% CI, 1.0–7.12). Older age was associated with anxiety (OR 0.94; 95% CI, 0.90–0.98) and depression (OR 0.96; 95% CI, 0.93–0.99).

Changes in daily/leisure activities due to COVID-19

About the changes in their daily/leisure activities (Table S2), the specialists will never again: hang out with someone they do not know well (24.81%), ride a subway/bus (19.55%), attend a church/other religious service (19.55%), exercise at a gym/fitness studio (16.554%), work in a shared office (13.53%), or attend to a wedding or a funeral (11.28%).

TABLE 1 Prevalence of psychological outcomes and variables related to COVID-19

	Allergists (n = 51)			Otolaryngologists (n = 82)			Total (n = 133)		
Condition	n	%	95% CI	n	%	95% CI	n	%	95% CI
Anxiety	39	76.47	63.09–86.13	46	56.10	45.31–66.32	85	63.91	55.45–71.58
Depression	22	43.14	30.49–56.74	23	28.05	19.42–38.63	45	33.83	26.33–42.24
Stress	25	49.02	35.85–62.31	23	28.05	19.42–38.63	48	36.09	28.41–44.54
Presence of anxiety in comorbidity with									
Depression	21	41.18	28.74–54.84	23	28.05	19.42–38.63	44	33.08	25.64–46.46
Stress	22	43.14	30.49–56.74	22	26.83	18.38–37.35	44	33.08	25.64–46.46
Presence of depression in comorbidity with stress	15	29.41	18.62–43.07	17	20.73	13.27–30.81	32	24.06	17.55–32.02
GAD7: Anxiety severity scores61									
Mild	21	41.18		30	36.59		51	38.35	
Moderate	10	19.61		12	14.63		22	16.54	
Severe	8	15.69		4	4.88		12	9.02	
PHQ9: Major depression severity scores									
Mild	17	33.33		14	17.07		31	23.31	
Moderate	4	7.84		6	7.32		10	7.52	
Moderate-severe	1	1.96		2	2.44		3	2.26	
Severe	0	0.00		1	1.22		1	0.75	
PSS-10: Stress severity scores261									
Low	26	50.98		59	71.95		85	63.91	
Moderate	23	45.10		23	28.05		46	34.59	
High	2	3.92		0	0.00		2	1.50	
Variables related to COVID-19									
Have you been afraid of contagion by COVID-19? Yes	42	82.35		71	86.59		113	84.96	
Have you been afraid of the possibility of a negative outcome (death/sequelae) due to COVID-19? Yes	42	82.35		68	82.93		110	82.71	
Have you been afraid of the possibility of infecting your family and/or friends with COVID-19? Yes	48	94.12		74	90.24		122	91.73	
COVID-19 Fear score (on a scale from 1 to 5)									
Fear of contagion[a]	4 (3–4)			3 (3–4)			4 (3–4)		
Negative outcome (death/sequelae)[a]	4 (3–5)			3 (3–4)			3.5 (3–4)		
Infect a family member and/or friends[a]	4.5 (4–5)			4 (3–5)			4 (3–5)		

[a]Values are expressed as median (p25–p75).

DISCUSSION

The highest rates of psychological outcomes in healthcare workers from all over the world during the pandemic were reported in Spain.[1] These high rates of psychological outcomes are similar to the prevalence found in our population. Nevertheless, there is no data about the levels of depression/anxiety/stress in Colombian healthcare workers before the pandemic to compare with. The National Mental Health Survey from Colombia reported a prevalence of depression and anxiety disorders of 7.7% to 10.1% in adult populations.[9] The prevalence of depression

TABLE 2 Factors associated with symptoms of anxiety, depression, and stress

	Anxiety						Depression						Stress					
	Bivariate model		Multivariate model		Reduced model[a]		Bivariate model		Multivariate model		Reduced model[a]		Bivariate model		Multivariate model		Reduced model[a]	
**Variable**	OR	95% CI	OR	95% CI	OR	95% CI	OR	95% CI	OR	95% CI	OR	95% CI	OR	95% CI	OR	95% CI	OR	95% CI
Age (years)	0.96	0.93–0.99	0.95	0.91–0.99	**0.94**	**0.90–0.98**	0.97	0.94–0.99	0.99	0.95–1.02	**0.96**	**0.93–0.99**	0.98	0.95–1.01	1.00	0.96–1.04	0.99	0.95–1.02
Female	0.88	0.44–1.78	0.47	0.18–1.22	0.47	0.19–1.13	1.05	0.51–2.15	0.74	0.31–1.78	–	–	1.00	0.49–2.02	0.97	0.39–2.40	–	–
Marital status																		
Divorced/widowed	0.41	0.10–1.66	0.92	0.19–4.53	–	–	0.45	0.07–2.74	0.60	0.09–3.97	–	–	1.51	0.37–6.17	1.40	0.26–7.46	–	–
Single	2.95	0.71–12.30	2.22	0.39–12.54	–	–	2.60	0.83–8.10	2.03	0.56–7.30	–	–	2.73	0.88–8.53	3.60	0.92–14.13	–	–
Free union	1.92	0.61–6.03	0.68	0.17–2.66	–	–	2.02	0.73–5.60	0.98	0.30–3.23	–	–	3.32	1.18–9.33	2.25	0.67–7.54	–	–
Otolaryngologists	0.40	0.19–0.87	0.48	0.18–1.28	**0.34**	**0.14–0.81**	0.52	0.25–1.07	0.56	0.23–1.35	–	–	0.41	0.20–0.85	0.42	0.17–0.99	**0.38**	**0.16–0.90**
Are the personal protection elements enough? Yes	1.55	0.70–3.40	1.33	0.53–3.32	–	–	1.12	0.50–2.53	1.10	0.45–2.68	–	–	0.80	0.36–1.78	0.70	0.28–1.74	0.62	0.26–1.48
Have you been afraid of contagion by COVID-19? Yes	1.96	0.77–5.02	1.11	0.28–4.46	–	–	2.10	0.69–6.37	2.02	0.43–9.49	–	–	3.32	0.99–11.11	1.80	0.35–9.41	3.33	0.95–11.65
Have you been afraid of the possibility of a negative outcome (death/sequelae) due to COVID-19? Yes	2.73	1.11–6.72	2.32	0.64–8.45	**2.65**	**1.0–7.12**	1.93	0.69–5.40	1.17	0.29–4.81	–	–	2.90	0.97–8.65	1.93	0.42–8.89	–	–
Are you working in telemedicine? Yes	1.37	0.68–2.78	1.18	0.49–2.88	–	–	1.14	0.57–2.33	1.03	0.45–2.37	–	–	1.58	0.78–3.20	1.93	0.42–8.89	–	–
Number of hours worked per week	0.99	0.98–1.00	1.00	0.98–1.01	0.99	0.98–1.01	1.00	0.98–1.01	1.00	0.98–1.01	0.99	0.98–1.01	0.99	0.98–1.00	0.99	0.98–1.01	–	–
Substantial reduction in consultation during the pandemic	1.01	1.00–1.03	1.03	1.00–1.05	**1.03**	**1.01–1.05**	1.00	0.98–1.02	1.00	0.98–1.02	1.00	0.98–1.02	1.00	0.98–1.02	1.01	0.98–1.03	1.02	0.99–1.04

[a]The reduced model was based on the Furnival-Wilson leaps-and-bounds algorithm.
[b]Bolded numbers highlight the significant associations between the variables.

and anxiety in the general population has significantly raised worldwide due to the COVID-19 pandemic,[1] these differences could be related to this scenario.

Healthcare workers are particularly vulnerable to develop these psychological outcomes due to overburdened workload, inefficiencies in medical records, and broken healthcare systems.[1,4] Among the factors associated with anxiety, we stand out that the reduction in consultations/surgeries during the pandemic (OR 1.03; 95% CI, 1.01–1.05) could be improved with financial support. Urgent financial and psychological/psychiatric interventions should be granted by healthcare institutions.

Among the limitations of the study, we highlight that the specialist were contacted through the otolaryngology/allergy societies, which may not be representative of all available specialists in Colombia; and despite we send the invitation to all the specialists, we did not achieve a complete response. Prior authors state that there are 584 otolaryngologists in Colombia,[10] which would account for a participation rate of 14.04%. Therefore, these associations should be analyzed from an exploratory perspective. We did not expect to reach the entire population as there was no information about the prevalence of these outcomes prior the pandemic. Thus, we previously established a sample size to achieve statistical significance. Due to the cross-sectional design of the study, we can display associations but no causal relationship between the variables. Further studies in Latin American countries at different time points of the pandemic are needed.

ACKNOWLEDGMENTS

This work was supported by the Unidad Medico Quirúrgica de Otorrinolaringología UNIMEQ-ORL from Bogotá, Colombia. Special thanks to the "Asociación Colombiana de de Otorrinolaringología, Cirugía de cabeza y Cuello, Maxilofacial y Estética Facial (ACORL)", and the "Asociación Colombiana de Alergias Asma e Inmunología (ACAAI)" for their support in collecting data.

CONFLICTS OF INTEREST

The authors have declared no conflicts of interest.

ORCID

Lucia C. Pérez-Herrera MD https://orcid.org/0000-0001-8041-6433
Augusto Peñaranda MD, MSc https://orcid.org/0000-0003-1598-8472

REFERENCES

1. Vizheh M, Qorbani M, Arzaghi SM, Muhidin S, Javanmard Z, Esmaeili M. The mental health of healthcare workers in the COVID-19 pandemic: a systematic review. *J Diabetes Metab Disord*. 2020;19:1967–1978.
2. Workman AD, Welling DB, Carter BS, et al. Endonasal instrumentation and aerosolization risk in the era of COVID-19: simulation, literature review, and proposed mitigation strategies. *Int Forum Allergy Rhinol*. 2020;10(7): 798-805.
3. Zou L, Ruan F, Huang M, et al. SARS-CoV-2 viral load in upper respiratory specimens of infected patients. *N Engl J Med*. 2020;382(12):1177-1179.
4. Pappa S, Ntella V, Giannakas T, Giannakoulis VG, Papoutsi E, Katsaounou P. Prevalence of depression, anxiety, and insomnia among healthcare workers during the COVID-19 pandemic: a systematic review and meta-analysis. *Brain Behav Immun*. 2020;88:901–907.
5. García-Campayo J, Zamorano E, Ruíz MA, et al. Cultural adaptation into Spanish of the Generalized Anxiety Disorder-7 (GAD-7) scale as a screening tool. *Eur Psychiatry*. 2009;24: S538.
6. Saldivia S, Aslan J, Cova F, Vicente B, Inostroza C, Rincón P. Propiedades psicométricas del PHQ-9 (Patient Health Questionnaire) en centros de atención primaria de Chile. *Revista Medica De Chile*. 2019;147(1):53-60.
7. Trujillo HM, González-Cabrera JM. Psychometric properties of the Spanish version of the Perceived Stress Scale (PSS). *Psicol Conductual*. 2007;15(3):457-477.
8. Sanger-Katz M, Cain Miller C, Bui Q. When 511 epidemiologists expect to fly, hug and do 18 other everyday activities again. The New York Times. June 8, 2020.
9. Gómez-Restrepo C, Tamayo Martínez N, Bohórquez A, et al. Depression and anxiety disorders and associated factors in the adult Colombian population, 2015 National Mental Health Survey. *Rev Colomb Psiquiatr*. 2016;45(Suppl 1): 58-67.
10. Bright T, Mújica OJ, Ramke J, et al. Inequality in the distribution of ear, nose and throat specialists in 15 Latin American countries: an ecological study. *BMJ Open*. 2019;9(7):5-12.

SUPPORTING INFORMATION

Additional supporting information may be found online in the Supporting Information section at the end of the article.

How to cite this article: Pérez-Herrera LC, Moreno-López S, Peñaranda D, et al. Effect of the COVID-19 pandemic on the mental health, daily and occupational activities of otolaryngologists and allergists in Colombia: a national study. *Int Forum Allergy Rhinol*. 2021;11:1599–1603.
https://doi.org/10.1002/alr.22831
