# Supplementary material for: Mental health status and related factors influencing healthcare workers during the COVID-19 pandemic: A systematic review and meta-analysis
Source: PLoS One. 2024 Jan 19;19(1):e0289454. doi: 10.1371/journal.pone.0289454 (PMC10798549; doi:10.1371/journal.pone.0289454)
Supplement: S1 Data — (ZIP) [file pone.0289454.s011.zip › literatures/138.pdf]

Neurological Sciences (2022) 43:2241–2251
https://doi.org/10.1007/s10072-021-05813-y

COVID-19

Sleep disorders and mental health in hospital workers during the COVID-19 pandemic: a cross-sectional multicenter study in Northern Italy

Paola Proserpio[1] · Elena Zambrelli[2] · Andrea Lanza[1] · Ambra Dominese[3] · Roberta Di Giacomo[3] · Rui Quintas[3] · Irene Tramacere[4] · Annalisa Rubino[1] · Katherine Turner[2] · Claudio Colosio[5] · Federica Cattaneo[1] · Maria Paola Canevini[2,6] · Armando D'Agostino[6,7] · Elio Clemente Agostoni[1] · Giuseppe Didato[3]

Received: 6 October 2021 / Accepted: 6 December 2021 / Published online: 13 January 2022
© Fondazione Società Italiana di Neurologia 2021

Abstract

Introduction From the beginning of the COVID-19 pandemic, healthcare workers had to face unprecedented emergency needs associated with an extraordinary amount of psychological distress. In this cross-sectional multicenter study, we investigated sleep disturbances, and the level of anxiety and depression among the healthcare and non-healthcare staff of three hospitals in Milan (Italy) during the COVID-19 outbreak. Moreover, we explored potential predisposing factors for affective symptoms and poor sleep.

Methods Between June and July 2020, we administered an online questionnaire to evaluate the presence of sleep disorders (Pittsburgh Sleep Quality Index), insomnia (Sleep Condition Indicator), anxiety (State Trait Anxiety Inventory), and depression (Beck Depression Inventory-II). We used univariate and multivariate analysis to evaluate the association between the personal conditions and sleep and affective disorders.

Results The 964 participants reported high rates of sleep disorders (80.3%)—mainly insomnia (30.5%)—anxiety (69.7%), and depression (32.8%). The multivariate analysis showed a strong association of sleep disorders, especially insomnia, with female gender ($p=0.004$), divorced marital status ($p=0.015$), self-isolation ($p=0.037$), and chronic diseases ($p=0.003$). Anxiety was significantly associated with teleworking ($p=0.001$), while depressive symptoms were associated with self-isolation ($p=0.028$), modified work schedules ($p=0.03$), and chronic diseases ($p=0.027$).

Conclusion In hospital workers, the high prevalence of sleep and psychiatric symptoms during the COVID-19 outbreak appears to be determined mainly by modifications of personal or work habits. Teleworking was associated with increased anxiety. An accurate planning of hospital activities and a psychological support are needed to prevent and manage sleep and mental disorders.

Keywords COVID-19 pandemic · Sleep disorders · Mental health · Hospital workers · Teleworking

Paola Proserpio and Elena Zambrelli equally contributed as first authors.

✉ Giuseppe Didato
giuseppe.didato@istituto-besta.it

1 Department of Neuroscience, Sleep Medicine Centre, ASST Grande Ospedale Metropolitano Niguarda, Piazza Ospedale Maggiore, 20162 Milan, Italy

2 Epilepsy Centre, Sleep Medicine Centre, Childhood and Adolescence Neuropsychiatry Unit, ASST Santi Paolo E Carlo, San Paolo Hospital, Via Antonio di Rudinì, 8, 20142 Milan, Italy

3 Epilepsy Unit, Sleep Disorders Unit, Fondazione IRCCS Istituto Neurologico Carlo Besta, Via Celoria 11, 20133 Milan, Italy

4 Department of Research and Clinical Development, Scientific Directorate, Fondazione IRCCS Istituto Neurologico Carlo Besta, Via Celoria 11, 20133 Milan, Italy

5 Occupational Health Unit, ASST Santi Paolo E Carlo, San Paolo Hospital, Via Antonio di Rudinì, 8, 20142 Milan, Italy

6 Department of Health Sciences, Università Degli Studi, Milan, Italy

7 Department of Mental Health and Addiction, ASST Santi Paolo E Carlo, Milan, Italy

Introduction

In December 2019, an outbreak of a highly infectious acute respiratory syndrome caused by a novel coronavirus named the SARS-CoV-2 originated in Wuhan, China, and rapidly spread worldwide leading to the declaration of the "COVID-19 pandemic" in March 2020 [1].

The management of the sudden onset of a serious and immediately life-threatening infection with a high risk of transmission led to an extreme pressure and stress on healthcare workers. Indeed, the increased workload, together with the need to wear airtight protective equipment, often caused physical exhaustion. The scarce availability of protective materials, the concern for their own health and that of their relatives, and the pressure towards frequent ethically troublesome decisions on the rationing of care all contributed to an extraordinary amount of psychological distress among medical workers. Moreover, health personnel frequently decided to self-isolate from relatives, and with the aim of lowering the risk for nosocomial infections, interaction with colleagues was also discouraged, further contributing to loneliness and loss of psychological support. To date, different studies analyzed the effect of this current unprecedented crisis on the psychological well-being of healthcare staff showing a high prevalence of affective symptoms such as anxiety (23.2%) and depression (22.8%) [2]. Less is known on the impact of the outbreak on non-healthcare hospital workers, who are also likely to have experienced relevant psychological distress due to the intense transformation of all hospital activities.

Psychological distress is considered one of the primary causes of insomnia, and any stressful life event has the potential to precipitate sleep disturbances [3]. In this perspective, the COVID-19 pandemic could have likely intensified a pre-existing condition in healthcare providers. These workers seem to be particularly prone to sleep disorders, and recent evidence suggests that poor sleep quality is a frequent complaint [4]. Several studies reported a further increase of fatigue, poor sleep, anxiety, and depression among the medical staff during the epidemics of previous coronavirus variants [5]. The fast spread of COVID-19 placed a similar stress on healthcare workers, making them more vulnerable to insomnia also during this outbreak. Indeed, surveys conducted among Chinese health staff since the rise of the COVID-19 pandemic revealed very high rates of insomnia (34–36%), frequently associated with the presence of anxiety and depression [6, 7].

Sleep is an essential process to maintain homeostasis of different systems. Indeed, sleep plays a fundamental role in emotion regulation and immune functions [8]. Recent evidence showed how sleep disorders could increase the risk of infectious disease and the incidence of psychiatric disorders. Thus, investigating sleep quality and identifying strategic measures to prevent or minimize sleep disturbances among healthcare workers is essential to enhance their immunity against coronavirus. Moreover, considering the effect of poor sleep on mental health, the treatment of sleep disorders is also crucial to prevent long-term psychiatric illness. For this purpose, a tailored psychological approach (by means of cognitive-behavioral therapy for insomnia) has been proposed during the COVID-19 outbreak to improve sleep in different specific populations, i.e., patients with anxiety, students during home confinement, but also people facing changed work schedules and requirements [9].

In this study, we aimed to investigate the prevalence of sleep disturbances and the level of anxiety and depression among the healthcare and non-healthcare staff of 3 hospitals in Milan (Italy) and to recognize predisposing factors of affective symptoms and poor sleep during COVID-19 outbreak. The findings of this study can also give some insights into the mechanisms underlying the onset of sleep disturbances, especially insomnia, and their relationship with mental health symptoms, under acute precipitating conditions.

Materials and methods

Study design and participants

An anonymous cross-sectional multicenter study was performed among the healthcare and non-healthcare workers of 3 hospitals in Milan, Italy (ASST Grande Ospedale Metropolitano Niguarda, ASST Santi Paolo e Carlo, and Fondazione IRCCS Istituto Neurologico Carlo Besta) through an online questionnaire. The questionnaire was distributed through the institutional webpages or emails of the centers involved between June 15 and July 15, 2020. Participation was voluntary, without compensation, and all subjects gave their informed consent for inclusion before completing the questionnaire. The study was conducted in accordance with current national privacy legislation and was approved by the ethics committee of the hospitals involved.

Questionnaires

The self-administered questionnaire was composed of 2 sections. The first section investigated the main sociodemographic, occupational, and clinical information of the participants. In the second section, the subject was asked to fill in four questionnaires in their validated Italian version. Specifically, the Pittsburgh Sleep Quality Index (PSQI) evaluates the quality of sleep, and a cutoff score of 5 discriminates

between those with "good" and "poor" sleep [10, 11]. The Sleep Condition Indicator (SCI) was applied to assess the presence of insomnia; a score under 17 is used to indicate the presence of insomnia [12, 13]. The State Trait Anxiety Inventory (STAI-Y) is a commonly used measure of state anxiety. A cutoff score of 40 is commonly used to define probable clinical levels of anxiety [14]. Finally, the Beck Depression Inventory-II (BDI-II) was used for measuring the severity of depression; the standardized cutoff used to define a probable mild depression state is 13 [15]. The time spent completing the questionnaire was about 15 min. Therefore, the variables under study have been dichotomized in normal and pathological, grouping the participants in either one of the two conditions for each scale administered.

Study objectives

The primary aim was to assess the prevalence of subjects with sleep disorders by analyzing the scores of the PSQI and SCI questionnaires (percentage of subjects with PSQI scores > 5 and percentage of subjects with SCI ≤ 16). Secondary aims consisted in assessing (1) the prevalence of subjects with anxiety and depression by analyzing the scores of the STAI-Y and BDI-II (percentage of subjects with pathological scores) and (2) the association between the PSQI, SCI, STAI-Y, and BDI-II scores and the demographic, personal, and work variables collected in the first section of the questionnaire to explore potential influencing factors for sleep disturbances, insomnia, depression, and anxiety.

Statistical analysis

Descriptive analyses were performed for all variables. Chi-square tests and multivariate logistic regression models were used to assess the association between risk factors and the presence of insomnia, sleep disorders, depression, and anxiety. Odds ratio (OR) and 95% CI were obtained from logistic regression models. Two-tailed *p*-values < 0.05 were considered statistically significant. STATA statistical software, version 16 (StataCorp. 2019. Stata Statistical Software: Release 16. College Station, TX: StataCorp LLC) was used for the statistical analysis.

Results

Demographic characteristics and descriptive analysis

Nine hundred sixty-four hospital workers (79.6% females) completed the online questionnaire. Their main sociodemographic, occupational, and clinical data are summarized in Table 1. In particular, 94 subjects (9.8%) had been diagnosed with COVID-19, and 309 (32.1%) underwent self-isolation as a preventive measure to protect their relatives against the risk of transmission of COVID-19 infection.

Table 1 Demographic characteristics and descriptive analysis

	Participants *n* (%)
Overall	964
Gender (female)	767 (79.6%)
Age (years)	
21–30	112 (11.6%)
31–40	193 (20.0%)
41–50	250 (25.9%)
51–60	335 (34.8%)
> 61	74 (7.7%)
Chronic diseases	277 (28.7%)
Marital status	
Unmarried	335 (34.8%)
Married	512 (53.1%)
Divorced	109 (11.3%)
Widowed	8 (0.8%)
COVID-19 infection	94 (9.8%)
Flatmates with COVID-19 infection	129 (13.4%)
Isolation	
No	655 (67.9%)
Yes, self-isolated at home	228 (23.7%)
Yes, moved to a different accommodation	81 (8.4%)
Hospital	
COVID-19 wards (*Niguarda and Ss. Paolo e Carlo*)	757 (81.2%)
No COVID-19 wards (*Besta*)	175 (18.8%)
Occupation	
Frontline healthcare workers	770 (79.9%)
Non-frontline healthcare workers	92 (9.6%)
Non-healthcare workers	101 (10.5%)
Site of work	
Ward (clinical or surgical)	568 (59.0%)
Outpatient's department	203 (21.1%)
Laboratory	80 (8.3%)
Offices	111 (11.5%)
Shift workers	439 (45.5%)
Workers in a COVID-19 ward	283 (29.4%)
Modified work schedules	409 (42.4%)
Increased hours at work	279 (28.9%)
Modified duties	328 (34.0%)
Teleworking	136 (14.1%)
Sleep disorders before COVID-19 pandemic	280 (29.1%)
Sleep disorders during COVID-19 pandemic	704 (73.0%)
With drug use	176 (18.3%)
Without drug use	528 (54.8%)
New-onset sleep disorders	451 (64.1%)
Pre-existing sleep disorders	253 (35.9%)

Two hundred eighty participants (29.1%) declared they suffered from sleep disorders before COVID-19 pandemic, while 704 (73.0%) had sleep disturbances during the pandemic outbreak, and 176 (18.3%) needed to take medication. In the last group of subjects, 451 (64.1%) had new-onset sleep disorders. According to the scales administered (Fig. 1), (1) at PSQI, 774 subjects (80.3%) had pathological scores (i.e., bad sleep quality or disturbed sleep); (2) at SCI, insomnia was present in 294 (30.5%); (3) at STAI-Y, anxiety was present in 672 (69.7%); and (4) at BDI-II, scores indicating a depressive disorder were present in 316 (32.8%).

The scores of each component of PSQI and of each subitem of SCI are shown in Table 2. Many participants had an increase in sleep latency, but most of them reported a reduction in sleep duration and maintenance, as well as a decrease in sleep quality and efficiency and significant daytime dysfunction. Nightmares were reported by many participants.

Univariate analysis

At univariate analysis, the scores of the sleep scales administered showed the following significant associations.

Pathological PSQI scores (i.e., a sleep disorder was present) were significantly associated with female gender ($p < 0.0001$), chronic diseases ($p = 0.015$), self-isolation ($p < 0.001$), working at a hospital with COVID-19 wards ($p = 0.004$), being a shift worker ($p = 0.003$), working in a COVID-19 ward ($p < 0.001$), modified work schedules ($p = 0.039$), modified duties at work during COVID-19 outbreak ($p = 0.007$), being affected by sleep disorders before ($p < 0.001$) and during COVID-19 outbreak ($p < 0.001$), especially when drug therapy was necessary, feeling depressed ($p < 0.001$), and suffering from anxiety ($p = 0.002$; Table 3).

Pathological SCI scores (i.e., insomnia was present) were significantly associated with female gender ($p = 0.001$), age ($p = 0.023$), suffering from chronic diseases ($p < 0.001$), marital status ($p = 0.004$), self-isolation ($p = 0.008$), being affected by sleep disorders before ($p < 0.001$) and during COVID-19 outbreak ($p < 0.001$), especially when drug therapy was necessary, and feeling depressed ($p < 0.001$; Table 3).

Pathological STAI-Y scores (i.e., anxiety status) were significantly associated with age ($p = 0.04$), no self-isolation ($p = 0.044$), and teleworking ($p = 0.004$; Table 4).

Pathological BDI-II scores (i.e., depression status) were significantly associated with age ($p = 0.02$), being affected by sleep disorders before ($p < 0.001$) and during COVID-19 outbreak ($p < 0.001$), self-isolation ($p = 0.002$), modified work schedules ($p = 0.027$), and suffering from chronic diseases ($p = 0.006$; Table 4).

Among subjects who already suffered from sleep disorders before COVID-19 outbreak, 99.2% had pathological PSQI scores and 56.9% had pathological SCI scores as compared respectively to 91.4% and 29.7% of subjects who had new-onset sleep disorders ($p < 0.001$; Table 3).

Moreover, 47.4% of subjects who already suffered from sleep disorders before COVID-19 outbreak had pathological BDI-II scores as compared to 36.6% of subjects who had new-onset sleep disorders ($p = 0.005$; Table 4).

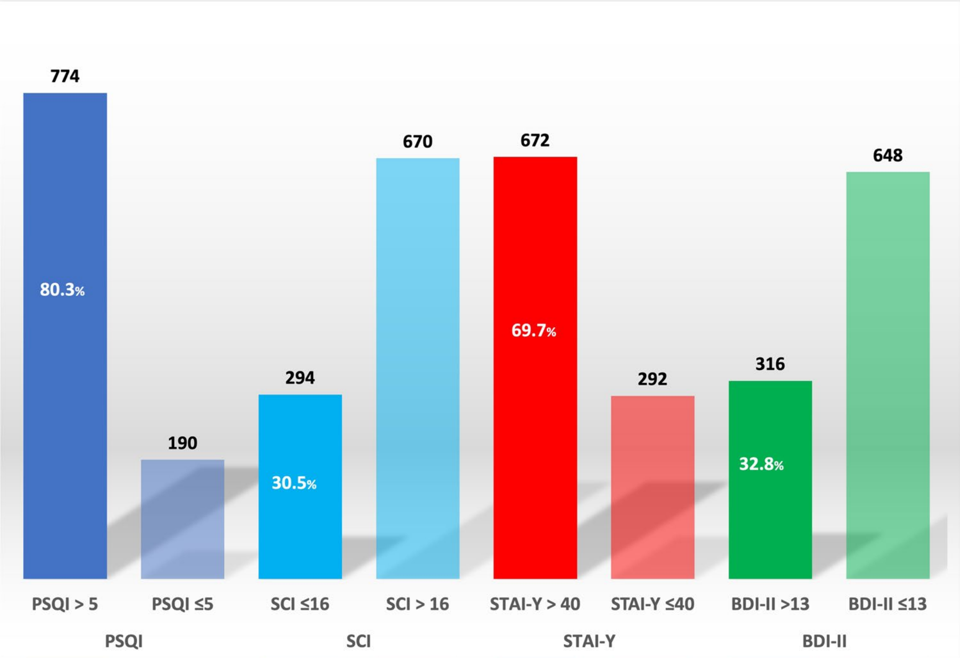

Fig. 1 Scores of the four administered scales: Pittsburgh Sleep Quality Index (PSQI), Sleep Condition Indicator (SCI), State Trait Anxiety Inventory (STAI-Y), Beck Depression Inventory-II (BDI-II)

Table 2 Subitems of Pittsburgh Sleep Quality Index (PSQI) and Sleep Condition Indicator (SCI)

	Overall	PSQI > 5		Overall	SCI ≤ 16
PSQI components	**964 (100.0%)**	**774 (80.3%)**	SCI subitems	964 (100.0%)	**294 (30.5%)**
Sleep quality			Sleep quality		
Normal or mild reduction	414 (42.9%)	227 (29.3%)	Normal or mild reduction	305 (31.6%)	3 (1.1%)
Moderate to severe reduction	550 (57.1%)	547 (70.7%)	Moderate to severe reduction	659 (68.4%)	291 (98.9%)
Sleep latency			Sleep latency		
< 30 min	485 (50.3%)	299 (38.6%)	< 30 min	667 (69.2%)	111 (37.7%)
> 30 min at least once weekly	479 (49.7%)	475 (61.4%)	> 30 min	297 (30.8%)	183 (62.3%)
Sleep duration			WASO		
> 6 h per night	313 (32.5%)	150 (19.4%)	< 30 min	582 (60.4%)	65 (22.1%)
≤6 h per night	651 (67.5%)	624 (80.6%)	> 30 min	382 (39.6%)	229 (77.9%)
Habitual sleep efficiency			Sleep disorder onset		
> 85%	455 (47.2%)	277 (35.8%)	Last month/no disorder	371(38.5%)	19 (6.5%)
75–84%	243 (25.2%)	231 (29.8%)	Last 6 months	288 (29.9%)	124 (42.2%)
< 75%	266 (27.6%)	266 (34.4%)	> 6 months	305 (31.6%)	151 (51.4%)
Sleep disturbances			Sleep disturbances		
Less than once weekly	397 (41.2%)	146 (19.0%)	< 3 days weekly	564 (58.5%)	33 (11.2%)
At least once weekly	567 (58.8%)	555 (71.7%)	At least 3 days weekly	400 (41.5%)	261 (88.8%)
Maintenance insomnia	*746 (77.4%)*	*694 (89.7%)*			
Nightmares	*435 (45.1%)*	*409 (52.8%)*			
Daytime dysfunction			Daytime dysfunction		
Less than once weekly	646 (67.1%)	469 (60.6%)	Mood, energy, relationships		
At least once weekly	318 (32.9%)	305 (39.4%)	Moderate to severe	475 (49.3%)	262 (89.1%)
Use of sleeping medications (last month)			Concentration, productivity, vigilance		
No	695 (72.1%)	511 (66.1%)	Moderate to severe	377 (39.1%)	218 (74.1%)
At least once	269 (27.9%)	263 (33.9%)	General dysfunction		
			Moderate to severe	366 (37.9%)	222 (75.5%)

WASO wake time after sleep onset

Multivariate analysis

At multivariate analysis, including significant variables at univariate analysis, the pathological scores of the administered scales were significantly associated with the following variables (Table 5):

- Pathological PSQI scores were significantly associated with female gender ($p < 0.001$), self-isolation ($p < 0.001$), modified duties during COVID-19 outbreak ($p = 0.017$), and suffering from chronic diseases ($p = 0.028$).
- Pathological SCI scores were significantly associated with female gender ($p = 0.004$), divorced marital status ($p = 0.015$), self-isolation ($p = 0.037$), and suffering from chronic diseases ($p = 0.003$).
- Pathological STAI-Y scores were significantly associated with teleworking ($p = 0.001$).
- Pathological BDI-II scores were significantly associated with self-isolation ($p = 0.028$), modified work schedules ($p = 0.03$), and suffering from chronic diseases ($p = 0.027$).

Discussion

In this questionnaire-based cross-sectional multicenter study, we evaluated the prevalence and potential predictive factors of sleep disorders, mainly insomnia, and mental health disturbances—i.e., anxiety and depression—among a wide population of hospital workers in Milan, during the first wave of the COVID-19 pandemic in 2020. Several studies and reviews have already highlighted multiple psychological effects of COVID-19 outbreak on the mental well-being of health personnel, including depression and anxiety but also sleep problems, burnout, and posttraumatic stress disorders [2]. In 2020, some authors stated the need to collect high-quality data on the mental health effects of the COVID-19 pandemic across the whole population and vulnerable groups [16]. In line with this call, we decided to investigate sleep and psychiatric disorders in the entire population of hospital workers,

Table 3 Univariate analysis of Pittsburgh Sleep Quality Index (PSQI) and Sleep Condition Indicator (SCI) scores

	PSQI > 5	PSQI ≤ 5	*p* value*	SCI ≤ 16	SCI > 16	*p* value*
Overall	**774 (80.3%)**	**190 (19.7%)**		**294 (30.5%)**	**670 (69.5%)**	
Gender			**< 0.001**			**0.001**
Female	639 (83.3%)	128 (16.7%)		253 (33.0%)	514 (67.0%)	
Male	135 (68.5%)	62 (31.5%)		41 (20.8%)	156 (79.2%)	
Age (years)			0.14			**0.023**
21–30	89 (79.5%)	23 (20.5%)		28 (25.0%)	84 (75.0%)	
31–40	147 (76.2%)	46 (23.8%)		48 (24.9%)	145 (75.1%)	
41–50	201 (80.4%)	49 (19.6%)		81 (32.4%)	169 (67.6%)	
51–60	282 (84.2%)	53 (15.8%)		120 (35.8%)	215 (64.2%)	
> 61	55 (74.3%)	19 (25.7%)		17 (23.0%)	57 (77.0%)	
Chronic diseases	236 (85.2%)	41 (14.8%)	**0.015**	107 (38.6%)	170 (61.4%)	**< 0.001**
Marital status			0.47			**0.004**
Unmarried	264 (78.8%)	71 (21.2%)		85 (25.4%)	250 (75.6%)	
Married	414 (80.9%)	98 (19.1%)		160 (31.2%)	352 (68.8%)	
Divorced	88 (80.7%)	21 (19.3%)		44 (40.4%)	65 (59.6%)	
Widowed	8 (100%)	0 (0.0%)		5 (62.5%)	3 (37.5%)	
Living with someone at home	650 (80.6%)	157 (19.4%)	0.65	245 (30.4%)	562 (69.6%)	0.83
COVID-19 infection	82 (87.2%)	12 (12.8)	0.08	29 (30.9%)	65 (69.1%)	0.94
Flatmates with COVID-19 infection	101 (78.3%)	28 (21.7%)	0.54	40 (31.0%)	89 (69.0%)	0.89
Self-isolation	281 (90.9%)	28 (9.1%)	**< 0.001**	112 (36.2%)	197 (63.8%)	**0.008**
Hospital			**0.004**			0.54
COVID-19 wards	618 (81.6%)	139 (18.4%)		234 (30.9%)	523 (69.1%)	
No COVID-19 wards	126 (72.0%)	49 (28.0%)		50 (28.6%)	125 (71.4%)	
Occupation			0.46			0.86
Frontline healthcare workers	624 (81.0%)	146 (19.0%)		236 (30.7%)	534 (69.3%)	
Non-frontline healthcare workers	70 (76.1%)	22 (23.9%)		26 (28.3%)	66 (71.7%)	
Non-healthcare workers	79 (78.2%)	22 (21.8%)		32 (31.7%)	69 (68.3%)	
Site of work			0.79			0.16
Ward (clinical or surgical)	462 (81.3%)	106 (18.7%)		159 (28.0%)	409 (72.0%)	
Outpatient's department	160 (78.8%)	43 (21.2%)		74 (36.4%)	129 (63.6%)	
Laboratory	63 (78.8%)	17 (21.2%)		25 (31.2%)	55 (68.8%)	
Offices	87 (78.4%)	24 (21.6%)		35 (31.5%)	76 (68.5%)	
Shift workers	371 (84.5%)	68 (15.5%)	**0.003**	139 (31.7%)	300 (68.3%)	0.47
Workers in COVID-19 ward	238 (84.1%)	45 (15.9%)	**< 0.001**	81 (28.6%)	202 (71.4%)	0.14
Modified work schedules	341 (83.4%)	68 (16.6%)	**0.039**	127 (31.1%)	282 (68.9%)	0.75
Increased hours at work	235 (84.2%)	44 (15.8%)	0.05	96 (34.4%)	183 (65.6%)	0.09
Modified duties at work	279 (85.1%)	49 (14.9%)	**0.007**	102 (31.1%)	226 (68.9%)	0.77
Teleworking	100 (73.5%)	36 (26.5%)	**0.032**	39 (28.7%)	97 (71.3%)	0.62
Sleep disorders before the COVID-19 pandemic	274 (97.9%)	6 (2.1%)	**< 0.001**	150 (53.6%)	130 (46.4%)	**< 0.001**
Sleep disorders during the COVID-19 pandemic						
With drug use	175 (99.4%)	1 (0.6%)	**< 0.001**	112 (63.6%)	64 (36.4%)	**< 0.001**
Without drug use	488 (92.4%)	40 (7.6%)		166 (31.4%)	362 (68.6%)	
New-onset sleep disorders	412 (91.4%)	39 (8.6%)	**< 0.001**	134 (29.7%)	317 (70.3%)	**< 0.001**
Pre-existing sleep disorders	251 (99.2%)	2 (0.8%)		144 (56.9%)	109 (43.1%)	
Depression (BDI-II)	307 (97.1%)	9 (2.9%)	**< 0.001**	187 (59.2%)	129 (40.8%)	**< 0.001**
Anxiety (STAI-Y)	552 (77.7%)	150 (22.3%)	**0.002**	200 (29.8%)	472 (70.2%)	0.45

**p*-values from chi-square tests

BDI Beck Depression Inventory, *STAI* State Trait Anxiety Inventory

Table 4 Univariate analysis of State Trait Anxiety Inventory (STAI-Y) and Beck Depression Inventory (BDI-II) scores

	STAI > 40	STAI ≤ 40	*p* value*	BDI > 13	BDI ≤ 13	*p* value*
Overall	**672 (69.7%)**	**292 (30.3%)**		**316 (32.8%)**	**648 (67.2%)**	
Gender			0.13			0.19
Female	526 (68.6%)	241 (31.4%)		259 (33.8%)	508 (66.2%)	
Male	146 (74.1%)	51 (25.9%)		57 (28.9%)	140 (71.1%)	
Age (years)			**0.04**			**0.016**
21–30	79 (70.5%)	33 (29.5%)		32 (28.6%)	80 (71.4%)	
31–40	142 (73.6%)	51 (26.4%)		54 (28.0%)	139 (72.0%)	
41–50	187 (74.8%)	63 (25.2%)		82 (32.8%)	168 (67.2%)	
51–60	214 (63.9%)	121 (36.1%)		131 (39.1%)	204 (60.9%)	
> 61	50 (67.6%)	24 (32.4%)		17 (23.0%)	57 (77.0%)	
Chronic diseases	188 (67.9%)	89 (32.1%)	0.43	109 (39.3%)	168 (60.7%)	**0.006**
Marital status			0.15			0.20
Unmarried	239 (71.3%)	96 (28.7%)		109 (32.5%)	226 (67.5%)	
Married	362 (70.7%)	150 (29.3%)		159 (31.1%)	353 (68.9%)	
Divorced	66 (60.5%)	43 (39.5%)		44 (40.4%)	65 (59.6%)	
Widowed	5 (62.5%)	3 (37.5%)		4 (50.0%)	4 (50.0%)	
Living with someone at home	564 (69.9%)	243 (30.1%)	0.78	256 (31.7%)	551 (68.3%)	0.11
COVID-19 infection	66 (70.2%)	28 (29.8%)	0.91	32 (34.0%)	62 (66.0%)	0.78
Flatmates with COVID-19 infection	96 (74.4%)	33 (25.6%)	0.21	48 (37.2%)	81 (62.8%)	0.25
Self-isolation	202 (65.4%)	107 (34.6%)	**0.044**	122 (39.5%)	187 (60.5%)	**0.002**
Hospital			0.38			0.70
COVID-19 wards	524 (69.2%)	233 (30.8%)		245 (32.4%)	512 (67.6%)	
No COVID-19 wards	127 (72.6%)	48 (27.4%)		54 (30.9%)	121 (69.1%)	
Occupation			0.25			0.62
Frontline healthcare workers	530 (68.8%)	240 (31.2%)		251 (32.6%)	519 (67.4%)	
Non-frontline healthcare workers	71 (77.2%)	21 (22.8%)		34 (37.0%)	58 (63.0%)	
Non-healthcare workers	71 (70.3%)	30 (29.7%)		31 (30.7%)	70 (69.3%)	
Site of work			0.57			0.10
Ward (clinical or surgical)	397 (69.9%)	171 (30.1%)		170 (29.9%)	398 (70.1%)	
Outpatient's department	139 (68.5%)	64 (31.5%)		74 (36.4%)	129 (63.6%)	
Laboratory	61 (76.3%)	19 (23.7%)		33 (41.2%)	47 (58.8%)	
Offices	75 (67.6%)	36 (32.4%)		38 (34.2%)	73 (65.8%)	
Shift workers	295 (67.2%)	144 (32.8%)	0.12	153 (34.8%)	286 (65.2%)	0.21
Workers in COVID-19 ward	198 (69.9%)	85 (30.1%)	0.81	91 (32.2%)	192 (67.8%)	0.25
Modified work schedules	287 (70.2%)	122 (29.8%)	0.78	150 (36.7%)	259 (63.3%)	**0.027**
Increased hours at work	189 (67.7%)	90 (32.3%)	0.39	102 (36.6%)	177 (63.4%)	0.11
Modified duties at work	235 (71.7%)	93 (28.3%)	0.34	119 (36.3%)	209 (63.7%)	0.10
Teleworking	109 (80.2%)	27 (19.8%)	**0.004**	37 (27.2%)	99 (72.8%)	0.13
Sleep disorders before the COVID-19 pandemic	194 (69.3%)	86 (30.7%)	0.85	128 (45.7%)	152 (54.3%)	**< 0.001**
Sleep disorders during the COVID-19 pandemic						
With drug use	120 (68.2%)	56 (31.8%)	0.46	98 (55.7%)	78 (44.3%)	**< 0.001**
Without drug use	363 (68.8%)	165 (31.2%)		187 (35.4%)	341 (64.6%)	
New-onset sleep disorders	308 (68.3%)	143 (31.7%)	0.81	165 (36.6%)	286 (63.4%)	**0.005**
Pre-existing sleep disorders	175 (69.2%)	78 (30.8%)		120 (47.4%)	133 (52.6%)	

*p-values from chi-square tests

including frontline and non-frontline healthcare providers but also non-healthcare workers. Moreover, we enrolled workers of hospitals both with and without COVID-19 wards. This study design allowed us to differentiate sleep and mental health disturbances directly associated with pandemic-related healthcare issues from those that were more extensively related to a sudden work and lifestyle transformation in hospital staff.

Table 5 Multivariate analysis including significant variables at univariate analysis.*

Variables	OR	95% CI	*p* value
PSQI > 5 vs ≤ 5			
Gender (F vs M)	2.42	1.66–3.55	< 0.001
Self-isolation (yes vs no)	2.71	1.73–4.25	< 0.001
Duties at work (modified vs unchanged)	1.61	1.09–2.39	0.017
Chronic diseases (yes vs no)	1.58	1.05–2.38	0.028
SCI ≤ 16 vs > 16			
Gender (F vs M)	1.76	1.19–2.60	0.004
Marital status (divorced vs unmarried)	1.93	1.14–3.27	0.015
Self-isolation (yes vs no)	1.39	1.02–1.89	0.037
Chronic diseases (yes vs no)	1.62	1.18–2.22	0.003
STAI > 40 vs ≤ 40			
Teleworking (yes vs no)	2.29	1.39–3.79	0.001
BDI > 13 vs ≤ 13			
Self-isolation (yes vs no)	1.40	1.04–1.91	0.028
Modified work schedules (yes vs no)	1.40	1.03–1.90	0.03
Chronic diseases (yes vs no)	1.42	1.04–1.95	0.027

BDI Beck Depression Inventory, *CI* confidence interval, *OR* odds ratio, *PSQI* Pittsburgh Sleep Quality Index, *SCI* Sleep Condition Indicator, *STAI* State Trait Anxiety Inventory

*Only significant variables at multivariate analysis are reported

In line with the previous studies, in our population, we documented alarming rates of sleep disorders (80.3%), mainly insomnia (30.5%), and anxiety (69.7%), but also a high prevalence of depression (32.8%). In terms of sleep quality, 29.1% of subjects reported having sleep disturbances prior to the COVID-19 pandemic. Out of the total 73.0% who complained of sleep disturbances during the outbreak, 64.1% had a new onset, and 18.3% needed to take medication.

According to PSQI and SCI scores, sleep problems and insomnia were present in 80.3% and 30.5%, respectively. Similarly, previous studies conducted in healthcare workers during the COVID-19 pandemic reported a prevalence of insomnia of 27–41%[2] and of sleep disorders (according to PSQI) of 61.6–71.7% [17, 18]. Moreover, our data confirm that—independently from the pandemic—health professionals seem to be more prone to poor quality of sleep, probably because of the general stressors they are generally exposed to [4]. However, the prevalence of their sleep complaints highly increased during the COVID-19 outbreak.

Prolonged sleep latency but mainly reduced sleep duration and continuity were reported by most participants in our study. Furthermore, many of them complained of sleep quality and sleep efficiency reduction and expressed concerns about daytime dysfunction and waking performance. Interestingly, many subjects reported nightmares, thus suggesting a relationship between daily mental activity during the pandemic and sleep disturbances [19].

The multivariate analysis showed a strong association of the presence of sleep disorders, especially insomnia, with female gender, divorced marital status, self-isolation, modified duties during the COVID-19 outbreak, and chronic diseases. Other studies during the COVID-19 pandemic found that female health workers had worse sleep quality than male ones [17, 20]. Previous epidemiological studies revealed that female sex represents a risk factor for insomnia, with a hazard ratio of 1.41 for women versus men regarding insomnia [21]. One of the first survey conducted in China among medical staff involved in the COVID-19 outbreak found that factors related to insomnia included perceived lack of psychological support, having high levels of uncertainty and being worried about infection [6]. Analogously, Xiao et al. found that social support for medical staff correlated significantly with both self-efficacy and quality of sleep [22]. In accordance with these findings, we suggest that in our population, divorced and isolated people were at higher risk of insomnia in our sample due to a lack of social or family support systems to stabilize emotions, share experiences, and maintain social connections. A possible explanation of a higher prevalence of insomnia in subjects with chronic diseases might be related to their worries about being affected by COVID-19. Indeed, since the beginning of the pandemic, it was evident that patients with comorbidities had an increased risk to develop a more severe disease. Thus, the concern about personal health may impact sleep quality induced by anxiety symptoms [23]. Finally, especially during the first peak of the COVID-19 outbreak, many healthcare workers were asked to change their duties, and after a brief training, they were included into the frontline battle against COVID-19. Moreover, frequent policy changes and unclear case management standards induced an increased level of stress and anxiety, favoring the onset or the worsening of sleep disorders.

Other than sleep disorders, as stated above, our results provide evidence that a high proportion of hospital workers also experienced significant levels of anxiety (69.7%) and depression (32.8%) during the COVID-19 pandemic. These data are in line with recent literature results although the different scales and population selection criteria adopted by each study caused great heterogeneity of anxiety and depression prevalence [2, 24]. In this study, depressive symptoms were significantly associated with self-isolation, modified work schedules, and chronic diseases, while anxiety showed a strong association with teleworking. We already discussed how isolation and chronic diseases could favor psychological burden in hospital workers because of lack of psychological support and greater concerns about the risk of infection. Moreover, during the COVID-19 outbreak, healthcare workers were frequently asked to change their work schedules and underwent an overwhelming workload pressure. Previous studies showed that work overload represents a critical cause

of exhaustion, which led health professionals to burnout and which in turn may affect hospital outcomes such as the quality and safety of provided care [25].

Furthermore, in our study, we found that sleep disorders, insomnia, and depression during the pandemic were more prevalent among subjects who already suffered from sleep disorders before the COVID-19 outbreak. The bidirectional link between sleep problems and mental health disturbances is well known, and insomnia has recently been identified as an independent clinical predictor of depression and suicide risk [26, 27].

Interestingly, we found that the participants who had been diagnosed with COVID-19 or who worked in a COVID-19 ward or in a COVID-19 hospital did not show a higher prevalence of sleep disorders, nor anxiety or depression. The same lack of association was encountered for the type of occupation because frontline healthcare workers were not more affected than non-frontline healthcare workers or non-healthcare workers. Data from the literature on this topic are discordant. Indeed, most studies found that being on the frontline represented a risk factor for developing the mental health concerns and that medical staff had greater fear, anxiety, and depression levels than administrative staff or general population [24]. However, a minority of studies revealed opposite results, as we found in our population. In particular, Li et al. found that frontline nurses had lower levels of trauma than both the general public and non-frontline nurses, hypothesizing that nurses have better training to face up to emergency [28]. Similarly, Tan BYQ et al. showed that non-medical workers had greater anxiety and stress compared to medical workers [29]. Therefore, according to our data, we can hypothesize that non-healthcare hospital workers faced the same general stressors as frontline and non-frontline health professionals during the pandemic. We can exclude that the presence of a previous sleep disorder contributed to level responses to the outbreak across the three groups, considering that only a minority reported prior sleep disturbances and that most were of new onset. The high rate of transmission of SARS-CoV-2 infection and the need for a thorough and fast reorganization of most hospital activities might have triggered similar levels of stress for both administrative and healthcare hospital staff. It should be also considered that the latter, especially if on the frontline of COVID-19 management, had easier availability of protective equipment, thus reducing the feelings of danger and exposure to unsafe conditions.

Another finding of our study is that teleworking was significantly associated with higher anxiety scores at STAI-Y. Although this has been previously reported [30], it remains a matter of debate given the mounting evidence of healthier quality of life and emotional state among people working remotely from home [31]. Of note, responders on teleworking did not report a higher percentage of sleep disorder complaints nor of depression. Teleworkers reported higher levels of anxiety, stress, and fatigue compared to their office-based counterpart, even prior to the pandemic, which is perhaps associated with technological overload and pervasive use of online surveillance and monitoring of work. Considering the increased popularity and prospective intensification of this work modality, not only among hospital workers, the adequate prevention of its possible risks is mandatory. Despite promoting flexible work scheduling, teleworking may indeed enhance affective distress due to an increase in the amount of hours spent working online at home as compared to working at the office and due to the limitation of interpersonal relationships with colleagues [30].

Therefore, clinicians and health authorities should be alert to the possibility of hospital staff developing sleep and mental health disorders while facing the COVID-19 pandemic and even after the long-awaited conclusion of this outbreak. The findings of this study should direct future research to establish the role of psychological distress during emergency conditions, such as during a pandemic, in the development of sleep and mental disorders so that preventive and clinical management strategies can be developed. Prospective cohort studies and longer-term follow-up studies are needed to support and extend the findings of our study. Furthermore, enhanced psychiatric follow-up and sleep disorder screening should be considered for hospital staff (both frontline/non-frontline healthcare and non-healthcare personnel) who has worked, and still works, during the COVID-19 pandemic.

This study has some limitations. Firstly, its cross-sectional design did not allow us to compare the pre-existing mental health status of our subjects with their psychological profiles during the outbreak and to investigate any possible long-term effects of pandemic, such as posttraumatic stress disorder. Moreover, an online survey is inherently prone to self-report bias of sociodemographic and other information; the lack of objective sleep measures, such as actigraphy or polysomnography, and clinical interviewing reduces the accuracy of sleep quality and mental health complaints.

The higher number of female participants as compared to males could have determined a higher prevalence of sleep disorders among the population of responders. On the other hand, the responders suffering from sleep disturbances, both pre-existing or of new onset during the pandemic, could have been more prone to participate and fill in the questionnaire.

In conclusion, in this large and heterogeneous population study on hospital workers, we confirm a high prevalence of sleep and psychiatric symptoms during the COVID-19 outbreak. The conditions more strongly associated with insomnia or mood disorders were the ones that denoted a significant modification of personal and work habits. These results suggest interesting hypotheses on the pathophysiology of sleep and mood disorders and their impact on work productivity in emergency conditions such as a pandemic. Moreover,

improved knowledge on the relevance of diagnosing and treating sleep disturbances to adequately manage the mental health of hospital workers, as well as the need to accurately plan healthcare activities ahead of emergency conditions, clearly emerges from our findings. For this reason, different individual or organizational strategies have been provided by researchers [24]. Psychological support for all hospital workers should empower their emotional resilience, reduce detrimental long-term effects on mental health, and increase their work efficiency. Furthermore, the findings of this study suggest that even independently from a pandemic, sleep and related mood disturbances can arise after an unplanned and incautious modification of work duties and schedules. A timely identification and effective treatment of sleep disorders can reduce the risk of mood disorders, especially when facing an emergency, enabling the actions needed and enhancing the outcomes. Finally, management of sleep disorders (such as through cognitive-behavioral strategies) [32] should be recommended considering the impact of sleep not only on well-being and mental health but also on the waking performance and on the immunological system [8].

Acknowledgements We acknowledge all the hospital workers who participated in the study.

Author contribution Paola Proserpio, Giuseppe Didato, Elena Zambrelli, and Andrea Lanza: Conceptualization, data curation, methodology, and writing (original draft, review, and editing). Irene Tramacere: Methodology, statistical analysis, and writing (original draft, review, and editing). Annalisa Rubino and Federica Cattaneo: Data curation. Ambra Dominese, Roberta Di Giacomo, Rui Quintas, Katherine Turner, Claudio Colosio, Maira Paola Canevini, Armando D'Agostino, and Elio Clemente Agostoni: Writing (review and editing). Paola Proserpio, Elena Zambrelli, and Giuseppe Didato: Supervision and validation. The first draft of the manuscript was written by Paola Proserpio, Giuseppe Didato, Elena Zambrelli, and Andrea Lanza, and all coauthors commented on previous versions of the manuscript. All authors read and approved the final manuscript.

Data availability Any data and material associated with this article will be made available by reasonable request to the corresponding author.

Declarations

Ethics approval This study was conducted in accordance with current national privacy legislation and was approved by the ethics committee of the hospitals involved.

Informed consent Informed consent was obtained from all individual participants prior to the online questionnaire.

Conflict of interest The authors declare no competing interests.

References

1. Huang C, Wang Y, Li X et al (2020) Clinical features of patients infected with 2019 novel coronavirus in Wuhan, China. Lancet Lond Engl 395:497–506. https://doi.org/10.1016/S0140-6736(20)30183-5
2. Pappa S, Ntella V, Giannakas T et al (2020) Prevalence of depression, anxiety, and insomnia among healthcare workers during the COVID-19 pandemic: a systematic review and meta-analysis. Brain Behav Immun 88:901–907. https://doi.org/10.1016/j.bbi.2020.05.026
3. Morin CM, Rodrigue S, Ivers H (2003) Role of stress, arousal, and coping skills in primary insomnia. Psychosom Med 65:259–267. https://doi.org/10.1097/01.psy.0000030391.09558.a3
4. Zeng L-N, Yang Y, Wang C et al (2020) Prevalence of poor sleep quality in nursing staff: a meta-analysis of observational studies. Behav Sleep Med 18:746–759. https://doi.org/10.1080/15402002.2019.1677233
5. Brooks SK, Dunn R, Amlôt R et al (2018) A systematic, thematic review of social and occupational factors associated with psychological outcomes in healthcare employees during an infectious disease outbreak. J Occup Environ Med 60:248–257. https://doi.org/10.1097/JOM.0000000000001235
6. Zhang C, Yang L, Liu S et al (2020) Survey of insomnia and related social psychological factors among medical staff involved in the 2019 novel coronavirus disease outbreak. Front Psychiatry 11. https://doi.org/10.3389/fpsyt.2020.00306
7. Wang S, Xie L, Xu Y et al (2020) Sleep disturbances among medical workers during the outbreak of COVID-2019. Occup Med. https://doi.org/10.1093/occmed/kqaa074
8. Irwin MR (2015) Why sleep is important for health: a psychoneuroimmunology perspective. Annu Rev Psychol 66:143–172. https://doi.org/10.1146/annurev-psych-010213-115205
9. Altena E, Baglioni C, Espie CA et al (2020) Dealing with sleep problems during home confinement due to the COVID-19 outbreak: practical recommendations from a task force of the European CBT-I Academy. J Sleep Res 29:e13052. https://doi.org/10.1111/jsr.13052
10. Buysse DJ, Reynolds CF, Monk TH et al (1989) The Pittsburgh Sleep Quality Index: a new instrument for psychiatric practice and research. Psychiatry Res 28:193–213. https://doi.org/10.1016/0165-1781(89)90047-4
11. Curcio G, Tempesta D, Scarlata S et al (2013) Validity of the Italian version of the Pittsburgh Sleep Quality Index (PSQI). Neurol Sci Off J Ital Neurol Soc Ital Soc Clin Neurophysiol 34:511–519. https://doi.org/10.1007/s10072-012-1085-y
12. Espie CA, Kyle SD, Hames P et al (2014) The Sleep Condition Indicator: a clinical screening tool to evaluate insomnia disorder. BMJ Open 4:e004183. https://doi.org/10.1136/bmjopen-2013-004183
13. Palagini L, Ragno G, Caccavale L et al (2015) Italian validation of the Sleep Condition Indicator: a clinical screening tool to evaluate insomnia disorder according to DSM-5 criteria. Int J Psychophysiol Off J Int Organ Psychophysiol 98:435–440. https://doi.org/10.1016/j.ijpsycho.2015.08.008
14. Spielberger CD, Gorsuch RL, lushene RE, (1983) Manual for the state-trait anxiety inventory (Form Y). Consulting Psychologists Press Inc, Palo Alto, CA
15. Beck AT, Ward CH, Mendelson M et al (1961) An inventory for measuring depression. Arch Gen Psychiatry 4:561–571. https://doi.org/10.1001/archpsyc.1961.01710120031004
16. Holmes EA, O'Connor RC, Perry VH et al (2020) Multidisciplinary research priorities for the COVID-19 pandemic: a call for action for mental health science. Lancet Psychiatry 7:547–560. https://doi.org/10.1016/S2215-0366(20)30168-1
17. Qi J, Xu J, Li B-Z, et al (2020) The evaluation of sleep disturbances for Chinese frontline medical workers under the outbreak of COVID-19. Sleep Med 5

18. Wang W (2020) Sleep disturbance and psychological profiles of medical staff and non-medical staff during the early outbreak of COVID-19 in Hubei Province. China Front Psychiatry 11:8
19. Gorgoni M, Scarpelli S, Alfonsi V et al (2021) Pandemic dreams: quantitative and qualitative features of the oneiric activity during the lockdown due to COVID-19 in Italy. Sleep Med 81:20–32. https://doi.org/10.1016/j.sleep.2021.02.006
20. Zhang W, Wang K, Yin L, et al Mental health and psychosocial problems of medical health workers during the COVID-19 epidemic in China. Psychother Psychosom 9
21. Zhang B, Wing Y-K (2006) Sex differences in insomnia: a meta-analysis. Sleep 29:85–93
22. Xiao H, Zhang Y, Kong D et al (2020) The effects of social support on sleep quality of medical staff treating patients with coronavirus disease 2019 (COVID-19) in January and February 2020 in China. Med Sci Monit Int Med J Exp Clin Res 26:e923549–e923551
23. Kirwan M, Pickett SM, Jarrett NL (2017) Emotion regulation as a moderator between anxiety symptoms and insomnia symptom severity. Psychiatry Res 254:40–47. https://doi.org/10.1016/j.psychres.2017.04.028
24. Shreffler J, Petrey J, Huecker M (2020) The impact of COVID-19 on healthcare worker wellness: a scoping review. West J Emerg Med 21:8
25. Portoghese I, Galletta M, Coppola RC et al (2014) Burnout and workload among health care workers: the moderating role of job control. Saf Health Work 5:152–157. https://doi.org/10.1016/j.shaw.2014.05.004
26. Baglioni C, Battagliese G, Feige B et al (2011) Insomnia as a predictor of depression: a meta-analytic evaluation of longitudinal epidemiological studies. J Affect Disord 135:10–19. https://doi.org/10.1016/j.jad.2011.01.011
27. Pigeon WR, Pinquart M, Conner K (2012) Meta-analysis of sleep disturbance and suicidal thoughts and behaviors. J Clin Psychiatry 73:e1160-1167. https://doi.org/10.4088/JCP.11r07586
28. Li Z, Ge J, Yang M et al (2020) Vicarious traumatization in the general public, members, and non-members of medical teams aiding in COVID-19 control. Brain Behav Immun 88:916–919. https://doi.org/10.1016/j.bbi.2020.03.007
29. Tan BYQ, Chew NWS, Lee GKH et al (2020) Psychological impact of the COVID-19 pandemic on health care workers in Singapore. Ann Intern Med 173:317–320. https://doi.org/10.7326/M20-1083
30. Morilla-Luchena A, Muñoz-Moreno R, Chaves-Montero A, Vázquez-Aguado O (2021) Telework and social services in Spain during the COVID-19 pandemic. Int J Environ Res Public Health 18. https://doi.org/10.3390/ijerph18020725
31. Hallman DM, Januario LB, Mathiassen SE et al (2021) Working from home during the COVID-19 outbreak in Sweden: effects on 24-h time-use in office workers. BMC Public Health 21:528. https://doi.org/10.1186/s12889-021-10582-6
32. Crew EC, Baron KG, Grandner MA et al (2020) The Society of Behavioral Sleep Medicine (SBSM) COVID-19 Task Force: objectives and summary recommendations for managing sleep during a pandemic. Behav Sleep Med 18:570–572. https://doi.org/10.1080/15402002.2020.1776288

Publisher's Note Springer Nature remains neutral with regard to jurisdictional claims in published maps and institutional affiliations.
