# Supplementary material for: Mental health status and related factors influencing healthcare workers during the COVID-19 pandemic: A systematic review and meta-analysis
Source: PLoS One. 2024 Jan 19;19(1):e0289454. doi: 10.1371/journal.pone.0289454 (PMC10798549; doi:10.1371/journal.pone.0289454)
Supplement: S1 Data — (ZIP) [file pone.0289454.s011.zip › literatures/14.pdf]

Internal Medicine
The Japanese Society of Internal Medicine
doi: 10.2169/internalmedicine.5694-20
Intern Med 59: 2693-2699, 2020
http://internmed.jp

【 ORIGINAL ARTICLE 】

Anxiety, Depression, and Resilience of Healthcare Workers in Japan During the Coronavirus Disease 2019 Outbreak

Nobuyasu Awano[1], Nene Oyama[2], Keiko Akiyama[2], Minoru Inomata[1], Naoyuki Kuse[1], Mari Tone[1], Kohei Takada[1], Yutaka Muto[1], Kazushi Fujimoto[1], Yu Akagi[3], Momoko Mawatari[3], Akihiro Ueda[3], Junko Kawakami[4], Junko Komatsu[5] and Takehiro Izumo[1]

Abstract:
Objective Coronavirus disease 2019 (COVID-19) is spreading around the world. The aim of this study was to assess the degree of anxiety, depression, resilience, and other psychiatric symptoms among healthcare workers in Japan during the COVID-19 pandemic.
Methods This survey involved medical healthcare workers at the Japanese Red Cross Medical Center (Tokyo, Japan) between April 22 and May 15, 2020. The degree of symptoms of anxiety, depression, and resilience was assessed using the Japanese versions of the 7-item Generalized Anxiety Disorder Scale (GAD-7), Center for Epidemiologic Studies Depression Scale (CES-D), and 10-item Connor-Davidson Resilience Scale. Furthermore, we added original questionnaires comprising three factors: (i) anxiety and fear of infection and death; (ii) isolation and unreasonable treatment; and (iii) motivation and escape behavior at work.
Results In total, 848 healthcare workers participated in this survey: 104 doctors, 461 nurses, 184 other co-medical staff, and 99 office workers. Among all participants, 85 (10.0%) developed moderate-to-severe anxiety disorder, and 237 (27.9%) developed depression. Problems with anxiety and fear of infection and death, isolation and unreasonable treatment, and motivation and escape from work were higher in the depression group than in the non-depression group (total CES-D score ≥ 16 points). Being a nurse and high total GAD-7 scores were risk factors of depression. Older workers and those with higher resilience were less likely to develop depression than others.
Conclusion During the COVID-19 epidemic, many healthcare workers suffered from psychiatric symptoms. Psychological support and interventions for protecting the mental health of them are needed.

Key words: anxiety, coronavirus disease 2019, depression, healthcare worker, resilience

(Intern Med 59: 2693-2699, 2020)
(DOI: 10.2169/internalmedicine.5694-20)

Introduction

Coronavirus disease 2019 (COVID-19), which is an infectious disease caused by severe acute respiratory syndrome coronavirus 2, has spread worldwide (1). In Japan, there have been more than 16,000 infected patients and 800 deaths as of the end of May 2020 (2). The World Health Organization declared COVID-19 a pandemic on January 30, 2020, and the Japanese government declared a state of emergency on April 7, 2020 (2). Despite the implementation of several preventive measures and countermeasures in countries worldwide, the pandemic has yet to be controlled.

Since February 2020, our hospital, which is located in central Tokyo, has allocated dozens of beds in its general ward and intensive-care unit for the treatment of patients with COVID-19. We treated a total of 54 patients with COVID-19 between February 1, 2020 and May 15, 2020. As

[1]Department of Respiratory Medicine, Japanese Red Cross Medical Center, Japan, [2]Department of Mental Health, Japanese Red Cross Medical Center, Japan, [3]Department of Infectious Disease, Japanese Red Cross Medical Center, Japan, [4]Department of Nursing, Japanese Red Cross Medical Center, Japan and [5]Health Care Center, Japanese Red Cross Medical Center, Japan
Received for publication June 24, 2020; Accepted for publication August 5, 2020
Correspondence to Dr. Nobuyasu Awano, awanobu0606@hotmail.co.jp

the number of infected patients increases, the physical and mental fatigue of healthcare workers also rapidly increases, possibly resulting in fear regarding infection of themselves and their families, distress over losing their patients and colleagues, anxiety due to the lack of personal protective equipment (PPE), and depression due to the persistence of the current situation. It has been established that being isolated, working in high-risk positions, and coming in contact with infected individuals causes psychiatric symptoms (3). A previous report demonstrated that many healthcare workers had suffered from anxiety, depression, and isolation during the severe acute respiratory syndrome (SARS) epidemic (4). In addition, approximately 10% of healthcare workers had experienced high levels of post-traumatic stress disorder (PTSD) after the SARS outbreak (5).

Generalized anxiety disorder is one of the most common anxiety disorders in adults. The 7-item Generalized Anxiety Disorder Scale (GAD-7), comprising seven questions, is commonly used to assess anxiety symptoms (6). The Center for Epidemiologic Studies Depression Scale (CES-D) is an established, simple, and self-reporting tool that has been used to evaluate depressive symptoms (7). These scales are reliable and available to the Japanese population (8, 9). Resilience is the concept of a dynamic system to withstand or recover from serious challenges that threaten stability, viability, or development (10). It is a multidimensional property that changes with circumstances, time, age, sex, origin, and living environment (11, 12). The 10-item Connor-Davidson Resilience Scale (CD-RISC 10) is frequently used to assess resilience (11). Higher scores on this scale are interpreted as higher resilience (11, 13). This scale has also been confirmed to be reliable and valid for general adults and university students in Japan (13).

There have been several reports overseas regarding the mental health of healthcare workers involved in the treatment of patients with COVID-19 (14, 15). However, to date, there has been no such study in Japan. Therefore, in this study, we conducted in-hospital questionnaires to investigate the actual state of anxiety, depression, resilience, and other psychiatric symptoms.

Materials and Methods

Participants

This survey was conducted between April 22 and May 15, 2020, and involved all healthcare workers employed at the Japanese Red Cross Medical Center. The total number of workers was 1964, which included 411 doctors, 1,024 nurses, 328 other co-medical staff (such as pharmacists, laboratory technologists, radiological technologists, nutritionists, physical therapists, clinical psychologists, medical engineers, public health nurses, nursing assistants, and medical social workers), and 201 office workers. Those who were directly engaged in the treatment of patients with COVID-19 were defined as frontline workers.

Questionnaire

The survey was anonymous and was conducted on the institute website using an electronic medical record. The contents of the questionnaire included occupation type; age; sex; presence or absence of direct engagement in the treatment for patients with COVID-19; and the Japanese versions of the GAD-7, CES-D, and CD-RISC 10. The GAD-7 measures the frequency with which the seven symptoms of anxiety occurred in the previous two weeks (range: 0-21). The CES-D comprises 20 items and assesses depression symptoms from the total score (range: 0-60). The total scores of these scales were interpreted as follows: GAD-7, no/minimal (0-4), mild (5-9), moderate (10-14), and severe (15-21) anxiety (6, 16); CES-D, normal (0-15) and depression (16-60) (7). The CD-RISC 10 evaluates resilience using 10 items (range: 0-40), and the cut-off value varies depending on the situation and population. The use of the CD-RISC 10 in this study was permitted by Jonathan R.T. Davidson. In addition, we added the original questionnaires in Japanese to examine three factors: (i) anxiety and fear of infection and death (10 items); (ii) isolation and unreasonable treatment (5 items); (iii) motivation and escape behavior at work (3 items) (Table 1). Each question had four possible answers: 0 (Not applicable at all); 1 (A little applicable); 2 (Almost applicable); and 3 (Applicable).

Statistical analyses

The results for continuous variables were shown as medians with interquartile ranges (IQRs). The chi-squared test was performed on categorical variables. The Mann-Whitney U test and Kruskal-Wallis test were applied to compare continuous variables between two or more groups. A Spearman's correlation analysis was used to evaluate the correlation of each scale. Participants were divided into two groups according to the previously reported total CES-D cut-off value (16 points), and the factors were compared. A multivariable logistic regression analysis was performed to determine the potential risk factors of the symptoms of depression (total CES-D score ≥ 16 points). The association between risk factors and outcomes was presented as odds ratios (ORs) and 95% confidence intervals (CIs).

All statistical analyses were performed using EZR (Saitama Medical Center, Jichi Medical University, Saitama, Japan), a graphical user interface for R (The R Foundation for Statistical Computing, Vienna, Austria). A two-tailed $p<0.05$ denoted statistically significant difference.

Ethics statement

This study was approved by the Ethics Committee for Clinical Studies, Japanese Red Cross Medical Center. Informed consent was provided by the participants in accordance with the principles of the Declaration of Helsinki.

Table 1. The Contents of the Original Questionnaire.

		Not applicable at all	A little applicable	Almost applicable	Applicable
Questions on anxiety and fear of infection and death (Total score: 30)					
1	I am worried that I will catch COVID-19	0	1	2	3
2	I am worried that I will die from COVID-19	0	1	2	3
3	I am worried that I will transfer COVID-19 to my family	0	1	2	3
4	I am worried that I will transfer COVID-19 to my colleagues	0	1	2	3
5	I am worried that my family will catch COVID-19	0	1	2	3
6	I am worried that my family will transfer COVID-19 to me	0	1	2	3
7	I am worried that my family will die from COVID-19	0	1	2	3
8	I am worried that my colleagues will catch COVID-19	0	1	2	3
9	I am worried that my colleagues will transfer COVID-19 to me	0	1	2	3
10	I am worried that my colleagues will die from COVID-19	0	1	2	3
Questions on isolation and unreasonable treatment (Total score: 15)					
1	I am isolated at home	0	1	2	3
2	I am isolated at the hospital	0	1	2	3
3	I have been treated unreasonably at work	0	1	2	3
4	My family is isolated from others	0	1	2	3
5	My family has been treated unreasonably by others	0	1	2	3
Questions on motivation and escape behavior at work (Total score: 9)					
1	I am afraid to go to work	0	1	2	3
2	I do not want to work	0	1	2	3
3	I want to take a break from work	0	1	2	3

COVID-19: coronavirus disease 2019

Table 2. Demographic Characteristics of the Participants.

	Overall	Doctors	Nurses	Other co-medical staff*	Office workers
Workers, n	1,964	411	1,024	328	201
Participants, n (%)	848 (43.2)	104 (25.3)	461 (45.0)	184 (56.1)	99 (49.3)
Age, median (IQR), years	37 (28–47)	43 (36–53)	35 (27–44)	37 (29–49)	43 (31–50)
Males, n	213	79	28	79	27
Frontline workers, n	232	39	151	36	6

Continuous variables are presented as the median with interquartile range (IQR).
*pharmacists, laboratory technologists, radiological technologists, nutritionists, physical therapists, clinical psychologists, medical engineers, public health nurses, nursing assistants, and medical social workers

Results

Demographic characteristics

The demographic characteristics are shown in Table 2. Among the 1,964 workers to whom the questionnaire was distributed, 848 (43.2%) participated in the survey: 104 doctors (response rate: 25.3%); 461 nurses (response rate: 45.0%); 184 other co-medical staff (response rate: 56.1%); and 99 office workers (response rate: 49.3%). The median age of all participants was 37 years old, and there were 213 men and 635 women. A total of 232 participants were frontline workers, and the majority of them were nurses (151 nurses).

Measurement scores

The answers to the questionnaires are described in Table 3. The median GAD-7 score for all participants was 4 (IQR: 1-7); 763 workers (90.0%) had no or mild anxiety, whereas 85 workers (10.0%) had moderate-to-severe anxiety. The total GAD-7 score was significantly higher in older workers than in younger workers (p=0.034). Likewise, frontline workers scored higher than non-frontline workers (p< 0.001).

The median CES-D score was 12 (IQR: 7-16), and 237 healthcare workers (27.9%) developed symptoms of depression. Nurses were most likely to develop depressive symptoms, with 34.9% of them having a total CES-D score ≥ 16 points. In addition, the total CES-D score was significantly higher among younger workers, women, and frontline workers than others.

The median CD-RISC 10 score was 22 (IQR: 18-27). There was no significant difference in the total CD-RISC 10 score according to occupation type, age, sex, or working position. The Spearman's correlation analysis revealed a posi-

Table 3. Mental Health Measurements in the Total Cohort and Subgroups.

	Occupation type					
	Overall (n=848)	Doctors (n=104)	Nurses (n=461)	Other co-medical staff* (n=184)	Office workers (n=99)	p value
Total GAD-7 score, median (IQR)	4 (1–7)	3 (2–5)	4 (2–7)	4 (2–6)	3 (1–6)	0.25
No/minimal and mild anxiety (0–9 points), n (%)	763 (90.0)	93 (89.4)	410 (88.9)	169 (91.8)	91 (91.9)	0.67
Moderate and severe anxiety (10–21 points), n (%)	85 (10.0)	11 (10.6)	51 (11.1)	15 (8.2)	8 (8.1)	
Total CES-D score, median (IQR)	12 (7–16)	8 (3–12)	13 (9–18)	10 (6–15)	12 (7–15)	<0.001
Normal (<16 points), n (%)	611 (72.1)	92 (88.5)	300 (65.1)	143 (77.7)	76 (76.8)	<0.001
Depression (≥16 points), n (%)	237 (27.9)	12 (11.5)	161 (34.9)	41 (22.3)	23 (23.2)	
Total CD-RISC 10 score, median (IQR)	22 (18–27)	28 (20–31)	23 (19–28)	19 (14–23)	18 (14–23)	0.11
Original questionnaires, median (IQR)						
Questions on anxiety and fear of infection and death	12 (8–19)	9 (6–19)	12 (8–19)	12 (9–21)	11 (8–20)	0.039
Questions on isolation and unreasonable treatment	0 (0–1)	0 (0–1)	0 (0–1)	0 (0–1)	0 (0–1)	0.14
Questions on motivation and escape behavior at work	2 (0–3)	0 (0–2)	2 (0–3)	2 (0–3)	1 (0–3)	<0.001

	Age			Sex		
	Younger workers (<38 years) (n=446)	Older workers (≥38 years) (n=402)	p value	Males (n=213)	Females (n=635)	p value
Total GAD-7 score, median (IQR)	3 (1–6)	4 (2–7)	0.034	3 (2–6)	4 (1–7)	0.31
No/minimal and mild anxiety (0–9 points), n (%)	401 (89.9)	362 (90.0)	1	192 (90.1)	571 (89.9)	1
Moderate and severe anxiety (10–21 points), n (%)	45 (10.1)	40 (10.0)		21 (9.9)	64 (10.1)	
Total CES-D score, median (IQR)	12 (8–18)	10 (6–15)	<0.001	10 (5–14)	12 (8–18)	<0.001
Normal (<16 points), n (%)	296 (66.4)	315 (78.4)	<0.001	171 (80.3)	440 (69.3)	0.002
Depression (≥16 points), n (%)	150 (33.6)	87 (21.6)		42 (19.7)	195 (30.7)	
Total CD-RISC 10 score, median (IQR)	20 (16–26)	23 (19–29)	0.091	23 (18–29)	21 (18–27)	0.19
Original questionnaires, median (IQR)						
Questions on anxiety and fear of infection and death	13 (9–21)	10 (8–17)	<0.001	11 (7–19)	12 (9–19)	0.10
Questions on isolation and unreasonable treatment	0 (0–1)	0 (0–1)	0.014	0 (0–1)	0 (0–1)	0.49
Questions on motivation and escape behavior at work	2 (0–3)	1 (0–3)	<0.001	1 (0–3)	2 (0–3)	<0.001

	Working position		
	Frontline workers (n=232)	Non-frontline workers (n=616)	p value
Total GAD-7 score, median (IQR)	4 (2–8)	3 (1–6)	<0.001
No/minimal and mild anxiety (0–9 points), n (%)	197 (84.9)	566 (91.9)	0.004
Moderate and severe anxiety (10–21 points), n (%)	35 (15.1)	50 (8.1)	
Total CES-D score, median (IQR)	12 (8–19)	11.5 (7–16)	0.029
Normal (<16 points), n (%)	155 (66.8)	456 (74.0)	0.040
Depression (≥16 points), n (%)	77 (33.2)	160 (26.0)	
Total CD-RISC 10 score, median (IQR)	18 (13–22)	23 (19–29)	0.061
Original questionnaires, median (IQR)			
Questions on anxiety and fear of infection and death	13 (8–19)	11 (8–19)	0.30
Questions on isolation and unreasonable treatment	0 (0–2)	0 (0–1)	0.002
Questions on motivation and escape behavior at work	2 (0–3)	1.5 (0–3)	0.18

Continuous variables are presented as the median with interquartile range (IQR).

*pharmacists, laboratory technologists, radiological technologists, nutritionists, physical therapists, clinical psychologists, medical engineers, public health nurses, nursing assistants, and medical social workers

GAD-7: 7-item Generalized Anxiety Disorder Scale, CES-D: Center for Epidemiologic Studies Depression Scale, CD-RISC 10: 10-item Connor–Davidson Resilience Scale

tive correlation between the total GAD-7 score and total CES-D score and a negative correlation between the total GAD-7 score and total CD-RISC10 score and between the total CES-D score and total CD-RISC10 score (r=0.52, p<

Table 4. Comparison of Demographic Characteristics and Mental Health Measurements at the Cutoff Point of the CED-D Scale.

	Healthy group Total CES-D score < 16 (n=611)	Depression group Total CES-D score ≥ 16 (n=237)	p value
Total GAD-7 score, median (IQR)	3 (1–5)	7 (5–10)	<0.001
No/minimal and mild anxiety (0–9 points), n (%)	588 (96.2)	175 (73.8)	<0.001
Moderate and severe anxiety (10–21 points), n (%)	23 (3.8)	62 (26.2)	
Total CD-RISC 10 score, median (IQR)	23 (19–29)	18 (13–22)	<0.001
Original questionnaires, median (IQR)			
Questions on anxiety and fear of infection and death	10 (8–17)	16 (10–23)	<0.001
Questions on isolation and unreasonable treatment	0 (0–0)	1 (0–3)	<0.001
Questions on motivation and escape behavior at work	1 (0–2)	3 (2–5)	<0.001

Continuous variables are presented as the median with interquartile ranges (IQR).
CES-D: Center for Epidemiologic Studies Depression Scale, GAD-7: 7-item Generalized Anxiety Disorder Scale, CD-RISC 10: 10-item Connor–Davidson Resilience Scale

Table 5. Risk Factors for Depression (Total CES-D Score ≥ 16 Points) Identified by Multivariable Logistic Regression Analysis.

	OR	95% CI	p value
Occupation type			
Doctors	Reference		
Nurses	3.40	1.39–8.30	0.007
Other co-medical staff*	2.05	0.83–5.07	0.12
Office workers	2.41	0.87–6.69	0.090
Age, years	0.95	0.93–0.97	<0.001
Females	1.25	0.72–2.17	0.42
Frontline workers	1.07	0.69–1.65	0.77
Total GAD-7 score	1.43	1.34–1.52	<0.001
Total CD-RISC 10 score	0.93	0.91–0.96	<0.001

*pharmacists, laboratory technologists, radiological technologists, nutritionists, physical therapists, clinical psychologists, medical engineers, public health nurses, nursing assistants, and medical social workers
CES-D: Center for Epidemiologic Studies Depression Scale, GAD-7: 7-item Generalized Anxiety Disorder Scale, CD-RISC 10: 10-item Connor–Davidson Resilience Scale, OR: odds ratio, CI: confidence interval

0.001; r=–0.27, p<0.001; and r=–0.43, p<0.001, respectively).

In all participants, the median scores of the original questionnaires on anxiety and fear of infection and death, isolation and unreasonable treatment, and motivation and escape behavior at work were 12 (IQR: 8-19), 0 (IQR: 0-1), and 2 (IQR: 0-3), respectively. Doctors had lower scores for questionnaires on anxiety and fear of infection and death than other healthcare workers. In contrast, younger workers had significantly higher scores for the questionnaires than older workers (p<0.001). Nurses, other co-medical staff, younger workers, and women had higher scores for questionnaires on motivation and escape behavior at work than others. The scores for questionnaires on isolation and unreasonable treatment were very low in all populations.

Based on the CES-D cut-off value (16 points), we divided the participants into healthy and depression groups and compared their measurement scores (Table 4). The total GAD-7 score and the score of each type of the original questionnaire were significantly higher in the depression group than in the healthy group (all p values <0.001). There were significantly more participants in the depression group who complained of moderate-to-severe anxiety than in the healthy group (p<0.001). In contrast, the total CD-RISC 10 score was significantly lower in the depression group than in the healthy group. (p<0.001).

Risk factors of the symptoms of depression

The potential risk factors of depression (total CES-D score ≥ 16 points) were examined using a multivariable logistic regression analysis (Table 5). The original questionnaire scores were not adopted as explanatory factors because their reliability and validity were not guaranteed. Being a nurse and having high total GAD-7 scores were significantly associated with symptoms of depression (OR: 3.40; 95% CI: 1.39-8.30; p=0.007 and OR: 1.43; 95% CI: 1.34-1.52; p<0.001, respectively). In contrast, older workers and those with high total CD-RISC 10 scores were significantly less likely to develop symptoms of depression than others (OR: 0.95; 95% CI: 0.93-0.97; p<0.001 and OR: 0.93; 95% CI: 0.91-0.96; p<0.001, respectively).

Discussion

We conducted a large-scale questionnaire survey on the mental health of healthcare workers in our hospital during the COVID-19 pandemic. Among the 848 participants, 85 (10.0%) developed moderate-to-severe anxiety disorder, and 237 (27.9%) developed symptoms of depression. Problems with anxiety and fear of infection and death, isolation and unreasonable treatment, and motivation and escape from work were all greater in the depression group (total CES-D score ≥16) than in the healthy group (total CES-D score < 16). Being a nurse and having higher total GAD-7 scores were risk factors of depression, whereas older workers and those with higher total CD-RISC 10 scores were less likely to develop symptoms of depression than others.

There have been several studies on mental burden during the COVID-19 pandemic. A survey from China targeting the general population reported that 53.8% of the participants rated the psychological impact as moderate-to-severe, 28.8% reported moderate-to-severe anxiety (17), and 14.6% developed symptoms of depression (18). The mental health of healthcare workers has also been investigated overseas. In China, healthcare workers reported high rates of anxiety (44.6%), depression (50.4%), and insomnia (34.0%) (16). At the time of the survey, COVID-19 was rapidly spreading in China, and the majority of participants resided in Wuhan, the center of the outbreak. It is thought that these differences in the situation and location led to a difference in the frequency of mental illness between the previous report and the present study. In this study, the total GAD-7 score was higher in older and frontline workers than in others. The tendency for COVID-19 to be severe in elderly individuals (19) and the stress of directly treating patients with COVID-19 may aggravate anxiety. In addition, a previous survey revealed that working on the frontline was an independent risk factor of worse mental health outcomes, such as symptoms of depression, anxiety, insomnia, and distress (14). The increased likelihood of mental symptoms among frontline workers may be attributed to their inability to rest outside of the workplace due to preventive restrictions and reduced means of entertainment. Furthermore, because mass media releases a large amount of information on COVID-19 daily, frontline workers are constantly exposed to negative news on COVID-19 outside their workplace. It is considered that these situations may impose a large mental burden on them. However, in the present study, being a frontline worker was not an independent risk factor of depression, and 26.0% of non-frontline workers developed symptoms of depression. We observed that not only frontline workers but also non-frontline workers have suffered a mental burden during the ongoing COVID-19 pandemic.

Regarding the original questionnaire, anxiety and fear of infection and death were the most frequently observed conditions in our survey. Although there were few problems related to isolation, unreasonable treatment, motivation, and escape behavior at work, social isolation and discrimination against healthcare workers were found likely to occur during the current COVID-19 pandemic. In fact, discrimination and prejudice against healthcare workers and their families have been reported in Japan (2). If the pandemic continues, some of them may consider taking leave or retiring. In addition, repeated negative experiences or witnessing death can result in PTSD. Notably, 7.4% of healthcare workers in Singapore and India developed PTSD during the ongoing COVID-19 pandemic (20). Therefore, continuous surveillance of psychological disorders and long-term intervention are essential to support the mental health of healthcare workers.

Depression can cause a variety of symptoms, such as sleep disturbance, fatigue, reduced appetite, loss of interest, and feelings of worthlessness, and may eventually lead to suicide. We used the CES-D to assess the symptoms of depression among the participants. The survey showed that the total CES-D score was significantly higher among nurses, younger workers, women, and frontline workers than others. These findings are consistent with those reported in previous studies (14, 15). Furthermore, being a nurse, being young, and having a high total GAD-7 score were independent risk factors of depression. The accumulation of anxiety regarding infection and death of self, family, or colleagues; lack of PPE; and prolongation of the pandemic may be associated with the development of depression. Therefore, it is important to manage anxiety among healthcare workers, who are a high-risk group for the occurrence of depression.

This study demonstrated that the total CD-RISC 10 score was lower in the depression group than in the healthy group and that healthcare workers with higher CD-RISC 10 scores were less likely to develop depression than others. These results mean that healthcare workers with lower resilience are at a higher risk of developing depression than others. These workers should be more proactive in receiving psychological intervention. Several studies have reported the effectiveness of psychosocial resilience training. These approaches have led to improvements in resilience (12, 21). In addition, the effects of such interventions have been reported to be long-lasting and may be useful for preventing the recurrence of psychological symptoms and PTSD (12, 22). Recently, guidelines regarding psychological crisis intervention for both patients and healthcare workers have been published by several countries and institutions (23-25). These guidelines emphasize the importance of ensuring sufficient PPE and resting, preventing stigmatization, and providing psychological care to protect the mental health of healthcare workers. They also suggest utilizing online mental interventions to avoid close contact. It is necessary to enhance psychological resources and establish a system for easy access to mental support.

Limitations

Several limitations associated with the present study warrant mention. First, owing to the low response rate (43.2%), response bias may exist if non-respondents were either too stressed to respond or not stressed at all and not interested in this survey. Second, this was a one-time survey; thus, longitudinal data are lacking. In addition, changes in psychiatric symptoms with or without mental healthcare interventions remain unclear. Third, this study involved healthcare workers in a single institution; thus, the results may not be representative of all institutions in Japan. Larger prospective nationwide studies are warranted to verify these findings.

Conclusion

In conclusion, this study indicated that among the healthcare workers in the hospital, 10.0% had moderate-to-severe anxiety, and 27.9% developed symptoms of depression during the COVID-19 pandemic. Being a nurse, high levels of anxiety, younger age, and lower resilience were risk factors

of depression. Further psychological support and interventions for protecting the mental health of healthcare workers are warranted to win the long-term fight with COVID-19.

The authors state that they have no Conflict of Interest (COI).

Acknowledgement

The authors thank Dr. Rinmei Fukuda from the Japanese Red Cross Medical Center, Tokyo, Japan, for his general support.

References

1. Zhu N, Zhang D, Wang W, et al. A novel coronavirus from patients with pneumonia in China, 2019. N Engl J Med **382**: 727-733, 2020.
2. Japanese Ministry of Health, Labor and Welfare. About coronavirus Disease 2019 (COVID-19). Tokyo, Japan [Internet]. 2020 [cited 2020 May 31]. Available from: https://www.mhlw.go.jp/english/
3. Maunder R, Hunter J, Vincent L, et al. The immediate psychological and occupational impact of the 2003 SARS outbreak in a teaching hospital. CMAJ **168**: 1245-1251, 2003.
4. Su TP, Lien TC, Yang CY, et al. Prevalence of psychiatric morbidity and psychological adaptation of the nurses in a structured SARS caring unit during outbreak: a prospective and periodic assessment study in Taiwan. J Psychiatr Res **41**: 119-1130, 2007.
5. Wu P, Fang Y, Guan Z, et al. The psychological impact of the SARS epidemic on hospital employees in China: exposure, risk perception, and altruistic acceptance of risk. Can J Psychiatry **54**: 302-311, 2009.
6. Spitzer RL, Kroenke K, Williams JBW, Löwe B. A brief measure for assessing generalized anxiety disorder: the GAD-7. Arch Intern Med **166**: 1092-1097, 2006.
7. Radloff LS. The CES-D scale: a self-report depression scale for research in the general population. Appl Psychol Meas **1**: 385-401, 1977.
8. Shima S, Shikano T, Kitamura T. A new self-report depression scale. Psychiatry **27**: 717-723, 1985 (in Japanese).
9. Demura S, Sato S. Problems with the Japanese version of the Center for Epidemiologic Studies Depression (CES-D) scale when applied to Japanese independent community-dwelling elderly: an examination of the factor structure. Environ Health Prev Med **8**: 184-190, 2003.
10. Masten AS. Resilience in children threatened by extreme adversity: frameworks for research, practice, and translational synergy. Dev Psychopathol **23**: 493-506, 2011.
11. Connor KM, Davidson JRT. Development of a new resilience scale: the Connor-Davidson Resilience scale (CD-RISC). Depress Anxiety **18**: 76-82, 2003.
12. Konradt CE, Cardoso TA, Mondin TC, et al. Impact of resilience on the improvement of depressive symptoms after cognitive therapies for depression in a sample of young adults. Trends Psychiatry Psychother **40**: 226-231, 2018.
13. Ito M, Nakajima S, Shirai A. Reliability and validity of Japanese version Conner-Davidson Recovery Power Scale: consideration for general adults and university students. National Center for Mental and Nervous Center Mental Health Research Institute Annual Report **22**: 294, 2010 (in Japanese).
14. Lai J, Ma S, Wang Y, et al. Factors associated with mental health outcomes among health care workers exposed to coronavirus Disease 2019. JAMA Netw Open **3**: e203976, 2020.
15. Kisely S, Warren N, McMahon L, Dalais C, Henry I, Siskind D. Occurrence, prevention, and management of the psychological effects of emerging virus outbreaks on healthcare workers: rapid review and meta-analysis. BMJ **369**: m1642, 2020.
16. Löwe B, Decker O, Müller S, et al. Validation and standardization of the generalized anxiety disorder screener (GAD-7) in the general population. Med Care **46**: 266-274, 2008.
17. Wang C, Pan R, Wan X, et al. Immediate psychological responses and associated factors during the initial stage of the 2019 coronavirus disease (COVID-19) epidemic among the general population in China. Int J Environ Res Public Health **17**: 1729, 2020.
18. Lei L, Huang X, Zhang S, Yang J, Yang L, Xu M. Comparison of prevalence and associated factors of anxiety and depression among people affected by versus people unaffected by quarantine during the COVID-19 epidemic in Southwestern China. Med Sci Monit **26**: e924609, 2020.
19. Wu Z, McGoogan JM. Characteristics of and important lessons from the coronavirus disease 2019 (COVID-19) outbreak in China: summary of a report of 72314 cases from the Chinese Center for Disease Control and Prevention. JAMA **323**: 1239-1242, 2020.
20. Chew NWS, Lee GKH, Tan BYQ, et al. A multinational, multicentre study on the psychological outcomes and associated physical symptoms amongst healthcare workers during COVID-19 outbreak. Brain Behav Immun. Forthcoming.
21. Burton NW, Pakenham KI, Brown WJ. Feasibility and effectiveness of psychosocial resilience training: a pilot study of the READY program. Psychol Health Med **15**: 266-277, 2010.
22. Horn SR, Charney DS, Feder A. Understanding resilience: new approaches for preventing and treating PTSD. Exp Neurol **284**: 119-132, 2016.
23. The British Psychological Society. The psychological needs of healthcare staff as a result of the coronavirus pandemic. UK [Internet]. 2020 [cited 2020 May 31]. Available from: https://www.bps.org.uk/sites/www.bps.org.uk/files/News/News%20-%20Files/Psychological%20needs%20of%20healthcare%20staff.pdf
24. United Nations. Policy brief: COVID-19 and the need for action on mental health. USA [Internet]. 2020 [cited 2020 May 31]. Available from: https://www.un.org/sites/un2.un.org/files/un_policy_brief-covid_and_mental_health_final.pdf
25. Li W, Yang Y, Liu ZH, et al. Progression of mental health services during the COVID-19 outbreak in China. Int J Biol Sci **16**: 1732-1738, 2020.

The Internal Medicine is an Open Access journal distributed under the Creative Commons Attribution-NonCommercial-NoDerivatives 4.0 International License. To view the details of this license, please visit (https://creativecommons.org/licenses/by-nc-nd/4.0/).

© 2020 The Japanese Society of Internal Medicine
