# Supplementary material for: Mental health status and related factors influencing healthcare workers during the COVID-19 pandemic: A systematic review and meta-analysis
Source: PLoS One. 2024 Jan 19;19(1):e0289454. doi: 10.1371/journal.pone.0289454 (PMC10798549; doi:10.1371/journal.pone.0289454)
Supplement: S1 Data — (ZIP) [file pone.0289454.s011.zip › literatures/140.pdf]

BMC Psychiatry

RESEARCH

Open Access

Mental health condition of physicians working frontline with COVID-19 patients in Bangladesh

Aminur Rahman[1*], Farah Deeba[2], Sadika Akhter[1], Farzana Bashar[1], Dilruba Nomani[1], Jaap Koot[3], Kamrun Nahar Koly[1], Faysal Bin Salah[4], Kim Haverlag[3] and Iqbal Anwar[1]

Abstract

Background: The impact of the unpredictable COVID-19 pandemic had triggered new challenges for mental health. This quick survey aimed to identify the mental health status of physicians who served the people during COVID-19 in Bangladesh.

Methodology: The cross sectional survey was conducted adopting a quantitative approach and using an online questionnaire through Facebook Platform Group. Data was collected from August-October, 2020, on socio-demographic status, information on COVID-19 and questionnaires about Depression Anxiety Stress Scale (DASS-21). A total of 395 participants were enrolled from all eight administrative divisions of Bangladesh.

Result: Our study reported a higher prevalence of depression (55.3%), anxiety (35.2%), and stress (48.4%) among 347 participants. Female physicians were found to have more stress (OR = 2.16, 95% CI: 1.09 – 4.30) compared to the male. Physicians who were previously diagnosed as mentally ill were found to be significantly more depressed (OR = 3.45, 95% CI: 1.07 – 11.10) and stressed (OR = 4.22, 95% CI: 1.48 – 12.02) compared to them who did not. Along with that, having a chronic disease, working in non-government and COVID hospitals significantly contributed to poor mental health outcomes.

Conclusion: The study findings denoted that, the mental health of physicians was deeply affected by the pandemic situation. The availability of appropriate mental health support will help foster resilience by giving them the ability and confidence to manage crisis moments like the COVID-19 pandemic.

Keywords: Anxiety, Depression, Stress, Physician, COVID-19, Bangladesh

Introduction

Currently the world is facing a "global disaster" called Corona Virus Disease 2019 (COVID-19) for which many countries are in a stage of "epidemiological emergency" [1]. There have been 131,487,572 positive cases confirmed as well as 2,857,702 deaths reported until 7 April, 2021, making this pandemic spread across 213 countries [2]. Bangladesh has reported approximately 1,000,543 confirmed positive cases along with 16,004 deaths so far in this pandemic, which began on 8 July, 2021 [3]. According to different projections, this number of death cases may cross 41 million throughout the world by 23 July, 2021 [4]. This pandemic has created unanticipated disarray in the global health system since the global health governance has not responded adequately during the COVID-19 outbreak [5]. The disease spread rapidly across the globe due to some unique properties of the

*Correspondence: draminur@icddrb.org
[1] International Center for Diarrhoeal Disease and Research Bangladesh (icddr,b), 68 Shaheed Tajuddin Ahmed Sarani, Mohakhali, Dhaka 1205, Bangladesh
Full list of author information is available at the end of the article

© The Author(s) 2021. **Open Access** This article is licensed under a Creative Commons Attribution 4.0 International License, which permits use, sharing, adaptation, distribution and reproduction in any medium or format, as long as you give appropriate credit to the original author(s) and the source, provide a link to the Creative Commons licence, and indicate if changes were made. The images or other third party material in this article are included in the article's Creative Commons licence, unless indicated otherwise in a credit line to the material. If material is not included in the article's Creative Commons licence and your intended use is not permitted by statutory regulation or exceeds the permitted use, you will need to obtain permission directly from the copyright holder. To view a copy of this licence, visit http://creativecommons.org/licenses/by/4.0/. The Creative Commons Public Domain Dedication waiver (http://creativecommons.org/publicdomain/zero/1.0/) applies to the data made available in this article, unless otherwise stated in a credit line to the data.

virus (highly contagious, frequent mutations, easy mode of transmission, relatively unaffected by climatic variations) [6]. During any uncertain situation it is common for an individual to feel anxious, out of control, bewildered, etc. [7]. So during this crisis period, individuals are expected to encounter several mental health challenges ranging from panic attacks, phobia, anxiety, depression, sleep disturbances to dissociative like symptoms [8]. Fortunately, most people have the option to keep themselves safe through maintaining social distancing and other preventive measures. However, the healthcare professionals, especially physicians working in the frontline have an increased risk of getting infected by COVID-19 as they so not have the opportunities to work from home [9]. They treat the patients with confirmed and or suspected COVID-19 on a daily basis. Some have to stay separated from families due to self-quarantine after their hospital duties. As a result, preliminary research among physicians demonstrated that they are suffering from fear of being stigmatised and various stress reactions [10]. Lower middle-income countries have higher burden of mental disorders than economically developed countries in the normal situation, which could be worsened by COVID-19 situation [10–12].

In a densely populated and resource constrained setting like Bangladesh, the health systems are coping with the pandemic with several limitations. Similar to other countries, physician belong to the most vulnerable groups to be infected by this contagious virus. Bangladesh was identified as one of the countries with highest physician' mortality in the crisis (4%) [13]. According to a published report by Bangladesh Medical Association (BMA), on 25 July 2021, more than 3058 physicians were infected with COVID-19 and 172 have passed away due to complications [14]. Such facts reflect the vulnerable situation of the physicians in the country. If the physicians' mental condition is affected, it may hamper the service delivery to the community during this crisis period.

Mental disorders are generally not perceived as serious health problems and are not within the priorities in the health care delivery system in resource-poor settings like Bangladesh. Moreover there is chance of being stigmatised if someone reveals the news of having any mental diseases [10]. Study suggest that mental disorder with co-morbidities may reduce life expectancy by about 20 years [15]. So, to ensure continuity of medical services, maintaining the mental health wellbeing of these front liners should be the top priority of medical authorities. To support the physician's community, it is imperative to understand the mental health status of Bangladeshi physicians in this ongoing COVID-19 crisis. Till date, there is scarcity of reliable information on the mental health of Bangladeshi physicians with an appropriate sample size [16–18]. So, we have designed a quick survey, with a cross sectional study design and distributed this among physicians of Bangladesh to assess their mental health status that might be helpful to the policy makers to intervene in appropriate manner at this current COVID-19 period.

Generic definition

Stress is the body's reaction to a threat, whereas anxiety is the body's reaction to the stress. People can manage their stress and anxiety with relaxation techniques, such as breathing exercises, physical activity, and talking about their worries. Sometimes, stress and anxiety can overwhelm people. Depression is a mood disorder that causes a persistent feeling of sadness and loss of interest when exist more than two weeks. Depression has many possible causes, such as genetics, brain chemicals and your life situation [19].

Methods and materials

Study setting

Physicians who were the members of "Facebook platform group" in Bangladesh and when one confirmed their phone number from a Bangladeshi telecommunication vendor, were invited for this study. The Facebook Platform group is an organization of Bangladeshi physicians, medical and dental students established on 26 September 2013. It has 114,000 members who are Bangladeshi registered physicians living around the globe. It has been working to establish the rights, promoting support and working for quality improvement of the physician's community who are put in place in all parts of the country. It aims to build a positive Bangladesh by improving the overall health sector of Bangladesh [20]. In Bangladesh there were around 87,000 registered physicians in July 2019 statistics [21], this means that nearly all the Bangladeshi physicians are the members in this group. We had only selected those who were working in Bangladesh during our study period. There were periodical posting of a banner in the Facebook group by the group administrator and there was equal chance to be included in the study. This might help the study to avoid the selection bias following the online platform. Our study shows that the participants age group are nearly equal below and over 27 years age. This also reflects that all ages are there within the participants.

Selection criteria:

Inclusion criteria: 1. The Bangladeshi physicians who are Facebook platform group members. 2. Who own a mobile phone which belongs to Bangladesh legal mobile operator (Grameen, Robi, Bangla link and Tele Talk) for providing services in Bangladesh. 3. Who gave consent for participation.

Exclusion criteria: Who didn't provide consent to participate in the study.

Study design and data collection method

It was a cross sectional survey. A total of 395 participants were enrolled from all the eight administrative divisions of Bangladesh. Quantitative methods were adopted to measure the depression, anxiety and stress status of physicians.

A data collection tool was developed combining socio-demographic variables, information on COVID-19 and Depression Anxiety Stress Scale (DASS-21) [22, 23]. Tools were developed with access to a Google form doc which was free for everyone to approach from anywhere in the country. After developing the draft of the online tool, pre-testing was done among 20 physicians who were not included in the main study, to finalize the tool. No modifications were needed in questionnaire after pre-test. The online process was highly useful during this COVID-19 period as it helped us to get the data directly from the study participants within a short period of time, without face to face interaction.

Questionnaire on demographic information

Demographic information was obtained from the participants on various variables, such as: age, sex, place of working, marital status.

Depression anxiety stress Scale-21 (DASS-21)

The DASS-21 was derived from the 42-item developed by the researchers at the University of New South Wales, Australia [24] There are seven items to identify depression, seven items for anxiety and seven items for stress overall scale score indicates no specific psychological problems but indicated disturbance or distress in an individual. The higher the score the higher the level of distress is. The items are rated on a 4-point Likert-type scale where, 0 = Did not apply to me at all, 1 = Applied to me to some degree, or some of the time, 2 = Applied to me to a considerable degree, or a good part of time and 3 = Applied to me very much, or most of the time. The obtained scores are interpreted in the following ways, for depression, the levels are stated as normal if scores are within 0-4, Mild =5-6, Moderate = 7-10, Severe = 1-13, Extremely Severe= > 14+; for Anxiety the levels are stated as Normal = 0-3, Mild = 4-5, Moderate = 6-7, Severe = 8-9, Extremely Severe = > 10+, and for Stress, Normal = 0-7, Mild = 8-9, Moderate = 10-12, Sever = 13-16 and Extremely Severe= > 17+. In our study the Bengali version of the DASS-21 was used. The Bangla DASS 21 was translated and validated by [25]. The correlation between English and Bangla scale was found for depression subscale was 0.98, anxiety subscale was 0.92 and stress subscale was 0.93. The Cronbach's Alpha for depression, anxiety and stress subscales were found 0.99, 0.96 and 0.97 respectively.

Data collection procedure

An online data collection form along with consent was sent to each participant. A banner was created for the Facebook group platform to invite all Bangladeshi physicians to participate in this study. Only physicians who had confirmed their phone number from a Bangladeshi telecommunication vendor, were included. The platform administrative team helped the Principal Investigator to get the participants' list including the mobile number and email addresses of each eligible participant. To honour their participation, we offered each participant 3.5 GB internet data on their phones. The research team recharged their internet data within three days after completing the survey. Participants were selected on a first come first serve basis until reaching the required sample size.

Sample size calculation

We took as reference a national survey on mental health in Bangladesh which showed 16% population were in depression [26]. We wanted to detect 16% depression among physicians with 4% margin of error and 95% CI. Therefore, a minimum of 323 physicians were needed to be assessed. To compensate for 20% non-response, 395 physicians were needed for the study.

Data analysis

After completion of data collection, data were thoroughly checked for consistencies, completeness and then cleaned and edited manually. 48 cases having missing information on DASS-21 items were removed from the data and then a total of 347 cases were retained during for analysis. Statistical Package for Social Sciences (SPSS) version 23 was used to analyse the data. Frequencies and percentages were calculated for demographic and other indicators. Based on scores obtained on DASS-21, we labelled the cases that were within the range of normal and mild as 'normal', kept 'moderate' as it is. We summarised 'severe' and 'extremely severe' into one single category and named it as 'severe' for all three variables, that are depression, anxiety and stress. We then categorized 'normal' and 'moderate' as 'not stressed' 'not depressed' 'not anxious' and 'severe' and 'extremely severe' as stressed', 'depressed' and 'anxious' for further bivariate and multivariate analysis. We stratified working in COVID and non-COVID hospitals [27]. Significances of relationship of different categorical indicators with different outcomes were measured through chi-square tests. All tests were considered significant at $p < 0.05$ level. All

the covariates which found to have significant relations with outcome variables from bivariate findings as well as important covariates identified from literature, were adjusted through binary logistic regression. Multi-collinearity was also checked before doing regression analysis using Variation Inflation Factor (VIF). No multicollinearity among covariates was found as VIF < 10.

Outcome variables were: Depression, Anxiety and Stress. Exposure variables were: socio-demographic, health and work-related characteristics of the physician's that are age, sex, marital status, previously diagnosed chronic diseases, previous history of diagnosed mental health issues, history of availing or receiving psychotherapy, place of work, types of hospital, direct contact with COVID-19 patient, satisfied with available Personal Protective Equipment (PPE).

Ethical approval

Ethical approval for the study (protocol number PR-20046) was obtained from Institutional Review Board of International Center for Diarrhoeal Disease Research, Bangladesh (icddr,b).

Results

Demographic characteristics of the physicians

The socio-demographic, health condition and work environment related characteristics of physicians are described in Table 1.

Among the 347 physicians, 82 (23.6%) were working at COVID hospitals and 265 (76.4%) at non-COVID hospitals. Among them, it was observed that for the both groups more than 60% of the respondents were male physicians. In COVID hospitals, 61.0% of physicians were 27 years of age whereas for non-COVID hospitals the percentage for the group was only 45.7. Around 90% of physicians had MBBS degree for both types of hospitals. 34% of physicians were previously diagnosed with chronic diseases, more than 80% had no previous history of diagnosed mental health issues, and only 3% of COVID-19 hospitals and 11% of non-COVID hospitals had history of availing or receiving psychotherapy. 57.3% of physician's work at government hospitals which were providing COVID-19 related health services whereas 42.7% of then provide service at non-government COVID 19 hospitals. 98% of the physicians of COVID hospitals were working in direct contact with COVID patients whereas 80% of non-COVID hospitals physicians were doing the same. Based on the data from the Government COVID-19 designated hospitals whereas 26.8% physicians were satisfied with PPE (Not representative hospitals in general as this was dependent on data only from government hospital's physicians) (Table 1).

Based on the DASS-21 scale definition, we found that the prevalence of depression was 55.3%, anxiety was 35.2% and stress was 48.4% among the physicians (Fig. 1). The study findings revealed that physicians of COVID-19 hospitals were found to be more severely depressed, stressed and anxious than physicians of non-COVID-19 hospitals physicians (Fig. 2).

Male physicians were found to be more depressed irrespective of place of work and type of hospitals compared to females. Similarly, male physicians of government and COVID-19 hospitals were found to be more stressed and anxious. Male physicians of non-government hospitals were also more anxious (Fig. 3).

Table 2 shows the significant results from bivariate and multivariate analysis to identify the determinants associated with depression, stress and anxiety.

Sex was seemed to be an important factor for development of depression, anxiety and stress. After adjustment, only females were found to be significantly more stressed (OR = 2.16, 95% CI: 1.09 - 4.30) compared to males.

Table 2 shows that place of work was a significant factor for stress. The physicians working at government hospitals were significantly more stressed (OR = 4.08, 95% CI: 1.77 - 9.41) compared to their counterparts.

Physicians whose age was less than or equal to 27 years were found to develop depression, anxiety and stress more than their counterparts; though the results were not significant.

Bivariate findings revealed also that, previously diagnosed chronic diseases were found to be significant for stress. Moreover, physicians who had previous history of chronic disease were found to develop depression (OR = 2.61, 95% CI: 1.14 – 5.99) and stress more than their counterparts (OR = 2.51, 95% CI: 1.22 – 5.18). Similarly, physicians who had previous history of diagnosed mental health issues were prone to develop depression (OR = 3.45, 95% CI: 1.07 – 11.10) and stress (OR = 4.22, 95% CI: 1.48 – 12.02) more than those who don't have such previous history. Moreover, history of availing or receiving psychotherapy was an important factor behind development of both depression and anxiety. We found 127 physicians were severely depressed, stressed or anxious. As per ethical guideline we immediately inform (after analysis) them to contact with the psychiatrist if they were not done already (data was not shown).

Discussion

In the recent years, prevalence of mental health conditions among the physicians has been observed to be higher compared to the general population under similar circumstances [28]. Unfortunately, the current COVID-19 circumstances have proved to be a catalyst in further

Table 1 Socio-demographic, health and work related characteristics of physicians by COVID and Non-COVID hospitals

Socio-demographic characteristics	COVID Hospitals(*N* = 82) n (%)	Non-COVID Hospitals (*N* = 265) n (%)	*p*-value
**Sex**			
Male	57 (69.5)	164 (61.9)	0.210
Female	25 (30.5)	101 (38.1)	
**Age (in years)**			
<=27	32 (39.0)	144 (54.3)	0.015
> 27	50 (61.0)	121 (45.7)	
**Education**			
MBBS	73 (89.0)	240 (90.6)	0.682
Postgraduate	9 (11.0)	25 (9.4)	
**Marital status**			
Married	45 (54.9)	135 (50.9)	0.533
Unmarried or Others	37 (45.1)	130 (49.1)	
**Health conditions related characteristics**			
**Previously diagnosed chronic diseases**			
Yes	28 (34.1)	89 (33.6)	0.925
No	54 (65.9)	176 (66.4)	
**Previous history of diagnosed mental health issues**			
Yes	15 (18.3)	28 (10.6)	0.063
No	67 (81.7)	237 (89.4)	
**History of availing/receiving psychotherapy**			
Yes	3 (3.7)	29 (10.9)	0.046
No	79 (96.3)	236 (89.1)	
**Work environment related characteristics**			
**Place of work**			
Government Hospital	47 (57.3)	66 (24.9)	< 0.001*
Non-government Hospital	35 (42.7)	199 (75.1)	
**Direct contact with COVID-19 patient**			
Yes	81 (98.8)	211 (79.6)	< 0.001
No	1 (1.2)	54 (20.4)	
**Satisfied with available PPE (n = 82)**			
Yes	22 (26.8)	–	–
No	60 (73.2)	–	

Note: Total of Satisfied with PPE is not equal to total sample (N = 347) as this was dependent on data only from Government hospitals physicians

deterioration the mental health status of the health care professionals [10].

Hence, in line with the above statement, the findings in the current study reveal that between 35% and 55% of the physicians were found to develop depression, anxiety and stress. Also, the study showed that around 20% of physicians had severe form of depression, anxiety and stress. Depression was more prevalent than anxiety and stress during the COVID-19 pandemic, as was found in other studies in Bangladesh and Middle East [29–31]. On the other hand, in China, where this pandemic started, anxiety was the leading mental health condition among the physicians [32]. In South Asian countries, slight differences were found between the prevalence of anxiety, depression and stress. In Bangladesh anxiety was more prevalent than depressive symptoms with a difference of huge margin [31, 33, 34]. Most of the participants of our study were young and manifestation of depression in these young physicians more than other feelings, like anxiety and stress, indicates that dealing with this highly infectious disease created feelings of uncertainty around progression in life and future during and beyond this unprecedented situation of the pandemic. Well-trained physicians were better able to cope with the situation. The differences with findings of other studies can be explained by differences in research methodology. In

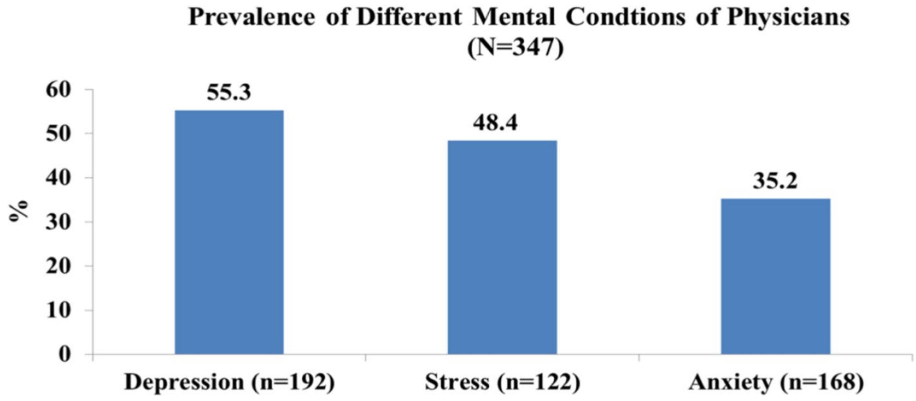

Fig. 1 Distribution of prevalence of different mental conditions of physicians

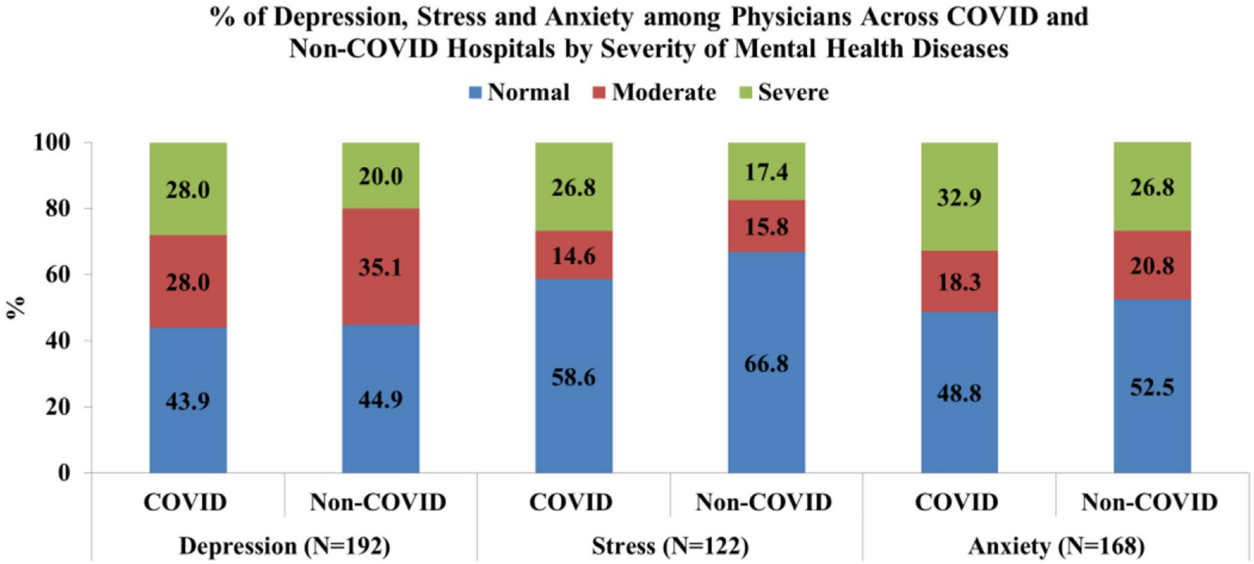

Fig. 2 Distribution of Depression, Stress and Anxiety among Physicians across COVID and Non-COVID Hospitals by Severity of Mental Health Diseases

another study conducted in Bangladesh among COVID-19 patients found 56%-87% proportion of depression and anxiety [35].

Sex

Furthermore, bivariate findings of the current study show that depression, anxiety and stress to be more prevalent among female physicians in comparison to male physicians. Psychological vulnerability of female physicians during the COVID-19 was also reported in majority of studies conducted globally and specially in South Asia including Bangladesh [29–31, 36, 37]. Generally, women manifest other psychological symptoms compared to men [38], for instance, responses to stress [39], build-up of lifelong stress reactions [39], or coping strategies [40]. Female physicians in Bangladesh have dual household and professional roles during pandemic. They experience stress due to the fear of transmission of the disease to their family members [41], (especially to young children who are more dependent on mothers), isolation from family by living in hotels on top of their (females'). This may explain the higher prevalence of depression, anxiety and stress [10, 30, 42–45].

Age

Physicians whose age was less than or equal to 27 years were found to have depression, anxiety and stress higher than the physicians aged more than 27 years;

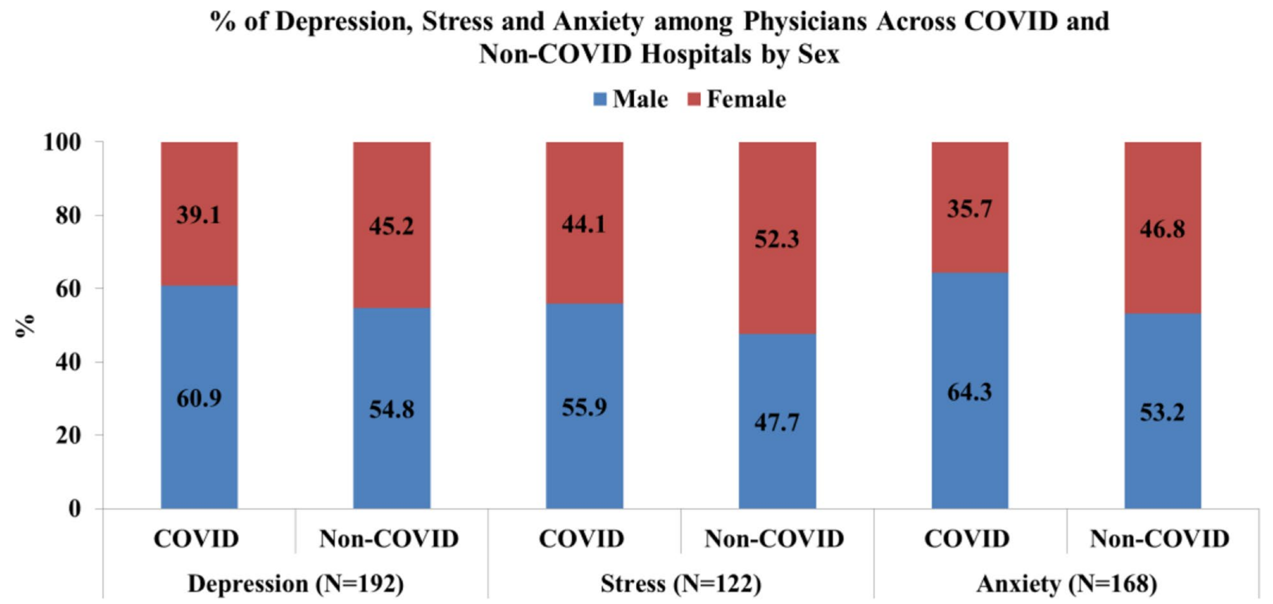

Fig. 3 Distribution of Depression, Stress and Anxiety among Physicians across COVID and Non-COVID Hospitals by Sex

though the results were not significant. Thus, in relation to age, previous studies conducted in the Middle Eastern Countries during the COVID-19 have demonstrated higher prevalence of mental health conditions among the age group younger than 50 years [29, 30]. Likewise, in China the middle aged (30-49 years) physicians were more resilient to mental health illness compared to younger aged (18-30 yrs) physicians [36]. Since, the findings of the current study are somewhat consistent with findings of other countries, it can be assumed that in Bangladesh, younger inexperienced physicians with limited skills, training and sudden Intensive Care Unit (ICU) deployments are less resilient than to older physicians, leading to strain on psychological health among the younger physicians [36, 46].

Government and non- government

Previous research studies have evidently proved the negative impact of COVID-19 on the psychological health of physicians, however no clear stratification among government and non-government physicians have been pointed out [18, 31–33, 41, 42, 47–49]. The physicians working at government hospitals were significantly more stressed (OR = 4.08, 95% CI: 1.77 - 9.41) compared to their counterparts.

Higher prevalence of anxiety and depression among non-government physicians can be assumed due to job insecurity at non-government hospitals compared to government hospitals and limited patient access due to the policies of the non- government hospital against patient admission without non COVID certification [43, 50]. On the other hand, denial of private hospitals to admit patients during the initial phase of the pandemic has led to more fear of being overwhelmed by patients and more fear of lack of materials and PPE, which might have contributed to the higher prevalence of stress among the government hospital physicians [43, 50, 51].

COVID-19 and non-COVID-19

Our study has revealed that physicians of COVID-19 hospitals were found to be more severely depressed, stressed and anxious than physicians of non-COVID-19 hospitals. In multivariate analysis, physicians of COVID-19 hospitals were found to be more depressed and anxious but less stressed than those from non-COVID-19 hospitals; though these results were not significant. In coherence with the current study, a few systemic reviews from other parts of the world have also found that COVID-19 is negatively impacting the psychological health of front line health workers [48, 49]. In China where the first case of COVID-19 was discovered, frontline physicians were prone to higher severity of mental health conditions than the non-front line physicians [18, 32, 41]. Studies from neighbouring South Asian countries like Pakistan and India, have also yielded higher mental health impact on COVID-19 frontline physicians [33, 52].

Similarly, previous studies from Bangladesh have shown that the physicians to be psychologically burdened due to the COVID-19 pandemic [37, 42]. In a low resource country like Bangladesh, personal protective

Table 2 Factors associated with depression, anxiety and stress among physicians: results from bivariate and multivariate analysis

Factors	Depression (*N* = 347)			Anxiety (N = 347)			Stress (N = 347)		
	Yes (*N* = 192) n (%)	*P*-value	Adjusted OR(95% CI)	Yes (*N* = 168) n (%)	*P*-value	Adjusted OR(95% CI)	Yes (*N* = 122) n (%)	*P*-value	Adjusted OR(95% CI)
**Place of work**									
Government Hospital	55 (48.7)	0.083	0.59 (0.25 - 1.39)	47 (41.6)	0.077	0.62 (0.29 - 1.30)	48 (42.5)	0.047	4.08 (1.77 - 9.41)
Non-government Hospital	137 (58.5)		Ref	121 (51.7)		Ref	74 (31.6)		Ref
**Type of hospital**									
COVID hospital	46 (56.1)	0.873	1.25 (0.49 - 3.22)	42 (51.2)	0.561	1.87 (0.85 - 4.15)	34 (41.5)	0.171	0.69 (0.30 - 1.59)
Non-COVID hospital	146 (55.1)		Ref	126 (47.5)		Ref	88 (33.2)		Ref
**Sex**									
Male	108 (48.9)	0.001*	Ref	94 (42.5)	0.004*	Ref	61 (27.6)	< 0.001	Ref
Female	84 (66.7)		1.54 (0.69 - 3.44)	74 (58.7)		1.11 (0.56 - 2.19)	61 (48.4)		2.16 (1.09 - 4.30)
**Age (in years)**									
<= 27	94 (53.4)	0.465	2.06 (0.92 - 4.59)	82 (46.6)	0.49	1.58 (0.79 - 3.16)	59 (33.5)	0.517	1.03 (0.52 - 2.05)
> 27	98 (57.3)		Ref	86 (50.3)		Ref	63 (36.8)		Ref
**Marital status**									
Married	99 (55.0)	0.897	Ref	87 (48.3)	0.975	Ref	63 (35.0)	0.949	Ref
Unmarried or Others	93 (55.7)		2.17 (0.95 - 4.99)	81 (48.5)		1.28 (0.63- 2.62)	59 (35.3)		1.23 (0.61- 2.47)
**Previously diagnosed chronic diseases**									
Yes	69 (59.0)	0.33	2.61 (1.14 – 5.99)	62 (53.0)	0.224	1.88 (0.93 - 3.79)	50 (42.7)	0.035	2.51 (1.22 - 5.18)
No	123 (53.5)		Ref	106 (46.1)		Ref	72 (31.3)		Ref
**Previous history of diagnosed mental health issues**									
Yes	33 (76.7)	0.003*	3.45 (1.07 - 11.10)	27 (62.8)	0.044	0.98 (0.37 - 2.59)	28 (65.1)	< 0.001	4.22 (1.48 - 12.02)
No	159 (52.3)		Ref	141 (46.4)		Ref	91 (30.9)		Ref
**History of availing/receiving psychotherapy**									
Yes	25 (78.1)	0.006*	0.97 (0.24 - 3.95)	25 (78.1)	< 0.001*	2.87 (0.82 - 10.05)	14 (43.8)	0.285	0.68 (0.25 -1.85)
No	167 (53.0)		Ref	143 (45.4)		Ref	108 (34.3)		Ref
**Direct contact with COVID-19 patient**									
Yes	158 (54.1)	0.291	Ref	135 (46.2)	0.061	Ref	103 (35.3)	0.917	Ref
No	34 (61.8)		0.90 (0.30 - 2.69)	33 (60.0)		2.15 (0.83 - 5.55)	19 (34.5)		0.43 (0.18 - 1.05)
**Satisfied with PPE (*N* = 46)**									
Yes	13 (59.1)	0.741		11 (50.0)	0.894		8 (36.4)	0.57	
No	33 (55.0)			31 (51.7)			26 (43.3)		
**Multiple mental health issues present**									
Yes	168 (98.2)	< 0.001*	0.01 (0.00 - 0.02)	149 (87.1)	< 0.001*	0.02 (0.01 - 0.04)	115 (67.3)	< 0.001	0.01 (0.01 - 0.03)
No	24 (13.6)		Ref	19 (10.8)		Ref			Ref

equipment shortages for frontline workers, shortage of human and infrastructural resources (qualified physicians, ICU beds, low oxygen supply, ventilators etc.) for managing the increasing cases of infected patients have posed as challenges to the physicians [51, 53, 54].

Other factors

Physicians having previous history of chronic disease were found to develop depression and stress more than their counterparts. Moreover, history of availing or receiving psychotherapy was an important factor behind development of both depression and

anxiety. As expectedly this finding is in line with previous studies, as the COVID-19 has posed new sets of healthcare challenges leading to deteriorating of already prevalent mental health and physical health condition of physicians [18, 31–33, 41, 42, 48, 49].

The constantly changing landscape of the COVID-19 pandemic, critical patient care decision making, no specific lifesaving treatment, witnessing high numbers of deaths and deteriorating health of co-workers, isolation from family members and assuming unfamiliar clinical roles and expanded workload for COVID-19 patient care has psychologically burdened the physicians [54–56].

Strengths and limitations

Along with different crucial findings, we consider the limitations of this study too. A general shortcoming of a cross-sectional study is that, the study cannot be used to draw a causal relationship among the exposure and outcome variables. Also, being a web based study, meant exclusion of physicians with no internet access or inactivity on social media indicating sampling bias [31]. In addition, no COVID-19 specific tools were used to assess the outcome variables.

In spite of all the limitations, the strength of our study is its attempt to stratify the mental health conditions among COVID-19 and non-COVID-19 hospital, government and non-government physicians, gender and age differences, which is a much-needed addition to the previously done research studies. Most importantly, the findings can be used by policy makers and hospital administrator to implement psychological first aid, a tailored psychological intervention based on the needs of individual staff to mitigate risks effectively [57].

Conclusion

The study findings denoted that, the mental health of physicians was deeply affected by the pandemic. Availability of appropriate mental health support will help to foster resilience by giving them the ability and confidence to manage stressors in crisis moments like COVID-19. It is an interesting finding of our study has demonstrated that heightened depression in younger physicians compared to the older physicians and it needs to be explored further to understand the reasons to take policy action.

Abbreviations

CI: Confidence Interval; Covid: Corona Virus Disease; DASS: Depression Anxiety Stress Scale; icddr,b: International Center for Diarrhoeal Disease Research, Bangladesh; ICU: Intensive Care Unit; MBBS: Bachelor of medicine and Bachelor of Surgery; OR: Odds Ratio; PPE: Personal Protective Equipment; PR: Protocol Number; SPSS: Statistical Package for Social Sciences; UK: United Kingdom; VIF: Variation Inflation Factor.

Acknowledgements

Platform of Facebook group in Bangladesh.

Authors' contributions

AR, FD, JK conceptualized the study, designed, AR and DN managed and analysed the data, and interpreted the results. AR, drafted the article. AR, FD, JK, FB, KK, DN,SA, FBS and IA reviewed, edited and updated the manuscript. All authors reviewed and approved the final version of the manuscript. The corresponding author accepts responsibility as guarantor.

Funding

The principal author would like to acknowledge European Union for providing funding for this. The principal author would like to acknowledge European Union for providing funding for this. The authors would also like to acknowledge the contribution of the current donors providing unrestricted support to icddr, b that include the Government of Bangladesh, Canada, Sweden and the UK. We gratefully acknowledge these donors for their support and commitment to icddr,b's research efforts.

Availability of data and materials

Data contain potentially identifying or sensitive information from delivering women. However, "data can be available on request". The data request should be submitted to the Research Administration (RA) of icddr,b and will be assessed by the corresponding ethics committee, the Institutional Review Board of icddr,b.

Declarations

Ethics approval and consent to participate

Ethical approval for the study (protocol number PR-20046) was obtained on 19 July 2020 from Institutional Review Board of International Center for Diarrhoeal Disease Research, Bangladesh (icddr,b). All methods were carried out in accordance with relevant guidelines and regulations. Informed written consent was obtained from each study participant.

Consent for publication

Not Applicable.

Competing interests

The Author declare no conflict of Interest.

Author details

[1]International Center for Diarrhoeal Disease and Research Bangladesh (icddr,b), 68 Shaheed Tajuddin Ahmed Sarani, Mohakhali, Dhaka 1205, Bangladesh. [2]Department of Clinical Psychology, University of Dhaka, Dhaka 1000, Bangladesh. [3]Department of Health Sciences, University Medical Center Groningen, Groningen, Netherlands. [4]Central Executive Council 2020-21 at Platform, Facebook, Dhaka, Bangladesh.

Received: 25 July 2021 Accepted: 29 November 2021
Published online: 09 December 2021

References

1. Dash B: Can India's emergency response to Covid-19 be more systematic? In., vol. 2020. India: DownToEarth; 2020
2. WHO Coronavirus (COVID-19) Dashboard. https://covid19.who.int.
3. Corona Virus Cases, Bangladesh. https://www.worldometers.info/coronavirus/country/bangladesh/.
4. WHO Coronavirus Disease (COVID-19) Dashboard. https://covid19.who.int/.
5. Covid19 and implications on global health governance. https://www.orfonline.org/expert-speak/covid19-and-implications-on-global-health-governance-66174/.
6. Mackenzie JS, Smith DW. COVID-19: a novel zoonotic disease caused by a coronavirus from China: what we know and what we don't. Microbiol Aust. 2020;41(1):45–50.

7. Grupe DW, Nitschke JB. Uncertainty and anticipation in anxiety: an integrated neurobiological and psychological perspective. Nat Rev Neurosci. 2013;14(7):488–501.
8. Banerjee D. The COVID-19 outbreak: crucial role the psychiatrists can play. Asian J Psychiatr. 2020;50:102014.
9. McCauley L, Hayes R: Taking responsibility for front-line health-care workers. Lancet Public Health. 2020;5(9):e461–e462.
10. Galbraith N, Boyda D, McFeeters D, Hassan T. The mental health of doctors during the COVID-19 pandemic. BJPsych Bull. 2021;45(2):93–97.
11. Bass JK, Bornemann TH, Burkey M, Chehil S, Chen L, Copeland JR, Eaton WW, Ganju V, Hayward E, Hock RS. A United Nations General Assembly Special Session for mental, neurological, and substance use disorders: the time has come. PLoS Med. 2012;9(1):e1001159.
12. Hock RS, Or F, Kolappa K, Burkey MD, Surkan PJ, Eaton WW. A new resolution for global mental health. Lancet (London, England). 2012;379(9824):1367.
13. Tribune D. Coronavirus: Doctors' mortality rate in Bangladesh 'highest in the world'. In. Dhaka: Dhaka Tribune; 2021.
14. COVID-19 update. https://bma.org.bd/.
15. Colton C, Manderscheid R. Congruencies in Increased Mortality Rates, Years of Potential Life Lost, and Causes of Death among Public Mental Health Clients in Eight States [accessed on January 20, 2009]. Prev Chronic Dis. 2006;3(2):1–14 2011.
16. Sadiq MS, Morshed NM, Rahman W, Chowdhury NF, Arafat SY, Mullick MS. Depression, anxiety, stress among postgraduate medical residents: a cross sectional observation in Bangladesh. Iran J Psychiatry. 2019;14(3):192.
17. Khan S, Akter S, Khan T, Shariar G, Awal Miah MA. Psychological distress among Bangladeshi physicians: roles of perceived stigma, fear of infection and resilience in the context of Covid-19 pandemic. J Soc Distress Homelessness. 2021:1–10.
18. Cai Q, Feng H, Huang J, Wang M, Wang Q, Lu X, Xie Y, Wang X, Liu Z, Hou B. The mental health of frontline and non-frontline medical workers during the coronavirus disease 2019 (COVID-19) outbreak in China: A case-control study. J Affec Disord. 2020;275:210–15.
19. Depression care at Mayo Clinic. https://www.mayoclinic.org/diseases-conditions/depression/care-at-mayo-clinic/mac-20356018.
20. Platform. In.; 2021.
21. Tribune D. Patient, doctors, nurses ratio: Bangladesh lags far behind its neighbours. In. Dhaka: Dhaka Tribune; 2021.
22. Lovibond PF, Lovibond SH. The structure of negative emotional states: comparison of the depression anxiety stress scales (DASS) with the Beck depression and anxiety inventories. Behav Res Ther. 1995;33(3):335–43.
23. Cohen S, Kamarck T, Mermelstein R. A global measure of perceived stress. J Health Soc Behav. 1983:385–396.
24. Antony MM, Bieling PJ, Cox BJ, Enns MW, Swinson RP. Psychometric properties of the 42-item and 21-item versions of the Depression Anxiety Stress Scales in clinical groups and a community sample. Psychol Assess. 1998;10(2):176.
25. Alim SAHM, Rabbani MG, Karim E, Mullick MSI, Al Mamun A, Khan MZR. Assessment of depression, anxiety and stress among first year MBBS students of a public medical college, Bangladesh. Bangladesh J Psychiatry. 2015;29(1):23–29.
26. Firoz A, Karim M, Alam M, Rahman A, Zaman M. Prevalence, medical care, awareness and attitude towards mental illness in Bangladesh. Bangladesh J Psychiatry. 2006;20(1):9–32.
27. Service DGoH: how to run a COVID-19 Hospital in Bangladesh. Dhaka; 2020.
28. Cole TR, Carlin N. The suffering of physicians. Lancet. 2009;374(9699):1414–15.
29. AlAteeq DA, Aljhani S, Althiyabi I, Majzoub S. Mental health among healthcare providers during coronavirus disease (COVID-19) outbreak in Saudi Arabia. J Infect Public Health. 2020;13(10):1432–7.
30. Naser AY, Dahmash EZ, Al-Rousan R, Alwafi H, Alrawashdeh HM, Ghoul I, Abidine A, Bokhary MA, AL-Hadithi HT, Ali D. Mental health status of the general population, healthcare professionals, and university students during 2019 coronavirus disease outbreak in Jordan: a cross-sectional study. Brain Behav. 2020;10(8):e01730.
31. Hasan MT, Hossain S, Safa F, Anjum A, Khan AH, Koly KN, Alam SF, Rafi MA, Podder V, Trisa TI. Prevalence of anxiety and depressive symptoms among physicians during the COVID-19 pandemic in Bangladesh: a cross-sectional study. medRxiv. 2020.
32. Que J, Le Shi JD, Liu J, Zhang L, Wu S, Gong Y, Huang W, Yuan K, Yan W, Sun Y. Psychological impact of the COVID-19 pandemic on healthcare workers: a cross-sectional study in China. Gen Psychiatry. 2020;33(3).
33. Chatterjee SS, Bhattacharyya R, Bhattacharyya S, Gupta S, Das S, Banerjee BB. Attitude, practice, behavior, and mental health impact of COVID-19 on doctors. Indian J Psychiatry. 2020;62(3):257.
34. Amin F, Sharif S, Saeed R, Durrani N, Jilani D. COVID-19 pandemic-knowledge, perception, anxiety and depression among frontline doctors of Pakistan. BMC Psychiatry. 2020;20(1):1–9.
35. Hasan M, Tabssum T, Ambia N, Zaman M, Rahman M, Khan A. Mental Health of the COVID-19 Patients in Bangladesh. MMJ. 2021;30(1):189–95.
36. Hou T, Zhang T, Cai W, Song X, Chen A, Deng G, Ni C. Social support and mental health among health care workers during Coronavirus Disease 2019 outbreak: A moderated mediation model. Plos one. 2020;15(5):e0233831.
37. Urooj U, Ansari A, Siraj A, Khan S, Tariq H. Expectations, fears and perceptions of doctors during Covid-19 pandemic. Pak J Med Sci. 2020;36(COVID19-S4):S37.
38. Chaplin TM, Hong K, Bergquist K, Sinha R. Gender differences in response to emotional stress: an assessment across subjective, behavioral, and physiological domains and relations to alcohol craving. Alcohol Clin Exp Res. 2008;32(7):1242–50.
39. Tolin DF, Foa EB. Sex differences in trauma and posttraumatic stress disorder: a quantitative review of 25 years of research; 2008.
40. Saha A, Deeba F. Gender differences in coping styles of patients with depression: a comparative study. Dhaka Univ J Biol Sci. 2018;27(2):145–54.
41. Lai J, Ma S, Wang Y, Cai Z, Hu J, Wei N, et al. Factors associated with mental health outcomes among health care workers exposed to coronavirus disease 2019. JAMA Netw Open. 2020;3(3):e203976.
42. Hassan MZ, Monjur MR, Styczynski AR, Rahman M, Banu S. Protecting frontline healthcare workers should be the top priority in low-resource health systems: Bangladesh and COVID-19. Infect Control Hosp Epidemiol. 2021;42(1):121–22.
43. Podder I, Agarwal K, Datta S. Comparative analysis of perceived stress in dermatologists and other physicians during national lock-down and COVID-19 pandemic with exploration of possible risk factors: A web-based cross-sectional study from Eastern India. Dermatol Ther. 2020;33(4):e13788.
44. Halley MC, Mathews KS, Diamond LC, Linos E, Sarkar U, Mangurian C, Sabry H, Goyal MK, Olazo K, Miller EG. The Intersection of Work and Home Challenges Faced by Physician Mothers During the Coronavirus Disease 2019 Pandemic: A Mixed-Methods Analysis. J Women's Health. 2021;30(4):514–24.
45. Linos E, Halley MC, Sarkar U, Mangurian C, Sabry H, Olazo K, Mathews KS, Diamond L, Goyal MK, Linos E. Anxiety Levels Among Physician Mothers During the COVID-19 Pandemic. Am J Psychiatry. 2021;178(2):203–204.
46. Coughlan C, Nafde C, Khodatars S, Jeanes AL, Habib S, Donaldson E, Besi C, Kooner GK: COVID-19: lessons for junior doctors redeployed to critical care. Postgrad Med J. 2021;97(1145):188–191.
47. Purohit B, Bandyopadhyay T: Beyond job security and money: driving factors of motivation for government doctors in India. Hum Resour Health. 2014;12(1):1–13.
48. Muller RAE, Stensland RSØ, van de Velde RS. The mental health impact of the covid-19 pandemic on healthcare workers, and interventions to help them: a rapid systematic review. Psychiattry Res. 2020:113441.
49. Pappa S, Ntella V, Giannakas T, Giannakoulis VG, Papoutsi E, Katsaounou P: Prevalence of depression, anxiety, and insomnia among healthcare workers during the COVID-19 pandemic: A systematic review and meta-analysis. Brain Behav Immun. 2020;88:901–7.
50. Santarone K, McKenney M, Elkbuli A. Preserving mental health and resilience in frontline healthcare workers during. COVID-19. 2020;38(7):1530–1.
51. Khan HR, Howlader T, Islam MM: Battling the COVID-19 pandemic: is Bangladesh prepared? Medrxiv. 2020.
52. Dasgupta H, Kumar S: Role stress among doctors working in a government hospital in Shimla (India). Eur J Soc Sci. 2009;9(3):356–70.
53. Ayanian JZ. Mental health needs of health care workers providing frontline COVID-19 care. In: JAMA Health Forum: 2020: American Medical Association; 2020. p. e200397.

54. Smereka J, Szarpak L. The use of personal protective equipment in the COVID-19 pandemic era. Am J Emerg Med. 2020;38(7):1529–30.
55. Barba R, Rosado C, Pardo-Moreno J, Rey-Biel J: Managing people, roles, and resources during covid-19 surge how the Rey Juan carlos university hospital in Madrid quickly modified its spaces, staffing levels, and roles to confront a surge of covid-19 patients. In.; 2020.
56. Salam A, Yousuf R, Bakar SMA, Haque M. Stress among medical students in Malaysia: A systematic review of literatures. Int Med J. 2013;20(6):649–55.
57. Gold JA. Covid-19: adverse mental health outcomes for healthcare workers: British Medical Journal Publishing Group; 2020.

Publisher's Note

Springer Nature remains neutral with regard to jurisdictional claims in published maps and institutional affiliations.

Ready to submit your research? Choose BMC and benefit from:

- fast, convenient online submission
- thorough peer review by experienced researchers in your field
- rapid publication on acceptance
- support for research data, including large and complex data types
- gold Open Access which fosters wider collaboration and increased citations
- maximum visibility for your research: over 100M website views per year

At BMC, research is always in progress.

Learn more biomedcentral.com/submissions

BMC
