# Supplementary material for: Mental health status and related factors influencing healthcare workers during the COVID-19 pandemic: A systematic review and meta-analysis
Source: PLoS One. 2024 Jan 19;19(1):e0289454. doi: 10.1371/journal.pone.0289454 (PMC10798549; doi:10.1371/journal.pone.0289454)
Supplement: S1 Data — (ZIP) [file pone.0289454.s011.zip › literatures/142.pdf]

ELSEVIER

Since January 2020 Elsevier has created a COVID-19 resource centre with free information in English and Mandarin on the novel coronavirus COVID-19. The COVID-19 resource centre is hosted on Elsevier Connect, the company's public news and information website.

Elsevier hereby grants permission to make all its COVID-19-related research that is available on the COVID-19 resource centre - including this research content - immediately available in PubMed Central and other publicly funded repositories, such as the WHO COVID database with rights for unrestricted research re-use and analyses in any form or by any means with acknowledgement of the original source. These permissions are granted for free by Elsevier for as long as the COVID-19 resource centre remains active.

Asian Journal of Psychiatry 51 (2020) 102080

Contents lists available at ScienceDirect

Asian Journal of Psychiatry

journal homepage: www.elsevier.com/locate/ajp

Letter to the Editor

Mental health of medical workers in Pakistan during the pandemic COVID-19 outbreak

On January 30, 2020, WHO (World Health Organization) announced the occurrence of the novel coronavirus and declared a PHEIC (Public Health Emergency of International Concern), which is the sixth PHEIC under the IHR (International Health Regulations) after H1N1 Influenza (2009), Polio (2014), Ebola in West Africa (2014), Zika (2016), Ebola in DRC (2019) (Euro-surveillance Editorial, 2020). On February 11, 2020, novel coronavirus was officially named by WHO as Corona Virus Disease 2019 – COVID-19. The pandemic not only brought the high mortality rate from the viral infection but also psychological rest and mental catastrophe to the rest of the world (Xiao, 2020). Such uncertainty and unpredictability of pandemic outbreak of infectious disease from its clinical presentation, infectious causes, epidemiological features, fast transmission pattern, seriousness of public health impact, novelty, scale, implication for international public health, and underprepared health facilities to address the pandemic outbreak of COVID-19 have considerably high potential for psychological fear contagion as well and often result in prevalent multitude of psychological problems such as fear, anxiety, stigma, prejudice, marginalization towards the disease and its relation of all people ranging from healthy to at-risk individuals to care-workers (Mak et al., 2009). Mass quarantine could cause a sense of collective hysteria, fear, and anxiety in health workers working in hospitals, inpatient and outpatient care, large tertiary care centers, community based hospitals, primary care settings, nursing homes, assisted living facilities and all isolation units. The medical health-care workers who are exposed and in direct contact with the confirmed and suspected coronavirus cases are vulnerable to both high risk infection and mental health problems – worried, scared, experiencing bereavement and trauma. With the advent of COVID-19 in Pakistan, medical workers have been under physical and psychological pressure including high risk of infection, inadequate equipment for safety from contagion, isolation, exhaustion, and lack of contact with family. The severity is causing further mental health problems which not only effect medical workers' decision making ability but could also have long term detrimental effect on their overall well-being. The unremitting stress medical health-care workers is experiencing could trigger psychological issues of anxiety, fear, panic attacks, posttraumatic stress symptoms, psychological distress, stigma and avoidance of contact, depressive tendencies, sleep disturbances, helplessness, interpersonal social isolation from family social support and concern regarding contagion exposure to their friends and family. The sudden role reversal from a healthcare provider to the COVID-19 confirmed or suspected patient potentially lead to sense of frustration, helplessness, and adjustment challenges in healthcare professionals. Fear of labeling, stigmatization and discrimination potentially impede healthcare workers intent to seek counselling and psychotherapeutic interventions (Zheng, 2020). Despite the common mental health problems and psychosocial issues among healthcare workers in such settings, most health professionals do not often seek or receive a systematic mental health care (Xiang et al., 2020).

Healthcare workers and professionals' –who work under high stress environment – emotional and behavioral responses are naturally adaptive in the face of extreme (unpredictable and uncertain) stress, and thus counselling and psychotherapy based on the stress-adaptation model might act as early and prompt intervention. Addressing the mental health issues in medical workers is thus important for the better prevention and control of the pandemic (Banerjee, 2020). Medical workers usually are rotated within the state's provinces to care for confirmed or suspected cases, strengthen logistic support and ease the pressure on health-care personnel. In such case, online and electronic media broadcast medical advice on how to prevent the risk of transmission between patients and medical workers in medical setting could reduce the pressure on medical workers.

A detailed psychological crisis intervention plan should be developed: a) by building a mental health intervention medical team to provide online courses for awareness of psychological impact of stressful events to guide medical workers, b) and a psychological assistance hotline intervention for medical workers to discuss their psychological concerns with the trained and specialized team of mental health practitioners. Hospitals in this regard should provide frequent shift-system, guarantee food and living supplies, offer pre-job training to address identification and responses to psychological issues in patients, families, and themselves. Moreover, psychological counselors/counselling psychologists should regularly visit medical workers to listen to their stories for their catharsis and provide support.

To deal with the secondary mental health problems involved in the COVID-19 pandemic, urgent psychological crisis intervention model (PCIM) should be developed and implemented through the medium of internet technology. This PCIM integrate teams of physicians, psychiatrists, psychologists/mental health practitioners, and social workers to deliver early psychological intervention to patients, families and medical staff. Diverse range of measures implemented across various health-care settings would assist swift, smooth and safer early screening and intervention and later rehabilitation. Epidemiological data on mental health consequences, psychological impact, psychiatric morbidity and psychosocial issues with the advent of COVID-19 and their screening, assessment, control, treatment plans, management, progress reports, health status updates, prevention and intervention has yet to be explored to respond to these challenges. This publication marks the

https://doi.org/10.1016/j.ajp.2020.102080
Received 2 April 2020; Accepted 3 April 2020
1876-2018/ © 2020 Elsevier B.V. All rights reserved.

preliminary initiation of guidance to provide multifaceted mental health dynamics and psychological intervention of medical workers in Pakistan.

Financial disclosure

The authors declare no financial disclosure related to the submission.

Declaration of Competing Interest

The authors declare no conflicts of interest

Acknowledgment

None.

Waleed Rana*
Medical Officer at Alkhidmat Hospital Mansura, Lahore and Alumnus of Hainan Medical, University, China

Sonia Mukhtar
Independent Researcher and Practitioner Counselling Psychologist and Alumnus of University of Management and Technology, Lahore, Pakistan

Shamim Mukhtar,
Compliance Officer at Texpak Pvt Ltd and Alumnus of University of the Punjab, Lahore, Pakistan
E-mail addresses: waleedrana312@gmail.com (W. Rana), sonia.mukhtar12@gmail.com (S. Mukhtar), shamim.mukhtar13@gmail.com (S. Mukhtar),

References

Banerjee, D., 2020. The COVID-19 outbreak: crucial role the psychiatrists can play. Asian J. Psychiatr. 102014. https://doi.org/10.1016/j.ajp.2020.102014.
Mak, I.W., Chu, C.M., Pan, P.C., Yiu, M.G., Chan, V.L., 2009. Long-term psychiatric morbidities among SARS survivors. Gen. Hosp. Psychiatr. 31, 318–326.
Xiang, Y.T., Yang, Y., Li, W., Zhang, L., Zhang, Q., Cheung, T., Ng, C.H., 2020. Timely mental health care for the 2019 novel Coronavirus outbreak is urgently needed. Lancet Psychiatr. 7, 228–229. https://doi.org/10.1016/S2215-0366(20)30046-8.
Xiao, C., 2020. A novel approach of consultation on 2019 novel coronavirus (COVID-19)-Related psychological and mental problems: structured letter therapy. Psychiatr. Invest. 17 (2), 175–176.
Zheng, Wei, 2020. Mental health and a novel coronavirus (2019-nCoV) in China. J. Aff. Disord. https://doi.org/10.1016/j.jad.2020.03.041. published online 21 March 2020.

* Corresponding author.
