# Supplementary material for: Mental health status and related factors influencing healthcare workers during the COVID-19 pandemic: A systematic review and meta-analysis
Source: PLoS One. 2024 Jan 19;19(1):e0289454. doi: 10.1371/journal.pone.0289454 (PMC10798549; doi:10.1371/journal.pone.0289454)
Supplement: S1 Data — (ZIP) [file pone.0289454.s011.zip › literatures/145.pdf]

ELSEVIER

Since January 2020 Elsevier has created a COVID-19 resource centre with free information in English and Mandarin on the novel coronavirus COVID-19. The COVID-19 resource centre is hosted on Elsevier Connect, the company's public news and information website.

Elsevier hereby grants permission to make all its COVID-19-related research that is available on the COVID-19 resource centre - including this research content - immediately available in PubMed Central and other publicly funded repositories, such as the WHO COVID database with rights for unrestricted research re-use and analyses in any form or by any means with acknowledgement of the original source. These permissions are granted for free by Elsevier for as long as the COVID-19 resource centre remains active.

REV COLOMB PSIQUIAT. 2021;xxx(xx):xxx–xxx

Asociación Colombiana de Psiquiatría ACP

Revista Colombiana de Psiquiatría

www.elsevier.es/rcp

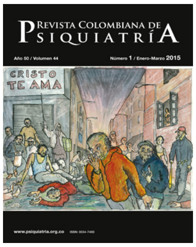

Artículo de actualización

Prevalencia y características clínicas de los síntomas depresivos y ansiosos de los trabajadores de una institución de salud en Medellín durante la pandemia por COVID-19

Miguel Restrepo-Martínez[a,*], *Marcela Escobar*[a], *Luz Aida Marín*[a] y *Diana Restrepo*[b]

[a] *Departamento de Salud Mental, Clínica Las Américas Auna, Medellín, Colombia*
[b] *Universidad CES, Medellín, Colombia*

INFORMACIÓN DEL ARTÍCULO

Historia del artículo:
Recibido el 14 de septiembre de 2020
Aceptado el 15 de febrero de 2021
On-line el xxx

Palabras clave:
Síntomas
Depresión
Ansiedad
Sector sanitario
Personal sanitario

RESUMEN

Introducción: Afrontar las circunstancias de la vida y el trabajo que la pandemia por COVID-19 ha impuesto al personal sanitario implica un reto extraordinario: cuidar y atender a otros, exponiéndose a sí mismos al contagio. A médicos, enfermeras, terapeutas y demás personal asistencial y no asistencial en clínicas y hospitales, se les pide estar preparados para trabajar en situaciones extraordinariamente complejas y estresantes, lo que los hace vulnerables a problemas mentales.

Objetivo: Determinar la prevalencia y las características clínicas de los síntomas ansiosos y depresivos del personal que trabaja en una institución de salud en Medellín, Colombia.

Métodos: Estudio observacional, descriptivo y transversal, a partir de una encuesta diseñada al efecto, la cual incluyó 2 escalas para tamizar síntomas depresivos y ansiosos, además de variables sociodemográficas.

Resultados: Se incluyó a 1.247 trabajadores de la institución de salud. El 14,6% reportó síntomas depresivos y el 18,5%, ansiosos clínicamente significativos. Se encontró una mayor proporción de síntomas depresivos y ansiosos moderados-graves en la modalidad de trabajo presencial.

Conclusiones: Los síntomas ansiosos y depresivos son muy prevalentes entre los trabajadores de una institución de salud de Medellín durante la pandemia de COVID-19. Dado el papel vital del sector salud en tiempos de pandemia, el desarrollo de programas de salud mental que aborden los problemas de esta población debería considerarse una prioridad.

© 2021 Asociación Colombiana de Psiquiatría. Publicado por Elsevier España, S.L.U. Todos los derechos reservados.

* *Autor para correspondencia.*
Correo electrónico: mrm87356@hotmail.com (M. Restrepo-Martínez).

https://doi.org/10.1016/j.rcp.2021.02.001
0034-7450/© 2021 Asociación Colombiana de Psiquiatría. Publicado por Elsevier España, S.L.U. Todos los derechos reservados.

Cómo citar este artículo: Restrepo-Martínez M, et al. Prevalencia y características clínicas de los síntomas depresivos y ansiosos de los trabajadores de una institución de salud en Medellín durante la pandemia por COVID-19. Rev Colomb Psiquiat. 2021. https://doi.org/10.1016/j.rcp.2021.02.001

Prevalence and Clinical Characteristics of Depression and Anxiety Symptoms in Staff at a Health Institution in Medellin During the COVID-19 Pandemic

Keywords:
Symptoms
Depression
Anxiety
Health sector
Health workers

A B S T R A C T

Introduction: The COVID-19 pandemic has represented an extraordinary challenge for health workers as they care for others while exposing themselves to contagion. Doctors, nurses, therapists and other non-care staff in clinics and hospitals are asked to be prepared to work in particularly complex and stressful situations, which makes them vulnerable to mental health problems.

Objective: To determine the prevalence and clinical characteristics of anxiety and depression symptoms in staff working at a health institution in Medellin, Colombia.

Methods: Observational, descriptive and cross-sectional study, based on a survey designed for the investigation, which included two scales to screen depression and anxiety symptoms, as well as sociodemographic variables.

Results: A total of 1,247 workers from the health institution were included. Of these, 14.6% reported symptoms of depression and 18.5% of clinically significant anxiety. A higher proportion of moderate to severe depression and anxiety symptoms was found in those working face to face.

Conclusions: Anxiety and depression symptoms are highly prevalent among staff at a health institution in Medellin, Colombia, during the COVID-19 pandemic. Given the vital role of the health sector in times of pandemic, the development of mental health programmes that address the problems of this population should be considered a priority.

© 2021 Asociación Colombiana de Psiquiatría. Published by Elsevier España, S.L.U. All rights reserved.

Introducción

El 30 de enero de 2020, la Organización Mundial de la Salud (OMS) declararó estado de emergencia mundial por el sugimiento de una nueva cepa de coronavirus, el SARS-CoV-2[1], reportado 1 mes antes en Wuhan, China, a partir de un brote de neumonía[2]. En general, el modo de vida de cada persona se ha visto afectada, de diversas maneras, en todos los continentes, en todos los países y en todos los grupos socioeconómicos.

Más de 6 meses después del primer caso reportado, aún no se dispone de una vacuna o un tratamiento específico para la COVID-19 y no se vislumbra el fin de la propagación de la enfermedad[3]. La implementación de cuarentenas prolongadas, el distanciamiento físico y otras estrategias de salud pública destinadas a disminuir la tasa de contagio al parecer tienen un impacto negativo en la salud mental de un grupo importante de la población[4].

Desde esta perspectiva, el personal de salud se enfrenta a un reto extraordinario: cuidar de otros, exponiéndose a sí mismos al contagio. A médicos, enfermeras, terapeutas y demás personal asistencial y no asistencial en clínicas y hospitales, se les pide que estén preparados para trabajar en situaciones extraordinariamente complejas de exposición permanente a la COVID-19[5-7]. Así, el personal de salud parece ser la categoría más expuesta a los desafíos psicológicos de una nueva normalidad. Es probable que esta carga psicológica se deba a la reorganización del personal, el aumento de la intensidad del trabajo y la ansiedad de estar expuesto al virus en el hospital y, a su vez, de llevar la infección a casa[8].

Poco tiempo después de declararse la pandemia, comenzaron a publicarse estudios que reportaban altos niveles de estrés en personal de salud, denominado ahora «la primera línea» de atención, lo cual se ha visto reflejado en una mayor prevalencia de insomnio y síntomas ansiosos y depresivos, además de un probable incremento en las tasas de suicidio en esta población[5,9]. Continuar investigando sobre este tema permite generar información que ayude a fortalecer la capacidad de respuesta de los sistemas sanitarios, además de monitorizar e influir positivamente en los descelaces de salud mental de los trabajadores de la salud[10]. El objetivo de este estudio es determinar la prevalencia y las características clínicas de los síntomas depresivos y ansiosos de los trabajadores de una institución de salud en Medellín, Colombia.

Métodos

Se realizó un estudio observacional, descriptivo y transversal. Auna es un grupo de salud de ámbito regional, con cerca de 6.000 colaboradores, que cuenta con una red de clínicas y centros médicos en Perú y Colombia[11]. La Clínica Las Américas Auna es una institución sanitaria ubicada en Medellín y dispone de 304 camas para atender a niños y adultos que requieren servicios de urgencias, hospitalización y cuidados intensivos para pacientes con enfermedades médicas y quirúrgicas de alta complejidad.

A partir de la declaración de la pandemia de COVID-19[12], el área de salud mental de la Clínica Las Américas Auna quiso alinear sus esfuerzos con las propuestas de la *New Health Foundation* (institución sin ánimo de lucro de observación y

Cómo citar este artículo: Restrepo-Martínez M, et al. Prevalencia y características clínicas de los síntomas depresivos y ansiosos de los trabajadores de una institución de salud en Medellín durante la pandemia por COVID-19. Rev Colomb Psiquiat. 2021. https://doi.org/10.1016/j.rcp.2021.02.001

optimización de los sistemas de salud)[13] para fortalecer, apoyar e intervenir al personal sanitario en tiempo de crisis.

Este programa incluyó: *a)* formación sobre cuidados para prevenir el contagio del coronavirus y técnicas de regulación emocional; *b)* identificación de personas con síntomas ansiosos y depresivos de importancia clínica, a quienes se ofrece intervención individual por psicología y psiquiatría, y *c)* sesiones cortas de acupuntura y realización de pautas activas como promoción de bienestar.

Participantes

Se invitó a participar en el estudio a los 2.100 empleados de la institución de las áreas asistenciales y administrativas. Entre el 1 de marzo y el 31 de mayo de 2020, el Departamento de Comunicadiones, a través de los diferentes canales de comunicación internos como la intranet, carteleras, voz a voz, líderes de grupos de trabajo y el correo electrónico de cada empleado, motivó la participación en el estudio. La encuesta estuvo disponible durante 8 semanas, tiempo que duró la recolección de la muestra; 1.247 empleados realizaron las encuestas y aceptaron participar en el estudio. Se tuvo un criterio de inclusión: ser empleado de la institución durante el tiempo de recolección de la muestra. Se excluyó a quienes no dieron su consentimiento informado.

Variables

Se incluyeron variables sociodemográficas: edad, sexo, oficio —asistencial, administrativo—, modalidad de trabajo —presencial, teletrabajo— y clínicas psiquiátricas: 9 síntomas depresivos, 7 síntomas ansiosos y puntuaciones de las escalas Cuestionario de Salud del Paciente-9 (PHQ-9) y Trastorno de Ansiedad Generalizada (GAD-7).

Fuentes de datos

La información se recogió de una encuesta, diseñada por los investigadores para este estudio, que incluyó 22 preguntas con opciones de respuesta tipo Likert de fácil comprensión y respuesta; para completarla se necesitaba 10-15 min. No se realizó prueba piloto.

Instrumentos empleados

Este estudio incluyó 2 escalas psicométricas disponibles para aplicar a población colombiana y de uso libre[14]: el PHQ-9 y la GAD-7. El PHQ-9 consta de 9 preguntas tipo Likert con respuestas entre 0 y 3 puntos. Las puntuaciones posibles van de 0 a 27. Los puntos de corte 5, 10, 15 y 20 son para depresión leve, moderada, moderadamente grave y grave. Con un punto de corte > 10 se tiene una sensiblidad del 88% y una especificidad del 88%[15]. La GAD-7 consta de 7 preguntas tipo Likert, con respuestas entre 0 y 3 puntos. Las puntuacines posibles van de 0 a 21. Los puntos de corte propuestos de 5, 10 y 15 son para síntomas leves, moderados y graves; se recomienda evaluación clínica con puntuaciones > 10. Con estos puntos de corte, la escala tiene una sensibilidad del 89% y una especificidad del 82%[16]. Ambos cuestionarios cuentan con validación al español[17-19].

Sesgos

Los investigadores tuvieron en cuenta los siguientes sesgos: *a)* de selección; se realizó una amplia campaña de divulgación de la investigación a través de los canales de comunicación de la institución y a cada persona se le brindaron numerosas oportunidades para participar del estudio; *b)* de información; los datos fueron anonimizados y solo los trataron los investigadores garantizando privacidad y uso de la información con fines estrictamente clínicos y de investigación; además se emplearon instrumentos válidos, que exploraron síntomas mentales ocurridos en las últimas 2 semanas, lo que disminuye el sesgo de memoria, y *c)* confusión; el alcance del estudio es de tipo descriptivo, por lo que su objetivo no es buscar asociaciones. Sin embargo, no se incluyeron otras variables que clasifiquen según el encuestado la causa o el origen de los síntomas ansiosos y depresivos reportados.

Tamaño muestral

El tamaño de la muestra fue 1.247 individuos. El número de personas que respondieron a la encuesta durante el periodo de estudio determinó el tamaño muestral, de modo que se trata de una muestra no probabilística, no representativa, en la cual no todos los individuos tuvieron la misma probabilidad de ser elegidos.

Análisis de la información

La información se analizó con el *software* estadístico SPSS® versión 21 (SPSS Inc.; Estados Unidos), licencia amparada. Las variables cualitativas categóricas se presentaron mediante frecuencias absolutas y relativas. Las variables cuantitativas se presentan como media ± desviación estándar, la mediana [intervalo intercuartílico], según los supuestos de normalidad (prueba de Shapiro-Wilk). Se hizo análisis por subgrupos de edad, sexo y oficio mediante el estadístico χ^2 de Pearson o Fisher, asumiendo diferencias significativas a partir de $p < 0{,}05$. Se determinó la prevalencia de síntomas depresivos (PHQ-9 > 10) y ansiosos (GAD-7 > 5) clínicamente significativos. Se completaron todas las encuestas, por lo que no hubo datos ausentes.

Aspectos éticos

Esta investigación fue aprobada por el Comité de Investigación Institucional. Según la normativa colombiana, se clasifica con un riesgo menor que el mínimo[20]. Se tomó el consentimiento informado a cada participante. Se tuvieron en cuenta los principios de la Declaración de Helsinki de la Asociación Médica Mundial[21]. Se garantizó la confidencialidad y el respeto por la información brindada. Este manuscrito se construyó siguiendo las directrices de la Declaración STROBE[22].

Resultados

El 59,09% de las personas invitadas a participar respondieron a la encuesta. De las 1.291 encuestas diligenciadas, el 3,5% (n = 44) no autorizó el uso de la información para investigación, por lo que se las excluyó de la muestra. Se presentan resultados a partir de 1.247 encuestas diligenciadas en su tota-

Cómo citar este artículo: Restrepo-Martínez M, et al. Prevalencia y características clínicas de los síntomas depresivos y ansiosos de los trabajadores de una institución de salud en Medellín durante la pandemia por COVID-19. Rev Colomb Psiquiat. 2021. https://doi.org/10.1016/j.rcp.2021.02.001

Tabla 1 – Características sociodemográficas y laborales de la población de estudio

Edad (años)	37,1 ± 10,5<br>36,0 [15]
Sexo	
Varones	288 (23,1)
Mujeres	959 (76,9)
Tipo de trabajo	
Relacionado con el cuidado de pacientes	842 (67,5)
Administrativo	405 (32,5)
Forma de trabajar durante pandemia	
Presencial	998 (80,0)
A distancia	249 (20,0)
Tipo de personal asistencial	
Auxiliar de enfermería	404 (32,4)
Enfermero profesional	114 (9,1)
Médico	156 (28,3)
Terapeuta respiratorio	15 (1,2)
Otros	335 (26,9)

Los valores expresan n (%), media ± desviación estándar o mediana [intervalo intercuartílico].

lidad. La mediana de edad fue 36 [15] (intervalo, 18-80) años; las demás características se presentan en la tabla 1.

Prevalencia de síntomas ansiosos

Se indagó por la presencia de síntomas ansiosos en la población de estudio en las últimas 2 semanas. El 49,9% de la población estudiada no tuvo síntomas ansiosos de importancia clínica. Se identificaron síntomas ansiosos leves (GAD-7 > 5) en el 31,7%, síntomas moderados (GAD-7 > 10) en el 13,4% y graves (GAD-7 > 15) en el 5,1% de los participanes. En la tabla 2 se presentan los síntomas ansiosos agrupados en síntomas de menor importancia clínica (ningún día-algunos días) y de mayor importancia clínica (más de la mitad de los días-casi todos los días).

Prevalencia de síntomas depresivos

Se indagó por síntomas depresivos en la población de estudio en las últimas 2 semanas. El 59,4% de los participantes no tuvo síntomas depresivos; se identicaron síntomas leves (PHQ-9 = 5-9) en el 26,0%, moderados (PHQ-9 = 10-14) en el 8,2%, moderadamente graves (PHQ-9 = 15-19) en el 4,7% y graves (PHQ-7 > 20) en el 1,7%. En la tabla 3 se presentan los síntomas depresivos agrupados en síntomas de menor importancia clínica (ningún día-algunos días) y de mayor importancia clínica (más de la mitad de los días-casi todos los días).

Síntomas neurovegetativos e ideación suicida según sexo

Se encontró que el 19,3% de las mujeres y el 12,5% de los varones presentaban problemas relacionados con el apetito. En tanto que el 25,7% de las mujeres y el 19,8% de los varones tenían problemas relacionados con el sueño. En cuanto a la ideación de muerte o suicido, 62 mujeres (6,4%) y 19 varones (6,6%) admitieron este tipo de ideas en las últimas 2 semanas.

Síntomas depresivos y ansiosos entre moderados y graves

El 14,6 y el 18,5% de los encuestados reportaron, respectivamente, síntomas depresivos y ansiosos clínicamente significativos (de intensidad moderada y grave). Estos se compararon según sexo, franjas de edad y modalidad de trabajo (a distancia-virtual). Se encontró mayor proporción una de síntomas depresivos y ansiosos moderados a graves en mujeres, modalidad de trabajo presencial y en los más jóvenes (18-25 años) (figs. 1-3).

Se exploraron posibles asociaciones de los síntomas depresivos y ansiosos con las demás variables de estudio. La presencia de síntomas ansiosos se asoció con el sexo femenino (p = 0,004) y la modalidad de trabajo presencial (p = 0,004). Los síntomas depresivos se asociaron con sexo femenino (p = ,005), pero no con la modalidad de trabajo presencial (p = 0,1 33). Asimismo la presencia de ansiedad moderada fue mayor en el personal que desempeñaba labores asociadas con el cuidado de la salud que en aquellos con labores administrativas (p = 0,046).

Discusión

Durante 2020 los trabajadores del sector salud han afrontado situaciones inéditas: atender a pacientes con COVID-19, sortear los cambios sociales, afrontar fuertes estresores como la muerte de sus pares y tomar decisiones en escenarios clínicos de gran incertidumbre[8]. Esto ha hecho que los profesionales de la salud sean descritos en términos heroicos. Sin embargo, la potencial crisis de salud mental que podría desarrollarse entre los trabajadores de salud no ha recibido suficiente atención[9]. El estrés al que se exponen actualmente puede afectar a la

Tabla 2 – Síntomas ansiosos en las últimas 2 semanas

Síntomas	Ningún día-varios días, n (%)	Más de la mitad de los días-casi todos los días, n (%)
Se ha sentido nervioso	997 (79,9)	250 (20,0)
No ha sido capaz de parar o controlar sus preocupaciones	1.051 (84,3)	196 (15,8)
Se ha preocupado demasiado por motivos diferentes	1.005 (80,6)	242 (19,5)
Ha tenido dificultad para relajarse	1.015 (81,4)	232 (18,6)
Se ha sentido tan inquieto que no se puede quedar quieto	1.114 (89,3)	133 (10,6)
Se ha molestado o irritado fácilmente	1.079 (86,5)	168 (13,4)
Ha tenido miedo de que algo terrible fuera a pasar	994 (79,7)	253 (20,3)

Cómo citar este artículo: Restrepo-Martínez M, et al. Prevalencia y características clínicas de los síntomas depresivos y ansiosos de los trabajadores de una institución de salud en Medellín durante la pandemia por COVID-19. Rev Colomb Psiquiat. 2021. https://doi.org/10.1016/j.rcp.2021.02.001

Tabla 3 – Síntomas depresivos en las últimas 2 semanas

Síntomas	Ningún día-algunos días, n (%)	Más de la mitad de los días-casi todos los días, n (%)
Poco interés o placer en hacer cosas	1.139 (90,9)	114 (9,1)
Se ha sentido decaído, deprimido, sin esperanzas	1.132 (90,8)	115 (9,2)
Ha tenido dificultad para dormirse o permanecer dormido o ha dormido demasiado	943 (75,6)	304 (24,4)
Se ha sentido cansado o con poca energía	1.011 (81,1)	304 (18,9)
Sin apetito o ha comido en exceso	1.026 (82,3)	221 (17,7)
Se ha sentido mal con usted mismo o que es un fracaso o que ha quedado mal con usted o su familia	1.146 (91,9)	101 (8,1)
Ha tenido dificultad para concentrarse en actividades como leer periódico o ver televisión	1.120 (89,8)	127 (10,1)
Se ha movido o hablado tan lento que otras personas podrían haberlo notado o tan inquieto o agitado que ha estado moviéndose mucho más de lo normal	1.177 (94,4)	70 (5,6)
Pensamientos que estaría mejor muerto o de lastimarse de alguna manera	1.227 (98,4)	20 (1,6)

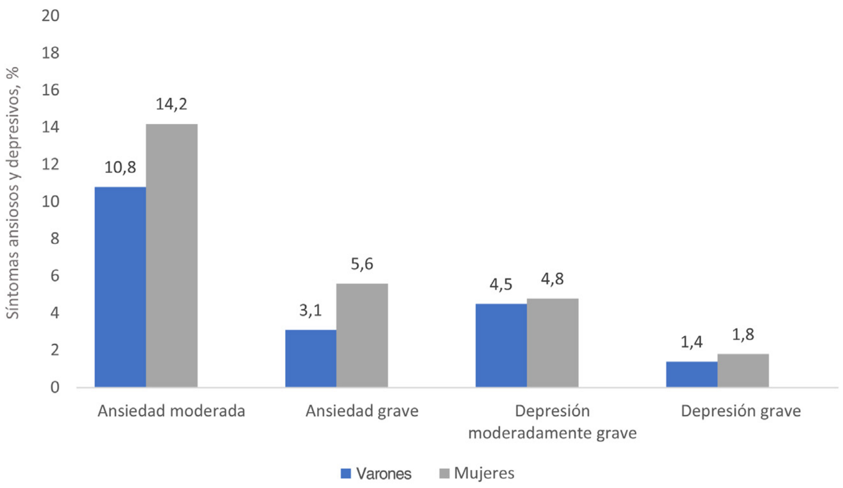

Figura 1 – Proporción de personas con síntomas ansiosos y depresivos moderados-graves según sexo.

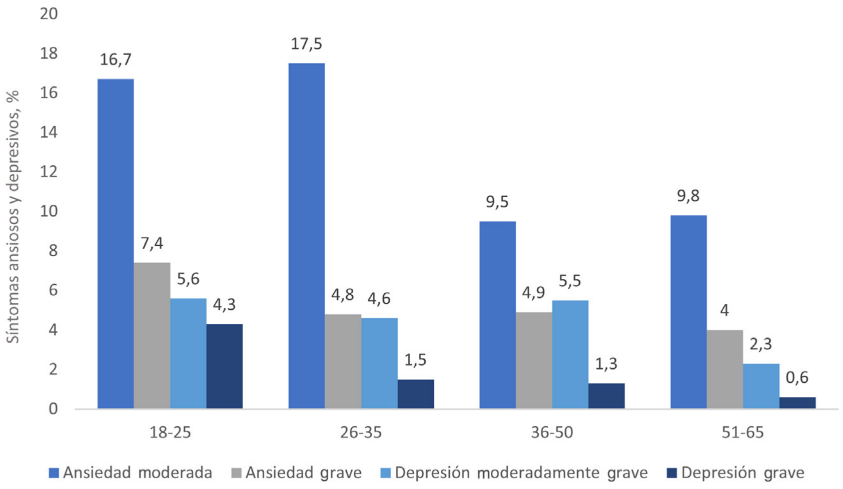

Figura 2 – Proporción de personas con síntomas ansiosos y depresivos moderados-graves según franjas de edad.

Cómo citar este artículo: Restrepo-Martínez M, et al. Prevalencia y características clínicas de los síntomas depresivos y ansiosos de los trabajadores de una institución de salud en Medellín durante la pandemia por COVID-19. Rev Colomb Psiquiat. 2021. https://doi.org/10.1016/j.rcp.2021.02.001

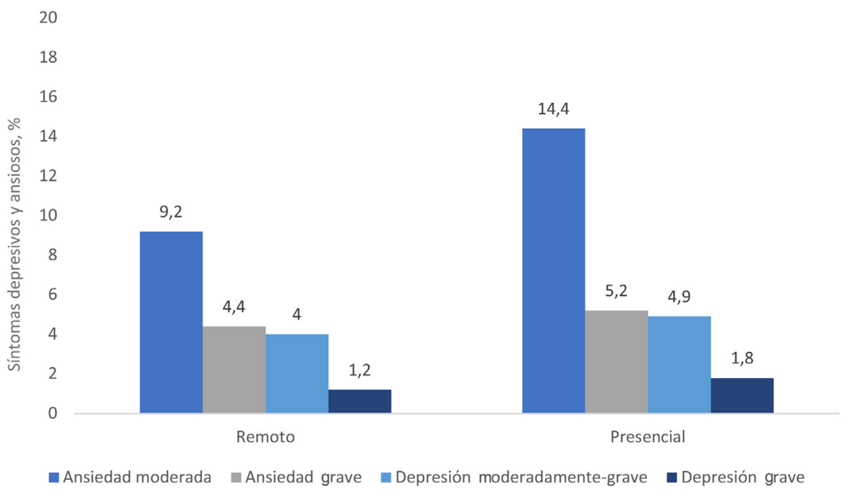

Figura 3 – Proporción de personas con síntomas de ansiedad y depresivos moderados-graves según modalidad de trabajo.

salud física y mental de la fuerza laboral sanitaria y, a su vez, reducir su capacidad para enfrentarse eficazmente a la actual emergencia de salud[8].

El principal hallazgo de este estudio es que 1 de cada 5 personas encuestadas mostraba síntomas ansiosos y 1 de cada 6, síntomas depresivos de importancia clínica. En en la población general de Medellín, la prevalencia de trastorno depresivo mayor en los últimos 30 días es del 2,20% (la de cualquier trastorno del estado del ánimo, el 2,82%) y la del trastorno de ansiedad generalizada, del 0,46% (cualquier trastorno de ansiedad, el 5,08%)[23]. Por lo tanto, las prevalencias de síntomas depresivos y ansiosos en nuestro estudio con población del sector salud fueron 7 veces mayores para depresión y los de ansiedad, hasta 40 veces.

Nuestros hallazgos coinciden con estudios recientes realizados en otros países, que han arrojado resultados alarmantes respecto a la salud mental del personal sanitario[24,25]. Por ejemplo, la encuesta realizada por el equipo de Jianbo Lia et al.[24], en 1.257 sujetos pertenecientes al personal de salud de China, encontró síntomas depresivos y ansiosos (entre moderados y graves) en un 14,8 y un 12.,3% de los encuestados respectivamente. Asimismo, en una revisión sistemática y metanalítica reciente que incluyó 13 estudios y 33.062 participantes, Pappa et al.[25] encontraron unas prevalencias de ansiedad del 23,2%, de depresión del 22,8% y de insomnio del 38,9%, lo cual muestra la elevada prevalencia de estos síntomas mentales en los trabajadores de la salud.

Otros autores han querido determinar cómo se relacionan algunos síntomas de salud mental según el trabajo realizado durante la pandemia. En China, Zhang et al.[7] realizaron un estudio con 2.182 personas con el objetivo de comparar los problemas psicosociales y de salud mental durante el brote de la COVID-19 en 2 grupos de trabajadores. Encontraron mayores prevalencias de insomnio (el 38,4 frente al 30,5%; $p < 0,01$), ansiedad (el 13,0 frente al 8,5%; $p < 0,01$), depresión (el 12,2 frente al 9,5%; $p < 0,04$), somatización (el 1,6 frente al 0,4%; $p < 0,01$) y síntomas obsesivos compulsivos (el 5,3 frente al 2,2%; $p < 0,01$) en el grupo médico. De modo similar, en nuestro estudio se observó que los individuos con trabajos relacionados con el cuidado de la salud reportaron síntomas ansiosos de intensidad moderada con mayor frecuencia que los trabajadores de áreas no clínicas. Así, los diferentes factores estresantes durante las largas horas de trabajo, la privación de sueño, además de los síntomas ansiosos y depresivos, podrían constituir una fuente importante de fatiga y mal juicio clínico, lo cual tiene impacto negativo no solo en la calidad de vida del personal de salud, sino también en la adecuada atención de los pacientes en necesidad.

Además, es importante tener el cuenta que, aun en tiempo previos a la pandemia de COVID-19, el personal de salud constituye una población vulnerable en sí misma en cuanto a problemas de salud mental. De hecho, la tasa de suicidio de los médicos es un 44% mayor que la de la población general; el personal de enfermería y otros trabajadores de la salud también están en alto riesgo[26]. Algunos autores señalan que este riesgo podría aumentar ante la situación actual[9]. En concordancia, el 6,5% de los individuos de nuestro estudio admitieron haber tenido ideas de muerte o suicidio en las últimas 2 semanas. Otros estudios no han reportado hallazgos en este aspecto en particular, que se debe abordar lo antes posible[27].

Limitaciones

Entre las limitaciones de este estudio, se encuentran las siguientes: primero, por tratarse de un estudio de tipo transversal, no se puede establecer con exactitud la relación temporal entre la exposición y la enfermedad; segundo, los instrumentos empleados para identificar tanto los síntomas depresivos como los ansiosos, si bien son instrumentos válidos y de amplio uso en investigación, no permiten hacer los diagnósticos de trastorno depresivo o ansioso; tercero, los datos que se presentan solo describen lo ocurrido en un tiempo específico y no son generalizables a otro tiempo incluso en la misma población de estudio; cuarto, los sesgos de memoria son posbiles en este tipo de estudio; quinto, la participación de los empleados estuvo cerca pero fue < 60%, y sexto, los criterios de inclusión y exclusión mencionados previamente no tuvieron en cuenta factores como el tiempo de contrato laboral, las incapacidades o el tiempo vacacional, lo se reflejará en una muestra más heterogénea y por sí misma

Cómo citar este artículo: Restrepo-Martínez M, et al. Prevalencia y características clínicas de los síntomas depresivos y ansiosos de los trabajadores de una institución de salud en Medellín durante la pandemia por COVID-19. Rev Colomb Psiquiat. 2021. https://doi.org/10.1016/j.rcp.2021.02.001

no representativa. Sin embargo, este estudio aporta información importante frente a síntomas depresivos y ansiosos en un grupo grande de empleados de una institución de salud en la ciudad de Medellín durante la pandemia de COVID-19.

Conclusiones

Se identificaron síntomas ansiosos y depresivos de alta prevalencia en trabajadores de una institución de salud durante la pandemia de COVID-19 en la ciudad de Medellín, Colombia. No se observó un claro predominio de síntomas ansiosos y depresivos según el sexo como es habitual en la población general. Si bien son necesarios más estudios para medir el impacto de estos síntomas en el personal del sector salud, su alta prevelencia ya es preocupante.

Los ejecutivos y gerentes de atención médica deben conocer el potencial aumento del estrés psicológico al que se enfrenta el personal de salud durante los tiempos de la COVID-19. El apoyo al personal del sector salud y sus familias podría proteger de los resultados psicológicos adversos. Dado el papel vital de esta población en tiempos de pandemia, el desarrollo de programas de salud mental que aborden los problemas de sector salud debería considerarse una prioridad.

Conflicto de intereses

Ninguno

BIBLIOGRAFÍA

1. Organización Mundial de la Salud. Coronavirus disease 2019 (COVID-19) Situation Report – 11. WHO Bull. 2020;(January):1-7.
2. Wuhan Municipal Health Commission. Report of clustering pneumonia of unknown etiology in Wuhan City. 2019. [Internet]. Disponible en: http://wjw.wuhan.gov.cn/%0Dfront/web/showDetail/2019123108989. Citado 10 May 2020.
3. Vaccines and treatment of COVID-19 [Internet]. European Centre for Disease Prevention and Control. Disponible en: https://www.ecdc.europa.eu/en/covid-19/latest-evidence/vaccines-and-treatment. Citado 4 Sep 2020.
4. Pfefferbaum B, North CS. Mental health and the COVID-19 pandemic. N Engl J Med. 2020:3.
5. Chen Q, Liang M, Li Y, Gou J, Fei D, Wang L, et al. Mental health care for medical staff in China during the COVID-19 outbreak. Lancet Psychiatry. 2020;7:e15–6.
6. Montes-Arcón PS. General practitioners and mental health in the COVID-19 pandemic. Duazary. 2020;17:17–9.
7. Zhang WR, Wang K, Yin L, Zhao W, Xue Q, Peng M, et al. Mental health and psychosocial problems of medical health workers during the COVID-19 epidemic in China. Psychother Psychosom. 2020;100053(45.).
8. Shanafelt T, Ripp J, Trockel M. Understanding and addressing sources of anxiety among health care professionals during the COVID-19 pandemic. JAMA. 2020;2019:2019–20.
9. Reger MA, Piccirillo ML, Buchman-Schmitt JM. COVID-19, mental health, and suicide risk among health care workers: looking beyond the crisis. J Clin Psychiatry. 2020;81(5.).
10. Tricco AC, Langlois EV, Straus SE. World Health Organization, Alliance for Health Policy and Systems Research. Rapid reviews to strengthen health policy and systems: a practical guide [Internet]. 2017. Disponible en: http://apps.who.int/iris/bitstream/10665/258698/1/9789241512763-eng.pdf. Citado 1 Sep 2020.
11. Las Americas Auna [Internet]. Disponible en: https://www.lasamericas.com.co/. Citado 4 Sep 2020.
12. Burki TK. Coronavirus in China. Lancet Respir Med. 2020;8:238.
13. New Health Foundation [Internet]. Disponible en: http://www.newhealthfoundation.org/. Citado 1 Sep 2020.
14. Patient Health Questionnaire (PHQ) Screener s. Free Download [Internet]. Disponible en: /select-screener/. Citado 1 Sep 2020.
15. Kroenke K, Spitzer RL, Williams JBW. The PHQ-9: Validity of a brief depression severity measure. J Gen Intern Med. 2001;16:606–13.
16. Spitzer RL, Kroenke K, Williams JBW, Löwe B. A brief measure for assessing generalized anxiety disorder: the GAD-7. Arch Intern Med. 2006;166:1092.
17. Tomas Baader M, José Luis Molina F, Silvia Venezian B, Rojas C, Renata Farías S, Fierro-Freixenet C, et al. Validación y utilidad de la encuesta PHQ-9 (Patient Health Questionnaire) en el diagnóstico de depresión en pacientes usuarios de atención primaria en Chile. Rev Chil Neuro-Psiquiatr. 2012;50:10–22.
18. Cassiani-Miranda CA, Vargas-Hernández MC, Pérez-Anibal E, Herazo-Bustos MI, Hernández-Carrillo M. Reliability and dimensionality of PHQ-9 in screening symptoms of depression among health science students in Cartagena, 2014. Biomédica. 2016:37.
19. García-Campayo J, Zamorano E, Ruiz MA, Pardo A, Pérez-Páramo M, López-Gómez V, et al. Cultural adaptation into Spanish of the generalized anxiety disorder-7 (GAD-7) scale as a screening tool. Health Qual Life Outcomes. 2010;8:1–11.
20. Resolucion 8430 de 1993, Octubre 4, por la cual se establecen las normas cientifico tecnicas y administrativas para la investigacion en salud. Bogotá: Ministerio de Salud de Colombia; 1993.
21. WMA - The World Medical Association. Declaración de Helsinki de la AMM. Principios éticos para las investigaciones médicas en seres humanos [Internet]. Disponible en: https://www.wma.net/es/policies-post/declaracion-de-helsinki-de-la-amm-principios-eticos-para-las-investigaciones-medicas-en-seres-humanos/. Citado 1 Sep 2020.
22. Vandenbroucke JP, Von Elm E, Altman DG, Gøtzsche PC, Mulrow CD, Pocock SJ, et al. Mejorar la comunicación de estudios observacionales en epidemiología (STROBE): explicación y elaboración. Gac Sanit. 2009;23:158, e1-158.e28.
23. de Galvis YT, Pérez GC, Hincapié GMS, Zapata CS, Silval JB. Estudio de Salud Mental Medellín 2019. 1.a ed Medellín: CES; 2020.
24. Lai J, Ma S, Wang Y, Zhongxiang C, Jianbo H, Ning W, et al. Factors associated with mental health outcomes among health care workers exposed to coronavirus disease 2019. JAMA Netw Open. 2020;3:e203976.
25. Pappa S, Ntella V, Giannakas T, Giannakoulis VG, Papoutsi E, Katsaounou P. Prevalence of depression, anxiety, and insomnia among healthcare workers during the COVID-19 pandemic: A systematic review and meta-analysis. Brain Behav Immun. 2020;88:901–7.
26. Hawton K, Agerbo E, Simkin S, Platt B, Mellanby RJ. Risk of suicide in medical and related occupational groups: A national study based on Danish case population-based registers. J Affect Disord. 2011;134:320–6.
27. Galbraith N, Boyda D, McFeeters D, Hassan T. The mental health of doctors during the COVID-19 pandemic. Br J Psych Bull. 2020:1–4.

Cómo citar este artículo: Restrepo-Martínez M, et al. Prevalencia y características clínicas de los síntomas depresivos y ansiosos de los trabajadores de una institución de salud en Medellín durante la pandemia por COVID-19. Rev Colomb Psiquiat. 2021. https://doi.org/10.1016/j.rcp.2021.02.001
