# Supplementary material for: Mental health status and related factors influencing healthcare workers during the COVID-19 pandemic: A systematic review and meta-analysis
Source: PLoS One. 2024 Jan 19;19(1):e0289454. doi: 10.1371/journal.pone.0289454 (PMC10798549; doi:10.1371/journal.pone.0289454)
Supplement: S1 Data — (ZIP) [file pone.0289454.s011.zip › literatures/146.pdf]

WORKFORCE

The psychological impact of COVID-19 on healthcare workers in Pakistan

Authors: Babar Riaz,[A] Wajid A Rafai,[A] Ahmad Ussaid,[A] Atif Masood,[B] Sohail Anwar,[C] Faisal A Baig,[B] Khurram Saleem,[D] Shumaila A Nasir,[E] Zaima Firdous[E] and Farrukh Iqbal[D]

ABSTRACT

Introduction

The COVID-19 pandemic has challenged healthcare facilities and healthcare professionals' stamina and wellbeing. This study examines the psychological impact of COVID-19 on healthcare professionals.

Methods

This analytical cross-sectional study was conducted in July 2020 after institutional review board approval at a tertiary care institution in Lahore, Pakistan. A total of 175 healthcare workers participated following an online Depression, Anxiety and Stress Scale-21 (DASS-21) questionnaire invitation and 41 were excluded following pre-existing mental health conditions. Data was analysed using MS Excel and SPSS Amos 23. Chi-squared test and regression were applied for comparison and impact of confounding variables respectively ($p<0.05$ was considered significant).

Results

Out of 134, 66 (49%) were doctors, 24 (18%) were nurses and 44 (33%) were non-medical professionals. Ninety-five (70%) with age 21–30 years. Male to female ratio was 2:1. Overall mean depression score accounted for 6.89 ± 6.64; anxiety score was 7.28 ± 6.74 and stress score was 8.83 ± 6.93. Mild depression, anxiety and stress was noted in 21 (15.6%), eight (6%) and 27 (20.1%) healthcare workers, respectively. A statistically significant difference ($p<0.05$) was observed among healthcare workers for depression, anxiety and stress.

Conclusion

This study demonstrated considerable impact of COVID-19 on mental health of healthcare workers. A well-structured targeted mental health support programme is needed urgently to support and reduce the long-term impact on healthcare workers' mental health and wellbeing.

Authors: [A]senior registrar, University College of Medicine and Dentistry, Lahore, Pakistan; [B]associate professor of medicine, University College of Medicine and Dentistry, Lahore, Pakistan; [C]assistant professor of pulmonology, University College of Medicine and Dentistry, Lahore, Pakistan; [D]professor of medicine, University College of Medicine and Dentistry, Lahore, Pakistan; [E]postgraduate resident trainee, University College of Medicine and Dentistry, Lahore, Pakistan

KEYWORDS: depression, anxiety, stress, COVID-19, healthcare workers

DOI: 10.7861/fhj.2020-0193

Introduction

SARS-CoV-2 enrooted itself in different parts of the world as international travel was still in its full flow. According to the studies published during previous SARS or Ebola epidemics, the beginning of an unexpected and promptly dangerous sickness could prompt exceptionally demanding duty on healthcare workers.[1] Increased workload, physical weariness, insufficient personal protective equipment, nosocomial transmission and the need to settle on morally troublesome choices on proportionality of health services could seriously affect physical and mental health. Seclusion, social isolation and risk of disease transmission to friends and family members can further subvert their flexibility, just as extreme, frequently disrupting fluctuations in work approach. Healthcare workers are, in particular, helpless against emotional wellness issues, including fear, uneasiness, anxiety and depression.[2] The COVID-19 pandemic emerged as significant worldwide general health crisis. There were 465,915 established cases in 199 nation, and 21,031 individuals had lost their lives by late March 2020. This disease has tested worldwide public medical services frameworks, and the wellbeing of patient-facing healthcare workers (HCWs).

Confronting this alarming situation, patient-facing HCWs in Pakistan were in danger of developing psychological distress. The incremental trend of confirmed and suspected cases, overwhelming remaining tasks at hand, consumption of personal protective equipment (PPE), broad media inclusion, absence of explicit medication and feelings of being ineffectual may all add to the psychological burden. In a developing country like Pakistan, where the majority are below the poverty line and struggling daily for survival, mental health in general has never been dealt with appropriately and, rather, has been considered a taboo. Low health sector budget, unemployment, low literacy rate and failure to deliver impartial health services at all sectors add cumulatively to misery especially during the current pandemic. Initial response of the general population in Pakistan to COVID-19 awareness was poor as many labelled it conspiracy by the government and healthcare sector, and this attitude of the public led to immense pressure on healthcare workers while they were taking care of

© Royal College of Physicians 2021. All rights reserved.

COVID-19 patients with limited resources and at the same time dealing with public denial.

Studies indicated that those healthcare providers who were infected with virus and feared transmission to their family, companions and associates felt vulnerable and stigmatised, reported reluctance to work, and revealed encountering significant levels of pressure, uneasiness and depression that could have long-term physical and mental health problems. Comparable concerns about the emotional well-being, mental health alteration and recovery of healthcare providers treating and thinking about COVID-19 patients are currently emerging.[3–5]

Healthcare professionals of all specialties are dealing with patients with COVID-19. The fast spread of this infection and the serious manifestations pushed numerous nations to the furthest limits of their medical care frameworks. They needed to manage deficiency of ventilators and emergency unit beds to think about the flood of sick patients, and an absence of PPE. While confronting a high volume of sick patients inside a short timeframe, medical services experts had to perform to their maximum capacity. They expected to deal with this shift and associated stressors while confronting a serious danger of contracting infection, remaining tasks at hand, moral predicaments and a rapidly developing practice condition that contrasted significantly from conventional medical practice.[6]

HCWs came across astonishingly traumatic encounters during the COVID-19 pandemic in different countries, particularly in Pakistan, who had not experienced such outbreaks. The circumstances deteriorated further following the infection of numerous HCWs who were then isolated, hence lessening the workforce. There had been a significantly high percentage of HCWs who acquired COVID-19 in Spain, followed by hospitalisation.[7] More than 10,000 HCWs got infected in between 23 March 2020 to 09 April 2020 in Italy.[8] In Pakistan as well, many healthcare providers suffered from COVID-19. To date, there had not been studies of note in Pakistan where any comparisons have been done among medical and non–medical professionals regarding the severity of COVID-19 psychological impact.

Considering utilisation of the healthcare provider in hospital in the era of a pandemic, particularly for communicable diseases, it is necessary to monitor mental health. It could only be taken into policy matter if there are ample data regarding the effects of a pandemic like COVID-19 on medical and non-medical healthcare workers. Therefore, this study was conducted to gauge psychological impact of COVID-19 with probable underlying predisposing reasons among medical and non-medical professionals in healthcare setting.

Material and methods

This analytical cross-sectional study was conducted in July 2020 at tertiary care hospital in Lahore, Pakistan. Healthcare workers were invited to participate through self-administered online questionnaire after approval from institutional review board. Non-probability convenient sampling technique was used. A total of 175 participants, including medical and non-medical professionals, working in hospital took part in the study, out of which, 41 were excluded due to pre-existing mental health conditions making 134 the total number of respondents for final data analysis. An online form was set up, which included Depression Anxiety Stress Scale – 21 (DASS-21).[9] DASS-21 is a 21-component aid drafted to gauge the cardinal psychic states: depression, anxiety and stress. Scores for each individual were calculated based upon their answers to each question related to the three psychological states and final score for each was obtained by multiplying observed score by two. Healthcare workers were divided into two groups; 'medical' (physicians and nurses) and 'non-medical' (allied health professionals, pharmacists, technicians, administration and clerical support staff). In addition to the DASS-21 questionnaire, the online form also included information on informed consent, demographic characteristics and previous mental health history. The primary outcome was prevalence of anxiety, depression and stress, and the predisposing reasons among healthcare workers. Secondary outcomes were a comparison and impact of workplace on prevalence and severity of depression, anxiety and stress among the medical and non-medical professionals.

The collected data was analysed using MS Excel and SPSS Amos software (version 23.0; SPSS, Armonk, USA). Continuous parameters were computed as mean ± standard deviation (SD) and categorical parameters were presented as frequencies and percentages. Regression analysis was carried out to check the impact of higher age, gender and type of profession on the presence of depression, anxiety and stress among the participants. Chi-squared test was used to compare severity of depression, anxiety and stress in the group. Statistical significance was considered at $p<0.05$.

Results

Out of 134 participants, 67.9% were medical professionals and 32.1% non-medical professionals. Male to female ratio was 2:1 with 85 (63.4%) men and 49 (36.6%) women. There were 95 (70.9%) in the age bracket 21–30 years, 30 (22.4%) in 31–40 years bracket, seven (5.2%) in the 41–50 years age bracket, and one (0.7%) in each age group of 51–60 years and 61–70 years (Fig 1). Sixty-six (49.3%) were doctors, 24 (17.9%) were nurses and 44 (32.1%) were non-medical professionals. The overall mean depression score was 6.89 ± 6.64; anxiety score was 7.28 ± 6.74 and stress score was 8.83 ± 6.93. Mild depression was noted in 21 (15.7%), mild anxiety in eight (6.0%) and mild stress in 27 (20.1%) healthcare workers. A statistically significant difference noted in medical and non-medical healthcare workers for the depression, anxiety and stress

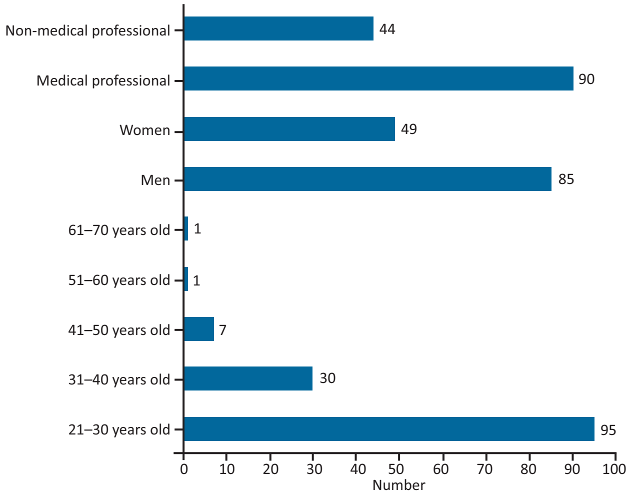

Fig 1. Demographics of study participants. n=134.

© Royal College of Physicians 2021. All rights reserved.

Table 1. Depression, anxiety and stress among healthcare workers

Psychological conditions	DASS-21 severity	Medical professionals, n=90		Non-medical professionals, n=44	p-value
		Doctors, n=66	Nurses, n=24		
**Depression, n (%)**	Normal	44 (46.3)	23 (24.2)	28 (29.5)	**0.017**
	Mild	8 (38.1)	1 (4.8)	12 (57.1)	
	Moderate	7 (63.6)	0 (0.0)	4 (36.4)	
	Severe	5 (100.0)	0 (0.0)	0 (0.0)	
	Extremely severe	2 (100.0)	0 (0.0)	0 (0.0)	
**Anxiety, n (%)**	Normal	43 (55.8)	23 (29.9)	11 (14.3)	**0.001**
	Mild	3 (37.5)	1 (12.5)	4 (50.0)	
	Moderate	11 (35.5)	0 (0.0)	20 (64.5)	
	Severe	2 (22.2)	0 (0.0)	7 (77.8)	
	Extremely severe	7 (77.8)	0 (0.0)	2 (22.2)	
**Stress, n (%)**	Normal	53 (53.0)	24 (24.0)	23 (23.0)	**<0.001**
	Mild	11 (40.7)	0 (0.0)	16 (59.3)	
	Moderate	1 (16.7)	0 (0.0)	5 (83.3)	
	Severe	1 (100.0)	0 (0.0)	0 (0.0)	
	Extremely severe	0 (0.0)	0 (0.0)	0 (0.0)	

p<0.05 considered significant. DASS-21 = Depression, Anxiety and Stress Scale-21.

(Table 1). Thirty-six (26.9%) were concerned about the possibility of contracting COVID-19, 19 (14.2%) reporting lack of training, five (3.7%) presented increased workload, 59 (44.0%) feared infecting a family member and 15 (11.2%) quoted lack of awareness in general population.

A statistically significant difference was observed regarding predisposing reasons for anxiety, stress and depression (Table 2). A statistically significant difference was noted among healthcare workers regarding the impact of the workplace on the frequency of anxiety, stress and depression (Table 3). The statistically significant effect of confounding variables such as age, gender and types of professionals on prevalence of psychological conditions is shown in Fig 3 (P-P plot of regression standardised residual).

Discussion

The study has clearly demonstrated significant negative psychological impact of COVID-19 on healthcare workers. It has also highlighted the probable underlying predisposing reasons for such mental health harm. Many factors such as vulnerability to infection, lack of control of the situation, viral spread, health of family members and isolation can play an influential role on psychological response of HCWs especially in a country like Pakistan. Among others, reluctance to seek help when needed; shortage of PPE, medications and equipment; and rising level of suspected and confirmed cases are major cause for concern. Self-medication, denial and rationalisation may be initial defence mechanisms used to confront stressful situations but may result in failure to seek help when being exposed to mental health harm.[10] Feeling of solitude and despair arise following immense pressure from these factors and lead to mental agony, impatience, anxiety, depression and mental fatigue.[11]

In this study, we found that a significant number of HCWs fell prey to anxiety, stress and depression. Inappropriate health reforms for patient management, paucity of understanding among the general population and low adherence with safety precautions could warrant to such incremented frequency of anxiety, depression and stress in Pakistan. A large number of studies noted exorbitant apprehension, despondency, nervous tension and even post-traumatic stress disorder among HCWs, even after a considerable time had elapsed after this pandemic wave; and our study findings are consistent with other published literature.[12]

Our study showed that overall, 39 (29.1%) healthcare workers (medical and non-medical) had depression, 57 (42.5%) suffered from anxiety and 34 (25.3%) reported stress which was relatively low in comparison to study conducted by Lai *et al* in China, which

Table 2. Predisposing reasons for depression, anxiety and stress

	Medical professionals, n=90	Non-medical professionals, n=44 (%)	p-value
**Possibility of contracting COVID-19, n (%)**	20 (22.0)	16 (37.2)	p<0.001
**Lack of appropriate training, n (%)**	4 (4.4)	15 (34.9)	
**Increased workload, n (%)**	5 (5.5)	1 (2.27)	
**Possibility of infecting a family member, n (%)**	53 (58.2)	6 (14.0)	
**Lack of awareness in general population, n (%)**	9 (10.0)	6 (14.0)	

p<0.05 considered significant.

© Royal College of Physicians 2021. All rights reserved.

Table 3. Impact of workplace on prevalence of depression and anxiety on healthcare workers

Factor	Workplace	Category	Medical professional, n=90		Non-medical professional, n=44	p-value
			Doctors, n=66	Nurses, n=24		
Depression, n (%)	Daily rounds	Normal	14 (62.4)	3 (17.6)	0 (0.0)	<0.001
		Mild	0 (0.0)	0 (0.0)	1 (100.0)	
		Moderate	17 (82.4)	0 (0.0)	0 (0.0)	
		Severe	0 (0.0)	0 (0.0)	0 (0.0)	
		Extremely severe	0 (0.0)	0 (0.0)	0 (0.0)	
Anxiety, n (%)	COVID-19 ICU	Normal	9 (45.0)	9 (45.0)	2 (10.0)	0.035
		Mild	1 (100.0)	0 (0.0)	0 (0.0)	
		Moderate	1 (20.0)	0 (0.0)	4 (80.0)	
		Severe	1 (33.3)	0 (0.0)	2 (66.7)	
		Extremely severe	0 (0.0)	0 (0.0)	1 (100)	
	Daily rounds	Normal	13 (86.7)	2 (13.3)	0 (0.0)	0.023
		Mild	0 (0.0)	1 (50.0)	1 (50.0)	
		Moderate	0 (0.0)	0 (0.0)	0 (0.0)	
		Severe	1 (100)	0 (0.0)	0 (0.0)	
		Extremely severe	0 (0.0)	0 (0.0)	0 (0.0)	
	COVID-19 general/ private ward	Normal	15 (53.6)	10 (35.7)	3 (10.7)	<0.001
		Mild	2 (50.0)	0 (0.0)	2 (50.0)	
		Moderate	7 (41.2)	0 (0.0)	10 (58.0)	
		Severe	0 (0.0)	0 (0.0)	3 (100.0)	
		Extremely severe	7 (87.5)	0 (0.0)	1 (12.5)	

p<0.05 considered significant. ICU = intensive care unit.

reported that 50.4% had depression, 44.6% suffered from anxiety and 71.5% reported stress. The major reasons for the observed low impact was the timing of the study (tail end of first wave of COVID-19), improvised personal protection and general stigma in acknowledging mental health issues in the majority of the population in Pakistan based on social taboo. Our study reported the majority of the respondents (63.4%) were men, perhaps due to male oriented society and overall participants aged 21–30 years, whereas Lai *et al* reported majority of respondents as females and aged 26–40 years. Healthcare workers in the direct care of COVID-19 patients were at a higher risk of depression (odds ratio (OR) 1.52; 95% confidence interval (CI) 1.11–2.09; p=0.01), anxiety (OR 1.57; 95% CI 1.22–2.02; p<0.001) and distress (OR 1.60; 95% CI 1.25–2.04; p<0.001) which correlates with findings in our study (p=0.01 for depression, p=0.001 for anxiety and p<0.001 for stress).[13]

With regards to the reasons for the mental health conditions, we found that majority of the medical professionals were worried

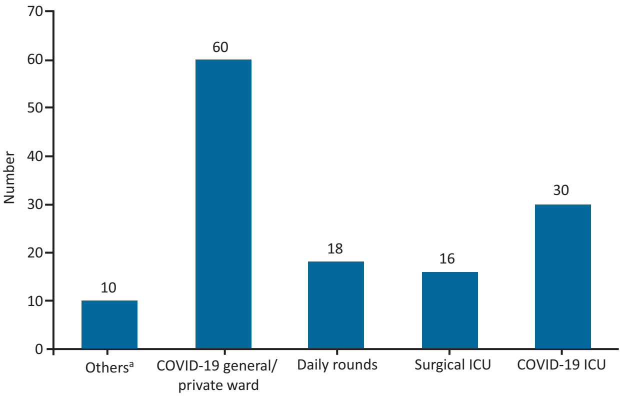

Fig 2. Workplace of healthcare workers (medical and non-medical). [a] COVID-19 assessment unit and main emergency department. ICU = intensive care unit.

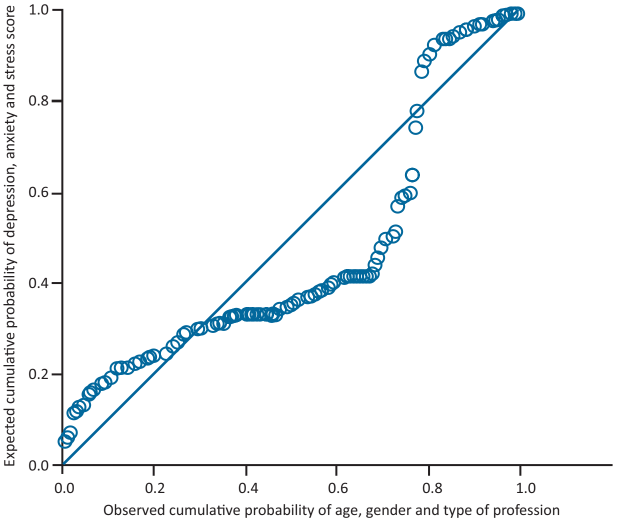

Fig 3. Effect of the confounding variables on the prevalence of depression, anxiety and stress in healthcare workers. Depression p=0.001; anxiety p=0.007; stress p=0.29.

© Royal College of Physicians 2021. All rights reserved.

about infecting their family reported by 53 (58.2%) followed by contracting COVID-19 themselves (20; 22.0%), with statistically significant difference p<0.001, which is consistent with the previously published literature.[14] A greater reason for worry about family is that often the extended family is living as one unit in Pakistan. Furthermore, of note, we found that non-medical professionals were worried about contracting COVID-19 themselves (16; 37.2%), followed by lack of appropriate training reported by 15 (34.9%) participants. Reasons for this may be persistent fear and reluctance despite adequate measures and mass causalities worldwide being broadcasted on media. Of note, non-medical professionals had higher prevalence of anxiety reported by 33 (75.0%) participants followed by stress among 21 (47.7%) and depression in 16 (36.3%) participants, which is comparable to other published studies.[15]

Our study highlighted that majority of the healthcare workers suffered from mild depression reported by 21 (15.3%) participants, mild anxiety observed in eight (6.0%) participants and mild stress among 27 (20.0%) participants which was comparable to a study conducted in Wuhan.[16] Disease transmission to the society and family members had been major concerns of healthcare providers in Pakistan in addition to losing their lives while being patient facing. A considerable number in Pakistan acquired infection and many lost their lives.[17,18] Similarly, during this pandemic, a significant number of healthcare workers acquired infection and died from COVID-19 in multiple highly resourced countries of the world.[13,19,20] In a study from China, anxiety and depression accounted for 22.6–36.3% and 16.5–48.3%, respectively, broadly comparable to rates of 23.2% and 22.8% among HCWs during COVID-19. It depicts the considerable impact on population, and the results were comparable in our current study.[21,22]

One the basis of our findings, it is recommended that the all healthcare workers (irrespective of medical or non-medical) should be encouraged to seek mental health support. The stress and anxiety levels could reduce if we define achievable rostering and ensure the availability of suitable medical equipment. Governments must ensure the proper deliberation of the knowledge and ensure efforts to reduce the percentage of the patients' attendants to the healthcare setups amid a crisis. Health professionals involved in direct care of COVID-19 patients should have their mental health regularly screened and monitored in order to protect them from nervous breakdown. At the same time, it is essential to identify secondary psychosocial risk factors (such as living with minors and elderly family members), which could be a potential source for mental health disturbance. All these measures become inevitable in a developing country like Pakistan where mass awareness regarding mental health is required through use of electronic, print and social media in order to remove the hesitation and taboo of being labelled on anyone who seeks help for mental health.

Our study has a few limitations, it has been conducted during the tail end of first wave of COVID-19 which could possibly explain a reduced severity of mental health issues among healthcare workers. Another limiting factor is that it is single centre study which binds it to relatively small sample size in comparison to multi-centre studies.

Conclusion

The results of our study significantly highlight psychological afflictions such as depression, anxiety and stress among healthcare workers and predisposing factors, which is worrisome. Targeted interventions must be used to enhance psychological wellbeing of healthcare workers and reinforce the capacity of healthcare systems for future outbreaks. Healthcare institutions should ensure adequate help, deliver customised education and training, and corroborate reasonable assets. As a supportive measure, telemedicine, for instance, could play an important role in psychosocial needs monitoring and service delivery. Similarly, communal support can play an important role to increase competence of healthcare workers and diminish the degree of anxiety and stress. More research studies of qualitative nature would open the door to much better understanding of the reasons and practical solutions in future. ■

Acknowledgements

We acknowledge the uninterrupted support from the University College of Medicine and Dentistry, and the University of Lahore Teaching Hospital's clinical administration team and concerned authorities during the pandemic's first wave which paved the way for this study.

References

1 Mudroch DR, Howie SR. The global burden of lower respiratory infections: making progress, but we need to do better. *Lancet Infect Dis* 2018;18:1162–3.
2 Lu Y-C, Chang Y-Y, Shu B-C. Mental symptoms in different health professionals during the SARS attack: A follow-up study. *Psychiatric Quarterly* 2009;80:107.
3 Maunder R, Hunter J, Vincent L *et al*. The immediate psychological and occupational impact of the 2003 SARS outbreak in a teaching hospital. *CMAJ* 2003;168:1245–51.
4 Bai Y, Lin C-C, Lin C-Y *et al*. Survey of stress reactions among health care workers involved with SARS outbreak. *Psychiatric Services* 2004;55:1055–7.
5 Lee AM, Wong JG, McAlonan GM *et al*. Stress and psychological distress among SARS survivors 1 year after the outbreak. *Can J Psychiatry* 2007;52:233–40.
6 Shanafelt T, Ripp J, Trockel M. Understanding and addressing sources of anxiety among health care professionals during the COVID-19 pandemic. *JAMA* 2020;323:2133–4.
7 García-Fernández L, Romero-Ferreiro V, López-Roldán PD *et al*. Mental health impact of COVID-19 pandemic on Spanish healthcare workers. *Psychol Med* 2020 [Epub ahead of print].
8 Bettinsoli ML, Di Riso D, Napier JL *et al*. Mental health conditions of italian healthcare professionals during the COVID-19 disease outbreak. *Appl Psychol Health Well Being* 2020;12:1054–73.
9 Lovibond SH, Lovibond PF. *Manual for the depression anxiety and stress scales*, 2nd edn. Sydney: Psychology Foundation, 1995.
10 Rimmer A, Chatfield C. What organisations around the world are doing to help improve doctors' wellbeing. *BMJ* 2020;369:m1541.
11 Braquehais MD, Sher L. Posttraumatic stress disorder in war veterans: A discussion of the Neuroevolutionary Time-depth Principle. *J Affect Disord* 2010;125:1–9.
12 Braquehais MD, Vargas-Cáceres S, Gómez-Durán E *et al*. The impact of the COVID-19 pandemic on the mental health of healthcare professionals. *QJM* 2020:hcaa207.
13 Lai J, Ma S, Wang Y *et al*. Factors associated with mental health outcomes among health care workers exposed to coronavirus disease 2019. *JAMA Network Open* 2020;3:e203976-e.
14 Sandesh R, Shahid W, Dev K *et al*. Impact of COVID-19 on the mental health of healthcare professionals in Pakistan. *Cureus* 2020;12:e8974.
15 Tan BYQ, Chew NWS, Lee GKH *et al*. Psychological impact of the COVID-19 pandemic on health care workers in Singapore. *Ann Intern Med* 2020;173:317–20.

© Royal College of Physicians 2021. All rights reserved.

16 Hu D, Kong Y, Li W *et al*. Frontline nurses' burnout, anxiety, depression, and fear statuses and their associated factors during the COVID-19 outbreak in Wuhan, China: A large-scale cross-sectional study. *EClinicalMedicine* 2020;24:100424.
17 Saqlain M, Munir MM, ur Rehman S *et al*. Knowledge, attitude, practice and perceived barriers among healthcare professionals regarding COVID-19: A Cross-sectional survey from Pakistan. *J Hosp Infect* 2020;105:419–23.
18 Saleem Z, Majeed MM, Rafique S *et al*. COVID-19 pandemic fear and anxiety among healthcare professionals in Pakistan. *Research Square* 2020 [pre-print].
19 Chen W, Huang Y. To protect health care workers better, to save more lives with COVID-19. *Anesth Anal* 2020;131:97–101.
20 Ersoy A. The frontline of the COVID-19 pandemic: Healthcare workers. *Turkish Journal of Internal Medicine* 2020;2:31–2.
21 Wang C, Pan R, Wan X *et al*. A longitudinal study on the mental health of general population during the COVID-19 epidemic in China. *Brain Behav Immun* 2020;87:40–8.
22 Gao J, Zheng P, Jia Y *et al*. Mental health problems and social media exposure during COVID-19 outbreak. *PLoS One* 2020;15:e0231924.

Address for correspondence: Dr Wajid Ali Rafai, Department of Internal Medicine, University College of Medicine and Dentistry, University of Lahore, Defence Road, Lahore, Pakistan.
Email: wajidalirafai@gmail.com

© Royal College of Physicians 2021. All rights reserved.
