# Supplementary material for: Mental health status and related factors influencing healthcare workers during the COVID-19 pandemic: A systematic review and meta-analysis
Source: PLoS One. 2024 Jan 19;19(1):e0289454. doi: 10.1371/journal.pone.0289454 (PMC10798549; doi:10.1371/journal.pone.0289454)
Supplement: S1 Data — (ZIP) [file pone.0289454.s011.zip › literatures/148.pdf]

ELSEVIER

Since January 2020 Elsevier has created a COVID-19 resource centre with free information in English and Mandarin on the novel coronavirus COVID-19. The COVID-19 resource centre is hosted on Elsevier Connect, the company's public news and information website.

Elsevier hereby grants permission to make all its COVID-19-related research that is available on the COVID-19 resource centre - including this research content - immediately available in PubMed Central and other publicly funded repositories, such as the WHO COVID database with rights for unrestricted research re-use and analyses in any form or by any means with acknowledgement of the original source. These permissions are granted for free by Elsevier for as long as the COVID-19 resource centre remains active.

P.863

The impact of COVID-19 on mental health of healthcare workers and police/army forces in the Republic of North Macedonia

G. Ristevska-Dimitrovska [1], D. Batic [2]

[1] *University St. Kliment Ohridski, Higher Medical School Bitola, Bitola, Macedonia;* [2] *University St. Kliment Ohridski, Faculty of Security, Skopje, Macedonia*

Background: Soon after detection of first COVID-19 positive case in February 2020, the government of Republic of North Macedonia enforced restrictive measures that included long police hours, and complete quarantine of the population during the weekends until middle of May 2020. The restrictive strategy gave effects, the curve was flattened with around 20 cases per day. In June 2020, right after religious holidays, the second, more severe wave began with around 150 cases per day.

The aim: of the study is to estimate the prevalence of psychological problems and level of resilience in healthcare workers and police/army forces who have been on the front line in the COVID-19 pandemic and to compare the results with the general population.

Methods: Self-reported, web-based survey was conducted in the end of May 2020, right before the second wave begun. Anxiety, depression and PTSD symptoms and level of resilience were measured with Generalized Anxiety Disorder 7-item scale (GAD-7), Patient Health Questionnaire (PHQ-9), Short PTSD Rating Interview (SPRINT -8) and Connor-Davidson Resilience Scale 10 (CD-RISC-10).

Results: The 304 participants completed the survey, with a mean age of 35 years (min 18, max 66), 69.1% female, 30.9% male. 102 (33.6%) were healthcare workers (81.4% female/18.6% male); 78 (25.7%) were police/army forces who were engaged to enact the restrictive measures (37.2% female/62.8% male); 124 (40.7%) were general population (79% female/21% male) who were not directly involved with COVID-19 infected or suspects.

The anxiety levels (GAD-7 cut-off $\geq$10) were significantly higher in healthcare workers 26.5% than general population 17.7% and police/army group 2.6% ($\chi 2$ =18.233; df 2, p<0.001). Depressive symptoms were most prevalent in the general population 52.4%, followed by the healthcare workers 49%, and police/army forces 25.6%, but more severe depressive symptomatology was most prevalent in healthcare workers 10.7%, followed by general population 5.6% and police/army forces 3.8% ($\chi 2$ =18.316; df 4, p=0.001). Healthcare workers also had significantly more frequent manifestation of PTSD symptoms (SPRINT-8 cut-off $\geq$14) 33.3%, followed by general population 30.6% and police/army force 16.7% ($\chi 2$ =6.845; df 2, p=0.033). The levels of resilience were not significantly different in the 3 groups.

Conclusions: The COVID-19 pandemic, restrictive measures, social isolation [1], fear to be infected or to infect loved ones, tremendous workload are some of the variety of factors that have negative impact on the mental health of the healthcare workers [2]. The police and army forces seem to handle the situation with less deterioration on their mental health, perhaps because of their training to deal with stressful and violent situations. The healthcare workers on the other hand, suffer greatly, because the battle is on their ground, and the impact of the COVID-19 pandemic disturbs their personal, social and professional environment the most. In the face of the second wave of the pandemic, and expected third wave in Autumn 2020, the authorities should take measures to prevent long term deterioration of mental health of healthcare workers, while the police and army force may not be in an immediate risk.

No conflict of interest

References

[1] Luo, M., Guo, L., Yu, M., Jiang, W., Wang, H., 2020. The psychological and mental impact of coronavirus disease 2019 (COVID-19) on medical staff and general public - A systematic review and meta-analysis. Psychiatry research 291, 113190 Advance online publication.

[2] Talevi, D, Socci, V, Carai, M, Carnaghi, G, Faleri, S, Trebbi, E, di Bernardo, A, Capelli, F, Pacitti, F., 2020. Mental health outcomes of the CoViD-19 pandemic. Riv Psichiatr.May-Jun 55 (3), 137–144.

doi: 10.1016/j.euroneuro.2020.09.622

P.865

Consequences of a single social defeat on accumbal dopamine measures: in vivo voltammetric study

V. Nemets [1], A. Deal [2], R. Gainetdinov [1], E. Budygin [2]

[1] *Saint Petersburg State University, Institute of Translational Biomedicine and Saint Petersburg State University Hospital, Saint Petersburg, Russia;* [2] *Wake Forest School of Medicine, Neurobiology and Anatomy, Winston Salem, USA*

Stress is considered a trigger for the development of several serious psychopathological conditions, including anxiety, depression and drug and alcohol addictions. Undoubtedly, multiple neurobiological mechanisms contribute to this process, while the etiological nature of the relationship is unclear still. Recent findings in our lab suggest that repeated social defeat stress may contribute to alcohol addictive behaviors through overlapped adaptive changes in accumbal dopamine (DA) release [1].

The present study explores the consequences of a single exposure to social defeat on presynaptic DA dynamics in rat the nucleus accumbens (NAcc).

Adult male Sprague Dawley rats (300-350g) served as the stressed subjects and were introduced as intruder rats to the home cage of a resident aggressor Long Evans rat (>450g) for a single session [1]. The following day, DA measurements were performed in the rat NAcc using voltammetry in vivo [2,3]. The rat was anesthetized via urethane (1.5mg/kg i.p.) and then stereotaxic surgery was performed. A stimulating electrode was lowered into the VTA and a carbon fiber recording electrode into the NAcc. Once a baseline was established, raclopride (D2 receptor antagonist; 2mg/kg i.p.) was injected and measurements were taken for 90 minutes.
