# Supplementary material for: Mental health status and related factors influencing healthcare workers during the COVID-19 pandemic: A systematic review and meta-analysis
Source: PLoS One. 2024 Jan 19;19(1):e0289454. doi: 10.1371/journal.pone.0289454 (PMC10798549; doi:10.1371/journal.pone.0289454)
Supplement: S1 Data — (ZIP) [file pone.0289454.s011.zip › literatures/149.pdf]

JAMA Network Open

Original Investigation | Psychiatry

Mental Health Outcomes Among Italian Health Care Workers During the COVID-19 Pandemic

Rodolfo Rossi, MD, PhD; Valentina Socci, PhD; Tommaso Benedetto Jannini, MD; Francesca Pacitti, MD; Alberto Siracusano, MD; Alessandro Rossi, MD; Giorgio Di Lorenzo, MD, PhD

Abstract

IMPORTANCE Health care workers (HCWs) exposed to COVID-19 have high rates of mental health issues. However, longitudinal data on the evolution of mental health outcomes in HCWs are lacking.

OBJECTIVE To evaluate the mental health outcomes among Italian HCWs 14 months after the beginning of the COVID-19 pandemic.

DESIGN, SETTING, AND PARTICIPANTS This longitudinal cohort study collected data from March 1 to April 30, 2020 (T1) and from April 1 to May 31, 2021 (T2), from 2856 Italian HCWs aged 18 years or older who responded to an online questionnaire. Participants were also recruited via snowballing, a technique in which someone who receives the invitation to participate forwards it to his or her contacts.

EXPOSURES Frontline vs second-line position, job type, hospitalization for COVID-19, and colleagues or family members affected by COVID-19.

MAIN OUTCOMES AND MEASURES Outcomes are depression symptoms, anxiety symptoms, insomnia symptoms, and posttraumatic stress symptoms (PTSSs). Four different trajectories are described for each condition: resilient, remittent, incident, and persistent.

RESULTS Of the 2856 HCWs, 997 (34.9%) responded to the follow-up assessment (mean [SD] age, 42.92 [10.66] years; 816 [82.0%] female). Depression symptoms (b = −2.88; 95% CI, −4.05 to −1.71), anxiety symptoms (b = −2.01; 95% CI, −3.13 to −0.88), and PTSSs (b = −0.77; 95% CI, −1.13 to −0.42) decreased over time; insomnia symptoms increased (b = 3.05; 95% CI, 1.63-4.47). Serving as a frontline HCW at T1 was associated with decreased symptoms of depression (b = −1.04; 95% CI, −2.01 to −0.07), and hospitalization for COVID-19 was associated with increased depression symptoms (b = 5.96; 95% CI, 2.01-9.91); younger age (b = −0.36; 95% CI, −0.70 to −0.03) and serving as a frontline HCW at T1 (b = −1.04; 95% CI, −1.98 to −0.11) were associated with decreased anxiety symptoms. Male sex was associated with increase in insomnia symptoms (b = 1.46; 95% CI, 0.39-2.53). Serving as a frontline HCW at T1 (b = −0.42; 95% CI, −0.71 to −0.13) and being a physician (b = −0.52; 95% CI, −0.81 to −0.24) were associated with a decrease in PTSSs, whereas younger age (b = 0.35; 95% CI, 0.09-0.61) and male sex (b = 0.12; 95% CI, 0.01-0.22) were associated with an increase in PTSSs. Depression trajectories were 629 resilient (65.5%), 181 remittent (18.8%), 58 incident (6.0%), and 92 persistent (9.6%). Anxiety trajectories were 701 resilient (73.3%), 149 remittent (15.6%), 45 incident (4.7%), and 61 persistent (6.4%). Insomnia trajectories were 858 resilient (88.9%), 77 remittent (8.0%), 20 incident (2.1%), and 10 persistent (1.0%). The PTSS trajectories were 363 resilient (38.5%), 267 remittent (28.3%), 86 incident (9.1%), and 226 persistent (24.0%).

(continued)

Key Points

Question What are the mental health outcomes among Italian health care workers during the COVID-19 pandemic?

Findings In this longitudinal cohort study of 2856 health care workers in Italy during the COVID-19 pandemic the prevalence of depression symptoms, anxiety symptoms, insomnia symptoms, and posttraumatic stress symptoms decreased among Italian health care workers 14 months after the beginning of the COVID-19 pandemic. Prolonged work with patients with COVID-19 was significantly associated with mental health outcomes, whereas quitting work as a frontline health care worker was significantly associated with decrease in mental health issues.

Meaning These findings help to identify the potential risk factors for health care workers exposed to direct contact with patients with COVID-19 and could help inform better preventive policies regarding mental health in this particular population.

Author affiliations and article information are listed at the end of this article.

Open Access. This is an open access article distributed under the terms of the CC-BY License.

Downloaded From: https://jamanetwork.com/ on 12/21/2022

Abstract (continued)

CONCLUSIONS AND RELEVANCE In this cohort study, relative to the beginning of the COVID-19 pandemic, mental health among HCWs has improved. Factors associated with change in mental health outcomes could help in the design of prevention strategies for HCWs.

JAMA Network Open. 2021;4(11):e2136143. doi:10.1001/jamanetworkopen.2021.36143

Introduction

In the early stages of the COVID-19 pandemic, frontline health care workers (HCWs) experienced high levels of psychological distress and adverse mental health outcomes, including depression, anxiety, insomnia, and posttraumatic stress symptoms (PTSSs).[1] Furthermore, frontline HCWs had higher levels of mental health symptoms compared with the general population.[2] Common risk factors included being female and younger, being a nurse, having a lower socioeconomic status or a lower educational level, having high risks of contracting COVID-19, and being socially isolated.[3,4] Protective factors included having sufficient medical resources, having up-to-date and accurate information about COVID-19, and taking preventive measures.

Although numerous cross-sectional survey studies have been published,[5] a relatively small number of long-term longitudinal investigations have been conducted, with mixed results regarding overall trends in mental health outcomes in HCWs.[6] In this article, we report on a 14-month longitudinal cohort study among Italian HCWs. This study aimed to assess depression symptoms, anxiety symptoms, insomnia symptoms, and PTSSs, the change in prevalence of the selected conditions, and the associated risk or protective factors.

Methods

Study Design

This longitudinal, observational cohort study includes data collected at baseline between March 1 and April 30, 2020 (T1) and follow-up data collected between April 1 and May 31, 2021 (T2). Data collection was conducted using an online questionnaire disseminated via sponsored advertisements on Facebook and snowballing (a common dissemination technique used in observational studies in which someone who receives the invitation to participate in the study forwards the invitation to his or her contacts) via short message service starting from the investigators' personal acquaintances. Data were collected using Google Forms at T1 and SurveyMonkey at T2. No prevention of double entries was adopted; however, cases with duplicate emails or telephone numbers were eliminated. Of 2856 initial participants, 1904 individuals provided consent to be contacted for the follow-up, 1615 via email and 289 using short message service. A total of 997 (34.91%) responded to the second assessment. The T1 and T2 answers were linked using email or telephone contact. Eligibility criteria included age of 18 or older and online consent. Participants were not offered any incentive or compensation for participating to this study. Extended details on sampling and cohort have been published elsewhere.[2] Approval for this study was obtained from the local institutional review board at the University of L'Aquila. Online consent was obtained from the participants after a brief presentation of the study aim. No personal information was collected for this study. The study followed the Strengthening the Reporting of Observational Studies in Epidemiology (STROBE) reporting guideline.

Exposure Measures

Information about the following exposures was collected at both time points. First, being a frontline or second-line worker was assessed by the questions, "Are you currently working with COVID-19 patients?" at T1 and "Have you been working with COVID-19 patients in the last year?" at T2.

Downloaded From: https://jamanetwork.com/ on 12/21/2022

Responses from T1 and T2 were joined in a single categorical variable with 4 levels: frontline at T1 and T2, not frontline at T1 or T2, frontline at T2 only, and frontline at T1 only. Second, having any colleagues affected, hospitalized, or deceased because of COVID-19 in the last month (T1) or during the previous year (T2). Third, having any family member affected, hospitalized, or deceased because of COVID-19 in the last month (T1) or during the previous year (T2). Fourth, being infected with SARS-CoV-2, without symptoms, with symptoms treated at home, or being hospitalized. Fifth, job type (ie, being a physician, general practitioner, nurse, health care assistant, or other HCW).

Outcomes

Key mental health outcomes were depression symptoms, anxiety symptoms, insomnia symptoms, and PTSSs, assessed using the Italian version of the following instruments. First, the 9-item Patient Health Questionnaire (PHQ-9)[7] assesses 9 depression symptoms rated on a 4-point Likert scale, with 1 indicating not at all and 4 indicating nearly every day. The total score was accounted for with a categorical variable defined according to a cut-off score of 15 or higher. In our sample, internal consistency was $\alpha = .87$. Second, the 7-item Generalized Anxiety Disorder scale (GAD-7)[8] assesses 7 anxiety symptoms on a 4-point Likert scale, with 1 indicating not at all and 4 indicating nearly every day. The total score was accounted for with a categorical variable defined according to a cut-off score of 15 or higher. In our sample, internal consistency was $\alpha = .91$. Third, the 7-item Insomnia Severity Index (ISI)[9] is a 7-item self-report questionnaire that assesses the nature, severity, and consequences of insomnia on a 5-point Likert scale, with higher scores indicating higher severity of insomnia symptoms. The total score was accounted for with a categorical variable defined according to a cut-off score of 22 or higher. In our sample, internal consistency was $\alpha = .90$. Fourth is the Global Psychotrauma Screen–posttraumatic symptoms subscale (GPS-PTSS).[10,11] The GPS is a 22-item self-report instrument with a dichotomous answer that covers PTSSs, disturbances in self-organization, anxiety, depression, sleep problems, dissociation, self-harm, substance abuse, and other physical, emotional, or social problems, with yes indicating the presence of a symptom and no indicating the absence of symptoms. The 5-item PTSS subscale covers core posttraumatic stress disorder symptoms, including reexperiencing, hypervigilance, and avoidance. The PTSSs were considered of clinical relevance if more than 3 of 5 symptoms were reported. Consistent with previous research,[6] conditions for each individual were classified as resilient (below cut-off at both T1 and T2), persistent (above cut-off at both T1 and T2), incident (above cut-off at T2 only), and remitting (above cut-off at T1 only).

Covariates

Sex, age, and personal history of mental health treatment before the COVID-19 pandemic were selected as established variables that might moderate the association between the selected risk factors and outcomes. Age was standardized and reversed to express increased risk associated with younger age.

Statistical Analysis

In a preliminary stage, age, sex, PHQ-9 score, GAD-7 score, GPS-PTSS score, and ISI score were modeled as factors associated with participation to the follow-up in a logistic regression model. Main analyses were conducted in 3 stages. First, the key characteristics of the sample were described using descriptive statistics. Repeated-measures 2-tailed *t* test and χ^2 test were conducted to explore changes in the overall symptoms score and prevalence of conditions in the sample. Second, a mixed-effect regression was conducted to explore heterogeneity among subgroup variables in the outcomes' change over time, with sex, age, frontline working position, occupation, and self- and colleagues' exposure to contagion as subgroup variables. Third, a multinomial logistic regression was performed to explore the association between each condition's trajectory (ie, resilient, persistent, incident and remitting) and key COVID-19–related risk factors at T1 and T2.

Downloaded From: https://jamanetwork.com/ on 12/21/2022

Analyses were conducted in Stata statistical software, version 16 (StataCorp). Statistical significance was set at a 2-sided $P < .05$.

Results

A total of 2856 individuals (mean [SD] age, 42.92 [10.66] years; 816 [82.0%] female) responded to the first assessment. Of these individuals 2064 (72.3%) provided consent for the second assessment and were thus invited to participate in the study. Of these, 997 of the 2856 (34.9%) responded to the second assessment, independently of sex (odds ratio [OR], 0.86; 95% CI, 0.71-1.05) and age (OR, 0.99; 95% CI, 0.99-1.00). Baseline PHQ-9 (OR, 1.01; 95% CI, 0.99-1.02]), GAD-7 (OR, 1.01; 95% CI, 1.00-1.02), ISI (OR, 1.00; 95% CI, 0.99-1.01), and GPS-PTSS (OR, 1.07; 95% CI, 1.00-1.12) scores did not substantially affect attendance to the follow-up assessment.

Characteristics of the sample are reported in **Table 1**. Compared with baseline, all outcomes except ISI score had a significant decrease in total score (PHQ-9: mean [SD] score at baseline, 10.62 [5.83]; mean [SD] score in the last month, 8.85 [5.28]; mean difference, −1.76; 95% CI, −1.41 to −2.09; GAD-7: mean [SD] score at baseline, 9.35 [5.62]; mean [SD] score in the last month, 7.81 [4.83]; mean difference, −1.55; 95% CI, −1.22 to −1.87; ISI: mean [SD] score at baseline, 11.22 [7.26]; mean [SD] score in the last month, 13.41 [3.92]; mean difference, +2.18; 95% CI, 1.77-2.60; GPS-PTSS: mean [SD] score at baseline, 2.40 [1.36]; mean [SD] score in the last month, 1.78 [1.52]; mean difference, −0.61; 95% CI, −0.51 to −0.71) and prevalence (PHQ-9: 281 [28.2%] patients at baseline and 150 [15.6%] in the last month; −12.56; $\chi^2_1 = 94.53$; $P < .001$; GAD-7: 218 [21.9%] at baseline and 106 [11.1%] in the last month; −10.78; $\chi^2_1 = 88.05$; $P < .001$; ISI: 91 [9.1%] at baseline and 30 [3.1%] in the last month; −6.02; $\chi^2_1 = 22.32$; $P < .001$; GPS-PTSS: 519 [52.2%] at baseline and 312 [33.0%] in the last month; −19.11; $\chi^2_1 = 75.55$; $P < .001$).

Linear mixed-models results (**Table 2**) indicate a decrease of depression symptoms (b = −2.88; 95% CI, −4.05 to −1.71), anxiety symptoms (b = −2.01; 95% CI, −3.13 to −0.88), and PTSSs (b = −0.77; 95% CI, −1.13 to −0.42) over time and an increase in the overall insomnia score (b = 3.05; 95% CI, 1.63-4.47). Interaction terms of the independent variables by time indicate that factors associated with decrease over time in depression symptoms were being a frontline HCW at T1 only (b = −1.04; 95% CI, −2.01 to −0.07); on the contrary, having been hospitalized because of COVID-19 (b = 5.96; 95% CI, 2.01-9.91) and having a history of psychiatric or psychological treatment before the pandemic (b = 0.83; 95% CI, 0.06-1.60) were associated with an increase over time in depression

Table 1. Characteristics of the Sample and Outcomes[a]

Characteristic	Baseline	Follow-up
Sex		
Female	816/997 (82.0)	816/997 (82.0)
Male	181/997 (18.0)	181/997 (18.0)
Age, mean (SD), y	41.96 (10.65)	42.92 (10.66)
Working position		
Frontline	516/997 (51.8)	435/997 (43.6)
Second line	481/997 (48.2)	562/997 (56.4)
Occupation		
Nurse	366/997 (36.7)	365/997 (36.6)
Physician	249/997 (25.0)	251/997 (25.2)
General practitioner	24/997 (2.4)	28/997 (2.8)
Health care assistant	100/997 (10.0)	100/997 (10.0)
Other	258/997 (25.9)	253/997 (25.4)
Educational level		
Undergraduate	212/991 (21.4)	212/991 (21.4)
Postgraduate	779/991 (78.6)	779/991 (78.6)

[a] Data are presented as number/total number (percentage) of study participants unless otherwise indicated.

Downloaded From: https://jamanetwork.com/ on 12/21/2022

symptoms. Factors associated with a decrease over time in anxiety symptoms were younger age (b = −0.36; 95% CI, −0.70 to −0.03) and being a frontline HCW at T1 only (b = −1.04; 95% CI, −1.98 to −0.11).

Male sex was associated with an increase over time in insomnia symptoms (b = 1.46; 95% CI, 0.39-2.53). Decrease over time of PTSSs was associated with being a frontline HCW at T1 only (b = −0.42; 95% CI, −0.71 to −0.13) and being a physician (b = −0.52; 95% CI, −0.81 to −0.24), whereas younger age (b = 0.35; 95% CI, 0.09-0.61) and male sex (b = 0.12; 95% CI, 0.01-0.22) were associated with increase over time in PTSSs. Because these results are from multivariable linear mixed models, all changes were identified after correcting for the effects of covariates

The trajectories of each condition are reported in **Table 3** and the **Figure**. Multinomial regression analyses are reported in **Table 4**. Regarding depression, 629 individuals (65.5%) had resilient conditions, 181 (18.8%) had remittent conditions, 58 (6.0%) had incident conditions, and 92 (9.6%) had persistent conditions. Regarding anxiety, 701 (73.3%) individuals had resilient conditions, 149 (15.6%) had remittent conditions, 45 (4.7%) had incident conditions, and 61 (6.4%) had persistent conditions. Regarding insomnia, 858 individuals (88.9%) had resilient conditions, 77

Table 2. Linear Mixed-Model Results

	β (95% CI)			
Variable	PHQ-9	GAD-7	ISI	GPS-PTSS
Time	−2.88 (−4.05 to −1.71)	−2.01 (−3.13 to −0.88)	3.05 (1.63 to 4.47)	−0.77 (−1.13 to −0.42)
Sex				
Female × time				
Male × time	−0.06 (−0.94 to 0.82)	−0.17 (−1.02 to 0.67)	1.46 (0.39 to 2.53)	0.35 (0.09 to 0.61)
Age × time[a]	−0.20 (−0.55 to 0.14)	−0.36 (−0.70 to −0.03)	−0.17 (−0.58 to 0.25)	0.12 (0.01 to 0.22)
Frontline status × time[b]				
At T1 only	−1.04 (−2.01 to −0.07)	−1.04 (−1.98 to −0.11)	−1.80 (−2.98 to −0.62)	−0.42 (−0.71 to −0.13)
At T2 only	0.45 (−0.77 to 1.68)	0.27 (−0.91 to 1.45)	−0.99 (−2.47 to 0.50)	0.11 (−0.26 to 0.48)
At T1 and T2	−0.22 (−1.08 to 0.64)	−0.09 (−0.92 to 0.73)	−0.25 (−1.29 to 0.79)	−0.11 (−0.37 to 0.15)
Occupation × time[c]				
Nurse	−0.29 (−1.19 to 0.61)	−0.22 (−1.09 to 0.65)	−0.74 (−1.83 to 0.36)	−0.18 (−0.45 to 0.09)
Physician	0.64 (−0.31 to 1.60)	−0.19 (−1.11 to 0.74)	0.78 (−0.38 to 1.95)	−0.52 (−0.81 to −0.24)
General practitioner	−0.35 (−2.76 to 2.07)	0.46 (−1.87 to 2.79)	0.36 (−2.57 to 3.30)	−0.32 (−1.04 to 0.40)
Health care assistant	0.36 (−0.91 to 1.63)	−0.09 (−1.32 to 1.14)	−0.29 (−1.83 to 1.25)	−0.05 (−0.43 to 0.33)
Pandemic-related variables				
Has had COVID-19 × time				
Yes	0.06 (−0.90 to 1.02)	−0.04 (−0.97 to 0.88)	−0.15 (−1.31 to 1.02)	0.15 (−0.14 to 0.44)
Hospitalized	5.96 (2.01 to 9.91)	2.35 (−1.44 to 6.15)	1.24 (−3.55 to 6.03)	0.71 (−0.47 to 1.89)
Asymptomatic	−1.66 (−3.46 to 0.14)	−0.92 (−2.64 to 0.81)	0.03 (−2.15 to 2.20)	−0.09 (−0.63 to 0.44)
Colleagues have had COVID-19 × time				
Deceased from COVID-19	0.11 (−1.73 to 1.96)	−0.05 (−1.82 to 1.72)	−1.60 (−3.84 to 0.63)	0.33 (−0.22 to 0.88)
Yes	0.52 (−0.38 to 1.43)	0.28 (−0.59 to 1.15)	−0.36 (−1.45 to 0.74)	0.18 (−0.09 to 0.46)
Yes, hospitalized	0.54 (−0.65 to 1.72)	0.15 (−0.99 to 1.29)	−0.92 (−2.35 to 0.52)	0.05 (−0.30 to 0.41)
Family member has had COVID-19 × time				
Yes, deceased	−0.20 (−1.75 to 1.35)	0.31 (−1.18 to 1.80)	0.96 (−0.92 to 2.85)	0.39 (−0.08 to 0.85)
Yes	−0.61 (−1.38 to 0.17)	−0.28 (−1.03 to 0.46)	−0.82 (−1.76 to 0.12)	−0.03 (−0.26 to 0.21)
Yes, hospitalized	1.21 (−0.06 to 2.47)	0.83 (−0.39 to 2.04)	−0.30 (−1.83 to 1.23)	0.18 (−0.20 to 0.56)
Mental health history				
Mental health treatment during pandemic × time	−0.06 (−0.90 to 0.78)	0.25 (−0.55 to 1.06)	−0.49 (−1.50 to 0.52)	0.18 (−0.07 to 0.43)
Mental health treatment before pandemic × time	0.83 (0.06 to 1.60)	−0.02 (−0.76 to 0.73)	0.42 (−0.51 to 1.36)	−0.01 (−0.24 to 0.22)

Abbreviations: GAD-7, 7-item Generalized Anxiety Disorder scale; GPS-PTSS, Global Psychotrauma Screen-posttraumatic symptoms; ISI, Insomnia Severity Index; PHQ-9, 9-item Patient Health Questionnaire; T1, time 1 (March 1 to April 30, 2020); T2, time 2 (April 1 to May 31, 2021).

[a] Age is standardized and reversed.

[b] Reference category is never frontline.

[c] Reference category is other health care worker.

Downloaded From: https://jamanetwork.com/ on 12/21/2022

(8.0%) had remittent conditions, 20 (2.1%) had incident conditions, and 10 (1.0%) had persistent conditions. Regarding PTSSs, 363 individuals (38.5%) had resilient conditions, 267 (28.3%) had remittent conditions, 86 (9.1%) had incident conditions, and 226 (24.0%) had persistent conditions.

Multinomial logistic regression found that female sex was positively associated with persistent depression symptoms (OR, 3.69; 95% CI, 1.54-8.82), anxiety symptoms (OR, 6.50; 95% CI, 1.55-27.26), and remittent (OR, 2.99; 95% CI, 1.89-4.73) and incident (OR, 2.60; 95% CI, 1.60-4.20) PTSSs. Younger age was positively associated with remittent depression (OR, 1.22; 95% CI, 1.02-1.45) and incident (OR, 1.46; 95% CI, 1.13-1.88) and persistent (OR, 1.27; 95% CI, 1.06-1.53) PTSSs.

Regarding frontline status history, being a frontline HCW at T1 only was positively associated with remittent depression symptoms (OR, 1.74; 95% CI, 1.10-2.76), insomnia symptoms (OR, 2.26; 95% CI, 1.09-4.67), and PTSSs (OR, 1.87; 95% CI, 1.18-2.97); being a frontline HCW at T2 only was positively associated with persistent depression (OR, 2.43; 95% CI, 1.07-5.52) and with remittent (OR, 3.59; 95% CI, 1.58-8.14) and persistent (OR, 20.50; 95% CI, 1.84-228.27) insomnia symptoms; being a frontline HCW at T1 and T2 was positively associated with persistent depression (OR, 2.18; 95% CI, 1.17-4.04), remittent insomnia symptoms (OR, 2.08; 95% CI, 1.06-4.09), and remittent (OR, 1.57; 95% CI, 1.03-2.39) and persistent (OR, 1.75; 95% CI, 1.12-2.72) PTSSs.

Regarding occupation, being a physician was negatively associated with persistent anxiety symptoms (OR, 0.37; 95% CI, 0.15-0.92). Regarding pandemic-related variables, having been hospitalized for COVID-19 was positively associated with persistent depression symptoms (OR, 1.70;

Table 3. Trajectories of the Selected Outcomes

	No./No. (%)			
Trajectory	PHQ-9	GAD-7	ISI	GPS-PTSS
Resilient	629/960 (65.5)	701/956 (73.3)	858/965 (88.9)	363/942 (38.5)
Remittent	181/960 (18.8)	149/956 (15.6)	77/965 (8.0)	267/942 (28.3)
Incident	58/960 (6.0)	45/956 (4.7)	20/965 (2.1)	86/942 (9.1)
Persistent	92/960 (9.6)	61/956 (6.4)	10/965 (1.0)	226/942 (24.0)

Abbreviations: GAD-7, 7-item Generalized Anxiety Disorder scale; GPS-PTSS, Global Psychotrauma Screen–posttraumatic symptoms; ISI, Insomnia Severity Index; PHQ-9, 9-item Patient Health Questionnaire.

Figure. Trajectory of Selected Outcomes

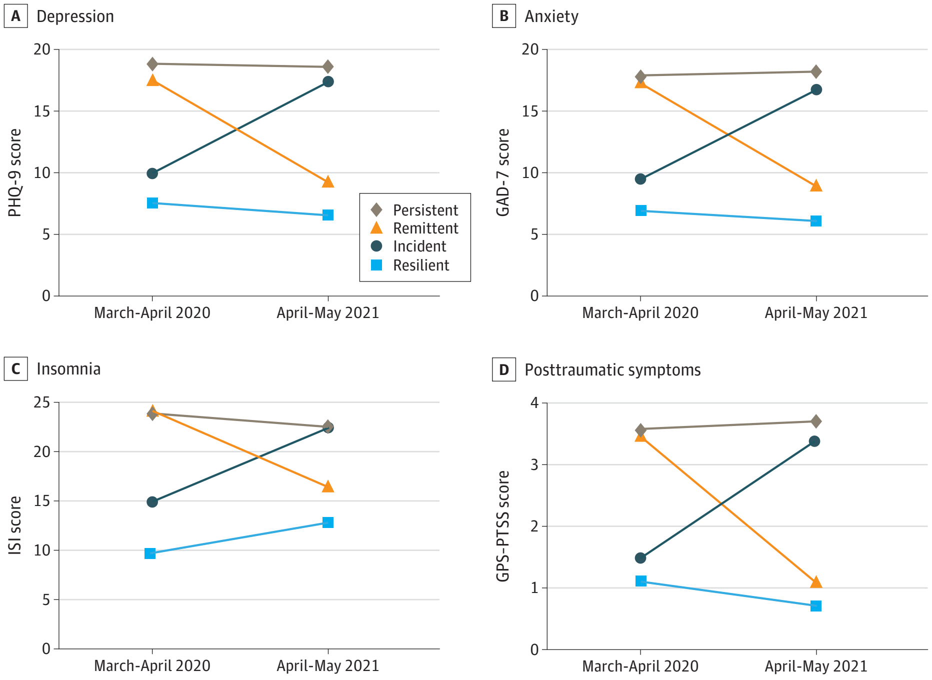

GAD-7 indicates 7-item Generalized Anxiety Disorder scale; GPS-PTSS, Global Psychotrauma Screen–posttraumatic symptoms subscale; ISI, Insomnia Severity Index; PHQ-9, 9-item Patient Health Questionnaire.

Downloaded From: https://jamanetwork.com/ on 12/21/2022

Table 4. Multinomial Logistic Regression

Variable	OR (95% CI)		
	Remittent	Incident	Persistent
**Depression**			
Male	1 [Reference]	NA	NA
Female	1.58 (0.98-2.56)	1.08 (0.53-2.21)	3.69 (1.54-8.82)
Age[a]	1.22 (1.02-1.45)	0.99 (0.74-1.33)	1.07 (0.84-1.36)
Frontline status history[b]			
At T1 only	1.74 (1.10-2.76)	1.38 (0.60-3.19)	1.97 (0.98-3.96)
At T2 only	0.83 (0.42-1.64)	1.52 (0.58-3.96)	2.43 (1.07-5.52)
At T1 and T2	1.00 (0.65-1.55)	1.37 (0.68-2.77)	2.18 (1.17-4.04)
Occupation[c]			
Nurse	1.53 (0.96-2.43)	1.28 (0.59-2.77)	1.28 (0.70-2.34)
Physician	1.07 (0.64-1.77)	1.12 (0.49-2.55)	0.61 (0.29-1.29)
General practitioner	2.30 (0.73-7.28)	1.66 (0.31-8.94)	2.40 (0.64-8.96)
Health care assistant	1.59 (0.85-2.97)	1.77 (0.64-4.87)	0.93 (0.38-2.30)
Pandemic-related variables			
Has had COVID-19	0.88 (0.56-1.37)	0.57 (0.24-1.32)	1.70 (1.01-2.86)
Colleagues infected, hospitalized, or deceased	0.84 (0.54-1.30)	1.15 (0.52-2.51)	1.21 (0.61-2.38)
Family infected, hospitalized, or deceased	1.21 (0.85-1.71)	0.72 (0.40-1.29)	1.24 (0.78-1.99)
Mental health history			
Psychiatric treatment			
During pandemic	1.38 (0.91-2.09)	1.98 (1.08-3.65)	1.70 (1.01-2.88)
Before pandemic	1.07 (0.73-1.58)	2.30 (1.27-4.16)	1.65 (1.00-2.72)
**Anxiety**			
Male	1 [Reference]	NA	NA
Female	0.96 (0.61-1.51)	1.70 (0.64-4.54)	6.50 (1.55-27.26)
Age[a]	1.28 (1.06-1.54)	0.95 (0.68-1.32)	1.29 (0.97-1.72)
Frontline status[b]			
At T1 only	1.28 (0.77-2.12)	1.14 (0.47-2.80)	1.63 (0.77-3.45)
At T2 only	1.38 (0.75-2.54)	1.72 (0.61-4.82)	0.47 (0.10-2.12)
At T1 and T2	0.87 (0.54-1.39)	0.93 (0.41-2.09)	1.59 (0.80-3.17)
Occupation[c]			
Nurse	1.23 (0.75-2.02)	1.43 (0.63-3.27)	0.74 (0.38-1.46)
Physician	1.16 (0.69-1.94)	0.70 (0.25-1.94)	0.37 (0.15-0.92)
General practitioner	1.11 (0.29-4.20)	1.96 (0.36-10.63)	1.34 (0.25-7.01)
Health care assistant	1.18 (0.58-2.37)	1.03 (0.30-3.51)	1.01 (0.40-2.52)
Pandemic-related variables			
Has had COVID-19	0.77 (0.48-1.25)	0.96 (0.43-2.12)	1.39 (0.75-2.58)
Colleagues infected, hospitalized, or deceased	1.02 (0.63-1.65)	1.22 (0.51-2.93)	1.22 (0.56-2.67)
Family infected, hospitalized, or deceased	1.43 (0.99-2.07)	1.03 (0.54-1.95)	1.48 (0.85-2.57)
Mental health history			
Psychiatric treatment			
During pandemic	0.92 (0.59-1.45)	2.27 (1.15-4.48)	1.70 (0.92-3.15)
Before pandemic	1.23 (0.82-1.85)	1.94 (0.99-3.81)	1.07 (0.59-1.96)
**Insomnia**			
Male	1 [Reference]	NA	NA
Female	1.36 (0.67-2.77)	1.82 (0.40-8.23)	NA
Age[a]	0.99 (0.77-1.27)	0.82 (0.49-1.35)	0.61 (0.28-1.32)
Frontline status[b]			
At T1 only	2.26 (1.09-4.67)	2.92 (0.73-11.65)	4.00 (0.33-48.12)
At T2 only	3.59 (1.58-8.14)	0.96 (0.10-8.94)	20.50 (1.84-228.27)
At T1 and T2	2.08 (1.06-4.09)	2.21 (0.63-7.77)	3.85 (0.35-42.61)

(continued)

Downloaded From: https://jamanetwork.com/ on 12/21/2022

Table 4. Multinomial Logistic Regression (continued)

Variable	OR (95% CI)		
	Remittent	Incident	Persistent
Occupation[c]			
Nurse	1.77 (0.92-3.40)	1.55 (0.40-5.96)	2.66 (0.27-26.38)
Physician	0.43 (0.17-1.08)	0.77 (0.15-3.99)	3.29 (0.31-34.90)
General practitioner	1.92 (0.48-7.66)	1.69 (0.15-19.16)	NA
Health care assistant	1.75 (0.74-4.16)	1.34 (0.21-8.47)	3.44 (0.19-62.86)
Pandemic-related variables			
Has had COVID-19	1.03 (0.57-1.85)	0.92 (0.29-2.96)	0.94 (0.18-5.00)
Colleagues infected, hospitalized, or deceased	0.99 (0.51-1.92)	0.82 (0.22-3.09)	NA
Family infected, hospitalized, or deceased	0.96 (0.58-1.58)	1.15 (0.45-2.97)	2.73 (0.63-11.78)
Mental health history			
Psychiatric treatment			
During pandemic	1.25 (0.70-2.21)	0.49 (0.15-1.62)	1.17 (0.25-5.60)
Before pandemic	1.06 (0.62-1.84)	2.87 (1.08-7.62)	1.71 (0.38-7.76)
**Posttraumatic symptoms**			
Male	1 [Reference]	NA	NA
Female	2.99 (1.89-4.73)	1.49 (0.81-2.71)	2.60 (1.60-4.20)
Age[a]	0.98 (0.82-1.16)	1.46 (1.13-1.88)	1.27 (1.06-1.53)
Frontline status[b]			
At T1 only	1.87 (1.18-2.97)	0.67 (0.29-1.52)	1.66 (1.00-2.77)
At T2 only	0.59 (0.31-1.14)	1.26 (0.59-2.68)	0.79 (0.41-1.52)
At T1 and T2	1.57 (1.03-2.39)	1.29 (0.70-2.37)	1.75 (1.12-2.72)
Occupation[c]			
Nurse	1.24 (0.80-1.94)	0.76 (0.40-1.45)	1.44 (0.90-2.31)
Physician	1.42 (0.89-2.26)	0.79 (0.40-1.56)	1.17 (0.70-1.97)
General practitioner	1.64 (0.54-4.95)	0.94 (0.18-4.90)	1.23 (0.36-4.19)
Health care assistant	1.70 (0.90-3.21)	1.84 (0.82-4.15)	1.63 (0.81-3.25)
Pandemic-related variables			
Has had COVID-19	0.78 (0.50-1.20)	0.94 (0.51-1.75)	0.94 (0.61-1.45)
Colleagues infected, hospitalized, or deceased	0.97 (0.64-1.47)	0.98 (0.53-1.80)	1.90 (1.13-3.21)
Family infected, hospitalized, or deceased	1.38 (0.98-1.94)	1.55 (0.94-2.55)	1.47 (1.02-2.11)
Mental health history			
Psychiatric treatment			
During pandemic	1.25 (0.82-1.90)	1.63 (0.90-2.95)	1.59 (1.03-2.44)
Before pandemic	0.87 (0.59-1.27)	0.85 (0.49-1.48)	0.95 (0.64-1.41)

Abbreviations: NA, not applicable; OR, odds ratio.

[a] Age is standardized and reversed.

[b] Reference category is never frontline.

[c] Reference category is other health care worker.

95% CI, 1.01-2.86), whereas having colleagues (OR, 1.90; 95% CI, 1.13-3.21) or a family member (OR, 1.47; 95% CI, 1.02-2.11) with COVID-19 was positively associated with persistent PTSSs.

Having seen a mental health professional during the pandemic was positively associated with incident (OR, 1.98; 95% CI, 1.08-3.65) or persistent (OR, 1.70; 95% CI, 1.01-2.88) depression symptoms, incident anxiety symptoms (OR, 2.27; 95% CI, 1.15-4.48), and persistent PTSSs (OR, 1.59; 95% CI, 1.03-2.44). Because these results are from multivariable regression models, all changes were identified after correcting for the effects of covariates.

Discussion

To our knowledge, this is the first study to report on longitudinal mental health data in an Italian sample of health care workers 14 months after the beginning of the COVID-19 pandemic. We report on longitudinal data collected at baseline during the first wave of the COVID-19 pandemic (March to April 2020) and at a single follow-up 14 months later, with a response rate at follow-up of nearly one-third. In our sample, we found an overall decrease in depression symptoms, anxiety symptoms,

Downloaded From: https://jamanetwork.com/ on 12/21/2022

and PTSSs, together with a mean score increase in insomnia symptoms. The prevalence of the selected outcomes, defined according to cut-off scores widely used in the literature, decreased between 6% for insomnia symptoms and 19% for PTSSs.

Taking into account the presence or absence of the outcomes at the 2 time points, we could explore how the different outcomes evolved over time. In our sample, most participants never developed clinically relevant depression, anxiety, or insomnia symptoms. However, nearly two-thirds of the sample developed PTSSs at some point during the pandemic. Of those who had developed clinically relevant depression, anxiety, or insomnia symptoms at the first time point, only a few developed a persistent condition throughout the pandemic. In contrast, in most participants who had developed a condition in the early stages of the pandemic, their symptoms had remitted 1 year later, although PTSSs continued to affect nearly one-fourth of our sample. This finding could be explained by the different sensibility and psychometric properties of the GPS- PTSS compared with the PHQ-9, GAD-7 and ISI; however, it could also be that a large proportion of the HCW population has actually experienced mild PTSSs with little or no functional consequences. Another possible explanation is that, because of the timing of the data collection, the PTSSs that we detected at T1 could be better explained as an acute stress reaction, whereas the PTSSs at T2 could actually indicate posttraumatic stress disorder. Consistent with our findings, a recent meta-analysis[3] found that the prevalence of acute stress was nearly 3 times higher than that of posttraumatic stress disorder.

Regarding insomnia symptoms, our results indicate that the mean symptom score has been increasing by 20% in the overall sample. However, this finding did not result in a corresponding increase in the prevalence of clinically relevant insomnia symptoms, suggesting an overall worsening of insomnia in the sample, although below the limits of clinical relevance.

In line with previous results from the early stages of the pandemic,[1,2] working as a frontline HCW was confirmed as a relevant risk factor for several mental health outcomes. In particular, stopping work as a frontline HCW (ie, being a frontline HCW at T1 only) was associated with remission in depression symptoms and PTSSs and a decrease in all the considered outcome scores, whereas being a frontline HCW throughout the pandemic (ie, being a frontline HCW at both T1 and T2) was associated with persistent depression symptoms and PTSSs. These results should be taken with caution, given the association between being a frontline HCW at T1 and T2 and remittent insomnia symptoms and PTSSs. In this regard, further research on putative moderators of this association is warranted.

According to recent meta-analyses,[4,12] female HCWs were more likely to experience poor mental health outcomes. In our study, we found that female sex was associated with persistence of depression symptoms, anxiety symptoms, and PTSSs, even though male participants had a larger increase over time in PTSS and insomnia.

Contrary to early findings of younger age being associated with worse outcomes,[13] at follow-up young age was associated with remission of depression and anxiety symptoms. However, younger age was also associated with incident PTSS and insomnia.

To date, a relatively small number of studies have addressed the longitudinal trends in mental health outcomes among HCWs. A Canadian study[6] of 373 HCWs during 5 months found similar rates of the trajectories of mental conditions. Contrary to our results, a Japanese longitudinal study[14] (8 months) found an increase in psychological distress among HCWs. However, this study evaluated psychological distress using an occupational medicine tool for job-related stress and did not distinguish between frontline and second-line HCWs. Finally, a Belgian study[15] on a small sample of nurses found a decreasing trend over time of psychological distress.

Strengths and Limitations

This study has several strengths. In particular, its longitudinal nature and prompt data collection make it a one-of-a-kind study in the currently available literature. Our study also has several limitations. First, the online sampling technique could have introduced a relevant self-selection bias, and the design did not allow for the estimation of response rate at baseline. Second, the relatively

Downloaded From: https://jamanetwork.com/ on 12/21/2022

small sample size warrants caution in the generalizability of the results. Third, this study is based on self-report measures that inherently convey a systematic bias in estimating the target outcome.

Conclusions

The results of this study highlight a decreasing trend of mental health symptoms in the Italian HCWs. Age, sex, and frontline working position were relevant risk factors for the persistence of conditions over time. These results could inform working policies that should avoid overexposure of HCWs to frontline working positions in the future.

ARTICLE INFORMATION

Accepted for Publication: September 30, 2021.

Published: November 24, 2021. doi:10.1001/jamanetworkopen.2021.36143

Open Access: This is an open access article distributed under the terms of the CC-BY License. © 2021 Rossi R et al. *JAMA Network Open*.

Corresponding Author: Rodolfo Rossi, MD, Department of Systems Medicine, University of Rome Tor Vergata, Via Montpellier 1, 00133, Rome, Italy (rudy86.rossi@gmail.com).

Author Affiliations: Department of Systems Medicine, University of Rome Tor Vergata, Rome, Italy (R. Rossi, Jannini, Siracusano, Di Lorenzo); Department of Biotechnological and Applied Clinical Sciences, University of L'Aquila, L'Aquila, Italy (Socci, Pacitti, A. Rossi); IRCSS Fondazione Santa Lucia, Rome, Italy (Di Lorenzo).

Author Contributions: Dr Rossi had full access to all the data in the study and takes responsibility for the integrity of the data and the accuracy of the data analysis.

Concept and design: R. Rossi, Pacitti, A. Rossi, Di Lorenzo.

Acquisition, analysis, or interpretation of data: R. Rossi, Socci, Jannini, Pacitti, Siracusano, Di Lorenzo.

Drafting of the manuscript: R. Rossi, Pacitti, Di Lorenzo.

Critical revision of the manuscript for important intellectual content: All authors.

Statistical analysis: R. Rossi, Di Lorenzo.

Obtained funding: Pacitti, A. Rossi, Di Lorenzo.

Administrative, technical, or material support: Jannini, Pacitti, A. Rossi, Di Lorenzo.

Supervision: Pacitti, Siracusano, A. Rossi, Di Lorenzo.

Conflict of Interest Disclosures: None reported.

REFERENCES

1. Rossi R, Socci V, Pacitti F, et al. Mental health outcomes among frontline and second-line health care workers during the coronavirus disease 2019 (COVID-19) pandemic in Italy. *JAMA Netw Open*. 2020;3(5):e2010185. doi:10.1001/jamanetworkopen.2020.10185

2. Rossi R, Socci V, Pacitti F, et al. Mental health outcomes among healthcare workers and the general population during the COVID-19 in Italy. *Front Psychol*. 2020;11:608986. doi:10.3389/fpsyg.2020.608986

3. Marvaldi M, Mallet J, Dubertret C, Moro MR, Guessoum SB. Anxiety, depression, trauma-related, and sleep disorders among healthcare workers during the COVID-19 pandemic: a systematic review and meta-analysis. *Neurosci Biobehav Rev*. 2021;126:252-264. doi:10.1016/j.neubiorev.2021.03.024

4. Luo M, Guo L, Yu M, Jiang W, Wang H. The psychological and mental impact of coronavirus disease 2019 (COVID-19) on medical staff and general public—a systematic review and meta-analysis. *Psychiatry Res*. 2020;291:113190. doi:10.1016/j.psychres.2020.113190

5. Saragih ID, Tonapa SI, Saragih IS, et al. Global prevalence of mental health problems among healthcare workers during the Covid-19 pandemic: A systematic review and meta-analysis. *Int J Nurs Stud*. 2021;121:104002. doi:10.1016/j.ijnurstu.2021.104002

6. Dufour M-M, Bergeron N, Rabasa A, Guay S, Geoffrion S. Assessment of psychological distress in health-care workers during and after the first wave of COVID-19: a Canadian longitudinal study. *Can J Psychiatry*. 2021;66(9):807-814. doi:10.1177/07067437211025217

Downloaded From: https://jamanetwork.com/ on 12/21/2022

7. Spitzer RL, Kroenke K, Williams JB; Primary Care Evaluation of Mental Disorders. Validation and utility of a self-report version of PRIME-MD: the PHQ primary care study: primary care evaluation of mental disorders; patient health questionnaire. *JAMA*. 1999;282(18):1737-1744. doi:10.1001/jama.282.18.1737

8. Spitzer RL, Kroenke K, Williams JBW, Löwe B. A brief measure for assessing generalized anxiety disorder: the GAD-7. *Arch Intern Med*. 2006;166(10):1092-1097. doi:10.1001/archinte.166.10.1092

9. Morin CM, Belleville G, Bélanger L, Ivers H. The Insomnia Severity Index: psychometric indicators to detect insomnia cases and evaluate treatment response. *Sleep*. 2011;34(5):601-608. doi:10.1093/sleep/34.5.601

10. Olff M, Bakker A, Frewen P, et al. Screening for consequences of trauma—an update on the global collaboration on traumatic stress. *Eur J Psychotraumatol*. 2020;11(1):1752504. doi:10.1080/20008198.2020.1752504

11. Rossi R, Socci V, Talevi D, et al. Trauma-spectrum symptoms among the Italian general population in the time of the COVID-19. *Eur J Psychotraumatol*. 2021;12(1). doi:10.1080/20008198.2020.1855888

12. Pappa S, Ntella V, Giannakas T, Giannakoulis VG, Papoutsi E, Katsaounou P. Prevalence of depression, anxiety, and insomnia among healthcare workers during the COVID-19 pandemic: a systematic review and meta-analysis. *Brain Behav Immun*. 2020;88:901-907. doi:10.1016/j.bbi.2020.05.026

13. Rossi R, Jannini TB, Socci V, Pacitti F, Lorenzo GD. Stressful life events and resilience during the COVID-19 lockdown measures in Italy: association with mental health outcomes and age. *Front Psychiatry*. 2021;12:635832. doi:10.3389/fpsyt.2021.635832

14. Sasaki N, Asaoka H, Kuroda R, Tsuno K, Imamura K, Kawakami N. Sustained poor mental health among healthcare workers in COVID-19 pandemic: a longitudinal analysis of the four-wave panel survey over 8 months in Japan. *J Occup Health*. 2021;63(1):e12227. doi:10.1002/1348-9585.12227

15. Van Steenkiste E, Schoofs J, Gilis S, Messiaen P. Mental health impact of COVID-19 in frontline healthcare workers in a Belgian Tertiary care hospital: a prospective longitudinal study. *Acta Clin Belg*. 2021;(March):1-8. doi: 10.1080/17843286.2021.1903660

Downloaded From: https://jamanetwork.com/ on 12/21/2022
