# Supplementary material for: Mental health status and related factors influencing healthcare workers during the COVID-19 pandemic: A systematic review and meta-analysis
Source: PLoS One. 2024 Jan 19;19(1):e0289454. doi: 10.1371/journal.pone.0289454 (PMC10798549; doi:10.1371/journal.pone.0289454)
Supplement: S1 Data — (ZIP) [file pone.0289454.s011.zip › literatures/15.pdf]

Received: 9 May 2021 | Revised: 2 July 2021 | Accepted: 12 July 2021

DOI: 10.1002/brb3.2304

Brain and Behavior Open Access WILEY

ORIGINAL RESEARCH

Assessing mental health status among Iranian healthcare workers in times of the COVID-19 pandemic: A web-based cross-sectional study

Marzieh Azizi[1] | Mahsa Kamali[2] | Mahmood Moosazadeh[3,4] | Mohsen Aarabi[5] | Roya Ghasemian[6] | Maryam Hasannezhad Reskati[7] | Forouzan Elyasi[8]

[1] Department of Reproductive Health and Midwifery, School of Nursing and Midwifery, Tehran University of Medical Sciences, Tehran, Iran

[2] Pediatric Infectious Diseases Research Center, Communicable Diseases Institute, Mazandaran University of Medical Sciences, Sari, Iran

[3] Gastrointestinal Cancer Research Center, Non-communicable Diseases InstituteMazandaran University of Medical Sciences, Sari, Iran

[4] Health Sciences Research Center, Addiction Institute, Mazandaran University of Medical Sciences, Sari, Iran

[5] Department of Family Medicine, School of Medicine, Mazandaran University of Medical Sciences, Sari, Iran

[6] Antimicrobial Resistance Research Center, Department of Infectious Diseases, Mazandaran University of Medical Sciences, Sari, Iran

[7] Research Ethics Committee, Imam khomeini Hospital, Mazandaran, University of Medical Sciences, Sari, Iran

[8] Psychiatry and Behavioral Sciences Research Center, Sexual and Reproductive Health Research Center, Imam Khomeini General Hospital, Addiction Institute, Mazandaran University of Medical Sciences, Sari, Iran

Correspondence
Forouzan Elyasi, Psychosomatic Ward, Imam Khomeini General Hospital, Razi Ave., Sari, Mazandaran, IR 48157-33971, Iran.
Email: forouzan.elyasi@gmail.com, F.elyasi@mazums.ac.ir

The manuscript is being submitted to: social psychiatry and epidemiology, general topics in psychiatry and related fields

This research project was approved by the **Ethics Committee of Mazandaran University of Medical Sciences (IR.MAZUMS.REC.1399.7574), Sari, Iran**.

Funding information
Mazandaran University of Medical Sciences, Grant/Award Number: 7574

Abstract

Objectives: The present study was conducted to assess 3(HCWs) during the COVID-19 pandemic.

Methods: A total number of 7626 HCWs were included in this web-based cross-sectional study, via the convenience sampling technique. To collect the required data, the sociodemographic characteristics information form, the Depression Anxiety Stress Scale-21 (DASS-21), and the Corona Disease Anxiety Scale (CDAS) were also employed. In addition, data analysis was performed using the SPSS Statistics software (ver. 24), as well as descriptive statistics, Chi-square test (χ^2), and univariate/multivariate logistic regression models.

Results: The CDAS results revealed that 47.9% and 70.5% of the HCWs had experienced moderate levels of physical and psychological anxiety, respectively. Based on the DASS results, 44.8%, 43%, and 34.8% of the HCWs had been subjected to depression, anxiety, and stress symptoms during the COVID-19 pandemic, respectively. The logistic regression models correspondingly showed that depression among the HCWs was significantly correlated with risk factors, such as the age groups of 20–30 years

Abbreviations: CDAS, the Corona Disease Anxiety Scale; DASS-21, the Depression Anxiety Stress Scale-21; HCWs, healthcare workers; MHME, Ministry of Health and Medical Education; SD, standard deviation; WHO, World Health Organization

This is an open access article under the terms of the Creative Commons Attribution License, which permits use, distribution and reproduction in any medium, provided the original work is properly cited.
© 2021 The Authors. *Brain and Behavior* published by Wiley Periodicals LLC

($p = .001$), 31–40 years ($p = .006$), female HCWs ($p > .001$), history of physical illnesses ($p = .004$), and history of psychiatric disorders ($p > .001$). Moreover, factors including the age groups of 20–30 years ($p < .001$), 31–40 years ($p < .001$), 41–50 years ($p < .001$), female HCWs ($p > .001$), history of physical illnesses ($p < .001$), and history of psychiatric disorders ($p > .001$) were assumed as significant predictors of anxiety in these individuals. Besides, factors such as the age groups of 20–30 years ($p = .002$), 31–40 years ($p = .004$), female HCWs ($p > .001$), occupation ($p = .016$), history of physical illnesses ($p < .001$), and history of psychiatric disorders ($p > .001$) could significantly predict the prevalence rate of stress in the HCWs in times of this crisis.

Conclusion: Given the importance of mental health status among HCWs during the COVID-19 pandemic, health administrators and policymakers of the Ministry of Health and Medical Education in Iran are suggested to provide psychological screening and supportive care programs for HCWs with the aim of enhancing their mental health and successful coping with critical circumstances.

KEYWORDS
anxiety, COVID-19, depression, healthcare workers, stress

1 | INTRODUCTION

Severe acute respiratory syndrome coronavirus 2 (SARS-CoV-2), commonly presented as coronavirus disease 2019 (COVID-19), emerged since late 2019 in the city of Wuhan, Hubei Province, China (Hall, 2020; Vindegaard & Benros, 2020). The rapid spread of the disease, the delayed onset of its symptoms, as well as the high asymptomatic carriers of this condition correspondingly transformed this disease into the main challenge facing healthcare systems worldwide (Hall, 2020; Li et al., 2020; Liu et al., 2020). The World Health Organization (WHO) introduced COVID-19 as a public health emergency of international concern (Hall, 2020; Mohindra et al., 2020; Wong et al., 2020). Within approximately 8 months from the beginning of the COVID-19 pandemic, WHO statistics on September 20, 2020, reported 30,675,675 confirmed cases of COVID-19 and 954,417 deaths all over the world (WHO Coronavirus Disease (COVID-19), 2020). The WHO statistics about Iran revealed that 422,140 confirmed cases of COVID-19, 24,301 deaths, and 359,570 recovered cases had been identified until September 20, 2020 (WHO Coronavirus Disease (COVID-19), 2020).

Healthcare workers (HCWs) are vital resources in each country whose health and safety are crucial not only for providing continuous and effective care services to patients, but also keeping health warning situations, such as outbreaks under control (Liu et al., 2020). Although HCWs are responsible for patient care during the COVID-19 pandemic, they are playing against numerous health concerns, such as personal protective equipment shortages, fear of mortality and morbidity caused by COVID-19, fear of infection for themselves and its transmission to family members, along with loss of coworkers due to this condition (Chew et al., 2020; Galbraith et al., 2020; Walton et al., 2020). These risk factors have potentially increased the possibility of occurrence of short- and long-term mental health problems, including depressive symptoms, anxiety, insomnia, and grief, as well as the development of post-traumatic stress disorders among these individuals (Galbraith et al., 2020; Hall, 2020; Liu et al., 2020; Muller et al., 2020; Shechter et al., 2020). The WHO has further put emphasis on the extremely high burden of the pandemic on different aspects of HCWs' well-being and has even called for taking the necessary actions to address their immediate needs to save their lives and to prevent a serious impact on their physical and mental health status (World Health Organization, 2020).

The prevalence of mental health problems among HCWs during the COVID-19 pandemic has been so far investigated in several studies worldwide (Gold, 2020; Hall, 2020; Liu et al., 2020; Lu et al., 2020; Shechter et al., 2020). In a survey on frontline HCWs in China, 50% of these individuals had levels of depression, 45% of them were suffering from anxiety, and 34% of the cases had experienced insomnia in times of this crisis (Lai et al., 2020). A large-scale study in this country, exploring 7236 individuals, including 2250 HCWs, had further aimed at examining mental health burden through measuring anxiety, depression, and poor sleep symptoms and had found that 34.7% of the HCWs had symptoms of anxiety, 19.8% of them had shown depressive symptoms, and 23.6% had reported sleep disturbance (Huang & Zhao, 2020). Another survey in China had similarly demonstrated that 57% of the HCWs had acute stress, 48% of them had experienced depressive symptoms, and 33% of the cases had felt anxiety symptoms (Shechter et al., 2020). According to the results of several studies in Iranian context, 39.6–65.6% of hospital staff had been reported with moderate-to-severe levels of anxiety and 42.3% of the them had shown moderate-to-severe depressive symptoms during the COVID-19 pandemic (Kaveh et al., 2020; Taghizadeh et al., 2020).

Given the long-term period of the COVID-19 pandemic, the ongoing high prevalence rate of new confirmed cases, and the added high workload of HCWs and its considerable negative effect on their mental health, the present study aimed to investigate mental health status among HCWs in times of this crisis in Iran.

2 | MATERIALS AND METHODS

2.1 | Study design and participants

This web-based cross-sectional survey was performed on 7626 HCWs selected through the convenience sampling technique, from April 6 to May 19, 2020, in all cities of Iran and during the COVID-19 pandemic. The HCWs included in this study were comprised of physicians (viz. general practitioners, specialists, medical residents, and medical students), nurses, dentists, pharmacists, midwives, laboratory personnel, radiologists, and other staff, having direct or indirect interactions with patients infected with COVID-19 hospitalized in inpatient and outpatient care wards, fever clinics, prehospital emergencies, and primary healthcare centers. The inclusion criteria were all HCWs who had experienced direct or indirect contacts with COVID-19 patients, showing willingness to participate in this study. The individuals who were not as HCWs or therapeutic personnel were excluded from this study.

2.2 | Ethical considerations

This research project was approved by the Ethics Committee of Mazandaran University of Medical Sciences (IR.MAZUMS.REC.1399.7574), Sari, Iran. All the procedures performed in the study involving human participants were also in accordance with the ethical standards of the institutional and/or national research committee and the Declaration of Helsinki (DoH) 1964 and its later amendments or comparable ethical guidelines. To adhere to the ethical considerations, an online informed consent form was obtained from the individuals participating in this survey after explaining research objectives, and they were then assured about the confidentiality of their information.

2.3 | Instruments and data collection

The Persian version of the questionnaires was completed using an online platform, developed by Sadra Rayaneh Novin Tabarestan Engineering Co., based in Mazandaran provinces, Iran (http://psych.mazandums.ir). After designing, the web-based questionnaires were checked by this study researchers regarding its correction and then self-report web-based questionnaires were distributed via the cyberspace, including popular messaging apps, that is, WhatsApp, Telegram, Instagram, and Short Message Service among HCW groups that working in the different therapeutic settings during COVID-19 pandemic. The time for filling the questionnaires by participants was considered approximately 15–20 min. These web-based questionnaires were distributed among different groups of HCWs and they also distributed them among other groups they knew. On the first page of the online questionnaire, researchers wrote a text for participants regarding the research title and aim of this study, the confidentiality of their information and identity. Also, they explained briefly the questionnaires content and the methods of responding to the questions. Only those questionnaires were considered filled that participants filled them until the end-stage based on the instruction of the designed questionnaire. The corresponding author of this study had a username and password for checking the numbers of filled questionnaires.

In this study, three main instruments were also completed by the participants, namely, the sociodemographic characteristics information form, the Depression Anxiety Stress Scale-21 (DASS-21), and the Corona Disease Anxiety Scale (CDAS). Upon completing the questionnaires by the participants, the scores obtained from the participants' responses were automatically calculated and tabulated. Moreover, the HCWs achieving high scores (namely, unfavorable mental health status) could contact the 4030 phone number dedicated to free consultations by the Ministry of Health and Medical Education (MHME).

2.3.1 | Sociodemographic characteristics information form

The sociodemographic characteristics of the participants included gender, age, marital status, place of living, level of education, field of study, working position, years of work experience, history of mental and physical problems, number of children, working units (i.e., inpatient or outpatient care wards), as well as some items about the COVID-19 pandemic, evaluated in this study.

2.3.2 | Depression Anxiety Stress Scale-21

The DASS-21, as the shortened version of the DASS-42, was developed by Lovibond et al. to assess the symptoms of depression, anxiety, and stress among adults (Le et al., 2017) in clinical or nonclinical settings (Musa et al., 2007). The DASS-21 was a self-report measure by which the participants could rate the frequency and the severity of their experiences of negative emotions over the past week on a four-point Likert-type scale from 0, which meant "did not apply to me at all," to 3 denoting "applied to me very much or most of the time." Overall, in each subscale, the score could range from 0 to 63 and the greater the score, the higher the severity of depression, anxiety, and stress (Asghari et al., 2008; Norton, 2007). Scoring for the depression subscale was also divided into 0–9 representing normal mental health status (namely, without depressive symptoms), 10–13 showing mild depressive symptoms, 14–20 standing for moderate depressive symptoms, 21–27 denoting severe depressive symptoms, and ≥28 considered as extremely severe symptoms. In terms of rating the anxiety subscale, scores 0–7 were categorized as normal mental health status (viz. without anxiety), scores 8–9 referring to mild anxiety, and

the scores of 10–14, 15–19, and ≥28 implying moderate, severe, and extremely severe anxiety among participants, respectively. Moreover, the stress subscale was scored as follows, 0–14 representing normal or without stress, scores 15–18 showing mild stress, and scores 19–25, 26–33, and ≥34 denoting moderate, severe, and extremely severe stress, respectively. The internal consistency of this scale had been estimated to be good-to-excellent (Antony et al., 1998). The psychometric properties of the Malaysian version of this questionnaire had further shown its appropriate Cronbach's alpha values of 0.84, 0.74, and 0.79, respectively, for depression, anxiety, and stress (Musa et al., 2007). In another study, the reliability of this scale in the subscales of depression, anxiety, and stress had been approved with Cronbach's alpha values of 0.82, 0.90, and 0.93, respectively (Henry & Crawford, 2005). Based on the results of a study in Iran, the reliability of this instrument had been reported by 0.86, 0.76, and 0.79 for the three subscales of depression, anxiety, and stress, respectively (Jafari et al., 2017).

2.3.3 | Corona Disease Anxiety Scale

The CDAS, designed and validated by Alipour et al., was to measure the prevalence of anxiety caused by the COVID-19 pandemic. This scale included 18 items and two subscales of psychological and physical symptoms scored on a four-point Likert-type scale from 0 meaning never or no anxiety to 3 representing always with anxiety. The total score of this scale also ranged from 0 to 54. In the subscale of psychological symptoms, scores 0–5, 6–19, and 20–27 were considered as mild, moderate, and severe psychological symptoms, respectively. As well, physical symptoms scored 0–1 showed no or mild symptoms, 2–9 indicated moderate symptoms, and 10–27 reflected severe physical symptoms. The higher scores on this questionnaire could suggest the higher level of anxiety. The reliability of this scale had been measured using the Cronbach's alpha coefficient for the first factor ($\alpha = 0.879$), the second factor ($\alpha = 0.861$), and the whole questionnaire ($\alpha = 0.919$) (Alipour et al., 2020).

2.4 | Statistical analysis

Data analysis in this study was performed using the SPSS Statistics software (ver. 24) (SPSS Inc., IBM Corp., USA). To identify the missing data, they were also checked and cleaned before analysis. To describe the quantitative variables, descriptive analyses, such as mean± standard deviation (SD), were employed. Moreover, frequency and percentage were applied to explain the qualitative variables. The prevalence rates of depression, anxiety, and stress caused by COVID-19 were correspondingly reported and the Chi-square test (χ^2) was utilized to compare between-group differences. Univariate/multivariate logistic regression models were further used to explore the potential predictors of anxiety in times of this crisis. *p*-values less than .05 were also considered statistically significant.

3 | RESULTS

3.1 | Sociodemographic characteristics of participants

The sociodemographic characteristics of HCWs are presented in Table 1. A total number of 7626 HCWs were accordingly included in this study and the results revealed that most of the participants (56.2%) aged 20–30 years and approximately two-thirds (73.2%) of them were females. More than half of the participants were also married and 64.7% of the cases had no children. Nearly, 70% of the HCWs were holding Bachelor's degrees and nurses were the most frequent individuals participating in this study. Within the first 2 months of the COVID-19 pandemic, nearly 45% of the participants had been working in isolation wards related to inpatient COVID-19 patients. Among different settings involved with COVID-19, more than 50% of the HCWs had been working in hospitals and 40% of them had worked 4–8 h/day in contact with COVID-19. Among the participants, 19.6% and 8.4% of them, respectively, had history of physical illnesses and psychiatric disorders based on their self-report.

3.2 | Prevalence of anxiety based on the CDAS

The CDAS results are given in Tables 2 and 3. According to the CDAS, the prevalence of physical and psychological anxiety symptoms was assessed among the HCWs and the results showed that most of the participants (47.9%) had experienced moderate physical anxiety symptoms and also more than 70% of them had suffered from moderate psychological anxiety symptoms. The results also demonstrated that anxiety caused by COVID-19 was significantly correlated with gender ($p < .001$), age ($p < .001$), marital status ($p = .005$), level of education ($p = .001$), occupation ($p < .001$), working in isolation wards ($p = .014$), working units ($p < .001$), working hours with COVID-19 patients ($p < .001$), history of physical illnesses ($p < .001$), and history of psychiatric disorders ($p < .001$).

3.3 | Prevalence of depression, anxiety, and stress based on the DASS-21

The DASS-21 results are listed in Tables 2 and 4. In this regard, 44.8% of the HCWs had experienced levels of depression during this pandemic and most of them had reported moderate depression (15.4%). In addition, depression was significantly correlated with age ($p < .001$), gender ($p < .001$), marital status ($p < .001$), having children ($p < .001$), level of education ($p = .006$), occupation ($p = .020$), working hours with COVID-19 patients ($p = .022$), history of physical illnesses ($p < .001$), and history of psychiatric disorders ($p < .001$).

In this study, the prevalence of anxiety in the HCWs was estimated by 43%, and among them, most participants had experienced moderate anxiety (17.4%). Anxiety in the HCWs was significantly associated

TABLE 1 Sociodemographic characteristics of participants

Characteristics	Frequency (%), N = 7626
Age groups (years)	
20–30	4287 (56.2)
31–40	2286 (30.0)
41–50	829 (10.9)
51–60	204 (2.7)
> 60	20 (0.3)
Gender	
Male	2044 (26.8)
Female	5582 (73.2)
Marital status	
Single	3176 (41.6)
Married	4257 (55.8)
Divorced or widowed	193 (2.5)
Having children	
Yes	2694 (35.3)
No	4932 (64.7)
Level of education	
Undergraduate	888 (11.6)
Bachelor's degree	5323 (69.8)
Master's and PhD degree	628 (8.2)
General and special professional doctorate	787 (10.3)
Occupation	
Clinician	1098 (14.4)
Nurse	4409 (57.8)
Midwife	1036 (13.6)
Others	1083 (14.2)
Working in isolated units	
Yes	3420 (44.8)
No	4206 (55.2)
Working units	
Hospitals	4157 (54.5)
Outpatient clinics	751 (9.8)
Laboratories	210 (2.8)
Imaging centers	99 (1.3)
Others	2409 (31.6)
Working hours with COVID-19 patients	
0	1121 (14.7)
1–4	2075 (27.2)
4–8	3074 (40.3)
> 8	1356 (17.8)
History of physical illness	
Yes	1497 (19.6)
No	6129 (80.4)
History of psychiatric disorders	
Yes	639 (8.4)
No	6987 (91.6)

with factors, such as age ($p < .001$), gender ($p < .001$), having children ($p < .001$), level of education ($p < .001$), occupation ($p = .016$), working in isolation wards ($p < .001$), working hours with COVID-19 patients ($p < .001$), history of physical illnesses ($p < .001$), and history of psychiatric disorders ($p < .001$).

The study results similarly revealed that 34.8% of the HCWs had experienced stress during this crisis and mild levels of stress (11.1%) were reported in most cases. Stress in the HCWs was significantly correlated with age ($p < .001$), gender ($p < .001$), marital status ($p < .001$), having children ($p < .001$), level of education ($p = .037$), occupation ($p = .023$), working in isolation wards ($p = .006$), working hours with COVID-19 patients ($p = .029$), history of physical illnesses ($p < .001$), and history of psychiatric disorders ($p < .001$).

3.4 | Logistic regression models for predictors of depression, anxiety, and stress in HCWs during COVID-19 pandemic

The predictors of depression, anxiety, and stress among the HCWs are shown in Table 5. In this regard, the multivariate logistic regression indicated that depression among the HCWs was significantly correlated with risk factors, such as age groups of 20–30 years (odds ratio [OR] = 2.26, 95% confidence interval [CI], 1.38–3.71, $p = .001$), 31–40 years (OR = 1.99, 95% CI, 1.22–3.24, $p = .006$), female HCWs (OR = 1.40, 95% CI, 1.20–1.63, $p > .001$), history of physical illnesses (OR = 1.23, 95% CI, 1.06–1.43, $p = .004$), and history of psychiatric disorders (OR = 2.90, 95% CI, 2.42–3.46, $p > .001$). Factors such as working 1–4 h (OR = 0.79, 95% CI, 0.66—0.95, $p = .013$) and 4–8 h (OR = 0.81, 95% CI, 0.68—0.95, $p = .012$) compared with working more than 8 h were additionally among the predictors of depression in the HCWs. All the mentioned factors could predict 6.3% variance of depression among the HCWs (R-squared [R^2]: 3.9%, adjusted $R^2 = 6.3\%$, $p < .001$).

Besides, factors such as age groups of 20–30 years (OR = 4.25, 95% CI, 2.25–8.04, $p < .001$), 31–40 years (OR = 3.42, 95% CI, 1.82–6.43, $p < .001$), 41–50 years (OR = 2.31, 95% CI, 1.20–4.44, $p < .001$), female HCWs (OR = 1.55, 95% CI, 1.32–1.82, $p > .001$), history of physical illnesses (OR = 1.76, 95% CI, 1.52–2.03, $p < .001$), and history of psychiatric disorders (OR = 2.72, 95% CI, 2.27–3.27, $p > .001$) were assumed as significant predictors of anxiety among the HCWs. Working 0 h (OR = 0.78, 95% CI, 0.61—0.98, $p = .037$) and 1–4 h (OR = 0.69, 95% CI, 0.57—0.84, $p < .001$) compared with working more than 8 h with COVID-19 patients were accordingly taken into account as the significant predictors of anxiety in the HCWs. These factors could predict 6.6% variance of anxiety in the individuals (R^2: 3.9%, adjusted $R^2 = 6.6\%$, $p < .001$).

Furthermore, factors such as age groups of 20–30 years (OR = 3.50, 95% CI, 1.59–7.72, $p = .002$), 31–40 years (OR = 3.13, 95% CI, 1.43–6.48, $p = .004$), female HCWs (OR = 1.64, 95% CI, 1.33–2.02, $p > .001$), $p = .016$), history of physical illnesses (OR = 1.51, 95% CI, 1.25–1.81, $p < .001$), and history of psychiatric disorders (OR = 2.80, 95% CI, 2.25–3.47, $p > .001$) could significantly predict the prevalence of stress among the HCWs (R^2: 2.4%, adjusted $R^2 = 6.6\%$, $p < .001$). In addition, factors such as working 1–4 h (OR = 0.69, 95% CI, 0.54—0.89, $p = .004$) and 4–8 h (OR = 0.79, 95% CI, 0.64—0.98, $p = .035$) compared with working more than 8 h and working in hospitals in comparison with other working units (OR = 0.77, 95% CI, 0.62—0.95, $p = .016$) were

TABLE 2 Prevalence of anxiety based on CDAS and DASS

Questionnaire domains and severity					
	Severity of anxiety, $N = 7626$ (%)				
CDAS	Without or mild	Moderate	Severe		
Physical symptoms	2449 (32.1)	3653 (47.9)	1524 (20.0)		
Psychological symptoms	567 (7.4)	5380 (70.5)	1679 (22.1)		
	Severity of depression, anxiety, and stress, $N = 7626$ (%)				
DASS	Normal	Mild	Moderate	Severe	Very severe
Depression	4208 (55.2)	1055 (13.8)	1171 (15.4)	496 (6.5)	696 (9.1)
Anxiety	4352 (57.0)	661 (8.7)	1327 (17.4)	500 (6.6)	786 (10.3)
Stress	4969 (65.2)	850 (11.1)	750 (9.8)	625 (8.2)	432 (5.7)

deemed as significant predictors of stress among the HCWs during this crisis.

4 | DISCUSSION

This study aimed to investigate mental health status among HCWs in all cities of Iran. Accordingly, the prevalence rates of depression, anxiety, and stress in these individuals were assessed through the DASS-21, whose results showed that 44.8% of the HCWs had experienced depression, 43% of them had reported anxiety, and 34.8% had also suffered from stress during the COVID-19 pandemic. It is obvious that frontline HCWs engaged in direct diagnosis, treatment, and care of patients with COVID-19 and physically and psychologically challenged when committing themselves to providing high-quality care for such patients are at higher risks of psychiatric disorders (Lai et al., 2020). Consistent with the present study, the results of a survey in Nepal, assessing mental health status among HCWs during the COVID-19 pandemic, had revealed that the prevalence of anxiety and depressive symptoms in the HCWs was by 42% and 37.5%, respectively (Khanal et al., 2020). In this study, the researchers had declared that high levels of anxiety and depression among the HCWs could be attributed to factors, such as personal protective equipment shortages and resultant fear of getting infected. One other survey in agreement with the present study had also investigated mental health status and quality of life among Indian HCWs during the COVID-19 pandemic and had reported that 47% of the HCWs had shown depressive symptoms and 50% of them had experienced anxiety symptoms (Suryavanshi et al., 2020). Similar findings had been additionally reported in a study in China, showing the prevalence rates of the symptoms of anxiety (46.04%), depression (46.04%), insomnia (44.37%), and psychological problems (28.75%) in HCWs during the pandemic in this country (Que et al., 2020). Another survey in China, investigating mental health status among the HCWs, had also reported that 50.4% of the HCWs had depression, 44.6% had experienced anxiety, and 71.5% of them had suffered from stress (Lai et al., 2020). Although the levels of depression and anxiety in this survey were approximately similar to the ones in the present study, the level of stress among Chinese HCWs was significantly higher because China was the first country wherein COVID-19 emerged, so it is obvious that stress among the HCWs was significantly higher than that in other countries. One other survey in China, assessing the prevalence rates of anxiety, depression, and stress among pediatric nurses, had further demonstrated that 15.4% of the nurses had depressive symptoms, 32.6% of them had experienced anxiety, and 18.0% of the cases had reported stress (Zheng et al., 2020), which were in conflict with the findings in the present study. The discrepancy in the prevalence rates of psychological symptoms in the given surveys might be attributed to cultural differences in the levels of supportive services for Iranian and Chinese societies. Moreover, the results of a study in Turkey had revealed that 77.6% and 60% of the HCWs had experienced depressive and anxiety symptoms (Şahin et al., 2020). The Ministry of Health in this country had also considered psychiatric support and counseling units for HCWs during the COVID-19 pandemic (Şahin et al., 2020). In this respect, the MHME in Iran had similarly implemented specific programs for psychological counseling not only for HCWs but also for the general population through certain phone lines. In this survey, the prevalence rates of depression and anxiety had been reported to be higher than those in the present study, probably due to different sample sizes and research instruments.

The results of this study also showed that depression, anxiety, and stress among the HCWs were significantly correlated with risk factors, such as age, female gender, history of physical illnesses, and history of psychiatric disorders. Based on the results reported in some studies, history of psychiatric disorders (Khanal et al., 2020; Şahin et al., 2020), female gender (Aiyer et al., 2020; Şahin et al., 2020; Suryavanshi et al., 2020), and age (Suryavanshi et al., 2020) had been identified as risk factors for mental health problems, such as anxiety and depression among HCWs in different settings and countries. Moreover, factors such as marital status and stressful events in work environments had been assessed in the related literature, which were not significant in the present study (Suryavanshi et al., 2020). Consistently, the findings of a survey in China had reported that increased workload, physical symptoms, such as respiratory and digestive symptoms, negative coping styles, and job burnout, could be significantly associated with anxiety and depression (Chen et al., 2020).

TABLE 3 CDAS in participants during COVID-19 outbreak in Iranian population stratified by sociodemographic variables ($N = 7626$)

Variables	Physical symptoms of anxiety					Psychological symptoms of anxiety					Total score of CDAS			
	Without to mild, *n* (%)	Moderate to severe, *n* (%)	Very severe, *n* (%)	X^2	*p*-value	Without to mild, *n* (%)	Moderate to severe	Very severe	X^2	*p*-value	Without anxiety	With anxiety	X^2	*p*-value
Gender														
Male	861 (11.3)	907 (11.9)	276 (3.6)	153.07	<.001	244 (3.2)	1482 (19.4)	318 (4.2)	130.56	<.001	1171 (15.4)	873 (11.4)	104.82	<.001
Female	1588 (20.8)	2746 (36.0)	1248 (22.4)			323 (4.2)	3898 (51.1)	1361 17.8)			2460 (32.3)	3122 40.9)		
Age groups														
20–30	1384 (18.1)	2076 (27.2)	827 (10.8)	28.11	<.001	272 (3.6)	3050 (40.0)	965 (12.7)	52.31	<.001	1991 (26.1)	2296 (30.1)	32.83	<.001
31–40	684 (9.0)	1094 (14.3)	508 (6.7)			181 (2.4)	1570 (20.6)	535 (7.0)			1067 (14.0)	1219 (16.0)		
41–50	289 (3.8)	378 (5.0)	162 (2.1)			84 (1.1)	587 (7.7)	158 (2.1)			430 (5.6)	399 (5.2)		
51–60	83 (1.1)	94 (1.2)	27 (0.4)			27 (0.4)	156 (2.0)	21 (0.3)			130 (1.7)	74 (1.0)		
>60	9 (0.1)	11 (0.1)	0 (0.0)			3 (0.0)	17 (0.2)	0 (0.0)			13 (0.2)	7 (0.1)		
Marital status														
Single	1084 (14.2)	1488 (19.5)	604 (7.9)	14.81	**.005**	241 (3.2)	2261 (29.6)	674 (8.8)	10.55	**.032**	1572 (20.6)	1604 (21.0)	10.70	**.005**
Married	1293 (17.0)	2079 (27.3)	885 (11.6)			303 (4.0)	2982 (39.1)	972 (12.7)			1958 (25.7)	2299 (30.1)		
Divorced or widowed	72 (0.9)	86 (1.1)	35 (0.5)			23 (0.3)	137 (1.8)	33 (0.4)			101 (1.3)	92 (1.2)		
Having children														
Yes	812 (10.6)	1331 (17.5)	551(7.2)	7.47	**.024**	229 (3.0)	1851(24.3)	614 (8.1)	9.492	**.009**	1256 (16.5)	1438 (18.9)	1.641	.200
No	1637 (21.5)	2322 (30.4)	973 (12.8)			338 (4.4)	3529 (46.3)	1065 (14.0)			2375 (31.1)	2557 (33.5)		
Level of education														
Undergraduate	333 (4.4)	380 (5.0)	175 (2.3)	28.750	<.001	97 (1.3)	621 (8.1)	170 (2.2)	46.338	<.001	456 (6.0)	432 (5.7)	33.274	**.001**
Bachelor's degree	1627 (21.3)	2590 (34.0)	1106 (14.5)			338 (4.4)	3753 (49.2)	1232 (16.2)			2422 (31.8)	2901 (38.0)		
Master's and PhD degree	211 (2.8)	301 (3.9)	116 (1.5)			70 (0.9)	431 (5.7)	127 (1.7)			325 (4.3)	303 (4.0)		
General and special professional doctorate	278 (3.6)	382 (5.0)	127 (1.7)			62 (0.8)	575 (7.5)	150 (2.0)			428 (5.8)	359 (4.7)		
Occupation														
Clinician	391 (5.1)	509 (6.7)	198 (2.6)		**.015**	98 (1.3)	786 (10.3)	214 (2.8)		**.001**	587 (7.7)	511 (6.7)		
Nurse	1369 (18.0)	2163 (28.4)	877 (11.5)			286 (3.8)	3135 (41.1)	988 (13.0)			2044 (26.8)	2365 (31.0)		
Midwife	356 (4.7)	469 (6.2)	211 (2.8)			99 (1.3)	721 (9.5)	216 (2.8)			509 (6.7)	527 (6.9)		
Others	333 (4.4)	512 (6.7)	238 (3.1)	15.697		84 (1.1)	738 (9.7)	261 (3.4)	22.680		491 (6.4)	592 (7.8)	21.037	<.001
Working in isolated wards														
Yes	1026 (13.5)	1689 (22.1)	705 (9.2)	12.709	**.002**	266 (3.5)	2376 (31.2)	778 (10.2)	3.502	.174	1575 (20.7)	1845 (24.2)	6.056	**.014**
No	1423 (33.8)	1964 (25.8)	819 (10.7)			301 (3.9)	3004 (39.4)	901 (11.8)			2056 (27.0)	2150 (28.2)		

(Continues)

TABLE 3 (Continued)

Variables	Physical symptoms of anxiety: Without to mild, *n* (%)	Moderate to severe, *n* (%)	Very severe, *n* (%)	X^2	*p*-value	Psychological symptoms of anxiety: Without to mild, *n* (%)	Moderate to severe	Very severe	X^2	*p*-value	Total score of CDAS: Without anxiety	With anxiety	X^2	*p*-value
Working units														
Hospitals	1256 (16.5)	2042 (26.8)	859 (11.3)	39.943	<.001	266 (3.5)	2942 (38.6)	949 (12.4)	28.932	.001	1914 (25.1)	2243 (29.4)	23.765	<.001
Outpatient clinics	252 (3.3)	361 (4.7)	138 (1.8)			58 (0.8)	543 (7.1)	150 (2.0)			364 (4.8)	387 (5.1)		
Laboratories	54 (0.7)	116 (1.5)	40 (0.5)			11 (0.1)	148 (1.9)	51 (0.7)			86 (1.1)	124 (1.6)		
Imaging centers	27 (0.4)	48 (0.6)	24 (0.3)			6 (0.1)	67 (0.9)	26 (0.3)			38 (0.5)	61 (0.8)		
Other	670 (11.2)	1086 (14.2)	463 (6.1)			226 (2.9)	1680 (22.1)	503 (6.0)			1229 (16.1)	1180 (15.5)		
Working hours with COVID-19 patients														
0	426 (5.6)	472 (6.2)	223 (2.9)		<.001	124 (1.6)	771 (10.1)	226 (3.0)		<.001	608 (8.0)	513 (6.7)		
1–2	459 (6.0)	598 (7.8)	211 (2.8)			113 (1.5)	926 (12.1)	229 (3.0)			673 (8.8)	595 (7.8)		
2–4	256 (3.4)	408 (5.4)	143 (1.9)			56 (0.7)	596 (7.8)	155 (2.0)			396 (5.2)	411 (5.4)		
4–6	349 (4.6)	549 (7.2)	188 (2.5)			81 (1.1)	777 (10.2)	228 (3.0)			543 (7.1)	543 (7.1)		
6–8	557 (7.3)	994 (13.0)	437 (5.7)			97 (1.3)	1397 (18.3)	494 (6.5)			832 (10.9)	1156 (15.2)		
>8	402 (5.3)	632 (8.3)	322 (4.2)	71.401		96 (1.3)	913 (12.0)	347 (4.6)	75.817		579 (7.6)	777 (10.2)	77.647	<.001
History of physical illnesses														
Yes	396 (5.2)	691 (9.1)	410 (5.4)	70.861	<.001	93 (1.2)	984 (12.9)	420 (5.5)	40.727	<.001	607 (8.0)	890 (11.7)	37.281	<.001
No	2053 (26.9)	2962 (38.8)	1114 (14.6)			474 (6.2)	4396 (57.6)	1259 (16.5)			3024 (39.7)	3105 (40.7)		
History of psychiatric disorders														
Yes	160 (2.1)	305 (4.0)	174 (2.3)	29.197	<.001	45 (0.6)	404 (5.3)	190 (2.5)	24.314	<.001	259 (3.4)	380 (5.0)	14.021	<.001
No	2289 (30.0)	3348 (43.9)	1350 (17.7)			522 (6.8)	4976 (65.3)	1489 (19.5)			3372 (44.2)	3615 (47.4)		

TABLE 4 Prevalence of depression, anxiety, and stress based on demographic variables

	Severity of depression							Severity of anxiety							Severity of stress						
Variables	Normal	Mild	Moderate	Severe	Very severe	X^2	*p*-value	Normal	Mild	Moderate	Severe	Very severe	X^2	*p*-value	Normal	Mild	Moderate	Severe	Very severe	X^2	*p*-value
Gender																					
Male	1252 (16.4)	252 (3.3)	274 (3.6)	118 (1.5)	148 (1.9)	43.333	<.001	1371 (18.0)	135 (1.8)	282 (3.7)	95 (1.2)	161 (2.1)	115.075	<.001	1519 (19.9)	168 (2.2)	148 (1.9)	131 (1.7)	78 (1.0)	404.225	<.001
Female	2956 (38.8)	803 (10.5)	897 (11.8)	378 (5.0)	548 (7.2)			2981 (39.1)	526 (6.9)	1045 (13.7)	405 (5.3)	625 (8.2)			3450 (45.2)	682 (8.9)	602 (7.9)	494 (6.5)	354 (4.6)		
Age groups																					
20–30	2254 (29.6)	579 (7.6)	698 (9.2)	324 (4.2)	432 (5.7)	84.709	<.001	2341 (30.7)	388 (5.1)	758 (9.9)	324 (4.2)	476 (6.2)	72.376	<.001	2660 (34.9)	500 (6.6)	483 (6.3)	382 (5.0)	262 (3.4)	86.510	<.001
31–40	1268 (16.6)	327 (4.3)	356 (4.7)	137 (1.8)	198 (2.6)			1309 (17.2)	196 (2.6)	407 (5.3)	133 (1.7)	241 (3.2)			1511 (19.8)	249 (3.3)	205 (2.7)	193 (2.5)	128 (1.7)		
41–50	532 (7.0)	119 (1.6)	93 (1.2)	25 (0.3)	60 (0.8)			545 (7.1)	55 (0.7)	128 (1.7)	39 (0.5)	62 (0.8)			616 (8.1)	80 (1.0)	51 (0.7)	46 (0.6)	36 (0.5)		
51–60	140 (1.8)	28 (0.4)	21 (0.3)	9 (0.1)	6 (0.1)			144 (1.9)	19 (0.2)	31 (0.4)	3 (0.0)	7 (0.1)			165 (2.2)	19 (0.2)	10 (0.1)	4 (0.1)	6 (0.1)		
>60	14 (0. 2)	2 (0.0)	3 (0.0)	1 (0.0)	0 (0.0)			13 (0.2)	3 (0.0)	3 (0.0)	1 (0.0)	0 (0.0)			17 (0.2)	2 (0.0)	1 (0.0)	0 (0.0)	0 (0.0)		
Marital status																					
Single	1540 (20.2)	443 (5.8)	566 (7.4)	275 (3.6)	352 (4.6)	140.264	<.001	1749 (22.9)	271 (3.6)	578 (7.6)	230 (3.0)	348 (4.6)	13.300	.102	1941(25.5)	358 (4.7)	362 (4.7)	312 (4.1)	203 (2.7)	53.994	<.001
Married	2571 (33.7)	589 (7.7)	566 (7.4)	207 (2.7)	324 (4.2)			2491 (32.7)	375 (4.9)	718 (9.4)	255 (3.3)	418 (5.5)			2909 (38.1)	469 (6.2)	366 (4.8)	296 (3.9)	217 (2.8)		
Divorced or widowed	97 (1.3)	23 (0.3)	39 (0.5)	14 (0.2)	20 (0.3)			112 (1.5)	15 (0.2)	31 (0.4)	15 (0.2)	20 (0.3)			119 (1.6)	23 (0.3)	22 (0.3)	17 (0.2)	12 (0.2)		
Having children																					
Yes	1651 (21.6)	379 (5.0)	344 (4.5)	119 (1.6)	201 (2.6)	86.997	<.001	1626 (21.3)	239 (3.1)	440 (5.8)	138 (1.8)	251 (3.3)	27.850	<.001	1899 (24.9)	289 (3.8)	199 (2.6)	174 (2.3)	133 (1.7)	63.436	<.001
No	2557 (33.5)	676 (8.9)	827 (10.8)	377 (4.9)	495 (6.5)			2726 (35.7)	422 (5.5)	887 (11.6)	362 (4.7)	535 (7.0)			3070 (40.3)	561 (7.4)	551 (7.2)	451 (5.9)	299 (3.9)		
Level of education																					
Undergraduate	515 (6.8)	123 (1.6)	113 (1.5)	47 (0.6)	90 (1.2)	27.546	.006	540 (7.1)	73 (1.0)	129 (1.7)	55 (0.7)	91 (1.2)	36.630	<.001	625 (8.2)	83 (1.1)	66 (0.9)	65 (0.9)	49 (0.6)	22.070	.037
BSC	2904 (38.1)	721 (9.5)	866 (11.4)	350 (4.6)	482 (6.3)			2939 (38.5)	489 (6.4)	966 (12.7)	360 (4.7)	569 (7.5)			3429 (45.0)	606 (7.9)	442 (7.1)	442 (5.8)	301 (3.9)		
MSC and PhD	368 (4.8)	101 (1.3)	74 (1.0)	33 (0.4)	52 (0.7)			363 (4.8)	49 (0.6)	117 (1.5)	37 (0.5)	62 (0.8)			422 (5.5)	68 (0.9)	58 (0.8)	40 (0.5)	40 (0.5)		
General and special professional doctorate	421 (5.5)	110 (1.4)	118 (1.5)	66 (0.9)	72 (0.9)			510 (6.7)	50 (0.7)	115 (1.5)	48 (0.6)	64 (0.8)			493 (6.5)	93 (1.2)	81 (1.1)	78 (1.0)	42 (0.6)		
Occupation																					
Clinician	580 (7.6)	139 (1.8)	173 (2.3)	97 (1.3)	109 (1.4)	24.132	.020	668 (8.8)	78 (1.0)	162 (2.1)	74 (1.0)	116 (1.5)	24.746	.016	697 (8.9)	123 (1.6)	119 (1.6)	110 (1.4)	67 (0.9)	23.663	.023
Nurse	2427 (31.8)	618 (8.1)	698 (9.2)	281 (3.7)	385 (5.0)			2443 (32.0)	411 (5.4)	799 (10.5)	304 (4.0)	452 (5.9)			2848 (37.3)	513 (6.7)	447 (5.9)	363 (4.8)	238 (3.1)		
Midwife	609 (8.0)	141 (1.8)	137 (1.8)	52 (0.7)	97 (1.3)			624 (8.2)	75 (1.0)	182 (2.4)	55 (0.7)	100 (1.3)			718 (9.4)	95 (1.2)	91 (1.2)	75 (1.0)	57 (0.7)		
Others	592 (7.8)	157 (2.1)	163 (2.1)	66 (0.9)	105 (1.4)			617 (8.1)	97 (1.3)	184 (2.4)	67 (0.9)	118 (1.5)			724 (9.5)	119 (1.6)	93 (1.2)	77 (1.0)	70 (0.9)		
Working in isolated wards																					
Yes	1853 (24.3)	464 (6.1)	556 (7.3)	232 (3.0)	315 (4.1)	5.518	.238	1863 (24.4)	292 (3.8)	639 (8.4)	252 (3.3)	374 (4.9)	21.914	<.001	2170 (28.5)	377 (4.9)	372 (4.9)	307 (4.0)	194 (2.5)	14.328	.006
No	2355 (30.9)	591(6.8)	615 (8.1)	264 (3.5)	381 (5.0)			2489 (32.6)	369 (4.8)	688 (9.0)	248 (3.3)	412 (5.4)			2799 (36.7)	473 (6.2)	378 (5.0)	318 (4.2)	238 (3.1)		

(Continues)

TABLE 4 (Continued)

Variables	Severity of depression							Severity of anxiety							Severity of stress						
	Normal	Mild	Moderate	Severe	Very severe	X^2	*p*-value	Normal	Mild	Moderate	Severe	Very severe	X^2	*p*-value	Normal	Mild	Moderate	Severe	Very severe	X^2	*p*-value
Working hours with COVID-19 patients																					
0	630 (8.3)	156 (2.0)	174 (2.3)	65 (0.9)	96 (1.3)	34.624	.022	677 (8.9)	92 (1.2)	176 (2.3)	62 (0.8)	114 (1.5)	36.362	<.001	760 (10.0)	111 (1.5)	99 (1.3)	84 (1.1)	67 (0.9)	33.642	.029
1–2	744 (9.8)	177 (2.3)	157 (2.1)	85 (1.1)	105 (1.4)			781 (10.2)	96 (1.3)	212 (2.8)	68 (0.9)	111 (1.5)			869 (11.4)	129 (1.7)	108 (1.4)	94 (1.2)	68 (0.9)		
2–4	427 (5.6)	123 (1.6)	135 (1.8)	55 (0.7)	67 (0.9)			496 (6.5)	65 (0.9)	128 (1.7)	48 (0.6)	7(0.9)			525 (6.9)	98 (1.3)	81 (1.1)	71 (0.9)	32 (0.4)		
4–6	615 (8.1)	154 (2.0)	164 (2.2)	62 (0.8)	91 (1.2)			638 (8.4)	92 (1.2)	196 (2.6)	62 (0.8)	98 (1.3)			721 (9.5)	114 (1.5)	114 (1.5)	82 (1.1)	55 (0.7)		
6–8	1059 (13.9)	288 (3.8)	325 (4.3)	128 (1.7)	188 (2.5)			1051 (13.8)	185 (2.4)	378 (5.0)	156 (2.0)	218 (2.9)			1252 (16.4)	250 (3.3)	203 (2.7)	165 (2.2)	118 (1.5)		
>8	733 (9.6)	157 (2.1)	216 (2.8)	101 (1.3)	149 (2.0)			709 (9.3)	131 (1.7)	237 (3.1)	104 (1.4)	175 (2.3)			842 (11.0)	148 (1.9)	145 (1.9)	129 (1.7)	92 (1.2)		
History of physical illness																					
Yes	730 (9.6)	218 (2.9)	281 (3.7)	97 (1.3)	171 (2.2)	39.520	<.001	711 (9.3)	134 (1.8)	294 (3.9)	119 (1.6)	239 (3.1)	98.819	<.001	903 (11.8)	177 (2.3)	142 (1.9)	156 (2.0)	119 (1.6)	36.101	<.001
No	3478 (45.6)	837 (11.0)	890 (11.7)	399 (5.2)	525 (6.9)			3641 (47.7)	527 (6.9)	1033 (13.5)	381 (5.0)	547 (7.2)			4066 (53.3)	673 (8.8)	608 (8.0)	469 (6.2)	313 (4.1)		
History of psychiatric disorders																					
Yes	187 (2.5)	97 (1.3)	154 (2.0)	71 (0.9)	130 (1.7)	239.452	<.001	225 (3.0)	55 (0.7)	143 (1.9)	83 (1.1)	133 (1.7)	187.033	<.001	259 (3.4)	94 (1.2)	96 (1.3)	103 (1.4)	87 (1.1)	223.198	<.001
No	4021 (52.7)	958 (12.6)	1017(13.3)	425 (5.6)	566 (7.4)			4127 (54.1)	606 (7.9)	1184 (15.5)	417 (5.5)	653 (6.8)			4710 (61.8)	756 (9.9)	654 (8.6)	522 (6.8)	345 (4.5)		

In this study, working less than 8 h/day was a predictor of depression and anxiety among HCWs and also working less than 8 h/day and working in hospitals compared with other working units were assumed as significant predictors of stress among HCWs during the COVID-19 pandemic. The results of a study investigating the prevalence and associated factor of anxiety among HCWs in China had found that, after adjusting for sociodemographic characteristics, such as gender, age, level of education, and marital status, the cases who had experienced direct contacts in treatment of infected patients in hospitals had suffered from higher anxiety than those who had not done so (Liu et al., 2020). Besides, the results of another survey in China had shown that working in an isolation ward or fever clinic could be an independent risk factor of depression, anxiety, and stress (Zheng et al., 2020). These studies had not compared clinical settings and had not even assessed hours of working with COVID-19 patients; however, in these two mentioned studies, working in hospitals and being engaged with COVID-19 patients had been associated with high levels of anxiety and stress in HCWs. In a survey, factors such as self-efficacy, resilience, along with social support had been introduced as factors of psychological symptoms among nurses (Deying et al., 2020). Although these variables were not assessed in the present study, supportive services were obviously associated with better mental health status among the HCWs.

A comprehensive literature search showed that this study had the highest number of sample sizes compared to other published Southwest Asian studies in this regard. Also, because HCWs of all provinces in Iran participated in this study, so the generalizability of the results is increased. Although this study was conducted on a large sample size, which boosted the generalizability of its results to Iranian HCWs, it was merely limited to a group of individuals having access to the internet and social media.

5 | CONCLUSION

The results of this study showed that the HCWs had partially experienced a high prevalence rate of psychological symptoms during the COVID-19 pandemic. As psychological well-being and mental health status among HCWs are important issues associated with quality of care given to COVID-19 patients, health administrators and policymakers of the MHME are suggested to provide psychological screening and supportive care programs for HCWs with the aim of enhancing their mental health status and successful coping with critical circumstances.

ACKNOWLEDGMENTS

This study was funded by Mazandaran University of Medical Sciences (No. 7574), Sari, Iran. We declare that the funder had no role in the study design, collection, analysis, or interpretation of the data, writing of the manuscript, or the decision to submit the manuscript for publication.

CONFLICTS OF INTEREST

All authors report no conflicts of interest.

TABLE 5 Logistic regression for predictors of DASS among participants

	Variables	Depression (adjusted R^2 = 6.3%)				Anxiety (adjusted R^2 = 6.6%)				Stress = (adjusted R^2 = 6.6%)			
		Univariate		Multivariate		Univariate		Multivariate		Univariate		Multivariate	
		OR (95% CI)	*p*-value	OR (95% CI)	*p*-value	OR (95% CI)	*p*-value	OR (95% CI)	*p*-value	OR (95% CI)	*p*-value	OR (95% CI)	*p*-value
Age	20–30	2.71 (1.70–4.31)	<.001	2.26 (1.38–3.71)	.001	4.46 (2.41–8.19)	<.001	4.25 (2.25–8.04)	<.001	3.40 (1.59–7.27)	.002	3.50 (1.59–7.72)	.002
	31–40	2.16 (1.35–3.47)	.001	1.99 (1.22–3.24)	.006	3.79 (2.04–7.02)	<.001	3.42 (1.82–6.43)	<.001	3.18 (1.48–6.85)	.003	3.13 (1.43–6.84)	.004
	41–50	1.43 (0.86–2.36)	.163	1.32 (0.79–2.21)	.288	2.68 (1.41–5.09)	.003	2.31 (1.20–4.44)	.011	2.28 (1.02–5.09)	.042	2.11 (0.94–4.75)	.070
	>50	Ref.	Ref.	Ref.	Ref.	Ref.	Ref.	Ref.	Ref.	Ref.	Ref.	Ref.	Ref.
Gender	Male	Ref.	Ref.	Ref.	Ref.	Ref.	Ref.	Ref.	Ref.	Ref.	Ref.	Ref.	Ref.
	Female	1.39 (1.21–1.59)	<.001	1.40 (1.20–1.63)	<.001	1.58 (1.36–1.83)	<.001	1.55 (1.32–1.82)	<.001	1.61 (1.32–1.95)	<.001	1.64 (1.33–2.02)	<.001
Marital status	Single	1.15 (0.80–1.64)	.431	1.13 (0.77–1.64)	.521	1.05 (0.68–1.46)	.979	0.99 (0.66–1.48)	.982	1.08 (0.66–1.76)	.745	1.19 (071–2.01)	.489
	Married	0.64 (0.45–0.91)	.014	0.72 (0.49–1.04)	.083	0.84 (0.58–1.23)	.391	0.99 (0.67–1.47)	.990	0.81 (0.49–1.31)	.398	0.97 (0.58–1.60)	.916
	Divorced or widowed	Ref.	Ref.	Ref.	Ref.	Ref.	Ref.	Ref	Ref	Ref	Ref	Ref	Ref
Having children	Yes	0.60 (0.52–0.68)	<.001	0.92 (0.76–1.10)	.385	0.75 (0.66–0.86)	<.001	0.90 (0.75–1.08)	.288	0.78 (0.66–0.92)	.004	1.01 (0.79–1.28)	.915
	No	Ref.	Ref.	Ref.	Ref.	Ref.	Ref.	Ref.	Ref.	Ref.	Ref.	Ref.	Ref.
Level of education	Undergraduate	0.83 (0.65–1.07)	.155	1.09 (0.76–1.57)	.620	1.18 (0.90–1.55)	.208	1.65 (1.12–2.44)	.011	0.94 (0.67–1.31)	.725	1.34 (0.84–2.16)	.216
	BSc	0.93 (0.77–1.12)	.460	1.10 (0.79–1.55)	.544	1.27 (1.03–1.57)	.025	1.49 (1.04–2.14)	.029	0.97 (0.75–1.25)	.835	1.22 (0.79–1.90)	.358
	MSc and PhD	0.76 (0.58–1.007)	.056	0.94 (0.64–1.38)	.755	1.13 (0.84–1.51)	.414	1.43 (0.95–2.14)	.084	1.02 (0.71–1.45)	.915	1.35 (0.82–2.21)	.229
	General and special professional doctorate	Ref.	Ref.	Ref.	Ref.	Ref.	Ref.	Ref.	Ref.	Ref.	Ref.	Ref.	Ref.
Occupation	Clinician	1.02 (0.82–1.27)	.844	1.38 (0.99–1.93)	.053	0.81 (0.64–1.02)	.080	1.31 (0.92–1.86)	.133	0.98 (0.73–1.31)	.923	1.33 (0.87–2.05)	.182
	Nurse	0.89 (0.76–1.03)	.139	0.90 (0.75–1.09)	.318	0.92 (0.79–1.08)	.343	0.87 (0.72–1.06)	.172	0.87 (0.71–1.07)	.210	0.95 (0.74–1.22)	.715
	Midwife	0.84 (0.68–1.03)	.107	1.05 (0.83–1.33)	.660	0.78 (0.63–0.98)	.033	0.92 (0.72–1.17)	.503	0.75 (0.56–0.99)	.049	0.78 (0.57–1.07)	.129
	Others	Ref.	Ref.	Ref.	Ref.	Ref.	Ref.	Ref.	Ref.	Ref.	Ref.	Ref.	Ref.
Working in isolated wards	Yes	1.07 (0.95–1.20)	.227	0.97 (0.85–1.11)	.743	1.20 (1.06–1.3)	.002	1.07 (0.94–1.23)	.281	1.08 (0.92–1.26)	.312	1.05 (0.88–1.25)	.584
	No	Ref.	Ref.	Ref.	Ref.	Ref.	Ref.	Ref.	Ref.	Ref.	Ref.	Ref.	Ref.
Working hours with COVID-19 patients	0	0.78 (0.64–0.95)	.018	0.81 (0.65–1.01)	.069	0.72 (0.58–0.88)	.002	0.78 (0.61–0.98)	.037	0.83 (0.64–1.08)	.186	0.84 (0.63–1.13)	.264
	1–4	0.77 (0.65–0.91)	.003	0.79 (0.66–0.95)	.013	0.64 (0.53–0.77)	<.001	0.69 (0.57–0.84)	<.001	0.68 (0.54–0.86)	.001	0.69 (0.54–0.89)	.004
	4–8	0.77 (0.66–0.90)	.002	0.81 (0.68–0.95)	.012	0.81 (0.69–0.95)	.012	0.85 (0.72–1.04)	.056	0.77 (0.62–0.94)	.014	0.79 (0.64–0.98)	.035
	>8	Ref.	Ref.	Ref.	Ref.	Ref.	Ref.	Ref.	Ref.	Ref.	Ref.	Ref.	Ref.
Working units	Hospitals	1.08 (0.95–1.23)	.239	1.05 (0.89–1.23)	.529	1.16 (1.01–1.33)	.025	0.99 (0.84–1.18)	.972	0.92 (0.77–1.09)	.349	0.77 (0.62–0.95)	.016
	Outpatient clinics	0.88 (0.71–1.10)	.279	0.81 (0.64–1.03)	.099	0.91 (0.72-1.15)	.454	0.83 (0.65–1.06)	.148	0.91 (0.68–1.21)	.538	0.81 (0.59–1.09)	.173
	Laboratories	1.23 (0.87–1.74)	.234	1.14 (0.78-1.67)	.487	0.95 (0.64–1.42)	.836	0.80 (0.52–1.23)	.326	1.14 (0.72–1.80)	.551	0.98 (0.59–1.61)	.941
	Imaging centers	0.93 (0.55–1.59)	.813	0.88 (0.50–1.4)	.673	1.26 (0.76–2.11)	.364	1.10 (0.64–1.90)	.710	0.49 (0.20–1.23)	.131	0.42 (0.16–1.08)	.072
	Other	Ref.	Ref.	Ref.	Ref.	Ref.	Ref.	Ref.	Ref.	Ref.	Ref.	Ref.	Ref.
History of physical illness	Yes	1.23 (1.07–1.41)	.003	1.23 (1.06–1.43)	.004	1.76 (1.53–2.02)	<.001	1.76 (1.52–2.03)	<.001	1.57 (1.31–1.87)	<.001	1.51 (1.25–1.81)	<.001
	No	Ref.	Ref.	Ref.	Ref.	Ref.	Ref.	Ref.	Ref.	Ref.	Ref.	Ref.	Ref.
History of psychological illness	Yes	2.95 (2.48–3.50)	<.001	2.90 (2.42–3.46)	<.001	2.82 (2.36–3.36)	<.001	2.72 (2.27–3.27)	<.001	3.03 (2.46–3.74)	<.001	2.80 (2.25–3.47)	<.001
	No	Ref.	Ref.	Ref.	Ref.	Ref.	Ref.	Ref.	Ref.	Ref.	Ref.	Ref.	Ref.

AUTHORS' CONTRIBUTION

FE designed the study and collected the data. MA and MK collected the data, wrote the paper, and contributed to the study design. MM analyzed the data. All authors made a substantial contribution to writing of the paper draft and met the four criteria for authorship recommended by the International Committee of Medical Journal Editors.

ETHICAL STATEMENT

All procedures performed in the study involving human participants were in accordance with the ethical standards of the institutional and/or national research committee and with the 1964 Helsinki declaration and its later amendments or comparable ethical standards. The Ethics Committee of MAZUMS approved the study (Ethical code: IR.MAZUMS.REC.1399.7574).

DATA AVAILABILITY STATEMENT

The datasets used and/or analyzed during the current study are available from the corresponding author on reasonable request.

PEER REVIEW

The peer review history for this article is available at https://publons.com/publon/10.1002/brb3.2304.

ORCID

Marzieh Azizi https://orcid.org/0000-0002-0835-3201
Forouzan Elyasi https://orcid.org/0000-0002-6872-481X

REFERENCES

Aiyer, A., Surani, S., Ratnani, I., & Surani, S. (2020). Mental health impact of COVID-19 on healthcare workers in the USA: A cross-sectional web-based survey. *Journal of Depression and Anxiety*, *9*(4), 1–8. https://doi.org/10.35248/2167-1044.20.9.373

Alipour, A., Ghadami, A., Alipour, Z., & Abdollahzadeh, H. (2020). Preliminary validation of the Corona Disease Anxiety Scale (CDAS) in the Iranian sample. *Quarterly Journal of Health Psychology*, *8*(4), 163–175. https://doi.org/10.30473/HPJ.2020.52023.4756

Antony, M. M., Bieling, P. J., Cox, B. J., Enns, M. W., & Swinson, R. P. (1998). Psychometric properties of the 42-item and 21-item versions of the Depression Anxiety Stress Scales in clinical groups and a community sample. *Psychological Assessment*, *10*(2), 176–181. https://doi.org/10.1037/1040-3590.10.2.176

Asghari, A., Saed, F., & Dibajnia, P. (2008). Psychometric properties of the Depression Anxiety Stress Scales-21 (DASS-21) in a non-clinical Iranian sample. *International Journal of Psychology*, *2*(2), 82–102.

Chen, J., Liu, X., Wang, D., Jin, Y., He, M., Ma, Y., …, & Xiang, X. (2020). Risk factors for depression and anxiety in healthcare workers deployed during the COVID-19 outbreak in China. *Social Psychiatry and Psychiatric Epidemiology*, *56*, 47–55. https://doi.org/10.1007/s00127-020-01954-1

Chew, N. W., Lee, G. K., Tan, B. Y., Jing, M., Goh, Y., Ngiam, N. J., & Shanmugam, G. N. (2020). A multinational, multicentre study on the psychological outcomes and associated physical symptoms amongst healthcare workers during COVID-19 outbreak. *Brain, Behavior, and Immunity*, *88*, 559–565. https://doi.org/10.1016/j.bbi.2020.04.049

Deying, H., Yue, K., Wengang, L., Qiuying, H., Xin Zhang, L. X. Z., Su Wei, W., & Hong-Gu, H. (2020). Frontline nurses' burnout, anxiety, depression, and fear statuses and their associated factors during the COVID-19 outbreak in Wuhan, China: A large-scale cross-sectional study. *EClinicalMedicine*, *24*, 100424. https://doi.org/10.1016/j.eclinm.2020.100424

Galbraith, N., Boyda, D., McFeeters, D., & Hassan, T. (2020). The mental health of doctors during the Covid-19 pandemic. *BJPsych Bulletin*, *45*(2), 93–97. https://doi.org/10.1192/bjb.2020.44

Gold, J. A. (2020). Covid-19: Adverse mental health outcomes for healthcare workers. *Bmj (Clinical Research Ed.)*, *369*, m1815. https://doi.org/10.1136/bmj.m1815

Hall, H. (2020). The effect of the COVID-19 pandemic on healthcare workers' mental health. *Journal of the American Academy of PAs*, *33*(7), 45–48. https://doi.org/10.1097/01.JAA.0000669772.78848.8c

Hassannia, L., Taghizadeh, F., Moosazadeh, M., Zarghami, M., Taghizadeh, H., Dooki, A. F., et al. (2021). Anxiety and Depression in Health Workers and General Population During COVID–19 in IRAN: A Cross–Sectional Study. *Neuropsychopharmacology Reports*, *41*(1), 40–49. https://doi.org/10.1002/npr2.12153

Henry, J. D., & Crawford, J. R. (2005). The short-form version of the Depression Anxiety Stress Scales (DASS-21): Construct validity and normative data in a large non-clinical sample. *British Journal of Clinical Psychology*, *44*(2), 227–239. https://doi.org/10.1348/014466505X29657

Huang, Y., & Zhao, N. (2020). Mental health burden for the public affected by the COVID-19 outbreak in China: Who will be the high-risk group? *Psychology, Health & Medicine*, *26*(1), 23–34. https://doi.org/10.1080/13548506.2020.1754438

Jafari, P., Nozari, F., Ahrari, F., & Bagheri, Z. (2017). Measurement invariance of the Depression Anxiety Stress Scales-21 across medical student genders. *International Journal of Medical Education*, *9*(8), 116–122. https://doi.org/10.3389/fpsyg.2018.02064

Kaveh, M., Davari-tanha, F., Varaei, S., Shirali, E., Shokouhi, N., Nazemi, P., & Ashraf, M. A. (2020). Anxiety levels among Iranian health care workers during the COVID-19 surge: A cross-sectional study. MedRxiv. https://doi.org/10.1101/2020.05.02.20089045

Khanal, P., Devkota, N., Dahal, M., Paudel, K., & Joshi, D. (2020). Mental health impacts among health workers during COVID-19 in a low resource setting: A cross-sectional survey from Nepal. *Globalization and Health*, *16*(1), 1–12. https://doi.org/10.1186/s12992-020-00621-z

Lai, J., Ma, S., Wang, Y., Cai, Z., Hu, J., Wei, N., & Li, R. (2020). Factors associated with mental health outcomes among health care workers exposed to coronavirus disease 2019. *JAMA Network Open*, *3*(3), e203976. https://doi.org/10.1001/jamanetworkopen.2020.3976

Le, M. T. H., Tran, T. D., Holton, S., Nguyen, H. T., Wolfe, R., & Fisher, J. (2017). Reliability, convergent validity and factor structure of the DASS-21 in a sample of Vietnamese adolescents. *PLoS One*, *12*(7), e0180557. https://doi.org/10.1371/journal.pone.0180557

Li, W., Yang, Y., Liu, Z.-H., Zhao, Y.-J., Zhang, Q., Zhang, L., & Xiang, Y.-T. (2020). Progression of mental health services during the COVID-19 outbreak in China. *International Journal of Biological Sciences*, *16*(10), 1732–1738. https://doi.org/10.7150/ijbs.45120

Liu, C.-Y., Yang, Y.-Z., Zhang, X.-M., Xu, X., Dou, Q.-L., Zhang, W.-W., & Cheng, A. S. (2020). The prevalence and influencing factors in anxiety in medical workers fighting COVID-19 in China: A cross-sectional survey. *Epidemiology & Infection*, *148*, e98. https://doi.org/10.1017/S0950268820001107

Liu, Q., Luo, D., Haase, J. E., Guo, Q., Wang, X. Q., Liu, S., & Yang, B. X. (2020). The experiences of health-care providers during the COVID-19 crisis in China: A qualitative study. *Lancet Global Health*, *8*(6), e790–e798. https://doi.org/10.1016/S2214-109X(20)30204-7

Lu, W., Wang, H., Lin, Y., & Li, L. (2020). Psychological status of medical workforce during the COVID-19 pandemic: A cross-sectional study. *Psychiatry Research*, *288*, 112936. https://doi.org/10.1016/j.psychres.2020.112936

Mohindra, R., Ravaki, R., Suri, V., Bhalla, A., & Singh, S. M. (2020). Issues relevant to mental health promotion in frontline health care providers managing quarantined/isolated COVID19 patients. *Asian Journal of Psychiatry*, *51*, 102084. https://doi.org/10.1016/j.ajp.2020.102084

Muller, R. A. E., Stensland, R. S. Ø., & van de Velde, R. S. (2020). The mental health impact of the covid-19 pandemic on healthcare workers,

and interventions to help them: A rapid systematic review. *Psychiatry Research*, *293*, 113441. https://doi.org/10.1016/j.psychres.2020

Musa, R., Fadzil, M. A., & Zain, Z. (2007). Translation, validation and psychometric properties of Bahasa Malaysia version of the Depression Anxiety and Stress Scales (DASS). *ASEAN Journal of Psychiatry*, *8*(2), 82–89.

Norton, P. J. (2007). Depression Anxiety and Stress Scales (DASS-21): Psychometric analysis across four racial groups. *Anxiety, Stress, and Coping*, *20*(3), 253–265. https://doi.org/10.1080/10615800701309279

World Health Organization. (2020). Coronavirus disease 2019 (COVID-19): situation report.

Que, J., Le Shi, J. D., Liu, J., Zhang, L., Wu, S., Gong, Y., & Sun, Y. (2020). Psychological impact of the COVID-19 pandemic on healthcare workers: A cross-sectional study in China. *General Psychiatry*, *33*(3), e100259. https://doi.org/10.1136/gpsych-2020-100259

Şahin, M. K., Aker, S., Şahin, G., & Karabekiroğlu, A. (2020). Prevalence of depression, anxiety, distress and insomnia and related factors in healthcare workers during COVID-19 pandemic in Turkey. *Journal of Community Health*, *45*(6), 1168–1177. https://doi.org/10.1007/s10900-020-00921-w

Shechter, A., Diaz, F., Moise, N., Anstey, D. E., Ye, S., Agarwal, S., & Chang, B. (2020). Psychological distress, coping behaviors, and preferences for support among New York healthcare workers during the COVID-19 pandemic. *General Hospital Psychiatry*, *66*, 1–8. https://doi.org/10.1016/j.genhosppsych.2020.06.007

Suryavanshi, N., Kadam, A., Dhumal, G., Nimkar, S., Mave, V., Gupta, A., & Gupte, N. (2020). Mental health and quality of life among healthcare professionals during the COVID-19 pandemic in India. *Brain and Behavior*, *10*, e01837. https://doi.org/10.1002/brb3.1837

Taghizadeh, F., Hassannia, L., Moosazadeh, M., Zarghami, M., Taghizadeh, H., Dooki, A. F., & Hedayatizadeh-Omran, A. (2020). Anxiety and depression in health workers and general population during COVID-19 epidemic in Iran: A web-based cross-sectional study. https://doi.org/10.1101/2020.05.05.20089292

Vindegaard, N., & Benros, M. E. (2020). COVID-19 pandemic and mental health consequences: Systematic review of the current evidence. *Brain, Behavior, and Immunity*, *89*, 531–542. https://doi.org/10.1016/j.bbi.2020.05.048

Walton, M., Murray, E., & Christian, M. D. (2020). Mental health care for medical staff and affiliated healthcare workers during the COVID-19 pandemic. *European Heart Journal: Acute Cardiovascular Care*, *9*(3), 241–247. https://doi.org/10.1177/2048872620922795

WHO Coronavirus Disease (COVID-19) Dashboard Data. 2020.

WHO Coronavirus Disease (COVID-19) Dashboard Data. 2020.

Wong, A. H., Pacella-LaBarbara, M. L., Ray, J. M., Ranney, M. L., & Chang, B. P. (2020). Healing the healer: Protecting emergency health care workers' mental health during COVID-19. *Annals of Emergency Medicine*, *76*(4), 379–384. https://doi.org/10.1016/j.annemergmed.2020.04.041

Zheng, R., Zhou, Y., Qiu, M., Yan, Y., Yue, J., Yu, L., & Hu, Y. (2020). Prevalence and associated factors of depression, anxiety, and stress among Hubei pediatric nurses during COVID-19 pandemic. *Comprehensive Psychiatry*, *104*, 152217. https://doi.org/10.1016/j.comppsych.2020.152217

How to cite this article: Azizi, M., Kamali, M., Moosazadeh, M., Aarabi, M., Ghasemian, R., Hasannezhad Reskati, M., & Elyasi, F. (2021). Assessing mental health status among Iranian healthcare workers in times of the COVID-19 pandemic: A web-based cross-sectional study. *Brain and Behavior*, *11*, e2304. https://doi.org/10.1002/brb3.2304
