# Supplementary material for: Mental health status and related factors influencing healthcare workers during the COVID-19 pandemic: A systematic review and meta-analysis
Source: PLoS One. 2024 Jan 19;19(1):e0289454. doi: 10.1371/journal.pone.0289454 (PMC10798549; doi:10.1371/journal.pone.0289454)
Supplement: S1 Data — (ZIP) [file pone.0289454.s011.zip › literatures/150.pdf]

JAMA Network | Open

Research Letter | Psychiatry

Mental Health Outcomes Among Frontline and Second-Line Health Care Workers During the Coronavirus Disease 2019 (COVID-19) Pandemic in Italy

Rodolfo Rossi, MD; Valentina Socci, PhD; Francesca Pacitti, MD; Giorgio Di Lorenzo, MD; Antinisca Di Marco, PhD; Alberto Siracusano, MD; Alessandro Rossi, MD

Author affiliations and article information are listed at the end of this article.

Introduction

Health care workers (HCWs) involved in the coronavirus disease 2019 (COVID-19) pandemic are exposed to high levels of stressful or traumatic events and express substantial negative mental health outcomes,[1] including stress-related symptoms and symptoms of depression, anxiety, and insomnia. In this cross-sectional study, we report on mental health outcomes among HCWs in Italy.

Methods

This cross-sectional, web-based study collected data between March 27 and March 31, 2020, using an online questionnaire spread via social networks using a snowball technique and sponsored social network advertisements. Approval for this study was obtained from the local institutional review board at University of L'Aquila. Online consent was obtained from the participants.

The sampling period corresponded to the days immediately preceding the COVID-19 contagion peak, associated with the highest level of health care system utilization. All HCWs reporting that they work in Italy were eligible. Because of the self-selected and nonprobabilistic nature of the sample, invitations and response rates could not be quantifiable, as reported by American Association for Public Opinion Research (AAPOR) reporting guideline. The questionnaire investigated demographic variables, workplace characteristics (ie, being a frontline or second-line worker), and information regarding the direct consequences of COVID-19, including having colleagues infected or deceased. Frontline and second-line HCWs were defined by a single yes or no question, "Are you currently working with COVID-19 patients?" Key mental health outcomes were posttraumatic stress symptoms (PTSS), symptoms of depression, anxiety, insomnia, and perceived stress, assessed using the Italian version of the Global Psychotrauma Screen (GPS),[2] the 9-item Patient Health Questionnaire (PHQ-9),[3] the 7-item Generalized Anxiety Disorder scale (GAD-7),[4] the 7-item Insomnia Severity Index (ISI),[5] and the 10-item Perceived Stress Scale (PSS).[6] Participants were classified as endorsing the previously listed symptoms according to the following cutoffs: at least 3 on the 5 item GPS–posttraumatic stress disorder subscale, at least 15 on the PHQ-9, at least 15 on the GAD-7, and at least 22 on the ISI. Cutoffs were extracted from the original articles describing each measure. Because no official cutoff for the PSS was available, a quartile split was used. Age was standardized to a mean (SD) of 0 (1) and then reversed (ie, multiplied by −1) to show a positive coefficient.

A multivariable logistic regression model was fitted to explore the association of the selected outcomes with sex, age, frontline working position, occupation, and self and colleagues' exposure to contagion. The association between outcomes and potential risk factors was assessed by seemingly unrelated regression models that allow joint modelling of correlated outcomes. Analyses were conducted in Stata version 16 (StataCorp). Statistical significance was set at $P < .05$, and all tests were 2-tailed.

Results

A total of 1379 HCWs completed the questionnaire; the response rate could not be calculated. Sample characteristics are reported in **Table 1**. A total of 681 respondents (49.38%) endorsed PTSS;

Open Access. This is an open access article distributed under the terms of the CC-BY License.

Downloaded From: https://jamanetwork.com/ on 12/21/2022

Table 1. Sample Characteristics

Characteristic	No./total No. (%) Overall (N = 1379)	Northern Italy (n = 667)[a]	Central Italy (n = 412)[b]	Southern Italy (n = 280)[c]
Sex				
Women	1064/1379 (77.2)	533/667 (79.9)	321/412 (77.9)	194/280 (69.3)
Men	315/1379 (22.8)	134/667 (20.1)	91/412 (22.1)	86/280 (30.7)
Age, mean (SD), y	39.0 (16.0)	38.0 (16.0)	41.0 (16.0)	38.0 (15.0)
Working position				
Frontline	725/1378 (52.57)	448/667 (67.17)	181/412 (43.93)	88/279 (31.43)
Second-line	653/1378 (47.35)	219/667 (32.83)	231/412 (56.07)	191/279 (68.21)
Occupation				
Nurse	472/1378 (34.23)	265/667 (39.7)	131/412 (31.8)	67/279 (23.93)
Physician	433/1378 (31.40)	163/667 (24.44)	164/412 (39.81)	100/279 (35.71)
General practitioner	86/1378 (6.24)	35/667 (5.25)	22/412 (5.34)	28/279 (10.00)
Other[d]	275/1378 (19.94)	137/667 (20.54)	77/412 (18.69)	58/279 (20.71)
HCA	112/1378 (8.12)	67/667 (10.04)	18/412 (4.37)	26/279 (9.29)
Education level				
Undergraduate	222/1373 (16.10)	131/667 (19.64)	49/410 (11.95)	36/276 (13.04)
Postgraduate	1151/1373 (83.47)	536/667 (80.36)	361/410 (88.05)	240/276 (86.96)
GPS-PTSD score ≥3	681/1376 (49.38)	352 (52.77)	193 (46.84)	127/280 (45.85)
PHQ-9 score ≥15	341/1378 (24.73)	182/666 (27.29)	99/412 (24.03)	55/280 (19.64)
GAD-7 score ≥15	273/1378 (19.80)	130/666 (19.49)	84/412 (20.39)	55/280 (19.64)
ISI score ≥22	114/1378 (8.27)	66/667 (9.90)	28/412 (6.80)	17/280 (6.07)
PSS score, first quartile	302/1378 (21.90)	160/667 (23.99)	86/412 (20.87)	52/280 (18.57)
Total score, median (IQR)				
GPS	9 (6-12)	9 (6-12)	9 (6-12)	8 (5-12)
PHQ-9	10 (5-14)	10 (6-15)	10 (5-14)	8 (5-13)
GAD-7	9 (4-13)	9 (5-13)	9 (4-14)	8 (3.5-13)
ISI	10 (4-16)	11 (5-17)	10 (4.5-15)	8 (3-16)
PSS	24 (18-29)	24 (19-29)	24 (19-28)	22 (16-28)

Abbreviations: GAD-7, 7-item Generalized Anxiety Disorder Scale; GPS-PTSD, Global Psychotrauma Scale–posttraumatic stress disorder subscale; HCA, health care assistant; IQR, interquartile range; ISI, Insomnia Severity Index; PHQ-9, 9-item Patient Health Questionnaire; PSS, Perceived Stress Scale.

[a] Northern Italy includes Aosta Valley, Piedmont, Liguria, Lombardy, Trentino-Alto Adige, Veneto, Friuli-Venezia Giulia, and Emilia Romagna.

[b] Central Italy includes Tuscany, Marche, Umbria, and Lazio.

[c] Southern Italy includes Abruzzo, Molise, Campania, Apulia, Basilicata, Calabria, Sicily, and Sardinia.

[d] Other includes professionals such as laboratory technicians, radiology technicians, and physiotherapists, among others.

Table 2. Seemingly Unrelated Logistic Regression Analysis[a]

Variable	GPS-PTSD OR (95% CI)	GPS-PTSD *P* value	PHQ-9 OR (95% CI)	PHQ-9 *P* value	GAD-7 OR (95% CI)	GAD-7 *P* value	ISI OR (95% CI)	ISI *P* value	PSS OR (95% CI)	PSS *P* value
Standardized age	0.69 (0.53-0.88)	.003	0.74 (0.56-0.98)	.04	0.60 (0.44-0.82)	.001	0.71 (0.46-1.09)	.12	0.63 (0.46-0.85)	.002
Sex										
Men	1 [Reference]	NA	1 [Reference]	NA	1 [Reference]	NA	1 [Reference]	NA	1 [Reference]	NA
Women	2.31 (1.76-3.05)	<.001	2.03 (1.44-2.87)	<.001	2.18 (1.49-3.19)	<.001	1.38 (0.82-2.33)	.23	2.64 (1.80-3.87)	<.001
Working position										
Second-line	1 [Reference]	NA	1 [Reference]	NA	1 [Reference]	NA	1 [Reference]	NA	1 [Reference]	NA
Frontline	1.37 (1.05-1.80)	.03	1.04 (0.76-1.42)	.92	1.13 (0.80-1.59)	.69	1.27 (0.78-2.07)	.41	1.16 (0.84-1.60)	.49
Occupation										
Other[b]	1 [Reference]	NA	1 [Reference]	NA	1 [Reference]	NA	1 [Reference]	NA	1 [Reference]	NA
Nurse	1.12 (0.81-1.55)	.48	1.36 (0.95-1.96)	.16	1.09 (0.74-1.61)	.82	2.03 (1.14-3.59)	.02	0.74 (0.51-1.08)	.09
Physician	1.20 (0.86-1.67)	.28	0.71 (0.48-1.05)	.06	0.96 (0.64-1.44)	.77	0.89 (0.46-1.72)	.70	0.75 (0.51-1.11)	.13
GP	1.75 (1.03-2.97)	.04	0.98 (0.53-1.82)	>.99	1.05 (0.53-2.08)	.85	1.47 (0.56-3.87)	.42	1.18 (0.66-2.11)	.57
HCA	0.95 (0.60-1.52)	.84	1.18 (0.70-1.98)	.57	1.05 (0.60-1.84)	.91	2.34 (1.06-5.18)	.04	0.59 (0.33-1.05)	.07

(continued)

Downloaded From: https://jamanetwork.com/ on 12/21/2022

Table 2. Seemingly Unrelated Logistic Regression Analysis[a] (continued)

	GPS-PTSD		PHQ-9		GAD-7		ISI		PSS	
Variable	OR (95% CI)	P value	OR (95% CI)	P value	OR (95% CI)	P value	OR (95% CI)	P value	OR (95% CI)	P value
Colleagues affected										
None	1 [Reference]	NA	1 [Reference]	NA	1 [Reference]	NA	1 [Reference]	NA	1 [Reference]	NA
Deceased	2.60 (1.30-5.19)	.007	2.07 (1.05-4.07)	.04	0.97 (0.41-2.29)	.91	2.94 (1.21-7.18)	.02	1.84 (0.88-3.87)	.11
Infected and hospitalized	1.54 (1.10-2.16)	.01	1.39 (0.95-2.03)	.12	1.18 (0.78-1.77)	.49	1.14 (0.66-1.96)	.68	1.93 (1.30-2.85)	.001
Infected and in quarantine	1.59 (1.21-2.09)	.001	1.38 (1.00-1.90)	.047	1.19 (0.85-1.67)	.29	0.88 (0.54-1.45)	.63	1.66 (1.19-2.32)	.002
Exposure										
Not exposed to contagion	1 [Reference]	NA	1 [Reference]	NA	1 [Reference]	NA	1 [Reference]	NA	1 [Reference]	NA
Exposed to contagion	1.23 (0.93-1.62)	.14	1.54 (1.11-2.14)	.01	1.14 (0.81-1.62)	.44	1.45 (0.88-2.39)	.14	1.01 (0.73-1.41)	.93

Abbreviations: HCA, health care assistant; GAD-7, 7-item Generalized Anxiety Disorder Scale; GP, general practitioner; GPS-PTSD, Global Psychotrauma Scale–posttraumatic stress disorder subscale; HCA, health care assistant; ISI, Insomnia Severity Index; PHQ-9, 9-item Patient Health Questionnaire; PSS, Perceived Stress Scale; OR, odds ratio.

[a] In this model, independent variables and covariates are entered all at the same time, so every variable is controlled for all others.

[b] Other includes professionals such as laboratory technicians, radiology technicians, and physiotherapists, among others.

341 (24.73%), symptoms of depression; 273 (19.80%), symptoms of anxiety; 114 (8.27%), insomnia; and 302 (21.90%), high perceived stress (Table 1).

A total of 18 participants (1.31%) were excluded from regression analysis because of missing data. Younger age and female sex were associated with all investigated outcomes except insomnia (eg, anxiety for standardized age: odds ratio [OR], 0.60; 95% CI, 0.44-0.82; $P = .001$; perceived stress for standardized age: OR, 0.63; 95% CI, 0.46-0.85; $P = .002$; PTSS among women: OR, 2.31; 95% CI, 1.76-3.05; $P < .001$; depression among women: OR, 2.03; 95% CI, 1.44-2.87; $P < .001$). Being a frontline HCW was associated with PTSS (OR, 1.37; 95% CI, 1.05-1.80; $P = .03$). General practitioners were more likely to endorse PTSS than other HCWs (OR, 1.75; 95% CI, 1.03-2.08; $P = .04$), while nurses and health care assistants were more likely to endorse severe insomnia (nurses: OR, 2.03; 95% CI, 1.14-3.59; $P = .02$; health care assistants: OR, 2.34; 95% CI, 1.06-5.18; $P = .04$). Having a colleague deceased was associated with PTSS (OR, 2.60; 95% CI, 1.30-5.19; $P = .007$) and symptoms of depression (OR, 2.07; 95% CI, 1.05-4.07; $P = .04$) and insomnia (OR, 2.94; 95% CI, 1.21-7.18; $P = .02$); having a colleague hospitalized was associated with PTSS (OR, 1.54; 95% CI, 1.10-2.16; $P = .01$) and higher perceived stress (OR, 1.93; 95% CI, 1.30-2.85; $P = .001$); and having a colleague in quarantine was associated with PTSS (OR, 1.59; 95% CI, 1.21-2.09; $P = .001$), symptoms of depression (OR, 1.38; 95% CI, 1.00-1.90; $P = .047$), and higher perceived stress (OR, 1.66; 95% CI, 1.19-2.32; $P = .002$). Being exposed to contagion was associated with symptoms of depression (OR, 1.54; 95% CI, 1.11-2.14; $P = .01$) (**Table 2**).

Discussion

To our knowledge, this is the first report on mental health outcomes and associated risk factors among HCWs in Italy during the COVID-19 pandemic. These results are in line with previous reports from China,[1] confirming a substantial proportion of mental health issues, particularly among young women and frontline HCWs. The main limitation is the impossibility of determining the sampling error or making inferences about populations because of the sampling technique. Our results warrant further monitoring and specific interventions for HCWs throughout the COVID-19 pandemic to prevent long-term mental health–related disabilities.

Downloaded From: https://jamanetwork.com/ on 12/21/2022

ARTICLE INFORMATION

Accepted for Publication: May 1, 2020.

Published: May 28, 2020. doi:10.1001/jamanetworkopen.2020.10185

Open Access: This is an open access article distributed under the terms of the CC-BY License. © 2020 Rossi R et al. *JAMA Network Open*.

Corresponding Author: Rodolfo Rossi, MD, Department of Systems Medicine, University of Rome Tor Vergata, Via Montpellier, 1, 00133 Rome, Italy (rudy86.rossi@gmail.com).

Author Affiliations: Department of Systems Medicine, University of Rome Tor Vergata, Rome, Italy (R. Rossi, Di Lorenzo, Siracusano); Department of Applied Clinical Sciences and Biotechnologies, University of L'Aquila, L'Aquila, Italy (Socci, Pacitti, A. Rossi); Psychiatry and Clinical Psychology Unit, Fondazione Policlinico Tor Vergata, Rome, Italy (Di Lorenzo, Siracusano); IRCCS Fondazione Santa Lucia, Rome, Italy (Di Lorenzo); Department of Information Engineering, Computer Science, and Mathematics, University of L'Aquila, L'Aquila, Italy (Di Marco).

Author Contributions: Drs R. Rossi and Pacitti had full access to all the data in the study and take responsibility for the integrity of the data and the accuracy of the data analysis.

Concept and design: R. Rossi, Socci, Pacitti, Di Lorenzo, Siracusano.

Acquisition, analysis, or interpretation of data: R. Rossi, Socci, Pacitti, Di Lorenzo, Di Marco, A. Rossi.

Drafting of the manuscript: R. Rossi, Socci, Pacitti, Di Lorenzo, Di Marco.

Critical revision of the manuscript for important intellectual content: R. Rossi, Socci, Pacitti, Di Lorenzo, Siracusano, A. Rossi.

Statistical analysis: R. Rossi, Pacitti, Di Lorenzo.

Obtained funding: R. Rossi, Pacitti, Di Lorenzo, Di Marco.

Administrative, technical, or material support: Pacitti, Di Lorenzo.

Supervision: R. Rossi, Socci, Pacitti, Di Lorenzo, Siracusano.

Conflict of Interest Disclosures: None reported.

Funding/Support: This study was supported by Territori Aperti, a project funded by the Fondo Territori Lavoro e Conoscenza of the Confederazione Generale Italiana del Lavoro, the Confederazione Italiana Sindacati Lavoratori, and the Unione Italiana del Lavoro.

Role of the Funder/Sponsor: The funder had no role in the design and conduct of the study; collection, management, analysis, and interpretation of the data; preparation, review, or approval of the manuscript; and decision to submit the manuscript for publication.

REFERENCES

1. Lai J, Ma S, Wang Y, et al. Factors associated with mental health outcomes among health care workers exposed to coronavirus disease 2019. *JAMA Netw Open*. 2020;3(3):e203976. doi:10.1001/jamanetworkopen.2020.3976

2. Olff M, Bakker A, Frewen P, et al. Screening for consequences of trauma: an update on the global collaboration on traumatic stress. *Eur J Psychotraumatol*. 2020;11(1):1752504. doi:10.1080/20008198.2020.1752504

3. Spitzer RL, Kroenke K, Williams JB; Primary Care Evaluation of Mental Disorders. Validation and utility of a self-report version of PRIME-MD: the PHQ primary care study: Primary Care Evaluation of Mental Disorders, Patient Health Questionnaire. *JAMA*. 1999;282(18):1737-1744. doi:10.1001/jama.282.18.1737

4. Spitzer RL, Kroenke K, Williams JBW, Löwe B. A brief measure for assessing generalized anxiety disorder: the GAD-7. *Arch Intern Med*. 2006;166(10):1092-1097. doi:10.1001/archinte.166.10.1092

5. Morin CM, Belleville G, Bélanger L, Ivers H. The Insomnia Severity Index: psychometric indicators to detect insomnia cases and evaluate treatment response. *Sleep*. 2011;34(5):601-608. doi:10.1093/sleep/34.5.601

6. Cohen S, Hoberman HM. Positive events and social supports as buffers of life change stress. *J Appl Soc Psychol*. 1983;13(2):99-125. doi:10.1111/j.1559-1816.1983.tb02325.x

Downloaded From: https://jamanetwork.com/ on 12/21/2022
