# Supplementary material for: Mental health status and related factors influencing healthcare workers during the COVID-19 pandemic: A systematic review and meta-analysis
Source: PLoS One. 2024 Jan 19;19(1):e0289454. doi: 10.1371/journal.pone.0289454 (PMC10798549; doi:10.1371/journal.pone.0289454)
Supplement: S1 Data — (ZIP) [file pone.0289454.s011.zip › literatures/151.pdf]

BMC Emergency Medicine

RESEARCH

Open Access

Investigation of stress, anxiety, and depression levels of Pre-Hospital Emergency Medicine personnel in eastern Iran during the Covid-19 pandemic

Mohammadreza Sabbaghi[1,2], Kheizaran Miri[3,2], Reza Kahi[3,2] and Mohammad Namazi Nia[3,2*]

Abstract

Background: Pre-hospital emergency medicine (PHEM) personnel are at risk of developing psychological disorders during the Covid-19 pandemic. This study aimed to investigate depression, anxiety, and stress levels of the Iranian PHEM personnel during the Covid-19 pandemic.

Methods: This descriptive cross-sectional study was performed on 544 PHEM personnel chosen by purposive sampling in North Khorasan, Khorasan-Razavi, South Khorasan, Sistan-Baluchestan, and Kerman provinces in eastern Iran from August to September 2021. Data collection tools included a demographic information questionnaire and the standardized 21-item Depression, Anxiety, and Stress Scale (DASS-21). Data were analyzed in SPSS 16 using one-way analysis of variance and linear regression.

Results: The mean scores of depression, anxiety, and stress were 8.7 ± 9.2, 7.0 ± 7.8, and 11.6 ± 9.2, respectively. Depression, stress, and anxiety were more prevalent in the age group of 41-55 years, people with master's and higher degrees, people with a history of underlying diseases, and people with over 10 years of work experience($p < 0.05$). Depression and stress also showed a significant relationship with the type of employment. Stress alone was also significantly associated with working less than 35 hours a week and living separately from family($p < 0.05$).

Conclusions: PHEM personnel suffer from significant levels of depression, anxiety, and stress during the Covid-19 pandemic. Therefore, in order to improve the mental condition, it is recommended that the work schedule and services provided to these people be designed in such a way that they have more time for rest and communication with their family members. The personnel should also have easier access to the expert team in the fields of counseling and psychiatry.

Keywords: Stress, Anxiety, Depression, Pre-Hospital Emergency Medicine

Background

The Covid-19 pandemic started in Wuhan, China on December 31, 2019 [1]. On January 30, 2020, the World Health Organization declared the emerging global epidemic a Public Health Emergency of International Concern (PHEIC). The first positive case (SARS-CoV-2) was reported in Qom, Iran on February 18, 2020. Given the high contagiousness of the virus, other cases were soon

*Correspondence: mnamazi99@gmail.com

[2] Health Sciences Research Center, Torbat Heydariyeh University of Medical Sciences, Torbat Heydariyeh, Iran
Full list of author information is available at the end of the article

© The Author(s) 2022. **Open Access** This article is licensed under a Creative Commons Attribution 4.0 International License, which permits use, sharing, adaptation, distribution and reproduction in any medium or format, as long as you give appropriate credit to the original author(s) and the source, provide a link to the Creative Commons licence, and indicate if changes were made. The images or other third party material in this article are included in the article's Creative Commons licence, unless indicated otherwise in a credit line to the material. If material is not included in the article's Creative Commons licence and your intended use is not permitted by statutory regulation or exceeds the permitted use, you will need to obtain permission directly from the copyright holder. To view a copy of this licence, visit http://creativecommons.org/licenses/by/4.0/. The Creative Commons Public Domain Dedication waiver (http://creativecommons.org/publicdomain/zero/1.0/) applies to the data made available in this article, unless otherwise stated in a credit line to the data.

detected based on definitive diagnostic criteria in other parts of Iran [2, 3]. According to epidemiological reports, the countries with the highest number of cases are the United States, India, and Brazil. As of April 16, 2022, this virus had infected over 503,889,335 people and killed 6,220,830 people worldwide. In Iran, the number of cases was 7,205,064 and the number of deaths was 140,800 [4].

SARS-CoV-2 is a group of viruses originating in bats that cause varying degrees of disease in animals and humans. It is transmitted through inhalation or contact with contaminated droplets and has an incubation period of 2 to 14 days [5]. In mild cases, it causes symptoms such as fever, dry cough, sore throat, shortness of breath, and fatigue; however, in more severe cases, it causes organ failure, septic shock, pulmonary edema, pneumonia, etc. [6, 7].

Since its onset, the Covid-19 pandemic has caused a wave of stress, anxiety, and depression across the world [8]. Despite their ongoing efforts, researchers have yet to find an antiviral drug for Covid-19 that has been clinically proven to be 100% effective in humans [5, 6]. On the other hand with the emergence of new mutations, ethere are some debates against the protective effects of the vaccines [9]. As this disease is difficult to prevent and treat, it has caused a lot of stress and anxiety in many communities [7, 10]. Continuous mutation of the virus into new strains, shocking news about infections and deaths, and the difficulty in controlling and fighting Covid-19 can all contribute to psychological disorders and fear and anxiety among those who have been exposed, especially healthcare workers [11, 12].

Pre-Hospital Emergency Medicine (PHEM) personnel are an important part of the health care system. These personnel, at both administrative and operational levels, provide services to people of different nationalities, ethnicities and cultures in urban, road, air, rail and sea emergency bases, both in normal conditions and in the event of accidents and disasters. Iran, with a population of about 80 million people, is located in East Asia. At present, nursing accounts for 10 to 12% of the different categories of pre-hospital emergency services [13]. Evidence suggests that these healthcare professionals are particularly vulnerable to stress, anxiety, and depression during the pandemic because they have to wear heavy protective clothing and N-95 masks and endure the mental pressure ofwhile dealing with patients and their families daily [10, 11]. The risk of being infected and transmitting the disease to their own families, longer working hours, poor sleep quality, high number of missions, fatigue, and the poorly known nature of the virus all have physical and mental health implications for PHEM personnel [12, 14].

Vujanovic et al. (2021) conducted a study on 189 employees, including firefighters, pre-hospital emergency personnel, etc., and showed that pre-hospital emergency personnel had a higher level of depression and anxiety than other groups because they confronted with public more [11]. Another study was conducted on the mental health of 822 family members of the staff of five hospitals in China, and showed that 37.73% of them had anxiety while 29.35% f them had signs of depression, which could be an important consequence of psychological disorders [15].

Although, the role of PHEM personnel is to link between pre-hospital care and hospital care, their physical and mental health has not received much attention [16]. In addition, no research has been conducted on the mental health of pre-hospital emergency personnel during the Covid-19 pandemic in Iran. This descriptive study aimed to assess the mental health of PHEM personnel in order to lay the groundwork for more thoughtful management, care, and protection programs for this group of healthcare professionals.

Methods

Setting and study design

This descriptive cross-sectional study was performed on 544 Iranian PHEM personnel selected by purposive sampling from August to October 2021.

Populations, inclusion, and exclusion criteria

The inclusion criteria were PHEM professionals in any emergency station during the fifth peak of the Covid-19 pandemic in eastern Iran and consent to participate in the study.

Instruments of measurement

Data collection tools were a demographic information and the standardized 21-item Depression, Anxiety, and Stress Scale (DASS-21). demographic information inventory with 10 items, including age, gender, marital status, level of education, work experience (as a PHEM professional in years), type of employment, workplace, type of station, average working hours per week,and the quarantine status (at the end of each shift).

The DASS-21 developed by Lovibond and Lovibond in 1995, [17], consists of 21 questions on a 4-point Likert scale. Out of these 21 questions, 7 are related to stress (difficulty to wind down, over-reacting to situations, nervous energy, feeling agitated, difficulty to relax, feeling intolerant of obstacles, feeling quick-tempered), 7 are related to anxiety (dryness of mouth, breathing difficulty, trembling, worry of panicking, feeling close to panic, palpitations, feeling scared without reason), and 7 are related to depression (lack of positive feelings, difficulty to start work, feeling indifferent,

feeling despondent, inability to become enthusiastic, feeling worthless, seeing life as meaningless).

The final score of each subscale is obtained by summing the scores of the questions related to that subscale. Each question is scored from zero (does not apply to me at all) to three (applies to me very much or most of the time). Since this questionnaire is a shortened version of the original 42-item scale, the final score of each subscale was doubled in the present study. The severity of symptoms was then classified according to Table 1 [18].

The reliability of this tool has been established for the general population of Mashhad with a coefficient of 0.70 for the depression subscale, 0.66 for the anxiety subscale, and 0.76 for the stress subscale [19]. The validity and reliability of this tool have also been approved by Mehdipour and Najafi [18, 20]. In the present study, the validity of DASS-21 was verified by the qualitative content validity method. For this purpose, 10 faculty members of the Department of Nursing and Emergency Medicine of Torbat Heydariyeh University of Medical Sciences were asked to evaluate the tool, which was then corrected and modified according to their feedbacks. The reliability of DASS-21 was verified by 10 subjects, who showed its internal consistency with a Cronbach's alpha of 0.81.

Sampling

After obtaining a permission from the School of Nursing and Midwifery of Torbat-e Heydarieh University of Medical Sciences, The first author of the present study, considering the pre-hospital emergency employment during telephone calls and virtual meetings with the heads of each region, gave sufficient explanations about the research and provided them with the link of the mentioned questionnaire, and then the heads of each region from Provided PHEM to each region via internal messengers and SMS. The informed consent form was inserted at the first section of the online questionnaire, which was designed to be completed only with consent.

Table 1 Severity of Depressive, Anxiety and Stress Disorders based on DASS-42 rating

Severity	Depression	Anxiety	Stress
**Normal**	0-9	0-7	0-14
**Little**	10-13	8-9	15-18
**Mediocre**	14-20	10-14	19-25
**Severe**	21-28	15-19	26-33
**Very severe**	> 28	> 20	> 33

Statistical analysis

The collected data were analyzed using the SPSS16. Descriptive statistics (frequency distribution, mean, and standard deviation) were used to describe and categorize the data, and one-way analysis of variance and linear regression were used to analyze the relationship between demographic variables and DASS-21 scores. The normality of quantitative variables was assessed by the Kolmogorov-Smirnov test. All tests were performed at the 95% confidence level and the significance level of 0.05.

Results

The mean age of PHEM personnel ($N=544$) was 34.1 ± 7.4 years, ranging from 20 to 55 years. Fifty-four percent of them ($N=294$) had an associate's degree, and 49.8% ($N=271$) had over 10 years of work experience. The mean work experience was 9.68 ± 6.30 years. In addition, 54.8% of the PHEM personnel ($N=298$) were working over 75 hours per week and 48.0% of them ($N=261$) were working in urban emergency stations (Table 2).

The mean depression, anxiety, and stress scores of the subjects were 8.7 ± 9.2, 7.0 ± 7.8, and 11.6 ± 9.2, respectively. The prevalence of depression was 36.2%, of which 13.2% ($N=72$) had moderate depression. The prevalence of anxiety was 36.7%, of which 14.7% ($N=80$) had moderate anxiety. Furthermore, the prevalence of stress was 31.0%, of which 11.4% ($N=62$) had moderate stress (Table 3).

When the depression scores of different demographic groups were compared, the age group of 41-55 years, people with master's and higher degrees, people with a history of underlying diseases, people with over 10 years of work experience, and hired staff all had showed significantly higher depression scores ($p < 0.05$).

A similar comparison for anxiety scores showed significantly higher anxiety scores for the age group of 41-55 years, people with master's and higher degrees, people with a history of underlying diseases, people with over 10 years of work experience, people working less than 35 hours per week on average, and people living separately from their family ($p < 0.05$).

Comparison of stress scores based on demographic variables also showed significantly higher stress scores for the age group of 41-55 years, people with master's and higher degrees, people with a history of underlying diseases, people with over 10 years of work experience, and hired staff ($p < 0.05$) (Table 2).

The linear regression analysis of the relationship between depression and demographic variables showed that depression was significantly more prevalent among women, people with a history of underlying diseases, and hired staff than other groups. AlsoIn addition, married

Table 2 Mean scores of depression, anxiety and stress based on the studied variables in pre-hospital emergency personnel

Variable		N (%)	Depression		Anxiety		Stress	
			Mean ± SD	*P*-value	Mean ± SD	*P*-value	Mean ± SD	*P*-value
Age	20- 30 years	167 (30.6)	8.3 ± 9.1	0.029	6.1 ± 7.5	0.002	10.5 ± 9.2	0.000
	31- 40 years	250 (45.9)	8.0 ± 8.5		6.4 ± 7.2		10.9 ± 8.7	
	41- 55 years	127 (23.3)	10.6 ± 10.6		9.1 ± 9.1		14.6 ± 9.8	
Gender	Man	512 (94.1)	8.3 ± 8.9	0.000	6.6 ± 7.5	0.000	11.2 ± 9.0	0.000
	Woman	32 (5.8)	14.8 ± 12.1		13.1 ± 11.0		17.8 ± 10.1	
Marital status	Single	88 (16.1)	10.3 ± 10.1	0.182	7.0 ± 8.0	0.936	12.1 ± 9.8	0.760
	Married	454 (83.4)	8.4 ± 9.0		7.0 ± 7.8		11.6 ± 9.1	
	Widowed	2 (0.3)	4.0 ± 0.0		5.0 ± 7.0		8.0 ± 8.4	
Level of Education	Technician	294 (54.0)	7.7 ± 8.4	0.012	6.0 ± 6.7	0.006	10.4 ± 8.6	0.002
	Expert	228 (41.9)	9.8 ± 9.6		7.9 ± 8.5		13.0 ± 9.6	
	Master and above	22 (4.0)	11.2 ± 13.1		9.8 ± 12.3		14.6 ± 11.2	
History of disease	Yes	129 (23.7)	11.7 ± 10.3	0.000	10.3 ± 8.7	0.000	15.4 ± 9.9	0.000
	No	415 (76.2)	7.8 ± 8.7		6.0 ± 7.3		10.5 ± 8.7	
Work experience	1- 5 years	138 (25.3)	8.4 ± 9.4	0.032	6.2 ± 7.8	0.002	10.7 ± 9.4	0.000
	6- 10 years	135 (24.8)	7.2 ± 8.0		5.4 ± 6.2		9.4 ± 8.0	
	Over 10 years	271 (49.8)	9.7 ± 9.6		8.1 ± 8.4		13.2 ± 9.5	
Employment Status	Commitment	79 (14.5)	8.1 ± 8.4	0.001	5.4 ± 6.9	0.000	9.2 ± 7.8	0.000
	Contract	91 (16.7)	7.8 ± 8.1		6.4 ± 6.9		11.5 ± 8.4	
	Peyman	132 (24.2)	8.9 ± 8.9		7.0 ± 7.6		12.0 ± 9.4	
	Official	140 (25.7)	11.3 ± 11.1		9.6 ± 9.6		14.4 ± 10.5	
	Remark	20 (3.6)	5.8 ± 5.7		4.0 ± 4.1		10.9 ± 6.9	
	Company	82 (15.0)	6.3 ± 8.0		5.4 ± 6.4		8.9 ± 8.0	
Average working hours per week	Less than 35 hours	21 (3.8)	11.7 ± 12.7	0.088	11.2 ± 11.9	0.024	13.5 ± 12.3	0.099
	Between 35 and 75 hours	225 (41.3)	9.4 ± 9.4		7.2 ± 7.7		12.5 ± 9.1	
	More than 75 hours	298 (54.7)	8.0 ± 8.9		6.5 ± 7.5		10.9 ± 9.0	
After the end of the work shift	I return home and keep in touch with family members	490 (90.0)	8.5 ± 9.0	0.110	6.8 ± 7.7	0.030	11.5 ± 9.2	0.565
	I'm going home and I'm in the quarantine room	40 (7.3)	10.2 ± 10.7		7.8 ± 8.4		12.9 ± 9.7	
	I live in a new house away from my family	14 (2.5)	13.1 ± 11.8		12.2 ± 10.0		13.1 ± 9.1	
Rate of study to increase disease prognosis	Less than an hour a day	287 (52.7)	9.2 ± 9.5	0.523	7.0 ± 7.9	0.710	11.9 ± 9.6	0.470
	Between one and 2 hours a day	177 (32.5)	8.0 ± 8.6		6.7 ± 7.4		11.4 ± 8.5	
	Between two and 3 hours a day	44 (8.0)	7.9 ± 9.1		7.0 ± 7.9		9.9 ± 7.3	
	More than 3 hours a day	36 (6.6)	8.8 ± 10.4		8.4 ± 9.2		12.8 ± 11.9	
Place of service	Inside the city	261 (47.9)	9.1 ± 9.3	0.331	7.8 ± 8.2	0.067	12.3 ± 9.3	0.124
	Between cities	224 (41.1)	7.9 ± 8.7		5.9 ± 6.4		10.6 ± 8.9	
	Communication Center	30 (5.5)	9.6 ± 11.2		6.8 ± 10.4		12.1 ± 11.1	
	Headquarters	25 (4.5)	11.2 ± 10.5		8.8 ± 10.6		13.8 ± 9.1	
	Mcmc	4 (0.7)	6.5 ± 7.8		5.0 ± 10.0		5.5 ± 8.5	

ANOVA Test

people had lower depression scores than single people (Table 4).

The linear regression analysis of the relationship between anxiety and demographic variables showed the significantly higher prevalence of anxiety among women, people with longer working hours, people with a history of underlying diseases, and hired staff. Among different groups of PHEM professionals, those working in road emergency stations had lower anxiety scores than others (Table 5).

The linear regression analysis of the relationship between stress and demographic variables showed that

Table 3 Frequency distribution of depression, anxiety and stress in prehospital emergency personnel

Type of degree disorder	Depression N (%)	Anxiety N (%)	Stress N (%)
**Normal**	347 (63.8)	344 (63.2)	375 (68.9)
**Little**	67 (12.3)	45 (8.3)	62 (11.4)
**Medium**	72 (13.2)	80 (14.7)	54 (9.9)
**Severe**	26 (4.8)	28 (5.1)	38 (7.0)
**Very severe**	32 (5.9)	47 (8.6)	15 (2.8)

stress was more prevalent among people with a history of underlying diseases. Among different groups of PHEM professionals, those working in MCMC had lower stress scores than others (Table 6).

Discussion

This study aimed to assess the mental health of Iranian PHEM personnel. This assessment showed that the subjects were mostly suffering from moderate levels of stress, anxiety, and depression. Overall, the prevalence of depression, anxiety, and stress was estimated to be 36.2, 36.7, and 31.1%, respectively. These are similar to the rates of reported for the general Iranian population [21], but lower than the rate reported by Sarbozi et al. (2020). for the healthcare teams working under pandemic conditions [22]. Given that the present study coincided with the fifth peak of the Covid-19 pandemic in Iran, most of the participants and their families were already vaccinated, and they had fairly better access to protective equipment (e.g. masks, personal protective clothing, and disinfectants). This lower rate of depression, anxiety, and

Table 4 Multiple linear regression results of the effect of the studied variables on depression

Variable	Unstandardized B	Coefficients Std. Error	Standardized Coefficients Beta	t	Df	confidence interval		p
						Lower Bound	Upper Bound	
Gender	4.161	1.735	0.106	2.398	9	0.752	7.569	0.017
Marital status	−2.504	1.136	−0.100	− 2.204	9	−4.735	− 0.272	0.028
Level of Education	0.801	0.843	0.043	0.950	9	−0.856	2.457	0.343
History of disease	3.312	0.985	0.152	3.361	9	1.376	5.247	0.001
Work experience	−0.008	0.082	− 0.005	− 0.093	9	−1.169	0.153	0.926
Interurban emergency service	−1.369	0.831	−0.073	−1.648	9	−3.000	0.263	0.100
Official employment situation	2.410	1.038	0.114	2.321	9	0.370	4.449	0.021
Employment status of the company	−0.989	1.176	−0.038	− 0.841	9	−3.299	1.321	0.401
After the end of each shift	1.951	1.252	0.066	1.559	9	−0.508	4.410	0.120
Base	5.218	3.553	–	1.516	9	−1.545	11.981	0.130

Table 5 Multiple linear regression results of the effect of the studied variables on anxiety

Variable	Unstandardized B	Coefficients Std. Error	Standardized Coefficients Beta	t	Df	confidence interval		p
						Lower Bound	Upper Bound	
Gender	4.474	1.491	0.133	3.001	12	1.545	7.403	0.003
Age	−0.035	0.086	− 0.032	− 0.402	12	− 0.204	0.134	0.688
Level of Education	0.0304	0.720	0.019	0.422	12	−1.111	1.718	0.673
History of disease	3.406	9.829	0.184	4.108	12	1.777	5.035	0.000
Work experience	0.061	0.107	0.049	0.573	12	−0.149	0.272	0.567
Interurban emergency service	−1.820	0.701	−0.115	−2.297	12	−3.197	−0.443	0.010
Employment status of commitments	−0.511	1.127	−0.023	−0.454	12	− 2.725	1.702	0.650
Official employment situation	1.845	0.886	0.102	2.082	12	0.104	3.586	0.038
Employment status of Remark	−2.690	1.753	−0.064	−1.534	12	−6.135	0.754	0.126
Employment status of the company	−0.206	1.070	−0.009	−1.192	12	−2.308	1.897	0.848
After the end of each shift	0.007	0.016	0.018	0.411	12	−0.025	0.039	0.681
Average working hours per week	2.418	1.029	0.097	2.350	12	0.397	4.440	0.019
Base	−0.755	3.633	–	− 0.208	12	−7.891	6.382	0.835

Table 6 The result of multiple linear regression The effect of the studied variables on stress

Variable	Unstandardized B	Coefficients Std. Error	Standardized Coefficients Beta	t	Df	confidence interval		p
						Lower Bound	Upper Bound	
Gender	4.350	1.764	0.110	2.467	11	0.886	7.815	0.063
Age	−0.072	0.101	−0.057	− 0.708	11	− 0.271	0.127	0.479
Level of Education	1.051	0.848	0.056	1.239	11	−0.615	2.717	0.216
History of disease	3.941	0.976	0.181	4.037	11	2.023	5.859	0.000
Work experience	0.098	0.0127	0.067	0.774	11	−0.151	0.347	0.439
Interurban emergency service	−1.593	0.829	−0.085	−1.921	11	−3.222	0.036	0.055
MCMC place of service	−8.481	2.998	−0.117	−2.829	11	−14.371	−2.592	0.005
Employment status of commitments	−1.713	1.323	−0.065	−1.295	11	−4.311	0.886	0.196
Official employment situation	1.370	1.031	0.065	1.328	11	0.656	3.396	0.185
Employment status of the company	−1.338	1.254	−0.052	−1.068	11	−3.801	1.125	0.286
Average working hours per week	−0.003	0.019	−0.006	−0.138	11	−0.040	0.035	0.890
Base	7.414	3.979	–	1.863	11	−0.402	15.230	0.063

stress may indicate that the psychological impact of the COVID-19 pandemic on PHEM personnel is diminishing [23].

The results of a similar study on the outbreaks of SARS and Ebola (2015) showed the higher prevalence of psychological disorders such as anxiety, stress, and depression among healthcare staff and PHEM personnel working in affected areas, which was attributed to the psychological pressure of living in those areas [24]. However, in the present study, subjects were working under the Covid-19 pandemic, which has affected a huge number of people across the world.

Dadashzadeh et al. (2017) found that PHEM personnel in Iran suffered from high levels of stress and anxiety [25]. Lai et al. (2020). discovered high levels of depression (50.4%) and anxiety (44.6%) in medical staff working in Wuhan, China, during the outbreak of Covid-19 [26]. The present study; however, showed lower levels of depression, anxiety, and stress in PHEM personnel compared to the above two studies. One possible explanation for this discrepancy is that Lai's study was performed near the onset of the COVID-19 pandemic, when medical personnel encountered the first peak, which may have caused them to have more severe psychological reactions. Our study, on the other hand, was performed when most medical personnel had been vaccinated or had already infected with the disease, which may have affected their level of stress and anxiety in the face of the pandemic.

A study on the SARS epidemic in Hong Kong (2004) reported that only 30% of staff, including pre-hospital emergency personnel had contact with SARS patients due to concerns about illness, disability and death of family members. They experienced more anxiety than other personnel [27], which was consistent with our findings because the two studies were conducted in the same stage of the pandemic.

This study found a significant relationship between age and depression, as people aged 41-55 years were more depressed. With increasing age and approaching retirement, it seems that the incidence of burnout due to activity on holidays and away from family increases. On the other hand, due to lack of manpower and intensive shift work in the morning, evening, night, sleep patterns are disrupted, indicating physical problems and underlying diseases that are interrelated with psychological problems, and can explain the relationship between age and depression [28].

The present study also found a significant relationship between age and anxiety, which was consistent with the findings of Khamseh et al. (2011). One study examined five stressors among 413 people from different nursing groups and showed a significant relationship between age and anxiety. Interpersonal stressors, patient care, and physical environment were the causes of anxiety among the personnel of different nursing groups. Since, pre-hospital emergency personnel encounter various accidents during their shift work, it is consistent with the present study [29].

The present study found a significant relationship between age and stress, which was consistent with the results of Ghasemi et al. (2011). Findings of Ghasemi study showed that there is a significant relationship between nurses' job stress and age variable and 43.5% of nurses over 40 years of age had high job stress. It seems that high work pressure, the presence of the patient's relatives on her bed and their high expectations, hard work, exposure to violence and threats in the workplace are the reasons for the relationship between age and stress [30].

This study also found a significant relationship between the level of education and the levels of depression, anxiety, and stress, which was consistent with similar results reported from China [31]. It appears that PHEM personnel with higher levels of education experience higher levels of depression, anxiety, and stress because of poor life-work balance, the gap between reality and expectations, and higher engagement with media, scientific journals, the internet, etc .[32].

The present study indicated a significant relationship between the history of underlying diseases, stress, anxiety, and depression, which was consistent with the findings of Hosseinabadi that were performed on nurses during the Covid-19 pandemic [33]. Various studies revealed that PHEM personnel experienced elevated levels of catecholamine and cortisol because of emotional stress and missions in unfamiliar environments and different shift work [34], which might affect their mental health state. Furthermore, PHEM personnel cannot control their stress and anxiety problems for prolonged periods, which will increase the likelihood of stress, anxiety, and depression among them (poor mental health) [35].

Our study found a significant relationship between work experience and stress, anxiety, and depression; the more experienced the PHEM personnel, the higher the levels of stress, anxiety, and depression. According to Sakko Onsri et al. (2016), 33% of PHEM personnel with over 10 years of work experience had a high level of stress [36], which was consistent with our findings. One of the reasons for this finding is that people with more work experience are more likely to have underlying diseases and family issues, which are in turn associated with psychological problems [37].

The present study reported,there was a significant relationship between employment type stress, anxiety, and depression, as hired employees had higher levels of stress, anxiety, and depression than people with other types of employment did. This finding is consistent with the study of Bahrami et al. [38], but not consistent with the study of Ghasemi et al. [30]. It appears that while the PHEM is a stressful profession, the hiring process, the higher level of organizational competition among these employees, and their higher awareness of work conditions and environment can aggravate the stressful work conditions [38].

In this study, PHEM personnel, who quarantined themselves at the end of each shift work showed higher levels of anxiety, which was consistent with the results of Wester et al. (2019) [39], because these people were more likely to experience psychological disorders due to being isolated from their families or infected with the virus [40, 41]. Therefore, the development of applications for social support of pre-hospital emergency personnel can play an important role in providing quality services in the community.

The cross-sectional nature of this study is considered one limitation, meaning that longer studies may yield different or more accurate results. In addition, the study only focused on the eastern parts of Iran, which could raise the possibility of bias due to inadequate distribution of facilities and human resources. Another limitation was the use of electronic questionnaires because of the guidelines issued by Iran's national anti-corona headquarters to prevent the spread of COVID-19, meaning that sampling was voluntary and could therefore be biased. It is suggested that future studies examine depression, anxiety and stress in other parts of Iran. It is also essential that there are practical studies to promote PHEM mental health.

Conclusion

The findings of this study suggested that PHEM personnel experienced significant levels of depression, anxiety, and stress during the Covid-19 pandemic. In order to improve the levels of stress and anxiety of the pre-hospital personnel, it is recommended that the work schedule and services provided to these people be designed in such a way that they have more time for rest and communication with their family members. In addition, the personnel should also have easier access to the expert team in the fields of counseling and psychiatry to facilitate international standards of workplace health and to provide better services to society.

Abbreviations

PHEM: Pre-hospital emergency medicine; DASS-21: DEPRESSION, Anxiety, and Stress Scale; PHEIC: Public Health Emergency of International Concern; DASS-21: Depression, Anxiety and Stress Scale.

Acknowledgments

The findings of this study suggested that PHEM personnel experienced significant levels of depression, anxiety, and stress during the Covid-19 pandemic. Thus, it is essential to create an independent mental health and counseling unit in the organizational chart of Iran's National Medical Emergency Organization.

Authors' contributions

All authors have read and approved the manuscript. Study design: MNN, KM, MS; data collection and analysis: RK, MS; manuscript preparation: MNN, KM, MS, RK.

Funding

This research was supported by a grant from Torbat Heydariyeh University of Medical Sciences.

Availability of data and materials

The datasets generated during the current study are available in Figshare repository, [https://doi.org/10.6084/m9.figshare.19170248].

Declarations

Ethics approval and consent to participate
This research was carried out after approval of the regional research ethics committee with the code IR.THUMS.REC.1400.045, presentation of the research design to relevant officials, and coordination with the accident and emergency medicine centers of medical universities in the study area (eastern Iran). The research purpose was explained verbally, online informed consent was in the first part of the online questionnaire, and the PHEM professional completed the questionnaire only after registration of the informed consent. The research units were explained about the information confidentiality and the voluntary nature of the participation in the study. All methods were performed in accordance with the relevant guidelines and regulations, which were aligned in accordance with the Declaration.

Consent for publication
Not applicable.

Competing interests
No competing fnancial interests exist.

Author details
[1]Department of Medical Emergency, School of Nursing and Midwifery, Torbat Heydariyeh University of Medical Sciences, Torbat Heydariyeh, Iran. [2]Health Sciences Research Center, Torbat Heydariyeh University of Medical Sciences, Torbat Heydariyeh, Iran. [3]Department of Nursing, School of Nursing and Midwifery, Torbat Heydariyeh University of Medical Sciences, Torbat Heydariyeh, Iran.

Received: 1 February 2022 Accepted: 12 May 2022
Published online: 03 June 2022

References
1. Wu JT, Leung K, Leung GM. Nowcasting and forecasting the potential domestic and international spread of the 2019-nCoV outbreak originating in Wuhan, China: a modelling study. Lancet. 2020;395(10225):689–97.
2. Gorbalenya AE, Baker SC, Baric R, Groot RJ, Drosten C, Gulyaeva AA, et al. Severe acute respiratory syndrome-related coronavirus: The species and its viruses–a statement of the Coronavirus Study. Group. 2020.
3. Organization WH. Novel Coronavirus (2019-nCoV); Situation Report 3; 2020. URL https://www.who.int/docs/default-source/coronaviruse/situation-reports/20200121-sitrep-1-2019-ncov.pdf. 2020.
4. https://www.worldometers.info/coronavirus/
5. Singhal T. A review of coronavirus disease-2019 (COVID-19). Indian J Pediatr. 2020;87(4):281–6.
6. Son C, Hegde S, Smith A, Wang X, Sasangohar F. Effects of COVID-19 on college students' mental health in the United States: Interview survey study. J Med Internet Res. 2020;22(9):e21279.
7. Sohrabi C, Alsafi Z, O'neill N, Khan M, Kerwan A, Al-Jabir A, et al. World Health Organization declares global emergency: A review of the 2019 novel coronavirus (COVID-19). Int J Surg. 2020;76:71–6.
8. Nicola M, Alsafi Z, Sohrabi C, Kerwan A, Al-Jabir A, Iosifidis C, et al. The socio-economic implications of the coronavirus pandemic (COVID-19): A review. Int J Surg. 2020;78:185–93.
9. Wang P, Nair MS, Liu L, Iketani S, Luo Y, Guo Y, et al. Antibody resistance of SARS-CoV-2 variants B. 1.351 and B. 1.1. 7. Nature. 2021;593(7857):130–5.
10. Taylor WD, Blackford JU. Mental health treatment for front-line clinicians during and after the coronavirus disease 2019 (COVID-19) pandemic: a plea to the medical community. Am College Phys. 2020:574–5.
11. Vujanovic AA, Lebeaut A, Leonard S. Exploring the impact of the COVID-19 pandemic on the mental health of first responders. Cogn Behav Ther. 2021;50(4):320–35.
12. Sabetian G, Moghadami M, Haghighi LHF, Shahriarirad R, Fallahi MJ, Asmarian N, et al. COVID-19 infection among healthcare workers: a cross-sectional study in southwest Iran. Virol J. 2021;18(1):1–8.
13. Bahadori M, Ghardashi F, Izadi AR, Ravangard R, Mirhashemi S, Hosseini SM. Pre-hospital emergency in Iran: A systematic review. Trauma monthly. 2016;21(2).
14. Shoja E, Aghamohammadi V, Bazyar H, Moghaddam HR, Nasiri K, Dashti M, et al. Covid-19 effects on the workload of Iranian healthcare workers. BMC Public Health. 2020;20(1):1–7.
15. Ying Y, Ruan L, Kong F, Zhu B, Ji Y, Lou Z. Mental health status among family members of health care workers in Ningbo, China, during the coronavirus disease 2019 (COVID-19) outbreak: a cross-sectional study. BMC psychiatry. 2020;20(1):1–10.
16. Kalantari S, Hosseinzadeh M. An assessment of general health of operational staff of pre-hospital emergency in Golestan province. J Health Care. 2017;18(4):359–67.
17. Lovibond PF, Lovibond SH. The structure of negative emotional states: Comparison of the Depression Anxiety Stress Scales (DASS) with the Beck Depression and Anxiety Inventories. Behav Res Ther. 1995;33(3):335–43.
18. Najafi Kalyani M, Pourjam E, Jamshidi N, Karimi S, Najafi KV. Survey of stress, anxiety, depression and self-concept of students of Fasa University of medical sciences, 2010. J Fasa University Med Sci. 2013;3(3):235–40.
19. Sahebi A, Asghari MJ, Salari RS. Validation of depression anxiety and stress scale (DASS-21) for an. Iranian population. 2005.
20. Mehdipour-Rabori R, Nematollahi M. The effect of recommended Azkar on anxiety, stress, and depression in families of patients undergoing open heart surgery. Iran J Nurs Midwifery Res. 2014;19(3):238.
21. Mohammadi SM, Ashtari S, Khosh FM. The psychological impact of COVID-19 pandemic on mental health of Iranian population. Int J Travel Med Global Health. 2020;9(1):19–24.
22. Sarboozi-Hoseinabadi T, Askari M, Miri K, Namazi Nia M. Depression, Anxiety, and Stress of Nurses' Family Members in Covid–19 Pandemic; a Case Study of Torbat-e Heydariyeh Hospitals. Iran J War Public Health. 2020;12(4):241–8.
23. Bilge Y, Keles E, Baydili KN. The impact of COVID-19 vaccination on mental health. J Loss Trauma. 2022;27(3):285–8.
24. Li L, Wan C, Ding R, Liu Y, Chen J, Wu Z, et al. Mental distress among Liberian medical staff working at the China Ebola Treatment Unit: a cross sectional study. Health Qual Life Outcomes. 2015;13(1):1–6.
25. Dadashzadeh A, Rahmani A. Exposure to stressors among emergency medical technicians in pre-hospital emergency departments of East Azerbaijan Province. Iran. 2017.
26. Lai J, Ma S, Wang Y, Cai Z, Hu J, Wei N, et al. Factors associated with mental health outcomes among health care workers exposed to coronavirus disease 2019. JAMA Netw Open. 2020;3(3):e203976–e.
27. Cheong D, Lee C. Impact of severe acute respiratory syndrome on anxiety levels of front-line health care workers. Hong Kong Med J. 2004;10(5):325–30.
28. Muntaner C, Van Dussen DJ, Li Y, Zimmerman S, Chung H, Benach J. Work organization, economic inequality, and depression among nursing assistants: A multilevel modeling approach. Psychol Rep. 2006;98(2):585–601.
29. Khamseh F, Roohi H, Ebaady A, Hajiamini Z, Salimi H, Radfar S. Survey Relationship between demographic factors and stress, anxiety and depression in nurses working in selected hospitals in Tehran city. J Holistic Nursing Midwifery. 2011;21(1):13–21.
30. MORTAGHI GM. Nurses job stress in therapeutic educational centers in Zanjan; 2011.
31. Wang C, Pan R, Wan X, Tan Y, Xu L, Ho CS, et al. Immediate psychological responses and associated factors during the initial stage of the 2019 coronavirus disease (COVID-19) epidemic among the general population in China. Int J Environ Res Public Health. 2020;17(5):1729.
32. Maghsoudi J. The effect of a gratitude program on job stress & burnout in Esfahan prehospital technicians. Iran Occupational Health. 2020;17(1):208–18.
33. Hosseinabadi MB, Etemadinezhad S. Evaluating the relationship between job stress and job satisfaction among female hospital nurses in Babol: An application of structural equation modeling. Health Promot Perspect. 2018;8(2):102.
34. Field T, Diego M. Cortisol: the culprit prenatal stress variable. Int J Neurosci. 2008;118(8):1181–205.
35. Malhi GS, Mann JJ. Depression. Lancet. 2018;392(10161):2299–312.
36. Sakkomonsri J, Suwan-Ampai P, Kaewboonchoo O. Factors Associated with Job Stress among Ambulance Nurses in Bangkok. Bangkok Med J. 2016;12:33.
37. Effati-Daryani F, Zarei S, Mohammadi A, Hemmati E, Yngyknd SG, Mirghafourvand M. Depression, stress, anxiety and their predictors in Iranian

pregnant women during the outbreak of COVID-19. BMC psychology. 2020;8(1):1–10.
38. Bahrami A, Akbari H, Mousavi GA, Hannani M, Ramezani Y. Job stress among the nursing staff of Kashan hospitals. Feyz J Kashan Univ Med Sci. 2012;16(1).
39. Wester M, Giesecke J. Ebola and healthcare worker stigma. Scandinavian J Public Health. 2019;47(2):99–104.
40. Guo J, Liao L, Wang B, Li X, Guo L, Tong Z, et al. Psychological Effects of COVID-19 on Hospital Staff: A National Cross-Sectional Survey of China Mainland. Available at SSRN 3550050. 2020.
41. Khodabakhshi-koolaee A. Living in home quarantine: analyzing psychological experiences of college students during Covid-19 pandemic. J Military Med. 2020;22(2):130–8.

Publisher's Note

Springer Nature remains neutral with regard to jurisdictional claims in published maps and institutional affiliations.

Ready to submit your research? Choose BMC and benefit from:

- fast, convenient online submission
- thorough peer review by experienced researchers in your field
- rapid publication on acceptance
- support for research data, including large and complex data types
- gold Open Access which fosters wider collaboration and increased citations
- maximum visibility for your research: over 100M website views per year

At BMC, research is always in progress.

Learn more biomedcentral.com/submissions

BMC
