# Supplementary material for: Mental health status and related factors influencing healthcare workers during the COVID-19 pandemic: A systematic review and meta-analysis
Source: PLoS One. 2024 Jan 19;19(1):e0289454. doi: 10.1371/journal.pone.0289454 (PMC10798549; doi:10.1371/journal.pone.0289454)
Supplement: S1 Data — (ZIP) [file pone.0289454.s011.zip › literatures/152.pdf]

Received: 23 January 2021 | Revised: 19 March 2021

DOI: 10.1002/npr2.12179

ORIGINAL ARTICLE

NEUROPSYCHOPHARMACOLOGY REPORTS

WILEY

Prevalence of corona-associated anxiety and mental health disorder among dentists during the COVID-19 pandemic

Hamid Salehiniya[1] | Hamid Abbaszadeh[2]

[1]Social Determinants of Health Research Center, Birjand University of Medical Sciences, Birjand, Iran

[2]Department of Oral and Maxillofacial Pathology, Faculty of Dentistry, Birjand University of Medical Sciences, Birjand, Iran

Correspondence
Hamid Abbaszadeh, Department of Oral and Maxillofacial Pathology, Faculty of Dentistry, Birjand University of Medical Sciences, Birjand, Iran.
Email: hamidabbaszade@yahoo.com

Abstract

Aim: The aim of this study was to investigate the prevalence and severity of corona-associated anxiety and mental health disorder among Iranian dentists during the COVID-19 pandemic.

Methods: A study was conducted using online survey from May 2nd to 14th, 2020. The questionnaire consisted of three sections: demographic information, anxiety (18 questions), and general health (GH) (GHQ-28). Scoring system was based on Likert scale. The questionnaire was registered at Porsline website. Data were analyzed using *t* test and Pearson's correlation coefficient statistical tests.

Results: A total of 320 dentists from all over the country fully completed questionnaires. 42.5% of dentist had corona-associated anxiety and 32.5% out of them had mild severity and no severe anxiety was observed. 62.5% of dentists were nonpsychiatric according to GHQ-28, 35% had mild disorders in GH, and no one had severe GH disorders. There were significant relationships between gender, marital status, and family history of psychiatric disorders with GH status. There was a significant relationship between history of physical illness with corona-associated anxiety. There was a significant relationship between history of psychiatric disorders with corona-associated anxiety and GH status.

Conclusion: The prevalence of corona-associated anxiety and mental disorders in dentists was moderate; by holding psychological workshops to maintain and strengthen the morale of dentists during the corona pandemic, along with teaching them the correct way to use personal protective equipment, while maintaining the mental health of dentists, we will help them return to work and provide dental services.

KEYWORDS

anxiety, COVID-19, dentists, Iran, mental health disorder

1 | INTRODUCTION

The 2019 novel coronavirus (2019-nCoV) epidemic has spread around the world. The coronavirus disease 2019 (COVID-19) not only poses risk of death, but also puts a lot of psychological pressure on people around the world. There are reports of the psychological impact of the disease on a wide range of people, from patients to medical staffs.[1] The virus can be transmitted through sneezing,

This is an open access article under the terms of the Creative Commons Attribution-NonCommercial-NoDerivs License, which permits use and distribution in any medium, provided the original work is properly cited, the use is non-commercial and no modifications or adaptations are made.
© 2021 The Authors. *Neuropsychopharmacology* Reports published by John Wiley & Sons Australia, Ltd on behalf of the Japanese Society of Neuropsychopharmacology.

coughing, contact with contaminated surfaces, blood, saliva, and aerosols, and can lead to corona disease. Because transmission is possible through all of these pathways in the dental environment, and dentists are in close contact with patients and are particularly vulnerable to aerosols, dentistry is one of the most risky occupations for COVID-19.[2] Dentists, in addition to the general fear of infection with this disease at the community level, like other people, have a persistent fear and anxiety caused by the high probability of transmitting the virus during dental procedures, especially in the absence or limitation of personal protective equipment. To this fear must be added the concern about the long-term financial impact of the disease caused by reduced working hours and the number of patients.[3] On the other hand, no standard antiviral treatment has been provided for this disease.[4] Also, since nurses are in close contact with infected people, they are the main part of the infection transmission chain. Some studies showed that nurses had anxiety for themselves and their family affliction with COVID-19.[4,5] All of these stresses and worries can pose serious threats to the general health of dentists.[5] The aim of this study was to investigate the prevalence and severity of corona-associated anxiety and mental health disorder among Iranian dentists during the COVID-19 pandemic.

2 | METHODS

In this cross-sectional descriptive-analytical study, conducted May 2nd to 14th, 2020, the study population included all general and specialist dentists working in public and private centers in Iran who are satisfied to enter the study. Dentists working abroad, and dentists who are not currently working in dentistry, did not enter the study. Also, dentists who did not complete the conscious consent form were excluded from the study. Questionnaires that had duplicated internet protocol address (IP address) or took less than ten minutes to complete were also excluded from the study. Sampling method in this study was online and available.

After the approval of the study in the university's ethics committee, a questionnaire consisting of three sections was designed. The first part consisted of demographic data including age, gender, marital status, education degree, working experience (in year), history of physical illness (such as diabetes mellitus, hypertension, etc), history of psychiatric disorders (such as anxiety, depression, etc), and family history of psychiatric disorders (Wang et al 2020). The second part included a researcher-made questionnaire consisting of 18 questions related to corona-associated anxiety (Appendix S1). The validity of this questionnaire was confirmed through the content validity index method and its reliability through the Cronbach's alpha method (Cronbach's alpha was 0.92). The scoring system used in this study for corona-associated anxiety was based on the Likert scale 0, 1, 2, 3. The minimum score was 0, and the maximum was 54. Higher anxiety scores indicated higher levels of corona-associated anxiety. In the total score of the questionnaire, a score of less than 15 indicates lack of corona-associated anxiety or the minimum. A score of 15-27 indicates a mild corona-associated anxiety, a score of 28-41 indicates a moderate corona-associated anxiety, and a score of 42-54 indicates a severe corona-associated anxiety.

The third part consisted of 28 questions related to GH using the general health questionnaire-28 [GHQ-28]) (Appendix S2); the validity and reliability of which have been confirmed in previous studies. Via GHQ-28,[6] we assessed somatic symptoms (from questions 1-7), anxiety and sleep disorder (from questions 8-14), social function (from questions 15-21), and depression symptoms (from questions 22-28). The scoring system used in this study for GHQ-28 was based on the Likert scale 0, 1, 2, 3. The minimum score was 0, and the maximum was 84. Higher GHQ-28 scores indicated higher levels of distress. On subscales, a score of 0-6 means no psychological burden or the minimum. A score of 7-11 indicates a mild psychological burden, a score of 12-16 indicates a moderate psychological burden, and a score of 17-21 indicates a severe psychological burden. In the total score of the questionnaire, a score of less than 23 indicates a person's general health (lack of psychiatric disorder or nonpsychiatric) or the minimum psychological burden. A score of 23 to 40 indicates a mild psychological burden, a score of 41 to 60 indicates a moderate psychological burden, and a score of 61 to 84 indicates a severe psychological burden.[7]

Due to the conditions of the corona crisis in the country, observance of social distance, and also the national nature of the study, the research was done online. The study questionnaire was prepared and uploaded on the Porsline website, and then, the link of the questionnaire along with the necessary explanations (including conscious consent to enter the study) was prepared and shared on the Dental Society channel in Telegram Messenger (link: https://survey.porsline.ir/s/vdLKbas/). Relevant links were also sent to other dental groups and channels, and this process continued until the sample size was reached.

After completing the questionnaires, the obtained information was entered into the SPSS software, version 16, and was analyzed using *t* test and Pearson's correlation coefficient statistical tests. In order to identify factors which predict corona-associated anxiety backward regression method was used.

3 | RESULTS

Out of a total of 325 questionnaires received, 320 questionnaires were fully completed, which were included in the study, and 5 other incomplete questionnaires were excluded from the study. The average age of the participants was 44.38 ± 10.66. Of the 320 participants, 53.8% were males and 46.2% were females. Also, out of 320 participants, 72.5% were general dentists and 27.5% were specialist dentists. 76.3% of respondents were married and 23.7% were single. The average working experience of the participants was 17.31 ± 10.62. 20% had history of physical illness and 80% were healthy. 12.5% reported history of psychiatric disorders. Table 1 shows the distribution of dentists in terms of corona-associated anxiety and dentists in terms of GH (Table 1). Table 2 shows distribution of dentists in subscales of GH (Table 2).

TABLE 1 Distribution of dentists in terms of corona-associated anxiety and general health

Variable	Sub variable	N	Percent
Anxiety	Healthy	184	57.5
	Mild	104	32.5
	Moderate	32	10
	Severe	0	0
General health	Healthy	200	62.5
	Mild	112	35.0
	Moderate	8	2.5
	Severe	0	0

TABLE 2 Distribution of dentists in subscales of general health

	Frequency	
General health	n	Percent
Somatic symptoms		
Healthy	240	75
Mild	72	22.5
Moderate	8	2.5
Severe	0	0
Anxiety and sleep disorder		
Healthy	228	71.3
Mild	52	16.2
Moderate	32	10
Severe	8	2.5
Social function		
Healthy	68	21.2
Mild	204	63.8
Moderate	48	15
Severe	0	0
Depression symptoms		
Healthy	288	90
Mild	20	6.2
Moderate	8	2.5
Severe	4	1.3

Table 3 shows the result of Pearson's correlation coefficient analysis which shows correlations between GHQ-28 scores with age, working experience, and corona-associated anxiety score and between corona-associated anxiety scores with factors such as age and working experience (Table 3; Figures 1 and 2).

According to Table 4, although females had more corona-associated anxiety than males, the difference was not significant (*P*-value >.05). Also, there was a significant difference between the mean total GHQ-28 scores of male and female dentists (*P*-value <.05). There was not any significant difference between the mean total corona-associated anxiety scores and the mean total GHQ-28 scores of general and specialist dentists (*P*-value >.05). There was not any significant difference between the mean total corona-associated anxiety scores of single and married dentists (*P*-value >.05). There was a significant difference between the mean total GHQ-28 scores of single and married dentists (*P*-value <.05). There was a significant difference between the mean total corona-associated anxiety scores of dentists without and with history of physical illness (*P*-value <.05). There was not any significant difference between the mean total GHQ-28 scores of dentists with and without history of physical illness (*P*-value >.05). There was a significant difference between the mean total corona-associated anxiety scores of dentists without and with history of psychiatric disorders (*P*-value <.05). There was a significant difference between the mean total GHQ-28 scores of dentists with and without history of psychiatric disorders (*P*-value = .0001). There was not any significant difference between the mean total corona-associated anxiety scores of dentists without and with family history of psychiatric disorders (*P*-value >.05). There was a significant difference between the mean total GHQ-28 scores of dentists with and without family history of psychiatric disorders (*P*-value <.05) (Table 4).

TABLE 3 The result of Pearson's correlation coefficient analysis showing correlations between general health questionnaire-28 scores and corona-associated anxiety scores with other studied factors

	Score	
Factor	General health questionnaire-28 scores	Corona-associated anxiety score
Age		
Pearson's *r*	−.17	.02
P-value	.003	.75
Correlation	Negative and significant	Positive but not significant
Working experience		
Pearson's *r*	−.14	.04
P-value	.01	.53
Correlation	Negative and significant	Positive but not significant
Corona-associated anxiety		
Pearson's *r*	.52	
P-value	<.001	
Correlation	Positive and significant	

In order to identify factors which predict corona-associated anxiety, backward regression method was conducted. The results of adapted analysis showed that a history of physical illness and a history of psychiatric disorders were significantly associated with corona-associated anxiety (*P*-values <.05) (Table 5). Other studied factors were not significantly associated with corona-associated anxiety and do not predict it (*P*-values >.05). The model for prediction of Corona-associated anxiety is as follows: Corona-associated

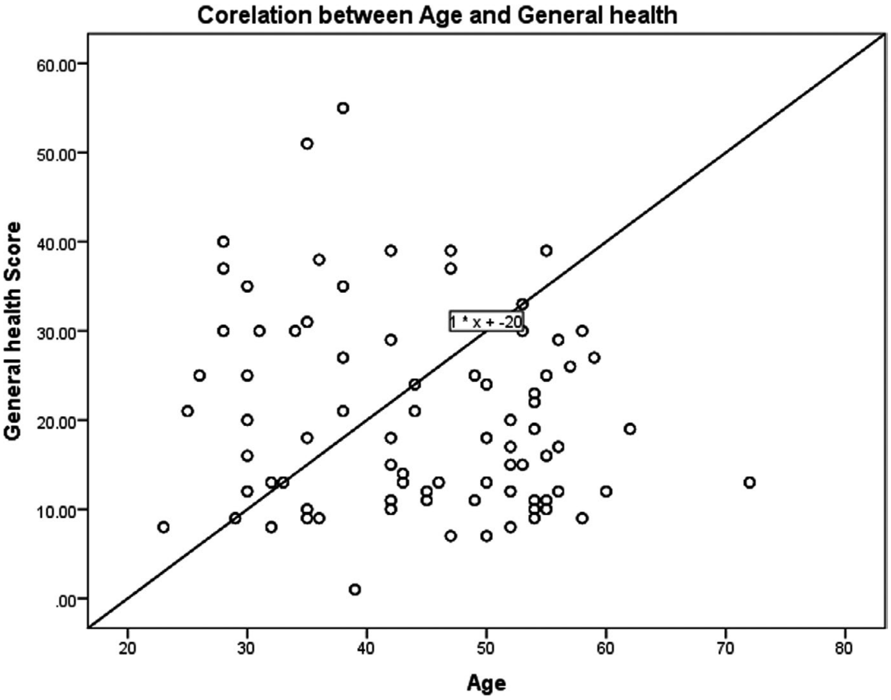

FIGURE 1 Correlation between general health score and age

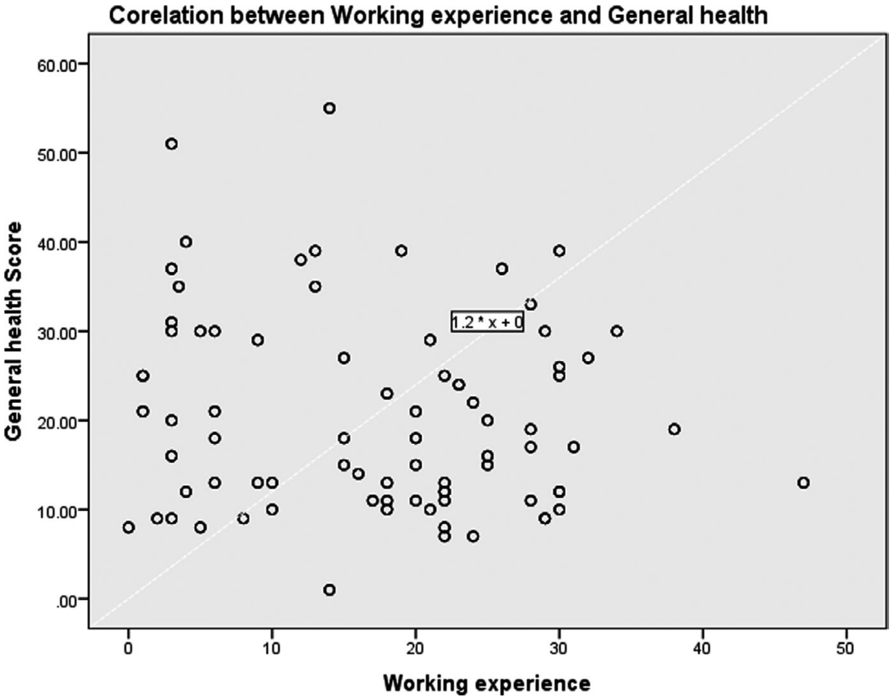

FIGURE 2 Correlation between general health score and working experience

anxiety = 4.188 + 2.41 (History of physical illness) + 6.91 History of mental illness.

4 | DISCUSSION

In general, COVID-19 has affected all aspects of life in society and can be associated with fear, anxiety, and worry, especially for dentists.

According to the results, as the dentist's age decreases, the GHQ-28 score increases and the dentist is in a more unfavorable general health condition. As the dental work experience decreases, the GHQ-28 score increases and the dentist is in a more unfavorable general health condition. The results showed that with increasing corona-associated anxiety, the dentist's GHQ-28 increased and the more anxious dentist had a more unfavorable general health status. Although the relationship was not significant, the dentist's corona-associated anxiety increased with age. Although the relationship was

TABLE 4 Comparison of Corona-associated anxiety scores and General health questionnaire-28 scores among different studied group

Variable	Sub Variable	Score: Mean ± standard deviation (SD)	Score: Statistic result
Corona-associated anxiety scores			
Sex	Male	13.93 ± 9.11	T = −1.81
	Female	15.75 ± 8.83	DF = 318
			.07
Degree	General	15.13 ± 9.57	T = 1.17
	Specialist	13.81 ± 7.33	DF = 318
			.24
Marital status	Single	14.89 ± 8.88	T = 0.13
	Married	14.73 ± 9.07	DF = 318
			.89
History of physical illness	No	14.07 ± 7.90	T = −2.79
	Yes	17.56 ± 12.20	DF = 318
			.006
Family history of psychiatric disorder	No	14.55 ± 9.1	T = −0.91
	Yes	15.73 ± 8.67	DF = 318
			.36
History of psychiatric disorder	No	13.84 ± 8.42	T = −5.08
	Yes	21.30 ± 10.38	DF = 318
			<.001
General health questionnaire-28 scores			
Sex	Male	18.27 ± 8.44	T = −3.70
	Female	22.72 ± 12.92	T = 318
			<.001
Degree	General	20.13 ± 10.01	T = −0.53
	Specialist	20.86 ± 13.17	DF = 318
			.59
Marital status	Single	22.52 ± 12.81	T = 2.00
	Married	19.65 ± 10.24	DF = 318
			.04
History of physical illness	No	20.18 ± 11.20	T = −0.49
	Yes	20.93 ± 9.98	DF = 318
			.62
Family history of psychiatric disorder	No	19.42 ± 10.87	T = −3.18
	Yes	24.33 ± 10.47	DF = 318
			<.001
History of psychiatric disorder	No	18.98 ± 10.5	T = −6.17
	Yes	29.80 ± 9.27	DF = 318
			<.001

not significant, the dentist's corona-associated anxiety increased with working experience.

In our study, female dentists had higher mean total corona-associated anxiety scores than male dentists. This means that female dentists have experienced more anxiety during the corona crisis and need more psychological support. Female dentists had significantly higher mean total GHQ-28 scores than male dentists. This means that female dentists have recently experienced more psychological burden, and in terms of general health, they were in a worse condition than men in the last month and need more help to improve their psychological condition and improve their general health.

In our study, the mean total corona-associated anxiety scores and mean total GHQ-28 scores were not significantly different between general and specialist dentists. This means that education degree did not affect anxiety and general health status of dentists during the corona crisis.

In our study, the mean total corona-associated anxiety scores were not significantly different between single and married dentists. It means that marital status did not affect dentists' anxiety during corona. The mean of total GHQ-28 scores was significantly higher in single dentists. It means that single dentists have recently experienced more psychological burden, and in terms of general health, they were in a worse condition than married dentists in the last month and need more help to improve their psychological condition and improve their general health.

In our study, the mean of total corona-associated anxiety scores was significantly higher in dentists with history of physical illness. It means that history of physical illness affected corona-associated anxiety, and dentists with history of physical illness have recently experienced more anxiety during corona crisis, and they were in a worse condition than dentists without history of physical illness in the last month and need more psychological support. The mean of total GHQ-28 scores was not significantly different between dentists without and with history of physical illness. It means that history of physical illness did not affect recent general health status of dentists.

In our study, the mean of total corona-associated anxiety scores and total GHQ-28 scores was significantly higher in dentists with history of psychiatric disorder. It means that history of psychiatric disorder affected corona-associated anxiety and recent general health status, and dentists with history of psychiatric disorder have recently experienced more anxiety during corona crisis, and they were in a worse condition in terms of recent general health status than dentists without history of psychiatric disorder.

In the study by Ahmed et al,[8] more than two-thirds of the general dental practitioners (78%) were anxious and had been feared from COVID-19. They concluded that dental practitioners around the world are in a state of anxiety and fear due to the COVID-19 pandemic. In our study, 42.5 of dentists had some degree of corona-associated anxiety. This difference in the percentage of reported anxiety of dentists may be related to inherent differences of studied populations. Most of the participants in their study were from Pakistan and Saudi Arabia. This may indicate a greater resilience of Iranian dentists in the face of environmental stress than dentists in these two countries, and this in itself can be rooted in the strong religious beliefs of Iranians.[8]

In the study by Shacham et al, results revealed that elevated psychological distress was found among dentists who have

TABLE 5 Result of backward regression showing correlation of studied factors which predict corona-associated anxiety

Variable	Coefficient	Confidence interval	Statistical result
Constant	4.188	0.44-8.19	T = 1.99<br>*P* = .4
History of physical illness	2.41	0.1-4.83	T = 1.96<br>*P* = .05
History of mental illness	6.91	3.97-9.83	T = 5.63<br>*P* < .001

Note: P-value for the model: $P < .001$, F = 14.91, Tolerance = 0.96, VIF = 1.03, $R = .29$, $R^2 = .086$, Adjusted $R^2 = .08$. The model: Corona-associated anxiety = 4.188 + 2.41 (History of physical illness) + 6.91 History of mental illness.

background illness. This finding is consistent with the findings of our study because in our study as well corona-associated anxiety was significantly higher in dentists with history of physical illness. The findings of our study and their study indicate that the presence of physical illnesses such as cardiovascular disease, hypertension, diabetes mellitus, etc can affect the tolerance threshold of dentists and expose them to more psychological distress than healthy dentists. In their study, lower psychological distress was associated with being in a committed relationship. This finding is also consistent with the findings of our study because in our study single dentists had been experienced more psychiatric disorders and conversely, married dentists were in a better general health status and had less psychological distress. This means that a marital relationship and having a spouse or an emotional relationship with commitment can be effective in reducing the anxiety of dentists and establishing peace of mind.[9]

In the study by Consolo et al,[10] dentists reported feelings of concern (70.2%), anxiety (46.4%), and fear (42.4%). The prevalence of reported anxiety for Italian dentists during the corona crisis is almost the same as in our study. The reason for this can be related to the similarity of mood and culture between the people of Iran and Italy. They reported that 9% of dentists in their study had a severe anxiety. In our study, dentists had no severe anxiety. The reason for this difference could be related to the more patience and tolerance of Iranian dentists over Italian dentists, which makes them less anxious about external psychological pressures, including stress during the corona crisis.

Nemati et al[4] study on 85 nurses showed their anxiety for themselves and their family affliction with COVID-19.

In general, during corona crisis, the prevalence of corona-associated anxiety and mental disorders in dentists is moderate; therefore, planning to reduce corona-associated anxiety in this group is essential. By holding psychological workshops to maintain and strengthen the morale of dentists during the corona pandemic, along with teaching them the correct way to use personal protective equipment, while maintaining the mental health of dentists, we will help them return to work and provide dental services.

ACKNOWLEDGMENTS

The authors thank Vice-Chancellor for Research of Birjand University of medical sciences for their support of this project.

CONFLICT OF INTEREST

The authors have no conflicts of interest to report.

AUTHOR CONTRIBUTIONS

Both authors have participated in providing the draft of the manuscript, revising the manuscript, and confirming the final version of the manuscript, and have participated in the acquisition of data. Both authors have participated in the production of the idea of the research; Dr HS has participated in the analysis and interpretation of data.

APPROVAL OF THE RESEARCH PROTOCOL BY AN INSTITUTIONAL REVIEWER BOARD

The research protocol of this cross-sectional study was approved by the Human Research Ethics Committee of the Birjand University of Medical Sciences (BUMS) (approval number: IR.BUMS.REC.1399.050).

INFORMED CONSENT

Informed consent was obtained from all individual participants (or their legal guardians) included in the study.

REGISTRY AND THE REGISTRATION NO. OF THE STUDY/TRIAL

The study has been registered in the university research system with the registration No. of the study is #5386.

DATA AVAILABILITY STATEMENT

The raw data belonged to this study cannot be made publicly available, because the disclosure of personal data was not included in the research protocol of the present study.

ORCID

Hamid Abbaszadeh https://orcid.org/0000-0002-7443-0607

REFERENCES

1. Cao W, Fang Z, Hou G, Han M, Xu X, Dong J, et al. The psychological impact of the COVID-19 epidemic on college students in China. Psychiatry Res. 2020;287:e112934.
2. Duruk G, Gümüşboğa Z. Investigation of Turkish dentists' clinical attitudes and behaviors towards the COVID-19 pandemic: a survey study. Brazilian Oral Res. 2020;34:e054.

3. Nair AKR, Chellaswamy KS, Kattula D, Thavarajah R, Mohandoss AA. Perceived stress and psychological (Dis) stress among Indian endodontists during COVID19 pandemic lock down. medRxiv. 2020.
4. Nemati M, Ebrahimi B, Nemati F. Assessment of Iranian nurses' knowledge and anxiety toward COVID-19 during the current outbreak in Iran. Archives of Clin Infect Dis. 2020;15:e102848.
5. Qiu J, Shen B, Zhao M. A nationwide survey of psychological distress among Chinese people in the COVID-19 epidemic: implications and policy recommendations. Gen Psychiatr. 2020;33(2):e100213.
6. Malakouti SK, Fatollahi P, Mirabzadeh A, Zandi T. Reliability, validity and factor structure of the GHQ-28 used among elderly Iranians. Int Psychogeriatr. 2007;19(4):623–34.
7. Matud MP, García MC. Psychological distress and social functioning in elderly Spanish people: a gender analysis. Int J Environ Res Public Health. 2019;16(3):341.
8. Ahmed MA, Jouhar R. Fear and practice modifications among dentists to combat novel coronavirus disease (COVID-19) outbreak. Int J Environ Res Public Health. 2020;17(8):2821.
9. Shacham M, Hamama-Raz Y, Kolerman R, Mijiritsky O, Ben-Ezra M, Mijiritsky E. COVID-19 factors and psychological factors associated with elevated psychological distress among dentists and dental hygienists in Israel. Int J Environ Res Public Health. 2020;17(8):2900.
10. Consolo U, Bellini P, Bencivenni D, Iani C, Checchi V. Epidemiological aspects and psychological reactions to COVID-19 of dental practitioners in the northern Italy Districts of Modena and Reggio Emilia. Int J Environ Res Public Health. 2020;17(10):3459.

SUPPORTING INFORMATION

Additional supporting information may be found online in the Supporting Information section.

How to cite this article: Salehiniya H, Abbaszadeh H. Prevalence of corona-associated anxiety and mental health disorder among dentists during the COVID-19 pandemic. *Neuropsychopharmacol Rep*. 2021;41:223–229. https://doi.org/10.1002/npr2.12179
