# Supplementary material for: Mental health status and related factors influencing healthcare workers during the COVID-19 pandemic: A systematic review and meta-analysis
Source: PLoS One. 2024 Jan 19;19(1):e0289454. doi: 10.1371/journal.pone.0289454 (PMC10798549; doi:10.1371/journal.pone.0289454)
Supplement: S1 Data — (ZIP) [file pone.0289454.s011.zip › literatures/153.pdf]

*Disaster Medicine and Public Health Preparedness*

**www.cambridge.org/dmp**

**Brief Report**

**Cite this article:** Salman M, Mustafa ZU, Raza MH, *et al*. Psychological effects of COVID-19 among health care workers, and how they are coping: A web-based, cross-sectional study during the first wave of COVID-19 in Pakistan. *Disaster Med Public Health Prep*. doi: https://doi.org/10.1017/dmp.2022.4.

**Keywords:**
anxiety; coping; COVID-19; depression; health care workers; Pakistan

**Corresponding author:**
Muhammad Salman,
Emails: msk5012@gmail.com or muhammad.salman@pharm.uol.edu.pk

© The Author(s), 2022. Published by Cambridge University Press on behalf of Society for Disaster Medicine and Public Health, Inc.

# Psychological Effects of COVID-19 Among Health Care Workers, and How They Are Coping: A Web-Based, Cross-Sectional Study During the First Wave of COVID-19 in Pakistan

Muhammad Salman[1], Zia Ul Mustafa[2], Muhammad Husnnain Raza[3], Tahir Mehmood Khan[4,5], Noman Asif[6], Humera Tahir[7], Naureen Shehzadi[6], Tauqeer Hussain Mallhi[8], Yusra Habib Khan[8], Kishwar Sultana[1], Fahad Saleem[9] and Khalid Hussain[6]

[1]Faculty of Pharmacy, The University of Lahore, Lahore, Pakistan; [2]Department of Pharmacy Services, District Headquarter Hospital, Pakpattan, Pakistan; [3]Faisalabad Institute of Cardiology, Faisalabad, Pakistan; [4]Institute of Pharmaceutical Science, University of Veterinary and Animal Sciences, Lahore, Pakistan; [5]School of Pharmacy, Monash University, Bandar Sunway, Selangor, Malaysia; [6]Punjab University College of Pharmacy, University of the Punjab, Lahore, Pakistan; [7]Ruth Pfau College of Nutrition Sciences, Lahore Medical and Dental College, Lahore, Pakistan; [8]Department of Clinical Pharmacy, College of Pharmacy, Jouf University, Sakaka, Al-Jouf, Kingdom of Saudi Arabia and [9]Faculty of Pharmacy and Health Sciences, University of Balochistan, Quetta, Pakistan

## Abstract

**Objective:** The aim of this study is to ascertain the psychological impacts of coronavirus disease (COVID-19) among the Pakistani health care workers (HCWs) and their coping strategies.
**Methods:** This web-based, cross-sectional study was conducted among HCWs (N = 398) from Punjab Province of Pakistan. The generalized anxiety scale (GAD-7), patient health questionnaire (PHQ-9), and Brief-COPE were used to assess anxiety, depression, and coping strategies, respectively.
**Results:** The average age of respondents was 28.67 years (SD = 4.15), with the majority being medical doctors (52%). Prevalences of anxiety and depression were 21.4% and 21.9%, respectively. There was no significant difference in anxiety and depression scores among doctors, nurses, and pharmacists. Females had significantly higher anxiety ($P = 0.003$) and depression ($P = 0.001$) scores than males. Moreover, frontline HCWs had significantly higher depression scores ($P = 0.010$) than others. The depression, not anxiety, score was significantly higher among those who did not receive the infection prevention training ($P = 0.004$). The most frequently adopted coping strategies were religious coping (M = 5.98, SD = 1.73), acceptance (M = 5.59, SD = 1.55), and coping planning (M = 4.91, SD = 1.85).
**Conclusion:** A considerable proportion of HCWs are having generalized anxiety and depression during the ongoing COVID-19 pandemic. Our findings call for interventions to mitigate mental health risks in HCWs.

Fighting at the forefronts, providing medical care to patients, health care workers (HCWs) are under constant threat of contracting COVID-19.[1] Data across the globe show that HCWs are increasingly being infected with COVID-19. In Pakistan, more than 10 000 HCWs have been infected with COVID-19, and many lost their lives to it.[2]

The continuous spread of COVID-19, risk of getting infection and transmitting it to loved ones, increased work load, physical exhaustion, shortage of personal protective equipment, and the need to make ethically difficult decisions on rationing of care can profoundly influence the physical as well as mental health of HCWs. This constant stress can trigger a variety of psychological manifestations such as anxiety, depression, sleep disturbances, panic attacks, posttraumatic stress symptoms, and helplessness.[3] People adopt various coping methods to deal with adversity or traumatic experiences. Positive coping can evoke positive emotions and behaviors leading to better responses to adversity, whereas negative coping styles are not ideal at managing anxiety and stress.[4]

To initiate necessary measures to protect the mental well-being and mitigate vulnerability of HCWs amid the COVID-19 pandemic, the current study was aimed to assess generalized anxiety and depression among Pakistani HCWs during the ongoing pandemic. Furthermore, we also evaluated coping strategies adopted by them to manage aforementioned psychological distress.

SDMPH
SOCIETY FOR DISASTER MEDICINE & PUBLIC HEALTH

## Methods

### Study Design, Settings, and Population

A web-based, cross-sectional survey was conducted among HCWs (doctors, nurses, and pharmacists) of the Punjab Province of Pakistan. Those who were unwilling to participate, could not understand the English language, health care assistants, and other hospital staff were excluded.

### Ethical Approval

This study was approved by the Research Ethics Committee of the Department of Pharmacy Practice, Faculty of Pharmacy, The University of Lahore (REC/DPP/FOP/17). An online informed consent was obtained from participants. There was no identifying information on the questionnaire. In addition, survey respondents were provided a clear explanation about the confidentiality of their responses.

### Sampling

The sample size for this study was calculated using the Daniel sample size equation: $n = Z^2P(1\text{-}P)/d^2$.[5] By keeping a 95% confidence interval, 50% expected proportion/prevalence, and 5% precision, a minimum sample of 384 HCWs was required. We used an exponential non-discriminative snowball sampling for data collection (April 15–May 20, 2020). The initial set of invitees included 10 health professionals (4 medical doctors, 3 pharmacists, and 3 nurses). This set of invitees forwarded the questionnaire to their colleagues/coworkers whom they considered suitable for the study, and this second set forwarded the questionnaire in the same way, and so on.

### Data Collection Tool

Google Forms were used to disseminate (via WhatsApp and Facebook messenger) the online self-completed questionnaire to assess the psychological effects of COVID-19 among HCWs and coping strategies adopted by them. As the medical, pharmacy, and nursing education in Pakistan is entirely English-medium, there was no need to translate the questionnaire into the Urdu language.[1,6,7] The questionnaire consisted of 2 parts (Supplementary file). Part I collected demographic data of the responders (eg, age, gender, occupation, hospital name, experience, duty during the pandemic), whereas Part II contained the below-mentioned scales to determine anxiety, depression, and coping strategies.

## Outcome Measure

We used generalized anxiety scale (GAD-7) to assess anxiety among respondents.[8] It had 7 items, each of which was scored 0 ("not at all") to 3 ("nearly every day"), providing a 0 to 21 score. The total score was categorized into 4 severity groups: minimal-none (≤ 4), mild (5–9), moderate (10–14), and severe (≥ 15). The patient health questionnaire (PHQ-9) was used to assess depression. It contained 9 items, each of which was scored 0 (not at all) to 3 (nearly every day), providing a 0–27 score. The total score was categorized into 5 severity groups: minimal-none (≤ 4), mild (5–9), moderate (10–14), moderately severe (15–19), and severe (≥ 20).[9] In the present study, respondents' achieving scores ≥ 10 on GAD-7 and PHQ-9 were considered as having anxiety and depression, respectively.

The Brief-COPE scale was used to assess the coping strategies adopted by HCWs during the COVID-19 pandemic.[10] It contained 28 items, each of which was scored from 1 ("I have not been doing this at all") to 4 ("I have been doing this a lot"). This scale explored the 14 coping methods: self-distraction, active coping, denial, substance use, use of emotional support, use of instrumental support, behavioral disengagement, venting, positive reframing, planning, humor, acceptance, religion, and self-blame. Possible scores for each subscale ranged from 2 to 8, with higher scores indicating a higher tendency to utilize the corresponding coping style.

### Data Analysis

All the data were entered and analyzed using IBM SPSS version 22 for Windows (IBM Corp, Armonk, NY).[11] Continuous data were presented as mean (M) and standard deviation (SD), whereas categorical data were expressed as number and percentages. Age was classified into young adulthood (18–35 years), middle age (36–55 years), and seniors (≥ 56 years).[12] Moreover, HCWs were grouped into frontline or second-line workers according to their duties during the pandemic. HCWs who were directly engaged (diagnoses, treatment, and care of patients) with COVID-19 patients were defined as frontline workers.[13] The independent t-test and analysis of variance (ANOVA) test were performed, where applicable, to compare difference of anxiety, depression, and coping strategy scores among demographic variables. Welch's ANOVA was performed instead of the classic ANOVA when the assumption of homogeneity of variances was violated. Moreover, for trichotomous or polychotomous variables, a series of post-hoc analysis (Tukey's HSD and Games-Howell test, where applicable) were performed to assess significance among intergroup variables. A $P$-value of less than 0.05 was considered statistically significant.

## Results

A total of 428 HCWs responded to the survey, with 398 HCWs providing consent and being included in the study (participation rate 93%). Demographics of the respondents are shown in Table 1. Mean age of respondents was 28.67 years (SD = 4.15), with female preponderance (56%). About 52% were physicians, whereas nurses and pharmacists accounted for 33.4% and 15.1%, respectively. Around 33% (isolation wards/quarantine facility = 28% and COVID-19 intensive care units = 5%) were directly engaged in managing COVID-19 patients, and 32.9% reported they have received COVID-19 infection prevention training. There was no significant difference ($P > 0.05$) of demographics (age, gender, experience, and occupation) among frontline and second-line HCWs. Additionally, no statistically significant difference of demographics was seen among HCWs who received infection prevention training and those who did not.

The mean anxiety and depression scores were 6.83 (SD = 4.44) and 6.72 (SD = 5.14), respectively. The frequencies of respondents having none–mild, moderate, and severe anxiety were 78.7%, 13.1%, and 8.3%, respectively. About 22% met the PHQ-9 scale's criteria for depression (minimal–none 35.9%, mild 42.2%, moderate 12.8%, moderately severe 7.3%, and severe 1.8%). A comparison of anxiety and depression scores among respondents' demographics is shown in Table 1. Female respondents were found to have significantly higher anxiety ($P = 0.003$) and depression ($P = 0.001$) scores than male respondents. Significant differences of anxiety score were observed in young and middle age HCWs ($P = 0.032$). Depression score was significantly higher among frontline workers and those who did not receive infection prevention training (Table 2).

**Table 1.** Anxiety and depression assessments based on respondents' demographics

| Variable | N (%) | Mean score ± SD | | | |
|---|---|---|---|---|---|
| | | Anxiety | *P*-value | Depression | *P*-value |
| **Age** | | | **0.032** | | 0.191 |
| Young adulthood | 378 (95.0) | 6.94 ± 4.49 | | 6.80 ± 5.20 | |
| Middle age | 20 (5.0) | 4.75 ± 2.81 | | 5.25 ± 3.29 | |
| **Gender** | | | **0.003** | | **0.001** |
| Male | 183 (46.0) | 6.10 ± 4.28 | | 5.81 ± 5.13 | |
| Female | 215 (54.0) | 7.44 ± 4.50 | | 7.48 ± 5.02 | |
| **Occupation** | | | 0.349 | | 0.255* |
| Doctor | 205 (51.5) | 6.52 ± 4.38 | | 6.60 ± 5.45 | |
| Nurse | 133 (33.4) | 7.23 ± 4.27 | | 7.22 ± 4.06 | |
| Pharmacist | 60 (15.1) | 6.98 ± 5.01 | | 6.00 ± 6.03 | |
| **Experience (years)** | | | 0.393 | | 0.270 |
| ≤ 5 | 302 (75.9) | 6.67 ± 4.37 | | 6.62 ± 5.19 | |
| 6-10 | 77 (19.3) | 7.44 ± 4.75 | | 7.39 ± 5.20 | |
| > 10 | 19 (4.8) | 6.89 ± 4.37 | | 5.42 ± 3.42 | |

*Welch's ANOVA was used instead of classic ANOVA, as the assumption of homogeneity of variances was violated.

**Table 2.** Analysis of anxiety and depression among frontline and second-line workers

| Psychological effects | Mean score ± SD | | | | | |
|---|---|---|---|---|---|---|
| | | | | Infection prevention training for COVID-19 | | |
| | Frontline HCWs | Second line HCWs | *P*-value | Yes | No | *P*-value |
| Anxiety | 7.12 ± 4.84 | 6.68 ± 4.24 | 0.372 | 6.61 ± 4.26 | 6.93 ± 4.54 | 0.498 |
| Depression | 7.74 ± 5.98 | 6.20 ± 4.58 | **0.010** | 5.71 ± 4.58 | 7.21 ± 2.33 | **0.004** |

HCWs, health care workers.

As shown in Table 3, the mean score was highest for religious coping (M = 5.98, SD = 1.73) followed by acceptance (M = 5.59, SD = 1.55) and coping planning (M = 4.91, SD = 1.85), whereas it was the lowest for substance use (M = 2.59, SD = 1.06) followed by self-blame (M = 2.90, SD = 1.29). The relationship of demographics with various coping strategies is described in Table 3. There was no significant difference of coping styles among age categories except for self-blame ($P = 0.041$). Females were observed to have significantly higher scores for behavioral disengagement ($P = 0.040$), venting ($P = 0.015$), and religious/spiritual coping ($P = 0.003$) than males. Amongst HCWs categories, statistically significant difference was seen for self-distraction ($P = 0.023$), denial ($P < 0.001$), substance use ($P = 0.003$), seeking emotional support ($P = 0.033$), and behavioral disengagement ($P = 0.030$). Furthermore, as shown in Table 4, there was no significant difference of all the coping styles among frontline and second-line HCWs except for denial ($P = 0.033$) and positive reframing ($P = 0.012$).

A subgroup analysis was carried out to determine significance among intergroup variables. Nurses had significantly higher coping style scores on denial ($P < 0.001$), substance use ($P = 0.001$), and behavioral disengagement ($P = 0.046$) than doctors, and higher scores on emotional support ($P = 0.049$) than pharmacists.

## Discussion

A recent position paper has underscored the dire need of quality data on the psychological effects of the COVID-19 pandemic across the whole population and vulnerable groups, and on brain function, cognition, and mental health of patients with COVID-19.[14] Since HCWs are amongst the high-risk groups for getting COVID-19 and are particularly vulnerable to a variety of mental health problems, the present study was undertaken to provide insight on the impact of the COVID-19 pandemic on mental well-being of Pakistani doctors, nurses, and pharmacists fighting at the forefronts. Our findings revealed that prevalences of anxiety and depression were 21.4% and 21.9%, respectively. A recent review article pooled the data on the prevalence of depression, anxiety, and posttraumatic stress disorder (PTSD) from 65 studies involving 97 333 health care workers across 21 countries.[15] The calculated pooled prevalence of depression was 21.7% (95% CI: 18.3-25.2); of anxiety, 22.1% (95% CI: 18.2-26.3); and of PTSD, 21.5% (95% CI: 10.5-34.9).

The European and American quantitative studies have shown moderate and high levels of stress, anxiety, depression, sleep disturbance, and burnout, with diverse coping strategies and more frequent and intense symptoms among women and nurses, without conclusive results by age.[16] Lai et al. observed that the nurses, women, frontline workers, and those working in Wuhan, China, had more severe degrees of all measurements of mental health symptoms (depression, anxiety, insomnia, and distress) than other HCWs.[17] Consistent with these findings, we also found that females had significantly higher depression and generalized anxiety scores than males. Frontline workers and those who did not receive COVID-19 infection prevention training had significantly greater depression scores than others. However, contrary to

**Table 3.** Coping strategies adopted by the study participants

| Variable | Mean ± SD | | | | | | | | | | | | | |
|---|---|---|---|---|---|---|---|---|---|---|---|---|---|---|
| | Self-distraction | Active coping | Denial | Substance use | Emotional support | Informational support | Behavioral disengagement | Venting | Positive reframing | Planning | Humor | Acceptance | Religion | Self-blame |
| **Overall** | 4.41 ± 1.53 | 4.59 ± 1.69 | 3.03 ± 1.23 | 2.59 ± 1.06 | 4.18 ± 1.79 | 4.48 ± 1.89 | 3.03 ± 1.49 | 3.74 ± 1.53 | 4.54 ± 1.62 | 4.91 ± 1.85 | 3.12 ± 1.43 | 5.59 ± 1.55 | 5.98 ± 1.73 | 2.90 ± 1.29 |
| **Age** | | | | | | | | | | | | | | |
| Young adulthood | 4.43 ± 1.55 | 4.60 ± 1.69 | 3.02 ± 1.23 | 2.57 ± 1.04 | 4.18 ± 1.79 | 4.48 ± 1.89 | 3.05 ± 1.52 | 3.77 ± 1.54 | 4.53 ± 1.63 | 4.93 ± 1.83 | 3.11 ± 1.43 | 5.60 ± 1.55 | 6.00 ± 1.74 | 2.93 ± 1.28* |
| Middle age | 4.05 ± 1.19 | 4.30 ± 1.63 | 3.10 ± 1.25 | 2.95 ± 1.36 | 4.15 ± 1.87 | 4.35 ± 1.87 | 2.70 ± 0.92 | 3.20 ± 1.32 | 4.60 ± 1.63 | 4.40 ± 2.26 | 3.30 ± 1.49 | 5.40 ± 1.54 | 5.75 ± 1.55 | 2.45 ± 0.95 |
| **Gender** | | | | | | | | | | | | | | |
| Male | 4.49 ± 1.56 | 4.71 ± 1.63 | 2.92 ± 1.23 | 2.54 ± 1.05 | 4.01 ± 1.75 | 4.32 ± 1.82 | 2.87 ± 1.32* | 3.54 ± 1.47* | 4.62 ± 1.70 | 4.89 ± 1.85 | 3.18 ± 1.47 | 5.62 ± 1.62 | 5.71 ± 1.72† | 2.85 ± 1.31 |
| Female | 4.34 ± 1.51 | 4.48 ± 1.73 | 3.12 ± 1.22 | 2.63 ± 1.06 | 4.33 ± 1.82 | 4.61 ± 1.94 | 3.17 ± 1.61 | 3.92 ± 1.57 | 4.47± 1.56 | 4.93 ± 1.86 | 3.07 ± 1.40 | 5.57 ± 1.49 | 6.22 ± 1.71 | 2.95 ± 1.23 |
| **Occupation** | | | | | | | | | | | | | | |
| Doctor | 4.52 ± 1.57* | 4.60 ± 1.65 | 2.81 ± 1.19¥ | 2.41 ± 0.87† | 4.08 ± 1.80* | 4.33 ± 1.87 | 2.84 ± 1.32* | 3.66 ± 1.51 | 4.57 ± 1.70 | 4.81 ± 1.86 | 3.09 ± 1.42 | 5.71 ± 1.54 | 5.90 ± 1.63 | 2.91 ± 1.33 |
| Nurse | 4.13 ± 1.40 | 4.40 ± 1.79 | 3.35 ± 1.26 | 2.84 ± 1.25 | 4.49 ± 1.87 | 4.76 ± 1.92 | 3.24 ± 1.72 | 3.90 ± 1.58 | 4.49 ± 1.48 | 5.09 ± 1.88 | 3.24 ± 1.48 | 5.34 ± 1.49 | 6.15 ± 1.83 | 2.96 ± 1.16 |
| Pharmacist | 4.65 ± 1.60 | 4.93 ± 1.54 | 3.03 ± 1.13 | 2.62 ± 1.02 | 3.83 ± 1.50 | 4.35 ± 1.87 | 3.23 ± 1.44 | 3.67 ± 1.49 | 4.55 ± 1.68 | 4.83 ± 1.76 | 2.97 ± 1.38 | 5.75 ± 1.66 | 5.92 ± 1.85 | 2.77 ± 1.28 |
| **Experience** | | | | | | | | | | | | | | |
| ≤ 5 years | 4.45 ± 1.56 | 4.56 ± 1.65 | 2.99 ± 1.25 | 2.53 ± 1.04 | 4.08 ± 1.71 | 4.47 ± 1.93* | 3.00 ± 1.39 | 3.75 ± 1.54 | 4.54 ± 1.65 | 4.87 ± 1.83 | 3.16 ± 1.47 | 5.59 ± 1.54 | 5.93 ± 1.71 | 2.95 ± 1.32 |
| 6-10 years | 4.34 ± 1.50 | 4.78 ± 1.84 | 3.16 ± 1.17 | 2.74 ± 1.08 | 4.45 ± 2.02 | 4.48 ± 1.79 | 3.22 ± 1.87 | 3.73 ± 1.48 | 4.43 ± 1.56 | 5.09 ± 1.90 | 2.97 ± 1.29 | 5.66 ± 1.55 | 6.10 ± 1.85 | 2.79 ± 1.12 |
| > 10 years | 4.11 ± 1.24 | 4.16 ± 1.54 | 3.00 ± 1.00 | 2.95 ± 1.13 | 4.68 ± 2.03 | 4.53 ± 1.71 | 2.79 ± 1.31 | 3.68 ± 1.67 | 4.89 ± 1.49 | 4.74 ± 2.05 | 3.05 ± 1.43 | 5.42 ± 1.71 | 6.32 ± 1.57 | 2.63 ± 1.07 |

*$P < 0.05$; †$P < 0.01$; ¥$P < 0.001$.

**Table 4.** Comparison of coping strategies between frontline and second-line health care workers

| | Mean ± SD | | | | | |
|---|---|---|---|---|---|---|
| | | | | Infection prevention training for COVID-19 | | |
| Coping strategies | Frontline HCWs | Second-line HCWs | *P*-value | Yes | No | *P*-value |
| Self-distraction | 4.48 ± 1.55 | 4.37 ± 1.52 | 0.509 | 4.52 ±1.55 | 4.36 ± 1.52 | 0.318 |
| Active coping | 4.78 ± 1.69 | 4.49 ± 1.68 | 0.100 | 4.66 ± 1.70 | 4.55 ± 1.68 | 0.557 |
| Denial | 3.21 ± 1.23 | 2.93 ± 1.18 | **0.033** | 2.95 ± 1.10 | 3.06 ± 1.29 | 0.420 |
| Substance use | 2.74 ± 1.19 | 2.51 ± 0.97 | 0.050 | 2.53 ± 0.99 | 2.61 ± 1.09 | 0.479 |
| Emotional support | 4.42 ± 1.83 | 4.06 ± 1.77 | 0.058 | 4.40 ± 1.09 | 4.07 ± 1.73 | 0.092 |
| Informational support | 4.70 ± 1.89 | 4.37 ± 1.88 | 0.097 | 4.63 ± 2.02 | 4.40 ± 1.82 | 0.290 |
| Behavioral disengagement | 3.12 ± 1.54 | 2.99 ± 1.47 | 0.407 | 3.19 ± 1.72 | 2.96 ± 1.37 | 0.171 |
| Venting | 3.90 ± 1.54 | 3.66 ± 1.52 | 0.144 | 3.86 ± 1.61 | 3.69 ± 1.49 | 0.279 |
| Positive reframing | 4.83 ± 1.64 | 4.39 ± 1.60 | **0.012** | 4.74 ± 1.72 | 4.44 ± 1.57 | 0.081 |
| Planning | 5.09 ± 1.80 | 4.82 ± 1.87 | 0.162 | 5.17 ± 1.89 | 4.78 ± 1.82 | **0.049** |
| Humor | 3.23 ± 1.59 | 3.07 ± 1.35 | 0.328 | 3.09 ± 1.40 | 3.13 ± 1.45 | 0.778 |
| Acceptance | 5.66 ± 1.60 | 5.58 ± 1.52 | 0.532 | 5.79 ± 1.66 | 5.50 ± 1.49 | 0.081 |
| Religion | 6.09 ± 1.60 | 5.93 ± 1.76 | 0.391 | 6.24 ± 1.70 | 5.86 ± 1.74 | **0.036** |
| Self-blame | 2.85 ± 1.20 | 2.93 ± 1.30 | 0.541 | 2.95 ± 1.28 | 2.88 ± 1.27 | 0.644 |

HCWs, health care workers.

previous studies,[16–18] nurses were not found to have a higher degree of anxiety and depression than other HCWs. Similar to the results of Elbay et al.[19] and Rossi et al.,[13] older in years was a protective factor for anxiety in our study.

Regarding the coping strategies, Eisenberg et al. have reported 2 major components, namely "avoidant coping" and "approach coping" in the Brief-COPE.[4] The humor and religion subscales were not included in either component as they did not exclusively load on either of the abovementioned components. Avoidant coping was described by the subscales of denial, substance use, venting, behavioral disengagement, self-distraction, and self-blame. These coping styles are not ideal at managing anxiety and stress.[4] On the other hand, approach coping is characterized by the subscales of active coping, positive reframing, planning, acceptance, seeking emotional, and informational support. Compared to avoidant coping, these have been associated with better responses to adversity, including adaptive practical adjustment, better physical health outcomes, and more stable emotional responding. Meyer categorized the strategies measured by the Brief-COPE into maladaptive and adaptive coping.[20] In addition to other subscales, religion and humor were considered as adaptive coping. In the present study, it was encouraging to see that our respondents' scores for positive coping strategies were greater than avoidant or maladaptive coping (Table 3). Moreover, we found a moderate positive association between maladaptive coping and anxiety ($r = 0.324$; $P < 0.001$) and depression ($r = 0.377$; $P < 0.001$). Adaptive coping had a low degree association with anxiety ($r = 0.269$; $P < 0.001$) and depression ($r = 0.146$; $P = 0.003$). Our findings regarding the commonly used coping strategies to deal with stress, anxiety, and depression during the COVID-19 pandemic were consistent with the results of an earlier study among medical, pharmacy, allied health sciences, and other university students.[21]

Although we achieved our study objectives, our study had a few shortcomings. First, this study was conducted among HCWs from the Punjab Province of Pakistan, so our findings may not be generalized to the other provinces of the country. Second, this was an online survey administered using snowball sampling method, therefore, the issue of coverage error, referral bias, and selective participation may exist. Third, we computed the response rate by dividing the number of HCWs who agreed to participate ($n = 398$) with the total number of respondents ($n = 428$). As IP addresses and/or cookies were not used to assign a unique user identifier, we could not determine exact response rates (view rate [unique survey visitors divided by unique site visitors] and participation rate [agreed to participate divided by unique first survey page visitors]).[22] However, multiple entries from the same individual were prevented by checking "limit to one response" option in the "Google Forms" settings. Fourth, we used a self-administered questionnaire so disadvantages associated with self-report data (eg, introspective ability, response bias, and sampling bias) may be present. Fifth, the data of non-participants were not available for comparison. Last, the clinical assessment for the diagnosis of depression and generalized anxiety disorders as per criteria of the Diagnostic and Statistical Manual of Mental Disorders was not done. However, our findings offer valuable insight about the psychological impact of COVID-19 among frontline medical forces and their coping strategies.

## Conclusions

Our study revealed that 21.4% and 21.9% of health care professionals had generalized anxiety and depression, respectively, during the first wave of the COVID-19 pandemic in Pakistan. Most frequently adopted coping strategies were religious/spiritual coping, acceptance, planning, active coping, and positive reframing. Findings draw attention to proactively take steps to protect the mental well-being, enhance resilience, and mitigate vulnerability of health care forces during the COVID-19 pandemic.

**Supplementary material.** To view supplementary material for this article, please visit https://doi.org/10.1017/dmp.2022.4

**Acknowledgments.** Authors are grateful to all of the health care workers for sparing their valuable time to fill out the study instrument. Moreover, as it was a COVID-19-related research article, this manuscript was uploaded as a preprint on the *medRxiv*.[23]

**Author contributions.** MS, TMK, and MHR conceived and designed the study. MS, ZUM, and NA conducted literature review and designed the study questionnaire. MS, NS, TMK, and HT conducted statistical analyses, with additional advice regarding analyses contributed by KH, THM, KS, FS, and YHK. MS drafted the manuscript, and all authors contributed to editing it and approved the final manuscript.

**Conflict(s) of interest.** None declared.

## References

1. **Salman M, Mustafa Z, Asif N, *et al.*** Knowledge, attitude and preventive practices related to COVID-19 among health professionals of Punjab province of Pakistan. *J Infect Dev Ctries.* 2020;14(7):707-712.
2. Death Toll of Healthcare Workers in Pakistan Due to COVID-19 Reaches 100. ANI. Published 2020. Accessed April 28, 2020. https://www.aninews.in/news/world/asia/death-toll-of-healthcare-workers-in-pakistan-due-to-covid-19-reaches-10020201201185445/
3. **Pappa S, Ntella V, Giannakas T, *et al.*** Prevalence of depression, anxiety, and insomnia among healthcare workers during the COVID-19 pandemic: a systematic review and meta-analysis. *Brain Behav Immun*. 2020;88:901-907. https://doi.org/10.1016/j.bbi.2020.05.026
4. **Eisenberg SA, Shen BJ, Schwarz ER, Mallon S.** Avoidant coping moderates the association between anxiety and patient-rated physical functioning in heart failure patients. *J Behav Med.* 2012;35(3):253-261. https://doi.org/10.1007/s10865-011-9358-0
5. **Daniel WW**, Ed. *Biostatistics: a foundation for analysis in the health sciences*. 7th ed. John Wiley & Sons; 1999.
6. **Salman M, Mustafa ZU, Asif N, *et al.*** Knowledge, attitude and preventive practices related to COVID-19: a cross-sectional study in two Pakistani university populations. *Drugs Ther Perspect.* 2020;36(7):319-325.
7. **Salman M, Mustafa ZU, Rao AZ, *et al.*** Serious inadequacies in high alert medication-related knowledge among Pakistani nurses: findings of a large, multicenter, cross-sectional survey. *Front Pharmacol.* 2020;11:1026.
8. **Spitzer RL, Kroenke K, Williams JB.** Generalized anxiety disorder 7-item (GAD-7) scale. *Arch Intern Med.* 2006;166:1092-1097.
9. **Kroenke K, Spitzer RL, Williams JB.** The PHQ-9: validity of a brief depression severity measure. *J Gen Intern Med.* 2001;16(9):606-613. https://doi.org/10.1046/j.1525-1497.2001.016009606.x
10. **Carver CS.** You want to measure coping but your protocol' too long: consider the brief cope. *Int J Behav Med.* 1997;4(1): 92-100. https://doi.org/10.1207/s15327558ijbm0401_6
11. IBM SPSS Statistics for Windows, Version 22.0. IBM Corp; 2013.
12. **Petry NM.** A comparison of young, middle-aged, and older adult treatment-seeking pathological gamblers. *Gerontologist.* 2002;42(1):92-99
13. **Rossi R, Socci V, Pacitti F, *et al.*** Mental health outcomes among frontline and second-line health care workers during the coronavirus disease 2019 (COVID-19) pandemic in Italy. *JAMA.* 2020;3(5):e2010185.
14. **Holmes EA, O'Connor RC, Perry VH, *et al.*** Multidisciplinary research priorities for the COVID-19 pandemic: a call for action for mental health science. *Lancet Psychiatry.* 2020;7(6):547-560. https://doi.org/10.1016/S2215-0366(20)30168-1
15. **Li Y, Scherer N, Felix L, Kuper H.** Prevalence of depression, anxiety and post-traumatic stress disorder in health care workers during the COVID-19 pandemic: a systematic review and meta-analysis. *PLoS One.* 2021;16(3): e0246454.
16. **Danet AD.** Psychological impact of COVID-19 pandemic in Western frontline healthcare professionals. A systematic review. *Med Clín.* 2021;156:449-458.
17. **Lai J, Ma S, Wang Y, *et al.*** Factors associated with mental health outcomes among health care workers exposed to coronavirus disease 2019. *JAMA.* 2020;3(3):e203976.
18. **Hayat K, Arshed M, Fiaz I, *et al.*** Impact of COVID-19 on the mental health of healthcare workers: a cross-sectional study from Pakistan. *Public Health Front.* 2021;9:410.
19. **Elbay RY, Kurtulmuş A, Arpacıoğlu S, Karadere E.** Depression, anxiety, stress levels of physicians and associated factors in COVID-19 pandemics. *Psychiatry Res.* 2020;290:113130.
20. **Meyer B.** Coping with severe mental illness: relations of the Brief COPE with symptoms, functioning, and well-being. *J Psychopathol Behav Assess.* 2001;23(4):265-277. https://doi.org/10.1023/A:1012731520781
21. **Salman M, Asif N, Mustafa ZU, *et al.*** Psychological impairment and coping strategies during the COVID-19 pandemic among students in Pakistan: a cross-sectional analysis. *Disaster Med Public Health Prep*. Published online October 22, 2020. Accessed October 23, 2020. https://doi.org/10.1017/dmp.2020.397
22. **Eysenbach G.** Improving the quality of Web surveys: the Checklist for Reporting Results of Internet E-Surveys (CHERRIES). *J Med Internet Res.* 2004;6(3):e34.
23. **Salman M, Raza MH, Mustafa ZU, *et al.*** The psychological effects of COVID-19 on frontline healthcare workers and how they are coping: a web-based, cross-sectional study from Pakistan. *medRxiv*. Preprint posted online June 5, 2020. Accessed June 5, 2020. https://doi.org/10.1101/2020.06.03.20119867
