# Supplementary material for: Mental health status and related factors influencing healthcare workers during the COVID-19 pandemic: A systematic review and meta-analysis
Source: PLoS One. 2024 Jan 19;19(1):e0289454. doi: 10.1371/journal.pone.0289454 (PMC10798549; doi:10.1371/journal.pone.0289454)
Supplement: S1 Data — (ZIP) [file pone.0289454.s011.zip › literatures/154.pdf]

Saudi Pharmaceutical Journal 30 (2022) 1009–1017

Contents lists available at ScienceDirect

Saudi Pharmaceutical Journal

journal homepage: www.sciencedirect.com

Original article

Mental health, burnout and resilience in community pharmacists during the COVID-19 pandemic: A cross-sectional study

Zeana Samir AlKudsi [a,1], Nadin Hany Kamel [a,1], Alla El-Awaisi [a], Mujahed Shraim [b], Maguy Saffouh El Hajj [a,*]

[a] College of Pharmacy, QU Health, Qatar University, Doha, Qatar
[b] College of Health Sciences, QU Health, Qatar University, Doha, Qatar

ARTICLE INFO

Article history:
Received 6 February 2022
Accepted 26 April 2022
Available online 30 April 2022

Keywords:
Mental health
Burnout
Resilience
Depression
Anxiety
Stress
Community pharmacist
Qatar
Middle East
COVID-19

ABSTRACT

Background: The study aimed to assess burnout, resilience, and levels of depression, anxiety, stress and fear among community pharmacists during the pandemic, and examine if fear of COVID-19 is associated with these outcomes of interest.

Methods: A cross-sectional survey of community pharmacists in Qatar was conducted. Pharmacists' burnout was measured by the Maslach Burnout Inventory: Human Services Survey for Medical Personnel (MBI-HSS™ for MP-Mindgarden). Resilience was assessed using the Connor-Davidson Resilience Scale-10 (CD-RISC-10). Depression, anxiety, and stress were measured by the Depression, Anxiety, and Stress Scale (DASS-21). Fear of COVID-19 was assessed by the Fear of COVID-19 Scale (FCV-19S).

Results: 256 respondents completed the survey and were included in the final study analysis (response rate: 42.7%). Overall, participants reported a moderate level of burnout as illustrated in the mean scores of the three burnout dimensions; 20.54 (SD = 12.37) for emotional exhaustion, 6.76 (SD = 6.22) for depersonalization, and 36.57 (SD = 9.95) for personal accomplishment. Moreover, depression, anxiety and stress were reported by 44.8%, 53.2% and 25.4% of particiants respectively. Participants had shown moderate resilience (mean score: 27.64 (SD = 8.31)) and their mean score fear of COVID19 was 15.67 (SD = 6.54). Fear of COVID-19 was a statistically significant and an independent predictor of depression, anxiety, and stress levels.

Conclusions: The pharmacists experienced moderate burnout but moderate resilience, which indicates their potential to overcome difficulties. Future interventions at the personal, national and organizational levels are required to enhance the pharmacists' wellbeing by decreasing stress, improving self-efficacy and resilience, and preventing burnout.

© 2022 The Author(s). Published by Elsevier B.V. on behalf of King Saud University. This is an open access article under the CC BY license (http://creativecommons.org/licenses/by/4.0/).

1. Introduction

Coronavirus Disease (COVID-19) has spread globally since 2019 and has been declared as a pandemic by the World Health Organization (WHO) on March 11, 2020 (Dhama et al., 2020; Han et al., 2020; Lu et al., 2020). COVID-19 is caused by the severe acute respiratory syndrome coronavirus 2 (SARS-CoV-2) which, as of April 13, 2022, has affected over 500 million persons globally and has caused over six million deaths (WHO, 2022). In the State of Qatar, 362,007 have tested positive for COVID-19 as of April 4, 2022 (Qatar Ministry of Public Health, 2022). This international health crisis has put the entire healthcare system under high pressure compromising the wellbeing of frontline healthcare workers, as new cases and exacerbations of old cases of depression, anxiety, physical and mental fatigue, stress, and burnout have been witnessed among healthcare providers (AlAteeq et al., 2020, Adams and Walls, 2020; Alshekaili et al., 2020; Ghahramani et al., 2021, Hu et al., 2020; Lai et al., 2020; Panagioti et al., 2018; Pappa et al., 2020; Şahin et al., 2020). A systematic review and meta-analysis of burnout among healthcare workers (HCWs) during COVID-19 included 13 studies and reported a pooled overall

* Corresponding author.
E-mail addresses: za1609629@student.qu.edu.qa (Z. Samir AlKudsi), nk1703048@student.qu.edu.qa (N. Hany Kamel), elawaisi@qu.edu.qa (A. El-Awaisi), mshraim@qu.edu.qa (M. Shraim), maguyh@qu.edu.qa (M. Saffouh El Hajj).

[1] Both authors contributed equally to the study.

Peer review under responsibility of King Saud University.

ELSEVIER Production and hosting by Elsevier

https://doi.org/10.1016/j.jsps.2022.04.015
1319-0164/© 2022 The Author(s). Published by Elsevier B.V. on behalf of King Saud University.
This is an open access article under the CC BY license (http://creativecommons.org/licenses/by/4.0/).

prevalence of burnout of 52% (Ghahramani et al., 2021). Papa et al conducted a systematic review and meta-analysis to synthesize existing evidence on the prevalence of depression, anxiety and insomnia among HCWs during the COVID-19 outbreak. Anxiety was assessed in 12 studies, with a pooled prevalence of 23.2% and depression in 10 studies, with a prevalence rate of 22·8% (Pappa et al., 2020). Moreover, a study conducted in in Turkey among HCWs reported that 77.6%, 60.2% and 50.4% of HCWs exhibited depression, anxiety and distress respectively during the COVID-19 pandemic (Şahin et al., 2020). In another study in Oman among frontline HCWs, 32.3%, 34.1%, 23.8% and 18.5% were reported to have depression, anxiety, stress and insomnia, respectively during the COVID-19 pandemic (Alshekaili et al., 2020). Alateeq et al explored depression and anxiety levels among HCWs during the COVID-19 outbreak in Saudi Arabia, over 50% had depressive disorder (55.2%), and generalized anxiety disorder (51.4%) (AlAteeq et al., 2020).

Burnout is a syndrome that has gained increased attention recently especially after the COVID-19 outbreak (Fessell and Cherniss, 2020). Burnout has several definitions and is assessed using a variety of methods. The World Health Organization defines burnout as chronic workplace stress (WHO, 2019). Moreover, Christina Maslach an American social psychologist and professor emerita of psychology at the University of California defines burnout as a psychological syndrome characterized by three dimensions including emotional exhaustion, depersonalization, and low personal accomplishment (Maslach et al., 1997). Emotional exhaustion (EE) describes feelings of being emotionally overextended and exhausted by work (Maslach and Jackson, 1981; Maslach et al., 1997). Depersonalization (DP) designates unfeeling and negative behaviors towards recipients of one's care and detachment from caring. The final aspect of burnout is a low personal accomplishment (PA) which is characterized by feelings of being incompetent and unable to fulfill the job responsibilities (Maslach and Jackson, 1981; Maslach et al., 1997). The most widely used gold standard measure of burnout is the Maslach Burnout Inventory (MBI) (Maslach and Jackson, 1981; Maslach et al., 1997; Whatmore, 2000).

Physical and emotional stress, anxiety depression and burnout among healthcare professionals are associated with negative physical, psychological, and occupational consequences (Parker and Kulik, 1995). These consequences include job dissatisfaction, poor quality of care, an increase in medical errors, a high prevalence of absenteeism as well as a high chance of giving up the profession (Parker and Kulik, 1995). Burnout syndrome has also been found to decrease motivation and performance (Parker and Kulik, 1995), to cause depression (Bianchi et al., 2015), sleep disturbances (Ekstedt et al., 2006; Vela-Bueno et al., 2008), anxiety (Ding et al., 2014), fatigue (Ekstedt et al., 2006), alcohol abuse (Oreskovich et al., 2012), cardiovascular diseases (May et al., 2014; de Vente et al., 2015), and suicidal thoughts (Shanafelt et al., 2011).

On the other hand, promoting resilience in healthcare providers is very essential (Fertleman and Carroll, 2013; Bowden et al., 2015). Resilience is defined by the American Psychological Association as the "process of adapting and the ability to bounce back when facing adversity, tragedy, trauma, or stress (American Psychological Association, 2002)." Resilience has a complex nature and is determined by a mixture of personal, social and cultural factors (Southwick et al., 2014). Resilience can be nurtured and strengthened with training and education (Jackson et al., 2007). Developing strategies to improve resilience and integrating them in the healthcare system can help healthcare providers to build strength and confidence, and to mitigate burnout and stress instead of looking at deficits and weaknesses (American Psychological Association, 2002).

Burnout in the pharmacy profession has been assessed by several previous studies pre-COVID-19. A study conducted in the United States (US) indicated that 53.2% of health-system pharmacists have a high level of burnout on at least one subscale of the MBI tool (Durham et al., 2018). Community pharmacists are also at high risk of burnout. A cross-sectional Turkish study by Calgan et al on community pharmacists revealed that 71.3% had high levels of inefficacy or reduced personal accomplishment (Calgan et al., 2011). Another study on community pharmacists in France found that 56.2% of respondents to the MBI questionnaire experienced burnout syndrome and 10.5% of them had severe burnout (Balayssac et al., 2017). In addition, a Serbian study by Jočić and Krajnović showed that 60.3% and 55.8% of community pharmacists had a high degree of anxiety and stress levels respectively and 44.4% suffered from a high level of burnout syndrome (Jocic and Krajnovic, 2014). Furthermore, 74.9% of community pharmacists experience burnout in at least one of the three subscales of the MBI in a study in the US (Patel et al., 2021).

Moreover, the COVID-19 pandemic has put community pharmacists at an even higher risk of experiencing stress and other mental health problems. The induced diversion of patients away from hospitals and the fear of contracting the virus had made community pharmacists the most accessible healthcare providers during the pandemic (Elbeddini et al., 2020). Community pharmacists have played a great role in fighting and preventing the COVID-19 pandemic from spreading. Their responsibilities have included: advising, counseling, and educating the community on precautionary measures, making the appropriate referral for suspected cases, providing the public with pharmaceutical care, medications, and personal protective equipment (PPE) in addition to managing minor ailments to decrease unnecessary hospital visits (Ung, 2020; Al-Quteimat and Amer, 2021). For community pharmacists to assume these new and demanding roles, various interventions should be implemented to improve their work engagement and resilience and decrease their work pressure and stress (Ung, 2020).

To our knowledge, no information is available in relation to mental health, burnout and resilience among community pharmacists in Qatar during COVID-19. Therefore, the primary objectives of the study were to assess the level of burnout, depression, anxiety, stress, fear, and resilience of community pharmacists during the COVID-19 pandemic. The secondary objectives were to assess the relationship between fear of COVID-19 with the different mental health outcomes among community pharmacists.

2. Methods

2.1. Study design and study participants

A cross-sectional survey of community pharmacists was conducted in Qatar. Pharmacists who were licensed and practicing in community pharmacies in Qatar were eligible to participate in the study.

2.2. Questionnaire implementation

Qatar Ministry of Public Health (MoPH) database was used to randomly select a sample of 385 community pharmacists in Qatar using the random generator function in Statistical Package for Social Sciences (SPSS®) version 27. Selected pharmacists were contacted by phone to invite them to participate in the study and to seek their consent for participation. An email containing the questionnaire URL through the SurveyMonkey® platform (https://www.surveymonkey.com) was sent to consenting pharmacists. Participants were informed of the details of this study including

the length of the questionnaire and the time needed for its completion, storage of data, study purpose and investigators in the study informed consent which was accessible in the first section of the questionnaire. Three survey reminders were sent via email at 2-week intervals. No incentives were given to pharmacists to fill the questionnaire.

2.3. Sample size calculation

Approximately 1200 community pharmacists practice in Qatar. Considering a margin of error of 5% and a confidence level of 95%, and an estimated prevalence of 50% of burnout among community pharmacists in Qatar, the minimal sample size was calculated as 385 pharmacists. To account for potential loss of follow-ups 600 pharmacists were invited to participate.

2.4. Survey instrument

A self-administered questionnaire, that was kept open from October 2020 to February 2021, was used to solicit anonymous responses from pharmacists. An extensive literature review was conducted to search for and select the best, most suitable, reliable, and validated questionnaires/scales that can answer the study objectives. The draft questionnaire was evaluated for face and content validity by experienced pharmacy faculty at Qatar University, and among 15 community pharmacists for content clarity, the relevance of items, and the time needed to complete the survey. Modifications were made to the questionnaire accordingly. The final questionnaire was written in English and comprised 31 closed and open-ended questions that could be completed within an average of 20–25 min. The questionnaire had six sections. The first section of the questionnaire collected information about the pharmacists' sociodemographic and practice characteristics including age, gender, marital status, medical history, pharmacy position and type, living arrangements among others. The second section of the questionnaire contained questions in relation to COVID-19 including the history of exposure to COVID-19 infected patients, history of contracting the virus including the severity of symptoms, satisfaction with safety precautions applied in the pharmacy, and salary changes during the pandemic. Pharmacists' burnout was measured in the third section by the Maslach Burnout Inventory: Human Services Survey for Medical Personnel (MBI-HSS™ for MP-Mindgarden) (Maslach et al., 1997). The scale contains 22 items covering three domains: emotional exhaustion (EE, 9 items), depersonalization (DP, 5 items), and personal accomplishment (PA, 8 items). The items were measured using a seven-point Likert scale, ranging from zero (never) to six (every day). When interpreting the results, the higher the EE and DP mean scores the higher the burnout, however, the higher the scores for the PA the lower the burnout. The cut-off points used for each domain were; no or mild EE (≤18), moderate EE (19–26), high EE (≥27), no or mild DP (≤5), moderate DP (6–9), high DP (≥10), high PA indicating low burnout (≥40), moderate PA indicating moderate burnout (34–39), no or mild PA indicating high burnout (≤33) (Hu et al., 2020). The Cronbach's alpha value was computed for the MBI-HSS for the MP scale. Community pharmacists' resilience was measured by the Connor-Davidson Resilience Scale-10 (CD-RISC-10) in the fourth section of the survey (Connor and Davidson, 2003). The 10-item version of CD-RISC consists of items 1, 4, 6, 7, 8, 11, 14, 16, 17, and 19 from the original CD-RISC 25 scale. This modified version was confirmed to perform well in exploratory and confirmatory factor analyses. It has excellent internal consistency, test re-test reliability, and construct validity (Davidson, 2018). The scale's items are scored with a five-point Likert scale ranging from one (not: true at all to five: true nearly all the time). The total score of the scale ranges from 0 to 40. Higher mean scores indicate better resilience (Connor and Davidson, 2003). Furthermore, Cronbach's alpha value was computed for the CD-RISC-10 scale. Pharmacists' depression, anxiety, and stress were measured by the Depression, Anxiety, and Stress Scale (DASS-21) in the fifth section (Osman et al., 2012). The DASS-21 has 21 items that assess depression (7 items), anxiety (7 items), and stress (7 items). Each item was measured by four-point Likert scale ranging from zero (never) to three (almost always). The total scores of the depression axes range from 0 to 21 with (0–4. 5–6, 7–10, 11–13, and ≥14) as cut-off points indicating normal, mild, moderate, severe, and extremely severe depression, respectively. Moreover, the total scores of the anxiety axes range from 0 to 21 with (0–3,4–5, 6–7, 8–9, and ≥10) as cut-off points indicating normal, mild, moderate, severe, and extremely severe anxiety, respectively. While the total scores of the stress axes range from 0 to 21 with (0–7, 8–9, 10–12, 13–16, and ≥17) as cut-off points indicating normal, mild, moderate, severe, and extremely severe stress, respectively. Similarly, Cronbach's alpha value was computed for the DASS-21 scale (Osman et al., 2012). Pharmacists' fear of COVID-19 was assessed in the last section by the Fear of COVID-19 Scale (FCV-19S). The scale has seven items. Each item was measured by a five-point Likert scale ranging from 1 (strongly disagree) to 5 (strongly agree), with the score ranging from 7 to 35. The higher the total mean score, the higher is the fear of COVID-19 (Ahorsu et al., 2020). The Cronbach's alpha value was also computed for the Fear of COVID-19 scale. Reliability analysis was conducted to assess the internal consistency of each scale. The Cronbach's alpha values were 0.813, 0.928, 0.941 and 0.932 for the MBI-HSS for MP scale, the CD-RISC-10 scale, the DASS-21 scale, and the Fear of COVID-19 scale, respectively.

2.5. Data analysis

Descriptive statistics were used to summarize included variables using numbers with percentages for categorical variables and means with standard deviation (SD) for continuous variables. Multivariable ordinal regression was used to estimate the relationship between fear of COVID-19 scores and ordinal outcome variables, burnout domains (EE, DP, and PA), and DASS-21 domains (depression, anxiety, and stress), while adjusting for other predictor variables independently associated with each outcome variable and/or contributing to the best-fitting regression model. Multivariable linear regression was used to estimate the relationship between fear of COVID-19 scores and resilience scores (a continuous variable). Odds ratios (OR) with 95% confidence intervals (CI) were used as a measure of association. All inferential statistical tests were two-sided. A P-value less than 0.05 was considered statistically significant. All analyses were performed using IBM Statistics (version 27.0, IBM Corp).

2.6. Ethics approval and consent

The study proposal was ethically approved by the Qatar University International Review Board with the approval number: QU-IRB 1319-EA/20. Participation was entirely voluntary, and the participants' confidentiality was assured throughout the study.

3. Results

The study results are reported in accordance with the Checklist for Reporting Results of Internet E-Surveys (CHERRIES) (Eysenbach, 2004).

During the data collection period, 337 surveys were collected. Eighty-one survey attempts did not have answers to some of the survey questions and were thus excluded. Consequently, 256

respondents completed the full survey and were included in the final study analysis (response rate: 42.7%).

Table 1 shows the community pharmacists' sociodemographic and practice characteristics. The mean age of pharmacists was 35.8 years (SD = 7.5) and the majority were males (56.3%), married (75.7%) and live with family (60.9%). The majority of pharmacists were from India (41.6%), didn't have either chronic medical conditions (84.0%) nor mental health problems (98.0%). In addition, most

Table 1
Sociodemographic and Community Pharmacists Characteristics.

Characteristic	Mean (SD)	Frequency (Percent)
Age (N = 241)	35.8 (7.5)	
Gender (N = 256)		
Male		144(56.3%)
Female		112(43.7%)
Country of origin (N = 255)		
India		106(41.6%)
Philippines		28(11.0%)
Pakistan		6 (2.3%)
Middle Eastern countries		28(11.0%)
African countries		84(32.9%)
Other		3 (1.2%)
Marital status (N = 256)		
Single		48 (18.8%)
Married		194 (75.7%)
Other		14 (5.5%)
Number of children (N = 193)	1.8(1.1)	
Current living arrangement (N = 256)		
Alone		63 (24.6%)
With family		156 (60.9%)
With coworkers		37 (14.5%)
Chronic medical conditions (N = 256)		
No disease		215 (84.0%)
With chronic disease		41 (16.0%)
Cardiovascular		14 (34.2%)
Diabetes		17 (41.5%)
Hypothyroidism		3 (7.3%)
Asthma		2 (4.8%)
Other[α]		7 (17.1%)
History of mental health problems (N = 256)		
Yes		5 (2.0%)
No		251 (98.0%)
Highest pharmacy degree (N = 256)		
BPharm/BSc Pharm		191 (74.6%)
PharmD		19 (7.4%)
MSc		7 (2.7%)
Mpharm		38 (14.9%)
PhD		1 (0.4%)
Years of practice as community pharmacists in Qatar? (N = 256)		
Less than 1 year		15 (5.9%)
1–5 years		113 (44.1%)
6–10 years		62 (24.2%)
More than 10 years		66 (25.8%)
Position in the pharmacy (N = 256)		
Staff pharmacist		157 (61.3%)
Pharmacy supervisor		24 (9.4%)
Pharmacy manager		75 (29.3%)
Community pharmacy type (N = 256)		
Independent single pharmacy		42 (16.4%)
Chain pharmacy		193 (75.4%)
Other[†]		21 (8.2%)
Community pharmacy location (N = 256)		
Pharmacy located in a shopping mall or supermarket		77 (30.1%)
Pharmacy located in a private clinic		52 (20.3%)
Community pharmacy located in other places		127 (49.6%)
Type of employment (N = 256)		
Part time		18 (7.0%)
Full time		238 (93.0%)
Average hours of work per week in pharmacy (N = 240)	45.6(14.9)	
Average total monthly income (N = 256)		
Less than 5000 QR		39 (15.2%)
5000–9999 QR		162 (63.3%)
10000–14999 QR		32 (12.5%)
Prefer not to disclose		23 (9.0%)
The approximate number of patients per day (N = 245)	80.4(101.8)	
The approximate number of prescriptions filled per day (N = 243)	21.2(31.5)	
Average personally rewarding hours per day (N = 244)	4.1(2.8)	

[†] Clinic pharmacy, warehouse.
[α] Hypothyroidism, infantile hemiplegia, obesity, Crohn' s disease, ulcerative colitis.

Table 2
Community Pharmacists' COVID-19 Related Information.

Characteristic	Frequency (Percent)
Salary changes during COVID-19 (N = 256)	
Same salary or higher	210 (82.0%)
Reduction in salary	46 (18.0%)
Working hours changes during COVID-19 (N = 256)	
No change	203 (79.3%)
Reduction in hours	18 (7%)
Increase in hours	35 (13.7%)
Changes in tasks and responsibilities during COVID-19 (N = 256)	
No change	199 (77.7%)
Yes there is change	57 (22.3%)
Fact-to-Face Contact with COVID Patients (N = 256)	
No	87 (34.0%)
Yes	83 (32.4%)
Do not Know	86 (33.6%)
Infected by COVID-19 (N = 256)	
Yes, confirmed with a COVID-19 test	21 (8.2%)
No	219 (85.5%)
Not sure (had symptoms but no test done)	16 (6.3%)
Symptoms Severity (N = 37)	
Severe (e.g. high fever of 39.4 degrees C or higher, difficulty breathing)	5 (13.5%)
Moderate (e.g. cough, fever of 38 degrees C or higher, some shortness of breath)	11 (29.7%)
Mild (e.g. cold-like symptoms)	21 (56.8%)
Hospitalization (N = 37)	
Yes (in regular patient ward)	9 (24.3%)
Yes (in intensive care unit)	0 (0%)
No	28 (75.7%)
Satisfaction with safety measures by pharmacy against COVID-19 (N = 256)	
Very satisfied	71 (27.8%)
Satisfied	125 (48.8%)
Neither satisfied nor dissatisfied	45 (17.6%)
Dissatisfied	8 (3.1%)
Very dissatisfied	7 (2.7%)

of them had BSc Pharm as their highest pharmacy degree (74.6%) and an experience duration of 1 to 5 years as a community pharmacist (44.1%). The majority worked in a chain pharmacy (75.4%) with a position of staff pharmacist (61.3%) working full time (93.0%) with an average of 45.6 working hours (SD = 14.9) per week. The pharmacists reported providing service for approximately 80.4 patients per day (SD = 101.8) and processed 21.2 prescriptions per day (SD = 31.5).

Pharmacists' COVID-19 related information and work changes during the pandemic are presented in Table 2. Most pharmacists (82.0%) had the same or a higher salary during the COVID-19 pandemic. Only 21 pharmacists were infected by COVID-19 (8.2%). Of those infected, 56.8% reported having mild symptoms. The majority of pharmacists were satisfied or highly satisfied with the COVID-19 safety measures (76.6%).

Table 3 summarizes the burnout, resilience, depression, anxiety and stress, and fear of COVID-19 levels of surveyed community pharmacists in Qatar. Most of the participants reported moderate or high emotional exhaustion (53.1%) with a mean score of 20.5 (SD = 12.4). Over half of participants (50.8%) reported moderate or high depersonalization with a mean score of 6.8 (SD = 6.2), and 52.7% reported no or mild personal accomplishment scores with a mean score of 36.6 (SD = 10.0). The mean resilience score was 27.6 (SD = 8.3). Moreover, 44.8% of participants reported moderate to extremely severe depression, 53.2% reported mild to extremely severe anxiety, and 25.4% reported mild to extremely severe stress (Table 3). The mean fear of COVID-19 score was 15.7 (SD = 6.5).

The relationships between fear of COVID-19 with mental health, burnout, and resilience are shown in Table 4. Fear of COVID-19 was a statistically significant and independent predictor of depression, anxiety, and stress levels. An increase in fear of COVID-19 by 1 unit was associated with increased odds of having a higher level of depression by 11% (95% CI 6%, 18%), higher level of anxiety by 20% (95 CI 13%, 28%), and a higher level of stress by 9% (95% CI 2%, 17%). An increase in fear of COVID-19 by 1 unit was associated with increased odds of higher EE and DP levels by 8% (95% CI 2%, 15%) and 9% (95% CI 3%, 15%), respectively. Similarly, an increase in fear of COVID-19 by 1 unit was associated with increased odds of higher PA levels by 4%, but this was not statistically significant (95% −2%, 9%). In addition, an increase in fear of COVID-19 by 1 unit was associated with lower odds of higher resilience score by 18% (95% CI 2%, 31%).

4. Discussion

To the best of our knowledge, this study is the first to examine the level of community pharmacists' burnout, resilience, and other mental health issues including depression, anxiety, and stress during the COVID-19 pandemic in Qatar and its association with fear of COVID-19. In this study, community pharmacists have reported moderate levels of burnout during the COVID-19 pandemic. For instance, 53.1%, 50.8%, and 69.5% of surveyed pharmacists reported moderate-high EE and DP, and moderate-low PA, respectively. These findings are consistent with a study done in China by Zhao et al which showed that 48.2% and 48.0% of Chinese hospital pharmacists had high and moderate EE, respectively, 87.2% and 12.0% had high and moderate DP, respectively and 0.6% and 99.4% had moderate/low PA during the pandemic (Zhao et al., 2020). Another study that was conducted with French community pharmacists reported that the pharmacists had a mean score of 23.0 ± 11.4 for EE, 10.9 ± 5.5 for DP, and 48.1 ± 7.2 for PA. These scores are very close to the mean scores obtained in this study (Lange et al., 2020). Furthermore, a study that included 647 Australian pharmacists by Johnston et al demonstrated very similar results to the current

Table 3
Mental Health outcomes, burnout and resilience of community pharmacists.

	Mean (SD)	N(%)	Possible Range
**MBI:EE** [1a]	20.5 (12.4)		0–54
No or Mild EE (≤18)		120 (46.9%)	
Moderate EE (19–26)		50 (19.5%)	
High EE (≥27)		86 (33.6%)	
**MBI:DP** [2a]	6.8 (6.2)		0–30
No or Mild DP (≤5)		126 (49.2%)	
Moderate DP (6–9)		56 (21.9%)	
High DP (≥10)		74 (28.9%)	
**MBI:PA** [3a]	36.6 (10.0)		0–48
High PA indicating low burnout (≥40)		78 (30.5%)	
Moderate PA indicating moderate burnout (34–39)		43 (16.8%)	
No or Mild PA indicating high burnout (≤33)		135 (52.7%)	
**Resilience** [b]	27.6 (8.3)		0–40
**DASS-21: Depression** [c]	4.9 (4.5)		0–21
Normal depression (0–4)		122 (55.2%)	
Mild depression (5–6)		32 (14.5%)	
Moderate depression (7–10)		38 (17.2%)	
Severe depression (11–13)		15 (6.8%)	
Extremely severe depression (≥14)		14 (6.3%)	
**DASS-21: Anxiety** [c]	4.7 (4.1)		0–19
Normal anxiety (0–3)		101 (46.8%)	
Mild anxiety (4–5)		42 (19.4%)	
Moderate anxiety (6–7)		32 (14.8%)	
Severe anxiety (8–9)		17 (7.9%)	
Extremely severe anxiety (≥10)		24 (11.1%)	
**DASS-21: Stress** [c]	5.7 (4.2)		0–21
Normal stress (0–7)		164 (74.6%)	
Mild stress (8–9)		22 (10.0%)	
Moderate stress (10–12)		20 (9.1%)	
Severe stress (13–16)		10 (4.5%)	
Extremely severe stress (≥17)		4 (1.8)	
**Fear** [d]	15.7 (6.5)		7–35

[a] MBI-HSS for MP: Maslach Burnout Inventory: Human Services Survey for Medical Personnel (N = 256).[1]EE = emotional exhaustion. [2]DP = depersonalization. [3]PA = personal accomplishment.
[b] Resilience: CD-RISC-10: Connor-Davidson Resilience Scale-10 (N = 234).
[c] DASS-21: The Depression, Anxiety and Stress Scale (N = 221 for depression, N = 216 for anxiety N = 220 for stress).
[d] Fear of COVID-19 Scale (N = 218).

Table 4
Crude and adjusted associations between fear of COVID-19 with mental health, burnout, and resilience among 218 community pharmacists.

Outcome variable	Crude association				Adjusted association			
	β[d]	SE[e]	OR[f] (95% CI[g])	p-value	β	SE	OR (95% CI)	p-value
**DASS-21 Depression**[a]	0.08	0.02	1.08 (1.04, 1.12)	<0.001	0.11	0.03	1.11 (1.06, 1.18)[h]	<0.001
**DASS-21 Anxiety**[a]	0.14	0.02	1.15 (1.10, 1.20)	<0.001	0.18	0.03	1.20 (1.13, 1.28)[i]	<0.001
**DASS-21 Stress**[a]	0.06	0.02	1.06 (1.01, 1.11)	0.020	0.09	0.03	1.09 (1.02, 1.17)[j]	0.013
**MBI: EE** [b1]	0.05	0.02	1.06 (1.02, 1.10)	0.007	0.08	0.03	1.08 (1.02, 1.15)[k]	0.007
**MBI: DP** [b2]	0.05	0.02	1.05 (1.01, 1.09)	0.013	0.08	0.03	1.09 (1.03, 1.15)[l]	0.004
**MBI: PA** [b3]	0.03	0.02	1.03 (0.99, 1.07)	0.097	0.04	0.03	1.04 (0.98, 1.09)[m]	0.205
**Resilience**[c]	−0.15	0.08	0.86 (0.72, 1.03)	0.092	−0.20	0.09	0.82 (0.69, 0.98)[n]	0.032

[a] DASS-21: The Depression, Anxiety and Stress Scale.
[b] MBI-HSS for MP: Maslach Burnout Inventory: Human Services Survey for Medical Personnel.[1]EE = emotional exhaustion. [2]DP = depersonalization. [3]PA = personal accomplishment.
[c] Resilience: CD-RISC-10: Connor-Davidson Resilience Scale-10.
[d] Regression coefficient.
[e] Standard error.
[f] Odds ratio.
[g] Confidence interval.
[h] Adjusted for age, gender, position in the pharmacy, community pharmacy type, and change in working hours during COVID-19.
[i] Adjusted for age, gender, current living arrangements, position in the pharmacy, monthly income, number of personally rewarding hours, and change in tasks and responsibilities during COVID-19.
[j] Adjusted for age, gender, current living arrangements, number of personally rewarding hours, number of prescriptions filled per day, and fact-to-face contact with COVID-19 patients.
[k] Adjusted for age, gender, country of origin, monthly income, number of personally rewarding hours, and change in working hours during COVID-19.
[l] Adjusted for age, gender, country of origin, change in income, number of patients seen per day, and number of personally rewarding hours.
[m] Adjusted for age, gender, country of origin, and monthly income.
[n] Adjusted for age, gender, monthly income, type of employment, and current living arrangements.

study. The mean EE, DP, and PA scores were 28.5 ± 13.4, 7.98 ± 5.6, and 36.6 ± 7.6, respectively (Johnston et al., 2021). There are several potential causes of burnout in our study. These causes were explored and discussed in details in a qualitative study that we conducted using the Job Demands Resouce Model among community pharmacists during the pandemic (Abdelsadig Mohammed

et al., 2022). Causes include the job and emotional demands (including fear of infection) that community pharmacists had during the pandemic in Qatar. Community pharmacists were serving as frontline healthcare providers, as many patients avoided hospitals and clinics and shifted for perceived safer options "the community pharmacies" to receive care. These demands in turn caused an increase in the workload and pressure on community pharmacists and potentially led to their burnout.

Moreover, this study showed that the levels of depression and anxiety among community pharmacists during the pandemic were moderate with 44.8% of the participants had moderate to severe depression and 53.2% had mild to severe anxiety. In comparison, in a study conducted among health care workers in China during COVID-19 using Patient Health Questionnaire (PHQ-9) and the seven-item Generalized Anxiety Disorder Scale (GAD-7), one-third of participants had mild to severe depressive symptoms, whereas nearly one-quarter of participants had mild to severe anxiety symptoms (Yan et al., 2021). Furthermore, a recent study conducted in Egypt among healthcare workers during COVID-19 reported that 94%, 90.5%, and 98.5% of participants showed different degrees of depression, anxiety, and stress respectively (Aly et al., 2021). Rossi et al reported the levels of depression and anxiety among Italian frontline and second-line healthcare workers during the COVID-19 pandemic with 24.7% of participants having depression, 19.8% having anxiety, and 21.9% having stress using the PHQ-9, GAD-7 and the Perceived Stress Scale (PSS-10) scales respectively (Rossi et al., 2020). The depression and anxiety symptoms reported among community pharmacists in this study are expected. These healthcare workers were serving on the frontline during the pandemic and several factors may have played a major role in increasing their risk of experiencing symptoms of depression, anxiety, and stress. These include their feelings of uncertainty and vulnerability, their fear of personally contracting the virus, and unknowingly passing it to their family and friends. This is accompanied by the sudden changes in their daily work routine including working overtime under stressful, constrained conditions and their emergent obligation to adhere to the newly imposed COVID-19 safety precautions (Wong et al., 2005). In fact, pharmacists in this study expressed a moderate level of fear of COVID-19 as measured by the Fear of COVID-19 scale. However, this was a significant predictor of lower resilience scores and higher depression, anxiety, stress symptoms severity. This is in line with the literature where fear was a positive predictor of nurses' psychological distress and job dissatisfaction (Labrague and de Los Santos, 2021). Addressing the fear of COVID-19 among community pharmacists in Qatar through appropriate family, peer, and organizational support and by keeping the pharmacists informed about the latest information related to COVID-19 may improve their mental wellbeing and potentially their job satisfaction. Organizational support includes, but is not limited to, managerial support, by offering a safe work environment through the supply of PPE and infection control procedures.

The mean resilience score using CD-RISC-10 was 27.6 which indicates a relatively moderate resilience. Similarly, a recent systematic review by Baskin and Bartlett showed an overall moderate resilience (CD-RISC range = 35.5–92.8) among healthcare workers during the COVID-19 pandemic across different countries (Baskin and Bartlett, 2021). The resiliency shown among the community pharmacists in Qatar highlights their ability to tolerate uncertainty. It also sheds the light on the important role of personal and individual resources in defeating work-related distress and in overcoming mental health problems presented during unforeseen situations. This resilience also aids in maintaining the quality of pharmacist-provided patient-centered services and pharmacist-patient relationships (Di Trani et al., 2021). Yet the individual promising resilience of community pharmacists is not enough, system resilience is a key. Qatar community pharmacies should be very well prepared and equipped for disaster management and outbreaks. A very structured and detailed protocol should be set in place including having: policies for safe and effective interaction with patients, task delegation and allocation between the pharmacy staff, reduction in the number of working hours, open communication between the pharmacy staff and management, staff autonomy and involvement in decision-making. In addition, organizational support mechanisms to detect and mitigate early burnout and to conserve and sustain the wellbeing of pharmacists are needed. Psychotherapy and psychological treatment through telemedicine and hotline phone number should be available and accessible to support pharmacists during these difficult times. Moreover, offering pharmacists psychological resources and materials (e.g. printed or online pamphlets, journals, etc.) on mental and psychological well-being, coping and stress management, and self-care practices are also vital. Moreover, adequate training of pharmacists and pharmacy technicians for disaster management is vital to ensure their emotional and physical readiness to combat any potential disaster or outbreak. This training can be facilitated by utilizing diverse platforms including webinars, social media platforms, or other virtual technologies.

A potential limitation to our study is that despite the advantages of using an online survey in case pharmacists are highly stressed they may not be interested in completing the survey, and this may have led to response bias. Moreover, due to the lack of previous studies that assessed the mental health status of community pharmacists in Qatar, comparing the study results before and during the COVID-19 pandemic to assess the magnitude of the pandemic impact on the wellbeing of pharmacists was not plausible. In addition, a long-term follow-up to assess the long-term psychological impact of the pandemic on pharmacists is needed.

5. Conclusion

Based on the survey results, community pharmacists reported moderate levels of burnout, depression, anxiety, and fear of COVID-19. Nonetheless, they demonstrated moderate resilience. Moreover, the fear of COVID-19 was shown to be a positive predictor of burnout and other mental health outcomes. These results can be generalizable to other community pharmacists in the region and elsewhere who have been at the front-line caring for patients during the pandemic. Community pharmacists have a crucial role in preventing and limiting the spread of the coronavirus. Their responsibilities do not only lie in advising, counseling, and educating the public but also in the screening of suspected cases and in making referrals when needed. Hence, implementing interventions on the personal, organizational and national levels to promote the psychological well-being of community pharmacists during this pandemic or other future emergencies is essential.

Disclosure

The preliminary results of this study were presented as a poster at Qatar University Annual Research Forum 2021.

Declaration of Competing Interest

The authors declare that they have no known competing financial interests or personal relationships that could have appeared to influence the work reported in this paper.

Acknowledgements

We would like to thank all pharmacists who filled out the survey.

Data availability statement

Data is available upon request from the study lead investigator.

Funding

This study was funded by student grants [QUST-2-CPH-2020-21] from the Qatar University Office of Research and Graduate Studies. Its contents are solely the responsibility of the authors and do not necessarily represent the official views of Qatar University.

References

Abdelsadig Mohammed, H., Elamin, S. A., El-Awaisi, A., & El Hajj, M. S. (2022). Use of the job demands-resource model to understand community pharmacists' burnout during the COVID-19 pandemic. Res. Soc. Administr. Pharm. : RSAP, S1551-7411(22)00087-0. Advance online publication. https://doi.org/10.1016/j.sapharm.2022.03.011.

Adams, J.G., Walls, R.M., 2020. Supporting the Health care workforce during the COVID-19 global epidemic. JAMA 323 (15), 1439–1440. https://doi.org/10.1001/jama.2020.3972.

Ahorsu, D.K., Lin, C.-Y., Imani, V., Saffari, M., Griffiths, M.D., Pakpour, A.H., 2020. The fear of COVID-19 scale: development and initial validation. Int. J. Mental Health Addict. 1–9. https://doi.org/10.1007/s11469-020-00270-8.

AlAteeq, D.A., Aljhani, S., Althiyabi, I., Majzoub, S., 2020. Mental health among healthcare providers during coronavirus disease (COVID-19) outbreak in Saudi Arabia. J. Infect. Public Health 13 (10), 1432–1437. https://doi.org/10.1016/j.jiph.2020.08.013.

Al-Quteimat MSc BCOP, O.M., Amer RPh MSc, A. M. (2021). SARS-CoV-2 outbreak: How can pharmacists help? Res. Soc. Administr. Pharm.: RSAP, 17(2), 480–482. https://doi.org/10.1016/j.sapharm.2020.03.018.

Alshekaili, M., Hassan, W., Al Said, N., Al Sulaimani, F., Jayapal, S.K., Al-Mawali, A., Chan, M.F., Mahadevan, S., Al-Adawi, S., 2020. Factors associated with mental health outcomes across healthcare settings in Oman during COVID-19: frontline versus non-frontline healthcare workers. BMJ Open 10 (10), e042030. https://doi.org/10.1136/bmjopen-2020-042030.

Aly, H.M., Nemr, N.A., Kishk, R.M., Elsaid, N.M.A.B., 2021. Stress, anxiety and depression among healthcare workers facing COVID-19 pandemic in Egypt: a cross-sectional online-based study. BMJ Open 11 (4), e045281. https://doi.org/10.1136/bmjopen-2020-045281.

American Psychological Association. (2002). Investigating the road to resilience. Monitor Psychol., 33(9). https://www.apa.org/monitor/oct02/pp.

Balayssac, D., Pereira, B., Virot, J., Collin, A., Alapini, D., Cuny, D., Gagnaire, J.-M., Authier, N., Vennat, B., Quaini, F., 2017. Burnout, associated comorbidities and coping strategies in French community pharmacies—BOP study: a nationwide cross-sectional study. PLoS One 12 (8), e0182956. https://doi.org/10.1371/journal.pone.0182956.

Baskin, R.G., Bartlett, R., 2021. Healthcare worker resilience during the COVID-19 pandemic: an integrative review. J. Nurs. Manag. 29 (8), 2329–2342. https://doi.org/10.1111/jonm.13395.

Bianchi, R., Schonfeld, I.S., Laurent, E., 2015. Burnout–depression overlap: a review. Clin. Psychol. Rev. 36, 28–41. https://doi.org/10.1016/j.cpr.2015.01.004.

Bowden, G.E., Smith, J.C.E., Parker, P.A., Boxall, M.J.C., 2015. Working on the edge: Stresses and rewards of work in a front-line mental health service. Clin. Psychol. Psychother. 22 (6), 488–501. https://doi.org/10.2146/ajhp170818.

Calgan, Z., Aslan, D., Yegenoglu, S., 2011. Community pharmacists' burnout levels and related factors: an example from Turkey. Int. J. Clin. Pharm. 33 (1), 92–100.

Connor, K.M., Davidson, J.R.T., 2003. Development of a new resilience scale: The Connor-Davidson Resilience Scale (CD-RISC). Depression Anxiety 18 (2), 76–82. https://doi.org/10.1002/da.10113.

Davidson JRT. Connor-Davidson Resilience Scale (CD-RISC) Manual. Unpublished. 08-19-2018. https://www.cd-risc.com.

de Vente, W., van Amsterdam, J.G.C., Olff, M., Kamphuis, J.H., Emmelkamp, P.M.G., 2015. Burnout is associated with reduced parasympathetic activity and reduced HPA axis responsiveness, predominantly in males. Biomed Res. Int. 2015, 1–13. https://doi.org/10.1155/2015/431725.

Dhama, K., Khan, S., Tiwari, R., Sircar, S., Bhat, S., Malik, Y.S., Singh, K.P., Chaicumpa, W., Bonilla-Aldana, D.K., Rodriguez-Morales, A.J., 2020. Coronavirus disease 2019–COVID-19. Clin. Microbiol. Rev. 33 (4), e00028–e120. https://doi.org/10.1128/CMR.00028-20.

di Trani, M., Mariani, R., Ferri, R., de Berardinis, D., Frigo, M.G. (2021). From resilience to burnout in healthcare workers during the COVID-19 emergency: the role of the ability to tolerate uncertainty. Front. Psychol., 12. https://www.frontiersin.org/article/10.3389/fpsyg.2021.646435.

Ding, Y., Qu, J., Yu, X., Wang, S. (2014). The mediating effects of burnout on the relationship between anxiety symptoms and occupational stress among community healthcare workers in China: a cross-sectional study. PloS One, 9 (9), e107130.

Durham, M.E., Bush, P.W., Ball, A.M., 2018. Evidence of burnout in health-system pharmacists. Am. J. Health-System Pharm. 75 (23_Supplement_4), S93–S100. https://doi.org/10.2146/ajhp170818.

Ekstedt, M., Söderström, M., Åkerstedt, T., Nilsson, J., Søndergaard, H.-P., Aleksander, P., 2006. Disturbed sleep and fatigue in occupational burnout. Scand. J. Work Environ. Health 32 (2), 121–131. https://doi.org/10.5271/sjweh.987.

Elbeddini, A., Wen, C.X., Tayefehchamani, Y., To, A., 2020. Mental health issues impacting pharmacists during COVID-19. J. Pharm. Policy Pract. 13 (1), 1–6. https://doi.org/10.1186/s40545-020-00252-0.

Eysenbach, G., 2004. Improving the quality of Web surveys: the Checklist for Reporting Results of Internet E-Surveys (CHERRIES). J. Med. Int. Res. 6, (3). https://doi.org/10.2196/jmir.6.3.e34 e34.

Fertleman, C., Carroll, W., 2013. Protecting students and promoting resilience. BMJ 347 (sep02 3), f5266.

Fessell, D., Cherniss, C., 2020. Coronavirus disease 2019 (COVID-19) and beyond: micropractices for burnout prevention and emotional wellness. J. Am. College Radiol. 17 (6), 746–748. https://doi.org/10.1016/j.jacr.2020.03.013.

Ghahramani, S., Lankarani, K.B., Yousefi, M., Heydari, K., Shahabi, S., Azmand, S., 2021. A systematic review and meta-analysis of burnout among healthcare workers during COVID-19. Front. Psychiatry 12,. https://doi.org/10.3389/fpsyt.2021.758849 758849.

Han, Q., Lin, Q., Jin, S., You, L., 2020. Coronavirus 2019-nCoV: A brief perspective from the front line. J. Infect. 80 (4), 373–377. https://doi.org/10.1016/j.jinf.2020.02.010.

Hu, D., Kong, Y., Li, W., Han, Q., Zhang, X., Zhu, L.X., Wan, S.W., Liu, Z., Shen, Q.u., Yang, J., He, H.-G., Zhu, J., 2020. Frontline nurses' burnout, anxiety, depression, and fear statuses and their associated factors during the COVID-19 outbreak in Wuhan, China: A large-scale cross-sectional study. EClinicalMedicine 24, 100424. https://doi.org/10.1016/j.eclinm.2020.100424.

Jackson, D., Firtko, A., Edenborough, M., 2007. Personal resilience as a strategy for surviving and thriving in the face of workplace adversity: a literature review. J. Adv. Nurs. 60 (1), 1–9. https://doi.org/10.1111/j.1365-2648.2007.04412.x.

Jocic, D., Krajnovic, D., 2014. State anxiety, stress and burnout syndrome among community pharmacists: relation with pharmacists' attitudes and beliefs. Ind. J. Pharm. Educat. Res. 48 (2), 9–15. https://doi.org/10.5530/ijper.48.2.3.

Johnston, K., O'Reilly, C.L., Scholz, B., Georgousopoulou, E.N., Mitchell, I., 2021. Burnout and the challenges facing pharmacists during COVID-19: results of a national survey. Int. J. Clin. Pharm. 43 (3), 716–725. https://doi.org/10.1007/s11096-021-01268-5.

Labrague, L.J., Santos, J.A.A., 2021. Fear of COVID-19, psychological distress, work satisfaction and turnover intention among frontline nurses. J. Nurs. Manag. 29 (3), 395–403. https://doi.org/10.1111/jonm.13168.

Lai, J., Ma, S., Wang, Y., Cai, Z., Hu, J., Wei, N., Wu, J., Du, H., Chen, T., Li, R., Tan, H., Kang, L., Yao, L., Huang, M., Wang, H., Wang, G., Liu, Z., Hu, S., 2020. Factors associated with mental health outcomes among health care workers exposed to coronavirus disease 2019. JAMA Network Open 3 (3), e203976. https://doi.org/10.1001/jamanetworkopen.2020.3976.

Lange, M., Joo, S., Couette, P.-A., de Jaegher, S., Joly, F., Humbert, X., 2020. Impact on mental health of the COVID-19 outbreak among community pharmacists during the sanitary lockdown period. Ann. Pharm. Fr. 78 (6), 459–463. https://doi.org/10.1016/j.pharma.2020.09.002.

Lu, H., Stratton, C.W., Tang, Y.-W., 2020. Outbreak of pneumonia of unknown etiology in Wuhan, China: The mystery and the miracle. J. Med. Virol. 92 (4), 401–402. https://doi.org/10.1002/jmv.25678.

Maslach, C., Jackson, S.E., 1981. The measurement of experienced burnout. J. Organiz. Behav. 2 (2), 99–113. https://doi.org/10.1002/job.4030020205.

Maslach, C., Jackson, S., Leiter, M., 1997. The Maslach Burnout inventory manual. In: Evaluating Stress: A Book of Resources vol. 3, pp. 191–218.

May, R.W., Sanchez-Gonzalez, M.A., Brown, P.C., Koutnik, A.P., Fincham, F.D., 2014. School burnout and cardiovascular functioning in young adult males: a hemodynamic perspective. Stress 17 (1), 79–87. https://doi.org/10.3109/10253890.2013.872618.

Oreskovich, M.R., Kaups, K.L., Balch, C.M., Hanks, J.B., Satele, D., Sloan, J., Meredith, C., Buhl, A., Dyrbye, L.N., Shanafelt, T.D. (2012). Prevalence of alcohol use disorders among American surgeons. Arch. Surg. 147(2), 168–174. https://doi-org/10.1001/archsurg.2011.148.

Osman, A., Wong, J.L., Bagge, C.L., Freedenthal, S., Gutierrez, P.M., Lozano, G., 2012. The Depression Anxiety Stress Scales—21 (DASS-21): Further Examination of Dimensions, Scale Reliability, and Correlates. J. Clin. Psychol. 68 (12), 1322–1338. https://doi.org/10.1002/jclp.21908.

Panagioti, M., Geraghty, K., Johnson, J., Zhou, A., Panagopoulou, E., Chew-Graham, C., Peters, D., Hodkinson, A., Riley, R., Esmail, A., 2018. Association Between Physician Burnout and Patient Safety, Professionalism, and Patient Satisfaction: A Systematic Review and Meta-analysis. JAMA Intern. Med. 178 (10), 1317–1331. https://doi.org/10.1001/jamainternmed.2018.3713.

Pappa, S., Ntella, V., Giannakas, T., Giannakoulis, V.G., Papoutsi, E., Katsaounou, P., 2020. Prevalence of depression, anxiety, and insomnia among healthcare workers during the COVID-19 pandemic: A systematic review and meta-analysis. Brain Behav. Immun. 88, 901–907. https://doi.org/10.1016/j.bbi.2020.05.026.

Parker, P.A., Kulik, J.A., 1995. Burnout, self-and supervisor-rated job performance, and absenteeism among nurses. J. Behav. Med. 18 (6), 581–599. https://doi.org/10.1007/BF01857897.

Patel, S.K., Kelm, M.J., Bush, P.W., Lee, H.-J., Ball, A.M., 2021. Prevalence and risk factors of burnout in community pharmacists. J. Am. Pharm. Assoc. 61 (2), 145–150. https://doi.org/10.1016/j.japh.2020.09.022.

Qatar Ministry of Public Health. (2022). COVID-19 [cited 2022May5]. Available from: https://covid19.moph.gov.qa/EN/Pages/default.aspx.

Rossi, R., Socci, V., Pacitti, F., di Lorenzo, G., di Marco, A., Siracusano, A., Rossi, A., 2020. Mental Health Outcomes Among Frontline and Second-Line Health Care Workers During the Coronavirus Disease 2019 (COVID-19) Pandemic in Italy. JAMA Network Open 3 (5), e2010185. https://doi.org/10.1001/jamanetworkopen.2020.10185.

Şahin, M.K., Aker, S., Şahin, G., Karabekiroğlu, A., 2020. Prevalence of Depression, Anxiety, Distress and Insomnia and Related Factors in Healthcare Workers During COVID-19 Pandemic in Turkey. J. Community Health 45 (6), 1168–1177. https://doi.org/10.1007/s10900-020-00921-w.

Shanafelt, T.D., Balch, C.M., Dyrbye, L., Bechamps, G., Russell, T., Satele, D., Rummans, T., Swartz, K., Novotny, P.J., Sloan, J., Oreskovich, M.R., 2011. Special report: suicidal ideation among American surgeons. Arch. Surg. 146 (1), 54–62. https://doi.org/10.1001/archsurg.2010.292.

Southwick, S.M., Bonanno, G.A., Masten, A.S., Panter-Brick, C., Yehuda, R., 2014. Resilience definitions, theory, and challenges: interdisciplinary perspectives. Eur. J. Psychotraumatol. 5 (1), 25338. https://doi.org/10.3402/ejpt.v5.25338.

Ung, C.O.L., 2020. Community pharmacist in public health emergencies: Quick to action against the coronavirus 2019-nCoV outbreak. Res. Soc. Administr. Pharm. : RSAP 16 (4), 583–586. https://doi.org/10.1016/j.sapharm.2020.02.003.

Vela-Bueno, A., Moreno-Jiménez, B., Rodríguez-Muñoz, A., Olavarrieta-Bernardino, S., Fernández-Mendoza, J., de la Cruz-Troca, J.J., Bixler, E.O., Vgontzas, A.N., 2008. Insomnia and sleep quality among primary care physicians with low and high burnout levels. J. Psychosom. Res. 64 (4), 435–442. https://doi.org/10.1016/j.jpsychores.2007.10.014.

Whatmore, L. (2000). Stress in health professionals. Jenny Firth-Cozens and Roy Payne, John Wiley & Sons, Chichester, 1999, pp. xviii+ 264. 0-471-99876-1 (paper), 0-471-99875-2 (cloth). Stress and Health, 16(5), 327–329. 10.1002/1099-1700(200010)16:5%3C327::AID-SMI877%3E3.0.CO;2-L.

Wong, T.W., Yau, J.K.Y., Chan, C.L.W., Kwong, R.S.Y., Ho, S.M.Y., Lau, C.C., Lau, F.L., Lit, C.H., 2005. The psychological impact of severe acute respiratory syndrome outbreak on healthcare workers in emergency departments and how they cope. Eur. J. Emerg. Med. 12 (1), 13–18.

World Health Organization. (2019). Burnout an "occupational phenomenon": International Classification of Diseases. https://www.who.int/news/item/28-05-2019-burn-out-an-occupational-phenomenon- international-classification-of-diseases.

World Health Organization. (2022). WHO Coronavirus (COVID-19) Dashboard. https://covid19.who.int/.

Yan, T., Zhizhong, W., Jianzhong, Z., Yubo, Y., Jie, L., Junjun, Z., Guangtian, L., 2021. Depressive and Anxiety Symptoms Among People Under Quarantine During the COVID-19 Epidemic in China: A Cross-Sectional Study. Front. Psychiatry 12,. https://doi.org/10.3389/fpsyt.2021.566241 566241.

Zhao, J., Zhang, X., Du, S., 2020. Factors associated with burnout and job satisfaction in Chinese hospital pharmacists. Medicine 99 (35), e21919. https://doi.org/10.1097/MD.0000000000021919.
