# Supplementary material for: Mental health status and related factors influencing healthcare workers during the COVID-19 pandemic: A systematic review and meta-analysis
Source: PLoS One. 2024 Jan 19;19(1):e0289454. doi: 10.1371/journal.pone.0289454 (PMC10798549; doi:10.1371/journal.pone.0289454)
Supplement: S1 Data — (ZIP) [file pone.0289454.s011.zip › literatures/155.pdf]

ELSEVIER

Since January 2020 Elsevier has created a COVID-19 resource centre with free information in English and Mandarin on the novel coronavirus COVID-19. The COVID-19 resource centre is hosted on Elsevier Connect, the company's public news and information website.

Elsevier hereby grants permission to make all its COVID-19-related research that is available on the COVID-19 resource centre - including this research content - immediately available in PubMed Central and other publicly funded repositories, such as the WHO COVID database with rights for unrestricted research re-use and analyses in any form or by any means with acknowledgement of the original source. These permissions are granted for free by Elsevier for as long as the COVID-19 resource centre remains active.

Medicina Clínica 159 (2022) 268–277

ELSEVIER

MEDICINA CLINICA

www.elsevier.es/medicinaclinica

Original article

Mental health assessment of Spanish frontline healthcare workers during the SARS-CoV-2 pandemic

Pau Sobregrau Sangrà[a,b,j,*], Thaís Castro Ribeiro[c,d], Silvia Esteban-Sepúlveda[e,f], Esther García Pagès[d], Beatriz López Barbeito[a], Jordi Aguiló Llobet[g], José Luís Pomar Moya-Prats[h], Luís Pintor Pérez[b,i,j], Sira Aguiló Mir[a]

[a] *Emergency Department, Hospital Clinic of Barcelona, Barcelona 08036, Spain*
[b] *Faculty of Psychology, University of Barcelona (UB), Barcelona 080035, Spain*
[c] *Psychiatry Department, Hospital Clinic of Barcelona, Barcelona 08036, Spain*
[d] *Networking Biomedical Research Center: Bioengineering, Biomaterials and Nanomedicine (CIBER-BBN), Universitat Autònoma de Barcelona (UAB), Bellaterra 08193, Spain*
[e] *Research Group in Nursing Care (GRECI), Hospital del Mar Institute of Medical Research (IMIM), Barcelona 08003, Spain*
[f] *Consorci Parc de Salut MAR de Barcelona, Hospital del Mar of Barcelona, Barcelona 08003, Spain*
[g] *Microelectronics and Electronic Systems Department, Universitat Autònoma de Barcelona (UAB), Bellaterra 08193, Spain*
[h] *Cardiac Surgery Department, The Thorax Institute, Hospital Clinic of Barcelona, Barcelona 08036, Spain*
[i] *Clinical Institute of Neurosciences (ICN), Hospital Clinic of Barcelona, Barcelona 08036, Spain*
[j] *Biomedical Research Institute August Pi i Sunyer (IDIBAPS), Hospital Clinic of Barcelona, Barcelona 08036, Spain*

ARTICLE INFO

Article history:
Received 3 September 2021
Accepted 12 November 2021
Available online 17 December 2021

Keywords:
SARS-CoV-2 pandemic
Anxiety symptoms
Acute stress symptoms
Depression symptoms
Posttraumatic stress disorder
Healthcare workers

ABSTRACT

Background and objective: The Covid-19 pandemic continues challenging health systems globally, exposing healthcare workers to constant physical and psychological stressors. To date, several studies have already shown the catastrophic impact on the mental health of medical personnel during the early period of the pandemic. Nevertheless, literature evidences the dearth of works that evaluate the effect over time, understanding the pandemic as a sustained extreme stressor. The present study examines the effect of the pandemic on the mental health of Covid-19 frontline healthcare workers at six months follow-up.
Material and methods: A total of 141 frontline healthcare workers from two tertiary hospitals were recruited between July and November 2020. Healthcare workers were evaluated psychologically at baseline and six months follow-up (January to May 2021) using psychometric tests for the assessment of acute stress (VASS, PSS-10, PCL-5), anxiety (STAI) and depression (PHQ-2)
Results: Overall, there was a general worsening of the mental health between the two psychological assessments, especially regarding depression and predisposition to perceiving the situations as a threat. Nurses and nurse aides showed poorer mental health while physicians improved over time. Reduced working hours and higher physical exercise resulted in better mental health among healthcare workers. Women and nursing staff were the most affected by psychological distress at baseline and six months follow-up.
Conclusion: Reduced working hours, adequate resting periods, physical exercise, and efficient intervention strategies are of utmost importance in preventing, controlling, and reducing psychological distress among healthcare workers when coping with critical scenarios such as the current pandemic.
© 2021 The Authors. Published by Elsevier España, S.L.U. This is an open access article under the CC BY license (http://creativecommons.org/licenses/by/4.0/).

* Corresponding author.
E-mail address: sobregrau@clinic.es (P.S. Sangrà).

https://doi.org/10.1016/j.medcli.2021.11.007
0025-7753/© 2021 The Authors. Published by Elsevier España, S.L.U. This is an open access article under the CC BY license (http://creativecommons.org/licenses/by/4.0/).

Análisis de la salud mental de trabajadores sanitarios españoles de primera línea durante la pandemia por SARS-CoV-2

Palabras clave:
Pandemia SARS-CoV-2
Ansiedad
Estrés agudo
Depresión
Estrés postraumático
Personal sanitario

R E S U M E N

Antecedentes y objetivo: La pandemia Covid-19 sigue desafiando a los sistemas sanitarios, exponiendo al personal asistencial a estresores físicos y psicológicos. Actualmente, varios estudios han demostrado el impacto catastrófico en la salud mental del personal asistencial durante la primera etapa de la pandemia, pero pocos han considerado el seguimiento de los síntomas. El presente estudio examina el efecto de la pandemia en la salud mental del personal sanitario de primera línea a los 6 meses de seguimiento.
Material y métodos: Se evaluó psicológicamente a 141 trabajadores sanitarios de primera línea de 2 hospitales terciarios al inicio del estudio (julio-noviembre, 2020) y a los 6 meses (enero-mayo, 2021) mediante pruebas psicométricas para el estrés agudo (VASS, PSS-10, PCL-5), la ansiedad (STAI) y la depresión (PHQ-2).
Resultados: En general, se observó un empeoramiento de la salud mental entre las 2 evaluaciones psicológicas, especialmente en depresión y predisposición a percibir las situaciones como una amenaza. La salud mental del personal de enfermería empeoró con el tiempo, mientras que los médicos mejoraron. La reducción de la jornada laboral y el aumento del ejercicio físico mejoraron la salud mental. Las mujeres y el personal de enfermería fueron los más afectados por el malestar psicológico al inicio y a los 6 meses de seguimiento.
Conclusión: Jornadas laborales reducidas, períodos de descanso adecuados, ejercicio físico y estrategias de intervención eficientes son de suma importancia para prevenir, controlar y reducir el malestar psicológico entre el personal sanitario ante escenarios críticos como la pandemia actual.
© 2021 Los Autores. Publicado por Elsevier España, S.L.U. Este es un artículo Open Access bajo la licencia CC BY (http://creativecommons.org/licenses/by/4.0/).

Introduction

Since first detected in Wuhan (China) in December 2019, the rapid worldwide spread of the SARS-CoV-2 virus has had a catastrophic effect, compromising the mental health and wellbeing of the general population, and especially of those on the frontline fighting against the virus.[1]

In frontline healthcare workers, the lack of resources alongside the overcrowded care wards, quarantines, increased workload, use of personal protective equipment (PPE), physical exhaustion, and fear of transmitting the disease, among others, have become risk factors for increased stress, anxiety, depression, sleep problems, and even suicide rate.[2] In other words, the wide range of physical, psychological and emotional stressors to which healthcare workers are constantly exposed when coping with the day-to-day situations of the Covid-19 pandemic have challenged and endangered their mental health and life quality,[3] thus interfering with the care quality and efficiency of health systems.[4]

Although the clinical and socio-economic implications of high psychological distress are well documented in the literature, assessing mental health in unprecedented care scenarios such as the current pandemic remains a challenge within the clinical practice. On the one hand, no precise tools objectively measure the intensity with which a stressor affects an individual.[3] Physiological evaluation of symptoms is both complex and invasive, making follow-up difficult. On the other hand, psychometric questionnaires cannot determine the effect of the stressor on the person's health or even whether the stressor is being overcome.[3,5] Furthermore, learning effects and the desirability response bias often limit psychometric questionnaire results, as with online surveys.[6]

To date, despite the complexity of assessing psychological distress, especially in critical scenarios, several works have conducted cross-sectional evaluations of the healthcare worker's mental health during the first wave of the Covid-19 pandemic. In this regard, study findings relate to the psychological impact on healthcare workers of the 2003 SARS outbreak.[7,8] Severe anxiety, depression and posttraumatic stress symptoms (PTSS), with prevalence of these disorders across medical personnel ranging from 9 to 90%, 5 to 21% and 11% to 16%, respectively.[3,9,10]

Nevertheless, far from the meaningful prevalence of psychological distress during the early stages of the Covid-19 pandemic and the future clinical and occupational implications of a late and poor diagnosis across healthcare workers,[11] the literature evidences the dearth of studies that have considered the follow-up of the symptoms to shed light on whether the elevated anxiety, depression and PTSS symptoms are sustained, reduced or increased over time.[12]

The present study aimed to examine the effect of the Covid-19 pandemic on the mental health of frontline healthcare workers at six months follow-up, understanding this worldwide care crisis as a persistent extreme stressor.

Methods

Study design

A prospective, longitudinal and multicentre study with Spanish Covid-19 frontline healthcare workers from two tertiary hospitals, the Hospital Clinic of Barcelona and the Hospital del Mar of Barcelona.

All study procedures complied with the Helsinki declaration for research and received approval from the Ethics Committee Board of both hospitals.

Setting and subjects

This study was conducted with 141 Covid-19 frontline healthcare workers from two Spanish tertiary hospitals. The recruitment process comprised the months of July and November 2020 (both inclusive). Then, healthcare workers were scheduled consecutively at six months for a follow-up assessment (until May 2021). Signed informed consent was required to take part in the study.

The recruitment process was conducted through internal diffusion, using the institutional email, across the medical units designated for the care of Covid-19 patients. The recruitment process also considered healthcare workers from the external units (i.e., health hotels) enabled for Covid-19 hospitalisations.

Healthcare workers recruited for the study were grouped according to their professional category: physicians, nurses, and nurse aides. The medical units where the recruitment process was

considered were the Intensive Care Unit (ICU), Emergency Service and Covid-19 hospitalisation wards. Covid-19 hospitalisation wards included Internal Medicine, Infections, Pneumology, Gastroenterology, and health hotels.

The inclusion criteria were to be a healthcare worker, have worked at any of the medical services mentioned above, be directly involved in managing SARS-CoV-2 patients, and have accepted participation by signing the informed consent.

All frontline healthcare workers recruited for the study performed similar duties regarding the care of Covid-19 patients regardless of the medical unit worked during the period evaluated. Likewise, all healthcare workers from the two hospitals were assessed under the same premises to reduce performance bias.

The flowchart in Fig. 1 shows the recruitment and follow-up of healthcare workers.

Assessment instruments

- *Subjective perceived stress*: assessed with the Visual Analogue Scale for stress (VASS).[13] The VASS is a visual 100-point scale (0, not at all; 100, absolutely stressed). Although it has shown reliable discriminative sensitivity and construct validity, the VASS test is not a diagnostic tool.[13] Accordingly, we used the cut-off points obtained in a previous work conducted by our research evaluating caregivers of chronic patients (high-stress levels) and controls (low-stress levels).[5] A VASS score equal to or below 30 and equal or above 31 and 70 indicated low, moderate and high perceived stress, respectively.
- *Stress appraisal*: assessed with the 10-item Perceived Stress Scale (PSS-10).[14] The PSS-10 is based on a Likert scale, with responses ranging from 0 (never) to 4 (very often). A PSS-10 score equal to or below 13, equal to or above 14 and higher than 26 indicated low, moderate and high-stress appraisal, respectively. The PSS-10 cut-offs used in this work were in line with those observed in other Covid-19 studies.[15]
- *Posttraumatic Stress Symptoms (PTSS)*: assessed with the Posttraumatic Stress Disorder Checklist for DSM-5 (PCL-5),[16] which has high internal consistency in measuring posttraumatic stress (PTSS) symptoms (Cronbach's alpha: 0.94). A total PCL-5 score equal to or higher than 31 was indicative of a possible posttraumatic stress disorder (PTSD).
- *Anxiety symptoms*: assessed with the State-Trait Anxiety Inventory (STAI).[17] The STAI-State (STAI-S) subscale evaluates the current anxiety, while the STAI-Trait (STAI-T) subscale indicates the propensity to be anxious on a personality basis. There are no normative STAI test values for medical staff. Accordingly, we used the STAI cut-off scores defined in previous research conducted with caregivers of chronic patients and controls.[5] An STAI-S total score of 10 suggested low anxiety, 37 moderate anxiety, and higher than 36 severe anxiety. On the other hand, an STAI-T cut-off point of 14, 26 and above 26 indicated low, moderate and high predisposition to perceive situations as a threat, respectively.
- *Depression symptoms*: assessed with the Patient Health Questionnaire-2 (PHQ-2).[18] A PHQ-2 score equal to or above 3 suggested possible Major Depressive Disorder (MDD), with a sensitivity of 83% and a specificity of 92%.
- *Sociodemographic variables*: assessed with the clinical and sociodemographic form. The form included data regarding the gender, age, psychiatric history (i.e., affective and anxiety disorders), physical activity levels, professional category, regular working shift, weekly working hours (i.e., part-time and full-time), working hours increase, medical service performed during the pandemic, to be off work due to Covid-19 infection (i.e., sick leave), requested help and taken stress-related medication due to high psychological burden, and substance abuse (tobacco, alcohol or other drugs).
- *Electrophysiological signals*: assessed with medical-grade devices: the NeXus-10 MKII (Mind Media B.V., CD Herten, Netherlands) system and the E4 wrist-worn (Empatica Inc., Cambridge, MA) wearable. Together, these devices registered breathing (Resp), electrodermal activity (EDA), electrocardiography (ECG), photoplethysmography (PPG) and body temperature (Temp).

Psychometric questionnaires required, on average, 15–20 min to be completed. Healthcare workers needed no special education or training to complete them independently.

Procedure

Participants were recruited between July and November 2020 (both inclusive) at the medical units designated for the care of Covid-19 patients. After signing the informed consent, healthcare workers were scheduled for a first assessment [A1] (Fig. 1). The estimated time between recruitment and the first evaluation was no more than seven days, and always respecting the availability of each healthcare worker.

The first assessment was comprised of two different stages: the psychological assessment and the physiological assessment.

On the one hand, the psychological assessment consisted of administering the five psychometric questionnaires described in the previous section. Psychometric tests were applied in person and in the following order: VASS, PSS-10, PCL-5, STAI and PHQ-2.

On the other hand, the physiological assessment consisted of evaluating different stress-related physiological variables using medical-grade technology (i.e., NeXus-10 MKII & E4 wrist-worn). The physiological assessment lasted approximately 25 min, and the data collected is meant to be analysed in future work.

The psychological assessment always preceded the physiological assessment, and a break was given between the two assessments.

Once finalised the first assessment, healthcare workers were scheduled at six months for a second assessment [A2]. The second assessment included the psychological and physiological procedures performed in the first assessment (Fig. 1).

Statistical analysis

First, a description of all sociodemographic variables and psychological results were performed. Then, statistic tests were applied accordingly.

The Paired Samples *T*-Test and One-Way repeated measures ANOVA were used to examine differences over time for the sample and within groups, respectively. The Wilcoxon signed-rank test was applied when assumptions for the Paired Samples *T*-Test were not met. The Independent Samples *T*-Test and One-Way ANOVA were used to examine for differences between groups in mental health at baseline and at six months follow-up, independently. The Mann–Whitney *U* test and the Kruskal–Wallis test were used when assumptions for the parametric form of the above tests were not met. The Bonferroni correction was applied when needed.

Participants lost to follow-up (23.4%) were not considered in the final analysis. To exclude the possibility that the loss of participants had occurred selectively and thus skewing the final results, a set of sensitive analyses (i.e., Chi-square tests for comparison of proportions and the Independent Samples *T*-Test, One-Way ANOVA and their respective parametric forms when needed) were conducted comparing the 21 participants lost to follow-up against the sample finally included in the study.

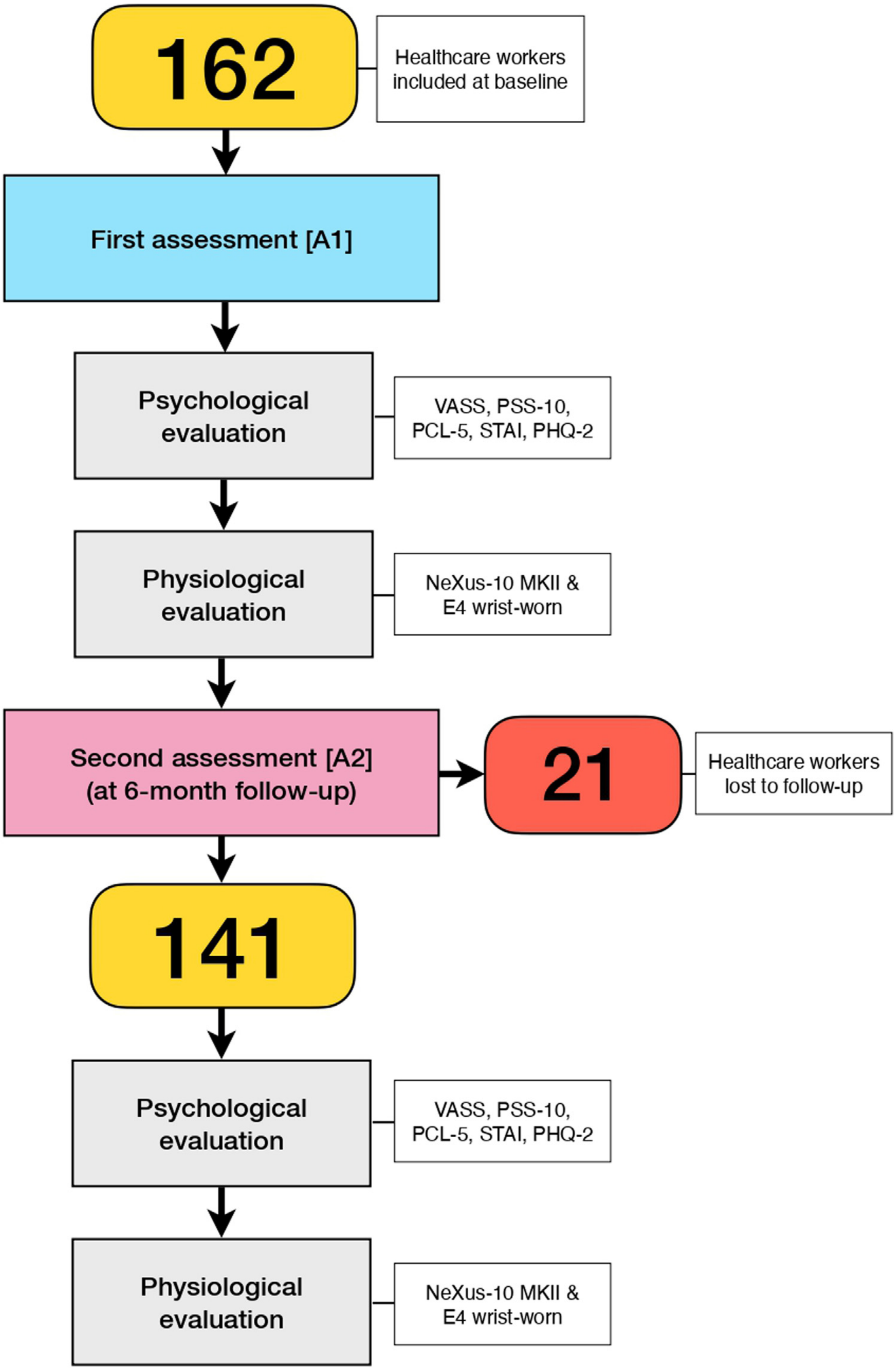

Fig. 1. Flowchart of the recruitment and follow-up of healthcare workers.

SPSS v.26 for Windows was used for all data analyses. All results were interpreted with a 95% confidence interval (CI) and a significance level (p-value) of 0.05.

Results

Demographic and clinical characteristics of the sample

A total of 141 healthcare workers, 77 from the Hospital Clinic of Barcelona and 64 from the Hospital del Mar of Barcelona, were finally included in the study. Twenty-one (1489%) healthcare workers were lost to follow-up. Nevertheless, sensitive analyses conducted in this regard revealed no significant differences between those who completed the study and those lost to follow-up ($p > .05$). A description of the clinical and sociodemographic characteristics of the sample is shown in Table 1.

According to Table 1, the majority of healthcare workers were women (87.9%), middle-age (39.7%), nurses (60.3%), from the morning shift (45.4%) and the Emergency service (42.6%). A total of 84 (59.6%) healthcare workers reported having increased the

Table 1
Clinical and sociodemographic characteristics of the 141 healthcare workers included in the study.

	Healthcare workers ($n = 141$)		
	n	%	Mean (S.D.)
Age (years)			38.3 (11.3)
Gender			
Men	17	12.10	
Women	124	87.90	
Age (groups in years)			
18–30	47	33.30	
31–45	56	39.70	
46–62	38	27.00	
Psychiatric history			
No	127	90.10	
Yes	14	9.90	
Professional category			
Physicians	35	24.80	
Nurses	85	60.30	
Nurse aides	21	14.90	
Working shift			
Mornings	64	45.40	
Afternoons	21	14.90	
Nights	37	26.20	
On-call	19	13.50	
Medical service working			
Covid-19 Wards	50	35.50	
Emergency Service	60	42.60	
ICU	31	22.00	
Weekly working hours (hours)			
18.5–30	13	9.22	
31–40	124	87.90	
Over 40	4	2.84	
Working hours increase			
No	57	40.40	
Yes	84	59.60	
Off work due to Covid-19			
No	78	55.30	
Yes	63	44.70	
Physical activity			
Low	17	12.10	
Medium	77	54.60	
High	47	33.30	
Request help			
No	121	85.80	
Yes	20	14.20	
Under stress-related medication			
No	111	78.70	
Yes	30	21.30	
Smoker			
No	97	68.80	
Yes	44	31.20	
Substance abuse increase (tobacco, alcohol, others)			
No	96	68.10	
Yes	45	31.90	

working hours during the period evaluated, while 63 (44.7%) had been off work due to Covid-19 infection. Only 14% reported to have requested help to overcome the day-to-day situations of the pandemic, and another 31.2% were prescribed stress-related medication due to high psychological distress. Almost 10% of the sample reported a previous psychiatric disorder.

Longitudinal quantitative psychological assessment, at baseline and six months follow-up

Overall, there was an increase in the psychometric tests' mean scores from the first to the second psychological assessment conducted at six months follow-up. Nevertheless, the analysis conducted on the data revealed only significant differences in the STAI-T ($T = 3358$, $Z = -2.20$, $p = .026$) subscale and the PHQ-2 ($T = 206.5$, $Z = -3.32$, $p < .001$) test. Table 2 shows the results of the quantitative psychological evaluation for the sample at the first and second assessments and the statistical difference between the two.

When changes in mental health were analysed over time within each group, the professional category involved significant differences in the VASS ($F(2,138) = 6.477$, $p = .002$) and PSS-10 ($F(2,138) = 5.62$, $p = .005$) tests, and both STAI-S ($F(2,138) = 5.33$, $p = .006$) and STAI-T ($F(2,138) = 4.40$, $p = .014$) subscales. Nurses showed significantly worse perceived stress ($p = .002$), stress appraisal ($p = .034$), anxiety ($p = .040$) and predisposition to perceiving situations as a threat ($p = .001$) from the first to the second assessment. On the contrary, physicians showed a significant improvement in perceived stress ($p = .027$) and anxiety ($p = .012$) at six months follow-up.

The hours per week worked also showed significant differences over time in the VASS ($F(1,139) = 3.89$, $p = .050$), PCL-5 ($F(1,139) = 7.92$, $p = .006$) and PSS-10 ($F(1, 139) = 5.88$, $p = .017$) tests, and the STAI-S ($F(1, 139) = 6.33$, $p = .013$) subscale. Part-time healthcare workers significantly improved PTSS ($p = .012$), stress appraisal ($p = 023$) and anxiety ($p = .026$) from the first to the second psychological assessment. In contrast, full-time healthcare workers significantly worsened the perceived stress ($p = .040$) at six months follow-up. A psychiatric history also suggested increased perceived stress and depression, without statistically significant differences ($p > .05$).

Cross-sectionally, the analysis conducted on the psychological data obtained from the baseline (Table 3) and six months follow-up (Table 4) assessments, separately, also revealed statistically significant differences.

On the one hand, at baseline (Table 3), women had statistically significant higher levels of anxiety ($t(139) = 2.78$, $p = .006$), predisposition in perceiving situations as a threat ($U = 744.5$, $p = .050$) and depression ($U = 686$, $p = .012$) than men. The professional category significantly differed in the PHQ-2 ($\chi^2(2) = 11.64$, $p = .003$) test, with the nursing staff showing higher depression than physicians ($p < .05$).

The PSS-10 ($F(2, 138) = 6.61$, $p = .003$) and PHQ-2 ($\chi^2(2) = 6.12$, $p = .047$) tests, and the STAI-S ($F(2, 138) = 5.83$, $p = .017$) and STAI-T ($F(2, 138) = 7.60$, $p = .006$) subscales also showed significant differences depending on the levels of physical activity. Healthcare workers doing medium and high levels of exercise showed statistically significant lower stress appraisal, anxiety, predisposition to perceiving situations as a threat and depression than those doing low physical activity ($p < .05$).

Healthcare workers who had been off work due to the Covid-19 infection ($U = 1958.5$, $p = .025$) and requested help to overcome the day-to-day pandemic ($U = 758.5$, $p = .004$) showed significantly higher depression than those who were not on sick leave and requested help, respectively. Healthcare workers who sought help also showed greater PTSS ($U = 715.6$, $p = .004$), anxiety ($U = 872.5$, $p = .046$) and predisposition to perceiving situations as a threat ($U = 840$, $p = .029$). The same results were observed between medicated and not medicated healthcare workers, with

Table 2
Results in the quantitative psychological assessment for the sample at baseline and six months follow-up, and differences over time.

	Healthcare workers ($n = 141$)				
	Mean	Median	SD	SE	p-Value
VASS					
First assessment [A1]	49.60	50.00	22.42	1.89	
Second assessment [A2]	52.67	58.00	22.74	1.91	
PCL-5					
First assessment [A1]	21.38	20.00	12.79	1.08	
Second assessment [A2]	21.85	20.00	14.39	1.21	
PSS-10					
First assessment [A1]	16.97	16.00	5.93	0.50	
Second assessment [A2]	16.99	17.00	6.00	0.50	
STAI-S					
First assessment [A1]	25.77	25.00	9.93	0.84	
Second assessment [A2]	26.11	25.00	10.92	0.92	
STAI-T					
First assessment [A1]	20.33	19.00	8.38	0.71	*
Second assessment [A2]	21.54	20.00	9.37	0.79	
PHQ-2					
First assessment [A1]	1.01	1.00	1.28	0.11	***
Second assessment [A2]	1.50	1.66	1.14	0.10	

Note.
* $p < .05$.
** $p < .01$.
*** $p < .001$.

those under stress-related medication having significantly higher mean scores in the following tests: PCL-5 (U= 1407, $p = .002$), PHQ-2 ($U = 989.5$, $p < .001$), STAI-S ($U = 1212$, $p = .022$), and STAI-T ($U = 1061.5$, $p = .002$).

Healthcare workers with smoking habits and substance abuse had significantly higher PTSS than non-smokers ($U = 1623.50$, $p = .023$) and without substance abuse ($U = 1500$, $p = .003$). A psychiatric history suggested greater psychological distress without statistically significant differences ($p > .05$).

On the other hand, at six months follow-up (Table 4), women continued having significantly higher mean scores than men in all the psychometric questionnaires ($p < .05$), except in the PCL-5 test ($p > .05$).

The professional category also involved significant differences in the PCL-5 ($\chi^2(2) = 12.26$, $p = .002$) and PHQ-2 ($\chi^2(2) = 4.22$, $p = .023$) tests, and STAI-S ($\chi^2(2) = 6.51$, $p = .039$) and STAI-T ($\chi^2(2) = 6.14$, $p = .046$) subscales. Nurses had higher PTSS ($p = .002$), anxiety ($p = .042$), depression ($p = .018$), and predisposition in perceiving situations as a threat ($p = .038$) than physicians. Likewise, full-time healthcare workers significantly increased perceived stress (U = 534.5, $p = .033$) and PTSS ($U = 559.5$, $p = .050$) than those working part-time.

Similar to the results obtained in the first assessment, the levels of physical activity entailed significant differences in both the STAI-S ($\chi^2(2) = 8.25$, $p = .016$) and STAI-T ($\chi^2(2) = 7.85$, $p = .020$) subscales. Healthcare workers with low physical activity levels had significantly higher anxiety ($p = .033$) and a predisposition to perceive situations as a threat ($p = .026$) than those with high physical activity levels.

At six months follow-up, significant differences in the VASS ($\chi^2(2) = 6.42$, $p = .040$) and PSS-10 ($\chi^2(2) = 6.57$, $p = .037$) tests were found depending on the medical unit healthcare workers worked. Healthcare workers from Covid-19 hospitalisation wards showed significantly higher perceived stress than those working at the Emergency Service ($p = .044$). Likewise, healthcare workers from the ICU service showed worse stress appraisal than those working at the Emergency Service ($p = .036$).

Healthcare workers who sought help showed significantly higher stress appraisal ($\chi^2(2) = 4.35$, $p = .037$), anxiety ($\chi^2(2) = 7.72$, $p = .005$), depression ($\chi^2(2) = 7.51$, $p = .002$) and predisposition to perceiving situations as a threat ($\chi^2(2) = 8.43$, $p = .004$) than those that did not have sought help. Healthcare workers taking stress-related medication also had significantly greater perceived stress ($\chi^2(2) = 5.24$, $p = .022$), anxiety ($\chi^2(2) = 3.95$, $p = .047$) and predisposition to perceiving situations as a threat ($\chi^2(2) = 6.05$, $p = .014$) than healthcare workers not medicated due to high psychological distress.

Lastly, healthcare workers with tobacco consumption and substance abuse also had significantly higher depression ($\chi^2(2) = 4.50$, $p = .034$) and PTSS ($\chi^2(2) = 7.21$, $p = .007$) than non-smokers and without substance abuse habits. At six months follow-up, a psychiatric history also seemed to involve worse overall psychological outcomes without statistically significant differences ($p > .05$).

Discussion

The current study aimed to investigate the effect of the Covid-19 pandemic over time in frontline Spanish healthcare workers. To the best of our knowledge, our study is the first to provide consistent results from face-to-face psychological assessments, with the main finding being the overall worsening of healthcare workers' mental health between the first and second psychological assessments conducted at six months follow-up, especially regarding depression and predisposition to perceiving situations as a threat.[19]

Another relevant finding was that nurses showed a meaningful worsening of stress, anxiety and depression between the first and second psychological assessment. Nurse aides also appeared to show the same tendency as nurses, without differences over time being significant. Instead, physicians presented a general improvement at six months follow-up, significantly decreasing perceived stress and anxiety. A possible contributing factor to these differences in mental health depending on the professional category might be the added care and contact nurses and nurse aides usually have with the ill patient compared to physicians.

Table 3
Results of the quantitative psychological assessment at baseline.

		VASS		PCL-5		PSS-10		STAI-S		STAI-T		PHQ-2	
	N	Mean	S.D.	Mean	S.D.	Mean	S.D.	Mean	S.D.	Mean	S.D.	Mean	S.D.
Gender													
Men	17	40.53	21.57	17.82	12.39	14.24	4.88	19.65	8.60	16.18	7.46	.35	.79
Women	124	50.84	22.33	21.87	12.81	17.35	5.98	26.61	9.84	20.90	8.37	1.10	1.31
p-Value					**	*	*						
Psychiatric history													
No	127	48.80	23.53	20.22	12.42	16.05	6.35	24.45	10.89	17.34	7.39	.073	.93
Yes	14	56.77	15.88	23.69	13.31	17.31	6.45	26.23	10.51	24.31	7.41	1.38	1.66
p-Value													
Professional category													
Physicians	35	53.83	22.77	18.00	13.12	17.46	7.45	25.23	12.53	19.51	9.33	.46	.95
Nurses	85	47.78	21.65	22.65	12.51	16.62	5.43	25.59	8.73	20.71	8.36	1.16	1.33
Nurse aides	21	49.90	24.93	21.90	12.92	17.57	5.13	27.43	10.03	20.14	6.98	1.29	1.35
p-Value							**						
Weekly working hours													
Part-time	13	48.00	31.26	23.69	16.46	19.62	6.74	28.00	13.19	23.38	11.42	1.08	1.50
Full-time	128	49.76	21.48	21.15	12.41	16.70	5.80	25.55	9.58	20.02	8.01	1.00	1.26
p-Value													
Working shift													
Mornings	64	50.98	21.88	20.52	13.03	17.05	5.88	24.89	9.84	20.19	8.53	.91	1.16
Afternoons	21	54.86	19.43	24.29	14.21	15.90	5.61	28.86	11.21	21.43	6.79	1.29	1.49
Nights	37	43.59	23.74	22.68	11.02	17.08	5.52	25.38	8.46	21.05	8.11	1.30	1.49
On-call	19	50.79	23.90	18.58	13.61	17.68	7.39	26.11	11.48	18.16	10.09	.47	.70
p-Value													
Physical activity													
Low	17	60.00	24.10	27.35	15.43	21.47	6.34	33.18	11.70	26.82	10.32	1.71	1.65
Medium	77	49.16	21.57	21.16	12.15	16.79	5.54	25.05	8.97	20.32	7.86	1.06	1.33
High	47	46.55	22.56	19.60	12.43	15.64	5.73	24.28	9.82	17.98	7.32	.66	.89
p-Value				**	*	**	*						
Medical service working													
Covid-19 wards	50	52.92	19.44	18.82	11.99	17.06	6.41	25.32	9.26	20.98	8.87	1.24	1.33
Emergency service	60	47.42	24.45	21.93	12.04	16.67	5.81	25.10	10.02	19.80	8.08	.88	1.14
ICU	31	48.45	22.88	24.45	14.89	17.42	5.48	27.81	10.84	20.29	8.37	.87	1.43
p-Value													
Off work due to Covid-19													
No	78	49.18	24.15	21.41	13.43	16.65	6.19	24.96	10.74	19.54	8.11	.85	1.29
Yes	63	50.11	20.25	21.35	12.05	17.37	5.60	26.78	8.82	21.30	8.67	1.21	1.25
p-Value							*						
Request help													
No	121	49.00	23.25	20.11	12.41	16.64	5.96	25.15	10.14	19.73	8.31	.87	1.15
Yes	20	53.20	16.52	29.10	12.62	18.95	5.43	29.55	7.78	23.95	8.13	1.85	1.66
p-Value			**		*	*	**						
Under stress-related medication													
No	111	49.00	22.37	19.80	12.69	16.49	5.99	24.97	10.27	19.25	8.22	.77	1.04
Yes	30	51.80	22.82	27.23	11.56	18.77	5.41	28.73	8.05	24.30	7.90	1.87	1.68
p-Value			**		*	**	***						
Working hours increase													
No	57	48.96	22.52	22.04	13.18	16.93	5.80	26.26	10.51	20.75	8.24	1.07	1.21
Yes	84	50.02	22.47	20.94	12.57	17.00	6.04	25.44	9.57	20.04	8.52	.96	1.33
p-Value													
Smoker													
No	97	50.04	22.69	19.71	12.33	16.71	6.02	25.29	10.24	19.98	8.52	.90	1.14
Yes	44	48.61	22.04	25.07	13.15	17.55	5.75	26.84	9.26	21.09	8.13	1.25	1.53
p-Value			*										
Substance abuse													
No	96	49.64	22.65	19.01	11.15	17.06	6.03	25.32	9.87	20.33	8.05	.95	1.16
Yes	45	49.51	22.17	26.44	14.61	16.78	5.76	26.73	10.11	20.31	9.15	1.13	1.52
p-Value			**										

Note.
* $p < .05$.
** $p < .01$.
*** $p < .001$.

Table 4
Results of the quantitative psychological assessment at six months follow-up.

		VASS		PCL-5		PSS-10		STAI-S		STAI-T		PHQ-2	
	N	Mean	S.D.	Mean	S.D.	Mean	S.D.	Mean	S.D.	Mean	S.D.	Mean	S.D.
Gender													
Men	17	38.71	16.21	15.41	9.80	12.71	3.79	17.06	7.43	14.82	5.90	.89	.73
Women	124	54.58	22.88	22.73	14.72	17.58	6.01	27.35	10.76	22.46	9.40	1.59	1.16
p-Value		**		**	***	**	*						
Psychiatric history													
No	127	48.02	20.79	18.69	11.57	14.46	5.87	22.54	11.38	19.11	8.64	1.33	1.22
Yes	14	48.85	25.91	21.66	15.33	15.92	5.96	23.39	10.90	22.00	9.59	1.46	1.20
p-Value													
Professional category													
Physicians	35	45.14	23.16	15.29	13.18	15.86	6.39	21.77	10.80	18.11	8.43	1.06	1.12
Nurses	85	55.68	22.04	24.16	14.12	17.79	5.82	27.40	10.66	23.02	9.85	1.69	1.15
Nurse aides	21	53.00	22.99	23.43	14.67	15.67	5.76	28.14	10.73	21.24	7.56	1.51	.95
p-Value			**		*	*	*						
Weekly working hours													
Part-time	13	38.77	26.25	15.38	15.29	16.38	7.42	22.92	12.37	22.00	11.70	.075	.089
Full-time	124	54.08	21.98	22.51	14.19	17.05	5.86	26.44	10.76	21.49	9.16	1.42	1.23
p-Value		*	*										
Working shift													
Mornings	64	52.20	24.69	19.69	15.93	16.56	6.00	24.19	11.30	20.56	9.57	1.37	1.16
Afternoons	21	52.76	21.16	23.57	12.99	17.29	5.91	28.52	12.34	22.43	10.61	1.58	1.34
Nights	37	53.32	21.72	25.30	12.28	17.95	6.00	28.05	9.66	23.68	8.36	1.68	1.01
On-call	19	52.84	21.09	20.53	13.65	16.26	6.31	26.16	9.88	19.68	8.98	1.53	1.09
p-Value													
Physical activity													
Low	17	59.88	22.95	22.82	17.78	20.35	6.96	32.12	12.40	25.06	11.61	1.79	1.29
Medium	77	54.73	21.06	23.00	13.29	16.86	5.63	26.34	9.93	22.23	8.46	1.53	1.02
High	47	46.68	24.38	19.62	14.85	16.00	5.92	23.57	11.25	19.13	9.54	1.36	1.26
p-Value					*	*							
Medical service working													
Covid-19 wards	50	57.58	22.83	21.08	15.23	17.72	6.62	26.74	11.63	21.70	9.62	1.49	1.15
Emergency service	60	47.55	20.36	21.77	12.40	15.55	5.37	24.83	9.95	20.97	8.67	1.51	1.09
ICU	31	54.65	25.50	23.26	16.79	18.61	5.64	27.58	11.62	22.39	10.47	1.50	1.25
p-Value		*		*									
Off work due to Covid-19													
No	78	51.79	23.29	21.18	15.09	16.94	6.12	25.42	11.15	20.97	9.21	1.42	1.21
Yes	63	53.75	22.18	22.68	13.54	17.06	5.88	26.97	10.66	22.24	9.60	1.61	1.04
p-value													
Request help													
No	121	51.26	22.89	21.18	14.63	16.55	6.00	25.07	10.82	20.60	9.08	1.37	1.05
Yes	20	61.15	20.26	25.90	12.39	19.70	5.31	32.40	9.51	27.20	9.34	2.29	1.34
p-value				*	**	**	**						
Under stress-related medication													
No	111	50.61	22.31	21.03	14.81	16.81	6.15	25.14	11.13	20.71	9.57	1.51	1.16
Yes	30	60.27	23.08	24.90	12.43	17.67	5.44	29.70	9.43	24.60	8.04	1.47	1.06
p-value			*			*	*						
Working hours increase													
No	57	49.72	21.16	21.54	13.91	16.25	5.33	25.44	10.77	21.58	9.90	1.54	1.14
Yes	84	54.67	23.67	22.06	14.78	17.50	6.39	26.57	11.06	21.51	9.06	1.48	1.15
p-value													
Smoker													
No	97	52.16	23.88	20.95	15.16	16.74	5.96	25.41	11.36	21.21	9.55	1.37	1.18
Yes	44	53.77	20.21	23.84	12.45	17.55	6.10	27.66	9.82	22.27	9.03	1.80	.98
p-value							*						
Substance abuse													
No	96	51.05	23.38	19.42	12.83	16.80	5.54	25.22	10.72	21.50	9.19	1.41	1.16
Yes	45	56.11	21.14	27.04	16.21	17.40	6.92	28.02	11.22	21.62	9.87	1.71	1.07
p-value			**										

Note.
* $p < .05$.
** $p < .01$.
*** $p < .001$.

Consistent with early studies in which reduced working hours appeared as a resilience factor in critical contexts,[21,22] our study showed that healthcare workers on a part-time basis had a general improvement over time in psychological distress. On the contrary, full-time healthcare workers appeared to deteriorate their mental health between the first and second psychological assessment, having significantly higher stress appraisal. Together, these results emphasise the importance of maintaining managing standards in medical centres to foster shorter shifts and guarantee enough resting periods to reduce the risk and vulnerability of healthcare workers against psychological burden and professional burnout in critical care scenarios as the current pandemic.[4,23]

The literature also extensively shows in a wide range of clinical contexts that a psychiatric history predisposes to future psychiatric disorders and comorbidities, especially when an extreme life event (i.e., Covid-19 pandemic) triggers it.[24] In line with this, our work seemed to reinforce this argument by suggesting a notable exacerbation of stress, anxiety and depression from the first to the second assessment in healthcare workers with a previous psychiatric disorder. Despite this, the differences in psychological distress over time were not significant for this group. A possible explanation for the lack of statistical significance could be an insufficiently long follow-up. Another explanation could be using psychological assessment instruments not designed to assess mental health in the context of the pandemic as a sustained stressor over time. In either case, these results call for an increment in the control and follow-up of healthcare workers to prevent their mental health exacerbation and consequently compromising their efficiency and care quality in future pandemics.

On the other hand, the levels of psychological distress found in the cross-sectional analysis were within the range of previously published works conducted in medical personnel.[25] In other words, at baseline, women showed worse psychological distress than men and nurses and nurse aides were at higher risk of poorer mental health than physicians, with depression being significantly worse across the nursing staff. Higher physical activity levels positively influenced healthcare workers' mental health, improving stress, anxiety, depression.[26] Healthcare workers who were off due to Covid-19 infection, requested help and took stress-related medication showed worse mental health deterioration.[27] Tobacco consumption and substance abuse followed the same tendency, worsening healthcare workers' mental health,[28] namely PTSS. A history of psychiatric disorder also seemed to involve poorer mental health, without observing significant differences.

Also consistent with early investigations,[29–31] women continued having greater psychological distress at six months follow-up than men. Nurses and nurse aides also had poorer mental health than physicians, being these differences better explained due to the closer contact and longer care nursing staff usually have with patients.[20] Not surprisingly, we found that working full-time (i.e., longer hours) significantly increased the PTSS and perceived stress compared to working part-time. Again, these results emphasise the need to reduce the working hours to guarantee adequate resting periods to decrease the impact of prolonged stress exposure at both personal and care levels.[30]

Just as in the baseline psychological assessment, the less physical activity healthcare workers reported daily, the poorer mental health they presented.[26] Working in Covid-19 hospitalisation wards and ICUs was related overall to greater psychological distress than working at the Emergency Service, being the levels of acute stress significantly lower in this latter group.[32] These differences between Covid-19 hospitalisation wards and ICU and Emergency Services are probably because, in most cases, the latter refers to the connecting unit between the initial phase of the disease and the patient's worsening (i.e., the Covid-19 ward and ICU services), which would entail a relatively lower emotional burden. In addition, the Emergency Service generally attends patients only during the first 24 h after being medically admitted, as opposed to days or weeks (or even months) as in the Covid-19 and ICU hospitalisation units, which may also lead to a lower psychological burden.

At six months follow-up, to have requested help and been taking stress-related medication continued increasing the overall levels of psychological distress,[28] and the psychiatric history also suggested worse psychological outcomes without statistically significant differences. These latter results added to the fact that substance abuse resulting from the pandemic accounted for almost 32% of the sample, increasing PTSS significantly, underlines the crucial importance of designing new effective intervention strategies to control, reduce and prevent the worsening of mental health of all healthcare workers when coping with critical scenarios as the current pandemic.

Nevertheless, considering the lack of specialists the current pandemic has brought with it, together with the cost-benefit of individualised sessions and mental health stigmatisation among healthcare workers, online psychotherapies could improve the accessibility and availability of mental health services in high-demand contexts. In addition, online psychotherapies also aid in reducing the spread of the virus that naturally occurs in face-to-face therapies.[33] Online Cognitive Behaviour Therapy (ICBT) has shown efficacy in a broad range of clinical settings: from psychiatric patients with substance abuse or severe disorders to patients with chronic pain, among others.[34,35] Accordingly, ICBT may be an interesting mental health strategy to combat the psychological burden associated with unprecedented health contexts as the current pandemic.

This study has some limitations. Firstly, the lack of a control group prevented comparing front-line healthcare workers and second-line healthcare workers (not directly involved in the care of Covid-19 patients). Secondly, using psychometric questionnaires that were not designed to evaluate anxiety, depression and acute stress in the context of the pandemic as a stressor maintained over time and without diagnostic capacity, as in the VASS test, may limit the interpretation of the results as some cases of psychological distress may have been underdiagnosed. Lastly, the inability to assess off work healthcare workers may hinder illustrating the real impact of the pandemic on healthcare workers' mental health. Even with these limitations, our findings are consistent with previously published works. In addition, our work sheds light on likely upcoming mental health consequences on healthcare workers and their worsening in the long-term if the situation is not rapidly resolved or coped efficiently. We strongly encourage future research to include long comprehensive follow-ups of healthcare workers. Also, to consider including a control group and other relevant study cohorts (i.e., second front-line healthcare workers and those in quarantine due to Covid-19 infection and could not be assessed) that allow additional comparisons.

Conclusion

In line with results obtained in previous cross-sectional investigations, the present work shows that the yet ongoing Covid-19 pandemic continues to cause a general deterioration of the mental health of frontline healthcare workers, especially regarding depression and the predisposition to perceiving the day-to-day experiences of the pandemic as a threat. In addition, while nurses and nurse aides showed poorer psychological outcomes at six months follow-up, physicians showed overall greater mental health, significantly reducing perceived stress and anxiety. Working part-time during the follow-up period enhanced healthcare workers' wellbeing more than those working full-time. At baseline

and six months follow-up, women, nurses, and nurse aides were at higher risk of psychological burden.

Understanding the need to enforce management standards to ensure reduced working hours and thus adequate resting periods, and foster the practice of physical exercise while together with new intervention strategies for all healthcare workers is of great value in reducing risk and vulnerability to psychological burden and professional burnout in unprecedented care scenarios such as the current pandemic.

Conflict of interest

None.

Acknowledgements

To first-line healthcare workers who, despite the care pressure, agreed to participate.

References

1. García-Iglesias JJ, Gómez-Salgado J, Martín-Pereira J, Fagundo-Rivera J, Ayuso-Murillo D, Martínez-Riera JR, et al. Impact of SARS-CoV-2 (Covid-19) on the mental health of healthcare professionals: a systematic review. Rev Esp Salud Publica. 2020;94, e202007088.
2. González-Sanguino C, Ausín B, Castellanos MÁ, Saiz J, López-Gómez A, Ugidos C, et al. Mental health consequences during the initial stage of the 2020 Coronavirus pandemic (COVID-19) in Spain. Brain Behav Immun. 2020;87:172–6, http://dx.doi.org/10.1016/j.bbi.2020.05.040.
3. Sobregrau Sangrà P, Aguiló Mir S, Castro Ribeiro T, Esteban-Sepúlveda S, García Pagès E, López Barbeito B, et al. Mental health assessment of Spanish healthcare workers during the SARS-CoV-2 pandemic. A cross-sectional study. Compr Psychiatry. 2022;112, http://dx.doi.org/10.1016/j.comppsych.2021.152278, 152278.
4. Albott CS, Wozniak JR, McGlinch BP, Wall MH, Gold BS, Vinogradov S. Battle buddies: rapid deployment of a psychological resilience intervention for health care workers during the COVID-19 pandemic. Anesth Analg. 2020;131:43–54, http://dx.doi.org/10.1213/ANE.0000000000004912.
5. Aguiló Mir S, García Pagès E, López Barbeito B, Ribeiro TC, Garzón-Rey JM, Aguiló Llobet J. Design and validation of an electrophysiological based tool to assess chronic stress. Case study: burnout syndrome in caregivers. Stress. 2021;24:384–93, http://dx.doi.org/10.1080/10253890.2020.1807512.
6. Andrade C. The limitations of online surveys. Indian J Psychol Med. 2020;42:575–6, http://dx.doi.org/10.1177/0253717620957496.
7. Maunder R. The experience of the 2003 SARS outbreak as a traumatic stress among frontline healthcare workers in Toronto: lessons learned. Philos Trans R Soc Lond B: Biol Sci. 2004;359:1117–25, http://dx.doi.org/10.1098/rstb.2004.1483.
8. Wu P, Fang Y, Guan Z, Fan B, Kong J, Yao Z, Liu X, Fuller CJ, Susser E, Lu J, Hoven CW. The psychological impact of the SARS epidemic on hospital employees in China: exposure, risk perception, and altruistic acceptance of risk. Can J Psychiatry. 2009;54:302–11, http://dx.doi.org/10.1177/070674370905400504.
9. Muller AE, Hafstad EV, Himmels JPW, Smedslund G, Flottorp S, Stensland SØ, et al. The mental health impact of the covid-19 pandemic on healthcare workers, and interventions to help them: a rapid systematic review. Psychiatry Res. 2020;293, http://dx.doi.org/10.1016/j.psychres.20113441, 113441.
10. Krishnamoorthy Y, Nagarajan R, Saya GK, Menon V. Prevalence of psychological morbidities among general population, healthcare workers and COVID-19 patients amidst the COVID-19 pandemic: a systematic review and meta-analysis. Psychiatry Res. 2020;293:113382, http://dx.doi.org/10.1016/j.psychres.20113382.
11. Maunder RG, Lancee WJ, Balderson KE, Bennett JP, Borgundvaag B, Evans S, Fernandes CM, Goldbloom DS, Gupta M, Hunter JJ, McGillis Hall L, Nagle LM, Pain C, Peczeniuk SS, Raymond G, Read N, Rourke SB, Steinberg RJ, Stewart TE, VanDeVelde-Coke S, Veldhorst GG, Wasylenki DA. Long-term psychological and occupational effects of providing hospital healthcare during SARS outbreak. Emerg Infect Dis. 2006;12:1924–32, http://dx.doi.org/10.3201/eid1212.060584.
12. Pierce M, McManus S, Jessop C, John A, Hotopf M, Ford T, et al. Says who? The significance of sampling in mental health surveys during COVID-19. Lancet Psychiatry. 2020;7:567–8, http://dx.doi.org/10.1016/S2215-0366(20)30237-6.
13. Lesage FX, Berjot S, Deschamps F. Clinical stress assessment using a visual analogue scale. Occu Med (Lond). 2012;62:600–5, http://dx.doi.org/10.1093/occmed/kqs140.
14. Cohen S, Kamarck T, Mermelstein R. A global measure of perceived stress. J Health Soc Behav. 1983;24:385–96, http://dx.doi.org/10.2307/2136404.
15. Pedrozo-Pupo JC, Pedrozo-Cortés MJ, Campo-Arias A. Perceived stress associated with COVID-19 epidemic in Colombia: an online survey. Cad Saude Publica. 2020;36, http://dx.doi.org/10.1590/0102-311x00090520, e00090520.
16. Weathers FW, Litz BT, Keane TM, Palmieri PA, Marx BP, Schnurr PP. PTSD checklist for DSM-5 (PCL-5); 2013. www.ptsd.va.gov [accessed 23.7.21].
17. Spielberger CD, Gorsuch RL, Lushene R, Vagg PR, Jacobs GA. Manual for the State-Trait Anxiety Inventory. Palo Alto, CA: Consulting Psychologists Press; 1983.
18. Kroenke K, Spitzer RL, Williams JBW. The patient health questionnaire-2. Med Care. 2003;41:1284–92, http://dx.doi.org/10.1097/01.MLR.0000093487.78664.3C.
19. Sasaki N, Asaoka H, Kuroda R, Tsuno K, Imamura K, Kawakami N. Sustained poor mental health among healthcare workers in COVID-19 pandemic: a longitudinal analysis of the four-wave panel survey over 8 months in Japan. J Occup Health. 2021;63:e12227, http://dx.doi.org/10.1002/1348-9585.12227.
20. Zhu Z, Xu S, Wang H, Liu Z, Wu J, Li G, et al. COVID-19 in Wuhan: sociodemographic characteristics and hospital support measures associated with the immediate psychological impact on healthcare workers. EClinicalMedicine. 2020;24, http://dx.doi.org/10.1016/j.eclinm.2020.100443, 100443.
21. Wong K, Chan AHS, Ngan SC. The effect of long working hours and overtime on occupational health: a meta-analysis of evidence from 1998 to 2018. Int J Environ Res Public Health. 2019;16:2102, http://dx.doi.org/10.3390/ijerph16122102.
22. Zerbini G, Ebigbo A, Reicherts P, Kunz M, Messman H. Psychosocial burden of healthcare professionals in times of covid-19 – a survey conducted at the university hospital Augsburg. Ger Med Sci. 2020;18:1–9, http://dx.doi.org/10.3205/000281.
23. Sato K, Kuroda S, Owan H. Mental health effects of long work hours, night and weekend work, and short rest periods. Soc Sci Med. 2020;246:112774, http://dx.doi.org/10.1016/j.socscimed.2019.112774.
24. Sobregrau P, Andreu C, Carreño M, Donaire A, Rumià J, Boget T, et al. Psychiatric disorders in patients with resistant temporal lobe epilepsy two years after undergoing elective surgery. A longitudinal study. Epilepsy Behav. 2021;118, http://dx.doi.org/10.1016/j.yebeh.2021.107921, 107921.
25. Cénat JM, Blais-Rochette C, Kokou-Kpolou CK, Noorishad PG, Mukunzi JN, McIntee SE, et al. Prevalence of symptoms of depression, anxiety, insomnia, posttraumatic stress disorder, and psychological distress among populations affected by the COVID-19 pandemic: a systematic review and meta-analysis. Psychiatry Res. 2021;295:113599, http://dx.doi.org/10.1016/j.psychres.2020.113599.
26. Biddle S. Physical activity and mental health: evidence is growing. World Psychiatry. 2016;15:176–7, http://dx.doi.org/10.1002/wps.20331.
27. Alonso J, Vilagut G, Mortier P, Ferrer M, Alayo I, Aragón-Peña A, et al. Mental health impact of the first wave of COVID-19 pandemic on Spanish healthcare workers: a large cross-sectional survey. Rev Psiquiatr Salud Ment. 2021;14:90–105, http://dx.doi.org/10.1016/j.rpsm.2020.12.001.
28. Mccauley JL, Killeen T, Gros DF, Brady KT, Back SE. Posttraumatic stress disorder and co-occurring substance use disorders: advances in assessment and treatment. Clin Psychol (N Y). 2012;19, http://dx.doi.org/10.1111/cpsp.12006.
29. Erquicia J, Valls L, Barja A, Gil S, Miquel J, Leal-Blanquet J, et al. Emotional impact of the Covid-19 pandemic on healthcare workers in one of the most important infection outbreaks in Europe. Med Clin. 2020;155:434–40, http://dx.doi.org/10.1016/j.medcli.2020.07.006.
30. Kang L, Ma S, Chen M, Yang J, Wang Y, Li R, et al. Impact on mental health and perceptions of psychological care among medical and nursing staff in Wuhan during the 2019 novel coronavirus disease outbreak: a cross-sectional study. Brain Behav Immun. 2020;87:11–7, http://dx.doi.org/10.1016/j.bbi.2020.03.028.
31. Rodríguez-Rey R, Garrido-Hernansaiz H, Collado S. Psychological impact of COVID-19 in Spain: early data report. Psychol Trauma. 2020;12:550–2, http://dx.doi.org/10.1037/tra0000943.
32. Lasalvia A, Bonetto C, Porru S, Carta A, Tardivo S, Bovo C, et al. Psychological impact of COVID-19 pandemic on healthcare workers in a highly burdened area of north-east Italy. Epidemiol Psychiatr Sci. 2021;30:1–13, http://dx.doi.org/10.1017/S2045796020001158.
33. Ho CS, Chee CY, Ho RC. Mental health strategies to combat the psychological impact of COVID-19 beyond paranoia and panic. Ann Acad Med. 2020;49:155–60.
34. Takano A, Miyamoto Y, Kawakami N, Matsumoto T. Web-based cognitive behavioral relapse prevention program with tailored feedback for people with methamphetamine and other drug use problems: development and usability study. JMIR Ment Health. 2016;3:e1, http://dx.doi.org/10.2196/mental.4875.
35. Soh HL, Ho RC, Ho CS, Tam WW. Efficacy of digital cognitive behavioural therapy for insomnia: a meta-analysis of randomised controlled trials. Sleep Med. 2020;75:315–25, http://dx.doi.org/10.1016/j.sleep.2020.08.020.
