# Supplementary material for: Mental health status and related factors influencing healthcare workers during the COVID-19 pandemic: A systematic review and meta-analysis
Source: PLoS One. 2024 Jan 19;19(1):e0289454. doi: 10.1371/journal.pone.0289454 (PMC10798549; doi:10.1371/journal.pone.0289454)
Supplement: S1 Data — (ZIP) [file pone.0289454.s011.zip › literatures/156.pdf]

The Impact of COVID-19 on the Mental Health of Healthcare Professionals

Sir,

The coronavirus disease (COVID-19) has been declared a pandemic of respiratory illness, posing a serious public health risk by spreading through various means. While information on its transmissibility is still limited, attention is being paid to minimise the harm by emphasising the public on hygiene, social distancing, and by implementing nationwide lockdown. Healthcare workers (HCWs) remain highly exposed to the disease through contact with their patients, where conventional protection with face masks has proved insufficient. For instance, a patient undergoing surgery in a hospital in Wuhan, China, infected 14 healthcare workers even before manifesting any symptoms.[1] There are estimates that 3.5% of all COVID-19 positive cases in China were HCWs, 14.8% of whom were severe or critical (WHO novel coronavirus situation, March 21, 2020). The figures from Italy reveal that 9% of all COVID-19 cases are healthcare workers.

According to a 2019 British survey, approximately 80% of physicians were found to be at high risk of burnout.[2] The burnout phenomenon, in turn, correlates with clinical depression, anxiety, sleep disturbance and substance misuse. Recent figures suggest a two-fold risk of suicidal ideation among doctors as compared to the general population in the US.[3] Though data from Pakistan is scarce, one study quotes the rates of depression and anxiety among hospital doctors to be 25% and 40%, respectively.[4] Most public hospitals in Pakistan lack the infrastructure and human resource to manage the current crisis, with poorly equipped system and staff unversed in health and safety principles. HCWs, already subject to burnout and mental stress, are now exposed to a novel virus that puts them at risk of contracting a fatal disease and creates a state of uncertainty, panic, apprehensiveness, and insecurity regarding their wellbeing as well as that of their family. Recently, two reports of committing suicide have emerged of European nurses dealing with COVID-19 patients. Medical mistrust is yet another challenge faced by HCWs, since the emergence of various unsupervised media platforms, and certain circulating rumors among the public, such as the coronavirus being a made-up disease or a conspiracy. This potentially raises rage and denial among the public, who therefore defy the rules set by authorities and refuse to comply with precautionary measures, increasing the risk of spread of the infection, consequently adding to the hospital workload. Since there is no independent legislation on occupational safety and health issues in the country, the physical and mental wellbeing of staff remains at stake.

Finally, the strained healthcare system offers insufficient mental health services, as only 0.4% of healthcare expenditures by the government health department are devoted to mental health, in contrast with 11.9% of its global burden of disease (WHO, 2008). Moreover, the nationwide lockdown and the stigma of having a mental illness drive the HCWs further away from seeking mental healthcare.

The ability of self-medication among doctors during mental distress may also potentially worsen their mental health struggles. Therefore, along with the implementation of preventive strategies against the disease, provisions of short-term and long-term mental health support including psychological first-aid, counselling and specialised psychiatric services, as well as occupational health services to healthcare workers need to be part of a comprehensive plan against the crisis.

CONFLICT OF INTEREST:
Authors declared no conflict of interest.

AUTHORS' CONTRIBUTION:
MAAS: Conceived the idea.
HS: Shared insights.
Both authors co-wrote and reviewed the final manuscript.

REFERENCES

1. Chang D, Xu H, Rebaza A, Sharma L, Cruz CSD. Protecting healthcare workers from subclinical coronavirus infection. *Lancet Respir* Med 2020; **8(3)**:e13.
2. Sykes C, Borthwick C, Baker E. Mental health and wellbeing in the medical profession. The British Medical Association 2019.
3. WebMD. (2018). Doctors Suicide Rate Highest of Any Profession. Retrieved from: http://www.webmd.com/mental-health/news/20180508/doctors-suicide-rate-highest-ofany-profession#1
4. Atif K, Khan HU, Ullah MZ, Shah FS, Latif A. Prevalence of anxiety and depression among doctors; The unscreened and undiagnosed clientele in Lahore, Pakistan. *Pak J Med Sci* 2016; **32(2)**:294-8.

Muhammad Ali Awab Sarwar[1] and Huda Sarwar[2]

[1]Akhtar Saeed Medical and Dental College, Lahore, Pakistan
[2]Institute of Public Health, Lahore, Pakistan

Correspondence to: Dr. Muhammad Ali Awab Sarwar, Akhtar Saeed Medical and Dental College, Lahore, Pakistan
E-mail: aliawab7@gmail.com

Received: April 27, 2020; Revised: May 24, 2020;
Accepted: July 02, 2020
DOI: https://doi.org/10.29271/jcpsp.2020.Supp.S83
