# Supplementary material for: Mental health status and related factors influencing healthcare workers during the COVID-19 pandemic: A systematic review and meta-analysis
Source: PLoS One. 2024 Jan 19;19(1):e0289454. doi: 10.1371/journal.pone.0289454 (PMC10798549; doi:10.1371/journal.pone.0289454)
Supplement: S1 Data — (ZIP) [file pone.0289454.s011.zip › literatures/157.pdf]

ELSEVIER

Since January 2020 Elsevier has created a COVID-19 resource centre with free information in English and Mandarin on the novel coronavirus COVID-19. The COVID-19 resource centre is hosted on Elsevier Connect, the company's public news and information website.

Elsevier hereby grants permission to make all its COVID-19-related research that is available on the COVID-19 resource centre - including this research content - immediately available in PubMed Central and other publicly funded repositories, such as the WHO COVID database with rights for unrestricted research re-use and analyses in any form or by any means with acknowledgement of the original source. These permissions are granted for free by Elsevier for as long as the COVID-19 resource centre remains active.

5. Bullard J, Dust K, Funk D, et al. Predicting infectious SARS-CoV-2 from diagnostic samples. Clin Infect Dis 2020.
6. Centers for Disease Control and Prevention. Testing guidelines for nursing homes. Interim SARS-CoV-2 testing guidelines for nursing home residents and healthcare personnel. Atlanta: Centers for Disease Control and Prevention; 2020.
7. Dosa D, Jump RL, LaPlante K, Gravenstein S. Long-term care facilities and the coronavirus epidemic: Practical guidelines for a population at highest risk. J Am Med Dir Assoc 2020;21:569−571.
8. Guglielmi G. The explosion of new coronavirus tests that could help to end the pandemic. Nature 2020;583:506−509.
9. Paltiel AD, Zheng A, Walensky RP. Assessment of SARS-CoV-2 screening strategies to permit the safe reopening of college campuses in the United States. JAMA Netw Open 2020;3:e2016818.
10. Larremore DB, Wilder B, Lester E, et al. Test sensitivity is secondary to frequency and turnaround time for COVID-19 surveillance. medRxiv 2020.

Charlotte Lanièce Delaunay, MPH
Department of Epidemiology
Biostatistics, and Occupational Health
School of Population and Global Health
McGill University
Montreal, Canada

Department of Medicine
McGill University Health Center
Montreal, Canada

Sahar Saeed, PhD
Department of Internal Medicine - Infectious Disease
Washington University School of Medicine
Institute for Public Health
Center for Dissemination and Implementation
St. Louis, MO, USA

Quoc Dinh Nguyen, MD, MA, MPH
Department of Epidemiology, Biostatistics, and Occupational Health
School of Population and Global Health
McGill University
Montreal, Canada

Division of Geriatric Medicine
Centre Hospitalier de l'Université de Montréal
Montreal, Canada

Centre de Recherche du Centre Hospitalier de l'Université de Montréal
Montreal
Canada

http://doi.org/10.1016/j.jamda.2020.08.022

Mental Health Impact of SARS-COV-2 Pandemic on Long-Term Care Facility Personnel in Poland

Dear Editor:

The COVID-19 pandemic has had a disproportionate impact on long-term care facility (LTCF) residents worldwide, with 19% to 72% of COVID-19-—related deaths occurring in LTCFs.[1] While facing this critical situation, LTCF personnel have to cope with an overwhelming workload, a depletion of adequate personal protection equipment (PPE), and deaths caused by SARS-CoV-2 infections.[2,3] As of this writing, there are few scientific studies addressing epidemiologic data and intervention models focused on LTCF and COVID-19. Therefore, the aim of this study was to assess psychological consequences (somatic symptoms, anxiety and insomnia, social dysfunction, and depression) among LTCF employees exposed to the SARS-CoV-2 coronavirus pandemic crisis. In addition, we investigated if factors such as PPE availability, safety guidelines, or access to psychiatric and psychological support at the workplace correlated with the level of psychological distress experienced by personnel.

Data were collected through an anonymous online survey between May 25 and June 25, 2020, among personnel of Polish LTCFs. The survey was accessed 242 times, and 12 participants' responses were rejected for leaving >70% questions unanswered. The response rate was 73.5% (n = 178 completed surveys); participants included LTCF personnel, including managers, administrative and maintenance staff, nurses, medical doctors, medical caregivers, social workers, physiotherapists, occupational therapists, and psychologists.

The survey consisted of 3 sections: (1) the sociodemographic section; (2) the authors' questionnaire with questions related to COVID-19 exposure, working conditions, access to PPE and mental health services; and (3) the General Health Questionnaire (GHQ-28),[4] which consists of 28 questions scored on a 4-point Likert-type scale, illustrating the frequency of specific psychopathological symptoms such as somatic symptoms, anxiety and insomnia, social dysfunction, and depression experiences over the preceding 4 weeks. Higher GHQ-28 scores indicate higher levels of distress. The study obtained ethical clearance (KB-365/2020) and was performed in accordance with the Declaration of Helsinki.

The statistical analysis was performed with the R for Windows package (version 4.0.2). The normality of data was analyzed using the D'Agostino-Pearson test and visual assessment. Comparisons of qualitative variables were performed using the chi-squared test. Qualitative and quantitative variables were compared using the Mann-Whitney or Kruskal-Wallis test. The level of statistical significance was set at 0.05.

The study findings demonstrate the importance of institutional factors of LTCF on mental health of employees (Table 1). First, LTCF staff who had PPE access at the workplace received lower scores in the GHQ-28 social dysfunction subscale ($P = .018$); especially important were disposable aprons ($P = .02$) and full-body protection suits ($P = .006$). These findings are in accordance with recent studies by Zhang et al[5] and Maciaszek et al[6] in which PPE access predicted better physical health and lower distress. Implementation of a mandatory face mask policy in Poland significantly reduced scores on all GHQ-28 subscales in the general population,[7] indicating the significance of PPE for both physical and mental health.

Second, the results suggest that the availability of workplace safety guidelines reduced anxiety symptoms. Participants whose workplaces had well-developed guidelines scored lower in the GHQ-28 anxiety and insomnia subscale ($P = .031$). It is also in line with the Medicare & Medicaid Services perspective that sets quality and safety standards in the health care system and defined one of its goals as prevention of COVID-19 transmission through issuing guidance and recommendations, providing PPE and testing needs recommendations in LTC facilities, and increasing payment for COVID-19 testing.[8]

Third, working conditions were crucial for the mental health of the respondents, as LTCF shift workers scored higher in the GHQ-28 somatic symptoms subscale ($P = .05$). The feeling that there were too few people in the workplace during the pandemic was related to the greater severity of psychopathological symptoms as evaluated with the GHQ-28 total score ($P = .009$).

Finally, availability of psychological support and care was also a crucial factor associated with better coping with the pandemic situation. Our study presents evidence that people who knew that they

The authors declare no conflicts of interest.

Table 1
Factors Related to the GHQ-28 Total Score and Its Subscales

Variable, Answer	n (%)	Score, Mean (SD)	*P* value
GHQ-28 total score			
Access to psychological support at workplace			
Yes	105 (59)	20.09 (8.07)	<.001*
No	74 (41)	26.16 (9.51)	
Too few employees compared to the workload due to COVID-19			
Strongly disagree	0	—	.009†
Disagree	5 (2.8)	24.00 (12.39)	
Neutral	61 (34.3)	20.50 (7.83)	
Agree	68 (38.2)	21.39 (7.59)	
Strongly agree	44 (24.7)	27.14 (11.27)	
Access to PPE: disposable aprons			
Yes	149 (83.7)	21.75 (8.36)	.02*
No	29 (16.3)	26.86 (11.81)	
Access to PPE: protection suits			
Yes	110 (61.8)	24.95 (9.97)	.006*
No	68 (38.2)	21.11 (8.35)	
GHQ-28 Social Dysfunction subscale			
Access to PPE in general			
Yes	173 (97.2)	6.65 (2.26)	.018*
No	5 (2.8)	9.75 (3.59)	
GHQ-28 Somatic Symptoms subscale			
Work in a shift system			
Yes	55 (30.9)	6.85 (2.71)	.05*
No	123 (69.1)	6.03 (2.40)	
GHQ-28 Anxiety and Insomnia subscale			
Availability of workplace safety guidelines during COVID-19			
Yes	173 (97.2)	5.45 (2.15)	.031*
No	5 (2.8)	6.70 (2.23)	

*Univariate analysis: Mann-Whitney *U* test.
†Univariate analysis: Kruskal-Wallis test.

have the opportunity to talk to a psychologist at their workplace (not necessarily use it) received significantly lower GHQ-28 total scores than personnel who did not have access to such services ($P < .01$).

In summary, our study argues that psychopathologic manifestations may be modifiable through workplace factors such as access to PPE, safety guidelines, and psychological support. As far as we are aware, no specific guidelines have been developed for managing personnel well-being in LTCF. Findings from this study create grounds for effective interventions aiming to restore psychological health in this group. Therefore, to avoid significant mental health crisis among LTCF personnel, availability of protective measures and psychological support should become a recommended response to COVID-19 internationally.

References

1. Gordon AL, Goodman C, Achterberg W, et al. Commentary: COVID in care homes—challenges and dilemmas in healthcare delivery. Age Ageing 2020;49: 701–705.
2. Kruse F, Remers T, Jeurissen P. The impact of COVID-19 on long-term care in the Netherlands. Available at: https://www.limburger.nl/cnt/dmf20200308_00150905/eerste-limburgse-coronadode-woonde-in-; 2020. Accessed August 25, 2020.
3. Care fit for carers: Ensuring the safety and welfare of NHS and care workers during and after Covid-19. IPPR. Available at: https://www.ippr.org/research/publications/care-fit-for-carers. Accessed August 25, 2020.
4. Makowska Z, Merecz D, Mościcka A, Kolasa W. The validity of General Health Questionnaires, GHQ-12 and GHQ-28, in mental health studies of working people. Int J Occup Med Environ Health 2002;15:353–362.
5. Zhang SX, Liu J, Afshar Jahanshahi A, et al. At the height of the storm: Healthcare staff's health conditions and job satisfaction and their associated predictors during the epidemic peak of COVID-19. Brain Behav Immun 2020;87: 144–146.
6. Maciaszek J, Ciulkowicz M, Misiak B, et al. Mental health of medical and non-medical professionals during the peak of the COVID-19 pandemic: A cross-sectional nationwide study. J Clin Med 2020;9:2527.
7. Szczesniak D, Ciulkowicz M, Maciaszek J, et al. Psychopathological responses and face mask restrictions during the COVID-19 outbreak: Results from a nationwide survey. Brain Behav Immun 2020;87:161–162.
8. Levitt AF, Ling SM. COVID-19 in the long-term care setting: The CMS perspective. J Am Geriatr Soc 2020;68:1366–1369.

Adrianna Senczyszyn, MSc
Department of Psychiatry
Wroclaw Medical University
Wroclaw, Poland

Katarzyna M. Lion, PhD
Menzies Health Institute Queensland
Griffith University
Brisbane, Australia

Dorota Szcześniak, PhD, Elżbieta Trypka, PhD
Department of Psychiatry
Wroclaw Medical University
Wroclaw, Poland

Justyna Mazurek, PhD
Department and Division of Medical Rehabilitation
Wroclaw Medical University
Wroclaw, Poland

Marta Ciułkowicz, MD, Maria Maćkowiak, MSc
Department of Psychiatry
Wroclaw Medical University
Wroclaw, Poland

Marta Duda-Sikuła, MBA
International Scientific Project Department
Wroclaw Medical University
Wroclaw, Poland

Renata Wallner, PhD, Joanna Rymaszewska
Department of Psychiatry
Wroclaw Medical University
Wroclaw, Poland

http://doi.org/10.1016/j.jamda.2020.09.020
