# Supplementary material for: Mental health status and related factors influencing healthcare workers during the COVID-19 pandemic: A systematic review and meta-analysis
Source: PLoS One. 2024 Jan 19;19(1):e0289454. doi: 10.1371/journal.pone.0289454 (PMC10798549; doi:10.1371/journal.pone.0289454)
Supplement: S1 Data — (ZIP) [file pone.0289454.s011.zip › literatures/158.pdf]

RESEARCH ARTICLE Turkish Journal of Psychiatry 2021;32(2):75-86

https://doi.org/10.5080/u25964

Burnout in Healthcare Professionals During the Covid-19 Pandemic in a Tertiary Care University Hospital: Evaluation of the Need for Psychological Support

Özen ÖNEN SERTÖZ[1], Özlem KUMAN TUNÇEL[2], Nezih SERTÖZ[3], Cenan HEPDURGUN[4], Damla İŞMAN HAZNEDAROĞLU[5], Canan BOR[6]

SUMMARY

Objective: Anxiety, stress and burnout are observed in healthcare workers during the Covid-19 pandemic. This study aimed to determine the levels of anxiety and burnout and related risk factors in the healthcare workers and assistant personnel at a tertiary referral university hospital.

Method: Hospital Anxiety Depression Scale (HADS), Maslach Burnout Inventory (MBI) and a sociodemographic questionnaire were delivered on-line to all employees of our hospital. 683 individuals were to the study who completed and returned the forms between April 15-30, 2020.

Results: Stress, depression and anxiety levels were significantly higher in nurses. Emotional burnout and depersonalization symptoms were higher and personal accomplishment scores were lower in resident physicians and nurses in comparison to the other groups. Being single, having a child and living away from home were factors underlying the burnout severity. Frontline work in the Covid-19 clinics did not affect the levels of anxiety, stress and depression. On the basis of the cut off points of the HADS scores; anxiety disorder (OR: 7.19) and depression (OR 3.43) were the most relevant risk factors for emotional exhaustion. Anxiety disorder was also the main risk factor for depersonalization. Depersonalization was 2.7-fold more among the nurses complaining of being overworked. Social support from the work environment and the family was protective against depersonalization.

Conclusion: Covid-19 pandemic has adversely affected the wellbeing of healthcare workers. The presence of depression and anxiety increased the risk of burnout. Social support can be expected to protect against burnout. It is, therefore, of great importance to implement urgently effective psychosocial and organizational interventions in order to protect the mental health of healthcare workers and to prevent burnout.

Keywords: Anxiety, depression, healthcare worker, burnout

INTRODUCTION

On December 31, 2019, the World Health Organization (WHO) China Country Office reported cases of pneumonia of unknown aetiology in Wuhan city, Hubei province of China. On January 7, 2020, the infectious agent was identified as a new Coronavirus that was not previously detected in humans. The whole world is currently battling against this disease, which was defined as Covid-19 (Turkish Ministry of Health, 2020). Having been caught unprepared by the rapid contagion and the high incidences of death due to the serious disease course, the strategies worldwide to bring the pandemic under control have come to the forefront. However, in contrast to much emphasis put on vaccines and treatments, systematic serious steps have not yet been taken against the risk factors of burnout among healthcare workers. The psychosomatic syndrome of burnout caused by stressful work environment presents as the detachment from the specific meaning and purpose of a profession, the inability to pay the requisite attention to others expecting a service or the psychological withdrawal from a job as the reaction to excessive stress and professional dissatisfaction (Kaçmaz

Received: 20.08.2020, **Accepted:** 26.01.2021, **Available Online Date:** 04.06.2021

[1]Prof., [2, 4, 5]MD., Ege University Faculty of Medicine, Department of Psychiatry, [3, 6]Assoc. Prof., Ege University Faculty of Medicine, Department of Anesthesiology and Reanimation, İzmir, Turkey.

e-mail: onensertoz@gmail.com

2005). The Maslach Burnout Inventory (MBI) was created as a screening tool for burnout with respect to symptoms of emotional exhaustion, depersonalization and professional accomplishment (Maslach 1976, Maslach and Jackson 1986, Maslach et al. 2001).

The studies on healthcare professionals in Turkey demonstrated that whereas heavy workload, long working hours, being young and inexperienced increased burnout (Erol et al. 2012, Balci et al. 2013, Güven 2013, Gülbayrak 2015, Kama 2017), high social support and having children (Erol et al. 2012, Güven 2013) and having better mental health (Soncu 2010) decreased burnout. There are contradictory results on the impact of gender and marital status on burnout (Özyurt et al. 2006, Şahin et al. 2008, Erol et al. 2012, Gülbayrak 2015). A recent review reported the mean prevalences of burnout to be 10% in the European Union (EU) countries and 17% in non-EU countries, varying as, for example, 25% in Turkey, 13% in Albania, and that the Medscape National Physician Burnout and Suicide Report (2020) referred to 43% incidence of burnout with an overall higher incidence among females (De Hert 2020).

Possible consequences of burnout in the workplace include decreased job satisfaction, absenteeism, staff turnover, and distrust, with adverse effects on the lives of employees including unhappiness, anxiety, depression, isolation, substance abuse, impaired family relationships and divorce. In physicians, burnout may lead to more serious consequences compared to other professions, such as inadequate patient care and patient dissatisfaction. The negative outcomes of burnout in the healthcare area may put a serious financial burden on both the caregiver and the hospital by causing medical errors (De Hert 2020). Researchers have paid attention to the effects of the Covid-19 pandemic on the mental health and burnout among healthcare professionals.

As Covid-19 is primarily transmitted by droplets and close contact, healthcare workers have the highest risk of exposure to the disease. It has been shown that healthcare workers experience depressive and anxiety symptoms during the Covid-19 pandemic (Chen et al. 2020, Koh 2020) and that the level of anxiety, stress and self-efficacy in healthcare workers correlates with social support and sleep quality (Xiao et al. 2020). A review of 14 investigations made during the Covid-19 pandemic on the stress level of healthcare workers mainly comprising nurses and other medical personnel reported that the highest strain and stress complaints were associated with anxiety disorder and depression. The severity of mental symptoms correlated with age, gender, profession, specialization, activities carried out and working in close contact with Covid-19 patients. Personnel selection, resilience, preventive psychosocial interventions and social support were among the variables that moderated the stress experience (Bohlken et al. 2020). The risk factors affecting mental well-being were limited hospital resources, lack of personal protective equipment (PPE) and not being able to access PPE, fear of exposure to the virus as occupational risk, long working hours, disruption of sleep, work-private life imbalance involving responsibilities and duties towards the patient despite the fear of transmitting Covid-19 to family members, ignoring personal and family needs due to increased workload, lack of communication and access to up-to-date information (Raudenska et al. 2020, Sasangohar et al. 2020).

For successful management of the pandemic, which is expected to last long, and better provision of healthcare, it is important to maintain the well-being of the healthcare professionals by determining the risk factors related to burnout, initiating psychosocial interventions as early as possible to prevent burnout development and organize the working conditions, the workload and equipment availability. Although anxiety among healthcare workers was investigated and reviews were written about the burnout syndrome with the advent of the Covid-19 in Turkey; there are not yet any up-to-date published investigations on burnout in healthcare workers.

Considering the mental effects of the combat against the Covid-19; this study aimed to determine the severity of burnout and the related risk factors among healthcare workers and non-medical personnel working under the difficult conditions of the pandemic. It is also aimed to use the results of the study in planning measures to increase the mental well-being of healthcare professionals.

METHODS

The approval of Ege University Medical Research Ethics Committee 06.04.2020 dated and 99166796-050.06.04 numbered was obtained for this study registered with the protocol number 20-4T / 12. Subsequently, the necessary permissions were obtained from the chief physician and the Deanery of the Faculty of Medicine in order to distribute the study questionnaire to the hospital staff. Also, approval was received from the Ministry of Health COVID-19 Scientific Research Evaluation Commission (https://bilimselarastirma.saglik.gov.tr/BasvuruForms/Özen Önen Sertöz-2020-05-04T14_56_26.xml).

Participants

A link was sent via text message or e-mail to all staff working in Ege University Hospital to recruit participants. The Google questionnaire form included brief information about the study; informed consent form; sociodemographic data form; questions about the place of work, place of assignment, working conditions, medical and psychiatric history, sleep and appetite,

stress level; the Hospital Anxiety Depression Scale (HADS) and the MBI were included. When the recipients ticked the study consent checkbox, they were able to view and answer further questions. The study was carried out during April 15-30, 2020 when a total of 716 completed forms and consents were received. However, 33 were found to be repeated entries with data entries that were consecutive in time and with all the same answers indicating duplicate entries. The data of the first entry were included in the study, such that the total data analysed in this study belonged to 683 participants. Working in the services and intensive care units where Covid-19 patients were treated, in the emergency department where samples of suspected Covid-19 patients were taken, and in the services where Covid-19 suspicious patients were followed, were defined as "working at the frontline".

Data Acquisition Tools

The Sociodemographic Questionnaire (SDQ): This document comprised questions on sociodemographic and clinical information such as the working conditions, job position, sleep-appetite changes, medical and psychiatric history.

The Maslach Burnout Inventory (MBI): The MBI, developed to measure burnout (Maslach and Jackson 1981) consists of 22 items evaluating burnout in three subdimensions on Emotional Exhaustion (MBI-EE), Depersonalization (MBI-DP) and Personal Accomplishment (MBI-PA). The MBI-EE and the MBI-DP use negative expressions and the MBI-PA consists of positive expressions. Therefore, the score on each subdimension is evaluated separately. High scores on the MBI-EE and MBI-DP and low score on the MBI-PA indicate burnout (Çam, 1992). The validity and reliability of the MBI adapted to the Turkish language was reported by Ergin (1992, 1996).

The Hospital Anxiety Depression Scale (HADS): Developed by Zigmond and Snaith (1983) the HADS has 2 subscales for assessing the level, changes of severity and the risk of anxiety on the HADS-A and depression on the HADS-D. Validity and reliability of the HADS-TR in the Turkish language was reported by Aydemir et al. (1997) with score cut-off points of 10 and 7, respectively, for the HADS-A and HADS-D. The participants of our study who scored above these cut-off values were accepted as having "anxiety disorder" or "depression", respectively. The HADS is also used in participants from the general population as well as with physical diseases (Sanne et al. 2003).

In addition to the psychometric scales completed, the participants were asked to score personal stress level between 0 and 10.

Statistical Analysis

The IBM SPSS Statistics 25.0 was used for data analysis. The Kolmogorov Smirnov test ($n \geq 50$) was used to confirm normality of data distribution. The numerical variables with and without normal distribution were expressed, respectively, by the mean and standard deviation and the median, maximum and minimum values or the interquartile range (IQR). Correlations between numerical variables with normal and non-normal distribution were determined by, respectively, the Pearson and the Spearman tests. For comparing more than two groups, the Kruskal Wallis Test was used, followed by the Dunn test with Bonferroni correction in the cases of significance. The Mann Whitney U and Student T tests were used for two group comparisons according to the distribution characteristics of numerical variables and the chi-square test was used for comparison of the categorical variables. Logistic Regression Analysis was performed to determine the risk factors associated with burnout. Results are given as OR (odds ratio), 0.95% confidence interval and the p value, with $p<0.05$ accepted as the statistical significance level for all hypotheses. Since the MBI subdimensions do not have cut-off points, the best cut-off point for each was determined with the ROC curve in order to apply logistic regression analyses.

RESULTS

General announcements at the Faculty of Medicine Hospital are sent to all staff registered in the hospital database. The Google questionnaire with information about the study was sent to a total of 5566 individuals comprising 1205 academicians (336 professors, 62 associate professors, 25 lecturers, 782 resident physicians), 53 specialist physicians, 1474 nurses, 1701 cleaning staff and 1133 other-administrative staff. The responding 683 staff were included in the study, which was undertaken in April 2020, during the first peak of the pandemic between March and June 2020, when flexible working system was adopted in our hospital and staff over the age of 60 and with chronic medical conditions had administrative leave. The questionnaire was completed by four groups consisting of academic specialist physicians, nurses, resident physicians and the assistant staff. The response rate to the questionnaire was calculated to be 15.4%, based on the number of the individuals in this group registered in the system.

The participants included 475 (69.5%) females and 208 (30.5%) males, with median age of 38 (20-66, min-max) years, median professional experience of 12 (0-42, min-max) years, comprising 94 (13.8%) resident doctors, 100 (14.6%) specialist doctors or lecturer-faculty members, 200 (29.3%) nurses and 289 (42.3%) assistant staff. The sociodemographic

Table 1. Comparison of the Sociodemographic Characteristics and Working Conditions of Four Different Healthcare Profession Groups

Variables		Faculty Members		Residents		Nurses		Assistant Staff	
		n	%	n	%	n	%	n	%
Female		68	14.3	60	63.8	184	92	163	56.4
Marital Status	Single	7	7	64	68.1	53	26.5	40	13.8
	Married	80	80	29	30.9	138	69	217	75.1
	Divorced or widowed	13	13	1	1.1	9	4.5	32	11.1
Have children		77	77	6	6.4	115	57.5	214	74
Assignment +		10	10	29	30.9	32	16	54	18.8
Change in shift-work system +		72	72	86	91.5	90	45	217	75.6
Work-shifts	Daytime	74	74	8	8.5	56	28	169	58.9
	Night	13	13	47	50	144	72	93	32.4
	24 hours	13	13	39	41.5	0	0	25	8.7
Accommodating away from home		6	6	4	4.3	23	11.5	26	9.1
Past psychiatric history +		27	27	36	38.3	58	29	82	28.4
Suicide attempt at past +		2	2	1	1.1	18	9	14	4.8
**Difficulties related to working conditions in the pandemic**		n	%	n	%	n	%	n	%
Job satisfaction		37	37	27	28.7	61	30,5	61	21,1
Lack of equipment		42	42	45	47.9	105	52,5	155	53,6
Lack of medical knowledge		31	31	43	45.7	80	40	104	36
Work overload in the shifts		13	13	29	30.9	49	24.5	64	22.1
Inadequacy of physical conditions (resting area, shower, toilets etc.)		29	29	43	45.7	101	50.5	111	38.4
Lack of proper nutrition		45	45	56	59.6	136	68	179	61.9
Mobbing		7	7	15	16	33	16.5	37	12.8
Communication problems with colleagues		28	28	30	31.9	37	18.5	49	17
Communication problems with patients and their relatives		21	21	34	36.2	51	25.5	41	14.2
Chronic disease +		41	41	12	12.8	71	35.5	8	30.4
Smoking +		14	14	25	26.6	63	31.5	114	39.4
		M	IQR	M	IQR	M	IQR	M	IQR
		(n=100)		(n=94)		(n=200)		(n=289)	
Age		47	15	28	3	36	14	40	11
The age of the youngest individual at home		15.5	29	27	3	17	22	13	19
The age of the oldest individual at home		52	14	42	26	45	15	49	14
Years in the profession		21.5	18.5	3	2	12[a]	14	13	13.5
Weekly working hours		40[b]	20	48[c]	30	40	2	40[d]	24
Knowledge level on Covid-19*		8	2	6	2	7[a]	2	7[d]	2

a: n=198, b: n=99, c: n=91, d: n=285.
*: participants are required to rate between 0-10.
M: median, IQR: interquartile range

data on these four groups are presented comparatively in Table 1 and their psychosocial evaluations are presented in Table 2.

Correlations of the MBI, HADS subscale scores and the data on the personal stress level with the other variables were analyzed in the whole sample and presented in the Table 3.

The correlations of the MBI subscale scores with the other variables were examined separately for each occupation group. Among the resident doctors, there were significant correlations between MBI-EE and the weekly working hours (r=0.213, p=0.043); MBI-PA and the knowledge level about Covid-19 (r=0.330, p=0.001). Among the academic faculty members MBI-EE was significantly correlated with age (r=-0.413, p<0.0001), the youngest person's age living at home (p=0.026; r=-0.223), the oldest person's age living at home (p=0.041; r=-0.204), years in the profession (p<0.0001; r=-0.426), weekly working duration (p=0.001; r=0.318), and sleep duration (p=0.007; r=-0.270); MBI-PA was significantly correlated with age (p<0.0001; r=0.429), youngest person's age living at home (p=0.012; r=0.250), oldest person's age

Table 2. Comparison of The Psychosocial Assessments of The Four Different Healthcare Profession Groups

Variables		Faculty Members (FM)		Residents (R)		Nurses (N)		Assistant Staff (AS)		Statistics
		n	%	n	%	n	%	n	%	
Sleep duration	Increased	14	14	34	36.2	37	18.5	34	11.8	χ^2=37.722 p<0.0001
	Decreased	34	34	30	31.9	100	50	134	46.7	
	No change	52	52	30	31.9	63	31.5	119	41.5	
Appetite	Increased	24	24	39	41.5	85	42.5	76	26.5	χ^2=34.755 p<0.0001
	Decreased	25	25	17	18.1	30	55	23	145	
	No change	51	51	38	40.4	55	27.5	145	50.5	
Weight	Increased	32	32	41	43.6	89	44.5	97	33.8	χ^2=14.489 p=0.025
	Decreased	17	17	10	10.6	40	20	49	17.1	
	No change	51	51	43	45.7	71	35.5	141	49.1	
Social support at work	Insufficient	19	19	30	31.9	71	35.5	76	26.5	χ^2=28.708 p=0.00007
	Somewhat sufficient	37	37	47	50	90	45	119	41.5	
	Sufficient	44	44	17	18.1	39	19.5	92	32.1	
Social support from friends	Insufficient	9	9	14	14.9	39	19.5	39	13.6	χ^2=29.481 p=0.0005
	Somewhat sufficient	29	29	45	47.9	100	50	138	48.1	
	Sufficient	62	62	35	37.2	61	30.5	110	38.3	
Social support from the family	Insufficient	4	4	3	3	10	5	22	7.7	χ^2=17.544 p=0.007
	Somewhat sufficient	11	11	19	20.2	57	28.5	61	21.3	
	Sufficient	85	85	72	76.6	133	66.5	204	71.1	
Anxiety disorder		63	63	66	70.2	163	81.5	186	64	χ^2=19.793 p=0.0002
Depression		23	23	31	33	111	55.5	103	35.6	χ^2=36.357 p<0.0001
		**M**	**IQR**	**M**	**IQR**	**M**	**IQR**	**M**	**IQR**	
		**(n=100)**		**(n=94)**		**(n=200)**		**(n=289)**		
Daily sleep duration (hours)		7	2	7	2	6	3	7^a	2	p=0.079
Stress score*		7	4	7.5	2	9^b	2	8^c	3	p<0.0001<br>FM-R: p=1<br>FM-N: p<0.0001<br>FM-AS: p<0.0001<br>R- N: p<0.0001<br>R- AS: p=0.004<br>N- AS: p=0.024
Emotional exhaustion		16	8	22	10	24	11	16	12	p<0.0001<br>FM -R: p<0.0001<br>FM - N: p<0.0001<br>FM-AS: p=1<br>R-N: p=0.663<br>R-AS: p<0.0001<br>N-AS: p<0.0001
Depersonalization		4	5	8	5	7	6	4	5	p<0.0001<br>FM-R: p<0.0001<br>FM-N: p<0.0001<br>FM- AS: p=1<br>R- N: p=0.040<br>R-AS: p<0.0001<br>N-AS: p<0.0001
Personal accomplishment		22	6	18.5	5	20	5	22	7	p<0.0001<br>FM - R: p<0.001<br>FM - N: p<0.001<br>FM - AS: p=0.937<br>R- N: p=0.211<br>R- AS: p<0.001<br>N- AS: p=0.001
Hospital Anxiety Depression-Anxiety score		9	5	9	6	12	6	9	6	p<0.0001<br>FM -R: p=1<br>FM-N: p<0.0001<br>FM- AS: p=1<br>R- N: p<0.0001<br>R- AS: p=1<br>N- AS: p<0.0001
Hospital Anxiety Depression-Depression score		8	5	8.5	7	11	6	9	6	p<0.0001<br>FM-R: p=1<br>FM - N: p<0.0001<br>FM - AS: p=0,171<br>R- N: p<0.0001<br>R- AS: p=1<br>N- AS: p<0.0001

a: n=287, b: n=198, c: n=285
*: participants are required to rate between 0-10.
M: median, IQR: interquartile range
Bonferroni correction was applied in pairwise comparisons. Dunn test was performed as post-hoc test.

Table 3. Correlations of the MBI and Hads Scores and the Data on Stress Level With the Other Variables

Variables	Age	The age of the youngest individual at home	The age of the oldest individual at home	Years of professional experience	Knowledge level on COVID-19*	Weekly working hours	Sleep duration (hours)
MBI-EE	p<0.0001 r=-0.318	p=0.476 r=0.027	p<0.0001 r=-0.186	p<0.0001 r=-0.242	p<0.0001 r=-0.163	p=0.0002 r=0.142	p<0.0001 r=-0.229
MBI-DP	p<0.0001 r=-0.275	p=0.008 r=0.102	p<0.0001 r=-0.149	p<0.0001 r=-0.251	p=0.0001 r=-0.148	p=0.001 r=0.132	p=0.006 r=-0.105
MBI-PA	p<0.0001 r=0.282	p=0.604 r=-0.020	p<0.0001 r=0.167	p<0.0001 r=0.228	p<0.0001 r=0.288	p=0.045 r=-0.077	p=0.141 r=0.056
HADS-A	p<0.0001 r=-0.149	p=0.002 r=-0.119	p<0.0001 r=-0.159	p=0.052 r=-0.074	p=0.002 r=-0.120	p=0.356 r=0.036	p<0.0001 r=-0.283
HADS-D	p=0.0003 r=-0.138	p=0.019 r=-0.090	p=0.003 r=-0.113	p=0.210 r=-0.048	p=0.001 r=-0.130	p=0.176 r=0.052	p<0.0001 r=-0.265
Stress score*	p=0.057 r=-0.073	p=0.002 r=-0.117	p=0.052 r=-0.075	p=0.250 r=-0.044	p=0.167 r=0.053	p=0.116 r=0.061	p<0.0001 r=-0.261
Sleep duration (hours)	p=0.677 r=-0.016	p=0.002 r=0.119	p=0.098 r=0.064	p=0.799 r=-0.010	p=0.435 r=0.030	p=0.576 r=-0.022	
Weekly working hours	p<0.0001 r=-0.266	p=0.452 r=0.029	p=0.046 r=-0.077	p<0.0001 r=-0.302	p=0.110 r=-0.062		
Knowledge level on COVID-19*	p<0.0001 r=0.245	p=0.342 r=0.037	p=0.051 r=-0.075	p<0.0001 r=0.204			
Years of professional experience	p<0.0001 r=0.860	p=0.115 r=-0.060	p<0.0001 r=0.321				
The age of the oldest individual at home	p<0.0001 r=0.365	p=0.166 r=0.053					
The age of the youngest individual at home	p=0.304 r=-0.039						

MBI-EE: Maslach Burnout Inventory, emotional exhaustion subscale; MBI-DP: Maslach Burnout Inventory, depersonalization subscale; MBI-PA: Maslach Burnout Inventory, personal accomplishment subscale; HADS-A: Hospital Anxiety Depression Scale, anxiety subscale; HADS-D: Hospital Anxiety Depression Scale, depression subscale
* participants are required to rate between 0-10.

living at home (p=0.0004; r=0.347), years in the profession (p<0.0001; r=0.397), and the level of knowledge about Covid-19 (p=0.006; r=0.272); MBI-DP was significantly correlated with age (p<0.0001; r=-0.457), youngest person's age at home (p=0.046; r=-0.200), oldest person's age living at home (p=0.0002; r=-0.366) and the years in the profession (p<0.0001; r=-0.464). Among the nurses, MBI-EE was significantly correlated with age (p<0.0001; r=-0.278), oldest person's age at home (p=0.009; r=-0.185), years in the profession (p=0.0001; r=-0.268), knowledge level about Covid-19 (p=0.013; r=-0.177), and sleep duration (p<0.0001; r=-0.295); MBI-PA was significantly correlated with age (p=0.013; r=0.176), oldest person's age living at home (p=0.009; r=0.185), years in the profession (p=0.011; r=0.181) and knowledge level about Covid-19 (p<0.0001; r=0.276); also there is correlation between the MBI-DP and sleep duration (p=0.010; r=-0.181). Among assistant staff, MBI-EE was significantly correlated with age (p=0.004; r=-0.170), level of knowledge about Covid-19 (p=0.006; r=-0.162) and sleep duration (p<0.0001; r=-0.273); MBI-PA was significantly correlated with age (p=0.047; r=0.117), and knowledge level about Covid-19 (p=0.0001; r=0.226); MBI-DP was significantly correlated with level of knowledge about Covid-19 (p=0.022; r=-0.135) and sleep duration (p=0.015; r=-0.144).

The effects of the categorical variables on the MBI score were analysed. Being on assignment did not create a statistically significant difference in the entire participant group and the four professional groups. Gender factor had an effect only in the assistant staff group with higher MBI-EE scores among female assistant staff as compared to the males (Z=-2.014, p=0.044). The effect of marital status on the level of burnout did not differ significantly in the four professional groups analysed separately but was found to have an effect on the MBI-EE (p<0.0001) and MBI-DP (p=0.003) scores of the entire group of participants. The MBI-EE and MBI-DP scores were higher among the singles as compared to the married participants (p<0.0001 and p=0.005, respectively) and the divorced or widowed participants (p=0.001 and p=0.035, respectively). The scores of all participants with children were lower on MBI-EE (Z=-5.603, p<0.0001) and MBI-DP (Z=-6.188, p<0.0001) and higher on MBI-PA (Z=-4.822, p<0.0001). The scores of the academic faculty members on MBI-EE (Z=-2.113, p=0.035) and MBI-DP (Z=-2.190, p=0.029), and of the nurses on MBI-EE (Z=-3.450, p=0.001) were lower. Separation from home had

Table 4. The Impact of Social Support at Work or From Family, Friends on the MBI Scores

	Workplace social support						Family social support					
	InS	SS	S		p		InS	SS	S		p	
**Residents**	Median-IQR or Mean±SD			SxInS	SxSS	SSxInS	Median-IQR or Mean±SD			SxInS	SxSS	SSxInS
MBI-EE	26-11	21-4	15-9	<0.001	0.015	0.010	23.3±2.1	26±6.9	20.2±7.6	0.749	0.009	0.837
MBI-DP	9.2±4.7	8.8±3	6.2±4.1	0.029	0.040	0.923	9±1	10.8±3.9	7.8±3.8	0.855	0.008	0.724
MBI-PA	18-4	18-4	23-6	0.001	0.002	1	18±1	18.3±5.1	18.5±3.9		p=0.962; F=0.039	
**Faculty Members**												
MBI-EE	21.5±8.9	16.1±5.8	13.5±5.9	<0.001	0.167	0.012	18-15	16-11	15-10		p=0.103	
MBI-DP	5-7	6-5	3.5-3	0.017	0.285	1	6.5-9	8-7	4-4		p=0.060	
MBI-PA	21.5±3.5	20.7±4.3	23.6±3.7	0.129	0.003	0.734	19.3±4.2	17.8±2.9	22.8±3.8	0.153	<0.001	0.791
**Nurses**												
MBI-EE	27-10	22.5-12	20-12	<0.001	0.237	<0.001	29-9	25-12	23-13	0.046	0.392	0.314
MBI-DP	8.2±3,8	7±4.3	4.7±3.4	<0.001	0.011	0.086	7.5-8	8-6	7-7		p=0.150	
MBI-PA	18-6	18-4	20-6	0.023	0.398	0.373	19.8±4.5	19.2±3.3	20±4.6		p=0.531, F=0.635	
**Assistant Staff**												
MBI-EE	21-13	16-11	13-14	<0.001	0.002	0,006	24.5-13	18-12	15-12	<0.001	0.054	0.009
MBI-DP	6.3±4.6	5.5±3.7	3.3±2.7	<0.001	0.002	0,001	10-6	6-7	4-4	<0.001	0.030	0.008
MBI-PA	20.5-9	21-6	24-6	<0.001	0.002	0.857	19.9±4.8	19.4±5.3	22.1±5	0.135	0.001	0.917
**Residents**	Friend social support											
MBI-EE	28.7±5,8	22.1±6	17.7±8.1	<0.001	0.014	0.006						
MBI-DP	9.86±5,6	8.82±3.26	7.43±3.72		p=0.098, F=1.161							
MBI-PA	18.5-9	19-6	18-7		0.576							
**Faculty Members**												
MBI-EE	19±8.2	18.6±7.9	14.4±6.1	0.144	0.021	0.984						
MBI-DP	4-8	6-6	4-4	1	0.026	0.730						
MBI-PA	22.3±4.3	20.7±4	22.8±4		p=0.062, F=2.869							
**Nurses**												
MBI-EE	28-8	24.5-12	22-13	<0.001	0.094	0.015						
MBI-DP	9-5	7-6	5-6	0.002	0.033	0.411						
MBI-PA	18-5	20-5	21-5	0.028	0.222	0.591						
**Assistant Staff**												
MBI-EE	22-13	28-11	13.5-12	<0.001	<0.001	0.061						
MBI-DP	6-6	5-6	3-3	<0.001	0.001	0.230						
MBI-PA	19-9	21-6	23-7	0.007	0.017	0.732						

Normally distributed variables are given with mean and standard deviation levels, skewed distributed variables are given with median and interquartile change levels.
SxInS: comparison of the group with sufficient social support with the group that has insufficient social support.
SxSS: comparison of the group with sufficient social support with the group that has somewhat sufficient social support.
SSxInS: comparison of the group with somewhat sufficient social support with the group that has insufficient social support.
If there is no significant difference in the comparison analysis of three groups, the p value obtained from the analysis of three group comparison is presented.
IQR: interquartile range, SD: standard deviation, S: Sufficient, InS: insufficient, SS: Somewhat sufficient, MBI-EE: Maslach Burnout Inventory, emotional exhaustion subscale; MBI-DP: Maslach Burnout Inventory, depersonalization subscale; MBI-PA: Maslach Burnout Inventory, personal accomplishment subscale; HADS-A: Hospital Anxiety Depression Scale, anxiety subscale;

effects on the nurse group only, with raised score on MBI-EE (Z=-2.777, p=0.005). MBI-DP (Z=-2.318, p=0.020) scores of the faculty members and MBI-EE (Z=-4.120, p<0.0001) scores of the nurses those involved in the treatment of Covid-19 patients were found to be higher (assistant staff were not taken into consideration in this analysis). Treating Covid-19 patients did not significantly affect the MBI scoring by the resident doctors. In relation to partaking in the diagnosis process for Covid-19, the scores of the resident doctors on MBI-PA (t=2.219, p=0.029) were lower and the scores of the academic faculty members on MBI-EE (t=2.602, p=0.011) and MBI-DP (Z=-2.565, p=0.011) were higher (only physicians were taken into consideration in this analysis). In all four groups, the participants with a history of psychiatric disorder diagnosed before the pandemic had higher scores on MBI-EE (Z=-3.229, p=0.001) and lower scores on MBI-PA (Z=-2.997, p=0.003). The scores of the resident doctors were raised on MBI-DP (Z=-2.275, p=0.023) and MBI-EE (t=2.596, p=0.011), and the academic faculty members had raised scores on MBI-EE (Z=-3.181, p=0.001) and lowered scores on the MBI-PA (t=2.008, p=0.047). The nurses had lower scores on MBI-PA (t=2.122, p=0.035), and the assistant staff had higher scores on MBI-EE (Z=-2.334, p=0.020). Participants from all groups who had to work for 24 hours during the pandemic had higher scores on MBI-EE (Z=-2.125, p=0.034) and MBI-DP (Z=-3.212, p=0.001) and lower scores on MBI-PA (p=0.045; Z=-2.000). In the same category, academic faculty members had higher scores on MBI-EE (t=-2.774, p=0.007) and MBI-DP (Z=-2.452, p=0.014). As summarized in Table 4; support from work, friends and family has also been found to be effective on the MBI subscale scores.

For all participants, the highest complaint about working conditions was on the lack of proper nutrition (n=416, 61%) and the least made complaint was on mobbing incidents (n=92, 13.5%). The correlation of the MBI scores with the complaints made by the entire group of participants on lack of personal equipment, inadequacy of knowledge, work overload in the shifts, inadequacy of physical conditions, lack of proper nutrition, professional dissatisfaction, mobbing or by the participants who described communication problems with colleagues, were significant with raised scores on the MBI-EE (respectively, p=0.002; 0.007; <0.0001; <0.0001; <0.0001; <0.0001; <0.0001; 0.003) and the raised scores on the MBI-DP (respectively, p=0.042; 0.0003; <0.0001; <0.0001; 0.012; 0.001; 0.0001; 0.002) scores, and lower MBI-PA (respectively, p=0.005; <0.0001; 0.015; 0.004; 0.004; 0.006; 0.002; 0.003) scores. All participants experiencing problems in communicating with patients and their relatives had higher MBI-DP scores than those who did not have this difficulty (Z=-2.842, p=0.004). When evaluated on group basis, complaints on overloaded shift work raised the MBI-EE (t=0.311, p=0.001) scores of the residents, of the assistant staff (Z=-2.118, p=0.034) and of the nurses (Z=-3.379, p=0.001) who also had raised scores on MBI-DP (Z=-3.881, p<0.0001) and lowered scores on MBI-PA (t=2.020, p=0.045). The assistant staff complaining of personal equipment shortage had higher scores on MBI-EE (Z=-2.578, p=0.010) and lower scores on MBI-PA (Z=-2.899, p=0.004). Complaints on lack of knowledge were associated with lower MBI-PA (t=1.996, p=0.047) scores among nurses; and with higher scores on MBI-EE (Z=-2.576, p=0.010;) and MBI-DP (Z=-3.522, p<0.001;) and lower scores on MBI-PA (Z=-3.570, p<0.001;) among the assistant staff. Complaints on lack of knowledge did not affect the burnout level among physicians. Complaints related to the inadequacies of the physical conditions were associated with higher scores of the resident doctors on MBI-DP (t=2.471, p=0.015), of the assistant staff on MBI-EE (Z=-3.576, p<0.001) and on MBI-DP (Z=-1.965, p=0.049) scores, and of the nurses on MBI-EE (Z=-2.984, p=0.003) and MBI-DP (Z=-2.170, p=0.030). In relation to complaints on lack of proper nutrition, the MBI-PA scores of the residents (t=2.256, p=0.026) were lower; the MBI-EE scores of the academic faculty members (t=2.984, p=0.004) and of the assistant staff (Z=-3.457, p=0.001) were higher. Whereas problems on professional dissatisfaction did not affect the MBI scores of the physicians; the MBI-EE (Z=-4.307, p<0.001) and the MBI-DP (Z=-2.493, p=0.013) scores of nurses were raised, and the MBI-PA (t=1.985, p=0.049) scores were lowered; and the MBI-EE (Z=-2.309, p=0.021) scores of the assistant staff were raised. Nurses who complained about mobbing had higher MBI-EE (Z=-3.160, p=0.002) and MBI-DP (Z=-2.739, p=0.006) scores, and lower MBI-PA (Z=-2.589, p=0.010) scores; and the assistant staff who shared the problem also had higher MBI-EE (Z=-4.427, p<0.001) and MBI-DP (Z=-2.143, p=0.032) scores. Whereas "having problems in communication with colleagues" did not affect the MBI scores of physicians and nurses; the assistant staff had higher scores on MBI-DP (Z=-2.771, p=0.006) and lower scores on MBI-PA (Z=-2.779, p=0.005). Communication problems with patients and their relatives did not affect the MBI scores of physicians and nurses but raised the scores among assistant staff on MBI-DP (Z=-2.194, p=0.028).

Since there is not a cut off score for MBI, variables associated with the scores on the burnout subscale in group comparisons were evaluated by regression analysis using robust modeling but significant correlations were not determined. In order to carry out a logistic regression analysis, the best cut off points for the MBI subscale scores were evaluated by the area under curve (AUC) in the ROC curve analysis and were found to be 17.5 for MBI-EE (AUC=0.847, p<0.001) and 5.5 for MBI-DP (AUC=0.726, p<0.001). An optimal cut-off point could

Table 5. Logistic Regression Identified Risk Factors Related to Burnout

	p	OR	95% Confidence interval
**Emotional Exhaustion**			
All participants			
Anxiety disorder	<0.001	7.195	4.354-11.892
Depression	<0.001	3.434	2.186-5.393
Weekly working hours	0.015	1.018	1.003-1.032
Complaint about the lack of medical knowledge	0.031	0.606	0.384-0.956
Complaint about the inadequacy of physical conditions	0.030	1.653	1.050-2.603
Complaint about lack of proper nutrition	0.040	1.592	1.021-2.483
Faculty members			
Anxiety disorder	0.017	7.120	1.425-35.568
Depression	0.007	10.342	1.887-56.691
The age of the youngest individual at home	0.022	0.907	0.834-0.986
Complaint about lack of proper nutrition	0.016	6.346	1.407-28.630
Resident Doctors			
Anxiety disorder	0.001	23.837	3.530-160.984
Past psychiatric history +	0.003	24.642	2.885-210.513
Nurses			
Anxiety disorder	<0.001	25.267	6.822-93.582
Depression	0.048	2.778	1.008-7.655
The age of the youngest individual at home	0.024	1.089	1.011-1.172
Assistant Staff			
Anxiety disorder	<0.001	6.805	3.077-15.050
Depression	<0.001	4.856	2.463-9.576
Complaint about the lack of medical knowledge	0.003	0.341	0.166-0.700
Complaint about the inadequacy of physical conditions	0.022	2.273	1.127-4.584
**Depersonalization**			
All participants			
Anxiety disorder	<0.001	2.420	1.550-3.778
Depression	0.003	1.832	1.225-2.739
Workplace social support	0.018	1.774	1.105-2.848
Family social support	0.005	1.812	1.193-2.752
The age of the youngest individual at home	0,026	1.023	1.003-1.045
Nurses			
Complaint about the work overload in the shifts	0.029	2.689	1.106-6.540
Assistant Staff			
Anxiety disorder	0.002	3.037	1.492-6.181
Depression	0.043	1.887	1.019-3.493
Family social support	0.003	2.581	1.396-4.774

OR: Odds ratio

not be determined for MBI-PA (AUC=0303, p<0.001). The scores equal to or above the cut-off points were evaluated as burnout in the relevant fields, and binary logistic regression analyses were carried out for the entire participant group and the individual occupational groups. The variables associated with MBI-EE and MBI-DP scores including anxiety disorder, depression, marital status, having children, social support in the work environment, social support in the family environment, social support from friends, age, age of the oldest person at home, years in the profession, Covid-19 knowledge level, weekly working hours, sleep duration, lack of personal protective equipment, lack of knowledge about Covid-19, change in working hours (categorical), physical conditions, nutritional deficiency, professional satisfaction, mobbing, colleague communication, psychiatric history and the 24-hour work status were further evaluated in the logistic regression analysis. Also, the variables found to be correlated with the MBI subscales in the participant groups were included in the model. In these analyses the "partially adequate" and "insufficient" social support categories were combined. A statistically significant model could not be constructed for depersonalization among the resident doctors and academic faculty members. The risk factors identified by multiple logistic regression analysis to be associated with emotional exhaustion and depersonalization in the entire group of participants and the occupational groups are presented in Table 5.

DISCUSSION

To the best of our knowledge, this is the first investigation in Turkey on burnout and related psychosocial factors in a large group of participants composed of healthcare workers and assistant staff in a tertiary hospital during the first wave of the Covid-19 pandemic. In our study, the levels of stress, depression and anxiety were found to be significantly higher in nurses compared to the other groups. Burnout scores were higher on emotional exhaustion and depersonalization subscales and lower on personal accomplishment subscale among the resident doctors and nurses as compared to the other groups. The most prevalent complaint on the working conditions of the pandemic was lack of proper nutrition, and the least expressed concern was mobbing. Analysis of the correlations between the complaints and the scores on burnout subscales showed that the participants complaining of lack of knowledge, inadequacy of physical conditions and lack of proper nutrition had higher emotional exhaustion scores in the entire group. All complaints, apart from the problems associated with communication with the patients and their relatives, lowered the personal accomplishment scores of the participants.

Anxiety disorder (OR: 7.19) and depression (OR: 3.43) were the risk factors that caused the highest increase in emotional burnout in the entire group of participants. Complaint on the lack of proper nutrition (OR: 6.34) was also a risk factor for burnout among the academic faculty members. Anxiety disorder also constituted the highest risk for depersonalization in the entire group, but the nurses who complained about loaded working hours had 2.7-fold higher risk of depersonalisation as compared to the nurses who did not make this complaint.

We found significantly raised anxiety and depression levels among the nurses in comparison to the other participant groups. Reports from Turkey and the world have confirmed the increases in anxiety and depression among healthcare workers with nurses being the most affected group (Sakaoğlu et al. 2020, Çankaya 2020, Lai et al. 2020, Liu et al. 2020). The contagiousness of the Covid-19 causing increased morbidity and mortality creates a pressure and worry among healthcare workers. Nurses work for long periods in close and frequent contact with the patients. During the SARS pandemic emergency department nurses had experienced more stress as compared to the physicians (Wong et al. 2005, Shih et al 2007, Chan 2003). Observation of more psychological symptoms among the nurses in our study is in agreement with the literature.

Within the entire participant group and in each occupational group, statistically significant differences with respect to the levels of anxiety, depression and stress were not determined between the frontline workers and the other participants who continued to work in their usual workplaces during the pandemic. Other studies reported that during the Covid-19 pandemic, the frontline healthcare workers were more exhausted (Barello et al. 2020) or less exhausted (Wu et al. 2020) in comparison to those not working in the frontline. In a questionnaire which was completed by healthcare workers from 60 different countries including Turkey, 50% of the participants gave affirmative response to the question ‘’Do you feel exhausted?” on grounds of the increased workload of the Covid-19 pandemic. The relatively less reports of burnout from Turkey as compared to the developed countries were ascribed to the pre-peak early stage of the pandemic (Morgantini et al. 2020).

In contrast to our results, a review of 14 articles on the subject stated that mental symptom severity was affected by working closely with Covid-19 patients (Bohlken et al. 2020). Also, it was reported that healthcare workers caring for Covid-19 patients had approximately twice the anxiety and depression levels compared to healthcare workers who had almost no contact with these patients (Lu et al. 2020). In our study, resident doctors and nurses had the highest symptoms of depersonalization and emotional exhaustion as compared to the other occupational subgroups indicating that they were under greater risk for burnout. However, there are studies reporting that nurses experienced more burnout than doctors (Morgantini et al. 2020, Wu et al. 2020).

In this study, it was found that longer shift hours increased burnout levels, whereas support from family, work and friends, and professional experience decreased burnout levels. In a multinational study, long working hours were among the factors associated with burnout (Morgantini et al. 2020). In general, factors such as professional experience, working hours, frequency of working on weekends and the number of people working together in the workplace are associated with burnout (Kamal et al. 2019). A review reported that social support perceived by healthcare professionals protected against stress related anxiety during the Covid-19 pandemic (Bohlken et al. 2020). In our study, individuals in the entire participant group complaining of lack of personal protective equipment, lack of information about Covid-19, busy working hours, inadequacy of physical conditions, lack of proper nutrition, professional dissatisfaction, mobbing or communication problems with colleagues had significantly higher scores on emotional exhaustion and depersonalization scores and lower personal accomplishment scores as assessed by the MBI. It was found that only depersonalization scores were higher among the participants experiencing communication problems with patients and their relatives. Our results on the effects of workplace difficulties related to lack of personal protective equipment, lack of knowledge about Covid-19, work overload in the shifts and inadequacy of physical conditions have similarities with the results reported in other countries (Morgantini et al. 2020, Sasangohar et al. 2020, Raudenska et al. 2020).

In this study, the risk factors for burnout determined by logistic regression analysis included anxiety disorder, depression, weekly working hours, lack of knowledge, complaints about inadequate physical conditions and complaints about nutritional inadequacies. The factors that increased the risk of depersonalization included anxiety disorder, depression, lack of social support from the work and family environments, and the age of the youngest person at home. Also, having a psychiatric history increased the risk of emotional exhaustion among the resident doctors by 24 times; and having anxiety disorder increased the risk of emotional exhaustion among the nurses by 25 times. The risk of depersonalization among the nurses complaining about overload shiftwork increased by 2.7 times as compared to those who did not express this problem. Anxiety disorder (OR: 3.037) caused the highest depersonalization risk among the assistant staff. Morgantini et al. (2020) found that, among the risk factors in healthcare workers; work that prevents meeting the responsibilities at home (time pressure), lack of time for education (excessive workload), exposure to Covid-19 patients, having to make a life-death decision (work stress) are associated with burnout;

while the factor related to personal protective equipment (lack of organizational support) was found to be protective in terms of burnout. In this study burnout was evaluated with the response to a single question and measurements related to depression and anxiety were not made (Morgantini et al. 2020). It can be said, however, that this study with references to "*excessive workload", "time pressure"* and *"insufficient organizational support"* shows similarities with the complaints about weekly working hours, lack of knowledge, inadequate physical conditions and nutritional irregularities, identified in our study as risk factors for burnout.

The fact that, in our study, anxiety disorder, depression and history of psychiatric disorders were among the risk factors for burnout showing that the mental health of the individual is an important factor in relation to burnout. Other studies in Turkey have also shown the adverse effects of the Covid-19 pandemic on the mental health of healthcare workers (Sakaoğlu et al. 2020, Çankaya 2020).

Acquisition of self-report data online that could not be compared with clinical interviews is a limitation of our study. The results may have been affected by the curtailing of the response to our survey by the presence of too many online surveys addressing the health sector and also by the changes in working conditions due to the pandemic. The group participating in the study may have consisted of people who experienced more difficulties during the pandemic. Moreover, having based the data on a single tertiary pandemic hospital as against collecting data from other pandemic hospitals may be considered as a limitation. The above cited limitations lower the generalizability of our study results. Multi-centered follow-up studies are needed to obtain further results regarding burnout among healthcare professionals during the Covid-19 pandemic.

The strength of our study is in being the first in Turkey to assess the mental well-being and the risk factors associated with burnout among healthcare workers and other staff during the first wave peak period of the pandemic. One of the important aspects of the study was the inclusion of assistant staff with a majority consisting of the cleaning staff.

CONCLUSION

Currently, it is necessary to evaluate burnout among healthcare workers as an outcome of social, cultural and technological pressures and not as a personal inadequacy (Launer 2020). During the Covid-19 pandemic, maintaining the wellbeing of healthcare workers is important for increasing the quality of patient care. Our study has shown that nurses constitute the high-risk group for developing stress, anxiety and depression; also nurses and resident doctors experience more burnout. In the Covid-19 pandemic, which is expected to last for a long time, taking measures to protect healthcare workers against the burnout syndrome, planning preventive psychosocial supports and organizations are of great importance for the wellbeing of healthcare workers and providing quality patient care.

REFERENCES

Aydemir O (1997) Hastane anksiyete ve depresyon ölçeği Türkçe formunun geçerlilik ve güvenilirliği. Turk Psikiyatri Derg 8:187–280.

Balcı UG, Demir Ş, Kurdal Y et al (2013) Hemşirelerde Tükenmişlik ve Yorgunluk Semptomları. Tepecik Eğit Hast Derg 23:83-7.

Barello S, Palamenghi L, Graffigna G (2020) Burnout and somatic symptoms among frontline healthcare professionals at the peak of the Italian COVID-19 pandemic. Psychiatry Res 290:11312, downloaded from https://www.sciencedirect.com/science/article/pii/S0165178120311975?via%3Dihub on 19th August 2020.

Bohlken J, Schömig F, Lemke MR et al (2020) COVID-19 pandemic: Stress experience of healthcare workers - A Short Current Review. Psychiatr Prax 47:190–7.

Çam O (1993) Tükenmişlik envanterinin geçerlik ve güvenirliğinin araştırılması. VII. National Congress of Psychology: Ankara. Türk Psikologlar Derneği Yayını (p. 155-9).

Çankaya M (2020) Covid-19 pandemisi ve sağlık çalışanlarının iyilik hali değişimi. Atlas International Congress on Social Sciences 7 Abstract Book (ed: Nagy H, Huseynov R) ISPEC Publications, 23-25 September Budapest, Hungary, p:446-60.

Chan S (2003) Nurses fighting against severe acute respiratory syndrome (SARS) in Hong Kong. J. Nurs Scholarsh 35:209.

Chen Q, Liang M, Li Y et al (2020) Mental health care for medical staff in China during the COVID-19 outbreak. Lancet Psychiatry 7:e15-e16.

De Hert S (2020) Burnout in Healthcare workers: Prevalence, impact and preventative Sstrategies. Local Reg Anesth 28;13:171-83.

Dugani S, Afari H, Hirschhorn LR et al (2018) Prevalence and factors associated with burnout among frontline primary health care providers in low- and middle-income countries: A systematic review. Gates Open Res 11;2:4.

Ergin C (1992) Doktor ve hemşirelerde tükenmişlik ve Maslach tükenmişlik ölçeğinin uyarlanması. VII. National Congress of Psychology: Ankara. Türk Psikologlar Derneği Yayını (p. 143-54).

Ergin C (1996) Maslach tükenmişlik ölçeğinin Türkiye sağlık personeli normları. 3P Dergisi 4:28–33.

Erol A, Akarca F, Değerli V et al (2012) Acil servis çalışanlarında tükenmişlik ve iş doyumu. Klinik Psikiyatri 15:103-10.

Gülbayrak B (2015) Üniversite, Kamu ve özel hastanelerde çalışan kadın ve erkek hemşirelerin tükenmişlik düzeyleri (unpublished thesis). University of Turkish Aeronautical Association-Graduate School of Social Science, Ankara.

Güven S (2013) Cerrahi kliniklerde çalışan hemşirelerin mizah tarzları ile tükenmişlik düzeyleri arasındaki ilişkinin saptanması (Unpubelished thesis). Başkent University Institute of Health Sciences Nursing Department, Ankara.

Kaçmaz N (2005) Tükenmişlik (Burnout) sendromu. İst Tıp Fak Derg 68:29-32.

Kama A (2017) Özel ve devlet hastanelerinin acil servis personellerinin iş doyumu ve tükenmişlik düzeylerinin karşılaştırılmasına yönelik Kahramanmaraş ilinde bir araştırma (Unpublished thesis) Kahramanmaraş Sütçü İmam University Institute of Social Sciences Department of Busidess Administration, Kahramanmaraş.

Kamal AH, Bull JH, Wolf SP et al (2016) Prevalence and predictors of burnout among hospice and palliative care clinicians in the US. J Pain Symptom Manage 51:690-6.

Koh D (2020) Occupational risks for COVID-19 infection. Occup Med (Lond) 12;70:3-5.

Lai J, Ma S, Wang Y et al (2020) Factors Associated With Mental Health Outcomes Among Health Care Workers Exposed to Coronavirus Disease. JAMA Netw Open 2;3(3):e203976. downloaded from https://jamanetwork.com/journals/jamanetworkopen/fullarticle/10.1001/jamanetworkopen.2020.3976 on 19th August 2020.

Launer J (2020) Burnout in the age of COVID-19. Postgrad Med J 96(1136), 367–8.

Liu CY, Yang YZ, Zhang XM et al (2020) The prevalence and influencing factors in anxiety in medical workers fighting COVID-19 in China: a cross-sectional survey. Epidemiol Infect 148, e98. downloaded from https://www.cambridge.org/core/journals/epidemiology-and-infection/article/prevalence-and-influencing-factors-in-anxiety-in-medical-workers-fighting-covid19-in-china-a-crosssectional-survey/D493886C34C350A01D3D0501AC89B9CB on 19th August 2020.

Lu W, Wang H, Lin YL et al (2020) Psychological status of medical workforce during the COVID-19 pandemic: A cross-sectional study. Psychiatry Res 288, 112936, 1-5.

Maslach C, Jackson SE (1981) The measurement of experienced burnout. J Organ Behav 2:99–113.

Maslach C (1976) Burned-out. Human Behav 5:16–22.

Maslach C, Jackson SE (1986) Maslach Burnout Inventory Manual. 2nd ed. Consulting Psychologist Press, Palo Alto.

Maslach C, Schaufeli WB, Leiter MP (2001) Job burnout. Annu Rev Psychol 52:397–422.

Morgantini LA, Naha U, Wang H et al (2020) Factors contributing to healthcare professional burnout during the COVID-19 pandemic: A Rapid turnaround global survey. Preprint. downloaded from https://www.medrxiv.org/content/10.1101/2020.05.17.20101915v1 on 19th August 2020.

Özyurt A, Hayran O, Sur H (2006) Predictors of burnout and job satisfaction among Turkish physicians. Q J Med 99:161-9.

Raudenska J, Steinerova V, Javurkova A et al (2020) Occupational burnout syndrome and post traumatic stress among healthcare professionals during the novel coronavirus disease 2019 pandemic. Best Prac and Res Clin Anaest 34:553-60.

Sakaoğlu HH, Orbatu D, Emiroğlu M et al (2020) Covid-19 salgını sırasında sağlık çalışanlarında Spielberger Durumluk ve Sürekli Kaygı düzeyi. Tepecik Eğit Hast Derg 30:1-9.

Sanne B, Mykletun A, Dahl AA et al (2003) Occupational differences in levels of anxiety and depression: the Hordaland Health Study. J Occup Environ Med 45:628–38.

Sasangohar F, Jones SL, Masud FN et al (2020) Provider burnout and fatigue during the COVID-19 pandemic: lessons learned from a high-volume intensive care unit. Anesth Analg 131:106–11.

Shah K, Chaudhari G, Kamrai D et al (2020) How essential is to focus on physician's health and burnout in coronavirus (COVID-19) pandemic? Cureus 12:10–2.

Shih FJ, Gau ML, Kao CC et al (2007) Dying and caring on the edge: Taiwan's surviving nurses' reflections on taking care of patients with severe acute respiratory syndrome. Appl Nurs Res 20:171–80.

Soncu E (2010) Türkiye'deki ruh sağlığı çalışanlarının psikiyatrik semptom düzeyleri, bağlanma sitilleri ve tükenmişlik seviyeleri (Unpublished thesis). İstanbul Bilgi University Institute of Social Sciences, İstanbul.

Şahin D, Turan FN, Alparslan N et al (2008) Devlet hastanesinde çalışan sağlık personelinin tükenmişlik düzeyleri. Nöropsikiyatri Arşivi 45:116-21.

TR Ministry of Health (2020) Covid-19 (SARS-CoV-2 Enfeksiyonu) Genel bilgiler, epidemiyoloji ve tanı, Downloaded from https://covid19bilgi.saglik.gov.tr/depo/rehberler/covid-19-rehberi/COVID-19_REHBERI_GENEL_BILGILER_EPIDEMIYOLOJI_VE_TANI.pdf on 19th August 2020.

Wong TW, Yau JKY, Chan CLW et al (2005) The psychological impact of severe acute respiratory syndrome outbreak on healthcare workers in emergency departments and how they cope. Eur J Emerg Med 12:13–8.

Wu Y, Wang J, Luo C et al (2020) A comparison of burnout frequency among oncology physicians and nurses working on the frontline and usual wards during the COVID-19 epidemic in Wuhan, China. J Pain Symptom Manage 60:e60–e65.

Xiao H, Zhang Y, Kong D et al (2020) The effects of social support on sleep quality of medical staff treating patients with coronavirus disease 2019 (COVID-19) in January and February 2020 in China. Med Sci Monit. 5;26, e923549 downloaded from https://www.medscimonit.com/download/index/idArt/923549 on 19th August 2020.

Zigmond AS, Snaith RP (1983) The hospital anxiety and depression scale. Acta Psychiatr Scand 67(6), 361–70.
