# Supplementary material for: Mental health status and related factors influencing healthcare workers during the COVID-19 pandemic: A systematic review and meta-analysis
Source: PLoS One. 2024 Jan 19;19(1):e0289454. doi: 10.1371/journal.pone.0289454 (PMC10798549; doi:10.1371/journal.pone.0289454)
Supplement: S1 Data — (ZIP) [file pone.0289454.s011.zip › literatures/159.pdf]

Journal of Multidisciplinary Healthcare

Dovepress
open access to scientific and medical research

Open Access Full Text Article

ORIGINAL RESEARCH

Anxiety and Resilience of Healthcare Workers During COVID-19 Pandemic in Indonesia

This article was published in the following Dove Press journal:
Journal of Multidisciplinary Healthcare

Yunias Setiawati[1,2]
Joni Wahyuhadi[3,4]
Florentina Joestandari[5]
Margarita M Maramis[1,2]
Atika Atika[6]

[1]Department of Psychiatry, Faculty of Medicine, Universitas Airlangga, Surabaya, Jawa Timur, Indonesia; [2]Department of Psychiatry, Dr. Soetomo General Hospital, Surabaya, Jawa Timur, Indonesia; [3]Faculty of Medicine, Universitas Airlangga, Surabaya, Jawa Timur, Indonesia; [4]Department of Neuro Surgery, Dr. Soetomo General Hospital, Surabaya, Jawa Timur, Indonesia; [5]Human Resource Department, Dr Soetomo General Hospital, Surabaya, Jawa Timur, Indonesia; [6]Department of Public Health, Universitas Airlangga, Surabaya, Jawa Timur, Indonesia

Introduction: The COVID-19 pandemic has an impact on the physical health and mental health of the community, including healthcare workers. Several studies have shown symptoms of depression, anxiety, and sleep disorders in healthcare workers during this pandemic. However, not many studies have examined the resilience of healthcare workers during this pandemic. Resilience is a person's ability to rise and adapt when times are difficult and is considered to have a protective effect on mental problems.

Purpose: This study aims to determine the correlation between resilience and anxiety in healthcare workers during COVID-19 pandemic.

Materials and Methods: This research was a cross-sectional study with observational analytic methods. The respondents were healthcare workers at Dr. Soetomo Hospital as the COVID-19 referral hospital in Surabaya, East Java, Indonesia. Data were collected from 10 to 16 June 2020 by distributing online questionnaires through the Google form application. There were three questionnaires used: demographic data, the State-Trait Anxiety Inventory (STAI) questionnaire, and the Connor–Davidson Resilience Scale (CR-RISC) questionnaire.

Results: The 227 respondents had filled out the questionnaire online with 33% had high state anxiety and 26.9% had high trait anxiety. The mean score of the respondents' resilience was 69 ± 15.823. The Spearman correlation test showed a significant relationship between anxiety and resilience (p <0.05), both S-Anxiety and T-Anxiety.

Conclusion: A significant correlation was found between the level of resilience and anxiety experienced by healthcare workers during the COVID-19 pandemic. The lower the resilience, the higher the anxiety experienced.

Keywords: state anxiety, trait anxiety, mental health, mental illness

Introduction

Background

Cases of Coronavirus Disease (COVID-19) were first discovered in Wuhan, China at the end of December 2019. Since then, COVID-19 cases have spread rapidly to other countries until finally WHO established COVID-19 as a public health emergency of international concern.[1] Indonesia confirmed its first positive case on March 2, 2020, and continues to have an increase in the number of positive cases to 29,521 cases with a total death toll of 1770 (as of June 5, 2020).[2]

This COVID-19 pandemic not only impacts physical health but also affects mental health. COVID-19 is a new disease so researchers are still trying to identify the characteristics of this disease, such as the way it is transmitted, its treatment, and its prevention. Uncertainty about the things faced can increase anxiety in the community.[3,4] Several studies have shown that outbreaks affect the

Correspondence: Yunias Setiawati
Department of Psychiatry, Faculty of Medicine, Universitas Airlangga, Jl. Mayjen. Prof. Dr. Moestopo, 47, Surabaya, Jawa Timur 60286, Indonesia
Tel +62315501681 60286
Fax +62315022472
Email yunias.setiawati@gmail.com

submit your manuscript | www.dovepress.com
DovePress
http://doi.org/10.2147/JMDH.S276655

© 2021 Setiawati et al. This work is published and licensed by Dove Medical Press Limited. The full terms of this license are available at https://www.dovepress.com/terms.php and incorporate the Creative Commons Attribution – Non Commercial (unported, v3.0) License (http://creativecommons.org/licenses/by-nc/3.0/). By accessing the work you hereby accept the Terms. Non-commercial uses of the work are permitted without any further permission from Dove Medical Press Limited, provided the work is properly attributed. For permission for commercial use of this work, please see paragraphs 4.2 and 5 of our Terms (https://www.dovepress.com/terms.php).

psychological state of society. A study conducted in the early phases of the COVID-19 pandemic in China involved 1210 respondents from the general population and showed that more than half of the respondents experienced moderate to severe psychological impact. Besides, one-third of the respondents experienced moderate to severe anxiety.[5] Another study examined two aspects of anxiety: state anxiety and trait anxiety. State anxiety is the anxiety feeling that is felt when facing a difficult situation, while trait anxiety is the tendency of one's personality to experience state anxiety. This study found that the ratio of state anxiety in respondents was greater than the ratio of trait anxiety which showed that this pandemic could play a role in causing anxiety in the community.[6]

The impact on mental health also occurs in healthcare workers, especially those who are at the forefront in dealing with COVID-19. Healthcare workers have a high risk of becoming infected due to exposure to patients and they have heavy workloads so that it can affect their psychological status. A study in China showed symptoms of depression, somatization, and anxiety are higher in healthcare workers in COVID-19 referral hospitals compared to healthcare workers who worked in hospitals that were not COVID-19 referrals.[7] During the Severe Acute Respiratory Syndrome (SARS) outbreak in 2003, a study showed that anxiety in healthcare workers who made contact with SARS patients was significantly higher when compared to healthcare workers who did not make contact with SARS patients.[8]

In dealing with outbreaks, the ability to adapt and overcome existing difficulties is needed. This is known as resilience. A study showed that resilience can act as a protective factor for mental illness, such as anxiety and depression. This indicates that resilience is related to the level of anxiety of health workers, in the sense that: the more resilient a person is, the better his mental health is.[9]

Mahmood & Ghaffar (2014) explain that resilience is a good adaptation process in situations of trauma, tragedy, or other stressful events. Resilience is not a personality trait but rather something that involves behavior, thoughts, or actions that anyone can learn.[10] According to Bonanno, resilience is the ability of a person in normal circumstances when faced with an event that has the potential to interfere physically and mentally. In this case, resilience is one of the right ways to deal with an adverse event or one that can cause trauma, because resilience is an ability to maintain a stable balance.[11]

Although the references above indicate a link between the level of anxiety and resilience of health workers with regard to their work situation; and many researchers have studied anxiety, depression, and sleep problems in healthcare workers during this pandemic, but there has not been much research on resilience during this pandemic, prompting us to conduct this research. To deal with a long-term pandemic where definitive therapy has not yet been found, resilience is needed for healthcare workers to manage this COVID-19 pandemic.[12,13]

Purpose

This study aims to determine the level of resilience and anxiety in healthcare workers who represent important aspects in handling COVID-19 outbreaks and to find out whether there is a correlation between the level of resilience and anxiety in healthcare workers in Indonesia during the COVID-19 pandemic.

Materials and Methods

Time, Location and Design of the Study

This research was carried out in relation to the occurrence of the COVID-19 pandemic in Indonesia, especially at referral centers for affected patients. Therefore, Dr. Soetomo Hospital, Surabaya as one of the national referral hospitals in Indonesia was chosen as the research location. The study was conducted in early June to early August 2020. This research used a cross-sectional design to prove the correlation between resilience and anxiety levels of health workers during the COVID-19 pandemic era.

Subjects of the Study

The respondents of this study were healthcare workers at Dr. Soetomo Hospital as the COVID-19 referral hospital in Surabaya, East Java, Indonesia. This hospital has increased the capacity of its isolation room which was originally only 14 beds to 255 beds for handling COVID-19 cases. Healthcare workers who were the subjects of this study consisted of all components that support the functioning of the hospital from 11 categories, namely: pharmacists, pharmacist assistants, midwives, physiotherapist, nutritionists, nurses, medical record officers, laboratory staff, psychologists, electromedical technicians and supporting staff.

The total of all categories of health workers mentioned above was 227 people, and all of them were involved as

research subjects, so that the respondents were selected using the total sampling technique.

Data Collection and Instruments

Data were collected from 10 to 16 June 2020 by distributing online questionnaires through the Google form application. The respondents gave informed consent before taking part in this study. Although the data is collected online, the respondents actually only come from one hospital which is the national level referral in Indonesia. Thus, complete and valid information from the respondents can be clearly identified by the researcher. The implementation of online data collection is more intended to limit physical contact between researchers and respondents, as it is known that limiting physical contact is important to reduce the spread of COVID-19.

Three parts of the questionnaire must be filled out, namely demographic data, the State-Trait Anxiety Inventory (STAI) questionnaire, and the Connor–Davidson Resilience Scale (CR-RISC) questionnaire. In order for the research to be carried out easily, all parts of the questionnaire were written in Indonesian, as the respondent's native language, by testing its validity and reliability.

State-Trait Anxiety Inventory (STAI)

The State-Trait Anxiety Inventory (STAI) was created by Charles D. Spielberger in collaboration with Richard L. Gorsuch and Robert C. Lushene (1983). STAI is used to measure anxiety symptoms using the self-report method or questionnaire. This anxiety test aims to measure two concepts of anxiety which are momentary anxiety (State anxiety) and basic anxiety (Trait anxiety). State anxiety is an unpleasant emotion caused by something threatening and dangerous. State anxiety evaluates the current state of anxiety, asking how respondents are currently feeling using items that measure subjective feelings of fear, tension, nervousness, anxiety, and activation of autonomic nerves. Trait anxiety evaluates more stable aspects of anxiety including calmness, self-confidence, and a feeling of security.[14]

The 40 statements on the STAI questionnaire consist of 20 statements to evaluate state anxiety and 20 statements to evaluate trait anxiety. The responses for the S-Anxiety questionnaire are 1) Not at all, 2) Somewhat, 3) Moderately so, 4) Very much so. The responses for the T-Anxiety questionnaire are 1) Almost never, 2) Sometimes, 3) Often, 4) Almost always. The total score interpretation for each sub-test ranges from 20 to 80. Scores of 20–37 are categorized as no or low anxiety, scores of 38–44 are categorized as moderate anxiety, and scores of 45–80 are categorized as high anxiety.[14]

Several other studies in the COVID-19 pandemic era also used the same instrument, for example, research on the mental health of health workers in Italy,[15] research on anxiety among health workers and the general public in Turkey,[16] and research on anxiety from adolescents in Turkey.[17]

All items in the questionnaire were declared to have passed the content validity test based on references and the results of consultations with relevant experts from the Soetomo Hospital Surabaya, had passed the construct validity test with p-value >0.05 for all items of STAI I and STAI II, and declared reliable based on the results of the Cronbach-alpha test (STAI I = 0.940 and STAI II = 0.905).

Connor–Davidson Resilience Scale – 25 (CD RISC-25)

Connor–Davidson Resilience Scale – 25 (CD RISC-25) is a tool used to measure a person's resilience. CD RISC-25 consists of 25 statements originating from 17 domains of characteristics of people who have resilience, namely: the ability to see change or stress as a challenge/opportunity, commitment, the realization that not everything can be controlled, seeking support from others, closeness to others, self-efficacy, the reinforcing effects of stress, past success, realistic feelings about control and availability of choices, sense of humor, action-oriented approach, patience, tolerance for negative feelings, adaptation to change, optimism, and faith.[18]

Each statement on CD RISC-25 has five response options: 1) Strongly Disagree, 2) Disagree, 3) Slightly Agree, 4) Agree, 5) Strongly Agree. The answers obtained will be given a score of 0–4 with a minimum total value of zero and a maximum of 100. The score obtained illustrates the level of resilience of the subject.[19]

The RISC-25 CD was developed by Jonathan Davidson and Kathryn Connor as a measurement tool for assessing resilience in clinical and research situations. CD RISC-25 has a reliability of 0.87 which indicates that the CD RISC-25 is suitable to be used to assess resilience in this study.[19]

Several other studies in the COVID-19 pandemic era also used the same instruments, for example, research on

the anxiety and resilience of physicians in Israel,[20] as well as research on individual resilience in various countries.[21]

All items in the questionnaire were declared to have passed the content validity test based on references and the results of consultations with relevant experts from the Seotomo Hospital Surabaya, had passed the construct validity test with p-value >0.05 for all items, and declared reliable based on the results of the Cronbach-alpha test (0.968).

Data Management and Analysis

The data processing and analysis procedures were as follows: 1) data cleaning (checking the accuracy and completeness of the data; 2) tabulation of raw data for easy analysis; 3) data normality testing using the one-sample Kolmogorov–Smirnov test; 4) hypothesis testing based on the results of the normality test, namely the Pearson correlation test for normally distributed data or the Spearman correlation test for data not normally distributed.

Ethical Approval

In order to ensure that this research is free from ethical violations, ethical approval has been obtained from the Health Research Ethics Committee of Dr. Soetomo Hospital, Surabaya, Indonesia. All major aspects of research ethics have been examined, namely 1) respect for the autonomy of respondents, without neglecting informed consent which is accompanied by information about the objectives and the duration of the study, research procedures, risks and inconveniences, benefits for the respondent, anonymity and freedom of resignation at any time; 2) provide benefits for respondents; 3) ensure efficiency; and 4) ensure fairness for respondents.

Results

There were 227 respondents who had filled out the questionnaire online, consisting of 38 men (16.7%) and 189 women (83.3%) with an average age of 39.67 ± 9434 (Table 1). The mean value of the respondents' resilience was 69 ± 15,823 (Table 2). A total of 75 respondents (33%) had high state anxiety and 61 respondents (26.9%) had high trait anxiety (Table 3).

The one-sample Kolmogorov Smirnov test was used to determine the distribution of the data. The total anxiety score data were normally distributed (p> 0.05), both S-Anxiety and T-Anxiety, while the total resilience score

Table 1 Respondents' Characteristics

Characteristics	Frequency	Percentage
Sex		
Man	38	16.7
Woman	189	83.3
Age		
Mean ± Std. Deviation	39.67 ± 9.434	
Median (min – max)	38 (23–58)	
Marital Status		
Single	14	6.2
Married	206	90.7
Widower	7	3.1
Children in Family		
1	52	22.9
2	101	44.5
3	32	14.1
4	9	4
Others	33	14.5
Educational Status		
High School Degree	9	4
Diploma Degree	140	61.7
Bachelor Degree	70	30.8
Master Degree	8	3.5
Job Occupation		
Pharmacist	1	0.4
Pharmacist Assistant	8	3.5
Midwife	16	7
Physiotherapist	2	0.9
Nutritionist	34	15
Nurse	134	59
Medical Record Officer	16	7
Laboratory Staff	2	0.9
Psychologist	2	0.9
Electromedical Technician	1	0.4
Supporting Staff	11	4.8
The Income per Month (Rp)		
<3,000,000	19	8.4
3,000,000–6,000,000	89	39.2
6,000,000–9,000,000	65	28.6
9,000,000–12,000,000	29	12.8
12,000,000–15,000,000	19	8.4
>15,000,000	6	2.6
Distance from Home to Hospital (km)		
0–5	46	20.3
5–10	46	20.3
10–15	24	10.6
> 15	111	48.9

Table 2 Descriptive Values of Total S-Anxiety, T-Anxiety and Resilience Scores

Total Scores	n	Mean ± Std. Deviation	Median (Min–Max)
S-Anxiety	227	39.63 ± 11.540	40 (20–78)
T-Anxiety	227	39.42 ± 7.999	39 (20–63)
Resilience	227	69 ± 15.823	72 (25–100)

Table 3 Level of S-Anxiety and T-Anxiety

Anxiety	Frequency	Percentage
S-Anxiety		
No or Low Anxiety (20–37)	99	43.6
Moderate Anxiety (38–44)	53	23.3
High Anxiety (45–80)	75	33
T-Anxiety		
No or Low Anxiety (20–37)	90	39.6
Moderate Anxiety (38–44)	76	33.5
High Anxiety (45–80)	61	26.9

data were not normally distributed ($p < 0.05$) (Table 4). The correlation between anxiety and resilience was analyzed using the Spearman correlation test because the resilience data were not normally distributed.

The Spearman correlation test showed a significant relationship between anxiety and the total resilience score ($p < 0.05$), both S-Anxiety and T-Anxiety (Table 5). It can be seen that the correlation coefficient between resilience and S-Anxiety is −0.519; this indicates a strong correlation; Meanwhile, the correlation coefficient between resilience and S-Anxiety was −0.483 which indicates a moderate level of correlation. The direction of the relationship between anxiety and the total resilience score is negative with moderate relationship strength. This means that the higher the anxiety, the lower the total resilience score.

Table 4 Normal Distribution Tests for Anxiety and Resilience

Total Scores	N	P-Value
S-Anxiety	227	0.548
T-Anxiety	227	0.561
Resilience	227	0.017

Table 5 Correlation Between Anxiety and Resilience

Correlation	n	r_s	P-Value
S-Anxiety and Resilience	227	−0.519	< 0.001
T-Anxiety and Resilience	227	−0.483	< 0.001

Discussion

Healthcare workers as part of the healthcare system that handles COVID-19 are prone to experiencing anxiety. Many factors can be a source of anxiety for them, including limited personal protective equipment (PPE), fear of being a carrier for the people closed to them, fear of contracting COVID-19, limitations of rapid and swab examination facilities, limited healthcare facilities in handling the number of existing cases, high morbidity which is accompanied by a rapid increase in the number of cases.[22–24] In Indonesia, the increase of positive cases per day is still high, namely above 1000 cases per day (June 23–29, 2020) with a Case Fatality Rate (CFR) of 5.1%.[25]

More than half of the respondents in this study (56.3%) had moderate to high levels of state anxiety. This number is higher compared to some previous studies in other countries. In China, an online multicentre survey was conducted to determine the mental problems of healthcare workers and found 44.7% of them showed symptoms of anxiety.[26] A multinational study conducted in Singapore and India showed that only 15.7% of the total respondents had symptoms of anxiety.[27] A meta-analysis that examined 12 studies of the prevalence of anxiety in healthcare workers showed a pooled prevalence of 23.2%.[28]

The high level of anxiety of healthcare workers in this study can be influenced by the situation in East Java. East Java is the province with the highest positive COVID-19 cases in Indonesia with CFR of 7.3%, higher than the Indonesian CFR and global CFR (5.1%) (as of 30 June 2020).[25] This anxiety can also be caused by the large number of healthcare workers infected with COVID-19 in East Java. A total of 175 medical workers were tested positive for COVID-19 with a mortality rate of 3%. The majority of the infected people were doctors and nurses.[29]

In this study, the proportion of respondents with high state anxiety (33%) was greater than the proportion of respondents with high trait anxiety (26.9%). This supports previous research in China which showed more respondents with state anxiety (15.8%) compared to respondents with trait anxiety (4.0%). State anxiety that reflects the current anxiety shows that this pandemic can act as a source of anxiety in the community.[6]

The anxiety that occurs in healthcare workers needs to get treatment because anxiety can increase the risk of adverse events. A study showed that anxiety or depression in healthcare workers increased the risk of adverse events, such as traffic accidents, work accidents, or medical errors,

as much as 63%.[30] As a part that plays a role in handling the COVID-19 pandemic, mental health in healthcare workers needs attention because it will have an impact on the handling of this pandemic.

Healthcare facilities are a workplace full of challenges so resilience is an important aspect needed by healthcare workers, especially during a pandemic like this. Resilience is the ability of an individual to rise and adapt to conditions that cause distress. Resilient individuals have optimism and confidence to be able to control conditions even in unfavorable conditions.[9] Resilience is indicated by the presence of several characteristics in individuals, such as optimism, adaptability, self-confidence, positive self-image, empathy, and tolerance. Resilience is a combination of personality and life experiences that ultimately lead to the ability to adapt positively.[31,32]

This study showed there was a significant correlation between resilience and anxiety of the respondents, both state anxiety and trait anxiety. A high level of anxiety was associated with a low level of resilience. This study used a cross-sectional method so that it could not show a causal relationship between anxiety and resilience, but this supported previous studies. A meta-analysis showed high resilience associated with better mental health.[33] Resilience showed a protective effect on anxiety and depression.[9] This indicates that resilience is related to the level of anxiety of health workers, in the sense that: the more resilient a person is, the better his mental health is.[9]

The results of this study are evidence that strengthens that resilience is really related to the level of anxiety experienced by health workers in the era of the COVID-19 pandemic. In this case, the more resilient the health workers are, the better their anxiety level will be.

This is relevant to the Mahmood & Ghaffar statement that resilience is a good adaptation process in situations of trauma, tragedy, or other stressful events. Resilience is not a personality trait but rather something that involves behavior, thoughts, or actions that anyone can learn.[12]

Resilience is the ability of a person in normal circumstances when faced with an event that has the potential to interfere physically and mentally. In this case, resilience is one of the right ways to deal with an adverse event or one that can cause trauma, because resilience is an ability to maintain a stable balance.[13]

Mental health problems not only become a problem during the pandemic but also remain a burden after the pandemic ends. This can be seen from the SARS outbreak that occurred in 2003. A study in Canada was conducted 2 years after the SARS outbreak to compare psychological conditions and burnout between healthcare workers in Toronto hospitals that handled SARS cases and healthcare workers in Hamilton hospitals that did not handle SARS cases. There was higher psychological stress on healthcare workers in Toronto. Another finding was an increase in smoking behavior and alcohol consumption in healthcare workers in Toronto. Even so, the level of burnout and psychological stress on healthcare workers who worked in high-risk and high-intensity work settings tended to be lower. This is thought to be influenced by the high resilience gained from longer work experience.[34]

Some experts believe that resilience is an aspect that can be developed. Some ways that can be done to increase resilience are through experience, learning, and formal training.[31,32] In addition, several mechanisms of mature ego defense can increase resilience during a pandemic, including humor, altruism, anticipation, and self-observation.[35] The development of resilience needs to be made a priority to prepare healthcare workers to deal with crises and reduce mental health problems in the future.[36,37] As an effort to maintain the mental health of the healthcare workers, the hospital in this study provides a vent corner facility. The healthcare workers will get psychological assistance and treatment as needed.

Limitations

The anxiety of health workers in the era of the COVID-19 pandemic is certainly related to many factors, but in this study, it was only related to one factor, namely resilience. In further studies, this limitation should be overcome, so that it will be known more clearly what factors are most associated with anxiety among health workers during the COVID-19 pandemic era.

The results of this study are not compared with the results of studies in the period before the COVID-19 pandemic, so it is not clear whether there is an increase in the level of anxiety about health workers due to this pandemic. But apart from that, the results of this study have proven that the anxiety of health workers is significantly associated with resilience, an information that can be used as a basis for future studies.

Conclusion

In this study, a significant correlation was found between the level of resilience and anxiety experienced by healthcare workers. The lower the resilience, the higher the anxiety experienced. This can be used as a consideration for psychiatric assistance and procurement of policies to

increase the resilience of healthcare workers to avoid mental health problems.

Acknowledgments

Thanks and highest appreciation were conveyed to the Dean of Faculty of Medicine, Universitas Airlangga, who facilitated this research.

Disclosure

The authors report no conflicts of interest in this work.

References

1. Mahase E. China coronavirus: WHO declares international emergency as death toll exceeds 200. *BMJ*. 2020;368:1.
2. Indonesian Task Force for COVID-19. Infografis COVID-19 [homepage on the Internet]. Infografis for COVID-19; 2020. Available from: https://covid19.go.id/p/berita/infografis-covid-19-5-juni-2020. Accessed June 6, 2020.
3. Shigemura J, Ursano RJ, Morganstein JC, Kurosawa M, Benedek DM. Public responses to the novel 2019 coronavirus (2019-nCoV) in Japan: mental health consequences and target populations. *Psychiatry Clin Neurosci*. 2020;74(4):281–282. doi:10.1111/pcn.12988
4. Cheng C, Cheung MWL. Psychological responses to outbreak of severe acute respiratory syndrome: a prospective, multiple time-point study. *J Pers*. 2005;73(1):261–285. doi:10.1111/j.1467-6494.2004.00310.x
5. Wang C, Riyu P, Wan X, et al. Immediate psychological responses and associated factors during the initial stage of the 2019 coronavirus disease (COVID-19) epidemic among the general population in China. *Int J Environ Res Public Health*. 2020;17(5):1729. doi:10.3390/ijerph17051729
6. Liu X, Luo WT, Li Y, et al. Psychological status and behavior changes of the public during COVID-19 epidemic in China. *Infect Dis Poverty*. 2020;9:58. doi:10.1186/s40249-020-00678-3
7. Wu K, Wei X. Analysis of psychological and sleep status and exercise rehabilitation of front-line clinical staff in the fight against COVID-19 in China. *Med Sci Monit Basic Res*. 2020;26:e924085. doi:10.12659/MSMBR.924085
8. Poon E, Liu KS, Cheong DL, Lee CK, Yam LYC, Tang WN. Impact of severe acute respiratory syndrome on anxiety levels of front-line health care workers. *Hong Kong Med J*. 2004;10(5):325–330.
9. Gheshlagh RG, Sayehmiri K, Ebadi A, et al. The relationship between mental health and resilience: a systematic review and meta-analysis. *Iran Res Crescent Med J*. 2017;31.
10. Mahmood K, Ghaffar A. The relationship between resilience, psychological distress and subjective well-being among dengue fever survivors. *Global J Inc*. 2014;14(10):13–24.
11. Bonanno GA. Loss, trauma, and human resilience. Have we underestimated the human capacity to thrive after aversive events? *Am Psychol Assoc*. 2004;59(1):20–28. doi:10.1037/0003-066X.59.1.20
12. Cunningham AC, Goh HP, Koh D. Treatment of COVID-19: old tricks for new challenges. *Critical Care*. 2020;24:91. doi:10.1186/s13054-020-2818-6
13. Sadati AK, Lankarani MHB, Lankarani KB. Risk society, global vulnerability and fragile resilience; sociological view on the coronavirus outbreak. *Shiraz E-Med J*. 2020;21(4):e102263.
14. Kayikcioglu O, Bilgin S, Seymenoglu G, Deveci A. State and trait anxiety scores of patients receiving intravitreal injections. *Biomed Hub*. 2017;2:478993. doi:10.1159/000478993
15. Di Tella M, Romeo A, Benfante A, Castelli L. Mental health of healthcare workers during the COVID-19 pandemic in Italy. *J Eval Clin Pract*. 2020;26:1583–1587. doi:10.1111/jep.13444
16. Hacimusalar Y, Kahve AC, Yasar AB, Aydin MS. Anxiety and hopelessness levels in COVID-19 pandemic: a comparative study of healthcare professionals and other community sample in Turkey. *J Psychiatr Res*. 2020;129:181–188. doi:10.1016/j.jpsychires.2020.07.024
17. Kılınçel S, Muratdağı G, Aydın A, Usta MB. Factors affecting the anxiety levels of adolescents in home-quarantine during COVID-19 pandemic in Turkey. *Asia Paci Psychiatr*. 2020;e12406:1–6.
18. Connor KM, Davidson JRT. Development of a new resilience scale: the Connor-Davidson Resilience Scale (CD-RISC). *Depress Anxiety*. 2003;18(2):76–82. doi:10.1002/da.10113
19. Davidson JRT Connor-Davidson Resilience Scale (CD-RISC) manual. [homepage on the Internet]. CD-RISC; 2020. Available from: http://www.connordavidson-resiliencescale.com/CD-RISC%20Manual%2008-19-18.pdf. Accessed June 6, 2020.
20. Mosheva M, Hertz-Palmor N, Ilan SD, et al. Anxiety, pandemic-related stress and resilience among physicians during the COVID-19 pandemic. *Depress Anxiety*. 2020;37:965–971. doi:10.1002/da.23085
21. Ferreira RJ, Buttell F, Cannon C. COVID-19: immediate predictors of individual resilience. *Sustainability*. 2020;12(16):1–11. doi:10.3390/su12166495
22. Shanafelt T, Ripp J, Trockel M. Understanding and addressing sources of anxiety among health care professionals during the COVID-19 pandemic. *JAMA*. 2020;323(21):2133. doi:10.1001/jama.2020.5893
23. Lai J, Ma S, Wang Y, et al. Factors associated with mental health outcomes among health care workers exposed to coronavirus disease 2019. *JAMA Netw Open*. 2020;3(3):e203976. doi:10.1001/jamanetworkopen.2020.3976
24. Nemati M, Bahareh E, Nemati F. Assessment of Iranian nurses' knowledge and anxiety toward COVID-19 during the current outbreak in Iran. *Arch Clin Infest Dis*. 2020;(29):e102848.
25. Ministry of Health of The Republic of Indonesia. *Emerging Infection*. Jakarta: Ministry of Health of The Republic of Indonesia; 2020.
26. Liu S, Yang L, Zhang C, et al. Online mental health services in China during the COVID-19 outbreak. *Lancet Psychiatr*. 2020;7:e17–e18. doi:10.1016/S2215-0366(20)30077-8
27. Chew NWS, Lee GKH, Tan BYQ, et al. A multinational, multicentre study on the psychological outcomes and associated physical symptoms amongst healthcare workers during COVID-19 outbreak. *Brain Behav Immun*. 2020;88(21):559–565. doi:10.1016/j.bbi.2020.04.049
28. Pappa S, Ntella V, Giannakas T, Giannakoulis VG, Papoutsi E, Katsaounou P. Prevalence of depression, anxiety, and insomnia among healthcare workers during the COVID-19 pandemic: a systematic review and meta-analysis. *Brain Behav Immun*. 2020;(8).
29. Zahroh F 175 health workers in East Java exposed to Covid-19, 6 people dead, Surabaya city is the most (175 tenaga kesehatan di Jawa Timur terpapar Covid-19, 6 orang gugur, Kota Surabaya terbanyak) [homepage on the Internet]. Tribun Jatim; 2020. Available from: https://jatim.tribunnews.com/2020/06/16/175-tenaga-kesehatan-di-jawa-timur-terpapar-covid-19-6-orang-gugur-kota-surabaya-terbanyak?_ga=2.91830426.1215662172.1593414029-1537672028.1532780186. Accessed June 30, 2020.
30. Weaver MD, Vetter C, Rajratnam SMW, et al. Sleep disorders, depression and anxiety are associated with adverse safety outcomes in healthcare workers: a prospective cohort study. J Sleep Res. 2018;27(6):e12722. doi:10.1111/jsr.12722
31. Matheson C, Robertson HD, Elliott AM, Iversen L, Murchie P. Resilience of primary healthcare professionals working in challenging environments: a focus group study. *Br J Gen Pract*. 2016;66(648):e507–15. doi:10.3399/bjgp16X685285

32. McAllister M, McKinnon J. The importance of teaching and learning resilience in the health disciplines: a critical review of the literature. *Nurse Educ Today*. 2009;29:371–379. doi:10.1016/j.nedt.2008.10.011
33. Farber F, Rosendahl J. The Association between resilience and mental health in the somatically ill: a systematic review and meta-analysis. *Dtsch Arztebl Int*. 2018;115(38):621–627. doi:10.3238/arztebl.2018.0621
34. Maunder RG, Lancee WJ, Balderson KW, et al. Long-term psychological and occupational effects of providing hospital healthcare during SARS outbreak. *Emerg Infect Dis*. 2006;12(12):1924–1932. doi:10.3201/eid1212.060584
35. Marcinko D, Jakovljevic M, Jaksic N, Bjedov S, Mindoljevic Drakulic A. The importance of psychodynamic approach during COVID-19 pandemic. *Psychiatr Danub*. 2020;32(1):15–21. doi:10.24869/psyd.2020.15
36. Duncan D. What the COVID-19 pandemic tells us about the need to develop resilience in the nursing workforce. *Nurs Manage*. 2020;27(3):22–27. doi:10.7748/nm.2020.e1933
37. Santarone K, McKenney M, Elkbuli A. Preserving mental health and resilience in frontline healthcare workers during COVID-19. *Am J Emerg Med*. 2020;38(7):1530–1531. doi:10.1016/j.ajem.2020.04.030

Journal of Multidisciplinary Healthcare Dovepress

Publish your work in this journal

The Journal of Multidisciplinary Healthcare is an international, peer-reviewed open-access journal that aims to represent and publish research in healthcare areas delivered by practitioners of different disciplines. This includes studies and reviews conducted by multidisciplinary teams as well as research which evaluates the results or conduct of such teams or healthcare processes in general. The journal covers a very wide range of areas and welcomes submissions from practitioners at all levels, from all over the world. The manuscript management system is completely online and includes a very quick and fair peer-review system. Visit http://www.dovepress.com/testimonials.php to read real quotes from published authors.

Submit your manuscript here: https://www.dovepress.com/journal-of-inflammation-research-journal
