# Supplementary material for: Mental health status and related factors influencing healthcare workers during the COVID-19 pandemic: A systematic review and meta-analysis
Source: PLoS One. 2024 Jan 19;19(1):e0289454. doi: 10.1371/journal.pone.0289454 (PMC10798549; doi:10.1371/journal.pone.0289454)
Supplement: S1 Data — (ZIP) [file pone.0289454.s011.zip › literatures/16.pdf]

ELSEVIER

Since January 2020 Elsevier has created a COVID-19 resource centre with free information in English and Mandarin on the novel coronavirus COVID-19. The COVID-19 resource centre is hosted on Elsevier Connect, the company's public news and information website.

Elsevier hereby grants permission to make all its COVID-19-related research that is available on the COVID-19 resource centre - including this research content - immediately available in PubMed Central and other publicly funded repositories, such as the WHO COVID database with rights for unrestricted research re-use and analyses in any form or by any means with acknowledgement of the original source. These permissions are granted for free by Elsevier for as long as the COVID-19 resource centre remains active.

Symptoms of Mental Health Disorders in Critical Care Physicians Facing the Second COVID-19 Wave

A Cross-Sectional Study

Elie Azoulay, MD, PhD; Frédéric Pochard, MD, PhD; Jean Reignier, MD, PhD; Laurent Argaud, MD, PhD; Fabrice Bruneel, MD; Pascale Courbon, RN; Alain Cariou, MD, PhD; Kada Klouche, MD, PhD; Vincent Labbé, MD, PhD; François Barbier, MD, PhD; Christophe Guitton, MD, PhD; Alexandre Demoule, MD, PhD; Achille Kouatchet, MD; Olivier Guisset, MD; Mercé Jourdain, MD, PhD; Laurent Papazian, MD, PhD; Guillaume Van Der Meersch, MD; Danielle Reuter, MD; Virginie Souppart, RN; Matthieu Resche-Rigon, MD, PhD; Michael Darmon, MD, PhD; and Nancy Kentish-Barnes, PhD; on behalf of the FAMIREA Study Group

BACKGROUND: Working in the ICU during the first COVID-19 wave was associated with high levels of mental health disorders.

RESEARCH QUESTION: What are the mental health symptoms in health care providers (HCPs) facing the second wave?

STUDY DESIGN AND METHODS: A cross-sectional study (October 30-December 1, 2020) was conducted in 16 ICUs during the second wave in France. HCPs completed the Hospital Anxiety and Depression Scale, the Impact of Event Scale-Revised (for post-traumatic stress disorder), and the Maslach Burnout Inventory.

RESULTS: Of 1,203 HCPs, 845 responded (70%) (66% nursing staff, 32% medical staff, 2% other professionals); 487 (57.6%) had treated more than 10 new patients with COVID-19 in the previous week. Insomnia affected 320 (37.9%), and 7.7% were taking a psychotropic drug daily. Symptoms of anxiety, depression, post-traumatic stress disorder, and burnout were reported in 60.0% (95% CI, 56.6%-63.3%), 36.1% (95% CI, 32.9%-39.5%), 28.4% (95% CI, 25.4%-31.6%), and 45.1% (95% CI, 41.7%-48.5%) of respondents, respectively. Independent predictors of such symptoms included respondent characteristics (sex, profession, experience, personality traits), work organization (ability to rest and to care for family), and self-perceptions (fear of becoming infected or of infecting family and friends, feeling pressure related to the surge, intention to leave the ICU, lassitude, working conditions, feeling they had a high-risk profession, and "missing the clapping"). The number of patients with COVID-19 treated in the first wave or over the last week was not associated with symptoms of mental health disorders.

INTERPRETATION: The prevalence of symptoms of mental health disorders is high in ICU HCPs managing the second COVID-19 surge. The highest tiers of hospital management urgently need to provide psychological support, peer-support groups, and a communication structure that ensure the well-being of HCPs. CHEST 2021; 160(3):944-955

KEY WORDS: anxiety; burnout; COVID-19 pandemic; ICU clinicians; depression; post-traumatic stress disorder

FOR EDITORIAL COMMENT, SEE PAGE 797

ABBREVIATIONS: HADS = Hospital Anxiety and Depression Scale; HCP = health care professional; IES-R = Impact of Event Scale-Revised; MBI = Maslach Burnout Inventory; MBTI = Myers-Briggs Type Indicator; PTSD = post-traumatic stress disorder; VAS = visual analog scale

AFFILIATIONS: From the Medical Intensive Care Unit (E. Azoulay, F. Pochard, M. Darmon, V. Souppart, and N. Kentish-Barnes [FAMIREA Study Group]), AP-HP, Saint Louis University Hospital, Paris, France; the Medical Intensive Care Unit (P. Courbon and A. Cariou), Cochin

Take-home Points

Study Question: What are the mental health symptoms in frontline health care providers (HCPs) facing the second COVID-19 wave?

Results: Insomnia affected 320 of 845 respondents (37.9%). Symptoms of anxiety, depression, post-traumatic stress disorder, and burnout were reported in 60.0% (95% CI, 56.6%-63.3%), 36.1% (95% CI, 32.9%-39.5%), 28.4% (95% CI, 25.4%-31.6%), and 45.1% (95% CI, 41.7%-48.5%) of respondents, respectively.

Interpretation: With the successive COVID-19 waves, strategies to mitigate psychological burden are urgently warranted to both protect HCPs and ensure that the highest quality of care is maintained.

The second wave of the COVID-19 pandemic in France is being met by health care professionals (HCPs) who may not yet have recovered from the stress-related mental illness symptoms caused by the first wave.[1,2] Sources of stress include physical and mental exhaustion, anger about equipment shortages, shortages of beds resulting in a need for triage, communication challenges and isolation, grief from the unusually high number of deaths, sadness at not being able to let families of dying patients in to say goodbye, pain from losing colleagues to the disease, fear of transmitting the virus to their family and, in some cases, residual symptoms after having contracted the virus themselves. Insomnia, anxiety, and depression were prevalent among frontline HCPs during the first surge.[1,3,4] In accordance with their extensive exposure to the effects of the pandemic, the psychological burden among HCPs working in ICUs was particularly high.[5,6] These risk factors for mental health disorders came against a background of a high rate of burnout among ICU HCPs.

The fact that the pandemic is out of control and produces multiple surges may result in additional anxiety, compared with the first surge. In addition, information about COVID-19 that has spread through social media platforms has created fears that in some respects may not be justified.[7] Fear is a major concern,[6,8] as it causes severe stress, particularly among HCPs who come into contact with infected patients during their work.[9] Fear of contracting the virus, fear of dying, and fear of infecting loved ones can combine to worsen the psychological burden. In addition, the increased number of patients can be dealt with only by subjecting staff to longer hours, which has been shown to be associated with increased physical exhaustion and to contribute to the development of symptoms of severe burnout, together with the moral distress produced by having to make suboptimal decisions due to the large number of patients.[10-13] Obtaining sufficient rest may be impossible because of persistent insomnia.[1,5,6] Financial and other burdens also accumulate.

One-third of young adults surveyed after the initial declaration of a state of emergency in the United States had symptoms of post-traumatic stress disorder.[14] Emotional distress created by the pandemic may adversely impact patient safety and staff retention during and beyond the pandemic.[15] During a surge, the increased number of staff members on sick leave or leaving their profession results in staff shortages that cannot be corrected, as there is no reserve of trained ICU HCPs. These many factors may well overcome the personal resilience resources of HCPs, notably in ICUs.

The objective of this multicenter cross-sectional survey was to determine the prevalence of, and risk factors for, symptoms of anxiety, depression, post-traumatic stress disorder (PTSD), and severe burnout among ICU HCPs during the second COVID-19 surge in France. We hope the data thus obtained will serve to guide preventive and curative strategies for mental illness symptoms in this population.

University Hospital, AP-HP, Centre-Université de Paris, Paris, France; the Intensive Care Unit (F. Bruneel), André Mignot Hospital, Le Chesnay, France; AP-HP (A. Demoule), Groupe Hospitalier Universitaire AP-HP-Sorbonne Université, Pitié-Salpêtrière site, Service de Pneumologie, Médecine Intensive et Réanimation (Département R3S) and Sorbonne Université, INSERM, UMRS1158 Neurophysiologie Respiratoire Expérimentale et Clinique, Paris, France; the Medical Intensive Care Unit (A. Kouatchet), Angers Teaching Hospital, Angers, France; the Medical-Surgical Intensive Care Unit (D. Reuter), Centre Hospitalier Sud Francilien, Corbeil, France; the Department of Intensive Care Medicine (K. Klouche), Lapeyronie Hospital, Montpellier, France; the Medical Intensive Care Department (L. Argaud), Edouard Herriot Hospital, Hospices Civils de Lyon, Lyon, France; the Medical Intensive Care Unit (F. Barbier), La Source Hospital, Centre Hospitalier Régional d'Orléans, Orléans, France; Centre Hospitalier Universitaire Lille (M. Jourdain), Department of Intensive Care, Hôpital Roger Salengro, INSERM, Lille University, U1190, Lille, France; the Medical Intensive Care Unit (J. Reignier), University Hospital Center, Nantes, France; the Respiratory and Infectious Diseases ICU (L. Papazian), AP-HM Hôpital Nord, Marseille, France; the Clinical Research Unit (M. Resche-Rigon), AP-HP, Saint Louis University Hospital, Paris, France; the Medical Intensive Care Unit (O. Guisset), Saint-André Hospital, Bordeaux, France; the Medical Intensive Care Unit (V. Labbé), AP-HP, Tenon University Hospital, Paris, France; the Medical-Surgical Intensive Care Unit (G. Van Der Meersch), AP-HP, Avicenne University Hospital, Bobigny, France; and the Medical Intensive Care Unit (C. Guitton), Le Mans Hospital, Le Mans, France.

FUNDING/SUPPORT: This study was funded by the French Ministry of Health and sponsored by AP-HP (project AP-HP 200389)

CORRESPONDENCE TO: Elie Azoulay, MD, PhD; email: elie.azoulay@aphp.fr

Copyright © 2021 Published by Elsevier Inc under license from the American College of Chest Physicians.

DOI: https://doi.org/10.1016/j.chest.2021.05.023

Methods

The CPP Sud Méditerranée ethics committee approved this study on March 31, 2020 (2020-A00809-30; CNRIPH: 20.03.27.73019). A questionnaire was sent to bedside HCPs working in 16 ICUs that were part of the FAMIREA study group, during the second wave of the pandemic. The HCPs were invited to complete the online survey confidentially between October 30 and December 1, 2020.

The Survey Questionnaire

The data reported in Tables 1 through 3 and in Figures 1 through 5 were collected online. The questionnaire included items from the questionnaire used for a study during the first surge,[6] as well as new items identified from a literature review and semistructured interviews with HCPs working in the participating ICUs. The main components of the questionnaire included exposure to COVID-19 (number of patients treated, infection among family or friends); visitation policies for family members; professional and personal impact of the pandemic (fear of being infected or of infecting family and friends, ability to rest, family balance, ability to care for family, feeling of lassitude, working conditions, intention to leave the ICU); personal information (demographics and habits regarding alcohol, tobacco, and psychotropic drugs); and three scales, the Hospital Anxiety and Depression Scale (HADS), the Impact of Event Scale-Revised (IES-R), and the Maslach Burnout Inventory (MBI). Preparation of the questionnaire was conducted as for our study of the first surge.[6] An invitation to complete the survey was e-mailed to all HCPs working in the participating ICUs, through mailing lists, WhatsApp groups, a poster with QR (quick response) codes in each ICU, and local interventions by study investigators. HCPs were defined as nurses, nursing assistants, residents, interns, clinical fellows, senior intensivists, and other professionals working in the ICUs. The reasons for choosing the HADS and MBI scales, and a brief description of each, can be found in our earlier article.[6,12,13,16] To detect symptoms of PTSD, we used the Impact of Event Scale-Revised (IES-R),[14] a self-report scale whose 22 items assess subjective distress caused by traumatic events. The items correspond to 14 of the 17 *Diagnostic and Statistical Manual of Mental Disorders*, 4th ed. (DSM-IV) symptoms of PTSD. Respondents are asked to indicate their level of distress during the past week caused by difficulties related to a specific stressful life event. The total score can range from 0 to 88, and subscale scores can be calculated for the Intrusion, Avoidance, and Hyperarousal subscales.

For variables depicting the COVID-19 experience, the responses were either binary (yes or no) or made on a 0 to 10 visual analog scale (VAS; ie, for intention to leave the ICU, lassitude, or working conditions). VASs are convenient, easy, and rapid to administer and have been proved reliable for measuring a characteristic, subjective phenomenon, or attitude that is believed to range across a continuum of values and cannot easily be directly measured. Fear was identified and characterized through qualitative interviews.

Study Outcomes

Mental health symptoms included anxiety and depression, defined as scores > 7 on the relevant HADS subscales[6,17]; this cutoff has the advantage of corresponding to a Patient Health Questionnaire-9 score ≥ 5, strictly based on the DSM-IV. Symptoms of PTSD were defined as an IES-R score ≥ 26,[18] and severe burnout was defined as an MBI score > -9 as previously reported.[12]

Personality Assessment Using the Myers-Briggs Type Indicator

Because some personality traits have been associated with mental health outcomes, respondents self-explored their personal preferences by answering questions that differentiated opposite traits in four categories of the Myers-Briggs Type Indicator (MBTI)[19] (E/I, Extraversion or Introversion; S/N, Sensing or iNtuition; T/F, Thinking or Feeling; and J/P, Judgment or Perception). The test result was thus four letters (eg, INFJ). The 16 different combinations of these four letters correspond to 16 different personalities (Fig 1). In each personality, either Sensing or iNtuition and either Thinking or Feeling are dominant characteristics. The 16 personalities can be collapsed into four roles, namely, analysts (fiercely independent, open-minded, and strong-willed), explorers (masters of tools and techniques), diplomats (warm, tactful, and empathetic), and sentinels (hard-working, precise, and conventional). The role is believed to influence goals, interests, preferred activities, and leadership styles.

Statistical Analysis

Data are described as median and interquartile range (IQR) or as number and percentage. Categorical variables were compared by Fisher exact test, and continuous variables were compared by Kruskal-Wallis test.

Independent predictors for symptoms of anxiety, depression, PTSD, and severe burnout were assessed by logistic regression and mixed logistic models. First, we built a logistic regression model using variables identified by univariate analysis, as well as clinically relevant variables. We used conditional stepwise variable selection with .2 as the *P* value for entry into the model and .1 as the *P* value for removal. Interactions and correlations between the explanatory variables were carefully checked. Continuous variables for which log-linearity was not confirmed were transformed into categorical variables according to the median or IQR. Last, a mixed model was constructed on the basis of previously selected variables, with the center as a random effect on the intercept. This model adjusting for a center effect was preplanned to be the final model, and its results are reported in the article. All models were assessed for calibration and discrimination. Residuals were plotted and the distributions inspected. We did not perform statistical adjustments for multiple comparisons.

Interactions linking mental illness symptoms are depicted by Venn diagrams.

Interactions between MBTI personalities and mental illness symptoms were assessed by multiple correspondence analysis.

All tests were two-sided, and *P* values less than .05 were considered statistically significant. Analyses were done with R software version 3.6.2,[20] including the “lme4,” “lmerTest,” “FactoMineR,” and “Factoextra” packages.

Results

Respondents

The survey started at a time where France had between 30,000 and 45,000 new COVID-19 cases per day, about 300 admissions to the ICU every day, and overall 3,500 patients in the ICUs in France (8 to 15 patients per ICU). The number of deaths varied between 200 and 400 deaths per day. Among the 1,203 HCPs working in the 16 participating ICUs, 845 (70%) fully completed the survey. Table 1 details their characteristics. Twelve

TABLE 1] Respondent Characteristics[a]

Characteristic	No. (%) or Median (IQR)
Role in the ICU	
Nurse	412 (48.7%)
Nurse assistant	143 (16.9%)
Resident or intern	97 (11.5%)
Attending physician	175 (20.7%)
Other allied professional[b]	18 (2.2%)
Female sex	571 (67.5%)
Age, y	33 (28-41)
Worked in the ICU during the first wave	777 (92%)
Had COVID-19	123 (14.5%)
Number of colleagues who had COVID-19	6 (4-10)
Participated in team debriefing after the first wave	308 (36.4%)
Fear (0-10) of being infected in the first wave	5 (3-7)
Fear (0-10) of infecting family and friends in the first wave	8 (6-10)
Fear (0-10) of being infected in the second wave	4 (2-6)
Fear (0-10) of infecting family and friends in the second wave	7 (5-9)
Rank (0-10) the feeling of pressure generated by the second surge	7 (6-8)
Daily psychotropic drug consumption	
Started a treatment	56 (6.6%)
Increased a treatment	9 (1.1%)
Insomnia	320 (37.9%)
Able to rest during the surge	
Not at all	213 (25.1%)
From time to time	462 (54.8%)
Very often	170 (20.1%)
Able to care for family during the surge	
Not at all	194 (22.8%)
From time to time	448 (53.1%)
Very often	203 (24.1%)
Family visits in the ICU	
Less frequent than in the first wave	648 (76.7%)
As frequent as in the first wave	138 (16.3%)
More frequent than in the first wave	59 (7%)
Impact (0-10) of restricted visitation policies on:	
Patients	3 (2-5)
Health care providers	5 (4-7)
Quality of decision-making	5 (4-5)
Feeling (0-10) of lassitude	5 (3-8)
Finding the working conditions difficult (0-10)	4 (3-5)
Feeling they have a high-risk profession (0-10)	5 (2-7)
Intending to leave the ICU (0-10)	5 (3-8)

IQR = interquartile range.
[a]Number of respondents: 845.
[b]Physiotherapists, psychologists, and clinical pharmacists.

TABLE 2] Prevalence of Mental Health Disorders According to Function in the ICU

Symptoms	All Respondents (N = 845 [100%])	Nurses (n = 412 [48.7%])	Nursing Assistants (n = 143 [16.9%])	Residents and Interns (n = 97 [11.5%])	Attending Physicians (n = 175 [20.7%])	Allied Professionals[a] (n = 18 [2.2%])	*P* Value
Symptoms of anxiety	507 (60%)	264 (64%)	85 (59.4%)	64 (66%)	83 (47.4%)	11 (61.1%)	.003
Symptoms of depression	305 (36.1%)	154 (37.5%)	52 (36.3%)	39 (40.2%)	54 (30.8%)	6 (33.3%)	.52
PTSD-related symptoms	240 (28.4%)	132 (32%)	40 (28%)	26 (26.8%)	38 (21.7%)	4 (22.2%)	.13
Intrusion	6 (2-12)	7 (3-12)	8 (3-13)	6 (1-11)	4 (1-9)	5 (2-11)	.0001
Avoidance	4 (1-10)	5 (1-11)	6 (2-10)	3 (0-10)	2 (0-7)	2 (0-8)	< .0001
Hyperarousal	3 (1-7)	3 (1-7)	3 (1-7)	3 (0-7)	1 (0-6)	1.5 (1-6)	.004
Symptoms of severe burnout	381 (45.1%)	194 (47.1%)	61 (42.6%)	59 (60.8%)	57 (32.6%)	10 (55.6%)	.0002
Exhaustion	15 (8-27)	17 (9-29)	12 (6-27)	20 (13-31)	12 (7-21)	8 (5-25)	< .0001
Depersonalization	6 (3-12)	7 (3-13)	5 (2-10)	11 (6-16)	6 (3-12)	2 (0-6)	< .0001
Personal achievement	34 (26-39)	33 (27-38)	34 (25-42)	31 (25-36)	35 (29-41)	27 (12-42)	.006

PTSD = post-traumatic stress disorder.
[a]Physiotherapists, psychologists, and clinical pharmacists.

(75%) ICUs were university-affiliated. The median number of beds per ICU was 18 (14-22) before the pandemic and 30 (25-35) during the second wave. Most respondents (727; 86%) had treated more than 30 patients with COVID-19 in the first wave and 487 (57.6%) had treated more than 10 new patients with COVID-19 in the week preceding the survey. The study was performed at a time when the proportion of patients with COVID-19 in the participating ICUs was more than 50%.

Respondents' Experience Assessed Using Visual Analog Scale Scores

Most elements of the COVID-19 experience were negative, as shown in Table 1. However, 719 (85%) respondents reported being proud of the work done. Fear of being infected or of infecting family and friends was almost as marked during the second wave as during the first. Only a minority of respondents felt they were not at all able to get any rest or to care for their families.

Personality Types and Mental Illness Symptoms

Figure 1 reports the results of the MBTI evaluation, with the proportion of respondents having each personality type and the four roles. The most frequent role was the explorer (observation and prospection), followed by the sentinel (observation and judgment), the diplomat (intuition and feeling), and the analyst (intuition and thinking). The MBTI type could not be determined for 27 respondents. Figure 2 shows that the roles were differently distributed across functions in the ICU.

Scales for Symptoms of Mental Illness

Symptoms of anxiety, depression, PTSD, and burnout were found in 60.0% (95% CI, 56.6%-63.3%), 36.1% (95% CI, 32.9%-39.5%), 28.4% (95% CI, 25.4%-31.6%), and 45.1% (95% CI, 41.7%-48.5%) of the respondents, respectively. As shown in Table 2 and Figure 3, the presence of three IES-R dimensions (intrusion, avoidance, and hyperarousal) varied significantly across HCP functions, whereas the number of respondents with a score ≥ 26 did not. Regarding the MBI, the three domains (exhaustion, depersonalization, and personal achievement) and total score also varied significantly across HCP functions, with the prevalence of burnout being highest among residents, interns, and ICU allied professionals. Importantly, the number of patients with COVID-19 seen or treated in the first wave or over the previous 7 days was not associated with symptoms of mental health disorders. Figure 4 shows that the prevalence of symptoms of

TABLE 3] Multivariable Analysis: Factors Associated With the Presence of Symptoms of Anxiety, Depression, Post-Traumatic Stress Disorder, or Severe Burnout

Factor	OR Associated With:			
	Symptoms of Anxiety	Symptoms of Depression	PTSD-Related Symptoms	Symptoms of Severe Burnout
Female sex	1.90 (1.30-2.77)	...	...	...
Role in the ICU				
Resident or intern	1.81 (1.05-3.44)	...	2.91 (1.47-5.72)	...
Other allied professionals[a]	3.33 (1.05-10.6)	...	4.11 (1.25-13.5)	...
Number of years spent working in the ICU	...	...	0.96 (0.93-0.99)/y	0.96 (0.94-0.99)/y
Fear (0-10) of being infected in the first wave	1.11 (1.03-1.19)/point	1.09 (1.02-1.17)/point	1.09 (1.02-1.18)/point	1.10 (1.01-1.18)/point
Fear (0-10) of infecting family in the second wave	1.11 (1.04-1.19)/point	1.10 (1.02-1.18)/point	...	1.16 (1.07-1.26)/point
Feeling (0-10) of pressure related to the surge	...	...	...	1.28 (1.13-1.46)/point
Feeling of lassitude (0-10)	...	0.89 (0.83-0.96)/point	0.88 (0.81-0.95)/point	...
Ranking (0-10) of working conditions	0.86 (0.78-0.95)/point	...	0.85 (0.76-0.95)/point	...
Feel they have a high-risk profession (0-10)	1.09 (1.02-1.17)/point	...	1.17 (1.01-1.26)/point	...
Report (0-10) missing the first-wave clapping	...	...	...	1.07 (1.01-1.14)/point
Intend (0-10) to leave the ICU	1.12 (1.05-1.19)/point	1.17 (1.10-1.25)/point	1.16 (1.08-1.24)/point	1.14 (1.07-1.22)/point
Able to rest during the surge				
Not at all	Ref	Ref	Ref	Ref
From time to time	0.51 (0.33-0.79)	0.62 (0.42-0.90)	0.48 (0.32-0.72)	0.52 (0.35-0.77)
Very often	0.25 (0.14-0.42)	0.31 (0.18-0.57)	0.42 (0.25-0.73)	0.29 (0.16-0.55)
Able to care for family during the surge				
Not at all	...	Ref	...	...
From time to time	...	0.70 (0.47-1.03)	...	...

(Continued)

TABLE 3] *(Continued)*

Factor	OR Associated With: Symptoms of Anxiety	Symptoms of Depression	PTSD-Related Symptoms	Symptoms of Severe Burnout
Very often	…	0.31 (0.18-0.53)	…	…
Myers-Briggs personality role[b]				
Explorers	…	Ref	Ref	Ref
Sentinels	…	1.19 (0.78-1.82)	0.27 (0.81-1.99)	1.38 (0.86-2.22)
Diplomats	…	1.84 (1.11-3.03)	1.13 (1.66-1.93)	0.82 (0.47-1.83)
Analysts	…	1.58 (0.85-2.95)	1.76 (1.09-3.29)	2.34 (1.21-4.53)

PTSD = post-traumatic stress disorder.
[a]Physiotherapists, psychologists, and clinical pharmacists.
[b]See Figure 1 for details.

depression, PTSD, and severe burnout, but not of anxiety, varied significantly across the four MBTI roles. Figure 5 depicts the significant differences in HADS, IES-R, and MBI scores according to the respondents' ability to rest or to care for their own family.

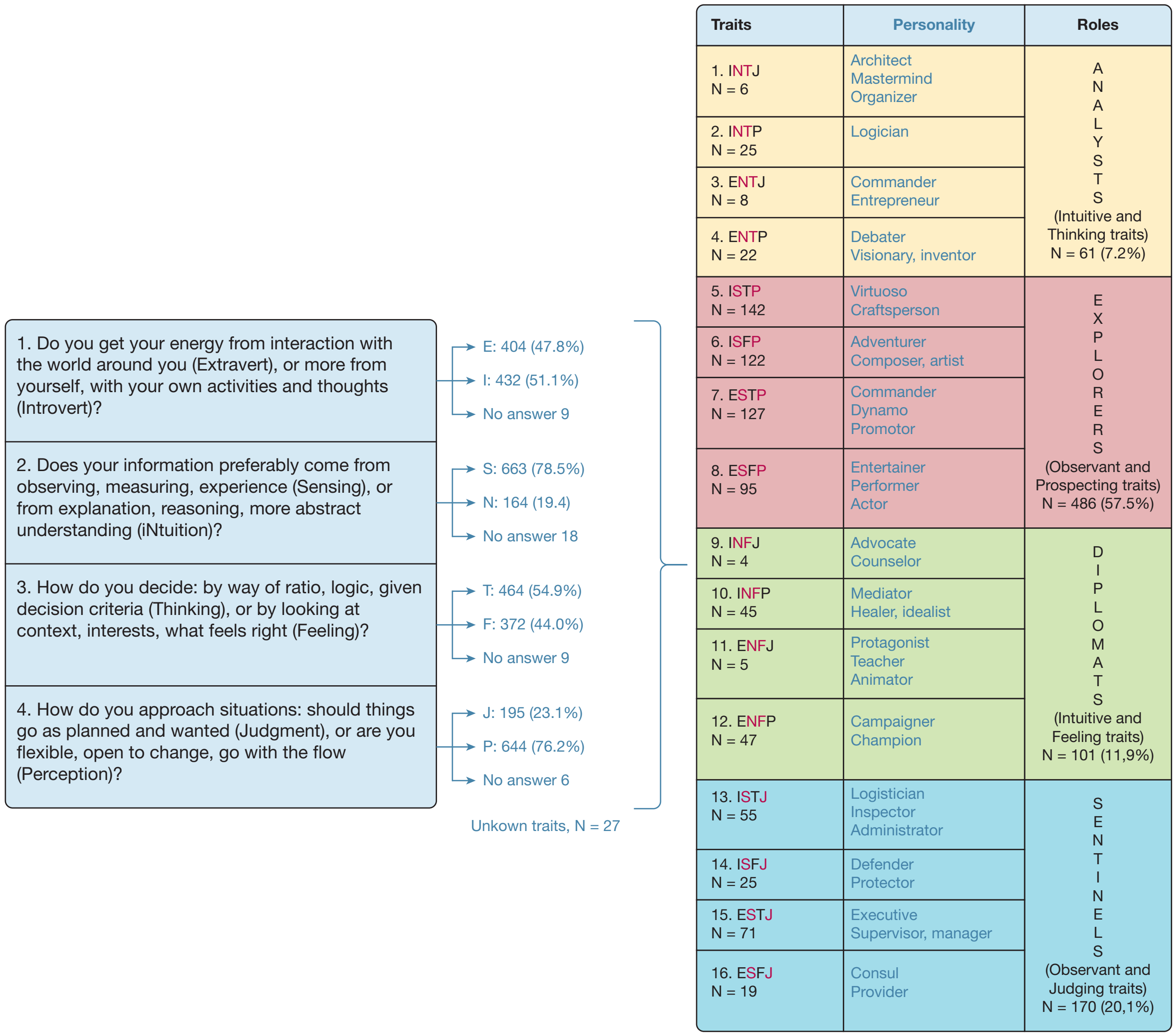

Figure 1 – *Myers and Briggs' personality types.*

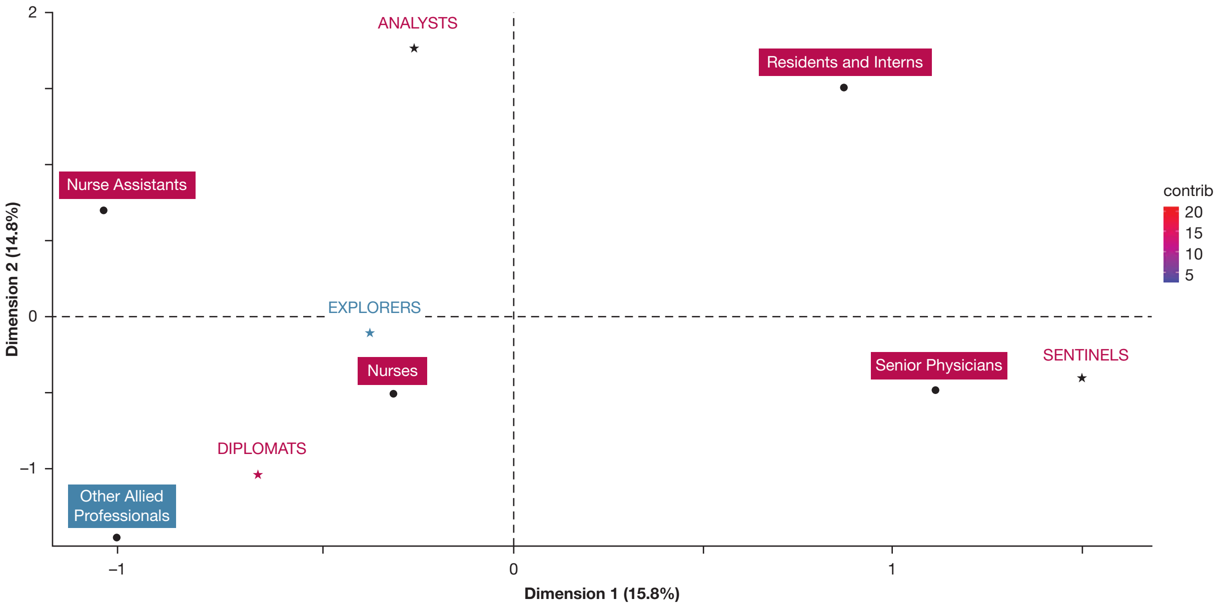

Figure 2 – *Principal component analysis displaying personality roles according to the Myers-Briggs Type Indicator and profession of ICU health care professionals.*

Multivariable Analysis

Table 3 reports the findings from the multivariable analysis. Few factors were significantly associated with all four types of symptoms: they included fear of being infected in the first wave, fear of infecting family and friends in the second wave, intention to leave the ICU, and inability to rest. Factors associated with anxiety were female sex; being a resident or intern; or being a physiotherapist, psychologist, or clinical pharmacist. Protective factors included an ability to rest, which protected against all four symptoms; a greater number of years of ICU experience, which protected against symptoms of PTSD and severe burnout; better working conditions, which protected against symptoms of anxiety and PTSD; and an ability to care for family, which protected against depression. In addition, a greater feeling of lassitude was associated with symptoms of depression and PTSD.

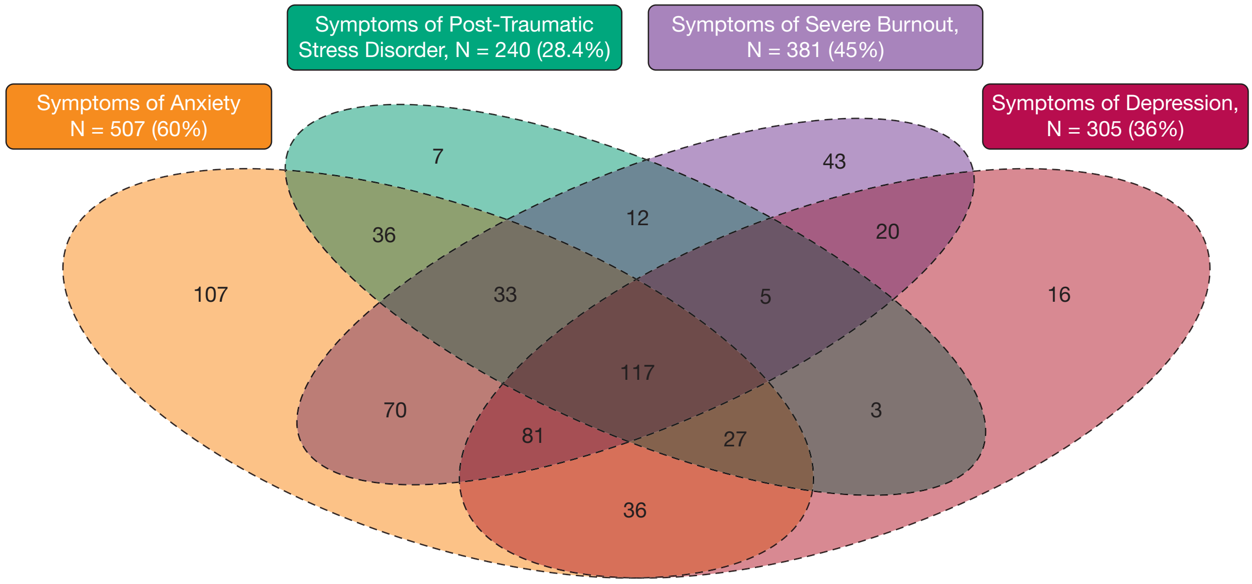

Figure 3 – *Venn diagram illustrating the overlapping of symptoms of anxiety, depression, post-traumatic stress disorder, and severe burnout.*

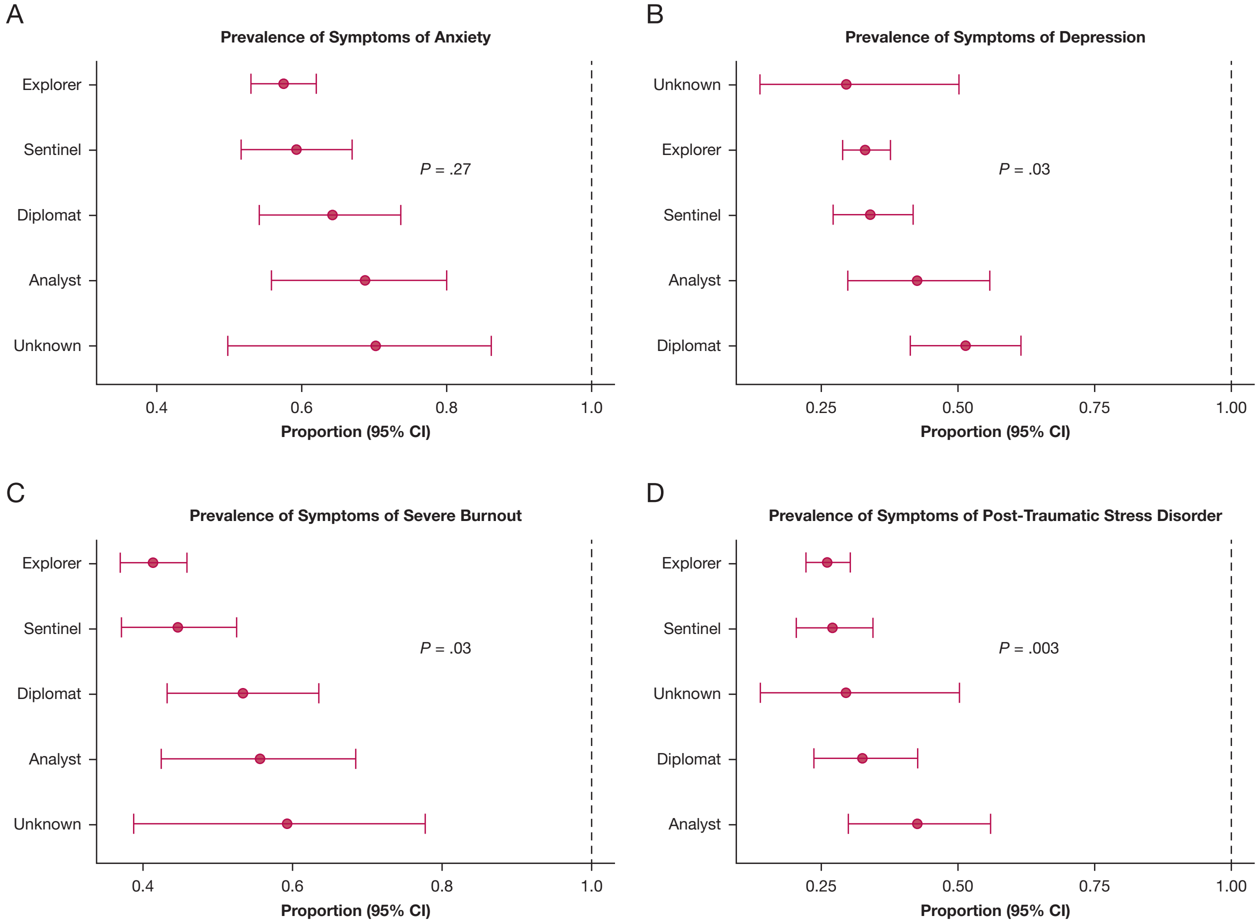

Figure 4 – *Prevalence of symptoms of mental illness for each personality role according to the Myers-Briggs Type Indicator.*

Last, among MBTI personality types, diplomats were at higher risk for depression, whereas analysts were at higher risk for PTSD and severe burnout.

Discussion

HCPs facing the second COVID-19 surge had high prevalences of symptoms of anxiety, depression, PTSD, and severe burnout. Insomnia affected nearly two-fifths of respondents. Fear of being infected or of infecting one's family, inability to rest, inability to care for one's family, feeling pressured, reporting working in more difficult conditions, and perceiving ICU work as difficult were independent predictors of mental illness symptoms.

Compared with the findings collected during the first wave in the same ICUs,[6] prevalence of symptoms of anxiety and depression increased, and the fear of infection or the inability to rest or to care for one's family persisted, showing that these HCPs have been subject to persistent pressure and strain.

Another important risk factor for mental illness concerns personality traits, which can be easily identified. This is the first study to assess personality in ICU HCPs. We chose the MBTI, whose results have been reported to shed light on communication styles within health care teams. The four MBTI roles differed regarding the risk of some of the mental illness symptoms. Personality types are stable factors (compared with workload) that are important predictors of individual perception and response to workplace requirements.[21] Personality influences both work performance and mental health.[22]

Our evidence of prevalent mental illness symptoms among ICU HCPs during a COVID-19 surge is consonant with other studies.[1-5,9-11,15,23] This body of evidence indicates a need to develop preventive and mitigating strategies. Mental distress has often been viewed as a weakness but should now be openly framed as a common occupational hazard by the institutional and department leadership.[24] Efforts should be directed

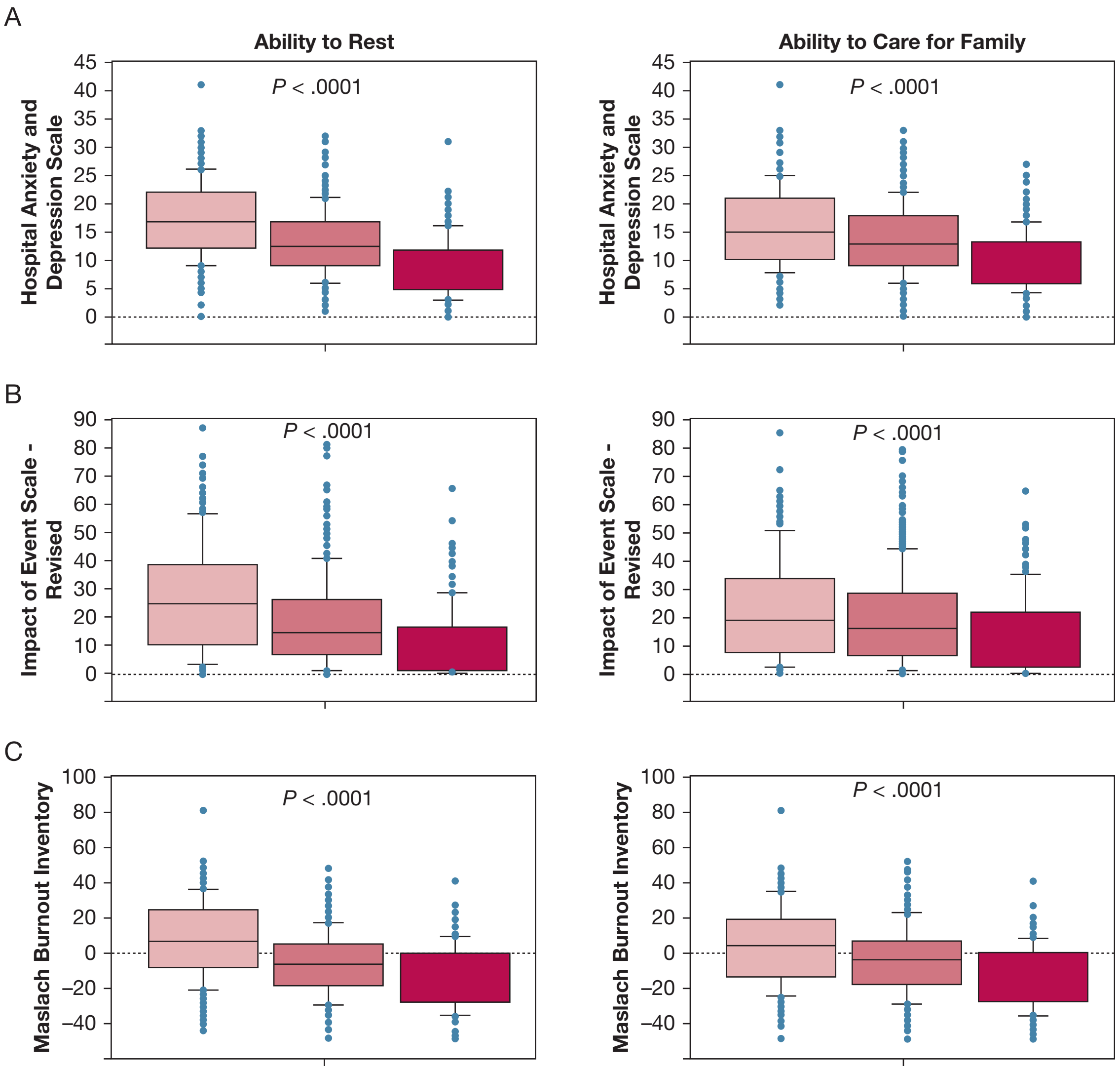

Figure 5 – *Box plots depicting respondents' symptoms of anxiety and depression (A, Hospital Anxiety and Depression Scale), post-traumatic stress disorder (B, Impact of Event Scale-Revised), and symptoms of severe burnout (C, Maslach Burnout Inventory). Light gray indicates an inability to rest or to care for family, dark gray an ability to rest or to care for family from time to time, and black an ability to rest or to care for family most of the time. P < .0001 for all comparisons (Kruskal-Wallis test).*

to all staff members.[6] In one study, less time spent in direct patient care was associated with more acute insomnia.[3]

We suggest that special efforts are needed in three domains. First, adequate provision of personal protective equipment, canceling nonessential events to prioritize resources, allowing time to rest, providing access to rapid SARS-CoV-2 testing, and family support are crucial to decrease fear and social isolation.[25,26] Second, the pressure that the additional patient numbers puts on ICU HCPs should be minimized.[27] Because recruiting new staff members is difficult, limiting absenteeism and retaining staff in the department may be more feasible. A pool of non-ICU nurses and physicians may be trained to provide some components of critical care to patients with COVID-19. The leadership should recognize the staff's extraordinary efforts, work alongside them, be on the alert for signs of mental distress they may exhibit, and offer solutions.[28] Good communication within the team is vital, as is trust that the institution will acknowledge mental difficulties as an occupational hazard and be consistently supportive. Thus, in addition to the department leaders, the hospital leadership must be fully committed to protecting the HCPs. One way to show such support is the provision of adequate supplies of personal protective equipment and of rapid testing.

The importance of good communication deserves to be emphasized. Among our respondents, only one-third reported having had debriefing sessions after the first wave. Debriefing sessions may provide opportunities to understand specific concerns of HCPs, identify sources of emotional distress, assure HCPs that their concerns have been heard, and develop means of answering those concerns to the greatest extent possible.[23] Another method of improving communication is known as tiered huddles, a method pioneered by the Cleveland Clinic.[29] Tiered huddles are series of brief conversations about issues that cannot be resolved in the department and often involve systems or processes. The issues raised are rapidly brought to the senior leadership levels through the different tiers, as each tier has a representative from the next tier (known as a boundary spanner). The presence of boundary spanners encourages HCPs to adopt a learning mindset (generation of new ideas to resolve issues or improve practices) rather than a performance mindset (application of protocols).[15] Thus, the issues receive hospital management attention within hours and are often resolved within 24 h. Last, education and training for both nurses and physicians should include courses on work organization, the reality of mental health symptoms, as well as screening tools and management strategies.

This study has several limitations. All participating ICUs were in France, raising concerns about the general applicability of our findings to other countries. However, in the first wave of the pandemic, symptoms of mental illness were reported in the same proportions as in other countries.[1,2,6,10,25] Moreover, we included a large number of ICUs that were at the front line of COVID-19 management. Second, the survey was anonymous, and there were insufficient data on individuals to recognize HCPs who also responded to our survey during the first wave. We were therefore not able to identify HCPs whose mental status improved, worsened, or remained unchanged between the two waves. However, the 70% response rate and the fact that more than 90% of the respondents worked in an ICU during the first wave suggest that the global trends are likely to apply to all respondents. Third, this study did not properly compare health care worker outcomes across COVID-19 waves. In addition, this study does not compare mental health symptoms in critical care health care providers exposed or not to the COVID-19 pandemic. For instance, symptoms of anxiety and depression have not been properly assessed in critical care nurses and physicians. However, several studies reported a high prevalence of symptoms of severe burnout, suggesting that the COVID-19 outbreak is a situation at high risk of burnout.[12,13,30] Fourth, we used a simple way to assess personality traits in health care workers. However, dichotomizing personality traits that are typically viewed as a continuum is a limitation that will need to be addressed in future qualitative studies. Last, between the two waves, senior physicians at our hospital made heartfelt pleas to urge the hospital management to provide support to frontline physicians. Some improvements may therefore have occurred between the two waves. However, the culture of efficiency and economy that permeates hospital management in France tends to be impervious to the types of interventions needed to protect the mental health of HCPs.

Interpretation

In summary, we found a high level of mental health symptoms among frontline ICU HCPs during the second COVID-19 wave. Determinants of this psychological burden included personal characteristics, the work environment, and self-perceptions. Strategies to prevent and mitigate this burden are warranted to protect our valuable HCPs and to ensure that the highest quality of care is maintained despite the considerable increase in patient numbers.

Acknowledgments

Author contributions: E. A. and N. K.-B. designed the study and obtained the funding and authorizations. M. D. and F. P. were part of the steering committee and helped to design the project and to select the metrics and mental health outcomes. A. C., F. Bruneel, A. D., A. K., D. R., K. K., L. A., F. Barbier, M. J., J. R., L. P., M. R.-R., O. G., V. L., G. V. D. M., V. S., and C. G. took part in designing the survey instrument, validated the pilot, and guided survey completion in their departments. All of the authors took part in analyzing the data, raising hypotheses, and guiding the analysis. All of the authors revised the text for important intellectual content and approved submission of the final version to *CHEST*.

Financial/nonfinancial disclosures: The authors have reported to *CHEST* the following: E. A. reports personal fees from Gilead, Baxter, Ablybx, Alexion, Pfizer, grants and nonfinancial support from Fisher & Payckle, MSD, Gilead, outside the submitted work; M. D. reports personal fees from Gilead, Astelas, MSD, grants and nonfinancial support from MSD, outside the submitted work; V. L. reports advisory board fees from Amomed unrelated with the present study; F. Barbier reports other from MSD, other from BioMérieux, outside the submitted work; L. P. reports personal fees from MSD, grants and nonfinancial support from Sedana, outside the submitted work; A. D. reports personal fees from Medtronic, grants, personal fees and nonfinancial support from Philips, personal fees from Baxter, personal fees from Hamilton, personal fees and nonfinancial support from Fisher & Paykel, grants from French Ministry of Health, personal fees from Getinge, grants, personal fees and nonfinancial support from Respinor, grants, personal fees and nonfinancial support from Lungpacer, personal fees from Lowenstein, outside the submitted work. None declared (F. P., J. R., L. A., F. Bruneel, P. C., A. C., K. K., C. G., A. K., O. G., M. J., G. V. D. M., D. R., V. S., M. R. R., and N. K. B.).

Role of sponsors: The sponsor had no role in the design of the study, the collection and analysis of the data, or the preparation of the manuscript.

References

1. Pappa S, Ntella V, Giannakas T, Gianznakoulis VG, Papoutsi E, Katsaounou P. Prevalence of depression, anxiety, and insomnia among healthcare workers during the COVID-19 pandemic: a systematic review and meta-analysis. *Brain Behav Immun.* 2020;88:901-907.
2. Wu T, Jia X, Shi H, et al. Prevalence of mental health problems during the COVID-19 pandemic: a systematic review and meta-analysis. *J Affect Disord.* 2020;281:91-98.
3. McCall WV, Mensah-Bonsu D, Withers AE, Gibson RW. Short-term insomnia disorder in health care workers in an academic medical center before and during COVID-19: rates and predictive factors. *J Clin Sleep Med.* 2021;17(4):749-755.
4. Wahlster S, Sharma M, Lewis AK, et al. The coronavirus disease 2019 pandemic's effect on critical care resources and health-care providers: a global survey. *Chest.* 2021;159(2):619-633.
5. Lai J, Ma S, Wang Y, et al. Factors associated with mental health outcomes among health care workers exposed to coronavirus disease 2019. *JAMA Netw Open.* 2020;3(3):e203976.
6. Azoulay E, Cariou A, Bruneel F, et al. Symptoms of anxiety, depression, and peritraumatic dissociation in critical care clinicians managing patients with COVID-19: a cross-sectional study. *Am J Respir Crit Care Med.* 2020;202(10):1388-1398.
7. Secosan I, Virga D, Crainiceanu ZP, Bratu LM, Bratu T. Infodemia: another enemy for Romanian frontline healthcare workers to fight during the COVID-19 outbreak. *Medicina (Kaunas).* 2020;56(12):679.
8. Shultz JM, Cooper JL, Baingana F, et al. The role of fear-related behaviors in the 2013-2016 West Africa Ebola virus disease outbreak. *Curr Psychiatry Rep.* 2016;18(11):104.
9. Arora A, Jha AK, Alat P, Das SS. Understanding coronaphobia. *Asian J Psychiatr.* 2020;54:102384.
10. Azoulay E, De Waele J, Ferrer R, et al. Symptoms of burnout in intensive care unit specialists facing the COVID-19 outbreak. *Ann Intensive Care.* 2020;10(1):110.
11. Barello S, Palamenghi L, Graffigna G. Burnout and somatic symptoms among frontline healthcare professionals at the peak of the Italian COVID-19 pandemic. *Psychiatry Res.* 2020;290:113129.
12. Poncet MC, Toullic P, Papazian L, et al. Burnout syndrome in critical care nursing staff. *Am J Respir Crit Care Med.* 2007;175(7):698-704.
13. Embriaco N, Azoulay E, Barrau K, et al. High level of burnout in intensivists: prevalence and associated factors. *Am J Respir Crit Care Med.* 2007;175(7):686-692.
14. Liu CH, Zhang E, Wong GTF, Hyun S, Hahm HC. Factors associated with depression, anxiety, and PTSD symptomatology during the COVID-19 pandemic: clinical implications for U.S. young adult mental health. *Psychiatry Res.* 2020;290:113172.
15. Rangachari P, Woods JL. Preserving organizational resilience, patient safety, and staff retention during COVID-19 requires a holistic consideration of the psychological safety of healthcare workers. *Int J Environ Res Public Health.* 2020;17(12):4267.
16. Zigmond AS, Snaith RP. The hospital anxiety and depression scale. *Acta Psychiatr Scand.* 1983;67(6):361-370.
17. Pochard F, Azoulay E, Chevret S, et al. Symptoms of anxiety and depression in family members of intensive care unit patients: ethical hypothesis regarding decision-making capacity. *Crit Care Med.* 2001;29(10):1893-1897.
18. Forbes D, Creamer M, Phelps A, et al. Australian guidelines for the treatment of adults with acute stress disorder and post-traumatic stress disorder. *Aust N Z J Psychiatry.* 2007;41(8):637-648.
19. Vande Walle KA, Pavuluri Quamme SR, Leverson GE, et al. Association of personality and thinking style with effective surgical coaching. *JAMA Surg.* 2020;155(6):480-485.
20. R Core Team. R: a language and environment for statistical computing. 2020. Vienna, Austria: R Foundation for Statistical Computing. https://www.R-project.org/. Accessed June 8, 2021.
21. Flesia L, Monaro M, Mazza C, et al. Predicting perceived stress related to the Covid-19 outbreak through stable psychological traits and machine learning models. *J Clin Med.* 2020;9(10):3350.
22. Duan-Porter W, Hatch D, Pendergast JF, et al. 12-month trajectories of depressive symptoms among nurses: contribution of personality, job characteristics, coping, and burnout. *J Affect Disord.* 2018;234:67-73.
23. Rakesh G, Pier K, Costales TL. A call for action: cultivating resilience in healthcare providers. *Am J Psychiatry Resid J.* 2017;12(4):3-5.
24. Tomlin J, Dalgleish-Warburton B, Lamph G. Psychosocial support for healthcare workers during the COVID-19 pandemic. *Front Psychol.* 2020;11:1960.
25. De Brier N, Stroobants S, Vandekerckhove P, De Buck E. Factors affecting mental health of health care workers during coronavirus disease outbreaks (SARS, MERS & COVID-19): a rapid systematic review. *PLoS One.* 2020;15(12):e0244052.
26. Coelho CM, Suttiwan P, Arato N, Zsido AN. On the nature of fear and anxiety triggered by COVID-19. *Front Psychol.* 2020;11:581314.
27. Barello S, Falcó-Pegueroles A, Rosa D, Tolotti A, Graffigna G, Bonetti L. The psychosocial impact of flu influenza pandemics on healthcare workers and lessons learnt for the COVID-19 emergency: a rapid review. *Int J Public Health.* 2020;65(7):1205-1216.
28. Shanafelt T, Ripp J, Trockel M. Understanding and addressing sources of anxiety among health care professionals during the COVID-19 pandemic. *JAMA.* 2020;323(21):2133-2134.
29. Mihaljevic T. Tiered daily huddles: the power of teamwork in managing large healthcare organisations. *BMJ Qual Saf.* 2020;29(12):1050-1052.
30. Merlani P, Verdon M, Businger A, Domenighetti G, Pargger H, Ricou B. Burnout in ICU caregivers: a multicenter study of factors associated to centers. *Am J Respir Crit Care Med.* 2011;184(10):1140-1146.
