# Supplementary material for: Mental health status and related factors influencing healthcare workers during the COVID-19 pandemic: A systematic review and meta-analysis
Source: PLoS One. 2024 Jan 19;19(1):e0289454. doi: 10.1371/journal.pone.0289454 (PMC10798549; doi:10.1371/journal.pone.0289454)
Supplement: S1 Data — (ZIP) [file pone.0289454.s011.zip › literatures/160.pdf]

Since January 2020 Elsevier has created a COVID-19 resource centre with free information in English and Mandarin on the novel coronavirus COVID-19. The COVID-19 resource centre is hosted on Elsevier Connect, the company's public news and information website.

Elsevier hereby grants permission to make all its COVID-19-related research that is available on the COVID-19 resource centre - including this research content - immediately available in PubMed Central and other publicly funded repositories, such as the WHO COVID database with rights for unrestricted research re-use and analyses in any form or by any means with acknowledgement of the original source. These permissions are granted for free by Elsevier for as long as the COVID-19 resource centre remains active.

Clinical Gastroenterology and Hepatology 2021;19:836–838

RESEARCH CORRESPONDENCE

Psychological Health Among Gastroenterologists During the COVID-19 Pandemic: A National Survey

Eric D. Shah,* Mohsen Pourmorteza,‡ B. Joseph Elmunzer,§ Sarah K. Ballou,|| Georgios I. Papachristou,‡ Luis F. Lara,‡ Uche Okafor,‡ Sheryl A. Pfeil,‡ Darwin L. Conwell,‡ and Somashekar G. Krishna,‡ On behalf of the North American Alliance for the Study of Digestive Manifestations of COVID-19

*Section of Gastroenterology and Hepatology, Dartmouth-Hitchcock Health, Lebanon, New Hampshire; ‡Division of Gastroenterology, Hepatology & Nutrition, The Ohio State Wexner Medical Center, Columbus, Ohio; §Division of Gastroenterology and Hepatology, Medical University of South Carolina, Charleston, South Carolina; and ||Division of Gastroenterology, Beth Israel Deaconess Medical Center, Boston, Massachusetts

The COVID-19 pandemic poses unprecedented and unique challenges to gastroenterologists eager to maintain clinical practice, patients' health, and their own physical/mental well-being. We aimed to estimate the prevalence and critical determinants of psychological distress in gastroenterologists during the COVID-19 pandemic.

Methods

Between May and June 2020, a national cross-sectional survey was distributed to gastroenterologists in the United States. The primary study outcomes were psychological distress (Patient Health Questionnaire-8, General Anxiety Disorder-7) and insomnia (Insomnia Severity Index-7). Over several electronic meetings and a validation process involving pilot testing, we developed domains representing personal challenges, practice-related challenges, and perceived exposure risks during the COVID-19 pandemic to identify specific determinants of psychological health outcomes. We assessed whether resilient coping skills (Brief Resilient Coping Scale) modified psychological health outcomes and assessed well-being (Physician Well-Being Index) as an indicator of poor psychological health. The study used logistic regression to evaluate the effects of personal challenges, professional challenges, and perceived COVID-19 exposure factors during the pandemic. The full development and validation process and statistical plan is reported in the Supplementary file.

Results

A total of 153 gastroenterologists completed the questionnaire (65.1% of respondents who started the survey; see Supplementary Table 1 for a full description of the cohort) representing 32 US states with a mean age of 46.1 ± 10.1 years and a mean of 13.0 ± 10.1 years in practice. A 22.7% of respondents were female.

Depression (8.5%) and anxiety (7.2%) were uncommon. However, 25.5% met clinical cutoff scores for insomnia. Eighty-five percent reported moderate-to-high well-being. Among the 15.0% of responders reporting low well-being, the Physician Well-Being Index instrument strongly predicted clinical depression, anxiety, and insomnia in univariate analyses ($P < .001$ for all outcomes).

No personal or practice-related factors and no perceived COVID-19-related exposure risks reliably predicted clinical depression in univariate analysis. A perceived lack of personal protective equipment was associated with clinical anxiety (odds ratio [OR], 4.398; 95% confidence interval [CI], 1.018–18.989; $P = .047$). Age older than 60 (OR, 4.216; 95% CI, 1.264–14.060; $P = .046$), years in practice (OR, 1.039; 95% CI, 1.003–1.077; $P = .035$), and isolation outside of the home were associated with clinical insomnia (OR, 4.611; 95% CI, 1.733–12.272; $P = .026$). Gastroenterologists who more frequently performed endoscopy were less likely to report clinical insomnia (OR, 0.792; 95% CI, 0.639–0.981; $P = .033$).

A 30.7% of gastroenterologists reported low resilient coping skills. Clinical insomnia (found in one-quarter of respondents) was significantly associated with low resilient coping (OR, 3.80; 95% CI, 1.16–12.46). After adjusting for age, gender, and resilient coping, most factors in univariate analyses were no longer associated with clinical insomnia. However, female gastroenterologists (OR, 5.61; 95% CI, 1.26–24.87; $P = .02$), gastroenterologists isolating from household members (OR, 5.63; 95% CI, 1.67–19.03; $P = .005$), and gastroenterologists in practices with fewer than 15 attending physicians (OR, 9.76; 95% CI, 2.25–42.28; $P = .002$) were more likely to report insomnia.

Most current article

© 2021 by the AGA Institute
1542-3565/$36.00
https://doi.org/10.1016/j.cgh.2020.11.043

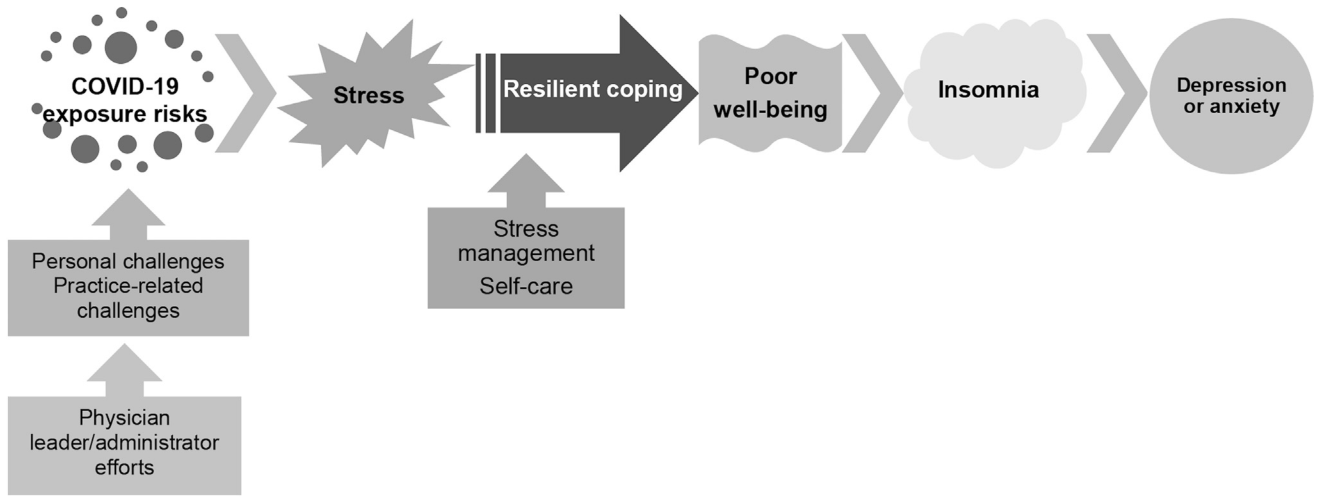

Figure 1. Framework of intervenable targets to improve psychological health among gastroenterologists during the COVID-19 pandemic.

Discussion

Identifying effective ways to mitigate psychological distress among gastroenterologists and other health care workers during the COVID-19 pandemic is critical to maintaining a healthy and productive health care workforce.[1] However, the complexity of mental health poses challenges to designing effective solutions to mitigate psychological distress. In the current study, we found that singular personal challenges, practice-related challenges, and perceived COVID-19-related exposure risks (eg, perception of personal protective equipment availability) had little association with important psychological health outcomes including depression or anxiety. What explains this discrepancy, and why might focused interventions, such as "improving personal protective equipment availability," alone be insufficient to improve psychological health?

Resilience is defined as the "mental processes and behaviors that a person uses to protect themselves from the potential negative effects of stressors."[2] Resilient coping skills allow individuals in stressful situations to avoid negative psychological health consequences, such as depression and anxiety (Figure 1). Importantly, resilience is a skill that can be learned and improved. Indeed, in an international survey some physicians reported positive effects of COVID-19 stress, especially pride of being a health care worker.[3]

Physician leaders and other administrators should consider strategies to maintain resilient coping skills among their colleagues, such as dedicated resilience training and self-care,[4,5] noting that low resilient coping skills are prevalent.[6] Practice leaders should continually engage gastroenterologists on addressing the multitude of challenges faced by our procedure-predominant and patient-centered specialty, rather than solely focusing on brief, short-term "silver bullet" interventions alone.[5] Professional societies and policymakers should work to support smaller gastroenterology practices, which may face outsized obstacles in stopping elective procedures while maintaining sufficient financial liquidity to support their practice, rapidly developing a health information technology infrastructure for telemedicine, obtaining adequate personal protective equipment, and other challenges.[7,8] Overall, these efforts will ensure that gastroenterologists can maintain a high degree of psychological health so that they remain effective in providing outstanding patient care during the COVID-19 pandemic.

Supplementary Material

Note: To access the supplementary material accompanying this article, visit the online version of *Clinical Gastroenterology and Hepatology* at www.cghjournal.org, and at https://doi.org/10.1016/j.cgh.2020.11.043.

References

1. Hartzband P, Groopman J. Physician burnout, interrupted. N Engl J Med 2020;382:2485–2487.
2. Robertson IT, Cooper CL, Sarkar M, et al. Resilience training in the workplace from 2003 to 2014: a systematic review. J Occup Organ Psychol 2015;88:533–562.
3. Medscape US and International Physicians' COVID-19 Experience Report. Available at: https://www.medscape.com/slideshow/2020-physician-covid-experience-6013151. Accessed November 2, 2020.
4. West CP, Dyrbye LN, Sinsky C, et al. Resilience and burnout among physicians and the general US working population. JAMA Netw Open 2020;3:e209385.
5. Greenberg N, Docherty M, Gnanapragasam S, et al. Managing mental health challenges faced by healthcare workers during COVID-19 pandemic. BMJ 2020;m1211.
6. Johns Hopkins Coronavirus Resource Center. Available at: https://coronavirus.jhu.edu/us-map. Accessed November 2, 2020.
7. Allen JI, Kaushal N. New models of gastroenterology practice. Clin Gastroenterol Hepatol 2018;16:3–6.

8. Shah ED, Amann ST, Karlitz JJ. The time is now: a guide to sustainable telemedicine during COVID-19 and beyond. Am J Gastroenterol 2020;115:1371–1375.

Reprint requests
Address requests for reprints to: Somashekar G. Krishna, MD, MPH, Division of Gastroenterology, Hepatology & Nutrition, Ohio State Wexner Medical Center, 395 West Twelfth Avenue, Columbus, Ohio 43210. e-mail: somashekar.krishna@osumc.edu.

CRediT Authorship Contributions

Shah (Conceptualization, Methodology, Visualization, Investigation, Resources, Writing- Original draft preparation, Writing - Review & Editing, Funding acquisition)

Krishna (Conceptualization, Visualization, Methodology, Resources, Investigation, Writing - Review & Editing, Supervision, Project administration)

Pourmorteza (Formal analysis, Data curation, Investigation, Visualization)

Elmunzer (Conceptualization, Resources, Methodology, Investigation, Writing - Review & Editing, Project administration)

Conwell (Conceptualization, Resources, Methodology, Investigation, Writing - Review & Editing, Project administration

Ballou (Methodology, Writing - Review & Editing)

Papachristou (Methodology, Writing - Review & Editing, Resources. Okafor: Methodology, Software, Investigation, Resources, Data curation)

Lara (Methodology, Writing - Review & Editing, Resources. Okafor: Methodology, Software, Investigation, Resources, Data curation)

Pfeil (Methodology, Writing - Review & Editing, Resources. Okafor: Methodology, Software, Investigation, Resources, Data curation)

Conflicts of interest

The authors disclose no conflicts.

Funding
Eric D. Shah is supported by the AGA Research Foundation's 2019 AGA-Shire Research Scholar Award in Functional GI and Motility Disorders.

Supplementary Material

Survey Instrument Development

The survey instrument was built over several electronic meetings among study authors and the members of the North American Alliance for the Study of Digestive Manifestations of COVID-19 to develop generalizable thematic domains of possible determinants, which have been proposed to affect psychological health during the COVID-19 and prior pandemics among health care providers incorporating validated coping and well-being scores focusing on anxiety, depression, and insomnia. Domains included personal challenges, practice-related challenges, and perceived exposure risks during the COVID-19 pandemic. We also assessed respondents' degree of resilient coping skills (Brief Resilient Coping Scale; score range, 4–20; low resilient coping, score $\leq$14) and well-being (Physician Well-Being Index; score range, 0–7; poor well-being, score $\geq$4), because scores are known to influence risks of developing clinical depression and anxiety. The instrument was then pilot tested among physician members of the North American Alliance for the Study of Digestive Manifestations of COVID-19 to establish content and face validity and to maximize test-retest reliability; these efforts were important to finalize the order of questions, maximize comprehension, and minimize completion time. The near-final version was reviewed by a trained gastroenterology clinical health psychologist (SB).

Study Outcomes

Study outcomes were depression (Patient Health Questionnaire-8; score range, 0–27; clinical depression, score $\geq$10), anxiety (General Anxiety Disorder-7; score range, 0–21; clinical anxiety, score $\geq$10), and insomnia (Insomnia Severity Index-7; score range, 0–28; clinical insomnia, score $\geq$15).

Survey Distribution

Considering the evolving nature of the COVID-19 pandemic and the need for timely dissemination of our findings, numerous means were used to rapidly distribute our cross-sectional survey to gastroenterologists across the United States using several outlets including mass email, promotion on social media, and direct contact by physician members of the North American Alliance for the Study of Digestive Manifestations of COVID-19 between May and June 2020, with a goal of maximizing representation in geography and types of gastroenterology practices (solo, physician-owned, and hospital-employed). The survey was performed, deidentified, housed, and analyzed at The Ohio State Wexner Medical Center and administered using RedCAP (https://redcap.bmi.osumc.edu). Institutional review board approval was obtained before study initiation and participants had to consent to participate.

Statistics

Descriptive statistics were reported using a Student *t* test or Fisher exact chi-square test as appropriate. Samples containing missing data were excluded from regression analysis. Univariate logistic regression was performed to assess effects of each demographic and practice-specific variable, with indicator variables representing insomnia, resilient coping, and well-being. Outcomes were reported using odds ratios and 95% confidence intervals. For insomnia, multivariable logistic regression was performed using a backward stepwise selection methodology to identify key determinants of insomnia. Age, gender, and resilient coping skills were forced into our final model. Forward stepwise selection was used to validate the final models (not shown). Because of the low frequency of clinical depression or anxiety among gastroenterologists in our survey, multivariable analyses were not performed on these outcomes. Analysis was performed using SAS/STAT version 9.4 (Cary, NC).

Supplementary Table 1. Baseline Demographics of Gastroenterology Physician Respondents

Characteristic	Descriptors	Overall cohort
Age	Mean ± SD	46.1 ± 10.1
Gender	Female	22.7%
	Male	74.6%
	Preferred not to answer	2.7%
Race	White	56.9%
	Asian	29.4%
	Black	2.0%
	Native American	0.0%
	Pacific Islander	0.7%
	More than 1 race	4.6%
	Other	6.5%
Ethnicity	Hispanic/Latino	8.1%
	Non-Hispanic/Latino	86.5%
	Prefer not to answer	5.4%
Specialty	General gastroenterology	37.9%
	Advanced endoscopy	37.9%
	Hepatology/transplant hepatology	7.2%
	Inflammatory bowel diseases	6.5%
	Other	10.5%
Number of years in practice	Mean ± SD	13.0 ± 10.1
Was the respondent on standby to provide inpatient care for COVID-19 patients	Yes	59.2%
	No	40.8%
Had the respondent provided direct gastroenterology-related care to COVID-19 patients	Yes	47.0%
	No	53.0%
Number of gastroenterology attendings in the practice	Mean ± SD	22.6 ± 24.5 gastroenterology attendings
Employment arrangement	Hospital/health-system employed	75.1%
	Physician-owned practice/other	24.9%
Hospital location	Rural	13.2%
	Urban	86.8%
Hospital bed size	1–149 beds	4.0%
	150–500 beds	35.5%
	>500 beds	60.5%
Does the practice train gastroenterology fellows	Yes	69.7%
	No	30.3%
Number of half-day endoscopy sessions conducted by the respondent per week during COVID-19 pandemic	Mean ± SD	2.1 ± 2.1
Administrative and/or leadership responsibilities	Yes	49.7%
	No	50.3%

Supplementary Table 1. Continued

Characteristic	Descriptors	Overall cohort
Change in compensation caused by COVID-19	Reduction in compensation	43.1%
	No reduction in compensation	38.4%
	Not yet, but cuts are being considered	18.5%
Perception of current status of the local COVID-19 surge curve	Ascending	19.2%
	Plateau	31.1%
	Descending	49.7%
Perception of personal protective equipment availability during COVID-19 pandemic	Adequate	90.7%
	Not adequate	9.3%
Perception of personal health risk for COVID-19 morbidity/mortality	High-risk	14.6%
	Not high-risk	73.5%
	Uncertain	9.9%
	Preferred not to answer	2.0%
Household with children	Yes	68.4%
	No	30.9%
	Other	0.7%
Was the childcare situation of the respondent affected by the pandemic	Yes	51.0%
	No	46.1%
	Not applicable	2.9%
Perception of whether household members were at high-risk for COVID-19 morbidity/mortality	Yes	20.1%
	No	77.0%
	Uncertain	2.9%
Whether the respondent isolated from other household members during the pandemic	Yes	33.5%
	No	66.5%
Depressive disorder (Patient Health Questionnaire-8 score >9)	Yes	8.5%
	No	91.5%
Clinical anxiety (Generalized Anxiety Disorder-7 score >9)	Yes	7.2%
	No	92.8%
Clinical insomnia (Insomnia Severity Index-7 score >14)	Yes	25.5%
	No	74.5%
Physician well-being	Moderate-to-high well-being	85.0%
	Low well-being	15.0%
Resilient coping skills (Brief Resilient Coping Scale)	High coping skills	69.3%
	Low coping skills	30.7%

SD, standard deviation.
