# Supplementary material for: Mental health status and related factors influencing healthcare workers during the COVID-19 pandemic: A systematic review and meta-analysis
Source: PLoS One. 2024 Jan 19;19(1):e0289454. doi: 10.1371/journal.pone.0289454 (PMC10798549; doi:10.1371/journal.pone.0289454)
Supplement: S1 Data — (ZIP) [file pone.0289454.s011.zip › literatures/162.pdf]

ELSEVIER

Since January 2020 Elsevier has created a COVID-19 resource centre with free information in English and Mandarin on the novel coronavirus COVID-19. The COVID-19 resource centre is hosted on Elsevier Connect, the company's public news and information website.

Elsevier hereby grants permission to make all its COVID-19-related research that is available on the COVID-19 resource centre - including this research content - immediately available in PubMed Central and other publicly funded repositories, such as the WHO COVID database with rights for unrestricted research re-use and analyses in any form or by any means with acknowledgement of the original source. These permissions are granted for free by Elsevier for as long as the COVID-19 resource centre remains active.

European Journal of Obstetrics & Gynecology and Reproductive Biology 253 (2020) 90–94

Contents lists available at ScienceDirect

European Journal of Obstetrics & Gynecology and Reproductive Biology

journal homepage: www.elsevier.com/locate/ejogrb

Full length article

Mental health amongst obstetrics and gynaecology doctors during the COVID-19 pandemic: Results of a UK-wide study

Neha Shah[a,*], Ali Raheem[b], Michail Sideris[c], Luxmi Velauthar[a], Ferha Saeed[a]

[a] Newham University Hospital NHS Trust, Glen Rd, London, E13 8SL, UK
[b] South London and Maudsley NHS Foundation Trust, Maudsley Hospital, Denmark Hill, London, Greater London, SE5 8AZ, UK
[c] Women's Health Research Unit, Queen Mary University of London, Yvonne Carter Building, 58 Turner Street, E1 2AB, UK

A R T I C L E I N F O

Article history:
Received 21 June 2020
Received in revised form 27 July 2020
Accepted 29 July 2020

Keywords:
Mental health
Obstetrics
Gynaecology
Education and training

A B S T R A C T

Objective: To explore the impact of the COVID-19 pandemic on the mental health of Obstetricians and Gynaecologists.
Study design: A cross-section survey-based study amongst doctors working within Obstetrics and Gynaecology in the United Kingdom.
Results: A total of 207 doctors completed the survey. Obstetricians and Gynaecologists reported significantly higher rates of both Major Depressive Disorder (versus, $p = 0.023$) and Generalised Anxiety Disorder (versus, $p = 0.044$) as compared to the UK-wide estimates. Sub-group analysis showed that anxiety was more common amongst female doctors as compared to males (versus, $p = 0.047$). Although the prevalence of GAD was higher amongst registrars compared to their Consultant and/or Senior House Officer counterparts, this was not statistically significant.
Respondents felt that the most significant factor for work-related changes to mental health was keeping up to date with frequently changing guidelines and protocols related to COVID-19. Only of respondents felt able to talk to colleagues about their mental health.
Conclusions: Key findings include the high prevalence of mental health conditions amongst doctors, demonstration of the persistent taboo that mental health carries within the speciality and the key contributory factors to poor mental health.
Further work should be done to assess if changes to the way new and updating guidelines, protocols and pathways are disseminated reduces the impact on the mental health of doctors. With the threat of a second COVID-19 peak looming, now more than ever, it is vital that steps are taken to break the stigmatisation of mental health amongst doctors, encouraging doctors to seek help when required.
© 2020 Elsevier B.V. All rights reserved.

Introduction

The mental health of healthcare professionals is an often-neglected subject, despite evidence to suggest that poor mental health has negative impacts not only on personal wellbeing but also reduced productivity, increase in sick leave, increased likelihood of human errors and lower patient satisfaction [1–5].

To date, there have been over 3.5 million cases of confirmed COVID-19 infection, with almost 250,000 deaths [6]. As a result, the COVID-19 pandemic has applied huge pressures on healthcare systems and workers. Obstetricians and Gynaecologists have had to manage pregnant patients with this novel condition with only a limited evidence-base. Rapidly changing protocols and guidelines related to COVID-19 management, staffing levels and shift patterns have added an additional dimension to these pressures. On top of that, healthcare workers are in fear of acquiring the infection themselves and spreading this further to their loved ones; some doctors even having to isolate from their families because of this.

It is therefore important to ascertain the impact of these stressors on the mental health of doctors and to identify the contributory factors. This understanding may help to support healthcare staff effectively, minimising the negative impact on

* Corresponding author.
E-mail address: Neha.shah16@nhs.net (N. Shah).

https://doi.org/10.1016/j.ejogrb.2020.07.060
0301-2115/© 2020 Elsevier B.V. All rights reserved.

mental health and improving clinical performance with a subsequent improvement in patient outcomes during future COVID-19 peaks.

Study Objectives

To explore the impact of the COVID-19 pandemic on the mental health of Obstetricians and Gynaecologists and identify significant contributory factors.

Methods

We performed a cross-sectional, survey-based study of doctors working in the field of Obstetrics and Gynaecology across all UK Hospitals. Surveys were distributed to doctors via a number of methods. This included dissemination of the survey to trainees via newsletters, directly emailing doctors to circulate amongst their hospitals and via social media. It was made clear to the respondents that their participation was voluntary and that responses would be anonymous. Informed consent was implied on return of the survey.

The survey was created in collaboration with both Obstetrics and Gynaecology and Psychiatry doctors. Surveyed information included demographics, history of past mental health conditions requiring treatment, screening for current symptoms of depression and anxiety, the significance of contributory factors and the effects of mental health on workplace behaviour. Screening for major depressive disorders (MDD) and generalised anxiety disorders (GAD) were performed using the GAD-2 and PHQ-2 questionnaires. A GAD-2 score of 3 or more was interpreted as likely GAD (sensitivity, specificity) [7]. A PHQ-2 score of 3 or more was interpreted as likely MDD (sensitivity, specificity) [8]. For the survey to be accepted, all questions had to be answered.

Data-analysis

We primarily used inferential descriptive statistics including one-sample to compare the prevalence of anxiety and depression in our study sample against UK-wide population estimates. Comparative population estimates were obtained from a 7 yearly UK-wide survey, last completed in 2016 [10]. We used chi-squared test to explore whether demographics were associated with likely MDD and GAD. Finally, we performed a binary logistic regression analysis to explore causative factors for changes in mental health [9]. Analyses were performed using IBM SPSS for Windows Version 26.

Results

Two-hundred and seven doctors completed the survey (female; male). Age distribution was as follows: (n = 93) aged years, (n = 93) aged years, (n = 21) were aged years. Regarding the clinical-grade, (n = 58) of respondents were Consultants, (n = 99) were Specialty Registrars and (n = 50) were Senior House Officers. Demographics of respondents is demonstrated in Table 1.

Of the respondents, (n = 46) of respondents stated that they had previously received treatment for a mental health condition. (n = 33) of respondents had a PHQ-2 score of 3 or more, suggestive of MDD while (n = 51) of respondents had a GAD-2 score of 3 or more suggestive of GAD.

The rates of both MDD (1 versus, p = 0.023) and GAD (versus, p = 0.044) were significantly higher in Obstetrics and Gynaecology doctors as compared to UK-wide population estimates [10]. GAD was more prevalent amongst female doctors as compared to males (prevalence versus, p = 0.047). Although the prevalence of GAD was higher amongst registrars compared to their Consultant and/or Senior House Officer counterparts, this was not statistically significant (Fig. 1). There was no statistically significant difference in the likelihood of MDD or GAD depending on if respondents had a previous history of mental health disorders requiring treatment (prevalence versus, p = 0.176).

Respondents felt that the most significant causative factor for work-related changes to mental health was keeping up to date with frequently changing guidelines, pathways and protocols related to COVID-19 practice in O&G. of respondents stated that this factor had caused at least a moderate effect on their mental health, with of respondents stating the effect was high or very high. Table 5 demonstrates the perceived contribution of causative factors to participants mental health status.

Multiple binary logistic regression showed that there was a positive correlation between the extent respondents felt that the rapidly evolving environment had an impact on their mental health and a PHQ-2 score suggestive of a MDD (OR = 1.825; CI: 1.241, 2.987; p = 0.003). There was also a positive correlation between the extent respondents were concerned about

Table 1
Respondent's demographics.

	Number of respondents (%)
**Gender**	
Male	39 (18.9 %)
Female	167 (81.1 %)
**Age**	
20−34 years	93 (44.9 %)
35−49 years	93 (44.9 %)
50−69 years	21 (10.1 %)
**Clinical Grade**	
Consultant	58 (28 %)
Registrar	99 (47.8 %)
SHO	50 (24.2 %)
**Ethnicity**	
White	98 (47.34 %)
Asian	66 (31.88 %)
Black	23 (11.11 %)
Mixed	6 (2.90 %)
Other	10 (4.83 %)
Not disclosed	4 (1.93 %)

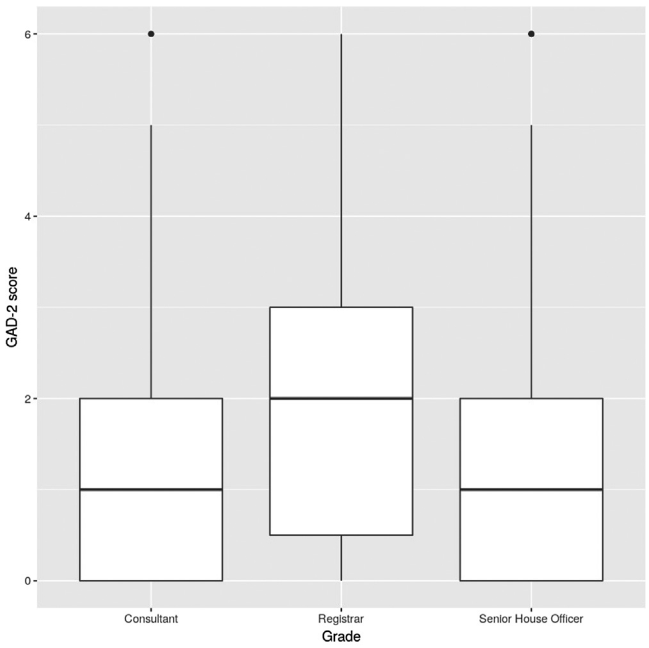

Fig. 1. Box plot depicting Clinical Grade and GAD-2 score.

Table 2
Proportion of respondents with scores suggestive of Major Depressive Disorder (MDD) and Generalised Anxiety Disorder (GAD).

	Number of respondents (%)
**GAD-2 score**	
≤2 (unlikely GAD)	156 (75.36 %)
≥3 (likely GAD)	51 (24.64 %)
**PHQ-2 score**	
≤2 (unlikely MDD)	174 (84.06 %)
PHQ-2 score ≤2 (unlikely MDD)	33 (15.94 %)

*GAD = Generalised Anxiety Disorder, MDD = Major Depressive Disorder.

contracting COVID-19 from the workplace environment and a GAD-2 score suggestive of GAD (OR = 1.671; CI: 1.058, 2.640; p = 0.028).

Assessed on a Likert scale, of respondents agreed that their current mental health status was negatively affected by their workplace behaviour. of respondents had considered taking time off work during the COVID-19 pandemic due to their mental state. of respondents felt well-supported in their workplace environment. were aware of wellbeing services available locally and nationally for healthcare staff. Only felt able to talk to their colleagues about their mental health.

Table 2 summarises the proportion of respondents with scores suggestive of Major Depressive Disorder (MDD) and Generalised Anxiety Disorder (GAD). Table 3 summarises sub-group analyses for Generalised Anxiety Disorder (GAD). Table 4 summarises sub-group analyses for Major Depressive Disorder (MDD).

Discussion

Our study shows a significantly higher prevalence of symptoms of MDD and GAD amongst Obstetrics and Gynaecology doctors during the COVID-19 pandemic, with this being highest amongst female doctors (Table 5).

Whilst there is a paucity of data regarding the impact of COVID-19 on the mental health of doctors, our results sadly resonate findings from two other similar studies performed in the UK. A study by British Medical Association (BMA) during the COVID-19 pandemic found that of doctors were dealing with depression, anxiety, stress, burnout, emotional distress, or another mental health condition related to or made worse by their work [11]. A

Table 3
Sub-group analyses for Generalised Anxiety Disorder (GAD).

	GAD-2 score ≤2 (unlikely GAD)	GAD-2 score ≥2 (likely GAD)	P value
**Gender**			P = 0.047
Male	35 (87.50 %)	5 (12.50 %)	
Female	121 (72.46 %)	46 (27.54 %)	
**Age**			P=0.894
20–34 years	71 (76.34 %)	22 (23.66 %)	
35–49 years	70 (75.27 %)	23 (24.73 %)	
50–69 years	15 (71.43 %)	6 (28.57 %)	
**Clinical Grade**			P = 0.184
Consultant	69 (69.70 %)	30 (30.30 %)	
Registrar	41 (82.0 %)	9 (18.0 %)	
Senior House Officer	46 (79.31 %)	12 (20.69 %)	
**Ethnicity**			P = 0.770
White	74 (75.51 %)	24 (24.49 %)	
Asian	48 (72.73 %)	18 (27.27 %)	
Black	19 (82.61 %)	4 (17.39 %)	
Mixed	4 (66.67 %)	2 (33.33 %)	
**Previous history of treatment for mental health disorder**			P = 0.155
Yes	31 (67.39 %)	15 (32.61 %)	
No	125 (77.64 %)	36 (22.36 %)	

Data reported as number of respondents (% of respondents).

Table 4
Sub-group analysis for Major Depressive Disorder (MDD).

	PHQ-2 score ≤2 (unlikely MDD)	PHQ-2 score ≥2 (likely MDD)	P value
**Gender**			P = 0.104
Male	37 (92.50 %)	3 (7.50 %)	
Female	137 (82.04 %)	30 (17.96 %)	
**Age**			P = 0.474
20–34 years	75 (80.65 %)	18 (19.35 %)	
35–49 years	81 (87.10 %)	12 (12.90 %)	
50–69 years	18 (85.71 %)	3 (14.29 %)	
**Clinical Grade**			P = 0.344
Consultant	80 (80.81 %)	19 (19.19 %)	
Registrar	42 (84.0 %)	8 (16 %)	
Senior House Officer	52 (89.66 %)	6 (10.34 %)	
**Ethnicity**			P = 0.130
White	85 (86.74 %)	13 (13.27 %)	
Asian	50 (75.76 %)	16 (24.24 %)	
Black	21 (91.30 %)	2 (9.52 %)	
Mixed	4 (66.67 %)	2 (33.33 %)	
**Previous history of treatment for mental health disorder**			P = 0.97
Yes	102 (63.35 %)	59 (36.65 %)	
No	29 (63.04 %)	17 (36.96 %)	

Data reported as number of respondents (% of respondents).

Table 5
The perceived contribution of causative factors to participants mental health status

Contributary factor	Moderate, high, or very high contribution to mental health status	High or very high contribution to mental health status
Keeping up to date with frequently changing guidelines, pathways, and protocols	129 (62.32 %)	174 (84.06 %)
Concern about being able to provide competent medical care if deployed to a new area	91 (43.95 %)	132 (63.77 %)
Uncertainty around the effects of COVID-19 on pregnancy and its management	79 (38.16 %)	131 (63.29 %)
Concerns about contracting COVID-19 from the workplace	82 (39.61 %)	130 (62.80 %)
Access to appropriate personal protective equipment	74 (35.75 %)	125 (60.39 %)
A rapidly evolving practice environment that differs greatly from what you are familiar with	69 (33.33 %)	125 (60.39 %)
Increased workload	69 (33.33 %)	108 (52.17 %)

Data reported as number of respondents (% of respondents) and ordered in terms of perceived contribution to mental health status.

mental health survey of orthopaedic team members in the United Kingdom during the COVID-19 pandemic found that the prevalence of GAD and MDD was almost three times and six times greater respectively than the general population [12].

These studies re-enforce the importance of providing mental health support to doctors during the COVID-19 pandemic.

The unknowns surrounding COVID-19 lead to vast volumes of research being conducted rapidly resulting in frequently updated guidelines, protocols and pathways. As the most significant factor to affect doctor's mental health status, it would be prudent to establish methods of mitigating the impact these changes have. Potential methods include having clear platforms for easy access to the most-updated guidelines and protocols with clear summaries of recommendations and changes from previous versions. In addition, having virtual online teaching sessions to disseminate updates on guidelines may be beneficial.

Less than of respondents felt that they were able to talk to their colleagues about their mental health, which demonstrates the persistent taboo that mental health carries within the speciality. It is known that fear of and discrimination potentially impede healthcare workers intent to seek support and psychotherapeutic interventions [13]. Changing attitudes and perceptions towards mental health has been an on-going challenge [14] with greater efforts needed to destigmatise mental health in the profession, thereby improving support for doctors.

Although this is not by any means an easy or quick endeavour, certain departmental and individual factors can help to gradually change cultures [15].

Departmental initiates include raising awareness of the high prevalence of mental health conditions amongst Obstetricians and Gynaecologists and the potential impact that these factors have on both doctor's wellbeing and patient care. Training staff, in particular those in leadership roles, so that they feel confident having conversations about mental health can be key in shaping how doctors cope and recover through difficult times. Identifying accessible mental health champions and support pathways sends a clear message that support is available if colleagues are experiencing a mental health problem. Having visible and accessible leadership when managing complex patients with novel conditions may also help to reduce the mental health impact on doctors.

On an individual level, we can all challenge stigma and prejudice by being approachable and confident about mental health and taking steps to normalise conversations and encourage open dialogues.

Strengths of this study included survey respondents from a range of ages, ethnicities and clinical grades with the survey being UK-wide. It is also the first reported study that has assessed the impact of COVID-19 on Obstetrics and Gynaecology doctors.

Limitations of the study include the relatively small study size as compared to the proportion of doctors working in the field of Obstetrics and Gynaecology in the United Kingdom. Online surveys may be more prone to selection and response bias. Snowball sampling was used to help mitigate selection bias and recruit hard to reach subjects. The cross-sectional design, whilst demonstrating associations cannot demonstrate significant causal relationships.

Of note, population estimates of the prevalence of both GAD and MDD were from before the COVID-19 pandemic. There has been significant evidence suggesting the increasing prevalence of these conditions during the COVID-19 pandemic [16]. However, at the time of this study, there was no available prevalence figures for MDD and GAD in the general population during the COVID-19 pandemic to compare to.

Conclusions

Key findings were the high prevalence of mental health conditions amongst doctors, demonstration of the persistent taboo that mental health carries within the speciality and the key contributory factors to poor mental health.

Further work should be done to assess if changes to the way new and updating guidelines, protocols and pathways are disseminated reduces the impact on the mental health of doctors. With the threat of a second COVID-19 peak looming, now more than ever, it is vital that steps are taken to break the stigmatisation of mental health amongst doctors, encouraging doctors to seek help when required.

Disclosure of Interests

None.

Contribution to authorship

NS, AR and FS created the survey. NS and FS were involved in disseminating the survey throughout the UK. Statistical analysis was completed by NS and confirmed by AR and MS. NS drafted the manuscript. FS, AR, MS, LV contributed to editorial changes.

Ethics approval

Not required.

This project did not go through the formal regulatory process, however, was reviewed by the Joint Research Management Office.

Funding

None

Completing interests

None.

References

[1] Anagnostopoulos F, Liolios E, Persefonis G, Slater J, Kafetsios K, Niakas D. Physician burnout and patient satisfaction with consultation in primary health care settings: evidence of relationships from a one-with-many design. J Clin Psychol Med Sett 2012;19(Dec (4)):401–10.
[2] Dewa CS, Loong D, Bonato S, Thanh NX, Jacobs P. How does burnout affect physician productivity? A systematic literature review. BMC Health Serv Res 2014;28(Jul 14):325.
[3] Fahrenkopf AM, Sectish TC, Barger LK, Sharek PJ, Lewin D, Chiang VW, et al. Rates of medication errors among depressed and burnt out residents: prospective cohort study. BMJ 2008;336(Mar (7642)):488–91.
[4] Lu Dw, Dresden S, McCloskey C, Branzetti J, Gisondi Ma. Impact of burnout on self-reported patient care among emergency physicians. West J Emerg Med 2015;16(Dec (7)):996–1001.
[5] Shanafelt TD, Mungo M, Schmitgen J, Storz KA, Reeves D, Hayes SN, et al. Longitudinal study evaluating the association between physician burnout and changes in professional work effort. Mayo Clin Proc 2016;91(Apr (4)):422–31.
[6] John Hopkins University of Medicine. Johns Hopkins Coronavirus Resource Center. Available from: https://coronavirus.jhu.edu [Accessed 2 April 2020].
[7] Skapinakis P. The 2-item Generalized Anxiety Disorder scale had high sensitivity and specificity for detecting GAD in primary care. Evid Based Med 2007;12(Oct (5)):149.
[8] Arroll B, Goodyear-Smith F, Crengle S, Gunn J, Kerse N, Fishman T, et al. Validation of PHQ-2 and PHQ-9 to screen for major depression in the primary care population. Ann Fam Med 2010;8(Jul-Aug (4)):348–53.
[9] Beaumont R. Health science statistics using R and R commander. Banbury, Oxfordshire: Scion Publishing 2014; 2015.
[10] NHS Digital. Adult Psychiatric Morbidity Survey: Survey of Mental Health and Wellbeing, England. 2014 Available from: https://webarchive.nationalarchives.gov.uk/20180328140249/http://digital.nhs.uk/catalogue/PUB21748 [Accessed 4 April 2020].
[11] British Medical Journal. Covid-19: Doctors need proper mental health support, says BMA. Available from: https://www.bmj.com/content/369/bmj.m2192 [Accessed 24 July 2020].
[12] Thakrar A, Raheem A, Chui K, Karam E, Wickramarachchi Chin K. Trauma and orthopaedic team members' mental health during the COVID-19 pandemic. Bone Joint Open 2020;6(June (1)):316–25.
[13] Wei Zheng. Mental health and a novel coronavirus (2019-nCoV) in China. J Aff Disord 2020;269(Mar (1)) 201-2020.
[14] Bianchi EF, Bhattacharyya MR, Meakin R. Exploring senior doctors' beliefs and attitudes regarding mental illness within the medical profession: a qualitative study. BMJ Open 2016;6(9)e012598.
[15] Mental Health at Work. Supporting healthcare workers' mental health – Mental Health at Work. Available from: https://www.mentalhealthatwork.org.uk/toolkit/supporting-healthcare-workers-mental-health [Accessed 24 July 2020].
[16] National Institute for Health Research (NIHR). The potential impact of COVID-19 on mental health outcomes and the implications for service solutions. Available from: https://arc-w.nihr.ac.uk/research-and-implementation/covid-19-response/potential-impact-of-covid-19-on-mental-health-outcomes-and-the-implications-for-service-solutions [Accessed 15 April 2020].
