# Supplementary material for: Mental health status and related factors influencing healthcare workers during the COVID-19 pandemic: A systematic review and meta-analysis
Source: PLoS One. 2024 Jan 19;19(1):e0289454. doi: 10.1371/journal.pone.0289454 (PMC10798549; doi:10.1371/journal.pone.0289454)
Supplement: S1 Data — (ZIP) [file pone.0289454.s011.zip › literatures/164.pdf]

Since January 2020 Elsevier has created a COVID-19 resource centre with free information in English and Mandarin on the novel coronavirus COVID-19. The COVID-19 resource centre is hosted on Elsevier Connect, the company's public news and information website.

Elsevier hereby grants permission to make all its COVID-19-related research that is available on the COVID-19 resource centre - including this research content - immediately available in PubMed Central and other publicly funded repositories, such as the WHO COVID database with rights for unrestricted research re-use and analyses in any form or by any means with acknowledgement of the original source. These permissions are granted for free by Elsevier for as long as the COVID-19 resource centre remains active.

General Hospital Psychiatry 66 (2020) 1–8

Contents lists available at ScienceDirect

General Hospital Psychiatry

journal homepage: www.elsevier.com/locate/genhospsych

Psychological distress, coping behaviors, and preferences for support among New York healthcare workers during the COVID-19 pandemic

Ari Shechter[a], Franchesca Diaz[a], Nathalie Moise[a], D. Edmund Anstey[a], Siqin Ye[a], Sachin Agarwal[b], Jeffrey L. Birk[a], Daniel Brodie[a], Diane E. Cannone[a], Bernard Chang[c], Jan Claassen[b], Talea Cornelius[a], Lilly Derby[a], Melissa Dong[a], Raymond C. Givens[a], Beth Hochman[d], Shunichi Homma[a], Ian M. Kronish[a], Sung A.J. Lee[a], Wilhelmina Manzano[e,h], Laurel E.S. Mayer[f], Cara L. McMurry[a], Vivek Moitra[g], Patrick Pham[a], LeRoy Rabbani[a], Reynaldo R. Rivera[e,h], Allan Schwartz[a], Joseph E. Schwartz[a,i], Peter A. Shapiro[f], Kaitlin Shaw[a], Alexandra M. Sullivan[a], Courtney Vose[e,h], Lauren Wasson[a], Donald Edmondson[a], Marwah Abdalla[a,*]

[a] Department of Medicine, Columbia University Irving Medical Center, New York, NY, United States of America
[b] Department of Neurology, Columbia University Irving Medical Center, New York, NY, United States of America
[c] Department of Emergency Medicine, Columbia University Irving Medical Center, New York, NY, United States of America
[d] Department of Surgery, Columbia University Irving Medical Center, New York, NY, United States of America
[e] NewYork-Presbyterian Hospital, New York, NY, United States of America
[f] Department of Psychiatry, Columbia University Irving Medical Center, New York, NY, United States of America
[g] Department of Anesthesiology, Columbia University Irving Medical Center, New York, NY, United States of America
[h] School of Nursing, Columbia University, New York, NY, United States of America
[i] Department of Psychiatry and Behavioral Sciences, Stony Brook University, Stony Brook, NY, United States of America

ARTICLE INFO

Keywords:
COVID-19
Distress
Anxiety
Depression
Insomnia
Coping
Healthcare worker

ABSTRACT

Objective: The mental health toll of COVID-19 on healthcare workers (HCW) is not yet fully described. We characterized distress, coping, and preferences for support among NYC HCWs during the COVID-19 pandemic.
Methods: This was a cross-sectional web survey of physicians, advanced practice providers, residents/fellows, and nurses, conducted during a peak of inpatient admissions for COVID-19 in NYC (April 9th–April 24th 2020) at a large medical center in NYC (n = 657).
Results: Positive screens for psychological symptoms were common; 57% for acute stress, 48% for depressive, and 33% for anxiety symptoms. For each, a higher percent of nurses/advanced practice providers screened positive vs. attending physicians, though housestaff's rates for acute stress and depression did not differ from either. Sixty-one percent of participants reported increased sense of meaning/purpose since the COVID-19 outbreak. Physical activity/exercise was the most common coping behavior (59%), and access to an individual therapist with online self-guided counseling (33%) garnered the most interest.
Conclusions: NYC HCWs, especially nurses and advanced practice providers, are experiencing COVID-19-related psychological distress. Participants reported using empirically-supported coping behaviors, and endorsed indicators of resilience, but they also reported interest in additional wellness resources. Programs developed to mitigate stress among HCWs during the COVID-19 pandemic should integrate HCW preferences.

1. Introduction

Coronavirus disease 2019 (COVID-19) is a global pandemic caused by Severe Acute Respiratory Syndrome Coronavirus-2 (SARS-CoV-2) [1]. As of May 8, New York City (NYC) is a global epicenter of the pandemic, with > 180,000 confirmed cases [2,3]. Consequently, NYC

* Corresponding author at: Center for Behavioral Cardiovascular Health, 622 West 168th Street, PH 9-301, Columbia University Irving Medical Center, New York, NY 10032, United States of America.
E-mail address: ma2947@cumc.columbia.edu (M. Abdalla).
@marwahabdalla (M. Abdalla)

https://doi.org/10.1016/j.genhosppsych.2020.06.007
Received 19 May 2020; Received in revised form 1 June 2020; Accepted 2 June 2020
0163-8343/ © 2020 Elsevier Inc. All rights reserved.

hospitals have had to rapidly reconfigure clinical spaces and restructure clinical teams to address the surge of patients with COVID-19. Many healthcare workers have therefore been redeployed to areas outside their usual clinical specialty and/or expertise, often working extra shifts and longer hours to meet high volume patient demand. As SARS-CoV-2 is highly infectious, healthcare workers are at increased risk for acquiring and potentially transmitting COVID-19 to patients, co-workers, and family/friends.

Healthcare workers are facing unprecedented amounts of COVID-19-related psychological stress across professional and personal domains [4]. A cross-sectional study [5] of Chinese healthcare workers found that > 50% reported depression and > 70% reported distress during the COVID-19 pandemic. In NYC, death by suicide has already been reported in frontline providers taking care of patients with COVID-19 [6]. Quantitative data about the types and levels of COVID-19-related stress among United States (US) healthcare workers are lacking. Additionally, there is a dearth of quantitative data about US healthcare workers' 1) coping/stress reduction behaviors, 2) optimism and sense of purpose, and 3) interest in specific wellness resources to mitigate adverse psychological consequences of the COVID-19 pandemic. Understanding the needs and desires of healthcare workers is particularly important as hospitals develop and implement support measures for clinicians. This study reports the sources and degrees of COVID-19-related distress that healthcare workers are experiencing, their current coping behaviors, and the wellness resources they believe may help.

2. Methods

2.1. Study design

Data came from the first wave of the COVID-19 Healthcare Provider Study, an ongoing cross-sectional survey of healthcare workers within a large medical center in NYC. Participants were given the choice to enter a longitudinal follow-up which entails completing additional questionnaires every 2 weeks, for up to 3 months. Participants were eligible if they were physicians (including residents/fellows), advanced practice providers, or registered nurses, and provided care at the medical center. Participants were invited using a standardized recruitment email with a link to an electronic Qualtrics survey. This email was sent to listservs for physicians and advanced practice providers: nurse practitioners and physician assistants ($n = 1870$), housestaff (interns/residents/fellows: $n = 974$), and nurses ($n = 4273$). The first email was sent on April 9, 2020 (a peak of the COVID-19 pandemic in NYC [7,8]), and the first participant enrolled on that date. A total of 974 participants enrolled and began the survey (response rate 13.7%). Of the 974 participants, 657 (67%) completed all questions on the survey between April 9–24, 2020 and are included in this cross-sectional analysis. We compared scores on the first survey items presented between survey completers and those who had not yet completed the survey, to explore potential differences between the two. The Columbia University Irving Medical Center Institutional Review Board approved the study protocol. All participants provided electronic informed consent.

2.2. Study survey

The study survey included questions about demographics, recent clinical roles, a brief psychological screen, assessment of distress due to COVID-19-specific stressors, meaning/purpose, coping behaviors, and wellness resources desired by healthcare workers.

2.2.1. Psychological screening

We used brief screening tools to measure acute stress symptoms, anxiety, and depressive symptoms. As less than a month had passed since the start of the pandemic and first participant enrollment in the survey, we used the 4-item Primary Care PTSD screen [9] (PC-PTSD, range 0–4; score ≥ 3 indicates a positive screen) as a marker for acute stress; if symptoms persist, participants may develop post-traumatic stress disorder (PTSD). Other psychological screens included: the Patient Health Questionnaire-2 [10] (PHQ-2, range 0–6; score ≥ 3 indicates a positive screen for depression), and the 2-item Generalized Anxiety Disorder [11] scale (GAD-2, range 0–6; score ≥ 3 indicates a positive screen for anxiety). We also assessed loneliness with a single-item measure [12–14], optimism with a single item from the Life Orientation Test-Revised [15], and single items for sleep duration and disturbances (e.g., poor quality sleep, difficulty falling or staying asleep, waking up too early, and/or feeling that sleep is not refreshing), with questions modified from the Pittsburgh Sleep Quality Index [16] and Insomnia Severity Index [17].

2.2.2. COVID-19-specific sources of distress

As there are no COVID-19-specific validated questionnaires assessing distress, we developed an instrument based on consensus from a multidisciplinary group of providers, supplemented by review of the literature [5,18] regarding COVID-19-related sources of distress in healthcare workers. Using a 5-point Likert scale (1, *no distress*; 5, *extreme distress*), participants were asked to rate how much distress they experienced on items within the following categories: 1) National policies and guidelines regarding COVID-19, 2) Family and concerns outside the clinical environment, and 3) Personal and clinical work/environment. Participants also had the option to describe other stressors/concerns. The full list of potential stressors is in the eMethods. We categorized stressors as highly distressing if rated as either 4 (*very distressing*) or 5 (*extremely distressing*).

2.2.3. Coping behaviors and potential wellness resources

We asked participants whether they were currently engaging in any of six types of coping behaviors (e.g., exercise, meditation), with the option to describe other coping or stress reduction activities they were using (eMethods). Using a 4-point Likert scale (0, *not interested*; 1, *somewhat interested*; 2, *quite a bit interested*; 3, *extremely interested*), participants were asked to rate their interest in any of five types of psychological wellness resources, with the option to describe others that would be helpful (eMethods). We categorized participants as being interested in receiving a wellness resource if they reported at least "quite a bit" of interest.

2.3. Statistical analysis

We report the percentages of participants who rated each source of distress as highly distressing. We also report the percentages of participants who endorsed each of the coping behaviors, and those with interest in the various types of wellness resources. We generated word clouds using Tableau [19] and Google word cloud generator software [20] based on participants' free-text responses describing any other psychological, cognitive, or emotional symptoms, additional coping behaviors being used, and additional wellness resources participants desired (**eFigures 1** and **2**).

Based on prior research on COVID-19-related distress [5], we compared the proportion of participants who screened positive for acute stress, depressive symptoms, and anxiety using a chi-squared test with post-hoc z-test comparisons across groups (1) attending physicians, (2) housestaff, and (3) nurses. Since only 48 advanced practice providers completed the study, this group was combined with nurses. **eFigures 3** and **4** provide a comparison of estimated marginal means for acute stress symptoms by group. Nurse practitioners and physician assistants were very similar to nurses overall. For continuous measures of psychological and behavioral variables, we compared mean ratings across clinical roles using a one-way analysis of variance (ANOVA) with post-hoc Bonferroni test for group comparisons. All analyses were performed in IBM SPSS Statistics version 26.

Table 1
Participant characteristics ($N = 657$).

Demographic characteristics	N (%)
Age range	
18–24	11 (1.5)
25–34	336 (46.8)
35–44	150 (20.9)
45–54	86 (12.0)
55–64	64 (8.9)
65–74	3 (0.4)
≥75	2 (0.3)
No answer	5 (0.7)
Gender	
Woman	509 (70.9)
Man	143 (19.9)
Genderqueer	1 (0.1)
Prefer not to answer	4 (0.4)
Race	
White	428 (59.6)
Asian	103 (14.3)
Black	58 (8.1)
Hawaiian/Pacific Islander	4 (0.6)
American Indian/Native American	2 (0.3)
Other	17 (2.4)
More than one race	22 (3.1)
Prefer not to answer	45 (6.3)
Ethnicity	
Non-Hispanic or Latino	539 (71.1)
Hispanic or Latino	80 (11.1)
Prefer not to answer	38 (5.3)
Number of household members (including participant)	
1	132 (18.4)
2	244 (34.0)
≥3	281 (39.1)
Living with a family member who is a healthcare provider	171 (23.8)
Clinical role	
Attending physician	141 (21.5)
Resident or fellow	141 (21.5)
Nurse	313 (47.6)
Advanced practice provider	48 (7.3)
Other	14 (2.1)
Practice setting for majority of shifts since March 1, 2020	
Emergency department	74 (11.2)
Intensive care unit	262 (39.9)
Inpatient (non-ICU): COVID-19 focused	126 (19.2)
Inpatient (non-ICU): not COVID-19 focused	61 (9.3)
Outpatient: COVID-19 focused	25 (3.8)
Outpatient: not COVID-19 focused	74 (11.2)
Other	35 (5.9)

Abbreviations: COVID-19 = Coronavirus disease 2019; ED = Emergency Department; ICU = Intensive Care Unit.
Advanced practice providers included nurse practitioners and physician assistants.

3. Results

Demographic and clinical role characteristics are shown in Table 1. By April 24, 2020, $n = 974$ enrolled and began the survey, and $n = 657$ (67%) completed all survey questions. Women comprised 70.9% of the sample. All attending physicians ($n = 141$) and enrolled housestaff ($n = 141$) completed the survey. Of 691 nurses and advanced practice providers, 54% ($n = 375$) completed the survey prior to data being locked for the present analysis. We compared ratings of sources of distress between those who completed the study and those who began but did not complete the survey, because sources of distress were presented first in the survey.

3.1. Identification of distress

Fig. 1 shows the percentage of participants who rated each source of distress as highly distressing. Seventy-four percent of participants reported that concerns about transmitting COVID-19 to family and loved ones was highly distressing. Over 60% of participants rated the following concerns as highly distressing: the health of family/friends, maintaining social distancing from family, lack of control and/or uncertainty, and uncertainty about colleagues' COVID-19 status. Other major sources of distress pertained to national shortages of personal protective equipment (PPE), testing, and lack of national guidelines regarding treatment for COVID-19. Relative to other sources, fewer participants reported high distress about lack of national discharge guidelines for patients with COVID-19, their number of clinical hours, or disturbed sleep. Participants who completed the survey differed from those who had not yet completed the survey only in that, for the $n = 21$ noncompleters who responded, 24% were highly distressed by treating another healthcare worker for COVID-19 (vs 49% in the full sample, $p = 0.02$).

3.2. Psychological screening

A large proportion of participants screened positive for psychological impacts of COVID-19 (Fig. 2). Fifty-seven percent of participants screened positive for acute stress, 48% screened positive for depressive symptoms, and 33% screened positive for anxiety. There were statistically significant differences among groups for each domain of psychological impact (acute stress $p < 0.001$; depressive symptoms $p = 0.004$; anxiety $p < 0.001$). Nurses/advanced practice providers were significantly more likely than attending physicians to screen positive for acute stress (64% vs. 40%, $p < 0.001$) and depressive symptoms (53% vs. 38%, $p = 0.004$). Nurses/advanced practice providers were also more likely than attending physicians and housestaff to screen positive for anxiety (40% vs. 15% [$p = 0.001$] and 17% [$p = 0.001$], respectively). The proportion of housestaff with positive screens for acute stress and depression did not differ from either attending physicians or nurses/advanced practice providers.

3.3. Sleep

The mean (± SD) daily sleep duration for all participants was 5.89 ± 1.21 h/day. Sleep duration for attending physicians, housestaff, and nurses/advanced practice providers was 6.24 ± 0.10, 6.30 ± 0.10, and 5.62 ± 0.06 h/day, respectively, and was longer in attending physicians and housestaff vs. nurses/advanced practice providers ($F_{2,654} = 26.44$, $p < 0.001$). Sleep disturbances were common, with 26% reporting severe or very severe sleep problems and an additional 45% reporting moderate sleep problems. Severity of sleep disturbances differed by group ($F_{2,654} = 17.51$, $p < 0.001$), with nurses/advanced practice providers reporting the worst sleep problems and housestaff not differing significantly from attending physicians.

3.4. Loneliness

Participants reported substantial loneliness, with 65% feeling lonely at least several days in the past week. Groups differed significantly ($F_{2,654} = 6.54$, $p = 0.002$), with nurses/advanced practice providers and housestaff reporting more loneliness than attending physicians, but not differing from each other.

3.5. Optimism and sense of meaning/purpose

There were no group differences in optimism ($F_{2,654} = 1.26$, $p = 0.29$), with 48% of the full sample either agreeing or strongly agreeing that they “expect more good things than bad to happen” to them. Sixty-one percent of all participants reported an increased sense of meaning/purpose during this time, with no between-group differences ($F_{2,654} = 1.53$, $p = 0.22$).

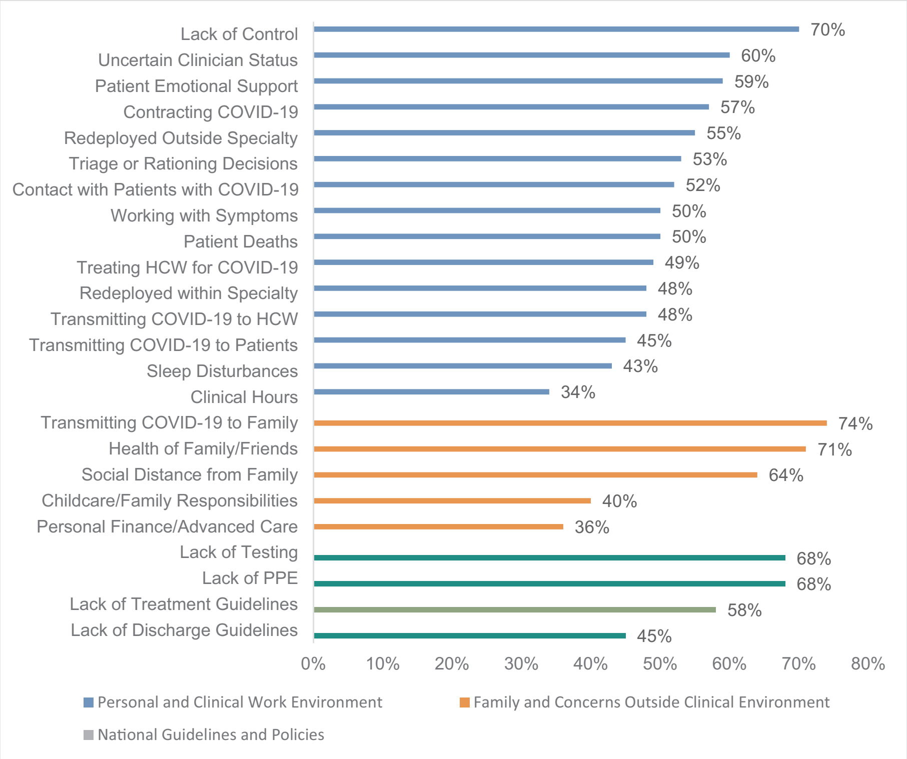

Fig. 1. Percent of participants who endorsed distress due to clinical, family, national, and personal concerns.

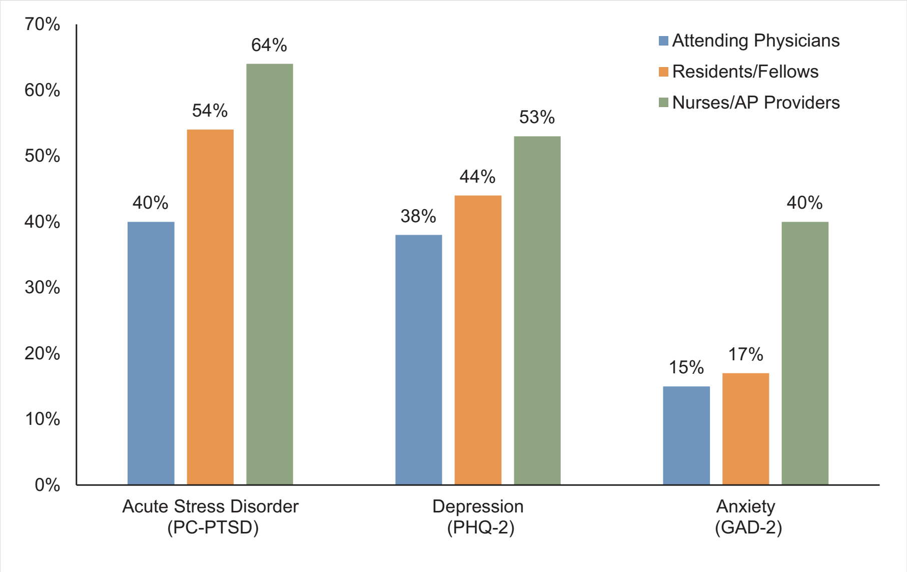

Fig. 2. Percentage of participants who screened positive for acute stress, depressive symptoms, and anxiety by clinical role.

3.6. Coping behaviors

Eighty percent of participants reported engaging in at least one type of coping behavior to manage stress (1.7 ± 1.3 types, Fig. 3). Physical activity/exercise was the most commonly endorsed behavior (59%), followed by engaging with faith-based religion and/or spirituality (23%), yoga (25%), and/or meditation (23%). Participants also reported engaging with talk therapy (26%) and virtual provider support groups (16%). Sixteen percent endorsed "other," and 14% reported engaging in none of the coping behaviors listed. Participants who screened positive for acute stress reported engaging in more coping behaviors (2.1 ± 1.3) than those who screened negative (1.6 ± 1.2, $F_{1,655} = 4.84$ $p < 0.001$). Similar differences were found for those who screened positive vs. negative for depressive symptoms.

3.7. Potential wellness resources

Fifty-one percent of participants expressed "quite a bit" or "extreme" interest in at least one of the five proposed types of support, 20% reported interest in only one of the options, and 30% in two or more options. The number of resources that each participant expressed interest in was positively correlated with the number of wellness behaviors in which they reported engaging, r_{655} (656) = 0.18, $p < 0.001$. Among the suggested support types, the two options that

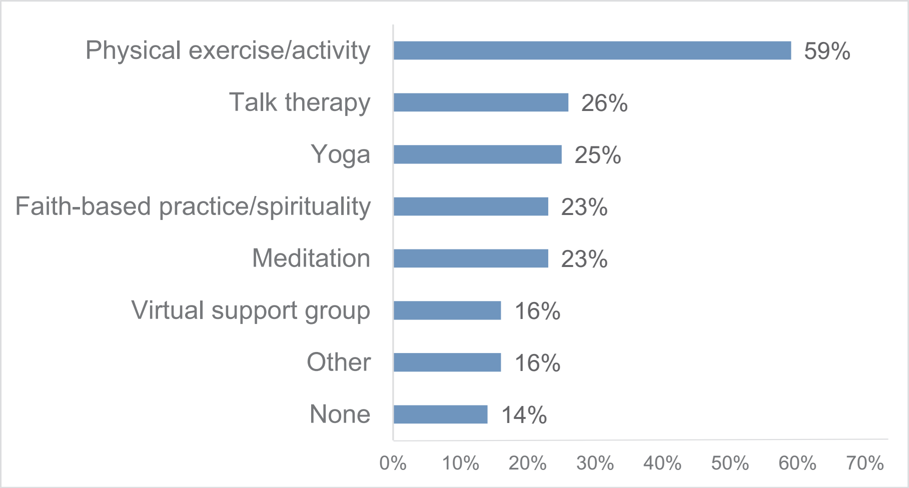

Fig. 3. Coping behaviors endorsed by participants by clinical role.

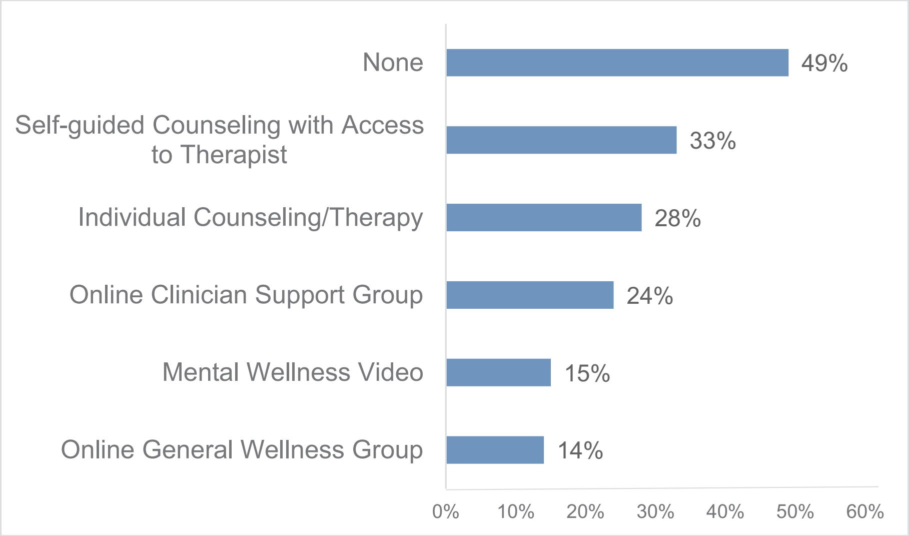

Fig. 4. Participant interest in proposed wellness resources by clinical role.

included access to an individual therapist garnered the most interest, with online self-guided counseling with access to a therapist (33%) slightly preferred over traditional individual counseling/therapy (28%) (Fig. 4). Participants who screened positive for acute stress reported interest in more wellness resources (1.3 ± 1.5) than those who screened negative (0.9 ± 1.3, $F_{1,655}$ = 3.21, p = 0.001). Similar differences were found for those who screened positive vs. negative for depressive symptoms and anxiety.

4. Discussion

Our cross-sectional survey of levels and sources of distress in healthcare workers treating patients with COVID-19 in NYC is, to our knowledge, the largest such study conducted to date in the US. More than half of healthcare workers screened positive for acute stress (PTSD symptoms within 1 month of trauma), almost half screened positive for depression, and one-third screened positive for anxiety. Nearly 75% of participants reported at least moderate insomnia symptoms, and the average daily self-reported sleep duration was < 6 h.

Three of every 4 healthcare workers were highly distressed by fears about transmitting COVID-19 to family or friends, and most were highly distressed by having to maintain “social distance” from family. In the clinical environment, perceived lack of control/uncertainty, treating other healthcare workers for COVID-19, and uncertainty about colleagues' COVID-19 status were the most common sources of high distress. Concerning national issues that affect healthcare workers locally, the national unavailability of COVID-19 testing capabilities, limited PPE, and lack of national treatment guidelines for patients were considerable sources of distress.

Although all healthcare workers are experiencing psychological toll, nurses were particularly affected. The different responsibilities of nurses may partly explain the higher rates of positive acute stress screens and other impacts, as they are spending more time delivering direct patient care. In the context of COVID-19, those responsibilities increase the likelihood of vicarious traumatization, including having to provide direct social support or emotional labor for patients in place of patients' family who are not allowed inside the hospital due to transmission concerns. Although these PTSD-like symptoms are normal and expected during traumatic events, and symptoms are expected to decline for many who currently screen positive for acute stress, a substantial proportion are likely to subsequently meet diagnostic criteria for PTSD [21].

Current findings are consistent with other reports of mental health impacts of COVID-19 on healthcare workers. In the only other large study, a cross-sectional survey of 1257 healthcare workers in China during the COVID-19 pandemic, over 70% reported distress, with 50%

reporting depression and 34% reporting insomnia [5]. More severe symptoms were generally seen in nurses, women, those working in Wuhan (the epicenter of the outbreak in China), and in frontline workers [5].

Qualitative reports based on interviews [18] and listening sessions [4] informed the sources of distress assessed in this study. In a study of 69 healthcare workers [4], sources of distress included access to PPE and testing, propagation by the individual of COVID-19 at home or work, lack of organizational support, lack of access to childcare, being unable to provide competent medical care, and limited up-to-date information and communication. A substantial psychological toll, including heightened depression, anxiety, and insomnia, was also reported among healthcare workers during the Middle East Respiratory Syndrome (MERS) [22] and Severe Acute Respiratory Syndrome (SARS) [23–25] outbreaks. Working in a high-risk environment and more direct patient care were among the factors related to poor mental health outcomes in healthcare workers during infectious disease outbreaks [26].

Although this was a cross-sectional survey, distress can persist beyond the pandemic's surge period. In a study comparing healthcare workers with high- and low-risk exposure during the peak of the 2003 SARS outbreak, both groups showed similarly elevated levels of perceived stress [27]. In a 1-year follow-up after the crisis subsided, perceived stress decreased in the low-risk group, but increased in the high-risk group. High-risk workers had significantly greater depression ratings at 1-year follow-up, which was partially mediated by stress related to contact with SARS [27]. In the 1–2 years following the outbreak, healthcare workers from hospitals treating SARS patients had higher levels of distress and PTSD vs. healthcare workers from matched neighboring hospitals that did not treat SARS patients [28].

Sustained COVID-19-related psychological distress is expected to have downstream impacts on healthcare workers' physical health. There is an association between clinical workplace environmental stressors and long-term cardiometabolic risk [29–32], and stress can influence health in ways that are both direct (systemic inflammation, arterial damage, increased blood pressure) and indirect (maladaptive coping such as substance use, poor sleep). Sustained psychological distress and poor sleep may disturb the body's physiological stress response system, thereby contributing to further health risk [33].

We also assessed current coping behaviors and the types of wellness resources that participants might find useful. Many reported already engaging in stress reduction activities, especially physical activity/exercise, but also talk therapy, virtual support groups, and religious/spiritual practices. However, 1 in 7 participants reported engaging in no stress-reducing activities. Our findings highlight the need for rapid interventions (psychological or organizational) to reduce psychological distress in healthcare workers. It is also important to respect workers' desires concerning the type, timing, and content of such interventions. Some workers indicated interest in structured psychological wellness resources, including individual counseling with a therapist or support group with other healthcare workers, while others offered suggestions for workplace innovations. Despite current challenges, many respondents reported being optimistic, and over half reported an increased sense of meaning or purpose during this period.

There are several strengths in this study. This is a comprehensive study of psychological wellness among healthcare workers actively treating patients with COVID-19 in NYC, an epicenter of the pandemic. The survey began during a peak of the COVID-19 outbreak in NYC. The timing of assessments allowed us to describe the acute stressors facing healthcare workers, their current coping behaviors and desired wellness resources. There were also limitations, including a low initial response rate, uneven gender distribution, and relatively small sample size, compared to the entire population of healthcare workers. The healthcare workers who chose to participate may not be representative of non-participating healthcare workers (e.g., some who did not participate may have been too overwhelmed especially as this survey was administered during a peak of the COVID-19 pandemic and healthcare workers may not have had time to participate in the survey due to competing clinical demands; others may not have experienced COVID-19 as a major stressor). Participants were recruited from a large medical center in NYC, and may not be representative of all hospitals.

Finally, our reliance on only subjective measures to assess sleep is another limitation, since self-reports of sleep duration and quality can show low agreement with objectively-assessed sleep [34].

5. Conclusions

We surveyed healthcare workers treating patients with COVID-19 in NYC, a global epicenter of the pandemic. We quantified several sources of distress, ranging from fears of COVID-19 transmission, clinical challenges, and perceived lack of control, to concerns about family and home life. Workers are experiencing substantial distress with large proportions screening positive for acute stress, depressive symptoms, and sleep disturbances. These findings should inform the development and implementation of interventions to mitigate the impact of sustained psychological distress on long-term mental and physical wellbeing in healthcare workers. The lessons learned from the COVID-19 pandemic should help decision-makers at all levels of government, hospital management, and the community to promote readiness to protect healthcare workers as we navigate this and future public health crises. We hope that these findings will influence policies at other institutions that face a rapid rise in patients with COVID-19 in the future.

Author statements

Ari Shechter PhD: Conceptualization, methodology, writing - original draft preparation, writing - reviewing and editing. Franchesca Diaz BA: Methodology, software, data curation, writing - reviewing and editing. Nathalie Moise MD MS: Conceptualization, methodology, writing - reviewing and editing. David E. Anstey MD: Conceptualization, methodology, writing - reviewing and editing. Siqin Ye MD: Conceptualization, methodology, writing - reviewing and editing. Sachin Agarwal MD MPH: Conceptualization, methodology, writing - reviewing and editing. Jeffrey L. Birk PhD: Conceptualization, methodology, writing - reviewing and editing. Daniel Brodie MD: Writing - reviewing and editing. Diane E. Cannone MPH: Conceptualization, methodology. Bernard Chang MD PhD: Methodology, writing - reviewing and editing. Jan Claassen MD PhD: Writing - reviewing and editing. Talea Cornelius PhD, MSW: Conceptualization, methodology, writing - reviewing and editing. Lilly Derby BS: Writing - reviewing and editing. Melissa Dong BA: Methodology, software, data curation, writing - reviewing and editing. Raymond C. Givens MD PhD: Writing - reviewing and editing. Beth Hochman MD: Writing - reviewing and editing. Shunichi Homma MD: Methodology, writing - reviewing and editing. Ian M. Kronish MD: Conceptualization, methodology, writing - reviewing and editing. Sung A.J. Lee MPH: Methodology, software, data curation, writing - reviewing and editing. Wilhelmina Manzano MA, RN, NEA-BC, FAAN: Methodology, writing - reviewing and editing. Laurel E.S. Mayer MD: Conceptualization, methodology, writing - reviewing and editing. Cara L. McMurry MPH: Methodology, software, data curation, writing - reviewing and editing. Vivek Moitra MD: Writing - reviewing and editing. Patrick Pham BS: Methodology, data curation, writing - reviewing and editing. LeRoy Rabbani MD: Writing - reviewing and editing. Reynaldo R. Rivera DNP, RN, NEA-BC, FAAN: Methodology, writing - reviewing and editing. Allan Schwartz MD: Methodology, writing - reviewing and editing. Joseph Schwartz PhD: Conceptualization, methodology, writing - reviewing and editing. Peter Shapiro MD: Conceptualization, methodology, writing - reviewing and editing. Kaitlin Shaw MPH: Methodology, software, data curation, writing - reviewing and editing. Alexandra M. Sullivan MPH: Methodology, writing - reviewing and editing. Courtney Vose DNP, MBA, RN, APRN, NEA-BC: Methodology,

writing - reviewing and editing. Lauren Wasson MD: Methodology, writing - reviewing and editing. Donald Edmondson PhD MPH: Conceptualization, methodology, data curation, formal analysis, writing - original draft preparation, writing - reviewing and editing. Marwah Abdalla MD MPH: Conceptualization, methodology, data curation, writing - original draft preparation, writing - reviewing and editing, project administration, supervision.

Funding sources

Dr. Abdalla receives support through 18AMFDP34380732 from the American Heart Association and the NIH/NHLBI (K23HL141682-01A1 and R01HL146636-01A1). Dr. Shechter receives support from NIH/NHLBI (R01HL141494 and R01HL146911). Dr. Edmondson receives support from NIH through NIA U24AG052175 and P30AG064198, and NHLBI R01HL132347. Dr. Chang receives support from NIH/NHLBI (R01 HL 141811, R01 HL 146911). Dr. Kronish receives support from NIA (P30 AG064198), NIH/NHLBI (R01-HL123368 and R01-HL117832) and the Agency for Healthcare Research and Quality (AHRQ, R01-HS024262). Dr. Birk receives support from NIH/NHLBI (R21 HL 145970).

Data sharing

Data will be made available to interested individuals upon formal request made to authors.

Transparency statement

The lead author affirms that the manuscript is an honest, accurate, and transparent account of the study being reported; that no important aspects of the study have been omitted; and that any discrepancies from the study as originally planned (and, if relevant, registered) have been explained.

Declaration of competing interest

None of the authors have relevant disclosures.

Acknowledgments

We thank our colleagues for their time and invaluable contributions to this on-going study. We also wish to acknowledge the dedication and commitment of the staff, personnel, and healthcare workers at our institutions for their continued care of patients, families, and our community during the NYC COVID-19 pandemic.

Appendix A. Supplementary data

Supplementary data to this article can be found online at https://doi.org/10.1016/j.genhosppsych.2020.06.007.

References

[1] Andersen KG, Rambaut A, Lipkin WI, et al. The proximal origin of SARS-CoV-2. Nat Med 2020;26(4):450–2. https://doi.org/10.1038/s41591-020-0820-9. [published Online First: 2020/04/15].
[2] New York coronavirus map and case count Available from https://www.nytimes.com/interactive/2020/us/new-york-coronavirus-cases.html#county; 2020, Accessed date: 8 May 2020.
[3] NYSDOH. Available from https://covid19tracker.health.ny.gov/views/NYS-COVID19-Tracker/NYSDOHCOVID-19Tracker-DailyTracker?%3Aembed=yes&%3Atoolbar=no&%3Atabs=n; 2020, Accessed date: 30 April 2020.
[4] Shanafelt T, Ripp J, Trockel M. Understanding and addressing sources of anxiety among health care professionals during the COVID-19 pandemic. JAMA 2020. https://doi.org/10.1001/jama.2020.5893. [published Online First: 2020/04/08].
[5] Lai J, Ma S, Wang Y, et al. Factors associated with mental health outcomes among health care workers exposed to coronavirus disease 2019. JAMA Netw Open 2020;3(3):e203976https://doi.org/10.1001/jamanetworkopen.2020.3976. [published Online First: 2020/03/24].
[6] Board PE. Health care suicides: another tragic toll of the coronavirus pandemic. NY Post 2020https://nypost.com/2020/04/27/health-care-suicides-another-tragic-toll-of-coronavirus-pandemic/.
[7] Ellison A. COVID-19 peak dates: updated projections for each state. 2020 [Available from: COVID-19 peak dates: Updated projections for each state accessed 4/30/2020].
[8] Campo-Flores A. What does a coronavirus peak in New York mean? Wall Street J 2020https://www.wsj.com/articles/what-does-a-coronavirus-peak-in-new-york-mean-11586338201.
[9] Prins A, Ouimette P, Kimerling R, et al. The primary care PTSD screen (PC-PTSD): development and operating characteristics. Int J Psychiatry Clin Pract 2004;9:9–14. https://doi.org/10.1185/135525703125002360.
[10] Kroenke K, Spitzer RL, Williams JB. The Patient Health Questionnaire-2: validity of a two-item depression screener. Med Care 2003;41(11):1284–92. https://doi.org/10.1097/01.MLR.0000093487.78664.3C. [published Online First: 2003/10/30].
[11] Kroenke K, Spitzer RL, Williams JB, et al. Anxiety disorders in primary care: prevalence, impairment, comorbidity, and detection. Ann Intern Med 2007;146(5):317–25. https://doi.org/10.7326/0003-4819-146-5-200703060-00004. [published Online First: 2007/03/07].
[12] Aartsen M, Jylha M. Onset of loneliness in older adults: results of a 28 year prospective study. Eur J Ageing 2011;8(1):31–8. https://doi.org/10.1007/s10433-011-0175-7. [published Online First: 2011/04/09].
[13] Theeke LA. Predictors of loneliness in U.S. adults over age sixty-five. Arch Psychiatr Nurs 2009;23(5):387–96. https://doi.org/10.1016/j.apnu.2008.11.002. [published Online First: 2009/09/22].
[14] Gruenewald T, Crosswell A, Epel E, et al. Measures of stress in the Health and Retirement Study and the HRS Family of Studies: user guide Available from https://164f89be-6459-4649-bea5-b3d3fcc248ee.filesusr.com/ugd/890fda_66d3318e858045559cfd85cccbd92724.pdf; 2020, Accessed date: 6 May 2020.
[15] Scheier MF, Carver CS, Bridges MW. Distinguishing optimism from neuroticism (and trait anxiety, self-mastery, and self-esteem): a reevaluation of the Life Orientation Test. J Pers Soc Psychol 1994;67(6):1063–78. https://doi.org/10.1037//0022-3514.67.6.1063. [published Online First: 1994/12/01].
[16] Buysse DJ, Reynolds 3rd CF, Monk TH, et al. The Pittsburgh Sleep Quality Index: a new instrument for psychiatric practice and research. Psychiatry Res 1989;28(2):193–213. [published Online First: 1989/05/01].
[17] Morin CM, Belleville G, Belanger L, et al. The Insomnia Severity Index: psychometric indicators to detect insomnia cases and evaluate treatment response. Sleep 2011;34(5):601–8. https://doi.org/10.1093/sleep/34.5.601. [published Online First: 2011/05/03].
[18] Chen Q, Liang M, Li Y, et al. Mental health care for medical staff in China during the COVID-19 outbreak. Lancet Psychiatry 2020;7(4):e15–6. https://doi.org/10.1016/S2215-0366(20)30078-X. [published Online First: 2020/02/23].
[19] Tableau. Available from http://www.tableau.com4/28/2020.
[20] Word cloud generator Available from https://gsuite.google.com/marketplace/app/word_cloud_generator/360115564222, Accessed date: 28 April 2020.
[21] Manser SS, Houck K, Kramer MD, et al. Do screening and a randomized brief intervention at a level 1 trauma center impact acute stress reactions to prevent later development of posttraumatic stress disorder? J Trauma Acute Care Surg 2018;85(3):466–75. https://doi.org/10.1097/TA.0000000000001977. [published Online First: 2018/05/23].
[22] Lee SM, Kang WS, Cho AR, et al. Psychological impact of the 2015 MERS outbreak on hospital workers and quarantined hemodialysis patients. Compr Psychiatry 2018;87:123–7. https://doi.org/10.1016/j.comppsych.2018.10.003. [published Online First: 2018/10/22].
[23] Su TP, Lien TC, Yang CY, et al. Prevalence of psychiatric morbidity and psychological adaptation of the nurses in a structured SARS caring unit during outbreak: a prospective and periodic assessment study in Taiwan. J Psychiatr Res 2007;41(1–2):119–30. https://doi.org/10.1016/j.jpsychires.2005.12.006. [published Online First: 2006/02/08].
[24] Wu P, Fang Y, Guan Z, et al. The psychological impact of the SARS epidemic on hospital employees in China: exposure, risk perception, and altruistic acceptance of risk. Canadian Journal of Psychiatry Revue canadienne de psychiatrie 2009;54(5):302–11. https://doi.org/10.1177/070674370905400504. [published Online First: 2009/06/06].
[25] Lin CY, Peng YC, Wu YH, et al. The psychological effect of severe acute respiratory syndrome on emergency department staff. Emergency Medicine Journal: EMJ 2007;24(1):12–7. https://doi.org/10.1136/emj.2006.035089. [published Online First: 2006/12/22].
[26] Brooks SK, Dunn R, Amlot R, et al. A systematic, thematic review of social and occupational factors associated with psychological outcomes in healthcare employees during an infectious disease outbreak. J Occup Environ Med 2018;60(3):248–57. https://doi.org/10.1097/jom.0000000000001235. [published Online First: 2017/12/19].
[27] McAlonan GM, Lee AM, Cheung V, et al. Immediate and sustained psychological impact of an emerging infectious disease outbreak on health care workers. Canadian Journal of Psychiatry Revue canadienne de psychiatrie 2007;52(4):241–7. https://doi.org/10.1177/070674370705200406. [published Online First: 2007/05/16].
[28] Maunder RG, Lancee WJ, Balderson KE, et al. Long-term psychological and occupational effects of providing hospital healthcare during SARS outbreak. Emerg Infect Dis 2006;12(12):1924–32. https://doi.org/10.3201/eid1212.060584. [published Online First: 2007/03/01].
[29] Theorell T, Karasek RA. Current issues relating to psychosocial job strain and cardiovascular disease research. J Occup Health Psychol 1996;1(1):9.

[30] Honkonen T, Ahola K, Pertovaara M, et al. The association between burnout and physical illness in the general population–results from the Finnish Health 2000 study. J Psychosom Res 2006;61(1):59–66. https://doi.org/10.1016/j.jpsychores.2005.10.002. [published Online First: 2006/07/04].

[31] Melamed S, Shirom A, Toker S, et al. Burnout and risk of cardiovascular disease: evidence, possible causal paths, and promising research directions. Psychol Bull 2006;132(3):327–53. https://doi.org/10.1037/0033-2909.132.3.327. [published Online First: 2006/05/25].

[32] Melamed S, Shirom A, Toker S, et al. Burnout and risk of type 2 diabetes: a prospective study of apparently healthy employed persons. Psychosom Med 2006;68(6):863–9. https://doi.org/10.1097/01.psy.0000242860.24009.f0. [published Online First: 2006/11/30].

[33] Chang B. Can hospitalization be hazardous to your health? A nosocomial based stress model for hospitalization. Gen Hosp Psychiatry 2019;60:83.

[34] Girschik J, Fritschi L, Heyworth J, et al. Validation of self-reported sleep against actigraphy. J Epidemiol 2012;22(5):462–8.
