# Supplementary material for: Mental health status and related factors influencing healthcare workers during the COVID-19 pandemic: A systematic review and meta-analysis
Source: PLoS One. 2024 Jan 19;19(1):e0289454. doi: 10.1371/journal.pone.0289454 (PMC10798549; doi:10.1371/journal.pone.0289454)
Supplement: S1 Data — (ZIP) [file pone.0289454.s011.zip › literatures/165.pdf]

DOI:10.11817/j.issn.1672-7347.2020.200070
http://xbyxb.csu.edu.cn/xbwk/fileup/PDF/202006633.pdf

2019冠状病毒病疫情期间医护人员心理健康状况

申海艳[1,2]，王惠平[1,2]，周霏[1,2]，陈晋东[3]，邓露[1,3]

（1. 中南大学湘雅二医院临床护理学教研室，长沙 410011；2. 中南大学湘雅二医院手术部，长沙 410011；
3. 中南大学精神卫生研究所，长沙 410011）

[摘要] **目的：**评估医护人员在2019冠状病毒病(coronavirus disease 2019，COVID-19)疫情期间的心理健康状况，并分析其影响因素。**方法：**选取中南大学湘雅医院和湘雅二医院373名医护人员作为研究对象。分别采用一般社会学资料量表、症状自评量表(Symptom Check-List 90，SCL-90)及自制舆情应对问卷收集医护人员一般社会学资料、心理健康得分情况和应对COVID-19相关舆情信息能力情况；并进一步比较不同一般社会学特征及舆情信息应对能力的医护人员心理健康得分，分析医护人员心理健康状况的影响因素。**结果：**373名医护人员SCL-90中10个因子的平均分均<2，14.21%的医护人员存在1个及以上的因子得分≥2，其中恐怖(11.26%)、强迫症状(7.77%)、焦虑(5.63%)。医护人员获取COVID-19相关信息的主要来源包括微信、微博和今日头条、电视和广播；66.22%的医护人员会经常通过官方网站或正规渠道来证实有关COVID-19信息的真实性；微信里大量COVID-19相关信息会让医护人员感到紧张(34.05%)、焦虑(30.29%)、没有安全感(29.22%)；68.63%的医护人员有时会因为看到COVID-19相关信息而担心自己被感染。医护人员工作的科室，近期是否出现咳嗽、发热等症状，担心被感染的程度等对医护人员的SCL-90得分有影响，差异具有统计学意义(均$P<0.05$)。逐步回归分析发现微信中转发的大量COVID-19相关信息对生活的影响程度，近期有无咳嗽、发热等相关症状，微信中转发的大量COVID-19相关信息是否让自己难以入睡，工作的科室及担心被感染的程度是医护人员心理健康的影响因素(均$P<0.05$)。**结论：**COVID-19疫情期间医护人员会出现不同程度的心理问题，建议建立心理援助平台，并正确引导舆情方向，以促进医护人员心理健康。

[关键词] 2019冠状病毒病；肺炎；心理健康状况；医护人员；舆情信息

Mental health status of medical staff in the epidemic period of coronavirus disease 2019

SHEN Haiyan[1,2], WANG Huiping[1,2], ZHOU Fei[1,2], CHEN Jindong[3], DENG Lu[1,3]

(1. Teaching and Research Section of Clinical Nursing, Second Xiangya Hospital, Central South University, Changsha 410011; 2. Operating Room, Second Xiangya Hospital, Central South University, Changsha 410011; 3. Institute of Mental Health, Central South University, Changsha 410011, China)

ABSTRACT **Objective:** To explore the psychological status of medical staff in the epidemic period of coronavirus disease 2019 (COVID-19), and to analyze its influential factors.

收稿日期(Date of reception)：2020-02-08
第一作者(First author)：申海艳，Email: csshenhaiyan@csu.edu.cn, ORCID: 0000-0002-6455-2360
通信作者(Corresponding author)：邓露，Email: csdenglu1026@csu.edu.cn, ORCID: 0000-0001-8312-4292

©Journal of Central South University (Medical Science). All rights reserved.

Methods: A total of 373 medical staff from Xiangya Hospital and the Second Xiangya Hospital of Central South University were enrolled for this study. The General Sociological Data Questionnaire, Symptom Check-List 90 (SCL-90), and self-designed public opinion response questionnaire were used to assess general sociological data, mental health scores, and ability to respond to COVID-19 related public opinion information of medical staff. The mental health scores of medical staff with different general sociological data and public opinion information coping abilities were compared. Influential factors of mental health were analyzed.

Results: The average score of 10 factors in SCL-90 of 373 medical staff was less than 2 points. 14.21% medical staff had one or more factor scores more than two points, including 11.26% with terror symptoms, 7.77% with compulsive symptoms, and 5.63% with anxiety. The main sources of COVID-19 information for medical staff included WeChat, microblog, Jinri toutiao, TV and radio. 66.22% medical staff regularly verified information about COVID-19 through official websites or formal channels. A great deal of COVID-19 information in WeChat could make medical staff nervous (34.05%), anxious (30.29%), and insecure (29.22%). 68.63% medical staff sometimes were worried about getting infected because they knew information about COVID-19. Different departments of medical staff, getting cough or having a fever recently, and the degree of fear of infection had an impact on the SCL-90 score of medical staff, the differences were all statistically significant (all $P<0.05$). Stepwise regression analysis showed that the impact of COVID-19 information on their life in WeChat, getting cough or having a fever recently, insomnia-early caused by COVID-19 information in WeChat, different departments, and the degree of fear of infection COVID-19 were the influential factors for the mental health of medical staff (all $P<0.05$).

Conclusion: During the epidemic of COVID-19, medical staff suffered from psychological problems to various degrees. It is necessary to establish a psychological assistance platform and guide the direction of public opinion correctly to promote the mental health of medical staff.

KEY WORDS coronavirus disease 2019; pneumonia; mental health status; medical staff; public opinion

2019年12月以来，中国及全球多个国家和地区出现2019冠状病毒病(coronavirus disease 2019，COVID-19)疫情，其传播速度快、范围广，传染性强，且尚无特异性治疗药物，严重威胁人类的生命健康[1]。作为这一公共卫生危机事件的亲历者，医护人员遭受着巨大的心理压力，并伴有明显的不安全感和职业倦怠[2]。微信已成为全社会人际交往和信息分享的主要网络途径之一，因其具有半私密性的特点，人们往往乐于在微信中发表观点或转发信息[3]。在微信中有着海量信息，而这些信息的真实性不能得到及时有效地核实，加上医护人员群体较普通群体更容易通过微信朋友圈、公众号获取有关COVID-19的信息，如果处理不当，将增加医护人员的心理负担。本研究通过调查医护人员心理健康状况及舆情信息应对情况，分析其影响因素，以期为对医护人员开展有效的心理干预提供参考。

1 对象与方法

1.1 对象

采用便利抽样法，于2020年1月30日到2月1日选取中南大学湘雅医院和湘雅二医院医护人员作为研究对象，共发放问卷392份，回收问卷392份，有效问卷373份，有效回收率为95.15%。其中男48名，女325名；年龄18~56(29.55±6.93)岁；汉族346名，少数民族27名；医生98名，护士275名；本科及以

©Journal of Central South University (Medical Science). All rights reserved.

下学历189名，硕士102名，博士82名；初级职称172名，中级职称131名，高级职称70名；82名来自内科，外科166名，急诊科、ICU、感染科、发热门诊、呼吸科共65名，其他科室60名；独居40名，2人同住48名，3人同住65名，≥4人同住220名；近期出现咳嗽、发热等症状者8名，无症状365名；38名过去14 d内与确诊或者疑似COVID-19患者有过近距离接触，335名无接触。

1.2 研究工具

1.2.1 一般社会学资料量表

在参考文献[4]的基础上，自行设计一般社会学资料量表。量表内容主要包括：性别，年龄，民族，职业，学历，工作的科室，同住人数，近期是否出现咳嗽、发热等症状，过去14 d内是否与确诊或者疑似COVID-19患者有过近距离接触。

1.2.2 症状自评量表

症状自评量表(Symptom Check-List 90，SCL-90)是目前使用最广泛的精神障碍及心理疾病门诊检查量表，且适用于评估医护人员心理健康状况[5-6]。SCL-90包括躯体化、强迫症状、人际敏感、抑郁、焦虑、敌对、恐怖、偏执、精神病性、其他(主要反映睡眠和饮食状况)共10个因子，共包含90个条目，采用Likert 5级评分法，其中“没有”计1分，“较轻”计2分，“中等”计3分，“较重”计4分，“严重”计5分。因子中各条目得分相加除以条目数即为该因子均分，得分越高，表明自觉症状越严重。因子均分≥2即表明该因子的症状达轻度及以上。本研究中该总量表的Cronbach’s α系数为0.982，说明具有良好的信效度，适用于本研究。

1.2.3 舆情应对能力问卷

参考文献[7]自行设计舆情应对能力问卷，用于评估医护人员在COVID-19疫情期间对微信中COVID-19相关舆情信息的应对情况，包括获取信息途径、对信息的信任程度、是否会主动核实信息的真实性、转发相关信息的目的及转发频率、相关信息对心理和日常生活的影响。

1.3 问卷采集

通过网络平台发放问卷，使用统一、规范的指导语，向调查对象详细解释研究的目的、意义及问卷的填写方式。本研究为匿名调查，且所有数据资料仅供本研究使用。设置IP地址限制，每人只能填写1份问卷；设定提交标准，如出现答题方式错误或答题不完整的问卷均不能提交。问卷提交后，数据由2人进行核对，对于选项前后矛盾或答案均为同一选项的无效问卷进行剔除。本研究经中南大学湘雅二医院医学伦理委员会批准，调查对象均知情同意。

1.4 统计学处理

采用SPSS 19.0软件对数据进行分析，计量资料采用均数±标准差($\bar{x}\pm s$)进行描述；计数资料采用频数、百分比进行描述。以医护人员的一般社会学资料及舆情应对能力作为自变量，以心理健康得分作为因变量进行单因素分析，组间比较采用独立样本t检验，多组间比较采用单因素方差分析，影响因素采用逐步回归分析，$P<0.05$为差异具有统计学意义。

2 结　果

2.1 医护人员心理健康状况SCL-90得分情况

SCL-90的各因子得分情况、因子得分≥2的人数及占比见表1，其中有53名(14.21%)医护人员出现1个及以上的因子得分≥2，得分≥2的人数较多的因子依次为恐怖、强迫症状、焦虑。

表1 SCL-90得分情况(*n*=373)

Table 1 Score of SCL-90 (*n*=373)

因子	分值	分值≥2/[例(%)]
躯体化	1.00~3.50(1.13±0.32)	16(4.29)
强迫症状	1.00~3.90(1.27±0.45)	29(7.77)
人际敏感	1.00~3.33(1.20±0.36)	20(5.36)
抑郁	1.00~3.69(1.16±0.35)	16(4.29)
焦虑	1.00~3.20(1.19±0.35)	21(5.63)
敌对	1.00~4.17(1.12±0.35)	12(3.22)
恐怖	1.00~4.29(1.33±0.52)	42(11.26)
偏执	1.00~3.83(1.14±0.32)	16(4.29)
精神病性	1.00~3.40(1.10±0.29)	10(2.68)
其他(睡眠、饮食)	1.00~3.00(1.16±0.34)	17(4.56)

2.2 医护人员应对COVID-19相关舆情信息的能力

医护人员获取COVID-19相关信息的主要来源包括微信、微博和今日头条、电视和广播；57.91%的医护人员相信从微信获取的COVID-19相关信息但是不会转发；66.22%的医护人员会经常通过官方网站或正规渠道来证实有关COVID-19信息的真实性；37.80%的医护人员认为微信中转发的大量COVID-19相关信息对自己的生活有较大影响；微信里大量COVID-19相关信息会让医护人员感到紧张(34.05%)、焦虑(30.29%)、没有安全感(29.22%)；68.63%的医护人员有时会因为看到COVID-19相关信息而担心自己被感染(表2)。

©Journal of Central South University (Medical Science). All rights reserved.

表2 医护人员应对舆情信息的状况分析(n=373)

Table 2 Condition of coping ability of public opinion information for medical staff (n=373)

项目	人数	构成比/%	项目	人数	构成比/%
1. COVID-19最新信息的来源(多选)			5. 您在微信转发有关COVID-19信息的目的是		
电视、广播	315	84.45	传递正能量	267	71.58
微博、今日头条	308	82.57	支持朋友	4	1.07
微信	318	85.25	事关自身及好友利益	86	23.06
QQ	101	27.08	做消息灵通人士	6	1.61
他人电话告知	64	17.16	无意识转发	10	2.68
2. 您对从微信获取的COVID-19相关			6. 您认为微信转发的大量有关COVID-19的		
信息的信任行为			信息对您生活的影响？		
相信但不转发	216	57.91	没有影响	43	11.53
相信且直接转发	28	7.51	较小影响	62	16.62
相信且加工转发	15	4.02	中等影响	127	34.05
不相信不转发	44	11.80	较大影响	141	37.80
不相信且核实	70	18.76	7. 微信里大量有关COVID-19的信息会让您		
3. 您会通过官方网站或正规渠道来证实			恐惧	53	14.21
有关COVID-19信息的真实性吗？			焦虑	113	30.29
从不	12	3.22	难以入睡	19	5.09
有时	96	25.74	没有安全感	109	29.22
经常	247	66.22	感到紧张	127	34.05
总是	18	4.82	坐立不安	16	4.29
4. 您会在微信里转发有关COVID-19信息吗？			刷微信时间延长	173	46.38
几乎很少	152	40.75	8. 您会因为COVID-19信息担心自己被感染吗？		
有时	168	45.04	从不	62	16.62
经常	53	14.21	有时	256	68.63
			经常	38	10.19
			总是	17	4.56

2.3 不同一般社会学特征及舆情信息应对能力的医护人员心理健康得分比较

医护人员工作的科室，近期是否出现咳嗽、发热等症状，担心被感染的程度，是否通过官方网站或正规渠道来证实有关COVID-19信息的真实性，是否在微信里转发有关COVID-19信息，微信中转发的大量COVID-19相关信息是否让自己感到恐惧、焦虑、难以入睡、没有安全感或紧张，微信中转发的大量COVID-19相关信息对生活的影响程度对医护人员的SCL-90得分有影响，差异具有统计学意义(P<0.05，表3)。

表3 医护人员心理健康状况在一般社会学特征及舆情应对能力上的差异性检验(n=373)

Table 3 Difference test of different general sociological data and public opinion coping ability on mental health status of medical staff (n=373)

项目	n	SCL-90得分	$F/t/t'$	P
工作科室				
内科	82	95.78±8.33	62.309	<0.001
外科	166	99.33±12.85		
发热门诊、感染科或ICU	65	143.22±52.27		
其他科室	60	98.05±12.31		

©Journal of Central South University (Medical Science). All rights reserved.

表3(续)

项目	*n*	SCL-90得分	*F*/*t*/*t*'	*P*
近期有无咳嗽、发热等相关症状				
有	8	159.75±59.22	2.618	0.034
无	365	104.81±27.65		
您会通过官方网站或正规渠道来证实有关COVID-19信息的真实性吗?				
从不	12	129.58±60.92	2.817	0.039
有时	96	103.45±21.24		
经常	247	105.87±29.99		
总是	18	105.44±28.97		
您会在微信里转发有关COVID-19信息吗?				
几乎很少	152	102.69±21.66	4.464	0.012
有时	168	105.62±30.30		
经常	53	116.64±42.65		
微信中转发的大量COVID-19相关信息对生活的影响程度				
没有影响	43	96.58±9.40	17.893	<0.001
较小影响	62	95.97±10.41		
中等影响	127	99.15±13.23		
严重影响	141	119.43±42.48		
微信中转发的大量COVID-19相关信息让自己感到恐惧				
是	320	104.00±26.46	−2.329	0.023
否	53	118.04±42.55		
微信中转发的大量COVID-19相关信息让自己感到焦虑				
是	260	102.29±23.57	−3.089	0.002
否	113	114.50±39.05		
微信中转发的大量COVID-19相关信息让自己难以入睡				
是	354	104.37±27.50	−2.873	0.010
否	19	136.21±47.89		
微信中转发的大量COVID-19相关信息让自己没有安全感				
是	264	103.39±24.80	−2.237	0.027
否	109	112.28±38.32		
微信中转发的大量COVID-19相关信息让自己感到紧张				
是	246	103.11±26.69	−2.442	0.015
否	127	111.57±34.03		
您会因为看到COVID-19相关信息而担心自己被感染吗?				
从不	62	98.21±16.91	7.001	<0.001
有时	256	104.62±27.96		
经常	38	118.74±38.27		
总是	17	126.53±48.68		

2.4 医护人员心理健康状况的影响因素分析

以症状自评总分作为因变量，以有统计学意义的指标作为自变量，采用逐步回归分析发现：微信中转发的大量COVID-19相关信息对生活的影响程度，近期有无咳嗽、发热等相关症状，微信中转发的大量COVID-19相关信息是否让自己难以入睡，工作的科室及担心被感染的程度是影响医护人员心理健康状况的关键变量(表4)。

©Journal of Central South University (Medical Science). All rights reserved.

表4 心理健康状况影响因素的逐步回归分析

Table 4 Stepwise regression analysis of influencial factors of mental health status in medical staff

影响因素	b	b'	t	P	R^2	F
常量	107.145	11.889	9.012	<0.001	0.227	21.614
微信中转发的大量COVID-19相关信息对生活的影响程度	6.522	1.407	4.634	<0.001		
近期有无咳嗽、发热等症状	−44.282	9.554	−4.635	<0.001		
微信中转发的大量COVID-19相关信息是否让自己难以入睡	23.944	6.261	3.825	<0.001		
工作的科室	4.837	1.396	3.466	0.001		
担心被感染的程度	5.183	2.114	2.452	0.015		

3 讨 论

本研究结果显示：医护人员SCL-90中10个因子的平均分均<2，14.21%的医护人员出现1个及以上的因子得分≥2，其中11.26%的出现恐怖症状，7.77%的出现强迫症状，5.63%的出现焦虑症状，5.36%的出现人际敏感，4.56%的有其他睡眠和饮食问题，偏执、抑郁各占4.29%，说明在COVID-19疫情期间，部分医护人员存在不同程度的心理应激反应。危机事件对公众心理影响巨大，往往可导致人们在心理、认知、情感和行为上出现功能失调和社会混乱[8]。在甲型H_1N_1流行性感冒大流行期间，医护人员工作负荷加大，易出现疲惫、对疾病流行感到焦虑，且对甲型H_1N_1流行性感冒存在高水平的疾病不确定感[9-10]。在H_7N_9禽流感流行期间，护士的主要体验包括救治工作任务重、压力大，担忧自己和亲友的安全[11]。面对COVID-19疫情，恐慌是一种正常的心理现象，有助于人们采取积极的措施降低被感染的风险，但如果不加以冷静地理性思考，过度恐慌、紧张或失去理性控制，任恐惧心理发展，将导致严重的不良后果[12]。此次事件正好发生在中国的传统节日春节期间，由于病毒的传染性强且潜伏期长，国家号召公众尽量减少外出，尤其避免去公众场合。医护人员由于工作的特殊性，需要坚守在临床科室，外出上班以及为患者提供诊疗服务均会增加被感染的风险，从而可能会加重医护人员的恐慌心理和焦虑情绪。此次COVID-19主要是通过呼吸道飞沫传播和接触传播及消化道传播，大力提倡外出戴口罩、勤洗手等预防措施，因此强迫症状得分相对较高。

本研究结果显示：医护人员获取有关COVID-19疫情最新信息的主要来源依次为微信，电视、广播及微博、今日头条，占比均在80%以上；69.44%的医护人员相信微信中有关COVID-19疫情信息，但仅11.53%会转发相关信息；71.04%的医护人员经常或总是通过官方网站或正规渠道来证实有关COVID-19疫情信息的真实性；59.25%的医护人员有时或经常转发有关信息至微信，转发的主要目的是传递正能量(71.58%)，说明大部分医护人员能正确对待和处理微信中有关COVID-19疫情信息。2003年严重急性呼吸综合征(severe acute respiratory syndrome，SARS)流行期间，大众获取有关信息主要通过电视、广播及报纸等，网络信息对人们生活的影响程度还比较小。随着中国社会进入网络信息化新时代，微信已成为大众在线交流信息的重要平台[13]。2019年《第43次中国互联网络发展状况统计报告》[14]显示：中国网民数量达8.29亿，互联网普及率为59.67%，网络新闻用户达6.75亿，网民获取信息的渠道更加多元化。截至2018年底，微信已拥有10亿以上的用户，约400万公众号处于活跃状态，每月有7亿以上粉丝在公众号平台活跃[15]。COVID-19疫情信息会迅速地引起公众的高度关注，很容易且不自觉地给谣言传播提供了平台，同时也可能对疫情防控起积极作用[7]。基于此次事件的突发性，在微信中传播的很多信息的真实性和准确性不能及时、有效地被核实，这将加重公众的担心和猜疑，更容易引起恐慌[16]。医护人员比非医护人员更容易通过微信、公众号及医学相关网站获取到有关COVID-19疫情最新信息，且自身具有医学常识及通过医院组织有关COVID-19疫情专题培训获得的COVID-19诊疗方案、工作人员的自身防护、消毒隔离等相关专业知识，因此能够更理性地对待舆情信息，及时辨别谣言信息，转发真实信息，使微信朋友圈里非医学界的好友能获取准确信息，提高防范意识，传递正能量。

微信颠覆了传统的社交方式，人们的生活已经过度依赖和信任微信，但虚拟易产生欺骗，微信作为舆论的主要载体，传播信息存在蝴蝶效应，一条简单的微信能引发众多微信用户参与讨论、转发与分享，甚至形成社会舆论[17]，而谣言也很容易通过微信平台大范围地传播。本研究发现：在微信中大量有关感染COVID-19的信息影响医护人员生活或导致其难以入睡会造成医护人员心理健康方面的问题。此次公共危机事件发生以后，COVID-19疫情相关信

©Journal of Central South University (Medical Science). All rights reserved.

息通过微信、微博等自媒体平台迅速传播，同时由于事件的突发性，权威媒体无法即时获取最新信息或部分信息需通过核实后才能由权威媒体发布，这就更容易滋生谣言误导公众[7,18]。如果医护人员没有辨别舆情信息的能力，则会加重其心理负担，影响其睡眠质量甚至生活质量。医护人员应学习辨别有关舆情的基本知识，提高处理公众平台舆情信息的能力，同时医院相关管理部门应加强对舆情信息的管理，引导微信中舆论方向，防止舆情负面信息的不当扩散。

本研究发现担心自己被感染COVID-19或出现咳嗽、发热等症状的医护人员容易出现心理健康问题。医护人员在为患者进行治疗和护理时，被感染的风险增加。COVID-19感染潜伏期长，初期患者有发热、乏力、干咳的症状[19]，类似于感冒症状，不易识别且容易被忽视。一旦出现类似症状，医护人员的心理负担会明显增加。个体对危险的感知水平是创伤后应激综合征的关键预测因素，感知到高危环境的护士比暴露于高危环境的护士会表现出更严重的疲惫感及恐惧感[20-21]。

医护人员工作的科室是影响其心理健康的一个关键变量。本研究发现：在发热门诊、感染科或ICU工作的医护人员的SCL-90得分高于其他科室的医护人员，说明一线科室的医护人员更容易出现心理问题，与徐明川等[16]的研究结果相似。徐明川等[16]发现首批抗击COVID-19的临床一线支援护士中有92.68%出现不良情绪反应，主要原因是担心被感染、应对公共突发事件经验较少、工作负荷巨大等。在此次COVID-19疫情中，发热门诊、感染科或ICU的临床一线人员每日需为确诊或疑似患者提供治疗和护理，由于应对突发应急事件的经验较少，对COVID-19不了解，工作环境封闭式管理，工作负荷大，感染风险高，多名医护人员被确诊感染COVID-19，心理压力加重[22]。Liu等[23]在SARS疫情过去3年后，对抗击SARS的一线医护人员进行心理调查，发现单身、在暴发期间被隔离、在暴发前接触过其他创伤性事件会增加其3年后出现严重抑郁症状的发生率。对SARS时期一线医务人员远期心理健康状况进行调查，结果发现：在SARS结束后1年，医护人员的心理健康水平低于对照组，主要表现焦虑、抑郁等情绪的变化[24]。医院可增加心理护士岗位，让心理护士参与院内外公共卫生突发事件及灾难事件的心理救援[25]。医院管理者应重视医护人员的心理健康状况，及时给与疏导，如2020年1月31日中国医学救援协会心理救援分会、国家精神心理疾病临床医学研究中心联合学术支持下建立的应对COVID-19疫情相关心理危机的干预平台在中南大学湘雅二医院正式开通，通过热线电话及互联网在线咨询功能为医护人员及公众提供心理援助。

本研究的不足为：1)研究对象均来自湖南省，调查面窄，样本量小；2)仅探讨微信舆情信息处理对医护人员心理健康的影响，具有一定的局限性；3)只进行横断面调查，未对医护人员进行心理干预，后续将进一步完善研究。

利益冲突声明：作者声称无任何利益冲突。

参考文献

[1] 王琛, 王旋. 新型冠状病毒感染的流行、医院感染及心理预防[J]. 全科护理, 2020, 18(3): 1-4.
WANG Chen, WANG Xuan. The epidemic, nosocomial infection and psychological prevention of novel coronavirus infection[J]. Chinese General Practice Nursing, 2020, 18(3): 1-4.

[2] 龚会. 医护人员应对突发性事件的抗逆力及其EAP模式[J]. 成都师范学院学报, 2017, 33(5): 84-88.
GONG Hui. The model of employee assistant program on resilience of health care workers to cope with emergencies[J]. Journal of Sichuan College of Education, 2017, 33(5): 84-88.

[3] 邓银华. 微信朋友圈大学生用户信息分享意愿的影响因素研究[D]. 湘潭: 湘潭大学, 2015.
DENG Yinhua. Research on determined factors of Wechat friends circle college student users' information sharing intention[D]. Xiangtan: Xiangtan University, 2015.

[4] 中华人民共和国卫生健康委员会, 国家中医药管理局. 新型冠状病毒感染的肺炎诊疗方案(试行第5版)[EB/OL].(2020-02-05) [2020-02-14]. http://www. nhc. gov. cn/yzygj/s7653p/202002/d4b895337e19445f8d728fcaf1e3e13a/files/ab6bec7f93e64e7f998d802991203cd6.pdf.
National Health Commission of the People's Republic of China, National Administration of Traditional Chinese Medicine. Diagnosis and treatment protocol for novel coronavirus pneumonia (Trial Version 5) [EB/OL].(2020-02-05)[2020-02-14]. http://www.nhc.gov.cn/yzygj/s7653p/202002/d4b895337e19445f8d728fcaf1e3e13a/files/ab6bec7f93e64e7f998d802991203cd6.pdf.

[5] 王姿欢, 俞文兰, 沈壮, 等. 症状自评量表 (SCL-90) 应用于中国职业女性心理测评的信效度评价[J]. 中国工业医学杂志, 2017, 30(4): 247-250.
WANG Zihuan, YU Wenlan, SHEN Zhuang, et al. Reliability and validity of the symptom checklist 90 in Chinese professional females[J]. Chinese Journal of Industrial Medicine, 2017, 30(4): 247-250.

[6] 方必基, 谢云天, 刘彩霞. 近十一年医生症状自评量表 (SCL-90) 调查结果的Meta分析及常模确定[J]. 现代预防医学, 2017, 44(9): 1642-1646.
FANG Biji, XIE Yuntian, LIU Caixia. Reports on self-reporting inventory (SCL-90) for doctors in recent eleven years and construction of norm: a Meta-analysis[J]. Modern Preventive Medicine, 2017, 44(9): 1642-1646.

©Journal of Central South University (Medical Science). All rights reserved.

[7] 叶阿萍, 邵安. 舆情信息在微信的传播机制——基于426位大学生的实证分析[J]. 新媒体研究, 2018, 4(2): 1-4.
YE Aping, SHAO An. Communication mechanism of public opinion information in WeChat: based on empirical analysis of 426 college students[J]. New Media Research, 2018, 4(2): 1-4.
[8] 吕扬, 贾燕瑞, 高凤莉. 参与救治新发急性呼吸道传染病医护人员心理健康影响因素及应对策略[J]. 中国护理管理, 2019, 19(1): 83-86.
LÜ Yang, JIA Yanrui, GAO Fengli. A study review of psychological status of health care professionals engaged in emerging acute respiratory infectious diseases[J]. Chinese Nursing Management, 2019, 19(1): 83-86.
[9] Matsuishi K, Kawazoe A, Imai H, et al. Psychological impact of the pandemic (H1N1) 2009 on general hospital workers in Kobe[J]. Psychiatry Clin Neurosci, 2012, 66(4): 353-360.
[10] Taha S, Matheson K, Cronin T, et al. Intolerance of uncertainty, appraisals, coping, and anxiety: the case of the 2009 H1N1 pandemic[J]. Br J Health Psychol, 2014, 19(3):592-605.
[11] 苗琪琪, 刘铭扬, 郑沙沙, 等. 护士在救治人感染H7N9禽流感患者时的工作体验[J]. 中华护理杂志, 2018, 53(6): 720-723.
MIAO Qiqi, LIU Mingyang, ZHENG Shasha, et al. The lived experience of nurses during the treatment of patients infected with avian influenza A (H7N9) virus: a qualitative research[J]. Chinese Journal of Nursing, 2018, 53(6): 720-723.
[12] 刘允正, 康继祥. 非典型肺炎对社会生活、社会心理的影响及对策[J]. 华北煤炭医学院学报, 2003, 5(6): 796-797.
LIU Yunzheng, KANG Jixiang. The influence and countermeasures of SARS on social life and social psychology [J]. Journal of North China Coal Medical College, 2003, 5(6): 796-797.
[13] 吴仁献, 谢朝武, 谈天然. 突发事件政务舆情内部人际传播机制研究——以8.8九寨沟地震旅游政务微信传播为例[J]. 情报杂志, 2019, 38(9): 118-125.
WU Renxian, XIE Chaowu, TAN Tianran. Research on insider mechanism of government public opinion in emergencies—a case study of travel government WeChat dissemination in the 8.8 Jiuzhaigou earthquake[J]. Journal of Intelligence, 2019, 38 (9): 118-125.
[14] 中国互联网络信息中心(CNNIC). 第43次中国互联网络发展现状统计报告[EB/OL]. (2019-02-28) [2020-02-01]. http://www.cac.gov.cn/2019-02/28/c_1124175686.htm.
CNNIC. The 43rd China Statistical Report on internet development[EB/OL]. (2019-02-28) [2020-02-01]. http://www.cac.gov.cn/2019-02/28/c_1124175686.htm.
[15] 白岳. 基于微信生态构建电信运营商互联网运营体系的研究[J]. 现代经济信息, 2019(21): 321, 323.
BAI Yue. Research on the construction of telecom operators' Internet operation system based on WeChat ecology[J]. Modern Economic Information, 2019(21): 321, 323.
[16] 徐明川, 张悦. 首批抗击新型冠状病毒感染肺炎的临床一线支援护士的心理状况调查[J]. 护理研究, 2020, 34(3): 1-3.
XU Mingchuan, ZHANG Yue. Psychological status survey of first clinical first-line support nurses fighting against pneumonia caused by a 2019 novel coronavirus infection[J]. Chinese Nursing Research, 2020, 34(3): 1-3.
[17] 张宏, 李杰. 复杂网络的微信网络信息传播研究[J]. 科学技术与工程, 2014, 14(17): 244-247.
ZHANG Hong, LI Jie. Research on WeChat network information transmission in complex networks[J]. Science Technology and Engineering, 2014, 14(17): 244-247.
[18] 林志军. 论社会安全稳定视阈下的舆情传播特征及其管控[J]. 学校党建与思想教育, 2018, (17): 72-74.
LIN Zhijun. The characteristics and control of public opinion communication from the perspective of social security and stability[J]. The Party Building and Ideological Education in Schools, 2018, (17): 72-74.
[19] Huang CL, Wang YM, Li XW, et al. Clinical features of patients infected with 2019 novel coronavirus in Wuhan, China [J]. The Lancet, 2020, 395(10223): 497-506.
[20] Marjanovic Z, Greenglass ER, Coffey S. The relevance of psychosocial variables and working conditions in predicting nurses’ coping strategies during the SARS crisis: an online questionnaire survey[J]. Int J Nurs Stud, 2007, 44(6): 991-998.
[21] Wu P, Fang YY, Guan ZQ, et al. The psychological impact of the SARS epidemic on hospital employees in China: exposure, risk perception, and altruistic acceptance of risk[J]. Can J Psychiat, 2009, 54(5): 302-311.
[22] 尹平, 潘小平, 赵子文, 等. SARS病区医护人员的心理健康及心理干预影响的研究[J]. 中国健康心理学杂志, 2004, 12 (4): 257-259.
YIN Ping, PAN Xiaoping, ZHAO Ziwen, et al. Study of mental health status of physicians and nurses working at SARS area and the effect of psychological intervention[J]. Chinese Journal of Health Psychology, 2004, 12(4): 257-259.
[23] Liu XH, Kakade M, Fuller CJ, et al. Depression after exposure to stressful events: lessons learned from the severe acute respiratory syndrome epidemic[J]. Compr Psychiat, 2012, 53 (1): 15-23.
[24] 杨来启, 吴兴曲, 张彦, 等. 非典时期一线医务人员远期心理健康状况调查研究[J]. 中国健康心理学杂志, 2007, 15 (6): 567-569.
YANG Laiqi, WU Xingqu, ZHANG Yan, et al. Mental health status of medical staffs fighting SARS: a long-dated investigation[J]. China Journal of Health Psychology, 2007, 15 (6): 567-569.
[25] 王玉秀. 心理护士岗位的设立及实践[J]. 中华护理杂志, 2019, 54(1): 88-90.
WANG Yuxiu. The establishment and practice of full-time psychological nurses[J]. Chinese Journal of Nursing, 2019, 54 (1): 88-90.

（本文编辑 郭征）

本文引用：申海艳, 王惠平, 周霏, 陈晋东, 邓露. 2019冠状病毒病疫情期间医护人员心理健康状况[J]. 中南大学学报(医学版), 2020, 45(6): 633-640. DOI:10.11817/j.issn.1672-7347.2020.200070

Cite this article as: SHEN Haiyan, WANG Huiping, ZHOU Fei, CHEN Jindong, DENG Lu. Mental health status of medical staff in the epidemic period of coronavirus disease 2019[J]. Journal of Central South University. Medical Science, 2020, 45(6): 633-640. DOI:10.11817/j.issn.1672-7347.2020.200070

©Journal of Central South University (Medical Science). All rights reserved.
