# Supplementary material for: Mental health status and related factors influencing healthcare workers during the COVID-19 pandemic: A systematic review and meta-analysis
Source: PLoS One. 2024 Jan 19;19(1):e0289454. doi: 10.1371/journal.pone.0289454 (PMC10798549; doi:10.1371/journal.pone.0289454)
Supplement: S1 Data — (ZIP) [file pone.0289454.s011.zip › literatures/166.pdf]

 J Nepal Med Assoc 2020;58(228):550-3

OPEN ACCESS CC BY
doi: 10.31729/jnma.5087

Prevalence of Psychological Effect of COVID-19 on Medical Professionals in a Tertiary Care Center

Surendra Lal Shrestha[1]

[1]Department of Medicine, National Academy of Medical Science (NAMS), Bir Hospital, Kathmandu, Nepal.

ABSTRACT

Introduction: COVID-19 is a pandemic disease first detected in Wuhan, China on last December 2019. Many doctors and nurses, were infected and lost their life by COVID-19 around the world. Therefore COVID-19 brought unbearable psychological pressure on doctors, and nurses. The objective of this study is to find the prevalence of anxiety among medical doctors and nurses.

Methods: This is a descriptive cross-sectional study of 101 doctors and nurses carried out in a tertiary care center. Convenience sampling was done with the study period from April to May 2020. Ethical approval was taken from the institutional review board of NAMS (IRB reference no. 1076). The collected data stored and analyzed with statistical software (SPSS version 26.0). Point estimate at 95% Confidence Interval was calculated along with frequency and proportion for binary data.

Results: Out of 101 participants prevalence of anxiety was found to be 74 (73.3%) (64.68-81.33 at 95% Confidence Interval). Among them, 9 (8.9%) of participants experienced sever types of generalized anxiety disorder, 23 (22.8%) moderate, and 42 (41.6%) mild type. Similarly, 18 (17.8%) and 10 (9.9%) of participants felt very difficult and extreme difficulty at the workplace and home respectively.

Conclusions: The mental health of medical doctors and nurses is significantly affected during the COVID-19 pandemic. Hospital administration should conduct psychological preparedness training to the medical profession before posting on duty to provide quality health services to the patients.

Keywords: *COVID-19; doctor; generalized anxiety disorder; nurses.*

INTRODUCTION

COVID-19 is a pandemic and belief to be spread to humans via wild animals. The study showed that SARS-CoV-2 is a new member of the Coronaviridae family.[1,2] SARS-CoV-2 represents genetic similarity (96.3%) with the bat coronavirus.[3] WHO declared an outbreak of a novel coronavirus as a public health emergency of international concern and was followed by the declaration of a pandemic on March 11, 2020.[4,5]

To date, over 6 million are suffering from the COVID-19 and more than 350 thousand death occurred in the world. Many doctors and nurses, are infectedduring the treatment of COVID-19 positive patients and lost their life by COVID-19 around the world. In Study conducted in china, COVID positive health workers in Wuhan ,3.8%

Correspondence: Dr. Surendra Lal Shreshtha, Department of Medicine, National Academy of Medical Science (NAMS), Bir Hospital, Kathmandu, Nepal. Email: drsurendralal@gmail.com, Phone:+977-9851240716.

Free Full Text Articles are Available at www.jnma.com.np

of laboratory-confirmed cases of COVID-19 occurred in healthcare personnel, and 14.6% of these cases were either severe or critical.[6]Therefore doctors, and nurses facing unbearable psychological pressure. Still, there is a challenge to develop specific medicine, vaccines and the method to block further transmission.[7]

The objective of this study is to evaluate the degree of stress among the medical doctor and nurses using Generalized Anxiety Disorder (GAD)-7 score which contains 7 items of questions.[8]

METHODS

This is a descriptive cross-sectional study of a total of 101 medical doctors and nurses working in general wards, isolation ward, different departments, and ICU in a tertiary care center. Medical doctors and nurses were included in this study. Convenience sampling was donewith the study period from April to May 2020. Ethical approval was taken from the institutional review board of NAMS (IRB reference no. 1076).

The Generalized Anxiety Disorder Scale (GAD-7) is one of the most widely used scoring scales for the detection and screening of anxiety disorders. Each participant of different departments was given a set of seven questions to tick the answer and thereafter collected answer sheets for further processing. Verbal consent was taken from each participant after explaining the relevant details of the study. Those who did not give consent for participation were excluded from the study. Confidentiality maintained to the utmost. The sample size was calculated using the following formula

Sample size (n) = Z^2 x p x q /e^2

= $(1.96)^2$ x 0.07x 0.93/0.05^2

= 100.04

Therefore 101 participants were chosen for the study.

Where,

Z = 1.96 for confidence interval at 95%

p = prevalence 7 %

q = 1-p

e = margin of error 5%

The collected data stored and analyzed with statistical software (SPSS version 26.0) to get the final interpretation.

RESULTS

Out of 101 participants prevalence of anxiety was found to be 74 (73.3%) (64.68-81.33 at 95% Confidence Interval). Among them, 9 (8.9%) of participants experienced sever types of generalized anxiety disorder, 23 (22.8%) moderate, and 42 (41.6%) mild type (Table 1).

Table 1. The severity of GAD.

The severity of stress (anxiety)	Male	Female	n (%)
Normal	14	13	27 (26.7)
Mild	20	22	42 (41.6)
Moderate	6	17	23 (22.8)
Sever	3	6	9 (8.9)
Total	43	58	101 (100)

Similarly, 18 (17.8%) and 10 (9.9%) of participants felt very difficult and extreme difficulty at the workplace and home respectively (Table 2).

Table 2. The number of participants showing difficulty at work, and home.

Difficulty at work and home	n (%)
Not difficult at all	11 (10.9)
Somewhat difficult	62 (61.4)
Very difficult	18 (17.8)
Extremely difficult	10 (9.9)
Total	101 (100)

Among 101 participants, 60 (59.4%) doctors were having different specialties working in different departments, and 41 (40.6%) nurses working in the isolation ward, medical ward, and ICU (Table 3). Similarly, there were 43 (42.6%) males and 58 (57.4%) were female.

Table 3. Department wise distribution.

Departments	n (%)
Isolation	8 (7.9)
ICU	27(26.7)
Medical	27(26.7)
Emergency	12(11.9)
Ward	12 (11.9)
Surgery	5 (5)
Dental	10 (9.9)
Total	101(100)

DISCUSSION

In this study, the mental health of medical doctors and

nurses is significantly affected during public health emergencies like COVID-19. In this study, it is found that 8.9 % of participants experienced a severe type of anxiety disorder, 22.8% moderate, and 41.6% mild type.A similar studywas done by Cao W et al. in students which showed 0.9% were in severe anxiety, 2.7% in moderate anxiety, and 21.3% in mild anxiety states. Relatives or acquaintances infected with COVID-19 was a factor for increasing their anxiety (OR = 3.007, 95% CI = 2.377 - 3.804).[9]

Lu W et al. found the medical staff showed moderate and severe fear which was higher than the administrative staff group (70.6% VS 58.4%). Likewise, 22.6% of medical staff found to be shown mild to moderate anxiety and 2.9% were severe anxiety. Similarly, as compared to the non-medical staff, front line medical staff with close contact with infected patients, including working in the departments of respiratory, emergency, infectious disease, and ICU, showed higher scores on the fear scale, and they were 1.4 times more likely to feel fear, twice more likely to suffer anxiety and depression.[10]

During the treatment of COVID-19, medical workers in Wuhan faced enormous pressure, due to inadequate protection from infection, work overload, patients with negative emotions, loss of contact with their families. This leads to mental health problems such as stress, anxiety, depressive symptoms, insomnia, denial, anger, and fear. To cope with the situation, psychological intervention teams have been made by the Mental Health Centre of Wuhan, coordinating with other teams of experts to prepare and treat the health personnel. On Jan 27, 2020, the National Health Commission of China published a national guideline of psychological crisis intervention for COVID-19. This publication is the first to guide and provide psychological protection of the mental health of medical workers.[11]

According to Xiang et al. health professionals, working with confirmed or suspected COVID-19 pneumonia, are at risk of infection and mental health problems. Health workers in a Beijing hospital who were quarantined and worked in high-risk settings of SARS units, or had family or friends who were infected with SARS, had more post-traumatic stress symptoms, depression, anxiety, fear, and frustration. So mental health assessment, support, treatment, and services are necessary to cope with the 2019-nCoV outbreak.[12]

Maintaining staff mental health is essential to better control infectious diseases. It can be done by Psychological intervention medical team, which guide medical staff to deal with psychological problems and provides guidance and supervision to solve psychological problems performingactivities to release stress.[13]

CONCLUSIONS

The mental health of medical doctors and nurses is significantly affected during public health emergencies like COVID-19 and they require attention, help, and support of hospital administration, and senior medical colleagues. It is suggested that hospital administration should initiate psychological services and training to the medical profession to provide quality health services to the patients.

Conflict of Interest: None.

REFERENCES

1. Zhu N, Zhang D, Wang W, et al. A novel coronavirus from patients with pneumonia in China, 2019. N Engl J Med. 2020;382(8):727-33.[PubMed | Full Text | DOI]

2. Lu R, Zhao X, Li J, et al. Genomic characterisation and epidemiology of 2019 novel coronavirus: implications for virus origins and receptor binding. Lancet. 2020;395(10224):565-74. [PubMed | Full Text | DOI]

3. Paraskevis D, Kostaki EG, Magiorkinis G, et al. Full-genome evolutionary analysis of the novel corona virus (2019-nCoV) rejects the hypothesis of emergence as a result of a recent recombination event. Infect Genet Evol. 2020;79:104212. [PubMed | Full Text | DOI]

4. WHO. Statement on the second meeting of the International Health Regulations (2005) Emergency Committee regarding the outbreak of novel coronavirus (2019-nCoV) [Internet]. WHO Newsroom. 2020 Jan 30:News Room.Available from: https://www.who.int/news-room/detail/. [Full Text]

5. WHO. WHO Director-General's opening remarks at the media briefing on COVID-19 [Internet]. WHO Director-General Speeches.2020 Mar 11. Available from:https://www.who.int/dg/speeches/detail/. [Full Text]

6. Epidemiology Working Group for NCIP Epidemic Response, Chinese Center for Disease Control and Prevention. The epidemiological characteristics of an outbreak of 2019 novel coronavirus diseases (COVID-19) in China.Zhonghua Liu Xing Bing Xue Za Zhi. 2020;41(2):145-51. Chinese. [PubMed | Full Text | DOI]

7. Xiao C, Li X, Liu S, et al. HIV-1 did not contribute to the 2019-nCoV genome.Emerg Microbes Infect.2020;9(1):378-81. [PubMed | Full Text | DOI]

8. Spitzer RL, Kroenke K, Williams JB, Lowe B. A brief measure for assessing generalized anxiety disorder: the GAD-7. Arch Intern Med. 2006;166(10):1092-7. [PubMed | Full Text | DOI]

9. Cao W, Fang Z, Hou G, et al. The psychological impact of the COVID-19 epidemic on college students in China. Psychiatry Res.2020;287:112934. [PubMed | Full Text | DOI]

10. Lu W,Wang H, Lin Y,et al.Psychological status of medical workforce during the COVID-19pandemic:A cross-sectional study.Psychiatry Res. 2020;288:112936. [PubMed | Full Text | DOI]

11. Kang L, Li Y, Hu S, et al. The mental health of medical workers in Wuhan, China dealing with the 2019 novel coronavirus. The Lancet Psychiatry. 2020;7(3):e14. [PubMed | Full Text | DOI]

12. Xiang Y-T, Yang Y, Li W, et al. Timely mental health care for the 2019 novel coronavirus outbreak is urgently needed. The Lancet Psychiatry. 2020;7(3):228-9. [PubMed | Full Text | DOI]

13. Chen Q, Liang M, Li Y, et al. Mental health care for medical staff in China during the COVID-19 outbreak. The Lancet Psychiatry. 2020;7(4):e15-6. [PubMed | Full Text | DOI]

© The Author(s) 2018.

This work is licensed under a Creative Commons Attribution 4.0 International License. The images or other third party material in this article are included in the article's Creative Commons license, unless indicated otherwise in the credit line; if the material is not included under the Creative Commons license, users will need to obtain permission from the license holder to reproduce the material. To view a copy of this license, visit http://creativecommons.org/licenses/by/4.0/
