# Supplementary material for: Mental health status and related factors influencing healthcare workers during the COVID-19 pandemic: A systematic review and meta-analysis
Source: PLoS One. 2024 Jan 19;19(1):e0289454. doi: 10.1371/journal.pone.0289454 (PMC10798549; doi:10.1371/journal.pone.0289454)
Supplement: S1 Data — (ZIP) [file pone.0289454.s011.zip › literatures/169.pdf]

Received: 11 September 2020 | Revised: 16 January 2021 | Accepted: 27 January 2021

DOI: 10.1111/jocn.15685

ORIGINAL ARTICLE

Journal of Clinical Nursing WILEY

Anxiety, sleep disorders and self-efficacy among nurses during COVID-19 pandemic: A large cross-sectional study

Valentina Simonetti RN, MSN, PhD, Nurse Tutor[1] | Angela Durante RN, MSC, PhD, Post Doc Research Fellow[2] | Rossella Ambrosca RN, MSN, PhD Candidate[2] | Paola Arcadi RN, MSN, PhD Candidate[2] | Giusi Graziano PhD, Biostatistician[3,4] | Gianluca Pucciarelli RN, PhD, FAHA, Research Fellow[2] | Silvio Simeone RN, MSN, PhD, Research Fellow[2] | Ercole Vellone RN, PhD, FESC, Associate Professor[2] | Rosaria Alvaro RN, MSN, FESC, FAAN, Professor[2] | Giancarlo Cicolini RN, MSN, PhD, Researcher[4]

[1]Politecnica Delle Marche University, ASUR Marche, AV 5, Ascoli Piceno, Italy

[2]Department of Biomedicine and Prevention, University of Rome Tor Vergata, Rome, Italy

[3]Center for Outcomes Research and Clinical Epidemiology (CORESEARCH), Pescara, Italy

[4]Department of Biomedical Science and Human Oncology, University of Bari Aldo Moro, Bari, Italy

Correspondence
Valentina Simonetti, Politecnica delle Marche University, Ascoli Piceno, Italy.
Email: v.simonetti@staff.univpm.it

Abstract

Aim and objectives: To assess the prevalence of anxiety, sleep disorders and self-efficacy and their predicting factors among nurses facing COVID-19.

Background: The spread of COVID-19 throughout the world determined a series of modifications of several National Health Service organisations, with a potential series of psychological consequences among nurses, who were particularly afflicted by this situation of changes and precariousness.

Design: A cross-sectional study was carried out from February–April 2020.

Methods: A total of 1,005 nurses employed in different Italian hospital wards, during the COVID-19 pandemic, were recruited. Analyses were based on descriptive statistics and multivariate logistic regression. The STROBE checklist for cross-sectional studies was used in this study.

Results: The prevalence of sleep disturbances, moderate anxiety and low self-efficacy was 71.4%, 33.23% and 50.65%, respectively. We found a positive correlation between anxiety and sleep quality (0.408; $p < .0001$) and negative correlations between self-efficacy and anxiety (−0.217; $p < .0001$) and sleep quality and self-efficacy (−0.134; $p < .0001$).

The factor independently associated with all variables was gender. Females were more prone to sleep disturbances, anxiety and low levels of self-efficacy than males ($p < .05$).

Conclusions: The prevalence of anxiety, sleep disorders and low self-efficacy among Italian nurses during the COVID-19 pandemic was high. Healthcare managers should recognise and consider these results to reduce the risk of the onset of major mental problems that could result in post-traumatic stress disorder.

Relevance to clinical practice: Nurses facing major incidents as COVID-19 pandemic are among healthcare personnel exposed to a high risk to develop psychological disturbance that should be assessed and recognised, in order to find helpful coping

 © 2021 John Wiley & Sons Ltd

strategies to inform support services and avoid to hesitate in post-traumatic stress disorders.

KEYWORDS
anxiety, COVID-19 pandemic, nurses, nursing, self-efficacy, sleep disorders

1 | INTRODUCTION

The severe acute respiratory syndrome coronavirus 2 (SARS-CoV-2), also known as 2019 novel coronavirus (2019-nCoV), was identified for the first time in China in December 2019 at Wuhan, with a spread of more than 81,000 confirmed cases (World Health Organization [WHO], 2020).

In January 2020, this coronavirus (COVID-19) entered countries that are neighbours to China (Thailand, South Korea, Japan and Australia), and also spread to the United States and Europe, including Italy. On March 11, the WHO declared SARS-CoV-2 a pandemic (Cucinotta & Vanelli, 2020), having infected more than 100,000 people in 100 countries (Callaway, 2020). Data regarding this pandemic have rapidly evolved, with 2,094,069 confirmed cases in the United States (US), 867,624 in Brazil, 536,484 in Russia, 332,424 in India, 297,482 in the United Kingdom, 243,928 in Spain and 236,989 in Italy, with 115,732 deaths in the United States, followed by Brazil (43,332), United Kingdom (41,783) and Italy (34,385) as of 15 June 2020 (Center for System Science and Engineering [CSEE], 2020).

In order to counteract and contain the virus' spread and contagions, in the hope of not overwhelming national health services (NHS), the governments of many countries enacted extraordinary measures to limit viral transmission and minimise the likelihood that people who were not infected would come into contact with people who were infected, leading to a strain on the capacity of health services (Remuzzi & Remuzzi, 2020). Regions of Italy revised their healthcare organisational models to cope with the emergency, providing suitable paths for symptomatic and non-symptomatic patients by managing cases at home or in hospital according to clinical-care complexity. Healthcare systems also responded by increasing the number of beds in both intensive and sub-intensive units and also equipping new hospitals when necessary (Spina et al., 2020). These measures were aimed at effectively responding to the needs of those who were already infected and required admission to an intensive care unit (ICU) for acute respiratory distress syndrome largely due to COVID-19 pneumonia (Remuzzi & Remuzzi, 2020).

In this situation of precariousness and changes, healthcare workers directly involved in the diagnosis, treatment and care of patients with COVID-19 have been forced to work under higher workload pressure. This resulted in sustained pressing schedules and personnel often being relocated by their home departments to meet the needs of ICU patients to help staff already reduced to a minimum, and sometimes, this work was done without the availability of appropriate personal protective equipment (PPE; Nacoti et al., 2020). In addition, the factors associated with social distancing and isolation are known to lead to an increased risk of psychological distress and other mental health symptoms (Bansal et al., 2020). In particular, stress (Huang et al.2020; Kang, Li, et al., 2020; Lai et al., 2020), insomnia (Kang, Li, et al., 2020; Kang, Ma, et al., 2020; Lai et al., 2020) and anxious states (Huang, & Zhou2020; Kang, Li, et al., 2020; Lai et al., 2020) affected healthcare workers especially if nurses, women, and directly involved at the front line, caring for COVID-19 patients (Lai et al., 2020). Moreover, during the 2003 SARS outbreak, it was reported that the fear of transmitting the infection to relatives, friends and colleagues caused negative psychological reactions among healthcare workers (Bai et al., 2004; Chua et al., 2004; Lee et al., 2007; Maunder et al., 2003) and appeared to be a factor in growing anxiety levels (Nacoti et al., 2020).

What does this paper contribute to the wider global clinical community?

- Anxiety and sleep disorders have a great impact on the psychophysical health of nurses, affecting professional performance and patient safety. Conversely, better self-efficacy may play an important role in reducing anxiety and stress.
- This study found that the prevalence of anxiety, sleep disorders and low levels of self-efficacy was consistent among Italian nurses providing care to patients with suspected or confirmed COVID-19. Further, there was a correlation among these factors. Gender was an independent predictive factor, with females more likely to suffer these effects.
- Assessing the risk factors associated with psychological disturbances could aid in finding helpful coping strategies to inform support services.

2 | BACKGROUND

The workloads of healthcare workers during a pandemic involve exhausting shifts necessary to both manage the health emergency and overcome long-standing shortages of healthcare personnel, particularly nurses, and can cause negative outcomes for the safety of patients (in terms of mortality and morbidity) and health professionals (in lower job satisfaction, burnout and intention to leave; Griffiths et al., 2018; Sasso et al., 2017). This, in turn, negatively affects these workers' sleep quality (Dong et al., 2017; Han et al., 2016), increasing the risk of distressed psychophysical health revealed in symptoms such as memory loss, low reactivity, irritability, worsening depression and

suicidal tendencies (Mieda & Sakurai, 2013; Rosado et al., 2015). In China, during this current pandemic, more than 70% of healthcare workers reported psychological issues, including insomnia, anxiety and depression (Lai et al., 2020), and they were at a higher risk of developing these outcomes than the general population (Huang & Zhao, 2020). In particular, chronic sleep disturbances have been associated with absenteeism (Kuppermann et al., 1995), increased medical errors, malpractice risk and early retirement (Kessler et al., 2011; Kling et al., 2010; Nanda et al., 2017; Ohayon & Paiva, 2005). Xiao et al. (2020) conducted a study among medical staff treating patients with COVID-19 and reported how levels of anxiety, stress and self-efficacy were strictly dependent on sleep quality and social support. It is reasonable to assume that when faced with these psychosocial health issues, protective psychosocial shields or strengths like resilience, belief in self-efficacy, and coping strategies could limit the severity of disease in these individuals (Farber et al., 2000). In particular, self-efficacy is an important factor that influences an individual's ability to exert self-control (Bray et al., 2015; Najmi et al., 2013),which represents, in turn, the prime factor for the cultivation of resilience and other psychosocial strengths among individuals (Morrison & Pidgeon, 2017). Previous research has reported significant associations between self-efficacy and mental health (Hu et al., 2020) and well-being (Yuksel et al., 2019), and it is an important element in promoting health-related intentions and behaviours (Sheeran et al., 2016). These aspects should be recognised, considered and adequately addressed by healthcare managers (Dong et al., 2017) to reduce the risk of the onset of major mental disorders that could result in post-traumatic stress disorder.

Among healthcare workers, nurses play a pivotal role in public health and, as a result, are faced with fears of virus contagion during direct contact with patients and the potentially ensuing series of psychological consequences, especially among those providing care in contexts characterised by change and precariousness (Fernandez et al., 2020). Additional sources of stress for nurses are the separation from their family, sleep deprivation and heavy workloads created by health system demands and staff shortages (Fernandez et al., 2020), as well as being relocated to units outside of their usual practice areas, such as specialised pandemic clinics (Seale et al., 2009).

However, to our current knowledge, evidence addressing the factors that influence sleep quality, anxiety and self-efficacy levels among nurses on the front line against COVID-19 is limited; thus, our study aimed to investigate these effects in order to provide insight into these risk factors.

3 | METHODS

3.1 | Objectives

The aim of this study was to describe the prevalence of anxiety, sleep disorders and low levels of self-efficacy among Italian nurses during the COVID-19 pandemic. We also investigated the potential predictors of these symptoms with respect to the demographic and occupational characteristics of the study sample.

3.2 | Design

A multicentre, cross-sectional study was conducted in all Italian regions.

In order to grant adequate and complete reporting of research, we used the Strengthening the Reporting of Observational Studies in Epidemiology (STROBE) guideline (von Elm et al., 2008) for cross-sectional studies (File S1).

3.3 | Participants

We first asked for the participation of at least two hospitals (between public, private or affiliated) for each Italian Region. No exclusion criteria were applied for hospitals: each hospital who accepted to participate was included as centre of data collection. Finally, a total of 42 Italian hospitals gave their agreement to take part of the survey. We did require the appropriate ethics committee to approve the study (the initial approval was granted by the Coordinating Centre of Policlinico of Rome "Tor Vergata").

A previously trained nurse researchers' team administered a questionnaire to a convenience sample of nurses who were employed in different hospital wards during the COVID-19 pandemic. Nurses who assisted COVID-19 patients during the study period (both in hospital and in territorial health services), able to read and understand the Italian language and who, after understanding the purpose of the study, agreed to participate and provided informed consent, were enrolled.

3.4 | Data collection

The following instruments were used for data collection between February–April 2020:

The Pittsburgh Sleep Quality Index (PSQI; Buysse et al., 1989). The 19-item PSQI is an instrument for measuring sleep quality in seven domains: subjective sleep quality, sleep latency, sleep duration, habitual sleep efficiency, sleep disturbances, use of sleep medication and daytime dysfunction. Respondents were asked to self-rate each of these seven domains for the past month using a 4-point Likert format with responses from 0–3, where a higher score meant worse sleep quality in each domain. A global score from 0–5 can be obtained from the PSQI, with a higher score indicating poorer sleep. Items asking a bed mate or roommate to evaluate the respondent's sleep were not used in this study.

The Self-Rating Anxiety Scale (SAS; Zung, 1971) is a 20-item instrument used to self-assess anxiety. Each item uses a 4-point Likert scale for responses, from "almost never" to "very often." Final scores of 0 to 20 indicate a probable very low level of anxiety, 21–40 indicate low levels of anxiety, 41–60 mean moderate anxiety levels and 61–80 indicate high levels.

The General Self-Efficacy Scale (GSE; Schwarzer & Jerusalem, 1995) is a 10-item instrument with a 4-point Likert scale response from 1 ("not at all true") to 4 ("completely true"). The total score, calculated by finding the sum of the individual item responses, ranges from 10–40, with a higher score indicating better self-efficacy levels.

We also used a questionnaire developed by the research team to collect participants' socio-demographic and job data, including age, gender, marital status, number of children, education (basic/post-basic/training programme in maxi-emergencies) and professional and work information that encompassed the type of facility (public or private), job title, income, job role, work area and work experience.

Previous researchers have tested the psychometric proprieties of the scales we used for data collection, demonstrating their validity and reliability. Results included a Cronbach's alpha of 0.835 for the Italian version of the PSQI (Curcio et al., 2013), a Cronbach's alpha of 0.835 for the SAS (Zung, 1971) and a Cronbach's alpha between 0.76–0.90 for the GSE (Sibilia et al., 1997).

However, to confirm the accuracy and content validity of all scales, we estimated the internal consistency of the scales used in this study. The Cronbach's alpha measured for the PSQI, SAS and GSE were 0.75, 0.76 and 0.92, respectively.

3.5 | Ethical considerations

The study protocol was approved by the Ethics Committee of the Coordinating Centre in Rome. We also obtained approval from the nursing department of each hospital. Nurses were enrolled after giving their informed consent. Data confidentiality, which was in line with the Personal Data Act (523/1999), was ensured in the data collection and data analysis phases.

3.6 | Data analysis

Quantitative variables were summarised as median and interquartile range (IQR) according to their distribution. Categorical variables were summarised as frequency and percentage. A chi-square test was performed to compare categorical variables. The Mann–Whitney U-test or Kruskall–Wallis test was performed to evaluate differences in score results among study groups, as appropriate. Spearman's correlation coefficient was used to assess correlation among scores. Multiple logistic regression analyses were performed separately to evaluate the factors associated with each score categorised (PSQI: low quality VS good quality; SAS: very low/low VS moderate/high; GSE: low level VS high level). The variables considered in the models were all those significantly associated with at least one score in the univariate analyses: gender, age, working area, years of work experience, post-registration courses, number of children, reallocation and nursing role. The results are presented as odds ratios (OR) with 95% confidence intervals (95% CI). All tests were considered statistically significant for a *p*-value less than .05. All statistical analyses were performed using SAS software (version 9.4; Cary, NC, USA).

3.7 | Sample size

The primary aim of the study was to assess sleep disturbances, anxiety and self-efficacy levels among nurses assisting patients during the COVID-19 pandemic. As we did not know the percentage of participation and in order to ensure that all regions of the north, centre and south of Italy were represented, the minimum required sample size (n = 357) was calculated considering a population size of at least 5000 subjects, with an error margin of 5%, a 95% confidence interval and an estimated response rate of 50%.

4 | RESULTS

4.1 | Characteristics of the study sample

A total of 1200 questionnaires were administered, and 1005 nurses, equally distributed among regions of the north, centre and south of Italy (24.08%, 37.11% and 38.81%, respectively), completed the structured questionnaire, for a response rate of 83.75%. The mean age of the sample was 40.2 ± 10.80 years (range: 22–65 years), and about half of them were married (47.66%). Most participants were female (65.97%), with no children (49.75%), employed in general hospital wards (42.19%) of a public hospital (81.89%), engaged in a clinical role (86.47%) and had a first-level degree (83.78%). Less than half (37.21%) attended a course on major incidents or were relocated after the COVID-19 emergency (Table 1).

4.2 | Scores of PSQI, SAS and GSE

Table 2 shows the median (IQR) scores on the PSQI, SAS and GSE, which were 9 (6–11), 37 (32–43) and 29 (26–32), respectively. The percentage of nurses with a PSQI > 5 was 75.72%, indicating poor sleep quality. More than 65.37% of the sample had low levels of anxiety (21–40), and almost half of them were distributed between the two groups of high and low levels of self-efficacy.

4.3 | Scores correlation

Table 3 reports the correlation found among the scores for the PSQI, SAS and GSE, with a strong positive correlation between the SAS and PSQI (0.408; $p < .001$) and negative correlations between the GSE and SAS (−0.217; $p < .001$) and the PSQI and GSE (−0.134; $p < .001$).

4.4 | Association among variables

As reported in Table 4, there was a statistically significant difference in the PSQI, SAS and GSE scores between males and females, with higher levels of sleep disturbance and anxiety reported by females ($p < .05$).

Older age, male gender, attending post-registration courses and a longer length of working experience were associated with higher levels of self-efficacy ($p = .0193$, $p = .0023$, $p = .0155$ and $p = .0168$, respectively). Conversely, being relocated seemed to be a factor not associated with sleep disturbances, anxiety or self-efficacy.

Anxiety levels were higher among clinical nurses working in general hospital wards (median = 38; IQR, 33–43), territorial health services (median = 37.5; IQR, 32–42) and emergency (median = 37; IQR, 32–43) areas than with those involved in outpatient services (median = 36; IQR, 31–42) or management/education/research. The levels were also higher among those who had more than two children (median = 37; IQR, 32–41) or none (median = 38; IQR, 32–43) than those with one child (median = 35; IQR, 31–41) or two children (median = 36; IQR, 32–43).

TABLE 1 Socio-demographic characteristics and working data of the study sample (*n* = 1005)

Age mean ± SD	40.2 ± 10.80
Working region *n* (%)	
North	242 (24.08)
Centre	373 (37.11)
South and island	390 (38.81)
Type of hospital *n* (%)	
Public	823 (81.89)
Private	182 (18.11)
Gender *n* (%)	
Male	342 (34.03)
Female	663 (65.97)
Marital status *n* (%)	
Unmarried	526 (52.34)
Married	479 (47.66)
Number of children *n* (%)	
0	500 (49.75)
1	161 (16.02)
2	275 (27.36)
>2	69 (6.87)
Level of education *n* (%)	
First-level degree (bachelor in nursing or equivalent title)	842 (83.78)
Second-level degree (MSN or PhD)	163 (16.22)
Post-registration courses (specialist university programmes) *n* (%)	490 (48.76)
Years of work experience *n* (%)	
<10	408 (40.6)
10–20	268 (26.67)
>20	329 (32.74)
Working area *n* (%)	
Emergency	388 (38.61)
General hospital ward	424 (42.19)
Outpatient	91 (9.05)
Territorial Health Services	54 (5.37)
Management/education/research	48 (4.78)
Nursing role *n* (%)	
Clinical	369 (86.47)
Management	262 (26.07)
Relocated after COVID-19 emergency *n* (%)	262 (26.07)
Attending major incidents training *n* (%)	374 (37.21)

4.5 | Multiple logistic regression analysis

Multiple logistic regression (Table 5) showed that after controlling for confounders, the factor of gender was independently associated with anxiety, sleep disorders and self-efficacy. Females were more prone to developing disturbances than males, with low sleep quality OR = 1.36; 95% CI: 1.00–1.86; $p = .04$; moderate/high anxiety levels OR = 1.96; 95% CI: 1.45–2.64; $p < .0001$; and low self-efficacy levels OR = 1.36; 95% CI: 1.04–1.78; $p = .02$.

TABLE 2 Scores of the Pittsburgh Sleep Quality Index (PSQI), Self-Rating Anxiety Scale (SAS) and General Self-Efficacy Scale (GSE) in the total cohort

PSQI median (IQR)	9 (6–11)
PSQI (>5: low quality) *n* (%)	761 (75.72)
PSQI (≤5: good quality) *n* (%)	244 (24.28)
SAS median (IQR)	37 (32–43)
SAS (0–20: very low level) *n* (%)	4 (0.4)
SAS (21–40: low level) *n* (%)	657 (65.37)
SAS (41–60: moderate level) *n* (%)	334 (33.23)
SAS (61–80: high level) *n* (%)	10 (1)
GSE median (IQR)	29 (26–32)
GSE (<29: low level) *n* (%)	509 (50.65)
GSE (>30: high level) *n* (%)	496 (49.35)

TABLE 3 Score correlations measured with Spearman's correlation coefficient

	PSQI	SAS	GSE
PSQI	1	0.408<br><0.0001	−0.134<br><0.0001
SAS	0.408<br><0.0001	1	−0.217<br><0.0001
GSE	−0.134<br><0.0001	−0.217<br><0.0001	1

N = 1005.

TABLE 4 Association between PSQI, SAS and GSE scores and characteristics of the sample (*N* = 1005)

	N	PSQI	SAS	GSE
Gender				
Female	663	9 (6–12)	38 (33–43)	29 (25–32)
Male	342	8 (5–11)	36 (31–40)	30 (26–34)
p-value		**.0042**	**<.0001**	**.0023**
Age				
<33 years	356	8 (6–11)	37 (32–43)	29 (25–31)
34–46 years	323	9 (5–12)	37 (32–44)	30 (26–33)
>46 years	325	8 (6–11)	36 (32–42)	30 (26–34)
p-value		.9014	.071	**.0193**
Working area				
Emergency	388	8 (5–10)	37 (32–43)	30 (26–33)
General hospital	424	8 (6–10)	38 (33–43)	29 (25–32)
Outpatient	91	7 (5–9)	36 (31–42)	29 (25–32)
Territorial Health Services	54	7 (5–11)	37.5 (32–42)	30 (26–34)
Management/education/research	48	6 (3–9)	33.5 (30–39.5)	30 (26–36)
p-value		**.0002**	**.0466**	.464
Years of work experience				
<10	408	9 (6–11)	38 (32–43)	29 (25–32)
10–20	268	8 (5–11)	37 (32–43.5)	30 (26.5–32)
>20	329	8 (6–11)	36 (32–42)	30 (26–34)
p-value		.5807	.196	**.0168**
Attending major incidents training				
Yes	374	9 (6–12)	37 (33–43)	29 (25–32)
No	631	8 (6–11)	37 (32–43)	29 (25–32)
p-value		.4408	.694	.540
Level of education				
First-level degree	842	9 (6–12)	37 (32–43)	29 (26–32)
Second-level degree	163	8 (5–11)	36 (31–43)	30 (26–33)
p-value		.1332	.376	.425
Post-registration courses				
Yes	490	8 (6–12)	37 (32–43)	30 (26–33)
No	515	9 (5–11)	37 (32–43)	29 (25–32)
p-value		.9968	.911	**.0155**
Number of children				
0	500	9 (6–11)	38 (32–43)	29 (26–32)
1	161	9 (6–12)	35 (31–41)	29 (25–33)
2	275	8 (6–11)	36 (32–43)	30 (26–34)
>2	69	8 (5–11)	37 (32–41)	29 (25–33)
p-value		.669	**.0392**	.308
Relocated				
No	743	8 (6–11)	37 (32–43)	29 (26–33)
Yes	262	9 (6–12)	38 (33–44)	29 (26–32)
p-value		.0568	.0535	.684
Nursing role				
Clinical	869	9 (6–11)	37 (32–43)	29 (26–32)
Management	136	8.5 (5.–11.5)	35 (31–41)	30 (26–34)

(Continues)

TABLE 4 (Continued)

	N	PSQI	SAS	GSE
p-value		.4141	**.0045**	.207
Working region				
North	242	9 (6–12)	36 (32–43)	29.5 (25–32)
Centre	373	8 (5–11)	37 (32–43)	29 (26–33)
South and island	390	8 (6–11)	37 (32–43)	29 (26–32)
p-value		.2499	.944	.791
Marital status				
Unmarried	526	9 (6–12)	37 (32–43)	29 (25–32)
Married	479	8 (6–11)	36 (32–42)	30 (26–33)
p-value		.2863	.206	.120
Type of hospital				
Public	823	8 (6–11)	37 (32–43)	30 (26–33)
Private	182	9 (6–11)	37 (32–43)	29 (25–32)
p-value		.7232	.907	.287

Bold values indicate significance ($p < .05$).

Working in the management/education/research area seemed to be a protective factor associated with a lower risk of developing sleep disturbances compared to those working in territorial health services (OR = .40; 95% CI, 0.17–0.96; $p = .042$).

5 | DISCUSSION

The purpose of this study was to measure levels of sleep disorders, anxiety and perceived self-efficacy among Italian nurses working during the COVID-19 pandemic and to evaluate the potential predictive factors associated with them.

The prevalence of anxiety, sleep disorders and low levels of self-efficacy was consistent among Italian nurses providing care to patients with suspected or confirmed COVID-19.

The results showed alarming rates of sleep disturbances (75.72%) and moderate anxiety (33.23%), and 50.65% of the participants reported low general self-efficacy levels in this study. Most of the respondents were female clinical nurses aged 22–65 years, working in a general ward of a public hospital, and they were equally distributed throughout the northern, central and southern regions of Italy.

Gender was an independent predictive factor for anxiety, poor sleep quality and low self-efficacy. Being female was associated with higher levels of anxiety and sleep deprivation, and in addition, working in a territorial health services setting seemed to be an additional risk factor for poor sleep quality. Women experienced generally lower levels of self-efficacy than men.

The scores from the SAS, PSQI and GSE were all correlated with each other, underlining that self-efficacy has a great impact on anxiety and sleep disturbances, with positive or negative effects occurring when the perceived levels of self-efficacy increased or decreased, respectively.

5.1 | Sleep quality

In our sample, being a woman and working on the front lines were associated with poor sleep quality. The median PSQI score of front-line nurses (involved in emergency or general hospital) in this study was 8 (IQR, 5–10 and 6–10, respectively) which was similar to the average of 8.48 ± 3.63 of front-line nurses in Wuhan measured during their COVID-19 outbreak, as reported by Tu et al. (2020). However, the prevalence of poor sleep quality (75.72%) reported in our study is higher than the 60% reported by Tu et al. (2020). One possible explanation for the higher prevalence of sleep disturbance among Italian nurses in comparison with the Wuhan frontline nurses could be related to the period during which we collected data, at the beginning of the COVID-19 outbreak. In fact, although we had information on the experience of China starting to face the pandemic before all other countries, the Italian NHS found itself unprepared, and many front-line workers were forced to fight the virus without adequate PPEs (Cicolini et al., 2020). In addition, nurses' shifts were longer and more exhausting in Italy because of the shortage of nurses and physicians, which was more evident in the Italian context during the emergency situation (Cicolini et al., 2020). This would be expected to result in sleep deprivation (Gillet et al., 2020) that could have a great impact on patient safety and work injuries as largely documented by previous research (Kling et al., 2010; Yuh, 2011).

Also, the fear for personal safety and the risk of passing the infection to relatives, as well as the general lack of knowledge about virus at that time might also result in sleep disturbances, as reported by Su et al. (2007).

Contrary to the results of similar research (Lai et al. 2020; Su et al. 2007), no increasing reports of sleep disturbances were seen in our study among females and nurses who were relocated from their original work area.

5.2 | Anxiety symptoms

The prevalence of moderate anxiety in our study sample was considerable (33.23%) but not higher than in the study by Tuet al. (2020). Working area, female gender, clinical role and number of children (more than two or none) were associated with severe anxiety. This finding is consistent with studies showing that in the general population, the prevalence of anxiety disorders in women is approximately twice as high as in men. Psychosocial contributors (e.g. stressors) have been discussed as possible causes for the higher rates of anxiety in women (Bandelow & Michaelis, 2015). In this emergency health context, anxiety symptoms could be due to fear of contracting the virus, stress at work, direct exposure to COVID-19 patient care, perceived negative feelings, lack of social support (Su et al., 2007) or worries about infecting their children. In a recent qualitative study conducted at Wuhan Union Hospital, most front-line clinical nurses experienced great psychological pressure when caring for COVID-19 patients, and they were prone to fear and anxiety (Tan et al., 2020), thus explaining our results underlining how working area (frontline or territorial health services) and clinical role are associated with anxiety disorders.

5.3 | Self-efficacy

Respondents in our study were divided between those who reported high levels of self-efficacy (49.35%) and those who reported low levels (50.65%), indicating that half of them believed they had inadequate abilities to cope with the added stress factors during the pandemic. Males, older nurses, those with longer term working experience and those who attended post-registration courses experienced higher levels of self-efficacy, helping them in promoting personal performance in the face of adversity (Amini & Noroozi, 2018).

Our results are all in agreement with those of studies in the literature, where males in our study had higher median values of self-efficacy than females (Handiyani et al., 2019), possibly because of their ability to solve problems and find solutions (Imus et al., 2017). Many researchers have suggested that age could influence self-efficacy given that, in general, a mature person has developed better abilities in thinking and performing a task (Molinari & Monserud, 2009; Robbins & Judge, 2017). Working experience (longer service) was also related to better self-efficacy, as supported by evidence (Gloudemans et al., 2013; Lee & Ko, 2010). Bandura (1997) theorised that individuals with a higher level of education (post-registration courses, in this case) will learn more through formal education so that they get more opportunities to learn to cope with problems.

The findings of a recent study suggested that self-efficacy was a significant predictor of mental health (Yıldırım & Güler, 2020). For this reason, new insight into factors associated with this important element in promoting health-related intentions and behaviours is of paramount importance.

TABLE 5 Multiple logistic regression analysis predicting PSQI, SAS and GSE scores

Variable	OR	95%CI	*p*-value
PSQI			
Gender			
Female vs Male	1.36	1.00–1.86	**.04**
Working Area			
Outpatient vs Territorial Health Services	0.92	0.43–1.95	.838
Emergency Care vs Territorial Health Services	1.42	0.74–2.71	.287
General Hospital vs Territorial Health Services	1.48	0.78–2.82	.224
Management/Edu/Res vs Territorial Health Services	0.40	0.17–0.96	**.042**
SAS			
Gender			
Female vs Male	1.96	1.45–2.64	**<.0001**
GSE			
Gender			
Female vs Male	1.36	1.04–1.78	**.0241**

Note:: The variables considered in the multiple logistic models were gender, age, working area, years of work experience, post-registration courses, number of children, reallocation and nursing role.
Bold values indicate significance ($p < .05$).

5.4 | Predicting factors

Multiple logistic regression analysis showed that working in management/education/research areas could be considered a protective predictive factor against developing sleep disturbances. These results could be explained by the fact that this group of nurses is not involved in front line and delivering patient care. Also, as noted, males in our study experienced better self-efficacy levels than women. These findings indicated that, during the pandemic, women were more vulnerable to symptoms of anxiety and sleep disorders, as already demonstrated by previous research (Lai et al., 2020; Luo et al., 2020; Xiao et al., 2020). Also, nurses working in territorial health services were more prone to developing insomnia. In particular, when we correlated the scores of the scales, we observed a strong positive correlation between the SAS and PSQI (0.418; $p < .001$), revealing that when sleep quality was reduced, anxiety levels increased. The concept that anxiety affects sleep quality is confirmed by reports in the literature (Jarrin et al., 2014; Reeth et al., 2000; Xiao et al. 2020), where some researchers have shown that anxious people generally struggle during sleep and experience frequent awakenings during sleep (Alvaro et al., 2013).

We also observed a negative correlation between the GSE and SAS (−0.217; $p < .001$) and between the PSQI and GSE (−0.159; $p < .001$), suggesting that as sleep and anxiety scores decreased, self-efficacy grew, and vice versa ($p < .001$), which means that when nurses have better self-efficacy, they may experience fewer health problems. These data are consistent with that from Hu et al.

(2020). It is also consistent with the concept that self-efficacy is a positive mental state that enhances sleep quality (Rutledge et al., 2013; Xiao et al., 2020) and that anxiety has a negative effect on self-efficacy because it reduces positive behaviours and initiative (Bandura & Adams, 1977; Miller et al., 2006). These findings highlighted the importance of designing interventions specifically targeted towards female nurses facing the COVID-19 pandemic in Italy, who were found to be suffering anxiety and sleep disorders. Also, because working in territorial health services seems to be a resultant risk factor negatively affecting sleep quality, we suggest further investigations to better understand aspects potentially associated with this disturbance. In this regard, a recent review of De Sio et al. (2020) recommended to implement effective preventive measures aimed at fighting anxiety and insomnia (e.g. mindfulness-based strategies, cognitive behavioural counselling, support groups and debriefing sessions) in the workplace. Of note, cases of suicide among healthcare workers are sometimes reported as an extreme reaction to chronic exposure to these risk factors (Goyal et al., 2020; Sher, 2020). A professional team comprising mental health personnel (e.g. psychotherapists, social workers and psychological counsellors) could be a resource both for the general public and healthcare workers, to help them to deal with the emotional distress and other mental disorders caused by epidemics and other public health emergencies (Duan & Zhu, 2020).

5.5 | Limitations

The strength of this study is represented by the large sample size of nurses in Italy during the pandemic, but it also presents some limitations that should be discussed. First, the convenience sample of nurses who agreed to participate could limit the generalisability of the results. Second, because of the cross-sectional design of the study, we could not determine causal relationships, only associations in the analysis of predictors of anxiety, sleep disorders and self-efficacy. Third, the present study used the PSQI to measure sleep quality among nurses, but it should be considered that nurses are usually shift workers, so the results could be overestimated with respect to the worse levels reported.

6 | CONCLUSIONS

In conclusion, our study reported a high prevalence of anxiety, sleep disorders and low self-efficacy levels among Italian nurses. Specific interventions to prevent exacerbation of these symptoms should be considered in order to promote mental well-being and coping strategies among those who take care of patients in emergency situations and are exposed to a high risk of contagion. This requires clear communication and leadership structures, communication training, psychosocial support, identification of individual attributes and organisational resources to build self-efficacy, which will help achieve improvements in the mental health of nurses. More importantly, it will also help with creating an optimal framework of conditions for clinical work, for example, through the correct allocation and management of human resources to reduce nursing work intensity and job pressures. It is also important for managers to address the correct supply of material resources as PPEs to promote safety in clinical settings.

6.1 | Relevance to clinical practice

This study highlights the importance to assess and recognise psychological disturbances and related risk factors among nurses facing major incidents, as COVID-19 pandemic, that are exposed to a high risk to develop high levels of anxiety and sleep disturbance, especially if females, working in territorial health services.

ACKNOWLEDGEMENT

We thank Giuseppe Di Martino for their support in data management.

CONFLICT OF INTEREST

No conflict of interest has been declared by the authors.

AUTHORS' CONTRIBUTIONS

Project design and direction: VS, GC; data collection: AD, RoA, PA, GP, SS, EV, RA, GC, VS; statistical analysis: GG; writing the article: VS, GC; planning and supervision: AD, RoA, PA, GP, SS, RA, EV; all authors discussed the results and commented on the manuscript.

ORCID

Valentina Simonetti https://orcid.org/0000-0002-7185-4850
Angela Durante https://orcid.org/0000-0003-1034-5988
Paola Arcadi https://orcid.org/0000-0003-1050-4707
Giusi Graziano https://orcid.org/0000-0002-3347-8049
Gianluca Pucciarelli https://orcid.org/0000-0001-6915-6802
Silvio Simeone https://orcid.org/0000-0001-9266-0185
Ercole Vellone https://orcid.org/0000-0003-4673-7473
Rosaria Alvaro https://orcid.org/0000-0002-4659-1569
Giancarlo Cicolini https://orcid.org/0000-0002-2736-1792

REFERENCES

Alvaro, P., Roberts, R. M., & Harris, J. K. (2013). A systematic review assessing bidirectionality between sleep disturbances, anxiety, and depression. *Sleep*, *36*(7), 1059–1068. https://doi.org/10.5665/sleep.2810.

Amini, M. T., & Noroozi, R. (2018). Relationship between self-management strategy and self-efficacy among staff of Ardabil disaster and emergency medical management centers. *Health in Emergencies and Disasters Quarterly*, *3*(2), 85–90. https://doi.org/10.29252/nrip.hdq.3.2.85.

Bai, Y., Lin, C. C., Lin, C. Y., Chen, J. Y., Chue, C. M., & Chou, P. (2004). Survey of stress reactions among health care workers involved with the SARS outbreak. *Psychiatric Services*, *55*(9), 1055–1057. https://doi.org/10.1176/appi.ps.55.9.1055.

Bandelow, B., & Michaelis, S. (2015). Epidemiology of anxiety disorders in the 21st century. *Dialogues in Clinical Neuroscience*, *17*, 327.

Bandura, A. (1997). *Self-efficacy: the exercise of control*. W.H. Freeman and Company.

Bandura, A., & Adams, N. E. (1977). Analysis of self-efficacy theory of behaviour change. *Cognitive Therapy and Research*, *1*, 287–310. https://doi.org/10.1007/BF01663995.

Bansal, P., Bingemann, T. A., Greenhawt, M., Mosnaim, G., Nanda, A., Oppenheimer, J., Sharma, H., Stukus, D., & Shaker, M. (2020). Clinician wellness during the COVID-19 pandemic: extraordinary times and unusual challenges for the allergist/immunologist. *The Journal of Allergy and Clinical Immunology in Practice*, *8*(6), 1781–1790.e3. https://doi.org/10.1016/j.jaip.2020.04.001.

Bray, S. R., Graham, J. D., & Saville, P. D. (2015). Self-control training leads to enhanced cardiovascular exercise performance. *Journal of Sports Sciences*, *33*(5), 534–543. https://doi.org/10.1080/02640414.2014.949830.

Buysse, D. J., Reynolds, C. F., Monk, T. H., Berman, S. R., & Kupfer, D. J. (1989). The Pittsburgh Sleep Quality Index: A new instrument for psychiatric practice and research. *Psychiatry Research*, *28*(2), 193–213. https://doi.org/10.1016/0165-1781(89)90047-4.

Callaway, E. (2020). Time to use the p-word? Coronavirus enter dangerous new phase. *Nature*, *579*(12), https://doi.org/10.1038/d41586-020-00551-1.

Center for System Science and Engineering (CSEE) at Johns Hopkins University (JHU) (2020). *2020 Coronavirus COVID-19 Global Cases*. Retrieved from https://gisanddata.maps.arcgis.com/apps/opsdashboard/index.html#/bda7594740fd40299423467b48e9ecf6

Chua, S. E., Cheung, V., Cheung, C., McAlonan, G. M., Wong, J. W. S., Cheung, E. P. T., Chan, M. T. Y., Wong, M. M. C., Tang, S. W., Choy, K. M., Wong, M. K., Chu, C. M., & Tsang, K. W. T. (2004). Psychological effects of the SARS outbreak in Hong Kong on high-risk health care workers. *Canadian Journal of Psychiatry*, *49*(6), 391–393. https://doi.org/10.1177/070674370404900609.

Cicolini, G., Arcadi, P., & Simonetti, V. (2020). Retrieved from http://journalofadvancednursing.blogspot.com/2020/04/covid-19-there-is-no-more-time-alert.html

Cucinotta, D., & Vanelli, M. (2020). WHO Declares COVID-19 a Pandemic. *Acta Biomedica*, *91*(1), 157–160. https://doi.org/10.23750/abm.v91i1.9397.

Curcio, G., Tempesta, D., Scarlata, S., Marzano, C., Moroni, F., Rossini, P. M., Ferrara, M., & De Gennaro, L. (2013). Validity of the Italian version of the Pittsburgh Sleep Quality Index (PSQI). *NeurologicalSciences*, *34*(4), 511–519. https://doi.org/10.1007/s10072-012-1085-y.

De Sio, S., Buomprisco, G., Perri, R., Bruno, G., Mucci, N., Nieto, H. A., Battagliola, T., & Cedrone, F. (2020). Work-related stress risk and preventive measures of mental disorders in the medical environment: an umbrella review European Review for Medical and Pharmacological. *Science*, *24*(2), 821–830. https://doi.org/10.26355/eurrev_202001_20065.

Dong, H., Zhang, Q., Sun, Z., Sang, F., & Xu, Y. (2017). Sleep disturbances among Chinese clinical nurses in general hospitals and its influencing factors. *BMC Psychiatry*, *17*(1), 241. https://doi.org/10.1186/s12888-017-1402-3.

Duan, L., & Zhu, G. (2020). Psychological interventions for people affected by the COVID-19 epidemic. *Lancet Psychiatry*, *7*(4), 300–302. https://doi.org/10.1016/S2215-0366(20)30073-0.

Farber, E. W., Schwartz, J. A., Schaper, P. E., Moonen, D. J., & McDaniel, J. S. (2000). Resilience factors associated with adaptation to HIV disease. *Psychosomatics*, *41*(2), 140–146. https://doi.org/10.1176/appi.psy.41.2.140.

Fernandez, R., Lord, H., Halcomb, E., Moxham, L., Middleton, R., Alananzeh, I., & Ellwood, L. (2020). Implications for COVID-19: a systematic review of nurses' experiences of working in acute care hospital settings during a respiratory pandemic. *International Journal of Nursing Studies*, *8*, 103637. https://doi.org/10.1016/j.ijnurstu.2020.103637. [Epub ahead of print].

Gillet, N., Huyghebaert-Zouaghi, T., Réveillère, C., Colombat, P., & Fouquereau, E. (2020). The effects of job demands on nurses' burnout and presenteeism through sleep quality and relaxation. *Journal of Clinical Nursing*, *29*, 583–592. https://doi.org/10.1111/jocn.15116.

Gloudemans, H. A., Schalk, R. M., & Reynaert, W. (2013). The relationship between critical thinking skills and self-efficacy beliefs in mental health nurses. *Nurse Education Today*, *33*(3), 275–280. https://doi.org/10.1016/j.nedt.2012.05.006.

Goyal, K., Chauhan, P., Chhikara, K., Gupta, P., & Singh, M. P. (2020). Fear of COVID 2019: First suicidal case in India!. *Asian Journal Psychiatry*, *49*, 101989. https://doi.org/10.1016/j.ajp.2020.101989.

Griffiths, P., Ball, J., Bloor, K., Böhning, D., Briggs, J., Dall'Ora, C., Iongh, A. D., Jones, J., Kovacs, C., Maruotti, A., Meredith, P., Prytherch, D., Saucedo, A. R., Redfern, O., Schmidt, P., Sinden, N., & Smith, G. (2018). *Nurse staffing levels, missed vital signs and mortality in hospitals: retrospective longitudinal observational study*. NIHR Journals Library. https://doi.org/10.3310/hsdr06380.

Han, Y., Yuan, Y., Zhang, L., & Fu, Y. (2016). Sleep disorder status of nurses in general hospitals and its influencing factors. *Psychiatria Danubina*, *28*(2), 176–183.

Handiyani, H., Kusumawati, A. S., Karmila, R., Wagiono, A., Silowati, T., Lusiyana, A., & Widyana, R. (2019). Nurses' self-efficacy in Indonesia. *Enfermieria Clinica*, *29*(S2), 252–256. https://doi.org/10.1016/j.enfcli.2019.04.030.

Hu, D., Kong, Y., Li, W., Han, Q., Zhang, X., Zhu, L. X., & He, H. G. (2020). Frontline nurses' burnout, anxiety, depression, and fear statuses and their associated factors during the COVID-19 outbreak in Wuhan, China: A big-scale cross-sectional study. Anxiety, depression, and fear statuses and their associated factors during the COVID-19 outbreak in Wuhan, China: A big-scale cross-sectional study. *The Lancet*, *24*, 100424. https://doi.org/10.2139/ssrn.3566144.

Huang, J. Z., Han, M. F., Luo, T. D., Ren, A. K., & Zhou, X. P. (2020). Mental health survey of 230 medical staff in a tertiary infectious disease hospital for CoViD-19. *Chinese Journal of Industrial Hygiene and Occupational Diseases*, *38*(3), 192–195. https://doi.org/10.3760/cma.j.cn121094-20200219-00063.

Huang, Y., & Zhao, N. (2020). Generalized anxiety disorder, depressive symptoms and sleep quality during COVID-19 outbreak in China: a web-based cross-sectional survey. *Psychiatry Research*, *288*, 112954. https://doi.org/10.1016/j.psychres.2020.112954.

Imus, F. S., Burns, S., & Weglarz, D. M. (2017). Self-efficacy and graduate education in a nurse anesthesia program: a pilot study. *AANA Journal*, *85*(3), 205–216.

Jarrin, D. C., Chen, I. Y., Ivers, H., & Morin, C. M. (2014). The role of vulnerability in stress-related insomnia, social support and coping styles on incidence and persistence of insomnia. *Journal of Sleep Research*, *23*, 681–688. https://doi.org/10.1111/jsr.12172.

Kang, L., Li, Y., Hu, S., Chen, M., Yang, C., Xiang Yang, B., Wang, Y., Hu, J., Lai, J., Ma, X., Chen, J., Guan, L., Wang, G., Ma, H., & Liu, Z. (2020). The mental health of medical workers in Wuhan, China dealing with the 2019 novel coronavirus. *The Lancet Psychiatry*, *7*(3), e14. https://doi.org/10.1016/S2215-0366(20)30047-X.

Kang, L., Ma, S., Chen, M., Yang, Y., Wang, Y., Li, R., Yao, L., Bai, H., Cai, Z., Xiang Yang, B., Hu, S., Zhang, K., Wang, G., Ma, C., & Liu, Z. (2020). Impact on mental health and perceptions of psychological care among medical and nursing staff in Wuhan during the 2019 novel coronavirus disease outbreak: A cross-sectional study. *Brain, Behavioural and Immunity*, *87*, 11–17. https://doi.org/10.1016/j.bbi.2020.03.028.

Kessler, R. C., Berglund, P. A., Coulouvrat, C., Hajak, G., Roth, T., Shahly, V., Shillington, A. C., Stephenson, J. J., & Walsh, J. K. (2011). Insomnia and the performance of US workers: results from the America insomnia survey. *Sleep*, *34*(9), 1161–1171. https://doi.org/10.5665/SLEEP.1230.

Kling, R. N., McLeod, C. B., & Koehoorn, M. (2010). Sleep problems and workplace injuries in Canada. *Sleep*, *33*(5), 611–618. https://doi.org/10.1093/sleep/33.5.611.

Kuppermann, M., Lubeck, D. P., Mazonson, P. D., Patrick, D. L., Stewart, A. L., Buesching, D. P., & Fifer, S. K. (1995). Sleep problems and their correlates in a working population. *Journal of General Internal Medicine*, *10*(1), 25–32. https://doi.org/10.1007/BF02599573.

Lai, J., Ma, S., Wang, Y., Cai, Z., Hu, J., Wei, N., Wu, J., Du, H., Chen, T., Li, R., Tan, H., Kang, L., Yao, L., Huang, M., Wang, H., Wang, G., Liu, Z., & Hu, S. (2020). Factors associated with mental health outcomes among health care workers exposed to Coronavirus Disease 2019. *JAMA Network Open*, *3*(3), e203976. https://doi.org/10.1001/jamanetworkopen.2020.3976.

Lee, A. M., Wong, J. G., McAlonan, G. M., Cheung, V., Cheung, C., Sham, P. C., Chu, C., Wong, P., Tsang, K. W. T., & Chua, S. E. (2007). Stress and psychological distress among SARS survivors 1 year after the outbreak. *Canadian Journal of Psychiatry*, *52*(4), 233–240. https://doi.org/10.1177/070674370705200405.

Lee, T. W., & Ko, Y. K. (2010). Effects of self-efficacy, affectivity and collective efficacy on nursing performance of hospital nurses. *Journal of Advanced Nursing*, *66*(4), 839–848. https://doi.org/10.1111/j.1365-2648.2009.05244.x.

Luo, M., Guo, L., Yu, M., Jiang, W., & Wang, H. (2020). The psychological and mental impact of coronavirus disease 2019 (COVID-19) on medical staff and general public - A systematic review and meta-analysis. *Psychiatry Research*, *291*, 113190. https://doi.org/10.1016/j.psychres.2020.113190.

Maunder, R., Hunter, J., Vincent, L., Bennett, J., Peladeau, N., Leszcz, M., Sadavoy, J., Verhaeghe, L. M., Steinberg, R., & Mazzulli, T. (2003). The immediate psychological and occupational impact of the 2003 SARS outbreak in a teaching hospital. *Canadian Medical Association Journal*, *168*(10), 1245–1251.

Mieda, M., & Sakurai, T. (2013). Orexin (hypocretin) receptor agonists and antagonists for treatment of sleep disorders. Rationale for development and current status. *CNS Drugs*, *27*(2), 83–90. https://doi.org/10.1007/s40263-012-0036-8.

Miller, R. L., Pallant, J. F., & Negri, L. M. (2006). Anxiety and stress in the postpartum: Is there more to postnatal distress than depression? *BMC Psychiatry*, *6*, 12. https://doi.org/10.1186/1471-244X-6-12.

Molinari, D. L., & Monserud, M. (2009). Rural nurse cultural self-efficacy and job satisfaction. *Journal of Transcultural Nursing*, *20*(2), 211–218. https://doi.org/10.1177/1043659608330350.

Morrison, R. M., & Pidgeon, A. (2017). Cultivating resilience and self-control among university students: an experimental study. *Universal Journal of Psychology*, *5*(1), 1–7. https://doi.org/10.13189/ujp.2017.050101.

Nacoti, M., Ciocca, A., Giupponi, A., Brambillasca, P., Lussana, F., Pisano, M., Goisis, G., Bonacina, D., Fazzi, F., Naspro, R., Longhi, L., Cereda, M., & Montaguti, C. (2020). At the epicenter of the Covid-19 pandemic and humanitarian crises in Italy: Changing perspectives on preparation and mitigation. *NJEM Catalyst Innovation in Care Delivery*, *1*(2), 1–5. https://doi.org/10.1056/CAT.20.0080.

Najmi, S. B., Marasi, M. R., Hashemipour, M., Hovsepian, S., & Ghasemi, M. (2013). The perceived self-efficacy and its interrelation with communication in family and glycemic control in adolescents with type 1 diabetes. *Pakistan Journal of Medical Sciences*, *29*(1 Suppl), 334–339. https://doi.org/10.12669/PJMS.291(SUPPL).3528.

Nanda, A., Wasan, A., & Sussman, J. (2017). Provider health and wellness. *The Journal of Allergy and Clinical Immunology in Practice*, *5*(6), 1543–1548. https://doi.org/10.1016/j.jaip.2017.05.025.

Ohayon, M. M., & Paiva, T. (2005). Global sleep dissatisfaction for the assessment of insomnia severity in the general population of Portugal. *Sleep Medicine*, *6*(5), 435–441. https://doi.org/10.1016/j.sleep.2005.03.006.

Reeth, O. V., Weibel, L., Spiegel, K., Leproult, R., Dugovic, C., & Maccari, S. (2000). Interactions between stress and sleep: From basic research to clinical situations. *Sleep Medicine Reviews*, *4*(2), 201–219. https://doi.org/10.1053/smrv.1999.0097.

Remuzzi, A., & Remuzzi, G. (2020). COVID-19 and Italy: what next? *Lancet*, *395*(10231), 1225–1228. https://doi.org/10.1016/S0140-6736(20)30627-9.

Robbins, S. P., & Judge, T. A. (2017). *Organizational behavior*, 17th ed. Pearson Education, Inc., publishing as Prentice Hall.

Rosado, I. V., Russo, G. H., & Maia, E. M. (2015). Produzirsaúdesuscitaadoecimento? As contradições do trabalhoemhospitaispúblicos de urgência e emergência [Generating health elicits illness? The contradictions of work performed in emergency care units of public hospitals]. *Ciencia & Saude Coletiva*, *20*(10), 3021–3032. https://doi.org/10.1590/1413-812320152010.13202014.

Rutledge, C. M., La Guardia, A. C., & Bluestein, D. (2013). Predictors of self-efficacy for sleep in primary care. *Journal of Clinical Nursing*, *22*(9–10), 1254–1261. https://doi.org/10.1111/jocn.12005.

Sasso, L., Bagnasco, A., Zanini, M., Catania, G., Aleo, G., Santullo, A., Spandonaro, F., Icardi, G., Watson, R., & Sermeus, W. (2017). The general results of the RN4CAST survey in Italy. *Journal of Advanced Nursing*, *73*(9), 2028–2030. https://doi.org/10.1111/jan.13066.

Schwarzer, R., & Jerusalem, M. (1995). Generalized Self-Efficacy scale. In I. J. Weinman, S. Wright, & M. Johnston (Eds.), *Measures in health psychology: A user's portfolio. Causal and control beliefs* (pp. 35–37). NFER-NELSON.

Seale, H., Leask, J., Po, K., & MacIntyre, C. R. (2009). "Will they just pack up and leave?" - attitudes and intended behaviour of hospital health care workers during an influenza pandemic. *BMC Health Services Research*, *9*, 30. https://doi.org/10.1186/1472-6963-9-30.

Sheeran, P., Maki, A., Montanaro, E., Avishai-Yitshak, A., Bryan, A., Klein, W. M. P., Miles, E., & Rothman, A. J. (2016). The impact of changing attitudes, norms, and self-efficacy on health-related intentions and behavior: A meta-analysis. *HealthPsychology*, *35*(11), 1178–1188. https://doi.org/10.1037/hea0000387.

Sher, L. (2020). COVID-19, anxiety, sleep disturbances and suicide. *Sleep Medicine*, *70*, 124. https://doi.org/10.1016/j.sleep.2020.04.019.

Sibilia, L., Schwarzer, R., & Jerusalem, M. (1997). *Italian Adaptation of the General Self-Efficacy Scale - Self-Efficacy Generalizzata*. Retrieved from http://userpage.fu-berlin.de/~health/italian.htm.

Spina, S., Marrazzo, F., Migliari, M., Stucchi, R., Sforza, A., & Fumagalli, R. (2020). The response of Milan's Emergency Medical System to the COVID-19 outbreak in Italy. *Lancet*, *395*(10227), e49–e50. https://doi.org/10.1016/S0140-6736(20)30493-1.

Su, T. P., Lien, T. C., Yang, C. Y., Su, Y. L., Wang, J., Tsai, S., & Yin, J. (2007). Prevalence of psychiatric morbidity and psychological adaptation of the nurses in a structured SARS caring unit during outbreak: a prospective and periodic assessment study in Taiwan. *Journal of Psychiatric Research*, *41*(1–2), 119–130. https://doi.org/10.1016/j.jpsychires.2005.12.006.

Tan, R., Yu, T., Luo, K., Teng, F., Liu, Y., Luo, J., & Hu, D. (2020). Experiences of clinical first-line nurses treating patients with COVID-19: A qualitative study. *Journal of Nursing Management*. *28*, 1381–1390.

Tu, Z. H., He, J. W., & Zhou, N. (2020). Sleep quality and mood symptoms in conscripted frontline nurse in Wuhan, China during COVID-19 outbreak: A cross-sectional study. *Medicine (Baltimore)*, *99*(26), e20769. https://doi.org/10.1097/MD.0000000000020769.

von Elm, E., Altman, D. G., Egger, M., Pocock, S. J., Gøtzsche, P. C., Vandenbroucke, J. P., & Initiative, S. T. R. O. B. E. (2008). The Strengthening the Reporting of Observational Studies in Epidemiology (STROBE) statement: guidelines for reporting observational studies. *Journal of Clinical Epidemiology*, *61*(4), 344–349. https://doi.org/10.1016/j.jclinepi.2007.11.008. PMID: 18313558.

World Health Organisation (WHO). (2020). *Novel Coronavirus (2019-nCoV) Situation Reports-62*. Retrieved from https://www.who.int/emergencies/diseases/novel-coronavirus-2019/situation-reports

Xiao, H., Zhang, Y., Kong, D., Li, S., & Yang, N. (2020). The Effects of Social Support on Sleep Quality of Medical Staff Treating Patients with Coronavirus Disease 2019 (COVID-19) in January and February 2020 in China. *Medical Science Monitor: International Medical Journal of Experimental and Clinical Research*, *5*(26), e923549. https://doi.org/10.12659/MSM.923549. Published 2020 Mar 5.

Yıldırım, M., & Güler, A. (2020). COVID-19 severity, self-efficacy, knowledge, preventive behaviors, and mental health in Turkey. *Death Studies*, 1–8. https://doi.org/10.1080/07481187.2020.1793434. [Epub ahead of print].

Yuh, D. D. (2011). Effect of sleep deprivation on patient safety: new awakening or tired argument? *Archives of Surgery*, *146*(9), 1085.

Yuksel, A., Bayrakci, H., & Bahadır-Yilmaz, E. (2019). Self-efficacy, psychological well-being and perceived social support levels in pregnant women. *International Journal of Caring Sciences*, *12*(2), 1–10.

Zung, W. W. (1971). A rating instrument for anxiety disorders. *Psychosomatics*, *12*(6), 371–379. https://doi.org/10.1016/S0033-3182(71)71479-0.

SUPPORTING INFORMATION

Additional supporting information may be found online in the Supporting Information section.

How to cite this article: Simonetti V, Durante A, Ambrosca R, et al. Anxiety, sleep disorders and self-efficacy among nurses during COVID-19 pandemic: A large cross-sectional study. *J Clin Nurs*. 2021;30:1360–1371. https://doi.org/10.1111/jocn.15685
