# Supplementary material for: Mental health status and related factors influencing healthcare workers during the COVID-19 pandemic: A systematic review and meta-analysis
Source: PLoS One. 2024 Jan 19;19(1):e0289454. doi: 10.1371/journal.pone.0289454 (PMC10798549; doi:10.1371/journal.pone.0289454)
Supplement: S1 Data — (ZIP) [file pone.0289454.s011.zip › literatures/17.pdf]

Iran J Psychiatry 2022; 17: 2: 136-143

**Original Article**

# Burnout and Mental Health of COVID-19 Frontline Healthcare Workers: Results from an Online Survey

**Mohammad Babamiri[1], Saeid Bashirian[2], Salman Khazaei[3], Mohammad Sadegh Sohrabi[4], Rashid Heidarimoghadam[1*], Alireza Mortezapoor[4], Sepide Zareian[3]**

**Abstract**

**Objective:** The COVID-19 pandemic has been prolonged and healthcare workers have become exhausted. The purpose of this study was to investigate burnout and its relationship with mental health in COVID-19 frontline healthcare workers.
**Method:** This cross-sectional study was carried out in all hospitals where patients with COVID-19 were admitted in Hamedan, Iran. With the census method and considering the inclusion criteria, 924 COVID-19 frontline healthcare workers participated in this study. Data were collected using a web-based survey consisting of demographic characteristics, GHQ-28, and Maslach Burnout Inventory. Statistical analysis was performed using Stata 14.
**Results:** The results showed that the main cause of concern and stress in employees was that it was not clear how long this situation would continue. The results regarding burnout and mental health showed that 29.33% of participants were high in emotional exhaustion (EE), 10.93% were high in depersonalization (DP), 34.31% were low in personal accomplishment (PA), 50.4% had physical symptoms, 50.2% had anxiety and insomnia symptoms, 62.2% had social dysfunction and 17.5% had depression symptoms. The results of the multivariate logistic regression showed that EE had the greatest role in reducing mental health of employees with OR = 6.92 for moderate EE and OR = 39.42 for high EE ($P < 0.001$).
**Conclusion:** COVID-19 frontline healthcare workers are at risk for burnout and poor mental health. Health policies should be implemented to help reduce burnout in healthcare workers. Also, person-directed and organizational-directed interventions to rejuvenate these employees seem necessary.

**Key words:** *Burnout; COVID-19; Healthcare; Mental Health*

**1.** Department of Ergonomics, Research Center for Health Sciences, School of Public Health, Hamadan University of Medical Sciences, Hamadan, Iran.
**2.** Department of Public Health, School of Health, Social Determinants of Health Research Center, Hamadan University of Medical Sciences, Hamadan, Iran.
**3.** Research Center for Health Sciences, Hamadan University of Medical Sciences, Hamadan, Iran.
**4.** Department of Ergonomics, School of Public Health, Hamadan University of Medical Sciences, Hamadan, Iran.

***Corresponding Author:**
Address: Research Center for Health Sciences and Department of Ergonomics, School of Public Health, Hamadan University of Medical Sciences, Hamadan, Iran, Postal Code: 6517838736.
Tel: 98-81 38380398, Fax: 98-81 38380398, Email: dr_haidari@umsha.ac.ir

**Article Information:**
Received Date: 2020/12/14, Revised Date: 2021/04/28, Accepted Date: 2021/06/09

Copyright © 2022 Tehran University of Medical Sciences. Published by Tehran University of Medical Sciences.
This work is licensed under a Creative Commons Attribution-NonCommercial 4.0 International license (https://creativecommons.org/licenses/by-nc/4.0/). Noncommercial uses of the work are permitted, provided the original work is properly cited

With the outbreak of COVID-19, almost all countries are involved in the fight against this disease. The pandemic has directly affected the medical staff of countries, and healthcare workers are at the frontline of the fight against this virus (COVID-19 frontline healthcare workers) and at the same time are at the highest risk of contracting the virus. COVID-19 can cause multiple short-term stressors for physicians, nurses, and all medical staff (1, 2), and, in the long run and similar to previous cases of infectious diseases such as severe acute respiratory syndrome (SARS), can cause burnout (3, 4).

Studies conducted during the MERS and SARS virus epidemic in this field also indicate existence of stress and tense conditions for medical frontline staff facing the disease and there is need to pay attention to the health of these employees (5-10). Short-term risk factors for frontline staff are multiple and concurrent physical, psychological, emotional and psychological stresses that can cause anxiety, insomnia, increased error rates, decreased ability and fatigue in the short term and burnout, underlying illness, and posttraumatic stress disorder (PTSD) in the long term (11). If stress is not controlled during the outbreak of disease for medical staff, it can hurt the quality of work and psychological health of healthcare workers at the forefront of the fight against COVID-19. Burnout and deterioration of employee health ultimately reduces quality of service and health of patients with the disease (7, 8, 12) and thus creating a vicious cycle that reduces the country’s ability to deal with COVID-19. Research in the medical sector shows that high workload, insufficient support, lack of adequate equipment, and death of patients can cause burnout and eventually mental and physical illness in employees (13-15). Therefore, burnout is one of the first issues that should be considered especially when the disease is continuous and prolonged.

Previous studies reported that symptoms of job burnout appear after people are constantly experiencing anxiety and stress (16, 17) and is defined as a type of exhaustion that makes a person unable to experience good feelings about their job and cannot not understand its value (18). According to Maslach *et al.* (19), burnout has three main dimensions: Emotional Exhaustion (EE), Depersonalization (DP), and Personal Accomplishment (PA). Job burnout also can have many side effects. For example, it has a negative impact on employee mental health (20). Therefore, it can be understood that anxiety can lead to burnout and it can have more serious consequences by affecting mental health. These series of psychological complications, like dominoes, are generated one after another, and each has a significant impact on productivity and job performance as well as increase in intention to leave and absenteeism (21). Therefore, in the context of the outbreak of COVID-19 disease, hospitals are facing a shortage of staff. If, as a result of difficult and complex working conditions, the rate of burnout in employees increases and their mental health is negatively affected, the situation will certainly become more difficult and complex and the possibility of dealing with this disease will suffer. Therefore, it is necessary to examine the current situation of health workers in terms of burnout and mental health.

Therefore, in this study, the rate of burnout and mental health of employees during the outbreak of COVID-19 has been investigated. The main hypotheses of this study are the existence of an effective relationship between burnout and the mental health of employees. Determining the effective dimensions that cause burnout in these conditions is the practical purpose of this study. Investigating these hypotheses can direct efforts of specialists to perform interventions to reduce burnout and increase mental health in COVID-19 frontline healthcare workers.

## Materials and Methods

### *Participants*

This is a cross-sectional study carried out for seven days from May 16 to May 22, 2020. The research plan was approved by the Research Ethics Committee. The collected data were anonymous and treated as confidential. The study population included healthcare workers in all hospitals where patients with COVID-19 were admitted in Hamedan, Iran. No physical or mental illness was considered as the entry criterion. Before starting the questionnaire, individuals were asked if they had any physical or mental illness. Only those who had no mental or physical illness were entered into this study. Questionnaires were designed electronically and sent to individuals using internal intranet systems. In an official letter, participants were asked to participate in the study. Sampling was done by the census method. The total population was 1200 and finally, 924 people answered the research questionnaires (response rate: 77%). The following questionnaires were used in this study:

**GHQ-22:** The General Health Questionnaire measures the following four dimensions with 28 items: physical symptoms, depression, anxiety and insomnia, and social dysfunction. Each subscale has seven questions that are graded by the 4-choice Likert method (from never with a score of zero to always with a score of 4). The range of scores in each subscale is from 0 to 21. A higher score indicates poorer mental health. Nazifi *et al.* showed that GHQ has an appropriate internal consistency for assessing general health. Cronbach's alpha for subscales was between 0.74 and 0.89. The validity of the GHQ was confirmed by Factor analysis (22). In the present study, Cronbach's alpha of the subscales was; physical symptoms (0.71), depression (0.70), anxiety and insomnia (0.76), and social dysfunction (0.82).

### *Maslach Burnout Inventory*

To measure job burnout, the Maslach Burnout Inventory (MBI) was employed. It contains 22 items that are

graded by the seven-choice Likert method (from never with a score of zero to always with a score of six). The questionnaire measures three dimensions of burnout: emotional exhaustion, depersonalization, and personal accomplishment (23). Reliability of MBI with Cronbach's alpha for 3 dimensions greater than 0.7 was reported in the Iranian sample and validity was confirmed by exploratory factor analysis (24). Based on Maslach *et al.* (25) cutoffs for moderate and severe emotional exhaustion being ≥ 17 and ≥ 27, moderate and severe depersonalization being ≥ 7 and ≥ 13, and moderate and severe reduced personal accomplishment being ≤ 38 and ≤ 21.

***Statistical method***

Descriptive statistics were reported as a number (%) for categorical variables and mean (SD) for continuous variables across participant background. The Chi-square test was used to investigate the association between participant demographic variables and their psychological characteristics. Multivariable logistic regression was performed to explore the association of demographic characteristics and categories of burnout (EE, DP, and PA) with the risk of poor mental health. Hosmer and Lemeshow's approach were used for model building and variables with p values less than 0.05 in the crude model were entered into the multivariable model. The results of the logistic regression model were presented with an odds ratio (OR) and 95% confidence interval (CI). All analyses were performed using Stata 14.

***Ethical considerations***

This article presents the results of a project confirmed by the ethics committee of Hamedan University of Medical Sciences with the ethics number of IR.UMSHA.REC.1399.028.

## Results

According to Table 1, most members of the sample are females (57.8%). Based on marital status and age, most participants are single between 31-40 years of age. 49% of the participants had direct contact with COVID-19 patients. Most participants (23%) reported that the fear of being infected with coronavirus was more stressful than anything else.

Based on results in Table 2, 29.33% of participants were high in EE, 10.93% were high in DP and 34.31% were low in PA. Of the total participants, 169 had burnout in two subscales, and 59 had burnout in all three subscales. 50.4% of participants had physical symptoms, 50.2% had anxiety and insomnia symptoms, 62.2% had social dysfunction and 17.5% had depression symptoms.

Results in Table 3 showed that there is a significant relationship between gender and burnout and mental health ($P \leq 0.05$) and the number of women who are exhausted and have low mental health is higher than number of men. There was no significant relationship between age and burnout, but in terms of mental health, the lowest level of mental health was seen in the 20 to 30 years old group ($P \leq 0.05$). In terms of type of job, physicians had the highest rate of burnout and the lowest level of mental health. Participants who had direct contact with a COVID-19 patient were more burned out and were less healthy mentally ($P \leq 0.05$). The relation between marital status and burnout was not significant.

The results of the multivariate logistic regression are shown in Table 4. After adjusting for other variables in the model, females had 2.4 fold higher odds of low mental health compared to men ([OR = 2.40, 95% CI: 1.66-3.43)], $P < 0.001$). Those who had direct contact with COVID-19 patients had 2.74 fold higher odds of low mental health compared to staff that did not have direct contact with COVID-19 patients ([OR = 2.74, 95% CI: 1.93-3.90)], $P < 0.001$). Staff with moderate and high EE had 6.27 and 35.98 fold higher odds of low mental health compared to staff with low EE, respectively ($P < 0.001$). Health staff with moderate and low PA had 1.55 and 1.59 fold higher odds of low mental health compared to staff with high PA ($P < 0.05$).

**Table 1. Demographic Characteristics of Study Participants**

| Variable | Category | No (%) |
|---|---|---|
| Gender | Male | 390 (42.2) |
| | Female | 534 (57.8) |
| Age | 20-30 | 166 (18.00) |
| | 31-40 | 366 (39.6) |
| | 41-50 | 285 (30.8) |
| | 51-60 | 107 (11.6) |
| Marital status | Single | 594 (64.3) |
| | Married | 330 (35.7) |
| Job | Nurse assistant | 80 (8.7) |
| | Nurse | 86 (9.3) |
| | Physician | 76 (8.2) |
| | Service staff | 77 (8.3) |

| | | |
|---|---|---|
| | Other staff | 605 (65.5) |
| Direct contact with a COVID-19 | Yes | 453 (49.00) |
| | No | 471 (51.00) |
| The main cause of stress and anxiety | It is not known how long the disease will last. | 627 (67.8) |
| | Fear of getting sick | 473 (51.1) |
| | Impaired daily activities and reduced social communication | 407 (44) |
| | Lack of facilities and equipment | 531 (57.4) |
| | Increase in workload | 403 (43.6) |
| | Insufficient and vague information about the disease | 211 (22.8) |
| | Non-observance of hygienic principles by colleagues | 126 (13.6) |
| | Non-observance of hygienic principles by others | 589 (63.7) |
| | Financial problems caused by quarantine | 224 (24.2) |
| | Consequences after quarantine | 230 (24.2) |

**Table 2. Frequency of Subjects Based on Burnout and Mental Health during COVID-19 Pandemic among Frontline Healthcare Workers**

| | Variable | Category | No (%) |
|---|---|---|---|
| Burnout | EE* | Low | 388 (41.99) |
| | | Moderate | 265 (28.68) |
| | | High | 271 (29.33) |
| | DP** | Low | 499 (54.00) |
| | | Moderate | 324 (35.05) |
| | | High | 101 (10.93) |
| | PA*** | High | 252 (27.27) |
| | | Moderate | 355 (38.42) |
| | | Low | 317 (34.31) |
| Mental health | Physical symptoms | No | 458 (49.6) |
| | | Low | 268 (29.00) |
| | | Moderate | 146 (15.8) |
| | | High | 52 (5.6) |
| | Anxiety and insomnia | No | 460(49.8) |
| | | Low | 199 (21.5) |
| | | Moderate | 178 (19.3) |
| | | High | 87 (9.4) |
| | Social dysfunction | No | 349 (37.8) |
| | | Low | 371 (40.2) |
| | | Moderate | 163 (17.6) |
| | | High | 41 (4.4) |
| | Depression | No | 762 (82.5) |
| | | Low | 40 (4.3) |
| | | Moderate | 108 (11.7) |
| | | High | 14 (1.5) |

*Emotional Exhaustion, ** Depersonalization, *** Personal Accomplishment

**Table 3. Psychological Characteristics of Research Sample based on Demographic Variables during COVID-19 Pandemic among Frontline Healthcare Workers**

| Variable | Category | Burnout No (%) | Burnout Yes (%) | P-value | Mental Health Healthy (%) | Mental Health Suspected (%) | P-value |
|---|---|---|---|---|---|---|---|
| Gender | Male | 225 (57.69) | 165 (42.31) | 0.000 | 221 (56.67) | 169 (43.33) | 0.000 |
| | Female | 243 (45.51) | 291 (54.49) | | 193 (36.14) | 341 (63.86) | |
| Age | 20-30 | 81 (48.80) | 85 (51.20) | 0.393 | 60 (36.14) | 106 (63.86) | 0.002 |
| | 31-40 | 179 (48.91) | 187 (51.09) | | 151 (41.26) | 215 (58.74) | |
| | 41-50 | 146 (51.23) | 139 (48.77) | | 149 (52.28) | 136 (47.72) | |
| | 51-60 | 62 (57.94) | 45 (42.06) | | 54 (50.47) | 53 (49.53) | |
| Marital status | Single | 297 (50.00) | 297 (50.00) | 0.596 | 274 (46.13) | 320 (53.87) | 0.278 |
| | Married | 171 (51.82) | 159 (48.18) | | 140 (42.42) | 190 (57.58) | |
| Job | Nurse assistant | 50 (62.50) | 30 (37.50) | 0.038 | 34 (42.50) | 46 (57.50) | 0.004 |
| | Nurse | 40 (46.51) | 46 (53.49) | | 33 (38.37) | 53 (61.63) | |
| | Physician | 32 (42.11) | 44 (57.89) | | 23 (30.26) | 53 (69.74) | |
| | Service staff | 46 (59.74) | 31 (40.26) | | 46 (59.74) | 31 (40.26) | |
| | Office staff | 300 (49.59) | 305 (50.41) | | 278 (45.95) | 327 (54.05) | |
| Contact | Yes | 197 (43.49) | 256 (56.51) | 0.000 | 138 (30.46) | 315 (69.54) | 0.000 |
| | No | 271 (57.54) | 200 (42.46) | | 276 (58.60) | 195 (41.40) | |

**Table 4. Multivariable Analysis of Categories of Emotional Exhaustion, Depersonalization and Personal Accomplishment in Association with Low Mental Health during COVID-19 Pandemic among Frontline Healthcare Workers**

| Variable | Category | Crude Model OR (95% CI) | Crude Model P-value | Adjusted Model* OR (95% CI) | Adjusted Model* P-value |
|---|---|---|---|---|---|
| Gender | male | 1 | | 1 | |
| | female | 2.31 (1.77-3.02) | ≤0.001 | 2.40 (1.66-3.43) | ≤0.001 |
| Age | 51-60 | 1 | | | |
| | 20-30 | 1.9 (1.1-3.09) | 0.06 | | |
| | 31-40 | 1.45 (0.94-2.24) | 0.09 | | |
| | 41-50 | 0.93 (0.59-1.45) | 0.75 | | |
| Marital status | Married | 1 | | | |
| | Single | 1.1 (0.81-1.49) | 0.53 | | |
| Job | Nurse assistant | 1 | | | |
| | Nurse | 1.87 (0.64-2.20) | 0.58 | | |
| | Physician | 1.7 (0.88-3.3) | 0.11 | | |
| | Service staff | 0.49 (0.26-0.99) | 0.05 | | |
| | Office staff | 0.86 (0.54-1.39) | 0.56 | | |
| Contact | No | 1 | | 1 | |
| | Yes | 3.23 (2.46-4.24) | ≤0.001 | 2.74 (1.93-3.90) | ≤0.001 |
| EE | Low | 1 | | 1 | |
| | Moderate | 7.75 (5.43-11.05) | ≤0.001 | 6.27 (4.23-9.30) | ≤0.001 |
| | High | 47.56 (28.36-79.76) | ≤0.001 | 35.98 (19.91-65.05) | ≤0.001 |
| DP | Low | 1 | | | |
| | Moderate | 2.74 (2.05-3.66) | ≤0.001 | | |
| | High | 7.96 (4.47-14.2) | ≤0.001 | | |
| PA | High | 1 | | 1 | |
| | Moderate | 2.72 (1.94-3.81) | ≤0.001 | 1.55 (1.00-2.38) | 0.04 |
| | Low | 4.9 (3.43-7.01) | ≤0.001 | 1.59 (1.00-2.54) | 0.04 |

* Adjusted for the variables included to the multivariable model

## Discussion

Due to the increased workload of healthcare providers during the outbreak of the Coronavirus, the rate of burnout and consequently the mental health of these employees have been affected. Therefore, in this study, the rate of burnout and mental health in the COVID-19 frontline healthcare workers was investigated and their relationship with demographic variables was also examined. The highest rates of burnout were in PA (34.31%), EE (29.33%), and DP (10.93%), respectively. 18% of participants had burnout in two subscales, and 6% had burnout in all three subscales.

About burnout, other studies have shown that in the face of this pandemic, the personal accomplishment of the healthcare workers decreases (26). Hu *et al.*, who investigated burnout in front-line nurses in China, concluded that the highest rate of burnout occurred in the personal accomplishment dimension (27). In burnout, personal accomplishment is the tendency to negatively evaluate the worth of one's work, feeling incompetent in performing one's job, and a generalized poor professional self-esteem representing a failure to achieve goals (28, 29). Reduced personal accomplishment is what we expected. Long-term exposure to pandemics, lack of definitive treatment for the disease, and lack of facilities at the height of the outbreak are issues that cause a sense of reduced personal accomplishment in the treating staff. COVID-19 frontline healthcare workers are witnessing deaths of people whose lives cannot have been saved by medical science, and, as a result, their professional self-confidence is declining and they may underestimate the value of their work. It should also be noted that those who work in the healthcare system under quarantine and lock-down conditions must respect social distancing as much as possible, and the result is social isolation in these people. Social isolation reduces degree of social support (30) that is one of the main sources of stress reduction (31). The result of this is reduction in mental health and increase in burnout in healthcare workers. Increased burnout (especially in the form of reduced personal accomplishment) has many implications for healthcare systems. The negative consequences of increased burnout in previous epidemics have also been shown (32). Employees who become exhausted and deem their work worthless or undervalued lose their desire to work overtime, their work efficiency is reduced, and sick leave and absence also increase (32, 33). If the corona pandemic continues, the consequences of burnout will reduce the healthcare system's ability to cope with the disease (34, 35).

In terms of mental health, moderate and high levels of physical symptoms, anxiety, insomnia, social dysfunction, and depression were 21.4%, 28.7%, 22%, and 13.2%, respectively. A meta-analysis on 12 studies that were performed in China and in Singapore found that anxiety, depression, and insomnia prevalence among healthcare workers during the COVID-19 outbreak was 23.2%, 22.8%, and 38.9% (36). Combining these results, we found that the impact of this pandemic on the mental health of healthcare workers was significant. Anxiety and insomnia demonstrated a high rate in participants and it was due to factors such as increased workload during the pandemic, constant contact with COVID-19 patients, sleep disturbance, fatigue, and the possibility of getting an infection (37).

The results of our study confirmed that there is a relationship between burnout and mental health in employees. Based on the results of a study conducted by Eurofound in 2018, it was found that there is a relationship between burnout and having anxiety or depression (38). In addition, the relationship between mental health indicators and burnout in frontline employees has also been shown in the study by Hu *et al.* (27). Also, in a meta-analysis study, researchers concluded that there is a strong relationship between burnout, depression and anxiety (39). Based on logistic regression, it can be said that emotional exhaustion has the greatest role in reducing the mental health of COVID-19 frontline healthcare workers. Previous studies showed that critical emergencies like the COVID-19 pandemic can easily trigger emotional exhaustion (40). Emotional exhaustion is the feeling of being tired and emotionally worn down and occurs in the form of lack of energy and feeling of being overloaded on the job (38) which eventually causes anxiety, depression, and reduced mental health.

These negative consequences become even more important when results showed that the rate of burnout is higher in females than males (41). This result was also seen in mental health and the results showed that the level of mental health in females is lower than in males. Studies conducted on the Iranian population during the COVID-19 outbreak have generally shown that the prevalence of depression and anxiety is higher in females compared to males (42). Therefore, it is not unreasonable to expect this result in women working in the health sector. Women are generally more vulnerable to stress, and if we consider the possibility of getting sick as a chronic stressor, then women's mental health suffers more from this chronic stress. Although in some studies, the rate of burnout was reported equally in females and males (38), but since a large amount of the burden of the healthcare system is on women, it is necessary to consider special interventions to reduce burnout in women (41). The results also showed that a direct relationship with COVID-19 patients was strongly associated with rate of burnout and reduced mental health. Having direct contact with COVID-19 patients greatly increased the risk of developing the disease; therefore, these people are exposed to a severe health threat that can reduce mental health. Providing personal care equipment could reduce the rate of burnout and enhance mental health. The results also showed that although the rate of burnout did not change significantly with age, the rate of mental health decreased. This may

be because the COVID-19 pandemic is more dangerous for older people. This research has conducted at Hamadan University of Medical Sciences because the course of COVID-19 disease and also the possibilities for dealing with this disease are different in different provinces. Thus, we should be careful in generalizing the results.

## Limitation

One of the limitations of the present study was that it was a cross-sectional study and the studied variables were collected as a self-report. Also, due to the focus of this study on COVID-19 frontline healthcare workers, there is a possibility for bias in the results because there is potential for these risk factors to occur in other healthcare settings that directly and indirectly deal with COVID-19 patients.

## Conclusion

Finally, it can be concluded that COVID-19 frontline healthcare workers are at risk of burnout and reduced mental health due to the stress of facing this particular condition. This risk is higher in women who are directly exposed to patients with COVID-19 and in older employees. Therefore, person-directed and organizational-directed interventions (32) and multicomponent intervention programs (43) seem necessary in order to rejuvenate these employees.

## Acknowledgment

The authors of this article would like to thank all the participants in this study. We also thank the Vice Chancellor for Research and Technology of Hamadan University of Medical Sciences for financial support of this research (grant number: 990119150).

## Conflict of Interest

None.

## References

1. Dewey C, Hingle S, Goelz E, Linzer M. Supporting Clinicians During the COVID-19 Pandemic. Ann Intern Med. 2020;172(11):752-3.
2. Santarone K, McKenney M, Elkbuli A. Preserving mental health and resilience in frontline healthcare workers during COVID-19. Am J Emerg Med. 2020;38(7):1530-1.
3. Lee SH, Juang YY, Su YJ, Lee HL, Lin YH, Chao CC. Facing SARS: psychological impacts on SARS team nurses and psychiatric services in a Taiwan general hospital. Gen Hosp Psychiatry. 2005;27(5):352-8.
4. Styra R, Hawryluck L, Robinson S, Kasapinovic S, Fones C, Gold WL. Impact on health care workers employed in high-risk areas during the Toronto SARS outbreak. J Psychosom Res. 2008;64(2):177-83.
5. Chan SS, Leung GM, Tiwari AF, Salili F, Leung SS, Wong DC, et al. The impact of work-related risk on nurses during the SARS outbreak in Hong Kong. Fam Community Health. 2005;28(3):274-87.
6. Chong MY, Wang WC, Hsieh WC, Lee CY, Chiu NM, Yeh WC, et al. Psychological impact of severe acute respiratory syndrome on health workers in a tertiary hospital. Br J Psychiatry. 2004;185:127-33.
7. Khalid I, Khalid TJ, Qabajah MR, Barnard AG, Qushmaq IA. Healthcare Workers Emotions, Perceived Stressors and Coping Strategies During a MERS-CoV Outbreak. Clin Med Res. 2016;14(1):7-14.
8. Kim Y, Seo E, Seo Y, Dee V, Hong E. Effects of Middle East Respiratory Syndrome Coronavirus on post-traumatic stress disorder and burnout among registered nurses in South Korea. Int J Healthc. 2018;4(2):27-33.
9. Maunder R, Hunter J, Vincent L, Bennett J, Peladeau N, Leszcz M, et al. The immediate psychological and occupational impact of the 2003 SARS outbreak in a teaching hospital. Cmaj. 2003;168(10):1245-51.
10. Maunder RG, Lancee WJ, Balderson KE, Bennett JP, Borgundvaag B, Evans S, et al. Long-term psychological and occupational effects of providing hospital healthcare during SARS outbreak. Emerg Infect Dis. 2006;12(12):1924-32.
11. Wu P, Fang Y, Guan Z, Fan B, Kong J, Yao Z, et al. The psychological impact of the SARS epidemic on hospital employees in China: exposure, risk perception, and altruistic acceptance of risk. Can J Psychiatry. 2009;54(5):302-11.
12. Zhu Z, Xu S, Wang H, Liu Z, Wu J, Li G, Miao J, Zhang C, Yang Y, Sun W, Zhu S. COVID-19 in Wuhan: Sociodemographic characteristics and hospital support measures associated with the immediate psychological impact on healthcare workers. EClinicalMedicine. 2020;24:100443.
13. Mirkamali K, Ahmadizad A, Kazemzadeh S, Varmaghani M. Determining the Relationship Between Job Burnout and Employee Productivity. Iran J Ergon. 2019;7(1):37-44.
14. Visser MR, Smets EM, Oort FJ, De Haes HC. Stress, satisfaction and burnout among Dutch medical specialists. Cmaj. 2003;168(3):271-5.
15. Heidarimoghadam R, Saidnia H, Joudaki J, Mohammadi Y, Babamiri M. Does mental workload can lead to musculoskeletal disorders in healthcare office workers? Suggest and investigate a path. Cogent Psychol. 2019;6(1):1-8.
16. Koutsimani P, Montgomery A, Georganta K. The Relationship Between Burnout, Depression, and Anxiety: A Systematic Review and Meta-Analysis. Front Psychol. 2019;10:284.
17. Babamiri M, Siegrist J, Zemestani M. The Factorial Structure and Psychometric Properties

of the Persian Effort-Reward Imbalance Questionnaire. Saf Health Work. 2018;9(3):334-8.
18. Maslach C, Leiter MP. Understanding the burnout experience: recent research and its implications for psychiatry. World Psychiatry. 2016;15(2):103-11.
19. Maslach C, Jackson SE, Leiter MP. MBI: Maslach burnout inventory: CPP, Incorporated Sunnyvale, CA; 1996.
20. Abdi masooleh F, Kaviani H, Khaghanizade M, Momeni Araghi A. The relationship between burnout and mental health among nurses. Tehran Univ Med J. 2007;65(6):65-75.
21. Ghaderi S, Rezagholy P, Tawana H, Nouri B. The Relationship between Occupational Burnout and Intention to Leave in Nurses Working in Training Hospitals in Sanandaj, Iran. J Nursing, Midwifery and Paramedical Faculty. 2019;4(3):25-34.
22. Nazifi M, Mokarami HR, Akbaritabar A, Faraji Kujerdi M, Tabrizi R, Rahi A. Reliability, validity and factor structure of the persian translation of general health questionnire (ghq-28) in hospitals of kerman university of medical sciences. Journal of Fasa University of Medical Sciences. 2014;3(4):336-42.
23. Maslach C, Jackson SE, Leiter MP. MBI: Maslach burnout inventory. Sunnyvale, CA: CPP, Incorporated; 1996.
24. Moalemi S, Kavosi Z, Beygi N, Deghan A, Karimi A, Parvizi MM. Evaluation of the Persian Version of Maslach Burnout Inventory-Human Services Survey among Iranian Nurses: Validity and Reliability. Galen Med J. 2018;7:e995.
25. Maslach C, Jackson S, Leiter M, Zalaquett C, Wood R. Evaluating stress: a book of resources, Vol 2. USA: Scarecrow education; 1998.
26. Giusti EM, Pedroli E, D'Aniello GE, Stramba Badiale C, Pietrabissa G, Manna C, et al. The Psychological Impact of the COVID-19 Outbreak on Health Professionals: A Cross-Sectional Study. Front Psychol. 2020;11:1684.
27. Hu D, Kong Y, Li W, Han Q, Zhang X, Zhu LX, et al. Frontline nurses' burnout, anxiety, depression, and fear statuses and their associated factors during the COVID-19 outbreak in Wuhan, China: A large-scale cross-sectional study. EClinicalMedicine. 2020;24:100424.
28. López-López IM, Gómez-Urquiza JL, Cañadas GR, De la Fuente EI, Albendín-García L, Cañadas-De la Fuente GA. Prevalence of burnout in mental health nurses and related factors: a systematic review and meta-analysis. Int J Ment Health Nurs. 2019;28(5):1032-41.
29. Gulalp B, Karcioglu O, Sari A, Koseoglu Z. Burnout: need help? J Occup Med Toxicol. 2008;3:32.
30. Evans M, Fisher EB. Social Isolation and Mental Health: The Role of Nondirective and Directive Social Support. Community Ment Health J. 2022;58(1):20-40.
31. Ye Z, Yang X, Zeng C, Wang Y, Shen Z, Li X, et al. Resilience, Social Support, and Coping as Mediators between COVID-19-related Stressful Experiences and Acute Stress Disorder among College Students in China. Appl Psychol Health Well Being. 2020;12(4):1074-94.
32. Babamiri M, Alipour N, Heidarimoghadam R. Research on reducing burnout in health care workers in critical situations such as the COVID-19 outbreak. Work. 2020;66(2):379-80.
33. Salvagioni DAJ, Melanda FN, Mesas AE, González AD, Gabani FL, Andrade SM. Physical, psychological and occupational consequences of job burnout: A systematic review of prospective studies. PLoS One. 2017;12(10):e0185781.
34. Liebensteiner MC, Khosravi I, Hirschmann MT, Heuberer PR, Thaler M. Massive cutback in orthopaedic healthcare services due to the COVID-19 pandemic. Knee Surg Sports Traumatol Arthrosc. 2020;28(6):1705-11.
35. Leocani L, Diserens K, Moccia M, Caltagirone C. Disability through COVID-19 pandemic: neurorehabilitation cannot wait. Eur J Neurol. 2020;27(9):e50-e1.
36. Pappa S, Ntella V, Giannakas T, Giannakoulis VG, Papoutsi E, Katsaounou P. Prevalence of depression, anxiety, and insomnia among healthcare workers during the COVID-19 pandemic: A systematic review and meta-analysis. Brain Behav Immun. 2020;88:901-7.
37. Moazzami B, Razavi-Khorasani N, Dooghaie Moghadam A, Farokhi E, Rezaei N. COVID-19 and telemedicine: Immediate action required for maintaining healthcare providers well-being. J Clin Virol. 2020;126:104345.
38. Dinibutun SR. Factors Associated with Burnout Among Physicians: An Evaluation During a Period of COVID-19 Pandemic. J Healthc Leadersh. 2020;12:85-94.
39. Koutsimani P, Montgomery A, Georganta K. The Relationship Between Burnout, Depression, and Anxiety: A Systematic Review and Meta-Analysis. Front Psychol. 2019;10:284.
40. Kim JS, Choi JS. Factors Influencing Emergency Nurses' Burnout During an Outbreak of Middle East Respiratory Syndrome Coronavirus in Korea. Asian Nurs Res (Korean Soc Nurs Sci). 2016;10(4):295-9.
41. Templeton K, Bernstein CA, Sukhera J, Nora LM, Newman C, Burstin H, et al. Gender-based differences in burnout: Issues faced by women physicians. NAM Perspect. 2019;5(1):1-16.
42. Shahriarirad R, Erfani A, Ranjbar K, Bazrafshan A, Mirahmadizadeh A. The mental health impact of COVID-19 outbreak: a Nationwide Survey in Iran. Int J Ment Health Syst. 2021;15(1):19.
43. Heidarimoghadam R, Mohammadfam I, Babamiri M, Soltanian AR, Khotanlou H, Sohrabi MS. What do the different ergonomic interventions accomplish in the workplace? A systematic review. Int J Occup Saf Ergon. 2022;28(1):600-24.
