# Supplementary material for: Mental health status and related factors influencing healthcare workers during the COVID-19 pandemic: A systematic review and meta-analysis
Source: PLoS One. 2024 Jan 19;19(1):e0289454. doi: 10.1371/journal.pone.0289454 (PMC10798549; doi:10.1371/journal.pone.0289454)
Supplement: S1 Data — (ZIP) [file pone.0289454.s011.zip › literatures/170.pdf]

Since January 2020 Elsevier has created a COVID-19 resource centre with free information in English and Mandarin on the novel coronavirus COVID-19. The COVID-19 resource centre is hosted on Elsevier Connect, the company's public news and information website.

Elsevier hereby grants permission to make all its COVID-19-related research that is available on the COVID-19 resource centre - including this research content - immediately available in PubMed Central and other publicly funded repositories, such as the WHO COVID database with rights for unrestricted research re-use and analyses in any form or by any means with acknowledgement of the original source. These permissions are granted for free by Elsevier for as long as the COVID-19 resource centre remains active.

Asian Journal of Psychiatry 61 (2021) 102685

Contents lists available at ScienceDirect

Asian Journal of Psychiatry

journal homepage: www.elsevier.com/locate/ajp

Short communication

Mental health issues and coping among health care workers during COVID19 pandemic: Indian perspective

Jawahar Singh [a], Mamta Sood [b,*], Rakesh K. Chadda [b], Vishwajeet Singh [c], Dheeraj Kattula [d]

[a] Ex Senior Resident, Department of Psychiatry, All India Institute of Medical Sciences, Ansari Nagar, New Delhi, 110029, India
[b] Professor, Department of Psychiatry, All India Institute of Medical Sciences, Ansari Nagar, New Delhi, 110029, India
[c] Scientist III (Biostatistics), Department of Geriatric Medicine, All India Institute of Medical Sciences, Ansari Nagar, New Delhi, 110029, India
[d] Senior Resident, Department of Psychiatry, All India Institute of Medical Sciences, Ansari Nagar, New Delhi, 110029, India

ARTICLE INFO

Keywords:
Health care workers
COVID 19
Depression
Somatic symptoms
Anxiety
Coping

ABSTRACT

Objectives: We assessed mental health issues among Indian health care workers (HCWs) and their coping strategies during COVID 19 pandemic.
Methodology: An online survey was conducted in 348 HCWs using PHQ-SADS and Brief-COPE inventory.
Results: Depression, anxiety and somatic symptoms were present in 54 %, 44.3 % and 54.6 % of HCWs and were more in those working in COVID19 areas. The nurses and female HCWs had more moderate to severe somatic symptoms. HCWs with moderate to severe symptoms used simultaneously both maladaptive and adaptive coping strategies.
Conclusion: The HCWs during COVID19 pandemic have significant mental health issues and use multiple coping strategies.

1. Introduction

Healthcare workers (HCWs) including doctors, nursing professionals, and support staff have played a crucial role in the COVID 19 pandemic. They are directly involved in the care of patients and are at increased risk of exposure to COVID19. Inadequate protection from high contagion virus may add to the risk of infection (Herron et al., 2020).

Mental health issues like depression, insomnia, anxiety and stress among HCWs have been reported (Lai et al., 2020; Kang et al., 2020; Zhang et al., 2020; Huang et al., 2020; Spoorthy et al., 2020) in multiple studies. HCWs are not only worried about their safety but also are worried about transmitting the infection to their family members. These can cause significant impairment in the quality and productivity of work.

A number of studies from India have reported increased prevalence of psychiatric symptoms like anxiety, depression, stress and insomnia in HCWs (Selvaraj et al., 2020; Mathur et al., 2020; Suryavanshi et al., 2020; Tomar et al., 2020; Parthasarathy et al., 2021) and have studied their relationship with quality of life (Suryavanshi et al., 2020; Tomar et al., 2020). Guo et al. (2010) have highlighted association of maladaptive coping with poor mental health outcomes in general population. However, there is lack of information about coping strategies used by HCWs to deal with psychiatric problems during the COVID19 pandemic. Therefore, we planned to address this knowledge gap by assessing mental health issues among HCWs and their coping during COIVD19 pandemic.

2. Methodology

The present study was conducted on health care workers working in the hospitals providing COVID19 care in the national capital region of India. The study was cross-sectional. We decided to do an online survey as it was not possible to meet the HCWs in person due to rapid surge in COVID19 cases. Online survey form using google forms was designed in English. Online survey was conducted from 16th July to 5th August 2020 almost 4 months after the pandemic started in India.

The survey had four sections: consent form, sociodemographic performa, patient health questionnaire: somatic, anxiety, and depressive symptom scales (PHQ-SADS) and brief COPE. Consent form had participant information sheet and consent form. Basic sociodemographic data including age, sex, occupation, marital status and living status were collected. PHQ-SADS (Kroenke et al., 2010) is a

* Corresponding author.
E-mail addresses: drjawaharaiims@gmail.com (J. Singh), soodmamta@gmail.com (M. Sood), drrakeshchadda@gmail.com (R.K. Chadda), vishu.bhu1@gmail.com (V. Singh), askdheeraj@gmail.com (D. Kattula).

https://doi.org/10.1016/j.ajp.2021.102685
Received 17 January 2021; Received in revised form 23 April 2021; Accepted 6 May 2021
Available online 14 May 2021
1876-2018/© 2021 Elsevier B.V. All rights reserved.

self-reported instrument and includes PHQ-9, GAD-7, and PHQ-15 to assess depression, anxiety and somatic symptoms respectively. The scores on PHQ-9 are categorized as minimal/no depression (0–4), mild depression (5–9), moderate depression (10–14), or severe depression (15–21). On GAD-7 scores are categorized as minimal/no anxiety (0–4), mild anxiety (5–9), moderate anxiety (10–14), or severe anxiety (15–21). The PHQ-15 scores are categorized as minimal/no somatic symptoms (0–4), mild somatic symptoms (5–9), moderate somatic symptoms (10–14), or severe somatic symptoms (15–21). The final item on each scale is the respondent's global rating of symptom-related difficulty. Cut-off scores of 5, 10, and 15 or above indicate increasing levels of severity from mild, moderate and severe on all three scales. Brief-COPE is abbreviated version of the COPE (Coping Orientation to Problems Experienced) Inventory and has 28 items that measure 14 factors of 2 items on a 4-point Likert scale ranging from 0 to 3 (Carver, 1997). Coping strategies have been classified as adaptive (active coping, planning, emotional support, instrumental support, positive reframing, religion, humour, and acceptance) and maladaptive (venting, denial, substance use, self-blame, behavioural disengagement, and self-distraction) (Meyer, 2001).

Approval of the institute ethic committee (IEC-663/03.07.2020, RP-40/2020) was obtained. The link of survey was sent to HCWs through social media or emails. Each participant of the survey provided electronic informed consent before participation. Confidentiality of participants was ensured during and after the data collection.

Assuming prevalence of depression to be 12.3 %, anxiety to be 13 % and 1.6 % for somatization as in study from China (Zhang et al., 2020), at 95 % confidence interval and 5% margin of error we get a sample size requirement of 165, 174 and 25 respectively. Our study included 348 participants. Depression, somatic symptoms, and anxiety were seen in two meaningful categories of 'normal and mild' vs 'moderate and severe'. This was also done to avoid losing the effect due to multiple categories with small sample entries. A p-value of less than 0.05 was considered as statistically significant. Statistical software, STATA/SE version 14.2 (StataCorp LP, College Station, TX, USA), was used for the analysis.

3. Results

Three hundred and forty-eight health care workers completed the survey, among them 242 were doctors and 106 were nurses. Table 1 details their socio-demographic characteristics.

About half (n = 188, 54.02 %) of the HCWs had depression; 107 (30.75 %) had mild, 45(12.93 %) moderate and 36(10.34 %) severe depression. One hundred and fifty-four subjects had anxiety (44.25 %); 89(25.97 %) had mild, 34(9.77 %) had moderate and 31(8.91 %) had severe anxiety. One hundred and ninety subjects had somatic symptoms (54.60 %); 118 (33.91 %) had mild, 50 (14.37 %) had moderate and 22 (6.32 %) had severe somatic symptoms. Those working in COVID areas had significantly more moderate to severe depression, anxiety and somatic symptoms compared to those working in non-COVID areas. Nurses had significantly more moderate to severe somatic symptoms as compared to doctors. Females had significantly more moderate to severe somatic symptoms compared to males (Table 2).

HCWs with moderate to severe depression, anxiety and somatic symptoms used all the maladaptive coping strategies like self-destruction, denial, substance use, behaviour disengagement, venting and self-blame They also used multiple adaptive coping strategies, these differed between the groups. Instrumental support and planning were used by all the HCWs. However, those with moderate to severe depression used additionally humour, those with anxiety used emotional support whereas those with somatic symptoms used acceptance and religion (Table 3).

Table 1
Socio-demographic Characteristics of Health Care Workers.

Demographic variable	Health care workers N (%)	Doctors N (%)	Nurses N(%)
Number	348	242 (69.5)	106 (30.5)
**Age** (years) Mean (±SD)	31.8 (7.1)	31.15 (6.1)	33.2 (8.9)
**Sex**			
Male	194 (55.8)	150 (62)	44 (41.5)
Female	154 (44.3)	92 (38)	62 (58.5)
**Marital status**			
Married	185 (53.2)	114 (47.1)	71 (67)
Unmarried	159 (45.7)	125 (51.7)	34 (32.1)
Divorced/separated	04 (1.2)	03 (1.2)	01 (0.9)
**Living status**			
Alone	113 (32.5)	100 (41.3)	13 (12.3)
With family/Co-workers	235 (67.5)	142 (58.7)	93 (87.7)
**Working area**			
COVID	236 (67.8)	173 (71.5)	63 (59.4)
Non-COVID	112 (32.2)	69 (28.5)	43 (40.6)
**Setting of work**			
Govt	302 (86.8)	200 (82.6)	102 (96.2)
Private	46 (13.2)	42 (17.4)	04 (3.8)

4. Discussion

In a cross-sectional online survey conducted on 348 HCWs, using PHQ-SADS, we found depression, anxiety and somatic symptoms to be 54 %, 54.6 % and 44.3 % in them. The level of anxiety and depression reported are in line with those reported by Selvaraj et al. (2020); Suryavanshi et al. (2020); Lai et al. (2020) and Kang et al. (2020). However, our results are in variance with those reported by Parthasarathy et al. (2021); Huang et al. (2020) and Zhang et al. (2020). Zang et al. had reported somatization to be 1.6 % as assessed on symptom checklist 90 R. These differences could be due to differences in the populations chosen, their working conditions, and resources available for dealing with COVID 19 and time of assessment. As the time passed, there was greater workload with increasing number of COVID19 cases. Working in COVID19 designated areas was significantly a higher risk for moderate to severe depression, somatic symptoms and anxiety symptoms. This could be attributed to high risk of COVID19 exposure, associated fear of being contagious to others, apprehension and stress; similar findings reported in earlier studies. (Zhang et al., 2020; Lai et al., 2020). It highlights the need for HCWs at frontline of COVID19 patients care to receive special attention and support. Somatic symptoms were experienced significantly more by female HCWs. This is in line with the extant literature (Kroenke and Spitzer, 1998; Zhang et al., 2020). Preponderance of females amongst the nurses may also explain significantly higher somatic symptoms in them.

HCWs with moderate to severe depression, somatic symptoms and anxiety used significantly more maladaptive coping strategies. It was interesting to note the simultaneous use of adaptive coping strategies along with maladaptive coping strategies. Use of maladaptive coping skills could have perpetuated or worsened the mental health conditions but the use of adaptive coping strategies at the same time also helped them to continue functioning adequately. Probably these adaptive coping strategies safeguarded the HCWs while working during such stressful situations (Babore et al., 2020). Assessment of coping strategies can be a good guide for specific interventions to boost adaptive coping and lower maladaptive coping strategies, thus imparting skills to HCWs for dealing with mental health issues (Kang et al., 2020).

This study has a few limitations. It was conducted in the national capital region of India; the findings may not be generalizable for the entire country due to difference in available resources and number of cases. There may be a selection bias that HCWs with poor knowledge of English language and those with severe problems may not have participated.

5. Conclusion

HCWs experienced significant mental health issues and they used

Table 2
Depression, Anxiety and Somatic symptoms in Health Care Workers.

Severity of Symptom	Occupation No. (%)		OR (95 % CI)	Sex No. (%)		OR (95 % CI)	Work area No. (%)		OR (95 % CI)	Living status No. (%)		OR (95 % CI)
	Doctor	Nurses		Male	Female		Non-COVID	COVID		Workers/ Family	Alone	
**Depression severity (on PHQ9)**												
Normal-Mild	186 (76.9)	81 (76.4)	1.03 (0.60−1.76)	150 (77.3)	117 (75.97)	1.08 (0.65−1.78)	99 (88.4)	168 (71.2)	3.08 (1.62−5.86)	186 (79.2)	81 (71.7)	0.66 (0.39−1.11)
Moderate-Severe	56 (23.1)	25 (23.6)		44 (22.7)	37 (24.03)		13 (11.6)	68 (28.8)		49 (20.8)	32 (28.3)	
**Somatic symptoms severity (on PHQ15)**												
Normal-Mild	199 (82.2)	77 (72.6)	1.74 (1.02−2.99)	168 (86.6)	108 (70.13)	2.75 (1.61−4.71)	98 (87.5)	178 (75.4)	2.28 (1.21−4.29)	186 (79.2)	54 (47.8)	1.03 (0.59−1.79)
Moderate-Severe	43 (17.8)	29 (27.9)		26 (13.4)	46 (29.87)		14 (12.5)	58 (24.6)		49 (20.8)	36 (31.9)	
**Anxiety symptoms severity (on GAD7)**												
Normal-Mild	193 (79.8)	90 (84.9)	0.70 (0.38−1.30)	163 (84)	120 (77.9)	1.49 (0.87−2.56)	100 (89.3)	183 (77.5)	2.41 (1.23−4.72)	196 (83.4)	87 (77)	0.66 (0.38−1.16)
Moderate-Severe	49 (20.3)	16 (15.1)		31 (16)	34 (22.1)		12 (10.7)	53 (22.5)		39 (16.6)	26 (23)	

(*p < 0.05, **p < 0.01), OR-Odds Ratio, CI-Confidence Interval, COVID-Corona Virus Disease, PHQ-Patient Health Questionnaire, GAD-Generalised Anxiety Disorder.

Table 3
Association between Coping Strategies and Depression, Anxiety and Somatic Symptoms (Moderate to Severe Symptoms).

Variable	Depressive symptoms		Student T-test (P value)	Somatic symptoms		Student T-test (P value)	Anxiety symptoms		Student T-test (P value)
Coping strategies	No Mean (±SD)	Yes Mean (±SD)		No Mean (±SD)	Yes Mean (±SD)		No Mean (±SD)	Yes Mean (±SD)	
Self-destruction (maladaptive)	4.2 (1.6)	5.1 (1.5)	<0.001**	4.3 (1.6)	5 (1.7)	<0.001**	4.2 (1.6)	5.4 (1.6)	<0.001**
Active coping (adaptive)	4.6 (1.7)	4.9 (1.6)	0.15	4.6 (1.7)	5 (1.6)	0.09	4.6 (1.7)	4.9 (1.7)	0.19
Denial (maladaptive)	2.7 (1.8)	3.3 (1.5)	<0.001**	2.7 (1.2)	3.3 (1.5)	<0.001**	2.7 (1.2)	3.4 (1.6)	<0.001**
Substance use (maladaptive)	2.4 (0.9)	3.03 (1.8)	<0.001**	2.5 (1.1)	2.7 (1.6)	0.18	2.4 (0.95)	3.1 (1.9)	<0.001**
Emotional support (adaptive)	4.4 (1.8)	4.72 (1.7)	0.16	4.4 (1.8)	4.8 (1.7)	0.06	4.4 (1.7)	5.1 (1.9)	<0.001**
Behavioural disengagement (maladaptive)	3 (1.5)	4.29 (1.6)	<0.001**	3.1 (1.5)	4.18 (1.6)	<0.001**	3 (1.5)	4.5 (1.6)	<0.001**
Venting (maladaptive)	3.5 (1.4)	4.87 (1.5)	<0.001**	3.6 (1.5)	4.68 (1.6)	<0.001**	3.6 (1.5)	4.9 (1.5)	<0.001**
Instrumental support (adaptive)	4 (1.7)	4.67 (1.8)	<0.001**	4 (1.7)	4.63 (1.9)	<0.001**	4 (1.7)	4.9 (1.9)	<0.001**
Positive reframing	4.6 (1.8)	4.64 (1.6)	0.73	4.5 (1.7)	4.79 (1.8)	0.25	4.5 (1.8)	4.9 (1.6)	0.15
Self-blame (maladaptive)	2.7 (1.1)	4.59 (1.9)	<0.001**	2.9 (1.4)	4.04 (1.9)	<0.001**	2.7 (1.1)	4.9 (1.9)	<0.001**
Planning (adaptive)	4.5 (1.7)	5.06 (1.6)	0.01*	4.5 (1.8)	5.09 (1.6)	0.01*	4.5 (1.7)	5.2 (1.6)	<0.001**
Humour (adaptive)	3.3 (1.5)	3.7 (1.8)	0.02*	3.4 (1.6)	3.43 (1.6)	0.76	3.3 (1.6)	3.6 (1.8)	0.1
Acceptance (adaptive)	5.5 (1.8)	5.7 (1.7)	0.30	5.4 (1.8)	6 (1.7)	0.01*	5.5 (1.8)	5.6 (1.7)	0.7
Religion (adaptive)	4.1 (2)	4.1 (2)	0.94	4 (1.9)	4.7 (2.1)	<0.001**	4.1 (1.9)	4.2 (2.1)	0.8

(*p < 0.05, **p < 0.01), SD-Standard deviation.

both maladaptive and adaptive coping strategies to deal with mental health issues. Our findings suggest that intervention package for HCWs aimed at handling mental health issues should include strategies to enhance coping skills.

Role of funding source

Nil.

Declaration of Competing Interest

The authors report no declarations of interest.

Acknowledgments

Nil.

References

Babore, A., Lombardi, L., Viceconti, M.L., Pignataro, S., Marino, V., Crudele, M., Candelori, C., Bramanti, S.M., Trumello, C., 2020. Psychological effects of the COVID-2019 pandemic: perceived stress and coping strategies among healthcare professionals. Psychiatry Res. 293 (November), 113366.

Carver, C.S., 1997. You want to measure coping but your protocol too long: consider the brief cope. Int. J. Behav. Med. 4, 92–100.

Guo, J., Feng, X.L., Wang, X.H., van IJzendoorn, M.H., 2010. Coping with COVID-19: exposure to COVID-19 and negative impact on livelihood predict elevated mental health problems in Chinese adults. Int. J. Environ. Res. Public Health 17 (11), 3857.

Herron, J.B.T., Hay-David, A.G.C., Gilliam, A.D., Brennan, P.A., 2020. Personal protective equipment and Covid 19-a risk to healthcare staff? Br. J. Oral Maxillofac. Surg. 58, 500–502.

Huang, C., Wang, Y., Li, X., Ren, L., Zhao, J., Hu, Y., Zhang, L., Fan, G., Xu, J., Gu, X., Cheng, Z., 2020. Clinical features of patients infected with 2019 novel coronavirus in Wuhan, China. Lancet 395, 497–506, 2020.

Kang, L., Li, Y., Hu, S., Chen, M., Yang, C., Yang, B.X., Wang, Y., Hu, J., Lai, J., Ma, X., Chen, J., 2020. The mental health of medical workers in Wuhan, China dealing with the 2019 novel coronavirus. Lancet Psychiatry 7 (3), e14.

Kroenke, K., Spitzer, R.L., 1998. Gender differences in the reporting of physical and somatoform symptoms. Psychosom. Med. 60, 150–155.

Kroenke, K., Spitzer, R.L., Williams, J.B., Löwe, B., 2010. The patient health questionnaire somatic, anxiety, and depressive symptom scales: a systematic review. Gen. Hosp. Psych. 32, 345–359.

Lai, J., Ma, S., Wang, Y., Cai, Z., Hu, J., Wei, N., Wu, J., Du, H., Chen, T., Li, R., Tan, H., 2020. Factors associated with mental health outcomes among health care workers exposed to coronavirus disease 2019. JAMA Netw. Open 3 (3), e203976.

Meyer, B., 2001. Coping with severe mental illness: relations of the Brief COPE with symptoms, functioning, and well-being. J. Psychopathol. Behav. Assess. 23, 265–277.

Parthasarathy, R., Jaisurya, T.S., Thennarasu, K., Murthy, P., 2021. Mental health issues among health care workers during the COVID-19 pandemic - A study from India. Asian J. Psychiatr. 58, 102626.

Selvaraj, P., Muthukanagaraj, P., Saluja, B., Jeyaraman, M., Anudeep, T.C., Gulati, A., et al., 2020. Psychological impact of COVID-19 pandemic on health-care professionals in India – A multicentric cross-sectional study. Indian. J. Med. Sci. https://doi.org/10.25259/IJMS_193_2020.

Spoorthy, M.S., Pratapa, S.K., Mahant, S., 2020. Mental health problems faced by healthcare workers due to the COVID-19 pandemic–A review. Asian J. Psychiatr. 51, 102119.

Suryavanshi, N., Kadam, A., Dhumal, G., Nimkar, S., Maye, V., Gupta, A., Cox, S.R., Gupte, N., 2020. Mental health and quality of life among healthcare professionals during the COVID-19 pandemic in India. Brain Behav. 10, e01837.

Tomar, B.S., Suman, S., Singh, P., Raj, P., Nathiya, D., 2020. Mental health outcome and professional quality of life among healthcare workers during COVID-19 pandemic: a frontline-COVID survey. Hamdan. Med. J. 13, 196–202.

Zhang, W.R., Wang, K., Yin, L., Zhao, W.F., Xue, Q., Peng, M., Min, B.Q., Tian, Q., Leng, H.X., Du, J.L., Chang, H., 2020. Mental health and psychosocial problems of medical health workers during the COVID-19 epidemic in China. Psychother. Psychosom. 89, 242–250.
