# Supplementary material for: Mental health status and related factors influencing healthcare workers during the COVID-19 pandemic: A systematic review and meta-analysis
Source: PLoS One. 2024 Jan 19;19(1):e0289454. doi: 10.1371/journal.pone.0289454 (PMC10798549; doi:10.1371/journal.pone.0289454)
Supplement: S1 Data — (ZIP) [file pone.0289454.s011.zip › literatures/171.pdf]

Risk Management and Healthcare Policy

Dovepress
open access to scientific and medical research

Open Access Full Text Article

ORIGINAL RESEARCH

Determinants of Mental Health and Practice Behaviors of General Practitioners During COVID-19 Pandemic in Bali, Indonesia: A Cross-sectional Study

Firman Parulian Sitanggang[1]
Gede Benny Setia Wirawan[2]
I Md Ady Wirawan[3]
Cokorda Bagus Jaya Lesmana[4]
Pande Putu Januraga[2]

[1]Department of Radiology, Faculty of Medicine, Udayana University/Sanglah General Hospital, Denpasar, Bali, Indonesia; [2]Center for Public Health Innovation, Faculty of Medicine, Udayana University, Denpasar, Bali, Indonesia; [3]Department of Public Health and Preventive Medicine, Faculty of Medicine, Udayana University, Denpasar, Bali, Indonesia; [4]Department of Psychiatry, Faculty of Medicine, Udayana University/Sanglah General Hospital, Denpasar, Bali, Indonesia

Purpose: We aim to study the level of mental health distress and COVID-19 prevention in practice behaviors among general practitioners (GPs) in Bali, Indonesia, as well as their determinants.

Methods: We conducted a cross-sectional online survey. Survey recruitment material was disseminated by purposive snowballing through regional professional association as well as research team's personal acquaintances. The survey measured mental health status by DASS-21 questionnaire and practice behavior by a questionnaire based on WHO recommendations for hand hygiene and PPE use during the COVID-19 pandemic. We conducted multivariate analyses to identify independent determinants for mental health and practice behavior.

Results: Analyses included 635 (41.75%) of GPs in Bali. Mental health status was relatively good with prevalence of depression, anxiety, and stress of 13.2%, 19.7%, and 11% respectively, lower than previous studies in Indonesia and elsewhere. Practice behavior, however, was not considerably lower with only 65.4% and 32.1% reported consistent hand hygiene and recommended PPE use respectively. Routine optional PPE use was reported by 23.6% of respondents. Long working hours and fear of COVID-19 was identified as detrimental to mental health while consistent hand hygiene improved it. Meanwhile, workplace, work setting, and fear of COVID-19, were identified as determinants for PPE use. GPs working in primary health centers and private hospitals were also found to have less adherence to hand hygiene protocols.

Conclusion: Our results showed relatively good mental health status along with inadequate infection prevention in practice behavior of GPs in Bali, Indonesia. Intervention should be made to improve practice behavior. Determinants of practice behavior identified in this study could help to pinpoint intervention targets.

Keywords: COVID-19, mental health, prevention behavior, health-care workers, general practitioners, Indonesia

Introduction

The COVID-19 pandemic has put a special strain on health-care systems worldwide. In the early stages of the pandemic in China, the health-care system was near to collapse due to high hospitalization rate. During the peak of the outbreak in Wuhan, China, 24.5 out of 10,000 adults were hospitalized, with nearly half of them in a critical condition. Moreover, around 2.6 out of 10,000 adults needed critical care every day.[1]

Correspondence: Gede Benny Setia Wirawan
Gedung PS IKM, Fakultas Kedokteran Universitas Udayana, Jl. PB Sudirman, Denpasar, Bali, 80234, Indonesia
Email benny.wirawan007@gmail.com

© 2021 Sitanggang et al. This work is published and licensed by Dove Medical Press Limited. The full terms of this license are available at https://www.dovepress.com/terms.php and incorporate the Creative Commons Attribution – Non Commercial (unported, v3.0) License (http://creativecommons.org/licenses/by-nc/3.0/). By accessing the work you hereby accept the Terms. Non-commercial uses of the work are permitted without any further permission from Dove Medical Press Limited, provided the work is properly attributed. For permission for commercial use of this work, please see paragraphs 4.2 and 5 of our Terms (https://www.dovepress.com/terms.php).

A similar situation also developed in Indonesia, although much less visible due to noted lack of transparency in Indonesian pandemic-related data.[2] Moreover, the currently available data may also be an underestimation as regional variation of health care availability affect detection rate.[3] However, even with lack of a clear picture of the situation, experts still noted the potential shortcomings of the Indonesian health-care system. A forecast of the situation in one Indonesian province predicted a potential overcapacity of the health-care system within months into the pandemic if it went unmitigated.[4]

The strain it put on the health-care system has caused larger workload for health-care workers. This includes general practitioners (GPs), in Indonesia, who worked in various settings in the health-care system, ranging from private primary care practice to hospital emergency rooms, taking a role as gatekeeper to the health-care system.[5] Increased workload due to the COVID-19 pandemic, compounded by the required changes to workplace safety, such as personal protective equipment (PPE) usage, may cause mental distress to health-care workers.[6–8] At the same time, there has been evidence where mental health and compliance to recommended COVID-19 preventive behavior was associated with each other, creating a feedback loop.[8,9]

Behavioral changes require discipline, which is precluded by good mental health. At the same time, rapid changes may potentially cause mental distress to health-care workers.[8] It may lead to a potential feedback loop between health-care workers' mental health and preventive behavior adherence, as well as with other various other factors that may affect both outcomes.[9]

As the situation surrounding COVID-19 continued to develop, new information and data points would be necessary to keep us updated on the current situation in the field. Thus, this research is aimed at updating the health-care community and policymakers on determinants of mental health and preventive behaviors to better increase compliance and better protect the gatekeepers to our health-care system.

Methods

Study Settings

Data collection of this study took place in July 1 to 14, 2020. During the same period, COVID-19 cases in Indonesia rose from 57,770 cases to 78,572 with average of around 1300 new cases each day. In Bali, during the same period, cases rose from 1520 to 2346 with average of 70 new cases daily.[10]

There was no publicly available data that tracked health-care capacity and hospital occupancy during the pandemic. However, available 2019 reports from Indonesian health ministry estimated hospital bed availability of 1.18 beds per 1000 residents nationwide. Regional data in Bali was slightly better with 1.59 beds per 1000 residents.[11]

Of health-care workers, there were 51,398 actively practicing GPs in Indonesia, 1521 practiced medicine in Bali. In the Indonesian context, GPs are not limited to practicing medicine in primary health-care facilities. Instead, they regularly worked in various health-care institutions from private practice to clinics, and hospitals. GPs, and physicians in general, are also allowed to work in up to three institutions.[5] In hospitals, they worked in various settings from emergency rooms, inpatient wards, to outpatient facilities. Available 2019 data showed that from 1521 GPs in Bali, 462 worked in public health centers (ie, primary health centers) and 831 worked in hospitals, both public and private.[11] As previously noted, these numbers do not exclude the possibility of GPs who worked in multiple institutions.

Study Design and Data Collection

We conducted a cross-sectional survey online to learn about mental health and practice behavior of GPs during the COVID-19 pandemic. Data was collected by online form developed on the KoBo Toolbox platform in the period July 1to 14, 2020. Respondent recruitment was conducted by nonprobability purposive snowball sampling with recruitment material disseminated through GPs' professional association regional branches, the Indonesian Medical Association (Ikatan Dokter Indonesia/IDI), and personal messages to GPs known to the research team. Respondents were encouraged to forward the message and recruit more respondents in their community. Inclusion criteria for analysis was GPs who practiced medicine in Bali, Indonesia during the survey period and completed the online survey.

Variables and Measurement

The online survey form collected data on demographic, work experience, workplace and work setting, COVID-19 knowledge, fear of COVID-19, mental health, and practice behavior. Knowledge was measured using a questionnaire we developed based on the WHO Europe COVID-19

survey tool and guidelines[12] with a score range from 0 to 26. Fear of COVID-19 was measured using an instrument developed by Ahorsu et al[13] consisting of seven questions with a score range from 0 to 28.

Mental health of GPs was measured using standard Depression, Anxiety, and Stress Scale (DASS-21). DASS-21 has previously been translated into an Indonesian version.[14] Validation and reliability analysis showed the measurement was reliable and valid in an Indonesian sample.[15] It has three distinct subtest scores for depression, anxiety, and stress. Score range for each scale was 0 to 42. Further, respondents were classified based on each scale with cutoff point of ≥10 for depression, ≥8 for anxiety, and ≥15 for stress.[16] In our data, internal reliability (Cronbach α) value for depression, anxiety, and stress subscales of DASS-21 was 0.853, 0.797, and 0.868, respectively. Cronbach α value for overall questionnaire was 0.934.

Practice behavior was measured by three different scales, each to measure adherence to hand hygiene guidelines, adherence to recommended PPE use, and use of optional PPE. Hand hygiene guidelines were based on recommended "Five Moment" hand hygiene promoted by WHO since before the COVID-19 pandemic,[17] which consisted of five items measuring adherence to each hand hygiene moment. Recommended PPE use was based on WHO recommendation for rational PPE use which consisted of four items: medical mask, goggles, medical gown, and medical gloves.[18] Meanwhile, an optional PPE list was based on our observation of PPE used by health-care workers in Bali, Indonesia except those included in the recommended list. Optional PPE items in the survey included head cap, apron, boots, and hazmat suits.

Each item for practice behavior was measured with a four-item Likert scale ranging from "never", "sometimes", "often", to "always", corresponding for score of 0 to 3 respectively. As such, score range for hand hygiene was 0 to 15, and for each PPE use measures, both recommended and optional, was 0 to 12. Furthermore, each practice behavior measure was classified as consistent if its score averaged 3, or answering "always" for all item, and classified as routine if its score averaged <3 but ≥2.

Data Analysis

We conducted multivariate binomial logistic regression to identify independent determinants of mental health status as well as practice behavior adherence. Categorical variables for depression, anxiety, and stress were established as dependent variables for mental health. Meanwhile, consistency of hand hygiene practice and recommended PPE use, as well as routine optional PPE use, were established as dependent variables for practice behavior. All analyses were conducted on IBM SPSS 23.0.

Ethical Consideration

Respondents were given an electronic informed consent form at the landing page of the online survey form. To participate, respondents had to answer in the affirmative to giving their consent. As incentive for participation, respondents were given a souvenir worth IDR 100,000. Respondents could retract their consent by discontinuing the online survey before completion. The method of this study was reviewed and approved by Udayana University Faculty of Medicine/Sanglah General Hospital Ethical Committee with letter no. 1403/UN14.2.2.VII.14/LT/2020.

Results

We received 687 responses. From these, 35 were ineligible, one refused to complete the survey, and 16 were duplicates. We analyzed responses from the remaining 635 respondents who are GPs working in Bali, Indonesia with characteristics detailed in Table 1. Among them, 45.5% were male and median age was 30 years old (IQR 27–34). Median working experience as a GP was five years (IQR 2–10) with the majority (46.1%) working in public hospitals with median working hours of 36 h (IQR 18–43.50) per week. There was even distribution among other workplaces, which included private practices, private clinics, as well as private hospitals. There were also GPs working in primary health centers, a public facility serving both community preventive and individual curative health services. By work setting, most reported working in emergency rooms (40.6%) or outpatient clinics (39.5%). A minority of 8.3% reported working in inpatient wards and 11.5% reported working in other settings (eg, clinical laboratory, rotation system, etc).

On COVID-19, the majority of respondents reported having had COVID-19 patients in their practice and average COVID-19 knowledge score was adequate with median of 22 (IQR 20–24) out of maximum 26. Meanwhile the average respondent was moderately afraid of COVID-19 with a median value for fear of COVID-19 score of 9 (IQR 6–13).

Table 1 Sociodemographic Characteristics of Respondents

Variables	Total (n=635)	Male (n=288)	Female (n=347)
**Age (years)**			
Mean (±SD)	32.3 (±7.7)	32.7 (±8.6)	31.9 (±6.8)
Median (IQR)	30.0 (27.0–34.0)	30.0 (27.5–34.0)	30.0 (37.0–34.0)
**Professional experience (years)**			
Mean (±SD)	7.2 (±6.9)	7.6 (±7.6)	6.9 (±6.3)
Median (IQR)	5.0 (2.0–10.0)	5.0 (2.5–10.0)	5.0 (2.0–10.0)
**Workplace, n (%)**			
Private practice	67 (10.6)	38 (13.2)	29 (8.4)
Private clinic	99 (15.6)	34 (11.8)	65 (18.7)
Primary health center	78 (12.3)	30 (10.4)	48 (13.8)
Private hospital	98 (15.4)	35 (15.6)	53 (15.3)
Public hospital	293 (46.1)	141 (49.0)	152 (143.8)
**Work setting, n (%)**			
Emergency rooms	258 (40.6)	125 (43.4)	133 (38.3)
Outpatient clinics	251 (39.5)	102 (35.4)	149 (42.9)
Inpatient wards	53 (8.3)	23 (8.0)	30 (8.6)
Others (eg, laboratories)	73 (11.5)	38 (13.2)	35 (10.1)
**Weekly work hours (hours)**			
Mean (±SD)	33.5 (±19.2)	34.7 (±18.8)	32.6 (±19.5)
Median (IQR)	36.0 (18.0–43.5)	36.0 (20.0–47.5)	36.0 (16.0–42.0)
**Fear of COVID-19 score (range 0–28)**			
Mean (±SD)	9.0 (±4.9)	8.1 (±4.8)	9.8 (±4.9)
Median (IQR)	9.0 (6.0–13.0)	8 (4.5–11.0)	10.0 (7.0–13.5)
**COVID-19 knowledge (range 0–26)**			
Mean (±SD)	21.37 (±2.96)	21.1 (±3.1)	21.6 (±2.8)
Median (IQR)	22.0 (20.0–24.0)	22.0 (19.0–23.5)	22.0 (20.0–24.0)
**Have you ever had COVID-19 patient?**			
No	174 (27.4)	78 (27.1)	96 (27.7)
Yes	461 (72.6)	210 (72.9)	251 (72.3)

Mental health characteristics visible in Table 2, based on DASS-21 questionnaire, showed the average respondent was in good mental health condition. From maximum score of 42, median score for depression, anxiety, and stress among respondents was 2 (IQR 0–6), 2 (IQR 0–6), and 6 (IQR 2–10) respectively. Proportion of depression, anxiety, and stress were 13.2%, 19.7%, and 11% respectively, which indicates overall good mental health among respondents.

On health behavior, visible in Table 2, adherence to hand hygiene protocol was found to be relatively high with median score of 15 (IQR 14–15) in a 0 to 15 scale. Adherence to recommended PPE use (medical mask, goggle/face shield, disposable gown, and medical gloves) was also relatively high with median score of 11 (IQR 9–12) in a scale of 0 to 12. However, as a maximum score was necessary to qualify with adequate adherence on both parameters, only 65.4% and 32.1% of respondents were classified as adequately in compliance to hand hygiene and PPE-use protocols, respectively. At the same time, all respondents reported use of at least one optional PPE (eg, head cap, apron, boots, and, hazmat suit) with a median score of 6 (IQR 4–7) and 23.6% were classified as routine users of optional PPE.

More detail on practice behavior, as visible in Table 3, adherence to "Five Moments" hand hygiene guidelines was in the range of 73.5% to 94.8%. The least adhered to hand hygiene moment was before touching

Table 2 Mental Health and Preventive Behaviors of Respondents

Variables	Total (n=635)	Male (n=288)	Female (n=347)
**Mental health (DASS-21), n (%)**			
Depression	84 (13.2)	31 (10.8)	53 (15.3)
Anxiety	125 (19.7)	50 (17.4)	25 (21.6)
Stress	70 (11.0)	31 (10.8)	39 (11.2)
**Hand hygiene, n (%)**			
Consistent	415 (65.4)	183 (63.5)	232 (66.9)
Inconsistent	220 (34.6)	105 (36.5)	115 (33.1)
**PPE-use adherence, n (%)**			
Consistent	204 (32.1)	78 (27.1)	126 (36.3)
Inconsistent	431 (67.9)	210 (72.9)	221 (63.7)
**Extra PPE-use, n (%)**			
Routine	150 (23.6)	66 (22.9)	84 (24.2)
Not routine	485 (76.4)	222 (77.1)	263 (75.8)

a patient, only consistently adhered to by 73.5% of respondents, while the most adhered to was after exposure to bodily fluid, consistently adhered to by 94.8% of respondents.

Adherence to recommended PPE use was as low as 49% for medical gown usage. However, nearly all respondents reported to consistently wearing medical mask when practicing medicine, adhering to guidelines. Most respondents, 81.9% of them, also reported consistently wearing head cap or hairnet, an optional PPE. Other optional PPE, such as apron, boots, and hazmat suits, was not as routinely or consistently used.

On determinants of mental health (Table 4), working hours and fear of COVID-19 were consistently found as independent determinants of depression, anxiety, and stress on multivariate analyses. More weekly working hours and more intense fear of COVID-19 incrementally increased risk for depression, anxiety, and stress. Consistent hand hygiene behavior was also found as an independent protective factor for anxiety and depression.

Multivariate analyses for determinants of preventive behavior (Table 5) identified workplace and work setting as consistent independent factors for hand hygiene and recommended PPE use. Workplace was associated as independent determinant for hand hygiene with GPs working in a primary health center and private hospital were found less likely to practice consistent hand hygiene behavior. At the same time, GPs working in a private hospital were more likely to use optional PPE.

Meanwhile, various factors were independently associated with recommended PPE use, which include sex, work setting, and fear of COVID-19. Female GPs were found more likely to consistently wear recommended PPE. Working in an emergency room was also associated with consistent recommended PPE use, compared to other work

Table 3 Detail on Practice Behaviors Characteristics of Respondents

	Never	Sometimes	Often	Always
**Handwashing**				
Before touching patient	3 (0.5)	33 (5.2)	132 (20.8)	467 (73.5)
Before performing procedure	1 (0.2)	8 (1.3)	91 (14.3)	535 (84.3)
After exposed to bodily fluids	5 (0.8)	5 (0.8)	23 (3.6)	602 (94.8)
After touching patients	1 (0.2)	3 (0.5)	44 (6.9)	587 (92.4)
After touching medical equipment	1 (0.2)	9 (1.4)	98 (15.4)	527 (83.0)
**Recommended PPE**				
Goggle/face shield	5 (0.8)	41 (6.5)	158 (24.9)	431 (67.9)
Medical mask	0 (0.0)	1 (0.2)	8 (1.3)	626 (98.6)
Medical gown	44 (6.9)	92 (14.5)	188 (29.6)	311 (49.0)
Medical gloves	14 (2.2)	87 (13.7)	195 (30.7)	339 (53.4)
**Optional PPE**				
Head cap/hairnet	5 (0.8)	26 (4.1)	84 (13.2)	520 (81.9)
Apron	178 (28.0)	244 (38.4)	116 (18.3)	97 (15.3)
Boots	225 (35.4)	280 (44.1)	72 (11.3)	58 (9.1)
Hazmat suit	177 (27.9)	287 (45.2)	92 (14.5)	79 (12.4)

Table 4 Multivariate Analyses for Determinants of Mental Health

Variables	OR (95%CI)		
	Depression	Anxiety	Stress
Female sex	1.24 (0.73–2.11)	1.03 (0.65–1.61)	0.71 (0.40–1.26)
Age (1 year increment)	1.07 (0.95–1.20)	1.01 (0.92–1.12)	0.98 (0.87–1.11)
Work experience (1 year increment)	0.93 (0.82–1.06)	0.96 (0.86–1.07)	1.00 (0.87–1.13)
**Workplace**			
Public hospital	1	1	1
Private practice	0.98 (0.31–3.09)	1.11 (0.41–2.98)	0.92 (0.26–3.22)
Private clinic	1.10 (0.42–2.89)	0.91 (0.40–2.06)	1.01 (0.36–2.79)
Primary health center	0.53 (0.19–1.49)	0.72 (0.32–1.64)	0.56 (0.19–1.65)
Private hospital	1.74 (0.89–3.41)	0.94 (0.51–1.74)	1.05 (0.49–2.23)
**Work setting**			
Emergency room	1	1	1
Outpatient clinic	1.21 (0.56–2.62)	0.93 (0.48–1.79)	1.88 (0.82–4.32)
Inpatient ward	0.52 (0.18–1.54)	0.34 (0.13–0.90)**	0.57 (0.17–1.89)
Others	1.25 (0.49–3.20)	0.64 (0.27–1.52)	1.15 (0.38–3.52)
Work hour (1 hour increment)	1.02 (1.01–1.04)**	1.01 (1.00–1.03)*	1.02 (1.00–1.04)*
Fear of COVID-19 score (1 point increment)	1.25 (1.18–1.33)**	1.24 (1.17–1.30)**	1.27 (1.18–1.36)**
COVID-19 knowledge (1 point increment)	1.08 (0.98–1.18)	1.06 (0.98–1.15)	1.16 (1.05–1.29)
Have had COVID-19 patient	1.60 (0.78–3.25)	1.57 (0.86–2.84)	1.75 (0.80–3.80)
Consistent hand hygiene	0.60 (0.35–1.03)	0.58 (0.37–0.92)*	0.52 (0.29–0.92)*
Consistent PPE-use	0.74 (0.42–1.31)	0.75 (0.46–1.22)	1.02 (0.55–1.88)

Notes: **p*<0.05; ***p*<0.01.

settings. Likelihood to consistently wear recommended PPE was also associated with more intense fear of COVID-19.

Discussion

Our results showed that GPs in Bali, Indonesia, had overall good mental health condition and practice behavior. Prevalence of depression, anxiety, and stress among our respondents was 13.2%, 19.7%, and 11.0%, respectively. At the same time, reported adherence to hand hygiene and PPE use in practice was relatively high although only a 65.4% and 32.1% of respondents were classified consistently adhering to hand hygiene and recommended PPE use recommendation, respectively. However, 23.6% respondents also reported routine use of additional PPE, such as head cap, apron, boots, and hazmat suit.

The data also identified different determinants for mental health and COVID-19 preventive behavior among GPs in Bali, Indonesia. A pattern emerges where mental health was negatively affected by long working hours and intense fear of COVID-19, itself a mental state.

Workplace and work setting seems to be the main determinant for hand hygiene and PPE use. GPs working in emergency rooms and with intense fear of COVID-19 are more likely to consistently wear recommended PPE, indicating risk of exposure, or perceived risk, seems to be a factor in practice behavior. Although recommended PPE use was mostly affected by work setting, GPs in private hospital were found more likely to use additional optional PPE. Moreover, GPs working in primary health centers and private hospitals were less likely to report consistent hand hygiene behavior.

Prevalence of mental health problems reported in this study is lower compared to previous studies in various settings. It is compared to previous reports from other countries reported in a previous systematic review.[6] Similarly, another meta-analysis reported pooled prevalence

Table 5 Multivariate Analyses for Determinants of COVID-19 Preventive Behavior

Variables	OR (95%CI)		
	**Hand Hygiene**	**Recommended PPE**	**Optional PPE**
Female sex	1.22 (0.87–1.73)	1.54 (1.07–2.22)*	1.13 (0.76–1.67)
Age (1 year increment)	1.04 (0.96–1.12)	1.01 (0.93–1.09)	1.06 (0.97–1.16)
Work experience (1 year increment)	0.97 (0.89–1.05)	1.01 (0.92–1.10)	0.94 (0.85–1.03)
**Workplace**			
Public hospital	1	1	1
Private practice	1.19 (0.58–2.47)	0.97 (0.43–2.20)	0.51 (0.19–1.32)
Private clinic	0.86 (0.46–1.61)	1.30 (0.66–2.57)	0.70 (0.32–1.52)
Primary health center	0.38 (0.21–0.68)**	1.11 (0.59–2.09)	1.27 (0.66–2.45)
Private hospital	0.56 (0.34–0.92)*	1.61 (0.98–2.65)	1.95 (1.17–3.23)*
**Work setting**			
Emergency room	1	1	1
Outpatient clinic	0.75 (0.45–1.22)	0.37 (0.22–0.64)**	0.73 (0.42–1.27)
Inpatient ward	0.94 (0.47–1.88)	0.40 (0.19–0.81)**	0.46 (0.21–1.03)
Others	0.64 (0.36–1.16)	0.24 (0.11–0.49)**	0.86 (0.44–1.65)
Work hour (1 hour increment)	1.00 (0.99–1.01)	0.99 (0.98–1.00)	1.01 (0.99–1.02)
Fear of COVID-19 score (1 point increment)	1.01 (0.98–1.05)	1.06 (1.02–1.11)**	0.98 (0.94–1.03)
COVID-19 knowledge (1 point increment)	1.03 (0.97–1.09)	0.96 (0.90–1.02)	1.03 (0.97–1.11)
Have had COVID-19 patient	0.87 (0.56–1.35)	1.39 (0.86–2.24)	1.24 (0.73–2.09)
Depression	0.92 (0.48–1.77)	0.76 (0.38–1.53)	0.99 (0.48–2.05)
Anxiety	0.68 (0.40–1.18)	0.74 (0.41–1.32)	1.29 (0.71–2.35)
Stress	0.76 (0.38–1.51)	1.40 (0.68–2.90)	0.90 (0.41–1.97)

Notes: *p<0.05; **p<0.01.

of 22.8% for depression and 23.2% for anxiety, higher than our current findings.[19] However, it should be noted that few of included studies in these reviews used DASS-21 as a mental health measurement tool. The only two included studies utilizing DASS-21 reported widely different prevalence of depression and anxiety, ranging from 8.9% to 55.9% for depression and 14.5% to 67.5% for anxiety.[6,19]

Similar studies from Indonesia, which generally included all health-care workers, reported prevalence of depression ranging from 22.8% to 52.1%[20,21] while reported prevalence of anxiety ranged from 28.1% to 57.6%.[20–22] No studies reported prevalence of stress. However, some studies reported prevalence of burnout (26.8%)[21] and insomniac symptoms (47.9%).[20] All reported prevalence was higher than our findings.

Geography and timing seem to factor in the difference of reported prevalence between our study and previous results. Closer reading of included studies in previous systematic reviews showed that earlier studies, with data collected earlier in the pandemic, reported higher prevalence of mental health problems.[6,19] Meanwhile, previous studies in Indonesia were conducted in Java[21,22] and Borneo[20] which experienced a heavier burden of COVID-19 cases during the time of their respective surveys compared to that experienced in Bali during the course of ours. These studies also use nonprobability online-survey-based data collection method which imply comparability with our results.

Population difference could also played a role as our studies included GPs specifically while other studies included all health-care workers, including specialist physicians, nurses, and others.[19,21,22] A study comparing mental health between physicians, nurses, and dentists previously reported lower level of anxiety for

physicians,[23] which may help explain the low level of mental health problems found in our survey which only included GPs.

Risk factors for adverse mental health condition were similar to previously known risk factors. Working longer hours has been known as risk factors for depression, anxiety, and stress.[24,25] Long working hours has also been attributed to burnout syndrome reported by health-care workers.[26] Meanwhile, the protective effect of consistent hand hygiene can be attributed to perceived control. Being able to control one's risk, such as by practicing good preventive behavior, have been associated with better mental health.[27]

Basic protective measure for COVID-19 transmission was hand hygiene which has been previously disseminated as the "Five Moments" protocol, mandating hand-washing in circumstances before and after health-care workers physically touched a patient's body, bodily fluid, or physical surroundings.[18] Our data showed adequate adherence based on scoring with 65.4% classified as consistently practicing good hand hygiene. While high, it was lower compared to other studies which reported around 95% compliance to hand hygiene guidelines.[28,29] In fact, this level of compliance matched pre-COVID-19 pandemic level of compliance reported in another study.[29]

Additional protective measures were PPE use, recommended by WHO, which included goggles, medical masks, gown, and medical gloves.[18] This recommendation is supported by previous reviews based on the SARS-CoV-2 mode of transmission and routinely available PPEs.[30] Usage of recommended PPE based on our data averaged around 75–95% with medical masks being the most consistently used while less than half reported consistent use of medical gowns.

Similar to hand hygiene practice, however, despite high most respondents routinely wear recommended PPE, with only a minority classified as consistent in their use. This is much lower compared to other similar surveys in other settings which reported around 80% adherence to recommended use of gloves, medical masks, goggles, and gowns.[31] Another later study reported an even higher level of adherence, averaging at 90%.[32]

Factors affecting usage of recommended PPE are similar, however. A previous study reported different working stations showed different levels of adherence for PPE use, especially for previously nonroutine PPE such as goggles and gowns.[31] Another study reported difference between those working in COVID-19 wards and non-COVID-19 wards.[32] Findings that more consistent recommended PPE use was reported among GPs working in emergency rooms may be attributed to higher perceived risk of exposure in that work setting. This is supported by the finding that fear of COVID-19 was another independent determinant of PPE use. While this is true,[33] it was not a justification to loosen our protective behavior.

Gender was also a factor affecting recommended PPE use adherence with female GPs found more likely to consistently wear recommended PPE. This is in line with various other findings. Although rarely observed affecting PPE use among health-care workers, females in the general population have repeatedly beenfound to have better adherence to COVID-19 preventive behaviors.[34–36] This difference has been attributed to higher perceived risk and fear of COVID-19 observed among women.[34]

This study identified weak parts of our effort to protect frontline health-care workers, especially GPs. Inconsistent adherence to recommended PPE use should be addressed appropriately, beginning with further study to investigate the reason behind the low adherence. Our findings of work settings, which reported lower adherence should allow policy makers to specifically target their intervention to the places that needed it the most. Similar interventions should also be conducted to address low adherence to hand hygiene guidelines in some workplaces.

The strength of this study is the sample size. We managed to collect data from over 600 GPs in Bali, Indonesia. This is a considerable number as according to Ministry of Health report of 2019, there was a total of 1521 GPs in said province.[11] Thus, our respondents represent 41.75% of GPs in the province. This study is also one of few to specifically study GPs during the COVID-19 pandemic.

At the same time, our inclusion criteria which was limited to GPs means generalization of our results would be narrow. Adherence to safety protocol should be promoted by a "culture of safety".[37] As GPs usually work in teams with other health-care workers, their inclusion would be important to assess "culture of safety" in their respective workplaces. Meanwhile, determinants of mental health problems in this study were not exhaustive and there could be non-COVID-19 mental health determinants that may affect mental health of our respondents. Employment of the nonprobability snowball sampling method may also limit generalizability of our data, although this is somewhat offset by the sheer proportion of the sample compared to the target population.

Conclusion

Our study revealed low level of adverse mental health conditions among GPs in Bali, Indonesia, mostly associated with working hours and fear of COVID-19. At the same time, we identified issues in adherence to COVID-19 prevention in practice behaviors of GPs. There was a moderate level of adherence to hand hygiene guidelines and relatively low adherence to recommended PPE use. Workplace, work setting, and fear of COVID-19 were found as determinants for PPE use. These findings infer the need of further intervention to improve adherence to COVID-19 prevention, especially PPE use, among GPs.

Acknowledgments

We would like to acknowledge the contribution of our colleague in various Balinese regional chapters of Indonesian Medical Association in helping to disseminate the survey recruitment materials of this study.

Disclosure

The authors report no conflicts of interest in this work.

References

1. Li R, Rivers C, Tan Q, Murray MB, Toner E, Lipsitch M. The demand for inpatient and ICU beds for COVID-19 in the US: lessons from Chinese cities. *medRxiv*. 2020.
2. Januraga PP, Harjana NPA. Improving public access to COVID-19 pandemic data in Indonesia for better public health response. *Front Public Health*. 2020;8(November):8–11.
3. Wirawan GBS, Januraga PP. Correlation of demographic, health care availability, and COVID-19 outcome: Indonesian Ecological Study. *Front Public Health*. 2021;9(605290).
4. Wirawan A, Januraga PP. Forecasting COVID-19 transmission and healthcare capacity in Bali, Indonesia. *J Prev Med Public Health*. 2020;53(3):158–163. doi:10.3961/jpmph.20.152
5. Mahendradhata Y, Trisnantoro L, Listyadewi S, et al. *The Republic of Indonesia Health System Review*. Hort K, Patcharanarumol W, editors. New Delhi: World Health Organization; 2017. Available from: https://apps.who.int/iris/handle/10665/254716. Accessed May 10, 2021.
6. Vizheh M, Qorbani M, Arzaghi SM, Muhidin S, Javanmard Z, Esmaeili M. The mental health of healthcare workers in the COVID-19 pandemic: a systematic review. *J Diabetes Metab Disord*. 2020. doi:10.1007/s40200-020-00643-9
7. Muller AE, Hafstad EV, Himmels JPW, et al. The mental health impact of the COVID-19 pandemic on healthcare workers, and interventions to help them: a rapid systematic review. *Psychiatry Res*. 2020;293(September):113441. doi:10.1016/j.psychres.2020.113441
8. Cawcutt KA, Starlin R, Rupp ME. Fighting fear in healthcare workers during the COVID-19 pandemic. *Infect Control Hosp Epidemiol*. 2020;41(10):1192–1193. doi:10.1017/ice.2020.315
9. Ejeh FE, Owoicho S, Saleh AS, Madukaji L, Okon KO. Factors associated with preventive behaviors, anxiety among healthcare workers and response preparedness against COVID-19 outbreak: a one health approach. *Clin Epidemiol Glob Health*. 2021;10 (December 2020):100671. doi:10.1016/j.cegh.2020.11.004
10. COVID-19 response acceleration task force. distribution maps (Indonesian) [Internet]; 2020 [cited January 31, 2021]. Available from: https://covid19.go.id/peta-sebaran. Accessed May 10, 2021.
11. Indonesian Ministry of Health. Indonesian health profile 2019 [Internet]. Jakarta; 2020. Available from: https://pusdatin.kemkes.go.id/folder/view/01/structure-publikasi-data-pusat-data-dan-informasi.html. Accessed May 10, 2021.
12. World Health Organization. Survey tool and guidance: rapid, simple, flexible behavioural insights on COVID-19 [Internet]. Copenhagen; 2020. Available from: https://apps.who.int/iris/handle/10665/333549. Accessed May 10, 2021.
13. Ahorsu DK, Lin C-Y, Imani V, Saffari M, Griffiths MD, Pakpour AH. The fear of COVID-19 scale: development and initial validation. *Int J Ment Health Addict*. 2020. doi:10.1007/s11469-020-00270-8
14. Indira IE. Stress questionnaire: stress investigation from dermatologist perspective. In: *Psychoneuroimmunology in Dermatology. Kelompok Studi Imunodermatologi Indonesia*. 2016:141–142.
15. Onie S, Kirana AC, Alfian A, Mustika NP, Adesla V, Ibrahim R. Assessing the predictive validity and reliability of the DASS-21, PHQ-9 and GAD-7 in an Indonesian sample. *psyArXiv*. 2020.
16. Lovibond SH, Lovibond PF. *Manual for the Depression Anxiety Stress Scales*. 2nd ed. Sydney: Psychology Foundation of Australia; 1996.
17. World Health Organization. WHO guidelines on hand hygiene in health care: a summary first global patient safety challenge clean care is safer care [Internet]. Geneva; 2009. Available from: https://www.who.int/gpsc/5may/tools/who_guidelines-handhygiene_summary.pdf. Accessed May 10, 2021.
18. World Health Organization. Risk assessment and management of exposure of health care workers in the context of COVID-19: interim guidance 19 March 2020 [Internet]. Geneva; 2020. Available from: https://apps.who.int/iris/handle/10665/331496. Accessed May 10, 2021.
19. Pappa S, Ntella V, Giannakas T, Giannakoulis VG, Papoutsi E, Katsaounou P. Prevalence of depression, anxiety, and insomnia among healthcare workers during the COVID-19 pandemic: a systematic review and meta-analysis. *Brain Behav Immun*. 2020;88(May):901–907. doi:10.1016/j.bbi.2020.05.026
20. Hanggoro AY, Suwarni L, Selviana M. Dampak psikologis pandemi COVID-19 pada petugas layanan kesehatan: studi. *J Kesehat Masy Nas*. 2020;15(2):13–18.
21. Sunjaya DK, Herawati DMD, Siregar AY. Depressive, anxiety, and burnout symptoms on health care personnel at a month after COVID-19 outbreak in Indonesia: a documentary research using Rasch model analysis. *Res Sq*. 2020;1–13.
22. Setiawati Y, Wahyuhadi J, Joestandari F, Maramis MM, Atika A. Anxiety and resilience of healthcare workers during COVID-19 pandemic in Indonesia. *J Multidiscip Healthc*. 2021;14:1–8. doi:10.2147/JMDH.S276655
23. Cotrin P, Moura W, Gambardela-Tkacz CM, Pelloso FC. Healthcare workers in Brazil during the COVID-19 pandemic: a cross-sectional online survey. *Inquiry*. 2020;57.
24. Shaukat N, Ali DM, Razzak J. Physical and mental health impacts of COVID-19 on healthcare workers: a scoping review. *Int J Emerg Med*. 2020;13(1):1–8. doi:10.1186/s12245-020-00299-5
25. Li Z, Dai J, Wu N, Jia Y, Gao J, Fu H. Effect of long working hours on depression and mental well-being among employees in Shanghai: the role of having leisure hobbies. *Int J Environ Res Public Health*. 2019;16(24):24. doi:10.3390/ijerph16244980
26. De Hert S. Burnout in healthcare workers: prevalence, impact and preventative strategies. *Local Reg Anesth*. 2020;13:171–183. doi:10.2147/LRA.S240564
27. Sigurvinsdottir R, Thorisdottir IE, Gylfason HF. The impact of COVID-19 on mental health: the role of locus on control and internet use. *Int J Environ Res Public Health*. 2020;17(19):1–15. doi:10.3390/ijerph17196985

28. Derksen C, Keller FM, Lippke S. Obstetric healthcare workers' adherence to hand hygiene recommendations during the COVID-19 pandemic: observations and social-cognitive determinants. *Appl Psychol Health Well Being*. 2020;12(4):1286–1305. doi:10.1111/aphw.12240
29. Roshan R, Feroz AS, Rafique Z, Virani N. Rigorous hand hygiene practices among health care workers reduce hospital-associated infections during the COVID-19 pandemic. *J Prim Care Community Health*. 2020;11:10–13.
30. Park SH. Personal protective equipment for healthcare workers during the COVID-19 pandemic. *Infect Chemother.* 2020;52(2):165–182. doi:10.3947/ic.2020.52.2.165
31. Zhao Y, Liang W, Luo Y, et al. Personal protective equipment protecting healthcare workers in the Chinese epicentre of COVID-19. *Clin Microbiol Infect*. 2020;26(12):1716–1718. doi:10.1016/j.cmi.2020.07.029
32. Neuwirth MM, Mattner F, Otchwemah R. Adherence to personal protective equipment use among healthcare workers caring for confirmed COVID-19 and alleged non-COVID-19 patients. *Antimicrob Resist Infect Control*. 2020;9(1):1–5. doi:10.1186/s13756-020-00864-w
33. Nguyen LH, Drew DA, Graham MS, et al. Risk of COVID-19 among front-line health-care workers and the general community: a Prospective Cohort Study. *Lancet Public Health*. 2020;5(9):e475–83. doi:10.1016/S2468-2667(20)30164-X
34. Galasso V, Pons V, Profeta P, Becher M, Brouard S, Foucault M. Gender differences in COVID-19 attitudes and behavior: panel evidence from eight countries. *Proc Natl Acad Sci U S A*. 2020;117(44):27285–27291. doi:10.1073/pnas.2012520117
35. Ali KF, Whitebridge S, Jamal MH, Alsafy M, Atkin SL. Perceptions, knowledge, and behaviors related to COVID-19 among social media users: Cross-Sectional Study. *J Med Internet Res*. 2020;22(9):1–9. doi:10.2196/19913
36. Alshammary F, Siddiqui AA, Amin J, et al. Prevention knowledge and its practice towards COVID-19 among general population of saudi arabia: a gender-based perspective. *Curr Pharm Des*. 2020. doi:10.2174/1381612826666200818213558
37. Haji JY, Subramaniam A, Kumar P, Ramanathan K, Rajamani A. State of personal protective equipment practice in indian intensive care units amidst COVID-19 pandemic: a nationwide survey. *Indian J Crit Care Med*. 2020;24(9):809–816. doi:10.5005/jp-journals-10071-23550

Risk Management and Healthcare Policy Dovepress

Publish your work in this journal

Risk Management and Healthcare Policy is an international, peer-reviewed, open access journal focusing on all aspects of public health, policy, and preventative measures to promote good health and improve morbidity and mortality in the population. The journal welcomes submitted papers covering original research, basic science, clinical & epidemiological studies, reviews and evaluations, guidelines, expert opinion and commentary, case reports and extended reports. The manuscript management system is completely online and includes a very quick and fair peer-review system, which is all easy to use. Visit http://www.dovepress.com/testimonials.php to read real quotes from published authors.

Submit your manuscript here: https://www.dovepress.com/risk-management-and-healthcare-policy-journal
