# Supplementary material for: Mental health status and related factors influencing healthcare workers during the COVID-19 pandemic: A systematic review and meta-analysis
Source: PLoS One. 2024 Jan 19;19(1):e0289454. doi: 10.1371/journal.pone.0289454 (PMC10798549; doi:10.1371/journal.pone.0289454)
Supplement: S1 Data — (ZIP) [file pone.0289454.s011.zip › literatures/173.pdf]

Received: 3 August 2021 | Accepted: 25 August 2021

DOI: 10.1111/resp.14143

COMMENTARY

Official Journal of the Asian Pacific Society of Respirology Respirology APSR WILEY

Mental health among healthcare workers during the COVID-19 pandemic

The novel coronavirus SARS-CoV-2 (COVID-19) has infected over 200 million people globally with 4.4 million deaths (as of August 2021). Efforts to contain the pandemic have included rapid adoption of border closures, social distancing restrictions, stay-at-home orders and major changes to the delivery and accessibility of health care. The impacts of these measures on the psychosocial well-being and mental health of the general public are well documented (with over 4000 papers on mental health published during the pandemic). There has been less attention to the impacts on healthcare workers (HCWs), despite awareness that doctors and nurses already suffer from poor mental health and increased rates of occupational burnout, anxiety, depression and suicide than other occupations.[1–3] The pandemic has brought increased workplace demands and stressors, with HCWs having to quickly adapt to increased workloads, large volumes of new information, new work practices including the widespread use of telehealth and personal protective equipment (PPE), redeployment or job insecurity, social change and increased risks to their own lives and family members.

The prevalence of and risk factors for mental illness in HCWs during the pandemic have been examined in many countries, mostly by cross-sectional, single time-point, online surveys. Meta-analyses of data from some HCW surveys undertaken early during the pandemic suggest that approximately one fifth to one quarter experienced anxiety, depression or post-traumatic stress disorder.[4,5] A survey of 26,174 HCWs in the United States by the Centre for Disease Control indicated that 53% had symptoms of at least one mental health condition, therefore the overall mental illness burden is high.[6] The Australian COVID-19 Frontline Health Workers Study, which was conducted from August to October 2020 during the Australian second wave of the pandemic, is the largest multi-professional survey ($n = 7846$ complete responses) on this topic that has included both primary and secondary care HCWs.[7] Data from this study identified higher prevalence of mental health conditions, including symptoms of anxiety 59.8%, burnout (emotional exhaustion) 70.9% and depression 57.3%, despite participants having very high resilience scores.[7] Limitations of many of the studies to date include selection bias due to voluntary participation, either low or incalculable response rates, studies being undertaken very early in the pandemic and single time-point measurements of outcomes. There is also a need to understand the longitudinal mental health effects of the pandemic on HCWs.

Independent risk factors associated with increased mental illness in HCWs have been explored, with many occurring consistently across studies and relating to either personal or workplace factors. Independent risk factors associated with anxiety, depression and burnout included younger age, female sex, nursing background, inadequate protection against COVID-19 or high risk of exposure to COVID-19, having a family member with suspected COVID-19, pre-existing mental illness or chronic physical health condition, reduced social support, lower levels of specialized training or experience and perceived stigma associated with working in frontline areas.[7–9] While working in high-risk environments with patients infected with COVID-19 has been identified as a risk factor for mental illness,[9] HCWs have experienced high levels of mental health symptoms irrespective of caseload of COVID-19 patients.[7] Importantly, access to sufficient medical resources (including PPE), up to date and accurate information, and taking precautionary measures to reduce infection risk have been identified as protective factors for maintaining psychological well-being during the COVID-19 pandemic.[5,7,8]

The prevalence of suicidal and self-harm ideation among HCWs is concerning. A meta-analysis, comprising 61 studies, identified the prevalence of suicide attempts was 1.0% and of suicidal ideation was up to 17% in physicians during non-pandemic times, with insufficient data for other professions.[10] The prevalence of suicidal ideation has been reported as between 3.6% and 8.4% in Belgian and Spanish HCWs during the COVID-19 pandemic, and a UK study found that 13% of 709 intensive care unit workers in English hospitals had thoughts of self-harm during the pandemic.[11–13] Risk factors for suicidal ideation or thoughts of self-harm during this pandemic include current or prior mental illness, having been hospitalized with COVID-19 infection, family members infected with COVID-19, self-rated probability of contracting COVID-19, perceived stress, lack of organizational communication and coordination, lack of personnel or supervision in the workplace and financial stress.[11,13] By contrast, those who reported increased social support, had higher self-rated health, were more willing to work with COVID-19 patients, felt supported and were confident in defeating COVID-19 were at lower risk.[11] Concerningly, HCWs often felt poorly supported by their workplaces during the pandemic.[7]

The COVID-19 pandemic has been associated with significant mental health symptoms in HCWs working in both primary and secondary care. Meta-analyses comparing the impacts of the COVID-19 pandemic on HCWs compared

© 2021 Asian Pacific Society of Respirology.

to the general population[8,14,15] have found that the prevalence of anxiety, depression and other mental illnesses were significantly higher in HCWs than the general public. Many personal and workplace mental health risk factors have been identified. Concerns regarding inadequate resources (particularly PPE), lack of workplace training, organization and support, inaccurate and inconsistent information and poor organizational preparedness are common and highly concerning.

The World Health Organization has designated 2021 the 'International Year of the Health Worker'. Crises affecting HCWs and healthcare provision are becoming the 'new normal', as events associated with climate change and globalization become more frequent and more severe. The COVID-19 pandemic is therefore a call for organizations and leaders to be well prepared for these events and have clear polices, practices and support services that actively protect psychological well-being and prevent occupational mental illness. These issues have repercussions not only for individuals, but also for quality of care, patient safety and workforce retention and engagement. Better understanding of the long-term impacts of crises on mental health and implementation of evidence-based approaches to preserve psychological well-being are crucial in responding to the current pandemic and future crises.

KEYWORDS
COVID-19, healthcare workers, mental heath, occupational health, pandemic

CONFLICT OF INTEREST

None declared.

Natasha Smallwood BMedSci, MBBS, SpecCertPallCare, MSc, FRCP, FRACP, PhD[1,2]
Karen Willis BA, MA (Hons), GradDipHealthProm, PhD[3,4]

[1]*Department of Respiratory Medicine, The Alfred Hospital, Prahran, Victoria, Australia*
[2]*Department of Allergy, Immunology and Respiratory Medicine, Central Clinical School, The Alfred Hospital, Monash University, Melbourne, Victoria, Australia*
[3]*Professor of Public Health, College of Health and Biomedicine, Victoria University, Melbourne, Victoria, Australia*
[4]*Division of Critical Care and Investigative Services, Royal Melbourne Hospital, Parkville, Victoria, Australia*

ORCID

Natasha Smallwood https://orcid.org/0000-0002-3403-3586
Karen Willis https://orcid.org/0000-0001-8036-8814

REFERENCES

1. De Cieri H, Shea T, Cooper B, Oldenburg B. Effects of work-related stressors and mindfulness on mental and physical health among Australian nurses and healthcare workers. J Nurs Scholarsh. 2019;51: 580–9.
2. Imo UO. Burnout and psychiatric morbidity among doctors in the UK: a systematic literature review of prevalence and associated factors. BJPsych Bull. 2017;41:197–204.
3. Milner AJ, Maheen H, Bismark MM, Spittal MJ. Suicide by health professionals: a retrospective mortality study in Australia, 2001-2012. Med J Aust. 2016;205:260–5.
4. Li Y, Scherer N, Felix L, Kuper H. Prevalence of depression, anxiety and post-traumatic stress disorder in health care workers during the COVID-19 pandemic: a systematic review and meta-analysis. PLoS One. 2021;16:e0246454.
5. Pappa S, Ntella V, Giannakas T, Giannakoulis VG, Papoutsi E, Katsaounou P. Prevalence of depression, anxiety, and insomnia among healthcare workers during the COVID-19 pandemic: a systematic review and meta-analysis. Brain Behav Immun. 2020;88:901–7.
6. Bryant-Genevier J, Rao CY, Lopes-Cardozo B, Kone A, Rose C, Thomas I, et al. Symptoms of depression, anxiety, post-traumatic stress disorder, and suicidal ideation among state, tribal, local, and territorial public health workers during the COVID-19 pandemic – United States, March-April 2021. MMWR Morb Mortal Wkly Rep. 2021;70:947–52.
7. Smallwood N, Karimi L, Bismark M, Putland M, Johnson D, Dharmage S, et al. High levels of psychosocial distress among Australian frontline health workers during the COVID-19 pandemic. Gen Psychiatry. 2021;72:124–30.
8. Luo M, Guo L, Yu M, Jiang W, Wang H. The psychological and mental impact of coronavirus disease 2019 (COVID-19) on medical staff and general public – a systematic review and meta-analysis. Psychiatry Res. 2020;291:113190.
9. Serrano-Ripoll MJ, Meneses-Echavez JF, Ricci-Cabello I, Fraile-Navarro D, Fiol-deRoque MA, Pastor-Moreno G, et al. Impact of viral epidemic outbreaks on mental health of healthcare workers: a rapid systematic review and meta-analysis. J Affect Disord. 2020;277: 347–57.
10. Dutheil F, Aubert C, Pereira B, Dambrun M, Moustafa F, Mermillod M, et al. Suicide among physicians and health-care workers: a systematic review and meta-analysis. PLoS One. 2019;14: e0226361.
11. Bruffaerts R, Voorspoels W, Jansen L, Kessler RC, Mortier P, Vilagut G, et al. Suicidality among healthcare professionals during the first COVID19 wave. J Affect Disord. 2021;283:66–70.
12. Greenberg N, Weston D, Hall C, Caulfield T, Williamson V, Fong K. Mental health of staff working in intensive care during Covid-19. Occup Med (Lond). 2021;71:62–7.
13. Mortier P, Vilagut G, Ferrer M, Serra C, Molina JD, Lopez-Fresnena N, et al. Thirty-day suicidal thoughts and behaviors among hospital workers during the first wave of the Spain COVID-19 outbreak. Depress Anxiety. 2021;38:528–44.
14. Deng Y, Chen Y, Zhang B. Different prevalence trend of depression and anxiety among healthcare workers and general public before and after the peak of COVID-19 occurred in China: a meta-analysis. Asian J Psychiatr. 2021;56:102547.
15. Krishnamoorthy Y, Nagarajan R, Saya GK, Menon V. Prevalence of psychological morbidities among general population, healthcare workers and COVID-19 patients amidst the COVID-19 pandemic: a systematic review and meta-analysis. Psychiatry Res. 2020;293:113382.

How to cite this article: Smallwood N, Willis K. Mental health among healthcare workers during the COVID-19 pandemic. Respirology. 2021;26:1016–7. https://doi.org/10.1111/resp.14143
