# Supplementary material for: Mental health status and related factors influencing healthcare workers during the COVID-19 pandemic: A systematic review and meta-analysis
Source: PLoS One. 2024 Jan 19;19(1):e0289454. doi: 10.1371/journal.pone.0289454 (PMC10798549; doi:10.1371/journal.pone.0289454)
Supplement: S1 Data — (ZIP) [file pone.0289454.s011.zip › literatures/174.pdf]

Since January 2020 Elsevier has created a COVID-19 resource centre with free information in English and Mandarin on the novel coronavirus COVID-19. The COVID-19 resource centre is hosted on Elsevier Connect, the company's public news and information website.

Elsevier hereby grants permission to make all its COVID-19-related research that is available on the COVID-19 resource centre - including this research content - immediately available in PubMed Central and other publicly funded repositories, such as the WHO COVID database with rights for unrestricted research re-use and analyses in any form or by any means with acknowledgement of the original source. These permissions are granted for free by Elsevier for as long as the COVID-19 resource centre remains active.

Comprehensive Psychiatry 112 (2022) 152278

Contents lists available at ScienceDirect

Comprehensive Psychiatry

journal homepage: www.elsevier.com/locate/comppsych

Mental health assessment of Spanish healthcare workers during the SARS-CoV-2 pandemic. A cross-sectional study

Pau Sobregrau Sangrà [a,b,*], Sira Aguiló Mir [a], Thaís Castro Ribeiro [c], Silvia Esteban-Sepúlveda [d,e], Esther García Pagès [c], Beatriz López Barbeito [a], José Luís Pomar Moya-Prats [f], Luís Pintor Pérez [b], Jordi Aguiló Llobet [g]

[a] Emergency Department, Hospital Clinic of Barcelona, Barcelona, 08036, Spain
[b] Psychiatry Department, Hospital Clinic of Barcelona, Barcelona 08036, Spain
[c] Networking Biomedical Research Center: Bioengineering, Biomaterials and Nanomedicine (CIBER-BBN), Universitat Autònoma de Barcelona (UAB), Bellaterra 08193, Spain
[d] Research Group in Nursing Care (GRECI), Hospital del Mar Institute of Medical Research (IMIM), Barcelona 08003, Spain
[e] Consorci Parc de Salut MAR de Barcelona, Hospital del Mar of Barcelona, Barcelona 08003, Spain
[f] Cardiac Surgery Department, The Thorax Institute, Hospital Clinic of Barcelona, Barcelona 08036, Spain
[g] Microelectronics and Electronic Systems Department, Universitat Autònoma de Barcelona (UAB), Bellaterra 08193, Spain

A R T I C L E I N F O

Keywords:
SARS-CoV-2 pandemic
Anxiety symptoms
Acute stress symptoms
Depression symptoms
Posttraumatic stress disorder
Healthcare workers

A B S T R A C T

Introduction: The SARS-CoV-2 outbreak is posing unprecedented care scenarios, increasing the psychological distress among healthcare workers while reducing the efficiency of health systems. This work evaluated the psychological impact of the Covid-19 pandemic on Spanish frontline healthcare workers of two tertiary hospitals.
Material and methods: Healthcare workers were recruited from the medical units designated for the care of Covid-19 patients. The psychological assessment consisted of an individual, face-to-face session where gold-standard psychometric tests were administered to assess stress (VASS & PSS-10), anxiety (STAI), depression (PHQ-2) and posttraumatic stress disorder (PCL-5). Regression models were also fitted to identify predictors of psychological distress.
Results: Overall, almost 13% of healthcare workers showed severe anxiety, while more than 26% had high levels of perceived stress. More than 23% presented severe posttraumatic stress symptoms, and another 13% had PHQ-2 scores equal to or above 3, compatible with Major Depressive Disorder (MDD) diagnosis, respectively. Women, stress-related medication, overworking, performing in Covid-19 wards, and substance abuse were risk factors for increased psychological distress. Instead, practising exercise reduced the burden.
Conclusion: This study outlines the severe psychological impact of the Covid-19 pandemic on Spanish frontline healthcare workers. The stress, depression and anxiety levels found were similar to those reported in similar works but much higher than in Wuhan healthcare workers. Knowledge of risk factors for increased psychological distress may help to develop comprehensive intervention strategies to prevent, control and reduce the mental health exacerbation of healthcare workers, thereby maintaining the effectiveness of health systems in critical scenarios.

1. Introduction

The rapid worldwide spread of the current Covid-19 outbreak is causing unprecedented social and care situations, leading to increased levels of stress, anxiety, and depression, along with insomnia, poor-quality life and even increased suicide rate, among the population [1–5].

When coping with this scenario, healthcare workers are exposed to a broad range of sustained physical, psychological and emotional stressors, becoming at risk for chronic stress (i.e., maladaptive continued physiological and psychological responses resulting from prolonged exposure to a stressful event) [6,7] and professional burnout. In other words, these stressors compromise healthcare workers' wellbeing and mental health, thus endangering the care quality and efficiency of health systems in critical contexts [8,9]. Examples of the day-to-day stressors of

* Corresponding author at: Psychiatry Department, Hospital Clínic of Barcelona, C/Roselló 140, 08036 Barcelona, Spain.
E-mail address: sobregrau@clinic.es (P. Sobregrau Sangrà).

https://doi.org/10.1016/j.comppsych.2021.152278

Available online 9 October 2021
0010-440X/© 2021 Published by Elsevier Inc. This is an open access article under the CC BY-NC-ND license (http://creativecommons.org/licenses/by-nc-nd/4.0/).

the Covid-19 pandemic are the increased workload, physical exhaustion, nosocomial transmission and the need to take ethically challenging decisions on the care rationing [10,11].

Although the clinical and socio-economic implications of high and persistent stress levels are well documented in the literature, the diagnosis of chronic stress remains a challenge within the clinical practice today. On the one hand, there are no adequate and precise tools that objectively measure the intensity of a stressor on an individual. The available tools do not allow either non-invasive monitoring of the stress levels at a physiological level or the follow-up of the symptoms. On the other hand, the available psychometric questionnaires cannot determine whether the stressor is undermining health or, on the contrary, it is being overcome [12–15]. Furthermore, learning effects and the desirability response bias often limit psychometric tools, as in online surveys [16–18].

Despite the difficulty of assessing stress, especially when understanding the Covid-19 pandemic as a sustained extreme stressor, several published works based exclusively on online psychological assessments design have already shown high stress, anxiety and depression prevalence across the care community due to the pandemic [19–23].

The current study aims to quantify the immediate psychological impact of the SARS-CoV-2 pandemic in Spanish Covid-19 frontline healthcare workers, based on face-to-face psychological assessments to cross-check the results obtained in similar studies conducted using online evaluations. The results derived from this quantitative psychological assessment will be used in a second and more extensive work led by our research team for the design and validation, together with different stress-related physiological variables, of an easy-to-use assessment tool for the early diagnosis and monitoring of chronic stress in medical personnel [13].

2. Material and methods

2.1. Study design

A cross-sectional study with Covid-19 frontline healthcare workers from two Spanish tertiary hospitals, the Hospital Clinic of Barcelona and the Hospital del Mar of Barcelona, working in the direct care of SARS-CoV-2 patients.

All the study procedures complied with the Helsinki declaration for research and received approval from the Ethics Committee Board of the Hospital Clinic of Barcelona and Hospital del Mar of Barcelona.

2.2. Setting and subjects

A total of 184 Covid-19 frontline healthcare workers from two Spanish tertiary hospitals, the Hospital Clinic of Barcelona ($N = 90$) and Hospital del Mar of Barcelona ($N = 94$), were recruited for the study between July and October 2020 (both inclusive).

The recruitment process was carried out through internal dissemination of the study, using the institutional email, across all the medical units of both hospitals designated for the care of Covid-19 patients. The recruitment of healthcare workers also included the external facilities (i.e., health hotels) set up to hospitalise patients affected by the same illness. Signed informed consent was required to take part in the study.

The recruitment process was conducted in the Intensive Care Unit (ICU), Emergency Service and Covid-19 hospitalisation wards. Covid-19 hospitalisation wards comprised the units of Internal Medicine, Infection, Pneumology and Gastroenterology, and the external facilities specifically enabled for SARS-CoV-2 hospitalisations. The categories of frontline healthcare workers included in the study were physicians, nurses, and nurse aides.

The study inclusion criteria were: a) being a frontline healthcare worker, b) having worked at any of the medical services mentioned above, c) being directly involved in managing SARS-CoV-2 patients, and d) having accepted participation by signing the informed consent. On the other hand, healthcare workers with any of the following criteria were excluded from the study: a history of severe psychiatric disorder (e.g., psychotic disorder), ongoing psychiatric disorder at baseline (e.g., Major Depressive Disorder or Generalised Anxiety Disorder), and intellectual disability that would prevent from answering all questionnaires independently.

All frontline healthcare workers in the study performed similar duties regarding Covid-19 patients, except the Emergency Service medical personnel who only treated these patients the first 24 h from the medical admission. Examples of the care duties performed according to the professional category were: 1) Physicians: care and initial diagnosis of the disease by Covid-19, examination and assessment of the evolution and decision making; 2) Nurses: taking of vital signs, vascular access and taking of samples and airway control; 3) Nurse aides: hygiene, postural changes and diets of the patients.

All healthcare workers were managed and assessed under the same premises across the two hospitals to reduce performance bias.

2.3. Assessment instruments

- Subjective perceived stress: evaluated with the Visual Analogue Scale for stress (VASS) [24]. Although it has shown reliable discriminative sensitivity and construct validity, the VASS test is not a diagnostic tool [24]. Accordingly, we used the cut-off points obtained from previous research conducted by our research team to distinguish between caregivers of chronic patients (expected with high-stress levels) and controls (non-caregivers [expected with low-stress levels]) [13]. A VASS score equal to or below 30 and equal or above 31 and 70 indicated low, moderate and high perceived stress levels, respectively.
- Stress appraisal: evaluated with the 10-item Perceived Stress Scale (PSS-10) [25]. A PSS-10 score equal to or below 13, equal or above 14 and equal or higher than 27 indicated low, moderate and high-stress appraisal. The PSS-10 cut-offs used were similar to those observed in other Covid-19 studies [26,27].
- Posttraumatic Stress Disorder (PTSD): evaluated with the Posttraumatic Stress Disorder Checklist for DSM-5 (PCL-5) [28], which has high internal consistency in measuring posttraumatic stress symptoms (PTSS) (Cronbach's alpha: 0.94). A total PCL-5 score equal to or higher than 31 was indicative of a possible PTSD diagnosis.
- Anxiety: evaluated with the State-Trait Anxiety Inventory (STAI) [29], with the STAI-State (STAI-S) subscale evaluating the current levels of anxiety and the STAI-Trait (STAI-T) subscale the propensity to be anxious on a personality basis. There are no normative STAI test values for medical staff. Accordingly, we used the STAI cut-off scores defined in previous research conducted with caregivers of chronic patients and controls [13]. An STAI-S cut-off point of 10 suggested low anxiety levels, 37 moderate anxiety levels, and scores above 37 high anxiety levels. On the other hand, an STAI-T cut-off point of 14, 26 and above 26 indicated low, moderate and high predisposition to perceive situations as a threat, respectively. These cut-off points are similar to those observed in Covid-19 studies [30].
- Depression: evaluated with the Patient Health Questionnaire-2 (PHQ-2) [31]. A PHQ-2 score of 3 or higher indicates a positive result for Major Depressive Disorder (MDD), with a sensitivity of 83% and a specificity of 92%.
- Individual differences: evaluated with the clinical and sociodemographic form. This assessed gender, age, psychiatric history (i.e., affective and anxiety disorders), living with people at high risk of COVID-19 infection, physical activity levels, professional and employment category, regular working shift, weekly working hours, working hours increase, and medical service worked during the pandemic. The form also included questions regarding leave off work due to Covid-19 infection (i.e., quarantine), requesting help due to high psychological burden, taking stress-related medication (i.e., benzodiazepines, analogues and antidepressants such as serotonin

reuptake inhibitors and serotonin and adrenaline reuptake inhibitors) and substance abuse (tobacco, alcohol or other drugs).

Psychometric tests required, on average, 15 to 20 min to be completed, and participants did not need any special education or training to complete them.

2.4. Procedures

After signing the informed consent, healthcare workers were scheduled for a first assessment. The first assessment was divided into two different stages: the psychological and physiological evaluation.

Firstly, the psychological evaluation consisted of the five psychometric questionnaires described in the previous section to comprehensively estimate the current stress levels, including PTSS, anxiety, and depression (Fig. 1). Unlike many other Covid-19 works, the psychological questionnaires were administered in person and in the following order: VASS, PSS-10, PCL-5, STAI (STAI-S & STAI-T) and PHQ-2. After the psychological assessment, healthcare workers continued with the physiological assessment.

Secondly, the physiological evaluation consisted of evaluating different stress-related physiological variables such as heart rate variability (HRV) and electrodermal activity (EDA) using medical-grade technology (i.e., NeXus-10 MKII device and the E4 wrist-worn) (Fig. 1). The physiological assessment lasted approximately 25 min, and the data collected will be analysed in future work.

Once finalised the first assessment, healthcare workers were scheduled again at six months for a second assessment. The second assessment included both the same psychological and physiological evaluations performed during the first assessment. As with the physiological data of the first assessment, the results of the follow-up assessment will also be discussed in future work.

2.5. Statistical analysis

A descriptive analysis of all variables of interest was first conducted. Then, statistic tests were applied accordingly.

Means of continuous variables with two levels were compared using Student's *t*-tests for independent samples. Independent-Samples Mann-Whitney *U* tests were used when the parametric assumptions for comparing continuous variables were not met. The Kruskal-Wallis test of variance was used to examine the differences across groups in continuous variables with three or more levels. The Bonferroni correction was applied in the non-parametric ANOVA analysis.

Lastly, multiple stepwise regression models were fitted to examine the independent effects of several variables on healthcare workers' mental health according to the quantitative psychological assessment, considering both the STAI-S and STAI-T subscales independently. The VASS test was excluded from the regression analysis since the PSS-10 addresses the subjectivity bias from measuring a multidimensional construct such as stress on a visual scale [32–34]. Gender, age (in groups), professional category, psychiatric history, employment category, weekly working hours, working shift, physical activity, medical service worked during the pandemic, having been off work during the pandemic, having requested help, having taken stress-related medication, working hours increase, smoking habits, and substance abuse were introduced in the initial model as predictors. The Backwards Elimination, in terms of the lowest AIC value, was used to fit the models.

The SPSS v.26 for Windows was used for all data analyses. All results were interpreted with a 95% confidence interval (CI) and a significance level (*p*-value) of 0.05.

3. Results

3.1. Demographic and clinical characteristics of the sample

A total of 184 Covid-19 frontline healthcare workers were included in the study. A description of all the clinical and sociodemographic characteristics of the sample is provided in Table 1.

As Table 1 shows, the majority of healthcare workers were women (84.8%), working as nurses (56.5%), of the morning shift (42.9%) and from the Emergency Service. Regarding the pandemic, more than 60% of healthcare workers reported an increase in the weekly working hours, while almost half (44%) were temporarily off work due to Covid-19 infection. Only 25 (13.5%) healthcare workers reported a history of a psychiatric disorder before the Covid-19 outbreak.

3.2. Quantitative psychological assessment

Table 2 shows the psychological assessment outcome for the sample and the distribution of healthcare workers according to their mean score across the different cut-off points of each psychometric test [13,26–28,31].

Following the results of Table 2, nearly 69% of healthcare workers reported moderate (42.39%) and high (26.09) levels of perceived stress. Consistent with this, 72% had moderate- (65.76%) and high-stress (6.52%) appraisals. Another 23.37% had PCL-5 scores equal to and above 30, suggesting possible PTSD diagnosis, while 13.04% obtained PHQ-2 scores equal to or higher than 3, compatible with MDD diagnosis. Lastly, more than 90% also reported moderate (77.72%) and high (12.50) anxiety levels, and over 70% showed a moderate (43.48%) and high (26.63%) predisposition to predisposition to perceiving situations as a threat.

Table 3 shows the mean scores and the comparative analysis outcome for each group across the psychometric tests applied at baseline.

Based on the results for each group and psychometric test (Table 3), women healthcare workers had significantly higher mean scores than men healthcare workers in all the psychometric questionnaires (PCL-5: $U = 1589.5, p = .022$; PSS-10: $U = 1257, p < .001$; STAI-S: $U = 1053.5$, p $< .001$; STAI-T: $U = 1322.5$, $p = .001$; PHQ-2: $U = 1313.5$, $p = .006$), except in the VASS test. In this latter test, differences across gender were

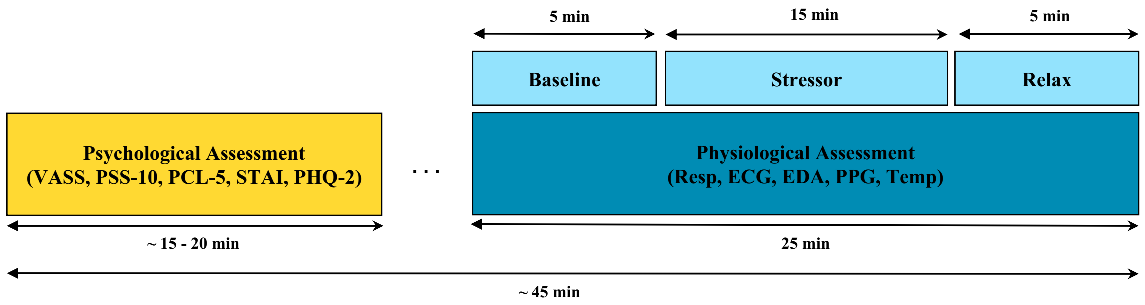

Fig. 1. Flowchart of the psychological and physiological evaluations at first assessment.
Note. Resp: Respiration rate; ECG: Electrocardiogram; EDA: Electrodermal activity; PPG: Photoplethysmography; Temp: Body temperature.

Table 1
Clinical and sociodemographic characteristics of the 184 healthcare workers included in the study.

	Healthcare workers (n = 184)		
	n	%	*Mean (S.D.)*
**Age (years)**			37.5 (11.0)
**Gender**			
Male	28	15.20	
Female	156	84.80	
**Psychiatric history**			
No	159	86.40	
Yes	25	13.50	
**Living with people at high risk of Covid-19 infection**			
No	120	65.20	
Yes	64	34.80	
**Professional category**			
Physicians	43	23.40	
Nurses	104	56.50	
Nurse aides	37	20.10	
**Employment category**			
Fixed-term	81	44.00	
Open-ended	103	56.00	
**Working shift**			
Mornings	79	42.90	
Afternoons	28	15.20	
Nights	52	28.30	
On-call	25	13.60	
**Weekly working hours (h)**			
15 to 30 h	17	9.24	
31 to 40 h	163	88.60	
Over 40 h	4	2.17	
**Physical activity**			
Low	26	14.10	
Medium	92	50.00	
High	66	35.90	
**Working unit**			
Covid-19 hospitalisation wards	67	36.40	
Emergency service	74	40.20	
Intensive Care Unit (ICU)	43	23.40	
**Off work due to Covid-19**			
No	103	56.00	
Yes	81	44.00	
**Request help**			
No	153	83.20	
Yes	31	16.80	
**Current stress-related medication**			
No	143	77.70	
Yes	41	22.30	
**Working hours increase**			
No	73	39.70	
Yes	111	60.30	
**Substance abuse increase (any)**			
No	124	67.40	
Yes	60	32.60	

not significant even though women healthcare workers continued showing higher mean scores than men healthcare workers (*U = 1730, p = .079)*.

Regarding the professional category, nurses and nurse aides appeared to have overall poorer psychological outcomes than physicians. Nevertheless, further analysis conducted on the data revealed only significant differences in the PCL-5 [$\chi^2(2) = 8.15$, $p = .017$] and PHQ-2 [$\chi^2(2) = 19.45$, $p < .001$] tests. On the one hand, nurses had significantly greater PTSS than physicians ($p = .011$). On the other hand, both nurses (p < .001) and nurse aides (p < .001) showed significantly worse depression than physicians.

Longer weekly working hours also indicated overall higher mean scores across all the psychometric tests. Nevertheless, significant differences were only found in the PSS-10 [F(2, 181) = 4.32, $p = .015$] test. Healthcare workers working over 40 h per week had significantly higher stress appraisal ($p = .026$) than those working 31 to 40 h per week.

Table 2
Quantitative psychological assessment and sample distribution according to the different cut-off points of each psychometric test.

	Healthcare workers (n = 184)		
	n	%	Mean (S.D.)
**VASS (perceived stress)**			48.40 (22.8)
≤ 30, *low*	58	31.52	20.30 (10.20)
> 30, *moderate*	78	42.39	53.10 (9.13)
≥ 70, *high*	48	26.09	74.80 (6.60)
**PCL-5 (posttraumatic stress symptoms)**			20.50 (12.6)
≤ 30, *low*	141	76.63	15.10 (8.17)
>30, severe – *suspected PTSD diagnosis*	43	23.37	38.20 (6.95)
**PSS-10 (stress appraisal)**			16.80 (5.79)
≤ 13, *low*	51	27.72	10.20 (2.22)
> 13, *moderate*	121	65.76	18.40 (3.50)
≥ 27, *high*	12	6.52	28.90 (2.57)
**STAI-S (current anxiety symptoms)**			24.80 (10.0)
≤ 10, *low*	18	9.78	8.39 (1.75)
>10, *moderate*	143	77.72	24.10 (6.75)
≥ 37, *high*	23	12.50	41.90 (3.13)
**STAI-T (predisposition to perceiving situations as a threat)**			20.00 (8.37)
≤ 14, *low*	55	29.89	10.53 (3.05)
> 15, *moderate*	80	43.48	19.88 (3.04)
≥ 26, *high*	49	26.63	30.74 (4.58)
**PHQ-2 (depression symptoms)**			1.08 (1.26)
≤ 2, *low*	160	86.96	0.64 (0.77)
> 2, severe – *suspected DM diagnosis*	24	13.04	3.54 (0.83)

Note. VASS: Visual Analogue Scale for Stress; PCL-5: The Posttraumatic Stress Disorder Checklist for DSM-5; PSS-10: 10-item Perceived Stress Scale; STAI-S; State subscale of the State-Trait Anxiety Inventory; STAI-T: Trait subscale of the State-Trait Anxiety Inventory; PHQ-2: The Patient Health Questionnaire-2.

Despite observing the same tendency between healthcare workers working over 40 h per week and those under 31 h per week, the differences between these two groups were not significant ($p = .203$).

In general, healthcare workers from the afternoon and night shifts also appeared to have worse psychological outcomes than those from the morning and on-call shifts. Nevertheless, the analysis conducted for the data only showed significant differences in the PHQ-2 [$\chi^2(3) = 10.22$, $p = .017$] test. Healthcare workers from the night shift had significantly greater depression than those working on-call ($p = .015$) and morning ($p = .021$) shifts. Likewise, healthcare workers from the afternoon shift also showed more significant depression than those from the on-call ($p = .027$) and morning ($p = .042$) shifts.

The analysis also showed that healthcare workers who requested help to get through the everyday pandemic experiences had significantly greater PTSS ($U = 1415.5$, $p < .001$), stress appraisal ($U = 1759.5$, $p = .023$), anxiety ($U = 1717.5$, $p = .016$), predisposition to perceiving situations as a threat ($U = 1658$, $p = .008$), and depression ($U = 1600.5$, $p < .032$) than those who had not requested any support. The same results were observed when comparing health workers according to whether they were taking stress-related medication. Healthcare workers taking stress-related medication showed significantly worse psychological

Table 3
Quantitative psychological assessment across study groups.

			VASS test	PCL-5 test	PSS-10 test	STAI-S subscale	STAI-T subscale	PHQ-2 test
		n	Mean (S.D.)	Mean (S.D.)	Mean (S.D.)	Mean (S.D.)	Mean (S.D.)	Mean (S.D.)
Gender	Men	28	41.89 (22.39)	15.61 (12.15)	13.36 (4.36)	17.14 (8.56)	14.86 (7.79)	0.50 (0.86)
	Women	156	49.61 (22.79)	21.42 (12.47)	17.46 (5.80)	26.14 (9.67)	20.89 (8.16)	1.18 (1.29)
	p-value			*	***	***	**	**
Age (groups; years old)	18 to 30	64	48.31 (23.24)	22.31 (13.90)	17.58 (5.61)	24.48 (9.83)	21.06 (8.24)	1.03 (1.33)
	31 to 45	76	47.07 (22.78)	19.58 (11.87)	16.32 (6.26)	24.55 (10.17)	18.82 (8.81)	1.05 (1.26)
	46 to 65	44	50.98 (22.65)	19.61 (11.65)	16.64 (5.19)	25.57 (10.24)	20.39 (7.68)	0.95 (1.12)
	p-value							
Psychiatric history	No	159	49.96 (23.37)	19.04 (12.35)	15.82 (6.26)	23.16 (11.01)	17.07 (7.31)	0.75 (0.93)
	Yes	25	55.21 (16.33)	22.93 (13.10)	17.29 (6.19)	26.36 (10.10)	20.86 (7.32)	1.43 (1.60)
	p-value							
Professional Category	Physician	43	53.23 (22.17)	16.21 (12.86)	16.51 (7.12)	23.28 (12.34)	18.40 (9.27)	0.45 (0.90)
	Nurses	104	47.57 (21.80)	22.22 (12.41)	16.67 (5.31)	25.02 (8.83)	20.58 (8.37)	1.21 (1.33)
	Nurse aides	37	45.30 (26.04)	20.84 (11.76)	17.65 (5.44)	25.81 (10.31)	20.11 (7.17)	1.47 (1.16)
	p-value			**				***
Medical Service	Covid-19 hospitalisation wards	67	48.73 (20.41)	18.61 (12.12)	17.13 (6.08)	24.58 (9.28)	20.96 (9.01)	1.29 (1.32)
	Emergency service	74	47.42 (24.86)	21.23 (11.68)	16.70 (5.81)	24.23 (10.44)	19.39 (7.82)	0.99 (1.13)
	Intensive Care Unit (ICU)	43	49.72 (23.23)	22.35 (14.48)	16.58 (5.38)	26.00 (10.54)	19.44 (8.30)	0.90 (1.35)
	p-value							
Weekly Working Hours	Up to 30	17	46.71 (30.30)	21.24 (15.57)	18.59 (6.51)	25.53 (13.01)	20.94 (11.21)	1.06 (1.34)
	31 to 40	163	48.39 (21.93)	20.53 (12.20)	16.47 (5.54)	24.59 (9.58)	19.80 (8.10)	1.08 (1.27)
	+40	4	57.50 (28.43)	18.00 (16.79)	24.00 (8.04)	29.00 (15.64)	23.00 (6.68)	1.00 (0.82)
	p-value				*			
Working Hours Increase	No	73	49.42 (23.45)	20.82 (12.75)	16.66 (5.51)	25.18 (10.69)	20.32 (7.82)	1.13 (1.20)
	Yes	111	47.78 (22.5)	20.35 (12.49)	16.95 (5.98)	24.50 (9.60)	19.75 (8.74)	1.05 (1.30)
	p-value							
Working Shift	Morning	79	50.35 (22.62)	18.82 (12.70)	16.62 (5.75)	23.58 (9.92)	19.29 (8.45)	0.87 (1.11)
	Afternoon	28	50.96 (22.48)	23.82 (12.96)	16.32 (4.93)	27.89 (10.09)	21.11 (6.47)	1.43 (1.45)
	Night	52	43.02 (23.61)	21.58 (11.16)	17.10 (5.90)	24.52 (9.61)	20.65 (8.37)	1.46 (1.44)
	On-call	25	50.80 (21.70)	20.12 (14.19)	17.52 (6.75)	25.56 (10.91)	19.44 (10.09)	0.57 (0.66)
	p-value							*
Risk people at home	No	120	48.60 (23.48)	21.38 (12.65)	16.87 (5.98)	24.63 (10.02)	20.02 (7.89)	1.05 (1.16)
	Yes	64	48.12 (21.76)	18.95 (12.35)	16.77 (5.46)	25.03 (10.12)	19.88 (9.27)	1.14 (1.46)
	p-value							
Physical Activity	Low	26	52.42 (24.91)	24.35 (15.20)	20.73 (6.61)	30.58 (11.19)	24.92 (9.95)	1.44 (1.47)
	Medium	92	48.87 (21.65)	20.97 (11.81)	16.68 (5.42)	24.45 (9.04)	20.15 (8.01)	1.19 (1.33)
	High	66	46.26 (23.71)	18.44 (12.24)	15.50 (5.33)	22.94 (10.17)	17.77 (7.39)	0.80 (1.01)
	p-value				**	**	***	
Off Work	No	102	48.73 (24.09)	20.07 (13.23)	16.58 (6.03)	23.95 (10.63)	19.30 (8.03)	1.00 (1.30)
	Yes	82	48.07 (21.31)	21.12 (11.74)	17.15 (5.49)	25.79 (9.18)	20.80 (8.75)	1.18 (1.21)
	p-value							
Request of Help/Support	No	153	48.17 (23.74)	19.08 (12.20)	16.39 (5.73)	24.04 (10.34)	19.27 (8.34)	0.97 (1.16)
	Yes	31	49.74 (17.97)	27.74 (11.99)	19.03 (5.63)	28.39 (7.41)	23.42 (7.74)	1.62 (1.59)
	p-value			***	*	*	**	*
Stress-Related Medication	No	143	48.11 (22.61)	18.58 (12.29)	16.15 (5.68)	23.69 (10.26)	18.64 (8.02)	0.84 (1.04)
	Yes	41	49.56 (23.85)	27.37 (11.18)	19.20 (5.60)	28.56 (8.18)	24.63 (7.96)	1.90 (1.60)
	p-value			***	**	**	***	***
Substance Abuse	No	123	48.50 (22.95)	18.43 (11.29)	16.76 (5.76)	24.54 (9.64)	19.85 (8.01)	0.96 (1.12)
	Yes	61	48.30 (22.78)	24.79 (13.96)	16.97 (5.89)	25.25 (10.83)	20.23 (9.11)	1.33 (1.48)
	p-value			**				

Note. * $p < .05$, ** $p < .01$, *** $p < .001$. VASS: Visual Analogue Scale for Stress; PCL-5: The Posttraumatic Stress Disorder Checklist for DSM-5; PSS-10: 10-item Perceived Stress Scale; STAI-S; State subscale of the State-Trait Anxiety Inventory; STAI-T: Trait subscale of the State-Trait Anxiety Inventory; PHQ-2: The Patient Health Questionnaire-2.

outcomes than those who were not: PCL-5: $U = 1666$, $p < .001$; PSS-10: $U = 2049.5$, $p = .003$; STAI-S: $U = 1971$, $p = .001$; STAI-T: $U = 1681$, p $< .001$; PHQ-2: $U = 1580.5$, $p < .001$. The perceived stress levels also appeared to follow the same trend across healthcare workers who requested help ($U = 2329.5$, $p = .876$) and took stress-related medication ($U = 2751.5$, $p = .548$), but without finding statistical significance.

In line with the above results, substance abuse also suggested more unsatisfactory overall psychological results. Nevertheless, the analysis conducted on the data revealed only significant differences in the PCL-5 ($U = 2737.5$, $p = .003$) test. Healthcare workers who abused substances showed significantly higher PTSS than those without abuse habits.

In contrast, performing higher levels of physical exercise seemed to improve the psychological outcome, observing significant differences in the PSS-10 [$F(2, 181) = 7.32$, $p = .001$] test and both STAI-S [$F(2, 181) = 5.80$, $p = .004$] and STAI-T [$F(2, 181) = 7.32$, $p < .001$] subscales. Healthcare workers who reported high levels of exercise daily had significantly lower stress appraisal (p $< .001$), anxiety ($p = .003$) and predisposition to perceiving situations as a threat (p $< .001$) compared to those having low levels of physical activity. Consistent with this, healthcare workers with medium levels of physical activity also showed significantly greater stress appraisal ($p = .004$), anxiety ($p = .014$) and predisposition to perceiving situations as a threat ($p = .023$) compared to those experiencing low levels of physical activity.

Having a psychiatric history and being off work due to Covid-19 infection also suggested a greater psychological burden than not having any previous psychiatric disorder or being off work due to Covid-19 infection. Nevertheless, no significant differences were found in these two latter groups, respectively. Neither in any of the other study variables analysed ($p > .05$).

3.3. Risk factors of acute stress, anxiety and depression symptoms across the sample

Table 4 shows the significant predictors for greater psychological distress (i.e., acute stress, anxiety, depression and PTSD) according to each psychometric test separately.

After adjusting for age and psychiatric history (Table 4), it was found that being a woman was associated with greater PTSS (β = 0.191, p = .006), stress appraisal (β = 0.239, p = .001), anxiety (β = 0.310, p < .001), depression (β = 0.170, p = .014) and predisposition to perceiving situations as a threat (β = 0.244, p = .001). Similarly, being under stress-related medication was also associated with higher PTSS (β = 0.257, p < .001), stress appraisal (β = 0.183, p = .009), anxiety (β = 0.174, p = .012), depression (β = 0.348, p < .001) and predisposition to perceiving situations as a risk (β = 0.265, p < .001). Overworking (i.e., working over 40 h per week) was associated with worse stress appraisal (β = 0.162, p = .002) and substance abuse was associated with higher PTSS (β = 0.223, p = .002).

Table 4
Regression coefficients for the significant predictors of psychological distress based on the quantitative psychological assessment.

		B	*95% CI*	*β*	*t*	*p-value*
PCL-5[a]	Stress-related medication[g]	7.727	[3.621, 11.834]	0.257	3.71	***
	Substance abuse[h]	5.939	[2.283, 9.595]	0.223	3.21	**
	Gender[f]	6.668	[1.930, 11.406]	0.191	2.77	**
PSS-10[b]	Physical activity[i]	−1.896	[−3.069, −0.724]	−0.221	−3.19	**
	Gender[f]	3.840	[1.669, 6.010]	0.239	3.49	**
	Stress-related medication[g]	2.542	[0.649, 4.434]	0.183	2.65	**
	Weekly working hours[j], >40 h/w	6.547	[0.883, 12.262]	0.162	2.26	**
STAI-S[c]	Gender[f]	8.641	[4.911, 12.371]	0.310	4.57	***
	Stress-related medication[g]	4.174	[0.923, 7.425]	0.174	2.53	*
	Physical activity[i]	−2.565	[−4.580, −0.550]	−0.173	−2.51	*
STAI-T[d]	Stress-related medication[g]	5.313	[2.639, 7.987]	0.265	3.92	***
	Gender[f]	5.663	[2.595, 8.731]	0.244	3.64	**
	Physical activity[i]	−2.585	[−4.243, −0.928]	−0.208	−3.08	**
PHQ-2[e]	Stress-related medication[g]	1.044	[0.640, 1.447]	0.348	5.11	***
	Gender[f]	0.590	[0.121, 1.058]	0.170	2.49	*
	Medical Service[k]	−0.241	[−0.462, −0.019]	−0.147	−2.14	*

Note. * **p < .05,** ** **p < .01,** *** **p < .001**. CI = confidence interval for B. PCL-5: The Posttraumatic Stress Disorder Checklist for DSM-5; PSS-10: 10-item Perceived Stress Scale; STAI-S; State subscale of the State-Trait Anxiety Inventory; STAI-T: Trait subscale of the State-Trait Anxiety Inventory; PHQ-2: The Patient Health Questionnaire-2.

[a] $F(5, 178) = 8.176$, $p < .001$, $R^2_{adj} = 0.164$.
[b] $F(5, 178) = 9.703$, $p < .001$, $R^2_{adj} = 0.192$.
[c] $F(3, 180) = 12.60$, $p < .001$, $R^2_{adj} = 0.199$.
[d] $F(6, 177) = 8.893$, $p < .001$, $R^2_{adj} = 0.160$.
[e] $F(9, 174) = 6.054$, $p < .001$, $R^2_{adj} = 0.206$.
[f] = reference category: men.
[g] reference category: no medication.
[h] reference category: no substance abuse.
[i] reference category: low physical activity.
[j] reference category: up to 30 h per week.
[k] reference category: Covid-19 hospitalisation wards.

On the contrary, higher levels of physical exercise were associated with lower stress appraisal (β = −0.221, p = .002), anxiety (β = −0.173, p = .013) and predisposition to perceiving situations as a threat (β = −0.208, p = .001). Similarly, working in Emergency Services and ICUs was associated with lower depression (β = −0.147, p = .033) than in Covid-19 hospitalisation wards.

4. Discussion

Since being detected in December 2019 in Wuhan (China), the SARS-CoV-2 virus has challenged more than 110 countries and entire health systems, posing immense pressure on healthcare workers' wellbeing and mental health [35,36].

Accordingly, the purpose of our study was to quantify the immediate psychological impact of the SARS-CoV-2 pandemic in Spanish Covid-19 frontline healthcare workers based on face-to-face psychological assessments to cross-check the results obtained in similar studies conducted using online evaluations. In this regard, the main finding was that more than 77% of healthcare workers had moderate anxiety levels, while almost 13% showed severe anxiety. Nearly 45% and 30% of healthcare workers also presented a moderate and high predisposition to perceiving the everyday pandemic situations as threatening, respectively, thus exacerbating the base anxious symptomatology. Furthermore, almost 13% had PHQ-2 scores consistent with MDD diagnosis. These results align with previously published works conducted on medical personnel during the pandemic [37–39]. Nevertheless, they also contrast sharply with the low prevalence of anxiety observed in South and Southeast Asian workers during the same period [40].

Another relevant finding was that over half (65.76%) of healthcare workers showed a moderate stress appraisal, which appeared to be consistent with the moderate levels of perceived stress (42.39%) resulting from the visual analogue scale. More importantly, almost a quarter (23.37%) of the sample presented severe PTSS, compatible with PTSD diagnosis. As with the prevalence of anxiety and depression discussed above, the levels of acute stress encountered in our study are comparable to those observed in Italian [41] and Spanish [37] healthcare workers but much higher than those reported in Asian healthcare workers [40]. A possible contributing factor to these differences between European and Asian Covid-19 works could be using different psychometric tests to evaluate the same psychological constructs. The differences in assessment times may have also played a role in determining the prevalence of psychological distress across healthcare workers from different regions.

Interestingly, the prevalence of acute stress, depression and anxiety observed in our study was also higher than what has been reported in Wuhan healthcare workers at the beginning of the pandemic [42–44]. As in the Covid-19 Asian works discussed above [40], differences between healthcare workers from different regions could be due to using different psychometric tests to assess the same psychological characteristics or performing the assessments at different times of the pandemic. The differences in psychological distress could also be caused by the resulting anticipatory effect between what was reported from China (area of origin) and what was potentially expected to happen shortly after in Spain. From the rapid spread of the disease to the increased mortality rates in both the general population and health workers [44].

On the other hand, the psychological burden fell more notable across women healthcare workers than men healthcare workers [37,38,41–43,45]. Conversely, the medical unit of work did not seem to have much influence on psychological distress. As already described in Section 2.2. (Setting and subjects), this study focused only on recruiting Covid-19 frontline healthcare workers from medical units dedicated to the care and hospitalisation of patients with this disease. Therefore, both the pressure in medical care and the tasks performed by healthcare workers were relatively the same, with minor exceptions regarding the length of hospitalisation in the Emergency Service. At the Emergency

Service, the rotation of Covid-19 patients was approximately every 24 h instead of days or weeks (or even months) as in the ICUs and Covid-19 hospitalisation wards.

The results of our study also showed that nurses and nurse aides had greater PTSS and depression than physicians [38,41,42,46,47]. The stress appraisal, anxiety, and predisposition to perceiving situations as threatening across nurses and nurse aides also appeared to follow the same tendency compared to physicians, although results were not significantly different. These findings align with similar research conducted to evaluate the psychosocial effects of the previous 2003 SARS outbreak on healthcare workers. In this study, the authors reported that the nursing staff showed greater psychological distress than other categories [48] due to the added care and contact with ill patients [42].

Worsened psychological distress resulting from longer working hours is widely documented in the literature [49,50]. Consistent with this, our study showed that healthcare workers working more than 40 h per week (i.e., overworking) had worse stress appraisal than working between 31 and 40 h per week. The results of comparing healthcare workers working more than 40 h per week against those working less than 30 h per week also seemed to point to this tendency. That is, the perceived stress, anxiety and predisposition to perceiving situations as a threat appeared to be markedly higher when working over 40 h. Nevertheless, no significant differences were found in this regard, nor between healthcare workers working 31 to 40 h per week and those working less than 30 h. Together, these results underline the importance of facilitating shorter working hours and thus longer resting periods to reduce the risk and vulnerability of all healthcare workers against psychological distress in the event of future pandemics [51].

Contrary to what was reasonably expected, an increase in the working hours due to the relentless increment of medical care demand did not involve significant differences regarding psychological distress [38]. A plausible explanation for these results may be the proper maintenance of the hospital's management standards. In other words, despite the continuous changes in medical care procedures, the opening up of new Covid-19 hospitalisation wards and the continuous transfer of care staff as needed, the length of usual working shifts throughout the pandemic remained relatively stable without major variations. Moreover, the healthcare workers recruited for the study were professionals who were coming from medical units with a regular heavy care workload (regardless of the pandemic), which could have been a factor that has facilitated their adaptation to the increased care demand, lowering their psychological distress even when working longer hours.

Nonetheless, the work shift did seem to have a substantial effect on the mental health of health care workers. Especially the night and afternoon shifts, worsening the depressive symptomatology compared to morning and on-call shifts [52,53]. The severe impact of late working hours on physical and psychological health, combined with the burden resulting from the heavy care demand the pandemic poses, seriously threatens the performance and efficiency of healthcare workers and thus the quality of the medical treatment provided against the disease [54–56].

To our surprise, having lived with people at high risk of Covid-19 infection did not appear to significantly affect the emotional distress of healthcare workers [37]. On the contrary, it seemed to involve a slight improvement in PTSS. Living with relatives at high risk of Covid-19 infection also seemed to reduce the stress appraisal and the predisposition to perceiving situations as a threat. In line with what other authors have previously suggested, keeping in touch and living with loved ones can mitigate the adverse impact of the loneliness and social isolation caused by the pandemic [57,58].

Our study also showed that the more physical exercise healthcare workers reported doing daily, the lower the psychological distress was [59,60]. Accordingly, healthcare workers doing low physical activity showed worse stress, anxiety, and depression than those doing medium and high exercise levels. These results emphasise the importance of fostering and continuing (as much as the situation allows to do so) with the practice of physical activity and other sorts of leisure activities to cope and minimise the psychological impact of the most devastating events of future pandemics [42,47].

As expected, to have requested help to overcome the pandemic day-to-day situations and have been prescribed stress-related medication due to high emotional burden was related to significantly higher psychological distress. The levels of perceived stress were also higher in those healthcare professionals who had sought help and were taking medication for stress, without finding statistical significance. This lack of significance may be due to the limitations that visual scales present when attempting to objectify a multidimensional construct such as stress [32,34].

Although the results were not significant for this group, it is also important to underline that having had a psychiatric history suggested worse psychological distress across healthcare professionals when evaluated at baseline. As it has already been described in a broad range of clinical contexts, a history of psychiatric disorders increases the predisposition to future psychiatric pathologies and comorbidities, especially when an extreme life event (i.e., Covid-19 pandemic) triggers it [61–63].

Similarly, substance abuse across healthcare workers led to greater PTSS [64]. Substance abuse also involved higher anxiety and depression, predisposition to perceiving situations as a risk, stress appraisal and perceived stress. Nevertheless, these latter results were not significant in our study. Since there is plenty of literature on drug addiction's health and functioning consequences [64], substance abuse derived from psychological burden is a major concern within the medical practice. On the one hand, as it may exacerbate the ongoing psychological distress. On the other hand, as it may also compromise the care standards of healthcare workers at times of greatest health demands [65].

Lastly, the regression models also showed that women were at greater risk of psychological distress than men. Taking stress-related medication predicted poorer mental health, increasing PTSS, stress appraisal, anxiety, depression, and predisposition to perceiving situations as a threat. Substance abuse was related to greater PTSS, and working over 40 h per week increased the stress appraisal. Likewise, working in Covid-19 hospitalisation wards predicted worse depression than performing in Emergency Services and ICUs. Nevertheless, an increase in physical activity levels appeared to reduce the stress appraisal, anxiety and predisposition to perceiving situations as a threat. With minor differences, our regression results were similar to most of those results observed in other studies conducted around the novel coronavirus pandemic [37,41,42,45].

Given the results discussed in this work and the potential harm that undiagnosed and not-well addressed high psychological distress may cause to the life of healthcare workers and consequently to healthcare systems in critical scenarios, it is of vital importance the design and implementation of effective evidence-based intervention strategies to reduce the psychological impact of future pandemics. Nevertheless, considering the cost-benefit of conducting individual sessions with the low availability of therapists due to Covid-19 infection the current pandemic has brought with it, combined with the stigmatisation and lack of awareness among healthcare workers, the development of online psychotherapies could improve the accessibility and availability of mental health care services in high demand contexts [66,67]. To further benefit, online psychotherapies also reduce the spread of the virus that naturally occurs in face-to-face therapies [66]. Online-based Cognitive Behaviour Therapy (ICBT), for instance, has shown efficacy in a wide range of psychiatric settings, as well as in other medical conditions such as chronic pain [67–70]. Accordingly, it may be an interesting mental health strategy to be applied to all healthcare workers to combat the psychological burden associated with unprecedented health contexts.

4.1. Limitations

This study has some limitations. Firstly, the lack of a control group prevented comparing frontline healthcare workers and those not involved in the direct care of Covid-19 patients (i.e., second-line healthcare workers). Secondly, to use psychometric questionnaires not designed to evaluate stress, anxiety and depression in the context of the pandemic as an extreme stressor maintained over time. Likewise, to use psychometric material with no diagnostic capacity, such as the VASS test, despite being widely present in the research community [24]. Together, this may limit the interpretation of the results as some cases may have been underdiagnosed. Thirdly, the inability to assess off work healthcare workers may hinder illustrating the real impact of the pandemic on healthcare workers' mental health. Fourthly, the nature of cross-sectional study designs does not allow interpretation for causality. Following the 2003 SARS experience, studies have shown that the effect of sustained adverse events on mental health remains throughout time, even after the stressor has been resolved [10,71–73]. Even with these limitations, our findings are consistent with previously published works. Therefore, we encourage future research to include a follow-up of healthcare workers to examine for changes in mental health over time while inviting other study areas to design and validate a unified, fast and easy-to-use tool for the early detection of chronic stress.

5. Conclusion

This study evidenced the severe psychological impact of the coronavirus pandemic on healthcare workers of two Spanish tertiary hospitals of reference for the care and diagnosis of suspected cases of Covid-19. The acute stress, depression and anxiety levels encountered were similar to those observed in most published works conducted on medical personnel [19,74] but much higher than those obtained in Wuhan healthcare workers [42]. Furthermore, to be a woman, under stress-related medication, working over 40 h per week, in Covid-19 hospitalisation wards, and substance abuse were predictors for severe psychological distress. On the contrary, the practice of physical activity was related to a lower psychological burden.

While the possible long-term consequences of the coronavirus pandemic remain unknown, knowledge of the risk factors for increased risk of psychological distress may help develop comprehensive improvement strategies to prevent, control and reduce the mental health exacerbation of healthcare workers, thereby maintaining the efficiency of health systems in critical scenarios. Online psychological intervention strategies such as ICBT may be a promising option, together with shorter working shifts and the practice of physical exercise, as they have demonstrated evidence to strengthen mental health resilience and reduce the risk of developing psychiatric morbidities in a broad psychiatric and medical context [66,69,75].

Funding

This work was supported by the FONDO SUPERA COVID-19 [Grant number: PI046782]: Banco Santander, CRUE Universidades Españolas and Consejo Superior de Investigaciones Científicas (CSIC), Spain.

Declaration of competing interest

None.

Acknowledgements

To first-line healthcare workers who, despite the pressure of care, agreed to participate.

References

[1] González-Sanguino C, Ausín B, Castellanos MÁ, Saiz J, López-Gómez A, Ugidos C, et al. Mental health consequences during the initial stage of the 2020 coronavirus pandemic (COVID-19) in Spain. Brain Behav Immun 2020;87:172–6. https://doi.org/10.1016/j.bbi.2020.05.040.

[2] Lee Y, Lui LMW, Chen-Li D, Liao Y, Mansur RB, Brietzke E, et al. Government response moderates the mental health impact of COVID-19: a systematic review and meta-analysis of depression outcomes across countries. J Affect Disord 2021; 290:364–77. https://doi.org/10.1016/J.JAD.2021.04.050.

[3] Wang C, Tee M, Roy AE, Fardin MA, Srichokchatchawan W, Habib HA, et al. The impact of COVID-19 pandemic on physical and mental health of Asians: a study of seven middle-income countries in Asia. PLoS One 2021;16:e0246824. https://doi.org/10.1371/JOURNAL.PONE.0246824.

[4] Wang C, Chudzicka-Czupała A, Tee ML, Núñez MIL, Tripp C, Fardin MA, et al. A chain mediation model on COVID-19 symptoms and mental health outcomes in Americans, Asians and Europeans. Sci Rep 2021. https://doi.org/10.1038/s41598-021-85943-7. 11:1 2021;11:1–12.

[5] Xiong J, Lipsitz O, Nasri F, Lui LMW, Gill H, Phan L, et al. Impact of COVID-19 pandemic on mental health in the general population: a systematic review. J Affect Disord 2020;277:55–64. https://doi.org/10.1016/J.JAD.2020.08.001.

[6] Mariotti A. The effects of chronic stress on health: new insights into the molecular mechanisms of brain–body communication. Future Sci OA 2015;1:FSO23. https://doi.org/10.4155/FSO.15.21.

[7] Hammen C, Kim EY, Eberhart NK, Brennan PA. Chronic and acute stress and the prediction of major depression in women. Depress Anxiety 2009;26:718–23. https://doi.org/10.1002/DA.20571.

[8] Tan YQ, Wang Z, Yap QV, Chan YH, Ho RC, Hamid ARAH, et al. Psychological health of surgeons in a time of COVID-19: a global survey. Ann Surg 2021. https://doi.org/10.1097/SLA.0000000000004775.

[9] Chew NWS, Ngiam JN, Tan BY, Tham SM, Tan CY, Jing M, et al. Asian-Pacific perspective on the psychological well-being of healthcare workers during the evolution of the COVID-19 pandemic. BJPsych Open 2020;6:e116. https://doi.org/10.1192/BJO.2020.98.

[10] Maunder RG, Lancee WJ, Balderson KE, Bennett JP, Borgundvaag B, Evans S, et al. Long-term psychological and occupational effects of providing hospital healthcare during SARS outbreak. Emerg Infect Dis 2006;12:1924–32. https://doi.org/10.3201/eid1212.060584.

[11] Carmassi C, Malacarne P, Dell'oste V, Bertelloni CA, Cordone A, Foghi C, et al. Post-traumatic stress disorder, burnout and their impact on global functioning in Italian emergency healthcare workers. Minerva Anestesiol 2021;87:556–66. https://doi.org/10.23736/S0375-9393.20.14853-3.

[12] Aguiló J, Ferrer-Salvans P, García-Rozo A, Armario A, Corbí Á, Cambra FJ, et al. Project ES3: Attempting to quantify and measure the level of stress. Rev Neurol 2015;61:405–15. https://doi.org/10.33588/rn.6109.2015136.

[13] Aguiló Mir S, García Pagès E, López Barbeito B, Ribeiro TC, Garzón-Rey JM, Aguiló Llobet J. Design and validation of an electrophysiological based tool to assess chronic stress. Case study: burnout syndrome in caregivers. Stress 2020;31: 1–10. https://doi.org/10.1080/10253890.2020.1807512.

[14] Aguiló S, García E, Arza A, Garzón-Rey JM, Aguiló J. Evaluation of chronic stress indicators in geriatric and oncologic caregivers: a cross-sectional study. Stress 2018;21:36–42. https://doi.org/10.1080/10253890.2017.1391211.

[15] Whalen KJ, Buchholz SW. The reliability, validity and feasibility of tools used to screen for caregiver burden: a systematic review. JBI Libr Syst Rev 2009;7: 1373–430. https://doi.org/10.11124/01938924-200907320-00001.

[16] Latkin CA, Mai NVT, Ha TV, Sripaipan T, Zelaya C, le Minh N, et al. Social desirability response bias and other factors that may influence self-reports of substance use and HIV risk behaviors: a qualitative study of drug users in Vietnam. AIDS Educ Prev 2016;28:417–25. https://doi.org/10.1521/aeap.2016.28.5.417.

[17] Andrade C. The limitations of online surveys. Indian J Psychol Med 2020;42: 575–6. https://doi.org/10.1177/0253717620957496.

[18] Pierce M, McManus S, Jessop C, John A, Hotopf M, Ford T, et al. Says who? The significance of sampling in mental health surveys during COVID-19. Lancet Psychiatry 2020;7:567–8. https://doi.org/10.1016/S2215-0366(20)30237-6.

[19] Alonso J, Vilagut G, Mortier P, Ferrer M, Alayo I, Aragón-Peña A, et al. Mental health impact of the first wave of COVID-19 pandemic on Spanish healthcare workers: a large cross-sectional survey. Rev Psiquiatr Salud Ment 2020;14:90–105. https://doi.org/10.1016/j.rpsm.2020.12.001.

[20] Wang C, Chudzicka-Czupała A, Grabowski D, Pan R, Adamus K, Wan X, et al. The association between physical and mental health and face mask use during the COVID-19 pandemic: a comparison of two countries with different views and practices. Front Psych 2020;11:569981. https://doi.org/10.3389/FPSYT.2020.569981.

[21] Wang C, Pan R, Wan X, Tan Y, Xu L, Ho CS, et al. Immediate psychological responses and associated factors during the initial stage of the 2019 coronavirus disease (COVID-19) epidemic among the general population in China. Int J Environ Res Public Health 2020;17:1729. https://doi.org/10.3390/IJERPH17051729.

[22] Wang C, Tripp C, Sears SF, Xu L, Tan Y, Zhou D, et al. The impact of the COVID-19 pandemic on physical and mental health in the two largest economies in the world: a comparison between the United States and China. J Behav Med 2021;2021(1): 1–19. https://doi.org/10.1007/S10865-021-00237-7.

[23] Buselli R, Corsi M, Baldanzi S, Chiumiento M, del Lupo E, Dell'Oste V, et al. Professional quality of life and mental health outcomes among health care workers exposed to Sars-Cov-2 (Covid-19). Int J Environ Res Public Health 2020;17:6180. https://doi.org/10.3390/IJERPH17176180.

[24] Lesage F-X, Berjot S, Deschamps F. Clinical stress assessment using a visual analogue scale. Occup Med (Lond) 2012;62:600–5. https://doi.org/10.1093/occmed/kqs140.
[25] Cohen S, Kamarck T, Mermelstein R. A global measure of perceived stress. J Health Soc Behav 1983;24:385–96. https://doi.org/10.2307/2136404.
[26] Pedrozo-Pupo JC, Pedrozo-Cortés MJ, Campo-Arias A. Perceived stress associated with COVID-19 epidemic in Colombia: an online survey. Cad Saúde Pública 2020;36:e00090520. https://doi.org/10.1590/0102-311X00090520.
[27] Campo-Arias A, Pedrozo-Cortés MJ, Pedrozo-Pupo JC. Pandemic-related perceived stress scale of COVID-19: an exploration of online psychometric performance. Rev Colomb Psiquiatr 2020;49:229–30. https://doi.org/10.1016/j.rcp.2020.05.005.
[28] Weathers FW, Litz BT, Keane TM, Palmieri PA, Marx BP, Schnurr PP. PTSD checklist for DSM-5 (PCL-5). https://www.ptsd.va.gov/:; 2013.
[29] Spielberger CD, Gorsuch RL, Lushene R, Vagg PR, Jacobs GA. Manual for the state-trait anxiety inventory. Palo Alto, CA: Consulting Psychologists Press; 1983.
[30] Milgrom Y, Tal Y, Finestone AS. Comparison of hospital worker anxiety in COVID-19 treating and non-treating hospitals in the same city during the COVID-19 pandemic. Isr J Health Policy Res 2020;9:55. https://doi.org/10.1186/s13584-020-00413-1.
[31] Kroenke K, Spitzer RL, Williams JBW. The patient health Questionnaire-2. Med Care 2003;41:1284–92. https://doi.org/10.1097/01.MLR.0000093487.78664.3C.
[32] Correll DJ. The measurement of pain: objectifying the subjective. In: Pain management. vol. 1. Elsevier Inc; 2007. p. 197–211. https://doi.org/10.1016/B978-0-7216-0334-6.50022-4.
[33] Scott J, Huskisson EC. Graphic representation of pain. Pain 1976;2:175–84. https://doi.org/10.1016/0304-3959(76)90113-5.
[34] Closs SJ, Barr B, Briggs M, Cash K, Seers K. A comparison of five pain assessment scales for nursing home residents with varying degrees of cognitive impairment. J Pain Symptom Manage 2004;27:196–205. https://doi.org/10.1016/j.jpainsymman.2003.12.010.
[35] Montemurro N. The emotional impact of COVID-19: from medical staff to common people. Brain Behav Immun 2020;87:23–4. https://doi.org/10.1016/j.bbi.2020.03.032.
[36] Carmassi C, Foghi C, Dell'Oste V, Cordone A, Bertelloni CA. Bui Eric, et al. PTSD symptoms in healthcare workers facing the three coronavirus outbreaks: what can we expect after the COVID-19 pandemic. Psychiatry Res 2020;292:113312. https://doi.org/10.1016/j.psychres.2020.113312.
[37] Erquicia J, Valls L, Barja A, Gil S, Miquel J, Leal-Blanquet J, et al. Emotional impact of the Covid-19 pandemic on healthcare workers in one of the most important infection outbreaks in Europe. Med Clin 2020;155:434–40. https://doi.org/10.1016/j.medcle.2020.07.010.
[38] Hammond NE, Crowe L, Abbenbroek B, Elliott R, Tian DH, Donaldson LH, et al. Impact of the coronavirus disease 2019 pandemic on critical care healthcare workers' depression, anxiety, and stress levels. Aust Crit Care 2020;34:146–54. https://doi.org/10.1016/j.aucc.2020.12.004.
[39] Carmassi C, Gesi C, Corsi M, Cremone IM, Bertelloni CA, Massimetti E, et al. Exploring PTSD in emergency operators of a major University Hospital in Italy: a preliminary report on the role of gender, age, and education. Ann Gen Psychiatry 2018;17:17. https://doi.org/10.1186/s12991-018-0184-4.
[40] Chew NWS, Lee GKH, Tan BYQ, Jing M, Goh Y, Ngiam NJH, et al. A multinational, multicentre study on the psychological outcomes and associated physical symptoms amongst healthcare workers during COVID-19 outbreak. Brain Behav Immun 2020;88:559–65. https://doi.org/10.1016/J.BBI.2020.04.049.
[41] Rossi R, Socci V, Pacitti F, di Lorenzo G, di Marco A, Siracusano A, et al. Mental health outcomes among frontline and second-line health care workers during the coronavirus disease 2019 (COVID-19) pandemic in Italy. JAMA Netw Open 2020;3:e2010185. https://doi.org/10.1001/jamanetworkopen.2020.10185.
[42] Zhu Z, Xu S, Wang H, Liu Z, Wu J, Li G, et al. COVID-19 in Wuhan: Sociodemographic characteristics and hospital support measures associated with the immediate psychological impact on healthcare workers. EClinicalMedicine. 2020 Jul;24:100443. https://doi.org/10.1016/j.eclinm.2020.100443.
[43] Kang L, Ma S, Chen M, Yang J, Wang Y, Li R, et al. Impact on mental health and perceptions of psychological care among medical and nursing staff in Wuhan during the 2019 novel coronavirus disease outbreak: a cross-sectional study. Brain Behav Immun 2020;87:11–7. https://doi.org/10.1016/j.bbi.2020.03.028.
[44] Wang C, López-Núñez MI, Pan R, Wan X, Tan Y, Xu L, et al. The impact of the COVID-19 pandemic on physical and mental health in China and Spain: cross-sectional study. JMIR Form Res 2021;5:e27818. https://doi.org/10.2196/27818.
[45] Rodríguez-Rey R, Garrido-Hernansaiz H, Collado S. Psychological impact of COVID-19 in Spain: early data report. Psychol Trauma 2020;12:550. https://doi.org/10.1037/tra0000943.
[46] Lai J, Ma S, Wang Y, Cai Z, Hu J, Wei N, et al. Factors associated with mental health outcomes among health care workers exposed to coronavirus disease 2019. JAMA Netw Open 2020;3:e203976. https://doi.org/10.1001/jamanetworkopen.2020.3976.
[47] Chen Q, Liang M, Li Y, Guo J, Fei D, Wang L, et al. Mental health care for medical staff in China during the COVID-19 outbreak. Lancet Psychiatry 2020;7:e15–6. https://doi.org/10.1016/S2215-0366(20)30078-X.
[48] Wong TW, Yau JKY, Chan CLW, Kwong RSY, Ho SMY, Lau CC, et al. The psychological impact of severe acute respiratory syndrome outbreak on healthcare workers in emergency departments and how they cope. Eur J Emerg Med 2005;12. https://doi.org/10.1097/00063110-200502000-00005.
[49] Wong K, Chan AHS, Ngan SC. The effect of long working hours and overtime on occupational health: a Meta-analysis of evidence from 1998 to 2018. Int J Environ Res Public Health 2019;16:2102. https://doi.org/10.3390/ijerph16122102.
[50] Zerbini G, Ebigbo A, Reicherts P, Kunz M, Messman H. Psychosocial burden of healthcare professionals in times of covid-19 – a survey conducted at the University Hospital Augsburg. Ger Med Sci 2020;18. https://doi.org/10.3205/000281. Doc05.
[51] Sato K, Kuroda S, Owan H. Mental health effects of long work hours, night and weekend work, and short rest periods. Soc Sci Med 2020;246:112774. https://doi.org/10.1016/J.SOCSCIMED.2019.112774.
[52] Johnson AL, Jung L, Brown KC, Weaver MT, Richards KC. Sleep deprivation and error in nurses who work the night shift. J Nurs Adm 2014;44:17–22. https://doi.org/10.1097/NNA.0000000000000016.
[53] Parry DA, Oeppen RS, Amin MSA, Brennan PA. Sleep: its importance and the effects of deprivation on surgeons and other healthcare professionals. Br J Oral Maxillofac Surg 2018;56:663–6. https://doi.org/10.1016/j.bjoms.2018.08.001.
[54] Wu K, Wei X. Analysis of psychological and sleep status and exercise rehabilitation of front-line clinical staff in the fight against COVID-19 in China. Med Sci Monit Basic Res 2020;26:e924085. https://doi.org/10.12659/MSMBR.924085.
[55] Vera San Juan N, Aceituno D, Djellouli N, Sumray K, Regenold N, Syversen A, et al. Mental health and well-being of healthcare workers during the COVID-19 pandemic in the UK: contrasting guidelines with experiences in practice. BJPsych Open 2021;7:e15. https://doi.org/10.1192/bjo.2020.148.
[56] Belingheri M, Paladino ME, Riva MA. Working schedule, sleep quality and susceptibility to COVID-19 in healthcare workers. Clin Infect Dis 2020;72:1676. https://doi.org/10.1093/cid/ciaa499.
[57] Hwang TJ, Rabheru K, Peisah C, Reichman W, Ikeda M. Loneliness and social isolation during the COVID-19 pandemic. Int Psychogeriatr 2020;32:1217–20. https://doi.org/10.1017/S1041610220000988.
[58] Hiremath P, Suhas Kowshik CS, Manjunath M, Shettar M. COVID 19: impact of lock-down on mental health and tips to overcome. Asian J Psychiatr 2020;51:102088. https://doi.org/10.1016/j.ajp.2020.102088.
[59] Biddle S. Physical activity and mental health: evidence is growing. World Psychiatry 2016;15:176–7. https://doi.org/10.1002/wps.20331.
[60] Faulkner GEJ, Taylor AH. Exercise, health and mental health. Emerging relationships. London: Routledge Taylor & Francis Group; 2005. https://doi.org/10.4324/9780203415016.
[61] Sobregrau P, Andreu C, Carreño M, Donaire A, Rumià J, Boget T, et al. Psychiatric disorders in patients with resistant temporal lobe epilepsy two years after undergoing elective surgery. A longitudinal study. Epilepsy Behav 2021;118:107921. https://doi.org/10.1016/j.yebeh.2021.107921.
[62] Otto C, Reiss F, Voss C, Wüstner A, Meyrose A-K, Hölling H, et al. Mental health and well-being from childhood to adulthood: design, methods and results of the 11-year follow-up of the BELLA study. Eur Child Adolesc Psychiatry 2020. https://doi.org/10.1007/s00787-020-01630-4.
[63] Fryers T, Brugha T. Childhood determinants of adult psychiatric disorder. Clin Pract Epidemiol Ment Health 2013;9:1–50. https://doi.org/10.2174/1745017901309010001.
[64] Mccauley JL, Killeen T, Gros DF, Brady KT, Back SE. Posttraumatic stress disorder and co-occurring substance use disorders: advances in assessment and treatment. Clin Psychol (New York) 2012;19:283–304. https://doi.org/10.1111/cpsp.12006.
[65] Pollock A, Campbell P, Cheyne J, Cowie J, Davis B, McCallum J, et al. Interventions to support the resilience and mental health of frontline health and social care professionals during and after a disease outbreak, epidemic or pandemic: a mixed methods systematic review. Cochrane Database Syst Rev 2020;11:CD013779. https://doi.org/10.1002/14651858.CD013779.
[66] Ho CS, Chee CY, Ho RC. Mental health strategies to combat the psychological impact of coronavirus disease 2019 (COVID-19) beyond paranoia and panic. Ann Acad Med 2020;49:155–60.
[67] Zhang MWB, Ho RCM. Moodle: the cost effective solution for internet cognitive behavioral therapy (I-CBT) interventions. Technol Health Care 2017;25:163–5. https://doi.org/10.3233/THC-161261.
[68] Soh HL, Ho RC, Ho CS, Tam WW. Efficacy of digital cognitive behavioural therapy for insomnia: a meta-analysis of randomised controlled trials. Sleep Med 2020;75:315–25. https://doi.org/10.1016/J.SLEEP.2020.08.020.
[69] Solomon D, Proudfoot J, Clarke J, Christensen H. e-cbt (mycompass), antidepressant medication, and face-to-face psychological treatment for depression in Australia: a cost-effectiveness comparison. J Med Internet Res 2015;17:e255. https://doi.org/10.2196/JMIR.4207.
[70] Takano A, Miyamoto Y, Kawakami N, Matsumoto T. Web-based cognitive behavioral relapse prevention program with tailored feedback for people with methamphetamine and other drug use problems: development and usability study. JMIR Mental Health 2016;3:e1. https://doi.org/10.2196/MENTAL.4875.
[71] Robertson E, Hershenfield K, Grace SL, Stewart DE. The psychosocial effects of being quarantined following exposure to SARS: a qualitative study of Toronto health care workers. Can J Psychiatry 2004;49:403–7. https://doi.org/10.1177/070674370404900612.
[72] Wu P, Fang Y, Guan Z, Fan B, Kong J, Yao Z, et al. The psychological impact of the SARS epidemic on hospital employees in China: exposure, risk perception, and altruistic acceptance of risk. Can J Psychiatry 2009;54:302–11. https://doi.org/10.1177/070674370905400504.

[73] Wang C, Pan R, Wan X, Tan Y, Xu L, McIntyre RS, et al. A longitudinal study on the mental health of general population during the COVID-19 epidemic in China. Brain Behav Immun 2020;87:40–8. https://doi.org/10.1016/J.BBI.2020.04.028.

[74] Cénat JM, Blais-Rochette C, Kokou-Kpolou CK, Noorishad PG, Mukunzi JN, McIntee SE, et al. Prevalence of symptoms of depression, anxiety, insomnia, posttraumatic stress disorder, and psychological distress among populations affected by the COVID-19 pandemic: a systematic review and meta-analysis. Psychiatry Res 2021;295:113599. https://doi.org/10.1016/j.psychres.2020.113599.

[75] Kumar V, Sattar Y, Bseiso A, Khan S, Rutkofsky IH. The effectiveness of internet-based cognitive behavioral therapy in treatment of psychiatric disorders. Cureus 2017;9:e1626. https://doi.org/10.7759/CUREUS.1626.
