# Supplementary material for: Mental health status and related factors influencing healthcare workers during the COVID-19 pandemic: A systematic review and meta-analysis
Source: PLoS One. 2024 Jan 19;19(1):e0289454. doi: 10.1371/journal.pone.0289454 (PMC10798549; doi:10.1371/journal.pone.0289454)
Supplement: S1 Data — (ZIP) [file pone.0289454.s011.zip › literatures/175.pdf]

ELSEVIER

Since January 2020 Elsevier has created a COVID-19 resource centre with free information in English and Mandarin on the novel coronavirus COVID-19. The COVID-19 resource centre is hosted on Elsevier Connect, the company's public news and information website.

Elsevier hereby grants permission to make all its COVID-19-related research that is available on the COVID-19 resource centre - including this research content - immediately available in PubMed Central and other publicly funded repositories, such as the WHO COVID database with rights for unrestricted research re-use and analyses in any form or by any means with acknowledgement of the original source. These permissions are granted for free by Elsevier for as long as the COVID-19 resource centre remains active.

Brain, Behavior, and Immunity 88 (2020) 60–65

Contents lists available at ScienceDirect

Brain, Behavior, and Immunity

journal homepage: www.elsevier.com/locate/ybrbi

Mental health status of medical staff in emergency departments during the Coronavirus disease 2019 epidemic in China

Xingyue Song[a,1], Wenning Fu[b,1], Xiaoran Liu[c,d], Zhiqian Luo[d,e], Rixing Wang[f], Ning Zhou[g,*], Shijiao Yan[c,h,*], Chuanzhu Lv[e,f,i,*]

[a] Department of Epidemiology and Biostatistics, and Ministry of Education Key Laboratory of Environment and Health, and State Key Laboratory of Environmental Health (Incubating), School of Public Health, Tongji Medical College, Huazhong University of Science and Technology, Wuhan, Hubei Province, China
[b] School of Nursing, Tongji Medical College, Huazhong University of Science and Technology, Wuhan, China
[c] Research Unit of Island Emergency Medicine, Chinese Academy of Medical Sciences (No. 2019RU013), Hainan Medical University, Haikou, Hainan, China
[d] Trauma Center, The First Affiliated Hospital of Hainan Medical University, Haikou, Hainan, China
[e] Key Laboratory of Emergency and Trauma of Ministry of Education, Hainan Medical University, Haikou, Hainan, China
[f] Department of Emergency, Hainan Clinical Research Center for Acute and Critical Diseases, The Second Affiliated Hospital of Hainan Medical University, Haikou, Hainan, China
[g] Central People's Hospital of Zhanjiang, Guangdong, China
[h] School of Public Health, Hainan Medical University, Haikou, Hainan, China
[i] Emergency and Trauma College, Hainan Medical University, Haikou, Hainan, China

ARTICLE INFO

Keywords:
Mental health
Medical staff
Emergency department
COVID-19

ABSTRACT

Background: The emergency department is considered to be a high-risk area, as it is often the first stop for febrile patients who are subsequently diagnosed with coronavirus disease 2019. This study, which employed a cross-sectional design, aimed to assess the mental health of emergency department medical staff during the epidemic in China.
Methods: Demographic data and mental health measurements were collected by electronic questionnaires from February 28, 2020 to March 18, 2020.
Outcomes: A total of 14,825 doctors and nurses in 31 provinces of mainland China completed the survey. The prevalence rates of depressive symptoms and post-traumatic stress disorder (PTSD) were 25.2% and 9.1%, respectively. Men were more likely to have depressive symptoms and PTSD than women. Those who were middle aged, worked for fewer years, had longer daily work time, and had lower levels of social support were at a higher risk of developing depressive symptoms and PTSD. Working in the Hubei province was associated with a higher risk of depressive symptoms, while those working in the Hubei province but residing in another province had a lower risk of depressive symptoms and PTSD. Being a nurse was associated with a higher risk of PTSD.
Interpretation: The findings suggest that targeted psychological interventions to promote the mental health of medical staff with psychological problems need to be immediately implemented. Special attention should be paid to local medical staff in Hubei.

1. Introduction

Coronavirus disease 2019 (COVID-19), an infectious respiratory disease, was first detected in December 2019 in Wuhan, Hubei province, China (Zhu et al., 2020; Zhou et al., 2020). Since January 2020, COVID-19 has spread rapidly in 211 countries and regions. According to the official website of the World Health Organization (WHO), as of April 23, 2020, more than 2 million laboratory confirmed cases and 160,000 deaths were reported worldwide (World Health Organization. Coronavirus disease, 2019). On March 11, 2020, the WHO officially declared the COVID-19 outbreak a global pandemic (Cucinotta and Vanelli, 2020).

* Corresponding authors at: Central People's Hospital of Zhanjiang, Guangdong, No. 236 Yuen Chu Road, Chikan District, Zhanjiang 524000, Guangdong Province, China (N. Zhou). School of Public Health, Hainan Medical University, Haikou, Hainan, No. 3 Xueyuan Road, Longhua Zone, Haikou 571199, China (S. Yan). Department of Emergency, Hainan Clinical Research Center for Acute and Critical Diseases, The Second Affiliated Hospital of Hainan Medical University, No. 3 Xueyuan Road, Longhua Zone, Haikou 571199, China (C. Lv).
E-mail addresses: zjzhou121@163.com (N. Zhou), 569289357@qq.com (S. Yan), lvchuanzhu677@126.com (C. Lv).
[1] These authors contributed equally to this work.

https://doi.org/10.1016/j.bbi.2020.06.002
Received 15 May 2020; Received in revised form 30 May 2020; Accepted 2 June 2020
Available online 05 June 2020
0889-1591/ © 2020 Elsevier Inc. All rights reserved.

The primary transmission route of COVID-19 is aerosol and close contact, putting medical staff at high risk of exposure (Li et al., 2020). According to the China-WHO joint investigation report of COVID-19, as of February 20, 2020 a total of 2055 laboratory-confirmed cases of COVID-19 infection among medical staff were reported in 476 hospitals in China (Health, 2020). The increasing number of confirmed cases, insufficient psychological preparation, lack of understanding of COVID-19, overwhelming workload, and lack of personal protective equipment in the early stages of this disease may cause varying degrees of psychological pressure among medical staff. Medical staff, as the primary population dealing with public health emergencies, may experience destructive or unpleasant feelings in the initial emotional stages. The panic and anxiety caused by the stress response, separate from the emergency, are also common among medical staff (Maunder et al., 2003; Bai et al., 2004; Chua et al., 2004).

Since the outbreak of the COVID-19 pandemic, several studies have investigated the mental health status of medical staff and have found that medical staff experience psychological connditions such as anxiety and depression (Huang et al., 2020; Lai et al., 2020; Liu et al.). However, the sample sizes of existing surveys are relatively small (< 5000 people) due to the limited timeframe of the investigation. Moreover, the emergency department is considered to be a high-risk area in a hospital, as it is often the first stop for febrile patients who are subsequently diagnosed with COVID-19. Therefore, the current study aimed to assess the mental health of emergency department medical staff during the epidemic in China, allowing for the provision of theoretical evidence to inform psychological interventions for medical staff.

2. Methods

2.1. Study design and participants

A cross-sectional study was conducted from February 28, 2020 to March 18, 2020. Electronic questionnaires were sent to medical staff working in the emergency department in a hospital equipped with fever clinics or wards for COVID-19 throughout the country by the Emergency Medicine Branch of the Chinese Medical Association. Snowball and convenience sampling methods were used to recruit subjects and a total of 14,825 emergency department medical staff from 31 provinces in mainland China were surveyed. The questionnaires included general demographic characteristics (age, gender, marital status, occupation, education level, technical title, location, and the department worked in during the outbreak), work related information (years of working, daily working time), social support, and mental health (depressive symptoms, post-traumatic stress disorder). The study was approved by the institutional ethics board of the Second Affiliated Hospital of Hainan Medical University (No. 2020R011)

2.2. Measurement of social support

The social support of participants was measured by the Perceived Social Support Scale (PSSS). The SPSS is a psychometrically sound self-report instrument that allows individuals to subjectively estimate their social support level. It includes 12 items with response options ranging from 1 (very strongly disagree) to 7 (very strongly agree), rendering it particularly valuable when time limitations are present (Zimet et al., 1988). The total score of perceived social support is the sum of all items. Scores range from 12 to 84, with higher scores suggesting greater levels of perceived social support. Social support is categorized into three levels according to the total score; low (score ≤ 36), moderate (37 ≤ score ≤ 60), and high (score ≥ 61) (Gao et al., 2019). The Chinese version of the PSSS has shown good reliability and validity in various populations (Feng et al., 2018; Wang et al., 2020; Zhang et al., 2018).

2.3. Measurement of depressive symptoms

The Center for Epidemiologic Studies Depression Scale (CES-D) was used to assess depressive symptoms. The CES-D is a 20-item self-report inventory that assesses the frequency with which the respondents experienced symptoms during the previous week (Radloff, 1977). Each item is scored on a four-point Likert-type scale ranging from 0 (rarely or none of the time, less than one day) to 3 (all of the time, 5–7 days). Both the reliability and validity of the Chinese version has been confirmed (Sun et al., 2017). The current study used a cut-off score of 15/16, with participants who scored 16 or more defined as having depressive symptoms (Chin et al., 2015; Zhang et al., 2015).

2.4. Measurement of post-traumatic stress disorder (PTSD)

PTSD was assessed by the PTSD Checklist for DSM-5 (PCL-5) (Blevins et al., 2015). The PCL-5 is a 20-item self-report scale, with individual items rated on a five-point Likert-type scale ranging from 0 (not at all) to 4 (extremely). The total score of PTSD is calculated as the sum of the all the items. The PCL-5 determines a provisional diagnosis in two ways: a) the presence (endorsed as 2 or greater) of at least one re-experiencing symptom (Criterion B; questions 1–5), one avoidance symptom (Criterion C; questions 6–7), two negative alterations in cognition or mood symptoms (Criterion D; questions 8–14), and two arousal symptoms (Criterion E; questions 15–20) or b) the sum of the total score is greater than 33 points. The current study used the second criterion as the operational definition of PTSD. The Chinese version of the PCL-5 has been validated and is widely used in psychological research (Li et al., 2019; Wang et al., 2015).

2.5. Statistical analysis

The characteristics, by location, are presented using unadjusted means with standard deviations (SD) for continuous variables and unadjusted proportions for categorical variables. Logistic regression models were used to evaluate the association between various factors and depressive symptoms and PTSD. Model 1 includes age, gender, marital status, occupation, education level, technical title, location, and department working during the outbreak. Model 2 adds years of working, daily working time, and social support to the previous model.

Stratified analyses were conducted according to gender and occupation (physician and nurse). Tests for interaction were performed by adding interaction terms of the study factors and the stratifying variable in the final model. All analyses were performed using SAS 9.4 (SAS Institute Inc.), and a two-sided p value < 0.05 was considered statistically significant.

3. Results

3.1. Participant characteristics

Baseline characteristics stratified by location are shown in Table 1. The mean age (SD) was 34.0 (8.2) years. Of the 14,825 participants, 946 (6.4%) worked in Wuhan (732 came from other provinces) and 13,879 (93.6%) worked outside of the Hubei province. Among them, 41.1% of the participants were physicians and 58.9% were nurses. Most participants were women (64.3%), married (71.2%), and had a bachelor's degree or above (74.8%). Approximately half (51.1%) of the participants had junior technical titles, 29.1% had intermediate technical titles, and 13.0% had senior technical titles. Many participants experienced psychological symptoms, with 3733 (25.2%) and 1353 (9.1%) qualifying as having depressive symptoms and PTSD, respectively.

Table 1
Characteristics of the study participants by location.*

Variables	Total (n = 14825)	Hubei province (n = 214)	Other provinces (assisted Hubei province) (n = 732)	Outside Hubei province (n = 13879)
**Age, mean (SD), y**	34.0 (8.2)	35.1 (8.7)	34.3 (6.7)	33.9 (8.3)
**Age group**				
18–25 years	1949 (13.2)	23 (10.8)	32 (4.3)	1894 (13.6)
26–30 years	4324 (29.2)	63 (29.4)	227 (31.0)	4034 (29.1)
31–40 years	5610 (37.8)	72 (33.6)	351 (48.0)	5187 (37.4)
> 40 years	2942 (19.8)	56 (26.2)	122 (16.7)	2764 (19.9)
**Gender**				
Male	5289 (35.7)	81 (37.9)	311 (42.5)	4897 (35.3)
Female	9536 (64.3)	133 (62.1)	421 (57.5)	8982 (64.7)
**Marital status**				
Unmarried	3892 (26.3)	54 (25.2)	217 (29.6)	3621 (26.1)
Married	10,555 (71.2)	155 (72.5)	501 (68.5)	9899 (71.3)
Divorced/Widowed	378 (2.5)	5 (2.3)	14 (1.9)	359 (2.6)
**Educational level**				
Technical school/College	3741 (25.2)	37 (17.3)	131 (17.9)	3573 (25.7)
University	9468 (63.9)	132 (61.7)	456 (62.3)	8880 (64.0)
Graduate school	1616 (10.9)	45 (21.0)	145 (19.8)	1426 (10.3)
**Occupation**				
Physician	6093 (41.1)	108 (50.5)	277 (37.8)	5708 (41.1)
Nurse	8732 (58.9)	106 (49.5)	455 (62.2)	8171 (58.9)
**Technical title**				
None	1009 (6.8)	8 (3.7)	17 (2.3)	984 (7.1)
Junior	7580 (51.1)	104 (48.6)	312 (42.6)	7164 (51.6)
Intermediate	4309 (29.1)	63 (29.5)	269 (36.8)	3977 (28.7)
Senior	1927 (13.0)	39 (18.2)	134 (18.3)	1754 (12.6)
**Working department on this outbreak**				
Emergency department	11,713 (79.0)	151 (70.6)	4 (0.6)	11,558 (83.3)
Fever clinic/Isolation treatment area/Intensive Care Unit	2269 (15.3)	46 (21.4)	722 (98.6)	1501 (10.8)
Other department	843 (5.7)	17 (8.0)	6 (0.8)	820 (5.9)
**Years of working**				
< 10 years	8766 (59.1)	121 (56.6)	395 (54.0)	8250 (59.5)
11–15 years	2372 (16.0)	26 (12.1)	168 (23.0)	2178 (15.7)
16–20 years	1440 (9.7)	21 (9.8)	96 (13.0)	1323 (9.5)
> 20 years	2247 (15.2)	46 (21.5)	73 (10.0)	2128 (15.3)
**Daily working time**				
< 9 h	8692 (58.7)	131 (61.2)	648 (88.6)	7913 (57.0)
9–10 h	3578 (24.1)	52 (24.3)	61 (8.3)	3465 (25.0)
11–12 h	1736 (11.7)	20 (9.4)	20 (2.7)	1696 (12.2)
> 12 h	819 (5.5)	11 (5.1)	3 (0.4)	805 (5.8)
**Social Support**				
Low	230 (1.6)	4 (1.9)	9 (1.2)	217 (1.6)
Moderate	4077 (27.5)	55 (25.7)	104 (14.2)	3918 (28.2)
High	10,518 (70.9)	155 (72.4)	619 (84.6)	9744 (70.2)
**Depressive symptoms**				
No	11,092 (74.8)	140 (65.4)	646 (88.3)	10,306 (74.3)
Yes	3733 (25.2)	74 (34.6)	86 (11.7)	3573 (25.7)
**Post-traumatic stress disorder**				
No	13,472 (90.9)	190 (88.8)	710 (97.0)	12,572 (90.6)
Yes	1353 (9.1)	24 (11.2)	22 (3.0)	1307 (9.4)

* Data are presented as frequency (percentage) unless otherwise indicated.

3.2. Factors associated with depressive symptoms

Table 2 shows the relationship between demographics, work related factors, and social support with depressive symptoms. In the final model, compared to those between the ages of 18 and 25, the ORs (95% CI) were 1.49 (1.28, 1.72), 1.43 (1.19, 1.72), and 1.36 (1.02, 1.81) for those between the age of 26 and 30 years, 31 and 40 years, and older than 40 years, respectively. Men were more likely to have depressive symptoms than women (OR: 1.12, 95% CI: 1.01, 1.24). Compared to married people, the ORs (95% CI) were 1.12 (1.00, 1.26) and 1.35 (1.07, 1.71) for unmarried and divorced/widowed people, respectively. Those with a graduate degree were more likely to have depressive symptoms than those with a technical school or college degree (OR: 1.26, 95% CI: 1.06, 1.50). Compared to working outside of the Hubei province, working in the Hubei province was associated with a higher risk of depressive symptoms (OR: 1.70, 95% CI: 1.26, 2.29), while those working in the Hubei province but residing in another province had a lower risk of depressive symptoms (OR: 0.50, 95% CI: 0.39, 0.65). Compared to those who have worked for 20 years or more, the ORs (95% CI) were 1.43 (1.16, 1.76), 1.47 (1.14, 1.89), and 1.52 (1.17, 1.98) for those who have worked for between 16 and 20 years, 11 and 15 years, and less than 10 years, respectively. Compared to those who work less than 9 h a day, those who work 9–10 h, 11–12 h, and more than 12 h a day are more likely to have depressive symptoms; ORs (95% CI) were 1.44 (1.31, 1.58), 1.47 (1.30, 1.66), and 1.96 (1.67, 2.31), respectively. Low (OR: 9.59, 95% CI: 7.22, 12.74) and moderate (OR: 3.37, 95% CI: 3.11, 3.66) levels of social support were associated with higher risk of depressive symptoms compared to a high level of social support.

Similar findings were obtained in the stratified analyses by gender (Table A.1) and occupation (Table A.2), although the magnitude of the associations varied between the different strata.

Table 2
Multivariate adjusted risk factors of depressive symptoms.

Variables	Model 1		Model 2	
	OR	95% CI	OR	95% CI
**Age** (ref. = 18–25 years)				
26–30 years	1.40	1.21, 1.62	1.49	1.28, 1.73
31–40 years	1.40	1.18, 1.66	1.43	1.19, 1.72
> 40 years	1.03	0.83, 1.26	1.36	1.02, 1.81
**Gender** (ref. = Female)				
Male	1.20	1.08, 1.32	1.12	1.01, 1.24
**Marital status** (ref. = Married)				
Unmarried	1.16	1.04, 1.30	1.12	1.00, 1.26
Divorced/Widowed	1.51	1.21, 1.89	1.35	1.07, 1.71
**Educational level** (ref. = Technical school/College)				
University	1.05	0.95, 1.16	1.09	0.99, 1.22
Graduate school	1.17	1.00, 1.37	1.26	1.06, 1.50
**Occupation** (ref. = Physician)				
Nurse	0.99	0.88, 1.10	1.11	0.98, 1.25
**Technical title** (ref. = None)				
Junior	0.99	0.84, 1.17	0.97	0.82, 1.16
Intermediate	1.01	0.84, 1.23	1.06	0.86, 1.30
Senior	0.89	0.71, 1.13	1.02	0.79, 1.32
**Location** (ref. = Outside Hubei province)				
Other provinces (assisted Hubei province)	0.39	0.30, 0.50	0.50	0.39, 0.65
Hubei provinces	1.54	1.16, 2.05	1.70	1.26, 2.29
**Working department on this outbreak** (ref. = Emergency department)				
Fever clinic/Isolation treatment area/ Intensive Care Unit	0.92	0.81, 1.04	0.92	0.80, 1.04
Other department	0.95	0.80, 1.11	0.84	0.71, 1.00
**Years of working** (ref. = > 20 years)				
16–20 years			1.43	1.16, 1.76
11–15 years			1.47	1.14, 1.89
< 10 years			1.52	1.17, 1.98
**Daily working time** (ref. = < 9 h)				
9–10 h			1.44	1.31, 1.58
11–12 h			1.47	1.30, 1.66
> 12 h			1.96	1.67, 2.31
**Social Support** (ref. = High)				
Moderate			3.37	3.11, 3.66
Low			9.59	7.22, 12.74

3.3. Factors associated with PTSD

The associations between various factors and risk of PTSD are shown in Table 3. In the final model, compared to those between the ages of 18 and 25, the OR (95% CI) were 1.48 (1.18, 1.86) and 1.34 (1.02, 1.77) for those between the age of 26 and 30 years and 31 and 40 years, respectively. Men (OR: 1.75, 95% CI: 1.51, 2.03) and nurses (OR: 1.43, 95% CI: 1.20, 1.71) were more likely to have PTSD than women and physicians. Compared with those who were married and technical school or college graduates, unmarried and undergraduates had a lower risk of PTSD, with ORs (95% CI) of 0.76 (0.64, 0.91) and 0.83 (0.72, 0.97), respectively. Compared to working outside of the Hubei province, those who were working in the Hubei province but residing in another province had a lower risk of PTSD (OR: 0.39, 95% CI: 0.25, 0.63). Compared to those who have worked for 20 years or more, the ORs (95% CI) were 1.57 (1.17, 2.11), 1.73 (1.20, 2.48), and 1.62 (1.11, 2.36) for those who have worked for between 16 and 20 years, 11 and 15 years, and less than 10 years, respectively. Compared to those who work less than 9 h a day, those who work 9–10 h, 11–12 h, and more than 12 h a day were more likely to have PTSD, with ORs (95% CI) of 1.36 (1.18, 1.56), 1.69 (1.43, 2.01), and 2.42 (1.96, 2.99), respectively. Low (OR: 5.49, 95% CI: 4.04, 7.45) and moderate (OR: 2.73, 95% CI: 2.42, 3.07) levels of social support were associated with a higher risk of PTSD compared to high levels of social support.

Similar findings were obtained in the stratified analyses by gender (Table A.3) and occupation (Table A.4), although the magnitude of the associations varied between the different strata.

4. Discussion

In the current cross-sectional study of 14,825 medical staff, 25.2% and 9.1% of participants reported depressive symptoms and PTSD, respectively, during the COVID-19 epidemic in China. In general, the older and local male medical staff in the Hubei province were more likely to have depressive symptoms and PTSD. Shorter longevity of work status and longer daily work hours were both associated with an increased risk of depressive symptoms and PTSD. However, social support had the greatest impacts on the mental health of medical staff; the lower the levels of social support, the greater the risk of depressive symptoms and PTSD. Those who were unmarried were more likely to have depressive symptoms, but less likely to have PTSD. Nurses were at greater risk of PTSD than physicians.

Compared to previous research, (Huang et al., 2020; Lai et al., 2020; Liu et al.) the current study demonstrated lower prevalence rates of depressive symptoms and PTSD among emergency department medical staff. This may be related to the timing of our study. The current study was conducted from February 28, 2020 to March 18, 2020, during which time the epidemic in China had been controlled and the work pressure of the medical staff was significantly reduced compared to the peak period of the epidemic (Pan et al., 2020).

The current study also demonstrated that men are more likely to have depressive symptoms and PTSD, which is inconsistent with a previous study conducted from January 29, 2020, to February 3, 2020 (Lai et al., 2020). One possible explanation for this difference may again be the timing of the current study, which was later than the comparative study, and that the mental status of medical staff may gradually improve over time. Women tend to pay more attention to

Table 3
Multivariate adjusted risk factors of post-traumatic stress disorder.

Variables	Model 1		Model 2	
	OR	95% CI	OR	95% CI
**Age** (ref. = 18–25 years)				
26–30 years	1.44	1.15, 1.81	1.48	1.18, 1.86
31–40 years	1.40	1.08, 1.83	1.34	1.02, 1.77
> 40 years	1.01	0.74, 1.39	1.37	0.91, 2.07
**Gender** (ref. = Female)				
Male	1.87	1.61, 2.17	1.75	1.51, 2.03
**Marital status** (ref. = Married)				
Unmarried	0.80	0.67, 0.95	0.76	0.64, 0.91
Divorced/Widowed	1.10	0.77, 1.57	0.97	0.68, 1.39
**Educational level** (ref. = Technical school/College)				
University	0.81	0.70, 0.94	0.83	0.72, 0.97
Graduate school	0.75	0.59, 0.96	0.81	0.63, 1.05
**Occupation** (ref. = Physician)				
Nurse	1.26	1.06, 1.50	1.43	1.20, 1.71
**Technical title** (ref. = None)				
Junior	0.88	0.70, 1.12	0.87	0.68, 1.11
Intermediate	0.79	0.60, 1.05	0.82	0.61, 1.09
Senior	0.93	0.65, 1.31	1.08	0.75, 1.56
**Location** (ref. = Outside Hubei province)				
Other provinces (assisted Hubei province)	0.30	0.19, 0.47	0.39	0.25, 0.63
Hubei provinces	1.29	0.84, 1.98	1.43	0.92, 2.21
**Working department on this outbreak** (ref. = Emergency department)				
Fever clinic/Isolation treatment area/ Intensive Care Unit	0.93	0.77, 1.13	0.93	0.77, 1.12
Other department	0.86	0.68, 1.10	0.71	0.55, 0.91
**Years of working** (ref. = > 20 years)				
16–20 years			1.57	1.17, 2.11
11–15 years			1.73	1.20, 2.48
< 10 years			1.62	1.11, 2.36
**Daily working time** (ref. = < 9 h)				
9–10 h			1.36	1.18, 1.56
11–12 h			1.69	1.43, 2.01
> 12 h			2.42	1.96, 2.99
**Social Support** (ref. = High)				
Moderate			2.73	2.42, 3.07
Low			5.49	4.04, 7.45

their experiences and feelings and are more willing to express their emotions. This behavior is conducive to the self-regulation of emotions (Neitzke, 2016). Consistent with previous studies, the current study demonstrated that middle-aged as well as unmarried, divorced, or widowed medical staff were at a higher risk of depressive symptoms and PTSD (Liu et al.). Middle-aged medical staff typically have a higher family burden and may be more concerned about their family members, which may affect their mental health. Medical staff who are unmarried, divorced, or widowed receive less care and/or communication from their partner(s) and may experience less family support. Similarly, the current study showed that lower levels of social support were associated with an increased risk of depressive symptoms and PTSD. These findings further highlight the importance of family and social support for medical staff to maintain good mental health (Umberson and Montez, 2010).

The prevalence of PTSD in nurses was higher than in physicians. This finding may be because a nurse is more closely connected with the patient, with greater contact time (Chan, 2003). A previous study conducted among medical staff in emergency departments during the SARS outbreak also showed that nurses were more likely to develop distress and use behavioral disengagement than physicians (Wong et al., 2005). The current study demonstrated that the fewer the years that medical staff had been working, the greater the risk of depressive symptoms and PTSD. A longer work tenure often means more clinical experience when faced with an epidemic, which may be conducive to stronger self-regulation ability. The current study also found that longer daily work time was associated with a higher risk of depressive symptoms and PTSD. An overload of work frequently imposes both physical and psychological strains on medical staff. The current results further illustrate the importance of reasonable work arrangement for the mental health of medical staff.

Compared to those working outside of the Hubei province, local medical staff in the Hubei Province were more likely to have depressive symptoms and PTSD, which is in line with a previous study (Lai et al., 2020). As the center of the epidemic, most of the confirmed cases occurred in the Hubei province than other regions and medical staff were at a high risk of infection for a long time. A previous study also found that 88% of medical staff infection with COVID-19 came from the Hubei province in China (Health, 2020). The current study also showed that medical staff working in the Hubei province who came from other provinces were at lower risk of depressive symptoms and PTSD. Since the COVID-19 outbreak, more than 30,000 medical staff from other provinces have worked in Hubei (Pan et al., 2020). These individuals were selected after their own registration, usually with adequate psychological preparations, and also received training of infectious disease protection before going to Hubei. Previous research has also shown that none of these individuals were infected with COVID-19 while working in the Hubei province.

The present study has several strengths. First, to the best of our knowledge, this is the largest survey on the mental health of emergency department medical staff during the COVID-19 epidemic in China. Second, the current study indicated that a considerable number of medical staff had mental health problems during the epidemic and the findings of this study could provide a valuable reference point for preventing mental health problems of medical staff in other areas of China and other countries. The limitations of the current study include its cross-sectional design and as the epidemic changes, the mental health of the medical staff may also change. Further research is needed to track the dynamic changes of medical staff's mental health status. In addition, all participants in the current study are from the emergency department. Due to the diverse working environments and experience of medical staff in other departments, the generalizability of these results to other populations remains to be verified.

5. Conclusions

The current study demonstrated that a considerable number of medical staff in the emergency department suffered from depressive symptoms and PTSD. It is important to maintain the mental health of medical staff to help control the epidemic of COVID-19. Psychological skills training should be strengthened to better regulate the psychological status of medical staff as well as to mitigate the psychological problems of patients. Targeted psychological interventions to promote the mental health of medical staff with psychological problems needs to be immediately implemented. Furthermore, special attention should be paid to local medical staff in Hubei.

Declaration of Competing Interest

The authors declare that they have no known competing financial interests or personal relationships that could have appeared to influence the work reported in this paper.

Acknowledgements

This study was supported by the Chinese Academy of Medical Sciences Innovation Fund for Medical Sciences (2019-I2M-5-023), the Hainan Provincial Science and Technology Major Project (ZDKJ201804) and the Hainan Social Development Fund (ZDYFXGFY2020004). The funders had no role in study design, data collection and analysis, decision to publish, or preparation of the manuscript. We thank the Chinese Academy of Medical Sciences for the

support.

Data availability

A de-identified dataset can be shared with investigators who meet the criteria and fulfil justification for access to the data for verification of the current findings.

Appendix A. Supplementary data

Supplementary data to this article can be found online at https://doi.org/10.1016/j.bbi.2020.06.002.

References

Zhu, N., Zhang, D., Wang, W., et al., 2020. A novel coronavirus from patients with pneumonia in China, 2019. N. Engl. J. Med. 382 (8), 727–733.

Zhou, P., Yang, X.L., Wang, X.G., et al., 2020. A pneumonia outbreak associated with a new coronavirus of probable bat origin. Nature 579 (7798), 270–273.

World Health Organization. Coronavirus disease 2019 (COVID-19) Situation Report-93. Accessed April 22, 2020 (https://www.who.int/docs/default-source/coronaviruse/situation-reports/20200422-sitrep-93-covid-19.pdf?sfvrsn=35cf80d7_4).

Cucinotta, D., Vanelli, M., 2020. WHO declares COVID-19 a pandemic. Acta bio-medica: Atenei Parmensis 91 (1), 157–160.

Li, Q., Guan, X., Wu, P., et al., 2020. Early transmission dynamics in Wuhan, China, of novel coronavirus-infected pneumonia. N. Engl. J. Med. 382 (13), 1199–1207.

National Health Commission of the People's Republic of China.. China-WHO joint investigation report of Covid-19. Accessed April 23, 2020 (http://www.nhc.gov.cn/jkj/s3578/202002/87fd92510d094e4b9bad597608f5cc2c.shtml).

Maunder, R., Hunter, J., Vincent, L., et al., 2003. The immediate psychological and occupational impact of the 2003 SARS outbreak in a teaching hospital. CMAJ: Can. Med. Association J. 168 (10), 1245–1251.

Bai, Y., Lin, C.C., Lin, C.Y., Chen, J.Y., Chue, C.M., Chou, P., 2004. Survey of stress reactions among health care workers involved with the SARS outbreak. Psychiatric Services 55 (9), 1055–1057.

Chua, S.E., Cheung, V., Cheung, C., et al., 2004. Psychological effects of the SARS outbreak in Hong Kong on high-risk health care workers. Can. J. Psychiatry Revue Canadienne de Psychiatrie 49 (6), 391–393.

Huang, J.Z., Han, M.F., Luo, T.D., Ren, A.K., Zhou, X.P., 2020. Mental health survey of 230 medical staff in a tertiary infectious disease hospital for COVID-19. Chin. J. Industrial Hygiene Occupational Diseases 38, E001.

Lai, J., Ma, S., Wang, Y., et al., 2020. Factors associated with mental health outcomes among health care workers exposed to coronavirus disease 2019. JAMA Network Open 3 (3), e203976.

Liu, Z., Han, B., Jiang, R., et al. Mental health status of doctors and nurses during COVID-19 epidemic in China (3/4/2020). Available at SSRN: https://ssrn.com/abstract=3551329 or http://dx.doi.org/10.2139/ssrn.3551329.

Zimet, G.D., Dahlem, N.W., Zimet, S.G., Farley, G.K., 1988. The multidimensional scale of perceived social support. J. Pers. Assess. 52 (1), 30–41.

Gao, L., Qu, J., Wang, A.Y., 2019. Anxiety, depression and social support in pregnant women with a history of recurrent miscarriage: a cross-sectional study. J. Reprod. Infant Psychol. 1–12.

Feng, D., Su, S., Wang, L., Liu, F., 2018. The protective role of self-esteem, perceived social support and job satisfaction against psychological distress among Chinese nurses. J. Nurs. Manag. 26 (4), 366–372.

Wang, Q., Liu, H., Ren, Z., et al., 2020. Sex difference in the association of coping styles and social support with psychological distress among patients with end-stage renal disease. PeerJournal 8, e8713.

Zhang, M., Zhang, J., Zhang, F., Zhang, L., Feng, D., 2018. Prevalence of psychological distress and the effects of resilience and perceived social support among Chinese college students: does sex make a difference? Psychiatry Res. 267, 409–413.

Radloff, L.S., 1977. The CES-D scale: a self-report depression scale for research in the general population. Appl. Psychol. Meas. 1 (3), 385–401.

Sun, X.Y., Li, Y.X., Yu, C.Q., Li, L.M., [Reliability and validity of depression scales of Chinese version: a systematic review]. Zhonghua liu xing bing xue za zhi2017; 38(1): 110-6.

Chin, W.Y., Choi, E.P., Chan, K.T., Wong, C.K., 2015. The psychometric properties of the Center for Epidemiologic Studies Depression Scale in Chinese primary care patients: factor structure, construct validity, reliability, sensitivity and responsiveness. PLoS ONE 10 (8), e0135131.

Zhang, Y., Ting, R.Z., Lam, M.H., et al., 2015. Measuring depression with CES-D in Chinese patients with type 2 diabetes: the validity and its comparison to PHQ-9. BMC Psychiatry 15 (1), 198.

Blevins, C.A., Weathers, F.W., Davis, M.T., Witte, T.K., Domino, J.L., 2015. The Posttraumatic Stress Disorder Checklist for DSM-5 (PCL-5): Development and Initial Psychometric Evaluation. J. Trauma Stress 28 (6), 489–498.

Li, J., Zhang, W., Chen, W., et al., 2019. Applications of the Chinese version of the primary care PTSD screen for DSM-5 (PC-PTSD-5) for children. J. Affect. Disord. 254, 109–114.

Wang, L., Zhang, L., Armour, C., et al., 2015. Assessing the underlying dimensionality of DSM-5 PTSD symptoms in Chinese adolescents surviving the 2008 Wenchuan earthquake. J. Anxiety Disord. 31, 90–97.

Pan, A., Liu, L., Wang, C., et al., 2020. Association of public health interventions with the epidemiology of the COVID-19 outbreak in Wuhan, China. JAMA.

Neitzke, A.B., 2016. An illness of power: Sex and the social causes of depression. Cult. Med. Psychiatry 40 (1), 59–73.

Umberson, D., Montez, J.K., Social relationships and health: a flashpoint for health policy. J. Health Social Behav., 2010; 51 Suppl: S54-66.

Chan, S., 2003. Nurses fighting against severe acute respiratory syndrome (SARS) in Hong Kong. J. Nurs. Scholarsh. 35 (3), 209.

Wong, T.W., Yau, J.K., Chan, C.L., et al., 2005. The psychological impact of severe acute respiratory syndrome outbreak on healthcare workers in emergency departments and how they cope. Eur. J. Emergency Med. 12 (1), 13–18.
