# Supplementary material for: Mental health status and related factors influencing healthcare workers during the COVID-19 pandemic: A systematic review and meta-analysis
Source: PLoS One. 2024 Jan 19;19(1):e0289454. doi: 10.1371/journal.pone.0289454 (PMC10798549; doi:10.1371/journal.pone.0289454)
Supplement: S1 Data — (ZIP) [file pone.0289454.s011.zip › literatures/176.pdf]

ELSEVIER

Since January 2020 Elsevier has created a COVID-19 resource centre with free information in English and Mandarin on the novel coronavirus COVID-19. The COVID-19 resource centre is hosted on Elsevier Connect, the company's public news and information website.

Elsevier hereby grants permission to make all its COVID-19-related research that is available on the COVID-19 resource centre - including this research content - immediately available in PubMed Central and other publicly funded repositories, such as the WHO COVID database with rights for unrestricted research re-use and analyses in any form or by any means with acknowledgement of the original source. These permissions are granted for free by Elsevier for as long as the COVID-19 resource centre remains active.

General Hospital Psychiatry 71 (2021) 142–144

Contents lists available at ScienceDirect

General Hospital Psychiatry

journal homepage: www.elsevier.com/locate/genhospsych

Letter to the Editor

# Generalized anxiety, depression and posttraumatic stress disorder in a national sample of U.S. internal medicine physicians during the COVID-19 pandemic

ARTICLE INFO

*Keywords*
COVID-19
Pandemic
Anxiety
Posttraumatic stress disorder
Depression
Physician

*To the Editor:*

Systematic reviews have reported increased prevalence of anxiety, depression, and posttraumatic stress disorder (PTSD) among health workers during the COVID-19 pandemic [1]. However, few studies have been conducted among health workers from the United States and many of those used convenience sampling [2], focused exclusively on workers at one or a few institutions [3], or had response rates of 20% or less [4]. The goal of this study was to identify the prevalence of generalized anxiety, depression, and PTSD during the COVID-19 pandemic in a national sample of internal medicine physicians (internists) and assess associations between self-rated risk of COVID-19 and mental health outcomes.

We conducted a national survey in 2020 of internists who are members of an online panel maintained by the American College of Physicians (ACP), the largest medical specialty organization in the United States. The panel, representative of the ACP U.S. post-graduate membership, consists of ACP members who consider participating in periodic online surveys for modest remuneration. Panel members who provided patient care at least 10% of their time were invited to participate. The survey was open from September 15, 2020 to October 8, 2020. The study was deemed exempt by the University of North Carolina at Chapel Hill IRB.

Outcomes were measured with short screening scales. Positive generalized anxiety disorder screening was defined as a score of 3 or greater on the GAD-2 (range 0–6) [5]. Positive depression screening was defined as a score of 3 on the PHQ-2 (range, 0–6) [6]. Positive PTSD screening was defined as a score of 6 or greater on a four-item scale based on the PCL-5 (range, 0–16) [7].

Among the 2145 eligible ACP Panel members, 810 responded to the survey, a 37.8% response rate. We used post-stratification weighting and raking [8] to make the age-gender and age-race/ethnicity composition of the sample comparable to that of the ACP Panel (Table S1). All analyses were weighted.

Prevalence estimates are reported for the three outcomes. Associations between COVID-19 risks and outcomes were assessed using logistic regression in MPLUS 8.5. Less than 15% of respondents had missing data for self-rated risk of developing or dying from COVID-19. Missing data were addressed using full information maximum likelihood in the logistic regressions using Mplus 8.5.

Sample characteristics are shown in Table S2.

The prevalence of positive screening for generalized anxiety was 14.9% (95% CI, 12.4%–17.4%); for depression, 11.7% (95% CI, 9.4%–13.9%); and for PTSD, 12.8% (95% CI, 10.5%–15.1%).

The prevalence of previous COVID-19 infection was 13.4% (95% CI, 11.0%–15.8%). Among respondents who had not previously had COVID-19, 28.3% (95% CI, 25.0%–31.8%) rated themselves at high or very high risk of becoming infected with COVID-19 and 8.0% (95% CI, 6.0%–10.0%) rated themselves at high or very high risk of death if infected with COVID-19.

In multivariable logistic regression models, respondents who rated their risk of developing COVID-19 as high or very high had moderately elevated odds of screening positive for depression and PTSD but not anxiety (Table 1). Respondents who rated their risk of death, if they developed COVID-19, as high or very high had markedly higher odds of all three adverse outcomes (Table 1). Those who had previously had COVID-19 infection had moderately lower odds of screening positive for depression but not generalized anxiety or PTSD.

In this national study, the estimated prevalences of screening positive for generalized anxiety, depression and PTSD were between 10% and 15%. Our estimates are lower than most [1] prevalence estimates from meta-analyses of the psychosocial impact of the COVID-19 pandemic on health care workers. The use of convenience samples in most previous studies of the impact on health care workers of the COVID-19 pandemic, with likely greater participation from those more adversely affected, may explain the higher rates they reported [9]. The difference in prevalence of mental health outcomes between our study and most other studies is not due to differences in measurement because those studies also used short screening instruments.

Physicians who rated themselves at high risk of developing COVID-19 at work and those who rated themselves at high risk of dying if infected had substantially higher prevalence of mental health outcomes

https://doi.org/10.1016/j.genhosppsych.2021.05.004
Received 15 May 2021; Received in revised form 18 May 2021; Accepted 20 May 2021
Available online 24 May 2021
0163-8343/© 2021 Elsevier Inc. All rights reserved.

**Table 1**
Associations between self-rated COVID-19 risks or infection and mental health outcomes, adjusted for potential confounding factors.

| | Screen Positive for Generalized Anxiety | Screen Positive for Depression | Screen positive for PTSD |
|---|---|---|---|
| | $n = 112$ | $n = 95$ | $n = 103$ |
| COVID-19 Risks | Weighted aOR[a] (95% CI) | Weighted aOR (95% CI) | Weighted aOR (95% CI) |
| Risk of developing COVID-19 at work | | | |
| High or very | 1.52 (0.90–2.57) | 2.55 (1.46–4.47) | 1.90 (1.15–3.12) |
| Very low, low or moderate | Ref. | Ref. | Ref. |
| Risk of dying from COVID-19 | | | |
| High or very high | 5.85 (2.89–11.82) | 7.23 (3.57–14.63) | 7.05 (3.55–13.98) |
| Very low, low or moderate | Ref. | Ref. | Ref. |
| Previous COVID-19 infection | | | |
| Yes | 0.71 (0.39–1.30) | 0.50 (0.27–0.90) | 0.67 (0.37–1.20) |
| No | Ref. | Ref. | Ref. |

[a] aOR denotes odds ratio adjusted for age category, gender, race-ethnicity, work setting (outpatient only, outpatient and inpatient, inpatient only), clinical subspecialty at high risk of exposure to COVID-19 (Hospital Medicine, Infectious Disease, Pulmonary Medicine, Critical Care Medicine, Emergency Medicine) versus all other specialties), total clinical hours in previous week, number of patients with suspected or confirmed COVID-19 seen in previous two weeks, type of health care setting (hospital, outpatient practice, setting devoted to care for the underserved, and other), respondent's assessment of rate of COVID-19 infection in their local community in previous two weeks (higher than U.S. as a whole, the same as the U.S. as a whole, lower than the U.S. as a whole) All odd ratios are based on a logistic regression model with post-stratification weights as a weight variable, with estimation using full information maximum likelihood.

and may merit special attention. Respondents who had had COVID-19 infection prior to the survey were less likely to screen positive for depression, perhaps because surviving COVID-19 lowered concerns about the unknown [10].

This study has limitations. The response rate was 37.9%. We used post-stratification weights for age, gender and race/ethnicity but other differences between the sample and the panel could have affected the findings. Also, prevalence estimates are based on short screening tests, which do not perfectly predict rates of mental disorders. Finally, the prevalence estimates are based on a survey from autumn 2020.

In summary, we found that the prevalence of screening positive for anxiety, depression, and PTSD in this national sample of internists in the U.S. during the COVID-19 pandemic was lower than in many other studies, suggesting overall resilience. However, the odds of adverse mental health outcomes were much higher among those who rated themselves at high risk of developing COVID-19 or dying, if infected.

## Funding

This work was supported by funding from the Office of Research, University of North Carolina, School of Medicine.

## Declaration of Competing Interest

None.

## Data availability

Data will be made available on request.

## Acknowledgements

None.

## Appendix A. Supplementary data

Supplementary data to this article can be found online at https://doi.org/10.1016/j.genhosppsych.2021.05.004.

## References

[1] Muller AE, Hafstad EV, Himmels JPW, et al. The mental health impact of the covid-19 pandemic on healthcare workers, and interventions to help them: a rapid systematic review. Psychiatry Res 2020;293:113441. https://doi.org/10.1016/j.psychres.2020.113441.

[2] Firew T, Sano ED, Lee JW, et al. Protecting the front line: a cross-sectional survey analysis of the occupational factors contributing to healthcare workers' infection and psychological distress during the COVID-19 pandemic in the USA. BMJ Open 2020;10:e042752. https://doi.org/10.1136/bmjopen-2020-042752.

[3] Rodriguez RM, Medak AJ, Baumann BM, et al. Academic emergency medicine physicians' anxiety levels, stressors, and potential stress mitigation measures during the acceleration phase of the COVID-19 pandemic. Acad Emerg Med 2020;27:700–7. https://doi.org/10.1111/acem.14065.

[4] Khusid JA, Weinstein CS, Becerra AZ, et al. Well-being and education of urology residents during the COVID-19 pandemic: results of an American national survey. Int J Clin Pract 2020:e13559. https://doi.org/10.1111/ijcp.13559.

[5] Kroenke K, Spitzer RL, Williams JB, Monahan PO, Löwe B. Anxiety disorders in primary care: prevalence, impairment, comorbidity, and detection. Ann Intern Med 2007;146:317–25. https://doi.org/10.7326/0003-4819-146-5-200703060-00004.

[6] Kroenke K, Spitzer RL, Williams JB. The Patient Health Questionnaire-2: validity of a two-item depression screener. Med Care 2003;41:1284–92. https://doi.org/10.1097/01.Mlr.0000093487.78664.3c.

[7] Zuromski KL, Ustun B, Hwang I, et al. Developing an optimal short-form of the PTSD checklist for DSM-5 (PCL-5). Depress Anxiety 2019;36:790–800. https://doi.org/10.1002/da.22942.

[8] Gelman A, Carlin JB. Poststratification and weighting adjustments. In: Groves RM, Dillman DA, Eltinge JL, Little RJA, editors. Survey nonresponse. New York: John Wiley & Sons; 2002. p. 289–303.

[9] Steel Z, Chey T, Silove D, Marnane C, Bryant RA, van Ommeren M. Association of torture and other potentially traumatic events with mental health outcomes among populations exposed to mass conflict and displacement: a systematic review and meta-analysis. JAMA 2009;302(5):537–49.

[10] Cabarkapa S, Nadjidai SE, Murgier J, Ng CH. The psychological impact of COVID-19 and other viral epidemics on frontline healthcare workers and ways to address it: a rapid systematic review. Brain Behav Immun Health 2020;8:100144. https://doi.org/10.1016/j.bbih.2020.100144.

Jeffrey Sonis[a,b,*], Donald E. Pathman[b,c], Susan Read[d], Bradley N. Gaynes[e], Courtney Canter[f,g], Patrick Curran[h], Thomas Miller[i]

[a] *Department of Social Medicine, School of Medicine, University of North Carolina at Chapel Hill, CB#7240, Chapel Hill, NC 27599-7240, United States*

[b] *Department of Family Medicine, CB#7595, School of Medicine, University of North Carolina at Chapel Hill, Chapel Hill, NC 27599-7595, United States*

[c] *Cecil G. Sheps Center for Health Services Research, University of North Carolina at Chapel Hill, 725 M.L.K. Jr. Boulevard, Chapel Hill, NC 27516, United States*

[d] *Research Center, American College of Physicians, 190 N. Independence Mall West, Philadelphia, PA 19106-1572, United States*

[e] *Department of Psychiatry, University of North Carolina at Chapel Hill, 333 S. Columbia Street, Suite 304, MacNider Hall, Chapel Hill, NC 27514, United States*

[f] *School of Medicine, University of North Carolina at Chapel Hill, 321 S. Columbia Street, Chapel Hill, NC 27516, United States*

[g] *Department of Anthropology, University of North Carolina at Chapel Hill, CB# 3115, 301 Alumni Hall, 207 E. Cameron Avenue, Chapel Hill, NC 27599-3115, United States*

[h] *Department of Psychology and Neuroscience, University of North Carolina at Chapel Hill, CB#3270, 235 E. Cameron Avenue, Chapel Hill, NC 27599-3270, United States*

[i] *Department of Medicine, University of North Carolina at Chapel Hill, CB#7005, 125 MacNider Hall, Chapel Hill, NC 27599-7005, United States*

[*] Corresponding author at: Department of Social Medicine, School of Medicine, CB#7240, University of North Carolina at Chapel Hill, Chapel Hill, NC 27599-7240, United States.

*E-mail addresses:* jsonis@med.unc.edu, Sonisjsonis@med.unc.edu (J. Sonis), don_pathman@unc.edu (D.E. Pathman), sread@apconline.org (S. Read), bradley_gaynes@med.unc.edu (B.N. Gaynes), courtney_canter@med.unc.edu (C. Canter), curran@unc.edu (P. Curran), thomas_miller@med.unc.edu (T. Miller).
