# Supplementary material for: Mental health status and related factors influencing healthcare workers during the COVID-19 pandemic: A systematic review and meta-analysis
Source: PLoS One. 2024 Jan 19;19(1):e0289454. doi: 10.1371/journal.pone.0289454 (PMC10798549; doi:10.1371/journal.pone.0289454)
Supplement: S1 Data — (ZIP) [file pone.0289454.s011.zip › literatures/177.pdf]

Psychological Medicine

cambridge.org/psm

Correspondence

Cite this article: Spiller TR *et al* (2020). Development of health care workers' mental health during the SARS-CoV-2 pandemic in Switzerland: two cross-sectional studies. *Psychological Medicine* 1–4. https://doi.org/10.1017/S0033291720003128

Received: 3 July 2020
Revised: 25 July 2020
Accepted: 11 August 2020

Key words:
COVID-19; health care workers; mental health; pandemic; SARS-CoV-2; Switzerland

Author for correspondence:
Tobias R. Spiller,
E-mail: tobias.r.spiller@gmail.com

© The Author(s), 2020. Published by Cambridge University Press. This is an Open Access article, distributed under the terms of the Creative Commons Attribution licence (http://creativecommons.org/licenses/by/4.0/), which permits unrestricted re-use, distribution, and reproduction in any medium, provided the original work is properly cited.

Development of health care workers' mental health during the SARS-CoV-2 pandemic in Switzerland: two cross-sectional studies

Tobias R. Spiller[1,2], Marie Méan[3], Jutta Ernst[4], Onur Sazpinar[5], Samuel Gehrke[2], Francesca Paolercio[6], Heidi Petry[4], Monique C. Pfaltz[1,2], Naser Morina[1,2], Oriane Aebischer[7], David Gachoud[3,7], Roland von Känel[1,2] and Sonja Weilenmann[2,6]

[1]Department of Medicine, University of Zurich, Zurich, Switzerland; [2]University of Zurich and Department of Consultation-Liaison Psychiatry and Psychosomatic Medicine, University Hospital Zurich, Zurich, Switzerland; [3]Department of Internal Medicine, Lausanne University Hospital, Lausanne, Switzerland; [4]Center for Clinical Nursing Science, University Hospital Zurich, Zurich, Switzerland; [5]Department of Internal Medicine, Spital Zollikerberg, Zollikon, Switzerland; [6]Department of Psychology, University of Zurich, Zurich, Switzerland and [7]Educational Unit, School of Medicine, Faculty of Biology and Medicine, University of Lausanne, Lausanne, Switzerland

Abstract

Background. Virus outbreaks such as the current SARS-CoV-2 pandemic are challenging for health care workers (HCWs), affecting their workload and their mental health. Since both, workload and HCW's well-being are related to the quality of care, continuous monitoring of working hours and indicators of mental health in HCWs is of relevance during the current pandemic. The existing investigations, however, have been limited to a single study period. We examined changes in working hours and mental health in Swiss HCWs at the height of the pandemic (T1) and again after its flattening (T2).
Methods. We conducted two cross-sectional online studies among Swiss HCWs assessing working hours, depression, anxiety, and burnout. From each study, 812 demographics-matched participants were included into the analysis. Working hours and mental health were compared between the two samples.
Results. Compared to prior to the pandemic, the share of participants working less hours was the same in both samples, whereas the share of those working more hours was lower in the T2 sample. The level of depression did not differ between the samples. In the T2 sample, participants reported more anxiety, however, this difference was below the minimal clinically important difference. Levels of burnout were slightly higher in the T2 sample.
Conclusions. Two weeks after the health care system started to transition back to normal operations, HCWs' working hours still differed from their regular hours in non-pandemic times. Overall anxiety and depression among HCWs did not change substantially over the course of the current SARS-CoV-2 pandemic.

The SARS-CoV-2 pandemic poses a significant challenge to health care workers (HCWs) all around the globe, affecting their workload and mental health (Ayanian, 2020; Kisely et al., 2020). Since workload and HCWs' well-being are related to the quality of provided care (Scheepers, Boerebach, Arah, Heineman, & Lombarts, 2015), both play a crucial role in sustaining a high work performance of the medical work force during this pandemic. Several studies investigated the mental health of HCWs during the current pandemic but reported partly contradicting findings (e.g. Lai et al., 2020; Rossi et al., 2020; Tian et al., 2020). Moreover, all of these studies were conducted at only one time point.

With this study, we aimed to assess changes in working hours and mental health (assessed as symptoms of anxiety, depression, and burnout) in Swiss HCWs at the height of the SARS-CoV-2 pandemic (T1) and again after its flattening (T2). Since prior research demonstrated none or only modest changes in anxiety and depression among HCWs over the course of previous pandemics (Chen et al., 2006; Chong et al., 2004; Su et al., 2007), we hypothesized that the difference in anxiety and depression between T1 and T2 would be smaller than the minimal clinically important difference. With regard to burnout, no hypothesis was specified.

We conducted two independent, cross-sectional online studies. Inclusion criteria for this analysis were (a) working as a nurse or physician in Switzerland, (b) being at least 18 years old and, (c) having no missing data in the variables of interest. To adjust for the differing characteristics of the two samples, participants were matched one-to-one regarding their age, gender, and profession. Under Swiss federal law, anonymous surveys do not require approval of an

CAMBRIDGE
UNIVERSITY PRESS

Table 1. Demographics, work characteristics, and COVID-19 exposure of two age, gender, and profession matched samples of 812 HCWs each at T1 and T2

Variable	T1 ($n = 812$)		T2 ($n = 812$)			
	Median	IQR	Median	IQR	Statistic[a]	p
Demographics						
Age in years	35	30–44	35	30–44	329 291.0	0.968
Women, n (%)	580	71.4	577	71.1	0.012	0.913
Professional experience in years	10	4–20	10	4.25–18	330 553.0	0.892
Nurses, n (%)	342	42.1	358	44.1	0.565	0.452
Physicians, n (%)	470	57.9	454	55.9		
German speaking, n (%)	635	78.2	417	51.4	207.193	<0.001
French speaking, n (%)	88	10.8	334	42.4		
Italian speaking, n (%)	89	10.9	51	6.3		
Work characteristics						
Total working hours in the previous 7 days	45	36–54	44	35–50	362 558.0	<0.001
Total working hours per week prior to the pandemic	45	38–50	42.5	38–50	323 176.0	0.383
Working more during the pandemic than before, n (%)	359	44.2	229	28.2	16.531	<0.001
Working less during the pandemic than before, n (%)	278	34.2	245	30.2	0.670	0.413
Average number of sleep hours in the previous 7 days	7	6–7.5	7	6–7	324 777.5	0.594
Having access to medical equipment	5	3–6	6	4–7	246 276.0	<0.001
Perceived support by employer	6	4–7	6	4–7	319 117.0	0.253
Perceived support by authorities	5	3–6	5	4–6	291 979.5	<0.001
Perceived passage of information by employer	6	4–7	6	5–7	310 493.0	0.037
Perceived passage of information by authorities	5	4–6	6	4–6	317 392.5	0.184
COVID-19 exposure						
Had suspected COVID-19 symptoms or tested positive for SARS-CoV-2, n (%)	122	15.0	149	18.4	2.994	0.084
Was exposed to suspected or confirmed COVID-19 patients at work, n (%)	649	79.9	555	68.4	27.777	<0.001
Frontline worker, n (%)	416	51.2	419	51.6	0.010	0.921
Mental health						
Anxiety	6	3–10	5	2–9	370 831.5	<0.001
Depression	5	2–9	5	2–9	322 760.0	0.463
Burnout	4	2–6	5	2–7	294 374.5	<0.001

IQR = interquartile range; frontline = worked in at clinical unit designated to diagnosis and treatment of patients with suspected or confirmed COVID-19; burnout = burnout overall symptom score; anxiety = GAD-7 overall score; depression = PHQ-9 overall score.
a*U* or χ^2.

institutional review board. Participants were recruited with a snowball technique. Data were collected between 28 March and 4 April 2020 (T1) and between 9 May and 14 May 2020 (T2).

Symptoms of anxiety and depression were measured with the *Generalized Anxiety Disorder 7* (GAD-7; Spitzer, Kroenke, Williams, and Löwe, 2006) and the *Patient Health Questionnaire-9* (PHQ-9; Kroenke, Spitzer, and Williams, 2001), respectively. The minimal clinically important difference was defined as four points for the GAD-7 (Toussaint et al., 2020) and as five points for the PHQ-9 (Löwe, Unützer, Callahan, Perkins, & Kroenke, 2004). Two single items derived from the *Maslach Burnout Inventory* (West, Dyrbye, Sloan, & Shanafelt, 2009) were used to assess burnout. The period of reference for all questions was the past 7 days.

Sample characteristics and median levels of symptoms were compared using χ^2 and Mann–Whitney *U* tests and carried out in JASP version 0.12 (JASP Team, 2020). Associations between multiple variables were investigated using network analytic methods (Epskamp, Borsboom, & Fried, 2018). These analyses were conducted in the R statistical environment. The chosen significance level for all tests was $\alpha = 0.05$.

The demographics and the differences in anxiety, depression, and burnout of both samples are outlined in Table 1. The share of participants working fewer hours than prior to the pandemic did not differ between T1 and T2 ($\chi^2 = 0.670$, $p = 0.413$), however, less participants in the T2 sample were working longer hours than prior to the pandemic ($\chi^2 = 16.531$, $p \leq 0.001$). Anxiety of participants of the first study was higher than among participants of the

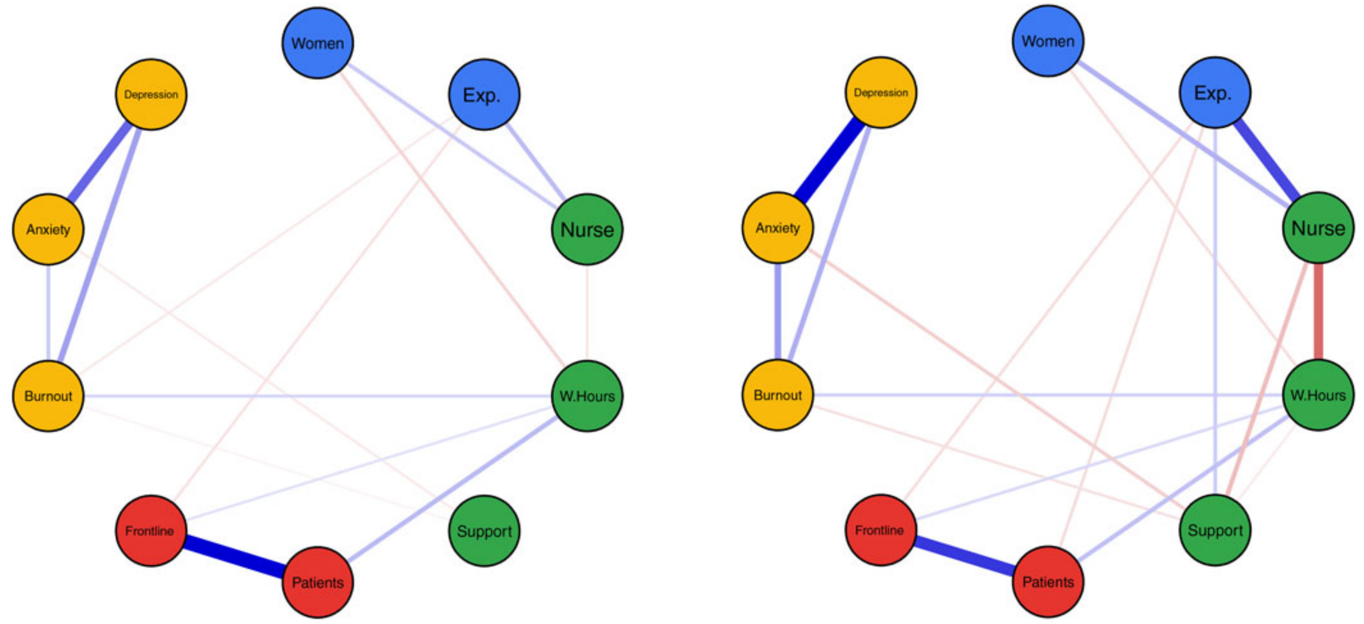

T1 **T2**

Fig. 1. Relationships between multiple variables for two matched samples of 812 HCWs each at T1 and T2. *Note.* Nodes represent variables. The coloring of the nodes indicates different groups of variables (demographics, workplace-related factors, exposure to COVID-19, and mental health); edges represent associations between the nodes (continuous /green= positive, dashed/red = negative, thickness = magnitude of the relationship); women = gender (levels: men = 1, women = 2); Exp. = professional experience in years; nurse = nursing staff (variable = *Profession*; levels: physician = 1, nurse = 2); W.Hours = total working hours in the previous 7 days; support = perceived support by employer; patients = exposure to suspected or confirmed COVID-19 patients at work (levels: No = 0, Yes = 1); ward = working in clinical unit designated to diagnosis and treatment of patients with suspected or confirmed COVID-19 (levels: No = 0, Yes = 1); burnout = overall burnout symptom score; anxiety = overall GAD-7 score; depression = overall PHQ-9 score.

second study ($U = 370\,831.5$, $p \le 0.001$, $r = 0.125$). Both samples had equal symptoms of depression ($U = 322\,760.0$, $p = 0.463$). Participants in the second study reported more burnout than participants in the first sample ($U = 294\,332.5$, $p \le 0.001$, $r = -0.107$). The results of the network analyses are presented in Fig. 1.

Two weeks after the Swiss health care systems started to transition back to normal operations, roughly 30% were working more and 30% were working less hours compared to their usual hours in non-pandemic times. This underscores the complex impact of the pandemic and the taken measures on HCWs' working hours. The increase in anxiety is in concordance with three studies conducted during earlier pandemics, whereas the nondifference in depressive symptoms contrasts them (Chen et al., 2006; Chong et al., 2004; Su et al., 2007). However, the differences in our study did not reach the minimal clinically relevant difference. In the case of burnout, it was of small magnitude. In the conducted network analyses burnout and anxiety were both independently related to lower perceived support by the employer in both studies, a well-described association also in non-pandemic contexts (Shanafelt & Noseworthy, 2017). The nontargeted recruitment, the non-representativeness of our sample, the adaptation of the questionnaires to cover the last 7 days, and the lack of data collected prior to the pandemic limit our study.

Taken together, our findings indicate that the course of the SARS-CoV-2 pandemic did not substantially impact the mental health of Swiss HCWs. In addition, they emphasize the employers influence on HCWs' mental health also during this ongoing pandemic.

Financial support. Tobias R. Spiller was supported by a Forschungskredit of the University of Zurich, grant no. FK-19-048.

Conflict of interest. We have no conflict of interest to declare.

References

Ayanian J. (2020). Mental health needs of health care workers providing frontline COVID-19 care. *JAMA Health Forum*, *1*(4), e200397–e200397.

Chen, R., Chou, K.-R., Huang, Y.-J., Wang, T.-S., Liu, S.-Y., & Ho, L.-Y. (2006). Effects of a SARS prevention programme in Taiwan on nursing staff's anxiety, depression and sleep quality: A longitudinal survey. *International Journal of Nursing Studies*, *43*(2), 215–225. doi:10.1016/j.ijnurstu.2005.03.006.

Chong, M.-Y., Wang, W.-C., Hsieh, W.-C., Lee, C.-Y., Chiu, N.-M., Yeh, W.-C., … Chen, C.-L. (2004). Psychological impact of severe acute respiratory syndrome on health workers in a tertiary hospital. *British Journal of Psychiatry*, *185*(2), 127–133. doi:10.1192/bjp.185.2.127.

Epskamp, S., Borsboom, D., & Fried, E. I. (2018). Estimating psychological networks and their accuracy: A tutorial paper. *Behavior Research Methods*, *50*(1), 195–212. doi:10.3758/s13428-017-0862-1.

JASP Team (2020). JASP (0.12.2) [Computer software]. https://jasp-stats.org/.

Kisely, S., Warren, N., McMahon, L., Dalais, C., Henry, I., & Siskind, D. (2020). Occurrence, prevention, and management of the psychological effects of emerging virus outbreaks on healthcare workers: Rapid review and meta-analysis. *BMJ*, *369*, m1642. doi:10/ggvcj4.

Kroenke, K., Spitzer, R. L., & Williams, J. B. W. (2001). The PHQ-9: Validity of a brief depression severity measure. *Journal of General Internal Medicine*, *16* (9), 606–613. doi:10.1046/j.1525-1497.2001.016009606.x.

Lai, J., Ma, S., Wang, Y., Cai, Z., Hu, J., Wei, N., … Hu, S. (2020). Factors associated with mental health outcomes among health care workers exposed to coronavirus disease 2019. *JAMA Network Open*, *3*(3), e203976. doi:10.1001/jamanetworkopen.2020.3976.

Löwe, B., Unützer, J., Callahan, C. M., Perkins, A. J., & Kroenke, K. (2004). Monitoring depression treatment outcomes with the patient health questionnaire-9. *Medical Care*, *42*(12), 1194–1201. doi: 10.1097/00005650-200412000-00006.

Rossi, R., Socci, V., Pacitti, F., Di Lorenzo, G., Di Marco, A., Siracusano, A., & Rossi, A. (2020). Mental health outcomes among frontline and second-line health care workers during the coronavirus disease 2019 (COVID-19) pandemic in Italy. *JAMA Network Open*, *3*(5), e2010185. doi:10/ggz7r9.

Scheepers, R. A., Boerebach, B. C. M., Arah, O. A., Heineman, M. J., & Lombarts, K. M. (2015). A systematic review of the impact of physicians' occupational well-being on the quality of patient care. *International Journal of Behavioral Medicine*, *22*(6), 683–698. doi:10.1007/s12529-015-9473-3.

Shanafelt, T. D., & Noseworthy, J. H. (2017). Executive leadership and physician well-being. *Mayo Clinic Proceedings*, *92*(1), 129–146. doi:10.1016/j.mayocp.2016.10.004.

Spitzer, R. L., Kroenke, K., Williams, J. B. W., & Löwe, B. (2006). A brief measure for assessing generalized anxiety disorder: The GAD-7. *Archives of Internal Medicine*, *166*(10), 1092. doi: 10.1001/archinte.166.10.1092.

Su, T., Lien, T., Yang, C., Su, Y., Wang, J., Tsai, S., & Yin, J. (2007). Prevalence of psychiatric morbidity and psychological adaptation of the nurses in a structured SARS caring unit during outbreak: A prospective and periodic assessment study in Taiwan. *Journal of Psychiatric Research*, *41*(1–2), 119–130. doi: 10.1016/j.jpsychires.2005.12.006.

Tian, T., Meng, F., Pan, W., Zhang, S., Cheung, T., Ng, C. H., … Xiang, Y.-T. (2020). Mental health burden of frontline health professionals treating imported patients with COVID-19 in China during the pandemic. *Psychological Medicine*, 1–2. doi:10/gg673s.

Toussaint, A., Hüsing, P., Gumz, A., Wingenfeld, K., Härter, M., Schramm, E., & Löwe, B. (2020). Sensitivity to change and minimal clinically important difference of the 7-item Generalized Anxiety Disorder Questionnaire (GAD-7). *Journal of Affective Disorders*, *265*, 395–401. doi:10.1016/j.jad.2020.01.032.

West, C. P., Dyrbye, L. N., Sloan, J. A., & Shanafelt, T. D. (2009). Single item measures of emotional exhaustion and depersonalization are useful for assessing burnout in medical professionals. *Journal of General Internal Medicine*, *24*(12), 1318–1321. doi:10/c3d9bb.
