# Supplementary material for: Mental health status and related factors influencing healthcare workers during the COVID-19 pandemic: A systematic review and meta-analysis
Source: PLoS One. 2024 Jan 19;19(1):e0289454. doi: 10.1371/journal.pone.0289454 (PMC10798549; doi:10.1371/journal.pone.0289454)
Supplement: S1 Data — (ZIP) [file pone.0289454.s011.zip › literatures/178.pdf]

Determinants of Stress, Depression, Quality of Life, and Intent to Leave in Washington State Emergency Medical Technicians During COVID-19

Pranav Srikanth, MS, Lily M. Monsey, BA, Hendrika W. Meischke, PhD, and Marissa G. Baker, PhD

Objectives: This study characterizes determinants of stress, depression, quality of life, and intent to leave among emergency medical technicians (EMTs) in the Puget Sound region, Washington, during the COVID-19 pandemic and identifies areas for intervention on these outcomes. **Methods:** A cross-sectional survey measured stress, depression, quality of life, and intent to leave among EMTs ($N = 123$). Regression models were developed for these outcomes. **Results:** A total of 23.8% of respondents were very likely to leave their position in the next 6 months. Job demands predicted stress and depression, and financial security predicted stress and quality of life. Intent to leave was predicted by stress, manager support, and length of employment. **Conclusions:** Increased exposure to hazards has impacted EMT mental health. Emergency medical technicians are vital to healthcare, so improving EMT health and well-being is important, as attrition during a pandemic could impact public health.

Keywords: occupational health, mental health, emergency medical technicians, stress, depression, quality of life, intent to leave

Emergency medical technicians (EMTs) are a vital component of an emergency medical services (EMS) system. Emergency medical technicians provide emergency and nonemergency transportation to hospitals, and out-of-hospital care ranging from basic first aid to patient stabilization and support during life-threatening emergencies. In the Washington State 2021 release of occupational employment and wage data, there were 3725 workers counted under SOC 29-2040 (EMTs and paramedics) with a median hourly wage of \$22.06.[1] However, paramedics, who have more advanced training, are higher paid than EMTs, making the EMT hourly pay rate often lower than the median for the whole SOC, an hourly rate that may not constitute a living wage for workers in major metropolitan areas of Washington, such as King County.[2]

Emergency medical technicians work demanding hours (including long or overnight shifts) and face stressful, demanding, and time-sensitive work conditions. Emergency medical technicians can encounter violence at work,[3,4] which can increase feelings of stress and intent to leave the profession.[4,5] During the COVID-19 pandemic, EMTs have worked in proximity with presumptive COVID-19 cases, which not only increases their risk of exposure to SARS-CoV-2 but also can contribute to adverse mental health outcomes.[6] The increased risk associated with working during the pandemic, coupled with pay that may be below the cost of living, has led to higher rates of attrition, especially among older EMS providers who have opted for early retirement.[7]

In King County, Washington, firefighters are also trained as paramedics or EMTs and receive higher pay and access to a suite of comprehensive benefits, as well as additional trainings and promotions,[8] while EMTs who work for private companies may not receive these same supports.[9] This can not only contribute to turnover[10] but also negatively impact well-being.[11] A recent survey of both EMTs and paramedics before the COVID-19 pandemic showed that 57% indicated a dependence on overtime, and 56% depended on working multiple jobs.[12] Dependence on extra work has been shown to increase the odds of job dissatisfaction and intent to leave EMS within 1 year.[12]

Previous occupational health research among EMTs has focused on stress, burnout, and turnover. Emergency medical technicians experience high levels of stress,[13] putting them at increased risk of posttraumatic stress reactions and the development of posttraumatic stress syndrome.[14] Chronic exposure to psychosocial stressors, such as lack of social support and high job demands, has previously been linked to job dissatisfaction,[15] and job dissatisfaction has been linked to adverse mental health outcomes[15]; however, limited research has investigated the direct relationship between psychosocial stressors and adverse mental health outcomes. Furthermore, research on attrition among EMTs has focused on attrition due to burnout or job dissatisfaction[16,17] and has not focused on attrition due to other workplace factors.

Here, we investigate factors related to stress, depression, quality of life, and intent to leave in a survey of 123 EMTs in the Puget Sound region of Washington during the COVID-19 pandemic. The goals of this study are to characterize and understand determinants of adverse mental health and well-being among EMTs and to identify areas for intervention to improve these outcomes. This study adds to the growing body of literature characterizing the impact of COVID-19 on frontline or essential workers and is one of the first studies to investigate how work factors such as pay and benefits, managerial support, and job demands influence the mental health, quality of life, and intent

Learning Objectives

- This study characterizes determinants of stress, depression, quality of life, and intent to leave among EMTs during the COVID-19 pandemic.
- Increased exposure to hazards has impacted EMT mental health and improving EMT health and well-being is important for occupational and public health.
- Finally this study identifies areas for intervention on these outcomes to reduce attrition during a pandemic which could impact public health.

From the Department of Environmental and Occupational Health Sciences, University of Washington School of Public Health, Seattle, Washington (Mr Srikanth, Ms Monsey, Dr Baker); and Department of Health Systems and Population Health, University of Washington School of Public Health, Seattle, Washington (Dr Meischke).

This study was supported by the Harry Bridges Center's Labor Studies Working Group, 2021.

Conflicts of interest: M.G.B. provided consulting on COVID-19 occupational hygiene protocols and received compensation through Mercer Total Healthcare Management; there was no overlap with the work presented here.

Funding sources: Research reported in this publication was supported by the Harry Bridges Center for Labor Studies Working Group and the National Institute for Occupational Safety and Health (NIOSH) under Federal Training Grant T42OH008433 The findings of this survey do not necessarily reflect the official views of the Harry Bridges Center for Labor Studies or NIOSH.

Baker, Srikanth, Monsey, and Meischke have no relationships/conditions/circumstances that present potential conflict of interest.

The JOEM editorial board and planners have no financial interest related to this research.

Address correspondence to: Marissa G. Baker, PhD, 4225 Roosevelt Way NE, Suite 100, Seattle, WA 98105 (bakermg@uw.edu).

Copyright © 2022 American College of Occupational and Environmental Medicine

DOI: 10.1097/JOM.0000000000002587

Copyright © 2022 American College of Occupational and Environmental Medicine. Unauthorized reproduction of this article is prohibited.

to leave among EMTs. This study is also among the first to investigate determinants of stress among EMTs during a pandemic. The role of EMTs in society is vital, particularly during a public health emergency, and the work summarized here is important to better identify how to support their health and well-being.

METHODS

Researchers collaborated with Teamsters Local 763 (Seattle, Wash) who represent transportation workers, including EMTs, in the Puget Sound region of Washington on a cross-sectional survey. The University of Washington Human Subjects Division determined this project to be exempt from review, as no identifying information was being collected by researchers.

Survey Development and Metrics Collected

A web-based survey was developed in REDCap.[18,19] The survey adapted questions from previously validated scales and was informed by conversations with EMT stakeholders to ensure that questions were appropriate and applicable for the population of interest. The survey was pilot tested by 2 EMTs and a union representative and revised based on their feedback. The survey took approximately 10 minutes to complete online and was open between August 16, 2021, and September 16, 2021.

To guide survey development and analysis, we created a conceptual model (Fig. 1), consisting of the outcomes of interest, and exposures related to those outcomes in literature. Relevant to this article, our survey assessed 4 outcomes of interest: the likelihood of leaving the occupation in the next 6 months (not at all likely, somewhat likely, very likely), quality of life (rated on a scale from 1 to 9, where 9 indicates excellent quality of life), depression (assessed via the Patient Health Questionnaire 2 [PHQ-2] validated scale[20]), and stress (assessed via the Perceived Stress Scale 4 [PSS-4] validated scale[21]).

We applied a cutoff score to the PHQ-2 and PSS-4 to make the variables into binary measures (low vs high). For depression measured via the PHQ-2, a score equal to or greater than 3 indicates that major depressive disorder is likely[22] and was used as a cutoff in subsequent analyses. For stress measured via the PSS-4, there is no validated cutoff score; however, a score of 6 has previously been used as the cutoff for high stress based on population norms,[23] so 6 was used as a cutoff score here.

The survey also evaluated pay and benefits, measured using a 5-question scale. These 5 questions asked respondents to rate whether they agree with statements using a four-point agreement scale (4 = strongly agree, 3 = agree, 2 = disagree, 1 = strongly disagree). Questions included whether their pay kept up with the cost of living, whether they had adequate money to cover an unexpected \$400 expense, whether they could afford to use employer-provided insurance to go to the doctor, whether they needed to work additional job(s) to afford the cost of living (reverse coded), and whether they could easily access benefits from their employee assistance program. Raw scores for all 5 questions were averaged for a composite pay and benefits scale; higher scores indicated more financial security. When combining the 5 questions, the composite pay and benefits scale had a Cronbach α value of 0.68, indicating moderate reliability between the measures.

Questions on job demands and supports were also included. Job demands were evaluated by averaging responses to 2 questions asking respondents whether their job demands interfere with their personal lives and how hard it is to take time off work for personal or family matters (Cronbach α = 0.55). The first of these questions was scored on a four-point frequency scale (1 = never, 2 = rarely, 3 = sometimes, 4 = often), and the second question was scored on a four-point

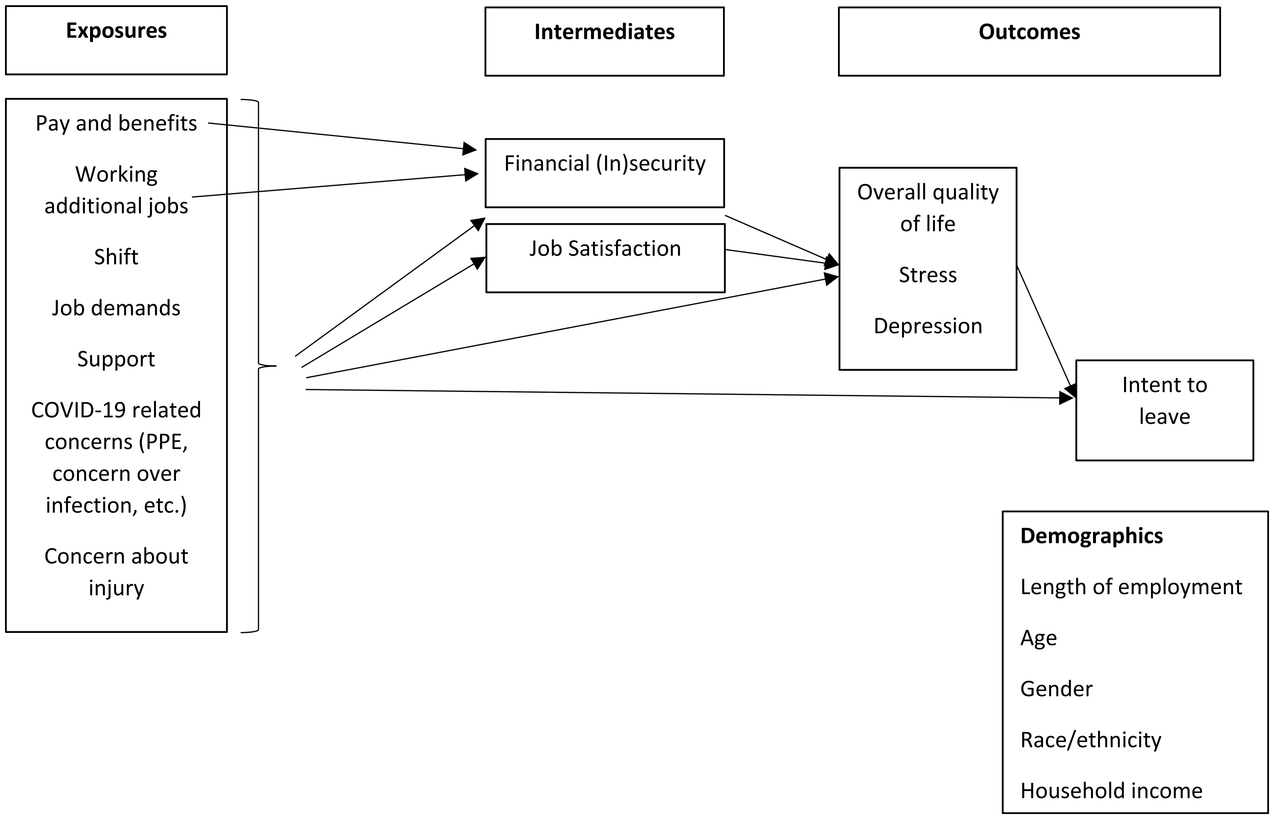

FIGURE 1. Conceptual model of the exposures and outcomes assessed in this study. An arrow indicates that a direct relationship is expected between the variables or groups of variables. Demographics can potentially impact all exposures and outcomes presented in this conceptual model.

© 2022 American College of Occupational and Environmental Medicine

Copyright © 2022 American College of Occupational and Environmental Medicine. Unauthorized reproduction of this article is prohibited.

difficulty scale (1 = not at all hard, 2 = not too hard, 3 = somewhat hard, 4 = very hard). Supports were measured as support received from their manager (averaging responses to one question on general support from manager and one question on support from manager during safety concerns; Cronbach $\alpha = 0.84$), support from coworkers (1 question), and support received outside of work (1 question). Questions on support were scored on a four-point agreement scale (1 = strongly disagree, 2 = disagree, 3 = agree, 4 = strongly agree). A higher score for both job demands and supports indicated more demands and supports, respectively; therefore, higher scores in supports are a positive finding, while higher scores in demands are a negative finding. Respondents answered 3 questions on job satisfaction (Cronbach $\alpha = 0.59$), which were adapted from Spector's 36 question Job Satisfaction Scale,[24] and were measured on a four-point agreement scale. Responses from the 3 questions were averaged, and a higher job satisfaction score indicated more job satisfaction.

Other questions were asked on concern over contracting COVID-19, whether their employer provided them with adequate personal protective equipment (PPE), whether they felt they may have had COVID-19 at some point since March 1, 2020, and whether they have concerns about injury while working. Additional variables collected on the survey are not discussed in this work.

Demographic data were also collected, including age, race/ethnicity, gender, years working in the profession, household income, education, and typical shift worked. Respondents were also asked whether they work additional jobs.

The survey was promoted to EMTs by Teamsters 763 staff. A total of 123 EMTs completed the survey.

Survey Data Analysis

Raw data were downloaded from REDCap (Nashville, TN). Questions were scored and combined to create scales, as explained previously. When questions were combined into a scale, some questions were reverse coded to ensure that all questions were in either the affirmative or negative to ease interpretation of the scale and results. Descriptive statistics were compiled for each measure.

A logistic regression model was developed for the outcomes of depression and stress, which was guided by the conceptual model shown in Figure 1, and included the pay and benefits scale, concern of COVID-19 infection at work, whether employers provided adequate PPE, whether respondents worked additional jobs, concern over injury at work, the job demands scale, manager support, and coworker support as predictors. Demographic data (age, gender, household income, length of employment, and shift) were also included in each model. Support outside of work and race/ethnicity were not included in the models because of the homogeneity of responses in this population. Job satisfaction was also not included in the models, because of homogeneity of responses in 2 of the 3 questions in the job satisfaction measure. A linear regression model was developed for the quality of life outcome, and an ordinal regression model was developed for the intent to leave outcome, both of which included the same predictors and demographics as the stress and depression models. The intent to leave model also included stress, depression, and quality of life as predictors, as shown in the conceptual model.

RESULTS

Table 1 summarizes demographics of the survey respondents. Emergency medical technicians who responded to the survey predominantly identified as male (56.9%) and White (82.6%). A total of 39.2% of the respondents have been employed for less than 3 years, and 41.5% reported a household income of less than $50,000.

Descriptive Results

Table 2 outlines the distribution of outcomes of interest and predictors.

TABLE 1. Characteristics of Survey Respondents (*N* = 123)

		***n* (%)**
Gender	Male	70 (56.9)
	Female	41 (33.3)
	Other	7 (5.6)
	Prefer not to answer	5 (4.1)
Race/ethnicity	White	86 (69.9)
	Hispanic/Latino/Spanish origin	<5
	Black/African	<5
	Asian	<5
	Native Hawaiian/Pacific Islander	<5
	American Indian/Alaska Native	0
	Middle Eastern/North African	0
	>1 response	17 (13.8)
	Prefer not to answer/missing	12 (9.7)
Age	18–24	23 (18.7)
	25–34	48 (39.0)
	34–44	27 (22.0)
	44–54	11 (8.9)
	>55	8 (6.5)
	Prefer not to answer	6 (4.9)
Shift	Rotating day shift	43 (36.1)
	Rotating night shift	14 (11.8)
	Regular day shift	44 (37.0)
	Regular night shift	18 (15.1)
	Missing	4 (3.2)
Length of employment	<1 yr	21 (16.8)
	1–3 yr	28 (22.4)
	3–5 yr	23 (18.4)
	5–10 yr	16 (12.8)
	>10 yr	33 (26.8)
	Prefer not to answer	2 (1.6)
Household income	<25,000	7 (5.7)
	25,000–50,000	44 (35.8)
	50,000–75,000	23 (17.7)
	75,000–100,000	14 (11.4)
	>100,000	21 (17.1)
	Prefer not to answer/missing	14 (11.4)
Education	Finished high school/GED	7 (5.7)
	Trade/vocational school	9 (7.3)
	Some college	51 (41.6)
	Finished college	50 (40.6)
	Masters/advanced degree	4 (3.2)
	Prefer not to answer	2 (1.6)

GED, General Educational Development Test.

A total of 23.8% of the respondents reported that they are very likely to leave their EMT position, and 35.2% reported that they are somewhat likely to leave their EMT position in the next 6 months.

A total of 67.5% of the respondents had a PSS-4 score indicating high stress (≥6). Thirty-five percent of the respondents had a PHQ-2 score indicating that major depressive disorder is likely (≥3).

A total of 16.3% of the respondents stated that they either agreed or strongly agreed that they are paid a fair wage (Table 2). Most respondents disagreed or strongly disagreed with all the questions on pay and benefits (percent agreement ranging from 6.5% to 43.3%).

A total of 83.7% of the EMT respondents reported being sometimes or often concerned about contracting COVID-19 at work. A total of 43.9% of the respondents indicated that they were not provided with adequate PPE over the past year. Thirty-nine percent of the EMT respondents felt that they might have had COVID-19 at some point since March 1, 2020.

Regression Models

Regression models were developed for stress, depression, quality of life, and intent to leave (Table 3).

 © 2022 American College of Occupational and Environmental Medicine

Copyright © 2022 American College of Occupational and Environmental Medicine. Unauthorized reproduction of this article is prohibited.

TABLE 2. Outcomes of Interest and Predictors

	n (%)	Mean (SD)
Overall stress (PSS-4: 0 = never, 1 = almost never, 2 = sometimes, 3 = fairly often, 4 = very often)		7.22 (3.43)
In the last month, how often have you felt that you were unable to control the important things in your life?*		2.03 (1.05)
In the last month, how often have you felt confident about your ability to handle your personal problems?		1.42 (1.07)
In the last month, how often have you felt that things were going your way?*		1.87 (1.04)
In the last month, how often have you felt difficulties were piling up so high that you could not overcome them?		1.88 (1.19)
Distribution of stress scores		
High stress (≥6)	83 (67.5)	
Low stress (<6)	37 (30.0)	
Missing	3 (2.4)	
Overall depression (PHQ-2: 0 = not at all, 1 = several days, 2 = more than half the days, 3 = nearly every day)		2.13 (1.86)
Little interest or pleasure in doing things		1.11 (0.97)
Feeling down, depressed, or hopeless		1.02 (0.98)
Distribution of depression scores		
Major depressive disorder likely (≥3)	43 (35.0)	
Major depressive disorder unlikely (<3)	78 (63.4)	
Missing	2 (1.6)	
Job satisfaction,† total average		2.43 (0.60)
No. respondents who agree or strongly agree that…		
I like my job	106 (86.2)	
I am paid a fair wage	20 (16.3)	
I feel I have good job security in this job	66 (53.7)	
Pay and benefits,† total average		2.29 (0.40)
No. respondents who agree or strongly agree that…		
My pay has kept up with the cost of living	8 (6.5)	
I need to work an additional job(s) in order to afford to live here*	78 (65.0)	
I have enough money to cover an unexpected $400 expense	55 (45.0)	
I can afford to use my employer-provided insurance to go to the doctor	49 (43.3)	
I can easily access benefits from my EAP	45 (39.2)	
I feel that I need to work overtime to make ends meet*	91 (75.3)	
Adequate sick leave,† total average		2.38 (0.95)
Employment		
Do you have any additional jobs right now (formal or informal, fulltime or part time)? (no. responses "yes")		53 (44.1)
Overall support (average of managerial, coworker, outside support)		2.82 (0.59)
Managerial support,† total average		2.69 (0.77)
No. respondents who agree or strongly agree that…		
I have access to managerial support if a safety concern arises.	81 (65.6)	
I have access to managerial support in general.	85 (69.1)	
Coworker support†		2.74 (0.83)
No. respondents who agree or strongly agree that…		
I have someone at work I can rely on when a difficult situation arises	80 (65.0)	
Outside support†		3.17 (0.89)
No. respondents who agree or strongly agree that…		
I have someone outside of work I can rely on for emotional help and support.	100 (82.5)	
Job demands, average of 2 questions (higher score indicates higher demands)		2.90 (0.67)
How hard is it to take time off during your work to take care of personal or family matters? (1 = not at all hard, 2 = not too hard, 3 = somewhat hard, 4 = very hard), number who responded "very hard" or "somewhat hard"*	73 (59.9)	
How often do the demands of your job interfere with your family life or personal time? (1 = never, 2 = rarely, 3 = sometimes, 4 = often), number who responded "sometimes" or "often"	99 (80.5)	
Overall well-being (nine-point scale)		5.65 (1.94)
Injury		
How often do you worry that you will get injured on the job? (1 = never, 2 = rarely, 3 = sometimes, 4 = often), number who responded "sometimes" or "often"	93 (75.6)	
COVID concerns		
In the past year, how concerned have you been about contracting COVID-19 due to your work? (1 = never concerned, 2 = rarely concerned, 3 = sometimes concerned, 4 = often concerned), number who responded "sometimes" or "often" concerned	103 (83.7)	
Over the past year, has your employer provided adequate PPE while you are at work? (no. responses "yes")	69 (56.1)	
At any time since March 1, 2020, have you felt that you might have had COVID-19? (no. responses "yes")	48 (39.0)	
Intent to leave		
Not at all likely	50 (41.0)	
Somewhat likely	43 (35.2)	
Very likely	29 (23.8)	

EAP, employee assistance program.
For all scales, higher scores indicate a more positive outcome.
*Indicates items that were reverse coded.
†Questions measured on a four-point agreement scale (1 = strongly disagree, 2 = disagree, 3 = agree, 4 = strongly agree).

© 2022 American College of Occupational and Environmental Medicine

Copyright © 2022 American College of Occupational and Environmental Medicine. Unauthorized reproduction of this article is prohibited.

TABLE 3. Regression Analyses for 4 Outcomes of Interest

	Stress (*n* = 101)		Depression (*n* = 102)		Quality of Life (*n* = 95)		Intent to Leave (*n* = 92)	
**Predictors**	**OR**	**95% CI**	**OR**	**95% CI**	**β**	**95% CI**	**OR**	**95% CI**
Pay and benefits	0.79*	0.66 to 0.96	0.85	0.69 to 1.03	1.35*	0.68 to 2.02	1.34	0.45 to 4.10
Concern of C19 infection	1.01	0.90 to 1.13	0.93	0.82 to 1.05	−0.04	−0.47 to 0.38	0.95	0.52 to 1.76
Employer providing PPE	1.02	0.84 to 1.23	0.95	0.76 to 1.15	0.42	−0.30 to 1.31	0.36*	0.12 to 1.00
Work additional jobs	1.13	0.95 to 1.35	1.15	0.96 to 1.39	−0.41	−1.06 to 0.23	1.18	0.46 to 2.95
Injury concern	1.03	0.91 to 1.17	0.98	0.86 to 1.13	0.45	−0.02 to 0.93	1.39	0.68 to 2.84
Job demands	1.18*	1.02 to 1.37	1.23*	1.05 to 1.44	−0.82*	−1.38 to −0.27	0.55	0.23 to 1.25
Manager support	1.07	0.93 to 1.23	1.00	0.86 to 1.16	0.05	−0.49 to 0.60	0.34*	0.14 to 0.78
Coworker support	0.91	0.82 to 1.02	0.90	0.80 to 1.01	0.36	−0.05 to 0.77	1.21	0.66 to 2.24
PSS-4 score (≥6 vs <6)	—	—	—	—	—	—	8.75*	2.44 to 34.55
PHQ-2 score (≥3 vs <3)	—	—	—	—	—	—	0.62	0.19 to 1.96
Quality of life	—	—	—	—	—	—	0.97	0.68 to 1.41
Age	0.97	0.90 to 1.04	0.99	0.91 to 1.07	−0.01	−0.27 to 0.26	0.91	0.63 to 1.30
Gender (male vs other)	1.06	0.89 to 1.26	0.94	0.78 to 1.13	0.28	−0.36 to 0.92	0.81	0.32 to 1.98
Household income (>$50,000 vs ≤$50,000)	1.02	0.85 to 1.22	1.06	0.87 to 1.29	0.63	−0.05 to 1.31	1.76	0.67 to 4.74
Shift (night vs day)	1.14	0.93 to 1.39	0.93	0.75 to 1.14	0.66	−0.09 to 1.40	0.77	0.26 to 2.26
Length of employment (≥3 vs < 3 yr)	1.16	0.95 to 1.43	1.05	0.85 to 1.30	−0.53	−1.27 to 0.21	0.25*	0.08 to 0.73

Models for stress and depression assessed using logistic regression.
Model for quality of life assessed using linear regression.
Model for intent to leave assessed using ordinal logistic regression.
*Indicates $P \leq 0.05$.

Results from the stress regression found that an increased demand score significantly increased the odds of high stress (odds ratio [OR], 1.18; confidence interval [CI], 1.02–1.37), while increases in pay and benefits significantly decreased the odds of high stress (OR, 0.79; CI, 0.66 to 0.96). This model explained 34% of the variance in stress.

Results from the depression regression found that an increased demand score significantly increased the odds of major depressive disorder being likely (OR, 1.23; CI, 1.05 to 1.44), while increases in coworker support was associated with decreased odds of likely major depressive disorder, and working additional jobs was associated with increased odds of likely major depressive disorder (OR, 0.90; CI, 0.80 to 1.01, and OR, 1.15; CI, 0.96 to 1.39, respectively). This model explained 30% of the variance in depression.

To investigate factors that were related to quality of life, we used a linear regression model (Table 3). Results from this regression model showed that for a 1-unit increase in the pay and benefits scale, the mean quality of life increased by 1.35 (CI, 0.68 to 2.02), and for a 1-unit increase in job demands, the mean quality of life decreased by 0.82 (CI, −1.38 to −0.27). This model explained 56% of the variance in quality of life.

Using an ordinal logistic regression model, we investigated factors that were associated with intent to leave in this population (Table 3). Results from this regression model showed that those who have PSS-4 scores greater than 6 (high stress) have significantly greater odds of expressing an intent to leave compared with those with PSS-4 scores less than 6 (OR, 8.75; CI, 2.44 to 34.55). Among those who were very or somewhat likely to leave, 12 had scores indicating low stress, while 58 had scores indicating high stress. A 1-unit increase in manager support significantly decreased the odds of expressing an intent to leave (OR, 0.34; CI, 0.14 to 0.78), and the respondents with more than 3 years of experience as an EMT had significantly decreased odds of expressing an intent to leave than those with less than 3 years of experience (OR, 0.25; CI, 0.08 to 0.73).

DISCUSSION

To date, limited research has characterized factors related to stress, depression, quality of life, and intent to leave among essential workers who were vital during the COVID-19 pandemic. Given the increased stressors due to the pandemic for first responders, and the limited supports that EMTs may have when compared with other first responders (such as firefighter/paramedics),[9] this study is important, as EMTs are crucial during a public health emergency, such as the COVID-19 pandemic. Maintaining and improving the safety and well-being of EMTs not only improves health among EMTs but also can have positive impacts on their ability to provide EMS to the community.

Results from our regression analyses found that increased job demands were significantly associated with increased odds of high stress and depression. This finding is consistent with previous research in other occupations that have shown relationships between high job demands and stress.[25,26] It may be possible to reduce job demands by developing programs at the union level for EMTs that reduce conflict between work and personal life. This could include increased flexibility in work schedules, more worker input in determining schedules, or changing the schedule bidding process so that EMTs receive schedules further in advance to provide time to plan around.

Most respondents indicated that their current pay and access to benefits were inadequate for their cost of living, and regression analyses found that increased financial security was significantly associated with decreased odds of high stress and intent to leave. Most EMTs also indicated that they do not believe they are paid a fair wage. Regression analyses also found that each additional job worked was associated with increased odds of depression. This finding indicates that there may be a relationship between additional jobs and depression, making increased pay important to ensure that EMTs do not need to work additional jobs to afford to live. Including these pay increases in city contracts, as is done for firefighters,[8,27] can guarantee these pay increases and reduce company-based variability in pay.

Like stress and depression, results for the regression model for quality life found that quality of life is significantly positively associated with pay and benefits and negatively associated with job demands. However, unlike stress and depression, quality of life may also be related to a lack of employer-provided PPE, concern over injury, coworker support, whether respondents work additional jobs, length of employment, household income, and work shift. Quality of life is a broad construct, consisting of a general assessment of one's life.[28] According to the Centers for Disease Control and Prevention, quality of life can be defined differently by individuals and can consist of health, jobs, living conditions, culture, social support, and socioeconomic

Copyright © 2022 American College of Occupational and Environmental Medicine. Unauthorized reproduction of this article is prohibited.

status.[29] While factors may influence quality of life by impacting one of these facets of quality of life, there may be no effect on outcomes, such as stress and depression. The regression model for quality of life provides information on factors that may not necessarily impact stress or depression among EMTs but impact overall well-being among these workers.

Nearly a quarter of EMTs reported that they were very likely to leave their EMT position within 6 months, and another 35% of EMTs reported that they were somewhat likely to leave. Previous research has indicated that one of the most notable contributors to intent to leave among EMTs is a desire for better pay and benefits,[30] which is further supported by our findings. In this study, stress was highly related with intent to leave, which is consistent with previous research that has identified a link between stress and intent to leave among other groups of healthcare workers.[31] While previous research has indicated that EMTs employed less than 5 years are more likely to leave,[32] our findings indicated that those who have been employed for less than 3 years were more likely to have an intent to leave, suggesting that attrition in the profession is primarily among new EMTs. In addition, EMTs who have been in the profession for longer are those who did not leave early on, which could be influenced by the healthy worker bias.[33]

One possible method to reduce attrition among less experienced employees is with employer or union-sponsored mentorship programs, partnering less experienced EMTs with more experienced EMTs.[34,35] To assess the effectiveness of these programs, they can subsequently be evaluated by using surveys to compare social support and mental health outcomes between EMTs who are involved in these programs and EMTs who are not involved and by comparing attrition among new EMTs stratified by program involvement.

Manager support significantly decreased the odds of a respondent expressing an intent to leave, which is consistent with research regarding intent to leave among other healthcare worker groups.[36,37] Previous investigations of managerial support in healthcare workers have indicated that in addition to more supportive work environments as a whole,[38,39] trust in managers,[38] increased worker involvement in manager-level decision making,[40] and worker empowerment[41] are associated with less of an intent to leave. Determining what managerial supports are needed by EMTs and ensuring they are enacted could help workplaces retain workers, which can reduce understaffing in workplaces and thereby alleviate workload.[42]

The EMTs reported being concerned about contracting COVID-19 at work. This concern could be exacerbated by the reported inadequacy in employer-provided PPE reported by the respondents. The lack of PPE and concerns about contracting COVID-19 at work suggest a lack of protection. Nearly 40% of the EMTs felt that they had COVID-19 at some point, which may suggest workplace exposure and can result in potential infection of other coworkers and community members. However, results from our regression analyses indicated that these concerns regarding workplace infection do not have significant associations with stress, depression, quality of life, or intent to leave, suggesting that the adverse mental health outcomes that affect this population are related to shortcomings of the workplace as a whole in supporting EMT mental health and not solely related to the challenges posed by the COVID-19 pandemic.

While concerns over COVID-19 infection were not significant predictors of any of the 4 outcomes of interest, it is still concerning from a public health perspective that 43.9% of the EMTs reported a lack of employer-provided PPE. Increasing the amount of employer-provided PPE available to EMTs not only can reduce concern regarding exposure to infectious diseases like COVID-19 but also may improve overall well-being. Providing training for EMTs through the union on their rights to PPE, as well as how to report unsafe work, can reduce workplace exposure concerns. These can have impacts both during the COVID-19 pandemic and afterward, as EMTs are at risk of exposure to infectious diseases in the community regardless of the pandemic.

Limitations

This study has several limitations. Because EMTs were recruited to take the survey by their local union, results may or may not be generalizable outside of the sampled population. This study used convenience sampling, which also impacts generalizability and can induce sampling bias. This study was further limited by its small sample size ($N = 123$) and had homogeneity in race and ethnicity, making it challenging to investigate the outcomes and needs of minority populations, who have had worse COVID-19 outcomes,[43,44] and have historically had worse occupational health outcomes. Furthermore, the small sample size may have contributed to the skewed distributions for outcomes, such as stress, where 67.5% of the respondents reported high stress group and only 30.0% reported low stress, and depression, where 63.4% of respondents reported low depression scores and only 35.0% reported high depression scores. This study also used the PSS-4 and PHQ-2 instead of the longer PSS-10 and PHQ-9 for stress and depression, respectively; while the shorter measures are validated, the longer measures allow for more discrimination in the measure, making differences easier to identify. This survey is cross-sectional and, therefore, only reflects attitudes toward COVID-19 at the time of the sample. This study was performed in the context of the COVID-19 pandemic and is, therefore, not generalizable to nonpandemic times. Emergency medical technicians may have concerns that are not adequately captured in surveys. Therefore, conducting focus group or key informant research can be useful to capture richer data on the needs of EMTs that may not be captured on a cross-sectional survey.

CONCLUSIONS

During the COVID-19 pandemic, EMTs have faced increased physical and mental hazards due to their inability to work from home and the stresses that come from working in proximity to others.[45] These hazards have not only impacted the mental health of EMTs but have also resulted in a high number of EMTs expressing an intent to leave, particularly among those with less experience.

Despite the limitations of this study, this study remains important to occupational health, because identifying and addressing factors that contribute to the mental health and quality of life of EMTs and reasons for attrition in the profession can have long-lasting positive impacts, both on EMTs and on other similar occupations. Findings from this study could also inform workplace interventions to improve well-being among EMTs, during COVID-19 and beyond. Furthermore, improving EMT health and well-being is important for public health, because EMTs play a vital role in the healthcare system, especially during a pandemic, and attrition of EMTs during a pandemic could impact public health.

ACKNOWLEDGMENTS

The authors gratefully acknowledge Teamsters Local 763 for their collaboration on this survey.

REFERENCES

1. Washington State Employment Security Department. Occupational employment and wage statistics (OEWS). 2021. Available at: https://esd.wa.gov/labormarketinfo/occupations. Accessed February 17, 2022.
2. Living Wage Calculator. Living wage calculation for King County, Washington. Available at: https://livingwage.mit.edu/counties/53033. Accessed October 28, 2021.
3. Oliver A, Levine R. Workplace violence: a survey of nationally registered emergency medical services professionals. *Epidemiology Research International*. 2015; 2015:e137246.
4. Murray RM, Davis AL, Shepler LJ, et al. A systematic review of workplace violence against emergency medical services responders. *New Solut*. 2020; 29:487–503.
5. Pozzi C. Exposure of prehospital providers to violence and abuse. *J Emerg Nurs*. 1998;24:320–323.
6. Shanafelt T, Ripp J, Trockel M. Understanding and addressing sources of anxiety among health care professionals during the COVID-19 pandemic. *JAMA*. 2020;323:2133–2134.

Copyright © 2022 American College of Occupational and Environmental Medicine. Unauthorized reproduction of this article is prohibited.

7. Cerullo M. Emergency medical technicians are quitting their jobs—COVID-19 makes it too dangerous. 2020. Available at: https://www.cbsnews.com/news/ems-workers-retiring-higher-rates-coronavirus-pandemic/. Accessed February 24, 2022.
8. Benefits—fire. Available at: https://www.seattle.gov/fire/jobs-and-opportunities/benefits. Accessed January 24, 2022.
9. Beekman D. Seattle City Council says contracted ambulance workers deserve better pay. *The Seattle Times*. 2018. Available at: https://www.seattletimes.com/seattle-news/politics/seattle-city-council-says-contracted-ambulance-workers-deserve-better-pay/. Accessed January 24, 2022.
10. Avesta Systems, Inc, American Ambulance Association. *AAA/Avesta 2019 EMS Employee Turnover Study Final*. 2019. Available at: https://ambulance.org/wp-content/uploads/2019/07/AAA-Avesta-2019-EMS-Employee-Turnover-Study-Final.pdf. Accessed January 20, 2022.
11. Beckman KL, Monsey LM, Archer MM, Errett NA, Bostrom A, Baker MG. Health and safety risk perceptions and needs of app-based drivers during COVID-19. *Am J Ind Med*. 2021;64:941–951.
12. Rivard MK, Cash RE, Chrzan K, Panchal AR. The impact of working overtime or multiple jobs in emergency medical services. *Prehosp Emerg Care*. 2020;24: 657–664.
13. Cydulka RK, Lyons J, Moy A, Shay K, Hammer J, Mathews J. A follow-up report of occupational stress in urban EMT-paramedics. *Ann Emerg Med*. 1989; 18:1151–6.
14. Donnelly E. Work-related stress and posttraumatic stress in emergency medical services. *Prehosp Emerg Care*. 2012;16:76–85.
15. Neale AV. Work stress in emergency medical technicians. *J Occup Med*. 1991; 33:991–997.
16. Boland LL, Kinzy TG, Myers RN, et al. Burnout and exposure to critical incidents in a cohort of emergency medical services workers from Minnesota. *West J Emerg Med*. 2018;19:987–995.
17. Whitley TW, Revicki DA, Allison EJ, Landis SS. Predictors of job satisfaction among rural emergency medical technicians. *Prehosp Disaster Med*. 1990; 5:217–223.
18. Harris PA, Taylor R, Thielke R, Payne J, Gonzalez N, Conde JG. Research Electronic Data Capture (REDCap)—a metadata-driven methodology and workflow process for providing translational research informatics support. *J Biomed Inform*. 2009;42:377–381.
19. Harris PA, Taylor R, Minor BL, et al. The REDCap consortium: building an international community of software platform partners. *J Biomed Inform*. 2019;95:103208.
20. Gilbody S, Richards D, Brealey S, Hewitt C. Screening for depression in medical settings with the Patient Health Questionnaire (PHQ): a diagnostic meta-analysis. *J Gen Intern Med*. 2007;22:1596–1602.
21. Cohen S. Perceived stress in a probability sample of the United States. In: *The Social Psychology of Health*. Thousand Oaks, CA: Sage Publications, Inc; 1988:31–67.
22. Kroenke K, Spitzer RL, Williams JB. The Patient Health Questionnaire-2: validity of a two-item depression screener. *Med Care*. 2003;41:1284–1292.
23. Malik AO, Peri-Okonny PA, Gosch K, et al. Abstract 13646: higher perceived stress levels are associated with an increased long-term mortality risk: a landmark analysis in patients with peripheral artery disease. *Circulation*. 2019;140:A13646.
24. Spector PE. *Job Satisfaction: Application, Assessment, Causes, and Consequences*. Thousand Oaks, CA: SAGE Publication, Inc; 1997.
25. Hansen AM, Blangsted AK, Hansen EA, Søgaard K, Sjøgaard G. Physical activity, job demand-control, perceived stress-energy, and salivary cortisol in white-collar workers. *Int Arch Occup Environ Health*. 2010;83:143–153. doi: 10.1007/s00420-009-0440-7.
26. Topcic M, Baum M, Kabst R. Are high-performance work practices related to individually perceived stress? A job demands-resources perspective. *Int J Hum Resour Manag*. 2016;27:45–66.
27. Mattison P. Agreement by and between the City of Seattle and International Association of Firefighters, Local 27 2019:75. Available at: https://www.seattle.gov/documents/Departments/HumanResources/Labor%20Relations/Local%2027%20CBA%20Effective%20through%20123121.pdf. Accessed January 20, 2022.
28. The World Health Organization Quality of Life Assessment (WHOQOL): development and general psychometric properties. *Soc Sci Med*. 1998;46:1569–1585.
29. Centers for Disease Control and Prevention. HRQOL concepts. 2018. Available at: https://www.cdc.gov/hrqol/concept.htm. Accessed February 3, 2022.
30. Rivard MK, Cash RE, Woodyard KC, Crowe RP, Panchal AR. Intentions and motivations for exiting the emergency medical services profession differ between emergency medical technicians and paramedics. *J Allied Health*. 2020;49:53–59.
31. Cartledge S. Factors influencing the turnover of intensive care nurses. *Intensive Crit Care Nurs*. 2001;17:348–355.
32. Patterson PD, Moore CG, Sanddal ND, Wingrove G, LaCroix B. Characterizing job satisfaction and intent to leave among nationally registered emergency medical technicians: an analysis of the 2005 LEADS Survey. *J Allied Health*. 2009;38:e84–91.
33. Eisen EA, Picciotto S, Robins JM. Healthy worker effect based in part on the article “healthy worker effect” by Ellen A. Eisen and James M. Robins, which appeared in the Encyclopedia of Environmetrics. In: *Encyclopedia of Environmetrics*. American Cancer Society; 2013.
34. Chen CM, Lou MF. The effectiveness and application of mentorship programmes for recently registered nurses: a systematic review. *J Nurs Manag*. 2014;22:433–42.
35. Blau G, Bentley MA, Eggerichs-Purcell J. Testing the impact of emotional labor on work exhaustion for three distinct emergency medical service (EMS) samples. *Career Dev Int*. 2012;17:626–45.
36. Strachota E, Normandin P, O’Brien N, Clary M, Krukow B. Reasons registered nurses leave or change employment status. *J Nurs Adm*. 2003;33:111–117.
37. Chen HC, Chu CI, Wang YH, Lin LC. Turnover factors revisited: a longitudinal study of Taiwan-based staff nurses. *Int J Nurs Stud*. 2008;45:277–85.
38. Gregory DM, Way CY, LeFort S, Barrett BJ, Parfrey PS. Predictors of registered nurses’ organizational commitment and intent to stay. *Health Care Manage Rev*. 2007;32:119–27.
39. Cowden T, Cummings G, Profetto-Mcgrath J. Leadership practices and staff nurses’ intent to stay: a systematic review. *J Nurs Manag*. 2011;19:461–477.
40. Boyle DK, Bott MJ, Hansen HE, Woods CQ, Taunton RL. Managers’ leadership and critical care nurses’ intent to stay. *Am J Crit Care*. 1999;8:361–371.
41. Larrabee JH, Janney MA, Ostrow CL, Withrow ML, Hobbs GR Jr., Burant C. Predicting registered nurse job satisfaction and intent to leave. *J Nurs Adm*. 2003;33:271–283.
42. Paterson JL, Sofianopoulos S, Williams B. What paramedics think about when they think about fatigue: contributing factors. *Emerg Med Australas*. 2014; 26:139–144.
43. Centers for Disease Control and Prevention. Disparities in COVID-19 illness. Centers for Disease Control and Prevention. 2020. Available at: https://www.cdc.gov/coronavirus/2019-ncov/community/health-equity/racial-ethnic-disparities/increased-risk-illness.html. Accessed February 19, 2022.
44. Washington State Department of Health. COVID-19 morbidity and mortality by race, ethnicity and spoken language in Washington state. 2022:37. Available at: https://doh.wa.gov/sites/default/files/2022-02/COVID-19MorbidityMortalityRaceEthnicityLanguageWAState.pdf. Accessed January 11, 2022.
45. Baker MG. Nonrelocatable occupations at increased risk during pandemics: United States, 2018. *Am J Public Health*. 2020;110:1126–32.

 © 2022 American College of Occupational and Environmental Medicine

Copyright © 2022 American College of Occupational and Environmental Medicine. Unauthorized reproduction of this article is prohibited.
