# Supplementary material for: Mental health status and related factors influencing healthcare workers during the COVID-19 pandemic: A systematic review and meta-analysis
Source: PLoS One. 2024 Jan 19;19(1):e0289454. doi: 10.1371/journal.pone.0289454 (PMC10798549; doi:10.1371/journal.pone.0289454)
Supplement: S1 Data — (ZIP) [file pone.0289454.s011.zip › literatures/18.pdf]

Impact of COVID-19 on the mental health of frontline and non-frontline healthcare workers in Sri Lanka

Anuradha Baminiwatta[1,2], Shamila De Silva[1,3], Aruni Hapangama[1,2], Kumarini Basnayake[1], Charani Abayaweera[1], Dinithi Kulasinghe[1], Dewmi Kaushalya[1], Shehan Williams[1,2]

(Index words: mental health; healthcare workers; frontline; depression; anxiety; stress)

Abstract

Introduction Healthcare workers (HCWs) are at risk of mental health problems during a pandemic. Being stationed at the frontline or not may have implications on their mental health.

Objectives The aims of this study were to assess depression, anxiety and stress among HCWs, to explore differences between frontline and non-frontline workers, and to investigate associated factors.

Methods In this cross-sectional study, frontline and non-frontline HCWs were recruited from a COVID-19 screening hospital in Sri Lanka. Mental health impact was assessed using Depression, Anxiety and Stress Scale (DASS-21). Sociodemographic data and perceptions of social and occupational circumstances were gathered. Categorical variables were analyzed using Chi square and logistic regression. Odds ratios were calculated for the effect of different perceptions on psychological morbidity.

Results A total of 467 HCWs participated, comprising 244 (52.2%) frontline and 223 (47.8%) non-frontline workers, with female preponderance (n=341, 77%). Prevalence of depression, anxiety and stress among HCWs were 19.5%, 20.6%, 11.8%, respectively. Non-frontline group showed a higher prevalence of depression (27% vs. 11%, $p<0.001$), anxiety (27% vs. 14%, $p=0.001$) and stress (15% vs. 8%, $p=0.026$). Being married, having children, living with family and higher income were associated with better psychological outcomes. Perceived lack of personal protective equipment, inadequate support from hospital authorities, greater discrimination, and lack of training to cope with the situation predicted poor mental health outcomes, and non-frontline HCWs were more likely to hold such perceptions.

Conclusion Addressing factors leading to negative psychological outcomes in HCWs should be a key concern during this pandemic.

Ceylon Medical Journal 2021; **66:** 16-31

DOI: http://doi.org/10.4038/cmj.v66i1.9351

[1]*North Colombo Teaching Hospital, Ragama, Sri Lanka,* [2]*Department of Psychiatry, Faculty of Medicine, University of Kelaniya, Sri Lanka,* [3]*Department of Medicine, Faculty of Medicine, University of Kelaniya, Sri Lanka.*

Correspondence: AB, *e-mail: <baminiwatta@kln.ac.lk>. Received 18 December 2020 and revised version 14 March 2020 accepted 20 March 2021*

This is an open-access article distributed under the terms of the Creative Commons Attribution License, which permits unrestricted use, distribution, and reproduction in any medium, provided the original author and source are credited.

Background

Since its initial identification from China in early January 2020, COVID-19 has been taking its unrelenting toll on myriad aspects of human lives worldwide [1]. The ever-rising numbers of cases and deaths announced repeatedly on media, often paired with cues of danger and despair, and the ensuing fear of contracting the disease, have understandably placed the human population at risk of mental health sequelae [2]. An increase in psychological morbidity in this background has already been reported from various places [2-4]. Since the first case in Sri Lanka was confirmed on 27 January 2020, the island has witnessed a steady rise in the number of cases, exceeding 2000 cases in June 2020, while this study was underway.

Healthcare workers (HCWs), whose services were brought to the limelight during this pandemic, are at a higher risk of psychological distress, as they interact with patients potentially having COVID-19 infection. Being a frontline (FL) HCW has been hypothesized to be a risk factor for adverse mental health outcomes, and a few studies from China[5] and Italy[6] have provided evidence of this. Even though not many HCWs in Sri Lanka had contracted COVID-19 at the time of this study, the staggering numbers of HCWs infected with COVID-19 as reported from other countries [7] may have led FL workers in Sri Lanka to vicariously experience this danger.

Recent reviews on risk factors for psychological morbidity during the pandemic have revealed that inadequacy of personal protective equipment (PPE), long working hours, poor social support and fear of transmitting the disease to family are associated with mental health problems [8,9].

Prolonged curfews and social distancing policies have imposed constraints on coping strategies usually adopted by people to alleviate stress. Religious activities, social gatherings, family outings, entertainment events, physical exercise and sport events have been all curtailed. Maladaptive coping strategies such as substance use were altered as well.

Investigating the psychological impact of the current pandemic on HCWs was important for many reasons. Good mental well-being is crucial for optimal occupational and social functioning of HCWs. Psychological consequences of a pandemic may persist even one year after the crisis among HCWs [10]. Therefore, appropriate psychological support must be provided to HCWs. To this end, the World Health Organization has issued instructions to healthcare leaders worldwide to ensure access to mental health services for HCWs [11]. However, in order to plan such strategies, it is important to gauge the prevailing nature and gravity of mental health issues among HCWs in the local setting. The objective of this study was to assess the psychological impact of COVID-19 in terms of depression, anxiety and stress among FL and NFL HCWs at a tertiary care hospital in Sri Lanka, and to investigate associated factors.

Method

Study design and setting

This cross-sectional study was conducted between June-August 2020 at North Colombo Teaching Hospital (NCTH), a screening centre for COVID-19 in the Western Province of Sri Lanka, deemed at 'high risk' for COVID-19 transmission.

Study participants

All categories of HCWs – doctors of all grades, nurses of all grades, ancillary staff (pharmacists, medical laboratory technologists, radiographers), and supporting staff were included.

Participant selection

Convenient sampling was used to recruit both FL and NFL participants. FL staff included HCWs who dealt with potentially COVID-19-infected patients being screened at the outpatient department, emergency treatment unit, fever corner, isolation wards, medical wards, pediatric wards, high dependency units and intensive care units. Employees of laboratories within the hospital who ran RT-PCR diagnostic tests for COVID-19 and ambulance drivers who transported suspected and confirmed patients were also considered as FL. All other HCWs employed at the hospital were considered NFL workers.

Sample size

Sample size was calculated using a standard equation used when proportions (prevalence rates) are compared between two groups (FL and NFL) [12].

An α of 0.05, a power of 80%, and proportion values ($p_1 = 0.58$; $p_2 = 0.45$) based on a prior study in China [5], were used for the calculation. This yielded a sample size of 225 for each group, for a total of 450 subjects. A final sample of 500 was deemed appropriate, considering possible non-responders.

Measures

Mental health status was assessed using Depression, Anxiety and Stress Scale (DASS-21). This 21-item, self-administered scale includes three 7-item subscales assessing depression, anxiety and stress separately, as experienced during the past week. Responses are provided on a 4-point Likert scale. Subscale scores are generated by summing the item scores in each subscale, and multiplying by two. DASS-21 has been translated and validated into Sinhala and Tamil [13,14]. Validity statistics of the Sinhala version, such as concurrent, criterion and construct validity, as well as reliability measures were comparable to the original English version. DASS-21 provides cut-offs to determine the severity of symptoms ('normal', 'mild', 'moderate', 'severe' and 'extremely severe') in each subscale.

A socio-demographic questionnaire was administered, alongside DASS-21. Perceptions about social and occupational circumstances and coping strategies were also assessed, using a 5-point Likert scale. This socio-demographic questionnaire was developed and refined by an expert panel that consisted of two psychiatrists and a physician. Previous literature was reviewed and local socio-cultural and administrative factors were considered in developing this questionnaire. The panel discussed and ensured its content and face validity.

2.6 Procedure

Ethics approval was obtained from the Institutional Ethics Review Committee. The permission of the Director of NCTH was also obtained. A list of FL and NFL units was prepared, and these units were visited in a planned and coordinated manner by the data collectors to invite potential participants. In order to ensure inclusiveness, different shifts were covered and staff leave was factored in, so that almost all members of staff in these identified units were invited to participate. Informed written consent was obtained. Participants filled the sociodemographic questionnaire and the DASS-21, on their own, in the language of their choice. Filling both questionnaires took approximately 15-20 minutes. In some instances, the participants from a certain unit were given a specified period to fill the questionnaires (up to 3 days), and the

filled questionnaires were collected at a later time; the respective units were contacted before visiting them to collect the filled forms. All hospital policies and guidelines for prevention of COVID-19 transmission, such as social distancing, wearing masks/visors, and hand hygiene, were adhered to, when interacting with participants.

2.7. Ethical issues

Ethical issues that may arise due to concerns of COVID-19 transmission were considered; however, we were able to minimize these risks by adhering to guidelines for prevention of COVID-19 transmission within the hospital. Considering the scientific importance of mental health research in the context of the COVID-19 pandemic and the potential impact on healthcare policy, benefits were deemed to outweigh risks.

Data analysis

IBM SPSS Version 21 was used for data analysis. Descriptive statistics were used to describe population characteristics. Prevalence rates were calculated based on DASS-21 cut-offs. Factors associated with binary outcomes (e.g. presence of depression) were assessed using Chi square test. Sociodemographic differences between FL and NFL groups were assessed using Chi square test; factors which were significant were fitted onto a logistic regression model, as confounders in the association between position (FL/NFL) and DASS-21 outcomes. To describe the effect of social and occupational perceptions on DASS-21 outcomes, the 5-point Likert scale was recoded into a binary variable; the affirmative responses, i.e. 'Agree' and 'Strongly agree', were combined into one category, and the negative responses i.e. 'Disagree' and 'Strongly disagree' were combined similarly; the neutral response was excluded from the analysis. Odds ratios were calculated to show the association between different perceptions and DASS-21 outcomes. Mann-Whitney U test compared the responses given by FL and NFL groups on the Likert scale.

Results

Sample characteristics

Of 500 HCWs invited to participate, 467 (93.4%) returned completed questionnaires. This comprised 244 (52%) FL workers and 223 (48%) NFL workers. Females (n=341, 77%) outnumbered males (n= 126, 23%).

Table 1 summarizes the socio-demographic characteristics of the whole sample and the differences between FL and NFL groups. The two groups differed significantly in occupation (χ^2 =14.5, p=0.002), income (χ^2 =20.5, p<0.001) and area of residence (χ^2 =7.6, p=0.022). FL group consisted of more doctors (38.4% vs. 29.4%) and supporting staff (25.2% vs. 16.9%), and less nurses (31% vs. 45.2%) and ancillary staff (5.4% vs. 8.7%), than NFL group. There were more HCWs of the highest income category (>150 000 Sri Lankan rupees per month) in the FL group than in the NFL group (32.2% vs. 17.6%). FL workers were more likely to live in urban areas (29.9% vs. 20.7%), and less likely to live in rural areas (24.1% vs. 33.8%), compared to NFL workers.

Table 1. Socio-demographic characteristics of the sample population and differences between the frontline and non-frontline groups

Characteristic	*Number (%)*			*Chi square (p value)*
	Overall	*Frontline*	*Non-frontline*	
**Sex**				0.64 (0.424)
Male	126 (27%)	62 (25.4%)	64 (28.7%)	
Female	341 (77%)	182 (74.6%)	159 (71.3%)	
**Age**				2.15 (0.542)
18-29 y	102 (21.8%)	56 (23.3%)	46 (20.8%)	
30-39 y	202 (43.3%)	109 (45.4%)	93 (42.1%)	
40-49 y	96 (20.6%)	44 (18.3%)	52 (23.5%)	
> 50 y	61 (13.1%)	31 (12.9%)	30 (13.6%)	
**Occupation**				14.5 (0.002)*
Doctor	157 (34.1%)	93 (38.4%)	64 (29.2%)	
Nurse / midwife	174 (37.7%)	75 (31%)	99 (45.2%)	
Supporting staff	98 (21.3%)	61 (25.2%)	37 (16.9%)	
Ancillary staff	32 (6.9%)	13 (5.4%)	19 (8.7%)	

(Continued)

Characteristic	*Number (%)*			*Chi square (p value)*
	Overall	*Frontline*	*Non-frontline*	
**Marital status**				0.623 (0.430)
Having a spouse currently	345 (73.9%)	184 (75.4%)	161 (72.2%)	
No spouse currently[a]	122 (26.1%)	60 (24.6%)	62 (27.8%)	
**Having children?**				0.198 (0.656)
Yes	280 (60.6%)	149 (61.6%)	131 (59.5%)	
No	182 (39.4%)	93 (38.4%)	89 (40.5%)	
**Education**				5.8 (0.209)
Up to ordinary level	52 (11.4%)	34 (14.3%)	18 (8.2%)	
Advanced level	153 (33.5%)	78 (32.8%)	75 (34.2%)	
Undergraduate degree	179 (39.2%)	89 (37.4%)	90 (41.1%)	
Postgraduate degree	40 (8.8%)	23 (9.7%)	17 (7.8%)	
Other (e.g. Diploma)	33 (7.2%)	14 (5.9%)	19 (8.7%)	
**Income (SL rupees)**				20.5 (<0.001)*
<25 000	24 (5.2%)	17 (7%)	7 (3.2%)	
< 50 000	132 (28.5%)	72 (29.7%)	60 (27.2%)	
50 000 – 150 000	214 (46.2%)	92 (38%)	122 (55%)	
> 150 000	117 (25.3%)	78 (32.2%)	39 (17.6%)	
**Area of residence**				7.6 (0.022)*
Urban	118 (25.5%)	72 (29.9 %)	46(20.7%)	
Semiurban	212 (45.8%)	111 (46.1 %)	101 (45.5%)	
Rural	133 (28.7%)	58 (24.1%)	75 (33.8%)	
**Presence of medical comorbidity**				3.59 (0.059)
Yes	88 (18.8%)	38 (15.6%)	50 (22.4%)	
No	379 (81.2%)	206 (84.4%)	173 (77.6%)	
**Living arrangement**				2.54 (0.281)
Living alone	47 (10.3%)	23 (9.6%)	24 (11.1%)	
Living with family	359 (78.9%)	195 (81.6%)	164 (75.9%)	
Shared apartment	49 (10.8%)	21 (8.8%)	28 (13%)	
**Presence of elderly family member**				2.32 (0.127)
Yes	135 (28.9%)	78 (32%)	57 (25.6%)	
No	332 (71.1%)	166 (68%)	166 (74.4%)	
**Presence of another family member working at the frontline**				
Yes	109 (24.6%)			
No	334 (75.4%)			
**Mode of travel to work**				2.72 (0.435)
Public transport	189 (40.8%)	92 (38.3%)	97 (43.5%)	
Own vehicle	187 (40.4%)	103 (42.9%)	84 (37.7%)	
Hired vehicle	7 (1.5%)	5 (2.1%)	2 (2.1%)	
On foot	80 (17.3%)	40 (16.7%)	40 (17.9%)	

Note: * The difference between the frontline and non-frontline groups is significant at 95% confidence

[a] Unmarried, widowed, divorced or separated individuals were combined into one category – 'No spouse currently' – to ensure an adequate expected count for each cell in the Chi square test.

Prevalence of depression, anxiety and stress

Table 2. Prevalence and severity of depression, anxiety and stress in the overall, frontline and non-frontline groups

	Depression N (%)			*Anxiety N (%)*			*Stress N (%)*		
	Total	*FL*	*NFL*	*Total*	*FL*	*NFL*	*Total*	*FL*	*NFL*
**Severity[a] Mild**	40 (8.6%)	17 (7%)	23 (10.3%)	31 (6.6%)	11 (4.5%)	20 (9%)	18 (3.9%)	11 (4.5%)	7 (3.1%)
**Moderate**	33 (7.1%)	8 (3.3%)	25 (11.2%)	46 (9.9%)	19 (7.8%)	27 (12.1%)	20 (4.3%)	6 (2.5%)	14 (6.3%)
**Severe**	7 (1.5%)	2 (0.8%)	5 (2.2%)	8 (1.7%)	2 (0.8%)	6 (2.7%)	10 (2.1%)	2 (0.8%)	8 (3.6%)
**Extreme**	11 (2.4%)	2 (0.8%)	9 (4%)	11 (2.4%)	3 (1.2%)	8 (3.6%)	7 (1.5%)	2 (0.8%)	5 (2.2%)
**Normal**	376 (80.5%)	215 (88.1%)	161 (72.2%)	371 (79.4%)	209 (85.7%)	162 (72.6%)	412 (88.2%)	223 (91.4%)	189 (84.8%)
**Abnormal[b]**	91 (19.5%)	29 (11.9%)	62 (27.8%)	96 (20.6%)	35 (14.3%)	61 (27.4%)	55 (11.8%)	21 (8.6%)	34 (15.2%)

Note: FL = Frontline; NFL = Non-frontline

[a]Cut-offs for depression severity on DASS-21 depression subscale were >9 for mild, >13 for moderate, >20 for severe, and >27 for extremely severe. For anxiety severity, cut-offs on DASS-21 anxiety subscale were >7 for mild, >9 for moderate, >14 for severe, and >19 for extremely severe. Cut-offs for stress severity on DASS-21 stress subscale were >14 for mild, >18 for moderate, >25 for severe, and >33 for extremely severe.

[b] 'Abnormal' category includes participants with any severity of depression/anxiety/stress (mild through extreme)

Table 2 summarizes the prevalence and severity of DASS-21 outcomes in the total, FL and NFL groups. The prevalence of depression, anxiety and stress among the HCWs in general was 19.5%, 20.6% and 11.8% respectively. NFL group showed a higher prevalence of depression (27% vs. 11%, p<0.001), anxiety (27% vs. 14%, p=0.001) and stress (15% vs. 8%, p=0.026) compared to the FL group. These differences were significant on both univariate and multivariate analyses. The adjusted odds ratios for depression, anxiety and stress in the FL group compared to NFL group, after controlling for sociodemographic differences (i.e. occupation, income and area of residence), were 0.34 (CI: 0.2-0.58), 0.48 (CI: 0.29-0.78), and 0.49 (CI: 0.27-0.93), respectively. The findings of this multivariate analysis are summarized in Table 3.

Sociodemographic factors associated with depression, anxiety and stress

The associations between socio-demographic factors and DASS-21 outcomes of depression, anxiety and stress are shown in Table 4. Having children (p=0.015), higher income (p=0.012) and living with family (p=0.002) were protective against depression. Being married (p=0.010), having children (p=0.009) and living with family (p=0.001) were associated with less anxiety. Being married (p=0.001) and having children (p=0.002) were protective for stress.

Table 3. Effect of being frontline or non-frontline on depression, anxiety and stress, after adjusting for socio-demographic factors [a]using binary logistic regression

Variable	*Depression*[b]			*Anxiety*[b]			*Stress*[b]		
	AOR	*95% CI*	*p value*	*AOR*	*95% CI*	*p value*	*AOR*	*95% CI*	*p value*
**Position**									
Frontline	0.34	0.205-0.580	<0.001*	0.48	0.295-0.783	0.003*	0.49	0.268-0.926	0.028*
Non-frontline	ref			ref			ref		
**Occupation**									
Doctor	0.97	0.354-2.675	0.957	0.53	0.204-1.38	0.196	3.76	0.727-19.4	0.114
Nurse/midwife	0.56	0.225-1.37	0.204	0.47	0.205-1.10	0.085	1.86	0.394-8.8	0.433
Supporting staff	1.11	0.403-3.07	0.836	0.85	0.316-2.26	0.738	2.7	0.532-13.6	0.331
Ancillary staff	ref			ref			ref		
**Area of residence**									
Urban	0.83	0.397-1.73	0.616	0.79	0.392-1.61	0.527	0.93	0.392-2.21	0.874
Semi-urban	1.22	0.677-2.21	0.503	1.06	0.597-1.89	0.832	1.06	0.505-2.2	0.887
Rural	ref			ref			ref		
**Income (SL rupees)**									
<25 000	4.46	1.13-17.5	0.033*	1.27	0.317-5.13	0.732	6.73	1.47-30.7	0.014*
25 000-50 000	2.73	0.96-7.77	0.060	1.04	0.377-2.86	0.942	2.51	0.742-8.52	0.139
50 000-150 000	2.53	1.09-5.87	0.030*	1.69	0.782-3.65	0.182	1.83	0.707-4.77	0.212
> 150 000	ref			ref			ref		

Note: AOR = Adjusted odds ratio; ref = reference category

[a]Only the socio-demographic variables which were found to be significantly different between frontline and non-frontline participants (see Table 1), were included in the logistic regression model as covariates.

[b]Cut-offs for depression, anxiety and stress on DASS-21 subscales were >9, >7 and >14, respectively.
*significant at 95% confidence

Perceptions of work environment, social circumstances and coping strategies, and their relationship with depression, anxiety and stress

The participants' responses regarding their social circumstances, support received at the hospital, and coping strategies used, are tabulated against DASS-21 outcomes, in Table 5. The risk of DASS-21 outcomes if one agrees with each statement is expressed using odds ratios. Workplace-related perceptions such as the fear of contracting COVID-19, inadequacy of PPE, lack of administrative support, lack of psychological support and constraints on delivering patient care were significantly associated with higher psychological morbidity. Perceptions related to personal circumstances including the fear of transmitting the infection to family members, discrimination experienced due to working in a COVID-19 screening hospital, and the difficulty in balancing professional and domestic lives were also associated with greater mental health burden. Among coping strategies, seeking help from family and friends, and spending time productively appeared to be protective. An increase in alcohol and other substance consumption was associated with depression, anxiety and stress. Practicing religion or taking up new hobbies were not associated with outcomes.

The differences in responses given by FL and NFL groups revealed that the NFL group was more likely to feel they were not provided adequate PPE, they were vulnerable to contract COVID-19 in spite of PPE, and they had not been provided adequate administrative and psychological support. NFL workers felt that they had not been trained to cope with workplace changes. Also, NFL workers had experienced greater discrimination due to their employment at a hospital, and were more worried about loss of employment or income. NFL workers were less likely to cope using the help of family and friends.

Table 4. Socio-demographic factors associated with depression, anxiety and stress

Variable	*Depression*		*Anxiety*		*Stress*	
	N out of total (%)	*Chi square (p value)*	*N out of total (%)*	*Chi square (p value)*	*N out of total (%)*	*Chi square (p value)*
Sex		0.145 (0.703)		1.11 (0.290)		1.04 (0.307)
Male	26/126 (20.6%)		30/126 (22.8%)		18/126(14.3%)	
Female	65/341 (19.1%)		66/341 (19.4%)		37/341 (10.9%)	
Age		3.25 (0.353)		2.46 (0.482)		5.83 (0.120)
18 - 29 years	19/102 (18.6%)		21/102 (20.6%)		16/102 (15.7%)	
30 - 39 years	44/202 (21.8%)		45/202 (22.3%)		24/202 (11.9%)	
40 - 49 years	18/96 (18.8%)		19/96 (19.8%)		11/96 (11.5%)	
> 50 years	7/61 (11.5%)		8/61 (13.1%)		2/61 (3.3%)	
Occupation		5.96 (0.114)		5.05 (0.168)		3.6 (0.308)
Doctor	26/157 (16.6%)		27/157 (17.2%)		19 (12.1%)	
Nurse/midwife	30/174 (17.2%)		35/174 (20.1%)		17 (9.8%)	
Supporting staff	26/98 (26.5%)		22/98 (22.4%)		16 (16.3%)	
Ancillary staff	9/32 (28.1%)		11/32 (34.4%)		2 (6.2%)	
Marital status		0.37 (0.055)		6.68 (0.010)*		12 (0.001)*
Having a spouse currently	60/345 (17.4%)		61/345(17.7%)		30/345 (8.7%)	
No spouse currently	31/122 (25.4%)		35/122 (28.7%)		25/122 (20.5%)	
Having children?		5.9 (0.015)*		6.88 (0.009)*		9.23 (0.002)*
Yes	45/280 (16.1%)		47/280 (16.8%)		23/280 (8.2%)	
No	46/182 (25.3%)		49/182 (26.9%)		32/182 (17.6%)	
Education		9.37 (0.95)		3.65 (0.599)		6.47 (0.263)
Primary education	0/4 (0%)		0/4 (0%)		0/4 (0%)	
Ordinary level	11/48 (22.9%)		9/48 (18.8%)		3/48 (6.2%)	
Advanced level	38/153 (24.8)		34/153 (24.2%)		23/153 (15%)	
Basic degree	29/179 (16.2%)		37/179 (19%)		20/179 (11.2%)	
Postgraduate	9/40 (22.5%)		10/40 (25%)		6/40 (15%)	
Other	2/33 (6.1%)		5/33 (15.2%)		1/33 (3%)	
Income (SL rupees)		11 (0.012)*		5.8 (0.121)		5.94 (0.115)
<25 000	7/24 (29.2%)		5/24(20.8%)		6/24 (25%)	
25 000 - 50 000	26/108 (24.1%)		21/108 (19.4%)		15/108 (13.9%)	
50 000 - 150 000	46/214 (21.5%)		53/214 (24.8%)		23/214 (10.7%)	
> 150 000	11/117 (9.4%)		16/117(13.4%)		10/117 (8.5%)	

(Continued)

Variable	Depression		Anxiety		Stress	
	N out of total (%)	*Chi square (p value)*	*N out of total (%)*	*Chi square (p value)*	*N out of total (%)*	*Chi square (p value)*
Area of residence		4.06 (0.131)		2.19 (0.333)		0.49 (0.781)
Urban	16/118 (13.6%)		19/118 (16.1%)		13/118 (11%)	
Semiurban	44/212 (20.8%)		46/212 (21.7%)		24/212 (11.3%)	
Rural	31/133 (22.3%)		31/133 (23.3%)		18/133 (13.5%)	
Presence of medical comorbidity		0.118 (0.732)		0.001 (0.979)		0.018 (0.894)
Yes	16/88 (18.2%)		18/88 (20.5%)		10/88 (11.4%)	
No	75/379 (19.8%)		78/379 (20.6%)		45/379 (11.9%)	
Living arrangement		12.5 (0.002)*		13 (0.001)*		4.5 (0.104)
Living alone	15/47 (31.9%)		17/47 (36.2%)		9/47 (19.1%)	
Living with family	58/359 (16.1%)		63/359 (17.5%)		36/359 (10%)	
Shared apartment	16/49 (32.7%)		16/49 (32.7%)		8/49 (16.3%)	
Presence of an elderly family member at home		0.006 (0.937)		0.099 (0.753)		0.081 (0.776)
Yes	26/135 (19.3%)		29/135 (21.5%)		15/135 (11.1%)	
No	65/332 (19.6%)		67/332 (20.2%)		40/332 (12%)	
Presence of another family member working at the frontline		2.95 (0.86)		0.010 (0.921)<br>0.005 (0.944)		
Yes	15/109 (13.8%)		23/109 (21.1%)		13/109 (11.9%)	
No	71/334 (21.3%)		69/334 (20.7%)		39/334 (11.7%)	
Mode of travel to work		3.24 (0.355)		4.2 (0.233)		5.3 (0.152)
Public transport	39/189 (20.6%)		42/189 (22.2%)		17/189 (9%)	
Own vehicle	30/187 (16%)		32/187 (17.1%)		21/187 (11.2%)	
Hired vehicle	1/7 (14.3%)		3/7 (42.9%)		1/7 (14.3%)	
On foot	20/80 (25%)		19/80 (23.8%)		15/80 (18.8%)	

Note: The prevalence of abnormal scores of depression (subscale score>9), anxiety (subscale score>7), and stress (subscale score>14), for each socio-demographic category has been listed. Significance of association was tested using Chi square test.

*Statistically significant at 95% confidence

Table 5. Participants' perceptions of work environment, social circumstances and coping strategies, and the associated risk of depression, anxiety and stress

Statement	*Depression*			*Anxiety*			*Stress*			*FL vs. NFL differences*[a]		
	+ve N (%)	*-ve N (%)*	*OR (CI)*	*+ve N (%)*	*-ve N (%)*	*OR (CI)*	*+ve N (%)*	*-ve N (%)*	*OR (CI)*	*Mean rank-FL*	*Mean rank-NFL*	*Mean Whitney U (p value)*
**Risk-increasing perceptions**												
**1. I worry that I might get infected during work**			3.4* (1.4-8.2)			5.7* (2-16.2)			3.5* (1.1-11.8)	229.2	234.1	2.6×10^4 (0.68)
Agree	65 (25.6%)	189 (77.4%)		69 (27.2%)	185 (72.8%)		37 (14.6%)	217 (85.4%)				
Disagree	6 (9.2%)	59 (90.8%)		4 (6.2%)	61 (93.8%)		3 (4.6%)	62 (95.4%)				
**2. I feel vulnerable to COVID-19 at my workplace despite the PPE provided**			1.57 (0.89-2.4)			2.4*(1.2-4.7)			2.1 (0.9-4.9)	210	251	2.2 (<0.001)*
Agree	57 (22.4%)	198 (77.6%)		190 (74.5%)	65 (25.5%)		219 (85.9%)	36 (14.1%)				
Disagree	15 (15.5%)	82 (84.5%)		85 (87.6%)	12 (12.4%)		90 (92.8%)	7 (7.2%)				
**3. I feel my work performance has become less due to worry about COVID-19**			2.8*(1.6-4.9)			1.7 (0.9-2.9)			2.4* (1.2-4.7)	220	239	2.6×10^4 (0.92)
Agree	41 (27.3%)	109 (72.7%)	34 (22.7%)	116 (77.3%)	24 (16%)		126 (84%)					
Disagree	24 (11.7%)	181 (88.3%)	30 (14.6%)	175 (85.4%)	15 (7.3%)		190 (92.7%)					
**4. Hospital staff should be screened periodically for COVID-19**			9.1[b]				9.6[b]		4.8[b]	225	230	2.5×10^4 (0.71)
Agree	81 (20.6%)	313 (79.4%)		85 (21.6%)	309 (78.4%)		48 (12.2%)	346 (87.8%)				
Disagree	0 (0%)	17 (100%)		0 (0%)	17 (100%)		0 (0%)	17 (100%)				

(Continued)

Statement	Depression			Anxiety			Stress			FL vs. NFL differences[a]		
	+ve N (%)	-ve N (%)	OR (CI)	+ve N (%)	-ve N (%)	OR (CI)	+ve N (%)	-ve N (%)	OR (CI)	Mean rank-FL	Mean rank-NFL	Mean Whitney U (p value)
**5. I worry I might infect my family**			1.2 (0.6-8.0)			2.6* (1.1-7.6)			1.8 (0.6-8.0)	232	229	2.6 × 10^4 (0.74)
Agree	57 (17.4%)	270 (82.6%)		81 (23.1%)	269 (76.9%)		46 (13.1%)	304 (86.9%)				
Disagree	5 (23.7%)	17 (76.3%)		4 (10.3%)	35 (89.7%)		3 (7.7%)	36 (92.3%)				
**6. If I get infected, there will be no one to take care of my family**			3.5* (1.5-7.9)			5.3* (2-13.6)			2.2 (0.9-5.2)	226	234	2.5×10^4 (0.50)
Agree	76 (25.2%)	225 (74.8%)	79 (26.2%)	222 (73.8%)		45 (15%)	256 (85%)					
Disagree	7 (8.8%)	73 (91.2%)	5 (6.2%)	75 (93.8%)		6 (7.5%)	74 (92.5%)					
**7. I worry that my family might be quarantined**			1.7 (0.9-3.4)			3.7* (1.6-8.5)			1.5 (0.8-4.0)	222	238	2.4×10^4 (0.17)
Agree	67 (23.8%)	214 (76.2%)	75 (26.7%)	206 (73.3%)			41 (14.6%)	240 (85.4%)				
Disagree	12 (15.2%)	67 (84.8%)	7 (8.9%)	72 (91.1%)			7 (8.9%)	72 (91.1%)				
**8. I worry about providing food/medicine/other needs for my family because of possible scarcities**			2.8* (1.5-5.1)			4.1* (2.2-8.2)			2.9* (1.4-6.3)	222	237	2.4×10^4 (0.21)
Agree	57 (27.4%)	151 (72.6%)		61 (29.3%)	147 (70.7%)		36 (17.3%)	172 (82.7%)				
Disagree	16 (11.9%)	119 (88.1%)		12 (8.9%)	123 (91.1%)		9 (6.7%)	126 (93.3%)				
**9. I have faced discrimination because I am employed at a COVID-19 screening hospital**			3.1* (1.7-5.4)			4.3* (2.4-7.8)			2.3* (1.1-4.4)	211	238	2.2×10^4 (0.025)*
Agree	47 (29.4%)	113 (70.6%)		53 (33.1%)	107 (76.9%)		26 (16.2%)	134 (83.8%)				
Disagree	21 (11.9%)	156 (88.1%)		18 (10.2%)	159 (89.8%)		14 (7.9%)	163 (92.1%)				
**10. I worry about loss of employment**		2.7* (1.5-4.8)			2.5* (1.4-4.3)				3.2* (1.5-6.9)	193	225	1.8×10^4 (0.004)*
Agree	29 (29.6%)	69 (70.4%)		30 (30.6%)	68 (69.4%)		17 (17.3%)	81 (82.7%)				
Disagree	31 (13.4%)	201 (86.6%)		35 (15.1%)	197 (84.9%)		14 (6%)	218 (94%)				

(Continued)

Statement	Depression			Anxiety			Stress			FL vs. NFL differences[a]		
	+ve N (%)	-ve N (%)	OR (CI)	+ve N (%)	-ve N (%)	OR (CI)	+ve N (%)	-ve N (%)	OR (CI)	Mean rank-FL	Mean rank-NFL	Mean Whitney U (p value)
**11. I have been required to live long periods away from my family**			1.6 (0.9-2.8)			1.8* (1.1-3.2)			3.1* (1.4-6.8)	219	228	2.4×10^4 (0.45)
Agree	56 (24.5%)	173 (75.5%)		56 (24.5%)	173 (75.5%)		38 (16.6%)	191 (83.4%)				
Disagree	22 (16.5%)	111 (83.5%)		20 (15%)	113 (85%)		8 (6%)	125 (94%)				
**12. I feel more challenged in balancing my work and domestic life**			2.4* (1.3-4.2)			2.5* (1.4-4.5)			3.0* (1.4-6.5)	212	219	2.2×10^4 (0.55)
Agree	46 (28.8%)	114 (71.2%)		48 (30%)	112 (70%)		31 (19.4%)	129 (80.6%)				
Disagree	20 (14.5%)	118 (85.5%)		20 (14.5%)	118 (85.5%)		19 (7.2%)	128 (92.8%)				
**13. I drink more alcohol/smoke more/abuse substances more since the lockdown started**			4.8* (1.8-12)			4.5* (1.7-11)			7.9* (2.9-21)	216	209	2.1×10^4 (0.53)
Agree	9 (50%)	9 (50%)		9 (50%)	9 (50%)		8 (44.4%)	10 (55.6%)				
Disagree	67 (17.2%)	323 (82.8%)		71 (18.2%)	319 (81.8%)		36 (9.2%)	354 (91.8%)				
**Protective perceptions**												
**1. I am provided with adequate PPE within the hospital**			0.40* (0.23-0.71)			0.48* (0.28-0.83)			0.47* (0.24-0.92)	261	203	2.2×10^4 (0.001)*
Agree	23 (11.8%)	172 (88.2%)		27 (13.8%)	168 (86.2%)		16 (8.2%)	179 (91.8%)				
Disagree	39 (24.8%)	118 (75.2%)		39 (24.8%)	118 (75.2%)		25 (15.9%)	132 (84.1%)				
**2. The hospital administration has provided me with adequate support**			0.76 (0.4-1.4)			0.64 (0.35-1.1)			0.46* (0.2-0.89)	240	214	2.3×10^4 (0.025)*
Agree	35 (16.6%)	176 (83.4%)		38 (18%)	173 (82%)		20 (9.5%)	191 (90.5%)				
Disagree	17 (20.7%)	65 (79.3%)		21 (25.6%)	61 (74.4%)		15 (18.3%)	67 (81.7%)				
**3. My supervisors are providing me adequate support**			0.51* (0.3-0.9)			0.6 (0.3-1.1)			0.55* (0.3-0.95)	247	204	2×10^4 (<0.001)*
Agree	36 (14.9%)	205 (85.1%)		41 (17%)	200 (83%)		25 (10.4%)	216 (89.6%)				
Disagree	16 (25.4%)	47 (74.6%)		16 (25.4%)	47 (74.6%)		11 (17.5%)	52 (82.5%)				

(Continued)

Statement	Depression			Anxiety			Stress			FL vs. NFL differences[a]		
	+ve N (%)	*-ve N (%)*	*OR (CI)*	*+ve N (%)*	*-ve N (%)*	*OR (CI)*	*+ve N (%)*	*-ve N (%)*	*OR (CI)*	*Mean rank-FL*	*Mean rank-NFL*	*Mean Whitney U (p value)*
**4. I am provided with adequate psychological support at my workplace**			0.32* (0.2-0.6)			0.44* (0.2-0.8)			0.49* (0.3-0.97)	241	209	2.1×10^4 (0.006)*
Agree	31 (14.4%)	184 (85.6%)		36 (16.7%)	179 (83.3%)		25 (11.6%)	190 (88.4%)				
Disagree	31 (34.4%)	59 (65.6%)		28 (31.1%)	62 (68.9%)		19 (21.1%)	71 (78.9%)				
**5. I have been adequately trained to handle the changes brought on by COVID-19**			0.55* (0.3-0.9)			0.58 (0.3-1.0)			0.48* (0.2-0.93)	245	206	2.1×10^4 (0.001)*
Agree	33 (16.3%)	170 (83.7%)		39 (19.2%)	164 (80.8%)		23 (11.3%)	180 (88.7%)				
Disagree	25 (26%)	71 (74%)		28 (29.2%)	68 (70.8%)		20 (20.8%)	76 (79.2%)				
**6. I think I spend my time productively despite COVID-19**			0.4* (0.2-0.9)			0.36* (0.2-0.8)			0.36 (0.1-0.91)	212	200	2×10^4 (0.26)
**7. I cope with the help of my family and friends**			0.5 (0.2-1.2)			0.37* (0.1-0.8)			0.36* (0.1-0.96)	237	215	2.3×10^4 (0.038)*
Agree	53 (18.2%)	239 (81.8%)		54 (18.5%)	238 (81.5%)		25 (8.6%)	267 (91.4%)				
Disagree	12 (35.3%)	22 (64.7%)		13 (38.2%)	21 (61.8%)		7 (20.6%)	27 (79.4%)				
**Equivocal perceptions**												
**1. I have been provided with enough support to meet my daily necessities**			1.3 (0.7-2.7)			1.2 (0.6-2.3)			1.6 (0.6-4.1)	228	225	2.5×10^4 (0.75)
Agree	47 (19.7%)	192 (80.3%)	49 (20.5%)	190 (79.5%)			28 (11.7%)	211 (88.3%)				
Disagree	12 (15%)	68 (85%)		14 (17.5%)	66 (82.5%)		6 (7.5%)	74 (92.5%)				
**2. I have adequate knowledge about COVID-19**			0.7 (0.2-2.0)			0.8 (0.3-2.2)			1.1 (0.3-4.8)	233	221	2.4×10^4 (0.28)
Agree	57 (17.4%)	270 (82.6%)		63 (19.3%)	264 (80.7%)		32 (9.8%)	295 (90.2%)				
Disagree	5 (22.7%)	17 (77.3%)		5 (22.7%)	17 (77.3%)		2 (9.1%)	20 (90.9%)				

(Continued)

Statement	Depression			Anxiety			Stress			FL vs. NFL differences[a]		
	+ve N (%)	-ve N (%)	OR (CI)	+ve N (%)	-ve N (%)	OR (CI)	+ve N (%)	-ve N (%)	OR (CI)	Mean rank-FL	Mean rank-NFL	Mean Whitney U (p value)
**3. I worry that media will invade my and my family's privacy if I get infected**			1.6 (0.9-2.7)			1.3 (0.7-2.2)			1.1 (0.6-2.1)	244	219	2.7×10^4 (0.036)*
Agree	45 (22.2%)	158 (77.8%)		45 (22.2%)	158 (77.8%)		25 (12.3%)	178 (87.7%)				
Disagree	23 (15.1%)	129 (84.9%)		27 (17.8%)	125 (82.2%)		17 (11.2%)	135 (88.8%)				
**4. I think I am coping with the current situation well**			0.7 (0.2-2.2)			0.6 (0.2-1.6)			1.7 (0.2-13)	235	219	2.3×10^4 (0.125)
Agree	56 (16.9%)	275 (83.1%)		60 (18.1%)	271 (81.9%)		30 (9.1%)	301 (90.9%)				
Disagree	4 (22.2%)	14 (87.8%)		5 (27.8%)	13 (72.2%)		1 (5.6%)	17 (94.4%)				
**5. I have taken up new hobbies**			0.8 (0.4-1.3)			0.8 (0.4-1.3)			0.7 (0.4-1.5)	231	216	2.3×10^4 (0.202)
Agree	35 (17.7%)	163 (82.3%)		34 (17.2%)	164 (82.8%)		18 (9.1%)	180 (90.9%)				
Disagree	28 (21.2%)	104 (78.8%)		28 (21.2%)	104 (78.8%)		16 (12.1%)	116 (87.9%)				
**6. I have developed better lifestyle modifications (healthier food, regular exercises, adequate sleep)**			0.7 (0.4-1.4)			0.95 (0.5-1.8)			1.7 (0.6-4.6)	235	224	2.5×10^4 (0.36)
Agree	42 (16.9%)	206 (83.1%)		45 (18.1%)	203 (81.9%)		26 (10.5%)	222 (88.5%)				
Disagree	17 (21.5%)	62 (78.5%)		15 (19%)	64 (81%)		5 (6.3%)	74 (93.7%)				
**7. I am practising my religion more than before**			1.04 (0.5-1.9)			1.2 (0.6-2.2)			1.4 (0.6-3.1)	222	210	2.2×10^4 (0.29)
Agree	23 (21.3%)	85 (88.7%)		26 (24.1%)	82 (75.9%)		15 (13.9%)	93 (86.1%)				
Disagree	24 (20.7%)	92 (79.3%)		24 (20.7%)	92 (79.3%)		12 (10.3%)	104 (89.7%)				

Note: OR = Odds ratio; FL = Frontline; NFL = Non-frontline; PPE = Personal Protective Equipment

Responses to statements are cross-tabulated with presence (+ve) or absence (-ve) of depression, anxiety and stress, respectively. For the purpose of this analysis, 'Strongly agree' and 'Agree' responses on the Likert scale have been combined into one'Agree' category, and 'Strongly disagree' and 'Disagree' have been combined into one 'Disagree' category. Those who responded with the 'neutral' response have been omitted. Cut-offs for depression, anxiety and stress were >9, >7 and >14, respectively. Statements where ORs are greater than 1 and significant for at least one outcome are listed under 'Risk-increasing perceptions' and those where ORs were less than 1 and significant for at least one outcome are listed under 'Protective perceptions'. Statements with no significant associations for any outcome are listed as Equivocal.

[a]Mann Whitney U test was used to compare the differences between FL and NFL groups in the responses to each statement. Higher mean ranks indicate a greater tendency to agree with each statement.

[b]Haldane-Anscombe correction was used to calculate the OR as one cell contained zero counts.

*Statistically significant at 95% confidence

Discussion

This study reports for the first time, higher depression, anxiety and stress among NFL HCWs in comparison to FL HCWs, during the COVID-19 pandemic, although both groups had higher psychological morbidity when compared to the general population. Having children, living with family, being married and higher income appeared to be protective. Perceived lack of PPE, administrative and psychological support, and discrimination were associated with psychological outcomes.

The study assessed HCWs from a COVID-19 screening centre located in a high-risk area. As the hospital was not a treating centre, FL HCWs who were at risk of being exposed to COVID-19 prior to testing, did not continue to treat patients who became positive. The NFL HCWs only ran the risk of unknowingly being exposed to COVID-19 patients. However, the criteria used to define the FL/NFL status of a HCW in this study may not accurately reflect their actual exposure to COVID-19. Nevertheless, we assumed that FL HCWs (as per present criteria) are at greater perceived risk of exposure to COVID-19 than their NFL counterparts. These two groups were dissimilar in perceptions about their social and work-related circumstances, which may have contributed to the disparity in psychological outcomes. For instance, the lack of PPE for NFL workers may have been a real concern as FL HCWs were given priority. Similarly, NFL workers may have been stigmatised unfairly by their communities for working in the hospital, despite having little risk of exposure to COVID-19. Adverse psychological effects of stigma during the pandemic have been similarly reported from Italy [15].

Consistent with previous observations [5,16], our study found perceived inadequacy of PPE to increase the risk of mental health disturbance. This highlights the need to ensure PPE for both FL and NFL HCWs. Also, NFL workers felt they had not been adequately trained to handle workplace changes caused by the pandemic. Such a lack of psychological preparedness would have increased their risk of mental health issues. These findings underscore the need to direct more attention to the mental health needs of NFL HCWs.

The prevalence of psychological morbidity in all HCWs is higher than the general population of Sri Lanka. A previous study [17] among non-HCWs in the country showed depression and anxiety disorder prevalence of 6.6% and 9.1%, respectively.

These prevalence rates among Sri Lankan HCWs seem to fall on the lower end of the range of findings from other countries. A review of studies on HCWs' mental health during this pandemic has reported a prevalence of 20-40% for depression and 30-70% for anxiety [9]. It should be borne in mind that these prevalence rates were derived using different instruments and cut-offs. A study from Singapore [18], not included in the foregoing review, used the same instrument and cut-offs as the current study, and found a relatively lower prevalence of depression (9%), anxiety (14.5%) and stress (6.6%) among HCWs.

Being a FL HCW was shown to elevate the risk of psychological sequelae in China [5,16,19] and Italy [6,20]. However, an absence of such a risk difference has also been reported from China [21]. Comparable research from other parts of the world is scarce.

Among Chinese HCWs[5], females exhibited greater levels of depression and anxiety. The present findings did not show a gender disparity. Female overrepresentation in the sample limits our ability to draw inferences about male HCWs. However, a preponderance of females in HCW samples is seen in other studies as well [18,22].

Higher mental health burden has been reported among nurses compared to other professional categories in China [5,23] and Japan [22], whereas in Singapore, non-medical professionals demonstrated greater levels of depression and anxiety [18]. However, no significant variation across professions was observed in the current study.

In line with established etiological understandings and empirical evidence [19], social support, in the form of being married or living with family, was shown to be protective against psychological problems. Having children also reduced the risk of depression, anxiety and stress; however, this could be the result of a confounding effect by marital status.

The fear of contracting COVID-19 and infecting family members being significant predictors of mental health issues in previous reports [5,9] was replicated in the present analysis. Those who felt it a challenge to balance their work and domestic life, and those who had been compelled to live away from their families demonstrated a higher prevalence of psychological issues. Coping with the help of family and friends was protective against mental health problems. A Chinese cohort similarly reported seeking support from family and friends as the most salient coping method [23]. These observations illustrate the impact of family-related concerns on HCWs' mental health during a pandemic. However, the directionality of this association cannot be verified from present findings, as those who are suffering from depression are in turn more likely to hold such negative cognitions.

The maladaptive nature of alcohol and other substance use is illustrated by the higher prevalence of depression, anxiety and stress among those who experienced an increase in this behaviour.

The high response rate of over 90%, which can be attributed to the convenience sampling and the different strategies used by the research team to ensure good response rate, is a strength of this study. However, non-probability convenience sampling would have introduced a sampling error. The study findings cannot be generalized to COVID-19 treating hospitals where FL HCWs may have constant exposure to confirmed cases. As the sample is

from a single centre, these context-specific issues are a limitation. Furthermore, the findings are from self-reported measures and cross sectional in nature and therefore do not establish clear morbidity or causality. Although mental health problems were assessed using a validated instrument, the sociodemographic questionnaire, which assessed social and occupational circumstances and coping methods, was not a formally validated tool, and therefore, may limit the validity of some inferences based on responses to this questionnaire.

Author contributions

All authors contributed to the conceptualization and design of the study. KB, DK, CAA and DSK contributed to the acquisition of data. AB and SW conducted the data analysis. AB, SW, STD and AH contributed to data interpretation and writing the manuscript. All authors read and approved the final manuscript.

Conflicts of interest

The authors declare that they have no conflicts of interest.

Acknowledgements

Authors would like to acknowledge the contributions made in data entry by Supipi Praneethi, Malika Gayathri Fernando, Thilini Abayabandara and Tharushika Amarasekara.

Ethics approval

Ethics approval was obtained from the Ethics Review Committee of the Faculty of Medicine, University of Kelaniya.

Funding

This research did not receive any specific grant from funding agencies in the public, commercial, or not-for-profit sectors.

Abbreviations

FL = frontline

NFL = non-frontline

HCW = health care worker

DASS-21 = Depression, anxiety and stress scale 21

References

1. World Health Organization. WHO Timeline COVID 19. 2020. https://www.who.int/news-room/detail/27-04-2020-who-timeline---covid-19 (Accessed on 03/05/2020)
2. Zandifar A, Badrfam R. Iranian mental health during the COVID-19 epidemic. *Asian J Psychiatr* 2020; **51**: 101990.
3. Wang C, Pan R, Wan X, *et al*. A longitudinal study on the mental health of general population during the COVID-19 epidemic in China. *Brain Behav Immun* 2020; **87**: 40-8.
4. Rajkumar RP. COVID-19 and mental health: A review of the existing literature. *Asian J Psychiatr* 2020; **52**: 102066.
5. Lai J, Ma S, Wang Y, *et al*. Factors Associated With Mental Health Outcomes Among Health Care Workers Exposed to Coronavirus Disease 2019. *JAMA Netw Open* 2020; **3**(3): e203976-e203976.
6. Rossi R, Socci V, Pacitti F, *et al*. Mental Health Outcomes Among Frontline and Second-Line Health Care Workers During the Coronavirus Disease 2019 (COVID-19) Pandemic in Italy. *JAMA Netw Open* 2020; **3**(5): e2010185-e2010185.
7. Ing EB, Xu Q, Salimi A, Torun N. Physician deaths from corona virus (COVID-19) disease. *Occup Med* (Chic Ill) 2020; **70**(5): 370-4.
8. Spoorthy MS, Pratapa SK, Mahant S. Mental health problems faced by healthcare workers due to the COVID-19 pandemic – A review. *Asian J Psychiatr* 2020; **51**: 102119.
9. Braquehais MD, Vargas-Cáceres S, Gómez-Durán, *et al*. The impact of the COVID-19 pandemic on the mental health of healthcare professionals. *QJM An Int J Med*. 2020; hcaa 207.
10. Lee AM, Wong JGWS, McAlonan GM, *et al*. Stress and Psychological Distress among SARS Survivors 1 Year after the Outbreak. *Can J Psychiatry* 2007; **52**(4): 233-40.
11. World Health Organization. Mental health and psychosocial considerations during the COVID-19 outbreak. World Health Organization, 2020. Available from: https://www.who.int/publications-detail/WHO-2019-nCoV-MentalHealth-2020.1(Accesed on 02/05/2020)
12. Wang H, Chow SC. Sample Size Calculation for Comparing Proportions. Wiley Encyclopedia of Clinical Trials. 2007. Available from: https://doi.org/10.1002/9780471462422.eoct005 (Accessed on 03/05/2020)
13. Rekha S. Adaptation and validation of the Depression, Anxiety and Stress Scale (DASS 21) among students of the University of Colombo. In: Annual Research Symposium, University of Colombo. 2012.
14. Sugirthan N. Tamil Translation of DASS-21. DASS translations.[Year unknown] http://www2.psy.unsw.edu.au/Groups/dass/Tamil/Sugirthan/Tamil Sugirthan.htm (Accessed on 30/04/2020).
15. Ramaci T, Barattucci M, Ledda C, Rapisarda V. Social stigma during COVID-19 and its impact on HCWs outcomes. *Sustainability* 2020; **12**: 13.
16. Dai Y, Hu G, Xiong H, Qiu H, Yuan X. Psychological impact

of the coronavirus disease 2019 (COVID-19) outbreak on healthcare workers in China. *medRxiv* 2020.

17. Dorrington S, Zavos H, Ball H, *et al.* Trauma, post-traumatic stress disorder and psychiatric disorders in a middle-income setting: prevalence and comorbidity. *Br J Psychiatry* 2014; **205**(5): 383-9.
18. Tan BYQ, Chew NWS, Lee GKH, *et al*. Psychological Impact of the COVID-19 Pandemic on Health Care Workers in Singapore. *Ann Intern Med* 2020; **173**(4): 317-20.
19. Liu X, Shao L, Zhang R, *et al*. Perceived Social Support and Its Impact on Psychological Status and Quality of Life of Medical Staffs After Outbreak of SARS-CoV-2 Pneumonia: A Cross-Sectional Study (Preprint). *Lancet* 2020.
20. Bettinsoli M, Riso D, Napier J, *et al.* Psychological Impact and Contextual Factors Associated With Physical and Mental Health Conditions of Italian Healthcare Professionals During the Covid-19 Disease Outbreak. *Appl Psychol Health Well-Being* 2020; **12**: 1054-73.
21. Liang Y, Chen M, Zheng X, Liu J. Screening for Chinese medical staff mental health by SDS and SAS during the outbreak of COVID-19. *J Psychosom Res*. 2020; **133**: 110102.
22. Matsuo T, Kobayashi D, Taki F, *et al*. Prevalence of Health Care Worker Burnout During the Coronavirus Disease 2019 (COVID-19) Pandemic in Japan. *JAMA Netw Open* 2020; **3**(8): e2017271-e2017271.
23. Cai H, Tu B, Ma J, *et al.* Psychological Impact and Coping Strategies of Frontline Medical Staff in Hunan Between January and March 2020 During the Outbreak of Coronavirus Disease 2019 (COVID-19) in Hubei, China. *Med Sci Monit Int Med J Exp Clin Res*. 2020; **26**: e924171.
