# Supplementary material for: Mental health status and related factors influencing healthcare workers during the COVID-19 pandemic: A systematic review and meta-analysis
Source: PLoS One. 2024 Jan 19;19(1):e0289454. doi: 10.1371/journal.pone.0289454 (PMC10798549; doi:10.1371/journal.pone.0289454)
Supplement: S1 Data — (ZIP) [file pone.0289454.s011.zip › literatures/181.pdf]

Journal of Multidisciplinary Healthcare

Dovepress
open access to scientific and medical research

Open Access Full Text Article

ORIGINAL RESEARCH

The correlation between professional quality of life and mental health outcomes among hospital personnel during the Covid-19 pandemic in Taiwan

Po-An Su[1]
Mu-Chen Lo[2]
Chiao-Ling Wang[2]
Ping-Chen Yang[3]
Chiao-I Chang[4]
Meng-Chuan Huang[4,5]
Ming-Kuo Huang[6]
Kuang-I Cheng[6]

[1]Department of Operation Management, Kaohsiung Medical University Hospital, Kaohsiung Medical University, Kaohsiung, Taiwan; [2]Department of Human Resource, Kaohsiung Medical University Hospital, Kaohsiung Medical University, Kaohsiung, Taiwan; [3]Department of Psychiatry, Kaohsiung Medical University Hospital, Kaohsiung Medical University, Kaohsiung, Taiwan; [4]Department of Public Health and Environmental Medicine, School of Medicine, College of Medicine, Kaohsiung Medical University, Kaohsiung, Taiwan; [5]Department of Nutrition and Dietetics, Kaohsiung Medical University Hospital, Kaohsiung, Taiwan; [6]Superintendent Office, Kaohsiung Medical University Hospital, Kaohsiung Medical University, Kaohsiung, Taiwan

Correspondence: Meng-Chuan Huang
Department of Public Health and Environmental Medicine, School of Medicine, College of Medicine, Kaohsiung Medical University, No. 100, Shih-Chuan 1st Road, Sanmin Dist., Kaohsiung, 80708, Taiwan
Tel +886 7-3121101 ext. 5341
Fax +886 7-3121101 ext. 5345
Email mechhu@kmu.edu.tw

Purpose: This study investigated the association between professional quality of life, working context, and mental health outcomes among hospital personnel in Taiwan during the worldwide upsurge in COVID-19 cases.

Patients and Methods: We recruited 503 hospital personnel to whom we administered online questionnaires containing items from the Professional Quality of Life (ProQoL) scale, which covers compassion satisfaction (CS), burnout (BO) and compassion fatigue (CF), the Depression, Anxiety and Stress Scale (DASS-21) and questions on work-related variables. Data were collected from 13 July to 19 August 2020.

Results: The participants generally reported moderate CS and BO and low CF. Overall prevalence of mild-to-extremely-severe stress, anxiety and depression was 24.5%, 39.6% and 31.2%, respectively. Multiple logistic regression revealed that moderate-to-high BO and CF correlated with increased risks of mild-to-extremely-severe stress (OR = 4.17 and 2.23, respectively), anxiety (OR = 4.86 and 2.81, respectively) and depression (OR = 5.83 and 3.01, respectively), while moderate-to-high CS correlated with reduced risks of stress (OR = 0.53) and depression (OR = 0.45) only. There were CS and BO differences in groups categorized by marital status and profession. Anxiety increased linearly by seniority <10, 10–19 and ≥20 years (p for trend <0.05).

Conclusion: In conclusion, the subscales of ProQOL, BO and CF appeared to be associated with increased risks of stress, anxiety and depression among hospital personnel during the COVID-19 epidemic. A long-term contingency program may be needed to adjust work context variables and support emotional well-being of these workers.

Keywords: COVID-19, depression, Anxiety and Stress Scale, DASS-21, hospital personnel, professional quality of life, ProQoL

Introduction

In recent years, the pandemic as a result of Coronavirus disease 2019 (COVID-19) has been one of the most serious situations for humanity. COVID-19's highly transmissible spread by asymptomatic carriers made its rapid rise a sudden challenge to the world's hospitals.[1,2] Medical healthcare workers (HCWs) (physicians, nurses) directly involved in the care of suspected or confirmed cases have been at risk of infection, themselves. Nonmedical personnel (allied health professionals, technicians, administrators, and maintenance workers) working in healthcare

Received: 26 July 2021
Accepted: 13 October 2021
Published: 21 December 2021

© 2021 Su et al. This work is published and licensed by Dove Medical Press Limited. The full terms of this license are available at https://www.dovepress.com/terms.php and incorporate the Creative Commons Attribution – Non Commercial (unported, v3.0) License (http://creativecommons.org/licenses/by-nc/3.0/). By accessing the work you hereby accept the Terms. Non-commercial uses of the work are permitted without any further permission from Dove Medical Press Limited, provided the work is properly attributed. For permission for commercial use of this work, please see paragraphs 4.2 and 5 of our Terms (https://www.dovepress.com/terms.php).

facilities have also been at high risk of infection. Thus, almost all hospital personnel have had to work intensively to care for the sudden onslaught of patients, prevent hospital infections, and manage their own risk of infection. This sudden intense and cautious focus at work has come with its own adverse effect on quality of life and psychological health.[3] One recent review of studies exploring Severe Acute Respiratory Syndrome (SARS)/Middle East Respiratory Syndrome (MERS)/ Severe acute respiratory syndrome coronavirus 2 (SARS-CoV-2) outbreaks and burnout syndrome among HCWs found that although there is still not enough data to draw clear conclusions about COVID-19, about one-third of HCWs manifested symptoms of burnout syndrome during previous SARS and MERS outbreaks.[4] The authors of that study found the prevalence rate in their review to be similar to those found in some categories of HCWs exposed to chronic occupational stress and poor work organization during non-epidemic periods.[4] One research team in Italy administered the same cross-sectional study[5] to the same frontline healthcare workers at one hospital twice, once during the first pandemic wave April 2020 and the second during the second wave December 2020. They reported widespread distress stemming from excessive and prolonged workloads, isolation, uncertainty about safety measures to be associated with various signs of impaired mental health including insomnia, anxiety and burnout. Importantly, they found a significant increase in number of workers manifesting symptoms of depression (49.4% vs 62.3%),[5] underscoring the possible consequences of prolonged stress.

There are relatively few studies investigating the impact of COVID-19 pandemic on mental health and quality of life in HCWs other than doctors and nurses. Two cross-sectional studies, administering Impact of Event Scale-Revised (IES-R)[6,7] and the Anxiety and Stress Scale (DASS-21),[7,8] reported mild stress among the general population during the early outbreak of COVID-19 in China. Using both scales, another study of personnel in one hospital in Singapore during the COVID-19 pandemic found that nonmedical HCWs had higher DASS-21 anxiety and stress subscale scores and higher IES-R total scores than medical HCWs.[9] Furthermore, two studies administering the Professional Quality of Life Scale (ProQOL), to HCWs in hospitals and primary care clinics in Europe found job satisfaction and quality of life to be decreased among staff members facing COVID-19 emergencies.[10,11] One of those studies reported that the subscales of QOL (burnout and secondary traumatization) correlated with anxiety.[11] Another study administering questionnaires to 595 HCWs in testing for SARS-CoV-2 in Italy found that HCWs positive for COVID-19 to be at significantly higher risk of anxiety, especially those with poor sleep quality,[12] a finding possibly suggesting a link between quality of life and mental health. Largely due to the intensive effort of hospital HCWs and public health measures, Taiwan was spared the brunt of possible consequences resulting from the COVID19 outbreak early on. This does not mean HCWs were not affected. The aim of this study was to assess the association between professional quality of life using ProQOL[13] and emotional well-being using DASS-21[8] among hospital personnel working directly with patients (physicians, nurses, allied health professionals) as well as hospital personnel usually not working directly with patients (technicians and administrators) in one large medical center located in southern Taiwan in mid-July 2020 during the COVID-19 pandemic.

Materials and Methods

Study Design and Subjects

A cross-sectional survey was designed to evaluate quality of life and psychological responses in hospital personnel serving at a 1600-bed medical center in southern Taiwan during the COVID-19 epidemic period. Questionnaires were designed and uploaded onto an online platform. An email with a link to the survey platform was sent along with a brief description explanation of survey via our hospital's internal email system to all members of our hospital staff aged above 20 year during 13 July to 19 August of 2020. The survey was strictly voluntary. Informed consent was waived because the questionnaire was filled out anonymously. The questionnaire could only be submitted after the participants had filled out all questionnaire items, so there were no incomplete questionnaires. Any questionnaires that had identical IP addresses, age, gender and occupation were excluded. G*Power was used estimate sample size needed to obtain 80% of power. Assuming a binary predictor in a multiple logistic regression and the H0 probability for outcome as 0.25, a sample size of 495 needed to achieve this. The research protocol was approved by the Ethics Review Committee of the Kaohsiung Medical University Hospital (IRB No 20200113).

Study Tools

Mandarin Chinese (traditional characters) was the language used to conduct this survey. The participants answered the questionnaires anonymously by the end of August. This questionnaire collected demographic data and job characteristic data, including occupation, department, shift work, years of experience, and service at a temporary quarantine station. The questionnaire also contained items from two survey tools, the ProQOL[13] and the DASS-21.[8] Currently, ProQOL IV or 5 version is translated into 26 languages including Mandarin Chinese in traditional characters.[13] For the administration of the survey in this study, we referenced and followed ProQOL Concise Manual.[13]

The ProQOL is used to evaluate negative and positive impacts of suffering and trauma in professionals working with people in care facilities and occupations. It consists of 30 items divided into three sub-scales: compassion fatigue (CF) with items like "I feel as though I am experiencing the trauma of someone I have [helped]", compassion satisfaction (CS) with items like "I get satisfaction from being able to [help] people", and burnout (BO) with items like "I feel overwhelmed because my case [work] load seems endless".[14] The items are generally scored from 1 (never) to 5 (very often). The ProQOL subscale scores total 50 points each: low (0–22), moderate (23–41), and high (≥42). The Cronbach's alpha (scale reliability) values for three subscales were 0.88 for Compassion Satisfaction, 0.75 for burnout and 0.81 for compassion fatigue.[13]

The DASS-21, a shortened version of the DASS-42,[15] has three subscales assessing the emotional states of depression, anxiety and stress. The scores for three subscales are categorized as follows. Stress is assessed as mild (15–18), moderate (19–25), severe (26–33), extremely severe (≥34). Anxiety is assessed as mild (8–9), moderate (10–14), severe (15–19), extremely severe (≥20). Depression is assessed as mild (10–13), moderate (14–20), severe (21–27), extremely severe (≥28). DASS-21 has been validated for its use in evaluating mental health statuses in Chinese and Singaporean HCWs and the general public during the SARS and COVID-19 pandemics.[9,16,17] The DASS–21 were originally administered to 1815 Chinese college students and reported Cronbach's alpha of 0.83, 0.80, and 0.82 for the Depression, Anxiety, and Stress subscales, respectively, and 0.92 for the total DASS.[18]

Statistical Analysis

Pearson chi-squared test and Student's *t*-test were used to compare differences between two groups, ANOVA and post-hoc test (Bonferroni test) to test differences among more than three groups. Logistic regression analysis was employed to examine relations between subscales of ProQOL and DASS-21 after adjusting for a range of covariates including age, gender, education, and working experience. A two-sided p-value <0.05 was considered significant. All statistical operations were performed using SPSS (version 22.0, SPSS Inc., Chicago, IL, USA).

Results

Our survey was administered via email to all hospital staff members mid-July, 2020. Five-hundred and three hospital members responded to this survey, giving us a 12% response rate. As can be seen in Table 1, a summary of the participants' demographic and job characteristics, of the 503 hospital personnel surveyed, 77 males (15.3%) and 426 females (84.7%). The distribution of the age ranges 21–30, 31–50, ≥51 years were 13.9%, 63.2% and 22.9%, respectively. Almost 28% of the responders (27.8%) had postgraduate degrees or above and 63.0% were married. Physicians, nurses, allied-health professionals, technicians and administrators made up 4.2%, 39.4%, 23.3%, 3%, and 30.2% of the participants. Approximately 57.7% of subjects (n = 290) were working day shifts, 39.6% (n = 199) rotating shifts, and 2.8% (n = 14) night shifts. Of the participants, 37.4%, 31.0%, and 31.6% had <10, 10–19 and ≥20 years of work experience, respectively. Two hundred fifty-one of the participants (49.9%) were assigned duty to work in Coronavirus quarantine stations in addition to performing their regular duties, from February to May, 2020.The mean scores for ProQOL and DASS-21 subscales scores and their distributions are summarized in Table 2. Mean ProQOL CS, BO and CF scores were 32.0 (±10.1), 24.9 (±6.6) and 20.9 (± 7.6), respectively. Based on the ProQOL results, participants were mostly found to have moderate CS levels (64.8%), moderate BO levels (66.6%) and low CF levels (63.2%). Mean DASS-21 stress, anxiety and depression subscale scores were 10.5 (± 8.2), 6.4 (±6.4), and 6.8 (±7.1) respectively. The overall prevalence of mild-to-extremely-severe depression, anxiety and stress was 24.5%, 39.6% and 31.2%, respectively.

Table 3 shows the results of our logistic regression analysis exploring relations between professional quality

Table 1 Demographic Characters of Hospital Personnel in Taiwan (n = 503)

Characteristics	Frequency (%)
Gender	
Male	77 (15.3)
Female	426 (84.7)
Age	
21–30	70(13.9)
31–50	318(63.2)
≥51	115(22.9)
Education Level	
Below college	363(72.2)
Postgraduate or above	140(27.8)
Marriage status	
Married	317(63.0)
Unmarried or others	186(37.0)
Occupation	
Physician	21(4.2)
Nurse	198(39.4)
Allied health professionals	117(23.3)
Technician	15(3.0)
Administrators	152(30.2)
Shift pattern	
Day shift	290(57.7)
Night shift	14(2.8)
Rotate shift	199(39.6)
Years of working	
<10 years	188(37.4)
10–19 years	156(31.0)
≥20 years	159(31.6)
Support Working position	
No	252(50.1)
Yes	251(49.9)

of life and mental health outcomes, controlling for potential confounders. A moderate-to-high CS was significantly associated with reduced risk of mild-to-extremely-severe stress (aOR: 0.53, 95% CI: 0.32–0.87) and depression (aOR: 0.45, 95% CI: 0.28–0.74), while a moderate-to-high BO correlated significantly with increased risk of mild-to-extremely-severe stress (aOR: 4.17, 95% CI: 2.34–7.43), anxiety (aOR: 4.86, 95% CI: 3.03–7.78) and depression (aOR: 5.83, 95% CI: 3.35–10.15). Similarly, a moderate-to-high CF also correlated significantly with increased risk of mild-to-extremely-severe stress (aOR: 2.23, 95% CI: 1.45–3.42), anxiety (aOR: 2.81, 95% CI: 1.90–4.15) and depression (aOR: 3.01, 95% CI: 2.03–4.62).As can be seen in Table 4, which categorizes ProQOL and mental health outcomes by demographic and job characteristics, participants who were married had significantly higher CS (33.2 ±9.8 vs 30.0 ± 10.2, P = 0.001) and lower BO (24.1 ± 6.6 vs 26.3 ± 6.3, P < 0.001), compared with their counterparts. The five identified professions had significantly different scores on CS (p = 0.001) and BO (p = 0.005), with technicians clearly reporting the lowest CS and highest BO scores. Pairwise post-hoc comparison (Bonferroni test), found the CS scores for technicians to be significantly lower than the physicians only group (20.6 ± 8.8 vs 35.2 ± 11.1, p < 0.001, data not shown), while their BO scores were different from allied health professionals (29.1 ± 7.0 vs 23.5 ± 6.6, p = 0.016, data not shown). Accumulated working experience (<10, 10–19, ≥20 years) was significantly and linearly correlated with increased anxiety (p for trends =0.002).

Discussion

This study of medical and non-medical hospital personnel serving in our large medical center in southern Taiwan found the participants to have moderate CS, BO and low CF. The overall prevalence of stress, anxiety and depression was 24.5%, 39.6% and 31.2%, respectively. After adjustment for confounding factors, moderate-to-high BO and CF appeared to associated with increased risk of mild-to-extremely-severe stress, anxiety and depression, while moderate-to-high CS was associated with reduced risks of stress and depression only (all p < 0.05). This study demonstrates the correlation between professional quality of life and mental health among hospital personnel.

The COVID-19 pandemic reached Taiwan in February 2020. Considering Wuhan's proximity to Taiwan and the large number of Taiwan citizens working in China and traveling back and forth from there, Taiwan could have easily been very seriously affected. Taiwan's first case of COVID-19 was detected on January 29, 2020. During this first wave of the epidemic in Taiwan, the number of active cases peaked on 6 April 2020. On that day, 307 cases were reported and were confirmed to be mostly imported. By April 2020, there were no new confirmed cases of community spread disease, a public health achievement made without need for locking down. By August 19, 2020, the end of this investigation, Taiwan had had a total of 486 confirmed cases. Taiwan's success was largely due to its initial rapid containment of COVID-19 pandemic through the implementation of border

Table 2 Distribution of Professional Quality of Life (ProQOL) and Depression Anxiety Stress Scales-21 (DASS-21) Among Hospital Personnel (N = 503) During COVID-19 Pandemic

ProQOL	Compassion Satisfaction (CS)	Burnout (BO)	Compassion Fatigue (CF)
Mean score	32.0 ± 10.1	24.9 ± 6.6	20.9 ± 7.6
Scoring category			
Low	98(19.5)	165(32.8)	318(63.2)
Moderate	326(64.8)	335(66.6)	181(36.0)
High	79(15.7)	3(0.6)	4(0.8)
DASS-21	Stress	Anxiety	Depression
Mean score	10.5 ± 8.2	6.4 ± 6.4	6.8 ± 7.1
Scoring category			
Normal	380(75.5)	304(60.4)	346(68.8)
Mild	50(9.9)	51(10.1)	57(11.3)
Moderate	47(9.3)	100(19.9)	79(15.7)
Severe	19(3.9)	25(5.0)	10(2.0)
Extremely severe	7(1.4)	23(4.6)	11(2.2)

Note: Data were presented as mean ± SD or N(%).

control, entry quarantine and protection of airport and harbour-related staff.[20,21] This was the situation under which this study was conducted. However, at the time of this writing,18 months after successfully keeping COVID-19 at bay, Taiwan is facing a surge in cases after an outbreak was discovered in mid-May, 2021. By 23th of September, 2021, The Taiwan Central Epidemic Command Center had announced that over 50% of people in Taiwan had received the first dose of a COVID-19 vaccine six months after the rollout began on March 22, and the total number of confirmed cases were 16,618 and deaths 841.[19]

The professional quality of life status among our participants was similar to those reported by a recent study in Spain, also performed under COVID-19 pandemic conditions,[22] both finding an overall predominance of moderate CS and BO. However, in the study conducted in Spain, 64.3% of hospital or primary HCWs (doctors, nurses and technicians) were found to have reported high CF.[22] Only 0.8% of our study subjects had high CF. This difference may be due in part to the fact that the investigation in Spain was conducted in their first wave of COVID-19 pandemic when, according to WHO, they had a maximum 6936 confirmed cases,[23] while our investigation was performed after Taiwan's first pandemic peak and before the recent upsurge. Therefore, the differences between Spain's high CF and Taiwan's low and medium CF might be due the differences in the time spent intensively caring for COVID-19 infected patients as well as the number of patients cared for. Although 63.2% of our hospital workers had low CF, 66.6% had medium BO and 36.0% medium CF. There are other studies reporting high rates of BO, CF and perceived stress among HCWs in more heavily hit countries around the world.[24,25]

The results of our DASS-21 showed the 31.2% of our participants had depression (11.3% mild and 19.9% moderate-extremely-severe), 39.6% had anxiety (10.1% mild and 29.5% moderate-to-extremely-severe), and 24.4% had signs of stress (9.9% mild and 14.5% moderate-to-extremely-severe stress). Two meta-analyses have assessed COVID-19's effect on the mental health of HCWs, one reporting an overall depression rate of 22.8% and the other increased manifestation of stress.[26,27] Our study found psychological distress in our participants to be comparable to that reported by a study of 1210 respondents belonging to the general population in 194 cities in China during the early days of the outbreak (January 31 to February 2, 2020).[7] The authors of that study performed a follow-up survey of 861 subjects and found no significant changes this distress.[8] One study administering DASS-21 online to primarily from the USA followed by Pakistan, Canada and UK between April 27th and May 13th, 2020 found that 58.6% had depression, 50.9% anxiety and 57.4% stress, the longer the duration, the higher the distress. These rates

Table 3 Association Between Professional Quality of Life (ProQOL) and Mental Health Outcomes Based on Depression Anxiety Stress Scales-21 (DASS-21) Among Hospital Personnel in Taiwan (N = 503) During COVID-19 Pandemic

	Stress				Anxiety				Depression			
	**Normal N(%)**	**Mild to Extremely Severe N (%)**	**OR (95% CI)**	**aOR* (95% CI)**	**Normal N(%)**	**Mild to Extremely Severe N (%)**	**OR (95% CI)**	**aOR* (95% CI)**	**Normal N(%)**	**Mild to Extremely Severe N (%)**	**OR (95% CI)**	**aOR* (95% CI)**
**Compassion satisfaction (CS)**												
Low	63 (16.6)	35 (28.5)	1	1	54 (17.8)	44 (22.1)	1	1	53 (15.3)	45 (28.7)	1	1
Moderate to high	317 (83.4)	88 (71.5)	0.50 (0.31–0.80)	0.53 (0.32–0.87)	250 (82.2)	155 (77.9)	0.76 (0.49–1.19)	0.69 (0.43–1.11)	293 (84.7)	112 (71.3)	0.45 (0.29–0.71)	0.45 (0.28–0.74)
**Burnout (BO)**												
Low	149 (39.2)	16 (13.0)	1	1	135 (44.4)	30 (15.1)	1	1	147 (42.5)	18 (11.5)	1	1
Moderate to high	231 (60.8)	107 (87.0)	4.31 (2.45–7.58)	4.17 (2.34–7.43)	169 (55.6)	169 (84.9)	4.50 (2.87–7.05)	4.86 (3.03–7.78)	199 (57.5)	139 (88.5)	5.70 (3.34–9.74)	5.83 (3.35–10.15)
**Compassion fatigue (CF)**												
Low	257 (67.6)	61 (49.6)	1	1	219 (72.0)	99 (49.7)	1	1	245 (70.8)	73 (46.5)	1	1
Moderate to high	123 (32.4)	62 (50.4)	2.12 (1.40–3.21)	2.23 (1.45–3.42)	85 (28.0)	100 (50.3)	2.60 (1.79–3.78)	2.81 (1.90–4.15)	101 (29.2)	84 (53.5)	2.79 (1.89–4.12)	3.01 (2.03–4.62)

Note: *Multiple logistic regression was adjusted for age (21–30, 31–50, ≥51 years), gender (male, female), education (below college, postgraduate or above), marriage status (married, unmarried/others), occupation (physician, nurse, allied health professionals, technician, administrators), years of working (<10, 10–19, ≥20 years).
Abbreviation: aOR, adjusted odds ratio.

Table 4 Characteristics in 503 Hospital Personnel Analyzed by Subscale Scores of Professional Quality of Life (ProQOL) and Depression Anxiety Stress Scales-21 (DASS-21) During COVID-19 Pandemic

Characteristics	ProQOL						DASS-21					
	Compassion Satisfaction (CS)	p*	Burnout (BO)	p*	Compassion Fatigue (CF)	p*	Stress	p*	Anxiety	p*	Depression	p*
Gender		0.746		0.750		0.133		0.373		0.600		0.718
Male	32.4 ± 11.4		24.7 ± 6.9		22.1 ± 8.8		9.7 ± 9.3		6.0 ± 6.7		7.1 ± 8.1	
Female	31.9 ± 9.8		25.0 ± 6.5		20.7 ± 7.4		10.7 ± 8.0		6.4 ± 6.3		6.8 ± 6.9	
Age		0.229		0.080		0.920		0.855		0.258		0.485
21–30	30.3 ± 10.2		26.4 ± 7.5		21.2 ± 7.2		10.3 ± 9.3		5.5 ± 6.8		6.9 ± 8.6	
31–50	32.1 ± 10.1		24.9 ± 6.4		20.9 ± 7.8		10.4 ± 8.2		6.3 ± 6.4		6.5 ± 7.0	
≥51	32.9 ± 9.9		24.2 ± 6.2		20.7 ± 7.4		10.9 ± 7.5		7.0 ± 6.0		7.5 ± 6.4	
P for trend**	0.097		0.031		0.690		0.593		0.101		0.463	
Education Level		0.419		0.991		0.170		0.870		0.073		0.286
Below college	31.0 ± 9.5		24.9 ± 5.8		19.6 ± 5.8		10.7 ± 8.4		7.8 ± 6.6		7.8 ± 7.3	
Postgraduate or above	32.1 ± 10.1		24.9 ± 6.7		21.1 ± 7.8		10.5 ± 8.2		6.2 ± 6.3		6.7 ± 7.1	
Marriage status		0.001		<0.001		0.660		0.237		0.586		0.081
Married	33.2 ± 9.8		24.1 ± 6.6		21.0 ± 7.4		10.2 ± 7.9		6.3 ± 6.2		6.4 ± 6.6	
Unmarried or others	30.0 ± 10.2		26.3 ± 6.3		20.7 ± 7.9		11.1 ± 8.7		6.6 ± 6.7		7.5 ± 7.8	
Occupation		<0.001		0.005		0.135		0.357		0.472		0.323
Physician	35.2 ± 11.1		23.4 ± 8.2		23.8 ± 8.5		10.6 ± 9.0		5.7 ± 6.0		6.6 ± 7.7	
Nurse	32.1 ± 9.5		25.5 ± 6.1		21.4 ± 7.3		11.0 ± 7.9		6.4 ± 6.4		7.0 ± 7.1	
Allied health professionals	34.1 ± 9.6		23.5 ± 6.6		20.4 ± 8.1		9.1 ± 7.5		5.6 ± 5.2		5.6 ± 5.8	
Technician	20.6 ± 8.8		29.1 ± 7.0		18.3 ± 5.4		10.9 ± 10.8		7.5 ± 9.3		8.0 ± 9.0	
Administrators	31.0 ± 10.3		25.1 ± 6.5		20.4 ± 7.6		10.9 ± 8.8		6.9 ± 7.0		7.3 ± 7.7	
Shift pattern		0.904		0.995		0.904		0.287		0.290		0.558
Day shift	32.1 ± 10.1		24.9 ± 6.5		20.9 ± 7.7		10.1 ± 8.0		6.2 ± 5.9		6.7 ± 6.8	
Night shift	33.1 ± 7.3		25.0 ± 7.6		21.7 ± 6.1		13.3 ± 9.5		8.9 ± 7.9		8.7 ± 10.2	
Rotate shift	31.9 ± 10.3		24.9 ± 6.6		20.8 ± 7.6		10.8 ± 8.4		6.5 ± 6.9		6.9 ± 7.4	
Years of working		0.351		0.671		0.509		0.220		0.010		0.091
<10 years	31.2 ± 10.7		25.2 ± 7.1		20.4 ± 7.8		9.7 ± 8.6		5.4 ± 6.0		6.3 ± 7.4	
10–19 years	32.2 ± 9.5		24.9 ± 6.2		21.4 ± 7.5		10.9 ± 7.9		6.5 ± 6.6		6.4 ± 6.8	
≥20 years	32.8 ± 9.8		24.6 ± 6.2		21.0 ± 7.5		11.1 ± 8.1		7.5 ± 6.5		7.8 ± 7.0	
P for trend**	0.152		0.383		0.447		0.103		0.002		0.050	

(*Continued*)

Table 4 (Continued).

Characteristics	ProQOL						DASS-21					
	Compassion Satisfaction (CS)	p*	Burnout (BO)	p*	Compassion Fatigue (CF)	p*	Stress	p*	Anxiety	p*	Depression	p*
Support Working position		0.057		0.221		0.906		0.361		0.604		0.760
No	31.2 ± 10.1		25.3 ± 6.1		20.9 ± 7.9		10.2 ± 8.1		6.2 ± 6.4		6.7 ± 6.9	
Yes	32.9 ± 10.0		24.6 ± 7.0		20.8 ± 7.3		10.8 ± 8.3		6.5 ± 6.4		6.9 ± 7.3	

Notes: *Independent *t*-test or ANOVA were used to determine the difference between the ProQOL or DASS-21 subscales scores and characteristics. **P for trend was performed by simple linear regression.

were lower in our study. One would think that people on the frontline of the pandemic would have higher rates of distress. However, people working in hospitals generally have much more knowledge regarding disease spread and prevention, and this could make them more confident about the pandemic than less knowledgeable populations.

In our study, compared to low BO scores, moderate-to-high BO scores correlated with multi-fold increases in risks of moderate-to-extremely-severe stress (OR: 4.17), anxiety (OR: 4.86) and depression (OR: 5.83). Moderate-to-high CF was also correlated with 2.23– to 3.01-fold increases in risks for stress, anxiety and depression, compared with low CF. In contrast, moderate-to-high CS was negatively associated with the three ProQOL subscales. These results are similar to those reported by two very recent surveys in Spain[14] and Italy,[11] both concluding professional quality of life can serve as a predictor for mental status among hospital workers. An study of 265 hospital workers serving in an outpatient clinic belonging to the Occupational Health Department of a major university hospital in Italy[11] found that ProQOL BO and secondary traumatization subscale scores correlated with worsening of depression (assessed using Patient Health Questionnaire-9, PHQ-9) and anxiety (assessed using General Anxiety Disorder-7, GAD-7). A survey of 537 hospital and primary care professionals working in health centers in Spain during the COVID-19 outbreak found CS, BO and CF to be directly associated with perceived stress by using Perceived Stress Scale-14 (PSS-14).[14] Considered together, that has been an association found between low professional quality of life and increased risk of mental health problems, including anxiety or depression, among hospital personnel during this pandemic.

In this study, married employees had significantly higher CS and lower BO scores than unmarried employees. This was not found to true for nurses Spain, though.[10] Marital status along with years of experiences, shift work and specialty has been correlated with CS or CF in nurses in specialized nursing units.[28,29] The marital status variable is relevant in that it can be considered a type of social support in addition to other types of support such as teammates and colleagues, and as such it can account for some of the variability in ProQOL. In general, however, studies have found nurses seek support more from co-workers[30] or spiritual beliefs[31] than personal relationships. The Singapore study found a higher prevalence anxiety in nonmedical workers than in medical workers in one hospital during the pandemic.[9] In China, frontline nurses had

significantly lower vicarious traumatization scores than non-frontline nurses and the general public in a recent COVID-19 study.[32] We found technicians to have the lowest CS scores and the highest BO scores, both positively associated with worse mental health status for these employees. High baseline burnout rates may exacerbate risks of mental health injury.[33] Therefore, there is a need for early interventions to reduce stress and mitigate the mental health impact.[34] Burnout has been reduced by the use of resting facilities, interaction with and support of family members and loved ones, and cooperative work relations.[34] Organizations can reduce burnout by improving workflow management, reducing workload, coaching staff on how to improve communication skills, increasing interoperability and providing practical training on protective interventions, which would improve sense of safety and assurance.[34]

The current study also observed that anxiety scores were linearly increased along with years of working experience (<10, 10–19, ≥20 years, p for trend = 0.010). This increase may be, in part, due to the fact that at our hospital there is a higher distribution of female employees and nurses with 20 years of working experience (87.4% female, 43.4% nurses, data not shown), compared to those with less than ten years (79.3% female, 29.8% nurses, data not shown). One very recent meta-analysis[26] just revealed that that over one-third of nurses have experienced stress, anxiety, depression and sleep disturbance during the COVID-19 outbreak. These results also highlight the need for early and appropriate interventions to reduce the psychological impacts on nurses.

This study has several limitations. The main limitation is that this study is a cross-sectional design study with small sample size. Additionally, the response rate of this study was 12% which is a commonly reported drawback of web-based studies compared to paper-based survey studies.[35,36] Such a limitation may impair the precision of estimates due to reduced sample size and the representativeness of the subject group, leading to selection bias. Another limitation is that the assessments of self-reported on-line questionnaire of psychological impact, anxiety, depression and stress may not always be align with face-to-face assessments by mental health professionals. Besides, this study was unable to distinguish whether these symptoms were a result of serving as a health care worker or simply a result of living under pandemic conditions, since we employed a convenience sampling. Still another limitation is that we did not have data on pre-pandemic occupational stress, which would allow us to better assess the pandemic's effect on profession quality of life and mental status in our employees.

Conclusion

ProQOL subscales, BO, CF and CS, appeared to be associated with mental health status among our hospital workers during this pandemic. Married participants were found to be more resilient, while technicians were found to be more vulnerable. Future longitudinal studies may be needed to further identify the context-specific role that pandemic conditions play on mental health of hospital personnel, delineating it from role of other occupational stressors. Future studies may also want to focus the development of workplace promotion programs aimed to prevent adverse mental health outcomes in hospital personnel under highly stressful pandemic situations.

Institutional Review Board Statement

The study was conducted according to the guidelines of the Declaration of Helsinki, and approved by the Institutional Review Board of Kaohsiung Medical University Hospital (protocol code: KMUHIRB-E(I)-20200113 and June 1, 2020 of approval). The informed consent requirement was waived due to the questionnaire of our study was anonymous, and the research involved no more than minimal risk to the participant and waiver of informed consent would not adversely affect the rights and welfare of the subjects.

Acknowledgments

We are grateful to all the subjects participating in this investigation. This study was supported by grants from Kaohsiung Medical University Hospital (KMUH-C34), and from the Ministry of Science and Technology, Taiwan (MOST 109-2320-B-037 -030 -MY3).

Author Contributions

All authors made a significant contribution to the work reported, whether that is in the conception, study design, execution, acquisition of data, analysis, and interpretation, or in all these areas; took part in drafting, revising, or critically reviewing the article; gave final approval of the version to be published; have agreed on the journal to which the article has been submitted; and agree to be accountable for all aspects of the work.

Disclosure

The authors report no conflicts of interest in this work.

References

1. Riou J, Althaus CL. Pattern of early human-to-human transmission of Wuhan 2019 novel coronavirus (2019-nCoV), December 2019 to January 2020. *Euro Surveill*. 2020;25(4). doi:10.2807/1560-7917.ES.2020.25.4.2000058
2. Rothe C, Schunk M, Sothmann P, et al. Transmission of 2019-nCoV infection from an asymptomatic contact in Germany. *N Engl J Med*. 2020;382(10):970–971. doi:10.1056/NEJMc2001468
3. Gold JA. Covid-19: adverse mental health outcomes for healthcare workers. *BMJ*. 2020;369:m1815. doi:10.1136/bmj.m1815
4. Magnavita N, Chirico F, Garbarino S, Bragazzi NL, Santacroce E, Zaffina S. SARS/MERS/SARS-CoV-2 outbreaks and burnout syndrome among healthcare workers. an umbrella systematic review. *Int J Environ Res Public Health*. 2021;18(8):4361. doi:10.3390/ijerph18084361
5. Magnavita N, Soave PM, Antonelli M. Prolonged stress causes depression in frontline workers facing the COVID-19 pandemic-a repeated cross-sectional study in a COVID-19 Hub-Hospital in central Italy. *Int J Environ Res Public Health*. 2021;18(14):7316. doi:10.3390/ijerph18147316
6. Zhang Y, Ma ZF. Impact of the COVID-19 pandemic on mental health and quality of life among local residents in Liaoning Province, China: a cross-sectional study. *Int J Environ Res Public Health*. 2020;17(7):2381.
7. Wang C, Pan R, Wan X, et al. A longitudinal study on the mental health of general population during the COVID-19 epidemic in China. *Brain Behav Immun*. 2020;87:40–48. doi:10.1016/j.bbi.2020.04.028
8. Wang C, Pan R, Wan X, et al. Immediate psychological responses and associated factors during the initial stage of the 2019 coronavirus disease (COVID-19) epidemic among the general population in China. *Int J Environ Res Public Health*. 2020;17(5):1729.
9. Tan BYQ, Chew NWS, Lee GKH, et al. Psychological impact of the COVID-19 pandemic on health care workers in Singapore. *Ann Intern Med*. 2020;173(4):317320. doi:10.7326/M20-1083
10. Ruiz-Fernandez MD, Perez-Garcia E, Ortega-Galan AM. Quality of life in nursing professionals: burnout, fatigue, and compassion satisfaction. *Int J Environ Res Public Health*. 2020;17(4):1253. doi:10.3390/ijerph17041253
11. Buselli R, Corsi M, Baldanzi S, et al. Professional quality of life and mental health outcomes among health care workers exposed to Sars-Cov-2 (Covid-19). *Int J Environ Res Public Health*. 2020;17(17):6180. doi:10.3390/ijerph17176180
12. Magnavita N, Tripepi G, Di Prinzio RR. Symptoms in health care workers during the COVID-19 epidemic. A cross-sectional survey. *Int J Environ Res Public Health*. 2020;17(14):5218. doi:10.3390/ijerph17145218
13. Stamm BH. *The Concise ProQOL Manual*. Vol ID: ProQOL.org. 2nd ed. Pocatello; 2010.
14. Ruiz-Fernandez MD, Ramos-Pichardo JD, Ibanez-Masero O, Cabrera-Troya J, Carmona-Rega MI, Ortega-Galan AM. Compassion fatigue, burnout, compassion satisfaction and perceived stress in healthcare professionals during the COVID-19 health crisis in Spain. *J Clin Nurs*. 2020;29(21–22):4321–4330. doi:10.1111/jocn.15469
15. Antony MM, Bieling PJ, Cox BJ, Enns MW, Swinson RP. Psychometric properties of the 42-item and 21-item versions of the Depression Anxiety Stress Scales in clinical groups and a community sample. *Psychological Assessment*. 1998;10(2):176–181. doi:10.1037/1040-3590.10.2.176
16. Lovibond PF, Lovibond SH. The structure of negative emotional states: comparison of the Depression Anxiety Stress Scales (DASS) with the Beck Depression and Anxiety Inventories. *Behav Res Ther*. 1995;33(3):335–343. doi:10.1016/0005-7967(94)00075-U
17. McAlonan GM, Lee AM, Cheung V, et al. Immediate and sustained psychological impact of an emerging infectious disease outbreak on health care workers. *Can J Psychiatry*. 2007;52(4):241–247. doi:10.1177/070674370705200406
18. Wang K, Shi HS, Geng FL, et al. Cross-cultural validation of the depression anxiety stress scale-21 in China. *Psychol Assess*. 2016;28(5):e88–e100. doi:10.1037/pas0000207
19. CDC Taiwan. COVID-19(SARS-CoV-2 infection). Available from: https://www.cdc.gov.tw/En. Accessed Oct, 07, 2021.
20. Wang CJ, Ng CY, Brook RH. Response to COVID-19 in Taiwan: big data analytics, new technology, and proactive testing. *JAMA*. 2020;323(14):1341–1342. doi:10.1001/jama.2020.3151
21. Cheng HY, Chueh YN, Chen CM, Jian SW, Lai SK, Liu DP. Taiwan's COVID-19 response: timely case detection and quarantine, January to June 2020. *J Formos Med Assoc*. 2021;120:1400–1404.
22. Ortega-Galan AM, Ruiz-Fernandez MD, Lirola MJ, et al. Professional quality of life and perceived stress in health professionals before COVID-19 in Spain: primary and hospital care. *Healthcare*. 2020;8(4):484.
23. WHO. Spain Situation in period of COVID-19. https://covid19.who.int/region/euro/country/es. Accessed February 17, 2021.
24. Zhang YY, Han WL, Qin W, et al. Extent of compassion satisfaction, compassion fatigue and burnout in nursing: a meta-analysis. *J Nurs Manag*. 2018;26(7):810–819. doi:10.1111/jonm.12589
25. Powell SK. Compassion fatigue. *Prof Case Manag*. 2020;25(2):53–55. doi:10.1097/NCM.0000000000000418
26. Pappa S, Ntella V, Giannakas T, Giannakoulis VG, Papoutsi E, Katsaounou P. Prevalence of depression, anxiety, and insomnia among healthcare workers during the COVID-19 pandemic: a systematic review and meta-analysis. *Brain Behav Immun*. 2020;88:901–907. doi:10.1016/j.bbi.2020.05.026
27. Bohlken J, Schomig F, Lemke MR, Pumberger M, Riedel-Heller SG. COVID-19-Pandemie: belastungen des medizinischen personals [COVID-19 pandemic: stress experience of healthcare workers - a short current review]. *Psychiatr Prax*. 2020;47(4):190–197. German.
28. Cavanagh N, Cockett G, Heinrich C, et al. Compassion fatigue in healthcare providers: a systematic review and meta-analysis. *Nurs Ethics*. 2020;27(3):639–665. doi:10.1177/0969733019889400
29. Xie W, Wang J, Zhang Y, et al. The levels, prevalence and related factors of compassion fatigue among oncology nurses: a systematic review and meta-analysis. *J Clin Nurs*. 2021;30(5–6):615–632. doi:10.1111/jocn.15565
30. Van der Heijden B, Mulder RH, Konig C, Anselmann V. Toward a mediation model for nurses' well-being and psychological distress effects of quality of leadership and social support at work. *Medicine*. 2017;96(15):e6505. doi:10.1097/MD.0000000000006505
31. Akbari M, Hossaini SM. The relationship of spiritual health with quality of life, mental health, and burnout: the mediating role of emotional regulation. *Iran J Psychiatry*. 2018;13(1):22–31.
32. Li Z, Ge J, Yang M, Feng J, Liu C, Yang C. Vicarious traumatization: a psychological problem that cannot be ignored during the COVID-19 pandemic. *Brain Behav Immun*. 2020;87:74. doi:10.1016/j.bbi.2020.04.047
33. Restauri N, Sheridan AD. Burnout and posttraumatic stress disorder in the coronavirus disease 2019 (COVID-19) pandemic: intersection, impact, and interventions. *J Am Coll Radiol*. 2020;17(7):921–926. doi:10.1016/j.jacr.2020.05.021
34. Sharifi M, Asadi-Pooya AA, Mousavi-Roknabadi RS. Burnout among healthcare providers of COVID-19; a systematic review of epidemiology and recommendations. *Arch Acad Emerg Med*. 2021;9(1):e7.

35. Dykema J, Jones NR, Piche T, Stevenson J. Surveying clinicians by web: current issues in design and administration. *Eval Health Prof*. 2013;36(3):352–381. doi:10.1177/0163278713496630

36. Cho YI, Johnson TP, Vangeest JB. Enhancing surveys of health care professionals: a meta-analysis of techniques to improve response. *Eval Health Prof*. 2013;36(3):382. doi:10.1177/0163278713496425

Journal of Multidisciplinary Healthcare Dovepress

Publish your work in this journal

The Journal of Multidisciplinary Healthcare is an international, peer-reviewed open-access journal that aims to represent and publish research in healthcare areas delivered by practitioners of different disciplines. This includes studies and reviews conducted by multidisciplinary teams as well as research which evaluates the results or conduct of such teams or healthcare processes in general. The journal covers a very wide range of areas and welcomes submissions from practitioners at all levels, from all over the world. The manuscript management system is completely online and includes a very quick and fair peer-review system. Visit http://www.dovepress.com/testimonials.php to read real quotes from published authors.

Submit your manuscript here: https://www.dovepress.com/journal-of-inflammation-research-journal
