# Supplementary material for: Mental health status and related factors influencing healthcare workers during the COVID-19 pandemic: A systematic review and meta-analysis
Source: PLoS One. 2024 Jan 19;19(1):e0289454. doi: 10.1371/journal.pone.0289454 (PMC10798549; doi:10.1371/journal.pone.0289454)
Supplement: S1 Data — (ZIP) [file pone.0289454.s011.zip › literatures/183.pdf]

Received: 27 January 2022 | Accepted: 27 January 2022
DOI: 10.1111/nicc.12759

CRITICAL COMMENTARY

BACCN Nursing in Critical Care WILEY

Impact of the COVID-19 pandemic on the work environment and mental health of intensive care unit nurses: Reflections from the United States

Sarah Sumner MSN, RN, NPD-BC, OCN, CHPN

Passan School of Nursing, Wilkes University, Wilkes-Barre, Pennsylvania, USA

Correspondence
Sarah Sumner, Wilkes University, Passan School of Nursing, Wilkes-Barre, Pennsylvania, USA.
Email: sarah.sumner@wilkes.edu

The coronavirus disease 2019 (COVID-19) pandemic has been the most disruptive force to the intensive care unit (ICU) work environment this century. That disruption has been profoundly evident over the last 2 years as ICU teams around the world were overwhelmed with surges of critically ill patients in need of respiratory support and other advanced interventions. The first major COVID-19 surge in the United States hit New York City, New York in March 2020. As hospitals became overwhelmed, the New York state governor put out a desperate plea to health care providers across the nation to help New York. As the surge continued, the work environment suffered and ICU nurses began to experience burnout, emotional exhaustion, compassion fatigue, moral distress, turnover and showed intent to leave.[1–3] Since then, hospitals in the United States have been competing to secure temporary ICU nurses – outbidding each other with skyrocketing weekly rates meant to incentivize nurses to work under the demanding and uncertain conditions.[4]

As ICU nurses cared for patients with the new, life-threatening severe acute respiratory syndrome coronavirus 2 (SARS-CoV 2) virus – often without adequate personal protective equipment (PPE) – many self-quarantined to prevent exposing their loved ones to the virus.[1,3] While at work, those same nurses witnessed the fear and suffering of their patients who were similarly separated from their families. When possible, ICU nurses used tablets to facilitate video calls between patients and their families, but even that mode of communication was challenging as nurses shifts were poorly staffed, and nurses were trying to keep up with near-daily changes to PPE protocols and treatment regimens.[3] Conditions were so chaotic during the first New York surge that some families of the deceased were not notified of their passing for hours to days, and hospitals had to place the dead in refrigerated trucks because mortuaries could not keep up with rate of death.[5] The COVID-19 pandemic exacted unprecedented physical, psychological and moral injuries on ICU nurses who were pressured to continue working despite experiencing fatigue, burnout and symptoms of the virus themselves.[3]

1 | WORK ENVIRONMENT AND MENTAL HEALTH

Evidence of COVID-19's impact on the mental health of ICU nurses is rapidly accumulating. A PubMed search conducted in December 2021 using the search terms: 'nurse', 'work environment', 'COVID' and 'mental health' yielded scores of citations reporting pandemic-associated anxiety, depressive symptoms, moral injury, burnout and the need for psychosocial support in nurses. These reports emerged from countries spanning the globe including China, Italy, Australia, Iran, Cyprus, the United Kingdom, the United States and the United Arab Emirates demonstrating the widespread mental health impact of the pandemic. In a large international study on the mental health impact of health care workers in 41 countries, Khajuria et al.[6] found that being a nurse, working in the ICU and lacking appropriate PPE were strongly associated with depressive symptoms. Conversely, health care workers who had received mental health support were less likely to report hopelessness or depressive symptoms (Khajuria).

The relationship between the work environment and nurse outcomes has been studied in nurses for decades.[7] The American Association of Critical Care Nurses (AACN) developed the Healthy Work Environment (HWE) model based on the best evidence linking aspects of the work environment to nurse outcomes.[8] This HWE model consists of six standards: skilled communication, true collaboration, effective decision-making, appropriate staffing, meaningful recognition and authentic leadership that can be measured with the Healthy Work Environment Assessment Tool [HWEAT].[8,9] The HWEAT can be used to correlate changes in the work environment with ICU nurse outcomes.[9]

 © 2022 British Association of Critical Care Nurses.

1.1 | Mental health

Adverse mental health outcomes including burnout, anxiety, depression, moral injury and risk for suicide have been reported in nurses during the COVID-19.[1,10–13] Female nurses were already more than twice as likely as women in the general population to commit suicide.[14] Even a single nurse suicide is likely to have profound negative consequences on the unit and institution where that nurse worked.[15]

1.2 | Moral distress and moral injury

Some of the mental health problems experienced by ICU nurses during the COVID-19 pandemic are the result of moral distress (MD) and moral injury (MI).[16] MD has been described often in the literature as the psychological distress nurses experience in relation to morally challenging situations.[2] MI in nurses is a relatively new concept. MI was first observed in military veterans after they were forced to take an action that violated their deeply held moral beliefs – often in high stakes situations such as combat.[12,17] MI in nurses is the distressing psychological, behavioural, social or spiritual aftermath that results when nurses violate, fail to act on, or witness a violation of their deeply held moral beliefs.[3,18–20] Nurses may have experienced MI as they bore witness to final goodbyes between a dying patient and their family via a videocall, were forced to uphold restrictive visitor policies or had to choose – sometimes with dire consequences – which patient to attend to first when multiple critically unstable patients demanded their attention.[3] When faced with these conditions, in the United States, hospitals may be forced to institute crisis standards of care to guide allocation of scarce resources.[21] Under those conditions, nurses must participate in the withholding or withdrawal of interventions that would have otherwise been provided, possibly violating their moral integrity and leading to the anxiety and depressive symptoms associated with MI. MI and MD threaten the stability of the ICU nursing workforce as nurses leave their jobs for less stressful positions or leave the nursing profession entirely.

1.3 | A tragic case: Dr. Lorna Breen

Although not a nurse, one of the earliest symbols of the mental health impact of the COVID-19 pandemic on health care workers in the United States was the suicide of Dr. Lorna Breen in April 2020.[22] Dr. Lorna Breen was an emergency room physician at the height of the surge in New York City. She contracted COVID-19 herself and tried to come back to work after a few days only to be overwhelmed with physical exhaustion and the distress of the chaos and suffering around her. Dr. Breen was reluctant to seek help as she was more concerned about the stigma of seeking psychological care than she was about her own well-being. After her death, her family lobbied the United States Congress to fund research on the impact of the COVID-19 pandemic on the mental health of healt hcare professionals. Beginning in 2022, grants totalling $140 million will be distributed to health systems and health training programs to identify strategies to prevent suicide, burnout and mental health conditions while promoting job satisfaction and the well-being of health care professionals. The funds will also raise awareness about the importance of mental health and fund research on the impact of COVID-19 on the mental health of health care professionals.[23]

As tragic as the death of Dr. Breen was, her suicide brought the need to acknowledge the strains of the work environment on the mental health of health care professionals into focus. The promise of her legacy is the integration of mental health as a measure of a healthy work environment. The risk for suicide is particularly prescient to the ICU nursing community as female nurses were already twice as likely women in the general population to commit suicide before the pandemic.[14] Two major risk factors for suicide are work-related stress and mental health problems, which ICU nurses seem to be experiencing disproportionately during COVID-19.[14,24] Creative and deliberate interventions must be implemented at the hospital and unit level to improve the work environment to support the mental health of ICU nurses.

2 | MENTAL HEALTH EXEMPLARS FROM THE UNITED STATES

Consider these three exemplars of programs in the United States to promote the mental health and well-being of health care professionals. The first two exemplars were in place before the pandemic, while the third one emerged during the pandemic.

2.1 | UCSD HEAR program

The University of California at San Diego (UCSD) launched the Healer Education Assessment and Referral (HEAR) Program in 2010 after a cluster of suicides within the medical school.[15] The program's objectives were to educate medical students about mental health issues and encourage their participation in a web-based, confidential mental health assessment and referral program. The assessment was developed in collaboration with the American Foundation for Suicide Prevention and has since been implemented at more than 60 medical teaching campuses across the country [AFSP].[15,25] The HEAR program at UCSD was extended to nurses in 2016 after the death of a nurse by suicide. A three-year retrospective evaluation of nurse participation in the program demonstrated that 527 nurses opted to participate in the online screening program. Of those, 48 were found to be at risk for suicide. One hundred seventy-six received just-in-time support from therapists, while 98 accepted referrals for additional follow-up treatment. A new addition to the program integrates real-time group emotional debrief sessions after difficult incidents, which are well attended by nurses.[15]

2.2 | Ohio State University BEST program

The Brief Emotional Support Team (BEST) training at the Ohio State University James Comprehensive Cancer Center grew out of a similar

program that had been established 2 years prior to recognize and support patients who had experienced trauma. The BEST program is facilitated by a psychiatric mental health clinical nurse specialist (CNS) and focusses on psychological first aid and crisis intervention peer-to-peer training.[26] By 2021, over 700 employees had completed BEST training. The workplace culture of wellness-first was credited with having a protective effect on staff as the pandemic unfolded.

2.3 | Providence my mental health matters

Providence Health System is a network of 52 hospitals in the Western United States with over 120 000 employees. Recognizing the stress of COVID-19 pandemic on the workforce, Providence launched an array of mental health services for all employees including access to individual telehealth visits.[27,28] Employees can access the care through an innovative digital application named Lyra that directly links employees with previously hard to access mental health care. After completing an online survey, employees are matched with either a mental health coach or a licensed mental health provider based on risk stratification. Appointments are generally available within 48 hours and covered with no out-of-pocket costs for up to 24 sessions.

3 | CONCLUSION

Although the cumulative effect of the COVID-19 pandemic on ICU nurses is unknown, it has clearly had a profound impact on the mental health of the ICU nursing workforce. The American Nurses Association (ANA) Code of Ethics requires nurses to adopt self-care as a duty to self.[29] It is possible that ICU nurses in the United States are leaving their positions over mental health concerns experienced during the pandemic. If so, administrators and clinical nurses must collaborate to address threats to mental health in the work environment to retain and sustain the ICU nursing workforce.[24] Even before the pandemic, the relationship between the work environment and nurse outcomes including burnout, emotional exhaustion, compassion fatigue, moral distress, turnover and intent to leave had been well established.[30]

Three innovative programs in the United States have been described that could be replicated across the country and around the world to assess, monitor and support nurse mental health and well-being. Further research is needed to understand the relationship between mental health-targeted programs and nurse perceptions of the work environment. When implemented according the AACN model, a healthy work environment can improve nurse satisfaction and retention.[31] The AACN has validated an instrument, the Healthy Work Environment Assessment Tool (HWEAT) that can be used for that kind of research.[8,32] Addressing the work environment and its effect on the mental health of ICU nurses will be crucial to attract and retain a skilled workforce. The importance of these efforts cannot be overstated – nurse mental health and well-being are the foundation for the strength and resiliency of the nursing profession.

REFERENCES

1. Kim S, Sloan C, Chechel L, Redila M, Ferguson J. Severe burnout and poor mental health among healthcare workers 6 months after covid-19 pandemic declaration. *J Nurs Adm*. 2021;51(11):554-560. doi:10.1097/nna.0000000000001063
2. Morley G, Sese D, Rajendram P, Horsburgh C. Addressing caregiver moral distress during the covid-19 pandemic. *Cleve Clin J Med*. 2020. doi:10.3949/ccjm.87a.ccc047
3. Rushton C, Turner K, Brock R, Braxton JM. Invisible moral wounds of the covid-19 pandemic: are we experiencing moral injury? *AACN Adv Crit Care*. 2021;32(1):119-125. doi:10.4037/aacnacc2021686
4. Hawryluk, M., & Bichell, R. E. (2020). Need a COVID-19 nurse? That'll be $8000 a week. KHN. Retrieved November 10, 2021, from https://khn.org/news/highly-paid-traveling-nurses-fill-staffing-shortages-during-covid-pandemic/
5. Baumbach, J., Clark, M., LaRocco, P., Peddie, S., & Schwartz, D. (2021). Crisis, care, and tragedy on LI. Newsday. Retrieved November 10, 2021, from https://projects.newsday.com/long-island/coronavirus-cold-spring-hills-nursing-home/
6. Khajuria A, Tomaszewski W, Liu Z, et al. Workplace factors associated with mental health of healthcare workers during the Covid-19 pandemic: an international cross-sectional study. *BMC Health Serv Res*. 2021;21(1):1–11. doi:10.1186/s12913-021-06279-6
7. Jennings BM. Work stress and burnout among nurses: role of the work environment and working conditions. In: Hughes RG, ed. *Patient Safety and Quality: an Evidence-Based Handbook for Nurses*. Agency for Healthcare Research and Quality; 2008.
8. American Association of Critical Care Nurses. (2021). Healthy Work Environments. Retrieved November 10, 2021, from https://www.aacn.org/nursing-excellence/healthy-work-environments
9. Ulrich B, Barden C, Cassidy L, Varn-Davis N. Critical care nurse work environments 2018: findings and implications. *Crit Care Nurse*. 2019;39(2):67-84. doi:10.4037/ccn2019605
10. Čartolovni A, Stolt M, Scott P, Suhonen R. Moral injury in healthcare professionals: a scoping review and discussion. *Nurs Ethics*. 2021;28(5):590–602. 96973302096677. doi:10.1177/0969733020966776
11. Lesley M. Psychoanalytic perspectives on moral injury in nurses on the frontlines of the COVID-19 pandemic. *J Am Psychiatr Nurses Assoc*. 2021;27(1):72-76. doi:10.1177/1078390320960535
12. Mantri S, Lawson J, Wang Z, Koenig HG. Identifying moral injury in healthcare professionals: the moral injury symptom scale-hp. *J Relig Health*. 2020;59(5):2323-2340. doi:10.1007/s10943-020-01065-w
13. Rahman A, Plummer V. Covid-19 related suicide among hospital nurses; case study evidence from worldwide media reports. *Psychiatry Res*. 2020;291:113272. doi:10.1016/j.psychres.2020.113272
14. Davis MA, Cher BY, Friese CR, Bynum JW. Association of US nurse and physician occupation with risk of suicide. *JAMA Psychiat*. 2021;78(6):651. doi:10.1001/jamapsychiatry.2021.0154
15. Davidson JE, Accardi R, Sanchez C, Zisook S, Hoffman LA. Sustainability and outcomes of a suicide prevention program for nurses. *Worldviews Evid Based Nurs*. 2020;17(1):24-31. doi:10.1111/wvn.12418
16. Hossain F, Clatty A. Self-care strategies in response to nurses' moral injury during covid-19 pandemic. *Nurs Ethics*. 2021;28(1):23-32. doi:10.1177/0969733020961825
17. U.S. Department of Veterans Affairs. (2020). Moral injury in health care workers. PTSD: National Center for PTSD. Retrieved August 13, 2021, from https://www.ptsd.va.gov/professional/treat/cooccurring/moral_injury_hcw.asp
18. Carey LB, Hodgson TJ. Chaplaincy, spiritual care and moral injury: considerations regarding screening and treatment. *Front Psych*. 2018;9:1–10. doi:10.3389/fpsyt.2018.00619
19. Dean W, Talbot SG, Caplan A. Clarifying the language of clinician distress. *Jama*. 2020;323(10):923. doi:10.1001/jama.2019.21576

20. Linzer M, Poplau S. Eliminating burnout and moral injury: bolder steps required. *EClinicalMedicine*. 2021;39:101090. doi:10.1016/j.eclinm.2021.101090
21. Emanuel EJ, Persad G, Upshur R, et al. Fair allocation of scarce medical resources in the time of Covid-19. *New Engl J Med*. 2020;382(21):2049-2055. doi:10.1056/nejmsb2005114
22. Knoll, C., Watkins, A., & Rothfeld, M. (2020). 'I couldn't do anything': The virus and an E.R. doctor's suicide. The New York Times.
23. Dr. Lorna Breen Heroes' Foundation. (2020). The legislation. Retrieved September 7, 2021, from https://drlornabreen.org/about-the-legislation/
24. De Kock JH, Latham H, Leslie SJ, et al. A rapid review of the impact of covid-19 on the mental health of healthcare workers: implications for supporting psychological well-being. *BMC Public Health*. 2021;21(1):1–18. doi:10.1186/s12889-020-10070-3
25. UC San Diego Health Sciences. (n.d.). About the Healer Education Assessment and Referral (HEAR) program. Retrieved November 11, 2021, from https://medschool.ucsd.edu/som/hear/about/Pages/default.aspx
26. The James: The Ohio State University Comprehensive Cancer Center. (2021). How Ohio State makes employee wellness a priority for health care workers. Retrieved November 11, 2021, from https://cancer.osu.edu/blog/how-ohio-state-makes-employee-wellness-a-priority-for-health-care-workers-at-the-james-cancer-hospital
27. Leventhal, R. (2020). Providence creates virtual behavioral health concierge service for employees. Healthcare Innovation. Retrieved November 11, 2021, from https://www.hcinnovationgroup.com/population-health-management/telehealth/news/21124677/providence-creates-virtual-behavioral-health-concierge-service-for-employees
28. Thompson J. Providence aims to address burnout with systemwide mental health survey. *Puget Sound Bus J*. 2021; Retrieved November 11, 2021, from https://www.bizjournals.com/seattle/news/2021/10/08/providence-burnout-survey.html
29. American Nurses Association. *Code of Ethics for Nurses with Interpretive Statements*. Second ed. Nursesbooks; 2015.
30. Wei H, Sewell KA, Woody G, Rose M. The state of the science of nurse work environments in the United States: a systematic review. *Int J Nurs Sci*. 2018;5(3):287-300. doi:10.1016/j.ijnss.2018.04.010
31. Barden C. *AACN Standards for Establishing and Sustaining Healthy Work Environments: A Journey to Excellence*. 2nd ed. American Association of Critical Care Nurses (AACN); 2016.
32. Connor J, Ziniel SI, Porter C, et al. Interprofessional use and validation of the AACN healthy work environment assessment tool. *Am J Crit Care*. 2018;27(5):363-371. doi:10.4037/ajcc2018179

How to cite this article: Sumner S. Impact of the COVID-19 pandemic on the work environment and mental health of intensive care unit nurses: Reflections from the United States. *Nurs Crit Care*. 2022;1-4. doi:10.1111/nicc.12759
