# Supplementary material for: Mental health status and related factors influencing healthcare workers during the COVID-19 pandemic: A systematic review and meta-analysis
Source: PLoS One. 2024 Jan 19;19(1):e0289454. doi: 10.1371/journal.pone.0289454 (PMC10798549; doi:10.1371/journal.pone.0289454)
Supplement: S1 Data — (ZIP) [file pone.0289454.s011.zip › literatures/185.pdf]

Received: 3 July 2020 | Revised: 12 August 2020 | Accepted: 23 August 2020

DOI: 10.1002/brb3.1837

ORIGINAL RESEARCH

Brain and Behavior Open Access WILEY

Mental health and quality of life among healthcare professionals during the COVID-19 pandemic in India

Nishi Suryavanshi[1,2] | Abhay Kadam[1,2] | Gauri Dhumal[1,2] | Smita Nimkar[1,2] | Vidya Mave[3] | Amita Gupta[3] | Samyra R. Cox[3] | Nikhil Gupte[1,3]

[1]Lakshya, Society for Public Health Education and Research, Pune, India

[2]Johns Hopkins University, Center for Clinical Global Health Education, Pune, India

[3]Johns Hopkins University, School of Medicine, Baltimore, MD, USA

Correspondence
Nishi Suryavanshi, Lakshya: Society for Public Health Education and Research, 307, Block II, Lloyd Chambers, Mangal war Peth., Pune, Maharashtra-411001, India.
Email: nishisuryavanshi@hotmail.com

Abstract

Background: The COVID-19 pandemic has placed healthcare professionals (HCP) in stressful circumstances with increased patient loads and a high risk of exposure. We sought to assess the mental health and quality of life (QoL) of Indian HCPs, the fourth highest-burden country for COVID-19.

Method: Using snowball sampling, we conducted an online survey in May 2020 among HCPs. Data were collected on demographics, depression, and anxiety using validated tools, quality of life, and perceived stressors. Multivariable logistic regression and principal component analysis were performed to assess risk factors associated with mental health symptoms.

Findings: Of 197 HCPs assessed, 157 (80%) were from Maharashtra, 130 (66%) from public hospitals, 47 (24%) nurses, 66 (34%) physicians, 101 (52%) females, and 81 (41%) ≤30 years. Eighty-seven percent provided direct COVID-19 care with 43% caring for >10 patients/day. A large proportion reported symptoms of depression (92, 47%), anxiety (98, 50%), and low QoL (89, 45%). Odds of combined depression and anxiety were 2.37 times higher among single HCPs compared to married (95% CI: 1.03–4.96). Work environment stressors were associated with 46% increased risk of combined depression and anxiety (95% CI: 1.15–1.85). Moderate to severe depression and anxiety were independently associated with increased risk of low QoL [OR: 3.19 (95% CI: 1.30–7.84), OR: 2.84 (95% CI: 1.29–6.29)].

Conclusion: Our study demonstrated a high prevalence of symptoms of depression and anxiety and low QoL among Indian HCPs during the COVID-19 pandemic. There is an urgent need to prevent and treat mental health symptoms among frontline HCPs.

KEYWORDS
COVID-19, healthcare professionals, India, mental health, quality of life, stressors

This is an open access article under the terms of the Creative Commons Attribution License, which permits use, distribution and reproduction in any medium, provided the original work is properly cited.
© 2020 The Authors. *Brain and Behavior* published by Wiley Periodicals LLC

1 | INTRODUCTION

The first laboratory-confirmed case of the novel coronavirus (COVID-19) was reported in India on 30 January 2020. Since then, there has been exponential growth in cases, with India now ranking fourth in the global burden of COVID-19 (https://coronavirus.jhu.edu/map.html; 2020; https://www.who.int; 2020). As of 18 June 2020, 366,946 COVID-19 cases and 12,237 deaths were reported in India, with Maharashtra state accounting for one-third of all of India's cases and 46% of deaths (https://www.mohfw.gov.in/index.php). The COVID-19 pandemic has placed healthcare professionals (HCPs) in stressful circumstances with increased patient loads, unprecedented disruptions to normal life, and high risk of exposure. According to the World Health Organization (WHO), over 22,000 HCPs across 52 countries were infected with COVID-19 by 8 April 2020. The WHO also highlighted that HCP infections are likely being underreported (www.statnews.com, 2020).

In India, over 200 HCPs including doctors and nurses have been diagnosed with COVID-19 (https://www.thehindu.com.). There are also reports of unsuspecting HCPs infected while caring for asymptomatic patients (https://www.newindianexpress.com). This pandemic has caused high levels of anxiety, stress, and depression in the general population (Wang et al., 2020), and HCPs may be disproportionately affected due to fear of infection, workplace stress, social isolation, and discrimination (https://www.livemint.com; 2020; Zhang & Ma, 2020).

However, the mental health status of Indian HCPs has not been formally assessed since the COVID-19 epidemic hit India. Globally, there is limited literature that investigated the effect of mental health symptoms among HCPs' on quality of life during an epidemic. Therefore, we sought to investigate the prevalence and severity of mental health symptoms among Indian HCPs during COVID-19, as well as its impact on quality of life. Such data are needed to inform healthcare administrators and policymakers to rapidly develop and implement mental health interventions among HCPs.

2 | MATERIALS AND METHODS

2.1 | Study design and participants

A structured survey questionnaire was designed in Google forms and made accessible online for self-administration. An informed consent script briefly explaining the objective of the study was provided at the beginning of the survey. HCPs who responded to the survey were assumed to have agreed to participate. To maintain confidentiality, personal details, and potential identifiers of HCPs were not collected. The survey was open from 5 May 2020 to 16 May 2020. We used snowball sampling methodology to send the survey to potential participants. This method is nonprobability sampling and entailed sending the survey link via e-mail, WhatsApp, and social media to physician and nurse listservs, social groups, and professional associations. We then requested recipients to disseminate the survey further among their networks of HCPs. On the survey, we clarified that HCPs directly or indirectly involved in caring for COVID-19 patients at public and private facilities in India were eligible to participate in the online survey. Due to the various techniques used to disseminate the survey, we were unable to precisely quantify recipients and track response rates as per American Association for Public Opinion Research (AAPOR) reporting guideline.

The study was approved by the ethics committee of Lakshya Society for Public Health Education and Research.

2.2 | Measures

Data were collected on demographic characteristics, mental health symptoms, quality of life, and perceived stressors.

2.2.1 | Demographic characteristics

Demographic data collected included HCP role (paraclinical, resident/intern, nurse, or physician), gender (male or female), age (18–24, 25–30, 31–40, 40–50 or >50 years), marital status (single [included unmarried, widowed/separated/divorced] and married), medical specialization (medicine, radiology, pediatrics, etc.), city of work, type of hospital (public or private), and years of experience. Participants were also asked if they were directly or indirectly engaged in diagnosing, treating, or caring for patients with confirmed or suspected COVID-19.

2.2.2 | Mental health symptoms

We used the Patient Health Questionnaire (PHQ-9) to assess the severity of symptoms of depression. PHQ-9 is a nine-item tool that has been validated in India (Indu et al., 2018). It has been used for measuring depression both in clinical and general population settings (Kroenke, Spitzer, & Williams, 2001). Each of the nine items is scored as 0 (not at all), 1 (several days), 2 (more than half of the days), or 3 (nearly every day). The total score for PHQ-9 ranges from 0 to 27. PHQ-9 scores were categorized using a cutoff score of ≥5 indicating the presence of any depressive symptoms and a cutoff score of ≥10 for the presence of moderate to severe depression (Kroenke et al., 2001).

We measured symptoms of anxiety using the Generalized Anxiety Disorder (GAD-7) questionnaire, a validated seven-item assessment (Spitzer, Kroenke, Williams, & Löwe, 2006), frequently used in India. Each of these seven items is scored as 0 (not at all), 1 (several days), 2 (more than half of the days), or 3 (nearly every day). The total score for the GAD-7 ranges from 0 to 21. We used a cutoff score of ≥5 indicating the presence of any anxiety symptoms and a cutoff score of >8 for the presence of moderate to severe anxiety (Obbarius et al., 2017).

2.2.3 | Quality of life

We used the validated one-item quality of life (QoL-1) visual analogue scale where QoL ranges from 1 (low or negative) to 7 (high or positive; de Boer et al., 2004; Siebens, Tsukerman, Adkins, Kahan, & Kemp, 2015). A midpoint is considered neither low nor high, but average. Respondents with scores below 4 were considered to have low QoL.

2.2.4 | Stressors

Participants were asked about the factors they perceive contributing to their mental stress. We used one multiple-choice question with 12 options for stressors based on a study conducted by Shwu-Hua Lee in Taiwan during the SARS outbreak (Lee et al., 2005). Based on the principal component analysis described below, stressors were categorized as either related to the work environment, work pressure, concerns about the epidemic, and family-related concerns. We also asked an open-ended question to solicit suggestions for stress-reduction strategies.

2.3 | Outcome

The outcomes of interest were the severity of symptoms of depression and anxiety, quality of life, and perceived stressors.

2.4 | Statistical analysis

Statistical analysis was done using STATA version 14.2. Demographic characteristics were summarized across mental health diagnoses using frequencies and percentages and compared using Fisher's exact test. Prevalence and 95% exact confidence interval (CI) for moderate to severe depression and anxiety, stressors, and low QoL was estimated for the overall group of HCPs and stratified by risk groups. Univariable and multivariable logistic regression was used to estimate unadjusted and adjusted odds ratios (OR) to assess independent risk factors associated with depression, anxiety, and QoL. The effect of depression and anxiety on low QoL was assessed using Fisher's exact test and logistic regression. Multivariable models were adjusted for variables significant in the univariable analysis, as well as for age, gender, and whether or not the HCP was directly involved in COVID-19 care. Since stressors are highly correlated, a principal component analysis was done to identify orthogonal components to assess association with moderate to severe depression and anxiety. To assess the effect of stressors, principal components were used as exposure variables in logistic regression analysis for mental health symptoms. Lastly, qualitative data from open-ended responses were analyzed to identify major themes with exemplary quotations.

3 | RESULTS

3.1 | Demographic characteristics of participants

An estimated 1,000 HCPs were contacted. The survey received 204 responses, of which six were duplicate entries and one was from outside of India. The analysis was performed on the remaining 197 HCPs. The demographic characteristics of participants are presented in Table 1. HCPs from 30 cities across 12 states responded to the survey with highest representation from Maharashtra (157, 80%; Figure 1); 66 (34%) were physicians, 47 (24%) nurses, 58 (29%) residents/interns and 26 (13%) were other types. Further, 101 (51%) were female, 81 (41%) were ≤30 years, and the majority (119, 60%) were married. Most (130, 66%) participants reported working in public hospitals and 92 (47%) had <5 years of experience. A total of 171 (87%) HCPs were directly engaged in diagnosing or treating patients with suspected or confirmed COVID-19, and 84 (43%) were caring for >10 patients per day.

3.2 | Depression and anxiety and associated characteristics

The overall prevalence of moderate to severe depression among respondents was 22% (95% CI: 17% – 29%), and 47% reported any symptoms of depression (95% CI: 40%–54%; Table S1). Proportion with moderate to severe depression was similar across different demographic characteristics, with the exception of marital status 25 (32%) of single HCPs had 2.5 times odds of moderate to severe depression versus 19 (16%) of married HCPs ($p < .01$) [OR (95% CI): 2.48 (1.24–4.94); $p = .01$]. The prevalence of moderate to severe depression was almost 30% among younger HCPs (≤30 years old) compared to 14% among older HCPs (>40 years old). Furthermore, older HCPs (>40 years old) were at lower odds of moderate to severe depression compared to the youngest group (OR: 0.39; 95% CI: 0.14–1.03). The associations between depression and gender, HCP role, direct involvement with COVID-19 care, and hospital setting were not statistically significant (Table 2).

Overall prevalence of moderate to severe anxiety among respondents was 29% (95% CI: 23% - 36%), and 50% reported any symptoms of anxiety (95% CI: 43%–57%; Table S1). The proportion with moderate to severe anxiety was similar across different demographic characteristics. The differences in odds of moderate to severe anxiety among the gender, HCP role, marital status, and hospital setting subgroups were not statistically significant (Table 2). Single marital status was independently associated with a two-fold increase in odds of moderate to severe depression and anxiety combined (OR: 2.37; 95% CI: 1.10–5.11; Table 2).

3.3 | Association of depression and anxiety with quality of life

Overall prevalence of low quality of life was 45% (95% CI: 38%–52%). Older (>40 years old) HCPs were more likely to report low

TABLE 1 Distribution of demographic characteristics and quality of life by mental health symptoms

Characteristics	Overall *n* (%)	Moderate to severe depression *n* (%)	Moderate to severe anxiety *n* (%)	Moderate to severe depression and anxiety combined *n* (%)
Overall	197 (100%)	44 (22%)	56 (29%)	33 (17%)
Gender				
Female	101 (51%)	23 (23%)	32 (32%)	17 (17%)
Male	96 (49%)	21 (22%)	24 (26%)	16 (17%)
Age (years)				
≤30	81 (41%)	24 (30%)	26 (33%)	18 (22%)
31–40	73 (37%)	14 (19%)	20 (28%)	10 (14%)
>40	43 (22%)	6 (14%)	10 (24%)	5 (12%)
Marital status				
Married	119 (60%)	19 (16%)	30 (26%)	14 (12%)
Single[a]	78 (40%)	25 (32%)	26 (34%)	19 (24%)
Direct COVID-19 care				
No	26 (13%)	3 (12%)	6 (24%)	3 (12%)
Yes	171 (87%)	41 (24%)	50 (30%)	30 (18%)
Avg. number of COVID-19 patients/day				
<10	113 (57%)	24 (21%)	28 (25%)	17 (15%)
>10	84 (43%)	20 (24%)	28 (33%)	16 (19%)
City				
Out of Pune	97 (49%)	19 (20%)	26 (27%)	19 (19%)
Pune	100 (51%)	25 (25%)	30 (30%)	14 (14%)
Hospital setting				
Private	67 (34%)	12 (18%)	15 (23%)	8 (12%)
Public	130 (66%)	32 (25%)	41 (32%)	25 (19%)
HCP role				
Paraclinical[b]	26 (13%)	4 (15%)	6 (11%)	2 (12%)
Resident/Intern	58 (29%)	15 (26%	19 (34%)	13 (22%)
Nurse	47 (24%)	13 (28%)	14 (25%)	7 (15%)
Physician	66 (34%)	12 (18%)	17 (30%)	10 (15%)
Years of experience				
<5	92 (47%)	25 (27%)	27 (30%)	19 (21%)
5–10	43 (22%)	9 (21%)	14 (33%)	7 (16%)
>10	62 (31%)	10 (16%)	15 (25%)	7 (11%)
Quality of life[c]				
Low	89 (45%)	32 (73%)	39 (70%)	24 (73%)
Average	53 (27%)	07 (16%)	08 (14%)	04 (12%)
High	55 (28%)	05 (11%)	09 (16%)	05 (15%)

Abbreviation: HCP, healthcare professional.

[a]Single included HCPs who are unmarried, widowed, separated, and divorced.

[b]Paraclinical HCPs included laboratory personnel, radiologists, X-ray technicians, and epidemiologists.

[c]Quality of life on global quality of life scale is reported as low if score is <4, average if score is = 4 and high if score is >4 and up to 7.

QoL compared to younger HCPs, though this did not reach statistical significance (50% vs. 42%, $p = .62$; Table S1). Risk of low QoL was approximately four times higher among moderate to severely depressed HCPs [73% (95% CI: 57%–85%) vs. 37% (95% CI: 30%–45%); OR: 4.49 (95% CI: 2.14–9.41); $p < .001$]. Similarly, risk of low QOL was four times higher among HCPs with moderate to severe anxiety [70% (95% CI: 56%–81%) vs. 36% (95% CI: 28%–45%); OR: 4.04 (95% CI: 2.07–7.87); $p < .001$] (Table 3; Figure 2). In a multivariable model

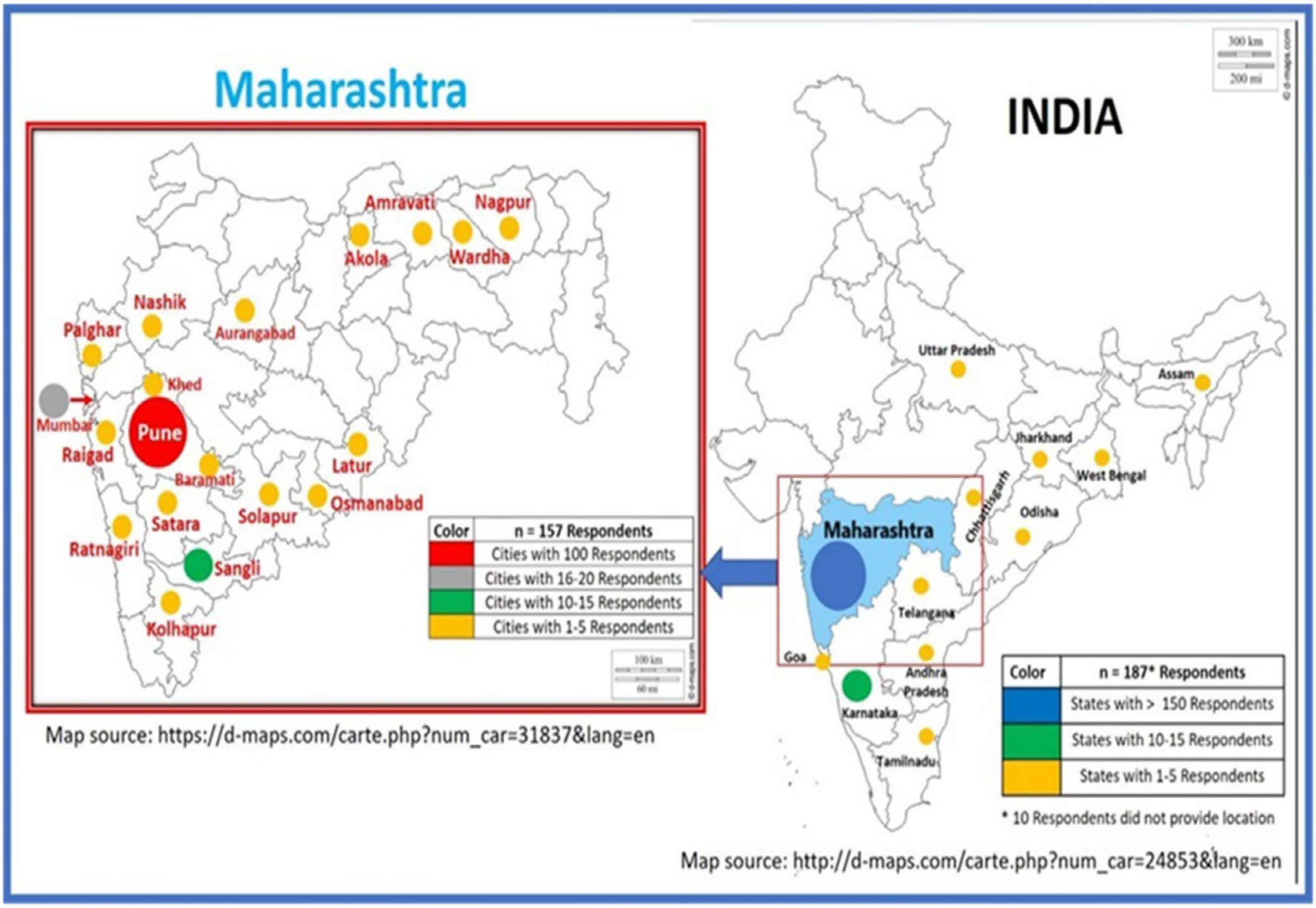

FIGURE 1 Map of India and Maharashtra showing geographical distribution of HCPs. Almost 80% of HCPs were from Maharashtra, epicenter of COVID-19 epidemic in India

adjusted for age, gender, marital status and direct involvement in COVID-19 care, moderate to severe depression [OR: 3.19 (95% CI: 1.30–7.84); $p = .01$], and moderate to severe anxiety [OR: 2.84 (95% CI: 1.29–6.29); $p = .01$] were independently associated with low QoL (Table 3).

3.4 | Perceived stressors contributing to moderate to severe depression and anxiety

Among the 12 perceived stressors assessed, we observed a comparatively high prevalence of moderate to severe depression among HCPs experiencing discrimination from co-workers or family members (47%) (Figure 3). The principal component analysis identified four components of independent stressors with Eigenvalues more than 1 that accounted for 62% of the variation. Based on the factor loadings, stressors primarily contributing to Component 1 (*work environment*) were lack of knowledge, lack of manpower, and fear of infection. Component 2 (*work pressure*) was more represented by pressure from seniors, pressure due to patient load, concerns about death rate among patients, and discrimination from co-workers or family members. Component 3 (*epidemic*) included stressors related to isolation and physical distancing, as well as the uncertainty of the epidemic control. Component 4 (*family-related*) included fear of infecting family members and loss of family members/relatives/friends (Figure 4a–c).

In a multivariable logistic regression analysis adjusting for age, gender, marital status, and direct involvement in COVID-19 care, Component 1 (work environment) was independently associated with a 46% increased risk of moderate to severe depression [OR: 1.46 (95% CI: 1.18–1.81); $p = .001$] (Figure 4a) and a 49% increased risk of moderate to severe anxiety [OR: 1.49 (95% CI: 1.22–1.83); $p < .001$] (Figure 4b). Component 1 (work environment) was also independently associated with 46% increased odds of moderate to severe depression and anxiety combined [OR: 1.46 (95% CI (1.15–1.85) $p < .01$; Figure 4c).

3.5 | Suggestions for stress-reduction strategies

In response to our open-ended question about stress-reduction strategies, the majority of respondents (133, 68%) suggested measures that HCPs could advocate for and hospital administration could implement. Three major themes emerged from the qualitative data: relaxation practices, improving work environment, and role of social media.

TABLE 2 Logistic regression estimates of severity of mental health symptoms across demographic characteristics

Characteristics	Moderate to severe depression OR, *p*-value	Moderate to severe anxiety OR (95% CI), *p*-value	Moderate to severe depression and anxiety combined OR, *p*-value
Gender			
Female	Ref	Ref	Ref
Male	0.95 (0.49–1.86), $p = .88$	0.71 (0.38–1.32), $p = .28$	0.99 (0.47–2.09), $p > .95$
Age (years)			
≤30	Ref	Ref	Ref
31–40	0.56 (0.27–1.20), $p = .13$	0.80 (0.40–1.60), $p = .53$	0.56 (0.24–1.30), $p = .17$
>40	0.39 (0.14–1.03), $p = .06$	0.65 (0.28–1.52), $p = .32$	0.46 (0.16–1.34), $p = .16$
Marital status			
Married	Ref	Ref	Ref
Single[a]	2.48 (1.24–4.94), $p = .01$	1.48 (0.78–2.79), $p = .23$	2.37 (1.10–5.11), $p = .03$
Direct COVID-19 care			
No	Ref	Ref	Ref
Yes	2.42 (0.69–8.47), $p = .17$	1.33 (0.50–3.53), $p = .57$	1.63 (0.46–5.79), $p = .45$
Avg. number of COVID-19 patients/day			
<10	Ref	Ref	Ref
>10	1.16 (0.59–2.28), $p = .67$	1.46 (0.78–2.73), $p = .23$	1.33 (0.63–2.81), $p = .46$
City			
Out of Pune	Ref	Ref	Ref
Pune	1.37 (0.70–2.69), $p = .36$	1.19 (0.64–2.22), $p = .59$	1.39 (0.65–2.96), $p = .39$
Hospital setting			
Private	Ref	Ref	Ref
Public	1.50 (0.71–3.14), $p = .29$	1.60 (0.81–3.18), $p = .18$	1.76 (0.74–4.14), $p = .20$
HCP role			
Paraclinical[b]	Ref	Ref	Ref
Resident/Intern	1.92 (0.57–6.48), $p = .29$	1.58 (0.54–4.62), $p = .40$	2.21 (0.57–8.56), $p = .25$
Nurse	2.10 (0.61–7.28), $p = .24$	1.39 (0.46–4.22), $p = .57$	1.34 (0.32–5.70), $p = .69$
Physician	1.22 (0.36–4.20), $p = .75$	1.10 (0.38 3.21), $p = .86$	1.37 (0.34–5.43), $p = .66$
Years of experience			
<5	Ref	Ref	Ref
5–10	1.41 (0.59–3.35), $p = .44$	1.13 (0.52–2.46), $p = .77$	0.75 (0.29–1.94), $p = .55$
>10	0.73 (0.27–1.97), $p = .53$	0.76 (0.36–1.59), $p = .47$	0.49 (0.19–1.25), $p = .13$

Abbreviation: HCP, healthcare professional
[a]Single included HCPs who are unmarried, widowed, separated, and divorced.
[b]Paraclinical HCPs included laboratory personnel, radiologists, X-ray technicians, and epidemiologists.

3.5.1 | Relaxation practices

Forty-four (33%) HCPs thought that practicing meditation or yoga, pursuing hobbies (e.g., reading, watching movies, and listening to music) could divert attention from hectic daily duties and reduce stress.

> Every day at least 1 hr, spend time in enjoying things like music, playing with kids, watching movie. That will reduce stress and make u feel better to proceed with work.
> (Female, <40 years old, physician, private hospital)

> Relaxation methods like deep breathing, yoga, engaging in family, etc would help to reduce stress.
> (Female, 31–40 years old, nurse, public hospital)

3.5.2 | Improving work environment

Forty-four (33%) HCPs expressed that their anxiety could be reduced by reducing patient load, proper training in COVID-19 management guidelines, adequate isolation wards, sufficient rest and good nutrition, and sufficient supply of personal protective equipment (PPE).

TABLE 3 Factors associated with low quality of life among healthcare professionals

Characteristics	N	Low QoL n (%)	Low QoL, univariable OR, p-value	Low QoL multivariable OR, p-value
Gender				
Female	101 (51%)	42 (42%)	Ref	Ref
Male	96 (49%)	47 (49%)	1.35 (0.77–2.37), $p = .30$	1.28 (0.68–2.41), $p = .44$
Age				
≤30	81 (41%)	34 (42%)	Ref	Ref
31–40	73 (37%)	33 (45%)	1.14 (0.60–2.16), $p = .69$	1.49 (0.64–3.51), $p = .35$
>40	43 (22%)	22 (51%)	1.45 (0.69–3.04), $p = .33$	2.09 (0.76–5.71), $p = .15$
Marital status				
Married	119 (60%)	55 (46%)	Ref	Ref
Single[a]	78 (40%)	34 (44%)	0.96 (0.54–1.72),$p = .90$	1.15 (0.50–2.64), $p = .75$
Direct COVID-19 care				
No	26 (13%)	13 (50%)	Ref	Ref
Yes	171 (87%)	76 (44%)	0.80 (0.35–1.83), $p = .60$	0.56 (0.22–1.43), $p = .22$
Avg. number of COVID-19 patients/day				Not included
<10	113 (57%)	48 (42%)	Ref	
>10	84 (43%)	41 (49%)	1.29 (0.73–2.28), $p = .38$	
City				
Out of Pune	97 (49%)	45 (46%)	Ref	Not included
Pune	100 (51%)	44 (44%)	0.91 (0.52–1.59), $p = .74$	
Hospital setting				
Private	67 (34%)	31 (46%)	Ref	Not included
Public	130 (66%)	58 (45%)	0.94 (0.52–1.69), $p = .83$	
HCP role				
Paraclinical[b]	26 (13%)	15 (58%)	Ref	Not included
Resident/Intern	58 (29%)	24 (41%)	0.52 (0.20–1.32), $p = .17$	
Nurse	47 (24%)	21 (45%)	0.59 (0.23–1.56), $p = .29$	
Physician	66 (34%)	29 (44%)	0.57 (0.23–1.44), $p = .24$	
Years of experience				
<5	92 (47%)	19 (44%)	Ref	Not included
5–10	43 (22%)	39 (42%)	1.08 (0.52–2.23), $p = .84$	
>10	62 (31%)	31 (50%)	1.36 (0.71–2.60), $p = .35$	
Moderate to severe depression				
Absent	153 (78%)	57 (37%)	Ref	Ref
Present	44 (22%)	32 (73%)	4.49 (2.14–9.41), $p < .001$	3.19 (1.30–7.84), $p = .01$
Moderate to severe anxiety				
Absent	138 (71%)	50 (36%)	Ref	Ref
Present	56 (29%)	39 (70%)	4.04 (2.07–7.87), $p < .001$	2.84 (1.29–7.84), $p = .01$

(Continues)

TABLE 3 (Continued)

Characteristics	*N*	Low QoL *n* (%)	Low QoL, univariable OR, *p*-value	Low QoL multivariable OR, *p*-value
Moderate to severe depression and anxiety combined				
Absent	164 (83%)	65 (40%)	Ref	Not included
Present	33 (17%)	24 (73%)	4.06 (1.78–9.29), $p = .001$	

Note: Multivariable model was adjusted for: gender, age, marital status, direct COVID-19 care, symptoms of moderate to severe anxiety, symptoms for moderate to severe depression. Moderate to severe depression and anxiety combined was derived from having moderate to severe depression and anxiety, it was not used in the multivariable modeling because of high collinearity.

Abbreviations: defined as low if a respondent scored <4 on global quality of life scale ranging from 1 to 7; HCP, healthcare professional; QOL, quality of life.

[a]Single included HCPs who are unmarried, widowed, separated, and divorced.

[b]Paraclinical HCPs included laboratory personnel, radiologists, X-ray technicians, and epidemiologists.

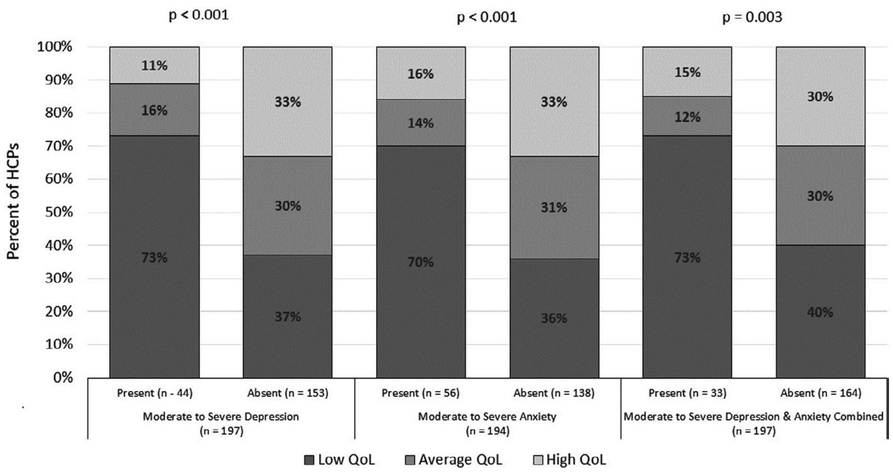

FIGURE 2 Association of moderate to severe depression and anxiety by quality of life. Quality of life (QoL) was measured on Likert scale ranging from 1 to 7; score >4 was considered as high QoL; score of 4 is average QoL; and score below 4 is low QoL. Effect of moderate to severe depression and anxiety on QoL using Fisher's exact test shows significant association. Moderate to severe depression is defined as depression score ≥10 on Patient health Questionnaire (PHQ-9), and presence of moderate to severe anxiety is defined as score ≥8 on General Anxiety Disorder (GAD-7) scale; presence of moderate to severe depression and anxiety combined included those HCPs who reported both moderate to severe depression and anxiety

> After long hours of work and risking our life salary deduction is very disgusting. Govt should increase remuneration. At least health drinks, protein food etc should be distributed to nurses, common people only sitting at home and praying will not boost our energy.
>
> (Female, 41–50 years old, nurse, public hospital)

> Proper planning by hospital management will help solve many issues we are currently facing. provision of proper PPE kits give confidence at workplace. Multidisciplinary approach will help treat patients better.
>
> (Male, 25–30 years old, resident, public hospital)

3.5.3 | Role of social media

Another major theme that emerged was the role of media in spreading fear among people and causing stigmatization and stress among HCPs. HCPs suggested that media should help build awareness instead of spreading myths.

> Proper guidance by seniors and avoidance of discrimination by the public against doctors and nurses who are treating COVID positive patients…This can be done via online awareness programs or via media like television given spreading awareness about this disease that it need not be stigmatized. Stop seeing news all day, better focus on positive out of this situation.
>
> (Male, 25–30 years old, physician, public hospital)

Other measures suggested were sending residents home periodically, offering counseling support and coping strategies (suggested by 15 HCPs), facilitating indoor recreational activities, and establishing a safe space for airing grievances.

> To create a platform where they (medical residents) can voice their worries.
>
> (Female, >50 years old, physician, public hospital)

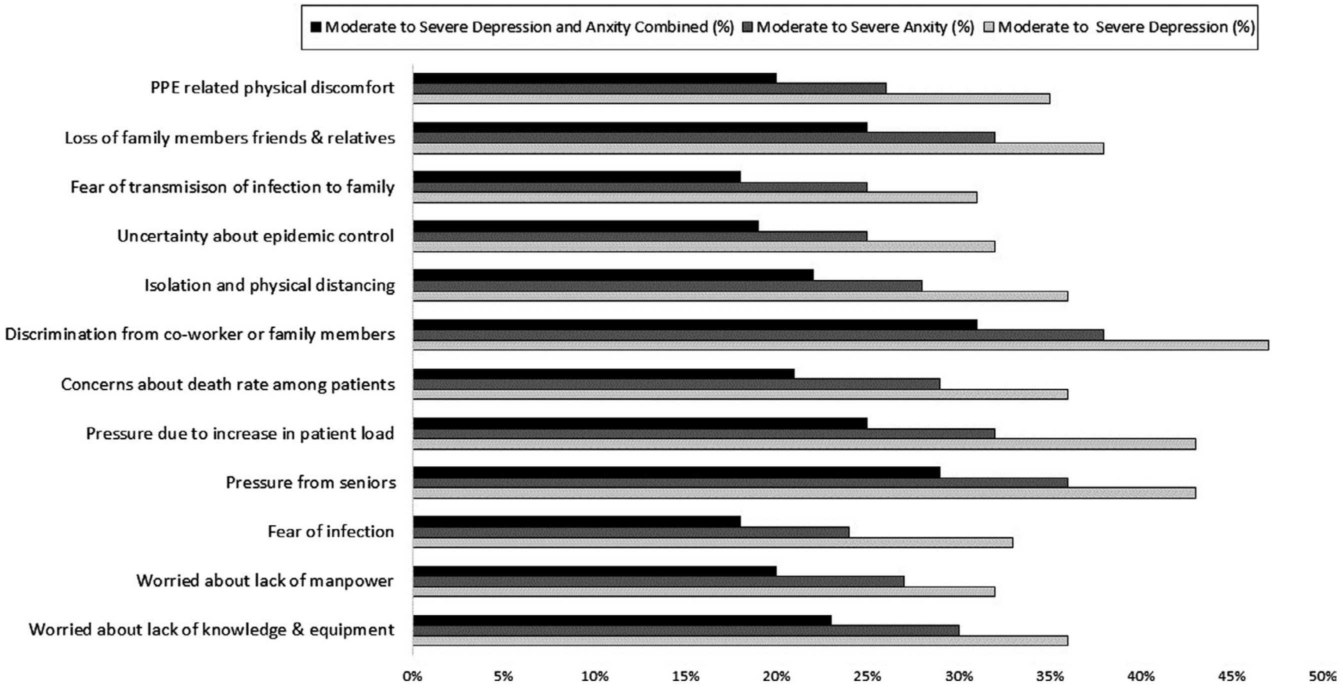

FIGURE 3 Distribution of stressors by mental health symptoms. There are 12 major stressors contributing to moderate to severe depression and anxiety. Moderate to severe depression is defined as depression score ≥10 on Patient health Questionnaire (PHQ-9), and presence of moderate to severe anxiety is defined as score ≥8 on General Anxiety Disorder (GAD-7) scale; presence of moderate to severe depression and anxiety combined included those HCPs who reported both moderate to severe depression and anxiety

4 | DISCUSSION

This study offers important insight into the mental health impact of COVID-19 on HCPs in India. First, we identified a very high prevalence of depression (47%) and anxiety (50%) symptoms among HCPs caring for COVID-19 patients. Second, in this context, younger and single HCPs may be at the highest risk of experiencing combined depression and anxiety—our study showed nearly a two-fold increased risk among single HCPs compared to their married counterparts. Third, stressors related to the work environment could be a key driver for combined depression and anxiety in this population. Lastly, moderate to severe depression and anxiety were both independently associated with low QoL, which was reported in nearly half of our respondents.

Our estimates of depression and anxiety symptoms among HCPs are consistent with earlier reports from China during the COVID-19 outbreak (Kang et al., 2020; Lai et al., 2020), but lower than estimates from China during the SARS outbreak (Chong et al., 2004). Our study observed much higher anxiety among HCPs than studies from the swine flu outbreak in India (Mishra et al., 2016) and from the recent COVID-19 outbreak in Italy (Rossi et al., 2020) where only about 20% of HCPs reported symptoms of depression and 8% reported anxiety. Mental health response to an epidemic may vary depending on the availability of clinical evidence, media reports, case fatality rates, the transmissibility of the disease, and isolation policies (Roy et al., 2020; Wang et al., 2020; Wong et al., 2005). Hence, the prevalence of anxiety observed in our study may be particularly high as the majority of our respondents were from Maharashtra, the epicenter of COVID-19 in India where patient load is high with limited resources.

Consistent with mental health investigations among medical students during COVID-19 in China (Kang et al., 2020; Liang, Chen, Zheng, & Liu, 2020), we found some association between age and self-reported depressive symptoms. However, our study also demonstrated a high risk of depression and anxiety among unmarried HCPs involved in COVID-19 care in India, which has not yet been reported in the literature. As opposed to another study of HCPs during COVID-19 in China, as well as the aforementioned study from Italy (Lai et al., 2020; Rossi et al., 2020), we did not find significant differences in the prevalence of mental health symptoms among HCPs from different cities. Furthermore, our study did not find any association between, gender or type of HCP (nurses vs. physicians), and mental health symptoms as opposed to these other studies conducted in China (Lai et al., 2020) and Italy (Rossi et al., 2020) where young female HCPs are reported be at higher risk of mental stress. This finding may be because most of our respondents were from public hospitals where HCPs across different gender could be experiencing similar stressors. This is further supported by our principal component analysis, which showed that work environment stressors were significantly associated with depression and anxiety among HCPs, irrespective of their gender. This analysis supports the urgent need for healthcare administrators to address work-related stressors including professional mental health intervention if required. This may be done by altering assignments and schedules, modifying expectations, and creating mechanisms to offer psychosocial support as needed (Pfefferbaum & North, 2020).

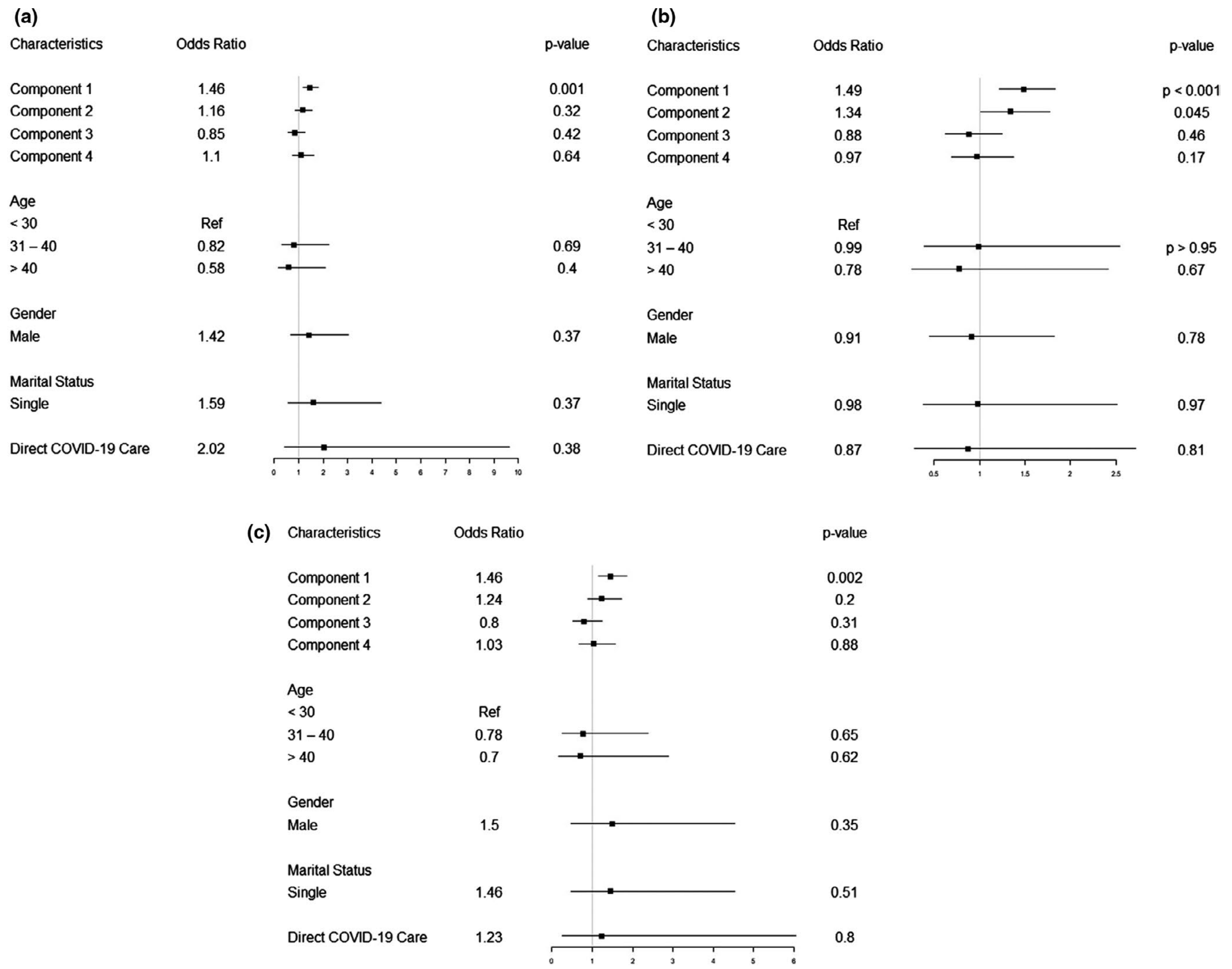

FIGURE 4 (a) Effect of perceived stressors on moderate to severe depression using principal components multivariable analysis. (b) Effect of moderate to severe anxiety using principal components multivariable analysis. (c) Effect of moderate to severe depression and anxiety combined using principal components multivariable analysis. Factor loading most represented in component 1 (Work Environment) are as follows: lack of knowledge and equipment, lack of manpower, fear of infection. Factor loading most represented in component 2 (Work Pressure) are as follows: pressure due to increase in patient load, pressure from seniors, discrimination from co-workers/family, worry about death rate among patients. Factor loading most represented in component 3 (Epidemic) are as follows: isolation and physical distancing, uncertainty of epidemic control, and factor loading most represented in component 4 (Family-related) are as follows: fear of transmission of infection to family, loss of family members/friends/relatives

There is a growing awareness of the need to protect HCPs from infection during the COVID-19 pandemic (Adams & Walls, 2020), but safeguarding the overall quality of life is also imperative. Forty-five percent of the HCPs in our study reported low QoL. A study in Vietnam also showed that people with suspected COVID-19 symptoms are more likely to be depressed and have a low quality of life (Nguyen et al., 2020). Evaluation of QoL in HCPs treating Ebola patients reported their feelings of social isolation and low quality of life (Lehmann et al., 2016). Moreover, COVID-related coverage in social media can be emotionally disturbing and HCPs may be experiencing social isolation, stigma, and anxiety, contributing to reduced quality of life. We found a significant association between combined depression and anxiety symptoms and quality of life. Importantly, our principal component analysis of stressors showed that overwhelming workload, lack of knowledge and training, and fear of contracting the disease may all contribute to poor mental health outcomes among HCPs. A similar finding was also observed during the 2003 SARS outbreak (Bai et al., 2004; Chua et al., 2004; Maunder et al., 2003).

To mitigate stress, the majority of respondents (133, 68%) in our study suggested measures that HCPs could advocate for and hospital administration could implement. Proper knowledge and training to manage COVID-19 patients, reducing patient workload, expanding isolation wards, allowing adequate breaks, and ensuring sufficient supply of PPE emerged as the most important issues that need immediate attention by authorities. These strategies are in line with the study conducted in China where strict protective measures, knowledge of virus prevention and transmission, social isolation measures, and positive self-attitude resulted in reducing

stress levels (Cai et al., 2020). Qualitative studies conducted in India (Mohindra, R, Suri, Bhalla, & Singh, 2020) and China also emphasized the importance of regular and intensive training for all HCPs to help effectively manage crises during COVID-19 pandemic (Liu et al., 2020).

Our study is the first to report on the mental health symptoms and its impact on quality of life among Indian HCPs during COVID-19 pandemic. However, our findings may not be generalizable as approximately 80% of respondents were from Maharashtra. Additionally, since our response rate was only about 20%, HCPs who were too inundated with work to respond may have been underrepresented. Secondly, while the vast majority of respondents were directly involved in COVID-19 care (87%), we did not assess pre-existing mental health symptoms among HCPs. Hence, we cannot comment on whether reported symptoms were triggered by the pandemic. It may also be possible that other psychosocial factors may have caused depression and anxiety among some of the participants which were not evaluated as part of this study. Hence, all the mental health symptoms cannot be attributed to only COVID related. A further longitudinal investigation of mental health outcomes using mixed-methods assessments is needed to provide an in-depth understanding of the short and long-term psychological implications of COVID-19 on HCPs.

5 | CONCLUSION

Our study demonstrated a high burden of depression and anxiety among young, unmarried HCPs serving COVID-19 patients in highly impacted regions of India. Further, we found that moderate to severe depression and anxiety among HCPs negatively impacted their overall quality of life during COVID pandemic. Protecting the mental health of frontline HCPs is paramount to COVID-19 response and control efforts. Rapid development and implementation of interventions to prevent and treat mental health conditions are urgently needed to support the growing number of HCPs caring for COVID-19 patients in India and worldwide.

DISCLAIMER

The findings and conclusions in this report are those of the author(s) and do not necessarily represent the official position of the Lakshya Society for Public Health Education and Research.

ACKNOWLEDGMENTS

We thank the staff of Lakshya and all who helped in distributing the survey to healthcare professionals. We thank all participants for taking the time to complete the survey.

CONFLICT OF INTEREST

None.

AUTHORS' CONTRIBUTIONS

NS, NG, AK, GD conceived the study. NS, AK, GD, and SN prepared data collection form. NS programmed the forms in Google forms platform. NS, VM, NG, AK, SN, and GD implemented the study, NG and NS performed data analyses and data interpretation. NS, NG, and SC drafted the initial manuscript. AG, VM, NS, NG, SRC, AK, GD, and SN critically reviewed the manuscript and provided inputs. All authors approved the manuscript.

PEER REVIEW

The peer review history for this article is available at https://publons.com/publon/10.1002/brb3.1837.

DATA AVAILABILITY STATEMENT

All the data required for this paper are presented in the manuscript.

ORCID

Nishi Suryavanshi https://orcid.org/0000-0001-8917-0302
Abhay Kadam https://orcid.org/0000-0002-6972-853X

REFERENCES

Adams, J. G., & Walls, R. M. (2020). Supporting the health care workforce during the COVID-19 global epidemic. *JAMA*, *323*(15), 1439–1440. https://doi.org/10.1001/jama.2020.3972

Bai, Y., Lin, C. C., Lin, C. Y., Chen, J. Y., Chue, C. M., & Chou, P. (2004). Survey of stress reactions among health care workers involved with the SARS outbreak. *Psychiatric Services*, *55*(9), 1055–1057. https://doi.org/10.1176/appi.ps.55.9.1055

Cai, H., Tu, B., Ma, J., Chen, L., Fu, L., Jiang, Y., & Zhuang, Q. (2020). Psychological impact and coping strategies of frontline medical staff in hunan between January and March 2020 during the outbreak of coronavirus disease 2019 (COVID-19) in Hubei, China. *Medical Science Monitor*, *26*, e924171–e924171. https://doi.org/10.12659/MSM.924171

Chong, M. Y., Wang, W. C., Hsieh, W. C., Lee, C. Y., Chiu, N. M., Yeh, W. C., … Chen, C. L. (2004). Psychological impact of severe acute respiratory syndrome on health workers in a tertiary hospital. *British Journal of Psychiatry*, *185*, 127–133. https://doi.org/10.1192/bjp.185.2.127

Chua, S. E., Cheung, V., Cheung, C., McAlonan, G. M., Wong, J. W. S., Cheung, E. P. T., … Tsang, K. W. T. (2004). Psychological effects of the SARS outbreak in Hong Kong on high-risk health care workers. *Canadian Journal of Psychiatry*, *49*(6), 391–393. https://doi.org/10.1177/070674370404900609

de Boer, A. G., van Lanschot, J. J., Stalmeier, P. F., van Sandick, J. W., Hulscher, J. B., de Haes, J. C., & Sprangers, M. A. (2004). Is a single-item visual analogue scale as valid, reliable and responsive as multi-item scales in measuring quality of life? *Quality of Life Research*, *13*(2), 311–320. https://doi.org/10.1023/B:QURE.0000018499.64574.1f

https://coronavirus.jhu.edu/map.html. (2020). COVID-19 Dashboard. Coronavirus Resource Center.

https://www.livemint.com./news/india/doctors-nurses-face-stigma-over-coronavirus-asked-to-vacate-rented-homes-11585074366997.html. Accesed on 10th May 2020.

https://www.mohfw.gov.in/index.php. Accessed on 18th June 2020.

https://www.newindianexpress.com./cities/delhi/2020/may/02/home-isolation-for-asymptomatic-patients-better-for-mental-health-delhi-healthcare-workers. Accesed on 10th May 2020.

https://www.thehindu.com./data/how-many-doctors-and-nurses-have-tested-positive-for-coronavirus-in-india/article31410464.ece. Accesed on 30th April 2020.

https://www.who.int. (2020). Coronavirus disease 2019 (COVID-19) Situation Report – 82. Accesed on 14th June 2020.

Indu, P. S., Anilkumar, T., Vijayakumar, K., Kumar, K., Sarma, P., Remadevi, S., & Andrade, C. (2018). Reliability and validity of PHQ-9 when administered by health workers for depression screening among women in primary care. *Asian Journal of Psychiatry*, *37*, 10–14. https://doi.org/10.1016/j.ajp.2018.07.021

Kang, L., Ma, S., Chen, M., Yang, J., Wang, Y., Li, R., … Liu, Z. (2020). Impact on mental health and perceptions of psychological care among medical and nursing staff in Wuhan during the 2019 novel coronavirus disease outbreak: A cross-sectional study. *Brain, Behavior, and Immunity*, *87*, 11–17. https://doi.org/10.1016/j.bbi.2020.03.028

Kroenke, K., Spitzer, R. L., & Williams, J. B. (2001). The PHQ-9: Validity of a brief depression severity measure. *Journal of General Internal Medicine*, *16*(9), 606–613. https://doi.org/10.1046/j.1525-1497.2001.016009606.x

Lai, J., Ma, S., Wang, Y., Cai, Z., Hu, J., Wei, N., … Hu, S. (2020). Factors associated with mental health outcomes among health care workers exposed to Coronavirus Disease 2019. *JAMA Network Open*, *3*(3), e203976. https://doi.org/10.1001/jamanetworkopen.2020.3976

Lee, S. H., Juang, Y. Y., Su, Y. J., Lee, H. L., Lin, Y. H., & Chao, C. C. (2005). Facing SARS: Psychological impacts on SARS team nurses and psychiatric services in a Taiwan general hospital. *General Hospital Psychiatry*, *27*(5), 352–358. https://doi.org/10.1016/j.genhosppsych.2005.04.007

Lehmann, M., Bruenahl, C. A., Addo, M. M., Becker, S., Schmiedel, S., Lohse, A. W., … Löwe, B. (2016). Acute Ebola virus disease patient treatment and health-related quality of life in health care professionals: A controlled study. *Journal of Psychosomatic Research*, *83*, 69–74. https://doi.org/10.1016/j.jpsychores.2015.09.002

Liang, Y., Chen, M., Zheng, X., & Liu, J. (2020). Screening for Chinese medical staff mental health by SDS and SAS during the outbreak of COVID-19. *Journal of Psychosomatic Research*, *133*, 110102. https://doi.org/10.1016/j.jpsychores.2020.110102

Liu, Q., Luo, D., Haase, J. E., Guo, Q., Wang, X. Q., Liu, S., … Yang, B. X. (2020). The experiences of health-care providers during the COVID-19 crisis in China: A qualitative study. *The Lancet. Global Health*, *8*(6), e790–e798. https://doi.org/10.1016/S2214-109X(20)30204-7

Maunder, R., Hunter, J., Vincent, L., Bennett, J., Peladeau, N., Leszcz, M., … Mazzulli, T. (2003). The immediate psychological and occupational impact of the 2003 SARS outbreak in a teaching hospital. *CMAJ*, *168*(10), 1245–1251.

Mishra, P., Bhadauria, U. S., Dasar, P. L., Sandesh, N., Kumar, S., Lalani, A., … Vyas, S. (2016). Knowledge, attitude and anxiety towards pandemic flu a potential bio weapon among health professionals in Indore City. *Przeglad Epidemiologiczny*, *70(1), 41–45*, 125–127.

Mohindra, R., R, R., Suri, V., Bhalla, A., & Singh, S. M. (2020). Issues relevant to mental health promotion in frontline health care providers managing quarantined/isolated COVID19 patients. *Asian Journal of Psychiatry*, *51*, 102084. https://doi.org/10.1016/j.ajp.2020.102084

Nguyen, H. C., Nguyen, M. H., Do, B. N., Tran, C. Q., Nguyen, T. T. P., Pham, K. M., … Duong, T. V. (2020). People with suspected COVID-19 symptoms were more likely depressed and had lower health-related quality of life: The potential benefit of health literacy. *Journal of Clinical Medicine*, *9*(4). https://doi.org/10.3390/jcm9040965

Obbarius, A., van Maasakkers, L., Baer, L., Clark, D. M., Crocker, A. G., de Beurs, E., … Rose, M. (2017). Standardization of health outcomes assessment for depression and anxiety: Recommendations from the ICHOM Depression and Anxiety Working Group. *Quality of Life Research*, *26*(12), 3211–3225. https://doi.org/10.1007/s11136-017-1659-5

Pfefferbaum, B., & North, C. S. (2020). Mental health and the Covid-19 pandemic. *New England Journal of Medicine*, *383*(6), 510–512. https://doi.org/10.1056/NEJMp2008017

Rossi, R., Socci, V., Pacitti, F., Di Lorenzo, G., Di Marco, A., Siracusano, A., & Rossi, A. (2020). Mental health outcomes among front-line and second-line health care workers during the coronavirus disease 2019 (COVID-19) pandemic in Italy. *JAMA Network Open*, *3*(5), e2010185. https://doi.org/10.1001/jamanetworkopen.2020.10185

Roy, D., Tripathy, S., Kar, S. K., Sharma, N., Verma, S. K., & Kaushal, V. (2020). Study of knowledge, attitude, anxiety & perceived mental healthcare need in Indian population during COVID-19 pandemic. *Asian Journal of Psychiatry*, *51*, 102083. https://doi.org/10.1016/j.ajp.2020.102083

Siebens, H. C., Tsukerman, D., Adkins, R. H., Kahan, J., & Kemp, B. (2015). Correlates of a single-item quality-of-life measure in people aging with disabilities. *American Journal of Physical Medicine & Rehabilitation*, *94*(12), 1065–1074. https://doi.org/10.1097/PHM.0000000000000298

Spitzer, R. L., Kroenke, K., Williams, J. B., & Löwe, B. (2006). A brief measure for assessing generalized anxiety disorder: The GAD-7. *Archives of Internal Medicine*, *166*(10), 1092–1097. https://doi.org/10.1001/archinte.166.10.1092

Wang, C., Pan, R., Wan, X., Tan, Y., Xu, L., Ho, C. S., & Ho, R. C. (2020). Immediate psychological responses and associated factors during the initial stage of the 2019 coronavirus disease (COVID-19) epidemic among the general population in China. *International Journal of Environmental Research and Public Health*, *17*(5). https://doi.org/10.3390/ijerph17051729

Wong, T. W., Yau, J. K. Y., Chan, C. L. W., Kwong, R. S. Y., Ho, S. M. Y., Lau, C. C., … Lit, C. H. (2005). The psychological impact of severe acute respiratory syndrome outbreak on healthcare workers in emergency departments and how they cope. *European Journal of Emergency Medicine*, *12*(1), 13–18. https://doi.org/10.1097/00063110-200502000-00005

www.statnews.com. (2020). The Covid-19 crisis too few are talking about: health care workers' mental health.

Zhang, Y., & Ma, Z. F. (2020). Impact of the COVID-19 pandemic on mental health and quality of life among local residents in Liaoning Province, China: A cross-sectional study. *International Journal of Environmental Research and Public Health*, *17*(7), 2381. https://doi.org/10.3390/ijerph17072381

SUPPORTING INFORMATION

Additional supporting information may be found online in the Supporting Information section.

How to cite this article: Suryavanshi N, Kadam A, Dhumal G, et al. Mental health and quality of life among healthcare professionals during the COVID-19 pandemic in India. *Brain Behav*. 2020;10:e01837. https://doi.org/10.1002/brb3.1837
