# Supplementary material for: Mental health status and related factors influencing healthcare workers during the COVID-19 pandemic: A systematic review and meta-analysis
Source: PLoS One. 2024 Jan 19;19(1):e0289454. doi: 10.1371/journal.pone.0289454 (PMC10798549; doi:10.1371/journal.pone.0289454)
Supplement: S1 Data — (ZIP) [file pone.0289454.s011.zip › literatures/187.pdf]

Observation: Brief Research Report

Psychological Impact of the COVID-19 Pandemic on Health Care Workers in Singapore

Background: In response to the coronavirus 2019 (COVID-19) pandemic, Singapore raised its Disease Outbreak Response System Condition alert to "orange," the second highest level. Between 19 February and 13 March 2020, confirmed cases rose from 84 to 200 (34.2 per 1 000 000 population), with an increase in patients in critical condition from 4 to 11 (5.5%) and no reported deaths in Singapore (1). Understanding the psychological impact of the COVID-19 outbreak among health care workers is crucial in guiding policies and interventions to maintain their psychological well-being.

Objective: We examined the psychological distress, depression, anxiety, and stress experienced by health care workers in Singapore in the midst of the outbreak, and compared these between medically and non–medically trained hospital personnel.

Methods and Findings: From 19 February to 13 March 2020, health care workers from 2 major tertiary institutions in Singapore who were caring for patients with COVID-19 were invited to participate with a self-administered questionnaire. In addition to information on demographic characteristics and medical history (**Table 1**), the questionnaire included the validated Depression, Anxiety, and Stress Scales (DASS-21) and the Impact of Events Scale–Revised (IES-R) instrument (2, 3). Health care workers included "medical" (physicians, nurses) and "nonmedical" personnel (allied health professionals, pharmacists, technicians, administrators, clerical staff, and maintenance workers). The primary outcome was the prevalence of depression, stress, anxiety, and posttraumatic stress disorder (PTSD) among all health care workers (**Table 2**). Secondary outcomes were comparison of the prevalence of depression, anxiety, stress, and PTSD, and mean DASS-21 and IES-R scores between medical and nonmedical health care workers. The Pearson χ^2 test and Student *t* test were used to compare categorical and continuous outcomes, respectively, between the 2 groups. Multivariable regression was used to adjust for the a priori defined confounders of age, sex, ethnicity, marital status, presence of comorbid conditions, and survey completion date.

Table 1. Participant Characteristics at Baseline

Characteristic	Overall (*N* = 470)	Nonmedical Health Care Personnel (*n* = 174)	Medical Health Care Personnel (*n* = 296)
**Sex, *n* (%)**			
Female	321 (68.3)	119 (68.4)	202 (68.2)
Male	149 (31.7)	55 (31.6)	94 (31.8)
**Median age (IQR), *y***	31 (28–36)	33 (28–39)	30 (28–35)
**Ethnicity, *n* (%)**			
Chinese	292 (62.1)	100 (57.5)	192 (64.9)
Indian	78 (16.6)	39 (22.4)	39 (13.2)
Malay	42 (8.9)	20 (11.5)	22 (7.4)
Other	58 (12.4)	15 (8.6)	43 (14.5)
**Marital status, n (%)**			
Single	228 (48.5)	83 (47.7)	145 (49.0)
Married	232 (49.4)	85 (48.9)	147 (49.7)
Divorced, separated, or widowed	10 (2.1)	6 (3.4)	4 (1.3)
**Occupation, *n* (%)**			
Physician	135 (28.7)	–	135 (45.6)
Nurse	161 (34.3)	–	161 (54.4)
Allied health care professional	65 (13.8)	65 (37.4)	–
Technician	10 (2.1)	10 (5.7)	–
Clerical staff	30 (6.4)	30 (17.2)	–
Administrator	33 (7.0)	33 (19.0)	–
Maintenance worker	36 (7.7)	36 (20.7)	–
**Medical history, *n* (%)**			
Hypertension	20 (4.3)	13 (7.5)	7 (2.4)
Hyperlipidemia	19 (4.0)	11 (6.3)	8 (2.7)
Diabetes mellitus	5 (1.1)	1 (0.6)	4 (1.4)
Asthma	26 (5.5)	10 (5.7)	16 (5.4)
Eczema	35 (7.4)	10 (5.7)	25 (8.4)
Migraine	58 (12.3)	27 (15.5)	31 (10.5)
Cigarette smoking	17 (3.6)	16 (9.2)	1 (0.3)
Ischemic heart disease	3 (0.6)	3 (1.7)	0
Stroke	1 (0.2)	1 (0.6)	0
Preexisting psychiatric illness	0	0	0
Other comorbid conditions	27 (5.7)	11 (6.3)	16 (5.4)

This article was published at Annals.org on 6 April 2020.

Annals of Internal Medicine © 2020 American College of Physicians

Table 2. Prevalence of Depression, Anxiety, Stress, and PTSD and Mean DASS-21 and IES-R Scores in Medical and Nonmedical Health Care Personnel (N = 470)

Outcome	Nonmedical Health Care Personnel (n = 174)	Medical Health Care Personnel (n = 296)	Crude Prevalence Ratio (95% CI)	Adjusted Prevalence Ratio (95% CI)*
**Prevalence, *n* (%)***				
Depression	18 (10.3%)	24 (8.1%)	1.28 (0.71 to 2.28)	1.12 (0.57 to 2.19)
Anxiety	36 (20.7%)	32 (10.8%)	1.91 (1.23 to 2.97)	1.85 (1.15 to 2.99)
Stress	12 (6.9%)	19 (6.4%)	1.07 (0.53 to 2.16)	1.01 (0.47 to 2.19)
PTSD	19 (10.9%)	17 (5.7%)	1.90 (1.02 to 3.56)	1.47 (0.71 to 3.04)
**Mean (SD) DASS-21 and IES-R scores**			**Crude Mean Difference (95% CI)**	**Adjusted Mean Difference (95% CI)†**
DASS depression	3.24 (5.07)	2.54 (5.23)	0.70 (−0.27 to 1.67)	0.46 (−0.62 to 1.54)
DASS anxiety	3.57 (3.91)	2.45 (4.28)	1.13 (0.35 to 1.91)	1.04 (0.15 to 1.94)
DASS stress	6.10 (5.95)	3.82 (5.74)	2.29 (1.19 to 3.38)	2.15 (0.88 to 3.41)
Total IES-R	9.40 (10.08)	5.85 (9.24)	3.55 (1.75 to 5.34)	3.35 (1.34 to 5.36)
IES-R Intrusion	0.47 (0.51)	0.31 (0.49)	0.16 (0.07 to 0.25)	0.15 (0.04 to 0.25)
IES-R Avoidance	0.46 (0.53)	0.27 (0.46)	0.19 (0.10 to 0.28)	0.18 (0.08 to 0.29)
IES-R Hyperarousal	0.35 (0.45)	0.22 (0.40)	0.13 (0.05 to 0.21)	0.12 (0.04 to 0.21)

DASS-21 = Depression, Anxiety, and Stress Scales; IES-R = Impact of Events Scale–Revised; PTSD = posttraumatic stress disorder.
* The DASS-21 is a 21-item system that provides independent measures of depression, stress, and anxiety with recommended severity thresholds. Cutoff scores >9, >7, and >14 indicate a positive screen for depression, anxiety, and stress respectively. The IES-R is a 22-item self-report instrument that measures the subjective distress caused by traumatic events. It has 3 subscales (intrusion, avoidance, and hyperarousal), which are closely affiliated with PTSD symptoms. A total IES-R cutoff score of 24 is used to classify PTSD as a clinical concern.
† Adjusted for age, sex, ethnicity, marital status, presence of comorbid conditions, and survey completion date. The adjusted prevalence ratio was derived from logistic regression models by calculating marginally adjusted prevalence for each group. The 95% CIs were derived by using the delta method. The adjusted mean difference was obtained by using linear regression.

Of 500 invited health care workers, 470 (94%) participated in the study; baseline characteristics are shown in Table 1. Sixty-eight (14.5%) participants screened positive for anxiety, 42 (8.9%) for depression, 31 (6.6%) for stress, and 36 (7.7%) for clinical concern of PTSD. The prevalence of anxiety was higher among nonmedical health care workers than medical personnel (20.7% versus 10.8%; adjusted prevalence ratio, 1.85 [95% CI, 1.15 to 2.99]; P = 0.011), after adjustment for age, sex, ethnicity, marital status, survey completion date, and presence of comorbid conditions. Similarly, higher mean DASS-21 anxiety and stress subscale scores and higher IES-R total and subscale scores were observed in nonmedical health care workers (Table 2).

Discussion: Overall mean DASS-21 and IES-R scores among health care workers were lower than those in the published literature from previous disease outbreaks, such as the severe acute respiratory syndrome (SARS). A previous study in Singapore found higher IES scores among physicians and nurses during the SARS outbreak, and an almost 3 times higher prevalence of PTSD, than those in our study (4). This could be attributed to increased mental preparedness and stringent infection control measures after Singapore's SARS experience.

Of note, nonmedical health care workers had higher prevalence of anxiety even after adjustment for possible confounders. Our findings are consistent with those of a recent COVID-19 study demonstrating that frontline nurses had significantly lower vicarious traumatization scores than non–frontline nurses and the general public (5). Reasons for this may include reduced accessibility to formal psychological support, less first-hand medical information on the outbreak, less intensive training on personal protective equipment and infection control measures.

As the pandemic continues, important clinical and policy strategies are needed to support health care workers. Our study identified a vulnerable group susceptible to psychological distress. Educational interventions should target nonmedical health care workers to ensure understanding and use of infectious control measures. Psychological support could include counseling services and development of support systems among colleagues.

Our study has limitations. First, data obtained from self-reported questionnaires were not verified with medical records. Second, the study did not assess socioeconomic status, which may be helpful in evaluating associations of outcomes and tailoring specific interventions. Finally, the study was performed early in the outbreak and only in Singapore, which may limit the generalizability of the findings. Follow-up studies could help assess for progression or even a potential rebound effect of psychological manifestations once the imminent threat of COVID-19 subsides.

In conclusion, our study highlights that nonmedical health care personnel are at highest risk for psychological distress during the COVID-19 outbreak. Early psychological interventions targeting this vulnerable group may be beneficial.

*Benjamin Y.Q. Tan, MD**
National University Health System and Yong Loo Lin School of Medicine, National University of Singapore
Singapore

*Nicholas W.S. Chew, MD**
National University Health System
Singapore

Grace K.H. Lee, MD
Yong Loo Lin School of Medicine, National University of Singapore

Mingxue Jing, MD
Yihui Goh, MD
National University Health System
Singapore

Leonard L.L. Yeo, MD
National University Health System and Yong Loo Lin School of Medicine, National University of Singapore
Singapore

Ka Zhang, MD
Howe-Keat Chin, MD
National University Health System
Singapore

Aftab Ahmad, MD
Faheem Ahmed Khan, MD
Ganesh Napolean Shanmugam, MBBCh
Ng Teng Fong General Hospital
Singapore

Bernard P.L. Chan, MD
Sibi Sunny, MD
Bharatendu Chandra, MD
Jonathan J.Y. Ong, MD
Prakash R. Paliwal, MD
Lily Y.H. Wong, BN
Renarebecca Sagayanathan, BSc
Jin Tao Chen, BN
Alison Ying Ying Ng, Dip
Hock Luen Teoh, MD
National University Health System
Singapore

Cyrus S. Ho, MD
National University of Singapore
Singapore

Roger C. Ho, MD
Institute of Health Innovation and Technology (iHealthtech), National University of Singapore
Singapore

Vijay K. Sharma, MD
National University Health System and Yong Loo Lin School of Medicine, National University of Singapore
Singapore

* Drs. Tan and Chew contributed equally to this work.

Disclosures: Disclosures can be viewed at www.acponline.org/authors/icmje/ConflictOfInterestForms.do?msNum=M20-1083.

Reproducible Research Statement: *Study protocol and statistical code:* Available from Dr. Sharma (e-mail, vijay_kumar_sharma@nuhs.edu.sg). *Data set:* Not available.

Corresponding Author: Vijay K. Sharma, MD, Division of Neurology, National University Health System, NUHS Tower Block, Level 10, 1 East Kent Ridge Road, Singapore 119228; e-mail, vijay_kumar_sharma@nuhs.edu.sg.

doi:10.7326/M20-1083

References

1. Ministry of Health Singapore. Updates on COVID-19 (coronavirus disease 2019) local situation. Ministry of Health, Singapore. Accessed at www.moh.gov.sg/covid-19 on 13 March 2020.
2. Lovibond SH. Lovibond PF. Manual for the Depression Anxiety Stress Scales. 2d ed. Sydney: Psychology Foundation of Australia; 1995.
3. Creamer M, Bell R, Failla S. Psychometric properties of the impact of event scale - revised. Behav Res Ther. 2003;41:1489-96. [PMID: 14705607]
4. Chan AO, Huak CY. Psychological impact of the 2003 severe acute respiratory syndrome outbreak on health care workers in a medium size regional general hospital in Singapore. Occup Med (Lond). 2004;54:190-6. [PMID: 15133143]
5. Li Z, Ge J, Yang M, et al. Vicarious traumatization in the general public, members, and non-members of medical teams aiding in COVID-19 control. Brain Behav Immun. 2020. [PMID: 32169498] doi:10.1016/j.bbi.2020.03.007
