# Supplementary material for: Mental health status and related factors influencing healthcare workers during the COVID-19 pandemic: A systematic review and meta-analysis
Source: PLoS One. 2024 Jan 19;19(1):e0289454. doi: 10.1371/journal.pone.0289454 (PMC10798549; doi:10.1371/journal.pone.0289454)
Supplement: S1 Data — (ZIP) [file pone.0289454.s011.zip › literatures/188.pdf]

A Global Survey of the Psychological Health of Surgeons during the COVID-19 Pandemic

1. Introduction

The World Health Organisation declared Coronavirus Disease-19 (COVID-19) a pandemic on 11th March 2020. As of 3rd August 2020, COVID-19 has affected more than 200 countries, resulting in more than 18 million cases and 692,000 deaths so far. The number of COVID-19 cases has been overwhelming and health care systems have been severely affected. In order to minimize the risk of infection and to divert resources to manage COVID-19 patients, the provision of urological services including outpatient clinics, investigations as well as operations have been significantly cut down[1, 2].

There has been a constant worry that endoscopic and laparoscopic procedures may be aerosol generating[3, 4], hence they may represent potential routes of viral transmission. Surgeons are at high risk of contracting COVID-19 despite the use of personal protective equipment. Moreover, many hospitals are experiencing manpower shortage, and even surgeons are often deployed to manage COVID-19 patients either in a voluntary or mandatory basis[1, 2]. COVID-19 is very contagious and such infective risk is imposed not only to surgeons themselves, but also to their family members who may be living together[5, 6]. Not only do surgeons have to face life and death situations in their clinical practice; their friends and family members may also suffer from severe consequences from COVID-19.

There have been a few local reports addressing the psychological impact of the COVID-19 pandemic on healthcare workers[7, 8]. However, the psychological impact on surgeons and surgery nurses remains largely unknown. Hence, we conducted an international survey to investigate the psychological impact of the COVID-19 pandemic on surgeons from a global perspective. We hypothesized that the degree of psychological impact would be higher for surgical staff deployed for COVID-19 work, certain surgical specialties that operate in the head, neck and thorax region, and for those who knew of someone who was diagnosed or who died of COVID-19.

2. Methods

2.1 Overview

We conducted a web-based survey to investigate the psychological impact of COVID-19 on surgeons and surgery nurses. Consultant/Attending surgeons, surgery registrars, residents, fellows and trainees, surgery nurse specialists and advanced practice providers were invited to participate in this survey. This study was approved by the National Healthcare Group Domain Specific Review Board, Singapore (Reference No. 2020/00782) and the Survey and Behavioural Research Ethics Committee of the Chinese University of Hong Kong (Reference No. SBRE-19-721).

2.2 Content development

The content of the survey was developed using a modified Delphi method[9]. The first draft of the questionnaire was developed by the steering committee (YQT, JM, JYCT, EC) after reviewing the current literature regarding the psychological impact of COVID-19. The questionnaire was sent out to the #SoMe4Surgery working group for their feedback. The questions were further refined by the steering committee until consensus was reached. The final set of questionnaire comprised 66 items, covering demographics (9 questions), the impact of the COVID-19 pandemic (14 questions), the Depression, Anxiety and Stress Scale (DASS-21) questionnaire (21 questions)[10], and the Impact of Events Scale-Revised questionnaire (IES-R) (22 questions)[11]. The complete set of survey is presented in the Supplementary Material.

The survey was primarily distributed via the #SoMe4Surgery Twitter platforms[12]. The invitation to participate in the study was sent out on 14th June 2020. The #SoMe4Surgery is a huge social network with multiple branches including #SoMe4Bariatrics, #SoMe4Breast, #SoMe4Endocrine, #SoMe4Endoscopy, #SoMe4IBD, #SoMe4PedSurg, #SoMe4Peritoneum, #SoMe4Precision, #SoMe4Proctology, #SoMe4SurgeryITeam, #SoMe4SurgeryPrehab and #UroSoMe[13]. Other organizations also disseminated the survey either via their Twitter accounts or mailing lists. Details on the number of followers of their Twitter accounts and the number of members in the mailing lists were summarized in the Supplementary Material.

2.3 Data collection

By clicking on the survey link (https://surveymonkey.com/r/COVIDPsych), participants would enter the introduction page of the survey. The purpose of the study and the target audience, i.e. “Surgery Nurse Specialists / Advanced Practice Providers”, “Surgery Trainees / Registrars / Fellows”, and “Consultant / Practising Surgeons”, were stated clearly in the introduction page. By proceeding to registration, participants were implied to consent to participate in the survey. In order to avoid missing data, responses to all questions were mandatory and the survey would not proceed unless all questions were completely answered. IP restriction was implemented, i.e. one IP address could only complete the survey once. Responses were collected via the Survey Monkey website and all data were only accessible by study investigators. The cumulative number of COVID-19 cases of each country was obtained from the European Centre for Disease Prevention and Control[14] and paired to each respondent based on the date they completed the survey.

2.4 Data synthesis and analysis

The primary outcomes were the overall DASS-21 and IES-R scores, and the Depression, Anxiety and Stress sub-scales of the DASS-21. For the DASS-21, cut-off scores of > 9,>7 and > 14 represent a positive screen of depression, anxiety and stress respectively. Depression subscale scores of 10–13 were deemed as “mild”, 14–20 as “moderate”, 21–27 as “severe”, and 28–42 as “extremely severe”. Anxiety subscale scores of 8-9 were deemed as “mild”, 10-14 as “moderate”, 15-19 as “severe”, and 20-42 as “extremely severe”. Stress subscale scores of 15-18 were deemed as “mild”, 19-25 as “moderate”, 26-33 as “severe”, and 34-42 as “extremely severe”. A cut off score of >24 for the IES-R score was deemed a positive screen for Post Traumatic Stress Disorder. Scores of 24-32 were deemed as “mild”, 33-36 as “moderate”, and >36 as “severe”.

Demographic data and the psychological impact of the COVID-19 pandemic were presented in a descriptive manner. We presented the DASS-21 sub-scale scores by varying levels of severity. Heat maps for the DASS-21 and IES-R scores were created to analyse the global psychological impact of the COVID-19 pandemic, by geographical location. For countries without any respondents, they were assumed to have similar DASS-21 and IES-R scores as the other countries within the same subgroup based on the WHO classification for global assessment of disease burden. Surgical specialities with the highest rates of respondents screening positive for psychological conditions were presented.

All statistical analyses were performed using SPSS for Windows version 26.0 (SPSS Inc, Chicago, IL, USA) and heatmaps were constructed using Tableau 2020.2. Continuous variables were presented as mean with standard deviation while frequencies and percentages for categorical variables. To determine the reliability and internal validity of questionnaire, Cronbach's alpha and Confirmatory Factor Analysis were used. Predictors for depression, anxiety, stress and PTSD were assessed using logistic regression. Statistical significance was set at 2-sided $p < 0.05$.

3. Results

Between 15 and 30 June 2020, a total of 4283 participants responded to the survey. Of which, 3391 completed the whole survey, with an overall complete response rate of 79.2%. The mean time required to complete the survey was 8 minutes.

3.1 Demographic Data

Demographic data was summarised in **Table 1**. Majority of the participants were in the 30 to 39 year-old age group, followed by the 40 to 49 year-old age group. 70% of respondents were male. The highest number of respondents were from Asia, followed by Europe, Africa, South America, North America and Australia/New Zealand. Six in ten respondents worked in teaching hospitals and academic institutions. 60.1% of respondents were of at least consultant/attending surgeon level and above, 34.1% were trainees/residents, and 5.8% were surgical nurse specialists. Classified by speciality, the top 5 specialities by participants were General Surgery (45.8%), Urology (22.9%), Trauma & Orthopaedic Surgery (6.3%), Otorhinolaryngology (4.5%), and Plastic Surgery (4.0%). One in four respondents had at least one pre-existing medical condition.

Table 1 - Demographics of survey respondents

	**Number (n)**	**Percentage (%)**
**Age**		
21-29 years	521	15.4
30-39 years	1360	40.1
40-49 years	863	25.4
50-59 years	463	13.7
60 years or above	184	5.4

**Gender**			
	Male	2377	70.1
	Female	1014	29.9
**Continent**			
	Asia	1546	45.6
	Australia & New Zealand	49	1.4
	Africa	344	10.1
	Europe	948	28.0
	North America	179	5.3
	South America	325	9.6
**Types of hospital/ institution**			
	Teaching hospital / Academic institution	2035	60.0
	Non-academic public hospital	398	11.7
	Private practice	491	14.5
	Mixture of public and private practices	467	13.8
**Level of training**			
	Surgery nurse specialists / Advanced practice providers	195	5.8
	Surgery trainees / Registrars / Fellows	1156	34.1
	Consultants / Practising urologists	2040	60.2
**Years of practice**			
	<5 years	1090	32.1
	6 to 10 years	803	23.7
	11- 15 years	538	15.9
	16-20 years	346	10.2
	>20 years	614	18.1
**Surgical specialty**			
	Cardiothoracic Surgery	84	2.5
	General Surgery	1552	45.8
	Neurosurgery	60	1.8
	Oral and Maxillofacial Surgery	104	3.1
	Otolaryngology	151	4.5
	Paediatric Surgery	119	3.5
	Plastic Surgery	137	4.0
	Trauma and Orthopaedic Surgery	214	6.3
	Urology	775	22.9
	Vascular Surgery	59	1.7
	Obstetrics and Gynaecology	110	3.2
	Ophthalmology	26	0.8
**History of Pre-existing Medical Co-morbidities**			
	Yes	850	25.1
	No	2541	74.9

3.2 Geographical Variation of DASS-21 and IES-R Scores

DASS-21 and IES-R Scores varied by geographical location. Overall, higher DASS-21 scores were seen Europe, Asia, North America and South America, compared to Africa and Australia/New Zealand. **Figure 1a** shows a global heat map of the mean total DASS-21 score. IES-R scores were higher in Europe, North America and South America, compared to Asia, Africa and Australia/New Zealand. **Figure 1b** shows a global heat map of the mean IES-R Score.

Figure 1a: Global Heat Map of the Mean DASS-21 Score

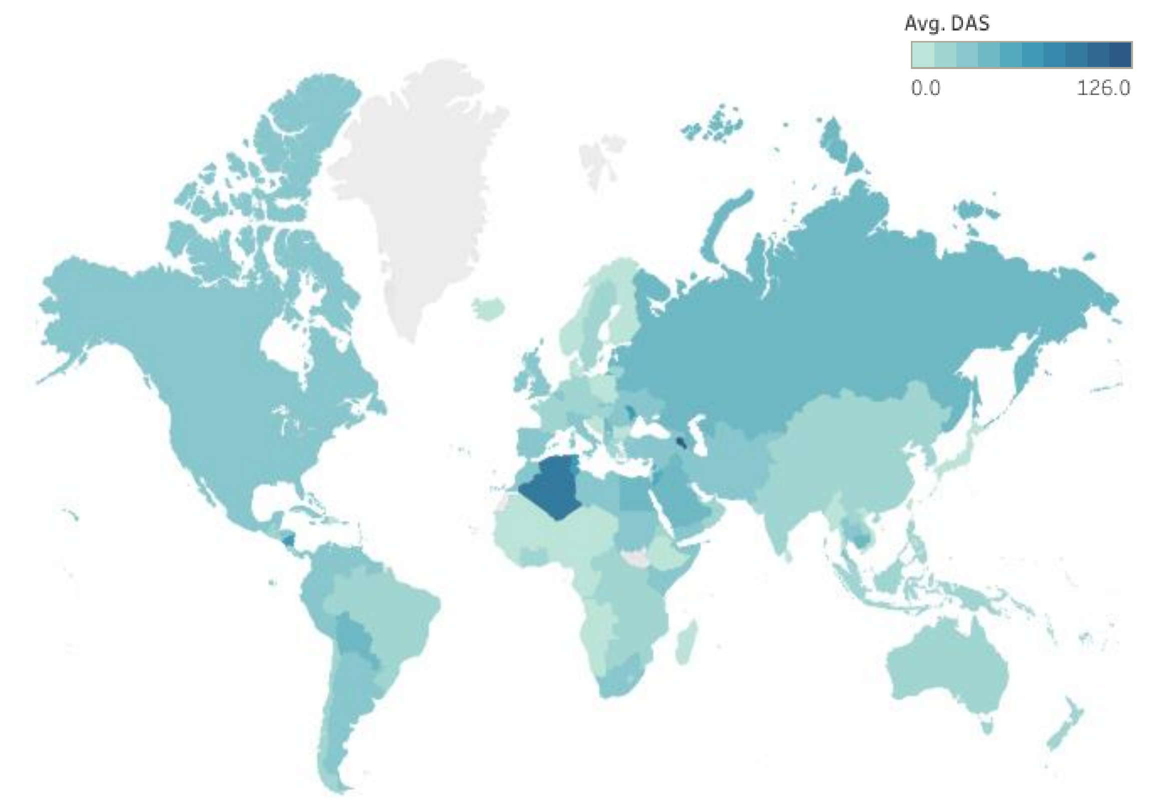

Figure 1b: Global Heat Map of the Overall IES-R Score

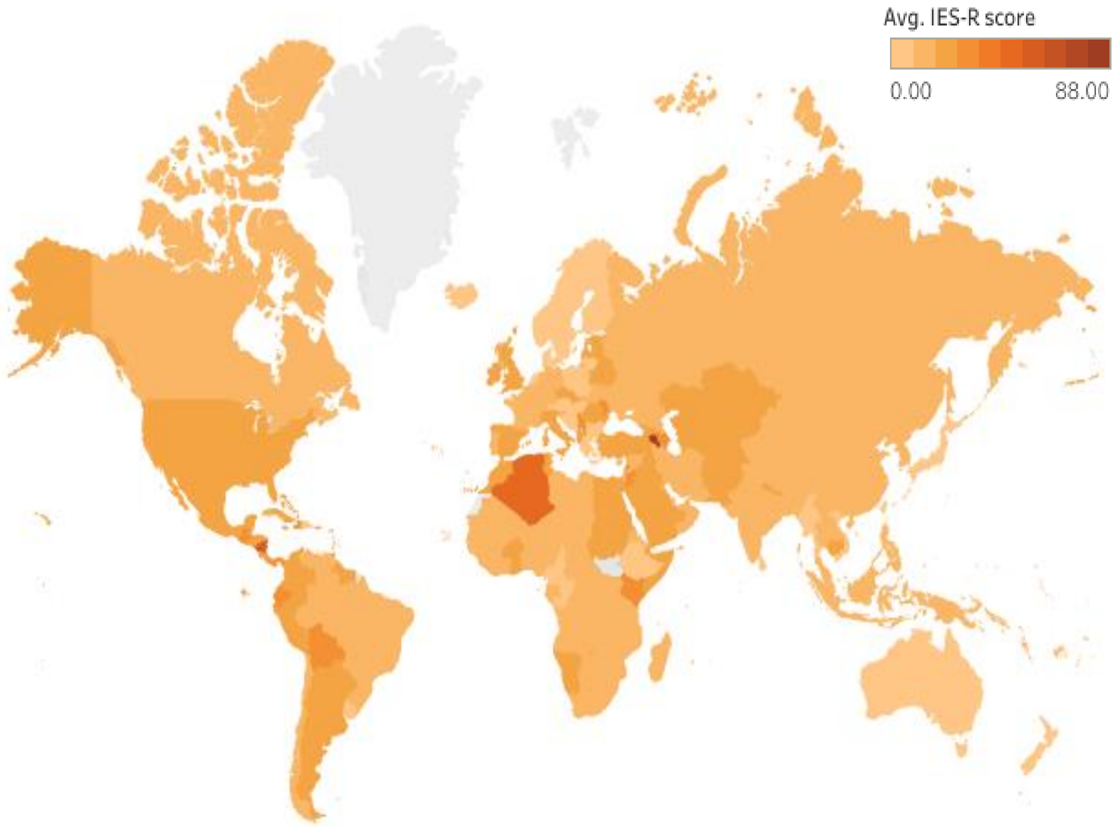

3.3 Psychological Impact of the COVID-19 Pandemic

The psychological impact of the COVID-19 pandemic on respondents was summarised in **Table 2**. 5.8% (195) of respondents had personal diagnoses of COVID-19. 8.6% (293) had family members diagnosed with COVID-19, and 4.5% (151) had family members who died from COVID-19. 58.7% of respondents had colleagues or friends who were diagnosed with COVID-19, and 27.7% had colleagues or friends who died from COVID-19. One in five respondents cared for patients who died from COVID-19.

Majority of respondents were deployed to care for COVID-19 patients. 24.8% and 58.8% of participants were deployed on a mandatory and voluntary basis respectively. Prior to this pandemic, 3.2%, 5.0% and 3.2% of respondents had been previously diagnosed with

depression, anxiety and stress respectively. Since the COVID-19 pandemic, an additional 1.6%, 4.3% and 3.8% of respondents were newly diagnosed with depression, anxiety and stress respectively. Overall, only 6.9% of respondents reported seeking psychological help. 37.4% of respondents reported having experienced insomnia since the start of the pandemic. 50.5% of respondents reported readily available mental health facilities at their workplaces, with the other half either reporting unavailability or difficulty accessing mental help. Nearly half (48.6%) of respondents did not discuss mental health issues with others.

Since the start of this pandemic, only 31.5% of respondents had taken vacation leave. The most commonly reported coping mechanisms for work stress were spending time with family/friends (66.9%), watching television (66.8%), social media (56.5%), exercising (46.4%), and reading non-medical books (34.4%).

Table 2 – Psychological Impact of the COVID-19 Pandemic

		Number (n)	Percentage (%)
**Diagnosis of COVID-19**			
	Personal Diagnosis	195	5.8
	Family Member	293	8.6
	Colleagues or Friends	1990	58.7
**Knowing Someone who Died from COVID-19**			
	Family Member	151	4.5
	Colleagues or Friends	940	27.7
	Patients	663	19.6
**Deployment to care for COVID-19 patients**			
	Yes, Mandatory	842	24.8
	Yes, Voluntary	1993	58.8
	No	556	16.4
**Pre-existing Psychological Health Conditions**			
	Depression	108	3.2
	Anxiety	169	5.0
	Stress	107	3.2
**New Diagnosis of Psychological Health Conditions**			
	Depression	54	1.6
	Anxiety	146	4.3
	Stress	128	3.8
**Sought Help for Psychological Health Conditions**			
	Yes	234	6.9
	No	3157	93.1
**Experienced Insomnia since the Pandemic Started**			
	Yes	1267	37.4
	No	2124	62.6
**Access to Mental Health Facilities at Workplace**			
	Readily available	1714	50.5

	Available but difficult to access	688	20.3
	Not available/Do not know	989	29.2
**Discussion of Mental Health Issues**			
	With Family	1052	31.0
	With Colleagues or Friends	1295	38.2
	Did not discuss	1648	48.6
**Taken a Vacation since the Start of COVID-19**			
	Yes	1068	31.5
	No	2323	68.5
**Coping Mechanisms for Work Stress**			
	Social Media	1916	56.5
	Reading Non-Medical Books	1168	34.4
	Exercising	1575	46.4
	Watching Television	2264	66.8
	Spending Time with Family and Friends	2269	66.9
	Meditation/Prayer	598	17.6

3.4 DASS-21 and IES-R Scores

The overall DASS-21 and IES-R scores by severity are summarised in **Table 3**. 32.8%, 30.8% and 25.9% of respondents screened positive for depression, anxiety and stress. 24.0% of respondents screened positive for PTSD.

Table 3: Overall DASS-21 and IES-R Scores by Severity

Severity	Depression	Anxiety	Stress	IES-R (PTSD)
**Normal**	67.2%	69.2%	74.1%	76.0%
**Mild**	9.9%	5.5%	9.1%	10.3%
**Moderate**	13.3%	12.6%	8.5%	2.7%
**Severe**	5.2%	5.4%	6.3%	11.0%
**Extremely Severe**	4.3%	7.2%	2.0%	N.A.

Tables S4.1 to S4.4 in the Supplementary Material summarizes the specialities with the highest proportion of respondents screening positive for psychological health conditions. For Depression, Cardiothoracic surgery had the highest proportion of respondents screening positive (38.1%), followed by Paediatric Surgery (36.1%), and General Surgery (35.3%). For Anxiety, Cardiothoracic surgery also had the highest proportion of respondents screening positive (39.3%), followed by Vascular Surgery (39.0%) and Neurosurgery (38.3%). For Stress, Ophthalmology had the highest proportion of respondents screening positive (34.6%),

followed by Neurosurgery (33.3%) and Cardiothoracic Surgery (32.1%). For PTSD, Ophthalmology had the highest proportion of respondents screening positive (34.6%), followed by Cardiothoracic Surgery (33.3%) and Neurosurgery (33.2%).

3.5 Multivariate Analysis of Predictive Factors for Psychological Health Conditions

Screen positive for depression

Variable	No	Yes	Unadjusted		Adjusted	
			OR (95%CI)	p-value	OR (95%CI)	p-value
Gender						
Male	1673(70.4%)	704(29.6%)	1.0		1.0	
Female	606(59.8%)	408(40.2%)	1.6(1.4-1.9)	<0.001	1.3(1.1-1.6)	0.003
Age						
21-29	321(61.6%)	200(38.4%)	3.2(2.1-4.9)	<0.001	3.2(1.7-6.2)	0.001
30-39	893(65.7%)	467(34.3%)	2.7(1.8-4.0)	<0.001	2.8(1.6-5.2)	0.001
40-49	560(64.9%)	303(35.1%)	2.8(1.8-4.2)	<0.001	2.7(1.5-4.7)	<0.001
50-59	351(75.8%)	112(24.2%)	1.6(1-2.6)	0.030	1.7(1.0-2.8)	0.031
60 and above	154(83.7%)	30(16.3%)	1.0		1.0	
Number of comorbids	0.33±0.68	0.34±0.71	1.0(0.9-1.1)	0.568	1.2(1.1-1.3)	0.005
Location of clinical practice						
Asia	1107(71.6%)	439(28.4%)	1.7(1.3-2.2)	0.001	1.6(1.1-2.2)	0.005
Australia or New Zealand	39(79.6%)	10(20.4%)	1.1(0.5-2.3)	0.839	1.0(0.4-2.3)	0.998
Africa	278(80.8%)	66(19.2%)	1.0		1.0	
Europe	547(57.7%)	401(42.3%)	3.1(2.3-4.2)	<0.001	2.6(1.9-3.6)	<0.001
North America	106(59.2%)	73(40.8%)	2.9(1.9-4.3)	<0.001	2.5(1.6-3.8)	<0.001
South America	202(62.2%)	123(37.8%)	2.6(1.8-3.6)	<0.001	2.2(1.5-3.3)	<0.001
Type of hospital						
Teaching hospital	1341(65.9%)	694(34.1%)	1.4(1.1-1.7)	0.004	1.1(0.8-1.4)	0.600
Non-academic	276(69.3%)	122(30.7%)	1.2(0.9-1.6)	0.271	0.90(0.65-1.24)	0.522
Private	357(72.7%)	134(27.3%)	1.0		1.0	
Mixture of public and private	305(65.3%)	162(34.7%)	1.4(1.1-1.9)	0.013	1.1(0.9-1.5)	0.370
Occupation						
Nurse	111(56.9%)	84(43.1%)	1.7(1.2-2.3)	0.001	1.3(0.9-1.9)	0.107
Trainee	764(66.1%)	392(33.9%)	1.1(1.0-1.3)	0.112	0.95(0.77-1.18)	0.665
Consultant	1404(68.8%)	636(31.2%)	1.0		1.0	
Years Practicing						
<5	707(64.9%)	383(35.1%)	1.9(1.5-2.4)	<0.001	1.3(0.8-2.0)	0.323
6-10	504(62.8%)	299(37.2%)	2.1(1.6-2.6)	<0.001	1.5(1.0-2.3)	0.049
11-15	366(68.0%)	172(32.0%)	1.6(1.3-2.1)	<0.001	1.2(0.8-1.7)	0.473
16-20	225(65.0%)	121(35.0%)	1.9(1.4-2.5)	<0.001	1.4(0.9-2.0)	0.106
>20	477(77.7%)	137(22.3%)	1.0		1.0	

Speciality<br>Cardiothoracic surgery<br>General surgery<br>Neurosurgery<br>Oral and maxillofacial surgery<br>Otolaryngology<br>Paediatric Surgery<br>Plastic surgery<br>Trauma and Orthopaedic Surgery<br>Urology<br>Vascular Surgery<br>O&G<br>Eye	52(61.9%)<br>1004(64.7%<br>)<br>39(65.0%)<br>71(68.3%)<br>109(72.2%)<br>76(63.9%)<br>94(68.6%)<br>166(77.6%)<br>530(68.4%)<br>39(66.1%)<br>82(74.5%)<br>17(65.4%)	32(38.1%)<br>548(35.3%)<br>21(35.0%)<br>33(31.7%)<br>42(27.8%)<br>43(36.1%)<br>43(31.4%)<br>48(22.4%)<br>245(31.6%)<br>20(33.9%)<br>28(25.5%)<br>9(34.6%)	2.1(1.2-3.7)<br>1.9(1.3-2.6)<br>1.9(1.0-3.5)<br>1.6(1.0-2.7)<br>1.3(0.8-2.2)<br>2.0(1.2-3.2)<br>1.6(1.0-2.6)<br>1.0<br>1.6(1.1-2.3)<br>1.8(0.9-3.3)<br>1.2(0.7-2.0)<br>1.8(0.8-4.4)	0.007<br><0.001<br>0.049<br>0.075<br>0.241<br>0.008<br>0.063<br><br>0.010<br>0.074<br>0.543<br>0.173	1.9(1.1-3.3)<br>1.5(1.0-2.1)<br>1.4(0.7-2.7)<br>1.4(0.8-2.5)<br>1.2(0.7-2.0)<br>1.5(0.9-2.6)<br>1.2(0.7-2.0)<br>1.0<br>1.3(0.9-1.9)<br>1.2(0.6-2.4)<br>1.0(0.6-1.9)<br>1.6(0.6-4.0)	0.030<br>0.040<br>0.378<br>0.203<br>0.472<br>0.125<br>0.511<br><br>0.150<br>0.555<br>0.870<br>0.318
Deployed for COVID-19<br>Yes<br>No	1910(67.4%<br>)<br>369(66.4%)	925(32.6%)<br>187(33.6%)	0.96(0.79-1.16)<br>1.0	0.644	1.2(0.9-1.4)<br>1.0	0.165
**Pre-existing psychological health conditions**<br>Depressive<br>Yes<br>No<br>Anxiety<br>Yes<br>No<br>Stress<br>Yes<br>No	30(27.8%)<br>2249(68.5%<br>)<br>64(37.9%)<br>2215(68.7%<br>)<br>33(30.8%)<br>2246(68.4%<br>)	78(72.2%)<br>1034(31.5%<br>)<br>105(62.1%)<br>1007(31.3%<br>)<br>74(69.2%)<br>1038(31.6%<br>)	5.7(3.7-8.7)<br>1.0<br>3.6(2.6-5.0)<br>1.0<br>4.9(3.2-7.4)<br>1.0	<0.001<br><0.001<br><0.001	3.6(2.3-5.8)<br>1.0<br>2.4(1.7-3.4)<br>1.0<br>3.8(2.4-6.0)<br>1.0	<0.001<br><0.001<br><0.001
**Knowing someone diagnosed with COVID-19**<br>Yes<br>No	1377(63.9%<br>)<br>902(73.0%)	778(36.1%)<br>334(27.0%)	1.5(1.3-1.8)<br>1.0	<0.001	1.2(1.0-1.4)<br>1.0	0.052
**Knowing someone died of COVID-19**<br>Yes<br>No	922(62.0%)<br>1357(71.2%<br>)	564(38.0%)<br>548(28.8%)	1.5(1.3-1.8)<br>1.0	<0.001	1.3(1.1-1.6)<br>1.0	0.002
**Took Leave**<br>Vacation<br>Yes<br>No<br>Sick, family care or compassionate<br>Yes<br>No	329(65.8%)<br>1950(67.5%<br>)<br>371(54.9%)<br>1908(70.3%<br>)	171(34.2%)<br>941(32.5%)<br>305(45.1%)<br>807(29.7%)	1.1(0.9-1.3)<br>1.0<br>1.9(1.6-2.3)<br>1.0	0.468<br><0.001	1.1(0.9-1.4)<br>1.0<br>1.5(1.3-1.9)<br>1.0	0.305<br><0.001

Screen positive for anxiety

Variable	No	Yes	Unadjusted		Adjusted	
			OR (95%CI)	p-value	OR (95%CI)	p-value
**Gender**						
Male	1728(72.7%)	649(27.3%)	1.0		1.0	
Female	620(61.1%)	394(38.9%)	1.7(1.4-2.0)	<0.001	1.4(1.1-1.6)	0.001
**Age**						
21-29	311(59.7%)	210(40.3%)	3.8(2.4-5.8)	<0.001	2.2(1.1-4.3)	0.025
30-39	940(69.1%)	420(30.9%)	2.5(1.6-3.8)	<0.001	1.7(0.9-3.1)	0.107
40-49	573(66.4%)	290(33.6%)	2.8(1.8-4.3)	<0.001	2.0(1.1-3.5)	0.021
50-59	368(79.5%)	95(20.5%)	1.4(0.9-2.3)	0.122	1.3(0.8-2.2)	0.271
60 and above	156(84.8%)	28(15.2%)	1.0		1.0	
Number of comorbids	0.33±0.68	0.34±0.73	1.0(0.9-1.1)	0.671	1.2(1.0-1.3)	0.026
**Location of clinical practice**						
Asia	1097(71.0%)	449(29.0%)	4.6(1.6-12.9)	0.004	4.9(1.6-15.2)	0.006
Australia or New Zealand	45(91.8%)	4(8.2%)	1.0		1.0	
Africa	280(81.4%)	64(18.6%)	2.6(0.9-7.4)	0.080	2.9(0.9-9.3)	0.074
Europe	598(63.1%)	350(36.9%)	6.6(2.3-18.5)	<0.001	6.3(2.0-19.8)	0.001
North America	113(63.1%)	66(36.9%)	6.6(2.3-19.1)	0.001	6.1(1.9-19.7)	0.003
South America	215(66.2%)	110(33.8%)	5.8(2.0-16.4)	0.001	5.4(1.7-17.1)	0.004
**Type of hospital**						
Teaching hospital	1389(68.3%)	646(31.7%)	1.3(1.0-1.6)	0.044	1.3(1.0-1.7)	0.030
Non-academic	292(73.4%)	106(26.6%)	1.0		1.0	
Private	359(73.1%)	132(26.9%)	1.0(0.8-1.4)	0.933	1.4(1.0-1.9)	0.060
Mixture of public and private	308(66.0%)	159(34.0%)	1.4(1.1-1.9)	0.019	1.6(1.1-2.2)	0.008
**Occupation**						
Nurse	107(54.9%)	88(45.1%)	2.1(1.6-2.8)	<0.001	1.7(1.2-2.4)	0.002
Trainee	775(67.0%)	381(33.0%)	1.3(1.1-1.5)	0.004	1.0(0.8-1.2)	0.974
Consultant	1466(71.9%)	574(28.1%)	1.0		1.0	
**Years Practicing**						
<5	696(63.9%)	394(36.1%)	2.5(2.0-3.2)	<0.001	2.2(1.4-3.5)	0.001
6-10	545(67.9%)	258(32.1%)	2.1(1.6-2.7)	<0.001	2.1(1.3-3.2)	0.001
11-15	367(68.2%)	171(31.8%)	2.1(1.6-2.7)	<0.001	1.9(1.2-2.8)	0.004
16-20	239(69.1%)	107(30.9%)	2.0(1.5-2.7)	<0.001	1.6(1.1-2.3)	0.024
>20	501(81.6%)	113(18.4%)	1.0		1.0	
**Speciality**						
Cardiothoracic surgery	51(60.7%)	33(39.3%)	2.3(1.3-4.0)	0.003	1.9(1.1-3.3)	0.032
General surgery	1046(67.4%)	506(32.6%)	1.7(1.2-2.4)	0.002	1.3(0.9-1.9)	0.167
Neurosurgery	37(61.7%)	23(38.3%)	2.2(1.2-4.1)	0.011	1.6(0.8-3.0)	0.200
Oral and maxillofacial surgery	70(67.3%)	34(32.7%)	1.7(1.0-2.9)	0.041	1.3(0.8-2.3)	0.322
Otolaryngology	107(70.9%)	44(29.1%)	1.5(0.9-2.4)	0.120	1.1(0.7-1.9)	0.615
Paediatric Surgery	77(64.7%)	42(35.3%)	1.9(1.2-3.2)	0.009	1.5(0.9-2.6)	0.135
Plastic surgery	98(71.5%)	39(28.5%)	1.4(0.9-2.3)	0.168	1.0(0.6-1.7)	0.928
Trauma and Orthopaedic Surgery	167(78.0%)	47(22.0%)	1.0		1.0	
Urology	564(72.8%)	211(27.2%)	1.3(0.9-1.9)	0.121	1.1(0.7-1.6)	0.728
Vascular Surgery	36(61.0%)	23(39.0%)	2.3(1.2-4.2)	0.009	1.4(0.7-2.7)	0.334
O&G	78(70.9%)	32(29.1%)	1.5(0.9-2.5)	0.158	1.4(0.8-2.4)	0.294
Eye	17(65.4%)	9(34.6%)	1.9(0.8-4.5)	0.155	1.6(0.6-4.1)	0.305

Deployed for COVID-19						
Yes	1990(70.2%)	845(29.8%)	0.77(0.63-0.93)	0.007	0.91(0.74-1.12)	0.365
No	358(64.4%)	198(35.6%)	1.0		1.0	
**Pre-existing psychological health conditions**						
Depressive						
Yes	40(37.0%)	68(63.0%)	4.0(2.7-6.0)	<0.001	2.5(1.6-3.9)	<0.001
No	2308(70.3%)	975(29.7%)	1.0		1.0	
Anxiety						
Yes	57(33.7%)	112(66.3%)	4.8(3.5-6.7)	<0.001	3.3(2.3-4.7)	<0.001
No	2291(71.1%)	931(28.9%)	1.0		1.0	
Stress						
Yes	40(37.4%)	67(62.6%)	4.0(2.7-5.9)	<0.001	3.1(2.0-4.9)	<0.001
No	2308(70.3%)	976(29.7%)	1.0		1.0	
**Knowing someone diagnosed with COVID-19**						
Yes	1422(66.0%)	733(34.0%)	1.5(1.3-1.8)	<0.001	1.1(1.0-1.4)	0.146
No	926(74.9%)	310(25.1%)	1.0		1.0	
**Knowing someone died of COVID-19**						
Yes	927(62.4%)	559(37.6%)	1.8(1.5-2.1)	<0.001	1.6(1.3-1.9)	<0.001
No	1421(74.6%)	484(25.4%)	1.0		1.0	
**Took Leave**						
Vacation						
Yes	330(66.0%)	170(34.0%)	1.2(1.0-1.5)	0.089	1.2(1.0-1.5)	0.048
No	2018(69.8%)	873(30.2%)	1.0		1.0	
Sick, family care or compassionate						
Yes	389(57.5%)	287(42.5%)	1.9(1.6-2.3)	<0.001	1.5(1.2-1.8)	<0.001
No	1959(72.2%)	756(27.8%)	1.0		1.0	

Screen positive for stress

Variable	No	Yes	Unadjusted		Adjusted	
			OR (95%CI)	p-value	OR (95%CI)	p-value
**Gender**						
Male	1853(78.0%)	524(22.0%)	1.0		1.0	
Female	659(65.0%)	355(35.0%)	1.9(1.6-2.2)	<0.001	1.6(1.3-1.9)	<0.001
**Age**						
21-29	355(68.1%)	166(31.9%)	3.8(2.3-6.3)	<0.001	3.3(1.6-6.9)	0.001
30-39	1003(73.8%)	357(26.3%)	2.9(1.8-4.7)	<0.001	2.9(1.5-5.7)	0.002
40-49	622(72.1%)	241(27.9%)	3.2(2.0-5.2)	<0.001	2.9(1.5-5.4)	0.001
50-59	368(79.5%)	95(20.5%)	2.1(1.3-3.5)	0.004	2.2(1.3-3.9)	0.005
60 and above	164(89.1%)	20(10.9%)	1.0		1.0	
Number of comorbids	0.34±0.72	0.31±0.60	0.94(0.84-1.05)	0.277	1.1(0.9-1.2)	0.248
**Location of clinical practice**						
Asia	1251(80.9%)	295(19.1%)	1.4(1.0-2.0)	0.036	1.3(0.9-1.9)	0.146
Australia or New Zealand	42(85.7%)	7(14.3%)	1.0(0.4-2.4)	0.994	0.96(0.38-2.44)	0.937
Africa	295(85.8%)	49(14.2%)	1.0		1.0	
Europe	596(62.9%)	352(37.1%)	3.6(2.6-4.9)	<0.001	3.0(2.1-4.3)	<0.001
North America	116(64.8%)	63(35.2%)	3.3(2.1-5.0)	<0.001	2.8(1.8-4.4)	<0.001
South America	212(65.2%)	113(34.8%)	3.2(2.2-4.7)	<0.001	2.7(1.8-4.2)	<0.001
**Type of hospital**						
Teaching hospital	1480(72.7%)	555(27.3%)	1.4(1.1-1.8)	0.002	1.1(0.8-1.4)	0.659
Non-academic	307(77.1%)	91(22.9%)	1.1(0.8-1.6)	0.409	0.84(0.59-1.19)	0.320
Private	390(79.4%)	101(20.6%)	1.0		1.0	
Mixture of public and private	335(71.7%)	132(28.3%)	1.5(1.1-2.0)	0.006	1.2(0.9-1.7)	0.266
**Occupation**						
Nurse	131(67.2%)	64(32.8%)	1.5(1.1-2.0)	0.013	1.1(0.8-1.6)	0.657
Trainee	845(73.1%)	311(26.9%)	1.1(1.0-1.3)	0.171	0.96(0.76-1.21)	0.725
Consultant	1536(75.3%)	504(24.7%)	1.0		1.0	
**Years Practicing**						
<5	777(71.3%)	313(28.7%)	1.8(1.4-2.4)	<0.001	1.4(0.8-2.2)	0.223
6-10	587(73.1%)	216(26.9%)	1.7(1.3-2.2)	<0.001	1.3(0.8-2.1)	0.218
11-15	394(73.2%)	144(26.8%)	1.7(1.3-2.2)	<0.001	1.3(0.8-1.9)	0.289
16-20	250(72.3%)	96(27.7%)	1.8(1.3-2.4)	<0.001	1.3(0.9-1.9)	0.239
>20	504(82.1%)	110(17.9%)	1.0		1.0	
**Speciality**						
Cardiothoracic surgery	57(67.9%)	27(32.1%)	2.1(1.2-3.8)	0.010	1.9(1.0-3.5)	0.035
General surgery	1113(71.7%)	439(28.3%)	1.8(1.2-2.5)	0.002	1.2(0.8-1.8)	0.286
Neurosurgery	40(66.7%)	20(33.3%)	2.2(1.2-4.3)	0.013	1.7(0.9-3.4)	0.132
Oral and maxillofacial surgery	84(80.8%)	20(19.2%)	1.1(0.6-1.9)	0.829	1.0(0.5-1.9)	0.999
Otolaryngology	118(78.1%)	33(21.9%)	1.3(0.7-2.1)	0.391	1.1(0.7-2.0)	0.625
Paediatric Surgery	88(73.9%)	31(26.1%)	1.6(0.9-2.7)	0.094	0.99(0.55-1.78)	0.973
Plastic surgery	98(71.5%)	39(28.5%)	1.8(1.1-3.0)	0.025	1.2(0.7-2.1)	0.498
Trauma and Orthopaedic Surgery	175(81.8%)	39(18.2%)	1.0		1.0	
Urology	593(76.5%)	182(23.5%)	1.4(0.9-2.0)	0.103	1.1(0.7-1.7)	0.667
Vascular Surgery	42(71.2%)	17(28.8%)	1.8(0.9-3.5)	0.077	1.3(0.6-2.6)	0.497
O&G	87(79.1%)	23(20.9%)	1.2(0.7-2.1)	0.561	0.92(0.49-1.70)	0.783
Eye	17(65.4%)	9(34.6%)	2.4(1.0-5.7)	0.054	1.8(0.7-4.6)	0.227

Deployed for COVID-19						
Yes	2101(74.1%)	734(25.9%)	0.99(0.81-1.22)	0.926	1.2(1.0-1.5)	0.084
No	411(73.9%)	145(26.1%)	1.0		1.0	
**Pre-existing psychological health conditions**						
Depressive						
Yes	40(37.0%)	68(63.0%)	5.2(3.5-7.7)	<0.001	3.2(2.1-5.0)	<0.001
No	2472(75.3%)	811(24.7%)	1.0		1.0	
Anxiety						
Yes	79(46.7%)	90(53.3%)	3.5(2.6-4.8)	<0.001	2.3(1.6-3.3)	<0.001
No	2433(75.5%)	789(24.5%)	1.0		1.0	
Stress						
Yes	49(45.8%)	58(54.2%)	3.6(2.4-5.2)	<0.001	2.6(1.7-4.0)	<0.001
No	2463(75.0%)	821(25.0%)	1.0		1.0	
**Knowing someone diagnosed with COVID-19**						
Yes	1524(70.7%)	631(29.3%)	1.6(1.4-2.0)	<0.001	1.2(1.0-1.5)	0.047
No	988(79.9%)	248(20.1%)	1.0		1.0	
**Knowing someone died of COVID-19**						
Yes	1024(68.9%)	462(31.1%)	1.6(1.4-1.9)	<0.001	1.4(1.1-1.6)	0.001
No	1488(78.1%)	417(21.9%)	1.0		1.0	
**Took Leave**						
Vacation						
Yes	373(74.6%)	127(25.4%)	0.97(0.78-1.2)	0.773	0.99(0.78-1.25)	0.926
No	2139(74.0%)	752(26.0%)	1.0		1.0	
Sick, family care or compassionate						
Yes	438(64.8%)	238(35.2%)	1.8(1.5-2.1)	<0.001	1.3(1.1-1.6)	0.003
No	2074(76.4%)	641(23.6%)	1.0		1.0	

Screen positive for Post-Traumatic Stress Disorder

Variable	No	Yes	Unadjusted		Adjusted	
			OR (95%CI)	p-value	OR (95%CI)	p-value
Gender						
Male	1888(79.4%)	489(20.6%)	1.0		1.0	
Female	690(68.0%)	324(32.0%)	1.8(1.5-2.1)	<0.001	1.6(1.3-1.9)	<0.001
Age						
21-29	361(69.3%)	160(30.7%)	3.8(2.3-6.4)	<0.001	1.9(0.9-4.0)	0.103
30-39	1033(76.0%)	327(24.0%)	2.7(1.7-4.5)	<0.001	1.5(0.7-3.0)	0.274
40-49	636(73.7%)	227(26.3%)	3.1(1.9-5.1)	<0.001	1.8(1.0-3.6)	0.069
50-59	383(82.7%)	80(17.3%)	1.8(1.1-3.1)	0.028	1.6(0.9-2.9)	0.100
60 and above	165(89.7%)	19(10.3%)	1.0		1.0	
Number of comorbids	0.33±0.68	0.34±0.74	1.0(0.9-1.1)	0.689	1.2(1.0-1.4)	0.010
Location of clinical practice						
Asia	1256(81.2%)	290(18.8%)	3.5(1.1-11.5)	0.035	3.4(0.9-12.4)	0.067
Australia or New Zealand	46(93.9%)	3(6.1%)	1.0		1.0	
Africa	285(82.8%)	59(17.2%)	3.2(1.0-10.6)	0.059	3.4(0.9-12.7)	0.075
Europe	648(68.4%)	300(31.6%)	7.1(2.2-23.0)	0.001	6.1(1.7-22.6)	0.006
North America	117(65.4%)	62(34.6%)	8.1(2.4-27.2)	0.001	7.0(1.8-26.5)	0.004
South America	226(69.5%)	99(30.5%)	6.7(2.0-22.1)	0.002	6.0(1.6-22.3)	0.008
Type of hospital						
Teaching hospital	1512(74.3%)	523(25.7%)	1.5(1.1-1.9)	0.005	1.5(1.1-2.0)	0.008
Non-academic	322(80.9%)	76(19.1%)	1.0		1.0	
Private	393(80.0%)	98(20.0%)	1.1(0.8-1.5)	0.747	1.4(1.0-2.1)	0.069
Mixture of public and private	351(75.2%)	116(24.8%)	1.4(1.0-1.9)	0.043	1.4(1.0-2.0)	0.071
Occupation						
Nurse	135(69.2%)	60(30.8%)	1.5(1.1-2.1)	0.011	1.1(0.8-1.6)	0.535
Trainee	866(74.9%)	290(25.1%)	1.1(1.0-1.4)	0.126	0.84(0.66-1.06)	0.149
Consultant	1577(77.3%)	463(22.7%)	1.0		1.0	
Years Practicing						
<5	787(72.2%)	303(27.8%)	2.4(1.9-3.2)	<0.001	2.9(1.7-4.8)	<0.001
6-10	592(73.7%)	211(26.3%)	2.2(1.7-3.0)	<0.001	2.7(1.7-4.4)	<0.001
11-15	410(76.2%)	128(23.8%)	2.0(1.5-2.7)	<0.001	2.0(1.3-3.2)	0.003
16-20	259(74.9%)	87(25.1%)	2.1(1.5-3.0)	<0.001	1.8(1.2-2.8)	0.007
>20	530(86.3%)	84(13.7%)	1.0		1.0	
Speciality						
Cardiothoracic surgery	56(66.7%)	28(33.3%)	2.6(1.5-4.7)	0.001	2.1(1.1-3.9)	0.017
General surgery	1161(74.8%)	391(25.2%)	1.8(1.2-2.6)	0.003	1.2(0.8-1.8)	0.395
Neurosurgery	40(66.7%)	20(33.3%)	2.6(1.4-5.1)	0.003	1.8(0.9-3.6)	0.122
Oral and maxillofacial surgery	80(76.9%)	24(23.1%)	1.6(0.9-2.9)	0.121	1.3(0.7-2.5)	0.351
Otolaryngology	123(81.5%)	28(18.5%)	1.2(0.7-2.1)	0.506	0.95(0.53-1.71)	0.864
Paediatric Surgery	88(73.9%)	31(26.1%)	1.9(1.1-3.2)	0.026	1.1(0.6-2.0)	0.745
Plastic surgery	99(72.3%)	38(27.7%)	2.0(1.2-3.4)	0.008	1.4(0.8-2.5)	0.240
Trauma and Orthopaedic Surgery	180(84.1%)	34(15.9%)	1.0		1.0	
Urology	607(78.3%)	168(21.7%)	1.5(1.0-2.2)	0.064	1.1(0.7-1.7)	0.609
Vascular Surgery	43(72.9%)	16(27.1%)	2.0(1.0-3.9)	0.051	1.2(0.6-2.6)	0.556
O&G	84(76.4%)	26(23.6%)	1.6(0.9-2.9)	0.091	1.2(0.7-2.3)	0.481
Eye	17(65.4%)	9(34.6%)	2.8(1.2-6.8)	0.023	2.1(0.8-5.5)	0.123

Deployed for COVID-19						
Yes	2169(76.5%)	666(23.5%)	0.85(0.69-1.05)	0.137	1.0(0.8-1.3)	0.775
No	409(73.6%)	147(26.4%)	1.0		1.0	
Pre-existing psychological health conditions						
Depressive						
Yes	41(38.0%)	67(62.0%)	5.6(3.7-8.3)	<0.001	3.5(2.3-5.5)	<0.001
No	2537(77.3%)	746(22.7%)	1.0		1.0	
Anxiety						
Yes	73(43.2%)	96(56.8%)	4.6(3.4-6.3)	<0.001	3.1(2.2-4.3)	<0.001
No	2505(77.7%)	717(22.3%)	1.0		1.0	
Stress						
Yes	55(51.4%)	52(48.6%)	3.1(2.1-4.6)	<0.001	2.3(1.5-3.5)	<0.001
No	2523(76.8%)	761(23.2%)	1.0		1.0	
Knowing someone diagnosed with COVID-19						
Yes	1563(72.5%)	592(27.5%)	1.7(1.5-2.1)	<0.001	1.2(1.0-1.5)	0.040
No	1015(82.1%)	221(17.9%)	1.0		1.0	
Knowing someone died of COVID-19						
Yes	1032(69.4%)	454(30.6%)	1.9(1.6-2.2)	<0.001	1.6(1.4-2.0)	<0.001
No	1546(81.2%)	359(18.8%)	1.0		1.0	
Took Leave						
Vacation						
Yes	377(75.4%)	123(24.6%)	1.0(0.8-1.3)	0.723	1.1(0.8-1.4)	0.576
No	2201(76.1%)	690(23.9%)	1.0		1.0	
Sick, family care or compassionate						
Yes	450(66.6%)	226(33.4%)	1.8(1.5-2.2)	<0.001	1.4(1.2-1.7)	0.001
No	2128(78.4%)	587(21.6%)	1.0		1.0	

Data expressed as mean ± SD for continuous variables; absolute numbers (percentage) for categorical variables. The factors that were found to be independently associated with PTSD were female, number of comorbids, Europe, North America, South America, teaching hospital, years of practising <5, years of practising 6-10, years of practising 11-15, years of practising 16-20, Cardiothoracic surgery, pre-existing depressive, pre-existing anxiety, pre-existing stress, knowing someone diagnosed with COVID-19, knowing someone died of COVID-19 and sick, family care or compassionate leave.

On multivariate analysis for the predictive factors for depression, females were 1.3 times as likely as males to screen positive for depression (95% CI: 1.1-1.5, $p=0.003$). Younger respondents were more likely to screen positive than older respondents ($p<0.05$). Respondents who knew someone diagnosed with COVID-19 were more likely to screen

positive for depression, OR 1.2 (p=0.045). Those who knew someone who died of COVID-19 were also significantly more likely to screen positive for depression, OR 1.3 (p=0.001). There were no statistically significant differences in depression rates when comparing location of clinical practice, type of hospital, speciality, occupation and deployment status.

On multivariate analysis for the predictive factors for anxiety, females were 1.4 times as likely as males to screen positive for anxiety (95% CI: 1.2-1.6, p=<0.001). Respondents with less years of practice were more likely to screen positive than those with more years in practice (p<0.05). Respondents who knew someone who died of COVID-19 were more likely to screen positive for anxiety, OR 1.6 (p<0.001). There were no statistically significant differences in depression rates when comparing location of clinical practice, type of hospital, speciality, occupation, deployment status, and knowing someone diagnosed with COVID-19.

On multivariate analysis for the predictive factors for stress, females were 1.6 times as likely as males to screen positive for stress (95% CI: 1.3-1.9, p<0.001). Younger respondents were more likely to screen positive than older respondents (p<0.05). Respondents who knew someone diagnosed with COVID-19 were more likely to screen positive for stress, OR 1.2 (p=0.034). Those who knew someone who died of COVID-19 were also more likely to screen positive for stress, OR 1.4 (p<0.001). There were no statistically significant differences in depression rates when comparing location of clinical practice, type of hospital, speciality, occupation and deployment status.

On multivariate analysis for the predictive factors for PTSD, females were 1.6 times as likely as males to screen positive for PTSD (95% CI: 1.3-1.9, p<0.001). Respondents with less years of practice were more likely to screen positive than those with more years in practice (p<0.05). Respondents who knew someone diagnosed with COVID-19 were more likely to screen positive for PTSD, OR 1.3 (p=0.03). Those who knew someone who died of COVID-19 were also more likely to screen positive for stress, OR 1.7 (p<0.001). There were no statistically significant differences in depression rates when comparing location of clinical practice, type of hospital, speciality, occupation and deployment status.

4. Discussion

The recent tragedies of Dr Lorna Breen and Yelena Nepomnyashchaya have cast a spotlight on mental health issues amongst doctors battling COVID-19 on the frontlines. Moral injury,

previously described by Litz et. al,[15] refers to the deleterious impact on military personnel when they fail to prevent, or simply watch, things that go against their sense of morality and identity. The lack of manpower, personal protective equipment and social support may have culminated moral injury, resulting in the premature termination of young promising lives. Medical health professionals have a higher suicide rate compared to that of the general public[16], and they are at an even more vulnerable position in the time of the COVID-19 pandemic[17].

The pandemic unearthed casualties on a scale rarely seen in recent times[18]. With the disruption of daily routines, halting of economies, diversion of personnel and resources to the healthcare frontline, one would be hard-pressed not to recognise the similarities between COVID-19 and combative warfare. It is estimated that within the United States, the deaths from COVID-19 has surpassed the deaths from armed conflict in recent times. There is sufficient literature documenting the psychological traumas of war, including that of the resolution of the World Health Assembly in 2005, which estimated that "10% of the people who experience traumatic events will have serious mental health problems and another 10% will develop behaviour that will hinder their ability to function effectively. The most common conditions are depression, anxiety and psychosomatic problems such as insomnia, or back and stomach aches"[19].

This is the first psychological health survey of surgical providers during COVID-19 pandemic, with a vigorous complete response rate of 79.2%. Our study sheds light on the acute stress brought about by the COVID-19 experience and the relevant psychological morbidities. Additional survey variables also reveal demographics that may be at greater risk of depression, anxiety, stress and post-traumatic stress disorder. These included gender, age, years of clinical practice as well as proximity to known COVID-19 cases. Interestingly, specialties which seemed the most affected involved those who operate in the head and neck and thorax region, such as neurosurgery, ophthalmology and cardiothoracic surgery. Ear Nose Throat surgery did not follow this trend notably, with recorded lower rates of psychological conditions. This may be due to a multitude of reasons, such as better baseline preparation for airway precautions and management, support and dedicated personal protective equipment for this specialty which may be deemed at high risk of exposure to respiratory aerosol.

With this knowledge of the pandemic's impact on surgical providers, there is impetus to identify those at risk of developing psychological morbidities and provide assistance. It should be noted that the DASS-21 is a screening tool for emotional and not a categorical measure of clinical diagnoses[20]. There is a demonstrated discrepancy between those who screened positive versus those who obtained formal diagnoses. Approximately 25-33% screened positive for depression, anxiety and stress on DASS-21 but only 6.9% sought psychological help. 4% were formally diagnosed since COVID started. Underutilisation of mental health care facilities has also similarly been established in other settings[21-24], such as spousal abuse victims and asylum seekers. Despite being healthcare providers, less than half of the survey respondents reported readily available and accessible mental health facilities at their workplaces. With resources diverted to address the physical toll of the COVID-19 pandemic, it is often easy to overlook the detrimental effects the pandemic has on the mental health[25, 26]. Interventions to reduce the stigma of seeking help and improving access to counsellors and mental health facilities should be implemented.

Our study also found that age was a protective factor when screening for psychological health conditions. There may be a multitude of reasons for this, such as younger doctors having more stress due to higher workload, increased uncertainty about training and disruptions to career progression due to COVID-19, as well as the presence of younger families. The apprenticeship model[27, 28] has been a long-adopted system for surgical training. This model may also lend structure to the support system for the younger surgical trainees, with the teachers sharing career advice and coping mechanisms with their disciples. A cultural change is also overdue to address negative perceptions of seeking help amongst both colleagues and seniors. The COVID-19 pandemic may have mental health impact outlasting its course, and may overwhelm many individuals' coping mechanisms. The experience and emotional resilience of older surgical providers may provide part of the answer to this burgeoning emergence of symptoms following traumatic stress exposure[29].

On a similar vein, the knowledge of someone who has died from COVID-19 and the absence of taking leave, vacation or otherwise, also contributes to an increased risk of psychological morbidity, based on our logistic regression analysis. Actively seeking out these factors in the risk stratification of healthcare workers may help to refine the approach to improving the mental health of the professionals as a whole. In addition, interventions to reduce the stigma of seeking help and improving access to counsellors and online mental health resources (e.g. online mindfulness-based therapy) should be implemented. These would include the

provision of psychologically safe spaces, education of self-care strategies, and management of emotions[30].

There were various limitations of this study. A majority of respondents were from the Europe and Asia continents. With unique stressors originating from each continent, the lack of representation from the United States of America may have resulted in an underestimation of the psychological health conditions, especially since the United States of America currently accounts for the highest number of COVID-19 cases and deaths worldwide. Moreover, the doctors who might have been truly pre-occupied with the COVID-19 response might not have had the opportunity to respond to this survey. Comparisons of mental stress incidence between individual countries was not performed due to non-uniform distribution of responses between countries.

Furthermore, this survey tool was only available in English, hence it might have limited responses from certain non-English literate countries. In addition, this was a cross sectional survey which captured a snapshot of the acute stress experienced by healthcare professionals. It was unable to reflect fluctuations which may be sensitive to the changing levels of COVID-19 outbreak.

In summary, surgical personnel demonstrated noteworthy levels of psychological trauma. Our findings suggest that being versed in the care of COVID-19 patients does not render one immune to the stresses of battling the disease on the frontline. The potential long drawn-out nature of the war against COVID-19 may also exacerbate the development of psychological disorders. The long-term impact of this ongoing traumatic event underscores the value of longitudinal and mental health care for healthcare personnel, with particular attention to those who know of someone diagnosed with, or who died of COVID-19.

References

1. Teoh JY, Ong WLK, Gonzalez-Padilla D, Castellani D, Dubin JM, Esperto F, et al. A Global Survey on the Impact of COVID-19 on Urological Services. Eur Urol. 2020;78(2):265-75.
2. Ong WLK, Lechmiannandan S, Loeb S, Teoh JY. Urologic Services in Public Hospitals Suffered a Greater Detriment Than Private Hospitals During the Battle of COVID-19. Urology. 2020.
3. Chan SM, Ma TW, Ka-Chun Chong M, Chan DL, Ng EKW, Chiu PWY. A proof of concept study: Esophagogastroduodenoscopy is an aerosol-generating procedure and

continuous oral suction during the procedure reduces the amount of aerosol generated. Gastroenterology. 2020.
4. de Leeuw RA, Burger NB, Ceccaroni M, Zhang J, Tuynman J, Mabrouk M, et al. COVID-19 and Laparoscopic Surgery: Scoping Review of Current Literature and Local Expertise. JMIR Public Health Surveill. 2020;6(2):e18928.
5. Nguyen LH, Drew DA, Graham MS, Joshi AD, Guo CG, Ma W, et al. Risk of COVID-19 among front-line health-care workers and the general community: a prospective cohort study. Lancet Public Health. 2020.
6. Yang MC, Hung PP, Wu YK, Peng MY, Chao YC, Su WL. A three-generation family cluster with COVID-19 infection: should quarantine be prolonged? Public Health. 2020;185:31-3.
7. Tan BYQ, Chew NWS, Lee GKH, Jing M, Goh Y, Yeo LLL, et al. Psychological Impact of the COVID-19 Pandemic on Health Care Workers in Singapore. Ann Intern Med. 2020;173(4):317-20.
8. Temsah MH, Al-Sohime F, Alamro N, Al-Eyadhy A, Al-Hasan K, Jamal A, et al. The psychological impact of COVID-19 pandemic on health care workers in a MERS-CoV endemic country. J Infect Public Health. 2020;13(6):877-82.
9. Normand SL, McNeil BJ, Peterson LE, Palmer RH. Eliciting expert opinion using the Delphi technique: identifying performance indicators for cardiovascular disease. Int J Qual Health Care. 1998;10(3):247-60.
10. Lovibond PF, Lovibond SH. The structure of negative emotional states: comparison of the Depression Anxiety Stress Scales (DASS) with the Beck Depression and Anxiety Inventories. Behav Res Ther. 1995;33(3):335-43.
11. Christianson S, Marren J. The Impact of Event Scale - Revised (IES-R). Medsurg Nurs. 2012;21(5):321-2.
12. Grossman RC, Mackenzie CS, Keller DS. #SoMe4Surgery: from inception to impact. BMJ Innovations. 2020;6:72-82.
13. Ioannidis A, Blanco-Colino R, Chand M, Pellino G, Nepogodiev D, Wexner SD, et al. How to make an impact in surgical research: a consensus summary from the #SoMe4Surgery community. Updates Surg. 2020.
14. Eurosurveillance Editorial T. Latest assessment on COVID-19 from the European Centre for Disease Prevention and Control (ECDC). Euro Surveill. 2020;25(8).
15. Litz BT, Stein N, Delaney E, Lebowitz L, Nash WP, Silva C, et al. Moral injury and moral repair in war veterans: a preliminary model and intervention strategy. Clin Psychol Rev. 2009;29(8):695-706.
16. Dutheil F, Aubert C, Pereira B, Dambrun M, Moustafa F, Mermillod M, et al. Suicide among physicians and health-care workers: A systematic review and meta-analysis. PLoS One. 2019;14(12):e0226361.
17. Serafini G, Parmigiani B, Amerio A, Aguglia A, Sher L, Amore M. The psychological impact of COVID-19 on the mental health in the general population. QJM. 2020.
18. Haider, II, Tiwana F, Tahir SM. Impact of the COVID-19 Pandemic on Adult Mental Health. Pak J Med Sci. 2020;36(COVID19-S4):S90-S4.
19. World Health O. Report to the 58th World Health Assembly: Health Action in Relation to Crises and Disasters. Prehosp Disaster Med. 2005;20(6):487-90.
20. Nieuwenhuijsen K, de Boer AG, Verbeek JH, Blonk RW, van Dijk FJ. The Depression Anxiety Stress Scales (DASS): detecting anxiety disorder and depression in employees absent from work because of mental health problems. Occup Environ Med. 2003;60 Suppl 1:i77-82.

21. Augsberger A, Yeung A, Dougher M, Hahm HC. Factors influencing the underutilization of mental health services among Asian American women with a history of depression and suicide. BMC Health Serv Res. 2015;15:542.
22. Chen H, Ma F. Underutilization of Mental Health Services among American Elders: Two-Dimensional Policy Evaluation Model. Soc Work Public Health. 2019;34(6):457-67.
23. Lichtenthal WG, Nilsson M, Kissane DW, Breitbart W, Kacel E, Jones EC, et al. Underutilization of mental health services among bereaved caregivers with prolonged grief disorder. Psychiatr Serv. 2011;62(10):1225-9.
24. Mackenzie CS, Gekoski WL, Knox VJ. Age, gender, and the underutilization of mental health services: the influence of help-seeking attitudes. Aging Ment Health. 2006;10(6):574-82.
25. Banna MHA, Sayeed A, Kundu S, Christopher E, Hasan MT, Begum MR, et al. The impact of the COVID-19 pandemic on the mental health of the adult population in Bangladesh: a nationwide cross-sectional study. Int J Environ Health Res. 2020:1-12.
26. El-Zoghby SM, Soltan EM, Salama HM. Impact of the COVID-19 Pandemic on Mental Health and Social Support among Adult Egyptians. J Community Health. 2020;45(4):689-95.
27. Butler BA, Butler CM, Peabody TD. Cognitive Apprenticeship in Orthopaedic Surgery: Updating a Classic Educational Model. J Surg Educ. 2019;76(4):931-5.
28. Mariette C. [Apprenticeship in laparoscopic surgery: Tools and methods for the surgeon in training]. J Chir (Paris). 2006;143(4):221-5.
29. Gooding PA, Hurst A, Johnson J, Tarrier N. Psychological resilience in young and older adults. Int J Geriatr Psychiatry. 2012;27(3):262-70.
30. Blake H, Bermingham F, Johnson G, Tabner A. Mitigating the Psychological Impact of COVID-19 on Healthcare Workers: A Digital Learning Package. Int J Environ Res Public Health. 2020;17(9).
