# Supplementary material for: Mental health status and related factors influencing healthcare workers during the COVID-19 pandemic: A systematic review and meta-analysis
Source: PLoS One. 2024 Jan 19;19(1):e0289454. doi: 10.1371/journal.pone.0289454 (PMC10798549; doi:10.1371/journal.pone.0289454)
Supplement: S1 Data — (ZIP) [file pone.0289454.s011.zip › literatures/19.pdf]

It is illegal to post this copyrighted PDF on any website.

Occupational Risk Factors and Mental Health Among Frontline Health Care Workers in a Large US Metropolitan Area During the COVID-19 Pandemic

Alison G. Cahill, MD, MSCI[a,*]; Megan E. Olshavsky, PhD[b]; D. Jeffrey Newport, MD, MS, MDiv[a,c]; Justin Benzer, PhD[c]; Kelly M. Chambers, RN, BSN[d]; James Custer, MS[e]; Paul J. Rathouz, PhD[e]; Stephanie Nutt, MA, MPA[a]; Sama Jwaied, BS[f]; Ryan Leslie, PhD[b]; and Elizabeth C. Matsui, MD, MHS[e]

ABSTRACT

Objective: To assess depression, anxiety, and burnout among health care workers using well-established validated scales and to examine associations of these mental health outcomes with personal protective equipment (PPE) and high-risk patient contact.

Methods: This prospective survey was conducted between August and October 2020 among 970 essential health care workers from 2 health systems in central Texas. The survey captured basic demographic, occupational, and baseline health information including history of mental health disorders. Depression, anxiety, and burnout were assessed with the 8-item Patient Health Questionnaire, 7-item Generalized Anxiety Disorder Scale, and 23-item Burnout Assessment Tool. Questions about clinical contact with patients with suspected or known COVID-19 were also incorporated.

Results: Approximately 24% of respondents had moderate or severe anxiety, 14% had moderate or severe depression, and 7% were at high risk for burnout. Statistically significant associations were found between perceived PPE adequacy and the 3 mental health outcomes, while accounting for age, gender, and education. Hours of contact with COVID-19 patients during aerosolizing procedures was positively correlated with measures of anxiety, burnout, and depression after adjustment for age, gender, and occupational role. Perception of PPE adequacy was inversely correlated with measures of depression, anxiety, and burnout among essential members of 2 health care systems, whose roles precluded working remotely during the pandemic.

Conclusion: This study highlights the correlations of perceptions of PPE adequacy and contact hours with COVID-19 patients undergoing aerosolizing procedures and employee mental well-being. Future work confirming the findings can help identify ways that systems can support their employees through similarly stressful and demanding events.

Prim Care Companion CNS Disord 2022;24(2):21m03166

To cite: Cahill AG, Olshavsky ME, Newport DJ, et al. Occupational risk factors and mental health among frontline health care workers in a large US metropolitan area during the COVID-19 pandemic. *Prim Care Companion CNS Disord.* 2022;24(2):21m03166.

To share: https://doi.org/10.4088/PCC.21m03166

© Copyright 2022 Physicians Postgraduate Press, Inc.

[a]Department of Women's Health, University of Texas at Austin, Dell Medical School, Austin, Texas
[b]Ascension, St Louis, Missouri
[c]Department of Psychiatry and Behavioral Sciences, University of Texas at Austin, Dell Medical School, Austin, Texas
[d]Department of Surgery and Perioperative Care, University of Texas at Austin, Dell Medical School, Austin, Texas
[e]Department of Population Health, University of Texas at Austin, Dell Medical School, Austin, Texas
[f]University of Texas at Austin, Austin, Texas
**Corresponding author:* Alison G. Cahill, MD, MSCI, Department of Women's Health, University of Texas at Austin, Dell Medical School, 1601 Trinity St, Austin, TX 78712 (alison.cahill@austin.utexas.edu).

The psychological strain on essential health care workers during the coronavirus disease 2019 (COVID-19) pandemic has been well documented in both the lay press and the scientific literature. Research from multiple countries has documented high burden of anxiety, depression, burnout, posttraumatic stress disorder (PTSD), and other measures of psychosocial stress among health care workers during the pandemic.[1] The literature is substantial enough to support the conduct of systematic reviews and meta-analyses,[1,2] which have documented 20%–30% pooled prevalence estimates for anxiety and depression.

Prior work on the psychological impact of pandemics on health care workers has been from lessons learned during influenza pandemics and prior outbreaks of earlier coronaviruses.[3–5] Existing literature to date on the impact of the COVID-19 pandemic has focused primarily on health care workers in countries outside of the United States, and the few studies among frontline health care workers in the United States estimated mental health outcome prevalence only during the earlier months of the pandemic.[6–9] Because there is scant information about the mental health toll among US frontline health care workers over the subsequent months of the pandemic, it is unclear whether the mental health distress documented in the earlier days of the pandemic has persisted throughout. In addition, the international literature has identified occupational risk factors for worse mental health, including reuse of personal protective equipment (PPE),[10] yet there is limited literature on occupational mental health risk factors among US health care workers.

We therefore conducted a survey of frontline health care workers from 2 health systems in central Texas to assess depression, anxiety, and burnout using well-established validated scales and to examine associations of these mental health outcomes with PPE and high-risk patient contact.

METHODS

Study Population

Staff, both clinical and nonclinical, of 2 health care systems in Texas were invited via e-mail to participate

You are prohibited from making this PDF publicly available.

For reprints or permissions, contact permissions@psychiatrist.com. ♦ © 2022 Copyright Physicians Postgraduate Press, Inc.

It is illegal to post this copyrighted PDF on any website.

Clinical Points

- Perceptions of adequacy of personal protective equipment can impact health care worker mental health.
- Number of hours in contact with patients with COVID-19 during aerosolizing procedures is correlated with health care worker anxiety.

in an online survey. E-mail invitations were sent in 4 waves between August and October 2020. Individuals who had not completed the survey received reminder e-mails 8 days after the first invitation and then again 8 days after that if they did not respond. A total of 17,125 invitations were sent, and 970 individuals completed at least part of the survey. Study team members were blind to participant identifiers and employers, which was explained to potential participants in the cover letter. The study was submitted to the University of Texas at Austin Institutional Review Board and was determined to be exempt.

Survey

The survey captured basic demographic, occupational, and baseline health information including a history of mental health disorders, in addition to the validated mental health scales described here.

Baseline PTSD symptoms were assessed using the PTSD Checklist for *DSM-5* (PCL-5).[11] The survey was available in both English and Spanish; 99.8% completed the English version and 0.2% completed the Spanish version.

Depression was assessed with the 8-item Patient Health Questionnaire (PHQ-8),[12] which is a validated diagnostic and severity assessment tool for depression in adults. Scores range from 0 to 20, with cut points for no depression, as well as mild, moderate, and severe depression of < 5, 5–9, 10–14, and ≥ 15, respectively.

Generalized anxiety was assessed with the 7-item Generalized Anxiety Disorder Scale (GAD-7),[13] which is a validated screening tool for generalized anxiety disorder. Scores < 5, 5–9, 10–14, and ≥ 15 are indicative of no, mild, moderate, and severe anxiety, respectively.

Burnout was evaluated using the 23-item Burnout Assessment Tool (BAT-23),[14] which is a validated tool for assessing risk of problematic burnout. Scores of 1–2.58, 2.59–3.01, and 3.02–5.0 represent no risk, at risk, and at very high risk of burnout.

Questions about clinical contact with patients with suspected or known COVID-19 were incorporated, including the number of hours in contact with such patients in the past week. Questions about provision of PPE and confidence in the adequacy of PPE were also included. These questions were as follows:

> "During the past 2 weeks at work, were you provided personal protective equipment?" Response options were 5-point Likert-type responses ranging from "always" to "never."

> "During the past 2 weeks at work, do you believe the provided personal protective equipment was adequate? Response options were 5-point Likert-type responses ranging from "very confident" to "very doubtful." There was also an option for "not applicable."

Statistical Analyses

Descriptive statistics were generated for participant demographics and characteristics. Both single and multiple regression models were fitted to the data to estimate associations of each of 3 mental health outcome scales (PHQ-8, GAD-7, and BAT-23) to reported confidence in adequacy of PPE and reported contact hours in the past week with COVID patients undergoing aerosol-generating procedures. The contact hours predictor variable was log-base-2 transformed so that the fitted coefficient represents the expected proportional increase in the mental health response variable for each doubling of the predictor. All regression analyses used a generalized linear density ratio model with identity link[15,16] fitted to the data for each of the 6 pairs of outcome and primary predictor. This model yields identical interpretations of regression coefficients as those in a standard linear regression model, with more statistically robust hypothesis tests and confidence intervals. Four different models were fitted to each outcome and primary predictor pair. First, a single predictor model tested for a linear trend between the outcomes and predictors of interest. Multiple regression models were fitted sequentially to test whether the addition of more adjusters modified the relationship of the outcome to the primary predictor. Model 1 included age, gender, and education as adjusters. Then, participant role and history of emotional or mental health problems were added subsequently in models 2 and 3, respectively.

Gender was categorized as male, female, and other, which included transgender male and female, nonbinary, other, and "prefer not to answer" responses. Race included White, Asian, more than 1 race, Black or African American, and other, which included American Indian or Alaska Native, Native Hawaiian or Other Pacific Islander, and "prefer not to answer" responses. For the mental health scales (PHQ-8, GAD-7, and BAT-23), if a participant completed only part of a particular scale, missing values were imputed by random sampling from the distribution of non-missing responses for that question; such item-level missingness was relatively rare. For the PHQ-8, GAD-7, and BAT-23, there were only 28, 26, and 59 participants who had at least 1 response imputed for totals of 30, 31, and 136 total item-level responses imputed for each scale, respectively. If a participant did not answer any of the questions for a given mental health scale, missing values were not imputed; rather, that participant was not included in analyses for that scale. Finally, to interpret the results in the setting of the COVID-19 admission census in central Texas, PHQ-8, GAD-7, and BAT-23 scores were plotted against the daily hospitalizations for COVID-19 in the Austin metropolitan

You are prohibited from making this PDF publicly available.

For reprints or permissions, contact permissions@psychiatrist.com. ♦ © 2022 Copyright Physicians Postgraduate Press, Inc.

It is illegal to post this copyrighted PDF on any website.

Table 1. Baseline Characteristics of the Sample

	Total (N = 970)	Patient Contact[a] (n = 716)	No Patient Contact[a] (n = 157)
**Demographic characteristics**			
Age, mean (SD) [n]	41.5 (11.1) [821]	40.7 (10.8) [605]	45.9 (11.3) [138]
Gender, n (%)			
Female	802 (83.5)	593 (83.1)	139 (89.1)
Race, n (%)			
White	784 (81.9)	586 (82.3)	126 (80.8)
Asian	56 (5.9)	47 (6.6)	4 (2.6)
> 1 race	(43) 4.5	34 (4.8)	6 (3.8)
Black or African American	38 (4.0)	21 (2.9)	11 (7.1)
Ethnicity, n (%)			
Hispanic	169 (17.5)	117 (16.3)	29 (18.5)
Education, n (%)			
High school diploma or GED	29 (3.1)	12 (1.7)	11 (7.0)
Some college or 2-year degree	163 (17.4)	119 (16.7)	27 (17.2)
4-year college graduate	276 (29.5)	222 (31.1)	42 (26.8)
Some school beyond college	43 (4.6)	28 (3.9)	13 (8.3)
Graduate or professional degree	426 (45.5)	332 (46.6)	64 (40.8)
**Baseline health conditions, n (%)**			
Emotional or mental health problems such as depression or anxiety	229 (23.6)	169 (23.6)	46 (29.3)
Asthma or other lung problems	149 (15.4)	117 (16.3)	23 (14.6)
Immune disorder	66 (6.8)	46 (6.4)	13 (8.3)
Diabetes or high blood sugar	58 (6.0)	36 (5.0)	16 (10.2)
Heart problems	52 (5.4)	38 (5.3)	14 (8.9)
Cancer	41 (4.2)	26 (3.6)	12 (7.6)
Kidney problems	20 (2.1)	16 (2.2)	3 (1.9)
**Occupational characteristics**			
Role, n (%)			
Nurse	379 (43.0)	321 (48.1)	37 (24.2)
Physician/advanced care practitioner	190 (21.5)	169 (25.3)	9 (5.9)
Administration/administrative support	84 (9.5)	34 (5.1)	45 (29.4)
Occupational/physical/respiratory therapy	41 (4.6)	34 (5.1)	3 (2.0)
Psychology and social work	26 (2.9)	17 (2.5)	7 (4.6)
Laboratory technician	22 (2.5)	9 (1.3)	9 (5.9)
Facilities/IT/security	10 (1.1)	4 (0.6)	4 (2.6)
Paramedic	6 (0.7)	6 (0.9)	0 (0.0)
Hours in contact with patients during initial evaluation of suspected COVID-19 in past week, mean (SD) [n]	…	3.4 (8.6) [691]	…
Hours in contact with patients with suspected or confirmed COVID-19 during aerosol generating procedures in past week, mean (SD) [n]	…	3.3 (9.5) [688]	…
During the <u>past 2 weeks</u> at work, were you provided PPE? [n (%)]			
Always		623 (87.3)	…
Usually	…	65 (9.1)	…
Sometimes	…	18 (2.5)	…
Rarely	…	5 (0.7)	…
Never	…	3 (0.4)	…
During the <u>past 2 weeks</u> at work, do you believe the provided personal protective equipment was adequate? [n (%)]			
Very confident	…	331 (46.5)	…
Somewhat confident	…	276 (38.8)	…
Uncertain	…	43 (6.0)	…
Somewhat doubtful	…	38 (5.3)	…
Very doubtful	…	21 (2.9)	…
Not applicable	…	3 (0.4)	…
**Mental health scales**			
GAD total score, mean (SD) [n]	6.4 (5.3) [838]	6.6 (5.2) [687]	5.8 (5.4) [150]
GAD categories, n (%)			
Minimal anxiety	370 (47.3)	292 (45.7	78 (54.9)
Mild anxiety	232 (29.7)	193 30.2	38 (26.8)
Moderate anxiety	106 (13.6)	94 14.7	12 (8.5)
Severe anxiety	74 (9.5)	60 9.4	14 (9.9)
PHQ total score, mean (SD) [n]	4.5 (4.5) [824]	4.5 (4.5) [675]	4.3 (4.5) [148]
PHQ categories, n (%)			
No depression	541 (66.1)	443 (66.1)	97 (66.0
Mild depression	169 (20.7)	137 (20.4)	32 (21.8)
Moderate depression	77 (9.4)	64 (9.6)	13 (8.8)
Severe depression	31 (3.8)	26 (3.9)	5 (3.4)

(continued)

You are prohibited from making this PDF publicly available.

For reprints or permissions, contact permissions@psychiatrist.com. ♦ © 2022 Copyright Physicians Postgraduate Press, Inc.

It is illegal to post this copyrighted PDF on any website.

Table 1 (continued).

	Total (N = 970)	Patient Contact[a] (n = 716)	No Patient Contact[a] (n = 157)
Mental health scales			
Burnout total score, mean (SD) [n]	1.9 (0.7) [798]	1.9 (0.7) [655]	1.8 (0.7) [142]
Burnout categories			
No risk of burnout	678 (85.0)	551 (84.1)	126 (88.7)
At risk for burnout	65 (8.1)	58 (8.9)	7 (4.9)
Very high risk of burnout	55 (6.9)	46 (7.0)	9 (6.3)
PCL-5, n (%)			
No	747 (91.5)	607 (90.9)	139 (94.6)
Yes	69 (8.5)	61 (9.1)	8 (5.4)

[a]Patient contact was determined by the participants' response to the question "During the past 2 weeks at work, did you come into contact with patients (ie, are you in the same room)?"
Abbreviations: BAT-23 = 23-item Burnout Assessment Tool, COVID-19 = coronavirus disease 2019, GAD-7 = 7-item Generalized Anxiety Disorder scale, PCL-5 = PTSD Checklist for *DSM-5*, PHQ = Patient Health Questionnaire.

Figure 1. Boxplots of PHQ-8, GAD-7, and BAT-23 Scores by Perceived Adequacy of PPE and COVID Patient Contact Hours[a]

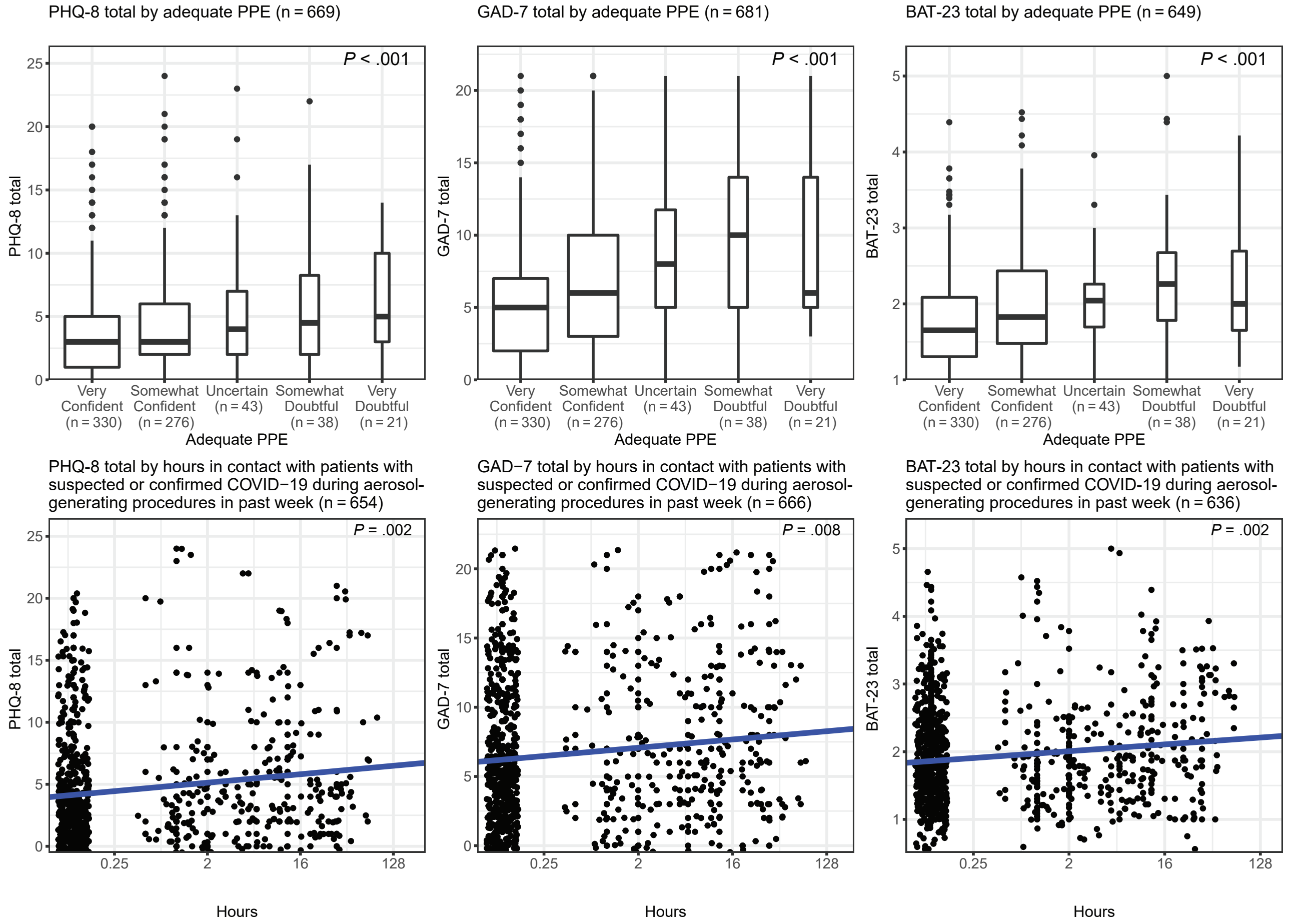

[a]Scatter plots have a jitter of ±0.5 in both the x and y direction.
Abbreviations: BAT-23 = 23-item Burnout Assessment Tool, COVID = coronavirus disease, GAD-7 = 7-item Generalized Anxiety Disorder Scale, PHQ-8 = 8-item Patient Health Questionnaire, PPE = personal protective equipment.

area. All statistical analysis was performed using R Statistical Software version 4.0.2.

RESULTS

A total of 970 people participated in the study. The mean age of the study population was 41.5 years (SD = 11.1), and 83.5% were female (n = 802). The distribution of race was 81.9% White (n = 784), 5.9% Asian (n = 56), 4.5% more than 1 race (n = 43), and 4.0% Black or African American (n = 38), and 17.5% were of Hispanic ethnicity (n = 169). The distribution of participant role was 43.0% nurses (n = 379), 21.5% physicians/advanced care practitioners (n = 190), 9.5% administration/administrative support (n = 84), 4.6%

You are prohibited from making this PDF publicly available.

For reprints or permissions, contact permissions@psychiatrist.com. ♦ © 2022 Copyright Physicians Postgraduate Press, Inc.

It is illegal to post this copyrighted PDF on any website.

Table 2. Associations Between Perceived Adequacy of PPE and COVID Patient Contact Hours and Mental Health Among Frontline Health Care Workers

	Model 1[a]			Model 2[b]			Model 3[c]		
	n	β	CI	n	β	CI	n	β	CI
PPE perceived adequate[d]									
PHQ-8 total score[e]	564	0.52	0.13–0.92	563	0.40	0.01–0.79	563	0.39	0.03–0.76
GAD-7 total score[e]	573	1.01	0.57–1.45	572	1.00	0.55–1.45	572	0.98	0.54–1.42
BAT-23 total score[e]	555	0.12	0.06–0.19	554	0.11	0.05–0.18	554	0.11	0.05–0.17
Contact hours[f]									
PHQ-8 total score[e]	553	0.14	−0.01–0.28	552	0.15	0.01–0.30	552	0.18	0.04–0.31
GAD-7 total score[e]	562	0.18	0.02–0.33	561	0.17	0.01–0.33	561	0.19	0.03–0.34
BAT-23 total score[e]	544	0.02	0.00–0.05	543	0.02	0.00–0.05	543	0.03	0.01–0.05

[a]Model adjusted for age, gender, and education.
[b]Model adjusted for age, gender, education, and role.
[c]Model adjusted for age, gender, education, role, and history of emotional or mental health problems.
[d]Adequate PPE is participant report of PPE adequacy scored from 1 being very confident to 5 being very doubtful.
[e]Mental health scales are the model outcomes.
[f]Contact hours with patients with suspected or confirmed COVID-19 during aerosol-generating procedures ($\log_2$ transformed).
Abbreviations: BAT-23 = 23-item Burnout Assessment Tool, COVID-19 = coronavirus disease 2019, GAD-7 = 7-item Generalized Anxiety Disorder scale, PCL-5 = PTSD Checklist for *DSM-5*, PHQ-8 = 8-item Patient Health Questionnaire, PPE = personal protective equipment.

occupational/physical/respiratory therapy (n = 41), 2.9% psychology and social work (n = 26), 2.5% laboratory technicians (n = 22), 1.1% facilities/IT/security (n = 10), and 0.7% paramedics (n = 6). Approximately 24% had moderate or severe anxiety, 14% had moderate or severe depression, and 7% were at high risk for burnout (Table 1).

Unadjusted analysis showed that the PHQ-8, GAD-7, and BAT-23 all have a statistically monotonic trend with confidence in PPE adequacy ($P < .001$, $P < .001$, $P < .001$, respectively) as well as with reported patient contact hours during aerosol-generating procedures ($P < .002$, $P < .008$, $P < .002$, respectively) (Figure 1).

For the predictor of perceived PPE adequacy (Table 2), model 1, which includes age, gender, and education as adjusters, showed statistically significant associations with all 3 mental health outcomes. The PHQ-8 score, on average, increased by 0.52 (95% CI, 0.13–0.92) for each level difference in perceived PPE inadequacy. The GAD-7 score, on average, increased by 1.01 (95% CI, 0.57–1.45) for each level difference in perceived PPE inadequacy. The BAT-23 score, on average, increased by 0.12 (95% CI, 0.06–0.19) for each level difference in perceived PPE inadequacy. These relationships were not significantly impacted with the sequential addition of health care role and history of emotional or mental health problems as adjuster variables in models 2 and 3 (Supplementary Figure).

The predictor of number of contact hours with patients during aerosol-generating procedures, model 1, which includes age, gender, and education as adjusters, showed statistically significant associations with the GAD-7 and BAT-23 scores (Table 2). On average, a 2:1 relative difference of contact hours was associated with an increase in GAD-7 score by 0.18 (95% CI, 0.02–0.33) and BAT-23 score by 0.02 (95% CI, 0.00–0.05). These relationships were not significantly impacted with the sequential addition of role and history of emotional or mental health problems as adjusters in models 2 and 3. In model 1, PHQ-8 score was not significantly associated with contact hours; however, this relationship was statistically significant in models 2 and 3 with the sequential addition of role and history of emotional or mental health problems as adjusters. Model 2 showed an average PHQ-8 increase of 0.15 (95% CI, 0.01–0.30) associated with a doubling of contact hours. The 3 mental health outcomes were strongly correlated with one another with correlation coefficients between 0.62 and 0.70 (Table 3).

Table 3. Correlation Among Mental Health Scales

Outcome	PHQ-8 Total Score	GAD-7 Total Score	BAT-23 Total Score
PHQ-8 total score	1.00	0.70	0.69
GAD-7 total score	0.70	1.00	0.62
BAT-23 total score	0.69	0.62	1.00

Abbreviations: BAT-23 = 23-item Burnout Assessment Tool, GAD-7 = 7-item Generalized Anxiety Disorder scale, PHQ-8 = 8-item Patient Health Questionnaire.

Finally, to interpret the results in the setting of the COVID-19 admission census in central Texas, PHQ-8, GAD-7, and BAT-23 scores were plotted against the daily hospitalizations for COVID-19 in the Austin metropolitan area (Figure 2).

DISCUSSION

Overall, the prevalence of moderate to severe anxiety was similar in this study population to that reported previously in other populations, but the prevalence rates of depression and burnout were lower. In addition, perception of PPE adequacy was inversely correlated with measures of depression, anxiety, and burnout among essential members of 2 health care systems whose roles precluded working remotely during the pandemic. These associations persisted even when adjusting for role in the health care system and level of education, as well as accounting for reported baseline mental health disorders preceding the pandemic. In addition, hours of contact with COVID-19 patients during aerosolizing procedures was positively correlated with measures of anxiety and burnout in all models and with depression after adjustment for age, gender, and occupational role. Among participants in our study, the

You are prohibited from making this PDF publicly available.

For reprints or permissions, contact permissions@psychiatrist.com. ♦ © 2022 Copyright Physicians Postgraduate Press, Inc.

It is illegal to post this copyrighted PDF on any website.

Figure 2. Scatterplots and Fitted Curves of Daily COVID Admissions in Austin Metropolitain Statistical Area vs Date and (A) PHQ–8 Scores, (B) GAD–7 Scores, and (C) BAT–23 Scores vs Date[a,b,c]

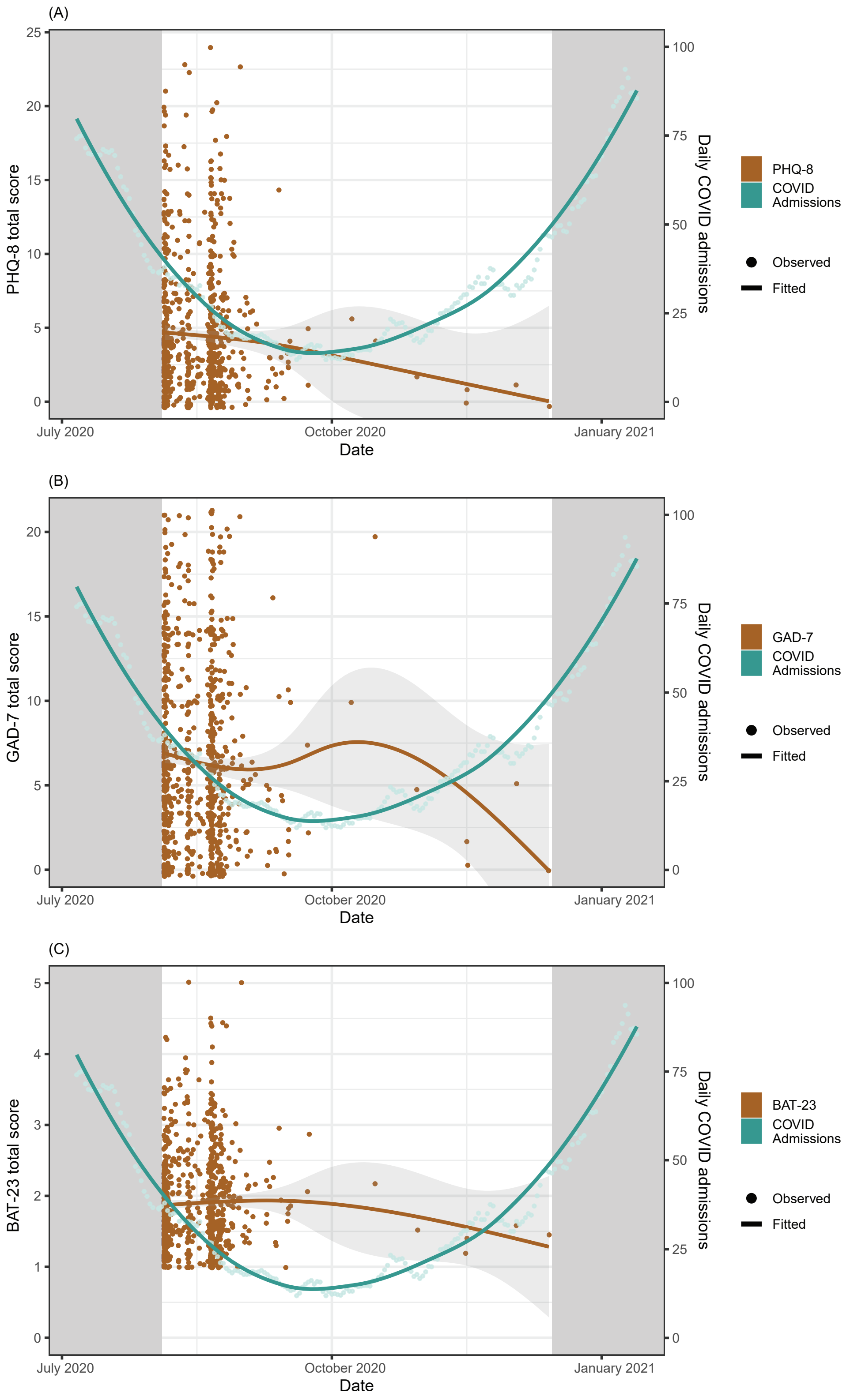

[a]Mental health outcome points are jittered to visualize response dispersion.
[b]Mental health outcome curve fitted by linear regression of outcome on a cubic natural spline of date with internal knots at 9/17/2020 and 09/30/2020.
[c]COVID admissions curve fitted by linear lowess smoothing.
Abbreviations: BAT-23 = 23-item Burnout Assessment Tool, COVID = coronavirus disease, GAD-7 = 7-item Generalized Anxiety Disorder scale, PHQ-8 = 8-item Patient Health Questionnaire.

You are prohibited from making this PDF publicly available.

For reprints or permissions, contact permissions@psychiatrist.com. ♦ © 2022 Copyright Physicians Postgraduate Press, Inc.

It is illegal to post this copyrighted PDF on any website.

majority were female, and the most common roles in the health care system were nurses, physicians, and advanced practitioners. However, we considered the health care system as a whole and defined essential workers broadly to include other essential roles such as facilities support and administration, which few prior studies have done.

An early study[17] of health care workers in China in January–February 2020 across 34 hospitals reported significant psychological burden during the COVID-19 pandemic. The authors[17] described evidence of depression, insomnia, and distress, particularly among nurses. A recent meta-analysis[2] of 25 cross-sectional studies found that nurses from around the world showed high rates of poor mental health outcomes, including depression, anxiety, and stress during the pandemic. However, the authors[2] were unable to comment on the impact of the pandemic on burnout because it was understudied. Our study similarly demonstrated high rates of depression and anxiety, as well as burnout, not only among nurses but also among workers in other essential health care roles.

A study[18] of health care workers during May 2020 explored occupational risks of adverse psychiatric outcomes in the northeast and southern United States. The researchers[18] found that providing care for inpatients or in the emergency department was a risk factor for COVID-19 infection, as well as depression, anxiety, and burnout, but they did not explore the impact of perceived PPE adequacy or contact with COVID-19 patients during aerosolizing procedures on mental health. A global study[19] of health care workers examined occupational factors that contributed to burnout. The authors[19] found that reported adequate PPE was only 54.8%, but that adequacy was positively correlated with a reduction in risk of burnout. However, only 30.8% of participants were from the United States, and the study was conducted in April 2020, which was early in the pandemic's impact on the United States. This study[19] also did not examine exposure to aerosolizing procedures as a mental health risk factor. A study[20] of 55 health care workers in March to April 2020 in urban Washington, DC identified lack of PPE as a risk factor for increased stress and burnout. As the pandemic continued into the summer of 2020 in the United States, PPE became more widely available to essential health care workers.[20]

In a large tertiary care hospital in New York, New York, during the initial COVID-19 surge, frontline health care workers reported that perceived support from hospital leadership reduced the psychological impact of the pandemic.[21] Similarly, a Cochrane meta-analysis[22] of 16 studies of factors impacting the mental well-being of essential workers during disease epidemics highlighted the importance of effective communication from organizations for whom they worked. The majority of participants in our study reported that they felt somewhat confident or very confident that their PPE was adequate, despite that it was conducted before the availability of COVID-19 vaccines in central Texas. Given the literature indicating the important role of workplace characteristics such as perceived support and effective communication in mitigating the mental health impact of COVID-19 on health care workers, we hypothesize that these findings may be due, at least in part, to the responses of these health care systems. For example, the health care systems prioritized providing PPE to their essential workers when there were national shortages and communicated regularly about ways their employees were being cared for including access to PPE and ongoing planning. These health systems also rapidly developed systems to train essential workers in the proper use of PPE. Additional studies examining the steps health care systems can take to mitigate the mental health toll on health care workers are needed to develop a better understanding of how health care systems can most effectively support essential workers during a pandemic.

Our study contributes to the existing literature regarding mental health of health care workers during the COVID-19 pandemic in the United States. The broad definition of essential workers allowed us to consider the impact on many members of the health care team, not just patient-facing members. The protections of participant privacy were put in place to encourage participation and honesty. Finally, we used validated tools to measure depression, anxiety, and burnout. There are also potential limitations to consider. Despite our attempt to survey across health care roles, most participants were patient-facing providers, and, thus, generalizability may be limited. While a large number of employees responded, they only represented 5.6% of the potential participants. Finally, in an effort to limit participant burden, we reduced the number of questions, but by doing so we were unable to ask about several other areas of interest including shifting roles and specific health care units (ie, intensive care unit) that might have impacted mental health.

Despite these potential limitations, this study contributes to our understanding of the impact of the COVID-19 pandemic on the mental health of essential health care workers. It also highlights the correlations of perceptions of PPE adequacy and contact hours with COVID-19 patients undergoing aerosolizing procedures and employee mental well-being. Future work confirming our findings across a diverse array of essential health care roles can help identify ways that systems can support their employees through similarly stressful and demanding events.

Submitted: October 1, 2021; accepted January 10, 2022.

Published online: March 8, 2022.

Relevant financial relationships: **Dr Newport** has received research support from Eli Lilly, GlaxoSmithKline, Janssen, the National Alliance for Research on Schizophrenia and Depression, the National Institutes of Health, Sage Therapeutics, Takeda, the Texas Health & Human Services Commission, and Wyeth; has served on speakers' bureaus and/or received honoraria from AstraZeneca, Eli Lilly, GlaxoSmithKline, Pfizer, and Wyeth; and has served on advisory boards for GlaxoSmithKline, Janssen, and Sage Therapeutics. The efforts of **Dr Rathouz** and **Mr Custer** on this project were supported by core funds of the Dell Medical School at the University of Texas at Austin. **Drs Cahill**, **Olshavsky**, **Benzer**, **Leslie**, and **Matsui** and **Mss Chambers**, **Nutt**, and **Jwaied** report no financial relationships relevant to the subject of this article.

Funding/support: None.

Supplementary material: See accompanying pages.

You are prohibited from making this PDF publicly available.

For reprints or permissions, contact permissions@psychiatrist.com. ♦ © 2022 Copyright Physicians Postgraduate Press, Inc.

It is illegal to post this copyrighted PDF on any website.

REFERENCES

1. Li Y, Scherer N, Felix L, et al. Prevalence of depression, anxiety and posttraumatic stress disorder in health care workers during the COVID-19 pandemic: a systematic review and meta-analysis. *PLoS One*. 2021;16(3):e0246454.
2. Varghese A, George G, Kondaguli SV, et al. Decline in the mental health of nurses across the globe during COVID-19: a systematic review and meta-analysis. *J Glob Health*. 2021;11:05009.
3. Barello S, Falcó-Pegueroles A, Rosa D, et al. The psychosocial impact of flu influenza pandemics on healthcare workers and lessons learnt for the COVID-19 emergency: a rapid review. *Int J Public Health*. 2020;65(7):1205–1216.
4. Kisely S, Warren N, McMahon L, et al. Occurrence, prevention, and management of the psychological effects of emerging virus outbreaks on healthcare workers: rapid review and meta-analysis. *BMJ*. 2020;369:m1642.
5. Carmassi C, Foghi C, Dell'Oste V, et al. PTSD symptoms in healthcare workers facing the three coronavirus outbreaks: what can we expect after the COVID-19 pandemic. *Psychiatry Res*. 2020;292:113312.
6. Hennein R, Mew EJ, Lowe SR. Socioecological predictors of mental health outcomes among healthcare workers during the COVID-19 pandemic in the United States. *PLoS One*. 2021;16(2):e0246602.
7. Benfante A, Di Tella M, Romeo A, et al. Traumatic stress in healthcare workers during COVID-19 pandemic: a review of the immediate impact. *Front Psychol*. 2020;11:569935.
8. Evanoff BA, Strickland JR, Dale AM, et al. Work-related and personal factors associated with mental well-being during the COVID-19 response: survey of health care and other workers. *J Med Internet Res*. 2020;22(8):e21366.
9. Greene T, Harju-Seppänen J, Adeniji M, et al. Predictors and rates of PTSD, depression and anxiety in UK frontline health and social care workers during COVID-19. *Eur J Psychotraumatol*. 2021;12(1):1882781.
10. Kea B, Johnson A, Lin A, et al. An international survey of healthcare workers use of personal protective equipment during the early stages of the COVID-19 pandemic. *J Am Coll Emerg Physicians Open*. 2021;2(2):e12392.
11. Weathers FW, Litz BT, Keane TM, et al. The PTSD Checklist for *DSM-5* (PCL-5). National Center for PTSD. US Dept of Veterans Affairs website. Accessed January 27, 2022. www.ptsd.va.gov
12. Kroenke K, Strine TW, Spitzer RL, et al. The PHQ-8 as a measure of current depression in the general population. *J Affect Disord*. 2009;114(1-3):163–173.
13. Spitzer RL, Kroenke K, Williams JBW, et al. A brief measure for assessing generalized anxiety disorder: the GAD-7. *Arch Intern Med*. 2006;166(10):1092–1097.
14. Schaufeli WB, Desart S, De Witte H. Burnout Assessment Tool (BAT)-development, validity, and reliability. *Int J Environ Res Public Health*. 2020;17(24):9495.
15. Rathouz PJ, Gao L. Generalized linear models with unspecified reference distribution. *Biostatistics*. 2009;10(2):205–218.
16. Wurm MJ, Rathouz PJ. Semiparametric generalized linear models with the gldrm package. *R J*. 2018;10(1):288–307.
17. Lai J, Ma S, Wang Y, et al. Factors associated with mental health outcomes among health care workers exposed to coronavirus disease 2019. *JAMA Netw Open*. 2020;3(3):e203976.
18. Firew T, Sano ED, Lee JW, et al. Protecting the front line: a cross-sectional survey analysis of the occupational factors contributing to healthcare workers' infection and psychological distress during the COVID-19 pandemic in the USA. *BMJ Open*. 2020;10(10):e042752.
19. Morgantini LA, Naha U, Wang H, et al. Factors contributing to healthcare professional burnout during the COVID-19 pandemic: a rapid turnaround global survey. *PLoS One*. 2020;15(9):e0238217.
20. Norful AA, Rosenfeld A, Schroeder K, et al. Primary drivers and psychological manifestations of stress in frontline healthcare workforce during the initial COVID-19 outbreak in the United States. *Gen Hosp Psychiatry*. 2021;69:20–26.
21. Feingold JH, Peccoralo L, Chan CC, et al. Psychological impact of the COVID-19 pandemic on frontline health care workers during the pandemic surge in New York City. *Chronic Stress (Thousand Oaks)*. 2021;5:2470547020977891.
22. Pollock A, Campbell P, Cheyne J, et al. Interventions to support the resilience and mental health of frontline health and social care professionals during and after a disease outbreak, epidemic or pandemic: a mixed methods systematic review. *Cochrane Database Syst Rev*. 2020;11:CD013779.

Supplementary material follows this article.

You are prohibited from making this PDF publicly available.

For reprints or permissions, contact permissions@psychiatrist.com. ♦ © 2022 Copyright Physicians Postgraduate Press, Inc.

THE PRIMARY CARE COMPANION FOR CNS DISORDERS

Supplementary Material

Article Title: Occupational Risk Factors and Mental Health Among Frontline Health Care Workers in a Large US Metropolitan Area During the COVID-19 Pandemic

Author(s): Alison G. Cahill, MD, MSCI; Megan E. Olshavsky, PhD; D. Jeffrey Newport, MD, MS, MDiv; Justin Benzer, PhD; Kelly M. Chambers, RN, BSN; James Custer, MS; Paul J. Rathouz, PhD;
Stephanie Nutt, MA, MPA; Sama Jwaied; Ryan Leslie, PhD; and Elizabeth C. Matsui, MD, MHS

DOI Number: https://doi.org/10.4088/PCC.21m03166

List of Supplementary Material for the article

1. Supplementary Figure. Boxplots of PHQ-8, GAD-7, BAT-23 Scores by Perceived Adequacy of PPE and COVID Patient Contact Hours

Disclaimer

This Supplementary Material has been provided by the author(s) as an enhancement to the published article. It has been approved by peer review; however, it has undergone neither editing nor formatting by in-house editorial staff. The material is presented in the manner supplied by the author.

It is illegal to post this copyrighted PDF on any website. ♦ © 2022 Copyright Physicians Postgraduate Press, Inc.
© Copyright 2022 Physicians Postgraduate Press, Inc.

Supplementary Figure. Boxplots of PHQ-8, GAD-7, BAT-23 Scores by Perceived Adequacy of PPE and COVID Patient Contact Hours

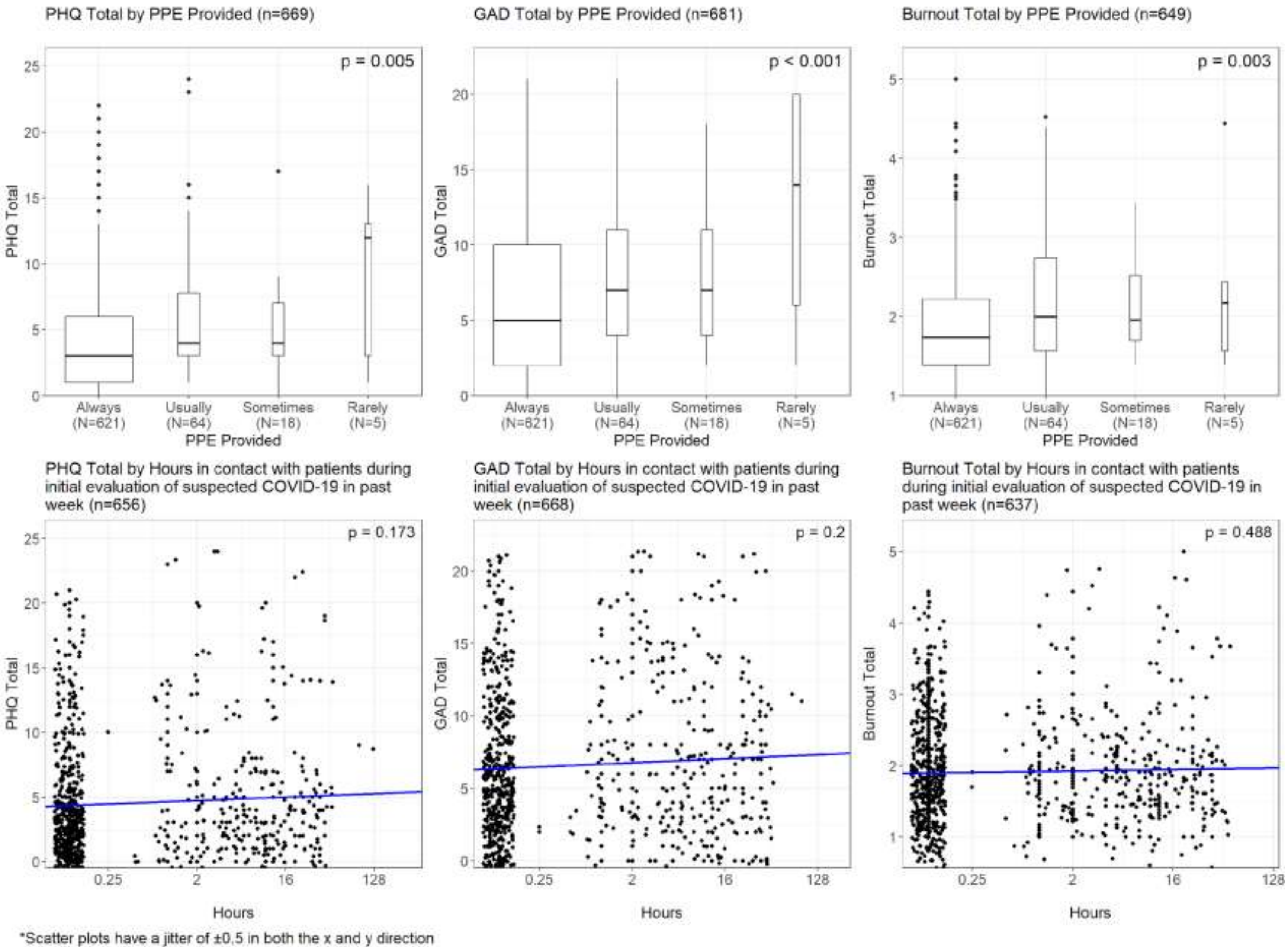

It is illegal to post this copyrighted PDF on any website. ♦ © 2022 Copyright Physicians Postgraduate Press, Inc.
