# Supplementary material for: Mental health status and related factors influencing healthcare workers during the COVID-19 pandemic: A systematic review and meta-analysis
Source: PLoS One. 2024 Jan 19;19(1):e0289454. doi: 10.1371/journal.pone.0289454 (PMC10798549; doi:10.1371/journal.pone.0289454)
Supplement: S1 Data — (ZIP) [file pone.0289454.s011.zip › literatures/190.pdf]

Scientific Research Report

Psychological Impact of the COVID-19 Pandemic on Emergency Dental Care Providers on the Front Lines in China

Jiang Tao [a], *Yueting Lin* [a], *Long Jiang* [a], *Zhuojun Zhou* [a], *Junjun Zhao* [a], *Donglin Qu* [a], *Wei Li* [b,c], *Yaqin Zhu* [a*]

[a] *Department of General Dentistry, Shanghai Ninth People's Hospital, College of Stomatology, Shanghai Jiao Tong University School of Medicine; National Clinical Research Center for Oral Diseases; Shanghai Key Laboratory of Stomatology and Shanghai Research Institute of Stomatology, Shanghai, China*
[b] *Department of Geriatric Psychiatry, Shanghai Mental Health Center, Shanghai Jiao Tong University School of Medicine, Shanghai, China*
[c] *Alzheimer's Disease and Related Disorders Center, Shanghai Jiao Tong University, Shanghai, China*

ARTICLE INFO

Article history:
Available online 9 December 2020

Key words:
Psychological disorder
Dental care providers
Infectious diseases
Mental health

ABSTRACT

Coronavirus disease 2019 (COVID-19) is an infectious disease that emerged at the end of 2019. On 30 January 2020, the World Health Organization (WHO) classified it as a pandemic. To examine the psychological effects on dental care providers in China in the midst of the COVID-19 outbreak and factors closely associated with those effects, we conducted a cross-sectional study online with 4 widely used self-administered questionnaires: the Patient Health Questionnaire-9, the General Anxiety Disorder-7, the Perceived Stress Scale-10, and the Acute Stress Disorder Scale. Univariate and multivariate analyses were performed to evaluate the variables that potentially affected the mental health of emergency dental care providers. As a result, 969 out of 1035 questionnaires were included in the analysis, with 642 respondents reporting more than 1 symptom (66.3%). The symptom of perceived stress was reported by the largest proportion of the respondents (66.2%, $n = 641$), and anxiety the least (7.1%, $n = 69$). After adjustment for confounders, it was found that dental practitioners with preexisting physical health conditions were at higher risk of depression (odds ratio [OR], 1.972; 95% CI, 1.128-3.448; $P = .017$), and perceived stress (odds ratio, 2.397 95% CI, 1.283-4.478; $P = .006$). Additionally, feelings of fear, helplessness, or terror resulting from the possibility of contracting COVID-19 were significantly associated with the prevalence of all the 4 psychological symptoms observed ($P < .05$). In the present study, we found that dental care providers suffered psychological depression, stress, anxiety, and posttraumatic stress disorder (PTSD) during COVID-19, which indicates the importance of psychological support at times of major epidemic outbreaks.
Chinese Clinical Trial Registry number: ChiCTR2000031538.
© 2021 The Authors. Published by Elsevier Inc. on behalf of FDI World Dental Federation. This is an open access article under the CC BY-NC-ND license (http://creativecommons.org/licenses/by-nc-nd/4.0/)

Introduction

Appearing at the end of 2019, coronavirus disease 2019 (COVID-19) overwhelmed medical systems in countries and regions worldwide. It is the highest priority to contain the outbreak; however, its psychological effects on health care workers should not be ignored. There is no doubt that health care workers who provide care for and treat patients on the front lines bear an enormous psychological burden. To prevent psychological problems from developing into secondary traumatization, attention to and knowledge of the psychological effects of the outbreak should be enhanced.

According to previous research on infectious diseases, including severe acute respiratory syndrome (SARS), Middle

Jiang Tao and Yueting Lin contributed equally to this work.
* *Corresponding author*. Department of General Dentistry, Shanghai Ninth People's Hospital, Shanghai Jiao Tong University School of Medicine, Shanghai 200011, China.
E-mail address: zyq1590@163.com (Y. Zhu).
https://doi.org/10.1016/j.identj.2020.12.001
0020-6539/© 2021 The Authors. Published by Elsevier Inc. on behalf of FDI World Dental Federation. This is an open access article under the CC BY-NC-ND license (http://creativecommons.org/licenses/by-nc-nd/4.0/)

East respiratory syndrome coronavirus (MERS-CoV), H1N1, Ebola viral disease (EVD), and COVID-19, medical staff showed physical symptoms of insomnia, exhaustion, and decreased appetite and suffered from the psychological symptoms of stress, depression, and anxiety.[1–5] The symptoms can persist for a long time, which possibly contributed to chronic psychological and physical health problems. [6,7] Dental practitioners in many countries also reported similar psychological distress following disaster exposure,[8] which is of continuing concern. Jun Shigemura et al,[9] who evaluated the psychological responses in dentists who conducted disaster victim identification after the 2011 Fukushima disaster, claimed that the dentist's psychological burden was associated with the disaster. Similarly, it was concluded that the COVID-19 emergency was having a highly negative impact on dental practitioners in 2 of the northern Italian districts, Israel, and some other parts of the world.[8,10,11]

Because severe acute respiratory syndrome coronavirus 2 (SARS-CoV-2) is transmitted through oral and nasal discharge from virus carriers,[12] the droplets generated in the course of coughing and exhaling are risk factors for disease transmission. Dental care providers are inevitably in close contact with patients during dental procedures when aerosols or droplets can settle on different surfaces, which makes dental clinics a high-risk place during an epidemic of respiratory infectious diseases. During the COVID-19 outbreak, dental care workers in China have remained in their posts out of a sense of duty despite uncertain dangers. However, there is little literature on the mental health status of emergency dental care providers in China. We aim to clarify the psychological impact of COVID-19 and to accelerate the development of a psychological support system for dental practitioners.

Methods

Study design and participants

This was a cross-sectional survey that was conducted from 3 April 2020 to 10 April 2020 using web-based anonymous questionnaires. Volunteers were recruited from about 100 medical institutions under the Emergency Committee of the Chinese Stomatological Association through snowball sampling.[13]

The inclusion criteria were (1) informed consent and participation on a voluntary basis and (2) Chinese citizenship. Participants were excluded if they (1) were younger than age 18 or older than age 65; (2) had a history of mental illness; or (3) answered the series of questions in the questionnaire identically or in a clear pattern (eg, choosing the same options).

The protocol and informed consent documents were submitted to and approved by the Medical Ethics Committee of Shanghai Ninth People's Hospital (SH9H-2020-T55-2) and Chinese Clinical Trial Registry (ChiCTR2000031538).

Sample size

Based on previously documented studies in a similar population,[14] we estimated that no more than 70% of the sample would manifest psychological symptoms. After controlling for type 1 error at 0.05 with a tolerance of 3%, a target sample size of 896 was calculated using the formula $N = Z_{1-\alpha^2/2}p(1-p)/d^2$.[15] Supposing that the response rate was above 90%, then at least 996 participants would be recruited.

Instruments and outcomes

The questionnaire was composed of 2 parts: demographic information and Chinese version self-rating scales. Data on respondents' gender, hospital type, age, marital status, level of education, parental status, history of mental illness, and physical health status were all collected. The scales part, which consists of Patient Health Questionnaire-9 (PHQ-9), General Anxiety Disorder-7 (GAD-7), Perceived Stress Scale-10 (PSS-10), and Acute Stress Disorder Scale (ASDS) evaluating depression, anxiety, perceived stress, and posttraumatic stress disorder (PTSD) symptoms, reported satisfying psychometric properties among many different Chinese populations.[16–19]

Patient Health Questionnaire-9 is one of the most commonly used depression screening tools in primary health care settings,[20] which has shown good validity and internal consistency (Cronbach's α: 0.56-0.94) in a previous study.[21] The performance characteristics are acceptable when the cutoff value is at or above 10.[22] The 7-item scale GAD-7 is a brief and convenient measure for identifying probable cases of anxiety. After validity and reliability analysis, prior study has supported its application in the general population.[23] A summary score of 10 for cutoff is recommended.[24] The Perceived Stress Scale-10 questionnaire is a psychological instrument assessing the perceived stress in one's life[25] with evidence showing appreciable validity and reliability (Cronbach's α at 0.72-0.91).[26] The cutoff point at 14 or greater indicates moderate to severe perceived stress.[27] Acute Stress Disorder Scale is a 19-item inventory using a cutoff score of 56 as the predictor of posttraumatic stress disorder.[28] Good validity and internal consistency were documented with the Cronbach's α from 0.76 to 0.96.[29]

Statistical analysis

The data were analysed with the software Statistical Package for Social Sciences (IBM SPSS Statistics 25). Based on the univariate analysis, specific variables were entered ($P < .10$) as independents in binary logistic regression models to further assess the impact of potential stressors. Dummy variables such as hospital types were defined. Confounding factors (ie, hospital types, gender, marital status, parental status, educational level, preexisting physical diseases, COVID-19–related experiences or thoughts) were adjusted when appropriate. The statistical significance level was set at .05 with a 2-tailed test.

Results

Participants and demographic characteristics

A total of 1035 questionnaires were returned, of which 66 with invalid data were excluded for certain reasons (Figure). The effective response rate was 93.6%.

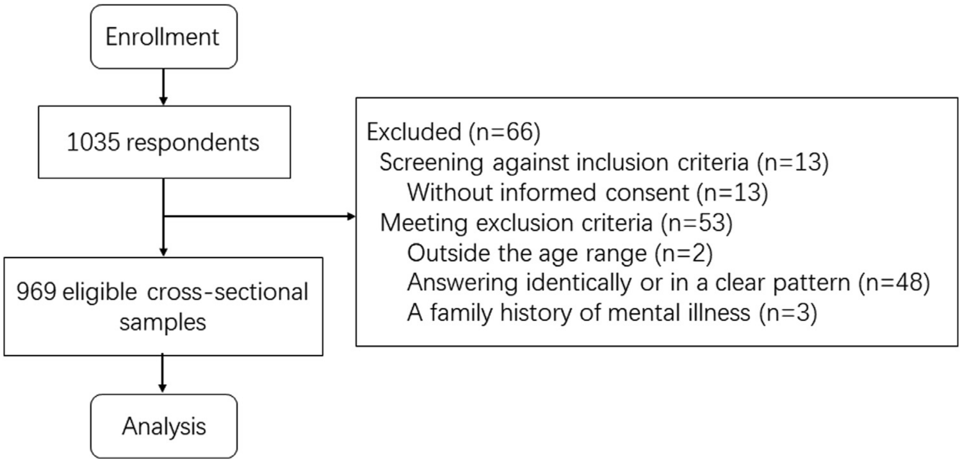

Fig. – Flow diagram of the survey progress.

Among the eligible participants, 496 worked in public stomatological hospitals (51.2%), 453 in public general hospitals (46.7%), and 20 in private hospitals (2.1%). A total of 79 suffered from physical diseases (8.2%). Women comprised 68.0% ($n = 659$) and men comprised 32.0% ($n = 310$) of the sample with a mean age of 35.55 years (SD = 8.259 years). The marital status was as follows: 72.4% of the respondents were married ($n = 702$), 25.9% ($n = 251$) were unmarried, and the rest was unspecified ($n = 16$, 1.7%). A total of 64.8% had a child or children ($n = 628$). In terms of educational levels, 391 participants had a master's degree or more education, 468 participants had a bachelor's degree, and 110 had an associate degree or less education. In total, 802 respondents worked in the initial stage of the outbreak.

Prevalence of psychological symptoms

Of all psychological symptoms, the most frequently reported was perceived stress, with a proportion of 66.2% ($n = 641$), followed by depression ($n = 134$, 13.8%), then PTSD ($n = 82$, 8.5%), and at last anxiety ($n = 69$, 7.1%). Overall, 327 respondents (33.7%) reported no symptoms, and 25 reported 4 symptoms (2.6%).

Associated and risk factors analysis

After adjusting for the confounding variables, preexisting physical diseases, having to face the possibility of treating a patient who later turned out to be a suspected or confirmed case, and the feelings of fear, helplessness, or terror resulting from the possibility of contracting COVID-19 were significantly associated with depression. Dental care providers who experienced these factors were at 1.972 (95% CI, 1.128-3.448; $P = .017$), 2.397 (95% CI, 1.283-4.478; $P = .006$), 2.640 (95% CI, 1.798-3.877; $P < .001$) times greater risk, respectively, than those who did not (Table 1).

As shown in Table 1, feelings of fear, helplessness, or terror resulting from the possibility of contracting COVID-19 (odds ratio [OR], 3.421; 95% CI, 2.026-5.776; $P < .001$) was a factor associated with more anxiety symptoms. Likewise, respondents that were subjected to the feelings of fear, helplessness, or terror resulting from the possibility of contracting COID-19 were at higher risk of developing PTSD (OR, 4.856; 95% CI, 2.904-8.120, $P < .001$) (Table 2).

Compared with dental care providers who worked in public stomatological hospitals, had a higher level of education, were without preexisting physical health conditions, and did not experience the feelings of fear, helplessness, or terror resulting from the possibility of contracting COVID-19, those who worked in public general hospitals (OR, 0.680; 95% CI, 0.512-0.903; $P = .008$) had a lower level of education (OR, 0.714, 95% CI, 0.575-0.887; $P = .002$), suffered from preexisting physical health conditions (OR, 2.442; 95% CI, 1.299-4.593; $P = .006$), and experienced feelings of fear, helplessness, or terror resulting from the possibility of contracting COVID-19 (OR, 3.052; 95% CI, 2.256-4.130; $P < .001$) showed a significantly higher level of association with high perceived stress (Table 2).

Discussion

In this cross-sectional study, the psychological responses of dental care providers to the COVID-19 outbreak was determined. Marital status, gender, educational levels, history of physical diseases, and other variables were assumed to be factors associated with the prevalence of psychological symptoms. Of all the variables observed, gender, marital status, and whether being on duty at the initial stage of the outbreak displayed no statistical differences, whereas the rest of the variables exhibited significant association with psychological symptoms.

Gender and marital status showed no differences between the observed symptoms, which is consistent with a study on the psychological effect of SARS on emergency department staff.[30] This finding might be because dental workers were exposed to similar risk despite their gender and had family responsibilities despite their marital status. However, some other studies on health care workers found that females and those unmarried were more vulnerable to psychological problems and attributed it to a nurses' longer contact time when caring for patients and younger age with less work

Table 1 – Univariate and multivariate analysis of participants with and without depression or anxiety.

Variables	PHQ-9					GAD-7				
	Univariate analysis			Multivariate analysis		Univariate analysis			Multivariate analysis	
	Without depression, *n* (%)	With depression, *n* (%)	*P* value	Adjusted OR (95% CI)	*P* value	Without anxiety, *n* (%)	With anxiety, *n* (%)	*P* value	Adjusted OR (95% CI)	*P* value
Hospital type			.153					.152		
Public general hospital	383 (84.5%)	70 (15.5%)				415 (91.6%)	38 (8.4%)			
Public stomatological hospital	434 (87.5%)	62 (12.5%)				466 (94.0%)	30 (6.0%)			
Private hospital	18 (90.0%)	2 (10.0%)				19 (95.0%)	1 (5.0%)			
Gender			.913					.492		
Male	269 (86.8%)	41 (13.2%)				290 (93.5%)	20 (6.5%)			
Female	566 (85.9%)	93 (14.1%)				610 (92.6%)	49 (7.4%)			
Marital status			.562					.476		
Married	612 (87.2%)	90 (12.8%)				650 (92.6%)	52 (7.4%)			
Unmarried	208 (82.9%)	43 (17.1%)				234 (93.2%)	17 (6.8%)			
Others	15 (93.8%)	1 (6.3%)				16 (100%)	0 (0.0%)			
Parental status			.558					.640		
No	288 (84.5%)	53 (15.5%)				318 (93.3%)	23 (6.7%)			
Yes	547 (87.1%)	81 (12.9%)				582 (92.7%)	46 (7.3%)			
Educational level			.496					.502		
≤An associate degree	96 (87.3%)	14 (12.7%)				102 (92.7%)	8 (7.3%)			
A bachelor's degree	406 (86.8%)	62 (13.2%)				440 (94.0%)	28 (6.0%			
≥A master's degree	333 (85.2%)	58 (14.8%)				358 (91.6%)	33 (8.4%))			
Jan. 23-Feb. 29 on duty			.559					.422		
No	147 (88.0%)	20 (12.0%)				159 (95.2%)	8 (4.8%)			
Yes	688 (85.8%)	114 (14.2%)				741 (92.4%)	61 (7.6%)			
Preexisting physical diseases			.014*	1.972 (1.128-3.448)	.017*			.691		
No	776 (87.2%)	114 (12.8%)				829 (93.1%)	61 (6.9%)			
Yes	59 (74.7%)	20 (25.3%)				71 (89.9%)	8 (10.1%)			
COVID-19–related experiences or thoughts										
a. I could be in direct contact with suspected or confirmed cases.			.604					.643		
No	392 (88.5%)	51 (11.5%)				421 (95.0%)	22 (5.0%)			
Yes	443 (84.2%)	83 (15.8%)				479 (91.1%)	47 (8.9%)			
b. I believed some of the patients I treated could turn out to be suspected or confirmed cases.			.019*	2.397 (1.283-4.478)	.006*			.298		
No	189 (94.0%)	12 (6.0%)				196 (97.5%)	5 (2.5%)			
Yes	646 (84.1%)	122 (15.9%)				704 (91.7%)	64 (8.3%)			

(*continued on next page*)

Table 1 (*Continued*)

	PHQ-9					GAD-7				
	Univariate analysis			Multivariate analysis		Univariate analysis			Multivariate analysis	
Variables	Without depression, *n* (%)	With depression, *n* (%)	*P* value	Adjusted OR (95% CI)	*P* value	Without anxiety, *n* (%)	With anxiety, *n* (%)	*P* value	Adjusted OR (95% CI)	*P* value
c. I believed some of the patients I treated could turn out to be asymptomatic cases.			.825				268			
No	85 (93.4%)	6 (6.6%)				90 (98.9%)	1 (1.1%)			
Yes	750 (85.4%)	128 (14.6%)				810 (92.3%)	68 (7.7%)			
d. I believed SARS-CoV-2 infection could be life-threatening to me or my family.			.221					.749		
No	130 (89.0%)	16 (11.0%)				140 (95.9%)	6 (4.1%)			
Yes	705 (85.7%)	118 (14.3%)				760 (92.3%)	63 (7.7%)			
e. I felt fear, helplessness, or terror because of the possibility of being contracted.			<.001*	2.640 (1.798-3.877)				<.001*	3.421 (2.026-5.776)	<.001*
No	527 (91.5%)	49 (8.5%)			<.001*	554 (96.2%)	22 (3.8%)			
Yes	308 (78.4%)	85 (21.6%)				346 (88.0%)	47 (12.0%)			
f. I was involved in medical relief efforts against SARS or avian influenza.			.836					.183		
No	784 (86.3%)	124 (13.7%)				847 (93.3%)	61 (6.7%)			
Yes	51 (83.6%)	10 (16.4%)				53 (86.9%)	8 (13.1%)			
Total	835 (86.2%)	134 (13.8%)				900 (92.9%)	69 (7.1%)			

COVID-19, coronavirus 2019; GAD-7, Generalized Anxiety Disorder; OR, odds ratio; PHQ-7, Patient Health Questionnaire-7; SARS, severe acute respiratory syndrome; SARS-CoV-2, severe acute respiratory syndrome coronavirus 2.

* $P < .05$.

Table 2 – Univariate and multivariate analysis of participants with and without PTSD or perceived stress.

Variables	ASDS					PSS-10				
	Univariate analysis			Multivariate analysis		Univariate analysis			Multivariate analysis	
	Without PTSD symptom, *n* (%)	With PTSD symptom, *n* (%)	*P* value	Adjusted OR (95% CI)	*P* value	Without perceived stress, *n* (%)	With perceived stress, *n* (%)	*P* value	Adjusted OR (95% CI)	*P* value
Hospital type			.265					.030		
Public general hospital	408 (90.1%)	45 (9.9%)				135 (29.8%)	318 (70.2%)		1 (reference)	.008
Public stomatological hospital	461 (92.9%)	35 (7.1%)				188 (37.9%)	308 (62.1%)		0.680 (0.512-0.903)	.565
Private hospital	18 (90.0%)	2 (10.0%)				5 (25.0%)	15 (75.0%)		1.366 (0.472-3.953)	
Gender			.229					.607		
Male	280 (90.3%)	30 (9.7%)				107 (34.5%)	203 (65.5%)			
Female	607 (92.1%)	52 (7.9%)				221 (33.5%)	438 (66.5%)			
Marital status			.741					.765		
Married	644 (91.7%)	58 (8.3%)				234 (33.3%)	468 (66.7%)			
Unmarried	227 (90.4%)	24 (9.6%)				88 (35.1%)	163 (64.9%)			
Others	16 (100.0%)	0 (0.0%)				6 (37.5%)	10 (62.5%)			
Parental status			.493					.093	1.336 (0.998-1.788)	.052
No	311 (91.2%)	30 (8.8%)				128 (37.5%)	213 (62.5%)			
Yes	576 (91.7%)	52 (8.3%)				200 (31.8%)	428 (68.2%)			
Educational level			.882					.002*	0.714 (0.575-0.887)	.002*
≤An associate degree	101 (91.8%)	9 (8.2%)				30 (27.3%)	80 (72.7%)			
A bachelor's degree	427 (91.2%)	41 (8.8%)				144 (30.8%)	324 (69.2%)			
≥A master's degree	359 (91.8%)	32 (8.2%)				154 (39.4%)	237 (60.9%)			
Jan. 23-Feb. 29 on duty			.316					.540		
No	150 (89.9%)	17 (10.2%)				52 (31.1%)	115 (68.9%)			
Yes	737 (91.9%)	65 (8.1%)				276 (34.4%)	526 (65.6%)			
Preexisting physical diseases			.360					.006*	2.442 (1.299-4.593)	.006*
No	819 (92.0%)	71 (8.0%)				315 (35.4%)	575 (64.6%)			
Yes	68 (86.1%)	11 (13.9%)				13 (16.5%)	66 (83.5%)			
COVID-19–related experiences or thoughts										
a. I could be in direct contact with suspected or confirmed cases.			.707					.146		
No	410 (92.6%)	33 (7.4%)				150 (33.9%)	293 (66.1%)			
Yes	477 (90.7%)	49 (9.3%)				178 (33.8%)	348 (66.2%)			
b. I believed some of the patients I treated could turn out to be suspected or confirmed cases.			.555							
No	190 (94.5%)	11 (5.5%)				80 (39.8%)	121 (60.2%)	.167		
Yes	697 (90.8%)	71 (9.2%)				248 (32.3%)	520 (67.7%)			
c. I believed some of the patients I treated could turn out to be asymptomatic cases.			868							
No	86 (94.5%)	5 (5.5%)				36 (39.6%)	55 (60.4%)	.736		

(continued on next page)

Table 2 (*Continued*)

	ASDS					PSS-10				
	Univariate analysis			Multivariate analysis		Univariate analysis			Multivariate analysis	
Variables	Without PTSD symptom, *n* (%)	With PTSD symptom, *n* (%)	*P* value	Adjusted OR (95% CI)	*P* value	Without perceived stress, *n* (%)	With perceived stress, *n* (%)	*P* value	Adjusted OR (95% CI)	*P* value
Yes	801 (91.2%)	77 (8.8%)				292 (33.3%)	586 (66.7%)			
d. I believed SARS-CoV-2 infection could be life-threatening to me or my family.			.744							
No	140 (95.9%)	6 (4.1%)				61 (41.8%)	85 (58.2%)	.878		
Yes	747 (90.8%)	76 (9.2%)				267 (32.4%)	556 (67.6%)			
e. I felt fear, helplessness, or terror because of the possibility of being contracted.			<.001*							
No	555 (96.4%)	21 (3.6%)		4.856 (2.904-8.120)	<.001*	249 (43.2%)	327 (56.8%)	<.001*	3.052 (2.256-4.130)	<.001*
Yes	332 (84.5%)	61 (15.5%)				79 (20.1%)	314 (79.9%)			
f. I was involved in medical relief efforts against SARS or avian influenza.			.162							
No	835 (92.0%)	73 (8.0%)				309 (34.0%)	599 (66.0%)	.705		
Yes	52 (85.2%)	9 (14.8%)				19 (31.1%)	42 (68.9%)			
Total	887 (91.5%)	82 (8.5%)				328 (33.8%)	641 (66.2%)			

ASDS, Acute Stress Disorder Scale; COVID-19, coronavirus 2019; OR, odds ratio; PSS-10, Perceived Stress Scale-10; PTSD, posttraumatic stress disorder; SARS, severe acute respiratory syndrome; SARS-CoV-2, severe acute respiratory syndrome coronavirus 2.

* $P < .05$.

experience.[31,32] Concerning the variable of whether one was on duty at the initial stage of the outbreak, adequate protections and prompt action might have played a role.

In agreement with the research regarding immediate psychological responses during the initial stage of COVID-19 among the general population in China,[32] our survey identified the preexisting physical disease as a factor associated with a higher risk of depression and anxiety. Previous studies on the psychological impact of SARS among frontline health care workers[31] also supported the variable of preexisting physical disease as a significant indicator of psychological morbidity.

Having the feelings of fear, helplessness, or terror resulting from the possibility of contracting COVID-19 presented statistically significant differences in the psychological outcomes of depression, anxiety, perceived stress, and PTSD. In a similar vein, several psychological studies on past outbreaks[1,33] showed the adverse effect of exposure to patients who were infected. Moreover, it is reported that fear for one's health mediated the relationship between outbreaks and psychological distress.[34]

Strengths of our study are as follows: this study added to the literature on the psychological impact of COVID-19 among Chinese dental care providers; the survey was conducted within 1 month of the initial stage, in which constant vigilance and response efforts remained; the sample was geographically representative with participants across provinces and cities in China.

However, there are several limitations to the present study. First, because of the nature of the cross-sectional study and the lack of normative data, causal inference cannot be made. Second, the self-reported data may be less accurate than clinical interviews. Third, other contributing factors such as workload[3] were not surveyed.

Further research on the long-term psychological effects of infectious diseases,[35] psychological resilience,[36] and altruism[37] exhibited during an outbreak and posttraumatic growth[38,39] induced by the outbreak remains to be conducted.

Conclusion

In conclusion, the emergence of COVID-19 affected dental care providers to a varying degree. In particular, poor physical health conditions and feelings of fear, helplessness, or terror resulting from the possibility of contracting COVID-19 require further attention.

This research fills a gap in the literature on the psychological reactions of Chinese dental workers during a virus outbreak and lays the foundation for the establishment of the causal relationship between infectious diseases and the prevalence of psychiatric morbidity. Additionally, it helps improve programs on psychological support for dental care workers and suggests a focus on preparation and interventions to aid psychological recovery after possible exposure to sources of infection.

Acknowledgements

The authors are grateful to the Emergency Committee of the Chinese Stomatological Association for their help in distributing the questionnaires.

Author contributions

Jiang Tao and Yueting Lin contributed to the concept and design of the study, acquisition of data, analysis and interpretation of data, and drafting and revising the article. Junjun Zhao, Zhuojun Zhou, Junli Han, Long Jiang, Rong Du, Hongtao Ma, and Donglin Qu contributed to the acquisition, analysis, and interpretation of data and critical revision of the manuscript. Wei Li and Yaqin Zhu contributed to the concept and design of the study, data acquisition, and critical revision of the manuscript for important intellectual content. All authors have approved the final version of the manuscript.

Funding

This research is funded by the General Research Project of Shanghai Ninth People's Hospital, Shanghai Jiao Tong University School of Medicine (grant no. 2020-19).

Conflict of interest

None disclosed.

REFERENCES

1. Maunder R, Hunter J, Vincent L, et al. The immediate psychological and occupational impact of the 2003 SARS outbreak in a teaching hospital. CMAJ 2003;168:1245–51.
2. Lee SM, Kang WS, Cho AR, et al. Psychological impact of the 2015 MERS outbreak on hospital workers and quarantined hemodialysis patients. Compr Psychiatry 2018;87:123–7.
3. Matsuishi K, Kawazoe A, Imai H, et al. Psychological impact of the pandemic (H1N1) 2009 on general hospital workers in Kobe. Psychiatry Clin Neurosci 2012;66:353–60.
4. Mohammed A, Sheikh TL, Gidado S, et al. An evaluation of psychological distress and social support of survivors and contacts of Ebola virus disease infection and their relatives in Lagos, Nigeria: a cross sectional study - 2014. BMC Public Health 2015;15:1–8.
5. Chew NWS, Lee GKH, Tan BYQ, et al. A multinational, multicentre study on the psychological outcomes and associated physical symptoms amongst healthcare workers during COVID-19 outbreak. Brain Behav Immun 2020;88:559–65.
6. Lee AM, Wong JGWS, McAlonan GM, et al. Stress and psychological distress among SARS survivors 1 year after the outbreak. Can J Psychiatry 2007;52:233–40.
7. McAlonan GM, Lee AM, Cheung V, et al. Immediate and sustained psychological impact of an emerging infectious disease outbreak on health care workers. Can J Psychiatry 2007;52:241–7.
8. Ahmed MA, Jouhar R, Ahmed N, et al. Fear and practice modifications among dentists to combat novel coronavirus disease (COVID-19) outbreak. Int J Environ Res Public Health 2020;17:2821. doi: 10.3390/ijerph17082821.
9. Shigemura J, Someda H, Tokuno S, et al. Disaster victim identification: psychological distress and posttraumatic stress in dentists after the 2011 Fukushima disaster. Psychiatry 2018;81:85–92.
10. Consolo U, Bellini P, Bencivenni D, et al. Epidemiological aspects and psychological reactions to COVID-19 of dental practitioners in the Northern Italy districts of Modena and

Reggio Emilia. Int J Environ Res Public Health 2020;17:3459. doi: 10.3390/ijerph17103459.
11. Shacham M, Hamama-Raz Y, Kolerman R, et al. COVID-19 factors and psychological factors associated with elevated psychological distress among dentists and dental hygienists in Israel. Int J Environ Res Public Health 2020;17:2900. doi: 10.3390/ijerph17082900.
12. World Health Organization. Advice on the use of masks in the context of COVID-19: interim guidance, 6 April 2020. World Health Organization. 2020. Available from: https://apps.who.int/iris/handle/10665/331693. Accessed April 29, 2020.
13. Wang C, Pan R, Wan X, et al. Immediate psychological responses and associated factors during the initial stage of the 2019 coronavirus disease (COVID-19) epidemic among the general population in China. Int J Environ Res Public Health 2020;17:1729. doi: 10.3390/ijerph17051729.
14. Kang L, Ma S, Chen M, et al. Impact on mental health and perceptions of psychological care among medical and nursing staff in Wuhan during the 2019 novel coronavirus disease outbreak: a cross-sectional study. Brain Behav Immun 2020;87:11–7.
15. Charan J, Biswas T. How to calculate sample size for different study designs in medical research? Indian J Psychol Med 2013;35:121–6.
16. Liu SI, Yeh ZT, Huang HC, et al. Validation of patient health questionnaire for depression screening among primary care patients in Taiwan. Compr Psychiatry 2011;52:96–101.
17. Tong X, An D, McGonigal A, et al. Validation of the generalized anxiety disorder-7 (GAD-7) among Chinese people with epilepsy. Epilepsy Res 2016;120:31–6.
18. Huang F, Wang H, Wang Z, et al. Psychometric properties of the perceived stress scale in a community sample of Chinese. BMC Psychiatry 2020;20:1–7.
19. Wang R, Wang L, Zhang J, et al. The structure of acute stress disorder among Chinese adults exposed to an earthquake: is dysphoric arousal a unique construct of acute posttraumatic responses? Scand J Psychol 2012;53:430–6.
20. Levis B, Benedetti A, Thombs BD. Accuracy of Patient Health Questionnaire-9 (PHQ-9) for screening to detect major depression: individual participant data meta-analysis. BMJ 2019;365:l1476. doi: 10.1136/bmj.l1476.
21. El-Den S, Chen TF, Gan YL, et al. The psychometric properties of depression screening tools in primary healthcare settings: a systematic review. J Affect Disord 2018;225:503–22.
22. Moriarty AS, Gilbody S, McMillan D, et al. Screening and case finding for major depressive disorder using the Patient Health Questionnaire (PHQ-9): a meta-analysis. Gen Hosp Psychiatry 2015;37:567–76.
23. Löwe B, Decker O, Müller S, et al. Validation and standardization of the Generalized Anxiety Disorder screener (GAD-7) in the general population. Med Care 2008;46:266–74.
24. Spitzer RL, Kroenke K, Williams JBW, et al. A brief measure for assessing generalized anxiety disorder: the GAD-7. Arch Intern Med 2006;166:1092–7.
25. Cohen S, Kamarck T, Mermelstein R. A global measure of perceived stress. J Health Soc Behav 1983;24:385–96.
26. Ng SM. Validation of the 10-item Chinese perceived stress scale in elderly service workers: one-factor versus two-factor structure. BMC Psychol 2013;1:1–8.
27. Wiriyakijja P, Porter S, Fedele S, et al. Validation of the HADS and PSS-10 and a cross-sectional study of psychological status in patients with recurrent aphthous stomatitis. J Oral Pathol Med 2020;49:260–70.
28. Bryant RA, Moulds ML, Guthrie RM. Acute Stress Disorder Scale: a self-report measure of acute stress disorder. Psychol Assess 2000;12:61–8.
29. Hansen M, Armour C, Wang L, et al. Assessing possible DSM-5 ASD subtypes in a sample of victims meeting caseness for DSM-5 ASD based on self-report following multiple forms of traumatic exposure. J Anxiety Disord 2015;31:84–9.
30. Lin CY, Peng YC, Wu YH, et al. The psychological effect of severe acute respiratory syndrome on emergency department staff. Emerg Med J 2007;24:12–7.
31. Tam CWC, Pang EPF, Lam LCW, et al. Severe acute respiratory syndrome (SARS) in Hongkong in 2003: stress and psychological impact among frontline healthcare workers. Psychol Med 2004;34:1197–204.
32. Lai J, Ma S, Wang Y, et al. Factors associated with mental health outcomes among health care workers exposed to coronavirus disease 2019. JAMA Netw Open 2020;3:e203976.
33. Lee SH, Juang YY, Su YJ, et al. Facing SARS: psychological impacts on SARS team nurses and psychiatric services in a Taiwan general hospital. Gen Hosp Psychiatry 2005;27:352–8.
34. Maunder RG, Lancee WJ, Rourke S, et al. Factors associated with the psychological impact of severe acute respiratory syndrome on nurses and other hospital workers in Toronto. Psychosom Med 2004;66:938–42.
35. Maunder RG, Lancee WJ, Balderson KE, et al. Long-term psychological and occupational effects of providing hospital healthcare during SARS outbreak. Emerg Infect Dis 2006;12:1924–32.
36. Itzhaki M, Peles-Bortz A, Kostistky H, et al. Exposure of mental health nurses to violence associated with job stress, life satisfaction, staff resilience, and post-traumatic growth. Int J Ment Health Nurs 2015;24:403–12.
37. Wu P, Fang Y, Guan Z, et al. The psychological impact of the SARS epidemic on hospital employees in China: exposure, risk perception, and altruistic acceptance of risk. Can J Psychiatry 2009;54:302–11.
38. Tedeschi RG, Calhoun LG. Posttraumatic growth: conceptual foundations and empirical evidence. Psychol Inq 2004;15:1–18.
39. Xu X, Hu ML, Song Y, et al. Effect of positive psychological intervention on posttraumatic growth among primary healthcare workers in China: a preliminary prospective study. Sci Rep 2016;6:1–7.
