# Supplementary material for: Mental health status and related factors influencing healthcare workers during the COVID-19 pandemic: A systematic review and meta-analysis
Source: PLoS One. 2024 Jan 19;19(1):e0289454. doi: 10.1371/journal.pone.0289454 (PMC10798549; doi:10.1371/journal.pone.0289454)
Supplement: S1 Data — (ZIP) [file pone.0289454.s011.zip › literatures/191.pdf]

NÖRO PSİKİYATRİ ARŞİVİ
ARCHIVES OF NEUROPSYCHIATRY

RESEARCH ARTICLE

Arch Neuropsychiatry 2022;59:133–138
https://doi.org/10.29399/npa.27785

Psychological Reactions of Turkish Healthcare Workers During Covid-19 Outbreak: The Impact of Stigmatization

Rümeysa TAŞDELEN[1], Batuhan AYIK[2], Hatice KAYA[3], Mete ERCİS[4], Erhan ERTEKİN[4]

[1]Department of Psychiatry, Marmara University İstanbul Pendik Education and Research Hospital, Pendik, İstanbul, Turkey
[2]Department of Psychiatry, İstanbul Erenkoy Education and Research Hospital, Erenkoy, İstanbul, Turkey
[3]Department of Psychiatry, İstanbul Sultanbeyli State Hospital, Sultanbeyli Community Mental Health Centre, Sultanbeyli, İstanbul, Turkey
[4]Department of Psychiatry, İstanbul University, İstanbul Faculty of Medicine, Fatih, İstanbul, Turkey

ABSTRACT

Introduction: The Coronavirus Disease 2019 (COVID-19) outbreak has resulted in huge psychological distress, especially for people working under risky conditions, such as healthcare workers. We aimed to investigate the psychological challenges of Turkish healthcare workers during the outbreak and discuss the factors that increase their burden, including stigmatization.

Methods: A cross-sectional online survey composed of sociodemographic data, questions about perceived stigma, and concerns about working conditions was completed. Depression Anxiety and Stress Scale-21 (DASS-21) was also used.

Results: 634 participants completed the survey and the prevalence of moderate to severe depression, anxiety, and stress-related symptoms were 36%, 35%, and 22%, respectively. Being female, working with close contact with patients with COVID-19 and having a psychiatric disorder history were risk factors for psychological distress. The healthcare workers with significantly higher depression, anxiety, and stress scores described having less amount of personal protective equipment, lower support from their supervisors, and more unsafe working conditions. Additionally, 7% of the participants stated that they perceived stigmatization by their families and close friends, 14% perceived stigmatization by their neighbours, relatives, and less intimate friends; and 7% perceived stigmatization by unfamiliar people. Healthcare workers who perceived other people as more dismissive had significantly higher depression and anxiety scores.

Conclusion: Our study shows that healthcare workers are at risk of developing psychological disturbances during infectious outbreaks. To minimize the psychological impact, additional risk factors such as having a previous psychiatric disorder, working under unsafe conditions, and stigmatization should be taken into account and a more supportive and safer environment should be provided.

Keywords: Anxiety, COVID-19, depression, healthcare workers, stigmatization

Cite this article as: Taşdelen R, Ayık B, Kaya H, Ercis M, Ertekin E. Psychological Reactions of Turkish Healthcare Workers During Covid-19 Outbreak: The Impact of Stigmatization. Arch Neuropsychiatry 2022;59:133-138.

INTRODUCTION

By the end of December 2019, cases of acute severe pneumonia were reported in Wuhan, China, which were caused by a novel form of coronavirus. The novel coronavirus has spread globally within months. On January 30, 2020, the World Health Organization declared the global outbreak a public health emergency of international concern and named it as "Coronavirus Disease 2019 (COVID-19)" (1).

Healthcare workers on the front line (henceforth referred to as HCWs), who are directly involved in the diagnosis, treatment, and care of patients with COVID-19, are at risk of developing psychological distress. Facing these war-like circumstances, overwhelmingly huge workload, depletion of personal protective equipment (PPE), worries about becoming infected or infecting family members, lack of specific drugs, widespread media coverage, witnessing the suffering and death of patients, feeling stigmatized and being inadequately supported may all contribute to the emotional and physical burnout of the HCWs. Previous studies about psychological reactions to the SARS outbreak among HCWs reported that they feared contagion and infection of their loved ones, felt uncertainty and stigmatization, reported reluctance to work or contemplating resignation, and experienced high levels of stress, anxiety, and depression (2–4). Similar concerns about the mental burden of the HCWs are now arising. A recent study from China revealed that the prevalence of symptoms of depression, anxiety, insomnia, and distress were found to be 50%, 44%, 34%, and 71%, respectively (5). Even if the number of

Highlights

- **COVID-19 pandemic has caused psychological symptoms in healthcare workers.**
- **Being female and having a previous psychiatric disorder are risk factors for distress.**
- **Working in close contact with patients is associated with psychological distress.**
- **Perceived stigma has a negative impact on the mental health of healthcare workers.**

Correspondence Address: Rümeysa Taşdelen, Department of Psychiatry, Marmara University İstanbul Pendik Education and Research Hospital, Fevzi Çakmak Quarter Muhsin Yazıcıoğlu Street No: 10 Üst Kaynarca -Pendik, İstanbul, Turkey • **E-mail:** drrtasdelen@gmail.com
Received: 10.03.2021, **Accepted:** 31.05.2021, **Available Online Date:** 03.05.2022
©Copyright 2021 by Turkish Association of Neuropsychiatry - Available online at www.noropskiyatriarsivi.com

cases decreases, it is still predicted that it will continue in the form of waves in the upcoming months and years. Therefore, the psychological symptoms of HCWs may continue to decrease their motivation, concentration, and efficacy of work (6). Previous clinical studies have also found that under stress, the neuroendocrine network regulated by the hypothalamic-pituitary-adrenal axis is dysregulated and stress hormone levels change (7). As a result, psychological symptoms inhibit immune defenses and increase the risk of autoimmune or inflammatory diseases. Enhanced susceptibility may result in a high risk of being infected. Also, these symptoms may affect the quality of life of the HCWs and lead to anxiety disorders, depression, and post-traumatic stress disorder in the long term. Additionally, in the outbreaks, a vulnerable group of people, such as the elderly, confirmed patients and their families, and HCWs, are labeled, stereotyped, discriminated against, and/or experience loss of status because of a perceived link with a disease. In May 2020, more than 200 incidents of COVID-19 related attacks on healthcare workers and health facilities during the ongoing pandemic as a result of stigmatization were reported (8). Facing with stigmatization and negative attitudes lead to isolation and enhance the psychological difficulties of HCWs (2, 3, 9). A recent study from India researching COVID-19-related stigma and stress of physicians found higher levels of stigma (62.1%) and stress (63.8%) (10). Another study which was investigating COVID-19-related perceived stigma and stress among dialysis staff showed that 54.6% of the participants had reported significant perceived stigma associated with their job and 36.1% of them perceived significant stress (11).

Consequently, it is highly important to acknowledge the psychological impact of this mounting threat on healthcare professionals working under stressful conditions as soon as possible and support them. In this study, we aim to provide an assessment of the mental health burden of Turkish HCWs and discuss the factors, including stigmatization, increasing their psychological distress.

METHODS

A cross-sectional survey was designed to assess the healthcare workers' psychological response during the COVID-19 outbreak using an online questionnaire. Data were collected from April 16 to May 16, 2020. Totally 69,392 confirmed cases and 1,518 deaths were declared by the government in Turkey on the 15th of April. Approximately 4000 new cases were being announced daily during those days. The number of confirmed cases was 146,457 on the 15th of May. To decrease contagion, the first curfew was announced between 10 and 12 April in 31 big cities by the Turkish government, and it was repeated on all weekends and holidays until June.

A snowball sampling strategy was utilized by focusing on recruiting doctors, nurses, other HCWs (e.g., dentists, laboratory personnel, psychologists, and social workers), and non-medical staff working at hospitals (e.g., technicians, secretaries, and cleaning staff). An online questionnaire created through Google Forms (Google Inc, Mountain View, CA, USA) was delivered to participants via social media groups of hospitals and online forums for healthcare professionals. All respondents provided informed consent at the beginning of the survey. Ethical approval for this study was obtained from the ethical committee of Istanbul Marmara University with the number 2020/499.

The study questionnaire includes seven main components as follows: socio-demographic characteristics (gender, age, marital status, occupation, living in cities with a curfew or not, working on the frontline or not), previous psychiatric or medical disorders, changes in nicotine, alcohol and social media use, Depression Anxiety Stress Scales (DASS-21), questions about perceived stigma and concerns about working conditions during the COVID-19 outbreak**.**

Psychological symptoms, such as depression, anxiety, and stress were assessed using DASS-21, which is a 4-point Likert-type screening instrument and demonstrated to be reliable and validated in the Turkish population (12, 13). The self-reported 21-item scale provides independent measures of depression, stress, and anxiety with recommended severity thresholds for depression, stress, and anxiety subscales. The scores for each of the three components were calculated by summing up the scores for the relevant items and multiplying by two to calculate the final score. Cut-off scores were 9, 7, and 14 for depression, anxiety, and stress, respectively. The severity of depression, anxiety, or stress is also assessed by subscale cut-off scores. The DASS-21 is a well-validated screening instrument, with excellent internal consistency in depression (Cronbach's α=0.87), anxiety (Cronbach's α=0.83), and stress (Cronbach's α=0.84) in the present sample.

Statistical Package for Social Sciences, version 20 (SPSS Inc., Chicago, IL) was used to analyze the data. Results were given as mean ± standard

Table 1. Sociodemographic variables and working conditions of the sample

	**n (%) / Mean ± SD**
Age	35.89±8.63
Gender	
Female	432 (68%)
Male	202 (32%)
Marital Status	
Single	220 (35%)
Married	414 (65%)
Having a child	
Yes	364 (58%)
No	270 (42%)
City of residence	
Cities with curfew	561 (89%)
Other cities	72 (11%)
Household status	
With parents	72 (12%)
With spouse and/or children	396 (62%)
Alone	140 (22%)
Other	26 (4%)
History of psychiatric disorder	
Yes	172 (27%)
No	462 (73%)
History of chronic illness or other risk factors	
Yes	134 (21%)
No	500 (79%)
Profession	
Doctor	426 (67%)
Nurse	118 (19%)
Other medical staff	27 (4%)
Non-medical staff	63 (10%)
Work experience (years)	10.85±85.59
Working with COVID-19 patients	
Yes	402 (63%)
No	232 (37%)
Working hours	
8–12 hour shifts	431 (68%)
24 hour shifts	114 (18%)
Other	89 (14%)
Presence of any COVID-19 symptoms	
Yes	209 (33%)
No	425 (67%)
COVID-19 testing situation	
No	492 (78%)
Yes, result is positive	19 (3%)
Yes, result is negative	123 (19%)

SD, Standard deviation; COVID-19, Coronavirus Disease 2019

Table 2. Depression, anxiety, and stress levels measured with DASS-21 (n=634)

	Mean ± SD	n (%)
Depression subscale score	5.69±4.85	
Anxiety subscale score	3.94±3.95	
Stress subscale score	6.12±4.52	
Moderate to extremely severe depression		226 (36%)
Moderate to extremely severe anxiety		222 (35%)
Moderate to extremely severe stress		140 (22%)
Total score	15.76±12.01	

DASS-21, Depression Anxiety Stress Scales; SD, Standard deviation

deviation for continuous variables and as count and percentages (%) for categorical variables. A p-value of less than 0.05 was considered statistically significant, and all analyses were two-tailed. Univariate analysis to compare continuous variables like DASS-21 scores was conducted using student t-test or one-way ANOVA. According to the homogeneity of the variance, Tukey or Tamhane's T2 test was used in the post-hoc pairwise comparison of the multiple groups. Pearson's correlation test was used for the correlation analysis. Later, multiple regression analysis was conducted to investigate the unique effects of different variables on total DASS-21 score.

RESULTS

In this study, a total of 634 participants' survey results were evaluated after excluding four subjects with missing data. The characteristics of the participants are presented in Table 1. The mean age was 35.89±8.63, and the vast majority were female (68%), married (65%), living with a spouse and/or children (62%), and living in the cities with a curfew (89%). Of the 634 responding participants, 426 (67%) were physicians, 118 (19%) were nurses, 27 (4%) were other medical staff members and 63 (10%) were non-medical staff members. A total of 402 (63%) participants were frontline HCWs (i.e., working with COVID-19 patients).

Mean depression, anxiety, stress levels and total DASS-21 scores are shown in Table 2. When we used predetermined cut-off values of the scale, a third of our sample had at least moderate depression and anxiety, and one-fourth had at least moderate stress. Comparison of the mean depression, anxiety, stress levels and total DASS-21 score according to sociodemographic variables and other associated factors are shown in Table 3. Higher DASS-21 scores were significantly correlated with being female and having a previous psychiatric disorder. However, it is also worthy of note that individuals who worked directly with patients with COVID-19 had higher total DASS-21 scores at a trend level. Additionally,

Table 3. Comparison and correlations of DASS-21 scores with sociodemographic variables and other factors related to COVID-19 pandemic

	DASS-21 Total			DASS-21 Depression			DASS-21 Anxiety			DASS-21 Stress		
	Mean ± SD	Test statistics	p	Mean ± SD	Test statistics	p	Mean ± SD	Test statistics	p	Mean ± SD	Test statistics	p
Age		r=-0.02	0.68		r=-0.05	0.22		r=0.00	0.99		r=0.01	0.81
Gender												
Female	17.41±12.4	t=5.500	<0.001	6.22±5.02	t=4.239	<0.001	4.56±4.11	t=6.453	<0.001	6.63±4.59	t=4.170	<0.001
Male	12.25±10.29			4.58±4.28			2.62±3.22			5.04±4.16		
Marital status												
Single	15.9±11.47	t=0.202	0.840	6.21±5.03	t=1.952	0.051	3.79±3.7	t=-0.736	0.462	5.9±4.16	t=-0.913	0.362
Married	15.69±12.29			5.42±4.74			4.03±4.08			6.24±4.7		
Profession												
Doctor	15.81±11.68	F=0.749	0.523	5.75±4.79	F=0.562	0.640	3.76±3.84	F=1.944	0.121	6.31±4.49	F=1.395	0.243
Nurse	15.51±12.8			5.68±5.09			4.12±4.19			5.70±4.59		
Other medical staff	13±11.56			4.51±4.2			3.7±4.15			4.78±4		
Non-medical staff	17.08±12.88			5.83±5.11			5±4.06			6.25±4.73		
Work experience (years)		r=-0.01	0.83		r=-0.03	0.39		r=0.01	0.73		r=-0.00	0.96
Working with COVID-19 patients												
Yes	16.43±12.26	t=1.831	0.068	5.96±4.98	t=1.787	0.074	4.11±4.04	t=1.404	0.161	6.36±4.66	t=1.717	0.087
No	14.62±11.49			5.24±4.6			3.66±3.77			5.72±4.23		
Lifetime psychiatric disorder												
Yes	19.04±11.67	t=4.250	<0.001	7.15±5.07	t=4.669	<0.001	4.49±3.77	t=2.121	0.034	7.40±4.39	t=4.425	<0.001
No	14.54±11.91			5.15±4.66			3.74±4			5.65±4.47		
Chronic illness or other risk factors												
Yes	17.37±12.33	t=1.743	0.082	6.13±5.03	t=1.163	0.245	4.64±4.13	t=2.309	0.021	6.60±4.61	t=1.364	0.173
No	15.33±11.89			5.58±4.8			3.76±3.88			6±4.49		
Had any COVID-19 symptoms												
Yes	19.09±12.47	t=4.978	<0.001	6.77±5.16	t=3.961	<0.001	5.18±4.1	t=5.644	<0.001	7.14±4.68	t=4.011	<0.001
No	14.13±11.43			5.16±4.61			3.34±3.73			5.63±4.35		
Had COVID-19 testing												
No	14.73±11.55	F=8.319	<0.001*	5.39±4.68	F=4.263	0.014*	3.62±3.77	F=7.823	<0.001*	5.72±4.29	F=9.176	<0.001*
Yes, result is positive	19.84±12.28			6.47±4.89			5.63±4.84			7.74±4.76		
Yes, result is negative	19.26±13.03			6.77±5.38			4.98±4.28			7.50±5.05		

*Post-hoc pairwise comparison showed that scores for individual with negative test results are higher than individual with no testing.
DASS-21, Depression Anxiety Stress Scales; COVID-19, Coronavirus Disease 2019; SD, Standard deviation

Table 4. Working environment and concerns and their association with DASS-21 Total scores

	n (%)	Mean ± SD	Test statistics	p
Factors related to working environment				
Having received professional training on COVID-19				
Yes	418 (66%)	15.77±12.21	*t*=0.01	p=0.995
No	216 (34%)	15.76±11.63		
Having adequate protective equipment				
Yes	459 (72%)	14.81±11.71	*t*=-3.25	p=0.001
No	175 (28%)	18.26±12.43		
Supervisors considering ideas, criticisms or contributions of healthcare workers				
Yes	379 (60%)	14.8±11.85	*t*=-2.47	p=0.014
No	255 (40%)	17.19±12.12		
Feeling safe while working				
Yes	205 (32%)	11.61±10.73	*t*=-6.45	p<0.001
No	429 (68%)	17.274±12.08		

DASS-21, Depression Anxiety Stress Scales; COVID-19, Coronavirus Disease 2019; SD, Standard deviation

Table 5. Perceived stigma in healthcare workers at association with DASS-21 Total scores

	Group 1 No change		Group 2 More supportive and positive		Group 3 More dismissive/ stigmatizing and negative			
	n (%)	Mean DASS-21 Total ± SD	n (%)	Mean DASS-21 Total ± SD	n (%)	Mean DASS-21 Total ± SD	p value*	Post-hoc pairwise comparison**
Reactions of families and close friends	185 (30%)	12.96±12.27	405 (63%)	16.26±11.32	44 (7%)	22.98±13.53	0.030	1<2, 1<3, 2<3
Reactions of neighbors, relatives and less intimate friends	271 (43%)	14.17±11.53	275 (43%)	15.76±11.73	88 (14%)	20.69±13.05	<0.001	1<3, 2<3
Reactions of unfamiliar people and from service sector	461 (73%)	15.02±11.67	127 (20%)	15.90±12.07	46 (7%)	22.87±13.04	<0.001	1<3, 2<3

*ANOVA, ** Tukey or Tamhane's T2.
DASS-21, Depression Anxiety Stress Scales; SD, Standard deviation

28% (n=179) of the overall sample were smokers. Among smokers, there was a difference between the change in smoking habits and total DASS-21 scores. Individuals who increased their smoking had higher scores (23.67±14.6) compared to the individuals who decreased smoking (15.48±9.17). However, we found no significant difference between alcohol consumption changes and total DASS-21 scores. When changes in social media usage were examined, there was a difference between the groups and total DASS-21 scores (p<0.001). The post-hoc comparison showed that individuals who increased their social media use "much more" had higher scores (20.48±11.22) compared to the individuals who used "more" (15.63±11.66) and the individuals who used "as usual" (13.7±12.25). However, their DASS-21 scores were not significantly different than that of the individuals who decreased their use (15.92±12).

Besides sociodemographic variables, other factors related to the psychological status of HCWs were also investigated. Associations with DASS-21 total and subscale scores and working conditions were presented in Table 4. The healthcare workers who described less availability of PPE, lower support from their supervisors, and more unsafe working conditions had significantly higher DASS-21 total scores.

Changes in total DASS-21 scores according to perceived stigma in HCWs are shown in Table 5. Most of the subjects stated that their family and friends became more supportive during the outbreak because they are HCWs. However, there was a significant difference between groups in their total DASS-21 scores (Table 5). Individuals who thought other people were more dismissive and displaying a negative attitude towards them had significantly higher total DASS-21 scores.

DISCUSSION

In an outbreak, since HCWs are highly likely to face a greater risk of infection and undertake a higher workload than the general population, they are at risk of a higher prevalence of psychological symptoms as described in previous studies (4, 9, 14, 15). According to our findings, 36% of the participants reported moderate to severe depressive symptoms, whereas 35% and 22% of them reported moderate to severe anxiety and stress levels, respectively. Our findings have similarities with the literature although the levels of psychological distress are lower in our study (5, 16–19). The studies conducted during the early period of the outbreak, before the end of March, have higher scores. The first study published from Turkey assessed the period between the 10th and 15th of March and indicated that the prevalences of depressive symptoms, anxiety and stress-related symptoms were 65%, 52%, and 41%, respectively (19). Similarly, another study from China reported that 50% of HCWs had symptoms of depression, 45% of them had anxiety, and 72% of them had distress in February. This seemingly decreased psychological burden of HCWs may be related to the government regulations about flexible and reduced working hours established at the end of March in Turkey. By the end of March, a rapid adaptation process started in the healthcare system, the number of outpatient units decreased, inpatient units converted to COVID-19 related wards, non-emergency admissions to hospitals were restrained, and HCWs started to work only in frontline positions with shifts to decrease confronted viral load. The anxiety about the uncertainty is relieved by the information that is given in both mainstream and social media daily. This may result in our lower depression, anxiety, and stress scores than previously reported.

Multivariate analysis showed that the sociodemographic characteristics associated with DASS-21 scores of the respondents were gender and a history of lifetime psychiatric disorder. Also, HCWs who worked directly with patients with COVID-19 had higher total DASS-21 scores. Similar to our findings, Lai et al. (5) indicated that women and frontline workers had a greater risk for developing adverse psychological outcomes, Zhang et al. observed being female, and being at risk of contact with patients with COVID-19 were the most common risk factors for insomnia, anxiety, and depression, and Lu et al. found that front line medical staff with close contact with infected patients were twice as likely to suffer anxiety and depression than non-clinical staff (5, 17, 18). Having a lifetime psychiatric disorder is a risk factor for relapses under stressful conditions. Both disruption of routine psychiatric examinations and working under the risk of infection may exacerbate the remitted symptoms. Unlike the previous studies, we could not find any correlation between having a previous chronic medical disease and depression or anxiety scores (17). This may be explained by the fact that since the end of March, the staff members with chronic diseases were allowed to not work in Turkey.

In addition to the factors affecting psychological status, our study revealed that the smoking habits of HCWs were related to depression and anxiety scores. Individuals who increased their smoking had higher DASS-21 scores. This may be explained by the precautions about smokers having a higher risk of COVID-19 morbidity and mortality, were not considered by HCWs under stressful conditions. Also, the changes in social media usage showed that individuals who increased their social media use much more had higher scores (20.48±11.22) compared to the individuals who used social media as usual (13.70±12.25). This information may show that uncertainty of conditions may result in anxiety and lead to more social media usage or the participants using more social media may become more anxious due to higher exposure to disaster scenarios, and vice versa. Psychological support and psycho-education should decrease the abuse and also the psychological symptoms.

Many countries experience various difficulties in healthcare management during the outbreak. Around the world, many HCWs got infected due to insufficient PPE availability (20). Additionally, factors such as excessive workload, the increasing number of cases over time, and the insufficiency of medications and intensive care units, all contributed to the increased mental burden of HCWs (6). In our study, the HCWs who described having inadequate PPE, lower support from supervisors, and more unsafe working conditions had significantly higher DASS-21 scores, which corroborates with a recent study (19). Specific preventions should be taken immediately to minimize the risk of contamination of healthcare professionals. The hospital administrators and supervisors should support them by considering their suggestions and complaints. Professional training about COVID-19 should be given to them to make them feel safer and more competent.

It has been revealed in previous studies that HCWs fear infecting their loved ones and relatives during the outbreak (5, 19, 21). In our study, HCWs experienced this fear most frequently: at a rate of 87%. As a result, they may be deprived of the social support they need, and the stress burden increases as indicated in previous studies (19, 22, 23). Some initiatives are needed to provide psychological care for HCWs with poor social support, such as creating environments where colleagues can share their concerns and establishing psychiatric counseling units specialized for them. Telephone, internet, or application-based psychiatric help programs have been developed in various countries of the world for this purpose (24, 25). Experienced mental health professionals started to provide peer support using social media (26). However, all these are still not common enough to serve the needs of HCWs and should be generalized.

Like the concerns of HCWs, the community also has the fear of being infected by HCWs. The fear and anxiety resulted in stigmatization and negative attitudes. In our study, although most of the participants perceive their family, friends, and other people as more supportive, 7% of participants stated that they perceive stigmatization by their families and close friends, 14% of them perceive stigmatization by their neighbors, relatives, and less intimate friends; and 7% of them perceive stigmatization by unfamiliar people like workers from the service sector. Similar to a study from Taiwan which reported that 20% of the participants felt stigmatized during the SARS outbreak (3), we found that HCWs who perceived other people as more dismissive and discriminating had significantly higher depression, anxiety, and stress scores. Inconsistent with the previous studies, our study also revealed that participants who had more support from their family and close friends had also significantly higher total DASS-21 scores (23, 27). Increased needs and demands of HCWs who have more depressive, anxiety, or stress symptoms may be associated with getting more support. Likewise, a recent study has shown that seeking help from families and friends is a common strategy to cope with psychological impacts (28).

Our cross-sectional study has several limitations. Firstly, as minimizing face-to-face interaction is recommended during COVID-19, an online survey was designed. Thus, psychiatric signs could not be verified by mental health professionals. Secondly, the study population may not represent all the HCWs because the snowballing sampling strategy does not give the opportunity to select the participants randomly, leading to selection bias. In addition, the causality is not clear. For example, while there is a strong association between psychiatric symptoms and perceived stigma, it is not possible to say psychiatric symptoms increase the perception of stigma or vice versa. Finally, since this study does not have a control group, and the outbreak has also affected the general population's mental health, the causality between providing healthcare and having psychiatric symptoms cannot be conclusively interpreted.

As a conclusion, the HCWs who are the backbone of this battle against the disease, are at high risk of developing psychological distress. In addition to being female, working in frontline positions and having a previous psychiatric disorder, having inadequate personal protective equipment, lower support from supervisors, unsafe working conditions, and feeling stigmatized increase depression, anxiety, and stress scores of HCWs. It is noteworthy that our findings may provide a reference point for understanding the difficulties of the HCWs during outbreaks and the need for developing prevention and treatment strategies considering related factors. More mental health services are required for the analysis of HCWs' psychological status and the improvements to make working conditions safer and supportive should be done.

Ethics Committee Approval: Ethical approval for this study was obtained from the ethical committee of Istanbul Marmara University with the number 2020/499.

Informed Consent: Participants gave informed consent about their data in the project, and the study has been carried out according to the Declaration of Helsinki.

Peer-review: Externally peer-reviewed.

Author Contributions: Concept and Design –RT, BA, HK; Supervision –EE; Resource –RT, BA, HK; Materials –ME; Data Collection and/or Processing – RT, BA, HK, ME, EE; Analysis and/or Interpretation – ME, BA; Literature Search – RT, HK; Writing – RT, BA, HK, ME, EE; Critical Reviews – EE

Conflict of Interest: The authors declared no potential conflict of interest for the research, authorship, and publication of this article.

Financial Disclosure: The authors received no financial support for the research, authorship, and publication of this article.

REFERENCES

1. WHO (World Health Organization). Statement on the meeting of the International Health Regulations (2005) Emergency Committee regarding the outbreak of novel coronavirus (2019-nCoV). WHO Newsletter 2020. p.1–6. https://www.who.int/news/item/30-01-2020-statement-on-the-second-meeting-of-the-international-health-regulations-(2005)-emergency-committee-regarding-the-outbreak-of-novel-coronavirus-(2019-ncov)
2. Maunder R, Hunter J, Vincent L, Bennett J, Peladeau N, Leszcz M, Sadavoy J, Verhaeghe LM, Steinberg R, Mazzulli T. The immediate psychological and occupational impact of the 2003 SARS outbreak in a teaching hospital. CMAJ 2003;168:1245–1251. https://www.ncbi.nlm.nih.gov/pmc/articles/PMC154178/
3. Bai YM, Lin C-C, Lin C-Y, Chen JY, Chue C-M, Chou P. Survey of stress reactions among health care workers involved with the SARS outbreak. Psychiatric Serv 2004;55:1055–1057. [Crossref]
4. Chua SE, Cheung V, Cheung C, McAlonan GM, Wong JWS, Cheung EPT, Chan MTY, Wong MMC, Tang SW, Choy KM, Wong MK, Chu CM, Tsang KWT. Psychological effects of the SARS outbreak in Hong Kong on high-risk health care workers. Can J Psychiatry 2004;49:391–393. [Crossref]
5. Lai J, Ma S, Wang Y, Cai Z, Hu J, Wei N, Wu J, Du H, Chen T, Li R, Tan H, Kang L, Yao L, Huang M, Wang H, Wang G, Liu Z, Hu S. Factors Associated With Mental Health Outcomes Among Health Care Workers Exposed to Coronavirus Disease 2019. JAMA Netw open 2020;3:e203976. [Crossref]
6. Imai R, Hori H, Itoh M, Lin M, Niwa M, Ino K, Ogawa S, Ishida M, Sekiguchi A, Matsui M, Kunugi H, Akechi T, Kamo T, Kim Y. Inflammatory markers and their possible effects on cognitive function in women with posttraumatic stress disorder. J Psychiatr Res 2018;102:192–200. [Crossref]
7. Passos IC, Vasconcelos-Moreno MP, Costa LG, Kunz M, Brietzke E, Quevedo J, Salum G, Magalhães PV, Kapczinski F, Kauer-Sant'Anna M. Inflammatory markers in post-traumatic stress disorder: A systematic review, meta-analysis, and meta-regression.Lancet Psychiatry 2015;2:1002–1012. [Crossref]
8. Bagcchi S. Stigma during the COVID-19 pandemic. Lancet Infect Dis 2020;20:782. [Crossref]
9. Lee SM, Kang WS, Cho AR, Kim T, Park JK. Psychological impact of the 2015 MERS outbreak on hospital workers and quarantined hemodialysis patients. Compr Psychiatry 2018;87:123–127. [Crossref]
10. Uvais NA, Shihabudheen P, Hafi NAB. Perceived Stress and Stigma Among Doctors Working in COVID-19-Designated Hospitals in India. Prim Care Companion CNS Disord; 2020;22:20br02724. [Crossref]
11. Uvais NA, Aziz F, Hafeeq B. COVID-19-related stigma and perceived stress among dialysis staff. J Nephrol 2020[Epub ahead of print]. [Crossref]
12. Lovibond SH, Lovibond PF. Manual for the Depression Anxiety Stress Scales, 2nd ed. Psychology Foundation of Australia; 1995. https://eprovide.mapi-trust.org/instruments/depression-anxiety-stress-scales
13. Yılmaz Ö, Boz H, Arslan A. The validity and reliability of depression stress and anxiety scale (Dass-21) Turkish short form. J Financ Econ Soc Res 2017;2:78–91. https://dergipark.org.tr/tr/download/article-file/354106
14. Tam CWC, Pang EPF, Lam LCW, Chiu HFK. Severe acute respiratory syndrome (SARS) in Hongkong in 2003: Stress and psychological impact among frontline healthcare workers. Psychol Med 2004;34:1197–1204. [Crossref]
15. Gershon R, Dernehl LA, Nwankwo E, Zhi Q, Qureshi K. Experiences and Psychosocial Impact of West Africa Ebola Deployment on US Health Care Volunteers. PLoS Curr 2016;8. [Crossref]
16. Chew NWS, Lee GKH, Tan BYQ, Jing M, Goh Y, Ngiam NJH, Yeo LLL, Ahmad A, Ahmed Khan F, Napolean Shanmugam G, Sharma AK, Komalkumar RN, Meenakshi P V., Shah K, Patel B, Chan BPL, Sunny S, Chandra B, Ong JJY, Paliwal PR, Wong LYH, Sagayanathan R, Chen JT, Ying Ng AY, Teoh HL, Tsivgoulis G, Ho CS, Ho RC, Sharma VK. A multinational, multicentre study on the psychological outcomes and associated physical symptoms amongst healthcare workers during COVID-19 outbreak. Brain Behav Immun 2020;88:559–565. [Crossref]
17. Zhang W-R, Wang K, Yin L, Zhao W-F, Xue Q, Peng M, Min B-Q, Tian Q, Leng H-X, Du J-L, Chang H, Yang Y, Li W, Shangguan F-F, Yan T-Y, Dong H-Q, Han Y, Wang Y-P, Cosci F, Wang H-X. Mental Health and Psychosocial Problems of Medical Health Workers during the COVID-19 Epidemic in China. Psychother Psychosom 2020;89:242–250. [Crossref]
18. Lu W, Wang H, Lin Y, Li L. Psychological status of medical workforce during the COVID-19 pandemic: A cross-sectional study. Psychiatry Res 2020;288:112936. [Crossref]
19. Elbay RY, Kurtulmuş A, Arpacıoğlu S, Karadere E. Depression, anxiety, stress levels of physicians and associated factors in Covid-19 pandemics. Psychiatry Res 2020;290:113130. [Crossref]
20. Ranney ML, Griffeth V, Jha AK. Critical supply shortages - The need for ventilators and personal protective equipment during the Covid-19 pandemic. New Engl J Med 2020;382 18:e41. [Crossref]
21. Tsamakis K, Rizos E, Manolis A, Chaidou S, Kympouropoulos S, Spartalis E, Spandidos D, Tsiptsios D, Triantafyllis A. [Comment] COVID-19 pandemic and its impact on mental health of healthcare professionals. Exp Ther Med 2020;19:3451–3453. [Crossref]
22. Huang JZ, Han MF, Luo TD, Ren AK, Zhou XP. Mental health survey of medical staff in a tertiary infectious disease hospital for COVID-19. Zhonghua Lao Dong Wei Sheng Zhi Ye Bing Za Zhi 2020;38:192–195. [Crossref]
23. Xiao H, Zhang Y, Kong D, Li S, Yang N. The Effects of Social Support on Sleep Quality of Medical Staff Treating Patients with Coronavirus Disease 2019 (COVID-19) in January and February 2020 in China. Med Sci Monit 2020;26:e923549-1–e923549-8. [Crossref]
24. Fagiolini A, Cuomo A, Frank E. COVID-19 Diary From a Psychiatry Department in Italy. J Clin Psychiatry 2020;81:20com13357. [Crossref]
25. Unützer J, Kimmel RJ, Snowden M. Psychiatry in the age of COVID-19. World Psychiatry 2020;19:130–131. [Crossref]
26. Cheng P, Xia G, Pang P, Wu B, Jiang W, Li Y-T, Wang M, Ling Q, Chang X, Wang J, Dai X, Lin X, Bi X. COVID-19 Epidemic Peer Support and Crisis Intervention Via Social Media. Community Ment Health J 2020;56:786–792. [Crossref]
27. Lv Y, Yao H, Xi Y, Zhang Z, Zhang Y, Chen J, Li J, Li J, Wang X, Luo GH. Social Support Protects Chinese Medical Staff from Suffering Psychological Symptoms in COVID-19 Defense. SSRN Electron J 2020. https://papers.ssrn.com/sol3/papers.cfm?abstract_id=3557996
28. Cai H, Tu B, Ma J, Chen L, Fu L, Jiang Y, Zhuang Q. Psychological impact and coping strategies of frontline medical staff in Hunan between January and March 2020 during the outbreak of coronavirus disease 2019 (COVID) in Hubei, China. Med Sci Monit 2020;26:e924171. [Crossref]
