# Supplementary material for: Mental health status and related factors influencing healthcare workers during the COVID-19 pandemic: A systematic review and meta-analysis
Source: PLoS One. 2024 Jan 19;19(1):e0289454. doi: 10.1371/journal.pone.0289454 (PMC10798549; doi:10.1371/journal.pone.0289454)
Supplement: S1 Data — (ZIP) [file pone.0289454.s011.zip › literatures/192.pdf]

·Hot topics on mental health in coronavirus disease 2019·　·2019冠状病毒病精神卫生专题·

DOI:10.11817/j.issn.1672-7347.2020.200241
http://xbyxb.csu.edu.cn/xbwk/fileup/PDF/202006613.pdf

2019冠状病毒病防控一线人员的心理状况

滕紫薇，黄兢，邱妍，谭羽希，钟秋平，唐慧，伍海姗，吴莹，陈晋东

（国家精神心理疾病临床医学研究中心，中南大学湘雅二医院精神病学科，长沙 410011）

[摘 要] 目的：2019冠状病毒病(coronavirus disease 2019，COVID-19)疫情为全球公共卫生危机，引发了不同人群的各种心理问题。本研究旨在了解COVID-19疫情对防控一线工作人员心理状况的影响。**方法：**在问卷星平台采用电子量表进行滚雪球抽样调查，使用抑郁症筛查量表(Patient Health Questionnare-9，PHQ-9)、焦虑自评量表(Self-Rating Anxiety Scale，SAS)、疲劳自评量表(Fatigue Self-assessment Scale，FSAS)评估一线工作人员抑郁、焦虑、疲劳度。**结果：**一线工作人员抑郁检出率49.1%，焦虑检出率21.8%，疲劳检出率76.0%，其中社区、乡镇工作人员抑郁、焦虑、疲劳检出率较医护人员、其他职业者高($P<0.01$)。一线工作人员的PHQ-9与年龄、家庭收入、家人的支持、服务对象是否满意、睡眠质量呈负相关(均$P<0.01$)，与文化程度、担心感染肺炎、每天关注疫情的时间以及疲劳呈正相关(均$P<0.01$)。SAS与年龄、家庭收入、家人的支持、服务对象是否满意、睡眠质量呈负相关(均$P<0.01$)，与性别、担心感染肺炎、每天关注疫情的时长以及疲劳呈正相关(均$P<0.01$)。**结论：**一线防控工作人员自身应合理安排工作和休息时间，及时调节不良情绪，缓解疲劳；政府部门和社会在关注COVID-19疫情时，也应多关心一线防控工作人员，尤其是社区、乡镇工作人员的心理状态，及时对他们采取调休、心理干预等措施。

[关键词] 2019冠状病毒病；肺炎；一线工作人员；抑郁；焦虑；疲劳

Mental health of front-line staff in prevention of coronavirus disease 2019

TENG Ziwei, HUANG Jing, QIU Yan, TAN Yuxi, ZHONG Qiuping,
TANG Hui, WU Haishan, WU Ying, CHEN Jindong

(National Clinical Research Center for Mental Disorders; Department of Psychaitry, Second Xiangya Hospital, Central South University, Changsha 410011, China)

ABSTRACT

Objective: The coronavirus disease 2019 (COVID-19) pandemic is a global public health crisis, which elicits psychological problems in different population**.** This study is to investigate the impact of COVID-19 on mental health in the front-line staff.

Methods: Patient Health Questionnare-9 (PHQ-9), Self-Rating Anxiety Scale (SAS), and

收稿日期(Date of reception)：2020-03-13
第一作者(First author)：滕紫薇，Email: zwaidc@csu.edu.cn, ORCID: 0000-0002-7566-777X
通信作者(Corresponding author)：陈晋东，Email: chenjindong@csu.edu.cn, ORCID: 0000-0003-4551-2344
基金项目(Foundation item)：国家自然科学基金(81971258，81901401)。This work was supported by the National Natural Science Foundation of China (81971258, 81901401).

©Journal of Central South University (Medical Science). All rights reserved.

Fatigue Self-assessment Scale (FSAS) were used to assess the depression, anxiety, and fatigue in front-line staff.

Results: The detection rates of depression, anxiety, and fatigue were 49.1%, 21.8%, and 76.0% among the front-line staff. The rates of depression, anxiety, and fatigue in community workers were higher than those in medical workers and other occupational staff (P<0.01). The PHQ-9 of front-line staffs was negatively correlated with age, family income, family members’ support, satisfaction of service objects, and sleep quality (all P< 0.01), while positively correlated with education level, fatigue, fear of pneumonia, and the duration of daily attention to the COVID-19 (all P<0.01). SAS was negatively correlated with age, family income, family support, satisfaction of objects service, and sleep quality (all P<0.01), while positively correlated with gender, fatigue, fear of pneumonia, and duration of daily attention to the COVID-19 (all P<0.01).

Conclusion: The front-line workers should manage work and rest time reasonably to adjust their negative mood and fatigue. The government and the society should pay more attention to the psychological state of the front-line staff, particularly for the staff working in the community or villages and towns in preventing the COVID-19 pandemic. Thus, front-line staff can be obtained mental intervention or be taken a rest from the high-intensive work.

KEY WORDS coronavirus disease 2019; pneumonia; front-line staff; depression; anxiety; fatigue

2019 冠状病毒病(coronavirus disease 2019，COVID-19)是指严重急性呼吸道综合征2型冠状病毒(severe acute respiratory syndrome coronavirus 2，SARS-CoV-2)感染导致的肺炎，其传播途径主要包括直接传播、气溶胶传播和接触传播，是一种高传染性的急性呼吸道传染病[1]。2020年2月，国家卫生健康委员会将COVID-19纳入《中华人民共和国传染病防治法》规定的乙类传染病，并采取甲类传染病的预防、控制措施[2]。

为有效控制COVID-19疫情，中国投入大量人力参与到抗疫一线。疫情期间，一线工作人员工作强度与精神压力倍增，关注他们的心理健康，及时进行心理干预，对于提升疫情防控效果和社会群体心理健康程度具有积极作用。最近，一项针对COVID-19疫情不同人群的心理状态的荟萃分析[3]显示其中有72.9%的调查对象存在心理不健康等问题。其调查人群大部分为医护人员、普通人或学生，没有疫情期间包括社区工作人员、警察、市场管理人员、志愿者等其他一线工作人员心理健康及疲劳程度的研究数据。随着国外疫情越来越严重，归国人群的增多，这些一线工作人员工作量明显加大，更易疲劳，其情绪也明显受影响。因此，本研究主要从抑郁、焦虑、疲劳3个方面对COVID-19疫情期间一线工作人员的心理健康状况进行分析，以了解其心理健康程度与职业、年龄、疲劳等方面的相关性，为改善疫情期间一线人员的心理健康状况提供理论依据。

1 对象与方法

1.1 对象

2020年3月2日至3月5日，选择COVID-19期间能读懂中文汉字的一线工作人员作为研究对象。通过互联网在问卷星平台以滚雪球抽样调查的方法收集相关问卷。

1.2 方法

1.2.1 一般资料收集

在受试者签署知情同意的基础上，收集其性别、年龄、婚姻、收入、受教育程度等一般人口学资料，以及对待疫情的态度、家人的支持、服务对象的满意程度等。

1.2.2 抑郁自评量表

采用抑郁症筛查量表(Patient Health Questionnare-9，PHQ-9)[4]评估一线工作人员抑郁情绪。PHQ-9中的每一个条目都对应抑郁症精神疾病诊断与统计手册-IV(the Diagnostic and Statistical Manual of Mental Disorders-IV，DSM-IV)的诊断标准，共含9个项目，4级评分，各项目得分之和为总分。其评分标准为：0~4分为无抑郁症状；5~9分为轻度抑郁；10~14分为中度抑郁；≥15分为重度抑郁。

©Journal of Central South University (Medical Science). All rights reserved.

1.2.3 焦虑自评量表

采用焦虑自评量表(Self-Rating Anxiety Scale，SAS)评估一线工作人员焦虑情绪。SAS由Zung[5]于1971年编制，广泛运用于各人群的焦虑测评。SAS包含20个项目，4级评分，其中有5项反向计分。各项目得分之和为初始分，再乘以1.25，四舍五入取整数得到标准分。其评判标准为：标准分<50为无焦虑；50~59为轻度焦虑；60~69为中度焦虑；70以上为重度焦虑。

1.2.4 疲劳自评量表

采用疲劳自评量表(Fatigue Self-assessment Scale，FSAS)[6]评估一线工作人员疲劳感。FSAS包含23个自评项目，前22项为5级评分，最后1项为自选分数评价自我疲劳感。此量表能较好地反映疲劳的类型及程度，其评判标准为：>6分为躯体疲劳、>6分为精神疲劳、>4分为疲劳后果、>3分为总体疲劳；在疲劳的特征中，>11分为睡眠/休息不能缓解疲劳的程度，>4分为疲劳的情境性明显。其具体评分标准可参考由中华中医药学会提出的疲劳的评定标准[6]。

1.3 统计学处理

采用SPSS 23.0软件进行数据分析。计量资料采用均数±标准差($\bar{x}\pm s$)表示，计数资料采用频数或率表示。计量资料比较采用非参数检验，计数资料的比较采用χ^2检验。各因素间的关系采用Spearman相关分析。$P<0.05$为差异有统计学意义。

2 结　果

2.1 一般资料

本次调查共收集1 049份调查问卷。其中63人为非防控一线工作人员，予以剔除，有效问卷为986份(表1)。其中女628人(63.7%)，男358人(36.3%)。年龄主要分布在青中年，25~34岁占37.7%，35~54岁占53.79%。91.16%的人口长期居住在城市，有慢性躯体或精神疾病者占15.4%，家庭收入10万元以下者占60.1%，已婚者占76.9%，4.8%为COVID-19密切接触者。33.7%一线工作人员关注疫情超过6 h，81.8%的一线工作人员担心在疫情期间被感染，10.3%的家人不是很支持或不支持受试者参加防疫工作，仅1.3%的一线工作人员认为自己的工作没有获得所服务对象的认可。20.5%的受试者不吸烟，5.3%的受试者有宗教信仰。

表1　一般资料情况

Table 1　Sociodemographic characteristics of the samples

组别	例数	构成比/%	组别	例数	构成比/%	组别	例数	构成比/%
性别	986		婚姻状态			家人支持您参与防疫工作吗?		
女	628	64.7	单身	228	23.1	不支持	8	0.8
男	358	36.3	已婚	758	76.9	不是很支持	94	9.5
年龄段/岁			学历			支持	697	70.7
18~24	52	5.3	初中及以下	9	0.9	非常支持	187	19.0
25~34	372	37.7	高中或中专	90	9.1	您所服务的对象对您的工作满意吗?		
35~54	533	54.1	大学专科或本科及以上	887	90.0	不满意	13	1.3
55~64	29	2.9	是COVID-19密切接触者	47	4.8	比较满意	286	29.0
>65	0		每天花多长时间关注疫情/h			满意	565	57.3
长期居住地			<1	217	22.0	特别满意	122	12.4
农村	86	8.7	1~3	344	34.9	有慢性身心疾病	152	15.4
城市	900	91.3	4~6	93	9.4	是否吸烟		
平时身心健康与否			>6	332	33.7	是	202	20.5
是	834	84.6	是否担心疫情期间会被感染			否	751	76.2
否	152	15.4	不会	179	18.2	曾经吸烟，现已戒烟	33	3.3
家庭年收入			有点担心	515	52.2	有否宗教信仰		
10万以下	593	60.1	担心	217	22.0	有	52	5.3
10万元以上	393	39.9	非常担心	75	7.6	否	934	94.7

婚姻状态：单身包括未婚、离异及丧偶

©Journal of Central South University (Medical Science). All rights reserved.

本次调查共调查了指挥协调者25人(2.5%)，街道(乡镇)工作人员67人(6.8%)，社区(村)工作人员234人(23.7%)，医护工作者338人(34.3%)，警察31人(3.1%)，志愿者45人(4.6%)，新闻工作者37人(3.8%)，市场管理人员93人(9.4%)，物业管理人员5人(0.5%)，其他参与疫情防控人员(快递员，蔬菜配送员等)111人(11.3%)。PHQ-9>5分有484人(49.1%)(表2)，其中有轻度抑郁症状278人(28.2%)，中度抑郁症状118人(12%)，重度抑郁症状88人(8.9%)。SAS>50分有215人(21.8%)，其中有轻度焦虑症状142(14.4%)，重度焦虑症状48(4.9%)，重度焦虑症状25(2.5%)。46.7%的工作人员存在睡眠问题，有664人(67.3%)感觉躯体疲劳，752人(76.3%)觉得精神疲劳，710人(72.0%)觉得自己的疲劳对情绪、工作与学习效率、日常生活、社交等方面存在影响，749人(76.0%)总体感觉疲劳，462人(46.2%)睡眠/休息不能缓解疲劳的程度，831人(84.3%)觉得日常活动、心理情绪、自然环境等因素产生了疲劳。

2.2 不同职业受试者之间抑郁、焦虑、疲劳的比较

将一线工作人员分为3大类：社区乡镇工作人员、医护人员、其他(表2)，可以明显观察到社区工作人员抑郁、焦虑、躯体疲劳、精神疲劳、疲劳后果、总体疲劳、睡眠/休息不能缓解疲劳的程度、疲劳的情境性较其他两者高(均 $P<0.01$)，而医护人员在三者评分中较低($P<0.01$)。

表2 不同职业受试者抑郁、焦虑、疲劳情况对比

Table 2 Comparison of depression, anxiety, and fatigue among different professional staffs

组别	例数	构成比/%	抑郁			焦虑		
			例数	构成比/%	评分	例数	构成比/%	评分
社区、乡镇工作人员	301	30.5	208	69.1	7.98±5.88	105	34.9	45.79±11.87
医护人员	338	34.3	117	34.6	4.00±4.61	45	13.3	38.89±9.61
其他*	347	35.2	159	45.8	5.43±5.91	65	18.7	41.19±11.44
χ^2				88.71			63.47	
P				<0.01			<0.01	

组别	躯体疲劳			精神疲劳			疲劳后果		
	例数	构成比/%	评分	例数	构成比/%	评分	例数	构成比/%	评分
社区、乡镇工作人员	248	82.4	31.76±27.33	265	88.0	33.37±25.81	262	87.0	46.26±39.80
医护人员	195	57.7	14.42±19.99	236	69.8	19.06±22.01	218	64.8	21.80±28.44
其他*	221	63.7	18.09±22.74	251	72.3	21.58±23.01	230	66.3	28.40±35.29
χ^2		92.58			72.21			86.68	
P		<0.01			<0.01			<0.01	

组别	总体疲劳			睡眠/休息不能缓解疲劳			疲劳的情境性		
	例数	构成比/%	评分	例数	构成比/%	评分	例数	构成比/%	评分
社区、乡镇工作人员	271	90.0	31.83±25.42	181	60.1	26.15±28.32	275	91.4	39.97±24.91
医护人员	228	67.5	15.79±19.29	122	36.1	11.98±20.26	268	79.3	25.10±22.97
其他*	250	72.0	19.44±22.01	159	45.8	17.33±25.29	288	83.0	29.90±24.699
χ^2		93.52			51.64			62.01	
P		<0.01			<0.01			<0.01	

*包括指挥协调者、警察、志愿者、新闻工作者、市场管理人员、物业管理人员等

2.3 一线工作人员抑郁、焦虑相关因素分析

Spearman相关分析结果显示：一线工作人员抑郁与年龄、家庭收入、家人的支持、服务对象是否满意、睡眠质量呈负相关(均 $P<0.01$，表3)，与文化程度、担心疫情期间会被感染、每天关注疫情的时长呈正相关(均 $P<0.01$)。焦虑与年龄、家庭收入、家人的支持、服务对象是否满意、睡眠质量呈负相关(均 $P<0.01$)，与性别、担心疫情期间会被感染、每天

©Journal of Central South University (Medical Science). All rights reserved.

关注疫情的时间呈正相关(均 P<0.01)。一线工作人员与躯体疲劳、精神疲劳、疲劳后果、总体疲劳、睡眠/休息不能缓解疲劳的程度、疲劳的情境性，以及疲劳的时间模式均呈正相关(均 P<0.01)。

表3　一线工作人员抑郁和焦虑的相关因素

Table 3　Correlated factors of depression and anxiety in front-line staff

因素	性别	年龄	家庭年收入	文化程度	家人的支持	服务对象是否满意	担心疫情期间会被感染
抑郁	0.057	−0.17**	−0.12**	0.10**	−0.29**	−0.34**	0.39**
焦虑	0.074*	−0.11**	−0.16**	0.24	−0.29**	−0.34**	0.39**

因素	每天关注疫情的时长	睡眠质量	躯体疲劳	精神疲劳	疲劳后果	总体疲劳
抑郁	0.14**	−0.57**	0.77**	0.81**	0.82**	0.84**
焦虑	0.15**	−0.49**	0.68**	0.65**	0.67**	0.69**

因素	睡眠/休息不能缓解疲劳的程度	疲劳的情境性	早上疲劳度	上午疲劳度	中午疲劳度	下午疲劳度	晚上疲劳度
抑郁	0.51**	0.44**	0.51**	0.56**	0.52**	0.54**	0.41**
焦虑	0.47**	0.31**	0.47**	0.51**	0.43**	0.41**	0.37**

**P<0.01

3 讨　论

本研究调查COVID-19疫情期间一线工作人员的心理健康状况，结果显示一线工作人员存在焦虑情绪(21.8%)、抑郁情绪(49.1%)以及疲劳状态(76%)。远远高于其他突发事件时一线工作人员表现出的心理健康问题。有17.3%的医护人员在严重急性呼吸综合征(severe acute respiratory syndrome，SARS)流行期间有明显的精神症状[7]，10.1%的难民工作志愿者有抑郁症状[8]。美国警方在经历了“9•11”事件后，其中24.7%的人有抑郁，5.8%有焦虑[9]。21.4%的记者在面临战争等极端危险情况时有严重抑郁[10]。这提示我们在COVID-19疫情中，应该更加关注一线工作人员的心理健康。

进一步分析表明社区、乡镇工作人员抑郁焦虑程度远远高于包括医护人员在内的其他职业者(P<0.01)。笔者认为：一方面是因为社区、乡镇工作人员相较于医护人员，在面对突发公共卫生事件时，更加缺乏专业的医学常识及医用物资，防护能力较弱，容易产生恐惧、紧张、抑郁等情绪；另一方面，社区、乡镇工作人员管辖范围大，工作人员数量相对较少，且疫情期间需长时间深入基层与各家各户交流，但社会理解度不够，有些人不予配合，工作量明显增加(包括人们隔离带来的烦躁、郁闷无处发泄，有时会以社区人员为情绪发泄出口)。而医护人员长期存在长时间连续工作的状态，可接受较高的工作强度。同时，本次疫情期间获得的精神肯定、物质支持(金钱奖励等)及制度保障(包括轮休制度)都比社区人员多。与疫情初期南方医院精神心理科牵头的一项针对医护人员的在线调查结果(抑郁和焦虑发病率分别为50.7%和44.7%)相比[11]，本次问卷调查医护人员的抑郁和焦虑发病率(分别为34.6%和13.3%)明显低于前者。笔者认为其原因有以下几方面：本次调查时疫情已得到有效控制，抗疫物资进行了有效补充，医护人员对于COVID-19的认识加深，且国家近期采取医生轮班制度，医护人员紧张工作状况得到有效缓解。

相关性分析发现家人支持程度、服务对象满意度、睡眠情况与一线工作人员的抑郁、焦虑水平呈负相关，这与之前在其他领域的研究[12]结果一致。因此家人的关怀、服务对象的认可有利于改善一线工作人员的心理健康状况。此外，家庭年收入较少的一线工作人员更容易产生负面情绪，这与王竞等[13]的研究结果相一致，即工资越高，其内心的安全感越强。赵丞智等[14]研究发现：获得物质支持多的震中地区的民众，比获得支持少的非震中地区的民众创伤症状少且轻。因此政府部门可通过提高工资、增加奖金、增大支持力度等措施来改善一线工作人员的心理健康。

本研究还揭示了疫情期间一线工作人员疲劳与抑郁、焦虑之间的正相关关系，即过度疲劳可能导致负面情绪，促进抑郁的发生[15]，这与Robinson等[16]的研究结果一致。同时，目前已发生多起因公殉职的案例，过度疲劳可能会诱发例如心脑血管急症[17]，导致猝死。疲劳是导致生活质量下降的重要原因，在帕金森病、癌症患者中疲劳是造成残疾的主

©Journal of Central South University (Medical Science). All rights reserved.

要原因之一[18-19]。本次调查发现患有慢性身心疾病者占15.4%，疲劳可能会进一步加重慢性病的病情，因此需要及时对一线工作人员进行心理或慢性病的干预及让其休息以调整身心状态。

2020年1月27日至3月5日，国务院、国家卫生健康委员会接连出台了14个与精神卫生、心理健康相关的文件。各地也采取了相应措施，但大部分针对的是医护工作人员、患者及患者家属，较少关注社区工作人员、警察等群体的心理健康。那么我们可以从以下几个方面采取相应措施。第一，进行心理健康教育。随着互联网时代的到来，可以通过微信、微博、抖音等途径针对性地发布如何在疫情期调整心理健康的视频、音频，或者进行线上讲座。第二，定期对一线工作人员进行心理量表的测定，若发现较严重的抑郁、焦虑或者疲劳患者，应及时告知其单位，使其能获得轮班、换班休息的机会。第三，对于严重抑郁、焦虑患者，可以使用热线电话、微信等途径进行线上心理干预，或者引导其去当地精神心理中心的门诊就诊，以获得面对面疏导等[11]。

本研究有一些不足之处：第一，医护人员及社区、乡镇工作人员收集的数据较多，其他职业如警察、志愿者等获取数据不足，应进一步扩大样本量。第二，此次问卷收集有地域局限性，在查看参与者地区分布时大部分受试者分布在湖南省，因此仅能代表湖南中高危地区一线工作人员的心理状况，不同地区，如湖北重灾区，又或者低危险度、感染较少的地区如西藏等，其一线工作人员心理状况会存在差异。

一线工作人员的抑郁、焦虑、恐慌、担心、害怕等负性情绪会影响其决策力、注意力、执行力，不利于疫情的防控；同时负性情绪也可能造成其永久的身心伤害[11]，给今后的生活带来严重影响。本研究发现精神疲劳、躯体疲劳等与抑郁焦虑发生率呈正相关，因此，一线防控工作人员自身应合理安排工作和休息，及时调节不良情绪，缓解疲劳；政府部门和社会在关注COVID-19疫情时，也应多关心一线防控工作人员，尤其是社区、乡镇工作人员的心理状态，及时对他们采取调休、心理干预等措施。

利益冲突声明：作者声称无任何利益冲突。

参考文献

[1] 国家卫生健康委员会. 新型冠状病毒感染的肺炎诊疗方案(试行第七版)[EB/OL]. (2020-03-03)[2020-03-13]. http://www. nhc. gov. cn/yzygj/s7653p/202003/46c9294a7dfe4cef80dc7f5912eb1989.shtml.
National Health Commission of the people’s Republic of China. Diagnosis and treatment of pneumonitis caused by new coronavirus (Trial version 7) [EB/OL]. (2020-03-03) [2020-03-13]. http://www. nhc. gov. cnyzygj/s7653p/202003/46c9294a7dfe4cef80dc7f5912eb1989.shtml.

[2] 国家卫生健康委员会. 中华人民共和国国家卫生健康委员会公告[EB/OL]. (2020-01-20)[2020-03-13]. http://www.nhc.gov.cn/xcs/zhengcwj/202001/44a3b8245e8049d2837a4f27529cd386.shtml.
National Health Commission of the people’s Republic of China. Announcement of the China National Health Commission of the People’s Republic of China[EB/OL]. (2020-01-20) [2020-03-13]. http://www. nhc. gov. cn/xcs/zhengcwj/202001/44a3b8245e8049d2837a4f27529cd386.shtml.

[3] 李伟, 张彩迪, 罗金晶, 等. 新型冠状病毒肺炎疫情期间不同人群心理状态的Meta分析[J]. 同济大学学报(医学版), 2020, 41(2): 147-154.
LI Wei, ZHANG Caidi, LUO Jinjing, et al. Psychological status among different populations during COVID-19 epidemic: A systematic review and Meta-analysis[J]. Journal of Tongji University. Medical Sciences, 2020, 41(2): 147-154.

[4] Kroenke K, Spitzer RL, Williams JB. The PHQ-9: validity of a brief depression severity measure[J]. J Gen Intern Med, 2001, 16(9): 606-613.

[5] Zung WW. A self-rating depression scale[J]. Arch Gen Psychiatry, 1965, 12(1): 63-70.

[6] 中华中医药学会团体标准.疲劳的评定标准[J]. 中华中医药杂志, 2019, 34(6): 2580-2583.
Group standard of Chinese Society of Traditional Chinese Medicine. Fatigue evaluation criteria[J]. Chinese Journal of Traditional Chinese Medicine, 2019, 34 (6): 2580-2583.

[7] Lu YC, Shu BC, Chang YY, et al. The mental health of hospital workers dealing with severe acute respiratory syndrome[J]. Psychother Psychosom, 2006, 75(6): 370-375.

[8] Orho A, Georgiadou E, Grimm T, et al. Professional and volunteer refugee aid workers-depressive symptoms and their predictors, experienced traumatic events, PTSD, burdens, engagement motivators and support needs[J]. Int J Environ Res Public Health, 2019, 16(22): 4542.

[9] Bowler RM, Kornblith ES, Li J, et al. Police officers who responded to 9/11: Comorbidity of PTSD, depression, and anxiety 10-11 years later[J]. Am J Ind Med, 2016, 59(6): 425-436.

[10] Feinstein A, Owen J, Blair N. A hazardous profession: war, journalists, and psychopathology[J]. Am J Psychiatry, 2002, 159(9): 1570-1575.

[11] Liu S, Yang L, Zhang C, et al. Online mental health services in China during the COVID-19 outbreak [J]. Lancet Psychiatry, 2020, 7(4): e17-e18.

[12] Wu SF, Young LS, Yeh FC, et al. Correlations among social support, depression, and anxiety in patients with type-2 diabetes [J]. J Nurs Res, 2013, 21(2):129-138.

©Journal of Central South University (Medical Science). All rights reserved.

[13] 王竞, 程雅倩, 周照, 等. 新型冠状病毒肺炎疫情对武汉市一线医护人员心理状况的影响[J]. 武汉大学学报(医学版), 2020, 41(4): 547-550.
WANG Jing, CHENG Yaqian, ZHOU Zhao, et al. Psychological status of Wuhan medical staff in fighting against COVID-19[J]. Medical Journal of Wuhan Univesity. Medical Science, 2020, 41(4): 547-550.
[14] 赵丞智, 汪向东, 高岚, 等. 张北尚义地震后创伤后应激障碍随访研究[J]. 中国心理卫生杂志, 2000, 14(6): 361-363.
ZHAO Chengzhi, WANG Xiangdong, GAO Lan, et al. Longitudinal study of earthquake-related PTSD in north China [J]. Chinese Journal of Mental Health, 2000, 14(6): 361-363.
[15] Corfield EC, Martin NG, Nyholt DR. Co-occurrence and symptomatology of fatigue and depression[J]. Compr Psychiatry, 2016, 71: 1-10.
[16] Robinson RL, Stephenson JJ, Dennehy EB, et al. The importance of unresolved fatigue in depression: costs and comorbidities[J]. Psychosomatics, 2015, 56(3): 274-285.
[17] Schnohr P, Marott JL, Kristensen TS, et al. Ranking of psychosocial and traditional risk factors by importance for coronary heart disease: the Copenhagen City Heart Study[J]. Eur Heart J, 2015, 36(22): 1385-1393.
[18] Bower JE. Cancer-related fatigue—mechanisms, risk factors, and treatments[J]. Nat Rev Clin Oncol, 2014, 11(10): 597-609.
[19] Kluger BM, Herlofson K, Chou KL, et al. Parkinson's disease-related fatigue: A case definition and recommendations for clinical research[J]. Mov Disord, 2016, 31(5): 625-631.

（本文编辑　陈丽文）

本文引用：滕紫薇, 黄兢, 邱妍, 谭羽希, 钟秋平, 唐慧, 伍海姗, 吴莹, 陈晋东. 2019冠状病毒病防控一线人员的心理状况[J]. 中南大学学报(医学版), 2020, 45(6): 613-619. DOI: 10.11817/j.issn.1672-7347.2020.200241

Cite this article as: TENG Ziwei, HUANG Jing, QIU Yan, TAN Yuxi, ZHONG Qiuping, TANG Hui, WU Haishan, WU Ying, CHEN Jindong. Mental health of front-line staff in prevention of coronavirus disease 2019[J]. Journal of Central South University. Medical Science, 2020, 45(6): 613-619. DOI:10.11817/j.issn.1672-7347.2020.200241

©Journal of Central South University (Medical Science). All rights reserved.
