# Supplementary material for: Mental health status and related factors influencing healthcare workers during the COVID-19 pandemic: A systematic review and meta-analysis
Source: PLoS One. 2024 Jan 19;19(1):e0289454. doi: 10.1371/journal.pone.0289454 (PMC10798549; doi:10.1371/journal.pone.0289454)
Supplement: S1 Data — (ZIP) [file pone.0289454.s011.zip › literatures/193.pdf]

Risk Management and Healthcare Policy

Dovepress
open access to scientific and medical research

Open Access Full Text Article

ORIGINAL RESEARCH

Mental Health and Health-Related Quality-of-Life Outcomes Among Frontline Health Workers During the Peak of COVID-19 Outbreak in Vietnam: A Cross-Sectional Study

This article was published in the following Dove Press journal:
Risk Management and Healthcare Policy

Hung Manh Than[1,*]
Vuong Minh Nong[2,*]
Cap Trung Nguyen[1]
Khiem Phu Dong[3]
Hoa Thi Ngo[4]
Tra Thu Doan[2]
Nga Thu Do[2]
Trang Huyen Thi Nguyen[5]
Thanh Van Do[2]
Co Xuan Dao[6]
Tuan Quang Nguyen[6]
Thach Ngoc Pham[3]
Cuong Duy Do[2]

[1]Emergency Department, National Hospital for Tropical Diseases, Hanoi, Vietnam; [2]Center for Tropical Diseases, Bach Mai Hospital, Hanoi, Vietnam; [3]National Hospital for Tropical Diseases, Hanoi, Vietnam; [4]Oxford University Clinical Research Unit, Hospital for Tropical Diseases, Ho Chi Minh City, Vietnam; [5]Department of Public Health, Thang Long University, Hanoi, Vietnam; [6]Bach Mai Hospital, Hanoi, Vietnam

*These authors contributed equally to this work

Purpose: Mental health is an important component of the protection strategy for healthcare workers (HCWs). However, it has not been well described in Vietnam during the COVID-19 outbreak. This study aims to measure the psychological distress and health-related quality-of-life among frontline healthcare workers during the peak of the outbreak in Vietnam.

Patients and Methods: We conducted a cross-sectional survey on 173 health workers at two national tertiary hospitals in Hanoi, Vietnam from March to April 2020. The psychological distress was measured by the Depression, Anxiety, and Stress Scale – 21 Items (DASS-21), Impact of Event Scale – Revised (IES-R), and the Insomnia Severity Index (ISI). EQ-5D-5L was used to determine the health-related quality-of-life (HRQoL) outcomes.

Results: Among 173 HCWs, the proportion of reported depression symptoms, anxiety symptoms, and stress was 20.2%, 33.5%, and 12.7%, respectively. The median EQ-5D-5L index score was 0.93 (IQR=0.85–0.94), and the anxiety/depression aspect had the highest reported problems. The most COVID-19-specific concerns among frontline HCWs were the reduction of income (59%) and the increase of living costs (54.3%). HCWs working in the COVID-19-designated hospital had a significantly higher rate of mental health problems and had a lower HRQoL outcome than those working in non-COVID-19-designated hospitals. Other factors associated with psychological distress and sleep problems include age, job title, income, chronic diseases status, and years of working in healthcare settings. HCWs who were ≥30 years old, had higher working years, had higher incomes, and had mental health and sleep problems were more likely to have lower HRQoL scores.

Conclusion: We reported a moderate rate of psychological distress and lower HRQoL outcomes among frontline HCWs during the COVID-19 outbreak in Vietnam. Various factors were found to be associated with mental health and HRQoL that might be useful for implementing appropriate interventions for HCWs in low-resource settings.

Keywords: COVID-19, SARS-CoV-2, mental health, psychological distress, health-related quality-of-life, health workers

Correspondence: Cuong Duy Do
Center for Tropical Diseases, Bach Mai Hospital, No. 78, Giai Phong Street, Dong Da District, Hanoi 100000, Vietnam
Tel +84 983 264 868
Email doduy.cuong@bachmai.edu.vn

Introduction

The global pandemic Coronavirus Disease 2019 (COVID-19) has been spread to over 200 countries and territories around the world, with the total number of infected cases at more than 26,000,000 and nearly 900,000 deaths, as of September 4, 2020.[1] In Vietnam, the government has issued a highly restricted

submit your manuscript | www.dovepress.com
DovePress
http://doi.org/10.2147/RMHP.S280749

© 2020 Manh Than et al. This work is published and licensed by Dove Medical Press Limited. The full terms of this license are available at https://www.dovepress.com/terms.php and incorporate the Creative Commons Attribution – Non Commercial (unported, v3.0) License (http://creativecommons.org/licenses/by-nc/3.0/). By accessing the work you hereby accept the Terms. Non-commercial uses of the work are permitted without any further permission from Dove Medical Press Limited, provided the work is properly attributed. For permission for commercial use of this work, please see paragraphs 4.2 and 5 of our Terms (https://www.dovepress.com/terms.php).

infection prevention and control (IPC) policy since early January 2020.[2,3,4] As of September 5, 2020, the total confirmed cases of COVID-19 exceeded 1,000, and 35 deaths had been reported. The COVID-19 outbreak in Vietnam has been experienced in two waves, the first wave was from January 25 to July 24, 2020, when the epicenters were imported cases from Asia-Pacific and European countries. The second wave started from July 25, 2020, and there was widespread community transmission in some localities.

The healthcare system in Vietnam has been significantly improved over recent years.[5] However, the overall capacity of health resources and the workforce are still limited when compared to other countries in the Asia-Pacific region.[6] The country reports only 8.3 medical doctors and 14.4 nurses per 10,000 population,[7] which causes a common situation of patient overcrowded in many hospitals and healthcare centers.[5] The peak-duration of the COVID-19 outbreak during the first wave in the country was from the 2nd-week of March to early April 2020. The highest number of active cases at a time was 178, and more than half of them were hospitalized at the National Hospital for Tropical Diseases (NHTD) in Hanoi. The psychological distress and associated mental health problems during the COVID-19 outbreak have been well-documented in frontline HCWs.[8,9] In Vietnam, availability and accessibility to psychiatry services are limited in both primary healthcare and occupational health areas. The HCWs are the major workforce and play the most crucial role in the COVID-19 containment and treatment strategy, and mental health support should be addressed in a comprehensive intervention to protect HCWs. During the second wave, the confirmed cases of SARS-CoV-2 have been rapidly growing, which doubled the total detected positives compared to the first wave after only 4 weeks. The second wave started with a nosocomial transmission in a major hospital, and a high proportion of non-COVID-19 critical ill patients and HCWs were infected.[10] It was estimated that thousands of HCWs were quarantined followed the containment measures, which significantly increased the burden for the healthcare system.

During recent years, health-related quality-of-life (HRQoL) has been widely used as a valuable health outcomes measurement in various healthcare settings and played an important role in interventions development and policy-making.[11–13] HRQoL could be assessed by generic instruments or disease-specific instruments.[14] Specific HRQoL tools were usually used in patients with certain conditions including chronic diseases such as HIV or cancer.[15,16] Generic HRQoL instruments could be applied in wider targets across many settings including healthy people. The advantages of generic HRQoL instruments were their simply characteristics, covering multiple health aspects, and the convenience of disseminating through patient's self-report, which help in rapid identification of health problems and comparability between different sub-groups among large populations.[14,17–19]

In Vietnam, evidence of the impact of the COVID-19 outbreak to mental health and HRQoL outcomes in HCWs were under researched.[20] In this study, we aim to compare the psychological distress, sleep disorder, and HRQoL outcomes between frontline healthcare workers at a COVID-19-designated hospital and a non-designated tertiary referral hospital in northern Vietnam during the peak of the outbreak in Vietnam, from March–April 2020.

Patients and Methods

Study Setting and Participants

We conducted a cross-sectional study among frontline HCWs working at the NHTD and the Center for Tropical Diseases (CTD), an affiliated department of Bach Mai Hospital (BMH), during March and April 2020. NHTD is the largest hospital for infectious and tropical diseases in Vietnam and has been designated as a COVID-19 treatment center for all confirmed SARS-CoV-2 cases in the Northern. BMH was one of the largest general tertiary hospital in the country. During the outbreak, the CTD of BMH was assigned for screening patients, detecting suspected cases, and transferring all suspicious persons to designated hospitals.

Convenience sampling was used to select participants if they were: 1) physicians, nurses, technicians, or pharmacists; 2) worked at the two hospitals during the study period; and 3) agreed to join the survey. A total of 173 HCWs participated in the survey, which accounted for 81% and 94% of total HCWs working at NHTD and CTD, respectively.

Data Collection and Measurements

Demographic Characteristics

We collected data about the demographics, years of work since graduation, and type of work of HCWs by using a structural self-reported questionnaire.

Mental Health Problems and Psychological Distress

The Depression, Anxiety, and Stress Scale – 21 Items (DASS-21) was used to measure the perceived stress,

anxiety, and depression symptoms during the COVID-19 outbreak in Asia.[21–25] The cut-off points for symptoms of depression, anxiety, and stress were ≥10, ≥8, and ≥15, respectively.[26] The DASS-21 has been previously validated in Vietnamese populations, and showed good sensitivity and reliability to detect mental health disorders.[27,28]

We applied the Impact of Event Scale – Revised (IES-R) and the Insomnia Severity Index (ISI) to assess the psychological distress and insomnia disorder among participants. For the IES-R, we used the cut-off of ≥24 to identifying a clinical concern of post-traumatic stress disorder (PTSD).[29] Respondents were considered as clinical insomnia if they reported the ISI score ≥15.[30] The Vietnamese version of IES-R and ISI scales were translated by experienced mental health professionals, and piloted in 10 HCWs for language validation before being disseminated.

An additional series of nine questions were used to identify the perceived distress in several aspects specifying for COVID-19. These items were retrieved from a pre-study focus discussion with eight HCWs at NHTD.

Health-Related Quality-of-Life

HRQoL was measured by European Quality of Life-Five Dimension-Five Level Scale (EQ-5D-5L). The EQ-5D-5L is a widely used HRQoL instrument consisting of five questions with responses on a five level Likert scale.[31] The EQ-5D-5L assessed five dimensions of HRQoL, including mobility, self-care, usual activities, pain or discomfort, and mental health condition. A health utility index was calculated from five items, and higher scores indicated higher HRQoL outcomes. In this study, we used a value set from Vietnamese populations to calculate health unity index scores (ranging from −0.5115 to 1).[32]

Statistical Analysis

Descriptive analysis was used to describe the study's variables. We used chi-square test and Mann–Whitney *U*-test to compare the qualitative and quantitative variables between designated hospital and non-designated hospital, respectively.

Multivariate logistic and linear regression were applied to determine associated factors with mental health and HRQoL outcomes among participants. We used the stepwise forward technique to obtain the appropriative model, which excluded the variable with significant level <0.2.

Results

In total 173 HCWs were enrolled in the study, the median age was 31, with 41.6% <30 years old; 68.2% female; and the majority of participants were nurses (63%). The median years of working in the medical field was 6 years (IQR=3–12); and nurses/other HCWs had a higher median years of experience working (6.5 years) than medical doctors (3 years). There was 19.7% of HCWs who had comorbidities, including cardiovascular diseases (2.3%), chronic obstructive pulmonary disease (1.2%), chronic hepatitis (10.4%), and musculoskeletal diseases (7.5%). The rate of current smokers was 8.1%, and overweight or obesity was 15% (Table 1).

The frequency of depression symptoms, anxiety symptoms, and stress, measured by DASS-21 scale, were 20.2%, 33.5%, and 12.7%, respectively. There were 12.1% and 20.2% of HCWs who had major PTSD symptoms and sleeping disorders, respectively. The proportion of mental health problems of HCWs from the designated hospital was significantly higher than those of HCWs from non-designated hospitals. Using EQ-5D-5L to measure HRQoL, we found that anxiety/depression dimension was the most reported problems among respondents (70.5%), followed by mobility (26.6%), pain/discomforts (24.3%), usual activities (19.7%), and self-care (6.4%), respectively. The median of EQ-5D-5L index score was 0.93 (ranging from 0.27–1.00; 22.0% had perfect HRQoL scores) which was significantly better among HCWs in non-designated hospitals (0.93 vs 0.87) (Table 2).

The most COVID-19 specific concerns among frontline HCWs were the reduction of income (59%) and increase of living costs (54.3%). In HCWs from COVID-19-designated hospitals, the proportion of reported concerns about the preventive measures were significantly higher than HCWs from non-designated hospitals, including risks of transmission (53.8% vs 26.9%; P=0.01), availability of personal protective equipment (PPE) (55.7% vs 25.4%; P<0.001), and overall IPC status at their hospital (60.4% vs 20.9%; P<0.001) (Table 3).

In multivariate logistic regression, HCWs working in a COVID-19-designated hospital were found to have more mental health problems than those working in non-designated hospitals (aOR=3.62; P<0.01). Other factors associated with having mental health problems were those having chronic diseases (aOR=2.61; P<0.05), having concerns of PPE availability (aOR=2.95; P<0.05), and having a shorter duration of working in medical field (aOR=0.17; P<0.05).

Table 1 General Characteristics of HCWs During the COVID-19 Outbreak in Vietnam

General Characteristics	Designated Hospital (N=106)	Non-Designated Hospital (N=67)	Total (N=173)	*P*-value
Gender, n (%)				
Male	42 (39.6%)	13 (19.4%)	55 (31.8%)	0.05
Female	64 (60.4%)	54 (80.6%)	118 (68.2%)	
Age, Median (IQR)	30 (27–36)	32 (29–38)	31 (27–36)	0.07
Major				
Physicians	33 (31.1%)	10 (14.9%)	43 (24.9%)	0.05
Nurses	62 (58.5%)	47 (70.2%)	109 (63%)	
Others	11 (10.4%)	10 (14.9%)	21 (12.1%)	
Years of working, Median (IQR)	6 (3–12)	7 (3–12)	6 (3–12)	0.29
Monthly incomes,* Median (IQR)	10,000 (6,000–10,000)	10,000 (7,000–14,000)	10,000 (7,000–12,000)	0.02
Having chronic diseases, n (%)	15 (14.2%)	19 (28.4%)	34 (19.7%)	0.02
Current smokers, n (%)	8 (7.6%)	9 (13.4%)	17 (9.8%)	0.20
Body mass index, n (%)				
Underweight	8 (7.5%)	6 (9.0%)	14 (8.1%)	0.64
Normal	84 (79.3%)	49 (73.1%)	133 (76.9%)	
Overweight/Obesity	14 (13.2%)	12 (17.9%)	26 (15.0%)	

Note: *In million Vietnam Dong.
Abbreviation: IQR, interquartile range.

Clinical doctors had less major PTSD symptoms and other HCWs (aOR=0.18; $P<0.05$). HCWs were concerned about the risk transmission of COVID-19 during work, were afraid of long-term serving at the hospital, and had higher working years were more likely to have clinical insomnia (Table 4).

HCWs who suffered from mental health problems (aCoeff=−0.06; $P<0.05$) and sleeping disorders symptoms (aCoeff=−0.04; $P<0.05$) had higher risk of having lower HRQoL index score than those who did not. Concerned about the long-term quarantine at hospital, longer years of working in a medical field and higher income were found to be associated with a lower score of EQ-5D-5L index (Table 5).

Discussion

To our knowledge, this is the first survey reported to assess the psychological impact of COVID-19 on mental health and HRQoL outcomes among frontline HCWs in Vietnam. We found a moderate rate of psychological problems in 173 staff under work at a COVID-19-designated hospital and a non-designated tertiary national hospital during the peak of the outbreak in the first wave of COVID-19. In overall, the data showed a slightly lower rate of perceived depression and distress symptoms among HCWs in Vietnam when compared to several reports in China, Italy, or the US.[8,33–35] The difference reflected the fact that Vietnam had a low number of COVID-19 active cases and the majority were in mild condition in the first wave from January to March 2020. In a setting of limited health resources such as Vietnam, flattening the curve of active cases through high-level and aggressive prevention measures could be the most appropriative approach to reduce the burden for the healthcare system, as well as minimize the work pressure and the overwhelmed situation among HCWs.[2,3]

A higher proportion of mental health problems and lower scores of HRQoL were found in HCWs from the COVID-19-designated hospital. These findings could be explained by the higher workloads among HCWs at the designated hospital, including screening for suspected cases, receiving quarantined people, as well as providing treatment for confirmed cases. Meanwhile, in non-designated hospitals, the main tasks were only screening and transferring. In China, a higher rate and more severe mental health problems were found among HCWs from designated hospitals in Wuhan compared to non-Wuhan-based and outside Hubei hospitals.[36,37]

The clinical doctors were less likely to have major PTSD symptoms than nurses and other HCWs. Studies showed mixed findings about the association between job title and level of distress during the COVID-19 outbreak.

Table 2 Mental Health and HRQoL Outcomes of HCWs During the COVID-19 Outbreak

	Designated Hospital (N=106)	Non-Designated Hospital (N=67)	Total (N=173)	*P*-value
Mental health problems				
Depression symptoms, n (%)	27 (25.5%)	8 (11.9%)	35 (20.2%)	0.03
Anxiety symptoms, n (%)	49 (46.2%)	9 (13.4%)	58 (33.5%)	<0.001
Stress, n (%)	19 (17.9%)	3 (4.5%)	22 (12.7%)	0.01
Major PTSD, n (%)	18 (17.0%)	3 (4.5%)	21 (12.1%)	0.01
Insomniac, n (%)	27 (25.5%)	8 (11.9%)	35 (20.2%)	0.03
DASS21 scores, Median (IQR)				
DASS21-Depression	4 (2–10)	2 (0–6)	4 (0–8)	0.004
DASS21-Anxiety	6 (2–12)	2 (0–4)	4 (2–10)	<0.001
DASS21-Stress	10 (4–14)	4 (2–10)	8 (4–14)	0.001
IES-R score, Median (IQR)	15 (8–21)	6 (3–12)	12 (5–19)	<0.001
ISI score, Median (IQR)	10 (8–15)	9 (7–12)	10 (7–14)	0.09
EQ-5D-5L profile				
Mobility,* n (%)	34 (32.1%)	12 (17.9%)	46 (26.6%)	0.04
Self-care,* n (%)	9 (8.5%)	2 (3.0%)	11 (6.4%)	0.15
Usual activities,* n (%)	22 (20.8%)	12 (17.9%)	34 (19.7%)	0.65
Pain/Discomfort,* n (%)	29 (27.4%)	13 (19.4%)	42 (24.3%)	0.24
Anxiety/Depression,* n (%)	84 (79.3%)	38 (56.7%)	122 (70.5%)	0.002
EQ-5D-5L index score, Median (IQR)	0.87 (0.80–0.93)	0.93 (0.88–1.00)	0.93 (0.85–0.94)	0.002
VAS score, Median (IQR)	95 (85–99)	95 (90–100)	95 (90–100)	0.20

Note: *Having problems.
Abbreviations: HRQoL, health-related quality-of-life; PTSD, post-traumatic stress disorder; DASS21, The Depression, Anxiety and Stress Scale – 21 Items; IQR, interquartile range; IES-R, Impact of Event Scale – Revised; ISI, Insomnia Severity Index; EQ-5D-5L, European Quality of Life-Five Dimension-Five Level Scale; VAS, Visual Analog Scale.

In Singapore, it was reported that non-medical HCWs had a significantly higher rate of distress than clinical staff.[38] In China, a recent meta-analysis study showed that doctors had a higher frequency of depression and anxiety symptoms than nurses.[8] In our experience, the higher level of psychological distress in nursing staff may be

Table 3 COVID-19-Related Concerns of HCWs During COVID-19 Outbreak

COVID-19-Related Concerns	Designated Hospital (N=106) n (%)	Non-Designated Hospital (N=67) n (%)	Total (N=173) n (%)	*P*-value
Concerned that they may get infected during work and care for patients	57 (53.8%)	18 (26.9%)	75 (43.4%)	0.01
Concerned that they may be able to transmit to family member(s)	52 (49.1%)	34 (50.8%)	86 (49.7%)	0.83
Concerned that they may not having adequately personal protective equipment	59 (55.7%)	17 (25.4%)	76 (43.9%)	<0.001
Concerned about the status of IPC at the hospital	64 (60.4%)	14 (20.9%)	78 (45.1%)	<0.001
Concerned that the outbreak may affect income	58 (54.7%)	44 (65.7%)	102 (59%)	0.15
Concerned that the outbreak may increase living costs	54 (50.9%)	40 (59.7%)	94 (54.3%)	0.26
Concerned about the possibility of being quarantined at hospital to serve continuously for long period of time	35 (33%)	26 (38.8%)	61 (35.3%)	0.44

Abbreviation: IPC, infection prevention and control.

Table 4 Multivariate Logistic Regression of Factors Associated with Mental Health Outcomes

	Mental Health Problems		Major PTSD		Clinical Insomnia	
	aOR	95% CI	aOR	95% CI	aOR	95% CI
Designated hospital vs non-designated hospital	3.62**	1.54–8.47	29.04**	2.91–289.81	1.33	0.49–3.65
Age (≥30 years vs <30 years)	0.55	0.26–1.17			0.25*	0.06–0.99
Job (Clinical doctor vs Others)			0.18*	0.04–0.93		
Income quintiles (Second vs First)			14.28*	1.25–163.18		
Chronic diseases (Yes vs No)	2.61*	1.05–6.48				
Current smokers (Yes vs No)			4.56	0.76–27.49		
BMI (Under/Overweight vs Normal)			0.32	0.06–1.78		
Concerned about may get infected during work (Yes vs No)			5.31*	1.22–23.03	4.37*	1.51–12.67
Concerned about may not having adequately PPE (Yes vs No)	2.93*	1.34–6.39	0.32	0.07–1.35		
Concerned about the status of IPC at the hospital (Yes vs No)			2.85	0.68–11.89	0.44	0.15–1.28
Concerned that the outbreak may affect income (Yes vs No)			3.76	0.96–14.72		
Concerned about the long-term quarantined (Yes vs No)	1.99	0.89–4.48			5.14*	1.6–16.47
Worked years quintiles (vs First)						
Second	0.17*	0.04–0.68			3.86*	1.05–14.22
Fourth					5.81*	1.25–27.03
Fifth					2.94	0.58–14.9

Notes: *P-value<0.05; **P-value<0.01.

Abbreviations: PTSD, post-traumatic stress disorder; aOR, adjusted odds ratio; 95% CI, 95% confidence interval; BMI, body mass index; PPE, personal protective equipment; IPC, infection prevention and control.

influenced by the nature of their work, when they were required to perform multiple tasks at the same time, including both administrative and medical professional work.[39,40]

The median score of EQ-5D-5L among HCWs was 0.95 (IQR=0.85–0.94), and 22.0% of them reported perfect health condition, which was not different from reports in the general population in Vietnam,[32,41] and in China during the COVID-19 outbreak.[42] However, the median HRQoL score was significantly lower in HCWs from the designated hospital compared to those from the non-designated hospital (0.87; IQR=0.80–0.93). The overall EQ-5D-5L score for HCWs in this study was higher than Vietnamese patients suffering from diabetes (0.8),[43] human immunodeficiency virus (HIV) (0.8),[44] skin diseases (0.73),[45] respiratory diseases (0.66),[46] dengue fever (0.66),[47] frail elderly (0.58),[48] and elderly after fall injury (0.46).[49] In addition, the anxiety/depression aspect was the most reported problem among HCWs from both hospitals (79.3% in the designated hospital and 56.7% in the non-designated hospital). This finding illustrated that mental health was the major contributor to the reduction of HRQoL in HCWs during the COVID-19 outbreak in Vietnam, and psychological interventions could both benefit mental health and the overall general health status of frontline HCWs. In many countries with a high burden of COVID-19, various mental health interventions have been deployed for frontier HCWs, such as online training, telehealth supports, behavioral group therapy, cognitive behavioral therapy (CBT), and mindfulness-based therapy.[50] Recent evidence has shown that electronically delivered CBT was a cost-effective intervention in various settings,[51] and mindfulness-based therapy was also a promising intervention.[52,53] However, in Vietnam, the inadequate mental healthcare system and lack of psychiatry services might be a major barrier for having effective interventions for HCWs.[54,55]

The most COVID-19 specific concerns among HCWs were that the outbreak might affect their income and

Table 5 Multivariate Linear Regression of Factors Associated with HRQoL Outcomes

	EQ-5D-5L Index Scores		VAS Scores	
	aCoeff	95% CI	aCoeff	95% CI
Designated hospital vs non-designated hospital	0.07	−0.01–0.15	0.83	−2.21–3.88
Age (≥30 years vs <30 years)	0.05*	0.01–0.08		
Years of working quintiles (vs First)				
Fourth	−0.04*	−0.08 - −0.005		
Fifth	−0.03	−0.08–0.01	2.71	−0.79–6.21
Income quintiles (vs First)				
Third	−0.11*	−0.2 - −0.03		
Fourth	−0.09*	−0.17 - −0.004		
Fifth	−0.09*	−0.17 - −0.01		
Chronic diseases (Yes vs No)			−3.2	−6.66–0.26
Concerned that the outbreak may affect income (Yes vs No)			3.39*	0.47–6.31
Concerned about the long-term quarantined (Yes vs No)	−0.04*	−0.07 - −0.004	−3.96*	−7.04–−0.89
Mental health problems (Yes vs No)	−0.06**	−0.1 - −0.03	−4.7**	−7.59–−1.81
Clinical insomnia (Yes vs No)	−0.04*	−0.09 - −0.004		
Constant	0.81**	0.7–0.93	92.56**	87.29–97.83

Notes: *P-value<0.05; **P-value<0.01.
Abbreviations: EQ-5D-5L, European Quality of Life–Five Dimension–Five Level Scale; VAS, Visual Analog Scale; aCoeff, adjusted coefficient; 95% CI, 95% confidence interval.

increase living costs. This situation was not only encountered in the medical area, but in all other fields, where the outbreak has significantly impacted on most socioeconomic aspects.[56] Our findings showed that community-level interventions could be a potential solution to reduce the external pressure on the work environment for HCWs. As they were the primary workforce in the outbreak, frontline HCWs may receive greater incentives for remuneration, fee waivers, living expenses, as well as other supports for their children. HCWs at the designated hospital had more concerns about the risk of transmission and protection status than those from the non-designated hospital. This was an understandable result when the designated hospitals received a larger number of COVID-19 patients, meaning HCWs there had a much higher frequency of direct contacts with infected cases. However, these factors contributed significantly to the increase of psychological distress, which suggested that the IPC measures in hospital still need to be better managed. The actual situation in Vietnam showed that nosocomial transmission in the hospital setting was the major driver of the two largest epicenters in the country, one that caused the second wave of the outbreak.[9] Other COVID-19-related factors associated with insomnia symptoms and lower HRQoL scores were the possibility of being quarantined at hospital to serve continuously for further long-term. Shift work has been proven to be associated with various health aspects of HCWs, including both physical and mental health outcomes.[57] This suggests that policy-makers must carefully review and developappropriate shift schedules, as well as transfer outreach HCWs from other hospitals to support COVID-19-designated hospitals if necessary.

The study has several limitations, including the small sample size, and non-availability of baseline data. In addition, data from NHTD and BMH were not representative for all HCWs in Vietnam, and a cross-sectional design might not be able to establish causal inference. We suggested further studies should be focused on the long-term impact of the outbreak on mental health status, as well as the urgent need of multi-level interventions to address the COVID-19-related psychological distress in healthcare workers.

Conclusion

The study showed a moderate level of psychological distress and HRQoL outcomes among HCWs in Vietnam during the peak of the first wave of COVID-19 outbreak from March to April 2020. HCWs from the designated hospital had a significantly higher burden of mental health and lower HRQoL score than those from non-designated

hospital. Mental health aspect was the primary contributor to the reduction of HRQoL among respondents, which suggested psychological interventions could both benefit the mental health and general health status of frontier HCWs. Various other factors were found to be associated with mental health and HRQoL outcomes that might be useful for policy-makers and hospital managers in low-resource settings during the COVID-19 outbreak.

Data Sharing Statement

The data that support the findings of this study are available on request from the corresponding author, CDD. The data are not publicly available due to restrictions, for example, their containing information that could compromise the privacy of research participants.

Ethics Approval and Informed Consent

The study was conducted in accordance with the Declaration of Helsinki, and was approved by the Ethical Committee of the National Hospital for Tropical Diseases and Bach Mai Hospital. All participants were provided informed consent.

Acknowledgments

The authors would like to thank all the health workers who participated in the study.

Author Contributions

All authors made a significant contribution to the work reported, whether that is in the conception, study design, execution, acquisition of data, analysis and interpretation, or in all these areas; took part in drafting, revising or critically reviewing the article; gave final approval of the version to be published; have agreed on the journal to which the article has been submitted; and agree to be accountable for all aspects of the work.

Funding

The authors did not receive any financial support for the survey work or for publication of the article.

Disclosure

The authors report no conflicts of interest in this work.

References

1. World Health Organization. *Coronavirus Disease (COVID-19) Weekly Epidemiological Update and Weekly Operational Update, 04 September 2020*; 2020.
2. Pham TQ, Rabaa M, Duong LH, et al. The first 100 days of SARS-CoV-2 control in Vietnam. *medRxiv*. 2020;2020.2005.2012.20099242.
3. Chen S, Zhang Z, Yang J, et al. Fangcang shelter hospitals: a novel concept for responding to public health emergencies. *Lancet*. 2020;395(10232):1305–1314. doi:10.1016/S0140-6736(20)30744-3
4. Duy C, Nong VM, Ngo A, et al. Nosocomial Coronavirus Disease Outbreak Containment, Hanoi, Vietnam, March-April 2020. *Emerg Infect Dis*. 2020;27(1). doi:10.3201/eid2701.202656
5. Takashima K, Wada K, Tra TT, Smith DR. A review of Vietnam's healthcare reform through the Direction of Healthcare Activities (DOHA). *Environ Health Prev Med*. 2017;22(1):74. doi:10.1186/s12199-017-0682-z
6. Electronic State Parties Self-Assessment Annual Reporting Tool (e-SPAR). Available from: https://extranet.who.int/e-spar#capacity-score. Accessed 05 May 2020.
7. World Health Organization. *Global Health Observatory Data Repository*; 2020.
8. Pappa S, Ntella V, Giannakas T, Giannakoulis VG, Papoutsi E, Katsaounou P. Prevalence of depression, anxiety, and insomnia among healthcare workers during the COVID-19 pandemic: a systematic review and meta-analysis. *Brain Behav Immun*. 2020;88:901–907. doi:10.1016/j.bbi.2020.05.026
9. Chew NWS, Lee GKH, Tan BYQ, et al. A multinational, multicentre study on the psychological outcomes and associated physical symptoms amongst healthcare workers during COVID-19 outbreak. *Brain Behav Immun*. 2020;88:559–565. doi:10.1016/j.bbi.2020.04.049
10. Vuong NM, Le Quyen NT, Tra DT, et al. The second wave of COVID-19 in a tourist hotspot in Vietnam. *J Travel Med*. 2020.
11. Guyatt GH, Feeny DH, Patrick DL. Measuring health-related quality of life. *Ann Intern Med*. 1993;118(8):622–629. doi:10.7326/0003-4819-118-8-199304150-00009
12. Crosby RD, Kolotkin RL, Williams GR. Defining clinically meaningful change in health-related quality of life. *J Clin Epidemiol*. 2003;56(5):395–407. doi:10.1016/S0895-4356(03)00044-1
13. Bakas T, McLennon SM, Carpenter JS, et al. Systematic review of health-related quality of life models. *Health Qual Life Outcomes*. 2012;10(1):134. doi:10.1186/1477-7525-10-134
14. Longworth L, Yang Y, Young T, et al. Use of generic and condition-specific measures of health-related quality of life in NICE decision-making: a systematic review, statistical modelling and survey. *Health Technol Assess (Rockv)*. 2014;18:1–224.
15. Clayson DJ, Wild DJ, Quarterman P, Duprat-Lomon I, Kubin M, Coons SJ. A comparative review of health-related quality-of-life measures for use in HIV/AIDS clinical trials. *Pharmacoeconomics*. 2006;24(8):751–765.
16. Montazeri A. Health-related quality of life in breast cancer patients: a bibliographic review of the literature from 1974 to 2007. *J Exp Clin Cancer Res*. 2008;27(1):32.
17. Ravens-Sieberer U, Erhart M, Wille N, Wetzel R, Nickel J, Bullinger M. Generic health-related quality-of-life assessment in children and adolescents. *PharmacoEconomics*. 2006;24(12):1199–1220.
18. Linde L, Sørensen J, Østergaard M, Hørslev-Petersen K, Hetland ML. Health-related quality of life: validity, reliability, and responsiveness of SF-36, EQ-15D, EQ-5D, RAQoL, and HAQ in patients with rheumatoid arthritis. *J Rheumatol*. 2008;35(8):1528–1537.
19. Tran BX, Nguyen LH, Ohinmaa A, Maher RM, Nong VM, Latkin CA. Longitudinal and cross sectional assessments of health utility in adults with HIV/AIDS: a systematic review and meta-analysis. *BMC Health Serv Res*. 2015;15(1):7. doi:10.1186/s12913-014-0640-z

20. Tran BX, Ha GH, Nguyen LH, et al. Studies of novel Coronavirus Disease 19 (COVID-19) pandemic: a global analysis of literature. *Int J Environ Res Public Health*. 2020;17(11):4095. doi:10.3390/ijerph17114095
21. Wang C, Pan R, Wan X, et al. Immediate psychological responses and associated factors during the initial stage of the 2019 Coronavirus Disease (COVID-19) epidemic among the general population in China. *Int J Environ Res Public Health*. 2020;17(5):1729. doi:10.3390/ijerph17051729
22. Wang C, Pan R, Wan X, et al. A longitudinal study on the mental health of general population during the COVID-19 epidemic in China. *Brain Behav Immun*. 2020;87:40–48. doi:10.1016/j.bbi.2020.04.028
23. Tee ML, Tee CA, Anlacan JP, et al. Psychological impact of COVID-19 pandemic in the Philippines. *J Affect Disord*. 2020;277:379–391.
24. Tan W, Hao F, McIntyre RS, et al. Is returning to work during the COVID-19 pandemic stressful? A study on immediate mental health status and psychoneuroimmunity prevention measures of Chinese workforce. *Brain Behav Immun*. 2020;87:84–92. doi:10.1016/j.bbi.2020.04.055
25. Hao F, Tan W, Jiang L, et al. Do psychiatric patients experience more psychiatric symptoms during COVID-19 pandemic and lockdown? A case-control study with service and research implications for immunopsychiatry. *Brain Behav Immun*. 2020;87:100–106. doi:10.1016/j.bbi.2020.04.069
26. Lovibond SH, Lovibond PF. *Manual for the Depression Anxiety Stress Scales*. Psychology Foundation of Australia; 1996.
27. Le MTH, Tran TD, Holton S, Nguyen HT, Wolfe R, Fisher J. Reliability, convergent validity and factor structure of the DASS-21 in a sample of Vietnamese adolescents. *PLoS One*. 2017;12(7): e0180557. doi:10.1371/journal.pone.0180557
28. Tran TD, Tran T, Fisher J. Validation of the depression anxiety stress scales (DASS) 21 as a screening instrument for depression and anxiety in a rural community-based cohort of northern Vietnamese women. *BMC Psychiatry*. 2013;13:24. doi:10.1186/1471-244X-13-24
29. Asukai N, Kato H, Kawamura N, et al. Reliability and validity of the Japanese-language version of the impact of event scale-revised (IES-R-J): four studies of different traumatic events. *J Nerv Ment Dis*. 2002;190(3):175–182. doi:10.1097/00005053-200203000-00006
30. Morin CM, Belleville G, Bélanger L, Ivers H. The insomnia severity index: psychometric indicators to detect insomnia cases and evaluate treatment response. *Sleep*. 2011;34(5):601–608. doi:10.1093/sleep/34.5.601
31. Herdman M, Gudex C, Lloyd A, et al. Development and preliminary testing of the new five-level version of EQ-5D (EQ-5D-5L). *Qual Life Res*. 2011;20(10):1727–1736. doi:10.1007/s11136-011-9903-x
32. Mai VQ, Sun S, Minh HV, et al. An EQ-5D-5L value set for Vietnam. *Qual Life Res*. 2020;29(7):1923–1933. doi:10.1007/s11136-020-02469-7
33. Rossi R, Socci V, Pacitti F, et al. Mental health outcomes among frontline and second-line health care workers during the Coronavirus Disease 2019 (COVID-19) Pandemic in Italy. *JAMA Netw Open*. 2020;3(5):e2010185–e2010185. doi:10.1001/jamanetworkopen.2020.10185
34. Giusti EM, Pedroli E, D'Aniello GE, et al. The psychological impact of the COVID-19 outbreak on health professionals: a Cross-Sectional Study. *Front Psychol*. 2020;11:1684. doi:10.3389/fpsyg.2020.01684
35. Shechter A, Diaz F, Moise N, et al. Psychological distress, coping behaviors, and preferences for support among New York healthcare workers during the COVID-19 pandemic. *Gen Hosp Psychiatry*. 2020;66:1–8.
36. Lai J, Ma S, Wang Y, et al. Factors associated with mental health outcomes among health care workers exposed to coronavirus disease 2019. *JAMA Netw open*. 2020;3(3):e203976–e203976. doi:10.1001/jamanetworkopen.2020.3976
37. Du J, Dong L, Wang T, et al. Psychological symptoms among frontline healthcare workers during COVID-19 outbreak in Wuhan. *Gen Hosp Psychiatry*. 2020;S0163-8343(0120)30045–30041.
38. Tan BYQ, Chew NWS, Lee GKH, et al. Psychological impact of the COVID-19 pandemic on health care workers in Singapore. *Ann Intern Med*. 2020;173(4):317–320. doi:10.7326/M20-1083
39. Tran TTT, Nguyen NB, Luong MA, et al. Stress, anxiety and depression in clinical nurses in Vietnam: a cross-sectional survey and cluster analysis. *Int J Ment Health Syst*. 2019;13:3. doi:10.1186/s13033-018-0257-4
40. Nguyen Ngoc A, Le Thi Thanh X, Le Thi H, Vu Tuan A, Nguyen Van T. Occupational stress among health worker in a National Dermatology Hospital in Vietnam, 2018. *Front Psychiatry*. 2020;10:950.
41. Nguyen LH, Tran BX, Hoang Le QN, Tran TT, Latkin CA. Quality of life profile of general Vietnamese population using EQ-5D-5L. *Health Qual Life Outcomes*. 2017;15(1):199. doi:10.1186/s12955-017-0771-0
42. Ping W, Zheng J, Niu X, et al. Evaluation of health-related quality of life using EQ-5D in China during the COVID-19 pandemic. *PLoS One*. 2020;15(6):e0234850–e0234850. doi:10.1371/journal.pone.0234850
43. Nguyen HTT, Moir MP, Nguyen TX, et al. Health-related quality of life in elderly diabetic outpatients in Vietnam. *Patient Prefer Adherence*. 2018;12:1347–1354. doi:10.2147/PPA.S162892
44. Tran BX, Dang AK, Truong NT, et al. Depression and quality of life among patients living with HIV/AIDS in the era of Universal Treatment Access in Vietnam. *Int J Environ Res Public Health*. 2018;15(12):2888.
45. Nguyen SH, Nguyen LH, Vu GT, et al. Health-related quality of life impairment among patients with different skin diseases in Vietnam: a Cross-Sectional Study. *Int J Environ Res Public Health*. 2019;16(3):305. doi:10.3390/ijerph16030305
46. Ngo CQ, Phan PT, Vu GV, et al. Effects of different comorbidities on health-related quality of life among respiratory patients in Vietnam. *J Clin Med*. 2019;8(2):214. doi:10.3390/jcm8020214
47. Tran BX, Thu Vu G, Hoang Nguyen L, et al. Cost-of-Illness and the health-related quality of life of patients in the dengue fever outbreak in Hanoi in 2017. *Int J Environ Res Public Health*. 2018;15(6):1174. doi:10.3390/ijerph15061174
48. Nguyen AT, Nguyen LH, Nguyen TX, et al. Frailty prevalence and association with health-related quality of life impairment among rural community-dwelling older adults in Vietnam. *Int J Environ Res Public Health*. 2019;16(20):3869.
49. Vu HM, Nguyen LH, Tran TH, et al. Effects of chronic comorbidities on the health-related quality of life among older patients after falls in Vietnamese Hospitals. *Int J Environ Res Public Health*. 2019;16(19):3623. doi:10.3390/ijerph16193623
50. Ho CS, Chee CY, Ho RC. Mental health strategies to combat the psychological impact of COVID-19 beyond paranoia and panic. *Ann Acad Med Singapore*. 2020;49(3):155–160.
51. Luo C, Sanger N, Singhal N, et al. A comparison of electronically-delivered and face to face cognitive behavioural therapies in depressive disorders: a systematic review and meta-analysis. *EClinicalMedicine*. 2020;24:100442.
52. Yüksel A, Bahadır Yılmaz E. The effects of group mindfulness-based cognitive therapy in nursing students: a quasi-experimental study. *Nurse Educ Today*. 2020;85:104268. doi:10.1016/j.nedt.2019.104268
53. Bäuerle A, Graf J, Jansen C, et al. An e-mental health intervention to support burdened people in times of the COVID-19 pandemic: coPE It. *J Public Health*. 2020;42(3):647–648. doi:10.1093/pubmed/fdaa058
54. Vuong DA, Van Ginneken E, Morris J, Ha ST, Busse R. Mental health in Vietnam: burden of disease and availability of services. *Asian J Psychiatr*. 2011;4(1):65–70. doi:10.1016/j.ajp.2011.01.005
55. Nguyen T, Tran T, Tran H, Tran T, Fisher J. Challenges in integrating mental health into primary care in Vietnam. In: Okpaku S, editor. *Innovations in Global Mental Health*. Cham: Springer International Publishing; 2019:1–21.

56. Nicola M, Alsafi Z, Sohrabi C, et al. The socio-economic implications of the coronavirus pandemic (COVID-19): a review. *Int J Surg*. 2020;78:185–193. doi:10.1016/j.ijsu.2020.04.018
57. Brown JP, Martin D, Nagaria Z, Verceles AC, Jobe SL, Wickwire EM. Mental health consequences of shift work: an updated review. *Curr Psychiatry Rep*. 2020;22(2):7.

Risk Management and Healthcare Policy Dovepress

Publish your work in this journal

Risk Management and Healthcare Policy is an international, peer-reviewed, open access journal focusing on all aspects of public health, policy, and preventative measures to promote good health and improve morbidity and mortality in the population. The journal welcomes submitted papers covering original research, basic science, clinical & epidemiological studies, reviews and evaluations, guidelines, expert opinion and commentary, case reports and extended reports. The manuscript management system is completely online and includes a very quick and fair peer-review system, which is all easy to use. Visit http://www.dovepress.com/testimonials.php to read real quotes from published authors.

Submit your manuscript here: https://www.dovepress.com/risk-management-and-healthcare-policy-journal
