# Supplementary material for: Mental health status and related factors influencing healthcare workers during the COVID-19 pandemic: A systematic review and meta-analysis
Source: PLoS One. 2024 Jan 19;19(1):e0289454. doi: 10.1371/journal.pone.0289454 (PMC10798549; doi:10.1371/journal.pone.0289454)
Supplement: S1 Data — (ZIP) [file pone.0289454.s011.zip › literatures/195.pdf]

Psychiatria Danubina, 2021; Vol. 33, Suppl. 13, pp S364-371
Medicina Academica Mostariensia, 2021; Vol. 9, No. 2, pp 364-371
© Medicinska naklada - Zagreb, Croatia
COVID-19 forum: Original paper

THE EFFECT OF COVID-19 OUTBREAK ON THE MENTAL STATUS OF HEALTH CARE PROFESSIONALS IN THE PEDIATRIC INTENSIVE CARE UNIT

Sevgi Topal[1], Onat Yilmaz,[2] Gokhan Ceylan[1], Ozlem Sarac Sandal[3], Utku Karaarslan[1], Gulhan Atakul[1], Mustafa Colak[1], Ekin Soydan[1], Pinar Seven[1], Arzu Caliskan Polat[1] & Hasan Agin[1]
[1]*University of Health Sciences, Dr. Behcet Uz Children's Hospital, Pediatric Intensive Care Unit, Izmir, Turkey*
[2]*University of Health Sciences, Ok Meydani Training and Research Hospital, Department of Psychiatry, Istanbul, Turkey*
[3]*Sivas Cumhuriyet University of Medicine, Pediatric Intensive Care Unit, Sivas, Turkey*

received: 20.9.2020; revised: 30.10.2020; accepted: 27.11.2020

SUMMARY

Background: *Coronavirus disease 2019 (COVID-19) may affect the mental status of health care professionals. The purpose of our study is to evaluate the mental health effects of the COVID-19 epidemic on health care professionals in the pediatric intensive care units (PICUs).*

Subjects and methods: *Our study was conducted prospectively between 01.04.20 and 10.04.20. The created questionnaire was applied to health care professionals through online platforms. Thus it was involved in 5 different institutions that participated from different regions of Turkey. With the questionnaire, we applied; the participants' age, gender, the general status of contamination and the level of COVID-19 knowledge were questioned. Besides; Beck Anxiety Scale, Acute Stress Scale (PCL-5), STAI-1 and STAI-2 (State and Trait Anxiety Inventory 1-2) scales were used to determine the anxiety levels.*

Results: *A survey of 210 participants, 86 (41%) doctors, 124 (59%) nurses, were included in our study. When we evaluate the Beck anxiety levels, the majority of the participants (44%) were normal, while about one third had mild anxiety. When we evaluated the acute stress scale, all participants had a certain amount of stress levels. The majority (80 people each (38%)) experienced mild and moderate acute stress. Being female and having chronic disease poses a high risk for anxiety (OR, 0.330; 95% CI, 0.087-1.250, p <0.05 and OR, 0.246; 95% CI, 0.068-1.116, p<0.05), preoccupation (OR, 0.603; 95% CI 0.261-1.395, p<0.05 and OR, 0.433; 95% CI, 0.122-1.538, p<0.05) and acute stress (OR, 0.294; 95% CI, 0.033-2.649, p<0.05 and OR, 0.317; 95 % CI 0.060-1.679, p<0.05). Professional definition, marital status and having a child do not pose any risk factors.*

Conclusion: *Our study has shown that the COVID-19 outbreak affects the mental status of health care professionals working at PICU at various levels.*

Key words: *acute stress scale – anxiety - COVID-19 outbreak - healthcare professionals*

* * * * *

INTRODUCTION

Seven of which were critical, 27 viral pneumonia cases were identified in Wuhan, China on December 12, 2019. The number of cases increased rapidly day by day (Li et al. 2020). On December 29, 2019, hospitals reported the patients with 'pneumonia of unknown etiology' using the surveillance mechanism, just like in the 2003 acute respiratory syndrome (SARS) epidemic (Xiang et al. 2013, Cheng & Shan 2019). It was revealed that the current situation was created by a new type of coronavirus which is named Coronavirus disease 2019 (COVID-19) (Cheng & Shan 2019). Over time, the infection spread to other cities in China and then to many countries around the world (Li et al. 2020). On January 30, 2020, the World Health Organization (WHO) declared the COVID-19 outbreak as an emerging global health problem and pandemic (World Health Organization. Emergency Committee regarding the outbreak of novel coronavirus (2019-nCoV) 2020).

Coronavirus disease 2019 is spreading all over the world in a short time and creating a pandemic and not having enough information about the disease may cause people to develop negative emotions and threaten their mental health. Recent studies in various countries of the world have shown that COVID-19 can cause negative mental problems such as reluctance, anxiety, sleep problems, avoidance behaviors, and depression in individuals (Shigemura et al. 2020, Brooks et al. 2020, Chinese Society of Psychiatry/ Novel Coronavirus Pneumonia 2020, Angus Reid Institute 2020, Asmundson & Taylor 2020, Li et al. 2020, Wang et al. 2020, Karaşar & Canli 2020, Al-Qahtani et al. 2020). Besides, health care professionals may experience more serious psychological effects due to both excessive workload of the pandemic and the higher risk of transmission when compared to the normal population (Chew et al. 2020). Although studies evaluating the mental health of the people in outbreaks are common, studies evaluating health workers are much more limited. When we look at the literature,

recent studies evaluating health workers have shown that the process might cause serious sleep problems and emotional distress in health professionals (Li et al. 2020, Xiang et al. 2020, Kang et al. 2020). Studies have been designed to evaluate depression, anxiety, and sleep problems of general health care professionals. The purpose of our study is to evaluate the mental health status of the health care professionals working in the pediatric intensive care units (PICUs) with anxiety levels and acute stress scales. Also, the determination of risk factors that can contribute to the anxiety levels in PICU workers is targeted.

SUBJECTS AND METHODS

Study Design

The study was conducted following the ethical standards stated in the 'Declaration of Helsinki', and was approved by the local ethics committee (2020/05-10).

Our study was conducted prospectively between 01.04.2020 and 10.04.2020 when the number of cases in our country exceeded 15000 and was seen in every city. The questionnaire was applied after the consent of the participants. Participants were given the chance to survey within the time frame they wanted. The questionnaire was designed not to contain identification information, and control was provided with the online database, allowing each participant to complete the questionnaire only once. The created questionnaire was applied on online platforms actively used by health care professionals working in PICU and by invitation via e-mail. Thus it was involved in 5 different institutions that participated from different regions of Turkey. All completed questionnaires are included in our study.

Participants

Health professionals with whom employees in PICU in 5 different cities in Turkey were invited as participants. The main criterion for the participants' inclusion in the study was their actively working since December 2019, when the COVID-19 epidemic began to appear worldwide, including at the time of the survey. The questionnaires of the participants who were not answered and completed all questions were not included in our study.

Outcomes and Covariates

With the questionnaire we applied; the participants' age, gender, the general status of contamination and thoughts were questioned. Then, after a comprehensive literature review and research, an 8-question questionnaire was created to determine the level of COVID-19 knowledge, which was designed in a simple way, where basic points were questioned.

In the last part of the questionnaire, Beck Anxiety Scale, Acute Stress Scale (PCL-5), State and Trait Anxiety Inventory-1 (STAI-1) for acute anxiety and State and Trait Anxiety Inventory-2 (STAI-2) for chronic anxiety were used to determine the anxiety levels. The Beck Anxiety Scale is a self-assessment scale. It is used to determine the frequency of anxiety symptoms experienced by individuals and it provides Likert type measurement. There are 4 options in each of the twenty-one symptom categories. Each item gets points between 0 and 3. The high score obtained from the scale indicates the severity of the anxiety experienced by the individual. While 0-7 points show a normal vital response, 8-15 points show mild, 16-25 points show medium level and 26-63 points show severe anxiety (Ulusoy et al. 1998). State and Trait Anxiety Scale (STAI-1 and STAI-2) is a Likert type scale that measures state and trait anxiety levels separately with 20 questions. High scores indicate high anxiety levels, low scores indicate low anxiety levels. Scores are between 20 and 80. Results under 36 points are accepted as no anxiety levels, mild anxiety between 36-42 points, score over 42 points are evaluated as high anxiety levels (Lecompte & Öner 1975). The Acute Stress Scale (PCL-5) is not used to make a diagnosis, but it is a guide for the clinician. In the scoring of the 7-question test in which acute stress symptoms are investigated, each question is scored between 0 and 4 points, and the score obtained by dividing the total score by 7 is accepted as the sign of the acute stress level. While evaluating, they are considered as; 0 point is no acute stress symptoms, 1 point is mild, 2 points are moderate, 3 points are serious, and 4 points are signs of excessive stress (Boysan et al. 2017).

Statistical Analysis

Statistical analysis was performed using Statistical Package for Social Sciences (SPSS) version 20.0 (SPSS Inc.; Chicago, IL, USA). The general information of the participants, their level of knowledge and their general views about COVID-19, and the results obtained from the anxiety scales were obtained by frequency and numerical analysis. Anxiety and preoccupation levels (severity) and conditions affecting acute stress conditions were evaluated between the groups by nonparametric Mann-Whitney U and Kruskal Wallis tests. Multivariable logistic regression analysis was used to determine possible risk factors for the anxiety levels expressed. Risk factor values for gender, occupational definition, marital status, having a child and having a chronic disease were presented as odds ratio (OR) and 95% CI for anxiety. Besides, the correlation between STAI-1 and PCL-5 and Beck anxiety scales was evaluated by Pearson correlation analysis to see if there was an acute effect due to COVID-19 in the participants. The significance level was taken as $p<0.05$.

RESULTS

A survey of 210 participants, 86 (41%) doctors, 124 (59%) nurses, were included in our study. One hundred fifty-two (28%) of the participants were female, 58 (72%) were male and their age was mean 30.4±7.7 (21-58). Eighty-seven (42%) were married, 123 (58%) were single and 63 (30%) had a child, 147 (70%) had no children. Thirty-five (18.1%) participants have chronic illnesses, mostly they have respiretory tract diseases, and 175 (83%) did not have any illness. At least 7 of the questions designed to evaluate the level of COVID-19 knowledge were answered correctly.

Table 1. General information of the participants and their level of knowledge about COVID-19

Demographic features and COVID-19 knowledge levels	N(%)
Age (years) (Mean ± SD) (min-max)	30.4±7.7 (21-58)
Gender	
Female	152 (72%)
Male	58 (28%)
Professional Definition	
Doctor	86 (41%)
Nurse	124 (59%)
Marital Status	
Married	87 (42%)
Single	123 (58%)
Having a Child	
Yes	63 (30%)
No	147 (70%)
Diagnosed Chronic Disease	
Heart disease	4 (2%)
Respiratory disease	14 (7%)
Diabetes mellitus	4 (2%)
Hypertension	4 (2%)
Other	9 (4%)
No	175 (83%)
Correct answering rates of questions measuring COVID knowledge level	
Coronavirus is transmitted through blood.	187 (89%)
The incubation period of the coronavirus takes 20-30 days.	170 (81%)
Fever, respiratory distress and cough are the most common symptoms of the disease.	210 (100%)
Frequent hand washing reduces the risk of disease transmission.	210 (100%)
Disinfectant use is not effective against coronavirus.	210 (100%)
At least 1-2 meters distance should be placed with a suspicious case.	210 (100%)
Most patients infected with coronavirus result in death.	195 (93%)
The coronavirus vaccine has started to be routinely administered.	210 (100%)

COVID-19: Coronavirus disease 2019, Max: Maximum, Min: Minimum, N: Number, SD: Standard deviation

Sixty-three percent of the participants answered all questions (8 questions) and 37% answered 7 questions correctly (Table 1).

In the section where we evaluated the general opinions of the participants about COVID-19, 77% of them thought that they would not be able to survive when they get infected, and the most common contamination place would be the hospital with 81%. They stated that their risk of getting coronavirus was 8 out of 10, their profession's impact on this issue was 8 out of 10, their anxiety about carrying the disease to their families was 7 out of 10, and their helplessness about this incident was 6 out of 10. The hygienic habits of 97% of participants had changed due to the COVID-19 pandemic. The most common protective measures used in the workplace were 93% frequent hand washing, 87% using a disinfectant, and 76% using masks or glasses. The most common methods preferred by the participants to cope with emotional difficulties were, 74% of them were to distract with other things and 67% to follow the recommendations of the authorities (Table 2).

When we evaluate the Beck anxiety levels, the majority of the participants (44%) had no symptoms of anxiety, while about one third of them expressed mild anxiety levels. When we evaluated the acute stress scale (PCL-5), all participants had a certain amount of stress levels. The majority (80 people each (38%)) experienced mild and moderate acute stress. According to the STAI-1 and STAI-2, a large part of the participants (71%) had severe anxiety (Table 3).

There was a significant correlation between STAI-1 and PCL-5 ($p<0.01$) and Beck anxiety levels ($p<0.01$). No correlation was observed between STAI-2 and PCL-5 ($p=0.290$) and Beck anxiety levels ($p=0.449$). Gender ($p=0.008$) and chronic disease ($p=0.004$) showed statistically significant effect on the anxiety levels of the participants. Professional definition ($p=0.239$), marital status ($p=0.544$), and having a child ($p=0.804$) do not affect. Also, gender ($p=0.010$) and chronic disease ($p=0.017$) had a statistically significant effect on acute stress state. Professional definition ($p=0.134$), marital status ($p=0.875$) and having a child ($p=0.323$) had no effect. Similarly, gender ($p=0.046$) and chronic disease ($p=0.012$) affect STAI levels statistically significant. Professional definition ($p=0.988$), marital status ($p=0.776$) and having a child ($p=0.766$) do not affect STAI levels (Table 4).

Being a female and having a chronic disease poses a high risk for anxiety (OR, 0.330; 95% CI, 0.087-1.250, $p<0.05$ and OR, 0.246; 95% CI, 0.068-1.116, $p<0.05$, respectively), preoccupation (OR, 0.603; 95% CI 0.261-1.395, $p<0.05$ and OR, 0.433; 95% CI, 0.122-1.538, $p<0.05$, respectively) and acute stress (OR, 0.294; 95% CI, 0.033-2.649, $p<0.05$ and OR, 0.317; 95 % CI 0.060-1.679, $p<0.05$, respectively).

Table 2. General opinions of the participants about COVID-19

	N (%)
Do you think you can survive the epidemic without catching coronavirus?	
Yes	48 (23%)
No	162 (77%)
How do you think coronavirus can infect you more?	
From workplace	171 (81%)
From shopping centers, markets, etc.	31 (15%)
From family members	6 (3%)
Other	2 (1)
Have your hygienic habits changes due to corona?	
Yes	203 (97%)
No	7 (3%)
What do you do as protective measures in your workplace? (You can choose more than one option)	
Using masks/glasses	160 (76%)
Using gloves	142 (68%)
Frequent hand washing	195 (93%)
Using disinfectant	182 (87%)
Avoiding crowded environments	167 (80%)
Not going out of where I work	159 (76%)
Dressing clothes for work	148 (71%)
Other	8 (4%)
How do you cope with the emotional difficulties you experienced in this process? (You can choose more than one option)	
I am not following the media.	28 (13%)
I am moving away from spoken environments about coronavirus	14 (7%)
I follow the recommendations of the authorities.	141 (67%)
I refuse to believe that coronavirus is a problem.	6 (3%)
I share my feelings with my friends and relatives.	107 (51%)
I try to relax with alcohol or smoking.	13 (6%)
I prefer to be alone.	37 (18%)
I'm trying to distract me from other things. (Ex: reading books, watching movies, listening to music, etc.)	155 (74%)
I'm making jokes about the subject.	57 (27%)
I do physical exercise/sports.	52 (25%)
I pray.	105 (50%)
I do meditation/yoga.	16 (8%)
I am making plans on what to do in case of illness.	64 (31%)
Whatever I do, I don't think I can handle it.	25 (12%)
Other	7 (4%)
Rate the suitability of the following sentences for you from 0 to 10 (0-none, 10-too many)	Mean (min-max)
Do you think you are at risk of getting coronavirus?	6 (1-10)
How risky do you find your job related to catching coronavirus?	8 (4-10)
Do you have concerns about transmitting the disease to your family?	7 (3-10)
Do you feel helpless about this incident?	6 (2-10)

COVID-19: Coronavirus disease 2019; Max: Maximum, Min: Minimum; N: Number

DISCUSSION

Our study has shown that the COVID-19 outbreak affects the mental status of health care professionals working at PICU at various levels. Various levels of stress were expressed by all of the participants and mild to moderate levels of anxiety in more than half of the participants. It has been revealed that all these conditions are observed more in females and those with chronic disease. Professional definition, marital status, and having a child do not pose any risk factors.

Coronavirus disease 2019 is rapidly transmitted by close contact with individuals (Cheng & Shan 2019). The disease causes respiratory diseases such as acute respiratory distress syndrome (ARDS) and acute respiratory failure, which is severe or even can result in death (Wang et al. 2020b). Due to the serious risks caused by the infection, strict quarantine measures have been taken almost throughout the country in China to keep the epidemic under control (World Health Organization/Novel Coronavirus 2020). One of the countries where the epidemic was most devastating after China was Italy.

Table 3. Evaluation of participants' anxiety scales

Scales	N (%)
Beck Anxiety Scale	
Normal	93 (44%)
Mild	62 (30%)
Moderate	38 (18%)
Severe	17 (8%)
PCL-5	
Normal	-
Mild	80 (38%)
Moderate	80 (38%)
Severe	41 (20%)
Extreme	9 (4%)
STAI-1 and STAI-2	
Normal	31 (15%)
Mild	30 (14%)
Severe	71 (71%)

Number, PCL-5: Acute Stress Scale; STAI: State and Trait Anxiety Inventory

In time, the epidemic spread has become so uncontrolled that environmental quarantine measures were introduced by stating that the services provided by the health system could not be sustained in this way and this increase would cause a collapse in the entire health system, especially in intensive care units (Grasselli et al. 2020). Afterward, as the infection spread, strict quarantine measures were taken in many countries, including Turkey. However, the epidemic exerted a heavy burden on the entire health system, especially in intensive care units. This workload may increase the psychological burden of health care workers due to the epidemic.

Rapid spreading, quarantine, negative contents from social media and increasing numbers of cases and deaths put people under psychological pressure and cause mental health problems (Li et al. 2020). In this critical process, there is an increased risk of developing psychological problems in health care professionals working in the front line by managing the diagnosis,

Table 4. Comparison of anxiety scales according to the characteristics of the participants (number (%))

	Gender			Professional Definition			Marital Status		
	Female	Male	p-value	Doctor	Nurse	p-value	Single	Married	p-value
Beck Anxiety Scale			*0.008*			0.239			0.544
Normal	57 (37)	36 (62)		37 (43)	56 (45)		56 (46)	37 (42)	
Mild	51 (33)	11 (19)		20 (23)	42 (34)		36 (29)	26 (30)	
Moderate	30 (20)	8 (14)		21 (25)	17 (14)		22 (18)	16 (19)	
Severe	14 (10)	3 (5)		8 (9)	9 (7)		9 (7)	8 (9)	
PCL-5			*0.010*			0.134			0.875
Normal	-	-							
Mild	52 (34)	31 (53)		29 (34)	54 (43)		48 (39)	35 (40)	
Moderate	62 (41)	18 (31)		34 (40)	46 (37)		50 (41)	30 (34)	
Severe	32 (21)	8 (14)		18 (20)	22 (18)		22 (18)	18 (20)	
Extreme	6 (4)	1 (2)		5 (6)	2 (2)		3 (2)	4 (6)	
STAI-1 and STAI-2			*0.046*			0.988			0.776
Normal	20 (13)	12 (21)		12 (14)	19 (15)		17 (14)	14 (16)	
Mild	19 (13)	12 (21)		13 (15)	17 (14)		20 (16)	10 (12)	
Severe	113 (74)	34 (58)		61 (71)	88 (71)		86 (70)	63 (72)	

	Having a Child			Diagnosed Chronic Disease		
Beck Anxiety Scale			0.804			*0.004*
Normal	28 (44)	65 (44)		9 (27)	85 (49)	
Mild	17 (27)	45 (30)		11 (31)	50 (29)	
Moderate	13 (21)	25 (17)		10 (28)	28 (16)	
Severe	5 (8)	12 (9)		5 (14)	12 (6)	
PCL-5			0.323			*0.017*
Normal				-		
Mild	27 (43)	56 (38)		17 (46)	64 (37)	
Moderate	21 (33)	59 (40)		9 (27)	72 (42)	
Severe	13 (20)	27 (18)		6 (18)	35 (19)	
Extreme	2 (4)	5 (4)		3 (9)	4 (2)	
STAI-1 and STAI-2			0.766			*0.012*
Normal	8 (13)	23 (16)		3 (9)	28 (16)	
Mild	7 (11)	23 (16)		3 (9)	27 (15)	
Severe	48(76)	101(68)		29(82)	120(69)	

PCL-5: Acute Stress Scale, STAI: State and Trait Anxiety Inventory

treatment, and care process of the patients. Each day increasing workload, depletion of protection equipment, and feelings of insufficient support cause a mental burden for health care professionals (Lai et al. 2020). In 2003, the SARS outbreak caused negative psychological reactions to health care workers. Health care workers were afraid of contamination and their families and friends being infected through themselves (Maunder et al. 2003). Besides, some patients experienced high levels of stress, anxiety, and depression symptoms (Lee et al. 2007). In the COVID-19 outbreak, health care professionals also experience physical fatigue, sleep problems, and emotional distress due to excessive workload and isolation (Kang et al. 2020). In a study including a great number of health care professionals, it was revealed that half of the participants had depressive symptoms, almost half were anxious and one third suffered from sleep problems (Liu et al. 2020). In our study, moderate and severe anxiety was observed in one fifth of the participants and more than half of them expressed serious and extreme stress, and one fourth and also stress in all, and preoccupation in 85% and three-quarters of them were severe. Relatively high levels of anxiety may be related to the fact that all the participants were intensive care workers. So far, the highest risk for transmission of COVID-19 is determined via tracheal intubation, and the procedure is somehow a routine daily activity in ICU settings. Hence, PICU workers encounter the highest viral load risk during tracheal intubation (Sorbello et al. 2020), and this may increase the mental depression experienced by intensive care workers.

Health professionals have a duty of caring for infected patients and fear of transmission of the disease as they have close contact with patients and their relatives (Xiang et al. 2020). This situation may increase the psychological pressure in the epidemic of health care professionals. In our study, three out of four health care workers stated that they would get coronavirus and 80% thinking of this would be at work. The participants stated that their profession poses a high risk of catching coronavirus and they feel helpless about the outbreak.

Having limited knowledge of COVID-19 can lead to anxiety and fear (Shigemura et al. 2020). Although seasonal influenza is more lethal than COVID-19, fear of COVID-19 is much higher. This may be related to the new definition of COVID-19 and inadequate information about it (Asmundson & Taylor 2020). In our study, we applied a simple questionnaire evaluating the knowledge levels of health care professionals. One-third of the participants answered only one question incurrectly, while the rest answered completely correctly. However, our survey questions consisted of simple information that individuals can learn through social media. On the other hand, the negative effect of the mental state in the participants may be that the follow-up and treatment process related to COVID-19 is not known medically enough.

Patients with chronic diseases such as hypertension and diabetes mellitus make regular hospital visits to arrange their medical treatment. Failure to access regular health care for these patients may create stress and cause psychological problems (Li et al. 2020). In our study, one-fifth of the participants had a chronic disease. Anxiety, stress, and preoccupation levels of those with chronic disease were found to be higher. However, this situation seen in health care workers may also be related to the fact that they have the idea of being infected and the infection progress being more severe when there is a comorbid disease.

In an epidemic, individuals may experience obsessive-compulsive symptoms by creating exaggerated reactions such as repetitive disinfection procedures due to fear of infection and transmission (Shigemura et al. 2020). In our study, the COVID-19 epidemic has shown that health care workers have changed their hygienic habits and the use of frequent handwashing and disinfectants has increased. We have not evaluated these habits whether they reached the compulsive level or not.

In a study where health care professionals were evaluated because of the COVID-19 outbreak, the female gender and being nurse were found significantly associated with the symptoms of depression, anxiety, and stress to be more severe. The most important reason for anxiety in nurses has been asserted that being young and having a less professional experience when compared to doctors (Lai et al 2020). Similarly, in our study, the female gender is a risk factor for higher levels of anxiety, stress development, and preoccupation. However, being a nurse did not constitute a risk factor statistically. This may only be related to the evaluation of PICU employees. Because PICU is a featured department and nurses mostly consist of a team with high professional experience.

According to our study, having a child does not have any effect on mental state, which was similar to previous study by Wang et al. (2020b). This may be related to knowing that the course of patients in pediatric patients is better than in adults. It may also be related to the fact that many of health care professionals change their housings with the fear that they will carry infection to their home. With this avoidance behavior, they may have moved away from the thought that they will infect their children.

An important point that has not been evaluated in previous studies is the mental problems of the participants that may exist before. In other words, underlying chronic anxiety or vulnerability to stressful situations may affect the mental status of the participants.

For this reason, a correlation was made between the tests and it was revealed that the stress and anxiety states experienced by the participants were acute.

When we evaluate the results of our study, we see that health care professionals working at PICU experience mental problems related to the epidemic. In case of an epidemic, not being aware of the mental problems that health care professionals might experience and not taking the necessary precautions may affect the working tempo by creating physical depression. Increasing workload, high risk of contamination, and negating the negative effects of social isolation and providing the necessary psychological support by professionals can not only reduce the mental influences of health care professionals but also prevent the loss of workforce and manage the process more positively.

Our study has some limitations. Comparative evaluation of health workers working in other units and those working in PICU can provide us with more information. Depression and sleep disturbance may also be other factors that can be evaluated.

CONCLUSIONS

During the outbreak of COVID-19 highly in actively working health professionals anxiety, preoccupation, and stress have been shown to develop in PICUs in Turkey. Especially having female gender and chronic disease constitutes a risk factor for mental effect. These mental problems experienced by health care professionals should not be ignored, necessary precautions should be taken to prevent them from occurring and necessary professional support should be provided quickly. Necessary interventions especially for females and with chronic disease should be done before the condition becomes chronic.

Acknowledgements: None.

Conflict of interest: None to declare.

Contribution of individual authors:

All the authors have significantly contributed to the manuscript, and they have all approved its final version.

References

1. *Al-Qahtani AM, Elgzar WT, Ibrahim HA. COVID-19 Pandemic: Psycho-social Consequences During the Social Distancing Period Among Najran City Population. Psychiatr Danub 2020; 32:280-6*
2. *Angus Reid Institute: Half of Canadians taking extra precautions as coronavirus continues to spread around the globe 2020. http://angusreid.org/wpcontent/uploads/2020/02/2020.02.04_Coronavirus.pdf*
3. *Asmundson GJG & Taylor S: Coronaphobia: Fear and the 2019-nCoV outbreak. J Anxiety Disord 2020; 70:102196*
4. *Boysan M, Ozdemir PG, Ozdemir O, Selvi Y, Yilmaz E, Kaya N: Psychometric properties of the Turkish version of the PTSD Checklist for Diagnostic and Statistical Manual of Mental Disorders, Fifth Edition (PCL-5). Psychiatr Clin Psychopharmacol 2017; 3:306-16*
5. *Brooks SK, Webster RK, Smith LE, Woodland L, Wessely S, Greenberg N, et al.: The psychological impact of quarantine and how to reduce it: rapid review of the evidence. Lancet 2020; 395:912-20*
6. *Cheng ZJ & Shan J: 2019 Novel coronavirus: where we are and what we know. Infection 2020; 48:155-63*
7. *Chew NWS, Lee GKH, Tan BYQ, Jing M, Goh Y, Ngiam NJH, et al.: A multinational, multicentre study on the psychological outcomes and associated physical symptoms amongst healthcare workers during COVID-19 outbreak. Brain Behav Immun 2020; 88:559-65*
8. *Chinese Society of Psychiatry: Expert Consensus on Managing Pathway and Coping Strategies for Patients with Mental Disorders during Prevention and Control of Infectious Disease Outbreak (Novel Coronavirus Pneumonia) 2020*
9. *Grasselli G, Pesenti A, Cecconi M: Critical Care Utilization for the COVID-19 Outbreak in Lombardy, Italy: Early Experience and Forecast During an Emergency Response. JAMA 2020 Mar 13*
10. *Kang L, Li Y, Hu S, Chen M, Yang C, Yang BX, et al.: The mental health of medical workers in Wuhan, China dealing with the 2019 novel coronavirus. Lancet Psychiatry 2020; 7:e14*
11. *Karaşar B, Canli D: Psychological Resilience and Depression during the Covid-19 Pandemic in Turkey. Psychiatr Danub 2020; 32:273-79*
12. *Lai J, Ma S, Wang Y, Cai Z, Hu J, Wei N, et al.: Factors Associated With Mental Health Outcomes Among Health Care Workers Exposed to Coronavirus Disease 2019. JAMA Netw Open 2020;3:e203976*
13. *Lecompte A & Öner N: Durumluk-Sürekli Kaygı Envanterinin Türkçe'ye Adaptasyon ve Standardizasyonu ile İlgili Bir Çalışma. IX. Milli Psikiyatri ve Nörolojik Bilimler Kongresi Çalışmaları 1975; 457-62. (Turkish)*
14. *Lee AM, Wong JG, McAlonan GM, Cheung V, Cheung C, Sham PC, et al.: Stress and psychological distress among SARS survivors 1 year after the outbreak. Can J Psychiatry 2007; 52:233-40*
15. *Li Q, Guan X, Wu P, Wang X, Zhou L, Tong Y, et al.: Early transmission dynamics in Wuhan, China, of novel coronavirus-infected pneumonia. N Engl J Med 2020; 382:1199-207*
16. *Li S, Wang Y, Xue J, Zhao N, Zhu T: The Impact of COVID-19 Epidemic Declaration on Psychological Consequences: A Study on Active Weibo Users. Int J Environ Res Public Health 2020; 17:pii: E2032*
17. *Li W, Yang Y, Liu ZH, Zhao YJ, Zhang Q, Zhang L, et al.: Progression of Mental Health Services during the COVID-19 Outbreak in China. Int J Biol Sci 2020; 16:1732-8*
18. *Liu S, Yang LL, Zhang CX, Xiang YT, Liu Z, Hu S, et al.: 2019 novel coronavirus: online mental health services. Lancet Psychiatry 2020; 7:17-8*
19. *Maunder R, Hunter J, Vincent L, Bennett J, Peladeau N, Leszcz M, et al.: The immediate psychological and occupational impact of the 2003 SARS outbreak in a teaching hospital. CMAJ 2003; 168:1245-51*

20. *Shigemura J, Ursano RJ, Morganstein JC, Kurosawa M, Benedek DM: Public responses to the novel 2019 coronavirus (2019-nCoV) in Japan: mental health consequences and target populations. Psychiatry Clin Neurosci 2020; 74:281-2*
21. *Sorbello M, El-Boghdadly K, Di-Giacinto I, Cataldo R, Esposito C, Falcetta S, et al.: Società Italiana di Anesthesia Analgesia Rianimazione e Terapia Intensiva (SIAARTI) Airway Research Group, and The European Airway Management Society. The Italian coronavirus disease 2019 outbreak: recommendations from clinical practice. Anesthesia 2020; 75:724-32*
22. *Ulusoy M, Şahin N, Erkmen H : Turkish Version of the Beck Anxiety Inventory: Psychometric Properties. J Cogn Psychotherap 1998; 12:163-72*
23. *Wang C, Pan R, Wan X, Tan Y, Xu L, Mclntyre RS, et al.: A Longitudinal Study on the Mental Health of General Population during the COVID-19 Epidemic in China. Brain Behav Immun. 2020a; 87:40-8*
24. *Wang D, Hu B, Hu C, Zhu F, Liu X, Zhang J, et al.: Clinical characteristics of 138 hospitalized patients with 2019 novel coronavirus-infected pneumonia in Wuhan, China. JAMA 2020b; 323:1061-9*
25. *World Health Organization: Statement on the second meeting of the International Health Regulations (2005) Emergency Committee regarding the outbreak of novel coronavirus (2019- nCoV). https://wwwwhoint/newsroom/detail/30-01-2020-statement-on-the-second-meetingof-the-international-health-regulations-(2005)-emergency-committee-regarding-the-outbreakof-novel-coronavirus-(2019-ncov) (access Feb 17th, 2020)*
26. *World Health Organization: Novel Coronavirus - China. https://www whoint/csr/don/12-January-2020-novel-coronavirus-china/en/ (access Feb 17, 2020)*
27. *Xiang N, Havers F, Chen T, Song Y, Tu W, Li L, et al.: Use of national pneumonia surveillance to describe influenza A(H7N9) virus epidemiology, China, 2004–2013. Emerg Infect Dis 2013; 19:1784-90*
28. *Xiang YT, Yang Y, Li W, Zhang L, Zhang Q, Cheung T, et al.: Timely mental health care for the 2019 novel coronavirus outbreak is urgently needed. Lancet Psychiatry 2020; 7:228-9*

Correspondence:
Sevgi Topal, MD, Pediatrician
University of Health Sciences, Dr. Behcet Uz Children's Hospital, Pediatric Intensive Care Unit
Ismet Kaptan Neighborhood, Sezer Doğan Street, 11, 35210, Izmir, Turkey
E-mail: sevgi_topal86@hotmail.com
