# Supplementary material for: Mental health status and related factors influencing healthcare workers during the COVID-19 pandemic: A systematic review and meta-analysis
Source: PLoS One. 2024 Jan 19;19(1):e0289454. doi: 10.1371/journal.pone.0289454 (PMC10798549; doi:10.1371/journal.pone.0289454)
Supplement: S1 Data — (ZIP) [file pone.0289454.s011.zip › literatures/197.pdf]

Rev Med Chile 2020; 148: 1121-1127

Salud mental en trabajadores de la salud durante la pandemia por COVID-19 en Chile

ALFONSO URZÚA[1,a], ANTONIO SAMANIEGO[2,b], ALEJANDRA CAQUEO-URÍZAR[3,a], ANTONIO ZAPATA PIZARRO[4], MATÍAS IRARRÁZAVAL DOMÍNGUEZ[5]

[1]Escuela de Psicología, Universidad Católica del Norte. Antofagasta, Chile.
[2]Universidad Nacional de Asunción. Asunción, Paraguay.
[3]Instituto de Alta Investigación, Universidad de Tarapacá. Arica, Chile.
[4]Facultad de Medicina, Universidad de Antofagasta y Hospital Regional de Antofagasta. Antofagasta, Chile.
[5]Facultad de Medicina, Universidad de Chile & Jefe de Salud Mental, Ministerio de Salud. Santiago, Chile.
[a]Psicólogo, Doctor en Psicología Clínica y de la Salud.
[b]Psicólogo, Magíster en Psicología Clínica.

Trabajo no recibió financiamiento.
Los autores declaran no tener conflictos de interés.

Recibido el 5 de mayo de 2020, aceptado el 21 de agosto de 2020.

Correspondencia a:
Dr. Alfonso Urzúa
Escuela de Psicología UCN,
Avenida Angamos 0610.
Antofagasta, Chile.
alurzua@ucn.cl

Mental health problems among health care workers during the COVID-19 pandemic

***Background**: COVID-19 has effects on the mental health of health care workers. **Aim**: To explore the presence of symptoms associated with mental health problems and associated risk factors in health workers. **Material and Methods**: The questionnaires PHQ-9 for depression, GAD-7 for anxiety, ISI-7 for insomnia and IES-R-22 for psychological distress were applied to 125 health care workers aged 18 to 67 years (32 physicians, 22 nurses and 71 of other professions) laboring in hospitals and primary care facilities along Chile. **Results**: Sixty five percent reported depression symptoms, 74% anxiety, 65% insomnia and 57% distress. Physicians had lower median scores in all scales than nurses and other health professionals. Professionals attending patients with respiratory infections or with COVID-19 had higher median scores in the scales that their counterparts. **Conclusions**: The frequency of mental health problems among these professionals is high and preventive measures should be taken.*

(Rev Med Chile 2020; 148: 1121-1127)

***Key words:** Anxiety; Coronavirus; Coronavirus Infections; Depression; Health Occupations; Mental Health.*

El 31 de diciembre de 2019, la Comisión Municipal de Salud de Wuhan (China) notificó la existencia de un conjunto de casos de neumonía en la ciudad, reportando tiempo después como causa un nuevo coronavirus, el SARS-CoV-2, causante de la enfermedad COVID-19. El 13 de enero de 2020 se confirmó el primer caso fuera de China[1]. Al 10 de agosto de 2020, la ya declarada pandemia había ocasionado más de 700 mil muertos y más de 19 millones de casos confirmados, de los cuales 10,5 millones son de las Américas y cerca de 370 mil en Chile[2].

A la fecha, las investigaciones principalmente han evidenciado los efectos del virus en la salud física, ya que alrededor de 1 de cada 5 personas que contraen el virus desarrollan una enfermedad grave y dificultades respiratorias[1], colapsando los sistemas de atención y generando mayores cargas de trabajo y estrés en los equipos de salud. Los efectos de esta sobrecarga y estrés, sumados a los de trabajar en constante riesgo de infección, evidentemente generan problemas de salud mental. Reportes iniciales en China han informado que cerca de 39% de los trabajadores sanitarios presentan problemas psicológicos, principalmente los que ya habían pasado por aislamiento y contagio a familiares o colegas[3]. Estudios que centren su interés en los efectos del COVID-19 en la salud mental de los equipos de salud aún son escasos, entre ellos uno realizado en China, donde se encontró que más de 50% de los encuestados reportó síntomas de depresión y 45% de ansiedad, siendo las enfermeras, las mujeres, los trabajadores de la salud de primera línea, y aquellos que trabajaba

en Wuhan, China los que informaron más severidad en todas las mediciones de salud mental[4]. En Latinoamérica, solo se conoce un estudio presentado en Paraguay, donde los porcentajes de participantes que reportaron síntomas clasificables como moderados y severos fueron de 32,2% para depresión, 41,3% en ansiedad, 27,8% en insomnio, 38,9% en distrés y 64,3% en fatiga por compasión[5].

En este contexto de urgencia sanitaria, esta investigación reporta datos exploratorios sobre la salud mental en funcionarios de salud en Chile.

Material y Método

Participantes

Se recolectaron datos de 125 trabajadores de Atención Secundaria (80%) y Atención Primaria en Salud (20%), entre 18 a 67 años ($M = 39$,59; $DE = 11$,08) a través de encuestas *online* realizadas durante las dos últimas semanas de abril de 2020.

De estos, 32 (25,6%) son médicos, 22 (17,6%) profesionales de enfermería y 71 (56,8%) correspondientes a otras profesiones. El 62,2% había atendido infecciones respiratorias y 36,8% casos de COVID-19. El 62,4% no contaba con equipos de protección individual. En su mayoría fueron mujeres (88%).

Instrumentos

Depresión

Se utilizó el PHQ-9 (9-ítem Patient Health Questionnaire, rango de 0 a 27 puntos), en su versión en castellano[6] y validado en Chile[7,8]. Este cuestionario permite categorizar en normal (0-4), leve (5-9), moderado (10-14) y severo (15-21).

Ansiedad

Para medir esta variable se aplicó el GAD-7 (7-item Generalized Anxiety Disorder, rango de 0 a 21 puntos)[9], en su versión en castellano[10]. Permite establecer cuatro categorías de acuerdo al puntaje obtenido: normal (0-4), leve (5-9), moderado (10-14) y severo (15-21).

Insomnio

Se utilizó el ISI (7-item Insomnia Severity Index, rango de 0 a 28 puntos)[11], en su versión en castellano[12]. El puntaje obtenido permite categorizar en normal (0-7), insomnio subumbral (8-14), insomnio clínico de moderada severidad (15-21) e insomnio clínico severo (22-28).

Distrés

Para evaluar síntomas de distrés se aplicó el IES-R (22-item Impact of Event Scale–Revised, rango de 0 a 88 puntos)[13], en su versión adaptada para Chile[14]. El puntaje total puede ser categorizado en normal (0-8), leve (9-25), moderado (26-43) y severo (44-88).

Procedimiento

Trabajo aprobado por el comité de ética institucional. Respetando la normativa de Helsinky, se solicitó el consentimiento informado *online* de los participantes previa recolección de datos, y su participación no incurrió en ningún tipo de riesgo. Los datos fueron completamente anónimos. Se calculó la proporción de participantes para los diferentes niveles de severidad de los síntomas de depresión, ansiedad, insomnio y distrés y se compararon las proporciones de los factores asociados por medio de la prueba χ^2 de Pearson. Se empleó la prueba U de Mann Whitney para la comparación de dos grupos. El puntaje de corte para detectar síntomas de depresión mayor, ansiedad, insomnio y distrés fue de 10,7,15 y 26, respectivamente, de acuerdo a lo utilizado en el estudio de Lai[4], basado en estudios previos con similares instrumentos[15-18].

Las puntuaciones obtenidas en los cuatro instrumentos aplicados se correlacionaron con la edad, la probabilidad de contagio en los próximos meses, la probabilidad de que otros se contagien y la cantidad de días desde el primer caso confirmado. Para este análisis se calculó el coeficiente de correlación de Spearman.

Resultados

La mediana (IQR) de las puntuaciones en el total de participantes, fue de 4 (1,0 a 5,5) para depresión, 6 (2,0 a 8,0) para ansiedad, 6 (3,0 a 12,0) para insomnio y 4 (0,0 a 14,0) para distrés. La Tabla 1 muestra la mediana obtenida en cada instrumento según los distintos grupos de análisis.

La mediana de las puntuaciones de las mujeres fue significativamente mayor que la de los hombres en depresión, ansiedad, insomnio y distrés (p = 0,000 en todas ellas).

La mediana de las puntuaciones de los profesionales de enfermería fue significativamente mayor que la de los médicos en depresión (p = 0,000), ansiedad (p = 0,001), insomnio (p = 0,000) y distrés (p = 0,002).Al comparar a los profesiona-

Tabla 1. Puntuaciones de las mediciones de depresión, ansiedad, insomnio y distrés en los subgrupos

	Sexo Mediana (IQR)			Profesión Mediana (IQR)					
	Hombres	Mujeres	p	Medicina	Enfermería	Otros	p[a]	p[b]	p[c]
PHQ-9	4 (1,0-5,5)	7 (4,0-12,0)	0,000	4 (2,0-5,0)	9 (6,0-12,0)	7 (4,0-12,0)	0,000	0,001	0,218
GAD-7	6 (2,0-8,0)	9 (5,0-13,0)	0,000	6 (3,0-9,0)	10,5 (6,0-13,0)	7 (4,0-12,0)	0,001	0,051	0,072
ISI	6 (3,0-12,0)	11 (7,0-16,0)	0,000	5,5 (3,0-8,0)	13,5 (9,0-16,0)	12 (7,0-16,0)	0,000	0,000	0,409
IES-R	4 (0,0-14,0)	21 (5,0-32,0)	0,000	5 (0,0-15,0)	22 (8,0-30,0)	16 (4,0-31,0)	0,001	0,002	0,420

	Atención de infecciones respiratorias Mediana (IQR)			Atención de casos COVID-19 Mediana (IQR)			Protección individual Mediana (IQR)		
	No	Sí	p	No	Sí	p	No	Sí	p
PHQ-9	5 (3,0-10,0)	7 (5,0-10,0)	0,057	5 (3,0-10,0)	8 (6,0-12,0)	0,025	5 (4,0-12,0)	7 (3,0-10,0)	0,688
GAD-7	7 (3,0-11,0)	8 (6,0-12,0)	0,258	7 (4,0-11,0)	9 (6,0-13,0)	0,106	9 (5,0-13,0)	6 (4,0-10,0)	0,013
ISI	9 (4,0-13,0)	12,5 (7,0-16,0)	0,035	9 (4,0-14,0)	14 (9,0-16,0)	0,027	10 (5,0-16,0)	9,5 (5,0-14,0)	0,525
IES-R	8 (2,0-22,0)	22 (5,0-28,0)	0,021	9,5 (2,0-26,0)	22 (20,0-26,0)	0,024	17 (4,0-31,0)	13 (1,0-24,0)	0,246

GAD-7: *7-item Generalized Anxiety Disorder*; IES-R: *22-item Impact of Event Scale-Revised*; IQR: Rango intercuartil; ISI: *7-item Insomnia Severity Index*; PHQ-9: *9-item Patient Health Questionnaire*.

les de medicina con los demás profesionales de la salud (odontología, paramédicos, kinesiología, entre otros), la mediana de las puntuaciones de estos fue significativamente menor en depresión (p = 0,001), insomnio (p = 0,000) y distrés (p = 0,002).

La mediana de las puntuaciones de quienes atendieron infecciones respiratorias versus los que no, fue significativamente mayor en insomnio (p = 0,0035) y distrés (p = 0,021). De igual manera, la mediana de las puntuaciones de quienes atendieron casos COVID versus los que no fue significativamente mayor en depresión (p = 0,025), insomnio (p = 0,027) y distrés (p = 0,024).

Por último, al comparar los profesionales que no contaban con protección individual con quienes sí, la mediana de estos fue significativamente mayor en ansiedad (p = 0,013).

Severidad de los síntomas

Del total de participantes, una mayor proporción presentó síntomas de depresión (82[65,6%]), ansiedad (93[74,4%]), insomnio (81[64,8%]) y distrés (71[56,8%]).

En comparación a los hombres, las mujeres presentaron una proporción significativamente mayor de casos con síntomas severos de depresión (p = 0,013), ansiedad (p = 0,000), insomnio (p = 0,004) y distrés (p = 0,001). Al comparar con los profesionales de medicina y enfermería, quienes pertenecían a las otras categorías (odontología, paramédicos, kinesiología, entre otros) presentaron mayor proporción de síntomas severos de depresión (p = 0,025), ansiedad (p = 0,033), insomnio (p = 0,001) y distrés (p = 0,045).

En comparación a quienes atendieron infecciones respiratorias, quienes no realizaron esta atención presentaron mayor proporción de síntomas severos de ansiedad (p = 0,012), insomnio (p = 0,029) y distrés (p = 0,022). Por otro lado, al comparar quienes atendieron casos de COVID-19 con quienes no atendieron estos casos, estos últimos presentaron mayor proporción de síntomas de insomnio (p = 0,037) y distrés (p = 0,005) (Tabla 2).

Tabla 2. Categorías de gravedad de las mediciones de depresión, ansiedad, insomnio y distrés en los subgrupos

			Sexo			Ocupación				Estado civil		
		Total	Hombres	Mujeres	p	Medicina	Enfermería	Otros	p	Solteros	Casados	p
PHQ-9, síntomas de depresión	Normal	43 (34,4)	19 (51,4)	24 (27,3)	0,013	19 (59,4)	5 (22,7)	19 (26,8)	0,025	11 (26,2)	32 (38,6)	0,271
	Leve	45 (36,0)	14 (37,8)	31 (35,2)		9 (28,1)	7 (31,8)	29 (40,8)		14 (33,3)	31 (37,3)	
	Moderada	20 (16,0)	2 (5,4)	18 (20,5)		2 (6,3)	6 (27,3)	12 (16,9)		9 (21,4)	11 (13,3)	
	Severa	17 (13,6)	2 (5,4)	15 (17,0)		2 (6,3)	4 (18,2)	11 (15,5)		8 (19,0)	9 (10,8)	
GAD-7, síntomas de ansiedad	Normal	32 (25,6)	15 (40,5)	17 (19,3)	0,000	12 (37,5)	2 (9,1)	18 (25,4)	0,033	10 (23,8)	22 (26,5)	,912
	Leve	48 (38,4)	19 (51,4)	29 (33,0)		14 (43,8)	7 (31,8)	27 (38,0)		18 (42,9)	30 (36,1)	
	Moderada	29 (23,2)	3 (8,1)	26 (29,5)		5 (15,6)	10 (45,5)	14 (19,7)		9 (21,4)	20 (24,1)	
	Severa	16 (12,8)	0 (0,0)	16 (18,2)		1 (3,1)	3 (13,6)	12 (16,9)		5 (11,9)	11 (13,3)	
ISI, síntomas de insomnio	Normal	44 (35,2)	21 (56,8)	23 (26,1)	0,004	22 (68,8)	4 (18,2)	18 (25,4)	0,001	13 (31,0)	31 (37,3)	,219
	Subumbral	50 (40,0)	13 (35,1)	37 (42,0)		8 (25,0)	10 (45,5)	32 (45,1)		15 (35,7)	35 (42,2)	
	Moderado	29 (23,2)	3 (8,1)	26 (29,5)		2 (6,3)	8 (35,4)	19 (26,8)		14 (33,3)	15 (18,1)	
	Severo	2 (1,6)	0 (0,0)	2 (2,3)		0 (0,0)	0 (0,0)	2 (2,8)		0 (0,0)	2 (2,4)	
IES-R, síntomas de distrés	Normal	54 (43,2)	25 (67,6)	29 (33,0)	0,001	21 (65,6)	6 (27,3)	27 (38)	0,045	17 (40,5)	37 (44,6)	0,972
	Leve	38 (30,4)	10 (27)	28 (31,8)		9 (28,1)	8 (36,4)	21 (29,6)		13 (31,0)	25 (30,1)	
	Moderada	19 (15,2)	2 (5,4)	17 (19,3)		1 (3,1)	4 (18,2)	14 (19,7)		7 (16,7)	12 (14,5)	
	Severa	14 (11,2)	0 (0,0)	14 (15,9)		1 (3,1)	4 (18,2)	9 (12,7)		5 (11,9)	9 (10,8)	

		Atención de infecciones respiratorias			Atención de casos COVID-19			Protección individual		
		No	Sí	p	No	Sí	p	No	Sí	p
PHQ-9	Normal	33 (41,8)	10 (21,7)	0,133	41 (39,4)	2 (9,5)	0,068	17 (36,2)	26 (33,3)	0,455
	Leve	26 (32,9)	19 (41,3)		34 (32,7)	11 (52,4)		15 (31,9)	30 (38,5)	
	Moderada	10 (12,7)	10 (21,7)		16 (15,4)	4 (19,0)		6 (12,8)	14 (17,9)	
	Severa	10 (12,7)	7 (15,2)		13 (12,5)	4 (19,0)		9 (19,1)	8 (10,3)	
GAD-7	Normal	26 (32,9)	6 (13,0)	0,012	30 (28,8)	2 (9,5)	0,079	8 (17,0)	24 (30,8)	0,060
	Leve	25 (31,6)	23 (50,0)		38 (36,5)	10 (47,6)		16 (34,0)}	32 (41,0)	
	Moderada	15 (19,0)	14 (30,4)		21 (20,2)	8 (38,1)		13 (27,7)	16 (20,5)	
	Severa	13 (16,5)	3 (6,5)		15 (14,4)	1 (4,8)		10 (21,3)	6 (7,7)	
ISI	Normal	32 (40,5)	12 (26,1)	0,029	42 (40,4)	2 (9,5)	0,037	16 (34,0)	28 (35,9)	0,782
	Subumbral	33 (41,8)	17 (37,0)		39 (37,5)	11 (52,4)		17 (36,2)	33 (42,3)	
	Moderado	12 (15,2)	17 (37,0)		21 (20,2)	8 (38,1)		13 (27,7)	16 (20,5)	
	Severo	2 (2,5)	0 (0,0)		2 (1,9)	0 (0,0)		1 (2,1)	1 (1,3)	
IES-R	Normal	41 (51,9)	13 (28,3)	0,022	51 (49,0)	3 (14,3)	0,005	19 (40,4)	35 (44,9)	0,491
	Leve	20 (25,3)	18 (39,1)		27 (26,0)	11 (52,4)		12 (25,5)	26 (33,3)	
	Moderada	8 (10,1)	11 (23,9)		13 (12,5)	6 (28,6)		9 (19,1)	10 (12,8)	
	Severa	10 (12,7)	4 (8,7)		13 (12,5)	1 (4,8)		7 (14,9)	7 (9,0)	

Datos expresan Frecuencia (%).

Discusión

Los efectos del COVID-19 han sido graves en el ámbito de la salud, ligados a millones de contagiados, miles de muertes e indicadores de colapso de los sistemas de atención sanitaria. Lamentablemente, muchas de las consecuencias empezamos a detectarlas recién ahora, después de afrontar las demandas más urgentes. Algunas de ellas serán los efectos a mediano y largo plazo de la salud mental de la población, pero también de los principales actores directos en la lucha contra la pandemia, como son los profesionales de la salud.

Los resultados del presente estudio muestran efectivamente que, al igual que otros estudios en personal de salud en otros países tan distantes como Paraguay[5] y China[19], un gran porcentaje del personal de salud encuestado presenta en la actualidad algún grado de sintomatología en ansiedad (74%), distrés (56%), depresión (66%) e insomnio (65%). Aun cuando es sabido que el equipo de salud podría tener mayor riesgo de problemas de salud mental por el tipo de trabajo, estos porcentajes superan ampliamente a lo reportado en otros estudios en personal de salud, como por ejemplo lo evidenciado por Ansoleaga[20] en trabajadores de un hospital pediátrico de alta complejidad (23% sintomatología depresiva y 34% distrés elevado) o en investigaciones anteriores en trabajadores no clínicos de un hospital público[21] (depresión 10%, ansiedad 30%). Aun cuando no ha sido posible encontrar datos actuales en salud mental previos a la pandemia en personal de salud, al compararla con estudios recientes, es indudable que hay un mayor reporte de casos con sintomatología tanto en estrés y ansiedad como en depresión, donde en esta última patología, es incluso muy superior a lo registrado en población general en el país, donde la sospecha de depresión alcanzaría 15,8% y la depresión como tal 6,2%[22].

En el caso específico del insomnio, 65% de la muestra reportó algún tipo de sintomatología asociada a trastornos del sueño, siendo este porcentaje muy superior al reportado a nivel de población general en Chile (26%), lo más probable debido a la alta relación existente entre trastornos del sueño y la presencia de sintomatología ansiosa y depresiva[23]. En estrés, sobre 57% de la muestra presentó alguna presencia entre leve a severa de síntomas asociados a este, posiblemente asociado al aumento del riesgo psicosocial laboral dado por las exigencias y consecuencias de la pandemia, ya que se ha aportado evidencia a que en personal de salud, una alta demanda psicológica asociada a una escasa latitud emocional sostenida en el tiempo conlleva a 2,5 veces mayor probabilidad de presentar sintomatología depresiva y 3,3 mayor chance de presentar distrés muy elevado[20].

Al estratificar el análisis, fue posible observar diferencias entre hombres y mujeres, donde estas últimas tenían medias más elevadas que los hombres en todas las variables evaluadas. El hecho de que las mujeres tengan prevalencias más altas de problemas de salud mental ya ha sido reportado previamente en estudios nacionales, donde por ejemplo las mujeres presentaban mayor sintomatología depresiva en un hospital de alta complejidad en Chile[20], o en estudios recientes en China que evalúan el impacto del coronavirus[4,19]. Aun cuando analizar esta diferencia escapa a los objetivos de este trabajo, esta diferencia probablemente tenga que ver con construcciones sociales y culturales, donde existen diferencias aprendidas en la manera de interiorizar y manifestar las emociones y los síntomas, además de una mayor vulnerabilidad de la mujer dada por las desigualdades del sistema, constituyéndose el género en una determinante estructural de la salud[24].

Igualmente, se encontró que los médicos tenían menor presencia de síntomas ansiosos y depresivos (carácter leve a severo), que las enfermeras y otros profesionales de la salud, al igual que menor sintomatología asociada a insomnio y estrés. Una posible explicación la sugiere Lai[4], quien ha encontrado que en el actual brote de COVID-19, las enfermeras presentaban mayor sintomatología, vinculado posiblemente a que su trabajo implicaba un contacto más cercano y frecuente con el paciente, además de una mayor sobrecarga de trabajo que implicaba muchas veces trabajar más horas de lo usual.

De igual manera, los datos aportan evidencia a que los profesionales expuestos al tratamiento y abordaje de pacientes con problemas respiratorios o con COVID-19, presentaron mayor sintomatología negativa que otros profesionales, especialmente en insomnio distrés. Similar tendencia es reportada en brotes similares como el SARS, por Lai[4] y Lin et al.[25], quienes reportaron que fueron los

profesionales que estaban en contacto directo con pacientes los que reportaban peores indicadores de salud mental y en estudios recientes en China, donde se ha reportado que aquellos médicos y enfermeras expuestos al coronavirus se veían más afectados en su salud mental, pudiendo incluso afectar su percepción de salud física[19], principalmente aquellos que trabajan en contacto estrecho con pacientes infectados[26].

Un análisis interesante sobre las posibles causas de ansiedad al interior del equipo de salud lo presenta Shanafelt et al.[27], siendo estas el acceso a equipo de protección personal apropiado, estar expuesto a COVID-19 en el trabajo y llevar la infección a casa y a la familia, no tener acceso rápido a las pruebas si desarrollan síntomas de COVID-19 y el miedo a propagar la infección en el trabajo, la incertidumbre de que su organización apoyará / se hará cargo de sus necesidades personales y familiares si desarrollan la infección, el acceso al cuidado de los niños durante el aumento de las horas de trabajo y el cierre de la escuela, el apoyo a otras personas y las necesidades de la familia a medida que aumentan las horas de trabajo y las demandas (comida, hidratación, alojamiento, transporte), poder brindar atención médica competente si se despliega a una nueva área (por ejemplo, enfermeras que no pertenecen a la UCI deben funcionar como enfermeras de la UCI), y la falta de acceso a información y comunicación actualizadas.

Es sabido que, si estas condiciones permanecen por un tiempo prolongado como ya ha ocurrido, empezarán a aumentar las condiciones severas en salud mental y salud física[28]. Peor aún, ya contamos con evidencia recientes de situaciones similares donde se ha reportado que los profesionales de la salud acuden menos a pedir ayuda psicológica o asesoramiento debido a los prejuicios y discriminación[29], a pesar de presentar problemas psicosociales entre los trabajadores de la salud en dichos entornos y con necesidad real de necesidad de apoyo en salud mental[30,31].

Los datos del presente estudio, más los reportados en la literatura sobre el tema, aportan evidencia a la necesidad urgente de implementar estrategias de abordaje y prevención específica para los profesionales de la salud con el fin de prevenir y aminorar consecuencias a mediano y largo plazo[32]. Un listado de recomendaciones puede ser revisadas en la literatura publicada[33,34].

Referencias

1. Organización Mundial de la Salud [Internet]. Emergencias: Nuevo coronavirus 2019 [citado el 1 de mayo de 2020]. Disponible en: https://www.who.int/es/emergencies/diseases/novel-coronavirus-2019
2. Organización Mundial de la Salud. [Internet]. Situation Report 202 [citado el 10 de agosto de 2020]. Disponible en: https://www.who.int/docs/default-source/coronaviruse/situation-reports/20200809-covid-19-sitrep-202.pdf?sfvrsn=2c7459f6_2
3. Dai Y, Hu G, Xiong H, Qiu H, Yuan X. Psychological impact of the coronavirus disease 2019 (COVID-19) outbreak on healthcare workers in China. MedRxiv 2020 (preprint). doi: 10.1101/2020.03.03.20030874.
4. Lai J, Ma S, Wang Y, Cai Z, Hu J, Wei N, et al. Factors Associated with Mental Health Outcomes Among Health Care Workers Exposed to Coronavirus Disease 2019. JAMA Network Open 2020; 3 (3): e203976. doi: 10.1001/jamanetworkopen.2020.3976.
5. Samaniego A, Urzúa A, Buenahora M, Vera-Villarroel P. Sintomatología asociada a trastornos de salud mental en trabajadores sanitarios en Paraguay: efecto COVID-19. Revista Interamericana de Psicología 2020; 54 (1): e1298.
6. Diez-Quevedo C, Rangil T, Sánchez-Planell L, Kroenke K, Spitzer R. Validation and Utility of the Patient Health Questionnaire in Diagnosing Mental Disorders in 1003 General Hospital Spanish Inpatients. Psychosom Med 2001; 63: 679-86.
7. Baader T, Molina JL, Venezian S, Rojas C, Farías R, Fierro-Freixenet C, et al. Validación y utilidad de la encuesta PHQ-9 (Patient Health Questionnaire) en el diagnóstico de depresión en pacientes usuarios de atención primaria en Chile. Rev chil neuro-psiquiatr 2012; 50 (1): 10-22.
8. Saldivia S, Aslan J, Cova F, Vicente B, Inostroza C, Rincón P. Propiedades psicométricas del PHQ-9 (Patient Health Questionnaire) en centros de atención primaria de Chile. Rev Med Chile 2019; 147 (1): 53-60.
9. Spitzer RL, Kroenke K, Williams JBW, Löwe B. A Brief Measure for Assessing Generalized Anxiety Disorder: The GAD-7. Arch Intern Med 2006; 166 (10): 1092-7.
10. García-Campayo J, Zamorano E, Ruiz MA, Pardo A, Pérez-Páramo M, López-Gómez V, et al. Cultural adaptation into Spanish of the generalized anxiety disorder-7 (GAD-7) scale as a screening tool. Health and Quality of Life Outcomes 2010; 8 (1): 8.
11. Bastien CH, Vallieres A, Morin CM. Validation of the Insomnia Severity Index as an outcome measure for insomnia research. Sleep Med 2001; 2 (4): 297-307.

12. Sierra JC, Guillén-Serrano V, Santos-Iglesias P. Insomnia Severity Index: some indicators about its reliability and validity on an older adults sample. Rev Neurol 2008; 47 (11): 566-70.
13. Weiss D. The impact of event scale-revised. En: Wilson J & Keane T. Editores. Assessing psychological trauma and PTSD. New York, NY: Guilford Press; 2004. p. 168-89.
14. Caamaño L, Fuentes D, González L, Melipillán R, Sepúlveda M, Valenzuela E. Adaptación y validación de la versión chilena de la escala de impacto de evento-revisada (EIE-R). Rev Med Chile 2011; 139 (9): 1163-8.
15. He XY, Li CB, Qian J, Cui HS, Wu WY. Reliability and validity of a generalized anxiety scale in general hospital outpatients. Shanghai Arch Psychiatry 2010; 22 (4): 200-3.
16. Wu KK, Chan KS. The development of the Chinese version of Impact of Event Scale–Revised (CIES-R). Soc Psychiatry Psychiatr Epidemiol 2003; 38 (2): 94-8.
17. Yu DS. Insomnia Severity Index: psychometric properties with Chinese community-dwelling older people. J Adv Nurs 2010; 66 (10): 2350-9.
18. Zhang YL, Liang W, Chen ZM, Zhang HM, Zhang JH, Weng XQ, et al. Validity and reliability of Patient Health Questionnaire-9 and Patient Health Questionnaire-2 to screen for depression among college students in China. Asia Pac Psychiatry 2013; 5(4): 268-75.
19. Kang L, Ma S, Chen N, Yang J, Li R, Yao L, et al. Impact on mental health and perceptions of psychological care among medical and nursing staff in Wuhan during the 2019 novel coronavirus disease outbreak: A cross-sectional study. Brain, Behavior and Immunity 2020; 87: 11-17.
20. Ansoleaga E. Indicadores de salud mental asociados a riesgo psicosocial laboral en un hospital público. Rev Med Chile 2015; 143: 47-55.
21. Ansoleaga E, Castillo-Carniglia A. Riesgo psicosocial laboral y patología mental en trabajadores de hospital. Rev Fac Nac Salud Publica 2011: 29: 372-9.
22. Ministerio de Salud. Gobierno de Chile. Encuesta Nacional De Salud 2016-2017. Segunda entrega de resultados. [Internet]. Santiago de Chile: Ministerio de Salud [Recuperado el 10 de agosto de 2020]. Disponible en: http://epi.minsal.cl/wp-content/uploads/2019/01/2%C2%BA-Resultados-ENS_DEPTO.EPIDEMIOLOGIA.MINSAL.14.01.2019.pdf.
23. Fritsch R, Lahsen P, Romero, R, Araya R, Rojas G. Trastornos del sueño en la población adulta de Santiago de Chile y su asociación con trastornos psiquiátricos comunes. Actas Esp Psiquiatr 2010; 38 (6): 358-64.
24. Sol-Pastorino M, Vanegas-López J, Florenzano-Urzúa R. Salud mental con perspectiva de género. Salud pública Méx 2017; 59 (6): 601-2.
25. Lin CY, Peng YC, Wu YH, Chang J, Chan CH, Yang DY. The psychological effect of severe acute respiratory syndrome on emergency department staff. Emergency Medicine Journal 2007; 24 (1): 12-17.
26. Lu W, Wang H, Lin Y, Li L. Psychological status of medical workforce during the COVID-19 pandemic: a cross sectional study. Psychiatry Research 2020; 288: 112936.
27. Shanafelt T, Ripp J, Trockel M. Understanding and Addressing Sources of Anxiety Among Health Care Professionals During the COVID-19 Pandemic. JAMA 2020; 323 (21): 2133-4.
28. Urzúa A, Vera-Villarroel P, Caqueo-Urízar A, Polanco-Carrasco R. La Psicología en la prevención y manejo del COVID-19. Aportes desde la evidencia inicial. Ter Psicol 2020; 38 (1): 103-18.
29. Zheng W. Mental health and a novel coronavirus (2019-nCoV) in China. J. A. Disord 2020. https://doi.org/10.1016/j.jad.2020.03.041.
30. Rana W, Mukhtar S, Mukhtar S Mental Health of Medical Workers in Pakistan during the 102 Pandemic COVID-19 Outbreak Asian J Psychiatr 2020; 51.
31. Xiang YT, Yang Y, Li W, Zhang L, Zhang Q, Cheung T, et al. Timely mental health care for the 2019 novel Coronavirus outbreak is urgently needed. Lancet Psychiatr 2020; 7: 228-9.
32. Caqueo-Urízar A, Urzúa A, Aragón-Caqueo D, Charles C, El-Khatib Z, Otu A, et al. Mental health and the COVID-19 pandemic in Chile. Psychological Trauma: Theory, Research, Practice, and Policy 2020; 12 (5): 521-3.
33. Walton M, Murray E, Christian M. Mental health care for medical staff and affiliated healthcare workers during the COVID-19 pandemic. European Heart Journal: Acute Cardiovascular Care 2020; 9: 241-7.
34. Ornell F, Chwartzmann S, Paim F, Correa de Magalhaes J. The impact of the COVID-19 pandemic on the mental health of health care professionals. Cad Saude Publica 2020; 36 (4): e00063520.
