# Supplementary material for: Mental health status and related factors influencing healthcare workers during the COVID-19 pandemic: A systematic review and meta-analysis
Source: PLoS One. 2024 Jan 19;19(1):e0289454. doi: 10.1371/journal.pone.0289454 (PMC10798549; doi:10.1371/journal.pone.0289454)
Supplement: S1 Data — (ZIP) [file pone.0289454.s011.zip › literatures/198.pdf]

Psychiatria Danubina, 2020; Vol. 32, No. 3-4, pp 563-569 https://doi.org/10.24869/psyd.2020.563 *Original paper*
© Medicinska naklada - Zagreb, Croatia

THE EFFECT OF THE COVID-19 PANDEMIC ON THE ANXIETY LEVEL OF EMERGENCY MEDICAL SERVICES PROFESSIONALS

Eren Usul[1], İshak Şan[2] & Burak Bekgöz[3]

[1]*Sincan Dr Nafiz Körez State Hospital, Emergency Service, Ankara, Turkey*
[2]*University of Health Sciences, Ankara City Hospital, Department of Emergency Medicine, Ankara, Turkey*
[3]*Ankara City Hospital, Department of Emergency Medicine, Ankara, Turkey*

received: 26.9.2020; revised: 18.10.2020; accepted: 25.11.2020

SUMMARY

Introduction: *Healthcare professionals are one of the groups most affected by a pandemic that affects the whole world. This study aimed to determine the anxiety level of emergency medical services professionals in Ankara, Turkey after the emergence of the COVID-19 pandemic.*

Subjects smd methods: *In the first part of the survey, the participants of the study were asked about their socio-demographic characteristics and their contact with the COVID-19 patients. In the second part, a survey with 20 questions that determined the state anxiety level derived from the State Anxiety Inventory was performed after obtaining verbal consent.*

Results: *The mean age of the participants was 33.1±6.9, while 52.7% of all participants were males. In this study, the mean STAI Anxiety Score was 50.7±11.6. Anxiety scores were higher in females and those who had family members at risk of COVID-19 infection ($p<0.05$). The majority of those who had family members at risk of the infection started to stay in guesthouses instead of going home. Participants were worried about transmitting the infection to their family members ($p<0.05$). They felt more anxious when treating COVID-19 diagnosed or other patients ($p<0.05$). In addition, they thought that their anxiety level increased in general ($p <0.05$).*

Conclusion: *The COVID-19 pandemic caused an anxiety increase in EMS workers in Turkey. Protecting the physical and mental health of the EMS employees who work at the front line against the COVID-19 pandemic and who have a high risk of infection, and ensuring their efficient work should be the main priority.*

Key words: *prehospital - COVID-19 - anxiety level - mental health*

* * * * *

INTRODUCTION

In December 2019, cases of unknown pneumonia began to emerge in Wuhan, China. The clinical course of these cases resembled that of viral pneumonia (World Health Organization 2020). The causative agent was identified to be a novel coronavirus initially named 2019-nCoV, which formed a clade within the *Sarbecovirus* subgenus of *Betacoronavirus* and had 86.9% nucleotide sequence identity to a previously reported virus, SARS-like CoV (Zhu et al. 2020). The virus is mainly transmitted by air (through respiratory droplets) or through the conjunctiva, nasal mucosa, mouth, or feces (Li et al. 2020).

This virus caused an epidemic, with 78,497 confirmed cases of infection and 2,744 deaths in China as of February 26, 2020, and then led to a pandemic covering more than 70 countries (Wu et al. 2020). The infection caused by 2019-nCoV, now known as SARS-CoV-2, is called COVID-19. The first case in Turkey was detected on March 11, 2020, and the number of cases has been increasing since then.

The sudden nature of the pandemic and the infectivity of the virus will inevitably cause stress, anxiety, and depression in healthcare workers. Anxiety is characterized by a state of fear and tension in response to a threat. (Öncül 2000) Unlike other researchers, Spielberger classified anxiety into two groups: state and trait anxiety (Spielberger 1966). State anxiety is characterized by distress, worry, and tension, indicative of an existing emergency (Konter 1996). It occurs before or during dangerous situations, often due to logical reasons; the reason for such anxiety can be understood by others. (Öğüt 2000) During pandemics that cause deaths across the world, healthcare professionals have to work extremely hard. The COVID-19 pandemic has therefore resulted in state anxiety in healthcare professionals.

In the case of an emergency, individuals first contact emergency medical services (EMS) Emergency intervention or inter-hospital transport of patients who are positive or are suspected to be positive is performed by EMS professionals. As EMS professionals are the first ones to respond to emergency cases, they are much more affected by pandemics than other healthcare professionals. This study was conducted to determine the state anxiety scores and related factors associated with the COVID-19 pandemic in EMS professionals in Ankara, Turkey.

SUBJECTS SMD METHODS

This was a cross-sectional study in which surveys were administered to EMS professionals in Ankara, Turkey. The necessary permissions for the study were

obtained from the Ankara Provincial Health Directorate, and the surveys were completed on a voluntary basis, meeting with every participant in person.

In the first part of the study, the study participants (n=402) were asked about their socio-demographic characteristics (age, gender, marital status, number of children, profession, work experience, and family) and about their contact with COVID-19 patients. In the second part, to determine the state anxiety level, a survey with 20 questions derived from the State Anxiety Inventory was administered to the participants after obtaining verbal consent.

The State-Trait Anxiety Inventory (STAI FORM TX-1, TX-2) was developed by Spielberger et al. (1970), while the Turkish validity and reliability of the scale were assessed by Öner and LeCompte (1983). The scale consists of two parts: the State Anxiety Scale and Trait Anxiety Scale. The State Anxiety Scale was used to test how an individual felt at a certain moment and under certain conditions. A high score indicated a high anxiety level.

All data were analyzed using IBM SPSS Statistics for Windows, Version 25.0 (IBM Corp. Released 2017. Armonk, NY: IBM Corp.). Frequency, percentage, mean ± standard deviation, median, and min–max were used as descriptive statistics. A chi-square test was used to compare categorical variables. The Kolmogorov–Smirnov test showed a normal distribution of continuous data. Independent samples t-test and one-way ANOVA were used for between-group comparisons. The Tukey HSD test was performed if there was a difference in multiple comparisons. Correlations between the variables were evaluated using Pearson's correlation coefficient. p-values < 0.05 were considered statistically significant.

RESULTS

Of the 402 EMS professionals who participated in our study, 47.3% (n=190) were females and 52.7% (n=212) were males. The mean age of the participants was 33.1±6.9 years (min: 20 years, max: 60 years). In total, 73.4% (n=295) of the participants were married. Moreover, 51.7% (n=208) of the participants were emergency medical technicians (EMTs), 27.4% (n=110) were paramedics, 12.4% (n=50) were drivers, 5% (n=20) were physicians, and 3.5% (n=14) were nurses. The mean work experience of the participants was 8.9±5.4 years, and their mean work hours were 47.3±7.8 hours/week. A total of 96.5% (n=388) of the participants did not have any previous mental disorder, 2.5% (n=10) had a previous mental disorder and had recovered, and 1% (n=4) had an existing mental disorder. The mean anxiety score of the participants was 50.7±11.6. Socio-demographic and professional characteristics of the participants are provided in Table 1.

In total, 35.8% (n=144) of the participants lived with family members at risk of COVID-19. In addition, 23.1% of the participants were worried about their family members getting infected, while 62.2% (n=250) were concerned about infecting their family members. Due to the COVID-19 pandemic, 14.4% (n=58) of the participants were staying in a guesthouse or in the hospital instead of going home. Moreover, 83.8% (n=337) of the participants had been treating COVID-19 patients since the emergence of the initial cases in Turkey. Of the participants, 67.7% thought that they had adequate personal protective equipment (PPE) during the interventions. However, 66.9% (n=269) of the participants still did not feel safe while treating COVID-19

Table 1. Socio-demographic and professional characteristics of the EMS staff

	n=402	%
Gender		
Female	190	47.3
Male	212	52.7
Age (Years) Mean ± SD/ Median (Min-Max)	33.1±6.9	32 (20-60)
Marital Status		
Single	107	26.6
Married	295	73.4
How many children do you have? Median (Min-Max)	1	
Profession		
Paramedics	110	27.4
EMTs	208	51.7
Nurses	14	3.5
Physicians	20	5.0
Drivers	50	12.4
Work experience (year) Mean ± SD / Median (Min-Max)	8.9±5.4	9 (1-30)
Medical history on mental health		
I did not have any mental disease	388	96.5
I've had mental disease, but recovered	10	2.5
I still have a mental disease	4	1.0
Depression	*3*	*0.7*
Obsessive-compulsive disorder (OCD)	*1*	*0.2*
Weekly working hours Mean ± SD / Median (Min-Max)	47.3±7.8	48 (10-96)

Table 2. The characteristics of EMS staff related to COVID-19

	n=402	%
Did you start staying somewhere else instead of your home after emerge of the COVID-19 pandemic?		
No	344	85.6
Yes	58	14.4
Have you ever treated a COVID-19 diagnosed patient?		
No	65	16.2
Yes	337	83.8
Do you think that you have the adequate PPE?		
No	130	32.3
Yes	272	67.7
Do you feel safe when treating the patients diagnosed or suspected of COVID-19?		
No	269	66.9
Yes	133	33.1
Did your anxiety level change when treating the patients diagnosed or suspected of COVID-19 than treating other patients.		
No	65	16.2
Yes, I felt more anxious when treating the patients diagnosed or suspected of COVID-19	306	76.1
Yes, I felt more anxious when treating other patients than those were diagnosed or suspected of COVID-19	31	7.7
Have there been any changes in your anxiety level in general?		
My anxiety level has not changed	51	12.7
My anxiety level increased	347	86.3
My anxiety level decreased	4	1.0
Do you have family members at risk of COVID-19 infection living with you?		
No	258	64.2
Yes	144	35.8
Are you currently more concerned about the health of family members you live with?		
No, I'm not more worried than before	59	14.7
Yes, I'm worried that they're going to be infected	93	23.1
Yes, I'm worried that I am going to transmit the infection to them	250	62.2

positive patients even though they thought they had adequate PPE. In addition, 76.1% (n=306) of the participants felt more anxious when treating COVID-19 patients than when treating other patients; 86.3% (n=347) of the participants stated that their anxiety levels had increased in general. The characteristics of the participants in relation to COVID-19 are presented in Table 2.

The mean anxiety score was 53.9±10.5 in females and 47.8±11.9 in males ($p<0.05$). The anxiety scores were found to decrease with an increase in the age of the participants ($r=-0.139$, $p<0.05$). The anxiety score was 41.2±13.8 in nurses, 51.7±11.1 in EMTs, 52.0±11.6 in paramedics, 49.3±12.7 in physicians, and 46.4±10.8 in drivers. Thus, nurses had a significantly lower anxiety score than the other participants ($p<0.05$). Moreover, the anxiety score of the participants who were concerned about infecting their family members (52.1±11.2) was significantly higher than that of the participants who did not have such a concern (46.3±12.6) ($p<0.05$).

The anxiety score of the participants who thought that they had adequate PPE (48.4±11.1) was significantly higher than that of the participants who thought that they did not have adequate PPE (55.4±11.2) ($p<0.05$). Furthermore, the anxiety score of the participants who did not feel safe when treating patients diagnosed with or suspected of having COVID-19 (53.3±10.4) was significantly different from that of the participants who felt safe when treating such patients (45.3±12.1, $p<0.05$). The anxiety score of the participants who felt more anxious when treating patients diagnosed with or suspected of having COVID-19 (51.3±10.5) as well as that of the participants who felt more anxious when treating other patients without COVID-19 (56.0±14.0) was significantly higher than that of the participants who did not feel any difference when treating patients with or without COVID-19 (45.2±13.2) ($p<0.05$). The participants who felt that their anxiety levels had increased in general had a significantly higher anxiety score (52.2±10.5) than those who felt that their anxiety levels had decreased (50.8±13.3) or remained unchanged (39.9±12.8) ($p<0.05$). The anxiety scores of the participants did not differ according to their marital status, number of children, work experience, and weekly working hours ($p>0.05$). In addition, the anxiety scores of the participants did not differ according to the presence of mental health issues, changes in accommodation, and treatment of COVID-19 patients. The state anxiety scores of the participants are provided in Table 3.

Table 3. State anxiety scores of the EMS personnel

	Anxiety Score	P
Gender		
Female	53.9±10.5	0.000[a]
Male (n=212)	47.8±11.9	
Marital Status		
Single	50.9±11.5	0.552[a]
Married	50.1±12.0	
Dou you have children?		
No	50.4±11.2	0.734[a]
Yes (n=264)	50.8±11.8	
Profession		
Paramedics (n=110)	52.0±11.6	0.001[c]
EMTs (n=208)	51.7±11.1	
Nurses (n=14)	41.2±13.8	Difference:
Physician (n=20)	49.3±12.7	Between 3 and
Driver (n=50)	46.4±10.8	1,2,4
Do you have a history of mental disease before?		
I did not have any mental disease (n=388)	50.5±11.4	
I've had mental disease, but recovered (n=10)	53.7±17.2	0.435[c]
I still have a mental disease (n=4)	56.3±12.6	
Depression		
Obsessive-compulsive disorder (OCD)		
Do you have family members at risk of COVID-19 infection living with you?		
No (n=258)	49.6±11.3	0.016[a]
Yes (n=144)	52.5±11.9	
Are you currently more concerned about the health of family members you live with?		
No, I'm not more worried than before (n=59)	46.3±12.6	0.002[c]
Yes, I'm worried that they're going to be infected (n=93)	49.7±11.4	Difference:
Yes, I'm worried that I am going to transmit the infection to them (n=250)	52.1±11.2	Between 1 and 3
Did you start staying somewhere else instead of your home after emerge of the COVID-19 pandemic?		
No (n=344)	50.5±11.7	0.502[a]
Yes, I started to stay in the guesthouse/ hospital instead of going home (n=58)	51.6±11.3	
Have you ever treated a COVID-19 diagnosed patient?		
No (n=65)	49.8±11.4	0.524[a]
Yes (n=337)	50.8±11.7	
Do you think that you have the adequate PPE?		
No (n=130)	55.4±11.2	0.000[a]
Yes (n=272)	48.4±11.1	
Do you feel safe when treating the patients diagnosed or suspected of COVID-19?		
No	53.3±10.4	0.000[a]
Yes	45.3±12.1	
Did your anxiety level change when treating the patients diagnosed or suspected of COVID-19 than treating other patients.		
No (n=65)	45.2±13.2	0.000[c]
Yes, i felt more anxious when treating the patients diagnosed or suspected of COVID-19 (n=306)	51.3±10.5	
Yes, i felt more anxious when treating other patients than those were diagnosed or suspected of COVID-19 (n=31)	56.0±14.0	Difference: Between 1 and 2,3
Have there been any changes in your anxiety level in general?		
My anxiety level has not changed (n=51)	39.9±12.8	0.000 [c]
My anxiety level increased (n=347)	52.2±10.5	
My anxiety level decreased (n=4)	50.8±13.3	Difference: Between 1 and 3

[a]: IndependentSapmles t Test, [b]: One-Way ANOVA

Among the participants who thought that they had adequate PPE, the anxiety score of those who felt safe when treating COVID-19 patients (44.8±11.9) was lower than the anxiety score of those who did not feel safe when treating such patients (51.4±9.6) ($p<0.05$). Among the participants who treated patients diagnosed with or suspected of having COVID-19, the anxiety score of those who felt more anxious when treating other patients with any other disease (57.2±13.1) was higher than the anxiety score of those who felt more anxious when treating COVID-19 positive patients (51.4±10.6). Moreover, the anxiety score of the participants who treated COVID-19 positive patients and felt an increase in their anxiety levels was 52.6±10.5 (Table 3).

While 58.6% (n=34) of the participants who started staying in a guesthouse or in the hospital after the COVID-19 pandemic had family members who were at risk of infection, 41.4% (n=24) did not have such at-risk family members.

DISCUSSION

EMS professionals who treat and transport patients diagnosed with or suspected of having COVID-19 tend to feel anxious. An increase in the number of diagnosed or suspected cases may increase the anxiety levels of the staff. Also, it is stated in a study by Lazzari et al. that even though the average population will show normal responses to pandemic stressors, others more mentally vulnerable to anxiety will have overstated emotional responses (Lazzari et al. 2020). Negative psychological reactions have been reported among healthcare workers during the 2003 SARS outbreak (Maunder et al. 2003). The anxiety and worries of EMS professionals working under heavy workloads and high stress levels may be affected by health and socio-demographic factors (Bennett et al. 2004).

This study aimed to determine the anxiety scores of 402 EMS professionals in Ankara, Turkey. The mean STAI anxiety score was found to be 50.7±11.6. In a study involving 282 EMS professionals in Elazig, Turkey, the mean state anxiety score was found to be 36.70±10.34. (Atıcı & Deveci 2019) The anxiety score due to COVID-19 was found to be high in our study. In another study conducted in China, healthcare workers dealing with COVID-19 reported high levels of depression, anxiety, insomnia, and distress (Lai et al. 2020). Increased stress, workload, and fear of transmission associated with COVID-19 may increase the anxiety scores of EMS professionals.

Anxiety disorders are common among individuals with mental health issues and are more common in women than in men (Gavin et al. 2005, Bourdon et al. 1988). Similarly, in our study, the anxiety level was found to be higher in women than in men. According to Badner, women have a higher anxiety level due to separation from their families (Badner et al. 1990). However, according to some other researchers, anxiety levels are higher in women because they express their concerns more easily than men. (Domar et al. 1989) Shevde and Panagopoulos also found higher anxiety levels in women and suggested that this was due to the inability of men to express their anxiety as much as women. (Shevde & Panagopoulos 1991) The results of our study are thus compatible with those of previous studies.

We found no correlation between anxiety scores and the number of children. The anxiety scores of the participants did not change depending on whether they had children or how many children they had. In their study involving nurses, Ocaktan et al. found no significant difference in anxiety scores between the study groups according to the number of children (Ocaktan et al. 2002). Thus, anxiety levels may be affected by individual differences rather than the number of children.

In our study, anxiety levels decreased as the age of the participants increased. Among the participants, the mean age of the nurses was the highest, but their anxiety level was the lowest. This may be due to an increase in maturity with age. However, we did not observe any correlation between work experience and anxiety scores.

There was no difference in anxiety scores among individuals who previously had a mental disorder and had recovered, those who had an existing mental disorder, and those who did not have any previous or existing mental disorder. Deveci et al. reported a high mean state anxiety score among individuals with mental disorders (Deveci et al. 2012). The number of participants with mental disorders was small in our study.

We also found that the anxiety score was higher in the participants who had family members at risk of COVID-19. COVID-19 causes more severe respiratory infections in the elderly and in individuals with chronic diseases (risk groups). (Lu et al. 2012) Hence, the anxiety scores of employees with such family members were higher. Moreover, of the participants (n=58) who had started staying in a guesthouse or in the hospital instead of going home, 58.6% (n=34) had family members at risk of infection.

Maunder et al. reported that the fear of transmitting SARS infection to family members, friends, and colleagues increased the anxiety of healthcare professionals. (Maunder et al. 2003) Similarly, in our study, the anxiety level was higher among the participants who were worried about transmitting the infection to family members. Of the participants who had family members at risk of infection, 69% were worried about transmitting the infection to them. It is normal to feel anxious during a pandemic, such as COVID-19, which threatens not only an individual's health but also the health of their loved ones.

Of the healthcare professionals who directly provide care and use the back cabinet of an ambulance when transporting COVID-19 patients, those who wear PPE have a lower risk of infection. (Centers for Disease Control and Prevention, 2020) In our study, the participants who thought that they had adequate PPE

had lower anxiety scores. We think that the news about the lack of PPE in the media could affect the anxiety levels of EMS professionals about PPE (Jacobs et al. 2020). Furthermore, healthcare professionals are not only at risk for anxiety but are also at risk to experience a wide range of emotions, including fear, fatigue, sleep problems, depression, or other emotional disturbances (Anjum et al. 2020).

In total, 83.8% (n=337) of the participants in our study had treated COVID-19 patients. In addition, 76.1% (n=306) of the participants felt more anxious when treating patients diagnosed with or suspected of having COVID-19. The participants who had treated patients diagnosed with COVID-19 had higher anxiety levels when treating patients without COVID-19. Bai et al. reported that COVID-19 patients are asymptomatic or have minimal symptoms at the onset of the disease. (Bai et al. 2020) Wei et al. reported that transmission in the presymptomatic period is an important issue. (Wei et al. 2020) We think that the high number of asymptomatic COVID-19 patients causes more anxiety in healthcare professionals.

In our study, 86.3% of the participants felt more anxious during the COVID-19 pandemic than before; their anxiety scores were also high according to the survey results. After the WHO declared the SARS-CoV-2 outbreak as a Public Health Emergency of International Concern (PHEIC), stress levels in healthcare workers increased. (World Health Organization, 2020) In a study by Salopek-Žiha et al., it was found that 67% of the medical staff was worried and very worried about the COVID-19 Pandemic (Salopek-Žiha et al. 2020). Furthermore, the same study found that while 17% of health workers reported having extremely severe anxiety, 10% reported moderate to extremely severe stress. (Salopek-Žiha et al. 2020) According to a study conducted in Wuhan, where the COVID-19 outbreak first emerged, mental health issues developed in healthcare workers immediately after the outbreak. (Kang et al. 2020) COVID-19 appears to be more contagious than SARS, and individuals can transmit the infection during the presymptomatic period and for several days after complete recovery. (Centers for Disease Control and Prevention, 2020) Unlike the controlled patient care environment in hospitals, EMS involve quick medical decision-making, intervention with limited information, and being in the enclosed confined space of an ambulance cabin; this increases the anxiety levels of the staff associated with COVID-19.

CONCLUSION

The COVID-19 pandemic has increased the anxiety levels of EMS professionals in Turkey. Physical and psychological protection of healthcare professionals is an important component of public health measures during the COVID-19 pandemic. Protection of the physical and mental health of EMS professionals who work at the front line against the COVID-19 pandemic and have a high risk of infection should be the main priority in order to ensure efficiency. Arrangements should be made to address the concerns of healthcare professionals regarding themselves and their families.

Limitations

This study was designed as a cross-sectional study, in which surveys were conducted to 402 EMS professionals. One limitation of the study was the total number of participants involved. With a larger sample size, a similar study of larger scale can be conducted with more accurate and satisfactory results. Also, not all EMS professionals have had contact with confirmed COVID-19 cases, which may have resulted in misleading survey outcomes.

Acknowledgements: None.

Conflict of interest: None to declare.

Contribution of individual authors:

Eren Usul: Constructing an idea or hypothesis for research and/or manuscript, planning methodology to reach the conclusion, taking responsibility in the construction of the whole or body of the manuscript, writer.

İshak Şan: Providing personnel, environmental and financial for the support and tools and instruments that are vital project, biological materials, reagents and referred patients.

Burak Bekgöz: Taking responsibility in logical interpretation and presentation of the results, taking responsibility in this necessary function, writer.

References

1. *Anjum S, Ullah R, Rana MS, Ali Khan H, Memon FS, Ahmed Y, et al: COVID-19 Pandemic: A Serious Threat for Public Mental Health Globally. Psychiatr Danub 2020; 32:245-50*
2. *Atıcı E & Deveci E: Investigation of State-Trait Anxiety Status of Emergency Health Service Stations Employees Affiliated to Elazig Center. ESTÜDAM Public Health Journal 2019; 4:301-13*
3. *Badner NH, Nielson WR, Munk S, Kwiatkowska C, Gelb AW: Preoperative anxiety: Detection and contributing factors. Can J Anaesth 1990; 37:444-7*
4. *Bai Y, Yao L, Wei T, Tian F, Jin DY, Chen L, et al: Presumed asymptomatic carrier transmission of COVID-19. JAMA 2020; 323:1406-1407*
5. *Bennett P, Williams Y, Page N, Hood K, Woollard M: Levels of mental health problems among UK emergency ambulance workers. Em Med J 2004; 21:235-36*
6. *Bourdon KH, Boyd JH, Rae DS, Burns BJ, Thompson JW, Locke BZ: Gender differences in phobias: results of the ECA community survey. J Anxiety Disorders 1988; 2:227–241*
7. *Centers for Disease Control and Prevention: First Responder Guidance. CDC, U.S. Department of Health &*

Human Services. 2020. Retrieved from https://www.cdc.gov/coronavirus/2019-ncov/hcp/guidance-for-ems.html

8. *Centers for Disease Control and Prevention: How It Spreads. CDC, U.S. Department of Health & Human Services. 2020. Retrieved from https://www.cdc.gov/coronavirus/2019-ncov/about/transmission.html*
9. *Deveci SE, Çalmaz A & Açık Y: The relationship between anxiety levels and health, social and demographic factors in students of a university opened in Eastern Anatolia. Dicle Med J 2012; 39:189-96*
10. *Domar AD, Everett LL & Keller MG: Preoperative anxiety: Is it a predictable entity? Anesth Analg l989; 69:763-7*
11. *Gavin NI, Gaynes BN, Lohr KN, Meltzer-Brody S, Gartlehner G, Swinson T: Perinatal depression: a systematic review of prevalence and incidence. Obstet Gynecol 2005; 106:1071-1083*
12. *Jacobs A, Richtel M & Baker M: 'At war with no ammo': doctors say shortage of protective gear is dire. New York Times. March 19, 2020; 1547-1548*
13. *Kang L, Ma S, Chen M, Yang J, Wang Y, Li R, et al: Impact on mental health and perceptions of psychological care among medical and nursing staff in Wuhan during the 2019 novel corona virus disease outbreak: a cross sectional study. Brain Behav Immun 2020; 87:11-17*
14. *Konter E: Stress and Performance in Sports. İzmir: Saray Medical Publishing, 1996*
15. *Lai J, Ma S, Wang Y, Cai Z, Hu J, Wei N, et al: Factors Associated with Mental Health Outcomes Among Health Care Workers Exposed to Coronavirus Disease 2019. JAMA Netw Open 2020; 3:e203976*
16. *Lazzari C, Shoka A, Nusair A, Rabottini M: Psychiatry in Time of COVID-19 Pandemic. Psychiatr Danub 2020; 32:229-35*
17. *Li Q, Guan X, Wu P, Wang X, Zhou L, Tong Y, et al: Early transmission dynamics in Wuhan, China, of novel coronavirus-infected pneumonia. N Engl J Med 2020; 382:1199–1207*
18. *Lu R, Yu X, Wang W, Duan X, Zhang L, Zhou W, et al: Characterization of human coronavirus etiology in Chinese adults with acute upper respiratory tract infection by real-time RT-PCR assays. PLoS one 2012; 7:e38638*
19. *Maunder R, Hunter J, Vincent L, Bennett J, Peladeau N, Leszcz M, et al: The immediate psychological and occupational impact of the 2003 SARS outbreak in a teaching hospital. CMAJ 2003; 168:1245-1251*
20. *Ocaktan ME, Keklik A & Çöl M: The Spielberger state and trait anxiety level in healthcare personnel working in health centers affiliated to the Abidinpaşa health group presidency. Journal of Ankara University Faculty of Medicine 2002; 55:21-8*
21. *Öğüt F: The Relationship Between Social Cohesion and Trait Anxiety. Master Thesis, Kocaeli: Kocaeli University, Institute of Health Sciences, 2000*
22. *Öncül R: Education and Educational Sciences Dictionary. MEB Publications, Ankara, 2000*
23. *Öner N & LeCompte A: State-Trait Anxiety Inventory Handbook. 1st Edition, Istanbul: Boğaziçi University Publications 1983; 1-26*
24. *Salopek-Žiha D, Hlavati M, Gvozdanović Z, Gašić M, Placento H, Jakić H, et al: Differences in Distress and Coping with the COVID-19 Stressor in Nurses and Physicians. Psychiatr Danub 2020; 32:287-93*
25. *Shevde K & Panagopoulos G: A Survey of 800 patients' knowledge, attitudes, and concerns regarding anesthesia. Anesth Analg 1991; 73:190-8*
26. *Spielberger CD: Theory and Research in Anxiety. (Ed: CD Spielberger) Anxiety and Behavior. Academic Press, 1966*
27. *Spielberger CD, Gorsuch RL & Lushene RE: Manual for State-Trait anxiety inventory, California: Consulting Psychologist Press, 1970*
28. *Wei WE, Li Z, Chiew CJ, Yong SE, Toh MP, Lee VJ: Presymptomatic Transmission of SARS-CoV-2 - Singapore, January 23–March 16, 2020. MMWR Morb Mortal Wkly Rep 2020; 69:411–415*
29. *World Health Organization. Statement on the second meeting of the International Health Regulations (2005) Emergency Committee regarding the outbreak of novel coronavirus (2019-nCoV). Published January 30, 2020. Accessed February 2, 2020*
30. *World Health Organization: Novel coronavirus – China. WHO 2020. Retrieved from http://www.who.int/csr/don/12-january-2020-novel-coronavirus-china/en*
31. *Wu JT, Leung K & Leung GM: Now casting and forecasting the potential domestic and international spread of the 2019-nCoV outbreak originating in Wuhan, China: a modeling study. Lancet 2020; 395:689-697*
32. *Zhu N, Zhang D, Wang W, Li X, Yang B, Song J, et al: A Novel Coronavirus from Patients with Pneumonia in China, 2019. N Engl J Med 2020; 382:727*

Correspondence:
Eren Usul, MD
Sincan Dr Nafiz Körez State Hospital, Emergency Service
İleri Mahallesi, Yıldırım Bulvarı, Leman Sokak, Sarp Sitesi 6/34, Pursaklar, Ankara, Turkey
E-mail: usuleren7@hotmail.com
