# Supplementary material for: Mental health status and related factors influencing healthcare workers during the COVID-19 pandemic: A systematic review and meta-analysis
Source: PLoS One. 2024 Jan 19;19(1):e0289454. doi: 10.1371/journal.pone.0289454 (PMC10798549; doi:10.1371/journal.pone.0289454)
Supplement: S1 Data — (ZIP) [file pone.0289454.s011.zip › literatures/199.pdf]

doi:10.1111/imj.14981

Evaluation of the effect of COVID-19 pandemic on anxiety severity of physicians working in the internal medicine department of a tertiary care hospital: a cross-sectional survey

Oğuz Abdullah Uyaroğlu,[1] Nursel Çalık Başaran,[1] Lale Ozisik,[1] Sevilay Karahan,[2] Mine Durusu Tanriover,[1] Gulay Sain Guven[1] and Serife Gul Oz[1]

Departments of [1]Internal Medicine, Division of General Internal Medicine, and [2]Biostatistics, Hacettepe University Faculty of Medicine, Ankara, Turkey

Key words
anxiety, COVID-19, female, internal medicine, internist.

Correspondence:
Oguz Abdullah Uyaroglu, Hacettepe University Faculty of Medicine, Department of Internal Medicine, Division of General Internal Medicine 06100 Sıhhıye, Ankara, Turkey.
Email: oguzuyaroglu@hotmail.com

Received 21 May 2020; accepted 3 July 2020.

Abstract

Background: Internists who have an important role in the global response to the COVID-19 pandemic are under both physical and psychological pressures.
Aims: To assess the anxiety among physicians working in the internal medicine department of a tertiary care hospital who are on the frontline of the COVID-19 pandemic.
Methods: This single-centre, non-intervention, cross-sectional descriptive study was conducted using an online survey questionnaire from 1 April to 14 April 2020. Physicians of the Department of Internal Medicine were invited to participate with a self-administered questionnaire. The degree of symptoms of anxiety was assessed by the Turkish versions of the 7-item Generalised Anxiety Disorder scale and Beck Anxiety Inventory, respectively.
Results: A total of 113 participants consented for the study and completed the questionnaire. The median age was 29 (IQR = 5) years and 53.1% were male. A total of 72 internists (63.7%) worked as 'frontline' healthcare workers directly engaged in diagnosing, treating or caring for patients with or suspected to have COVID-19. Female gender was significantly associated with high scores and levels in all scales compared to the male gender ($P < 0.005$). Having family members over 65 years old and with chronic diseases were significantly associated with high anxiety scores and levels ($P < 0.005$).
Conclusions: In this survey of internists in a university hospital equipped with clinics, wards and intensive care unit for patients with COVID-19, female gender and having family members over 65 years old and with chronic diseases were associated with increased anxiety levels.

Introduction

The World Health Organization (WHO) China Country Office was informed about accumulated pneumonia cases of unknown aetiology in Wuhan City, Hubei Province of China, on 31 December 2019.[1] The Chinese authorities isolated and identified a new type of coronavirus, named 2019 novel coronavirus (2019-nCoV, then SARS-CoV-2). The disease spread rapidly to many countries all over the world and WHO declared coronavirus disease 2019 (COVID-19) as a pandemic.[2] Since its identification in December 2019, COVID-19 has infected over 4 million people globally.[3]

Healthcare workers (HCW) have an important role in the global response against the COVID-19 pandemic. Since the start of the COVID-19 outbreak, concerns such as fear of being infected with coronavirus, anxiety of infecting loved ones, difficulty in access to personal protective equipment, delay in test results, lack of specific drugs, feelings of being inadequately supported and making quick decisions with limited information have increased. These concerns can increase the fragility of healthcare professionals and the healthcare delivery may worsen when their ability to stay calm and reassure the public is most needed. Maintaining an adequate healthcare workforce in this pandemic requires an adequate number of physicians, nurses, pharmacists and physical therapists, with abilities to care for a high volume of patients.

During the outbreak of severe acute respiratory syndrome (SARS) in 2003, significant emotional distress was present in 18–57% of HCW and was associated with quarantine, fear of contagion, concern for family, job stress, interpersonal isolation, perceived stigma and

Funding: None.
Conflict of interest: None.

© 2020 Royal Australasian College of Physicians

attachment insecurity.[4] In 2009 A/H1N1 influenza pandemic, 56.7% of HCW presented frequent concerns regarding their health and their families' health, high levels of psychological distress, worries about their functional ability and fears of stigmatisation during previous disease outbreaks.[5] Now, similar concerns about the emotional distress of HCW treating patients with COVID-19 are arising. Understanding the occupational and psychological effects of the COVID-19 outbreak is important because it comprises the well-being of physicians.

As of 11 March, after the detection of the first case in Turkey, certain hospitals were determined to serve as COVID-19 pandemic hospitals, as was our hospital. The disaster and the pandemic plans of the hospital, which is a Joint Commission Accredited 1200-bed, tertiary care, university hospital in the capital city of Turkey, have been activated. All policlinics were closed except the emergency department. All elective surgeries were cancelled. The hospital was organised entirely to take care of COVID-19 patients and emergent and urgent cases. Dedicated Internal Medicine Wards and Intensive Care Units (ICU) were reserved for follow up of COVID-19 patients. A COVID-19 initial evaluation outpatient clinic was established. Internal medicine department staff have been assigned to work in the COVID wards and outpatient clinic in alliance with the Departments of Chest Diseases, Cardiology and Infectious Diseases in accordance with the pandemic preparedness plan.

The objective of this study is to assess the psychological and occupational impact of COVID-19 pandemic on physicians working in the internal medicine department and to identify associated factors that increase vulnerability.

Methods

Study design and population

This single-centre, non-interventional, cross-sectional descriptive study was conducted in a tertiary care university hospital, using an online survey questionnaire from 1 April to 14 April 2020 when COVID-19 cases have recently been identified and trending towards the peak in Turkey. The first COVID-19 case was admitted to our hospital on 20 March 2020, and there were 147 confirmed cases until April 14. The personal protective equipment was distributed to the staff with regard to the guidance and the principles set by the Infection Control Unit and there were no shortages. The working hours of the doctors in the COVID-19 wards were amended to have 12-h working shifts every other day.

Physicians (academic staff and residents) of the Department of Internal Medicine were invited to participate with a self-administered questionnaire on a digital platform. Electronic informed consent of all participants was obtained. The study protocol was approved by the Institutional Ethics Board (Approval number: GO 20/353, date: 31.03.2020), and carried out in accordance with the ethical standards laid down in the 1964 Declaration of Helsinki as revised in 2000.

Screening questionnaire

The questionnaire consisted of three parts. The first part included questions about socio-demographic characteristics including age, sex (male, female), marriage status (married, single, divorced), family members status (having children, having family members over 65 years of age), working position (as a 'resident' or as an 'academic staff' that includes specialist, fellow, associate professor and professor), working department, working experience ('year of residency' for the residents and 'duration of working experience as a physician' for the academic staff), working area ('frontline' such as COVID-19 wards, ICU, outpatient clinics or 'non-COVID-19 areas' such as other wards and outpatient clinics) and medical history of the participants and their families.

In the second part, they were asked three questions which aimed to assess knowledge and fears about COVID-19 (Table 2).

In the third part, the degree of symptoms of anxiety was assessed by the 7-item Generalised Anxiety Disorder scale (GAD-7) and Beck Anxiety Inventory (BAI). All the scales used have previously been adapted to Turkish and were tested for their validity and reliability.[6,7] Participants who had scores greater than the cut-off threshold were characterised as having severe symptoms.

GAD-7 is one of the most frequently used and validated diagnostic self-report scales for screening, diagnosis and severity assessment of anxiety disorder. The total score of GAD-7 ranges from 0 to 21, with increasing scores indicating more severe functional impairments as a result of anxiety. The total scores of these measurement tools are interpreted as follows: GAD-7 level indicates minimal (score 0–4), mild (score 5–9), moderate (score 10–14) or severe anxiety (score 15–21). A score of 10 or greater in the original GAD-7 represents a reasonable cut-off point for identifying cases of general anxiety disorder.[8] We used '8' as the cut-off score for detecting symptoms of anxiety based on the Turkish version of GAD-7 scale.[6]

The BAI is used to determine the frequency of anxiety signs. There are 21 categories of signs, each consisting of four options, and each item is scored on a scale of 0–3; 0 – Not at all; 1 – Mildly (It did not bother me much); 2 – Moderately (It was very unpleasant, but I could stand it) and 3 – Severely (I could barely stand it). The total score has a minimum of 0 and a maximum of 63. The

© 2020 Royal Australasian College of Physicians

standardised score levels are as follows: 0–7, minimal anxiety level; 8–15, mild anxiety level; 16–25, moderate anxiety level; 26–63, severe anxiety level.[7]

Consequently, we used five main parameters in the assessment of the anxiety: anxiety levels according to scales ('GAD-7 level', 'BAI level'), total scores ('GAD-7 score', 'BAI score') and the cut-off score 8 for detecting symptoms of anxiety based on the Turkish version of GAD-7 scale ('GAD-7 Cut-off 8') (Tables 3, 4).

Statistical analysis

All statistical analyses were performed with SPSS statistical software package (SPSS, IBM, Armonk, NY, USA) version 25. In descriptive statistics, number and percentage were used for categorical variables. For continuous variables with normal distribution, mean and standard deviation (SD) were used; and for continuous variables that do not show normal distribution, interquartile range (IQR) was preferred. Pearson Chi-squared test or Fisher exact test were used in the evaluation of categorical variables. The suitability of variables to normal distribution was examined using visual and analytical methods. Non-normally distributed numerical data were analysed with Mann–Whitney *U*-test or Kruskal–Wallis test. For all comparisons, *P*-values less than 0.05 were considered as statistically significant.

Results

Demographic characteristics

A total of 113 participants consented for the study and completed the questionnaire. Median age was 29 (IQR = 5) years and 53.1% were male (Table 1). Seventy-seven (68.1%) of the participants were residents and the mean residency year was 2.3 (±1.1) years. Thirty-six (31.9%) of the participants were academic

Table 1 Demographic characteristics of participants, *n* = 113

Characteristics	Total (*n* = 113)	Residents (*n* = 77)	Academic staff (*n* = 36)
Age, median (IQR) (years)	29 (5)	27 (3)	36 (16)
Sex			
Male, *n* (%)	60 (53.1)	37 (48.1)	23 (63.1)
Female, *n* (%)	53 (46.9)	40 (51.9)	13 (36.9)
Marriage status			
Married, *n* (%)	56 (49.6)	25 (32.5)	31 (86.1)
Unmarried, *n* (%)	56 (49.6)	52 (67.5)	4 (11.1)
Divorced, *n* (%)	1 (0.6)	0	1 (2.8)
Children			
0, *n* (%)	86 (76.1)	74 (96.1)	12 (33.3)
1, *n* (%)	9 (8)	2 (2.6)	7 (19.4)
2, *n* (%)	15 (13.3)	1 (1.3)	14 (38.9)
3, *n* (%)	2 (1.8)	0	2 (5.6)
4, *n* (%)	1 (0.9)	0	1 (2.8)
Chronic disease			
No, *n* (%)	90 (79.6)	65 (84.4)	25 (69.4)
Yes, *n* (%)	23 (20.4)	12 (15.6)	11 (30.6)
Chronic disease in the family members			
No, *n* (%)	49 (43.4)	34 (44.2)	15 (41.7)
Yes, *n* (%)	64 (56.6)	43 (55.8)	21 (58.3)
≥ 65-year-old family member			
No, *n* (%)	72 (63.7)	57 (74)	15 (41.7)
Yes, *n* (%)	41(36.3)	20 (26)	21 (58.3)
Work experience†, (years)		**Mean (SD)** 2.3 (1.1)	**Median (IQR)** 7 (18)
Working area			
Frontline, *n* (%)	72 (63.1)	55 (71.4)	17 (47.2)
Intensive care unit, *n*	25	12	13
COVID-19 wards, *n*	35	32	3
COVID-19 outpatient clinic, *n*	12	11	1
Non-COVID-19 areas, *n* (%)	41 (36.9)	22 (28.6)	19 (52.8)

†Work experiences denotes the year of residency for the residents and the duration of working experience as a physician for the academic staff. IQR, interquartile range; SD, standard deviation.

© 2020 Royal Australasian College of Physicians

Table 2 Knowledge and fear assessment of the participants with regards to COVID-19

	Total (n = 113)			Residents (n = 77)			Academic staff (n = 36)			P-value
	No, n (%)	Partially, n (%)	Yes, n (%)	No, n (%)	Partially, n (%)	Yes, n (%)	No, n (%)	Partially, n (%)	Yes, n (%)	
Do you think you have enough knowledge about COVID-19?	11 (9.7)	57 (50.4)	45 (39.8)	10 (13)	38 (49.4)	29 (37.7)	1 (2.8)	19 (52.8)	16 (44.4)	0.227
Do you think you can manage the follow up and treatment process correctly when you have COVID-19 patients?	15 (13.3)	59 (52.2)	39 (34.5)	11 (14.3)	41 (53.2)	25 (32.5)	4 (11.1)	18 (50)	14 (38.9)	0.768
Are you anxious about following patients with COVID-19?	27 (23.9)	30 (26.5)	56 (49.6)	19 (24.7)	20 (26)	38 (49.4)	8 (22.2)	10 (50)	18 (27.8)	0.954

staff whose median working experience was 7 (IQR = 18) years. Fifty-two (67.5%) residents were unmarried and all but three had no children, whereas 31 (86.1%) academic staff were married and 24 of them (66.6%) had children. Among all participants, 23 (20.4%) had chronic diseases and 41 (36.3%) admitted that they had family members older than 65 years and 64 (56.6%) had family members suffering from chronic diseases.

A total of 72 internists (63.7%) worked as 'frontline' HCW directly engaged in diagnosing, treating or caring for patients with or suspected to have COVID-19. Remaining 41 physicians (36.3%) worked in areas that were deemed to be 'non-COVID-19 areas' such as general internal medicine, endocrinology, nephrology, rheumatology, gastroenterology, medical oncology, haematology, geriatric medicine, infectious disease, cardiology and pulmonology clinics.

Knowledge and fear

Table 2 lists the answers of the participants to questions with regard to the knowledge and fear towards COVID-19: 45 (39.8%) participants answered that they have enough knowledge about COVID-19, 39 (34.5%) participants stated that they can manage the follow up and treatment process correctly when they encounter COVID-19 patients, while 56 (49.6%) participants answered that they are anxious about the possibility of following COVID-19 patients. There was no statistically significant difference between residents and academic staff with regard to the answer categories (Table 2).

Anxiety scores and associated factors

The median (IQR) scores on the GAD-7 and the BAI for all internists were 4[5] and 6 (9.5) respectively. For the GAD-7 anxiety subscales, half of the internists, 57 (50.4%), had scores within the minimal level, while 35 (31%) internists were considered to suffer from mild anxiety (score: 5–9); 12 (10.6%) were considered to suffer from moderate anxiety (score: 10–14); and 9 (8%) were considered to suffer from severe anxiety (score: 15–21). Based on the Turkish version of GAD-7 scale most of the internists, 88 (77.9%), have normal scores, under the 'GAD-7 cut-off score 8', and did not require further assessment, whereas 25 (22.1%) internists scored ≥8 indicating the need for further assessment and/or referral to a mental health professional. BAI levels were also compatible with GAD-7 levels and the majority of internists were not found to have high anxiety scores (Table 3).

Female gender was significantly associated with high scores and levels in all five scales compared to male gender (GAD-7 level; $P = 0.001$, GAD-7 cut-off 8; $P = 0.009$, GAD-7 score; $P < 0.001$, BAI level; $P = 0.034$, BAI score; $P = 0.005$). Additionally, having family members over 65 years of age (GAD-7 level; $P = 0.013$, GAD-7 cut-off 8; $P = 0.037$, GAD-7 score; $P = 0.009$, BAI level; $P = 0.067$, BAI score; $P = 0.002$) and with chronic diseases (GAD-7 level; $P = 0.041$, GAD-7 cut-off 8; $P = 0.047$, GAD-7 score; $P = 0.005$, BAI level; $P = 0.038$, BAI score; $P = 0.001$) were significantly associated with high anxiety scores and levels in all 5 scales. Other sociodemographic variables including age, marriage status, children status were not associated with anxiety scores and levels (Table 3).

There was no statistically significant difference between residents and academic staff in terms of all five anxiety scores and levels. Working area was an insignificant factor in terms of anxiety, and working in either COVID-19 ICU or COVID-19 ward or COVID-19 outpatient clinic was not associated with higher anxiety scores and levels (Table 4).

Internists who answered 'yes' to the question of 'Are you anxious about following patients with COVID-19?'

© 2020 Royal Australasian College of Physicians

Table 3 Anxiety scores and associated factors

	Age (years)			Sex			Marriage status			Children status Having children?			Chronic disease			Chronic disease in family			Family member >65 years of age		
	<29	≥29	*P*-value	Male	Female	*P*-value	Married	Unmarried + Divorced	*P*-value	No	Yes	*P*-value	No	Yes	*P*-value	No	Yes	*P*-value	No	Yes	*P*-value
GAD-7 level																					
Minimal, *n*	27	30	0.649	40	17	**0.001**	30	27	0.891	44	13	0.045	50	7	0.083	32	25	**0.041**	43	14	**0.013**
Mild, *n*	17	38		15	20		17	18		24	11		25	10		12	23		20	15	
Moderate, *n*	8	4		3	9		5	7		12	0		10	2		3	9		7	5	
Severe, *n*	4	5		2	7		4	5		6	3		5	4		2	7		2	7	
GAD-7 cut-off 8																					
<8, *n*	41	57	0.339	53	35	**0.009**	44	44	1	65	23	0.434	71	17	0.817	43	45	**0.047**	61	27	**0.037**
≥8, *n*	15	10		7	18		12	13		21	4		19	6		6	19		11	14	
GAD-7 score, median (IQR)	6.01 (5.4)	5.4 (5.1)	0.580	3 (0–21)	6 (0–21)	<0.001	5.5 (5)	5.9 (5.5)	0.802	5.7 (5.2)	5.7 (5.5)	0.981	4 (0–21)	6 (0–21)	0.246	3 (0–21)	6 (0–21)	**0.005**	3.5 (0–21)	6 (0–21)	**0.009**
BAI level																					
Minimal, *n*	33	31	0.202	41	23	**0.034**	35	29	0.396	48	16	0.604	54	10	0.375	35	29	**0.038**	45	19	**0.067**
Mild, *n*	10	18		13	15		14	14		20	8		19	9		9	19		19	9	
Moderate, *n*	5	5		3	7		3	7		9	1		8	2		2	8		4	6	
Severe, *n*	8	3		3	8		4	7		9	2		9	2		3	8		4	7	
BAI score, median (IQR)	11.2 (13.5)	8.7 (10.1)	0.679	5 (0–63)	9 (0–60)	**0.005**	8.5 (10.9)	11.4 (12.8)	0.156	10.2 (11.7)	9.3 (12.7)	0.728	6 (0–60)	10 (0–63)	0.336	4 (0–49)	9 (0–63)	**0.001**	5 (0–43)	9 (1–63)	**0.002**

Bold values are statistically significant ($P < 0.05$). BAI, Beck Anxiety Inventory; GAD-7, 7-item Generalised Anxiety Disorder; IQR, interquartile range.

© 2020 Royal Australasian College of Physicians

Table 4 Anxiety scores and associated factors

	Working position			Working area			Working area Frontline (COVID-19 care areas)†				Question: 'Are you anxious about following patients with COVID-19?'				Total (n = 113), n (%)
	Resident	Academic Staff	*P*-value	Frontline	Non-COVID-19	*P*-value	ICU	Ward	Outpatient Clinic	*P*-value	No	Partially	Yes	*P*-value	
GAD-7 Level															
Minimal, *n*	39	18	0.181	35	22	0.879	12	19	4	0.772	20	18	19	**0.005**	57 (50.4)
Mild, *n*	21	14		24	11		8	12	4		6	9	20		35 (31)
Moderate, *n*	11	1		7	5		3	2	2		1	2	9		12 (10.6)
Severe, *n*	6	3		6	3		2	2	2		0	1	8		9 (8)
GAD-7 cut-off 8															
<8, *n*	57	31	0.231	57	31	0.661	19	31	7	0.075	26	25	37	**0.006**	88 (77.9)
≥8, *n*	20	5		15	10		6	4	5		1	5	19		25 (22.1)
GAD-7 score, mean (SD)	5.8 (5.4)	5.4 (5.0)	0.841	4 (0–21)	5 (0–21)	0.808	6.1 (5.6)	4.7 (4.6)	8.4 (6.2)	0.114	2 (0–10)	3,5 (0–19)	7 (0–21)	**<0.001**	Median (IQR) 4 (5)
BAI level															
Minimal, *n*	44	20	0.097	44	20	0.375	15	23	6	0.477	26	21	17	**<0.001**	64.4 (56.6)
Mild, *n*	15	13		15	13		6	7	2		1	6	21		28 (24.8)
Moderate, *n*	9	1		5	5		1	1	3		0	2	8		10 (8.8)
Severe, *n*	9	2		8	3		3	4	1		0	1	10		11 (9.7)
BAI score, mean (SD)	10.7 (12.9)	8.4 (11.2)	0.449	6 (0–60)	8 (0–63)	0.938	10.3 (11.4)	8.7 (10.8)	13.5 (16)	0.271	2 (0–12)	5 (0–34)	11 (0–63)	**<0.001**	Median (IQR) 6 (9.5)

†Analysis of the data of 72 participants who work in these areas. Bold values are statistically significant ($P < 0.05$). BAI, Beck Anxiety Inventory; GAD-7, 7-item Generalised Anxiety Disorder; ICU, intensive care unit; IQR, interquartile range; SD, standard deviation.

© 2020 Royal Australasian College of Physicians

were significantly associated with high anxiety scores and levels in all five scales (Table 4).

Discussion

COVID-19 outbreak caused considerable concern all around the world because of its speed of transmission and high mortality rate. A general public survey during the initial phase of the COVID-19 outbreak in China showed that 28.8% of the participants reported moderate-to-severe anxiety and 53.8% of respondents rated the psychological impact of the outbreak as moderate or severe.[9] Increasing anxiety and panic in all communities has also seriously affected HCW, who are under high-stress conditions due to increased risk of exposure to the virus, carrying the possibility of transmitting the virus to friends and family members, the lack of sufficient knowledge, resources, treatment or vaccine and the need to make new and quick decisions in critically ill patients. A cross-sectional study aiming to assess the mental health of Chinese public during COVID-19 outbreak identified that HCW were at high risk of mental illness.[10]

This study was carried out in the early stages of the pandemic when the number of COVID-19 cases were increasing rapidly towards the peak in Turkey. As the pandemic plan was activated, the command chain of the disaster plan was in place and all preparations were made quickly in our hospital. Staff was well provided with personal protective equipment in accordance to the guidance by the Infection Control Unit with best available evidence. Although routine work patterns of all staff have been disrupted, in order to limit viral exposure, working times of the HCW in COVID-19 areas have been reorganised in 12-h shifts every other day. No matter how resilient the healthcare system in the hospital is, the fear of an approaching disaster and the uncertainty ahead are strong factors for anxiety.

The overall median (IQR) scores on the GAD-7 and the BAI for all internists were 4[5] and 6 (9.5) respectively, reflecting minimal anxiety levels. More than half of the participants had minimal anxiety score levels (GAD-7; 50.4% and BAI; 56.6%). The prevalence of severe anxiety levels was 8% for GAD-7 and 9.7% for BAI respectively. In previous studies during the 2003 SARS outbreak, 2009 influenza A/H1N1 outbreak and 2014 Middle East Respiratory Syndrome outbreak, a significant proportion of HCW were reported to experience anxiety symptoms and to have high psychological distress.[5,11,12] Comparing different groups, doctors reported fewer worries than other HCW, while the anxiety level was significantly higher in nurses than in doctors.[13–16] Similarly, during the A/H1N1 influenza pandemic, greater proportion of nurses and auxiliary staff also had increased concerns and higher degree of worry compared to doctors.[5] In our study, the proportion of participants who developed anxiety and distress symptoms and the level of anxiety were lower than those reported in previous disease outbreaks, which could be attributed to the selection of participants only among physicians.

It has been shown that the lifetime prevalence of GAD is higher in women (6.6%) than in men (3.6%).[17] Similarly, anxiety levels of female physicians were reported to be higher than male physicians.[18,19] In a cross-sectional survey, female physicians had higher anxiety levels than males and have also been reported to be exposed to certain stress factors related to discrimination, lack of role models and support, role stress and overload.[20]

Biological factors, such as the variation in ovarian hormone levels, may contribute to the increased prevalence of depression and anxiety in women.[21] In our study, female physicians reported more severe symptoms of anxiety in all measurements, consistent with the results of previous studies.[22–25] Risk factors related to social life, differences in physical strength and personality traits might influence the risk of anxiety in women. Therefore, female doctors might need a closer follow up and higher level of support to maintain good mental health, while treating patients with COVID-19.

Studies during the SARS, influenza A/H1N1 and recent COVID-19 outbreaks showed that working in high-risk work environment was an independent risk factor for worse mental health outcomes.[26–29] On the other hand, there are studies showing that exposure to high-intensity and high-risk work environments is not the primary risk factor for psychological distress.[4] In the present study, working in a frontline COVID-19 area was not associated with higher anxiety scores and levels. Additionally, there was no significant difference between working either in COVID-19 ICU or COVID-19 ward or COVID-19 outpatient clinics.

Increasing age and chronic diseases have been reported to be risk factors for worse outcomes with regard to COVID-19. In this study, having family members over 65 years of age and with chronic medical conditions were significantly associated with high anxiety scores and levels in all measurements. Previous studies have also indicated that increased concerns about their family's health were important emotional stressors highly prevalent among HCW.[9,30,31] On the other hand, we could not demonstrate an association between having children and higher levels of anxiety, which might be due to the facts that children and adolescents are less likely to experience severe COVID-19. However, some studies indicated an association between high levels of

© 2020 Royal Australasian College of Physicians

concern about a child younger than 16 years getting COVID-19 and higher anxiety scores.[9,32,33]

This study has several limitations. First, it was limited in scope. All participants were internists in a Joint Commission International accredited, large, urban teaching hospital, which may limit the generalisation of our findings to all HCW and the community. Second, the duration of the study was the more stressful 2 weeks of the pandemic in our hospital, when the patient numbers increased sharply. This situation could have aggravated the mental health symptoms of HCW. We included only medical doctors and even only internists. The level of knowledge and perception may differ between internists and other medical specialists in terms of critical and difficult patient care, emergency patient approach experiences, and so on. We conducted a rapid survey due to the time limitation of the pandemic and a self-designed questionnaire was used. Gathering information regarding psychological distress by self-report is a further limitation.

Conclusion

It is important to understand the professional and psychological effects of practice during this COVID-19 outbreak, since the well-being of a large number of healthcare professionals is of concern. Female doctors and physicians who have elderly family members and family members with chronic medical conditions showed more severe symptoms of anxiety. We believe that the results of this study are valuable as it points out a vulnerable point in the healthcare system during the COVID-19 outbreak, that is the mental health. These findings need to be confirmed and investigated with follow-up studies that would be useful for the assessment of the progression of psychological manifestations.

References

1 Huang C, Wang Y, Li X, Ren L, Zhao J, Hu Y *et al*. Clinical features of patients infected with 2019 novel coronavirus in Wuhan, China. *Lancet* 2020; **395**: 497–506.

2 World Health Organization (WHO). Statement on the second meeting of the International Health Regulations (2005) Emergency Committee regarding the outbreak of novel coronavirus (2019-nCoV). Geneva, Switzerland: WHO; 2020.

3 World Health Organization (WHO). *Situation Report – 112. Data as Received by WHO from National Authorities by 10:00 CEST*. Geneva, Switzerland: WHO; 2020. Available from URL: https://www.who.int/docs/default-source/coronaviruse/situation-reports/20200511-covid-19-sitrep-112.pdf?sfvrsn=813f2669_2

4 Maunder RG, Lancee WJ, Balderson KE, Bennett JP, Borgundvaag B, Evans S *et al*. Long-term psychological and occupational effects of providing hospital healthcare during SARS outbreak. *Emerg Infect Dis* 2006; **12**: 1924–32.

5 Goulia P, Mantas C, Dimitroula D, Mantis D, Hyphantis T. General hospital staff worries, perceived sufficiency of information and associated psychological distress during the a/H1N1 influenza pandemic. *BMC Infect Dis* 2010; **10**: 322.

6 Konkan R, Şenormancı Ö, Güçlü O, Aydin E, Sungur MZ. Common Anxiety Disorder-7 (GAD-7) Test Turkish adaptation, validity and reliability. *Neuropsychiat Arch* 2013; **50**: 53–8.

7 Ulusoy M, Sahin NH, Erkmen H. The Beck anxiety inventory: psychometric properties. *J Cogn Psychother* 1998; **12**: 163–72.

8 Spitzer RL, Kroenke K, Williams JB, Löwe B. A brief measure for assessing generalized anxiety disorder: the GAD-7. *Arch Intern Med* 2006; **166**: 1092–7.

9 Wang C, Pan R, Wan X, Tan Y, Xu L, Ho CS *et al*. Immediate psychological responses and associated factors during the initial stage of the 2019 coronavirus disease (COVID-19) epidemic among the general population in China. *Int J Environ Res Public Health* 2020; **17**: 1729.

10 Huang Y, Zhao N. Generalized anxiety disorder, depressive symptoms and sleep quality during COVID-19 outbreak in China: a web-based cross-sectional survey. *Psychiatry Res* 2020; **288**: 112954.

11 Cabello IR, Echavez JFM, Serrano-Ripoll MJ, Fraile-Navarro D, de Roque MAF, Moreno GP *et al*. Impact of viral epidemic outbreaks on mental health of healthcare workers: a rapid systematic review. *medRxiv* 2020. doi: 10.1101/2020.04.02.20048892

12 Khalid I, Khalid TJ, Qabajah MR, Barnard AG, Qushmaq IA. Healthcare workers emotions, perceived stressors and coping strategies during a MERS-CoV outbreak. *Clin Med Res* 2016; **14**: 7–14.

13 Li L, Cheng S, Gu J. SARS infection among health care workers in Beijing, China. *JAMA* 2003; **290**: 2662–3.

14 Shih F-J, Gau M-L, Kao C-C, Yang C-Y, Lin Y-S, Liao Y-C *et al*. Dying and caring on the edge: Taiwan's surviving nurses' reflections on taking care of patients with severe acute respiratory syndrome. *Appl Nurs Res* 2007; **20**: 171–80.

15 Bukhari EE, Temsah MH, Aleyadhy AA, Alrabiaa AA, Alhboob AA, Jamal AA *et al*. Middle East respiratory syndrome coronavirus (MERS-CoV) outbreak perceptions of risk and stress evaluation in nurses. *J Infect Dev Countr* 2016; **10**: 845–50.

16 Wong TW, Yau JK, Chan CL, Kwong RS, Ho SM, Lau CC *et al*. The psychological impact of severe acute respiratory syndrome outbreak on healthcare workers in emergency departments and how they cope. *Eur J Emerg Med* 2005; **12**: 13–8.

17 Kessler RC, McGonagle KA, Zhao S, Nelson CB, Hughes M, Eshleman S *et al*. Lifetime and 12-month prevalence of DSM-III-R psychiatric disorders in the United States: results from the National Comorbidity Survey. *Arch Gen Psychiatry* 1994; **51**: 8–19.

18 Erdur B, Ergin A, Turkcuer I, Parlak I, Ergin N, Boz B. A study of depression and anxiety among doctors working in emergency units in Denizli, Turkey. *Emerg Med J* 2006; **23**: 759–63.

19 Alharthy N, Alrajeh OA, Almutairi M, Alhajri A. Assessment of anxiety level of emergency health-care workers by

© 2020 Royal Australasian College of Physicians

generalized anxiety disorder-7 tool. *Int J Appl Basic Med Res* 2017; **7**: 150–4.
20 Koçak M, Gül O, Aydın H, Aciksari K, Ozucelik D. Frequency of anxiety among physicians working in emergency departments and other clinics in Turkey: A cross-sectional survey. *Medeniyet Med J* 2019; **34**: 135–42.
21 Albert PR. Why is depression more prevalent in women. *J Psychiat Neurosci* 2015; **40**: 219–21.
22 Huang J, Han M, Luo T, Ren A, Zhou X. Mental health survey of 230 medical staff in a tertiary infectious disease hospital for COVID-19. *Zhonghua lao Dong wei Sheng zhi ye Bing za zhi= Zhonghua Laodong Weisheng Zhiyebing Zazhi = Chinese Journal of Industrial Hygiene and Occupational Diseases* 2020; **38**: 192–5.
23 Lai J, Ma S, Wang Y, Cai Z, Hu J, Wei N *et al*. Factors associated with mental health outcomes among health care workers exposed to coronavirus disease 2019. *JAMA Network Open* 2020; **3**: e203976-e.
24 Huang L, Rong Liu H. Emotional responses and coping strategies of nurses and nursing college students during COVID-19 outbreak. *medRxiv* 2020. doi: 10.1101/2020.03.05.20031898
25 Zhu Z, Xu S, Wang H, Liu Z, Wu J, Li G *et al*. COVID-19 in Wuhan: immediate psychological impact on 5062 health workers. *medRxiv* 2020. doi:10.1101/2020.02.20.20025338
26 Grace SL, Hershenfield K, Robertson E, Stewart DE. The occupational and psychosocial impact of SARS on academic physicians in three affected hospitals. *Psychosomatics* 2005; **46**: 385–91.
27 Matsuishi K, Kawazoe A, Imai H, Ito A, Mouri K, Kitamura N *et al*. Psychological impact of the pandemic (H1N1) 2009 on general hospital workers in Kobe. *Psychiatry Clin Neurosci* 2012; **66**: 353–60.
28 Oh N, Hong N, Ryu DH, Bae SG, Kam S, Kim K-Y. Exploring nursing intention, stress, and professionalism in response to infectious disease emergencies: the experience of local public hospital nurses during the 2015 MERS outbreak in South Korea. *Asian Nurs Res* 2017; **11**: 230–6.
29 Dai Y, Hu G, Xiong H, Qiu H, Yuan X. Psychological impact of the coronavirus disease 2019 (COVID-19) outbreak on healthcare workers in China. *medRxiv* 2020. doi:10.1101/2020.03.03.20030874
30 Kisely S, Warren N, McMahon L, Dalais C, Henry I, Siskind D. Occurrence, prevention, and management of the psychological effects of emerging virus outbreaks on healthcare workers: rapid review and meta-analysis. *BMJ* 2020; **369**: m1642.
31 Li S, Wang Y, Xue J, Zhao N, Zhu T. The impact of COVID-19 epidemic declaration on psychological consequences: a study on active Weibo users. *Int J Environ Res Public Health* 2020; **17**: 2032.
32 Koh D, Lim MK, Chia SE, Ko SM, Qian F, Ng V *et al*. Risk perception and impact of severe acute respiratory syndrome (SARS) on work and personal lives of healthcare Workers in Singapore What can we Learn? *Med Care* 2005; **43**: 676–82.
33 Maunder RG, Lancee WJ, Rourke S, Hunter JJ, Goldbloom D, Balderson K *et al*. Factors associated with the psychological impact of severe acute respiratory syndrome on nurses and other hospital workers in Toronto. *Psychosom Med* 2004; **66**: 938–42.

© 2020 Royal Australasian College of Physicians
