# Supplementary material for: Mental health status and related factors influencing healthcare workers during the COVID-19 pandemic: A systematic review and meta-analysis
Source: PLoS One. 2024 Jan 19;19(1):e0289454. doi: 10.1371/journal.pone.0289454 (PMC10798549; doi:10.1371/journal.pone.0289454)
Supplement: S1 Data — (ZIP) [file pone.0289454.s011.zip › literatures/20.pdf]

Submitted: 12 May, 2020 Accepted: 02 June, 2020 Published: 30 June, 2020 DOI:10.22514/sv.2020.16.0022

Open Access

Signa Vitae

ORIGINAL RESEARCH

The Evaluation of Knowledge, Attitudes, Depression and Anxiety Levels among Emergency Physicians during the COVID-19 Pandemic

Fatih ÇALIŞKAN[1,*], Burhan DOST[2]

[1]Department of Emergency Medicine, Faculty of Medicine, Ondokuz Mayıs University, Samsun,Turkey
[2]Department of Anesthesiology and Reanimation, Faculty of Medicine, Ondokuz Mayıs University, Samsun, Turkey

***Correspondence**
mdfcaliskan@gmail.com (Fatih Çalişkan)

Abstract

Background: The outbreak of COVID-19 has put enormous physical and psychological pressure on emergency physicians. The purpose of this study is to evaluate the levels of anxiety, depression, and related factors in this group during the COVID-19 pandemic. **Materials and Methods:** After obtaining ethics committee approval, we gathered the data for this descriptive study using an online questionnaire from March 14–20, 2020, from 290 emergency physicians in health care settings in Turkey. **Results:** The 290 emergency physicians who participated in the study had a high level of knowledge about COVID-19. According to their Hospital Anxiety Depression Scale (HADS) scores, depression was detected in 180 participants (62%) while anxiety was detected in 103 participants (35.5%), with the median depression and anxiety scores found to be 8 (0–21) and 7 (0–21), respectively. As to the requirement of a 14-day quarantine for an emergency physician performing an intubation, a statistically significant difference was found among job titles ($p = 0.039$). **Conclusion:** This study emphasizes the importance of recognizing psychological trauma caused by providing care during the COVID-19 pandemic and the necessity of providing psychological support for the protection of emergency physicians' mental health.

Keywords

Emergency, Health care, COVID-19, Anxiety, Depression

1. Introduction

On December 31, 2019, the World Health Organization (WHO) Country Office in China reported pneumonia cases of unknown etiology in the Hubei province, which contains the city of Wuhan. On January 7, 2020, the etiologic agent was identified as a novel coronavirus (2019-nCoV) that had not been previously detected in humans. Next, the disease caused by 2019-nCoV was named coronavirus disease 2019 (COVID-19), and the virus was named SARS-CoV-2 because of its close resemblance to SARS-CoV. After this date, the number of COVID-19 patients increased rapidly, and the illness was detected in health care workers. COVID-19 has spread quickly because of its high transmissibility. The number of COVID-19 cases has reached nearly two million worldwide, affecting approximately 185 countries about three months after this virus was first detected [1–3]. The first known COVID-19 case in Turkey was detected on March 11, 2020 [4]. This date coincides with the date the WHO declared COVID-19 a pandemic [5]. During our study (March 14–20, 2020), there were 670 confirmed COVID-19 cases and nine patient deaths from COVID-19 in Turkey [4].

Common symptoms of SARS-CoV-2 infection include fever, cough, and dyspnea; in more serious cases, pneumonia, severe acute respiratory infection, and kidney failure can develop, sometimes leading to death. SARS-CoV-2 is mainly transmitted via respiratory droplets. Asymptomatic people can be contagious as the virus has been detected in their respiratory secretions [6].

The battle against COVID-19 is continuing in many countries. To ensure success against the virus, every member of society, including health care professionals, must strictly adhere to control measures, the application of which is affected by their knowledge, attitudes, and practices (KAP) toward COVID-19 [7]. Lessons learned from previ-

This is an open access article under the CC BY-NC 4.0 license (https://creativecommons.org/licenses/by-nc/4.0/).
 ©2020 The Authors. Published by MRE Press.

TABLE 1. Evaluation of the attitudes of the participants about COVID-19 pandemic by job titles.

		Job Titles					
		Practitioner	EM Resident	EM specialist	Academician	Total	p
Q1	Disagree	76 (53.9)a	52 (73.2)b	33 (58.9)ab	18 (81.8)ab	179 (61.7)	0.039
	Undecided	42 (29.8)	9 (12.7)	15 (26.8)	3 (13.6)	69 (23.8)	
	Agree	23 (16.3)	10 (14.1)	8 (14.3)	1 (4.5)	42 (14.5)	
Q2	Disagree	1 (0.7)	2 (2.8)	0 (0)	0 (0)	3 (1)	0.628
	Undecided	2 (1.4)	2 (2.8)	1 (1.8)	1 (4.5)	6 (2.1)	
	Agree	138 (97.9)	67 (94.4)	55 (98.2)	21 (95.5)	281 (96.9)	
Q3	Disagree	21 (14.9)	8 (11.3)	4 (7.1)	3 (13.6)	36 (12.4)	0.704
	Undecided	15 (10.6)	9 (12.7)	5 (8.9)	1 (4.5)	30 (10.3)	
	Agree	105 (74.5)	54 (76.1)	47 (83.9)	18 (81.8)	224 (77.2)	
Q4	Disagree	84 (59.6)	45 (63.4)	36 (64.3)	14 (63.6)	179 (61.7)	0.422
	Undecided	27 (19.1)	7 (9.9)	6 (10.7)	5 (22.7)	45 (15.5)	
	Agree	30 (21.3)	19 (26.8)	14 (25)	3 (13.6)	66 (22.8)	
Q5	Disagree	15 (10.6)	8 (11.3)	11 (19.6)	3 (13.6)	37 (12.8)	0.481
	Undecided	18 (12.8)	12 (16.9)	6 (10.7)	1 (4.5)	37 (12.8)	
	Agree	108 (76.6)	51 (71.8)	39 (69.6)	18 (81.8)	216 (74.5)	
Q6	Disagree	12 (8.5)	8 (11.3)	5 (8.9)	1 (4.5)	26 (9)	0.615
	Undecided	6 (4.3)	3 (4.2)	5 (8.9)	0 (0)	14 (4.8)	
	Agree	123 (87.2)	60 (84.5)	46 (82.1)	21 (95.5)	250 (86.2)	
Q7	Disagree	31 (22)	20 (28.2)	11 (19.6)	3 (13.6)	65 (22.4)	0.802
	Undecided	54 (38.3)	23 (32.4)	21 (37.5)	8 (36.4)	106 (36.6)	
	Agree	56 (39.7)	28 (39.4)	24 (42.9)	11 (50)	119 (41)	
Q8	Disagree	24 (17)	13 (18.3)	9 (16.1)	1 (4.5)	47 (16.2)	0.811
	Undecided	48 (34)	23 (32.4)	20 (35.7)	10 (45.5)	101 (34.8)	
	Agree	69 (48.9)	35 (49.3)	27 (48.2)	11 (50)	142 (49)	

The results of comparisons are summarized as n (%).
Q1: Any person who has performed intubation should be quarantined for 14 days.
Q2: Working in emergency services carries a great risk of exposure to COVID-19.
Q3: I am afraid of being infected with COVID-19.
Q4: I want to change my job/department due to high risk of COVID-19.
Q5: I think that my workload has increased during COVID-19 pandemic.
Q6: I feel stressed at work.
Q7: I believe that the COVID-19 pandemic can be fully controlled worldwide.
Q8: I believe that our country will win the fight against COVID-19.

ous outbreaks, especially SARS in 2003, show that knowledge and attitudes toward infectious diseases are affected by the level of panic in the population, which can make attempts to prevent the spread of the disease more difficult. The cause of the infection is novel, and the duration of the disease is unknown. Because emergency services are the units to which all patients first apply during a pandemic, the workloads of emergency staffs have increased significantly. The high number of patients and the failure of health systems to respond to the increasing need for care during this acute period of this pandemic could lead to negative emotions among the emergency physicians on the front lines. For these reasons, we endeavored to determine the knowledge and attitudes of emergency physicians toward COVID-19 and to evaluate their levels of depression and anxiety in dealing with the pandemic.

2. Materials and Methods

2.1 Ethical Statement

This study was approved by the Clinical Research Ethics Committee, Faculty of Medicine, Ondokuz Mayis University (OMU CREC protocol no: 2020/241).

TABLE 2. Evaluation of the attitudes of the participants about COVID-19 pandemic according to the working experience.

		Working Experience in Emergency Service					
		1-5 year	6-10 years	11-15 years	>15 years	Total	p
Q1	Disagree	122 (61.9)	24 (66.7)	14 (60.9)	19 (55.9)	179 (61.7)	0.9
	Undecided	45 (22.8)	8 (22.2)	5 (21.7)	11 (32.4)	69 (23.8)	
	Agree	30 (15.2)	4 (11.1)	4 (17.4)	4 (11.8)	42 (14.5)	
Q2	Disagree	3 (1.5)	0 (0)	0 (0)	0 (0)	3 (1)	0.524
	Undecided	3 (1.5)	2 (5.6)	1 (4.3)	0 (0)	6 (2.1)	
	Agree	191 (97)	34 (94.4)	22 (95.7)	34 (100)	281 (96.9)	
Q3	Disagree	29 (14.7)	2 (5.6)	2 (8.7)	3 (8.8)	36 (12.4)	0.569
	Undecided	22 (11.2)	3 (8.3)	3 (13)	2 (5.9)	30 (10.3)	
	Agree	146 (74.1)	31 (86.1)	18 (78.3)	29 (85.3)	224 (77.2)	
Q4	Disagree	120 (60.9)	27 (75)	17 (73.9)	15 (44.1)	179 (61.7)	0.025
	Undecided	30 (15.2)ab	1 (2.8)a	3 (13)ab	11 (32.4)b	45 (15.5)	
	Agree	47 (23.9)	8 (22.2)	3 (13)	8 (23.5)	66 (22.8)	
Q5	Disagree	25 (12.7)	4 (11.1)	5 (21.7)	3 (8.8)	37 (12.8)	0.031
	Undecided	25 (12.7)ab	10 (27.8)a	1 (4.3)ab	1 (2.9)b	37 (12.8)	
	Agree	147 (74.6)	22 (61.1)	17 (73.9)	30 (88.2)	216 (74.5)	
Q6	Disagree	18 (9.1)	4 (11.1)	3 (13)	1 (2.9)	26 (9)	0.13
	Undecided	7 (3.6)	5 (13.9)	1 (4.3)	1 (2.9)	14 (4.8)	
	Agree	172 (87.3)	27 (75)	19 (82.6)	32 (94.1)	250 (86.2)	
Q7	Disagree	46 (23.4)	7 (19.4)	4 (17.4)	8 (23.5)	65 (22.4)	0.814
	Undecided	73 (37.1)	15 (41.7)	6 (26.1)	12 (35.3)	106 (36.6)	
	Agree	78 (39.6)	14 (38.9)	13 (56.5)	14 (41.2)	119 (41)	
Q8	Disagree	32 (16.2)	5 (13.9)	3 (13)	7 (20.6)	47 (16.2)	0.693
	Undecided	65 (33)	17 (47.2)	7 (30.4)	12 (35.3)	101 (34.8)	
	Agree	100 (50.8)	14 (38.9)	13 (56.5)	15 (44.1)	142 (49)	

The results of comparisons are summarized as n (%).
Q1: Any person who has performed intubation should be quarantined for 14 days.
Q2: Working in emergency services carries a great risk of exposure to COVID-19.
Q3: I am afraid of being infected with COVID-19.
Q4: I want to change my job/department due to high risk of COVID-19.
Q5: I think that my workload has increased during COVID-19 pandemic.
Q6: I feel stressed at work.
Q7: I believe that the COVID-19 pandemic can be fully controlled worldwide.
Q8: I believe that our country will win the fight against COVID-19.

2.2 Study Design and Population

We conducted our study using a sample of emergency physicians working in various health care institutions in Turkey. The emergency physicians participating in this study consisted of general practitioners, emergency medicine (EM) residents, EM specialists, and academicians from the Department of Emergency Medicine, Faculty of Medicine. We used a similar study as a point of reference to which we compared the anxiety and depression scores of the physicians; we based the average mean anxiety score in the anxiety group on 20.56 ± 4.85 and the average mean non-anxiety score in the non-anxiety group on 17.30 ± 4.54 [8]. According to a t-test power analysis with a 95% confidence interval and 95% test power, we determined that 55 participants should be included in each group. For the purpose of making statistical comparisons by creating subgroups within the study group, we determined that at least 110 participants should be recruited.

After receiving ethics committee approval, we gathered the data using an online questionnaire delivered via SurveyMonkey (SurveyMonkey, San Mateo, California, United States) from March 14-20, 2020. Responses were restricted to one per web browser (using the IP address) or email ad-

TABLE 3. Evaluation of the attitudes of the participants according to the COVID-19 training.

		COVID-19 Training			
		Present	Absent	Total	p
Q1	Disagree	113 (66 .5)	66 (55)	179 (61 .7)	0 .116
	Undecided	37 (21 .8)	32 (26 .7)	69 (23 .8)	
	Agree	20 (11 .8)	22 (18 .3)	42 (14 .5)	
Q2	Disagree	3 (1 .8)	0 (0)	3 (1)	0 .315
	Undecided	3 (1 .8)	3 (2 .5)	6 (2 .1)	
	Agree	164 (96 .5)	117 (97 .5)	281 (96 .9)	
Q3	Disagree	25 (14 .7)	11 (9 .2)	36 (12 .4)	0 .058
	Undecided	22 (12 .9)	8 (6 .7)	30 (10 .3)	
	Agree	123 (72 .4)	101 (84 .2)	224 (77 .2)	
Q4	Disagree	121 (71 .2)*	58 (48 .3)	179 (61 .7)	<0 .001
	Undecided	24 (14 .1)	21 (17 .5)	45 (15 .5)	
	Agree	25 (14 .7)	41 (34 .2)*	66 (22 .8)	
Q5	Disagree	20 (11 .8)	17 (14 .2)	37 (12 .8)	0 .774
	Undecided	23 (13 .5)	14 (11 .7)	37 (12 .8)	
	Agree	127 (74 .7)	89 (74 .2)	216 (74 .5)	
Q6	Disagree	15 (8 .8)	11 (9 .2)	26 (9)	0 .108
	Undecided	12 (7 .1)	2 (1 .7)	14 (4 .8)	
	Agree	143 (84 .1)	107 (89 .2)	250 (86 .2)	
Q7	Disagree	39 (22 .9)	26 (21 .7)	65 (22 .4)	0 .582
	Undecided	58 (34 .1)	48 (40)	106 (36 .6)	
	Agree	73 (42 .9)	46 (38 .3)	119 (41)	
Q8	Disagree	29 (17 .1)	18 (15)	47 (16 .2)	0 .707
	Undecided	56 (32 .9)	45 (37 .5)	101 (34 .8)	
	Agree	85 (50)	57 (47 .5)	142 (49)	

The results of comparisons are summarized as n (%).
Q1: Any person who has performed intubation should be quarantined for 14 days.
Q2: Working in emergency services carries a great risk of exposure to COVID-19.
Q3: I am afraid of being infected with COVID-19.
Q4: I want to change my job/department due to high risk of COVID-19.
Q5: I think that my workload has increased during COVID-19 pandemic.
Q6: I feel stressed at work.
Q7: I believe that the COVID-19 pandemic can be fully controlled worldwide.
Q8: I believe that our country will win the fight against COVID-19.

dress of the respondent. We recruited volunteer participants through social media (WhatsApp, Twitter, and LinkedIn). The questionnaire contained 45 questions to collect data on sociodemographic characteristics (12 items), workplace characteristics (six items), knowledge (five items), attitudes (eight items), and anxiety and depression using components of the HADS (14 items). The HADS is a self-assessment that measures anxiety and depression levels through a series of 14 questions, seven for anxiety and seven for depression, each scored between 0 and 3 [9]. The lowest possible score for depression and anxiety is 0, and the highest possible score is 21. The validity and reliability of this scale with a Turkish population was determined by Aydemir et al., with the cutoff score determined to be 7 for depression and 10 for anxiety [10].

2.3 Statistical Analysis

We used IBM® SPSS® Statistics V21 for statistical analysis of the data and expressed it as mean ± standard deviation, median (minimum–maximum) and number (%) after determining whether the data was parametric or non-parametric. We used the Kolmogorov–Smirnov test to evaluate the conformity of the quantitative data to a normal distribution, determining that it would be appropriate to use non-parametric tests for data analysis in this study. We used the Mann–Whitney U test for binary groups and the Kruskal–Wallis test for comparisons of more than two groups. We compared quantitative data using a chi-square test and accepted the statistical significance level as $p < 0.05$ for all tests.

3. Results

The average age of the 290 emergency physicians who participated in the study was 31.8 ± 6.9 years, and the median age was 30 (24–64) years. Of all participants, 61.7% were male. Of the emergency physicians, 67.9% declared that they had worked in emergency services for 1–5 years, 12.4% for 6–10 years, 7.9% for 11–15 years,

TABLE 4. Comparison of HADS score according to characteristic groups of participants.

Variables	Depression score Median (min-max)	Anxiety score Median (min-max)
Gender		
Male (n: 179)	8 (0-21)	8 (0-21)
Female (n: 111)	7 (0-21)	7 (0-21)
p value*	0 .458	0 .358
Marital status (n:)		
Married (n: 141)	7 (0-21)	7 (0-21)
Single (n: 149)	8 (0-21)	8 (0-21)
p value*	0 .117	0 .187
Having a child		
Absent (n: 179)	8 (0-21)	8 (0-21)
Present (n: 111)	7 (0-21)	7 (0-21)
p value*	0 .797	0 .506
Title		
Practitioner (n: 141)	7 (0-21)	7 (0-21)
EM Resident (n: 71)	7 (0-21)	7 (0-21)
EM specialist (n: 56)	9 (0-21)	9 (0-21)
Academician (n: 22)	8 (0-21)	8 (0-21)
p value**	0 .377	0 .885
Working experience in EM		
1-5 years (n: 197)	8 (0-21)	7 (0-21)
6-10 years (n: 36)	8 (0-16)	8 (0-19)
11-15 years (n: 23)	7 (1-17)	7 (1-19)
>15 years (n: 34)	8 (0-21)	7 (0-21)
p value**	0 .796	0 .994
Type of hospital		
Public hospital (n: 133)	8 (0-21)	8 (0-20)
Private (n: 7)	8 (2-14)	7 (0-21)
Educational center1 (n: 150)	8 (0-20)	7 (0-21)
p value**	0 .627	0 .744
Chronic illness		
Absent (n: 247)	8 (0-20)	7 (0-21)
Present (n: 43)	8 (0-21)	8 (0-20)
p value*	0 .383	0 .249
Cigarette habit		
No smoking (n: 191)	8 (0-20)	7 (0-21)
Increased (n:11)	12 (6-21)	12 (6-20)
No changed (n: 49)	7 (0-16)	7 (1-16)
Decreased (n: 39)	8 (3-18)	9 (3-18)
p değeri**	**0 .028**	**0 .005**
Alcohol consumption		
No drinking (n: 196)	7 (0-20)	7 (0-21)
No changed (n:47)	9 (1-20)	8 (1-20)
Decreased (n:35)	9 (1-18)	8 (3-18)
Increased (12)	12.5 (0-21)	12 (0-20)
p value**	0 .103	0 .205
Living elderly relatives at home		
Absent (n: 248)	7.5 (0-20)	7 (0-21)
Present (n: 42)	9 (0-21)	9.5 (0-20)
p value*	0 .130	0 .160
Contact with COVID-19 patients		
Absent (n: 134)	8 (0-20)	7 (0-21)
Present (n:156)	8 (0-21)	8 (0-20)
p value*	0 .938	0 .415
Mental illness		
Absent (n:255)	8 (0-21)	7 (0-20)
Present . not continue (n:16)	9 (0-15)	7 (0-16)
Present . continue (n:12)	9 (0-17)	8.5 (1-18)
Just started (n:7)	14 (2-20)	12 (1-21)
p value**	0 .152	0 .119

**Mann Whitney U test; **Kruskal Wallis test*

[1] *Educational centers defined as university and training-research hospitals.*

TABLE 5. Comparison of participants according to the presence of depression and anxiety. *

Variables	Depression		Anxiety	
	Absent	Present (n:180)	Absent	Present (n:103)
Gender				
Male	66 (36.9)	113 (63.1)	114 (63.7)	65 (36.3)
Female	44 (39.6)	67 (60.4)	73 (65.8)	38 (34.2)
p value	0.637		0.719	
Job Title				
Practitioner	55 (39)	86 (61)	88 (62.4)	53 (37.6)
EM resident	30 (42.3)	41 (57.7)	47 (66.2)	24 (33.8)
EM specialist	19 (33.9)	37 (66.1)	35 (62.5)	21 (37.5)
Academician	6 (27.3)	16 (72.1)	17 (77.3)	5 (22.7)
p value	0.557		0.568	
Working experience				
1-5 years	77 (39.1)	120 (60.9)	126 (64)	71 (36)
6-10 years	13 (36.1)	23 (63.9)	22 (61.1)	14 (38.9)
11-15 years	10 (43.5)	13 (56.5)	17 (73.9)	6 (26.1)
>15 years	10 (29.4)	24 (70.6)	22 (64.7)	12 (35.3)
p değeri	0.68		0.778	
Marital status				
Single	50 (33.6)	99 (66.4)	90 (60.4)	59 (39.6)
Married	60 (42.6)	81 (57.4)	97 (68.8)	44 (31.2)
p value	0.115		0.136	
Having a child				
Absent	65 (36.3)	114 (63.7)	113 (63.1)	66 (36.9)
Present	45 (40.5)	66 (59.5)	74 (66.7)	37 (33.3)
p value	0.471		0.541	
Chronic illness				
Absent	95 (38.5)	152 (61.5)	160 (64.8)	87 (35.2)
Present	15 (34.9)	28 (65.1)	27 (62.8)	16 (37.2)
p value	0.655		0.802	
Type of hospital				
Public hospital	50 (37.6)	83 (62.4)	82 (61.7)	51 (38.3)
Educational center[1]	57 (38)	93 (62)	101 (67.3)	49 (32.7)
Private hospital	3 (42.9)	4 (57.1)	4 (57.1)	3 (42.9)
p value	0.961		0.559	
COVID-19 protocol				
Unknown	15 (31.9)	32 (68.1)	26 (55.3)	21 (44.7)
Present	78 (43.3)	102 (56.7)	130 (72.2)	50 (27.8)
Absent	17 (27)	46 (73)	31 (49.2)	32 (50.8)
p value	0.046		0.002	
Cigarette habit				
Increased	1 (9.1)	10 (90.9)	4 (36.4)	7 (63.6)
Decreased	12 (30.8)	27 (69.2)	23 (59)	16 (41)
Unchanged	23 (46.9)	26 (53.1)	33 (67.3)	16 (32.7)
No smoking	74 (38.7)	117 (61.3)	127 (66.5)	64 (33.5)
p value	0.091		0.185	
Alcohol consumption				
Increased	4 (33.3)	8 (66.7)	5 (41.7)	7 (58.3)
Decreased	10 (28.6)	25 (71.4)	23 (65.7)	12 (34.3)
Unchanged	19 (40.4)	28 (59.6)	30 (63.8)	17 (36.2)
No drinking	77 (39.3)	119 (60.7)	129 (65.8)	67 (34.2)
p value	0.64		0.405	
Living elderly relatives at home				
Absent	96 (38.7)	152 (61.3)	166 (66.9)	82 (33.1)
Present	14 (33.3)	28 (66.7)	21 (50)	21 (50)
p value	0.507		0.034	
Contact with COVID-19 patients				
Absent	56 (41.8)	78 (58.2)	87 (64.9)	47 (35.1)
Present	54 (34.6)	102 (65.4)	100 (64.1)	56 (35.9)
p value	0.209		0.884	
Mental Illness				
Absent	100 (39.2)	155 (60.8)	168 (65.9)	87 (34.1)
Present. not continue	6 (37.5)	10 (62.5)	9 (56.2)	7 (43.8)
Present. continue	3 (25)	9 (75)	9 (56.2)	7 (43.8)
Just started	1(14.3)	6 (85.7)	1 (14.3)	6 (85.7)
p değeri	0.441		0.03	
COVID-19 training				
Present	79 (46.5)	91 (53.5)	118 (69.4)	52 (30.6)
Absent	31 (25.8)	89 (74.2)	69 (57.5)	51 (42.5)
p value	<0.001		0.037	

**Chi square test was used for analysis*

[1]*Educational centers defined as university and training-research hospitals.*

and 11.7% for more than 15 years. In terms of job titles, 48.6% of the participants were general practitioners, 24.5% were EM residents, 19.3% were EM specialists, and 7.6% were academicians. Of the emergency physicians, 45.9% worked in public hospitals, 26.2% worked in university hospitals, and 25.5% worked in training and research hospitals. Overall, emergency physicians who participated in the study represented 78 hospitals in 44 cities in Turkey.

The participants correctly answered questions about COVID-19 at a frequency ranging from 96.9% to 100%, and 58.6% answered that they had received training on COVID-19 from their employing institutions. Of the participants who reported having received COVID-19 training, 51.2% work in university or training and research hospitals, 44.7% work in public hospitals, and 4.1% work in private hospitals. For the question "What sources do you refer to concerning COVID-19?," 96.9% of the participants said the National COVID-19 Infection Guideline, 53.4% said academic publications, 51% said social media (e.g., WhatsApp, Twitter, and Instagram), and 46.9% said in-hospital training on COVID-19.

A total of 185 (63.8%) participants reported that they first evaluated suspected cases of COVID-19 in the emergency triage units of their hospitals. A total of 146 participants (50.3%) stated that triage was applied only by allied health personnel, such as paramedics and emergency nurses, in the emergency room, and 176 participants (60.7%) stated that a checklist was used for the correct evaluation of COVID-19 in the triage unit. Only 11 participants (3.8%) reported that there was no triage unit in their hospital, while 69 participants (23.8%) said they did not have a COVID-19 diagnostic checklist in their emergency triage unit. When asked about a standard protocol to be used in emergency rooms for the correct management of COVID-19, 62.1% of participants reported that there was a protocol in their hospitals.

Of all participants, 65.9% reported they did not have a habit of smoking. Of the 99 participants who did smoke, it was determined that 49 (16.9%) did not change their smoking habits, 39 (13.4%) decreased their smoking habits, and 11 (3.8%) increased their smoking habits since the start of the COVID-19 pandemic. Of all participants, 67.6% stated that they did not drink alcohol. Of the participants who reported consuming alcohol, it was determined that 94 (16.2%) did not change their alcohol consumption habits, 35 (12.1%) decreased their alcohol consumption, and 12 (4.1%) increased their alcohol consumption since the start of the COVID-19 pandemic. A total of 156 participants (53.8%) stated that they had been in contact with patients who tested positive for COVID-19. Only 42 (14.5%) of the emergency physicians stated that they lived with relatives over the age of 60.

We evaluated emergency physicians' attitudes toward COVID-19 and COVID-19 patients in the study. Comparisons of the participants' attitudes with their job titles, amounts of time spent working, and levels of COVID-19 training are summarized in Tables 1–3, respectively. For most of the questions, we found no statistically significant differences among the groups ($p > 0.05$; Tables 1–3). However, regarding the answers concerning whether "the person who performed intubation should be quarantined for 14 days," we found a statistically significant difference among different job title groups ($p = 0.039$). With respect to participants' agreement with the statement "I want to change my job/department due to high risk," we detected a statistically significant difference among different job title groups ($p = 0.025$). Of all participants, 71.1% stated that they did not want to change jobs/departments despite the high risk. With respect to participants' agreement with the statement "I think my workload has increased during this period," we found a statistically significant difference among different work experience groups ($p = 0.031$). Of all participants, 75.5% stated that COVID-19 did not increase their workloads. Similarly, we found a significant association between COVID-19 training and the effects of COVID-19 on workload ($p = 0.001$). According to the participants' response to the statement "I am afraid of being infected with COVID-19," there was no statistically significant difference among the work experience groups ($p > 0.05$). However, it is noteworthy that 66.7% of emergency physicians with less than five years of experience responded, "I am absolutely scared." A further 72% of the 170 participants who reported having received COVID-19 training stated that they feared becoming infected with COVID-19.

The median depression score of the participants in the study was 8 (0–21), while the median anxiety score was 7 (0–21). The distribution of scores obtained using the HADS are shown in Table 4 according to characteristics of the emergency physicians. We found no statistically significant differences between cigarette consumption and anxiety and depression scores ($p > 0.05$). However, it is noteworthy that the anxiety and depression scores of the emergency physicians who had increased their cigarette consumption were high, 12 (6–21) and 12 (6–20), respectively. Based on the HADS scores, 180 (62%) of the participants experienced depression and 103 (35.5%) experienced anxiety. The distribution of participants experiencing depression and anxiety is shown in Table 5. We observed a statistically significant difference between the presence of mental illness and that of anxiety ($p = 0.030$). Newly developed symptoms of anxiety appeared in 85% of emergency physicians who reported having no known mental illness. We also found a statistically significant difference between the presence of elderly relatives in participants' homes and participants' anxiety levels ($p = 0.034$). While 66.9% of emergency physicians who did not live with elderly relatives did not experience anxiety, anxiety was present in half of those emergency physicians who lived with relatives over 60 years of age.

We observed that health professionals who reported being well informed about pandemics had reduced anxiety rates ($p < 0.001$). Similarly, we found that depression levels were lower in health care workers who received COVID-

19 training ($p = 0.037$) (Table 5). We found statistically significant differences between the presence of a standard COVID-19 protocol in emergency departments and the frequency of depression and anxiety in participants ($p = 0.046$ and 0.002, respectively). We detected depression in 73% of participants who stated that there were no standard COVID-19 protocols in their hospitals. Similarly, we found anxiety in 50.8% of those who stated that there were no such protocols in their hospitals. Additionally, we found no statistical difference in the distribution of anxiety and depression rates among participants in relation to the presence of a triage unit and a COVID-19 diagnostic checklist ($p > 0.05$).

4. Discussion

The COVID-19 pandemic affects the lives of health care professionals both physically and psychologically. According to data from the SARS outbreak in 2003, health care workers accounted for 21% of SARS infections worldwide [11]. Health care professionals' worries about infecting their family and friends or becoming infected with SARS led to stress and depression [12]. Our study explored the anxiety and depression levels and related characteristics of emergency physicians working in the front lines of the COVID-19 pandemic.

In this study, we administered questions to measure emergency physicians' levels of knowledge concerning COVID-19. Although only 58.6% of the emergency physicians stated that they had received COVID-19 training from their workplaces, almost all the participants (96.9%–100%) answered questions concerning COVID-19 correctly. A previous study found high awareness of COVID-19 among allied health care staff and doctors [13]. The high levels of knowledge concerning COVID-19 among emergency physicians working in Turkey could be explained by the fact that the disease was detected in China four months before it was detected in Turkey. Given that COVID-19 is a current issue increasing in importance every day, it has attracted high levels of interest from emergency physicians fighting the disease as well as created a steady stream of information from social media and scientific sources. As highlighted by Johnson and Hariharan, the most effective method of managing epidemics and pandemics is to raise awareness of the disease and provide education concerning the disease for both health care workers and the public [14]. Emergency physicians appear to possess sufficient awareness and knowledge of COVID-19 according to the survey responses.

The WHO defined the situation of excessive correct and incorrect information on social media pertaining to the COVID-19 pandemic as an ”infodemic” [15]. This situation has made it difficult for people to access reliable, important information. Although 96.6% of the study participants stated that they were following the Ministry of Health's National COVID-19 Infection Guideline, 53% reported they were sourcing information from academic publications, and 51% said they were sourcing information from social media. During the course of the worldwide spread of COVID-19, the day-to-day worsening of the pandemic has caused people to experience increased negative emotions and despair. In our study, only 41% of emergency physicians believed that this pandemic would be completely controlled worldwide.

According to the HADS results in the study, 62% of participants had depression and 33.5% experienced anxiety; these values were remarkably higher than those reported in the literature. Lv et al. reported the overall incidence of anxiety among Chinese health care workers to be 34.7% [16]. In a study by Li et al., participants used significantly more words pertaining to negative emotions and anxiety, indicators of serious psychological effects, after the COVID-19 pandemic in China was explained to them [17]. Zhu et al. conducted a study of 79 health care workers struggling with pneumonia related to COVID-19 and found that the frequencies of depression and anxiety among these participants were 46.5% and 11.4%, respectively [8].

In terms of gender, 63.1% of the study participants with depression were male. Regarding the relationship between gender and depression, our data did not match that of Zhu et al. in which 19.4% of those with symptoms of depression were men, and being male was determined to be a protective factor against depression [8]. However, the data we obtained in this study showed differences in the prevalence rates of depression and anxiety between gender groups.

In the study, we found no significant differences between cigarette consumption and depression and anxiety scores ($p > 0.05$). However, emergency physicians who reported increased cigarette consumption since the start of the COVID-19 pandemic had the highest median depression and anxiety scores, 12 (6–21) and 12 (6–20), respectively. As emphasized by previous studies, public health emergencies such as the SARS and COVID-19 pandemics can trigger an emotional stress response involving high anxiety and other negative emotions [17]. Participants in the present study who reported increased smoking over the course of the pandemic period had higher anxiety and depression scores than others; this association could have been due to a negative emotional stress response. The cutoff value for depression score in the HADS scale was determined to be 7 for a Turkish population, and the median depression score of the participants in the present study was 8 (10). However, our study suggests that being well informed can decrease levels of anxiety and depression in health care professionals. Similarly, Zhong et al. reported that negative attitudes and practices decreased when health care professionals' knowledge level of COVID-19 increased [7]. Although we compared anxiety and depression scores with many factors—gender, marital status, job title, amount of time spent working in emergency services, alcohol consumption, smoking habits, chronic illness, mental illness, living with an elderly relative, triage unit existence, and diagnostic checklist availability—we did not find them to be significantly associated with most of these factors.

Our study had several limitations. First, the data and

relevant analyses presented herein were derived from a cross-sectional study design. Thus, it was difficult to make causal inferences in it. Second, we used a web-based survey method to avoid bias, necessitating the inclusion of volunteer participants; therefore, the possibility of selection bias should be considered.

5. Conclusion

This study analyzed data on Turkish emergency physicians' levels of knowledge concerning the COVID-19 pandemic, their attitudes about this pandemic, newly developed depression and anxiety in these physicians, and other related factors. As a result, we determined that emergency physicians are quite knowledgeable and aware of this pandemic. We also determined that this pandemic, which has caused anxiety and fear worldwide, has had an especially significant effect on emergency physicians, causing negative emotions such as anxiety and depression. These results suggest that in addition to the education, support, and precautions needed for emergency physicians to work more comfortably, psychological support measures are necessary for the protection of their mental health.

ACKNOWLEDGMENTS

English redaction of this article was performed by Scribendi® Editing Service.

CONFLICTS OF INTEREST

All the authors contributed sufficiently in the work to take responsibility for appropriate portions of the content. The authors have no competing interests to declare.

FUNDING INFORMATION

This research did not receive any specific grant from funding agencies in the public, commercial, or not-for-profit sectors.

REFERENCES

[1] Dong E, Du H, Gardner L. An interactive web-based dashboard to track COVID-19 in real time. The Lancet infectious diseases. 2020. Available at: https://doi.org/10.1016/S1473-3099(20)30120-1. Accesed time: 15 April 2020.

[2] COVID-19 Dashboard by the Center for Systems Science and Engineering (CSSE) at Johns Hopkins University (JHU). Available at: https://coronavirus.jhu.edu/map.html. Accesed time: 15 April 2020.

[3] Dost B, Koksal E, Terzi Ö, Bilgin S, Ustun YB, Arslan HN. Attitudes of Anesthesiology Specialists and Residents Toward Patients Infected with the Novel Coronavirus (COVID-19): A National Survey Study. Surgical Infections. 2020. Published Online :6 Apr 2020 Available at: https://doi.org/10.1089/sur.2020.097.

[4] Republic of Turkey Ministry of Health Public Health Divison. COVID-19 (SARS-CoV-2 Infectıon) Guideline. 2020; 11. Updated at: 14 April 2020. Available at: https://covid19bilgi.saglik.gov.tr/depo/rehberler/COVID-19_Rehberi.pdf.

[5] World Health Organization. WHO Director-General's opening remarks at the media briefing on COVID-19 - 11 March 2020. Geneva, Switzerland. 2020. Available at: https://www.who.int/dg/speeches/detail/who-director-general-s-opening-remarks-at-the\-media-briefing-on-covid-19---11-march-2020.

[6] Lum LHW, Tambyah PA. Outbreak of COVID-19–an urgent need for good science to silence our fears?. Singapore Medical Journal 2020; 61:55. Available at: http://doi.org/10.11622/smedj.2020018.

[7] Zhong, BL, Luo W, Li HM, Zhang QQ, Liu XG, Li WT, Li Y. Knowledge, attitudes, and practices towards COVID-19 among Chinese residents during the rapid rise period of the COVID-19 outbreak: a quick online cross-sectional survey. Int J Boil Sci. 2020;16:1745.

[8] Zhu J, Sun L, Zhang L, Wang H, Fan A, Yang B, et al. Prevalence and Influencing Factors of Anxiety and Depression Symptoms in the First-Line Medical Staff Fighting Against the COVID-19 in Gansu. The Lancet. Published online Available at: https://dx.doi.org/10.2139/ssrn.3550054.

[9] Zigmond AS, Snaith RP. The hospital anxiety and depression scale. Acta psychiatr scand. 1983;67:361-370.

[10] Aydemir Ö, Güvenir T, Küey L, Kultur S. Hospital Anxiety and Depression Scale Turkish Form: validation and reliability study. Turk Psikiyatri Derg. 1997;8:280e7.

[11] Maunder R, Hunter J, Vincent L, et al. The immediate psychological and occupational impact of the 2003 SARS outbreak in a teaching hospital. CMAJ. 2003;168:1245-1251.

[12] Bai Y, Lin CC, Lin CY, Chen JY, Chue CM, Chou P. Survey of stress reactions among health care workers involvedwith the SARS outbreak. Psychiatr Serv. 2004;55:1055-1057.

[13] Giao H, Han NTN, Van Khanh T, Ngan VK, Van Tam V, Le An P. Knowledge and attitude toward COVID-19 among healthcare workers at District 2 Hospital, Ho Chi Minh City. Asian Pac J Trop Med. 2020;13.

[14] Johnson EJ, Hariharan S. Public health awareness: knowledge, attitude and behaviour of the general public on health risks during the H1N1 influenza pandemic. J Public Health. 2017;25:333-7.

[15] World Health Organization. Novel coronavirus (2019-nCov):situation report-13. Available on: https://www.who.int/docs/default-source/coronaviruse/situation-reports/20200202-sitrep-13-ncov-v3.pdf?sfvrsn=195f4010_6.

[16] Lv Y, Zhang Z, Zeng W, Li J, Wang X, Luo G. Anxiety and Depression Survey of Chinese Medical Staff Before and During COVID-19 Defense. The Lancet 2020. Published online. Available at: https://dx.doi.org/10.2139/ssrn.3551350.

[17] Li S, Wang Y, Xue J, Zhao N, Zhu T. The impact of COVID-19 epidemic declaration on psychological consequences: a study on active weibo users. Int J Res Public Health. 2020;17:2032.

How to cite this article: Fatih Çalişkan, Burhan DOST. The Evaluation of Knowledge, Attitudes, Depression and Anxiety Levels among Emergency Physicians during the COVID-19 Pandemic. Signa Vitae.2020;16(1):163-171. doi:10.22514/sv.2020.16.0022.
