# Supplementary material for: Mental health status and related factors influencing healthcare workers during the COVID-19 pandemic: A systematic review and meta-analysis
Source: PLoS One. 2024 Jan 19;19(1):e0289454. doi: 10.1371/journal.pone.0289454 (PMC10798549; doi:10.1371/journal.pone.0289454)
Supplement: S1 Data — (ZIP) [file pone.0289454.s011.zip › literatures/200.pdf]

Original Article
https://doi.org/10.9758/cpn.2022.20.1.97 pISSN 1738-1088 / eISSN 2093-4327
Clinical Psychopharmacology and Neuroscience 2022;20(1):97-108 Copyrightⓒ 2022, Korean College of Neuropsychopharmacology

Anxiety, Depression and Burnout Levels of Turkish Healthcare Workers at the End of the First Period of COVID-19 Pandemic in Turkey

Burak Uz[1,2], **Esra Savaşan**[3], **Dila Soğancı**[4]

[1]Department of Internal Medicine, Division of Adult Hematology, Medicana International Istanbul Hospital, [2]Department of Internal Medicine, Division of Adult Hematology, Medicana International Samsun Hospital, [3]Medicana International Samsun Hospital, [4]Clinical Psychology, Medicana International Samsun Hospital, Istanbul, Turkey

Objective: COVID-19 pandemic affected the mental health of healthcare workers (HCWs) as well as their physical health. In this study, we aimed to evaluate the anxiety, depression and burnout levels of Turkish HCWs after the first period of the pandemic.
Methods: The participants filled sociodemographic data form, Hospital Anxiety and Depression Scale (HADS) and Maslach Burnout Inventory (MBI).
Results: In this study, 221 HCWs (68.8% female) with a median age of 28 (20−66) years were included. Concerning HADS cut-off points, 39.8% of the participants scored above the depression cut-off point, while 26.2% scored above the cut-off point for anxiety. The anxiety (HADS-A) and depression (HADS-D) scores of nurses and medical secretaries were significantly higher than the physicians. Also, the anxiety and depression rates of nurses were higher than both physicians and medical secretaries. Emotional exhaustion (MBI-EE) and depersonalization (MBI-D) scores were highest in nurses, followed by medical secretaries and physicians, respectively. In multivariate analysis, being a nurse (OR: 4.671, p = 0.044) or medical secretary (OR: 4.013, p = 0.048), requirement of using a mental health support line (OR: 4.641, p = 0.005), having any kind of addiction (OR: 2.562, p = 0.019) and being under antidepressant therapy (OR: 3.096, p = 0.036) significantly increased the risk of anxiety. However, in multivariate analyses, the only requirement of using a mental health support line significantly increased the risk of depression (OR: 8.542, p = 0.001).
Conclusion: Female HCWs, nurses and medical secretaries experienced higher levels of mental health symptoms than male HCWs and physicians.

KEY WORDS: COVID-19; Healthcare workers; Anxiety; Depression; Burnout; Mental health support line.

INTRODUCTION

The worldwide pandemic caused by severe acute respiratory syndrome Coronavirus 2 (SARS-CoV-2) disease (COVID-19) continues to be a major public health concern. The first validated case was diagnosed on March 11, 2020, in Turkey. As of 05 November, 386,820 individuals have been diagnosed with COVID-19, of whom 2,564 individuals (0.66%) are being seriously ill, and 10,639 individuals (2.75%) have lost their lives [1].

Similar to previous pandemics [2], owing to the disease itself or related lockdowns and isolation procedures, COVID-19 pandemic also resulted in many psychosocial impacts on healthcare workers (HCWs) both in the short and long-term periods. Mental health problems faced by medical staff during this devastating period highly varied from more frequently encountered effects, such as anxiety, depressive symptoms, insomnia, burnouts, increased stress, to less frequently exposed ones, like substance abuse or suicidal behavior [3]. Fundamentally, all these mentioned psychological responses can be seen as an escape or a natural defense mechanism given to a crisis. Facing an emerging and fast-spreading disease with many

Received: November 6, 2020 / **Revised:** December 6, 2020
Accepted: February 16, 2021
Address for correspondence: Burak Uz
Department of Internal Medicine, Division of Adult Hematology, Medicana International Istanbul Hospital, Beylikduzu, Istanbul 34520, Turkey
E-mail: burakuz78@gmail.com
ORCID: https://orcid.org/0000-0001-9326-4301

This is an Open-Access article distributed under the terms of the Creative Commons Attribution Non-Commercial License (http://creativecommons.org/licenses/by-nc/4.0) which permits unrestricted non-commercial use, distribution, and reproduction in any medium, provided the original work is properly cited.

unknown points (concerning contagion, etiopathogenesis and complications), but without an effective treatment, increased the uncertainty and anxiety levels of HCWs. The lack of preventive vaccine, ongoing outbreak and disease-related deaths, and particularly coping with the loss of their colleagues against the battle to COVID-19 further aggravated distress levels of medical staff. Fear of self-contamination or concern of transmitting the virus to family members and close relatives, especially to elderly people with chronic disorders or those with immune deficiency, increased the risk of profound anxiety [4]. Intense work environment under intense stress, lack of adequate medical staff or personal protective equipment, as well as high expectations from HCWs could trigger emotional outbursts resulting in a feeling of worthlessness, burnouts and withdrawal among HCWs [5,6].

The proximity of the medical staff to COVID-19 positive patients will increase their contamination risk accordingly. Nurses, physicians and patient caregivers working at emergency wards, infection or respiratory diseases, or isolation units are particularly involved in SARS-CoV-2 infected patient care. We can define those individuals as "frontline HCWs" who are the most vulnerable to mental health problems due to pandemic [7]. Most of these frontline HCWs are being isolated and quarantined who are likely to perceive greater stigmatization than the other HCWs (who are not involved directly in COVID-19 patient care) and consistently more affected psychologically. High-risk perception of HCWs due to increased disease awareness, inadequate social support arising from isolation and stigmatization, accompanied by previous or current psychiatric illnesses or possible financial problems due to flexible working hours and disgraceful reports on doctor assaults after deaths of COVID-19 patients may trigger psychological events at any time during the combat against pandemic [3].

A limited number of studies elucidating the inevitable effects of the COVID-19 outbreak on the mental health status of HCWs has been reported. We hypothesized that anxiety, depression and burnout levels in Turkish HCWs might increase during the recent pandemic. We, therefore, aimed to evaluate anxiety, depression and burnout levels of Turkish HCWs after the first peak of the COVID-19 outbreak and investigate potential risk factors during this hugely challenging period.

METHODS

Study Characteristics

This was a cross-sectional study conducted at a tertiary referral hospital in Samsun, Turkey. The present study aimed to measure the levels of anxiety, depression and burnout levels among HCWs (physicians, nurses, and medical secretaries). The participants who gave informed consent were able to fill sociodemographic data form, the Hospital Anxiety and Depression Scale (HADS) and the Maslach Burnout Inventory (MBI). This study was conducted in accordance with the rules of the Declaration of Helsinki.

Since our study was a single-center study and a limited number of healthcare professionals was employed, we planned to include all physicians and nurses in our study. On the other hand, although medical secretaries were not anticipated in the frontline setting of combat against COVID-19, they were deemed appropriate to be included in this study, as they were large in number (n = 97) and produced valuable support for frontline HCWs in the background. Sixty-four out of 97 physicians (65.9%), 87 out of 150 nurses (58.0%), and 70 out of 97 medical secretaries (72.2%) were included in this study; missing/drop-out participants are shown by a diagram (Fig. 1). The participants who were willing to participate in our study and signed the informed consent form were included. The exclusion criteria included the HCWs who did not sign the informed consent form and HCWs with missed or abandoned study forms. Besides, the personnel other than physicians, nurses, or medical secretaries (administrative staff, cleaning staff, security guards, technical service personnel, or other operators) were also excluded from this study. This study was conducted between 16 June 2020 and 31 August 2020.

Sociodemographic data form

This form was designed by the investigators and included two main parts: (a) Sociodemographic data of the participants and (b) Questionnaire dedicated for COVID-19 pandemic.

Hospital Anxiety and Depression Scale (HADS)

The HADS was developed by Zigmond and Snaith [8], which is used to investigate anxiety and depression levels in a certain society and hospital samples. It consists of two

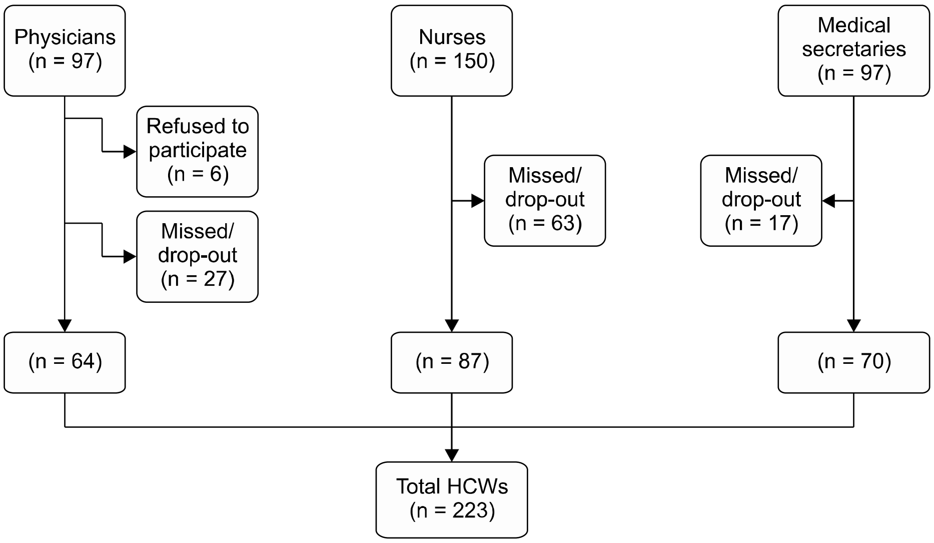

Fig. 1. Diagram showing the study population and recruitment process. HCWs, healthcare workers.

subscales: anxiety (HADS-A) and depression (HADS-D). There are 14 items in total, seven items in each scale. Each question is scored as 0−3 on the 4-point Likert scale, and higher scores indicate increased severity of anxiety and depression. The total score for each subscale ranges from 0 to 21. Reliability and validity of the Turkish version of the HADS were conducted by Aydemir *et al.* [9]. Using ROC analysis, cut-off scores for Turkish society have been determined as 10 for anxiety and 7 for depression subscale.

Maslach Burnout Inventory (MBI)

The MBI was developed by Maslach and Jackson [10], which is used to investigate occupational burnout levels of certain individuals or groups. It consists of 22 items in total and the MBI measures three dimensions of burnout: emotional exhaustion (MBI-EE, 9 items), depersonalization (MBI-D, 5 items) and personal accomplishment (MBI-PA, 8 items). Each question/proposition is scored between 0 and 4 on the 5-point Likert scale. Individuals with increased burnout levels are expected to have high MBI-EE and MBI-D scores but low MBI-PA scores. In contrast, lower scores of MBI-EE and MBI-D, but a high MBI-PA score indicates an individual with decreased levels of burnout. The adaptation of the MBI for Turkish society was performed by Ergin [11].

Maslach Burnout Inventory-Human Services Survey (MBI-HSS) is the original and most commonly used version of the MBI. MBI-HSS was developed to detect burnout levels of professionals working in the human services, including nurses, physicians, health aides or counselors [10,12]. In the present study, the MBI-HSS version was used to investigate burnout levels of HCWs.

Statistical Method

The Ministry of Health approval was obtained on June 12th, 2020 (form number: 2020-06-09T12_21_59). Institutional Academic and Ethical Committee approval was also obtained on June 18th, 2020, from Medicana International Samsun Hospital (no. 7106).

The data were analyzed using the SPSS version 25 (IBM Co., Armonk, NY, USA). Descriptive statistics of categorical variables were presented as number and percent. A chi-square test was used to compare categorical variables. The variables were investigated using the visual and analytical methods (Kolmogorov−Smirnov/Shapiro−Wilk's test) whether or not they were normally distributed. Descriptive statistics of numerical variables were presented as mean ± standard deviation for normally distributed or median (min−max) for non-normally distributed variables.

Independent Sample *t* test and one-way ANOVA analysis were used to compare groups for normally distributed variables. In the subgroup analysis of multiple groups, Tukey or Tamhane tests were used according to the homogeneity of the variances. Intergroup homogeneity of variance of normally distributed variables was evaluated with the Levene test. For non-normally distributed variables, Mann−Whitney *U* and Kruskal−Wallis tests were performed. Pairwise comparisons were used to compare multiple groups without normal distribution.

Table 1. Demographic data of the participants

Variables		Descriptive statistics
Age (yr)		28 (20−66)[a]
Gender	Woman	152 (68.8)
	Man	69 (31.2)
Residential area	City	177 (80.1)
	Town	42 (19)
	Village	2 (0.9)
Education	Secondary education	79 (35.7)
	Associate degree	52 (23.5)
	University	24 (10.9)
	Post-graduate	25 (11.3)
	PhD	41 (18.6)
Marital status	Single	80 (36.2)
	Married/have a partner	133 (60.2)
	Divorced	6 (2.7)
	Widow	2 (0.9)
Child	No	112 (50.7)
	Yes	109 (49.3)
Profession	Physician	64 (29.0)
	Nurse	87 (39.4)
	Medical Secretary	70 (31.7)
Total time of professional experience	< 5 years	72 (32.6)
	5−10 years	61 (27,6)
	11−20 years	36 (16.3)
	> 20 years	52 (23.5)
Number of shifts per month	None	142 (64.3)
	1−3	16 (7.2)
	4−6	9 (4.1)
	7−9	12 (5.4)
	≥ 10	42 (19)
Daily working time	≤ 8 hours	47 (21.3)
	9−11 hours	107 (48.4)
	≥ 12 hours	61 (27.6)
Previous or a current psychiatric disease	No	195 (88.2)
	Own	16 (7.2)
	Family	10 (4.5)
Usage of an antisepressant	No	30 (13.6)
	Yes	191 (86.4)
Addiction	None	137 (62.0)
	Cigarette	74 (33.5)
	Alcohol	7 (3.2)
	Substance	3 (1.4)
Beware of a mental health support line	Yes	95 (43)
	No	126 (57)
Requirement of using a mental health support line	Yes	23 (10.4)
	No	198 (89.6)
Suicidal behaviour	Suicidal idea	4 (1.8)
	Suicide attempt	1 (0.5)
	No	216 (97.7)

Values are presented as number (%).
PhD, doctor of philosopy.
[a]Numerical variables without normal distribution are presented as median (min−max).

The correlation coefficients for the numerical variables did not show a normal distribution; therefore, Spearman's rho coefficient was used. Univariate and multivariate logistic regression analyses were conducted to evaluate risk factors that could affect HADS. p values of < 0.05, < 0.01, and < 0.001 were considered as statistically significant.

RESULTS

Demographic Data

In this study, 221 HCWs (68.8% female) with a median age of 28 (20−66) years were included. Medical staff consisted of 64 (29.0%) doctors, 87 nurses (39.4%) and 70 medical secretaries (31.7%). The detailed demographic data of the participants are depicted in Tables 1, 2.

The average monthly household income of the study sample was as follows: over 8,000 Turkish Lira (TL) in 64 (29.0%) participants, 4,500−8,000 TL in 49 (22.2%) participants, and 2,000−4,500 TL in 108 (48.9%) participants.

Evaluation of HADS

Concerning HADS cut-off points [9], 39.8% (n = 88) of the participants scored above the depression cut-off point,

Table 2. Demographic data of the participants regarding COVID-19 pandemic

Demographic data		Descriptive statistics
Chronic disease	Yes	63 (28.5)
	No	158 (71.5)
Who do you live with?	Parents	46 (20.8)
	Spouse & child(s)	89 (40.3)
	Spouse	34 (15.4)
	Alone	30 (13.6)
	Other	22 (10.0)
Person aged > 60 at home	Yes	53 (24)
	No	168 (76)
Person with COVID-19 infection among friends and relatives	Yes	143 (64.7)
	No	78 (35.3)
Working status arrangement during the pandemic	No	90 (40.7)
	Flexible working	96 (43.4)
	Unpaid leave	16 (7.2)
	Other	14 (6.3)
Sufficiency of the measures about COVID-19 pandemic taken by the institution	Sufficient	66 (29.9)
	Partially sufficient	105 (47.5)
	Insufficient	50 (22.6)
Following current media	Yes	188 (85.1)
	Partially	26 (11.8)
	No	7 (3.2)

Values are presented as number (%).

while 26.2% (n = 58) scored above the cut-off point for anxiety. A statistically significant relationship was found between the profession of HCWs and whether they had depression or not ($p < 0.001$). Similarly, there was a significant relationship between the profession of HCWs and the presence of anxiety ($p < 0.001$). Both the rates of depression and anxiety were highest in nurses (51.1 and 40.2%), followed by medical secretaries (35.2 and 25.7%), and significantly lowest in physicians (13.6 and 7.8%). Nurses and medical secretaries had significantly higher median values of HADS-A and HADS-D scores than those values of physicians (Table 3). However, the median HADS-A and HADS-D scores were similar between nurses and medical secretaries ($p = 0.205$ and $p = 0.542$, respectively).

Evaluation of MBI

A comparison of MBI subscale scores concerning profession groups is given in Table 4. The mean MBI-EE score was highest in nurses (19.10 ± 9.17), followed by medical secretaries (14.37 ± 6.89) and lowest in physicians (10.81 ± 6.48). All p values were statistically significant between groups ($p_{\text{physician-nurse}} < 0.001$; $p_{\text{physician-medical secretary}} = 0.008$; $p_{\text{nurse-medical secretary}} = 0.001$). The mean MBI-D score was significantly higher in nurses than in medical secretaries ($p_{\text{nurse-medical secretary}} = 0.024$) and physicians ($p_{\text{physician-nurse}} = 0.004$), while the physicians and medical secretaries had similar MBI-D scores ($p_{\text{physician-medical secretary}} = 0.898$). However, MBI-PA scores were similar between study groups ($p = 0.238$).

Evaluation and Comparison of the Demographic Data Regarding HADS and MBI

HADS-A & HADS-D

HADS-A and HADS-D scores were significantly higher among women, individuals without a post-graduate or PhD education level, who were single and not having a child. Additionally, increased daily working time (at all levels), receiving antidepressant, the requirement of using a mental health support line and having suicidal behaviour were significantly associated with higher HADS-A and HADS-D scores.

HADS-A and HADS-D scores were significantly lower in those whose total professional experience was ≥ 20 years than those whose total professional experience was <

Table 3. Evaluation of HADS in terms of profession groups

Variables	Physician	Nurse	Medical secretary	p value
Depression subscale[a]	3 (0−12)	8 (0−21)	7 (0−12)	< 0.001[c]
Anxiety subscale[a]	3.5 (0−14)	9 (0−20)	8 (0−16)	< 0.001[c]
Depression[b]				
No	52 (39.1)	42 (31.6)	39 (29.3)	< 0.001[d]
Yes	12 (13.6)	45 (51.1)	31 (35.2)	
Anxiety[b]				
No	59 (36.2)	52 (31.9)	52 (31.9)	< 0.001[d]
Yes	5 (7.8)	35 (40.2)	18 (25.7)	

HADS, Hospital Anxiety and Depression Scale.
[a]Numerical variables without normal distribution are presented as median (min−max). [b]Categorical variables are presented as number (%, column percent). [c]Kruskal−Wallis test, [d]chi-square test, $p < 0.001$.

Table 4. Evaluation of MBI subscales in terms of profession groups

MBI subscales	Physician	Nurse	Medical secretary	p value[b]
Emotional exhaustion[a]	10.81 ± 6.48	19.10 ± 9.17	14.37 ± 6.89	< 0.001[c]
Desensitization[a]	3.80 ± 3.01	5.70 ± 4.20	4.13 ± 3.11	0.002[d]
Personal accomplishment[a]	23.61 ± 4.62	23.41 ± 4.30	22.40 ± 4.66	0.238

MBI, Maslach Burnout Inventory; EE, emotional exhaustion; D, desensitization.
[a]Numerical variables with normal distribution are expressed as mean ± standard deviation and compared by One-Way ANOVA test. [b]When the groups were evaluated according to MBI-EE, the mean score of at least one group was significantly different from others ($p < 0.001$). Similary, when the groups were evaluated according to MBI-D, the mean score of at least one group was significantly different from others ($p = 0.002$). [c]$p < 0.001$; [d]$p < 0.01$.

20 years ($p < 0.001$). HADS-D scores were significantly lower in those whose number of shifts per month was 0 or 1−3 than those whose number of shifts was ≥ 10 (5.6 ± 3.8, 5.4 ± 3.2, and 8.8 ± 4.6, respectively; $p < 0.001$). Similarly, HADS-A scores of individuals who did not have shifts were significantly low than those whose number of shifts per month was ≥ 10 (6.3 ± 4.2 vs. 10.8 ± 5.2; $p <$ 0.001).

Having a COVID-19 positive individual among friends or relatives, not to have any change in working status (as compared to flexible working) and finding the measures taken by the institution as partially sufficient or insufficient were significantly associated with higher scores of HADS-A and HADS-D. In addition, individuals living with only their spouse had significantly lower median HADS-A scores ($p = 0.020$).

MBI-EE

MBI-EE scores were significantly higher among women, individuals without a post-graduate or PhD educational status, who are single, without a child, and those with a higher daily working time schedules (at all levels). Additionally, the requirement of antidepressant treatment or a mental health support line and the presence of suicidal behaviour were also associated with increased MBI-EE scores. MBI-EE scores were significantly lower in those whose total professional experience was ≥ 20 years than those whose total professional experience was < 20 years ($p < 0.001$). Similarly, MBI-EE scores of individuals who did not have shifts were significantly low than those whose number of shifts per month was ≥ 10 (13.6 ± 7.7 vs. 19.7 ± 9.4; $p < 0.001$).

The presence of a patient with COVID-19 among friends or relatives, no change in working condition (as compared to flexible working), finding the measures taken against the pandemic partially sufficient or insufficient, and not following the current media were significantly associated with increased MBI-EE scores. In contrast, individuals living with only their spouse had significantly lower mean MBI-EE scores ($p = 0.014$).

MBI-D

MBI-D scores were significantly higher in participants with busier daily working time schedules (at every level) and participants required for a mental health support line. In addition, having an individual diagnosed with COVID-19 among friends/relatives and not having a change in working status (as compared to flexible working) were significantly associated with higher MBI-D scores. MBI-D scores of individuals who did not have shifts were significantly low than those whose number of shifts per month was ≥ 10 (4.1 ± 3.3 vs. 6.5 ± 4.5; $p = 0.002$). Other variables and the total time of professional experience did not affect MBI-D scores significantly.

MBI-PA

MBI-PA scores were identified as significantly higher among individuals with a child, without previous or current psychiatric illness, not receiving antidepressants, and without suicidal behaviour. In addition, finding the measures taken by the institution during the pandemic as sufficient and following current media significantly increased MBI-PA scores. Other factors, the total time of professional experience and number of shifts per month, did not affect MBI-PA scores significantly.

Logistic Regression Analyses

Risk factors affecting the participants' anxiety status were investigated using univariate and multivariate logistic regression analysis, and the results are presented in Table 5. According to the results of univariate logistic regression analysis, the independent risk factors affecting the anxiety status were gender, having children, profession, use of mental support line, adequacy of the measures taken by the institution, presence of the COVID-19 among friends or close environment, media follow-up and daily working hours. In multivariate analysis, being a nurse (odds ratio [OR]: 4.671, 95% confidence interval [95% CI]: 1.044−20.896, $p = 0.044$) or medical secretary (OR: 4.013, 95% CI: 1.001−16.086, $p = 0.048$) increased the risk of anxiety 4.671 and 4.013 folds, respectively, when compared to being a physician. Additionally, using a mental health support line (OR: 4.641, 95% CI: 1.604−13.427, $p = 0.005$), having any kind of addiction (OR: 2.562, 95% CI: 1.170−5.612, $p = 0.019$) and using antidepressant (OR: 3.096, 95% CI: 1.077−8.905, $p =$ 0.036) increased the risk of anxiety 4.641, 2.562, and 3.096 folds, respectively. Finally, a daily working time of ≥ 12 hours increased anxiety risk 3.544 folds compared to ≤ 8 hours (OR: 3.544, 95% CI: 0.886−14.184, $p =$ 0.074).

Risk factors affecting the participants' depression status

Table 5. Results of univariate and multivariate logistic regression analysis of factors affecting anxiety

Variables		Univariate logistic regression analysis		Multivariate logistic regression analysis	
		OR (95% CI)	p value	OR (95% CI)	p value
Gender	Man (reference)	1		1	
	Woman	2.36 (1.137−4.900)	0.021[c]	0.916 (0.327−2.567)	0.868
Child	Yes (reference)	1		1	
	No	1.83 (0.995−3.368)	0.052[d]	0.586 (0.242−1.419)	0.236
Profession	Physician (reference)	1		1	
	Nurse	7.942 (2.897−21.773)	< 0.001[a]	4.671 (1.044−20.896)	0.044[c]
	Medical secretary	4.085 (1.417−11.774)	0.009[b]	4.013 (1.001−16.086)	0.048[c]
Chronic disease	No (reference)	1			
	Yes	1.055 (0.545−2.041)	0.875		
Beware of a mental health support line	No (reference)	1			
	Yes	0.618 (0.331−1.151)	0.129		
Using a mental health support line	No (reference)	1		1	
	Yes	6.759 (2.688−16.996)	< 0.001[a]	4.641 (1.604−13.427)	0.005[b]
Antidepressant	No (reference)	1		1	
	Yes	2.101 (0.942−4.687)	0.070[d]	3.096 (1.077−8.905)	0.036[c]
Person aged above 60 at home	No (reference)	1			
	Yes	1.147 (0.575−2.290)	0.696		
Addiction	No (reference)	1		1	
	Yes (cigarette−alcohol-substance)	2.627 (1.423−4.852)	0.002[b]	2.562 (1.170−5.612)	0.019[c]
Previous or current psychiatric disease	No (reference)	1			
	Yes (own or family)	1.040 (0.413−2.620)	0.933		
Sufficieny of measures about COVID-19 taken by the institution	Sufficient (reference)	1		1	
	Partial	2.417 (1.061−5.503)	0.036[c]	0.539 (0.174−1.664)	0.282
	Insufficient	4.222 (1.712−10.410)	0.002[b]	0.949 (0.408−2.208)	0.904
Person with COVID-19 among friends or relatives	No (reference)	1		1	
	Yes	2.032 (1.031−4.005)	0.041[c]	1.052 (0.465−2.383)	0.903
Following media	Yes (reference)	1		1	
	Partial	7.5 (1.408−39.950)	0.018[c]	3.377 (0.393−29.011)	0.267
	No	0.9 (0.341−2.375)	0.831	1.366 (0.430−4.337)	0.597
Daily working time (hours/day)	≤ 8 (reference)	1		1	
	9−11	2.429 (0.865−6.821)	0.092	1.485 (0.465−4.749)	0.505
	≥ 12	6.671 (2.320−19.176)	< 0.001[a]	3.544 (0.886−14.184)	0.074[d]

OR, odds ratio; CI, confidence interval.
[a]$p < 0.001$; [b]$p < 0.01$; [c]$p < 0.05$; [d]$p < 0.10$.

were investigated using univariate and multivariate logistic regression analysis, and the results are shown in Table 6. According to the results of the univariate logistic regression analysis, the independent risk factors affecting the depression status were gender, having children, profession, the requirement of using a mental health support line, adequacy of the measures taken by the institution, presence of COVID-19 among friends or close relatives, presence of psychiatric disease and daily working hours. In multivariate analysis, only using a mental health support line increased depression risk 8.542 folds than non-users (OR: 8.542, 95% CI: 2.320−31.449, $p = 0.001$).

Reliability and Validity Analyses

Cronbach's alpha coefficient for the total HAD score, HADS-D and HADS-A subscales were 0.893, 0.776, and 0.868, respectively. On the other hand, Cronbach's alpha coefficient calculated for the total MBI, MBI-EE, MBI-D and MBI-PA subscales were 0.794, 0.905, 0.696, and 0.733, respectively. Consequently, the reliability of both scales and their subscales were considered adequate [13].

Concurrent validity analyses of the HADS and MBI subscales are depicted in Table 7. There was a positive and very good correlation between HADS-A and HADS-D (r^s: 0.720, $p < 0.001$).

Table 6. Results of univariate and multivariate logistic regression analysis of factors affecting depression

Variables		Univariate logistic regression analysis		Multivariate logistic regression analysis	
		OR (95% CI)	*p* value	OR (95% CI)	*p* value
Gender	Man (reference)	1		1	
	Woman	3.327 (1.729−6.402)	< 0.001[a]	2.030 (0.843−4.889)	0.114
Child	Yes (reference)	1		1	
	No	1.926 (1.115−3.326)	0.019[c]	0.754 (0.337−1.686)	0.492
Profession	Physician (reference)	1		1	
	Nurse	4.643 (2.181−9.883)	< 0.001[a]	2.079 (0.629−6.875)	0.230
	Medical secretary	3.444 (1.571−7.553)	0.002[b]	1.538 (0.515−4.594)	0.441
Chronic disease	No (reference)	1			
	Yes	0.823 (0.450−1.503)	0.526		
Beware of a mental health support line	No (reference)	1			
	Yes	0.868 (0.503−1.498)	0.612		
Using a mental health support line	No (reference)	1		1	
	Yes	12.75 (3.657−44.415)	< 0.001[a]	8.542 (2.320−31.449)	0.001[b]
Antidepressant	No (reference)	1			
	Yes	1.889 (0.871−4.099)	0.108		
Person aged above 60 at home	No (reference)	1			
	Yes	1.097 (0.585−2.055)	0.773		
Addiction	No (reference)	1			
	Yes (cigarette−alcohol-substance)	1.045 (0.600−1.819)	0.876		
Previous or current psychiatric disease	No (reference)	1		1	
	Yes (own or family)	2.279 (0.993−5.228)	0.052[d]	1.791 (0.623−5.155)	0.280
Sufficiency of measures about COVID-19 taken by the institution	Sufficient (reference)	1		1	
	Partial	2.62 (1.339−5.129)	0.005[b]	0.964 (0.365−2.544)	0.941
	Insufficient	2.087 (0.950−4.586)	0.067[d]	1.906 (0.848−4.282)	0.118
Person with COVID-19 among the friends or relatives	No (reference)	1		1	
	Yes	1.993 (1.107−3.586)	0.021[c]	1.302 (0.647−2.619)	0.459
Following media	Yes (reference)	1			
	Partial	2.197 (0.478−10.104)	0.312		
	No	1.648 (0.723−3.754)	0.234		
Daily working time (hours/day)	≤ 8 (reference)	1		1	
	9−11	2.122 (0.952−4.731)	0.066[d]	1.373 (0.553−3.411)	0.495
	≥ 12	4.361 (1.843−10.317)	0.001[b]	2.343 (0.734−7.481)	0.150

OR, odds ratio; CI, confidence interval.
[a]$p < 0.001$; [b]$p < 0.01$; [c]$p < 0.05$; [d]$p < 0.10$.

Table 7. Correlation analysis of HADS and MBI subscales

HADS and MBI subscales		Depression	Anxiety	Emotional exhaustion	Depersonalization	Personal accomplishment
Depression	r^s	1.000	0.720	0.589	0.408	−0.360
	p	-	< 0.001	< 0.001	< 0.001	< 0.001
Anxiety	r^s	0.720	1.000	0.685	0.463	−0.348
	p	< 0.001	-	< 0.001	< 0.001	< 0.001
Emotional exhaustion	r^s	0.589	0.685	1.000	0.632	−0.362
	p	< 0.001	< 0.001	-	< 0.001	< 0.001
Depersonalization	r^s	0.408	0.463	0.632	1.000	−0.405
	p	< 0.001	< 0.001	< 0.001	-	< 0.001
Personal accomplishment	r^s	−0.360	−0.348	−0.362	−0.405	-
	p	< 0.001	< 0.001	< 0.001	< 0.001	

HADS, Hospital Anxiety and Depression Scale; MBI, Maslach Burnout Inventory; r^s, Spearman's rho coefficient.
$p < 0.001$.

DISCUSSION

The present study was conducted following the first period of the pandemic in Turkey. Despite efforts taken for normalization, the overall anxiety and depression rates were considerably high (26.2% and 39.8%, respectively) among Turkish HCWs. In a Chinese study [14], the proportion of HCWs who experienced symptoms of anxiety and depression was much higher than our results (44.6% and 50.4%, respectively). Ayhan Başer *et al.* [15] reported that women, HCWs and people with psychiatric illness had significantly higher levels of anxiety. Mild, moderate and severe anxiety rates of HCWs were reported as 76.5%, 12.9%, and 11.5%, respectively. The increased rates of mental health symptoms among HCWs could be explained by the high-risk perception of medical staff compared to the general population [16], inevitable close contact with patients with COVID-19 and fear of contaminating their relatives. Uncertainty of the pandemic period also triggered anxiety and increased hopelessness levels of people during the first month of the pandemic in Turkey [17].

During the pandemic, the most affected profession group was nurses, followed by medical secretaries and physicians concerning mental health. Our findings were in accordance with the current literature [18]. In a cross-sectional study that was conducted in the first month of the pandemic in Turkey, the state anxiety levels of nurses were higher than both doctors and other HCWs [17]. Lai *et al.* [14] reported that women, nurses and front-line HCWs experienced more severe mental health symptoms of depression, anxiety, insomnia and distress than other HCWs. The susceptibility of nurses to mental health disorders could be explained by several factors: (a) The frequency of mental health disorders is higher in women among HCWs [18-20], and nurses are generally female. In our study, 88.5% of the nurses, 82.9% of the medical secretaries, and only 26.6% of the physicians were female. (b) Nurses were more directly participated in the treatment and patient care processes of high-risk patients compared to physicians [21]. (c) Hormonal differences might occur due to gender [22,23]. Although the physicians are anticipated to treat the COVID-19 patients at the frontline, the medical secretaries demonstrated worse mental health symptoms. This result could be explained by higher education status, awareness about COVID-19 and monthly income levels of the physicians. Similar to nurses, a great majority of the medical secretaries were female.

The anxiety levels of HCWs have increased with the increase in weekly working hours [17]. Our results are also compatible with this report. HADS-A, HADS-D, MBI-EE, and MBI-D scores were all higher in medical staff with a daily working time of $\geq$ 12 hours than those with $<$ 12 hours. In univariate regression analyses, it was shown that both anxiety and depression rates increased with the higher daily working time of $\geq$ 12 hours (6.67 and 4.36 folds, respectively) than those with $\leq$ 8 hours. A flexible working arrangement decreased anxiety, depression and burnout levels than a fixed working schedule.

Physician trainees who were exposed to patients with COVID-19 were more likely to be burned out [24]. In a study conducted at the peak period of the Italian COVID-19 pandemic, MBI-EE and gratification levels of Italian HCWs significantly increased. While Italy was one of the European countries most affected by the pandemic, higher gratification levels of Italian HCWs were considered a relative protective factor against increased levels of work-related distress and EE induced by the COVID-19 outbreak [25]. In Turkish adults, using a COVID-19 Burnout Scale, it was shown that the relationship between COVID-19 burnout and COVID-19 stress was partially mediated by resilience [26]. In the present study, women HCWs, nurses, unmarried individuals, HCWs working $\geq$ 12 hours per day had higher MBI-EE scores. On the other hand, staff whose total professional experience was $>$ 20 years had lower MBI-EE levels, which might be considered a "crystallized intelligence" with increasing experience, as demonstrated previously [27]. Yildirim *et al.* [28] also showed that young HCWs had higher health anxiety inventory scores than aged HCWs.

In the present study, the HCWs with higher educational levels had lower anxiety, depression and MBI-EE scores, which could suggest a positive effect of education on mental well-being. Our results were consistent with the current literature [20,29,30]. In contrast, university graduate nurses and anesthetic technicians had significantly higher anxiety scores, possibly due to increased awareness [30].

MBI-PA scores of the individuals without a current or previous psychiatric disorder were higher than those who had a psychiatric illness. Using antidepressants or having suicidal behaviour increased depression, anxiety and MBI-D subscores while decreased MBI-PA subscores.

Moreover, in multivariate regression analyses, we have shown that having an addiction or being under antidepressant treatment has increased the risk of anxiety. Similar to our results, the previous history of a psychiatric disorder has been reported as a risk factor for anxiety and health anxiety but not a risk factor for depression in a Turkish population sample [31]. Prevalence of anxiety, depression, insomnia and distress symptoms were significantly higher among Turkish HCWs with a psychiatric disorder and those HCWs receiving psychiatric support during the pandemic [32]. Suicidal behaviour, including suicidal ideation or suicide attempt, may further emerge in vulnerable populations during or after the peak period of the COVID-19 crisis. The elderly, medical staff, individuals with pre-existing or current psychiatric disorders, people without economic independence are at risk of suicidal behaviour. Stress-related mood and substance use disorders are also associated with suicidal behaviour [33,34]. In the present study, only five (2.3%) individuals stated suicidal behaviour with one (0.5%) suicide attempt, whereas 84 (38.0%) individuals had an addiction, mostly to cigarettes, followed by alcohol and substance.

Advanced age and accompanying chronic diseases are the most important predictors of mortality during the COVID-19 pandemic [35]. The individuals with chronic disease had increased levels of depression, anxiety and health anxiety levels [31]. State anxiety, trait anxiety and hopelessness levels were higher in individuals who live with an individual aged above 60 than those who did not [17]. Unexpectedly, in our study, anxiety, depression or burnout levels of HCWs did not alter significantly in terms of having a comorbid disease or living with an elderly individual at the same residence.

Our hospital was converted into a pandemic hospital towards the end of March 2020 by the Ministry of Health. To take the protective measures against COVID-19, an executive committee was set up by our institution. This team held regular meetings to solve the emerging challenges and collaborated with the local health authority. The participants who found the protective measures adequate (29.9%) had significantly low depression, anxiety and MBI-EE subscores, but significantly higher MBI-PA subscores than those who found these measures partially adequate or inadequate. Partly similar to our current results, the anxiety levels were high in people who stated that their close environment did not adhere to precautions taken for the pandemic, while people who stated that they adhered less to protective measures had low anxiety levels [15].

To adapt to the new conditions brought by COVID-19 and prevent acute stress disorders, a limited number of preventive mental health services has been offered to HCWs in Turkey. "Healthcare Professionals Mental Support Line," which offers 7/24 hours face-to-face live phone calls with volunteer psychiatrists, was opened on the 6th of April 2020 by the Psychiatric Association of Turkey. Unsurprisingly, the calls from HCWs considerably increased when the first physician loss occurred due to COVID-19, on the 1st of April 2020 [36]. A mobile application named "RUHSAD", which enables a remote support system by video-calls with experts, was also offered on 16th of April 2020 by the Republic of Turkey Ministry of Health [37]. In the present study, a considerable number of HCWs (43.0%) was aware of a mental health support line. However, only 10.4% of those required to use this line. Being aware of such a line did not relate with HADS-A, HADS-D or burnout scores, whereas the requirement of using such a line was associated with increased HADS-A, HADS-D, MBI-EE and MBI-D scores than those who did not. Moreover, in multivariate regression analysis, it was shown that the requirement of using a mental health support line was the only factor that increased the risk of depression among HCWs.

According to a web-based study conducted at the first period of the outbreak [15], the most common source of information about COVID-19 was internet/social media (99.7%), followed by TV (75.4%), inner circle (wife, relatives, neighbors, and friends; 24.4%), press (15.1%) and radio (10.3%). During the lockdown, the anxiety and distress levels increased due to a constant stream of information or excessive speculations on TV/social media platforms. In contrast, the prevalence of avoidance behaviour ranged from 4.3% to 4.9% during pandemics [31,38]. In the present study, seven (3.2%) participants did not follow media, which may indicate avoidance behaviour as a defense mechanism. These people who did not follow media had higher anxiety and MBI-EE scores but had lower MBI-PA scores than people who followed media at any level.

This study has several limitations. Firstly, due to its cross-sectional design, it is not possible to derive causal relationships from this research. Secondly, the study data were collected from a restricted population sample at a

single institution. The study sample has a selection bias because of its characteristics. Our sample is highly educated (Table 1), economically stable, and has good social support. Thus, the results should be interpreted with caution. Finally, there may be conflicting outcomes between our results and the existing literature concerning anxiety and depression rates, which could be related to using different scales and cut-off values. Besides these limitations, our study has some strengths. This study provided valuable aspects about the anxiety, depression and burnout levels of Turkish HCWs at the end of the first period of the pandemic. A relatively high number of individuals (n = 221), including three main groups (nurses, physicians, and medical secretaries), who can be in close contact during the management of patients with COVID-19, were evaluated. To our knowledge, medical secretaries have been evaluated for the first time as a separate group of the profession with our study. Additionally, no previous studies from Turkey have investigated burnout levels of HCWs during the pandemic, as well as anxiety and depression rates.

This study has drawn attention to high levels of anxiety, depression and burnout among Turkish HCWs after the first peak of the COVID-19 pandemic. By giving priority to high-risk groups (nurses, medical secretaries, HCWs who required telepsychiatric services, individuals with any kind of addiction and those under antidepressant treatment), it will be essential to provide psychiatric support to all HCWs in need. Switching to a flexible working schedule may decrease work-induce distress and therefore mitigate mental health symptoms induced by the COVID-19 pandemic. Local psychiatric teams can be established at an institutional base, which can determine risky staff periodically using simple and survey-based scales. HCWs can also be encouraged to use telepsychiatric services whenever they need them. The results of this study may further motivate mental healthcare professionals to provide timely interventions to prevent and reduce the long-term effects of the COVID-19 pandemic on the mental health status of HCWs.

■ Funding

None.

■ Acknowledgments

We would like to thank all of the individuals who have participated in this study, and especially Irem Tarhan, for her assistance with data collection.

■ Conflicts of Interest

No potential conflict of interest relevant to this article was reported.

■ Author Contributions

Conceptualization: Burak Uz, Esra Savaşan, Dila Soğancı. Methodology: Burak Uz, Dila Soğancı. Visualization: Burak Uz. Supervision: Burak Uz. Project administration: Burak Uz. Writing - original draft: Burak Uz. Writing - review & editing: Burak Uz.

■ ORCID

Burak Uz https://orcid.org/0000-0001-9326-4301
Esra Savaşan https://orcid.org/0000-0002-8882-2791
Dila Soğancı https://orcid.org/0000-0002-6814-4698

REFERENCES

1. Republic of Ministry of Health. *Table 19 patient Covidien-Turkey [Internet]. Ankara: Üniversiteler Mahallesi [cited at 2020 Nov 4]. Available from: https://covid19.saglik.gov.tr/.*
2. Robertson E, Hershenfield K, Grace SL, Stewart DE. *The psychosocial effects of being quarantined following exposure to SARS: a qualitative study of Toronto health care workers. Can J Psychiatry 2004;49:403-407.*
3. Dubey S, Biswas P, Ghosh R, Chatterjee S, Dubey MJ, Chatterjee S, *et al. Psychosocial impact of COVID-19. Diabetes Metab Syndr 2020;14:779-788.*
4. Chen Q, Liang M, Li Y, Guo J, Fei D, Wang L, *et al. Mental health care for medical staff in China during the COVID-19 outbreak. Lancet Psychiatry 2020;7:e15-e16.*
5. Wong TW, Yau JK, Chan CL, Kwong RS, Ho SM, Lau CC, *et al. The psychological impact of severe acute respiratory syndrome outbreak on healthcare workers in emergency departments and how they cope. Eur J Emerg Med 2005;12:13-18.*
6. Maunder R, Hunter J, Vincent L, Bennett J, Peladeau N, Leszcz M, *et al. The immediate psychological and occupational impact of the 2003 SARS outbreak in a teaching hospital. CMAJ 2003;168:1245-1251.*
7. Zhou Y, Ding H, Zhang Y, Zhang B, Guo Y, Cheung T, *et al. Prevalence of poor psychiatric status and sleep quality among frontline healthcare workers during and after the COVID-19 outbreak: a longitudinal study. Transl Psychiatry 2021;11:223.*
8. Zigmond AS, Snaith RP. *The hospital anxiety and depression scale. Acta Psychiatr Scand 1983;67:361-370.*
9. Aydemir O, Güvenir T, Küey L, Kültür S. *Validity and reliability of Turkish version of Hospital Anxiety and Depression Scale. Turk Psikiyatri Derg 1997;8:280-287.*

10. Maslach C, Jackson SE. *Maslach burnout inventory. 2nd ed. Palo Alto:Consulting Psychologists Press;1986. p.1-17.*
11. Ergin C. *[Maslach burnout inventory Turkey health personnel norms]. Psikiyatri Psikoloji Psikofarmakoloji (3P) Dergisi 1996;4:28-33. Turkish.*
12. Maslach C, Jackson SE, Leiter MP. *Maslach burnout inventory. 3rd ed. Palo Alto:Consulting Psychologists Press;1996. p.191-218.*
13. George D, Mallery P. *SPSS for Windows step by step: a simple guide and reference 11.0 update. 4th ed. Boston:Allyn & Bacon;2003.*
14. Lai J, Ma S, Wang Y, Cai Z, Hu J, Wei N, *et al. Factors associated with mental health outcomes among health care workers exposed to coronavirus disease 2019. JAMA Netw Open 2020;3:e203976.*
15. Ayhan Başer D, Çevik M, Gümüştakim Ş, Başara E. *Assessment of individuals' attitude, knowledge and anxiety towards COVID-19 at the first period of the outbreak in Turkey: a web-based cross-sectional survey. Int J Clin Pract 2020;74:e13622.*
16. Simione L, Gnagnarella C. *Differences between health workers and general population in risk perception, behaviors, and psychological distress related to COVID-19 spread in Italy. Front Psychol 2020;11:2166.*
17. Hacimusalar Y, Kahve AC, Yasar AB, Aydin MS. *Anxiety and hopelessness levels in COVID-19 pandemic: a comparative study of healthcare professionals and other community sample in Turkey. J Psychiatr Res 2020;129:181-188.*
18. Pappa S, Ntella V, Giannakas T, Giannakoulis VG, Papoutsi E, Katsaounou P. *Prevalence of depression, anxiety, and insomnia among healthcare workers during the COVID-19 pandemic: a systematic review and meta-analysis. Brain Behav Immun 2020;88:901-907.*
19. Kim MS, Kim T, Lee D, Yook JH, Hong YC, Lee SY, *et al. Mental disorders among workers in the healthcare industry: 2014 national health insurance data. Ann Occup Environ Med 2018;30:31.*
20. Wang Y, Di Y, Ye J, Wei W. *Study on the public psychological states and its related factors during the outbreak of coronavirus disease 2019 (COVID-19) in some regions of China. Psychol Health Med 2021;26:13-22.*
21. Saracoglu KT, Simsek T, Kahraman S, Bombaci E, Sezen Ö, Saracoglu A, *et al. The psychological impact of COVID-19 disease is more severe on intensive care unit healthcare providers: a cross-sectional study. Clin Psychopharmacol Neurosci 2020;18:607-615.*
22. Parker G, Brotchie H. *Gender differences in depression. Int Rev Psychiatry 2010;22:429-436.*
23. Li SH, Graham BM. *Why are women so vulnerable to anxiety, trauma-related and stress-related disorders? The potential role of sex hormones. Lancet Psychiatry 2017;4:73-82.*
24. Kannampallil TG, Goss CW, Evanoff BA, Strickland JR, McAlister RP, Duncan J. *Exposure to COVID-19 patients increases physician trainee stress and burnout. PLoS One 2020;15:e0237301.*
25. Barello S, Palamenghi L, Graffigna G. *Burnout and somatic symptoms among frontline healthcare professionals at the peak of the Italian COVID-19 pandemic. Psychiatry Res 2020;290:113129.*
26. Yıldırım M, Solmaz F. *COVID-19 burnout, COVID-19 stress and resilience: initial psychometric properties of COVID-19 Burnout Scale. Death Stud 2020. doi: 10.1080/07481187.2020.1818885. [Epub ahead of print]*
27. Cattell RB. *Theory of fluid and crystallized intelligence: a critical experiment. J Educ Psychol 1963;54:1-22.*
28. Yildirim TT, Atas O, Asafov A, Yildirim K, Balibey H. *Psychological status of healthcare workers during the COVID-19 pandemic. J Coll Physicians Surg Pak 2020;30:26-31.*
29. Zhong BL, Luo W, Li HM, Zhang QQ, Liu XG, Li WT, *et al. Knowledge, attitudes, and practices towards COVID-19 among Chinese residents during the rapid rise period of the COVID-19 outbreak: a quick online cross-sectional survey. Int J Biol Sci 2020;16:1745-1752.*
30. Koksal E, Dost B, Terzi Ö, Ustun YB, Özdin S, Bilgin S. *Evaluation of depression and anxiety levels and related factors among operating theater workers during the novel coronavirus (COVID-19) pandemic. J Perianesth Nurs 2020;35:472-477.*
31. Özdin S, Bayrak Özdin Ş. *Levels and predictors of anxiety, depression and health anxiety during COVID-19 pandemic in Turkish society: the importance of gender. Int J Soc Psychiatry 2020;66:504-511.*
32. Şahin MK, Aker S, Şahin G, Karabekiroğlu A. *Prevalence of depression, anxiety, distress and insomnia and related factors in healthcare workers during COVID-19 pandemic in Turkey. J Community Health 2020;45:1168-1177.*
33. Sher L. *The impact of the COVID-19 pandemic on suicide rates. QJM 2020;113:707-712.*
34. Conejero I, Berrouiguet S, Ducasse D, Leboyer M, Jardon V, Olié E, *et al. [Suicidal behavior in light of COVID-19 outbreak: clinical challenges and treatment perspectives]. Encephale 2020;46(3S):S66-S72. French.*
35. Zhou F, Yu T, Du R, Fan G, Liu Y, Liu Z, *et al. Clinical course and risk factors for mortality of adult inpatients with COVID-19 in Wuhan, China: a retrospective cohort study. Lancet 2020;395:1054-1062.*
36. Turkey Psychiatric Association. *COVID-19 and mental health [Internet]. Ankara: Turkey Psychiatric Association [cited at 2020 Oct 7]. Available from: https://www.psikiyatri.org.tr/menu/161/covid-19-ve-ruh-sagligi/.*
37. Mobile psychological support to healthcare professionals [Internet]. *İstanbul: TRT Haber; 2020 Apr 16 [cited at 2020 Oct 7]. Available from: https://www.trthaber.com/haber/saglik/saglik-personeline-mobil-psikolojik-destek-476610.html/.*
38. Rubin GJ, Amlôt R, Page L, Wessely S. *Public perceptions, anxiety, and behaviour change in relation to the swine flu outbreak: cross sectional telephone survey. BMJ 2009;339:b2651.*
