# Supplementary material for: Mental health status and related factors influencing healthcare workers during the COVID-19 pandemic: A systematic review and meta-analysis
Source: PLoS One. 2024 Jan 19;19(1):e0289454. doi: 10.1371/journal.pone.0289454 (PMC10798549; doi:10.1371/journal.pone.0289454)
Supplement: S1 Data — (ZIP) [file pone.0289454.s011.zip › literatures/201.pdf]

BRIEF RESEARCH COMMUNICATION

Mental Health Indices of Intensive Care Unit and Emergency Room Frontliners during the Severe Acute Respiratory Syndrome Coronavirus 2 Pandemic in India

Sonali Vadi[1], Sagar Shah[2], Sumiran Bajpe[3], Nimmi George[4], Arya Santhosh[5], Neha Sanwalka[6], Aparna Ramakrishnan[7]

Abstract

Background: The contagious coronavirus disease-2019 (COVID-19) pandemic has had a significant psychological impact on healthcare workers. Intensive care unit (ICU) and emergency room (ER) providers have functioned in an environment of fear for their health as well as their family well-being.

Aim and objective: The aim and objective of the article was to study mental health disorders (anxiety, depression, stress, post-traumatic stress disorder (PTSD), and sleep quality) in frontliners and to identify factors affecting mental health indices.

Materials and methods: A survey of 153 doctors and nurses working in ICU and ER was conducted from December 2020 to January 2021 using questionnaire with clinically validated scales: Acute Stress Disorder Scale, Perceived Stress Scale, Beck's Depression Inventory (BDI), Zung Self-rating Anxiety Scale (SAS), Global Pittsburgh Sleep Quality Index (PSQI), Multidimensional Scale of Perceived Social Support (MSPS), and New General Self-efficacy (NGSE) Scale. Analysis was performed to identify of gender, age, place of work, self-efficacy, and social support using logistic regression.

Results: Of the 153 participants, 21.6% had PTSD, 88.6% had moderate to high stress, 16.3% had anxiety, and 59.5% had poor sleep. Of these, the majority were females and those aged ≤30 years. Males had lesser depression, anxiety, and poor sleep compared to females ($p < 0.05$). Participants working in ER reported higher stress as compared to those working in ICU ($p < 0.05$). PTSD, perceived stress, depression, anxiety, and poor sleep were higher in participants with low-to-moderate perceived self-efficacy as compared to participants with high self-efficacy ($p < 0.05$).

Conclusion: This study reveals significant psychological distress among ER and ICU frontliners during severe acute respiratory syndrome *coronavirus 2* (SARS-CoV-2) pandemic, a typical high-acuity environment during the nonpandemic times.

Keywords: Emergency room, Frontline healthcare workers, Intensive care unit, Mental health survey, Severe acute respiratory syndrome *coronavirus 2* pandemic.

Indian Journal of Critical Care Medicine (2022): 10.5005/jp-journals-10071-24081

Introduction

Infectious outbreaks such as severe acute respiratory syndrome (SARS 2003), Ebola virus, Middle East respiratory syndrome, H1N1 influenza, and more recently coronavirus disease-2019 (COVID-19) have shown to negatively affect psychological health of frontline healthcare workers.[1,2] Critical care and emergency room (ER) have been the two workplaces where healthcare providers are closely exposed to pandemic patients. Disturbing aspect of a pandemic is that the effects of this stress will continue long after the exposure has ended. This can lead to acute stress disorder and post-traumatic stress disorder (PTSD).[3,4]

Given the prolonged pandemic, the aim of our study was to assess the prevalence of mental health disorders in frontline doctors and nurses working in ER and ICU in hospitals of the country using structured and clinically validated scales. We also aimed to identify the factors causing stress, PTSD, depression, anxiety, and sleep quality.

Materials and Methods

Study Design

A cross-sectional study was conducted from December 2020 to January 2021 in intensive care providers and ER frontline, high-risk group using purposive sampling. Respondents were invited to participate in this one-time self-administered online or paper questionnaire after an online or written informed consent, respectively. Research anonymity was assured.

[1,3–5]Department of Intensive Care Medicine, Kokilaben Dhirubhai Ambani Hospital and Medical Research Institute, Mumbai, Maharashtra, India

[2]Accident and Emergency Department, Kokilaben Dhirubhai Ambani Hospital and Medical Research Institute, Mumbai, Maharashtra, India

[6]Department of Nutrition and Biostatistics, NutriCanvas, Mumbai, Maharashtra, India

[7]Department of Psychiatry, Kokilaben Dhirubhai Ambani Hospital and Medical Research Institute, Mumbai, Maharashtra, India

Corresponding Author: Sonali Vadi, Department of Intensive Care Medicine, Kokilaben Dhirubhai Ambani Hospital and Medical Research Institute, Mumbai, Maharashtra, India, Phone: +91 9999999999, e-mail: sonalivadi@hotmail.com

How to cite this article: Vadi S, Shah S, Bajpe S, George N, Santhosh A, Sanwalka N, *et al.* Mental Health Indices of Intensive Care Unit and Emergency Room Frontliners during the Severe Acute Respiratory Syndrome Coronavirus 2 Pandemic in India. Indian J Crit Care Med 2022;26(1):100–107.

Source of support: Nil

Conflict of interest: None

© The Author(s). 2022 Open Access This article is distributed under the terms of the Creative Commons Attribution 4.0 International License (https://creativecommons.org/licenses/by-nc/4.0/), which permits unrestricted use, distribution, and non-commercial reproduction in any medium, provided you give appropriate credit to the original author(s) and the source, provide a link to the Creative Commons license, and indicate if changes were made. The Creative Commons Public Domain Dedication waiver (http://creativecommons.org/publicdomain/zero/1.0/) applies to the data made available in this article, unless otherwise stated.

Method of Data Collection

A close-ended questionnaire was used to collect data. Data were collected either with the help of physical forms or via Google forms of doctors and nurses from multiple institutions. Participants were also provided the option of sending their completed responses via email. All questionnaires were in English.

Demographic Information

Data regarding age, gender, and place of work were collected.

Research Instrument

Key mental health outcomes were measured using the validated acute stress disorder scale,[5] beck's depression inventory (BDI),[6] global Pittsburgh sleep quality index (PSQI),[7] Zung self-rating anxiety scale (SAS),[8] multidimensional scale of perceived social support (MSPSS),[9] perceived stress scale,[10] and new general self-efficacy (NGSE) Scale.[11]

Statistical Analysis

Data were presented mean ± SD or frequency (%). Chi-square test with cross-tabulations was used to compare differences in scales between genders, age groups, or places of work. Spearman's correlation was used to find a correlation between various scales. Multivariable odds logistic regression with proportional odds was run to determine the effect of gender, age, place of work, perceived self-efficacy (as measured by NGSE Scale), and perceived social support (as measured by MSPSS) on various mental health indices. Data of logistic regression are presented as B (parameter estimate (slope coefficient) represents the change in the log odds), adjusted odds ratio with 95% confidence interval (CI), and p value. $p < 0.05$ was statistically significant. Data were analyzed using SPSS for Windows (version 25, 2007, IBM Corporation, Armonk, New York, United States).

Results

Demographic Data

A total of 153 responses were obtained. Table 1 gives the demographic data of the study participants.

Table 1: Demographic data in study participants

Demographic data	*Frequency (%)*
**Age**	
21–25 years	47 (30.7%)
25–30 years	35 (22.9%)
31–35 years	45 (29.4%)
>40 years	26 (17%)
**Gender**	
Males	64 (41.8%)
Females	89 (58.2%)
**Place of work**	
ER	48 (31.4%)
ICU	105 (68.6%)

Acute Stress Disorder Scale

Minimum score obtained on Acute Stress Disorder Scale by a participant was 19, maximum was 84, and the mean total score was 40.6 ± 16.5. Overall, 33 (21.6%) respondents were at risk for PTSD (Fig. 1).

Perceived Stress Scale

The minimum score obtained on Perceived Stress Scale by a participant was 2, maximum was 39, and the mean total score was 19.7 ± 5.1. Overall, of the 153 participants, 18 (11.8%) reported low stress, 123 (80.4%) reported moderate stress, and 12 (7.8%) reported high stress (Fig. 2).

Beck's Depression Inventory

The minimum score obtained on BDI by a participant was 0, whereas maximum was 43. The mean total score obtained by the participants was 11.5 ± 9.6. Overall, 85 (55.6%) reported that the ups and downs are considered normal, 25 (16.3%) reported mild mood disturbance, 16 (10.5%) reported borderline clinical depression, 19 (12.4%) reported moderate depression, 6 (3.9%) reported severe depression, and 2 (1.3%) reported extreme depression. For further comparison, mild mood disturbance and borderline clinical depression were clubbed together whereas moderate to extreme depression were clubbed together. Significantly higher percentage of females had depression as compared to males ($p = 0.025$) (Fig. 3).

Zung Self-rating Anxiety Scale

The minimum score obtained on Zung SAS by a participant was 9, whereas maximum was 68. The mean total score obtained by the participants was 37.3 ± 8.6. Overall, of the 153 participants, 128 (83.7%) had normal levels, 24 (15.7%) had minimal to moderate anxiety, and 1 (0.7%) had marked anxiety. For further comparison, minimal to moderate anxiety and marked anxiety were combined together. Significantly higher percentage of females had anxiety as compared to males ($p = 0.001$). Significantly higher percentage of participants aged ≤30 years had more anxiety as compared to those aged ≥31 years ($p = 0.044$). Significantly higher percentage of participants who worked in ICU had anxiety as compared to those who worked in ER ($p = 0.022$) (Fig. 4).

Global Pittsburgh Sleep Quality Index

The minimum score obtained on Global PSQI by a participant was 0, whereas maximum was 18. The mean total Global PSQI score obtained by the participants was 7 ± 3.7. Of the 153 participants, 62 (40.5%) achieved a score of 5 or less indicating they were good sleepers, whereas 91 (59.5%) achieved a score of 6 or more indicating they were poor sleepers. Significantly higher percentage of females were poor sleepers as compared to males using Global PSQI ($p = 0.043$) (Fig. 5).

Multidimensional Scale of Perceived Social Support

The minimum score obtained on MSPSS by a participant was 1, whereas maximum was 7. The mean average score obtained by the participants was 5.7 ± 1.1. Overall, 2 (1.3%) reported low support, 40 (26.1%) reported moderate support, and 111 (72.5%) reported high support. For further comparison, low and moderate support were combined together. Lower percentage of participants aged ≥31 years reported high support as compared to participants aged ≤30 years ($p = 0.045$) (Fig. 6).

New General Self-efficacy Scale

The minimum score obtained on NGSE Scale by a participant was 1, whereas maximum was 5. The mean average NGSE score obtained

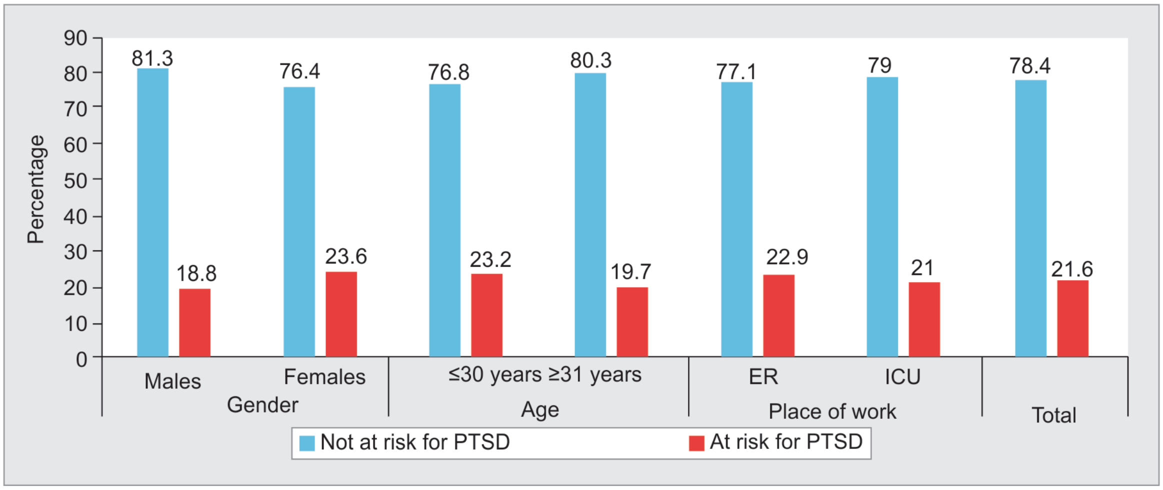

Fig. 1: Prevalence of post-traumatic stress disorder using acute stress disorder scale. Data presented as percentage. PTSD, post-traumatic stress disorder; ER, emergency room; ICU, intensive care unit

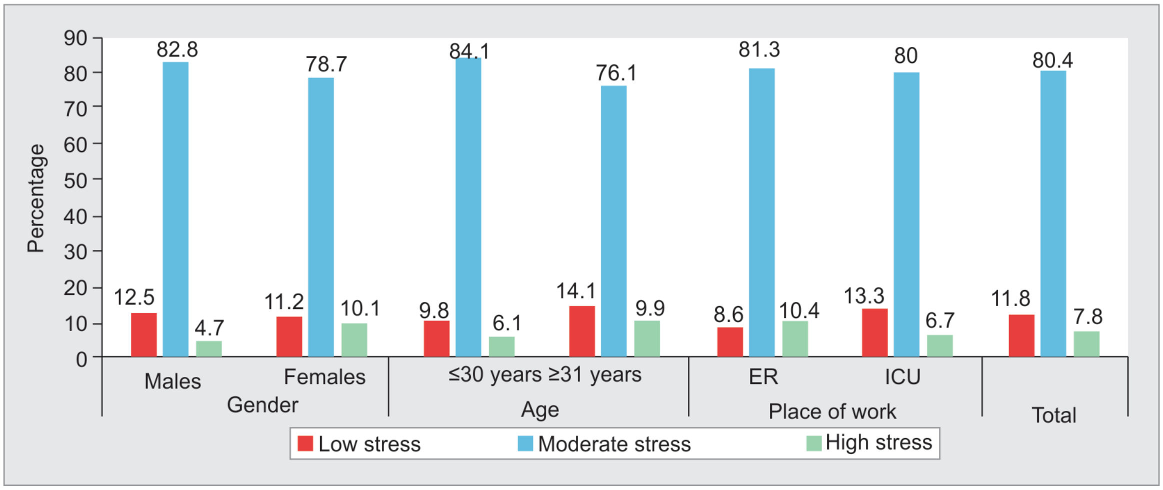

Fig. 2: Prevalence of stress using perceived stress scale. Data presented as percentage. ER, emergency room; ICU, intensive care unit

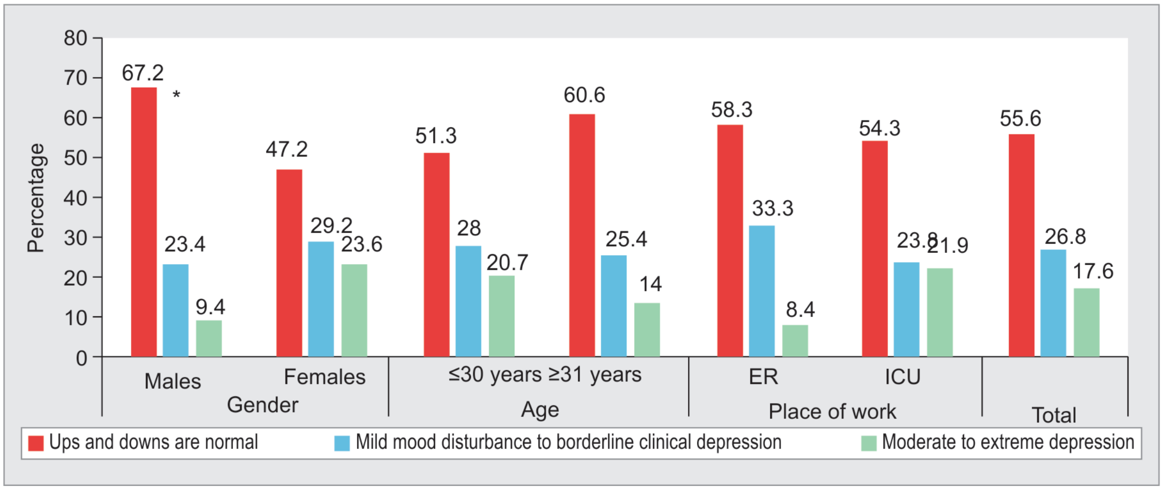

Fig. 3: Prevalence of depression using Beck's depression inventory. Data presented as percentage. ER, emergency room; ICU, intensive care unit. [*]p <0.05 for comparison between males and females

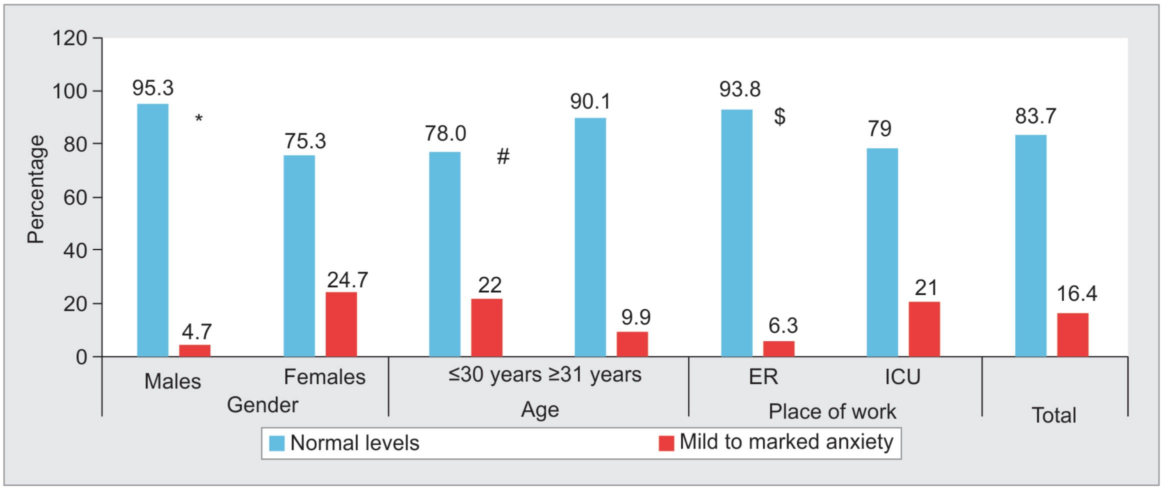

Fig. 4: Prevalence of anxiety using Zung SAS. Data presented as percentage. ER, emergency room; ICU, intensive care unit. $^{*}p < 0.05$ for comparison between genders, $^{\#}p < 0.05$ for comparison between age groups, and $^{\$}p < 0.05$ for comparison between places of work

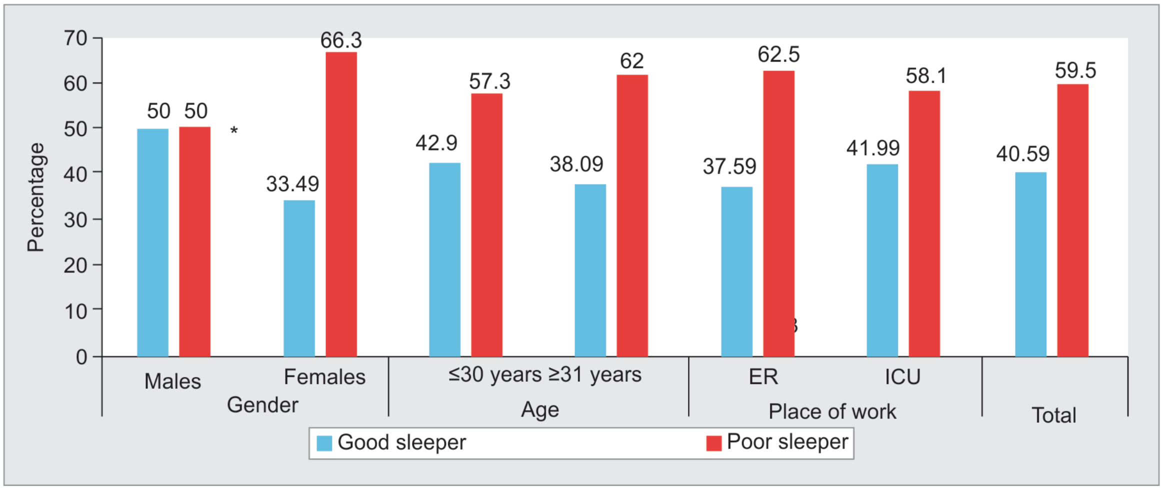

Fig. 5: Sleep quality using Global PSQI. Data presented as percentage. ER, emergency room; ICU, intensive care unit. $^{*}p < 0.05$ for comparison between genders

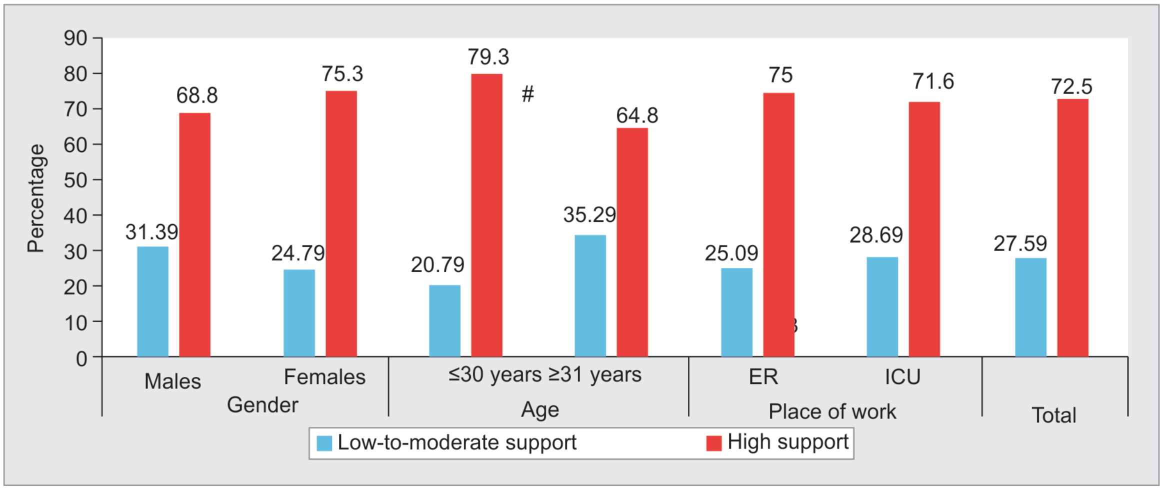

Fig. 6: Perceived social support using MSPSS. Data presented as percentage. ER, emergency room; ICU, intensive care unit. $^{\#}p < 0.05$ for comparison between age groups

by the participants was 3.7 ± 0.8. Overall, of the 153 participants, 82 (53.6%) reported low to moderate self-efficacy, whereas 71 (46.4%) reported good efficacy (Fig. 7).

Correlation between Various Scales

Table 2 gives correlations between various scales used in the study. NGSE Scale and MSPSS were significantly positively correlated with each other (p <0.001). NGSE Scale and MSPSS were significantly negatively correlated with BDI Scale, Zung SAS, and Perceived Stress Scale (p <0.001). NGSE Scale was also significantly negatively correlated with Global PSQI (p <0.001). Acute Stress Disorder Scale, BDI Scale, Zung SAS, Perceived Stress Scale, and Global PSQI were positively correlated with each other (p <0.001).

Multivariable odds logistic regression with proportional odds was run to determine the effect of gender, age, place of work, perceived self-efficacy (as measured by NGSE Scale), and perceived social support (as measured by MSPSS) on various mental health indices (Table 3). The odds of males having depression, anxiety, and poor sleep were lesser as compared to those of females (p <0.005). The odds of having poor sleep were lesser in the participants aged ≤30 years as compared to those in the participants aged ≥31 years (p <0.05). The odds of participants working in ER having stress were higher as compared to those of participants working in ICU (p <0.05). The odds of having PTSD, perceived stress, depression, anxiety, and poor sleep were higher in the participants with low to moderate perceived self-efficacy as compared to those in the participants with high self-efficacy (p <0.05).

Discussion

Interpretation of the Scales

Acute Stress Disorder Scale indexes acute stress disorder and predicts PTSD. BDI is an indicator of change in the level or degree of depression. *Zung SAS* screens anxiety disorders. *NGSE Scale* assesses an individual reaction to new or challenging tasks in various aspects of life. Self-efficacy is an individual's personal judgment of his/her capabilities in successfully carrying out a task.[12] *MSPSS* assesses three different sources of support—family, friends, and "significant other." *Perceived Stress Scale* aims to understand how different situations influence our mindsets and perceived stress. *Global PSQI* screens for sleep dysfunction.

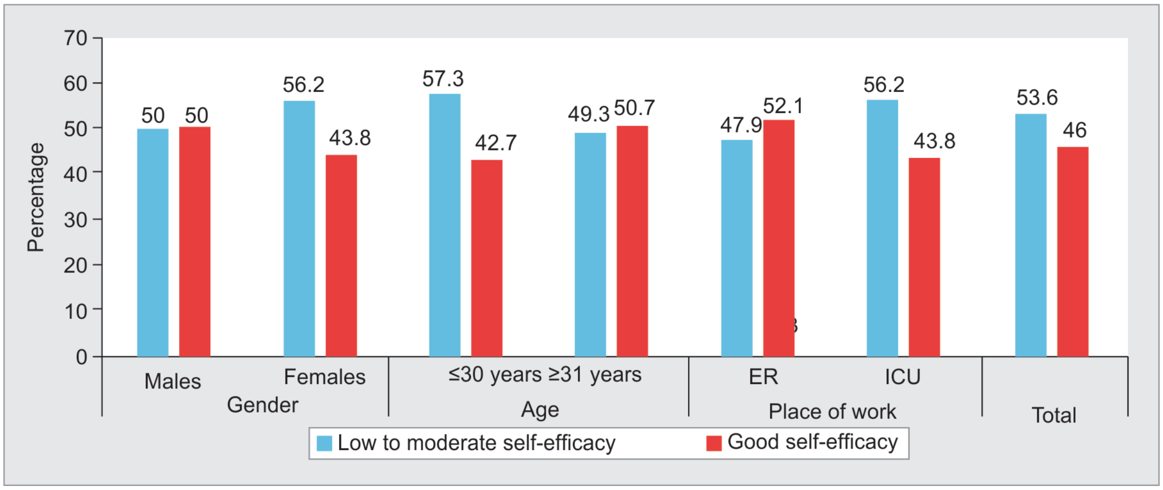

Fig. 7: Self-efficacy as reported by the participants using NGSE scale. Data presented as percentage. ER, emergency room; ICU, intensive care unit

Table 2: Correlation of various scales with each other

	Acute Stress Disorder Scale	*Beck's Depression Inventory*	*Zung Self-rating Anxiety Scale*	*New General Self-efficacy Scale*	*Multidimensional Scale of Perceived Social Support*	*Perceived Stress Scale*	*Global Pittsburgh Sleep Quality Index*
Acute Stress Disorder Scale	—	0.480*	0.460*	−0.127	−0.069	0.380*	0.386*
Beck's Depression Inventory	0.480*	—	0.678*	−0.396*	−0.230*	0.655*	0.580*
Zung Self-rating Anxiety Scale	0.460*	0.678*	—	−0.322*	−0.214*	0.486*	0.439*
New General Self-efficacy Scale	−0.127	−0.396*	−0.322*	—	0.391*	−0.354*	−0.215*
Multidimensional Scale of Perceived Social Support	−0.069	−0.230*	−0.214*	0.391*	—	−0.263*	−0.084
Perceived Stress Scale	0.380*	0.655*	0.486*	−0.354*	−0.263*	—	0.520*
Global Pittsburgh Sleep Quality Index	0.386*	0.580*	0.439*	−0.215*	−0.084	0.520*	—

Data presented as Spearman's Rho. *p <0.001 for statistical significance

Table 3: Risk factors for mental health indices identified by multivariable logistic regression

	Gender				*Age*				*Place of work*				*New General Self-efficacy Scale*				*Multidimensional Scale of Perceived Social Support*			
		Adjusted odds (95% CI)				*Adjusted odds (95% CI)*				*Adjusted odds (95% CI)*				*Adjusted odds (95% CI)*				*Adjusted odds (95% CI)*		
Mental health indices	*B*	*Males*	*Females*	*p value*	*B*	*≤30 years*	*≥31 years*	*p value*	*B*	*ER*	*ICU*	*p value*	*B*	*Low-to-moderate efficacy*	*High efficacy*	*p value*	*B*	*Low-to-moderate support*	*High support*	*p value*
Acute Stress Disorder Scale	−0.358	0.699 (0.261–1.874)	1 [ref]	0.477	−0.020	0.981 (0.393–0.248)	1 [ref]	0.967	0.319	1.376 (0.556–3.405)	1 [ref]	0.490	0.884	2.420 (1.022–5.730)	1 [ref]	*0.044*	−0.038	0.962 (0.392–2.365)	1 [ref]	0.933
Perceived Stress Scale	−0.822	0.439 (0.163–1.185)		0.104	−0.264	0.768 (0.303–1.945)		0.578	0.963	2.619 (1.007–6.810)		*0.048*	1.190	3.278 (1.279–8.444)		*0.013*	0.856	2.354 (0.854–6.485)		0.098
Beck's Depression Inventory	−0.929	0.395 (0.173–0.901)		*0.027*	−0.098	0.907 (0.424–1.938)		0.801	−0.018	0.982 (0.458–2.104)		0.962	1.502	4.489 (2.198–9.166)		*0.001*	0.185	1.203 (0.575–2.516)		0.623
Zung Self-rating Anxiety Scale	−1.547	0.213 (0.050–0.910)		*0.037*	0.218	1.244 (0.405–3.824)		0.703	−0.853	0.426 (0.112–1.614)		0.209	1.072	2.922 (1.010–8.454)		*0.048*	0.167	1.181 (0.414–3.372)		0.755
Global Pittsburgh Sleep Quality Index	−1.403	0.246 (0.098–0.148)		*0.003*	−1.047	0.351 (0.148–0.832)		*0.017*	0.825	2.281 (0.971–5.326)		0.059	1.285	3.616 (1.697–7.705)		*0.001*	−0.470	0.625 (0.272–1.438)		0.269

ER, emergency room; ICU, intensive care unit; B, parameter estimate (slope coefficient) represents the change in the log odds; adjusted odds, adjusted odds ratio; CI, confidence interval

Our Findings

Pandemic healthcare providers have dealt with a high level of responsibility as well as exposure to unusual patient outcomes. Post-traumatic symptoms include stress, depression, anxiety, and sleep disorders. Following exposure to a fatal contagion, our findings reveal that female gender, age <30 years, less work experience, insufficient sleep, and dearth of social support were positively associated with greater stress levels. Thirty-three (21.6%) respondents were at risk for PTSD. Of these, 23.6% were females, with a majority of these (23.2%) being of age <30 years. Twenty-one percent of those at risk were ICU care providers. About 59.5% of the respondents reported poor sleep.

Perceived social support has a definite connection with psychological well-being. Higher percentage of females as well as those aged <30 years reported high social support. It seems that younger adults are likely to divulge their problems to family, friends, and "significant other." Perceived social support reduces the occurrence of any psychopathology by acting as a safeguard between professional stressors and its unfavorable effects on the healthcare providers. In the current study, a significant negative correlation of MSPSS was observed with depression, anxiety, and stress. Also, when analyzing correlations at a cross-sectional level, results confirm a positive and significant correlation between perceived social support and self-efficacy. In turn, a good perception of self-efficacy has helped frontliners deal with adverse events such as stress, anxiety, and depression effectively. Male gender and self-efficacy were a better predictor of toughness toward psychological issues relative to perceived social support as the odds of developing various mental health disorders were lower in males and participants with higher self-efficacy.

Multiple facets of the pandemic as reported by the participants included—*contagion related*: rapid transmission, uncertain incubation period, and clinical manifestations; *family related*: self and family well-being; *workplace related*: seeing infection and deterioration of colleagues; witnessing high mortality rate related to the illness as well as in resource-limited settings, consistently reported stressor; providing care to patients in a unique setting where family visitation has been a challenge; lack of getting rest, restless sleep, somatic issues such as headache, irritability, overwhelming work-related pressure; *moral related*: demoralization; feeling of guilt following an inability to help; and *social related*: want of social support.

Implications of Our Study

Age, gender, personality trait, and level of social support impact an individual's coping ability.

Qualitative research recognizing the lived experiences of high-risk frontliners helps improve backing available at workplace. Psychological and practical support from supervisors and colleagues can have a protective effect against stress. Positive attitudes in workplace impact stress management. Increased self-efficacy improves sleep quality. Active psychosocial interventions, supportive administration, and planned strategies to deal with the psychological ramifications of the epidemic/pandemic lead to better stress management among the frontline workers. Thereby, it is imperative to improve self-efficacy and motivate even the family and friend circle of frontline workers to be supportive of the frontliners to reduce the mental health burden in this group.

Limitations of the Study

This survey was conducted later into the pandemic. Findings during the peak of the pandemic may have revealed a greater psychological distress among the frontline providers. We did not have any baseline pre-pandemic mental health status measurement. Timeline of onset of symptoms from the time of exposure may have helped diagnose those at-risk for PTSD. However, the objective of our study was to examine the presence of distressing symptoms in the frontliners of high-risk groups. Cross-sectional nature of the survey cannot analyze causal inferences. The employed self-reported questionnaire is at risk of responder bias.

Strengths of the Study

This study included respondents from various hospitals. We evaluated the effects of working in a stressful situation in real time to minimize recall bias. Psychological questionnaires employed in this survey have been used worldwide making our data comparable with results from other countries.

Conclusion

Managing mental health and psychosocial well-being is as important as managing physical health. Mental health of the frontline providers during this pandemic calls for screening of vulnerable individuals in high-risk groups, and the provision of timely psychological assistance to those in need.

Acknowledgments

The authors would like to thank all the frontline doctors and nurses who despite their busy schedules took time out to fill this survey.

Orcid

Sonali Vadi https://orcid.org/0000-0002-7341-2407
Sagar Shah https://orcid.org/0000-0003-4861-6755
Sumiran Bajpe https://orcid.org/0000-0001-6315-8436
Nimmi George https://orcid.org/0000-0002-5790-9632
Arya Santhosh https://orcid.org/0000-0001-9861-2520
Neha Sanwalka https://orcid.org/0000-0003-3428-3144
Aparna Ramakrishnan https://orcid.org/0000-0003-4752-3936

References

1. Fiest KM, Parsons Leigh J, Krewulak KD, Plotnikoff KM, Kemp LG, Ng-Kamstra J, et al. Experiences and management of physician psychological symptoms during infectious disease outbreaks: a rapid review. BMC Psychiatry 2021;21(1):91–104. DOI: 10.1186/s12888-021-03090-9.
2. Nikiforov SA, Allenov AM, Arestova AA, Lvova DP, Alehin SG. [The mental health and chronic infectious diseases]. Probl Sotsialnoi Gig Zdravookhranenniiai Istor Med 2020;28(6):1252–1258 [Russian]. DOI: 10.32687/0869-866X-2020-28-6-1252-1258.
3. Lee AM, Wong JG, McAlonan GM, Cheung V, Cheung C, Sham PC, et al. Stress and psychological distress among SARS survivors 1 year after the outbreak. Can J Psychiatry 2007;52(4):233–240. DOI: 10.1177/070674370705200405.
4. McAlonan GM, Lee AM, Cheung V, Cheung C, Tsang KW, Sham PC, et al. Immediate and sustained psychological impact of an

emerging infectious disease outbreak on health care workers. Can J Psychiatry 2007;52(4):241–247. DOI: 10.1177/070674370705200406.
5. Bryant RA, Moulds ML, Guthrie RM. Acute Stress Disorder Scale: a self-report measure of acute stress disorder. Psychol Assess 2000;12(1):61–68. PMID: 10752364.
6. Beck AT, Ward CH, Mendelson M, Mock J, Erbaugh J. An inventory for measuring depression. *Arch Gen Psychiatry 1961;4:*561–571. DOI: 10.1001/archpsyc.1961.01710120031004.
7. Buysse DJ, Reynolds CF 3rd, Monk TH, Berman SR, Kupfer DJ. The Pittsburgh Sleep Quality Index: a new instrument for psychiatric practice and research. Psychiatry Res 1989;28(2):193–213. DOI: 10.1016/0165-1781(89)90047-4.
8. Zung WWK. A rating instrument for anxiety disorders. Psychosomatics 1971;12(6):371–379. DOI: 10.1016/S0033-3182(71)71479-0.
9. Zimet GD, Dahlem NW, Zimet SG, Farley GK. The multidimensional scale of perceived social support. J Pers Assess 1988;52(1):30–41. DOI: 10.1207/s15327752jpa5201_2.
10. Cohen S, Kamarck T, Mermelstein R. A global measure of perceived stress. J Health Soc Behav 1983;24(4):386–396. DOI: 10.2307/2136404.
11. Chen G, Gully S, Eden D. Validation of a new general self-efficacy scale. Org Res Methods 2001;4(1):62–83. DOI: 10.1177/109442810141004.
12. Bandura A. Self-efficacy: the exercise of control. New York: Freeman; 1997.
