# Supplementary material for: Mental health status and related factors influencing healthcare workers during the COVID-19 pandemic: A systematic review and meta-analysis
Source: PLoS One. 2024 Jan 19;19(1):e0289454. doi: 10.1371/journal.pone.0289454 (PMC10798549; doi:10.1371/journal.pone.0289454)
Supplement: S1 Data — (ZIP) [file pone.0289454.s011.zip › literatures/202.pdf]

medicina

MDPI

Article

Mental Health and Associated Demographic and Occupational Factors among Health Care Workers during the COVID-19 Pandemic in Latvia

Laura Valaine [1,*], Gunta Ancāne [1], Artūrs Utināns [1] and Ģirts Briģis [2]

[1] Department of Psychosomatic medicine and Psychotherapy, Riga Stradiņš University, LV-1007 Riga, Latvia; gunta.ancane@rsu.lv (G.A.); arturs.utinans@rsu.lv (A.U.)
[2] Department of Public Health and Epidemiology, Riga Stradiņš University, LV-1007 Riga, Latvia; girts.brigis@rsu.lv
* Correspondence: laura.valaine@rsu.lv; Tel.: +371-28376456

Abstract: *Background and Objectives:* The COVID-19 pandemic has negatively impacted the health care system. Front-line health care workers (HCWs) are at a higher risk of mental health adverse outcomes. The aim of this study was to evaluate the frequency of the symptoms of depression and anxiety and associated demographic and occupational factors among front-line HCWs in Latvia. *Materials and Methods:* A cross-sectional quantitative study was performed in a population of HCWs during the first wave of the COVID-19 pandemic in Latvia. The participants were interviewed between 28 April 2020 and 2 June 2020. Answers from 864 HCWs were obtained. The participants reported their demographics, work-related information, contacts with COVID-19-positive patients and completed two standardised questionnaires that assessed the symptoms of anxiety (GAD-7) and depression (PHQ-9). The gathered data were analysed by a chi-squared test and binary logistic regression. The data analysis was performed using SPSS v25. *Results:* A total of 209 (24.8%) participants had depression symptoms and 145 (17.2%) had anxiety symptoms. Health care workers older than 50 had a lower risk of both depression (OR 0.422 (95% CI, 0.262–0.680)) and anxiety (OR 0.468 (95% CI, 0.270–0.813)). General practitioners had more frequent symptoms of depression and anxiety than participants who worked at hospitals (32.8% (n = 63) versus 19.4% (n = 73) and 27.1% (n = 52) versus 10.3% (n = 39), respectively (p = 0.037; p < 0.000)). Working more than 48 h during the week was associated with a higher risk of depression (OR 2.222 (95% CI, 1.315–3.753)) and anxiety (OR 2.416 (95% CI, 1.272–4.586)). *Conclusions:* The vulnerability of the health care system before the COVID-19 pandemic led to significant mental health adverse outcomes of HCWs during the COVID-19 pandemic in Latvia. A further cohort study is needed to evaluate the dynamics of mental health and other predisposing factors of HCWs.

Keywords: mental health; depression; anxiety; health care workers; COVID-19

check for updates

Citation: Valaine, L.; Ancāne, G.; Utināns, A.; Briģis, Ģ. Mental Health and Associated Demographic and Occupational Factors among Health Care Workers during the COVID-19 Pandemic in Latvia. *Medicina* **2021**, *57*, 1381. https://doi.org/10.3390/medicina57121381

Academic Editor: Jimmy T. Efird

Received: 9 November 2021
Accepted: 16 December 2021
Published: 18 December 2021

Publisher's Note: MDPI stays neutral with regard to jurisdictional claims in published maps and institutional affiliations.

Copyright: © 2021 by the authors. Licensee MDPI, Basel, Switzerland. This article is an open access article distributed under the terms and conditions of the Creative Commons Attribution (CC BY) license (https://creativecommons.org/licenses/by/4.0/).

1. Introduction

The World Health Organisation (WHO) on 11 March 2020 declared the novel coronavirus (COVID-19) outbreak a global pandemic [1]. An emergency situation due to the first wave of the COVID-19 infection in Latvia was declared on 12 March 2020 and it lasted until 10 June 2020 [2]. During the first COVID-19 wave, Latvia was called a success story in controlling COVID-19 infection among European Union countries because the number of COVID-19 patients was relatively small and the infection was well-controlled [3]. As of 15 June 2020, 1111 confirmed cases of COVID-19 and 30 deaths had been reported in Latvia [4].

The health care system in Latvia before the COVID-19 pandemic lagged behind the European average level regarding the health workforce. A shortage of doctors and especially nursing staff as well as underpayment, difficult working conditions and comparatively

low financing of the health system were just a few of the problems taking place for a long time [5]. It led to an increased burnout risk of health care workers (HCWs) even before the COVID-19 pandemic.

The importance of the mental health status of HCWs raises questions about how much it affects the health care system [6]. Poor mental health of HCWs is associated with a higher frequency of committed medical errors, which can lead to even worse outcomes in the mental health of HCWs [7].

The first studies stated that the mental health of HCWs was affected during the COVID-19 pandemic. Front-line HCWs were associated with a higher prevalence of depression and anxiety [8]. HCWs in low- and middle-income countries experienced considerable adverse mental health outcomes [9]. European and American quantitative studies also reported moderate and high levels of stress, anxiety, depression, sleep disturbance and burnout with diverse coping strategies and more frequent and intense symptoms among women and nurses without conclusive results by age [10]. The majority of research on the mental health of HCWs is from countries with a high COVID-19 impact on the health care system [8,11]. In countries with a lower COVID-19-caused health care burden (such as Cyprus), mental health issues were less frequent but still alarming [12]. The latest studies have confirmed that front-line HCWs are more likely to experience symptoms of depression and anxiety. Global anxiety rates are higher among nurses but depression rates among physicians and nurses are similar [13,14].

The aim of this study was to evaluate the frequency of the symptoms of depression and anxiety as well as the associated demographic and occupational factors among health care workers during the first wave of COVID-19 in Latvia.

2. Materials and Methods

2.1. Ethics

This study was approved on 20 April 2020 by the Research Ethics Committee of Riga Stradiņš University, Latvia, protocol number 6-1/04/1. Electronic or written informed consent for participation in the study was obtained from each participant. The participants could withdraw themselves from the survey at any time without providing any justification.

2.2. Study Design and Sampling

This was a cross-sectional quantitative study. The participants were interviewed at various timepoints between 28 April 2020 and 2 June 2020 during the first wave of COVID-19 in Latvia. We assumed that different timepoints during the relatively short period were not associated with changes over time. The participants were selected by a non-probability sampling approach. Physicians, physician assistants, nurses and other health care workers (nursing assistants, physiotherapists, dental technicians, medical students, etc.) were recruited from the intensive care departments and patient admission departments from three University hospitals and fourteen regional hospitals as well as from state emergency medical services, family physician practices and Riga Stradiņš University's Institute of Stomatology. All centres were in Latvia. Therefore, all areas of the health care system were included and represented to provide a generalised impression of the mental health status in various workplaces during the first wave of COVID-19. This study was carried out by interviews using self-addressed questionnaires in a paper format and a REDCap web-based format. The format depended on the internal rules of the specific medical institution based on the personal contact limitations because of COVID-19.

2.3. Inclusion Criteria

Only HCWs who worked as health care workers in the previously stated health care institutions during the first wave of the COVID-19 pandemic were voluntarily included in this study.

2.4. Measurement Tools

The participants reported their demographics, work-related information and contacts with COVID-19-positive patients and then filled in two standardised questionnaires that assessed the symptoms of anxiety (GAD-7) and depression (PHQ-9).

The survey included individual characteristics such as sex (male/female), age, education ($\leq$ undergraduate or $\geq$ postgraduate), relationship status (married, in a relationship, single), occupation (physician, physician assistant or nurse, other), work experience, workplace (hospital, state emergency medical service (SEMS), general practitioner practice (GP), Riga Stradiņš University Institute of Stomatology (Stomatology)) and working hours during the previous week.

The participants were asked whether they had been in contact with patients with a suspected or confirmed COVID-19 infection.

The current mental status was assessed by assessment tools in the Latvian language. A validated Latvian version of the 9 item Patient Health Questionnaire (PHQ-9; range, 0–27) was used to assess the symptoms of depression. The results of the questionnaire were categorised as follows: none (0), subclinical (1–4), mild (5–9), moderate (10–14), moderately severe (15–19) and severe (20–27) depression [15]. The cut-off score for clinically significant depression was 10 [16].

The 7 item Generalized Anxiety Disorder (GAD-7) scale (range, 0–21) was used to assess the symptoms of anxiety [17,18]. The results of the questionnaire were categorised as follows: normal (0–4), mild (5–9), moderate (10–14) and severe (15–21) anxiety. The cut-off score for clinically significant anxiety was 10 [19].

2.5. Study Population

Answers from 864 HCWs were obtained (age 19–82). Out of them, 20 HCWs were excluded from the analysis because they did not match the inclusion criteria (Table 1).

Table 1. Descriptive statistics of demographic characteristics and occupational information for the total sample.

	**No. (%)**	**Occupation**		
**Characteristic**	**Total**	**Physician**	**Nurse/Physician Assistant**	**Other**
Overall	844 (100)	350 (41.5; 95% CI, 38.2−44.8)	384 (45.5, 95% CI, 42.1−48.9)	110 (13.0; 95% CI, 10.7−15.3)
Sex				
Men	127 (15.0; 95% CI, 12.6−17.4)	56 (16.1, 95% CI, 12.2−20.0)	45 (11.8; 95% CI, 8.6−15.0)	26 (24.3, 95% CI 16.3−32.3)
Women	710 (84.1; 95% CI, 81.6−86.6)	292 (83.9; 95% CI, 80.0−87.8)	337 (88.2, 95% CI, 85.0−91.4)	81 (75.7, 95% CI, 67.7−83.7)
Age, y	40.0 (IQR 29.0−54.0)	46.0 (IQR 32.0−57.0)	38.0 (IQR 28.0−51.0)	32.0 (IQR 23.0−46.0)
Relationship status				
Married	360 (42.7, 95% CI, 39.4−46.0)	182 (52.4, 95% CI, 47.2−57.6)	150 (39.3, 95% CI, 34.4−44.2)	28 (25.7, 95% CI, 17.5−33.9)
In relationship, unmarried	281 (33.3, 95% CI, 30.1−36.5)	99 (28.5, 95% CI, 23.8−33.2)	140 (36.6, 95% CI, 31.8−41.4)	42 (38.5, 95% CI, 29.4−47.6)
Single	197 (23.3, 95% CI, 20.4−26.2)	66 (19.0, 95% CI, 14.9−23.1)	92 (24.1, 95% CI, 19.8−28.4)	39 (35.8, 95% CI, 26.8−44.8)
Education level				
Undergraduate	162 (19.2, 95% CI, 16.5−21.9)	0 (0.0)	81 (21.1, 95% CI, 17.0−25.2)	81 (73.6, 95% CI, 65.4−81.8)
Postgraduate	681 (80.7, 95% CI, 78.0−83.4)	350 (100)	302 (78.9, 95% CI, 74.8−83.0)	29 (26.4, 95% CI, 18.2−34.6)

Table 1. *Cont.*

	No. (%)	Occupation		
**Characteristic**	**Total**	**Physician**	**Nurse/Physician Assistant**	**Other**
Work experience, y	13.0 (IQR 5.0−30.0)	20.0 (IQR 6.5−33.0)	13.0 (IQR 5.0−30.0)	4.0 (IQR 1.0−7.0)
Working hours during previous week	48.0 (IQR 40.0−56.0)	40.0 (IQR 36.0−52.0)	48.0 (IQR 40.0−60.0)	48.0 (IQR 40.0−48.0)
Contact with COVID-19 patient				
Yes	497 (58.9, 95% CI, 55.6−62.2)	172 (49.3, 95% CI, 44.1−54.4)	253 (65.9, 95% CI, 61.2−70.6)	72 (65.5, 95% CI, 56.6−74.4)
No	346 (41.0, 95% CI, 37.7−44.3)	177 (50.7, 95% CI, 45.5−55.9)	131 (34.1, 95% CI, 29.4−38.8)	38 (34.5, 95% CI, 25.6−43.3)
Workplace				
Hospital	377 (44.7, 95% CI, 41.3−48.1)	131 (37.4, 95% CI, 32.3−42.5)	175 (45.6, 95% CI, 40.6−50.6)	71 (64.5, 95% CI, 55.6−73.4)
SEMS	194 (23.0, 95% CI, 20.2−25.8)	17 (4.9, 95% CI, 2.6−7.2)	151 (39.3, 95% CI, 34.4−44.2)	26 (23.6, 95% CI, 15.7−31.5)
Hospital and SEMS	43 (5.1, 95% CI, 3.6−6.6)	14 (4.0, 95% CI, 1.9−6.1)	23 (6.0, 95% CI, 3.6−8.4)	6 (5.5, 95% CI, 1.2−9.8)
GP	192 (22.7, 95% CI, 19.9−25.5)	165 (47.1, 95% CI, 41.−52.3)	27 (7.0, 95% CI, 4.4−9.6)	0 (0)
Stomatology	36 (4.3, 95% CI, 2.9−5.7)	21 (6.0, 95% CI, 3.5−8.5)	8 (2.1, 95% CI, 0.7−3.5)	7 (6.4, 95% CI, 1.8−11.0)

2.6. Data Analysis

A descriptive analysis of the demographic and work-related categorial factors (sex, relationship status, education level, contacts with COVID-19 patients, occupation and workplace) were reported as frequencies; scale factors (age, work experience, working hours during the previous week) were assessed using the Shapiro–Wilk normality test and were found not to be normally distributed so they were presented as medians with interquartile ranges (IQR). Age, work experience and working hours during the previous week were categorised for a further statistical analysis as follows: age 19–29, 30–39, 40–49, >50 years; work experience <5, 5–10, 11–20, 21–30, < 30 years; working hours during the previous week <40, 40–48, >48 h.

The categorical data were analysed using the chi-squared test and binary logistic regression. A data analysis was performed using SPSS v25. The significance level was set at 0.05 and all tests were two-tailed.

3. Results

3.1. Prevalence of the Symptoms of Depression and Anxiety

A total of 209 (24.8%) participants had clinically significant depression symptoms (PHQ-9 score of at least 10 points) and 145 (17.2%) had clinically significant anxiety symptoms (GAD-7 score of at least 10 points). Men had more frequent moderate depression (X^2 (5; n = 828) = 11.3; p = 0.045). Participants in the age group 19–21 experienced more frequent moderate depression (X^2 (15; n = 822) = 31.5; p = 0.008).

Education level was found to be associated with the symptoms of depression and anxiety. Postgraduate participants had more frequent depression and anxiety symptoms in contrast to undergraduate participants (X^2 (5; n = 834) = 25.4; p < 0.000; X^2 (3; n = 838) = 12.8; p = 0.005).

The participant group with work experience of 21–30 years was associated with more severe symptoms of anxiety than participants who had been working for more than 30 years (X^2 (12; n = 830) = 25.6; p = 0.012).

Participants who worked at a GP had more frequent symptoms of depression and anxiety than participants who worked at a hospital (32.8% versus 19.4% and 27.1% versus 10.3%, respectively) (X^2 (20; n = 833) =32.7; p = 0.037; X^2 (12; n = 837) = 40.3; p < 0.000).

Longer working hours were associated with the symptoms of depression; 34.1% participants who worked more than 48 h during the previous week had symptoms of depression versus 21.9% participants who worked 40–48 h during the previous week (X^2 (10; n = 807) = 31.8; p < 0.000). Participants who worked less than 40 h during the previous week had less anxiety symptoms (10.9% (X^2 (6; n = 810) = 21.4; p = 0.002)).

Contact with COVID-19 patients was not associated with a significant difference in mental health status or severity of the symptoms of depression and anxiety between the HCWs who were exposed to COVID-19 and those who were not exposed to COVID-19 (X^2 (5; n = 834) = 2.0; p = 0.850; X^2 (3; n = 838) = 0.03; p = 0.999) (Table 2).

3.2. Risk Factors of Clinically Significant Depression and Anxiety

A binary logistic regression was used in order to calculate the adjusted potential risk factors for depression and anxiety (presented in Table 3).

HCWs who worked in a GP were more likely to experience depression (OR = 2.312; 95% CI, 1.248–4.282; p = 0.008) compared with HCWs who worked at SEMS. Working more than 48 h per week was a risk factor for depression (OR = 2.222; 95% CI, 1.315–3.753; p = 0.003) compared with working less than 40 h per week. Male HCWs were less likely to experience anxiety (OR = 0.529; 95% CI, 0.288–0.970; p = 0.040) than females. HCWs aged 50 years or more had less chance of experiencing anxiety (OR = 0.468; 95% CI, 0.270–0.813; p = 0.007) than younger HCWs. HCWs who worked in a GP were more likely to experience anxiety (OR = 2.485; 95% CI, 1.256–4.917; p = 0.009) compared with HCWs who worked at SEMS. HCWs who worked 40 h per week or more were at a greater risk of anxiety (OR = 1.831; 95% CI, 1.017–3.296; p = 0.044) compared with working less than 40 h per week (Table 3).

Table 2. Severity of symptoms of depression and anxiety and associated demographic and occupational factors for the total sample.

	**No (%)**								**No (%)**					
	**Severity of Symptoms of Depression**								**Severity of Symptoms of Anxiety**					
	**None**	**Subclinical**	**Mild**	**Moderate**	**Moderately Severe**	**Severe**	**X^2 (df; *n*)**	***p* Value**	**Normal**	**Mild**	**Moderate**	**Severe**	**X^2 (df; *n*)**	***p* Value**
Overall	62 (7.4)	272 (32.6)	292 (35.0)	132 (15.8)	55 (6.5)	22 (2.6)			388 (46.2)	306 (36.5)	101(12.0)	44 (5.2)		
Sex														
Men	13 (10.2)	42 (33.1)	37 (29.1)	29 (22.8) *	5 (3.9)	1 (0.8)	11.3 (5; 828)	0.045	62 (48.8)	49 (38.6)	14 (11.0)	2 (1.6)	4.5 (3; 832)	0.213
Women	49 (7)	226 (32)	253 (36)	102 (14.6) *	50 (7.1)	21 (3.0)			324 (46.0)	252 (35.7)	87 (12.3)	42 (6.0)		
Age, y														
19–29	11 (4.6)	69 (29.1)	80 (33.8)	50 (21.1) *	22 (9.3)	5 (2.1)	31.5 (15; 822)	0.008	100 (41.8)	88 (36.8)	40 (16.7)	11 (4.6)	12.7 (9; 826)	0.177
30–39	4 (2.3) *	58 (33.7)	68 (39.5)	28 (16.3)	11 (6.4)	3 (1.7)			78 (45.3)	63 (36.6)	18 (10.5)	13 (7.6)		
40–49	15 (10.6)	44 (31.2)	52 (36.9)	17 (12.1)	8 (5.7)	5 (3.5)			63 (44.4)	56 (39.4)	18 (12.7)	5 (3.5)		
>50	29 (10.7) *	98 (36.0)	89 (32,7)	33 (12.1)	14 (5.1)	9 (3.3)			140 (51.3)	93 (34.1)	25 (9.2)	15 (5.5)		
Education level														
Undergraduate	22 (13.8) *	64 (40.0) *	53 (33.1)	14 (8.8) *	5 (3.1) *	2 (1.3)	25.4 (5; 834)	0.000	92 (57.9) *	48 (30.2)	16 (10.1)	3 (1.9) *	12.8 (3; 838)	0.005
Postgraduate	40 (5.9) *	208 (30.9) *	238 (35.3)	118 (17.5) *	50 (7.4) *	20 (3.0)			295 (43.4) *	258 (38.0)	85 (12.5)	41 (6.0) *		
Relationship status														
Married	27 (7.5)	115 (32.0)	127 (35.4)	63 (17.5)	20 (5.6)	7 (1.9)	11.1 (10; 829)	0.350	152 (42.3)	137 (38.2)	53 (14.8) *	17 (4.7)	13.0 (6; 833)	0.043
In relationship, unmarried	19 (6.9)	84 (30.7)	96 (35.0)	45 (16.4)	24 (8.8)	6 (2.2)			124 (44.4)	110 (39.4)	30 (10.8)	15 (5.4)		
Single	15 (7.7)	71 (36.2)	68 (34.7)	22 (11.2)	11 (5.6)	9 (4.6)			108 (55.4) *	57 (29.2) *	18 (9.2)	12 (6.2)		
Occupation														
Physician	17 (4.9) *	112 (32.1)	126 (36.1)	64 (18.3)	20 (5.7)	10 (2.9)	19.4 (10; 835)	0.035	149 (42.7)	138 (39.5)	42 (12.0)	20 (5.7)	6.8 (6; 839)	0.335
Nurse/physician assistant	33 (8.8)	111 (29.5)	136 (36.2)	57 (15.2)	29 (7.7)	10 (2.7)			177 (46.6)	135 (35.5)	48 (12.6)	20 (5.3)		
Other	12 (10.9)	49 (44.5) *	30 (27.3)	11 (10.0)	6 (5.5)	2 (1.8)			62 (56.4)	33 (30.0)	11 (10.0)	4 (3.6)		

Table 2. *Cont.*

	No (%)								No (%)					
	**Severity of Symptoms of Depression**								**Severity of Symptoms of Anxiety**					
	**None**	**Subclinical**	**Mild**	**Moderate**	**Moderately Severe**	**Severe**	**X^2 (df; *n*)**	***p* Value**	**Normal**	**Mild**	**Moderate**	**Severe**	**X^2 (df; *n*)**	***p* Value**
Work experience, y														
<5	10 (5.5)	59 (32.6)	59 (32.6)	36 (19.9)	14 (7.7)	3 (1.7)	28.3 (20; 825)	0.103	85 (47.0)	65 (35.9)	25 (13.8)	6 (3.3)	25.6 (12; 830)	0.012
5–10	9 (4.4)	64 (31.5)	73 (36.0)	39 (19.2)	14 (6.9)	4 (2.0)			87 (42.4)	77 (37.6)	27 (13.2)	14 (6.8)		
11–20	8 (6.6)	34 (28.1)	53 (43.8)	15 (12.4)	7 (5.8)	4 (3.3)			50 (41.0)	54 (44.3)	10 (8.2)	8 (6.6)		
21–30	13 (9.4)	41 (29.7)	54 (39.1)	16 (11.6)	11 (8.0)	3 (2.2)			63 (45.7)	45 (32.6)	27 (19.6) *	3 (2.2)		
>30	21 (11.5)	68 (37.4)	52 (28.6)	24 (13.2)	9 (4.9)	8 (4.4)			97 (52.7) *	62 (33.7)	12 (6.5) *	13 (7.1)		
Workplace														
Hospital	28 (7.6)	140 (37.8) *	129 (34.9)	48 (13.0) *	20 (5.4)	5 (1.4) *	32.7 (20; 833)	0.037	197 (52.7) *	138 (36.9)	27 (7.2) *	12 (3.2) *	40.3 (12; 837)	0.000
SEMS	15 (7.7)	48 (24.7) *	75 (38.7)	29 (14.9)	19 (9.8) *	8 (4.1)			73 (37.8) *	79 (40.9)	29 (15.0)	12 (6.2)		
Hospital and SEMS	2 (4.8)	16 (38.1)	13 (31.0)	8 (19.0)	3 (7.1)	0 (0)			23 (53.5)	10 (23.3)	8 (18.6)	2 (4.7)		
GP	11 (5.8)	55 (28.8)	62 (32.5)	41 (21.5) *	13 (6.8)	9 (4.7) *			76 (39.8) *	63 (33.0)	34 (17.8) *	18 (9.4) *		
Stomatology	4 (11.1)	13 (36.1)	13 (36.1)	6 (16.7)	0 (0)	0 (0)			17 (47.2)	16 (44.4)	3 (8.3)	0 (0)		
Working hours during previous week														
<40	17 (10.3)	52 (31.5)	63 (38.2)	23 (13.9)	5 (3.0) *	5 (3.0)	31.8 (10; 807)	0.000	86 (52.1)	61 (37.0)	12 (7.3) *	6 (3.6)	21.4 (6; 810)	0.002
40–48	32 (8.1)	144 (36.6)	131 (33.3)	56 (14.2)	19 (4.8)	11 (2.8)			196 (49.9) *	124 (31.6) *	47 (12.0)	26 (6.6)		
>48	7 (2.8) *	73 (29.3)	84 (33.7)	50 (20.1) *	29 (11.6) *	6 (0.4)			93 (26.9) *	107 (42.5) *	41 (16.3) *	11 (4.4)		
Contact with COVID−19 patient														
Yes	36 (7.3)	157 (31.9)	173 (35.2)	77 (15.7)	37 (7.5)	12 (2.4)	2.0 (5; 834)	0.850	230 (46.5)	179 (36.2)	60 (12.1)	26 (5.3)	0.03 (3; 838)	0.999
No	26 (7.6)	115 (33.6)	118 (34.5)	55 (16.1)	18 (5.3)	10 (2.9)			158 (46.1)	126 (36.7)	41 (12.0)	18 (5.2)		

* $p < 0.05$.

Table 3. Binary logistic regression analyses for occupational risk factors associated with symptoms of depression and anxiety.

	Depression				Anxiety			
	**Adjusted OR (95% CI) ***	***p* Value**	**Non-Adjusted OR (95% CI)**	***p* Value**	**Adjusted OR (95% CI) ***	***p* Value**	**Non-Adjusted OR (95% CI)**	***p* Value**
Sex								
Women	1 (reference)				1 (reference)			
Men	0.937 (0.585−1.501)	0.787	1.161 (0.759−1.777)	0.491	0.529 (0.288−0.970)	0.040	0.644 (0.368−1.124)	0.122
Age, y								
19–29	1 (reference)				1 (reference)			
30–39	0.557 (0.342−0.907)	0.019	0.671 (0.432−1.044)	0.077	0.749 (0.430−1.302)	0.305	0.810 (0.493−1.332)	0.407
40–49	0.497 (0.295−0.837)	0.009	0.562 (0.245−0.913)	0.020	0.647 (0.360−1.164)	0.146	0.712 (0.414−1.227)	0.221
>50	0.422 (0.262−0.680)	0.000	0.539 (0.361−0.804)	0.002	0.468 (0.270−0.813)	0.007	0.633 (0.401−0.999)	0.049
Occupation								
Physician	1 (reference)				1 (reference)			
Nurse/physician assistant	0.994 (0.644−1.534)	0.978	0.930 (0.668−1.295)	0.668	1.209 (0.727−2.012)	0.464	1.009 (0.690−1.475)	0.964
Other	0.710 (0.365−1.382)	0.313	0.566 (0.327−0.980)	0.042	1.296 (0.614−2.737)	0.497	0.731 (0.397−1.345)	0.314
Workplace								
SEMS	1 (reference)				1 (reference)			
GP	2.312 (1.248−4.282)	0.008	1.213 (0.787−1.870)	0.382	2.485 (1.256−4.917)	0.009	1.387 (0.867−2.218)	0.172
Stomatology	0.896 (0.317−2.533)	0.835	0.493 (0.194−1.249)	0.136	0.633 (0.167−2.406)	0.502	0.337 (0.098−1.154)	0.083
Hospital	0.721 (0.456−1.141)	0.163	0.606 (0.405−0.906)	0.015	0.476 (0.281−0.808)	0.006	0.432 (0.267−0.696)	0.001
Hospital and SEMS	0.684 (0.303−1.544)	0.360	0.874 (0.411−1.860)	0.727	1.112 (0.490−2.526)	0.800	1.123 (0.511−2.468)	0.772
Working hours during previous week								
<40	1 (reference)				1 (reference)			
40–48	1.073 (.0660−1.744)	0.777	1.121 (0.714−1.758)	0.620	1.831 (1.017−3.296)	0.044	1.863 (1.073−3.234)	0.027
>48	2.222 (1.315−3.753)	0.003	2.073 (1.305−3.564)	0.002	2.416 (1.272−4.486)	0.007	2.123 (1.193−3.780)	0.010
Contact with COVID−19 patient								
Yes	1 (reference)				1 (reference)			
No	0.821 (0.566−1.191)	0.299	0.931 (0.676−1.281)	0.660	0.820 (0.539−1.248)	0.354	0.998 (0.686−1.422)	0.948

* Adjusted for all the factors included in the table.

4. Discussion

This study revealed that 24.8% of HCWs had symptoms of depression and 17.2% had symptoms of anxiety during the first COVID-19 wave in Latvia. The pooled prevalence rate of depression in HCWs based on 57 cross-sectional studies was 24%, nearly the same as in our study [13]. The pooled prevalence rate of anxiety in HCWs based on 71 studies was higher than in our study at 25% [14]. Our results showed similar tendencies with other cross-sectional studies conducted during the early stages of the COVID-19 pandemic in the Europe region. In Italy, 24.73% HCWs had symptoms of depression and 19.80% had symptoms of anxiety; the cut-off score for both scales—PHQ-9 and GAD-7—was 15 not 10 as it was in our study [11]. The results from a study in Spain showed that the prevalence of depression among HCWs was 28.1% and anxiety was 22.5% [20]. A high prevalence of anxiety and depression was also reported in Belgium [21]. During the first COVID-19 wave, the number of COVID-19 patients was relatively small and the infection was well-controlled in Latvia [3]. However, the depression and anxiety rates were not far behind those countries that were hit hard by COVID-19. There are no data about the mental health of HCWs before the COVID-19 pandemic but there are data about the general adult population of Latvia. The prevalence of depressive episodes in patients of primary care in Latvia before COVID-19 was 10.2% [22]. In our study, we saw relatively high rates of depression and anxiety. It indicated a pre-existing overload of the health care system that possibly led to the poor mental health of HCWs before COVID-19. The Latvian public health care system is known by its severe underfunding and limited access to adequate quality care for the population. The number of HCWs per population in Latvia is below the European Union average and it leads to a high number of patients per one HCW. Low salaries and difficult working conditions are the main reasons why Latvian well-qualified HCWs seek workplaces elsewhere in Europe [5].

Early publications of the effects of the COVID-19 pandemic state that nurses, women and front-line workers suffered more from mental health issues [8,20,21,23]; a younger age was also a risk factor for poor mental health among HCWs in viral epidemic outbreaks [24]. In our study, the frequency of depression was the same among genders. An analysis of the severity of depression symptoms showed that men were more prone to moderate depression than woman. Women were at a higher risk of anxiety. In the general population of Latvia, poor mental health is associated with a higher age [22]. Our data showed an opposite tendency; HCWs at a younger age were more prone to the symptoms of depression. Similar data have been seen in other studies; a younger age was a risk factor for poor mental health in [20,23]. Older HCWs have more professional and personal experience as well as resilience and they have developed adaptive coping skills through the years of work [12]. Globally, nurses are more exposed to the symptoms of anxiety than physicians [14]. In our study, the occupation was not a statistically significant risk factor for both depression and anxiety. Our finding regarding depression was consistent with a recent meta-analysis; the prevalence of depression among nurses and physicians was similar [13]. In our study, the prevalence of depression and anxiety among physicians was 26.9% and 17.7%, respectively, but a globally pooled prevalence of depression and anxiety among physicians had an opposite tendency of 20.5% and 25.8%, respectively [25]. Due to an insufficient number of HCWs, the workload on all medical professions in the health care system in Latvia is high; therefore, the occupation by itself was not a risk factor. Health care workers who had contact with COVID-19 patients showed the same level of depression and anxiety; similar results were found in Belgium [21]. We could explain it by several factors: a low number of COVID-19 patients during the first COVID-19 wave in Latvia [3], a relatively short period of the first COVID-19 wave, an increase in workload, a fear of the contagion, direct and indirect confrontation with COVID-19 patients and identical safety measures in all departments [21].

Workplace and workload had an important role in the mental health of HCWs. Our study showed that working more than 48 h per week was associated with more severe

depression and anxiety symptoms. Another study showed a similar tendency; working more than 8 h per day was associated with anxiety symptoms [23]. Working in a GP was associated with the highest risk of depression and anxiety during the first wave of the COVID-19 pandemic in Latvia. Before COVID-19, general practitioners had a challenging work environment because of the model of the health care system in Latvia [5]. Workload and uncertainty about the health care system processes increased significantly in GPs during the first COVID-19 wave [26]. Uncertainty disrupts our ability to avoid or to mitigate threat and thus results in anxiety [27]. HCWs who worked in SEMS were exposed to a bigger uncertainty about the health of patients and COVID-19 status than HCWs who worked in a hospital; therefore, working in a hospital was associated with a lower risk of anxiety than working in SEMS. Hospitals were well-equipped and were exposed to a relatively low number of COVID-19 cases. In another study, the researchers compared the differences in PHQ-9 and GAD-7 responses between emergency medicine and non-emergency medicine HCWs. The results did not show any statistically significant differences in the anxiety or depression scores; emergency medicine workers reported significantly more coping skills than non-emergency medicine workers [28]. Raising awareness about distress in the workplace and training of coping skills could be a useful strategy to prevent HCW mental health problems.

Due to poor mental health among HCWs, it is important to assess the protective factors. Relationship status is one of them. Married physicians tend to experience fewer depression symptoms [23]. Another study reported a higher prevalence of any current mental disorder among unmarried HCWs [20]. Our study showed that relationship status by itself was not the main issue. The question was how satisfied people were in their relationship because satisfaction in relationships is associated with improved mental health [29]. Due to the workload and safety restrictions, there were limited opportunities to spend quality time with families. COVID-19 pandemic exposure was at a collective organisation level; interventions should also be group-oriented. Our study did not involve any interventions to improve the mental health of the study participants but another study stated that relaxation rooms for HCWs in health care institutions and group interventions showed a positive effect on the mental health of HCWs [30]. Hospitals that arranged early mental health interventions (such as psychological interventions, early introductions to the working environment and procedures where staff were encouraged to express their feelings and advised to communicate with colleagues or divided into certain professional groups with leadership with regular meetings set for professional and social support) experienced no adverse events during the first COVID-19 wave [31]. Emotional support and crisis interventions for HCWs in Latvia started only during the COVID-19 first wave [32]. Financial resources provided by the statutory health system for psychological interventions are still lacking in Latvia. New HCWs are constantly entering the healthcare system and they are exposed to the COVID-19 pandemic from their first day at the health care system. Such research enables health care institutions to be aware of the impact of a pandemic on the mental health of HCWs and to plan interventions to improve and maintain mental health, thereby improving the quality of health care.

The timing of the survey was a strength of this study whereas the limitations were the cross-sectional design, sampling, unknown mental health status and pre-existing psychiatric illness of HCWs in Latvia before the COVID-19 pandemic. The focus of the study was targeted only on the symptoms of depression and anxiety; however, it provided a good insight into the mental health of HCWs and we could compare results with similar studies. Due to the different safety measures in the health care institutions across Latvia, we used different survey distribution methods to gain access to HCWs across Latvia. The data may have been skewed by those willing and able to complete the survey. Our data showed different tendencies in comparison with other studies because of pre-existing problems in the health care system in Latvia. This is a part of a bigger longitudinal study of the mental health of HCWs in Latvia. This study is a reference point and a further assessment

is needed to identify the impact of the COVID-19 pandemic over time and different waves on the mental health of HCWs.

5. Conclusions

The weaknesses of the health care system before the COVID-19 pandemic explain the poor mental health of HCWs during the COVID-19 pandemic in Latvia. A further cohort study is needed to evaluate the dynamics of the mental health and other predisposing factors of HCWs.

Author Contributions: Methodology, L.V., G.A. and Ģ.B.; writing—original draft preparation, L.V. and A.U.; writing—review and editing, G.A. and Ģ.B.; supervision, Ģ.B. All authors have read and agreed to the published version of the manuscript.

Funding: This research received no external funding.

Institutional Review Board Statement: The study was conducted according to the guidelines of the Declaration of Helsinki and approved by the Research Ethics Committee of Riga Stradiņš University, Latvia, protocol number 6-1/04/1, date of approval 20 April 2020.

Informed Consent Statement: Informed consent was obtained from all subjects involved in the study.

Data Availability Statement: The data are not available due to privacy.

Conflicts of Interest: The authors declare no conflict of interest.

References

1. WHO. WHO Director-General's Opening Remarks at the Media Briefing on COVID-19. Available online: https://www.who.int/director-general/speeches/detail/who-director-general-s-opening-remarks-at-the-media-briefing-on-covid-19---11-march-2020 (accessed on 11 March 2020).
2. Par Ārkārtējās Situācijas Izsludināšanu. Minister Cabinet of Republic of Latvia. Available online: https://www.vestnesis.lv/op/2020/51A.1 (accessed on 17 October 2021).
3. Al-Salem, W.; Moraga, P.; Ghazi, H.; Madad, S.; Hotez, P.J. The emergence and transmission of COVID-19 in European countries, 2019–2020: A comprehensive review of timelines, cases and containment. *Int. Health* **2021**, *13*, 383–398. [CrossRef] [PubMed]
4. WHO. Latvia: WHO Coronovirus Disease (COVID-19) Dashboard with Vaccination Data, WHO Coronovirus (COVID-19) Dashboard With Vaccination Data. Available online: https://covid19.who.int/region/euro/country/lv (accessed on 5 December 2021).
5. Latvia: Country Health Profile 2017. State of Health in the EU: OECD Publishing, Paris/European Observatory on Health Systems and Policies, Brussels. 2017. Available online: https://www.oecd-ilibrary.org/social-issues-migration-health/latvia-country-health-profile-2017_9789264283466-en (accessed on 16 October 2021).
6. Dewa, C.S.; Loong, D.; Bonato, S.; Trojanowski, L. The relationship between physician burnout and quality of healthcare in terms of safety and acceptability: A systematic review. *BMJ Open* **2017**, *7*, e015141. [CrossRef]
7. Pereira-Lima, K.; Mata, D.A.; Loureiro, S.R.; Crippa, J.A.; Bolsoni, L.M.; Sen, S. Association Between Physician Depressive Symptoms and Medical Errors: A Systematic Review and Meta-analysis. *JAMA Netw. Open* **2019**, *2*, e1916097. [CrossRef] [PubMed]
8. Lai, J.; Ma, S.; Wang, Y.; Cai, Z.; Hu, J.; Wei, N.; Wu, J.; Du, H.; Chen, T.; Li, R.; et al. Factors Associated with Mental Health Outcomes Among Health Care Workers Exposed to Coronavirus Disease 2019. *JAMA Netw. Open* **2020**, *3*, e203976. [CrossRef]
9. Moitra, M.; Rahman, M.; Collins, P.Y.; Gohar, F.; Weaver, M.; Kinuthia, J.; Rössler, W.; Peterson, S.S.; Unutzer, J.; Saxena, S.; et al. Mental Health Consequences for Healthcare Workers During the COVID-19 Pandemic: A Scoping Review to Draw Lessons for LMICs. *Front. Psychiatry* **2021**, *12*, 602614. [CrossRef]
10. Danet, A.D. Psychological impact of COVID-19 pandemic in Western frontline healthcare professionals. A systematic review. *Med. Clin. (Engl. Ed.)* **2021**, *156*, 449–458. [CrossRef]
11. Rossi, R.; Socci, V.; Pacitti, F.; Di Lorenzo, G.; Di Marco, A.; Siracusano, A.; Rossi, A. Mental Health Outcomes Among Frontline and Second-Line Health Care Workers During the Coronavirus Disease 2019 (COVID-19) Pandemic in Italy. *JAMA Netw. Open* **2020**, *3*, e2010185. [CrossRef] [PubMed]
12. Chatzittofis, A.; Karanikola, M.; Michailidou, K.; Constantinidou, A. Impact of the COVID-19 Pandemic on the Mental Health of Healthcare Workers. *Int. J. Environ. Res. Public Health* **2021**, *18*, 1435. [CrossRef]
13. Olaya, B.; Pérez-Moreno, M.; Bueno-Notivol, J.; Gracia-García, P.; Lasheras, I.; Santabárbara, J. Prevalence of Depression among Healthcare Workers during the COVID-19 Outbreak: A Systematic Review and Meta-Analysis. *J. Clin. Med.* **2021**, *10*, 3406. [CrossRef]

14. Santabárbara, J.; Bueno-Notivol, J.; Lipnicki, D.M.; Olaya, B.; Pérez-Moreno, M.; Gracia-García, P.; Idoiaga-Mondragon, N.; Ozamiz-Etxebarria, N. Prevalence of anxiety in health care professionals during the COVID-19 pandemic: A rapid systematic review (on published articles in Medline) with meta-analysis. *Prog. Neuro-Psychopharmacol. Biol. Psychiatry* **2021**, *107*, 110244. [CrossRef] [PubMed]
15. Vrublevska, J.; Trapencieris, M.; Rancans, E. Adaptation and validation of the Patient Health Questionnaire-9 to evaluate major depression in a primary care sample in Latvia. *Nord. J. Psychiatry* **2018**, *72*, 112–118. [CrossRef] [PubMed]
16. Kroenke, K.; Spitzer, R.L.; Williams, J.B. The PHQ-9: Validity of a brief depression severity measure. *J. Gen. Intern. Med.* **2001**, *16*, 606–613. [CrossRef] [PubMed]
17. Spitzer, R.L.; Kroenke, K.; Williams, J.B.; Löwe, B. A brief measure for assessing generalized anxiety disorder: The GAD-7. *Arch. Intern. Med.* **2006**, *166*, 1092–1097. [CrossRef]
18. Swinson, R.P. The GAD-7 scale was accurate for diagnosing generalised anxiety disorder. *Evid. Based Med.* **2006**, *11*, 184. [CrossRef] [PubMed]
19. Kroenke, K.; Spitzer, R.L.; Williams, J.B.; Monahan, P.O.; Löwe, B. Anxiety disorders in primary care: Prevalence, impairment, comorbidity, and detection. *Ann. Intern. Med.* **2007**, *146*, 317–325. [CrossRef]
20. Alonso, J.; Vilagut, G.; Mortier, P.; Ferrer, M.; Alayo, I.; Aragón-Peña, A.; Aragonès, E.; Campos, M.; Cura-González, I.D.; Emparanza, J.I.; et al. Mental health impact of the first wave of COVID-19 pandemic on Spanish healthcare workers: A large cross-sectional survey. *Rev. Psiquiatr. Salud Ment.* **2021**, *14*, 90–105. [CrossRef] [PubMed]
21. Tiete, J.; Guatteri, M.; Lachaux, A.; Matossian, A.; Hougardy, J.M.; Loas, G.; Rotsaert, M. Mental Health Outcomes in Healthcare Workers in COVID-19 and Non-COVID-19 Care Units: A Cross-Sectional Survey in Belgium. *Front. Psychol.* **2020**, *11*, 612241. [CrossRef]
22. Rancans, E.; Renemane, L.; Kivite-Urtane, A.; Ziedonis, D. Prevalence and associated factors of mental disorders in the nationwide primary care population in Latvia: A cross-sectional study. *Ann. Gen. Psychiatry* **2020**, *19*, 25. [CrossRef]
23. Rashid, M.; Alam, M.; Talukder, A.; Rahman Razu, S.; Ward, P.R.; Ali, M. Mental Health of Physicians During COVID-19 Outbreak in Bangladesh: A Web-Based Cross-Sectional Survey. *Front. Public Health* **2021**, *9*, 592058.
24. Serrano-Ripoll, M.J.; Meneses-Echavez, J.F.; Ricci-Cabello, I.; Fraile-Navarro, D.; Fiol-deRoque, M.A.; Pastor-Moreno, G.; Castro, A.; Ruiz-Pérez, I.; Campos, R.Z.; Gonçalves-Bradley, D.C. Impact of viral epidemic outbreaks on mental health of healthcare workers: A rapid systematic review and meta-analysis. *J. Affect. Disord.* **2020**, *277*, 347–357. [CrossRef]
25. Johns, G.; Samuel, V.; Freemantle, L.; Lewis, J.; Waddington, L. The global prevalence of depression and anxiety among doctors during the covid-19 pandemic: Systematic review and meta-analysis. *J. Affect. Disord.* **2022**, *298 Pt A*, 431–441. [CrossRef]
26. Villeruša, A. COVID-19 Pandēmijas Ietekme uz Veselības Aprūpes Sistēmu Latvijā: Pieredze un Nākotnes Risinājumi. Riga Stradiņš University, 2021. Available online: https://www.rsu.lv/sites/default/files/imce/Dokumenti/prezentacijas/wp1_covid_ietekme_uz_va_zinatnieku-brokastis_03..2021_villerusa.pdf (accessed on 16 October 2021).
27. Grupe, D.W.; Nitschke, J.B. Uncertainty and anticipation in anxiety: An integrated neurobiological and psychological perspective. *Nat. Rev. Neurosci.* **2013**, *14*, 488–501. [CrossRef] [PubMed]
28. Young, K.P.; Kolcz, D.L.; O'Sullivan, D.M.; Ferrand, J.; Fried, J.; Robinson, K. Health Care Workers' Mental Health and Quality of Life During COVID-19: Results from a Mid-Pandemic, National Survey. *Psychiatr. Serv.* **2021**, *72*, 122–128. [CrossRef] [PubMed]
29. Valaine, L.; Zilgalve, K.L.; Lagzdiņa, L.; Ancāne, G.; Briģis, Ģ. *Association between Family Relationship Satisfaction and Symptoms of Depression and Anxiety among Health Care Workers during the First Emergency Situation of COVID-19 in Latvia*; RSU Research Week 2021: Knowledge for Use in Practice; Riga Stradiņš University: Rīga, Latvia, 2021; p. 245.
30. Rolling, J.; Mengin, A.C.; Palacio, C.; Mastelli, D.; Fath, M.; Gras, A.; Von Hunolstein, J.J.; Schröder, C.M.; Vidailhet, P. COVID-19: Mental Health Prevention and Care for Healthcare Professionals. *Front. Psychiatry* **2021**, *12*, 566740. [CrossRef] [PubMed]
31. Shen, X.; Zou, X.; Zhong, X.; Yan, J.; Li, L. Psychological stress of ICU nurses in the time of COVID-19. *Crit. Care* **2020**, *24*, 200. [CrossRef]
32. Medicīnas Darbiniekiem un Policistiem Pieejams Bezmaksas Psiholoģiskais Atbalsts. Ministry of Health of the Republic of Latvia, 2020. Available online: https://www.vm.gov.lv/lv/jaunums/medicinas-darbiniekiem-un-policistiem-pieejams-bezmaksas-psihologiskais-atbalsts (accessed on 15 October 2021).
