# Supplementary material for: Mental health status and related factors influencing healthcare workers during the COVID-19 pandemic: A systematic review and meta-analysis
Source: PLoS One. 2024 Jan 19;19(1):e0289454. doi: 10.1371/journal.pone.0289454 (PMC10798549; doi:10.1371/journal.pone.0289454)
Supplement: S1 Data — (ZIP) [file pone.0289454.s011.zip › literatures/203.pdf]

ELSEVIER

Since January 2020 Elsevier has created a COVID-19 resource centre with free information in English and Mandarin on the novel coronavirus COVID-19. The COVID-19 resource centre is hosted on Elsevier Connect, the company's public news and information website.

Elsevier hereby grants permission to make all its COVID-19-related research that is available on the COVID-19 resource centre - including this research content - immediately available in PubMed Central and other publicly funded repositories, such as the WHO COVID database with rights for unrestricted research re-use and analyses in any form or by any means with acknowledgement of the original source. These permissions are granted for free by Elsevier for as long as the COVID-19 resource centre remains active.

Journal of Medical Imaging and Radiation Sciences 52 (2021) 586–594

Journal of Medical Imaging and Radiation Sciences
Journal de l'imagerie médicale et des sciences de la radiation

www.elsevier.com/locate/jmir

Research Article

Coronavirus-related anxiety and fear among South African diagnostic radiographers working in the clinical setting during the pandemic

Riaan van de Venter[a,*], Razana Williams[a], Carmen Stindt[b] and Wilma ten Ham-Baloyi[c]

[a] Department of Radiography, School of Clinical Care Sciences, Faculty of Health Sciences, Nelson Mandela University, Port Elizabeth, Eastern Cape, South Africa
[b] Unit of Statistical Consultation, Nelson Mandela University, Port Elizabeth, Eastern Cape, South Africa
[c] Faculty of Health Sciences, Nelson Mandela University, Port Elizabeth, Eastern Cape, South Africa

ABSTRACT

Introduction: The current coronavirus pandemic has impacted the healthcare sector significantly. Policies and practices had to be amended to ensure maximum safety for both patients and healthcare professionals, including radiographers. This led to negative impacts on the occupational wellbeing and mental health of radiographers.

Purpose: The aim of this study was to fill the gap in knowledge about coronavirus-related anxiety and fear among diagnostic radiographers across South Africa in order to inform policy and practice so as to mitigate the negative influence the coronavirus pandemic conditions has on the occupational wellbeing of diagnostic radiographers working on the frontline.

Methodology: A quantitative, descriptive research design, using a cross-sectional approach, was employed. Two-hundred and forty-eight (n=248) South African diagnostic radiographers working in the clinical setting during the current coronavirus pandemic were recruited through social media. Data pertaining to their coronavirus-related anxiety and fear were collected through a digital questionnaire comprising three parts: demographics, coronavirus anxiety scale (CAS), and fear of coronavirus-19 scale (FCV-19S).

Results: Most of the participants' coronavirus anxiety scale scores are indicative of probable dysfunctional anxiety (69.8%). The participants had higher levels of coronavirus-related fear compared to anxiety. Anxiety levels were dependent on biological sex. For all other demographic variables anxiety and fear levels were independent.

Conclusion: Support strategies should be implemented to mitigate the negative impacts of a pandemic such as the coronavirus pandemic on the occupational wellbeing and mental health of diagnostic radiographers. More research in this area is recommended to inform future policy and workforce development as well as practice amendments.

RÉSUMÉ

Introduction : La pandémie actuelle de coronavirus a eu un impact considérable sur le secteur des soins de santé. Les politiques et les pratiques ont dû être modifiées pour garantir une sécurité maximale aux patients et aux professionnels de la santé, y compris les radiographes. Cela a eu des répercussions négatives sur le bien-être professionnel et la santé mentale des radiographes.

Objectif : L'objectif de cette étude était de combler le manque de connaissances sur l'anxiété et la peur liées au coronavirus chez les radiographes de diagnostic en Afrique du Sud afin de guider les politiques et les pratiques de manière à atténuer l'influence négative de la pandémie de coronavirus sur le bien-être professionnel des radiographes de diagnostic travaillant en premiére ligne.

Competing interests: All authors declare no existing relationships and/or activities that could have influenced the submitted work. The first author is an associate editor of the JMIRS and was not involved in any part of the review or decision processes as per the JMIRS conflict of interest policy.
Contributors: All authors contributed to the conception and/or design of the work, the acquisition, analysis, and/or interpretation of the data. All authors were involved in drafting and commenting on the paper and have approved the final version.
Funding: This study was funded by the Nelson Mandela University COVID-19 research grant. Ethical Approval: Ethics approval was obtained from the research ethics committee: human (REC-H) at the Nelson Mandela University. Informed consent was obtained from each participant.
* Corresponding author at: H-Block, Ground Floor, Ocean Sciences Campus, Nelson Mandela University, Gomery Avenue, Summerstrand, Port Elizabeth Eastern Cape, 6031 South Africa.

E-mail address: riaan.vandeventer@mandela.ac.za (R. van de Venter).

1939-8654/$ - see front matter © 2021 Published by Elsevier Inc. on behalf of Canadian Association of Medical Radiation Technologists.
https://doi.org/10.1016/j.jmir.2021.09.016

Méthodologie : Un plan de recherche quantitatif et descriptif, utilisant une approche transversale, a été utilisé. Deux cent quarante-huit (n=248) radiographes de diagnostic sud-africains travaillant en milieu clinique pendant la pandémie actuelle de coronavirus ont été recrutés par le biais des médias sociaux. Les données relatives à leur anxiété et à leur peur liées au coronavirus ont été recueillies par le biais d'un questionnaire numérique comprenant trois parties : données démographiques, échelle d'anxiété liée au coronavirus (CAS) et échelle de peur du coronavirus-19 (FCV-19S).

Résultats : Les scores de la plupart des participants à l'échelle d'anxiété liée au coronavirus indiquent une anxiété dysfonctionnelle probable (69,8 %). Les participants présentaient des niveaux plus élevés de peur liée au coronavirus que d'anxiété. Les niveaux d'anxiété dépendaient du sexe biologique. Pour toutes les autres variables démographiques, les niveaux d'anxiété et de peur étaient indépendants.

Conclusion : Des stratégies de soutien devraient être mises en œuvre pour atténuer les impacts négatifs d'une pandémie telle que celle du coronavirus sur le bien-être professionnel et la santé mentale des radiographes de diagnostic. Il est recommandé de poursuivre les recherches dans ce domaine afin d'éclairer les politiques futures et le développement de la main-d'œuvre ainsi que les modifications de la pratique.

Keywords: Novel coronavirus; COVID-19; Occupational stress; Workplace wellbeing; Personnel management

Introduction

Historically the world has grappled with many infectious epidemics, for example, Ebola, cholera, Zika, HIV/AIDS, H1N1 influenza, as well as severe acute respiratory syndrome (SARS), which is caused by a type of coronavirus. However, South Africa has not been affected previously, like other countries, with reference to respiratory system related pandemics [1]. The novel coronavirus (also referred to as SARS-CoV-2) outbreak was declared a global pandemic by the World Health Organization (WHO) on 11 March 2020 [2]. Early cases of SARS-CoV-2 were identified in Wuhan, Hubei province, China in the latter part of 2019 [3]. The SARS-CoV-2 leads to coronavirus disease (COVID-19) that presents primarily as an atypical viral pneumonia, but clinical manifestations affecting other body systems have also been reported [4–6].

In South Africa, a national state of disaster was declared on 15 March 2020 [7]. To mitigate the transmission of the SARS-CoV-2 the South African government implemented a five-level alert system, which is used to manage the gradual easing of the lockdown, where alert level five is most restrictive and level one the least restrictive. On 5 March 2020, South Africa had its first confirmed case, when an individual returned from a skiing trip in Italy [8]. The first wave peak of the current coronavirus pandemic was during epidemiological weeks 24 – 28 (early June 2020 to early July 2020); the peak of the second wave was during epidemiological week 47 of 2020 through to week 1 of 2021 (mid-November 2020 to early January 2021) [9]. On 10 June 2021, the third wave commenced in some provinces in South Africa [10]. The country is still grappling with the third wave as at 27 July 2021 although there is evidence, according to the acting minister of health, that some provinces have passed the peak of the third wave [11]. In South Africa, the third wave is dominated by the delta variant of the SARS-CoV-2, whilst the alpha, beta and eta variants also contribute to the infections [12]. The statistics available at 26 July 2021 indicate a 91% recovery rate, 144 020 active cases, and 70 018 as the total cumulative number of deaths [13]. Data from February 2021 for healthcare professionals (HCPs) indicate that they have a three to four times higher likelihood of contracting the SARS-CoV-2 and developing coronavirus disease (COVID-19). In February 2021 it was estimated that 40 000 HCPs in South Africa had been infected by the novel coronavirus thus far; 6 473 had been hospitalised, and 663 had succumbed to COVID-19 [14]. No updated statistics could be found. These numbers may be much higher as they do not include those of the third wave, which some provinces technically entered into in mid-June 2021.

The first phase of the COVID-19 vaccination programme commenced in South Africa on 17 February 2021, during which HCPs were vaccinated with the single-shot Johnson and Johnson (J&J) vaccine as part of the Sisonke clinical trial [14]. To date (26 July 2021), 6 613 704 vaccinations have been administered to South Africans. South Africans receive either the single dose J&J vaccine or the two-dose Pfizer vaccine [15].

Despite there being policies in place to mitigate the transmission of the SARS-CoV-2 among HCPs, many felt at risk of contracting the virus [16]. This also applied within a diagnostic radiography context. Literature globally and from the African continent highlights numerous challenges pertaining to changes in the workflow, working environments, policies, infection control practices, and personal hygiene and care. Some of these challenges include increased workloads, exhaustion, powerlessness, information overload, and the lack of personal protective equipment (PPE), as well as support and training to cope with the burden associated with the coronavirus pandemic. These challenges further complicate radiographers' working conditions. This makes for a very arduous and stressful environment where radiographers have to provide optimal patient-centred care whilst being mindful of mitigating and preventing cross-infections, optimising the limited resources available to provide the required services, and reducing the probability of suffering burnout [17–21]. The effect of this adapted working environment is increased occupational stress, anxiety and fear as radiographers feel that they have very limited control of the situation and challenges they face [17–19,22–23]. In terms of the respective findings of studies in the Middle East, North Africa and India (MENAIN) region [24], Ghana [25], and one province in South Africa [26] radiographers were both very anxious and fearful of the virus and its impact. Radiographers

were impacted negatively mentally, financially and physically, which impacted on their performance at work and their livelihood outside of the workplace. Some authors highlight that these challenges are context-dependent and therefore different settings may be impacted differently by these challenges and variations thereof [23].

A South African study, which pertained to one of the nine provinces of South Africa, was conducted among radiographers [26], but it was not specific to anxiety and fear associated with the coronavirus pandemic. Hence, there is still very little South African-related evidence, nationally, to inform policy and practice. The aim of this study was to fill the gap in knowledge about coronavirus-related anxiety and fear among diagnostic radiographers across South Africa to inform policy and practice so as to mitigate the negative influence the coronavirus pandemic conditions has on the occupational wellbeing of frontline diagnostic radiographers.

Methodology

Ethics approval was obtained from the research ethics committee: human (REC-H) at the Nelson Mandela University. Informed consent was obtained from each participant. Participants did not have to provide details of their names so as to ensure anonymity when completing the online questionnaire. Participant identifiable information, such as email and IP addresses, was not retrieved in order to maintain privacy. Participation was voluntary, and participants could complete the questionnaire online at a time that suited them which enhanced autonomy. The data were stored by the first author on a university laptop in a password protected folder due to the optimal security measures in place within the university network and which is according to the university's policy. Only the authors had access to the data for analysis and reporting purposes.

Research design

A quantitative, descriptive research design, using a cross-sectional approach, was used to determine and describe the coronavirus-related anxiety and fear levels of South African diagnostic radiographers working in the clinical setting during the pandemic. The association of the anxiety and fear levels with the participants' biological sex, years of clinical experience, and the province in which they worked during the ongoing pandemic, were also established.

Target population sampling and sample size

The target population for this study was all South African diagnostic radiographers that worked or were working in the clinical environment during the coronavirus pandemic, from the time that the national state of disaster was declared mid-March 2020 to March 2021, and were registered with the Health Professions Council of South Africa (HPCSA) on either the community service or independent practice registers. There was a total of 7584 (n=7584) diagnostic radiographers on these HPCSA registers (personal communication, HPCSA, 2021). The required representative diagnostic radiographers' sample size, using Slovin's formula with a 95% confidence interval and 5% margin of error, was 366 [27]. This method of sample size determination was deemed appropriate since there was no literature pertaining to the target population's coronavirus-related anxiety and fear [27]. Only 248 (n=248) diagnostic radiographers participated in the study instead of the required number (n=366).

Participant recruitment

A convenience, snowball sampling strategy was implemented to recruit participants. This was justified since diagnostic radiographers were already overburdened with work conditions which may have resulted in their unavailability during the data collection period. Therefore, using this sampling method countered this concern as intended participants that were readily available were targeted, with the assistance of other participants through word-of-mouth. This process also contributed to randomisation since any diagnostic radiographer that was available during the data collection period could participate if they were willing to. Each potential participant had an equal chance of participating which ensured their right to self-determination to participate was upheld [28]. Participants were recruited by the first two authors. This was done by posting a link containing the participant information letter, informed consent form, and questionnaire on their personal Facebook and LinkedIn pages, the Society of Radiographers of South Africa's (SORSA) Facebook page, the Association of Radiographers of South Africa's (ARSA) Facebook page as well as sending the link using WhatsApp to their personal professional networks. Data were collected over a four-week period during February and March 2021. The link to the questionnaire was shared via social media once a week for the four-week period. This time period was commensurate with the latter part of the second wave of the coronavirus pandemic in South Africa.

Data collection

A digital questionnaire was used and hosted on Google forms due to the ease of use, privacy and accessibility of the software [29]. The questionnaire contained a preamble that explained the study to participants as well as what is expected of them. Following the preamble was the informed consent form which required participants to answer questions in this regard. Only those that responded 'yes' were able to access the questionnaire. The questionnaire had three sections. Section A covered three, closed-ended items related to demographics (biological sex, years of clinical experience and the province in which each participant was working during the coronavirus pandemic between March 2020 and March 2021); section B covered five items regarding anxiety based on the coronavirus anxiety scale (CAS); [30] and section C covered seven items related to aspects of fear related to COVID-19 based on the fear of COVID-19 scale (FCV-19S) [31]. Sections B and C were five-point Lik-

Table 1
Confirmatory factor analysis (CFA) model fit.

FACTOR	PARAMETERS	CMIN/DF	GFI	SRMR	CFI	RMSEA
CAS	All significant ($p < 0.01$)	3.611	0.970	0.019	0.986	0.103
FCV-19S	All significant ($p < 0.01$)	4.712	0.946	0.041	0.965	0.123

ert scale type questions where participants could indicate their level of agreement with each statement posed. The set-up of the questionnaire precluded a participant from submitting more than one completed submission.

Data analysis

Data were analysed by the third author, using IBM SPSS Statistics 26. Descriptive statistical methods (mean, standard deviations, range, distribution frequencies and percentages) were employed to provide summative descriptions of the participants' coronavirus-related anxiety and fear levels. Inferential statistics (confirmatory factor analysis, correlations, independent sample t-tests and one-way ANOVAs) were performed to determine whether there were relationships between the participants' anxiety and fear levels and their biological sex, years of clinical experience and the province in which participants work/worked during the coronavirus pandemic from March 2020 when a national state of disaster was declared to March 2021 [32]. To determine the participants COVID-19 related anxiety, their responses were summated as they could select 1 to 5: (never = 1 and always = 5) per question and a minimum score of 5 and a maximum score of 25. A CAS score of ≥ 9 represents probable dysfunctional coronavirus-related anxiety [30]. To calculate the scores of participants' fear levels from the FCV-19S a total was obtained from strongly disagree scored 1 to strongly agree scored 5. A participant could thus core between 7 and 35. The higher their fear score, the greater their fear of COVID-19 [31]. Results were considered statistically significant when $p < 0.05$.

Reliability and validity

A pilot study was conducted with 20 participants (n=20) to ensure reliability and validity of the scales used and to determine whether the scales were applicable to the South African radiography context. The results of the pilot study were not included in the main study. Standardised scoring procedures were used to analyse and interpret the results and the questionnaire format and instructions were the same for all participants [33]. The available Cronbach's alpha scores for the CAS ($\alpha = 0.92$) [30] and FCV-19S ($\alpha = 0.82$) [31] points to these scales being highly reliable. Our study yielded a similar Cronbach's alpha score for the CAS (α =0.92) and a higher Cronbach's alpha score for the FCV-19S ($\alpha = 0.91$). To assess the validity of each scale, confirmatory factor analysis (CFA) was performed. The results of the CFAs are summarised in Table 1. According to Hair et al. [34], to assess model fit for CFAs, three to four goodness of fit indices should be assessed, and all model parameters should be statistically significant. The indices chosen for this study, along with the criteria for adequate model fit in parentheses, were based on the recommendations of Hair et al. [34] which include a minimum discrepancy per degree of freedom (CMIN/df) ratio value < 5, goodness-of-fit index (GFI) value > 0.90, standardised root-mean-square-residual (SRMR) value < 0.07, comparative fit index (CFI) value > 0.90, and root-mean-square-error of approximation (RMSEA) value < 0.08 for good model fit and < 0.1 for adequate model fit. Based on the results, both factor structures for the scales showed adequate model fit and were deemed to be valid (Table 1).

Results

The optimal sample size for a representative sample of the target population was 366 participants (n=366) but 248 participants (n=248) completed the online questionnaire and it was deemed a useable sample. According to Costello and Osborne (2005) [35] an adequate sample size can be determined using a sample-to-item ratio of a minimum of 5-to-1. But, a ratio of 20-to-1 is preferred. Considering the questionnaire used in our study, sections B and C comprised 12 items that measured anxiety and fear. Using the criterion above the minimum sample size should be between 60 and 240. Therefore, while the sample size obtained was below the desired sample size calculated for the specified statistical power, it is greater than the guidelines provided by Costello and Osborne (2005) and was therefore deemed adequate to use [35].

Table 2 presents demographic statistics. The majority of participants were female 89.9% and had clinical experience between 0 and 11 years (23% with 0 – 5 years and 28.2% with 6 – 11 years). Over a third (34.3%) of the participants (85/248) were from the Eastern Cape, and the next highest was 19.4% from the Gauteng province (48/248).

Coronavirus anxiety scale (CAS)

The CAS comprised five items. Fig. 1 shows the distribution of responses to the five items. It can be seen that the majority of participants indicated that they never (20% – 38%), seldom (16% – 22%) or sometimes (27% – 29%) experienced these anxiety behaviours. The percentage of participants that indicated that they experienced these anxiety behaviours often or always comprised the remaining 15% – 30%. This indicates that the participants did not perceive to frequently suffer from the anxiety behaviours measured by the CAS.

The summated scores for the CAS were determined. According to Lee [30] a CAS score of ≥ 9 indicates probable dysfunc-

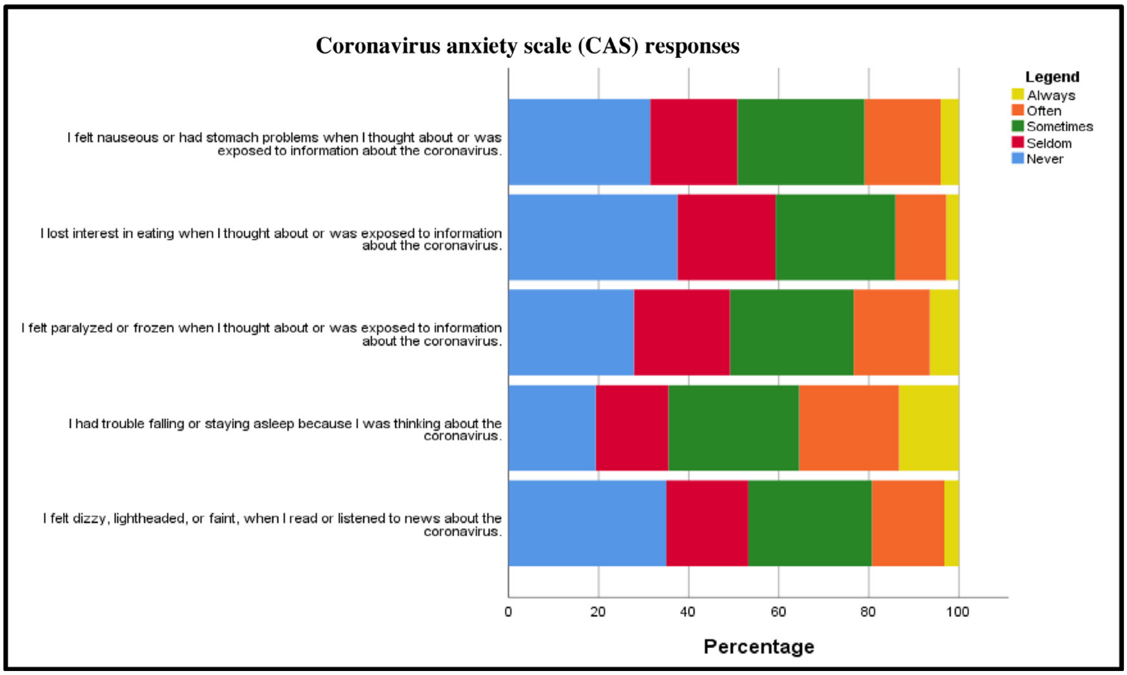

Fig. 1. Distribution of the CAS responses.

Table 2
Demographic statistics.

BIOLOGICAL SEX		N (%)
.	Female	223 (89.9%)
	Male	25 (10.1%)
CLINICAL EXPERIENCE		
	0 – 5 years	57 (23%)
	6 – 11 years	70 (28.2%)
	12 – 17 years	39 (15.7%)
	18 – 23 years	35 (14.1%)
	24 – 29 years	12 (4.8%)
	30 – 35 years	21 (8.5%)
	36 or more years	14 (5.6%)
PROVINCE		
	Eastern Cape	85 (34.3%)
	Free State	4 (1.6%)
	Gauteng	48 (19.4%)
	KwaZulu-Natal	38 (15.3%)
	Limpopo	12 (4.8%)
	Mpumalanga	17 (6.9%)
	North West	8 (3.2%)
	Northern Cape	4 (1.6%)
	Western Cape	32 (12.9%)

tional coronavirus-related anxiety. Our study results indicated that 30.2% of all participants had a score of < 9, and 69.8% had a score of ≥ 9. This means that the majority of the participants had CAS scores indicative of probable dysfunctional coronavirus-related anxiety.

Fear of coronavirus-19 scale (FCV-19S)

The FCV-19S comprised seven items. The distribution of the participants' responses to these items are summarised in

Table 3
Categorisation of CAS and FCV-19S scores.

	MEAN	SD	LOW (%)	MEDIUM (%)	HIGH (%)
CAS	12.44	5.32	46.80%	37.90%	15.30%
FCV-19S	22.72	6.86	19.80%	46.00%	34.30%

Fig. 2. It can be seen that the distribution of responses to the FCV-19S comprised mainly agree (13% – 35%) and strongly agree (8% – 40%) for the majority of FCV-19S items. The distribution of responses indicated that more participants had a higher degree of perceived fear of the coronavirus, relative to the anxiety related to the coronavirus.

The summated scores were determined for the CAS and FCV-19S scales. The average CAS score for all the participants was 12.44 (SD = 5.32). The average score for FCV-19S was 22.72 (SD = 6.86). As the two scales had different summated score ranges, the scores were categorised into three categories: low, medium, and high scores. The three category ranges for the CAS were low (5 – 11.667), medium (11.667 – 18.333) and high (18.333 – 25). For the FCV-19S the ranges for the three categories were low (7 – 16.333); medium (16.333 – 25.666); and high (25.666 – 35). This was done in order to facilitate the comparison between the two scales. The categorised responses are summarised in Table 3.

Relationship between coronavirus-related anxiety and fear

To ascertain whether there was a statistically significant relationship between the anxiety and fear levels of participants related to the coronavirus, a Pearson's correlation coefficient was

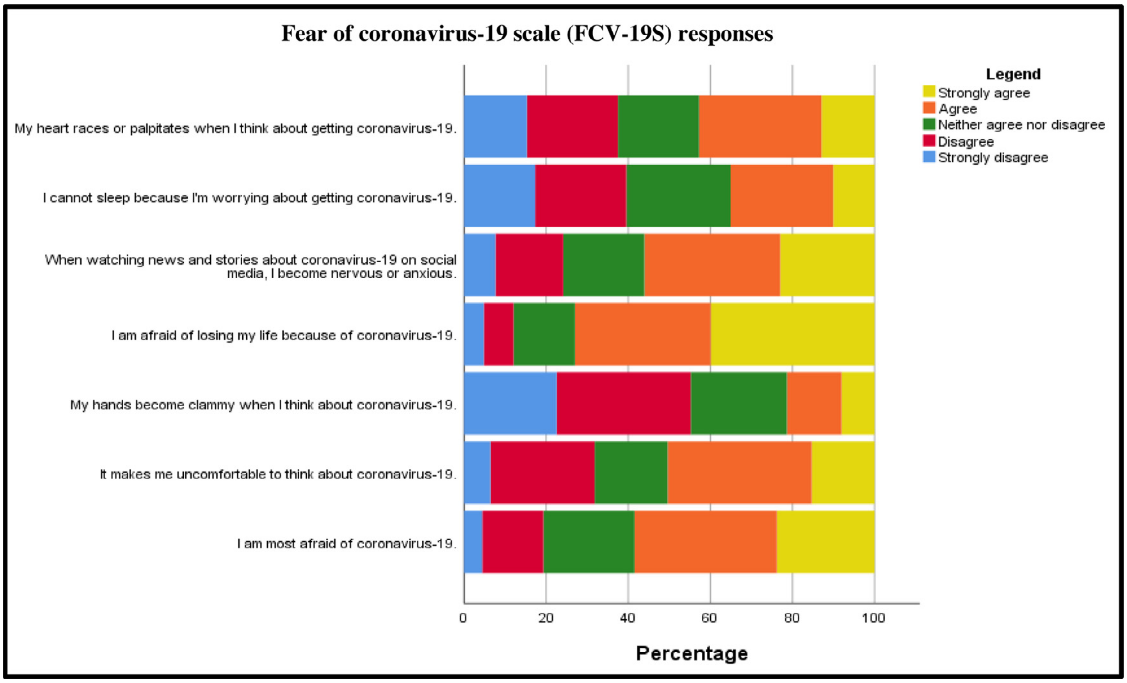

Fig. 2. Distribution of the FCV-19S responses.

calculated. A significant, strong positive relationship between CAS and FCV-19S scores was found ($r = 0.703$, $p = 0.000$).

Associations between participants' demographics and coronavirus-related anxiety and fear levels

Independent sample t-tests and one-way ANOVAs were performed to establish whether there was any statistically significant difference between the CAS and FCV-19S scores according to biological sex, clinical experience, and province where the respective participants worked or was working during March 2020 and March 2021 in South Africa. There were no statistically significant differences for clinical experience in either CAS {$F(4, 243) = 1.48$, $p = 0.209$} or FCV-19S {$F(4, 243) = 0.655$, $p = 0.624$} scores. Similarly, no statistically significant differences were found according to the province listed by the respective participants within either CAS {$F(6, 241) = 0.554$, $p = 0.767$} or FCV-19S {$F(6, 241) = 0.736$, $p = 0.621$}.

However, there was a statistically significant difference in terms of the CAS scores and the participants' biological sex ($t = 2.278$, $df = 246$, $p = 0.024$). It was found that the female participants' CAS score was significantly higher ($mean_{Female} = 12.695$, $SD_{Female} = 5.178$) than the males ($mean_{Male} = 10.16$, $SD_{Male} = 6.101$). Considering the participants' biological sex, relative to their coronavirus-related fear , no statistically significant difference in the FCV-19S scores and the participants' biological sex ($t = 1.323$, $df = 246$, $p = 0.187$) was found.

Discussion

This study set out to determine the coronavirus-related anxiety and fear levels among South African diagnostic radiographers that worked in the clinical setting during the current coronavirus pandemic. The time of participant recruitment and data collection was during the latter part of the second wave (February 2021 to March 2021) of the current pandemic. We consider this time period to be a possible influencing factor on the results since we appreciate that the findings may possibly have been different if the data were collected at the start or during the peak of the second wave of this pandemic.

Literature highlights that infectious disease pandemics, including the coronavirus pandemic, is a significant stressor for all people globally. This can thus impact their wellbeing. These pandemics can lead to depression, fear, anxiety, panic attacks, somatic symptoms associated with distress, psychosis, exacerbation or development of new psychiatric symptoms and suicidality [30,36–39]. Other aspects associated with pandemics, such as crisis communication methods, misinformation and media coverage concerning the coronavirus pandemic and COVID-19, as well as lockdowns, restricted movement, limited freedom, being separated from significant others and friends, loss of income and the fear associated with many uncertainties related to what the future holds post-pandemic, which can impact people's mental health negatively [40–41]. The impacts associated with the coronavirus pandemic conditions are no different in the healthcare context and for healthcare professionals. Compounding healthcare professionals lived experiences and occupational stress during the coronavirus pandemic even further is the many practice and policy changes and amendments, additional precautions that need to be put in place to provide quality healthcare to patients, and ensuring that they take the necessary precautions to protect themselves and others from contracting the SARS-CoV-2 [42–43]. Considering the results of our study,

there appears to be some convergence with literature in that being exposed to information about the coronavirus and COVID-19 may increase individuals' fear and anxiety since the majority of participants indicated agree or strongly agree (56.1%) in terms of the FCV-19S item on the impact of being exposed to information about the coronavirus through watching news and coming across stories on social media.

We also found that the participants had lower to medium levels of coronavirus-related anxiety (46.8% low and 37.9% medium) relative to the medium to higher levels of fear of the coronavirus (46% medium and 34.3% high). Therefore, their perception of fear of the coronavirus was greater than their perception of coronavirus-related anxiety. This may be ascribed to the CAS items relating to specific physiological responses to anxiety such as feeling dizzy, lightheaded, or faint when reading or listening to the news about the coronavirus and struggling to fall or stay asleep because they were thinking about the coronavirus, which may not have been experienced by all participants as opposed to a more common and general feeling of fear of the virus itself or the fear of losing one's life to COVID-19. Another plausible reason for this finding is that the participants may not have recalled having the specific physiological responses measured by the coronavirus anxiety scale (CAS). Bakioğlu et al. [44] describe anxiety as a future reaction to a new situation or looming threat and fear as a present reaction when experiencing a real or impending danger. Stress and anxiety are associated with fear which in turn increases stress and anxiety especially during a pandemic [45]. Although it is thought that fear and anxiety are separate emotional states, an overlap in the underlying behavioural mechanisms cannot be excluded as anxiety is merely fear in an elaborate form [46]. Another probable explanation for the abovementioned finding of the participants' higher fear levels compared to their lower anxiety levels is that they were presently experiencing/living with and through the threat posed by the coronavirus. They thus may develop anxiety at a later stage due to the increasing levels of stress and fear of working during the coronavirus pandemic. This finding in our study relates to other studies regarding fear and anxiety among radiographers globally, continentally and nationally: radiographers have higher levels of fear of the coronavirus and the effects of it compared to anxiety towards the coronavirus [24–26,39,47].

Although our study findings indicate that the participants had lower coronavirus-related anxiety, as measured by the CAS, indications have been found that the majority of the participants in our study did have symptoms of probable dysfunctional coronavirus-related anxiety (69.8%). This is a comparable finding with that of similar studies involving patients, healthcare workers, and the general population in Korea, Turkey and India respectively [30,48–52]. Dysfunctional anxiety is considered as the state in which individuals experience excessive and prolonged bouts of unease, fear and irrational apprehension, which debilitate individuals' normal functioning and daily life [53]. This finding, and the conclusion related to higher levels of fear experienced by the participants discussed above, in relation to the definition of dysfunctional anxiety points to the increased likelihood that the participants in our study were at risk of developing dysfunctional coronavirus-related anxiety at a later stage.

The strong positive relationship between CAS and FCV-19S, which indicated that there is a strong correlation between anxiety and fear of COVID-19 ($r = 0.703$, $p = 0.000$), further substantiates the above conclusion related to the increased risk of the participants developing dysfunctional coronavirus-related anxiety. The strong positive relationship also indicates that the participants in our study perceived some form of psychological effect due to COVID-19. Findings akin to ours were found in India ($r = 0.47$, $p = 0.000$) [51] and Turkey ($r = 0.54$, $p = 0.001$) [54]

Female participants in our study also had a significantly higher CAS score relative to the male participants ($p = 0.024$). Similarly, results of studies in Turkey, [52,54] Eastern Europe,[39] and India [51] showed higher coronavirus-related anxiety scores for female participants. However, the FCV-19S scores showed no statistically significant difference between female and male participants ($p = 0.187$). This finding is similar to a study conducted among the radiology workforce in the Middle East, North Africa and India, as it was found there was no statistically significant association between FCV-19s and participants' biological sex [24].

The results of our study indicated that there were no significant differences in the score for anxiety ($p = 0.209$) and fear ($p = 0.624$) according to clinical experience, indicating that experience did not have a significant effect on the perceptions of anxiety and fear of COVID-19. This is in keeping with the literature. For example, in their respective studies Elashmi et al. [24] and Hu et al. [55] documented no significant association between fear of the novel coronavirus and work experience. It was also shown in the findings in our study that the province in which the participants worked during March 2020 and March 2021 did not affect their coronavirus-related anxiety ($p = 0.767$) or fear ($p = 0.621$). Elashmi et al. [24] also found no statistically significant association between participants' place of work and coronavirus-related anxiety. They did however find a statistically significant association between participants' coronavirus-related fear levels and place of work. A possible explanation for this dissimilar finding of Elashmi et al. [24] relative to our study could be ascribed to the latter focussing on the province in which participants worked or were working during the current coronavirus pandemic and not specific sectors of work as Elashmi et al. [24] did. This argument can be upheld since various external factors can influence how the coronavirus pandemic influences different people in different settings [23].

Limitations

The social media recruitment and timing of data collection may have been possible limitations. The limited sizes of the researchers' social media networks and the platforms used may have had a possible influence on the reach of potential participants, and subsequently the sample size. Collection of data was

done during the latter part of the second wave of the pandemic. This may have contributed to not achieving the desired sample size as potential participants may have been under pressure at work whilst performing their duties at the frontline. Our study did not explore possible factors that contributed to the participants' coronavirus-related anxiety and fear .

Conclusions and recommendations

Participants experienced greater levels of coronavirus-related fear relative to coronavirus-related anxiety. Most may be at increased risk of developing anxiety due to the CAS score indicating probable dysfunctional anxiety for the majority of participants. A significant relationship between the participants' fear and anxiety contributes to the above conclusion. Although participants' biological sex had a statistically significant association with their anxiety levels it was not significant in terms of their fear levels. It was found that their anxiety and fear levels were independent from clinical experience and the province in which they were working or had worked during the current coronavirus pandemic from February 2021 and March 2021, which was commensurate with the latter part of the second wave of the pandemic in South Africa.

Given the findings it therefore becomes imperative to develop support strategies to mitigate the negative impacts of a pandemic, such as the coronavirus pandemic on the occupational wellbeing and mental health of radiographers. These support mechanisms could include effective, regular, controlled communication regarding a disease such as COVID-19; establishing and encouraging participation in employee wellness programmes or debriefing sessions; education and training regarding recognising the signs and symptoms of stress, fear, anxiety and psychological disorders, as well as how to adopt healthy coping mechanisms; and implementing a regular screening for mental wellness to prompt timeous intervention. The findings of our work contribute to understanding the impact of the coronavirus pandemic on South African diagnostic radiographers. Our findings could be used in policy development and practice amendment to inform workforce development.

Future research endeavours, in similar situations, should investigate radiographers' anxiety and fear longitudinally. A qualitative exploration of radiographers' experiences to understand possible reasons for their anxiety and fear is also recommended. A systematic review and meta-analysis of radiographers' anxiety, fear and related conditions globally should also be considered to get a more holistic perspective about the phenomenon.

References

[1] Council on Foreign Relations. (2021). Major Epidemics of the Modern Era 1899 –2021. Available at: https://www.cfr.org/timeline/major-epidemics-modern-era. Accessed July 27, 2021.

[2] World Health Organization. (2020). WHO Director-General's opening remarks at the media briefing on COVID-19 –11 March 2020. Available at: https://www.who.int/director-general/speeches/detail/who-director-general-s-opening-remarks-at-the-media-briefing-on-covid-19—11-march-2020. Accessed April 27, 2021.

[3] Rajkumar RP. COVID-19 and mental health: a review of the existing literature. *Asian J Psychiatry*. 2020;52(102066):1–5 Accessed May 10, 2021. doi:10.1016/j.ajp.2020.102066.

[4] Li X, Geng M, Peng Y, Meng L, Lu S. Molecular immune pathogenesis and diagnosis of COVID-19. *J Pharmaceut Anal.* 2020;10(2):102–108 Accessed April 21, 2021. doi:10.1016/j.jpha.2020.03.001.

[5] Rothan HA, Byrareddy SN. The epidemiology and pathogenesis of coronavirus disease (COVID-19) outbreak. *J Autoimmun*. 2020;109(102433):1–4 Accessed April 27, 2021. doi:10.1016/j.jaut.2020.102433.

[6] Yuki K, Fujiogi M, Koutsogiannaki S. COVID-19 pathophysiology: a review. *Clin Immunol.* 2020;215(108427):1–7 Accessed April 27, 2021. doi:10.1016/j.clim.2020.108427.

[7] Department of Corporate Governance and Traditional Affairs. *Declaration of a national state of disaster*. Government Gazette: Pretoria; 2020 Available at https://www.gov.za/documents/disaster-management-act-declaration-national-state-disaster-covid-19-coronavirus-16-mar. Accessed April 27, 2021.

[8] Mkhize, Z. (2020). First case of COVID-19 coronavirus reported in SA. Available at: https://www.nicd.ac.za/first-case-of-covid-19-coronavirus-reported-in-sa/. Accessed July 27, 2021.

[9] National Institute for Communicable Diseases. (2021). An update on COVID-19 outbreak in South Africa. The first and the second wave of COVID-19 cases in South Africa, January 2021. *Communicable Diseases Communiqué*, 20(1), 6–8. Available at: https://www.nicd.ac.za/wp-content/uploads/2021/01/NICD-Monthly-Communiqu%C3%A9-January-2021.pdf. Accessed July 27, 2021.

[10] Jimoh, S. (2021). Latest confirmed cases of COVID19 in South Africa (10 June 2021). Available at: https://www.nicd.ac.za/latest-confirmed-cases-of-covid-19-in-south-africa-10-june-2021/. Accessed July 27, 2021.

[11] South African Government News Agency. (2021). SA passes third wave peak, threat of a new surge looms. Available at: https://www.sanews.gov.za/south-africa/sa-passes-third-wave-peak-threat-new-surge-looms. Accessed July 27, 2021.

[12] National Institute for Communicable Diseases. (2021). Covid-19 update: delta variant in South Africa. Available at: https://www.nicd.ac.za/covid-19-update-delta-variant-in-south-africa/. Accessed July 13, 2021.

[13] National Department of Health. (2021). Update on Covid-19 (26th July 2021). Available at: https://sacoronavirus.co.za/2021/07/26/update-on-covid-19monday-26-july-2021/. Accessed July 27, 2021.

[14] National Department of Health. (2021). South Africa commences early access vaccine rollout to healthcare workers - Sisonke! Let's work together to protect our healthcare workers. Available at: http://www.health.gov.za/wp-content/uploads/2021/02/Media-Statement-South-Africa-rollout-Covid-19-Vaccine.pdf. Accessed April 27, 2021.

[15] National Department of Health. (2021). Latest vaccine statistics. Available at: https://sacoronavirus.co.za/latest-vaccine-statistics/. Accessed July 13, 2021.

[16] Rees K, Dunlop JL, Patel-Abrahams S, Struthers H, McIntyre JA. Primary healthcare workers at risk during COVID-19: an analysis of infections in HIV service providers in five districts of South Africa. *S Afr Med J.* 2021;111(4):309–314 http://dx.doi.org/10.7196%2FSAMJ.2021.v111i4.15434. Accessed April 27, 2021.

[17] Cherisch MF, Gray G, Fairlie L, et al. COVID-19 in Africa: care and protection for frontline healthcare workers. *Globaliz Health.* 2020;16(1):1–6 Available at: https://europepmc.org/article/med/32414379. Accessed May 10, 2021.

[18] Eastgate P, Neep MJ, Steffens T, Westerink A. COVID-19 pandemic – considerations and challenges for the management of medical imaging departments in Queensland. *J Med Radiat Sci.* 2020;67(4):345–351 Accessed May 10, 2021. doi:10.1002/jmrs.423.

[19] Liu Q, Luo D, Haase JE, et al. The experiences of health-care providers during the COVID-19 crisis in China: a qualitative study. *Lancet Global Health*. 2020;8(6):e790–e798 Accessed May 10, 2021. doi:10.1016/S2214-109X(20)30204-7.
[20] Maizlin NN, Ohorodnyk P. Investigating the initial effect of COVID-19 on the functioning of diagnostic imaging facilities. *J Med Imag Radiat Sci*. 2020;51(4):574–578.
[21] Mukherjee P, Lim TC, Chawla A, Chou H, Peh WCG. Adaptability and responsiveness: keys to operational measures in a regional hospital radiology department during the current COVID-19 pandemic. *Br J Radiol Open*. 2020;2(1):1–9 Accessed January 15, 2021. doi:10.1259/bjro.20200017.
[22] Akudjedu TN, Lawal O, Sharma M, et al. Impact of the COVID-19 pandemic on radiography practice: findings from a UK radiography workforce survey. *Br J Radiol Open*. 2020;2(1):1–11 Accessed January 15, 2021. doi:10.1259/bjro.20200023.
[23] Stogiannos N, Fotopoulos D, Woznitza N, Malamateniou C. COVID-19 in the radiology department: what radiographers need to know. *Radiography*. 2020;26(3):254–263.
[24] Elashmi W, Akudjedu TN, Abuzaid M, David LR, Tekin HO, Cavli B, Issa B. The radiology workforce's response to the COVID-19 pandemic in the Middle East, North Africa and India. *Radiography*. 2021;27(2):360–368.
[25] Akudjedu TN, Botwe BO, Wuni A-R, Mishio NA. Impact of the COVID-19 pandemic on clinical radiography practice in low resource settings: the Ghanaian radiographers' perspective. *Radiography*. 2021;27(2):443–452.
[26] Lewis S, Mulla F. Diagnostic radiographers' experience of COVID-19, Gauteng South Africa. *Radiography*. 2021;27(2):346–351.
[27] Glen, S. (2012). Slovin's formula: what is it and when do I use it? Available at: https://www.statisticshowto.com/how-to-use-slovins-formula/. Accessed May 10, 2021.
[28] Brink H, van der Walt C, van Rensburg G. *Fundamentals of research methodology for healthcare professionals*. 4th ed. Cape Town: Juta; 2018.
[29] Melo, S. (2018). Advantages and disadvantages of Google forms. https://mydatascope.com/blog/en/advantages-and-disadvantages-of-google-forms/. Accessed May 10, 2021.
[30] Lee SA. Coronavirus anxiety scale: a brief mental health screener for COVID-19 related anxiety. *Death Stud*. 2020;44(7):393–401 Accessed January 15, 2021. doi:10.1080/07481187.2020.1748481.
[31] Ahorsu DK, Lin C-Y, Imani V, Saffari M, Griffiths MD, Pakpour AH. The fear of COVID-19 scale: development and initial validation. *Int J Mental Health Addict*. 2020:1–9 March 2020. Accessed January 15, 2021. doi:10.1007/s11469-020-00270-8.
[32] Pietersen J, Maree K. *First steps in research*. 2nd ed. Overview of some of the most popular statistical techniques In Maree K (Ed). Pretoria: Van Schaik; 2016.
[33] Salkind NJ. *Exploring research*. 8th ed. Harlow: Pearson; 2014.
[34] Hair JF, Black WC, Babin BJ, Anderson RE. *Multivariate data analysis*. 7th ed. Harlow: Pearson Education Limited; 2014.
[35] Costello AB, Osborne J. Best practices in exploratory factor analysis: four recommendations for getting the most from your analysis. *Practical Assessment, Res Eval*. 2005;10(7):1–9.
[36] Sim K, Chan YH, Chong PN, Chua HC, Soon SW. Psychosocial and coping responses within the community health care setting towards a national outbreak of an infectious disease. *J Psychosom Res*. 2010;68:195–202 Accessed May 27, 2021. doi:10.1016/j.jpsychores.2009.04.004.
[37] Xiang YT, Yang Y, Li W, Zhang Q, Cheung T, Ng CH. Timely mental health care for the 2019 novel coronavirus outbreak is urgently needed. *Lancet Psychiatry*. 2020;7(3):228–229 Accessed May 29, 2021. doi:10.1016/S2215-0366(20)30046-8.
[38] Rana W, Mukhtar S, Mukhtar S. Mental health of medical workers in Pakistan during the pandemic COVID-19 outbreak. *Asian J Psychiatry*. 2020;51 102080. Accessed May 28, 2021. doi:10.1016/j.ajp.2020.102080.
[39] Reznik A, Gritsenko V, Konstantinov V, Khanmenka N, Isralowitz R. COVID-19 fear in Eastern Europe: Validation of the fear of COVID-19 scale. *Int J Mental Health Addict*. 2020 May 2020. Accessed May 27, 2021. doi:10.1007/s11469-020-00283-3.
[40] Su Z, McDonnell D, Wen J, et al. Mental health consequences of COVID-19 media coverage: the need for effective crisis communication practices. *Global Health*. 2021;17(4) Accessed July 13, 2021. doi:10.1186/s12992-020-00654-4.
[41] Al Dhaheri AS, Bataineh1 MF, Mohamad MN, et al. Impact of COVID-19 on mental health and quality of life: Is there any effect? A cross-sectional study of the MENA region. *PLoS One*. 2021;16(3) e0249107. Accessed July 13, 2021. doi:10.1371/journal.pone.0249107.
[42] Ness MM, Saylor J, Di Fusco LA, Evans K. Healthcare providers' challenges during the coronavirus disease (COVID-19) pandemic: a qualitative approach. *Nurs Health Sci*. 2021;23(2):389–397 Accessed July 13, 2021. doi:10.1111/nhs.12820.
[43] Ruiz C, Llopis D, Roman A, Alfayate E, Herrera-Peco I. Spanish radiographers' concerns about the COVID-19 pandemic. *Radiography*. 2021;27(2):414–418.
[44] Bakioğlu F, Korkmaz O, Ercan H. Fear of COVID-19 and positivity: mediating role of intolerance of uncertainty, depression, anxiety, and stress. *Int J Mental Health Addict*. 2020 May 2020. Accessed May 29, 2021. doi:10.1007/s11469-020-00331-y.
[45] Ornell F, Schuch JB, Sordi AO, Kessler FHP. Pandemic fear" and COVID-19: mental health burden and strategies. *Brazilian J Psychiatry*. 2020;42(3):232–235 Accessed May 29, 2021. doi:10.1590/1516-4446-2020-0008.
[46] Steimer T. The biology of fear- and anxiety-related behaviors. *Dialogues Clin Neurosci*. 2002;4(3):231–249 Accessed May 29, 2021. doi:10.31887/DCNS.2002.4.3/tsteimer.
[47] Zhou M, Tang F, Wang Y, Nie H, Zhang L, You G. Knowledge, attitude and practice regarding COVID-19 among health care workers in Henan, China. *J Hosp Infect*. 2020;105(2):183–187 Accessed May 27, 2021. doi:10.1016/j.jhin.2020.04.012.
[48] Lee SA. Replication analysis of the Coronavirus Anxiety Scale. *Dusunen Adam*. 2020;33:203–205 Accessed July 20, 2021. doi:10.14744/DAJPNS.2020.00079.
[49] Lee SA, Mathis AA, Jobe MC, Pappalardo EA. Clinically significant fear and anxiety of COVID-19: A psychometric examination of the Coronavirus Anxiety Scale. *Psychiatry Res*. 2020;290 Accessed July 20, 2021. doi:10.1016/j.psychres.2020.113112.
[50] Choi E, Lee J, Lee SA. Validation of the Korean version of the obsession with COVID-19 scale and the Coronavirus anxiety scale. *Death Stud*. 2020;14:1–7 Accessed July 20, 2021. doi:10.1080/07481187.2020.1833383.
[51] Srivastava A, Bala R, Srivastava AK, Mishra A, Shamim R, Sinha P. Anxiety, obsession and fear from coronavirus in Indian population: a web-based studying using COVID-19 specific scales. *Int J Commun Med Public Health*. 2020;7(11):4570–4577 Accessed July 20, 2021. doi:10.18203/2394-6040.ijcmph20204763.
[52] Yurttas B, Poyraz BC, Sut N, et al. Willingness to get the COVID-19 vaccine among patients with rheumatic diseases, healthcare workers and general population in Turkey: a web-based survey. *Rheumatol Int*. 2021;41:1105–1114 Accessed July 19, 2021. doi:10.1007/s00296-021-04841-3.
[53] Websiter S. Getting the basics on Dysfunctional Anxiety. *Nexus Psychol*. 2021 Available at: https://www.nexuspsychology.com.au/getting-the-basics-on-dysfunctional-an. Accessed July 20.
[54] Evren C, Evren B, Dalbudak E, Topcu M, Kutlu N. Measuring anxiety related to COVID-19: A Turkish validation study of the Coronavirus Anxiety Scale. *Death Stud*. 2020:1–7 June 2020. Accessed May 27, 2021. doi:10.1080/07481187.2020.1774969.
[55] Hu D, Kong Y, Li W, et al. Frontline nurses' burnout, anxiety, depression, and fear statuses and their associated factors during the COVID-19 outbreak in Wuhan, China: A large-scale cross-sectional study. *EClin Med*. 2020;24 Accessed July 20, 2021. doi:10.1016/j.eclinm.2020.100424.
