# Supplementary material for: Mental health status and related factors influencing healthcare workers during the COVID-19 pandemic: A systematic review and meta-analysis
Source: PLoS One. 2024 Jan 19;19(1):e0289454. doi: 10.1371/journal.pone.0289454 (PMC10798549; doi:10.1371/journal.pone.0289454)
Supplement: S1 Data — (ZIP) [file pone.0289454.s011.zip › literatures/204.pdf]

European Journal of Pediatrics (2019) 178:681–693
https://doi.org/10.1007/s00431-019-03334-7

ORIGINAL ARTICLE

Occupational well-being in pediatricians—a survey about work-related posttraumatic stress, depression, and anxiety

Minouk Esmée van Steijn [1,2] · Karel Willem Frank Scheepstra [3] · Gulfidan Yasar [4] · Miranda Olff [3,5] · Martine Charlotte de Vries [6,7] · Maria Gabriel van Pampus [1]

Received: 27 September 2018 / Revised: 14 January 2019 / Accepted: 28 January 2019 / Published online: 19 February 2019
© The Author(s) 2019

Abstract

The objective of this study was to study mental health, coping, and support after work-related adverse events among pediatricians. Physicians are frequently exposed to adverse events. It makes them at risk for posttraumatic stress disorder (PTSD), depression, and anxiety disorders. Besides the personal impact, physicians could pose a threat towards patients, as mental health problems are associated with medical errors. A questionnaire was sent to all members of the Pediatric Association of The Netherlands in October 2016. The questionnaire focused on adverse events, coping, and support. The Hospital Anxiety and Depression Scale and the Trauma Screening Questionnaire were included for evaluation of anxiety, depression, and posttraumatic stress. Four hundred ten questionnaires (18.9%) were eligible for analysis. Seventy-nine % ($n = 325$) of the respondents experienced adverse events, with "missing a diagnosis" having the most emotional impact and "aggressive behavior" as the most common adverse event. Nine (2.2%) pediatricians scored above the cut-off value on the Trauma Screening Questionnaire, indicative of PTSD. In total, 7.3% ($n = 30$) and 14.1% ($n = 58$) scored above the cut-off values in the Hospital Anxiety and Depression Scale, indicative of depression and anxiety. Only 26.3% reported to have a peer support protocol available for emotional support following adverse events.

Conclusion: Pediatricians experience a considerable amount of adverse and potentially traumatizing events associated with significantly higher mental health problems compared to the general high-income population. Aggression towards pediatricians seems to be a common problem. Protocolled (peer) support should be implemented.

Communicated by Nicole Ritz

✉ Minouk Esmée van Steijn
m.e.vansteijn@olvg.nl

Karel Willem Frank Scheepstra
k.w.scheepstra@amc.nl

Gulfidan Yasar
g.yasar@amc.nl

Miranda Olff
m.olff@amc.nl

Martine Charlotte de Vries
m.c.de_vries@lumc.nl

Maria Gabriel van Pampus
m.g.vanpampus@olvg.nl

1 Department of Obstetrics and Gynecology, OLVG, Oosterpark 9, 1091 AC Amsterdam, The Netherlands

2 Department of Obstetrics and Gynecology, Amsterdam UMC, University of Amsterdam, Amsterdam, The Netherlands

3 Department of Psychiatry, Amsterdam UMC, University of Amsterdam, Amsterdam, The Netherlands

4 Amsterdam UMC, University of Amsterdam, Amsterdam, The Netherlands

5 Arq Psychotrauma Expert Group, Diemen, The Netherlands

6 Department of Medical Ethics and Health Law, Leiden University Medical Center, Leiden, The Netherlands

7 Department of Pediatrics, Leiden University Medical Center, Leiden, The Netherlands

What is known:
• *Physicians are frequently exposed to adverse events. It makes physicians at risk for depression, anxiety, and posttraumatic stress.*
• *Physicians who are affected by these events pose a threat towards patients, as mental health problems are associated with medical errors.*

What is new:
• *Pediatricians experience a considerable amount of adverse and potentially traumatizing events associated with significantly higher mental health problems.*
• *It is advised that (peer) support after adverse events is protocolled and education on coping strategies is implemented, to improve mental well-being of pediatricians.*

Keywords Adverse events · Mental health · Occupational stress · Work-related posttraumatic stress disorder · Work-related depression · Work-related anxiety · Workplace aggression

Abbreviations

DSM-IV	Diagnostic and Statistical Manual of Mental Disorders, 4th Edition
EJ	Eva Jacobs
GY	Gulfidan Yasar
HADS	Hospital Anxiety and Depression Scale
MdV	M.C. de Vries
NVK	Pediatric Association of The Netherlands
PTSD	Posttraumatic stress disorder
TSQ	Trauma Screening Questionnaire

Introduction

Physicians are frequently exposed to adverse events, such as medical errors, patient safety incidents, violence, and complaints. Adverse events may lead to mental health problems including posttraumatic stress disorder (PTSD) [1, 2]. Apart from this personal impact, diminished occupational well-being among physicians is linked to decreased professionalism, more medical errors and poorer patient outcomes [3–6]. The Canadian Medical Education Directives for Specialists 2015 (CanMEDS 2015) specify that physicians should take responsibility for their own health and well-being and that of their colleagues in order to provide optimal patient care [7]. Therefore, it is important to identify which events are risk factors for physicians to develop mental health problems. Furthermore, it is important to know what kind of support is needed to cope with these events.

The 1-year prevalence of mood disorders (major depression, bipolar disorder, and dysthymia) among Dutch citizens with a high income is 3.0%, and the 1-year prevalence of anxiety disorders is 6.0% [8]. In physicians, the prevalence of both depression and anxiety disorders can be as high as 29% and 24%, respectively [9, 10]. Also, physicians have an elevated risk of developing PTSD as they are more frequently exposed to adverse events during their career [11]. Pediatricians experience repetitive stress when dealing with sick children and their emotional and desperate parents, which may be extra stressful compared to other specialties [12]. Physicians' way of coping and their personal resilience might be important in preventing depression, anxiety, and PTSD. Therefore, the aim of this study was to examine work-related stressors and associated mental health problems in pediatricians as well as their ways of coping and received emotional support in their institute.

Materials and methods

A cross-sectional questionnaire was conducted with members of the Pediatric Association of The Netherlands (NVK).

Among the members of the NVK are residents, attending, non-practicing, and retired pediatricians. At the time of the questionnaire, there were 2160 members in total. All members received an invitation from the NVK for an online questionnaire and three reminders over the course of a 3-month period from October 2016. The questionnaire was sent through SurveyMonkey® using an anonymous (non-traceable) link.

The questionnaire consisted of 56 questions and contained two validated screening instruments, the Trauma Screening Questionnaire (TSQ) and the Hospital Anxiety and Depression Scale (HADS, Appendix) [13–16]. The first draft of the questionnaire was reviewed by members of the Childbirth and Psychotrauma Research (CAPTURE) group of the hospital OLVG in Amsterdam, The Netherlands, as well as by MdV. Furthermore, this questionnaire was conducted with gynecologists in 2014 and with orthopedic surgeons in 2016 (accepted for publication) [17]. The questionnaires were kept very similar to make it possible to compare the different specialties.

The TSQ is a ten-item screening instrument based on items from the PTSD Symptom Scale-Self Report and has five items concerning re-experiencing and five items concerning arousal. Answers can be "yes" or "no" [18]. The cut-off score indicative of PTSD symptoms is six [14, 15]. Only respondents who answered yes to experiencing a traumatic event at least 4 weeks ago filled out the TSQ. Within 4 weeks after an adverse event, an acute stress reaction might trigger complaints comparable to those found with PTSD. However, if the complaints exist for

more than 4 weeks, the complaints are more likely to be due to PTSD.

The HADS is a 14-item screening instrument for depression and anxiety, where both subscales contain seven questions each. Each question contains an answer consisting of a four-point Likert scale. The cut-off value of the Dutch version for clinically relevant depressive symptoms (HADS-D) or anxiety symptoms (HADS-A) is equal to or bigger than eight. The total HADS cut-off value for psychological distress is equal to or bigger than 12.

Further questions were added to the questionnaire to explore demographics (age, employment, working experience, sub-specialism), assess adverse events at work, way of coping with adverse events and how pediatricians learned to cope, satisfaction with current support, and if and how the current support system should change (Appendix).

Statistical analysis was performed with IBM Statistical Package for the Social Sciences (SPSS, version 22). Only completed questionnaires were analyzed. Open questions were categorized by two independent contributors (GY and EJ), the overall inter-rater agreement was calculated with Cohen's kappa. Demographic data and multiple-choice questions were analyzed using descriptive statistics, and P values were calculated with binomial tests. Differences in categorical outcomes between residents and attending physicians were tested with either chi-square tests (χ^2) or Fisher's exact. In continuous data, independent t or Mann-Whitney U tests were used. A two-sided P value of 0.05 or smaller was considered statistically significant.

This study was exempted from ethical approval by the Medical Research Ethics Committees United (MEC-U) and registered under the number W18.096.

Results

Population characteristics

A total of 456 questionnaires (21.1%) were completed, of which 410 questionnaires (18.9%) were eligible. Figure 1 shows the inclusion diagram.

Table 1 shows the baseline characteristics of the respondents, compared with the members of the NVK, the reference group. Gender and the amount of residents were comparable between respondents and the reference group; however, there were more attending pediatricians, less retired pediatricians, and less pediatricians with a management function in our sample (Table 1). Table 2 shows the baseline characteristics divided in the subgroups resident, attending, retired, and non-practicing.

Selected quotes of the respondents are added in Table 3 to visualize the events they experience as adverse, divided in aggression by parents and death of a patient.

Work-related stressors

The following events were experienced as high emotional impact stressors at work by the respondents (multiple answers possible): missing a diagnosis (71.2%, $n = 292$), suspicion of child abuse (49.3%, $n = 202$), doubts about making the right decision (48.3%, $n = 198$), death of a patient (38.0%, $n = 156$), and critically ill children (26.3%, $n = 108$; Fig. 2). Almost 80% ($n = 325$) of the respondents indicated that they actually perceived an event as an adverse event, of which 277 described this event. Aggressive behavior of parents towards the physician was most commonly named as an adverse event (42.5%, $n = 118$).

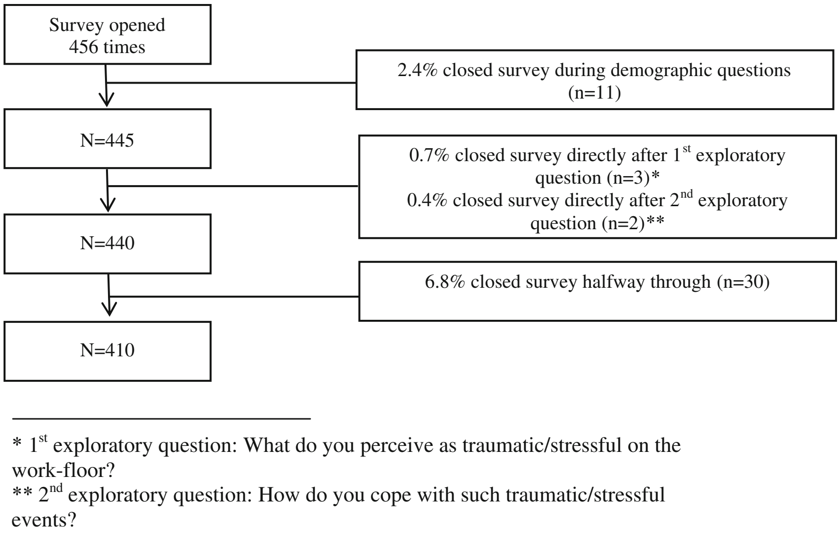

Fig. 1 Inclusion diagram of selecting the questionnaires

Table 1 Baseline characteristics respondents and NVK

	Responding pediatricians ($n = 410$)	Composition of membership NVK[a] ($n = 2160$)	*P* value
Gender			
Male	134 (32.7)	759 (35.1)	0.17
Female	276 (67.3)	1402 (64.9)	0.17
Position			
Resident	74 (18.0)	377 (17.5)	0.41
Attending physician	307 (74.9)	1307 (60.5)	< 0.001
Retired	23 (5.6)	254 (11.8)	< 0.001
Non-practicing	6 (1.5)	222 (10.3)	< 0.001
Age (in years)			
20–29	19 (4.6)	–	
30–39	108 (26.3)	–	
40–49	115 (28.0)	–	
50–59	110 (26.8)	–	
60–69	46 (11.2)	–	
≥ 70	12 (2.9)	–	
Years in practice			
≤ 5	40 (9.8)	–	
6–10	71 (17.3)	–	
11–15	57 (13.9)	–	
16–20	79 (19.3)	–	
21–25	57 (13.9)	–	
26–30	49 (12.0)	–	
> 30	57 (13.9)	–	

All values shown in *n* (%), *P* values calculated with binomial tests

– unknown, *NVK* Pediatric Association of The Netherlands

[a] Non-respondents plus respondents

Posttraumatic stress disorder

Table 4 shows the outcomes of the TSQ. Among the respondents, 79.3% experienced an adverse event at work, of which 34.9% ($n = 143$) reported having experienced this event during their work more than 4 weeks ago.

The mean score of the TSQ was significantly lower in the group of participants where a peer support protocol was present for adverse events (0.77 ± 1.06) compared to the group where no protocol was present or where it was not used (1.62 ± 2.10, $P = 0.02$).

Depression and anxiety

Outcomes of the HADS are shown in Table 4. Attending pediatricians have significantly more depressive symptoms according to the HADS-D compared to residents ($P = 0.03$).

Coping

The most common coping strategies after adverse events were (multiple answers possible) seeking support from colleagues (86.1%, $n = 353$), seeking support from friends and family (73.2%, $n = 300$), seeking some other form of distraction (32.7%, $n = 134$), or doing sports (22.4%, $n = 92$). Respondents learned their coping strategies (multiple answers possible) during residency (58.3%, $n = 239$), as an attending (55.1%, $n = 226$), during clerkships (20.2%, $n = 83$), and 21.0% ($n = 86$) reported to having never learnt to cope with adverse events.

Of the respondents, 41.0% ($n = 168$) has seriously considered quitting their job at some point in their career. Most common reasons for this were (multiple answers possible) disbalance between work and private life (75.0%, $n = 126$), high workload (68.5%, $n = 115$), disutility (working outside of working hours; 44.6%, $n = 75$), too much stress (38.7%, $n = 65$), and too much responsibility (37.5%, $n = 63$; Fig. 3).

Table 2 Baseline characteristics in subgroups

	Total ($n = 410$)	Resident ($n = 74$)	Attending ($n = 307$)	Retired ($n = 23$)	Non-practicing ($n = 6$)
Gender					
Male	134 (32.7)	11 (14.9)	103 (33.6)	16 (69.6)	4 (50.0)
Female	276 (67.3)	63 (85.1)	204 (66.4)	7 (30.4)	4 (50.0)
Age (in years)					
20–29	19 (4.6)	19 (25.7)	0 (0)	0 (0)	0 (0)
30–39	108 (26.3)	55 (74.3)	53 (17.3)	0 (0)	0 (0)
40–49	115 (28.0)	0 (0)	114 (37.1)	0 (0)	1 (16.7)
50–59	110 (26.8)	0 (0)	106 (34.5)	0 (0)	4 (66.7)
60–69	46 (11.2)	0 (0)	34 (11.1)	12 (52.2)	0 (0)
≥ 70	12 (2.9)	0 (0)	0 (0)	11 (47.8)	1 (16.7)
Years in practice					
≤ 5	40 (9.8)	37 (50.0)	3 (1.0)	0 (0)	0 (0)
6–10	71 (17.3)	40 (50.0)	34 (11.1)	0 (0)	0 (0)
11–15	57 (13.9)	0 (0)	56 (18.2)	0 (0)	1 (16.7)
16–20	79 (19.3)	0 (0)	76 (24.8)	2 (8.7)	1 (16.7)
21–25	57 (13.9)	0 (0)	54 (17.6)	1 (4.3)	2 (33.3)
26–30	49 (12.0)	0 (0)	47 (15.3)	1 (4.3)	1 (16.7)
> 30	57 (13.9)	0 (0)	37 (12.1)	19 (82.6)	1 (16.7)
Complaints at disciplinary board[a]	46 (11.2)	1 (1.4)	39 (12.7)	4 (17.4)	2 (33.3)

All values shown in *n* (%)

[a] Pediatricians who received complaints at the disciplinary board

Furthermore, 6.6% ($n = 27$) considered quitting because of a complaint to the disciplinary board.

Six percent ($n = 26$) of the respondents admitted that they were dealing with adverse events by drinking more alcohol and 1.2% ($n = 5$) by taking new medication. Professional psychological help was sought by 9.8% ($n = 44$) and 16.1% ($n = 66$) stifled emotions.

After being exposed to a work-related adverse event, 18.5% ($n = 76$) of the respondents adjusted their work. Most common ways to do this were (multiple answers possible) performing more diagnostic tests (51.9%, $n = 40$), calling a colleague earlier (36.4%, $n = 28$), work less (33.8%, $n = 26$), and starting treatment faster (20.8%, $n = 16$). Over time, 40.5% ($n = 166$) of the respondents reported to have become more defensive.

Support

Of the respondents, 26.3% ($n = 108$) indicated that there was a protocol for support in the case of an adverse event in their current working environment, 34.2% indicated that there was no protocol, and the remainder did not know whether there was a protocol. Furthermore, 50.2% ($n = 206$) thought that a culture change is necessary concerning coping with adverse events. When asked what the standardized support system involved (multiple answers possible), respondents indicated that discussing the situation with the present team (71.2%, $n = 292$) and self-initiated peer support with direct colleagues (64.9%, $n = 266$) were used. Of the respondents, 16.3% ($n = 67$) indicated that there was no support system at all and 41.5% ($n = 170$) confided that there is not enough opportunity to discuss adverse events and express emotions.

According to the respondents, the preferred form of support would be (multiple answers possible) discussing the situation with the present team (76.3%, $n = 313$), peer support from direct colleagues (72.0%, $n = 295$), and professionally organized peer support (43.2%, $n = 178$). Even though 28.5% ($n = 117$) would prefer to get help from a psychologist or coach, only 9.8% ($n = 40$) actually sought out this kind of help.

Discussion

The aim of this study was to examine work-related stressors and associated mental health problems in pediatricians as well as their ways of coping and received emotional support in their institute. First of all, among pediatricians, work-related stressors during their career were high. Suspicion of child abuse and critically ill children are two topics that distinguish pediatricians from other specialties and why this specific specialty can have high emotional burden. Notable is the high prevalence of aggressive behavior towards pediatricians, as

Table 3 Quotes of pediatricians*

Aggression
"During two periods in my career I was stalked, they phoned me, also during the night, either hanging up or telling me they knew where my family lived."
"We admitted a physically abused patient whose parents had severe psychiatric problems. I reported them to the Child Care and Protection Board and afterwards there were letters in the room of the patient telling me something would happen to me. These parents even went to my parents' house."
"I was held hostage twice, once by a desperate father, once by a drug-addicted father."
"I was threatened by parents during cardiopulmonary resuscitation, they kept yelling at me: 'You killed my child!'."
"I was threatened by parents with a gun after a patient died."
"At the outpatient clinic there was a big, strong father who grabbed me by the throat and pushed me in the corner"
Death of a patient
"The death of a patient due to a mistake with medication."
"The sudden death of a neonate whom I treated for half a year. It was hard to keep control during resuscitation and it took a long time for me to regain confidence during acute situations."
"A neonate who could not be intubated by anyone and who died."
"I sent a neonate with mild respiratory complaints back home. A day later he was presented with severe cardiomyopathy and despite maximum resuscitation at the ER, he died."
"Failed resuscitation of a neonate when my supervisor was not present."
"The death of a toddler due to drowning when my own children were toddlers. The parallelism and vulnerability had a huge emotional impact."

Question 23: Can you briefly describe the adverse event(s)?

These quotes are selected from all the answers

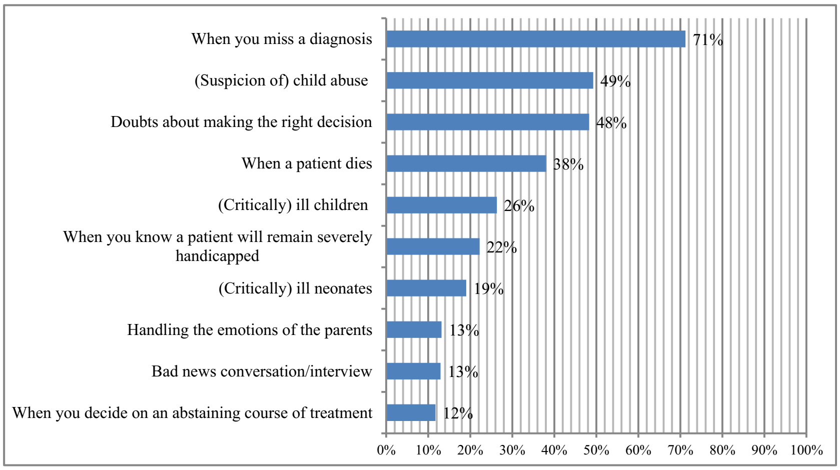

Fig. 2 Top 10 events pediatricians describe as an adverse event ($n = 410$), multiple answers possible

Table 4 HADS and TSQ scores

	Total ($n = 410$)	Resident ($n = 74$)	Attending ($n = 307$)	Retired ($n = 23$)	Non-practicing ($n = 6$)	*P* value**
Experienced potential psychotraumatic event at work as a physician	325 (79.3)					
Depression						
HADS-D score above cut-off	30 (7.3)	1 (1.4)	27 (8.8)	1 (4.3)	1 (16.7)	0.03
Anxiety						
HADS-A score above cut-off	58 (14.1)	9 (12.2)	47 (15.3)	0 (0)	2 (33.3)	0.49
Combined anxiety and depression						
Combined HADS score above cut-off	79 (19.3)	13 (17.6)	63 (20.5)	1 (4.3)	2 (33.3)	0.57
PTSD						
Traumatic experience (criterion A)	143 (34.9)	23 (31.1)	111 (36.2)	7 (30.4)	2 (33.3)	
TSQ score above cut-off[a]	9 (6.3)	2 (8.7)	6 (5.4)	1 (14.3)	0 (0.0)	0.63

All values shown in *n* (%)

HADS Hospital Anxiety and Depression Scale, *HADS-D* Hospital Anxiety and Depression Scale—Depression, *HADS-A* Hospital Anxiety and Depression Scale—Anxiety, *TSQ* Trauma Screening Questionnaire

**χ^2 test between residents and attending

[a] Measurements based on the following: total $n = 143$, resident $n = 23$, attending $n = 111$, retired $n = 7$, and non-practicing $n = 2$

stated in the quotes. Therefore, we think that it is necessary to not only develop a better support system after an adverse event but to also implement ways to teach pediatricians to cope with aggression. For example, certain training programs (conflict management and de-escalation (CMD)) focus on how to cope with aggression [19, 20]. Another stressor may be a complaint to the disciplinary board. This has a high impact on psychological well-being and is associated with defensive practice [21]. More than half of the pediatricians who received a complaint at the disciplinary board seriously considered quitting their job. More work-years corresponded with a higher chance to receive a complaint. Whether the non-practicing group stopped working because of complaints at the disciplinary board cannot be answered because of the small numbers. The amount pediatricians who received a complaint to the disciplinary board is low compared to gynecologists [17].

The point prevalence of PTSD in The Netherlands is 1.3%, making that the point prevalence of work-related PTSD is expected to be even lower [8]. In our questionnaire, when pediatricians experienced a traumatic event, we found a high point prevalence of symptoms indicative of work-related PTSD (2.2%). However, compared to the study of Ruitenburg et al., who found a prevalence of PTSD complaints of 15% in hospital physicians, the percentage we found seems low. Nonetheless, the study of Ruitenburg et al. used a much lower threshold than is normally used to assess PTSD complaints and does not use Criterion A [10]. Furthermore,

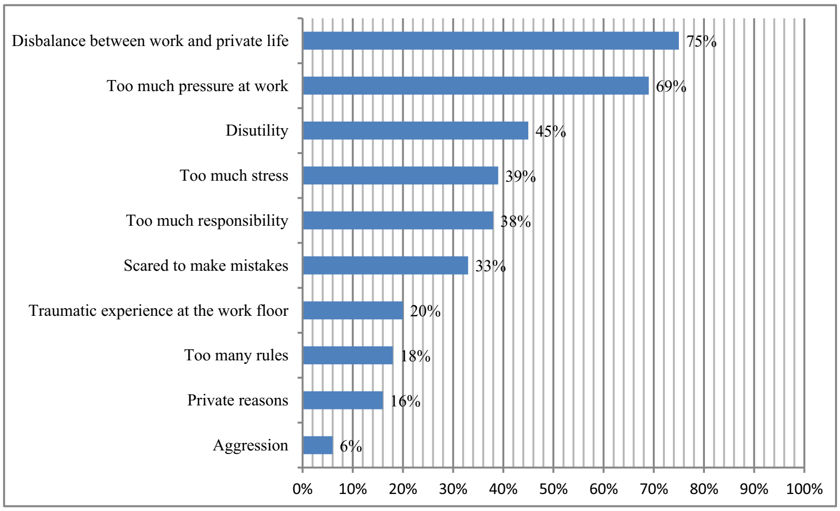

Fig. 3 Top 10 possible reasons pediatricians describe to stop working ($n = 168$). Multiple answers possible

we found more respondents with depressive and anxiety symptoms as compared to 1-year prevalences found in the general Dutch high-income population [8].

After experiencing an adverse event, a little over a quarter of all respondents indicated that there was a protocol regarding adverse events, but over half of the respondents do think a culture change is needed. Therefore, pediatricians have the need for a better support system. When more than half of the pediatricians perceive that care surrounding adverse events is not sufficient, this could lead to unnecessary stress. Physicians might experience more barriers than non-physicians to seek out professional help for mental health problems due to their fear of losing their license, denial of problems, and embarrassment [22]. When physicians are unfit, this may have a negative impact on their practice [23], whereas occupational well-being can positively contribute to patient satisfaction and the quality of interpersonal aspects of care [6]. In this questionnaire, coping strategies applied by the pediatricians were similar to coping strategies of gynecologists in the study by Baas et al. [17]. Almost 20% of the respondents adjusted their job and 40% seriously considered quitting their job completely. This is consistent with the findings of Hawkins et al. [24], who also found that physicians reduce work hours, retire, or quit medicine altogether because of a high work strain. Reported alcohol use to cope with adverse events seems to be quite low in our respondents with only 6%, especially since Hyman et al. found percentages of 6% (daily use) up to 25% (occasional use) of substances [25].

Thus far, little research has been done on this topic, specifically concerning mental health problems in relation to institutional or peer support. This study, which included validated questionnaires, allowed for detailed data collection. Allowing respondents to fill out examples of their experiences gives further insight in the way they experience their problems.

Limitations of the study are the response rate, with 18.9% lower than the average e-mail response rate of 25–30% [26]. Reasons for the low response rate could not only be the heavy workload of pediatricians but also the fact that the questionnaire was spread through a non-personal email account from the NVK, which people may not always read. With 410 completed questionnaires, however, we have a representative cohort comparable to the reference group. Another limitation is the risk of participation bias, because as with any questionnaire, pediatricians who are involved with this topic are more likely to participate. Concerning depression and anxiety, screening rates are generally an overestimation, especially when using self-report questionnaires. However, these are point prevalences, so when compared to 12-month prevalences, it can be an underestimation. Furthermore, depression and anxiety are not merely work-related.

In conclusion, work-related stressors in Dutch pediatricians are high and can subsequently lead to posttraumatic stress disorder. Parental aggression towards pediatricians seems to be a common problem, something that should be addressed, for example with CMD training programs [19]. In this study, the amount of pediatricians with PTSD complaints was higher compared to prevalences found in the general population and the same applies to depression and anxiety symptoms. There is no national standardized support after adverse events for pediatricians, while other occupations where there is an occupational hazard do have such support (e.g., in the police, military, firefighters). It is advised that evidence-based support (e.g., peer support) after adverse events is protocolled and education on coping strategies is implemented, to improve mental well-being of pediatricians.

Acknowledgments We would like to thank all members of the Pediatric Association of The Netherlands (NVK) who participated in this questionnaire for their interest in this topic. Furthermore, we would like to thank the Pediatric Association of The Netherlands (NVK) for their help. Lastly, we would like to thank Eva Jacobs (EJ) for categorizing all answers.

Authors' contributions Dr. M.G. van Pampus, K.W.F. Scheepstra and M.E. van Steijn conceptualized and designed the study, drafted the initial manuscript and reviewed and revised the manuscript.

G. Yasar collected data, carried out the initial analysis and reviewed and revised the manuscript.

Prof. dr. M.C. de Vries and prof. dr. Olff reviewed and revised the manuscript for important intellectual content.

All authors approved the final manuscript as submitted and agree to be accountable for all aspects of the work.

Compliance with ethical standards

This study was exempted from ethical approval by the Medical Research Ethics Committees United (MEC-U) and registered under the number W18.096.

Financial disclosure statement The authors have no financial relationships relevant to this article to disclose.

Conflict of interest The authors declare that they have no conflicts of interest.

Questionnaire Pediatricians

1. What is your gender?
1. Male
2. Female
2. What is your age?
1. 20–29 years
2. 30–39 years
3. 40–49 years
4. 50–59 years
5. 60–69 years
6. ≥ 70 years
3. Are you a member of the Dutch Society of Pediatricians (NVK)?
1. Yes
2. No
4. What is your current position?
1. Resident

2. Attending physician
3. Retired
4. Non-practicing/management
5. How many years have you been working in the pediatrics field as a physician (including as intern)?
1. ≤ 5 years
2. 6–10 years
3. 11–15 years
4. 16–20 years
5. 21–25 years
6. 26–30 years
7. > 30 years
6. At the moment, are you working in a general hospital or in an academic referral center?
1. General hospital
2. Academic referral center
3. Both
4. I do not work anymore, but I used to work in a general hospital
5. I do not work anymore, but I used to work in an academic referral center
7. At which department do you work the most (if you are not practicing anymore, fill in where you used to work)?
1. Neonatology
2. Emergency room
3. General pediatrician
4. NICU
5. Child intensive care
6. Policlinic
7. Children's oncology
8. Other…
8. What do you consider to be an adverse event on the work-floor (more than one answer possible)?
1. Not applicable
2. Bad news conversation/interview
3. (Critically) ill neonates
4. (Critically) ill children
5. When a patient dies
6. When you miss a diagnosis
7. When you have to refuse a patient
8. When you are in doubt whether you are making the right decision
9. When an inexperienced colleague is on call
10. When you decide on an abstaining course of treatment
11. Treatment of neonates
12. When you know a patient will remain severely handicapped
13. Handling the emotions of the parents
14. (Suspicion of) child abuse
15. Other …
9. How do you cope with adverse events on the work-floor (more than one answer possible)?
1. Not applicable/never experienced
2. Seeking professional help
3. Use new medication
4. Drink (more) alcohol
5. Smoke (more) cigarettes
6. Use (more) drugs
7. Work out (more)
8. Going home as soon as possible
9. Call in sick
10. Hide your emotions
11. Find a distraction
12. Praying or other religious activities
13. Talking to friends and/or family
14. Informally discussing the matter with peers/colleagues
15. Quitting
16. Work less
17. Not working any evening/night shifts
18. Other …
10. The current support organized by your institution after an adverse event is good:
1. Strongly disagree
2. Disagree
3. Agree
4. Strongly agree
11. There is plenty of room to informally discuss adverse events in the department/partnership:
1. Strongly disagree
2. Disagree
3. Agree
4. Strongly agree
12. There is plenty of room to informally discuss adverse events in the hospital:
1. Strongly disagree
2. Disagree
3. Agree
4. Strongly agree
13. There should be a culture change regarding support after an adverse event:
1. Strongly disagree
2. Disagree
3. Agree
4. Strongly agree
14. There is a protocol at your institution regarding support after an adverse event:
1. Yes
2. Yes, but nobody uses it
3. No, but there is one currently being created
4. No
5. I do not know
15. Support after an adverse event consisted of (more than one answer possible):
1. I have never experienced an adverse event
2. There was none
3. Professionally organized peer support

4. Self-initiated peer support with direct colleagues (own department)
5. Self-initiated peer support with indirect colleagues (different department)
6. Conversation(s) with a psychologist or coach
7. Evaluation with the present team
8. Help from the medical officer after a sick-leave
9. Other …

16. Your preferred support after an adverse event would be (more than one answer possible):
1. Not applicable
2. Support is unnecessary
3. Professionally organized peer support
4. Evaluation with the present team
5. Peer support from direct colleagues (own department)
6. Peer support from indirect colleagues (different department)
7. Mindfulness
8. Other …

17. It should be mandatory for the hospital to organize support after an adverse event:
1. Yes
2. No

18. You have learned to cope with adverse events through/during (more than one answer possible):
1. Med-school (without clerkships)
2. Clerkships
3. Internships
4. Residency
5. Attending physician
6. Other study
7. Psychological help
8. Specific course/training
9. Peer-review
10. Mindfulness
11. Never learnt
12. Other…

19. In the course of your career, you have become more defensive:
1. Strongly disagree
2. Disagree
3. Agree
4. Strongly agree

20. Have you changed your working-conditions (e.g., less shifts, more diagnostic tests) after experiencing a patient-related adverse event?
1. Yes
2. No

21. What did you change (more than one answer possible)?
1. Work less
2. Less evening/night-shifts
3. No evening/night-shifts anymore
4. Calling a colleague sooner
5. Quit my job
6. More diagnostic tests
7. Start treatment sooner
8. Other…

22. Sometimes people experience traumatic events, such as live-threatening situations as a cause of a natural disaster, high-impact trauma or fire; being attacked or raped; witness a murder, death or hear find out someone close to them experienced something terrible. As a physician, one can experience such events in patient-care: critical illness or death of a patients, severe injury, as well as violent behavior from a patient or their family.

Have you ever, during your work AS A PHYSICIAN experienced such (adverse) events/incidents?

1. Yes
2. No

23. Can you briefly describe the adverse event(s)?
1. Yes, please describe it…
2. No

24. Did (some part of) this/these adverse event(s) take place more than 4 weeks ago?
1. Yes
2. No

TSQ. Please indicate whether or not you have experienced any of the following at least twice in the past week:

25. Upsetting thoughts or memories about the event that have come into your mind against your will
1. Yes
2. No

26. Upsetting dreams about the event.
1. Yes
2. No

27. Acting or feeling as though the event were happening again.
1. Yes
2. No

28. Feeling upset by reminders of the event.
1. Yes
2. No

29. Bodily reactions (such as fast heartbeat, stomach churning, sweatiness, dizziness) when reminded of the event.
1. Yes
2. No

30. Difficulty falling or staying asleep.
1. Yes
2. No

31. Irritability of outbursts of anger.
1. Yes

2. No
32. Difficulty concentrating.
1. Yes
2. No
33. Heightened awareness of potential danger to yourself and others.
1. Yes
2. No
34. Being jumpy or being startled at something unexpected.
1. Yes
2. No
35. It might be that you did not experience the reactions and emotions as described on the previous pages, but that you recognize them from a past period in your life. For example, having disturbing thoughts or dreams, reliving the event, and getting upset. Do you recognize these reactions?
1. Yes
2. No
36. Which of these reactions do you recognize (more than one answer possible)?
1. Disturbing thoughts
2. Disturbing dreams
3. Reliving experiences
4. Getting upset
5. Physical responses
6. Insomnia
7. Irritability
8. Trouble focusing
9. Hyper arousal
10. Tensed
37. How long did these symptoms last?
1. < 4 weeks
2. > 4 weeks
3. > 6 months
4. > 1 year
38. Have you ever been diagnosed with PTSD (posttraumatic stress-disorder)?
1. Yes
2. No

HADS©. Tick the box beside the reply that is closest to how you have been feeling in the past week. Do not take too long over you replies: your immediate is best.*

39. I feel tense or "wound up":
1. Most of the time
2. A lot of the time
3. From time to time, occasionally
4. Not at all
40. I still enjoy the things I used to enjoy:
1. Definitely as much
2. Not quite so much
3. Only a little
4. Hardly at all
41. I get a sort of frightened feeling as if something awful is about to happen:
1. Very definitely and quite badly
2. Yes, but not so badly
3. A little, but it does not worry me
4. Not at all
42. I can laugh and see the funny side of things:
1. As much as I always could
2. Not quite so much now
3. Definitely not so much now
4. Not at all
43. Worrying thoughts go through my mind:
1. A great deal of the time
2. A lot of the time
3. From time to time, occasionally
4. Only occasionally
44. I feel cheerful:
1. Not at all
2. Not often
3. Sometimes
4. Most of the time
45. I can sit at ease and feel relaxed:
1. Definitely
2. Usually
3. Sometimes
4. Most of the time
46. I feel as if I am slowed down:
1. Nearly all the time
2. Very often
3. Sometimes
4. Not at all
47. I get a sort of frightened feeling like "butterflies" in the stomach:
1. Not at all
2. Occasionally
3. Quite often
4. Very often
48. I have lost interest in my appearance:
1. Definitely
2. I do not take as much care as I should
3. I may not take quite as much care
4. I take just as much care as ever
49. I feel restless as I have to be on the move:
1. Very much indeed
2. Quite a lot
3. Not very much
4. Not at all
50. I look forward with enjoyment to things:
1. As much as I ever did
2. Rather less than I used to
3. Definitely less than I used to

4. Hardly at all
51. I get sudden feelings of panic:
1. Very often indeed
2. Quite often
3. Not very often
4. Not at all
52. I can enjoy a good book or radio or TV program:
1. Often
2. Sometimes
3. Not often
4. Very seldom
53. Have you ever seriously considered quitting your job as a pediatrician? (e.g., by looking for a different job, talking to human resources about ending your contract)
1. Yes, absolutely
2. Yes, sometimes
3. No, never
54. What was the reason to consider quitting? (More than one answer possible)
1. Bad collaboration (working together) with a co-worker
2. Not challenging enough within the field
3. A lot of complications
4. Afraid to make mistakes
5. Too much responsibility
6. Traumatic experience on the work-floor
7. Complaint(s) from patients
8. One-sided
9. A lot of stress
10. A high workload
11. Disbalance between work and private life
12. Disagreements with work providers
13. Different interests
14. New challenge
15. Too much administration
16. Too much bureaucracy
17. Patient-violence
18. Too many rules
19. Insufficient guidance from supervisors
20. Problems with the partnership
21. Private reasons
22. Disutility
23. Other…
55. Did you ever face a complaint at the disciplinary board?
1. Yes
2. No

*Numbers before answers do not reflect the score corresponding with the answer.

Open Access This article is distributed under the terms of the Creative Commons Attribution 4.0 International License (http://creativecommons.org/licenses/by/4.0/), which permits unrestricted use, distribution, and reproduction in any medium, provided you give appropriate credit to the original author(s) and the source, provide a link to the Creative Commons license, and indicate if changes were made.

Publisher's note Springer Nature remains neutral with regard to jurisdictional claims in published maps and institutional affiliations.

References

1. Kessler RC, Aguilar-Gaxiola S, Alonso J, Benjet C, Bromet EJ, Cardoso G, Degenhardt L, de Girolamo G, Dinolova RV, Ferry F, Florescu S, Gureje O, Haro JM, Huang Y, Karam EG, Kawakami N, Lee S, Lepine JP, Levinson D, Navarro-Mateu F, Pennell BE, Piazza M, Posada-Villa J, Scott KM, Stein DJ, ten Have M, Torres Y, Viana MC, Petukhova MV, Sampson NA, Zaslavsky AM, Koenen KC (2017) Trauma and PTSD in the WHO world mental health surveys. Eur J Psychotraumatol 8:1353383
2. de Vries GJ, Olff M (2009) The lifetime prevalence of traumatic events and posttraumatic stress disorder in the Netherlands. J Trauma Stress 22:259–267
3. Brazeau CM, Schroeder R, Rovi S, Boyd L (2010) Relationships between medical student burnout, empathy, and professionalism climate. Acad Med 85:S33–S36
4. Shanafelt TD, Balch CM, Bechamps G, Russell T, Dyrbye L, Satele D, Collicott P, Novotny PJ, Sloan J, Freischlag J (2010) Burnout and medical errors among American surgeons. Ann Surg 251:995–1000
5. de Oliveira GS Jr, Chang R, Fitzgerald PC, Almeida MD, Castro-Alves LS, Ahmad S, McCarthy RJ (2013) The prevalence of burnout and depression and their association with adherence to safety and practice standards: a survey of United States anesthesiology trainees. Anesth Analg 117:182–193
6. Scheepers RA, Boerebach BC, Arah OA, Heineman MJ, Lombarts KM (2015) A systematic review of the impact of physicians' occupational well-being on the quality of patient care. Int J Behav Med 22:683–698
7. Frank SL Jr, Sherbino J (eds) (2015) CanMEDS 2015 Physician Competency Framework. Royal College of Physicians and Surgeons of Canada, Ottawa
8. de Graaf R, ten Have M, van Gool C, van Dorsselaer S (2012) Prevalence of mental disorders and trends from 1996 to 2009. Results from the Netherlands mental health survey and incidence Study-2. Soc Psychiatry Psychiatr Epidemiol 47:203–213
9. Bernburg M, Vitzthum K, Groneberg DA, Mache S (2016) Physicians' occupational stress, depressive symptoms and work ability in relation to their working environment: a cross-sectional study of differences among medical residents with various specialties working in German hospitals. BMJ Open 6:e011369
10. Ruitenburg MM, Frings-Dresen MH, Sluiter JK (2012) The prevalence of common mental disorders among hospital physicians and their association with self-reported work ability: a cross-sectional study. BMC Health Serv Res 12:292–298
11. Skogstad M, Skorstad M, Lie A, Conradi HS, Heir T, Weisaeth L (2013) Work-related post-traumatic stress disorder. Occup Med (Lond) 63:175–182
12. American Psychiatric Association (2013) Diagnostic and statistical manual of mental disorders: DSM-5. Arlington, VA, American Psychiatric Association, Washington, DC
13. Zigmond AS, Snaith RP (1983) The hospital anxiety and depression scale. Acta Psychiatr Scand 67:361–370

14. Brewin CR, Rose S, Andrews B, Green J, Tata P, McEvedy C, Turner S, Foa EB (2002) Brief screening instrument for post-traumatic stress disorder. Br J Psychiatry 181:158–162
15. Dekkers AM, Olff M, Naring GW (2010) Identifying persons at risk for PTSD after trauma with TSQ in the Netherlands. Community Ment Health J 46:20–25
16. Mouthaan J, Sijbrandij M, Reitsma JB, Gersons BP, Olff M (2014) Comparing screening instruments to predict posttraumatic stress disorder. PLoS One 9:e97183
17. Baas MAM, Scheepstra KWF, Stramrood CAI, Evers R, Dijksman LM, van Pampus MG (2018) Work-related adverse events leaving their mark: a cross-sectional study among Dutch gynecologists. BMC Psychiatry 18:73
18. Foa EB, Riggs DS, Dancu CV, Rothbaum BO (1993) Reliability and validity of a brief instrument for assessing post-traumatic stress disorder. J Trauma Stress 6:459–473
19. Hemati-Esmaeili M, Heshmati-Nabavi F, Pouresmail Z, Mazlom S, Reihani H (2018) Educational and managerial policy making to reduce workplace violence against nurses: an action research study. Iran J Nurs Midwifery Res 23:478–485
20. Rosenman ED, Vrablik MC, Charlton PW, Chipman AK, Fernandez R (2017) Promoting workplace safety: teaching conflict management and de-escalation skills in graduate medical education. J Grad Med Educ 9:562–566
21. Bourne T, Vanderhaegen J, Vranken R, Wynants L, De Cock B, Peters M, Timmerman D, Van Calster B, Jalmbrant M, Van Audenhove C (2016) Doctors' experiences and their perception of the most stressful aspects of complaints processes in the UK: an analysis of qualitative survey data. BMJ Open 6:e011711
22. Brooks E, Early SR, Gendel MH, Miller L, Gundersen DC (2018) Helping the healer: population informed workplace wellness recommendations for physician well-being. Occup Med (Lond) 68(4): 279–281
23. Wallace JE, Lemaire JB, Ghali WA (2009) Physician wellness: a missing quality indicator. Lancet 374:1714–1721
24. Hawkins M (2016) 2016 Survey of America's physicians. Practice patterns & perspectives. The Physicians Foundation, Memphis
25. Hyman SA, Shotwell MS, Michaels DR, Han X, Card EB, Morse JL, Weinger MB (2017) A survey evaluating burnout, health status, depression, reported alcohol and substance use, and social support of anesthesiologists. Anesth Analg 125:2009–2018
26. Yun GW, Trombo CW (2000) Comparative response to a survey executed by post, e-mail, & web form. Journal of Computer-Mediated Communication 6(1)
