# Supplementary material for: Mental health status and related factors influencing healthcare workers during the COVID-19 pandemic: A systematic review and meta-analysis
Source: PLoS One. 2024 Jan 19;19(1):e0289454. doi: 10.1371/journal.pone.0289454 (PMC10798549; doi:10.1371/journal.pone.0289454)
Supplement: S1 Data — (ZIP) [file pone.0289454.s011.zip › literatures/205.pdf]

Healthcare Worker Mental Health After the Initial Peak of the COVID-19 Pandemic: a US Medical Center Cross-Sectional Survey

Michael J. Van Wert, LCSW-C, MPH[1], *Sonal Gandhi, MD*[2], *Ishaan Gupta, MD*[2], *Amteshwar Singh, MD, MEHP, FACP*[2], *Shaker M. Eid, MD, MBA*[2], *M. Haroon Burhanullah, MD*[1], *Henry Michtalik, MD, MPH, MHS*[2,3,4], *and Mansoor Malik, MD, MBA*[1]

[1]Department of Psychiatry and Behavioral Sciences, Johns Hopkins University School of Medicine, Baltimore, MD, USA; [2]Department of Medicine, Johns Hopkins University School of Medicine, Baltimore, MD, USA; [3]Center for Health Services and Outcomes Research, Johns Hopkins Bloomberg School of Public Health, Baltimore, MD, USA; [4]Armstrong Institute for Patient Safety and Quality, Johns Hopkins Medicine, Baltimore, MD, USA.

BACKGROUND: There is a paucity of data on the mental health impact of the Coronavirus disease 2019 (COVID-19) pandemic on United States (US) healthcare workers (HCWs) after the first surge in the spring of 2020.
OBJECTIVE: To determine the impact of the pandemic on HCWs, and the relationship between HCW mental health and demographics, occupational factors, and COVID-19 concerns.
DESIGN: Cross-sectional survey in an urban medical center (September–November 2020) in Baltimore, MD, in the United States.
PARTICIPANTS: A total of 605 HCWs (physicians, nurse practitioners, nurses, physician assistants, patient care technicians, respiratory therapists, social workers, mental health therapists, and case managers).
MAIN MEASURES: Measures of mental health (Patient Health Questionnaire-2, Generalized Anxiety Disorder-7, PROMIS Sleep Disturbance 4a, Impact of Event Scale-Revised, Maslach Burnout Inventory-2 item, Connor-Davidson Resilience Scale-2 item), demographics, occupational factors, and COVID-19 related concerns.
KEY RESULTS: Fifty-two percent of 1198 HCWs responded to the survey and 14.2% reported depression, 43.1% mild or higher anxiety, 31.6% sleep disturbance, 22.3% posttraumatic stress symptoms, 21.6% depersonalization, 46.0% emotional exhaustion, and 23.1% lower resilience. Relative to HCWs providing in-person care to COVID-19 infected patients none of their working days, those doing so all or most days were more likely to experience worse depression (adjusted odds ratio, 3.9; 95% CI, 1.3–11.7), anxiety (aOR, 3.0; 95% CI, 1.4–6.3), possible PTSD symptoms (aOR, 2.6; 95% CI, 1.1–5.8), and higher burnout (aOR, 2.6; 95% CI, 1.1–6.0). Worse mental health in several domains was associated with higher health fear (aORs ranged from 2.2 to 5.0), job stressors (aORs ranged from 1.9 to 4.0), perceived social stigma/avoidance (aORs ranged from 1.8 to 2.9), and workplace safety concerns (aORs ranged from 1.8 to 2.8).
CONCLUSIONS: US HCWs experienced significant mental health symptoms eight months into the pandemic. More time spent providing in-person care to COVID-19-infected patients and greater COVID-19-related concerns were consistently associated with worse mental health.

KEY WORDS: Severe acute respiratory syndrome coronavirus 2 (SARS-CoV-2); COVID-19; Healthcare workers; Mental health; Pandemic.

J Gen Intern Med 37(5):1169–76
DOI: 10.1007/s11606-021-07251-0
© Society of General Internal Medicine 2021

Prior presentations *Gandhi, S.; Gupta, I.; Malik, M.; Singh, A.; Burhanullah, H.; Van Wert, M. Depression and anxiety among healthcare workers at Johns Hopkins Bayview Medical Center during the COVID-19 pandemic: a cross-sectional survey. March 12, 2021. Poster presented at the Johns Hopkins Department of Medicine and Whiting School of Engineering Research Retreat, Baltimore, MD.*

Van Wert, M. How are Bayview healthcare providers doing during COVID-19? A cross-sectional survey of depression, anxiety, PTSD, sleep disturbance, burnout, and resilience. April 7, 2021. Johns Hopkins Bayview Community Psychiatry Program in-service training series, Baltimore, MD.

Michael J. Van Wert and Sonal Gandhi are co-first authors.

Received April 29, 2021
Accepted October 27, 2021
Published online January 6, 2022

INTRODUCTION

The human toll of the Coronavirus disease 2019 (COVID-19) has been staggering, with over 200 million infections and 4 million deaths globally as of August 2021, and the largest proportion of these from any single country has come from the USA.[1] This has placed significant stress on the US healthcare system and the healthcare workers (HCWs) who constitute its backbone, and will likely continue to do so as the country struggles to vaccinate all Americans amidst new virus variants. The stakes are undoubtedly high as poor HCW well-being and burnout are associated with impaired cognitive function, depersonalization, dropout from work, and medical errors.[2,3]

As the pandemic has become more widespread, there has been a growing literature capturing the detrimental mental health impact on HCWs globally,[4–6] but relatively fewer studies have come from the US, where the burden of physical

illness has been disproportionately high. US studies have described the initial impact of COVID-19 on HCW mental health, including depression,[7–15] anxiety,[7–18] stress [7,11–15,17,19–23], and burnout.[9,10,21–24] Risk factors associated with poor US HCW mental health have included higher frequency of exposure to infected patients,[7,9,13,16,22] being a woman,[8,14,23,24] younger age,[9,14,20] and previous psychiatric history.[14,15]

Several gaps exist in the literature studying the mental health impact of COVID-19 on HCW. First, most of the studies are from outside the US or describe HCW mental health during the first peak of new cases and deaths in the US between March and May 2020. As the pandemic has persisted and HCWs have had to adapt, understanding how mental health has been impacted beyond the initial months, as well as between peak periods, is needed. Second, while some existing studies have examined risk factors for poorer mental health, there is a need to further investigate these associations. The present study sought to capture the mental health impact of COVID-19 on an intentionally diverse sample of HCWs 8 months into the pandemic and during a trough of new cases and deaths. It also examined the relationship between HCW mental health and demographics, occupational factors, and COVID-19 concerns. We hypothesized that more frequent occupational exposure to COVID-19 would be associated with worse mental health. Moreover, following Maunder et al.,[25] which found domains of HCW concerns to be associated with worse mental health during the SARS-CoV1 pandemic, we hypothesized that similar domains would be associated with HCW mental health during the COVID-19 pandemic. As the pandemic has persisted, understanding these factors is key to developing targeted interventions that best support the most vulnerable HCWs.

METHODS

Study Design

This cross-sectional survey was e-mailed via Qualtrics (Qualtrics, Provo, UT) to 1198 HCWs within a large urban medical center in Baltimore, Maryland. from 9/9/2020 to 11/26/2020, at which time there was a city mask mandate and many businesses allowed 50% capacity (Appendix 1). All department managers were contacted and HCW e-mails provided were invited, including those in the emergency room, intensive care units, intermediate medical care units, inpatient psychiatry, medical surgical floors, outpatient clinics, environmental services, and food services. Consent consisted of a response to the survey invitation. Seven e-mail reminders were sent. The study occurred between the July 2020 and January 2021 peaks of the second and third surges in Maryland. All responses were deidentified. The Johns Hopkins Medicine Institutional Review Board approved this protocol (IRB00246458).

Survey Components

Demographic and Occupational Factors. Respondent demographics included age, gender, race, and living situation. Occupational factors included role, setting, and amount of time providing in-person (e.g., in physical presence) care to COVID-19-infected patients.

Measures of Mental Health. Six measures were administered: the Patient Health Questionnaire-2(PHQ-2) (0–6, $\geq$ 3 indicates a likely positive screen for depression) measured depression;[26] the Generalized Anxiety Disorder-7(GAD-7) (0–21, 0–4: minimal anxiety, 5–9: mild anxiety, 10–14: moderate anxiety, 15–21: severe anxiety) measured anxiety;[27] the Adult PROMIS Short From v.1.0—Sleep Disturbance 4a (t-score from 32 to 73.3, 32–54: within normal limits, 55–60: mild, 61–70: moderate, 71–73.3: severe) measured sleep disturbance;[28–30] the Impact of Event Scale-Revised(IES-R) (0–88, higher = more symptoms) measured posttraumatic stress (PTSD);[31] the Maslach Burnout Inventory 2-item(MBI-2) (depersonalization, emotional exhaustion; each question ranges from 0 to 6, higher = more symptoms) measured burnout;[32,33] and the Connor-Davidson Resilience Scale 2-item (CD-RISC-2) (0–8, higher = more resilience) measured resilience.[34]

Measures of COVID-19-Related Concerns. Three yes/no statements adapted from Maunder et al.[25] were used to assess three domains of HCW concern: health fear, job stressors, and perceived social stigma and avoidance (Fig. 1). A response of "yes" was coded as 1 and "no" as 0, and responses were summed across the three statements (range of 0–3) in each domain, with higher scores representing a higher degree of concern.

To assess workplace safety concerns on a five-point Likert scale (definitely yes, probably yes, might/might not, probably no, definitely no), two statements were adapted from Maunder et al.[25] Responses were coded 0 (definitely yes), 1 (probably yes), 2 (might/might not), 3 (probably no), and 4 (definitely no), and responses were summed across the two statements (range of 0–8), with higher scores representing higher concern.

Statistical Analysis

To examine the relationship between mental health and HCW demographics, occupational factors, and COVID-19 concerns, logistic regression was used to estimate adjusted odds ratios and 95% confidence intervals (significance level of $p < 0.05$). Adjusted odds ratios reflect analyses which controlled for age, gender, race, living situation, occupational role, setting, and COVID-19 work exposure.

All measures of mental health were dichotomized guided by a goal of approximating meaningful screening cutoffs delineating symptomatic from non-symptomatic: PHQ-2 ($\geq$ 3 indicating likely depression vs. below 3;[8,12,26]) GAD-7

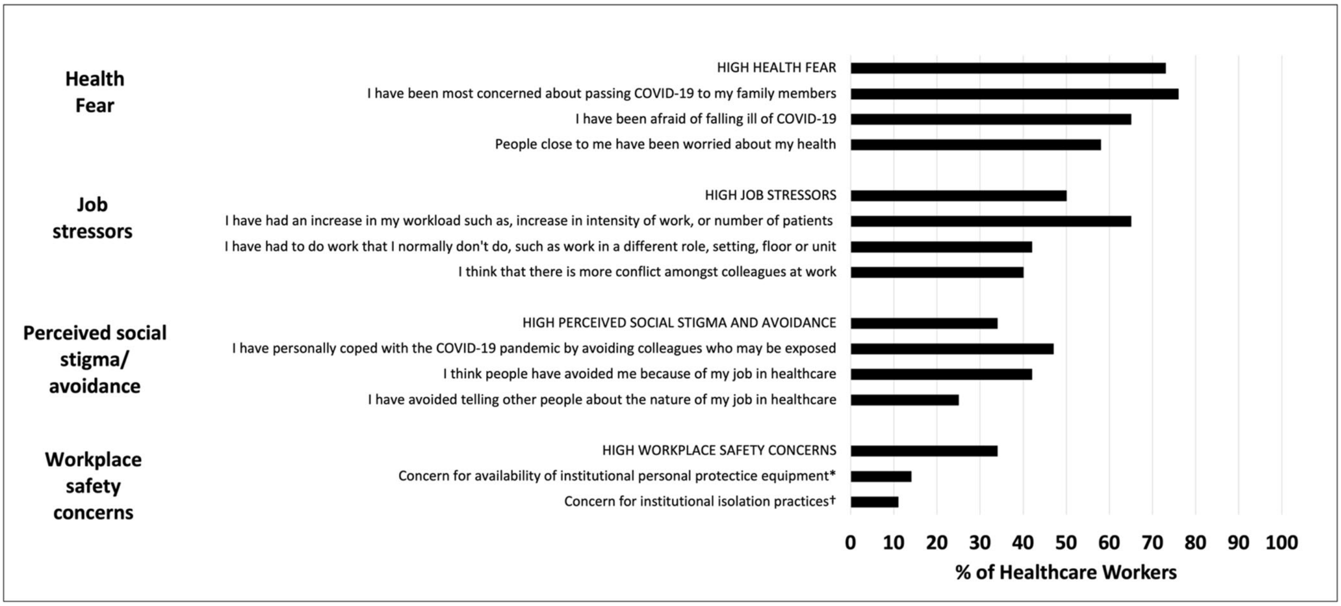

Figure 1 Healthcare worker concerns during the COVID-19 pandemic (% of sample, *n* = 605). Workplace safety concern statements were worded such that an affirmative response (e.g., yes) indicated no/low concern, whereas an affirmative response on statements in health fear, job stressors, and perceived social stigma/avoidance indicated concern. *The statement to which survey respondents answered was: There is enough PPE available to adequately protect yourself at your institution. †The statement to which survey respondents answered was: Current isolation practices at your institution protect you from contracting the COVID-19 infection

(minimal vs. mild or higher;[7,8,13–15,27]) Adult PROMIS Short Form v1.0—Sleep Disturbance 4a (within normal limits vs. any sleep disturbance;[28–30]) IES-R (≥ 22 indicating clinical concerns of PTSD vs. less than 22;[35]MBI-2 (low burnout: answering less than once a week on both questions vs. high burnout: answering once a week or more on either question;[36]) and the CD-RISC-2 (lower resilience: < 6 vs. higher resilience: ≥ 6.[34]).

For measures of health fear, job stressors, and perceived social stigma/avoidance, responses were dichotomized into "low" and "high" using a cutoff of 1 or below for "low" and 2 or above for "high," the latter indicating responding "yes" to a majority (at least 2 out of 3) of the concerns in the domain. For the measure of workplace safety concern, whose statements had different response options than those in the other domains, a score of 3 or above was considered "high," (< 3 = "low") indicating any disagreement in perceived efficacy of either of the two institutional safety precautions.

Demographics were categorized as follows: age (19–39 vs. 40 +), gender (men vs. women), race (White, Black/African American, Asian, other), living situation (alone vs. with others), occupational role (categorized by work task similarity: medication prescribers (physicians, residents, physician assistants, nurse practitioners), non-prescribing psychosocial health providers (social workers, mental health therapists, case managers, other related), non-prescribing physical health providers (nurses, patient care techs, respiratory therapists)), work setting (outpatient, emergency room, inpatient, multiple), and COVID-19 occupational exposure (collapsed into three groups based on the question, "During the COVID-19 pandemic, how often (working days) do you provide in-person clinical care to patients with the diagnosis of COVID-19?": None/Does not apply, Some/About half, and All/Most). Data not reported was excluded from analyses. Analyses were conducted using STATA version 13 (StataCorps, TX).

RESULTS

A total of 605 HCWs (52% of e-mailed HCWs responded) were included in the analysis (Table 1). Using measures (Table 2), 14.2% of HCWs reported depression, 43.1% mild or higher anxiety, 31.6% any sleep disturbance, 22.3% PTSD symptoms, and 23.1% lower resilience. On the burnout measure, 21.6% reported depersonalization and 46.0% reported emotional exhaustion at least a few times a month (Table 2).

Mental Health and Demographics

HCWs 40 years or older were less likely than those younger than 40 to report mild or higher anxiety (aOR, 0.5; 95% CI, 0.3–0.8) (Table 3). Asian HCWs were less likely than white HCWs to report sleep disturbance (aOR, 0.4; 95% CI, 0.2–0.8). Finally, HCWs who lived with someone were less likely to report depression (aOR, 0.3; 95% CI, 0.2–0.6) and lower resilience (aOR, 0.5; 95% CI, 0.3–0.9) compared to HCWs who lived alone.

Mental Health and COVID-19-Related Occupational Factors

HCWs who spent all/most their working days providing in-person care to COVID-19 patients were more likely to report depression (aOR, 3.9; 95% CI, 1.3–11.7), mild or higher

Table 1 Healthcare Worker Demographics and Occupational Factors ($n = 605$)

Demographic/occupational factor	N (%)
**Age (years)**	
19–29	139 (23.0)
30–39	170 (28.1)
40–49	124 (20.5)
50 +	158 (26.1)
Not reported	14 (2.3)
**Gender**	
Men	108 (17.9)
Women	475 (78.5)
Prefer to self-identify	2 (0.3)
Not reported	20 (3.3)
**Race**	
White	350 (57.9)
Black/African American	92 (15.2)
Asian	99 (16.4)
American Indian/Alaska Native	2 (0.3)
Native Hawaiian/Pacific Islander	2 (0.3)
Other	29 (4.8)
Not reported	12 (5.1)
**Living situation**	
Alone	86 (14.2)
With someone	507 (83.8)
Not reported	12 (2.0)
**Occupational role**	
Non-prescriber psychosocial health providers	
Social worker/mental health therapist/case manager/ other	166 (27.4)
Medication prescribers	
Physician/resident/physician assistant/nurse practitioner	139 (23.0)
Non-prescriber physical health providers	
Nurse/patient care tech/respiratory therapist	283 (46.8)
Not reported	17 (2.8)
**Work setting (not mutually exclusive)**	
Inpatient	
Medical surgical floor	214 (35.4)
Intensive care unit	85 (14.1)
Intermediate care unit	80 (13.2)
Inpatient psychiatry	36 (6.0)
Emergency room	141 (23.3)
Outpatient	134 (22.2)
Not reported	114 (18.4)
**Time providing in-person care to COVID-19-infected patients**	
All/most of the working days	147 (24.3)
Some/About half the working days	214 (35.4)
None of the working days/Does not apply	164 (27.1)
Not reported	80 (13.2)

anxiety (aOR, 3.0; 95% CI, 1.4–6.3), PTSD symptoms (aOR, 2.6; 95% CI, 1.1–5.8), and higher burnout (aOR, 2.6; 95% CI, 1.1–6.0) compared to those who spent none of their days doing so (Table 3). Similarly, HCWs who spent half/some of their days were more likely to report anxiety (aOR, 2.1; 95% CI, 1.1–4.1) than those who did none. Anxiety was the only mental health domain that was associated with occupational role, with non-prescribing physical health providers less likely to report anxiety than non-prescribing psychosocial health providers (aOR, 0.3; 95% CI, 0.1–0.9).

Table 2 Mental Health Impact of the COVID-19 Pandemic on Healthcare Workers ($n = 605$)

Measure of mental health	N (%)
**PHQ-2***	
Yes	86 (14.2)
No	475 (78.5)
Not reported	44 (7.3)
**GAD-7†**	
Minimal	299 (49.4)
Mild	162 (26.8)
Moderate	68 (11.2)
Severe	31 (5.1)
Not reported	45 (7.4)
**PROMIS Sleep Disturbance‡**	
Within normal limits	369 (61.0)
Mild	109 (18.0)
Moderate	75 (12.4)
Severe	7 (1.2)
Not reported	45 (7.4)
**IES-R§**	
Yes	135 (22.3)
No	409 (67.6)
Not reported	61 (10.1)
**MBI-2‖**	
Depersonalization	
Never	178 (39.2)
A few times a year or less	88 (19.4)
Once a month or less	45 (9.9)
A few times a month	37 (8.2)
Once a week	21 (4.6)
A few times a week	26 (5.7)
Every day	14 (3.1)
Not reported	45 (9.9)
Emotional exhaustion	
Never	51 (11.2)
A few times a year or less	87 (19.2)
Once a month or less	62 (13.7)
A few times a month	73 (16.1)
Once a week	41 (9.0)
A few times a week	50 (11.0)
Every day	45 (9.9)
Not reported	45 (9.9)
**CD-RISC-2¶**	
≥ 6	405 (66.9)
< 6	140 (23.1)
Not reported	60 (9.9)

**Yes (≥ 3 indicates a likely positive screen for depression) †minimal (0–4), mild (5–9), moderate (10–14), severe (15–21) ‡within normal limits (32–54), mild (55–60), moderate (61–70), severe (71–73.3) §yes (≥ 22, PTSD could be a clinical concern) ‖each question ranges from 0 to 6 (0 = Never to 6 = Every day) ¶higher (≥ 6), lower (< 6)*

Mental Health and COVID-19-Related Concerns

Overall, 72.4% of HCWs reported high health fear, 50.1% high job stressors, 33.6% high perceived social stigma and avoidance, and 33.6% high workplace safety concern (Fig. 1). HCWs reporting high health fear were more likely than those reporting low fear to report depression (aOR, 5.0; 95% CI, 1.5–16.8), mild or higher anxiety (aOR, 3.9; 95% CI, 2.1–7.0), sleep disturbance (aOR, 2.2; 95% CI, 1.2–3.9), PTSD symptoms (aOR, 5.7; 95% CI, 2.3–13.8), high burnout (aOR, 2.5; 95% CI, 1.3–5.0), and lower resilience (aOR, 2.8; 95% CI, 1.4–5.7) (Table 4). Similarly, those reporting high (relative to

Table 3 Associations Between Healthcare Worker Demographics/Occupational Factors and Mental Health (Adjusted Odds Ratios (aOR) and 95% CI)

Healthcare worker demographics and occupational factors	Measure of healthcare worker mental health											
	Depression (PHQ-2) (Ref = Unlikely depression)		Anxiety (GAD-7) (Ref = Minimal)		Sleep disturbance (PROMIS) (Ref = Within normal limits)		Posttraumatic stress (IES-R) (Ref = Unlikely PTSD)		Burnout (MBI-2) (Ref = Low burnout)		Lower resilience (CD-RISC-2) (Ref = Higher resilience)	
	aOR	95% CI	aOR	95% CI	aOR	95% CI	aOR	95% CI	aOR	95% CI	aOR	95% CI
Age (Ref = < 40)	0.6	(0.3–1.1)	0.5	(0.3–0.8)*	0.7	(0.5–1.1)	0.9	(0.5–1.4)	0.7	(0.5–1.2)	0.7	(0.4–1.1)
Gender (Ref = men)	0.9	(0.4–1.9)	1.3	(0.7–2.2)	0.9	(0.5–1.7)	1.4	(0.7–2.7)	1.7	(0.8–3.4)	0.7	(0.4–1.3)
Race (Ref = white)												
Black/African American	1.3	(0.6–2.7)	0.6	(0.3–1.1)	0.8	(0.4–1.4)	0.6	(0.3–1.2)	0.7	(0.3–1.3)	0.5	(0.2–1.1)
Asian	0.6	(0.2–1.4)	0.8	(0.5–1.5)	0.4	(0.2–0.8)*	0.6	(0.3–1.3)	0.7	(0.4–1.4)	1.2	(0.7–2.3)
Other	0.2	(0.02–1.6)	0.5	(0.2–1.3)	0.9	(0.4–2.3)	1.6	(0.7–4.1)	1.1	(0.4–3.1)	1.0	(0.4–2.5)
Living situation (Ref = alone)	0.3	(0.2–0.6)*	0.9	(0.5–1.6)	0.7	(0.4–1.3)	0.7	(0.4–1.3)	0.8	(0.4–1.5)	0.5	(0.3–0.9)*
Occupational role (Ref = SW/MHT/C.Mgmt/other)†												
MD/PA/NP‡	1.1	(0.3–4.6)	0.5	(0.2–1.4)	0.6	(0.2–1.5)	0.7	(0.2–1.9)	0.8	(0.3–2.5)	0.9	(0.3–2.6)
Nurse/PT/RT§	0.7	(0.2–3.0)	0.3	(0.1–0.9)*	0.8	(0.3–2.2)	0.8	(0.3–2.3)	0.6	(0.2–1.8)	1.1	(0.4–3.2)
Work setting (Ref = outpatient only)												
Emergency room only	0.7	(0.2–3.4)	1.7	(0.6–5.2)	1.6	(0.6–4.7)	0.8	(0.3–2.5)	1.4	(0.4–4.8)	0.5	(0.2–1.7)
Inpatient only	0.8	(0.2–4.1)	1.7	(0.6–5.2)	1.2	(0.4–3.6)	0.7	(0.2–2.3)	1.3	(0.4–4.5)	1.0	(0.3–3.1)
Multiple settings	0.8	(0.2–4.2)	1.5	(0.5–4.4)	1.1	(0.4–3.1)	0.7	(0.2–2.2)	1.1	(0.3–3.7)	1.4	(0.5–4.3)
Weekly days providing in-person care to COVID-infected patients (Ref = none)												
Some/half	2.6	(0.9–7.4)	2.1	(1.1–4.1)*	1.2	(0.6–2.3)	1.2	(0.5–2.5)	1.4	(0.6–3.0)	1.0	(0.5–2.1)
All/most	3.9	(1.3–11.7)*	3.0	(1.4–6.3)*	1.7	(0.8–3.6)	2.6	(1.1–5.8)*	2.6	(1.1–6.0)*	1.2	(0.6–2.6)

**$p < 0.05$; Adjusted odds ratios (aOR) controlled for age, race, gender, living situation, occupational role, work setting, and COVID-19 work exposure*
†Non-prescriber psychosocial health providers: Social workers, mental health therapists, case managers, and other related staff
‡ Medication prescribers: Physicians, physician assistants, nurse practitioners
§Non-prescriber physical health providers: Nurses, patient technicians, respiratory therapists

low) job stressors were more likely to report depression (aOR, 2.5; 95% CI, 1.3–4.8), anxiety (aOR, 2.8; 95% CI, 1.8–4.3), sleep disturbance (aOR, 2.1; 95% CI, 1.3–3.3), PTSD symptoms (aOR, 4.0; 95% CI, 2.3–6.9), high burnout (aOR, 4.0; 95% CI, 2.3–7.0), and lower resilience (aOR, 1.9; 95% CI, 1.2–3.0).

HCWs who reported high (relative to low) perceived social stigma and avoidance had a higher likelihood of sleep

Table 4 Associations Between Healthcare Worker COVID-19-Related Concerns and Mental Health (Adjusted Odds Ratios (aOR) and 95% CI)

Healthcare worker concerns	Measure of healthcare worker mental health											
	Depression (PHQ-2) (Ref = Unlikely depression)		Anxiety (GAD-7) (Ref = Minimal)		Sleep disturbance (PROMIS) (Ref = Within normal limits)		Posttraumatic stress (IES-R) (Ref = Unlikely PTSD)		Burnout (MBI-2) (Ref = Low burnout)		Lower resilience (CD-RISC-2) (Ref = Higher resilience)	
	aOR	95% CI	aOR	95% CI	aOR	95% CI	aOR	95% CI	aOR	95% CI	aOR	95% CI
Health fear (Ref = low)	5.0	(1.5–16.8)*	3.9	(2.1–7.0)*	2.2	(1.2—3.9)*	5.7	(2.3–13.8)*	2.5	(1.3–5.0)*	2.8	(1.4–5.7)*
Job stressors (Ref = low)	2.5	(1.3- 4.8)*	2.8	(1.8–4.3)*	2.1	(1.3—3.3)*	4.0	(2.3- 6.9)*	4.0	(2.3–7.0)*	1.9	(1.2–3.0)*
Perceived social stigma and avoidance (Ref = low)	1.4	(0.8- 2.6)	1.4	(0.9–2.2)	1.8	(1.1—2.8)*	2.2	(1.3- 3.5)*	2.9	(1.7–4.9)*	1.5	(0.9–2.4)
Workplace safety concern (Ref = low)	1.8	(1.02–3.3)*	1.8	(1.2–2.7)*	1.5	(0.95–2.3)	2.5	(1.5- 3.9)*	2.8	(1.7–4.7)*	1.3	(0.9–2.1)

**$p < 0.05$; adjusted odds ratios (AOR) controlled for age, race, gender, living situation, occupational role, work setting, and COVID-19 work exposure*

disturbance (aOR, 1.8; 95% CI, 1.1–2.8), PTSD symptoms (aOR, 2.2; 95% CI, 1.3–3.5), and high burnout (aOR, 2.9; 95% CI, 1.7–4.9). Respondents reporting high (relative to low) workplace safety concern were more likely to report depression (aOR, 1.8; 95% CI, 1.02–3.3), anxiety (aOR, 1.8; 95% CI, 1.2–2.7), PTSD symptoms (aOR, 2.5; 95% CI, 1.5–3.9), and high burnout (aOR, 2.8; 95% CI, 1.7–4.7).

DISCUSSION

To date, the present study is one of the first to describe the mental health impact of the COVID-19 pandemic on a diverse sample of US HCWs after the initial surge. Eight months into the pandemic, HCWs experienced significant depression, anxiety, sleep disturbance, posttraumatic stress, burnout, and lower resilience. Irrespective of age, gender, race, occupational role, work setting, and living situation, more time spent providing in-person care to COVID-19 patients was consistently associated with worse mental health. Furthermore, this is one of the first studies to report associations between grouped domains of HCW's COVID-19 concerns and their mental health.

The prevalence of depression in our study was comparable with that of larger multi-regional US studies[8,14,15] at the first peak of the pandemic ranging from 13.9 to 17.0%, but lower than a New York City sample[12] of 48.0%. Similarly, 16.3% of present HCWs reporting moderate or higher anxiety was comparable with two US studies reporting from 15.6 to 16.1%,[8,14] but lower than 33.0% reported in two other studies.[14,15] Although the present study was unable to assess symptom levels prior to the pandemic, it is plausible they worsened over time given that depression prevalence increased from 8.7% in 2018 to 14.4% in April 2020 in the adult general population,[37] and studies have found higher symptom rates in HCWs relative to the general population prior to the pandemic.[38–42] While our survey was administered between surges of new cases in Maryland, stress on the healthcare system was still significant. During the survey period, hospitals in Maryland met or exceeded 85% capacity of staffed intensive and acute care beds, a benchmark used to gauge stress on the healthcare system[43] (Appendix 2). Additionally, with the prediction of an upcoming surge in December 2020, it is likely that fear of rising hospitalizations impacted the mental health of HCWs.

We found that being 40 or older, Asian, and living with someone might place HCWs at lower risk for some mental health symptoms. Similar findings during COVID-19 related to age have been reported in other US studies, with higher anxiety[7–9,15] and stress[19,20] associated with younger age. Previous experience in a public health emergency is protective against distress,[44] and younger HCWs might be less likely to have experienced such an emergency. Younger HCWs might also spend more time on social media which might increase exposure to COVID-19-related news, including misinformation, and subsequently increase the likelihood of anxiety[14] and sleep disturbance. Presently, Asian HCWs were less likely to report sleep disturbance, but there were no significant associations with other mental health domains based on race. Other US studies have found that non-white HCWs were less likely to report anxiety[13,14] and depression.[9] These findings are notable given that racial and ethnic minorities have been at higher risk for worse COVID-19-related health outcomes.[45–48] Even pre-pandemic, however, non-whites have reported lower levels of anxiety[49] and sleep disturbance[50] than whites, possibly related to differential comfort expressing mental health or measurement factors. Regarding living situation, being in a committed relationship[51] is protective for HCWs, and a higher degree of social support is associated with lower stress and depression in the general population.[52] Higher resilience and lower depression associated with living together might be explained by easier access to in-person emotional support, which may have benefits over support via virtual means (e.g., Zoom).

In the present study, HCW occupational role was not associated with mental health, except for anxiety. Non-prescribing physical health providers in the present sample were less likely than non-prescribing psychosocial health providers to report anxiety. Our findings diverge from previous studies[12,14,44,53,54] where nurses have been more likely to experience worse mental health. Many outpatient mental health providers, who largely constituted the group more likely to report anxiety, had to abruptly adapt to practicing remotely, were redeployed, had work hours reduced, and feared layoffs. Additionally, it is possible that HCWs providing direct COVID-19 treatment had habituated after many months to higher work anxiety.

The present study demonstrated that HCWs providing care to COVID-19 patients all/most days of the week reported worse outcomes in all mental health domains, except sleep disturbance and resilience, compared to those providing it none of their days. Similar findings have been reported in other studies globally[53,55–57] and in the US, where more frequent occupational exposure to COVID-19 patients has been associated with depression,[7,13] anxiety,[7,13,16] posttraumatic stress/stress,[7,13,16] and burnout.[22] We also found a higher likelihood of anxiety among those who spent some/half of their days caring for COVID-19 patients compared to those who spent none of their days doing so. A difference between these two exposure groups, however, did not exist with other mental health domains. This suggests that there may be threshold levels of exposure when the effect on mental health becomes most detrimental. Moreover, mental health domains may have different thresholds, as anxiety was affected at lower exposure levels while resilience and sleep disturbance were not associated with exposure. These findings suggest a two-pronged approach for healthcare systems: providing broad supports to all HCWs to prevent adverse mental health outcomes, and providing targeted support interventions for HCWs informed by exposure level.

Our study identifies HCW concerns associated with worse mental health outcomes. Although we are unable to establish causality, Beck's cognitive model[58] suggests that these concerns are one primary mechanism through which emotional, physiological, and behavioral symptoms arise. Fear of becoming infected,[44,56,59] infecting family members,[8,17,19,44] increased workload,[20] concern about PPE,[20,44] and perceived healthcare worker stigma[14] have been identified in other samples. The majority (86.9%) of HCWs in the present sample reported a high degree of concern on at least one of the four domains, and more concerns within domains were associated with worse health. HCWs may have reported high health fear related to predicted holiday-related case surges. Work stressor concerns conceivably stemmed from constant changes to job descriptions, uncertainty of roles, and staffing constraints. Avoidance of co-workers among some HCW may have emerged from knowledge of transmission risk,[60] particularly prior to widespread vaccine availability. As such, hospital systems should assess HCW concerns regularly to guide interventions, adjust efforts dynamically, and engage in transparent communication of plans and policies, which may ultimately improve mental health outcomes.

Although the present study has multiple strengths, several limitations must be acknowledged. First, this was a single-center study which may limit the applicability of its results at other institutions. Nevertheless, our study had over 40% non-white participants and purposefully sought to represent a diverse group of HCWs. Second, given the nature of survey studies, there is always a possibility of non-response bias. To maximize wide HCW response, initial outreach was done with each surveyed department to increase engagement, and e-mail reminders were sent to HCWs over the course of the study period. Third, given the cross-sectional nature of the study, it is impossible to establish causal relationships between the risk factors and mental health domains. Finally, this cross-sectional survey did not account for baseline HCW mental health history, or the presence of symptoms or concerns prior to COVID-19.

The present study demonstrates that the COVID-19 pandemic continued to adversely affect the mental health of many US HCWs several months in and between surges of the pandemic. Both occupational factors and HCW concerns were associated with poorer mental health. Understanding these factors provides a basis for healthcare systems to develop and inform targeted evidence-based interventions, such as virtual town halls, scheduled weekly briefings, and check-ins with staff, to best support HCWs. Studies should further investigate the mental health impact of COVID-19 longitudinally, as well as the acceptability and efficacy of occupational mental health interventions for HCWs.

Corresponding Author: *Michael J. Van Wert, LCSW-C, MPH; Department of Psychiatry and Behavioral Sciences, Johns Hopkins University School of Medicine, Baltimore, MD, USA (e-mail: mvanwer1@jhmi.edu).*

Supplementary Information *The online version contains supplementary material available at https://doi.org/10.1007/s11606-021-07251-0.*

Declarations:

Conflict of Interest: *The authors declare that they do not have a conflict of interest.*

REFERENCES

1. coronoavirus.jhu.edu. Baltimore: Johns Hopkins University and Medicine Coronavirus Resource Center; c2021. Available from: https://coronavirus.jhu.edu/map.html. Accessed 6 Aug 2021.
2. **Hall LH, Johnson J, Watt I, Tsipa A, O'Connor DB.** Healthcare staff wellbeing, burnout, and patient safety: A systematic review. PLoS One. 2016;11(7):e0159015. https://doi.org/10.1371/journal.pone.0159015.
3. **Bridgeman PJ, Bridgeman MB, Barone J.** Burnout syndrome among healthcare professionals. Am J Health Syst Pharm. 2018; 75(3):147-152.
4. **Muller AE, Hafstad EV, Himmels JPW, et al.** The mental health impact of the covid-19 pandemic on healthcare workers, and interventions to help them: A rapid systematic review. Psychiatry Res. 2020; 298: 113441.
5. **De Kock JH, Latham HA, Leslie, SJ, et al.** A rapid review of the impact of COVID-19 on the mental health of healthcare workers: implications for supporting psychological well-being. BMC Public Health. 2021; 21:104.
6. **Preti E, Di Mattei V, Perego G, et al.** The psychological impact of epidemic and pandemic outbreaks on healthcare workers: rapid review of the evidence. Curr Psychiatry Rep. 2020;22(8):43.
7. **Arnetz JE, Goetz C, Sudan S, Arble E, Janisse J, Arnetz B.** Personal protective equipment and mental health symptoms among nurses during the COVID-19 pandemic. J Occup Environ Med. 2020;62(11):892-897.
8. **Barzilay R, Moore TM, Greenberg DM, et al.** Resilience, COVID-19-related stress, anxiety and depression during the pandemic in a large population enriched for healthcare providers. Transl Psychiatry. 2020;10:291.
9. **Evanoff BA, Strickland JR, Dale AM, et al.** Work-related and personal factors associated with mental well-being during the COVID-19 response: Survey of health care and other workers. J Med Internet Res. 2020;22(8):e21366.
10 **Firew T, Sano ED, Lee JW, et al.** Protecting the front line: a cross-sectional survey analysis of the occupational factors contributing to healthcare workers' infection and psychological distress during the COVID-19 pandemic in the USA BMJ Open. 2020;10:e042752.
11. **Pearman A, Hughes ML, Smith EL, Neupert SD.** Mental health challenges of United States healthcare professionals during COVID-19. Front Psychol. 2020;11:2065.
12. **Shechter A, Diaz F, Moise N, et al.** Psychological distress, coping behaviors, and preferences for support among New York healthcare workers during the COVID-19 pandemic. Gen Hosp Psychiatry. 2020;66:1-8.
13. **Gainer DM, Nahhas RW, Bhatt NV, Merrill A, McCormack J.** Association between proportion of workday treating COVID-19 and depression, anxiety, and PTSD outcomes in US physicians. J Occup Environ Med. 2021;63(2):89-97.
14. **Hennein R, Mew EJ, Lowe SR.** Socio-ecological predictors of mental health outcomes among healthcare workers during the COVID-19 pandemic in the United States. PLoS One. 2021;16(2):e0246602.
15. **Young KP, Kolcz DL, O'Sullivan DM, Ferrand J, Fried J, Robinson K.** Health care workers' mental health and quality of life during COVID-19: Results from a mid-pandemic, national survey. Psychiatr Serv. 2021;72(2):122-128.
16. **Demirjian NL, Fields BKK, Song C, et al.** Impacts of the coronavirus disease 2019 (COVID019) pandemic on healthcare workers: A nationwide survey of United States radiologists. Clin Imaging. 2020; 68:218-25.
17. **Huffman EM, Athanasiadis DI, Anton NE, et al.** How resilient is your team? Exploring healthcare providers' well-being during the COVID-19 pandemic. Am J Surg. 2020:S0002-9610(20)30570-5.
18. **Dugani, SB, Geyer HL, Maniaci MJ, Fischer KM, Coghan IT, Burton C.** Psychological wellness of internal medicine hospitalists during the COVID-10 pandemic. Hosp Pract. 2021;49(1):47-55.

19. **Ali H, Cole A, Ahmed A, Hamasha S, Panos G**. Major Stressors and Coping Strategies of Frontline Nursing Staff During the Outbreak of Coronavirus Disease 2020 (COVID-19) in Alabama. J Multidiscip Healthc. 2020;13:2057-2068.
20. **Marco CA, Larkin GL, Feeser VR, Monti JE, Vearrier L**. Post-traumatic stress and stress disorders during the COVID-19 pandemic: Survey of emergency physicians. JACEP Open. 2020;1(6): 1594-1601.
21. **Rodriguez RM, Medak AJ, Baumann BM, et al**. Academic emergency medicine physicians' anxiety levels, stressors, and potential stress mitigation measures during the acceleration phase of the COVID-19 pandemic. Acad Emerg Med. 2020;27:700-707.
22. **Sagherian K, Steege LM, Cobb SJ, Cho H**. Insomnia, fatigue and psychosocial well-being during COVID-19 pandemic: A cross-sectional survey of hospital nursing staff in the United States. J Clin Nurs. 2020;00:1-14.
23. **Baumann BM, Cooper RJ, Medak AJ, et al**. Emergency physician stressors, concerns, and behavioral changes during COVID-19: A longitudinal study. Acad Emerg Med. 2021;00:1-11.
24. **Kelker H, Yoder K, Musey P Jr, et al**. Prospective study of emergency medicine provider wellness across ten academic and community hospitals during the initial surge of the COVID-19 pandemic. BMC Emerg Med. 2021;21(1):36.
25. **Maunder RG, Lancee WJ, Rourke S, et al**. Factors associated with the psychological impact of severe acute respiratory syndrome on nurses and other hospital workers in Toronto. Psychosom Med. 2004;66(6):938–942.
26. **Kroenke K, Spitzer RL, Williams JB**. The Patient Health Questionnaire-2: validity of a two-item depression screener. Med Care. 2003;41(11):1284-92.
27. **Spitzer RL, Kroenke K, Williams JB, Löwe B**. A brief measure for assessing generalized anxiety disorder: the GAD-7. Arch Intern Med. 2006;166(10):1092-7.
28. **Buysse DJ, Yu L, Moul DE, et al**. Development and validation of patient-reported outcome measures for sleep disturbance and sleep-related impairments. Sleep. 2010;33(6):781-92.
29. **Yu L, Buysse DJ, Germain A, et al**. Development of short forms from the PROMIS™ sleep disturbance and Sleep-Related Impairment item banks. Behav Sleep Med. 2011;10(1):6-24.
30. Healthmeasures.net. U.S. Department of Health and Human Services: PROMIS, Patient-Reported Outcomes Measurement Information System; c2021. Available from: https://www.healthmeasures.net/score-and-interpret/interpret-scores/promis/promis-score-cut-points. Accessed 14 Apr 2021.
31. **Weiss DS, Marmar CR**. The Impact of Event Scale—Revised. In: Wilson JP, Keane TM, eds. Assessing psychological trauma and PTSD. New York: The Guilford Press; 1997:399-411.
32. **Maslach C, Jackson SE, Leiter MP**. Maslach Burnout Inventory Manual. In: Zalaquett CP, Wood RJ, eds. Evaluating stress: A book of resources. Michigan: Scarecrow Press; 1997:191-218.
33. **Li-Sauerwine S, Rebillot K, Melamed M, Addo N, Lin M**. A 2-question summative score correlates with the Maslach Burnout Inventory. West J Emerg Med. 2020;21(3):610-617.
34. **Vaishnavi S, Connor K, Davidson JR**. An abbreviated version of the Connor-Davidson Resilience Scale (CD-RISC), the CD-RISC2: psychometric properties and applications in psychopharmacological trials. Psychiatry Res. 2007;152(2-3):293-7.
35. **Rash CJ, Coffey SF, Baschnagel JS, Drobes DJ, Saladin ME**. Psychometric properties of the IES-R in traumatized substance dependent individuals with and without PTSD. Addict Behav. 2008;33(8):1039-47.
36. **West CP, Dyrbye LN, Sloan JA, Shanafelt TD**. Single item measures of emotional exhaustion and depersonalization are useful for assessing burnout in medical professionals. J Gen Intern Med. 2009;24(12):1318-21.
37. **Daly M, Sutin AR, Robinson E**. Depression reported by US adults in 2017-2018 and March and April 2020. J Affect Disord. 2021;278:131-135.
38. **Mata DA, Ramos MA, Bansal N, et al**. Prevalence of Depression and Depressive Symptoms Among Resident Physicians: A Systematic Review and Meta-analysis. JAMA. 2015;314(22):2373-83.
39 **Dyrbye LN, West CP, Satele D, et al**. Burnout among U.S. medical students, residents, and early career physicians relative to the general U.S. population. Acad Med. 2014;89(3):443-51.
40. **Shanafelt TD, Boone S, Tan L, et al**. Burnout and satisfaction with work-life balance among US physicians relative to the general US population. Arch Intern Med. 2012;172(18):1377-85.
41. **Letvak S, Ruhm CJ, McCoy T**. Depression in hospital-employed nurses. Clin Nurse Spec. 2012;26(3):177-82.
42. **Schernhammer E**. Taking their own lives – the high rate of physician suicide. **N Engl J Med**. 2005;352(24):2473-6.
43. coronavirus.maryland.gov. Maryland: Maryland Department of Health, Maryland COVID-19 Data Dashboard; c2021 [cited 2021 Apr 14].
44. **Cai W, Lian B, Song X, Hou T, Deng G, Li H**. A cross-sectional study on mental health among health care workers during the outbreak of Corona virus disease 2019. Asian J Psychiatr. 2020;51:102111.
45. **Price-Haygood EG, Burton J, Fort D, Seoane L**. Hospitalization and mortality among black patients and white patients with Covid-19. N Engl J Med. 2020; 382:2534-2543.
46 **Killerby ME, Link-Gelles R, Haight SC, et al**. COVID-19 Response Clinical Team. Characteristics associated with hospitalization among patients with COVID-19—Metropolitan Atlanta, Georgia, March-April 2020. MMWR Morb Mortal Wkly Rep. 2020;69:790-794.
47. **Hooper MW, Napoles AM, Perez-Stable EJ**. COVID-19 and racial/ethnic disparities. JAMA. 2020;323(24):2466-67.
48. 48. Fitzpatrick KM, Harris C, Drawve G. Living in the midst of fear: depressive symptomatology among US adults during the COVID-19 pandemic. Depress Anxiety. 2020;37(10):1-8.
49. **Asnaani A, Richey JA, Dimaite R, Hinton DE, Hofmann SG**. A cross-ethnic comparison of lifetime prevalence rates of anxiety disorders. J Nerv Ment Dis. 2010;198(8):551-5.
50. **Grandner MA, Patel NP, Gehrman PR, et al**. Who gets the best sleep? Ethnic and socioeconomic factors related to sleep complaints. Sleep Med. 2010;11(5):470-8.
51. **Shacham M, Hamama-Raz Y, Kolerman R, Mijiritsky O, Ben-Ezra M, Mijiritsky E**. COVID-19 factors and psychological factors associated with elevated psychological distress among dentists and dental hygienists in Israel. Int J Environ Res Public Health. 2020;17(8):2900
52. **Gloster AT, Lamnisos D, Lubenko J, et al**. Impact of COVID-19 pandemic on mental health: An international study. PLoS ONE. 2020;15(12): e0244809.
53. **Lai J, Ma S, Wang Y, et al**. Factors associated with mental health outcomes among health care workers exposed to coronavirus disease. JAMA Netw Open. 2020;3(3): e203976.
54. **Cao J, Wei J, Zhu H, Duan Y, Geng W, Hong X, et al**. A study of basic needs and psychological wellbeing of medical workers in the fever clinic of a tertiary general hospital in Beijing during the COVID-19 Outbreak. Psychother Psychosom. 2020;89(4):252–4.
55. **Kang L, Ma S, Chen M, Yang J, Wang Y, Li R, et al**. Impact on mental health and perceptions of psychological care among medical and nursing staff in Wuhan during the 2019 novel coronavirus disease outbreak: a crosssectional study. Brain Behav Immun. 2020;87:11–7.
56. **Lu W, Wang H, Lin Y, Li L**. Psychological status of medical workforce during the COVID-19 pandemic: a cross-sectional study. Psychiatry Res. 2020;288: 112936.
57. **Wang S, Xie L, Xu Y, Yu S, Yao B, Xiang D**. Sleep disturbances among medical workers during the outbreak of COVID-2019. Occup Med (Lond). 2020;70(5): 364–9.
58. **Beck AT**. Thinking and depression: Theory and therapy. Arch Gen Psychiatry. 1964;10: 561-571.
59. **Zhang W, Wang K, Yin L, Zhao W, Xue Q, Peng M, et al**. Mental health and psychosocial problems of medical health workers during the COVID-19 epidemic in China. Psychother Psychosom. 2020;89(4):1–9.
60. **Schneider S, Piening B, Nouri-Pasovsky PA et al**. SARS-Coronavirus-2 cases in healthcare workers may not regularly originate from patient care: lessons from a university hospital on the underestimated risk of healthcare worker to healthcare worker transmission. Antimicrob Resist Infect Control, 2020;9:192.

Publisher's Note *Springer Nature remains neutral with regard to jurisdictional claims in published maps and institutional affiliations.*
