# Supplementary material for: Mental health status and related factors influencing healthcare workers during the COVID-19 pandemic: A systematic review and meta-analysis
Source: PLoS One. 2024 Jan 19;19(1):e0289454. doi: 10.1371/journal.pone.0289454 (PMC10798549; doi:10.1371/journal.pone.0289454)
Supplement: S1 Data — (ZIP) [file pone.0289454.s011.zip › literatures/206.pdf]

frontiers in Psychiatry

ORIGINAL RESEARCH
published: 18 November 2021
doi: 10.3389/fpsyt.2021.701127

Psychological Impact of Exposure to the COVID-19 Sanitary Crisis on French Healthcare Workers: Risk Factors and Coping Strategies

Alexis Vancappel[1,2,3]*, *Eline Jansen*[1], *Nawal Ouhmad*[4], *Thomas Desmidt*[1,3], *Bruno Etain*[5], *Chantal Bergey*[6], *Marguerite d'Ussel*[7], *Marie-Odile Krebs*[8], *Claire Paquet*[5], *Christian Réveillère*[2], *Coraline Hingray*[9,10] *and Wissam El-Hage*[1,3,11]

[1] CHRU de Tours, Pôle de Psychiatrie-Addictologie, Centre Régional de Psychotraumatologie CVL, Tours, France, [2] Département de Psychologie, EE 1901 Qualipsy, Qualité de vie et Santé Psychologique, Université de Tours, Tours, France, [3] UMR 1253, iBrain, Université de Tours, INSERM, Tours, France, [4] Département de Psychologie, laboratoire EA 2114 PAVeA, Université de Tours, Tours, France, [5] Université de Paris, Assistance Publiques des Hôpitaux de Paris, APHP Nord, GH Lariboisière-Fernand Widal, DMU Neurosciences, Département de Psychiatrie et Médecine Addictologique, Paris, France, [6] CHRU de Bordeaux, CH Charles Perrens, Bordeaux, France, [7] Groupe Hospitalier Paris Saint-Joseph, Paris, France, [8] Centre Hospitalier Sainte Anne, Paris, France, [9] Pôle Hospitalo-Universitaire de Psychiatrie d'Adultes du Grand Nancy, Centre Psychothérapique de Nancy, Laxou, France, [10] CHRU de Nancy, Département de Neurologie, Nancy, France, [11] CIC 1415, CHRU de Tours, INSERM, Tours, France

OPEN ACCESS

Edited by:
Claudia Carmassi, University of Pisa, Italy

Reviewed by:
Nicola Magnavita, Catholic University of the Sacred Heart, Italy
Annalisa Cordone, University of Pisa, Italy

***Correspondence:**
Alexis Vancappel
a.vancappel@chu-tours.fr

Specialty section:
This article was submitted to Mood Disorders, a section of the journal Frontiers in Psychiatry

Received: 03 May 2021
Accepted: 30 September 2021
Published: 18 November 2021

Citation:
Vancappel A, Jansen E, Ouhmad N, Desmidt T, Etain B, Bergey C, d'Ussel M, Krebs M-O, Paquet C, Réveillère C, Hingray C and El-Hage W (2021) Psychological Impact of Exposure to the COVID-19 Sanitary Crisis on French Healthcare Workers: Risk Factors and Coping Strategies. Front. Psychiatry 12:701127. doi: 10.3389/fpsyt.2021.701127

Background: COVID-19 sanitary crisis is associated with emotional difficulties such as depression, anxiety and reactional post-traumatic symptoms among healthcare workers. Indeed, healthcare workers were particularly exposed to COVID-19 sanitary crisis. This study aimed to investigate the effects of exposure to COVID-19 sanitary crisis on affective symptoms (anxiety, post-traumatic stress, burnout) among French healthcare workers and the mediating role of cognitive emotion regulation strategies (positive re-evaluation and set in perspective) and coping strategies (active coping, planning, instrumental support, emotional support, emotional expression, positive reappraisal, acceptance, denial, blame, humor, religion, distraction, substance use, behavioral disengagement).

Method: This cross-sectional survey-based study collected demographic data and mental health measurements from 1,010 volunteers (838 women) who consented online to participate, from March 24 to June 28, 2020, in France. Participants filled out online questionnaires and visual analogic scales that evaluate affective symptoms related to the COVID-19 sanitary crisis, namely symptoms of post-traumatic stress, burnout, emotion regulation abilities, and coping abilities.

Results: The majority (57.8%) of the participants presented post-traumatic symptoms. Depending on the sub-dimensions evaluated, a proportion of participants reported moderate (25.9–31.2%) to severe (17.2–40.7%) burnout symptoms. We found a significant effect of the level of exposure to COVID-19 on affective symptoms. Being a woman, having a lower job position and having less experience were associated with higher level of affective symptoms. Moreover, coping strategies had a mediating effect on the relation between stress and burnout, supporting the coping reserve model.

Conclusion: Post-traumatic and burnout symptoms were highly prevalent among French healthcare workers at the beginning of the COVID-19 crisis. Exposure to COVID-19 is a determining factor. We can thus promote both coping training and a good environment to limit the emotional consequences of exposure to COVID-19.

Keywords: outbreak, COVID-19, health care workers, sanitary crisis, burnout, post-traumatic, mediation

INTRODUCTION

The 21st January 2020, the World Health Organization published its first report relating the existence of coronavirus disease 2019 (COVID-19). In order to slow down the spread of COVID-19, the French government imposed quarantine measures for 2 months, from March 17 to May 11, 2020. On July 7, 2020, the virus had spread in 216 different countries and 11,425,209 cases had been confirmed (1). France was strongly impacted with large and growing numbers of confirmed cases (168,335) and deaths (29,920) (2), between January and July 2020. The COVID-19 outbreak was considered as a sanitary crisis, meaning it is an urgent health-threatening situation (3).

A meta-analysis gathering the results from studies performed on past outbreaks and quarantines has set out the presence of multiple affective disorders such as anxiety, depressive, and post-traumatic symptoms. It was also claimed that studies should show a particular interest on the psychological effect of COVID-19 among healthcare workers (4). Indeed, healthcare workers are particularly exposed to COVID-19. They are exposed to death and risk of death that constitutes the first criteria for developing post-traumatic stress disorder (5). Studies performed during the past outbreaks have demonstrated an increased risk of multiple emotional disorders (anxiety, depression, anger) among healthcare workers (6–8). Therefore, multiple researchers focused on the psychological consequences of COVID-19 among healthcare workers. Cross-sectional studies demonstrated a high prevalence of emotional difficulties such as anxiety, insomnia, depressive, post-traumatic stress and burnout symptoms among healthcare workers across different countries [e.g., (9–13)]. Different meta-analysis confirmed an important prevalence of anxiety (23.2–33%), depressive (22.8–28%), post-traumatic (20.7%) and burnout (34.4%) symptoms (14–16). However, these cross-sectional studies lacked of control groups (17). Longitudinal studies were also performed but with inconsistent results. For instance, one study showed that healthcare workers had higher level of burnout after the crisis as compared to pre-COVID level of burnout (18); but, another study found a lower level of burnout after the COVID-19 crisis as compared to pre-COVID level (19). Finally, a repeated cross-sectional study set out an increased prevalence of depression among health workers over time after the crisis.

Some studies tried to understand and to conceptualize the psychological consequences of COVID-19 among healthcare workers. The first factors proposed to explain these emotional difficulties were increased time pressure and workload (20), as it increases work-life imbalance, known as a risk factor for burnout (21). Other authors have proposed that repeated stress could be the cause of burnout (22, 23). This is congruent with a previous developed model (24) suggesting that stress would reduce one's coping reserve, resulting in burnout. Indeed, a meta-analysis set out an association between coping strategies and emotional difficulties within healthcare workers during outbreaks (20). However, to our knowledge, no study evaluated the mediating role of coping strategies as proposed by Dunn et al. (24).

Therefore, the present study aimed to investigate the effects of exposure to COVID-19 sanitary crisis on mental health among French healthcare workers. Especially, we were interested in the effect of COVID-19 exposure on anxiety, post-traumatic stress and burnout symptoms. This study aimed also to identify the mediating role of cognitive emotion regulation strategies (positive re-evaluation and set in perspective) and coping strategies (active coping, planning, instrumental support, emotional support, emotional expression, positive reappraisal, acceptance, denial, blame, humor, religion, distraction, substance use, behavioral disengagement) on the relation between stress exposure and burnout.

METHOD

Participants

In this cross-sectional study, we recruited 1,010 participants using social media (Facebook, Twitter and LinkedIn) and mailing lists from ten main university hospitals across the country (e.g., Paris, Bordeaux, Tours, Nancy, ...). All healthcare workers working at university hospitals were eligible to answer the study. Participation required reading an information note online, ticking a box to consent to participate, and choosing to either continue with the study or decline to proceed. The experiment and consent procedures were approved by the ethics committee of the University (Comité d'Ethique de la Recherche Tours-Poitiers). Participants did not receive any compensation for the study.

Measures

Following consent, participants answered socio-demographic questions and indicated their level of exposure to COVID-19 with multiple-choice questions. We distinguished first-line workers who worked with patients infected by COVID-19, second-line workers who worked with potentially infected patients, and third-line workers who had no contact with infected or potentially infected patients. Participants also reported the number of suspected, confirmed and severe cases of coronavirus in their hospital unit and the number of deaths. We evaluated if the participants and their relatives were infected or had risk factors. Then, participants filled out successively a series of online

self-questionnaires. Data were collected between March 24 and June 28, 2020.

Maslach Burnout Inventory

The MBI (25) is a self-reported questionnaire, composed of 22 items, that evaluates burnout symptoms. Participants had to answer questions on a 7 point Likert scale from 0 (never) to 6 (every day). This scale is composed of 3 sub-dimensions: exhaustion, depersonalization and accomplishment. The French version has good psychometric properties and more specially a good internal consistency for each sub-dimension: exhaustion (Cronbach $\alpha = 0.90$), depersonalization ($\alpha = 0.64$) and accomplishment (0.74) (26). We also found a good internal consistency within our sample: exhaustion ($\alpha = 0.90$), depersonalization ($\alpha = 0.66$) and accomplishment ($\alpha = 0.74$). According to the same authors, an exhaustion-score under 18 is considered as low, under 27 as moderate and above 28 as high. A depersonalization-score under 3 is considered as low, under 7 as moderate and above 8 as high. An accomplishment-score under 34 is considered as low, under 35 as moderate and above 40 as high.

Cognitive Emotion Regulation Scale

The CERQ (27) is a self-reported questionnaire that evaluates cognitive emotion regulation strategies. We used two sub-dimensions of this scale: positive re-evaluation and set in perspective. Each sub-dimension is composed of 4 items. Participants had to answer on a Likert scale from 1 (almost never) to 5 (almost always). The French version demonstrated good psychometric properties for the different subscales (28) (Cronbach α between 0.68 and 0.87). We also found a good internal consistency in our sample for the two sub-dimensions (positive re-evaluation $\alpha = 0.73$, and set in perspective $\alpha = 0.76$).

Impact Event Scale 6 Items

The IES-6 (29, 30) is a self-reported questionnaire that was used to evaluate post-traumatic symptoms related to the COVID-19 outbreak. Participants had to answer questions related to the main events they identified, on a 5 points Likert scale. Experimental studies demonstrated good psychometric properties (Cronbach $\alpha = 0.80$), similar in our sample ($\alpha = 0.81$). The cut-off is 10.5 for post-traumatic stress disorder (29).

Visual Analogic Scales

Participants answered 30 analogic visual questions to assess the effect of COVID-19 on their mental health. Questions were developed by the authors based on a clinical consideration of symptoms experienced by healthcare workers during the crisis. Participants were asked to respond on a Likert scale from 0 to 10. The questions evaluated stress, worries, powerlessness, guilt, anger and other emotional difficulties related to the sanitary crisis (see **Supplementary Material 1**).

Brief COPE

This COPE (31) is a self-reported questionnaire evaluates coping strategies. It is composed of 28 items. It assesses active coping, planning, instrumental support, emotional support, emotional expression, positive reappraisal, acceptance, denial, blame, humor, religion, distraction, substance use, behavioral disengagement. Each dimension is evaluated with two questions. The scale demonstrated good psychometric properties.

Statistical Analysis

We used a 10.5 cutoff value for the IES-6 to evaluate the prevalence of PTSD, as suggested to have a good balance between sensitivity and specificity and the best overall efficiency (29, 30). We also used the categorization proposed for the French MBI (26) to describe moderate and important levels of emotional burnout. We performed multiple *t*-tests to evaluate the effect of sex and exposure to COVID-19. We used Bonferroni correction to adjust the alpha values. We divided alpha by the number of *t*-tests performed. We obtained a 0.0025 alpha value. Then, we performed correlational analyses to evaluate factors associated with affective symptoms depending on the age, professional experience, coping strategies and emotional regulation strategies. We performed one-way ANOVAs and *post-hoc* contrast analysis to evaluate the effect of the level of exposure and job position on emotional difficulties. Then, we performed multiple linear regressions to assess models of burnout. The first one evaluated the effect of IES-6 scores on MBI-exhaustion scores. Then, we added, with a step-by-step method, the different coping strategies that were significantly related to both IES-6 scores and MBI-exhaustion scores: emotional support, positive reinterpretation, denial, blame, acceptance, humor, substance use, and behavioral disengagement. Only significant predictors were progressively added to the model. Finally, we used bootstrapping analysis to assess the mediating effect of these strategies on the link between IES-6 scores and MBI-exhaustion scores. All the analysis were performed with SPSS software 23th version. Mediation analysis were performed with the 4th model of the complementary plug-in developed for SPSS (32).

RESULTS

Descriptive Results

General Information

We recruited 1,010 participants (838 women; mean age 39.24 ± 11.13). A majority (78%) of the participants worked for the public hospital. We mainly recruited nurses (35.6%), doctors (18.3%), and nursing assistants (17.9%) (complete demographics in **Table 1**). The mean number of years of experience was 14.47 ± 10.64. Participants worked all over the country. The two main regions represented are Ile de France (47.4%) and Centre Val de Loire (13.5%). The other regions represented each between 2.6 and 5.4% of the global sample. All the descriptive data of the quantitative variable are presented in **Table 1**.

Exposure to COVID-19

On the global sample, 43.9% of the participants had close relatives with suspected COVID-19 infection; 40.8% had close relatives with a risk factor disease (e.g., respiratory disease); 31.2% had close relatives tested positive for COVID-19; 13.9% were infected by coronavirus and 21.2% presented risk factor diseases for COVID-19. A majority (52.9%) of the participants

TABLE 1 | Descriptive data.

	First line	Second line	Third line	Total
**IES-6**				
Intrusion, 0–8	5.37 ± 2.33	5.02 ± 2.15	4.49 ± 2.09	5.16 ± 2.26
Avoidance, 0–8	3.13 ± 2.00	2.87 ± 1.84	2.83 ± 1.76	3.00 ± 1.93
Hyper-arousal, 0–8	3.54 ± 2.22	3.40 ± 2.11	2.94 ± 2.26	3.44 ± 2.19
Total, 0–24	12.03 ± 5.53	11.29 ± 4.96	10.26 ± 5.05	11.60 ± 5.30
**MBI**				
Exhaustion, 0–54	24.57 ± 12.66	23.10 ± 12.52	20.52 ± 13.03	23.68 ± 12.67
Depersonalization, 0–30	8.53 ± 5.35	7.30 ± 6.14	6.36 ± 5.61	7.88 ± 6.25
Accomplishment, 0–48	37.33 ± 7.01	37.96 ± 7.39	36.53 ± 8.44	37.52 ± 7.28
**CERQ**				
Positive reevaluation, 4–20	13.26 ± 3.47	13.28 ± 3.76	12.66 ± 3.34	13.22 ± 3.55
Set in perspective, 4–20	13.39 ± 3.71	13.47 ± 3.54	13.19 ± 3.68	13.40 ± 3.64
**COPE**				
Active coping, 2–8	5.46 ± 1.48	5.55 ± 1.42	5.12 ± 1.21	5.47 ± 1.44
Planning, 2–8	5.25 ± 1.59	5.39 ± 1.52	4.73 ± 1.25	5.27 ± 1.55
Instrumental support, 2–8	4.94 ± 1.62	4.98 ± 1.53	4.96 ± 1.39	4.96 ± 1.57
Emotional support, 2–8	4.88 ± 1.62	4.70 ± 1.50	4.55 ± 1.49	4.78 ± 1.56
Emotional expression, 2–8	5.02 ± 1.58	5.11 ± 1.56	5.00 ± 1.34	5.05 ± 1.55
Positive reinterpretation, 2–8	5.12 ± 1.64	5.31 ± 1.62	5.21 ± 1.32	5.20 ± 1.61
Acceptance, 2–8	5.74 ± 1.50	5.91 ± 1.54	5.74 ± 1.49	5.81 ± 1.52
Denial, 2–8	2.89 ± 1.32	2.88 ± 1.25	3.06 ± 1.31	2.90 ± 1.29
Blame, 2–8	4.20 ± 1.35	3.92 ± 1.50	3.91 ± 1.25	4.07 ± 1.31
Humor, 2–8	3.50 ± 1.35	3.73 ± 1.49	3.53 ± 1.40	3.60 ± 1.41
Religion, 2–8	2.88 ± 1.50	2.93 ± 1.47	2.75 ± 1.19	2.89 ± 1.46
Distraction, 2–8	5.08 ± 1.57	5.31 ± 1.47	3.36 ± 1.29	5.19 ± 1.51
Substance use, 2–8	2.78 ± 1.32	2.69 ± 1.25	2.70 ± 1.12	2.74 ± 1.28
Behavioral disengagement, 2–8	3.03 ± 1.21	2.90 ± 1.12	3.06 ± 1.12	2.98 ± 1.19
**Job positions**				
Nurse	242 (45.3%)	99 (24.8%)	19 (24.7%)	360 (35.6%)
Doctor	94 (17.6%)	76 (19%)	15 (19.5%)	185 (18.3%)
Nursing assistant	123 (23%)	48 (12%)	10 (13%)	181 (17.9%)
Health executive	13 (2.4%)	50 (12.5%)	3 (3.9%)	66 (6.5%)
Psychologist	8 (1.5%)	34 (8.5%)	8 (10.4%)	50 (5%)
Medical secretary	0 (0%)	13 (3.3%)	4 (5.2%)	17 (1.7%)
Pharmacist	2 (0.4%)	10 (2.5%)	3 (3.9%)	15 (1.5%)
Hospital public agent	5 (0.9%)	7 (1.8%)	0 (0%)	12 (1.2%)
Other	47 (8.8%)	62 (15.5%)	15 (19.5%)	124 (12.3%)

Mean ± Standard Deviation; number (percentage).
CERQ, Cognitive Emotion Regulation Questionnaire; COPE, Brief COPE questionnaire; IES-6, Impact of Events Scale 6 items; MBI, Maslach Burnout Inventor; First line, direct contact with COVID-19 patients; Second line, potential contact with infected patients; Third line, no contact with infected patients.

were in first-line with COVID-19 patients, 39.5% were in second-line with potential contact with infected patients, and 7.6% of the participants were in third-line (no contact with infected patients). The median numbers of COVID-19 cases reported by the participants in their hospital department were as follows: 3 suspected cases, 1 confirmed case, 1 serious case, 1 death due to COVID-19 and none non-accepted patient due to a lack of space.

Stress and Worries

For the next sections, we considered 7–10 as an important level, reported with visual analogic scales. A majority (77%) of the participants had an important level of worries about close relatives' health; 64.9% described an important level of fear to contaminate a close relative; 42.6% worried about their children health; 41.7% were afraid to contaminate their children; and 19.4% were worried about finding a baby-sitter.

Powerlessness

A quarter (27.3%) of the participants reported a high level of powerlessness at work, 31.9% reported a high level of powerlessness at home, 13.8% described lots of ethical dilemma, 24.4% had important feelings of not responding to patients demands, 51.6% reported an important lack of material, and 49,7% had an important feeling of putting lose relatives in danger.

Guilty

The percentages of high level of guilt feelings were 21.4% for guilt at work and 19.8% for guilt in the personal life. A quarter (24.6%) of the participants strongly blamed themselves for not being able to protect their close relations, 4.6% thought that they have strongly set patients' life in danger, 6.1% described an important increase of mistakes at work.

Anger

Over half (52.3%) of the participants reported high level of anger at work, 68.8% of the participants strongly blamed the state the delay in information, 69% thought firmly that the state did not manage the crisis as good as possible, 64.7% thought strongly that the citizens do not respect the quarantine, and 55.7% strongly blamed the state for not helping health workers. The great majority of the participants (80.8%) behaved respectfully toward the patients, 73.2% thought their colleagues had an important exemplarily behavior, 47.3% thought strongly that the hospital reacted as best as possible, 29.9% thought strongly that the hospital managed well its materials.

Emotional Impact

Participants reported an important level of stress since the beginning of the crisis (40.9%), a high level of sadness (36.4%), significant sleep disturbances (32.4%), significant appetite disturbances (22.6%), and important feeling of regret (15.1%). We found that 57.8% of the participants presented significant PTSD symptoms. Using MBI-scores, we found that 25.9% of the participants presented moderate exhaustion and 40.7% of the participants presented severe exhaustion. We also found 30.6% of moderate and 21.2% of severe depersonalization. Finally, we found 31.2% of moderate and 17.1% of severe lack of accomplishment.

The results of the different scales and subscales are presented in **Table 1**.

Group Comparisons

We found a significant effect of sex on IES-6 scores ($t = -5.96$; $p < 0.001$) but not on MBI scores. Women tend to have higher PTSD symptoms. We did not find any effect for having a close relative suspected of being infected. We found a positive effect of having a close relative infected by COVID-19 on IES-6 scores ($t = 3.06$; $p = 0.002$), but not on MBI scores. Participants with a close relative infected have a higher level of PTSD. We found a significant effect of having a close relative with a risk factor on IES-6 scores ($t = 4.91$; $p < 0.001$) and MBI-exhaustion scores ($t = 3.49$; $p = 0.001$), but not on the other MBI-sub-dimensions. Participants with a close relative with a risk factor tend to have more emotional difficulties. We did not find a significant effect of being infected on the IES-6 and MBI scores.

Correlational Analysis

We found different factors associated with the emotional difficulties evaluated through IES-6, MBI and visual analogic scores. We found that age, number of years of experience, positive reevaluation, set in perspective, active coping, planning, positive reinterpretation, acceptance, and humor were negatively related to emotional difficulties. On the opposite, we found that emotional support, emotional expression, denial, blame, substance use, and behavioral disengagement were positively related to emotional difficulties. Surprisingly, emotional support and feelings were positively related to accomplishment. We did not find significant results for instrumental support and distraction. All the results are presented in **Table 2**.

One-Way ANOVA

Effect of Level of Exposure

We performed a one-way ANOVA to evaluate the effect of level of exposure to COVID-19. We found a significant global effect on IES-6 scores ($F = 5.00$; $p < 0.007$); on exhaustion-MBI scores ($F = 4.14$; $p = 0.016$) and depersonalization scores ($F = 7.12$; $p = 0.001$) but not on accomplishment scores ($F = 1.63$; $p = 0.20$). We used Schéffé *post-hoc* to identify the contrast between the different conditions. We found a significant contrast between first-line and third-line health workers for the IES-6 scores (95% IC = 0.20–3.35), between first-line and third-line for exhaustion-MBI (95I% C = 0.27–7.82) and between first-line and second-line (95I C% = 0.22–2.24) and first-line and third-line (95I% = 0.36–4.08) for depersonalization. The scores of the different scales depending on the level of exposure are presented in **Figure 1**.

Effect of Job Position

We found a significant effect of job on IES-6 scores ($F = 6.84$; $p < 0.001$) and MBI scores: exhaustion (2.43; $p = 0.013$), depersonalization ($F = 5.80$; $p < 0.001$), and accomplishment ($F = 6.06$; $p < 0.001$). The descriptive results are presented in **Figure 2**. We completed the analysis by performing *post-hoc* contrast for job group with a sample size equal or superior to 50. For IES-6 scores, *post hoc* comparisons set out a significant contrast between doctors and nurses (95% IC = −3.99 to −0.29) and nursing assistants (95% IC = −5.26 to −0.98), and between psychologists and nursing assistants (95% IC = 0.63–7.16). We found a significant contrast between psychologists and nurses (95% IC = −7.81 to −0.50) and nursing assistants (95I% C = −8.29 to −0.56) for the depersonalization subscale. We found a significant contrast between psychologists and nurses (IC% 95 = 0.1–8.52) and doctors and nurses (95 IC = 0.12–5.22) for the accomplishment subscale. In general, doctors and psychologists tend to have less traumatic and burnout manifestations than nurses and nursing assistants.

Mediation Analysis

Step-by-Step Approach

We entered IES-6 scores in a linear regression to predict MBI-exhaustion scores, showing IES-6 scores as a significant predictor ($\beta = 0.9$; $p < 0.001$). When adding the different coping strategies, significantly associated with both IES-6 scores and MBI-exhaustion scores, we found a significant model ($R = 0.923$; $R^2 = 0.852$; $F = 823.232$; $ddl = 7$; $p < 0.001$). In this model, the beta value of IES-6 is lower than in the first model ($\beta =$

TABLE 2 | Correlational analysis.

	IES-6 Total	MBI-E	MBI-D	MBI-A	Increased anxiety	Increased sadness	Sleep disturbance	Appetite disturbance	Feeling of regret
Age	−0.108**	−0.104**	−0.196**	0.129**	−0.122**	−0.119**	−0.104**	−0.087**	−0.018
Years of experience	−0.082**	−0.083**	−0.168**	0.039	−0.088**	−0.070*	−0.075*	−0.060	0.015
**CERQ**									
Positive reevaluation	−0.116**	−0.179**	−0.063*	0.319**	−0.164**	−0.197**	−0.155**	−0.108**	−0.140**
Set in perspective	−0.115**	−0.117**	0.033	0.175**	−0.135**	−0.176**	−0.151**	−0.135**	−0.147**
**COPE**									
Active coping	−0.049	−0.105**	−0.104**	0.318**	−0.101**	−0.138**	−0.092**	−0.064*	−0.078*
Planning	−0.041	−0.076*	−0.084**	0.283**	−0.075*	−0.117**	−0.061	−0.058	−0.039
Instrumental support	0.047	0.047	0.007	0.164**	0.030	0.016	0.011	0.013	−0.046
Emotional support	0.192**	0.166**	0.056	0.083**	0.191**	0.181**	0.125**	0.105**	0.027
Emotional expression	0.073*	0.034	−0.032	0.137**	0.063*	0.024	0.034	−0.003	−0.038
Positive reinterpretation	−0.183**	−0.225**	−0.115**	0.257**	−0.251**	−0.252**	−0.220**	−0.193**	−0.177**
Acceptance	−0.226**	−0.245**	−0.202**	0.292**	−0.223**	−0.299**	−0.218**	−0.192**	−0.194**
Denial	0.266**	0.233**	0.212**	−0.094**	0.249**	0.252**	0.200**	0.198**	0.217**
Blame	0.155**	0.210**	0.184**	−0.028	0.167**	0.188**	0.118**	0.157**	0.169**
Humor	−0.199**	−0.158**	−0.012	0.134**	−0.233**	−0.231**	−0.225**	−0.176**	−0.143**
Religion	0.109**	0.033	−0.046	0.068*	0.064*	0.068*	0.049	0.061	0.065*
Distraction	0.037	−0.020	0.020	0.105**	0.024	−0.004	−0.006	−0.003	−0.052
Substance use	0.167**	0.195**	0.168**	−0.031	0.158**	0.121**	0.147**	0.106**	0.085**
Behavioral disengagement	0.155**	0.305**	0.269**	−0.211**	0.186**	0.243**	0.140**	0.184**	0.215**

*CERQ, Cognitive Emotion Regulation Questionnaire; COPE, Brief COPE questionnaire; IES-6, Impact of Events Scale; MBI, Maslach Burnout Inventory; MBI-E, Maslach Burnout Inventory-Exhaustion; MBI-D, Maslach Burnout Inventory-Depersonalization; MBI-A, Maslach Burnout Inventory-Accomplishment. *p < 0.05; **p < 0.01.*

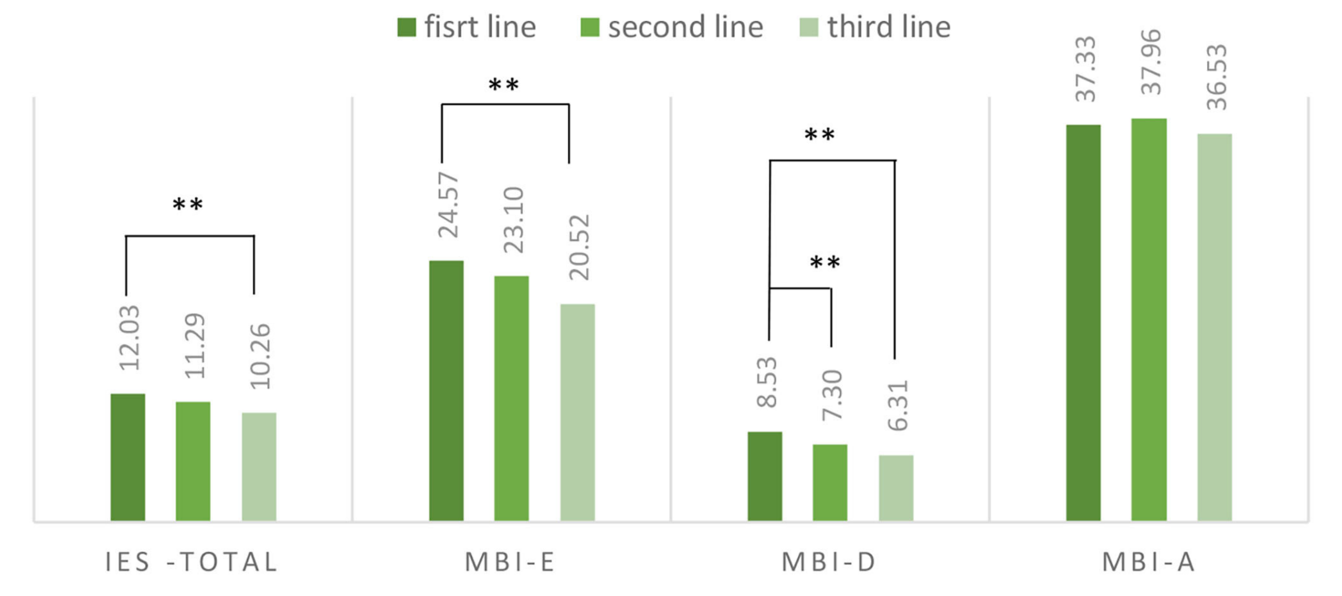

FIGURE 1 | Emotional difficulties depending on the level of exposure. MBI-E, Maslach Burnout Inventory-Exhaustion; MBI-D, Maslach Burnout Inventory-Depersonalization; MBI-A, Maslach Burnout Inventory-Accomplishment. **Significant difference.

0.457; $p < 0.001$). The other significant predictive factors are behavioral disengagement ($\beta = 0.247$; $p < 0.001$); blame ($\beta = 0.156$; $p < 0.001$); substance use ($\beta = 0.91$; $p = 0.001$); positive reinterpretation ($\beta = -148$; $p < 0.001$), emotional support ($\beta = 0.90$; $p = 0.022$) and denial ($\beta = 0.065$; $p = 0.038$).

Bootstrapping Analysis

Then, we performed a bootstrapping analysis with the remained predictors. We found a significant global model ($R = 0.58$; $R^2 = 0.33$; $F = 71.77$; $df = 7$; $p < 0.001$). However, the indirect effect of IES-6 scores on MBI-exhaustion scores through emotional

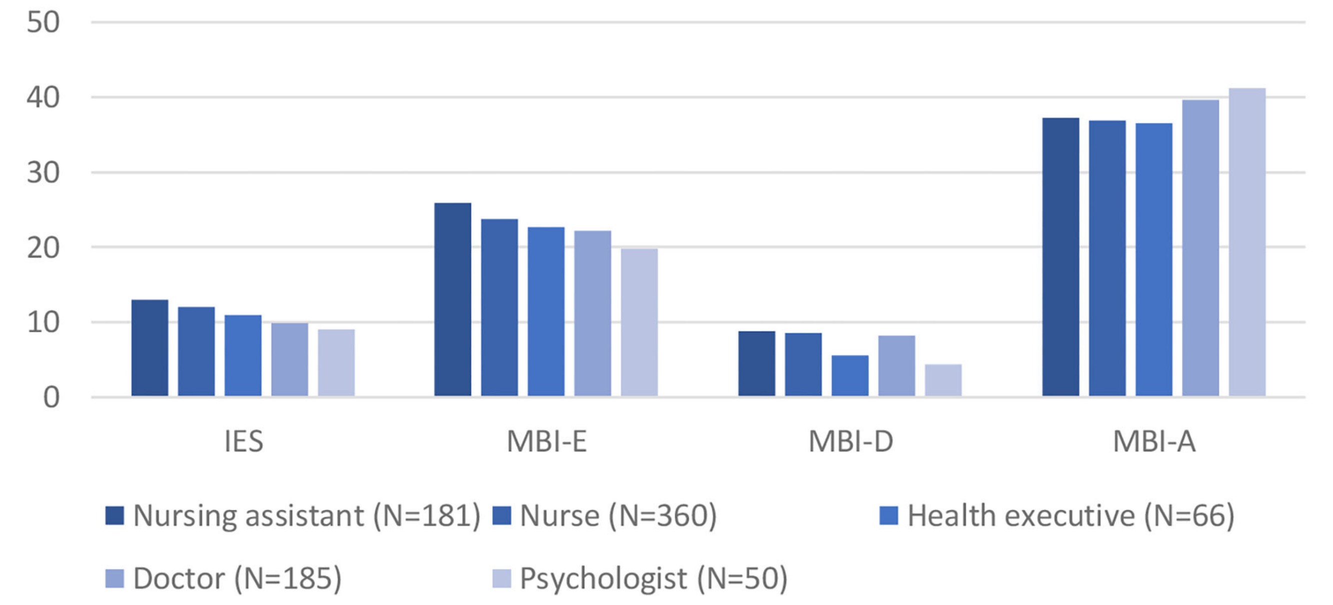

FIGURE 2 | Emotional difficulties depending on the job position. MBI-E, Maslach Burnout Inventory-Exhaustion; MBI-D, Maslach Burnout Inventory-Depersonalization; MBI-A, Maslach Burnout Inventory-Accomplishment.

support and denial were not significant, respectively, IC 95% = [−0.0007–0.0545] and [0.0003–0.680]. Therefore, we removed these two coping strategies and performed the analysis again. We obtained a significant model ($R = 0.573$; $R^2 = 0.0.33$; $F = 98.42$; $df = 5$; $p < 0.001$). Each of the coping strategy left demonstrated a significant indirect effect of IES-6 scores on MBI-exhaustion scores: blame (IC95% = [0.070–0.0682]); substance use (IC95% = [0.0099–0.059]; behavioral disengagement (IC95% = [0.040–0.111] and positive reinterpretation (IC95% = [0.0188–0.0764]. The final mediation model is presented in **Figure 3**.

DISCUSSION

We evaluated the psychological effect of exposure to COVID-19 sanitary crisis among French healthcare workers. We found a high prevalence of post-traumatic symptoms (57.8%). This prevalence is above the percentage identified by Tan et al. (13) who set out, using the IES-6, a prevalence of 17% of PTSD among medical workers. It is also above the prevalence of 20.7% identified in a meta-analysis (16). Using MBI-sub-dimensions, we also found moderate (25.9–31.2%) and severe (17.2–40.7%) emotional burnout. Those results are close to those observed in the Italian survey (9) that highlighted the following prevalence of burnout through the MBI-scale: 19.1–27.1% of moderate and 24.7–53.2% of high emotional burnout. However, the observed results are close to the results identified in another meta-analysis (34.4%, 13). Italian workers seem to have a higher percentage of severe burnout but a lower level of moderate burnout. This could be explained by the severity of the crisis that tended to be greater in Italy as compared to France.

Additionally, we found an effect of COVID-19 exposure on PTSD and burnout symptoms. This is congruent with previous results who identified that being first-line worker as a risk factor for emotional difficulties (11, 33). We can assume though that front-line workers have greater time pressure and workload, resulting in more important level of emotional difficulties (20).

We set out multiple factors associated with emotional difficulties (emotional burnout and post-traumatic symptoms). In congruence with previous studies (11, 15, 33), we found that being a woman is a significant risk factor. We found that nurses and nursing assistants tend to be more impacted than doctors or psychologists. This is also congruent with results presented in other countries (11, 13, 33). We highlighted that higher age and experience are associated with less emotional difficulties.

Positive re-evaluation, set in perspective, active coping, planning, positive reinterpretation, acceptance, and humor were negatively related to PTSD symptoms and emotional burnout. We also set out that emotional support, emotional expression, denial, blame, substance use, and behavioral disengagement were positively related to emotional difficulties. Surprisingly, emotional support and feelings were positively related to accomplishment. Those results are partly congruent with previous studies that set out a positive relation between passive coping and religion, and PTSD and a negative relationship between active coping and distraction, and PTSD (34). Indeed, emotional support and feelings are passive coping strategies, associated with increased level of emotional difficulties. On the contrary, the positive association between these strategies and accomplishment suggest an association with a positive feeling. Another study set out a positive relationship between PTSD and emotion-focused strategies (35) and self-blame (36). The association between coping strategies and burnout is also congruent with previous studies. Indeed, a positive relationship between escape avoidance and confronting and burnout; and a

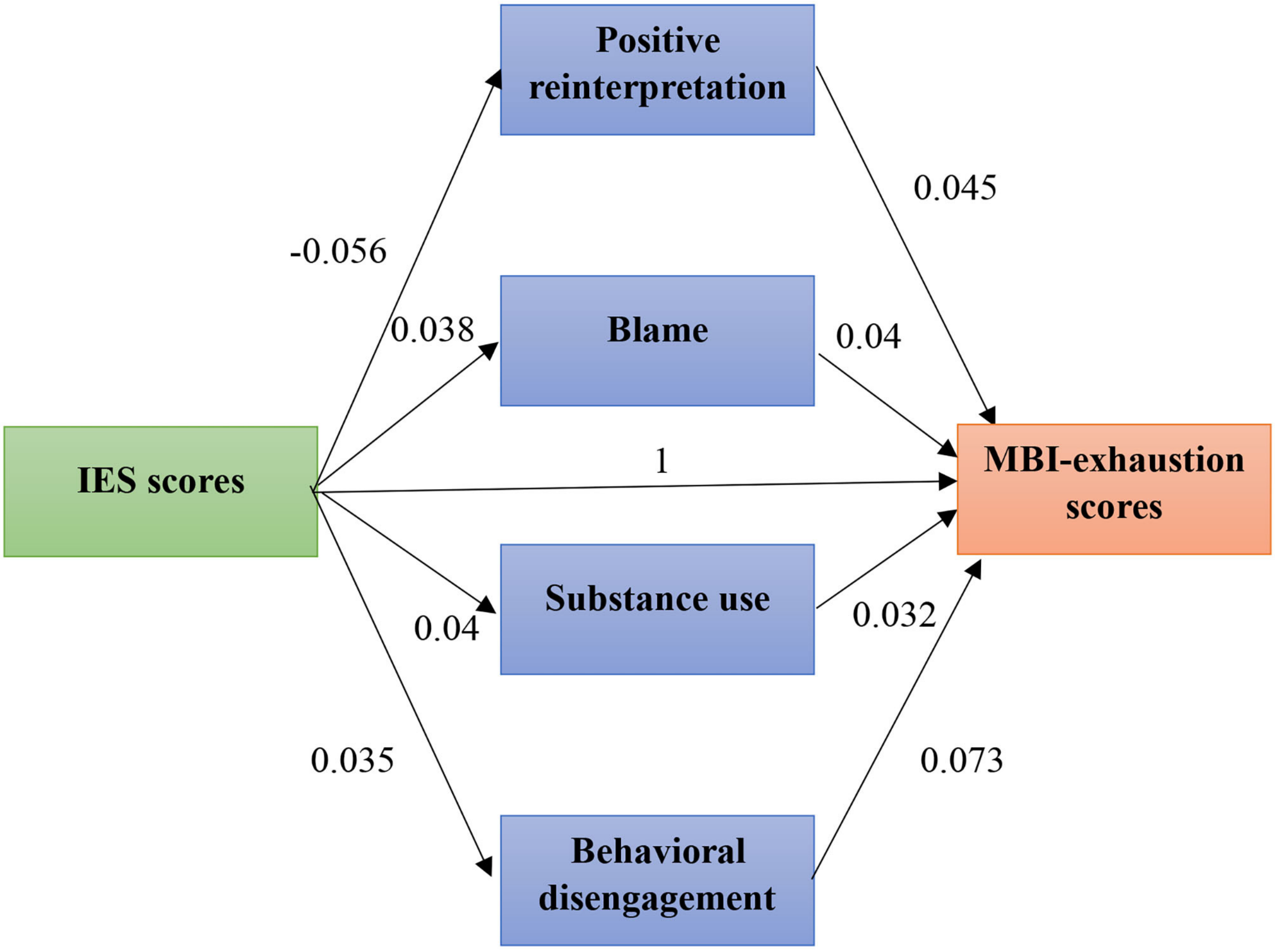

FIGURE 3 | Direct and indirect effect of stress on burnout.

negative relationship between planning/problem solving, positive reframing and seeking social support; and burnout have been set out (37). Another study has confirmed the relationship between cognitive emotion regulation, positive reinterpretation and burnout (38).

Finally, we assessed the mediation effect of coping strategies on the link between stress and burnout. We found that the relationship between stress and burnout is partly mediated by coping strategies. This is congruent with the coping reserve model (24) suggesting that stress drains coping abilities.

Altogether, our results highlight the relevance of coping/stress management training for healthcare workers. This training would fill the coping reserve and would prevent burnout that could be understood as a long-term effect of stress. Indeed a meta-analysis performed on 1,521 participants has demonstrated the positive effect of coping training on emotional burnout (39). Some researchers have published a proposition of coping preparation to help healthcare workers to cope with outbreak crisis (40).

This study has a few limitations. First, emotional difficulties have been evaluated with self-reported questionnaires. However, semi-structured interviews are gold standard evaluations to formally assess emotional difficulties. There is an imbalance between men and women that limits the extension of our conclusions. Furthermore, we did not get enough participants to evaluate the effect of work localization. Indeed, the French region "Grand Est" was especially affected by COVID-19, but we did not have enough participants from this region to perform special analysis on those participants. We do not know if participants suffered or had an history of psychiatric conditions. This could have influenced the results of the study. Data were gathered during the first months following the beginning of the crisis in France. Therefore, data reflects only the psychological state of healthcare workers during this period. We recruited a non-probability sample, because only participants who had access to the link of the study could take part in the study. Finally, this study is cross-sectional and cannot set out causal relationships.

CONCLUSION

There is an important prevalence of post-traumatic and emotional burnout symptoms among French healthcare workers

during COVID-19 crisis. Exposure to sanitary crisis seems to be a determining factor. The more healthcare workers are exposed to COVID-19, the more they present emotional difficulties. Being a woman, having a lower job position and having less experience are consistent significant risk factors for emotional difficulties across studies. We identified multiple coping strategies associated with emotional difficulties and the mediating effect of coping strategies on the link between stress and burnout. This suggests the relevance of both coping training and a good environment to prepare healthcare workers to future sanitary crisis and to limit the emotional consequences.

DATA AVAILABILITY STATEMENT

The raw data supporting the conclusions of this article will be made available by the authors, without undue reservation.

ETHICS STATEMENT

The studies involving human participants were reviewed and approved by CERNI (Comité de la recherche Tours-Poitiers). The patients/participants provided their written informed consent to participate in this study.

AUTHOR CONTRIBUTIONS

AV performed the statistical analysis and wrote the first version of the manuscript. All authors took part in the development of the methodology and data gathering process, added their modifications, and finally agreed the last version.

ACKNOWLEDGMENTS

We would like to thank the University of Tours for the material support. We are grateful to Association Française de Psychiatrie Biologique et de Neuropsychopharmacologie (AFPBN) who contributed to the collection of the survey data.

SUPPLEMENTARY MATERIAL

The Supplementary Material for this article can be found online at: https://www.frontiersin.org/articles/10.3389/fpsyt.2021.701127/full#supplementary-material

REFERENCES

1. World Health Organization. *COVID-19 Situation Reports*. (2020). Available online at: https://www.who.int/emergencies/diseases/novel-coronavirus-2019/situation-reports (accessed September 9, 2020).
2. Santé Publique France.s Infection au nouveau Coronavirus (SARS-CoV-2), COVID-19, *France et Monde*. (2020). Available online at: https://maladies-et-traumatismes/maladies-et-infections-respiratoires/infection-a-coronavirus/articles/infection-au-nouveau-coronavirus-sars-cov-2-covid-19-france-et-monde
3. Futura, la rédaction de. *Crise sanitaire*. Futura (2021). Available online at: https://www.futura-sciences.com/sante/definitions/pandemie-crise-sanitaire-19283/ (accessed September 9, 2020).
4. Brooks SK, Webster RK, Smith LE, Woodland L, Wessely S, Greenberg N, et al. The psychological impact of quarantine and how to reduce it: rapid review of the evidence. *Lancet.* (2020) 395:912-20. doi: 10.1016/S0140-6736(20)30460-8
5. American Psychiatric Association. *Diagnostic and Statistical Manual of Mental Disorders: Dsm-5 (5th Edn). American Psychiatric Publishing*. (2013). doi: 10.1176/appi.books.9780890425596
6. Bai Y, Lin CC, Lin CY, Chen JY, Chue CM, Chou P. Survey of stress reactions among health care workers involved with the SARS outbreak. *Psychiatr Serv.* (2004) 55:1055-7. doi: 10.1176/appi.ps.55.9.1055
7. Jung H, Jung SY, Lee MH, Kim MS. Assessing the presence of post-traumatic stress and turnover intention among nurses post-middle east respiratory syndrome outbreak: the importance of supervisor support. *Workplace Health Saf.* (2020) 68:337–45. doi: 10.1177/2165079919897693
8. Maunder R, Hunter J, Vincent L, Bennett J, Peladeau N, Leszcz M, et al. The immediate psychological and occupational impact of the 2003 SARS outbreak in a teaching hospital. *CMAJ*. (2003) 168:1245–51.
9. Barello S, Palamenghi L, Graffigna G. Burnout and somatic symptoms among frontline healthcare professionals at the peak of the Italian COVID-19 pandemic. *Psychiatry Res.* (2020) 290:113129. doi: 10.1016/j.psychres.2020.113129
10. Buselli R, Corsi M, Baldanzi S, Chiumiento M, Del Lupo E, Dell'Oste V, et al. Professional quality of life and mental health outcomes among health care workers exposed to Sars-Cov-2 (Covid-19). *Int J Environ Res Public Health.* (2020) 17:6180. doi: 10.3390/ijerph17176180
11. Lai J, Ma S, Wang Y, Cai Z, Hu J, Wei N, et al. Factors associated with mental health outcomes among health care workers exposed to coronavirus disease 2019. *JAMA Netw Open.* (2020) 3:e203976. doi: 10.1001/jamanetworkopen.2020.3976
12. Que J, Shi L, Deng J, Liu J, Zhang L, Wu S, et al. Psychological impact of the COVID-19 pandemic on healthcare workers: a cross-sectional study in China. *Gen Psychiatry.* (2020) 33:e100259. doi: 10.1136/gpsych-2020-100259
13. Tan B, Chew N, Lee G, Jing M, Goh Y, Yeo L, et al. Psychological impact of the COVID-19 pandemic on health care workers in Singapore. *Ann Intern Med.* (2020) 173:317–20. doi: 10.7326/M20-1083
14. Luo M, Guo L, Yu M, Jiang W, Wang H. The psychological and mental impact of coronavirus disease 2019 (COVID-19) on medical staff and general public - A systematic review and meta-analysis. *Psychiatry Res.* (2020) 291:113190. doi: 10.1016/j.psychres.2020.113190
15. Pappa S, Ntella V, Giannakas T, Giannakoulis VG, Papoutsi E, Katsaounou P. Prevalence of depression, anxiety, and insomnia among healthcare workers during the COVID-19 pandemic: a systematic review and meta-analysis. *Brain Behav Immun.* (2020) 88:901–7. doi: 10.1016/j.bbi.2020.05.026
16. Salazar de Pablo G, Vaquerizo-Serrano J, Catalan A, Arango C, Moreno C, Ferre F, et al. Impact of coronavirus syndromes on physical and mental health of health care workers: systematic review and meta-analysis. *J Affect Disord.* (2020) 275:48-57. doi: 10.1016/j.jad.2020.06.022
17. Magnavita N, Di Prinzio RR, Chirico F, Sacco A, Quintavalle G. COVID-19 and staff mental health: is there an evidence? An Italian field study. *Eur J Public Health*. (2020) 30:ckaa165.565. doi: 10.1093/eurpub/ckaa165.565
18. Kok N, van Gurp J, Teerenstra S, van der Hoeven H, Fuchs M, Hoedemaekers C, et al. Coronavirus disease 2019 immediately increases burnout symptoms in ICU professionals: a longitudinal cohort study*. *Crit Care Med.* (2021) 49:419–27. doi: 10.1097/CCM.0000000000004865
19. Khalafallah AM, Lam S, Gami A, Dornbos DL, Sivakumar W, Johnson JN, et al. Burnout and career satisfaction among attending neurosurgeons during the COVID-19 pandemic. *Clin Neurol Neurosurg.* (2020) 198:106193. doi: 10.1016/j.clineuro.2020.106193
20. Wang H, Zhou X, Jia X, Song C, Luo X, Zhang H, et al. Emotional exhaustion in front-line healthcare workers during the COVID-19 pandemic in Wuhan, China: the effects of time pressure, social sharing and cognitive appraisal. *BMC Public Health.* (2021) 21:829. doi: 10.1186/s12889-021-10891-w

21. Schwartz SP, Adair KC, Bae J, Rehder KJ, Shanafelt TD, Profit J, et al. Work-life balance behaviours cluster in work settings and relate to burnout and safety culture: a cross-sectional survey analysis. *BMJ Qual Saf.* (2019) 28:142–50. doi: 10.1136/bmjqs-2018-007933
22. Aronsson G, Theorell T, Grape T, Hammarström A, Hogstedt C, Marteinsdottir I, et al. A systematic review including meta-analysis of work environment and burnout symptoms. *BMC Public Health.* (2017) 17:264. doi: 10.1186/s12889-017-4153-7
23. Magnavita N, Chirico F, Garbarino S, Bragazzi NL, Santacroce E, Zaffina S. SARS/MERS/SARS-CoV-2 outbreaks and burnout syndrome among healthcare workers. An umbrella systematic review. *Int J Environ Res Public Health.* (2021) 18:4361. doi: 10.3390/ijerph18084361
24. Dunn L, Iglewicz A, Moutier C. A conceptual model of medical student well-being: promoting resilience and preventing burnout. *Acad Psychiatry.* (2008) 32:44-53. doi: 10.1176/appi.ap.32.1.44
25. Maslach C, Jackson SE. The measurement of experienced burnout. *J Organ Behav.* (1981) 2:99-113. doi: 10.1002/job.4030020205
26. Dion G, Tessier R. Validation de la traduction de l'Inventaire d'épuisement professionnel de Maslach et Jackson. / *Validation of a French translation of the Maslach Burnout Inventory (MBI). Can J Behav Sci.* (1994) 26:210-27. doi: 10.1037/0008-400X.26.2.210
27. Garnefski N, Kraaij V, Spinhoven P. Negative life events, cognitive emotion regulation and emotional problems. *Pers Individ Dif.* (2001) 30:1311-27. doi: 10.1016/S0191-8869(00)00113-6
28. Jermann F, Van der Linden M, D'acremont M, Acremont, Zermatten A. Cognitive Emotion Regulation Questionnaire (CERQ): confirmatory factor analysis and psychometric properties of the French Translation. *Eur J Psychol Assess.* (2006) 22:126-31. doi: 10.1027/1015-5759.22.2.126
29. Hosey MM, Leoutsakos JS, Li X, Dinglas VD, Bienvenu OJ, Parker AM, et al. Screening for posttraumatic stress disorder in ARDS survivors: validation of the Impact of Event Scale-6 (IES-6). *Crit Care.* (2019) 23:276. doi: 10.1186/s13054-019-2553-z
30. Thoresen S, Tambs K, Hussain A, Heir T, Johansen VA, Bisson JI. Brief measure of posttraumatic stress reactions: impact of Event Scale-6. *Soc Psychiatry Psychiatr Epidemiol.* (2010) 45:405–12. doi: 10.1007/s00127-009-0073-x
31. Muller L, Spitz E. Evaluation multidimensionnelle du coping: validation du Brief COPE sur une population française [Multidimensional assessment of coping: validation of the Brief COPE among French population]. *Encephale.* (2003) 29:507–18.
32. Preacher KJ, Hayes AF. Asymptotic and resampling strategies for assessing and comparing indirect effects in multiple mediator models. *Behav Res Methods.* (2008) 40:879-91. doi: 10.3758/BRM.40.3.879
33. Vizheh M, Qorbani M, Arzaghi SM, Muhidin S, Javanmard Z, Esmaeili M. The mental health of healthcare workers in the COVID-19 pandemic: a systematic review. *J Diabetes Metab Disord.* (2020) 19:1967–78. doi: 10.1007/s40200-020-00643-9
34. Adhikari Baral I, Bhagawati B. Post traumatic stress disorder and coping strategies among adult survivors of earthquake, Nepal. *BMC Psychiatry.* (2019) 19:118. doi: 10.1186/s12888-019-2090-y
35. Kucmin T, Kucmin A, Turska D, Turski A, Nogalski A. Coping styles and dispositional optimism as predictors of post-traumatic stress disorder (PTSD) symptoms intensity in paramedics. *Psychiatr Pol.* (2018) 52:557-71. doi: 10.12740/PP/68514
36. Tomsis Y, Gelkopf M, Yerushalmi H, Zipori Y. Different coping strategies influence the development of PTSD among first-time mothers. *J Matern Fetal Neonatal Med.* (2018) 31:1304–10. doi: 10.1080/14767058.2017.1315658
37. Ceslowitz SB. Burnout and coping strategies among hospital staff nurses. *J Adv Nurs.* (1989) 14:553-8. doi: 10.1111/j.1365-2648.1989.tb01590.x
38. Bamonti P, Conti E, Cavanagh C, Gerolimatos L, Gregg J, Goulet C, et al. Coping, cognitive emotion regulation, and burnout in long-term care nursing staff: a preliminary study. *J Appl Gerontol.* (2019) 38:92-111. doi: 10.1177/0733464817716970
39. Lee HF, Kuo CC, Chien TW, Wang YR. A meta-analysis of the effects of coping strategies on reducing nurse burnout. *Appl Nurs Res.* (2016) 31:100-10. doi: 10.1016/j.apnr.2016.01.001
40. Schreiber M, Cates DS, Formanski S, King M. Maximizing the resilience of healthcare workers in multi-hazard events: lessons from the 2014-2015 Ebola response in Africa. *Mil Med.* (2019) 184:114-20. doi: 10.1093/milmed/usy400

Conflict of Interest: The authors declare that the research was conducted in the absence of any commercial or financial relationships that could be construed as a potential conflict of interest.

Publisher's Note: All claims expressed in this article are solely those of the authors and do not necessarily represent those of their affiliated organizations, or those of the publisher, the editors and the reviewers. Any product that may be evaluated in this article, or claim that may be made by its manufacturer, is not guaranteed or endorsed by the publisher.

Copyright © 2021 Vancappel, Jansen, Ouhmad, Desmidt, Etain, Bergey, d'Ussel, Krebs, Paquet, Réveillère, Hingray and El-Hage. This is an open-access article distributed under the terms of the Creative Commons Attribution License (CC BY). The use, distribution or reproduction in other forums is permitted, provided the original author(s) and the copyright owner(s) are credited and that the original publication in this journal is cited, in accordance with accepted academic practice. No use, distribution or reproduction is permitted which does not comply with these terms.
