# Supplementary material for: Mental health status and related factors influencing healthcare workers during the COVID-19 pandemic: A systematic review and meta-analysis
Source: PLoS One. 2024 Jan 19;19(1):e0289454. doi: 10.1371/journal.pone.0289454 (PMC10798549; doi:10.1371/journal.pone.0289454)
Supplement: S1 Data — (ZIP) [file pone.0289454.s011.zip › literatures/208.pdf]

ELSEVIER

Since January 2020 Elsevier has created a COVID-19 resource centre with free information in English and Mandarin on the novel coronavirus COVID-19. The COVID-19 resource centre is hosted on Elsevier Connect, the company's public news and information website.

Elsevier hereby grants permission to make all its COVID-19-related research that is available on the COVID-19 resource centre - including this research content - immediately available in PubMed Central and other publicly funded repositories, such as the WHO COVID database with rights for unrestricted research re-use and analyses in any form or by any means with acknowledgement of the original source. These permissions are granted for free by Elsevier for as long as the COVID-19 resource centre remains active.

Asian Journal of Psychiatry 55 (2021) 102517

Contents lists available at ScienceDirect

Asian Journal of Psychiatry

journal homepage: www.elsevier.com/locate/ajp

Letter to the Editor

## Mental wellbeing of frontline healthcare workers during COVID-19 pandemic in Qatar

The Coronavirus disease 2019 (COVID-19) began with initial cases in China in December 2019 and by January 2020, World Health Organization (WHO) declared it as a Public Health Emergency of International Concern (WHO Director-General's opening remarks at the media briefing on COVID-19 - 11 March, 2020 n.d.). In the current pandemic concerns about vulnerability of healthcare workers was reported (Lai et al., 2020; Rajkumar, 2020). This is no different from research studies from previous epidemics, where healthcare workers exposed to the frontlines were reported to be vulnerable to poor mental health outcomes. The editorial of this journal (Tandon, 2020) reiterated the importance of health care systems identifying vulnerable groups and creating mitigation strategies to ensure psychological wellbeing of its health care workers.

There has been no systematic evaluation of the psychological impact of COVID-19 on healthcare workers in the Arabian Gulf region. The aim of the current study was to evaluate mental wellbeing of the front line healthcare workers in a well-resourced hospital designated to manage COVID-19 patients in the early phase of the outbreak in Qatar. The study is a single center, cross-sectional, exploratory web-based survey. The protocol for the research project was approved by Institutional Review Board. A total of 194 of 610 contacted staff members completed the survey, with a participation rate of 32 %. The primary outcome of interest in the study was mental wellbeing as measured by the Warwick-Edinburgh Mental Wellbeing Scale (WEMWBS) (Stewart-Brown et al., 2011). Demographic data were self-reported by the participants, including age, gender, ethnicity, occupation and living arrangement. A total of 142 (83 %) of participants were male. Of all the participants, 110 (64.3 %) were less than 35 years old, 27.5 % were aged 35–44 and 8.2 % were aged 45–65. The participants included 29 (17 %) physicians, 110 (64.3 %) nurses and 32 (18.7 %) allied healthcare workers. The participants were from diverse ethnic backgrounds with majority being Indian 64 (37.4 %) and Philippine 62 (36.3 %). Approximately 30 % (29.2 %, 95 % CI 22.9, 36.5 %) of the participants had wellbeing scores of less than 45 indicating sub-optimal wellbeing and high risk of psychological distress and depression. Out of these, 35 (20 %) had wellbeing scores of less than 40 indicating high risk of major depression. Chi-square statistical analysis showed participants in the age group of 34–44, females, not living with family, Arab ethnicity, and non-nurses to be positively associated with well-being outcomes, however most of these differences were statistically insignificant ($P > 0.05$). Similar trend was observed when we compared actual mean WEMWBS scores across various participants' characteristics. The results of univariate and multivariate logistic regression analysis testing for each predictor and their possible association with low WEMWBS scores (score cutoff value

**Table 1**
Logistic regression analysis to determine potential predictors and risk factors associated with low WEMWBS scores.

| Participants Characteristics | Univariate Logistic regression | | | Multivariate Logistic regression | | |
|---|---|---|---|---|---|---|
| | Unadjusted Odds ratio (OR) | 95 % CI for OR | P-value | Adjusted Odds ratio[a] (OR) | 95 % CI for OR | P-value |
| Age group | | | | | | |
| 25 to 34 years | 1. 0 (reference) | | | 1.0 (reference) | | |
| >34 to 44 years | 0.48 | 0.21, 1.10 | 0.083 | 0.74 | 0.26, 2.11 | 0.575 |
| >44 to 54 years | 0.90 | 0.26, 3.13 | 0.870 | 1.32 | 0.27, 6.44 | 0.730 |
| Gender | | | | | | |
| Male | 1.0 (reference) | | | 1.0 (reference) | | |
| Female | 0.81 | 0.32, 2.05 | 0.650 | 0.43 | 0.15, 1.20 | 0.106 |
| Ethnicity | | | | | | |
| Arab | 1.0 (reference) | | | 1.0 (reference) | | |
| South Asian | 2.81 | 0.87, 9.03 | 0.084 | 3.11 | 0.79, 12.23 | 0.105 |
| Philippines | 3.44 | 1.06, 11.15 | 0.040 | 5.58 | 1.30, 24.01 | 0.021 |
| Others | 2.50 | 0.36, 17.57 | 0.357 | 3.62 | 0.43, 30.21 | 0.235 |
| Profession | | | | | | |
| Doctor | 1.0 (reference) | | | 1.0 (reference) | | |
| Nurse | 1.72 | 0.68, 4.40 | 0.253 | 1.57 | 0.38, 6.43 | 0.534 |
| Others | 0.45 | 0.12, 1.73 | 0.245 | 0.38 | 0.07, 2.09 | 0.266 |
| Living status | | | | | | |
| Living with family | 1.0 (reference) | | . | 1.0 (reference) | | |
| Not living with family | 0.87 | 0.44, 1.74 | 0.693 | 0.66 | 0.27, 1.60 | 0.360 |

WEMWBS: Warwick-Edinburgh Mental Wellbeing Scale.
In dichotomous outcome variable: WEMWBS scores $\geq$ 45 was considered as reference group.
[a] Adjusted for predictors age, gender, ethnicity, profession and living status.

https://doi.org/10.1016/j.ajp.2020.102517
Received 26 October 2020
Available online 25 December 2020
1876-2018/© 2020 Elsevier B.V. All rights reserved.

<45) are presented in Table 1. The multivariable logistic regression analysis showed that participants with Philippine ethnicity remained significantly associated with increased risk (more than fivefold higher risk of having WEMWBS score <45) of adverse mental well-being (adjusted OR 5.58; 95 % CI 1.30, 24.01; P = 0.021) controlling and adjusting for all other potential confounder and predictors shown in Table 1. Finally, we computed a prediction model to evaluate the discriminative ability of potential significant variables with statistical P < 0.10 on the occurrence of lower score cutoff values (WEMWBS score <45 score) that inversely effect mental well-being. Multivariate logistic regression indicated that the final model demonstrated a modest fit (area under the curve (AUC) = 0.716, 95 % CI 0.63, 0.80) and included the potential predictors and risk factors as shown in Table 1. Ours is the first study to evaluate impact on healthcare workers from mental well-being perspective in the region. Qatar represents a unique state funded healthcare system, which made significant changes in its functioning in response to the pandemic (Wadoo et al., 2020; Karim et al., 2020). Approximately 30 % of the participants had wellbeing scores of less than 45 on WEMWBS indicating suboptimal wellbeing. This is consistent with the broader literature showing that most healthcare workers are resilient and do not succumb to overwhelming distress, however some are more vulnerable leading to suboptimal wellbeing and vulnerability to possible depressive or anxiety disorders. Psychological distress has been attributed to long working hours, risk of infection, and shortages of protective equipment, loneliness, physical fatigue, and separation from families. All the studies in this area including ours report nurses to be more vulnerable than physicians. This can be attributed to prolonged contact with patients. The factors like 'not living with the family' is associated with better outcomes and can be attributed to participants being less fearful of passing the infection to their loved ones. It was interesting to note in our study that participants belonging to Arab ethnicities fare well on mental wellbeing outcomes. Of importance to note is that unlike other international research, our study shows females to be less vulnerable than men.

## Contributors

All authors contributed to the design, data collection, data analysis and interpretation of the results.

YI & NC led data collection.

PK led data analysis.

OW, JL, MA, NM and MM led interpretation and presentation of results.

OW wrote the initial draft of the manuscript.

All the authors read and approved the final manuscript.

## Funding

The authors have not declared a specific grant for this research from any funding agency in the public, commercial or not-for-profit sectors.

## Acknowledgments

We are thankful to all healthcare workers who participated in this research during this unprecedented pandemic. We would also like to thank Mr. Yassine Khalladi, Head Nurse Mental Health Services, Hazm Mebaireek General Hospital, Hamad Medical Corporation for his support during this research project.

## References

Karim, M.A., Wadoo, O., Reagu, S.M., et al., 2020. Telepsychiatry in the Arabian Gulf region – implications beyond the Covid-19 pandemic. Asian J. Psychiatr. 54, 102397 https://doi.org/10.1016/j.ajp.2020.102397.

Lai, J., Ma, S., Wang, Y., et al., 2020. Factors associated with mental health outcomes among health care workers exposed to Coronavirus disease 2019. JAMA Netw Open. 3 (3), e203976 https://doi.org/10.1001/jamanetworkopen.2020.3976.

Rajkumar, R.P., 2020. COVID-19 and mental health: a review of the existing literature. Asian J. Psychiatr. https://doi.org/10.1016/j.ajp.2020.102066.

Stewart-Brown, S., Platt, S., Tennant, A., et al., 2011. The Warwick-Edinburgh MENTAL WELL-BEING SCALE (WEMWBS): a valid and reliable tool for measuring mental well-being in diverse populations and projects. J. Epidemiol. Commun. Health 65, A38–A39. https://doi.org/10.1136/jech.2011.143586.86.

Tandon, R., 2020. The COVID-19 pandemic, personal reflections on editorial responsibility. Asian J. Psychiatr. 50 https://doi.org/10.1016/j.ajp.2020.102100.

Wadoo, O., Latoo, J., Reagu, S.M., et al., 2020. Mental Health during COVID-19 in. Qatar 33 (6). https://doi.org/10.1136/gpsych-2020-100313.

WHO Director-General's opening remarks at the media briefing on COVID-19 -. 11 March 2020 [WWW Document], n.d. URL https://www.who.int/dg/speeches/detail/who-director-general-s-opening-remarks-at-the-media-briefing-on-covid-19-.11-march-2020 (accessed 7.1.20).

Ovais Wadoo[a,*], Javed Latoo[a], Yousaf Iqbal[a], Nirvana Swamy Kudlur Chandrappa[a], Prem Chandra[b], Naseer Ahmad Masoodi[c], Muna A. Rahman S. Al-Maslamani[d], Majid Alabdulla[a,e]

[a] *Mental Health Services, Hamad Medical Corporation, Doha, Qatar*

[b] *Medical Research Center, Hamad Medical Corporation, Doha, Qatar*

[c] *Ambulatory General Internal Medicine, Hamad Medical Corporation, Doha, Qatar*

[d] *Communicable Disease Center Hamad Medical Corporation, Doha, Qatar*

[e] *Qatar University, College of Medicine, Doha, Qatar*

[*] Corresponding author at: Mental Health Services, Hamad Medical Corporation, PO Box 3050, Qatar.

*E-mail address:* owadoo@hamad.qa (O. Wadoo).
