# Supplementary material for: Mental health status and related factors influencing healthcare workers during the COVID-19 pandemic: A systematic review and meta-analysis
Source: PLoS One. 2024 Jan 19;19(1):e0289454. doi: 10.1371/journal.pone.0289454 (PMC10798549; doi:10.1371/journal.pone.0289454)
Supplement: S1 Data — (ZIP) [file pone.0289454.s011.zip › literatures/209.pdf]

DANISH MEDICAL JOURNAL

Original Article

Dan Med J 2021;68(6):A01210073

The impact of the COVID-19 pandemic on mental health among healthcare workers in ear-nose-throat clinics

Lena Walvik[1], Maiken Wissing Brejnebøl[1], Andreas Tomaas Ravn[2], Ramon Gordon Jensen[1], Anders Korsgaard Christensen[3] & Christian von Buchwald[1]

1) Department of Otorhinolaryngology, Head & Neck Surgery and Audiology, Copenhagen University Hospital – Rigshospitalet, 2) The Ear, Nose and Throat Clinic, Frederiksberg, 3) Department of Crisis Psychology, Copenhagen University Hospital – Rigshospitalet, Denmark

Dan Med J 2021;68(6):A01210073

ABSTRACT

INTRODUCTION: During the first wave of the COVID-19 pandemic, it was established that otorhinolaryngologists were at a high risk of contracting the infection due to examinations of the upper airways. The aim of this study was to evaluate the impact of the COVID-19 pandemic on mental health among healthcare workers (HCWs) in primary ear-nose-throat (ENT) practices.

METHODS: This was a cross-sectional questionnaire study among HCWs assessing symptoms of anxiety (Generalized Anxiety Disorder, ten-item scale) and depression (Patients Health Questionnaire, nine-item scale). The survey targeted otolaryngologists and staff in primary private practices in the Capital Region of Denmark during the COVID-19 lock-down in May 2020.

RESULTS: For 30% of the participants, signs were observed of depressive symptoms and 13% had signs of anxiety symptoms. Seventy percent felt sufficiently protected by their available personal protective equipment. Fifty-two percent worried about becoming infected in relation to their work and 56% feared infecting their families.

CONCLUSIONS: HCWs in Danish primary ENT practices did not display extraordinary stress reactions during the first wave of the pandemic. Noticeably, participants were concerned about acquiring the infection or transmitting it to their household.

FUNDING: none.

TRIAL REGISTRATION: not relevant.

Because of the COVID-19 pandemic and its rapid development in Europe, the Danish government initiated several restrictions comprising lock down of schools and public institutions that culminated in a de facto closure of the Danish borders on 14 March 2020. In preparation of a possible severe course of the pandemic, the Danish health authorities consequently redistributed resources and work routines [1]. In early April 2020, the number of cases peaked at 467 new diagnosed cases/day (7.5% of 6,210 tested), 535 admitted to hospital and 153 in need of intensive care [2]. Within a month, the number of infected patients was again stable and gradually decreasing, leading the Danish government gradually to reopen society. This included resuming elective procedures and treatments in public and private healthcare, but with strict requirements to use of personal protective equipment (PPE) among staff and frequent testing for COVID-19 in patients [3].

In the initial stages of the current pandemic, it was established that healthcare workers (HCWs) were at a significant risk of contracting COVID-19 [4]. Otorhinolaryngologists were affected at higher rates than other medical doctors (MDs), which was acknowledged by the Confederation of European Otorhinolaryngology - Head and Neck Surgery [5]. A possible explanation was the close contact with patient's airway mucosa during routine ear-nose-throat (ENT) examinations, i.e. endoscopy and surgery in the upper airways can induce sneezing and coughing. COVID-19, caused by the novel coronavirus SARS-CoV-2, seemed to carry a high viral load especially in the nasopharynx, posing an increased risk for otorhinolaryngologists [6].

Initially, one of the main challenges related to combating the pandemic was the overall shortage of adequate PPE and access to COVID-19 testing. Denmark was no exception in this regard [7]. The structural changes and the ongoing risk of exposure were placing HCWs under potential physical and psychological stress. By the end of March 2020, Denmark received a large private donation, consisting of personal protective gear and test equipment, which also became accessible to primary healthcare.

A Chinese study of mental health among HCWs during the COVID-19 pandemic demonstrated that 50% of doctors and nurses were showing signs of depression. Personnel with direct patient contact had a significantly higher risk of moderate-to-severe depression, anxiety and difficulty of sleeping [8, 9]. Furthermore, a questionnaire study among HCWs in New York during the first wave of COVID-19 found positive screens for depressive (48%) and for anxiety symptoms (33%) [10].

The aim of this questionnaire study was to evaluate the impact of the first wave of the COVID-19 pandemic on mental health among healthcare professionals in primary ENT clinics in the Capital Region of Denmark.

METHODS

Setting

In Denmark, approximately 50% of otorhinolaryngologists are organised in a primary care setting as private practitioners. Patients with symptoms in the ENT region can seek direct medical advice in ENT practice without prior referral. The clinics are themselves responsible for procuring protective equipment.

Study design

This was a cross-sectional questionnaire study, assessing symptoms of anxiety and depression using validated questionnaires. The survey was addressed to otolaryngologists in private primary practices in the Capital Region of Denmark including their employees aged ≥ 25 years during the COVID-19 lock down (from 14 March to 14 April 2020). A synchronous and similar study was set up at the ENT Department at Rigshospitalet, Copenhagen (yet unpublished data).

The questionnaire was set up and made available through the Research Electronic Data Capture (REDCap) tool, hosted at our institution [11]. A direct link to the questionnaire was sent to the ENT clinics electronically by email on 4 May 2020. A reminder was sent out to all clinics by email on 12 May 2020, and the survey was concluded two days later.

The questionnaire consisted of a demographic part, including age, gender, profession and work experience. The second part concerned work organisation and exposure to COVID-19, and stigma in conjunction with work. The third part consisted of two separate and validated questionnaires regarding signs of depression and anxiety within the two preceding weeks: Patients Health Questionnaire, nine items (PHQ-9), objectifying the degree of depression, and the Generalized Anxiety Disorder scale, ten items (GAD-10). Both scales used ordinal Likert response scales, and data were handled as continuous data numbered 0-5 for the PHQ-9 and 0-3 for the GAD-10.

For the PHQ-9, a score of five or more was considered positive for signs of depression. For the PHQ-9, a score of five or more was considered positive for signs of depression. A GAD-10 score of eight or above was considered positive for signs of anxiety. Lastly, the respondents were asked to grade their level of self-perceived stress on a visual analogue scale (VAS). The participants had to fill in the demographic data and complete at least one of the questionnaires to be included.

The survey was fully anonymous and participants were not required to provide any contact information. Furthermore, age and work experience were indicated as intervals. Information encouraging contact to a general practitioner (GP) in case of self-perceived mental stress was provided at the end of the survey.

Data were analysed, and continuous variables are presented as median, range and interquartile range (IQR). To test difference in proportions, a Fisher's exact test was used.

The study was categorised as a quality assurance study. Under Danish law, no approval from an ethics committee or from the Danish Data Protection Agency was required.

Trial registration: not relevant.

RESULTS

We invited employees from all 57 ENT clinics in the Capital Region and received 75 individual responses in REDCap. One of the respondents only answered the demographic questions and none of the succeeding questionnaires, and this respondent was excluded from further analysis.

On average, each clinic was staffed by one otolaryngologist and 2.5 employees, estimating an approximate total of 57 otolaryngologists and 143 employees, yielding an estimated response rate of 37%. Among respondents, 51 (72%) were women and 23 (28%) men. Their median age range was 50-54 years. The MDs constituted the largest group (49%). Among respondents, 38% were tested for COVID-19 (throat swab for polymerase chain reaction analysis), of whom 7% tested positive (**Table 1**).

TABLE 1 Demographic data (N = 74).

Characteristics of respondents	
Man:woman, n (%)	23:51 (31:69)
Age, median range, yrs	50-54
Job position, n (%)	
Doctor	36 (49)
Nurse	8 (11)
Secretary	23 (31)
Other	7 (9)
Work experience: ENT-practice, median range, yrs	> 5
COVID-19 test status, n (%)	
Not tested	43 (58)
Positive	5 (7)
Negative	26 (35)

ENT = ear-nose-throat.

When answering questions about work organisation, exposure and stigma (https://ugeskriftet.dk/files/a01210073_-_supplementary.pdf), the majority of respondents reported many changes in their everyday life, but similar work routines and a decreased pace of work. Among respondents, 70% reported feeling sufficiently protected by the PPE made available. Furthermore, 74% had patient contact and the majority of these were either present during or themselves performed aerosol-producing procedures. More than half of the respondents were worried about becoming infected with COVID-19 in relation to their work and 52% feared infecting their families. Fifteen percent felt that others were distancing themselves from them, and 40% responded that they isolated themselves from others because of their work.

The GAD-10 questionnaire was completed by 71 (96%) respondents, with a median score of three (range: 0-24, IQR: 1-6). Among respondents, 13% scored positive for signs of mild-to-moderate anxiety (**Table 2**), and no significant gender difference was observed (p = 0.4). The PHQ-9 questionnaire was completed by 56 (74%), with a median score of three (range: 0-15, IQR: 2-5). Among respondents, 30% scored positive for signs of mild-to-moderate signs of depression (**Table 3**), and significantly more women than men scored positive for signs of depression (p = 0.01). No significant difference was observed in stress reactions (PHQ-9- and GAD-10 scores) between those with and those without direct patient contact (p = 0.74). The VAS for self-perceived stress was completed by 67 (89%) respondents with a median score of 12 (range: 0-78, IQR: 4-33) (**Figure 1**).

TABLE 2 Results of Generalized Anxiety Disorder scale, ten-item, (N = 71 (96%)).

	Anxiety: score				
	none: 0-7	mild: 8-14	moderate: 15-19	moderate-severe: 20-29	severe: 30-50
Total, n (%)	62 (87.3)	6 (8.5)	2 (2.8)	1 (1.4)	0
Man:woman, %	31:69	17:83	0:100	0:100	0:0
Profession, %					
Doctor	48.4	50.0	0.0	0.0	0.0
Nurse	8.1	50.0	0.0	0.0	0.0
Secretary	32.3	0.0	100.0	100.0	0.0
Other	11.3	0.0	0.0	0.0	0.0

TABLE 3 Results of Patients Health Questionnaire, nine-item (N = 56 (79%)).

	Signs of depression: score				
	none: 0-4	mild: 5-9	moderate: 10-14	moderate-severe: 15-19	severe: 20-27
Total, n (%)	39 (69.9)	14 (25.0)	2 (3.6)	1 (1.8)	0
Man:woman, %	31:69	0:100	0:100	0:100	0:0
Profession, %					
Doctor	44.0	21.0	100.0	0.0	0.0
Nurse	8.0	29.0	0.0	0.0	0.0
Secretary	38.0	43.0	0.0	100.0	0.0
Other	10.0	7.0	0.0	0.0	0.0

FIGURE 1 Visual analogue scale 0-100[a].

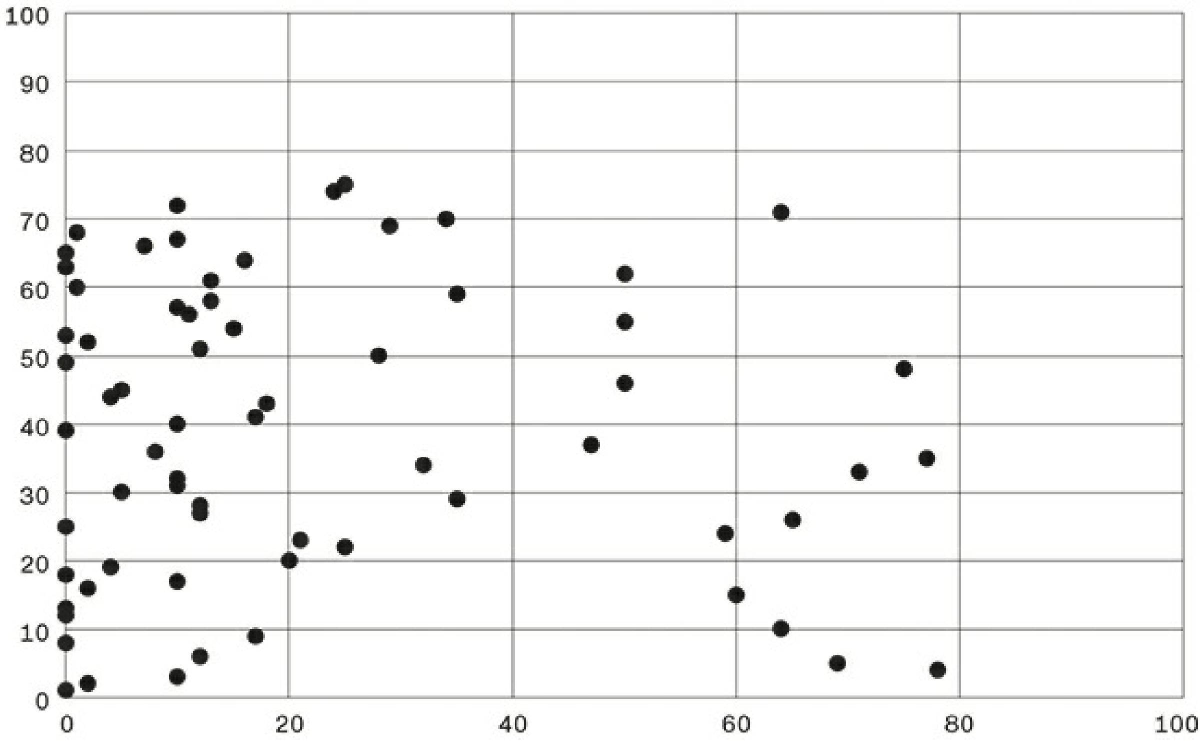

a) 0 = not feeling stressed, 100 = maximal self-perceived stress.

DISCUSSION

In this cross-sectional questionnaire study, we investigated the mental health of the staff in ENT practices in the Capital Region of Denmark during the first wave of the COVID-19 pandemic. In relation to the general population, a study on Danish mental health (2016) found that 29% of women and 21% of men had signs of stress. Furthermore, 31.6% reported signs of depression and 30% signs of anxiety during the two preceding weeks [12]. A study on mental well-being among Danish GPs reported 8.4% of GPs to have a poor overall mental health with poor general well-being, substantial burnout and high stress [13]. In comparison, our study found that 13% of respondents had experienced signs of anxiety and 30% signs of depression during the past two weeks. This may indicate that the HCWs in question did not display extraordinary stress reactions due to the COVID-19 pandemic. Certainly, the ideal basis for comparison would have been to conduct a baseline study on mental health prior to the pandemic.

During the epidemics/pandemics, HCWs are generally exposed to a significant amount of stress. Throughout the Ebola outbreak in 2014-2015 in Sierra Leone, HCWs who had direct patient contact exhibited psychological symptoms like obsession compulsion, paranoid ideation, interpersonal sensitivity and depression [14]. In 2003, during the SARS epidemic, multiple studies reported a significant psychological impact on HCWs, measuring significant emotional distress in up to 57% [15-17]. Although the specialty of otolaryngology was considered a high-risk field for contracting COVID-19, our study found none of the participants to have scores for signs of severe depression or anxiety during the survey period [5]. Furthermore, 7% of respondents reported having had a positive throat swab for COVID-19, which was a high percentage compared with the background population at the time of the study (0.5-1.8%) [18]. Various reasons may possibly help explain these findings; at the time, Denmark experienced a relatively mild course of the pandemic, the PPE supply had returned to normal and

COVID-19 testing was made widely available.

During the SARS outbreak, fear of contagion and concern for infecting family members were persistent worries among HCWs [16, 17]. This was consistent with our findings as most respondents reported fear of becoming infected at work (56%) and fear of infecting their household (56%). Although reporting risk of containing COVID-19 at work (52%), our study found that most respondents (70%) felt sufficiently protected by the PPE available.

The response rate for this questionnaire study was 37% of the estimated study population. The awareness of COVID-19 and the consequences the virus entailed were considered a potential stressor in a self-employed private practice setting. The doctors constituted the largest group of respondents (49%); and with only 57 clinics in the region, fear of being recognised may have kept subjects from participating. Furthermore, the questionnaire was sent out to each clinic's main email-address. Awareness of mental health with the potential risk of sick leave, and particularly the question "Do you feel well informed by your manager about COVID-19?", may possibly have stopped the employers (the MDs) from forwarding the questionnaire to their employees. Lastly, we do not have the exact number of closed clinics during the survey period: some clinics were closed due to COVID-19 among the staff, others because of low activity due to the lock-down and others for fear of becoming infected. Nevertheless, our response rate was consistent with those of similar studies, e.g., the nationwide questionnaire considering the mental health of Danes (35%) and a questionnaire considering the psychological effect of SARS on HCWs (39%) [12, 19].

The response rate was higher for the first questionnaires (GAD-10) than for the last listed questionnaire (PHQ-9), which is a classic result in questionnaire studies due to respondent fatigue [20]. In contrast, the VAS score for self-perceived stress was well responded. This is also a classic finding in questionnaires. A high VAS score corresponded well to high scores on both the GAD-10- and the PHQ-9 scale.

Limitations

The limited response rate increased the risk of bias, including non-response bias, i.e. the most stressed/depressed might not have participated. Furthermore, a potential source of bias is that baseline mental health (e.g., diagnosed depression or anxiety) among participants was unknown. Additionally, the exact response rate could not be estimated because no official record was available of either the number or the gender of employees in ENT practices. As stated above, the questionnaire was forwarded to the clinics' main email address and may not have been forwarded to the entire staff. The lower age limit was set to 25 years, possibly excluding some personnel. Some clinics had medical students employed in a part-time position, and we aimed this questionnaire towards permanent employees.

Future studies

Baseline mental health has, to our knowledge, never been investigated in ENT practices before. Hence, it would be interesting to repeat the surveys in an ordinary setting not affected by the pandemic. Furthermore, concern for the occupational safety of healthcare workers and their relatives could be an important focus point in health leadership during the further course of the pandemic.

CONCLUSIONS

This study suggests that the staff in ENT practices in the Danish Capital Region did not display extraordinary stress reactions due to the first wave of the COVID-19 pandemic. Noticeably, the participants were concerned about being infected themselves or transmitting COVID-19 to their household.

Correspondence *Lena Walvik*. E-mail: lena.walvik@regionh.dk

Accepted 27 April 2021

Conflicts of interest none. Disclosure forms provided by the authors are available with the full text of this article at ugeskriftet.dk/dmj

REFERENCES

1. Danish Health Authority. Notat om reduktion af hospitalsaktivitet ifm COVID-19. Danish Health Authority, 2020. www.sst.dk/-/media/Udgivelser/2020/Corona/Hospitalskapacitet/Notat-om-reduktion-af-hospitalsaktivitet-ifm-med-COVID-19.ashx?la=da&hash=C2EF7016267E5DF9250BD0568276A6792775A73E (1 Jun 2020).
2. Statistics Denmark. COVID-19 smittespredning mv. pr. dag i 2020 (eksperimentel statistik) efter nøgletal og tid . Statistics Denmark, 2020. www.statistikbanken.dk/statbank5a/selectvarval/define.asp?PLanguage=0&subword=tabsel&MainTable=SMIT1&PXSId=222029&tablestyle=&ST=SD&buttons=0 (21 Oct 2020).
3. Danish Health Authority. COVID-19: generelle retningslinjer for planlægning af aktivitet og forebyggelse af smittespredning i sundhedsvæsenet. Danish Health Authority, 2020. www.sst.dk/-/media/Udgivelser/2020/Corona/Retningslinjer/Generelle-retningslinjer-for-planlaegning-af-aktivitet-i-sundhedsvaesent.ashx?la=da&hash=9F3B08A0B72C4167F97D07DE114115A7AC61E665 (15 Jun 2020).
4. Wang J, Zhou M, Liu F. Reasons for healthcare workers becoming infected with novel coronavirus disease 2019 (COVID-19) in China. J Hosp Infect 2020;105:100-1.
5. Confederation of European ORL-HNS. CEORL-HNS Statement to COVID-19. CEORL-HNS, 2020 www.ceorlhns.org/covid-19 (27 May 2020).
6. Zou L, Ruan F, Huang M et al. SARS-CoV-2 Viral load in upper respiratory specimens of infected patients. N Engl J Med 2020;382:1177-9.
7. Wang X, Zhang X, He J. Challenges to the system of reserve medical supplies for public health emergencies: reflections on the outbreak of the severe acute respiratory syndrome coronavirus 2 (SARS-CoV-2) epidemic in China. Biosci Trends 2020;14:3-8.
8. Lai J, Ma S, Wang Y et al. Factors associated with mental health outcomes among health care workers exposed to coronavirus disease 2019. JAMA Netw Open 2020;3:e203976.
9. Vindegaard N, Benros ME. COVID-19 pandemic and mental health consequences: systematic review of the current evidence. Brain Behav Immun 2020;89:531-42.
10. Shechter A, Diaz F, Moise N et al. Psychological distress, coping behaviors, and preferences for support among New York healthcare workers during the COVID-19 pandemic. Gen Hosp Psychiatry 2020;66:1-8.
11. Harris PA, Taylor R, Thielke R, et al. Research electronic data capture (REDCap)--a metadata-driven methodology and workflow process for providing translational research informatics support. J Biomed Inform 2009;42:377-81.
12. Danish Health Authority. Danskernes sundhed den nationale sundhedsprofil 2017. Danish Health Authority, 2017. www.sst.dk/da/udgivelser/2018/~/media/73EADC242CDB46BD8ABF9DE895A6132C.ashx (15 Aug 2020).
13. Nørøxe KB, Pedersen AF, Bro F et al. Mental well-being and job satisfaction among general practitioners: a nationwide cross-sectional survey in Denmark. BMC Fam Pract 2018;19:130.
14. Ji D, Ji Y-J, Duan X-Z et al. Prevalence of psychological symptoms among Ebola survivors and healthcare workers during the 2014-2015 Ebola outbreak in Sierra Leone: a cross-sectional study. Oncotarget 2017;8:12784-91.
15. Tam CWC, Pang EPF, Lam LCW et al. Severe acute respiratory syndrome (SARS) in Hong Kong in 2003: stress and psychological impact among frontline healthcare workers. Psychol Med 2004;34:1197-204.
16. Maunder RG, Lancee WJ, Rourke S et al. Factors associated with the psychological impact of severe acute respiratory syndrome on nurses and other hospital workers in Toronto. Psychosom Med 2004;66:938-42.
17. Nickell LA, Crighton EJ, Tracy CS et al. Psychosocial effects of SARS on hospital staff: survey of a large tertiary care institution. CMAJ 2004;170:793-8.
18. Statens Serum Institut. Notat: foreløbige resultater fra den repræsenta- tive seroprævalensundersøgelse af COVID-19.

2020:1-2. https://files.ssi.dk/Forelobige resultater fra seropraevalensundersogelse af COVID19-20052020 (1 Jun 2020).

19. Maunder RG, Lancee WJ, Balderson KE et al. Long-term psychological and occupational effects of providing hospital healthcare during SARS outbreak. Emerg Infect Dis 2006;12:1924-32.

20. Lavrakas P, ed. Encyclopedia of survey research methods. California: Thousand Oaks, 2008. https://methods.sagepub.com/reference/encyclopedia-of-survey-research-methods (15 Aug 2020).
