# Supplementary material for: Mental health status and related factors influencing healthcare workers during the COVID-19 pandemic: A systematic review and meta-analysis
Source: PLoS One. 2024 Jan 19;19(1):e0289454. doi: 10.1371/journal.pone.0289454 (PMC10798549; doi:10.1371/journal.pone.0289454)
Supplement: S1 Data — (ZIP) [file pone.0289454.s011.zip › literatures/21.pdf]

International Journal of
Environmental Research and Public Health

MDPI

Article

Mental Health Symptoms among General Practitioners Facing the Acute Phase of the COVID-19 Pandemic: Detecting Different Reaction Groups

Claudia Carmassi [1], Valerio Dell'Oste [1,2,*], Filippo Maria Barberi [1], Carlo Antonio Bertelloni [1], Virginia Pedrinelli [1,2] and Liliana Dell'Osso [1]

[1] Department of Clinical and Experimental Medicine, University of Pisa, 56126 Pisa, Italy; claudia.carmassi@unipi.it (C.C.); fil.barberi@gmail.com (F.M.B.); carlo.ab@hotmail.it (C.A.B.); virginiapedrinelli@gmail.com (V.P.); liliana.dellosso@unipi.it (L.D.)
[2] Department of Biotechnology, Chemistry and Pharmacy, University of Siena, 53100 Siena, Italy
* Correspondence: valerio.delloste@gmail.com; Tel.: +39-050-2219760; Fax: +39-050-2219787

Abstract: During the 2020 first wave of the COVID-19 pandemic, general practitioners (GPs) represented the first line of primary care and were highly exposed to the pandemic risks, with a consequent risk of developing a wide range of mental health symptoms. However, scant data are still available on factors associated with a worse outcome. The aim of the present study was to investigate mental health symptoms in 139 GPs in the aftermath of the first COVID-19 national lockdown in Italy, detecting groups of subjects with different depressive, anxiety, and post-traumatic stress symptom severity. The impact of the mental health symptoms on quality of life and individual functioning were also evaluated. A cluster analysis identified three groups with mild (44.6%), moderate (35.3%), and severe psychopathological burden (20.1%). Higher symptom severity was related to younger age, fewer years in service as GPs, working in a high incidence area for the pandemic, having a relative at risk of medical complications due to COVID-19, besides more severe global functioning impairment, burnout, and secondary traumatic stress. The present findings showed that GPs, forced to perform their professional activity in extremely stressful conditions during the COVID-19 pandemic, were at high risk of developing mental health problems and a worse quality of life.

Keywords: post-traumatic stress disorder (PTSD); depression; anxiety; mental health burden; burnout; primary care; COVID-19 pandemic; global functioning

check for updates

Citation: Carmassi, C.; Dell'Oste, V.; Barberi, F.M.; Bertelloni, C.A.; Pedrinelli, V.; Dell'Osso, L. Mental Health Symptoms among General Practitioners Facing the Acute Phase of the COVID-19 Pandemic: Detecting Different Reaction Groups. *Int. J. Environ. Res. Public Health* **2022**, *19*, 4007. https://doi.org/10.3390/ijerph19074007

Academic Editors: María Jesús Irurtia, Jairo Rodríguez-Medina and Arthur L. Cantos

Received: 24 December 2021
Accepted: 24 March 2022
Published: 28 March 2022

Publisher's Note: MDPI stays neutral with regard to jurisdictional claims in published maps and institutional affiliations.

Copyright: © 2022 by the authors. Licensee MDPI, Basel, Switzerland. This article is an open access article distributed under the terms and conditions of the Creative Commons Attribution (CC BY) license (https://creativecommons.org/licenses/by/4.0/).

1. Introduction

The COVID-19 pandemic has not only affected the physical health and economic situation of billions of people around the world, it has also been a risk factor for the onset or worsening of many mental disorders [1]. In this case, healthcare workers (HCWs) have been particularly affected as they have found themselves at the forefront of fighting this exceptional global health problem. HCWs may be particularly vulnerable to developing mental disorders during a pandemic due to the high risk of contracting the pathogen, the increased work stress and fear of spreading the pathogen to family members and loved ones, and the need to constantly use personal protective equipment (PPE), with long-term psychopathological consequences [2]. During the COVID-19 pandemic, HCWs have been exposed to a high risk of infection, mortality, excessive workloads, and continuing uncertainty about the pandemic's course. This type of experience certainly consisted in a traumatic and stressful event, with several studies highlighting a negative impact on HCWs' mental health and an increased risk of developing psychopathological sequelae, including post-traumatic stress disorder (PTSD), depression, and anxiety [3–5].

In the context of healthcare professionals, general practitioners (GPs) have played a pivotal role as the first entry point to primary care for citizens [6], and this has been

particularly relevant in countries where the healthcare system is specifically tailored to a first line intervention for detecting critical cases across the country devoted to these professionals. Since the beginning of the pandemic, GPs have played a critical role in reporting infections, supporting assistance networks, and treating subjects with mild symptoms, confronting not only an extraordinary workload [7], but also exploring the need for new and challenging work changes [8,9]. Especially during the acute phase related to the so called "first wave", together with the HCWs of the emergency and intensive care departments [3,4], GPs were the most exposed to the risks of the pandemic and operating individually, outside of the hospital wards, in the context of primary care out-patient clinics and often without adequate PPE [10,11], facing emergency conditions (they usually do not) outside their usual work setting [12,13], leading to an increased risk of acute psychopathological burden.

In this regard, it is important to evaluate the impact of trauma and stress of the COVID-19 pandemic in this specific group of HCWs. Most of the literature on HCWs facing the COVID-19 pandemic revealed a considerable proportion of participants reporting a severe impact on depressive, anxiety, and posttraumatic stress symptoms [14–16]. Particularly, while there are studies analyzing the impact of the COVID-19 pandemic on GPs' mental health [17–19], no study specifically investigated the acute co-occurring burden of depressive, anxiety, and PTSD symptoms among professionals working in the first line in the acute phase of the pandemic. Results from a large multi-country study investigating mental health impacts of COVID-19 on HCWs in the eastern region in the first phase of the pandemic found that specialists and GPs had the highest job-related distress rates when compared to the other HCWs [20]. Another study on a sample of over 250 Singaporean GPs revealed that 21.4% of healthcare professionals met criteria for anxiety, 82.1% for burnout, 26.6% for depression, and 8.9% for PTSD, finding that working in a public primary care setting was associated with anxiety and depression, and they further pointed out changes to clinical and operational practices and increased workloads as possible stressors [21]. Lange et al. [17] analyzed the psychopathological impact of COVID-19 in a sample of French GPs during the first lockdown period, reporting significant PTSD symptoms (11%) and high burnout symptoms (42%), with women reporting more stress and burnout symptoms than men. Di Monte et al. [18] investigated the impact on GPs' work management during the COVID-19 emergency, showing how 40% of the sample was in a low burnout profile and about 5% was in high burnout profile. A recent study by Castelli et al. [19] explored the impact of COVID-19 on mental health of GPs in northern Italy. In the first wave of the COVID-19 pandemic, northern Italy was the most affected Italian region, while central and southern Italy were exposed to the pandemic to a lesser extent. The study showed clinically relevant PTSD symptoms in GPs who reported not receiving adequate information to protect their families and clear guidelines for COVID-19 diagnosis and treatment in order to do their job properly.

However, there is expanding literature reporting that exposure to traumatic events may increase the risk for multiple psychiatric disorders and the comorbidity between post-traumatic stress, depressive, and anxiety symptoms in this framework is well acknowledged [22–27]. Furthermore, the co-occurrence of these conditions can be related to underlying processes [28], and it is usually related to more severe psychopathological symptoms [28,29]. Against this backdrop, in the framework of the COVID-19 pandemic and similarly to previous infectious outbreaks, considerable rates of post-traumatic stress, depressive, and anxiety symptoms were shown among HCWs facing the emergency [30]. Interestingly, some authors pointed out the importance of the role of the association and interplay among different mental health symptoms, highlighting that co-occurrence was overall associated with a worse quality of life, work, and social adjustment [3,4,31,32]. Studies on the psychopathological impact of the COVID-19 pandemic have increased the relevance of the interplay among post-traumatic stress symptoms that may be particularly challenging due to the prolonged duration and variability of the traumatic exposure, as

well as anxious and depressive symptoms that may overlap or influence each other [33]. In this regard, we aimed to explore this complex relationship.

The aim of the present study was to investigate the presence of mental health symptoms in GPs in the immediate aftermath of the first COVID-19 national lockdown in Italy. In particular, we aimed at detecting groups of subjects with different psychopathological burdens, determined by depressive, anxiety, and post-traumatic stress symptom severity by means of a cluster analysis. The secondary aim was to examine possible socio-demographic and work factors associated with each group. Third, we investigated the impact of psychopathological symptoms on the quality of work life and individual functioning.

2. Materials and Methods

2.1. Sample Recruitment and Assessment

The sample of the present study included 139 GPs recruited in Italy from May 2020 to June 2020, in the immediate aftermath of the first acute phase of the COVID-19 outbreak. In fact, after the first cases of COVID-19 were detected in the Lombardy region, in northern Italy, on 21 February 2020, other outbreaks quickly occurred in the North of the country. Despite the drastic restrictions imposed by the Italian Government in those areas, several other outbreaks began in other regions, and in a few weeks Italy became the country with the greatest number of infected people after China, forcing the authorities to extend the previously adopted restrictions, enacting a nationwide COVID-19 lockdown on 9 March which lasted until 18 May 2020. The most affected regions were those in the north of the country with more than half of the total cases.

All participants filled out an online questionnaire created via the Google Forms platform, after being clearly informed about the study and providing an informed consent upon the opportunity to ask questions. GPs from all healthcare facilities in Italy were eligible for participation in this survey and a link was shared to WhatsApp groups of HCWs and through social media, asking GPs to participate in the study. The link was also shared with HCWs on the researchers' contact lists. All data were collected anonymously. The study was conducted in accordance with the Declaration of Helsinki and the Ethics Committee of the Azienda Ospedaliero-Universitaria Pisana (CEAVNO) approved all recruitment and assessment procedures (ID: 17151/2020).

2.2. Measures

Psychiatric assessments included the following questionnaires: the Impact of Event Scale-Revised (IES-R) to investigate the acute post-traumatic stress symptoms [34], the Generalized Anxiety Disorder 7-item [35] to investigate the presence of anxiety symptoms, the Patient Health Questionnaire 9-item [36,37] to detect the presence of depressive symptoms, the Work and Social Adjustment Scale [38] to examine the global impairment of functioning related to mental health burden, the Professional Quality of Life Scale-5 (ProQOL-5) [39] to examine compassion satisfaction (CS), and compassion fatigue (CF) related to work activities.

The 22-item Impact of Event Scale-Revised (IES-R) [34] was developed to assess probable post-traumatic stress disorder (PTSD) by covering three symptoms' clusters (intrusion, avoidance, and hyperarousal) and showed high internal consistency [40]. This questionnaire is one of the most commonly used scales to screen rescue workers for mental health problems [41,42]. The IES-R items are rated on a 5-point rating scale and individuals with a score equal or above 33 have probable PTSD diagnoses.

The Generalized Anxiety Disorder Assessment, 7-item version (GAD-7) [35] is part of the Patient Health Questionnaire (PHQ), a widely established screening instrument for common mental disorders. GAD-7 was shown to be a reliable and valid instrument with good test-retest reliability and high internal consistency. GAD-7 consists of seven items and is commonly used to screen for general anxiety disorders based on criteria from the DSM-IV-TR. The patient is asked to self-report how often he/she had been bothered by seven common anxiety disorder symptoms during the last two weeks. For each item, scores

of 0, 1, 2, and 3 must be given. Scores of 0–4, 5–9, 10–14, and 15–21 indicate minimal, mild, moderate, and severe anxiety symptoms, respectively [35,43].

The Patient Health Questionnaire, 9-item version (PHQ-9) [36,37] is used to assess the main criteria of major depressive disorder and it has shown high consistency with a diagnosis based on structured interviews. It has nine items that are self-rated on a scale from 0 (not at all) to 3 (nearly every day), so that the available range is 0–27. A score above 10 indicates moderate to severe depressive symptoms [44,45].

The Work and Social Adjustment Scale (WSAS) [38] is a test used to evaluate and measure work and social adjustment. It includes five items that assess the individual's ability to perform the activities of everyday life and how these are affected in the week prior to the assessment. Each of the five items is rated on a nine-point scale ranging from 0 (not at all) to 8 (severe interference), so that the total scores are between 0 and 40. The internal consistency of the instrument and the reliability of the test-retest were good [38].

The ProQOL-5 by Stamm [39] is a 30-item Likert scale ranging from 1 (never) to 5 (very often) that analyzes the professional quality of life. It integrates two aspects, namely compassion satisfaction (CS) and compassion fatigue (CF). CF has two factors: burnout (e.g., exhaustion, frustration, anger, and depression) and secondary traumatization (or secondary traumatic stress, ST). The ProQOL-5 category raw scores may range from 10 to 50. Score ranges are also available for each category.

2.3. Statistical Analyses

All statistical analyses were performed using the Statistical Package for Social Science (SPSS), version 26.0. Continuous variables were reported as mean ± standard deviation (SD), whereas categorical variables were reported as percentages. All tests were two-tailed and a p value < 0.05 was considered statistically significant.

We used a K-means cluster analysis based on the standardized IES-R, GAD-7, and PHQ-9 total scores in order to identify groups of subjects at different levels of psychopathological burden. We used squared Euclidean distance for the divergence measure between cases. To classify cases, the method of iterative updating of clustered centroids was chosen, with the new clusters centers to be calculated after all cases are assigned to a given cluster. To ensure maximum efficiency, the final cluster centers estimated from a random sample were utilized as initial centers to classify the entire file. To assess the stability of a given solution, we compared results on data sorted in different ways. After comparing the results obtained for different K values, we identified as the most satisfactory solution the one that involves 3 clusters (K = 3). This solution determined small within-cluster variability compared to the difference between clusters, and that all the cluster sizes were greater than 10% of the total sample size.

Chi-square was computed in order to evaluate differences in categorical variables among the three groups. One-way ANOVA was utilized to compare continuous normally distributed variables among the three groups, while the Kruskal–Wallis test was utilized for the non-parametric ones.

3. Results

3.1. Socio-Demographic and Work-Related Characteristics

The total sample included 139 GPs, 60 (43.2%) males and 79 females (56.8%). Eighty-nine subjects (64.0%) aged >55 years, 110 (79.1%) were married or cohabiting, 86 (61.9%) had a son, 79 (56.8%) had a postgraduate degree, 48 (36.9%) lived/worked in a COVID-19 high incidence area (Northern Italy), whilst 91 lived in COVID-19 low incidence areas (Central and Southern Italy). A psychiatric family history was present in 25 (18.1%) subjects. The mean years of service as GPs were 22.9 ± 13.9.

Concerning the impact of the COVID-19 pandemic on the GPs' work, 123 (88.5%) subjects reported a lack of PPE, 111 (80.4%) individuals took care of COVID-19 patients, and 49 (35.3%) assisted patients deceased due to the COVID-19. Moreover, twenty-five subjects (18.0%) reported to be at risk of medical complications in the case of COVID-19

infection, 28 (20.1%) had to be isolated from family, 16 (11.5%) were quarantined, and four (2.9%) were positive to COVID-19. Furthermore, 29 individuals (20.9%) had a close one at risk of medical complications in the case of COVID-19 infection, 54 (38.8%) a relative infected by COVID-19, and 16 (11.5%) a loss of a relative or a close one by the COVID-19.

3.2. Cluster Analysis

As initial clusters, we utilized the final centers estimated by a preliminary application of a k-means cluster analysis on a random sample of 50 subjects, in order to reduce the distance calculations and to select a good set of initial clusters. The second K-means cluster analysis applied to the entire data file met criterion 0 of convergence at the ninth iteration. We defined the three groups of subjects determined by the second K-means cluster analysis: the mild psychopathological burden group (n = 62, 44.6%), the moderate psychopathological burden group (n = 49, 35.3%), and the severe psychopathological burden group (n = 28, 20.1%), respectively. Table 1 shows the initial cluster centers, the iteration history, and the final cluster centers in the three groups.

Table 1. K-Means Cluster Analysis features. Initial cluster centers, Iteration History and Final Cluster Centers in the Mild psychopathological burden (n = 62), Moderate psychopathological burden (n = 49) and Severe psychopathological burden (n = 28) groups.

	**Mild Psychopathological Burden**	**Moderate Psychopathological Burden**	**Severe Psychopathological Burden**
	Initial Cluster centers		
IES-R Z score	1.19567	−0.03190	1.86689
GAD-7 Z score	−1.59421	1.70268	1.04330
	Iteration History		
1	1.325	1.688	1.627
2	0.037	0.238	0.313
3	0.041	0.112	0.046
4	0.091	0.121	0.000
5	0.013	0.018	0.000
6	0.017	0.022	0.000
7	0.014	0.019	0.000
8	0.015	0.019	0.000
9	0.000	0.000	0.000
	Final Cluster centers		
IES-R Z score	−0.79658	0.22244	1.49859
GAD-7 Z score	−0.74518	0.04935	1.54750
PHQ-9 Z score	−0.77139	0.10245	1.47696

The distances between the final cluster centers were: 1.560 between the mild psychopathological burden and the moderate psychopathological burden groups; 3.947 between the mild psychopathological burden and the severe psychopathological burden groups; 2.400 between the moderate psychopathological burden and the severe psychopathological burden groups. The average distance of cases from their classification cluster center was 0.83 ± 0.39. Finally, in the dispersion analysis, the IES-R scores presented a slightly higher influence in forming the clusters than the PHQ-9 and GAD-7 scores (see Table 2).

Table 2. K-Means Cluster Analysis features. Dispersion analysis.

	Cluster Mean Square (SE)	F	*p*
GAD-7 Z score	52.280 (0.300)	173.997	<0.001
IES-R Z score	50.800 (0.280)	181.185	<0.001
PHQ-9 Z score	49.236 (0.284)	173.342	<0.001

3.3. IES-R, GAD-7 and PHQ-9 Comparisons between the Three Groups

The IES-R total mean score was 21.5 ± 16.4, and 23.0% subjects (n = 32) reported a probable PTSD diagnosis. The GAD-7 total mean score was 7.4 ± 4.7, and 31.7% (n = 44) subjects rated moderate–severe anxiety symptoms. Moreover, the PHQ-9 total mean score was 7.0 ± 4.8, and 28.1% (n = 39) subjects presented moderate–severe depressive symptoms. The mild psychopathological burden group showed no subjects with PTSD, anxiety or moderate–severe depressive symptoms. The moderate psychopathological burden group reported 14.3%, 32.7% and 24.5% GPs with PTSD, anxiety, or depressive moderate–severe symptoms, respectively. Finally, the severe psychopathological burden group presented 89.3%, 100.0%, and 96.4% subjects with PTSD, anxiety, or depressive moderate-severe symptoms, respectively. Details on the psychopathological characteristics of the three cluster groups are shown in Figure 1. Comparisons of the mean scores of the IES-R, GAD-7, and PHQ-9 in the three groups are reported in Table 3.

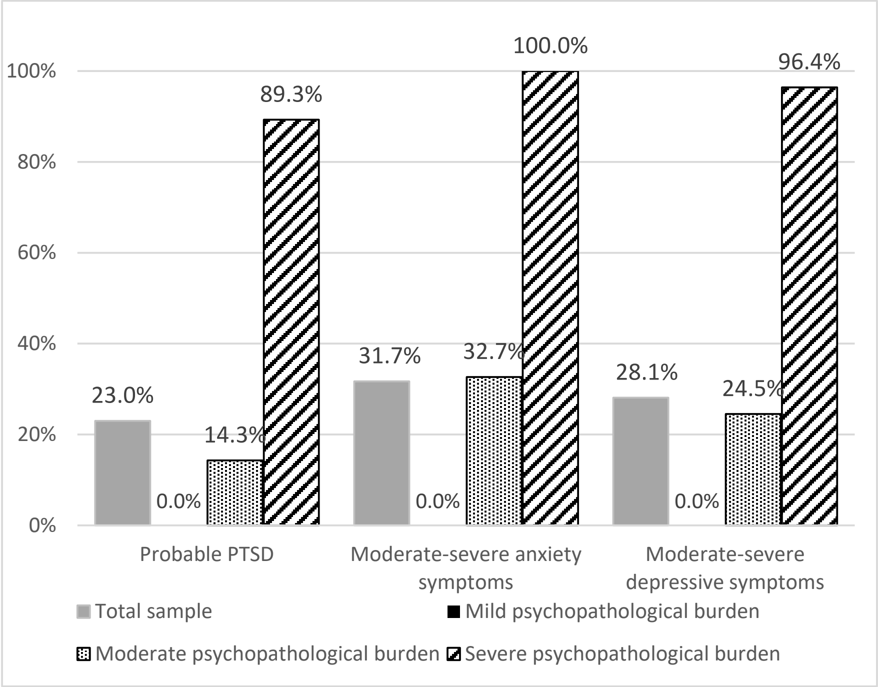

Figure 1. Prevalence of PTSD, Anxiety and Depressive symptoms by means of the IES-R, GAD-7 and PHQ-9 scores in the Mild psychopathological burden (n = 62), Moderate psychopathological burden (n = 49) and Severe psychopathological burden (n = 28) groups.

Table 3. Comparison of IES-R, GAD-7 and PHQ-9 scores in the total sample (n = 139) and in the Mild psychopathological burden (n = 62), Moderate psychopathological burden (n = 49) and Severe psychopathological burden (n = 28) groups.

	Total Sample (Mean ± SD)	Mild Psychopathological Burden (Mean ± SD)	Moderate Psychopathological Burden (Mean ± SD)	Severe Psychopathological Burden (Mean ± SD)	*p*	Post-Hoc *
IES-R total score	21.5 ± 16.4	9.4 ± 5.8	22.3 ± 9.3	46.8 ± 12.2	<0.001	c > a,b<br>b > a
Intrusion domain score	1.0 ± 0.9	0.4 ± 0.3	1.0 ± 0.6	2.3 ± 0.7	<0.001	c > a,b<br>b > a
Avoidance domain score	0.8 ± 0.7	0.4 ± 0.4	0.9 ± 0.5	1.7 ± 0.6	<0.001	c > a,b<br>b > a
Arousal domain score	1.2 ± 0.9	0.5 ± 0.3	1.2 ± 0.5	2.6 ± 0.6	<0.001	c > a,b<br>b > a
GAD-7 total score	7.4 ± 4.7	3.6 ± 1.9	8.3 ± 2.8	14.1 ± 3.0	<0.001	c > a,b<br>b > a
PHQ-9 total score	7.0 ± 4.8	3.4 ± 2.0	7.6 ± 2.8	14.2 ± 3.2	<0.001	c > a,b<br>b > a

* (p < 0.05). a: Mild Psycho-pathological Burden; b: Moderate Psy-chopathological Burden; c: Severe Psychopathological Burden.

3.4. Socio-Demographic and Work-Related Characteristics Comparison between the Three Groups

Table 4 shows the comparison of the socio-demographic and clinical characteristics between the three groups. The mild psychopathological burden group presented significantly more subjects aged over 55 years than the severe psychopathological burden one. Consistently, the severe psychopathological burden group included subjects with significantly fewer years in service as GPs, besides a higher number of subjects from a COVID-19 high incidence area than the other two groups. Finally, the moderate psychopathological burden group presented more subjects with a relative at risk for medical complications related to COVID-19 with respect to the mild psychopathological burden one.

Table 4. Sociodemographic and COVID-19 characteristics in the total sample (n = 139) and in the Mild psychopathological burden (n = 62), Moderate psychopathological burden (n = 49) and Severe psychopathological burden (n = 28) groups.

	Total Sample *n* (%)	Mild Psychopathological Burden *n* (%)	Moderate Psychopathological Burden *n* (%)	Severe Psychopathological Burden *n* (%)	*p*	Post-Hoc *
Females	79 (56.8)	30 (48.4)	31 (63.3)	18 (64.3)	-	-
Age > 55 years	71 (51.1)	39 (62.9)	26 (53.1)	6 (21.4)	0.024	a > c
Married	110 (79.1)	51 (82.3)	40 (81.6)	19 (67.9)	-	-
Having a son	86 (61.9)	38 (61.3)	32 (65.3)	16 (57.1)	-	-
Postgraduate degree	79 (56.8)	34 (54.8)	27 (55.1)	18 (64.3)	-	-
Living/working in a COVID-19 High Incidence Area	48 (36.9)	18 (31.0)	15 (32.6)	15 (57.7)	0.049	c > a,b
Years in service as GPs (mean ± SD)	22.9 ± 13.9	26.1 ± 13.5	23.2 ± 13.7	14.7 ± 12.0	0.001	a,b > c
Psychiatric Family History	25 (18.1)	11 (18.0)	9 (18.4)	5 (17.9)	-	-

Table 4. *Cont.*

	Total Sample *n* (%)	Mild Psy-chopathological Burden *n* (%)	Moderate Psychopathological Burden *n* (%)	Severe Psychopatho-logical Burden *n* (%)	*p*	Post-Hoc *
Professional variables related to COVID-19						
Lack of personal protective equipment	123 (88.5)	57 (91.9)	44 (89.8)	22 (78.6)	-	-
Taking care of COVID-19 patients	111 (80.4)	48 (78.7)	39 (79.6)	24 (85.7)	-	-
Taking care of patients deceased due to COVID-19	49 (35.3)	19 (30.6)	19 (38.8)	11 (39.3)	-	-
Personal variables related to COVID-19						
Being at risk for medical complications related to COVID-19 infection	25 (18.0)	10 (16.1)	10 (20.4)	5 (17.9)	-	-
Being quarantined	16 (11.5)	6 (9.7)	6 (12.2)	4 (14.3)	-	-
Isolation from family	28 (20.1)	11 (17.7)	8 (16.3)	9 (32.1)	-	-
Positive to COVID-19	4 (2.9)	3 (4.8)	0 (0.0)	1 (3.6)	-	-
A relative at risk for medical complications related toCOVID-19	29 (20.9)	8 (12.9)	16 (32.7)	5 (17.9)	0.036	b > a
A relative positive for COVID-19	54 (38.8)	23 (37.1)	19 (38.8)	12 (42.9)	-	-
Loss of a relative for the COVID-19	16 (11.5)	11 (17.7)	3 (6.1)	2 (7.1)	-	-

* (p < 0.05). a: Mild Psycho-pathological Burden; b: Moderate Psy-chopathological Burden; c: Severe Psy-chopatholog-ical Burden.

3.5. WSAS and ProQOL-5 Comparison between the Three Groups

The WSAS and ProQOL-5 total and domain scores across the three groups are reported in Table 5. The severe psychopathological burden group presented significantly higher total WSAS and individual item scores, besides lower ProQOL-5 Compassion Satisfaction and higher ProQOL-5 Burnout and Secondary Traumatic Stress scores than the other two groups. Further, the moderate psychopathological burden group presented significantly higher scores in the WSAS total and individual item scores, as well as ProQOL-5 Burnout and Secondary Traumatic Stress scores than the mild psychopathological burden one.

Table 5. Comparison of WSAS and ProQOL-5 scores in the total sample (n = 139) and in the Mild psychopathological burden (n = 62), Moderate psychopathological burden (n = 49) and Severe psychopathological burden (n = 28) groups.

	Total Sample (Mean ± SD)	Mild Psychopatho-logical Burden (Mean ± SD)	Moderate Psychopatho-logical Burden (Mean ± SD)	Severe Psychopatho-logical Burden (Mean ± SD)	*p*	Post-Hoc *
WSAS total score	13.2 ± 10.7	5.8 ± 6.0	15.2 ± 8.7	25.7 ± 8.4	<0.001	c > a,b<br>b > a
Ability to work impairment	2.3 ± 2.2	1.1 ± 1.4	2.5 ± 1.7	4.9 ± 2.4	<0.001	c > a,b<br>b > a
Home management impairment	1.9 ± 2.3	0.6 ± 0.8	2.2 ± 2.2	4.1 ± 2.6	<0.001	c > a,b<br>b > a

Table 5. *Cont.*

	Total Sample (Mean ± SD)	Mild Psychopatho-logical Burden (Mean ± SD)	Moderate Psychopatho-logical Burden (Mean ± SD)	Severe Psychopatho-logical Burden (Mean ± SD)	*p*	Post-Hoc *
Social leisure activities impairment	2.8 ± 2.7	1.3 ± 2.0	3.2 ± 2.6	5.1 ± 2.3	<0.001	c > a,b<br>b > a
Private leisure activities impairment	3.9 ± 3.2	2.2 ± 2.9	4.5 ± 2.9	6.4 ± 2.0	<0.001	c > a,b<br>b > a
Close relationships impairment	2.4 ± 2.5	0.8 ± 1.2	2.8 ± 2.4	5.2 ± 2.3	<0.001	c > a,b<br>b > a
ProQOL-5						
Compassion Satisfaction	35.4 ± 6.9	37.4 ± 5.9	34.5 ± 6.8	32.6 ± 8.2	0.005	a > c
Burnout	27.0 ± 6.2	23.1 ± 4.2	28.7 ± 5.5	32.9 ± 5.4	<0.001	c > a,b<br>b > a
Secondary Traumatic Stress	21.0 ± 7.0	17.2 ± 5.5	21.2 ± 5.2	28.7 ± 6.2	<0.001	c > a,b<br>b > a

* ($p < 0.05$). a: Mild Psycho-pathological Burden; b: Moderate Psy-chopathological Burden; c: Severe Psy-chopathological Burden.

4. Discussion

The present study first explored the variables related to a different psychopathological burden of the COVID-19 pandemic on GPs operating in the management of patients during the first acute phase of the emergency in Italy. Particularly, our results showed a prevalence of PTSD, anxiety, and depression in 23.0%, 31.7%, and 28.1% subjects, respectively. Further, subjects with severe psychopathological burden also showed higher impairment in work and social functioning, higher levels of burnout and secondary traumatic stress, and higher levels of peritraumatic distress with respect to the other two groups.

PTSD rates found by this study were in line with the incidence rates of PTSD observed in GPs [17,19,21,27] and emergency workers before the COVID-19 pandemic [46,47], ranging from 10% to 21% [48]. GPs adapted their practice methods to COVID-19 pandemic setting with new responsibilities, facing increased burdens of work and unedited stressful clinical and organizational conditions, such as additional safety protocols related to the limitation of contagion [49]. GPs represented the first line to a huge number of requests without clear prevention or screening instruments, lack of PPE, and uncertainty regarding the procedures and treatments required [50]. Particularly, Castelli et al. [19] evaluated the symptoms of anxiety, depression, and PTSD in a sample of 246 GPs in Italy. The survey found that 75% of subjects had clinically relevant anxiety symptoms, 32% post-traumatic stress symptoms, and 37% depressive symptoms. Interestingly, we reported rates for moderate to severe anxiety symptoms lower than those by Castelli et al. [19], however, methodological differences should be taken into account: these latter, in fact, adopted a different psychopathological assessment instrument, besides including mild symptoms in the threshold adopted, while we focused on moderate to severe symptoms. Furthermore, 41% of GPs interviewed reported that adequate PPE had not been provided, 48% that they had not received adequate information to protect their family, and 61% that they did not have clear diagnostic guidelines on COVID-19. Younger age, female gender, and fewer years of practice were significantly associated with higher levels of anxiety and depressive symptoms.

A cross-sectional study on a total of 531 GPs in Colombia [51] showed moderate to severe anxiety symptoms in 39.3% of subjects, with the highest prevalence among women. Social discrimination, distress, job disappointment, nightmares, stress, and other symptoms of fear about the pandemic correlated significantly with higher anxiety symptoms. Conversely, a greater sense of state or employer protection, higher job satisfaction, and confidence in government information were associated with lower anxiety symptoms. An increase in time spent looking for information on COVID-19, the perception that PPE was not adequate, and the greater number of infected in care patients correlated significantly

with the presence of moderate to severe depressive symptoms, leading to worse quality of life levels in a sample of Italian GPs [50].

Dividing the sample into three clusters (mild, moderate, and severe psychopathological burden) based on the level of psychopathological symptoms, the severe psychopathological burden group had a significantly higher number of younger subjects and with significantly fewer years of service as GPs than that with mild psychopathological symptom. This result is in line with the literature recognizing the younger age [52–54] and lesser work experience [14,55,56] as risk factors for psychiatric morbidity in HCWs during infectious outbreaks. The cluster group with severe psychopathological burden also had a significantly higher number of subjects from an area with a high COVID-19 incidence than the other two groups. As widely demonstrated in the literature, a greater traumatic exposure level and, consequently, greater psychopathological reactions occur more frequently in subjects employed in proximity to the most affected regions, suggesting an "epicenter effect" of pandemic [3].

Further, the moderate psychopathological burden group had more subjects with a relative at risk of medical complications related to COVID-19 than the mild psychopathological burden one. In fact, as evidenced by previous studies [57–59], not only the direct exposure to the consequences of the pandemic in the workplace, but also the health conditions of relatives or friends, represent a risk factor for the development of psychiatric disorders, including post-traumatic stress symptomatology. Interestingly, although numbers are small in order to lead to definitive conclusions, the loss of a relative showed a lower prevalence in the moderate and severe psychopathological burden with respect to the mild psychopathological one. A possible interpretation of this result would corroborate the loss of a loved one as a potential trigger for different psychopathological reactions than PTSD, such as prolonged grief disorder, when the loss event does not fulfil DSM-5 criteria for being considered traumatic [60,61]. Similarly, it is important to note the fact that living with a relative at risk for medical complications related to COVID-19 was mostly reported by GPs in the moderate psychopathological burden group. Thus, despite the highest prevalence emerges when merging the moderate with the severe psychopathological burden groups, with respect to the mild one, an increasing progression is not detected, suggesting a more complex interaction between potentially traumatic COVID-19 characteristics and the difference in their weight in contributing to psychopathology.

Regarding the impact on socio-occupational functioning, the group with severe psychopathological burden showed a significantly higher impairment than the other two groups, while the group with moderate psychopathological burden reported significantly higher work and social functional impairment than the group with milder symptoms. The present finding is in line with literature showing that psychopathological symptoms among HCWs, including post-traumatic stress, anxiety, and depressive symptoms, were associated with higher functional impairment. Particularly, some of us recently reported that higher levels of functioning impairment were found among HCWs facing COVID-19 emergency, with post-traumatic stress, depressive, and anxiety symptoms, with respect to those without [4]. In a sample of HCWs working in a major university hospital in Italy [31], significant correlations between depressive and anxiety symptoms and work and social functioning impairment were shown, with HCWs being at risk for both depressive and anxiety symptoms and related work and social impairment, while a study on 137 emergency department HCWs revealed that subjects with PTSD reported significantly higher work and social functioning impairment compared to those without [46].

Professionals with high rates of psychopathological symptoms reported higher burnout and secondary stress with respect to the other two groups. In a study on 332 French GPs, Lange et al. [17] found burnout symptoms in almost a quarter of participants, and almost 11% reported significant post-traumatic stress symptoms. Lum et al. [21] in a sample of 257 Singapore GPs showed a prevalence of burnout more than 80%, with a considerable comorbidity with moderate to severe anxiety, depressive, and posttraumatic symptoms (21.4%, 26.6% and 8.9%, respectively). Moreover, a study on 265 HCWs working in a major

university hospital and facing the acute phase of COVID-19 outbreak in Italy showed that burnout and secondary traumatization were positively associated with depressive and anxiety symptoms [31]. Another study, investigating the relationships between dimensions of burnout and various psychological features amongst Italian GPs during the COVID-19 emergency, revealed that 40% of participants had a low burnout profile and about 5% showed a high burnout profile [18]. The authors also highlighted how improving task-oriented problem management appeared to protect against burnout, also organizing both training and psychological interventions for GPs, with the aim of providing them greater skills in emotional regulation, with a positive impact on their quality of life [18,62,63].

When discussing the results of the present study, some limitations should be considered, including the cross-sectional design of the study, the relatively small sample size, as well as the use of self-administered assessment tools (that may not always be aligned with assessment by mental health professionals) and the methodological limitation related to the online data collection. Finally, the use of convenience sampling may limit the generalizability of results.

5. Conclusions

Adverse mental health outcomes emerged among Italian GPs on duty during the first wave of COVID-19 pandemic. The present findings also showed that GPs were forced to perform their professional activity in extremely stressful conditions and that it was associated with the development of psychopathological symptoms and higher functional impairment [64,65]. Thus, further longitudinal studies are needed to better explore the psychopathological burden of the COVID-19 pandemic on GPs, in order to assess and promote tailored prevention and intervention strategies for the current and future public health emergencies. Moreover, the association found in the literature between higher employer protection, job satisfaction, and confidence in government information with lower levels of psychopathological symptoms highlights the need for more concrete support to GPs from health authorities, both for GPs' mental wellness and for citizens assisted by them.

Author Contributions: Conceptualization, C.C., V.D., F.M.B. and L.D.; methodology, C.C., V.D. and F.M.B.; formal analysis, V.D. and C.A.B.; investigation, V.D., F.M.B. and V.P.; data curation, V.D., F.M.B. and V.P.; writing—original draft preparation, C.C., V.D., F.M.B. and V.P.; writing—review and editing, C.C., V.D., C.A.B. and V.P.; supervision, C.C. and L.D. All authors have read and agreed to the published version of the manuscript.

Funding: This research received no external funding.

Institutional Review Board Statement: The study was conducted in accordance with the Declaration of Helsinki and approved by the Ethics Committee of the Azienda Ospedaliero-Universitaria Pisana (CEAVNO) (ID: 17151/2020).

Informed Consent Statement: Informed consent was obtained from all subjects involved in the study.

Data Availability Statement: The data presented in this study are available on request from the corresponding author under the condition of the approval of the Ethics Committee of the Azienda Ospedaliero-Universitaria Pisana (CEAVNO). The data are not publicly available due to privacy/ethical restrictions.

Acknowledgments: We acknowledge the Italian College of General Practitioners, particularly the sections of the provinces of Lucca (Umberto Quiriconi) and Pisa (Giuseppe Figlini), for promoting the present study to their members.

Conflicts of Interest: The authors declare no conflict of interest.

References

1. World Health Organization (WHO). Mental Health and COVID-19: Early Evidence of the Pandemic's Impact: Scientific Brief. 2. 2022. Available online: https://www.who.int/publications/i/item/WHO-2019-nCoV-Sci_Brief-Mental_health-2022.1 (accessed on 11 March 2022).
2. Vindegaard, N.; Benros, M.E. COVID-19 pandemic and mental health consequences: Systematic review of the current evidence. *Brain Behav. Immun.* **2020**, *89*, 531–542. [CrossRef] [PubMed]
3. Carmassi, C.; Dell'Oste, V.; Bui, E.; Foghi, C.; Bertelloni, C.A.; Atti, A.R.; Buselli, R.; Di Paolo, M.; Goracci, A.; Malacarne, P.; et al. The interplay between acute post-traumatic stress, depressive and anxiety symptoms on healthcare workers functioning during the COVID-19 emergency: A multicenter study comparing regions with increasing pandemic incidence. *J. Affect. Disord.* **2022**, *298 Pt A*, 209–216. [CrossRef]
4. Carmassi, C.; Pedrinelli, V.; Dell'Oste, V.; Bertelloni, C.A.; Cordone, A.; Bouanani, S.; Corsi, M.; Baldanzi, S.; Malacarne, P.; Dell'Osso, L.; et al. Work and social functioning in frontline healthcare workers during the covid-19 pandemic in Italy: Role of acute post-traumatic stress, depressive and anxiety symptoms. *Riv. Psichiatr.* **2021**, *56*, 189–197. [PubMed]
5. Shanafelt, T.V.; Ripp, J.; Trockel, M. Understanding and Addressing Sources of Anxiety Among Health Care Professionals During the COVID-19 Pandemic. *JAMA* **2020**, *323*, 2133–2134. [CrossRef] [PubMed]
6. Cabarkapa, S.; King, J.A.; Ng, C.H. The psychiatric impact of COVID-19 on healthcare workers. *Aust. J. Gen. Pract.* **2020**, *49*, 791–795. [CrossRef] [PubMed]
7. Opstelten, W.; Cals, J.; van der Horst, H.E. Huisartsgeneeskunde: Continu in ontwikkeling [General Practice: Continuously developing]. *Ned. Tijdschr. Voor Geneeskd.* **2020**, *163*, D4459.
8. Chen, K.; Liang, C.; Ruihong, L. Contributions of general practitioners in China facing the COVID-19 outbreak. *Aust. J. Gen. Pract.* **2021**, *50* (Suppl. 51), 1–2. [CrossRef]
9. Davies, M.; Carr, D.; Dugan, J.; Hart, N.; Kirkpatrick, R.; Loughrey, C.; Loughrey, P.; O'Neill, G. Support for general practitoners during covid-19. *Ulst. Med. J.* **2021**, *90*, 151–156.
10. Rossi, R.; Socci, V.; Pacitti, F.; Di Lorenzo, G.; Di Marco, A.; Siracusano, A.; Rossi, A. Mental Health Outcomes Among Frontline and Second-Line Health Care Workers During the Coronavirus Disease 2019 (COVID-19) Pandemic in Italy. *JAMA Netw. Open* **2020**, *3*, e2010185. [CrossRef]
11. Vagni, M.; Maiorano, T.; Giostra, V.; Pajardi, D. Hardiness, stress and secondary trauma in Italian healthcare and emergency workers during the COVID-19 pandemic. *Sustainability* **2020**, *12*, 5592. [CrossRef]
12. de Sutter, A.; Llor, C.; Maier, M.; Mallen, C.; Tatsioni, A.; van Weert, H.; Windak, A.; Stoffers, J. Family medicine in times of 'COVID-19': A generalists' voice. *Eur. J. Gen. Pract.* **2020**, *26*, 58–60. [CrossRef] [PubMed]
13. Sarti, T.D.; Lazarini, W.S.; Fontenelle, L.F.; Almeida, A. [What is the role of Primary Health Care in the COVID-19 pandemic?]. Qual o papel da Atenção Primária à Saúde diante da pandemia provocada pela COVID-19? *Epidemiol. Serv. Saude Rev. Sist. Unico Saude Bras.* **2020**, *29*, e2020166.
14. Lai, J.; Ma, S.; Wang, Y.; Cai, Z.; Hu, J.; Wei, N.; Wu, J.; Du, H.; Chen, T.; Li, R.; et al. Factors Associated with Mental Health Outcomes Among Health Care Workers Exposed to Coronavirus Disease 2019. *JAMA Netw. Open* **2020**, *3*, e203976. [CrossRef] [PubMed]
15. Chew, N.; Lee, G.; Tan, B.; Jing, M.; Goh, Y.; Ngiam, N.; Yeo, L.; Ahmad, A.; Ahmed Khan, F.; Napolean Shanmugam, G.; et al. A multinational, multicentre study on the psychological outcomes and associated physical symptoms amongst healthcare workers during COVID-19 outbreak. *Brain Behav. Immun.* **2020**, *88*, 559–565. [CrossRef]
16. Wang, Y.; Ma, S.; Yang, C.; Cai, Z.; Hu, S.; Zhang, B.; Tang, S.; Bai, H.; Guo, X.; Wu, J.; et al. Acute psychological effects of Coronavirus Disease 2019 outbreak among healthcare workers in China: A cross-sectional study. *Transl. Psychiatry* **2020**, *10*, 348. [CrossRef]
17. Lange, M.; Joo, S.; Couette, P.A.; Le Bas, F.; Humbert, X. Impact on mental health of the COVID-19 outbreak among general practitioners during the sanitary lockdown period. *Ir. J. Med. Sci.* **2022**, *191*, 93–96. [CrossRef]
18. Di Monte, C.; Monaco, S.; Mariani, R.; Di Trani, M. From Resilience to Burnout: Psychological Features of Italian General Practitioners During COVID-19 Emergency. *Front. Psychol.* **2020**, *11*, 2476. [CrossRef]
19. Castelli, L.; Di Tella, M.; Benfante, A.; Taraschi, A.; Bonagura, G.; Pizzini, A.; Romeo, A. The psychological impact of COVID-19 on general practitioners in Piedmont, Italy. *J. Affect. Disord.* **2021**, *281*, 244–246. [CrossRef]
20. Ghaleb, Y.; Lami, F.; Al Nsour, M.; Rashak, H.A.; Samy, S.; Khader, Y.S.; Al Serouri, A.; Bahaa Eldin, H.; Afifi, S.; Elfadul, M.; et al. Mental health impacts of COVID-19 on healthcare workers in the Eastern Mediterranean Region: A multi-country study. *J. Public Health* **2021**, *43* (Suppl. 3), iii34–iii42. [CrossRef]
21. Lum, A.; Goh, Y.L.; Wong, K.S.; Seah, J.; Teo, G.; Ng, J.Q.; Abdin, E.; Hendricks, M.M.; Tham, J.; Nan, W.; et al. Impact of COVID-19 on the mental health of Singaporean GPs: A cross-sectional study. *BJGP Open* **2021**, *5*, BJGPO.2021.0072. [CrossRef]
22. Kessler, R.C.; Chiu, W.T.; Demler, O.; Merikangas, K.R.; Walters, E.E. Prevalence, severity, and comorbidity of 12-month DSM-IV disorders in the National Comorbidity Survey Replication. *Arch. Gen. Psychiatry* **2005**, *62*, 617–627. [CrossRef] [PubMed]
23. Hashemian, F.; Khoshnood, K.; Desai, M.M.; Falahati, F.; Kasl, S.; Southwick, S. Anxiety, depression, and posttraumatic stress in Iranian survivors of chemical warfare. *JAMA* **2006**, *296*, 560–566. [CrossRef] [PubMed]
24. Ginzburg, K.; Ein-Dor, T.; Solomon, Z. Comorbidity of posttraumatic stress disorder, anxiety and depression: A 20-year longitudinal study of war veterans. *J. Affect. Disord.* **2010**, *123*, 249–257. [CrossRef] [PubMed]

25. Kar, N.; Bastia, B.K. Post-traumatic stress disorder, depression and generalised anxiety disorder in adolescents after a natural disaster: A study of comorbidity. *Clin. Pract. Epidemiol. Ment. Health CP EMH* **2006**, *2*, 1–7. [CrossRef]
26. Carmassi, C.A.; Bertelloni, C.A.; Massimetti, G.; Miniati, M.; Stratta, P.; Rossi, A.; Dell'Osso, L. Impact of DSM-5 PTSD and gender on impaired eating behaviors in 512 Italian earthquake survivors. *Psychiatry Res.* **2015**, *225*, 64–69. [CrossRef] [PubMed]
27. Dell'Osso, L.; Stratta, P.; Conversano, C.; Massimetti, E.; Akiskal, K.K.; Akiskal, H.S.; Rossi, A.; Carmassi, C. Lifetime mania is related to post-traumatic stress symptoms in high school students exposed to the 2009 L'Aquila earthquake. *Compr. Psychiatry* **2014**, *55*, 357–362. [CrossRef]
28. Price, M.; Legrand, A.C.; Brier, Z.; Hébert-Dufresne, L. The symptoms at the center: Examining the comorbidity of posttraumatic stress disorder, generalized anxiety disorder, and depression with network analysis. *J. Psychiatr. Res.* **2019**, *109*, 52–58. [CrossRef]
29. Axelson, D.A.; Birmaher, B. Relation between anxiety and depressive disorders in childhood and adolescence. *Depress. Anxiety* **2001**, *14*, 67–78. [CrossRef]
30. Salazar de Pablo, G.; Vaquerizo-Serrano, J.; Catalan, A.; Arango, C.; Moreno, C.; Ferre, F.; Shin, J.I.; Sullivan, S.; Brondino, N.; Solmi, M.; et al. Impact of coronavirus syndromes on physical and mental health of health care workers: Systematic review and meta-analysis. *J. Affect. Disord.* **2020**, *275*, 48–57. [CrossRef]
31. Buselli, R.; Corsi, M.; Baldanzi, S.; Chiumiento, M.; Del Lupo, E.; Dell'Oste, V.; Bertelloni, C.A.; Massimetti, G.; Dell'Osso, L.; Cristaudo, A.; et al. Professional Quality of Life and Mental Health Outcomes among Health Care Workers Exposed to Sars-Cov-2 (Covid-19). *Int. J. Environ. Res. Public Health* **2020**, *17*, 6180. [CrossRef]
32. Carmassi, C.; Cerveri, G.; Bertelloni, C.A.; Marasco, M.; Dell'Oste, V.; Massimetti, E.; Gesi, C.; Dell'Osso, L. Mental health of frontline help-seeking healthcare workers during the COVID-19 outbreak in the first affected hospital in Lombardy, Italy. *Psychiatry Res.* **2021**, *298*, 113763. [CrossRef] [PubMed]
33. Barbano, A.C.; van der Mei, W.F.; deRoon-Cassini, T.A.; Grauer, E.; Lowe, S.R.; Matsuoka, Y.J.; O'Donnell, M.; Olff, M.; Qi, W.; Ratanatharathorn, A.; et al. International Consortium to Prevent PTSD. Differentiating PTSD from anxiety and depression: Lessons from the ICD-11 PTSD diagnostic criteria. *Depress. Anxiety* **2019**, *36*, 490–498. [CrossRef] [PubMed]
34. Weiss, D.S.; Marmar, C.R. The impact of event scale–revised. In *Assessing Psychological Trauma and PTSD*; Wilson, J.P., Keane, T.M., Eds.; Guilford Press: New York, NY, USA, 1997; pp. 399–411.
35. Spitzer, R.L.; Kroenke, K.; Williams, J.B.; Löwe, B. A brief measure for assessing generalized anxiety disorder: The GAD-7. *Arch. Intern. Med.* **2006**, *166*, 1092–1097. [CrossRef] [PubMed]
36. Spitzer, R.L.; Kroenke, K.; Williams, J.B. Validation and utility of a self-report version of PRIME-MD: The PHQ primary care study. Primary Care Evaluation of Mental Disorders. Patient Health Questionnaire. *JAMA* **1999**, *282*, 1737–1744. [CrossRef]
37. Kroenke, K.; Spitzer, R.L.; Williams, J.B. The PHQ-9: Validity of a brief depression severity measure. *J. Gen. Intern. Med.* **2001**, *16*, 606–613. [CrossRef]
38. Mundt, J.C.; Marks, I.M.; Shear, M.K.; Greist, J.H. The Work and Social Adjustment Scale: A simple measure of impairment in functioning. *Br. J. Psychiatry J. Ment. Sci.* **2002**, *180*, 461–464. [CrossRef]
39. Stamm, B.H. *The Concise ProQOL Manual*, 2nd ed.; The ProQOL.org: Pocatello, ID, USA, 2010.
40. Creamer, M.; Bell, R.; Failla, S. Psychometric properties of the Impact of Event Scale-Revised. *Behav. Res. Ther.* **2003**, *41*, 1489–1496. [CrossRef]
41. Cetin, M.; Kose, S.; Ebrinc, S.; Yigit, S.; Elhai, J.D.; Basoglu, C. Identification and posttraumatic stress disorder symptoms in rescue workers in the Marmara, Turkey, earthquake. *J. Trauma. Stress* **2005**, *18*, 485–489. [CrossRef]
42. Matsuoka, Y.; Nishi, D.; Nakaya, N.; Sone, T.; Noguchi, H.; Hamazaki, K.; Hamazaki, T.; Koido, Y. Concern over radiation exposure and psychological distress among rescue workers following the Great East Japan Earthquake. *BMC Public Health* **2012**, *12*, 249. [CrossRef]
43. Beard, C.; Björgvinsson, T. Beyond generalized anxiety disorder: Psychometric properties of the GAD-7 in a heterogeneous psychiatric sample. *J. Anxiety Disord.* **2014**, *28*, 547–552. [CrossRef]
44. Kroenke, K.; Spitzer, R.L.; Williams, J.B.; Löwe, B. The Patient Health Questionnaire Somatic, Anxiety, and Depressive Symptom Scales: A systematic review. *Gen. Hosp. Psychiatry* **2010**, *32*, 345–359. [CrossRef] [PubMed]
45. Beard, C.; Hsu, K.J.; Rifkin, L.S.; Busch, A.B.; Björgvinsson, T. Validation of the PHQ-9 in a psychiatric sample. *J. Affect. Disord.* **2016**, *193*, 267–273. [CrossRef] [PubMed]
46. Carmassi, C.; Malacarne, P.; Dell'Oste, V.; Bertelloni, C.A.; Cordone, A.; Foghi, C.; Dell'osso, L. Post-traumatic stress disorder, burnout and their impact on global functioning in Italian emergency healthcare workers. *Minerva Anestesiol.* **2021**, *87*, 556–566. [CrossRef] [PubMed]
47. Berger, W.; Coutinho, E.S.; Figueira, I.; Marques-Portella, C.; Luz, M.P.; Neylan, T.C.; Marmar, C.R.; Mendlowicz, M.V. Rescuers at risk: A systematic review and meta-regression analysis of the worldwide current prevalence and correlates of PTSD in rescue workers. *Soc. Psychiatry Psychiatr. Epidemiol.* **2012**, *47*, 1001–1011. [CrossRef]
48. Hegg-Deloye, S.; Brassard, P.; Jauvin, N.; Prairie, J.; Larouche, D.; Poirier, P.; Tremblay, A.; Corbeil, P. Current state of knowledge of post-traumatic stress, sleeping problems, obesity and cardiovascular disease in paramedics. *Emerg. Med. J.* **2014**, *31*, 242–247. [CrossRef]
49. Plagg, B.; Piccoliori, G.; Oschmann, J.; Engl, A.; Eisendle, K. Primary Health Care and Hospital Management During COVID-19: Lessons from Lombardy. *Risk Manag. Healthc. Policy* **2021**, *14*, 3987–3992. [CrossRef]

50. Amerio, A.; Bianchi, D.; Santi, F.; Costantini, L.; Odone, A.; Signorelli, C.; Costanza, A.; Serafini, G.; Amore, M.; Aguglia, A. Covid-19 pandemic impact on mental health: A web-based cross-sectional survey on a sample of Italian general practitioners. *Acta Bio-Med. Atenei Parm.* **2020**, *91*, 83–88.
51. Monterrosa-Castro, A.; Redondo-Mendoza, V.; Mercado-Lara, M. Psychosocial factors associated with symptoms of generalized anxiety disorder in general practitioners during the COVID-19 pandemic. *J. Investig. Med. Off. Publ. Am. Fed. Clin. Res.* **2020**, *68*, 1228–1234. [CrossRef]
52. Sim, K.; Chong, P.N.; Chan, Y.H.; Soon, W.S. Severe acute respiratory syndrome-related psychiatric and posttraumatic morbidities and coping responses in medical staff within a primary health care setting in Singapore. *J. Clin. Psychiatry* **2004**, *65*, 1120–1127. [CrossRef]
53. Su, T.P.; Lien, T.C.; Yang, C.Y.; Su, Y.L.; Wang, J.H.; Tsai, S.L.; Yin, J.C. Prevalence of psychiatric morbidity and psychological adaptation of the nurses in a structured SARS caring unit during outbreak: A prospective and periodic assessment study in Taiwan. *J. Psychiatr. Res.* **2007**, *41*, 119–130. [CrossRef]
54. Wu, P.; Fang, Y.; Guan, Z.; Fan, B.; Kong, J.; Yao, Z.; Liu, X.; Fuller, C.J.; Susser, E.; Lu, J.; et al. The psychological impact of the SARS epidemic on hospital employees in China: Exposure, risk perception, and altruistic acceptance of risk. *Can. J. Psychiatry Rev. Can. De Psychiatr.* **2009**, *54*, 302–311. [CrossRef] [PubMed]
55. Chong, M.Y.; Wang, W.C.; Hsieh, W.C.; Lee, C.Y.; Chiu, N.M.; Yeh, W.C.; Huang, O.L.; Wen, J.K.; Chen, C.L. Psychological impact of severe acute respiratory syndrome on health workers in a tertiary hospital. *Br. J. Psychiatry J. Ment. Sci.* **2004**, *185*, 127–133. [CrossRef] [PubMed]
56. Lancee, W.J.; Maunder, R.G.; Goldbloom, D.S. Prevalence of psychiatric disorders among Toronto hospital workers one to two years after the SARS outbreak. *Psychiatr. Serv.* **2008**, *59*, 91–95. [CrossRef] [PubMed]
57. Brewin, C.R.; Andrews, B.; Valentine, J.D. Meta-analysis of risk factors for posttraumatic stress disorder in trauma-exposed adults. *J. Consult. Clin. Psychol.* **2000**, *68*, 748–766. [CrossRef] [PubMed]
58. Kang, L.; Ma, S.; Chen, M.; Yang, J.; Wang, Y.; Li, R.; Yao, L.; Bai, H.; Cai, Z.; Xiang Yang, B.; et al. Impact on mental health and perceptions of psychological care among medical and nursing staff in Wuhan during the 2019 novel coronavirus disease outbreak: A cross-sectional study. *Brain Behav. Immun.* **2020**, *87*, 11–17. [CrossRef]
59. Modenese, A.; Gobba, F. Increased Risk of COVID-19-Related Deaths among General Practitioners in Italy. *Healthcare* **2020**, *8*, 155. [CrossRef]
60. Lenferink, L.; van den Munckhof, M.; de Keijser, J.; Boelen, P.A. DSM-5-TR prolonged grief disorder and DSM-5 posttraumatic stress disorder are related, yet distinct: Confirmatory factor analyses in traumatically bereaved people. *Eur. J. Psychotraumatol.* **2021**, *12*, 1–14. [CrossRef]
61. Carmassi, C.; Bertelloni, C.A.; Cordone, A.; Cappelli, A.; Massimetti, E.; Dell'Oste, V.; Dell'Osso, L. Exploring mood symptoms overlap in PTSD diagnosis: ICD-11 and DSM-5 criteria compared in a sample of subjects with Bipolar Disorder. *J. Affect. Disord.* **2020**, *276*, 205–211. [CrossRef]
62. Galleta-Williams, H.; Esmail, A.; Grigoroglou, C.; Zghebi, S.S.; Zhou, A.Y.; Hodkinson, A.; Panagioti, M. The importance of teamwork climate for preventing burnout in UK general practices. *Eur. J. Public Health* **2020**, *30* (Suppl. 4), iv36–iv38. [CrossRef]
63. Low, C.; Goh, T.J.; Chan, Y.H.; Fung, D.; Cheong, P.Y. Mental Resilience of Medical Practitioners in Singapore during COVID-19: Survey Results from a Webinar Course on Resilience. *Int. J. Environ. Res. Public Health* **2021**, *18*, 9801. [CrossRef]
64. Thornton, J. Covid-19: How coronavirus will change the face of general practice forever. *BMJ* **2020**, *368*, m1279. [CrossRef] [PubMed]
65. Kurotschka, P.K.; Serafini, A.; Demontis, M.; Serafini, A.; Mereu, A.; Moro, M.F.; Carta, M.G.; Ghirotto, L. General Practitioners' Experiences During the First Phase of the COVID-19 Pandemic in Italy: A Critical Incident Technique Study. *Front. Public Health* **2021**, *9*, 623904. [CrossRef] [PubMed]
