# Supplementary material for: Mental health status and related factors influencing healthcare workers during the COVID-19 pandemic: A systematic review and meta-analysis
Source: PLoS One. 2024 Jan 19;19(1):e0289454. doi: 10.1371/journal.pone.0289454 (PMC10798549; doi:10.1371/journal.pone.0289454)
Supplement: S1 Data — (ZIP) [file pone.0289454.s011.zip › literatures/210.pdf]

BJPsych open

BJPsych Open (2021)
7, e24, 1–7. doi: 10.1192/bjo.2020.150

Mental health symptoms in a cohort of hospital healthcare workers following the first peak of the COVID-19 pandemic in the UK

Kasun Wanigasooriya, Priyanka Palimar, David N. Naumann, Khalida Ismail, Jodie L. Fellows, Peter Logan, Christopher V. Thompson, Helen Bermingham, Andrew D. Beggs and Tariq Ismail

Background

The coronavirus disease 2019 (COVID-19) pandemic is likely to lead to a significant increase in mental health disorders among healthcare workers (HCW).

Aims

We evaluated the rates of anxiety, depressive and post-traumatic stress disorder (PTSD) symptoms in a population of HCW in the UK.

Method

An electronic survey was conducted between the 5 June 2020 and 31 July 2020 of all hospital HCW in the West Midlands, UK using clinically validated questionnaires: the 4-item Patient Health Questionnaire(PHQ-4) and the Impact of Event Scale-Revised (IES-R). Univariate analyses and adjusted logistic regression analyses were performed to estimate the strengths in associations between 24 independent variables and anxiety, depressive or PTSD symptoms.

Results

There were 2638 eligible participants who completed the survey (female: 79.5%, median age: 42 years, interquartile range: 32–51). The rates of clinically significant symptoms of anxiety, depression and PTSD were 34.3%, 31.2% and 24.5%, respectively. In adjusted analysis a history of mental health conditions was associated with clinically significant symptoms of anxiety (odds ratio (OR) = 2.3, 95% CI 1.9–2.7, $P < 0.001$), depression (OR = 2.5, 95% CI 2.1–3.0, $P < 0.001$) and PTSD (OR = 2.1, 95% CI 1.7–2.5, $P < 0.001$). The availability of adequate personal protective equipment (PPE), well-being support and lower exposure to moral dilemmas at work demonstrated significant negative associations with these symptoms ($P \leq 0.001$).

Conclusions

We report higher rates of clinically significant mental health symptoms among hospital HCW following the initial COVID-19 pandemic peak in the UK. Those with a history of mental health conditions were most at risk. Adequate PPE availability, access to well-being support and reduced exposure to moral dilemmas may protect hospital HCW from mental health symptoms.

Keywords

Anxiety; depression; post-traumatic stress disorder (PTSD); healthcare workers; COVID-19.

Copyright and usage

© The Author(s), 2020. Published by Cambridge University Press on behalf of the Royal College of Psychiatrists. This is an Open Access article, distributed under the terms of the Creative Commons Attribution licence (http://creativecommons.org/licenses/by/4.0/), which permits unrestricted re-use, distribution, and reproduction in any medium, provided the original work is properly cited.

Background

By September 2020, the coronavirus disease 2019 (COVID-19) pandemic caused by the novel SARS-CoV2 infection had claimed the lives of over 850 000 people worldwide.[1] The pandemic has stretched the limits of healthcare systems to beyond capacity.[2] Healthcare workers (HCW) have been exposed to numerous stressors and life events including: a rapid escalation in workload; sudden changes in roles and responsibilities including critical decision-making (also referred to as moral injury);[3] witnessing higher than the usual number of deaths and contracting the virus.[4,5] For many HCW, there has been a significant reduction in the usual sources of available social support because of changes in working schedules and social isolation measures.[3,5]

Psychological impact

Holmes et al recently highlighted the importance of addressing the psychological impact of the pandemic on hospital staff as a key multidisciplinary mental health research priority.[6] Several studies from Asia have evaluated the mental health impact of the COVID-19 pandemic on HCW.[7–11] The consensus from these studies was that symptoms of mental health conditions were frequent among HCW during and after the peak of the pandemic in each country.[12] This is similar to reports of increased anxiety, depression and post-traumatic stress disorder (PTSD) symptoms among HCW following the 2003 SARS outbreak in Asia.[13] There is also concern regarding the impact of PTSD symptoms on the National Health Service (NHS) workforce and the most effective interventions to support HCW.[6,14]

Aims

The mental health consequences of the COVID-19 pandemic on HCW in Western nations remains uncertain. As of August 2020, the UK had recorded the fifth highest number of deaths from COVID-19 worldwide.[1] The first peak of the pandemic in the UK occurred between March and May 2020.[15] More than 100 HCW in the UK lost their lives to COVID-19.[5,16] Several measures have been implemented to address the mental health sequelae of the pandemic, including the implementation of several staff well-being programmes and the allocation of over £5 million for mental health research.[17,18] This study aims to describe the rates of clinically significant symptoms of anxiety, depression and PTSD and associated occupational exposures, health and sociodemographic characteristics, in a cohort of hospital-based HCW from the UK, in the immediate aftermath of the COVID-19 pandemic peak.

Method

Study design and setting

A cross-sectional survey of HCW employed in ten NHS acute general and mental health hospitals set in the West Midlands, UK

was conducted between the 5 June 2020 and the 31 July 2020. The county has an ethnically and socioeconomically diverse population.[19] This region also observed a high incidence of COVID-19 cases and a high mortality rate.[20] The study was approved by the UK Health Research Authority (HRA, Reference: 20/HRA/2865). Research Ethics Committee approval was not required for this study and this was confirmed by the HRA. Site-specific approval was obtained from each of the research and development departments of all participating acute general ($n = 7$) and mental health ($n = 3$) NHS hospital Trusts. Informed consent was obtained from all participants and recorded electronically at the start of the study.

Study participation

Eligible participants included all staff who worked or volunteered on-site at one of the participating hospitals for over 50% of their working week during the initial peak of the COVID-19 pandemic in the UK. For this study, the latter was defined as the 23 March 2020 to the 23 May 2020. Staff who were on any type of leave for over 50% of the time during this period and those working from home or based in the community were not eligible to take part in this study. Eligible participants were invited to complete a confidential, voluntary electronic survey using the SurveyMonkey (San Mateo, California, USA) online survey administration and management platform. The survey was approximately 15 minutes long. It was distributed to participants via email, newsletters, posters, flyers and social media platforms to maximise reach and encourage participation.

Exposure variables

The survey collected self-reported data on 24 independent variables (Table 1). These included sociodemographic factors (age, gender, ethnicity, relationship status, number of dependents and immigration status); current health status (mental health conditions and physical illness); lifestyle factors (weekly smoking and alcohol consumption); employment factors (job title, total duration of employment in healthcare, the type of hospital, location of work within the hospital, whether infected patients were treated at the workplace, patient-facing duties, availability of adequate personal protective equipment (PPE), availability and use of well-being support). Data were also collected on the impact of COVID-19 on professional life (redeployment, increased working hours, morally uncomfortable changes in the way they worked as a subjective measure of moral dilemma or injury in their day-to-day practices in the workplace); impact on personal life (diagnosis of COVID-19 in either self or a cohabitant, admission to hospital with COVID-19 for either self, or close family or friend). The collected data were stored securely and processed confidentially in compliance with UK data protection law and the European Union General Data Protection Regulations.

Mental health symptoms

The 4-item Patient Health Questionnaire (PHQ-4) was used to assess symptoms of anxiety and depression.[21,22] The Impact of Event Scale-Revised (IES-R) score was used to assess symptoms of PTSD.[23]

The psychometric properties of the PHQ-4 were acceptable as a screening tool. Studies have reported a sensitivity of 86% and specificity of 83% for generalised anxiety disorder on the anxiety subscale for scores ≥ 3.[21] The corresponding figures for the depression subscale were 83% and 90%, respectively, for major depressive disorder.[22] The sensitivity and specificity of the IES-R has been reported as 91% and 82%, respectively, for a diagnosis of PTSD, where the cut-off score was ≥ 33.[23] In this study, we used a score of ≥ 3 for each subscale of the PHQ-4 as the threshold score to detect the presence of clinically significant symptoms of anxiety and depression, and a score of ≥ 33 on the IES-R as the threshold score to detect the presence of clinically significant symptoms of PTSD.

Data analysis

Data were collated using Microsoft Excel (Redmond, Washington, USA) and summarised as median (interquartile range) for non-normal data, and as proportions (percentage) for categorical data. Statistical analysis was performed using SPSS V.25 (IBM, New York, USA). The rates of mental health symptoms were calculated. For the categorical survey responses that were returned as ‘prefer not to say’ or ‘unsure,’ where combined analysis with the other responses was not possible, these were treated as missing data during statistical analysis. Univariate (unadjusted) analysis of measurements was performed for 24 predetermined, independent exposure variables using χ^2-tests to assess the significance of association. Adjusted analysis using binary logistic regression modelling was performed to obtain odds ratios (ORs) and 95% CIs.

The regression analysis was conducted separately for each dependent variable (anxiety (PHQ-4 anxiety subscale ≥ 3), depressive (PHQ-4 depression subscale ≥ 3) and PTSD (IES-R ≥ 33) symptoms), against all 24 measured independent variables to identify factors demonstrating a significant association for each symptom group. Multicollinearity of the 24 independent variables was assessed by calculating their variance inflation factors (VIFs). A *P*-value of less than 0.05 was assigned as the level of statistical significance.

Results

A total of 2706 participants completed the survey of whom only 2638 met the eligibility criteria. Their median age was 42 years (interquartile range (IQR) = 32–51). The majority ($n = 2096$, 79.5%) were women and 19.9% ($n = 524$) were men (Table 1). Eighteen ($n = 18$, <0.1%) respondents did not disclose their gender. Nearly a fifth ($n = 455$, 17.2%) belonged to Black, Asian and minority ethnic (BAME) groups. The majority ($n = 2016$, 76.4%) were in a relationship and nearly half ($n = 1298$, 49.2%) had dependents. In total, 84.0% ($n = 2216$) of participants were UK residents since childhood, the remainder emigrated as adults.

Around two-fifths ($n = 983$, 37.3%) reported a history of mental health conditions and 78.2% ($n = 769$) of these participants were prescribed medication or psychological therapies. Approximately a quarter ($n = 669$, 25.4%) reported a history of physical illness. Smokers accounted for 11.4% ($n = 301$) of the sample and 65.4% ($n = 1725$) consumed alcohol.

Most respondents were nurses ($n = 775$, 29.4%), followed by doctors ($n = 460$, 17.4%). Many staff ($n = 1403$, 53.2%) performing various other roles within the hospitals also took part in the survey (Supplementary Table 1 available at https://doi.org/10.1192/bjo.2020.150).

A third of participants ($n = 942$, 35.7%) had worked in healthcare for 10 years or less. The majority worked in acute general hospitals ($n = 2500$, 94.8%) and the remainder at mental health hospitals ($n = 91$, 3.5%). A minority ($n = 47$, 1.8%) were uncertain of their hospital type. Staff were based on in-patient wards ($n = 704$, 26.7%), on intensive therapy units (ITUs, $n = 382$, 14.5%), in emergency departments ($n = 123$, 4.7%) and other locations within their respective hospitals ($n = 1429$, 54.2%; see Supplementary Table 1). The majority of participants ($n = 1554$, 59%) reported that they undertook patient-facing duties (i.e. front-line clinical staff).

Table 1 Participant sociodemographic, lifestyle, health and employment factors[a]

	PHQ-4: anxiety subscale ≥ 3			PHQ-4: depression subscale ≥ 3			PTSD symptoms: IES-R ≥ 33		
	Yes, *n* (%)	No, *n* (%)	*P*[b]	Yes, *n* (%)	No, *n* (%)	*P*[b]	Yes, *n* (%)	No, *n* (%)	*P*[b]
Total	906 (34.3)	1732 (65.7)	–	824 (31.2)	1814 (68.8)	–	647 (24.5)	1991 (75.5)	–
Age (years)									
≤30	241 (41.4)	341 (58.6)	<0.001	209 (35.9)	373 (64.1)	0.001	161 (27.7)	421 (72.3)	0.013
>30–40	247 (37.1)	418 (62.9)		232 (34.9)	433 (65.1)		174 (26.2)	491 (73.8)	
>40–50	215 (29.7)	510 (70.3)		199 (27.4)	526 (72.6)		169 (23.3)	556 (76.7)	
>50–60	171 (29.8)	403 (70.2)		156 (27.2)	418 (72.8)		115 (20.0)	459 (80.0)	
>60	32 (34.8)	60 (65.2)		28 (30.4)	64 (69.6)		28 (30.4)	64 (69.6)	
Gender									
Female	762 (36.4)	1334 (63.6)	<0.001[c]	685 (32.7)	1411 (67.3)	<0.001[c]	553 (26.4)	1543 (73.6)	<0.001[c]
Male	134 (25.6)	390 (74.4)		130 (24.8)	394 (75.2)		84 (16)	440 (84)	
Prefer not to say	10 (55.6)	8 (44.4)		9 (50.0)	9 (50.0)		10 (55.6)	8 (44.4)	
Ethnicity									
BAME	154 (33.8)	301 (66.2)	0.806	135 (29.7)	320 (70.3)	0.428	129 (28.4)	326 (71.6)	0.037
White	752 (34.4)	1431 (65.6)		689 (31.6)	1494 (68.4)		518 (23.7)	1665 (76.3)	
Relationship status									
In a relationship	688 (34.1)	1328 (65.9)	0.672	601 (29.8)	1415 (70.2)	0.004	470 (23.3)	1546 (76.7)	0.009
Not in a relationship	218 (35.0)	404 (65.0)		223 (35.9)	399 (64.1)		177 (28.5)	445 (71.5)	
Dependents									
Yes	420 (32.4)	878 (67.6)	0.034	375 (28.9)	923 (71.1)	0.011	317 (24.4)	981 (75.6)	0.903
No	486 (36.3)	854 (63.7)		449 (33.5)	891 (66.5)		330 (24.6)	1010 (75.4)	
Immigration status									
Emigrated to the UK	128 (30.3)	294 (69.7)	0.058	115 (27.3)	307 (72.7)	0.054	109 (25.8)	313 (74.2)	0.497
UK resident from childhood	778 (35.1)	1438 (64.9)		709 (32.0)	1507 (68.0)		538 (24.3)	1678 (75.7)	
History of mental health conditions									
Yes	457 (46.5)	526 (53.5)	<0.001[c]	440 (44.8)	543 (55.2)	<0.0001[c]	327 (33.3)	656 (66.7)	<0.001[c]
No	418 (26.3)	1172 (73.7)		355 (22.3)	1235 (77.7)		294 (18.5)	1296 (81.5)	
Prefer not to say	31 (47.7)	34 (52.3)		29 (44.6)	36 (55.4)		26 (40.0)	39 (60.0)	
History of physical illness									
Yes	252 (37.7)	417 (62.3)	0.018[c]	245 (36.6)	424 (63.4)	<0.001[c]	200 (29.9)	469 (70.1)	<0.001[c]
No	632 (32.7)	1303 (67.3)		561 (29.0)	1374 (71.0)		432 (22.3)	1503 (77.7)	
Prefer not to say	22 (64.7)	12 (35.3)		18 (52.9)	16 (47.1)		15 (44.1)	19 (55.9)	
Smoker									
Yes	131 (43.5)	170 (56.5)	<0.001	132 (43.9)	169 (56.1)	<0.001	106 (35.2)	195 (64.8)	<0.001
No	775 (33.2)	1562 (66.8)		692 (29.6)	1645 (70.4)		541 (23.1)	1796 (76.9)	
Alcohol consumer									
Yes	561 (32.5)	1164 (67.5)	0.007	494 (28.6)	1231 (71.4)	<0.001	377 (21.9)	1348 (78.1)	<0.001
No	345 (37.8)	568 (62.2)		330 (36.1)	583 (63.9)		270 (29.6)	643 (70.4)	
Job title									
Doctors	101 (22.0)	359 (78.0)	<0.001[d]	82 (17.8)	378 (82.2)	<0.001[d]	61 (13.3)	399 (86.7)	0.149[d]
Nurses	279 (36.0)	496 (64.0)		255 (32.9)	520 (67.1)		226 (29.2)	549 (70.8)	
Other[e]	526 (37.5)	877 (62.5)		487 (34.7)	916 (65.3)		360 (25.7)	1043 (74.3)	
Duration of employment within healthcare									
≤10 years	360 (38.2)	582 (61.8)	0.002	350 (37.2)	592 (62.8)	<0.001	270 (28.7)	672 (71.3)	<0.001
>10 years	546 (32.2)	1150 (67.8)		474 (27.9)	1222 (72.1)		377 (22.2)	1319 (77.8)	
Based at an acute general hospital									
Yes	854 (34.2)	1646 (65.8)	0.396	786 (31.4)	1714 (68.6)	0.335	611 (24.4)	1889 (75.6)	0.662
No[f]	52 (37.7)	86 (62.3)		38 (27.5)	100 (72.5)		36 (26.1)	102 (73.9)	
Location within hospital									
In-patient ward/emergency department/ITU	435 (36.0)	774 (64.0)	0.104	379 (31.3)	830 (68.7)	0.909	355 (29.4)	854 (70.6)	<0.001
Other[e]	471 (33.0)	958 (67.0)		445 (31.1)	984 (68.9)		292 (20.4)	1137 (79.6)	
Hospital-treated COVID-19 patients									
Yes	872 (34.2)	1679 (65.8)	0.344	793 (31.1)	1758 (68.9)	0.368	630 (24.7)	1921 (75.3)	0.272
No[g]	34 (39.1)	53 (60.9)		31 (35.6)	56 (64.4)		17 (19.5)	70 (80.5)	
Patient-facing duties (i.e. front-line clinical staff)									
Yes	554 (35.6)	1000 (64.4)	0.091	465 (29.9)	1089 (70.1)	0.081	420 (27)	1134 (73)	<0.001
No	352 (32.5)	732 (67.5)		359 (33.1)	725 (66.9)		227 (20.9)	857 (79.1)	

PHQ-4, 4-item Patient Health Questionnaire; IES-R, Impact of Event Scale-Revised; PTSD, post-traumatic stress disorder; BAME, Black, Asian and minority ethnic; ITU, intensive therapy unit; COVID-19, coronavirus disease 2019.
a. Percentages are demonstrated in rows.
b. Pearson χ^2 statistical test used for univariate analysis to obtain *P*-values.
c. 2 × 2 χ^2-analysis – excludes the 'prefer not to say' group.
d. 2 × 2 χ^2-analysis of doctors and nurses (combined) versus other staff groups.
e. Supplementary Table 1 provides a distribution of all participant job titles and locations of work.
f. Includes participants based at acute mental health hospitals and those who were uncertain of the type of hospital.
g. Includes those who reported no and those who were uncertain.

Participant experiences

Just over a half ($n = 1452$, 55%) reported that adequate PPE was available at their workplace (Table 2). The remainder reported that this was not the case ($n = 812$, 30.8%), were unsure ($n = 322$, 12.2%) or preferred not to comment ($n = 52$, 2%). The majority were aware of well-being measures implemented by their employer ($n = 2064$, 78.2%) but only 15.4% ($n = 405$) accessed any form of psychological support during the study period. A third ($n = 873$,

Table 2 Participant experiences during the coronavirus disease 2019 (COVID-19) pandemic[a]

	PHQ-4: anxiety subscale ≥ 3			PHQ-4: depression subscale ≥ 3			PTSD symptoms: IES-R ≥ 33		
	Yes, *n* (%)	No, *n* (%)	*P*[b]	Yes, *n* (%)	No, *n* (%)	*P*[b]	Yes, *n* (%)	No, *n* (%)	*P*[b]
Total	906 (34.3)	1732 (65.7)	–	824 (31.2)	1814 (68.8)	–	647 (24.5)	1991 (75.5)	–
Adequate PPE availability									
Reported adequate PPE	406 (28.0)	1046 (72.0)	<0.001	371 (25.6)	1081 (74.4)	<0.001	272 (18.7)	1180 (81.3)	<0.001
Did not report adequate PPE[c]	500 (42.2)	686 (57.8)		453 (38.2)	733 (61.8)		375 (31.6)	811 (68.4)	
Well-being support available at work									
Reported availability	634 (30.7)	1430 (69.3)	<0.001	565 (27.4)	1499 (72.6)	<0.001	425 (20.6)	1639 (79.4)	<0.001
Did not report availability[d]	272 (47.4)	302 (52.6)		259 (45.1)	315 (54.9)		222 (38.7)	352 (61.3)	
Redeployment									
Reported redeployment[e]	320 (36.7)	553 (63.3)	0.079	276 (31.6)	597 (68.4)	0.767	274 (31.4)	599 (68.6)	<0.001
Did not report redeployment[f]	586 (33.2)	1179 (66.8)		548 (31)	1217 (69)		373 (21.1)	1392 (78.9)	
Increased working hours									
Reported increase	348 (34.3)	667 (65.7)	0.96	331 (32.6)	684 (67.4)	0.228	273 (26.9)	742 (73.1)	0.025
Did not report increase[g]	558 (34.4)	1065 (65.6)		493 (30.4)	1130 (69.6)		374 (23)	1249 (77)	
Morally uncomfortable changes in the way they worked (moral dilemmas)									
Denied experiencing	344 (25.5)	1007 (74.5)	<0.001	337 (24.9)	1014 (75.1)	<0.001	193 (14.3)	1158 (85.7)	<0.001
Did not deny experiencing[c]	562 (43.7)	725 (56.3)		487 (37.8)	800 (62.2)		454 (35.3)	833 (64.7)	
Developed COVID-19									
Yes[d]	262 (36.4)	458 (63.6)	0.175	238 (33.1)	482 (66.9)	0.217	209 (29)	511 (71)	0.001
No	644 (33.6)	1274 (66.4)		586 (30.6)	1332 (69.4)		438 (22.8)	1480 (77.2)	
A cohabitant developed COVID-19									
Yes	179 (34.3)	343 (65.7)	0.977	157 (30.1)	365 (69.9)	0.523	131 (25.1)	391 (74.9)	0.736
No	727 (34.4)	1389 (65.6)		667 (31.5)	1449 (68.5)		516 (24.4)	1600 (75.6)	
Admission to hospital with COVID-19									
Self, close family or friend[h]	189 (41.8)	263 (58.2)	<0.001	154 (34.1)	298 (65.9)	0.153	161 (35.6)	291 (64.4)	<0.001
No	717 (32.8)	1469 (67.2)		670 (30.6)	1516 (69.4)		486 (22.2)	1700 (77.8)	

PHQ-4, 4-item Patient Health Questionnaire; IES-R, Impact of Event Scale-Revised; PTSD, post-traumatic stress disorder; PPE, personal protective equipment.
a. Percentages are demonstrated in rows.
b. Pearson χ^2 statistical test used for univariate analysis to obtain *P*-values.
c. Includes those that reported yes and prefer not to say.
d. Includes all participants who had symptomatic COVID-19 illness diagnosed by polymerase chain reaction testing, clinician diagnosed or self-diagnosed as per Public Health England guidance.
e. Includes all forms of redeployment for example different speciality, department or hospital.
f. Includes no and not applicable.
g. Includes those that reported a decrease, no change and not applicable.
h. Close family member – nuclear family, first degree relative whom the respondent lived with or associated on at least once a week; a close friend: A friend whom the participant lived with or associated with at least once a week.

33.1%) of participants were redeployed as a result of the pandemic and 38.5% ($n = 1015$) reported increased working hours. In addition, 51.2% ($n = 1351$) reported morally uncomfortable changes in the way they worked.

Of those who reported a diagnosis of COVID-19 ($n = 720$, 27.3%), this was either confirmed on polymerase chain reaction testing of nasopharyngeal swabs ($n = 277$), diagnosed by a clinician ($n = 47$) or self-diagnosed based on symptoms and Public Health England guidance ($n = 396$). Approximately a fifth of staff ($n = 522$, 19.8%) also reported that a cohabitant had developed COVID-19 during the period in question. Only 17.1% ($n = 452$) reported an admission to hospital with COVID-19 for self, or a close family member or friend.

Significant mental health symptoms

On the anxiety subscale of the PHQ-4, 34.3% ($n = 906$) scored ≥3; on the depression subscale of the PHQ-4, 31.2% ($n = 824$) scored ≥3 and 24.5% ($n = 647$) scored ≥33 on the IES-R (Supplementary Fig. 1).

Univariate analysis

Participants who were women, those with a history of mental health or physical conditions, were smokers or consumed alcohol, worked as a doctor or a nurse, and had worked for 10 years or less, were significantly more likely to have higher rates of anxiety symptoms (Table 1). These independent variables were also significantly associated with higher rates of depressive and PTSD symptoms. On the other hand, participants who had adequate PPE and well-being support available, and did not experience morally uncomfortable changes in the way they worked, reported significantly lower rates of all mental health symptoms (Table 2). Being admitted or having a close family member or friend admitted with COVID-19 infection was associated with increased anxiety and PTSD symptoms but not depressive symptoms (Table 2).

Logistic regression analysis

The adjusted logistic regression analysis was performed for 2534 participants (excluding any missing data) for each of the dependent variables: anxiety, depression and PTSD symptoms using the clinically significant thresholds previously stated. All 24 independent variables considered in the univariate analysis were included in the regression analysis. An assessment of multicollinearity of the 24 independent variables revealed all the VIFs to be less than 2 (maximum 1.6), indicating the validity of including all these independent variables in the logistic regression modelling.

Anxiety symptoms

The statistically significant associations were as follows: younger participants, women and those reporting an admission to hospital with COVID-19 for self, or close family member or friend were around 50% more likely to report anxiety symptoms ($P \leq 0.001$) (Table 3).

A history of mental health conditions was also significantly associated with a greater than twofold odds of clinically significant anxiety symptoms (OR = 2.3, 95% CI 1.9–2.7, $P < 0.001$). Doctors and nurses were 20% less likely to report anxiety compared with other hospital HCW (OR = 0.8, 95% CI 0.6–0.9, $P = 0.009$). Furthermore, those who reported adequate PPE availability,

Table 3 Factors associated with significant mental health symptoms on adjusted analysis[a]

	PHQ-4: anxiety subscale score ≥ 3, OR (95% CI); *P*	PHQ-4: depression subscale score ≥ 3, OR (95% CI); *P*	PTSD symptoms: IES-R score ≥ 33, OR (95% CI); *P*
Age, years: ≤40	1.4 (1.1–1.7); 0.001	–	–
Female gender	1.5 (1.2–1.9); 0.001	–	1.7 (1.3–2.2); <0.001
A history of mental health conditions	2.3 (1.9–2.7); <0.001	2.5 (2.1–3.0); <0.001	2.1 (1.7–2.5); <0.001
A history of physical illness	–	–	1.4 (1.1–1.7); 0.009
Smoker	–	1.5 (1.1–1.9); 0.005	1.5 (1.1–1.9); 0.012
Alcohol consumer	–	0.8 (0.7–0.9); 0.028	0.8 (0.6–0.9); 0.038
Worked as a doctor or nurse	0.8 (0.6–0.9); 0.009	–	0.8 (0.6–0.9); 0.034
Based in an acute general hospital	–	1.8 (1.1–2.8); 0.016	–
Working on in-patient ward/in emergency department/on ITU	–	–	1.4 (1.1–1.8); 0.006
Reported adequate PPE at work	0.7 (0.5–0.8); <0.001	0.7 (0.6–0.9); 0.001	0.7 (0.5–0.8); <0.001
Well-being support available at work	0.6 (0.5–0.7); <0.001	0.5 (0.4–0.7); <0.001	0.5 (0.4–0.7); <0.001
Denied morally uncomfortable changes	0.5 (0.4–0.6); <0.001	0.6 (0.5–0.7); <0.001	0.4 (0.3–0.5); <0.001
Redeployed	–	–	1.5 (1.2–1.9); <0.001
Increased working hours	–	–	1.3 (1.1–1.6); 0.006
Admitted to hospital with COVID-19[b]	1.4 (1.1–1.7); 0.009	–	1.7 (1.3–2.2); <0.001

PHQ-4, 4-item Patient Health Questionnaire; PTSD, post-traumatic stress disorder; IES-R, Impact of Event Scale-Revised; ITU, intensive therapy unit; PPE, personal protective equipment; COVID-19, coronavirus disease 2019.
a. There are 2534 participants included in the analysis, excluding 104 who were treated as missing data where responses were ambiguous (i.e. prefer not to say) and could not therefore be combined or analysed as separate categories. A total of 24 independent variables (as displayed on Tables 1 and 2) were used in the analysis. Only the statistically significant ($P < 0.05$) results are displayed on Table 3. Please refer to Supplementary Table 2 for a full list of variables included in the regression analysis.
b. Admission to hospital of self, close family or friend.

well-being support availability and where no morally uncomfortable changes took place, were around 50% less likely to have anxiety symptoms ($P < 0.001$; see Table 3 and Supplementary Fig. 2). There were no significant associations between the remaining exposures and anxiety symptoms (Supplementary Table 2).

Depressive symptoms

Smokers were 50% more likely to report depressive symptoms (OR = 1.5, 95% CI 1.1–1.9, $P = 0.005$). A history of mental health conditions had the strongest association, with two and half times greater odds of hospital HCW reporting depressive symptoms (OR = 2.5, 95% CI 2.1–3.0, $P < 0.01$). There was an almost twofold increase in odds of depressive symptoms when the participant was based in an acute general hospital compared with a mental health setting (OR = 1.8, 95% CI 1.1–2.8, $P = 0.016$). On the other hand, alcohol consumers were 20% less likely to experience these symptoms (OR = 0.8, 95% CI 0.7–0.9, $P = 0.028$). Furthermore, staff with adequate PPE availability, adequate well-being support and those who did not report morally uncomfortable changes in the way they worked were up to 50% less likely to report depressive symptoms ($P \leq 0.001$; see Table 3 and Supplementary Fig. 3). There were no significant associations between the remaining exposures and depressive symptoms (Supplementary Table 2).

Symptoms of PTSD

A history of mental health conditions was associated with a twofold increased odds of clinically significant PTSD symptoms (OR = 2.1, 95% CI 1.7–2.5, $P < 0.001$). Several exposures were associated with increased likelihood of clinically significant PTSD symptoms: female gender, history of physical illness, smoking, being based on in-patient wards, in emergency departments or on ITUs, redeployment and admission to hospital for self, a close family or friend with COVID-19 ($P < 0.05$; see Table 3). The exposures of alcohol consumption and working as a doctor or a nurse were associated with a 20% lower likelihood of reporting clinically significant PTSD symptoms ($P < 0.05$; see Table 3).

There were 30–50% less odds of clinically significant PTSD symptoms when there were adequate PPE and well-being support available ($P < 0.001$; see Table 3 and Supplementary Fig. 4). Participants who reported that no morally uncomfortable changes took place in the way they worked demonstrated approximately 60% less odds of clinically significant PTSD symptoms (OR = 0.4, 95% CI 0.3–0.5, $P < 0.001$). There were no significant associations between the remaining exposures and PTSD symptoms (Supplementary Table 2).

Discussion

Main findings and comparison with findings from other studies

We found that around a third of hospital HCW reported clinically significant symptoms of anxiety and depression. A quarter reported clinically significant PTSD symptoms. Previous studies (pre-COVID-19) reported a baseline rate of clinically significant PTSD symptoms (also defined as an IES-R score ≥ 33) among 15–16% of HCW from the UK.[24,25] No comparable published data for UK HCW PHQ-4 scores were found. However, the rates of anxiety and depression among the UK general population has previously been reported to be around 19.7%.[26] A recent study found that around 20% of the UK general public experienced symptoms of anxiety and depression, and approximately 17% experienced PTSD symptoms during the COVID-19 pandemic.[27]

The higher rates of mental health symptoms identified in the current study might be associated with working in a hospital setting during the COVID-19 pandemic. Nevertheless, the rates of symptoms of anxiety and depression among HCW in this study is lower compared with data from China that reported rates of anxiety of 40.6%; of depression of 50.4%, of sleep disturbance of 34% and of distress of 71.5%.[7] Differences could be attributed to a multitude of factors including cultural, political and socioeconomic variations across the two study populations.

Interpretation of the data

In our adjusted analysis a previous history of mental health conditions consistently demonstrated odds of greater than two in participants reporting clinically significant symptoms of anxiety, depression and PTSD. In contrast, the availability of adequate PPE, access to well-being support and not experiencing morally

uncomfortable changes in the way they worked were all significantly negatively associated with participants reporting these symptoms.

Younger participants aged 40 or under demonstrated greater odds of reporting clinically significant anxiety symptoms. Smoking was associated with depression and PTSD symptoms but not anxiety. Female gender and a hospital admission for self, or a close family member or a friend were associated with anxiety and PTSD symptoms but not depression. Several other exposures were associated with PTSD symptoms but not anxiety or depression (for example redeployment, increased working hours and working on in-patient wards, in emergency departments or on ITUs).

Implications

Our findings may prompt healthcare employers to focus their attention on the provision of specific interventions that may protect HCW against an adverse impact on mental health during crises such as the COVID-19 pandemic. These may include the provision of greater access to well-being support for staff, ensuring the availability of adequate PPE and protection from exposure to moral dilemmas in the workplace.

Furthermore, careful workforce planning to mitigate the adverse effects of redeployment and minimising the risk of viral infection may reduce the risk of staff experiencing PTSD symptoms. Attention should also be given to staff at greater risk such as those with a history of mental health conditions, female staff and smokers. Special consideration and additional support in the workplace could also be considered for younger employees, redeployed staff members and those working in potentially high-risk areas (such as in-patient wards, emergency departments and ITUs)

There were some unexpected findings in this analysis. One such finding was the protective effect working as a doctor or a nurse had on participants reporting clinically significant symptoms of anxiety and PTSD. This could be attributed to factors such as training, experience and coping mechanisms or resilience from previous working practices in stressful healthcare environments. Furthermore, there was no statistically significant increase in odds of self-reported mental health symptoms among staff undertaking patient-facing duties compared with staff in other roles. Therefore, it is important to ensure support is available to staff in all job roles who may potentially be at risk and not just front-line clinical staff.

We also observed reduced odds of clinically significant symptoms of depression and PTSD reported by participants who consumed any amount of alcohol. The clinical significance and relevance of the latter association are unknown.

Limitations

There were some limitations in our study. The time elapsed between traumatic exposure and the onset of symptoms is key to making a diagnosis of PTSD. However, the aim of the study was not to make diagnoses of mental health disorders but to screen the target population for the presence of clinically concerning symptoms. The survey was conducted relatively close to the duration of the UK's COVID-19 pandemic peak. The elevated scores on the IES-R may be representative of an acute stress reaction that usually resolves within a few months. Further follow-up of these participants is required to ascertain the persistence of symptoms – a planned analysis by our study group. Our data is from a cross-sectional survey. Therefore, causal inferences cannot be made. Furthermore, the data were collected through a self-report questionnaire, which is at risk of responder bias.

There were also several strengths. This study is one of the first in the UK to report on the mental health impact on hospital staff of working during the COVID-19 pandemic. These findings may be generalisable to the wider UK population of hospital employees given the relatively large sample size and representative demographic sample (Supplementary Table 3).

In conclusion, during the COVID-19 pandemic, there were higher rates of common mental health symptoms in hospital HCW in the UK, especially among those with a history of mental health conditions. Occupational interventions such as adequate PPE and well-being support availability, and reduced exposure to moral dilemmas appear to protect hospital HCW against these symptoms.

Kasun Wanigasooriya, MBChB, Institute of Biomedical Research, College of Medical and Dental Science, University of Birmingham, UK; and University Hospitals Birmingham NHS Foundation Trust, UK; **Priyanka Palimar**, MRCPsych, Department of Child and Adolescent Psychiatry, Forward Thinking Birmingham, Birmingham Women's and Children's Hospital NHS Foundation Trust, UK; **David N. Naumann**, PhD, University Hospitals Birmingham NHS Foundation Trust, UK; **Khalida Ismail**, MRCPsych, Department of Psychological Medicine, Institute of Psychiatry, Psychology and Neurosciences, King's College London, Weston Education Centre, UK; **Jodie L. Fellows**, DClinPsy, University Hospitals Birmingham NHS Foundation Trust, UK; **Peter Logan**, MBChB, Walsall Healthcare NHS Trust, UK; **Christopher V. Thompson**, FRCS, Sandwell and West Birmingham Hospitals NHS Trust, UK; **Helen Bermingham**, MBChB, Institute of Biomedical Research, College of Medical and Dental Science, University of Birmingham, UK; and University Hospitals Birmingham NHS Foundation Trust, UK; **Andrew D. Beggs**, PhD, Institute of Biomedical Research, College of Medical and Dental Science, University of Birmingham, UK; and University Hospitals Birmingham NHS Foundation Trust, UK; **Tariq Ismail**, MD, University Hospitals Birmingham NHS Foundation Trust, UK

Correspondence: Kasun Wanigasooriya. Email: kasun.wanigasooriya@nhs.net

First received 19 Sep 2020, final revision 8 Nov 2020, accepted 11 Nov 2020

Supplementary material

Supplementary material is available online at https://doi.org/10.1192/bjo.2020.150.

Data availability

All relevant data and results included in this article have been published along with the article and its supplementary information files. Anonymised data can be obtained on reasonable request from the corresponding author at the end of the STAT-STRESS Covid-19 study.

Acknowledgements

We would like to acknowledge our study sponsor, Birmingham Women's and Children's Hospital NHS Foundation Trust. We are grateful for the support from the research and development teams and executive leadership of all ten acute National Health Service Hospital Trusts in the West Midlands, UK. We would further like to thank the West Midlands Research Collaborative and West Midlands Surgical Society for their endorsement of this study. We extend our gratitude to Mr S. Karandikar (Heartlands Hospital), Mr R. M. Tirumularaju (Good Hope Hospital), Dr E. Plunkett (Queen Elizabeth Hospital Birmingham) and Dr E. Jenkinson (New Cross Hospital) from the West Midlands, UK for their assistance with participant recruitment. The statistical analysis was overseen by Mr Peter Nightingale, statistician at the University Hospitals Birmingham NHS Foundation Trust, UK. Finally, we would like to acknowledge and thank all our participants for their time spent taking part in this study.

Author contributions

K.W. and P.P. designed the study, created the survey, analysed the data and wrote the main manuscript. D.N.N. and K.I. were involved in data interpretation and co-writing the main manuscript. J.L.F. was involved in survey design and participant recruitment. P.L., C.V.T., H.B. and A.D.B. were involved in study promotion, participant recruitment, reviewing and editing of the manuscript. T.I. was the most senior author. He provided supervision and guidance for the study. He was involved in the study design, management, data interpretation, reviewing and editing the final manuscript.

Funding

The University Hospitals Birmingham Charity covered the operational expenses incurred during this study (Remittance Reference No: C05976).

Declaration of interest

None.

References

1 John Hopkins University (JHU). *Mortality Analyses – Coronavirus Resource Centre*. JHU, 2020 (https://coronavirus.jhu.edu/data/mortality).

2 CovidSurg Collaborative. Elective surgery cancellations due to the COVID-19 pandemic: global predictive modelling to inform surgical recovery plans. *Br J Surg* 2020; **107**: 1440–9.

3 Rosa WE, Ferrell BR, Wiencek C. Increasing critical care nurse engagement of palliative care during the COVID-19 pandemic. *Crit Care Nurse*. [Epub ahead of print] 23 July 2020. Available from: https://doi.org/10.4037/ccn2020946.

4 Zhan M, Qin Y, Xue X, Zhu S. Death from Covid-19 of 23 health care workers in China. *N Engl J Med* 2020; **382**: 2267–68.

5 Cook T, Kursumovic E, Lennane S. Exclusive: deaths of NHS staff from Covid-19 analysed. *Health Serv J* 2020, April 22 (https://www.hsj.co.uk/exclusive-deaths-of-nhs-staff-from-covid-19-analysed/7027471.article).

6 Holmes EA, O'Connor RC, Perry VH, Tracey I, Wessely S, Arsenault L, et al. Multidisciplinary research priorities for the COVID-19 pandemic: a call for action for mental health science. *Lancet Psychiatry* 2020; **7**: 547–60.

7 Lai J, Ma S, Wang Y, Cai Z, Hu J, Wei N, et al. Factors associated with mental health outcomes among health care workers exposed to coronavirus disease 2019. *JAMA Network Open* 2020; **3**: e203976–88.

8 Cai H, Tu B, Ma J, Chen L, Fu L, Jiang Y, et al. Psychological impact and coping strategies of frontline medical staff in Hunan between January and March 2020 during the outbreak of Coronavirus Disease 2019 (COVID-19) in Hubei, China. *Med Sci Monit* 2020; **26**: e924171.

9 Xiao H, Zhang Y, Kong D, Li S, Yang N. The effects of social support on sleep quality of medical staff treating patients with coronavirus disease 2019 (COVID-19) in January and February 2020 in China. *Med Sci Monit* 2020; **26**: e923549.

10 Mohindra R, Ravaki R, Suri V, Bhalla A, Singh SM. Issues relevant to mental health promotion in frontline health care providers managing quarantined/isolated COVID19 patients. *Asian J Psychiatr* 2020; **51**: 102084.

11 Tan BY, Chew NW, Lee GK, Jing M, Goh Y, Yeo LL, et al. Psychological impact of the COVID-19 pandemic on health care workers in Singapore. *Ann Intern Med* 2020; **173**: 317–20.

12 Spoorthy MS, Pratapa SK, Mahant S. Mental health problems faced by healthcare workers due to the COVID-19 pandemic—a review. *Asian J Psychiatr* 2020; **51**: 102119.

13 Chan AO, Huak CY. Psychological impact of the 2003 severe acute respiratory syndrome outbreak on health care workers in a medium size regional general hospital in Singapore. *Occup Med* 2004; **54**: 190–6.

14 Dutheil F, Mondillon L, Navel V. PTSD as the second tsunami of the SARS-Cov-2 pandemic. *Psychol Med* [Epub ahead of print] 24 Apr 2020 (https://doi.org/10.1017/S0033291720001336).

15 UK Government (GOV.UK). *Coronavirus (COVID-19) in the UK*. GOV.UK, 2020 (https://coronavirus.data.gov.uk/cases).

16 Rimmer A. Covid-19: Two thirds of healthcare workers who have died were from ethnic minorities. *BMJ* 2020; **369**: m1621.

17 UK Parliament. *Approved: Mental Health Impacts of COVID-19 on Healthcare Workers and Carers*. UK Parliament, 2020 (https://post.parliament.uk/work-programme/approved-mental-health-impacts-of-covid-19-on-healthcare-workers-and-carers/).

18 UK Government. *£5 million Funding Given to Mental Health Community Projects*. GOV.UK, 2020 (https://www.gov.uk/government/news/5-million-funding-given-to-mental-health-community-projects).

19 UK Government. *Regional Competitiveness and Employment Objective 2007–2013. West Midlands European Regional Development Fund Operational Programme*. GOV.UK, 2012. (https://assets.publishing.service.gov.uk/government/uploads/system/uploads/attachment_data/file/315877/WM_OP_FINAL_v3.1.pdf).

20 Caul S. *Deaths Involving COVID-19 by Local Area and Socioeconomic Deprivation Statistical Bulletins*. Office of National Statistics, 2020 (https://www.ons.gov.uk/peoplepopulationandcommunity/birthsdeathsandmarriages/deaths/bulletins/deathsinvolvingcovid19bylocalareasanddeprivation/previousReleases).

21 Kroenke K, Spitzer RL, Williams JB, Monahan PO, Löwe B. Anxiety disorders in primary care: prevalence, impairment, comorbidity, and detection. *Ann Intern Med* 2007; **146**: 317–25.

22 Kroenke K, Spitzer RL, Williams JB. The Patient Health Questionnaire-2: validity of a two-item depression screener. *Med Care* 2003; **41**: 1284–92.

23 Creamer M, Bell R, Failla S. Psychometric properties of the impact of event scale—revised. *Behav Res Ther* 2003; **41**: 1489–96.

24 Naumann DN, McLaughlin A, Thompson CV, Hardy E, Fellows JL, Crombie NC. Acute stress and frontline healthcare providers. *J Paramed Pract* 2017; **9**: 516–21.

25 Thompson CV, Naumann DN, Fellows JL, Bowley DM, Suggett N. Post-traumatic stress disorder amongst surgical trainees: an unrecognised risk? *Surgeon* 2017; **15**: 123–30.

26 Evans J, Macrory I, Randall C. *Measuring National Well-Being: Life in the UK:* 2016. Office of National Statistics, 2016 (https://www.ons.gov.uk/peoplepopulationandcommunity/wellbeing/articles/measuringnationalwellbeing/2016#how-good-is-our-health).

27 Shevlin M, McBride O, Murphy J, Miller JG, Hartman TK, Levita L, et al. Anxiety, depression, traumatic stress, and COVID-19 related anxiety in the UK general population during the COVID-19 pandemic. *BJPsych Open* 2020; **6**: e125.

EXTRA CONTENT ONLINE
OPEN ACCESS
CC BY
