# Supplementary material for: Mental health status and related factors influencing healthcare workers during the COVID-19 pandemic: A systematic review and meta-analysis
Source: PLoS One. 2024 Jan 19;19(1):e0289454. doi: 10.1371/journal.pone.0289454 (PMC10798549; doi:10.1371/journal.pone.0289454)
Supplement: S1 Data — (ZIP) [file pone.0289454.s011.zip › literatures/212.pdf]

Epidemiology and Infection

cambridge.org/hyg

Original Paper

Cite this article: Weibelzahl S, Reiter J, Duden G (2021). Depression and anxiety in healthcare professionals during the COVID-19 pandemic. *Epidemiology and Infection* **149**, e46, 1–10. https://doi.org/10.1017/S0950268821000303

Received: 11 October 2020
Revised: 1 January 2021
Accepted: 1 February 2021

Key words:
COVID-19; help-seeking behaviour; mental health; nursing staff; occupational stress; pandemics

Author for correspondence:
S. Weibelzahl, E-mail: weibelzahl@pfh.de

© The Author(s), 2021. Published by Cambridge University Press. This is an Open Access article, distributed under the terms of the Creative Commons Attribution licence (http://creativecommons.org/licenses/by/4.0/), which permits unrestricted re-use, distribution, and reproduction in any medium, provided the original work is properly cited.

CAMBRIDGE UNIVERSITY PRESS

Depression and anxiety in healthcare professionals during the COVID-19 pandemic

S. Weibelzahl[1], J. Reiter[2] and G. Duden[3,4]

[1]PFH Private University of Applied Sciences Göttingen, Göttingen, Germany; [2]University of Vienna, Vienna, Austria; [3]University of Osnabrueck, Osnabrueck, Germany and [4]University of Leipzig, Leipzig, Germany

Abstract

Healthcare staff have been at the centre of the fight against the COVID-19 pandemic, facing diverse work-related stressors. Building upon studies from various countries, we aimed to investigate (1) the prevalence of various work-related stressors among healthcare professionals in Germany specific to the COVID-19 pandemic, (2) the psychological effects of these stressors in terms of clinical symptoms, and (3) the healthcare professionals' help-seeking behaviour. To this end, $N = 300$ healthcare professionals completed an online survey including the ICD-10 Symptom Rating checklist (ISR), event-sampling questions on pandemic-related stressors and self-formulated questions on help-seeking behaviour. Participants were recruited between 22 May and 22 July 2020. Findings were analysed using *t* tests, regressions and comparisons to large clinical and non-clinical samples assessed before and during the pandemic. Results show that healthcare professionals were most affected by protective measures at their workplace and changes in work procedures. Psychological symptoms, particularly anxiety and depression, were significantly more severe than in a non-clinical pre-pandemic sample and in the general population during the pandemic. At the same time, most professionals indicated that they would not seek help for psychological concerns. These findings indicate that healthcare employers need to pay greater attention to the mental health of their staff.

Introduction

Globally, healthcare workers are at the centre of the fight against the COVID-19 pandemic. In the initial phase of the pandemic, healthcare professionals received a lot of media attention and praise from the general public for their *heroic* work [1]. While many employees started working from home or were even barred from attending their workplace, employees in the healthcare sector were affected in a dual sense: not only were they required to continue working, they were also exposed to increased risks due to the nature of their jobs. This twofold burden has had a global economic and medical impact, while also increasing psychological strain on the affected workers [2].

As the pandemic spread from country to country, studies investigated the well-being of healthcare professionals in many places, including China, Singapore, Japan, Italy, Saudi Arabia and Switzerland [3]. The well-being of this target group is of high concern, as health systems depend on their workforces and a collapse would have severe consequences for the general public.

In this study, we set out to investigate whether healthcare professionals in Germany have been affected psychologically by the COVID-19 pandemic and, if so, what kind of work-related stress they have been facing in this new situation.

Evidence from other countries

As China was the first country to face high infection rates of COVID-19, the first studies exploring the well-being of healthcare staff were run in Wuhan and other regions of China from January 2020 onwards [4–7]. A systematic review of 14 studies on the impact on healthcare workers in different regions of China and in Singapore demonstrated an extensive rise in experienced stress as well as depression and anxiety symptoms. Across all studies, between 2.2% and 14.5% of respondents exhibited severe anxiety and depression symptoms. Influencing factors included age, gender, occupation, specialisation, type of activities performed and proximity to COVID-19 patients [8]. Some of these findings are based on large samples. For instance, Lai *et al.* [9] assessed $N = 1257$ healthcare workers in several regions of China and found high levels of depression (50.4%), anxiety (44.6%), insomnia (34.0%) and distress (71.5%).

Increased mental health risks were also observed in Japan [10] and later on in Switzerland [3]. Italian healthcare professionals showed symptoms of burnout 5 weeks after the local onset of the pandemic [11]. In Saudi Arabia, healthcare professionals displayed a higher level of

anxiety and worry compared to previous outbreaks of MERS-CoV in the country, despite the fact that no cases of COVID-19 had been registered at the time [12]. Using structural equation modelling on a variety of data sources, Miller [13] demonstrated that frontline staff, who come into contact with either possible or confirmed cases of COVID-19, are exposed to even higher mental and emotional risks than other healthcare workers.

While the COVID-19 pandemic is unprecedented with regard to its prevalence, its rapid spread [14] and the type of protective measures applied at large scale, similar effects on healthcare staff have been observed in previous outbreaks of other infectious diseases. A systematic review of 44 studies on former epidemics and pandemics showed that symptoms of mental disorders were common in healthcare workers both during and after the outbreaks [15]. Symptoms of depression were reported by 27.5–50.7%, symptoms of insomnia by 34–36.1% and symptoms of severe anxiety by 45% of participants. Also, between 11% and 73.4% of participants reported post-traumatic stress symptoms. After 1–3 years, the level was still at 10–40%.

Stress factors

Which individual and work-related factors are believed to increase or decrease the risk of developing clinical symptoms? Many healthcare professionals worry about infecting family members, experience fears and uncertainty concerning the mortality and morbidity of the disease, and some must face the death of colleagues [14, 16, 17]. Surprisingly, the fear of becoming infected oneself seems to be less prominent than the fear of infecting one's family [18]. Intolerance of uncertainty has a strong impact on mental well-being in a pandemic setting. This is significantly mediated by rumination and fear [19].

Key individual protective factors include social support and self-efficacy. Social support increases self-efficacy and sleep quality, which in turn reduces anxiety and stress [20]. It has been widely demonstrated that self-efficacy beliefs are an important moderator of the impact of work-related stressors [e.g. 21]. Self-efficacy is also one of the key concepts of the Health Belief Model [22] and a good predictor of health behaviour. Self-efficacy can reduce infection-related worries [23] and insomnia [20]. However, pandemic conditions are characterised by high levels of uncertainty and rapid changes in both work practices and social life. A general climate of wariness and uncertainty has been observed, as the emergence of mutations sporadically sets back knowledge gained about the disease and vaccination progress is slow. The treatment healthcare professionals can actually provide is still limited, as specific medications are not available yet. Healthcare workers have also raised concerns about not being able to provide competent care [14, 7, 17]. This kind of situation is detrimental to self-efficacy.

Work-related organisational factors that increase the risk of developing clinical symptoms among healthcare professionals include depletion of personal protective equipment [16], lack of other medical resources such as specific drugs, ventilators and intensive care unit beds, as well as communication issues resulting from rapidly changing information or a lack of up-to-date information [14]. Changes in work practices such as having to adjust to wearing personal protective equipment and redeployment are also key concerns [24]. Frontline staff working with (possibly) infected patients have been shown to be more affected by changes in work practices and stress than healthcare professionals who work with non-COVID-19 patients [15, 13]. For detailed information on protective factors for healthcare professionals during epidemics and pandemics, see Preti *et al.* [15].

Mental health support for healthcare professionals

Various intervention strategies to improve the mental health of healthcare professionals during the COVID-19 pandemic have been proposed. For instance, healthcare providers may establish an *emotional support plan*, including strategies for information and screening, providing emotional support and building support networks [24]. Regular interaction among teams can help workers discuss critical situations and check in on each other's well-being [25]. Healthcare workers and their team leaders can also learn to apply and support self-care strategies through e-learning programmes [26]. Several countries have established telephone hotlines to provide immediate crisis support [27–29].

However, despite the severe impact of the pandemic on healthcare professionals, some staff members have proven reluctant to accept mental health support. According to Chen *et al.* [18], professionals tend to claim that they do not have any problems and just need some uninterrupted rest.

Evidence from the German health sector

In Germany, the pandemic took a somewhat different course than in many other countries. A strategy of early and extensive testing [30] resulted in less drastic lockdown measures than in other European countries [31] and avoided an overload of the health system.

Nevertheless, research shows that the pandemic and the lockdown measures affected the mental health of the German population as well. For instance, Bäuerle *et al.* [32] found a significant increase in distress, anxiety and depression symptoms and a decrease in health status since the onset of the COVID-19 outbreak in a cross-sectional study with more than 15,000 participants. These mental health effects were predicted by pandemic-related fear, while trust in the government's actions and subjective level of information predicted a weaker increase in mental strain. Higher levels of psychosocial distress, depressive symptoms and anxiety were also found in a large cross-sectional survey ($N = 3545$) focusing on the lockdown in Germany between 1 and 15 April [33]. Furthermore, the survey revealed higher levels of irritability and a decrease in sense of coherence, sexual contentment, overall well-being and sleep quality.

In a longitudinal study with four time points and 979 participants, Zacher and Rudolph [2] showed that while there were no significant changes in positive and negative affect and life satisfaction between December 2019 and March 2020, there was a significant decrease in these constructs between March and May 2020. In this latter timespan, individual differences in life satisfaction were positively correlated with positive reframing, active coping and controllability appraisals and negatively correlated with centrality appraisals, planning and threat. Negative affect was positively related to denial, substance use, centrality appraisals, threat and self-blame, and negatively related to emotional support and controllability appraisals.

In contrast to most previous studies in Germany, our study aimed to focus on healthcare professionals and the impacts of the COVID-19 pandemic on their mental health. To our knowledge, only two studies so far have looked at the mental health of healthcare professionals in Germany during the pandemic: Zerbini *et al.* [34] investigated the psychosocial burden of

Table 1. IDs and sample sizes of ISR reference groups context

	before pandemic	during pandemic
clinical sample	R_{C+P-} $N = 12\,265$	–
non-clinical sample	R_{C-P-} $N = 2512$	R_{C-P+} $N = 1744$

Table 2. Distribution of participants' professions by gender

profession	*f*	*m*	*N*	rel (%)
inpatient nursing care	92	12	104	35
inpatient elder care	23	5	28	9
non-medical health sector	21	7	28	9
physical therapist	18	8	26	9
home care	24	0	24	8
social worker	15	6	21	7
inpatient physician	6	6	12	4
physician's assistant	12	0	12	4
psychotherapist	7	1	8	3
independent physician	5	2	7	2
pharmacist	7	0	7	2
dentist, dental nurse	4	2	6	2
paramedic	1	4	5	2
midwife	1	0	1	0
other	4	3	7	2
**Total**	**240**	**56**	**296**	**100**

f, female; *m*, male; *N*, total; rel, relative percentage.
$N = 4$ participants did not indicate their gender.

healthcare professionals in a hospital in Augsburg during the COVID-19 pandemic. In this study, nurses in COVID-19 hospital units reported more stress, depressive mood and exhaustion and less work-related fulfilment than nurses in regular units. Scores for physicians, on the other hand, were similar across the different units. Uncertainty about the future and job strain were reported as the most common reasons for psychosocial burden. Resources for coping that were mentioned were psychosocial support, leisure time as well as improved structural adjustments to the pandemic in the hospital such as keeping work schedules stable.

Surprisingly, Skoda *et al.* [35] found in a cross-sectional study conducted in March 2020 at the beginning of the lockdown period in Germany that $N = 2224$ healthcare professionals, including physicians, nursing staff and paramedics, showed significantly lower levels of depression, COVID-19-related fears, generalised anxiety and higher health status than their sample of non-healthcare professionals. Similarly to Zerbini *et al.* [34], they found that nurses were the most psychologically strained among the healthcare professionals. An explanation for the lower levels of generalised anxiety in healthcare professionals more generally could relate to the fact that subjective level of information correlated negatively with generalised anxiety levels and was higher among healthcare professionals than non-healthcare professionals.

Similarly to these two studies, our study aimed to determine the mental health effects of the COVID-19 pandemic on healthcare professionals. In contrast to Zerbini *et al.* [34], who focused on a single hospital, we aimed to investigate the mental health of healthcare professionals across professions and German states. We also extended the findings of the two prior studies by investigating the underlying reasons for healthcare professionals' psychological distress and the factors that shaped their help-seeking behaviour.

Method

We created a cross-sectional online survey to assess healthcare professionals' mental well-being and the perceived stress factors they are experiencing.

Instrument

The first section of the survey explored participants' demographics such as age, gender and occupation.

For the second section, a list of potential stress factors was compiled based on literature as well as informal discussions with nurses and healthcare administrative staff. Participants were asked how strongly they were affected by each stress factor on a five-point Likert scale from *not at all* to *extremely*. Participants could also indicate if the factor *does not apply* to their situation.

Psychological well-being was measured with the self-report questionnaire ICD-10 Symptom Rating (ISR) [36]. The ISR was originally developed based on the well-known ICD-10 [37] to assess the symptoms of psychological disorders in German-speaking countries, and includes subscales for depression, anxiety, eating disorders, obsessive-compulsive disorder and somatoform disorder symptoms as well as an *extra*-subscale with various additional symptoms. It comprises a total of 29 items, which are rated on a five-point Likert scale from 0 (*does not apply*) to 4 (*extremely*). Item ratings are averaged to compute subscale scores (three to four items for each disorder and 12 items for the extra subscale) as well as a total score. ISR total scores have been shown to have very good internal consistency (Cronbach's $\alpha = .92$). The internal consistency of the subscales is also good (Cronbach's $\alpha = .78–.86$) [38]. High retest reliability for the individual scales ranging from .70 to .94 has been reported in different clinical and non-clinical samples [39]. The scale exhibits a good ability to differentiate clinical groups ($N = 12\,265$, $M = 1.22$, s.d. $= 0.65$) from non-clinical control groups ($N = 2512$, $M = 0.40$, s.d. $= 0.45$), with 84–88% sensitivity and 71–75% specificity [40]. It also highly correlates with similar but longer instruments such as the symptom checklist SCL-90-R [41].

We chose the ISR because large clinical ($C+$) and non-clinical ($C-$) norm samples are available for Germany. These were assessed before the pandemic ($P-$) [36, 42] and are referred to as reference groups R_{C+P-} and R_{C-P-} in our study. We also included a large sample of the general public ($C-$) assessed at the peak of the first wave of the pandemic ($P+$) in Germany [43][1] which we will refer to as reference group R_{C-P+} (see Table 1).

[1]We would like to thank the authors of this study for making some of their data available to us for statistical comparisons.

In the third and final section of the survey, we explored whether participants would consider using different types of mental support and which barriers to seeking help for psychological strain they had experienced.

Sample

Participants were recruited through healthcare providers, a press release and personal contacts between 22 May and 22 July 2020[2]. At that time, the most severe lockdown measures to date had been lifted again across Germany, i.e. shops and restaurants were allowed to resume operation and schools were slowly re-opening, but social distancing and other restrictions were still in place. No incentives were given for participation and no personally identifiable information was collected. Neither IP addresses nor GPS data were stored. All participants gave their informed consent for participation and for their responses to be stored electronically. We obtained institutional review board (IRB) approval from the PFH Private University of Applied Sciences Göttingen for this procedure.

A total of 300 participants completed the survey. One case was removed previously as it was identified as a test answer. No outliers were detected. The majority of participants were female (81%). Participants' age ranged between 17 and 65 years old ($M = 40.65$, s.d. = 12.9). The majority lived in Lower Saxony (59%), North Rhine-Westphalia (9%) and Hesse (9%). Most participants worked in inpatient nursing care (35.7%). Table 2 provides an overview of the distribution of occupations among participants.

Using the statistics software R (Version 4.0.2) [44] in RStudio [45] and numerous helper packages, we computed basic descriptive statistics, followed by t-tests and χ^2-tests where group comparisons were warranted. The importance of stress factors was estimated via (multiple) linear regressions after checking for the test prerequisites. Odds ratios for help-seeking were estimated using binary logistic regression. Our anonymised dataset and codebook are available for download via the Open Science Framework (OSF) website at: https://doi.org/10.17605/OSF.IO/EHM67 [46]

Results

Stress factors

Participants reported that they were most affected by protective measures to avoid spreading the virus, as these impede both patient contact and work processes in general. The pandemic also led to various changes in work procedures. Table 3 provides an overview of the stress factors mentioned.

Table 3. How strongly are you affected by the following aspect during the COVID-19 pandemic at your workplace? (0 = not at all; 4 = extremely)

stress factor	*N*	*M*	s.d.
protective measures hinder patient contact	285	2.76	1.03
protective measures hinder work processes	297	2.58	1.00
changes in work procedures	298	2.57	1.04
need for childcare in own household[a]	91	2.36	1.51
anxiety about infection of family members	290	2.30	1.25
limited contact to colleagues	287	2.18	1.13
anxiety about self-infection	285	1.78	1.18
increasing number of serious illnesses and deaths	243	1.29	1.18
job insecurity	234	1.16	1.25

N, total; *M*, mean; s.d., std. deviation.
[a]This item was presented conditional on the response to a previous question about having children; number of children not assessed.

Mental health

When analysing the ISR scores, we first checked their internal consistency. The results were almost identical to previous findings, with Cronbach's α ranging between $\alpha = .78$ and $\alpha = .9$ for the subscales and $\alpha = .94$ for the total scale.

The observed severity of clinical symptoms was high on all five scales (see Table 4 and Fig. 1). In particular, the frequency of depression and anxiety symptoms was unexpectedly high, with 9% reporting severe depression symptoms and another 65% light to medium symptoms. A total of 41% reported at least light symptoms of anxiety, while in reference group R_{C-P-}, assessed before the pandemic, only 21% displayed these levels of symptoms. We split the sample by gender (see Table 5) and by age group (see Table 6), but did not find any systematic differences between the subgroups for any of the scales (gender by severity: $\chi^2(4, N = 296) \leq 6.89$, $p > .05$; age by severity: $\chi^2(12, N = 300) \leq 19$, $p > .05$).

For all symptom scales, healthcare professionals scored significantly higher than the reference group R_{C-P-} before the pandemic (df ≥ 331.53, t ≥ 5.36, $p < .001$), but lower than the clinical group R_{C+P-} (df ≥ 314.92, t ≤ −9.08, $p < .001$). However, the increase cannot be explained by the lockdown measures alone. Healthcare professionals also showed significantly more symptoms than the general population reference group during the pandemic (R_{C-P+}) on both the depression scale ($\Delta M = 0.5$, 95% CI [0.39, 0.61], $t(2072) = 8.88$, $p < .001$) and the anxiety scale ($\Delta M = 0.35$, 95% CI [0.23, 0.47], $t(383) = 5.83$, $p < .001$). Most notably, the rate of severe symptoms was significantly higher on both the depression scale ($\chi^2(1, N = 1960) = 1022.99$, $p < .001$) and the anxiety scale ($\chi^2(1, N = 2002) = 1024.29$, $p < .001$).

Which stress factors contributed to anxiety and depression? A multiple regression of ISR depression scores on the eight stress factors ($R^2 = 0.211$, $F(8, 284) = 9.47$, $p < .001$) revealed that *job insecurity* was the single most important predictor of depression symptoms (see Table 7). While on average, participants felt that job insecurity hardly affected them ($M = 1.15$, s.d. = 1.24 on a scale from 0 to 4), high levels of job insecurity were strongly associated with psychological symptoms. *Anxiety about infection of family members* and *protective measures that hinder work processes* were also good predictors of the level of depression symptoms. A second regression for ISR anxiety scores ($R^2 = 0.16$, $F(8, 284) = 6.75$, $p < .001$) revealed a similar picture. Again, *job insecurity* and *infection of family members* were associated with anxiety symptoms. An *increasing number of serious illnesses and deaths* also contributed to anxiety (see Table 8).

Professionals with direct patient contact ($M = 0.86$) did not report more severe symptoms than those in administration (categorised based on profession, $M = 1.03$, $t(50.1) = 1.36$, $p = .91$, 1 −

[2]We would like to thank the students from the MSc Psychology and MSc Business Psychology programmes at PFH Private University of Applied Sciences Göttingen who supported data collection for this study.

Table 4. Severity of symptoms in ISR compared to the three reference groups

scale	group	none (%)	suspected (%)	light (%)	medium (%)	severe (%)
anxiety	healthcare professionals	52.3	6.3	24.7	11.7	5
	C + P–	5.4	2.5	20.2	32.1	39.8
	C – P+	70.7	5.9	12.8	7.4	3.2
	C – P–	71.8	7.2	16.1	4.2	0.7
depression	healthcare professionals	18	7.3	40	25.3	9.3
	C + P–	6.5	3.3	24.2	35.5	30.5
	C – P+	42.3	10	29.1	13.7	4.8
	C – P–	68.1	8.9	17.1	4.8	1.1
compulsion	healthcare professionals	56.7	9	22	9	3.3
	C + P–	8.8	2.6	18.4	32.9	37.3
	C – P+	67.4	7.9	13.8	6.9	4
	C – P–	75.9	8	12.8	4.2	0.7
somatoform	healthcare professionals	42.3	30.3	9	15.3	3
	C + P–	22.7	10.4	38	17.3	11.6
	C – P+	69.4	18.9	4.6	5.1	2
	C – P–	62.3	12.1	22	3.2	0.4
eating disorder	healthcare professionals	30	12	31	20.3	6.7
	C + P–	16.9	7	24.1	27	25
	C – P+	43.1	11.8	25.1	13.6	6.3
	C – P–	52.8	12	22.6	11.2	1.4
ISR total	healthcare professionals	30.3	9	20	29.3	11.3
	C + P–	12.1	4.2	15	41.5	27.2
	C – P+	58.7	6.1	14.4	15.7	5
	C – P–	68	6.8	11.5	10.2	3.5

$\beta(d = 0.5) = .91$). However, as people with pre-existing medical conditions are at higher risk during the pandemic, those with a pre-existing condition were significantly more worried ($M_{yes} = 1.13$, $M_{no} = 0.75$, $t(253.5) = 3.36$, $p < .001$) and reported more severe symptoms overall ($M_{yes} = 1.04$, $M_{no} = 0.76$, $t(260.2) = 3.88$, $p < .001$).

Help-seeking

Overall, the majority of participants described themselves as experiencing symptoms of depression and anxiety. However, when asked whether they would like to receive psychological support to deal with the crisis, most participants declined (see Table 9). Out of the 300 participants, 209 (70%) scored 0.5 or higher on the ISR total scale which is considered a *suspected* clinical diagnosis or more. However, only 84 (40%) of these participants said that they would consider seeking psychological support. A binary logistic regression revealed that participants with higher ISR scores were more likely to seek help ($b = 1.61$, OR = 5.01, $z = 6.54$, $p < .001$).

Why are healthcare professionals who supposedly need help not seeking help? One hundred (48%) of the 209 participants who were supposedly in need claimed that others needed the support more urgently. Seventy (33%) were not aware of suitable support services or offerings. Sixty-six (32%) felt that they were not distressed enough to require support. Fifty-two (25%) claimed that they did not have time to seek help. The majority–82% of all participants and 79% of those who supposedly needed help–indicated that they had sufficient social support outside the workplace.

Discussion

Consistent with reports from other countries and with our expectations, healthcare professionals in Germany reported high levels of depression and anxiety during the pandemic. Severe levels of depression symptoms were reported by 9.3% and severe symptoms of anxiety by 5% of participants. These values were similar to those reported in another German sample [34] assessed 2 months earlier under more severe lockdown conditions. Moreover, comparisons show that mental stress levels of healthcare staff were consistently above those reported by a general population sample during the pandemic and below a clinical sample prior to the pandemic; nevertheless, reported help-seeking intentions were low.

Naturally, these findings are subject to certain limitations. First, our data are cross-sectional, which limits the extent to which causal claims are possible. While it is possible for us to report to what

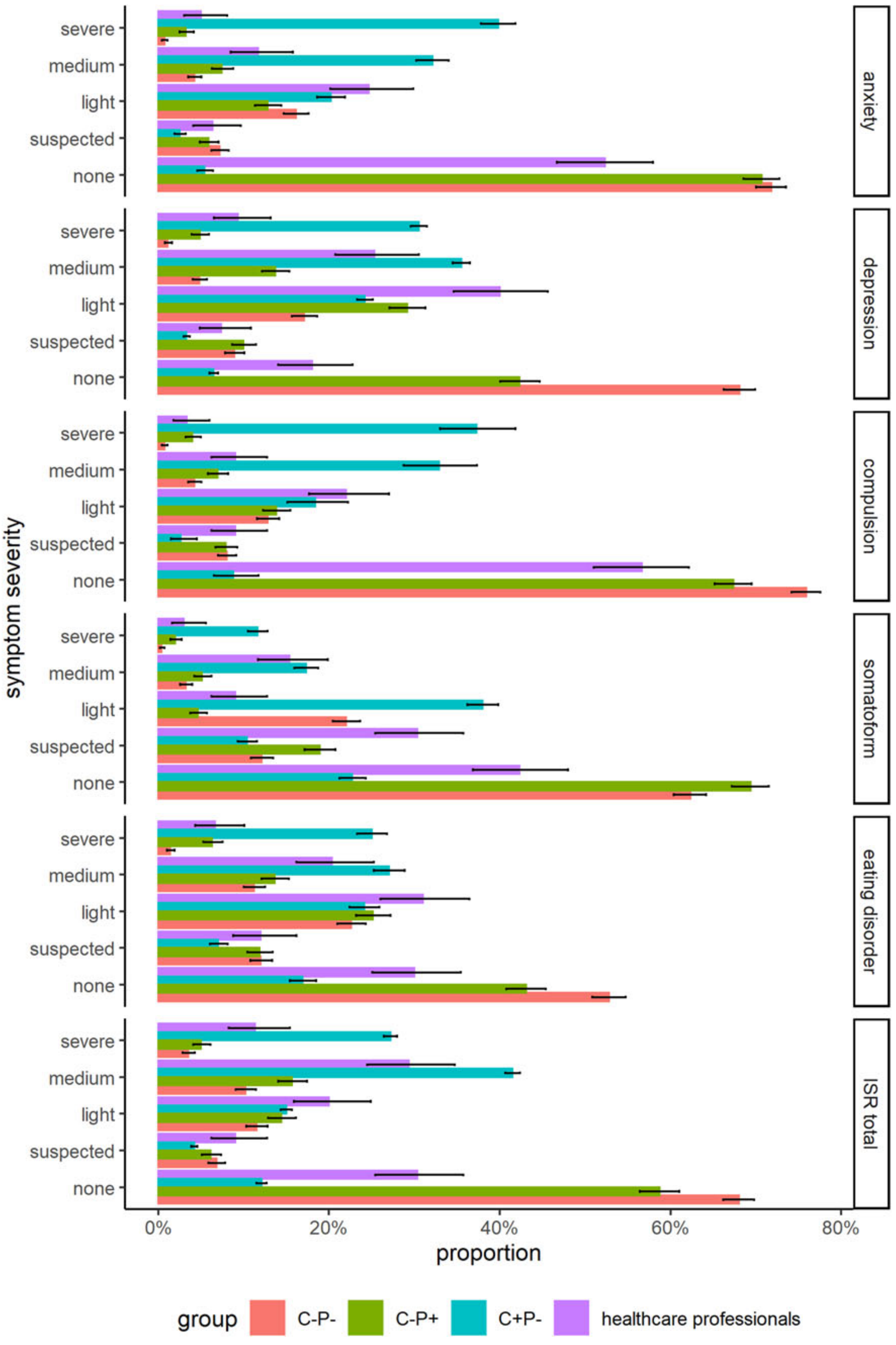

Fig. 1. Comparison of the relative frequency of severity levels for the ISR scales. Error bars indicate 95% confidence interval of the respective proportion.

Table 5. Severity of symptoms in ISR split by gender (4 participants did not indicate their gender)

scale	gender	*N*	none (%)	suspected (%)	light (%)	medium (%)	severe (%)
anxiety	female	240	50.4	7.5	23.8	13.3	5
	male	56	62.5	1.8	26.8	5.4	3.6
depression	female	240	16.7	6.7	40.8	27.5	8.3
	male	56	25	10.7	37.5	14.3	12.5
compulsion	female	240	57.5	8.3	22.1	8.8	3.3
	male	56	53.6	12.5	21.4	10.7	1.8
somatoform	female	240	43.8	29.2	8.8	15	3.3
	male	56	39.3	33.9	7.1	17.9	1.8
eating disorder	female	240	28.7	12.5	30.8	20.8	7.1
	male	56	33.9	10.7	33.9	16.1	5.4
ISR total	female	240	28.3	9.6	20.8	30	11.2
	male	56	41.1	7.1	16.1	23.2	12.5

Table 6. Severity of symptoms in ISR split by age

scale	age	*N*	none (%)	suspected (%)	light (%)	medium (%)	severe (%)
anxiety	<30	78	53.8	7.7	23.1	11.5	3.8
	30–41	74	56.8	5.4	18.9	13.5	5.4
	42–53	83	45.8	6	31.3	12	4.8
	>53	65	53.8	6.2	24.6	9.2	6.2
depression	<30	78	21.8	9	37.2	25.6	6.4
	30–41	74	12.2	9.5	41.9	24.3	12.2
	42–53	83	19.3	4.8	42.2	21.7	12
	>53	65	18.5	6.2	38.5	30.8	6.2
compulsion	<30	78	56.4	12.8	17.9	9	3.8
	30–41	74	59.5	8.1	21.6	8.1	2.7
	42–53	83	57.8	3.6	24.1	12	2.4
	>53	65	52.3	12.3	24.6	6.2	4.6
somatoform	<30	78	46.2	29.5	9	15.4	
	30–41	74	51.4	25.7	4.1	13.5	5.4
	42–53	83	36.1	33.7	14.5	12	3.6
	>53	65	35.4	32.3	7.7	21.5	3.1
eating disorder	<30	78	25.6	9	30.8	24.4	10.3
	30–41	74	37.8	9.5	27	16.2	9.5
	42–53	83	30.1	8.4	37.3	21.7	2.4
	>53	65	26.2	23.1	27.7	18.5	4.6
ISR total	<30	78	26.9	10.3	14.1	38.5	10.3
	30–41	74	33.8	8.1	20.3	21.6	16.2
	42–53	83	28.9	9.6	24.1	26.5	10.8
	>53	65	32.3	7.7	21.5	30.8	7.7

extent participants themselves think pandemic-related work-specific stressors caused deteriorations in their mental health, a true test of causality over time, both for work-related stressors and help-seeking behaviour, would require longitudinal data. Second, our data may be biased by self-selection. While the online survey was widely accessible and the survey was fairly short, thus

Table 7. Multiple regression of ISR depression scores on stress factors

predictor	*b*	95% CI	*t*(284)	
Intercept	0.45	[0.12, 0.78]	2.67	.008
changes in work procedures	0.03	[−0.07, 0.13]	0.61	.542
protective measures hinder work processes	0.12	[0.01, 0.23]	2.23	.026*
protective measures hinder patient contact	0.07	[−0.02, 0.16]	1.56	.119
limited contact to colleagues	−0.01	[−0.10, 0.07]	−0.31	.759
anxiety about self-infection	0.04	[−0.06, 0.13]	0.73	.465
anxiety about infection of family members	0.15	[0.05, 0.24]	3.03	.003**
job insecurity	0.16	[0.07, 0.24]	3.73	<.001***
increasing number of serious illnesses and deaths	0.03	[−0.05, 0.11]	0.67	.506

Note. b, unstandardised regression coefficient; CI, confidence interval; $^{*}p < .05$, $^{**}p < .01$, $^{***}p < .001$, $R^2 = 0.211$, adjusted $R^2 = 0.188$, $F(8, 284) = 9.47$, $p < .001$.

Table 8. Multiple regression of ISR anxiety scores on stress factors

predictor	*b*	95% CI	*t*(284)	*P*
Intercept	0.19	[−0.18, 0.55]	1.00	.316
changes in work procedures	−0.01	[−0.12, 0.10]	−0.17	.868
protective measures hinder work processes	0.03	[−0.09, 0.15]	0.45	.653
protective measures hinder patient contact	−0.01	[−0.11, 0.09]	−0.11	.910
limited contact to colleagues	0.03	[−0.07, 0.12]	0.60	.546
anxiety about self-infection	−0.02	[−0.12, 0.09]	−0.32	.751
anxiety about infection of family members	0.19	[0.08, 0.30]	3.55	<.001***
job insecurity	0.13	[0.04, 0.23]	2.91	.004**
increasing number of serious illnesses and deaths	0.12	[0.03, 0.21]	2.51	.012*

Note. b, unstandardised regression coefficient; CI, confidence interval; $^{*}p < .05$, $^{**}p < .01$, $^{***}p < .001$, $R^2 = 0.16$, adjusted $R^2 = 0.136$, $F(8, 284) = 6.75$, $p < .001$.

Table 9. Frequency of responses to ‘Would you like to receive psychological support to deal with the crisis?’ categorised by supposed need for support based on ISR scale

Would you seek psychological help?	not in need	in need	total
No, I am fine.	53	35	88
No, I get sufficient support.	25	54	79
No, I prefer to deal with it on my own.	6	36	42
I will consider it.	7	60	67
Yes, but not psychotherapy.	0	14	14
Yes, psychotherapy.	0	10	10
**All**	**91**	**209**	**300**

‘not in need’ means ISR < 0.5; ‘in need’ means ISR ≥ 0.5

lowering the cognitive load required to complete it, it is conceivable that the healthcare workers suffering the most did not participate because they could not muster the time or mental energy. This would imply an underestimation of actual psychological strain among healthcare staff. Finally, our findings are limited by the timing of the survey. The questionnaire was circulated after the first wave of the pandemic and after the end of the first lockdown measures, during early summer. At this point in time, a second wave was expected, but still in the distant future, and the first wave had largely died down. This, too, may bias the data towards under-representing the true strain healthcare staff were exposed to at the peak of the crisis. In countries that are currently experiencing a second wave, it has consistently hit harder than the first wave, with more hospitalisations and higher death counts. Therefore, current data should continue to be collected to examine the mental burden among healthcare professionals today and how the ongoing strain is affecting them.

The reported severity of symptoms is in line with previous studies on the mental health effects of the COVID-19 pandemic on healthcare staff from other countries [e.g. 7, 6], which, according to a review, place the prevalence of severe symptoms between 2.2% and 14.5% [8]. German healthcare workers’ level of strain is even more elevated than that of the general population during the pandemic assessed a few weeks before our sample. This is particularly concerning. As the pandemic reached its first peak across the globe, even countries that had been under-funding and neglecting their healthcare systems were made aware of the crucial importance of healthcare workers.

Politicians and members of the general public expressed their appreciation for *absolutely essential* workers–with frontline medical staff representing a key group. In light of this, it seems crucial to take measures to reduce psychological strain among these

workers–particularly because both the pandemic and the consequences of the psychological distress caused by it are expected to persist for the foreseeable future. To demonstrate the consequences by example: depression and fatigue are correlated with major medical errors. In a study with doctors in training, the odds ratio associated with a positive depression screening was OR = 2.22, i.e., those screened positively were twice as likely to report a major medical error [47].

However, in spite of the severity of reported psychological strain, reported help-seeking behaviour and help-seeking intentions were consistently low. Many healthcare professionals are not seeking help, citing either concerns about the distribution of resources (i.e. based on the assumption that a limited amount of psychological support is available, they stated that others needed it more than they did, they themselves were not distressed enough to require support, and/or they already had a sufficient support network) or accessibility issues (not having the time to seek help, not being aware of a service that meets their needs).

This points to two underlying issues: First, while numerous employers, public agencies and non-profit organisations hurried to create mental health services to support healthcare staff during the pandemic, e.g. telephone hotlines like *Talk2Us* [29] and free-of-charge psychotherapy provided to nursing staff by the German professional association for nursing staff [48], it seems that these were not accessible enough overall or did not meet healthcare staff's perceived needs. One issue was most likely awareness; since most of these support services were set up at the beginning of the pandemic and had thus only been active for a few months at the time we conducted our survey, they were not yet well-known and established among healthcare workers. This would explain why about one-third of our sample claimed to not be aware of a suitable service. Just over a quarter of participants stated they did not have time to seek help, which might indicate that current support offerings do not fit with demand. Future research should investigate how support services need to be designed to meet healthcare staff's needs, e.g. by being flexible to suit staff's schedules, being less time-consuming than current services are perceived to be, and being more easily accessible.

Second, the large portions of our sample claiming that they were not in need of support despite severely elevated levels of mental strain, that they already had sufficient support and that others needed it more urgently seem to be indicative of a climate that discourages help-seeking behaviour and speaking out about mental health issues in the healthcare community. A work culture pervaded by a general expectation to prioritise patient care before personal well-being and to refrain from acts that could be interpreted as displaying weakness, such as admitting to being overwhelmed or seeking professional help [49], could be a crucial factor inhibiting help-seeking behaviour.

It is crucial that future research also investigates the work climate and culture in the healthcare sector and the norms they set around mental health; exploratory qualitative studies seem warranted. While social norms of this kind are complex and slow to change, it is crucial they be identified and addressed, because if help-seeking behaviour truly is widely stigmatised in the healthcare community, improving the accessibility of support services alone is bound to have very limited effects on the rates of healthcare workers seeking help. What our data, alongside various other studies, have done is establish that there is a need to provide mental health support to the healthcare community; the question that research must target next is why and when this need does and does not translate into uptake of support.

Data availability statement

Our anonymised dataset and codebook are available for download via the Open Science Framework (OSF) website at: https://doi.org/10.17605/OSF.IO/EHM67

References

1. **Cox CL** (2020) Healthcare heroes: problems with media focus on heroism from healthcare workers during the COVID-19 pandemic. *Journal of Medical Ethics* **46**, 510–513.
2. **Zacher H and Rudolph CW** (2020) Individual differences and changes in subjective wellbeing during the early stages of the COVID-19 pandemic. *American Psychologist* **76**, 50–62. doi: 10.1037/amp0000702 .
3. **Krammer S *et al.*** (2020) Adjustment disorder, depression, stress symptoms, Corona related anxieties and coping strategies during the Corona pandemic (COVID-19) in Swiss medical staff. *PPmP – Psychotherapie Psychosomatik Medizinische Psychologie* **70**, 272–282.
4. **Du J *et al.*** (2020) Psychological symptoms among frontline healthcare workers during COVID-19 outbreak in Wuhan. *General Hospital Psychiatry* **67**, 144–145. doi: 10.1016/j.genhosppsych.2020.03.011 .
5. **Lu W *et al.*** (2020) Psychological status of medical workforce during the COVID-19 pandemic: a cross-sectional study. *Psychiatry Research* **288**, 112936.
6. **Liu CY *et al.*** (2020) The prevalence and influencing factors in anxiety in medical workers fighting COVID-19 in China: a cross-sectional survey. *Epidemiology & Infection* **148**, e98.
7. **Kang L *et al.*** (2020) Impact on mental health and perceptions of psychological care among medical and nursing staff in Wuhan during the 2019 novel coronavirus disease outbreak: a cross-sectional study. *Brain, Behavior, and Immunity* **87**, 11–17.
8. **Bohlken J *et al.*** (2020) COVID-19 pandemic: stress experience of healthcare workers – a short current review. *Psychiatrische Praxis* **47**, 190–197.
9. **Lai J *et al.*** (2020) Factors associated with mental health outcomes among health care workers exposed to coronavirus disease 2019. *JAMA Network Open* **3**, e203976.
10. **Makino M *et al.*** (2020) Mental health crisis of Japanese health care workers under COVID-19. Psychological trauma: theory, research. *Practice, and Policy* **12**, S136–S137.
11. **Barello S, Palamenghi L and Graffigna G** (2020) Burnout and somatic symptoms among frontline healthcare professionals at the peak of the Italian COVID-19 pandemic. *Psychiatry Research* **290**, 113129. doi: 10.1016/j.psychres.2020.113129 .
12. **Temsah MH *et al.*** (2020) The psychological impact of COVID-19 pandemic on health care workers in a MERS-CoV endemic country. *Journal of Infection and Public Health* **13**, 877–882.
13. **Miller K** (2020) Mental and emotional risks of frontline healthcare providers involved in responding to COVID-19 pandemic. *Psychosociological Issues in Human Resource Management* **8**, 19–24.
14. **El-Hage W *et al.*** (2020) Health professionals facing the coronavirus disease 2019 (COVID-19) pandemic: what are the mental health risks? *L'Encephale* **46**, S73–S80.
15. **Preti E *et al.*** (2020) The psychological impact of epidemic and pandemic outbreaks on healthcare workers: rapid review of the evidence. *Current Psychiatry Reports* **22**, 43.
16. **Hall H** (2020) The effect of the COVID-19 pandemic on healthcare workers' mental health. *Journal of the American Academy of Physician Assistants* **33**, 45–48.
17. **Shanafelt T, Ripp J and Trockel M** (2020) Understanding and addressing sources of anxiety among health care professionals during the COVID-19 pandemic. *Journal of the American Medical Association* **323**, 2133–2134.
18. **Chen Q *et al.*** (2020) Mental health care for medical staff in China during the COVID-19 outbreak. *The Lancet Psychiatry* **7**, e15–e16.
19. **Satici B *et al.*** (2020) Intolerance of uncertainty and mental wellbeing: serial mediation by rumination and fear of COVID-19. *International Journal of Mental Health and Addiction*. doi: 10.1007/s11469- 020-00305-0.

20. **Xiao H *et al.*** (2020) The effects of social support on sleep quality of medical staff treating patients with coronavirus disease 2019 (COVID-19) in January and February 2020 in China. *Medical Science Monitor: International Medical Journal of Experimental and Clinical Research* **26**, e923549–1.
21. **Jex SM and Bliese PD** (1999) Efficacy beliefs as a moderator of the impact of work-related stressors: a multilevel study. *Journal of Applied Psychology* **84**, 349.
22. **Champion VL and Skinner CS** (2008) The health belief model. In Glanz K, Rimer BK and Viswanath K (eds), *Health Behavior and Health Education: Theory, Research, and Practice*, 4th Edn. Hoboken, New Jersey: John Wiley & Sons, pp. 45–65.
23. **Ho SM *et al.*** (2005) Fear of severe acute respiratory syndrome (SARS) among health care workers. *Journal of Consulting and Clinical Psychology* **73**, 344.
24. **Miotto K *et al.*** (2020) Implementing an emotional support and mental health response plan for healthcare workers during the COVID-19 pandemic. *Psychological Trauma: Theory, Research, Practice, and Policy* **12**, 165–167.
25. **Greenberg N *et al.*** (2020) Managing mental health challenges faced by healthcare workers during COVID-19 pandemic. *BMJ* **368**, 1–4. doi: 10.1136/bmj.m1211 .
26. **Blake H *et al.*** (2020) Mitigating the psychological impact of COVID-19 on healthcare workers: a digital learning package. *International Journal of Environmental Research and Public Health* **17**, 1–15. doi: 10.3390/ijerph17092997 .
27. **Feinstein RE *et al.*** (2020) A health care workers mental health crisis line in the age of COVID-19. *Depression and Anxiety* **37**, 822–826. doi: 10.1002/da.23073.
28. **Gonzalez A *et al.*** (2020) Supporting health care workers during the COVID-19 pandemic: mental health support initiatives and lessons learned from an academic medical center. *Psychological Trauma: Theory, Research, Practice, and Policy* **12**, 168–170.
29. **Reiter J and Duden G** (2020) Talk2Us website. Available at https://www.talk2us.de/ (Accessed 2020 Oct 11).
30. **Cohen J and Kupferschmidt K** (2020) Countries test tactics in 'war' against COVID-19. *Science (New York, N.Y.)* **367**, 1287–1288.
31. **Torri E *et al.*** (2020) Italian public health response to the COVID-19 pandemic: case report from the field, insights and challenges for the department of prevention. *International Journal of Environmental Research and Public Health* **17**, 3666.
32. **Bäuerle A *et al.*** (2020) Mental health burden of the COVID-19 outbreak in Germany: predictors of mental health impairment. *Journal of Primary Care & Community Health* **11**, 1–8. doi: 10.1177/2150132720953682 .
33. **Jung S, Kneer J and Krüger THC** (2020) Mental health, sense of coherence, and interpersonal violence during the COVID-19 pandemic lockdown in Germany. *Journal of Clinical Medicine* **9**, 3708, 1–8.
34. **Zerbini G *et al.*** (2020) Psychosocial burden of healthcare professionals in times of COVID-19 – a survey conducted at the University Hospital Augsburg. *GMS German Medical Science* **18**, 1–9. doi: 10.3205/000281.
35. **Skoda EM *et al.*** (2020) Psychological burden of healthcare professionals in Germany during the acute phase of the COVID-19 pandemic: differences and similarities in the international context. *Journal of Public Health* **42**, 688–695.
36. **Tritt K *et al.*** (2008) Development of the ICD-10–Symptom-Rating (ISR) questionnaire. *Zeitschrift für Psychosomatische Medizin und Psychotherapie* **54**, 409–418.
37. **World Health Organization** (2004) *ICD-10: International Statistical Classification of Diseases and Related Health Problems*, vol. **1**. Geneva: World Health Organization.
38. **Fischer HF *et al.*** (2010) Factor structure and psychometric properties of the ICD-10-Symptom-Rating (ISR) in samples of psychosomatic patients. *PPmP – Psychotherapie Psychosomatik Medizinische Psychologie* **60**, 307–315.
39. **Fischer HF *et al.*** (2010) Retest-reliability and sensitivity to change of the ICD-10–Symptom-Rating (ISR) in different samples. *PPmP – Psychotherapie Psychosomatik Medizinische Psychologie* **61**, 162–169.
40. **Tritt K *et al.*** (2015) ICD-10–Symptom-Rating (ISR) – Das Handbuch zum Fragebogen. neobooks.
41. **Schmitz N *et al.*** (2000) The Symptom Check-List-90-R (SCL-90-R): a German validation study. *Quality of Life Research* **9**, 185–93.
42. **Tritt K *et al.*** (2010) Der Fragebogen ICD-10–Symptom-Rating (ISR) – Kurzdarstellung der Normierung. Available at http://www.iqp-online.de/index.php?page=download.
43. **Schelhorn I *et al.*** (2020) Depression symptoms during the COVID-19 pandemic in different regions in Germany. doi: 10.31234/osf.io/p9wz8 . Available at psyarxiv.com/p9wz8.
44. **R Core Team** (2020) *R: A Language and Environment for Statistical Computing*. Vienna, Austria: R Foundation for Statistical Computing. Available at https://www.R-project.org/.
45. **RStudio Team** (2020) *RStudio: Integrated Development Environment for R. RStudio*. Boston, MA: PBC. Available at http://www.rstudio.com/.
46. **Weibelzahl S, Duden G and Reiter J** (2020) Pandemic-induced psychological stress in healthcare professionals. doi: 10.17605/OSF.IO/EHM67 . Available at osf.io/ehm67.
47. **West CP *et al.*** (2009) Association of resident fatigue and distress with perceived medical errors. *JAMA* **302**, 1294–1300. eprint: https://jamanetwork.com/journals/jama/articlepdf/184625/joc90102_1294_1300.pdf. Available at https://doi.org/10.1001/jama.2009.1389 .
48. **Deutscher Berufsverband für Pflegeberufe [German professional association for nursing staff]** (2020) Psych4Nurses website. Available at https://www.dbfk.de/de/themen/psych4nurses.php (Accessed 2020 Oct 11).
49. **Galbraith ND, Brown KE and Clifton E** (2014) A survey of student nurses' attitudes toward help seeking for stress. *Nursing Forum* **49**, 171–181.
