# Supplementary material for: Mental health status and related factors influencing healthcare workers during the COVID-19 pandemic: A systematic review and meta-analysis
Source: PLoS One. 2024 Jan 19;19(1):e0289454. doi: 10.1371/journal.pone.0289454 (PMC10798549; doi:10.1371/journal.pone.0289454)
Supplement: S1 Data — (ZIP) [file pone.0289454.s011.zip › literatures/213.pdf]

LGBT Health
Volume 9, Number 5, 2022
© Mary Ann Liebert, Inc.
DOI: 10.1089/lgbt.2021.0276

Short Report

Open camera or QR reader and
scan code to access this article
and other resources online.

Mental Health Disparities Among Sexual and Gender Minority Frontline Health Care Workers During the Height of the COVID-19 Pandemic

Hailey Wojcik, MA,[1,2] Aaron S. Breslow, PhD,[2,3] Marla R. Fisher,[2,3] Caryn R.R. Rodgers, PhD,[2,4]
Patryk Kubiszewski,[2,3] and Vilma Gabbay, MD[3,5]

Abstract

Purpose: This study measured mental health disparities in a Bronx, New York sample of frontline health care workers collected May–July, 2020, during the first wave of the COVID-19 pandemic.
Methods: Using survey data ($N = 741$), we compared demographics, COVID-19 stressors, and adverse mental health outcomes between sexual and gender minority (SGM, $n = 102$) and non-SGM ($n = 639$) health care workers through chi-square/Kruskal–Wallis tests, crude/adjusted odds, and prevalence ratios.
Results: SGM frontline health care workers had significantly higher depression, anxiety, impact of COVID-19, and psychological distress. Income (lower), age (younger), and COVID-19 stressors accelerated differences.
Conclusion: Health care systems should support SGM frontline health care workers through affirming trauma-informed programming.

Keywords: gender identity, health disparities, mental health, sexual orientation

Introduction

Sexual and gender minority (SGM) communities are disproportionately burdened by adverse mental health outcomes, largely due to minority stress.[1–3] COVID-19 has likely exacerbated these disparities, yet we are only beginning to understand how this global pandemic may uniquely affect SGM adults, particularly those working in health care.[4,5] This inquiry is critical given increased prevalence of anxiety, depression, and alcohol use in this population when compared with cisgender heterosexual people (i.e., non-SGM people).[6]

These disparities were exacerbated due to the COVID-19 pandemic, particularly during the first wave in spring 2020, due to reduced capacity of SGM community centers[4,7] and inadequate sexuality- and gender-based workplace protections.[8] In emerging studies, SGM communities have reported unique stressors due to the COVID-19 pandemic, such as living with nonaffirming families,[9] interruptions to affirming mental health and gender care,[4] and a lack of safe affordable housing.[10,11] The impact is disparate within SGM communities, as Black SGM people were hardest hit by unemployment (18%) and reduced work hours (31%).[8]

These factors are an immediate concern given high suicide rates pre-COVID-19, with national attempt prevalence around 25% across adult sexual minority samples[12] and 30%–81% across adult gender minority samples (compared with an estimated 0.6% prevalence in the general population of adults in the United States).[13] Another vulnerable cohort for psychiatric sequelae is frontline health care workers,

[1]Department of Psychology, The City College of New York, New York, New York, USA.
[2]PRIME Center for Health Equity, Department of Psychiatry and Behavioral Sciences, Albert Einstein College of Medicine, Bronx, New York, USA.
[3]Psychiatry Research Institute at Montefiore Einstein, Department of Psychiatry and Behavioral Sciences, Albert Einstein College of Medicine, Bronx, New York, USA.
[4]Department of Pediatrics, Albert Einstein College of Medicine, Bronx, New York, USA.
[5]Nathan Kline Institute for Psychiatric Research, Orangeburg, New York, USA.
*These authors are co-first authors.

who face major stressors, including lack of autonomy, sudden redeployment, inadequate access to personal protective equipment (PPE), and fear of disease.[14] In one study, 33% of frontline health care workers had moderate anxiety, 17% had depression, and 5% reported suicidal ideation.[15]

Little is known about disparities between health care workers, including among SGM health care workers who live and work at the intersection of minority stress and vocational risk.[16] One recent study ($n = 277$) with SGM health care workers revealed workplace minority stressors, including working with politically and/or religiously conservative colleagues, lack of knowledge about SGM identities, and identity concealment from colleagues and patients.[17] Another study with gender minority health care workers found stressors unique to transphobic stigma, 78% censored themselves to avoid being outed at work, 69% heard colleagues disparage transgender patients, and a third witnessed a colleague discriminate against or refuse care to transgender patients.[18]

Concerns often emerge in medical school.[19] In a 2021 survey with medical school graduates, 29.5% of sexual minority and 60% of gender minority respondents reported hiding their identities due to fear of discrimination and lack of social support.[20]

SGM frontline health care workers may also demonstrate minority-specific protective factors (i.e., resilience and radical acceptance,[21,22] having a justice-oriented mindset)[23] that may buffer the impact of pandemic-related stress. For example, they may be better-equipped (or, conversely, affected by trauma-related stress) due to community proximity to the ongoing HIV/AIDS crisis.[24] Possibly as a result of having to navigate risk prepandemic, SGM people in the United States seem to be more accepting of COVID-19 vaccines than the general population.[25] As SGM presence in medicine is growing, with 0.7% of 2021 graduates identifying as trans, 4.3% gay/lesbian, and 5% bisexual,[20] it is important to assess how this future generation is faring during this ongoing global crisis.

This study thus aims to capture COVID-19-era differences between SGM and non-SGM frontline health care workers, using survey data collected during the height of the pandemic at a Bronx, NY hospital. Data were collected at Montefiore Medical Center (Montefiore), a large urban hospital system with >2.6 million annual ambulatory visits, 100 locations, and nearly 300,000 emergency visits in a typical year.[26] During the spring of 2020, Montefiore was hit hard by COVID-19, with the first affected patients admitted March 11, 2020.[26] The Bronx had the highest rates of COVID-19 infections, hospitalizations, and deaths of the five New York City boroughs, driven by systemic racism and economic inequality.[27] The Bronx is home to a large SGM population, and Montefiore is a site with an emerging SGM workforce.

This study thus aims to complement emerging data on SGM mental health during this crisis,[5] and inform strategies to support SGM people, who often work and live with multiple marginalized populations.[11,14]

Methods

Data

Data were collected May 4 through July 10, 2020 using an online Qualtrics.com survey. The sample included 741 health care workers at Montefiore, recruited on internal listservs through e-mails from clinical leads, department chairs, and union groups. Those who clicked on a survey link were informed of participant rights and clicked ''Yes'' to consent. Participants who consented completed a questionnaire (average completion time = 32.56 minutes) about demographics, COVID-19 stressors, and adverse mental health outcomes. Overall, 966 people consented; 741 (76.71%) answered all questions about items of interest, including adverse mental health outcomes and COVID-19 stressors. Procedures were approved by the Institutional Review Board at Albert Einstein College of Medicine, Protocol No. 2020-11469.

Measures

Three types of data were collected. First, we collected demographic characteristics, including gender, ''How would you describe your gender identity?''; sexual orientation, ''How do you identify your sexual orientation?''; race, ''How would you describe your race/ethnicity?''; income, ''What is your annual income?''; and age, ''How old are you?'' See Table 1 for response options.

Second, we measured experiences of COVID-19 stressors through five dichotomous (Yes/No) questions: ''Do you feel scared you may be sick with COVID-19?''; ''Do you feel scared you may infect your family, friends, and/or other people you live with?''; ''Were you deployed to an area different from where you usually work to work directly with COVID-19 patients?''; ''Was there a time when you felt a lack of autonomy in your work related to the COVID-19 crisis?''; and ''Was there a time when you did not have adequate access to PPE?''

Third, we captured seven adverse mental health outcomes: depression (using the 9-item Patient Health Questionnaire-9 [PHQ-9]),[28] anxiety (using the 7-item Generalized Anxiety Disorder-7 Scale [GAD-7])[29]; post-traumatic stress (using the 5-item Primary Care Posttraumatic Stress Disorder (PTSD) Screen for DSM-5 [PC-PTSD])[30]; impact of COVID-19 (using an adapted version of the 22-item Impact of Event Scale [IES])[31]; insomnia (using the 7-item Insomnia Severity Index [ISI])[32]; psychological distress (using the 6-item Kessler 6 [K-6])[33]; and alcohol use (using the 3-item Alcohol Use Disorders Identification Test-Concise [AUDIT-C]).[34]

Participants who met criteria for any adverse mental health outcome(s) were provided with referrals for web-based mental health resources and free confidential services at Montefiore, including an emotional support telephone line and an in-person Caregiver Support Center.[26]

Using self-reported sexual orientation and gender identity (SOGI) data, we split the sample into two subgroups: SGM, $n = 102$ and non-SGM, $n = 639$. We combined participants in these two categories to measure potential common experiences of SGM stressors among SGM participants. Although imperfect, this method led to the creation of a subsample of 102 SGM health care workers which enabled us to measure their experiences in comparison with non-SGM (i.e., cisgender and heterosexual) health care workers.

Participants were assigned to the SGM subgroup in the case they clicked at least one sexual minority identity (i.e., asexual, bisexual, lesbian/gay, pansexual, queer, same-gender loving, and unsure/questioning), and/or at least one gender minority identity (i.e., transgender man, transgender woman,

TABLE 1. DIFFERENCES IN DEMOGRAPHIC CHARACTERISTICS AND COVID-19 STRESSORS BETWEEN SEXUAL AND GENDER MINORITY AND NON SEXUAL AND GENDER MINORITY HEALTH CARE WORKERS

	SGM (n = 102)	*Non-SGM* (n = 639)	*Bivariate comparisons*
	n (%)	n (%)	χ^2
Demographic characteristics			
Gender			
Cisgender man	29 (17.2)	140 (21)	**92.178*****
Cisgender woman	43 (42.2)	454 (71)	
Genderqueer, nonbinary	6 (5.9)	—	
Other	24 (23.5)	32 (5)	
Chose not to answer	—	13 (2)	
Sexual orientation			
Asexual	25 (24.5)	—	**732.589*****
Bisexual	20 (19.6)	—	
Heterosexual	1 (0.2)	638 (99.8)	
Lesbian/gay	39 (38.2)	—	
Pansexual	2 (2)	—	
Queer	7 (6.9)	—	
Same-gender loving	1 (1)	—	
Unsure/questioning	6 (5.9)	—	
Chose not to answer	1 (1)	1 (0.2)	
Race			
White	48 (47.1)	354 (55.4)	4.303
Black/African American	11 (10.8)	58 (9.1)	
Latinx/Hispanic	18 (17.6)	81 (12.7)	
Asian/Asian American	15 (14.7)	103 (16.1)	
Multi/Biracial, Other	10 (9.8)	43 (6.7)	
Annual income			
$0–60,000	28 (27.7)	86 (13.6)	**13.942****
$60,001–80,000	21 (20.8)	133 (21)	
$80,001–100,000	12 (11.9)	85 (13.4)	
$100,000+	40 (39.6)	328 (51.9)	
Age,[a] median (IQR)	29 (20.25–42)	29 (20–44)	**4.341***
COVID-19 stressors			
1. Scared you may have COVID-19	38 (40.9)	179 (30.1)	**4.281***
2. Scared you will infect others	61 (59.8)	429 (67.1)	2.111
3. Redeployed to a COVID-19 unit	28 (27.7)	176 (27.8)	0
4. Lack of autonomy at work	56 (60.9)	322 (54.4)	1.351
5. Inadequate PPE	54 (58.7)	316 (53.3)	0.938

Some values are <741 due to missing data. % = valid percent within subsample. For gender, no participants selected transgender man, transgender woman, or Two-Spirit so these have been removed from the table. Bold = significance. *$p<0.05$; **$p<0.01$; ***$p<0.001$.

[a]Kruskal–Wallis test.

df, degrees of freedom; IQR, interquartile range; non-SGM, non sexual and gender minority; PPE, personal protective equipment; SGM, sexual and gender minority.

Two-Spirit, genderqueer/nonbinary). Participants who clicked ''Other'' or chose not to answer were only included in the SGM subsample if they also clicked one or more of the above sexual minority and/or gender minority identities. Participants who did not click any of these identities were included in the non-SGM subsample.

Statistical analyses

Data were analyzed using SPSS version 27 (IBM Corporation, Armonk, NY, 2020). First, we estimated frequencies of demographics and the five COVID-19 stressors (Table 1). Chi-square tests were used for categorical variable comparisons and Kruskal–Wallis test for age ($\alpha = 0.05$), followed by *z*-score *post hoc* tests to identify factor-level differences. Second, we created dichotomous variables for adverse mental health outcomes to indicate whether participants met the clinical cutoff for each scale (Table 2 and Fig. 1). We used standard cutoff scores with the exception of the AUDIT-C, which has a cutoff of 3 for women and 4 for men.

To analyze data in the most gender-inclusive way possible, we used the lower cutoff score of 3 for the AUDIT-C. We measured *n*/% within each subgroup ≥cutoff for each scale, and crude (unadjusted) odds ratios (ORs) regressing SGM. Third, we calculated adjusted ORs (aORs) for each mental health outcome (cutoff), regressing SGM and adjusting for non-SOGI demographics (i.e., race, income, and age) and COVID-19 stressors (Table 3 and Fig. 2). We did not include sexual orientation or gender identity as covariates because these variables' SOGI data were used to create separate SGM and non-SGM subsamples, and thus results of this analysis would have had no meaning.

Finally, we calculated adjusted prevalence ratios (APRs) by computing a count variable summing the number of

Table 2. Differences in Prevalence and Odds for Adverse Mental Health Outcomes Between Sexual and Gender Minority and Non Sexual and Gender Minority Health Care Workers

Adverse mental health outcome	*Scale*	*Cutoff*	*SGM* (n = 102) n *(%)* ≥*Cutoff*	*Non-SGM* (n = 639) n *(%)* ≥*Cutoff*	*Univariate unadjusted ORs among SGM (Ref: non-SGM)* *OR (95% CI)*
Depression	PHQ-9	10	33 (35.9)	123 (20.7)	**2.137**** **(1.336–3.420)**
Anxiety	GAD-7	7	45 (48.9)	191 (32.9)	**1.955**** **(1.254–3.047)**
Post-traumatic stress	PC-PTSD	3	38 (41.3)	195 (33.5)	1.397 (0.891–2.189)
Impact of COVID-19	IES	33	39 (43.3)	180 (31.6)	**1.657*** **(1.054–2.606)**
Insomnia	ISI	15	15 (16.7)	78 (13.7)	1.262 (0.69–2.307)
Psychological distress	K6	13	18 (20.0)	60 (10.5)	**2.125*** **(1.188–3.802)**
Alcohol use	AUDIT-C	3	47 (52.2)	260 (45.5)	1.307 (0.838–2.041)

This table contains descriptive statistics of each group's prevalence ≥cutoff score for each adverse mental health outcome, as well as unadjusted (crude) ORs and 95% confidence intervals regressing sexual and gender minority (SGM; ref: non-SGM) on each adverse mental health outcome. Bold indicates significance. *$p<0.05$; **$p<0.01$; ***$p<0.001$.

AUDIT-C, Alcohol Use Disorders Identification Test-Concise; CI, confidence interval; GAD-7, Generalized Anxiety Disorder-7 Scale; IES, Impact of Event Scale; ISI, Insomnia Severity Index; K6, Kessler 6; OR, odds ratio; PC-PTSD, Primary Care Posttraumatic Stress Disorder Screen for DSM-5; PHQ-9, Patient Health Questionnaire-9.

adverse mental health outcome scales on which participants met cutoff (range: 0–7). We ran a Poisson log-linear model with SGM as factor and non-SOGI demographics/COVID-19 stressors as covariates. We did not correct for multiple comparisons in our adjusted model. Given SGM health care workers are an understudied potentially vulnerable group, we prioritized avoiding Type II errors (i.e., missing potential disparities) over avoiding Type I errors. We present crude ORs, then aORs, controlling for demographics and COVID-19 stressors, then report corrected results using a Bonferroni correction ($\alpha = 0.006$, or 0.05/8 tests) in the text.

Results

Demographic characteristics and COVID-19 stressors

See Table 1 for descriptive statistics and group comparisons. We found significant bivariate associations between SGM status and gender ($p<0.001$); sexual orientation ($p<0.001$); annual income ($p=0.003$), with SGM health care workers significantly more likely to make $0–60,000 and less likely to make $100,000+; and age ($p=0.037$), with SGM participants skewing younger. Among COVID-19 stressors, SGM participants were significantly more likely to fear they may have COVID-19 ($p=0.039$).

Higher unadjusted odds

Descriptive statistics and crude ORs for each mental health outcome are listed in Table 2 and visualized in Figure 1. SGM participants had higher raw prevalence of all adverse mental health outcomes. Analyses revealed significantly higher unadjusted odds for depression (OR = 2.137, $p=0.002$, 35.9% vs. 20.7%), anxiety (OR = 1.955, $p=0.003$, 48.9% vs. 32.9%), impact of COVID-19 (OR = 1.657, $p=0.029$, 43.3% vs. 31.6%), and psychological distress (OR = 2.125, $p=0.011$, 20.0% vs. 10.5%).

Higher adjusted odds

aORs for each adverse mental health outcome, controlling for demographics and COVID-19 stressors, are presented in

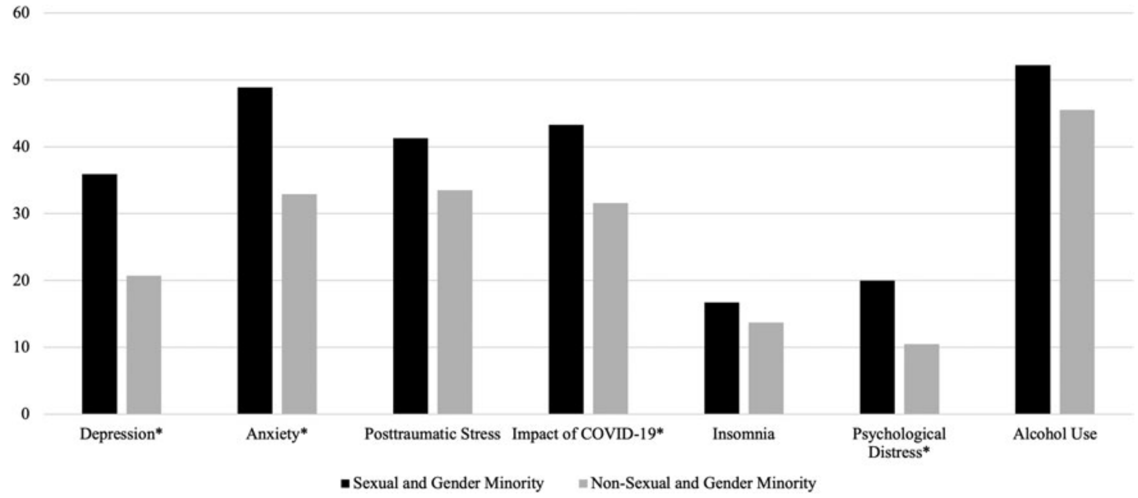

FIG. 1. Unadjusted prevalence of adverse mental health outcomes among sexual and gender minority and non sexual and gender minority frontline health care workers. *Indicates significant difference between groups at the 0.05 level in terms of univariate unadjusted odds ratios.

Table 3. Adjusted Odds Ratios and Adjusted Prevalence Ratios for Adverse Mental Health Outcomes

	Depression	Anxiety	Post-traumatic stress	Impact of COVID-19	Insomnia	Psychological distress	Alcohol use	Prevalence risk
				aOR (95% CI)				*APR (95% CI)*
SGM status (Reference: non-SGM)								**1.301**** **(1.100–1.471)**
SGM	**1.887**** **(1.127–3.161)**	**1.807**** **(1.096–2.976)**	1.197 (0.729–1.964)	1.539 (0.933–2.537)	1.149 (0.604–2.186)	1.848 (0.993–3.437)	1.355 (0.839–2.189)	
Race (Reference: White)								0.969 (0.910–1.032)
Black	0.727 (0.356–1.486)	0.681 (0.348–1.333)	1.042 (0.556–1.951)	1.446 (0.767–2.726)	0.781 (0.337–1.810)	0.584 (0.208–1.639)	**0.326***** **(0.174–0.611)**	
Latinx/Hispanic	0.940 (0.521–1.696)	1.516 (0.883–2.601)	1.524 (0.899–2.583)	1.629 (0.948–2.801)	1.221 (0.623–2.396)	1.311 (0.637–2.698)	**0.277***** **(0.160–0.478)**	
Asian	0.702 (0.387–1.273)	1.102 (0.664–1.826)	0.850 (0.514–1.406)	0.832 (0.494–1.399)	0.571 (0.268–1.220)	1.226 (0.601–2.499)	**0.348***** **(0.217–0.559)**	
Multi/Biracial	1.199 (0.576–2.495)	1.275 (0.631–2.579)	1.076 (0.544–2.128)	0.740 (0.356–1.541)	0.884 (0.356–2.195)	0.794 (0.2820–2.237)	**0.206***** **(0.099–0.430)**	
Income (Reference: $100k+)								**1.178**** **(1.040–1.334)**
$0–60k	**2.372**** **(1.299–4.330)**	**1.864*** **(1.060–3.276)**	1.105 (0.631–1.936)	1.520 (0.856–2.700)	1.510 (0.703–3.243)	**3.147**** **(1.511–6.556)**	1.103 (0.642–1.896)	
$60,001–80k	1.087 (0.621–1.901)	1.142 (0.695–1.876)	0.964 (0.593–1.566)	1.010 (0.611–1.668)	1.488 (0.768–2.884)	1.325 (0.638–2.749)	0.684 (0.428–1.093)	
$80,001–100k	1.656 (0.921–2.977)	**1.987*** **(1.153–3.425)**	1.179 (0.692–2.008)	1.699 (0.989–2.917)	**2.385*** **(1.221–4.660)**	1.593 (0.728–3.486)	0.719 (0.424–1.221)	
Age (Reference: younger)								**0.989***** **(0.983–0.995)**
Older	**0.981*** **(0.963–1.000)**	**0.979*** **(0.963–0.996)**	**0.973**** **(0.957–0.989)**	0.990 (0.973–1.006)	0.994 (0.973–1.017)	0.998 (0.975–1.022)	0.990 (0.975–1.004)	
COVID-19 Stressors (Reference: no)								
1. Scared you may have.	1.359 (0.884–2.090)	1.269 (0.854–1.885)	1.396 (0.952–2.049)	1.272 (0.859–1.886)	1.326 (0.795–2.214)	0.971 (0.552–1.709)	1.338 (0.909–1.970)	**1.192*** **(1.042–1.362)**
2. Scared you may infect.	1.153 (0.704–1.888)	**1.923**** **(1.224–3.020)**	**1.628*** **(1.048–2.530)**	**1.913**** **(1.209–3.026)**	1.043 (0.573–1.900)	0.916 (0.495–1.696)	0.890 (0.599–1.321)	**1.345**** **(1.136–1.593)**
3. Redeployed to COVID-19.	1.434 (0.943–2.180)	1.256 (0.853–1.850)	1.293 (0.886–1.887)	**1.478*** **(1.007–2.168)**	1.392 (0.850–2.281)	1.016 (0.583–1.773)	0.970 (0.670–1.405)	**1.186**** **(1.038–1.356)**
4. Lack of autonomy.	**2.658***** **(1.695–4.169)**	**3.010***** **(2.033–4.455)**	**1.551*** **(1.066–2.258)**	**1.957**** **(1.326–2.890)**	**2.756**** **(1.554–4.890)**	**1.986*** **(1.105–3.569)**	1.334 (0.928–1.916)	**1.767***** **(1.521–2.052)**
5. Inadequate PPE	1.360 (0.880–2.102)	0.966 (0.654–1.426)	**1.702**** **(1.164–2.489)**	1.416 (0.959–2.090)	1.135 (0.673–1.916)	1.555 (0.873–2.772)	0.733 (0.508–1.057)	**1.219**** **(1.056–1.407)**

aORs and APRs, adjusted for demographics and COVID-19 stressors. Bold = significance.
*$p < 0.05$; **$p < 0.01$; ***$p < 0.001$.
aORs, adjusted odds ratios; APRs, adjusted prevalence ratios.

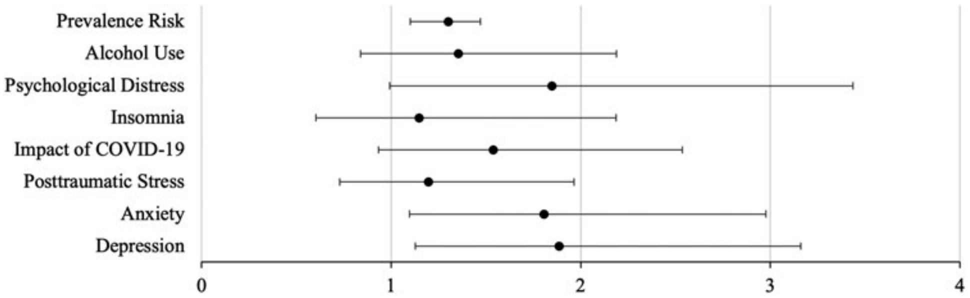

FIG. 2. Adjusted odds ratios of adverse mental health outcomes for sexual and gender minority (reference: non sexual and gender minority) frontline health care workers, adjusting for demographics and COVID-19 stressors.

Table 3 and visualized in Figure 2. After adjusting, SGM health care workers remained at significantly higher odds for depression (aOR = 1.887, $p = 0.016$) and anxiety (aOR = 1.807, $p = 0.02$), although no longer for impact of COVID-19 or psychological distress. Post-traumatic stress, insomnia, and alcohol use remained nonsignificant.

In *post hoc* analyses, we used the Bonferroni correction ($\alpha = 0.006$) to determine if SGM frontline health care workers had significantly higher odds for adverse mental health outcomes after correcting for multiple comparisons. SGM health care workers still had significantly higher prevalence risk for more adverse mental health outcomes overall ($p = 0.002$), although they no longer demonstrated significantly higher odds for depression ($p = 0.016$) or anxiety ($p = 0.02$) at this lower-threshold p-value for multiple comparisons. It thus may be that the SGM group had higher risk overall, although specific manifestations of disparities were unclear.

We also examined odds for each adverse mental health outcome by race, income, age, and COVID-19 stressors, and found multiple significant associations. Odds for depression were significantly higher among participants who earned $0–60,000 (aOR = 2.372, $p = 0.005$), were younger (aOR = 0.981, $p = 0.049$), and reported lack of autonomy at work (aOR = 2.658, $p < 0.001$). Odds for anxiety were significantly higher among those earning either $0–60,000 (aOR = 1.864, $p = 0.030$) or $80,001–100,000 (aOR = 1.987, $p = 0.013$), younger workers (aOR = 0.979, $p = 0.015$), those afraid of infecting others with COVID-19 (aOR = 1.923, $p = 0.005$) or reporting lack of autonomy at work (aOR = 3.010, $p < 0.001$).

Odds for post-traumatic stress were significantly higher for younger workers (aOR = 0.973, $p = 0.001$), and reporting fear of infecting others (aOR = 1.628, $p = 0.030$), lack of autonomy (aOR = 1.551, $p = 0.022$), and inadequate PPE (aOR = 1.702, $p = 0.006$). Post-traumatic stress was the only outcome for which inadequate access to PPE played a significant role. Odds for impact of COVID-19 were not significantly associated with any demographics, although were higher among those who endorsed fear of infecting others (aOR = 1.913, $p = 0.006$), redeployment (aOR = 1.478, $p = 0.046$), and lack of autonomy (aOR = 1.957, $p = 0.001$). Impact of COVID-19 was the only outcome for which redeployment played a significant role.

Odds for insomnia were significantly higher among workers earning $80,001–100,000 (aOR = 2.385, $p = 0.011$) and reporting lack of autonomy (aOR = 2.756, $p = 0.001$). Odds for psychological distress were significantly higher among workers earning $0–60,000 (aOR = 3.147, $p = 0.002$) and those reporting a lack of autonomy at work (aOR = 1.986, $p = 0.022$). Alcohol use was the only outcome with significant odds based on participant race. Specifically, health care workers of color, across groups, had significantly lower odds than White participants (Black aOR = 0.326; Latinx/Hispanic aOR = 0.277; Asian/Asian American aOR = 0.348; Multi/Biracial, Other aOR = 0.206, $p < 0.001$ for all).

Higher adjusted prevalence risk

APRs, controlling for demographics and COVID-19 stressors, are detailed in Table 3. The model was a good fit ($\chi^2 = 233.361$, $p < 0.001$); all factors except race met significance. SGM health care workers had higher risk for more adverse mental health outcomes overall, with an adjusted prevalence risk of 1.301 ($p = 0.002$). Earning below $100k was associated with higher risk when compared to earning $100k+ (APR = 1.178, $p = .010$), whereas older age was associated with lower risk (APR = 0.989, $p < 0.001$). All five COVID-19 stressors were significantly associated with higher risk.

Discussion

In this study, SGM frontline health care workers reported a disparately high burden of adverse mental health outcomes during the first wave of the COVID-19 pandemic. In particular, the SGM group had statistically higher odds for depression and anxiety, even when controlling for sociodemographic differences and COVID-19-related stressors. These disparities may be explained in part by differences in income (lower), age (younger), and experiencing COVID-19 stressors (increased likelihood of fear of infecting others with COVID-19 and lack of autonomy at work). Before adjusting, SGM frontline health care workers had 2.137 times higher odds for depression, 1.955 for anxiety, 1.657 for impact of COVID-19, and 2.125 for psychological distress.

After adjusting, higher odds for depression (1.887) and anxiety (1.807) remained significant. This was explained in part by differences in income, age, and lack of autonomy at work. Overall, corroborating recent data that SGM frontline health care workers may experience additional stress,[4,6,17] our SGM subgroup had a 1.301 times greater adjusted risk prevalence. Lower income, younger age, and all five COVID-19 stressors had increased risk. Despite recent findings that SGM health care workers may cope with workplace stigma and stress through alcohol use,[17] we did not find associations between SGM identity and AUDIT-C scores. However, White frontline health care workers had higher odds for alcohol use than their colleagues of color.

Two of the main findings (i.e., higher odds for depression and anxiety among SGM frontline health care workers) were

no longer significant after correcting for multiple comparisons. There are multiple explanations for this. First, this may be a result of Type I error. Second, the difference between uncorrected and correct results may be due to the small sample size of SGM frontline health care workers ($n = 102$). Third, COVID-19-related stress may have been so ubiquitously stressful for everyone in the sample[35] that statistically significant differences based on SOGI data were not easy to detect. We thus echo calls for further research with larger, more diverse samples to investigate how SGM health care workers are doing.[17]

This study adds to the growing literature on vulnerabilities for SGM people[22] and health care workers[14,15] during the pandemic. The results echo calls for affirming nuanced programming for SGM health care workers targeting depression, anxiety, impact of the pandemic, and psychological distress. This is especially urgent given both the continuing duration of the pandemic and the increase of SGM representation in medicine.[20]

Limitations and future directions

Our study procedure had multiple limitations. First, we did not capture information about potential SGM-specific mechanisms accelerating higher rates of adverse mental health outcomes.[8,10,11] We do not intend to frame SGM identity as a risk factor. Rather, we assume the preponderance of minority stressors within medicine (perhaps felt even more strongly during a pandemic) may drive differences between these two subgroups.[19] Second, survey administration by the participants' home institution may have muted or amplified social desirability bias.

Third, we included response options for transgender women and men, although no participants identified as such (5.9% identified as genderqueer, nonbinary; 28.5% as other), perhaps evidence of structural barriers to employment for transgender people in medicine.[18] Because our data are convenience sampled and cross-sectional, we cannot make causal or longitudinal claims. Fourth, we did not capture ameliorative processes unique to SGM frontline health care workers.[21]

Our analyses had limitations. First, participants did not say whether they were ''SGM,'' but were coded as such through SOGI data, reflecting researcher bias. The term ''SGM'' has inherent semantic and ideological limitations, given it is researcher-driven rather than a term that is commonly used in the community. We assume this term does not encapsulate participants' experiences.

Second, there are inherent limitations in how we aggregated multiple subgroups of SGM people (i.e., gay men, lesbian women, and nonbinary people) into a combined SGM subsample. Doing so increased power and enabled us to measure common impacts of minority stress. However, we were unable to report within-SGM disparities and hope future studies with larger samples will examine subgroup differences. We also may be inflating the significance of results that may not have been evident in disaggregated samples, per the Simpson's paradox.[36]

Third, we analyzed the AUDIT-C using the cutoff of 3 [to avoid binary cutoffs for men (4) vs. women (3)], perhaps inflating prevalence. Fourth, we found significant differences in terms of income, wherein SGM health care workers were significantly more likely to make less money. We did not control for role/position. That said, if there are existing role/position differences between SGM and non-SGM health care workers, these differences would be demonstrative of economic disparities worth measuring and mitigating.

Finally, we made the decision, in line with previous SGM studies,[37] not to be overly conservative in correcting for multiple comparisons in our primary analyses because we were more worried about false negatives (i.e., missing potentially important differences between SGM and non-SGM groups) than false positives. Given depression and anxiety did not remain significant after correcting for family-wise error rate in *post hoc* analyses, it is possible these results were spurious, or we lacked power due to sample size. We maintain the importance of the results given the paucity of data specific to SGM health care worker mental health.

Conclusion

Despite marketing campaigns declaring ''we're all in this together'' and naming hospital workers ''health care heroes,'' COVID-19 and associated stressors disproportionately impacted marginalized groups, even among providers. This includes SGM health care workers, who in this sample experienced significantly higher rates of adverse mental health outcomes when compared with cisgender, heterosexual health care workers in this study. These findings bolster calls for affirming interventions to support SGM health care workers at hard-hit hospitals.

Acknowledgments

We thank Dr. Benjamin Lê Cook, Xinyu Yang, and Brian Mullin for their help in the development of a statistical analysis plan. We thank Sherry Simkovic for her help preparing and submitting the Institutional Review Board proposal and assisting in building the survey tool. We also acknowledge Drs. Jonathan Alpert, Sriya Bhattacharyya, and Carol Bernstein for establishing COVID-19 Emotional Support services at Montefiore Medical Center and for their help conceptualizing this project. Finally, we thank all health care workers for their dedication to prevention, treatment, and care during this difficult time.

Authors' Contributions

Conceptualization, data curation, formal analysis, methodology, writing—original draft preparation, and writing—review and editing by H.W. Conceptualization, data curation, formal analysis, investigation, methodology, writing—original draft preparation, and writing—review and editing by A.S.B. Methodology, writing—original draft preparation, and writing—review and editing by M.R.F. Writing—original draft preparation and writing—review and editing by C.R.R. and P.K. Conceptualization, data curation, investigation, methodology, supervision, and writing—original draft preparation by V.G.

Disclaimer

The content is solely the responsibility of the authors and does not necessarily represent the official views of the National Institutes of Health.

Author Disclosure Statement

No competing financial interests exist.

Funding Information

A.S.B.'s contributions to the research reported in this publication were supported in part by the National Institute of Mental Health of the National Institutes of Health under award number K23MH128582 for which A.S.B. is principal investigator. V.G.'s contributions to the research reported in this publication were supported in part by the National Institutes of Mental Health of the National Institutes of Health under award numbers R01MH120601, R21MH121920, R21MH126501, and R01MH126821 for which V.G. is principal investigator, as well as by the Department of Psychiatry and Behavioral Sciences at Albert Einstein College of Medicine.

References

1. Meyer IH: Resilience in the study of minority stress and health of sexual and gender minorities. Psychol Sex Orientat Gend Divers 2015;2:209–213.
2. James SE, Herman JL, Rankin S, et al.: *The Report of the 2015 U.S. Transgender Survey*. Washington, DC: National Center for Transgender Equality, 2016.
3. Medley G, Lipari RN, Bose J, et al.: Sexual orientation and estimates of adult substance use and mental health: Results from the 2015 National Survey on Drug Use and Health. 2016. Available at www.samhsa.gov/data/sites/default/files/NSDUHSexualOrientation-2015/NSDUH-SexualOrientation-2015/NSDUH-SexualOrientation-2015.htm Accessed November 29, 2021.
4. Gato J, Barrientos J, Tasker F, et al.: Psychosocial effects of the COVID-19 pandemic and mental health among LGBTQ+ young adults: A cross-cultural comparison across six nations. J Homosex 2021;68:612–630.
5. Drabble L, Eliason M: Introduction to special issue: Impacts of the COVID-19 pandemic on LGBTQ+ health and well-being. J Homosex 2021;68:1–15.
6. Salerno JP, Williams ND, Gattamorta KA: LGBTQ populations: Psychologically vulnerable communities in the COVID-19 pandemic. Psychol Trauma 2020;12:S239–S242.
7. Pachankis JE, Clark KA, Jackson SD, et al.: Current capacity and future implementation of mental health services in US LGBTQ community centers. Psychiatr Serv 2021; 72:669–676.
8. Human Rights Campaign: The economic impact of COVID-19 on Black LGBTQ people. 2020. Available at www.hrc.org/resources/the-economic-impact-of-covid-19-on-black-lgbtq-people Accessed November 29, 2021.
9. Fish JN, McInroy LB, Paceley MS, et al.: "I'm kinda stuck at home with unsupportive parents right now": LGBTQ youths' experiences with COVID-19 and the importance of online support. J Adolesc Health 2020;67:450–452.
10. Chatterjee S, Biswas P, Guria RT: LGBTQ care at the time of COVID-19. Diabetes Metab Syndr 2020;14:1757–1758.
11. Human Rights Campaign: The lives and livelihoods of many in the LGBTQ community are at risk amidst COVID-19 crisis. 2020. Available at www.hrc.org/resources/the-lives-and-livelihoods-of-many-in-the-lgbtq-community-are-at-risk-amidst-covid-19-crisis Accessed November 29, 2021.
12. Meyer IH, Russell ST, Hammack PL, et al.: Minority stress, distress, and suicide attempts in three cohorts of sexual minority adults: A US probability sample. PLoS One 2021;16: e0246827.
13. Narang P, Sarai SK, Aldrin S, Lippmann S: Suicide among transgender and gender-nonconforming people. Prim Care Companion CNS Disord 2018;20:18nr02273.
14. Shanafelt T, Ripp J, Trockel M: Understanding and addressing sources of anxiety among health care professionals during the COVID-19 pandemic. JAMA 2020;323:2133–2134.
15. Young KP, Kolcz DL, O'Sullivan DM, et al.: Health care workers' mental health and quality of life during COVID-19: Results from a mid-pandemic, national survey. Psychiatr Serv 2021;72:122–128.
16. Gacita A, Gargus E, Uchida T, et al.: Introduction to safe space training: Interactive module for promoting a safe space learning environment for LGBT medical students. MedEdPORTAL 2017;13:10597.
17. Eliason MJ, Streed C Jr, Henne M: Coping with stress as an LGBTQ+ health care professional. J Homosex 2018;65: 561–578.
18. Dimant OE, Cook TE, Greene RE, Radix AE: Experiences of transgender and gender nonbinary medical students and physicians. Transgend Health 2019;4:209–216.
19. Mansh M, White W, Gee-Tong L, et al.: Sexual and gender minority identity disclosure during undergraduate medical education: "In the closet" in medical school. Acad Med 2015;90:634–644.
20. Association of American Medical Colleges: Medical school graduation questionnaire: 2021 all schools summary report. 2021. Available at www.aamc.org/media/55736/download Accessed November 29, 2021.
21. Gonzalez KA, Abreu RL, Arora S, et al.: "Previous resilience has taught me that I can survive anything": LGBTQ resilience during the COVID-19 pandemic. Psychol Sex Orientat Gend Divers 2021;8:133–144.
22. Goldbach C, Knutson D, Milton DC: LGBTQ+ people and COVID-19: The importance of resilience during a pandemic. Psychol Sex Orientat Gend Divers 2020;8:123–132.
23. Jen S, Stewart D, Woody I: Serving LGBTQ+/SGL elders during the Novel Corona Virus (COVID-19) pandemic: Striving for justice, recognizing resilience. J Gerontol Soc Work 2020;63:607–610.
24. Santo N: Reflections on the HIV/AIDS Crisis, COVID-19, and resilience in gay men: Ghosts of our past, demons of our present. In: *Shared Trauma, Shared Resilience During a Pandemic: Social Work in the Time of COVID-19*. Edited by Tosone C. Cham, Switzerland: Springer International Publishing, 2021, pp 127–133.
25. Tegan and Sara Foundation: US LGBTQ+ community and the COVID-19 vaccine: Attitudes and drivers. 2021. Available at www.teganandsarafoundation.org/tsf-covid-survey Accessed November 29, 2021.
26. Bernstein CA, Bhattacharyya S, Adler S, Alpert JE: Staff emotional support at Montefiore Medical Center during the COVID-19 pandemic. Jt Comm J Qual Patient Saf 2021;47:185–189.
27. Wadhera RK, Wadhera P, Gaba P, et al.: Variation in COVID-19 hospitalizations and deaths across New York City boroughs. JAMA 2020;323:2192–2195.
28. Kroenke K, Spitzer RL: The PHQ-9: A new depression diagnostic and severity measure. J Gen Intern Med 2001;16: 606–613.

29. Spitzer RL, Kroenke K, Williams JB, Löwe B: A brief measure for assessing generalized anxiety disorder: The GAD-7. Arch Intern Med 2006;166:1092–1097.
30. Prins A, Ouimette P, Kimerling R, et al.: The Primary Care PTSD Screen for DSM-5 (PC-PTSD-5): Development and evaluation within a veteran primary care sample. J Gen Intern Med 2016;31:1206–1211.
31. Horowitz M, Wilner N, Alvarez W: Impact of Event Scale: A measure of subjective stress. Psychosom Med 1979;41: 209–218.
32. Bastien CH, Vallières A, Morin CM: Validation of the Insomnia Severity Index as an outcome measure for insomnia research. Sleep Med 2001;2:297–307.
33. Kessler RC, Andrews G, Colpe LJ, et al.: Short screening scales to monitor population prevalences and trends in non-specific psychological distress. Psychol Med 2002;32: 959–976.
34. Bush K, Kivlahan DR, McDonell MB, et al.: The AUDIT alcohol consumption questions (AUDIT-C): An effective brief screening test for problem drinking. Ambulatory Care Quality Improvement Project (ACQUIP). Alcohol Use Disorders Identification Test. Arch Intern Med 1998; 158:1789–1795.
35. Shechter A, Diaz F, Moise N, et al.: Psychological distress, coping behaviors, and preferences for support among New York healthcare workers during the COVID-19 pandemic. Gen Hosp Psychiatry 2020;66:1–8.
36. Ashley F: Simpson's Paradox in LGBTQ+ policy: A case study. Sex Res Soc Policy 2021;18:800–804.
37. Braitman AL, Lewis RJ, Derlega VJ, Wilson SA: Minority stressors and dual identities: An analysis of lesbians' expressive writing journals. J Lesbian Stud 2008;12:501–517.

Address correspondence to:
Hailey Wojcik, MA
Department of Psychology
The City College of New York
160 Convent Avenue
North Academic Center, Room 101
New York, NY 10031
USA

E-mail: hwojcik000@citymail.cuny.edu
