# Supplementary material for: Mental health status and related factors influencing healthcare workers during the COVID-19 pandemic: A systematic review and meta-analysis
Source: PLoS One. 2024 Jan 19;19(1):e0289454. doi: 10.1371/journal.pone.0289454 (PMC10798549; doi:10.1371/journal.pone.0289454)
Supplement: S1 Data — (ZIP) [file pone.0289454.s011.zip › literatures/215.pdf]

Journal of Psychiatric Research 137 (2021) 673–680

Contents lists available at ScienceDirect

Journal of Psychiatric Research

journal homepage: www.elsevier.com/locate/jpsychires

Pandemic-related mental health risk among front line personnel

Hannah M. Wright [a], Brandon J. Griffin [b,c], Kotaro Shoji [d,e,f], Tiffany M. Love [a], Scott A. Langenecker [a], Charles C. Benight [e,f], Andrew J. Smith [a,f,g,*]

[a] University of Utah School of Medicine, USA
[b] Central Arkansas VA Health Care System, USA
[c] University of Arkansas for Medical Sciences, USA
[d] Gifu University of Medical Science, Japan
[e] University of Colorado Colorado Springs, USA
[f] National Institute for Human Resilience, USA
[g] Salt Lake City VA Healthcare System, USA

ARTICLE INFO

Keywords:
COVID-19
Frontline responders
Mental health
Acute traumatic stress

ABSTRACT

The mental health of frontline workers is critical to a community's ability to manage crises and disasters. This study assessed risks for mental health problems (traumatic stress, depression, anxiety, alcohol use, insomnia) in association with pandemic-related stressors in a sample of emergency and hospital personnel ($N = 571$). Respondents completed self-report surveys online from April 1st to May 7th, 2020 in the Rocky Mountain region of the United States. Results showed that roughly fifteen to thirty percent of respondents screened positive for each disorder. Odds of screening positive were similar between groups for probable acute traumatic stress, depressive disorder, anxiety disorder, and alcohol use disorder; emergency personnel reported significantly higher rates of insufficient sleep than healthcare workers. Logistic regressions showed that respondents who reported having an immunocompromised condition had higher odds of acute traumatic stress, anxiety, and depression. Having an immunocompromised household member was associated with higher odds of insufficient sleep and anxiety. Being in a direct care provision role was associated with higher odds of screening positive for risky alcohol use. Being in a management role over direct care providers was associated with higher odds of screening positive for anxiety, risky alcohol use, and insufficient sleep. There was an inverse relationship between number of positive COVID-19 cases and anxiety, such that as positive cases went up, anxiety decreased. Overall, the mental health risks that we observed early in the COVID-19 pandemic are elevated above previous viral outbreaks (SARS) and comparable to rates shown in disasters (9/11 attacks; Hurricane Katrina).

1. Introduction

On January 30th, 2020, the World Health Organization declared the COVID-19 pandemic to be a 'public health emergency of international concern' (World Health Organization, 2020). The virus threatened to overwhelm hospital systems across the U.S. with 206,000 confirmed cases (Johns Hopkins University, 2020) and 6200 deaths by April. During the pandemic, communities have relied on the support of frontline responders including emergency personnel (e.g., fire service, law enforcement, and emergency medical personnel) and healthcare workers (e.g., physicians, nurses, and mental health professionals) who are tasked with caring for and interacting with potentially infected persons. As such, the mental health of emergency and medical personnel is critical to public health and national adaptation and recovery efforts.

During typical non-crisis times, emergency personnel are at increased risk for psychiatric disorders, including depression (Berg et al., 2006; Chiu et al., 2010; Fullerton et al., 2004; Hartley et al., 2011; Stanley et al., 2018) and posttraumatic stress disorder (PTSD; Boffa et al., 2017; Boffa et al., 2018; Kim et al., 2018). These risks increase when emergency personnel respond to events that are extraordinarily stressful and potentially traumatic (Fullerton et al., 2004), such as natural disasters (Osofsky et al., 2011) and terrorist attacks (Soo et al., 2011; Luft et al., 2012). Healthcare professionals are also at increased risk of professional burnout (see Aiken et al., 2001; Aiken et al., 2002), but generally are at lower risk for mental health disorders relative to emergency responders. Certain medical specialties, however, carry a

* Corresponding author. University of Utah School of Medicine, Department of Psychiatry, 501 Chipeta Way, Salt Lake City, UT, 84018, USA.
E-mail address: andrew.james.smith@hsc.utah.edu (A.J. Smith).

https://doi.org/10.1016/j.jpsychires.2020.10.045
Received 28 August 2020; Received in revised form 28 October 2020; Accepted 30 October 2020
Available online 4 November 2020
0022-3956/© 2020 Elsevier Ltd. All rights reserved.

higher risk for stress-related disorders as a rational function of exposure to higher stress environments (e.g., emergency medicine; Mealer et al., 2009; Shanafelt et al., 2012).

Research conducted during previous viral crises (SARS epidemic) showed that hospital employees were at high risk for PTSD (Wu et al., 2009) and reported a high prevalence of general anxiety/worry and depression (Chong et al., 2004). Consistent with this literature, emergent research in the current COVID-19 pandemic shows that hospital personnel have increased risk for anxiety and depression (Baker et al., 2020; Cai et al., 2020; Lai et al., 2020; Mohindra et al., 2020; Walton et al., 2020). Notably, mental health among emergency personnel (fire and police service members) has not been studied in response to medical disasters such as a viral pandemic. Taken together, there is a clear need to assess mental health problems among emergency and healthcare personnel during the COVID-19 pandemic.

In the current study, we examined the likelihood of screening positive for acute traumatic stress, depression, anxiety, risky alcohol use, and insufficient sleep among emergency and hospital personnel. Based on empirical precedent summarized above from COVID-19 and SARS contexts, we hypothesized that a minority of respondents would demonstrate psychiatric risks, but that the psychiatric risks would be elevated compared to estimates obtained during non-crisis times. Additionally, we hypothesized that psychiatric risks would increase in association with four pandemic-related stressors, which were assessed based on a combination of previous empirical support and the phenomenology of stressors experienced in the midst of a dynamic pandemic environment (e.g., performing one's job as a frontline responder while lacking information and knowledge, protective and therapeutic resources, and rapidly changing guidelines from the Center for Disease Control). Those four pandemic stressors were: (1) having direct contact with potentially infected persons (Kang et al., 2020; Lai et al., 2020; Tam et al., 2004); (2) managing personnel who are in direct contact with potentially infected persons (based on the phenomenology of stress associated with making leadership decisions with potential health consequences for personnel); (3) being immunocompromised oneself (Novel Coronavirus Pneumonia Emergency Response Epidemiology Team, 2020), and; (4) having a household member who is immunocompromised (see Adams and Walls, 2020).

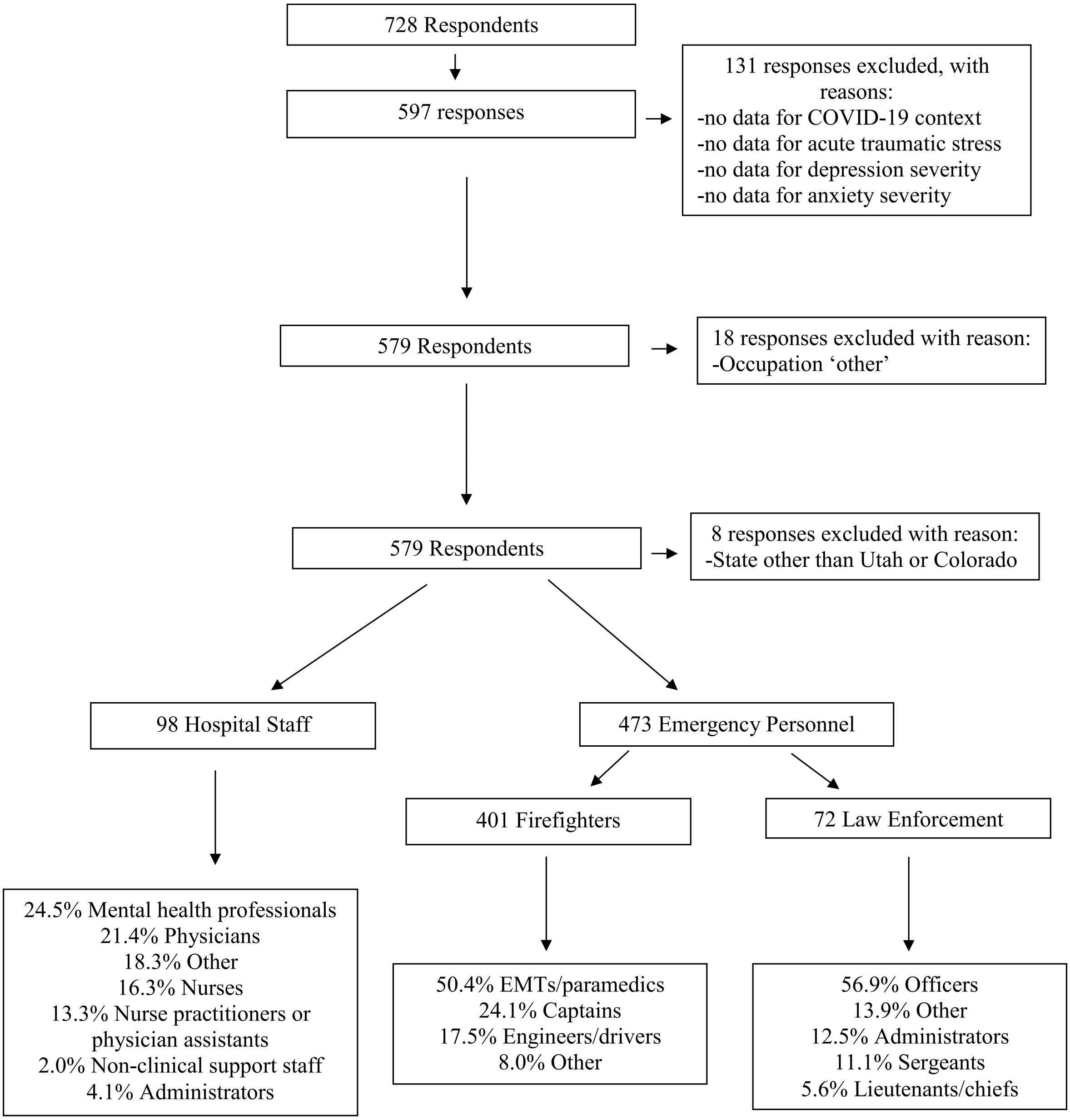

Fig. 1. Data cleaning/case removal processes and a breakdown of professional roles within each category of participants.

2. Methods

2.1. Subjects

A total of 571 participants (81.0% male, mean age = 42.93 [SD = 11.02]) were included for analyses, comprised of 82.8% emergency personnel (n = 473 [401 firefighters; 72 law enforcement]) and 17.2% hospital staff (n = 98). See Fig. 1 for data cleaning/case removal processes and a breakdown of professional roles within each category of participants.

2.2. Procedures

Study approval was obtained from IRBs at the University of Utah and University of Colorado Colorado Springs prior to survey administration. Respondents who completed the electronic survey between April 1st, 2020 to May 7th, 2020 were from three fire service agencies, one law enforcement agency, and one Veterans Affairs Medical Center. Fig. 2 displays the overall number of reported cases by date during the survey period in the two primary counties where respondents worked: Salt Lake County, UT and El Paso County, CO. During the time period in which the survey was active, total COVID-19 cases increased from 396 to 2912 in Salt Lake County and from 286 to 1055 in El Paso County, constituting less than 1% of the population positive in each country. Reported number of positive COVID-19 cases was obtained from the Johns Hopkins Coronavirus Resource Center (Johns Hopkins Univeristy, 2020; https://coronavirus.jhu.edu/map.html).

2.3. Materials

Acute traumatic stress. The PTSD Checklist for DSM-5 (PCL-5; Weathers et al., 2012) is a 20-item self-report measure answered on a 5-point Likert scale (1 = *not at all* to 5 = *extremely*). All 20 items were summed into a total score, and the recommended cutoff of 33 was used to indicate probable stress-related disorder (Blevins et al., 2015). Note that participants were not required to endorse a core trigger traumatic event (Criterion A) prior to completing this measure. Internal consistency was high for this sample (Cronbach's α = 0.95).

Depression. The Patient Health Questionnaire-8 (PHQ-8; Kroenke et al., 2009) is answered on a 4-point Likert scale (0 = *not at all* to 3 = *nearly every day*) and excludes the item that assesses suicidal ideation. For this study, the eight items were summed to obtain a total score, and a recommended clinical cutoff score of 10 was used to indicate probable (e.g., moderate) depressive disorder (Kroenke et al., 2009). Internal consistency in this study was high (Cronbach's α = 0.89).

Anxiety. The Generalized Anxiety Disorder scale (GAD-7; Spitzer et al., 2006) is a 7-item measure answered on a four-point Likert scale (0 = not at all to 3 = nearly every day). A total sum score was utilized and a recommended clinical cutoff of 10 was used to indicate probable anxiety disorder. Internal consistency in this study was high (Cronbach's α = 0.92).

Alcohol Use and Sleep. The Alcohol Use Disorders Identification Test - Consumption Questions (AUDIT-C; Bush et al., 1998) is a 3-item measure to assess frequency and quantity of alcohol use. The questions and answering scale were adapted to refer to alcohol use "within the past month" to prevent conflation in follow-up longitudinal research (assessments occurring monthly) as part of a larger study. A total sum score was utilized and a recommended clinical cutoff of 4 for men and 3 for women was used to indicate increased risk for alcohol use disorder (Bush et al., 1998). Internal consistency was acceptable for this sample (Cronbach's α = 0.71). For sleep, participants reported the number of hours of sleep per night when off duty, with insufficient sleep defined as fewer than 6 h of sleep per night (Soderstrom, Jeding, Ekstedt, Perski, Akerstedt, 2012).

Pandemic-related stressors. Four questions assessed exposure to pandemic-related stressors answered using a yes/no response format. Question 1 asked if respondents were "directly engaged in responding to people with elevated temperatures or people with confirmed COVID-19" (Lai et al., 2020). Question 2 asked if they 'manage personnel who have direct contact with patients who might be infected with COVID-19?' Questions 3 and 4 asked respondents 'do you have a compromised immune system due to a medical condition' and 'does someone in your household have a compromised immune system due to a medical condition.'

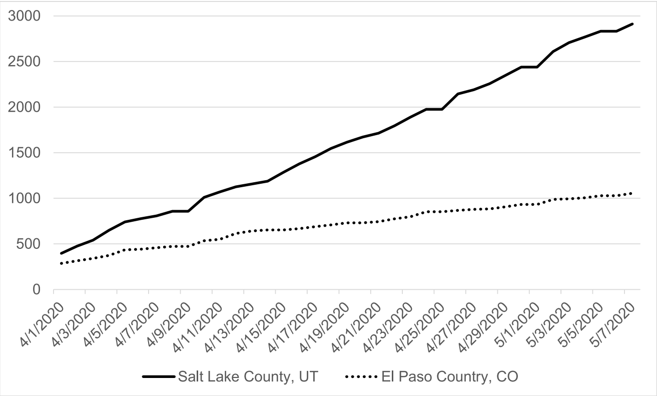

Fig. 2. Overall number of positive COVID-19 cases reported by county. Number of positive cases represents less than 1% of the overall population for each county.

2.4. Data analyses

Data Handling. Percentages of missing data for the main outcomes were 0.3% for acute traumatic stress reactions, 0.4% for depression severity, and 0.4% for anxiety severity. To evaluate patterns of missing data, we conducted tests of missing completely at random (MCAR; Jamshidian and Jalal, 2010); MCAR refers to the probability of missingness not depending on either observed variables nor unobserved variables (Schafer and Graham, 2002). Results of the MCAR test showed a significant Hawkins test ($p < .001$), but the non-parametric test was not significant ($p = .16$), indicating that the missing data for acute traumatic stress items were MCAR. For depression severity, the Hawkins test was significant ($p < .001$), and the non-parametric test was not significant ($p = .38$), indicating MCAR. For anxiety severity, the Hawkins test was significant ($p < .001$), but the non-parametric test was not significant ($p = .12$), indicating MCAR. Missing data were imputed by a singular value decomposition-based method (Troyanskaya et al., 2001). The singular value decomposition is a robust imputation method for relatively high percentage of missing data (up to 10–15%). Notably, alcohol (missing 0.2%) and sleep (missing 4.4%) were not imputed due to the small number of questions used to assess (3 for alcohol and 1 for sleep, respectively), as well as their behavioral/quantity nature ('how much' alcohol and sleep); listwise deletion was used to handle missingness for analyses examining these two outcomes.

Hypothesis Testing Analyses. All analyses were done using IBM SPSS v.26. Analyses 1 and 2 used chi-square tests of independence to examine percentages of those screening positive for acute traumatic stress, depression, anxiety, problematic alcohol use, and insufficient sleep and exposures to pandemic-related stressors by profession (emergency [reference group] v healthcare personnel). Analysis 3 combined emergency personnel and hospital workers into one sample and used binomial logistic regression to examine the relative contribution of exposure to the pandemic-related occupational stressors to psychological outcomes. All analyses also included the effects of two covariate controls (i.e., gender and profession) on criterion variables.

Finally, a variable (number of positive COVID-19 cases) was constructed and included in all logistic regression analyses to provide a measure of objective disease exposure based on data available in the

Johns Hopkins Coronavirus Resource Center (https://coronavirus.jhu.edu/map.html). Our variable was constructed based on the number of positive cases reported on the day that the survey was completed by a respondent by county of occupation (Salt Lake County, UT or El Paso County, CO). Each participant's 'number of positive cases' score was placed on a scale that ranged from 1 to 5, representing 500 positive case intervals (e.g., 1 = 0 to 500 cases; 2 = 501 to 1000 cases; up to 5 = 2001 + cases).

3. Results

Probable Mental Health Diagnosis by Profession. Rates of probable diagnosis are displayed in Table 1. Approximately fifteen to thirty percent of respondents screened positive for probable disorders: acute traumatic stress (15%), depression (20%), anxiety (17%), problematic alcohol use (31%), and insufficient sleep (21%). Tests of between group differences showed no evidence of difference by profession in acute traumatic stress, depression, anxiety, or alcohol use. Healthcare personnel had lower odds of reporting insufficient sleep than emergency personnel, χ^2 (1, $N = 571$) = 5.85, $p = .016$, OR = 0.46.

Exposure to Pandemic-Related Stressors by Profession. Rates of exposure to pandemic-related stressors are displayed in Table 2. Emergency personnel (78.6%) reported a higher rate of contact with patients suspected or confirmed for COVID-19, χ^2 (1, $N = 569$) = 56.01, $p < .001$), relative to healthcare personnel (41.2%). Hospital workers had a higher rate of being immunocompromised (17%) than emergency personnel (9%), χ^2 (1, $N = 570$) = 9.66, $p = .002$. Nearly 50% of respondents managed personnel and 20% reported living with someone who was immunocompromised, with no evidence of differences by profession.

Associations between Exposure to Pandemic-Related Stressors and Probable Mental Health Diagnosis. Exposure to pandemic-related stressors and the number of positive COVID-19 cases were entered to predict screening results (0 = negative, 1 = positive) for each of the mental health screening instruments (Table 3). Findings for each of the five prediction models were consistent after controlling for covariates (gender and occupation).

A test of the full model predicting acute traumatic stress was statistically significant, χ^2 (5, $N = 571$) = 15.00, $p = .010$. Respondents who were immunocompromised had higher odds of screening positive for acute traumatic stress. Overall, accurate classification of cases was fair (85.6%). For probable depressive disorder, the full model approached significance, χ^2 (5, $N = 571$) = 10.03, $p = .074$. Being immunocompromised was associated with higher odds of positive depression screen.

For probable anxiety disorder prediction, the full model was statistically significant, χ^2 (5, $N = 571$) = 25.03, $p < .001$. Being immunocompromised was associated with higher odds of screening positive for an anxiety disorder; managing personnel and having an immunocompromised household member were marginally associated with higher odds of screening positive for an anxiety disorder. An inverse association between number of COVID-19 cases and anxiety was observed, such that respondents who completed the survey during dates on which there were a greater number of confirmed COVID-19 cases had lower odds of screening positive for anxiety. Overall classification of cases was fair (82.6%).

For alcohol use, a test of the full model was statistically significant, χ^2 (5, $N = 571$) = 16.12, $p = .007$. Respondents in direct contact with patients and who manage personnel who are in direct contact with patients had higher odds of screening positive for risky alcohol use. Overall success rate of classification was fair (68.2%). For insufficient sleep (<6 h per night), a test of the full model was statistically significant, χ^2 (5, $N = 571$) = 13.17, $p = .022$. Respondents had higher odds of reporting insufficient sleep when they endorsed being in a management role over direct care providers or having an immunocompromised household member. Overall success rate of classification was fair (78.7%).

4. Discussion

This study provides a measure of mental health and pandemic stress among emergency personnel and healthcare staff. Overall, 56% of this sample demonstrated cumulative risk for any of the five mental health outcomes. Healthcare workers reported higher diagnostic risk rates in this study (15.3% acute traumatic stress; 20.1% depression; 23.5% anxiety) than during the SARS epidemic (i.e., 5% traumatic stress, 11% depression, and 10.2–12% anxiety; Bai et al., 2004; Poon et al., 2004). Acute traumatic stress, depression, and anxiety rates reported in this study were notably less than rates reported among healthcare workers responding to COVID-19 in the Wuhan region of China (depression 50.4%, anxiety 44.6%, and insomnia 34%; Lai et al., 2020). These findings suggest that proximity to environments with worse disease outbreaks may play a role in distress and future comparisons of distress, a vital feature for contextualizing healthcare workers' pandemic mental health and experiences. Additionally, approximately 36% of healthcare workers in this sample reported risky alcohol use compared to estimates that 12.9%–21.4% of physicians report risky alcohol use levels in non-crisis contexts (Oreskovich et al., 2015).

The picture for emergency personnel is just as concerning, characterized by higher rates of exceeding diagnostic risk thresholds (14.4%

Table 1
Rates of probable diagnosis by profession.

Item	Whole Sample ($N = 571$)	Emergency Personnel ($n = 473$)	Healthcare Personnel ($n = 98$)	χ^2	OR	95% CI
Acute Traumatic Stress				0.06	1.08	0.59, 1.98
Positive	14.5	14.4	15.3			
Negative	85.5	85.6	84.7			
Depression				0.01	1.02	0.59, 1.75
Positive	20.1	20.1	20.4			
Negative	79.9	79.9	79.6			
Anxiety				3.10	1.60	0.95, 2.72
Positive	17.3	16.1	23.5			
Negative	82.7	83.9	76.5			
Risky Alcohol Use				1.11	1.28	0.81, 2.02
Positive	31.6	30.6	36.1			
Negative	68.4	69.4	63.9			
Insufficient Sleep				5.85*	0.46	0.24, 0.87
Positive	21.4	23.3	12.2			
Negative	78.6	76.7	87.8			

Note. Acute Traumatic Stress risk threshold = PCL-5 ≥33, depression risk threshold = PHQ-8 ≥10; anxiety risk threshold = GAD-7 ≥ 10; hazardous alcohol use threshold, AUDIT-C ≥ 3 (for women), ≥4 (for men); insufficient sleep threshold ≤ 6 h. Values are given as percentages. Chi-square tests indicate a difference between healthcare personnel relative to emergency personnel (reference category). Abbreviations include: OR = odds ratio, 95% CI = 95% confidence interval. *$p < .05$, **$p < .01$, ***$p < .001$.

Table 2
Rates of exposure to pandemic-related stressors by profession.

Item	Whole Sample	Emergency Personnel	Healthcare Personnel	χ^2	OR	95% CI
Direct Patient Contact				56.00***	0.19	0.12, 0.30
Yes	72.2	78.6	41.2			
No (ref.)	27.8	21.4	58.8			
Manage Personnel				1.23	0.78	0.50, 1.21
Yes	45.8	46.8	40.6			
No (ref.)	54.2	53.2	59.4			
Immunocompromised Self				9.66**	2.62	1.40, 4.90
Yes	9.1	7.4	17.3			
No (ref.)	90.9	92.6	82.7			
Immunocompromised Other				1.42	1.37	0.81, 2.32
Yes	19.2	18.3	23.5			
No (ref.)	80.8	81.7	76.5			

Note. Values are given as percentages. Chi-square tests indicate a difference between healthcare personnel relative to emergency personnel (reference category). OR = odds ratio; 95% CI = 95% confidence intervals for an odds ratio and a *t*-test. Abbreviations include: OR = odds ratio, 95% CI = 95% confidence interval. $^*p < .05$, $^{**}p < .01$, $^{***}p < .001$.

for acute traumatic stress, 20.1% for depression, and 16.1% anxiety) than in typical non-crisis times (Del Ben, Scotti, Chen and Fortson, 2006; Carey et al., 2011; Hartley et al., 2011; Meyer et al., 2012; Robinson et al., 1997). Acute traumatic stress, depression, and anxiety rates in this sample are comparable/similar to rates in previous disasters (Hurricane Katrina and 9/11; CDC, 2006; Perrin et al., 2007). Additionally, approximately 30% of the emergency responders in this sample reported risky alcohol use, compared to estimates of risky alcohol use among 16.8%–23.5% of emergency personnel in non-crisis contexts (Smith et al., 2019; Lindsay, 2008).

Insufficient sleep was the only criterion variable on which emergency and hospital personnel significantly differed (23.3% of emergency personnel vs. 12.2% of healthcare personnel). This finding is consistent with longstanding knowledge of sleep problems among emergency personnel (Charles et al., 2007), the result of a combination of shift work that disrupts circadian rhythm and occupational trauma exposure (Neylan et al., 2002). Insufficient sleep is associated with adverse outcomes across diverse physical health, mental health, and cognitive health domains (Krause et al., 2017; Lieberman et al., 2006; Slaven et al., 2011; Zunszain et al., 2011). Long before the arrival of the pandemic, sleep was an area of critical intervention need for emergency personnel, and remains so (e.g., see Neylan et al., 2002).

Examination of how COVID-19 related stressors were associated with risk for probable diagnoses was done using logistic regressions, which yielded four primary sets of outcomes. First, having an immunocompromised condition (immunocompromised self) significantly increased the odds for exceeding risky levels of acute traumatic stress, anxiety, and depression. This result has face validity considering the nature of COVID-19 as a virus that places individuals with medical comorbidities at higher risk for adverse outcomes if infected. Immunocompromised individuals may benefit from being identified and offered alternate roles in their work that may reduce anxiety, fear, and helplessness associated with becoming infected.

Second, respondents involved in direct patient care for potentially infected patients or in management roles over direct care providers (respectively) had higher odds of screening positive for risky alcohol use. In stressful environments, alcohol can be used as a short-term coping strategy, exemplified by a culture of alcohol use that exists among emergency personnel (Arrigo and Garsky, 1997; Jeong et al., 2017). Although alcohol may provide short-term coping relief via avoidance of distress (Wardell et al., 2020), it carries risk for negative consequences for serious physical and mental health problems (e.g., Batey et al., 1992; Boffetta et al., 2006; Brennan et al., 2016; Mueller et al., 1994). As this pandemic continues to disrupt social and organizational systems, we must consider replacing risky alcohol use coping with coping solutions that are more health-sustaining, adapted to the boundaries set by the pandemic (e.g., in the context of social distancing), such as exercise (Abrantes et al., 2017), social support seeking/recruitment (Smith et al., 2015), and mindfulness practices (Weinstein et al., 2009). Further, given the high functioning groups that we surveyed in this study (i.e., employed and educated emergency and healthcare workers), perhaps a palatable and effective intervention entry point is availed through alcohol harm reduction education and strategies that tap into the knowledge and motivations of emergency and healthcare personnel (Charlet and Heinz, 2017; Marlatt and Witkiewitz, 2002). Finally, it will be important to increase access, technologies, and funding that makes evidence-based treatments available to frontline responders for whom acute alcohol abuse persists into a chronic problem (see Kiluk et al., 2019; Miller and Rollnick, 2013; Najavits, 2002). In sum, offsetting the downstream effects of the rise in risky alcohol use may be a critical area for longitudinal investigation, funding, and intervention investment.

Third, being in a management role over direct care providers or having an immune compromised household member were associated with increased odds of insufficient sleep and anxiety. This combination of findings suggests the potential impact of worry for others and/or one's role in determining others' health and safety within both personal (household; Cai et al., 2020; Mohindra et al., 2020) and professional spheres (e.g., making decisions that may place employees at risk for infection). Individuals in either of these pandemic stressor categories may benefit from preventatively seeking interventions that address anxiety and sleep from a combined cognitive, behavioral, and biological perspective (e.g., CBT for insomnia, Perlis et al., 2006; sleep hygiene, Lacks and Rotert, 1986; CBT-I Coach APP). Further, these particular exposure categories may place individuals in morally complex situations that incurs increased psychological risk that should be considered in future research and clinical care (Griffin et al. under review).

Fourth, we observed an inverse association between number of positive COVID-19 cases and anxiety (i.e., as the number of cases increased, anxiety decreased). This finding may be the result of the timing of this survey, conducted during the ramping up of resources, training, and infrastructure to prepare for increase in infections and patients (largely during the month of April 2020). During this anticipatory phase, uncertainty and initial distress may have occurred due to the ultimate passivity of not yet seeing the estimated influx of patients (in military parlance, the 'hurry up and wait' phase). Early in the pandemic healthcare workers may have experienced anticipatory anxiety knowing that the pandemic was coming, while witnessing few cases locally. During this initial phase, infection rates and hospitalizations remained low in the counties in which this study was being conducted amidst daily news about high death rates and combat-like reports from population centers like New York City. While infection rates remained low in the counties sampled, frontline workers may have experienced something akin to the 'Honeymoon' phase (DeWolfe, 2000) experienced in community recovery following a disaster as there was increased availability of resources in preparation for possible influx of patients as

Table 3
Logistic regressions predicting probable diagnosis by exposure to pandemic-related stressors.

	OR	OR 95% CI	*p*	AOR	AOR 95% CI	*p*
**Acute Traumatic Stress**						
Number of positive cases	.88	0.72, 1.08	.225	0.88	0.72, 1.08	.223
Direct Patient Contact	1.83	0.99, 3.39	.054	1.88	1.00, 3.54	.051
Manage Personnel	1.47	0.90, 2.39	.121	1.45	0.89, 2.37	.138
Immunocompromised Self	2.48	1.22, 5.05	.012	2.43	1.19, 4.96	.014
Immunocompromised Other	1.34	0.76, 2.37	.314	1.37	0.78, 2.43	.276
**Depression**						
Number of positive cases	0.85	0.71, 1.01	.067	0.85	0.72, 1.03	.095
Direct Patient Contact	1.50	0.90, 2.49	.120	1.50	0.89, 2.54	.131
Manage Personnel	0.98	0.34, 1.50	.915	1.07	0.69, 1.65	.769
Immunocompromised Self	2.12	1.10, 4.08	.024	2.18	1.13, 4.23	.021
Immunocompromised Other	1.11	0.66, 1.87	.689	1.05	0.61, 1.78	.869
**Anxiety**						
Number of positive cases	0.78	0.64, 0.94	.011	0.79	0.64, 0.96	.019
Direct Patient Contact	1.15	0.67, 1.94	.617	1.24	0.72, 2.14	.442
Manage Personnel	1.58	1.00, 2.50	.052	1.58	0.99, 2.52	.053
Immunocompromised Self	2.67	1.39, 5.15	.003	2.58	1.33, 4.98	.005
Immunocompromised Other	1.68	1.00, 2.82	.051	1.71	1.02, 2.88	.044
**Risky Alcohol Use**						
Number of positive cases	0.98	0.85, 1.14	.813	1.03	0.89, 1.20	.677
Direct Patient Contact	1.85	1.18, 2.90	.008	2.18	1.35, 3.52	.001
Manage Personnel	1.56	1.08, 2.25	.018	1.64	1.13, 2.38	.010
Immunocompromised Self	1.21	0.63, 2.31	.563	1.16	0.61, 2.23	.652
Immunocompromised Other	0.85	0.53, 1.36	.487	0.82	0.51 1.33	.425
**Insufficient Sleep**						
Number of positive cases	1.04	0.88, 1.22	.642	1.00	0.84, 1.18	.984
Direct Patient Contact	1.08	0.66, 1.77	.747	0.93	0.56, 1.56	.793
Manage Personnel	1.77	1.16, 2.68	.007	1.73	1.13, 2.64	.011
Immunocompromised Self	0.78	0.36, 1.68	.523	0.82	0.38, 1.80	.623
Immunocompromised Other	1.69	1.04, 2.74	.035	1.67	1.02, 2.75	.043

Note. Abbreviations include Odds Ratio (OR), Adjusted Odds Ratio (AOR), and 95% Confidence Interval (95% CI). An odds ratio greater than 1.00 indicates that participants who endorsed a given item were more likely to screen positive than were participants who did not endorse the item. Adjusted Odds Ratios account for variance in outcomes explained by gender and profession in addition to displayed variables. Number of cases was assessed using an ordinal variable representing discrete categories that increased by 500 cases.

well as a sense of community. The inverse association shown in our study may well be different in places with higher rates of infection and death. The relationship between mental health risk and disease spread is sure to evolve over time, likely to involve a complex interplay of socio-politics, occupational stress, and individual differences.

4.1. Limitations

This study has a number of limitations. First, these data are cross-sectional, which does not provide temporal precedence needed to understand predictive effects of pandemic stressors. If this pandemic is defined by anything, it is dynamic change, requiring a longitudinal approach to be fully understood (e.g., change in infection rates, CDC recommendations, current events such as civil demonstrations and protests, political turmoil, and economic uncertainty). Acute traumatic stress reactions can be considered normative in the initial stages of a crisis and should only be considered diagnosable if they persist and contribute to more generalized impairments. Longitudinal studies should examine whether/how/for whom morbidities persist or emerge. Second, larger samples are needed to be sufficiently statistically powered to test a variety of predictors of mental health problems in addition to the contributions of exposure to pandemic-related occupational stressors and a select few other factors (e.g., gender and profession). For example, personal and familial history of mental health problems, prior adversity and trauma, other sociodemographic variables (e.g., years of professional experience), etc. will be important to consider. Third, our sample of healthcare workers (~17% of the current study sample) was small relative to emergency responders. Moreover, given the composition of the healthcare subsample, our study does not represent healthcare workers in the most intense treatment settings (e.g., ER, ICU), and thus likely does not generalize to all healthcare workers (see Fig. 1 for a breakdown of sample characteristics). Future work that is able to distinguish between physical proximity based on healthcare setting will be important for distinguishing the mental health effects of more intense levels of objective exposure. Fourth, this study did not provide a comparative sample in a region more intensely affected with higher death rates and caseload, albeit that the data from this study will be useful for future efforts to contextualize the mental health of frontline responders from other regions. We used normalized measures (PCL-5; PHQ-8; GAD-7; AUDIT-C) to allow for such comparisons in the future.

Fifth, our construction of the variable 'number of positive cases' is an approximation of disease spread/evolution, and should be interpreted with caution given its collinearity with time and experience across this pandemic. Imprecision in reporting and testing protocols let to our choice to construct ordinal ranges (1 = 0 to 500 cases; 2 = 501 to 1000 cases, etc), in part to avoid implying precision. Notwithstanding this limitation, the 'number of positive cases' variable that we constructed can be replicated for comparison in future studies in different geographical locations, as long as data are available on number of positive cases. Undoubtedly, the quantification and operationalization of the disease spread/evolution is an area for future investigation that will ideally increase in sophistication and accuracy as better data emerge.

Additionally, there may be personal characteristics of individuals who enrolled in the study and filled out the survey early (e.g., more anxiety and hypervigilance) vs later in the study (e.g., sense of duty, non-anxiety features). Given the rapid deployment of the survey to a self-selected group, future research is needed to verify the generalizability of the findings. Also, depression may play more of a role in later stages of the pandemic than it did in these data, as the long-term lack of solution, political turmoil, new waves of infection, and relative global helplessness set in more apparently for some. These notions may explain why the prediction model only approached significance for

understanding depression in this assessment done early in the course of the pandemic.

4.2. Conclusions

Our overall findings suggest that a sizable proportion of frontline responders during the COVID-19 pandemic are at risk for psychiatric morbidity, at severity rates higher than previous viral outbreaks and similar to previous disasters (e.g., 9/11 and Hurricane Katrina). Several key differences to note, however, are that the COVID-19 disaster is more severe than previous viral outbreaks experienced in over 100 years, and that this is not a geographically contained disaster (such as a natural disaster or terrorist attack), albeit that geographical location in which frontline workers are embedded is likely to play a predictive and moderating role (relate to, for example, policies, preparedness, infection rates, and deaths). The global nature of this disaster means that many more frontline responders and hospital personnel are being affected simultaneously by this event than any event in modern history. The currently available mental health service offerings and access for frontline responders are inadequate (NAEMT, 2016). Many personnel have limited access to employee assistance programs, which are designed for short-term difficulties in life, and few seek treatment outside of their organization, especially during times of crisis. Our findings should be a wake-up call for administrators and relief agencies in developing plans for the on-going threat of COVID-19 and future crises. Further, longitudinal research is critical to understand the psychiatric impact of this pandemic and to inform services and policies that meet the needs of frontline responders.

Authors contributions

H. Wright: Conceptualization, Methodology, Writing-Original Draft, Project administration. B. Griffin: Methodology, Formal Analysis, Writing-Original Draft. K. Shoji: Methodology, Formal Analysis. T. Love: Verification, Writing-Review and Editing. S. Langenecker: Verification, Writing-Review and Editing, Supervision. C. Benight: Methodology, Verification, Writing-Review and Editing. A. Smith: Conceptualization, Methodology, Writing-Original Draft, Supervision, Project administration.

Declaration of competing interest

The authors confirm that there are no relevant financial or non-financial competing interests to report with this paper and no competing interests to declare.

Appendix A. Supplementary data

Supplementary data to this article can be found online at https://doi.org/10.1016/j.jpsychires.2020.10.045.

References

Aiken, L.H., Clarke, S.P., Sloane, D.M., Sochalski, J.A., Busse, R., Clarke, H., Giovanneti, P., Hunt, J., Rafferty, A.M., Shamian, J., 2001. Nurses' reports on hospital care in five countries. Health Aff. 20 (3), 43–53. https://doi.org/10.1377/hlthaff.20.3.43.

Abrantes, A., Scalco, M., O'Donnell, S., Minami, H., Read, J., 2017. Drinking and exercise behaviors among college students: between and within-person associations. Journal of behavioral medicine 40 (6).

Adams, Walls, 2020. Supporting the health care workforce during the COVID-19 global epidemic. Jama 323 (15).

Aiken, L.H., Clarke, S.P., Sloane, D.M., International Hospital Outcomes Research Consortium, 2002. Hospital staffing, organization, and quality of care: cross-national findings. Int. J. Qual. Health Care 14 (1), 5–13. https://doi.org/10.1093/intqhc/14.1.5.

Arrigo, B.A., Garsky, K., 1997. Police Suicide: A Glimpse behind the Badge. Critical Issues in Policing: Contemporary Readings, pp. 609–626.

Bai, Yamei, Lin, Chao-Cheng, Lin, Chih-Yuan, Chen, Jen-Yeu, Chue, Ching-Mo, Chou, Pesus, 2004. Survey of stress reactions among health care workers involved with the SARS outbreak. Psychiatr. Serv. 55 (9), 1055–1057. https://doi.org/10.1176/appi.ps.55.9.1055.

Baker, M.G., Peckham, T.K., Seixas, N.S., 2020. Estimating the burden of United States workers exposed to infection or disease: a key factor in containing risk of COVID-19 infection. PLoS One 15 (4), e0232452. https://doi.org/10.1371/journal.pone.0232452.

Batey, R.G., Burns, T., Benson, R.J., Byth, K., 1992. Alcohol consumption and the risk of cirrhosis. Med. J. Aust. 156 (6), 413–416.

Berg, A.M., Hem, E., Lau, B., Ekeberg, Ø., 2006. An exploration of job stress and health in the Norwegian police service: a cross sectional study. J. Occup. Med. Toxicol. 1 (1), 26.

Blevins, C.A., Weathers, F.W., Davis, M.T., Witte, T.K., Domino, J.L., 2015. The posttraumatic stress disorder checklist for DSM-5 (PCL-5): Development and initial psychometric evaluation. Journal of traumatic stress 28 (6), 489–498.

Boffa, J.W., Stanley, I.H., Hom, M.A., Norr, A.M., Joiner, T.E., Schmidt, N.B., 2017. PTSD symptoms and suicidal thoughts and behaviors among firefighters. J. Psychiatr. Res. 84, 277–283. https://doi.org/10.1016/j.jpsychires.2016.10.014.

Boffa, J.W., Stanley, I.H., Smith, L.J., Mathes, B.M., Tran, J.K., Buser, S.J., Vujanovic, A. A., 2018. Posttraumatic stress disorder symptoms and suicide risk in male firefighters: the mediating role of anxiety sensitivity. J. Nerv. Ment. Dis. 206, 179–186. https://doi.org/10.1097/nmd.0000000000000779.

Boffetta, Paolo, Hashibe, Mia, La Vecchia, Carlo, Zatonski, Witold, Rehm, Jürgen, 2006. The burden of cancer attributable to alcohol drinking. Int. J. Canc. 119 (4), 884–887.

Brennan, P.L., SooHoo, S., Lemke, S., Schutte, K.K., 2016. Alcohol use predicts 10-year depressive symptom trajectories in the health and retirement study. J. Aging Health 28 (5), 911–932.

Bush, K., Kivlahan, D.R., McDonell, M.B., Fihn, S.D., Bradley, K.A., 1998. The AUDIT alcohol consumption questions (AUDIT-C): an effective brief screening test for problem drinking. Arch. Intern. Med. 158 (16), 1789–1795. https://doi.org/10.1001/archinte.158.16.1789.

Cai, H., Tu, B., Ma, J., Chen, L., Fu, L., Jiang, Y., Zhuang, Q., 2020. Psychological impact and coping strategies of frontline medical staff in hunan between January and March 2020 during the outbreak of coronavirus disease 2019 (COVID-19) in hubei, China. Med. Sci. Mon. Int. Med. J. Exp. Clin. Res.: Int. Med. J. Exper. Clin. Res. 26, e924171 https://doi.org/10.12659/msm.924171.

Carey, M.G., Al-Zaiti, S.S., Dean, G.E., Sessanna, L., Finnell, D.S., 2011. Sleep problems, depression, substance use, social bonding, and quality of life in professional firefighters. J. Occup. Environ. Med. 53, 928–933. https://doi.org/10.1097/jom.0b013e318225898f.

Centers for Disease Control and Prevention CDC, 200). Health hazard evaluation of police officers and firefighters after Hurricane Katrina—new Orleans, Louisiana, October 17–28 and November 30–December 5, 2005. MMWR Morb. Mortal. Wkly. Rep., 55(16):456–458.

Charles, L., Burchfiel, C., Fekedulegn, D., Vila, B., Hartley, T., Slaven, J., Violanti, J., 2007. Shift work and sleep: the Buffalo Police health study. Polic. An Int. J. Police Strategies Manag. 30 (2), 215–227. https://doi.org/10.1108/13639510710753225.

Charlet, K., Heinz, A., 2017. Harm reduction—a systematic review on effects of alcohol reduction on physical and mental symptoms. Addiction Biol. 22 (5), 1119–1159.

Chiu, S., Webber, M.P., Zeig-Owens, R., Gustave, J., Lee, R., Kelly, K.J., Prezant, D.J., 2010. Validation of the Center for Epidemiologic Studies Depression Scale in screening for major depressive disorder among retired firefighters exposed to the World Trade Center disaster. J. Affect. Disord. 121, 212–219. https://doi.org/10.1016/j.jad.2009.05.028.

Chong, M.-Y., Wang, W.-C., Hsieh, W.-C., Lee, C.-Y., Chiu, N.-M., Yeh, W.-C., Huang, T.-L., Wen, J.-K., Chen, C.-L., 2004. Psychological impact of severe acute respiratory syndrome on health workers in a tertiary hospital. Br. J. Psychiatr. 185 (2), 127–133. https://doi.org/10.1192/bjp.185.2.127.

Del Ben, K.S., Scotti, J.R., Chen, Y., Fortson, B.L., 2006. Prevalence of posttraumatic stress disorder symptoms in firefighters. Work. Stress 20, 37–48. https://doi.org/10.1080/02678370600679512.

DeWolfe, D.J., 2000. Training Manual for Mental Health and Human Service Workers in Major Disasters. US Department of Health and Human Services, Substance Abuse and Mental Health Services Administration, Center for Mental Health Services.

Fullerton, C.S., Ursano, R.J., Wang, L., 2004. Acute stress disorder, posttraumatic stress disorder, and depression in disaster or rescue workers. Am. J. Psychiatr. 161, 1370–1376. https://doi.org/10.1176/appi.ajp.161.8.1370.

Griffin, B. J., Pyne, J. M., Smith, A. J., Usset, T., Harris, I. J., Cooney, N., Messias, E., Cucciare, M. A., & Maguen, S. (under review). Moral Injury Among First Responders during the Coronavirus Pandemic. Manuscript under editorial review.

Hartley, T.A., Burchfiel, C.M., Fekedulegn, D., Andrew, M.E., Violanti, J.M., 2011. Health disparities in police officers: comparisons to the US general population. Int. J. Emerg. Ment. Health 13 (4), 211–220.

Jamshidian, M., Jalal, S., 2010. Tests of homoscedasticity, normality, and missing completely at random for incomplete multivariate data. Psychometrika 75, 649–674. https://doi.org/10.1007/s11336-010-9175-3.

Jeong, H.S., Park, S., Lim, S.M., Ma, J., Kang, I., Kim, J., et al., 2017. Psychometric properties of the alcohol use disorders identification test-consumption (AUDIT-C) in public first responders. Subst. Use Misuse 52 (8), 1069–1075.

Johns Hopkins Univeristy, 2020. COVID-19 Dashboard.

Kang, L., Li, Y., Hu, S., Chen, M., Yang, C., Yang, B.X., et al., 2020. The mental health of medical workers in Wuhan, China dealing with the 2019 novel coronavirus. Lancet Psychiatr. 7 (3), e14.

Kiluk, B.D., Ray, L.A., Walthers, J., Bernstein, M., Tonigan, J.S., Magill, M., 2019. Technology-delivered cognitive-behavioral interventions for alcohol use: a meta-analysis. Alcohol Clin. Exp. Res. 43 (11), 2285–2295.

Kim, J.E., Dager, S.R., Jeong, H.S., Ma, J., Park, S., Kim, J., et al., 2018. Firefighters, posttraumatic stress disorder, and barriers to treatment: results from a nationwide total population survey. PLoS One 13, e0190630. https://doi.org/10.1371/journal.pone.0190630.

Krause, A.J., Simon, E.B., Mander, B.A., Greer, S.M., Saletin, J.M., Goldstein-Piekarski, A. N., Walker, M.P., 2017. The sleep-deprived human brain. Nat. Rev. Neurosci. 18 (7), 404.

Kroenke, K., Strine, T., Spitzer, R., Williams, J., Berry, J., Mokdad, A., 2009. The PHQ-8 as a measure of current depression in the general population. J. Affect. Disord. 114 (1–3), 163–173.

Lacks, P., Rotert, M., 1986. Knowledge and practice of sleep hygiene techniques in insomniacs and good sleepers. Behav. Res. Ther. 24 (3), 365–368.

Lai, J., Ma, S., Wang, Y., Cai, Z., Hu, J., Wei, N., Hu, S., 2020. Factors associated with mental health outcomes among health care workers exposed to coronavirus disease 2019. JAMA Netw. Open 3 (3), E203976. https://doi.org/10.1001/jamanetworkopen.2020.3976.

Lieberman, H.R., Niro, P., Tharion, W.J., Nindl, B.C., Castellani, J.W., Montain, S.J., 2006. Cognition during sustained operations: comparison of a laboratory simulation to field studies. Aviat Space Environ. Med. 77 (9), 929–935.

Lindsay, V., 2008. Police officers and their alcohol consumption. Police Q. 11 (1), 74–87. https://doi.org/10.1177/1098611107309564.

Luft, Schechter, Kotov, Broihier, Reissman, Guerrera, Bromet, 2012. Exposure, probable PTSD and lower respiratory illness among World Trade Center rescue, recovery and clean-up workers. Psychol. Med. 42 (5), 1069–1079. https://doi.org/10.1017/s003329171100256x.

Marlatt, G.A., Witkiewitz, K., 2002. Harm reduction approaches to alcohol use: health promotion, prevention, and treatment. Addict. Behav. 27 (6), 867–886.

Mealer, M., Burnham, E.L., Goode, C.J., Rothbaum, B., Moss, M., 2009. The prevalence and impact of post traumatic stress disorder and burnout syndrome in nurses. Depress. Anxiety 26 (12), 1118–1126. https://doi.org/10.1002/da.20631.

Meyer, E.C., Zimering, R., Daly, E., Knight, J., Kamholz, B.W., Gulliver, S.B., 2012. Predictors of posttraumatic stress disorder and other psychological symptoms in trauma-exposed firefighters. Psychol. Serv. 9 (1), 1–15. https://doi.org/10.1037/a0026414.

Miller, W.R., Rollnick, S., 2013. Motivational Interviewing, third ed. Guilford Publications, United Kingdom. Helping People Change.

Mohindra, R., Ravaki, R., Suri, V., Bhalla, A., Singh, S.M., 2020. Issues relevant to mental health promotion in frontline health care providers managing quarantined/isolated COVID19 patients. Asian J. Psychiatr. 51, 102084.

Mueller, T.I., Lavori, P.W., Keller, M.B., Swartz, A., Warshaw, M., Hasin, D., Akiskal, H., 1994. Prognostic effect of the variable course of alcoholism on the 10-year course of depression. Am. J. Psychiatr. 151 (5), 701–706.

NAEMT, 2016. National survey on EMS mental health services. Retrieved from: http://www.naemt.org/docs/default-source/ems-health-and-safety-documents/mental-healthgrid/2016-naemt-mental-health-report-8-14-16.pdf.

Najavits, L., 2002. Seeking Safety: A Treatment Manual for PTSD and Substance Abuse. Guilford Publications.

Neylan, Metzler, Best, Weiss, Fagan, Liberman, Marmar, 2002. Critical incident exposure and sleep quality in police officers. Psychosom. Med. 64 (2), 345–352.

Novel Coronavirus Pneumonia Emergency Response Epidemiology Team, 2020. Vital Surveillances: the Epidemiological Characteristics of an Outbreak of 2019 Novel Coronavirus Diseases (COVID-19)—China. China CDC Weekly.

Oreskovich, M.R., Shanafelt, T., Dyrbye, L.N., Tan, L., Sotile, W., Satele, D., West, C.P., Sloan, J., Boone, S., 2015. The prevalence of substance use disorders in American physicians. Am. J. Addict. 24 (1), 30–38. https://doi.org/10.1111/ajad.12173.

Osofsky, H.J., Osofsky, J.D., Arey, J., Kronenberg, M.E., Hansel, T., Many, M., 2011. Hurricane Katrina's first responders: the struggle to protect and serve in the aftermath of the disaster. Disaster Med. Public Health Prep. 5 (S2), S214–S219. https://doi.org/10.1001/dmp.2011.53.

Perlis, M.L., Jungquist, C., Smith, M.T., Posner, D., 2006. Cognitive Behavioral Treatment of Insomnia: A Session-By-Session Guide, vol. 1. Springer Science & Business Media.

Perrin, M., DiGrande, L., Wheeler, K., Thorpe, L., Farfel, M., Brackbill, R., 2007. Differences in PTSD prevalence and associated risk factors among World Trade Center disaster rescue and recovery workers. (post-traumatic stress disorder) (Report). Am. J. Psychiatr. 164 (9), 1385–1394. https://doi.org/10.1176/appi.ajp.2007.06101645.

Poon, E., Cheong, K., Lee, D., Yam, C., Liu, L., Tang, W., 2004. Impact of severe acute respiratory syndrome on anxiety levels of front line health care workers. Hong Kong Med. J. 10 (5), 325–330.

Robinson, H.M., Sigman, M.R., Wilson, J.P., 1997. Duty-related stressors and PTSD symptoms in suburban police officers. Psychol. Rep. 81 (3), 835–845. https://doi.org/10.2466/pr0.1997.81.3.835.

Schafer, J.L., Graham, J.W., 2002. Missing data: our view of the state of the art. Psychol. Methods 7, 147–177. https://doi.org/10.1037/1082-989X.7.2.147.

Shanafelt, T.D., Boone, S., Tan, L., Dyrbye, L.N., Sotile, W., Satele, D., West, C.P., Sloan, J., Oreskovich, M.R., 2012. Burnout and satisfaction with work-life balance among US physicians relative to the general US population. Arch. Intern. Med. 172 (18), 1377–1385. https://doi.org/10.1001/archinternmed.2012.3199.

Slaven, J.E., Mnatsakanova, A., Burchfield, C.M., Smith, L.M., Charles, L.E., Andrew, M. E., et al., 2011. Association of sleep quality with depression in police officers. Int. J. Emerg. Ment. Health 13 (4), 267–277.

Smith, A.J., Donlon, K., Anderson, S.R., Hughes, M., Jones, R.T., 2015. When seeking influences believing and promotes posttraumatic adaptation. Hist. Philos. Logic 28 (3), 340–356.

Smith, L.J., Bartlett, B.A., Tran, J.K., Gallagher, M.W., Alfano, C., Vujanovic, A.A., 2019. Sleep disturbance among firefighters: understanding associations with alcohol use and distress tolerance. Cognit. Ther. Res. 43 (1), 66–77. https://doi.org/10.1007/s10608-018-9955-0.

Söderström, M., Jeding, K., Ekstedt, M., Perski, A., Åkerstedt, T., 2012. Insufficient sleep predicts clinical burnout. J. Occup. Health Psychol. 17 (2), 175. https://doi.org/10.1037/a0027518.

Soo, J., Webber, M., Gustave, J., Lee, R., Hall, C., Cohen, H., Prezant, D., 2011. Trends in probable PTSD in firefighters exposed to the World Trade Center disaster, 2001-2010. Disaster Med. Public Health Prep. 5 (S2), S197–S203. https://doi.org/10.1001/dmp.2011.48.

Spitzer, R.L., Kroenke, K., Williams, J.B.W., Löwe, B., 2006. A brief measure for assessing generalized anxiety disorder: the GAD-7. Arch. Intern. Med. 166, 1092–1097. https://doi.org/10.1001/archinte.166.10.1092.

Stanley, I.H., Boffa, J.W., Smith, L.J., Tran, J.K., Schmidt, N.B., Joiner, T.E., Vujanovic, A.A., 2018. Occupational stress and suicidality among firefighters: examining the buffering role of distress tolerance. Psychiatr. Res. 266, 90–96. https://doi.org/10.1016/j.psychres.2018.05.058.

Tam, C., Pang, E., Lam, L., Chiu, H., 2004. Severe acute respiratory syndrome (SARS) in Hong Kong in 2003: stress and psychological impact among front line healthcare workers. Psychol. Med. 34 (7), 1197–1204. https://doi.org/10.1017/s0033291704002247.

Troyanskaya, O., Cantor, M., Sherlock, G., Brown, P., Hastie, T., Tibshirani, R., Botstein, D., Altman, R.B., 2001. Missing value estimation methods for DNA microarrays. Bioinformatics 17, 520–525. https://doi.org/10.1093/bioinformatics/17.6.520.

Walton, M., Murray, E., Christian, M., 2020. Mental health care for medical staff and affiliated healthcare workers during the COVID-19 pandemic. Eur. Heart J.: Acute Cardiovasc. Care 9 (3), 241–247.

Wardell, J., Kempe, T., Rapinda, K.K., Single, A., Bilevicius, E., Frohlich, J.R., et al., 2020. Drinking to Cope during the COVID-19 Pandemic: the Role of External and Internal Stress-Related Factors in Coping Motive Pathways to Alcohol Use, Solitary Drinking, and Alcohol Problems.

Weathers, Litz, Herman, Huska, Keane, 2012. The PTSD Checklist (PCL): Reliability, validity, and diagnostic utility, 1993.

Weinstein, N., Brown, K.W., Ryan, R.M., 2009. A multi-method examination of the effects of mindfulness on stress attribution, coping, and emotional well-being. J. Res. Pers. 43 (3), 374–385.

World Health Organization, 2020. Statement on the second meeting of the International Health Regulations (2005) Emergency Committee regarding the outbreak of novel coronavirus (2019-nCoV). Published on January 30. https://www.who.int/news-room/detail/30-01-2020-statement-on-the-secondmeeting-of-the-international-health-regulations-(2005)-emergency-committee-regarding-the-outbreakof-novel-coronavirus-(2019-ncov.

Wu, P., Fang, Y., Guan, Z., Fan, B., Kong, J., Yao, Z., et al., 2009. The psychological impact of the SARS epidemic on hospital employees in China: exposure, risk perception, and altruistic acceptance of risk. Can. J. Psychiatr. 54 (5), 302–311. https://doi.org/10.1177/070674370905400504.

Zunszain, P.A., Anacker, C., Cattaneo, A., Carvalho, L.A., Pariante, C.M., 2011. Glucocorticoids, cytokines and brain abnormalities in depression. Prog. Neuro Psychopharmacol. Biol. Psychiatr. 35 (3), 722–729.
