# Supplementary material for: Mental health status and related factors influencing healthcare workers during the COVID-19 pandemic: A systematic review and meta-analysis
Source: PLoS One. 2024 Jan 19;19(1):e0289454. doi: 10.1371/journal.pone.0289454 (PMC10798549; doi:10.1371/journal.pone.0289454)
Supplement: S1 Data — (ZIP) [file pone.0289454.s011.zip › literatures/216.pdf]

MEDICAL SCIENCE MONITOR BASIC RESEARCH

HUMAN STUDY

e-ISSN 2325-4416
© Med Sci Monit Basic Res, 2020; 26: e924085
DOI: 10.12659/MSMBR.924085

Received: 2020.03.07
Accepted: 2020.03.30
Published: 2020.05.11

Analysis of Psychological and Sleep Status and Exercise Rehabilitation of Front-Line Clinical Staff in the Fight Against COVID-19 in China

Authors' Contribution:
Study Design A
Data Collection B
Statistical Analysis C
Data Interpretation D
Manuscript Preparation E
Literature Search F
Funds Collection G

ABCDEG **Koulong Wu**
ABCFG **Xuemei Wei**

Physical Education Department, Wannan Medical College, Wuhu, Anhui, P.R. China

Corresponding Author: Xuemei Wei, e-mail: weixuemei@wnmc.edu.cn

Source of support: This study was supported by the Key Project of Humanities and Social Sciences of Anhui Provincial Department of Education (SK2018A0193)

Background: The aim of this study was to understand the changes in psychological factors and sleep status of front-line medical staff in the fight against COVID-19 and provide evidence of exercise interventions to relieve psychological stress and improve sleep status for medical staff.

Material/Methods: A survey study was conducted among 120 front-line medical staff in the fight against COVID-19, of which 60 medical staff worked at the designated hospital (experimental group) and 60 medical staff worked at the non-designated hospital (control group). The Symptom Checklist 90 (SCL-90), Self-rating Anxiety Scale (SAS), Self-rating Depression Scale (SDS), and PTSD Checklist-Civilian Version (PCL-C) were used to assess mental status. Sleep status was assessed using the Pittsburgh Sleep Quality Index (PSQI).

Results: SCL-90 scores of somatization, depression, anxiety, and terror were higher than normal in front-line medical staff at the designated hospital. The SAS (45.89±1.117), SDS (50.13±1.813), and PCL-C (50.13±1.813) scores in the experimental group were higher than the normal control group, and were significantly different from those in the control group on SDS and PCL-C scales ($P<0.05$). The total average PSQI of the experimental group was 16.07±3.761, indicating that the sleep quality was poor. Among them, participants with moderate insomnia reached 61.67%, and participants with severe insomnia reached 26.67%.

Conclusions: There are psychological symptoms and sleep symptoms in front-line medical staff who participate in the fight against COVID-19, and they affect each other. Hospitals should improve emergency management measures, strengthen psychological counseling for clinical front-line medical staff, strengthen exercise intervention, and improve their sleep quality and mental health.

MeSH Keywords: Adaptation, Psychological • Coronavirus Infections • Dyssomnias • Medical Staff, Hospital

Full-text PDF: https://www.basic.medscimonit.com/abstract/index/idArt/924085

2606 5 2 21

This work is licensed under Creative Common Attribution-NonCommercial-NoDerivatives 4.0 International (CC BY-NC-ND 4.0)

Indexed in: [Index Medicus/MEDLINE] [EMBASE/Excerpta Medica] [Chemical Abstracts/CAS]

Background

In recent years, infectious disease outbreaks such as H1N1 influenza, the Ebola outbreak in West Africa, and the Zika virus outbreaks have frequently occurred, which seriously threatened human survival and development. Since December 2019, pneumonia caused by a new coronavirus infection has been found in Wuhan, Hubei Province, China, and globally. At present, the China Centers for Disease Control has included this coronavirus in the category of infectious diseases in China and has adopted measures for the prevention and control of this new infectious disease. On January 31, 2020, the World Health Organization (WHO) officially recognized the epidemic as a public health emergency of international concern (PHEIC) and suggested that it be named "COVID-19".

With the development of the epidemic, the number of confirmed and suspected patients has continued to increase, and the workload and work pressure of front-line clinical staff to fight the epidemic have also increased. Front-line health care workers face not only heavy workloads, but also the risk of infection. Due to the special and high-risk work of clinical front-line medical staff, their psychological pressure is huge, which affects their sleep quality and physical and mental health. Appropriate physical exercise and exercise intensity can help improve sleep quality and physical and mental health. Good sleep quality can promote rapid recovery of body function, relieve work fatigue, and maintain sufficient energy, physical strength and a healthy mental state [1].

The purpose of this study was to understand the psychological status and sleep status of front-line medical staff in the fight against the COVID-19 epidemic. Normative and systematic assessments and observations are conducted with a view to early strengthening exercise intervention, providing reference basis and intervention strategies for improving sleep quality.

Material and Methods

Participants

The new coronavirus has human-to-human characteristics. For this study we designate an experimental group and a control group. The experimental group consisted of 60 cases of front-line medical staff from a designated hospital for COVID-19 infection were selected, that is, they were in close contact with patients, and there was a risk of infection at any time. The age range was 25–59 years (mean 33.5±12.4 years). The control group consisted of 60 cases of front-line medical staff from non-designated hospital were selected, and their age was similar to that of the experimental group. In this study, the inclusion criteria for the 2 groups were as follows: participation in the epidemic prevention and control work for more than 4 weeks. No history of neurological, psychiatric, or other systemic serious illnesses. No drug abuse and the ability to complete a neuropsychological scale examination. All the participants signed a test agreement that they would comply with the test requirements. There were no significant differences in age, gender or education between the 2 groups ($P>0.05$), (Table 1).

Assessment and testing of mental state

For the assessment and testing we used online system questionnaires and self-assessment scales and self-exercising rehabilitation exercise prescription. Study participants voluntarily filled out the forms and completed the assessment scales, and we recommend ed that medical staff exercise according to the exercise rehabilitation prescription.

The Symptom Checklist 90 (SCL-90)

The SCL-90 scale has been used internationally for many years, and the reliability and validity of each factor are stable. There is a total of 90 items, and 9 factor scores such as average scores for positive items, somatization, obsessive-compulsive symptoms, interpersonal relationships, depression, anxiety, hostility, terror, paranoia, and psychosis are calculated respectively [2-4].

The Self-rating Depression Scale (SDS)

The SDS was compiled by Zung et al. [5] in 1965. It is used to assess the subjective severity of depression in an individual. The scale has a total of 20 entries, 10 forward scores, 10 reverse points, and is a 4-level score. The cumulative score of each entry is multiplied by 1.25 to obtain the standard total score. A standard total score of <53 indicates absence of depression, 53–63 points indicate mild depression, 64–74 points indicate moderate depression, ≥75 points indicate severe depression, and the higher scores indicate more severe depression.

The Self-rating Anxiety Scale (SAS)

The SAS is used to assess the severity of anxiety in an individual [6–8]. The scale has a total of 20 entries, 15 forward scores, 5 reverse points, and is a 4-level score. The cumulative score of each entry is multiplied by 1.25 to obtain the standard total score. A total score of <50 points indicates the absence of anxiety, 50–59 points indicate mild anxiety, 60–69 points indicate moderate anxiety, and ≥70 points indicate severe anxiety. A higher score indicates a more severe degree of anxiety.

The Pittsburgh Sleep Quality Index (PSQI)

The PSQI is mainly aimed at the sleep quality of an individual in the recent 1 month [9,10]. There are 9 large entries in

This work is licensed under Creative Common Attribution-NonCommercial-NoDerivatives 4.0 International (CC BY-NC-ND 4.0)

Indexed in: [Index Medicus/MEDLINE] [EMBASE/Excerpta Medica] [Chemical Abstracts/CAS]

Table 1. Comparative statistics of age, gender and education of the 2 groups (n, %).

Species	Number	Gender		Age	Education		
		Male	Female		Bachelor	Master	PhD
Experimental group	60	16 (26.7%)	44 (73.3%)	33.5±12.4	39 (65.0%)	19 (31.7%)	2 (3.3%)
Control group	60	15 (25.0)	45 (75.0)	33.8±11.9	42 (70.0)	17 (28.3)	1 (1.7)
t/χ^2		–0.205		0.216	0.087		
P		0.785		0.624	0.964		

total, of which entry 5 is subdivided into 10 entries (a-j), so there are 18 entries in total. Eighteen items make up 7 components, each of which is scored on a 0 to 3 scale, respectively: sleep quality, time to sleep, sleep time, sleep efficiency, sleep symptoms, hypnotic drugs, and daytime dysfunction. PSQI scoring rules are as follows: each component is scored on a 0 to 3 scale, and the cumulative score of each component is the total PSQI score, with a total score ranging from 0 to 21. Higher scores indicate worse sleep quality. 0 to 5 points indicate good sleep quality, 6 to 10 points indicate average sleep quality, 11 to 15 points indicate poor sleep quality, and 16 to 21 points indicate very poor sleep quality. A total score >7 indicates sleep disturbance.

The PTSD Checklist-Civilian Version (PCL-C)

The PCL-C was developed by the American Center for Post-Traumatic Stress Disorders to assess the post-traumatic experience of individuals in non-war states. The evaluation items include avoidance/numbness, high alertness, and re-experience in 3 dimensions. There is a total of 17 items, and each item can be scored as 1 to 5 points. The higher the score is, the more severe the symptoms of stress disorder will be. PCL-C is often used to evaluate the effects of diagnosis, intervention and treatment of post-traumatic stress disorder. It has good reliability and validity and is one of the most widely used tools in this field [11–15].

Statistical analysis

Data analysis was performed using SPSS 20.0 statistical software. Measurement data are expressed as mean±standard deviation (M±SD), and count data are expressed as frequency or percentage. The *t*-test or analysis of variance (ANOVA) was used to test for differences in measurement data. Continuous variables such as sleep quality and mental state were analyzed using Pearson correlation analysis and linear regression analysis. The test level was α=0.05, that is, $P<0.05$ was considered statistically significant.

Results

SCL-90 scale

The results of SCL-90 scale in the experimental group showed that the scores of somatization, depression, anxiety, and terror were higher than those of the control group. Among these scores, there were significant differences in somatization, depression, terror factor, total scores, and total mean scores ($P<0.05$), as shown in Table 2.

SAS, SDS, and PCL-C scales

The SAS, SDS, and PCL-C scores in the experimental group were higher than normal. Compared with the control group, the differences in the scores of the SDS and PCL-C scales in the experimental group were statistically significant ($P<0.01$ and $P<0.05$ respectively), as shown in Table 3.

PSQI scales

The measurement results of the PSQI scale showed that of the 60 medical staff member in the experimental group (fever clinic), 7 staff members had mild insomnia (11.67%) with a total score of 7 to 11, 37 staff members had moderate insomnia with a total score of 12 to 16 (61.67%), and 16 staff members had severe insomnia (26.67%) with a total score of 17 to 21. The overall PSQI average score was (16.07±3.761), of which the sleep quality (2.69±0.098) accounted for the highest score. There were significant differences in the PSQI test between the experimental group and the control group with respect to other dimensions except for hypnotic drugs ($P<0.05$), and the differences in sleep time and sleep efficiency between the 2 groups were statistically significant ($P<0.01$). The PSQI total score was 16.07, which indicates that the sleep quality was poor, as shown in Table 4.

Exercise intervention

Medical staff who exercised according to the exercise prescriptions generally had better psychological stress and sleep status than other staff.

This work is licensed under Creative Common Attribution-NonCommercial-NoDerivatives 4.0 International (CC BY-NC-ND 4.0)

Indexed in: [Index Medicus/MEDLINE] [EMBASE/Excerpta Medica] [Chemical Abstracts/CAS]

Table 2. Comparison of factors in the SCL-90 scale (M±SD).

Species	Experimental group (n=60)	Control group (n=60)	*t*	*P*
Somatization	2.13±0.068	1.34±0.145	−0.246	0.014
Obsessive-compulsive	1.79±0.784	1.49±0.324	0.923	0.350
Interpersonal sensitivity	1.67±0.796	0.34±0.074	0.987	0.235
Depression	1.92±0.798	1.35±0.236	0.703	0.047
Anxiety	2.02±0.688	1.98±0.211	0.895	0.069
Hostility	1.61±0.806	1.56±0.136	0.274	0.765
Terror	2.28±0.773	1.11±0.057	1.012	0.030
Paranoid	1.53±0.701	1.45±0.058	0.301	0.734
Psychotic	1.49±0.679	1.28±0.063	1.153	0.243
Other (diet sleep)	1.71±0.801	1.67±0.179	0.278	0.801
Total score	144.67±56.344	94.87±6.367	0.692	0.047
Overall average	1.76±0.702	1.33±0.085	0.706	0.049

M±SD – mean±standard deviation; SCL-90 – Symptom Checklist 90.

Table 3. Comparison of factors in SAS, SDS, and PCL-C scales (M±SD).

Species	Experimental group (n=60)	Control group (n=60)	*t*	*P*
SAS	45.89±1.117	41.024±1.145	0.743	0.056
SDS	50.13±1.813	36.11±2.061	1.787	0.042
PCL-C	33.73±1.556	29.89±1.974	2.314	0.031

M±SD – mean±standard deviation; SAS – Self-rating Anxiety Scale; SDS – Self-rating Depression Scale; PCL-C – PTSD Checklist-Civilian Version.

Table 4. Comparison of factors in the PSQI (M±SD).

Species	Experimental group (n=60)	Control group (n=60)	*t*	*P*
Sleep quality	2.69±0.098	1.84±0.005	2.201	0.015
Sleep latency	2.46±0.188	1.23±0.024	1.321	0.021
Sleep time	2.35±0.094	1.14±0.077	2.087	0.004
Sleep efficiency	2.06±0.087	1.31±0.087	0.563	0.003
Sleep symptoms	2.25±0.085	1.90±0.051	0.985	0.029
Hypnotic drugs	1.95±0.096	1.26±0.006	0.574	0.665
Daytime dysfunction	2.31±0.113	1.81±0.047	2.111	0.039
Total score	16.07±3.761	10.49±3.135	0.915	0.036

PSQI – Pittsburgh Sleep Quality Index; M±SD – mean ± standard deviation.

Related analysis

The somatization, depression, terror, SDS, PCL-C, and other dimensions on the PSQI except for hypnotic drugs, sleep time, and sleep efficiency of the experimental group were significantly related ($P<0.01$), as shown in Table 5.

Discussions

When an acute infectious disease is prevalent in a certain area, medical personnel who are in close contact with patients with infectious diseases may experience various psychological problems. When the body encounters a major emergency,

This work is licensed under Creative Common Attribution-NonCommercial-NoDerivatives 4.0 International (CC BY-NC-ND 4.0)

Indexed in: [Index Medicus/MEDLINE] [EMBASE/Excerpta Medica] [Chemical Abstracts/CAS]

Table 5. Correlation analysis of mental state and sleep of experimental group staff (r).

Species	Somatization	Depression	Terror	SCL-90	SDS	PCL-C
Sleep quality	0.613*	0.596*	0.567*	0.633*	0.533*	0.683*
Sleep latency	0.613*	0.567*	0.489*	0.597*	0.467*	0.593*
Sleep time	0.021	0.054	–0.013	0.035	–0.038	0.042
Sleep efficiency	0.039	0.089	0.042	0.075	–0.033	0.081
Sleep symptoms	0.688*	0.644*	0.627*	0.701*	0.546*	0.717*
Hypnotic drugs	0.023	0.067	0.074	0.071	–0.032	0.065
Daytime dysfunction	0.493*	0.517*	0.433*	0.577*	0.403*	0.425*

* $P<0.01$. SCL-90 – Symptom Checklist 90; SDS – Self-rating Depression Scale; PCL-C – PTSD Checklist-Civilian Version.

when routine and normality are disrupted, a stress response will occur. Especially when people feel that their life is in danger, a series of internal psychological reactions including emotional, thinking, and behavior changes may occur. These include fear, anxiety, grief, depression, and psycho physiological reactions such as fatigue, pain, palpitation, chest tightness, and decreased appetite. In severe cases, post-traumatic stress disorder occurs. Providing positive self-exercising and psychological interventions is of great significance for the prevention and reduction of acute stress disorder, post-traumatic stress disorder, and other mental symptoms, and positive self-exercising and psychological interventions have an important promoting effect on physical therapy. But this all needs to be based on the understanding of changes in mental state.

In this COVID-19 epidemic, front-line medical staff must face the trauma from the epidemic setting, but also must face the risk of coronavirus infection, the fear of many unknown conditions that occur during providing treatment, and the possible threats to their well-being. The failure of treatments, coping with various misunderstandings from the affected side during treatment, etc., create a huge pressure that can easily cause mental and physical symptoms. From the SCL-90 scale in Table 2 combined with Figure 1, we can see that the scores of somatization, depression, anxiety, and terror of the experimental group were higher than those of the control group. Among them, the total score of SCL-90 and the scores of somatization, depression, and terror were significantly different between the experimental group and the control group. The difference in anxiety scores may reflect that the control group medical staff in an epidemic setting also had a certain degree of anxiety.

This study also found that the SAS, SDS, and PCL-C scores of medical staff in the medical experimental group were higher than normal, as shown in Table 3. The results showed that the medical staff in the experimental group had higher somatization symptoms, depression symptoms, and symptoms related to post-traumatic stress disorder. Clinical studies have found that under stress, the neuroendocrine network regulated by the HPA axis in the patient's body is disordered, the level of corticotropin-releasing factor is increased, and the level of cortisol is low, leading to the continuous activation of the adrenergic pathway [16]. As a result, emotions such as anxiety, fear, irritability, and hypersensitivity are more likely to occur. Numerous studies have suggested that changes in neuroanatomy and neuroendocrine under stress conditions can cause immune system symptoms. Several inflammatory factors, including tumor necrosis factor-α (TNF-α), interleukin (IL)-1β, IL-6, and interferon-γ are all higher than those of healthy people [17,18], which increases the risk of autoimmune or inflammatory diseases. Susceptibility, and the acceleration of the development of disease course, thereby increasing the burden of disease on these individuals.

This study evaluated the sleep status of front-line medical staff. The measurement results using the PSQI scale showed that among the 60 medical staff in the experimental group, the total PSQI score was 16.07±3.761, and the sleep quality accounted for the highest score. Compared with the control group, the medical staff of the experimental group had significant differences in other dimensions except for hypnotic drugs ($P<0.05$). From Table 4 and Figure 2, we can be see that the epidemic situation has a huge impact on the sleep of front-line medical staff, and the sleep quality of front-line medical staff at fever clinics is generally lower than that of medical staff at non-fever clinics. Further correlation analysis showed that somatization, depression, terror, SDS, and PCL-C of the medical staff in the experimental group were significantly related to other dimensions on the PSQI other than hypnotic drugs, sleep time, and sleep efficiency ($P<0.05$). Previous clinical studies have shown that the relationship between anxiety and depression and sleep symptoms is obvious. These negative emotions can increase the sleep latency of individuals to a certain extent, which makes it difficult to fall asleep, even wake up during the night, wake up early, and have more dreams.

This work is licensed under Creative Common Attribution-NonCommercial-NoDerivatives 4.0 International (CC BY-NC-ND 4.0)

Indexed in: [Index Medicus/MEDLINE] [EMBASE/Excerpta Medica] [Chemical Abstracts/CAS]

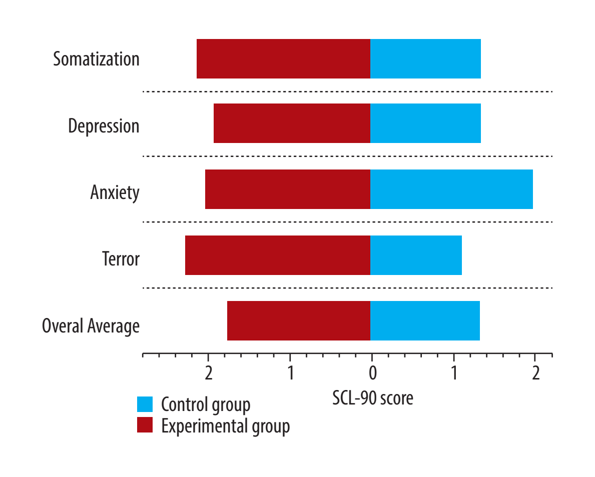

Figure 1. SCL-90 scores of front-line medical staff. SCL-90 – Symptom Checklist 90.

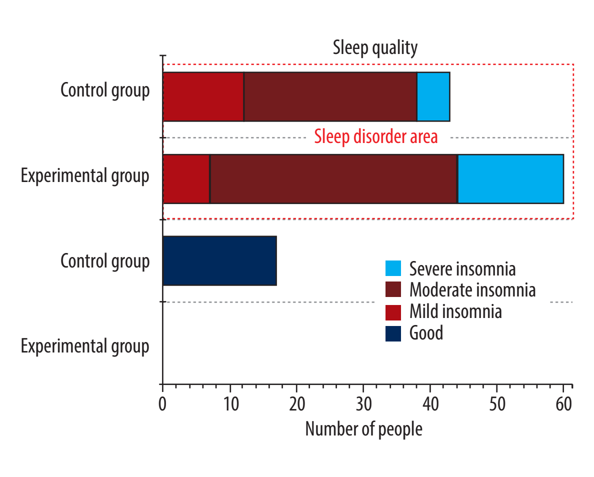

Figure 2. Impact of COVID-19 epidemic situation on sleep status of front-line medical staff.

As a result, sleep efficiency is reduced, and sleep symptoms such as sleep structure symptoms occur. Sleep helps to clear metabolic wastes in the brain, such as lactic acid, β-amyloid, etc. [19]. Sleep deprivation can affect all aspects of physical health, have a wide range of effects on emotional and mental performance, and physiological functions such as cardiovascular, endocrine, immune system, and energy metabolism [20], and can even cause irreversible damage [21]. And long-term recurrent episodes of insufficient sleep can lead to emotional symptoms. Emotional symptoms can also increase the barriers to various systems such as immunity, learning, and memory.

In studies of sleep symptoms, daytime functional status is one of the important reference indicators. Related findings in this study found that this important indicator was related to various psychological test indicators, indicating that these abnormal mental states have an important impact on daytime function. Therefore, reasonable rotation is not only a guarantee for the normal activities of daytime medical services, but also one of the important means to adjust the mental state of medical staff.

In addition, it is recommended that front-line medical personnel perform more physical exercises and exercise at home according to the rehabilitation exercise prescription; exercises such as yoga, tai chi, and qigong can greatly reduce psychological stress and improve sleep quality. According to the feedback results of medical staff, after exercising according to the exercise prescription, the effect of improving sleep was obvious. Aerobic rehabilitation exercise prescriptions can improve cardiopulmonary function and body health, relieve mental stress, and provide physical protection for good sleep.

Conclusions

In summary, the evaluation of the mental and sleep status of front-line medical staff in an epidemic setting shows that there is a certain degree of anxiety, depression, and stress disorder, and the sleep situation is also affected. Therefore, we should actively pay attention to the mental health and sleep status of front-line medical personnel and adopt sports intervention measures. Physical exercise with suitable intensity and quantity can relieve psychological pressure, eliminate mental tension, and achieve the effect of psychological stability. And even adopt drug intervention treatment strategies if necessary, which will play a positive role in overall treatment and prognosis of medical staff during an epidemic. The shortcomings of this study were: 1) the specific work roles of front-line medical staff, such as nurses, doctors, or other medical staff, may vary due to different job positions or nature, and have not been discussed; 2) specific data statistics and comparative analysis of exercise intervention was not done, and multifactor analysis was not performed in this study.

Acknowledgements

The authors thank Deputy Chief Physician Weiren Du of the Second People's Hospital of Wuhu City, Chief Physician Ruiquan Wang and Chief Physician Cegang Liu of the First Affiliated Hospital of Wannan Medical College, and Deputy Chief Physician Fulai Pei of the Second Affiliated Hospital for their care in questionnaire design, and survey and data analysis guidance and great help.

Conflict of interest

None.

This work is licensed under Creative Common Attribution-NonCommercial-NoDerivatives 4.0 International (CC BY-NC-ND 4.0)

Indexed in: [Index Medicus/MEDLINE] [EMBASE/Excerpta Medica] [Chemical Abstracts/CAS]

References:

1. Marine AD, Singh-Manoux A, Shipley MJ et al: Sleep duration and sleep disturbances partly explain the association between depressive symptoms and cardiovascular mortality: The Whitehall II cohort study. J Sleep Res, 2014; 23(1): 94–97
2. Derogatis LR, Lipman RS, Covi L: SCL-90: An outpatient psychiatric rating scale – preliminary report. Psychopharmacology Bulletin, 1973 ;9(1): 41–47
3. Derogatis LR, Rickels K, Rock AF: The SCL-90 and the MMPI: A step in the validation of a new self-report scale. Br J Psychiatry, 1976; 128(3): 280–89
4. Holi MM, Sammallahti PR, Aalberg VA: A finnish validation study of the SCL-90. Acta Psychiatr Scand, 1998; 97(1): 42–46
5. Zung WK: A self-rating depression scale. Archives of General Psychiatry, 1965; 12(1): 63–69
6. Jegede RO: Psychometric characteristics of Yoruba Version of Zung's Self-Rating Depression Scale and Self-Rating Anxiety Scale. Afr J Med Sci, 1979; 8(3-4): 133–37
7. Olatunji BO, Deacon BJ, Abramowitz JS et al: Dimensionality of somatic complaints: Factor structure and psychometric properties of the Self-Rating Anxiety Scale. J Anxiety Disord, 2006; 20(5): 550–61
8. Lindsay WR, Michie A: Adaptation of the Zung Self-Rating Anxiety Scale for people with a mental handicap. J Ment Defic Res, 1988; 32(Pt 6): 485–90
9. Buysse DJ, Reynolds CF, Monk TH et al: Quantification of subjective sleep quality in healthy elderly men and women using the Pittsburgh Sleep Quality Index (PSQI). Sleep, 1991;14(4): 331–38
10. Tsai PS, Wang SY, Wang MY et al: Psychometric evaluation of the Chinese version of the Pittsburgh Sleep Quality Index (CPSQI) in primary insomnia and control subjects. Qual Life Res, 2005; 14(8): 1943–52
11. Fodor K, Pozen J, Ntaganira J et al: The factor structure of posttraumatic stress disorder symptoms among Rwandans exposed to the 1994 genocide: A confirmatory factor analytic study using the PCL-C. J Anxiety Disord, 2015; 32: 8–16
12. Smith MY, Redd W, Duhamel K et al: Validation of the PTSD Checklist-Civilian Version in survivors of bone marrow transplantation. J Trauma Stress, 1999; 12(3): 485–99
13. Conybeare D, Behar E, Solomon A et al: The PTSD Checklist-Civilian Version: Reliability, validity, and factor structure in a nonclinical sample. J Clin Psychol, 2012; 68(6): 120–30
14. Miles JNV, Marshall GN, Schell TL: Spanish and English versions of the PTSD Checklist-Civilian Version (PCL-C): Testing for differential item functioning. J Trauma Stress, 2008; 21(4): 369–76
15. Karstoft KI, Andersen SB, Bertelsen M et al: Diagnostic accuracy of the Posttraumatic Stress Disorder Checklist – Civilian Version in a representative military sample. Psychol Assess, 2014; 26(1): 321–25
16. Sabban EL, Serova LI, Newman E et al: Changes in gene expression in the locus coeruleus-amygdala circuitry in inhibitory avoidance PTSD model. Cell Mol Neurobiol, 2018; 38(1): 273–80
17. Imai R, Hori H, Itoh M et al: Inflammatory markers and their possible effects on cognitive function in women with posttraumatic stress disorder. J Psychiatr Res, 2018; 102: 192–200
18. Passos IC, Vasconcelos-Moreno MP, Costa LG et al: Inflammatory markers in post-traumatic stress disorder: A systematic review, meta-analysis, and meta-regression. Lancet Psychiatry, 2015; 2(11): 1002–12
19. Xie L, Kang H, Xu Q et al: Sleep drives metabolite clearance from the adult brain. Science, 2013; 342(6156): 373–77
20. Grandner MA, Jackson NJ, Pak VM et al: Sleep disturbance is associated with cardiovascular and metabolic disorders. J Sleep Res, 2012; 21(4): 427–33
21. Mullington JM, Haack M, Toth M et al: Cardiovascular, inflammatory, and metabolic consequences of sleep deprivation. Prog Cardiovasc Dis, 2009; 51(4): 294–302

This work is licensed under Creative Common Attribution-NonCommercial-NoDerivatives 4.0 International (CC BY-NC-ND 4.0)

Indexed in: [Index Medicus/MEDLINE] [EMBASE/Excerpta Medica] [Chemical Abstracts/CAS]
