# Supplementary material for: Mental health status and related factors influencing healthcare workers during the COVID-19 pandemic: A systematic review and meta-analysis
Source: PLoS One. 2024 Jan 19;19(1):e0289454. doi: 10.1371/journal.pone.0289454 (PMC10798549; doi:10.1371/journal.pone.0289454)
Supplement: S1 Data — (ZIP) [file pone.0289454.s011.zip › literatures/217.pdf]

INQUIRY: The Journal of Health Care Organization, Provision, and Financing
Volume 57: 1–6
© The Author(s) 2020
Article reuse guidelines:
sagepub.com/journals-permissions
DOI: 10.1177/0046958020957114
journals.sagepub.com/home/inq

The Psychological Status and Self-Efficacy of Nurses During COVID-19 Outbreak: A Cross-Sectional Survey

Huan Xiong, MS[1], Shuanglian Yi, BS[1], and Yufen Lin, BS[1]

Abstract

A novel coronavirus pneumonia broke out and gradually developed into a global public health problem. Health care workers, especially nurses, suffered from great occupational pressure and psychological distress during the outbreak of infectious diseases. We performed a cross-sectional survey to investigate the psychological status and self-efficacy of nurses in public hospital during COVID-19 outbreak between 16th and 25th February 2020. A total of 223 nurses participated in this study. The prevalence of anxiety and depression symptoms was 40.8% (CI 95%: 34.4%-47.2%) and 26.4% (CI 95%: 20.6%-42.2%), respectively. There was no difference in the prevalence of anxiety symptoms among demographic variables. There was significant differences in the prevalence of depression symptoms according to professional titles ($P=.020$). The mean score of self-efficacy was 25.90 ± 7.55. The self-efficacy was negatively correlated with anxiety ($r=-0.161$, $P<.05$). The psychological status of nurses in public hospital during COVID-19 outbreak needs our attention. Improving nurses' self-efficacy in dealing with emerging infectious diseases may be helpful to their psychology.

Keywords

COVID-19, anxiety, depression, self-efficacy

What do we already know about this topic?
Health care workers, especially nurses suffered from great stress and psychological distress during the epidemic of infectious diseases.
How does your research contribute to the field?
We reported the psychological status and self-efficacy of nurses during COVID-19 outbreak.
What are your research's implications toward theory, practice, or policy?
We hope we can provide basis for psychological intervention of nurses.

Introduction

In December 2019, a novel coronavirus pneumonia case was reported in Wuhan, China, and then the novel coronavirus (COVID-19) broke out in China rapidly and spread to other countries. The novel coronavirus pneumonia soon attracted international concern and was listed as a public health emergencies by WHO.[1] The novel coronavirus pneumonia was confirmed to be a human-to-human disease, and the main source of transmission was symptomatic diagnosed patients.[2] Moreover, it was reported that asymptomatic carriers may also transfer the COVID-19 to others.[3] On February 25th, 78064 confirmed cases and 2715 deaths cases have been reported in mainland China.[4] As a result, the general public were likely to be stressed anxious and depressed during the outbreak and at the peak of the COVID-19 epidemic.[5,6] During the outbreak of COVID-19, medical staff in public hospital, especially nurses, provide health service for patients as usual. The patients they contact may be those being in incubation period, waiting to be diagnosed, or asymptomatic carriers. They are exposed to many known and unknown risk factors. As of February 11th, 3019 medical staff were infected

[1]Zhongshan Hospital Xiamen University, Fujian, P.R. China

Received 9 April 2020; revised 3 July 2020; revised manuscript accepted 23 July 2020

Corresponding Author:
Huan Xiong, Department of Gastroenterology, Zhongshan Hospital Xiamen University, No. 201, Hubin Road, Siming District, Xiamen, Fujian 361004, P.R. China.
Email: 18079140130@163.com

Creative Commons Non Commercial CC BY-NC: This article is distributed under the terms of the Creative Commons Attribution-NonCommercial 4.0 License (https://creativecommons.org/licenses/by-nc/4.0/) which permits non-commercial use, reproduction and distribution of the work without further permission provided the original work is attributed as specified on the SAGE and Open Access pages (https://us.sagepub.com/en-us/nam/open-access-at-sage).

with COVID-19 in mainland China, resulting in 5 death.[7] Coping with infectious diseases, the medical staff were under great occupational pressure and experienced psychological distress.[6] Little is known about psychological status of nurses during COVID-19 outbreak.

Health care workers suffered from great stress and psychological distress during the epidemic of infectious diseases. For example, health care workers reported disease related concern and suffered from somatic symptoms and psychological distress during SARS attack.[7,8] Middle East respiratory syndrome coronavirus caused worry, fear and PTSD-like symptoms among hospital workers.[9,10] A significant proportion of health care workers experienced worry and anxiety about influenza pandemic.[11]

Due to reduced accessibility to psychological support, less first-hand medical information, less intensive training on personal protective equipment and infection control measures during COVID-19 pandemic, health care workers were more likely to experience stress and psychological distress.[8] A multinational, multicentre study showed that out of 906 health care workers who participated in the survey during COVID-19 pandemic suffered from expansive range of physical symptoms, 5.3% was moderate to very-severe depression, and 8.7% was moderate to extremely-severe anxiety.[9] These psychological distress caused by acute infectious diseases may persist for a long time and even lead to posttraumatic stress disorder.[10,11] Compared with other hospital staff, nurses had a high proportion of psychological distress.[12] What's more, a study found that non-frontline nurses who worked in hospitals were more likely to suffer from psychological problems than front-line nurses.[13] Therefore, we intended to investigate the psychological status of these nurses.

Self-efficacy reflects a belief and a sense of confidence in whether individuals can use their capacity to achieve tasks.[14] The self-efficacy is related to the level of motivation, actions, and psychological state.[15] Schwarzer raised the concept of general self-efficacy, which referring to an individual's overall self-confidence in dealing with challenges of different environments contexts or burgeoning issues.[16] It can reflect individual's behavior and psychological status in different contexts. The self-efficacy of nurses correlated with mental health, resilience, and job burnout.[17-19] Studies found there was a negative correlation between general self-efficacy and anxiety.[20,21] During the outbreak of SARS, health care workers with low self-efficacy experienced higher fear.[22] In addition, self-efficacy was a important factor in predicting nurses' willingness to care for patients with emerging infectious disease.[23,24] This study was to examine the psychological status and self-efficacy of nurses still working in public hospital during COVID-19 outbreak, and explore the relationships among demographic variables, anxiety, depression, and self-efficacy.

Methods

Study Design and Data Collection

This study was a descriptive cross-sectional survey. The participants were from one of public tertiary hospitals in Xiamen, Fujian Province. This hospital is not responsible for the treatment of COVID-19, but it also admits fever and suspected patients. Once the patients are confirmed to be infected with COVID-19, they will be sent to another government designated hospital for treatment. Respondents was limited to registered nurses still working in the hospital during the outbreak of COVID-19. We searched the departments admitting patients as usual in the hospital's electronic medical record system. Then we explained the survey to the head nurses of each department and obtain their consent. We sent recruitment and the internet questionnaire to the head nurses every day, and they helped us to transmit questionnaires to nurses. Nurses choose whether to participate in the survey according to their own willingness. When no one participated in the survey for 3 days, we terminated the questionnaire. The survey was granted by the medical ethics committee of hospital (xmzsyyky Ethics No. 2020-006). Data were collected between 16th and 25th February 2020, and a total of 223 nurses participated in the study.

Measures

Demographic characteristics. The nurses' demographic characteristics in this study include gender, age, education level, professional titles, working experience, department, living status, the history of contact with confirmed, suspected or fever patient.

Psychological status

The Generalized Anxiety Disorder-7 (GAD-7). Spitzer developed a 7-item self-report instrument based on the DSM-IV criteria for screening generalized anxiety disorder.[25] It asked participants how often they were bothered by some typical anxiety symptoms during the last 2 weeks. Each item is scored from 0 to 3. When the total score is more than 5 points, it indicates that respondents may experience anxiety symptoms. GAD-7 had good reliability and validity in Chinese population.[26]

The Chinese Version of the Patient Health Questionnaire (PHQ-9). The PHQ-9 was developed by Kroenke et al based on the DSM-IV criteria, and it was to assess the severity of depression.[27] The PHQ-9 consists of 9 items, with a total score ranging from 0 to 27. Respondents whose score is over 5 points may have different degrees of depression symptoms or even depression disorder. This scale is brief and convenient, and it has excellent internal consistency reliability and

test-retest reliability. The Cronbach's α of the Chinese version of PHQ-9 in the general population was 0.86, test-retest reliability coefficient was 0.86.[28]

Self-efficacy. We used the Chinese Version of the General Self-Efficacy Scale to measure the confidence and belief of nurses when facing to COVID-19. The scale includes 10 items, ranging from 1 to 4. The higher scores indicate having greater confidence and self-efficacy in coping challenges and stress. The Cronbach's α coefficient, test-retest reliability coefficient, and split-half reliability were 0.87, 0.83 and 0.82, respectively.[29]

Data Analysis.

All the data were analyzed using SPSS22.0. Demographic information, and scores of anxiety, depression, and self-efficacy were described by number (n), percentage (%), mean and standard deviation. The relationship between anxiety, depression, and demographic variables were examined by chi-square test or Fisher exact tests. To explore the relationship between anxiety, depression, and self-efficacy, we used Pearson correlation analysis.

Results

A total of 361 nurses from 11 departments met our inclusion exclusion criteria, and finally 231 nurses participated in the study. The response rate was 61.8%. The majority of the participants were female (97.3%), between 26 and 45 years old (71.7%), and had baccalaureate degree. Almost half of the nurses had primary professional titles and less than 10 years of working experience. Fifty-nine nurses contacted with confirmed or suspected patients during outbreak, and 67.7% of nurses contacted with fever patients. Other demographic information is shown in Table 1.

The mean score of GAD-7 was 4.31 ± 4.52, and PHQ-9 was 3.24 ± 4.04 (Table 3). Table 2 shows the prevalence of anxiety and depression among nurses. The prevalence of anxiety and depression symptoms was 40.8% (CI 95%: 34.4%-47.2%) and 26.4% (CI 95%: 20.6%-42.2%), respectively. Ninety one nurses experienced different degree of anxiety whose GAD-7 scores were more than 5 points, including 64 mild anxiety, 19 moderate anxiety and 8 severe anxiety. A total of 59 nurses had depressive symptoms, and the proportion of mild, moderate, severe, and extremely severe depression was 74.6%, 18.6%, 5.1%, and 1.7%. There was no difference in the prevalence of anxiety symptoms in demographic variables. There was significant differences in the prevalence of depression symptoms according to professional titles ($P = .020$) (see Table 1).

The mean score of self-efficacy was 25.90 ± 7.55. And as shown in Table 3, the nurses' self-efficacy was negatively correlated with anxiety ($r = -0.161$, $P < .05$). There was no significant correlation between self-efficacy and depression ($r = -0.104$, $P > 0.05$).

Discussion

This survey was to investigate psychological status and self-efficacy of nurses in public hospital, still caring for patients during COVID-19 outbreak. We found the prevalence of anxiety and depression symptoms among nurses was 40.8% and 26.4%, respectively, which was higher than other similar studies on health care workers and non-healthcare workers.[8,9] Possible explanation may be that China was the first country facing a large-scale outbreak of COVID-19, health care workers suffered from great physical and psychological stress. According to other study, it was reported nurses in one of designated hospital were under great psychological stress and experienced psychological symptoms, such as compulsion, anxiety, fear, etc.[30] We found the prevalence of anxiety symptoms among nurses was 40.8%, and depression was 26.4%. Although this hospital is not government designated hospital, the results indicate that nurses have high levels of anxiety and depression. This hospital it admits fever patients and patients waiting to be diagnosed. Nurses in this hospital are also at high risk of exposure to the virus. Unlike nurses in designated hospital who are certain about the patients' diagnoses and wear a full set of protective equipments, they may have come across newly admitted patients without signs and symptoms to indicate whether they had infected COVID-19 and they may not have taken effective protective measures. In our study, the incidence of moderate and severe anxiety was 12.1%, and depression was 6.7%, which was lower than reported general population.[5] Similarly, a study found that the general public had higher level of vicarious traumatization than the front-line nurses.[13] This may be related to the prolonged lockdown affecting daily life and poor disease knowledge.[6,13] These results imply that nurses in this type of hospital have great psychological distress during outbreak. It suggests that managers should pay attention to nurses' psychological status and provide support to relieve their anxiety and depression.

In this study, nurses with intermediate professional titles have higher prevalence of depression symptoms. A possible explanation is that nurses with intermediate titles are well-experienced. They are an important part of the battle of epidemic prevention and control, and they can evaluate the risk of infection according to clinical experience. As a result, they may have greater psychological distress. We also found that there was no difference in the prevalence of anxiety symptoms among demographic variables. It may be that anxiety symptoms are widespread among nurses during COVID-19 outbreak. It needs to be further verified.

Table 1. The Prevalence of Anxiety and Depression Symptoms Among Nurses in Demographic Characteristics [*n*/(%)].

Variable	*n*	Anxiety		Depression	
		Yes	*P*	Yes	*P*
Gender			.690		.656
female	217 (97.3)	88 (40.6)		57 (26.3)	
male	6 (2.7)	3 (50)		2 (33.3)	
Age group (years old)			.127		.055
≤25	43 (19.3)	15 (34.9)		6 (14.0)	
26-35	103 (46.2)	37 (35.9)		25 (24.3)	
36-45	57 (25.5)	27 (47.4)		21 (36.8)	
46-55	20 (9)	12 (60.0)		7 (35.0)	
Educational level			.160		.051
Associate's degree	55 (24.7)	18 (32.7)		9 (16.4)	
Bachelor's degree and above	168 (75.3)	73 (43.5)		50 (29.8)	
Working experience (years)			.116		.070
<2	36 (16.1)	12 (33.3)		6 (16.7)	
2-5	31 (13.9)	10 (32.3)		6 (19.4)	
6-10	56 (25.1)	19 (33.9)		11 (19.6)	
11-15	51 (22.9)	23 (45.1)		19 (37.3)	
>15	49 (22.0)	27 (55.1)		17 (34.7)	
Professional titles			.113		.020
primary	113 (50.7)	39 (34.5)		21 (18.6)	
Intermediate	92 (41.2)	42 (45.7)		33 (35.9)	
senior	18 (8.1)	10 (55.6)		5 (27.8)	
Department			.501		.321
Infection and isolation wards	20 (9.0)	7 (35.0)		5 (25.0)	
Emergency room	14 (6.3)	6 (42.9)		5 (37.5)	
ICU	15 (6.7)	8 (53.3)		7 (46.7)	
Respiratory	14 (6.3)	9 (64.3)		4 (28.6)	
Gastroenterology	30 (13.4)	11 (36.7)		5 (16.7)	
Neurology	24 (10.8)	7 (29.2)		5 (20.8)	
Hematology	25 (11.2)	8 (32.0)		3 (12.0)	
Nephrology	21 (9.4)	8 (38.1)		6 (28.6)	
other medicine wards	60 (26.9)	27 (45.0)		19 (31.7)	
Living status			.485		.793
Live with family	147 (65.9)	64 (43.5)		41 (27.9)	
Live with friend or colleague	43 (19.3)	16 (37.2)		10 (23.3)	
Live alone	33 (14.8)	11 (33.3)		8 (24.2)	
Contact with confirmed or suspected patients during outbreak			.552		.632
Yes	59 (26.5)	26 (44.1)		17 (28.8)	
No	164 (73.5)	65 (39.6)		42 (25.6)	
Contact with fever patients during outbreak			.445		.758
Yes	151 (67.7)	59 (39.1)		39 (25.8)	
No	72 (32.3)	32 (44.4)		20 (27.8)	

Note. Other medicine wards include endocrinology, cardiology, and oncology department.

The mean score of self-efficacy was 25.90 ± 7.55, which was lower than previous studies.[31,32] Possible reasons may be due to the fact that COVID-19 is a novel coronavirus and causes an outbreak in a short time. Nurses lack experience and confidence to cope with it. The results demonstrated that self-efficacy was negatively correlated with anxiety ($r = -0.161$, $P < .05$), which was consistent with previous studies.[33] Nurses who were not confident in coping with the COVID-19 may feel more anxious. COVID-19 is a new coronavirus, and it causes a rapid outbreak of disease. Medical staff need to learn the knowledge of novel coronavirus pneumonia and adjust therapeutic plan continuously. Some nurses feel their knowledge is insufficient, which makes nurses feel incompetent and results in negative psychological experience.[34] This can provide a reference for the training of nurses' emergency ability in the future. Different

Table 2. The Prevalence of Anxiety and Depression Among Nurses [*n*/(%)].

	Mild	Moderate	Severe	Extremely severe	Total
Anxiety	64 (28.7)	19 (8.5)	8 (3.6)	-	91 (40.8)
Depression	44 (19.7)	11 (4.9)	3 (1.3)	1 (0.5)	59 (26.4)

Table 3. Correlation Analysis of Anxiety, Depression, and Self-Efficacy.

	GAD-7	PHQ-9	Self-efficacy
GAD-7	1		
PHQ-9	0.689**	1	
Self-efficacy	−0.161*	−0.104	1

*$P<.05$. **$P<.01$.

from previous studies, there was no significant correlation between self-efficacy and depression.[33] This may be due to the difference in population and situations.

In order to better respond to outbreaks and avoid psychological crisis on population, the Chinese government has taken a series of psychological assistance intervention, including releasing guiding principles for psychological intervention during COVID-19, publishing psychological guidelines books, creating psychological assistance hotlines, and providing online psychological counseling.[35] However, according to guidelines issued by the central health authority, the first targets of psychological intervention are confirmed patients, frontline health professionals and administrative staff.[36] This study found the nurse in this hospital had high incidence of anxiety and depression, and there was no significant difference in the incidence of anxiety and depression among departments and whether exposed to confirmed patients or not. These indicated that the psychological status of nurses who worked in non designed hospital or who didn't contact with confirmed patients during outbreak also needs attention and support. Online psychological self-help intervention system,[37] like online cognitive behavioral therapy and mindfulness-based interventions, may be helpful to promote nurses' mental health. Cognitive behavioral therapy may challenge cognitive bias when they overestimate the risk of contracting and suffering from COVID-19, and enhance confidence in the ability to take care of patients.[6,38] Mindfulness-based interventions is aimed at reducing stress through mindfulness meditation practices.[39] They have demonstrated efficacy in reducing depression and anxiety symptoms among university students during the epidemic of COVID-19.[40]

There were some limitations in this study. First, we only sent recruitment to nurses in 1 hospital, so this study failed to compare the differences among hospitals and groups of people, such as doctors, technician, administrative staff, etc. Thus, the findings should be further confirmed in different hospital and different health care workers. Second, this research lasted for 10 days, and a total of 223 nurses took part in it ultimately. Response bias may exist and sample size may affect data analysis results. Sample size needs to be expanded in the future. Third, this study was a cross-sectional survey, lacking follow-up, and the questionnaires were self-report. There were likely to lead to recall and reporting bias. Moreover, we couldn't distinguish if the anxiety or depression was caused by the COVID-19 or preexisting, and lacked follow-up care for nurses who had severe anxiety or depression.

In conclusion, our results show that nurses in non government designated hospital have great prevalence of anxiety and depression symptoms during COVID-19 outbreak. Nurses' professional titles are related to depression. Nurses with intermediate professional titles experience more depression symptoms. The self-efficacy coping to COVID-19 was negatively correlated with anxiety.

Acknowledgments

We express our gratitude to all the head nurses who assisted in the investigation. We show appreciation to all the nurses who participated in the investigation.

Declaration of Conflicting Interests

The author(s) declared no potential conflicts of interest with respect to the research, authorship, and/or publication of this article.

Funding

The author(s) received no financial support for the research, authorship, and/or publication of this article.

ORCID iD

Huan Xiong https://orcid.org/0000-0001-5106-167X

References

1. World Health Organization. Novel coronavirus (2019-nCoV) situation report. https://www.who.int/docs/default-source/coronaviruse/situation-reports/20200202-sitrep-13-ncov-v3.pdf?sfvrsn=195f4010_6. Published February 2, 2020. Accessed February 12, 2020.
2. Chan JF, Yuan S, Kok KH, et al. A familial cluster of pneumonia associated with the 2019 novel coronavirus indicating person-to-person transmission: a study of a family cluster. *The Lancet*. 2020;395(10223):514-523.
3. Bai Y, Yao LS, Wei T, et al. Presumed asymptomatic carrier transmission of COVID-19. *JAMA*. 2020;323:1406-1407. doi:10.1001/jama.2020.2565.
4. National Health Commission of the People's Republic of China. Up to 24 February 25th novel coronavirus pneumonia epidemic situation. http://www.nhc.gov.cn/xcs/yqtb/202002/741ce06130284a77bfbf699483c0fb60.shtml. Published February 26, 2020. Accessed February 26, 2020.
5. Wang C, Pan R, Wan XY, et al. A longitudinal study on the mental health of general population during the COVID-19 epidemic in China. *Brain Behav Immun*. 2020;87:40-48.
6. Wang C, Pan R, Wan XY, et al. Immediate psychological responses and associated factors during the initial stage of the 2019 coronavirus disease (COVID-19) epidemic

among the general population in China. *Brain Behav Immun*. 2020;17(5):1729. doi:10.3390/ijerph17051729.
7. Chinese Center for Disease Control and Prevention. Epidemiological characteristics of novel coronavirus pneumonia. *Chin J Epidemiol*. 2020;2(41):145-151.
8. Tan BYQ, Chew NWS, Lee GKH, et al. Psychological impact of the COVID-19 pandemic on health care workers in Singapore [published online ahead of print April 6, 2020]. *Ann Intern Med*. doi:10.7326/M20-1083.
9. Chew NWS, Lee GKH, Tan BYQ, et al. A multinational, multicentre study on the psychological outcomes and associated physical symptoms amongst healthcare workers during COVID-19 outbreak [published online ahead of print April 21, 2020]. *Brain Behav Immun*. doi:10.1016/j.bbi.2020.04.049.
10. Lancee WJ, Maunder RG, Goldbloom DS. Prevalence of psychiatric disorders among Toronto Hospital workers one to two years after the SARS outbreak. *Psychiatr Serv*. 2008;59(1):91-95.
11. Lung FW, Lu YC, Chang YY, Shu BC. Mental symptoms in different health professionals during the SARS attack: a follow-up study. *Psychiatr Q*. 2009;80(2):107-116.
12. Nickell LA. Psychosocial effects of SARS on hospital staff: survey of a large tertiary care institution. *Can Med Assoc J*. 2004;170(5):793-798.
13. Li Z, Ge J, Yang M, et al. Vicarious traumatization in the general public, members, and non-members of medical teams aiding in COVID-19 control [published online ahead of print March 10, 2020]. *Brain Behav Immun*. 2020;88:916-919. doi:10.1016/j.bbi.2020.03.007.
14. Bandura A. Self-efficacy: toward a unifying theory of behavioral change. *Psychol Rev*. 1977;84:191-215.
15. Bandura A. *Self-efficacy: The Exercise of Control*. New York: Freeman; 1997.
16. Schwarzer R, Aristi B. Optimistic self-beliefs: assessment of general perceived self-efficacy in thirteen cultures. *World Psychol*. 1997;3(12):177-190.
17. Hsieh YH, Wang HH, Ma SC. The mediating role of self-efficacy in the relationship between workplace bullying, mental health and an intention to leave among nurses in Taiwan. *Int J Occup Med Environ Health*. 2019;32(2):245-254.
18. Yu F, Raphael D, Mackay L, Smith M, King A. Personal and work-related factors associated with nurse resilience: a systematic review. *Int J Nurs Stud*. 2019;93:129-140.
19. Consiglio C, Borgogni L, Vecchione M, Maslach C. Self-efficacy, perceptions of context, and burnout: a multilevel study on nurses. *La Medicina del lavoro*. 2014;105(4): 255-268.
20. Schwarzer R, Mueller J, Greenglass E. Assessment of perceived general self-efficacy on the internet: data collection in cyberspace. *Anxiety Stress Copin*. 1999;12(2):145-161.
21. Wang CK, Hu ZF, Liu Y. The reliability and validity of general self-efficacy scale. *Chinese J Appl Psychol*. 2001;7(1):37-40.
22. Ho SMY, Kwong-Lo RSY, Mak CWY, Wong JS. Fear of severe acute respiratory syndrome (SARS) among health care workers. *J Consult Clin Psychol*. 2005;73(2):344-349.
23. Lee J, Kang SK. Factors influencing nurses' intention to care for patients with emerging infectious diseases: application of the theory of planned behavior. *Nurs Health Sci*. 2020;22: 82-90.
24. Natan MB, Zilberstein S, Alaev D. Willingness of future nursing workforce to report for duty during an avian influenza pandemic. *Res Theory Nurs Pract*. 2015;29(4):266-275.
25. Spitzer RL, Kroenke K, Williams JB, Löwe B. A brief measure for assessing generalized anxiety disorder: the GAD-7. *Arch Intern Med*. 2016;166:1092-1097.
26. Wang Y, Chen R, Zhang L. Evaluation of the reliability and validity of the generalized anxiety disorder 7-item scale among inpatients in general hospital. *J Clin Psychiatry*. 2018;28(3):168-171.
27. Kroenke K, Spitzer RL, Williams JB. The PHQ-9: validity of a brief depression severity measure. *J Gen Intern Med*. 2001;16:606-613.
28. Wang WZ, Bian Q, Zhao Y, et al. Reliability and validity of the Chinese version of the Patient Health Questionnaire (PHQ-9) in the general population. *Gen Hosp Psychiatry*. 2014;36(5): 539-544.
29. Zhang JX, Schwarzer R. Measuring optimistic self-beliefs: a Chinese adaptation of the General Self-Efficacy Scale. *Psychologia*. 1995;38:174-181.
30. Zhu HR, Chen F, Tao L, Xiong Q, He J, Tang M. The current status and influencing factors on psychological stress of clinical nurse in the designated hospitals during the period of prevention and control of novel coronavirus pneumonia. Modern Clinical Nursing. Published 2020. http://kns.cnki.net/kcms/detail/44.1570.R.20200224.1059.002.html.
31. Hu SH, Yu YM, Chang WY, Lin YK. Social support and factors associated with self-efficacy among acute- care nurse practitioners. *J Clin Nurs*. 2018;27(3):876-882.
32. Dan X, Liu JY, Xu SH, et al. Mediating effect of self-efficacy and colleague solidarity on innovative behavior and career success of nurses. *Chinese J Behav Med Brain Sci*. 2018;27(7):633-638.
33. Li L, Neili X, Lie W. Moderating role of self-efficacy on the associations of social support with depressive and anxiety symptoms in Chinese patients with rheumatoid arthritis. *Neuropsychiatr Dis Treat*. 2017;13:2141-2150.
34. Yang HH, Dai L, Chen QX, Lv GZ. A qualitative study on the psychological stress of first-line nurses in primary hospitals who participated in the fight against novel coronavirus pneumonia. J Nurs Admin. Published 2020. http://kns.cnki.net/kcms/detail/11.4716.C.20200220.1442.002.html.
35. Wang Y, Zhao X, Feng Q, Liu L, Yao Y, Shi J. Psychological assistance during the coronavirus disease 2019 Outbreak in China. *J Health Psychol*. 2020;25(6):733-737.
36. National Health Commission of China. Guiding principles for emergency psychological crisis intervention for the COVID-19 outbreak [in Chinese]. http://www.nhc.gov.cn/xcs/zhengcwj/202001/6adc08b966594253b2b791be5c3b9467.shtml. Published January 27, 2020. Accessed July 1, 2020.
37. Liu S, Yang L, Zhang C, et al. Online mental health services in China during the COVID-19 outbreak. *Lancet Psychiat*. 2020;7(4):17-18.
38. Ho CS, Chee CY, Ho RC. Mental health strategies to combat the psychological impact of COVID-19 beyond paranoia and panic. *Ann Acad Med Singap*. 2020;49(3):155-160.
39. Hofmann SG, Gómez AF. Mindfulness-based interventions for anxiety and depression. *Psychiatr Clin North Am*. 2017;40(4):739-749.
40. El Morr C, Ritvo P, Ahmad F, Moineddin R. Effectiveness of an eight-week web-based mindfulness virtual community intervention for university students on symptoms of stress, anxiety, and depression: a randomized controlled trial [published online ahead of print June 17, 2020]. *JMIR Ment Health*. doi:10.2196/18595.
