# Supplementary material for: Mental health status and related factors influencing healthcare workers during the COVID-19 pandemic: A systematic review and meta-analysis
Source: PLoS One. 2024 Jan 19;19(1):e0289454. doi: 10.1371/journal.pone.0289454 (PMC10798549; doi:10.1371/journal.pone.0289454)
Supplement: S1 Data — (ZIP) [file pone.0289454.s011.zip › literatures/22.pdf]

Original article

# The psychological impact of the Coronavirus emergency on physicians and nurses: an Italian observational study

*Maria Chiara Carriero[1]; Luana Conte[2–3]; Marica Calignano[4], Roberto Lupo[5]; Antonino Calabrò[6]; Pietro Santoro[7]; Giovanna Artioli[8]; Cosimo Caldararo[9]; Maurizio Ercolani[10]; Maicol Carvello[11]; Antonio Leo[12]*

[1]"Santa Chiara" Institute, Rome, Italy; [2]Laboratory of Biomedical Physics and Environment, Department of Mathematics and Physics "E. De Giorgi", University of Salento, Lecce, Italy; [3]Advanced Data Analysis in Medicine (ADAM), Laboratory of Interdisciplinary Research Applied to Medicine (DReAM), University of Salento and ASL (Local Health Authority) Lecce, Italy; [4]University-Hospital, Parma Italy; [5]"San Giuseppe da Copertino" Hospital, ASL (Local Health Authority) Lecce, Italy; [6]"Nuovo Ospedale degli Infermi" Hospital, ASL (Local Health Authority) Biella; [7]MathCounseling Lecce, Italy; [8]IRCCS, "Santa Maria Nuova" Hospital, USL (Local Health Authority) - Reggio Emilia, Italy; [9]University of Bari, Lecce, Italy; [10]"ASUR Marche Area Vasta 2" Health Mental Department, Ancona, Italy; [11]Brisighella Community Hospital, ASL (Local Health Authority) of Romagna, Brisighella, Italy; [12]"Santa Chiara" Institute, Lecce, Italy

**Abstract.** *Background and aim of the work*: The Coronavirus has put a strain on the response capacity of health systems and there are various psychological effects on health workers. *Aim of the study*: To investigate the psychological impact of the coronavirus emergency on physicians and nurses. *Methods*: A study was conducted on a sample of nurses and physicians (n=770), who were asked to fill in a questionnaire investigating physical and psychological problems. It also included the IES (Impact Event Scale), STAI (State Trait Anxiety Inventory) scale and BDI (Beck Depression Inventory). *Results*: 87.7% of the sample was represented by nurses (n=675), 12.3% (n=95) by physicians. 52.3% (n=403). Among the psychological symptoms, stress (76.2%; n=587), anxiety (59.4%; n=457) and depression (11.8%) prevailed and only 3.9% of the healthcare personnel sought help from a psychologist. The total score of the IES-R scale was 3.47. A significant association emerged between exposure and the risk of contagion (p-value = 0.003), stress was more present among nurses than among physicians (77.5% vs. 67.4%; p = 0.003). Among physical symptoms, headache (52.2%; n=402) and pressure injuries (24.8% n= 191) prevailed. *Conclusions*: The results of the study show that mental health monitoring of health workers, who are at risk of developing major psychological disorders, is a priority.

**Key words:** psychological intervention, COVID-19, stress, anxiety, depression, psychological impact, Health workers, Mental health, Pandemic, Post-traumatic stress disorder.

## Introduction

On 31 December 2019, Chinese health authorities notified an outbreak of pneumonia cases of unknown aetiology in Wuhan city (Hubei, China). On 9 January 2020, the China CDC (Centre for Disease Control and Prevention of China) identified a new Coronavirus (called 2019-nCoV) as the etiological cause of this disease. On 30 January 2020, the World Health Organization (WHO) designated the COVID-19 outbreak a "public health emergency of international concern" (1). From 23 March 2020, this new Coronavirus (Sars CoV-2) spread rapidly around the world, infecting more than 294,110 people in 187 countries and killing 12,944 people (2). Pandemic conditions require an immediate response in terms of medical assistance, with health and social care workers having to be at the forefront of the epidemic in the various

health service settings. It has affected and is drastically affecting all social and economic sectors of the world and, above all, has caused a number of adverse physical and psychological effects in the general population and among health workers (3). Several studies show that concern about high mortality rates and restrictions on people's lives have contributed to higher levels of anxiety, depression and sleep disorders in the general population. (4). Psychological disorders can also manifest themselves in non-functional attitudes, such as continuous medical consultations to obtain reassurance, distrust of public authorities, (5) or discrimination and stigma towards particular populations (6). In addition, many events such as the ever-increasing number of deaths and confirmed and suspected cases, the workload and physical fatigue, the exhaustion of protective equipment, the widespread media coverage, the lack of specific drugs, the choice among patients whom to treat/select for essential therapy due to the lack of medical supplies, the risk of infection, the feeling of not being supported are all factors that can contribute to the formation of important psychological symptoms (7). Health workers are therefore faced with critical situations that increase the risk of psychological distress. (8) and this could have serious repercussions not only on their quality of life but also on the quality of care provided to the patient. Studies conducted in Turkey, Iran and Spain confirm the prevalence of psychological symptoms among healthcare workers. A study conducted in China (9) showed that depression (50.4%), anxiety (44.6%), insomnia (34.0%) and stress (71.5%) were the most common psychological symptoms. Risk factors included being female, being a nurse, having a high risk of contracting COVID-19 or having at least one family member with COVID-19 (9) and social isolation are the most cited for the development of severe psychological symptoms (7).

The global spread of COVID-19 has therefore put the responsiveness of health systems to the test and numerous research studies are needed to assess the mental health of health workers, given their important role in responding to the situation. In addition, WHO also recommends that a large number of studies should be carried out in these circumstances (11), to provide guidelines that can help strengthen the response capacity of health systems. In Italy, there are still few studies that have analyzed the psychological impact of the pandemic, looking at levels of anxiety and depression among medical and nursing staff. Our study aims to provide empirical data on the psychological outcomes of the pandemic in health workers.

## Main aim

To investigate the psychological impact of COVID-19 emergency on a population of physicians and nurses.

## Secondary aim

To survey the lifestyle, physical and psychological health status and difficulties experienced by health workers during the period of the Coronavirus emergency, from April 2020 to June 2020.

## Methods

### *Design*

Observational, cross-sectional and multicentre study.

### *Samples*

The study, conducted from April to June 2020, was carried out through the online dissemination of a questionnaire distributed by means of a link to all the presidents of the provincial orders of nursing and medical professions in the Country. All the Presidents of the Orders were sent an e-mail presenting the study and formally requesting their participation in the survey. Some of them did not reply to the e-mail. Others, however, did not wish to participate in the study. Only a few Presidents of the Orders agreed to participate in the survey, specifically eight provincial Orders of Nursing Professions (Lecco, Mantua, Genoa, Varese, Como, Lecce, Brindisi and Trapani) and one Order of Physicians and Dentists in the province of Lecce. After having obtained the access authorizations from the

respective Presidents to the mailing list, each medical and nursing professional belonging to the Order was sent an e-mail containing a brief presentation of the survey and the link to access the online questionnaire. Medical and nursing professionals, including nursing coordinators and nursing managers, working in both the public and private sectors were included in the study. They aged between 20 and 70 years and agreed to participate in the study by signing the informed consent. All medical and nursing professionals waiting for their first job were excluded. N=770 health professionals agreed to participate in the study.

*Data collection*

The survey instrument consists of 4 sections. The first section is aimed at collecting social-demographic data from participants, the second section was created by the study managers and explores the pandemic-induced lifestyle (12 items) and potential physical and psychological problems arising during the COVID-19 emergency (25 items). The third section includes the Impact Event Scale (IES) instrument (12), validated in the Italian language (13), widely used test to assess through 21 items the psychological impact and stress reactions caused by traumatic events. It consists of two subscales measuring the experiences of intrusion (items 1, 4, 5, 6, 10, 11, 14) and avoidance (items 2, 3, 7, 8, 9, 12, 13, 15). The items are rated on a 4-point Likert scale ranging from 0 (“not at all”) to 4 (“extremely”). The fourth section includes the State Trait Anxiety Inventory scale consisting of 20 items (14) which assesses the level of Trait Anxiety, as a tendency to perceive stressful situations as dangerous and threatening. The items are rated on a 4-point scale (1 to 4) corresponding to “Not at all”, “A little”, “Somewhat” and “Very much”. Higher scores are positively correlated with higher levels of anxiety. The fifth section is the Beck Depression Inventory (BDI) scale (15-16), consisting of 13 items that measure the presence and severity of depressive symptoms. The scale was constructed to measure the behavioral manifestations of depression, favoring the cognitive correlates, namely: sadness, pessimism, failure, dissatisfaction, guilt, self-esteem, suicide, loss of interest, indecision, appearance, work, fatigue, appetite. The test can be answered with a score from 0 to 3, while the total score ranges from 0 to 63. Scores from 0 to 13 indicate no depressive content; scores from 14 to 19 mild depression; scores from 20 to 28 moderate depression; scores from 29 to 63 severe depression.

All sections of the questionnaire were computerized using a pre-set form from the Google Drive platform.

## Ethical considerations

Within the presentation of the questionnaire, the ethical characteristics of the study were stated. It was emphasized that participation was voluntary, and that the participant could refuse to take part in the protocol whenever he or she wished. Those interested in participating were given an informed consent form, which reminded them of the voluntary nature of participation, as well as the confidentiality and anonymous nature of the information. In addition, to ensure that the questionnaires were anonymous and to allow for identification of participants, a sequential identification (ID) number was given to each registered participant. Each questionnaire, therefore, had an ID number that corresponded to the database ID.

## Data analysis

Descriptive analyses were conducted for all qualitative and quantitative variables using R-Studio software version 3.6.1. Continuous variables were summarized by means of mean and standard deviation (SD) and categorical variables by means of frequencies and percentages. After the descriptive analysis of all variables, the correlation between the S.T.A.I.- Y2 and B.D.I. scales and between these and the characteristics of the sample was analyzed. The ANOVA test was used to evaluate the difference between mean values on the IES; S.T.A.I.-Y2 and BDI scales. The association between occupational profile and levels of anxiety and stress, between work area and the impact scale, between exposure and the onset of symptoms was analyzed using the Anova tests. For all inferential analyses, statistically significant results below the 5% threshold are reported.

## Results

### *Demographic characteristics*

The sample that took part in the survey consisted of 770 health workers with a prevalence of the female gender (74.3%; n=572). Of the 770 participants in the study 675 were nurses (87.7%) and 95 were physicians (12.3%). 31% of the respondents had work experience of 1 to 5 years (n=239) and 49.2% had a Bachelor's degree as their highest level of education (n=379). 51.7% (n=391) live in the North, 16.4% (n=124) in the Centre and 31.9% (n=241) in the South. 78.6% of the sample (n=594) were not located in an area other than their own residence. 77.2% (n=584) worked closely with COVID-19 patients (not necessarily in a COVID-19 department), 33.3% (n=252) had to change department/work area due to the COVID-19 emergency. The area of work most represented in the study was the Critical Emergency Area (emergency department, 118, emergency medicine, intensive care, intensive short observation) with a percentage of 34.4% (n=265) (Tab. 1).

| **Table 1. Sample characteristics (n=770)** | **N. (%)** |
|---|---|
| **Gender** | |
| Female | 572 (74.3%) |
| Male | 198 (25.7%) |
| **Professional profile** | |
| Nurse | 675 (87.7%) |
| Physicians | 95 (12.3%) |
| **Years of work experience** | |
| 1-5 | 239 (31.0%) |
| 6-10 | 102 (13.2%) |
| 11-15 | 86 (11.2%) |
| 16-20 | 67 (8.7%) |
| 21-25 | 82 (10.6%) |
| 26-30 | 83 (10.8%) |
| Over 30 | 111 (14.4%) |
| **Qualification** | |
| Regional diplom | 167 (21.7%) |
| University diploma | 62 (8.1%) |
| PhD | 4 (0.5%) |
| Medical degree | 86 (11.2%) |
| Master's degree | 72 (9.4%) |
| Bachelor's degree | 379 (49.2%) |
| **In which geographical area do you live?** | |
| North | 391 (51.7%) |
| Centre | 124 (16.4%) |
| South | 241 (31.9%) |
| **If you are in a different area from where you live, can you tell us why?** | |
| Other | |
| Work | 13 (1.7%) |
| I am not located in a different area | 139 (18.4%) |
| Study | 594 (78.6%) |
| Holiday | 8 (1.1%) |
| | 2 (0.3%) |
| **Marital status** | |
| Married | 342 (45.2%) |
| Single | 349 (46.2%) |
| Separated | 60 (7.9%) |
| Widowed | 5 (0.7%) |

| | |
|---|---|
| **Children** | |
| No | 401 (53.0%) |
| Yes, adults | 131 (17.3%) |
| Yes, minors | 160 (21.2%) |
| Yes, both minors and adults | 64 (8.5%) |
| **Lives with** | |
| Other | 12 (1.6%) |
| Roommates | 31 (13.2%) |
| Spouse | 100 (13.2%) |
| Cohabitants | 85 (11.2%) |
| Living alone | 128 (16.9%) |
| Family with children | 282 (37.3%) |
| Parents | 111 (14.7%) |
| Relatives | 7 (0.9%) |
| **Have you worked closely with COVID-19 patients (not necessarily in a COVID-19 ward)?** | |
| No | 172 (22.8%) |
| Yes | 584 (77.2%) |
| **Did you have you have to change departments/work areas due to the COVID-19 emergency?** | |
| No | 504 (66.7%) |
| Yes | 252 (33.3%) |
| **Please indicate your current work area** | |
| COVID-19 area | 110 (14.3%) |
| COVID-19 post-acute area | 48 (6.2%) |
| Surgical area | 56 (7.3%) |
| Critical care emergency area | 265 (34.4%) |
| Management/administrative area | 16 (2.1%) |
| Maternal and child area | 22 (2.9%) |
| Geriatric-rehabilitation medical area | 77 (10.0%) |
| Multi-specialist medical area | 79 (10.3%) |
| Territorial area | 2 (0.3%) |
| Territorial area (Territorial medicine) | 43 (5.6%) |
| Prevention and safety | 25 (3.2%) |
| Services | 23 (3.0%) |
| **Which of the following categories do you fall into? Please choose one:** | |
| Other | 45 (5.8%) |
| Relative or close person of someone who tested positive | 21 (2.7%) |
| Person who knows someone who tested positive | 85 (11.0%) |
| Person who has had no direct contact with someone who is positive | 99 (12.9%) |
| Person who is positive and in quarantine | 52 (6.8%) |
| Person who is positive and hospitalised | 8 (1.0%) |
| Rescuer or health worker who comes into contact with positive people or people who know someone who has tested positive | 460 (59.7%) |

*Exposure, contagions and Personal Protective Equipment (PPE)*

The study found that 17.5 % (n=135) of healthcare workers developed symptoms indicative of Sars CoV-2. However, 13.9 % (n=107) did not stop working, 31.6 % (n=246) were not tested, 19.5 % (n=150) had difficulty undergoing the screening test, just over half of the sample 55.2 % (n=425) underestimated the public health effects of the pandemic during the initial days of the pandemic. 52.3% of the sample (n=403) did not feel that they had received good training from their health authority on the correct use of Personal Protective Equipment (PPE) against SARS CoV-2. 51.3% (n=395) stated that these devices were insufficient. 18.2% (n=140) stated that they had experienced at least

one moment when they had to choose among patients whom to treat/select for essential treatment due to lack of medical supplies. 15.5% of the sample chose whom to treat by age (n=119). Among the main concerns experienced during the pandemic, fear of making loved ones ill prevailed in 64.9% (n=500) (Tab. 2).

## Analysis of physical and psychological health

In the second section the participant was asked to define their physical and psychological health status by means of a form with a detailed list (25 items) of potential physical and psychological problems that

| **Table 2. Exposure, infections and Personal Protective Equipment (PPE) (n=770)** | **N. (%)** |
|---|---|
| **How many people do you think you have been exposed to?** | |
| None | 69 (9.0%) |
| 1-10 | 232 (30.1%) |
| 11-100 | 309 (40.1%) |
| More than 100 | 160 (20.8%) |
| **Have you ever had symptoms indicative of COVID-19 infection during this period?** | |
| No | 635 (82.5%) |
| Yes | 135 (17.5%) |
| **What did you do after developing symptoms indicative of COVID-19? (Select all relevant answers)** | |
| I continued working | 39 (5.1%) |
| Started medical treatment | 5 (0.6%) |
| I physically left my family/ loved ones | 29 (3.8%) |
| Went to the emergency room | 12 (1.6%) |
| Went to the general practitioner | 11 (1.4%) |
| Voluntarily self-quarantined | 53 (6.9%) |
| I had no symptoms of COVID-19 | 621 (80.6%) |
| **Have you been tested for COVID-19?** | |
| No | 277 (36.0%) |
| Yes | 493 (64.0%) |
| **Did you have difficulty being tested for COVID-19?** | |
| No | 374 (48.6%) |
| I have not been tested | 246 (31.6%) |
| Yes | 150 (19.5%) |
| **How many times have you been tested for COVID-19?** | |
| 0 | |
| 1-2 | 271 (35.2%) 370 |
| 3-4 | (48.1%) 109 |
| 5+ | (14.2%) 20 (2.6%) |
| **Do you think you understimated the effects of Pandemic on public health during the initial days of Pandemic?** | |
| No | 345 (44.8%) |
| Yes | 425 (55.2%) |
| **Do you think you have received good training about the correct use of Personal Protecion Equipment against COVID-19 from your Hospital** | |
| No | 403 (52.3%) |
| Yes | 367 (47.7%) |
| **Do you think that these protective devices were sufficient?** | |
| No | 395 (51.3%) |
| Yes | 375 (48.7%) |

| | |
|---|---|
| **Have you been provided with adequate Personal Protecion Equipment by your employer?** | |
| No | 301 (39.1%) |
| Yes | 469 (60.9%) |
| **Which of the following factors would influence/have influenced your decision to prioritise the treatment of certain groups of COVID-19 patients over others due to the shortage of medical supplies?** | |
| Other | 169 (21.9%) |
| Clinic | 157 (20.4%) |
| Age | 119 (15.5%) |
| Did not treat COVID-19 patients | 244 (31.7%) |
| Chronic conditions | 81 (10.5%) |
| **What are the main concerns you feel during this period? (Choose all relevant answers)** | |
| Other | 42 (5.5%) |
| Falling ill | 84 (10.9%) |
| Making your loved ones ill Not yet having adequate skills to take care of COVID-19 patients | 500 (64.9%) |
| Not yet having adequate skills to take care of COVID-19 patients | 33 (4.3%) |
| Not having adequate tools to take care of COVID-19 patients | 72 (9.4%) |
| I have no concerns | 39 (5.1%) |
| **During this period of coronavirus emergency did you start taking antidepressant and/or neuroleptic drugs?** | |
| No | 713 (92.6%) |
| Si | 57 (7.4%) |
| **Who did you ask for help when you were most distressed?** | |
| Friends | 118 (15.3%) |
| Colleagues | 158 (20.5%) |
| Family | 241 (31.3%) |
| Psychologist/psychotherapist | 30 (3.9%) |
| None | 223 (29.0%) |

arose during the COVID-19 emergency. Among the psychological symptoms stress (76.2%; n=587), anxiety (59.4%; n=457) and depression (11.8%; n=91) prevailed. Among the physical symptoms, headache/headache prevailed (52.2%; n=402); decubitus injuries caused by PPE (24.8% n= 191) and eating disorders (18.6%= 143) (Tab. 3).

*Medians, means and SD for the sub-scales of the questionnaires*

The total scores of the event impact scale, its subscales, trait anxiety and depression levels are shown in Table 4. Mean and SD were calculated for the total score and the subscales of the IES questionnaires associated with the work areas. Considering a score ranging from 0 ("not at all") to 4 ("extremely"), it can be seen that the territorial work area already showed high scores in the early stages of the pandemic (IES_R 6.99) followed by the COVID-19 area (IES_R 3.86), indicating the presence of PTSD. The results are reported in Table 5. Statistically significant results emerged between those who worked in close contact with COVID-19 patients and those who developed symptoms indicative of COVID-19 infection, where among other things the percentage of those who had COVID-19 symptoms doubled (19.5% compared to 9.9%) (Tab. 6). From the association between the professional profile and the levels of stress and levels of anxiety it emerges respectively that among nurse's stress is more present than among physicians (77.5% compared to 67.4%; p = 0.030) (Tab. 7); as well as for the various levels of anxiety, where however the chi-square test is not significant below the 5% threshold, but is significant at 10% (p = 0.083) (Tab. 8)

| Table 3. Indication of a physical/psychological problem that has/has influenced work activities during the COVID-19 emergency period | N. (%) |
|---|---|
| **Health problem** | |
| No | 628 (81.6%) |
| Yes | 142 (18.4%) |
| **Dizziness** | |
| No | 687 (89.2%) |
| Yes | 83 (10.8%) |
| **Allergies/Rhinitis/Sinusitis** | |
| No | 574 (74.5%) |
| Yes | 196 (25.5%) |
| **Dermatitis** | |
| No | 621 (80.6%) |
| Yes | 149 (19.4%) |
| **Depression** | |
| No | 679 (88.2%) |
| Yes | 91 (11.8%) |
| **Stress** | |
| No | 183 (23.8%) |
| Yes | 587 (76.2%) |
| **Anxiety** | |
| No | 313 (40.6%) |
| Yes | 457 (59.4%) |
| **Headache** | |
| No | 368 (47.8%) |
| Yes | 402 (52.2%) |
| **Bone pain** | |
| No | 583 (75.7%) |
| Yes | 187 (24.3%) |
| **Asthma** | |
| **No** | 724 (94.0%) |
| **Yes** | 46 (6.0%) |
| **Gastro-oesophageal reflux, gastritis** | |
| No | 555 (72.1%) |
| Yes | 215 (27.9%) |
| **Lumbago** | |
| No | 511 (66.4%) |
| Yes | 259 (33.6%) |
| **Arterial hypertension** | |
| No | 706 (91.7%) |
| Yes | 64 (8.3%) |
| **Menstrual pain** | |
| No | 563 (73.1%) |
| Yes | 207 (8.3%) |
| **Urination-retention disorders with recurrent cystitis** | |
| No | 696 (90.4%) |
| Yes | 74 (9.6%) |
| **Dyspnoea** | |
| No | 715 (92.9%) |
| Yes | 55 (7.1%) |

| | |
|---|---|
| **Excessive sweating with dehydration syndrome** | |
| No | 621 (80.6%) |
| Yes | 149 (19.4%) |
| **Heart palpitation** | |
| No | 583 (75.5%) |
| Yes | 187 (24.3%) |
| **Eating disorders** | |
| No | 627 (81.4%) |
| Yes | 143 (18.6%) |
| **Pressure injuries caused by Personal Protective Equipment (PPE)** | |
| No | 579 (75.2%) |
| Yes | 191 (24.8%) |
| **Fever** | |
| No | 706 (91.7%) |
| Yes | 64 (8.3%) |
| **Dry cough** | |
| No | 656 (85.2%) |
| Yes | 114 (14.8%) |
| **Conjunctivitis** | |
| No | 682 (88.6%) |
| Yes | 88 (11%) |
| **Loss of sense of taste (ageusia)** | |
| No | 699 (90.8%) |
| Yes | 71 (9.2%) |
| **Loss of sense of smell (anosmia)** | |
| No | 693 (90.0%) |
| Yes | 77 (10.0%) |
| **Levels of trait anxiety (STAY-Y2)** | |
| Anxiety Absent | 334 (43.4%) |
| Mild anxiety | 263 (34.2%) |
| Moderate anxiety | 131(17.0%) |
| Severe anxiety | 42 (5.5%) |

**Tab.** 4 Total scale scores

| | Age | IES avoidance | IES Intrusiveness | IES Iperarousal | IES-R | STAY-Y2 | BDI |
|---|---|---|---|---|---|---|---|
| **N** | 756 | 770 | 770 | 770 | 770 | 770 | 770 |
| **Missing** | 14 | 0 | 0 | 0 | 0 | 0 | 0 |
| **Mean** | 2.44 | 1.09 | 1.16 | 1.23 | 3.47 | 43.1 | 5.72 |
| **Median** | 2.00 | 0.938 | 1.00 | 1.00 | 2.99 | 42.0 | 4.00 |
| **Minimum** | 1 | 0.00 | 0.00 | 0.00 | 0.00 | 20 | 0 |
| **Maximum** | 5 | 4.00 | 4.00 | 4.00 | 12.0 | 74 | 39 |

## Discussion

This study aims to investigate the psycho-physical impact of the COVID-19 emergency on the quality of life, work-related stress and psycho-physical well-being of health workers. The sample that took part in the study by filling in the questionnaire consisted of 770 participants including nurses and physicians, 74.3% of whom were female, with work experience of 1 to 5 years and a three-year degree. The data from this study is in line with the study by Kang et al., (17), which shows both that the majority of professionals

**Table 5.** Mean scores and SD work area/scale of impact (IES)

| **Mean and SD Work area/IES scale** | | | | | | | | |
|---|---|---|---|---|---|---|---|---|
| **Please indicate your current area of work** | **IES_Avoidance M (SD)** | | **IES_ Intrusiveness M (SD)** | | **IES_Iperarousal M (SD)** | | **IES_R M(SD)** | |
| **COVID-19 area** | 1.21 | (0.9419 | 1.32 | (1.000) | 1.33 | (1.07) | 3.86 | (2.89) |
| **Post-acute COVID-19 area** | 1.14 | (0.930) | 1.16 | (0.910) | 1.23 | (0.911) | 3.53 | (2.63) |
| **Surgical area** | 1.06 | (0.940) | 1.07 | (1.11) | 1.23 | (1.05) | 3.35 | (2.97) |
| **Critical care emergency area** | 1.13 | (0.841) | 1.26 | (0.956) | 1.29 | (0.986) | 3.69 | (2.62) |
| **Management/administrative area** | 1.12 | (0.943) | 0.781 | (0.653) | 0.875 | (0.888) | 2.77 | (2.36) |
| **Maternal and child area** | 0.966 | (0.714) | 0.955 | (0.861) | 1.12 | (0.876) | 3.04 | (2.33) |
| **Geriatric-rehabilitation medical area** | 0.953 | (0.816) | 1.06 | (0.922) | 1.23 | (0.976) | 3.25 | (2.53) |
| **Multi-specialist medical area** | 1.17 | (0.888) | 1.20 | (0.970) | 1.29 | (1.03) | 3.66 | (2.76) |
| **Territorial area** | 2.31 | (0.972) | 2.38 | (0.884) | 2.30 | (0.990) | 6.99 | (2.85) |
| **Prevention and safety** | 0.800 | (0.784) | 0.875 | (0.834) | 0.936 | (0.896) | 2.61 | (2.28) |
| **Services** | 0.688 | (0585) | 0.641 | (0610) | 0.550 | (0.418) | 1.88 | (1.50) |

**Table 6.** Association between exposure and infection levels

| | **Have you had any symptoms indicative of COVID-19 infection?** $\chi^2$= **(p-value) =861(0.003)** | | |
|---|---|---|---|
| **Have you worked closely with COVID-19 patients (not necessarily on a COVID-19 ward)** | **NO n. (%)** | **YES n. (%)** | **Total n. (%)** |
| **No** | 155 (90.1%) | 17 (9.9 | 172 (100) |
| **Yes** | 470 (80.5%) | 114 (19.5) | 584(100) |
| **Total** | 625 (82.7) | 131 (17.3) | 756 (100) |

**Table 7.** Association between professional profile and stress levels

| | **Stress Levels** $\chi^2$= **(p-value) =470(0.003)** | | |
|---|---|---|---|
| **Professional profile** | **NO n. (%)** | **YES n. (%)** | **Total n. (%)** |
| **Nurse** | 152 (22.5) | 523 (77.5) | 675 (100) |
| **Physicians** | 31 (32.6) | 64 (67.4) | 95(100) |
| **Total** | 183 (23.8) | 587 (76.2) | 770 (100) |

**Table 8.** Association of trait anxiety levels and professional profile

| **Professional profile** | **Trait anxiety levels** $\chi^2$= **(p-value) 6.69 (0.083)** | | | | |
|---|---|---|---|---|---|
| | **Absent anxiety** | **Mild anxiety** | **Moderante anxiety** | **Severe anxiety** | **Total** |
| **Nurse** | 284 | 235 | 115 | 41 | 675 |
| | 42.1% | 34.8% | 17.0% | 6.1% | 100.0% |
| **Physicians** | 50 | 28 | 16 | 1 | 95 |
| | 52.6% | 29.5% | 16.8% | 1.1% | 100.0% |

were female and that their work experience ranged from 3 months to 17 years. More than half of the participants (51.7%) reside in Northern Italy, compared to smaller percentages representative of those residing in Southern and Central Italy. 78.6% are not in an area other than their residence. Moreover, 46.2% of the participants are single, 53.0% have no children and 37.3% live with a family with children. The area of work most represented in the study is the Critical Emergency Area (first aid, 118, emergency medicine, intensive care, intensive short observation) with a percentage of 34.4%. This figure is perfectly in line with what has been claimed in several studies conducted in various hospitals and critical care departments, including emergency departments (18–23). The study showed that 77.2% of health care workers had worked closely with COVID-19 patients but not necessarily in a COVID-19 ward and that 66.7% had not had to change wards/work areas due to the COVID-19 emergency. Again, this is in line with the findings of the studies by Hope et al. (24) and Seale et al. (25): Both agree that nurses are at the forefront of the health system's response to both epidemics and pandemics. In addition, nurses provide care directly to patients in close physical proximity, are often directly exposed to these viruses and are at high risk of developing disease. 59.7% of the sample were rescuers or health workers who come into contact with positive people or people who know someone who has tested positive. 40.1% believed they had been exposed to 11-100 people, 64.0% were tested for COVID-19, 48.1% were tested for COVID-19 1-2 times, 82% had no flu-like symptoms or symptoms indicative of COVID-19 infection and 13.9% did not stop working after developing these symptoms. After developing symptoms indicative of COVID-19 5.1% continued to work, 0.6% started medical treatment, 3.8% physically moved away from family/loved ones, 1.6% went to the emergency room, 1.4% went to the general practitioner, 6.9% voluntarily quarantined themselves, consistent with the study by Lam and Hung, 2013 (18). The results of our study show a significant association between work area and risk of Post-Traumatic Stress Disorder (PTSD), in particular the territorial areas (community medicine) and COVID-19 area are those with higher scores (IES_R 6.99 & IES_R 3.86). The greatest concern that health workers feel in this period is that of making loved ones ill. This result is consistent with the findings of some studies (18,26–28), which found that the risk of being infected, transmission to family members, stigma about vulnerabilities in their work and restrictions on personal freedom were reported as key concerns (29). 55.2% thought they had underestimated the public health effects of the Pandemic during the initial days of the Pandemic, 52.3% thought they had not received good training from the health authority on the correct use of PPE against COVID-19. This contrasts with the study by Liu et al. (30) which showed that healthcare professionals received training in the correct use of PPE and in reducing their exposure to infection when caring for patients with COVID-19. Moreover, also Coia et al. (31) agreed that the selection and appropriate use of all PPE, including respiratory and face protection, should be supported by education and training of staff. 51.3% of the participants thought that PPE was not sufficient. This finding is fully in line with Kang et al. that participants were still concerned that PPE could not provide absolute protection (15). 92.6% of the participants had never taken antidepressant and/or neuroleptic drugs during this period. 31.3% of the participants asked their family for help in their moments of greatest distress, while only 3.9% of the health care personnel sought help from a psychologist/psychotherapist despite the presence of important psychological symptoms, such as anxiety (59.4%), stress (76.2%) and depression (11.8%) and despite the high risk of developing post-traumatic stress disorder. Preti et al. reported that among the psychopathological outcomes, anxiety and post-traumatic reactions were the most studied, and the results underlined the high prevalence of these areas of symptomatology in health professionals dealing with epidemic/pandemic outbreaks (8).

In addition, 34.2% presented mild anxiety, 17.0% presented moderate anxiety and 5.5% presented severe anxiety; this is in full agreement with several studies (15,28) which highlight the fact that nurses experienced greater anxiety about their health while caring for infected patients during a pandemic. (29). This does not deviate from what was previously stated by Pappa et al. (32) which showed that most experienced mild symptoms for both depression and anxiety, while

moderate and severe symptoms were less common among participants. The results of our study show that nurse practitioners experienced higher levels of stress in the early months of the pandemic than physicians (see Table VII). This underlines the need for early diagnosis and the importance of effectively collecting and treating psychological symptoms before they develop into more complex and lasting clinical pictures as shown by the results of a study conducted in Italy (33). Mental health monitoring and adequate psychological care and intervention must therefore be considered fundamental for the support of the whole community and, in particular, of the most fragile or exposed persons, such as health workers.

## Conclusions

The results of our study must be considered taking into account some limitations concerning the sample size, which consisted in the majority of nurses compared to physicians; the lack of follow-up of the psychological consequences and the lack of investigation of the long-term effects of the participants in the study and, finally, the choice of electronic dissemination of the questionnaire that may have excluded professionals Physicians and nurses with a low computer background. However, this could be considered as a preliminary study that could contribute to the understanding of psychological consequences among healthcare professionals involved in the SARS-CoV-2 epidemic.

This study aims to investigate the psychological impact of the Coronavirus emergency on healthcare professionals, physicians and nurses. Furthermore, it aims to return a greater awareness not only of the emotional and psychological consequences but also of the difficulties experienced by healthcare professionals during this period, particularly from April 2020 to June 2020. Looking to the future, further studies could investigate the psychological impact not only on healthcare professionals but also on social and health workers (OSS), who were also on the frontline during the Pandemic. In addition, it would be useful to analyses the long-term effects of this emergency in order to suggest appropriate interventions at both local and national levels. It might also be useful to investigate the effectiveness of psychological support in such delicate situations.

**Acknowledgements:** We would like to thank the medical and nursing professionals who contributed to this study. We would like to thank in particular Dr. Filippini Aurelio (President of order of nursing professions, province of Varese) and Dr. Marchisio Daniele (President of the scientific society "G.F.T - Triage Training Group) for having contributed to the realization of this contribution.

**Conflict of interest:** Each author declares that they have no commercial associations (e.g. consultancies, shares, patent/licence agreements, etc.) that could lead to a conflict of interest in relation to the submitted article.

## References

1. Garfin DR, Silver RC, Holman EA. The novel coronavirus (COVID-2019) outbreak: Amplification of public health consequences by media exposure. Health Psychol. maggio 2020;39(5):355–7.
2. Coronavirus Disease (COVID-19) Situation Reports [Internet]. [citato 21 gennaio 2021]. Disponibile su: https://www.who.int/emergencies/diseases/novel-coronavirus-2019/situation-reports
3. Brooks SK, Webster RK, Smith LE, Woodland L, Wessely S, Greenberg N, et al. The psychological impact of quarantine and how to reduce it: rapid review of the evidence. The Lancet. 14 marzo 2020;395(10227):912–20.
4. Banerjee D. The COVID-19 outbreak: Crucial role the psychiatrists can play. Asian Journal of Psychiatry. 1 marzo 2020;50:102014.
5. Lima CKT, Carvalho PM de M, Lima I de AAS, Nunes JVA de O, Saraiva JS, de Souza RI, et al. The emotional impact of Coronavirus 2019-nCoV (new Coronavirus disease). Psychiatry Res. maggio 2020;287:112915.
6. Hahad O, Gilan DA, Daiber A, Münzel T. [Public Mental Health as One of the Key Factors in Dealing with COVID-19]. Gesundheitswesen. maggio 2020;82(5):389–91.
7. Lai J, Ma S, Wang Y, Cai Z, Hu J, Wei N, et al. Factors Associated With Mental Health Outcomes Among Health Care Workers Exposed to Coronavirus Disease 2019. JAMA Netw Open. 2 marzo 2020;3(3):e203976.
8. Preti E, Di Mattei V, Perego G, Ferrari F, Mazzetti M, Taranto P, et al. The Psychological Impact of Epidemic and Pandemic Outbreaks on Healthcare Workers: Rapid Review of the Evidence. Curr Psychiatry Rep. 10 luglio 2020;22(8):43.
9. Cao W, Fang Z, Hou G, Han M, Xu X, Dong J, et al. The psychological impact of the COVID-19 epidemic

on college students in China. Psychiatry Res. maggio 2020;287:112934.
10. Luo M, Guo L, Yu M, Jiang W, Wang H. The psychological and mental impact of coronavirus disease 2019 (COVID-19) on medical staff and general public - A systematic review and meta-analysis. Psychiatry Res. settembre 2020;291:113190.
11. Rafforzamento dell'Organizzazione mondiale della sanità: l'UE è pronta ad assumere un ruolo di primo piano [Internet]. [citato 23 gennaio 2021]. Disponibile su: https://www.consilium.europa.eu/it/press/press-releases/2020/11/06/strengthening-the-world-health-organization-the-eu-is-ready-to-take-the-leading-role/
12. Horowitz M, Wilner N, Alvarez W. Impact of Event Scale: a measure of subjective stress. Psychosom Med. maggio 1979;41(3):209–18.
13. Pietrantonio F, De Gennaro L, Di Paolo MC, Solano L. The Impact of Event Scale: validazione di una versione italiana. J Psychosom Res. 2003 Ott;55(4):389-93. doi: 10.1016/s0022-3999(02)00638-4. PMID: 14507552.
14. STAI : state-trait anxiety inventory : forma Y : manuale / Charles D. Spielberger ; adattamento italiano a cura di Luigi Pedrabissi e Massimo Santinello [Internet]. [citato 21 gennaio 2021]. Disponibile su: http://www.cbt.biblioteche.provincia.tn.it/oseegenius/resource?uri=6131914
15. Beck AT, Ward CH, Mendelson M, Mock J, Erbaugh J. An inventory for measuring depression. Arch Gen Psychiatry. giugno 1961;4:561–71.
16. Sica, C., & Ghisi, M. (2007). The Italian versions of the Beck Anxiety Inventory and the Beck Depression Inventory-II: psychometric properties and discriminant power.
17. Kang HS, Son YD, Chae S-M, Corte C. Working experiences of nurses during the Middle East respiratory syndrome outbreak. International Journal of Nursing Practice. 2018;24(5):e12664.
18. Chung BPM, Wong TKS, Suen ESB, Chung JWY. SARS: caring for patients in Hong Kong. Journal of Clinical Nursing. 2005;14(4):510–7.
19. Holroyd E, McNaught C. The SARS crisis: reflections of Hong Kong nurses. International Nursing Review. 2008;55(1):27–33.
20. Lam KK, Hung SYM. Perceptions of emergency nurses during the human swine influenza outbreak: a qualitative study. Int Emerg Nurs. ottobre 2013;21(4):240–6.
21. Lam SKK, Kwong EWY, Hung MSY, Chien WT. Emergency nurses' perceptions regarding the risks appraisal of the threat of the emerging infectious disease situation in emergency departments. International Journal of Qualitative Studies on Health and Well-being. 1 gennaio 2020;15(1):1718468.
22. Liu H, Liehr P. Instructive messages from Chinese nurses' stories of caring for SARS patients. Journal of Clinical Nursing. 2009;18(20):2880–7.
23. Corley A, Hammond NE, Fraser JF. The experiences of health care workers employed in an Australian intensive care unit during the H1N1 Influenza pandemic of 2009: A phenomenological study. International Journal of Nursing Studies. 1 maggio 2010;47(5):577–85.
24. Hope D, Bates T, Penke L, Gow AJ, Starr JM, Deary IJ. Fluctuating Asymmetry and personality. Personality and Individual Differences. 1 gennaio 2011;50(1):49–52.
25. MacIntyre CR, Cauchemez S, Dwyer DE, Seale H, Cheung P, Browne G, et al. Face mask use and control of respiratory virus transmission in households. Emerg Infect Dis. febbraio 2009;15(2):233–41.
26. Chiang H-H, Chen M-B, Sue I-L. Self-state of nurses in caring for SARS survivors. Nurs Ethics. gennaio 2007;14(1):18–26.
27. Hope K, Massey P, Osbourn M, Durrheim D, Kewley C, Turner C. Senior clinical nurses effectively contribute to the pandemic public health response. Australian journal of advanced nursing : a quarterly publication of the Royal Australian Nursing Federation, The. 1 luglio 2011;28.
28. Koh D, Lim MK, Chia SE, Ko SM, Qian F, Ng V, et al. Risk perception and impact of Severe Acute Respiratory Syndrome (SARS) on work and personal lives of healthcare workers in Singapore: what can we learn? Med Care. luglio 2005;43(7):676–82.
29. Fernandez R, Lord H, Halcomb E, Moxham L, Middleton R, Alananzeh I, et al. Implications for COVID-19: A systematic review of nurses' experiences of working in acute care hospital settings during a respiratory pandemic. International Journal of Nursing Studies. 1 novembre 2020;111:103637.
30. Liu M, Cheng S-Z, Xu K-W, Yang Y, Zhu Q-T, Zhang H, et al. Use of personal protective equipment against coronavirus disease 2019 by healthcare professionals in Wuhan, China: cross sectional study. BMJ. 10 giugno 2020;369:m2195.
31. Coia J, Ritchie L, Adisesh A, Booth C, Bradley C, Bunyan D, et al. Guidance on the use of respiratory and facial protection equipment. Journal of Hospital Infection. 17 settembre 2013;85.
32. Pappa S, Ntella V, Giannakas T, Giannakoulis VG, Papoutsi E, Katsaounou P. Prevalence of depression, anxiety, and insomnia among healthcare workers during the COVID-19 pandemic: A systematic review and meta-analysis. Brain Behav Immun. agosto 2020;88:901–7.
33. Simonetti V, Durante A, Ambrosca R, Arcadi P, Graziano G, Pucciarelli G, Simeone S, Vellone E, Alvaro R, Cicolini G. Anxiety, sleep disorders and self-efficacy among nurses during COVID-19 pandemic: A cross-sectional study. J Clin Nurs. 2021 Feb 3. doi: 10.1111/jocn.15685.

**Correspondence:**
Received: 6 April 2021
Accepted: 22 June 2021
Luana Conte
University of Salento, Lecce, Italy
luana.conte@unisalento.it
