# Supplementary material for: Mental health status and related factors influencing healthcare workers during the COVID-19 pandemic: A systematic review and meta-analysis
Source: PLoS One. 2024 Jan 19;19(1):e0289454. doi: 10.1371/journal.pone.0289454 (PMC10798549; doi:10.1371/journal.pone.0289454)
Supplement: S1 Data — (ZIP) [file pone.0289454.s011.zip › literatures/220.pdf]

frontiers | Frontiers in Public Health

ORIGINAL RESEARCH
published: 19 July 2022
doi: 10.3389/fpubh.2022.904550

Prevalence of Post-traumatic Stress Disorder Status Among Healthcare Workers and Its Impact on Their Mental Health During the Crisis of COVID-19: A Cross-Sectional Study

Yue Yang[1†], Di Liu[2†], Bingshuo Liu[1†], Weiyan Ou[3], Licheng Wang[4], Yuanshuo Ma[4], Lihua Fan[4], Yu Shi[5*] and Lei Shi[3*]*

[1] Harbin Medical University Cancer Hospital, Harbin, China, [2] School of Marxism, Harbin Medical University, Harbin, China, [3] School of Health Management, Southern Medical University, Guangzhou, China, [4] School of Health Management, Harbin Medical University, Harbin, China, [5] Vanke School of Public Health, Tsinghua University, Beijing, China

Objective: After the unprecedented coronavirus disease 2019 (COVID-19) outbreak, the health status of the general population has suffered a huge threat, and the mental health of front-line healthcare providers has also encountered great challenges. Therefore, this study aims to: (1) investigate the prevalence and influencing factors of post-traumatic stress disorder (PTSD) among healthcare providers, and (2) verify the moderating role of self-efficacy in the influence of PTSD on mental health.

Methods: A cross-sectional study was conducted using an online survey of 1993 participants. The presence of depression, anxiety, self-efficacy, and PTSD was evaluated using screening tests from March 1. Sociodemographic and COVID-19-related data were also collected. A data analysis was performed using descriptive statistics, Pearson's correlation coefficient, and multiple linear regression.

Results: The prevalence of PTSD among healthcare providers was 9.3%. PTSD was negatively correlated with self-efficacy ($r = -0.265$, $P < 0.01$), anxiety ($r = -0.453$, $P < 0.01$), and depression ($r = 0.708$, $P < 0.01$). Profession, daily working hours, maximum continuous working days, and daily sleep time were influencing factors of PTSD. A binary logistic regression analysis showed that physicians (OR = 2.254, 95% CI = 1.298, 3.914) and nurses (OR = 2.176, 95% CI = 1.337, 3.541) were more likely to experience PTSD than other healthcare providers.

Conclusion: Self-efficacy has a moderating effect on the influence of PTSD on anxiety and depression. This suggests that health managers need to respond to the current psychological crisis of healthcare providers, implement appropriate psychological interventions, and minimize the psychological harm caused by COVID-19.

Keywords: COVID-19, PTSD, healthcare providers, self-efficacy, mental health

OPEN ACCESS

Edited by:
Francesco Chirico, Catholic University of the Sacred Heart, Italy

Reviewed by:
Pietro Crescenzo, Italian Red Cross, Italy
Valentina Lucia La Rosa, University of Catania, Italy

****Correspondence:***
Lei Shi
hydleishi@126.com
Yu Shi
hydshiyu@163.com
Lihua Fan
lihuafan@126.com

† These authors have contributed equally to this work

Specialty section:
This article was submitted to Public Mental Health, a section of the journal Frontiers in Public Health

Received: *25 March 2022*
Accepted: *21 June 2022*
Published: *19 July 2022*

Citation:
Yang Y, Liu D, Liu B, Ou W, Wang L, Ma Y, Fan L, Shi Y and Shi L (2022) Prevalence of Post-traumatic Stress Disorder Status Among Healthcare Workers and Its Impact on Their Mental Health During the Crisis of COVID-19: A Cross-Sectional Study. Front. Public Health 10:904550. doi: 10.3389/fpubh.2022.904550

INTRODUCTION

The outbreak of the coronavirus disease 2019 (COVID-19) has severely affected the medical and health service system (1). In the initial stage of the outbreak, the number of suspected and confirmed cases increased daily, and the number of medical staff was extremely insufficient (2), which led to inadequate rest time for medical staff (3). Moreover, medical materials were relatively scarce, so medical staff could only reuse protective facilities and rest continuously to increase the use efficiency of protective facilities (4). More seriously, medical staff have to conduct intensive front-line work and also conduct long-term pandemic prevention and control. Physical and mental fatigue can lead to mental exhaustion and psychological stress of staff, accompanied by symptoms of anxiety and depression (5, 6). Some researchers have also focused on the mental health problems of vulnerable groups such as pregnant women and students during COVID-19 (7, 8), and the online learning problems of the young population and medical needs of pregnant women are now worthy of attention (9). In Pakistan, medical workers have been under physical and psychological pressure, including a high risk of infection, inadequate equipment for safety from contagion, isolation, exhaustion, and lack of contact with family (10). German scholars have also confirmed that the incidence of mental health problems (stress, anxiety, depressive symptoms, sleep disturbance, and irritability) in Germany has increased owing to the impact of the COVID-19 pandemic (11). The latest Italian study of healthcare providers points out that healthcare workers involved in the COVID-19 pandemic are exposed to high levels of stressful or traumatic events and express substantial negative mental health outcomes (12). Similarly, data from Spain show that the impact of the pandemic on mental health during the first weeks is evident (13, 14). Research in India also shows that students' post-traumatic stress significantly contributed to depression during the lockdown of the COVID-19 pandemic (14, 15). Levels of anxiety and depression far above normal were also confirmed in the UK during the COVID-19 outbreak (16).

In summary, due to the lack of a comprehensive understanding of emerging infectious diseases, medical and healthcare providers inevitably experience some psychological panic when facing diseases, which may lead to psychological imbalances and thus cause psychological crises (17). In the event of a major infectious disease, the harm caused by psychological panic among healthcare providers is more difficult to predict than the disease itself. Healthcare providers bear great expectations from the general public, such as the moral support of society and the pressure on healthcare providers (18). The incomprehension and non-cooperation of patients leads to feelings of grievance, fear, and helplessness of medical staff, making them think that they should not only fight the virus but also prevent the patients from bringing harm to themselves. This causes great harm to both their bodies and minds, which cannot be eliminated easily (19). Under continuous high-intensity work and high stress psychological pressure, medical staff often focus on fighting the virus but often ignore the recovery of their own physical and psychological trauma (20). The symptoms of acute traumatic stress disorder may occur owing to the dual effects of body function and excessive psychological load (21).

Post-traumatic stress disorder (PTSD) refers to a delayed appearance and persistent mental disorder caused by one or more deaths, threat of death, serious injury, or threat of physical integrity (22). It can develop in response to exposure to an extremely stressful or traumatic event or an exceptionally threatening situation as a result of unusually threatening, catastrophic psychological trauma, resulting in delayed onset and long-term persistence of psychological disorders (23). PTSD is associated with a variety of factors (24), mainly divided into family and social psychological factors (such as economic conditions, social status, work status, education level, stressful life events, personality traits, childhood trauma, family violence, and war) and biological factors (25), such as genetic factors, neuroendocrine factors, nerves, and biochemical factors). Major traumatic events are basic conditions for the development of PTSD and are highly unpredictable. The outbreak of COVID-19 is a typical traumatic event, and healthcare providers are likely to develop a range of PTSD symptoms in the emotional, thinking, and behavioral dimensions 2 months after exposure to COVID-19, which can persist for at least a month without immediate psychological assistance. These symptoms can contribute to the development of mental health difficulties, including depression, PTSD, and suicidal ideation (26). Under the pandemic, healthcare providers are very prone to develop PTSD symptoms. A study from Norway showed that health workers and public service providers showed markedly high levels of PTSD symptoms, anxiety, and depression during the COVID-19 pandemic, and 27.7% of the sample had clinical or subclinical symptoms of PTSD (27). Unfortunately, long-term PTSD symptoms are likely to affect the mental health of healthcare providers. Previous studies have shown that PTSD has a negative impact on individuals' mental health (28); therefore, its symptoms in healthcare providers and its effects on anxiety, depression, and mental health are of great concern in this study. Nonetheless, self-efficacy can reportedly be used as an influencing factor of traumatic stress disorder and mental health (29), and can be regarded as one of the key variables of healthcare providers' self-healing and recovery.

"The speculation and judgment of an individual's ability to complete a certain behavior" has become the basic consensus of the definition of self-efficacy (30). While fighting COVID-19, healthcare providers' self-efficacy was expressed as "the degree of self-confidence to complete the work in fighting COVID-19 with their own comprehensive ability." It should be noted that the self-efficacy of healthcare providers is not only a buffer against the pressure of COVID-19 (31), but also the main channel for healthcare providers to overcome psychological stress when they have psychological stress reactions (32). Some scholars have pointed out that self-efficacy can alleviate an individual's psychological stress response and help them obtain post-traumatic growth from psychological stress, thus avoiding the occurrence of PTSD (33). The theory of self-efficacy suggests

Abbreviations: COVID-19, Corona Virus Disease 2019; PTSD, Post traumatic stress disorder; HCP, health care providers.

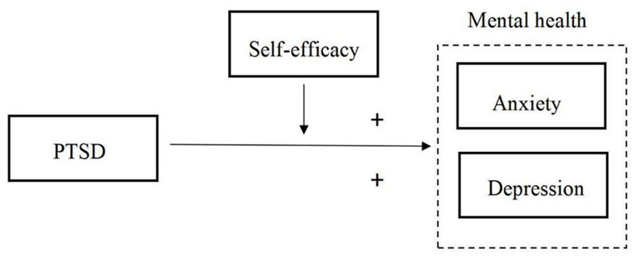

FIGURE 1 | Theoretical hypothesis model.

that in the face of possible danger, misfortune, and disaster, self-efficacy determines an individual's physical and mental reaction processes such as the degree of stress, anxiety, and depression (34). These emotional reactions affect individuals' mental health and behavior by changing the nature of their thinking process. Some studies have pointed out that people with a high level of coping efficacy are not anxious when dealing with emergencies. However, those who doubt their ability to deal with emergencies worry that the environment is dangerous, so they develop strong stress and anxiety and passively respond to the environment with various protective withdrawals or defensive behaviors (35, 36). These behaviors restrict the development of individual personalities and pose a potential threat to their mental health. Therefore, this study aimed to explore the mechanism of PTSD in healthcare providers regarding their mental health.

Hypothesis 1: PTSD of healthcare providers has a positive impact on anxiety

Hypothesis 2: PTSD of healthcare providers has a positive impact on depression.

Hypothesis 3: Self-efficacy of healthcare providers plays a mediating role in the effect of PTSD on their mental health, as shown in **Figure 1**.

METHODS

Ethics Approval

The Ethics Committee of the College of Public Health, Harbin Medical University approved the study, and individual consent was obtained from every participant's healthcare provider. On the front page of the questionnaire, we clearly indicated that the survey was anonymous. Hence, once the questionnaire was submitted successfully, we obtained the consent of the healthcare providers to participate in our investigation. All questionnaires were strictly managed by specialized personnel from the research team.

Participants and Settings

A pre-survey was conducted before the formal investigation to improve its scientific nature and reliability. A pre-survey of 20 healthcare providers (5 doctors, 10 nurses, 10 other healthcare providers) who were responding to COVID-19 was conducted using convenience sampling, and the average time to complete the questionnaire was 202–1,200 s. The confusing text was further modified to ensure that the items in the questionnaire were clearly understood by the healthcare providers.

A two-group large-sample normal approximation test of proportions with a one-sided 0.05 significance level would require a sample size of 792 in each group to have 80% power to reject the null hypothesis, assuming a non-inferiority margin of 10% and a rate of 80% in the control group. Healthcare providers across the country completed an online questionnaire from March to May 2020 in Heilongjiang Province, China. First, research team members contacted ∼30 hospital administrators in different hospitals and fully informed them of the purpose, contents, and methods of this survey to mobilize more healthcare providers to participate in this network survey. The middle and western regions were divided according to the geographical location of the Heilongjiang Province. These samples are from hospitals in seven cities of Heilongjiang Province in China, including Harbin, Qiqihar, Mudanjiang, Jiamusi, Yichun, Daqing and Mohe. A contact from each hospital was asked to investigate about 300 medical staff covering each department. To avoid social contact during the COVID-19 outbreak a web page link to our questionnaire survey (https://www.wjx.cn/) was sent to participants outside working hours by mobile phone. Once the questionnaire was completed, the data management platform received the corresponding records and recorded the participants' time spent answering it. A response time of <5 min was considered invalid because a pre-survey test to determine a valid questionnaire completion time was reported to be more than 5 min.

The inclusion criteria for the study were being an HCW and a regular employee of the hospital, having more than 1 year of experience as an HCW, and providing informed consent for voluntary participation. The exclusion criteria for the study was removing subjects with previous psychological or psychiatric problems. The data management platform showed that 2,260 questionnaires were distributed, and 1,993 participants completed valid questionnaires. The response rate was 88.19%.

Instruments

The questionnaire included demographic information, PTSD scale, self-efficacy scale, GAD-7 scale, and WHO-5 scale, which were distributed among Chinese healthcare providers.

PTSD (Post-Traumatic Stress Disorder) Scale

The PTSD checklist for the DSM-5 is a 20-item self-report measure that assesses the presence and severity of PTSD symptoms. Items on the PCL-5 correspond with the DSM-5 criteria for PTSD (37). Respondents were asked to rate how bothered they had been by each of the 20 items in the past month, with items rated on a 5-point Likert-type scale ranging from 0 to 4 (0 = seldom, 1 = fewer, 2 = sometimes, 3 = often, 4 = frequently). Positive standard: Summing all 20 items (range 0–80) and using a cut-off point score of 31–33 appears to be reasonable based on the current

psychometric work. A total score ≥31 was selected to indicate the possibility of PTSD. Items were summed to provide a total severity score; the Cronbach's alpha coefficient for this scale was 0.968.

Self-efficacy

Self-efficacy refers to the subjective judgment of whether one can succeed in a certain achievement behavior. In this study, it was introduced in the background of the COVID-19 outbreaks. The scale comprises three questions: (1) I believe that I can avoid COVID-19 infection. (2) I know how to avoid COVID-19. (3) I believe I can be cured even if I am unfortunately infected with COVID-19. A 5-point Likert scale (from 1 = strongly disagree to 5 = strongly agree) was used for assessment, and this instrument showed high reliability ($\alpha = 0.885$).

Mental Health

To quickly screen the mental health of healthcare providers, the Self-rating Anxiety and Depression Scale, which is widely used in China and has good reliability and validity, was used in this study.

Anxiety

The scale was used to screen for generalized anxiety and evaluate symptom severity (38). Seven items were graded from 0 (not at all) to 3 (almost every day), and the total score ranged from 0 to 21. Among them, 0–4 points were normal, 5–9 points, mild anxiety, 10–14 points, moderate anxiety, 15–21 points, severe anxiety. Cronbach's alpha coefficient for this scale was 0.947.

Depression

The five-item scale is one of the most widely used scales to evaluate subjective wellbeing (39), with five items. Each item adopts a 6-level scoring method with 0–5 points. The original score was the sum of five items, with a total score of 0–25 points. The higher the score, the higher the level of wellbeing of the patients. A total score ≤13 indicates that there may be depression, and the Cronbach's alpha coefficient for this scale was 0.945.

Statistical Analysis

Descriptive statistical analyses were used to determine demographic variables. A chi-squared test was used to analyze the relationship between the demographic characteristics of the respondents, their PTSD, self-efficacy, generalized anxiety symptoms, and subjective wellbeing. Taking the significant factors as independent variables and the experience of PTSD in the past month as the dependent variable, a binary logistic regression analysis was conducted to identify the risk factors for PTSD among healthcare providers. The level of significance was set at $P < 0.05$. Pearson's correlation coefficients were computed to examine the relationship between PTSD and the other variables. The data for this survey were obtained from a cross-sectional online questionnaire. All data analyses were performed using the SPSS version 25.0 (IBM Corp, BM SPSS Statistics for Windows, Armonk, NY).

RESULTS

Demographics and Characteristics

The demographic characteristics are shown in **Table 1**. The data indicates that the majority of the sample was female (81.3%), with an average age of 37.10 years. Nurses (60.1%) and physicians (19.8%) were the main occupational groups of respondents in this sample, and their service experience was mostly 6–10 years (29.6%). Approximately 44.5% of respondents were of intermediate title, 73.5% were undergraduates, and 78.3% were married. Among the participants surveyed, the departments of medical health workers were fever clinics (3.7%), respiratory medicine (3.0%), thoracic surgery (1.9%), infectious diseases (1.3%), and other departments (90.1%). Approximately 21.5% of the participants directly participated in the pandemic prevention and control work. Furthermore, 36.8% of the participants worked more than 8 h a day, and 39.2% of the longest continuous working days exceeded 14 days. In terms of daily sleep time, 14.1% of healthcare providers' daily sleep time was <4 h a day.

Differences of Prevalence of PTSD Among Participants With Different Demographic Characteristics

Table 2 shows the correlation between the demographic characteristics of the respondents, pandemic prevention participation, daily working hours, longest continuous working days, daily sleep time and whether PTSD had occurred. The results show that the profession of medical healthcare providers in China ($X^2 = 10.938$, P=0.004), daily working hours ($X^2 = 26.268$, $P < 0.001$), longest continuous working days ($X^2 = 34.635$, $P < 0.001$), and daily sleep time ($X^2 = 108.643$, $P < 0.001$) were significantly associated with PTSD (**Table 2**).

Factors Related to PTSD

Using binary logistic regression analysis, we found that physicians (OR = 2.254, 95% CI = 1.298, 3.914) and nurses (OR = 2.176, 95% CI = 1.337, 3.541) were more likely to experience PTSD than other healthcare providers. Meanwhile, the working hours per day were <4 h (OR = 0.283, 95% CI = 0.117, 0.685) and 5–8 h (OR = 0.410, 95% CI = 0.247, 0.683) were less likely to experience PTSD than healthcare providers who worked more than 13 h a day. The risk of PTSD for healthcare providers working 10–13 days (OR = 2.023, 95% CI = 1.093, 3.745) and continuously for more than 14 days (OR = 2.390, 95% CI = 1.539, 3.713) was much higher than for those who worked for <5 consecutive days. Moreover, healthcare providers who sleep for 5–7 h (OR = 0.240, 95% CI = 0.170, 0.338) and more than 8 h (OR = 0.111, 95% CI = 0.046, 0.263) a day were less likely to have PTSD symptoms than those who slept for <4h (**Table 3**).

TABLE 1 | Demographic characteristics of the respondents (*n* = 1,993).

Characteristic	Classes	*N*	%
Gender	Female	1,656	83.1
	Male	337	16.9
Age	20–30	518	26.0
	31–40	862	43.3
	41–50	457	22.9
	51–60	153	7.7
	>60	3	0.2
Degree of Education	Junior college or below	341	17.1
	Undergraduate	1,465	73.5
	Master degree or above	187	9.4
Marital status	Unmarried	344	17.3
	Married	1,560	78.3
	Widowhood/divorce	89	4.4
Profession	Physician	396	19.8
	Nurse	1,197	60.1
	Other health care providers	400	20.1
Work Department	Fever clinics	74	3.7
	Respiratory medicine	59	3.0
	Thoracic surgery	38	1.9
	Infectious diseases	25	1.3
	Other departments	1,797	90.1
Professional ranks and titles	Unrated	150	7.5
	Junior professional ranks	790	39.6
	Intermediate title	887	44.5
	Senior title	166	8.3
Service years	1–5 years	250	12.5
	6–10 years	589	29.6
	11–15 years	378	19.0
	16–20 years	286	14.4
	>20 years	490	24.6
Epidemic prevention participation	Yes	428	21.5
	No	1,565	78.5
Daily working hours	≤4 h	141	7.1
	5–8 h	1,120	56.2
	9–12 h	591	29.7
	≥13 h	141	7.1
Longest continuous working days	≤5 days	616	30.9
	6–9 days	424	21.3
	10–13 days	171	8.6
	≥14 days	782	39.2
Daily sleep time	≤4 h	281	14.1
	5–7 h	1,484	74.5
	≥8 h	228	11.4

Correlations Between Different Variables

Means, SD, and internal consistencies of the measures were computed. The Pearson correlation coefficients of the continuous variables are presented in **Table 4**. All variables were significantly correlated with each other. PTSD was negatively correlated with self-efficacy (r = −0.265, P< 0.01), anxiety (r = −0.453, P < 0.01), and depression (r = 0.708, P < 0.01). Self-efficacy was positively correlated with anxiety (r = 0.311, P < 0.01) and depression (r = −0.263, P < 0.01), and anxiety was positively correlated with depression (r = −0.525, P < 0.01).

TABLE 2 | Chi-square test for PTSD-related risk factors ($n = 1,993$).

Characteristic	Classes	PTSD		X^2	*P*
		**Yes**	**No**		
Gender	female	153	1,503	0.022	0.882
	male	32	305		
Age	20–30	43	475	3.05	0.550
	31–40	76	786		
	41–50	48	409		
	51–60	18	135		
	>60	0	3		
Degree of Education	Junior college or below	32	309	1.341	0.511
	Undergraduate	140	1,325		
	Master degree or above	13	174		
Marital status	Unmarried	29	315	1.202	0.548
	Married	146	1,414		
	Widowhood/divorce	10	71		
Profession	Physician	42	354	10.938	0.004
	Nurse	123	1,074		
	Other health care providers	20	380		
Work Department	Fever clinics	11	63	2.972	0.563
	Respiratory Medicine	5	54		
	Thoracic surgery	3	35		
	Infectious Diseases	2	23		
	Other departments	164	1,633		
Professional ranks and titles	Non rating	6	144	5.967	0.133
	Junior professional ranks	72	718		
	Intermediate title	64	563		
	Senior title	43	383		
Service years	1–5 years	13	237	8.833	0.065
	6–10 years	52	537		
	11–15 years	34	344		
	16–20 years	35	251		
	>20 years	51	439		
Epidemic prevention participation	Yes	39	389	0.019	0.891
	No	146	1,419		
Daily working hours	≤4 h	7	134	26.268	<0.001
	5–8 h	79	1,041		
	9–12 h	77	514		
	≥13 h	22	119		
Longest continuous working days	≤5 days	32	584	34.635	<0.001
	6–9 days	27	397		
	10–13 days	20	151		
	≥14 days	106	676		
Daily sleep time	≤4 h	72	209	108.643	<0.001
	5–7 h	107	1,377		
	≥8 h	6	222		
Incidence of PTSD	9.3%				

Moderator Analyses of Study Variables

The results of the moderator analyses are summarized in **Table 5**. First, profession, daily working hours, longest continuous working days, and daily sleep time were treated as control variables in the regression equations. A model was constructed using self-efficacy (M) as a moderator. In this model, PTSD was set as the predictor (X) and anxiety and depression were regarded as outcome variables (Y). We used 5,000 bootstrap samples and determined the moderating effect using 95% CIs. In the model of the effect of PTSD on anxiety ($\beta = 0.119$, $P < 0.001$), Hypothesis 1 was supported, and the interaction between PTSD and self-efficacy (X*W) was significant ($P < 0.001$). Therefore, self-efficacy moderates the relationship between PTSD and anxiety and the moderating

TABLE 3 | Binary logistic regression of risk factors associated with PTSD ($n = 1,993$).

Variables		B	OR	95%CI	*P*
Profession	Other health care providers	Ref	1.000		
	Physician	0.813	2.254	(1.298, 3.914)	0.004
	Nurse	0.777	2.176	(1.337, 3.541)	0.002
Daily working hours	≥13 h	Ref	1.000		
	≤4 h	−1.264	0.283	(0.117, 0.685)	0.005
	5–8 h	−0.890	0.410	(0.247, 0.683)	0.001
	9–12 h	−0.210	0.810	(0.485, 1.355)	0.423
Longest continuous working days	≤5 days	Ref	1.000		
	6–9 days	0.145	1.156	(0.669, 1.999)	0.603
	10–13 days	0.705	2.023	(1.093, 3.745)	0.025
	≥14 days	0.871	2.390	(1.539, 3.713)	<0.001
Daily sleep time	≤4 h	Ref	1.000		
	5–7 h	−1.427	0.240	(0.170, 0.338)	<0.001
	≥8 h	−2.203	0.111	(0.046, 0.263)	<0.001

TABLE 4 | The means, SD, and internal consistencies of variables.

	Variables	M	SD	α	1	2	3	4
1	PTSD	14.053	14.144	0.968	-			
2	Self-efficacy	11.28	2.845	0.885	−0.265**	-		
3	Anxiety	6.966	6.106	0.947	−0.453**	0.311**	-	
4	Depression	9.457	6.560	0.945	0.708**	−0.236**	−0.525**	-

TABLE 5 | Moderation analyses of PTSD.

Item (Model 1 in Model Templates)

		R^2	B	SE	t	*P*	LLCI	ULCI
Outcome variable: Anxiety								
	Constant	0.525	6.727	0.834	8.060	<0.001	5.090	8.364
Moderator variable	Self-efficacy		−0.579	0.133	−4.364	<0.001	0.840	−0.319
Independent variable	PTSD		0.199	0.022	8.939	<0.001	0.155	0.243
	PTSD*Self-efficacy		0.022	0.006	3.682	<0.001	0.010	0.034
Control variable	Profession		−0.275	0.151	−1.828	0.068	−0.571	0.020
	Daily working hours		0.434	0.138	3.149	0.002	0.164	0.704
	Longest continuous working days		0.233	0.077	3.048	0.002	0.083	0.383
	Daily sleep time		−1.286	0.199	−6.451	<0.001	−1.677	−0.895
Outcome variable: Depression								
	Constant	0.304	2.812	1.085	2.592	0.010	0.685	4.940
Moderator variable	Self-efficacy		1.822	0.173	10.556	<0.001	1.483	2.160
Independent variable	PTSD		0.015	0.029	−0.527	0.599	−0.072	0.042
	PTSD*Self-efficacy		−0.037	0.008	−4.718	<0.001	−0.052	−0.022
Control variable	Profession		0.055	0.196	0.281	0.779	−0.329	0.439
	Daily working hours		−0.806	0.179	−4.499	<0.001	−1.157	−0.454
	Longest continuous working days		−0.414	0.099	−4.162	<0.001	−0.609	−0.219
	Daily sleep time		2.370	0.259	9.148	<0.001	1.861	2.878

effect diagram is shown in **Figure 2**. In the model of the effect of PTSD on depression ($\beta = 0.015$, $P < 0.001$), Hypothesis 2 was supported, and the interaction between PTSD and self-efficacy (X*W) was also significant ($P < 0.001$). Therefore, self-efficacy moderates the relationship between PTSD and depression, thus supporting Hypothesis 3, as shown in **Figure 3**. The modified model and standardized path are shown in **Figure 4**.

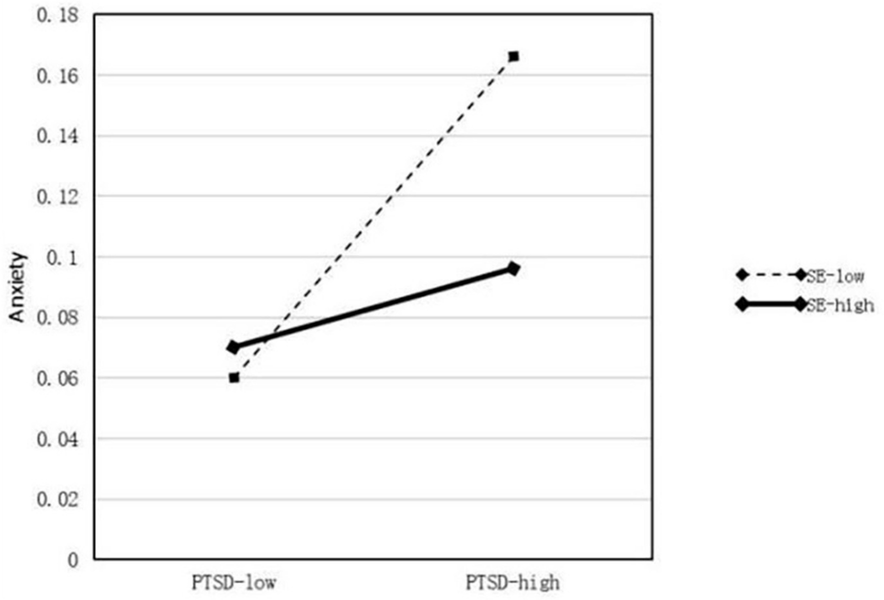

FIGURE 2 | Moderating role of self-efficacy in the effect of PTSD on anxiety.

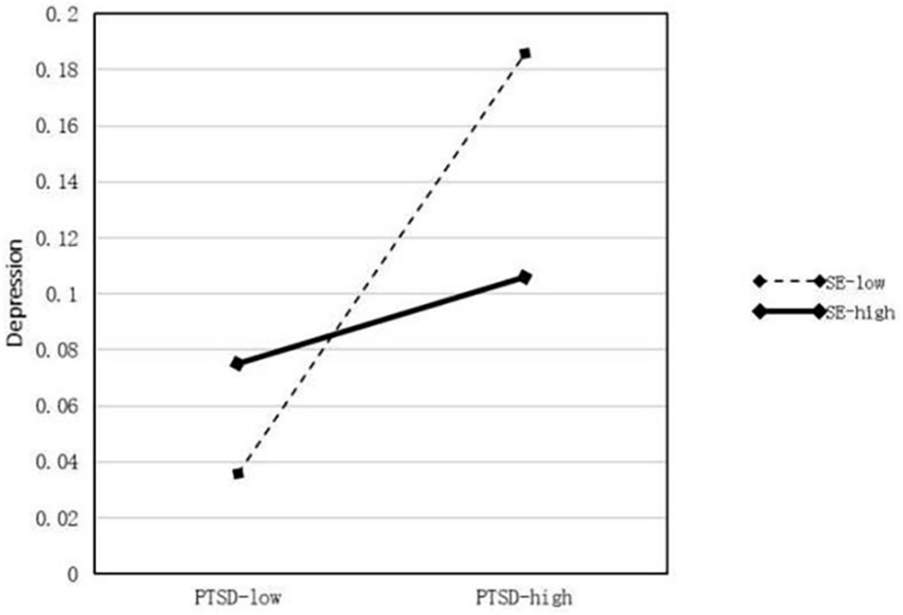

FIGURE 3 | Moderating role of self-efficacy in the effect of PTSD on depression.

DISCUSSION

Status of PTSD Among Health Care Providers and Its Influencing Factors

The COVID-19 pandemic not only magnified the vulnerability of the medical system but also highlighted the serious mental health crisis of healthcare providers (40). The results of this study clearly indicate that the incidence of PTSD among healthcare providers in Heilongjiang Province, China, during the COVID-19 outbreak was 9.3%, which is higher than that of the general population (41). Being exposed to COVID-19 cases in hospitals, being quarantined, the death or illness

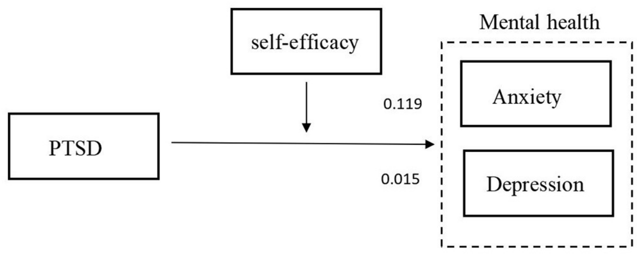

FIGURE 4 | Modified model and standardized path coefficients.

of a relative or friend from COVID-19, and heightened self-perception of danger by the lethality of the virus can all negatively impact the mental wellbeing of healthcare providers (42, 43). Unfortunately, inadequate protective resources reduce the job security of healthcare providers (40). Therefore, in response to the impact of COVID-19, some healthcare providers in all countries have developed a range of PTSD symptoms, such as irritability, hypervigilance, reckless behavior, nightmares, concentration problems and sleep disorders (44).

Our study clearly points out that the daily working hours and the longest continuous working days of healthcare providers are risk factors for PTSD, and it is obvious that the increase in working hours consumes more physical and mental energy from healthcare providers. Furthermore, COVID-19 bears the characteristics of direct contact transmission, aerosol transmission, strong infectivity, and fast transmission speed; latent period patients without clinical symptoms also have infectious characteristics (45). Therefore, the longer the exposure time of patients to COVID-19, the greater the probability of COVID-19 infection, which increases the psychological pressure on healthcare providers.

Sleep time is reduced by long-term continuous work, and healthcare providers cannot achieve adequate physical and mental recovery, which increases the risk of PTSD. These findings are consistent with those of other studies (46). However, physicians and nurses were more likely to experience PTSD than other healthcare providers. This result is consistent with the frequency of patients' direct contact with healthcare providers in hospitals. Physicians and nurses frequently interact with patients in the process of diagnosis and treatment of COVID-19 (20). Healthcare providers must wear airtight protective clothing to enter the isolation area for various medical care operations. Symptoms such as hypoxia and dizziness caused by masks, headaches, and vomiting caused by goggles challenge the physical and psychological limits of healthcare providers. Therefore, doctors and nurses have become a high-risk group for PTSD among medical and healthcare providers, which also suggests that health management departments should pay attention to the allocation and adjustment of their working hours and reduce the work pressure by shortening working hours, taking regular rest, and shifting shifts (47). Additionally, timely psychological intervention and assistance for doctors and nurses can reduce the risk of PTSD among healthcare providers.

Self-efficacy of Healthcare Providers Moderates the Effects of PTSD on Their Mental Health

During the COVID-19 pandemic, the focus of public health authorities was on the biological and physical repercussions of the outbreak rather than on mental health issues. However, with a growing number of reports on the increasing mental health burden caused by the COVID-19 outbreak, more calls were made for measures to enhance mental health support for the public (48). In particular, scholars have studied the need to increase appropriate psychoeducational interventions to prevent and curb harm to people's mental health during the COVID-19 pandemic (49). People's awareness of the risk of emerging infectious diseases is an important factor in public health crisis management (50), which needs greater attention from society and scholars.

Recent research has reviewed the impact of COVID-19 on social psychology (51). Therefore, this study clearly proposed that PTSD developed by healthcare workers while fighting against COVID-19, has a negative impact on their mental health level, which is mainly reflected in the four clinical symptom groups of PTSD, leading healthcare workers to a sub-health mental state, accumulate bad emotions, and aggravate the level of anxiety and depression. This is also consistent with the view pointed out by previous scholars (52), "re-experience symptoms," "avoidance/numbness symptoms," "increased alertness symptoms" and "plc-c" symptoms will reduce the physical and mental health level of individuals, and gradually induce individual negative coping, resulting in behavioral changes, such as job burnout (53), turnover intention and even suicidal tendency (54). Furthermore, scholarly studies have confirmed the importance of monitoring mental health and assessed the psychological consequences of unexpected and potentially traumatic events such as the COVID-19 pandemic (55). In particular, adequate preventive and psychological support measures must be ensured to avoid addictive behaviors, such as eating disorders risk (56), resulting from negative and unregulated emotions related to the traumatic experiences of the pandemic.

Furthermore, this study identified the mechanism of the impact of PTSD caused by the COVID-19 pandemic on healthcare providers' mental health, that is, self-efficacy regulates the impact of PTSD on anxiety and depression. The theory of motivation attribution suggests that self-efficacy can affect individual attribution patterns (57). Healthcare providers with high self-efficacy tend to attribute adversity to their own lack of effort, and then self-motivated individuals can actively complete the work of fighting against the pandemic (58). However, low-self-efficacy of healthcare providers can easily be attributed to their own lack of ability, resulting in many negative emotions such as self-doubt, anxiety, irritability, and indifference (59). Therefore, self-efficacy is the key factor in breaking this negative cycle in the process of healthcare providers' PTSD having a negative impact on their mental health. It is necessary to conduct a basic psychological assessment of healthcare providers affected by COVID-19 and determine whether they meet the criteria for

PTSD, anxiety, depression, and risk of suicide. For those who have not yet reached PTSD severity, active measures should be taken to prevent their transition.

Therefore, this study puts forward three policy suggestions. First, high-risk groups of healthcare providers should be identified. For people who are diagnosed with PTSD, hospital management should adopt measures to improve their self-efficacy and buffer the negative effects of PTSD on their mental health. Second, the method and content of psychological interventions should be clarified. To reduce the risk of COVID-19 infection, it is worthwhile to contemplate the introduction of online or phone-based psychoeducation on the outbreak to promote mental wellness and psychological interventions, such as training self-efficacy, cognitive behavioral therapy, and mindfulness-based cognitive therapy. Finally, we call on society to provide adequate understanding and care to healthcare providers; stigmatization and professional discrimination should be replaced by more care and cooperation.

LIMITATIONS

Although this study yielded significant results, several limitations must be acknowledged. First, the non-random sampling network survey method potentially causes a sample bias, which can affect the study results. The questionnaire distribution method used in this study may also lead to a potential but incalculable sample-size bias. Second, its cross-sectional nature prevented the establishment of a causal relationship between the variables. Therefore, one important suggestion is that longitudinal studies should be conducted in the future. Third, the data were self-reported, which may have led to errors caused by memory bias. These limitations should be addressed in future studies.

CONCLUSION

This study focused on clarifying mental health problems in the medical workplace. Hence, it verified that PTSD has a significant predictive function for anxiety and depression among healthcare providers. Moreover, the key variable "self-efficacy" was introduced as a new interpretation path to reveal the moderating mechanism of its effects on healthcare providers' mental health. Therefore, healthcare providers' self-efficacy as their own original psychological wealth is prone to generating interaction effects with PTSD. The findings suggest that there is potential value in training to improve the self-efficacy of healthcare workers. Undoubtedly, this study has provided new theoretical contributions and practical references, emphasizing the benefits of healthcare workers' mental health, which also encourages health managers' timely response to the secondary crisis caused by COVID-19, especially the protection of the physical and mental health of healthcare providers.

DATA AVAILABILITY STATEMENT

The original contributions presented in the study are included in the article/supplementary material, further inquiries can be directed to the corresponding authors.

ETHICS STATEMENT

The studies involving human participants were reviewed and approval was obtained from the Ethics Committee of the Southern Medical University (approval number 202132). The patients/participants provided their written informed consent to participate in this study.

AUTHOR CONTRIBUTIONS

YY, DL, and BL wrote the first draft of the manuscript. WO and YM edited the paper. LF, YS, and LS revised the manuscript. YY, YM, YS, and LS analyzed the research data. All authors have read and approved the manuscript.

FUNDING

This work was supported by the Guangdong Basic and Applied Basic Research Foundation (2020A1515110369), Project funded by China Postdoctoral Science Foundation (2021M701592), Directive Project of Medical Scientific Research Foundation in Guangdong (A2022379), Key Laboratory Development Project for Philosophy and Social Sciences in Guangdong (G620369695), and the Sanming project of Medicine in Shenzhen (SZSM202111001).

ACKNOWLEDGMENTS

The authors give our sincerely thanks to all participants who helped our collecting data and in distributing questionnaires to the other subjects.

REFERENCES

1. The Lancet. COVID-19: too little, too late? *Lancet*. (2020) 395:755. doi: 10.1016/S0140-6736(20)30522-5
2. Mack TM. The ghost of pandemics past. *Lancet*. (2005) 365:1370–2. doi: 10.1016/S0140-6736(05)66352-0
3. Hyder AA. Allocating medical resources in the time of Covid-19. *N Engl J Med*. (2020) 382:e79. doi: 10.1056/NEJMc2009666
4. Kampf G, Scheithauer S, Lemmen S, Saliou P, Suchomel M. COVID-19-associated shortage of alcohol-based hand rubs, face masks, medical gloves and gowns – proposal for a risk-adapted approach to ensure patient and healthcare worker safety. *J Hosp Infect*. (2020) 105:424–7. doi: 10.1016/j.jhin.2020.04.041
5. Montemurro N. The emotional impact of COVID-19: from medical staff to common people. *Brain Behav Immun*. (2020) 87:23–4. doi: 10.1016/j.bbi.2020.03.032
6. Chirico F, Ferrari G, Nucera G, Szarpak U, Ilesanmi O. Prevalence of anxiety, depression, burnout syndrome, and mental health disorders among healthcare workers during the COVID-19 pandemic: a rapid umbrella review of systematic reviews. *J Health Soc Sci.* (2021) 6:209–20. doi: 10.19204/2021/prvl7

7. Perceived Health Risk B. Psychological experiences and expectations for the future. *Front Psychol.* (2020) 11:559951. doi: 10.3389/fpsyg.2020.559951
8. Biviá-Roig G, Rosa V, Gómez-Tébar M, Serrano-Raya L, Amer-Cuenca JJ, Caruso S, et al. Analysis of the Impact of the Confinement Resulting from COVID-19 on the Lifestyle and Psychological Wellbeing of Spanish Pregnant Women: An Internet-Based Cross-Sectional Survey. *Int J Environ Res Public Health.* (2020) 17:5933. doi: 10.3390/ijerph17165933
9. Esposito S, Giannitto N, Squarcia A, Neglia C, Principi N. Development of psychological problems among adolescents during school closures because of the COVID-19 lockdown phase in Italy: a cross-sectional survey. *Front Pediatr.* (2021) 8:628072. doi: 10.3389/fped.2020.628072
10. Rana W, Mukhtar S, Mukhtar S: Mental health of medical workers in Pakistan during the pandemic COVID-19 outbreak. *Asian J Psychiatry*. (2020) 51:102080. doi: 10.1016/j.ajp.2020.102080
11. Stefanie Jung JKTHCK: The German COVID-19 survey on mental health: primary results. *medRxiv*. (2020) 25:6–15. doi: 10.1101/2020.05.06.20090340
12. Rodolfo Rossi VS, Pacitti F, et al. Mental health outcomes among front and second line health workers associated with the COVID-19 pandemic in Italy. *medRxiv*. (2020) 26:32–40.
13. Li K, Qin Y, Wu J, Yan J. *Containing the Virus or Reviving the Economy? Evidence from Individual Expectations during the COVID-19 Epidemic*. SSRN Electronic Journal. Available online at: https://ssrn.com/abstract=3563597 (accessed March 29, 2021).
14. Tarchi L, Crescenzo P, Talamonti K. Prevalence and predictors of mental distress among Italian Red Cross auxiliary corps: a cross-sectional evaluation after deployment in anti-COVID-19 operations. *Military Psychol*. (2021). doi: 10.1080/08995605.2022.2069983
15. Sinha D, Manna P. The effect of lockdown due to COVID-19 on post-traumatic stress and depression among college students of Kolkata, hotspot district of West Bengal, India. *J Hum Soc Sci.* (2020) 25:58–64.
16. Ru Jia KA, Chalder T, Massey A, Broadbent E, Coupland C, Vedhara K. *Mental Health in the UK During the COVID-19 Pandemic: Early Observations*. (2020). Available online at: https://www.medrxiv.org/content/10.1101/2020.05.14.20102012v1.full.pdf (accessed May 14, 2021).
17. Hsing A, Zhang JS, Peng K, Lin WK, Wu YH, Hsing JC, et al. *A Rapid Assessment of Psychological Distress and Well-Being: Impact of the COVID-19 Pandemic and Shelter-in-Place*. (2021). Available online at: https://ssrn.com/abstract=3578809(accessed May 24, 2021).
18. Li K, Qin Y, Wu J, Yan J. *Containing the Virus or Reviving the Economy? Evidence from Individual Expectations during the COVID-19 Epidemic*. (2020). Available online at: https://ssrn.com/abstract=3563597; http://dx.doi.org/10.2139/ssrn.3563597 (accessed March 29, 2020).
19. Restauri N, Sheridan AD. Burnout and Posttraumatic Stress Disorder in the Coronavirus Disease 2019 (COVID-19) pandemic: intersection, impact, and interventions. *J Am Coll Radiol.* (2020) 17:921–6. doi: 10.1016/j.jacr.2020.05.021
20. Chan AOM, Yiong HC. Psychological impact of the 2003 severe acute respiratory syndrome outbreak on health care workers in a medium size regional general hospital in Singapore. *Occup Med*. (2004) 54:190–6. doi: 10.1093/occmed/kqh027
21. Kisely S, Warren N, McMahon L, Dalais C, Henry I, Siskind D. Occurrence, prevention, and management of the psychological effects of emerging virus outbreaks on healthcare workers: rapid review and meta-analysis. *BMJ.* (2020) 369:m1642. doi: 10.1136/bmj.m1642
22. North CS, Surís AM, Smith RP, King RV. The evolution of PTSD criteria across editions of DSM. *Ann Clin Psychiatry.* (2016) 28:197–208.
23. Robertson N, Perry A. Institutionally based health care workers' exposure to traumatogenic events: systematic review of PTSD presentation. *J Trauma Stress.* (2010) 23:417–20. doi: 10.1002/jts.20537
24. Miller MW. Personality and the etiology and expression of PTSD: a three-factor model perspective. *Clin Psychol Sci Pract.* (2003) 10:373–93. doi: 10.1093/clipsy.bpg040
25. Cohen H, Matar MA, Richter-Levin G, Zohar J. The contribution of an animal model toward uncovering biological risk factors for PTSD. *Ann N Y Acad Sci.* (2006) 1071:335–50. doi: 10.1196/annals.1364.026
26. Williamson V, Stevelink SAM, Greenberg N. Occupational moral injury and mental health: systematic review and meta-analysis. *Br J Psychiatry*. (2018) 212:339–46. doi: 10.1192/bjp.2018.55
27. Johnson SU, Ebrahimi OV, Hoffart A. PTSD symptoms among health workers and public service providers during the COVID-19 outbreak. *PLoS ONE.* (2020) 15:e0241032. doi: 10.1371/journal.pone.0241032
28. Asnaani A, Reddy MK, Shea MT. The impact of PTSD symptoms on physical and mental health functioning in returning veterans. *J Anxiety Disord.* (2014) 28:310–7. doi: 10.1016/j.janxdis.2014.01.005
29. Tyrrell CJ, Shofer JB, Trittschuh EH. Cognitive Self-Efficacy and Mental Health Ratings after a Memory Skills Group for Older Veterans with PTSD. *Clin Gerontol.* (2019) 42:398–407. doi: 10.1080/07317115.2019.1569189
30. Bandura A, Freeman WH, Lightsey R: Self-Efficacy: The Exercise of Control. *J Cogn Psychotherapy*. (1999) 13:158–166. doi: 10.1891/0889-8391.13.2.158
31. Yu X, Wang P, Zhai X, Dai H. Yang Q.The Effect of Work Stress on Job Burnout Among Teachers: The Mediating Role of Self-efficacy. *Soc Indic Res.* (2015) 122:701–8. doi: 10.1007/s11205-014-0716-5
32. Eri, Yoshida, Kazuko, Yamada, Ikuharu, Morioka. Relationship between self-efficacy and stress reaction among nurses with 2-5 years of job experience. *J Jpn Soc Nurs Res*. (2011) 34:65–72. doi: 10.15065/jjsnr.20110524007
33. Lotfikashani F, Vaziri S, Akbari ME, Kazemizanjani N, Shamkoeyan L. Predicting post traumatic growth based upon self-efficacy and perceived social support in cancer patients. *Iran J Cancer Prev.* (2014) 7:115.
34. Bandura A. The explanatory and predictive scope of self-efficacy theory. *J Soc Clin Psychol.* (1986) 4:359–73. doi: 10.1521/jscp.1986.4.3.359
35. Lagerveld SE, Blonk RWB, Brenninkmeijer V, Schaufeli WB. Return to work among employees with mental health problems: Development and validation of a self-efficacy questionnaire. *Work Stress.* (2010) 24:359–75. doi: 10.1080/02678373.2010.532644
36. Aidan M, Roger M, Lelia F. Mental health workers' perception of role self-efficacy and the organisational climate regarding the ethos of recovery. *Br J Soc Work.* (2015) 46:737–55. doi: 10.1093/bjsw/bcv054
37. Blevins CA, Weathers FW, Davis MT, Witte TK, Domino JL. The posttraumatic stress disorder checklist for DSM-5 (PCL-5): development and initial psychometric evaluation. *J Trauma Stress.* (2015) 28:489–98. doi: 10.1002/jts.22059
38. He X, Li C, Qian J, Cui H, Wu W. Reliability and validity of a generalized anxiety scale in general hospitals. *Shanghai Arch Psychiatry.* (2010) 22:200–3. doi: 10.3969/j.issn.1002-0829.2010.04.002
39. Topp CW, Østergaard SD, Søndergaard S, Bech P. The WHO-5 Well-Being Index: a systematic review of the literature. *Psychother Psychosom.* (2015) 84:167–76. doi: 10.1159/000376585
40. Chen Q, Liang M, Li Y, Guo J, Fei D, Wang L, et al. Mental health care for medical staff in China during the COVID-19 outbreak. *Lancet Psychiatry.* (2020) 7:e15–6. doi: 10.1016/S2215-0366(20)30078-X
41. Roccella M. Children and Coronavirus Infection (Covid-19): What to Tell Children to Avoid Post-traumatic Stress Disorder (PTSD). *Open Pediatr Med J.* (2020) 10:1–2. doi: 10.2174/1874309902010010001
42. Wu P, Fang Y, Guan Z, Fan B, Kong J, Yao Z, et al. The psychological impact of the SARS epidemic on hospital employees in China: exposure, risk perception, and altruistic acceptance of risk. *Can J Psychiatry.* (2009) 54:302–11. doi: 10.1177/070674370905400504
43. Greenberg N, Docherty M, Gnanapragasam S, Wessely S. Managing mental health challenges faced by healthcare workers during covid-19 pandemic. *BMJ.* (2020) 368:m1211. doi: 10.1136/bmj.m1211
44. Lai J, Ma S, Wang Y, Cai Z, Hu S. Factors Associated With Mental Health Outcomes Among Health Care Workers Exposed to Coronavirus Disease 2019. *JAMA Netw Open.* (2020) 3:e203976. doi: 10.1001/jamanetworkopen.2020.3976
45. Gupta MK, Lipner SR. Personal protective equipment recommendations based on COVID-19 route of transmission. *J Am Acad Dermatol.* (2020) 83:e45–6. doi: 10.1016/j.jaad.2020.04.068
46. Li Y, Qin Q, Sun Q, Sanford LD, Vgontzas AN, Tang X. Insomnia and psychological reactions during the COVID-19 outbreak in China. *J Clin Sleep Med.* (2020) 16:1417–8. doi: 10.5664/jcsm.8524

47. Ho CS CC, Ho RC. Mental health strategies to combat the psychological impact of COVID-19 beyond paranoia and panic. *Ann Acad Med Singapore*. (2020) 49:1–3. doi: 10.47102/annals-acadmedsg.202043
48. Liu D, Ren Y, Yan F, Li Y, Xu X, Yu X, et al. *Psychological Impact and Predisposing Factors of the Coronavirus Disease 2019 (COVID-19) Pandemic on General Public in China*. Available online at: https://ssrn.com/abstract=3551415 or http://dx.doi.org/10.2139/ssrn.3551415 (access ed on 14 May 2021)
49. Commodari E, La Rosa VL, Coniglio MA. Health risk perceptions in the era of the new coronavirus: are the Italian people ready for a novel virus? A cross-sectional study on perceived personal and comparative susceptibility for infectious diseases. *Public Health.* (2020) 187:8–14. doi: 10.1016/j.puhe.2020.07.036
50. Ding Y, Xu J, Huang S, Li P, Lu C, Xie S. Risk perception and depression in public health crises: evidence from the COVID-19 crisis in China. *Int J Environ Res Public Health.* (2020) 17:5728. doi: 10.3390/ijerph17165728
51. Dubey S, Biswas P, Ghosh R, Chatterjee S, Lavie C. Psychosocial impact of COVID-19. *Diabetes Metabol Syndr*. (2020) 14:779–88. doi: 10.1016/j.dsx.2020.05.035
52. Yayan EH. Post-traumatic stress disorder and mental health states of refugee children. *Arch Psychiatr Nurs.* (2018) 32:885–9. doi: 10.1016/j.apnu.2018.07.003
53. Cosentino C, Foà C, Bertuol M, Cappi V, Riboni S, Rossi S, et al. The impact of the alterations in caring for COVID-19 patients on Compassion Satisfaction and Compassion Fatigue in Italian nurses: a multi method study. *Acta Biomed.* (2022) 93:e2022190. doi: 10.23750/abm.v93iS2.13053
54. Magnavita N, Chirico F, Garbarino S, Bragazzi NL, Santacroce E, Zaffina S. SARS/MERS/SARS-CoV-2 Outbreaks and Burnout Syndrome among Healthcare Workers. An umbrella systematic review. *Int J Environ Res Public Health.* (2021) 18:4361. doi: 10.3390/ijerph18084361
55. La Rosa VL, Gori A, Faraci P, Vicario CM, Craparo G. Traumatic distress, alexithymia, dissociation, and risk of addiction during the first wave of COVID-19 in Italy: results from a cross-sectional online survey on a non-clinical adult sample. *Int J Ment Health Addict*. (2021) 12:1–17. doi: 10.1007/s11469-021-00569-0
56. Rodgers RF, Lombardo C, Cerolini S, Franko DL, Omori M, Fuller-Tyszkiewicz M, et al. The impact of the COVID-19 pandemic on eating disorder risk and symptoms. *Int J Eat Disord.* (2020) 53:1166–70. doi: 10.1002/eat.23318
57. Weiner B. *An Attribution Theory of Motivation and Emotion*. (1986).
58. Bandura A. Self-efficacy: toward a unifying theory of behavioral change. *Psychol Rev*. (1977) 84:191–215. doi: 10.1037/0033-295X.84.2.191
59. Bandura A, Locke EA. Negative self-efficacy and goal effects revisited. *J Appl Psychol.* (2003) 88:87–99. doi: 10.1037/0021-9010.88.1.87

Conflict of Interest: The authors declare that the research was conducted in the absence of any commercial or financial relationships that could be construed as a potential conflict of interest.

Publisher's Note: All claims expressed in this article are solely those of the authors and do not necessarily represent those of their affiliated organizations, or those of the publisher, the editors and the reviewers. Any product that may be evaluated in this article, or claim that may be made by its manufacturer, is not guaranteed or endorsed by the publisher.

Copyright © 2022 Yang, Liu, Liu, Ou, Wang, Ma, Fan, Shi and Shi. This is an open-access article distributed under the terms of the Creative Commons Attribution License (CC BY). The use, distribution or reproduction in other forums is permitted, provided the original author(s) and the copyright owner(s) are credited and that the original publication in this journal is cited, in accordance with accepted academic practice. No use, distribution or reproduction is permitted which does not comply with these terms.
