# Supplementary material for: Mental health status and related factors influencing healthcare workers during the COVID-19 pandemic: A systematic review and meta-analysis
Source: PLoS One. 2024 Jan 19;19(1):e0289454. doi: 10.1371/journal.pone.0289454 (PMC10798549; doi:10.1371/journal.pone.0289454)
Supplement: S1 Data — (ZIP) [file pone.0289454.s011.zip › literatures/223.pdf]

Psychological Status of Healthcare Workers during the Covid-19 Pandemic

Tuba Talo Yildirim[1], Osman Atas[2], Azad Asafov[3], Kadir Yildirim[4] and Hakan Balibey[5]

[1]*Department of Periodontology, Faculty of Dentistry, Firat University, Elazig, Turkey*
[2]*Department of Pediatric Dentistry, Faculty of Dentistry, Firat University, Elazig, Turkey*
[3]*Department of Child Psychiatry, Medical Park Hospital, Elazig, Turkey*
[4]*Department of Urology, Medical Park Hospital, Elazig, Turkey*
[5]*Department of Psychiatry, Medical Park Hospital, Elazig, Turkey*

ABSTRACT

Objective: To assess the levels of health anxiety and Beck depression according to demographic variables among a sample of healthcare workers during the COVID-19 pandemic.
Study Design: Cross-sectional, analytical study.
Place and Duration of Study: Firat University and Medical Park Hosspital, Elazığ, Turkey, from March to April 2020.
Methodology: A total of 270 healthcare workers (100 males and 170 females) filled out health anxiety ınventory (HAI), Beck depression ınventory (BDI), questionnaires. Evaluations of HAI and BDI were conducted according to demographic variables (age, gender, marital status, jop, life style, smoking habits, sistemic disease).
Results: Female healthcare workers had higher HAI and BDI scores than male ($p < 0.001$, $p < 0.001$). Young healthcare workers had higher HAI scores than aged healthcare workers ($p=0.021$) Healthcare workers with more knowledge about COVID-19 had lower HAI and BDI in ($p=0.021$, $p=0.019$). There is statistical significant differences in HAI and BDI scores in marital status ($p = 0.022$, $p=0.002$). The HAI and BDI scores of participants, who had close contact, were significantly higher than those who had no contact ($p = 0.009$ $p=0.028$). There were significant correlations between the two scales ($p < 0.05$).
Conclusion: During the COVID-19 pandemic, psychological support should be provided to all healthcare workers and working conditions should be corrected to relieve the anxiety and worry.

Key Words: *COVID-19, Healthcare workers, Health anxiety, Beck depress.*

How to cite this article: Yildirim TT, Atas O, Asafov A, Yildirim K, Balibey H. Psychological Status of Healthcare Workers during the Covid-19 Pandemic. *J Coll Physicians Surg Pak* 2020; **30(Supp)**:S26-S31 https://doi.org/10.29271/jcpsp.2020.Supp.S26.

INTRODUCTION

The new type of coronavirus (COVID-19) first appeared in Wuhan, a city in China's Hubei province, in December of 2019. COVID-19 has spread throughout the world across all continents except for Antarctica. With the increase of coronavirus cases around the world, the World Health Organization (WHO) declared that COVID-19 is a pandemic March, 2020.[1]

A coronavirus is a type of virus that can be transmitted from animals to humans; in such cases, this virus mutates when it passes to humans, further leading to human-to-human spread.[2]

Correspondence to: Tuba Talo Yildirim, Department of Periodontology, Faculty of Dentistry, Firat University, Elazig, 23100, Turkey
E-mail: dt_talo@hotmail.com
....
Received: May 08, 2020; Revised: June 15, 2020;
Accepted: June 24, 2020
DOI: https://doi.org/10.29271/jcpsp.2020.Supp.S26

A coronavirus can progress in different stages, such as mild, moderate, and severe; and such viruses are in the same group as severe acute respiratory syndrome (SARS) of 2002 and Middle-East respiratory syndrome (MERS) of 2012.[3] The new coronavirus is generally a disease that manifests in symptoms of high fever and cough; and in advanced cases, patients may endure respiratory distress. In addition, it has been shown that different symptoms such as nausea, vomiting, diarrhea, muscle-joint pain, and loss of appetite may occur. In severe cases, pneumonia, severe respiratory failure, kidney failure, and death may occur.[4]

The new coronavirus infection, COVID-19, is transmitted by respiratory secretions just as with other coronavirus infections. Respiratory secretion droplets released from infected individuals when coughing, sneezing, laughing, and speaking can spread throughout the environment, and if they come into contact with the mucous membranes of healthy people, this could cause the latter to become sick.[5] Close contact (less than one meter) is necessary for the disease to be transmitted from person to person in this way. For people infected with COVID-19, the asymptomatic incubation period has been reported to be

one to 14 days, or even 24 days later; thus, it has been confirmed that those without symptoms can also spread the virus.[6]

OSHA (Occupational Safety and Health Administration) put healthcare workers in a very high risk group in terms of the risk of COVID-19 infection.Those who apply aerosol producing procedures (intubation, cough induction, bronchoscopy, mouth-throat-nose examination, ophthalmological examinations, central catheter insertion, nebulizer use, cardiopulmonary resuscitation, oxygen therapy, non-invasive ventilation, examinations with some dental procedures, or invasive sample collection procedures), laboratory workers, patient care physicians, nurses and assistant health care workers are defined as risk groups.[6]

COVID-19 pandemic can cause high levels of anxiety among healthcare workers (*e.g.,* doctors, dentists, and nurses) due to the risk of disease transmission.

Therefore, the aim of the present study was to assess the health anxiety inventory (HAI) and Beck depression inventory (BDI) scores of healthcare workers to see whether the psychiatric parameters are differentiated in terms of each participant's gender, age, smoking status, chronic disease status, and lifestyle during COVID-19 pandemic.

METHODOLOGY

This cross-sectional, analytical study was conducted with a total of 270 healthcare workers (170 females and 100 males) in Elazığ, Turkey from March to April 2020. Participants with a history of mental illness or those taking anxiolytic, sedative, or antidepressant agents were excluded.

The Institutional Review Board of Fırat University approved this cross-sectional study (approval number: 2020/07-28). All participants voluntarily participated in this study. Participants were informed about the nature of the study through verbal communication. Informed consents were obtained from all participants. Later, the participants, who agreed to take part in the study, filled out the questionnaires. Demographic data for all of the participants were recorded. HAI and BDI scores were used to assess their anxiety and depression levels.

HAI was developed by Salkovskis *et al*. to evaluate levels of health anxiety.[15] The HAI is a self-report questionnaire consisting of 18 items.[7] The first 14 items of the scale consist of statements designed to assess the mental state of the patient. The remaining four questions ask the patients to provide an idea of what their mental state might be with the assumption that they have a serious illness. Each item is scored on a scale between 0 and 3, and higher values are considered to be indicative of serious anxiety.

BDI was developed and later modified by Beck *et al*.[8] The scale consists of 21 items designed to measure depressive symptoms.[8] The items on the BDI are assessed on a scale ranging from 0 to 3, and the total score ranges from 0–63. A higher total score is indicative of a more severe level of depression.

SPSS 23.0 for Windows was used for statistical analysis of the data. The data were obtained from the participants after all three measures were scored in accordance with the scoring directive. Qualitative data were expressed as numbers and percentages, while quantitative data as mean ± standard deviation (*SD*). In the analysis of the difference between the two groups, an independent Student's t-test was used, and one-way ANOVA testing along with Tukey HSD Post-hoc test was used for more than two groups; the Pearson correlation coefficient was used to determine the relationship between the scales. $p < 0.05$ was considered statistically significant.

RESULTS

A total of 270 healthcare workers, including 100 males (37.0%) and 170 females (63%), were included in the study. The age of the participants ranged between 21 – 60; the average age of females was 33.57 ±7.78 years, and the average age for males was 35.80 ±9.05 years. In terms of education level, 15 (5.6%) participants had completed high school, 144 (53.3%) had completed university, and 111 (41.1) were educated at the post-graduate level. When the marital status of the participants was evaluated, it was determined that 61.5% (n=166) were married, 36.3% (n=98) were single, and 2.2% (n=6) were divorced or widowed. Of these healthcare workers, 127 (47.0%) had children.

The study consisted of 104 doctors (38.5%), 88 dentists (32.6%), 39 nurses (14.4%), 12 health technicians (4.4%), 10 secretaries (3.7%), nine staff assistants (3.3%), and eight physiotherapists (3.0%). Regarding the workplace of the participants, 127 (47.0%) worked in public institutions, 102 (37.8%) worked in universities, and 41 (15.2%) worked in private institutions. The great majority of the participants (169) were working in outpatient clinics (62.6%), followed by 37 in service (13.7%), 24 in emergency rooms (8.9%), 12 in radiology departments (4.4%), 10 in operating rooms (3.7%), 10 in laboratories (3.7%), and eight in intensive care (3.0%).

The average HAI and BDI scores were 12.53 ±7.08 and 10.35 ±8.11 for males ($p<0.001$), respectively; and 17.11 ±7.70 ($p<0.001$) and 14.89 ±8.76 for females, respectively. There was a statistically significant difference between the anxiety and depression levels of females and males. A statistically significant relationship was also determined between the HAI scores of those in different age groups ($p=0.021$, Table I).

The married participants' mean HAI and BDI scores were 14.55 ±7.66 and 12.02 ±8.34 respectively. The HAI and BDI scores for single participants were 16.49 ±7.72 and 14.69 ±9.14, respectively, and the scores for those who were divorced or widowed were 21.50 ±8.80 and 22.00 ±7.85 respectively. Statistically significant differences were found for the HAI and BDI scores in the marital status ($p=0.022$; $p=0.002$). No statistically significant difference was found in terms of education level and the HAI and BDI scores ($p=0.730$; $p=0.261$). There was, however, a statistical significant difference in the HAI scores of those who had children ($p=0.014$, Table I).

Table I: The relationship between demographic variables and HAI, BDI level of participant.

	n (%)	HAI	p	BDI	p
**Gender**			<0.001*		<0.001*
Male	100 (37.0%)	12.53 ±7.08		10.35 ±8.11	
Female	170 (63.0%)	17.11 ±7.70		14.89 ±8.76	
**Age group**			0.021**		0.089
20-30	121 (44.8%)	15.96 ±7.87[a]		14.18 ±9.23	
31-40	92 (34.1%)	15.58 ±7.99[b]		13.33 ±8.81	
41-50	40 (14.8%)	15.78 ±7.04[c]		11.83 ±7.30	
41-60	17 (6.3%)	9.76 ±5.70[a,b,c]		8.94 ±7.64	
**Education level**			0.730		0.261
High School	15 (5.6%)	15.00 ±13.07		16.67 ±12.30	
University	144 (53.3%)	15.76 ±7.55		13.24 ±8.50	
Postgraduate	111 (41.1%)	15.01 ±7.20		12.70 ±8.60	
**Marital status**			0.022**		0.002**
Married	166 (61.5%)	14.55 ±7.66[a]		12.02 ±8.34[a,b]	
Single	98 (36.3%)	16.49 ±7.72		14.69 ±9.14[a]	
Divorced / widowed	6 (2.2%)	21.50 ±8.80[a]		22.00 ±7.85[b]	
**Have a child**			0.014*		0.088
No	127 (47.0%)	16.65 ±8.35		14.18 ±9.45	
Yes	143 (53.0%)	14.31±7.09		12.35 ±8.10	

Student's t test p<0.05; Shows one-way ANOVA p<0.05.
[a,b,c] Within the same measurement category, values with the same lower letter are statistically different by Tukey's post hoc analysis.

Table II: The relationship professional variables and HAI, BDI level of participant.

	n (%)	HAI	p	BDI	P
**Job**			0.088		0.467
Doctor	104 (38.5%)	14.12 ±7.54		13.32 ±9.44	
Dentist	88 (32.6%)	16.11 ±7.10		12.63 ±8.09	
Nurse	39 (14.4%)	16.46 ±7.71		13.61 ±8.14	
Physiotherapist	8 (3.0%)	18.75 ±6.32		10.38 ±5.24	
Technician	12 (4.4%)	11.58 ±7.13		11.42 ±10.12	
Secretary	10 (3.7%)	18.20 ±8.03		18.30 ±3.77	
Assistant staff	9 (3.3%)	18.00 ±14.62		15.22 ±13.81	
**Working place**			0.421		0.445
State institutions	127(47.0%)	15.02 ±7.11		12.49 ±8.14	
University	102 (37.8%)	16.20 ±7.74		13.81 ±8.67	
Private institutions	41 (15.2%)	14.66 ±9.71		13.95 ±10.87	
**Working area**			0.138		0.698
Outpatient clinic	169 (62.6%)	16.31 ±7.89		13.55 ±9.13	
Service	37 (13.7%)	14.35 ±7.86		12.76 ±7.32	
Emergency room	24 (8.9%)	15.25 ±7.14		14.25 ±10.31	
Operating room	10 (3.7%)	13.90 ±6.98		10.40 ±9.69	
Intensive care	8 (3.0%)	15.13 ±10.96		15.25 ±6.71	
Laboratory	10 (3.7%)	11.90 ±6.28		11.20 ±7.04	
Radiology process area	12 (4.4%)	10.75 ±4.52		10.42 ±6.76	
**Working year**			0.299		0.500
0-5	90 (33.3%)	16.41 ±7.96		14.58 ±8.78	
6-10	71 (26.3%)	14.85 ±8.48		12.65 ±9.79	
11-15	47 (17.4%)	15.11 ±7.33		12.53 ±8.60	
16-20	22 (8.1%)	17.00 ±5.59		11.95 ±6.67	
20 and above	40 (14.8%)	13.65 ±7.52		12.63 ±8.21	

The results of this study have shown that one's occupation, work place, working years, and institution had no significant effect on HAI or BDI scores (p >0.05, Table II).

Of the healthcare workers who participated in the study, 78.5% (n=212) lived with family, 11.1% (n=30) lived alone, 5.6% (n=15) lived with friends, and 4.8 % (n=13) lived with extended family. The mean HAI and BDI scores were 17.60 ±6.93 and 14.27 ±8.69 for those who lived alone, 17.53 ±8.43 nd 15.73 ±11.03 for those who lived with friends, 15.04 ±7.78 and 12.85 ±8.63 for those who lived with family, and 14.00 ±8.52 and 13.77 ±9.25 for those who lived with extended family. There was no statistically significant association between HAI and BDI scores and lifestyle (p=0.559; p=0.221, Table III).

Table III: The relationship between other variables and HAI, BDI level of participant.

	n (%)	HAI	p	BDI	P

**Life style**					
Single	30 (11.1%)	17.60 ±6.93	0.221	14.27 ±8.69	0.559
Friend	15 (5.6%)	17.53 ±8.43		15.73 ±11.03	
Family	212 (78.5%)	15.04 ±7.78		12.85 ±8.63	
Extended family	13 (4.8%)	14.00 ±8.52		13.77 ±9.25	
**Isolation**					
Yes	144 (53.3%)	15.19 ±7.28	0.626	13.00 ±8.56	0.674
No	126 (46.7%)	15.66 ±8.35		13.45 ±9.07	
**Close contact with person Who have foreign country entry and exit**					
Yes	26 (9.6%)	13.46 ±6.85[a]	0.009**	14.19 ±8.84	0.028**
No	163 (60.4%)	14.65 ±7.52[b]		12.07 ±8.04[a]	
Not know	81 (30%)	17.57 ±8.21[a,b]		15.19 ±9.88[a]	
**Knowledge Level**					
Never	10 (3.7%)	13.90 ±6.62	0.021**	12.00 ±10.08	0.019**
Little	135 (50%)	16.69 ±8.32[a]		14.41±8.80[a]	
Good	103 (38.1%)	14.64 ±7.06		12.82 ±8.74	
Very good	22 (8.1%)	11.86 ±6.76[a]		8.27 ±6.76[a]	

Student's t test p<0.05; Shows one-way ANOVA p<0.05.
[a,b,c]*Within the same measurement category, values with the same lower letter are statistically different by Tukey's post hoc analysis.*

Table IV: The relationship between cronic disease, smoking habits and HAI, BDI level of participant.

	n (%)	HAI	p	BDI	P
**Have a cronic disease**					
Yes	48 (17.8%)	17.69 ±8.65	0.025*	14.25 ±9.55	0.368
No	222 (82.2%)	14.92 ±7.51		12.99 ±8.63	
**Smoking habits**					
Yes	53 (19.6%)	15.51 ±7.20	0.919	15.17 ±8.58	0.070
No	217 (80.4%)	15.39 ±7.93		12.73 ±8.80	

Student's t test p<0.05.

Interestingly, a total of 114 (42.2%) of the individuals had put themselves under isolation because of COVID-19. The average HAI and BDI scores of the participants who were under isolation were 15.19 ±7.28 and 13.00 ±8.56, while the scores of those who had not put themselves under isolation were 15.66 ±8.35 and 13.45 ±9.07, respectively (p=0.626; p=0.674).

None of the participants had entered or exited a foreign country in the last month. However, 45 (16.7%) individuals were found to have been in close contact with people who entered or exited a foreign country. For the participants who had such close contact, the mean HAI and BDI scores were 13.46 ±6.85 and 14.19 ±8.84 respectively. For those who had not contact with such individuals, the mean scores were 14.65 ±7.52 and 12.07 ±8.04, respectively, and for those who did not know if they had, the mean scores were 17.57 ±8.21 and 15.19 ±9.88, respectively. The HAI and BDI scores for those who had close contact and or those who did not know if they had were significantly higher than those who did not have such contact (p=0.009; p=0.028, Table III). There was a statistically significant relationship between participants' levels of knowledge of COVID-19 and their HAI and BDI scores (p=0.021; p=0.019, Table III).

A total of 53 (19.6%) of the healthcare workers in the study smoked, 217 (80.4%) of the healthcare workers were non-smokers, and 42 (15.6%) of the participants had a chronic disease. There was no statistically significant differences between the HAI and BDI scores with respect to those who were smoking (p=0.919; p=0.070). However, individuals with chronic diseases had higher HAI scores than those without them, and this difference was statistically significant (p=0.025, Table IV). There was a significant correlation between the scales (r=0.583, p<0.001).

DISCUSSION

Coronaviruses envelope RNA viruses with many subtypes, and the diseases they can cause range in severity from the onset of simple respiratory symptoms to more serious clinical manifestations.[5] The cause of the disease called COVID-19 is a subtype called SARS-CoV-2.[5] The silent period between the contraction of the virus and the onset of disease symptoms is between two and 14 days (median four days).[9] Although it is known that the most important transmission path is through droplets, it is thought that the disease is transmitted by the incubation of the virus through mucosal surfaces in the body (such as the eyes or mouth) after direct contact.[9] After the onset of the COVID-19 pandemic, many healthcare workers' working hours were extended, and mortality rate also increased. Accordingly,

the number of patients examined daily and the time spent with each patient also increased. All healthcare workers carry the risk of COVID-19 contamination.[4] All of these situations negatively affect the psychology of healthcare professionals. In this study, we examined the HAI and BDI of the healthcare workers in this context to confirm our beliefs in this respect.

The results of this study revealed a significant correlation between the scores of the two designated psychiatric scales (HAI and BDI) after the COVID-19 pandemic among healthcare workers. The HAI and BDI scores were higher for females than males, and this finding overlaps with those of other studies.[10-12] However, age only influenced HAI scores and not BDI scores. This difference between genders is related to different anatomical structures as well as biological factors, such as sex hormones. In addition, such a difference can be explained by the fact that females have to take on the roles of mothers, wives, and professionals all at the same time. Psycho-social factors, such as women being exposed to more stress because they have more duties and responsibilities in life, are associated with higher levels of anxiety and depression.[13]

According to the results of our study, there was a negative relationship between age and HAI scores. This result may be related to the fact that young healthcare professionals have encountered pandemics for the first time, and thus they may be inexperienced in this respect.[14]

The lower HAI and BDI scores among married people could be related to the life experience gained by taking responsibility for others; married people may be more advanced in their abilities to establish interpersonal relationships and both deal with and resolve problems.[15] Interestingly, the findings of this study have shown that participants with children had higher HAI scores. Generally, such healthcare workers who have children are afraid of being infected and infecting their families. Moreover, children need their parents to survive; and accordingly, such individuals feel responsible for their children. Thus, they may have more anxiety about becoming infected.

The results of this study have shown that one's profession, work place, year of study, and institution had no significant effect on HAI and BDI scores ($p>0.05$, Table 2). In contrast to this work, Jianbo Lai *et al*.[16] reported that among healthcare workers in hospitals equipped with fever clinics or wards designated for patients with COVID-19 in Wuhan and other regions in China, participants reported experiencing psychological burdens, especially nurses, women, those in Wuhan, and other frontline healthcare workers directly engaged in the diagnosis, treatment, and care of patients with COVID-19.[16] This difference is related to the fact that COVID-19 is very easily transmitted by the respiratory tract, and that patients are more likely to infect the environment in the asymptomatic period. With respect to the latter claim, healthcare workers generally do not know who is positive at first. In fact, all patients coming to the hospital during this period may be assumed to have asymptomatic cases of COVID-19. Consequently, all employees who meet patients are at risk. Therefore, no difference has been detected between the healthcare professionals and their workplace with respect to HAI and BDI scores.

Individuals with chronic diseases had higher HAI scores than those without them. Zu *et al*.[17] reported that approximately half of the critically ill patients (49.0%) were affected by pre-existing chronic diseases (including, for example, cardiovascular disease, diabetes, chronic respiratory disease, and oncological diseases).[17] Recent studies have shown that there is a presence of aggravating factors in the most severe cases.[18, 19] With respect to critically ill patients, the most significant factor is hypertension, affecting 23.7% of patients in critical condition, and the second most significant is diabetes (without a distinction of the type being made), which affects 16.2% of the most severe cases.[20] Therefore, the host's predisposing factors significantly determine the progression, and the outcome of COVID-19. People with chronic diseases have a higher risk of succumbing to the more serious effects of COVİD-19 than healthy people. Accordingly, healthcare workers with chronic health diseases exhibit higher levels of anxiety.

HAI and BDI scores in those who have close contacts were significantly higher than those who are not in contact, because COVID-19 was first seen in Wuhan and spread to the rest of the world. People from abroad are more likely to become infected with COVID-19. The first case in our country was observed in a person who had close contact with people who had foreign country entry. After the first case, another important contributor to the rapid increase of COVID-19 was individuals who had returned from performing religious duties in Saudi Arabia. Persons who are in contact with those who have been abroad are a risk group due to COVID-19. Accordingly, healthcare professionals, who are in contact with people who had foreign country entry and exit, have high HAI and BDI scores.

According to the results of this study, individuals with high levels of knowledge about COVID-19 had lower HAI and BDI scores. People with more knowledge of COVID-19 can act more consciously to protect themselves, as they know more about how the disease is transmitted; and they may be less vulnerable to unnecessary worries.

While acknowledging the limitations of this study; further studies are needed to clarify whether COVID-19 affects the psychological status of healthcare workers. Additionally a larger sample size is needed to verify the results.

CONCLUSION

After the onset of the COVID-19 pandemic, HAI and BDI

scores were higher for females working in healthcare. Younger healthcare workers had higher HAI scores. Married individuals had lower HAI and BDI scores than those who were single, divorced, or widowed. Individuals who had close contact with people who had entered or exited a foreign country had higher HAI and BDI scores. Healthcare workers with more knowledge about COVID-19 had lower HAI and BDI scores.

ETHICAL APPROVAL:
Ethics Committee approval was received for this study from the local Ethics Commitee (2020-28-7).

PARTICIPANTS' CONSENT:
Informed consents were obtained from all participants.

CONFLICT OF INTEREST:
The authors declared that they have no conflicts of interest and no funding.

AUTHORS' CONTRIBUTION:
TTY: Conception, design, article writing.
OA: Data collection, literature review, conception.
AA: Data collection, reviewing the article.
KY: Statistics, data collection.
HB: Design, conception reviewing the article.

REFERENCES

1. Neher RA, Dyrdak R, Druelle V, Hodcroft EB, Albert J. Potential impact of seasonal forcing on a SARS-CoV-2 pandemic. *Swiss Med Wkly* 2020; **150**:w20224.
2. Chan JF, Yuan S, Kok KH, Wang To KK, Chu H, Yang J, *et al*. A familial cluster of pneumonia associated with the 2019 novel coronavirus indicating person-to-person transmission: A study of a family cluster. *Lancet* 2020; **395(10223)**: 514-23.
3. Huang C, Wang Y, Li X, Ren L, Zhao J, Hu Y, *et al*. Clinical features of patients infected with 2019 novel coronavirus in Wuhan, China. *Lancet* 2020; **395(10223)**:497-506.
4. Sabino-Silva R, Jardim ACG, Siqueira WL. Coronavirus COVID-19 impacts to dentistry and potential salivary diagnosis. *Clin Oral Investig* 2020; **24(4):**1619-21.
5. Peng X, Xu X, Li Y, Cheng L, Zhou X, Ren B. Transmission routes of 2019-nCoV and controls in dental practice. *Int J Oral Sci* 2020; **3(12):**9.
6. Kampf G, Todt D, Pfaender S, Steinmann E. Persistence of coronaviruses on inanimate surfaces and their inactivation with biocidal agents. *J Hosp Infect* 2020; **104(3):**246-51.
7. Salkovskis PM, Rimes KA, Warwick HM, Clark DM. The health anxiety inventory: development and validation of scales for the measurement of health anxiety and hypochondriasis. *Psychol Med* 2002; **32(5):**843-53.
8. Beck AT, Ward CH, Mendelson M, Mock J, Erbaugh J. An inventory for measuring depression. *Arch Gen Psychiatry* 1961; **4(6)**:561-71.
9. Wujtewicz M, Dylczyk-Sommer A, Aszkielowicz A, Zdanowski S, Piwowarczyk S, Owczuk R. COVID-19: What should anaethesiologists and intensivists know about it? *Anaesthesiol Intensive Ther* 2020; **52(1):**34-41.
10. Özer SK, Demir B, Tuğal Ö, KABAKÇI E. Montgomery-Asberg Depresyon Değerlendirme Ölçeği: Değerlendiriciler Arası Güvenilirlik ve Geçerlik Çalışması. *Türk Psikiyatri Dergisi* 2001; **12(3):**185-94.
11. MO Ö. Ruh sağlığı ve bozuklukları. Basım, Ankara: Feryal Matbaası 2002.
12. Talo Yildirim T, Dundar S, Bozoglan A, Karaman T, Dildes N, Kaya FA, *et al*. Is there a relation between dental anxiety, fear and general psychological status? *PeerJ* 2017; **5**:e2978.
13. Meana M. The meeting of pain and depression: Pomorbidity in women. *Can J Psychiatry* 1998; **43(9):**893-9.
14. McAlonan GM, Lee AM, Cheung V, Cheung C, Tsang KWT, Sham PC, *et al*. Immediate and sustained psychological impact of an emerging infectious disease outbreak on health care workers. *Can J Psychiatry* 2007; **52(4):**241-7.
15. Maslach C, Jackson S. Manual of maslach burnout inventory. İkinci baskı. In: California, Consulting Psychologists Press 1981.
16. Lai J, Ma S, Wang Y, Cai Z, Hu Z, Wei N, *et al*. Factors associated with mental health outcomes among health care workers exposed to coronavirus disease 2019. *JAMA Netw Open* 2020; **3(3):**e203976.
17. Zhu N, Zhang D, Wang W, Li X, Yang B, Song J, *et al*. A novel coronavirus from patients with pneumonia in China 2019. *N Engl J Med* 2020; **382(8):**727-33.
18. Liu K, Fang YY, Deng Y, Liu W, Wang MF, Ma JP, *et al*. Clinical characteristics of novel coronavirus cases in tertiary hospitals in Hubei Province. *Chin Med J (Engl)* 2020; **133 (9):**1025-31.
19. Wang D, Hu B, Hu C, Zhu F, Liu X, Zhang J, *et al*. Clinical characteristics of 138 hospitalised patients with 2019 novel coronavirus-infected pneumonia in Wuhan, China. *Jama* 2020; **323(11):**1061-9.
20. Kamer E, Çolak T. What to do when a patient infected with COVID-19 needs an operation: A pre-surgery, peri-surgery and post-surgery guide. *Turk J Colorectal Dis* 2020; **30**:1-8.

• • • • • • • • • • •
