# Supplementary material for: Mental health status and related factors influencing healthcare workers during the COVID-19 pandemic: A systematic review and meta-analysis
Source: PLoS One. 2024 Jan 19;19(1):e0289454. doi: 10.1371/journal.pone.0289454 (PMC10798549; doi:10.1371/journal.pone.0289454)
Supplement: S1 Data — (ZIP) [file pone.0289454.s011.zip › literatures/224.pdf]

Article

PanAfrican Medical Journal

Research

Perception of mental health and professional quality of life in Tunisian doctors during the COVID-19 pandemic: a descriptive cross-sectional study

Imen Youssfi, Najla Mechergui, Irtyah Merchaoui, Faten Bouden, Hanene Ben Said, Imen Youssef, Nizar Ladhari

Corresponding author: Imen Yousfi, Occupational Health Department, Charles Nicolle University Hospital of Tunis, Tunis, Tunisia. docteur.yousfi1990@gmail.com

Received: 16 Jun 2021 - **Accepted:** 10 Sep 2021 - **Published:** 05 Nov 2021

Keywords: Mental health, COVID-19, healthcare workers

Copyright: Imen Youssfi et al. Pan African Medical Journal (ISSN: 1937-8688). This is an Open Access article distributed under the terms of the Creative Commons Attribution International 4.0 License (https://creativecommons.org/licenses/by/4.0/), which permits unrestricted use, distribution, and reproduction in any medium, provided the original work is properly cited.

Cite this article: Imen Youssfi et al. Perception of mental health and professional quality of life in Tunisian doctors during the COVID-19 pandemic: a descriptive cross-sectional study. Pan African Medical Journal. 2021;40(139). 10.11604/pamj.2021.40.139.30358

Available online at: https://www.panafrican-med-journal.com//content/article/40/139/full

Perception of mental health and professional quality of life in Tunisian doctors during the COVID-19 pandemic: a descriptive cross-sectional study

Imen Youssfi[1,&], Najla Mechergui[1,2], Irtyah Merchaoui[3,4], Faten Bouden[1], Hanene Ben Said[1,2], Imen Youssef[1,2], Nizar Ladhari[1,2]

[1]Occupational Health Department, Charles Nicolle University Hospital of Tunis, Tunis, Tunisia, [2]Faculty of Medicine of Tunis, University of Tunis El Manar, Tunis, Tunisia, [3]Occupational Health Department, University Hospital of Monastir, Monastir, Tunisia, [4]Faculty of Medicine of Monastir, University of Monastir, Monastir, Tunisia

[&]Corresponding author
Imen Youssfi, Occupational Health Department, Charles Nicolle University Hospital of Tunis, Tunis, Tunisia

Abstract

***Introduction:** few research studies about mental health problems in medical staff during the first wave of the COVID-19 pandemic have been reported. The Aim of the study is to assess the prevalence of anxiety and insomnia, affecting the professional quality of life of physicians during COVID-19 pandemic. **Methods:** doctors answered an online questionnaire regarding their perception of insomnia, anxiety and professional quality of life during COVID-19 pandemic with psychological parameters including the Generalized Anxiety Disorder (GAD-7), Insomnia Severity Index (ISI) and Professional quality of life version 5 (ProQOL5). **Results:** anxiety was found in 64.8% of the participants. This disorder was respectively moderate and severe in 12.4% and 6.7% of cases. Insomnia was found in 51.4% of respondents, 29.5% of whom worked in the COVID circuit (p=0.17). Insomnia was assessed as mild, moderate and severe in respectively 38.1%, 11.4% and 1.9% of cases. Compassion satisfaction was moderate in 72.4 of cases and high in 24.8% of cases. The entire population with low CS belonged to the 20-29 age group (p=0.019). Compassion satisfaction was statistically higher in married people (32.7%) (p=0.004). This entity varied significantly with occupational grade (p=0.003), seniority in grade (p=0.011) and working in the private health sector (p=0.046). Burnout was moderate in 73.3% and low in 26.7% of cases. Burnout was significantly higher among single people (p=0.03) and statistically altered in the staff working in the COVID unit (p=0.028). Secondary traumatic disorder was above moderate in 69.6%. **Conclusion:** a high prevalence of psychological symptoms was found among doctors during COVID-19. Medical health workers are in need of health protection and adequate working conditions.*

Introduction

WHO declared on March 11th 2020, the COVID-19 outbreak as a 'Pandemic´ due to the increasing spread of the virus worldwide [1]. Outside Tunisia, COVID-19 has affected more than 200 countries and regions worldwide. A total of 2 954 222 cases were confirmed. Cumulative deaths reached 202 597 by 28th April 2020 [2]. Cumulative deaths reached 202 597 by 28th April 2020 [1]. As of 28 April 2020, a total of 967 COVID-19 cases in Tunisia have been confirmed, out of which 128 were health workers, and 40 Tunisians died from the illness.

In addition to physical damage, COVID-19 also caused serious psychological impact. In fact, people have shown fear and anxiety behaviours, leading to a great consumption and a significant shortage of masks and safety. Besides, most frontline healthcare staff faced with huge stress at work and prolonged working hours. The heavy workload might lead to poor sleep [3]. Medical health workers are first-line fighters treating patients with COVID-19. Every day, they face a high risk of SARS Cov2 infection and long and distressing work shifts to meet health requirements. In fact, they are exposed to a protracted source of distress which may exceed their individual adaptation skills [4]. Despite worldwide warning messages about mental health of healthcare workers during the COVID-19 pandemic [5], few research studies about mental health problems in medical and paramedical staff during the first wave of the COVID-19 pandemic in Tunisia have been reported.

Objectives: the aim of the present research is to assess the prevalence of anxiety, and insomnia, affecting professional quality of life of physicians during COVID-19 pandemic.

Methods

Study design and setting: this is a descriptive cross-sectional study performed via an online survey from April 28th to May 26th, 2020. The study began 8 weeks after the COVID-19 first case declaration in Tunisia. This survey period matched the decline of the first COVID-19 pandemic wave in Tunisia [6]. The online survey included questions on socio-demographic and clinical variables.

Participants: the online platform did not give warnings to those who gave up. As a result, participants were those who completed all questions of the online survey. Responses were anonymously collected.

Variables: demographic data such as gender, age, professional grade (trainee doctors, academic or senior doctors and specialist doctors), marital status, number of children, occupational data, title, speciality, health care sector and structure and seniority were collected as well as an assessment of COVID-19 contamination risk level. Participants were also asked whether they have worked in a COVID circuit and if they were confined after their shift. In addition, insomnia, anxiety and professional quality of life were assessed through standardized questionnaires.

Data resources/Measurements: anxious symptoms were assessed via the Generalized Anxiety Disorder (GAD-7) including seven items based on seven core symptoms and evaluating the frequency of symptoms within the last two weeks. Respondents report their symptoms using a 4-item-Likert-rating scale ranging from 0 (not at all) to 3 (almost every day) [7]. High scores indicate sharper anxiety impairments [8]. For the purposes of the feasibility study, we defined a GAD-total score threshold of at least 5 points for the presence of anxiety symptoms [9]. In screening of anxiety, a subscale/cut-off ≥3 in GAD-7 is recommended [8].

Insomnia was assessed via a 7-item self-report questionnaire (the Insomnia Severity Index (ISI)), assessing the nature, severity, and impact of insomnia. A 5-point Likert scale is used to rate each item (0 = no problem; 4 = very severe problem), yielding a total score ranging from 0 to 28 [10]. An ISI total score >10 indicates that insomnia is present. A cut-off score of 10 appears to be the best compromise to achieve optimal balance between sensitivity and specificity in a population-based sample [11]. Professional quality of life was measured via the Professional Quality of Life version 5 (ProQOL 5) [12, 13]. Subscale scores 3 items Compassion Scale (CS), Secondary Traumatic Disorder (STD), Burnout Scale (BS) were measured by calculation of corresponding questions points. The French versions of ProQOL 5 [13], GAD-7 [14], and ISI [15] were used; they were validated and showed excellent psychometric properties.

Bias: the non-response bias was encountered in our survey with an online questionnaire.

Study size: the study size was arrived at 105 complete responses.

Statistical analyses: the frequencies and percentages were computed for categorical variables and the means were calculated for numerical variables. χ^2 tests were used to compare proportions. Univariable analysis was performed to explore independent influence for the different mental health dimensions, such as insomnia, anxiety and quality of life. All hypotheses were tested at a significance level of 0.05. Data analyses were run via SPSS software, version 25.

Results

Participants: a total of 105 physicians responded to the questionnaire.

Descriptive data: seventy-seven of the respondents (73.3%) were female and 49(46.7%) were married. Eleven percent of them had at least 2. The 20-to-29-year-old age range was the most represented (41.9%). Our participants were medical residents in 53.3% and specialists in 9.5% of cases. They were working in public health sector in 87.6% of cases. Professional seniority was between 5 and 10 years in 65.7% of cases, while Job tenure in the current grade was less than 5 years in 80% of cases. They were trained in a medical speciality in 69.5 % and in a surgical speciality in 18.1% of cases. Eighty percent of the respondents worked in a university hospital, and 19% worked more than 8 hours per day. Working in COVID units concerned 50.5% of participants. Of these physicians, 43.4% were isolated after working in COVID units, but none was isolated after working in the inpatient units (Table 1).

Main results

Screening for Generalized Anxiety Disorder (GAD) and sleep quality: the mean score of the GAD was 6.36±4.55 (Table 2). Anxiety was found in 64.8% of the participants, 34.3% of whom worked in the COVID circuit, with no statistically positive link (p=0.54). This disorder was respectively moderate and severe in 12.4% and 6.7% of cases (Table 3). No socio-demographic or occupational factors varied significantly with the presence of anxiety, nor the severity of this disorder. The mean Insomnia Severity Index was 8.43±5.40 (Table 2). Insomnia was found in 51.4% of respondents, 29.5% of whom worked in the COVID circuit (p=0.17). It was assessed as mild, moderate and severe in respectively 38.1%, 11.4% and 1.9% of cases (Table 3). No socio-demographic or occupational factors varied significantly with the presence of insomnia.

Evaluation of the professional quality of life:*Compassion satisfaction (CS) was moderate in 72.4 of cases and high in 24.8% of cases. The entire population with low CS belonged to the 20-29 age group (p=0.019). Compassion satisfaction was statistically higher in married people (32.7%) (p=0.004). This entity varied significantly with occupational grade (p=.003), seniority in grade (p=0.011) and working in the private health sector (p=0.046). *Burnout Scale (BS) was moderate in 73.3% and low in 26.7% of cases. Burnout was significantly higher among single people (p=0.03) and altered in the staff working in the COVID unit (p=0.028). *Secondary Traumatic Disorder (STD) was above moderate in 69.6%. No socio-demographic or occupational factors varied significantly with this entity (Table 4).

Other analyses

Socio-demographics and occupational variables associated with mental health status of the Tunisians doctors during the COVID-19 epidemic: as for the anxiety disorder, respondents who were the most senior in grade (p=0.04) and those who were quarantined after their shifts had significantly higher GAD (p=0.009). Insomnia varied significantly with age group (p=0.025), seniority (p=0.05), speciality (p=0.02) and quarantine after work (p=0.01). Professional quality of life varied significantly with gender (p=0.023), marital status (p=0.013), children number (p=0.019), grade (p=0.008), working in a university hospital (p=0.003) and in COVID units (p=0.023). Professional quality of life was statistically altered in doctors suffering from a severe insomnia disorder (p=0.038) (Table 5).

Discussion

Key results: in our study, a high prevalence of psychological symptoms was found among medical health workers during COVID-19, as well as risk factors for them. Anxiety was found in 64.8% of the participants, 34.3% of whom worked in the COVID circuit. Insomnia was found in 51.4% of respondents. Burnout was moderate in 73.3% of the respondents. Our report found potential risk factors for medical health workers to develop insomnia, anxiety and burnout.

Limitations: the present study has limitations. A cross-sectional design was applied, although a longitudinal approach might help verify whether psychiatric disorders, especially post-traumatic stress disorder, might occur with the COVID-19 progression. Psychological assessment was based on an online survey and on self-report tools. Third, it is not possible to assess the participation rate, since it is unclear how many subjects received the link for the survey.

Interpretation: the risk of doctors´ infection with COVID-19 has significantly increased. In fact, as of the 20th March, no doctors have been infected with COVID-19 among about 40,000 medical personnel from the nation supporting Hubei medical services, then 5 medical health workers in one hospital of Wuhan died due to being infected with COVID-19 [4]. Nearly one hundred medical health workers in Tunisia were infected with COVID-19 at a very early stage (by April 2020). Medical health workers during the COVID-19 first outbreak had high prevalence rates of severe insomnia and anxiety.

Medical health workers might cope with many difficulties such as the insufficient understanding of the virus, the lack of prevention and control knowledge, the long-term workload, the high risk of exposure to patients with COVID-19, the shortage of medical safety equipment [5, 16], the lack of getting rest, and the exposure to critical life events such as death [17]. Undoubtedly, several risk factors might facilitate the development of psychopathology, including chronic insomnia [18]. Independent factors (younger age group, marital status, working in private health sector, being at risk of contact with COVID-19 patients in COVID units) were common risk factors for insomnia and anxiety among medical health workers.

Marital status was associated with mental health status among individuals experiencing mental health problems during COVID-19. Li and colleagues found that insomnia was related to marital status (OR = 0.57, p = 0.046, 95% CI: 0.33-0.99) among medical staff in Ningbo, China [19]. Another study by Tan and colleagues reported that the severity of psychiatric symptoms in the workforce returning to the workplace was significantly associated with marital status ($p < 0.05$) [20]. Liu and colleagues demonstrated a relationship between self-reported sleep condition and post-traumatic stress (PTS) prevalence, showing participants with worse sleep quality had higher post-traumatic stress prevalence [21]. This is in line with previous studies, indicating that poor sleep quality has been linked to both the onset and maintenance of PTS [22]. When faced with the same COVID-19 during the fight against the epidemic, medical health workers in private health sector might worry about being infected due to a different working place involving differences in availability of protection equipment and in medical conditions. Thus, different directions on caring for the medical health workers might be possible.

Adequate working conditions and recovery programs, such as programs favouring activities required to ensure the best physical, mental, and social conditions so that medical workers may progress towards an optimal state of health [23], seem necessary. This may support medical staff quickly adapting to the working environment and, maintaining a better mental and health balance to be able to work. Lowering job demands and workload [24], while increasing job control and reward, might help to protect medical health workers. Individual adequate interventions for medical staff in the current situation, where they wear medical protective equipment which cannot be removed during work time, are not sufficient in themselves. Story sharing would be important as well as reinforcing the positive assets of persons [25]. Simple, easy, practical methods are needed. Electronic devices, such as mobile phones and computers, may help reduce working hours. Later, continuously updated guidelines on how to handle with the patients with COVID-19 are needed, with rest in shifts for medical staff, rapid supply of medical protective items (including masks, glasses, and suits), and training on the COVID-19 diagnosis and treatment plan for all medical staff.

Generalisability: the use of clinical interviews is encouraged in future studies to draw a more comprehensive assessment of the problem.

Conclusion

In conclusion, a high prevalence of psychological symptoms was found among medical health workers during COVID-19 as well as risk factors for them. Medical health workers are in need of health protection and adequate working conditions, such as provision of necessary and sufficient medical protective equipment, arrangement of adequate rest, as well as recovery programs aimed at empowering resilience and psychological well-being [5].

What is known about this topic

- *Most frontline healthcare staff faced with huge stress at work and prolonged working hours;*
- *The heavy workload might lead to poor sleep.*

What this study adds

- *A high prevalence of psychological symptoms was found among medical health workers during COVID-19 as well as risk factors for them;*
- *Medical health workers are in need of health protection and adequate working conditions while COVID-19 pandemic.*

Competing interests

The authors declare no competing interests.

Authors' contributions

Concept and design: Imen Youssfi, Najla Mechergui. Acquisition, analysis, and interpretation of data: Imen Youssfi, Faten Bouden. Drafting of the manuscript: Hanene Ben Said. Critical revision of the manuscript: Najla Mechergui, Irtyah Merchaoui, Imen Youssef, Nizar Ladhari. Statistical analysis: Imen Youssfi, Faten Bouden. All authors read and approved the final version of manuscript.

Acknowledgments

The authors would like to thank all participants for their time and excellent cooperation.

Tables

Table 1: socio-demographic and occupational characteristics of the population
Table 2: anxiety and insomnia questionnaires
Table 3: severity levels of anxiety and insomnia
Table 4: PROQOL subscales severity according to socio-demographic and occupational variables
Table 5: anxiety, insomnia and professional quality of life severity according to socio-demographic and occupational variables

References

1. World Health Organization (WHO). WHO Director-General's opening remarks at the media briefing on COVID-19 - 11 March 2020. Accessed 23rd February 2021.
2. World Health Organization (WHO). Coronavirus disease 2019 (COVID-19) Situation Report – 99. Accessed 23rd February 2021.
3. Gillet N, Huyghebaert-Zouaghi T, Réveillère C, Colombat P, Fouquereau E. The effects of job demands on nurses´ burnout and presenteeism through sleep quality and relaxation. J Clin Nurs. 2020 Feb;29(3-4): 583-592. **PubMed** | **Google Scholar**
4. Fava GA, McEwen BS, Guidi J, Gostoli S, Offidani E, Sonino N. Clinical characterization of allostatic overload. Psychoneuroendocrinology. 2019 Oct;108: 94-101. **PubMed** | **Google Scholar**
5. Zhang W-R, Wang K, Yin L, Zhao W-F, Xue Q, Peng M *et al.*Mental Health and Psychosocial Problems of Medical Health Workers during the COVID-19 Epidemic in China. Psychother Psychosom . 2020;89(4): 242-250. **PubMed** | **Google Scholar**
6. République de Tunisienne, Ministère de la santé. COVID-19 En Chiffres. Accessed 23rd February 2021.
7. Toussaint A, Hüsing P, Gumz A, Wingenfeld K, Härter M, Schramm E *et al.*Sensitivity to change and minimal clinically important difference of the 7-item Generalized Anxiety Disorder Questionnaire (GAD-7). J Affect Disord. 2020 Mar 15;265: 395-401. **PubMed** | **Google Scholar**
8. Spitzer RL, Kroenke K, Williams JBW, Löwe B. A brief measure for assessing generalized anxiety disorder: the GAD-7. Arch Intern Med. 2006 May 22;166(10): 1092-7. **PubMed** | **Google Scholar**
9. Huang Y, Zhao N. Generalized anxiety disorder, depressive symptoms and sleep quality during COVID-19 outbreak in China: a web-based cross-sectional survey. Psychiatry Res. 2020 Jun;288: 112954. **PubMed** | **Google Scholar**

10. Morin CM, Belleville G, Bélanger L, Ivers H. The Insomnia Severity Index: psychometric indicators to detect insomnia cases and evaluate treatment response. Sleep. 2011 May 1;34(5): 601-8. **PubMed**| **Google Scholar**
11. Yu DSF. Insomnia Severity Index: psychometric properties with Chinese community-dwelling older people. J Adv Nurs. 2010 Oct;66(10): 2350-9. **PubMed**| **Google Scholar**
12. The ProQol. The ProQol Measure In English and Non-English Translations. Accessed 23rd February 2021.
13. ProQOL assessment. Satisfaction par la compassion et usure de la compassion (ProQOL) Version 5 (2009). Accessed 23rd February 2021.
14. eSanteMentale. Troubles anxieux (Dépistage avec 7 questions [GAD-7]). Accessed 23rd February 2021.
15. Apprendre les TCC. Index de Sévérité de l'Insomnie (ISI). Accessed 23rd February 2021.
16. World Health Organization (WHO). Shortage of personal protective equipment endangering health workers worldwide. Accessed 23rd February 2021.
17. Theorell T. Evaluating life events and chronic stressors in relation to health: stressors and health in clinical work. Adv Psychosom Med. 2012;32: 58-71. **PubMed**| **Google Scholar**
18. Wang HX, Wang L, Zhang WR, Xue Q, Peng M, Sun ZC *et al.*Effect of Transcranial Alternating Current Stimulation for the Treatment of Chronic Insomnia: A Randomized, Double-Blind, Parallel-Group, Placebo-Controlled Clinical Trial. Psychother Psychosom. 2020;89(1): 38-47. **PubMed**| **Google Scholar**
19. Li S, Wang Y, Xue J, Zhao N, Zhu T. The Impact of COVID-19 Epidemic Declaration on Psychological Consequences: A Study on Active Weibo Users. Int J Environ Res Public Health. 2020 Mar 19;17(6): 2032. **PubMed**| **Google Scholar**
20. Tan W, Hao F, McIntyre RS, Jiang L, Jiang X, Zhang L *et al.*Is returning to work during the COVID-19 pandemic stressful? A study on immediate mental health status and psychoneuroimmunity prevention measures of Chinese workforce. Brain Behav Immun. 2020 Jul;87: 84-92. **PubMed**| **Google Scholar**
21. Liu N, Zhang F, Wei C, Jia Y, Shang Z, Sun L *et al.*Prevalence and predictors of PTSS during COVID-19 outbreak in China hardest-hit areas: Gender differences matter. Psychiatry Res. 2020 May;287: 112921. **PubMed**| **Google Scholar**
22. Richards A, Kanady JC, Neylan TC. Sleep disturbance in PTSD and other anxiety-related disorders: an updated review of clinical features, physiological characteristics, and psychological and neurobiological mechanisms. Neuropsychopharmacology. 2020 Jan;45(1): 240-241. **PubMed**| **Google Scholar**
23. Sonino N, Fava GA. Rehabilitation in endocrine patients: a novel psychosomatic approach. Psychother Psychosom. 2007;76(6): 319-24. **PubMed**| **Google Scholar**
24. Cummings J. Sharing a traumatic event: the experience of the listener and the storyteller within the dyad. Nurs Res. Nov-Dec 2011;60(6): 386-92. **PubMed**| **Google Scholar**
25. Fava GA, Cosci F, Sonino N. Current Psychosomatic Practice. Psychother Psychosom. 2017;86(1): 13-30. **PubMed**| **Google Scholar**

**Table 1:** socio-demographic and occupational characteristics of the population	
**Variable**	**N(%)**
**Age**	
20-29 years	44(41.9)
30-39 years	37(35.2)
40-49 years	12(11.4)
>= 50 years	12(11.4)
**Gender**	
Male	28(26.7)
Female	77(73.3)
**Marital status**	
Single	54(51.4)
Married	49(46.7)
Divorced	2(1.9)
**Number of children**	
0	62(59)
1	31(29.5)
>=2	12(11.4)
**Total seniority**	
<5ans	9(8.6)
5-10 years	69(65.7)
>10 years	27(25.7)
**Job Tenure (in the current grade)**	
<5ans	84(80)
5-10 years	11(10.5)
>10 years	10(9.5)
**Specialty**	
Medical	73(69.5)
Surgical	19(18.1)
Fundamental	10(9.5)
Emergency-Intensive care	3(2.9)
**Work units**	
Covid Units	53(50.5)
Isolation	23(43.4)
No Isolation	30(56.4)
Inpatients Units	52(49.5)
Isolation	0
No Isolation	52(49.5)

Table 2: anxiety and insomnia questionnaires	
**Questionnaire Items**	**Median±SD**
**Generalized Anxiety Disorder (GAD)**	
Item 1: Feeling nervous, anxious or on edge	1±0.86
Item 2: Not being able to stop or control worrying	1±0.82
Item 3: Worrying too much about different things	1±0.83
Item 4: Trouble relaxing	1±0.87
Item 5: Being so restless that it is hard to sit still	1±0.78
Item 6: Becoming easily annoyed or irritable	1±0.81
Item 7: Feeling afraid as if something awful might happen	1±0.93
Total GAD	6.36±4.55
**Insomnia Severity Index (ISI)**	
Item 1: Falling asleep	1±1.02
Item 2: Staying asleep	1±0.92
Item 3: Early awakening	1±1.18
Item 4: Satisfaction	2±0.99
Item 5: Interference	1±0.99
Item 6: Noticeable	1±1.01
Item 7: Worry	1±0.89
Total ISI	8.43±5.40

Table 3: severity levels of anxiety and insomnia		
**Subscales**		N(%)
**GAD**	**subscales**	
Minimal	(0-4)	37(35.2)
Mild	(5-9)	48(45.7)
Moderate	(10-14)	13(12.4)
Severe	(15-21)	7(6.7)
**ISI subscales**		
Minimal	(0-7)	51(48.6)
Mild	(8-14)	40(38.1)
Moderate	(15-20)	12(11.4)
Severe	(21-26)	2(1.9)

**Table 4**: PROQOL subscales severity according to socio-demographic and occupational variables								
**Variables**	**Low CS**	**Moderate CS**	**High CS**	**Low BS**	**Moderate BS**	**Low STD**	**Moderate STD**	**High STD**
**Gender**								
Male	1.9%	16.2%	8.6%	9.5%	17.1%	7.6%	19%	0
Female	1%	56.2%	16.2%	17.1%	56.1%	22.9%	49.5%	1%
**Age Group**								
20-29	2.8%	33.5%	5.7%	8.6%	33.3%	18.1%	22.9%	1%
30-39	0	27.6%	7.7%	9.5%	25.7%	7.6%	27.6%	0
40-49	0	5.7%	5.7%	3.8%	7.6%	1.9%	9.5%	0
>=50	0	5.7%	5.7%	4.8%	6.7%	2.9	8.6%	0
**Marital Status**								
Single	2.9%	41%	7.6%	8.6%	42.9%	19%	31.4%	1%
Married	0	31.4%	15.2%	17.1%	29.5%	11.4%	35.2%	0
Divorced	0	0	1.9%	1%	1%	0	1.9%	0
**Children Number**								
0	2.9%	46.7%	9.5%	9.5%	49.5%	19%	39%	1%
1-2	0	18.1%	11.4%	13.3%	16.2%	9.5%	20%	0
>2	0	7.6%	3.8%	3.8%	7.6%	1.9%	9.5%	0
**Occupational Grade**								
Intern	0	4.8%	3.8%	1.9%	6.7%	2.9%	5.7%	0
Resident	2.9%	46.7%	3.8%	11.4%	41.9%	19%	33.3%	1%
Assistant	0	6.7%	1.9%	1%	7.6%	0%	8.6%	0
Associate Professor	0	3.8%	4.8%	3.8%	4.8%	2.9%	5.7%	0
Professor	0	2.9%	1.9%	2.9%	1.9%	1%	3.8%	0
General Practionner	0	3.8%	2.9%	1%	5.7%	0%	6.7%	0
Specialist	0	3.8%	5.7%	4.8%	4.8%	4.8%	4.8%	0
**Seniority**								
<5	0	4.8%	3.8%	1.9%	6.7%	2.9%	5.7%	0
5-10	2.9%	53.3%	9.5%	15.2%	50.5%	21%	43.8%	1%
>10	0	14.3%	11.4%	9.5%	16.2%	6.7%	19%	0
**Health Sector**								
Public	2.9	66.7%	18.1%	21%	66.7%	25.7%	61%	1%
Private	0	5.7%	6.7%	5.7%	6.7%	4.8%	7.6%	0
CS: compassion scale; BS: Burnout scale; STD: secondary traumatic disorder								

**Table 5**: anxiety, insomnia and professional quality of life severity according to socio-demographic and occupational variables			
**Variables**	**GAD**	**ISI**	**ProQOL**
**Gender**	0.17	-0.09	0.02
**Age Group**	-0.13	0.02	0.64
**Marital Status**	-0.15	-0.51	0.01
**Children Number**	0.06	0.13	0.41
**Occupational Grade**	0.11	0.05	0.11
**Seniority**	0.01	-0.03	-0.01
**Health Sector**	-0.02	-0.01	-0.76
**Specialty**	0.09	0.02	-0.55
**Quarantine after work**	0.009	0.05	-0.35
**Work in University Hospital**	0.07	0.18	0.05
**Work in COVID units**	-0.14	-0.05	0.37
