# Supplementary material for: Mental health status and related factors influencing healthcare workers during the COVID-19 pandemic: A systematic review and meta-analysis
Source: PLoS One. 2024 Jan 19;19(1):e0289454. doi: 10.1371/journal.pone.0289454 (PMC10798549; doi:10.1371/journal.pone.0289454)
Supplement: S1 Data — (ZIP) [file pone.0289454.s011.zip › literatures/226.pdf]

2020 and a pandemic on 11 March. Japan was the third country to register a first case of COVID-19 on 16 January 2020, after China and Thailand. The number of reported COVID-19 cases rapidly increased from the end of February 2020. Therefore, the Japanese government declared closing all schools from the 1st through 12th grades on 2 March and declared a PHEIC on 7 April. School and other education facilities' closures substantially disrupt the usual daily life and add stress for children and their families. At the time of writing this paper, the Japanese government continues to have all schools closed and insists that citizens stay at home as much as possible.

It is challenging for children with autism spectrum disorder (ASD) to understand the situation and to stay at home.[1] In addition, care for ASD children could be more difficult than that for children without ASD as the condition is characterized by difficulties in reciprocal social interaction skills; deficits in communication skills; stereotypic, obsessive, or repetitive behaviors; and restricted patterns of interests and activities.[2] Under the PHEIC, child psychiatrists must consider what can be done for ASD children in this stressful situation.

As for the situation above, we developed a supportive brochure about COVID-19 for ASD children and their family members who could be reached by online communication. The main benefit we sought was to help ASD children understand COVID-19 with visual supporting methods.

The brochure was made of three parts. The first part explains the purpose of the brochure and characteristics of ASD. The second explains coronavirus infection and how to avoid it. The third shows how to manage life while staying indoors. All content is accompanied by original hand-drawn illustrations (cartoons) because visual support helps in the understanding of content, especially for ASD children. This brochure can be downloaded by everyone from the homepage[3] and the URL address was sent to parents of 262 ASD children by a letter.

First part: The purpose of the brochure

With COVID-19 infection, all schools are closed and ASD children should stay at home, so the circumstances are different from usual, and they may have much friction with parents and other household members. Children with ASD have limited social cognitive ability and difficulty understanding only for words information. In this part, we made cartoons that explain the characteristics of ASD and problems that ASD children may have with the COVID-19 situation (Fig. 1a).

Second part: COVID-19 infection

It is difficult for children in elementary school to understand the COVID-19 infection. In order for ASD children to understand correctly, we made cartoons that explain COVID-19 infection and the methods for preventing it (Fig. 1b).

Third part: Eight small tips for helping ASD children stay at home

In this part, we indicate eight methods for ASD children and their family members. For example, how to structure daily life activities (time and place management) is indicated (Fig. 1c). Playing games also helps ASD children and family members to remain calm at home.[4] It may be valuable for them to play by themselves, too. Sharing feelings like anxiety and restricted interest of ASD children with family members will sometimes make their relationship better in the abnormal situation created by the forced staying at home (Fig. 1d). However, some patients and their family members are anxious about COVID-19 and would like to have professional support. Others who stay at home together for the whole day may experience stress. In that situation, child psychiatrists and support staff may provide medical advice over the telephone, as part of telemedicine services.

It is also important for children with ASD and their parents to connect with friends, teachers, and health-care practitioners. Parents of children with ASD showed significantly elevated parenting-related stress levels compared to those with typically developing children.[5] Providing greater parental leeway has a good influence on children with ASD. The social utility of mental health professionals in the management of the COVID-19 outbreak is confirmed.[6] Therefore, we should provide mental health support not only to children with ASD but also their families.

In conclusion, COVID-19 is an emerging illness that is rapidly spreading through Japan and the rest of the world. This brochure could aid parents by providing interventions for children with ASD. We have not collected the parents' comments on the brochure yet. We will assess the effect of this trial in future studies.

Disclosure statement

The authors declare no conflicts of interest.

References

1. Narzisi A. Handle the autism spectrum condition during coronavirus (COVID-19) *stay at home* period: Ten tips for helping parents and caregivers of young children. *Brain Sci*. 2020; **10**: E207.
2. *American Psychiatric Association. Diagnostic and Statistical Manual of Mental Disorders*, 5th edn. American Psychiatric Association, Washington, DC, 2013.
3. Center for Child Health, Behavior and Development, Ehime University Hospital. *Let's think about how to prevent COVID-19!* [Cited 13 June 2020.] Available from URL: https://www.m.ehime-u.ac.jp/school/neuropsychiatry/news/information/covid-19
4. Oppenheim-Leaf ML, Leaf JB, Call NA. Teaching board games to two children with an autism spectrum disorder. *J. Dev. Phys. Disabil.* 2012; **24**: 347–358.
5. Hoffman CD, Sweeney DP, Hodge D, Lopez-Wagner MC, Looney L. Parenting stress and closeness: Mothers of typically developing children and mothers of children with autism. *Focus Autism Other Dev. Disabil.* 2009; **24**: 178–187.
6. Sani G, Janiri D, Di Nicola M, Janiri L, Ferretti S, Chieffo D. Mental health during and after the COVID-19 emergency in Italy. *Psychiatry Clin. Neurosci.* 2020; **74**: 372.

Kentaro Kawabe, MD, PhD[1,2] Rie Hosokawa, MD,[1,2] Kiwamu Nakachi, MD,[1,2] Ayumi Yoshino, CP,[1,2] Fumie Horiuchi, MD, PhD[1,2] and Shu-ichi Ueno, MD, PhD[1]
[1]Department of Neuropsychiatry, Ehime University Graduate School of Medicine, and [2]Center for Child Health, Behavior and Development, Ehime University Hospital, Toon City, Japan
Email: matsufu@m.ehime-u.ac.jp
Received 11 May 2020; revised 23 May 2020; accepted 16 June 2020.

COVID-19 and medical staff's mental health in educational hospitals in Alborz Province, Iran

doi:10.1111/pcn.13098

The COVID-19 pandemic is ongoing. To date (1 June 2020), more than 6 million people around the world have been infected with the disease, and more than 370 000 have died.[1] COVID-19 is a very different condition to previous conditions experienced by most societies and this novel disease has created especially perplexing conditions for many members of the public and health-care workers (HCW). HCW have many concerns, including: (i) safety and personal protective equipment; (ii) assigned tasks in the inpatient department of COVID-19 patients; (iii) the potential need to quarantine after work; (iv) getting infected and transmitting the infection to their families; and (v) stigma.[2,3]

PCN Psychiatry and Clinical Neurosciences

Table 1 Association of demographic characteristics with depression, anxiety and stress in logistic regression analysis

		Anxiety		Stress		Depression	
		Cases with outcome/ total cases	Adjusted OR	Cases with outcome/ total cases	Adjusted OR	Cases with outcome/ total cases	Adjusted OR
Sex	Male	109/250	1	68/249	1	92/245	1
	Female	352/619	1.53 (1.089–2.149)*	237/621	1.857 (1.257–2.744)*	283/620	1.435 (1.012–2.035)*
Education	<Diploma	49/124	1	32/123	1	38/121	1
	Bachelor	314/575	1.341 (0.854–2.107)	200/575	1.278 (0.759–2.154)	252/573	1.513 (0.941–2.433)
	>Bachelor	94/159	1.269 (0.681–2.367)	71/162	0.93 (0.457–1.889)	83/162	1.248 (0.66–2.359)
Hiring	Official	237/387	1	155/386	1	188/389	1
	Unofficial	186/434	0.566 (0.416–0.771)*	111/436	0.55 (0.393–0.770)*	154/430	0.629 (0.461–0.859)*
	Medical resident	32/41	3.266 (1.117–9.549)*	32/41	4.398 (1.419–13.625)*	28/40	1.541 (0.55–4.319)
Age (years)	<30	152/293	1	117/293	1	132/292	1
	30–40	197/364	1.248 (0.883–1.764)	128/367	1.026 (0.709–1.485)	160/365	1.085 (0.767–1.534)
	>40	110/205	1.216 (0.805–1.837)	55/199	0.701 (0.443–1.111)	79/197	0.869 (0.572–1.322)
Occupation	Physician	50/78	1	45/80	1	48/79	1
	Nurse	303/533	1.213 (0.528–2.788)	191/530	0.611 (0.246–1.517)	228/531	0.487 (0.211–0.252)
	Technician	100/244	0.857 (0.375–1.959)	62/245	0.627 (0.252–1.560)	92/241	0.577 (0.252–1.321)
Front line staff	No	227/463	1	126/460	1	180/456	1
	Yes	235/408	1.282 (0.945–1.740)	180/412	1.985 (1.43–2.755)*	196/411	1.377 (1.015–1.867)*

*Statistically significant.
OR, odds ratio.

In the early days of the spread of the disease at the community level, there were other concerns for HCW. Restrictions on financial and human resources and hospital beds, drug shortages and problems related to restrictions on the possibility of using special hospital facilities (such as the use of intensive care units) in managing the situation caused many problems.[4, 5] Another problem that arose from the limited resources and spread of the disease at the community level was the use of physicians and other personnel of unrelated specialized disciplines in the field of COVID-19 to help advance diagnostic and therapeutic processes. This seemed inevitable to health policy-makers and raised other concerns about the abilities of staff groups to manage the difficult conditions of hospitalized patients with COVID-19 diagnosis.[6]

In this situation, in addition to the need to pay attention to the state of mental health among the masses,[7,8] who may experience fear, anxiety, depression, sleep disorders, suicidal thoughts, and other related disorders,[9,10] it is important to pay attention to the mental health status of the HCW of COVID-19 patients.

Due to the need to determine the mental health status of HCW for further diagnostic, therapeutic, and supportive measures and related planning, in this study, we examined the mental health status of HCW during the COVID-19 pandemic at the level of all university hospitals in Alborz Province, Iran. The ethics committee of Alborz University of Medical Sciences, Iran, has confirmed this study (IR.ABZUMS.REC.1399.011, date: 06-04-2020). The patients have given informed consent, and their anonymity has been preserved.

This was a cross-sectional, multicenter study conducted in nine educational hospitals in Alborz Province that had accepted COVID-19 patients. The study was conducted by sending an online questionnaire to the staff and collecting and evaluating its results. We used the 21-item Depression, Anxiety and Stress Scale to assess the depression, anxiety, and stress of HCW.

Among HCW, 41.7%, 51.2%, and 33.9%, respectively, had degrees of depression, anxiety, and stress. Depression was more common in women, those with a bachelor's degree, medical residents, those aged over 40 years, and doctors and staff in direct contact with patients. Anxiety was more prevalent in women, those with bachelor and higher education, medical residents, those aged 30 to 40 years, nurses, those with a bachelor's degree, and personnel and staff in direct contact with patients. Also, stress was most prevalent in women, those with a bachelor's degree, medical residents, those aged 30 to 40 years, and doctors and staff in direct contact with patients (Table 1). There was a positive linear correlation between depression and anxiety, depression and stress, and stress and anxiety among HCW.

This study found a high prevalence of depression, anxiety, and stress among COVID-19-related HCW in Iran. Nearly half of them had some degree of depression. About half of them suffered from anxiety and one-third experienced stress. Physicians and nurses, especially those in the front line, experienced a greater prevalence of these disorders. In terms of employment relations, medical residents experienced a higher prevalence of anxiety, stress, and depression than other employment groups (official and unofficial). In terms of sex, we also saw a higher prevalence of anxiety, stress, and depression in women than in men. The high correlation between these mental disorders also emphasizes the need to pay attention to other disorders when identifying one of them.

Health policy-makers should pay special attention to the mental health of HCW, as their roles are crucial, especially during the COVID-19 pandemic. The provision of adequate personnel in accordance with the capabilities of health systems, the prioritization of personal protective equipment, and the establishment of appropriate mental health-care systems, such as online systems and face-to-face counseling, could play an important role in improving the mental health of HCW.

Acknowledgments

We would like to thank the HCW who are responsible for the care and treatment of COVID-19 patients. We thank all those who contributed to the various stages of this project. We also thank Emam Ali and Shahid Rajaei Research Development Unit for their administrative contribution.

Disclosure statement

The authors declare that they have no conflict of interest.

References

1. World Health Organization. Coronavirus Disease (COVID-19) Situation Report – 133, 1 June 2020. [Cited 8 July 2020]. Available from URL: https://www.who.int/docs/default-source/coronaviruse/situation-reports/20200601-covid-19-sitrep-133.pdf?sfvrsn=9a56f2ac_4
2. Grover S, Dua D, Sahoo S, Mehra A, Nehra R, Chakrabarti S. Why all COVID-19 hospitals should have mental health professionals: The importance of mental health in a worldwide crisis! *Asian J. Psychiatry* 2020; **51**: 102147.
3. Badrfam R, Zandifar A. Stigma over COVID-19; New conception beyond individual sense. *Arch. Med. Res.* 2020. https://doi.org/10.1016/j.arcmed.2020.05.006
4. Zandifar A, Badrfam R. Fighting COVID-19 in Iran: Economic challenges ahead. *Arch. Iran. Med.* 2020; **23**: 284.
5. Badrfam R, Zandifar A. Coronavirus disease 2019 in Iran: The need for more attention to primary health care. *Public Health* 2020; **182**: 187.
6. Shariati B, Ardebili ME, Shalbafan M. Working in the emergency and inpatient COVID-19 special wards: A different experience for Iranian psychiatric trainees amid the outbreak. *Asian J. Psychiatry* 2020; **51**: 102157.
7. Zandifar A, Badrfam R. Iranian mental health during the COVID-19 epidemic. *Asian J. Psychiatry* 2020; **51**: 101990.
8. Zandifar A, Badrfam R. COVID-19: Considering the prevalence of schizophrenia in the coming decades. *Psychiatry Res.* 2020; **288**: 112982.
9. Ransing R, Adiukwu F, Pereira-Sanchez V *et al.* Early career psychiatrists' perspectives on the mental health impact and care of the COVID-19 pandemic across the world. *Asian J. Psychiatry* 2020; **51**: 102085.
10. Goyal K, Chauhan P, Chhikara K, Gupta P, Singh MP. Fear of COVID 2019: First suicidal case in India! *Asian J. Psychiatry* 2020; **49**: 101989.

Supporting information

Additional Supporting Information may be found in the online version of this article at the publisher's web-site:

Table S1. Frequency and missing percentage of demographic and psychiatric characteristics.

Table S2. Median (IQR) of Depression, Anxiety, and Stress Scale score according to demographic characteristics.

Atefeh Zandifar, MD,[1] Rahim Badrfam, MD,[2] Nami Mohammadian Khonsari,[3] Marzieh Assareh, MD,[4] Hossein Karim, MD,[5] Mehdi Azimzadeh, PhD,[6,7] Mohammad Noori Sepehr, PhD,[8] Ramin Tajbakhsh, MD,[9] Fatemeh Rahimi,[10] Nima Ghanipour,[3] Arash Agoushi,[3] Saeed Hassani Gelsefid,[3] Fateme Etemadi[11] and Mostafa Qorbani, PhD[6,7]
[1]Social Determinants of Health Research Center, Alborz University of Medical Sciences, Karaj, [2]Department of Psychiatry, Roozbeh Hospital, Tehran University of Medical Sciences, Tehran, [3]Student Research Committee, Alborz University of Medical Sciences, [4]Department of Psychiatry, Imam Hossein Hospital, [5]Department of Cardiology, School of Medicine, [6]Non-Communicable Diseases Research Center, Alborz University of Medical Sciences, Karaj, [7]Chronic Diseases Research Center, Endocrinology and Metabolism Population Sciences Institute, Tehran University of Medical Sciences, Tehran, [8]Department of Environmental Health, School of Health Research Center for Health, Safety and Environment, [9]Department of Internal Medicine, School of Medicine, Non-Communicable Diseases Research Center, [10]Cardiovascular Research Center, Shahid Rajaei Educational & Medical Center, and [11]Physical Therapy Ward, Alborz University of Medical Sciences, Karaj, Iran
Email: mqorbani1379@yahoo.com
Received 4 June 2020; revised 16 June 2020; accepted 18 June 2020.

Exposure to media and fear and worry about COVID-19

doi:10.1111/pcn.13095

Too much exposure to the media is known to be associated with greater fear and worry and poorer mental health in a health crisis.[1, 2] Repeated media exposure was reported to have a negative impact on mental health after the New York terrorist attack on 11 September 2001,[3] the Ebola outbreak,[4] and the Great East Japan Earthquake and Tsunami.[5] The use of social media as an information resource was associated with greater fear and worry about adverse health effects of possible radiation exposure after the Fukushima Nuclear Power Plant accident.[6] It has been argued that the same pattern may be observed in the outbreak of the new coronavirus disease 2019 (COVID-19),[2] and it is recommended to limit the frequency of access to the media and to choose a reliable information source to keep better mental health.[7] A study from Wuhan, China reported that social media exposure (such as Weibo and WeChat) was associated with depression and anxiety during the COVID-19 outbreak.[8] However, evidence is still limited in the current outbreak of COVID-19. Here, we report additional evidence based on a secondary analysis of data collected from a sample of employees in Japan in mid-March 2020 during the early phase of the COVID-19 outbreak in Japan.

The authors conducted a cross-sectional online survey to examine the relation between the type of media use and fear and worry about COVID-19 by using a sample from the cohort of full-time employees in February 2019 ($n = 4120$). Participants were assured that their anonymity would be preserved and provided online informed consent. This study was approved by the Research Ethics Committee of The University of Tokyo (No. 10856-(2)). Participants ($n = 1420$) completed an online self-report questionnaire during 19–22 March 2020. Participants included managers (8.8%), white-collar workers (62.8%), and blue-collar workers (28.4%); 13.5% of participants engaged in the medical and welfare sector. Detail information is available elsewhere.[9] The type of media as information source about COVID-19 was requested by using a list of 14 sources (e.g., television, radio, newspaper, Web media, any social network service [SNS; e.g., YouTube, Twitter, Facebook, and other SNS], governmental or organizational websites, chat with family or friends, workplace, medical organization or staff, academic papers, and others) with each item rated *Yes* (= 1) or *No* (= 0). Fear and worry about COVID-19 were measured by asking one item ('Do you feel anxiety over COVID-19?') on a 6-point Likert-type scale (ranging from 1 [*Not at all*] to 6 [*Feel strongly*]). The prevalence rates of media use were: television (89.9%), radio (11.3%), newspaper (27.7%), Web media (66.1%), any SNS (17.5%), governmental or organizational websites (15.6%), chat with family or friends (30.1%), workplace (27.7%), medical organization or staff (9.5%), academic papers (1.1%), and others (0.1%). A linear regression analysis revealed that the use of television ($\beta = 0.133$, $P = 0.0000005$) and Web media ($\beta = 0.113$, $P = 0.000031$) as information sources about COVID-19 significantly and positively correlated with fear and worry about COVID-19, after adjusting for sex, age, marital status, having at least one child, and occupational type. None of the other media use types significantly correlated with fear and worry about COVID-19.

Our survey was limited in many ways: the cross-sectional study design, nonrepresentative sample of employees, and lack of information about duration and frequency of the media use. However, our findings suggest that television and Web media as information sources about COVID-19 are associated with greater fear and worry about the disease in the general working population in Japan. Television broadcasts news and provides information about COVID-19 almost all day. Web media is
