# Supplementary material for: Mental health status and related factors influencing healthcare workers during the COVID-19 pandemic: A systematic review and meta-analysis
Source: PLoS One. 2024 Jan 19;19(1):e0289454. doi: 10.1371/journal.pone.0289454 (PMC10798549; doi:10.1371/journal.pone.0289454)
Supplement: S1 Data — (ZIP) [file pone.0289454.s011.zip › literatures/227.pdf]

ELSEVIER

Since January 2020 Elsevier has created a COVID-19 resource centre with free information in English and Mandarin on the novel coronavirus COVID-19. The COVID-19 resource centre is hosted on Elsevier Connect, the company's public news and information website.

Elsevier hereby grants permission to make all its COVID-19-related research that is available on the COVID-19 resource centre - including this research content - immediately available in PubMed Central and other publicly funded repositories, such as the WHO COVID database with rights for unrestricted research re-use and analyses in any form or by any means with acknowledgement of the original source. These permissions are granted for free by Elsevier for as long as the COVID-19 resource centre remains active.

L'Encéphale 48 (2022) 496–503

Disponible en ligne sur
ScienceDirect
www.sciencedirect.com

Elsevier Masson France
EM|consulte
www.em-consulte.com

Original article

Anxiety and sleep quality in a sample of Lebanese healthcare workers during the COVID-19 outbreak

Anxiété et qualité du sommeil dans un échantillon de personnels de santé au Liban pendant l'épidémie du Coronavirus

M. Zarzour[a,e,*], C. Hachem[a,e], H. Kerbage[a,e], S. Richa[a,e], D. El Choueifaty[a,f], G. Saliba[b,e], E. Haddad[b,e], E. Melki[c,e], G. Sleilaty[d,e], R. Saliby[e], C. Sabbagh[c,e], J. Choucair[b,e]

[a] *Department of Psychiatry, Hôtel-Dieu de France Hospital, Alfred Naccache, Beirut, 166830, Lebanon*
[b] *Department of Infectious Disease, Hôtel-Dieu de France, Alfred Naccache, Beirut, 166830, Lebanon*
[c] *Emergency Department, Hôtel-Dieu de France, Alfred Naccache, Beirut, 166830, Lebanon*
[d] *Clinical Research Centre, Hôtel-Dieu de France, Alfred Naccache, Beirut, 1104 2020, Lebanon*
[e] *Saint Joseph University, Faculty of Medicine, Damas Rd, Beirut, 1104 2020, Lebanon*
[f] *Saint Joseph University, Faculty of Nursing, Damas Rd, Beirut, 1104 2020, Lebanon*

ARTICLE INFO

Article history:
Received 6 April 2021
Accepted 30 June 2021
Available online 23 September 2021

Keywords:
Anxiety
Sleep
Healthcare workers
COVID-19
Pandemic
Lebanon

Mots clés :
Anxiété
Sommeil
Personnel de santé
COVID-19
Pandémie
Liban

ABSTRACT

A cross-sectional, survey based study was conducted in order to assess mental health outcomes among healthcare workers in a private university hospital involved in the COVID-19 response in Lebanon. The main objective was to quantify symptoms of anxiety and sleep quality using self-rating scales (the State-Trait Anxiety Inventory (STAI) and Pittsburgh Sleep Quality Index (PSQI) respectively), while identifying factors that might affect those symptoms. A total of 628 healthcare workers completed the survey; 409 (66.2%) were younger than 40 years, and 441 (71.4%) were women. Of all participants, 503 (81.4%) were nurses, 52 (8.4%) were physicians and 63 (10.2%) were residents. Registered nurses, residents, women, and younger participants presented higher scores on both scales than other categories of participants. Among factors related with COVID-19, those associated with higher scores were having relatives affected by the virus (22.2%), being excessively exposed to media (12.9%), and increasing the consumption of substances/alcohol (31.2%) during this period. Factors associated with higher risk of anxiety symptoms after multivariable logistic regression analysis were: female sex, young age, poor sleep quality, and living with elderly. Our findings contribute to the understanding of the psychological wellbeing of health care workers involved in the acute COVID-19 outbreak in Lebanon.

© 2021 L'Encéphale, Paris.

RÉSUMÉ

Une étude transversale, a été menée afin d'évaluer l'impact de la pandémie du coronavirus sur le niveau d'anxiété et la qualité du sommeil du personnel hospitalier d'un hôpital universitaire privé impliqué dans la réponse contre la pandémie au Liban, tout en identifiant les facteurs qui pourraient affecter ces symptômes. L'évaluation s'est effectuée à l'aide de questionnaires auto-administrés; un auto-questionnaire qui inclut les données sociodémographiques, la nature du travail exercé à l'hôpital, des questions sur les facteurs affectant le niveau de stress, et des questions sur la consommation de substances. Des échelles d'auto-évaluation ont été utilisées ; l'Inventaire d'anxiété d'État-Trait (STAI) pour le dépistage des symptômes anxieux, et l'indice de qualité du sommeil de Pittsburgh (PSQI) pour mesurer la qualité du sommeil. Un total de 628 personnels de santé a répondu au questionnaire. Parmi tous les participants, 503 (81,4 %) étaient des infirmiers/infirmières, 52 (8,4 %) des médecins et 63 (10,2 %) des internes. En ce qui concerne les caractéristiques des participants: 409 (66,2 %) avaient moins de 40 ans, 441 (71,4 %) étaient des

* Corresponding author. Hôtel-Dieu de France Hospital, Alfred Naccache, Beirut, 166830, Lebanon.
E-mail address: myzarzour@gmail.com (M. Zarzour).

https://doi.org/10.1016/j.encep.2021.06.016
0013-7006/© 2021 L'Encéphale, Paris.

femmes, 309 (55 %) étaient mariés, 333 (53,9 %) avaient un seul enfant, 428 (69,3 %) avaient un niveau universitaire, et 591 (95,6 %) ne présentaient pas une histoire psychiatrique. Les moyennes obtenues aux deux échelles étaient de 44,5 au STAI, et de 6,0 au PSQI. Parmi le total des participants, 61,5 % auront un score au STAI supérieur à 40, indiquant des symptômes anxieux modérés à sévères, et 48,4 % auront un score au PSQI supérieur à 5, indiquant une mauvaise qualité de sommeil. Les infirmiers/infirmières diplômés, les internes, les femmes et les participants plus jeunes présentent des scores significativement plus élevés au niveau des deux échelles que les autres catégories de participants. Les participants sans enfants, ceux ayant un niveau académique universitaire, et ceux ayant des antécédents psychiatriques présentent des scores significativement plus élevés uniquement en ce qui concerne la PSQI. Une proportion de 31,2 % de nos participants aura augmenté leur consommation d'alcool ou de substances (tabac, caféine, tranquillisants, cannabis ou autres). Parmi les facteurs liés à la COVID-19, le fait d'avoir des proches touchés par le virus (22,2 %), d'être excessivement exposé aux médias (12,9 %) et d'avoir augmenté la consommation de substances/alcool pendant la période de la pandémie (31,2 %) a été associé à des scores significativement plus élevés au niveau des deux échelles d'évaluation. Parmi les sources de stress liées à la pandémie rapportées, nous trouvons la peur d'être infecté ou d'infecter les autres dans 61,7 %, la peur que quelqu'un de leurs proches soit infecté des 45,6 %, la peur d'avoir un accès limité aux équipements médicaux dans 17 %, et la perte financière dans 16,3 %. Une analyse de régression logistique multivariée a permis d'identifier les facteurs suivants comme étant associés à un risque plus élevé de symptômes anxieux: sexe féminin, jeune âge, mauvaise qualité de sommeil et le fait de vivre avec des personnes âgées. La prévalence de symptômes anxieux et de troubles du sommeil dans notre étude s'avère supérieure à celles retrouvées dans la littérature, d'où la possibilité que nos résultats soient influencés de même par d'autres facteurs comme la crise socioéconomique que le Liban traverse. Par ailleurs, l'étude actuelle a été faite pendant une période où la pandémie était toujours considérée comme « contenue », ce qui peut expliquer l'absence de différence entre les soignants exposés directement (travaillant avec des personnes atteintes du virus) et ceux exposés indirectement. Nos résultats contribuent à la meilleure compréhension de l'état psychologique des professionnels de santé impliqués dans la réponse aiguë à la pandémie du coronavirus au Liban. Des études prospectives sont nécessaires afin d'évaluer le possible impact psychologique à long terme dans cette population.

© 2021 L'Encéphale, Paris.

1. Introduction

An outbreak of a new coronavirus disease, COVID-19, was detected in mainland China in December 2019 [1]. On January 30, 2020, the World Health Organization (WHO) declared the global COVID-19 outbreak a public health emergency of international concern and announced on March 11 that it can be characterized as a pandemic [2].

In the midst of this global pandemic, international and local public health authorities are addressing the urgent need for immediate actions to care for patients and contain the Covid-19 outbreak. Nonetheless, the current crisis has a major impact on global mental health, with reported high rates of negative psychological consequences in the general population including fear, anger, anxiety, depression, insomnia and a reduction in life satisfaction [3–6].

These observations are consistent with the findings of previous research that reveal significant mental health burden during outbreaks of infection, such as the SARS [7–10], H1N1 [10,11] and Equine Influenza outbreaks [12]. Further, a recent review on the psychological impact of quarantine, suggests that post-traumatic stress symptoms, confusion, and anger can be considerable and long lasting [13].

Dealing with this crisis situation, frontline health care professionals are facing numerous challenges. Studies from previous infectious epidemics such as SARS, H1N1 and Ebola outbreaks show that such extraordinary public health crises have a substantive mental burden on health care workers [14–24].

These studies report a broad spectrum of psychological difficulties among healthcare workers (HCWs), including high rates of anxiety, depressive, and posttraumatic symptoms, as well as fatigue, high stress levels and sleep difficulties. Moreover, Wu and colleagues observed that exposure to a severe infectious outbreak can lead to alcohol abuse/dependence particularly among HCWs that were quarantined or worked in high-risk wards [25]. Studies also show that psychological symptoms experienced by HCWs may develop or even worsen years after the outbreak [14,23,26].

Early studies suggest that not all HCWs are impacted equally during outbreaks. Nurses are reportedly the most affected in this group [20,21]. Other identified common risk factors that may contribute to the development of psychological distress in this population include: younger age [17], living with children [20], working in high risk environments (such as the ICU, emergency departments or isolations wards) and fear of spreading the virus [15,17,21–23], being socially rejected or stigmatized for working in a hospital [18,20,22], having been quarantined [14,18,25], or having a history of mood disorders [17].

Comparable to the SARS and MERS outbreaks, COVID-19 shares similar infectious and epidemiological characteristics as well as comparable psychological impact HCWs. This has been confirmed by the few studies that were done mainly in China after the COVID-19 outbreak. These studies show that HCWs, predominantly those with close contact with infected patients, are vulnerable to high risk of fear, insomnia, anxiety and depression [27–30].

Xiao and associates describe high levels of anxiety and stress in this population, that are directly related to sleep quality and social support [31].

In Lebanon, the government declared a state of general mobilization and health emergency on March 15th 2020, in an effort to contain the spread of the COVID-19 outbreak [32]. This health crisis is occurring in a country already struggling with economic, social and political instability.

A university medical hospital, was the first non-governmental hospital in Lebanon that quickly responded to the imposed emergency and got set to treat COVID-19 cases. An isolation ward as well as intensive care unit (ICU) beds were reserved for this purpose. In addition, a "Flu-clinic" was opened, as a separate part of the emergency department, for outpatient screening.

In this study, we assessed mental health outcomes among the above mentioned hospital staff during the COVID-19 outbreak in Lebanon. We aimed at quantifying symptoms of anxiety and sleep disturbance, while identifying factors that might affect those symptoms.

2. Methods

2.1. Study design

This study is a cross-sectional, hospital-based survey conducted at our hospital between April 1 and 22, 2020. The ethics committee of the hospital approved the study protocol.

2.2. Participants

This study included medical staff from different departments working at the hospital. Physicians, post-graduate residents, registered and practical nurses were invited to participate.

We distributed anonymous surveys along with explanatory letters to all registered and practical nurses present at the hospital during the period of the study. They were asked to return the completed form in a sealed envelope, for guaranteed confidentiality, to the supervisor of their unit. The researcher then collected the sealed envelopes.

Concerning physicians and residents, the same assessment tools were used, and they were sent to them as online anonymous forms.

2.3. Instruments

We used a self-administered survey that consisted of 3 main sections:

- a self-administered questionnaire that included questions about the following;

  - sociodemographic characteristics; sex, age, education level, marital status, number of children;
  - occupation and work history;
  - past psychiatric history;
  - potential stressful factors during the pandemic; working in COVID-19 units, being quarantined, having relatives infected or quarantined, living with children or elderly people, and media exposure related to the pandemic (via television/radio/newspapers/social networks);
  - impact of the pandemic on consumption patterns of the following substances: tobacco, alcohol, caffeine, anxiolytics, cannabis or other substances;
  - the participants' sources of stress regarding the pandemic and usual strategies used to reduce stress. Respondents could put multiple options to these questions.

2.3.1. The State-Trait Anxiety Inventory (STAI)

The STAI was developed by Spielberger to assess via a self-report scale the presence and severity of anxiety symptoms. It consists of two 20-item subscales; the State Anxiety Scale (S-Anxiety) and the Trait Anxiety Scale (T-Anxiety) [33,34].

In our study, we used the S-Anxiety subscale, which evaluates the current state of anxiety, asking subjects to report how they feel "right now, at this moment". Range of scores for each subtest is 20–80, the higher score indicating greater anxiety. Following the example of numerous authors [34], we used a threshold score of greater than 40 on the STAI (S-Anxiety) to identify the presence of moderate to severe anxiety symptoms.

2.3.2. The Pittsburgh Sleep Quality Index (PSQI)

The PSQI is a self-rated questionnaire which assesses, according to Buysse, sleep quality and sleep disturbances over a 1-month time interval [35]. Higher scores indicate worse sleep quality. A cut-off score of > 5 is used to distinguish poor sleepers from good sleepers [35].

Theses scales were chosen for being self-administered, widely used measures of anxiety and sleep quality. Also, for being validated in French [36,37], the main language used by the hospital employees.

The questionnaire was pilot-tested among a sample of HCWs to evaluate its face validity.

2.4. Statistical analysis

Data analysis was performed using SPSS statistical software version 21.0 (IBM Corp). The significance level was set at $\alpha = .05$, and all tests were 2-tailed.

Two-sample t test was used for continuous variables.

One-way ANOVA with post hoc Tukey examination between different groups was used to investigate differences.

Pearson's linear correlations of anxiety and sleep symptoms were made.

Qualitative variables were described by frequency distribution, while quantitative variables were described by the mean and standard deviation.

Multivariable logistic regression analysis was performed to identify factors associated with anxiety and altered sleep quality. The model was assessed using Nagelkerke R Square, and its adequacy assessed with the Hosmer-Lemeshow statistic. Adjusted odds ratio (OR) and a 95% confidence interval (CI) are reported.

3. Results

3.1. Participants' characteristics

In total, 618 HCWs completed the survey, including 52 physicians, 63 residents and 503 nurses (6.1% head nurses, 41.3% registered nurses and 34% practical nurses). The response rate for physicians, residents and nurses were 19.4%, 63% and 84.2% respectively.

The participants tended to be females, aged less than 30 years, in a relationship or married, with no children, and have a university educational level, as shown in Table 1. The majority of participants stated having no psychiatric history (Table 1).

3.2. Anxiety and sleep quality outcomes

The mean (SD) scores on the STAI for anxiety, and the PSQI for sleep quality for all respondents were 44.5 (12.2), and 6.0 (3.7), respectively.

Of all participants, 61.5% had STAI scores higher than 40, which indicates a moderate to severe anxiety symptoms, and 48.4% were "poor sleepers" as they got PSQI scores higher than 5.

3.3. Correlations to sociodemographic characteristics

Sex, age and occupational status were the three variables where a significant difference was observed in both the anxiety and sleep quality scores.

In fact, women had significantly higher scores compared with men in both scales ($P < 0.001$ for STAI and PSQI). Post hoc test showed that participants who were aged more than 50 had lower anxiety and sleep quality scores than those aged between 41 and 50 and significantly lower scores than whose younger than

Table 1
Sociodemographic characteristics and psychiatric history.

Characteristic	Number (n = 618)	Percentage (%)
Sex		
Male	177	28.6
Female	441	71.4
Age (years)		
< 30	262	42.4
31–40	147	23.8
41–50	128	20.7
> 50	81	13.1
Marital status		
Single	278	45.0
In a relationship/married	309	55.0
Divorced	25	4.0
Widowed	6	1.0
Number of children		
0	333	53.9
1	56	9.1
2	144	23.3
3 or more	85	13.8
Education level		
Elementary school	16	2.6
Middle school	78	12.6
High school	96	15.5
University studies	428	69.3
Occupational status		
Physician	52	8.4
Resident	63	10.2
Head nurse	38	6.1
Registered nurse	255	41.3
Practical nurse	210	34.0
Years of experience (years)		
< 5	190	30.7
5–10	133	21.5
10–20	154	24.9
> 20	141	22.8
Psychiatric history		
Yes	27	4.4
No	591	95.6

40 (P < 0.001 (STAI) and P = 0.005 (PSQI)). Concerning the occupational status, post hoc test showed that residents and registered nurses had greater scores on both scales; residents had significantly higher anxiety scores than physicians (P = 0.001) and higher sleep disturbances scores than practical nurses (P = 0.016). Registered nurses compared to physicians and practical nurses had significantly higher anxiety (P < 0.001 and P = 0.034, respectively) and sleep quality scores (P = 0.003 and P < 0.001, respectively) (Table 2).

3.4. Substance use patterns

During the pandemic, 31.2% of participants reported an increase in the consumption patterns of one or more of the substances listed in Table 3.

3.5. Correlations to COVID-19 related variables characteristics

In Table 4, we represent variables related to the pandemic and their influence on STAI and PSQI scores.

Participants who reported having relatives or friends who got infected or quarantined had higher anxiety and sleep disturbances scores than those who had not (P = 0.013 for STAI and P = 0.019 for PSQI). Post hoc test showed that participants who were exposed to COVID-19 related media for more than 2 hours per day, were significantly more anxious (P = 0.003) and had poorer sleep quality (P = 0.011) than those who were exposed to media less than 2 hours per day and to those who were not exposed at all. The increase in consumption patterns of alcohol and each of the substances listed in Table 3 except "other psychoactive drugs" was significantly correlated with higher scores in both scales (P < 0.001).

Scores of participants working in COVID-19 units were not significantly higher than those who were working in ordinary units. Likewise, participants who were quarantined or living with children/elderly did not show significantly higher scores than those who were not (Table 4).

A Pearson correlation was done to determine the relationship between STAI and PSQI scores. A statistically significant positive correlation was found between the two scores (r = .459, P < 0.001).

3.6. Sources of stress and coping strategies

Participants' answers to two multiple choice questions on sources of fear/stress related to the pandemic and strategies normally used to deal with stress are summarized in Tables 5 and 6. Greater number of stress sources was positively correlated to higher scores on anxiety and sleep disturbances (r = 0.316, P <0.001; r = 0.129, P = 0.001, respectively). Further, participants were asked whether they were still able to practice the strategies they normally use to deal with stress. Those who were still able to do it (60.4%) had lower anxiety and significantly lower sleep disturbances scores than those who were not (P = 0.096 for STAI and P = 0.011 for PSQI).

3.7. Logistic regression analyses

Multiple logistic regression analyses showed that, after controlling for covariates, female sex (OR 3.33, 95% CI: 2.00–5.53), young age (OR 1.55, 95% CI: 1.05–2.29), poor sleep (OR 3.23, 95% CI: 2.10–4.95) quality, as well as living with elderly (OR 1.65, 95% CI: 1.02–2.65), were associated with higher risk of presenting moderate to severe anxiety symptoms during the COVID-19 outbreak (Table 7).

4. Discussion

This cross-sectional survey examined mental health outcomes during the COVID-19 outbreak among a sample of HCWs from a private university hospital in Lebanon.

Findings from this study revealed that the anxiety level of HCWs during this period was high with a mean STAI score of 44.5. Besides their sleep quality was low with a mean PSQI score of 6.0. Overall, 61.5%, and 48.4% of all responders reported moderate to severe symptoms of anxiety and sleep disturbances, respectively.

To our knowledge, this is the first study to examine HCWs' mental health in Lebanon during the current pandemic. The prevalence of anxiety and sleep disturbances symptoms found in our sample was greater than that found in other samples of HCWs during infectious outbreaks [17,27,28]. High rates of anxiety and sleep difficulties were previously reported by studies done in different samples of the Lebanese general population [38,39]. In fact, the high scores of anxiety and sleep disturbances found in our study may also be explained by the economic, social and political instability that the country has been facing during the past 6 months. Therefore, with the COVID-19 outbreak, the country is undergoing a "two-in-one crisis" [40].

Moreover, the high rates found in your study can be explained by the high risk of burnout and emotional exhaustion in this population [41–43].

Anxiety and sleep difficulties are strongly correlated as shown in other studies [31]; anxiety can negatively impact the quality of sleep, while poor sleep negatively affects anxiety. Accordingly, it is essential to advise HCWs on the importance of maintaining a good sleep hygiene, and to provide them with the basic recommendations on this subject.

Similar to findings in the literature, women, participants with intermediate occupational status such as registered nurses and residents, younger responders (aged less than 40), and those who

Table 2
Scores of Anxiety and Sleep quality correlated to different sociodemographic characteristics.

Characteristic	No. (%)	STAI		PSQI	
		Scale mean (SD)	*P*-value	Scale mean (SD)	*P*-value
Overall	618 (100)	44.5 (12.2)		6.0 (3.7)	
Sex					
Male	177 (28.6)	40.1 (13.2)	< 0.001	4.8 (3.6)	< 0.001
Female	441 (71.4)	46.3 (11.4)		6.5 (3.7)	
Age (years)					
< 30	262 (42.4)	45.8 (11.9)	< 0.001	6.6 (3.7)	0.005
31–40	147 (23.8)	46.0 (13.1)		6.0 (4.0)	
41–50	128 (20.7)	43.3 (11.9)		5.4 (3.5)	
> 50	81 (13.1)	39.5 (10.7)		5.2 (3.7)	
Number of children					
0	333 (53.9)	44.9 (11.9)	0.513	6.4 (3.6)	0.007
1	56 (9.1)	44.3 (11.8)		6.1 (3.8)	
2	144 (23.3)	44.8 (13.4)		5.3 (3.6)	
3 or more	85 (13.8)	42.7 (11.9)		5.4 (4.2)	
Education level					
Elementary school	16 (2.6)	43.3 (15.4)	0.325	3.8 (2.1)	< 0.001
Middle school	78 (12.6)	44.1 (14.8)		5.1 (4.0)	
High school	96 (15.5)	42.6 (14.6)		4.9 (3.9)	
University studies	428 (69.3)	45.0 (10.9)		6.5 (3.6)	
Occupational status					
Physician	52 (8.4)	38.4 (10.7)	< 0.001	4.8 (3.8)	< 0.001
Resident	63 (10.2)	47.3 (12.2)		6.7 (3.2)	
Head nurse	38 (6.1)	41.8 (10.7)		5.9 (3.5)	
Registered nurse	255 (41.3)	46.5 (9.9)		6.9 (3.6)	
Practical nurse	210 (34)	43.2 (14.5)		5.0 (3.9)	
Psychiatric history					
Yes	27 (4.4)	47.1 (13.3)	0.252	8.2 (4.3)	0.002
No	591 (95.6)	44.4 (12.2)		5.9 (3.7)	
No	425 (68.8)	41.7 (11.4)		5.2 (3.4)	

STAI: State-Trait Anxiety Inventory; PSQI: Pittsburgh Sleep Quality Index.

Table 3
Change in consumption patterns during the COVID-19 pandemic.

Substance	Increased (%)	Unchanged (%)
Any substance	31.2	68.8
Tobacco	12.8	87.2
Caffeine	22.8	77.2
Alcohol	5.3	94.7
Tranquilizers/Hypnotics	3.2	96.8
Cannabis	1.0	99.0
Other psychoactive substances/drugs	0.3	99.7

had relatives or friends infected/quarantined reported more severe symptoms on both measurements [17,21,26,27]. A high level of anxiety among registered nurses and residents could be attributed to their closer and more frequent contact with patients, and their longer working schedules compared with other HCWs.

In particular, findings of our study emphasize the necessity to warrant the appropriate attention regarding women' mental health and well-being; women are known to be affected to a greater extent than men by depression, anxiety and psychological distress [44,45].

Consistent with previous findings, sources of fear/distress in our sample of HCWs included: fear of getting infected or spreading the virus, health of relatives and friends, limited access to medical equipment, and financial losses [21,24,27].

Interestingly, the most common strategy used to deal with distress was praying. This finding can be interpreted in the light of a cultural construction of health, especially in the highly religious and multiconfessional context of Lebanon [46]. In addition, strategies described in other studies such as seeking social support and doing physical activity were also reported among our participants [47].

Remarkably, working directly with COVID-19 patients and being quarantined were not predictive of higher scores which contradicts findings from the literature [16,18,48]. This may be justified by three facts. First, high scores can be explained not only by the pandemic, but also by the economic and socio-political crisis. Second, to date, the outbreak in Lebanon is considered to be "contained" according to the reports of the ministry of public health [32]. Third, at the hospital where this study was conducted, nurses were given the choice to work or not in COVID-19 units.

There are few studies reporting the effect that can similar outbreaks have on alcohol and substance use behavior among HCWs. One study described a relationship between exposure to the SARS outbreak and alcohol abuse/dependence among hospital employees 3 years later [25]. In our study, 18% of participants considered that "having a drink/smoking/taking a tranquilizer" were strategies they use to adapt with stress. Also, 31.2% of hospital staff reported having increased their consumption of alcohol, tobacco, caffeine, tranquilizers, cannabis or other drugs, during the current situation. These participants had higher anxiety and lower sleep quality scores. However, these results were not found after multivariate logistic regression. Comorbidity of substance use disorders with anxiety and sleep disorders is known and well described in the literature [49,50]. Seeking alcohol/substances as a coping strategy is harmful and can worsen anxiety and sleep disturbances symptoms. Raising awareness on this issue among HCWs is much needed, particularly during stressful periods such as infectious outbreaks.

Another interesting finding in our study was the impact of COVID-19 related media exposure on anxiety and sleep quality. Participants who followed the news concerning the Coronavirus pandemic via television/radio/newspapers/social networks for more than 2 hours per day were found to be more anxious and have poorer sleep quality than those who did not. Mental health

Table 4
Scores of Anxiety and Sleep quality correlated to the COVID-19 related variables.

Variable	Number (%)	STAI		PSQI	
		Scale mean (SD)	*P*-value	Scale mean (SD)	*P*-value
Department					
COVID-19 unit	256 (41.4)	44.6 (12.5)	0.898	6.1 (4.1)	0.519
Ordinary unit	362 (58.6)	44.4 (12.1)		5.9 (3.5)	
Any quarantining					
Yes	84 (13.6)	45.8 (13.9)	0.311	6.4 (4.4)	0.265
No	534 (86.4)	44.3 (11.9)		5.9 (3.6)	
Relative or friend got quarantined or infected					
Yes	137 (22.2)	46.8 (12.2)	0.013	6.7 (4.2)	0.019
No	481 (77.8)	43.8 (12.2)		5.8 (3.6)	
Living with children					
Yes	285 (46.1)	44.4 (12.3)	0.874	5.7 (3.8)	0.100
No	333 (53.9)	44.6 (12.2)		6.28 (3.7)	
Living with elderly					
Yes	177 (28.6)	45.4 (12.6)	0.236	6.1 (4.0)	0.579
No	441 (71.4)	44.1 (12.1)		5.9 (3.7)	
Media exposure					
> 2 hours per day	179 (29)	46.9 (12.9)	0.003	6.5 (4.3)	0.011
< 2 hours per day	401 (64.9)	43.8 (11.5)		5.9 (3.4)	
None	38 (6.1)	40.6 (14.4)		4.5 (3.6)	
Increase in consumption patterns of any substances or alcohol[a]					
Yes	193 (31.2)	50.7 (11.8)	< 0.001	7.8 (4.0)	< 0.001
No	425 (68.8)	41.7 (11.4)		5.2 (3.4)	

STAI: State-Trait Anxiety Inventory; PSQI: Pittsburgh Sleep Quality Index.
[a] Listed in Table 3.

Table 5
Reported sources of fear/distress related to the COVID-19 pandemic.

Source of fear/stress	Prevalence (%)
Fear of getting infected and infecting others	61.7
Fear that someone I know gets infected	45.6
Having limited access to medical equipment	17
Financial losses	16.3
Being quarantined	7.9
Being stigmatized	7.8
None	12.8
Other	3.2

Table 6
Strategies normally used to deal with stress.

Strategies used to deal with stress	Prevalence (%)
Praying	57.4
Watching TV/going to the movies	46.9
Doing a physical activity	38.9
Going out with friends	33.5
Reading a book	19.1
Having a drink/smoking/taking a tranquilizer	18
Taking regular breaks at work	11.7
Meditation/Yoga	3.4
None	5.7
Other	0.6

Table 7
Multiple logistic regression analysis of moderate to severe anxiety scores.

Variables	OR	95% CI	*P*-value
Female sex	3.33	2.00–5.53	< 0.001
Poor sleep	3.23	2.10–4.95	< 0.001
Young age	1.55	1.05–2.29	0.026
Living with elderly	1.65	1.02–2.65	0.039

OR: multivariate adjusted odds ratio; 95% CI: 95% confidence interval. Model's Nagelkerke R square = 0.39. Hosmer-Lemeshow *P*-value = 0.96.

experts have reported the negative impact of excessive media exposure on anxiety and obsessive symptoms during the covid-19 crisis [51–53]. In our study, it was not demonstrated to be associated with severe anxiety symptoms after multiple logistic regression analysis. However, it is essential to acknowledge the damaging impact that constant exposure to negative news may have on our mental health. Therefore, staying up to date on main local and national news is important but should not be excessive, as recommended by the CDC [54].

As for mental health care facilities and support that were made available to these HCW in Lebanon, in this particular hospital, psychological support was offered to HCWs by the psychiatry department, whether through support groups or individual care. It should be noted that along with the questionnaire distributed at the beginning, an explanatory leaflet was also distributed explaining the purpose of the study and giving the contact details of the people/services to refer to if severe anxiety symptoms were present (these people would benefit from free psychiatric consultations if they desire so).

On a national level, few measures have been implemented. Due to economic crisis in the country, financial compensation or crisis premium were unlikely during this period. However, the National Mental Health Programme at the Ministry of Public Health has been responding efficiently and proactively through the launching of a number of awareness campaigns addressing how to cope during the sanitary crisis [55]. In addition, the order of nurses in Lebanon, along with a Non-Governmental Organization (Embrace) provided mental health sessions for nurses, sharing helpful resources with the ones in need [56].

5. Limitations

Our study had several limitations.

First, it is a cross-sectional study, and conclusive causal relationships may not be established.

Second, the use of subjective self-reported questionnaires might have lacked the validity of face-to-face interviews.

Other limitations were a moderate response rate, which indicates that selection/response bias may still exist particularly if non-responders were too stressed to participate or did not feel concerned by this study.

Finally, caution should be practiced in generalizing the results to all medical staff in Lebanon, since it was done in one general hospital.

6. Conclusion

In summary, our findings contribute to the understanding of the psychological well-being of HCWs involved in the acute COVID-19 outbreak in Lebanon. Detecting the psychological impact among this population in the acute phase is crucial in order to implement the required assistance measures and encourage adaptive coping strategies. Prospective studies are necessary to assess the potential long-term psychological responses in this population.

Funding

There was no funding needed for this study.

Disclosure of interest

The authors declare that they have no competing interest.

Acknowledgments

We express our gratitude to all healthcare workers for their time and participation and for their hard work fighting this pandemic. We would also like to thank Mr Nicolas El Choueifaty for his contribution to the statistical analysis of this study.

Permission to use the French validated version of the State-Trait Anxiety Inventory (STAI) was given by Professor Stéphane Bouchard (Université du Québec en Outaouais).

References

[1] Coronavirus news, funding and resources for global health researchers - Fogarty International Center @ NIH. https://www.fic.nih.gov/ResearchTopics/Pages/infectiousdiseases-coronavirus-cov.aspx. Accessed April 7, 2020.

[2] Coronavirus disease (COVID-19) outbreak. https://www.who.int/westernpacific/emergencies/covid-19. Accessed April 7, 2020.

[3] Torales J, O'Higgins M, Castaldelli-Maia JM, et al. The outbreak of COVID-19 coronavirus and its impact on global mental health. Int J Soc Psychiatry 2020;66:317–20, http://dx.doi.org/10.1177/0020764020915212 [20764020915212].

[4] Wang C, Pan R, Wan X, et al. Immediate psychological responses and associated factors during the initial stage of the 2019 Coronavirus Disease (COVID-19) epidemic among the general population in China. Int J Environ Res Public Health 2020;17(5):1729, http://dx.doi.org/10.3390/ijerph17051729.

[5] Cao W, Fang Z, Hou G, et al. The psychological impact of the COVID-19 epidemic on college students in China. Psychiatry Res 2020;287:112934, http://dx.doi.org/10.1016/j.psychres.2020.112934.

[6] Li S, Wang Y, Xue J, et al. The impact of COVID-19 epidemic declaration on psychological consequences: a study on active Weibo users. Int J Environ Res Public Health 2020;17(6):2032, http://dx.doi.org/10.3390/ijerph17062032.

[7] Ko C-H, Yen C-F, Yen J-Y, et al. Psychosocial impact among the public of the severe acute respiratory syndrome epidemic in Taiwan. Psychiatry Clin Neurosci 2006;60(4):397–403, http://dx.doi.org/10.1111/j.1440-1819.2006.01522.x.

[8] Sim K, Chua HC. The psychological impact of SARS: a matter of heart and mind. CMAJ 2004;170(5):811–2, http://dx.doi.org/10.1503/cmaj.1032003.

[9] Leung GM, Lam T-H, Ho L-M, et al. The impact of community psychological responses on outbreak control for severe acute respiratory syndrome in Hong Kong. J Epidemiol Community Health 2003;57(11):857–63, http://dx.doi.org/10.1136/jech.57.11.857.

[10] Sprang G, Silman M. Posttraumatic stress disorder in parents and youth after health-related disasters. Disaster Med Public Health Prep 2013;7(1):105–10, http://dx.doi.org/10.1017/dmp.2013.22.

[11] Lau JTF, Griffiths S, Choi KC, et al. Avoidance behaviors and negative psychological responses in the general population in the initial stage of the H1N1 pandemic in Hong Kong. BMC Infect Dis 2010;10(1):139, http://dx.doi.org/10.1186/1471-2334-10-139.

[12] Taylor MR, Agho KE, Stevens GJ, et al. Factors influencing psychological distress during a disease epidemic: data from Australia's first outbreak of equine influenza. BMC Public Health 2008;8(1):347, http://dx.doi.org/10.1186/1471-2458-8-347.

[13] Brooks SK, Webster RK, Smith LE, et al. The psychological impact of quarantine and how to reduce it: rapid review of the evidence. Lancet 2020;395(10227):912–20, http://dx.doi.org/10.1016/S0140-6736(20)30460-8.

[14] Liu X, Kakade M, Fuller CJ, et al. Depression after exposure to stressful events: lessons learned from the severe acute respiratory syndrome epidemic. Compr Psychiatry 2012;53(1):15–23, http://dx.doi.org/10.1016/j.comppsych.2011.02.003.

[15] McAlonan GM, Lee AM, Cheung V, et al. Immediate and sustained psychological impact of an emerging infectious disease outbreak on health care workers. Can J Psychiatry 2007;52(4):241–7, http://dx.doi.org/10.1177/070674370705200406.

[16] Chen R, Chou KR, Huang YJ, et al. Effects of a SARS prevention programme in Taiwan on nursing staff's anxiety, depression and sleep quality: a longitudinal survey. Int J Nurs Stud 2006;43(2):215–25, http://dx.doi.org/10.1016/j.ijnurstu.2005.03.006.

[17] Su TP, Lien TC, Yang CY, et al. Prevalence of psychiatric morbidity and psychological adaptation of the nurses in a structured SARS caring unit during outbreak: a prospective and periodic assessment study in Taiwan. J Psychiatr Res 2007;41(1–2):119–30, http://dx.doi.org/10.1016/j.jpsychires.2005.12.006.

[18] Bai Y, Lin C-C, Lin C-Y, et al. Survey of stress reactions among health care workers involved with the SARS outbreak. Psychiatr Serv 2004;55(9):1055–7, http://dx.doi.org/10.1176/appi.ps.55.9.1055.

[19] Chan AOM, Huak CY. Psychological impact of the 2003 severe acute respiratory syndrome outbreak on health care workers in a medium size regional general hospital in Singapore. Occup Med (Lond) 2004;54(3):190–6, http://dx.doi.org/10.1093/occmed/kqh027.

[20] Nickell LA, Crighton EJ, Tracy CS, et al. Psychosocial effects of SARS on hospital staff: survey of a large tertiary care institution. CMAJ 2004;170(5):793–8, http://dx.doi.org/10.1503/cmaj.1031077.

[21] Maunder RG, Lancee WJ, Rourke S, et al. Factors associated with the psychological impact of severe acute respiratory syndrome on nurses and other hospital workers in Toronto. Psychosom Med 2004;66(6):938–42, http://dx.doi.org/10.1097/01.psy.0000145673.84698.18.

[22] Grace SL, Hershenfield K, Robertson E, et al. The occupational and psychosocial impact of SARS on academic physicians in three affected hospitals. Psychosomatics 2005;46(5):385–91, http://dx.doi.org/10.1176/appi.psy.46.5.385.

[23] Lee SM, Kang WS, Cho A-R, et al. Psychological impact of the 2015 MERS outbreak on hospital workers and quarantined hemodialysis patients. Compr Psychiatry 2018;87:123–7, http://dx.doi.org/10.1016/j.comppsych.2018.10.003.

[24] Abolfotouh MA, AlQarni AA, Al-Ghamdi SM, et al. An assessment of the level of concern among hospital-based health-care workers regarding MERS outbreaks in Saudi Arabia. BMC Infect Dis 2017;17(1):4, http://dx.doi.org/10.1186/s12879-016-2096-8.

[25] Wu P, Liu X, Fang Y, et al. Alcohol abuse/dependence symptoms among hospital employees exposed to a SARS outbreak. Alcohol Alcohol 2008;43(6):706–12, http://dx.doi.org/10.1093/alcalc/agn073.

[26] Wu P, Fang Y, Guan Z, et al. The psychological impact of the SARS epidemic on hospital employees in China: exposure, risk perception, and altruistic acceptance of risk. Can J Psychiatry 2009;54(5):302–11, http://dx.doi.org/10.1177/070674370905400504.

[27] Lai J, Ma S, Wang Y, et al. Factors associated with mental health outcomes among health care workers exposed to Coronavirus Disease 2019. JAMA Netw Open 2020;3(3):e203976, http://dx.doi.org/10.1001/jamanetworkopen.2020.3976.

[28] Lu W, Wang H, Lin Y, et al. Psychological status of medical workforce during the COVID-19 pandemic: a cross-sectional study. Psychiatry Res 2020;288:112936, http://dx.doi.org/10.1016/j.psychres.2020.112936.

[29] Kang L, Ma S, Chen M, et al. Impact on mental health and perceptions of psychological care among medical and nursing staff in Wuhan during the 2019 novel coronavirus disease outbreak: a cross-sectional study. Brain Behav Immun 2020;87:11–7, http://dx.doi.org/10.1016/j.bbi.2020.03.028.
[30] Huang JZ, Han MF, Luo TD, et al. [Mental health survey of 230 medical staff in a tertiary infectious disease hospital for COVID-19]. Zhonghua Lao Dong Wei Sheng Zhi Ye Bing Za Zhi 2020;38(0):E001, http://dx.doi.org/10.3760/cma.j.cn121094-20200219-00063.
[31] Xiao H, Zhang Y, Kong D, et al. The effects of social support on sleep quality of medical staff treating patients with Coronavirus Disease 2019 (COVID-19) in January and February 2020 in China. Med Sci Monit 2020;26:e923549, http://dx.doi.org/10.12659/MSM.923549.
[32] Coronavirus COVID-19 Lebanon Cases. https://www.moph.gov.lb/en/Pages/0/27788/coronavirus-lebanon-cases-. Accessed April 7, 2020.
[33] Spielberger CD. State-Trait Anxiety Inventory. In: The Corsini Encyclopedia of Psychology. American Cancer Society; 2010, http://dx.doi.org/10.1002/9780470479216.corpsy0943, 1-1.
[34] Julian LJ. Measures of anxiety: State-Trait Anxiety Inventory (STAI), Beck Anxiety Inventory (BAI), and Hospital Anxiety and Depression Scale-Anxiety (HADS-A). Arthritis Care Res (Hoboken) 2011;63(S11):S467–72, http://dx.doi.org/10.1002/acr.20561.
[35] Buysse DJ, Reynolds CF, Monk TH, et al. The Pittsburgh Sleep Quality Index: a new instrument for psychiatric practice and research. Psychiatry Res 1989;28(2):193–213, http://dx.doi.org/10.1016/0165-1781(89)90047-4.
[36] Gauthier J, Bouchard S. Adaptation canadienne-française de la forme révisée du State – Trait Anxiety Inventory de Spielberger. Can J Behav Sci 1993;25(4):559–78, http://dx.doi.org/10.1037/h0078881.
[37] Blais FC, Gendron L, Mimeault V, et al. [Evaluation of insomnia: validity of 3 questionnaires]. Encephale 1997;23(6):447–53.
[38] Chami HA, Bechnak A, Isma'eel H, et al. Sleepless in Beirut: sleep difficulties in an urban environment with chronic psychosocial stress. J Clin Sleep Med 2019;15(4):603–14, http://dx.doi.org/10.5664/jcsm.7724.
[39] Karam EG, Mneimneh ZN, Dimassi H, et al. Lifetime prevalence of mental disorders in Lebanon: first onset, treatment, and exposure to war. PLoS Med 2008;5(4):e61, http://dx.doi.org/10.1371/journal.pmed.0050061.
[40] Lebanon's economy and industry suffer under coronavirus impact - Executive Magazine. Accessed May 11, 2020. https://www.executive-magazine.com/economics-policy/healthcare/coronavirus/lebanons-economy-and-industry-suffer-under-coronavirus-impact.
[41] Chemali Z, Ezzeddine FL, Gelaye B, et al. Burnout among healthcare providers in the complex environment of the Middle East: a systematic review. BMC Public Health 2019;19(1):1337, http://dx.doi.org/10.1186/s12889-019-7713-1.
[42] Habis C, Maalouf RG. Impact of stress and burnout on the sexual desire of trainee doctors at Hôtel-Dieu de France hospital: a single-institution survey. Encephale 2019;45(5):371–5, http://dx.doi.org/10.1016/j.encep.2019.02.004.
[43] Alameddine M, Mourad Y, Dimassi H. A national study on nurses' exposure to occupational violence in lebanon: prevalence, consequences and associated factors. PLoS ONE 2015;10(9):e0137105, http://dx.doi.org/10.1371/journal.pone.0137105.
[44] Riecher-Rössler A. Sex and gender differences in mental disorders. Lancet Psychiatry 2017;4(1):8–9, http://dx.doi.org/10.1016/S2215-0366(16)30348-0.
[45] WHO | Gender and women's mental health. Accessed May 19, 2020. https://www.who.int/mental_health/prevention/genderwomen/en/.
[46] Yehya NA, Dutta MJ. Health, religion, and meaning: a culture-centered study of Druze women. Qual Health Res 2010;20(6):845–58, http://dx.doi.org/10.1177/1049732310362400.
[47] Chew QH, Wei KC, Vasoo S, Chua HC, Sim K. Narrative synthesis of psychological and coping responses towards emerging infectious disease outbreaks in the general population: practical considerations for the COVID-19 pandemic. Singapore Med J. Published online April 3, 2020. 10.11622/smedj.2020046.
[48] Marjanovic Z, Greenglass ER, Coffey S. The relevance of psychosocial variables and working conditions in predicting nurses' coping strategies during the SARS crisis: an online questionnaire survey. Int J Nurs Stud 2007;44(6):991–8, http://dx.doi.org/10.1016/j.ijnurstu.2006.02.012.
[49] Smith JP, Book SW. Anxiety and substance use disorders: a review. Psychiatr Times 2008;25(10):19–23.
[50] Rosenblum M. Substance abuse and insomnia. Minn Med 2017;100(3):38–9.
[51] French I, Lyne J. Acute exacerbation of OCD symptoms precipitated by media reports of COVID-19. Ir J Psychol Med. Published online May 21, 2020:1-14. 10.1017/ipm.2020.61.
[52] Kumar A, Somani A. Dealing with Corona virus anxiety and OCD. Asian Journal of Psychiatry 2020;51:102053, http://dx.doi.org/10.1016/j.ajp.2020.102053.
[53] Kearsley R, Duffy CC. The COVID-19 information pandemic: how have we managed the surge? Anaesthesia. Published online May 14, 2020. 10.1111/anae.15121.
[54] Coping with a Disaster or Traumatic Event. Accessed May 25, 2020. https://emergency.cdc.gov/coping/selfcare.asp.
[55] The national mental health programme (NMHP) https://www.moph.gov.lb/en/Pages/6/553/the-national-mental-health-program.
[56] Embrace Lebanon https://embracelebanon.org/.
