# Supplementary material for: Mental health status and related factors influencing healthcare workers during the COVID-19 pandemic: A systematic review and meta-analysis
Source: PLoS One. 2024 Jan 19;19(1):e0289454. doi: 10.1371/journal.pone.0289454 (PMC10798549; doi:10.1371/journal.pone.0289454)
Supplement: S1 Data — (ZIP) [file pone.0289454.s011.zip › literatures/229.pdf]

ELSEVIER

Since January 2020 Elsevier has created a COVID-19 resource centre with free information in English and Mandarin on the novel coronavirus COVID-19. The COVID-19 resource centre is hosted on Elsevier Connect, the company's public news and information website.

Elsevier hereby grants permission to make all its COVID-19-related research that is available on the COVID-19 resource centre - including this research content - immediately available in PubMed Central and other publicly funded repositories, such as the WHO COVID database with rights for unrestricted research re-use and analyses in any form or by any means with acknowledgement of the original source. These permissions are granted for free by Elsevier for as long as the COVID-19 resource centre remains active.

Psychiatry Research 294 (2020) 113541

Contents lists available at ScienceDirect

Psychiatry Research

journal homepage: www.elsevier.com/locate/psychres

Posttraumatic stress disorder symptoms in healthcare workers after the peak of the COVID-19 outbreak: A survey of a large tertiary care hospital in Wuhan

Hongyi Zhang [a,d], Yuqin Shi [b], Ping Jing [c,d], Peiyan Zhan [c,d], Yue Fang [c,d,**], Fang Wang [c,d,*]

[a] Department of Pediatric Surgery, Tongji Hospital, Wuhan 430030, China
[b] Department of Epidemiology and Health Statistics, School of Public Health, Medical College, Wuhan University of Science and Technology, Wuhan 430030, China
[c] Department of Neurology, The Central Hospital of Wuhan, Wuhan 430030, China
[d] Tongji Medical College and Huazhong University of Science and Technology, Wuhan 430030, China

ARTICLE INFO

Keywords:
Healthcare workers
Posttraumatic stress disorder
Epidemic disease
COVID-19
Mental illness
Multivariate regression analysis
Wuhan

ABSTRACT

This study examined the prevalence of posttraumatic stress disorder (PTSD) symptoms and assessed mental illness via an online survey among healthcare workers (HCWs) at the Central Hospital of Wuhan after the peak of the COVID-19 outbreak. PTSD symptoms were measured using the PTSD Checklist Civilian Version (PCL-C), with a cutoff score of 50. Among the 642 HCWs, the prevalence of probable PTSD was 20.87%. Additionally, 88.88%, 82.09%, 100%, and 95.52% of HCWs with probable PTSD reported varying degrees of anxiety, depression, somatic symptoms, and insomnia, respectively. HCWs with probable PTSD scored higher on the Hospital Anxiety and Depression Scale (HADS), Patient Health questionnaire-15 (PHQ-15), and Insomnia Severity Index (ISI) than non-PTSD HCWs (all $p < 0.05$). Multivariate regression analysis revealed that HCWs with negative COVID-19 tests (OR, 0.35; 95% CI, 0.21–0.58; $p < 0.00$), those with high Social Support Self-Rating Scale (SSRS) scores (OR, 0.30; 95% CI, 0.17–0.52; $p < 0.00$), and HCWs whose family members tested negative (OR, 0.64; 95% CI, 0.42–0.96; $p = 0.03$) were less likely to have probable PTSD. This study found a high prevalence of probable PTSD and severe mental illness among local HCWs. Our finding emphasizes the need to provide mental health support for HCWs.

1. Introduction

The coronavirus disease 2019 (COVID-19) first emerged during the end of December 2019 in Wuhan, and subsequently became a global pandemic (Pan et al., 2020; Ahorsu et al., 2020). Healthcare workers (HCWs) paid a heavy price in the battle against COVID-19, among whom the daily rate of cases (130.5 per million people) was higher than that in the general population (41.5 per million people) (Pan et al., 2020). In China, a total of 3387 (4.4%) of the 77,262 patients with COVID-19 were either medical professionals or worked in medical facilities. As of April 3, 2020, 18 medical professionals died from COVID-19 in Wuhan (Zhan et al., 2020).

Previous studies on severe acute respiratory syndrome (SARS) reported that the onset of highly contagious viral pneumonia is associated with significant challenges and shock to HCWs, causing mental illness and leading to posttraumatic stress disorder (PTSD) (Hong et al., 2009; Lancee et al., 2008; Chan and Huak, 2004; Wu et al., 2009; Maunder et al., 2006). COVID-19 is a new type of mass trauma due to its unfamiliarity and uncontrollability. The months-long COVID-19 outbreak, during which HCWs were continuously exposed to the trauma, could increase the adverse mental health impact of the COVID-19 outbreak (Rajkumar, 2020; Zhang et al., 2020; Kang et al., 2020; Cai et al., 2020).

In this study, we performed an online survey among HCWs at the Central Hospital of Wuhan to assess the prevalence of PTSD symptoms and identify predictive factors associated with PTSD. The Central Hospital of Wuhan was the first and hardest hit hospital by COVID-19 among the 27 tertiary hospitals in Wuhan. In January 2020, the hospital became a key facility for treating both COVID-19 and non-COVID-19 patients. On 6th April, the hospital discharged its last group of recovered COVID-19 patients and reopened as a non-COVID-19 patient care facility

* Corresponding author at: Department of Neurology, The Central Hospital of Wuhan, 26 Shengli Street, Wuhan, China, 430014.
** Co-Corresponding author.
E-mail address: wangfangdoc@tom.com (F. Wang).

https://doi.org/10.1016/j.psychres.2020.113541
Received 15 July 2020; Accepted 22 October 2020
Available online 25 October 2020
0165-1781/© 2020 Elsevier B.V. All rights reserved.

after thorough disinfection. All hospital staff underwent COVID-19 screening tests before serving non-COVID-19 patients. During the battle against the virus, more than 285 of the 4300 HCWs at the Central Hospital of Wuhan were infected with COVID-19, compared to a total of 1496 confirmed cases in local HCWs (Zhan et al., 2020; Pan et al., 2020). The hospital suffered more staff deaths than any other hospital in Wuhan. High psychological burdens and PTSD morbidity were expected. This study examined the levels of PTSD symptoms among HCWs at the Central Hospital of Wuhan. The morbidity and severity of anxiety and depression, somatic symptoms, and insomnia were also assessed. We hope that our findings will increase our understanding of the psychological impact of COVID-19 and improve the psychological well-being of local HCWs.

2. Methods

2.1. Study design

This cross-sectional study was conducted online between June 6, 2020 and June 13, 2020. The study was designed to survey the levels of PTSD and psychological distress among the staff of the Central Hospital of Wuhan. We recruited participants via WeChat using app-based, self-administered questionnaires. The questionnaires were forwarded to different WeChat hospital staff groups to recruit participants. People who completed the questionnaire were also encouraged to forward the survey to others.

Ethics approval was obtained from the Institutional Review Board of the Central Hospital of Wuhan (CHW-IRB-2020-041). All participants were informed of the purpose of the study before completing the online questionnaires. To protect the privacy of respondents, the survey was conducted anonymously.

2.2. Participants

Inclusion criteria: (1) staff of the Central Hospital of Wuhan, (2) WeChat users, (3) volunteers for the survey, and (4) people who could submit survey responses using the same IP address only once.

Exclusion criteria: (1) completion of the questionnaires within three minutes, (2) the existence of a medical history of PTSD, depressive disorders, or sleep disorders; (3) the existence of a history of mental disorder; or those receiving pharmacological or psychological treatments; (4) the existence of a history of unstable medical illness such as uncontrolled cardiac disease, hypertension, severe headaches, glaucoma, or seizures within the last year; and (5) the existence of one of the following conditions after April 6, 2020: respondents or their family members (HCWs' spouse, parents, siblings, and children) being COVID-19 positive, being hospitalized for COVID-19, working at a fever clinic, or being under quarantine.

The participants' medical histories of PTSD, mental disorder, pharmacological or psychological treatments, and unstable medical illness were collected via self-report during the online survey. History of a positive COVID-19 test was also collected via self-report. Participants were considered to be positive for COVID-19 if they had a positive result for SARS-CoV-2 virus by real-time reverse transcriptase–polymerase chain reaction assay or high-throughput sequencing of nasal and pharyngeal swab specimens (Lai et al., 2020; Pan et al., 2020).

2.3. Measurements

2.3.1. Posttraumatic stress disorder (PTSD)

Probable PTSD was assessed using the PTSD Checklist Civilian Version (PCL-C), with one item anchored to each of the 17 key symptoms required for determination of PTSD from the Diagnostic and Statistical Manual of Mental Disorders (Fourth Edition) (DSM-IV) (Sonis et al., 2009). It requires respondents to indicate the degree to which they have been affected during the past month by the PTSD symptoms. The 17 items of the PCL-C include three factors: reexperiencing, avoidance and numbing, and hyperarousal (Table 1). Probable PTSD was considered when an HCW had a total PCL-C score $\geq$50 (Wilberforce et al., 2010; Schlenger et al., 2002; Forbes et al., 2001). We used the term probable PTSD because the PCL-C, despite its excellent evaluation properties, is not a diagnostic interview (Sonis et al., 2009).

2.3.2. Depression and anxiety

The Hospital Anxiety and Depression Scale (HADS) is valid for the measurement of depression and anxiety (Falk et al., 2020). It excludes somatic symptoms of emotional distress that could be caused by the illness itself. It is composed of 14 items to which participants respond through a four-point scale (0–3) referring to overt symptoms within the last week. It is composed of two scales: anxiety (seven items) and depression (seven items). A summary score was calculated for both the anxiety and depression subscales. A score of 8 is indicative of anxiety or depression.

2.3.3. Somatization symptoms

The Patient Health Questionnaire-15 (PHQ-15) is one of the well-established and most frequently used instruments to identify people at risk for somatization (Kocalevent et al., 2013). It assesses the presence and severity of common somatic symptoms using 15 items. A summary score reflects the self-rated symptom burden, with higher scores indicating higher burden.

2.3.4. Subjective sleep quality

The Insomnia Severity Index (ISI), a valid self-report questionnaire of sleep, is used to measure the current perception in terms of symptom severity, stress, and impairment within the last two weeks (Morin et al., 2011). The ISI consists of seven sleep-related questions ranked on a five-point Likert scale. A summary score of the ISI ranges from 0 to 28. A score above or below the cutoff score of 8 is indicative of insomnia.

2.3.5. Social support

The Social Support Self-Rating Scale (SSRS) is one of the most commonly used instruments for measuring social support in China (Xiao, 1994). The high reliability and validity of this tool was proven in previous studies (Dai et al., 2016; Liu et al., 2018). It comprises of 10 items, which measure three subscales of social support: three items that assess objective support, four items that assess subjective support, and three items that assess social support availability. Higher scores indicate higher individual social support. High social support was defined as a total SSRS score $\geq$30 (Liu et al., 2018).

2.4. Statistical method

Percentages and standard deviations were calculated during descriptive analysis. Enumeration data were compared using the Chi-square test, and measurement data were compared using the Student's t-test. Multiple regression analyses were used to examine the relationship between the predictor variables and PTSD. Statistical analysis was conducted using SPSS 22.0 (SPSS Inc., Chicago, IL). The significance level was set at $p < 0.05$.

3. Results

3.1. Demographic characteristics

A total of 721 hospital staff participated in the online survey. However, 43 respondents were excluded from the study because they fulfilled at least one exclusion criteria. Among them, 16 completed the questionnaires within three minutes; 19 had a medical history of sleep disorders; and 13 respondents or their family members were COVID-19 positive, were hospitalized for COVID-19, were working at fever a clinic, or were under quarantine after April 6, 2020.

Table 1.
The 17-item PCL-C checklist scale.

The 17-item PCL-C checklist scale		PTSD (PCL-C scores≥50) $n = 134$ No. (and%) of respondents					Non-PTSD (PCL-C scores<50) $n = 508$ No. (and%) of respondents				
		1	2	3	4	5	1	2	3	4	5
**Re-experiencing**	1.Repeated disturbing memories, thoughts, or images	0 (0.00)	5(3.80)	78 (58.21)	36 (26.87)	15 (11.12)	120 (23.62)	223 (43.90)	154 (30.31)	11 (2.17)	0 (0.00)
	2.Repeated disturbing dreams	0 (0.00)	12 (8.95)	81 (60.45)	30 (22.39)	11 (8.21)	168 (33.07)	228 (44.88)	100 (19.68)	11 (2.17)	1 (0.20)
	3.Suddenly acting or feeling as if a stressful experience were happening again	1 (0.75)	26 (19.40)	81 (60.45)	21 (15.60)	5(3.80)	223 (43.90)	218 (42.91)	66 (12.99)	1(0.20)	0 (0.00)
	4.Feeling very upset at reminders	1 (0.75)	8(5.97)	81 (60.45)	30 (22.39)	14 (10.44)	169 (33.27)	227 (44.69)	109 (21.45)	3(0.59)	0 (0.00)
	5.Physical reactions to reminder	4 (3.00)	27 (20.15)	71 (52.97)	25 (18.66)	7(5.22)	239 (47.05)	195 (38.39)	67 (13.18)	7(1.38)	0 (0.00)
**Avoidance/ emotional numbness**	6.Avoid thinking or talking about it	6 (4.48)	22 (16.42)	69 (51.49)	27 (20.15)	10 (7.46)	226 (44.49)	194 (38.19)	74 (14.57)	13 (2.55)	1 (0.20)
	7.Avoid activities or situations	6 (4.48)	25 (18.66)	61 (45.52)	26 (19.40)	16 (11.94)	231 (45.47)	197 (38.78)	69 (13.39)	10 (2.16)	1 (0.20)
	8.Trouble remembering important parts	12 (8.95)	35 (26.12)	64 (47.76)	17 (12.69)	6(4.48)	194 (38.19)	210 (41.34)	84 (16.54)	15 (2.95)	5 (0.98)
	9.Loss interest	6 (4.48)	14 (10.45)	73 (54.47)	27 (20.15)	14 (10.45)	191 (37.60)	190 (37.40)	117 (23.03)	8(1.57)	2 (0.40)
	10.Feeling distant or cut off from others	1 (0.75)	13 (9.70)	59 (44.03)	43 (32.09)	18 (13.43)	172 (33.86)	161 (31.69)	125 (24.61)	41 (8.07)	9 (1.77)
	11.Feeling emotionally numb	10 (7.46)	25 (18.66)	64 (47.76)	25 (18.66)	10 (7.46)	258 (50.79)	167 (32.87)	64 (12.60)	18 (3.54)	1 (0.20)
	12.Feeling as if your future will somehow be cut short	3 (2.24)	11 (8.21)	82 (61.27)	23 (17.16)	15 (11.12)	233 (45.87)	177 (34.84)	89 (17.52)	8(1.57)	1 (0.20)
**Hyperarousal**	13.Trouble falling or staying asleep	0 (0.00)	3(2.24)	29 (21.64)	41 (30.60)	61 (45.52)	82 (16.14)	117 (23.03)	156 (30.71)	111 (21.85)	42 (8.27)
	14. Feeling irritable or having angry outbursts	1 (0.75)	6(4.48)	46 (34.33)	48 (35.82)	33 (24.62)	81 (15.94)	152 (29.92)	205 (40.35)	61 (12.02)	9 (1.77)
	15.Difficulty concentrating	2 (1.49)	5(3.80)	47 (35.07)	49 (36.57)	31 (23.07)	83 (16.34)	172 (33.86)	196 (38.58)	48 (9.45)	9 (1.77)
	16.Being super alert or watchful on guard	1 (0.75)	6(4.48)	50 (37.31)	48 (35.82)	29 (21.64)	131 (25.79)	187 (36.81)	146 (28.74)	39 (7.68)	5 (0.98)
	17.Feeling jumpy or easily startled	0 (0.00)	8(5.97)	63 (47.01)	35 (26.12)	28 (20.9)	170 (33.46)	187 (36.81)	126 (24.80)	24 (4.73)	1 (0.20)

PTSD, Posttraumatic stress disorder; PCL-C, PTSD Checklist Civilian Version; Re-experiencing: items #1–5; Avoidance/emotional numbness: items # 6–12; Hyperarousal: items # 13–17.
Respondents rate each item from 1 to 5 (1, never; 2, rare; 3, occasionally; 4, frequently; 5, constantly) to indicate the degree to which they have been bothered by the symptom over the past month.

The 678 respondents included 174 (25.66%) doctors, 468 (69.03%) nurses, 20 (3.10%) medical technicians, and 16 (2.21%) administrative staff. The data of 642 doctors and nurses (healthcare workers, HCWs) were analyzed in this study (Table 2). Of the HCWs, 96 (14.95%) were males and 546 (85.05%) were females; 274 (42.68%) were <30 years old; 299 (44.86%) were between 30 and 40 years old; 69 (12.46%) were 40 years or older; 421 (65.58%) respondents were frontline HCWs directly engaged in diagnosing, treating, or caring for patients with or suspected to have COVID-19; 335 (52.18%) were previously quarantined, 90 (14.02%) tested positive for COVID-19, and 330 HCWs (51.40%) reported that their family members were diagnosed with COVID-19.

3.2. Prevalence of probable PTSD

The entire study population was divided into two subgroups: "high-scoring respondents" at increased risk of probable PTSD (PTSD group, scores ≥50; $n = 134$), and "low-scoring respondents" (non-PTSD group, scores <50; $n = 508$).

Among the 642 HCWs, the prevalence of probable PTSD was 20.87%. The symptoms were considered severe if they were assigned a score of 4 or 5. Of the 314 HCWs with probable PTSD, five hyperarousal symptoms were most frequently assigned scores of 4 or 5 (items #13–17: 76.12%, 60.44%, 59.64%, 57.46%, and 47.02%, respectively), suggesting hyperarousal was the most common severe PTSD symptom (Table 1).

The 508 (79.13%) HCWs of the non-PTSD group scored less than 50. These HCWs also reported some PTSD symptoms, although they scored less than 50 (Table 1). It is worth noting that cutoff scores of 30 and 44 have been used to screen PTSD (Walker et al., 2002; Sonis et al., 2009). We chose a cutoff value of 50 because of its high specificity and sensitivity in predicting a clinical diagnosis of PTSD. If the cutoff PCL-C score had been decreased to 44, 240 (37.38%) HCWs would have been considered to be positive for probable PTSD. The HCWs with probable PTSD would have increased to 479 (74.61%) if 30 was chosen as the cutoff score. These HCWs should be followed-up to investigate their long-term outcomes.

In the PTSD group, compared with the non-PTSD group, there were more nurses (84.33% vs. 62.99%, $p<0.00$), more females (91.79% vs. 83.27%, $p = 0.01$), more COVID-19 positive HCWs (26.12% vs. 10.83%, $p < 0.00$), and more HCWs who reported that a family member tested positive (60.45% vs. 49.12%, $p = 0.02$). No differences in age, marital status, and education level were found between the two groups (all $p > 0.05$). No differences in the percentages of frontline HCWs who were quarantined and the percentages of frontline experience during the outbreak of SARS were found between the two groups (all $p > 0.05$) (Table 2).

There were fewer HCWs with high total SSRS in the PTSD group than in the non-PTSD group (12.69% vs. 32.28%; $p < 0.00$) (Table 2). Moreover, the females with PTSD had significantly lower subdimension scores for objective support (7.18 ± 2.66 vs. 8.39 ± 3.07), subjective support (10.38 ± 1.98 vs. 11.20 ± 1.95), and utilization of social support (7.00 ± 1.56 vs. 7.54 ± 1.60) than non-PTSD females (all $p < 0.00$)

Table 2.
Demographic characteristics of the respondents and risk factors associated with the presence of probable PTSD.

	Total respondents *n* = 642	PTSD (PCL-C scores≥50) *n* = 134	Non-PTSD (PCL-C scores <50) *n* = 508	*P* value
	No. (and%) of respondents			Chi-square test
**Sex**				
Female	546(85.05)	123(91.79)	423(83.27)	0.01
Male	96(14.95)	11(8.21)	85(16.73)	
**Age, years**				
<30	274(42.68)	57(42.54)	225(44.29)	0.29
30–40	299(44.86)	65(48.51)	220(43.31)	
>40	69(12.46)	12(8.95)	63(12.40)	
**Marital status**				
Married	387(60.28)	81(60.45)	306(60.24)	1.00
Single, divorced or separated	255(39.72)	53(39.55)	202(39.76)	
**Education level**				
Bachelor's Degree or higher	566(88.16)	122(91.04)	444(87.40)	0.29
Secondary/ Vocational-High Certificate	76(11.84)	12(8.96)	64(12.60)	
**Profession**				
Doctor	174(27.10)	23(15.67)	151(37.01)	0.00
Nurse	468(72.90)	111(84.33)	357(62.99)	
**Frontline healthcare workers**				
Yes	421(65.58)	95(70.90)	326(64.17)	0.15
No	221(34.42)	39(29.10)	182(35.83)	
**Quarantining**				
Yes	335(52.18)	68(50.75)	267(52.56)	0.38
No	307(47.82)	66(49.25)	241(47.44)	
**COVID-19 test**				
Positive	90(14.02)	35(26.12)	55(10.83)	0.00
Negative	552(85.98)	99(73.88)	453(89.17)	
**COVID-19 tests of family members**				
Positive	330(51.40)	81(60.45)	249(49.12)	0.02
Negative	312(48.60)	53(39.55)	259(50.88)	
**Frontline experience of SARS**				
Yes	180(28.04)	46(34.33)	134(26.38)	0.08
No	462(72.19)	88(65.67)	374(73.62)	
**Social support**				
Low to moderate total SSRS	461(71.81)	117(87.31)	344(67.72)	0.00
High total SSRS	181(28.19)	17(12.69)	164(32.28)	

PTSD, Posttraumatic stress disorder; PCL-C, PTSD Checklist Civilian Version; SSRS, Social Support Self-Rating Scale. Low to moderate total SSRS, scores<30; high total SSRS, scores ≥30. Family members refer to healthcare workers' (HCWs) spouses, parents, siblings, and children.

(independent samples *t*-test). However, no difference in scores for objective support (8.45 ± 2.94 vs. 9.64 ± 3.30), subjective support (10.36 ± 2.54 vs 11.26 ± 1.86), and utilization of social support (6.27 ± 1.85 vs. 7.29 ± 1.97) was found among males in the two groups (all $p >$ 0.05) (independent samples *t*-test).

3.3. Factors associated with probable PTSD

To elucidate the relationship among outbreak event exposures, risk perception, and level of PTSD, logistic regression analyses were conducted (Table 3). Negative COVID-19 tests among the HCWs were found to be negatively correlated with PTSD levels. The adjusted odds ratio was 0.35 (95% CI, 0.21–0.58; $p < 0.00$). Negative COVID-19 tests among the family members of HCWs were also protective against high PTSD levels in HCWs (OR, 0.64; 95% CI, 0.42–0.96; $p = 0.03$). Moreover, high social support scores were also found to be an independent protective factor against high PTSD levels (OR, 0.30; 95% CI, 0.17–0.52; $p < 0.00$). Multivariable logistic regression analysis revealed that the HCWs who tested negative for COVID-19 or those whose family members tested negative and those with high social support were less likely to have probable PTSD (Table 3).

Table 3.
Multiple regression analysis of factors associated with probable PTSD.

Factors	Estimated regression coefficient, β	Std. Error	Wald	*P* value	OR	95% CI
**Negative COVID-19 test**	−1.05	0.26	16.87	0.00	0.35	0.21–0.58
**High total SSRS**	−1.22	0.37	18.22	0.00	0.30	0.17–0.52
**Negative COVID-19 tests of HCWs' family members**	−0.45	0.21	4.77	0.03	0.64	0.42–0.96

PTSD, Posttraumatic stress disorder; HCWs, healthcare workers; SSRS, Social Support Self-Rating Scale.

3.4. Prevalence of anxiety and depression

With regards to HADS, 119 (88.88%) and 110 (82.09%) of the HCWs with probable PTSD had scores of 8 or higher for anxiety and depression symptoms, respectively. In the non-PTSD group, fewer HCWs exhibited anxiety (156, 30.71%, $p = 0.01$) and depression symptoms (362, 71.26%; $p < 0.01$) (Table 4).

The mean HADS scores of female HCWs with probable PTSD were significantly higher (anxiety: 10.66 ± 3.47 vs. 6.33 ± 2.98, $p < 0.00$; depression: 9.52 ± 2.19 vs. 8.45 ± 1.89, $p < 0.00$; total HADS score: 20.18 ± 4.54 vs. 14.79 ± 3.79; $p < 0.00$) than those of non-PTSD HCWs, confirming the higher prevalence of more severe anxiety and depression in female HCWs with PTSD (Table 5). In male HCWs, higher anxiety scores (9.55 ± 2.91 vs. 4.95 ± 2.54; $p < 0.00$) and higher total HADS scores (18.55 ± 4.46 vs. 13.36 ± 3.35; $p < 0.00$) were observed in PTSD HCWs than in those without PTSD. However, their depression scores (9.00 ± 2.10 vs. 8.41 ± 1.75; $p = 0.31$) were not significantly different (Table 5).

3.5. Prevalence of somatic symptoms

Of the respondents with probable PTSD, 134 (100%) had scores of 5 or higher and were considered to have positive somatic symptoms. Fewer HCWs (91.14%) in the non-PTSD group had positive somatic symptoms ($p < 0.00$) (Table 4). Among both female and male HCWs, the PHQ-15 score of the PTSD group was higher than that of the non-PTSD group (female, 19.41 ± 5.01 vs. 13.07 ± 5.42, $p < 0.00$; male, 17.09 ± 4.09 vs. 8.67 ± 4.40, $p < 0.00$) (Table 5). These results suggest higher rates of somatic symptoms and more severe somatic illness in HCWs with probable PTSD.

3.6. Sleep quality

Sleep quality and its components are reported in Table 4. According to the ISI score, 95.52% of HCWs with probable PTSD, compared with 40.16% of the non-PTSD respondents, reported poor sleep quality (ISI score>7) ($p < 0.00$). Moreover, more HCWs with probable PTSD were considered to have clinical insomnia (44.03% with moderate insomnia

Table 4.
Prevalence of anxiety, depression, somatic symptoms, and insomnia.

	PTSD (n = 134)	Non-PTSD (n = 508)	P value
	No. (and%) of respondents		Chi-square test
HADS			
Anxiety			
Normal (0–7)	15(11.12)	352(69.29)	0.00[a]
Borderline abnormal (8–10)	63(47.01)	118(23.23)	
Abnormal (11–21)	56(41.87)	38(7.48)	
Depression			
Normal (0–7)	24(17.91)	146(28.74)	0.01[b]
Borderline abnormal (8–10)	73(54.48)	295(58.07)	
Abnormal (11–21)	37(27.61)	67(13.19)	
PHQ-15			
No somatic symptom (0–4)	0(0.00)	45(8.86)	0.00[c]
Somatic symptom	134 (100.00)	463(91.14)	
Mild symptom (5–9)	4(2.98)	107(21.06)	
Moderate symptom (10–14)	19(14.18)	173(34.06)	
Severe symptom (≥15)	111(82.84)	183(36.02)	
ISI			
No clinically significant insomnia (0–7)	6(4.48)	304(59.84)	0.00[d]
Insomnia	128(95.52)	204(40.16)	
Subthreshold insomnia (8–14)	54(40.30)	167(32.87)	
Moderate clinical insomnia (15–21)	59(44.03)	34(6.69)	
Severe clinical insomnia (22–28)	15(11.19)	3(0.60)	

HADS, Hospital Anxiety and Depression Scale; PHQ-15, Patient Health Questionnaire-15; ISI, Insomnia Severity Index.
Two-tailed P value was calculated using Chi-square test; PTSD, Posttraumatic stress disorder.
P value[a] refers to the P value for Normal vs the Borderline abnormal+ Abnormal.
P value[b] refers to the P value for Normal vs the Borderline abnormal+ Abnormal.
P value[c] refers to the P value for no somatic symptoms vs. somatic symptoms (mild+moderate+severe somatic symptom).
P value[d] refers to the P value for no clinically significant insomnia vs. insomnia (subthreshold+moderate+severe clinical insomnia).

Table 5.
Scores of anxiety, depression, somatic symptoms, and insomnia (Mean ± SD).

	Female PTSD (n = 123)	Non-PTSD(n = 423)	P value	Male PTSD(n = 11)	Non-PTSD(n = 85)	P value
**Anxiety score**	10.66 ± 3.47	6.33 ± 2.98	0.00	9.55 ± 2.91	4.95 ± 2.54	0.00
**Depression score**	9.52 ± 2.19	8.45 ± 1.89	0.00	9.00 ± 2.10	8.41 ± 1.75	0.31
**Total HADS score**	20.18 ± 4.54	14.79 ± 3.79	0.00	18.55 ± 4.46	13.36 ± 3.35	0.00
**PHQ-15 score**	19.41 ± 5.01	13.07 ± 5.42	0.00	17.09 ± 4.09	8.67 ± 4.40	0.00
**ISI score**	15.46 ± 5.21	7.46 ± 4.68	0.00	15.45 ± 4.80	6.03 ± 4.69	0.00

The scores for depression and anxiety, somatization symptoms, subjective sleep quality, and social support were sub-grouped by gender.
An independent-samples t-test was conducted to compare the scores between PTSD and non-PTSD HCWs with the same gender.

and 11.19% with severe insomnia), compared with those without PTSD (6.69% with moderate insomnia and 0.60% with severe insomnia) ($p < 0.00$) (Table 4). Among both females and males, the ISI score of the PTSD group was higher than that of the non-PTSD group (female, 15.46 ± 5.21 vs. 7.46 ± 4.68, $p < 0.00$; male, 15.45 ± 4.80 vs. 6.03 ± 4.69, $p < 0.00$) (Table 5). These results suggest higher rates of insomnia and poorer sleep quality in HCWs with probable PTSD.

4. Discussion

In the last two decades, China experienced two waves of viral pneumonia pandemic: SARS and COVID-19 (Pan et al., 2020). Significant emotional distress was found in 18% to 57% of HCWs and SARS survivors during and after the SARS outbreak (Chan and Huak, 2004; Tam et al., 2004; Nickell et al., 2004; Maunder et al., 2006). Four to five years after the resolution of the SARS epidemic, new incidences of psychiatric disorder and high levels of PTSD were found in 5–10% of HCWs (Wu et al., 2009; Lancee et al., 2008; Hong et al., 2009). Due to the ease of transmission, lack of population immunity, lack of personal protective equipment, and the challenges of treating the disease, a higher rate of psychiatric morbidity related to COVID-19 is expected among HCWs (Pan et al., 2020; Cai et al., 2020; Rajkumar, 2020).

The clinical diagnosis of PTSD needs a structured clinical interview conducted by experienced clinical interviewers according to the Diagnostic and Statistical Manual of Mental Disorders (Neria et al., 2010). In this study, PTSD was assessed using the PTSD Checklist Civilian Version (PCL-C) (Sonis et al., 2009). PCL-C has been used extensively as a screening tool for PTSD, has been tested among HCWs, and has demonstrated a high level of diagnostic accuracy in the correspondence with clinical diagnosis of PTSD (Wilberforce et al., 2010; Joseph et al., 2014; Tang et al., 2016).

The reported PCL-C cutoff scores for screening PTSD were 30 (Walker et al., 2002), 44 (Sonis et al., 2009), and 50 (Schlenger et al., 2002; Wilberforce et al., 2010; Forbes et al., 2001). Previous studies revealed that a cutoff score of 50 yielded a sensitivity of 0.78–0.82, and a specificity of 0.83–0.86 for the diagnosis of PTSD using a structured clinical interview or a clinician-administered PTSD scale (Spitzer et al., 1992; Blanchard et al., 1996). We chose a cutoff value of 50 to identify probable PTSD. A total of 134 (20.87%) HCWs with probable PTSD were screened. These HCWs had significantly higher HAD, PHQ-15, and ISI scores, suggesting that severe anxiety, depression, somatic symptoms, and insomnia are associated with probable PTSD.

In this study, the female sex and marital status were not predictive of high levels of PTSD symptoms. These findings are inconsistent with previous studies that examined the mental health effects of the SARS outbreak (Mak et al., 2010; Hong et al., 2009; Sim et al., 2010). This might be explained by the fact that majority of the HCWs in this study were female (85.05%) and married (60.28%). Moreover, 52.18% of HCWs were quarantined at government-designated quarantine hotels. Complete separation reduced their fear of spreading the virus to their family members (Pan et al., 2020). Previous experience with SARS was not helpful in reducing the prevalence of probable PTSD. Although HCWs who were involved in the management of SARS patients knew how to protect themselves, they seemed to be overwhelmed by the impact of COVID-19.

It is worth noting that working on the frontline was not predictive of probable PTSD. This suggests that both the frontline and second-line HCWs faced similar threats due to COVID-19 during the pandemic. The Central Hospital of Wuhan was among the first hospitals that dealt with COVID-19 cases. In the early weeks after the COVID-19 outbreak, the hospital was overcrowded with patients with fever or respiratory symptoms. Before the first announcement of human-to-human transmission on January 20, 2020, it was not unusual for fever patients to be admitted to ordinary wards for treatment of their underlying medical conditions (Pan et al., 2020). As a result, it is not surprising that seven hospital staff (6 HCWs and 1 administrative staff), who did not work in isolation wards or fever clinics, died from COVID-19 (Zhan et al., 2020). On the other hand, the frontline HCWs were better protected. They were provided with hospital-designated hotel rooms near the hospital. These measures reduced the mental burden of frontline HCWs.

A meta-analysis revealed that low social support was one of the most important risk factors among the 25 potential risk factors of PTSD (Trickey et al., 2012; Hong et al., 2009). In this study, we found that high social support was an independent predictive factor for lower levels of

PTSD symptoms. The findings suggest that adequate access to economic assistance, psychological intervention, and sufficient utilization of available social support might help alleviate PTSD symptoms in HCWs.

The epidemic situation in the Central Hospital of Wuhan was more serious than in other local hospitals. The prevalence of PTSD-related symptoms in HCWs was expected to be higher. A study by Lai et al., which involved 1257 HCWs who treated patients exposed to COVID-19 in multiple regions of China, revealed that HCWs reported symptoms of depression (50.4%), anxiety (44.6%), and distress (71.5%) (Lai et al., 2020). Another study surveyed 14,825 HCWs in 31 provinces of mainland China and found that the prevalence rates of depressive symptoms and PTSD were 25.2% and 9.1%, respectively. Working in the Hubei province was associated with a higher risk of depressive symptoms (Song et al., 2020). By using different measurement scales, we found that 642 HCWs reported symptoms of depression (73.52%) and anxiety (42.83%). In the study by Lai and our study, the seven-item ISI was used to measure the severity of insomnia. In our study, 51.71% of all HCWs, including 95.52% of the probable PTSD HCWs and 40.16% of the non-PTSD HCWs, reported insomnia, which is much higher than that reported by Lia et al. (51.71% vs. 34.0%).

5. Limitations and future direction

This study has several limitations. First, this is a cross-sectional study, therefore no causal relationship between risk perception and PTSD can be established. Second, the PCL-C evaluates the level of PTSD symptoms, but cannot be used to diagnose PTSD itself (Sonis et al., 2009). HCWs with PCL-C scores <50 also presented with PTSD symptoms. These HCWs may develop chronic PTSD and may need long-term follow-up. Third, the HCWs' self-reports were prone to recall bias. Any self-reported information the participants provided might be incorrect despite their best effort to be honest and accurate. Moreover, the predominance of female nurses in the respondents could introduce some bias in the sample and reduce the generalizability of the results.

Future studies should involve a larger sample and multicenter randomized controlled trials and should use objective and quantitative measurements to investigate the long-term mental health effects of COVID-19. Studying the longitudinal trajectory of PTSD levels in HCWs will increase our understanding of the mental health impact of COVID-19.

6. Conclusions

This study highlighted several important findings. First, we found significant rates of probable PTSD (20.65%) in HCWs at the Central Hospital of Wuhan, about six months after the first local outbreak of COVID-19. Second, high levels of psychiatric and somatic illness and insomnia were associated with probable PTSD. Lastly, the HCWs with negative COVID-19 tests, those whose family members tested negative, and those with high social support were less likely to have probable PTSD. These factors must be understood in order to develop a theoretical account of PTSD in this population and to organize prevention programs that can help these HCWs to cope with future epidemics.

Ethics approval

Ethics approval was obtained from the Institutional Review Board of the Central Hospital of Wuhan (CHW-IRB-2020-041).

Author statement

H.Z. collected all the data and wrote the paper. Y.S. performed the statistical analysis. P.J. and P.Z. helped supervise the project. Y.F. and F. W. conceived the original idea and supervised the project. All authors discussed the results and contributed to the final manuscript.

Declaration of Competing Interest

The authors declare that they have no conflicts of interest.

Funding

This research did not receive any specific grant from funding agencies in the public, commercial, or not-for-profit sectors.

References

Ahorsu, D., Lin, C., Imani, V., Saffari, M., Griffiths, M., Pakpour, A., 2020. The fear of COVID-19 scale: development and initial validation. Int. J. Ment. Health Addict. 27, 1–9. https://doi.org/10.1007/s11469-020-00270-8. Mar.

Blanchard, E., Jones-Alexander, J., Buckley, T., Forneris, C., 1996. Psychometric properties of the PTSD Checklist (PCL). Behav. Res. Ther. 34, 669–673. https://doi.org/10.1016/0005-7967(96)00033-2.

Cai, W., Lian, B., Song, X., Hou, T., Deng, G., Li, H., 2020. A cross-sectional study on mental health among health care workers during the outbreak of Corona Virus Disease 2019. Asian J. Psychiatr. 24, 102111 https://doi.org/10.1016/j.ajp.2020.102111. Apr.

Chan, A., Huak, C., 2004. Psychological impact of the 2003 severe acute respiratory syndrome outbreak on health care workers in a medium size regional general hospital in Singapore. Occup. Med. (Lond) 54, 190–196.

Dai, W., Chen, L., Tan, H., Wang, J., Lai, Z., Kaminga, A., Li, Y., Liu, A., 2016. Association between social support and recovery from post-traumatic stress disorder after flood: a 13-14 year follow-up study in Hunan, China. BMC Public Health 194. https://doi.org/10.1186/s12889-016-2871-x. Feb 29.

Falk, A., Eriksson, M., Stenman, M., 2020. Depressive and/or anxiety scoring instruments used as screening tools for predicting postoperative delirium after cardiac surgery: a pilot study. Intensive Crit. Care Nurs. 26, 102851 https://doi.org/10.1016/j.iccn.2020.102851. Mar.

Forbes, D., Creamer, M., Biddle, D., 2001. The validity of the PTSD checklist as a measure of symptomatic change in combat-related PTSD. Behav. Res. Ther. 39, 977–986. https://doi.org/10.1016/s0005-7967(00)00084-x.

Hong, X., Currier, G., Zhao, X., Jiang, Y., Zhou, W., Wei, J., 2009. Posttraumatic stress disorder in convalescent severe acute respiratory syndrome patients: a 4-year follow-up study. Gen. Hosp. Psychiatry 31, 546–554. https://doi.org/10.1016/j.genhosppsych.2009.06.008.

Joseph, B., Pandit, V., Hadeed, G., Kulvatunyou, N., Zangbar, B., Tang, A., O'Keeffe, T., Wynne, J., Green, D., Friese, R., Rhee, P., 2014. Unveiling posttraumatic stress disorder in trauma surgeons: a national survey. J. Trauma Acute Care Surg. 77, 148–154. https://doi.org/10.1097/TA.0000000000000271.

Kang, L., Ma, S., Chen, M., Yang, J., Wang, Y., Li, R., Yao, L., Bai, H., Cai, Z., Xiang Yang, B., Hu, S., Zhang, K., Wang, G., Ma, C., Liu, Z., 2020. Impact on mental health and perceptions of psychological care among medical and nursing staff in Wuhan during the 2019 novel coronavirus disease outbreak: a cross-sectional study. Brain Behav. Immun. https://doi.org/10.1016/j.bbi.2020.03.028. Mar 30pii: S0889-1591(0820)30348-30342.

Kocalevent, R., Hinz, A., Brähler, E., 2013. Standardization of a screening instrument (PHQ-15) for somatization syndromes in the general population. BMC Psychiatry. 91 https://doi.org/10.1186/1471-244X-13-91. Mar 20.

Lai, J., Ma, S., Wang, Y., Cai, Z., Hu, J., Wei, N., Wu, J., Du, H., Chen, T., Li, R., Tan, H., Kang, L., Yao, L., Huang, M., Wang, H., Wang, G., Liu, Z., Hu, S., 2020. Factors associated with mental health outcomes among health care workers exposed to Coronavirus Disease 2019. JAMA Netw. Open 3, e203976. https://doi.org/10.1001/jamanetworkopen.2020.3976.

Lancee, W., Maunder, R., Goldbloom, D., 2008. Prevalence of psychiatric disorders among Toronto hospital workers one to two years after the SARS outbreak. Psychiatr. Serv. 59, 91–95. https://doi.org/10.1176/ps.2008.59.1.91.

Liu, X.-X., Liu, W.-H., Ping, M., Li, C.-Y., Liu, X.-Y., Song, P., 2018. Correlation between resilience and social support and anxiety in obstetric nurses. Front. Nurs. 5, 311–315. https://doi.org/10.1515/fon-2018-0040.

Mak, I., Chu, C., Pan, P., Yiu, M., Ho, S., Chan, V., 2010. Risk factors for chronic post-traumatic stress disorder (PTSD) in SARS survivors. Gen. Hosp. Psychiatry 32, 590–598. https://doi.org/10.1016/j.genhosppsych.2010.07.007.

Maunder, R., Lancee, W., Balderson, K., Bennett, J., Borgundvaag, B., Evans, S., Fernandes, C., Goldbloom, D., Gupta, M., Hunter, J., McGillis Hall, L., Nagle, L., Pain, C., Peczeniuk, S., Raymond, G., Read, N., Rourke, S., Steinberg, R., Stewart, T., VanDeVelde-Coke, S., Veldhorst, G., Wasylenki, D., 2006. Long-term psychological and occupational effects of providing hospital healthcare during SARS outbreak. Emerg. Infect. Dis. 12.

Morin, C., Belleville, G., Bélanger, L., Ivers, H., 2011. The Insomnia Severity Index: psychometric indicators to detect insomnia cases and evaluate treatment response. Sleep 34, 601–608. https://doi.org/10.1093/sleep/34.5.601.

Neria, Y., Olfson, M., Gameroff, M.J., DiGrande, L., Wickramaratne, P., Gross, R., Pilowsky, D.J., Neugebaur, R., Manetti-Cusa, J., Lewis-Fernandez, R., Lantigua, R., Shea, S., Weissman, M.M., 2010. Long-term course of probable PTSD after the 9/11 attacks: a study in urban primary care. J. Trauma Stress. 23 https://doi.org/10.1002/jts.20544.

Nickell, L., Crighton, E., Tracy, C., Al-Enazy, H., Bolaji, Y., Hanjrah, S., Hussain, A., Makhlouf, S., Upshur, R., 2004. Psychosocial effects of SARS on hospital staff: survey

of a large tertiary care institution. CMAJ 170, 793–798. https://doi.org/10.1503/cmaj.1031077.

Pan, A., Liu, L., Wang, C., Guo, H., Hao, X., Wang, Q., Huang, J., He, N., Yu, H., Lin, X., Wei, S., Wu, T., 2020. Association of public health interventions with the epidemiology of the COVID-19 outbreak in Wuhan, China. JAMA. https://doi.org/10.1001/jama.2020.6130. Apr 10.

Rajkumar, R., 2020. COVID-19 and mental health: a review of the existing literature. Asian J. Psychiatr. 10, 102066 https://doi.org/10.1016/j.ajp.2020.102066. Apr.

Schlenger, W.E., Caddell, J.M., Ebert, L., Jordan, B.K., Rourke, K.M., Wilson, D., Thalji, L., Dennis, J.M., Fairbank, J.A., Kulka, R.A., 2002. Psychological reactions to terrorist attacks: findings from the National Study of Americans' reactions to September 11. JAMA 288, 581–588. https://doi.org/10.1001/jama.288.5.581.

Sim, K., Huak Chan, Y., Chong, P., Chua, H., Wen Soon, S., 2010. Psychosocial and coping responses within the community health care setting towards a national outbreak of an infectious disease. J. Psychosom. Res. 68, 195–202. https://doi.org/10.1016/j.jpsychores.2009.04.004.

Song, X., Fu, W., Liu, X., Luo, Z., Wang, R., Zhou, N., Yan, S., Lv, C., 2020. Mental health status of medical staff in emergency departments during the Coronavirus disease 2019 epidemic in China. Brain Behav. Immun. 88, 60–65. https://doi.org/10.1016/j.bbi.2020.06.002.

Sonis, J., Gibson, J., de Jong, J., Field, N., Hean, S., Komproe, I., 2009. Probable posttraumatic stress disorder and disability in Cambodia: associations with perceived justice, desire for revenge, and attitudes toward the Khmer Rouge trials. JAMA 302, 527–536. https://doi.org/10.1001/jama.2009.1085.

Spitzer, R., Williams, J., Gibbon, M., First, M., 1992. The structured clinical interview for DSM-III-R (SCID). I: history, rationale, and description. Arch. Gen. Psychiatry 49, 624–629. https://doi.org/10.1001/archpsyc.1992.01820080032005.

Tam, C., Pang, E., Lam, L., Chiu, H., 2004. Severe acute respiratory syndrome (SARS) in Hong Kong in 2003: stress and psychological impact among frontline healthcare workers. Psychol. Med. 34, 1197–1204.

Tang, L., Pan, L., Yuan, L., Zha, L., 2016. Prevalence and related factors of post-traumatic stress disorder among medical staff members exposed to H7N9 patients. Int. J. Nurs. Sci. 4, 63–67. https://doi.org/10.1016/j.ijnss.2016.12.002.

Trickey, D., Siddaway, A., Meiser-Stedman, R., Serpell, L., Field, A., 2012. A meta-analysis of risk factors for post-traumatic stress disorder in children and adolescents. Clin. Psychol. Rev. 32, 122–138. https://doi.org/10.1016/j.cpr.2011.12.001.

Walker, E.A., Newman, E., Dobie, D.J., Paul, C., Wayne, K., 2002. Validation of the PTSD checklist in an HMO sample of women. Gen Hosp Psychiatry 24. https://doi.org/10.1016/s0163-8343(02)00203-7.

Wilberforce, N., Wilberforce, K., Aubrey-Bassler, F., 2010. Post-traumatic stress disorder in physicians from an underserviced area. Fam. Pract. 27, 339–343. https://doi.org/10.1093/fampra/cmq002.

Wu, P., Fang, Y., Guan, Z., Fan, B., Kong, J., Yao, Z., Liu, X., Fuller, C., Susser, E., Lu, J., Hoven, C., 2009. The psychological impact of the SARS epidemic on hospital employees in China: exposure, risk perception, and altruistic acceptance of risk. Can. J. Psychiatry 54, 302–311. https://doi.org/10.1177/070674370905400504.

Xiao, S., 1994. The theoretical basis and applications of social support rating scale (SSRS). (in Chinese). J. Clin. Psychiatry 4, 98–100.

Zhan, M., Qin, Y., Xue, X., Zhu, S., 2020. Death from COVID-19 of 23 health care workers in China. N Engl J Med. https://doi.org/10.1056/NEJMc2005696. Apr 15.

Zhang, W., Wang, K., Yin, L., Zhao, W., Xue, Q., Peng, M., Min, B., Tian, Q., Leng, H., Du, J., Chang, H., Yang, Y., Li, W., Shangguan, F., Yan, T., Dong, H., Han, Y., Wang, Y., Cosci, F., Wang, H., 2020. Mental health and psychosocial problems of medical health workers during the COVID-19 epidemic in China. Psychother. Psychosom. 9, 1–9. https://doi.org/10.1159/000507639. Apr.
