# Supplementary material for: Mental health status and related factors influencing healthcare workers during the COVID-19 pandemic: A systematic review and meta-analysis
Source: PLoS One. 2024 Jan 19;19(1):e0289454. doi: 10.1371/journal.pone.0289454 (PMC10798549; doi:10.1371/journal.pone.0289454)
Supplement: S1 Data — (ZIP) [file pone.0289454.s011.zip › literatures/23.pdf]

required ICU care or died were more likely to experience altered mental status (aOR 3.8, 95% CI: 2.1, 6.6), but were less likely to report history of fever (0.5, 95% CI: 0.3, 0.8).

Conclusions: In ED patients with acute COVID-19, complaints of sore throat, myalgias, headache or smell/taste disturbances were associated with discharge and convalescence at home. Patients who were ≥ age 65, Black/African American, experiencing dyspnea, diarrhea, or altered mental status were more likely to undergo hospital admission. Among all admitted patients, altered mental status was positively associated with ICU care or death, and a history of fever was negatively associated with ICU care or death. COVID-19 presents with a heterogeneous constellation of symptoms, and an understanding of the association of the presenting symptoms with the ultimate patient outcome may be useful for allocating resources and targeting management plans.

**Table 1.** Association of Initial Clinical Symptoms with Clinical Severity of ED Patients Presenting with COVID-19

| **Logistic Regression Models for Hospital Admission vs. Convalescence at Home** | OR (95% CI) | *p*-value | aOR (95% CI)† | *p*-value |
|---|---|---|---|---|
| **Age group** | | | | |
| < 65 years | 1.0 [Referent] | | 1.0 [Referent] | |
| ≥ 65 years | 9.0 [6.3, 12.7] | <0.0001 | 7.4 [5.0, 10.8] | <0.0001 |
| **Race** | | | | |
| Black/African American | 2.9 [1.7, 4.8] | <0.0001 | 3.0 [1.6, 5.8] | 0.0007 |
| White | 1.0 [Referent] | | 1.0 [Referent] | |
| Asian/American Indian/Alaska Native/Other | 1.5 [0.9, 2.5] | 0.1633 | 2.2 [1.1, 4.3] | 0.0251 |
| **Sore throat** | | | | |
| Yes | 0.3 [0.2, 0.5] | <0.0001 | 0.4 [0.2, 0.6] | 0.0006 |
| No | 1.0 [Referent] | | 1.0 [Referent] | |
| **Shortness of breath/Dyspnea** | | | | |
| Yes | 2.6 [2.0, 3.4] | <0.0001 | 2.7 [2.0, 3.7] | <0.0001 |
| No | 1.0 [Referent] | | 1.0 [Referent] | |
| **Muscle aches/Myalgia** | | | | |
| Yes | 0.4 [0.3, 0.5] | <0.0001 | 0.5 [0.4, 0.7] | 0.0002 |
| No | 1.0 [Referent] | | 1.0 [Referent] | |
| **Headache** | | | | |
| Yes | 0.3 [0.2, 0.4] | <0.0001 | 0.5 [0.4, 0.8] | 0.0018 |
| No | 1.0 [Referent] | | 1.0 [Referent] | |
| **Diarrhea** | | | | |
| Yes | 1.4 [1.1, 1.9] | 0.0257 | 1.6 [1.1, 2.2] | 0.0113 |
| No | 1.0 [Referent] | | 1.0 [Referent] | |
| **Olfactory/ Taste disturbance** | | | | |
| Yes | 0.3 [0.2, 0.5] | <0.0001 | 0.5 [0.3, 0.8] | 0.0070 |
| No | 1.0 [Referent] | | 1.0 [Referent] | |

| **Logistic Regression Models for ICU Care/Death vs. General Ward Admission** | OR (95% CI) | *p*-value | aOR (95% CI)‡ | *p*-value |
|---|---|---|---|---|
| **History of fever** | | | | |
| Yes | 0.6 [0.4, 0.9] | 0.0073 | 0.5 [0.3, 0.8] | 0.0067 |
| No | 1.0 [Referent] | | 1.0 [Referent] | |
| **Altered mental status/Confusion** | | | | |
| Yes | 3.5 [2.2, 5.7] | <0.0001 | 3.8 [2.1, 6.6] | <0.0001 |
| No | 1.0 [Referent] | | 1.0 [Referent] | |

† Adjusted for age, race, sore throat, shortness of breath/dyspnea, muscle aches/myalgia, headache, diarrhea, olfactory taste disturbance, using stepwise selection in logistic regression.
‡ Adjusted for history of fever and altered mental status/confusion, using stepwise selection in logistic regression.
ED, emergency department; OR, unadjusted odds ratio; aOR, adjusted odds ratio; CI, confidence interval; ICU, intensive care unit.

## 5 It's Time to Rethink How We Screen for Communicable Diseases in the Emergency Department: Lessons Learned From COVID-19

DiLorenzo MA, Davis MR, Dugas JN, Nelson KP, Grochow Mishuris R, Ingalls RR, Hochberg NS, Schechter-Perkins E/Boston Medical Center, Boston, Massachusetts

Study Objectives: COVID-19 symptom severity varies between patients, and some remain asymptomatic. During early April 2020, 70% of patients admitted to the emergency department (ED) of a major hospital in New England had COVID-19, many of whom required treatment in the intensive care unit. As the volume of COVID-19 cases presenting to the ED increased, it became essential to develop accurate triage protocols to separate COVID-positive from COVID-negative patients. This study assessed which of three different clinical screening tools – a nursing triage screen (NTS), an ED clinician Review of Systems (ROS), and a standardized ED attending physician COVID-19 probability assessment – was best at identifying patients who had COVID-19 (based on subsequent PCR confirmation).

Methods: All 748 patients admitted from the ED between April 27, 2020, and May 17, 2020 were included. Sensitivity, specificity, and positive and negative predictive values were calculated for each screening tool. Logistic regression was used to assess each tool's performance. A principal components analysis (PCA) was performed; the resulting factors were used to model COVID-19 positivity.

Results: The emergency physician's probability assessment yielded higher sensitivity (0.62, 95% confidence interval [CI] 0.53-0.71, Table 1) than the NTS (0.46, 95% CI 0.37-0.56), and had higher specificity (0.76, 95% CI 0.72-0.80) than the NTS (0.71, 95% CI 0.66-0.75) and the emergency clinician ROS (0.62, 95% CI 0.58-0.67). Categorization as moderate or high probability on the emergency physician's probability assessment was also associated with the highest odds of having COVID-19 in regression analyses (adjusted odds ratio=4.61, 95% CI 3.01-7.06). Moderate agreement (kappa 0.41-0.60) was observed between the NTS and ED clinician ROS for fever, cough, shortness of breath, and diarrhea; fair agreement (kappa 0.21-0.40) for sore throat, headache, abdominal pain, and vomiting; and poor agreement (kappa 0.00-0.20) for myalgias and chills. The 323 patients who had a response recorded for every symptom were included in the PCA. Only Factor 1 (fever, chills, fatigue, sore throat, rhinorrhea, and cough) was associated with increased odds of testing positive for COVID-19.

Conclusion: While the emergency physician's probability assessment had higher sensitivity and specificity than the other two tools, none of the tools evaluated in this study was sufficiently accurate enough to replace a COVID-19 PCR test on a patient entering a clinical setting where transmission control is crucial. These findings suggest that hospitals not rely on symptom or probability assessment in determining infection status but continue to utilize widespread testing. We recommend that providers in other countries experiencing COVID-19 surges consider the relevance of these findings and that as the pandemic develops (with the potential for continued new variant strains), diagnostic testing efforts should supersede the use of clinical screening tools.

**Table 1. Comparison of performance of three different ED screening tools**

| | Nursing Triage Screen | ED Provider Review of Systems | Attending Physician Probability Assessment |
|---|---|---|---|
| Sensitivity | 0.46 (0.37-0.56) | 0.53 (0.43-0.62) | 0.62 (0.53-0.71) |
| Specificity | 0.71 (0.66-0.75) | 0.62 (0.58-0.67) | 0.76 (0.72-0.80) |
| Positive Predictive Value | 0.29 (0.23-0.36) | 0.26 (0.21-0.33) | 0.40 (0.33-0.47) |
| Negative Predictive Value | 0.84 (0.80-0.87) | 0.84 (0.79-0.88) | 0.89 (0.85-0.92) |

## 6 Health Care Worker Psychological and Physiological Health During the COVID-19 Pandemic

Chang B, Shechter A/Columbia University

Study Objective: Previous work has established that frontline health care workers (HCWs), such as emergency physicians and nurses, are vulnerable to the development of adverse behavioral, psychological, and physical sequelae, which may persist long after the disaster. We examine the prevalence and predictors of psychological distress in ED clinicians working during the COVID-19 pandemic. We examined psychological and physiological (sleep, resting heart rate, blood pressure) of a sample of frontline providers during the COVID-19 pandemic

Methods: This was a sample of 52 clinicians (physicians, residents, nurses, PAs, NPs) who were frontline HCWs during the COVID-19 pandemic across a diverse (academic, community, urban, and suburban) range of four emergency departments in the New York Metropolitan area during July 2020-September 2020. Study design is a

longitudinal prospective cohort design. At baseline, we conducted a psychological test battery including measures of COVID-19 related stress, PTSD (PCL-5), anxiety (GAD-5), depression (PHQ-9), and burnout (Maslach Burnout Inventory). We also assessed home blood pressure at wake/sleep, resting heart rate, and sleep duration using an accelerometer watch device (Fitbit).

Results: Baseline demographics in our sample had more self-identified women participants (62%), caucasian (67%), with median age of 42. Sample was diverse, containing physicians/advanced practice providers (45%) nurses (43%) and residents (12%) At baseline, positive screens for psychological symptoms were common; 48% for acute stress, 37% for depressive, and 30% for anxiety symptoms. Overall, housestaff rates for acute stress and depression did not differ from attendings or nurses. Overall participants had elevated levels of emotional exhaustion on burnout surveys (median 24, SD 3.5). Average sleep duration was 6.2 hours (SD 1.3), resting heart rate of 86 (SD 18.2), and home blood pressure of 128/76. Increased levels of emotional exhaustion was positively associated with elevated resting blood pressure (Pearson r= .32), and resting heart rate (r=.38), while negatively associated with sleep duration (r=.23).

Conclusion: Our preliminary work and others have highlighted that HCWs are experiencing significant COVID-19-related psychological and physical distress. Future work and data will address key questions such as whether such elevated distress symptoms remain persistent with the evolution of the pandemic. This work and others emphasize the need for continued mental health support measures for HCWs both during and in the aftermath of the pandemic.

## 7 Withdrawn

## 8 Resident Physician Perspectives on the Impact Of COVID-19 on Professional Interpersonal Relationships and Workplace Social Capital: A Qualitative Study

Querin LB, Allen A, Hamm R, Flynn H/University of North Carolina, Chapel Hill, North Carolina

Study Objectives: Interpersonal relationships between emergency medicine resident physicians and their colleagues is key for resident wellness and for optimizing patient care. Studies have shown that the COVID-19 pandemic has reduced social connectedness and negatively impacted workplace social capital in many non-medical professions, yet studies are limited in the medical field, particularly with physicians. This project sought to uncover resident physician perspectives on the impact of COVID-19 on professional relationships and to gain ideas on how to optimize workplace social capital despite and beyond the current limitations of the pandemic.

Methods: This study was conducted at a single academic hospital in the United States between March-April 2021. Data was collected from emergency medicine residents via open-ended response online surveys and a semi-structured focus group discussion. A team of three investigators independently analyzed data from open-ended survey responses and the focus group transcription, using a grounded-theory approach and consensus of the independent analyses was subsequently generated to identify final themes and subthemes.

Results: Three main themes regarding resident perspective on professional interpersonal relationships and social capital were identified: team, trust, and support. We also report the 6 core factors discussed by residents that have changed secondary to the COVID-19 pandemic. EM resident physicians feel that the COVID-19 pandemic has impacted environment, socialization, staff turnover, teaching/debriefing, capacity limits and approach to education, which have demonstrated an overall negative impact on interpersonal relationships and social capital. It has also, however, positively impacted a sense of unity within the emergency department team that residents feel has enhanced relationships and social capital.

Conclusions: The COVID-19 pandemic will have lasting impacts on the way our hospitals and residencies function. The findings of this study may help to ignite discussion on how we can build on the positive, while limit the negative impacting features that have been shaped from the COVID-19 pandemic.

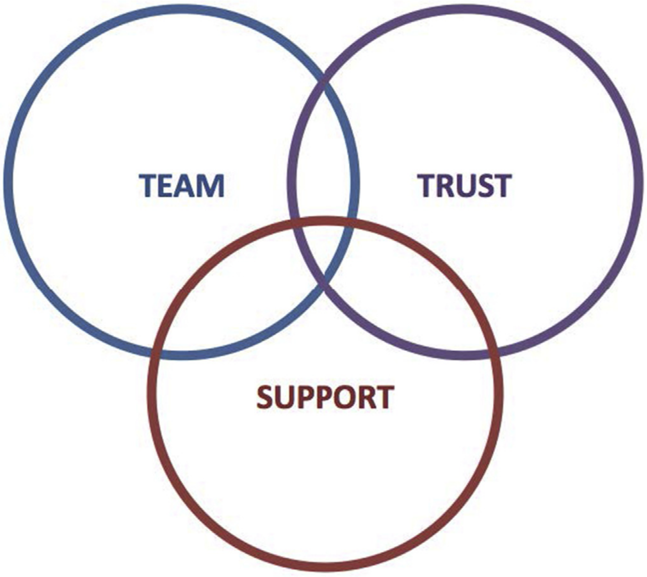


Figure 1. Three primary themes identified as key features of strong workplace interpersonal relationships.

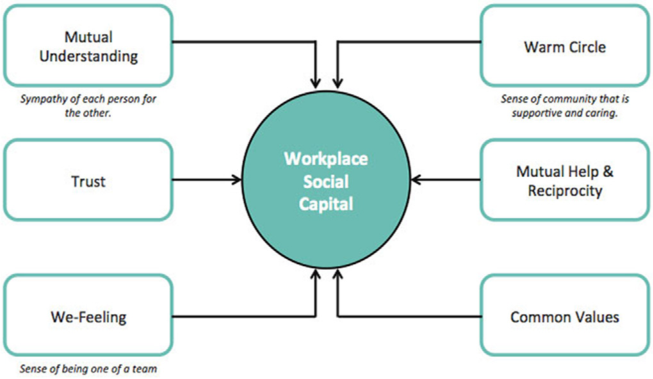


Figure 2. The 6 core features of workplace social capital.

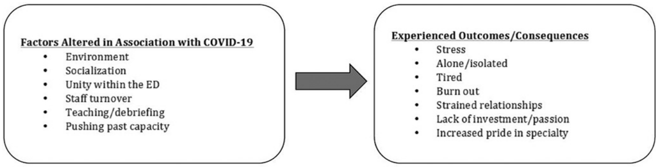


Figure 3. Factors changed during the COVID-19 pandemic and experience outcomes/consequences.
