# Supplementary material for: Mental health status and related factors influencing healthcare workers during the COVID-19 pandemic: A systematic review and meta-analysis
Source: PLoS One. 2024 Jan 19;19(1):e0289454. doi: 10.1371/journal.pone.0289454 (PMC10798549; doi:10.1371/journal.pone.0289454)
Supplement: S1 Data — (ZIP) [file pone.0289454.s011.zip › literatures/230.pdf]

Received: 26 November 2020 | Accepted: 2 September 2021

DOI: 10.1111/ijcp.14783

THE INTERNATIONAL JOURNAL OF CLINICAL PRACTICE WILEY

ORIGINAL PAPER

Primary care

Influencing factors for mental health of general practitioners in Hebei Province under the outbreak of COVID-19: A cross-sectional study

Jinjia Zhang | Shibin Song | Min Zhang | Rongying Wang

Department of General Practice, Second Hospital of Hebei Medical University, Shijiazhuang, China

Correspondence
Rongying Wang, Department of General Practice, Second Hospital of Hebei Medical University, Heping Western Road, No. 215, Shijiazhuang 050000, Hebei, China.
Email: wangrongying2017@sina.com

Abstract

Objective: This study aimed to understand the mental health status of general practitioners (GPs) in Hebei Province during the outbreak of coronavirus disease 2019, analyse influencing factors, and establish and evaluate the risk prediction model.

Methods: During February 25-29, 2020, a self-designed questionnaire was used to conduct an online survey of GPs in Hebei Province. The survey included a questionnaire on GPs' basic information, a questionnaire on GPs' working hardware and software facilities, and a questionnaire on GPs' mental health condition. A total of 1040 participants returned the completely filled valid questionnaire, and the answers were analyzed using the χ^2 test, Wilcoxon rank-sum test and logistic regression with SPSS 20.0 software. Based on the results of binary logistic regression analysis, a risk prediction model was established, and the receiver operating characteristic curve was used to evaluate the model.

Results: The results showed that 44.2% (460/1040) of GPs expressed anxiety after the outbreak. Absence of prescreening clinics, fever clinics or isolated observation rooms in primary medical institutions; persons in the administrative area required to be isolated; low sleep quality of GPs and less than 6 hours of sleep per day of GPs were risk factors affecting the mental health status of GPs. Also, epidemic-related training and adequate protective equipment were the protective factors for the mental health status of GPs.

Conclusion: The government should strengthen the infrastructure construction of community institutions, equip them with sufficient epidemic protection equipment, ensure the rest time of GPs and strengthen mental health training to ensure the mental and physical health of GPs.

1 | INTRODUCTION

The severe acute respiratory syndrome (SARS) epidemic in 2003,[1] the Middle East respiratory syndrome epidemic in 2012,[2] and the outbreak of Ebola disease in 2014[3] have caused extensive loss of human lives and property worldwide, as well as serious psychological trauma. Since COVID-19 was discovered in Wuhan in December 2019[4] and announced by the World Health Organization as a "public health emergency of international concern" at the end of January,[5] it has impacted global health, economics, culture and politics.[6,7]

COVID-19 can be transmitted from person to person. It is highly infectious.[8] Specific drugs are lacking, and hence the disease can be life-threatening if not treated in time. Owing to the suddenness, severity and unpredictability of the disease, public health emergencies serve as great stimulation to participants.[9] Once the stimulation exceeds individual's tolerance to negative emotions, it has serious

 © 2021 John Wiley & Sons Ltd

consequences, including cognitive changes, emotional changes, physical responses and behavioural changes.[10] In addition, the overload of epidemic information has a huge impact on public psychology, causing anxiety, worry and depression. Front-line medical staff face excessive work pressure and also are prone to psychological disorders, severe psychological stress response and post-traumatic stress disorder because of the uncertainty of disease risk and inadequate protective measures in the early stage.

The importance of primary healthcare has been recognised in the context of SARS.[11] Major public health incidents indicate that maximising primary healthcare capacity and protecting primary healthcare workers are the key to ensuring the maximum impact of primary health institutions.[12] Whenever a public health emergency occurs, general practitioners (GPs) are likely to be on the front lines of the response. Several studies focused on the mental health of medical staff in general hospitals during COVID-19.[13-15] However, no study explored the mental health of GPs. The purpose of this study was to understand the mental health status of GPs and related influencing factors in Hebei Province during the outbreak of COVID-19, thus providing a theoretical basis for improving the mental health of GPs in special periods and the lessons for other regions and future outbreaks.

What's known

- Several studies focused on the mental health of medical staff in general hospitals during COVID-19. However, no study explored the mental health of GPs.

What's new

- The anxiety of GPs is obvious during the outbreak of COVID-19.
- It is necessary to strengthen working hardware and software facilities, ensure the quality and time of sleep to improve the mental health of GPs.

2 | PARTICIPANTS AND METHODS

2.1 | Research participants

A cross-sectional survey was conducted during February 25-29, 2020, in which GPs in Hebei Province were selected to participate in the survey. A total of 1040 participants returned the completely filled questionnaire.

2.2 | Research methods

The survey was designed in accordance with the ethical principles set by the Declaration of Helsinki. The questionnaire was anonymous. The informed consent form is located on the introduction page, and participants express their consent by clicking the "Start" button.

The questionnaire used in this study was based on research purposes and designed after consulting the relevant literature. The questionnaire was entered into the Internet platform (star network platform) powered by www.wjx.cn. The entry settings of the questionnaire were modified after the pre-survey and then the questionnaire was published in the WeChat group, which was the most widely used social networking software in China. The same internet protocol address could only be answered once. The questionnaire consisted of three main parts: a questionnaire on GPs' basic information, a questionnaire on the working hardware and software facilities and a questionnaire on GPs' mental health condition.

This study used the Self-Assessed Anxiety Scale (SAS) to assess the mental health of GPs. SAS is a clinical measurement tool developed by Zung in 1971 to assess subjective symptoms of anxiety in patients.[16] The Chinese version of SAS has good reliability and validity.[17] It consists of 20 items. The evaluation time span is 1 week. Each item is divided into 1-4 grades according to the feeling of the latest week. The cumulative score of each item is the SAS total score. The higher the total score, the higher the anxiety level. The sum of the scores of the 20 items is the total score. The integral part of the total score × 1.25 is taken as the standard score, and the value is divided by the standard score 50; a score of ≥50 indicates the existence of anxiety.[18]

2.3 | Statistical analysis

Statistical analysis was performed using SPSS 20.0 statistical software. Enumeration data were expressed in relative numbers. The χ^2 test was used for intergroup comparison, while the Wilcoxon rank-sum test was used for rank data comparison. The logistic regression analysis was used for multivariate analysis. According to the results of binary logistic regression analysis, the risk prediction model was established and the joint predictors were obtained. The receiver operating characteristic (ROC) curve was used to evaluate the model. A P value less than .05 was considered statistically significant.

3 | RESULTS

3.1 | General characteristics of participants

A total of 1040 participants in 11 cities in Hebei Province received the valid questionnaire. Furthermore, 405 participants (38.9%) were aged 36-45 years, 635 were female (61.1%), 413 (39.7%) worked in urban community health service centres and 855 (82.2%) obtained epidemic information through the Internet. Other general characteristics were showed in Table 1.

TABLE 1 Comparison of the mental health status of GPs in different situations

	Mental health status of GPs			
	**Nonanxiety group (n = 580)**	**Anxiety group (n = 460)**	χ^2/Z	*P*
Age (year)				
25 and below	5 (50%)	5 (50%)	7.872	.163
26-35	160 (54.24%)	135 (45.76%)		
36-45	230 (56.79%)	175 (43.21%)		
46-55	145 (54.72%)	120 (45.28%)		
56-65	35 (70.00%)	15 (30.00%)		
66 and above	5 (33.33%)	10 (66.67%)		
Gender				
Male	215 (53.09%)	190 (46.91%)	1.935	.164
Female	365 (57.48%)	270 (42.52%)		
Professional title				
Chief physician	13 (86.67%)	2 (13.33%)	6.171	.104
Associate chief physician	62 (53.91%)	53 (46.09%)		
Attending physician	205 (54.67%)	170 (45.33%)		
Physician	300 (56.07%)	235 (43.93%)		
Unit				
Urban Community Health Service Center	213 (51.57%)	200 (48.43%)	5.273	.153
Urban Community Health Service Station	58 (59.18%)	40 (40.82%)		
Township Health Center	232 (59.18%)	160 (40.82%)		
Village Health Center	77 (56.20%)	60 (43.80%)		
Outbreak information access				
WeChat, Weibo, and Internet	482 (56.37%)	373 (43.63%)	2.522	.641
TV, radio	2 (28.57%)	5 (71.43%)		
Relatives, friends, and neighbors	2 (50.00%)	2 (50.00%)		
Unit notice	92 (54.12%)	78 (45.88%)		
Other	2 (50.00%)	2 (50.00%)		
Whether preview triage area, fever clinics or isolation room set was complete				
Complete setup	50 (62.50%)	30 (37.50%)	45.989	<.001
Set up some of them	200 (43.96%)	255 (56.04%)		
Not set	330 (65.35%)	175 (34.65%)		
Whether "Internet+" was used for epidemic management and monitoring				
Yes	470 (60.26%)	310 (39.74%)	25.467	<.001
No	110 (42.31%)	150 (57.69%)		
Whether joint prevention and control were implemented				
Yes	545 (56.77%)	415 (43.23%)	4.060	.044
No	35 (44.75%)	45 (55.25%)		
Whether protective supplies were sufficient				
Adequacy	465 (59.62%)	315 (40.38%)	52.123	<.001
Partial adequacy	85 (60.71%)	55 (39.29%)		
Inadequacy	30 (25.00%)	90 (75.00%)		
Whether anyone needed isolation observation in the area under the jurisdiction				
Yes	170 (45.95%)	200 (54.05%)	22.467	<.001
No	410 (61.19%)	260 (38.81%)		

(Continues)

TABLE 1 (Continued)

	Mental health status of GPs			
	**Nonanxiety group (n = 580)**	**Anxiety group (n = 460)**	χ^2/Z	***P***
Whether health education was provided to residents				
Yes	535 (55.21%)	434 (44.79%)	1.790	.181
No	45 (63.38%)	26 (36.62%)		
Whether training related to the outbreak was provided				
Yes	575 (57.21%)	430 (42.79%)	25.268	<.001
No	5 (14.29%)	30 (85.71%)		
Sleep quality after breakout				
Good	310 (66.67%)	155 (33.33%)	61.673	<.001
Not good	155 (40.26%)	230 (59.74%)		
Like the past	115 (60.53%)	75 (39.47%)		
Sleep time after breakout				
<6 hours	90 (37.50%)	150 (62.50%)	45.438	<.001
6-8 hours	440 (60.27%)	290 (39.73%)		
More than 8 hours	50 (71.43%)	20 (28.57%)		

3.2 | Comparison of the mental health status of GPs in different situations

GPs were divided into two groups based on their anxiety scores: anxiety group and nonanxiety group. Combined with professional knowledge and related literature, a single-factor analysis was performed on the general situation of GPs, the working software and hardware facilities and the sleep situation. The results showed that the mental health status of GPs was related to the following factors: whether the unit was equipped with precheck triage office, fever clinic or isolation observation room; whether the unit applied “Internet+” for epidemic management; whether the unit implemented joint prevention and control; whether the protective equipment of the unit was sufficient; whether anyone needed isolation observation in the area under jurisdiction; whether GPs received epidemic-related training; sleep quality after the outbreak; and sleep time after the outbreak ($P < .05$, Table 1).

3.3 | Logistic regression analysis of influencing factors for the mental health of GPs in Hebei Province under COVID-19

GPs' emotions were used as the dependent variable (assignment: no anxiety = 0; anxiety = 1) for binary logistic regression analysis. The results showed that no prescreening clinics, fever clinics or isolated observation rooms in the facilities of primary medical institutions (OR = 3.405, 95% CI: 1.889-6.139); persons in the administrative area who needed to be isolated (OR = 1.520, 95% CI: 1.132-2.040); low sleep quality of GPs (OR = 2.619, 95% CI: 1.856-3.694) and less than 6 hours of sleep per day of GPs (OR = 3.201, 95% CI: 1.597-6.413) were the risk factors affecting the mental health status of GPs ($P < .05$). Epidemic-related training (OR = .222, 95% CI: 0.080-0.614) and adequate protective equipment (OR = .163, 95% CI: 0.099-0.267) were the protective factors affecting the mental health status of GPs ($P < .05$, Table 2).

3.4 | Establishment and evaluation of risk prediction models of mental health of GPs in Hebei Province under COVID-19

Establishment of risk prediction model: Based on the results of binary logistic regression analysis, the risk prediction model was obtained by logit transformation: $P = \frac{1}{1+e^{-(1.496+1.225X_1-1.815X_2+0.419X_3-1.505X_4+0.963X_5+1.163X_6)}}$, and the joint predictor (L) was obtained as follows: $L = X_1 - 1.482X_2 + 0.342X_3 - 1.229X_4 + 0.786X_5 + 0.949X_6$, where X_1 denotes whether preview triage area, fever clinics or isolation room set was complete; X_2 denotes whether protective supplies were sufficient; X_3 denotes whether anyone needed isolation observation in the area under jurisdiction; X_4 denotes whether epidemic-related training was provided; X_5 denotes the sleep quality after the epidemic and X_6 denotes the sleep time after the epidemic.

Evaluation of risk prediction model: The ROC curve showed that the combined predictors were more effective compared with a single factor in predicting mental health disorders among GPs in Hebei Province in the case of COVID-19, and the area under the curve of this model was 0.768 (95% CI: 0.736 −0.800, $P < .001$). According to the Youden index (sensitivity + specificity −1), the threshold value of the best prediction was 0.350, the sensitivity was 71.4% and the specificity was 88.3%, as shown in Figure 1.

TABLE 2 Logistic regression analysis of influencing factors for the mental health of GPs

Independent variable	*B*	SE	Wald χ^2 value	*P* value	OR (95% CI)
Whether preview triage area, fever clinics or isolation room set was complete (taking the complete set as the reference)					
Set up some of them	0.306	0.294	1.079	.299	1.358 (0.763-2.417)
Not set	1.225	0.301	16.604	<.001	3.405 (1.889-6.139)
Whether "Internet+" was used for epidemic management and monitoring (taking no as the reference)					
Yes	−0.303	0.173	3.075	.080	0.739 (0.526-1.036)
Whether joint prevention and control was implemented (taking no as the reference)					
Yes	−0.575	0.300	3.674	.055	0.562 (0.312-1.013)
Whether protective supplies were sufficient (taking inadequacy as the reference)					
Partial adequacy	−1.075	0.294	13.366	<.001	0.341 (0.192-0.607)
Adequacy	−1.815	0.253	51.528	<.001	0.163 (0.099-0.267)
Whether anyone needed isolation observation in the area under jurisdiction (taking no as the reference)					
Yes	0.419	0.150	7.759	.005	1.520 (1.132-2.040)
Whether health education was provided to residents (taking no as the reference)					
Yes	−1.505	0.519	8.410	.004	0.222 (0.080-0.614)
Sleep quality after breakout (taking good as the reference)					
Like the past	0.533	0.196	7.380	.007	1.704 (1.160-2.502)
Not good	0.963	0.176	30.068	<.001	2.619 (1.856-0.694)
Sleep time after breakout (taking >8 hours as the reference)					
<6 hours	1.163	0.355	10.764	.001	3.201 (1.597-6.413)
6-8 hours	0.616	0.316	3.808	.051	1.851 (0.997-3.437)
Quantity	1.496	0.700	4.570	.033	4.464

4 | DISCUSSION

COVID-19 is a public health emergency of international concern,[5] which can cause huge physical and psychological damage to people. The psychological trauma lasts for a long time, and stress disorder even appears after the event.[19] As the foundation of the health service system, primary medical institutions are the first line of defense for the prevention and control of this public health concern. Compared with general population, GPs have more chances to be exposed to patients infected with COVID-19; hence, their psychological trauma and fear of being infected are more serious. This was confirmed during the SARS and HINI outbreaks. During the disease outbreaks, medical workers were more worried about them and their families being infected.[20,21] The present study had similar findings, showing that 44.2% (460/1040) of GPs reported anxiety after the outbreak. This rate is significantly higher than that reported in general adult population of China.[22]

The logistic regression analysis showed that the lack of prescreening clinics, fever clinics or isolated observation rooms in the facilities of primary medical institutions; persons in the administrative area who needed to be isolated; low sleep quality of GPs and less than 6 hours of sleep per day of GPs were the risk factors affecting the mental health status of GPs. Furthermore, epidemic-related training and adequate protective equipment were the protective factors. This was similar to previous results. A Canadian study showed[23] that GPs who had not received training on the prevention and control of infectious diseases during the SARS epidemic were more likely to have no confidence in understanding and treating SARS.

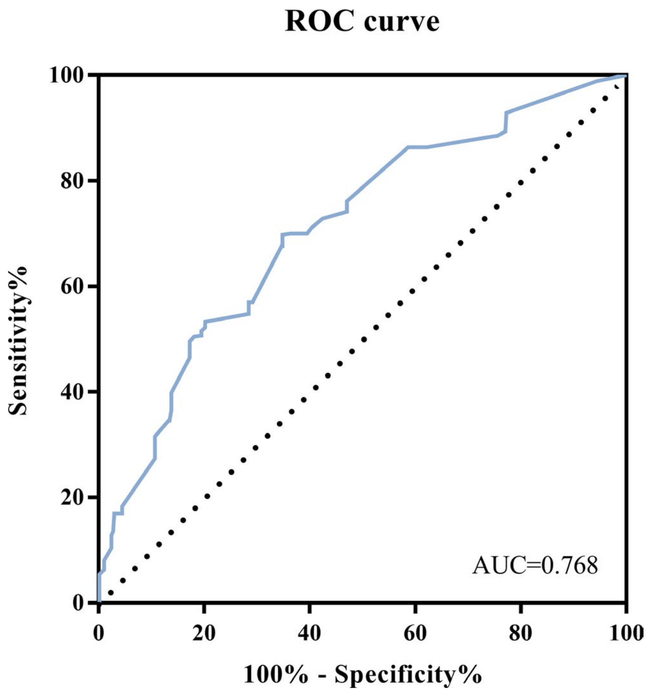

FIGURE 1 ROC curve of mental health disorders in GPs

Canadian doctors believed that the lack of training was a cause of anxiety among them. Similar findings were reported in Australia,[12] where GPs were eager to receive further information and training on pandemic preparedness to relieve stress during the pandemic; face-to-face guidance and training was considered the most appropriate method of use. A study in Singapore[24] showed that the clinic infrastructure and resources were limited during the SARS outbreak, with a severe shortage of personal protective equipment for family doctors, which made them anxious about the risk of infection and prompted them to buy and use personal protective equipment at their own expense regardless of cost. During the pandemic, GPs were most concerned about personal protective equipment. Of the 60 GPs interviewed, 55 said that they would stop working without personal protective equipment.[12] A study in Singapore showed that people in contact with patients with SARS and suspected patients had significant anxiety levels.[25] In the early stage of COVID-19, hospitals and primary health institutions played a major role in the epidemic. Besides basic diagnosis and treatment during the epidemic, GPs were also responsible for providing knowledge on the prevention and control of the disease and guidance on home-based medical isolation. GPs face high-intensity workloads; some even have a severe lack of sleep. A long-term exposure to unknown risks may lead to nervous tension, resulting in emotional reactions such as anxiety, depression, and fear, and subsequently, psychological problems. Severe cases may lead to post-traumatic stress disorder.[19,26]

Our study showed that low sleep quality and short sleep duration were risk factors affecting the mental health status of GPs. However, Sleep can be a cause or an effect of stress. Sleep disturbance is a primary symptom of major depressive disorder.[27] Stress negatively affect the body's ability to fall and stay asleep.[28] So more prospective longitudinal studies are needed to explore the independent bidirectional association between sleep disturbance and mental health.

Single factor assessment of the probability of the occurrence of mental health disorders in GPs has some limitations. Based on the results of binary logistic regression analysis, a risk prediction model was constructed and the joint predictors were determined. In this study, the area under the ROC curve for the occurrence of mental health disorders in GPs was 0.768. When the best prediction threshold was 0.350, the sensitivity and specificity of the prediction were 71.4% and 88.3%, respectively. The area under the ROC curve was greater than 0.7, indicating that the model had acceptable accuracy, sensitivity and specificity, but it needed further improvement. The factors affecting mental health are complex and diverse, including internal subjective factors and external objective factors, which are commonly referred to as individual factors and environmental factors, respectively. The state of mental health is the result of comprehensive action.[29,30] This study paid more attention to environmental factors. Future studies should pay more attention to personal factors, such as biological factors, psychological state factors and life events.

The results of this study showed that the more complete the hardware and software facilities of the medical unit of GPs, the higher the quality of relevant training and the better the sleep quality; sufficient sleep could alleviate the anxiety of GPs. Therefore, the government should strengthen the infrastructure construction of primary health institutions, equip them with adequate protective equipment, strengthen related training for GPs, reduce stress load, ensure the quality and time of sleep for GPs, strengthen mental health training, and relieve the psychological pressure of GPs. After the outbreak, it is necessary to continue to pay attention to the mental health status of GPs and carry out effective intervention strategies for high-risk groups to avoid the occurrence of post-traumatic stress disorder.

This study had some limitations. First, causal inferences could not be made because of the cross-sectional design of the study. The lack of SAS scores prior to the outbreak of COVID-19 limited the ability to draw more effective inferences from the data. Furthermore, there was relatively little about the specifics related to mental health consequences. Future studies should pay more attention to social factors, life events and mental health consequences.

In short, the anxiety of GPs is obvious during the outbreak of COVID-19. It is necessary to strengthen working hardware and software facilities, ensure the quality and time of sleep to improve the mental health of GPs.

ACKNOWLEDGEMENTS

The authors are grateful to Huadong Wu for proofreading this manuscript.

DISCLOSURES

The authors declared that they have no conflicts of interest to this paper.

DATA AVAILABILITY STATEMENT

The data used to support the findings of this study are available from the corresponding author upon request.

REFERENCES

1. Chowell G, Abdirizak F, Lee S, et al. Transmission characteristics of MERS and SARS in the healthcare setting: a comparative study. *BMC Med*. 2015;13(1):210-222. 10.1186/s12916-015-0450-0
2. Kucharski AJ, Althaus CL. The role of superspreading in Middle East respiratory syndrome coronavirus (MERS-CoV) transmission. *Euro Surveill*. 2015;20(25):14-18. 10.2807/1560-7917.es2015.20.25.21167
3. Breban R, Riou J, Fontanet A. Interhuman transmissibility of Middle East respiratory syndrome coronavirus: estimation of pandemic risk. *Lancet*. 2013;382(9893):694-699. 10.1016/S0140-6736(13)61492-0
4. Zhu NA, Zhang D, Wang W, et al. A novel coronavirus from patients with pneumonia in China, 2019. *N Engl J Med*. 2020;382(8):727-733. 10.1056/NEJMoa2001017
5. World Health Organization. *Statement on the Second Meeting of the International Health Regulations (2005) Emergency Committee Regarding the Outbreak of Novel Coronavirus (2019-nCoV) [EB/OL] [2020.1.30]*. https://www.who.int/news-room/detail/30-01-2020-statement-on-the-second-meeting-of-the-international-health-regulations-(2005)-emergency-committee-regarding-the-outbreak-of-novel-coronavirus-(2019-ncov)
6. Hui DS, I Azhar E, Madani TA, et al. The continuing 2019-nCoV epidemic threat of novel coronaviruses to global health—the latest 2019 novel coronavirus outbreak in Wuhan, China. *Int J Infect Dis*. 2020;93(91):264-266. 10.1016/j.ijid.2020.01.009
7. World Health Organization. *Novel Coronavirus (2019-nCoV) Situation Report-17 [EB/OL] [2020.2.6]*. https://www.who.int/docs/default-source/coronaviruse/situation-reports/20200206-sitrep-17-ncov.pdf?sfvrsn=17f0dca_4
8. Chan JF, Yuan S, Kok KH, et al. A familial cluster of pneumonia associated with the 2019 novel coronavirus indicating person-to-person transmission:a sdudy of a family cluster. *Lancet*. 2020;395(10223):514-523. 10.1016/S0140-6736(20)30154-9
9. Wu P, Fang Y, Guan Z, et al. The psychological impact of the SARS epidemic on hospital employees in China: exposure, risk perception, and altruistic acceptance of risk. *Can J Psychiatry*. 2009;54:302-311. 10.1177/070674370905400504
10. Wilson JP. Theoretical perspectives of traumatic stress and debriefings. *Int J Emerg Ment Health*. 1999;1:267-273.
11. Kort R, Stuart A, Bontovics E. Ensuring a broad and inclusive approach: a provincial perspective on pandemic preparedness. *Can J Public Health*. 2005;96:409-411. 10.1007/BF03405178
12. Shaw KA, Chilcott A, Hansen E, et al. The GP's response to pandemic influenza:a qualitative study. *Fam Pract*. 2006;23(3):267-272. 10.1093/fampra/cml014
13. Xiao H, Zhang Y, Kong D. The effects of social support on sleep quality of medical staff treating patients with coronavirus disease 2019 (COVID-19) in January and February 2020 in China. *Med Sci Monit*. 2020;5(26):e923549. 10.12659/MSM.923549
14. Zhang Y, Xie S, Wang PU, et al. Factors influencing mental health of medical workers during the COVID-19 outbreak. *Front Public Health*. 2020;22(8):491. 10.3389/fpubh.2020.00491
15. Cao J, Wei J, Zhu H, et al. A Study of basic needs and psychological wellbeing of medical workers in the fever clinic of a Tertiary General Hospital in Beijing during the COVID-19 outbreak. *Psychother Psychosom*. 2020;89(4):252-254. 10.1159/000507453
16. Zung WW. A rating instrument for anxiety disorders. *Psychosomatics*. 1971;12:371-379. 10.1016/S0033-3182(71)71479-0
17. Chan RCK, Xu T, Huang J, et al. Extending the utility of the depression anxiety stress scale by examining its psychometric properties in Chinese settings. *Psychiatry Res*. 2012;200:879-883. 10.1016/j.psychres.2012.06.041
18. Forlani G, Nuccitelli C, Caselli C, et al. A psychological support program for individuals with type 1 diabetes. *Acta Diabetol*. 2013;50:209-216. 10.1007/s00592-011-0269-x
19. Li Z, Ge J, Yang M, et al. Vicarious traumatization in the general public, members, and non-members of medical teams aiding in COVID-19 control. *Brain Beha Immun*. 2020;88:916-919. 10.1101/2020.02.29.20029322
20. Xiang Y-T, Yang Y, Li W, et al. Timely mental health care for the 2019 novel coronavirus outbreak is urgently needed. *Lancet Psychiatr*. 2020;7:228-229. 10.1016/S2215-0366(20)30046-8
21. Li Z, Ge J, Yang M, et al. Vicarious traumatization in the general public, members, and non-members of medical teams aiding in COVID-19 control. *Brain Beha Immun*. 2020;88:916-919. 10.1101/2020.02.29.20029322
22. Ran L, Wang WO, Ai M, et al. Psychological resilience, depression, anxiety, and somatization symptoms in response to COVID-19: a study of the general population in China at the peak of its epidemic. *Soc Sci Med*. 2020;262:113261. 10.1016/j.socscimed.2020.113261.
23. Jaakkimainen RL, Bondy SJ, Parkovnick M, et al. How infectious disease outbreaks affect community-based primary care physicians: comparing the SARS and H1N1 epidemics. *Can Fam Physician*. 2014;60(10):917-925.
24. Tan NC, Goh LG, Lee SS. Family physicians' experiences, behaviour, and use of personal protection equipment during the SARS outbreak in Singapore: do they fit the Becker Health Belief Model? *Asia Pac J Public Health*. 2006;18(3):49-56. 10.1177/10105395060180030901
25. Koh D, Lim MK, Chia SE, et al. Risk perception and impact of Severe Acute Respiratory Syndrome (SARS) on work and personal lives of healthcare workers in Singapore: what can we learn? *Med Care*. 2005;43:676-682. 10.1097/01.mlr.0000167181.36730.cc
26. Chit MIW, Ming CC, Chyou PP, Chung YMG, Lee CV. Long-term psychiatric morbidities among SARS survivors. *Gene Hosp Psychiatr*. 2009;31:318-326. 10.1016/j.genhosppsych.2009.03.001
27. Jindal RD, Thase ME. Treatment of insomnia associated with clinical depression. *Sleep Med Rev*. 2004;8(1):19-30. 10.1016/S1087-0792(03)00025-X
28. Kalmbach DA, Anderson JR, Drake CL. The impact of stress on sleep: pathogenic sleep reactivity as a vulnerability to insomnia and circadian disorders. *J Sleep Res*. 2018;27(6):e12710. 10.1111/jsr.12710
29. Baron M. Manic-depression genes and the new millennium: poised for discovery. *Mol Psychiatry*. 2002;7(4):342-358. 10.1038/sj.mp.4000998
30. Lopresti AL, Hood SD, Drummond PD. A review of lifestyle factors that contribute to important pathways associated with major depression: diet, sleep and exercise. *J Affect Disord*. 2013;148(1):12-27. 10.1016/j.jad.2013.01.014

How to cite this article: Zhang J, Song S, Zhang M, Wang R. Influencing factors for mental health of general practitioners in Hebei Province under the outbreak of COVID-19: A cross-sectional study. *Int J Clin Pract*. 2021;75:e14783. https://doi.org/10.1111/ijcp.14783
