# Supplementary material for: Mental health status and related factors influencing healthcare workers during the COVID-19 pandemic: A systematic review and meta-analysis
Source: PLoS One. 2024 Jan 19;19(1):e0289454. doi: 10.1371/journal.pone.0289454 (PMC10798549; doi:10.1371/journal.pone.0289454)
Supplement: S1 Data — (ZIP) [file pone.0289454.s011.zip › literatures/231.pdf]

Since January 2020 Elsevier has created a COVID-19 resource centre with free information in English and Mandarin on the novel coronavirus COVID-19. The COVID-19 resource centre is hosted on Elsevier Connect, the company's public news and information website.

Elsevier hereby grants permission to make all its COVID-19-related research that is available on the COVID-19 resource centre - including this research content - immediately available in PubMed Central and other publicly funded repositories, such as the WHO COVID database with rights for unrestricted research re-use and analyses in any form or by any means with acknowledgement of the original source. These permissions are granted for free by Elsevier for as long as the COVID-19 resource centre remains active.

Journal of Affective Disorders 297 (2022) 348–352

Contents lists available at ScienceDirect

Journal of Affective Disorders

journal homepage: www.elsevier.com/locate/jad

Research paper

Mental health outcome and resilience among aiding Wuhan nurses: One year after the COVID-19 outbreak in China

Ruili Zhang [a,1], Jianbo Lai [a,b,c,1], Ying Wang [d,1], Jinwen Huang [a], Shaohua Hu [a,b,c,**], Huafen Wang [e,*]

[a] Department of Psychiatry, First Affiliated Hospital, Zhejiang University School of Medicine, Hangzhou, China
[b] The Key Laboratory of Mental Disorder's Management of Zhejiang Province, Hangzhou 310003, China
[c] Brain Research Institute of Zhejiang University, Hangzhou 310003, China
[d] Department of Psychiatry, RenMin Hospital of Wuhan University, Wuhan, China
[e] Department of Nursing, First Affiliated Hospital, Zhejiang University School of Medicine, Hangzhou, China

ARTICLE INFO

Keywords:
Covid-19
Nurse
Aiding Wuhan
Mental health
Resilience

ABSTRACT

Objective: This study aimed to explore whether aiding Wuhan experience of nurses was associated with adverse mental health outcome one year after the COVID-19 outbreak in China.
Methods: In this study, 100 nurses with and 100 nurses without aiding Wuhan experience a year ago were enrolled from February 1, 2021 to March 31, 2021 in Zhejiang Province, China. Depression, anxiety, insomnia, distress and psychological resilience of participants was assessed and analyzed.
Results: A total of 100 participants from 112 aiding Wuhan nurses completed the survey, with a response rate of 89.3%. Another 100 nurses from the same hospitals without aiding Wuhan experience were enrolled as controls. In both groups, a considerable proportion of participants reported symptoms of depression (46.0% for the aiding Wuhan group vs. 49.0% for the controls, similarly hereinafter), anxiety (40.0% vs. 38.0%), and PTSD (61.0% vs. 56.0%). Aiding Wuhan nurses were more likely to suffer from insomnia (41.0% vs. 29.0%, $P = 0.041$). Multivariable logistic regression analysis showed that aiding Wuhan experience was not associated with depression (adjusted OR (AOR) 0.22; 95%CI, 0.05–1.01), anxiety (AOR 0.53; 95%CI, 0.12–2.43), insomnia (AOR 1.52; 95% CI, 0.76–3.02), PTSD (AOR 0.50; 95%CI, 0.19–1.34), or resilience (AOR 1.59; 95%CI, 0.78–3.26). Resilience was negatively correlated with depression, anxiety, insomnia, and PTSD.
Conclusions: This survey indicated that aiding Wuhan experience a year ago did not cause additional adverse mental health outcomes in nurses, expect for insomnia. The psychological status of nurses in general calls for more attention.

1. Introduction

Although the COVID-19 pandemic enters its second year, the turning point still seems unpredictable. In addition to the surge in infections and deaths, COVID-related mental stress has elicited another striking crisis in people's well-being. Adverse factors link to changes in mental health are complex, but not limited to, economic strife, social isolation and fear of infection (Abbott, 2021). Among all groups of population involved, nurses at the forefront of global fight against COVID-19 are confronted with special risks on psychological burdens. As early as March in 2020, we reported the mental health outcomes and related factors in health care workers (HCWs) exposed to COVID-19 (Lai et al., 2020). Nurses and other HCWs working in Wuhan, the epicenter of COVID-19 outbreak in China, experienced worse symptoms of depression, anxiety, insomnia and distress (Lai et al., 2020). With respect to their pivotal contributions, WHO has designated 2020–21 as the International Year of Nurses and Midwives (Guilamo-Ramos et al., 2021). However, the influence of coronavirus stress on mental health may linger long after the pandemic has subsided (Abbott, 2021). In a recently published meta-analysis, it has been reported that, from 1 to the 3 years after previous coronavirus

* Corresponding author at: Department of Nursing, First Affiliated Hospital, Zhejiang University School of Medicine, Hangzhou, China
** Corresponding author at: Department of Psychiatry, First Affiliated Hospital, Zhejiang University School of Medicine, Hangzhou, China
E-mail addresses: dorhushaohua@zju.edu.cn (S. Hu), 2185015@zju.edu.cn (H. Wang).
[1] These authors contributed equally to this work

https://doi.org/10.1016/j.jad.2021.10.050
Received 3 September 2021; Received in revised form 2 October 2021; Accepted 23 October 2021
Available online 25 October 2021
0165-0327/© 2021 Elsevier B.V. All rights reserved.

outbreaks, there were still 2–19% of HCWs experiencing post-traumatic stress disorder (PTSD) and 5–90% suffering from depression or anxiety symptoms (Galli et al., 2020). Therefore, the psychological impact of an emerging infectious disease outbreak may occur immediately (Lai et al., 2020), but also be sustained after a period interval (Galli et al., 2020; McAlonan et al., 2007). Monitoring the dynamic mental health impacts of this crisis seems to be necessary, especially among nurses exposed to the COVID-19 outbreak. Notably, most studies on sustained psychological impact of coronavirus outbreaks did not focus on nurses alone, but also included physicians, pharmacists, medical technicians, administrators and other HCWs (Galli et al., 2020). Nevertheless, the differences of psychological status among different healthcare professionals were not further analyzed.

To date, however, few study has ever reported the long-term psychological impacts of COVID-19 on frontline nurses. To address this issue, we performed a cross-sectional survey of mental health outcome and associated factors among nurses one year after their participation in the Aiding Hunan Campaign in the Spring of 2020. Another cohort of nurses was recruited as controls to alleviate the influence of the profession factor. This study helps to understand the current psychological status of aiding Wuhan (the provincial capital of Hunan) nurses and provides a reference for psychological care in this population.

2. Methods

2.1. Study design

This cross-sectional study was in line with the American Association for Public Opinion Research (AAPOR) reporting guideline. The study was a hospital-based survey sampling from February 1, 2021 to March 31, 2021, nearly one year after the Aiding Hunan Campaign for COVID-19 outbreak in China. Hospitals in Zhejiang Province with nurses participating in this campaign were eligible to participate. Finally, 14 hospitals (12 tertiary and 2 secondary hospitals) were included. Nurses with aiding Wuhan experience from the midmonth of February 2020 to the end of March 2020 were recruited from these hospitals and those without aiding Wuhan experience were recruited as controls.

This study received approval from the Institutional Ethical Review Board of the First Affiliated Hospital, Zhejiang University School of Medicine. Oral informed consent was obtained from all participants prior to the final enrollment. All subjects were allowed to withdraw from the survey at any time or from any item on their own desire. The survey was anonymous and confidentiality of all information was assured.

2.2. Outcomes and covariates

In this cross-sectional study, we focused on symptoms of depression, anxiety, insomnia, PTSD and resilience of all participants using the Chinese versions of validated psychometric tools (Connor and Davidson, 2003, He, 2010, Wu and Chan, 2003, Yu, 2010, Zhang et al., 2013), including the 9-item Patient Health Questionnaire (PHQ-9) (Zhang et al., 2013), the 7-item Generalized Anxiety Disorder (GAD-7) (He et al., 2010), the 7-item Insomnia Severity Index (ISI) (Yu, 2010), the 22-item Impact of Event Scale-Revised (IES-R) (Wu and Chan, 2003), and the Connor-Davidson Resilience Scale (CD-RISC) (Connor and Davidson, 2003).

The PHQ-9 was used to assess the severity of depressive symptoms. It is a 4-point Likert scale (0–3) with a total score ranging from 0 to 27 points. Scores of 0–4, 5–9, 10–14, and 15–27 indicate none, mild, moderate, and severe depressive symptoms, respectively (Kroenke et al., 2001). A PHQ-9 score higher than 9 was classified as depression.

The GAD-7 was used to measure the degree of anxiety. It is a 4-point Likert scale including 7 items, with a total score ranging from 0 to 21. Scores of 5, 10, and 15 are the cut-off points for mild, moderate, and severe anxiety, respectively (Spitzer et al., 2006). A score of 10 or greater was defined as anxiety.

The ISI was used to assess the severity of insomnia. Items are rated on a 5-point Likert scale ranging from 0 to 4, and the total score ranges from 0 to 28. A score of 0–7, 8–14, 15–21, and 22–28 indicates none, subthreshold, moderate, and severe insomnia, respectively (Bastien et al., 2001). A total score greater than 8 was considered to have symptoms of insomnia (Zhang et al., 2020).

The IES-R was used to measure the presence of psychological distress. It is a 5-point Likert scale composed of three sub-scales including intrusion, avoidance, and hyperarousal (Hyer and Brown, 2008). The total score ranges from 0 to 88, and participants can be classified to have none (0–8), mild (9–25), moderate (26–43), and severe (44–88) psychological distress, accordingly (Yang et al., 2021). A cut-off score of 33 was considered to define participants at risk of psychological distress (Reynolds et al., 2008).

The CD-RISC is a 5-point Likert scale (0–4) that consists of 25 items. It was used to distinguish resilient from non-resilient participants. The total score ranges from 0 to 100, and those with higher scores are considered to have better resilience (Connor and Davidson, 2003). According to a previous study, the cut-off point for identifying resilient participants in this study was set at 81 (Afshari et al., 2021).

Demographic profiles were self-reported by the respondents, including age (year-old), gender (male, or female), working years, marital status (unmarried, married with offspring, married without offspring, or divorced), educational level (technical secondary school, junior college, bachelor, master, or others), technical title (junior, intermediate, or senior), type of hospital (secondary, or tertiary), working department (emergency, intensive care unit, respiratory, infectious disease, or others), the night shift (yes, or no), and taking hypnotics (yes, or no). The technical title of respondents referred to the professional title certificated by the hospital.

2.3. Statistical analysis

All statistical analyses were performed using SPSS version 20 (IBM Co. LTD, Chicago, IL, USA). Continuous variables were presented as mean (standard deviation), whereas categorical variables were reported as count (percentage). Chi-square test and the non-parametric Mann-Whitney test were used to compute categorical data and the non-normal distributed data. Odds ratio (OR) and corresponding 95% confidence interval (CI) was estimated from logistic regression model to study the association between aiding Wuhan experience and the psychological status. The covariates included age, gender, working years, marital status, education, technique title, hospital level, department, night shift, and taking hypnotics. The Spearman rank correlation coefficient was used to assess the correlation between resilience and depression, anxiety, insomnia, and PTSD. All tests were two-tailed and the significance level was 0.05 ($P < 0.05$).

3. Results

3.1. Demographic characteristics

In this study, the aiding Wuhan group and the control group each has 100 nurses enrolled. In the aiding Wuhan group, 112 individuals were invited to participate and the response rate was 89.3%. In the control group, 127 individuals were invited and the response rate was 78.7%. Table 1 presents the demographics and occupational characteristics of nurses in the aiding Wuhan group and the control group. The average age of nurses aiding Wuhan was older than that of the control group (32.8 ±4.3 years vs. 31.3± 5.7 years). Participants in the aiding Wuhan group were more likely to have a higher technique title and less likely to have night shift work compared with that of the control group. No significant differences were found in gender, working years, marital status, education, hospital level, department, or taking hypnotics between the aiding Wuhan nurses and controls ($P > 0.05$).

Table 1
Demographics and occupational characteristics of participants.

Variables	Aiding Wuhan(%), $n = 100$	Control(%), $n = 100$	*P*
Age, years, mean(SD)	32.8(4.3)	31.3(5.7)	0.019
Female, n(%)	87(87.0)	89(89.0)	0.663
Working years, mean (SD)	10.3(5.0)	9.0(5.4)	0.094
Marital status, n(%)			0.935
Unmarried	24(24.0)	28(28.0)	
Married without offspring	13(13.0)	12(12.0)	
Married with offspring	61(61.0)	58(58.0)	
Divorced	2(2.0)	2(2.0)	
Education, n(%)			0.058
Technical secondary school	0(0.0)	1(1.0)	
Junior college	4(4.0)	11(11.0)	
Bachelor	90(90.0)	87(87.0)	
Master	1(1.0)	0(0.0)	
Others	5(5.0)	1(1.0)	
Technique title, n(%)			0.027
Junior	43(43.0)	60(60.0)	
Intermediate	48(48.0)	37(37.0)	
Senior	9(9.0)	3(3.0)	
Hospital level, n(%)			0.106
Tertiary hospital	93(93.0)	86(86.0)	
Secondary hospital	7(7.0)	14(14.0)	
Department, n(%)			0.559
Emergency	8(8.0)	12(12.0)	
ICU	18(18.0)	12(12.0)	
Respiratory	13(13.0)	11(11.0)	
Infectious disease	5(5.0)	3(3.0)	
Others	56(56.0)	62(62.0)	
Night shift, n(%)	74(74.0)	87(87.0)	0.020
Taking hypnotics, n(%)	15(15.0)	7(7.0)	0.071

3.2. Psychological status of two groups

Table 2 shows the levels of depression, anxiety, insomnia, PTSD and resilience in the aiding Wuhan and control groups. 46.0%, 40.0%, 41.0%, and 61.0% of the aiding Wuhan nurses reported symptoms of depression, anxiety, insomnia, and PTSD, respectively, while 49.0%, 38.0%, 29.0%, and 56.0% of the controls experienced these psychological symptoms. It was indicated that the aiding Wuhan nurses were more likely to suffer from insomnia compared with controls ($P = 0.041$). Of note, 12 (12.0%) participants in the aiding Wuhan group reported moderate degree of insomnia, while only 3 (3.0%) nurses in the control group suffered from moderate insomnia. There was no statistical difference between the aiding Wuhan and control groups in terms of resilience (27.0% vs. 21.0%, $P = 0.321$).

Table 2
Levels of depression, anxiety, insomnia, PTSD and resilience in aiding Wuhan and control groups.

Variables	Aiding Wuhan(%), $n = 100$	Control(%), $n = 100$	*P*
Depression			
None (0–4)	54(54.0)	51(51.0)	0.504
Mild (5–9)	39(39.0)	37(37.0)	
Moderate (10–14)	3(3.0)	8(8.0)	
Severe (15–27)	4(4.0)	4(4.0)	
Anxiety			
None (0–4)	60(60.0)	62(62.0)	0.823
Mild (5–9)	35(35.0)	32(32.0)	
Moderate (10–14)	3(3.0)	5(5.0)	
Severe (15–21)	2(2.0)	1(1.0)	
Insomnia			
None (0–7)	59(59.0)	71(71.0)	0.041
Sub threshold (8–14)	28(28.0)	25(25.0)	
Moderate (15–21)	12(12.0)	3(3.0)	
Severe (22–28)	1(1.0)	1(1.0)	
PTSD			
None (0–8)	39(39.0)	44(44.0)	0.835
Mild (9–25)	37(37.0)	28(28.0)	
Moderate (26–43)	18(18.0)	22(22.0)	
Severe (44–88)	6(6.0)	6(6.0)	
Resilience			
No (0–79)	73(73.0)	79(79.0)	0.321
Yes (80–100)	27(27.0)	21(21.0)	

3.3. Association of aiding Wuhan experience with mental health outcome

Table 3 presents the results of association between the aiding Wuhan experience and depression, anxiety, insomnia, PTSD and resilience. It was indicated that aiding Wuhan was not associated with depression (adjusted OR (AOR) 0.22; 95%CI, 0.05–1.01), anxiety (AOR 0.53; 95% CI, 0.12–2.43), insomnia (AOR 1.52; 95%CI, 0.76–3.02), PTSD (AOR 0.50; 95%CI, 0.19–1.34), or resilience (AOR 1.59; 95%CI, 0.78–3.26).

3.4. Correlation between resilience and mental health outcome

Table 4 examines the correlation between resilience and depression, anxiety, insomnia, and PTSD. Totally, resilience was negatively correlated with depression ($r = -0.434$), anxiety ($r = -0.324$), insomnia ($r = -0.362$), and PTSD (intrusion, $r = -0.397$; avoidance, $r = -0.325$; hyperarousal, $r = -0.369$; total IES-R, $r = -0.377$) in regardless of aiding Wuhan experience. High resilience was related to low levels of depression, anxiety, insomnia, and PTSD in both aiding Wuhan nurses and the controls.

4. Discussion

In this study, we first reported the current psychological status of aiding Wuhan nurses one year after the COVID-19 outbreak in China. Although a significant proportion of nurses presented symptoms of depression, anxiety, insomnia and PTSD, aiding Wuhan nurses did not report more prevalent psychological burden except for insomnia. Moreover, aiding Wuhan experience of nurses was not associated with the current psychological status. Psychological resilience was negatively related to symptoms of depression, anxiety, insomnia and PTSD in all participants. These findings provide a reference for future psychological care among this population.

Nurses play a central role in suppressing the COVID-19 pandemic. However, the pandemic's second wave among HCWs, especially those in the frontline, has emerged as an intractable issue and weakens the nursing workforce worldwide. As shown in accumulating studies, unmanageable psychological stress and emotional exhaustion was prevalent in nurses during the pandemic (Galanis et al., 2021; Lai et al., 2020; Stelnicki et al., 2020). Risk factors associated with poorer mental health and functioning among nurses were extensive and complicated, including female, younger age, lack of social support, longer working time, increased workload, inadequate personal protective equipment, fear of infection, perceived hospital safety climate, isolation from family and others (Cho et al., 2021; Galanis et al., 2021; Stelnicki et al., 2020). Previous studies have indicated that working in an area dedicated to COVID-19 patients was associated with worse acute mental health outcome (Ferry et al., 2021; Lai et al., 2020). Research on the long-term mental health outcome of COVID-19 pandemic on frontline nurses, however, is rare. In a recently published study with 4237 nurses from Hunan Province, China, researchers found that after nearly half a year from the domestic COVID-19 outbreak, 17.5%, 10.3% and 12.3% participants experienced moderate to severe levels of depression, anxiety and insomnia, respectively (Liang et al., 2021). The psychometric scales used in this study was consistent with ours. In our study, regardless of aiding Wuhan experience, the proportion of moderate to severe depression, anxiety and insomnia was 9.5%, 5.5% and 8.5%, respectively.

In the current study, on the one hand, the percentage of aiding

Table 3
Associations between aiding Wuhan experience and depression, anxiety, insomnia, PTSD and resilience.

	Aiding Wuhan, n(%)	Control, n(%)	UnadjustedOR(95%CI)	*P*	Adjusted*OR(95%CI)	*P*
Depression	7(7.0)	12(12.0)	0.55(0.21,1.47)	0.233	0.22(0.05,1.01)	0.051
Anxiety	5(5.0)	6(6.0)	0.83(0.24,2.80)	0.757	0.53(0.12,2.43)	0.415
Insomnia	41(41.0)	29(29.0)	1.70(0.95,3.06)	0.076	1.52(0.76,3.02)	0.238
PTSD	16(16.0)	18(18.0)	0.87(0.14,1.82)	0.707	0.50(0.19,1.34)	0.170
Resilience	27(27.0)	21(21.0)	1.39(0.72,2.67)	0.322	1.59(0.78,3.26)	0.204

Note: Cut-off points for determining depression, anxiety, insomnia, PTSD, and resilience were 10, 10, 8, 33, and 80, respectively.
*Adjusted for age, gender, working years, marital status, education, technique title, hospital level, department, night shift, and taking hypnotics.

Table 4
Correlation between resilience and depression, anxiety, insomnia, and PTSD.

	CD-RISC score		
	Total	Aiding Wuhan	Control
PHQ-9	−0.434**	−0.399**	−0.461**
GAD-7	−0.324**	−0.332**	−0.309**
ISI	−0.362**	−0.407**	−0.343**
IES-R total	−0.377**	−0.429**	−0.366**
IES-R Avoidance	−0.325**	−0.342**	−0.337**
IES-R Intrusion	−0.397**	−0.473**	−0.359**
IES-R Hyper-arousal	−0.369**	−0.387**	−0.367**

**$P < 0.01$.

Wuhan nurses with symptoms of depression, anxiety, insomnia and distress was as high as 46.0%, 40.0%, 41.0%, and 61.0%, respectively. An unfavorable psychological status amongst these nurses was related to occupational burnout (Ferry et al., 2021), which may ultimately result in future nursing shortage (Fauteux, 2021), thus calling for special support interventions. On the other hand, the psychological status of nurses in general seems to be especially worrying. Nearly half of the controls reported symptoms of depression and PTSD. In a recent published meta-analysis, the overall prevalence of depressive symptom was 43.83% (95% CI: 40.26−47.42%) among Chinese nurses (Xie et al., 2020). In addition, the prevalence of depression in Chinese nurses have showed an increased trend in recent years (Xie et al., 2020). Moreover, the mental health of nurses across the globe also declined during the COVID-19 pandemic (Varghese et al., 2021). Symptoms of depression, anxiety, insomnia and PTSD were reported by 32% (95% CI: 21−44%), 32% (95% CI: 21−44%), 38.3% (95% CI: 5.8−78.6%) and 18.6% (95% CI: 4.8−38%) of nurses worldwide (Varghese et al., 2021). These findings together indicate an emergency in the general mental health of nurses, suggesting more actions should be urgently implemented by health policy-makers.

In this study, we did not find more prevalent psychological symptoms except for insomnia in aiding Wuhan nurses than that of controls one year after the COVID-19 pandemic in China. Insomnia is a prevalent health problem among frontline HCWs (Ferry et al., 2021; Lai et al., 2020; Liang et al., 2021). Factors such as female, length of frontline service, technical title, hospital level, night shifts, educational years, and family income were associated with the severity of insomnia (Liang et al., 2021). In addition, we found that resilience of nurses was a protective factor for different psychological symptoms, including depression, anxiety, insomnia and PTSD. The effects of resilience on reduced personal accomplishment, depersonalization, burnout, emotional exhaustion in front-line nurses may be fully mediated by their positive or negative affect (Zhang et al., 2021). Therefore, policy-makers should deploy effective and accessible interventions to strengthen psychological resilience of nurses. In previous studies, different interventions aimed at supporting resilience or mental health of frontline HCWs have been developed, including workplace interventions (e.g., training, structure and communication), psychological support interventions (e.g., counselling and psychology services), pharmacological interventions and multifaceted interventions (Pollock et al., 2020). However, due to the lack of both quantitative and qualitative evidence from these studies, it was still unclear whether these interventions were beneficial to improve the resilience or mental health of frontline HCWs. In the future, studies focused on the effectiveness of these interventions remain to be the top priority.

4.1. Limitations

Some limitations in this study should be mentioned. First, the sample size was relatively small, and the participants were only from a single province of China, thus limiting the generalization of our findings. Given the varying and dynamic situation of COVID-19 epidemic in different countries, the psychological impacts on nurses may also be different. Second, the baseline psychological status of these aiding Wuhan nurses was not evaluated, and we could not directly analyze the aiding experience on their mental health. Third, although a significant of nurses reported psychological symptoms, we did not collect data regarding their accessibility and use of mental health service, which is also an important indicator of psychological status. Fourth, due to the cross-sectional design of this study, the dynamic change of mental health amongst aiding Wuhan nurses was not explored.

5. Conclusions

In summary, the current study presented a scenario of the mental health outcomes of nurses one year after the aiding Wuhan experience. Although aiding Wuhan nurses were more likely to suffer from insomnia, the common problems of psychological burden among nurses are another thorny issue that needs to be addressed. Approaches to strengthen the psychological resilience may help to alleviate symptoms of depression, anxiety, insomnia and PTSD of nurses.

6. Author contributions

R.Z., J.L., and Y.W. collected the data, analyzed the data and drafted the manuscript; J.H. collected the data; S.H. and H.W. conceived and designed the research, and edited the manuscript. S.H. and H.W. had full access to all of the data in the study and take responsibility for the integrity of the data and the accuracy of the data analysis.

Declaration of Competing Interest

None.

Acknowledgment

This study was supported by the Program from the Health and Family Planning Commission of Zhejiang Province (Grant No.2021KY682). We acknowledge all the participants who contributed to our work.

Financial disclosure

The funder had no role in the design and conduct of the study; collection, management, analysis, and interpretation of the data; preparation, review, or approval of the manuscript; and decision to submit

the manuscript for publication.

References

Abbott, A., 2021. COVID's mental-health toll: how scientists are tracking a surge in depression. Nature 590 (7845), 194–195.

Afshari, D., Nourollahi-Darabad, M., Chinisaz, N., 2021. Demographic predictors of resilience among nurses during the COVID-19 pandemic. Work 68 (2), 297–303.

Bastien, C.H., Vallieres, A., Morin, C.M., 2001. Validation of the Insomnia Severity Index as an outcome measure for insomnia research. Sleep Med. 2 (4), 297–307.

Cho, M., Kim, O., Pang, Y., Kim, B., Jeong, H., Lee, J., Jung, H., Jeong, S.Y., Park, H.Y., Choi, H., Dan, H., 2021. Factors affecting frontline Korean nurses' mental health during the COVID-19 pandemic. Int. Nurs. Rev. 68 (2), 256–265.

Connor, K.M., Davidson, J.R., 2003. Development of a new resilience scale: the Connor-Davidson Resilience Scale (CD-RISC). Depress Anxiety 18 (2), 76–82.

Fauteux, N., 2021. COVID-19: impact on nurses and nursing. Am. J. Nurs. 121 (5), 19–21.

Ferry, A.V., Wereski, R., Strachan, F.E., Mills, N.L., 2021. Predictors of UK healthcare worker burnout during the COVID-19 pandemic. QJM. 114 (6), 374–380.

Galanis, P., Vraka, I., Fragkou, D., Bilali, A., Kaitelidou, D., 2021. Nurses' burnout and associated risk factors during the COVID-19 pandemic: a systematic review and meta-analysis. J. Adv. Nurs. 77 (8), 3286–3302.

Galli, F., Pozzi, G., Ruggiero, F., Mameli, F., Cavicchioli, M., Barbieri, S., Canevini, M.P., Priori, A., Pravettoni, G., Sani, G., Ferrucci, R., 2020. A systematic review and provisional metanalysis on psychopathologic burden on health care workers of coronavirus outbreaks. Front. Psychiatry 11, 568664.

Guilamo-Ramos, V., Thimm-Kaiser, M., Benzekri, A., Hidalgo, A., Lanier, Y., Tlou, S., de Lourdes Rosas Lopez, M., Soletti, A.B., Hagan, H., 2021. Nurses at the frontline of public health emergency preparedness and response: lessons learned from the HIV/AIDS pandemic and emerging infectious disease outbreaks. Lancet Infect Dis. 21 (10), e326–e333.

He, X.Y., Li, C.B., Qian, J., Cui, H.S., Wu, W.Y., 2010. Reliability and validity of a generalized anxiety scale in general hospital outpatients. Shanghai Arch. Psychiatry 22 (4), 200–203.

Hyer, K., Brown, L.M., 2008. The impact of event scale–revised: a quick measure of a patient's response to trauma. Am. J. Nurs. 108 (11), 60–68 quiz 68-69.

Kroenke, K., Spitzer, R.L., Williams, J.B., 2001. The PHQ-9: validity of a brief depression severity measure. J. Gen. Intern. Med. 16 (9), 606–613.

Lai, J., Ma, S., Wang, Y., Cai, Z., Hu, J., Wei, N., Wu, J., Du, H., Chen, T., Li, R., Tan, H., Kang, L., Yao, L., Huang, M., Wang, H., Wang, G., Liu, Z., Hu, S., 2020. Factors associated with mental health outcomes among health care workers exposed to coronavirus disease 2019. JAMA Netw. Open 3 (3), e203976.

Liang, M., Chen, Q., Li, Y., Fan, X., Wang, Q., Wang, J., He, L., Li, X., Tao, H., Xie, L., Nie, H., 2021. Status quo and influencing factors for anxiety, depression, and insomnia among 4 237 nurses in Hunan Province. Zhong Nan Da Xue Xue Bao Yi Xue Ban 46 (8), 822–830.

McAlonan, G.M., Lee, A.M., Cheung, V., Cheung, C., Tsang, K.W., Sham, P.C., Chua, S.E., Wong, J.G., 2007. Immediate and sustained psychological impact of an emerging infectious disease outbreak on health care workers. Can. J. Psychiatry 52 (4), 241–247.

Pollock, A., Campbell, P., Cheyne, J., Cowie, J., Davis, B., McCallum, J., McGill, K., Elders, A., Hagen, S., McClurg, D., Torrens, C., Maxwell, M., 2020. Interventions to support the resilience and mental health of frontline health and social care professionals during and after a disease outbreak, epidemic or pandemic: a mixed methods systematic review. Cochr. Database Syst. Rev. 11, CD013779.

Reynolds, D.L., Garay, J.R., Deamond, S.L., Moran, M.K., Gold, W., Styra, R., 2008. Understanding, compliance and psychological impact of the SARS quarantine experience. Epidemiol. Infect. 136 (7), 997–1007.

Spitzer, R.L., Kroenke, K., Williams, J.B., Lowe, B., 2006. A brief measure for assessing generalized anxiety disorder: the GAD-7. Arch. Intern. Med. 166 (10), 1092–1097.

Stelnicki, A.M., Carleton, R.N., Reichert, C., 2020. Nurses' mental health and well-being: COVID-19 impacts. Can. J. Nurs. Res. 52 (3), 237–239.

Varghese, A., George, G., Kondaguli, S.V., Naser, A.Y., Khakha, D.C., Chatterji, R., 2021. Decline in the mental health of nurses across the globe during COVID-19: a systematic review and meta-analysis. J. Glob. Health 11, 05009.

Wu, K.K., Chan, K.S., 2003. The development of the Chinese version of Impact of Event Scale–Revised (CIES-R). Soc. Psychiatry Psychiatr. Epidemiol. 38 (2), 94–98.

Xie, N., Qin, Y., Wang, T., Zeng, Y., Deng, X., Guan, L., 2020. Prevalence of depressive symptoms among nurses in China: a systematic review and meta-analysis. PLoS One 15 (7), e0235448.

Yang, B.X., Xia, L., Huang, R., Chen, P., Luo, D., Liu, Q., Kang, L.J., Zhang, Z.J., Liu, Z., Yu, S., Li, X., Wang, X.Q., 2021. Relationship between eHealth literacy and psychological status during COVID-19 pandemic: a survey of Chinese residents. J. Nurs. Manag. 29 (4), 805–812.

Yu, D.S., 2010. Insomnia Severity Index: psychometric properties with Chinese community-dwelling older people. J. Adv. Nurs. 66 (10), 2350–2359.

Zhang, C., Yang, L., Liu, S., Ma, S., Wang, Y., Cai, Z., Du, H., Li, R., Kang, L., Su, M., Zhang, J., Liu, Z., Zhang, B., 2020. Survey of insomnia and related social psychological factors among medical staff involved in the 2019 Novel coronavirus disease outbreak. Front. Psychiatry 11, 306.

Zhang, X., Jiang, X., Ni, P., Li, H., Li, C., Zhou, Q., Ou, Z., Guo, Y., Cao, J., 2021. Association between resilience and burnout of front-line nurses at the peak of the COVID-19 pandemic: positive and negative affect as mediators in Wuhan. Int. J. Ment. Health Nurs. 30 (4), 939–954.

Zhang, Y.L., Liang, W., Chen, Z.M., Zhang, H.M., Zhang, J.H., Weng, X.Q., Yang, S.C., Zhang, L., Shen, L.J., Zhang, Y.L., 2013. Validity and reliability of patient health questionnaire-9 and patient health questionnaire-2 to screen for depression among college students in China. Asia Pac. Psychiatry 5 (4), 268–275.
