# Supplementary material for: Mental health status and related factors influencing healthcare workers during the COVID-19 pandemic: A systematic review and meta-analysis
Source: PLoS One. 2024 Jan 19;19(1):e0289454. doi: 10.1371/journal.pone.0289454 (PMC10798549; doi:10.1371/journal.pone.0289454)
Supplement: S1 Data — (ZIP) [file pone.0289454.s011.zip › literatures/232.pdf]

frontiers in Psychiatry

ORIGINAL RESEARCH
published: 21 January 2021
doi: 10.3389/fpsyt.2020.588008

Perceived Stress, Hope, and Health Outcomes Among Medical Staff in China During the COVID-19 Pandemic

Xin Zhang[1*†], *Rong Zou*[2†], *Xiaoxing Liao*[3], *Allan B. I. Bernardo*[4], *Hongfei Du*[5], *Zhechen Wang*[6], *Yu Cheng*[1,7*] and *Yulong He*[8*]

[1] Department of Medical Humanities, The Seventh Affiliated Hospital of Sun Yat-sen University, Shenzhen, China, [2] Hubei Key Laboratory of Sport Training and Monitoring, Department of Psychology, College of Health Science, Wuhan Sports University, Wuhan, China, [3] The Emergency and Disaster Rescue Medical Center, The Seventh Affiliated Hospital of Sun Yat-sen University, Shenzhen, China, [4] Psychology Department, De La Salle University, Manila, Philippines, [5] Institute of Advanced Studies in Humanities and Social Sciences, Beijing Normal University at Zhuhai, Zhuhai, China, [6] Department of Psychology, School of Social Development and Public Policy, Fudan University, Shanghai, China, [7] Department of Anthropology, School of Sociology and Anthropology, Sun Yat-sen University, Guangzhou, China, [8] Center for Digestive Disease, The Seventh Affiliated Hospital of Sun Yat-sen University, Shenzhen, China

OPEN ACCESS

Edited by:
Julian Chuk-ling Lai,
City University of Hong Kong,
Hong Kong

Reviewed by:
Lawrence T. Lam,
University of Technology
Sydney, Australia
Su Lu,
De Montfort University,
United Kingdom

***Correspondence:**
Xin Zhang
zhangx666@mail.sysu.edu.cn
Yu Cheng
chengyu@mail.sysu.edu.cn
Yulong He
heyulong@mail.sysu.edu.cn

† These authors have contributed equally to this work

Specialty section:
This article was submitted to Public Mental Health, a section of the journal Frontiers in Psychiatry

Received: 28 July 2020
Accepted: 24 December 2020
Published: 21 January 2021

Citation:
Zhang X, Zou R, Liao XX, Bernardo ABI, Du H, Wang Z, Cheng Y and He Y (2021) Perceived Stress, Hope, and Health Outcomes Among Medical Staff in China During the COVID-19 Pandemic. Front. Psychiatry 11:588008. doi: 10.3389/fpsyt.2020.588008

This study investigated the buffering role of hope between perceived stress and health outcomes among front-line medical staff treating patients with suspected COVID-19 infection in Shenzhen, China. In the cross-sectional study with online questionnaires, medical staff's perceived stress, anxiety, depression, sleep quality, and hope were measured by the 10-item Chinese Perceived Stress Scale, Hospital Anxiety and Depression Scale, the Pittsburgh Sleep Quality Index, and the Locus-of-Hope Scale, respectively. A total of 319 eligible front-line medical staff participated. The prevalence of anxiety (29.70%), depression (28.80%), poor sleep quality (38.90%) indicated that a considerable proportion of medical staff experienced mood and sleep disturbances during the COVID-19 pandemic. Internal locus-of-hope significantly moderated the effects of stress on anxiety, depression, and sleep quality. Moreover, external family locus-of-hope and external peer locus-of-hope significantly moderated the association between perceived stress and depression. The prevalence of symptoms indicates that both mental and physical health outcomes of front-line medical staff deserve more attention. Internal and external locus-of-hope functioned differently as protective factors for medical staffs' health and might be promising targets for intervention.

Keywords: perceived stress, locus-of-hope, anxiety, depression, sleep quality

INTRODUCTION

The Coronavirus Disease 2019 (COVID-19) has been spreading in many parts of the world since December 2019, including in some provinces of China. In January 2020, the government of Guangdong Province launched the level one response toward this major public health emergency. Shenzhen, as a city in Guangdong Province with a large number of migrant workers moving from other cities in China, responded rapidly and formulated emergency plans for epidemic control. Chinese central government further issued a number of documents calling for attention to the mental health of medical staff (1).

Our department of medical humanities had been providing on-site psychological support for front-line medical staff in a tertiary hospital in Shenzhen from the end of January to the end of March of 2020. This tertiary hospital is a designated hospital treating patients with suspected COVID-19 infection in Shenzhen. Once the patients waiting in the quarantine ward were further diagnosed as COVID-19 pneumonia, they would be immediately sent to the only one infectious disease hospital in Shenzhen. Front-line medical staff in this tertiary hospital have been exposed to multiple stress sources, such as the risk of contracting COVID-19, wearing protective equipment for continuously 4–6 h, increased workload, shift work together with social isolation during the rest period.

In interviews with the front-line medical staff in this tertiary hospital, anxiety, depression and poor sleep quality were three main themes reported by most of the staff. This is consistent with previous research that high prevalence rates of depression, anxiety, and poor sleep quality existed among front-line medical staff (2–6). A meta-analysis focusing on depression, anxiety, and insomnia among medical staff during the COVID-19 pandemics extracted thirteen studies, of which twelve were undertaken in China and one in Singapore (7). This study revealed that researchers utilized various measures in evaluating mood and sleep disturbances of medical staff fighting COVID-19 pandemic. A pooled prevalence of anxiety, depression, and insomnia was reported as 23.2% in 12 studies, 22.8% in 10 studies, and 38.9% across four studies, respectively. Based upon the findings of interviews and the COVID-19 related empirical literature, it could be concluded that anxiety, depression, and sleep quality are three common health indicators. Furthermore, we want to explore whether it is stress caused by COVID-19 that predicts depression, anxiety, and sleep.

Both front-line battle and quarantine are stressful life events for healthcare workers (8). Anxiety and depression often develop following stressful life events (9–11). Previous research shows that stressful situations at work contributes to anxiety and depression among hospital staff (12). Therefore, it is logical to speculate that perceived stress will be positively associated with anxiety and depression among the front-line medical staff in the context of COVID-19 pandemic.

Sleep problem has been identified as another health consequence of stress (13, 14). A longitudinal study reveals that reductions in perceived stress correlate significantly with improvements in sleep quality (15). During the COVID-19 pandemic, worldwide researchers focus on sleep quality as an important health indicator [e.g., (5, 16–18)]. Yet, these studies hardly directly tested the correlation between stress and sleep quality in the population of front-line medical staff. We aim to explore the relation between stress and sleep quality among the front-line medical staff. And we posit that perceived stress will predict medical staff's poor sleep quality.

More importantly, it is worth noting that there are also some medical staff who did not report poor sleep quality nor feelings of anxiety/depression. Individual differences in psychological strengths may explain the variability in how medical staff had been coping with the perceived stress and thereby influence their physical and mental health. Of the many psychological strengths, hope has often been researched in connection with levels of stress (19). In the present research, we examined one important psychological strength, hope (20–22) as a potential moderator of the association between perceived stress and health outcomes (i.e., anxiety, depression, and sleep quality) in front-line medical staff fighting against COVID-19.

Hope has long been considered as a critical trait of people confronting serious life events (23). Snyder's theory of hope has emerged as the most dominant paradigm for understanding individuals' hope (24, 25). According to Snyder (26), trait hope is an enduring cognitive-motivational and goal-oriented construct composed of two distinct yet related elements, that is agency and pathways. Agency refers to one's initiating and sustaining the motivation toward goal attainment, and pathways refer to one's sense of being able to make plans to achieve goals. Snyder's hope theory suggests that low hope persons yield more easily to stressors; whereas high hope persons view stressors as motivating challenges that enable them to achieve their goals (26).

However, scholars critically pointed out that a limitation of this theory is its individualistic origin (20). In collectivist cultures, agency may refer to the commitment and support of external agents; pathways to goal attainment may involve action of external agents (20, 27). Bernardo (20) proposed the locus-of-hope theory as an extension of Snyder' hope theory through integrating external locus-of-hope dimensions (i.e., family, peer, spiritual). External-family locus-of-hope refers to positive thoughts related to how goals can be achieved through the help of family. External-peers locus-of-hope pertains to thoughts that the degree to which friends or peers may operate as catalysts of goal attainment.

Higher levels of internal locus-of-hope was associated with less depression and anxiety (24, 28). Longitudinal studies also find statistically significant long term effect of internal locus-of-hope on future anxiety and depression (29). The protective effect of hope in attenuating the relationship between negative life events and depressive symptoms was attested to in an ethnically diverse sample of college students (30). Similar stress-buffering effects of internal locus-of-hope were demonstrated in adult patients (31). Internal locus-of-hope also reduced the effects of various adverse factors on anxiety and depression in adolescents (32), young adults (33), and adults (34). Consistent with conservation of resources theory of stress (35), these results show how hope functions like a resource and that the maintenance of this resource protects individuals for experiencing high levels of stress and its consequences; it is when hope is low, that individuals are driven to experiences the negative syndromes of stress. While there has been evidence for the role of internal locus-of-hope in reducing symptoms of anxiety and depression, there have not been studies inquiring into its relationship with physical symptoms like sleep quality.

There has also not been direct evidence of this stress-buffering effect related to external locus-of-hope, as external locus-of-hope is a relatively new construct. The evidence so far is that external locus-of-hope dimensions predict measures of coping (36, 37) and well-being in adolescents (38, 39), university students (40), and adults (41). One recent study found consistently negative associations between external locus-of-hope dimensions and

anxiety during the COVID-19 pandemic (42). But no prior research has investigated how external locus-of-hope moderates the relationship between perceived stress and mental health (i.e., depression, anxiety) or physical health (i.e., sleep quality), although there is some research on the buffering effect of external locus-of-hope on stressors and positive psychological outcome (43, 44). External locus-of-hope can also be considered a resource that protects individuals from stress and its psychological and physical consequence, but the direct evidence for the stress-buffering role of external locus-of-hope is not yet established.

In summary, this study examines whether hope serves as a protective moderator in the association between perceived stress and health outcomes (i.e., anxiety, depression, and sleep quality). Bernardo (20) posited that the internal and external dimensions are required for the full realization of hope under the context of collectivist cultures. Based upon previous work, the present research proposes that internal locus-of-hope might buffer the relationship between stress and health outcomes of front-line medical staff in China (see the hypotheses below). Moreover, the role of external locus-of-hope as a potential moderator is explored as well.

Hypothesis 1: Perceived stress will be positively associated with anxiety/depression.
Hypothesis 2: Perceived stress will be negatively associated with sleep quality.
Hypothesis 3: Internal locus-of-hope will moderate the relationship between perceived stress and anxiety/depression.
Hypothesis 4: Internal locus-of-hope will moderate the relationship between perceived stress and sleep quality.

MATERIALS AND METHODS

Participants and Procedure

Participants were 319 medical staff (age range: 22–54 years old, $M_{age} = 30.42$ years, $SD = 5.16$; 37.90% men) from a tertiary hospital designated treating suspected patients with COVID-19 in Shenzhen, China. These medical staff included 113 doctors (35.40%), 57 medical technicians (17.90%; i.e., pharmacist, radiation technician, and clinical laboratory examiner), and 149 nurses (46.70%). They all had college degree or above, and their working years ranged from 0.5 to 31 years ($M_{workingyears} = 6.66$ years, $SD = 5.40$). All participants provided informed consent before completing the measures.

Participants were asked to complete the online questionnaires during their spare time. In the introduction of survey, they were told that they were engaging in a psychological investigation in which there were no correct or incorrect answers. Data collection was from mid-February to late March 2020, the most serious period of the COVID-19 in China. The participants must be the medical staff who worked in the quarantine ward. Administration staff and medical staff who continued working in their own wards were excluded from this study. The survey was distributed via the hospital's online communication platform (i.e., Enterprise Wechat). In total, 385 front-line medical staff were approached, and the response rate was 83.5%. As there were emergencies during the pandemic period, some medical staff's rest time was irregular, and they reported that it was impossible to estimate their sleep time. In such cases, sleep time was encoded as missing data. There was <0.1% missing data and the missing data were estimated with regression procedure in SPSS. The research procedures were approved by the Sun Yat-sen University ethics committee (Approval Number: I0RG0003827).

Measures

Perceived Stress

Perceived stress is measured using the Chinese version (45) of the 10-item Perceived Stress Scale (CPSS) (46). Items are rated from 0 (*never have*) to 4 (*have a lot*). To reflect the perceived stress triggered by the pandemic, each item emphasizes that all the responses are based on the feelings since the outbreak of COVID-19 (e.g., "Since the COVID-19 has occurred, how often have you been upset because of something that happened unexpectedly?") Scale scores were the sum of items with reverse coding of relevant items. Higher scores reflected a higher perceived stress brought by the pandemic (Cronbach $\alpha = 0.75$).

Locus-of-Hope

Locus-of-Hope Scale [LOHS; example items are "My parents have lots of ways of helping me attain my goals" and "I have been able to meet my goals because of my friends' help," (20)] was used to measure the trait hope of medical staff. Three of its four sub-scales were used for the current study: internal, external-family, and external-peer LOH. The external-spiritual LOH was not included as a majority of the population in China have no religious affiliation. Each sub-scale comprises eight items, with a four-point Likert-type scale ranging from 1 (*definitely false*) to 4 (*definitely true*) for scoring each item. The Chinese version has been validated previously (27, 47). For the present study, the Cronbach α were 0.90 for internal LOH, 0.91 for external-family LOH, and 0.90 for external-peer LOH.

Anxiety and Depression

Hospital Anxiety and Depression Scale [HADS; (48)] was used to measure anxiety and depression. This scale includes 14 items making up two 7-item sub-scales, one measuring anxiety (HADS-A) and the other depression (HADS-D). Items are rated from 0 (*not a problem*) to 3 (*high level of problems*). A higher total score ranging from 0 to 21 of each sub-scale represents higher levels of anxiety and depression. A score of 7 or lower indicates no signs of anxiety or depression, 8–10 a borderline case of anxiety or depression, 11 or higher a definite case of anxiety or depression (49). The Chinese version of HADS has been validated (50, 51). Cronbach α in this sample were 0.81 for anxiety and 0.80 for depression.

Sleep Quality

Sleep quality during the latest 1 month was assessed by the Chinese version of Pittsburgh Sleep Quality Index (PSQI) (52, 53). It includes 19 items, which are combined into seven clinically-derived component scores-subjective sleep quality, sleep latency, sleep duration, habitual sleep efficiency, sleep disturbance, sleep medication and daytime dysfunction. The score of each component ranges from 0 (*no difficulty*) to 3 (*severe*

TABLE 1 | Univariate and bivariate statistics for all study variables ($N = 319$).

Variables	*M* (*SD*)	1	2	3	4	5	6	7	8	9
1 Sex	0.62 (0.49)									
2 Age	30.42 (5.16)									
3 Work years	6.66 (5.40)	−0.03	0.90***							
4 Stress	15.35 (5.40)	0.17**	−0.04	0.02						
5 INT	24.54 (3.53)	−0.07	−0.01	0.00	−0.50***					
6 EXF	23.42 (4.28)	−0.03	0.00	0.02	−0.33***	0.69***				
7 EXP	22.88 (3.88)	−0.08	−0.03	−0.01	−0.24***	0.65***	0.74***			
8 Anxiety	5.62 (3.63)	0.06	0.04	0.04	0.66***	−0.46***	−0.31***	−0.29***		
9 Depression	5.08 (3.77)	0.08	0.08	0.09	0.66***	−0.49***	−0.30***	−0.28***	0.76***	
10 Sleep	6.66 (3.49)	0.12*	−0.01	0.03	0.49***	−0.37***	−0.19***	−0.15**	0.54***	0.54***

*Sex was dummy coded such that 0 = male, 1 = female. INT, internal locus-of-hope; EXF, external-family locus-of-hope; EXP, external-peer locus-of-hope; Sleep, sleep quality. *p < 0.05, **p < 0.01, ***p < 0.001.*

difficulty). A total score is produced by summing the seven component scores, with a higher score indicating worse sleep quality. Previous research [e.g., (6)] has suggested a cut-off of the total score at 8 or above for the signs of poor sleep quality. Cronbach α in current study was 0.76.

Demographic Variables

In addition to the above research instruments, participants completed a questionnaire soliciting information about sex, age, and work years.

Data Analysis

We hypothesized that locus-of-hope would moderate the associations between stress and health (i.e., anxiety, depression, sleep quality). To test the moderation hypotheses, we used the PROCESS macro for SPSS [Model 1; (54)]. PROCESS calculates bias-corrected and accelerated bootstrapped confidence intervals (10,000 re-samples) for the size of each direct or conditional effect, with a significant effect indicated by a confidence interval that does not contain zero. To yield standardized coefficients, all variables (excluding sex) were converted to z-scores prior to analysis.

RESULTS

Preliminary Analyses

The prevalence of anxiety (HADS-A score ≥8) was 29.70%, and depression (HADS-D score ≥8) was 28.80%; 38.90% had poor sleep quality (PSQI score≥8). **Table 1** presents the descriptive statistics for all variables in this study. Perceived stress was negatively correlated with each dimension of locus-of-hope, and positively correlated with health outcomes (i.e., anxiety, depression, and sleep quality) of medical staff. Dimensions of locus-of-hope were negatively correlated with anxiety, depression and sleep quality. There were no significant differences in perceived stress, each dimension of locus-of-hope and health outcomes ($Fs = 0.03 \sim 1.55$, $ps > 0.05$) among the three types of medical staff (doctors, medical technicians, nurses). Among the demographic variables, only sex was significantly related to stress ($M_{\text{male}} = 14.18$, $SD = 5.47$; $M_{\text{female}} = 16.06$, $SD = 5.23$; $t = -3.05$, $p < 0.01$) and sleep quality ($M_{\text{male}} = 6.15$, $SD = 3.23$; $M_{\text{female}} = 6.98$, $SD = 3.61$; $t = -2.06$, $p < 0.05$), so sex was included as control variable in subsequent analyses.

Test of Moderation Model

For the present purposes, moderation was established if the interaction effect of stress and locus-of-hope existed (54). Following the principles of selecting control variables (55) when testing the interaction effect of stress and each locus-of-hope dimension, the other two dimensions were included as covariates due to the significant associations between each dimension of hope and health outcomes of the medical staff. As **Table 2** shows, only internal locus-of-hope moderated the association between perceived stress and anxiety. Perceived stress was positively associated with anxiety among medical staff with different levels of internal locus-of-hope. But simple effects analysis showed that for medical staff with low internal locus-of-hope, this positive relationship was stronger as indicated by the higher beta ($B_{\text{simple}} = 0.50$, $t = 11.62$, $p < 0.001$), compared to medical staff with high internal locus-of-hope, where the beta ($B_{\text{simple}} = 0.33$, $t = 8.56$, $p < 0.001$) was still positive but smaller. The comparison of the relationship between perceived stress and anxiety for low and high internal locus-of-hope medical staff is shown in **Figure 1A**.

Internal locus-of-hope, external-family locus-of-hope, and external-peer locus-of-hope moderated the association between perceived stress and depression. Perceived stress was positively associated with depression among medical staff with different levels of internal and external locus-of-hope. But simple effects analysis showed that for medical staff with low internal locus-of-hope, this positive relationship was stronger as indicated by the high beta ($B_{\text{simple}} = 0.54$, $t = 12.35$, $p < 0.001$), while for those with high internal locus-of-hope, the beta was still positive but weaker ($B_{\text{simple}} = 0.28$, $t = 7.43$, $p < 0.001$). The comparison of the relationship between perceived stress and depression for low and high internal locus-of-hope medical staff is shown in **Figure 1B**. For medical staff with low external-family locus-of-hope, this positive relationship was stronger as indicated by the higher beta ($B_{\text{simple}} = 0.48$, $t = 11.00$, $p < 0.001$), while for those with high external-family locus-of-hope,

TABLE 2 | Testing the moderation models of stress on health outcomes ($N = 319$).

Outcomes	Predictors	R^2	F	β	T	95% *CI*
Anxiety	Sex			−0.08	−0.93	[−0.25, 0.09]
	EXF			0.06	0.98	[−0.07, 0.20]
	EXP			−0.10	−1.53	[−0.23, 0.03]
	Stress			0.61	12.71***	[0.52, 0.71]
	INT			−0.15	−2.38*	[−0.28, −0.03]
	Stress × INT	0.49	49.01***	−0.12	−3.32**	**[−0.20, −0.05]**
	Sex			−0.08	−0.88	[−0.25, 0.10]
	INT			−0.14	−2.16*	[−0.27, −0.01]
	EXP			−0.07	−1.11	[−0.20, 0.06]
	Stress			0.60	12.33***	[0.50, 0.69]
	EXF			0.03	0.43	[−0.10, 0.16]
	Stress × EXF	0.47	46.40***	−0.06	−1.63	[−0.14, 0.01]
	Sex			−0.08	−0.88	[−0.25, 0.10]
	INT			−0.15	−2.31*	[−0.28, −0.02]
	EXF			0.06	0.90	[−0.07, 0.19]
	Stress			0.60	12.32***	[0.50, 0.69]
	EXP			−0.10	−1.47	[−0.23, 0.03]
	Stress × EXP	0.47	46.38***	−0.06	−1.61	[−0.13, 0.01]
Depression	Sex			−0.04	−0.45	[−0.20, 0.13]
	EXF			0.09	1.42	[−0.04, 0.22]
	EXP			−0.08	−1.23	[−0.20, 0.05]
	Stress			0.59	12.40***	[0.49, 0.68]
	INT			−0.23	−3.66***	[−0.36, −0.11]
	Stress × INT	0.50	52.90***	−0.18	−4.89***	**[−0.25, −0.11]**
	Sex			−0.03	−0.34	[−0.20, 0.14]
	INT			−0.21	−3.23**	[−0.34, −0.08]
	EXP			−0.04	−0.60	[−0.17, 0.09]
	Stress			0.57	11.83***	[0.48, 0.66]
	EXF			0.04	0.54	[−0.10, 0.17]
	Stress × EXF	0.46	56.74***	−0.11	−2.94**	**[−0.18, −0.04]**
	Sex			−0.04	−0.42	[−0.21, 0.14]
	INT			−0.23	−3.50***	[−0.36, −0.10]
	EXF			0.08	1.24	[−0.05, 0.22]
	Stress			0.57	11.65***	[0.47, 0.66]
	EXP			−0.07	−1.09	[−0.21, 0.06]
	Stress × EXP	0.45	58.10***	−0.08	−2.05*	**[−0.15, −0.003]**
Sleep	Sex			0.07	0.65	[−0.13, 0.27]
	EXF			0.11	1.41	[−0.04, 0.27]
	EXP			0.02	0.32	[−0.13, 0.18]
	Stress			0.41	7.27***	[0.30, 0.52]
	INT			−0.27	−3.54***	[−0.42, −0.12]
	Stress × INT	0.29	20.78***	−0.10	−2.23*	**[−0.19, −0.01]**
	Sex			0.06	0.55	[−0.15, 0.26]
	INT			−0.27	−3.50***	[−0.42, −0.12]
	EXP			0.05	0.59	[−0.10, 0.20]
	Stress			0.40	6.94***	[0.28, 0.51]
	EXF			0.10	1.21	[−0.06, 0.25]
	Stress × EXF	0.27	19.64***	0.004	0.08	[−0.08, 0.09]
	Sex			0.07	0.71	[−0.13, 0.27]
	INT			−0.27	−3.48***	[−0.42, −0.12]
	EXF			0.11	1.42	[−0.04, 0.27]
	Stress			0.40	7.13***	[0.29, 0.52]
	EXP			0.02	0.20	[−0.14, 0.17]
	Stress × EXP	0.25	27.38***	−0.07	−1.54	[−0.15, 0.02]

*CI, bootstrapped confidence interval; INT, internal locus-of-hope; EXF, external-family locus-of-hope; EXP, external-peer locus-of-hope; Sleep, sleep quality; Stress × INT/EXF/EXP, interactions of stress and INT/EXF/EXP. The bolded 95% CI indicated significant interaction effects. *p < 0.05, **p < 0.01, ***p < 0.001.*

the beta was still positive but weaker ($B_{\text{simple}} = 0.32$, $t = 8.31$, $p < 0.001$). The comparison of the relationship between perceived stress and depression for low and high external family locus-of-hope medical staff is shown in **Figure 1C**. For medical staff with low external-peer locus-of-hope, this positive relationship was stronger as indicated by the higher beta ($B_{\text{simple}} = 0.45$, $t = 10.40$, $p < 0.001$), while for those with high external-peer locus-of-hope, the beta was still positive but weaker ($B_{\text{simple}} = 0.34$, $t = 8.76$, $p < 0.001$). The comparison of the relationship between perceived stress and depression for low and high external peer locus-of-hope medical staff is shown in **Figure 1D**.

Only internal locus-of-hope moderated the association between perceived stress and sleep quality. Perceived stress was positively associated with sleep quality among medical staff with different levels of internal locus-of-hope. But simple effects analysis showed that for medical staff with low internal locus-of-hope, this positive relationship was stronger as indicated by the higher beta ($B_{\text{simple}} = 0.33$, $t = 7.21$, $p < 0.001$), compared to medical staff with high internal locus-of-hope, where the beta ($B_{\text{simple}} = 0.20$, $t = 5.16$, $p < 0.001$) was still positive but smaller. The comparison of the relationship between perceived stress and sleep quality for low and high internal locus-of-hope medical staff is shown in **Figure 1E**.

DISCUSSION

This was the first study to directly investigate the relationship between perceived stress and health outcomes (i.e., anxiety, depression, and sleep quality) among front-line medical staff from the perspective of positive psychology during the outbreak of COVID-19 in China. The prevalence of anxiety (29.70%), depression (28.80%), poor sleep quality (38.9%) is high and similar to the result of a meta-analysis study focusing on front-line medical staff during COVID-19 pandemic, that is 23.2% for anxiety, 22.8% for depression, and 38.9% for insomnia (7). Furthermore, the perceived stress was significantly associated with anxiety, depression, and sleep quality. The deleterious effects of stress on anxiety, depression, and sleep quality have been documented by abundant research [e.g., (9, 11, 12, 56)].

Recently published research has focused on the psychological impacts of COVID-19 on medical staff yet ignoring one's personal agency in improving one's own psychological well-being [e.g., (2, 3, 6)]. Our research paid attention to the protective role of hope in both mental and physical health of front-line medical staff during

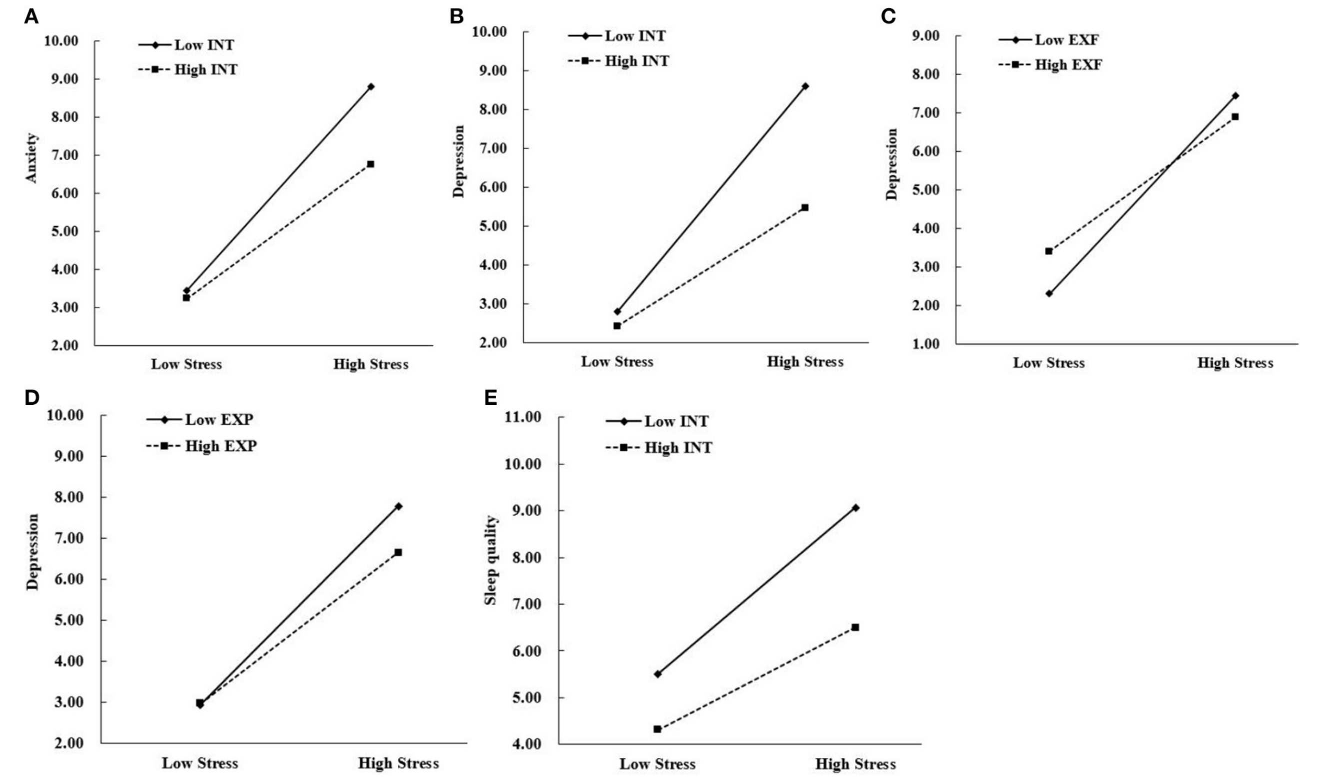

FIGURE 1 | (A,B,E) Illustrate internal locus-of-hope as a moderator of relationships between stress and anxiety, depression, and sleep quality, respectively. **(C,D)** Illustrate external family locus-of-hope and external peer locus-of-hope, respectively, as moderator of the relationship between stress and depression. INT, internal locus-of-hope; EXF, external-family locus-of-hope; EXP, external-peer locus-of-hope.

the COVID-19 pandemic. In line with our assumptions and also consistent with past research, internal locus-of-hope was shown to buffer the effect of perceived stress on anxiety and depression (24, 28–30). Furthermore, internal locus-of-hope moderated the relationship between perceived stress and sleep quality.

Maintaining hope during times of stress may promote medical staff to perceive such stressful events as challenges to be addressed or goals to be attained. Ultimately, this may reduce anxiety, depression, and improve sleep quality. Due to the nature of public health emergency, front-line medical staff had to work and rest in the isolated environment. Working in the closed medical ward, wearing protective equipment, and self-isolating in the designated hotel resulted in medical staff's being alone for most time. Under this specific circumstance, medical staff have to rely more on themselves than on external agents to alleviate perceived stress. Medical staff may also be accustomed to being self-reliant (e.g., relying on his or her own medical knowledge) instead of relying on their family members or friends to achieve medical goals as decreasing the risk of getting infected.

Moreover, external locus-of-hope (i.e., family and peer) buffered the effect of perceived stress on depression, but not anxiety. One possible explanation might be that hopelessness constitutes a major part of depression (57), thus both internal and external locus-of-hope significantly buffered the effect of perceived stress on depression. Anxiety represents anticipatory concerns regarding the negative outcome of a stressful event (58). In this study, medical staff's anxiety may be mainly manifested as worries regarding the risk of infection when treating patients with suspected COVID-19 pneumonia. Social support had been proven to buffer the effect of perceived stress on anxiety [e.g., (59, 60)]. Yet, medical staff are likely to be away from family during this period, which could mitigate the possible role of family as sources of hope; similarly, as one's medical staff peers are also under stress, they may not be potent hope sources, too. As such, internal locus-of-hope, that is relying on oneself (e.g., medical knowledge and clinical practice) rather than relying on the support of external agents (i.e., family and peer) may better mitigate the anxiety response to risk infection as one source of perceived stress. Further research is needed to explore the reason external locus-of-hope functions differently from internal locus-of-hope in its moderation role between perceived stress and various health outcomes.

This study contributes to our knowledge of hope by confirming its moderation role between perceived stress and health outcomes. In particular, the study reveals the difference between internal and external locus-of-hope in moderating the

relationships between perceived stress and health outcomes. This study also has implications for interventions: medical staff who experienced mood and sleep disturbances may benefit from hope-focused preventive interventions. The interventions would be to help foster in medical staff both internal locus-of-hope and external locus-of-hope. A single-session 90 min hope intervention (61), could be applicable to the front-line medical staff without occupying too much of their rest time. Medical staff could benefit from this short-term hope intervention by reconsidering their personal goals, potential obstacles and alternative pathways for goal attainment in the workplace. Our department should also encourage medical staff to call their family members and interact with their peers online as routine tasks after work. Acquiring support from peers or family members in achieving goals could prevent medical staff from developing depressive mood. Future research should examine how best to foster both external and internal locus-of-hope in the population of front-line medical staff.

Our findings should be interpreted in light of several limitations. First, the present study was exploratory and employed a cross-sectional design, which prohibited causal conclusions. Prospective research is necessary to determine the causal interrelationships between the variables in our study. Second, all medical staff were from one tertiary hospital, so caution should be exercised when generalizing our results to medical staff in other regions of China. Third, as we used self-report measures for all model variables, a common-method bias might exist which may impact validity. Multiple data collecting methods should be used in further research to improve internal validity.

DATA AVAILABILITY STATEMENT

The raw data supporting the conclusions of this article will be made available by the authors, without undue reservation.

ETHICS STATEMENT

The studies involving human participants were reviewed and approved by Sun Yat-sen University ethics committee (Approval Number: I0RG0003827). The patients/participants provided their written informed consent to participate in this study.

AUTHOR CONTRIBUTIONS

XZ made all the contacts with the participants and distributed the questionnaires via the hospital's online communication platform. RZ led the analytic process and analyzed the results. XZ, RZ, XL, AB, HD, YC, and YH contributed to the study design. ZW verified the findings of the analysis. All authors contributed to writing the paper.

FUNDING

This research was funded by China Post-doctoral Science Foundation (2020M672916) and Hubei Provincial Department of Education (B2018219). Research Start-up Fund of Post-doctoral of SAHSYSU (ZSQYRSFPD0004).

ACKNOWLEDGMENTS

The authors thank all participants who completed the survey despite their heavy workload.

REFERENCES

1. Chen Q, Liang M, Li Y, Guo J, Fei D, Wang L, et al. Mental health care for medical staff in China during the COVID-19 outbreak. *Lancet Psychiatry*. (2020) 7:e15–6. doi: 10.1016/S2215-0366(20)30078-X
2. Lai J, Ma S, Wang Y, Cai Z, Hu J, Wei N, et al. Factors associated with mental health outcomes among health care workers exposed to coronavirus disease 2019. *JAMA Network Open*. (2020) 3:e203976. doi: 10.1001/jamanetworkopen.2020.3976
3. Liu S, Yang L, Zhang C, Xiang YT, Liu Z, Hu S, et al. Online mental health services in China during the COVID-19 outbreak. *Lancet Psychiatry*. (2020) 7:e17–8. doi: 10.1016/S2215-0366(20)30077-8
4. Lu W, Wang H, Lin Y, Li L. Psychological status of medical workforce during the COVID-19 pandemic: a cross-sectional study. *Psychiatry Res*. (2020) 288:112936. doi: 10.1016/j.psychres.2020.112936
5. Xiao H, Zhang Y, Kong D, Li S, Yang N. The effects of social support on sleep quality of medical staff treating patients with coronavirus disease 2019 (COVID-19) in January and February 2020 in China. *Med Sci Monitor*. (2020) 26:e923549. doi: 10.12659/MSM.923921
6. Zhou YF, Zhou Y, Song Y, Ren L, Ng CH, Xiang YT, et al. Tackling the mental health burden of front-line healthcare staff in the COVID-19 pandemic: China's experiences. *Psychol Med*. (2020). doi: 10.1017/S0033291720001622
7. Pappa S, Ntella V, Giannakas T, Giannakoulis VG, Papoutsi E, Katsaounou P. Prevalence of depression, anxiety, and insomnia among healthcare workers during the COVID-19 pandemic: a systematic review and meta-analysis. *Brain Behav Immun*. (2020) 88:901–7. doi: 10.2139/ssrn.3594632
8. Gómez-Durán EL, Martin-Fumadó C, Forero CG. Psychological impact of quarantine on healthcare workers. *J Occup Environ Med*. (2020) 77:666–74. doi: 10.1136/oemed-2020-106587
9. Kessler RC. The effects of stressful life events on depression. *Annu Rev Psychol*. (1997) 48:191–214. doi: 10.1146/annurev.psych.48.1.191
10. Schneiderman N, Ironson G, Siegel SD. Stress and health: psychological, behavioral, and biological determinants. *Annu Rev Clin Psychol*. (2005) 1:607–28. doi: 10.1146/annurev.clinpsy.1.102803.144141
11. Tennant C. Life events, stress, and depression: a review of recent findings. *Aust. N. Z. J. Psychiatry*. (2002) 36:173–82. doi: 10.1046/j.1440-1614.2002.01007.x
12. Weinberg A, Creed F. Stress and psychiatric disorder in healthcare professionals and hospital staff. *Lancet*. (2000) 355:533–7. doi: 10.1016/S0140-6736(99)07366-3
13. Harvey AG, Jones C, Schmidt DA. Sleep and posttraumatic stress disorder: a review. *Clin Psychol Rev*. (2003) 23:377–407. doi: 10.1016/S0272-7358(03)00032-1
14. Stachele T, Domes G, Wekenborg M, Penz M, Kirschbaum C, Heinrichs M. Effects of a 6-week internet-based stress management program on perceived stress, subjective coping skills, and sleep quality. *Front Psychiatry*. (2020) 11:463. doi: 10.3389/fpsyt.2020.00463
15. Eliasson AH, Kashani M, Mayhew M, Assumpta U, Jacqueline H, Marina V. Reducing perceived stress improves sleep quality: a longitudinal outcomes study. *Chest*. (2010) 138:913A. doi: 10.1378/chest.10417
16. Grey I, Arora T, Thomas J, Saneh A, Tohme P, Abi-Habib R. The role of perceived social support on depression and sleep during the

COVID-19 pandemic. *Psychiat Res*. (2020) 293:1–6. doi: 10.1016/j.psychres.2020.113452
17. Kutana S, Lau PH. The impact of the 2019 coronavirus disease (COVID-19) pandemic on sleep health. *Can Psychol*. (2020). doi: 10.1037/cap0000256
18. Zhao X, Lan M, Li H, Yang J. Perceived stress and sleep quality during the 2019 coronavirus disease: a moderated mediation model. *Sleep Med*. (2020). doi: 10.1016/j.sleep.2020.05.021
19. O'Sullivan G. The relationship between hope, eustress, self-efficacy, and life satisfaction among undergraduates. *Soc Indic Res*. (2011) 101:155–72. doi: 10.1007/s11205-010-9662-z
20. Bernardo ABI. Extending hope theory: internal and external locus of trait hope. *Pers Indiv Differ*. (2010) 49:944–9. doi: 10.1016/j.paid.2010.07.036
21. Seligman MEP, Csikszentmihalyi M. Positive psychology: an introduction. *Am Psychol*. (2000) 55:5–14. doi: 10.1037/0003-066X.55.1.5
22. Snyder CR, Feldman DB, Taylor JD, Schroeder LL, Adams VH. The roles of hopeful thinking in preventing problems and enhancing strengths. *Appl Prev Psychol*. (2000) 9:249–70. doi: 10.1016/S0962-1849(00)80003-7
23. Scioli A, MacNeil S, Partridge V, Tinker E, Hawkins E. Hope, HIV, and health: a prospective study. *AIDS Care*. (2012) 24:149–56. doi: 10.1080/09540121.2011.597943
24. Berendes D, Keefe FJ, Somers TJ, Kothadia SM, Porter LS, Cheaveans JS. Hope in the context of lung cancer: relationship of hope to symptoms and psychological distress. *J Pain Symptom Manag*. (2010) 40:174–82. doi: 10.1016/j.jpainsymman.2010.01.014
25. Snyder CR. The past and possible futures of hope. *J Soc Clin Psychol*. (2000) 19:11–28. doi: 10.1521/jscp.2000.19.1.11
26. Snyder CR. Hope theory: rainbows in the mind. *Psychol Inq*. (2002) 13:249–75. doi: 10.1207/S15327965PLI1304_01
27. Du H, King RB. Placing hope in self and others: Exploring the relationships among self-construals, locus-of-hope and adjustment. *Pers Indiv Differ*. (2013) 54:332–7. doi: 10.1016/j.paid.2012.09.015
28. Peh CX, Liu J, Bishop GD, Chan HY, Chua SM, Kua EH, et al. Emotion regulation and emotional distress: the mediating role of hope on reappraisal and anxiety/depression in newly diagnosed cancer patients. *Psychooncology*. (2016) 26:1191–7. doi: 10.1002/pon.4297
29. Arnau R, Rosen D, Finch J, Rhudy J, Fortunato V. Longitudinal effects of hope on depression and anxiety: a latent variable analysis. *J Pers*. (2007) 75:43–63. doi: 10.1111/j.1467-6494.2006.00432.x
30. Visser PL, Loess P, Jeglic EL, Hirsch JK. Hope as moderator of negative life events and depressive symptoms in a diverse sample. *Stress Health*. (2013) 29:82–8. doi: 10.1002/smi.2433
31. Madan S, Pakenham KI. The stress-buffering effects of hope on adjustment to multiple sclerosis. *Int J Behav Med*. (2014) 21:877–90. doi: 10.1007/s12529-013-9384-0
32. Karababa A. The moderating role of hope in the relationship between maladaptive perfectionism and anxiety among early adolescents. *J. Genet. Psychol*. (2020) 181:159–70. doi: 10.1080/00221325.2020.1745745
33. Chang EC, Chang OD, Martos T, Sallay V, Zettler I, Steca P, et al. The positive role of hope on the relationship between loneliness and unhappy conditions in Hungarian young adults: how pathways thinking matters! *J Posit Psychol*. (2019) 14:724–33. doi: 10.1080/17439760.2018.1545042
34. Muyan M, Chang EC, Jilani Z, Yu T, Lin J, Hirsch JK. Loneliness and negative affective conditions in adults: is there any room for hope in predicting anxiety and depressive symptoms? *J Psychol*. (2016) 150:333–41. doi: 10.1080/00223980.2015.1039474
35. Hobfoll SE. Conservation of resources: a new attempt at conceptualizing stress. *Am Psychol*. (1989) 44:513–24. doi: 10.1037/0003-066X.44.3.513
36. Bernardo ABI, Wang TY, Pesigan IJA, Yeung SS. Pathways from collectivist coping to life satisfaction among Chinese: the role of locus-of-hope. *Pers Indiv Differ*. (2017) 106:253–6. doi: 10.1016/j.paid.2016.10.059
37. Bernardo ABI, Yeung SS, Resurreccion KF, Resurreccion RR, Khan A. External locus-of-hope, well-being, and coping of students: a cross-cultural investigation within Asia. *Psychol Schools*. (2018) 55:908–23. doi: 10.1002/pits.22155
38. Bernardo ABI. Hope in early adolescence: measuring internal and external locus-of-hope. *Child Indic Res*. (2015) 8:699–715. doi: 10.1007/s12187-014-9254-6
39. Munoz RT, Quinton KA, Worley JA, Hellman CM. Locus of hope: External hope in parents/guardians as an antecedent of adolescents' internal hope and life satisfaction. *Child Indic Res*. (2019) 12:1107–24. doi: 10.1007/s12187-018-9566-z
40. Du H, Bernardo ABI, Yeung SS. Locus-of-hope and life satisfaction: the mediating roles of personal self-esteem and relational self-esteem. *Pers Indiv Differ*. (2015) 83:228–33. doi: 10.1016/j.paid.2015.04.026
41. Bernardo ABI, Estrellado AF. Well-being of Filipino women who experienced intimate partner violence: a person-centered analysis. *Int J Adv Counsel*. (2017) 39:360–76. doi: 10.1007/s10447-017-9303-1
42. Bernardo ABI, Mendoza NB. Measuring hope during the COVID-19 outbreak in the Philippines: development and validation of the state Locus-of-Hope scale short form in Filipino. *Curr Psychol*. (2020). doi: 10.1007/s12144-020-00887-x
43. Bernardo ABI, Resurreccion KF. Financial stress and well-being of Filipino students: the moderating role of external locus-of-hope. *Philippine J Psychol*. (2018) 51:33–61. doi: 10.31710/pjp/0051.01.03
44. Datu JAD, Mateo NJ. How to combat the negative impact of discrimination in a collectivist context? The safeguarding function of peer-oriented hope. *Psychol Health Med*. (2017) 22:345–51. doi: 10.1080/13548506.2016.1164875
45. Wang Z, Chen J, Boyd JE, Zhang H, Jia X, Qiu J, et al. Psychometric properties of the Chinese version of the Perceived Stress Scale in policewomen. *PLoS ONE*. (2011) 6:e28610. doi: 10.1371/journal.pone.0028610
46. Cohen S, Kamarck T, Mermelstein R. A global measure of perceived stress. *J Health Soc Behav*. (1983) 24:385–96. doi: 10.2307/2136404
47. Bernardo ABI, Nalipay MJN. Social axioms as social foundations of locus-of-hope: a study in three Asian cultural groups. *Pers Indiv Differ*. (2016) 95:110–3. doi: 10.1016/j.paid.2016.02.046
48. Zigmond AS, Snaith RP. The hospital anxiety and depression scale. *Acta Psychiat Scand*. (1983) 67:361–70. doi: 10.1111/j.1600-0447.1983.tb09716.x
49. Arving C, Glimelius B, Brandberg Y. Four weeks of daily assessments of anxiety, depression and activity compared to a point assessment with the Hospital Anxiety and Depression Scale. *Qual Life Res*. (2008) 17:95–104. doi: 10.1007/s11136-007-9275-4
50. Li Q, Lin Y, Hu C, Xu Y, Zhou H, Yang L, et al. The Chinese version of hospital anxiety and depression scale: psychometric properties in Chinese cancer patients and their family caregivers. *Eur J Oncol Nurs*. (2016) 25:16–23. doi: 10.1016/j.ejon.2016.09.004
51. Wang H. Perceived stress, post-traumatic stress symptoms, anxiety and depression in medical staff after blood-induced occupational exposure. *Chinese J Health Psychol*. (2018) 26:1367–70. doi: 10.1093/ecam/neh127
52. Buysse DJ, Reynolds CF, Monk TH, Berman SR, Kupfer DJ. The Pittsburgh Sleep Quality Index-a new instrument for psychiatric practice and research. *Psychiatry Res*. (1989) 28:193–213. doi: 10.1016/0165-1781(89)90047-4
53. Liu XC, Tang MQ, Hu L, Wang AZ, Wu HX, Zhao GF, et al. Reliability and validity of the Pittsburgh sleep quality index. *Chinese J Psychiatry*. (1996) 29:103–7.
54. Hayes AF. *Introduction to Mediation, Moderation, and Conditional Process Analysis: A Regression-Based Approach*. New York, NY: Guilford Press (2013).
55. Bernerth JB, Aguinis H. A critical review and best-practice recommendations for control variable usage. *Pers Psychol*. (2016) 69:229–83. doi: 10.1111/peps.12103
56. Yan YW, Lin RM, Su YK. The relationship between adolescent academic stress and sleep quality: a multiple mediation model. *Soc Behav Pers*. (2018) 46:63–77. doi: 10.2224/sbp.6530
57. Abramson LY, Alloy LB, Metalsky GI. Hopelessness depression, a theory-based subtype of depression. *Psychol Rev*. (1989) 96:358–72. doi: 10.1037/0033-295X.96.2.358
58. Nezu AM, Nezu CM, Blissett SE. Sense of humor as a moderator of the relation between stressful events and psychological distress: a prospective analysis. *J Pers Soc Psychol*. (1988) 54:520–5. doi: 10.1037/0022-3514.54.3.520
59. Lau Y, Wong DFK, Wang Y, Kwong DHK, Wang Y.The roles of social support in helping Chinese women with antenatal depressive and anxiety

symptoms cope with perceived stress. *Arch Psychiat Nurs*. (2014) 28:305–13. doi: 10.1016/j.apnu.2014.05.009

60. Zhang H, Tang L, Ye Z, Zou P, Shao J, Wu M, et al. The role of social support and emotional exhaustion in the association between work-family conflict and anxiety symptoms among female medical staff: a moderated mediation model. *BMC Psychiatry*. (2020) 20:266. doi: 10.1186/s12888-020-02673-2
61. Feldman DB, Dreher DE. Can hope be changed in 90 minutes? Testing the efficacy of a single-session goal-pursuit intervention for college students. *J Happiness Stud*. (2012) 13:745–59. doi: 10.1007/s10902-011-9292-4

Conflict of Interest: The authors declare that the research was conducted in the absence of any commercial or financial relationships that could be construed as a potential conflict of interest.

Copyright © 2021 Zhang, Zou, Liao, Bernardo, Du, Wang, Cheng and He. This is an open-access article distributed under the terms of the Creative Commons Attribution License (CC BY). The use, distribution or reproduction in other forums is permitted, provided the original author(s) and the copyright owner(s) are credited and that the original publication in this journal is cited, in accordance with accepted academic practice. No use, distribution or reproduction is permitted which does not comply with these terms.
