# Supplementary material for: Mental health status and related factors influencing healthcare workers during the COVID-19 pandemic: A systematic review and meta-analysis
Source: PLoS One. 2024 Jan 19;19(1):e0289454. doi: 10.1371/journal.pone.0289454 (PMC10798549; doi:10.1371/journal.pone.0289454)
Supplement: S1 Data — (ZIP) [file pone.0289454.s011.zip › literatures/234.pdf]

Observational Study

Medicine®

OPEN

Mental health status among Chinese healthcare-associated infection control professionals during the outbreak of coronavirus disease 2019

A national cross-sectional survey

Ying Zhang, MSN[a,b], Lingyun Tian, MSN[c,d], Wan Li, BSN[c], Ximao Wen, BSN[a,b], Hongman Wu, BSN[a,b], Ruie Gong, BSN[a,b], Lanman Zeng, BSN[a,b], Feng Zhou, BSN[a,b], Zhenru Liu, BSN[a,b], Ziyuan Tang, BSN[a,b], Anhua Wu, PhD[b], Xun Huang, PhD[b,*]

Abstract

Recently, the coronavirus disease 2019 (COVID-19) epidemic has greatly threatened global public health. The responsibility of healthcare-associated infection control professionals (ICPs) is to prevent and control the nosocomial infections. The mental health status of ICPs deserves more attention, however, the correlational research is still lacking. This study aims to investigate the incidence and risk factors of mental health status among ICPs in China during the outbreak of COVID-19.

A national cross-sectional survey was performed. The online questionnaire was completed by 9228 ICPs from 3776 hospitals throughout China. Data collection tools were used, including demographics data questionnaire, the Chinese version of the 12-item general health questionnaire (GHQ-12) and the Chinese version of the psychological capital questionnaire (PCQ) for medical staff. Univariate and multivariable analyses were conducted.

The total score of mental health of Chinese ICPs was 3.45 ± 2.57. 5608 (60.77%) ICPs might have mental health problems. The psychological capital was in the upper-middle level with an average score of 3.72 ± 0.38. An increased mental health problem risk was associated with the greater self-efficacy and working in the public hospital; a significantly lower risk was obtained by working in the second-class hospital rather than in the third-class hospitals. Besides, mental health problem risk of ICPs working in hospitals of the western economic region or northeast economic region was more significant than that in hospitals of the central economic region. However, a lower risk was caused by the unmarried than married, and working years in department $\leq$1 year contributed to the lower risk than that >20 years. Moreover, fewer working hours per week, higher values of hope, and optimism each were contributed to a lower risk.

Chinese healthcare-associated ICPs were under different levels of mental health problems in fighting against COVID-19. More importantly, we should actively deal with the mental health problem of ICPs and help them get rid of psychological disorders.

Abbreviations: CI = confidence interval, COVID-19 = coronavirus disease 2019, GHQ-12 = the 12-item general health questionnaire, ICPs = infection control professionals, OR = odds ratio, PCQ = the psychological capital questionnaire, SARS-CoV-2 = severe acute respiratory syndrome coronavirus 2, SD = standard deviation.

Keywords: coronavirus disease 2019, infection control professionals, mental health, nosocomial infection control, psychological capital

Editor: Zeng-Jie Ye.

This work was supported by the Emergency Project of Prevention and Control for COVID-19 of Central South University [grant number 160260003] and the Surface Project of Natural Science Fund of Hunan province [grant number 2019JJ40403].

Competing interests: None declared.

The authors have no conflicts of interest to disclose.

The datasets generated during and/or analyzed during the current study are available from the corresponding author on reasonable request.

[a] Teaching and Research Section of Clinical Nursing, [b] Infection Control Center, Xiangya Hospital, Central South University, [c] Xiangya School of Nursing, Central South University, Changsha, [d] School of Nursing, Anhui University of Chinese Medicine, Hefei, China.

[*] Correspondence: Xun Huang, Infection Control Center, Xiangya Hospital, Central South University, 87 Xiangya Road, Kaifusi District, Changsha, Hunan 410008, China (e-mail: huangxun@csu.edu.cn).

Copyright © 2021 the Author(s). Published by Wolters Kluwer Health, Inc.
This is an open access article distributed under the terms of the Creative Commons Attribution-Non Commercial License 4.0 (CCBY-NC), where it is permissible to download, share, remix, transform, and buildup the work provided it is properly cited. The work cannot be used commercially without permission from the journal.

How to cite this article: Zhang Y, Tian L, Li W, Wen X, Wu H, Gong R, Zeng L, Zhou F, Liu Z, Tang Z, Wu A, Huang X. Mental health status among Chinese healthcare-associated infection control professionals during the outbreak of coronavirus disease 2019: A national cross-sectional survey. Medicine 2021;100:5(e24503).

Received: 11 September 2020 / Received in final form: 5 January 2021 / Accepted: 7 January 2021

http://dx.doi.org/10.1097/MD.0000000000024503

1. Introduction

Coronavirus disease 2019 (COVID-19) is an infectious disease caused by a newly discovered novel coronavirus (severe acute respiratory syndrome coronavirus 2 [SARS-CoV-2]), and causes a global epidemic in 2020.[1] To fight against COVID-19, 31 provinces, autonomous regions, and municipalities of China launched a level I response to public health emergencies by the end of January 2020. The population is generally susceptible to SARS-CoV-2. After infection, the patients may suffer from fever, dry cough, fatigue, chest distress, and other symptoms, even death.[2] Statistics have shown that the COVID-19 in Hubei province has a mortality rate of 5.9%, and that in other provinces is 0.98%.[3] The study has pointed out that the age-adjusted mortality ratio among all infected people in China was 0.66%.[4]

The situation of nosocomial infection control is severe. Since the COVID-19 outbreak, 3387 medical staff have been infected with COVID-19 in 476 medical institutions nationwide (2055 confirmed cases, 1070 clinically diagnosed cases, and 157 suspected cases); and this is an unignorable problem in fighting against COVID-19.[5] The State Council attaches great importance to the hospital infection control, and clearly declare that nosocomial infection control and scientific protection and training for medical staff should be strengthened further. The healthcare-associated infection control professionals (ICPs) are the primary link and core force to ensure the effective implementation of hospital infection control measures and the efficient completion of hospital infection control tasks.[6] They also provide scientific advice, support, and guidance to medical staff about the management of nosocomial infection control in medical institutions, and identify new and recurrent infectious diseases. In a word, the ICPs play a crucial role in improving medical safety and quality.[7]

The study has pointed out that the onset of sudden and immediate life-threatening diseases can put medical staff under tremendous pressure.[8] After the outbreak of COVID-19, medical staff not only rescue the infected patients, but also witness the whole fighting process of public health events. In addition to the pressure of the occupation itself, the conflict between their own safety needs and occupational requirements may make them more vulnerable to anxiety, depression, insomnia, and other psychological disorders.[9] As members of medical staff, the ICPs are not directly involved in the front-line diagnosis and treatment of COVID-19, and their mental health status is easy to be ignored. Due to the suddenness, strong infectivity, rapid spread, complexity, and unpredictability of COVID-19, the ICPs may encounter greater challenges and pressures in their work. Moreover, the infection of medical staff in the medical system, the collapse of the social support system, and concern about the risk of infection for family members and themselves will further increase the pressure on the ICPs. The constant high levels of pressure will lead to the exhaustion of the body's adaptation and adjustment to pressure, resulting in an imbalance of the body, even the physical and mental illness.[10] Therefore, it is crucial to investigate the mental health status of the ICPs in the outbreak period of COVID-19 for maintaining their health and consolidating the prevention and control of the "post-epidemic" period in China. However, research on the mental health of ICPs under the COVID-19 epidemic is rarely performed. To this end, the psychological status of ICPs during the outbreak of COVID-19 is explored, and the influencing factors are obtained, so as to assist the formulation of the psychological crisis intervention program for the individuals under the public health emergency.

2. Methods

2.1. Participants and sampling

This study is a national cross-sectional survey using convenient cluster sampling. The subjects were all healthcare-associated ICPs (>18 years old) from hospitals of different levels in China and voluntarily participated in the survey with informed consent. The ICPs refer to the full-time staff specialized in the monitoring, supervising, guidance, training and management of nosocomial infection control, undertaking the monitoring of risk factors related to hospital infection in medical activities, implementation, disinfection and isolation of safety protection measures, and disposal and management of medical waste in the medical institutions.[11] ICPs who are not on the job for sick leave, maternity leave, or other reasons are not included. Finally, 9326 ICPs from 3776 hospitals in 20 provinces participated in the survey, and 9228 (98.95%) valid questionnaires were confirmed.

2.2. Measurements

2.2.1. Demographic data. Demographic data include age, sex, marital status, highest degree, professional background, average monthly income, post category, work part-time in other departments, working years in the department, average working hours per week, hospital location (the specific economic region), nature of the hospital, level of hospital, and number of beds in a hospital.

2.2.2. Chinese version of the 12-item general health questionnaire (GHQ-12). Goldberg designed the GHQ-12 to reflect the mental health status of the respondents through 12-item self-evaluation results, among which 6 items are positively worded and the other are negatively worded.[12] GHQ-12 is widely used in the psychological evaluation of clinical patients and the self-evaluation of the general population.[13] The Chinese version of the GHQ-12 questionnaire, which has been sinicized by Cheng et al,[14] has good reliability and validity and is used as a screening tool for the identification of psychological disorders in Chinese psychiatric epidemiological investigation and community health services.[15] Each item in the GHQ-12 is evaluated by 4 indicators: better than usual, same as usual, less than usual, and much less than usual. Then respondents were asked to report their conditions and feelings over the past 4 weeks, including problems with sleep and appetite, decision-making ability, mastering of daily issues, self-esteem and subjective experiences of stress, tension, or sadness.[16] The bi-modal (0-0-1-1) and Likert scoring method (0-1-2-3) are the most commonly used scoring methods.[15] In this study, the bi-modal scoring method was adopted, that is, the first 2 options were marked as 0 point, and the second 2 options as 1 point. As a result, the scores for the 12 items range from 0 to 12 points. A score ≥ 3 indicates the poor mental health, and the higher the score, the more serious the mental problem.[17] The Cronbach α in this study is 0.810.

2.2.3. Chinese version of the psychological capital questionnaire (PCQ) for medical staff. Luthans originally compiled the PCQ to assess psychological capital.[18] The Chinese version of the PCQ for medical staff composed of 24 items and 4 dimensions, namely self-efficacy, hope, optimism, and resilience.

And each item was rated on a 5-point Likert scale (1=strongly disagree, 5=strongly agree); a higher total score indicates the higher level of psychological capital.[19] It has good validity and reliability, and the Cronbach α ranges from 0.768 to 0.879.[19] In this study, the Cronbach α of the total scale and 4 dimensions (self-efficacy, hope, optimism, and resilience) were 0.906, 0.879, 0.621, 0.868, and 0.691, respectively.

2.3. Data collection

The study was authorized by the Ethic Committee of the Xiangya Hospital of Central South University (Certificate: IRB202002010). With the help of the National Hospital Infection Monitoring and Management Training Base and the Platform of the Hospital Infection Control Branch of the Chinese Preventive Medicine Association, data were collected through Wenjuanxing (www.wjx.cn). The network link of the questionnaires was sent to the directors of hospital infection control departments in all hospitals through the Internet, then directors distributed the questionnaires website link to ICPs in the departments. The information on purpose, confidentiality, and anonymity were also included in the invitation of the online survey. Electronic informed consent with 2 options (yes/no) was provided to the ICPs prior to the investigation. ICPs who choose yes can answer the questionnaire, and they can quit at any time during the survey. The data was collected between March 27, 2020 and March 30, 2020 to investigate the mental health problems of ICPs during the COVID-19 outbreak in China in late February 2020.

2.4. Statistical analysis

The continuous variables were expressed by mean values ± standard deviation (SD), and the counting variables were expressed by frequency (percentage). To compare the differences between 2 groups of continuous variables, the Levene test for homogeneity of variance was first performed. If the variance was homogeneous, t test is used; if the variance was heterogeneous, t' test is used. Besides, Chi-square test was employed to identify 2 groups' differences in counting variables. To explore the influencing factors of mental health status, the backward stepwise regression of the dichotomous multivariate logistic model was used for the analysis. All statistical analysis was conducted by IBM SPSS Statistics 20.0, and 2-tailed $P<.05$ was considered to be statistically significant.

3. Results

3.1. Basic information of ICPs

According to the division of economic region in China, 3244 (35.15%) ICPs come from hospitals in the eastern economic region, 2732 (29.61%) come from hospitals in the central economic region, 2508 (27.18%) come from hospitals in the western economic region, and 744 (8.06%) come from hospitals in the northeast economic region. ICPs were composed of 623 men (6.75%) and 8605 women (93.25%) with a mean age of 42.26 ± 8.77 years; most of them were married (8384, 90.85%); the number of bachelor degree was the most (5692, 61.68%); the number of nursing professional background accounted for the most (7382, 80.00%); the average monthly income of 4876 (52.84%) ICPs was 3000 to 6000 yuan; in terms of post category distribution, the number of professional and technical post is the largest (5213, 56.49%); 4999 ICPs (54.17%) worked part-time in other departments; 3739 (40.52%) worked in his/her department for 1 to 5 years (including 5 years); 5199 (56.34%) usually worked about 35 to 45 hours per week on average; 7705 (83.50%) came from public hospitals; in terms of hospital level, there was the highest number of ICPs from secondary hospitals (4479, 48.54%); as for the number of hospital beds, there was the highest proportion of 100 to 499 beds (3415, 37.01%). Details of basic information of ICPs are shown in Table 1.

3.2. Mental health status in the past month of ICPs

The total score of the mental health of ICPs was 3.45 ± 2.57. The total score of 3 was taken as the threshold, that is, the score ≥3 suggests the individual with the possibility of poor mental health. Among 9228 ICPs, 5608 (60.77%) had mental health problems. According to the GHQ-12 scoring standard, each item has 4 options, which are assigned as "0–0-1-1" points. According to the scores of each ICP, the number of ICPs with 0 and 1 score for each item is summarized (Table 2). The results showed that there was the largest number of ICPs who scored 1 in item 5 "Felt constantly under strain," item 2 "Loss of sleep over worry," item 7 "Able to enjoy day-to-day activities," and item 9 "Feeling unhappy and depressed," which were 7125 (77.21%), 6258 (67.82%), 5012 (54.31%), and 4695 (50.88%), respectively. There was the largest number of ICPs who scored 0 in item 3 "Playing a useful part," item 6 "Couldn't overcome difficulties," item 4 "Capable of making decisions," and item 8 "Able to face problems," which were 8766 (94.99%), 8442 (91.48%), 8365 (90.65%), and 8362 (90.62%), respectively.

3.3. Psychological capital of ICPs

The total score of the psychological capital of ICPs was 89.24 ± 9.23, with an average score of 3.72 ± 0.38. As the average score range is 1 to 5, the psychological capital is in the upper-middle level. The scores of all dimensions of psychological capital are ranked in descending order as self-efficacy, optimism, resilience, and hope (Table 3).

3.4. Univariate analysis of mental health status on ICPs

In this study, the score <3 of GHQ-12 was considered as the negative group, that is, the ICP had no mental health problems; and the score ≥3 as the positive group, that is, the ICP was suspected of mental health problems. The univariate analysis showed that there were significant differences among the different ages, sexes, marital status, highest degree, professional background, average monthly income, work part-time in other departments, working years in department, average working hours per week, hospital location (the specific economic region), the nature of hospital, level of hospital, and the number of beds in hospital between the negative group and the positive group ($P<.05$) (Table 1). Moreover, the differences of psychological capital, self-efficacy, hope, optimism, and resilience between the negative group and the positive group were statistically significant ($P<.05$) (Table 3).

3.5. Multiple factors analysis of mental health status on ICPs

The negative group and positive group of mental health status on ICPs were taken as the dichotomous dependent variable. All the

Table 1

Basic information and distribution of negative (<3 score) and positive (≥3 score) mental health status on ICPs.

Demographics	All (n=9228)	Negative (n=3620)	Positive (n=5608)	t/X^2	P
Age, y	42.26±8.77	41.62±9.53	42.68±8.22	–5.494*	.000
Gender				5.112	.024
Male	623 (6.75%)	271 (43.50%)	352 (56.50%)		
Female	8605 (93.25%)	3349 (38.92%)	5256 (61.08%)		
Marital status				45.906	.000
Unmarried	564 (6.11%)	297 (52.66%)	267 (47.34%)		
Married	8384 (90.85%)	3221 (38.42%)	5163 (61.58%)		
Divorce or bereavement	280 (3.03%)	102 (36.43%)	178 (63.57%)		
Highest degree				18.888	.000
College degree or below	3110 (33.70%)	1292 (41.54%)	1818 (58.46%)		
Bachelor degree	5692 (61.68%)	2139 (37.58%)	3553 (62.42%)		
Master degree	394 (4.27%)	177 (44.92%)	217 (55.08%)		
Doctoral degree	32 (0.35%)	12 (37.50%)	20 (62.50%)		
Professional background				18.025	.003
Clinical medicine	872 (9.45%)	370 (42.43%)	502 (57.57%)		
Nursing	7382 (80.00%)	2833 (38.38%)	4549 (61.62%)		
Public health and preventive medicine	560 (6.07%)	250 (44.64%)	310 (55.36%)		
Medical laboratory science	182 (1.97%)	64 (35.16%)	118 (64.84%)		
Pharmacy	55 (0.60%)	28 (50.91%)	27 (49.09%)		
Others	177 (1.92%)	75 (42.37%)	102 (57.63%)		
Average monthly income (yuan)				14.309	.006
Less than 3000	555 (6.01%)	246 (44.32%)	309 (55.68%)		
3000–6000	4876 (52.84%)	1949 (39.97%)	2927 (60.03%)		
6001–9000	2535 (27.47%)	929 (36.65%)	1606 (63.35%)		
9001–12000	872 (9.45%)	341 (39.11%)	531 (60.89%)		
More than 12,000	390 (4.23%)	155 (39.74%)	235 (60.26%)		
Post category				2.237	.327
Professional and technical posts	5213 (56.49%)	2075 (39.80%)	3138 (60.20%)		
Management post	3882 (42.07%)	1498 (38.59%)	2384 (61.41%)		
Work skill post	133 (1.44%)	47 (35.34%)	86 (64.66%)		
Work part-time in other department				5.345	.021
No	4999 (54.17%)	1907 (38.15%)	3092 (61.85%)		
Yes	4229 (45.83%)	1713 (40.51%)	2516 (59.49%)		
Working years in department				78.679	.000
Less than 1 year	1661 (18.00%)	794 (47.80%)	867 (52.18%)		
1–5 year (including 5 years)	3739 (40.52%)	1458 (38.99%)	2281 (61.01%)		
5–10 years (including 10 years)	2357 (25.54%)	833 (35.34%)	1524 (64.66%)		
10–15 years (including 15 years)	878 (9.51%)	299 (34.05%)	579 (65.95%)		
15–20 years (including 20 years)	330 (3.58%)	122 (36.97%)	208 (63.03%)		
More than 20 years	263 (2.85%)	114 (43.35%)	149 (56.65%)		
Average working hours per week				76.995	.000
Less than 35 hours	376 (4.07%)	188 (50.00%)	188 (50.00%)		
35–45 hours	5199 (56.34%)	2176 (41.85%)	3023 (58.15%)		
46–60 hours	3075 (33.32%)	1087 (35.35%)	1988 (64.65%)		
61–80 hours	452 (4.90%)	131 (28.98%)	321 (71.02%)		
More than 80 hours	126 (1.37%)	38 (30.16%)	88 (69.84%)		
Hospital location				103.283	.000
Eastern economic region	3244 (35.15%)	1466 (45.19%)	1778 (54.81%)		
Central economic region	2732 (29.61%)	1063 (38.91%)	1669 (61.09%)		
Western economic region	2508 (27.18%)	803 (32.02%)	1705 (67.98%)		
Northeast economic region	744 (8.06%)	288 (38.71%)	456 (61.29%)		
Nature of hospital				121.033	.000
Private hospital	1523 (16.50%)	789 (51.81%)	734 (48.19%)		
Public hospital	7705 (83.50%)	2831 (36.74%)	4874 (63.26%)		
Level of hospital				139.607	.000
Unranked	819 (8.88%)	418 (51.04%)	401 (48.96%)		
First-class hospital	1386 (15.02%)	668 (48.20%)	718 (51.80%)		
Second-class hospital	4479 (48.54%)	1538 (34.34%)	2941 (65.66%)		
Third-class hospital	2544 (27.57%)	996 (39.15%)	1548 (60.85%)		
Number of beds in hospital				154.967	.000
Less than 100	2446 (26.51%)	1211 (49.51%)	1235 (50.49%)		
100–499	3415 (37.01%)	1173 (34.35%)	2242 (65.65%)		
500–1000	2121 (22.98%)	755 (35.60%)	1366 (64.40%)		
1001–2000	885 (9.59%)	341 (38.53%)	544 (61.47%)		
2001–3000	227 (2.46%)	85 (37.44%)	142 (62.56%)		
More than 3000	134 (1.45%)	55 (41.04%)	79 (58.96%)		

Values are presented as mean±standard error or number (%).

* Obtained using a t test for age; the chi-squared test was used for other variables.

Table 2

Score distribution of each item of GHQ-12 on the mental health status in the past month of ICPs.

Item	0 score (n/%)	1 score (n/%)
Able to concentrate	8195 (88.81)	1033 (11.19)
Loss of sleep over worry	2970 (32.18)	6258 (67.82)
Playing a useful part	8766 (94.99)	462 (5.01)
Capable of making decisions	8365 (90.65)	863 (9.35)
Felt constantly under strain	2103 (22.79)	7125 (77.21)
Couldn't overcome difficulties	8442 (91.48)	786 (8.52)
Able to enjoy day-to-day activities	4216 (45.69)	5012 (54.31)
Able to face problems	8362 (90.62)	866 (9.38)
Feeling unhappy and depressed	4533 (49.12)	4695 (50.88)
Losing confidence	7915 (85.77)	1313 (14.23)
Thinking of self as worthless	8132 (88.12)	1096 (11.88)
Feeling reasonably happy	6875 (74.50)	2353 (25.50)

GHQ-12 = 12-item general health questionnaire; ICP = infection control professional.

variables of basic information and 4 dimensions of psychological capital, a total of 18 variables, were included as independent variables. The results of multiple factors analysis showed that risk factors for mental health status of ICPs included the self-efficacy (odds ratio (OR) = 1.044, 95% confidence interval (CI): 1.020–1.068), public hospital versus private hospital (OR = 1.596, 95% CI: 1.403–1.814), second-class hospital versus third-class hospital (OR = 1.331, 95% CI: 1.153, 1.535), hospitals in western economic region versus hospitals in central economic region (OR = 1.297, 95% CI: 1.147–1.466), hospitals in northeast economic region versus hospitals in central economic region (OR = 1.201, 95% CI: 1.002–1.439); however, protective factors for mental health status of ICPs included unmarried versus married (OR = 0.709, 95% CI: 0.582–0.862), working years in department ≤1 year versus >20 years (OR = 0.751, 95% CI: 0.565–0.998), <35 working hours per week versus >80 hours (OR = 0.355, 95% CI: 0.223–0.566), 35 to 45 hours versus >80 hours (OR = 0.392, 95% CI: 0.258–0.595), 46–60 hours versus >80 hours (OR = 0.565, 95% CI: 0.371–0.861), hope (OR = 0.977, 95% CI: 0.957–0.998), and optimism (OR = 0.812, 95% CI: 0.794–0.831) (Table 4).

4. Discussion

To our knowledge, this is the first large-scale mental health survey of healthcare-associated ICPs during the COVID-19 epidemic in China as well as all around the world. The results of this study showed that 60.77% of ICPs might have mental health problems in the context of fighting against the COVID-19 epidemic. In particular, 77.21% of the subjects constantly felt under strain, 67.82% had insomnia, more than half could not enjoy day-to-day activities, and felt unhappy or depressed. It is pointed out that >98% of the medical staff strongly hope that ICPs can give guidance and help in their clinical work during the COVID-19 epidemic.[20] Therefore, the ICPs must, at the first time, assist in the formulation of the hospital infection control plan and work flow; conduct the training, supervision, inspection, and reporting on the hospital infection control; organize the distribution of protective materials.[21,22] As a result, ICPs had a heavy workload and tremendous pressure, which can easily induce mental health problems.

It is found that the married ICPs were more susceptible that of unmarried ones to mental health problems. In this study, 93.25% and 80.00% of the subjects were women and infection control nurses, respectively. This result may be correlated with the work-family conflict.[23,24] During the epidemic, they have to assume various roles in the family, such as wife, daughter-in-law, and mother, as well as the responsibilities of educating their children and supporting the elderly.[25] At the same time, they are always in a high-intensity working state of the front-line hospital infection control. More importantly, they have to bear the risk of the possible virus infection, and worry about whether their family members will be infected by themselves when they return home from the hospital. As the backbone of the family, the married ICPs might be under greater psychological pressure when there are conflicts among multiple roles.

ICPs with working years ≤1 significantly contributed to a lower mental health problem risk than that with working years >20. For the ICPs who have been working for <1 year, they are still in the initial stage of work, learning, and adapting to the working environment of the department. They do not have sufficient capacity and experience as the main staff to participate in the fight against the epidemic. In line with the protection of junior staff, they will not undertake high-risk work, therefore, they are less likely to develop mental health problems due to the low-risk work and the very few work responsibilities. However, senior staffs with working years >20 are generally experts in the field of hospital infection control. They are fully responsible for all aspects of the nosocomial infection control and devoted to high-intensity work to ensure the safety of patients and "zero" infection of medical staff. Therefore, ICPs with working years >20 years are under the unimaginable pressure, and mental health problems are inevitably caused for them.

The longer working hours a week increase the risk of mental health problems, which is consistent with other studies.[26,27] During the epidemic outbreak, with the increasing number of confirmed cases of patients and medical staff, the workload of ICPs has been increased furiously in the whole country. In addition, the other factors such as the possibility of long-term

Table 3

Score and distribution of psychological capital and each dimension between negative (<3 score) and positive (≥3 score) mental health status on ICPs.

Item	Total score	Average score	Negative (n = 3620)	Positive (n = 5608)	t/t'	P
Self-efficacy	23.87 ± 2.65	3.98 ± 0.44	24.30 ± 2.65	23.59 ± 2.62	12.690	.000
Hope	21.53 ± 2.75	3.59 ± 0.46	22.17 ± 2.76	21.12 ± 2.67	18.258*	.000
Optimism	22.26 ± 3.17	3.71 ± 0.53	23.31 ± 2.95	21.58 ± 3.12	26.887	.000
Resilience	21.59 ± 2.62	3.60 ± 0.44	22.18 ± 2.69	21.20 ± 2.50	17.884	.000
Total score of psychological capital	89.24 ± 9.23	3.72 ± 0.38	91.96 ± 9.36	87.49 ± 8.71	23.398	.000

Values are presented as mean ± standard error. ICP = infection control professional.
* Obtained using a t' test for hope; the t test was used for other variables.

Table 4

Multiple factors analysis of mental health status on ICPs.

Variable	B	SE	Walds	*P*	OR (95% CI)
Working years in department					
Less than 1 year	–0.286	0.145	3.887	.049	0.751 (0.565–0.998)
1–5 year (including 5 years)	–0.019	0.138	0.020	.889	0.981 (0.748–1.287)
5–10 years (including 10 years)	0.092	0.140	0.425	.514	1.096 (0.832–1.443)
10–15 years (including 15 years)	0.198	0.152	1.696	.193	1.219 (0.905–1.642)
15–20 years (including 20 years)	0.183	0.179	1.051	.305	1.201 (0.846–1.704)
More than 20 years					1[Reference]
Number of beds in hospital					
Less than 100	–0.344	0.208	2.735	.098	0.709 (0.472–1.066)
100–499	–0.003	0.200	0.000	.990	0.997 (0.674–1.476)
500–1000	0.060	0.194	0.095	.758	1.062 (0.725–1.554)
1001–2000	0.093	0.199	0.220	.639	1.098 (0.743–1.623)
2001–3000	0.231	0.235	0.970	.325	1.260 (0.795–1.996)
More than 3000					1[Reference]
Hospital location					
Eastern economic region	–0.072	0.057	1.576	.209	0.931 (0.832–1.041)
Western economic region	0.260	0.063	17.201	.000	1.297 (1.147–1.466)
Northeast economic region	0.183	0.092	3.946	.047	1.201 (1.002–1.439)
Central economic region					1[Reference]
Professional background					
Clinical medicine	–0.158	0.182	0.748	.387	0.854 (0.597–1.221)
Nursing	0.084	0.169	0.249	.618	1.088 (0.781–1.516)
Public health and preventive medicine	–0.349	0.191	3.347	.067	0.706 (0.486–1.025)
Medical laboratory science	0.166	0.235	0.501	.479	1.181 (0.745–1.872)
Pharmacy	–0.303	0.336	0.813	.367	0.739 (0.383–1.426)
Others					1[Reference]
Post category					
Professional and technical posts	–0.288	0.204	1.986	.159	0.750 (0.503–1.119)
Management post	–0.177	0.205	0.741	.389	0.838 (0.561–1.253)
Work skill post					1[Reference]
Average working hours per week					
Less than 35 hours	–1.036	0.238	18.968	.000	0.355 (0.223–0.566)
35–45 hours	–0.936	0.213	19.280	.000	0.392 (0.258–0.595)
46–60 hours	–0.570	0.214	7.071	.008	0.565 (0.371–0.861)
61–80 hours	–0.186	0.237	0.617	.432	0.830 (0.522–1.321)
More than 80 hours					1[Reference]
Self-efficacy	0.043	0.012	13.238	.000	1.044 (1.020–1.068)
Hope	–0.023	0.011	4.613	.032	0.977 (0.957–0.998)
Optimism	–0.208	0.012	322.022	.000	0.812 (0.794–0.831)
Nature of hospital					
Public hospital	0.467	0.066	50.796	.000	1.596 (1.403–1.814)
Private hospital					1[Reference]
Marital status					
Unmarried	–0.344	0.100	11.815	.001	0.709 (0.582–0.862)
Divorce or bereavement	0.004	0.135	0.001	.976	1.004 (0.771, 1.308)
Married					1[Reference]
Level of hospital					
Unranked	0.016	0.111	0.021	.885	1.016 (0.818–1.263)
First-class hospital	0.087	0.106	0.675	.411	1.091 (0.886, 1.344)
Second-class hospital	0.286	0.073	15.365	.000	1.331 (1.153, 1.535)
Third-class hospital					1[Reference]

large-scale exposure to high-risk infection and irregular diet weaken their own immunity, and make them become more susceptible to the infection.[5] Based on the above factors, the extension of working hours makes mental health problems of ICPs worse. Thus, the integration of the above factors was aggravated by the long working hours, resulting in an increased risk of mental health problems among ICPs.

ICPs in public hospitals are more prone to mental health problems. Public hospitals have played a leading role in reducing the mortality rate and improving the cure rate. Thus, ICPs in public hospitals have to face greater work pressure are more likely to have psychological problems than those in private hospitals. At the same time, it is also found that ICPs from secondary hospitals are more prone to mental health problems than those from tertiary hospitals. On March 13, 2020, the National Health Commission of the People's Republic of China issued a notice on further strengthening the hospital infection control during the epidemic, and made specific requirements for the hospital infection control from 7 aspects. Among them, it is required that the general hospitals at the second level and above

should establish infectious disease departments, which are specifically responsible for the organization and management of the pre-examination and triage of infectious diseases in hospital.[28] However, compared with tertiary hospitals, secondary hospitals have encountered many problems, including the insufficient number of ICPs for infection management, irregular reserves of health protection materials, and insufficient smooth transition of medical institutions from a steady state to an emergency state. Consequently, greater challenges and more severe stress are caused for the ICPs in their anti-epidemic work.

We took the central economic region headed by Hubei province as a reference and found a strange phenomenon: the ICPs in the western and northeastern economic regions with lighter epidemics are more likely to have mental health problems than those in the central region with severe epidemics. The reasons are speculated as follows: according to the requirements of our country's nosocomial infection control monitoring standards, one infection control professional is allocated for every 200 to 250 beds in the hospital.[29] However, the phenomenon of insufficient ICPs in our country is very common. Here Chongqing in the western region is taken as an example. In 2019, a total of 113 hospitals in Chongqing were surveyed. There were 160 ICPs in the surveyed hospitals, with an average of <2 ICPs in each hospital. In addition, there were <20 hospitals with the professional structure of 4 majors in nursing, clinical medicine, microbiological testing, and preventive medicine.[30] Unreasonable professional structure directly affects the hospital infection monitoring, risk identification, intervention, evaluation, and continuous quality improvement.[31] The lack of ICPs and weak abilities caused by the unreasonable professional background will inevitably increase their work resistance; the building layout of the key departments in most hospitals cannot meet the requirements of infection control, mainly reflected in the unclear zoning; Moreover, there is no real-time monitoring platform for the infection control behavior. In addition, the construction of microbiology rooms in hospitals is inadequate, especially the lack of pathogen nucleic acid detection platforms, and most laboratories do not have special facilities that meet the 3-level protective wear-off.[30] The above hardware facilities configuration requires a large amount of economic investment, and the western and northeastern regions are less developed than the central economic regions, so the above configuration is hardly achieved by the western and northeastern regions. To some extent, medical institutions have limited expenditures on hospital infection control, leaving the ICPs in a more dangerous working environment.

Psychological capital refers to the state of individual's positive psychological development, which is characterized by: have confidence (self-efficacy) to undertake and make necessary efforts to successfully complete challenging tasks; make positive attribution (optimism) for present and future success; adhere to the goal and redirect to the goal (hope) if necessary to achieve success; when encountering problems and adversities, keep and rebound or even surpass (resilience) to achieve success.[32] The results of this study show that the average score of mental capital and its 4 dimensions all are at a upper-middle level. In the univariate analysis, the higher the level of psychological capital or each dimension, the better the mental health status of the study population and the less likely the mental health problems will occur. When stress events occur, individuals with rich psychological capital tend to meet challenges with a proactive attitude, deal with the difficulties and frustrations they face, and have the confidence to solve problems and be able to adapt well to the surrounding changes. And being hopeful about the future makes it easier to avoid the effects of negative emotions and maintain a high level of life satisfaction.[33] Indeed, >90.00% ICPs felt able to make decisions on their own, face the problems encountered, overcome difficulties in work/life, and be a useful person. However, an interesting phenomenon was discovered in the multiple factors analysis, namely optimism and hope are independent protective factors, and self-efficacy turned out to be a risk factor. In other words, the more optimistic or hope, the less likely it is to have mental health problems; the higher the self-efficacy, the more likely to have mental health problems. Self-efficacy refers to an individual's ability expectations for implementing a specific behavior or the behavior required to produce a certain result, and it can be used to reflect a belief that the individual can take appropriate behavior to face environmental pressure.[34] Whether a person can successfully engage in an activity is influenced by his sense of self-efficacy, because the individual's feelings about his self-efficacy restrict or motivate his motivation level, behavior style, and various psychological levels.[35] The previous study has pointed out that people with high self-efficacy tend to attribute their failures to insufficient efforts, while those with comparable abilities but low self-efficacy attribute failures to their inability.[36] Based on this, we speculate that in the face of nosocomial infection control during the COVID-19 epidemic, individuals with high self-efficacy expect and believe that they can be good "gatekeepers" to protect the health of frontline medical workers and patients, even in an unprecedented high-pressure work environment. However, due to the complexity and uncertainty of the epidemic, it may cause them to be greatly challenged or even frustrated in their work, which in turn encourages them to work harder to achieve their goals. To some extent, higher self-efficacy may increase their work pressure and easily induce mental health problems.

There is an urgent to pay more attention to the mental health status of ICPs. For hospital managers in second-level public hospitals in the western economic region, they need fully in-depth understand the indirect economic and social benefits of infection control output.[37] Next, relevant funds should be invested to improve the configuration of hardware facilities related to hospital infection control management. Then, all hospital administrators need to implement more humane management and rationally arrange human resources and working hours. Finally, the married ICPs should be paid more attention as well. It is worth trying to reduce mental health problems by increasing psychological capital. For instance, the active-constructive response method in capitalization support can be considered for its positive effect on psychological capital. For instance, the active-constructive response method in capitalization support can be considered for its positive effect on psychological capital.[38] For example, the experience sharing of the positive events of the ICPs can be added in the regular work meeting, with the following advantages: the memory of the positive events of the sharers and responders can be strengthened; the enthusiastic response given by the responders can remind the value of their work, enhance professional identity, and reduce psychological pressure; and it is also conducive to the cultivation of the professional ability of the responders. Some studies have pointed out that group psychological counseling can be used to effectively improve the psychological capital of research subjects and adjust their immune function, so as to ease their sub-healthy state and relieve psychological pressure in the future work and life.[32,39]

Furthermore, acceptance, active coping, building cognitive behavior skills, stress reduction strategies, mindfulness, deep breathing, and gratitude are successful strategies proven by the research to improve mental health.[40]

However, there are still some limitations in our research. Firstly, the questionnaire is distributed non-randomly through the Wenjuanxing, so our research has a selective bias. Secondly, as a cross-sectional design, this study can only assess the mental health problems at that time, but cannot longitudinally observe the psychological health changes of ICPs. Finally, the other important outcomes related to mental health issues, such as anxiety, depression, and posttraumatic stress disorder have not been investigated. Therefore, we are unable to provide detailed information about specific mental health problems of the ICPs, and future research is required to explore these possible outcome indicators. As the crisis continues, research is recommended to screen specific mental health problems and evidence-based interventions for ICPs.

5. Conclusions

COVID-19 poses huge public health challenges to the whole world. Healthcare-associated ICPs are devoted to providing professional assistance and support to patients and all medical staff. They have made great contributions in fighting against COVID-19 at the cost of prevalent mental health problems. Nationwide, mental health problems are common among ICPs. Marital status, working hours per week, working years, hospital location (the specific economic region), grade and nature of hospital, self-efficacy, optimism and hope are the main factors that affect the mental health status of ICPs. In this study, the mental health status of Chinese ICPs is investigated during the COVID-19 epidemic, the influencing factors are analyzed, and the measures are proposed to alleviate mental health problems. This study provides a reference for mental health maintenance for ICPs in other countries.

Acknowledgments

The authors thank all frontline ICNs who are fighting COVID-19. They sincerely appreciate their participation in our study.

Author contributions

Conceptualization: Ying Zhang, Lingyun Tian, Anhua Wu, Xun Huang.
Data curation: Ying Zhang, Lingyun Tian, Anhua Wu, Xun Huang.
Formal analysis: Ying Zhang, Lingyun Tian.
Investigation: Ying Zhang, Wan Li, Ximao Wen, Hongman Wu, Ruie Gong, Lanman Zeng, Feng Zhou, Zhenru Liu, Ziyuan Tang, Xun Huang.
Methodology: Lingyun Tian, Wan Li, Anhua Wu, Xun Huang.
Project administration: Xun Huang.
Software: Ying Zhang, Lingyun Tian.
Supervision: Anhua Wu.
Writing – original draft: Ying Zhang, Lingyun Tian, Wan Li.
Writing – review & editing: Ying Zhang, Lingyun Tian, Wan Li, Ximao Wen, Hongman Wu, Ruie Gong, Lanman Zeng, Feng Zhou, Zhenru Liu, Ziyuan Tang, Anhua Wu, Xun Huang.

References

[1] Li ZJ, Chen QL, Feng LZ, et al. Active case finding with case management: the key to tackling the COVID-19 pandemic. Lancet 2020;396:63–70.
[2] National Health Commission of the People's Republic of China. Chinese management guideline for COVID-19 (Version 7.0). Available at: http://www.nhc.gov.cn/yzygj/s7653p/202003/46c9294a7dfe4cef80dc7f5912eb1989.shtml. Accessed June 10, 2020.
[3] Leung K, Wu JT, Liu D, et al. First-wave COVID-19 transmissibility and severity in China outside Hubei after control measures, and second-wave scenario planning: a modelling impact assessment. Lancet 2020;395:1382–93.
[4] Verity R, Okell LC, Dorigatti I, et al. Estimates of the severity of coronavirus disease 2019: a model-based analysis. Lancet Infect Dis 2020;20:669–77.
[5] Fu Q. Consideration on infection prevention and control in medical institutions based on the response to COVID-19. Chin J Nosocomiol 2020;30:1121–4.
[6] Qiao F, Zong ZY, Yin WJ. Applying management methods to improve the efficiency of hospital infection management. West China Med J 2015;30:201–3.
[7] Outcome competences for practitioners in infection prevention and control. J Hosp Infect 2011;79:278–88.
[8] Liu XH, Kakade M, Fuller CJ, et al. Depression after exposure to stressful events: lessons learned from the severe acute respiratory syndrome epidemic. Compr Psychiatry 2012;53:15–23.
[9] Pappa S, Ntella V, Giannakas T, et al. Prevalence of depression, anxiety, and insomnia among healthcare workers during the COVID-19 pandemic: a systematic review and meta-analysis. Brain Behav Immun 2020;88:901–7.
[10] Tang HH, Lu XY, Cai SX, et al. Investigation and analysis of mental health status of nurses supporting Wuhan during the COVID-19. Infect Int 2020;9:296–7.
[11] Chen CM, Zhao XZ, Fu WL. Thinking and experience of professional composition in nosocomial infection management team. Chin J Nosocomiol 2010;20:1574–5.
[12] Goldberg DP, Hillier VF. A scaled version of the General Health Questionnaire. Psychol Med 1979;9:139–45.
[13] Liang Y, Wang L, Yin XC. The factor structure of the 12-item general health questionnaire (GHQ-12) in young Chinese civil servants. Health Qual Life Outcomes 2016;14:136–45.
[14] Cheng TA, Wu JT, Chong MY, et al. Internal consistency and factor structure of the Chinese Health Questionnaire. Acta Psychiatr Scand 1990;82:304–8.
[15] Yang TZ, Huang L, Wu ZY. The application of Chinese health questionnaire for mental disorder screening in community settings in mainland China. Chin J Epidemiol 2003;24:20–4.
[16] Gelaye B, Tadesse MG, Lohsoonthorn V, et al. Psychometric properties and factor structure of the General Health Questionnaire as a screening tool for anxiety and depressive symptoms in a multi-national study of young adults. J Affect Disord 2015;187:197–202.
[17] Wang JL. Study on the mental health status and influencing factors of Chinese medical team members. Zhonghua Liu Xing Bing Xue Za Zhi 2019;40:574–9.
[18] Luthans F, Youssef CM, Avolio BJ. Psychological capital: developing the human competitive edge. J Asian Econ 2007;8:315–32.
[19] Meng XM. Study on Relationship Between Perceived Organizational Support, Psychological Capital and Job Stress of Medical Staff. Yanshan University; 2016.
[20] Zhao H, Xie SQ, Yang HZ, et al. Infection prevention and control-relevant demand of health care workers in medical team during fighting against COVID-19. Chin J Infect Control 2020;19:398–403.
[21] Ou YQ, Deng ZD, Chen HL, et al. Application of infection control supervisor system in a designated hospital for COVID-19. Chin J Nosocomiol 2020;30:1932–6.
[22] Zhu SC, Xiang Q, Yang C, et al. Experience of healthcare-associated infection monitoring from medical team for aiding Hubei province during COVID-19 epidemic period. Chin J Infect Control 2020;19:504–9.
[23] Yavas U, Babakus E, Karatepe OM. Attitudinal and behavioral consequences of work-family conflict and family-work conflict. Does gender matter? Int J Serv Ind Manag 2008;19:7–31.

[24] Wang Y, Chang Y, Fu JL, et al. Work-family conflict and burnout among Chinese female nurses: the mediating effect of psychological capital. BMC Public Health 2012;12:915–23.
[25] Yang SY. The Research of Present Occupation Female's Work-Family Role Conflicts. Shanxi University; 2013.
[26] Mo YY, Deng L, Zhang LY, et al. Work stress among Chinese nurses to support Wuhan in fighting against COVID-19 epidemic. J Nurs Manag 2020;28:1002–9.
[27] Wu JJ, Rong X, Chen F, et al. Investigation on sleep quality of first-line nurses in fighting against corona virus disease 2019 and its influencing factors. Chin Nurs Res 2020;34:558–62.
[28] National Health Commission of the People's Republic of China. Circular of the general office of the National Health Commission of the People's Republic of China on further strengthening the prevention and control of infection in medical institutions during the epidemic. Available at: http://www.nhc.gov.cn/yzygj/s7659/202003/0c85996bb762437581e98317365fa01c.shtml. Accessed June 10, 2020.
[29] Health industry standard of the people's Republic of China WS/T312-2009 - Code for hospital infection monitoring. Chin J Nosocomiol 2009;19:I0002.
[30] He L, Liu D. Challenges and reflections of COVID-19 on modern nosocomial infection management. Chin J Nosocomiol 2020;30:1601–5.
[31] Wu AH. Challenges and strategies of healthcare-associated infection control. West China Med J 2019;34:227–32.
[32] Luthans F, Youssef-Morgan CM. Psychological capital: an evidence-based positive approach. Annu Rev Organ Psych 2017;4:339–66.
[33] Fan LM, Jia LZ. The mediating effect of psychological capital on college students' negative life events and multiple happiness. J Heilongjiang Vocat Instit Ecol Eng 2020;33:119–22.
[34] Bandura A. Self-efficacy: toward a unifying theory of behavioral change. Psychol Rev 1977;84:191–215.
[35] Wang HB, Zhang JJ, Yao BX. Studying on the relationship among college students' general self-efficacy, security and mental health. Chin Health Serv Manag 2011;28:785–7.
[36] Li H. Research on students' self-efficacy and related concepts. J Hubei Univ Educat 2010;27:97–100.
[37] Wu YH, Lin SH, Liu R. Economic benefit analysis of nosocomial infection control. Chin J Nosocomiol 2013;23:5004–6.
[38] Jiang QQ, Chen YH, Diao QX, et al. Study on the impact of psychological capital and capitalization support of ICU specialist nurses on job burnout. J Nurs (China) 2020;27:74–8.
[39] Bai QQ, Wei X. The effect of group psychological counseling on alleviating the psychological pressure of pediatric nurses and improving their sub-health. Capital Food Med 2020;27:109–10.
[40] Zhong GL, Song ZY, Zhang JK, et al. Enlightenment of COVID-19 prevention and control on the systematic construction of epidemic prevention ability in medical institutions. Med J Air Force 2020;36:158–61.
