# Supplementary material for: Mental health status and related factors influencing healthcare workers during the COVID-19 pandemic: A systematic review and meta-analysis
Source: PLoS One. 2024 Jan 19;19(1):e0289454. doi: 10.1371/journal.pone.0289454 (PMC10798549; doi:10.1371/journal.pone.0289454)
Supplement: S1 Data — (ZIP) [file pone.0289454.s011.zip › literatures/235.pdf]

ELSEVIER

Since January 2020 Elsevier has created a COVID-19 resource centre with free information in English and Mandarin on the novel coronavirus COVID-19. The COVID-19 resource centre is hosted on Elsevier Connect, the company's public news and information website.

Elsevier hereby grants permission to make all its COVID-19-related research that is available on the COVID-19 resource centre - including this research content - immediately available in PubMed Central and other publicly funded repositories, such as the WHO COVID database with rights for unrestricted research re-use and analyses in any form or by any means with acknowledgement of the original source. These permissions are granted for free by Elsevier for as long as the COVID-19 resource centre remains active.

Comprehensive Psychiatry 104 (2021) 152217

Contents lists available at ScienceDirect

Comprehensive Psychiatry

journal homepage: www.elsevier.com/locate/comppsych

Prevalence and associated factors of depression, anxiety, and stress among Hubei pediatric nurses during COVID-19 pandemic

Ronghao Zheng [a,1], Yuren Zhou [b,1], Ming Qiu [a], Yiwen Yan [b], Jing Yue [c], Liping Yu [b], Xinyun Lei [d], Danna Tu [a], Yongqun Hu [a,*]

[a] Department of Pediatric, Maternal and Child Health Hospital of Hubei Province, Tongji Medical College, Huazhong University of Science and Technology, Wuhan, Hubei province, People's Republic of China
[b] Wuhan University School Of Health Sciences, Wuhan, Hubei province, People's Republic of China
[c] Emergency Department, Maternal and Child Health Hospital of Hubei Province, Wuhan, Hubei province, People's Republic of China
[d] Department of Nursing, Maternal and Child Health Hospital of Hubei Province, Tongji Medical College, Huazhong University of Science and Technology, Wuhan, Hubei province, People's Republic of China

ARTICLE INFO

Keywords:
COVID-19
pandemic
factor
depression
anxiety
stress

ABSTRACT

Background: The COVID-19 pandemic is putting healthcare workers across the world in an unprecedented situation. The purpose of this study was to evaluate the levels of depression, anxiety, and stress among Hubei pediatric nurses during the COVID-19 pandemic and to analyze the potential factors associated with them.
Materials and methods: A self-designed online questionnaire survey, which consisted of the demographic and selected features, the occupational protection knowledge, attitudes, and practices of COVID-19, and the Chinese version of Depression, Anxiety, and Stress Scale, were used to assess the levels of depression, anxiety, and stress among Hubei pediatric nurses during COVID-19 pandemic. The logistic regression analyses were performed to analyze the potential factors associated with depression, anxiety, and stress.
Results: A total of 617 pediatric nurses were included in the survey. A considerable proportion of pediatric nurses reported symptoms of depression (95 [15.4%]), anxiety (201 [32.6%]), and stress (111 [18.0%]). Results of multivariable logistic regression analyses indicated that the good occupational protection practices (for depression: OR = 0.455, 95%CI: 0.281 to 0.739; for anxiety: OR = 0.597, 95%CI: 0.419 to 0.851; for stress: OR = 0.269, 95%CI: 0.166 to 0.438) and the personal protective equipment (PPE) meeting work requirements (for depression: OR = 0.438, 95% CI: 0.246 to 0.778; for anxiety: OR = 0.581, 95%CI: 0.352 to 0.959; for stress: OR = 0.504, 95%CI: 0.283 to 0.898) were independent protective factors against depression, anxiety, and stress, respectively. Yet, working in an isolation ward or fever clinic was an independent risk factor associated with depression, anxiety, and stress, respectively (for depression: OR = 1.809, 95%CI: 1.103 to 2.966; for anxiety: OR = 1.864, 95%CI: 1.221 to 2.846; for stress: OR = 2.974, 95%CI: 1.866 to 4.741). Having suspected or confirmed COVID-19 patients in the departments (OR = 1.554, 95%CI: 1.053 to 2.294) and coming in contact with the patient's bodily fluids or blood (OR = 1.469, 95%CI: 1.031 to 2.095) were independent risk factors for anxiety, while >3 times of training for COVID-19 related information was an independent protective factor for depression (OR = 0.592, 95%CI: 0.360 to 0.974). Moreover, >10 years of working was an independent risk factor for stress (OR = 1.678, 95%CI: 1.075 to 2.618).
Conclusion: During the COVID-19 outbreak, a considerable proportion of Hubei pediatric nurses had psychological problems. The pediatric nurses endorsing the higher number of risk factors should be given special attention and necessary psychological intervention. Improving the levels of PPE so as to meet the work requirements and intensifying occupational protection practices might help safeguard pediatric nurses from depression, anxiety, and stress.
© 2020 The Authors. Published by Elsevier Inc. This is an open access article under the CC BY-NC-ND license (http://creativecommons.org/licenses/by-nc-nd/4.0/).

1. Introduction

The unprecedented crisis of coronavirus disease 2019 (COVID-19) pandemic has been profoundly affecting peoples' lives around the globe [1]. The measures taken to combat COVID-19, including isolation, contract restrictions, and economic shutdown, have imposed a complete change to the psychosocial environment in affected countries [1]. Hubei

* Corresponding author at: Department of Pediatric, Maternal and Child Health Hospital of Hubei Province, Tongji Medical College, Huazhong University of Science and Technology, 745 Wu Luo Road, Jie Dao Kou, Wuhan 430070, Hubei province, People's Republic of China.
E-mail address: hbfyek2020@126.com (Y. Hu).
[1] These authors contributed equally to this work.

https://doi.org/10.1016/j.comppsych.2020.152217
0010-440X/© 2020 The Authors. Published by Elsevier Inc. This is an open access article under the CC BY-NC-ND license (http://creativecommons.org/licenses/by-nc-nd/4.0/).

province, in particular, Wuhan city, which was the epicenter of the pandemic in China, had been seriously affected [2]. Several studies from China suggested that more than 25% of the general population experienced moderate to severe levels of stress- or anxiety-related symptoms during the COVID-19 pandemic [3,4]. According to previous studies on SARS or Ebola epidemics, healthcare workers could be experiencing extraordinary amounts of pressure. They might be at higher risk of anxiety and depression induced by this sudden life-threatening illness [5–7]. Moreover, SARS survivors, including healthcare workers, could have long-term psychiatric symptoms such as depression, anxiety, and post-traumatic stress disorder [7,8]. It has been shown that a high proportion of healthcare workers are at greater risk for developing Posttraumatic Stress Disorder (PTSD) and Posttraumatic Stress Symptoms (PTSS) after the three coronavirus outbreaks [9,10]. Exposure levels, occupational roles, years of work experience, marital status, and previous psychiatric disorders were identified as the risk factors associated with PTSD and/or PTSS. At the same time, family and social support, supervisors and colleagues support, training, and positive coping strategies were reported as the resilience or protective factors against PTSD and/or PTSS among healthcare workers facing the three coronavirus outbreaks [9]. Regarding the association between age and education level and mental health problems, inconsistent results have been reported in recent COVID-19 studies [10–13]. Nonetheless, it is of utmost importance to accounting for these factors when planning effective intervention strategies to reduce the risk of psychological disorders [9,11].

Recent studies consistently suggested that females tend to be more significantly affected by psychological distress compared to their male counterparts during the COVID-19 pandemic [11,14,15]. Nurses, who make up the largest number among female medical workers, were the most directly involved in the care and treatment of COVID-19 patients around the world; thus, their psychological status should be paid special attention. Few studies also reported that nurses exhibited more frequently mental health problems than physicians during this COVID-19 pandemic [16,17].

With the rapid spread of the epidemic, more and more cases of children affected with COVID-19 were reported all over the world [18–20]. Cases of severe or even critically ill children have also been reported [20–22]. Although children with COVID-19 had milder clinical symptoms and better clinical outcomes, the incubation period of the highly contagious virus for children was longer than that for adults [23]. Therefore, it is vital to understand the psychological status of pediatric nurses and provide them timely with psychological intervention in order to give better services to COVID-19 children. However, no research targeting mental health problems of Hubei pediatric nurses during the COVID-19 pandemic has been reported.

The aim of the current study was to evaluate mental health outcomes among pediatric nurses in Hubei province during the COVID-19 pandemic by quantifying the magnitude of symptoms of depression, anxiety, and stress using the Chinese version of Depression, Anxiety, and Stress Scale (DASS-21). We also analyzed potentially associated factors with depression, anxiety, and stress. Furthermore, we especially explore the influence of the occupational protection knowledge-attitude-practice (KAP) of pediatric nurses on mental health during the COVID-19 pandemic. Our study will provide important evidence that can be used to direct the promotion of mental well-being among pediatric nurses during the COVID-19 pandemic. The results may also help the healthcare system administration agencies to inform tailored interventions targeting the post-traumatic nature of the distress among pediatric nurses during the COVID-19 pandemic.

2. Materials and methods

2.1. Study design

This cross-sectional study was performed using an online questionnaire survey. A snowball sampling strategy was employed to recruit pediatric nurses working in Hubei province from March 6, 2020, to March 9, 2020. The online survey was first disseminated through the WeChat group to pediatric head nurses of Hubei hospitals, who were encouraged to pass it on to other pediatric nurses at Hubei hospitals. During this period, the cumulative total number of confirmed cases was 67,760 in Hubei province [24]. The study was approved by the clinical research ethics committee of the Maternal and Health Hospital of Hubei Province (2020ZECLLW049). Electronically informed consent was provided by all survey participants prior to their enrollment. Participants were asked to take at least one second to answer each item and were allowed to terminate the survey at any time. The participants' IP addresses were valid in Hubei province, and each IP address was allowed to complete the questionnaire only once. Data were anonymously collected through Wenjuanxing (www.wjx.cn), and confidentiality of information was assured.

2.2. Participants

Only the nurses who were working in the department of pediatric in Hubei province were asked to participate in this study. We excluded the participants who had a previous history of psychiatric diseases. Our questionnaire included a choice question on whether participants had psychiatric disorders prior to COVID-19; those who replied positively were automatically excluded by the platform.

2.3. Questionnaire

The questionnaire was self-designed and consisted of the demographic and selected features, the occupational protection KAP of COVID-19, and the Chinese version of DASS-21.

The basic demographic and selected features data, which were self-reported by the participants, included 20 items such as gender (male or female), age (≤40 years or > 40 years), educational level (< undergraduate or ≥ undergraduate), living with family or colleagues or alone, having underlying disease, type of hospital (no tertiary or tertiary), working at specialist children's hospital, working at designated hospital for COVID-19 patients, working at the hospital in Wuhan, years of working (≤10 years or > 10 years), working in an isolation ward or fever clinic, technical title (junior or intermediate or senior), duration of fighting against the epidemic (≤2 weeks or 3–5 weeks or > 5 weeks), number of patients assigned to nurse while on duty (≤8 or > 8), duration of duty (≤8 h or > 8 h), whether she was the primary nurse on duty, times of training for COVID-19 related information (0–3 or > 3), having suspected or confirmed COVID-19 patients at the department, coming in contact with patients' bodily fluids or blood, and whether personal protective equipment (PPE) met work requirements.

The occupational protection KAP of COVID-19 was designed by the five nurses with senior titles and one master of statistics according to the national health commission of the people's republic of China COVID-19 prevention and control scheme (fifth edition) [25] to assess nurses' response to COVID-19 from three dimensions. The part of occupational protection knowledge was divided into 16 questions, which included characteristics of the virus, ward disinfection, medical waste management, and other prevention and control measures of COVID-19. Each question had multiple choices, and only one choice was supposed to be made. 2 points were recorded for the correct answer, and 0 points were calculated for the incorrect answer. The part of occupational protection attitudes was assessed using 3 questions about the agreement on the equally important role among nurses and physicians, in-service nurses routinely learning about the prevention and control of infectious diseases, and nurses timely pointing out nonstandard prevention and control of the hospital. The assessment of occupational protection practices included 9 questions on timely hand hygiene, standard use of PPE, and standard ward disinfection and medical waste management, etc. The 5-point Likert scale was used for each question related to occupational protection attitudes and practices. Detailed contents of the

occupational protection KAP are outlined in Supplementary Table 1. The total score of each part was calculated as the final score. We further calculated the 80th percentile value (P_{80}) of the KAP final score. The final score ≥ P_{80} was defined as good occupational protection KAP, while the final score < P_{80} was deemed as regular occupational protection KAP. The Cronbach's alpha coefficient of the subscales of the occupational protection attitudes and practices was 0.799 and 0.803 in our sample, indicating high reliability. The reliability test for the occupational protection knowledge questionnaire was not applicable because these were not the scale data. As mentioned above, our occupational protection knowledge questionnaire should have good reliability because it was designed by five nurses with senior titles and one master of statistics, and it conformed to the national guideline.

Mental health status was measured utilizing the Chinese version of DASS-21, which was retrieved from the DASS website [26]. DASS-21 was proven to be a valid screening instrument for assessing depression, anxiety, and stress among patients and general populations [11,27–30]. To date, the DSAA has been translated into 53 languages [26], which makes it widely accessible for practitioners and researchers across the globe. The Chinese version of DASS-21 has been shown to have good reliability in the Chinese population, including hospital workers, healthcare workers, university students, and adult residents in China [29–33]. The Chinese version of DASS-21 includes depression, anxiety, and stress subscales, each one containing 7 items for a total of 21 items. All subscales were rated on a four-point Likert scale ranging from 0 (never) to 3 (almost always). Higher scores indicated more severe and negative emotional symptoms. The scores for each subscale were calculated by summing up the scores for the relevant items and then multiplying by two to obtain the final score. Cut-off scores of >9, >7, and > 14 represented a positive screen of depression, anxiety, and stress, respectively. The scores for judging depression were categorized as follows: mild (10–13), moderate (14–20), severe (21–27), extremely severe (28–42). The criteria for anxiety were deemed as follows: mild (8–9), moderate (10–14), severe (15–19), extremely severe (28–42). The stress subscale score was assessed as follows: mild (15–18), moderate (19–25), severe (26–33), extremely severe (34–42) [28]. The Chinese version of DASS-21 obtained high reliabilities in our sample, with Cronbach's alpha of 0.891, 0.862, and 0.895 for depression, anxiety, and stress subscales, respectively; Cronbach's alphas for the total scales was 0.941.

2.4. Study outcomes

The prevalence of depression, anxiety, and stress among the Hubei pediatric nurses at the critical period of the epidemic in China were evaluated. Additionally, the potential factors associated with the psychological outcomes above were also explored.

2.5. Statistical analysis

Data analysis was performed using IBM SPSS statistical software version 25.0. An analysis of descriptive statistics was performed to explore the demographic and selected features of the participants. The scores of occupational protection KAP in this study were not normally distributed and thus were presented as medians with interquartile ranges (IQRs). The ranked data were derived from the counts of each level for symptoms of depression, anxiety, and stress and were presented as numbers and percentages. A single factor logistic regression analysis was conducted to explore the significant associations between demographic and selected features and occupational protection KAP and mental health outcomes. Multivariable logistic regression analyses were employed to identify the independent factors associated with mental health outcomes. The associations between factors and outcomes were presented as odds ratios (ORs) and 95% confidence intervals (CIs). A P-value of <0.05 was considered statistically significant.

3. Results

3.1. Demographic and selected features

Among a total of 617 participants who were included in the questionnaire, 614 (99.5%) were women, and only 35 (5.7%) had the underlying disease. The majority were ≤ 40 years old (518 [84.0%]), from the tertiary hospital (487 [78.9%]), with >3 times of training for COVID-19 related information (482 [78.1%]). Overall, 334 (54.1%) pediatric nurses reported having suspected or confirmed COVID-19 patients in their departments, 147 (23.8%) pediatric nurses worked in the isolation ward or fever clinic, and 314 (50.9%) pediatric nurses had been fighting against the epidemic more than 5 weeks. Importantly, 538 (87.2%) pediatric nurses claimed that their PPE met their work requirements (Table 1).

3.2. The scores of occupational protection KAP

Overall, the participants had good performances of occupational protection KAP. Most participants (470 [76.2%]) scored more than $P80$ on occupational protection attitudes. Approximately half of the

Table 1
Demographic and selected features of participants.

Variables	Category	No.	Percentage (%)
Total		617	100
Age	≤40 years	518	84.0
	>40 years	99	16.0
Gender	Male	3	0.5
	Female	614	99.5
Educational level	<Undergraduate	178	28.8
	≥ Undergraduate	439	71.2
Living with family or colleagues	Solitary	209	33.9
	Living with family	295	47.8
	Living with colleagues	113	18.3
Underlying disease	No	582	94.3
	Yes	35	5.7
Type of hospital	Non-tertiary	130	21.1
	Tertiary	487	78.9
Specialist children's hospital	No	323	52.4
	Yes	294	47.6
Designated hospital for COVID-19 patients	No	330	53.5
	Yes	287	46.5
Hospital in Wuhan	No	282	45.7
	Yes	335	54.3
Years of working	≤10 years	395	64.0
	>10 years	222	36.0
Working in an isolation ward or fever clinic	No	470	76.2
	Yes	147	23.8
Technical title	Junior	147	23.8
	Intermediate	288	46.7
	Senior	182	29.5
Duration of fighting against the epidemic	≤2 weeks	119	19.3
	3-5 weeks	184	29.8
	>5 weeks	314	50.9
Number of patients assigned to nurse while on duty	≤8	448	72.6
	>8	169	37.4
Duration of duty	≤8 h	425	68.9
	>8 h	192	31.1
Primary nurse on duty	No	140	22.7
	Yes	477	77.3
Times of training for COVID-19 related information	0–3	135	21.9
	>3	482	78.1
Having suspected or confirmed COVID-19 patients in the department	No	283	45.9
	Yes	334	54.1
Coming in Contact with the patient's bodily fluids or blood	No	330	53.5
	Yes	287	46.5
PPE meeting work requirements	No	79	12.8
	Yes	538	87.2

Abbreviations: COVID-19, Coronavirus disease 2019; PPE, personal protective equipment.

participants (292 [47.3%]) scored more than *P*80 on occupational protection practices (Table 2).

3.3. The prevalence of mental health outcomes

Table 3 shows the various degrees of mental health outcomes during the COVID-19 pandemic. According to our predefined cut-offs for the DASS-21 scoring system, depression was found in 95 (15.4%), anxiety in 201 (32.6%), and stress in 111 (18.0%) participants. Overall, a considerable proportion of participants (247[40.0%]) had mental health problems.

Table 3
The prevalence of depression, anxiety, and stress among the participants.

	Probable depression		Probable anxiety		Probable stress		Probable mental health outcomes	
	No.	%	No.	%	No.	%	No.	%
Normal	522	84.6	416	67.4	506	82.0	370	60.0
Abnormal	95	15.4	201	32.6	111	18.0	247	40.0
Mild	43	7.0	52	8.4	42	6.8		
Moderate	39	6.3	90	14.6	44	7.1		
Severe	6	1.0	29	4.7	19	3.1		
Extremely severe	7	1.1	30	4.9	6	1.0		

3.4. Factors influencing depression, anxiety, and stress

The male participants were not included in both univariate analysis and multivariable logistic regression analysis due to only 3 male participants in our study. These 3 male participants did not have the depression, anxiety, and stress; the P_{80} values of the KAP remained the same after excluding the 3 male participants. Hence, excluding them from univariate and multivariable logistic regression analysis seemed reasonable.

3.4.1. Univariate analysis

Table 4 presents the relationships between the selected variables and mental health outcomes. The underlying diseases had a significant effect on depression, anxiety, and stress, respectively. However, the pediatric nurses who attended >3 times of training for COVID-19 related information or who reported PPE meeting work requirements or who had good occupational protection practices, were less susceptible to depression, anxiety, and stress, respectively. The pediatric nurses working in the isolation ward or fever clinic were more likely to suffer from depression, anxiety, and stress, compared with those working in other places. More pediatric nurses working in the department, which had suspected or confirmed COVID-19 patients, had the symptoms of anxiety and stress compared to other departments. Coming in contact with the patient's bodily fluids or blood had a significant effect on anxiety. Working at the designated hospital for COVID-19 patients had a significant association with stress. The positive occupational protection attitudes only had a significant effect on depression, whereas it did not have any significant effect on anxiety and stress. The pediatric nurses working in Wuhan hospitals had a significant association with stress. Compared with the junior pediatric nurses, the intermediate pediatric nurses were more prone to anxiety. On the other hand, the age, the educational level, living with family or colleagues, working at specialist children's hospital, the years of working, the duration of fighting against the epidemic, number of patients assigned to nurse while on duty, duration of duty, whether one was a primary nurse on duty, and the occupational protection knowledge did not have any significant effect on the mental health outcomes.

3.4.2. Multivariable logistic regression analysis

In order to adjust for more confounders, variables that had a *P*-value of <0.1 in univariate analysis or that were considered clinically relevant were entered into multivariable logistic regression analysis so as to identify the independent factors associated with mental health outcomes. Variables for inclusion were carefully chosen, given the number of available events, to ensure the parsimony of the final model. Given several previous studies [11–13,34,35], age, educational level, and knowledge towards infectious diseases were associated with mental health problems. Therefore, they were also included in the multivariable logistic regression model, although they did not show any significant differences in univariate analysis. Based on the result of a recent study [36], which revealed a relationship between days of working in isolation wards and psychological impacts among the medical staff and administrative staff; the duration of fighting against the epidemic was included in the multiple analyses. In addition, we included the variable of working at specialist children's hospital in multiple analyses since our study focused on pediatric nurses. Ultimately, 19 variables including age, educational level, living with family or colleagues or alone, underlying disease, type of hospital, working at specialist children's hospital, working at a designated hospital for COVID-19 patients, working at the hospital in Wuhan, years of working, working in an isolation ward or fever clinic, technical title, duration of fighting against the epidemic, times of training for COVID-19 related information, having suspected or confirmed COVID-19 patients in the department, coming in contact with the patient's bodily fluids or blood, whether PPE met work requirements and the occupational protection KAP, were analyzed in multivariable logistic regression models using the method of forwarding stepwise. The results of multivariable logistic regression analysis are presented in Table 5.

In the depression model, working in isolation ward or fever clinic (OR = 1.809, 95%CI: 1.103 to 2.966), times of training for COVID-19 related information (OR = 0.592, 95%CI: 0.360 to 0.974), PPE meeting work requirements (OR = 0.438, 95%CI: 0.246 to 0.778), and occupational protection practices (OR = 0.455, 95%CI: 0.281 to 0.739) were selected as independent factors associated with depression. For anxiety, PPE meeting work requirements (OR = 0.581, 95%CI: 0.352 to 0.959) and good occupational protection practices (OR = 0.597, 95%CI: 0.419 to 0.851) were independent protective factors while working in an isolation ward or fever clinic (OR = 1.864, 95%CI: 1.221 to 2.846), having suspected or confirmed COVID-19 patients in the department (OR = 1.554, 95%CI: 1.053 to 2.294), and coming in contact with the patient's bodily fluids or blood (OR = 1.469, 95%CI: 1.031 to 2.095) were independent risk factors. Four variables were associated with stress among pediatric nurses: years of working (OR = 1.678, 95%CI: 1.075 to 2.618), working in an isolation ward or fever clinic (OR = 2.974, 95% CI: 1.866 to 4.741), PPE meeting work requirements (OR = 0.504, 95% CI: 0.283 to 0.898), and occupational protection practices (OR = 0.269, 95%CI: 0.166 to 0.438).

Overall, the results indicated that after adjusting for the confounders, the good occupational protection practices and the PPE meeting work requirements resulted as independent protective factors against depression, anxiety, and stress, respectively. Yet, working in an isolation ward or fever clinic was an independent risk factor associated with depression, anxiety, and stress, respectively.

Table 2
The scores of occupational protection KAP of COVID-19 among the participants.

	Scores range	Median ± IQR	P_{80} (n)	≥ P_{80} [n (%)]
Knowledge	6–32	22 ± 6	26	134 (21.7%)
Attitudes	3–15	15 ± 0	15	470 (76.2%)
Practices	27–45	44 ± 3	45	292 (47.3%)
Total KAP	58–92	79 ± 7.5	84	137 (22.2%)

Abbreviations: KAP, knowledge-attitude-practice; COVID-19, Coronavirus disease 2019; ICQ, interquartile range; P_{80}, the 80th percentile value.

Table 4
Univariate analyses on factors associated with depression, anxiety, and stress.

Variables	Category (N)	Depression VS Non depression			Anxiety VS Non Anxiety			Stress VS Non Stress		
		No. (%)	OR(95%CI)	P	No. (%)	OR(95%CI)	P	No. (%)	OR(95%CI)	P
Age	≤40 years (516)	84(16.3)			173(33.5)			92(17.8)		
	>40 years (98)	11(11.2)	0.650 (0.333, 1.270)	0.208	28(28.6)	0.793 (0.493, 1.275)	0.339	19(19.4)	1.108 (0.640, 1.920)	0.713
Educational level	<Undergraduate (178)	26(14.6)			59(33.1)			28(15.7)		
	≥ Undergraduate (436)	69(15.8)	1.099 (0.674, 1.72)	0.705	142(32.6)	0.974(0.672, 1.412)	0.890	83(19.0)	1.260 (0.788, 2.013)	0.335
Living with family or colleagues	Solitary (207)	28(13.5)			69(33.3)			35(16.9)		
	Living with family (294)	42(14.3)	1.065 (0.637, 1.783)	0.809	87(29.6)	0.841 (0.573, 1.232)	0.373	48(16.3)	0.959 (0.595, 1.545)	0.863
	Living with colleagues (113)	25(22.1)	1.816 (1.000, 3.298)	0.050	45(39.8)	1.324 (0.823, 2.128)	0.247	28(24.8)	1.619 (0.924, 2.836)	0.092
Underlying disease	No (579)	85(14.7)			184(31.8)			100(17.3)		
	Yes (35)	10(28.6)	2.325 (1.078, 5.014)	0.031	17(48.6)	2.027 (1.021, 4.024)	0.043	11 (31.4)	2.195 (1.042, 4.627)	0.039
Type of hospital	Non-tertiary (128)	19(14.8)			49(38.3)			35 (27.3)		
	Tertiary (486)	76(15.6)	1.063 (0.616, 1.835)	0.825	152 (31.3)	0.734 (0.490, 1.100)	0.134	76 (15.6)	0.493 (0.311, 0.780)	0.003
Specialist Children's Hospital	No (321)	44(13.7)			102(31.8)			59 (18.4)		
	Yes (293)	51 (17.4)	0.754 (0.486, 1.169)	0.206	99 (33.8)	0.913 (0.651, 1.279)	0.596	52(13.2)	1.044 (0.691, 1.575)	0.839
Designated hospital for COVID-19 patients	No (328)	50(15.2)			103(31.4)			46(14.0)		
	Yes (286)	45 (15.7)	1.038 (0.670, 1.609)	0.867	98 (34.3)	1.139 (0.812, 1.596)	0.451	65 (22.7)	1.803 (1.189, 2.735)	0.006
Hospital in Wuhan	No (280)	41(14.6)			89(31.8)			60(21.4)		
	Yes (334)	54 (16.2)	1.124 (0.723, 1.748)	0.603	112(33.5)	1.083 (0.771, 1.520)	0.646	51 (15.3)	0.661(0.437, 0.998)	0.049
Years of working	≤10 years (393)	57(14.5)			123 (31.3)			63(16.0)		
	>10 years (221)	38 (17.2)	1.224 (0.782, 1.916)	0.377	78 (35.3)	1.197(0.845, 1.697)	0.311	48 (21.7)	1.453(0.957, 2.208)	0.080
Working in an isolation ward or fever clinic	No (468)	63(13.5)			133(28.4)			67(14.3)		
	Yes (146)	32 (21.9)	1.217 (1.040, 1.426)	0.015	68 (46.6)	1.300 (1.144, 1.476)	<0.001	44 (30.1)	1.372 (1.185, 1.588)	<0.001
Technical title	Junior (147)	19(12.9)			38(25.9)			22(15.0)		
	Intermediate (286)	46(16.1)	1.291 (0.726, 2.297)	0.384	101(35.3)	1.566(1.007, 2.436)	0.047	49(17.1)	1.175 (0.679, 2.031)	0.564
	Senior (181)	30(16.6)	1.338 (0.719, 2.491)	0.358	62(34.3)	1.494(0.925, 2.416)	0.101	40(22.1)	1.612 (0.909, 2.860)	0.103
Duration of fighting against the epidemic	≤2 weeks (118)	21(17.8)			37(31.3)			20(16.9)		
	3–5 weeks (182)	27(14.8)	0.805(0.431, 1.502)	0.495	54(29.7)	0.924 (0.559, 1.526)	0.756	30(16.5)	0.967 (0.520, 1.798)	0.916
	>5 weeks (314)	47(15.0)	0.813 (0.462, 1.430)	0.472	110(35.0)	1.180 (0.751, 1.856)	0.473	61(19.4)	1.181 (0.677, 2.061)	0.557
Number of patients assigned to nurse while on duty	≤8 (445)	71(16.0)			142(31.9)			77(17.3)		
	>8 (169)	24(14.2)	0.872 (0.528, 1.439)	0.592	59(34.9)	1.144 (0.788, 1.663)	0.479	34(20.1)	1.204 (0.768, 1.886)	0.419
Duration of duty	≤8 h (423)	68(16.1)			138(32.6)			81(19.1)		
	>8 h (191)	27(14.1)	0.859 (0.530, 1.393)	0.539	63(33.0)	1.016 (0.707, 1.462)	0.930	30(15.7)	0.787(0.497, 1.245)	0.306
Primary nurse on duty	No (140)	24(17.1)			47(33.6)			31(22.1)		
	Yes (474)	71(15.0)	0.852 (0.513, 1.413)	0.534	154(32.5)	0.952 (0.638, 1.421)	0.811	80(16.9)	0.714 (0.448, 1.137)	0.156
Times of training for COVID-19 related information	0–3 (135)	33(24.4)			58(43.0)			33(24.4)		
	>3 (479)	62(12.9)	0.460 (0.286, 0.739)	0.001	143(29.9)	0.565 (0.381, 0.837)	0.004	78(16.3)	0.601 (0.379, 0.954)	0.031
Having suspected or confirmed COVID-19 patients in the department	No (282)	35(12.4)			68(24.1)			39(13.8)		
	Yes (332)	60(18.1)	1.557(0.992, 2.444)	0.054	133(40.1)	2.103 (1.482, 2.985)	<0.001	72(21.6)	1.725(1.126, 2.645)	0.012
Coming in Contact with the patient's bodily fluids or blood	No (328)	52(15.9)			90(27.4)			56(17.1)		
	Yes (286)	43(15.0)	0.939(0.605, 1.457)	0.780	111(38.8)	1.677(1.194, 2.356)	0.003	55(19.2)	1.156(0.766, 1.745)	0.489
PPE meeting work requirements	No (79)	22(27.8)			35(44.3)			22(27.8)		
	Yes (535)	73(13.6)	0.409 (0.236, 0.710)	0.001	166(31.0)	0.566 (0.350, 0.914)	0.020	89(16.6)	0.517 (0.301, 0.889)	0.017
Occupational protection knowledge scores	$< P_{80}$ (480)	75(15.6)			163(34.0)			87(18.1)		
	$\geq P_{80}$ (134)	20(14.9)	0.947 (0.555, 1.618)	0.843	38(28.4)	0.770 (0.506, 1.172)	0.223	24(17.9)	0.986 (0.598, 1.623)	0.954
Occupational protection attitudes scores	$< P_{80}$ (147)	31(21.1)			57(38.8)			34(23.1)		
	$\geq P_{80}$ (467)	64(13.7)	0.594(0.369, 0.957)	0.032	144(30.8)	0.704 (0.479, 1.035)	0.074	77(16.5)	0.656 (0.416, 1.034)	0.069
Occupational protection practices scores	$< P_{80}$ (325)	67(20.6)			125(38.5)			85(26.2)		
	$\geq P_{80}$ (289)	28(9.7)	0.413 (0.257, 0.663)	<0.001	76(26.3)	0.571 (0.405, 0.806)	0.001	26(9.0)	0.279 (0.174, 0.448)	<0.001

Abbreviations: COVID-19, Coronavirus disease 2019; PPE, personal protective equipment; CI, confidence interval; P_{80}, the 80th percentile value.
Note: No. represents the number of depression, anxiety and stress in this category; % represents the ratio of the number of depression, anxiety and stress in this category to the total of this category.

4. Discussion

To the best of our knowledge, the present study represents the first attempt to provide data focusing on the mental health problems of Hubei pediatric nurses during the COVID-19 pandemic. Our study demonstrated that a considerable proportion of pediatric nurses were afflicted with depression (95 [15.4%]), anxiety (201 [32.6%]), and stress (111 [18.0%]) during this pandemic. Moreover, the current study identified certain independent protective and risk factors associated with depression, anxiety, and stress among Hubei pediatric nurses. Our survey

Table 5
Multivariate Logistic regression analyses on factors associated with depression, anxiety, and stress.

		Model (Forward stepwise: Likelihood Ratio)				
		B	S.E.	Adjusted OR	95%CI	P
Depression VS Non depression						
Working in an isolation ward or fever clinic	Yes (146) VS No (468)	0.593	0.252	1.809	1.103,2.966	0.019
Times of training for COVID-19 related information	>3 (479) VS 0–3 (135)	−0.524	0.254	0.592	0.360,0.974	0.039
PPE meeting work requirements	Yes (535) VS No (79)	−0.826	0.294	0.438	0.246,0.778	0.005
Occupational protection practices scores	≥ P_{80} (289) VS < P_{80} (325)	−0.787	0.247	0.455	0.281,0.739	0.001
Anxiety VS Non Anxiety						
Working in an isolation ward or fever clinic	Yes (146) VS No (468)	0.623	0.216	1.864	1.221,2.846	0.004
Having suspected or confirmed COVID-19 patients in the department	Yes (332) VS No (282)	0.441	0.199	1.554	1.053,2.294	0.027
Coming in Contact with the patient's bodily fluids or blood	Yes (286) VS No (328)	0.385	0.181	1.469	1.031,2.095	0.033
PPE meeting work requirements	Yes (535) VS No (79)	−0.543	0.255	0.581	0.352,0.959	0.034
Occupational protection practices scores	≥ P_{80} (289) VS < P_{80} (325)	−0.516	0.181	0.597	0.419,0.851	0.004
Stress VS Non Stress						
Working in an isolation ward or fever clinic	Yes (146) VS No (468)	1.090	0.238	2.974	1.866,4.741	<0.001
Years of working	>10 years (221) VS ≤10 years (393)	0.517	0.227	1.678	1.075,2.618	0.023
PPE meeting work requirements	Yes (535) VS No (79)	−0.685	0.295	0.504	0.283,0.898	0.020
Occupational protection practices scores	≥ P_{80} (289) VS < P_{80} (325)	−1.312	0.248	0.269	0.166,0.438	<0.001

Abbreviations: COVID-19, Coronavirus disease 2019; PPE, personal protective equipment; CI, confidence interval; P_{80}, the 80th percentile value.

was conducted at the critical period of this pandemic in China and targeted participants in Hubei province, which was the epicenter of the pandemic in China. Consequently, our findings might have certain implications and reference values for the pediatric nurses in regions and countries currently experiencing the COVID-19 pandemic.

The psychological impacts of COVID-19 on healthcare workers are already apparent. A previous study that used the same scale (DASS-21) as we did, reported that the prevalence rates of depression, anxiety, and stress among the healthcare workers in Singapore and India during the COVID-19 pandemic were 10.6%, 15.7%, and 5.2%, respectively [28], which was lower than in our study. Additionally, compared with Chinese medical health workers in a previous study [37], our study demonstrated that pediatric nurses working at Hubei hospitals experienced symptoms of depression and anxiety more frequently during this pandemic. A previous study showed high levels and rates of depression (50.4%) and anxiety (44.6%) among healthcare workers treating patients exposed to COVID-19 and indicated that nurses thereof had more severe mental health symptoms than other healthcare workers [16]. With the rapid spread of the epidemic, more and more pediatric nurses might be directly exposed to children with COVID-19. Accordingly, the mental health of pediatric nurses should be paid special attention to, and they should be provided with the necessary psychological intervention. Consistent with several previous studies [28,36], the majority of healthcare workers, including Hubei pediatric nurses in this current study, experienced mild and moderate symptoms of depression, anxiety, and/or stress. In contrast, severe symptoms were less common during the COVID-19 pandemic. These results highlighted the need for early identification and the importance of effectively recognizing and treating the milder symptoms or sub-threshold syndromes before they evolve to more complex and severe psychological responses.

Our study identified certain independent risk factors associated with depression, anxiety, and stress among Hubei pediatric nurses. Our finding indicated that working in an isolation ward or fever clinic, which means working in the frontline, was an independent risk factor for worse mental health outcomes in all dimensions of interest. This finding was consistent with several previous studies [16,36] arguing that compared with working in the second line, working in the frontline was associated with an increased risk of depression and/or anxiety [16,36]. As expected, having suspected or confirmed COVID-19 patients in the department and coming in contact with the patients' bodily fluids or blood were risk factors for anxiety in our study, which might be explained by the fact that COVID-19 is a human-to-human transmissible, highly morbid, and potentially fatal disease. A study also reported that the degree of contact with confirmed or suspected cases was directly related to the proportion of mental health disturbances [38]. Similar with the previous study [16], our study also demonstrated that >10 years of working had a significant adverse reaction to stress, which might be explained by the fact that healthcare workers with longer working experience were routinely assigned to care and treat more and/or more severe COVID-19 patients in hospitals of China, whereas the effective treatments of COVID-19 remain unknown or unclear, thus adding further stress.

Our study specially examined the effect of occupational protection KAP on mental health. KAP is a theoretical model that has been used to change human health-related behaviors [39]. Overall, Hubei pediatric nurses had a good performance on occupational protection KAP, which might be primarily due to the nature of the occupation, strict training and management of the hospital, and the overwhelming news reports on this public health emergency. Currently, no study has explored the association between occupational protection KAP and mental health during this COVID-19 pandemic. Lessons learned from the SARS outbreak suggested that knowledge and attitudes towards infectious diseases might affect the levels of panic-related emotions, which could further complicate attempts to prevent the spread of the disease [34,35]. Therefore, we included the occupational protection knowledge in the multivariable logistic regression model, although it was not significant in univariate analysis. Interestingly, after adjusting for the confound factors, the occupational protection knowledge and attitudes did not result as the independent factors associated with depression, anxiety, and stress, while the good occupational protection practices, as expected, resulted as an independent protective factor against these mental health problems. It is possible that when facing such a sudden and highly contagious disease, occupational protection practices are one of the most direct and most quick strategies used to avoid contracting the disease. Thus, healthcare workers might mainly focus on occupational protection practices and/or rapidly transform the effect of knowledge and attitudes into the result of practices. These findings suggested that strengthening occupational protection practices should be given priority when facing the pandemic outbreak.

In the current study, PPE meeting work requirements resulted as an independent protective factor associated with all dimensions of interesting psychological aspects, which indicated that adequate PPE not only protected Hubei pediatric nurses from infectious disease but also reduced the psychological impacts of the pandemic. Due to the complicated procedures of putting on and removing the PPE, training should be regularly performed. In our study, the training for COVID-19 related

information included the use of PPE, hand hygiene, management of occupational exposure, and relevant knowledge, diagnosis, and treatment for COVID-19. The pediatric nurses who attended >3 times of training for COVID-19 related information experienced symptoms of depression, anxiety, and stress less frequently, compared to those who attended ≤3 times of training for COVID-19 related information. Nevertheless, occupational protection knowledge did not have a significant effect on depression, anxiety, and stress. As aforementioned, we speculated that pediatric nurses might pay more attention to occupational protection practice than the occupational protection knowledge during training. Nevertheless, after adjusting for the confounders, times of training for COVID-19 related information only had a significant effect on depression; the reasons might be complicated, and further studies are required to interpret this phenomenon. Regarding the age and education level, inconsistent results have been reported in recent studies [11–13]. In the present study, these factors did not have a significant association with mental health outcomes both in univariate analysis and multiple analyses, which suggested that age and education level might have no significant effect on depression, anxiety, and stress among the cohort of Hubei pediatric nurses during this pandemic. Several studies indicated that the past medical history was associated with/increased risk of depression and/or anxiety [11,15,40,41]; however, the underlying disease was excluded from the multivariable logistic regression model, although it did have a significant association with depression, anxiety, and stress in univariate analysis. These results implied that underlying disease might be an important confounder in our study. On the other hand, statistical influence should be taken into account for the results of multiple analyses due to the small number of participants having underlying diseases.

The present study has some limitations that need to be pointed out. First, this was a cross-section study; thus, it is difficult to draw certain conclusions regarding its long-term effect. Yet, the long-term psychological implications of this population should be further investigated. Second, applying a more strict sampling method was difficult during the pandemic; the snowball sampling method was therefore utilized to recruit the participants. Third, there might be a selection bias because of individuals who were unable or unwilling to attend the online survey even though they received the survey link. Furthermore, the results of logistic regression were only based on female participants. Forth, the use of self-report questionnaires might lead to deviation and/or false information, e.g., the participants who declared not to suffer from a prior psychiatric disorder, which was not investigated more accurately. Fifth, the psychological responses to an epidemic of infectious disease are extremely complicated and might be associated with comprehensive factors. As mentioned above, the duration of fighting against the epidemic might be related to psychological problems, whereas it was not shown to have a significant effect according to the results of univariate and multiple analyses. Hence, findings in our study should be interpreted with caution; further longitudinal studies with large sample sizes need to validate the present findings.

5. Conclusions

A considerable proportion of Hubei pediatric nurses were found to have psychological problems, including depression, anxiety, and stress. Special attention and necessary psychological intervention should be provided to the pediatric nurses, who worked in an isolation ward or fever clinic and/or came in contact with the patient's bodily fluids or blood and/or had suspected or confirmed COVID-19 patients in their departments and/or had >10 years of working. Improving the levels of PPE so that they meet the work requirements and intensify the occupational protection practices might be helpful in safeguarding pediatric nurses from depression, anxiety, and stress.

Supplementary data to this article can be found online at https://doi.org/10.1016/j.comppsych.2020.152217.

Funding

This work was supported by Wuhan Young and Middle-aged Medical Backbone Talent Training Project ([2018]116).

Author contributions

Y.H. designed the study. Y.Z., Y.Y, L.Y., M.Q. and X.L. collected the data and prepared the supplementary material. R.Z., Y.Z., and Y.Y analyzed the data. R.Z., Y.H., and J.Y interpreted the data. R.Z., Y.H., and D.T. drafted the manuscript. R.Z., Y.H., and J.Y reviewed the data analysis and manuscript. All authors approved the final version for publication.

Declaration of Competing Interest

The authors declare there are no conflicts of interest regarding the publication of this paper.

Acknowledgements

The authors acknowledge the help of all the study participants.

References

[1] Fegert JM, Vitiello B, Plener PL, Clemens V. Challenges and burden of the coronavirus 2019 (COVID-19) pandemic for child and adolescent mental health: a narrative review to highlight clinical and research needs in the acute phase and the long return to normality. Child Adolesc Psychiatry Ment Health. 2020;14:20. https://doi.org/10.1186/s13034-020-00329-3.

[2] Ben-Ezra M, Sun S, Hou WK, Goodwin R. The association of being in quarantine and related COVID-19 recommended and non-recommended behaviors with psychological distress in Chinese population. J Affect Disord. 2020;275:66–8. https://doi.org/10.1016/j.jad.2020.06.026.

[3] Qiu J, Shen B, Zhao M, Wang Z, Xie B, Xu Y. A nationwide survey of psychological distress among Chinese people in the COVID-19 epidemic: implications and policy recommendations. Gen Psychiatry. 2020;33:e100213. https://doi.org/10.1136/gpsych-2020-100213.

[4] Wang C, Pan R, Wan X, Tan Y, Xu L, Ho CS, et al. Immediate psychological responses and associated factors during the initial stage of the 2019 coronavirus disease (COVID-19) epidemic among the general population in China. Int J Environ Res Public Health. 2020;17. https://doi.org/10.3390/ijerph17051729.

[5] Liu X, Kakade M, Fuller CJ, Fan B, Fang Y, Kong J, et al. Depression after exposure to stressful events: lessons learned from the severe acute respiratory syndrome epidemic. Compr Psychiatry. 2012;53:15–23. https://doi.org/10.1016/j.comppsych.2011.02.003.

[6] Lung FW, Lu YC, Chang YY, Shu BC. Mental symptoms in different health professionals during the SARS attack: a follow-up study. Psychiatry Q. 2009;80:107–16. https://doi.org/10.1007/s11126-009-9095-5.

[7] Vindegaard N, Eriksen Benros M. COVID-19 pandemic and mental health consequences: Systematic review of the current evidence. Brain Behav Immun. 2020. https://doi.org/10.1016/j.bbi.2020.05.048.

[8] Lee AM, Wong JG, McAlonan GM, Cheung V, Cheung C, Sham PC, et al. Stress and psychological distress among SARS survivors 1 year after the outbreak. Can J Psychiatry Revue Can Psychiatrie. 2007;52:233–40. https://doi.org/10.1177/070674370705200405.

[9] Carmassi C, Foghi C, Dell'Oste V, Cordone A, Bertelloni CA, Bui E, et al. PTSD symptoms in healthcare workers facing the three coronavirus outbreaks: what can we expect after the COVID-19 pandemic. Psychiatry Res. 2020;292:113312. https://doi.org/10.1016/j.psychres.2020.113312.

[10] Braquehais MD, Vargas-Cáceres S, Gómez-Durán E, Nieva G, Valero S, Casas M, et al. The impact of the COVID-19 pandemic on the mental health of healthcare professionals. QJM. 2020. https://doi.org/10.1093/qjmed/hcaa207.

[11] Mazza C, Ricci E, Biondi S, Colasanti M, Ferracuti S, Napoli C, et al. A Nationwide survey of psychological distress among Italian people during the COVID-19 pandemic: immediate psychological responses and associated factors. Int J Environ Res Public Health. 2020;17. https://doi.org/10.3390/ijerph17093165.

[12] Gao J, Zheng P, Jia Y, Chen H, Mao Y, Chen S, et al. Mental health problems and social media exposure during COVID-19 outbreak. PLoS One. 2020;15:e0231924. https://doi.org/10.1371/journal.pone.0231924.

[13] Huang J, Liu F, Teng Z, Chen J, Zhao J, Wang X, et al. Care for the psychological status of frontline medical staff fighting against COVID-19. Clin Infect Dis. 2020. https://doi.org/10.1093/cid/ciaa385.

[14] Ho CS, Chee CY, Ho RC. Mental health strategies to combat the psychological impact of COVID-19 beyond paranoia and panic. Ann Acad Med Singapore. 2020;49:155–60.

[15] Özdin S, Bayrak Özdin Ş. Levels and predictors of anxiety, depression and health anxiety during COVID-19 pandemic in Turkish society: the importance of

gender. Int J Soc Psychiatry. 2020;20764020927051. https://doi.org/10.1177/0020764020927051.

[16] Lai J, Ma S, Wang Y, Cai Z, Hu J, Wei N, et al. Factors associated with mental health outcomes among health care workers exposed to coronavirus disease 2019. JAMA Netw Open. 2020;3:e203976. https://doi.org/10.1001/jamanetworkopen.2020.3976.

[17] Huang JZ, Han MF, Luo TD, Ren AK, Zhou XP. Mental health survey of medical staff in a tertiary infectious disease hospital for COVID-19. Zhonghua Lao Dong Wei Sheng Zhi Ye Bing Za Zhi. 2020;38:192–5. https://doi.org/10.3760/cma.j.cn121094-20200219-00063.

[18] Zhang C, Gu J, Chen Q, Deng N, Li J, Huang L, et al. Clinical and epidemiological characteristics of pediatric SARS-CoV-2 infections in China: a multicenter case series. PLoS Med. 2020;17:e1003130. https://doi.org/10.1371/journal.pmed.1003130.

[19] Zimmermann P, Curtis N. Coronavirus infections in children including COVID-19: an overview of the epidemiology, clinical features, diagnosis, treatment and prevention options in children. Pediatr Infect Dis J. 2020;39:355–68. https://doi.org/10.1097/inf.0000000000002660.

[20] Morand A, Fabre A, Minodier P, Boutin A, Vanel N, Bosdure E, et al. COVID-19 virus and children: what do we know? Archives Pediatrie. 2020;27:117–8. https://doi.org/10.1016/j.arcped.2020.03.001.

[21] Recommendations for the diagnosis, prevention and control of the 2019 novel coronavirus infection in children (first interim edition). Zhonghua er ke za zhi = Chin J Pediatrics. 2020;58:169–74. https://doi.org/10.3760/cma.j.issn.0578-1310.2020.03.001.

[22] Kache S, Chisti MJ, Gumbo F, Mupere E, Zhi X, Nallasamy K, et al. COVID-19 PICU guidelines: for high- and limited-resource settings. Pediatr Res. 2020. https://doi.org/10.1038/s41390-020-1053-9.

[23] Hua CZ, Miao ZP, Zheng JS, Huang Q, Sun QF, Lu HP, et al. Epidemiological features and viral shedding in children with SARS-CoV-2 infection. J Med Virol. 2020. https://doi.org/10.1002/jmv.26180.

[24] National health commission of the people"s republic of China. Updates on the novel coronavirus outbreak up to March 9, 2020. http://www.nhc.gov.cn/xcs/yqtb/202003/948a03ad76f54d3583a018785efd7be9.shtml; 2020. [Accessed 10 March 2020].

[25] National health commission of the people"s republic of China. Prevention and control scheme of COVID-195th ed.. ; 2020http://www.nhc.gov.cn/jkj/s3577/202002/a5d6f7b8c48c451c87dba14889b30147.shtml [Accessed 22 February 2020].

[26] Lovibond PF. DASS translations. http://www2.psy.unsw.edu.au/groups/dass/translations.htm; 2018.

[27] Quek TC, Ho CS, Choo CC, Nguyen LH, Tran BX, Ho RC. Misophonia in Singaporean psychiatric patients: a cross-sectional study. Int J Environ Res Public Health. 2018; 15. https://doi.org/10.3390/ijerph15071410.

[28] Chew NWS, Lee GKH, Tan BYQ, Jing M, Goh Y, Ngiam NJH, et al. A multinational, multicentre study on the psychological outcomes and associated physical symptoms amongst healthcare workers during COVID-19 outbreak. Brain Behav Immun. 2020. https://doi.org/10.1016/j.bbi.2020.04.049.

[29] Lu S, Hu S, Guan Y, Xiao J, Cai D, Gao Z, et al. Measurement invariance of the depression anxiety stress scales-21 across gender in a sample of Chinese University Students. Front Psychol. 2018;9:2064. https://doi.org/10.3389/fpsyg.2018.02064.

[30] Jiang LC, Yan YJ, Jin ZS, Hu ML, Wang L, Song Y, et al. The depression anxiety stress Scale-21 in Chinese hospital workers: reliability, latent structure, and measurement invariance across genders. Front Psychol. 2020;11:247. https://doi.org/10.3389/fpsyg.2020.00247.

[31] Chan RCK, Xu T, Huang J, Wang Y, Zhao Q, Shum DHK, et al. Extending the utility of the depression anxiety stress scale by examining its psychometric properties in Chinese settings. Psychiatry Res. 2012;200:879–83. https://doi.org/10.1016/j.psychres.2012.06.041.

[32] McAlonan GM, Lee AM, Cheung V, Cheung C, Tsang KW, Sham PC, et al. Immediate and sustained psychological impact of an emerging infectious disease outbreak on health care workers. Can J Psychiatry Revue Can Psychiatrie. 2007;52:241–7. https://doi.org/10.1177/070674370705200406.

[33] Wen Y, Wu D, Lv X, Li H, Liu X, Yang Y, et al. Psychometric properties of the Chinese short version of depression anxiety and stress scale in Chinese adults. Chin J Public Health. 2012;28:1436–8.

[34] Person B, Sy F, Holton K, Govert B, Liang A. Fear and stigma: the epidemic within the SARS outbreak. Emerg Infect Dis. 2004;10:358–63. https://doi.org/10.3201/eid1002.030750.

[35] Tan N. An analysis on reasons of SARS-induced psychological panic among students. J Anhui Institute Edu. 2003;21:78–9.

[36] Lu W, Wang H, Lin Y, Li L. Psychological status of medical workforce during the COVID-19 pandemic: a cross-sectional study. Psychiatry Res. 2020;288:112936. https://doi.org/10.1016/j.psychres.2020.112936.

[37] Zhang WR, Wang K, Yin L, Zhao WF, Xue Q, Peng M, et al. Mental health and psychosocial problems of medical health workers during the COVID-19 epidemic in China. Psychother Psychosom. 2020:1–9. https://doi.org/10.1159/000507639.

[38] Kang L, Li Y, Hu S, Chen M, Yang C, Yang BX, et al. The mental health of medical workers in Wuhan, China dealing with the 2019 novel coronavirus. Lancet Psychiatry. 2020;7:e14. https://doi.org/10.1016/s2215-0366(20)30047-x.

[39] Zhong BL, Luo W, Li HM, Zhang QQ, Liu XG, Li WT, et al. Knowledge, attitudes, and practices towards COVID-19 among Chinese residents during the rapid rise period of the COVID-19 outbreak: a quick online cross-sectional survey. Int J Biol Sci. 2020;16:1745–52. https://doi.org/10.7150/ijbs.45221.

[40] Wang C, Pan R, Wan X, Tan Y, Xu L, McIntyre RS, et al. A longitudinal study on the mental health of general population during the COVID-19 epidemic in China. Brain Behav Immun. 2020;87:40–8. https://doi.org/10.1016/j.bbi.2020.04.028.

[41] Li G, Miao J, Wang H, Xu S, Sun W, Fan Y, et al. Psychological impact on women health workers involved in COVID-19 outbreak in Wuhan: a cross-sectional study. J Neurol Neurosurg Psychiatry. 2020. https://doi.org/10.1136/jnnp-2020-323134.
