# Supplementary material for: Mental health status and related factors influencing healthcare workers during the COVID-19 pandemic: A systematic review and meta-analysis
Source: PLoS One. 2024 Jan 19;19(1):e0289454. doi: 10.1371/journal.pone.0289454 (PMC10798549; doi:10.1371/journal.pone.0289454)
Supplement: S1 Data — (ZIP) [file pone.0289454.s011.zip › literatures/237.pdf]

Annals of Work Exposures and Health, 2021, 1–10
doi: 10.1093/annweh/wxab001
Original Article

The Chartered Society for Worker Health Protection

OXFORD

Original Article

Investigation on the Influencing Factors of Mental Health of Healthcare Workers for Aid in Hubei during the Outbreak of COVID-19

Peng Zhou[1,#], Na Du[2,*,#], Dongmei Diao[1], Yingjie OuYang[2] and Heshan Sameera Kankanam Pathiranage[3]

[1]West China Hospital of Sichuan University, Disaster Medical Center of Sichuan University, Stroke Center of Sichuan University, Emergency Department, Chengdu, Sichuan Province, 610041, China; [2]The Fourth People's Hospital of Chengdu, Clinical Psychology Department, Chengdu, Sichuan Province, 610039, China; [3]Southwestern University of Finance and Economics, Chengdu, Sichuan Province, 610074, China

[#]Peng Zhou and Na Du contribute equally to this work.
*Author to whom correspondence should be addressed. Tel: +0086-28-69515823; email: 13920785057@163.com

Submitted 1 August 2020; revised 26 December 2020; editorial decision 29 December 2020; revised version accepted 2 January 2021.

Abstract

Background: This study aimed to determine the factors that were related to the psychological health status of healthcare workers aid for Hubei after the COVID-19 outbreak.
Methods: A total of 1260 participants completed the Self-Rating Scale of Sleep (SRSS), the Generalized Anxiety Scale (GAD-7), and the 9-item patient health questionnaire (PHQ-9) via the on-line questionnaires, and their related experiences with COVID-19 were collected.

Results: The average SRSS score of all participants (25.13 ± 6.41) indicated a mild sleep problem, and the factors that influenced their sleep were the respondent's gender, whether they had patients who died under their care, their history of psychosis and whether their family members were infected with COVID-19. The average GAD-7 score of all participants (12.37 ± 4.89) indicated a moderate anxiety level. The main factors that influenced anxiety were the respondent's gender, years of work, history of psychosis, self-perceived health status, and whether their family members were infected with COVID-19. The average PHQ-9 score of all participants (8.90 ± 5.42) indicated a mild depression level. The primary factors that influenced depression were whether the respondent had nursed/treated severely ill patients in Hubei and whether they had a history of psychosis.
Conclusions: During the outbreak of COVID-19, the symptoms of anxiety were prominent among healthcare workers in Hubei. Moreover, male workers, those whose patients died during treatment, those with a history of anxiety disorders and those whose family members were infected with

© The Author(s) 2021. Published by Oxford University Press on behalf of the British Occupational Hygiene Society.

What's important about this paper?

Providing care to COVID-19 patients overwhelmed many healthcare systems, adversely affecting the psychological health of healthcare workers. Healthcare workers in Hubei, China, experienced mild sleep problems, moderate anxiety, and mild depression. All of these outcomes were associated with infection of family members by COVID-19 or experiences providing care to COVID-19 patients. Psychological support must be provided to healthcare workers to maintain their health and well-being when faced with crises like the COVID-19 pandemic.

COVID-19 reported more serious problems. Therefore, this particular group of healthcare workers needs to be monitored and provided with tailored psychological support.

Keywords: psychology; stress

Background

At the end of 2019, an outbreak of COVID-19 occurred in Wuhan, Hubei Province, China. The new type of virus is highly contagious. Currently, no effective preventive or curative therapeutic drug is available, which has led to massive public panic. When the virus was raging, a group of people risked infection when they applied to provide medical assistance in Hubei Province. Some people can choose to cancel, delay, or alter the way they work, but healthcare workers choose to take on more heavy work and fight against danger. Thus, this group of healthcare specialists in Hubei may have faced work pressure and tremendous mental pressure.

Relevant studies found that during the SARS outbreak in 2003, frontline healthcare workers experienced substantial mental pressure and work pressure (Maunder *et al.*, 2003; Maunder *et al.*, 2004; Wong *et al.*, 2005). Simultaneously, during the outbreak of the 2015 Middle East respiratory syndrome (MERS) Co-V, hospital workers in South Korea also experienced various negative emotions and stress (Son *et al.*, 2019). Frontline medical workers reported a great deal of emotional stress related to their work during the novel swine-origin influenza A (H1N1) outbreak, which originated in Mexico in 2009, and during the Ebola outbreak, which originated in Guinea in 2013 (Wheeler, 1997). However, during the outbreak of COVID-19 in Hubei Province, a large number of healthcare workers from other provinces and cities provided medical support. They were stationed at the centre of the outbreak for nearly 2 months, and this long-term separation from family members and having to adapt to a new place may have further increased the perceived pressure, as being assigned to unfamiliar workgroups could reduce collegial interactions (Maunder, 2004). A related report suggested that healthcare workers were at the forefront of a "special battle" against infectious diseases, thus placing them at a higher risk of infection (Grobler *et al.*, 2016). Therefore, the potential risk may have further increased their mental burden.

A recent report on COVID-19 revealed that frontline healthcare workers are vulnerable to the emotional impact of COVID-19 (Xiang *et al.*, 2020). However, until now, there has been little information on the mental health of healthcare workers who went to Hubei to provide assistance. Therefore, the purpose of this study is to understand the menal statue of these special healthcare workers in the terms of sleep, anxiety, and depression, and to analyse the factors that may affect their psychological conditions through the method of cross-sectional online survey. Identifying the characteristics of these healthcare workers and the factors impacting their mental health would be useful for screening out those who were at high risk of suffering mental problems and developing tailored psychological interventions for them.

Methodology

Subjects

On 8 February 2020, approximately 3 weeks after the World Health Organization announced the emergence of a new coronavirus (2019-nCoV) as a public health emergency of international concern (PHEIC), we conducted this cross-sectional study. We used a snowball sampling approach to distribute questionnaires online. The first questionnaire was distributed to a group with 105 people in WeChat (the members of this group were all healthcare workers from West China Hospital, who were sent to Hubei Province for medical assistance from exterior Hubei Area), when a participant completed the

questionnaire, they forwarded it to other similar groups to expand the sample size (we encouraged them to send this link to other WeChat groups that were also made up of healthcare workers from all over the country aiding for Hubei). The duration of this survey was only 15 days, because we had to guarantee they were still under their aid mission after finishing our survey. The principle of voluntary filling was adopted, and informed consent was provided on the first page of the questionnaire. The research has got the institutional review board (IRB) approval of West China Hospital. One thousand three hundred and fifty-two volunteers took part in our survey. After deleting incomplete and random responses and also the subjects who were not sent to Hubei for assistance were excluded (there is a question asking them about whether they took part in this assistance mission, and if they choose no, they were excluded), a total of 1260 valid questionnaires were analysed in this study, and the recuperation rate of the questionnaire was 93.2%.

Measures

The research tool (scale) was a self-reported questionnaire, which included four parts:

(1) The questionnaire about demographic characteristics: To assess their gender, age, working years, marital status, education level, whether they were physicians (if they chose no, which meant they were nurses), Professional technical title (the subjects chose their title level according to their registration certificate) and whether they had a history of psychosis (which means they had a diagnosis of mental illness in the past, including the diagnosis of anxiety, depression, bipolar disorder, etc.).

(2) Their related experiences with COVID-19: The participants' experiences regarding the epidemic were assessed by asking whether they nursed/treated seriously ill patients infected with COVID-19, whether the patients they treated had died, and whether their family members had been infected with COVID-19.

(3) Their self-perceived health status: It was rated using a 5-point scale: 1 point indicates that their physical condition is very good; 2 points indicates that their physical condition is good; 3 points indicates that their physical condition is average; 4 points indicates that their physical condition is poor; 5 points indicates that their physical condition is very poor.

(4) The scale on sleep, anxiety and depression: (a) The Self-Rating Scale of Sleep (SRSS) (Li, 2000) is composed of 10 items, and the response options for each item range from 1 to 5 points. The final score is the sum of each item, and the total score ranges from 10 to 50 points. It measures sleep from three aspects: sleep time, sleep quality, and sleep attitude. Higher scores indicate more serious sleep problems. Scores of 0–22 points indicate no sleep problem, 23–29 points indicate mild sleep problem, 30–39 points indicate moderate sleep problem, and 40–50 points indicate severe sleep problem. The scale's test–retest reliability = 0.5625, Cronbach' α = 0.6418 (Li, 2012). (b) The Generalized Anxiety Scale (GAD-7) (He *et al.*, 2010) consists of 7 items, and the response options for each item range from 0 to 3 points. The total score ranges from 0 to 21 points. Scores of 0–4 points indicate no anxiety, 5–9 points indicate mild anxiety, 10–14 points indicate moderate anxiety, and 15–21 points indicate severe anxiety. The Cronbach' α coefficient of this scale in Chinese version was 0.898 and the test–retest reliability was 0.856 (He *et al.*, 2010). (c) The nine-item Patient Health Questionnaire (PHQ-9) (Martin *et al.*, 2006) is composed of nine items, and the response options for each item range from 0 to 3 points. The total score ranges from 0 to 27 points. Scores of 0–4 points indicate no depression; 5–9 points indicate mild depression; 10–14 points indicate moderate depression; 15–19 points indicate moderate and severe depression; and 20–27 points indicate severe depression. The Chinese version of this scale was proved to have good reliability and validity (test–retest reliability = 0.934, Cronbach' α = 0.832) (Xu *et al.*, 2007).

Statistical analyses

All data were analyzed using Statistical Package for Social Sciences (SPSS) version 21.0. For each scale, the scores were summed. The mean scores of SRSS, GAD-7, and PHQ-9 were then used for comparisons between groups using a *T*-test or analysis of variance (ANOVA). All potential factors including social-demographic, experiences with COVID-19, and self-perceived health status were incorporated into a multiple linear regression model using the method of Enter to screen out risky factors of the total scores of the above three scales. A *P*-value of less than 0.05 was considered statistically significant.

Results

General situation

Demographics

A total of 1260 participants, including 585 men and 675 women, were surveyed. Among them, 882 were nurses

(70%), and 378 were physicians (30%). The average age of all participants was 30.47 ± 4.53 years old. The average working years of all participants was 8.61 ± 4.37 years. The distribution of their education levels was as follows: 84 workers graduated from secondary school (6.7%), 462 graduated from junior colleges (36.7%), 630 graduated from undergraduate programs (50%), and 84 graduated from graduate schools (6.7%). The distribution of marital status was as follows: 588 unmarried (46.7%); 672 married (53.3%).

Epidemic-related information

Among all the participants, 1176 people had nursed/treated seriously ill patients in Hubei (93.3%); 425 people had experienced the death of patients during their nursing/treatment (33.7%); and 85 respondents reported that they had family members who were infected with COVID-19 (6.7%). One hundred and twenty-four people had a history of an anxiety disorder (9.8%). The self-reported physical health status of the participants was follows: 294 persons thought they were in very good health (23.3%), 798 individuals considered themselves to be in good health (63.3%), and 168 individuals reported that they had average health (13.3%).

The SRSS, GAD-7, PHQ-9 score and their influencing factors

The score of SRSS, GAD-7, and PHQ-9

The average SRSS score of all participants was 25.13 ± 6.41 points, which indicated mild sleep problem. The average GAD-7 score and PHQ-9 score of all participants were 12.37 ± 4.89 and 8.90 ± 5.42, which indicated moderate anxiety and mild depression, respectively. This result indicates that healthcare workers who provided aid in Hubei may have prominent anxiety and a certain degree of depression.

The univariate analysis of scales on sleep, anxiety, and depression

The scores of patients were compared based on different demographic characteristics and epidemic-related experiences. The results showed that there were statistically significant differences in sleep quality ($P < 0.05$) based on gender, whether the respondent had patients who died under their care, history of mental illness, whether their family members were infected with COVID-19 and self-perceived physical health. There were statistically significant differences in symptoms of anxiety among different groups ($P < 0.05$) in terms of gender, marital status, educational background, title, whether they had nursed/treated seriously ill patients, whether they had a history of psychosis, whether their family members were infected with COVID-19 and their self-perceived health status. When coming to symptoms of depression, different groups in terms of educational backgrounds, whether the respondent had nursed/treated seriously ill patients, and whether they had a history of psychosis had significant discrepancy (see Table 1 for details).

Multiple stepwise regression analysis of the factors related to the sleep quality, anxiety, and depression among health care workers

Though we found there were some differences between different groups (Table 1), we wanted to know how these factors together affect the total scores of the SRSS, GAD-7, and PHQ-9, respectively, and we also wanted to establish the best predictive equations for these dependent variables. After verifying the normal distribution of the three dependent variables (the total scores of the above three scales are all close to the normal distribution), all measures including social-demographic (gender, age, working years, marital status, education level, occupation, title and whether they had a history of psychosis), experiences with COVID-19, and self-perceived health status were considered as independent variable. The assignments of the variables are shown in Table 1 (the numbers in brackets after each variable represent specific assignments of value). The results of the analysis indicated that gender, whether the respondent had patients who passed away under their care/treatment, whether participants had previously suffered from psychiatric-related diseases, and whether they had family members who were infected with COVID-19 during the outbreak ($P < 0.05$) are the primary factors that affect the participant's sleep quality (see Table 2 for details), and also the gender, working years, self-perceived health status, whether participants had previously suffered from psychiatric-related diseases, and whether their family members were infected with COVID-19 during the outbreak ($P < 0.05$) are the primary factors that affect their level of anxiety (see Table 3 for details), and whether participants had previously suffered from psychiatric-related diseases and whether they nursed/treated seriously ill patients during the outbreak ($P <$ 0. 05) are the primary factors that affect the participants' degree of depression (see Table 4 for details). The F-values (8, 1251; 8, 1251; 6, 1253) is 25.235, 21.395, and 6.769 ($P < 0.001$), which indicates that the fitted multiple linear progressive regression equations were statistically significant. After evaluating the regression equation models, the multiple correlation coefficient ($R = 0.708, 0.678, 0.408$) and the coefficient of determination ($R^2 = 0.481, 0.438, 0.142$) were calculated. Thus, these independent variables can effectively explain the 48.1%, 43.8%, and 14.2% of the variance in the sleep quality, anxiety, and depression of the participants, respectively.

Table 1. The univariate analysis of SRSS, GAD-7 and PHQ-9 ($x \pm s$, $n = 1260$).

Scales	Variable and assignments	Cases	Means	*t*	*F*	*P*
SRSS	**Gender**			3.397	—	0.001
	Male (1)	585	26.60 ± 6.38			
	Female (2)	675	23.67 ± 6.12			
	**Marriage**			1.037	—	0.301
	No (1)	588	24.64 ± 6.33			
	Yes (2)	672	25.56 ± 6.48			
	**Physician or not**			1.351	—	0.178
	No (1)	882	25.52 ± 6.96			
	Yes (2)	378	24.22 ± 4.82			
	**Whether nursed/treated seriously ill patients with COVID-19**			0.684	—	0.495
	Yes (1)	1176	25.21 ± 6.58			
	No (2)	84	24.00 ± 3.11			
	**Whether the patients they treated had died**			2.481	—	0.014
	Yes (1)	425	25.90 ± 6.28			
	No (2)	835	23.60 ± 6.44			
	**History of psychosis**			11.879	—	0.000
	Yes (1)	124	37.33 ± 0.48			
	No (2)	1136	23.78 ± 5.22			
	**Whether their family members had been infected with COVID-19**			2.049	—	0.042
	Yes (1)	85	28.50 ± 3.63			
	No (2)	1175	24.89 ± 6.50			
	**Education**			—	2.367	0.072
	Secondary (1)	84	24.00 ± 3.11			
	Junior (2)	462	23.82 ± 5.56			
	Undergraduate (3)	630	26.27 ± 7.34			
	Graduate(4)	84	25.00 ± 6.41			
	**Professional technical title**			—	0.521	0.595
	Novice (1)	630	25.53 ± 6.98			
	Middle (2)	504	24.58 ± 6.26			
	Senior (3)	126	25.33 ± 3.38			
	**Self-perceived health conditions**			—	4.201	0.016
	Very good (1)	294	24.21 ± 6.24			
	Good (2)	798	26.29 ± 6.78			
	Average (3)	168	27.50 ± 5.83			
GAD-7	**Gender**			6.832	—	0.000
	male (1)	585	14.46 ± 3.80			
	female (2)	675	10.28 ± 4.99			
	**Marriage**			2.576	—	0.011
	No (1)	588	11.45 ± 5.45			
	Yes (2)	672	13.17 ± 4.21			
	**Physician or not**			1.540	—	0.125
	No (1)	882	12.03 ± 5.42			
	Yes (2)	378	13.16 ± 3.25			
	**Whether nursed/treated seriously ill patients with COVID-19**			3.736	—	0.000
	Yes (1)	1176	12.69 ± 4.90			
	No (2)	84	7.79 ± 0.80			
	**Whether the patients they treated had died**			1.338	—	0.182
	Yes (1)	425	12.69 ± 4.77			
	No (2)	835	11.73 ± 5.11			

Table 1. Continued

Scales	Variable and assignments	Cases	Means	*t*	*F*	*P*
	**History of psychosis**			6.871	—	0.000
	Yes(1)	124	18.67 ± 2.11			
	No (2)	1136	11.67 ± 4.61			
	**Whether their family members had been infected with COVID-19**			2.927	—	0.004
	Yes (1)	85	16.00 ± 3.11			
	No (2)	1175	12.11 ± 4.90			
	**Education**			—	14.208	0.000
	Secondary (1)	84	13.50 ± 1.56			
	Junior (2)	462	10.22 ± 4.34			
	Undergraduate (3)	6 30	14.20 ± 5.04			
	Graduate (4)	84	9.29 ± 1.94			
	**Professional technical title**			—	10.428	0.000
	Novice (1)	630	11.30 ± 5.32			
	Middle (2)	504	14.15 ± 4.24			
	Senior (3)	126	10.52 ± 2.38			
	**Self-perceived health conditions**			—	4.366	0.014
	Very good (1)	294	11.24 ± 5.53			
	Good (2)	798	12.31 ± 4.38			
	Average (3)	168	14.61 ± 5.44			
PHQ-9	**Gender**			1.611	—	0.109
	Male(1)	585	9.50 ± 5.37			
	Female(2)	675	8.30 ± 5.42			
	**Marriage**			0.348	—	0.728
	No(1)	588	8.77 ± 5.23			
	Yes(2)	672	9.03 ± 5.60			
	**Physician or Not**			1.477	—	0.141
	No (1)	882	9.27 ± 5.68			
	Yes (2)	378	8.06 ± 4.69			
	**Whether nursed/treated seriously ill patients with COVID-19**			3.885	—	0.000
	Yes (1)	1176	9.28 ± 5.33			
	No (2)	84	3.64 ± 3.65			
	**Whether the patients they treated had died**			1.008	—	0.314
	Yes(1)	425	9.17 ± 5.60			
	No (2)	835	8.37 ± 5.03			
	**History of psychosis**			4.625	—	0.000
	Yes (1)	124	13.86 ± 5.58			
	No (2)	1136	8.35 ± 5.13			
	**Whether their family members had been infected with COVID-19**			0.391	—	0.697
	Yes (1)	85	8.36 ± 5.18			
	No(2)	1175	8.94 ± 5.45			
	**Education**			—	2.820	0.040
	Secondary(1)	84	6.93 ± 4.16			
	Junior (2)	462	8.17 ± 4.97			
	Undergraduate(3)	630	9.92 ± 5.74			
	Graduate (4)	84	7.29 ± 5.28			
	**Professional technical title**			—	0.663	0.516
	Novice (1)	630	8.70 ± 5.88			
	Middle(2)	504	9.38 ± 4.90			
	Senior(3)	126	8.05 ± 5.02			

Table 1. Continued

Scales	Variable and assignments	Cases	Means	*t*	*F*	*P*
	**Self-perceived health conditions**			—	0.911	0.404
	Very good (1)	294	8.41 ± 5.56			
	Good (2)	798	9.28 ± 5.45			
	Average (3)	168	8.23 ± 5.03			

Table 2. The multiple stepwise regression analysis of influencing factors of SRSS.

Variable	Regression coefficients	Standard error of regression coefficient	Standardized regression coefficient	*t*	*P*
Constant	69.850	5.927			
Gender	−1.652	0.740	−0.129	−2.233	0.027
Death[a]	−2.163	0.925	−0.159	−2.339	0.020
History of psychosis	−11.871	1.194	−0.557	−9.946	0.000
Family-infected[b]	−7.315	1.622	−0.285	−4.510	0.000
Age	1.583	0.968	0.117	1.635	0.104
Working years	1.042	0.663	0.097	1.571	0.118
Marriage	0.561	0.780	0.044	0.719	0.473
Physician or not	−1.640	1.184	−0.117	−1.385	0.168
Education	0.929	0.673	0.104	1.381	0.169
Professional technical title	−0.128	0.133	−0.087	−0.959	0.339
Self-perceived health conditions	1.248	1.474	0.049	0.846	0.395
Treated seriously ill patients[c]	−0.511	0.268	−0.127	−1.908	0.058

F (8, 1251) = 25.235 (P < 0.001), R = 0.708, R^2 = 0.481.
[a]Stands for whether the patients they treated had died.
[b]Stands for whether their family members had been infected with COVID-19.
[c]Stands for whether they had nursed/treated seriously ill patients infected with COVID-19.

Discussion

This study used an online questionnaire to assess the mental health status of healthcare workers who provided aid in Hubei during the COVID-19 outbreak. The relevant influencing factors that may affect the psychological conditions of the respondents were also examined, so that we can timely provide tailored psychological assistance services for them during their aid tasks, including psychological intervention through the Internet, telephone, etc., to improve their adaptability in unfamiliar circumstances and help them return to normal life.

What the demographic characteristics tell us

A total of 1260 healthcare workers were recruited in this study. Among them, the number of nurses was higher than that of doctors, which indicated that the shortage of nurses in the outbreak site was worse than that of doctors. Every day, nurses must perform the daily care for the patients. Only with the appropriate number of healthcare workers and effective collaboration can the work be optimized. In addition, 93.3% of the participants reported that they had nursed or treated seriously ill patients. Moreover, some healthcare workers also reported that they had family members who were infected. However, these workers had to continue to provide medical assistance even under great pressure, which may have contributed to their stress. Only 23.3% of the respondents reported that physical health was excellent. These phenomena indicate that China's medical workers are facing substantial work pressure. This suggests that more attention should be paid to the psychological health status of special populations.

The mental health of healthcare workers and their risk factors

The study revealed that our participants had mild sleep problems, and their average anxiety and depression were at moderate and mild level, respectively. Similarly, some studies also reported that the health care workers who

Table 3. The multiple stepwise regression analysis of influencing factors of GAD-7.

Variable	Regression coefficient	Standard error of regression coefficient	Standardized regression coefficient	*t*	*P*
Constant	31.630	4.708			
Gender	−2.906	0.588	−0.298	−4.945	0.000
Working years	0.274	0.099	0.245	2.785	0.006
History of psychosis	−6.817	0.948	−0.419	−7.192	0.000
Self-perceived health conditions	2.588	0.469	0.317	5.514	0.000
Family-infected [b]	−4.835	1.288	−0.247	−3.753	0.000
Death[a]	−1.440	0.892	−0.135	−1.614	0.108
Age	0.340	0.810	0.035	0.420	0.675
Marriage	0.346	0.507	0.051	0.683	0.496
Physician or not	−0.037	0.202	−0.012	−0.185	0.854
Education	1.248	1.474	0.049	0.846	0.398
Professional technical title	−0.170	0.164	−0.137	−1.037	0.301
Treated seriously ill patients [c]	−3.383	1.919	−0.156	−1.763	0.080

F (8, 1251) = 21.395 (P < 0.001), R = 0.678, R^2 = 0.438.
[a]Stands for whether the patients they treated had died.
[b]Stands for whether their family members had been infected with COVID-19.
[c]Stands for whether they had nursed/treated seriously ill patients infected with COVID-19.

Table 4. The multiple stepwise regression analysis of influencing factors of PHQ-9.

Variable	Regression coefficient	Standard error of regression coefficient	Standardized regression coefficient	*t*	*P*
Constant	18.827	3.883			
History of psychosis	−4.877	1.244	−0.271	−3.920	0.000
Treated seriously ill patients[c]	−5.597	1.542	−0.257	−3.619	0.000
Gender	−2.150	1.184	−0.198	−1.816	0.071
Working years	0.307	0.860	0.028	0.357	0.721
Self-perceived health conditions	2.343	1.305	0.199	1.796	0.074
Family-infected[b]	−1.928	1.067	−0.168	−1.808	0.072
Death[a]	−2.046	2.114	−0.094	−0.968	0.334
Age	1.094	1.304	0.134	0.839	0.403
Marriage	0.113	0.147	0.092	0.772	0.441
Physician or not	−0.722	0.730	−0.080	−0.989	0.324
Education	0.221	0.295	0.065	0.749	0.455
Professional technical title	−1.799	1.573	−0.221	−1.144	0.254

F (6,1253) = 6.769 (P < 0.001), R = 0.408, R^2 = 0.142.
[a]Stands for whether the patients they treated had died.
[b]Stands for whether their family members had been infected with COVID-19.
[c]Stands for whether they had nursed/treated seriously ill patients infected with COVID-19.

treated COVID-19 patients showed a significantly low self-assessment of their mental status (Stojanov *et al.*, 2020; Wańkowicz *et al.*, 2020). In Zhang's (2020) study, they also found medical health workers had a higher prevalence of insomnia than nonmedical health workers during the COVID-19 outbreak. However, the subjects in their researches came from different areas with heterogeneity. Our study focused on the relatively homogeneous group, which was more conducive to understand their sleep situation and mental status. Through the analysis of risk factors, we found that those who cared for or treated patients who eventually died were more likely to have sleep problems. And Zhang (2020) found being at risk of contact with COVID-19 patients in hospitals was

risk factor for insomnia among medical health workers. Our result was different from theirs, because our subjects needed to face the infected patients rather than having the risk of contacting with COVID-19 patients. Many of them cured/ treated critically ill patients, so the death of patients might become the key risk factor. Witnessing the death of a patient can lead to a certain amount of stress. Gao *et al.* (2014) found that the stress level of individuals who had seen death during an earthquake significantly increases. Therefore, it is understandable when medical workers have insomnia after experiencing the death of their patients. Additionally, those who considered their health worse were prone to develop anxiety symptoms, just as Zhang's (2020) finding that medical workers with organic diseases showed more anxiety. We also found the new result that the longer the participants have worked, the easier they are likely to suffer anxiety. The explanation might be that the healthcare workers with longer working years, although they are more experienced, tend to be older, and most of them are already married. Sudden aid tasks will greatly increase their risk of infection. They had to face worries about whether the virus will be transmitted to their family members after returning home. However, the younger ones may live alone, so this worry will be relatively reduced. In addition, our survey found that males who had a history of anxiety disorders and whose family members had been infected with COVID-19 would have more sleep problems and anxiety symptoms. In other's study, they discovered that being female was the risk factor of anxiety (Zhang *et al.*, 2020), which was contrary to our result. We thought that men are more likely to take a repressive approach when facing changes in the environment and stress events. However, this approach does not lead to the decline of negative emotions. Conversely, the repressed negative emotions may be expressed via another form of physical discomfort, such as insomnia, which is one of the most common symptoms of anxiety. The above screening of the relevant factors suggests that psychological interventions should be tailored based on the worker's circumstances. For those with special experience, offering regular psychological support and additional guidance for their sleep habits should be considered. Only good sleep can ensure that workers have the full mental capacity to cope with medical work. At the same time, it may also be necessary to design short-term, fast-acting psychological assistance programmes for this particular group of people to relieve anxiety, including focus-solving therapy and music therapy. At last, we also explored the healthcare workers' opinions on COVID-19, and made a little discussion. For detailed information, please refer to the supplementary material.

Strengths and limitations

In our study, the anonymous self-assessment method and the way of collecting questionnaires through the Internet help the subjects to expose their true inner feelings, instead of giving false answers taking other factors into account. In addition, this approach minimizes the risk of spreading the virus. The questionnaires we selected are all questionnaires with a small number of items so that it won't waste them a lot of time, which could guarantee enough time for them to rest. Although our study has the above-mentioned advantages, it still has certain shortcomings. To collect the time-sensitive data, our study has to use the snowball method to obtain the largest possible sample size, which is likely to lead to bias. Since all questionnaires use the method of self-assessment, the results obtained are subjective and can only reflect their feelings at that time. However, this survey only uses a cross-sectional study, and it is impossible to know the changes in the mental state of these participants after the assistance task. Therefore, if we can combine longitudinal surveys and telephone assessments in the future, we will understand the trend of their psychological changes more objectively.

Conclusions

Finally, through this study, we can discover that there is a specific lopsidedness in mental health level and sleep of healthcare workers aid for Hubei Province. When coming to the influencing factor, we also found that male workers, those whose patients have died during their treatment, with previous anxiety disorders and whose family members infected with COVID-19 were facing more serious problems. Therefore, this special group needs to be strengthened follow-up psychological support individually.

Acknowledgements

We sincerely thank all the healthcare workers who took part in our survey for their assistance in this study.

Funding

None to declare.

Conflicts of interest

None to declare.

Ethics approval and consent to participate

The research has got the institutional review board (IRB) approval of West China Hospital, and all the subjects were recruited in the principle of voluntary.

References

Gao HY, Yang JH, HuangYL *et al*. (2014) Investigations of the hospitalized wounded psychosomatic state in a general hospital of Sichuan Province after the Lushan earthquake. *Sichuan Mental Health*; **27**: 234–236.

Grobler L, Mehtar S, Dheda K *et al*. (2016) The epidemiology of tuberculosis in health care workers in South Africa: a systematic review. *BMC Health Serv Res*; **16**: 416.

He XX, Li CB, Qian J, *et al*. (2010) Reliability and validity of a generalized anxiety disorder scale in general hospital outpatients. *Shanghai Arch Psychiatry*; **22**: 200–203.

Li JM. (2012) Seff-Rating Scale of Sleep (SRSS). *China J Health Psychol*; **20**: 1851.

Li JM, Yin SF, Duan JX, *et al*. (2000) Analysis rating of sleep state of 13273 normal persons. *Health Psychol J*; **8**: 351–3.

Martin A, Rief W, Klaiberg A *et al*. (2006) Validity of the Brief Patient Health Questionnaire Mood Scale (PHQ-9) in the general population. *Gen Hosp Psychiatry*; **28**: 71–7.

Maunder R. (2004) The experience of the 2003 SARS outbreak as a traumatic stress among frontline healthcare workers in Toronto: lessons learned. *Philos Trans R Soc Lond B Biol Sci*; **359**: 1117–25.

Maunder R, Hunter J, Vincent L *et al*. (2003) The immediate psychological and occupational impact of the 2003 SARS outbreak in a teaching hospital. *CMAJ*; **168**: 1245–51.

Maunder RG, Lancee WJ, Rourke S *et al*. (2004) Factors associated with the psychological impact of severe acute respiratory syndrome on nurses and other hospital workers in Toronto. *Psychosom Med*; **66**: 938–42.

Son H, Lee WJ, Kim HS *et al*. (2019) Examination of hospital workers' emotional responses to an infectious disease outbreak: lessons from the 2015 MERS Co-V Outbreak in South Korea. *Disaster Med Public Health Prep*; **13**: 504–10.

Stojanov J, Malobabic M, Stanojevic G *et al*. (2020) Quality of sleep and health-related quality of life among health care professionals treating patients with coronavirus disease-19. *Int J Soc Psychiatry;* 0020764020942800. doi:10.1177/0020764020942800.

Wańkowicz P, Szylińska A, Rotter I. (2020) Assessment of mental health factors among health professionals depending on their contact with COVID-19 Patients. *Int J Environ Res Public Health*; **17**: 5849.

Wheeler HH. (1997) A review of nurse occupational stress research: 1. *Br J Nurs*; **6**: 642–5.

Wong TW, Yau JK, Chan CL *et al*. (2005) The psychological impact of severe acute respiratory syndrome outbreak on healthcare workers in emergency departments and how they cope. *Eur J Emerg Med*; **12**: 13–8.

Xiang YT, Yang Y, Li W *et al*. (2020) Timely mental health care for the 2019 novel coronavirus outbreak is urgently needed. *Lancet Psychiatry*; **7**: 228–9.

Xu Y, Wu HS, Xu YF. (2007) The reliability and validity of patient health questionnaire depression module (PHQ-9) in Chinese elderly. *Shanghai Psychiatr Med*; **19**: 257–8.

Zhang WR, Wang K, Yin L *et al*. (2020) Mental health and psychosocial problems of medical health workers during the COVID-19 Epidemic in China. *Psychother Psychosom*; **89**: 242–50.
