# Supplementary material for: Mental health status and related factors influencing healthcare workers during the COVID-19 pandemic: A systematic review and meta-analysis
Source: PLoS One. 2024 Jan 19;19(1):e0289454. doi: 10.1371/journal.pone.0289454 (PMC10798549; doi:10.1371/journal.pone.0289454)
Supplement: S1 Data — (ZIP) [file pone.0289454.s011.zip › literatures/238.pdf]

EClinicalMedicine 24 (2020) 100443

Contents lists available at ScienceDirect

EClinicalMedicine

journal homepage: https://www.journals.elsevier.com/eclinicalmedicine

Research Paper

COVID-19 in Wuhan: Sociodemographic characteristics and hospital support measures associated with the immediate psychological impact on healthcare workers

Zhou Zhu[a,1], Shabei Xu[a,1], Hui Wang[b,1], Zheng Liu[c,1], Jianhong Wu[d,1], Guo Li[a,1], Jinfeng Miao[a,1], Chenyan Zhang[e], Yuan Yang[e], Wenzhe Sun[a], Suiqiang Zhu[a], Yebin Fan[f], Yuxi Chen[g], Junbo Hu[d,*], Jihong Liu[h,*], Wei Wang[a,*]

[a] Department of Neurology, Tongji Hospital, Tongji Medical College, Huazhong University of Science and Technology, No. 1095 Jiefang Avenue, Qiaokou District, Wuhan 430030, Hubei, China
[b] Nursing Department, Tongji Hospital, Tongji Medical College, Huazhong University of Science and Technology, Wuhan, Hubei, China
[c] Department of Otolaryngology–Head and Neck Surgery, Tongji Hospital, Tongji Medical College, Huazhong University of Science and Technology, Wuhan, Hubei, China
[d] Department of Gastrointestinal Surgery, Tongji Hospital, Tongji Medical College, Huazhong University of Science and Technology, Wuhan, Hubei, China
[e] Department of Psychiatry, Tongji Hospital, Tongji Medical College, Huazhong University of Science and Technology, Wuhan, Hubei, China
[f] School of Computer Science and Technology, Huazhong University of Science and Technology, 1037 Luoyu Road, Wuhan 430074, Hubei, China
[g] Solomon H. Snyder Department of Neuroscience, Johns Hopkins University, Baltimore, MD 21218, USA
[h] Department of Urology, Tongji Hospital, Tongji Medical College, Huazhong University of Science and Technology, Wuhan, Hubei, China

ARTICLE INFO

Article History:
Received 16 April 2020
Revised 12 June 2020
Accepted 12 June 2020
Available online 24 June 2020

ABSTRACT

Background: The outbreak of COVID-19 has laid unprecedented threats and challenges to health workers (HWs) in Wuhan, China. We aimed to assess the sociodemographic characteristics and hospital support measures associated with the immediate psychological impact on HWs at Tongji Hospital in Wuhan during COVID-19 outbreak.

Methods: We conducted a single-center, cross-sectional survey of HWs via online questionnaires between February 8th and 10th, 2020. We evaluated stress, depression and anxiety by IES-R, PHQ-9, and GAD-7, respectively. We also designed a questionnaire to assess the perceptions of threat of COVID-19, and the satisfactions of the hospital's support measures. Multivariate logistic regressions were used to identify associated variables of acute stress, depression, and anxiety.

Findings: We received 5062 completed questionnaires (response rate, 77.1%). 29.8%, 13.5% and 24.1% HWs reported stress, depression and anxiety symptoms. Women (odds ratio [OR], 1.31; 95% CI, 0.47–0.97; $p = 0.032$), years of working >10 years (OR, 2.02; 95% CI, 1.47–2.79; $p<0.001$), concomitant chronic diseases (OR, 1.51; 95% CI, 1.27–1.80; $p<0.001$), history of mental disorders (OR, 3.27; 95% CI, 1.77–6.05; $p<0.001$), family members or relatives confirmed or suspected (OR, 1.23; 95% CI, 1.02–1.48; $p = 0.03$), hospital-based and department-based care (OR, 0.76; 95% CI, 0.60–0.97; $p = 0.024$) and full coverage of all departments for avoiding nosocomial infection (OR, 0.69; 95% CI, 0.53–0.89; $p = 0.004$) were associated with stress.

Interpretation: Women and those who have more than 10 years of working, concomitant chronic diseases, history of mental disorders, and family members or relatives confirmed or suspected are susceptible to stress, depression and anxiety among HWs during the pandemic. In addition, since HWs often have a greater stigma against mental problems than the general public, it is worthwhile to address the needs of the HWs during this pandemic and to provide appropriate psychological supports for those people at high risk of mental problems.

© 2020 The Author(s). Published by Elsevier Ltd. This is an open access article under the CC BY-NC-ND license. (http://creativecommons.org/licenses/by-nc-nd/4.0/)

1. Introduction

The rapid escalation of COVID-19 pandemic has resulted in central health crises all around the world. As the first outbreak area of COVID-19 pandemic, Wuhan presented the earliest reports of a large number of confirmed cases and high mortality, and were put on

* Corresponding authors.
E-mail addresses: jbhu@tjh.tjmu.edu.cn (J. Hu), jhliu@tjh.tjmu.edu.cn (J. Liu), wwang@vip.126.com (W. Wang).

1 These authors contributed equally to this work.

https://doi.org/10.1016/j.eclinm.2020.100443
2589-5370/© 2020 The Author(s). Published by Elsevier Ltd. This is an open access article under the CC BY-NC-ND license. (http://creativecommons.org/licenses/by-nc-nd/4.0/)

Research in context

Evidence before this study

We searched Google Scholar, Medline, Embase, Scopus, PubMed, Science Direct, Global Health Database, and the WHO library with the following key or title word [("new coronavirus pneumonia" OR "2019 novel coronavirus" OR "COVID-19″ OR "SARS-CoV-2″) AND ("medical workers"OR "medical profession" OR "medical personnel" OR "health workers" OR "health care workers") AND ("mental health" OR "psychological impact" OR "stress" OR "depression" OR "depressive" OR "anxiety" OR "anxious" OR "psychiatric symptoms")], up to April 15, 2020. Our search did not impose language restrictions. We examined all articles and reference lists of available after COVID-19 outbreak, and there are two studies on the risk factors of mental health problems of HWs in Wuhan city. However, the sampling designs of them are not very convincing and no research has explored the relationship between hospital support measures and the psychological status of HWs. Health workers in the outbreak epicenter presented high levels of psychological distress over the previous SARS outbreak and it is not known whether the previous experience is representative or unique.

Added value of this study

The COVID-19 outbreak is unique in recent history in its rapidity of transmission and the large number of SARS-CoV-2 infected people. Hence, HWs in Wuhan were facing immediate psychological impact. In this study, health workers from Tongji Hospital presented high rates of depression, anxiety and stress, and the psychological problems are highly associated with sociodemographic characteristics, the perceptions of threat of the COVID-19, and hospital support measures. In addition, we developed effective, easy-to-use clinical screening tools to identify high-risk groups of depression, anxiety and stress in HWs.

Implications of all the available evidence

Research on the factors of health workers' psychological problems may be helpful to develop policies and practices to minimize the risk of mental health problems affected by COVID-19 among health workers, thereby improving their work efficiency and promoting their long-term health.

unprecedented lockdowns all of sudden. Residents in Wuhan were generally isolated and immersed in tremendous reports from social media, which might exacerbate public fear, panic, and distress. Health workers (HWs) in Wuhan were saving lives while encountering an increasing workload and risk of infection. In the early stage of COVID-19 pandemic, it was reported that infected HWs accounted for 29% of all hospitalized COVID-19 patients [1]. Also, quarantined HWs might be facing potential social isolation, and unquarantined HWs experiencing social discrimination. Therefore, they are susceptible to complex emotional reactions and psychological distress [2].

The mental health problems of HWs would impair their attention, cognitive functioning, and clinical decision-making [3,4], consequently increase the occurrence of medical errors and incidents, and ultimately put patients at risk. It was also well known that acute stress in disasters could have a lasting effect on the overall wellbeing [5–7]. Moreover, acute stress in disasters could lead to resignation thoughts in HWs, further aggravating shortage of health care workforce. Hence, to explore the sociodemographic characteristics, sources of psychological stress and hospital support measures associated with the mental health problems of HWs in the COVID-19 pandemic have become an urgent public health concern.

Tongji Hospital, the biggest tertiary hospitals in Wuhan, has been designated by the government as "the specific hospital for the treatment of severe patients with COVID-19 in Wuhan". Since the outbreak of COVID-19, Tongji hospital has gradually opened 2000 beds for severe COVID-19 patients, and continually transferred HWs from all departments to fight the COVID-19. The management of Tongji Hospital was initially alert to the mental health problems that HWs might encounter, and therefore implemented a series of hospital support measures, including organizing WeChat Balint group, providing hospital-based and department-based care, full coverage of all departments for avoiding nosocomial infection, reasonable work shift arrangement, and sufficient logistical support and lodging support for HWs living apart from their families.

On Jan 26, 2020, China's National Health Commission (NHC) issued Guidelines for Emergency Psychological Crisis Interventions [8]. To date, research of large sample size on the immediate psychological impact of COVID-19 on HWs of Wuhan in response to the COVID-19 outbreak is still lacking. Meanwhile, few studies investigated the relationship between hospital support measures and the psychological status of HWs, although the psychological state under special events may be greatly affected by the hospital support measures [9]. We aimed to evaluate the immediate psychological impact on HWs at Tongji Hospital, to determine the associated variables of acute stress, depression, and anxiety, to assess the associations between hospital support measures and mental health, and to develop effective, easy-to-use clinical screening tools to identify high-risk individuals of acute stress, depression, and anxiety among HWs.

2. Methods

2.1. Study design and participants

The study was a single-center, cross-sectional survey. All the doctors, nurses and clinical technicians who were employed in Tongji Hospital at present were included in this study, and those who are engaged in administration, logistics, finance, etc. were excluded. The study was conducted between February 8th and 10th, 2020, two weeks after the authority in Wuhan suspended all public transport on January 23. The questionnaire consisted of five parts: online informed consent, sociodemographic information, perceptions of threat of COVID-19, the hospital's support measures and rating scales including Impact of Event Scale-Revised Questionnaires (IES-R), Patient Health Questionnaire-9 (PHQ-9), and Generalized Anxiety Disorder 7-item (GAD-7).

WJX online survey platform was used (https://www.wjx.cn/), and data were collected through an anonymous online questionnaire which were distributed to all HWs via WeChat. Only one response per person to the questionnaire was permitted. The database was exported from WJX survey platform. The senior investigators performed quality control by checking the collected questionnaires daily. Two researchers entered data into the database double-blindly using Epidata.3.0 to ensure accuracy. The study was approved by the institutional ethics board of Tongji Hospital, Tongji Medical College of Huazhong University of Science and Technology (ID: TJ-C20200129). The data analyses were done on unidentified datasets. This study conforms to the STROBE guidelines.

2.2. Measures

The sociodemographic information was collected. The frontline HWs are those directly providing services to confirmed or suspected COVID-19 patients. Exercise habits are defined as meeting the WHO physical activity recommendations for adults aged 18–64 years old [10]. We provide five items to assess the perceptions of threat of COVID-19: (1) Do you feel that you have a history of exposure to SARS-Cov-2? (2) Have you ever thought of resigning because of COVID-19 outbreak? (3) Have you

worried about the life-threatening once infected? (4) Do you feel that families and friends have avoided contact with you because of your work? (5) Have you worried about yourself or your family members being infected by SARS-Cov-2? The support measures included the following 5 items: (1) Have you joined Tongji WeChat Balint Group? (2) Have you received care provided by hospital and department administrators? (3) Are you satisfied with full coverage of all departments for avoiding nosocomial infection? (4) Are you satisfied with your work shift arrangement? (5) Are you satisfied with the logistical support and accommodations provided by Tongji Hospital? A pilot study was conducted in 85 doctors and nurses on January 30, 2020 using this questionnaire. We retested the same subjects after 7 days. The test-retest reliability of the designed questionnaire was 0.78 which could be widely accepted.

Hospital-based and department-based care included additional allowances for frontline HWs, preferential policies on the promotion of professional title to frontline HWs, regular greetings and reassurance from hospital executives, nursing leaders and department chairs for all HWs. In addition, when HWs are infected, such incidents should be treated as work-related injuries.

Administrators of Tongji Hospital have prepared hotels or dormitories for HWs to avoid the staffs' fear of transmitting the virus to family members. As all public transport were suspended, shuttle bus, healthy meals and hydration while working were prepared for HWs as logistical support. Full coverage of the departments was defined that secondary protective measures [11], including medical protective clothing, N95 filtering facepiece respirator, goggles or disposable full-face shield, medical latex gloves, boot covers, medical working cap and gown, need to be adopted in all clinic departments, no matter whether the patients were infected or not. Moreover, disinfection of workplace, shutdown central air-conditioning, and dissemination of facts about COVID-19 prevention were also included in the full coverage protective measures.

2.3. Outcomes

PHQ-9 Scale was used to measure the depression symptoms. A cutoff of ≥10 has been recommended for diagnosis of major depression, which provides adequate sensitivity (88.0%) and specificity (88.0%) [12]. GAD-7 Scale was used to identify anxiety disorders. A cutoff score ≥8 is recommended to identify anxiety symptoms, with adequate specificity (82.0%) and sensitivity (77.0%) [13]. IES-R is a 22-item self-reported measure applied to assess subjective stress caused by traumatic event, describing the three symptoms including avoidance, intrusion, and hyperarousal. In this study, COVID-19 was given as the specific event in the questionnaire (Appendix Part V). The IES-R was validated in previous COVID-19 research involving HWs [14–16]. IES-R scores>33 points were used to identify outcome of stress in this study, which provided adequate specificity (91.0%) and sensitivity (82.0%) [17,18]. The selected 3 questionnaires had good internal consistency with Cronbach's α coefficients of more than 0.80. To further explore the associated variables of depression, anxiety and acute stress in men HWs (758 subjects) and women HWs (4304 subjects) groups, we also attempt to complete gender-specific regression analyses.

2.4. Statistical analysis

Continuous variables were divided as categorical variables firstly and all variables were shown as the counts and percentages. Depressive, anxiety and acute stress symptoms were defined as the outcomes of multivariate logistic regression analyses. We considered the potential interactions between ten items of perceptions of threat of the COVID-19 and satisfactions of the support measures before variables selection using univariate analysis. There were no potential interactions between these ten co-variates based on the variance inflation factor (all VIF values <5) and tolerance (all tolerances >0.2) in multiple collinearity diagnosis for depression, anxiety and acute stress outcomes. We also considered the potential interactions between co-variates before variables screening, including age, years of working, annual household income, educational level and professional title level. There were also no potential interactions between these five co-variates based on the variance inflation factor (all VIF values <5) and tolerance (all tolerances >0.2) in multiple collinearity diagnosis for depression, anxiety and acute stress outcomes. Variables with p values <0.05 in univariate analyses were subjected to multivariate logistic regression analysis with a stepwise backwards elimination procedure. Odds ratios (ORs) and 95% confidence intervals (CIs) for depression, anxiety and acute stress were estimated. The OR is a way of comparing whether the probability of a certain event is the same for two groups. An OR of 1 implies that the event is equally likely in both groups. An OR greater than 1 implies that the event is more likely in the first group. An OR less than 1 implies that the event is less likely in the first group. Statistical analyses to identify associated variables were performed using SPSS 22.0. The Depression, Anxiety and Acute stress nomograms were formulated based on the results of multivariate logistic regression analysis using the R packages "rms", "Hmisc" and "ggplot2" in R 3.5.2 (http://www.r-project.org/). The performances of the nomograms were measured by Concordance statistics (C-statistics) and assessed by calibration curves. Bootstraps with 1000 resamples were applied to these activities. The higher C-statistics indicated better ability to distinguish HWs with different risks of three outcomes. We considered the risk screening models as a useful clinical tool particularly when the C-statistic is higher than 0.70 [19]. Each variable was projected upward to the value of the Points line to get the score of each parameter. The total score was calculated by adding each score and located on the Total Points line. The risk was obtained according to the total score by drawing vertical lines to the Risk line. The calibration curves were used to compare the observed probability with the predicted probability. Dots on the calibration plot would be close to a 45° diagonal line if the model calibration is correct.

3. Results

6568 individuals were surveyed and 5281 individuals completed the online questionnaire. 5062 HWs were included in the final analysis after excluding the 219 questionnaires with incomplete information (response rate, 77.1%, Fig. 1). The response rate of women and men HWs were 82.4% and 71.8%. Most subjects are in the age intervals of 19–29 (40.1%) and 30–49 years old (56.4%). The women HWs accounted for 85.0% of the current study. Overall characteristics for the total sample (n = 5062), those depressive HWs (n = 681, 13.5%) vs. non-depressive ones (n = 4381), anxious HWs (n = 1218, 24.1%) vs. non-anxious ones (n = 3844), psychological stress (n = 1509, 29.8%) vs. non-psychological stress (n = 3553) were presented (Table 1; Fig. 2). We also summarized the perceptions of the threat of COVID-19 among HWs and satisfactions of support measures provided by the hospital, as shown in Table 2. Our data showed 56.93% HWs worried about life-threatening once infected, 19.50% HWs felt that families and friends have avoided contact with them, and 10.1% HWs had thought of resigning during the COVID-19 outbreak.

Several variables were associated with the occurrence of depression, anxiety, and stress (all p values<0.05, Table 3). Take stress outcome as example, the associated variables were as following: women (OR, 1.31; 95% CI, 1.02–1.66; p = 0.03), years of working> 10 years (OR, 2.02; 95% CI, 1.47–2.79; p<0.001), concomitant chronic diseases (OR, 1.51; 95% CI, 1.27–1.80; p<0.001), history of mental disorders (OR, 3.27; 95% CI, 1.77–6.05; p<0.001), and family members or relatives confirmed or suspected (OR, 1.23; 95% CI, 1.02–1.48; p = 0.03); having a history of exposure to the 2019 novel coronavirus (SARS-Cov-2) (OR, 2.31; 95% CI, 1.83–2.91; p<0.001); having thought of resigning (OR, 2.27; 95% CI, 1.83–2.82; p<0.001); unsure thought of resigning (OR, 1.93; 95% CI,

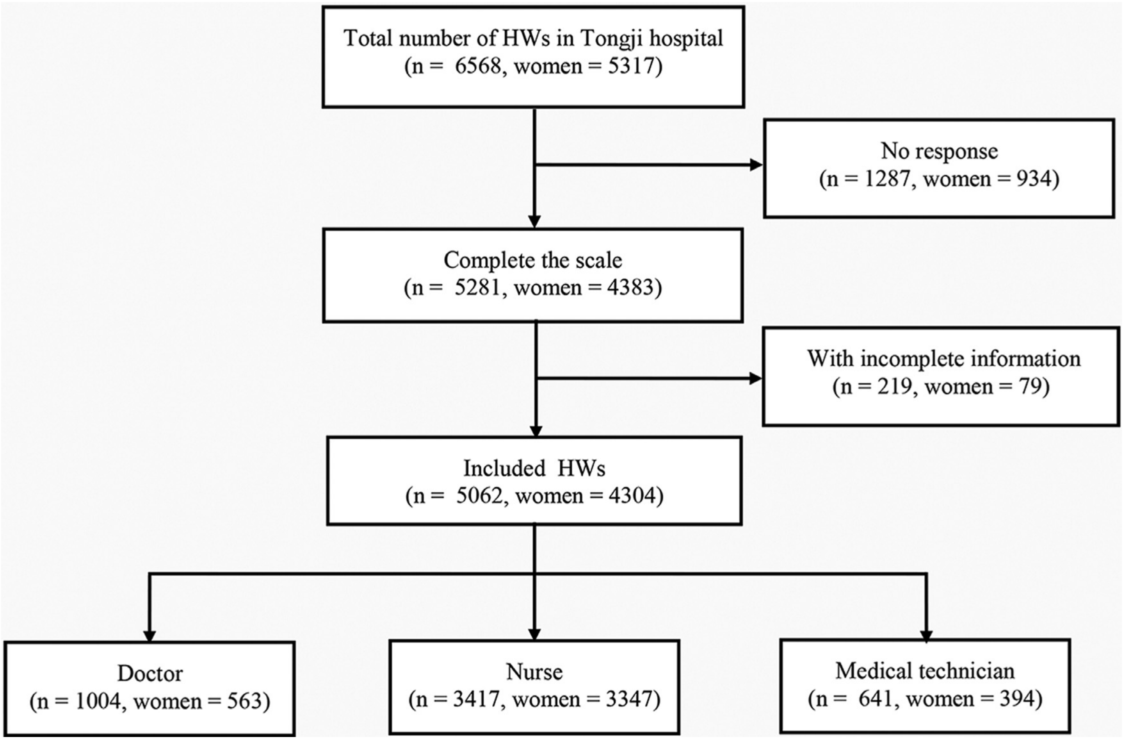

Fig 1. Flowchart.

1.56–2.38; p<0.001); worry about life-threatening (OR, 2.77; 95% CI, 2.24–3.42; p<0.001); feel of family members and friends' avoidance (OR, 2.01; 95% CI, 1.69–2.40; p<0.001). Other associated variables in the three outcomes were also presented (all p values<0.05, Table 3). Take stress outcome for example: care provided by hospital and department administrators (OR, 0.76; 95% CI, 0.60–0.97; p = 0.02) and satisfaction of full coverage for avoiding nosocomial infection (OR, 0.69; 95% CI, 0.53–0.89; p = 0.004).

Moreover, satisfaction of work shift arrangement, sufficient logistical support and accommodations provided by hospital, drinking history and suspected or confirmed COVID-19 were associated with depression symptoms. Living with family members, worry about myself or my family members being infected by SARS-Cov-2, exercise habit and satisfaction with logistical support and accommodations arranged by hospital were associated with anxiety symptoms.

The results of gender-specific logistic regression analysis were presented in Appendix ST 1-2.

3.1. Nomogram construction and internal validation

We created three nomograms to predict the risk of three outcomes (Fig. 3). The C-statistics for depression, anxiety and psychological stress nomograms were 0.779 (95% CI, 0.775–0.783), 0.765 (95% CI, 0.761–0.769) and 0.766 (95% CI, 0.762–0.770), respectively. The calibration curves were plotted in SF 2. Dots on the plots were very close to the 45° diagonal line, which suggested that the three nomograms were well-calibrated.

4. Discussion

Wuhan was the first hit area during the outbreak of COVID-19. The rising numbers of cases and deaths, coupled with the unprecedented lockdown of Wuhan, might create and spread public fear, panic, and distress. HWs at Tongji Hospital, officially designated as "the specific hospital for the treatment of severe patients with COVID-19 in Wuhan", might be facing a serious psychological challenge.

Our single-center, cross-sectional survey showed that 1509 (29.8%), 681 (13.5%) and 1218 (24.1%) HWs reported acute stress, depression, and anxiety symptoms. Consistently, a previous psychological survey of Chinese HWs from January 29, 2020 to February 3, 2020 involving 34 hospitals in China showed that 35% HWs reported moderate to severe stress (IES-$R \geq 26$), 14.8% HWs reported moderate to severe depression (PHQ-9 $\geq$ 10), 12.3% HWs reported moderate to severe anxiety (GAD-7 $\geq$ 10) [16]. However, a study of HWs during Singapore's COVID-19 outbreak from 19 February to 13 March 2020, showed much lower prevalence (7.7%) of stress (IES-$R \geq 24$) than that in our study [15], which may be related to the period and place that posed maximal stress on HWs in our study.

The management of Tongji Hospital implemented a series of support measures. 91.0% of HWs reported they had received hospital-based and department-based care, which was associated with acute stress, depression and anxiety. A previous study has reported that support from colleagues and supervisors and clear communication of directives and precautionary measures can reduce psychiatric symptoms [20]. In this study, hospital-based and department-based care, including the incentive of extra bonus policy and reassurance, understanding and communication from hospital executives, nursing leaders and department chairs, were associated with the mental health of HWs during the outbreak. However, social desirability may contribute to the high percentage of satisfaction in hospital-based and department-based care, since the questionnaire survey was conducted by the hospital administration department. Moreover, it was possible that HWs who did not feel care from hospitals and departments may be those with serious mental problems.

Confidence in infection control measures may also mitigate and facilitate mental problems of HWs. A recent study found that more intensive training on personal protective equipment and infection control measures were found to be helpful to support HW [15] and develop psychological resilience during COVID-19 pandemic [21]. This study showed that 79.5% HWs in Tongji hospital were satisfied with the full coverage of all departments for avoiding nosocomial infection, and full coverage of all departments was associated with

Table 1
Sociodemographic characteristics of Wuhan HWs dealing with the COVID-19.

Characteristics	Total (*N* = 5062)	Depression (*N* = 681)	Non-depression (*N* = 4381)	*p*	Anxiety (*N* = 1218)	Non-anxiety (*N* = 3844)	*p*	Psychological stress (*N* = 1509)	Non-psychological stress (*N* = 3553)	*p*
Age				0.006			<0.001			<0.001
19–29 years	2030 (40.1%)	235 (34.5%)	1795 (41.0%)		385 (31.6%)	1645 (42.8%)		518 (34.3%)	1512 (42.6%)	
30–49 years	2854 (56.4%)	421 (61.8%)	2433 (55.5%)		794 (65.2%)	2060 (53.6%)		944 (62.6%)	1910 (53.8%)	
> 49 years	178 (3.5%)	25 (3.7%)	153 (3.5%)		39 (3.2%)	139 (3.6%)		47 (3.1%)	131 (3.7%)	
Women	4304 (85.0%)	610 (89.6%)	3694 (84.3%)	<0.001	1081 (88.8%)	3223 (83.8%)	<0.001	1356 (89.9%)	2948 (83.0%)	<0.001
Marital status				0.04			<0.001			<0.001
Married	3356 (66.3%)	479 (70.3%)	2877 (65.7%)		895 (73.5%)	2461 (64.0%)		1072 (71.0%)	2284 (64.3%)	
Unmarried	1576 (31.1%)	184 (27.0%)	1392 (31.8%)		287 (23.6%)	1289 (33.5%)		394 (26.1%)	1182 (33.3%)	
Divorced/Widowed/ Separated	130 (2.6%)	18 (2.6%)	112 (2.6%)		36 (3.0%)	94 (2.5%)		43 (2.9%)	87 (2.5%)	
Master degree or higher level	1235 (24.4%)	150 (22.0%)	1085 (24.8%)	0.12	293 (24.1%)	942 (24.5%)	0.75	325 (21.5%)	910 (25.6%)	0.002
Occupation				0.03			0.003			<0.001
Doctor	1004 (19.8%)	114 (16.7%)	890 (20.3%)		232 (19.0%)	772 (20.1%)		243 (16.1%)	761 (21.4%)	
Nurse	3417 (67.5%)	489 (71.8%)	2928 (66.8%)		863 (70.9%)	2554 (66.4%)		1130 (74.9%)	2287 (64.4%)	
Medical technician	641 (12.7%)	78 (11.5%)	563 (12.9%)		123 (10.1%)	518 (13.5%)		136 (9.0%)	505 (14.2%)	
Professional title level				<0.001			<0.001			<0.001
Junior	3010 (59.5%)	359 (52.7%)	2651 (60.5%)		634 (52.1%)	2376 (61.8%)		843 (55.9%)	2167 (61.0%)	
Intermediate	1664 (32.9%)	270 (39.7%)	1394 (31.8%)		478 (39.2%)	1186 (30.9%)		563 (37.3%)	1101 (31.0%)	
Senior	388 (7.7%)	52 (7.6%)	336 (7.7%)		106 (8.7%)	282 (7.3%)		103 (6.8%)	285 (8.0%)	
Years of working				<0.001			<0.001			<0.001
<2 years	505 (10.0%)	45 (6.6%)	460 (10.5%)		77 (6.3%)	428 (11.1%)		92 (6.1%)	413 (11.6%)	
2–5 years	1724 (34.1%)	201 (29.5%)	1523 (34.8%)		338 (27.8%)	1386 (36.1%)		454 (30.1%)	1270 (35.7%)	
6–10 years	1376 (27.2%)	180 (26.4%)	1196 (27.3%)		354 (29.1%)	1022 (26.6%)		455 (30.2%)	921 (25.9%)	
>10 years	1457 (28.8%)	255 (37.4%)	1202 (27.4%)		449 (36.9%)	1008 (26.2%)		508 (33.7%)	949 (26.7%)	
Frontline at present	1736 (34.3%)	240 (35.2%)	1496 (34.2%)	0.58	416 (34.2%)	1320 (34.3%)	0.91	582 (38.6%)	1154 (32.5%)	<0.001
Current work place				0.48			0.64			<0.001
Non-isolation Ward	3240 (64.0%)	426 (62.6%)	2814 (64.2%)		767 (63.0%)	2473 (64.3%)		907 (60.1%)	2333 (65.7%)	
Isolation Ward	1607 (31.7%)	229 (33.6%)	1378 (31.5%)		400 (32.8%)	1207 (31.4%)		553 (36.7%)	1054 (29.7%)	
Off work or in isolation	215 (4.3%)	26 (3.8%)	189 (4.3%)		51 (4.2%)	164 (4.3%)		49 (3.2%)	166 (4.7%)	
Department				0.85			0.31			<0.001
Fever clinic, ED or isolation wards	1741 (34.4%)	239 (35.1%)	1502 (34.3%)		412 (33.8%)	1329 (34.6%)		582 (38.6%)	1159 (32.6%)	
Non-isolation wards	2614 (51.6%)	351 (51.5%)	2263 (51.7%)		649 (53.3%)	1965 (51.1%)		749 (49.6%)	1865 (52.5%)	
Other department	707 (14.0%)	91 (13.4%)	616 (14.1%)		157 (12.9%)	550 (14.3%)		178 (11.8%)	529 (14.9%)	
Annual household income				0.65			0.001			0.046
30–100 thousand Yuan	850 (16.8%)	104 (15.3%)	746 (17.0%)		170 (14.0%)	680 (17.7%)		219 (14.5%)	631 (17.8%)	
100–200 thousand Yuan	2276 (45.0%)	314 (46.1%)	1962 (44.8%)		529 (43.4%)	1747 (45.4%)		698 (46.3%)	1578 (44.4%)	
200–300 thousand Yuan	1237 (24.4%)	172 (25.3%)	1065 (24.3%)		328 (26.9%)	909 (23.6%)		378 (25.1%)	859 (24.2%)	
>300 thousand Yuan	699 (13.8%)	91 (13.4%)	608 (13.9%)		191 (15.7%)	508 (13.2%)		214 (14.2%)	485 (13.7%)	
Living at home	3758 (74.2%)	504 (74.0%)	3254 (74.3%)	0.89	908 (74.6%)	2850 (74.1%)	0.76	1073 (71.1%)	2685 (75.6%)	0.001
Living with family member	2514 (49.7%)	346 (50.8%)	2168 (49.5%)	0.52	646 (53.0%)	1868 (48.6%)	0.007	721(47.8%)	1793 (50.5%)	0.08
Past medical history				<0.001			<0.001			<0.001
In good health	4158 (82.1%)	488 (71.7%)	3670 (83.8%)		899 (73.8%)	3259 (84.8%)		1132 (75.0%)	3026 (85.2%)	
Have chronic non-communicable diseases	848 (16.8%)	161 (23.6%)	687 (15.7%)		281 (23.1%)	567 (14.8%)		340 (22.5%)	508 (14.3%)	
Have history of mental disorders	56 (1.1%)	32 (4.7%)	24 (0.5%)		38 (3.1%)	18 (0.5%)		37 (2.5%)	19 (0.5%)	
Smoking	148 (2.9%)	17 (2.5%)	131(3.0%)	0.48	25 (2.1%)	123 (3.2%)	0.04	31(2.1%)	117 (3.3%)	0.02
Drinking	426 (8.4%)	73 (10.7%)	353 (8.1%)	0.02	113 (9.3%)	313 (8.1%)	0.21	126 (8.3%)	300 (8.4%)	0.91
Exercise habit	857 (16.9%)	93 (13.7%)	764 (17.4%)	0.01	151 (12.4%)	706 (18.4%)	<0.001	209 (13.9%)	648 (18.2%)	<0.001
Parent status				<0.001			<0.001			<0.001
No child	1950 (38.5%)	224 (32.9%)	1726 (39.4%)		366 (30.0%)	1584 (41.2%)		479 (31.7%)	1471 (41.4%)	
One child	2444 (48.3%)	340 (49.9%)	2104 (48.0%)		640 (52.6%)	1804 (46.9%)		775 (51.4%)	1669 (47.0%)	
Two or more children	668 (13.2%)	117 (17.2%)	551 (12.6%)		212 (17.4%)	456 (11.9%)		255 (16.9%)	413 (11.6%)	
Families or relatives suspected or confirmed	763 (15.1%)	146 (21.4%)	617 (14.1%)	<0.001	259 (21.3%)	504 (13.1%)	<0.001	291 (19.3%)	472 (13.3%)	<0.001
Suspected or confirmed COVID-19	158 (3.1%)	41 (6.0%)	117 (2.7%)	<0.001	56 (4.6%)	102 (2.7%)	0.001	59 (3.9%)	99 (2.8%)	0.04

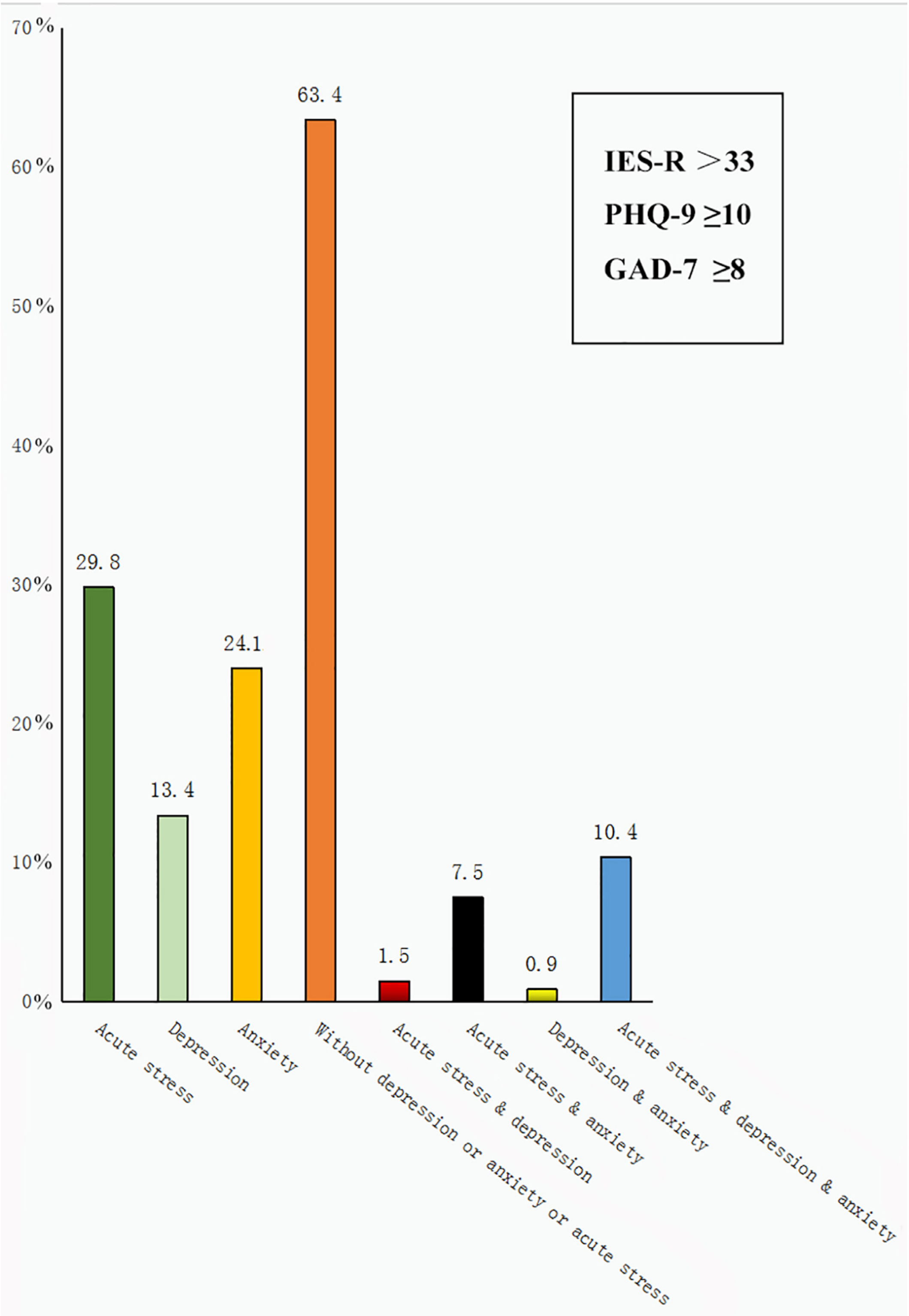

Fig 2. Distribution the prevalence of depression, anxiety and acute stress and their respective overlap.

acute stress, depression and anxiety. However, although adequate protective measures were provided, a significant proportion of HWs felt exposed or unsure exposure to the virus, and exaggerated the risk of contracting and dying from COVID-19 in this study. Consequently, dedicated counseling need be arranged to allay their fear of the infection and to challenge their cognitive biases, as well as to boost the confidence and morale of our HWs.

Reasonable work shift arrangement was associated with acute stress and depression, and satisfaction with logistical support and accommodations was associated with anxiety and depression in this study. It was also reported that the workforce could benefit from support measures including scheduled rest periods, regular exercise, nutritional meals, flexible staffing resources [9,22]. Hence, health facilities may consider shorter work hours, regular rest periods and rotating shifts for staff who work in high-risk jobs.

Table 2
Perceptions of threat of the COVID-19 and satisfactions of the support measures among Wuhan HWs dealing with the COVID-19.

Characteristics	Total (N = 5062)	Depression (N = 681)	Non-depression (N = 4381)	p	Anxiety (N = 1218)	Non-anxiety (N = 3844)	p	Psychological stress (N = 1509)	Non-psychological stress (N = 3553)	p
Do you feel that you have a history of exposure to the SARS-Cov-2?				<0.001			<0.001			<0.001
Disagree	822(16.2%)	50 (7.3%)	772 (17.6%)		73 (6.0%)	749 (19.5%)		125 (8.3%)	697 (19.6%)	
Agree	2000(39.5%)	386 (56.7%)	1614 (36.8%)		661(54.3%)	1339 (34.8%)		796 (52.8%)	1204 (33.9%)	
Unsure	2240 (44.3%)	245 (36.0%)	1995 (45.5%)		484(39.7%)	1756 (45.7%)		588 (39.0%)	1652 (46.5%)	
Have you ever thought of resigning because of the COVID-19 outbreak?				<0.001			<0.001			<0.001
Disagree	4057 (80.2%)	404 (59.3%)	3653 (83.4%)		773(63.5%)	3284 (85.4%)		979 (64.9%)	3078 (86.6%)	
Agree	511 (10.1%)	160 (23.5%)	351 (8.0%)		250(20.5%)	261 (6.8%)		290 (19.2%)	221 (6.2%)	
Unsure	494 (9.8%)	117 (17.2%)	377 (8.6%)		195(16.0%)	299 (7.8%)		240 (15.9%)	254 (7.1%)	
Have you worried about the life-threatening once infected?				<0.001			<0.001			<0.001
Disagree	1176 (23.2%)	62 (9.1%)	1114 (25.4%)		105 (8.6%)	1071 (27.9%)		140 (9.2%)	1036 (29.2%)	
Agree	2882 (56.9%)	533 (78.3%)	2349 (53.6%)		958(78.7%)	1924 (50.1%)		1154 (76.5%)	1728 (48.6%)	
Unsure	1004 (19.8%)	86 (12.6%)	918 (21.0%)		155(12.7%)	849 (22.1%)		215 (14.3%)	789 (22.2%)	
Worry about myself or my family members being infected by SARS-Cov-2	4663 (92.1%)	653(95.9%)	4010 (91.5%)	<0.001	1182(97.0%)	3481 (90.6%)	<0.001	1451(96.1%)	3212 (90.4%)	<0.001
Do you feel that family members and friends have avoided contact with you because of your work?				<0.001			<0.001			<0.001
Disagree	2513 (49.6%)	215 (31.6%)	2298 (52.5%)		414 (34.0%)	2099 (54.6%)		510 (33.8%)	2003 (56.4%)	
Agree	987 (19.5%)	240 (35.2%)	747 (17.1%)		389 (31.9%)	598 (15.6%)		463 (30.7%)	524 (14.8%)	
Unsure	1562 (30.9%)	226 (33.2%)	1336 (30.5%)		415 (34.1%)	1147 (29.8%)		536 (35.5%)	1026 (28.9%)	
Have joined in Tongji WeChat Balint Group	248 (4.9%)	28 (4.1%)	220 (5.0%)	0.31	68 (5.6%)	180 (4.7%)	0.21	81 (5.4%)	167 (4.7%)	0.31
Have received care provided by hospital and department administrators	4581 (90.5%)	559 (82.1%)	4022 (91.8%)	<0.001	1039(85.3%)	3542 (92.1%)	<0.001	1308 (86.7%)	3273 (92.1%)	<0.001
Are you satisfied with the full coverage of all departments for avoiding nosocomial infection?				<0.001			<0.001			<0.001
Satisfied	4024 (79.5%)	426 (62.6%)	3598 (82.1%)		805 (66.1%)	3219 (83.7%)		1059 (70.2%)	2965 (83.5%)	
Not satisfied	390 (7.7%)	107 (15.7%)	283 (6.5%)		163 (13.4%)	227 (5.9%)		186 (12.3%)	204 (5.7%)	
Unsure	648 (12.8%)	148 (21.7%)	500 (11.4%)		250 (20.5%)	398 (10.4%)		264 (17.5%)	384 (10.8%)	
Are you satisfied with your work shift arrangement?				<0.001			<0.001			<0.001
Satisfied	4198 (82.9%)	448 (65.8%)	3750 (85.6%)		873 (71.7%)	3325 (86.5%)		1087 (72.0%)	3111 (87.6%)	
Not satisfied	219 (4.3%)	80 (11.8%)	139 (3.2%)		99 (8.1%)	120 (3.1%)		130 (8.6%)	89 (2.5%)	
Unsure	645 (12.7%)	153 (22.5%)	492 (11.2%)		246 (20.2%)	399 (10.4%)		292 (19.4%)	353 (9.9%)	
Are you satisfied with the logistical support and accommodations provided by hospital?				<0.001			<0.001			<0.001
Satisfied	4234 (83.6%)	472 (69.3%)	3762 (85.9%)		889 (73.0%)	3345 (87.0%)		1149 (76.1%)	3085 (86.8%)	
Not satisfied	237 (4.7%)	70 (10.3%)	167 (3.8%)		101 (8.3%)	136 (3.5%)		117 (7.8%)	120 (3.4%)	
Unsure	591 (11.7%)	139 (20.4%)	452 (10.3%)		228 (18.7%)	363 (9.4%)		243 (16.1%)	348 (9.8%)	

Table 3
Factors associated with depression, anxiety and psychological stress in Wuhan HWs dealing with the COVID-19.

Characteristics	Subjects with and without depression		Subjects with and without anxiety		Subjects with and without psychological stress	
	OR (95% CI)	*p*	OR (95% CI)	*p*	OR (95% CI)	*p*
Women	1.75 (1.29–2.38)	<0.001	1.33 (1.07–1.67)	0.01	1.31 (1.02–1.66)	0.03
Master degree or higher level					1.55 (1.16–2.07)	0.003
Occupation						<0.001
Doctor						Ref
Nurse					2.24 (1.61–3.12)	<0.001
Medical technician					1.57 (1.12–2.21)	0.01
Years of working		<0.001		<0.001		<0.001
<2 years		Ref		Ref		Ref
2–5 years	1.17 (0.81–1.68)	0.40	1.10 (0.81–1.48)	0.54	1.25 (0.95–1.66)	0.12
6–10 years	1.36 (0.94–1.96)	0.11	1.57 (1.13–2.18)	0.007	1.71 (1.25–2.30)	0.001
>10 years	2.17 (1.51–3.14)	<0.001	2.32 (1.65–3.27)	<0.001	2.02 (1.47–2.79)	<0.001
Current work place						0.008
Non-isolation Ward					1.32 (1.10–1.59)	Ref
Isolation Ward						0.002
Off work or in isolation					1.21 (0.83–1.74)	0.32
Living with family members			1.18 (1.01–1.38)	0.04		
Past medical history		<0.001		<0.001		<0.001
In good health		Ref		Ref		Ref
Have chronic non-communicable diseases	1.35 (1.09–1.69)	0.007	1.38 (1.15–1.66)	0.001	1.51 (1.27–1.80)	<0.001
Have history of mental disorders	6.78 (3.73–12.32)	<0.001	5.03 (2.68–9.42)	<0.001	3.27 (1.77–6.05)	<0.001
Drinking	1.63 (1.18–2.25)	0.003				
Exercise habit			0.71 (0.57–0.88)	0.001		
Parent status						0.002
No child						Ref
One child					1.17 (0.97–1.41)	0.10
Two or more children					1.56 (1.22–1.99)	<0.001
Family members or relatives suspected or confirmed	1.26 (1.01–1.59)	0.045	1.35 (1.12–1.64)	0.002	1.23 (1.02–1.48)	0.03
Suspected or confirmed COVID-19	1.70 (1.13–2.57)	0.01				
Do you feel that you have a history of exposure to the SARS-Cov-2?		<0.001		<0.001		<0.001
Disagree		Ref		Ref		Ref
Agree	2.03 (1.46–2.81)	<0.001	3.23 (2.45–4.26)	<0.001	2.31 (1.83–2.91)	<0.001
Unsure	1.30 (0.93–1.81)	0.12	2.09 (1.58–2.76)	<0.001	1.46 (1.16–1.84)	0.001
Have you ever thought of resigning because of the COVID-19 outbreak?		<0.001		<0.001		<0.001
Disagree		Ref		Ref		Ref
Agree	2.28 (1.78–2.93)	<0.001	2.56 (2.06–3.18)	<0.001	2.27 (1.83–2.82)	<0.001
Unsure	1.77 (1.37–2.30)	<0.001	1.87 (1.50–2.34)	<0.001	1.93 (1.56–2.38)	<0.001
Have you worried about the life-threatening once infected?		<0.001		<0.001		<0.001
Disagree		Ref		Ref		Ref
Agree	2.20 (1.64–2.96)	<0.001	2.83 (2.23–3.59)	<0.001	2.77 (2.24–3.42)	<0.001
Unsure	1.20 (0.84–1.71)	0.32	1.27 (0.96–1.68)	0.09	1.44 (1.12–1.84)	0.004
Worry about myself or my family members being infected by SARS-Cov-2.			1.70 (1.16–2.50)	0.006		
Do you feel that family members and friends have avoided contact with you because of your work?		<0.001		<0.001		<0.001
Don't avoid		Ref		Ref		Ref
Avoid	1.94 (1.55–2.42)	<0.001	1.83 (1.52–2.21)	<00.001	2.01 (1.69–2.40)	<0.001
Unsure	1.21 (0.97–1.50)	0.09	1.22 (1.03–1.45)	0.02	1.44 (1.23–1.69)	<0.001
Have received care provided by hospital and department administrators	0.69 (0.52–0.90)	0.007	0.76 (0.60–0.97)	0.03	0.76 (0.60–0.97)	0.02
Are you satisfied with the full coverage of all departments for avoiding nosocomial infection?		0.02		<0.001		0.01
Not satisfied		Ref		Ref		Ref
Satisfied	0.70 (0.51–0.95)	0.02	0.65 (0.50–0.85)	0.002	0.69 (0.53–0.89)	0.004
Unsure	0.94 (0.68–1.32)	0.74	0.99 (0.74–1.33)	0.94	0.82 (0.61–1.10)	0.18

(*continued on next page*)

Table 3 (*Continued*)

Characteristics	Subjects with and without depression		Subjects with and without anxiety		Subjects with and without psychological stress	
	OR (95% CI)	*p*	OR (95% CI)	*p*	OR (95% CI)	*p*
Are you satisfied with your work shift arrangement?		<0.001				<0.001
Not satisfied		Ref				Ref
Satisfied	0.48 (0.34–0.67)	<0.001			0.45 (0.33–0.63)	<0.001
Unsure	0.61 (0.42–0.89)	0.01			0.65 (0.46–0.92)	0.02
Are you satisfied with logistical support and accommodations arranged by the hospital?		0.006		<0.001		
Not Satisfied		Ref		Ref		
Satisfied	0.67 (0.47–0.97)	0.03	0.69 (0.50–0.96)	0.03		
Unsure	1.00 (0.681.48)	0.997	1.09 (0.76–1.38)	0.64		

Early intervention could prevent the establishment of maladaptive cognitive or behavioral patterns among HWs in the COVID-19 outbreak. Given the traffic shutdown in Wuhan and the constraints of social distancing, the hospital's psychiatric team had tried to support staff through online WeChat Balint group. WeChat was used to promote the QR code of the online Balint group to each department to allow HWs to enter group for consultation. However, few HWs (5.0%) joined this group, and it was not associated with anxiety, depression and stress. Reasons for this may be as follows: 1) Doctors often have a greater stigma against mental problems than the general public [23]. A Study from Hunan Province during this COVID-19 pandemic also reported that HWs were reluctant to participate in the group or individual psychology interventions provided to them, and refused to admit that they had any psychological problems [24]. 2) The security and confidentiality of the online Balint Group may also be of concern of HWs. Therefore, it is worthwhile to address the needs of the HWs during this pandemic and to provide more appropriate psychological support for those people at high risk of mental problems.

Among the sociodemographic characteristics, the associated variables of acute stress, depression, and anxiety symptoms were women, with history of mental disorders, history of physiological chronic non-communicable diseases, and family members or relatives suspected or confirmed COVID-19, and years of working >10 years. The proportion of women HWs is 81.0% in Tongji hospital. Due to the higher response rate of women, the proportion of women in our study was as high as 85%. A recent general public study also found that woman was associated with greater psychological impact and PTSD symptoms in the crisis of COVID-19 [25]. Moreover, the situation of women HWs in Wuhan was even more difficult. Owing to entrenched traditional social roles in China, they were placed at a considerable dilemma, which existed between working and family care and between the family care and avoidance of contact with family members [26]. Longer years of working in the hospital was associated with psychological impact. This result could be possibly explained that most HWs with more than 10 working years had more occupational exhaustion and family responsibilities, since most HWs with more than 10 working years were married (91.97%, 1340/1457), and with one or more children (94.30%, 1374/1457). In addition, this study also identified vulnerable groups who are at high risk of psychological morbidities, including HWs with more children, with concomitant chronic diseases, nurse, HWs in isolation wards and infected HWs. Early psychological interventions targeting these vulnerable groups may be beneficial. In our study, the exercise habit was associated with a lower risk of anxiety symptoms. Nevertheless, only 857 (16.9%) participants had exercise habits, indicating that the importance of exercise should be highlighted among HWs in China.

The main strength of our study is the large sample of HWs in a hospital directly managing COVID-19 patients and performed at the peak of COVID-19 outbreak. The large sample size generated sufficient statistical power. Secondly, we examined and identified the association between hospital support measures and the immediate psychological impact on healthcare workers. Thirdly, this study identified vulnerable groups who are at high risk of acute stress, depression, and anxiety and developed screening nomograms with good discrimination ability which could be used in clinical practice. Some limitations of the study must also be acknowledged. First, the cross-sectional nature of the study does not allow appropriate testing of the direction of the effects. Second, the study was performed early in the outbreak and only in one hospital, which may limit the generalizability of the findings. Third, although all our questionnaires were conducted anonymously, HWs still may answer with social desirability which may have biased the results, since the questionnaire survey was conducted by the hospital administration department. Fourth, due to the difference of response rates between men and women HWs and the use of self-report scales, the biases are inevitable.

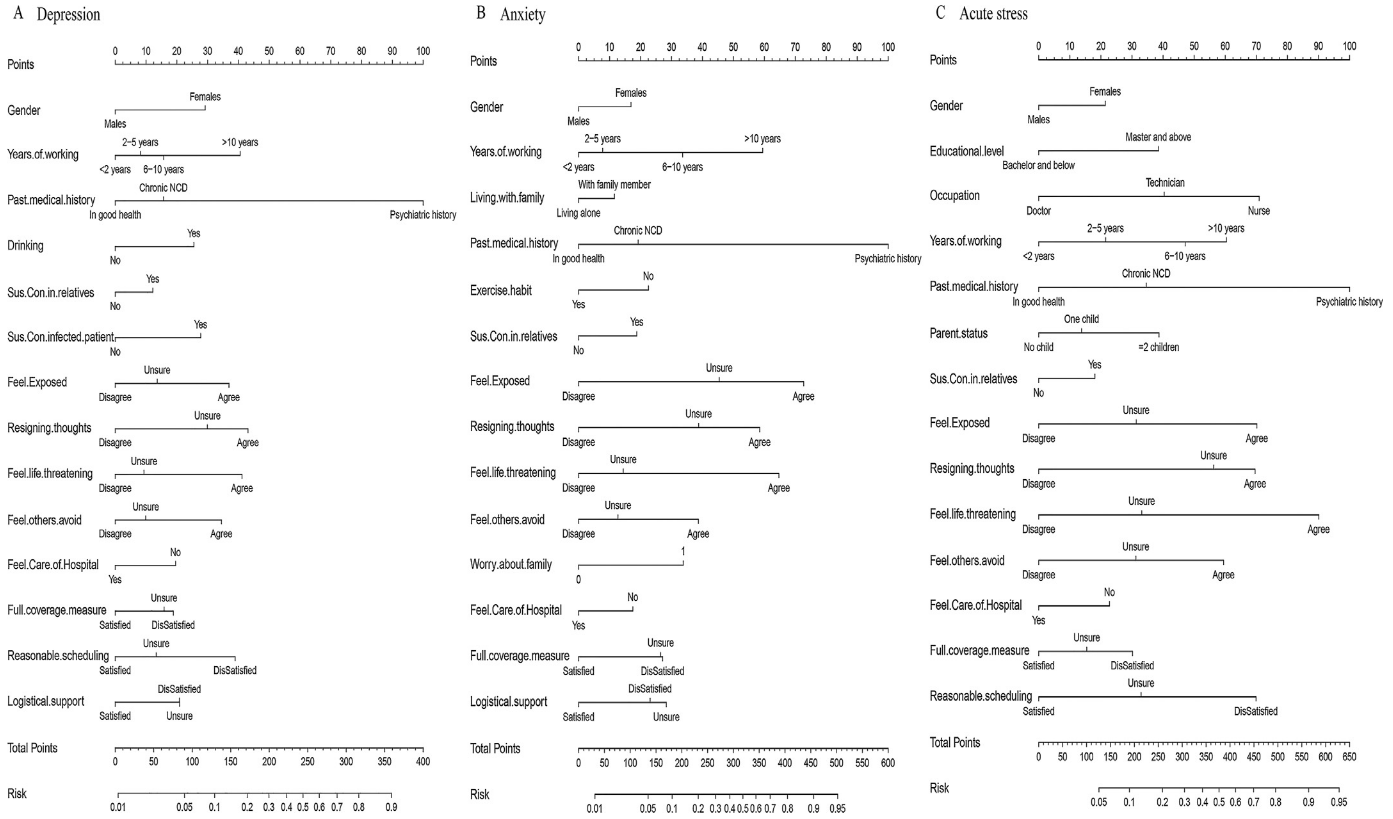

Fig 3. Nomograms of Depression, Anxiety and Acute stress. (A) Nomogram A to predict Depression symptom in HWs of Wuhan. (B) Nomogram B to predict Anxiety symptom in HWs of Wuhan. (C) Nomogram C to predict Acute stress symptom in HWs of Wuhan.

In summary, the cross-sectional survey showed that early hospital support measures were associated with the immediate psychological impact on healthcare workers in response to the COVID-19 outbreak. This study also identified vulnerable groups who are at high risk of psychological morbidities, and developed three effective, easy-to-use clinical screening tools to identify high-risk individuals of acute stress, depression, and anxiety among HWs. Nevertheless, although hospital support measures were provided, HWs still felt exposed to SARS-Cov-2, and exaggerated threat of COVID-19. Consequently, guiding policies and psychological interventions to maintain their psychological well-being is crucial. Follow-up studies on HWs are needed to assess for progression or even a potential rebound effect of psychological manifestations when the imminent threat of COVID-19 subsides.

Declaration of Competing Interest

The authors declare that they have no competing interests.

Acknowledgments

The authors thank the research personnel who assisted with data collection.

Funding section

Supported by the grants 2017YFC1310000 from the National Key Research and Development Program of the Ministry of Science and Technology of China, 2018KFYXMPT015 from Research and Development Office of Huazhong University of Science and Technology and 20200204010100096 from Wuhan Science and Technology Bureau. The study sponsors had no role in the design of the study protocol, data collection, data analysis, data interpretation, writing of the report, or the decision to submit the manuscript for publication.

Supplementary materials

Supplementary material associated with this article can be found in the online version at doi:10.1016/j.eclinm.2020.100443.

References

[1] Wang D, Hu B, Hu C, et al. Clinical characteristics of 138 hospitalized patients with 2019 novel coronavirus-infected pneumonia in Wuhan, China. JAMA 2020;323:1061–9.

[2] Kang L, Li Y, Hu S, et al. The mental health of medical workers in Wuhan, China dealing with the 2019 novel coronavirus. Lancet Psychiatry 2020;7:e14.

[3] LeBlanc VR. The effects of acute stress on performance: implications for health professions education. Acad Med: J Assoc Am Med Colleges 2009;84:S25–33.

[4] Panagioti M, Geraghty K, Johnson J, et al. Association between physician burnout and patient safety, professionalism, and patient satisfaction: a systematic review and meta-analysis. JAMA Intern Med 2018;178:1317–30.

[5] Grace MK, VanHeuvelen JS. Occupational variation in burnout among medical staff: evidence for the stress of higher status. Soc Sci Med 2019;232:199–208.

[6] Grassi L, Magnani K. Psychiatric morbidity and burnout in the medical profession: an Italian study of general practitioners and hospital physicians. Psychother Psychosom 2000;69:329–34.

[7] Mulfinger N, Sander A, Stuber F, et al. Cluster-randomised trial evaluating a complex intervention to improve mental health and well-being of employees working in hospital – a protocol for the SEEGEN trial. BMC Public Health 2019;19:1694.

[8] National Health Commission of the People's Republic of China. Guidelines for emergency psychological crisis interventions during the COVID-19 pandemic (in Chinese). 2020. http://www.gov.cn/zhengce/zhengceku/2020-01/27/content_5472433.htm. Accessed April 16, 2020

[9] Tan W, Hao F, McIntyre RS, et al. Is returning to work during the COVID-19 pandemic stressful? A study on immediate mental health status and psychoneuroimmunity prevention measures of Chinese workforce. Brain Behav Immun 2020;87:84–92.

[10] World Health Organization. WHO global strategy on diet, physical activity and health. 2018. http://www.who.int/dietphysicalactivity/factsheet_recommendations/en/. Accessed April 16, 2020

[11] National Health Commission of the People's Republic of China. Prevention and control of nosocomial infection by air borne diseases (in Chinese). 2020. http://www.nhc.gov.cn/wjw/s9496/201701/7e0e8fc6725843aabba8f841f2f585d2.shtml. Accessed April 16, 2020

[12] Kroenke K, Spitzer RL, Williams JB. The PHQ-9: validity of a brief depression severity measure. J Gen Intern Med 2001;16:606–13.

[13] Kroenke K, Spitzer RL, Williams JB, Monahan PO, Löwe B. Anxiety disorders in primary care: prevalence, impairment, comorbidity, and detection. Ann Intern Med 2007;146:317–25.
[14] Chew NWS, Lee GKH, Tan BYQ, et al. A multinational, multicentre study on the psychological outcomes and associated physical symptoms amongst healthcare workers during COVID-19 outbreak. Brain Behav Immun 2020. doi: 10.1016/j.bbi.2020.04.049.
[15] Tan BYQ, Chew NWS, Lee GKH, et al. Psychological impact of the COVID-19 pandemic on health care workers in Singapore. Ann Intern Med 2020. doi: 10.7326/M20-1083.
[16] Lai J, Ma S, Wang Y, et al. Factors associated with mental health outcomes among health care workers exposed to coronavirus disease 2019. JAMA Netw Open 2020;3:e203976.
[17] Asif IM, Price DE, Ewing A, Rao AL, Harmon KG, Drezner JA. The impact of diagnosis: measuring the psychological response to being diagnosed with serious or potentially lethal cardiac disease in young competitive athletes. Br J Sports Med 2016;50:163–6.
[18] Creamer M, Bell R, Failla S. Psychometric properties of the impact of event scale – revised. Behav Res Ther 2003;41:1489–96.
[19] James MT, Pannu N, Hemmelgarn BR, et al. Derivation and external validation of prediction models for advanced chronic kidney disease following acute kidney injury. JAMA 2017;318:1787–97.
[20] Chan AO, Huak CY. Psychological impact of the 2003 severe acute respiratory syndrome outbreak on health care workers in a medium size regional general hospital in Singapore. Occup Med 2004;54:190–6.
[21] Ho CS, Chee CY, Ho RC. Mental health strategies to combat the psychological impact of COVID-19 beyond paranoia and panic. Ann Acad Med Singap 2020;49:155–60.
[22] Kim SW, Su KP. Using psychoneuroimmunity against COVID-19. Brain Behav Immun 2020;87:4–5.
[23] Garelick A. Doctors' health: stigma and the professional discomfort in seeking help. Psychiatrist 2012;36:81–4.
[24] Chen Q, Liang M, Li Y, et al. Mental health care for medical staff in China during the COVID-19 outbreak. Lancet Psychiatry 2020;7:e15–e6.
[25] Wang C, Pan R, Wan X, et al. Immediate psychological responses and associated factors during the initial stage of the 2019 coronavirus disease (COVID-19) epidemic among the general population in China. Int J Environ Res Public Health 2020;17:1729.
[26] Li G, Miao J, Wang H, et al. Psychological impact on women health workers involved in COVID-19 outbreak in Wuhan: a cross-sectional study. J Neurol Neurosurg Psychiatr 2020. doi: 10.1136/jnnp-2020-323134.
