# Supplementary material for: Mental health status and related factors influencing healthcare workers during the COVID-19 pandemic: A systematic review and meta-analysis
Source: PLoS One. 2024 Jan 19;19(1):e0289454. doi: 10.1371/journal.pone.0289454 (PMC10798549; doi:10.1371/journal.pone.0289454)
Supplement: S1 Data — (ZIP) [file pone.0289454.s011.zip › literatures/25.pdf]

International Journal of
Environmental Research and Public Health

MDPI

Article

Impact of the COVID-19 Pandemic on the Mental Health of Healthcare Workers

Andreas Chatzittofis [1,2,*], Maria Karanikola [3], Kyriaki Michailidou [4] and Anastasia Constantinidou [1]

[1] Medical School, University of Cyprus, Nicosia 1678, Cyprus; constantinidou.anastasia@ucy.ac.cy
[2] Department of Clinical Sciences/Psychiatry, Umeå University, 90187 Umeå, Sweden
[3] Department of Nursing, School of Health Sciences, Cyprus University of Technology, Limassol 3041, Cyprus; maria.karanikola@cut.ac.cy
[4] Biostatistics Unit, The Cyprus Institute of Neurology and Genetics and Cyprus School of Molecular Medicine, Nicosia 1683, Cyprus; kyriakimi@cing.ac.cy
* Correspondence: Chatzittofis.andreas@ucy.ac.cy

Abstract: The coronavirus disease 2019 (COVID-19) has a great impact on healthcare workers (HCWs) that includes negative mental health outcomes, such as post-traumatic stress, anxiety and depressive symptoms. In this cross-sectional study, we report on mental health outcomes among HCWs in Cyprus. Data were collected between 3 May and 27 May 2020, with the use of an online questionnaire that included demographics (sex, age, occupation, education, work sector, years of work experience), the 9-item Patient Health Questionnaire (PHQ-9) which assesses depressive symptoms, the Impact of Events Scale Revised (IES-R), which measures post-traumatic stress disorder (PTSD) symptoms, and the-10 item Perceived Stress Scale (PSS-10) which quantifies stress responses. Participants (42% physicians, 24% nurses, 18% physiotherapists, 16% classified as "other") were 58% of female gender and aged 21–76. A total of 79 (18.6%) and 62 HCWs (14.6%) reported clinically significant depressive (PHQ-9 $\geq$ 10) and post-traumatic stress (IES-R > 33) symptoms respectively. Nurses were more likely than physicians to suffer from depression (adjusted prevalence ratio 1.7 (1.06–2.73); $p = 0.035$) and PTSD (adjusted prevalence ratio 2.51 (1.49–4.23); $p = 0.001$). Even in a country with a rather low spread of the COVID-19, such as Cyprus, HCWs reported a substantial mental health burden, with nurses reporting increased depressive and PTSD symptoms compared to other HCWs.

Keywords: COVID-19; post-traumatic stress; depression; healthcare workers

check for updates

Citation: Chatzittofis, A.; Karanikola, M.; Michailidou, K.; Constantinidou, A. Impact of the COVID-19 Pandemic on the Mental Health of Healthcare Workers. *Int. J. Environ. Res. Public Health* **2021**, *18*, 1435. https://doi.org/10.3390/ijerph18041435

Received: 24 January 2021
Accepted: 1 February 2021
Published: 3 February 2021

Publisher's Note: MDPI stays neutral with regard to jurisdictional claims in published maps and institutional affiliations.

Copyright: © 2021 by the authors. Licensee MDPI, Basel, Switzerland. This article is an open access article distributed under the terms and conditions of the Creative Commons Attribution (CC BY) license (https://creativecommons.org/licenses/by/4.0/).

1. Introduction

Coping with the coronavirus disease (COVID-19) pandemic has been a challenge for healthcare systems and especially healthcare workers (HCWs) worldwide. HCWs are confronted with a demanding work environment that impacts on their physical and mental health. It is well documented that during the Severe Acute Respiratory Syndrome (SARS) 2003 epidemic, a significant proportion of HCWs reported symptoms of post-traumatic syndrome, depression, anxiety and fear [1]. An early study in the course of the COVID-19 pandemic highlighted the importance of meeting HCWs' specific needs in order to reduce the stress they face and better cope with the pandemic [2]. A recent empirical study in China reported symptoms of indirect psychological trauma in both the general population and HCWs; however, frontline employees were not found to be more prone to these symptoms [3]. Previous studies on the psychological burden associated with the pandemic have mainly focused on frontline HCWs, those that are directly involved in COVID-19 patient care, such as those working in emergency departments and intensive care units. In addition, most of the studies on HCWs were conducted in countries with a high impact of the pandemic on the general population and the healthcare system, such as China and Italy [3–5].

Nevertheless, there are scant data on the COVID-19-related psychological distress in HCWs in countries with a decreased burden on their healthcare systems due to early

measures [6]. The Republic of Cyprus (RC) is a small island country (total population of 840,000) with a relatively limited impact of COVID-19 on the population, and subsequently on the healthcare system compared to other countries so far (November 2020). Specifically, a case fatality rate of 2.6% was reported in the RC until June 2020 [7], while at the beginning of May, the Cyprus Ministry of Health announced a total of 872 cases of COVID-19, with patients' median age at 46 years, and 15 deaths due to COVID-19.

At the same time, a small-scale study in a sample of the general population (n = 216) showed that 8.33% to 13.89% of participants experienced clinical anxiety and depressive symptoms [8]. However, the web-based study by Solomou and Constantinidou (2020) on 1642 adults of the general population revealed that more than one out of five reported mentally distressing symptoms [9]. The degree to which HCWs have been affected by the social isolation measures and the work-related challenges due to the pandemic remains understudied. Therefore, the aim of this study was to assess the mental distress of HCWs during the COVID-19 pandemic in the RC, particularly the presence of post-traumatic stress, depressive and anxiety symptoms.

2. Material and Methods

2.1. Ethics

Participants submitted their informed consent for participation in the study over a web-based platform. The study protocol was approved by the National Bioethics Committee of Cyprus (Protocol number: 2020.01.89).

2.2. Study Design and Sampling

This was a descriptive, correlational and cross-sectional study. Data were collected between 3 May and 7 May 2020 using an online questionnaire. This was disseminated via personal email to the members of the Cyprus Medical Association and the professional association of physiotherapists. Moreover, a targeted dissemination strategy through social networks to nurses, occupational therapists and pharmacists was applied using a snowball technique to recruit additional groups of HCWs. The sampling period was one week after the peak of the first wave of the pandemic in the RC, and just before the first phase of the gradual easing of restrictions after the general lockdown; the healthcare system was receiving the highest numbers of service users at that time.

2.3. Measurement Tools

The questionnaire included (a) demographic variables (sex, age, occupation, education, work sector, years of work experience), (b) personal history of mental health problems (*Have you ever been diagnosed with an anxiety disorder? Have you ever been diagnosed with a depressive disorder?*), and (c) the following validated assessment tools in Greek, which is the most widely spoken language in the RC:

1. The 9-item Patient Health Questionnaire (PHQ-9) for the assessment of depressive symptom intensity, during the preceding two weeks. The total scale score ranges between 0 and 27; values equal to 10 or greater were used herein as the cut off for clinically relevant depressive symptoms [10,11]. Since suicidality is the most alarming among depressive symptoms, we deemed PHQ-9 item [9]: (*Have you thoughts that you would be better off dead, or of hurting yourself? Not at all (0), Several Days (1), More than half of the days (2), Nearly every day (3)*), as indicative of a severe mental disturbance. Thus, scores $\geq$ 1 were considered highly indicative of suicide ideation and were reported in the results.
2. The 22-item Impact of Events Scale Revised (IES-R) for the assessment of post-traumatic stress symptoms during the last 7 days. Each item is scored 0–4. The total scale score ranges between 0 and 88; according to Mystakidou et al. [12], values greater than the cutoff of 33 denote clinically relevant symptoms.
3. The 10-item Perceived Stress Scale (PSS-10) for the measurement of self-perceived stress intensity. Each item is scored 0–4. The total scale score ranges between 0 and

40; higher scores reflect high stress levels [13,14]. As there is no consensus regarding cutoff values for PSS10 [13,14], we considered the upper quartile of the distribution of PSS-10 scores to denote clinically meaningful stress levels [15].

2.4. Data Analysis

Descriptive statistics of demographic, educational, employment and clinical variables were reported as mean (M) and standard deviations (SD), or frequencies for continuous (age, years of work experience, PHQ-9, IES-R, PSS-10 scores) and categorical (sex, education, work sector, personal mental health history, professional group, care for COVID-19 patients) variables, respectively. Skewness and kurtosis of the distribution of the continuous variables were evaluated by the Shapiro–Wilk test to confirm normality. The overall score of the PHQ 9, IES-R and PSS 10 scales was calculated as the sum of component items' scores.

We used the non-parametric Kruskal–Wallis test to investigate group differences in continuous variables. Univariate analyses were performed using linear (scores were used as continuous numerical variables) and logistic regression models. For the logistic regression models, the total score of all three scales was treated as a dichotomous categoric variable accounting for either absence (score bellow the cutoff, i.e., PHQ-9 < 10, IES-R < 33, and 1st to 3rd quartile for PSS-10), or presence (score equal or higher of the cut off, i.e., PHQ-9 $\geq$ 10, IES-R > 33, and upper quartile for PSS-10) of any clinical symptoms. We also developed multivariable linear and logistic regression models in order to adjust for potential confounders (age and gender). Prevalence rate ratios were calculated using logistic regression [16]. Statistical significance was set at $p < 0.05$, and all tests were 2-tailed. Data analyses were performed using the statistical software R [17].

3. Results

3.1. Participants' Characteristics

A total of 424 HCWs completed the questionnaire. In total, 248 (58.5%) were female and 176 (41.5%) were male, with a mean age of 38.78 years (range: 21–76 (SD: 11.40)). Male participants were older on average (mean (SD): 42.1 (11.9)) than females (mean (SD): 36.4 (10.4)). A total of 178 (42%) participants were physicians, 103 (24%) were nurses, 75 (18%) were physiotherapists and 68 (16%) were classified as "other" (i.e., occupational therapists, pharmacists, clinical psychologists). In total, 193 (46%) were employed in public inpatient settings, 142 (33%) in private outpatient settings, 67 (16%) in private inpatient settings, 14 (3.3%) in COVID-19 units and 8 (1.9%) in emergency departments. More than 50% of participants had at least 10 years of work experience. The characteristics of participants are presented in Table 1.

3.2. Depressive, PTSD and Stress Symptoms

Of the participants, 79 (19%) screened positive for clinical depressive symptoms (PHQ-9 score $\geq$ 10) and 62 (15%) for PTSD symptoms (IES-R score > 33). In total, 106 participants scored above 22.5 in the PSS-10 scale, which corresponds to the top 25% percentile, and were thus classified as experiencing high stress (Table 1). Additionally, 24 participants (5.7%) scored above 1 on the PHQ-9 question on suicide ideation and were thus classified as experiencing suicidality.

3.3. Univariate Associations of Depressive, PTSD and Stress Symptoms with Sex, Age, Personal History of Mental Health Problems and Years of Work Experience

Increased age and male gender were statistically significantly associated with reduced scores in PHQ-9 (beta = -0.09, $p = 1.83 \times 10^{-5}$ and beta = -1.46, $p = 0.003$ respectively) and IES-R scales (beta = -0.17, $p = 0.005$ and beta = -6.79, $p = 1.7 \times 10^{-6}$, respectively). A positive personal history of depression was associated with increased intensity of both PTSD and depressive symptoms (beta = 6.41, $p = 0.0003$ and beta = 3.23, $p = 0.02$, respectively). A positive personal history of an anxiety disorder did not associate with either

PTSD or depressive symptoms intensity. Increased self-perceived stress levels were positively associated with a positive personal history of an anxiety (beta = 4.7, $p = 1.1 \times 10^{-5}$) or depressive disorder (beta = 6.4, $p = 1.1 \times 10^{-8}$). Additionally, an adverse association was noted between years of work experience and PHQ-9 score (beta = −0.1, $p = 2.6 \times 10^{-6}$), IES-R score (beta = −0.24, p = 0.0003) and PSS scores (beta = −0.16, $p = 5 \times 10^{-7}$). Kruskal–Wallis tests were also performed and showed comparably significant *p*-values.

Table 1. Characteristics of study participants.

		Occupation			
**Variable**	**Total Sample N = 424** [1]	**Physicians N = 178** [1]	**Nurses N = 103** [1]	**Physiotherapists N = 75** [1]	**Other N = 68** [1]
Sex					
Female	248 (58%)	90 (51%)	64 (62%)	40 (53%)	54 (79%)
Male	176 (42%)	88 (49%)	39 (38%)	35 (47%)	14 (21%)
Age	38.8 (11.4)	43.9 (12.6)	34.7 (7.59)	38.2 (9.24)	32.2 (8.9)
Work sector					
Inpatient public sector	193 (46%)	87 (49%)	67 (65%)	10 (13%)	29 (43%)
Private outpatient sector	142 (33%)	54 (30%)	4 (3.9%)	58 (77%)	26 (38%)
Inpatient private sector	67 (16%)	31 (17%)	20 (19%)	7 (9.3%)	9 (13%)
COVID-19 unit	8 (1.9%)	1 (0.6%)	5 (4.9%)	0 (0%)	2 (2.9%)
Emergency department	14 (3.3%)	5 (2.8%)	7 (6.8%)	0 (0%)	2 (2.9%)
Years of work experience					
<5	84 (20%)	29 (16%)	15 (15%)	9 (12%)	31 (46%)
≥5 <10	96 (23%)	27 (15%)	43 (42%)	16 (21%)	10 (15%)
>10	244 (58%)	122 (69%)	45 (44%)	50 (67%)	27 (40%)
Educational level					
Associate Degree	18 (4.2%)	3 (1.7%)	1 (1.0%)	0 (0%)	14 (21%)
Bachelor's	176 (42%)	71 (40%)	37 (36%)	38 (51%)	30 (44%)
Master's	181 (43%)	63 (35%)	64 (62%)	36 (48%)	18 (26%)
Doctoral	49 (12%)	41 (23%)	1 (1.0%)	1 (1.3%)	6 (8.8%)
PHQ-9 score ≥ 10	79 (19%)	21 (12%)	27 (26%)	12 (16%)	19 (28%)
IES-R score > 33	62 (15%)	13 (7.3%)	26 (25%)	8 (11%)	15 (22%)
IES-R total	17 (14)	14 (13)	22 (16)	16 (12)	21 (15)
PSS-10 score	18 (7)	16 (7)	19 (6)	18 (7)	21 (8)
Stress levels (IQR)					
low	109 (26%)	60 (34%)	17 (17%)	20 (27%)	12 (18%)
medium	209 (49%)	9 (52%)	55 (53%)	32 (43%)	29 (43%)
high	106 (25%)	25 (14%)	31 (30%)	23 (31%)	27 (40%)

[1] Statistics presented: n/N (%); mean (SD), PHQ-9: the 9-item Patient Health Questionnaire, IES-R: the Impact of Events Scale Revised, PSS-10: the-10 item Perceived Stress Scale, IQR: Interquartile range.

3.4. Regression Analysis for Predicting Prevalence of Depressive, PTSD and Stress Symptoms

We performed logistic regression analyses, adjusted for sex and age, in order to calculate the prevalence ratio of depressive and PTSD symptoms and perceived stress level (we used the dichotomized categorial variables using the cutoff threshold of PHQ-9 ≥ 10, IES-R > 33 and upper quartile for PSS-10, respectively). Covariates evaluated were occupation, if participants provided care for COVID-19 patients and work setting (private vs. public) (Table 2). Adjusted prevalence ratio ((OR (95% CI)) was higher for nurses when compared to physicians, for depression 1.7 (1.06–2.73); p = 0.035, and PTSD 2.51 (1.49–4.23); p = 0.001.

Table 2. Logistic regression analyses for predicting prevalence of depressive, PTSD and stress symptoms.

	PHQ-9 Score ≥ 10		IES-R > 33		PSS-10 Score Upper Quartile	
	OR (95% CI) Adjusted *	*p*-Value *	OR (95% CI) Adjusted	*p*-Value *	OR (95% CI) Adjusted	*p*-Value *
Physicians (reference)	1		1		1	
Nurses	1.70 (1.06–2.73)	0.035	2.51 (1.49–4.23)	0.00146	1.17 (0.57–2.39)	0.68
Physiotherapist	1.18 (0.66–2.1)	0.58	1.27 (0.64–2.52)	0.52	1.29 (0.6–2.8)	0.52
Other	1.59 (0.93–2.7)	0.1	1.8 (0.99–3.25)	0.07	1.66 (0.8–3.43)	0.18
Care for COVID-19 patients						
No (reference)	1		1		1	
Yes	1.08 (0.91–1.28)	0.37	1.22 (1.01–1.48)	0.03	0.99 (0.79–1.25)	0.96
Work setting						
Public (reference)	1		1		1	
Private	1.29 (0.76–2.19)	0.35	0.70 (0.44–1.11)	0.12	0.97 (0.66–1.44)	0.88

OR (95% CI): Odds Ratio and 95% Confidence Interval, * adjusted for age and sex.

4. Discussion

In this report, we presented HCWs' mental health and associated risk factors in the Republic of Cyprus during the COVID-19 pandemic. Most of the published studies on HCWs' mental distress were conducted in Asian countries, mainly China, as well as in Italy and Spain [5]. To our knowledge, this is the first study in HCWs in a country with a relatively low SARS-CoV-2 burden. Specifically, approximately 19% of participants reported clinically relevant depressive symptoms, while 15% reported PTSD symptoms. Predictors of increased intensity of depressive and PTSD symptoms were female sex (estimates between 1.46 to 6.79 lower for men), younger age (estimates between 0.09 to 0.17 lower for each year decrease) and being a nurse versus other healthcare professions (ORs between 1.7–2.51).

These results are in line with previous reports from countries with a high prevalence of SARS-CoV-2. In particular, the magnitude of the pandemic was associated with increased psychological distress in HCWs in the Hubei province of China, who were reported to be at a high risk for depressive symptoms [4]. However, the impact on their mental health was comparable to the one reported herein, as a prevalence of approximately 14.8% of clinical depressive symptoms and 10% of severe PTSD symptoms in Chinese WCHs using the same assessment tools (PHQ 9 and IES-R) was reported, respectively [4].

Other researchers in China reported, in a large sample of physicians and nurses, a frequency of depressive symptoms up to 25%, and of approximately 9% for PTSD symptoms [18]. This study, however, was conducted in emergency department personnel when the pandemic was under control, not at the peak. Interestingly, a study in Singapore reported higher mental distress in non-medical personnel compared to medical (nurses and physicians), and a lower overall prevalence of mental distress than the one reported in previous outbreaks (Tan et al., 2020) [19]. The authors attributed this difference to the readiness of the healthcare system acquired during the previous SARS outbreak [19]. Thus, the high levels of depressive and PTSD symptoms reported in our study might be related to the fact that this was the first outbreak of a contagious disease in Cyprus with no previous experience on a viral epidemic.

One of the few European studies was conducted in 330 HCWs in Italy, via an online questionnaire, and found that female sex, nursing profession and employment in COVID-19 units were associated with increased risk for emotional exhaustion [5]. Moreover, although the researchers used a shorter version of the IES-R tool, they reported higher levels of PTSD symptoms, i.e., up to 36% compared to the present findings. This difference may be attributed to the fact that in this Italian study, the majority of participants were employed in COVID-19 units and thus directly confronted with the COVID-19 disease.

The present study showed that females and nurses reported increased mental distress during the pandemic. This is in line with data from the three major viral outbreaks of the last 20 years [5,18,20]. A possible explanation for this may be provided by the fact that nurses have direct and longer contact with patients. A review and meta-analysis by Serrano-Ripoll et al. (2020) [21] identified female sex, younger age, lack of support, stigma and occupational parameters as risk factors for mental health deterioration in HCWs during epidemic outbreaks [21]. Nevertheless, previous data associate the proximity of care during the pandemic with mental distress in HCWs, since frontline personnel with direct contact with patients with COVID-19 has been identified as being more vulnerable to anxiety and depressive symptoms [22]. Moreover, personal characteristics of HCWs, i.e., fear of infection, personal psychiatric history, limited time to rest, diminished ability to care for family and challenging decision-making, have been previously associated with increased risk for developing stress, depressive and trauma-like symptoms [5,22]. Some of the aforementioned factors were not included in the present study. Nevertheless, future quantitative studies need to include data on distress due to family concerns, workload, degree of satisfaction with personal protective equipment or education provided in relation to pandemic management.

We also found that approximately 6% of responders exhibited suicide ideation, one of the most alarming symptoms of mental distress. Increased suicide rates were reported in the general population during the SARS epidemic [23,24] as well as in HCWs during the COVID-19 pandemic [25,26]. It is already known that HCWs are in high risk for suicidal behavior [27]. In fact, the only study published on suicide during the COVID-19 pandemic reported that 6.5% of 8817 hospital HCWs in China reported suicidal and self-harm ideation [26], yet the impact of the COVID-19 pandemic on suicidal ideation remains to be clarified as there are no longitudinal data. Nevertheless, based on the results of the present study, one may underline the necessity of screening tests and preventive strategies toward vulnerable HCWs to secure mental wellbeing among them. A comprehensive approach that considers multiple health priorities at the mental, psychological and physical, level during this crisis is proposed to empower the entire healthcare work group.

Another finding of the present study was that HCWs with fewer years of work experience exhibited PTSD and depressive symptoms more frequently. This finding suggests that the more experienced the HCWs, the less mentally distressing experiences they report; this is supported by previous literature [20] and might be explained as the result of acquisition of resilience and development of adaptive coping mechanisms through the years of work.

The timing of data collection at the peak of the epidemic is a strength of the study, whereas the limitations include the cross-sectional design, the self-assessment of the HCWs' mental health, the post-hoc and arbitrary definition of stress levels (based on quartiles of PSS-10 scores), the dynamic nature of the pandemic and the fact that additional confounders may exist which have not been considered in the present study, such as pre-existing burnout or high moral stress. The recruitment methods cannot exclude the introduction of some form of selection bias in the study sample, which could jeopardize generalizability of results. Specifically, non-uniform sampling methods were used for different categories of HCWs. However, the characteristics of the HCWs in this study are similar to those reported in previous studies in isolated professional groups in Cyprus (i.e., association of nurses, Cyprus Medical Association, professional body physiotherapists), in terms of male/female ratio, age, educational level and years of experience [28–30], which allows us to suggest that our findings could well be generalizable to the HCWs in Cyprus. Finally, as symptoms

of PTSD usually manifest themselves after the traumatic experience occurs, the current study cannot identify the full impact of the COVID-19 pandemic, and longitudinal studies are needed. This was previously reported in the SARS epidemic [31]. Thus, further research is warranted to track the dynamic changes of HCWs' mental health status. Nevertheless, the present data do provide a robust view of the mental health status of HCWs during the peak of the pandemic.

5. Conclusions

In conclusion, these findings contribute to the growing literature on the mental health distress of HCWs during the COVID-19 pandemic. The current study demonstrated that a considerable number of HCWs reported clinical depressive and PTSD symptoms, revealing the necessity for preventive and supportive measures toward HCWs, even in countries with a relatively SARS-CoV-2 burden, such as the Republic of Cyprus.

Author Contributions: A.C. (Andreas Chatzittofis), A.C. (Anastasia Constantinidou) and M.K., conceptualized the study. All the authors were responsible for the design of the study. A.C. (Andreas Chatzittofis) was responsible for the data collection, coordination and supervision of the data collection. K.M. and A.C. (Andreas Chatzittofis) carried out the statistical analyses. All the authors were responsible for the interpretation of the data. K.M. and A.C. (Andreas Chatzittofis) wrote the first draft of the manuscript. All authors revised the paper critically for important intellectual content. All authors have read and agreed to the published version of the manuscript.

Funding: This research received no external funding.

Institutional Review Board Statement: The study protocols were approved by the National (RC) Bioethics Committee 30.04.2020 (Protocol 2020.01.89).

Informed Consent Statement: Informed consent was obtained from all subjects involved in the study.

Data Availability Statement: Data not available due to restrictions according to the IRB.

Conflicts of Interest: The authors declare no conflict of interest.

References

1. Maunder, R.; Hunter, J.; Vincent, L.; Bennett, J.; Peladeau, N.; Leszcz, M.; Sadavoy, J.; Verhaeghe, L.M.; Steinberg, R.; Mazzulli, T. The immediate psychological and occupational impact of the 2003 SARS outbreak in a teaching hospital. *CMAJ* **2003**, *168*, 1245–1251. [PubMed]
2. Shanafelt, T.; Ripp, J.; Trockel, M. Understanding and Addressing Sources of Anxiety among Health Care Professionals during the COVID-19 Pandemic. *JAMA* **2020**, *323*, 2133–2134. [CrossRef] [PubMed]
3. Li, Z.; Ge, J.; Yang, M.; Feng, J.; Qiao, M.; Jiang, R.; Bi, J.; Zhan, G.; Xu, X.; Wang, L.; et al. Vicarious traumatization in the general public, members, and non-members of medical teams aiding in COVID-19 control. *Brain Behav. Immun.* **2020**, *88*, 916–919. [CrossRef] [PubMed]
4. Lai, J.; Ma, S.; Wang, Y.; Cai, Z.; Hu, J.; Wei, N.; Wu, J.; Du, H.; Chen, T.; Li, R.; et al. Factors Associated With Mental Health Outcomes Among Health Care Workers Exposed to Coronavirus Disease 2019. *JAMA Netw. Open* **2020**, *3*, e203976. [CrossRef]
5. Giusti, E.M.; Pedroli, E.; D'Aniello, G.E.; Badiale, C.S.; Pietrabissa, G.; Manna, C.; Badiale, M.S.; Riva, G.; Castelnuovo, G.; Molinari, E. The Psychological Impact of the COVID-19 Outbreak on Health Professionals: A Cross-Sectional Study. *Front. Psychol.* **2020**, *11*, 1684. [CrossRef]
6. Zervides, C.; Sassi, M.; Kefala-Karli, P.; Sassis, L. Impact of COVID-19 pandemic on radiographers in the Republic of Cyprus. A questionnaire survey. *Radiography* **2020**. [CrossRef] [PubMed]
7. World Health Organization. *National Situation Report. Coronavirus Disease 2019 (COVID-19)*; Press and Information Office: Nicosia, Cyprus, 2020.
8. Stylianou, N.; Samouti, G.; Samoutis, G. Mental Health Disorders During the COVID-19 Outbreak in Cyprus. *J. Med. Life* **2020**, *13*, 300–305. [CrossRef]
9. Solomou, I.; Constantinidou, F. Prevalence and Predictors of Anxiety and Depression Symptoms during the COVID-19 Pandemic and Compliance with Precautionary Measures: Age and Sex Matter. *Int. J. Environ. Res. Public Health* **2020**, *17*, 4924. [CrossRef]
10. Hyphantis, T.; Kotsis, K.; Voulgari, P.V.; Tsifetaki, N.; Creed, F.; Drosos, A.A. Diagnostic accuracy, internal consistency, and convergent validity of the Greek version of the patient health questionnaire 9 in diagnosing depression in rheumatologic disorders. *Arthritis Care Res.* **2011**, *63*, 1313–1321. [CrossRef]
11. Kroenke, K.; Spitzer, R.L.; Williams, J.B. The PHQ-9: Validity of a brief depression severity measure. *J. Gen. Intern. Med.* **2001**, *16*, 606–613. [CrossRef]

12. Mystakidou, K.; Tsilika, E.; Parpa, E.; Galanos, A.; Vlahos, L. Psychometric properties of the Impact of Event Scale in Greek cancer patients. *J. Pain Symptom. Manag.* **2007**, *33*, 454–461. [CrossRef] [PubMed]
13. Cohen, S.; Kamarck, T.; Mermelstein, R. A global measure of perceived stress. *J. Health Soc. Behav.* **1983**, *24*, 385–396. [CrossRef] [PubMed]
14. Andreou, E.; Alexopoulos, E.C.; Lionis, C.; Varvogli, L.; Gnardellis, C.; Chrousos, G.P.; Darviri, C. Perceived Stress Scale: Reliability and validity study in Greece. *Int. J. Environ. Res. Public Health* **2011**, *8*, 3287–3298. [CrossRef]
15. Cohen, S. Perceived stress in a probability sample of the United States. In *The Social Psychology of Health*; Sage Publications, Inc.: Thousand Oaks, CA, USA, 1988; pp. 31–67.
16. Bastos, L.S.; Oliveira, R.D.V.C.D.; Velasque, L.D.S. Obtaining adjusted prevalence ratios from logistic regression models in cross-sectional studies. *Cad. Saúde Pública* **2015**, *31*, 487–495. [CrossRef] [PubMed]
17. R Core Team. *R: A Language and Environment for Statistical Computing*; R Foundation for Statistical Computing: Vienna, Austria, 2020.
18. Song, X.; Fu, W.; Liu, X.; Luo, Z.; Wang, R.; Zhou, N.; Yan, S.; Lv, C. Mental health status of medical staff in emergency departments during the Coronavirus disease 2019 epidemic in China. *Brain Behav. Immun.* **2020**, *88*, 60–65. [CrossRef]
19. Tan, B.Y.Q.; Chew, N.W.S.; Lee, G.K.H.; Jing, M.; Goh, Y.; Yeo, L.L.L.; Zhang, K.; Chin, H.K.; Ahmad, A.; Khan, F.A.; et al. Psychological Impact of the COVID-19 Pandemic on Health Care Workers in Singapore. *Ann. Intern. Med.* **2020**, *173*, 317–320. [CrossRef]
20. Carmassi, C.; Foghi, C.; Dell'Oste, V.; Cordone, A.; Bertelloni, C.A.; Bui, E.; Dell'Osso, L. PTSD symptoms in healthcare workers facing the three coronavirus outbreaks: What can we expect after the COVID-19 pandemic. *Psychiatry Res.* **2020**, *292*, 113312. [CrossRef]
21. Serrano-Ripoll, M.J.; Meneses-Echavez, J.F.; Ricci-Cabello, I.; Fraile-Navarro, D.; Fiol-deRoque, M.A.; Pastor-Moreno, G.; Castro, A.; Ruiz-Pérez, I.; Zamanillo Campos, R.; Gonçalves-Bradley, D.C. Impact of viral epidemic outbreaks on mental health of healthcare workers: A rapid systematic review and meta-analysis. *J. Affect. Disord.* **2020**, *277*, 347–357. [CrossRef]
22. Azoulay, E.; Cariou, A.; Bruneel, F.; Demoule, A.; Kouatchet, A.; Reuter, D.; Souppart, V.; Combes, A.; Klouche, K.; Argaud, L.; et al. Symptoms of Anxiety, Depression and Peritraumatic Dissociation in Critical Care Clinicians Managing COVID-19 Patients: A Cross-Sectional Study. *Am. J. Respir. Crit. Care Med.* **2020**, *202*, 1388–1398. [CrossRef]
23. Cheung, Y.T.; Chau, P.H.; Yip, P.S. A revisit on older adults suicides and Severe Acute Respiratory Syndrome (SARS) epidemic in Hong Kong. *Int. J. Geriatr. Psychiatry* **2008**, *23*, 1231–1238. [CrossRef]
24. Yip, P.S.; Cheung, Y.T.; Chau, P.H.; Law, Y.W. The impact of epidemic outbreak: The case of severe acute respiratory syndrome (SARS) and suicide among older adults in Hong Kong. *Crisis* **2010**, *31*, 86–92. [CrossRef] [PubMed]
25. Jun, J.; Tucker, S.; Melnyk, B.M. Clinician Mental Health and Well-Being During Global Healthcare Crises: Evidence Learned From Prior Epidemics for COVID-19 Pandemic. *Worldviews Evid. Based Nurs.* **2020**, *17*, 182–184. [CrossRef]
26. Xiaoming, X.; Ming, A.; Su, H.; Wo, W.; Jianmei, C.; Qi, Z.; Hua, H.; Xuemei, L.; Lixia, W.; Jun, C.; et al. The psychological status of 8817 hospital workers during COVID-19 Epidemic: A cross-sectional study in Chongqing. *J. Affect. Disord.* **2020**, *276*, 555–561. [CrossRef] [PubMed]
27. Dutheil, F.; Aubert, C.; Pereira, B.; Dambrun, M.; Moustafa, F.; Mermillod, M.; Baker, J.S.; Trousselard, M.; Lesage, F.X.; Navel, V. Suicide among physicians and health-care workers: A systematic review and meta-analysis. *PLoS ONE* **2019**, *14*, e0226361. [CrossRef]
28. Aristidou, L.; Mpouzika, M.; Papathanassoglou, E.D.E.; Middleton, N.; Karanikola, M.N.K. Association Between Workplace Bullying Occurrence and Trauma Symptoms Among Healthcare Professionals in Cyprus. *Front. Psychol.* **2020**, *11*, 575623. [CrossRef] [PubMed]
29. Pavlakis, A.; Raftopoulos, V.; Theodorou, M. Burnout syndrome in Cypriot physiotherapists: A national survey. *BMC Health Serv. Res.* **2010**, *10*, 63. [CrossRef]
30. Middleton, N.; Andreou, P.; Karanikola, M.; Kouta, C.; Kolokotroni, O.; Papastavrou, E. Investigation into the metric properties of the workplace social capital questionnaire and its association with self-rated health and psychological distress amongst Greek-Cypriot registered nurses: Cross-sectional descriptive study. *BMC Public Health* **2018**, *18*, 1061. [CrossRef]
31. Chong, M.Y.; Wang, W.C.; Hsieh, W.C.; Lee, C.Y.; Chiu, N.M.; Yeh, W.C.; Huang, O.L.; Wen, J.K.; Chen, C.L. Psychological impact of severe acute respiratory syndrome on health workers in a tertiary hospital. *Br. J. Psychiatry* **2004**, *185*, 127–133. [CrossRef]
