# Supplementary material for: Mental health status and related factors influencing healthcare workers during the COVID-19 pandemic: A systematic review and meta-analysis
Source: PLoS One. 2024 Jan 19;19(1):e0289454. doi: 10.1371/journal.pone.0289454 (PMC10798549; doi:10.1371/journal.pone.0289454)
Supplement: S1 Data — (ZIP) [file pone.0289454.s011.zip › literatures/26.pdf]

Social Psychiatry and Psychiatric Epidemiology (2021) 56:47–55
https://doi.org/10.1007/s00127-020-01954-1

ORIGINAL PAPER

Risk factors for depression and anxiety in healthcare workers deployed during the COVID-19 outbreak in China

Jie Chen[1] · Xinghuang Liu[1] · Dongke Wang[1] · Yan Jin[2] · Miao He[2] · Yanling Ma[3] · Xiaolong Zhao[4] · Shuangning Song[1] · Lei Zhang[1] · Xuelian Xiang[1] · Ling Yang[1] · Jun Song[1] · Tao Bai[1] · Xiaohua Hou[1]

Received: 18 March 2020 / Accepted: 1 September 2020 / Published online: 10 September 2020
© Springer-Verlag GmbH Germany, part of Springer Nature 2020

Abstract

Purpose This study was conducted to evaluate the status of depression and anxiety of healthcare workers and to explore the risk factors during the outbreak of COVID-19 in China.

Methods A cross-sectional study was designed using convenience sampling to obtain a sample of healthcare workers. A structured questionnaire was designed to collect the information of the basic characteristics, workload, and the health condition. Burnout, coping style, anxiety, and depression were measured by specific scales. Multiple logistic regression model was performed to explore the risk factors of anxiety or depression.

Results There were 902 questionnaires received between February 9, 2020 and February 11, 2020. The proportion of healthcare workers with symptoms of moderate/severe anxiety and moderate/severe depression were 16.63% and 18.29%, respectively. There were 24.50% healthcare workers experiencing moderate/severe anxiety and depression at the same time. The increased workload, respiratory symptoms, digestive symptoms, having done specific test(s) related to COVID-19, having family member needs to be taken care of, negative coping style, and job burnout were the independent risk factors of anxiety. Furthermore, the increased workload, respiratory symptoms, digestive symptoms, having done specific test(s) related to COVID-19, negative coping style, and job burnout were the independent risk factors of depression.

Conclusion More attention should be paid to the mental health of frontline healthcare workers at the outbreak of COVID-19 in China. Taking steps to reduce the intensity of the work and burnout will be effective to stabilize the mental state of them.

Keywords Healthcare workers · Depression · Anxiety · COVID-19

Introduction

A novel coronavirus, which was named SARS-CoV-2 by The International Committee on Taxonomy of Viruses (ICTV), broke out in Wuhan, Hubei province, China [1]. By March 4, 2020, the virus had infected at least 80,270 people in China, 2981 of them died. In Hubei province alone, 67,332 people have been diagnosed with the virus [2]. By February 20, 2020, 476 hospitals across the country had reported 2,055 laboratory-confirmed cases among healthcare workers, mostly from Hubei province (88%) [3]. This is the largest outbreak of the virus in China since SARS in 2003. Thousands of healthcare workers are fighting the virus across the country, especially in Hubei province.

Healthcare workers in such circumstances are faced with multiple sources of stress, including work intensity, workplace changes relative to daily work, as well as family responsibilities, which may affect mental health [4, 5].

Jie Chen, Xinghuang Liu and Dongke Wang contributed equally to this study.

Electronic supplementary material The online version of this article (https://doi.org/10.1007/s00127-020-01954-1) contains supplementary material, which is available to authorized users.

✉ Jun Song
song111jun@126.com

✉ Tao Bai
drbaitao@126.com

✉ Xiaohua Hou
houxh@hust.edu.cn

Extended author information available on the last page of the article

Moreover, personal coping style and the presence of job-related burnout might also have an important impact on levels of anxiety and depression among healthcare workers [6–8]. It is believed that preventing the negative effect on psychological state should be an important objective to defend the disasters [9, 10]. However, the impact on healthcare workers at the outbreak of COVID-19 was not clear. We felt it important to explore the risk factors associated with psychological problems in healthcare staff in order to provide evidence to support an appropriate intervention program.

Therefore, we conducted this cross-sectional study to evaluate the prevalence of symptoms of anxiety and depression of healthcare workers during COVID-19 epidemic and to identify associated risk factors.

Method

Study design and participants

We conducted a cross-sectional survey of psychological state on Chinese healthcare workers by delivering online questionnaires through WeChat platform (https://www.wechat.com/en/), a Chinese APP for communication among friends and colleagues, between February 9 and February 11, 2020. Data were collected by designed online questionnaires containing demographic characteristics, working and health conditions of participants, as well as four self-rated psychological scales. The purpose and use of this research were explained at the beginning of the online questionnaire. All the participants agreed the online informed consent before entering the online questionnaire. Only one response to the questionnaire per person was permitted. The questionnaire was filled out voluntarily and the personal information was kept confidential. This study was approved by the Ethics Committee of Union Hospital, Tongji Medical College.

Questionnaire

A structured questionnaire was designed to collect information on the basic characteristics, workload, and health condition. Subjective assessment of workload was evaluated by asking the question "How do you evaluate your workload in recent days? 5 = significantly higher than before, 4 = higher than before, 3 = the same as before, 2 = lower than before, 1 = significantly lower than before" [4]. If participants chose answers 5 and 4, then workload (subjective) would be evaluated as increased workload, and if they chose answers 3, 2, and 1, it would be evaluated as non-increased workload. Objective assessment of workload is working hours per week. Respiratory symptoms in the study included runny nose, cough, expectoration, dyspnea, and chest pain. Systemic symptoms include fatigue, muscle aches, headaches, sleep disturbances and others. Meanwhile, gastrointestinal symptoms include acid reflux, diarrhea, constipation, abdominal discomfort, bloating, abdominal pain and other. Even if the respondents were medical professionals, the explanation of those symptoms was added to the questionnaire. Specific tests related to COVID-19 included chest computed tomographic (CT) scans and real-time reverse transcription polymerase chain reaction assay (RT-PCR) for SARS-CoV-2 RNA of nasopharyngeal swab. The results were considered as positive if chest CT images showed bilateral patchy shadows or ground glass opacity in the lungs, with or without positive result of RT-PCR for SARS-CoV-2 RNA [11]. By definition, the frontline working environment included fever clinics and isolation wards.

Job burnout

The 15-item Chinese version of Maslach Burnout Inventory (CMBI) was used to assess job burnout in health care workers participated in this study. The CMBI scale consists of three dimensions of burnout: emotional exhaustion (5 items), depersonalization (4 items), and personal accomplishment (6 items). Each item scores from 1 (never) to 7 (every day) and items of personal accomplishment are reverse-scored. The CMBI scale has satisfying reliability and validity in Chinese population [12–15]. Cronbach's alpha for the total scale is 0.816, and Cronbach's alpha for three dimensions are 0.871, 0.807, and 0.787, respectively [14]. The cut-off scores of the three dimensions were larger than 25, 11, and 16, respectively, and no burnout is defined as all three dimensions under the cut-off scores [12, 14]

Coping style

The 20-item Trait Coping Style Questionnaire (TCSQ) was employed in this study to evaluate the coping style-positive coping (PC) or negative coping (NC) of participants. There are 10 items for each coping style, and each item scores from 1 to 5. The Chinese version of TCSQ was proved to be valid and reliable in Chinese population, with Cronbach's alpha for each coping style dimension at 0.790 and 0.776, respectively [14, 16]. The highest scoring between positive and negative coping style indicates a person's more usual coping style [12, 14].

Anxiety

The generalized anxiety disorder 7-item scale (GAD-7) was applied to evaluate participants' anxiety symptoms in the last 2 weeks. With each item ranging from 0 to 3, the total score of GAD-7 can be divided into four severity levels: severe ($>$ 14), moderate (10–14), mild (5–9), and no ($\le$ 4) anxiety

[17]. This study employed the Chinese version of GAD-7, which has been proven to have good reliability and validity in Chinese population [18, 19]. Cronbach's alpha coefficient of GAD-7 was 0.898 and Kappa value was 0.825 [18, 19]. Statistical analyses were done between moderate/severe and none/mild anxiety distinguished by the recommended cut-off value (total score = 10) [18–20].

Depression

Depression symptoms in the last 2 weeks were assessed by the 9-item Patient Health Questionnaire (PHQ-9) with each item ranging from 0 to 3. According to the total score, the severity of depression is defined as follows: severe ($>$ 20), moderate to severe (15–20), moderate (10–14), mild (5–9), and no (≤ 4) depression [17, 21]. The Chinese version of PHQ-9 applied in this study has high reliability and validity in general Chinese population, whose Cronbach's alpha equals to 0.86 [21, 22]. Statistical analyses were done between moderate/severe and none/mild depression distinguished by the recommended cut-off value (total score = 10) [20–22].

Statistical analyses

Difference in each characteristic described in our online questionnaire according to anxiety and depression status was evaluated by the chi-square (χ^2) test or Student's t test. Multiple logistic regression was done to eliminate the interference of confounding factors and find the independent risk factors of anxiety and depression. And the odds ratios (ORs) of anxiety and depression status were also calculated by step-by-step multiple logistic regression model. The C statistic (the area under the receiver operating characteristic (ROC) curve) was calculated to evaluate the discrimination of the logistic model. And a value larger than 0.7 is considered as clinically useful [23]. All statistical analyses in this study were performed by R 3.6.0 (https://www.r-project.org/). And double-tailed p value < 0.05 was considered as statistically significant.

Results

Characteristics of participants

There were 902 questionnaires received in total by convenience sampling, all of which were completed and included in the final statistical analysis. Questionnaires were filled by 902 healthcare workers: 543 (60.20%) doctors, 311 (34.48%) nurses, and 48 (5.32%) other occupations including the administrative and management staff of the hospital. Among all the participants, there were 619 (68.63%) females and 283 (31.37%) males. The information of titles of occupation were asked among doctors and nurses, and 532 (62.30%) of them had primary titles of occupation (Interns, residents, attending doctors, junior nurses and primary nurses), while 322 (37.70%) with senior titles (associate chief physician/associate professor, chief physician/professor, nurse-in-charge, deputy chief nurse, and senior nurse). There were 417 (46.23%) of participants assessed their workload as increased. However, only 247 (27.38%) healthcare workers fought on the front line (fever clinic, isolation ward or other work area with direct contact with patients of COVID-19), and rest 655 (72.62%) of them worked on second line (general clinics and wards or other places that do not have direct contact with the patients). There were 254 (28.16%) participants had done specific test(s) related to COVID-19 and 648 (71.84%) of them had not. Positive results of specific test(s) related to COVID-19 were detected in 14 (5.51%) participants.

In total, 681(75.50%) healthcare workers had none/mild anxiety and depression, and 221(24.50%) had moderate/severe anxiety and depression (Fig. 1). According to the GAD-7 score, 274 (30.38%) healthcare workers experienced mild anxiety, while 150 (16.63%) experienced moderate/

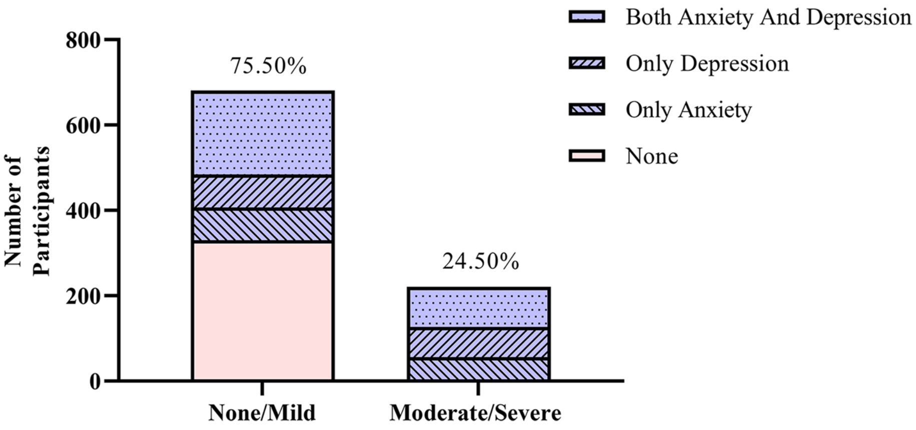

Fig. 1 Distribution of anxiety and depression. Most healthcare workers had none/mild anxiety or depression. About a quarter of healthcare workers had moderate/severe anxiety and depression. Anxiety and depression can be independent or combined in participants. Number of participants with both of anxiety and depression was more than those with the single

severe anxiety. On the other hand, there were 274 (30.38%) healthcare workers with mild depression and 165 (18.29%) with moderate/severe depression based on the score of PHQ-9. The remaining 330 (36.59%) healthcare workers were free from anxiety and depression symptoms. Anxiety and depression were significantly concomitant in our respondents, since 291(32.26%) healthcare workers had anxiety and depression at the same time.

The univariate analysis showed that some variables did have effects on anxiety and depression to different extend (Table 1). The occurrence rates of moderate/severe anxiety and depression were significantly higher in those who experienced increased workload (χ^2 test, 23.74% vs. 10.52%, $p<0.0001$ and 25.42% vs. 12.16%, $p<0.0001$ respectively) and in those who had respiratory (χ^2 test, 30.77% vs. 13.06%, $p<0.001$ and 32.97% vs. 14.58%, $p<0.0001$ respectively), digestive (χ^2 test, 29.75% vs. 11.82%, $p<0.0001$ and 31.40% vs. 13.48%, $p<0.0001$ respectively) or systemic symptoms (χ^2 test, 44.90% vs. 15.00%, $p<0.0001$ and 38.78% vs. 17.12%, $p=0.0003$ respectively) in the past 2 weeks. Negative coping style accessed by TCSQ (χ^2 test, 31.01% vs. 9.92%, $p<0.0001$ and 36.93% vs. 9.59%, $p<0.0001$ respectively), job burnout by CMBI (χ^2 test, 21.98% vs. 9.82%, $p<0.0001$ and 26.14% vs. 8.31%, $p<0.0001$ respectively) were also likely to be related to anxiety and depression in healthcare workers. There was no statistical difference in occurrence of moderate/severe anxiety and depression compared between participant with positive results of specific test(s) related to COVID-19 and those with negative results (Supplementary Table). Then comparison was performed between participants who have done specific test(s) and those who have not. It showed that participants who have done specific test(s) were more likely to have moderate/severe anxiety and depression symptoms than those who have not (23.62% vs. 13.89%, $p=0.0006$ and 25.59% vs. 15.43%, $p=0.0006$ respectively) (Table1). There was no evidence to show that sex (χ^2 test, 15.90% vs. 16.96%, $p=0.763$ and 16.25% vs. 19.22%, $p=0.328$ respectively), age (Student's t test, 37.19 vs. 36.41, $p=0.309$ and 36.68 vs. 36.50, $p=0.806$ respectively), occupation (χ^2 test, 17.68%, 14.47% vs. 18.75%, $p=0.442$ and 18.60%, 17.04% vs. 22.92%, $p=0.593$ respectively), had effect on the occurrence of moderate/severe anxiety and depression, respectively.

Independent risk factors of anxiety or depression

Then, step-by-step multiple logistic regression was performed to establish the best regression model and find the independent risk factors of anxiety and depression (Tables 2 and 3). These two models were clinically useful according to C statistic (0.815 and 0.848, respectively). Feeling of increase in workload (OR 2.238, 95% CI 1.503–3.361), respiratory symptoms (OR 1.897, 95% CI 1.212–2.944), digestive symptoms (OR 1.973, 95% CI 1.279–3.029), having done specific test(s) related to COVID-19 (OR 1.574, 95% CI 1.043–2.362), having family member needs to be taken care of (OR 1.546, 95% CI 1.016–2.390), negative coping style (OR 3.495, 95% CI 2.364–5.196), and job burnout (OR 1.939, 95% CI 1.276–2.988) were the independent risk factors of anxiety. At the same time, the following factors: feeling of increase in workload (OR 1.997, 95% CI 1.329–3.019), respiratory symptoms (OR 1.874, 95% CI 1.200–2.909), digestive symptoms (OR 2.101, 95% CI 1.387–3.178), having done specific test(s) related to COVID-19 (OR 1.610, 95% CI 1.069–2.415), negative coping style (OR 4.801, 95% CI 3.266–7.121), and job burnout (OR 3.121, 95% CI 2.028–4.913), were the independent risk factors of depression.

Working environment

The comparison between different working environments (front line or second line) was performed (Fig. 2). Feeling of increase in workload was more common in front line than second line (χ^2 test, 73.28% vs. 36.03%, $p<0.0001$). The proportion of healthcare workers who got respiratory (χ^2 test, 25.51% vs. 18.17%, $p=0.018$) and digestive (χ^2 test, 37.25% vs. 22.90%, $p<0.0001$) symptoms is higher in front line. More healthcare workers in front line had done specific test(s) related to COVID-19 (χ^2 test, 34.41% vs. 25.80%, $p=0.013$) than those in second line. But statistical significance was not detected on the negative coping style (χ^2 test, 33.60% vs. 31.15%, $p=0.530$) and job burnout (χ^2 test, 54.25% vs. 56.64%, $p=0.569$).

Discussion

Our study, a nationwide, observational study, was conducted from February 9 to February 11, 2020, which was the peak period since COVID-19 outbreak. We found that self-rated increase in workload, having respiratory symptoms, having digestive symptoms, having done specific test(s) related to COVID-19, negative coping style, and job burnout were independent risk factors for both anxiety and depression.

We call that attention should be paid to the psychological state of healthcare workers. When people are faced with a pressure source, they need to adapt or cope with it. When their coping ability cannot meet this need, they will produce a stress response, causing changes in physiology, psychology, and behavior [24]. The pathogen causing this new type of pneumonia is very similar to SARS, whose mortality rate is as high as 9.6% [25–27]. COVID-19, as a stress source, inevitably causes anxiety and tension among healthcare workers. In our study, we also found that healthcare workers in this outbreak were more prone to anxiety and depression,

Table 1 General characteristics of participants according to anxiety/depression symptoms ($n = 902$)[a]

Characteristics	Total	None/Mild Anxiety (%)	Moderate/Severe Anxiety (%)	Test value (χ^2/t)	*p* value	None/Mild Depression (%)	Moderate/Severe Depression (%)	Test value (χ^2/t)	*p* value
Sex				0.091	0.763			0.956	0.328
Male	283	238 (84.10)	45 (15.90)			237 (83.75)	46 (16.25)		
Female	619	514 (83.04)	105 (16.96)			500 (80.78)	119 (19.22)		
Age	902	36.41 ± 8.56	37.19 ± 8.64	− 1.018	0.309	36.50 ± 8.61	36.68 ± 8.42	− 0.246	0.806
Occupation				1.634	0.442			1.047	0.593
Doctor	543	447 (82.32)	96 (17.68)			442 (81.40)	101 (18.60)		
Nurse	311	266 (85.53)	45 (14.47)			258 (82.96)	53 (17.04)		
Others[b]	48	39 (81.25)	9 (18.75)			37 (77.08)	11 (22.92)		
Titles of occupation[c]				2.010	0.366			1.277	0.528
Primary title	532	437 (82.14)	95 (17.86)			432 (81.20)	100 (18.80)		
Senior title	322	276 (85.71)	46 (14.29)			268 (83.23)	54 (16.77)		
Working hours per week/h	902	46.32 ± 23.41	47.52 ± 23.84	−0.573	0.567	46.09 ± 230.05	48.42 ± 25.27	− 1.151	0.250
Workload (subjective)				27.342	< 0.0001***			25.476	< 0.0001***
Increased	417	318 (76.26)	99 (23.74)			311 (74.58)	106 (25.42)		
Non-increased	485	434 (89.48)	51 (10.52)			426 (87.84)	59 (12.16)		
Working environment				10.848	0.001***			12.515	0.0004***
Front line	247	189 (76.52)	58 (23.48)			183 (740.09)	64 (25.91)		
Second line	655	563 (85.95)	92 (14.05)			554 (84.58)	101 (15.42)		
Fever in last 2 weeks				4.982	0.026*			3.714	0.054
Yes	26	17 (65.38)	9 (34.62)			17 (65.38)	9 (34.62)		
No	876	735 (83.90)	141 (16.10)			720 (82.19)	156 (17.81)		
Respiratory symptoms in last 2 weeks				31.614	< 0.0001***			31.631	< 0.0001***
Yes	182	126 (69.23)	56 (30.77)			122 (67.03)	60 (32.97)		
No	720	626 (86.94)	94 (13.06)			615 (85.42)	105 (14.58)		
Systemic symptoms in last 2 weeks				27.747	< 0.0001***			13.131	0.0003***
Yes	49	27 (55.10)	22 (44.90)			30 (61.22)	19 (38.78)		
No	853	725 (85.00)	128 (15.00)			707 (82.88)	146 (17.12)		
Digestive symptoms in last 2 weeks				39.794	< 0.0001***			36.855	< 0.0001***
Yes	242	170 (70.25)	72 (29.75)			166 (68.60)	76 (31.40)		
No	660	582 (88.18)	78 (11.82)			571 (86.52)	89 (13.48)		
Specific test(s) related to COVID-19				11.776	0.0006***			11.928	0.0006***
Have done	254	194 (76.38)	60 (23.62)			189 (74.41)	65 (25.59)		
Have not done	648	558 (86.11)	90 (13.89)			548 (84.57)	100 (15.43)		
Family member needs to be taken care of				6.4627	0.011*			2.161	0.142
Yes	570	461 (80.88)	109 (19.12)			457 (80.18)	113 (19.82)		
No	332	291 (87.65)	41 (12.35)			280 (84.34)	52 (15.66)		
TCSQ[d]				61.276	< 0.0001***			96.042	< 0.0001***
NC	287	198 (68.99)	89 (31.01)			181 (63.07)	106 (36.93)		
PC	615	554 (90.08)	61 (9.92)			556 (90.41)	59 (9.59)		
Burnout (based on CMBI)				22.823	< 0.0001***			46.071	< 0.0001***
Yes	505	394 (78.02)	111 (21.98)			373 (73.86)	132 (26.14)		
No	397	358 (90.18)	39 (9.82)			364 (91.69)	33 (8.31)		

[a]Values were expressed as *n* (%) (appropriate for χ^2 test) or mean ± standard deviation (appropriate for Student's *t* test)

[b]Other occupation included the administrative and management staff of the hospital

[c]Primary title: interns, residents, attending doctors, junior nurses, and primary nurses; Senior title: associate chief physician/associate professor, chief physician/professor, nurse-in-charge, deputy chief nurse, and senior nurse. We did not evaluate the titles of occupation for other occupation except doctor and nurse

[d]TCSQ: the 20-item trait coping style questionnaire; *PC* positive coping, *NC* negative coping

Table 2 Multiple logistic regression analysis with risk factors for anxiety (C statistic: .815)

Characteristics	Estimate	SE[a]	*Z* value	*p* (>|z|)	OR (95% CI)
Workload					
Increased (vs. non-increased)	0.805	0.205	3.930	0.001***	2.238 (1.503, 3.361)
Respiratory symptoms in last 2 weeks					
Yes (vs. no)	0.640	0.226	2.833	0.005**	1.897 (1.212, 2.944)
Systemic symptoms in the past 2 weeks					
Yes (vs. No)	0.620	0.356	1.742	0.082	1.859 (0.918, 3.724)
Digestive tract symptoms in the past 2 weeks					
Yes (vs. no)	0.680	0.220	3.095	0.002**	1.973 (1.279, 3.029)
Specific tests related to COVID-19					
Yes (vs. no)	0.453	0.208	2.177	0.030*	1.574 (1.043, 2.362)
Family member needs to be taken care of					
Yes (vs. no)	0.413	0.231	1.785	0.045*	1.546 (1.016, 2.390)
TCSQ[b]					
NC (vs. PC)	1.251	0.200	6.239	< 0.0001***	3.495 (2.364, 5.196)
Burnout (based on CMBI)					
Yes (vs. no)	0.662	0.217	3.056	0.002**	1.939 (1.276, 2.988)

[a]*SE* standard error

[b]*TCSQ* the 20-item trait coping style questionnaire; *PC* positive coping, *NC* negative coping

Table 3 Multiple logistic regression analysis with risk factors for depression (C statistic: .848)

Characteristics	Estimate	SE[a]	*Z* value	*p* (>|z|)	OR (95% CI)
Working environment					
Front line (vs. second line)	0.359	0.217	1.656	0.098	1.431 (0.934, 2.185)
Workload					
Increased (vs. non-increased)	0.692	0.209	3.309	0.0009***	1.997 (1.329, 3.019)
Respiratory symptoms in the past 2 weeks					
Yes (vs. no)	0.628	0.226	2.784	0.005**	1.874 (1.200, 2.909)
Digestive tract symptoms in the past 2 weeks					
Yes (vs. no)	0.743	0.211	3.514	0.0004***	2.101 (1.387, 3.178)
Specific tests related to COVID-19					
Yes (vs. no)	0.476	0.208	2.292	0.022*	1.610 (1.069, 2.415)
TCSQ[b]					
NC (vs. PC)	1.569	0.199	7.902	< 0.0001***	4.801 (3.266, 7.121)
Burnout (based on CMBI)					
Yes (vs. no)	1.138	0.225	5.055	< 0.0001***	3.121 (2.028, 4.913)

[a]*SE* standard error

[b]*TCSQ* the 20-item trait coping style questionnaire, *PC* positive coping, *NC* negative coping

especially those in the front line. We have shown that in the battle against COVID-19, moderate percentages of healthcare workers experience anxiety and depression. Social support is the core of psychological intervention. Studies have shown that social support is important resources for stress coping [25]. In studies that have investigated the correlates of these psychological outcomes, social support is found to be one of the most reliable factors associated with fewer negative and more positive outcomes [28, 29]. We suggest that healthcare managers should pay full attention to the response of healthcare staff in the face of stress events and provide more social supports and resources to help them [10, 30]. They need to be able to acutely detect signs of psychological injury in healthcare staff, such as avoidance, moody, guilty, and help staff go through mental distress. Professional and evidence-based psychological care is needed, if the distress is severe and persistent [31].

In our survey, there was a positive association between anxiety or depression and job burnout, as well as with coping style. Healthcare workers who tend to have negative

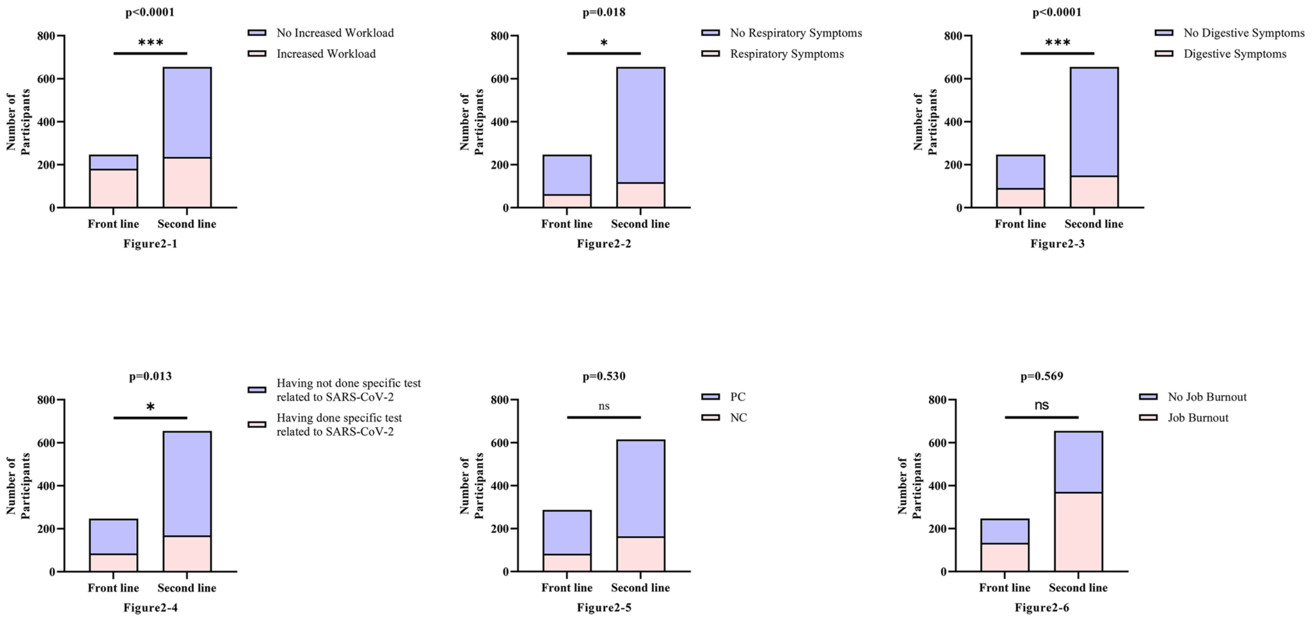

Fig. 2 Different characteristics between the front line and the second line. **2–1** More healthcare workers in front line had increased workload than the second line. **2–2** The proportion of respiratory symptoms among participants working in front line is higher than that of second line. **2–3** The proportion of digestive symptoms among participants working in front line is higher than that of second line. **2–4** A higher proportion of healthcare workers on the front line did specific test(s) related to COVID-19. **2–5** There was no significant difference in coping styles between the front line and the second line. **2–6** There was no significant difference in job burnout between the front line and the second line

coping style or job burnout are more likely to develop anxiety and depression. A similar phenomenon has been found in many studies [6–8]. So, it is reasonable to believe that taking certain measures to help alleviate job burnout and guide healthcare workers from negative coping to positive coping will effectively improve the psychological state of them.

From the result of step-by-step multiple logistic regression, we can get that working hours per week did not affect the incidence of anxiety and depression, while the subjective feeling of increase in workload did. It may support that the feeling of increase in workload during the outbreak can increase the occurrence of both anxiety and depression. Thus, our survey data indicate reducing the risk of anxiety and depression in health care workers by reducing the intensity of work per unit of time. Specific measures may include maximizing the number of healthcare workers involved in the disaster and batch management based on the patient's basic condition and severity of symptoms.

Usually, people who have illness or afraid of getting infected are more likely to have adverse psychological reactions. This phenomenon is in line with the findings in our study that the presence of respiratory and gastrointestinal symptoms, as well as having done specific test(s) related to COVID-19 was independent risk factor for anxiety and depression. Interestingly, fever, which is considered a very important first symptom of COVID-19 [11], did not affect the occurrence of anxiety or depression. We suspect this may be due to the small sample size of participants with fever ($n=26$), resulting in statistical instability. Above all, we speculate that the fear of getting infected with the virus might cause anxiety and depression in healthcare workers. We agree that healthcare managers should provide detailed and comprehensive information about this epidemic and pay more attention to the protection of healthcare staff who worked on front line, such as providing effective and adequate personal protective equipment (PPE) [31].

Based on the univariate analysis (Table 1), working environment (front line or second line) had an influence on moderate/severe anxiety or depression, while it was not thought to be an independent predictor factor for moderate/severe anxiety or depression according to step-by-step multiple logistic regression (Tables 2 and 3). This indicated that the effect of working environment on anxiety or depression might be eliminated by other factors in the multiple logistic regression analysis. According to the comparison between front line and second line (Fig. 2), an appropriate explanation is that the significant difference in the occurrence of moderate/severe anxiety or depression between front line and second line might be due to differences in certain factors between them, such as workload, respiratory symptoms, digestive symptoms, and whether specific test(s) related to COVID-19 had been performed.

We measured the intensity of the acute traumatic impact of the COVID-19 outbreak on hospital workers, as

characterized by anxiety, and depression. Some characteristics (sex, age, occupation, and titles of occupation) had been shown not to be factors that contribute to anxiety or depression in healthcare workers in this study (not included in step-by-step multiple logistic regression). Coincidentally, all these factors could not be affected by the outbreak of the virus. The indirect implication is that anxiety or depression in healthcare workers was more likely due to exposure to the risk of infection.

This study is an urgently needed original study investigating risk factors for depression and anxiety in healthcare staff working during the peak period of the SARS COV-2 outbreak and providing evidence in support of the need for steps to be taken to protect the psychological wellbeing of staff. However, there are some limitations about this study: Firstly, considering that it is difficult to directly investigate participants during the pandemic, we choose convenience sampling via online questionnaire. So, there is selection bias in this network research. Furthermore, it was impossible to define any characteristics of non-respondents due to the limitation of this method. But despite this, we found some independent risk factors, and the regression model has a good fitting degree. Secondly, although causality is uncertain in cross-sectional studies, we believe anxiety and depression are more likely to be the result, since there were evidences that stressful life events are very likely to lead to these mental states [32–34]. Finally, due to the lack of knowledge about the prevalence of anxiety and depression in this population before the outbreak, it is difficult to say with certainty to what extent these findings can be attributed to working circumstances during the coronavirus pandemic, although some risk factors of anxiety and depression mentioned in this study do have association with the outbreak of COVID-19, such as self-rated increase in workload, having respiratory and digestive symptoms.

Conclusion

Multiple factors, including having respiratory or digestive symptoms, having done specific test(s) related to COVID-19, self-reported increase in work intensity, negative coping style, and job burnout, participate in the anxiety or depression of healthcare workers. Taking steps to reduce work intensity and burnout will be effective to stabilize the mental state of them, especially for whom with negative coping style. More attention should be paid to the mental health of frontline healthcare workers at the outbreak of COVID-19 in China.

Author contributions JC, XL, DW, and TB were responsible for draft writing, conceived the idea for the Article, analyzed the data of this study and wrote the final manuscript. YJ, XZ, MH, YM and SS contributed to data collection in the fieldwork and gave suggestions for data analysis. XX, LY, JS, TB and XH contributed to the study design, supervised and checked the analyses, and critically reviewed the manuscript. All authors contributed to this study have approved the final report.

Funding Urgent projects of scientific and technological research on COVID-19 funded by Hubei province (2020FCA014) supported this work.

Availability of data and material The original data have been uploaded as electronic supplementary material.

Code availability R 3.6.0 (https://www.r-project.org/).

Compliance with ethical standards

Conflict of interest On behalf of all authors, the corresponding author states that there is no conflict of interest.

Ethics approval This study was approved by the Ethics Committee of Union Hospital, Tongji Medical College. This study was performed in accordance with the ethical standards laid down in the 1964 Declaration of Helsinki and its later amendments

Consent to participate All the participants agreed the online informed consent before entering the online questionnaire. The questionnaire was filled out voluntarily.

Consent for publication All authors have seen the manuscript and approved to submit to your journal.

References

1. Wang C, Horby PW, Hayden FG, Gao GF (2020) A novel coronavirus outbreak of global health concern. Lancet 395:470–473. https://doi.org/10.1016/S0140-6736(20)30185-9
2. National Health Commission of the People's Republic of China (2020–03–04) Daily briefing on novel coronavirus cases in China. https://en.nhc.gov.cn/2020-03/04/c_77270.htm. Accessed 3 June 2020
3. WHO (2020–02–28) Report of the WHO-China Joint Mission on Coronavirus Disease 2019 (COVID-19). https://www.who.int/publications/i/item/report-of-the-who-china-joint-mission-on-coronavirus-disease-2019-(covid-19). Accessed 3 June 2020
4. Tam CW, Pang EP, Lam LC, Chiu HF (2004) Severe acute respiratory syndrome (SARS) in Hong Kong in 2003: stress and psychological impact among frontline healthcare workers. Psychol Med 34:1197–1204. https://doi.org/10.1017/s0033291704002247
5. Kang L, Li Y, Hu S et al (2020) The mental health of medical workers in Wuhan, China dealing with the 2019 novel coronavirus. Lancet Psychiatry 7:e14. https://doi.org/10.1016/S2215-0366(20)30047-X
6. Folkman S et al (1986) Dynamics of a stressful encounter: Cognitive appraisal, coping, and encounter outcomes. J Pers Soc Psychol 50(5):992–1003. https://doi.org/10.1037/0022-3514.50.5.992
7. Koutsimani P, Anthony M, Georganta K (2019) The relationship between burnout, depression and anxiety: a systematic review and meta-analysis. Front Psychol 10:284. https://doi.org/10.3389/fpsyg.20190.00284
8. Creedy DK, Sidebotham M, Gamble J, Pallant J, Fenwick J (2017) Prevalence of burnout, depression, anxiety and stress in Australian

midwives: a cross-sectional survey. BMC Preg Childb 17(1):13. https://doi.org/10.1186/s12884-016-1212-5
9. Krystal JH, McNeil RL Jr (2020) Responding to the hidden pandemic for healthcare workers: stress. Nat Med 26:639. https://doi.org/10.1038/s41591-020-0878-4
10. Kisely S, Warren N, McMahon L, Dalais C, Henry I, Siskind D (2020) Occurrence, prevention, and management of the psychological effects of emerging virus outbreaks on healthcare workers: rapid review and meta-analysis. BMJ 369:m1642. https://doi.org/10.1136/bmj.m1642
11. Wang D, Hu B, Hu C et al (2020) Clinical characteristics of 138 hospitalized patients with 2019 novel coronavirus-infected pneumonia in Wuhan. China. JAMA. 323(11):1061–1069. https://doi.org/10.1001/jama.2020.1585
12. Zhou J et al (2016) Relationship between anxiety and burnout among Chinese physicians: a moderated mediation model. PLoS ONE 11:e0157013
13. Wang Y, Chang Y, Fu J, Wang L (2012) Work-family conflict and burnout among Chinese female nurses: the mediating effect of psychological capital. BMC Public Health 12:915
14. Ding Y et al (2015) The mediating role of coping style in the relationship between psychological capital and burnout among Chinese Nurses. PLoS ONE 10:e0122128
15. Xie Z, Wang A, Chen B (2011) Nurse burnout and its association with occupational stress in a cross-sectional study in Shanghai. J Adv Nurs 67:1537–1546
16. Qiao Z et al (2016) Prevalence and factors associated with occupational burnout among HIV/AIDS healthcare workers in China: a cross-sectional study. BMC Public Health 16:335
17. Yang Y et al (2019) Associations between erectile dysfunction and psychological disorders (depression and anxiety): a cross-sectional study in a Chinese population. Andrologia 51:e13395
18. He X, Li C, Qian J, Cui HS, Wu W (2010) Reliability and validity of a generalized anxiety scale in general hospital outpatients. J Shanghai Arch Psychiatry 22:200–203
19. Tong X, An D, McGonigal A, Park SP, Zhou D (2016) Validation of the generalized anxiety disorder-7 (GAD-7) among Chinese people with epilepsy. Epilepsy Res 120:31–36
20. Kroenke K, Rl S, Williams JB, Lowe B (2010) The patient health questionnaire somatic, anxiety, and depressive symptom scales: a systematic review. Gen Hosp Psychiatry 32:345–359
21. Wang W et al (2014) Reliability and validity of the Chinese version of the patient health questionnaire (PHQ-9) in the general population. Gen Hosp Psychiatry 36:539–544
22. Du N, Yu K, Ye Y, Chen S (2017) Validity study of Patient Health Questionnaire-9 items for Internet screening in depression among Chinese university students. LID. https://doi.org/10.1111/appy.12266 Asia Pac Psychiatry 9:e12266
23. Meurer WJ, Tolles J (2017) Logistic regression diagnostics: understanding how well a model predicts outcomes. JAMA 317:1068–1069
24. Cooper J (1999) Managing workplace stress in outpatient nursing. Prof Nurse 14:540–543
25. Xu X et al (2020) Evolution of the novel coronavirus from the ongoing Wuhan outbreak and modeling of its spike protein for risk of human transmission. Sci China Life Sci 63:457–460. https://doi.org/10.1007/s11427-020-1637-5
26. Roujian L et al (2020) Genomic characterisation and epidemiology of 2019 novel. The Lancet. https://doi.org/10.1016/S0140-6736(20)30251-8
27. WHO (2004) Summary of probable SARS cases with onset of illness from1 November 2002 to 31 July 2003. https://www.who.int/csr/sars/country/table2004_04_21/en/. Accessed 3 June 2020
28. Hobfoll SE, Stokes JP (1988) The process and mechanics of social support. In: Duck S, Hay DF, Hobfoll SE, Ickes W, Montgomery BM (eds) Handbook of personal relationships: Theory, research and interventions. John Wiley, Oxford, pp 497–517
29. Kaniasty K, Norris FH (2009) Distinctions that matter: received social support, perceived social support, and social embeddedness after disasters. In: Neria Y, Galea S, Norris FH (eds) Mental health and disasters. Cambridge University Press, New York, pp 175–200
30. Johnrev G et al (2018) Psychological outcomes in disaster responders a systematic review and meta-analysis on the effect of social support. Int J Disaster Risk Sci 9:344–358. https://doi.org/10.1007/s13753-018-0184-7
31. Greenberg N, Docherty M, Gnanapragasam S, Wessely S (2020) Managing mental health challenges faced by healthcare workers during covid-19 pandemic. BMJ 368:m1211. https://doi.org/10.1136/bmj.m1211
32. Kendler KS, Karkowski LM, Prescott CA (1999) Causal relationship between stressful life events and the onset of major depression. Am J Psychiatry 156:837–841. https://doi.org/10.1176/ajp.156.6.837
33. Craske MG, Stein MB, Eley TC et al (2017) Anxiety disorders. Nat Rev Dis Primers 3:17024. https://doi.org/10.1038/nrdp.2017.24
34. Faravelli C, Lo Sauro C, Lelli L et al (2012) The role of life events and HPA axis in anxiety disorders: a review. Curr Pharm Des 18:5663–5674. https://doi.org/10.2174/138161212803530907

Affiliations

Jie Chen[1] · Xinghuang Liu[1] · Dongke Wang[1] · Yan Jin[2] · Miao He[2] · Yanling Ma[3] · Xiaolong Zhao[4] · Shuangning Song[1] · Lei Zhang[1] · Xuelian Xiang[1] · Ling Yang[1] · Jun Song[1] · Tao Bai[1] · Xiaohua Hou[1]

[1] Division of Gastroenterology, Wuhan Union Hospital, Tongji Medical College, Huazhong University of Science and Technology, 1277 Jiefang Road, Wuhan 430022, China

[2] Wuhan Union Hospital, Tongji Medical College, Huazhong University of Science and Technology, 1277 Jiefang Road, Wuhan 430022, China

[3] Department of Respiratory and Critical Care Medicine, Wuhan Union Hospital, Tongji Medical College, Huazhong University of Science and Technology, 1277 Jiefang Road, Wuhan 430022, China

[4] Wuhan Hospital of Traditional Chinese and Western Medicine, 215 Zhongshan Road, Wuhan 430022, China
