# Supplementary material for: Mental health status and related factors influencing healthcare workers during the COVID-19 pandemic: A systematic review and meta-analysis
Source: PLoS One. 2024 Jan 19;19(1):e0289454. doi: 10.1371/journal.pone.0289454 (PMC10798549; doi:10.1371/journal.pone.0289454)
Supplement: S1 Data — (ZIP) [file pone.0289454.s011.zip › literatures/27.pdf]

MEDICAL SCIENCE MONITOR

CLINICAL RESEARCH

e-ISSN 1643-3750
© Med Sci Monit, 2021; 27: e929454
DOI: 10.12659/MSM.929454

Received: 2020.10.27
Accepted: 2021.04.24
Available online: 2021.05.24
Published: 2021.06.30

An Investigation of Mental Health Status Among Medical Staff Following COVID-19 Outbreaks: A Cross-Sectional Study

Authors' Contribution:
Study Design A
Data Collection B
Statistical Analysis C
Data Interpretation D
Manuscript Preparation E
Literature Search F
Funds Collection G

AE 1 **Liwen Chen**
BCD 2 **Dongmei Lin**
BC 3 **Haishan Feng**

1 Department of Neurology, Affiliated Zhongshan Hospital of Xiamen University, Xiamen, Fujian, P.R. China
2 Department of Gynaecology and Obstetrics, Affiliated Zhongshan Hospital of Xiamen University, Xiamen, Fujian, P.R. China
3 Department of Emergency Medicine, Affiliated Zhongshan Hospital of Xiamen University, Xiamen, Fujian, P.R. China

Corresponding Author: Haishan Feng, e-mail: fhshan123456@163.com
Source of support: Departmental sources

Background: COVID-19 (coronavirus disease 2019) broke out in China. This study was to investigate the situation of mental health status among medical staff following COVID-19.

Material/Methods: A cross-sectional study was conducted through structured questionnaires to collect the demographical information of the participating medical staff via WeChat following COVID-19 crisis. The Center for Epidemiologic Studies-Depression Scale (CES-D), impact of events scale revised (IES-R), and Pittsburgh Sleep Quality Index (PSQI) scale were used to evaluate depression, post-traumatic stress disorder (PTSD) symptoms, and sleep quality, respectively. 95% confidence intervals (CI) were calculated.

Results: A total of 597 medical staff's information was included for the statistical analysis, and found 45.23% of subjects had PTSD symptoms, the mean PSQI score was 6.320±3.587. The results of multivariable analysis implied that medical workers who did not participate in the Hubei aid program (β=4.128; 95% CI, 0.983-7.272; *P*=0.010) and PTSD symptoms (β=7.212; 95% CI, 4.807-9.616; *P*<0.001) were associated with a higher tendency to depression. The PSQI score was linearly related to the CES-D score (β=1.125; 95% CI, 0.804-1.445; *P*<0.001). Subgroup analysis showed that medical workers who did not participate in the Hubei aid program, no traumatic experience before COVID-19 outbreaks, and PTSD symptoms may affect the tendency to depression in females, but not in males. PSQI score was linearly related to the CES-D score both in males and females.

Conclusions: The medical staff with PTSD symptoms and higher PSQI score may have a higher tendency to depression following COVID-19 outbreaks.

Keywords: **COVID-19 • Depression • Medical Staff, Hospital**

Full-text PDF: https://www.medscimonit.com/abstract/index/idArt/929454

2806 4 — 28

This work is licensed under Creative Common Attribution-NonCommercial-NoDerivatives 4.0 International (CC BY-NC-ND 4.0)

Indexed in: [Current Contents/Clinical Medicine] [SCI Expanded] [ISI Alerting System] [ISI Journals Master List] [Index Medicus/MEDLINE] [EMBASE/Excerpta Medica] [Chemical Abstracts/CAS]

Background

In December 2019 the first pneumonia case of unknown causes was found in Wuhan, Hubei Province, China, and later proved to be caused by a novel coronavirus through sequencing of metagenomic RNA in bronchoalveolar lavage fluid samples, which has somewhat similar in phylogeny to severe acute respiratory syndrome associated coronavirus (SARS-CoV), thus it was named SARS-CoV-2 [1,2]. With the rapid spread of SARS-CoV-2, COVID-19 (coronavirus disease 2019) broke out and soon became prevalent nationwide; it was classified a public health emergency that needed international concern by the WHO [3]. As of early March 2020, the total number of confirmed cases in China had passed 80 000, which included not only patients from the general public, but also a number of medical staff.

According to a report by experts of the China-WHO joint inspection group, 3300 medical staff had been infected as of late February 2020. Previous studies revealed that during epidemic periods of infectious diseases, many medical staff, especially those who served on the front line, were prone to mental health problems. For example, during the outbreak of SARS, 27% of the medical staff in a hospital in Singapore were diagnosed with psychiatric symptoms [4]. A Chinese randomized sampling survey showed that front-line medical staff and SARS patients all (100%) had depression to some extent [5]. When Middle-East respiratory syndrome (MERS) broke out in South Korea, nurses were found to have severe psychological problems [6]. Post-traumatic stress disorder (PTSD) is the most common chronic mental illness after disasters or accidents, while depression is the most common complication, and its severity determines whether PTSD can be relieved within 1 year [7,8]. Additionally, studies also indicated that within 1-3 years following the SARS epidemic, the medical staff who had related experiences still had serious depressive disorders [9,10]. Hence, it is important to know about the medical staff's mental health status after COVID-19 outbreaks, and to determine the relevant risk factors. We assumed that the mental health status of medical staff was poor after the COVID-19 outbreaks.

In this study, we analyzed the mental health status of these medical staff, their PTSD symptoms, and quality of sleeping to identify the risk factors of depression, in order to provide efficient interventions to them according to their individual conditions, and to maintain their psychological and physical well-being.

Material and Methods

Research Strategy and Participants

This was a cross-sectional study with medical staff as the target population, using snowball sampling methods. A structured questionnaire survey was organized including 3 common sets of rating scales, demographic information required, exposures to COVID-19, perception levels of COVID-19-related risks, sleep quality, and PTSD symptoms.

The research was conducted in accordance with the Declaration of Helsinki and national clinical research regulations. Prior to the commencement of the study, the protocol was approved by the Ethics Committee of Zhongshan affiliated Hospital of Xiamen University. Relevant data collection has not been affected by other interventions. The only potential risk came from the protection of privacy. Hence, all research materials had the identifying information about the respondents removed. The distributed questionnaire also provided a detailed description of the study before participants responded, and informed consent was required.

The inclusion criteria of the study participants were: 1) age ≥18 years; 2) medical staff on duty during COVID-19 outbreaks; and 3) who expressed informed consent and willingness to participate. The exclusion criteria were: 1) refusing to be interviewed or providing incomplete answers; 2) diagnosed with mental or psychiatric disorders before the COVID-19 epidemic; or 3) clinically diagnosed or suspected COVID-19 infected cases. Finally, this research surveyed 597 medical staff.

Measurement of the Factors and Quality Assessment

General Information

The general information of the medical staff, including their ages, gender, ethnicities, marital status, educational levels, job positions, years of service, professional titles, COVID-19 exposure (work exposure, receiving quarantine, infection of relatives or friends), any painful experience before COVID-19 outbreaks (such as severe violent injury, witnessed serious injury/illness or death of a friend or family member, experienced a serious disaster), current diseases, and past medical history, were gathered for later statistical analysis.

Perception Levels of COVID-19-Related Risks

A scale of perception levels of COVID-19-related risks was designed with reference to previous studies [10,11], which consisted of 12 items, such as 'my job having putting me in great danger', 'a sense of extra pressure', and 'afraid of being infected with COVID-19', and the last item was used to measure the influence of altruism on perception of risks. Each item was answered by 'yes/no', and 'yes' was recorded as 1 point, while 'No' was recorded as 0 points. Higher scores indicated higher risk perception levels.

This work is licensed under Creative Common Attribution-NonCommercial-NoDerivatives 4.0 International (CC BY-NC-ND 4.0)

Indexed in: [Current Contents/Clinical Medicine] [SCI Expanded] [ISI Alerting System] [ISI Journals Master List] [Index Medicus/MEDLINE] [EMBASE/Excerpta Medica] [Chemical Abstracts/CAS]

Depression

Depressive symptoms of the participants in the latest week prior to the research were assessed by using the Center for Epidemiologic Studies-Depression Scale (CES-D) [12] with 20 items, like 'trifles making me annoyed', 'having a poor appetite', and 'still immersed in the anguish in heart in spite of my family and friends' help'. Each item had 4 frequency grades: 'occasionally or none (less than 1 day)' was given 0 points; 'sometimes' (1 to 2 days) was given 1 point; often or half of the time' (3 to 4 days) was given 2 points; and 'most of the time or continuously' (5 to 7 days) was given 3 points. Total scores ranged from 0 to 60, with higher scores indicating more severe depression. Scores ≥16 implied the occurrence of depression, and scores of 25 or higher were strongly associated with major depressive disorder [13], which has been regarded as the cut-off point for severe depression [14].

PTSD Symptoms

PTSD was assessed by the impact of events scale revised (IES-R) [10] with 22 items; for instance, 'anything relevant to COVID-19 will trigger my feeling at the time of the epidemic outbreak', 'I can hardly sleep steadily to the dawn', and 'other things will also make me think of the epidemic'. Each item was graded on a 5-point scale, with never occurring given 0 points, rarely occurring given 1 point, sometimes occurring given 2 points, often occurring given 3 points, and always occurring given 4 points. The total score was 0 to 88, and a score of ≥20 was considered as indicating the presence of PTSD symptoms [15].

Sleep Quality

The Pittsburgh Sleep Quality Index (PSQI) scale was used to assess the sleep status [16] among the study subjects in the past 1 month, which contained 15 optional items and 4 write-in items and was divided into 7 subscales: subjective sleep quality, sleep latency, sleep duration, sleep efficiency, sleep disturbances, use of sleeping medication, and daytime dysfunction. Total scores were 0-21 points, and higher scores indicate poorer sleep quality [17].

Statistical Analysis

The CES-D score was used as the outcome variable. The Shapiro test was used to test the normality of the quantitative variables. If normality was met, group comparisons were performed via one-way analysis of variance and expressed as mean±standard deviation (Mean±SD); if not, the Kruskal-Wallis H test was used and the results were expressed as median (quartiles) [Median (Q1, Q3)]. Between-group comparisons of qualitative independent variables were performed through the χ^2 test or Fisher's exact test and displayed as numbers of cases and percentages [n (%)]. Risk factors of depression were explored using stepwise ordered multiple logistic regression and included independent variables with *P* values <0.05 from the univariate analysis above to conduct the parallelism test. If parallelism was not satisfied, stepwise unordered multiple logistic regression analysis was used instead. All statistical tests were two-sided, and the differences were judged to be statistically significant at *P*<0.05. All statistical analyses were performed using SPSS 23.0 (IBM, Armonk, NY, USA) and SAS 9.4 (SAS Institute, Cary, North Carolina). In addition, the sample-size and power-calculation results calculated by PASS software showed that the statistical power of the total sample size, male sample size, and female sample size were 1.000, 0.997, and 1.000, respectively.

Results

Characteristics of the Participants

Among 597 study subjects, 72 (12.06%) were males, and 525 were females (87.94%). There were 410 subjects ≤35 years (68.68%), 143 were 36-45 years (23.95%), and 44 were 46 years or above (7.37%). There were 392 (65.67%) with an educational background of bachelor degree or above. Only 213 (35.68%) were public staff of the hospital, 295 (49.41%) with intermediate, deputy senior, or higher professional titles. There were 447 (74.87%) directly involved in the prevention and treatment of COVID-19, of which 84 (14.07%) were staff for aid in Hubei and 133 (22.28%) were at the departments for diagnosis and treatment of COVID-19. There were 270 (45.23%) who had PTSD symptoms (IES-R scores ≥20), and the mean PSQI score was 6.320±3.587 (**Table 1**). On the PSQI scale, the mean score of subjective sleep quality, sleep latency, sleep duration, sleep disturbances, and daytime dysfunction were 1.21±0.77, 1.31±0.94, 1.34±0.83, 0.98±0.66, and 1.56±0.98, respectively (**Table 2**).

Univariate Analysis

Univariate analysis showed that the CES-D score of medical workers not in the Hubei aid program was higher than that of those in the program (β=3.786; 95% CI, 0.337-7.234; *P*=0.032), and the CES-D score of PTSD symptoms subjects was higher than that of no PTSD symptoms subjects (β=10.487; 95% CI, 8.221-12.754; *P*<0.001). Subjects with no previous medical history (β=-8.178; 95% CI, -16.114--0.242; *P*=0.043), healthy (β=-9.439; 95% CI, -17.660--1.218; *P*=0.025), and no painful experience before COVID-19 outbreaks (β=-7.522; 95% CI, -12.543--2.501; *P*=0.003) had lower CES-D scores. Furthermore, the PSQI score was linearly correlated to the CES-D score (β=1.564; 95% CI, 1.409-1.718; *P*<0.001). The factors mentioned above were then introduced into the stepwise unordered multinomial logistic regression for analysis (**Table 1**).

This work is licensed under Creative Common Attribution-NonCommercial-NoDerivatives 4.0 International (CC BY-NC-ND 4.0)

Indexed in: [Current Contents/Clinical Medicine] [SCI Expanded] [ISI Alerting System] [ISI Journals Master List] [Index Medicus/MEDLINE] [EMBASE/Excerpta Medica] [Chemical Abstracts/CAS]

Table 1. Association between participants characteristics and CES-D score.

Variables	All (n=597)		β (95% CI)	*P*-value
Gender				
Males	72	(12.06)	Ref	
Females	525	(87.94)	-2.415 (-6.106, 1.276)	0.199
Age (years)				
≤35	410	(68.68)	Ref	
36-	143	(23.95)	0.262 (-2.560, 3.085)	0.855
46-	44	(7.37)	4.394 (-0.470, 9.257)	0.077
Nation				
Han	589	(98.66)	Ref	
Minority	8	(1.34)	5.322 (-3.120, 17.786)	0.169
Marital status				
Married	381	(63.82)	Ref	
Single	211	(35.34)	1.530 (-0.986, 4.047)	0.233
Divorced	5	(0.84)	12.430 (-0.771, 25.631)	0.065
Education				
Junior College and below	205	(34.33)	Ref	
Undergraduate	366	(61.31)	3.749 (-8.396, 15.893)	0.545
Postgraduate	26	(4.36)	4.079 (-7.984, 16.143)	0.507
Hospital type				
Public	578	(96.82)	Ref	
Private	19	(3.18)	5.149 (-1.700, 11.994)	0.140
Hospital level				
Tertiary hospital	561	(93.97)	Ref	
Secondary hospital	33	(5.53)	0.216 (-5.054, 5.486)	0.936
Community hospital	3	(0.50)	7.367 (-9.664, 24.398)	0.396
Employment status				
Staff on the establishment	213	(35.68)	Ref	
Contract staff	363	(60.80)	0.913 (-1.624, 3.45)	0.480
Other	21	(3.52)	-3.734 (-10.455, 2.986)	0.276
Post of duty				
Doctor	41	(6.87)	Ref	
Nurse	549	(91.96)	-3.454 (-8.212, 1.303)	0.154
Other	7	(1.17)	-2.362 (-14.381, 9.656)	0.700

This work is licensed under Creative Common Attribution-NonCommercial-NoDerivatives 4.0 International (CC BY-NC-ND 4.0)

Indexed in: [Current Contents/Clinical Medicine] [SCI Expanded] [ISI Alerting System] [ISI Journals Master List] [Index Medicus/MEDLINE] [EMBASE/Excerpta Medica] [Chemical Abstracts/CAS]

Table 1 continued. Association between participants characteristics and CES-D score.

Variables	All (n=597)		β (95% CI)	*P*-value
Technical post				
Primary	302	(50.59)	Ref	
Intermediate	230	(38.52)	-0.424 (-2.997, 2.149)	0.746
Senior vice	48	(8.04)	3.382 (-1.186, 7.951)	0.147
Senior	9	(1.51)	4.244 (-5.703, 14.190)	0.402
Other	8	(1.34)	-1.868 (-12.400, 8.665)	0.728
Participated in the epidemic prevention and treatment				
Yes	447	(74.87)	Ref	
No	150	(25.13)	1.048 (-1.726, 3.823)	0.458
Aid worker in Hubei				
Yes	84	(14.07)	Ref	
No	513	(85.93)	3.786 (0.337, 7.234)	0.032
The department where you worked during COVID-19				
Fever clinic	51	(8.54)	Ref	
Departments for the diagnosis and treatment of COVID-19 patients	133	(22.28)	-1.266 (-6.121, 3.588)	0.609
Departments for pre-triage and temperature screening	70	(11.73)	-0.658 (-6.084, 4.768)	0.812
General outpatient, emergency and ward	327	(54.77)	-0.230 (-4.667, 4.208)	0.919
Other	16	(2.68)	-1.092 (-9.538, 7.354)	0.800
Direct contact with confirmed or suspected COVID-19 patients or their biological samples				
Yes	322	(53.94)	Ref	
No	275	(46.06)	0.026 (-2.389, 2.441)	0.983
Live alone during COVID-19				
Yes	223	(37.35)	Ref	
No	374	(62.65)	-2.016 (-4.500, 0.467)	0.111
Medical history				
Yes	14	(2.35)	Ref	
No	574	(96.15)	-8.178 (-16.114, -0.242)	0.043
Unknown	9	(1.51)	-8.111 (-20.645, 4.423)	0.204
Diseases				
Yes	13	(2.18)	Ref	
No	576	(96.48)	-9.439 (-17.660, -1.218)	0.025
Unknown	8	(1.34)	-8.106 (-21.277, 5.066)	0.227
A confirmed or suspected case of COVID-19				
Yes	15	(2.51)	Ref	
No	582	(97.49)	-2.788 (-10.477, 4.900)	0.477

This work is licensed under Creative Common Attribution-NonCommercial-NoDerivatives 4.0 International (CC BY-NC-ND 4.0)

Indexed in: [Current Contents/Clinical Medicine] [SCI Expanded] [ISI Alerting System] [ISI Journals Master List] [Index Medicus/MEDLINE] [EMBASE/Excerpta Medica] [Chemical Abstracts/CAS]

Table 1 continued. Association between participants characteristics and CES-D score.

Variables	All (n=597)		β (95% CI)	*P*-value
Any quarantined (confirmed not infected)				
Yes	80	(13.40)	Ref	
No	517	(86.60)	0.797 (-2.736, 4.330)	0.658
A relative or friend infected with COVID-19				
Yes	1	(0.17)	Ref	
No	596	(99.83)	10.032 (-19.395, 39.459)	0.503
Before the outbreak of coVID-19, whether there was any painful experience				
Yes	36	(6.03)	Ref	
No	561	(93.97)	-7.522 (-12.543, -2.501)	0.003
PTSD				
No	327	(54.77)	Ref	
Yes	270	(45.23)	10.487 (8.221, 12.754)	<0.001
PSQI, Mean±SD	6.320±3.587		1.564 (1.409, 1.718)	<0.001

Table 2. Characteristics of subscales of Pittsburgh sleep quality index.

Variables	Value
PSQI, mean±SD	6.320±3.587
Subjective sleep quality, mean±SD	1.21±0.77
Sleep latency, mean±SD	1.31±0.94
Sleep duration, mean±SD	1.34±0.83
Sleep efficiency, M (Q1, Q3)	0 (0, 0)
Sleep disturbances, mean±SD	0.98±0.66
Use of sleeping medication, M (Q1, Q3)	0 (0, 0)
Daytime dysfunction, mean±SD	1.56±0.98

Multivariable Regression Analysis

The findings of the multivariable analysis in **Table 3** showed that the CES-D score of medical workers not in the Hubei aid program was also higher than that of those in the program (β=4.128; 95% CI, 0.983-7.272; *P*=0.010), and the CES-D score of PTSD symptoms subjects was also higher than that of subjects with no PTSD symptoms (β=7.212; 95% CI, 4.807-9.616; *P*<0.001). In addition, the PSQI score was still linearly correlated to the CES-D score (β=1.125; 95% CI, 0.804-1.445; *P*<0.001).

Subgroup analysis was performed based on gender to explore influencing factors in different populations. In males, the PSQI score was linearly correlated to the CES-D score (β=1.897; 95% CI, 0.804-1.445; *P*<0.001), and no significant difference was observed in other factors (*P*>0.05). In females, medical workers who did not participate in the Hubei aid program (β=4.988; 95% CI, 1.382-8.593; *P*=0.007), no traumatic experience before COVID-19 outbreaks (β=-6.360; 95% CI, -11.680- -1.040; *P*=0.019), and PTSD symptoms (β=7.856; 95% CI, 5.326-10.385; *P*<0.001) had higher CES-D scores. The PSQI score was linearly correlated to the CES-D score (β=0.890; 95% CI, 0.530-1.249; *P*<0.001) in females (**Table 4**).

Discussion

The COVID-19 pandemic is a global public health event which has a huge impact on people's lives and social economy. Countries around the world have invested a large amount of material and human resources into epidemic prevention and control. Since the outbreak of COVID-19, medical workers across the country have given up their holidays and been overloaded with work as the front-line force. Their physical and mental health during the epidemic has attracted the attention of all sectors of society and the country.

Medical staff needed to bear the high work demands and risk of infection during the epidemic, and sometimes they might not be understood by patients when treating them. Fatigue and stress can cause medical workers anxiety, sadness, helplessness, depression, and a series of bad moods [18]. Previous studies have reported that medical staff working in Hubei have

This work is licensed under Creative Common Attribution-NonCommercial-NoDerivatives 4.0 International (CC BY-NC-ND 4.0)

Indexed in: [Current Contents/Clinical Medicine] [SCI Expanded] [ISI Alerting System] [ISI Journals Master List] [Index Medicus/MEDLINE] [EMBASE/Excerpta Medica] [Chemical Abstracts/CAS]

Table 3. Influencing factors associated with depression among medical staff following COVID-19 outbreaks by the stepwise multinomial logistic model.

Variables	β (95% CI)	*P*
Aid worker in Hubei		
Yes	Ref	
No	4.128 (0.983, 7.272)	0.010
Medical history		
Yes	Ref	
No	4.334 (-4.895, 12.131)	0.963
Unknown	6.742 (-13.173, 13.308)	0.777
Diseases		
Yes	Ref	
No	4.626 (-12.440, 5.733)	0.469
Unknown	7.191 (-17.259, 10.985)	0.663
Before the outbreak of COVID-19, whether there was any painful experience		
Yes	Ref	
No	-2.155 (-5.773, 1.463)	0.243
PTSD		
No	Ref	
Yes	7.212 (4.807, 9.616)	<0.001
PSQI	1.125 (0.804, 1.445)	<0.001

Table 4. Subgroup analysis of the influencing factors related to depression based on gender.

Variables	Males		Females	
	β (95% CI)	*P*	β (95% CI)	*P*
Aid worker in Hubei				
Yes	Ref		Ref	
No	0.301 (-7.459, 8.060)	0.939	4.988 (1.382, 8.593)	0.007
Medical history				
Yes	Ref		Ref	
No	-6.318 (-31.066, 18.430)	0.612	2.662 (-6.706, 12.031)	0.577
Unknown	-2.572 (-41.681, 36.537)	0.896	-0.935 (-15.202, 13.331)	0.898
Diseases				
Yes	Ref		Ref	
No	3.966 (-17.802, 25.734)	0.717	-8.619 (-19.480, 2.241)	0.120
Unknown	-	–	-7.634 (-22.956, 7.688)	0.328
Before the outbreak of COVID-19, whether there was any painful experience				
Yes	Ref		Ref	
No	0.301 (-7.459, 8.060)	0.939	-6.360 (-11.680, -1.040)	0.019
PTSD				
No	Ref		Ref	
Yes	2.748 (-5.079, 10.575)	0.486	7.856 (5.326, 10.385)	<0.001
PSQI	1.897 (0.926, 2.867)	<0.001	0.890 (0.530, 1.249)	<0.001

This work is licensed under Creative Common Attribution-NonCommercial-NoDerivatives 4.0 International (CC BY-NC-ND 4.0)

Indexed in: [Current Contents/Clinical Medicine] [SCI Expanded] [ISI Alerting System] [ISI Journals Master List] [Index Medicus/MEDLINE] [EMBASE/Excerpta Medica] [Chemical Abstracts/CAS]

a higher tendency to depression [19,20]. However, our study showed that medical workers who did not participate in the Hubei aid program may have a higher tendency to depression. A possible explanation was that different populations and working environments may have potential effects on the occurrence of depression. Wang et al indicated that the higher the probability and intensity of exposure to COVID-19 patients, the greater the risk that medical staff will have depression [19]. The subgroup analysis indicated that whether they were Hubei aid workers had no effect on the occurrence of depression in males, but had an effect in females.

According to relevant studies, for a long period of time after the SARS epidemic, first-line medical workers had obvious depression and anxiety, and the PTSD symptoms were significantly higher than that of those who were second- or third-line workers [5,21], which is consistent with the conclusion of the present study, as medical staff with PTSD symptoms had higher CES-D scores. For those who were both medical care providers and who experienced the pandemic, occupational pressure and the conflict between personal safety and professional roles may lead to chronic symptoms of PTSD. A recent study by Guo et al showed that healthcare workers in Wuhan were more likely to suffer from anxiety and PTSD than those in other cities in Hubei province during the COVID-19 outbreak [22]. A cross-sectional survey conducted by Si et al, from February 23 to March 5, 2020 among 863 medical personnel in 7 provinces in China, found a 40.2% prevalence of significant PTSD symptoms [23]. In a systematic review by Carmassi et al that included 19 studies on SARS in 2003, 2 on MERS 2012, and 3 on the ongoing COVID-19 pandemic, level of exposure, working role, years of service, social and work support, job organization, quarantine, age, gender, marital status, and coping styles were found to be of relevance both as risk factors and resilience factors [24]. Song et al also found in the medical personnel they surveyed those who worked for fewer years, had longer daily work time, and lower levels of social support were at higher risk of developing PTSD during the COVID-19 crisis [25]. It is crucial to take these factors into account when planning effective intervention strategies [24], to avoid the reduction in medical personnel either due to sick leave or not being able to provide certain health services [26] after the pandemic.

This study also found that the PSQI score was linearly correlated with the CES-D score. Sleep quality was an important factor affecting medical staff, which was mainly due to the sudden outbreak of the epidemic, the number of infected people, and infections among doctors and nurses in local hospitals that created stress for front-line medical staff. Ferini-Strambi et al found that sleep disorders, especially insomnia, are often accompanied by depressive and anxiety symptoms [27]. As COVID-19 is a new disease, it creates new challenges for medical care workers who had not participated in a major outbreak, while medical staff who went through H1N1, SARS, H7N9, and other epidemics might show more anxiety and nervousness in the spread of the epidemic and the destructive effects of the virus based on their previous experiences [28]. For those who had previously had a traumatic experience like severe violent assault, disaster, or witnessing a traumatic event or illness or deaths of friends or family members, the outbreak created a double stress.

This research included information from 597 participants from different backgrounds (eg, different working environment, postgraduate educational level or others, any painful experience before COVID-19 epidemic or not) that were to some extent representative of the medical staff population. Factors such as PTSD symptoms, sleep quality, and the participants' demographic data were taken into account and provided a comprehensive analysis of the risk factors associated with depression among medical workers. The study protocol and questionnaire were subjected to evaluations and on-the-spot rehearsals several times by experts to ensure precision. However, our research has some limitations. For example, as the medical personnel we surveyed were all Chinese and they were surveyed randomly through an online questionnaire, their psychological states may be restricted by the temporal specific circumstances in china, and there may also be selection bias. In addition, some factors may have interplay with each other; for instance, any painful experience before the epidemic outbreak and a higher level of COVID-19-related risk perception may have reduced the sleep quality of the study subjects. Thus, future studies are needed to collect information from medical workers in other countries and from other populations for comparisons, enabling a more generalized and exact finding of the potential risk factors.

Conclusions

This study revealed that medical workers who did not participate in the Hubei aid program and PTSD symptoms were associated with a higher tendency to depression. The PSQI score was linearly related to the CES-D score. Subgroup analysis showed that medical workers who did not participate in the Hubei aid program, no painful experience before COVID-19 outbreaks, and PTSD symptoms may affect the tendency to depression in females, but not in males. Managers of medical institutions should pay close attention to the psychological changes of their workers; daily lives, undertake timely and appropriate interventions, and improve the stress management system of medical staff for public health emergencies, to be better prepared for the medical work during special events.

Conflict of Interest

None.

This work is licensed under Creative Common Attribution-NonCommercial-NoDerivatives 4.0 International (CC BY-NC-ND 4.0)

Indexed in: [Current Contents/Clinical Medicine] [SCI Expanded] [ISI Alerting System] [ISI Journals Master List] [Index Medicus/MEDLINE] [EMBASE/Excerpta Medica] [Chemical Abstracts/CAS]

References:

1. Huang C, Wang Y, Li X, et al. Clinical features of patients infected with 2019 novel coronavirus in Wuhan, China. Lancet (London, England), 2020;395(10223):497-506
2. Wu F, Zhao S, Yu B, et al. A new coronavirus associated with human respiratory disease in China. Nature. 2020;579(7798):265-69 Erratum in: Nature. 2020;580(7803):E7
3. Eurosurveillance Editorial Team. Note from the editors: World Health Organization declares novel coronavirus (2019-nCoV) sixth public health emergency of international concern. Euro Surveill. 2020:25(5):200131e
4. Chan AO, Huak CY. Psychological impact of the 2003 severe acute respiratory syndrome outbreak on health care workers in a medium size regional general hospital in Singapore. Occup Med (Lond). 2004;54(3):190-96
5. Wang FQ, Xu JY, Liu QA, et al. [Comparison of psychological status between patients with SARS and physicians. Nurses Treating SARS.] Chinese Mental Health Journal. 2003:(08):532-33 [in Chinese]
6. Park JS, Lee EH, Park NR, Choi YH. Mental health of nurses working at a government-designated hospital during a MERS-CoV outbreak: A cross-sectional study. Arch Psychiat Nurs. 2018;32(1):2-6
7. North CS, Kawasaki A, Spitznagel EL, Hong BA. The course of PTSD, major depression, substance abuse, and somatization after a natural disaster. J Nerv Ment Dis. 2004;192(12):823-29
8. Mak IW, Chu CM, Pan PC, et al. Risk factors for chronic post-traumatic stress disorder (PTSD) in SARS survivors. Gen Hosp Psychiatry. 2010;32(6):590-98
9. McAlonan GM, Lee AM, Cheung V, et al. Immediate and sustained psychological impact of an emerging infectious disease outbreak on health care workers. Can J Psychiatry. 2007;52(4):241-47
10. Liu X, Kakade M, Fuller CJ, et al. Depression after exposure to stressful events: Lessons learned from the severe acute respiratory syndrome epidemic. Compr Psychiatry. 2012;53(1):15-23
11. Chong MY, Wang WC, Hsieh WC, et al. Psychological impact of severe acute respiratory syndrome on health workers in a tertiary hospital. Br J Psychiatry. 2004;185:127-33
12. Radloff LS. The CES-D Scale: A self-report depression scale for research in the general population. Applied Psychological Measurement.1977;1(3):385-401
13. Craig TJ, Natta PAV. Recognition of depressed affect in hospitalized psychiatric patients: staff and patient perceptions. Dis Nerv Syst. 1976;37(10):561-66
14. Song Y, Huang Y, Liu D, et al. Depression in college: Depressive symptoms and personality factors in Beijing and Hong Kong college freshmen. Compr Psychiatry. 2008;49(5):496-502
15. Hawryluck L, Gold WL, Robinson S, et al. SARS control and psychological effects of quarantine, Toronto, Canada. Emerg Infect Dis. 2004;10(7):1206-12
16. Buysse DJ, Reynolds CF, Monk TH, et al. The Pittsburgh Sleep Quality Index: A new instrument for psychiatric practice and research. Psychiatry Res. 1989;28(2):193-213
17. Niu J, Han H, Wang Y, et al. Sleep quality and cognitive decline in a community of older adults in Daqing City, China. Sleep Med. 2016;17:69-74
18. Huang L, Dai J, Zhang H, et al. [The relationship between occupational burnout and health productivity impairment.] Journal of Environmental & Occupational Medicine. 2013;30(5):321-27 [in Chnese]
19. Wang LQ, Zhang M, Liu GM, et al. Psychological impact of coronavirus disease (2019) (COVID-19) epidemic on medical staff in different posts in China: A multicenter study. J Psychiat Res. 2020;129:198-205
20. Kang L, Ma S, Chen M, et al. Impact on mental health and perceptions of psychological care among medical and nursing staff in Wuhan during the 2019 novel coronavirus disease outbreak: A cross-sectional study. Brain Behav Immun. 2020;87:11-17
21. Wang C, Pan R, Wan X, et al. A longitudinal study on the mental health of general population during the COVID-19 epidemic in China. Brain Behav Immun. 2020;87:40-48
22. Guo WP, Min Q, Gu WW, et al. Prevalence of mental health problems in frontline healthcare workers after the first outbreak of COVID-19 in China: A cross-sectional study. Health Qual Life Outcomes. 2021;19(1):103
23. Si MY, Su XY, Jiang Y, et al. Psychological impact of COVID-19 on medical care workers in China. Infect Dis Poverty. 2020;9:113
24. Carmassi C, Foghi C, Dell'Oste V, et al. PTSD symptoms in healthcare workers facing the three coronavirus outbreaks: What can we expect after the COVID-19 pandemic. Psychiatry Res. 2020;292:113312
25. Song X, Fu W, Liu X, et al. Mental health status of medical staff in emergency departments during the Coronavirus disease 2019 epidemic in China. Brain Behav Immun. 2020;88:60-65
26. Rodriguez BO, Sanchez TL. The psychosocial impact of COVID-19 on health care workers. Int Braz J Urol. 2020;46(Suppl.1):195-200
27. Ferini-Strambi L, Zucconi M, et al. COVID-19 and sleep in medical staff: Reflections, clinical evidences, and perspectives. Curr Treat Options Neurol. 2020;22:29
28. Kisely S, Warren N, McMahon L, et al. Occurrence, prevention, and management of the psychological effects of emerging virus outbreaks on healthcare workers: Rapid review and meta-analysis. BMJ. 2020;369:m1642

This work is licensed under Creative Common Attribution-NonCommercial-NoDerivatives 4.0 International (CC BY-NC-ND 4.0)

Indexed in: [Current Contents/Clinical Medicine] [SCI Expanded] [ISI Alerting System] [ISI Journals Master List] [Index Medicus/MEDLINE] [EMBASE/Excerpta Medica] [Chemical Abstracts/CAS]
