# Supplementary material for: Mental health status and related factors influencing healthcare workers during the COVID-19 pandemic: A systematic review and meta-analysis
Source: PLoS One. 2024 Jan 19;19(1):e0289454. doi: 10.1371/journal.pone.0289454 (PMC10798549; doi:10.1371/journal.pone.0289454)
Supplement: S1 Data — (ZIP) [file pone.0289454.s011.zip › literatures/28.pdf]

International Journal of
Mental Health Nursing

International Journal of Mental Health Nursing (2021) *30*, 975–987 doi: 10.1111/inm.12852

ORIGINAL ARTICLE

The mental health status among nurses from low-risk areas under normalized COVID-19 pandemic prevention and control in China: A cross-sectional study

***Xian Chen,*[1] *Anne Arber,*[2] *Junyu Gao,*[3] *Li Zhang,*[4] *Meili Ji,*[5] *Dan Wang,*[6] *Jinfeng Wu*[7] *and Junjie Du*[8]**

[1]*Jiangsu Nursing Association, Nanjing, China,* [2]*Faculty of Health and Medical Sciences, The University of Surrey, Guildford, UK,* [3]*Emergency Department, The First Affiliated Hospital of Nanjing Medical University, Nanjing,* [4]*Nephrology Department, Zhongda Hospital Southeast University, Nanjing,* [5]*Department of Geriatrics, Nanjing First Hospital Nanjing Medical University, Nanjing,* [6]*Oncology Department, The Affiliated Hospital of Xuzhou Medical University, Xuzhou,* [7]*Department of Geriatrics, The First Affiliated Hospital of Nanjing Medical University, Nanjing, and* [8]*Department of Cardiovascular Surgery, The First Affiliated Hospital of Nanjing Medical University, Nanjing, China*

ABSTRACT: *This study aimed to investigate the mental health status of nurses from low-risk areas of novel coronavirus (COVID-19) pandemic, its potential impact factors, and the main stressors under the normalized prevention and control in China. A mobile phone app-based survey was conducted among registered nurses in Jiangsu province via a region-stratified sampling method. The questionnaire consisted of items on the demographic characteristics of the nursing staff and their Depression, Anxiety, Stress Scale-21 (DASS-21) along with questions for self-assessment of stressors that are associated with COVID-19. STROBE guideline was used. Among 1803 nurses who were working in the low-risk areas in Jiangsu, 22.0%, 29.8%, and 16.1% of them reported moderate to extreme levels of depression, anxiety, and stress, respectively. Having 11–15 years of working experience and being a fixed-term contract nurse were associated with experiencing worse mental health outcomes while supporting-Wuhan working experience and having mental health preparation course training were independent factors that had beneficial impact on their psychological well-being afterward. In terms of source of pressure, a key finding of this study is that the main stressor among these nurses was the lack of patient's understanding and cooperation (71.2%) which calls for better psychosocial communication between nurses and*

Corresponding authors: Jinfeng Wu, Department of Geriatrics, The First Affiliated Hospital of Nanjing Medical University, No. 300 Guangzhou Road, Nanjing 210029, China. Email: hlxhcx@sina.com
and
Junjie Du, Department of Cardiovascular Surgery, The First Affiliated Hospital of Nanjing Medical University, No. 300 Guangzhou Road, Nanjing 210029, China. Email: junjie.du@njmu.edu.cn
Funding statement: This article has none founding support.
Conflict of interest: The authors declare there was no conflict of interest.
Xian Chen, MSc, RN.
Anne Arber, PhD, MSc, RN.
Junyu Gao, BSc, RN.
Li Zhang, BSc, RN.
Meili Ji, BSc, RN.
Dan Wang, BSc, RN.
Jinfeng Wu, BSc, RN.
Junjie Du, MD, PhD.
Accepted February 03 2021.

© 2021 John Wiley & Sons Australia, Ltd

patients. The present findings would provide information for other regions at low risk of COVID-19 and may aid the provision of support and interventions for the benefit of the psychological well-being of nurses who are exposed to life-threatening occupational risks and are more vulnerable to the pandemic than others.

KEY WORDS: *anxiety, China, COVID-19, cross-sectional, depression, health status, mental health, nursing staff.*

BACKGROUND

The coronavirus disease 2019 (COVID-19) caused by the novel coronavirus strain SARS-CoV-2 is currently a global pandemic (Wang *et al.*, 2020a). In the beginning, the epidemic broke out on a large scale in Wuhan and its surrounding areas. With the strict administrational orders and extreme measures of the Chinese government authorities, the epidemic situation has been effectively controlled (Xinhua Press, 2020b). Although there are still scattered cases reported all over the country, the number of domestic cases has been greatly reduced. China classified all counties as low risk for COVID-19 from 7 May 2020 as no domestic cases had been reported on the Chinese mainland for four consecutive days, with no new deaths for 22 consecutive days (Xinhua Press, 2020a). Correspondingly, the national epidemic prevention and control policy has been changed from the blockade policy at the beginning of the outbreak to the gradual opening policy in public places under orderly control. However, hospitals in low-risk areas around the country still maintain a certain intensity of measures such as staff wearing personal protective equipment, having virus nucleic acid testing and chest X-rays for all hospitalized patients, which refers to 'normalized COVID-19 epidemic prevention and control' (The State Council, 2020).

In the battle with the COVID-19 epidemic, Chinese healthcare workers around the country experienced an elevated psychological burden (Chen *et al.*, 2020; Kuo *et al.*, 2020; Ning *et al.*, 2020; Tan *et al.*, 2020). On one hand, several research studies have validated the negative physical and psychological effect on the medical staff who were sent to Wuhan during the outbreak of COVID-19 (Hu *et al.*, 2020; Kang *et al.*, 2020; Mo *et al.*, 2020; Ni *et al.*, 2020; Tu *et al.*, 2020). Those who directly engaged in the diagnosis, treatment, and care for patients with COVID-19 are known to have experienced high psychological distress (Lai *et al.*, 2019). Among them, nurses who participated in front-line work in high-risk areas experienced great pressure on their mental health (Huang *et al.*, 2020; Zhan *et al.*, 2020; Zhao *et al.*, 2020; Zhu *et al.*, 2020). A previous study on the experience of medical staff responding to the SARS outbreak shows that effects on mental health have both short-term and long-term impacts (Maunder *et al.*, 2006). Therefore, it is desirable to understand the impact on supporting-Wuhan nurses' mental health status after they finished their tasks in high-risk areas for COVID-19 and went back to work in low-risk areas. On the other hand, there are a majority of nurses nationwide who had never worked in high-risk areas and since the outbreak are facing a long period of normalized epidemic prevention and control. These nurses have been experiencing unprecedented times to deliver safe and equitable care to all populations in need. It is also important to understand their psychological pressures and whether they have different experiences compared with their supporting-Wuhan counterparts in terms of mental health status. However, evidence-based evaluation of the psychological effects on nurses from low-risk areas under normalized COVID-19 epidemic prevention is still missing. It is also unclear to what extent supporting-Wuhan nurses experience stress during normalized COVID-19 measures and to what extent this impacts on their psychological well-being afterward. It is vital to understand these questions as they may have a great impact on evidence-based mental health support services and policy to support nurses working during and after the pandemic under normalized COVID-19 regulations.

Jiangsu province has been defined as a low-risk area of the COVID-19 pandemic since the beginning of the outbreak (Jiangsu Commission of Health, 2020). It is a highly developed region in eastern China with a population of 80 million and a 20 million floating population. Since the outbreak of the COVID-19 epidemic, Jiangsu had taken a series of measures with the highest level of strictness to control the spread of the virus. Check points were set at the main entrances of all public areas to take travellers' body temperature and check their recent itineraries. Suspected or confirmed

© 2021 John Wiley & Sons Australia, Ltd

patients with COVID-19 were sent to local fever clinics or assigned to a special hospital for separation and treatment. Jiangsu is the only place in China's five major economic provinces with a total number of confirmed cases not exceeding 700 (by the time of conducting of this survey). Among China's 34 provincial administrative regions, only Jiangsu, Tibet, Qinghai, Ningxia, and Macao have achieved zero mortality, in which only Jiangsu is in China's top 10 economic provinces. In addition, all 13 prefecture-level cities in Jiangsu have sent medical teams to Hubei province to support Wuhan and its surrounding areas in fighting the epidemic. In total, Jiangsu has sent more than 2800 medical staff including 1935 nurses, which counted the largest number from a single province in China. Therefore, in this study we conducted a regional-stratified sampling survey among nurses in Jiangsu province to investigate the mental health status of nurses from low-risk areas, the stressors for nurses, and their psychological care needs during normalized epidemic prevention and control of COVID-19.

METHODS

Study design and participants

A cross-sectional study was conducted with registered nurses in Jiangsu province. The study employs a mobile phone app-based anonymous survey questionnaire (Wenjuanxing, www.wjx.cn). The data collection period was from 29 July to 9 August 2020 (i.e. within a period of 8 weekdays and 4 weekends). The STROBE guideline for cross-sectional studies was used.

Upon approval by the Ethics Committee of the First Affiliated Hospital of Nanjing Medical University (approval number: 2020-SRFA-340) and the Jiangsu Nursing Association (JNA), JNA distributed an anonymous, self-rated questionnaire to all current members in Jiangsu via a WeChat applet and invited them to participate in the study. All study participants provided informed consent electronically prior to participating in the survey. The informed consent page presented two options (yes/ no). Only subjects who chose yes were guided to the questionnaire pages, and participants could quit the process at any time.

In order to represent the situation of nurses proportionally in the whole province, a regional-stratified sampling method was applied. Registered nurses located in 13 prefecture-level cities in Jiangsu province were divided into 13 groups (regions) based on their geographic locations. The questionnaire was distributed to all 13 regions and 150 filled questionnaires from each region were randomly selected if there were more than 150 responses in that region; otherwise all questionnaires from one region were included in case there were less than 150 responses in that region. Finally, a total of 1803 valid questionnaires were enrolled in the study (Figure 1).

Instruments

The questionnaire consists of three parts: basic demographic and professional data, Chinese version of Depression Anxiety Stress Scale 21 (DASS-21) questionnaire, and self-assessment of stressors that are associated with the COVID-19.

Demographic and professional data

The first domain of the questionnaire collected demographic and professional data including gender (male or female), age (divided into 5 ranks from 18 years to >51 years), marital status (single, married, or divorced), educational levels (technical secondary school diploma, college degree, bachelor degree, or master degree or above), working experience (divided into 5 ranks based on their working years), levels of nursing specialties (primary nurses, senior nurses, managers or deputy chief nurses or higher), employment type (permanent contract employees or fixed-term contract employees), hospital classification (tertiary, secondary or primary hospitals/surgeries), nurses that had supporting-Wuhan experience or had been at a local fever clinic, and whether they had received any mental health training preparation.

Mental health questionnaire

Mental health status was measured using the validated DASS-21 questionnaire (Norton, 2007). The DASS-21 questionnaire is a 21-item self-report tool to assess negative emotions of depression, anxiety, and stress, respectively. The participants were asked to rate their experience on each symptom on the DASS-21 over the past week from 'never' to 'most of time' on a 4-point severity scale. Scores of each scale were later added and categorized as normal, mild, moderate, severe, and extremely severe according to the DASS manual. The DASS-21 questionnaire has been translated into multiple languages including Mandarin which has been validated for its use (Chew *et al.*, 2020). The DASS-21 has been demonstrated to be a valid measure in assessing mental health in the Chinese population during the COVID-19 epidemic (Tan *et al.*, 2020).

© 2021 John Wiley & Sons Australia, Ltd

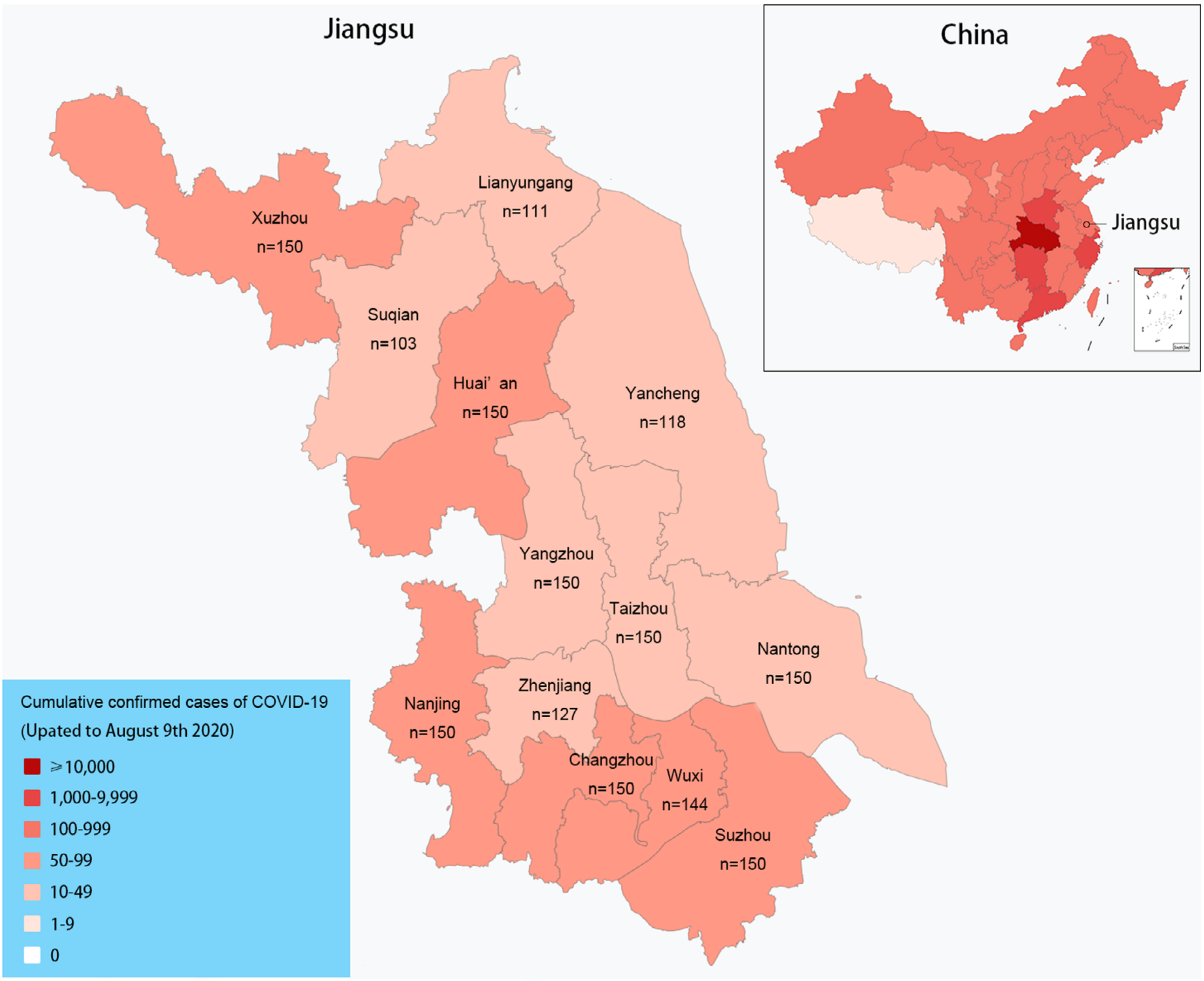

FIGURE 1 Survey sampling map.

Self-assessment of stressors that are associated with the COVID-19

A self-designed questionnaire of stressors was used to identify the personal, social, and work-related stressors of the nurses. The stressor questionnaire contains 12 items which was adapted from Kuo and colleagues' recent study investigating healthcare workers' stress during the COVID-19 pandemic (Kuo *et al.*, 2020). The participants were asked to give their opinions on each item as to whether it is a stressor based on their own experience. An attitudinal scale was applied to these items which required participants to make ratings along a five-point ranging from 'strongly agree' (score 1) to 'strongly disagree' (score 5).

The items in the stressor questionnaire were developed based on the description of stressors of COVID-19 in the literature and expert consultation (Kuo *et al.*, 2020). The stressor items are divided in to three themes: personal factors with three items (worried about being infected; stressed about decreasing income; worried about insufficient knowledge of COVID-19), social factors with four items (worried about lack of friends and families' support; worried about friends and families getting infected; discrimination by others; worried about the COVID-19 pandemic spread), and work factors with five items (worried about the lack of essential medical supplies; stressed about discomfort when wearing personal protective equipment; worried about the increased workload since COVID-19 restrictions began; stressed about the lack of patients' understanding and cooperation; worried about lack of management team's support). The content validity index of the scale was 0.92 in the original study by Kuo *et al.* (2020). The Cronbach's alpha value

© 2021 John Wiley & Sons Australia, Ltd

of the complete scale was 0.86 and for the three subscales was 0.73 to 0.82.

There were eight nurses from the Jiangsu Province Hospital who were asked to complete the questionnaire as a pilot study before the present survey to evaluate the feasibility and clarity of the questions. It took average 5 min for participants to fill out the questionnaire and they reported no barriers to completing the questionnaire and found it was easy to understand.

Statistical analysis

Data were analysed using IBM SPSS (version 22.0). Descriptive analysis was used to describe the general data. For count data, frequencies and percentages were used. The Mann–Whitney U-test was used to compare the differences for categorical variables with two groups and the Kruskal–Wallis test was used when having more than two groups. To determine potential risk factors for the development of anxiety, stress, and depression, ordinal logistic regression analysis was performed, and the associations between risk factors and outcomes are presented as odds ratios (ORs) and 95% CIs, after adjustment for confounders. P value < 0.05 indicated that a difference was statistically significant.

RESULTS

Demographic characteristics

A total of 1803 valid questionnaires were analysed. The demographic data are shown in Table 1. Among the sample of 1803 nurses, 1762 (97.7%) were women and 41 (2.3%) were men. Most of the participants were married (72.4%), 454 (25.2%) were single, and 43 (2.4%) were divorced. The sample comprised nurses aged from 18–25 (14.6%), 26-30 (29.17%), 31–40 (40.43%), 41–50 (12.65), and 51–60 (3.05%). The majority of nurses had bachelor degrees (81%), followed by college degrees (17.3%). Five hundred and fifty-five (30.8%) nurses had 6-10 years of working experience and 433 (24%) reported less than 6 years of nursing working experience. Among all the participants, 1224 (67.9%) were fixed-term contract nursing staff and 1591 (88.2%) were from tertiary hospitals. One hundred and twenty-nine (7.2%) nurses reported that they had supported Wuhan hospitals during the outbreak, and 422 (23.4%) had worked at a fever clinic department in local hospitals. A total of 586 (32.5%) participants stated that they had received mental health training courses.

TABLE 1 *Demographic characteristics of nurses participating in this study* (n = *1803)*

variables	Number	Percentage (%)
Total	1803	100
Gender		
Male	41	2.3
Female	1762	97.7
Age (years)		
18–25	264	14.6
26–30	526	29.2
31–40	729	40.4
41–50	228	12.6
≥51	56	3.1
Marriage		
Single	454	25.2
Married	1306	72.4
Divorced	43	2.4
Education level		
Technical secondary school diploma	23	1.3
College degree	312	17.3
Bachelor degree	1460	81
Master degree or above	8	0.4
Working experience (years)		
0–5	433	24
6–10	555	30.8
11–15	344	19.1
16–20	207	11.5
>20	264	14.6
Level of nursing specialties		
Primary nurse	309	17.1
Senior nurse	721	40
Manager	582	32.3
Deputy chief nurse or higher	191	10.6
Employment type		
permanent contract employee	579	32.1
Fixed-term contract employee	1224	67.9
Hospital classification		
Tertiary hospital	1591	88.2
Secondary hospital	202	11.2
Primary hospital/Surgery	10	0.6
Supporting-Wuhan experience		
Yes	129	7.2
No	1674	92.8
Had been at a local fever clinic		
Yes	422	23.4
No	1381	76.6
Received mental health course training		
Yes	586	32.5
No	1217	67.5

Mental health status among nurses at low-risk areas

In general, a considerable proportion of participants had demonstrated moderate to extreme levels of depression (396, 22%), anxiety (538, 29.8%), and stress (289, 16.1%). Fixed-term contract nurses reported

© 2021 John Wiley & Sons Australia, Ltd

experiencing more severe levels of depression ($P = 0.026$), anxiety ($P = 0.001$), and stress ($P = 0.026$) compared with permanent contract nurses. Nurses who had experience of supporting-Wuhan or had received mental health training courses were more likely to report less severe level of depression, anxiety, and stress than those who had not such experience or had not received any training courses (all $P < 0.01$). Otherwise, the levels of depression, anxiety, and stress reported by nurses in this study showed no significant differences when the nurses were categorized by demographic characteristics such as gender, age, marital status, educational level, working experience, levels of nursing specialties, hospital classification, and whether they had fever clinic experience (Table 2).

Risk factors for mental health outcomes identified by ordinal logistic regression analysis

Results of ordinal logistic regression analysis listed in the Table 3 showed that having 11–15 years working experience was associated with a higher level of depression (OR, 1.744, 95%CI, 1.042–2.918; $P = 0.034$) and anxiety (OR, 1.758, 95%CI, 1.076–2.869; $P = 0.024$). Compared with the fixed-term contract nurses, permanent contract nurses had a lower level of depression (OR, 0.678, 95%CI, 0.518–0.889; $P = 0.005$), anxiety (OR, 0.621, 95%CI, 0.480–0.804; $P < 0.001$), and stress (OR, 0.687, 95%CI, 0.509–0.927; $P = 0.014$). Nurses who had worked supporting-Wuhan hospitals were associated with a lower level of depression (OR, 0.541, 95%CI, 0.356–0.820; $P = 0.004$) and anxiety (OR, 0.503, 95%CI, 0.340–0.744; $P = 0.001$). Moreover, nurses who reported that had received mental health training courses showed the lowest levels of depression (OR, 0.629, 95%CI, 0.511–0.773; $P < 0.001$), anxiety (OR, 0.708, 95%CI, 0.583–0.860; $P = 0.001$), and work pressure levels (OR, 0.642, 95% CI, 0.510–0.808; $P < 0.001$).

Stressor factors

The answers for the stressor questionnaire were summarized in Table 4. Among the personal factors, the majority of nurses (65.6%) reported (strongly agree and agree) feeling stressed about their decreasing incomes. Among the social factors, most nurses (68.7%) worried about the COVID-19 pandemic spread. Regarding the work factors, a majority of nurses (71.2%) in this study reported feeling stressed about lack of patient's understanding and cooperation and 70.3% reported discomfort when wearing personal protective equipment followed by feeling stressed about increased workload (69.8%). In addition, approximately half of nurses (49%) felt that they did not get enough support and understanding from the hospital authorities.

DISCUSSION

To our best of knowledge, this is the first study to assess the mental health status of nurses from low-risk areas under normalized COVID-19 epidemic prevention and its associated factors. This cross-sectional survey recruited 1803 registered nurses from Jiangsu province and revealed a considerable high prevalence of depression, anxiety and stress among them under normalized COVID-19 epidemic prevention and control in China. Overall, 22.0%, 29.8%, and 16.1% of all participants reported moderate to extreme levels of depression, anxiety, and stress, respectively. Our study further indicated that having 11–15 years of working experience and being a fixed-term contract nurse were associated with experiencing worse mental health outcomes. Interestingly and informatively, supporting-Wuhan working experience and having mental health preparation course training were independent factors that had beneficial impact on their psychological well-being afterward.

The outbreak of COVID-19 puts enormous pressure on the Chinese healthcare system and its staff, leading to increased levels of stress and burnout among healthcare workers (Liu *et al.*, 2020a; Zhang *et al.*, 2020a; Zhang *et al.*, 2020b). A number of studies have showed that medical staff had suffered negative psychological outcomes during the COVID-19 pandemic and the mental health status of health care was worse than that of the general public (Li *et al.*, 2020; Qiu *et al.*, 2020; Wang *et al.*, 2020c; Zhu *et al.*, 2020). A previous study used the same instrument (DASS-21) to assess the psychological impact among 1210 average people from 194 cities in China during the COVID-19 outbreak (Wang *et al.*, 2020b). Their findings showed that the general population had moderate to extreme depression in 16.5%, anxiety in 28.8%, and stress in 8.1%, which are less than the results of the nurses from our study. Furthermore, studies showed that nurses had higher psychological distress compared with other medical staff and were more vulnerable to stress (Chew *et al.*, 2020; Salopek-Žiha *et al.*, 2020; Zhu *et al.*, 2020). Therefore, it is important to find ways to reduce their psychological stress and to increase their well-being to successfully combat COVID-19 (Maben & Bridges, 2020; Stelnicki *et al.*, 2020).

© 2021 John Wiley & Sons Australia, Ltd

TABLE 2 *Depression, anxiety, and stress levels of the nurses categorized by their demographic characteristics*

	Depression level						Anxiety level						Stress level					
	Normal N (%)	Mild N (%)	Moderate N (%)	Severe N (%)	Extreme N (%)	*P* value	Normal N (%)	Mild N (%)	Moderate N (%)	Severe N (%)	Extreme N (%)	*P* value	Normal N (%)	Mild N (%)	Moderate N (%)	Severe N (%)	Extreme N (%)	*P* value
Total number	1078 (59.8)	329 (18.2)	279 (15.5)	63 (3.5)	54 (3.0)		936 (51.9)	329 (18.2)	263 (14.6)	130 (7.2)	145 (8.0)		1282 (71.1)	232 (12.9)	157 (8.7)	95 (5.3)	37 (2.1)	
Gender						0.686						0.463						0.230
Male	26 (63.4)	6 (14.6)	7 (17.1)	1 (2.4)	1 (2.4)		22 (53.7)	10 (24.4)	6 (14.6)	1 (2.4)	2 (4.9)		32 (78.0)	7 (17.1)	0 (0)	1 (2.4)	1 (2.4)	
Female	1052 (59.7)	323 (18.3)	272 (15.4)	62 (3.5)	53 (3.0)		914 (51.9)	319 (18.1)	257 (14.6)	129 (7.3)	143 (8.1)		1250 (70.9)	225 (12.8)	157 (8.9)	94 (5.3)	36 (2.0)	
Age						0.095						0.057						0.128
18–25	165 (62.5)	41 (15.5)	38 (14.4)	7 (2.7)	13 (4.9)		140 (53.0)	43 (16.3)	37 (14.0)	20 (7.6)	24 (9.1)		188 (71.2)	34 (12.9)	19 (7.2)	15 (5.7)	8 (3.0)	
26–30	314 (59.7)	99 (18.8)	80 (15.2)	15 (2.9)	18 (3.4)		276 (52.5)	87 (16.5)	75 (14.3)	47 (8.9)	41 (7.8)		373 (70.9)	67 (12.7)	46 (8.7)	31 (5.9)	9 (1.7)	
31–40	430 (59.0)	126 (17.3)	120 (16.5)	33 (4.5)	20 (2.7)		366 (50.2)	139 (19.1)	111 (15.2)	48 (6.6)	65 (8.9)		515 (70.6)	83 (11.4)	73 (10.0)	42 (5.8)	16 (2.2)	
41–50	128 (56.1)	51 (22.4)	38 (16.7)	8 (3.5)	3 (1.3)		117 (51.3)	46 (20.2)	37 (16.2)	13 (5.7)	15 (6.6)		158 (69.3)	42 (18.4)	17 (7.5)	7 (3.1)	4 (1.8)	
≥51	41 (73.2)	12 (21.4)	3 (5.4)	0 (0)	0 (0)		37 (66.1)	14 (25.0)	3 (5.4)	2 (3.6)	0 (0)		48 (85.7)	6 (10.7)	2 (3.6)	0 (0)	0 (0)	
Marriage status						0.445						0.965						0.170
Single	278 (61.2)	74 (16.3)	66 (14.5)	15 (3.3)	21 (4.6)		239 (52.6)	78 (17.2)	61 (13.4)	39 (8.6)	37 (8.1)		321 (70.7)	56 (12.3)	41 (9.0)	25 (5.5)	11 (2.4)	
Married	778 (59.6)	246 (18.8)	206 (15.8)	44 (3.4)	32 (2.5)		674 (51.6)	242 (18.5)	198 (15.2)	88 (6.7)	104 (8.0)		936 (71.7)	169 (12.9)	107 (8.2)	69 (5.3)	25 (1.9)	
Divorced	22 (51.2)	9 (20.9)	7 (16.3)	4 (9.3)	1 (2.3)		23 (53.5)	9 (20.9)	4 (9.3)	3 (7.0)	4 (9.3)		25 (58.1)	7 (16.3)	9 (20.9)	1 (2.3)	1 (2.3)	
Educational level						0.684						0.233						0.107
Technical secondary school diploma	13 (56.5)	4 (17.4)	4 (17.4)	2 (8.7)	0 (0)		9 (39.1%)	7 (30.4)	3 (13.0)	2 (8.7)	2 (8.7)		13 (56.5)	5 (21.7)	1 (4.3)	3 (13.0)	1 (4.3)	
College degree	186 (59.6)	62 (19.9)	43 (13.8)	9 (2.9)	12 (3.8)		159 (51.0)	61 (19.6)	50 (16.0)	25 (8.0)	17 (5.4)		224 (71.8)	49 (15.7)	23 (7.4)	13 (4.2)	3 (1.0)	
Bachelor degree	873 (59.8)	261 (17.9)	232 (15.9)	52 (3.6)	42 (2.9)		761 (52.1)	261 (17.9)	209 (14.3)	103 (7.1)	126 (8.6)		1037 (71.0)	178 (12.2)	133 (9.1)	79 (5.4)	33 (2.3)	
Master degree or above	6 (75.0)	2 (25.0)	0 (0)	0 (0)	0 (0)		7 (87.5)	0 (0)	1 (12.5)	0 (0)	0 (0)		8 (100)	0 (0)	0 (0)	0 (0)	0 (0)	
Working experience						0.405						0.342						0.813
0–5	276 (63.7)	64 (14.8)	61 (14.1)	14 (3.2)	18 (4.2)		239 (55.2)	66 (15.2)	56 (12.9)	37 (8.5)	35 (8.1)		311 (71.8)	56 (12.9)	30 (6.9)	24 (5.5)	12 (2.8)	
6–10	323 (58.2)	113 (20.4)	89 (16.0)	14 (2.5)	16 (2.9)		280 (50.5)	108 (19.5)	78 (14.1)	48 (8.6)	41 (7.4)		394 (71.0)	67 (12.1)	55 (9.9)	29 (5.2)	10 (1.8)	
11–15	196 (57.0)	60 (17.4)	62 (18.0)	16 (4.7)	10 (2.9)		167 (48.5)	60 (17.4)	64 (18.6)	19 (5.5)	34 (9.9)		242 (70.3)	38 (11.0)	38 (11.0)	20 (5.8)	6 (1.7)	
16–20	122 (58.9)	38 (18.4)	33 (15.9)	9 (4.3)	5 (2.4)		108 (52.2)	37 (17.9)	32 (15.5)	13 (6.3)	17 (8.2)		142 (68.6)	33 (15.9)	16 (7.7)	13 (6.3)	3 (1.4)	
>21	161 (61.0)	54 (20.5)	34 (12.9)	10 (3.8)	5 (1.9)		142 (53.8)	58 (22.0)	33 (12.5)	13 (4.9)	18 (6.8)		193 (73.1)	38 (14.4)	18 (6.8)	9 (3.4)	6 (2.3)	
Level of nursing specialties						0.783						0.425						0.926
Primary nurse	192 (62.1)	54 (17.5)	39 (12.6)	10 (3.2)	14 (4.5)		158 (51.1)	58 (18.8)	41 (15.6)	28 (9.1)	24 (7.8)		216 (69.9)	46 (14.9)	22 (14.0)	17 (5.5)	8 (2.6)	
Senior nurse	429 (59.5)	122 (16.9)	119 (16.5)	24 (3.3)	27 (3.7)		384 (53.3)	117 (16.2)	96 (13.3)	57 (7.9)	67 (9.3)		514 (71.3)	78 (10.8)	68 (9.4)	41 (5.7)	20 (2.8)	
Manager	341 (58.6)	115 (19.8)	94 (16.2)	22 (3.8)	10 (1.7)		288 (49.5)	114 (19.6)	103 (17.7)	34 (5.8)	43 (7.4)		417 (71.6)	77 (13.2)	53 (9.1)	30 (5.2)	5 (0.9)	
Deputy chief nurse or higher	116 (60.7)	38 (19.9)	27 (14.1)	7 (3.7)	3 (1.6)		106 (55.5)	40 (20.9)	23 (12.0)	11 (5.8)	11 (5.8)		135 (70.7)	31 (16.2)	14 (7.3)	7 (3.7)	4 (2.1)	
Employment type						0.026						0.001						0.026
Permanent contract employee	363 (62.7)	109 (18.8)	81 (14.0)	15 (2.6)	11 (1.9)		324 (56.0)	118 (20.4)	76 (13.1)	23 (4.0)	38 (6.6)		430 (74.3)	71 (12.3)	46 (7.9)	24 (4.1)	8 (1.4)	
Fixed-term contract employee	715 (58.4)	220 (18.0)	198 (16.2)	48 (3.9)	43 (3.5)		612 (50.0)	211 (17.2)	187 (15.3)	107 (8.7)	107 (8.7)		852 (69.6)	161 (13.2)	111 (9.1)	71 (5.8)	29 (2.4)	
Hospital classification						0.444						0.406						0.407
Tertiary hospital	950 (59.7)	283 (17.8)	251 (15.8)	58 (3.6)	49 (3.1)		826 (51.9)	286 (18.0)	232 (14.6)	117 (7.4)	130 (8.2)		1130 (71.0)	199 (12.5)	142 (8.9)	85 (5.3)	35 (2.2)	
Secondary hospital	123 (60.9)	45 (22.3)	25 (12.4)	5 (2.5)	4 (2.0)		106 (52.5)	42 (20.8)	29 (14.4)	12 (5.9)	13 (6.4)		146 (72.3)	33 (16.3)	13 (6.4)	8 (44.0)	2 (1.0)	
Primary hospital	5 (50.0)	1 (10.0)	3 (30.0)	0 (0)	1 (10.0)		4 (40.0)	1 (10.0)	2 (20.0)	1 (10.0)	2 (20.0)		6 (60.0)	0 (0)	2 (20.0)	2 (20.0)	0 (0)	
Supporting-Wuhan experience						0.001						0.000						0.019
Yes	96 (74.4)	14 (10.9)	15 (11.6)	3 (2.3)	1 (0.8)		87 (67.4)	19 (14.7)	16 (12.4)	5 (3.9)	2 (1.6)		102 (79.1)	16 (12.4)	8 (6.2)	3 (2.3)	0 (0)	
No	982 (58.7)	315 (18.8)	264 (15.8)	60 (3.6)	53 (3.2)		849 (50.7)	310 (18.5)	247 (14.8)	125 (7.5)	143 (8.5)		1180 (70.5)	216 (12.9)	149 (8.9)	92 (5.5)	37 (2.2)	

(*Continued*)

© 2021 John Wiley & Sons Australia, Ltd

TABLE 2 (Continued)

	Depression level						Anxiety level						Stress level					
	Normal N (%)	Mild N (%)	Moderate N (%)	Severe N (%)	Extreme N (%)	*P* value	Normal N (%)	Mild N (%)	Moderate N (%)	Severe N (%)	Extreme N (%)	*P* value	Normal N (%)	Mild N (%)	Moderate N (%)	Severe N (%)	Extreme N (%)	*P* value
Fever clinic experience						0.550						0.809						0.082
Yes	247 (58.5)	79 (18.7)	68 (16.1)	14 (3.3)	14 (3.3)		222 (52.6)	68 (16.1)	61 (14.5)	31 (7.3)	40 (9.5)		286 (67.8)	57 (13.5)	46 (10.9)	26 (6.2)	7 (1.7)	
No	831 (60.2)	250 (18.1)	211 (15.3)	49 (3.5)	40 (2.9)		714 (51.7)	261 (18.9)	202 (14.6)	99 (7.2)	105 (7.6)		996 (72.1)	175 (12.7)	111 (8.0)	69 (5.0)	30 (2.2)	
Mental health course training						0.000						0.000						0.000
Yes	397 (67.7)	84 (14.3)	79 (13.5)	17 (2.9)	9 (1.5)		341 (58.2)	97 (16.6)	74 (12.6)	38 (6.5)	36 (6.1)		452 (77.1)	59 (10.1)	41 (7.0)	28 (4.8)	6 (1.0)	
No	681 (56.0)	245 (20.1)	200 (16.4)	46 (3.8)	45 (3.7)		595 (48.9)	232 (19.1)	189 (15.5)	92 (7.6)	109 (9.0)		830 (68.2)	173 (14.2)	116 (9.5)	67 (5.5)	31 (2.5)	

Our study found some factors that had a beneficial impact on the nurses' psychological well-being under normalized COVID-19 regulations. These factors included supporting-Wuhan working experience and having mental health preparation training courses. We found that 129 nurses who had experience going to Wuhan as part of supporting medical teams during the pandemic had lower levels of anxiety, work stress and depression than their nursing colleagues who did not go to Wuhan area. Some previous studies have shown that the front-line nurses who were working in Wuhan had more severe symptoms of anxiety, depression, and stress during the outbreak (Hu *et al.,* 2020; Lai *et al.,* 2019). However, when they finished their difficult tasks in Wuhan, these supporting-Wuhan nurses may feel relieved to be back in a low-risk environment but had also got the necessary confidence and experience in infection control procedures. Most of the supporting-Wuhan nurses were more knowledgeable about the epidemic as most of them were selected from infectious diseases departments such as the respiratory department, intensive care unit, and infection control department or had working experience at fever clinics. They had more experience of dealing with critical medical conditions and developed proper coping skills, therefore, felt more confident in infection control procedures (Hu *et al.,* 2020). In addition, the selected supporting-Wuhan nurses mostly were mostly from the main middle-level backbone staff from each hospital with sufficient working experience and psychological capacity (Li *et al.,* 2020). An official data showed that there were zero infections and zero mortality among the Jiangsu medical team who went to support Hubei province, which indicates they were well trained and prepared for this situation (Jiangsu Commission of Health, 2020). In the present survey, 586 nurses (35.5%) reported that they had received at least one mental health training course and this group demonstrated lower levels of anxiety, work pressure, and depression compared with those who never had such training during the pandemic. This result is consistent with other studies showing that mental health training is an effective measure to reduce the psychological pressure of the medical staff (Adams & Walls, 2020). Although such mental health training courses are usually available in many hospitals, they are most likely to have been neglected by a few, if not many, hospital administrators. Therefore, organizations should undertake awareness as well as interventional strategies, such as group interventions and counselling sessions in mental health on a regular basis, to minimize the stress and behavioural burden during the pandemic scare.

© 2021 John Wiley & Sons Australia, Ltd

TABLE 3 *Risk factors for mental health outcomes identified by ordinal logistic regression analysis*

	Depression			Anxiety			Stress		
Variable	OR	95% CI	P	OR	95% CI	P	OR	95% CI	P
Gender									
Male	1 [Reference]			1 [Reference]				1 [Reference]	
Female	0.766	0.401–1.465	0.421	0.888	0.477–1.652	0.707	1.210	0.55–2.641	0.632
Age									
18–25	1 [Reference]			1 [Reference]				1 [Reference]	
26–30	0.890	0.570–1.391	0.610	0.921	0.605–1.404	0.703	0.987	0.608–1.602	0.958
31–40	0.832	0.464–1.492	0.538	0.814	0.468–1.415	0.465	0.951	0.502–1.802	0.878
41–50	1.036	0.434–2.472	0.938	0.664	0.287–1.537	0.338	0.828	0.314–2.181	0.702
≥51	0.464	0.160–1.347	0.158	0.313	0.1130.863	0.025	0.333	0.096–1.149	0.082
Marriage									
Married	1 [Reference]			1 [Reference]				1 [Reference]	
Single	1.131	0.839–1.525	0.422	1.084	0.815–1.441	0.579	1.112	0.801–1.542	0.528
Divorced	1.363	0.763–2.435	0.296	0.877	0.489–1.573	0.660	1.654	0.899–3.040	0.105
Educational level									
Technical secondary school diploma	1 [Reference]			1 [Reference]				1 [Reference]	
College degree	0.696	0.300–1.614	0.398	0.654	0.293–1.464	0.302	0.386	0.161–0.929	0.033
Bachelor degree	0.647	0.282–1.486	0.304	0.660	0.298–1.462	0.306	0.433	0.183–1.025	0.057
Master degree or above	0.300	0.047–1.931	0.205	0.112	0.012–1.034	0.053	0.000	0.000–0.000	
Working experience									
0–5	1 [Reference]			1 [Reference]				1 [Reference]	
6–10	1.380	0.941–2.022	0.099	1.425	0.991–2.048	0.056	1.214	0.799–1.844	0.364
11–15	1.744	1.042–2.918	0.034	1.758	1.076–2.869	0.024	1.433	0.814–2.527	0.213
16–20	1.616	0.914–2.858	0.099	1.650	0.959–2.838	0.070	1.534	0.822–2.863	0.179
>20	1.501	0.638–3.529	0.353	2.312	1.014–5.275	0.046	1.629	0.628–4.221	0.316
Nursing specialties									
Primary nurse	1 [Reference]			1 [Reference]				1 [Reference]	
Senior nurse	1.075	0.724–1.594	0.722	0.869	0.599–1.261	0.460	0.837	0.547–1.281	0.413
Manager	1.100	0.672–1.797	0.706	1.037	0.651–1.654	0.879	0.815	0.478–1.391	0.453
Deputy chief nurse or higher	1.146	0.606–2.164	0.676	0.955	0.520–1.752	0.881	1.020	0.509–2.044	0.956
Employment type									
Fixed-term contract employee	1 [Reference]			1 [Reference]				1 [Reference]	
Permanent contract employee	0.678	0.518–0.889	0.005	0.621	0.480–0.804	0.000	0.687	0.509–0.927	0.014
Hospital classification									
Tertiary hospital	1 [Reference]			1 [Reference]				1 [Reference]	
Secondary hospital	0.862	0.636–1.168	0.338	0.899	0.674–1.200	0.470	0.856	0.611–1.201	0.370
Primary hospital	1.754	0.550–5.601	0.342	2.214	0.723–6.794	0.164	2.370	0.720–7.807	0.156
Supporting-Wuhan experience									
No	1 [Reference]			1 [Reference]				1 [Reference]	
Yes	0.541	0.356–0.820	0.004	0.503	0.340–0.744	0.001	0.694	0.439–1.097	0.118
Had been at a local fever clinic									
No	1 [Reference]			1 [Reference]				1 [Reference]	
Yes	1.097	0.882–1.363	0.404	1.060	0.861–1.306	0.582	1.250	0.987–1.582	0.064
Mental health course training									
No	1 [Reference]			1 [Reference]				1 [Reference]	
Yes	0.629	0.511–0.773	0.000	0.708	0.583–0.860	0.001	0.642	0.510–0.808	0.000

Our study also showed that being a nurse on a fixed-term contract was associated with more severe depression, anxiety, and work stress compared with permanent contract nurses. There are several reasons why this may happen but the issue of nursing pay, which is a legacy of Chinese healthcare system reform, remains its major cause. In fact, more than 65% of nurses in this survey reported feeling stressed because of decreasing incomes. Nursing pay policy in China includes three main parts: basic wage, task-related pay, and monthly bonuses (Cooke & Zhan, 2013). The amount of the basic wage is decided by the nurse's working experience, professional titles, job classification and his/her contract type. In China, fixed-term contract

© 2021 John Wiley & Sons Australia, Ltd

TABLE 4 *Stressor factors*

Stressors associated with COVID-19	Number	Percentage (%)
Personal factors		
1. Worried about being infected		
Strongly agree and agree	865	48.0
Not sure	379	21.0
Disagree and strongly disagree	559	31.0
2. Stressed of decreasing income		
Strongly agree and agree	1182	65.6
Not sure	294	16.3
Disagree and strongly disagree	327	18.1
3. Worried about the insufficient knowledge of Covid-19		
Strongly agree and agree	357	19.8
Not sure	377	20.9
Disagree and strongly disagree	1069	59.3
Social factors		
4. Worried about lack of friends and families' support		
Strongly agree and agree	258	14.3
Not sure	450	25.0
Disagree and strongly disagree	856	47.5
5. Worried friends and families getting infected		
Strongly agree and agree	1087	60.3
Not sure	277	15.4
Disagree and strongly disagree	439	24.3
6. Discrimination by others		
Strongly agree and agree	449	24.9
Not sure	360	20.0
Disagree and strongly disagree	994	55.1
7. Worried about the COVID-19 pandemic spread		
Strongly agree and agree	1239	68.7
Not sure	263	14.6
Disagree and strongly disagree	301	16.7
Work-related factors		
8. Worried about the lack of essential medical supplies		
Strongly agree and agree	497	27.6
Not sure	450	25.0
Disagree and strongly disagree	856	47.5
9. Stressed about the discomfort to wear the protective equipment		
Strongly agree and agree	1268	70.3
Not sure	223	12.4
Disagree and strongly disagree	312	17.3
10. Worried about the increased workload since COVID-19 restrictions began		
Strongly agree and agree	1259	69.8
Not sure	255	14.1
Disagree and strongly disagree	289	16.0
11. Stressed about the lack of patient's understanding and cooperation		
Strongly agree and agree	1283	71.2
Not sure	286	15.9
Disagree and strongly disagree	234	13.0
12. Worried about lack of manage team's support		
Strongly agree and agree	883	49.0
Not sure	355	19.7
Disagree and strongly disagree	565	31.3

nurses usually receive lower pay, have less job security, and are reported to have less job satisfaction than their counterparts with permanent contracts (Cooke & Zhan, 2013). Although the China Ministry of Health carried out the Nurse Regulations in 2008 which emphasized a need for 'equal pay for equal work' despite the nurse employment type, this regulation has produced very little effect and fixed-term contract nurses continue to experience a lower level of pay (Ministry of Human Resources & Social Security, 2020). The different incomes between fixed-term contract and permanent contract nurses have become more noteworthy due to the COVID-19 pandemic. In most hospitals in China, healthcare workers' monthly bonuses are heavily dependent on their individual departments. During the COVID-19 pandemic, many departments had fewer patients than usual and some departments had been locked down when confirmed COVID-19 cases were identified in the department. These situations ended up with nurses having few bonuses or even no bonuses adding to financial insecurity for some nurses, especially for fixed-term contract nurses.

In terms of a source of pressure, a key finding of this study is that the main stressor among the nurses at low-risk areas was the lack of patient's understanding and cooperation (71.2%). Several studies have mentioned the importance of psychosocial communication skills for medical and nursing staff during the early stage of pandemic when patients are frightened and anxious due to a lack of knowledge about the disease (Chen *et al.*, 2020; Liu *et al.*, 2020b). There could be issues related to trust whether nurses are unable to deal with patients' anxiety, panic, and other emotional problems properly which will inevitably have a pernicious influence on nurses' mental health. Under the normalized epidemic prevention and control of COVID-19, although the knowledge of COVID-19 and prevention measures are well distributed among societies, the general public, especially those in low-risk areas, may not take the prevention and control measures seriously enough. In hospitals, such misconduct of patients and their family members includes unwilling to wear face masks all the time, walking in and out of the wards at their own will, frequent rotation of family members to look after the patients. Medical staff, especially nurses, have to deal with improper behaviours with increasing chances of disadvising or stopping patients and their relatives, which means that the

© 2021 John Wiley & Sons Australia, Ltd

nurses experience high levels of misunderstanding among their patients and the public. Therefore, there is a need for effective psychosocial communication and understanding to contribute to the trust among patients, caregivers and nurses to reduce their anxiety.

IMPLICATIONS

The present findings would certainly provide information for all regions at low risk of COVID-19 in China which may aid the provision of support and interventions, such as providing mental health training courses or counselling sessions, for the benefit of the psychological well-being of nurses who are facing the unprecedented challenges at the moment and are more vulnerable to the pandemic than others. It also addresses the needs for enacting supportive legislation and regulation for nursing staff to improve their mental health in the workplace (Rosa *et al.,* 2020). This information may be taken as a source of reference by healthcare policy makers from other countries with caution due to the different nature of nursing in different countries.

LIMITATIONS

A limitation for this study was that all participants were from a single province which may not represent the situations in all low-risk areas nationwide. When designing the sampling, we considered to issue the questionnaire from different regions in the province and try our best to sample within the maximum range. In addition, for the timeliness of the survey, we did not spend much time to reach nurses from primary hospitals which may result in the uneven composition of the sample as 88.2% nurses came from tertiary hospitals, 11.2% from secondary hospitals and only 0.6% from primary hospitals. Therefore, the results may not be generalized to all nurses in Jiangsu province.

CONCLUSIONS

This study investigated the mental health status of nurses from low-risk areas in Jiangsu China. The results showed that a large portion of nurses from low-risk area were suffering from mental health disturbance under the normalized COVID-19 epidemic prevention and control. Having supporting-Wuhan experience and receiving mental health preparation courses had a positive impact on their psychological well-being afterward. Nurses with fixed-term contracts reported high rates of symptoms of anxiety, depression, and stress which requires special attention. The challenge of communication between these nurses and patients was one of the main sources of psychosocial stress.

RELEVANCE FOR CLINICAL PRACTICE

Our findings provide information that nurses from low-risk area were suffering from mental health disturbance under the normalized COVID-19 epidemic prevention and control. Such information may aid government or hospital authorities to take the necessary measures providing support and interventions for the benefit of the psychological well-being of nurses who are more vulnerable to the pandemic than others.

ACKNOWLEDGEMENTS

The authors thank the research participants for their participation in this research study and people who distributed the questionnaire to the nurses. We also would like to express our sincere appreciation to Dr. Liu Jin for his kindness and knowledge to help with statistics.

ETHICAL APPROVAL

The ethical approval was given by the Ethics Committee of the First Affiliated Hospital of Nanjing Medical University (approval number: 2020-SRFA-340).

REFERENCES

Adams, J.G. & Walls, R.M. (2020). Supporting the health care workforce during the COVID-19 global epidemic. *JAMA*, *323* (15), 1439.

Chen, Q., Liang, M., Li, Y. *et al*. (2020). Mental health care for medical staff in China during the COVID-19 outbreak. *The Lancet Psychiatry*, *7* (4), e15–e16.

Chew, N.W.S., Lee, G.K.H., Tan, B.Y.Q. *et al*. (2020). A multinational, multicentre study on the psychological outcomes and associated physical symptoms amongst healthcare workers during COVID-19 outbreak. *Brain, Behavior, and Immunity*, *88*, 559–565.

Cooke, F.L. & Zhan, C. (2013). Between market and bureaucracy: public healthcare reforms in China and nurses' terms and conditions. *The International Journal of Human Resource Management*, *24* (16), 3178–3195.

Hu, D., Kong, Y., Li, W. *et al*. (2020). Frontline nurses' burnout, anxiety, depression, and fear statuses and their associated factors during the COVID-19 outbreak in Wuhan, China: A large-scale cross-sectional study. *EClinicalMedicine*, *24*, 100424.

© 2021 John Wiley & Sons Australia, Ltd

Huang, H.-P., Zhao, W.-J. & Li, G.-R. (2020). Survey on knowledge and psychological crisis related to COVID-19 among nursing staff: a cross-sectional study. *JMIR Formative Research*, *4* (9), e20606.

Jiangsu Commission of Health (2020). Available from: http://www.jiangsu.gov.cn/jrobot/search.do?q=%E6%96%B0%E5%86%A0&webid=31&pg=10&p=1&tpl=2&category= [Accessed 18 October 2020.555Z].

Kang, L., Ma, S., Chen, M. *et al*. (2020). Impact on mental health and perceptions of psychological care among medical and nursing staff in Wuhan during the 2019 novel coronavirus disease outbreak: A cross-sectional study. *Brain, Behavior, and Immunity*, *87*, 11–17.

Kuo, F.L., Yang, P.H., Hsu, H.T. *et al*. (2020). Survey on perceived work stress and its influencing factors among hospital staff during the COVID-19 pandemic in Taiwan. *The Kaohsiung Journal of Medical Sciences*. *36* (11), 944–952.

Lai, J., Ma, S., Wang, Y. *et al*. (2020). Factors associated with mental health outcomes among health care workers exposed to coronavirus disease 2019. *JAMA Network Open*, *3* (3), e203976.

Li, Z., Ge, J., Yang, M. *et al*. (2020). Vicarious traumatization in the general public, members, and non-members of medical teams aiding in COVID-19 control. *Brain, Behavior, and Immunity*, *88*, 916–919.

Liu, C.-Y., Yang, Y.-Z., Zhang, X.-M. *et al*. (2020a). The prevalence and influencing factors in anxiety in medical workers fighting COVID-19 in China: a cross-sectional survey. *Epidemiology and Infection*, *148*, e98.

Liu, Q., Luo, D., Haase, J.E. *et al*. (2020b). The experiences of health-care providers during the COVID-19 crisis in China: a qualitative study. *The Lancet Global Health*, *8* (6), e790–e798.

Maben, J. & Bridges, J. (2020) Covid-19: Supporting nurses' psychological and mental health. *Journal of Clinical Nursing*, *29* (15-16), 2742–2750.

Maunder, R.G., Lancee, W.J., Balderson, K.E. *et al*. (2006). Long-term psychological and occupational effects of providing hospital healthcare during SARS outbreak. *Emerging Infectious Diseases*, *12* (12), 1924–1932.

Ministry of Human Resources and Social Security. (2020). Available from: http://www.mohrss.gov.cn/SYrlzyhshbzb/zcfg/flfg/fl/201605/t20160509_239643.html [Accessed 18 October 2020.884Z].

Mo, Y., Deng, L., Zhang, L. *et al*. (2020). Work stress among Chinese nurses to support Wuhan in fighting against COVID-19 epidemic. *Journal of Nursing Management*, *28* (5), 1002–1009.

Ni, M.Y., Yang, L., Leung, C.M.C. *et al*. (2020). Mental health, risk factors, and social media use during the COVID-19 epidemic and cordon sanitaire among the community and health professionals in Wuhan, China: Cross-Sectional Survey. *JMIR Mental Health*, *7* (5), e19009.

Ning, X., Yu, F., Huang, Q. *et al*. (2020) The mental health of neurological doctors and nurses in Hunan Province, China during the initial stages of the COVID-19 outbreak. *BMC Psychiatry*, *20* (1), 436.

Norton, P.J. (2007). Depression anxiety and stress scales (DASS-21): Psychometric analysis across four racial groups. *Anxiety, Stress & Coping*, *20* (3), 253–265.

Qiu, J., Shen, B., Zhao, M., Wang, Z., Xie, B. & Xu, Y. ((2020). A nationwide survey of psychological distress among Chinese people in the COVID-19 epidemic: implications and policy recommendations. *General Psychiatry*, *33* (2), e100213.

Rosa, W.E., Binagwaho, A., Catton, H. *et al*. (2020). Rapid investment in nursing to strengthen the global COVID-19 response. *International Journal of Nursing Studies*, *109*, 103668.

Salopek-Žiha, D., Hlavati, M., Gvozdanović, Z. *et al*. (2020). Differences in distress and coping with the covid-19 stressor in nurses and physicians. *Psychiatria Danubina*, *32* (2), 287–293.

Stelnicki, A.M., Carleton, R.N. & Reichert, C. (2020). Nurses' mental health and well-being: COVID-19 impacts. *Canadian Journal of Nursing Research*, *52* (3), 237–239.

Tan, W., Hao, F., McIntyre, R.S. *et al*. (2020). Is returning to work during the COVID-19 pandemic stressful? A study on immediate mental health status and psychoneuroimmunity prevention measures of Chinese workforce. *Brain, Behavior, and Immunity*, *87*, 84–92.

The State Council (2020). *Guiding opinions on novel coronavirus pneumonia epidemic prevention and control work by the State Council*. Available from: http://www.gov.cn/zhengce/content/2020-05/08/content_5509896.htm Accessed 18 October 2020.260Z.

Tu, Z.-H., He, J.-W. & Zhou, N. (2020). Sleep quality and mood symptoms in conscripted frontline nurse in Wuhan, China during COVID-19 outbreak: A cross-sectional study. *Medicine*, *99* (26), e20769. https://doi.org/10.1097/MD.0000000000020769

Wang, C., Horby, P.W., Hayden, F.G. & Gao, G.F. (2020a). A novel coronavirus outbreak of global health concern. *The Lancet*, *395* (10223), 470–473.

Wang, C., Pan, R., Wan, X. *et al*. (2020b). Immediate Psychological Responses and Associated Factors during the Initial Stage of the 2019 Coronavirus Disease (COVID-19) Epidemic among the General Population in China. *International Journal of Environmental Research and Public Health*, *17* (5), 1729.

Wang, C., Pan, R., Wan, X. *et al*. (2020c). A longitudinal study on the mental health of general population during the COVID-19 epidemic in China. *Brain, Behavior, and Immunity*, *87*, 40–48.

Xinhua Press (2020a). *Latest developments in epidemic control on May 8*. Available from: http://english.www.gov.cn/news/topnews/202005/08/content_WS5eb4914cc6d0b3f0e949729c.html Accessed 18 October 2020.626Z.

Xinhua Press (2020b). *Timeline of China's fight against the novel coronavirus*. Available from: http://english.www.gov.cn/news/topnews/202003/19/content_WS5e736ce7c6d0c201c2cbef8f.html Accessed 25 October 2020.

© 2021 John Wiley & Sons Australia, Ltd

Zhan, Y.-X., Zhao, S.-Y., Yuan, J. *et al*. (2020). Prevalence and influencing factors on fatigue of first-line nurses combating with COVID-19 in China: A descriptive cross-sectional study. *Current Medical Science*, *40* (4), 625–635.

Zhang, S.X., Liu, J., Afshar Jahanshahi, A. *et al*. (2020a) At the height of the storm: Healthcare staff's health conditions and job satisfaction and their associated predictors during the epidemic peak of COVID-19. *Brain, Behavior, and Immunity*, *87*, 144–146.

Zhang, W.-R., Wang, K., Yin, L. *et al*. (2020b). Mental health and psychosocial problems of medical health workers during the COVID-19 Epidemic in China. *Psychotherapy and Psychosomatics*, *89* (4), 242–250.

Zhao, F., Ahmed, F. & Faraz, N.A. (2020). Caring for the caregiver during COVID-19 outbreak: Does inclusive leadership improve psychological safety and curb psychological distress? A cross-sectional study. *International Journal of Nursing Studies*, *110*, 103725.

Zhu, J., Sun, cd/cd T:/issues/W3G/INM/30_4/12852/K:\sps-soft\WileyML_3G\suser\zip.exe APPSupport.zip *.td *.3f *.hyp *.m2t *.bak *.batdel AppSup.batL., Zhang, L. *et al*. (2020). Prevalence and influencing factors of anxiety and depression symptoms in the first-line medical staff fighting against COVID-19 in Gansu. *Frontiers in Psychiatry*, *11*, 386.

© 2021 John Wiley & Sons Australia, Ltd
