# Supplementary material for: Mental health status and related factors influencing healthcare workers during the COVID-19 pandemic: A systematic review and meta-analysis
Source: PLoS One. 2024 Jan 19;19(1):e0289454. doi: 10.1371/journal.pone.0289454 (PMC10798549; doi:10.1371/journal.pone.0289454)
Supplement: S1 Data — (ZIP) [file pone.0289454.s011.zip › literatures/3.pdf]

BMJ Open

Psychological impact of the COVID-19 pandemic on healthcare workers at acute hospital settings in the South-East of Ireland: an observational cohort multicentre study

Saied Ali ,[1] Sinead Maguire,[2] Eleanor Marks,[3] Maeve Doyle,[4] Claire Sheehy[5]

To cite: Ali S, Maguire S, Marks E, *et al*. Psychological impact of the COVID-19 pandemic on healthcare workers at acute hospital settings in the South-East of Ireland: an observational cohort multicentre study. *BMJ Open* 2020;**10**:e042930. doi:10.1136/bmjopen-2020-042930

► Prepublication history for this paper is available online. To view these files, please visit the journal online (http://dx.doi.org/10.1136/bmjopen-2020-042930).

Received 22 July 2020
Revised 04 November 2020
Accepted 30 November 2020

Check for updates

© Author(s) (or their employer(s)) 2020. Re-use permitted under CC BY-NC. No commercial re-use. See rights and permissions. Published by BMJ.

[1]Department of General Medicine, University Hospital Waterford, Waterford, Ireland
[2]Department of Rheumatology, St James' Hospital, Dublin, Ireland
[3]Department of Geriatric Medicine, St. Luke's General Hospital, Kilkenny, Ireland
[4]Department of Microbiology, University Hospital Waterford, Waterford, Ireland
[5]Department of Rheumatology, University Hospital Waterford, Waterford, Ireland

Correspondence to
Dr Saied Ali; saiedali@rcsi.ie

ABSTRACT

Objective Our study aims to understand the psychological impact of the COVID-19 pandemic among healthcare workers (HCWs) at acute hospital settings in the South-East of Ireland, as a crucial step in guiding policies and interventions to maintain their psychological well-being.

Design Observational cohort study.

Participants and setting 472 HCWs participated from two distinct acute hospital settings, A and B, in the South-East of Ireland.

Primary and secondary outcome measures Measures of psychological distress—depression, anxiety, acute and post-traumatic stress disorder (PTSD)—as dictated by the Depression, Anxiety and Stress Scale (DASS-21) and Impact of Event Scale-Revised (IES-R). An independent sample t-test and a Mann-Whitney U test was used to determine significance of difference in continuous variables between groups. Categorical variables were assessed for significance with a χ^2 test for independence.

Results The DASS-21 provided independent measures of depression (mean 4.57, IQR 2–7), anxiety (mean 3.87, IQR 1–6) and stress (mean 7.41, IQR 4–10). Positive scores were reflected in 201 workers (42.6%) for depression and 213 (45.1%) for both anxiety and stress. The IES-R measured subjective distress on three subscales: intrusion (mean 1.085, IQR 0.375–1.72), avoidance (mean 1.008, IQR 0.375–1.5) and hyperarousal (mean 1.084, IQR 0.5–1.667). Overall, 195 cases (41.3%) were concerning for PTSD. Site B scored significantly higher across all parameters of depression (5.24 vs 4.08, p<0.01), anxiety (4.66 vs 3.3, p<0.01), stress (8.91 vs 6.33, p<0.01) and PTSD (0.058 vs 0.043, p<0.01). Worse outcomes were also noted in HCWs with underlying medical ailments.

Conclusion Psychological distress is prevalent among HCWs during the COVID-19 pandemic; screening for adverse mental and emotional outcomes and developing timely tailored preventative measures with effective feedback are vital to protect their psychological well-being, both in the immediate and long-term.

Strengths and limitations of this study

- ► The study assessed the psychological impact of the pandemic on healthcare workers through validated questionnaires; one of few studies to address this topic to date for Irish hospital settings; and with access to data covering different worker cohorts and acute hospital settings enabled the use of comparative groups, strengthening statistical analyses.
- ► Findings relied on a self-reported survey which may question the authenticity of responses and give consideration to recall bias.
- ► Socioeconomic status was not recorded, which may be important in evaluating associations of outcomes and tailoring specific interventions.
- ► The study was conducted towards the end of the initial outbreak and at settings only in the south-east which impairs the generalisability of the findings and lends to selection bias.
- ► There was no pre-COVID-19 and post-COVID-19 pandemic study conducted, therefore it is difficult to ascertain whether it is truly COVID-19 or other intrinsic or extrinsic factors accountable for the psychological impact seen.

INTRODUCTION

In December 2019, Wuhan, China became the centre of an outbreak of pneumonia of unknown cause, later identified as SARS-CoV-2, and designated the 2019 novel coronavirus (COVID-19) by the World Health Organisation (WHO). This particular strain showed marked virulence and mortality; being declared a global pandemic on 11 March 2020.[1 2]

The Republic of Ireland (ROI) saw its first SARS-CoV-2 case on 29 February 2020, with its first attributed death on 11 March 2020. Initial projections predicted up to 1.9 million infected individuals unless drastic action from government officials and the public was taken.[3]

Hospitals experienced an extreme restructuring, with a swift change in team allocations and dynamics and ensuing bombardment with protocols and information. These were

untested waters, and healthcare workers (HCWs) were thrown into this new, frightening environment. They were facing a new viral strain with no recognised evidence-based antiviral therapy and finite resources.

With Public Health interventions, as of 28 October 2020, the total number of confirmed cases in the ROI was 58 047, with a plateauing of the cumulative epidemiological curve. Of those cases, 1889 represented fatalities, 8 of which were HCWs, with 2 employed in an acute hospital in the South-East. Altogether, 10 203 cases were among HCWs, with worrying rates of HCW transmission noted in one of the two institutions studied.[4]

Self-care is a familiar concept but HCWs have an intrinsic sense of duty of care toward their patients. They dedicate their time and energy, and on many occasions put the well-being of patients above their own. They may not feel empowered to exercise their right to self-care when facing a health crisis affecting those they are called to aid.

Undoubtedly, working in such unprecedented times would affect one's mental well-being.[5] A study undertaken in Singapore found HCWs suffered from a high level of psychological distress and represent a vulnerable group where psychological intervention may be beneficial.[6]

With this in mind, our study aims to understand the psychological impact of the COVID-19 pandemic among HCWs at acute hospital settings in the South-East of Ireland, as a crucial step in guiding policies and interventions to maintain their psychological well-being.

METHODOLOGY

Design and setting

This is an observational cohort multicentre study of HCWs at acute hospital settings in the South-East of Ireland.

Data collection

From 8 June 2020 to 22 June 2020, HCWs at two acute hospital settings, A and B, from the aforesaid region were invited to participate with a self-administered electronic survey. The survey was created via Smart Survey and distributed via the Health Service Executive Secure Web Mail service; permission was secured from all relevant stakeholders. Assuming a 5% statistical significance in a population of 2112 HCWs, a sample size of at least 326 HCWs is sought. In addition to demographic characteristics, including age, gender, relationship status, occupation, medical history and direct involvement with COVID-19 patients, the questionnaire included the validated Depression, Anxiety and Stress Scale (DASS-21) and the Impact of Events Scale-Revised (IES-R) instrument.[7 8] Participants were also asked to offer suggestions on how working through the COVID-19 pandemic could have been improved in terms of support of HCWs. Only one response per person to the survey was permitted with quality control performed daily through Smart Surveys inherent coding. Information on available support services were also provided to participants.

Validated rating scales

The depression scale assesses dysphoria, hopelessness, devaluation of life, self-deprecation, anhedonia and inertia. The anxiety scale assesses autonomic arousal, situational anxiety and subjective experience of anxious affect. The stress scale is sensitive to levels of chronic non-specific arousal. It assesses difficulty relaxing, nervous arousal, and being easily upset or agitated. Scores greater than 4, 3 and 7 are clinically concerning for depression, anxiety and stress, respectively. The DASS-21 is based on a dimensional rather than a categorical conception of psychological disorder, that is, it assumes that differences between depression, anxiety and stress experienced by normal subjects and clinical populations are differences of degree. The DASS-21 therefore has no direct implications for the allocation of patients to discrete diagnostic categories postulated in classificatory systems such as the International Classification of Diseases and Diagnostic and Statistical Manual of Mental Disorders.[7]

The IES-R is a self-report measure that assesses subjective distress caused by traumatic events and represents a measure of PTSD symptoms. There are three subscales: intrusion (intrusive thoughts, feelings and imagery, nightmares, dissociative-like re-experiencing), avoidance (numbing of responsiveness, avoidance of feelings, situations and ideas) and hyperarousal (anger, irritability, hypervigilance, difficulty concentrating, heightened startle), as well as a total subjective stress IES-R score. A gross total IES-R score greater than 24 or an overall mean of 1.09 is clinically concerning for PTSD.[8]

Both scales demonstrate excellent internal consistency, discriminative and convergent validities with Cronbach's alpha coefficient values of 0.81, 0.89 and 0.78 for the subscales of depression, anxiety and stress, respectively and 0.95 for total IES-R scores.[7 8]

Study outcomes

The primary outcome was the prevalence of depression, anxiety, stress and post-traumatic stress disorder (PTSD) among all HCWs.

Statistical analyses

Data analysis was performed using IBM SPSS V.26. Continuous variables were summarised as means within each group; categorical variables were described as percentages with frequency counts. An independent sample t-test and a Mann-Whitney U test was used to determine significance of difference in continuous variables between groups. For categorical variables, a χ^2 test for independence was used to test for statistical significance. A p value of <0.05 was deemed significant.

Patient and public involvement

There was no patient or public involvement in the production of this study.

Ali S, *et al*. *BMJ Open* 2020;**10**:e042930. doi:10.1136/bmjopen-2020-042930

RESULTS

In total, 472 HCWs participated in the study from two distinct hospital settings—58.3% (275) HCWs at site A and 41.7% (197) HCWs at site B, representing a response rate of 24.5% and 19.9%, respectively. Overall, 69.1% of respondents were women, with an average age of 40.7 years. There was a relatively even distribution between medical and non-medical staff, 47.9% versus 52.1%, with 57.6% HCWs reporting direct interaction with COVID-19 patients. 36.2% of participants had underlying health conditions (table 1).

The DASS-21 provided independent measures of depression (mean 4.57, IQR 2–7), anxiety (mean 3.87, IQR 1–6) and stress (mean 7.41, IQR 4–10) with scores greater than 4, 3 and 7 indicating positive screens, respectively.[7] This was detected in 42.6% workers for depression and 45.1% for both anxiety and stress (table 2).

The IES-R measured subjective distress on three subscales: intrusion (mean 1.085, IQR 0.375–1.72), avoidance (mean 1.008, IQR 0.375–1.5) and hyperarousal (mean 1.084, IQR 0.5–1.667). These subscales are closely associated with PTSD. A gross total IES-R score greater than 24 or an overall mean of 1.09 is clinically concerning for PTSD[8] which was present in 41.3% of participants in this study (table 2).

Above-mentioned scoring tools were also compared between defined groups—genders, occupations, hospital sites, COVID-19 interactions, relationships and morbidity (table 3). Significant differences were noted between hospital sites, with site B reporting worse mean scores in depression (5.24 vs 4.08, p<0.01), anxiety (4.66 vs 3.3, p<0.01), stress (8.91 vs 6.33, p<0.01) and PTSD (0.058 vs 0.043, p<0.01) as compared with hospital site A. Similarly, HCWs with underlying medical ailments scored significantly higher across the DASS-21 and IES-R (table 3).

63.8% participants offered suggestions on how working through the pandemic could have been improved, in terms of support of HCWs. Common responses included, but were not limited to, inclusiveness and involvement in decision making (86%), succinct and timely communication (83%), facilitating rest areas and staggered rosters (78%), improvement of staffing levels to facilitate effective patient care yet allow leave entitlements and social distancing (69%), on site mental health supports with a degree of personal interaction (66%), increased frequency of multidisciplinary educational and training sessions (66%) and development of childcare facilities (51%).

DISCUSSION

The ongoing COVID-19 pandemic is a global challenge which has resulted in significant mortality and morbidity worldwide, with more than 40 million cases and over 1 million deaths as of 4 October 2020.[9] As the disease spreads at a rapid pace, most affected countries have had difficulties in meeting the demands for supply of personal protective equipment and infrastructure. It has ravaged

Table 1 Participant characteristics at baseline

Average age (IQR), years	40.7 (32–48)
Gender, % (n)	
Male	30.93 (146)
Female	69.07 (326)
Relationship status, % (n)*	
Married	55.08 (260)
In a committed relationship	19.92 (94)
Single	18.22 (86)
Divorced/separated	6.14 (29)
Widowed	0.64 (3)
Place of employment, % (n)	
Hospital site A	58.26 (275)
Hospital site B	41.74 (197)
Occupation, % (n)†	
Doctor	19.28 (91)
Nurse	29.03 (137)
Clerical or administrative staff	13.77 (65)
Catering	1.48 (7)
Pharmacy	2.54 (12)
Security	4.87 (23)
Portering	1.27 (6)
Domestic	2.54 (12)
Laboratory	4.45 (21)
Maintenance	2.12 (10)
Healthcare assistant	4.66 (22)
Allied healthcare	10.38 (49)
Management	0.64 (3)
Audiologist	0.42 (2)
Radiographer	2.54 (12)
Interaction with COVID-19 patients, % (n)	
Yes	57.63 (272)
No	42.37 (200)
Underlying medical conditions, % (n)	
None	63.77 (301)
Hypertension	13.13 (62)
Dyslipidaemia	5.30 (25)
Elevated body mass index	4.87 (23)
Diabetes	1.91 (9)
Obstructive airway disease	7.63 (36)
Heart disease	0.63 (3)
Smoking	10.81 (51)
Other	4.45 (21)

*For comparative analysis, participants either 'married' or 'in a committed relationship' was classified as having relationship support, whilst 'single', 'divorced/separated' and 'widowed' were classified as without relationship support.
†Occupations were further grouped into medical (doctors and nurses) and non-medical (all others).

Table 2 Prevalence of depression, anxiety, stress and PTSD and mean scores of the DASS-21 and IES-R

Mean Depression, Anxiety and Stress Scale-21 scores, (IQR)	
DASS depression	4.57 (2–7)
DASS anxiety	3.87 (1–6)
DASS stress	7.41 (4–10)
Depression, anxiety and stress prevalence, n (%)	
Depression	201 (42.58)
Anxiety	213 (45.13)
Stress	213 (45.13)
Mean Impact of Event Scale-Revised scores, (IQR)	
IES-R avoidance	1.01 (0.38–1.50)
IES-R intrusion	1.09 (0.38–1.72)
IES-R hyperarousal	1.08 (0.50–1.67)
IES-R total	0.05 (0.02–0.08)
PTSD prevalence, n (%)	195 (41.31)

DASS-21, depression, anxiety and stress scale-21; IES-R, impact of events scale-revised; PTSD, post-traumatic stress disorder.

economies and social integrity, with a rising concern about mental health challenges.[10] With increased demands on an already taut healthcare sector, HCWs are faced with increased workload—with the ever-present risk of infection and the fear of transmission to their loved ones.[5 11] Often times they require self-quarantine when in contact with COVID-19 confirmed patients; and when coupled with social isolation and discrimination—results in complex emotional reactions.[12] Furthermore, the conflict between professionalism and personal fear for oneself has been linked to burnout with physical and psychological manifestations.

A recent study involving 1563 health professionals reported that 50.7% of participants reported depressive symptoms, 44.7% anxiety and 36.1% sleep disturbance.[13] Similar findings were uncovered in our study; with 42.58% of our populace scoring positive for depression, 45.13% for anxiety and 45.13% for stress, as per the DASS-21.

Table 3 shows a significant difference between the hospital sites, with site B demonstrating higher scores across all disciplines. It is important to note that site B experienced significant nosocomial transmission and mortality related to COVID-19 compared with site A. This demonstrates how experiences truly shape emotions.[14] Likewise, individuals suffering from medical conditions presented with higher statistically significant scores, likely attributed to the fact that those with comorbidity experienced poorer outcomes if COVID-19 was contracted.[15]

Scores were largely statistically insignificant between other groups, but it is interesting to note that the mean scores were higher overall than scores obtained from Singaporean HCWs, where a comparable study was conducted.[6] This could reflect varied pandemic responses, resource allocation and other environmental circumstances.

A recent systematic review examined 61 viral epidemic outbreak studies and concluded that the prevalence of anxiety, depression, acute and PTSD, and burnout was high during and after outbreaks; with problems having a long-lasting effect on the mental health of HCWs.[16] The prevalence of distress was higher than those being reported in our study, perhaps due to increased metal preparedness and rigorous infection control principles, as learnt from previous outbreaks.[6] The review also assessed the impact of educational and training initiatives employed to boost resilience and promote pandemic self-efficacy and interpersonal problem solving, of which had low evidentiary certainty.[15] In our study, more than 50% of sampled HCWs suggested the need for more education and training with a multidisciplinary approach, together with involvement in decision making. To boost morale, it has also been suggested to introduce shorter working periods, regular breaks and rotating shifts.

HCWs also recognised the need for psychological supports and we acknowledge the inadequacy of services to provide counselling for HCWs who have been dealing with infected persons; further limited by social distancing.

Our study is a mere snapshot of the present psychological state of our HCWs but we must prepare for future challenges. Many HCWs may develop PTSD, depression, anxiety and burnout *after* the cessation of the pandemic, which becomes an urgent public health concern.[10] This is particularly worrying while facing a likely resurgence of SARS-CoV-2 infection during the winter influenza season, when HCW resilience may be low.[17]

The liaison mechanism of mental health services needs to be strengthened and improved with a management system which promotes collaboration among organisations. Furthermore, the team of mental health professionals needs to be expanded and strengthened. In an age of technological advancement, online emergency psychological intervention based on artificial intelligence, community based scientific dissemination and social bond enhancement, virtual reality and neuromodulation-based intervention and human resources training for emergency intercession become more relevant, as proposed by Chinese psychologists.[18]

We recognise limitations in our study. Our findings rely on a self-reported survey which may question the authenticity of response as well as give consideration to recall bias. A past medical history of psychiatric disorders was not reported by participants, but we do recognise that this would be a significant risk factor in scoring positively on our assessments. Socioeconomic status was also not recorded, which may be important in evaluating associations of outcomes and tailoring specific interventions. Furthermore, the study was conducted towards the end of the outbreak and at settings only in the south-east which impairs the generalisability of the findings and lends to selection bias. Lastly, there was no pre-COVID-19 and post-COVID-19 pandemic study conducted, therefore it is

Table 3 Comparison of DASS-21 and IES-R scores among subgroups of sample population and statistical significance

Characteristic	Proportion, %	DASS-21 depression (mean)	P value	DASS-21 anxiety (mean)	P value	DASS-21 stress (mean)	P value	IES-R avoidance (Mean)	P value	IES-R intrusion (Mean)	P value	IES-R hyperarousal (Mean)	P value	IES-R total (Mean)	P value
Gender			0.38		0.07		0.15		0.06		0.08		0.02		0.02
Male	30.9	4.34		3.44		6.99		0.91		0.98		0.96		0.043	
Female	69.1	4.67		4.06		7.6		1.05		1.13		1.14		0.051	
Relationship status			0.28		0.86		0.9		0.28		0.66		0.39		0.39
Relationship support	75	4.46		3.89		7.42		0.99		1.07		1.06		0.048	
Without relationship support	25	4.88		3.82		7.36		1.07		1.11		1.14		0.052	
Place of employment			<0.01		<0.01		<0.01		<0.01		<0.01		<0.01		<0.01
Hospital site A	58.3	4.08		3.3		6.33		0.89		0.93		0.95		0.043	
Hospital site B	41.7	5.24		4.66		8.91		1.17		1.3		1.26		0.058	
Occupation			0.9		0.15		0.99		0.71		0.48		0.34		0.34
Medical	47.9	4.54		4.11		7.41		0.99		1.06		1.05		0.048	
Non-medical	52.1	4.59		3.65		7.41		1.02		1.11		1.12		0.051	
Direct interaction with COVID-19 patients			0.91		0.05		0.96		0.61		0.67		0.73		0.73
Yes	57.6	4.57		4.13		7.39		0.99		1.07		1.07		0.049	
No	42.4	4.53		3.49		7.41		1.03		1.1		1.1		0.05	
Underlying medical condition			<0.01		<0.01		<0.01		<0.01		<0.01		<0.01		<0.01
Yes	36.2	5.56		5.05		8.47		1.2		1.36		1.32		0.06	
No	63.8	4		3.2		6.8		0.9		0.93		0.95		0.043	

DASS-21, Depression, Anxiety and Stress Scale-21; IES-R, Impact of Events Scale-Revised.

difficult to ascertain whether it is truly COVID-19 or other intrinsic or extrinsic factors accountable for the psychological impact seen.

CONCLUSION

COVID-19 carries significant mental health hazards; to date there is a paucity of research addressing the mental health issues during the pandemic. Psychological distress was found to be prevalent among HCWs at acute hospital settings in the South-East of Ireland. Screening for adverse mental and emotional outcomes and exploring the feasibility and efficacy of psychotherapeutic interventions, together with provision of support mechanisms for HCWs, are vital to protect their psychological well-being. It is important to note that matters can be both immediate and remote, and it will be important to examine the long-term sequelae of this contagion.

Correction notice This article has been corrected since it first published. The provenance and peer review statement has been included.

Contributors The corresponding author attests that all listed authors meet authorship criteria and that no others meeting the criteria have been omitted. SA is the guarantor and had conceptualised and designed the study with support from MD and CS. Data were collected by SA and EM with SM performing all our data analysis and interpretation. SA prepared the drafted article which was critically revised by CS, SM and MD, with the final approval of the version to be published given by all authors.

Funding The authors have not declared a specific grant for this research from any funding agency in the public, commercial or not-for-profit sectors.

Conflicts of Interest No competing interests. All authors have completed the ICMJE uniform disclosure form at www.icmje.org/coi_disclosure.pdf and declare: no support from any organisation for the submitted work; no financial relationships with any organisations that might have an interest in the submitted work in the previous three years; no other relationships or activities that could appear to have influenced the submitted work.

Competing interests None declared.

Patient and public involvement Patients and/or the public were not involved in the design, or conduct, or reporting, or dissemination plans of this research.

Patient consent for publication Not required.

Ethics approval The study has been reviewed and approved by the Research Ethics Committee, Health Service Executive (HSE), South-East, Reference Number 033/2020.

Provenance and peer review Not commissioned; externally peer reviewed.

Data availability statement The datasets generated and analysed during the current study are available from the corresponding author on reasonable request.

Open access This is an open access article distributed in accordance with the Creative Commons Attribution Non Commercial (CC BY-NC 4.0) license, which permits others to distribute, remix, adapt, build upon this work non-commercially, and license their derivative works on different terms, provided the original work is properly cited, appropriate credit is given, any changes made indicated, and the use is non-commercial. See: http://creativecommons.org/licenses/by-nc/4.0/.

ORCID iD

Saied Ali http://orcid.org/0000-0002-1207-4352

REFERENCES

1 National Institute of Allergy and Infectious Diseases, U.S. Department of Health and Human Services. Coronaviruses, 2020. Available: www.niaid.nih.gov/diseases-conditions/coronaviruses
2 Zhou F, Yu T, Du R, *et al*. Clinical course and risk factors for mortality of adult inpatients with COVID-19 in Wuhan, China: a retrospective cohort study. *Lancet* 2020;395:1054–62.
3 Department of Health. Ireland's response to COVID-19 (Coronavirus), 2020. Available: www.gov.ie/en/publication/a02c5a-what-is-happening/
4 HPSC. "Epidemiology of COVID-19 in Ireland - daily reports, October 2020." Epidemiology of COVID-19 in Ireland - health protection surveillance centre, 2020. Available: www.hpsc.ie/a-z/respiratory/coronavirus/novelcoronavirus/casesinireland/epidemiologyofcovid-19inireland/
5 Silva Junior FJGda, Sales JCES, Monteiro CFdeS, Junior S, *et al*. Impact of COVID-19 pandemic on mental health of young people and adults: a systematic review protocol of observational studies. *BMJ Open* 2020;10:e039426.
6 Tan BYQ, Chew NWS, Lee GKH, *et al*. Psychological impact of the COVID-19 pandemic on health care workers in Singapore. *Ann Intern Med* 2020;173:317–20.
7 Lovibond SH, Lovibond PF. *Manual for the depression anxiety stress scales*. 2nd edn. Sydney: Psychology Foundation, 1995.
8 Christianson S, Marren J. The impact of event scale - revised (IES-R). *Medsurg Nurs* 2018;21:321–2.
9 WorldOMeter. COVID-19 coronavirus pandemic, 2020. Available: www.worldometers.info/coronavirus/
10 Saxena SK. Coping with Mental Health Challenges During COVID-19. In: Saxena SK, ed. *Coronavirus disease 2019 (COVID-19)*. Medical Virology: From Pathogenesis to Disease Control. 1. Singapore: Springer, 2020: 1. 199–211. ISBN: 978-981-15-4814-7.
11 The Lancet. COVID-19: protecting health-care workers. *The Lancet* 2020;395:922.
12 Zhu Z, Xu S, Wang H. COVID-19 in Wuhan: immediate psychological impact on 5062 health workers. *EClinicalMedicine* 2020;24:4–10 https://www.thelancet.com/journals/eclinm/article/PIIS2589-5370(20)30187-5/fulltext
13 Ho CS, Chee CY, Ho RC. Mental health strategies to combat the psychological impact of COVID-19 beyond paranoia and panic. *Ann Acad Med Singap* 2020;49:155–60.
14 Barrett LF, Mesquita B, Ochsner KN, *et al*. The experience of emotion. *Annu Rev Psychol* 2007;58:373–403.
15 Guan W-J, Liang W-H, Zhao Y, *et al*. Comorbidity and its impact on 1590 patients with COVID-19 in China: a nationwide analysis. *Eur Respir J* 2020;55:2000547.
16 Serrano-Ripoll MJ, Meneses-Echavez JF, Ricci-Cabello I, *et al*. Impact of viral epidemic outbreaks on mental health of healthcare workers: a rapid systematic review and meta-analysis. *J Affect Disord* 2020;277:347–57.
17 Kluge H. Statement – preparing for the autumn is a priority now at the WHO regional office for Europe, 2020. Available: www.euro.who.int/en/health-topics/health-emergencies/coronavirus-covid-19/statements/statement-preparing-for-the-autumn-is-a-priority-now-at-the-who-regional-office-for-europe
18 Qiu J-Y, Zhou D-S, Liu J, *et al*. Mental wellness system for COVID-19. *Brain Behav Immun* 2020;87:51–2.
