# Supplementary material for: Mental health status and related factors influencing healthcare workers during the COVID-19 pandemic: A systematic review and meta-analysis
Source: PLoS One. 2024 Jan 19;19(1):e0289454. doi: 10.1371/journal.pone.0289454 (PMC10798549; doi:10.1371/journal.pone.0289454)
Supplement: S1 Data — (ZIP) [file pone.0289454.s011.zip › literatures/30.pdf]

ELSEVIER

Since January 2020 Elsevier has created a COVID-19 resource centre with free information in English and Mandarin on the novel coronavirus COVID-19. The COVID-19 resource centre is hosted on Elsevier Connect, the company's public news and information website.

Elsevier hereby grants permission to make all its COVID-19-related research that is available on the COVID-19 resource centre - including this research content - immediately available in PubMed Central and other publicly funded repositories, such as the WHO COVID database with rights for unrestricted research re-use and analyses in any form or by any means with acknowledgement of the original source. These permissions are granted for free by Elsevier for as long as the COVID-19 resource centre remains active.

Am J Otolaryngol 41 (2020) 102694

Contents lists available at ScienceDirect

Am J Otolaryngol

journal homepage: www.elsevier.com/locate/amjoto

Mental health among head and neck surgeons in Brazil during the COVID-19 pandemic: A national study

Alyssa M. Civantos[a], Antonio Bertelli[b,1], Antonio Gonçalves[b], Emily Getzen[c], Changgee Chang[c], Qi Long[c], Karthik Rajasekaran[d,*,1]

[a] Perelman School of Medicine, University of Pennsylvania, Philadelphia, PA, USA
[b] Department of Surgery, Santa Casa De São Paulo Medical School, São Paulo, Brazil
[c] Department of Biostatistics, Epidemiology and Informatics, University of Pennsylvania, Philadelphia, PA, USA
[d] Department of Otorhinolaryngology – Head and Neck Surgery, University of Pennsylvania, Philadelphia, PA, USA

ARTICLE INFO

Keywords:
COVID-19
Mental health
Mental wellness
Head and neck surgeon
Psychiatric distress

ABSTRACT

Purpose: Head and neck surgeons are among the highest risk for COVID-19 exposure, which also brings great risk to their mental wellbeing. In this study, we aim to evaluate mental health symptoms among head and neck surgeons in Brazil surrounding the time it was declared the epicenter of the virus.
Materials and methods: A cross-sectional, survey-based study evaluating burnout, anxiety, distress, and depression among head and neck surgeons in Brazil, assessed through the single-item Mini-Z burnout assessment, 7-item Generalized Anxiety Disorder scale, 22-item Impact of Event Scale-Revised, and 2-item Patient Health Questionnaire, respectively.
Results: 163 physicians completed the survey (74.2% males). Anxiety, distress, burnout, and depression symptoms were reported in 74 (45.5%), 43 (26.3%), 24 (14.7%), and 26 (16.0%) physicians, respectively. On multivariable analysis, female physicians were more likely to report a positive screening for burnout compared to males (OR 2.88, CI [1.07–7.74]). Physicians 45 years or older were less likely to experience anxiety symptoms than those younger than 45 years (OR 0.40, CI [0.20–0.81]). Physicians with no self-reported prior psychiatric conditions were less likely to have symptoms of distress compared to those with such history (OR 0.11, CI [0.33–0.38]).
Conclusion: Head and neck surgeons in Brazil reported symptoms of burnout, anxiety, distress and depression during our study period within the COVID-19 pandemic. Institutions should monitor these symptoms throughout the pandemic. Further study is required to assess the long-term implications for physician wellness.

1. Introduction

The COVID-19 pandemic has spread across the globe within a matter of months. The epicenter of the pandemic recently shifted to South America, and specifically Brazil, in mid-May 2020 [1]. This region follows in the footsteps of the pandemic in the United States (US), Italy, and China, among others. Anecdotal reports from the preceding epicenters indicate that the virus poses great risk for exposure and infection to healthcare workers, especially those treating patients undergoing aerosol-generating procedures, such as head and neck surgeons [2–8]. Thus, there are several factors weighing on a head and neck surgeon's mind during these times, including fear of infection to self and family, concern for ability to provide high-quality patient care, and continuous changes to workplace procedures.

Impacts on physician wellbeing have already been demonstrated in prior epicenters of the COVID-19 pandemic. Studies from Wuhan, China, as well as in Singapore and India have shown elevated mental health symptoms of healthcare workers, reflected through validated surveys on anxiety, depression, insomnia, and distress [9–11]. Later, a national study in the US by our group analyzed mental health outcomes specifically in otolaryngologists, again finding symptom elevations [12]. It is not clear at this point whether these effects will have long-lasting implications. Some evidence from the 2003 SARS pandemic demonstrated continued effects in the following years, but a causal relationship is not yet established [13–15].

Given the increasing cases of COVID-19 in Brazil, we believe it is

* Corresponding author at: Department of Otorhinolaryngology-Head and Neck Surgery, 800 Walnut St, 18th Floor, Philadelphia, PA 19107, USA.
E-mail address: Karthik.Rajasekaran@pennmedicine.upenn.edu (K. Rajasekaran).
[1] Denotes co-senior authorship.

https://doi.org/10.1016/j.amjoto.2020.102694
Received 18 July 2020
Available online 21 August 2020
0196-0709/ © 2020 Elsevier Inc. All rights reserved.

Table 1
Demographic characteristics of the study population.

Characteristic	Total, N (%)	Location, N (%)					Prior psych. condition, N (%)		Comparison to baseline, N (%)		
		North	Northeast	Central-West	Southeast	South	Yes	No	Better	Worse	Same
Overall	163 (100)	10 (6.1)	38 (23.3)	13 (8.0)	91 (55.8)	11 (6.7)	18 (11.0)	145 (89.0)	19 (11.7)	54 (33.1)	90 (55.2)
Sex											
Men	121 (74.2)	7 (70.0)	30 (78.9)	10 (76.9)	65 (71.4)	9 (81.8)	6 (33.3)	115 (79.3)	12 (63.2)	33 (61.1)	76 (84.4)
Women	42 (25.8)	3 (30.0)	8 (21.1)	3 (23.1)	26 (28.6)	2 (18.2)	12 (66.7)	30 (20.7)	7 (36.8)	21 (38.9)	14 (15.6)
Age											
25–44	91 (55.8)	7 (70.0)	23 (60.5)	5 (38.5)	47 (51.6)	9 (81.8)	17 (94.4)	74 (51.0)	13 (68.4)	41 (75.9)	37 (41.1)
45 +	72 (44.2)	3 (30.0)	15 (39.5)	8 (61.5)	44 (48.4)	2 (18.2)	1 (5.6)	71 (49.0)	6 (31.6)	13 (24.1)	53 (58.9)

important to measure the mental health landscape among their head and neck surgeons. Thus, the aim of this study is to use validated surveys of burnout, anxiety, depression, and distress to assess mental health outcomes among Brazilian head and neck surgeons as they approach the height of the pandemic.

2. Material and methods

2.1. Study sample

This is a cross-sectional, survey-based, national study conducted during the COVID-19 pandemic in Brazil from May 14, 2020 to May 31, 2020. The self-administered, anonymous online survey was sent to all 700 physicians in the Brazilian Society of Head and Neck Surgery. Participation was voluntary, and participants could terminate the survey at any time. A REDCap (Research Electronic Data Capture) database was developed to capture survey data and was accessible only to study personnel. This project was determined to qualify as quality improvement by the University of Pennsylvania's Institutional Review Board.

2.2. Study measures

We examined symptoms of burnout, anxiety, distress, and depression for all participants, using validated measurement tools in Portuguese [16–21]. The single-item Mini-Z burnout assessment (range, 1–5) was used to assess burnout, with burnout defined as ≥3 [17,18]. The 7-item Generalized Anxiety Disorder scale (GAD-7; range, 0–21) was used to assess symptoms of anxiety over the past two weeks, with a scale of normal (0–4), mild (5–9), moderate (10–14), and severe (15–21) anxiety [16,21]. A score of 10 has been reported as a cut-off point for identifying cases of GAD. The GAD-7 included a final question assessing the "difficulty [these problems] made it for you to do your work, take care of things at home, or get along with other people" (range, 0–3). The 22-item Impact of Event Scale – Revised (IES-R; range, 0–88) was used to assess symptoms of distress over the past seven days [19,22], with a scale of subclinical (0 – 23), clinical concern for post-traumatic stress disorder (PTSD) (24–32), probable PTSD (33–36), and probable PTSD with immune suppression (37–88) [23–25]. The IES-R total score was also divided into three sub-scores: mean intrusion sub-score (0–4), mean avoidance sub-score (0–4), and mean hyperarousal sub-score (0–4) [22]. The intrusion sub-score assessed symptoms of "unbidden thoughts and images, troubled dreams, strong pangs or waves of feelings, and repetitive behavior" [19]; the avoidance sub-score measured "ideational constriction, behavioral inhibition and counterphobic activity, and awareness of emotional numbness;" [19] and finally the hyperarousal sub-score measured the "phenomena of hypervigilance, angry outbursts, and exaggerated startle response" [22]. The 2-item Patient Health Questionnaire (PHQ-2; range, 0–6), was used to assess symptoms of depression over the past two weeks, with a score of 3 as the cut-off for a positive depression screening requiring further evaluation with the more in-depth PHQ-9 [20]. All these tools were validated in a Brazilian cohort [21,26,27], with the exception of the Mini-Z burnout assessment, which was translated by a Brazilian head and neck surgeon, who is a native Portuguese speaker.

Demographic data were self-reported. Respondents selected one of 27 states in Brazil, which were later grouped into five regions: North, Northeast, Central-West, Southeast, and South. Respondents were also asked about a history of any psychiatric condition. Lastly, they categorized their current mental health as better, worse, or the same relative to their pre-pandemic baseline.

2.3. Statistical analysis

Data analysis was performed using R software version 3.6.3. The difference in distribution of symptoms across multiple groups was tested by the chi-square independence test (Table 2) and by the non-parametric Wilcoxon rank sum test and Kruskal-Wallis test (Table 3). To determine risk factors for severity of burnout, anxiety, distress, and depression, we used multiple logistic regression models (Table 4). The binary outcome variables were created for anxiety (normal vs other categories) and for distress (subclinical vs other categories). Sex, age, history of prior psychiatric condition, and location grouped by population size were included in the model. Given the increased prevalence of COVID-19 in areas with greater population density in the previous epicenters, our analysis evaluated respondents from the Southeast region, the most populated region, compared to the other four regions combined. All tests were two-sided and the significance level $\alpha = 0.05$ was applied. 95% confidence intervals were constructed, where applicable.

3. Results

3.1. Baseline characteristics

163 head and neck surgeons completed the survey (Table 1). Our response rate was 23.3%. Most participants were men (121 [74.2%]). 55.8% of respondents were between 25 and 44 years of age, and 44.2% were between 45 and 74 years of age. 91 (55.8%) participants worked in the Southeast region, and 72 (44.2%) worked in one of the other four regions (North: 10; Northeast: 38; Central-West: 13; South: 11). 18 (11%) of respondents reported a prior psychiatric condition. 55.2% reported that their mental health was currently the same as their baseline, 33.3% reported that it was worse, and 11.7% reported that it was better.

3.2. Mini-Z measure of burnout

Burnout was reported in 24 (14.7%) participants. A significantly greater number of females reported burnout compared to males ($p = 0.007$) (Table 2). The median (interquartile range, IQR) scores for all participants was 2.0 (1.0–2.0). Analysis of the median scores also found females to have significantly increased scores for burnout

Table 2
Symptom severity of burnout, anxiety, distress, and depression measurements in total cohort and subgroups.

Severity category	Total, N (%)	Sex, N (%)			Age, N (%)			Location, N (%)			Prior psych. condition, N (%)		
		Men	Women	*p* value	25–44	45 +	*p* value	Southeast	Other	*p* value	Yes	No	*p* value
Mini-Z: burnout symptoms													
Negative	139 (85.3)	109 (90.1)	30 (71.4)	**0.007**	74 (81.3)	65 (90.3)	0.167	75 (82.4)	64 (88.9)	0.350	13 (72.2)	126 (86.9)	0.192
Positive	24 (14.7)	12 (9.9)	12 (28.6)		17 (18.7)	7 (9.7)		16 (17.6)	8 (11.1)		5 (27.8)	19 (13.1)	
GAD-7: anxiety symptoms													
Normal	89 (54.6)	73 (60.3)	16 (38.1)	**0.001**	39 (42.9)	50 (69.4)	**0.005**	46 (50.5)	43 (59.7)	0.595	4 (22.2)	85 (58.6)	< **0.0005**
Mild	42 (25.8)	32 (26.4)	10 (23.8)		27 (29.7)	15 (20.8)		27 (29.7)	15 (20.8)		2 (11.1)	40 (27.6)	
Moderate	19 (11.7)	12 (9.9)	7 (16.7)		15 (16.5)	4 (5.6)		11 (12.1)	8 (11.1)		9 (50.0)	10 (6.9)	
Severe	13 (8.0)	4 (3.3)	9 (21.4)		10 (11.0)	3 (4.2)		7 (7.7)	6 (8.3)		3 (16.7)	10 (6.9)	
GAD-7: difficulty functioning													
Not difficult	61 (37.4)	53 (43.8)	8 (19.0)	**0.001**	26 (28.6)	35 (48.6)	**0.036**	33 (36.3)	28 (38.9)	0.971	2 (11.1)	59 (40.7)	0.065
Somewhat difficult	83 (50.9)	55 (45.5)	28 (66.7)		52 (57.1)	31 (43.1)		47 (51.6)	36 (50.0)		12 (66.7)	71 (49.0)	
Very difficult	16 (9.8)	13 (10.7)	3 (7.1)		10 (11.0)	6 (8.3)		9 (9.9)	7 (9.7)		3 (16.7)	13 (9.0)	
Extremely difficult	3 (1.8)	0 (0.0)	3 (7.1)		3 (3.3)	0 (0.0)		2 (2.2)	1 (1.4)		1 (5.6)	2 (1.4)	
IES-R: distress symptoms													
Subclinical	120 (73.6)	97 (80.2)	23 (54.8)	**0.006**	59 (64.8)	61 (84.7)	**0.042**	67 (73.6)	53 (73.6)	0.938	4 (22.2)	116 (80.0)	< **0.0005**
Clinical concern	11 (6.7)	6 (5.0)	5 (11.9)		8 (8.8)	3 (4.2)		7 (7.7)	4 (5.6)		3 (16.7)	8 (5.5)	
Probable PTSD	8 (4.9)	6 (5.0)	2 (4.8)		6 (6.6)	2 (2.8)		4 (4.4)	4 (5.6)		1 (5.6)	7 (4.8)	
Severe	24 (14.7)	12 (9.9)	12 (28.6)		18 (19.8)	6 (8.3)		13 (14.3)	11 (15.3)		10 (55.6)	14 (9.7)	
PHQ-2: depression symptoms													
Negative	137 (84.0)	105 (86.8)	32 (76.2)	0.171	73 (80.2)	64 (88.9)	0.199	74 (81.3)	63 (87.5)	0.393	11 (61.1)	126 (86.9)	**0.013**
Positive	26 (16.0)	16 (13.2)	10 (23.8)		18 (19.8)	8 (11.1)		17 (18.7)	9 (12.5)		7 (38.9)	19 (13.1)	

Abbreviations: GAD-7, 7-item Generalized Anxiety Disorder Scale; IES-R, Impact of Events Scale-Revised; IES-R "severe" level, probable PTSD with concern for immune suppression; PHQ-2, 2-item Patient Health Questionnaire.
Bold represents that it is statistically significant.

compared to males ($p = 0.002$) (Table 3). Multivariable logistic regression analysis also showed that female physicians were more likely to report a positive screening for burnout ($p = 0.036$) (Table 4).

3.3. GAD-7 measure of anxiety symptoms

Many participants had symptoms of anxiety (74 [45.5%]), with 25.8% of all participants in the mild range, 11.7% in the moderate range, and 8.0% in the severe range. For the final question asking "how difficult have these [symptoms] made it for you to do your work, take care of things at home, or get along with other people?," 50.9% of participants reported "somewhat difficult," 9.8% reported "very difficult," and 1.8% reported "extremely difficult." Females reported increased symptoms of anxiety ($p = 0.001$) and increased difficulty with the getting work done, tasks at home, or getting along with other people (p = 0.001) (Table 2). Physicians had significantly different responses to the GAD-7 between age groups ($p = 0.005$), with higher scores observed in the younger age group (age 25–44). Furthermore, respondents reporting a prior psychiatric condition had higher anxiety scores ($p < 0.0005$). The median (IQR) score on the GAD-7 was 4.0 (2.0–8.0). Similar to findings in severity of symptoms, females ($p = 0.004$), younger individuals ($p = 0.002$), and physicians with prior psychiatric conditions ($p \leq 0.0005$) had higher scores compared to their counterparts (Table 3). On multivariable analysis, physicians 45 years or older were less likely to experience anxiety symptoms than those younger than 45 years ($p = 0.011$) (Table 4).

3.4. IES-R measure of distress symptoms

Many participants had clinically concerning symptoms of distress (43 [26.3%]), with 6.7% of all participants in the clinically concerning range ("clinical concern"), 4.9% in the "probable PTSD" range, and 14.7% in the probable PTSD with immune suppression range ("severe"). Females experienced higher symptoms of distress ($p = 0.006$). Younger physicians experienced higher symptoms ($p = 0.042$). Additionally, physicians with a history of psychiatric condition had higher scores ($p < 0.0005$) (Table 2). The median (IQR) score on the IES-R for distress was 12.0 (5.0–25.0). The median (IQR) of the mean scores for the three sub-scores that comprise the distress score were 0.5 (0.1–1.0) for intrusive symptoms, 0.6 (0.2–1.2) for avoidance symptoms, and 0.5 (0.2–1.2) for hyperarousal symptoms. Similar to findings split into levels of severity of symptoms, the median scores showed females ($p = 0.001$); younger participants (p = 0.001); and those with a prior psychiatric history (p < 0.0005) to have higher scores (Table 3). The distress sub-scores demonstrated consistent results. Multivariable logistic regression analysis also showed that respondents with no prior psychiatric condition were less likely to report clinically concerning symptoms of distress (p = 0.001) (Table 4).

3.5. PHQ-2 measure of depression symptoms

26 (16.0%) participants had a positive screening on the depression tool. In a clinical setting, patients who screen positive on this depression instrument would then require a more detailed screening to assess depression. The median (IQR) score on the PHQ-2 for depression for all participants was 1.0 (0.0–2.0). Analysis of the median scores found females ($p = 0.007$), younger physicians ($p < 0.0005$), and respondents with a prior psychiatric history ($p < 0.0005$) to have significantly increased scores for depression (Table 3). Furthermore, physicians in the Southeast, had significantly higher depression scores than those in the other regions ($p = 0.009$). When adjusted for variables on multivariable analysis, there were no significant results.

4. Discussion

In this national survey-based study, we evaluated mental health outcomes among head and neck surgeons in Brazil during the period surrounding its identification as the newest epicenter of the COVID-19 pandemic. The importance of this issue cannot be overstated. Interventions worldwide have focused on efforts to contain the surge of the virus. Few countries have integrated mental health care in the emergency policies, despite the importance of this issue being

Table 3
Median scores of burnout, anxiety, distress, and depression measurements in total cohort and subgroups.

Scale	Total, median (IQR)	Sex, median (IQR)			Age, median (IQR)			Location, median (IQR)			Prior psych. condition, median (IQR)		
		Men	Women	P value	25–44	45+	*p* value	South-east	Other	*p* value	Yes	No	*p* value
Mini-Z: burnout symptoms	2.0 (1.0–2.0)	2.0 (1.0–2.0)	2.0 (2.0–3.0)	**0.002**	2.0 (1.0–2.0)	2.0 (1.0–2.0)	0.058	2.0 (1.0–2.0)	2.0 (1.0–2.0)	0.355	2.0 (2.0–2.8)	2.0 (1.0–2.0)	0.066
GAD-7: anxiety symptoms	4.0 (2.0–8.0)	3.0 (2.0–6.0)	7.0 (2.0–12.0)	**0.004**	6.0 (2.0–10.0)	3.0 (2.0–5.2)	**0.002**	4.0 (2.0–8.0)	3.0 (2.0–8.0)	0.397	12.0 (6.0–12.0)	4.0 (2.0–7.0)	**< 0.0005**
IES-R: intrusion symptoms	0.5 (0.1–1.0)	0.4 (0.1–0.9)	0.7 (0.4–2.1)	**0.001**	0.6 (0.3–1.6)	0.2 (0.1–0.7)	**0.001**	0.5 (0.2–1.0)	0.4 (0.1–1.0)	0.607	2.1 (1.0–2.8)	0.4 (0.1–0.9)	**< 0.0005**
IES-R: avoidance symptoms	0.6 (0.2–1.2)	0.5 (0.1–1.0)	1.2 (0.5–2.0)	**0.001**	0.8 (0.2–1.4)	0.4 (0.1–0.9)	**0.004**	0.8 (0.2–1.2)	0.5 (0.1–1.2)	0.673	1.6 (1.2–2.0)	0.5 (0.1–1.0)	**< 0.0005**
IES-R: hyperarousal symptoms	0.5 (0.2–1.2)	0.3 (0.2–1.0)	0.8 (0.3–2.1)	**0.005**	0.7 (0.3–1.5)	0.3 (0.2–0.8)	**0.004**	0.7 (0.2–1.2)	0.3 (0.2–1.0)	0.172	2.2 (1.2–2.8)	0.3 (0.2–1.0)	**< 0.0005**
IES-R: distress symptoms	12.0 (5.0–25.0)	10.0 (4.0–20.0)	20.0 (10.0–48.5)	**0.001**	15.0 (8.5–33.5)	7.0 (3.0–17.2)	**0.001**	12.0 (5.0–24.0)	9.5 (4.8–26.2)	0.394	43.5 (30.0–55.5)	10.0 (4.0–20.0)	**< 0.0005**
PHQ-2: depression symptoms	1.0 (0.0–2.0)	1.0 (0.0–2.0)	2.0 (0.2–2.0)	**0.007**	1.0 (0.0–2.0)	0.0 (0.0–1.0)	**< 0.0005**	1.0 (0.0–2.0)	0.0 (0.0–2.0)	**0.009**	2.0 (1.2–4.0)	1.0 (0.0–2.0)	**< 0.0005**

Abbreviations: GAD-7, 7-item Generalized Anxiety Disorder Scale; IES-R, Impact of Events Scale-Revised; PHQ-2, 2-item Patient Health Questionnaire.
Bold represents that it is statistically significant.

Table 4
Factors associated with symptoms of burnout, anxiety, distress, and depression following multivariable logistic regression.

	Participants with symptoms/ Total (%)	Adjusted OR (95%CI)	*p* value
Mini-Z: burnout symptoms			
Sex			
Men	12/121 (9.9)	1 [Reference]	
Women	12/42 (28.6)	2.88 (1.07–7.74)	**0.036**
Age			
25–44	17/91 (18.7)	1 [Reference]	
> 45	7/72 (9.7)	0.65 (0.23–1.82)	0.409
Location			
Southeast	16/91 (17.6)	1 [Reference]	
Other	8/72 (11.1)	0.61 (0.24–1.58)	0.310
Prior condition			
Yes	5/18 (27.8)	1 [Reference]	
No	19/145 (13.1)	0.78 (0.22–2.76)	0.700
GAD-7: anxiety symptoms			
Sex			
Men	48/121 (39.7)	1 [Reference]	
Women	26/42 (61.9)	1.49 (0.67–3.31)	0.333
Age			
25–44	52/91 (57.1)	1 [Reference]	
> 45	22/72 (30.6)	0.40 (0.20–0.81)	**0.011**
Location			
Southeast	45/91 (49.5)	1 [Reference]	
Other	29/72 (40.3)	0.65 (0.33–1.27)	0.207
Prior condition			
Yes	14/18 (77.8)	1 [Reference]	
No	60/145 (41.4)	0.36 (0.10–1.23)	0.104
IES-R: distress symptoms			
Sex			
Men	24/121 (19.8)	1 [Reference]	
Women	19/42 (45.2)	1.86 (0.77–4.47)	0.166
Age			
25–44	32/91 (35.2)	1 [Reference]	
> 45	11/72 (15.3)	0.58 (0.25–1.38)	0.220
Location			
Southeast	24/91 (26.4)	1 [Reference]	
Other	19/72 (26.4)	1.16 (0.53–2.56)	0.706
Prior condition			
Yes	14/18 (77.8)	1 [Reference]	
No	29/145 (20.0)	0.11 (0.03–0.38)	**0.001**
PHQ-2: depression symptoms			
Sex			
Men	16/121 (13.2)	1 [Reference]	
Women	10/42 (23.8)	1.27 (0.46–3.48)	0.639
Age			
25–44	18/91 (19.8)	1 [Reference]	
> 45	8/72 (11.1)	0.67 (0.25–1.80)	0.425
Location			
Southeast	17/91 (18.7)	1 [Reference]	
Other	9/72 (12.5)	0.65 (0.26–1.61)	0.351
Prior condition			
Yes	7/18 (38.9)	1 [Reference]	
No	19/145 (13.1)	0.32 (0.10–1.05)	0.061

Abbreviations: GAD-7, 7-item Generalized Anxiety Disorder Scale; IES-R, Impact of Events Scale-Revised; PHQ-2, 2-item Patient Health Questionnaire.
Bold represents that it is statistically significant.

highlighted by China [28]. Our prior work demonstrated elevated levels of anxiety and distress among head and neck surgeons in the US during the pandemic, and we sought to evaluate if this were true in Brazil as well at the early stage of it being the epicenter.

All respondents practiced in Brazil and were grouped by age, sex, location's population size, and self-reported prior psychiatric condition. Our male-to-female breakdown was representative of the national sample, of which 15% of the Brazilian Society of Head and Neck Surgery members are females. Our findings demonstrate varying proportions of participants with symptoms of burnout, anxiety, distress, and depression, with a third reporting their current mental health as

worse than their pre-pandemic baseline.

Nearly half of the participants reported experiencing symptoms of anxiety. A little over half of these respondents had anxiety symptoms in the "mild" range of the scale. The rest, about 20% of the total population, had a score in the moderate and severe range, which has been correlated with a diagnosis of generalized anxiety disorder [16]. Another common symptom was distress, which was at a clinically concerning level in a quarter of respondents. This was measured using the IES-R scale, which has been correlated with a risk of PTSD. In total, 20% of physicians had scores concerning for probable PTSD [24]. Of those 20%, more than half are at a level with concern for suppressed immune system as a result of the inciting event, which can last for years [25].

The other two symptoms, depression and burnout, were identified in a smaller number of physicians. Specifically, 16% of physicians had a positive score on the PHQ-2 screening tool. We can only conclude that these individuals experienced depressed mood and anhedonia over the past two weeks; they require further evaluation with the more detailed PHQ-9 to make any conclusions regarding a diagnosis of depression. Burnout was measured using the Mini-Z scale, which is a marker of the emotional exhaustion component of burnout [17,18]. This was noted in about 15% of the physicians. Burnout is characterized by three components: emotional exhaustion, depersonalization, and sense of personal accomplishment. Though emotional exhaustion is thought to be the best single measure of physician burnout, we cannot exclude the possibility that measures of the other two components, depersonalization and sense of personal accomplishment, would differ in their results.

With regards to risk factors, certain patient demographics were identified as potentially playing a role in the development of mental health symptoms during the pandemic. One such factor was age. Analysis of the symptom severity levels and median scores found significant differences in the less than 45 years old group. Specifically, the younger respondents had a higher percentage of participants with non-"normal" reported scores for anxiety and distress. On multivariable analysis, age remained significantly different for anxiety alone. The explanation for this finding is undoubtedly multifactorial but may reflect age-related differences in a physician's stage of life and career. Specifically, the less than 45 years old group are more likely to be starting families or having children at home. This may cause a heightened level of stress for infecting family members, which is well-described as a major concern for physicians at this time [29,30]. Furthermore, the younger surgeons are at a less established time in their careers, with less clinical experience. They are still building their practices and shaping the trajectory of their careers, which is also affected by the pandemic.

Another risk factor that was identified for potentially developing mental health symptoms was the respondent's sex. Our results support the frequent finding that females tend to have higher risk of mood and anxiety disorders. While these findings are still important to keep in mind, they are likely manifestations of the biases of our study tools and clinical medicine. Males are thought to be less likely to report any symptoms, even if they are experiencing them [31–33]. Furthermore, the symptoms they are experiencing may not fit into the standard measurement tools [32]. Given the risk of underdiagnosis, we should not over-interpret these sex-based findings.

Not surprisingly, a self-reported history of a prior psychiatric condition, present in 11% of respondents, was a significant risk factor for scores above a subclinical range. It is unclear whether these results reveal pre-existing baseline symptoms or an increased susceptibility to return of symptoms during stressful experiences. Interestingly, though, distress was the only score that participants with prior psychiatric condition were at significant risk for in the multivariable model. Thus, a prior reported condition is not necessarily driving the scores in general.

Our results must be interpreted in the context of the prevalence of COVID-19 cases in Brazil during our study period, which occurred at a time when cases were still greatly rising and continued to rise in the following weeks. The daily number of confirmed cases from the beginning of our study period to the end went from 11,385 on May 14, 2020 to 33,274 on May 31, 2020, according to data sourced from the European Centre for Disease Prevention and Control [34]. Of note, the daily confirmed case number had increased to 54,771 on June 20, 2020. Prior research suggests that administrators should be mindful and vigilant of physician well-being as this number increases. In the study of otolaryngologists in the US, higher case numbers correlated with higher distress scores [12]. We attempted to capture if case numbers were affecting mental health in our Brazilian population by evaluating the Southeast region compared to the other four regions combined, since the Southeast includes the major cities. We found that this was not the case on multivariable analysis. It is possible, though, that as cases increase and personal protective equipment diminish, physicians working in the more urban, densely crowded areas may in fact develop regional differences in symptoms. On the other hand, there are concerns that, although more rural areas are less populated, they have less resources and are more likely to be overwhelmed [35]. Furthermore, poorer living conditions may allow for quick spread. Thus, risk of infection spread, and risk of developing mental health symptoms, may be more nuanced in South American countries than simply population numbers.

There are several limitations to this study that must be considered. Importantly, there was no comparison to a control group and thus we cannot determine if these findings are reflective of symptoms of a Brazilian civilian, or specifically a head and neck surgeon. A prior study of healthcare workers during this pandemic in China did find mental health scores to be significantly increased compared to nonmedical health care workers though [10]. In addition, though we did ask subjects to indicate any prior history of psychiatric conditions, which we controlled for, there is risk of a response bias where certain subjects may be less likely to report given the stigma. The study was completely anonymous to help mitigate this. Given our response rate, there is a possibility of a non-response bias, though it is unclear in which direction the bias lies. Providers who did not respond to the survey may not have been interested due to lack of any personal mental health concerns. Conversely, they could have been experiencing an overwhelming symptom burden inhibiting them from engaging with the survey. Lastly, our study had a small sample size and thus could potentially be underpowered.

5. Conclusions

With continuous shifts in the prevalence of COVID-19 and higher concern for exposure with aerosolizing procedures, head and neck surgeons' mental health is at risk for disruption. We present here a cross-sectional analysis of multiple mental health symptoms among head and neck surgeons during the pandemic in Brazil, the most recent epicenter. Overall, the reported symptoms indicate that impacts on mental health may already be present at this relatively early phase (pre-peak), though at a low level. Brazilian institutions should take this information in the context of the experiences of epicenter nations that preceded them. This also applies more broadly to future waves of the pandemic. We urge institutions to use prior data to anticipate changes in the physician mental health climate as trends in COVID-19 continue to evolve. Early implementation of institutional measures can help combat overwhelming stressors. Further studies are needed to delineate the duration and extent of these symptoms, and whether they will cause clinical implications.

Funding and conflict of interests

This work was supported by the NIH P30-CA016520 grant (QL, CC). The authors have no relevant conflicts of interest.

Acknowledgements

None.

References

[1] Reuters. South America has become new COVID-19 epicenter, WHO says. Business insider May 22 https://www.businessinsider.com/south-america-has-become-new-covid-19-epicenter-who-says-2020-5; 2020, Accessed date: 10 June 2020.
[2] Givi B, Schiff BA, Chinn SB, et al. Safety recommendations for evaluation and surgery of the head and neck during the COVID-19 pandemic. JAMA Otolaryngology–Head & Neck Surgery 2020. https://doi.org/10.1001/jamaoto.2020.0780. Published online March 31.
[3] Tran K, Cimon K, Severn M, Pessoa-Silva CL, Conly J. Aerosol generating procedures and risk of transmission of acute respiratory infections to healthcare workers: a systematic review. PLoS ONE 2012;7(4):e35797. (doi:10.1371%2Fjournal.pone.0035797).
[4] Mick P, Murphy R. Aerosol-generating otolaryngology procedures and the need for enhanced PPE during the COVID-19 pandemic: a literature review. Journal of Otolaryngology-Head & Neck Surgery 2020;49(1):29. https://doi.org/10.1186/s40463-020-00424-7.
[5] Workman AD, Welling DB, Carter BS, et al. Endonasal instrumentation and aerosolization risk in the era of COVID-19: simulation, literature review, and proposed mitigation strategies. International Forum of Allergy & Rhinology 2020. https://doi.org/10.1002/alr.22577.
[6] Kowalski LP. COVID-19 pandemic: effects and evidence-based recommendations for otolaryngology and head and neck surgery practice. Head Neck 2020;42:1259–67. https://doi.org/10.1002/hed.26164.
[7] Husain IA. Why COVID-19 is a special danger to otolaryngologists. Scientific American; 2020 April 24 https://blogs.scientificamerican.com/observations/why-covid-19-is-a-special-danger-to-otolaryngologists/, Accessed date: 6 May 2020.
[8] Kulcsar MA, Montenegro FL, Arap SS, Tavares MR, Kowalski LP. High risk of COVID-19 infection for head and neck surgeons. International Archives of Otorhinolaryngology 2020;24(02). https://doi.org/10.1055/s-0040-1709725. (e129-e30).
[9] Lai J, Ma S, Wang Y, et al. Factors associated with mental health outcomes among health care workers exposed to coronavirus disease 2019. JAMA Netw Open 2020;3(3):e203976. https://doi.org/10.1001/jamanetworkopen.2020.3976.
[10] Zhang W-R, Wang K, Yin L, et al. Mental health and psychosocial problems of medical health workers during the COVID-19 epidemic in China. Psychother Psychosom 2020:1–9. https://doi.org/10.1159/000507639.
[11] Chew NWS, Lee GKH, Tan BYQ, et al. A multinational, multicentre study on the psychological outcomes and associated physical symptoms amongst healthcare workers during COVID-19 outbreak. Brain Behav Immun 2020. https://doi.org/10.1016/j.bbi.2020.04.049.
[12] Civantos AM, Byrnes Y, Chang C, et al. Mental health among otolaryngology resident and attending physicians during the COVID -19 pandemic: national study. Head Neck 2020. https://doi.org/10.1002/hed.26292.
[13] Lee AM, Wong JG, McAlonan GM, et al. Stress and psychological distress among SARS survivors 1 year after the outbreak. Can J Psychiatry 2007;52(4):233–40. https://doi.org/10.1177/070674370705200405.
[14] Liu X, Kakade M, Fuller CJ, et al. Depression after exposure to stressful events: lessons learned from the severe acute respiratory syndrome epidemic. Compr Psychiatry 2012;53(1):15–23. https://doi.org/10.1016/j.comppsych.2011.02.003.
[15] Lung F-W, Lu Y-C, Chang Y-Y, Shu B-C. Mental symptoms in different health professionals during the SARS attack: a follow-up study. Psychiatric Quarterly 2009;80(2):107–16. https://doi.org/10.1007/s11126-009-9095-5.
[16] Spitzer RL, Kroenke K, Williams JBW, Löwe B. A brief measure for assessing generalized anxiety disorder. Arch Intern Med 2006;166(10):1092. https://doi.org/10.1001/archinte.166.10.1092.
[17] Dolan ED, Mohr D, Lempa M, et al. Using a single item to measure burnout in primary care staff: a psychometric evaluation. J Gen Intern Med 2015;30(5):582–7. https://doi.org/10.1007/s11606-014-3112-6.
[18] Rohland BM, Kruse GR, Rohrer JE. Validation of a single-item measure of burnout against the Maslach Burnout Inventory among physicians. Stress and Health 2004;20(2):75–9. https://doi.org/10.1002/smi.1002.
[19] Horowitz M, Wilner N, Alvarez W. Impact of event scale: a measure of subjective stress. Psychosom Med 1979;41(3):209–18. https://doi.org/10.1097/00006842-197905000-00004.
[20] Kroenke K, Spitzer RL, Williams JBW. The patient health questionnaire-2. Med Care 2003;41(11):1284–92. https://doi.org/10.1097/01.mlr.0000093487.78664.3c.
[21] Moreno AL, Desousa DA, Souza AMFLP, et al. Factor structure, reliability, and item parameters of the Brazilian-Portuguese version of the GAD-7 questionnaire. Temas em Psicologia 2016;24(1):367–76. https://doi.org/10.9788/tp2016.1-25.
[22] Weiss DS, Marmar CR. The impact of event scale-revised. In: Wilson JP, Keane TM, editors. Assessing psychological trauma and PTSD. New York: Guilford; 1997. p. 399–411.
[23] Asukai N, Kato H, Kawamura N, et al. Reliability and validity of the Japanese-language version of the impact of event scale-revised (IES-R-J): four studies of different traumatic events. J Nerv Ment Dis 2002;190(3):175–82. https://doi.org/10.1097/00005053-200203000-00006.
[24] Creamer M, Bell R, Failla S. Psychometric properties of the impact of event scale—revised. Behav Res Ther 2003;41(12):1489–96. https://doi.org/10.1016/j.brat.2003.07.010.
[25] Kawamura N, Kim Y, Asukai N. Suppression of cellular immunity in men with a past history of posttraumatic stress disorder. Am J Psychiatry 2001;158(3):484–6. https://doi.org/10.1176/appi.ajp.158.3.484.
[26] De Lima Osório F, Vilela Mendes A, Crippa JA, Loureiro SR. Study of the discriminative validity of the PHQ-9 and PHQ-2 in a sample of Brazilian women in the context of primary health care. Perspect Psychiatr Care 2009;45(3):216–27. https://doi.org/10.1111/j.1744-6163.2009.00224.x.
[27] Caiuby AVS, Lacerda SS, Quintana MI, Torii TS, Andreoli SB. Adaptação transcultural da versão brasileira da Escala do Impacto do Evento-Revisada (IES-R). Cad Saude Publica 2012;28(3):597–603. https://doi.org/10.1590/s0102-311x2012000300019.
[28] Bao Y, Sun Y, Meng S, Shi J, Lu L. 2019-nCoV epidemic: address mental health care to empower society. The Lancet 2020;395(10224):e37–8. https://doi.org/10.1016/s0140-6736(20)30309-3.
[29] Pfefferbaum B, North CS. Mental health and the Covid-19 pandemic. N Engl J Med 2020. https://doi.org/10.1056/NEJMp2008017.
[30] Holmes EA, O'Connor RC, Perry VH, et al. Multidisciplinary research priorities for the COVID-19 pandemic: a call for action for mental health science. Lancet Psychiatry 2020. https://doi.org/10.1016/S2215-0366(20)30168-1.
[31] Courtenay WH. Constructions of masculinity and their influence on men's well-being: a theory of gender and health. Soc Sci Med 2000;50(10):1385–401. https://doi.org/10.1016/s0277-9536(99)00390-1.
[32] Smith DT, Mouzon DM, Elliott M. Reviewing the assumptions about men's mental health: an exploration of the gender binary. Am J Mens Health 2018;12(1):78–89. https://doi.org/10.1177/1557988316630953.
[33] Martin LA, Neighbors HW, Griffith DM. The experience of symptoms of depression in men vs women. JAMA Psychiat 2013;70(10):1100. https://doi.org/10.1001/jamapsychiatry.2013.1985.
[34] Roser M, Ritchie H, Ortiz-Ospina E, Hasell J. Coronavirus pandemic (COVID-19). OurWorldInData.org Updated on June 9 https://ourworldindata.org/coronavirus; 2020, Accessed date: 9 June 2020.
[35] Sandy M, Milhorance F. We are not even close to our peak yet.' Brazil risks being overwhelmed by coronavirus outbreak. Time May 15 https://time.com/5837434/brazil-coronavirus-covid-19-latin-america/; 2020, Accessed date: 11 June 2020.
