# Supplementary material for: Mental health status and related factors influencing healthcare workers during the COVID-19 pandemic: A systematic review and meta-analysis
Source: PLoS One. 2024 Jan 19;19(1):e0289454. doi: 10.1371/journal.pone.0289454 (PMC10798549; doi:10.1371/journal.pone.0289454)
Supplement: S1 Data — (ZIP) [file pone.0289454.s011.zip › literatures/31.pdf]

Erciyes Med J 2021; 43(6): 560–5 • DOI: 10.14744/etd.2021.03453
ORIGINAL ARTICLE – OPEN ACCESS

This work is licensed under a Creative Commons Attribution-NonCommercial 4.0 International License.

The Effects of COVID-19 Pandemic on the Mental Health of Healthcare Workers and Recommendations for Preventing Loss of Work Efficiency

Fatma Çölkesen[1], Fatih Çölkesen[2]

ABSTRACT

Objective: We aimed to evaluate the effect of the coronavirus disease 2019 (COVID-19) pandemic on the mental health conditions of healthcare workers and raise awareness regarding the measures to be taken in this regard.

Materials and Methods: A group of total 435 healthcare professionals, including 52.2% women, who worked in the pandemic clinics, were enrolled. The Hospital Anxiety and Depression Scale (HADS) was administered using a web-based questionnaire system. We divided the healthcare workers into the following three groups: physicians, nurses, and other auxiliary healthcare workers, and compared their HADS-A and HADS-D scores. We grouped the healthcare workers as per their departments and years of professional experience and compared the HADS-A and HADS-D scores of the groups.

Results: The mean patient age was 34.34±8.34 y. Further, 25.7% had received professional mental support, and 18.6% started undergoing professional psychological therapy for the first time in their lives owing to the psychological effects of the COVID-19 pandemic process. Healthcare workers were assessed for anxiety and depression using the HADS; 43.4% of them had anxiety and 65.1% had depression.

Conclusion: The present study stated that <50% of healthcare workers with mental disorders who were serving as frontline healthcare providers for COVID-19 patients receive professional mental support. The authorities should take precautions to prevent healthcare services from being interrupted and the negative impacts on healthcare workers' mental health during the COVID-19 pandemic.

Keywords: Anxiety, COVID-19, depression, healthcare, mental health, pandemic, work efficiency

Cite this article as: Çölkesen F, Çölkesen F. The Effects of COVID-19 Pandemic on the Mental Health of Healthcare Workers and Recommendations for Preventing Loss of Work Efficiency. Erciyes Med J 2021; 43(6): 560-5.

[1]Department of Infectious Diseases and Clinical Microbiology, University of Health Sciences, Konya Training and Research Hospital, Konya, Turkey
[2]Division of Clinical Immunology and Allergy, Department of Internal Medicine, Necmettin Erbakan University Meram Faculty of Medicine, Konya, Turkey

Submitted 14.10.2020

Accepted 18.02.2021

Available Online 05.03.2021

Correspondence Fatma Çölkesen, University of Health Sciences, Konya Training and Research Hospital, Department of Infectious Diseases and Clinical Microbiology, Konya, Turkey
Phone: +90 332 221 00 00
e-mail: fatma.derin@hotmail.com

©Copyright 2021 by Erciyes University Faculty of Medicine - Available online at www.erciyesmedj.com

INTRODUCTION

In December 2019, pneumonia cases of unknown etiology were detected in Wuhan, Hubei province, China. These cases could not be managed well; thus, the infection spread to other regions of China in a short span of time and then to the entire world, causing a pandemic (1). The cause of this pandemic was a new coronavirus, called SARS-CoV-2, that was similar to Serious Acute Respiratory Syndrome-Coronavirus (SARS-CoV). The disease is characterized by high fever, normal or decreased leukocyte count, lymphopenia, sudden development of respiratory distress, and radiological abnormalities that do not respond to 3–5 d of antibiotic treatment (2). The World Health Organization (WHO) named the disease "coronavirus disease 2019 (COVID-19)" (3). COVID-19 demonstrates a broad clinical spectrum, including asymptomatic infections, mild upper respiratory disease, respiratory failure, and even severe viral pneumonia, resulting in death (3). On January 30, 2020, the WHO declared the COVID-19 outbreak a global emergency (4).

Health professionals who manage COVID-19 patients are at a high risk of psychological stress and mental problems. The constant rise in the number of cases and death, increased workload, insufficient personal protective equipment, lack of virus-specific treatment and vaccine, and inadequate psychological support, raise the mental stress levels of healthcare professionals (5). Ensuring good mental health of frontline medical workers in charge of patient treatment is essential for good productivity of the health workforce (6). In particular, it is noteworthy that providing psychological support to frontline healthcare professionals is critical for ensuring good public and mental health in the era of this pandemic (7). Elkholy et al. (8) evaluated the mental health outcomes among Egyptian healthcare workers treating patients with confirmed or suspected COVID-19. A considerable proportion of the healthcare workers had symptoms of anxiety, depression, insomnia, and stress. Emergencies, such as pandemics, can expose healthcare workers to severe stress, increasing the risk of secondary trauma. As per a recent study, healthcare workers involved in COVID-19 treatment are exposed to considerable stress and are at a high risk of developing secondary trauma (9). Furthermore, healthcare workers have experienced high levels of stress, anxiety, and depression in previous pandemics (H1N1 influenza, SARS). Thus, these emotional reactions are expected to occur during a pandemic (10–12). However, these

Table 1. Distribution of the numbers and mean ages of the health care workers of both the sexes

Job title	Female (n=227)		Male (n=208)		p
	n	%	n	%	
Age (year), Mean±SD	34.64±8.57		34.01±8.09		0.437*
Doctors	112	50.8	118	49.2	
Nurses	89	54.9	73	45.1	0.596†
Other auxiliary healthcare workers	16	48.4	17	51.6	

SD: Standard deviation; *: Independent-Samples T-Test (data represent mean±standard deviation values); †: Chi Square test (data represent numbers and percentages)

emotional reactions experienced by healthcare workers not only affect their quality of life, but also jeopardize the long-term sustainability of health services (13). The present study aimed to evaluate the effect of the COVID-19 pandemic on the mental health conditions of healthcare workers and raise awareness for the measures to be taken in this regard.

MATERIALS and METHODS

Study Design

This research was a descriptive, comparative, and cross-sectional study. Healthcare professionals who were actively working to combat the COVID-19 pandemic were enrolled. We started the survey on May 5 and completed it on May 30. Verbal informed consent was obtained from all the study subjects. The Hospital Anxiety and Depression Scale (HADS) was applied to the groups with web-based survey systems. A form prepared by the researchers was used to obtain the sociodemographic data of the subjects. The questionnaire was used to collect demographic data and included items about how the pandemic process affected their social lives. We divided healthcare workers into the following three groups: doctors, nurses, and other auxiliary health workers. Moreover, we compared the subjects' HADS-A and HADS-D scores. We classified the healthcare workers as per their departments and years of professional experience. We compared the HADS-A and HADS-D scores of the groups.

Setting and Samples

A total of 435 healthcare workers (240 doctors, 162 nurses, and 33 other auxiliary healthcare workers) who volunteered to participate in this study were enrolled. Other auxiliary healthcare workers included midwives, paramedics, emergency medical technicians, anesthesia technicians, radiology technicians, lab technicians, and cleaning staff. Healthcare workers who fulfilled the research criteria (those working at the forefront in combating the COVID-19 pandemic) at the specified dates were enrolled via quota random sampling method and simple random sampling method (using web-based survey number). The proportions of participants with high HADS scores were compared using the chi square test among the healthcare worker subgroups (doctors, nurses, and other auxiliary healthcare workers). The sample size was determined using the G*Power 3.1 program. The minimum sample size was calculated to be 57 (sample size for one group: 19) subjects with 90% power at a 95% confidence interval with 2-tailed alpha <0.05 and a large (0.50) effect size. Difference between six independent means (departments and experience years range of healthcare workers) was used for the HADS scores (F-tests, one-way ANOVA). The minimum sample size was calculated to be 114 (sample size for one group: 19) subjects with 90% power at a 95% confidence interval with 2-tailed alpha <0.05 and a large (0.40) effect size. Thus, these sample sizes were larger than those estimated using the power calculation analysis.

Hospital Anxiety and Depression Scale

The scale was developed by Zigmond and Snaith (14). It is used to screen depression and anxiety in subjects with medical illnesses. The scale consists of 14 items; 7 of these assess anxiety and 7 assess depression. Responses are scored between 0 and 3 in quadruple Likert formats. The lowest score that a patient can get on both the subscales (anxiety and depression subscale) is 0, and the highest score is 21. Turkish reliability and validity of this scale were performed by Aydemir et al. (15) The Turkish version of the HAD scale was valid and reliable in patients (Cronbach's α of 0.8525 and 0.7784 for the HAD anxiety subscale and depression subscales, respectively). In the Turkish version of the HAD scale, the cut-off score for the anxiety subscale was 10 and that for the depression subscale was 7.

Statistical Analyses

SPSS version 22.0 statistical package software (IBM Corp., Armonk, NY, United States) was used for the statistical analyses. The Kolmogorov–Smirnov test was used to evaluate the normality of the distribution. Continuous variables are demonstrated as mean±standard deviation values, and categorical variables are presented as numbers and percentages. The mean values of the continuous variables were compared between independent groups using the independent-samples t-test and one-way ANOVA test, as appropriate. The chi square test was performed to compare the categorical variables of the study groups. The threshold for significance was defined at p<0.05.

RESULTS

A total of 435 healthcare workers participated in the study; 52.2% (227/435) of them were women. There was no significant differences in the healthcare workers in terms of sex and age (p=0.437 and 0.596, respectively); 55.2% (240/435) of the healthcare workers were doctors, 37.2% (162/435) were nurses, and 7.6% (33/435) were other auxiliary healthcare workers. The average patient age was 34.34±8.34 y (Table 1).

Table 2. Healthcare workers subgroups' Hospital Anxiety and Depression Scale (HADS) Scores

	Job title				
	**Doctor**	**Nurse**	**Other auxiliary healthcare workers**	**Total**	**p**
HADS-A score					
Normal range					
n	140	88	18	246	
%	58.4	54.4	54.5	56.6	
High					
n	100	74	15	189	0.656*
%	41.6	45.6	45.5	**43.4**	
Total					
n	240	162	33	435	
%	100.0	100.0	100.0	100.0	
HADS-D score					
Normal range					
n	81	58	13	152	
%	33.8	35.8	39.4	34.9	
High					
n	159	104	20	283	0.756*
%	66.2	64.2	60.6	**65.1**	
Total					
n	240	162	33	435	
%	100.0	100.0	100.0	100.0	

*: Chi Square test (data were shown as number and percentages); HADS-A: Hospital Anxiety and Depression Scale, anxiety subscale; HADS-D: Hospital Anxiety and Depression Scale, depression subscale

"Where do you get information about the COVID-19 pandemic?" The highest response (18.4%) to this question was "Doctors + Medical Literature + Television + Social Media." The lowest response rate (1.8%) was for "television only." Social media (12.2%) and medical literature (12%) had the highest rate in the single options. Total 97.7% of the participants reported that the frequency of face-to-face interaction with their relatives decreased during the COVID-19 pandemic process, 1.8% reported no change, and 0.5% reported an increase in interaction. Further, 95.2% of the participants stated that the frequency of handwashing increased during the COVID-19 pandemic; 3.2% reported no change, and 1.6% reported a decrease in handwashing. Of the total population, 73.1% stated that they entirely complied with the rules and measures announced by the Ministry of Health to prevent the transmission and spread of the COVID-19 pandemic, while 26.9% said that they complied with some of the rules. Further, 24.4% (106/435) of the participants reported that they had received professional mental support treatment (drug therapy, psychotherapy, etc.) at some point before the COVID-19 pandemic. Only 29.2% (31/106) of the subjects stated that their psychiatric treatment was ongoing. The proportion of healthcare professionals who started to receive professional support for the first time in their lives due to the mental effects of the COVID-19 pandemic process was 18.6% (81/435). A total of 25.7% (112/435) of healthcare professionals were actively undergoing psychological support therapy.

The proportion of healthcare workers whose HADS-A score was significant for anxiety was 43.4% and that of those whose HADS-D score was significant for depression was 65.1%. There were no significant differences in the anxiety and depression rates among the healthcare workers subgroups (p=0.656 and 0.756, respectively) (Table 2). When health care workers were grouped as per their departments; there was no significant difference in their anxiety and depression scores (p=0.400 and 0.355, respectively). There was no significant difference between the groups of healthcare workers' experience years in terms of HADS-A and HADS-D scores (p=0.750 and p=0.414 respectively) (Table 3).

DISCUSSION

We enrolled 435 healthcare workers in the present study. The HADS was used to evaluate the anxiety and depression symptoms. As per the HADS, the score of 43.4% of the participants was significant for anxiety and that of 65.1% was significant for depression. Further, 18.6% of the participants stated that they had started to receive professional mental support therapy for the first time in their lives owing to the harmful mental effects of the COVID-19 pandemic. Total 25.7% healthcare workers received professional mental health support. Although most of the participants revealed signs of anxiety and depression, only a small percentage of them

Table 3. The departments of healthcare workers, years of experience, and its impact on Hospital Anxiety and Depression Scale (HADS) Scores

	HADS-A score		HADS-D score	
	**Mean±SD**	**p**	**Mean±SD**	**p**
Departments				
Infectious diseases (n=55)	9.45±4.19		8.55±3.69	
Internal medicine (n=66)	10.57±4.43		8.82±4.05	
Intensive care unit (n=77)	10.73±4.27	0.400*	9.19±4.03	0.355*
Emergency services (n=58)	9.31±4.65		7.98±3.84	
Pulmonary medicine (n=38)	10.45±5.01		8.66±4.48	
Other pandemic clinics (n=141)	9.89±5.16		9.43±5.00	
Experience (years)				
0–3 (n=84)	9.93±4.33		8.76±4.08	
3–5 (n=49)	10.71±4.09		8.90±3.10	
5–10 (n=94)	9.51±5.19	0.750*	8.19±4.70	0.414*
10–15 (n=71)	10.08±4.37		9.20±4.19	
15–20 (n=64)	10.37±5.60		9.23±5.07	
>20 (n=73)	10.25±4.41		9.58±4.36	
Total (n=435)				

*: One-Way ANOVA Test (data represent mean±standard deviation values); HADS-A: Hospital Anxiety and Depression Scale, anxiety subscale; HADS-D: Hospital Anxiety and Depression Scale, depression subscale; SD: Standard deviation

wanted to receive treatment. There was no correlation between the HADS scores and the departments and professional experience periods of the healthcare workers.

The prevalence of anxiety, depression, insomnia, and distress symptoms among the healthcare workers in the study was 60.2%, 77.6%, 50.4%, and 76.4%, respectively, in Turkey (16). In another study, the anxiety and hopelessness levels of healthcare workers and non-healthcare workers and the factors affecting these were evaluated in Turkey. The hopelessness and state anxiety levels of healthcare workers were higher than those of non-healthcare workers (17).

In a study on healthcare workers in China during the COVID-19 pandemic, the prevalence of anxiety, depression, and stress disorder was 44.6%, 50.4%, and 71.5%, respectively (5). In another study on healthcare workers, 64.7% of the participants had symptoms of depression, 51.6% had symptoms of anxiety, and 41.2% had symptoms of stress (18). One of the leading causes of this distress in healthcare workers is the fear of becoming infected with the virus and infecting their family members (19). This fear requires isolation from their families, and they are also deprived of family support. Issues, such as childcare, make this situation more complicated. The work order changes, increasing the working hours. Higher workload and uncertainty of job descriptions are other factors that negatively affect the mental health of health workers (13). The arrangements made at the institutions of the healthcare workers for managing the pandemic cause health workers to change their workplaces and colleagues. This results in isolation from colleagues and familiar co-workers. While the increase in the number of cases and death toll fuels pessimism, witnessing the critical illnesses and deaths of their colleagues increases the mental breakdown (20). Along with the increasing number of cases, the absence of disease-specific antiviral therapy and vaccines is also concerning. Personal protective equipment (PPE) and material shortages are other reasons that increase contamination. These complex situations can cause ethical dilemmas, such as choosing between patient care and ensuring their safety (21). Social stigmatization and exclusion behaviors toward health workers commonly practiced by the public contribute to mental stress (13). Healthcare workers involved in COVID-19 treatment experience significant stress and may experience secondary trauma. Therefore, it is crucial to plan prevention strategies for future pandemic situations. Moreover, it can be a protective strategy against individual activity, stress, and secondary trauma in preventing negative emotions and thoughts. In emergency situations, high stress levels can cause healthcare workers to experience shortness of breath, impotence, cognitive difficulties, and difficulty in managing emotional responses and making decisions, resulting in anger. The lack of knowledge and PPE add to these factors. In such cases, even professionals can perceive a loss of self-efficacy in coping and, at the same time, develop incompatible responses owing to an inability to channel their skills more effectively (22).

Mental health disorders not only exert a negative effect on the health of medical personnel, but also adversely affect patient care and healthcare institutions (23). These adverse effects include conditions, such as insufficient work efficiency, low professional satisfaction level, deterioration in patient care quality and medical errors, resignations, and disruption in the health system (24).

Previous pandemics have also caused mental stress in healthcare workers. The SARS outbreak was associated with clinically significant distress in one-third to half of all healthcare workers. Quarantine practices, treating colleagues with SARS, fear of contamina-

tion, family health concern, job stress, interpersonal isolation, and stigma were factors that caused this distress. Two years after the epidemic ended, healthcare workers in hospitals that treated SARS patients had significantly higher rates of chronic stress symptoms than employees at other similar hospitals (25). In a study that examined the psychological effects of the outbreak of the influenza pandemic on healthcare workers in Kobe, high stress and anxiety were found in the participants (26).

The stress that occurred in healthcare workers during the SARS epidemic affected their mental health, performance, and behavior. A study conducted one year after the SARS epidemic in 2003 shows that the stress in healthcare workers who were providing medical care during the outbreak persisted even during the following year (27). Mental disorders seen during viral outbreaks may continue to have more severe extent, such as posttraumatic stress disorder (PTSD) (28). It is crucial to take urgent measures to preserve the mental health of healthcare professionals and ensure the smooth running of health services. The measures required to be taken in this regard are as follows:

- Working hours should be arranged, and breaks should be planned, considering the physical and mental health of the professionals.
- Restrooms should be arranged within the framework of social isolation rules.
- Ensure that the number of healthcare workers is sufficient in the unit studied.
- PPE and other materials should be supplied in sufficient numbers, and a sense of confidence should be created in the healthcare workers.
- Frontline healthcare workers should be replaced periodically and risk sharing should be performed.
- Protection methods should be explained clearly to novice healthcare workers.
- Rewards should be offered.
- Mental health scans of healthcare workers should be performed.
- Mood changes identified should be handled and treated at an early stage, without letting them progress to permanent psychological disorders.
- Professional support teams should be established to preserve mental health.
- Consultancy services should be organized via telephone, internet, and social media.
- Governments should provide assistance to healthcare workers in terms of childcare or family care
- Healthcare workers should be provided with support for family, friends, and other relatives, even if they are not in face-to-face interaction (education should be provided to the relatives of healthcare workers).

The measures to prevent the mental health of healthcare workers are vital in pandemic management. The fact that their governments and institution managers care about this issue shall ensure the long-term quality of health care services.

CONCLUSION

Preserving the mental status of healthcare workers is essential for the sustainability of healthcare systems. In the present study, we observed that healthcare professionals who have been working in the diagnosis, treatment, and care of patients with COVID-19 had a high prevalence of anxiety and depression symptoms. In addition, most of the healthcare professionals with symptoms of anxiety and depression did not receive mental health support treatment. The factors that caused this complex situation are also mentioned. Moreover, our suggestions to protect and treat the mental well-being of healthcare professionals are presented. We think that our study will raise awareness regarding the mental health of healthcare professionals during the pandemic and contribute to the adoption of the necessary precautions.

Limitations

The current study has certain limitations. First, owing to the isolation measures, a web-based questionnaire system was used, that is, the survey was not performed face-to-face. Second, the study was conducted in <1 month and provided cross-sectional information. The alterations in the psychological status of the participants that could have occurred in the long-term were not observed. Finally, only some participants were officially diagnosed obtained by examining mental health professionals. Healthcare workers who had mental health disorders as per the HADS were referred to the psychiatry clinic. However, due owing to the harmful effects of the pandemic process, feedbacks were not received.

Acknowledgements: We would like to thank all healthcare professionals working in the COVID-19 pandemic process and all scientists who have contributed to the diagnosis, treatment, and management of the pandemic through clinical trials.

Ethics Committee Approval: The Necmettin Erbakan University Clinical Research Ethics Committee granted approval for this study (date: 06.06.2020, number: 2020/2587).

Informed Consent: Written informed consent was obtained from patients who participated in this study.

Peer-review: Externally peer-reviewed.

Author Contributions: Concept – Fatma Ç, Fatih Ç; Design – Fatma Ç, Fatih Ç; Supervision – Fatma Ç, Fatih Ç; Resource – Fatma Ç; Materials – Fatih Ç; Data Collection and/or Processing – Fatma Ç, Fatih Ç; Analysis and/or Interpretation – Fatma Ç, Fatih Ç; Literature Search – Fatma Ç, Fatih Ç; Writing – Fatma Ç, Fatih Ç; Critical Reviews – Fatma Ç, Fatih Ç.

Conflict of Interest: The authors have no conflict of interest to declare.

Financial Disclosure: The authors declared that this study has received no financial support.

REFERENCES

1. Wang D, Hu B, Hu C, Zhu F, Liu X, Zhang J, et al. Clinical characteristics of 138 hospitalized patients with 2019 novel Coronavirus-infected pneumonia in Wuhan, China.. JAMA 2020; 323(11): 1061–9.
2. Lake MA. What we know so far: COVID-19 current clinical knowledge and research. Clin Med (Lond) 2020; 20(2): 124–7. [CrossRef]
3. Yılmazbaş P, Terzi Ö, Özçeker D. Did COVID-19 pandemic changed parents' approach to vaccination? Erciyes Med J 2021; 43(2): 130–4.
4. Maukayeva S, Tokayeva A, Issabekova Z, Kiyebayeva A, Karimova S.

COVID-19 in Kazakhstan: A Fatal Case Report. Erciyes Med J 2020; 42(4): 483–5. [CrossRef]
5. Lai J, Ma S, Wang Y, Cai Z, Hu J, Wei N, et al. Factors associated with mental health outcomes among health care workers exposed to Coronavirus disease 2019. JAMA Netw Open 2020; 3(3): e203976.
6. World Health Organization. Mental health and psychosocial considerations during the COVID-19 outbreak, 6 March 2020. Available from: URL: https://dochub.com/avolion/5lae27DR5Enlxl7RmqjZv1/mental-health-considerations-during-the-covid-19-outbreak-3-6-2020-pdf?dt=3HwrDtnj9_dTApGYUsrK.
7. Holmes EA, O'Connor RC, Perry VH, Tracey I, Wessely S, Arseneault L, et al. Multidisciplinary research priorities for the COVID-19 pandemic: a call for action for mental health science. Lancet Psychiatry 2020; 7(6): 547–60. [CrossRef]
8. Elkholy H, Tawfik F, Ibrahim I, Salah El-Din W, Sabry M, Mohammed S, et al. Mental health of frontline healthcare workers exposed to COVID-19 in Egypt: A call for action. Int J Soc Psychiatry. 2020 Sep 24:20764020960192. doi: 10.1177/0020764020960192. [Epub ahead of print]. [CrossRef]
9. Vagni M, Maiorano T, Giostra V, Pajardi D. Hardiness, stress and secondary trauma in Italian healthcare and Emergency workers during the COVID-19 pandemic. Sustainability 2020; 12(14): 5592. [CrossRef]
10. Goulia P, Mantas C, Dimitroula D, Mantis D, Hyphantis T. General hospital staff worries, perceived sufficiency of information and associated psychological distress during the A/H1N1 influenza pandemic. BMC Infect Dis 2010; 10: 322. [CrossRef]
11. Chan AO, Huak CY. Psychological impact of the 2003 severe acute respiratory syndrome outbreak on health care workers in a medium size regional general hospital in Singapore. Occup Med (Lond) 2004; 54(3): 190–6. [CrossRef]
12. Goh SSN, Chia MYC. Anxiety and morale in front-line healthcare workers during the coronavirus disease 2019 (COVID-19) outbreak at the National Screening Centre in Singapore. Ann Acad Med Singap 2020; 49(4): 259–62. [CrossRef]
13. Blake H, Bermingham F, Johnson G, Tabner A. Mitigating the psychological impact of COVID-19 on healthcare workers: a digital learning package. Int J Environ Res Public Health 2020; 17(9): 2997. [CrossRef]
14. Zigmond AS, Snaith RP. The hospital anxiety and depression scale. Acta Psychiatr Scand 1983; 67(6): 361–70. [CrossRef]
15. Aydemir Ö, Guvenir T, Kuey L, Kultur S. Validity and reliability of Turkish version of hospital anxiety and depression scale. Turk Psikiyatri Derg 1997; 8(4): 280–7.
16. Şahin MK, Aker S, Şahin G, Karabekiroğlu A. Prevalence of depression, anxiety, distress and insomnia and related factors in healthcare workers during COVID-19 pandemic in Turkey. J Community Health 2020; 45(6): 1168–77. [CrossRef]
17. Hacimusalar Y, Kahve AC, Yasar AB, Aydin MS. Anxiety and hopelessness levels in COVID-19 pandemic: A comparative study of healthcare professionals and other community sample in Turkey. J Psychiatr Res 2020; 129: 181–8. [CrossRef]
18. Elbay RY, Kurtulmuş A, Arpacıoğlu S, Karadere E. Depression, anxiety, stress levels of physicians and associated factors in Covid-19 pandemics. Psychiatry Res 2020; 290: 113130. [CrossRef]
19. Ho CS, Chee CY, Ho RC. Mental health strategies to combat the psychological impact of COVID-19 beyond paranoia and panic. Ann Acad Med Singap 2020; 49(3): 155–60. [CrossRef]
20. Ayanian J. Mental health needs of health care workers providing frontline COVID-19 care. JAMA Health Forum 2020. Available from: URL: https://jamanetwork.com/channels/health-forum/fullarticle/2764228. [CrossRef]
21. Vagni M, Maiorano T, Giostra V, Pajardi D. Coping With COVID-19: Emergency Stress, Secondary Trauma and Self-Efficacy in Healthcare and Emergency Workers in Italy. Front Psychol 2020; 11: 566912.
22. Shryock T. COVID-19 Raises Ethical Dilemmas for Many Physicians. 17 April 2020. Available from: URL: https://www.medicaleconomics.com/news/covid-19-raises-ethical-dilemmas-many-physicians.
23. Patel RS, Bachu R, Adikey A, Malik M, Shah M. Factors Related to Physician Burnout and Its Consequences: A Review. Behav Sci (Basel) 2018; 8(11): 98.[CrossRef]
24. Maunder RG, Leszcz M, Savage D, Adam MA, Peladeau N, Romano D, et al. Applying the lessons of SARS to pandemic influenza: an evidence-based approach to mitigating the stress experienced by healthcare workers. Can J Public Health 2008; 99(6): 486–8. [CrossRef]
25. Matsuishi K, Kawazoe A, Imai H, Ito A, Mouri K, Kitamura N, et al. Psychological impact of the pandemic (H1N1) 2009 on general hospital workers in Kobe. Psychiatry Clin Neurosci 2012; 66(4): 353–60.
26. West CP, Dyrbye LN, Shanafelt TD. Physician burnout: contributors, consequences and solutions. J Intern Med 2018; 283(6): 516–29.
27. Chong MY, Wang WC, Hsieh WC, Lee CY, Chiu NM, Yeh WC, et al. Psychological impact of severe acute respiratory syndrome on health workers in a tertiary hospital. Br J Psychiatry 2004; 185: 127–33.
28. Lee SM, Kang WS, Cho AR, Kim T, Park JK. Psychological impact of the 2015 MERS outbreak on hospital workers and quarantined hemodialysis patients. Compr Psychiatry 2018; 87: 123–7. [CrossRef]
