# Supplementary material for: Mental health status and related factors influencing healthcare workers during the COVID-19 pandemic: A systematic review and meta-analysis
Source: PLoS One. 2024 Jan 19;19(1):e0289454. doi: 10.1371/journal.pone.0289454 (PMC10798549; doi:10.1371/journal.pone.0289454)
Supplement: S1 Data — (ZIP) [file pone.0289454.s011.zip › literatures/32.pdf]

The Cost of Caring: Emotion, Burnout, and Psychological Distress in Critical Care Clinicians

Deena Kelly Costa[1,2] and Marc Moss[3]

[1]Department of Systems, Populations, and Leadership, School of Nursing, and [2]Institute for Healthcare Policy and Innovation, University of Michigan, Ann Arbor, Michigan; and [3]Division of Pulmonary Sciences and Critical Care, University of Colorado School of Medicine, Aurora, Colorado

In this issue of *AnnalsATS*, Jennifer Leckie (a critical care nurse) (pp. 785–786) describes a painful and heartbreaking story of caring for a patient in the intensive care unit (ICU) (1). Caring is a fundamental component of high-quality critical care. Yet, in the often myopic view to maximize the lives of others, we fail to recognize the chronic and insidious undoing critical care can have on clinicians. Leckie's essay alludes to several costs ICU clinicians pay when providing direct patient care. First, it raises awareness to the growing and alarming trend of burnout, and sheds light on other forms of psychological distress. In addition to burnout, these conditions warrant further attention and discussion. Second, it highlights the lack of support for the act of caring, which limits the ability to meet the needs of the current critical care workforce. Third, Leckie's personal story touches upon the stigma surrounding mental health issues in critical care clinicians, and alludes to the need for multipronged approaches to address and prevent psychological distress and burnout in the ICU workforce.

Emotion, Burnout, and Psychological Distress

Various emotions are natural responses to working in intensive care. These range from feelings of guilt, sadness, anger, and grief to emotional exhaustion and depersonalization, as seen in burnout syndrome. Burnout syndrome is defined as an individual response to particular work-related events that manifest in people that do not have baseline psychological disorders (2). Burnout usually develops gradually, and is typified by three symptoms—emotional exhaustion, depersonalization, and reduced personal accomplishment. It presents when there are divergences between one's own expectations of oneself and one's role and employer/organizational expectations of one's role. Critical care clinicians have some of the highest rates of burnout syndrome in healthcare. ICU nurses are reported to have higher rates of burnout compared with general care nurses (3), and nearly 86% could be classified as having burnout syndrome (4). Furthermore, a systematic review reports that the prevalence of burnout across studies of ICU clinicians ranges as high as 70% (5). However, burnout is not only damaging to clinicians. Studies suggest that burnout negatively impacts patient care and satisfaction (6). As such, there is growing interest in understanding and preventing burnout in critical care. In 2016, the Critical Care Collaborative Societies published a call to action about ICU clinician burnout, urging clinicians, administrators, and healthcare systems to acknowledge, address, and work to prevent burnout (2).

Although it is important that awareness to burnout syndrome is increasing, ICU clinicians are also dealing with more serious forms of psychological distress (7). Psychological distress can manifest in a variety of ways (Table 1). As evidenced in *I Will Not Cry*, normal emotional responses can intensify and transition to post-traumatic stress disorder (PTSD), depression, suicidal ideation, and even suicide. These conditions can be in response to experiencing "vicarious trauma" (8), and can occur regardless of an individual's predisposition. Indeed, estimates indicate that nearly one-quarter of all intensivists have symptoms of depression (9). In one study of 800 ICU nurses, 22% had symptoms of PTSD, and about one-fifth of those were classified as meeting PTSD diagnostic criteria (4). Even more alarming, nurses are reported to have the highest rates of suicide of all healthcare professionals (10). These statistics suggest that, in addition to burnout, understanding depression, PTSD, and other severe forms of psychological distress in ICU clinicians is critical to supporting the current critical care workforce.

Current Management and Prevention Approaches Fall Short

The current management approaches for burnout and other forms of psychological distress focus on individual coping, resting

(Received in original form April 22, 2018; accepted in final form May 4, 2018)

Supported in part by Agency for Healthcare Research and Quality grant K08HS024552, National Institute for Nursing Reseach (P20-NR015331) and the Center for Complexity and Self-management of Chronic Disease, and National Center for Complementary and Integrative Health grant R34AT009181 (M.M.).

Resources: United States—National Suicide Prevention Line 1-800-273-8255 (www.suicidepreventionlifeline.org); international—International Association for Suicide Prevention (www.iasp.info/resources/crises_centres).

Correspondence and requests for reprints should be addressed to Deena Kelly Costa, Ph.D., R.N., 400 North Ingalls #4351, Ann Arbor, MI 48108. E-mail: dkcosta@umich.edu.

Ann Am Thorac Soc Vol 15, No 7, pp 787–790, Jul 2018
Copyright © 2018 by the American Thoracic Society
DOI: 10.1513/AnnalsATS.201804-269PS
Internet address: www.atsjournals.org

Table 1. Possible reactions in response to a challenging or upsetting patient situation

Emotion	Manifested as
Frustration	Exhaustion/fatigue
Anger	Insomnia
Sadness	Withdrawal; frequent absenteeism
Grief	Withdrawal; inappropriate humor
Anxiety	Headaches; GI distress
PTSD	Hyperarousal (e.g., irritability, difficulty concentrating, startled reactions); avoidance; re-experiencing the traumatic event (e.g., nightmares or flashbacks)

Definition of abbreviations: GI = gastrointestinal; PTSD = post-traumatic stress disorder.
Partially adapted from Reference 2 and Reference 3.

much of the responsibility on the clinicians. There are a variety of coping strategies used in difficult situations to reduce stress. Unfortunately, some coping strategies may be effective in the short term, but can have long-term negative consequences. These unhealthy coping strategies include denial, depersonalization, compartmentalization, suppression, social isolation, and substance abuse. Indeed, the title and last few lines of Leckie's essay imply a denial and burial of emotion—"*I will not cry*." Clinicians unable to put aside their emotions and grief can feel as if this is a character flaw, which creates more stigma on an already stigmatized mental health condition. Sadly, such stigma can perpetuate the cycle of guilt, sadness, depersonalization, and loss of accomplishment that are hallmark signs of burnout or psychological distress. However, by continuing to bury one's emotions, or worse, stigmatizing those that are unable to, it implicitly teaches the future generation of critical care clinicians that this is the way to survive in critical care. Unaddressed mental health conditions and the surrounding stigma will, over time, create a workforce of clinicians that no longer care; creating cohorts of ICU clinicians with severe forms of psychological distress that will eventually leave the field, leading to the loss of the future workforce.

Although most of the current prevention strategies focus nearly exclusively on the individual clinician (11), the emphasis on individual interventions alone fuels subtle messaging that the individuals are at fault for not being resilient enough. Individual-focused interventions are necessary, but insufficient. Without concomitant team and system interventions to allow clinicians the opportunity and time to feel or process their own emotions, to give permission and time to take care of oneself, and to protect each other, the current approaches to addressing burnout and psychological distress are likely to fall short.

Steps Forward

The emotional impact of critical care is inevitable, but the organization of our current healthcare system can sometimes amplify this emotional impact, perpetuating feelings of isolation. Although collaboration is highly encouraged in the ICU, the systems to support such practices are limited, as practice occurs primarily in silos and clinicians rotate frequently, shift-to-shift and month-to-month. There is less opportunity to foster a community among team members. In the ICU, where care provided by a diverse interprofessional team is vital to high-quality patient care (12), the absence of such community can have many consequences, including to affect the clinicians' ability to manage emotions and symptoms of psychological distress. Focusing on strategies to manage the emotions before they progress is one key way to help develop a supportive community for individuals, interprofessional teams, and healthcare systems. To do so, solutions and interventions must be multipronged, addressing and supporting the individual and the team, and appropriately targeting system issues to minimize the impact on individuals (Figure 1).

Individual Solutions

A range of individual coping interventions are available, and include mindfulness techniques, stress-reduction exercises, and overall efforts to support self-care (11). In a systematic review and meta-analysis of interventions to address physician burnout, the majority focused on individual interventions, and were noted to be effective in reducing burnout, emotional exhaustion, and depersonalization (11). As suggested in the review, individual interventions, in conjunction with interprofessional team and system approaches, can be helpful in preventing and managing psychological distress and, potentially, burnout. However, virtually no studies have examined the combined effects of individual and system approaches to manage burnout and psychological distress.

Interprofessional Team Approaches

From an interprofesional team perspective, debriefings are one approach that can be helpful. However, debriefings need to be timely and include all the direct care providers. As the nurse describes in *I Will Not Cry*, she was not present for the debriefing that did occur. Teams, and particularly critical care attendings, nurse managers, and clinical nurse specialists, need to work together to facilitate routine debriefings, following a structured format or including an individual trained in debriefing, after any emotionally charged patient experience.

Another interprofessional team approach that has become increasingly more common is the medical pause. This is a purposeful pause, initiated by any team member in response to a challenging patient care situation (13). It is a team ritual that allows clinicians to support one another and acknowledge and recognize a patient, while simultaneously giving the team time, albeit brief, to process their emotions.

Storytelling has been used as a purposeful way to process information, allowing individuals to integrate a particular situation into one's individual life narrative (14). In fact, when individuals are able to incorporate a traumatic event into their personal narratives, they are more likely to grow from the trauma, referred to as post-traumatic growth (15). Post-traumatic growth is an individual's attempt to survive psychologically and make meaning of the situation (15). Storytelling and weaving narratives, oral or written, could be a powerful way for teams to process their emotions in response to critical care.

Virtual or in-person communities for ICU clinicians to share their thoughts, feelings, and emotions could be built into interprofessional team debriefings, unit huddles, morning rounds, or even in to sessions at professional critical care society meetings. Creating opportunities for written narratives, such as Leckie's essay, is another way for critical care journals to support and give voice to the emotional impact of critical care. Sharing stories also has the added benefit of educating trainees that feeling and caring is acceptable, and is part of the role of critical care clinicians.

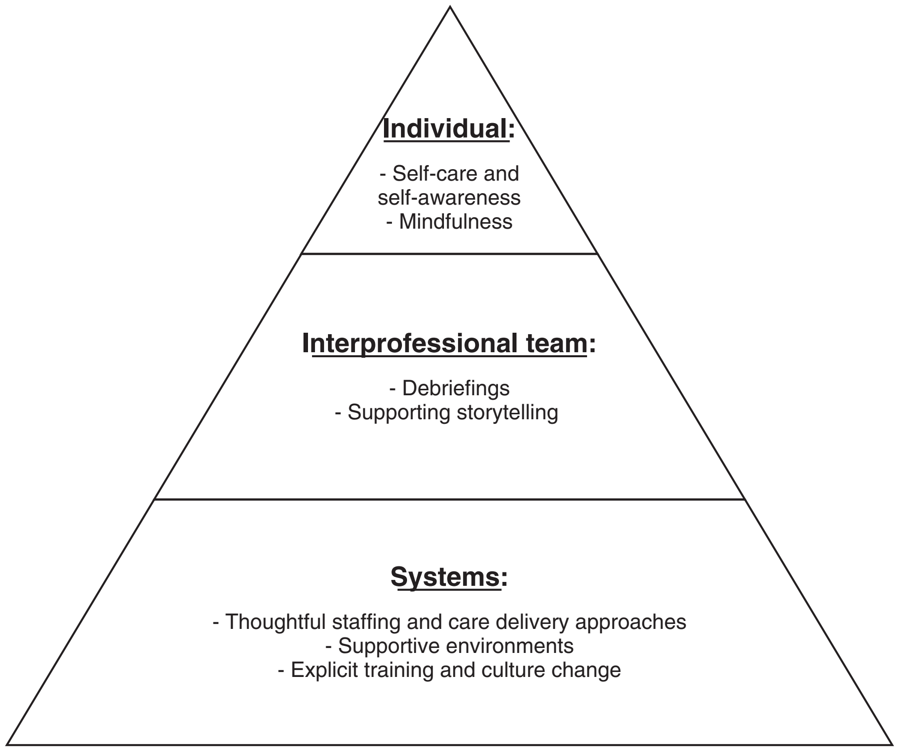

Figure 1. Multipronged solutions to address emotions, psychological distress, and burnout in critical care.

These are some examples of interprofessional team approaches to prevent and manage burnout and psychological distress. The overarching theme across all these approaches is the goal of fostering a supportive team and community, preventing social or emotional isolation, and destigmatizing the natural emotional responses to critical care.
As we continue to champion efforts of interprofessional care combined with efforts to increase patient and family engagement in the ICU, we must be better prepared to support, identify, and leverage resources when we observe colleagues in distress.

System Solutions

In addition to the individual and interprofessional team solutions, system solutions are integral.

First, though difficult patient care situations are common in the ICU, there are positive outcomes or "saves." However, these positive outcomes are rarely celebrated, and rarer still do clinicians ever see how ICU survivors fare. Growing efforts to improve ICU survivorship, such as post-ICU clinics, create opportunities for patients or families that had positive outcomes to potentially connect with their ICU caregivers. These interactions reinforce the benefit of critical care.

Second, interprofessional leaders and administrators should work together to discuss and develop thoughtful staffing approaches to manage challenging or distressing patient situations. High workloads are often reported as a main driver of burnout and psychological distress (11). As such, repeated exposure and high workload, without concomitant emotional support, can adversely impact clinicians. Understanding and asking when clinicians need a break is crucial to preventing negative consequences. For example, opportunities for personal days or "mental health days" need to be supplemental time for staff. Indeed, there was a statistically significant difference in number of days off in ICU physicians with depressive symptoms compared with those without depressive symptoms, suggesting that more days off may be a protective factor against depression and burnout (9). This observation underscores the need for organizational support and financial resources to cover personal days and other approaches to facilitating time off.

Finally, critical care research and clinical work need to move away from considering solely the absence of burnout as a desirable outcome. Instead, working toward developing joy in work (16), moving from absence of the negative to presence of the positive, is necessary to stimulate a culture shift. This will require efforts at multiple levels in each healthcare system, and will likely entail explicit training and education of staff and supervisors.

Conclusions

Caring for others is a vital component of high-quality critical care and is not without cost to the clinicians. However, it is naive to think that any solution to address the impact of emotion and burnout on critical care clinicians would involve no longer caring. The current emphasis on burnout syndrome in critical care is an important step, but recognition and awareness of all the psychological symptoms experienced by clinicians are crucial. Solutions must be multipronged and need to honor and respect the act of caring, recognize and support those that care, and work to improve the healthcare systems to allow clinicians to provide "high touch and high tech" care. If not, critical care runs the risk of losing a substantial portion of the workforce and potentially losing the most valuable part of critical care—the caring. ■

Author disclosures are available with the text of this article at www.atsjournals.org.

References

1 Leckie JD. I will not cry. *Ann Am Thorac Soc* 2018;15:785–786.
2 Moss M, Good VS, Gozal D, Kleinpell R, Sessler CN. A Critical Care Societies collaborative statement: burnout syndrome in critical care health-care professionals: a call for action. *Am J Respir Crit Care Med* 2016;194:106–113.
3 Mealer ML, Shelton A, Berg B, Rothbaum B, Moss M. Increased prevalence of post-traumatic stress disorder symptoms in critical care nurses. *Am J Respir Crit Care Med* 2007;175:693–697.

4 Mealer M, Burnham EL, Goode CJ, Rothbaum B, Moss M. The prevalence and impact of post traumatic stress disorder and burnout syndrome in nurses. *Depress Anxiety* 2009;26:1118–1126.
5 van Mol MM, Kompanje EJ, Benoit DD, Bakker J, Nijkamp MD, Seedat S. The prevalence of compassion fatigue and burnout among healthcare professionals in intensive care units: a systematic review. *PLoS One* 2015;10:e0136955.
6 Vahey DC, Aiken LH, Sloane DM, Clarke SP, Vargas D. Nurse burnout and patient satisfaction. *Med Care* 2004;42(2 suppl):II57–II66.
7 Weinstein MS. Out of the straitjacket. *N Engl J Med* 2018;378:793–795.
8 Newell JM, MacNeil GA. Professional burnout, vicarious trauma, secondary traumatic stress, and compassion fatigue: a review of theoretical terms, risk factors, and preventive methods for clinicians and researchers. *Best Pract Ment Health* 2010;6:57–68.
9 Embriaco N, Hraiech S, Azoulay E, Baumstarck-Barrau K, Forel JM, Kentish-Barnes N, *et al*. Symptoms of depression in ICU physicians. *Ann Intensive Care* 2012;2:34.
10 Office for National Statistics. Suicide by occupation, England : 2011 to 2015. Newport, South Wales: Office for National Statistics; 2017 [accessed 2018 Apr 18]. Available from: https://www.ons.gov.uk/releases/suicidesbyoccupationengland2011to2015.
11 West CP, Dyrbye LN, Erwin PJ, Shanafelt TD. Interventions to prevent and reduce physician burnout: a systematic review and meta-analysis. *Lancet* 2016;388:2272–2281.
12 Costa DK, Valley TS, Miller MA, Manojlovich M, Watson SR, McLellan P, *et al*. ICU team composition and its association with ABCDE implementation in a quality collaborative. *J Crit Care* 2018;44:1–6.
13 Bartels JB. The pause. *Crit Care Nurse* 2014;34:74–75.
14 Wheeler PL, Butell SS, Epeneter BJ, Langford CA, Taylor JD. Storytelling: a guided reflection activity. *J Nurs Educ* 2016;55:172–176.
15 Tedeschi RG, Calhoun LC. Posttraumatic growth: conceptual foundations and empirical evidence. *Psychol Inq* 2009;15:1–18.
16 Perlo J, Feeley D. Why focusing on professional burnout is not enough. *J Healthc Manag* 2018;63:85–89.
