# Supplementary material for: Mental health status and related factors influencing healthcare workers during the COVID-19 pandemic: A systematic review and meta-analysis
Source: PLoS One. 2024 Jan 19;19(1):e0289454. doi: 10.1371/journal.pone.0289454 (PMC10798549; doi:10.1371/journal.pone.0289454)
Supplement: S1 Data — (ZIP) [file pone.0289454.s011.zip › literatures/33.pdf]

frontiers | Frontiers in Public Health

ORIGINAL RESEARCH
published: 10 June 2022
doi: 10.3389/fpubh.2022.896843

# The Association Between Exposure to COVID-19 and Mental Health Outcomes Among Healthcare Workers

*Diana Czepiel[1,2], Hans W. Hoek[1,3,4], Afra van der Markt[5], Bart P. F. Rutten[6], Wim Veling[3], Frederike Schirmbeck[7], Franco Mascayano[4,8], Ezra S. Susser[4,8] and Els van der Ven[2]**

*[1] Parnassia Groep, Parnassia Psychiatric Institute, The Hague, Netherlands, [2] Faculty of Behavioral and Movement Sciences, Vrije Universiteit Amsterdam, Amsterdam, Netherlands, [3] University of Groningen, University Medical Center Groningen, University Center of Psychiatry, Groningen, Netherlands, [4] Department of Epidemiology, Mailman School of Public Health, Columbia University, New York, NY, United States, [5] Department of Psychiatry, Amsterdam Public Health Research Institute, Amsterdam University Medical Center, Vrije Universiteit Amsterdam, Amsterdam, Netherlands, [6] Department of Psychiatry and Neuropsychology, School for Mental Health and Neuroscience, Maastricht University Medical Centre, Maastricht, Netherlands, [7] Academic Medical Center, Department of Psychiatry, University of Amsterdam, Amsterdam, Netherlands, [8] Division of Behavioral Health Services and Policies, New York State Psychiatric Institute, New York, NY, United States*

OPEN ACCESS

**Edited by:**
*Rahul Shidhaye, Pravara Institute of Medical Sciences, India*

**Reviewed by:**
*Ivana Stasevic Karlicic, Clinic for Mental Disorders "Dr Laza Lazarevic", Serbia*
*Ameya Prasad Bondre, Indian Institute of Technology Bombay, India*

***Correspondence:**
*Els van der Ven*
*e.m.a.vander.ven@vu.nl*

**Specialty section:**
*This article was submitted to Public Mental Health, a section of the journal Frontiers in Public Health*

***Received:*** *15 March 2022*
***Accepted:*** *06 May 2022*
***Published:*** *10 June 2022*

**Citation:**
*Czepiel D, Hoek HW, van der Markt A, Rutten BPF, Veling W, Schirmbeck F, Mascayano F, Susser ES and van der Ven E (2022) The Association Between Exposure to COVID-19 and Mental Health Outcomes Among Healthcare Workers. Front. Public Health 10:896843. doi: 10.3389/fpubh.2022.896843*

Due to the unprecedented impact of the COVID-19 pandemic on health care systems, there has been great interest in the mental wellbeing of healthcare workers. While most studies investigated mental health outcomes among frontline vs. non-frontline healthcare workers, little is known about the impact of various work-related variables. The present study aimed to examine the association between work-related [i.e., having contact with COVID-19 patients, being redeployed due to the pandemic and availability of sufficient personal protective equipment (PPE)] and subjective (i.e., worries about getting infected or infecting others) exposures and self-reported mental health outcomes (i.e., psychological distress, depressive symptoms, and posttraumatic stress symptoms). Between February and May 2021, 994 healthcare workers employed at a variety of healthcare settings in the Netherlands filled out an online survey as part of the COVID-19 HEalth caRe wOrkErS (HEROES) study. Mental health outcomes were measured using the General Health Questionnaire-12, the Patient Health Questionnaire-9, and the Primary Care PTSD Screen for DSM-5. Approximately 13% reported depressive symptoms, 37% experienced psychological distress, and 20% reported posttraumatic stress symptoms. Multilevel linear models consisted of three levels: individual (work-related and subjective exposures), healthcare center (aggregated redeployment and availability of sufficient PPE), and regional (cumulative COVID-19 infection and death rates). Worries about infection were associated with all three mental health outcomes, whereas insufficient PPE was associated with psychological distress and depressive symptoms. There were no differences in outcomes between healthcare centers or provinces with different COVID-19 infection and death rates. Our findings highlight the importance of adequate PPE provision and the subjective experience of the COVID-19 pandemic. These factors should be part of interventions aimed at mitigating adverse mental health outcomes among healthcare workers during the COVID-19 pandemic.

**Keywords: COVID-19, depression, healthcare workers (HCWs), mental health, personal protective equipment (PPE), posttraumatic stress, psychological distress**

## INTRODUCTION

The COVID-19 pandemic has had an unprecedented worldwide impact on the social, economic, and psychological domain. Several studies have demonstrated high levels of adverse mental health outcomes globally, including symptoms of anxiety, psychological distress, depression and posttraumatic stress disorder (PTSD), in particular among groups who have been highly affected by the pandemic such as COVID-19 patients, patients with mental health problems and healthcare workers (HCW's) (1–5).

Evidence from previous pandemics, such as the Severe Acute Respiratory Syndrome (SARS) outbreak in 2003, as well as from the current COVID-19 pandemic, suggests that various exposures related to the pandemic put HCW's at an increased risk of manifesting mental health problems compared to the general population. Objective factors include for instance contact with COVID-19 patients, insufficient availability of personal protective equipment (PPE), and redeployment (5–12). The availability of sufficient PPE is a prerequisite for HCW's to be able to carry out their work-related tasks safely. Nevertheless, great shortages of PPE have been reported since the beginning of the pandemic (13). Previous research suggests that HCW's are more likely to exhibit mental health problems when they perceive the provided PPE to be insufficient (11), while sufficient availability of PPE has been shown to have a protective role against symptoms of depression, anxiety and PTSD (11, 14). Redeployment has been identified as another significant risk factor for adverse mental health outcomes (11) particularly when combined with insufficient support and PPE (15).

Besides objective exposures of this nature, it is of potential importance to examine subjective exposures in order to understand mental health outcomes during the COVID-19 pandemic. It has been found that perceived risk related to a high-impact event, such as an epidemic, is more strongly associated with mental health problems, compared to the direct exposure to the event (16). Similar findings have been reported during the COVID-19 pandemic, with fear of getting infected and of infecting loved ones being significant factors related to mental health problems (5, 7, 9, 17–19).

Few studies, however, have investigated how these objective and subjective exposures relate to mental health outcomes among different groups of HCW's. A large number of studies have treated HCW's as one homogenous group, despite that the nature of their work and the degree of their exposure to COVID-19 can differ significantly. Various studies have shown that frontline workers, namely those having direct contact with COVID-19 patients, such as HCW's working in emergency, intensive care and infectious disease units, have a greater chance of manifesting mental health problems during the COVID-19 pandemic compared to non-frontline colleagues (1, 7, 20). Other studies, however, have found that frontline HCW's are not at an increased risk for adverse mental health outcomes (5), as well as that frontline and non-frontline HCW's report similar levels of psychological distress (21). It has been also demonstrated that non-clinical HCW's have been experiencing mental health problems since the beginning of the pandemic, some of which at higher levels compared to frontline HCW's or clinical HCW's (9, 22, 23). This emphasizes the need to examine the effects of the COVID-19 pandemic among HCW's working at different positions and in various health care settings.

There is also limited research about the putative role of region-level factors. Regional differences have been reported in the prevalence of mental health problems in HCW's. For example, HCW's in African and in Latin American countries have been found to report higher rates of depression, anxiety and psychological distress compared to those in the US, Europe and Asian countries (20, 24). HCW's in Australia reported low rates of depression and anxiety compared to the general population or other essential workers during the pandemic (25). In addition, it has been demonstrated that HCW's working in regions with high COVID-19 infection rates may have more mental health problems than those working in regions with relatively low rates (9, 26, 27). Nevertheless, findings from general population surveys suggest that local infection rates are merely modestly associated with mental health outcomes (28). These findings highlight the regional variations in the impact of the COVID-19 pandemic on the mental health of HCW's.

It is of great importance that HCW's have access to the material and psychosocial resources needed to navigate their high-stress working environment, even more so during a pandemic, in order for the healthcare sector to continue functioning properly. It is essential to understand which work-related factors are associated with adverse mental health outcomes among HCW's during different phases of the pandemic. Such insights may inform interventions that aim to mitigate adverse psychological outcomes following the COVID-19 pandemic and promote mental wellbeing of HCW's during the pandemic and during future crises of a similar nature.

The current study therefore aims to investigate the relationship between work-related exposures (i.e., having contact with COVID-19 patients, availability of sufficient PPE, and redeployment), subjective exposures (i.e., worries about infection), and mental health outcomes among HCW's in the Netherlands, a country heavily impacted by the COVID-19 pandemic at the time of data collection. We include clinical HCW's working at the front line and in other departments, as well as non-clinical HCW's. We also explore whether individual differences in mental health outcomes can be explained by higher-level factors such as type of healthcare center or regional COVID-19 infection and/or death rates. We expect HCW's who report worrying about infection, having had contact with COVID-19 patients, considering the available PPE to be insufficient and being redeployed as a result of the pandemic to report higher levels of depressive symptoms, psychological distress and posttraumatic stress symptoms (PTSS). Furthermore, we anticipate that HCW's employed at healthcare centers with higher rates of redeployment and poorer availability of PPE, and those working in regions with higher COVID-19 infection and/or death rates to report more adverse mental health outcomes.

## MATERIALS AND METHODS

### Setting

The study was conducted in the Netherlands, which has a public healthcare system financed by healthcare insurances (29). During participant recruitment the Netherlands was facing the third wave of the pandemic. The Dutch government has followed a relatively liberal policy at the beginning of the pandemic to control the spread of COVID-19 (29). However, in December 2020 there was a complete lockdown in place including a curfew. In January 2021, the government launched its vaccination program against COVID-19. In March 2021 the COVID-19 infections reached a peak and they began to recede in May 2021 (30). Furthermore, there were substantial regional differences in terms of infection, hospitalization, and mortality rates. During the period of data collection, the provinces in the South and South-West of the country (including South Holland, Overijssel, Limburg and North Brabant) were the ones most heavily impacted, whereas the provinces of Groningen and Friesland were the least impacted. An overview of the COVID-19 infection and death rates for the months of January, March and May 2021 per province can be found in **Supplementary Table 1** (31).

### Study Design and Participants

This cross-sectional study forms part of the international COVID-19 HEalth caRe wOrkErS (HEROES) study aimed to evaluate the impact of the COVID-19 pandemic on the mental health of HCW's (32). The current study used data from a sample consisting of clinical and non-clinical workers (e.g., nurses, physicians, psychologists, dentists, managers and administrative stuff, security and cleaning stuff) employed at a wide range of healthcare centers in the Netherlands (e.g., hospitals, elderly homes, rehabilitation centers, ambulance service). The inclusion criteria were: being of legal age and working in a healthcare center that provided care to suspected (patients with symptoms similar to COVID-19 but without a positive test issued by the municipal health service) or confirmed cases of COVID-19 (patients with a positive test issued by the municipal health service). The sample (N=994) was recruited between February and May 2021. Most HCW's included in the study worked in Friesland (province in North Netherlands), Limburg (province in South Netherlands) or South Holland (province in West Netherlands).

Participants were recruited based on non-probability sampling either directly through the healthcare center at which they were employed or through health organization networks. More specifically, the study coordinator approached healthcare settings, explaining the specific aims and general procedures of the study and asked for assistance in recruiting potential participants. Ten healthcare facilities in three different regions of the Netherlands were recruited in order to ensure variation in local COVID-19 infection rates and/or COVID-19 deaths. These were not randomly selected within each region, but included based on two conditions. Participating healthcare centers were required to provide data on denominators, so that response rates could be calculated, and to have a contact person within the facility who would facilitate the distribution of the questionnaires among its HCW's. The requested data on denominators included the number of employed clinical and non-clinical workers, in total and stratified by gender. If the contact person agreed to support the study, a link to the digital platform describing the study and voluntary participation was forwarded to all workers of the facility *via* their work email address or *via* the healthcare center's internal communication system. The target population included both clinical and non-clinical HCW's. All HCW's registered within the facility would receive the questionnaire. However, the quick turnover of personnel could result in HCW's with a temporary contract or those employed *via* a recruitment agency being less likely to receive the questionnaire. More details about the recruitment method are provided in the HEROES protocol paper (32).

### Instruments

#### Psychological Distress

For the assessment of general psychological distress we used the Dutch version of the GHQ-12 (33), a well-validated scale which is often used as a screening instrument for psychiatric disorders (34). It is a self-report, unidimensional measure, consisting of 12 items evaluating the presence of symptoms during the past week. Half of the items are positively phrased (e.g., "During the past week, have you lost sleep due to being worried?"), whereas the other half is negatively phrased (e.g., "During the past week, have you felt capable of making decisions?"). All items are rated on a four-point Likert scale, ranging from 0 ("not at all"/ "much less than usual") to 3 ["(much) more than usual"]. Participants' score was calculated by reverse coding the negatively phrased items and summing up all items using the Likert scoring method (0–1–2–3). The internal consistency in the current study was good ($\alpha = 0.89$) (35).

#### Depressive Symptoms

To assess depressive symptoms we used the Dutch version of the Patient Health Questionnaire (PHQ)-9, a well-validated self-report measure often used as a screening instrument for depression (36). It contains 9 items corresponding to the symptoms of major depressive disorder according to the Diagnostic and Statistical Manual of Mental Disorders-IV (37). The items are rated on a four-point Likert scale based on how often participants have experienced the given symptom over the last 2 weeks (e.g., "During the past 2 weeks, have you felt little interest or pleasure in doing things?"). Answers range from 0 ("not at all") to 3 ("nearly every day"). All items were summed up to calculate a total score, which is regularly used as a severity measure (38). The internal consistency in the current study was good ($\alpha = 0.85$) (35).

#### Posttraumatic Stress Symptoms (PTSS)

In order to assess PTSS related to COVID-19 we used the Dutch version of a validated screening instrument, i.e., the Primary Care PTSD Screen for DSM-5 (PC-PTSD-5) (39) with one main alteration. The introductory item on exposure to traumatic events was omitted, given that the traumatic event of interest was the COVID-19 pandemic, and the wording of the remaining 5 items was changed to reflect that [i.e., replacing the word "event(s)" with the word "pandemic"]. Participants were asked

to respond to 5 items measuring the presence of PTSS over the past month (e.g., "In the past month, have you had nightmares about the pandemic or thoughts about the pandemic when you did not want to?"). Each item is rated on a dichotomous scale (yes/no). The total score of the questionnaire was used in order to capture symptom severity and maximize variation. The internal consistency in the current study was acceptable ($\alpha = 0.63$) (35).

### COVID-19 Work-Related Exposures

*Ad-hoc* items were used to measure work-related exposure to COVID-19. Having contact with COVID-19 patients was measured with the following item: "During the past week, have you been close to patients who were suspected or confirmed cases of COVID-19?" (dichotomous variable: yes/no). Availability of sufficient PPE was measured with the following item: "Do you believe that the personal protective equipment you have access to are sufficient to avoid getting the virus?" (0 = "No, they are completely insufficient" to 3 "Yes, they are sufficient"). Redeployment was measured with the following item: "In the past 3 months, have you been assigned to a new team and/or assigned new functions?" (dichotomous variable: yes/no).

Other relevant workplace-related variables included healthcare center type (i.e., emergency care, programmed care, non-hospital intramural care, patient transportation, support & auxiliary services, other), province where the workplace is situated and participant's current job (i.e., physicians, nursing staff, other clinical specialists and managers, support, and ancillary staff, and other HCW's). The specific grouping of HCW's professions in broader job categories can be found in **Supplementary Table 2**. Finally, region-level data regarding COVID-19 infection and death rates per province were obtained through online resources (40). We included cumulative rates reported on the start date of the recruitment (February 15th, 2021) to capture the cumulative burden that HCW's experienced since the beginning of the pandemic.

### COVID-19 Subjective Exposures (Worries About Infection)

Subjective exposure to COVID-19 (worries about infection) included the following items: (1) "In the past 3 months, how worried have you been about getting COVID-19?" (0 = "not at all" to 3 = "extremely"); (2) "In the past 3 months, how worried have you been about infecting your loved ones with COVID-19?" (0 = "not at all" to 3 = "extremely"). In the current study these two items were collapsed to create one composite score.

## Statistical Analyses

Intercorrelations among the main study variables were explored using Spearman's ranked-order correlation coefficients. Complete and non-complete cases were compared in terms of confounding variables and mental health outcomes. Wilcoxon rank-sum tests were used for continuous variables and Pearson's chi-square tests with Bonferroni-adjusted significance tests for categorical variables. Potential confounding variables were determined using directed acyclic graphs (DAG's), following the methodology suggested by Ferguson and colleagues (41). The open-source platform dagitty.net (42) was used to create the DAG's, which can be found in the **Supplementary Material**. The identified potential confounding variables which were included at the first level of the multilevel analyses were age, gender, completed education, current job, having someone under care, as well as the existence of a previously known chronic physical illness and the existence of previously known mental health problems.

We used multiple imputation by chained equations (MICE) to deal with missing data (43). The number of imputations was set to 20, based on the suggestions by Graham and colleagues (44), while the maximum number of iterations was set to 20 based on reached convergence. Predictors of missingness were added to the model according the guidelines for MICE (45). Visual and numerical inspection of the imputed data did not show deviations any problematic variables according to the rule of thumb described by Stuart and colleagues (46).

We used multilevel linear regression models to examine the association between having contact with COVID-19 patients, worries about infection, redeployment, availability of sufficient PPE, and mental health outcomes among HCW's, as well as to explore whether individual differences in mental health outcomes could be explained by higher-level factors. Prior to performing the multilevel analyses, lower-level continuous predictors were group-mean centered, whereas the third-level predictors were grand-mean centered. In addition, redeployment and availability of sufficient PPE were aggregated at the healthcare center level to be used as healthcare center-level variables. For each of the three mental health outcomes, a multilevel model was estimated, with healthcare center type and workplace location as random effects. At the individual level (level 1) the identified potential confounders and the individual predictors (contact with COVID-19 patients, worries about infection, being redeployed and availability of sufficient PPE) were added as fixed effects, at the healthcare center-level (level 2) aggregated redeployment and aggregated sufficient PPE were added as fixed effects, and at the work location level (level 3) the cumulative COVID-19 infection and death rates were added as fixed effects. All analyses were conducted using IBM SPSS Statistics software (version 26).

## Ethical Considerations

This study was performed in accordance with the guidelines outlined by Dutch legislation. The Medical Ethical boards of the Maastricht University Medical Center and the Amsterdam Medical Center assessed the study protocol. Both concluded that the study was exempt from ethical approval in the Netherlands given that the participants were not considered patients or identifiable individuals providing sensitive information following the Medical Research Involving Human Subjects Act (WMO). All participants provided written informed consent to participate in the study. At completion of the questionnaire detailed resources were provided on services (e.g., helplines, mental health services) for psychological support. Participants were urged to contact their general practitioner in case they reported mental health problems. It must be noted that the Netherlands has a public healthcare system in which it is mandatory to be registered with a general practitioner. In addition, Dutch healthcare centers are legally obliged to hire a physician caring for employees.

**TABLE 1 |** Descriptive statistics for confounding and outcome variables for complete cases and non-complete cases.

| | Complete cases ($n$ = 581) | Non-complete cases ($n$ = 403) | $\chi^2$/*Ws* |
|---|---|---|---|
| Age, Mdn (IQR) | 45 (33–55) | 47 (36–55) | 108,765.000, $p = 0.058$ |
| Gender, $n$ (%) | | | 4.523, $p = 0.104^{\dagger}$ |
| Female | 466 (80.2)[a] | 339 (82.5)[a] | |
| Male | 114 (19.6)[a] | 68 (16.5)[a] | |
| Other | 1 (0.2)[a] | 4 (1)[a] | |
| Completed education, $n$ (%) | | | 29.519, $p < 0.001$ |
| (Incomplete) primary school | 2 (0.3)[a] | 7 (1.8)[b] | |
| Secondary school | 14 (2.4)[a] | 25 (6.5)[b] | |
| Technical-professional training | 152 (26.6)[a] | 136 (35.3)[b] | |
| Undergraduate degree | 262 (45.8)[a] | 153 (39.7)[a] | |
| Postgraduate studies | 142 (24.8)[a] | 64 (16.6)[b] | |
| Someone under care, $n$ (%) | | | 0.003, $p = 0.957$ |
| No | 360 (62.4)[a] | 239 (62.6)[a] | |
| Yes | 217 (37.6)[a] | 143 (37.4)[a] | |
| Current job, $n$ (%) | | | 6.144, $p = 0.189$ |
| Physicians | 109 (18.8)[a] | 44 (16.7)[a] | |
| Nursing staff | 228 (39.2)[a] | 116 (44.1)[a] | |
| Other clinical specialists and managers | 117 (20.1)[a] | 40 (15.2)[a] | |
| Support & ancillary staff | 93 (16)[a] | 40 (15.2)[a] | |
| Other HCW's | 34 (5.9)[a] | 23 (8.7)[a] | |
| Chronic physical illness, $n$ (%) | | | 4.646, $p = 0.031$ |
| No | 455 (81)[a] | 117 (73.1)[b] | |
| Yes | 107 (19)[a] | 43 (26.9)[b] | |
| Previous mental health problems, $n$ (%) | | | 0.234, $p = 0.628$ |
| No | 528 (94)[a] | 151 (95)[a] | |
| Yes | 34 (6)[a] | 8 (5)[a] | |
| Psychological distress, Mdn (IQR) | 11 (8–16) | 12 (8.5–17) | 50,045.000, $p = 0.067$ |
| Depressive symptoms, Mdn (IQR) | 3 (1–6) | 4 (1–7) | 44,523.500, $p = 0.241$ |
| PTSS, Mdn (IQR) | 1 (0–2) | 2 (0–3) | 42,634.000, $p = 0.007$ |

*someone under care, having a minor; older adult; or individual with a disability under care; Values not sharing the same subscript are significantly different according to post hoc Bonferroni corrections.*

$^{\dagger}$*Likelihood ratio is reported instead of chi square, because more than 20% of cells had an expected count <5.*

# RESULTS

The response rate in the current study ranged from 2 to 13% among healthcare centers with higher observed response rates among women compared to men. Upon inspection of the data for systematic errors, participants who did not have an ID-number (n = 59) and who gave informed consent but left the study before responding to the first item (n = 29) were removed. This was considered to be due to technical errors and thus missing at random, because several healthcare centers have protected servers which were initially blocking the HEROES questionnaire. Compared to complete cases (participants who had no missing data on the main variables of interest), non-complete cases (participants who had at least one missing item in any of the main variables of interest) had a lower completed education level, were more likely to have a chronic physical illness and had a significantly higher score on the PTSD screening instrument (see **Table 1**). The percentage of missing values across variables ranged from 0.20% to 33.90%. Denominator data indicated that women, nurses, and physicians were more likely to participate in the study.

Participants' median age was 46 years (IQR = 34–55). Among HCW's in the current study, 13% reported symptoms of depression (cutoff score $\geq$ 10), 37% experienced psychological distress (cutoff score $\geq$ 14), and 20% reported PTSS (cutoff score $\geq$ 3). Remaining characteristics for the whole sample and stratified by the exposure variables can be found in **Table 2**, whereas intercorrelations between the main study variables and their score ranges can be found in **Table 3**.

Multilevel regression analyses indicated a significant association between worries about infection and all mental health outcomes, including self-reported psychological distress ($\beta = 0.80$, $p < 0.001$), symptoms of depression ($\beta = 0.79$, $p < 0.001$), and PTSS ($\beta = 0.33$, $p < 0.001$). Further, availability of sufficient PPE was significantly negatively associated

**TABLE 2 |** Participants' characteristics [*n* (%)] for the whole sample and stratified by reported contact with COVID-19 patients, worries about infection, sufficient PPE, and redeployment.

| | | Contact with COVID-19 patients | Worries about infection | Sufficient PPE | Redeployment |
|---|---|---|---|---|---|
| | All (*N* = 994) | Yes (*n* = 351) | Yes (*n* = 263) | Yes (*n* = 512) | Yes (*n* = 143) |
| Gender [a] | | | | | |
| Female | 805 (81) | 260 (74.1) | 215 (81.7) | 414 (80.9) | 118 (82.5) |
| Male | 182 (18.3) | 91 (25.9) | 47 (17.9) | 97 (18.9) | 24 (16.8) |
| Other | 5 (0.5) | 0 (0) | 1 (0.4) | 1 (0.2) | 1 (0.7) |
| Missing | 2 (0.2) | 0 (0) | 0 (0) | 0 (0) | 0 (0) |
| Completed education | | | | | |
| (Incomplete) primary schooling | 9 (0.9) | 1 (0.3) | 2 (0.8) | 2 (0.4) | 3 (2.1) |
| Secondary school | 39 (3.9) | 6 (1.7) | 8 (3) | 9 (1.8) | 1 (0.7) |
| Technical-professional training | 288 (29) | 88 (25.1) | 76 (28.9) | 144 (28.1) | 24 (16.8) |
| Undergraduate degree | 415 (41.8) | 174 (49.6) | 126 (47.9) | 229 (44.7) | 70 (49) |
| Postgraduate studies | 206 (20.7) | 75 (21.4) | 49 (18.6) | 114 (22.3) | 44 (30.8) |
| Missing | 37 (3.7) | 7 (2) | 2 (0.8) | 14 (2.7) | 1 (0.7) |
| Healthcare center type | | | | | |
| Emergency care | 88 (8.9) | 68 (19.4) | 28 (10.6) | 61 (11.9) | 18 (12.6) |
| Programmed care | 381 (38.3) | 168 (47.9) | 122 (46.4) | 215 (42) | 69 (48.3) |
| Non-hospital intramural care | 46 (4.6) | 8 (2.3) | 18 (6.8) | 26 (5.1) | 10 (7) |
| Patient transportation | 98 (9.9) | 67 (19.1) | 26 (9.9) | 64 (12.5) | 7 (4.9) |
| Support & auxiliary services | 124 (12.5) | 21 (6) | 33 (12.5) | 72 (14.1) | 13 (9.1) |
| Other | 110 (11.1) | 19 (5.4) | 34 (12.9) | 70 (13.7) | 23 (16.1) |
| Missing | 147 (14.8) | 0 (0) | 2 (0.8) | 4 (8) | 3 (2.1) |
| Current job | | | | | |
| Physicians | 153 (15.4) | 57 (16.2) | 36 (13.7) | 96 (18.8) | 39 (27.3) |
| Nursing staff | 344 (34.6) | 175 (49.9) | 117 (44.5) | 205 (40) | 61 (42.7) |
| Other clinical specialties & managers | 157 (15.8) | 80 (22.8) | 54 (20.5) | 102 (19.9) | 20 (14) |
| Support & ancillary staff | 133 (13.4) | 32 (9.1) | 39 (14.8) | 76 (14.8) | 16 (11.2) |
| Other HCW's | 57 (5.7) | 7 (2) | 17 (6.5) | 33 (6.4) | 7 (4.9) |
| Missing | 150 (15.1) | 0 (0) | 0 (0) | 0 (0) | 0 (0) |
| Work location[a] | | | | | |
| Friesland | 144 (14.5) | 61 (17.4) | 52 (19.8) | 79 (15.4) | 18 (12.6) |
| South Holland | 210 (21.1) | 111 (31.6) | 66 (25.1) | 127 (24.8) | 38 (26.6) |
| Limburg | 246 (24.7) | 52 (14.8) | 71 (27) | 115 (22.5) | 40 (28) |
| Other | 394 (39.6) | 127 (36.2) | 74 (28.1) | 191 (37.3) | 47 (32.9) |
| Missing | 0 (0) | 0 (0) | 0 (0) | 0 (0) | 0 (0) |
| Chronic physical illness | | | | | |
| No | 572 (57.5) | 268 (76.4) | 191 (72.6) | 385 (75.2) | 99 (69.2) |
| Yes | 150 (15.1) | 63 (17.9) | 61 (23.2) | 101 (19.7) | 28 (19.6) |
| Missing | 272 (27.4) | 20 (5.7) | 11 (4.2) | 26 (5.1) | 16 (11.2) |
| Previous mental health problems | | | | | |
| No | 679 (68.3) | 311 (88.6) | 236 (89.7) | 456 (89.1) | 115 (80.4) |
| Yes | 42 (4.2) | 20 (5.7) | 15 (5.7) | 29 (5.7) | 12 (8.4) |
| Missing | 273 (27.5) | 20 (5.7) | 12 (4.6) | 27 (5.3) | 16 (11.2) |

*All percentages are valid percentages.*

*For ease of interpretation the continuous exposure variables were classified as yes/no. The classification was performed as follows: participants were classified as worrying about infection if they had a score* $\geq 2$ *on either worries about getting infected and/or worries about infecting others; participants were classified as considering the provided PPE as sufficient if they had answered "Yes, they are sufficient". In the current table only the "yes" category is reported due to space limitations.*

[a]*It was chosen to report descriptive statistics for three of the twelve provinces in the Netherlands, namely for one with the lowest (Friesland) and two with the highest (South Holland and Limburg) cumulative infection rate at the time when participant recruitment begun.*

**TABLE 3 |** Intercorrelations and ranges for the main study variables.

| | 1 | 2 | 3 | 4 | 5 | 6 | 7 | Range |
|---|---|---|---|---|---|---|---|---|
| 1. Contact with COVID-19 patient(s) | – | | | | | | | Yes/no |
| 2. Worries about infection | 0.09* | – | | | | | | 0–6 |
| 3. Sufficient PPE | 0.03 | −0.17** | – | | | | | 0–3 |
| 4. Redeployment | −0.04 | 0.03 | 0.01 | – | | | | Yes/no |
| 5. Psychological distress | 0.02 | 0.20** | −0.17** | 0.02 | – | | | 0–36 |
| 6. Depressive symptoms | −0.01 | 0.24** | −0.17* | 0.07 | 0.75** | – | | 0–27 |
| 7. PTSS | 0.06 | 0.28** | −0.15** | 0.03 | 0.37** | 0.38** | – | 1–5 |

*$p < 0.005$, **$p < 0.001$.

**TABLE 4 |** Three-level multilevel models for psychological distress, depressive symptoms and PTSS.

| | Psychological distress | | Depressive symptoms | | PTSS | |
|---|---|---|---|---|---|---|
| **Variables** | **β (95% CI)** | **SE** | **β (95% CI)** | **SE** | **β (95% CI)** | **SE** |
| Intercept | 15* (2.02–27.97) | 6.57 | 7.25 (−1.58–16.07) | 4.48 | 3.08* (0.49–5.68) | 1.31 |
| Individual level | | | | | | |
| Female | 0.50 (−0.63–1.63) | 0.57 | 0.54 (−0.28–1.36) | 0.42 | 0.18 (−0.05–0.42) | 0.12 |
| Other gender | 0.28 (−6.68–7.24) | 3.52 | −91 (−5.99–4.18) | 2.57 | 1.00 (−0.85–2.84) | 0.92 |
| Postgraduate studies | 0.39 (−4.24–5.01) | 2.32 | 0.86 (−2.49–4.20) | 1.68 | −31 (−1.40–0.77) | 0.54 |
| Undergraduate degree | −0.00 (−4.37–4.36) | 2.20 | 0.78 (−2.54–4.10) | 1.66 | −0.02 (−1.13–1.08) | 0.55 |
| Technical- professional training | −0.30 (−4.80–4.20) | 2.26 | 0.80 (−2.61–4.21) | 1.71 | −0.16 (−1.25–0.93) | 0.54 |
| Secondary school | 2.08 (−2.85–7.00) | 2.48 | 2.56 (−0.82–5.94) | 1.71 | −0.07 (−1.26–1.12) | 0.60 |
| Physicians | 0.70 (−1.482.88) | 1.10 | −1.46 (−2.93–0.00) | 0.74 | −0.45 (−0.92–0.02) | 0.24 |
| Nursing staff | 1.39 (−0.36–3.13) | 0.89 | −0.77 (−2.04–0.50) | 0.65 | −0.23 (−0.63–0.14) | 0.20 |
| Other clinical specialists and managers | 1.07 (−0.70–2.85) | 0.90 | −0.56 (−1.85–0.74) | 0.66 | −0.33 (−0.74–0.08) | 0.21 |
| Support & auxiliary staff | 0.69 (−1.10–2.48) | 0.91 | −0.92 (−2.28–0.43) | 0.69 | −0.19 (−0.63–0.25) | 0.22 |
| Having someone under care | −0.25 (−1.04–0.54) | 0.40 | −0.01 (−0.62–0.59) | 0.31 | 0.06 (−0.12–0.24) | 0.09 |
| Chronic physical illness | 1.63** (0.63–2.62) | 0.51 | 0.77 (−0.01–1.56) | 0.40 | 0.18 (−0.04–0.41) | 0.11 |
| Previous mental health problems | 0.80 (−0.87–2.47) | 0.83 | 1.18 (−0.42–2.78) | 0.79 | −0.05 (−0.47–0.36) | 0.20 |
| Age | −0.01 (−0.05–0.02) | 0.02 | −0.01 (−0.04–0.02) | 0.01 | −0.00 (−0.01–0.01) | 0.00 |
| Contact with COVID patient(s) | −1.25 (−1.13–0.88) | 0.51 | −0.10 (−0.84–0.63) | 0.37 | 0.14 (−0.07–0.36) | 0.11 |
| Being worried about infection | 0.80** (0.49–1.11) | 0.16 | 0.79 ** (0.57–1.00) | 0.11 | 0.33 ** (0.26–0.40) | 0.03 |
| Sufficient PPE | −0.80* (−1.37-−0.23) | 0.29 | −0.48 * (−0.93–0.04) | 0.23 | −0.10 (−0.22–0.02) | 0.06 |
| Redeployed | −0.07 (−1.16–1.03) | 0.56 | 0.53 (−0.26–1.32) | 0.40 | 0.04 (−0.20–0.29) | 0.12 |
| Healthcare center level | | | | | | |
| Sufficient PPE aggregated | −1.02 (−5.04–2.99) | 2.03 | −1.27 (−3.87–1.33) | 1.32 | −0.59 (−1.33–0.14) | 0.37 |
| Redeployment aggregated | −7.42 (−21.07–6.23) | 6.92 | −0.80 (−10.79–9.19) | 5.06 | 0.09 (−2.68–2.87) | 1.40 |
| Regional level | | | | | | |
| Infection rates | −0.06 (−0.15–0.04) | 0.05 | 0.00 (−0.06–0.07) | 0.03 | −0.01 (−0.02–0.01) | 0.01 |
| Death rates | 1.98 (−1.69–5.64) | 1.87 | 0.69 (−1.67–3.06) | 1.20 | 0.23 (−0.29–0.75) | 0.26 |

*Having someone care, having a minor; older adult; or individual with a disability under care.*
*$p < 0.05$, **$p < 0.001$.

with psychological distress (β = −0.80, $p$ = 0.006) and symptoms of depression (β = −0.48, $p$ = 0.033), whereas having a physical illness was only significantly related to self-reported psychological distress (β = 1.63, $p$ = 0.001). We did not find more adverse mental health outcomes among HCW's who were redeployed or in contact with COVID-19 patients. Regarding higher-level exposures, we found no major impact of aggregated redeployment, aggregated availability of sufficient PPE, COVID-19 infection rates or COVID-19 death rates on any of the mental health outcomes. Also, no differences in mental health outcomes were found in terms of the examined confounders or between HCW's employed at different positions.

The three-level multilevel models are presented in **Table 4**, whereas the most parsimonious multilevel models (with merely the confounders

and individual level variables) are presented in **Supplementary Table 3**.

## DISCUSSION

In this Dutch cohort of HCW's assessed between February and May 2021 during the third wave of the COVID-19 pandemic, 13% of the HCW's reported symptoms of depression, 37% reported psychological distress and 20% reported PTSS. We observed strong associations between worries about infection and all mental health outcomes, including self-reported psychological distress, symptoms of depression and PTSS. Availability of sufficient PPE was negatively related to self-reported psychological distress and symptoms of depression. We did not find more adverse mental health outcomes among HCW's who were redeployed or in contact with COVID-19 patients. We also did not find any differences in mental health outcomes among HCW's employed in different healthcare centers or at different positions. Regarding higher-level exposures, we found no strong association of neither aggregated redeployment or availability of sufficient PPE at the healthcare center level, nor of COVID-19 infection or death rates at the regional-level with any of the mental health outcomes.

The proportion of mental health problems among HCW's reported in the current study are in line with the ranges reported in other European countries (10, 47) and in several meta-analyses of studies conducted in different global regions [13.3–41.1% for mild anxiety/psychological distress (8, 20, 21, 48–51), 16–31.4% for depressive symptoms (8, 21, 48–51), and 20.2–21.9% for PTSS (48, 50, 51)]. Previous studies reported an ∼26% increase in depressive and anxiety symptoms among the general population globally since the beginning of the pandemic (52), highlighting the widespread impact of the COVID-19 pandemic on mental health. It is noteworthy, however, that there are substantial regional differences in the extent to which mental health problems have increased, which varies from 12% in Southeast Asia, East Asia, and Oceania to 37% in North Africa and Middle East in the case of depressive symptoms, and from 14% in Southeast Asia, East Asia, and Oceania to 35% in South Asia in the case of anxiety symptoms (52).

The pattern of findings suggests that in this study, the subjective experience of the pandemic, had the largest impact on the mental wellbeing of HCW's. This contradicts studies demonstrating that frontline HCW's, who have direct contact with COVID-19 patients, experience more mental health problems compared to other HCW's (1, 7, 20). It is, nevertheless, in line with studies that have highlighted fear of getting infected and of infecting loved ones as risk factors for mental health problems among HCW's (14, 22). The current findings also coincide with a wider body of literature highlighting the subjective appraisal of crises and traumatic life events as drivers of negative outcomes (53).

Another salient finding was that insufficient availability of PPE was associated with more psychological distress and depressive symptoms. This coincides with several studies illustrating the negative association between PPE provision and mental health problems, such as anxiety, depression, and PTSS (8, 11, 14, 22, 27). It has also been found that HCW's who report insufficient availability of PPE worry more about getting infected and about infecting their loved ones (54, 55). This implies that availability of sufficient PPE might protect against the effect of worries about infection on the mental wellbeing of HCW's, as they feel safer and properly protected. Together with our findings highlighting the role of the subjective experience, we may hypothesize that HCW's who have insufficient access to PPE experience a lack of control increasing worries of infection which may, in turn, exacerbate psychological distress and symptoms of depression.

We also found that chronic physical illness is related to psychological distress. This is in line with literature suggesting that it constitutes a risk factor for poor mental health during the pandemic (22), as well as with studies showing that HCW's (5), COVID-19 patients (27) and the general population (56) with a pre-existing chronic physical illness are experiencing high levels of anxiety. Chronic physical illness may be considered a significant daily life burden, which is in itself associated with mental health problems (57). However, it can lead to a cumulative burden, considering that HCW's with a chronic physical illness belong to a high-risk group for COVID-19.

Contrary to our hypotheses, no differences in mental health outcomes were found between different healthcare centers or provinces. This seems consistent with the finding that only worries about infection, as opposed to being in contact with COVID-19 patients, was associated with psychological distress, depressive symptoms, and PTSS. Mental health problems did not appear to be directly related to exposure to higher local infection and death rates. This does not support studies in which HCW's working in areas with high infection rates reported more stress and anxiety (26). These findings were, however, mainly reported at the beginning of the pandemic, whereas it has been found that for instance local infection rates in the US are merely modestly associated with mental health outcomes (28).

This is one of the first studies investigating mental health outcomes among HCW's in a multilevel model with various objective and subjective COVID-19 related exposures at the individual, institutional and regional level. We advanced the current evidence on the impact of the COVID-19 pandemic on the mental health of HCW's by demonstrating that the subjective experience of the pandemic, as expressed by worries about infection and considering PPE to be sufficient, more so than objective COVID-19 exposures, were important factors for various adverse mental health outcomes. In the interpretation of these findings, there are several limitations that need to be considered. First, the current study was based on convenience sampling and had low response rates which may suggest selection of the population and limited generalizability to the broader group of HCW's. The limited available information on the denominator population indicated that women, nurses, and physicians were more likely to participate in the study. Second, there was a substantial percentage of missingness for our variables of interest which could have obscured the relations between the studied variables. Third, the study's design was cross-sectional, and we had no available data about the mental health of HCW's prior to the pandemic. Because of the study design

we were unable to establish any causal relations. Fourth, several non-standardized questions were created *Ad-hoc* to measure the main exposures in the current study and we adjusted the PTSS instrument to make it specific for the pandemic. Consequently, our results cannot be directly compared to other studies. Fifth, we had access to data on COVID-19 infection and death rates at the provincial level. These may conceal pronounced differences in impact of COVID-19 between healthcare centers in the same province. Lastly, we cannot draw any conclusions about possible presence of PTSD as we only used a screening instrument to examine PTSS. Importantly, the percentage of HCW's reporting PTSS may be overestimated considering that the pandemic was still ongoing during the recruitment period. Such symptoms should be monitored in the course of time to assess the possible manifestation of PTSD symptoms. Future studies may use a longitudinal design and structured interviews to determine the presence of clinically significant mental health problems. In addition, new research should include healthcare center-specific data that illustrate the burden at the institutional level.

## Conclusion and Implications

The current study demonstrated that worries about infection and availability of sufficient PPE was more strongly associated with adverse mental health outcomes among HCW's than the objective exposure to the COVID-19 pandemic in terms of being in contact with COVID-19 patients and being redeployed due to the pandemic. Being alert of early symptoms of mental health problems might guide the implementation of preventive and treatment interventions. At the individual level, screening for HCW's who are worrying about infection could help identify individuals who might profit from evidence-based psychological interventions. At the institutional and public health level, sufficient PPE may constitute a modifiable factor for potentially protecting against the adverse effects of the pandemic on HCW's. Stockpiling PPE and putting adequate and fitting PPE at the disposal of HCW's may protect not only their physical, but also their mental wellbeing.

# DATA AVAILABILITY STATEMENT

Code used for the analyses in this paper is available on request. For use of the HEROES-NL data, proposals can be sent to e.m.a.vander.ven@vu.nl.

# ETHICS STATEMENT

Ethical approval was not provided for this study on human participants because both the Medical Ethical Boards of the Maastricht University Medical Center and the Amsterdam Medical Center, who assessed the study protocol, concluded that the study was exempt from ethical approval in the Netherlands, given that the participants were not considered patients or identifiable individuals providing sensitive information, following the Medical Research Involving Human Subjects Act (WMO). The patients/participants provided their written informed consent to participate in this study.

# AUTHOR CONTRIBUTIONS

DC, HH, and EV were responsible for the study conceptualization, supervision, and writing the original manuscript draft. AM, WV, HH, BR, FS, and EV were involved in collecting and coordinating data collection at different healthcare centers. ES and FM initiated the international HEROES study and set up the study framework. All authors contributed to the interpretation of results, reviewed preliminary versions of the manuscript, and approved the final version.

# ACKNOWLEDGMENTS

We thank Dr. Liesbeth Visser from Medisch Centum Leeuwarden, Charlotte Smeenk-Noomen from Franciscus Gasthuis, Lizette Nieste, Clarien Schoeren-Langenkamp from Netwerk Acute Zorg Limburg and Johnatan Martis for their invaluable assistance with data collection, as well as Mathijs Deen for providing advice about the performed statistical analyses. We also acknowledge all health care workers who participated in the survey.

# SUPPLEMENTARY MATERIAL

The Supplementary Material for this article can be found online at: https://www.frontiersin.org/articles/10.3389/fpubh.2022.896843/full#supplementary-material

# REFERENCES


1. Phiri P, Ramakrishnan R, Rathod S, Elliot K, Thayanandan T, Sandle N, et al. An evaluation of the mental health impact of SARS-CoV-2 on patients, general public and healthcare professionals: a systematic review and meta-analysis. *EClinicalMedicine.* (2021) 34:100806. doi: 10.1016/j.eclinm.2021.100806
2. Vindegaard N, Benros ME. COVID-19 pandemic and mental health consequences: systematic review of the current evidence. *Brain Behav Immun.* (2020) 89:531–42. doi: 10.1016/j.bbi.2020.05.048
3. Saragih ID, Tonapa SI, Saragih IS, Advani S, Batubara SO, Suarilah I, et al. Global prevalence of mental health problems among healthcare workers during the COVID-19 pandemic: a systematic review and meta-analysis. *Int J Nurs Stud.* (2021) 121:104002. doi: 10.1016/j.ijnurstu.2021.104002
4. Dobson H, Malpas CB, Burrell AJC, Gurvich C, Chen L, Kulkarni J, et al. Burnout and psychological distress amongst Australian healthcare workers during the COVID-19 pandemic. *Australas Psychiatry.* (2021) 29:26–30. doi: 10.1177/1039856220965045
5. Olashore A, Akanni O, Fela-Thomas A, Khutsafalo K. The psychological impact of COVID-19 on health-care workers in African countries: a systematic review. *Asian J Soc Heal Behav.* (2021) 4:85–97. doi: 10.4103/shb.shb_32_21
6. Sahebi A, Nejati-Zarnaqi B, Moayedi S, Yousefi K, Torres M. The prevalence of anxiety and depression among healthcare workers during the COVID-19 pandemic: an umbrella review of meta-analyses. *Prog Neuropsychopharmacol Biol Psychiatr.* (2021) 107:110247. doi: 10.1016/j.pnpbp.2021.110247

7. Spoorthy MS, Pratapa SK, Mahant S. Mental health problems faced by healthcare workers due to the COVID-19 pandemic–a review. *Asian J Psychiatr*. (2020) 51:2018–21. doi: 10.1016/j.ajp.2020.102119
8. Thatrimontrichai A, Weber DJ, Apisarnthanarak A. Mental health among healthcare personnel during COVID-19 in Asia: a systematic review. *J Formos Med Assoc*. (2021) 120:1296–304. doi: 10.1016/j.jfma.2021.01.023
9. Rosales Vaca KM, Cruz Barrientos OI, Girón López S, Noriega S, More Árias A, Guariente SMM, et al. Mental health of healthcare workers of Latin American countries: A review of studies published during the first year of COVID-19 pandemic. *Psychiatry Res*. (2022) 311:114501. doi: 10.1016/j.psychres.2022.114501
10. Mediavilla R, Fernández-Jiménez E, Martínez-Alés G, Moreno-Küstner B, Martinez-Morata I, Jaramillo F, et al. Role of access to personal protective equipment, treatment prioritization decisions, and changes in job functions on health workers' mental health outcomes during the initial outbreak of the COVID-19 pandemic. *J Affect Disord*. (2021) 295:405–9. doi: 10.1016/j.jad.2021.08.059
11. Khajuria A, Tomaszewski W, Liu Z, Chen J. hua, Mehdian R, Fleming S, et al. Workplace factors associated with mental health of healthcare workers during the COVID-19 pandemic: an international cross-sectional study. *BMC Health Serv Res*. (2021) 21:262. doi: 10.1186/s12913-021-06279-6
12. Li TM, Pien LC, Kao CC, Kubo T, Cheng WJ. Effects of work conditions and organisational strategies on nurses' mental health during the COVID-19 pandemic. *J Nurs Manag*. (2022) 30:71–8. doi: 10.1111/jonm.13485
13. Burki T. Global shortage of personal protective equipment. *Lancet Infect Dis*. (2020) 20:785–6. doi: 10.1016/S1473-3099(20)30501-6
14. Luceño L, Talavera B, Yolanda G, Martín J. Symptoms of posttraumatic stress, anxiety, depression, levels of resilience and burnout in spanish health personnel during the COVID-19 pandemic. *Int J Environ Res Public Health*. (2020) 17:5514. doi: 10.3390/ijerph17155514
15. Vera San Juan N, Clark SE, Camilleri M, Jeans JP, Monkhouse A, Chisnall G, et al. Training and redeployment of healthcare workers to intensive care units (ICUs) during the COVID-19 pandemic: a systematic review. *BMJ Open*. (2022) 12:e050038. doi: 10.1136/bmjopen-2021-050038
16. Wu P, Fang Y, Guan Z, Fan B, Kong J, Yao Z, et al. The psychological impact of the SARS epidemic on hospital employees in China: Exposure, risk perception, and altruistic acceptance of risk. *Can J Psychiatry*. (2009) 54:302–11. doi: 10.1177/070674370905400504
17. Fleuren BPI, Poesen LT, Gifford RE, Zijlstra FRH, Ruwaard D, van de Baan FC, et al. We're not gonna fall: depressive complaints, personal resilience, team social climate, and worries about infections among hospital workers during a pandemic. *Int J Environ Res Public Health*. (2021) 18:4701. doi: 10.3390/ijerph18094701
18. Han Q, Zheng B, Agostini M, Jocelyn JB, Gützkow B, Kreienkamp J. et al. Associations of risk perception of COVID-19 with emotion and mental health during the pandemic. *Jama*. (2021) 284:247–55. doi: 10.1016/j.jad.2021.01.049
19. Gorini A, Fiabane E, Sommaruga M, Barbieri S, Sottotetti F, La Rovere MT, et al. Mental health and risk perception among Italian healthcare workers during the second month of the COVID-19 pandemic. *Arch Psychiatr Nurs*. (2020) 34:537–44. doi: 10.1016/j.apnu.2020.10.007
20. Zhang SX, Batra K, Liu T, Dong RK, Xu W, Yin A, et al. Meta-analytical evidence on mental disorder symptoms during the COVID-19 pandemic in Latin America. *medRxiv [Preprint]*. (2022). Available online at: https://pesquisa.bvsalud.org/portal/resource/e/ppmedrxiv-21259299
21. Pappa S, Chen J, Barnett J, Chang A, Dong RK, Xu W, et al. A systematic review and meta-analysis of the mental health symptoms during the COVID-19 pandemic in Southeast Asia. *Psychiatry Clin Neurosci*. (2022) 76:41–50. doi: 10.1111/pcn.13306
22. De Kock JH, Latham HA, Leslie SJ, Grindle M, Munoz SA, Ellis L, et al. A rapid review of the impact of COVID-19 on the mental health of healthcare workers: implications for supporting psychological well-being. *BMC Public Health*. (2021) 21:104. doi: 10.1186/s12889-020-10070-3
23. Rathod S, Pallikadavath S, Young AH, Graves L, Rahman MM, Brooks A, et al. Psychological impact of COVID-19 pandemic: Protocol and results of first three weeks from an international cross-section survey—focus on health professionals. *J Affect Disord Reports*. (2020) 1:100005. doi: 10.1016/j.jadr.2020.100005
24. Chen J, Farah N, Dong RK, Chen RZ, Xu W, Yin J, et al. Mental health during the COVID-19 crisis in Africa: a systematic review and meta-analysis. *Int J Environ Res Public Health*. (2021) 18:10604. doi: 10.3390/ijerph182010604
25. Toh WL, Meyer D, Phillipou A, Tan EJ, Van Rheenen TE, Neill E, et al. Mental health status of healthcare vs. other essential workers in Australia amidst the COVID-19 pandemic: initial results from the collate project. *Psychiatry Res*. (2021) 298:113822. doi: 10.1016/j.psychres.2021.113822
26. Vizheh M, Qorbani M, Arzaghi SM, Muhidin S, Javanmard Z, Esmaeili M. The mental health of healthcare workers in the COVID-19 pandemic: a systematic review. *J Diabetes Metab Disord*. (2020) 19:1967–78. doi: 10.1007/s40200-020-00643-9
27. Luo M, Lixia G, Minghzhou Y, Wenying J, Haiyan W. The psychological and mental impact of coronavirus disease 2019 (COVID- 19) on medical staff and general public—a systematic review and meta- analysis. *Psychiatry Res*. (2020) 291:113190. doi: 10.1016/j.psychres.2020.113190
28. Swaziek Z, Wozniak A. Disparities Old and New in US Mental Health during the COVID-19 pandemic. *Fisc Stud*. (2020) 41:709–32. doi: 10.1111/1475-5890.12244
29. Hoekman LM, Smits MMV, Koolman X. The Dutch COVID-19 approach: regional differences in a small country. *Heal polic Technol*. (2020) 9:613–22. doi: 10.1016/j.hlpt.2020.08.008
30. Rijksoverheid. *Coronavirus tijdlijn*. The Hague: Rijksoverheid (2021). Available online at: https://www.rijksoverheid.nl/onderwerpen/ (accessed August 27, 2021).
31. Rijksinstituut voor Volksgezondheid en Milieu (RIVM). *Ontwikkeling SARS-CoV-2 in grafieken*. The Hague: RIVM (2021). Available online at: https://www.rivm.nl/coronavirus-covid-19/grafieken (accessed August 27, 2021).
32. Mascayano F, van der Ven E, Moro MF, Schilling S, Alarcón S, Al Barathie J, et al. The impact of the COVID-19 pandemic on the mental health of healthcare workers: Study protocol for the COVID-19 HEalth caRe wOrkErS (HEROES) study. *Soc Psychiatry Psychiatr Epidemiol*. (2022). doi: 10.1007/s00127-021-02211-9
33. Koeter MWJ, Ormel J. *General Health Questionnaire, Nederlandse bewerking: Handleiding*. Lisse: Swets Test Services (1991).
34. Goldberg DP, Gater R, Sartorius N, Ustun TB, Piccinelli M, Gureje O, et al. The validity of two versions of the GHQ in the WHO study of mental illness in general health care. *Psychol Med*. (1997) 27:191–7. doi: 10.1017/S0033291796004242
35. Taber KS. The use of Cronbach's alpha when developing and reporting research instruments in science education. *Res Sci Educ*. (2018) 48:1273–96. doi: 10.1007/s11165-016-9602-2
36. Persoons P, Luyckx K, Desloovere C, Vandenberghe J, Fischler B. Anxiety and mood disorders in otorhinolaryngology outpatients presenting with dizziness: validation of the self-administered PRIME-MD Patient Health Questionnaire and epidemiology. *Gen Hosp Psychiatry*. (2003) 25:316–23. doi: 10.1016/S0163-8343(03)00072-0
37. American Psychiatric Association. *Diagnostic and Statistical Manual of Mental Disorders*. (1994).
38. Kroenke K, Spitzer RL, Williams JBW. The PHQ-9: Validity of a brief depression severity measure. *J Gen Intern Med*. (2001) 16:606–13. doi: 10.1046/j.1525-1497.2001.016009606.x
39. Bakker A, van der Meer CAI, Olff M. *Primary care PTSD screen for DSM-5 (PC-PTSD-5)– Nederlandstalige versie*. Amsterdam: Academisch Medisch Centrum (2014).
40. AlleCijfers.nl. *Statistieken over het Coronavirus en COVID-19*. AlleCijfers.nl (2021). Available online at: https://allecijfers.nl/nieuws/statistieken-over-het-corona-virus-en-covid19/ (accessed August 27, 2021).
41. Ferguson KD, McCann M, Katikireddi SV, Thomson H, Green MJ, Smith DJ, et al. Evidence synthesis for constructing directed acyclic graphs (ESC-DAGs): a novel and systematic method for building directed acyclic graphs. *Int J Epidemiol*. (2020) 49:322–9. doi: 10.1093/ije/dyz150
42. Textor J, van der Zander B, Gilthorpe MS, Liśkiewicz M, Ellison GT. Robust causal inference using directed acyclic graphs: The R package "dagitty". *Int J Epidemiol*. (2016) 45:1887–94. doi: 10.1093/ije/dyw341
43. Raghunathan T, Lepkowski J, Van Hoewyk J, Solenberger P. A multivariate technique for multiply imputing missing values using a sequence of regression models. *Surv Methodol*. (2001) 27:85–96.

44. Graham JW, Olchowski AE, Gilreath TD. How many imputations are really needed? some practical clarifications of multiple imputation theory. *Prev Sci.* (2007) 8:206–13. doi: 10.1007/s11121-007-0070-9
45. Schafer JL. Multiple imputation in multivariate problems when the imputation and analysis models differ. *Stat Neerl.* (2003) 57:19–35. doi: 10.1111/1467-9574.00218
46. Stuart EA, Azur M, Frangakis C, Leaf P. Multiple imputation with large data sets: a case study of the children's mental health initiative. *Am J Epidemiol.* (2009) 169:1133–9. doi: 10.1002/mpr.329
47. Moro MF, Calamandrei G, Poli R, Mattei V Di, Perra A, Kurotschka PK, et al. The impact of the COVID-19 pandemic on the mental health of healthcare workers in Italy: analyzing the role of individual and workplace-level factors in the reopening phase after lockdown. *Front Psychiatry Forthcoming.* (2022).
48. Li Y, Scherer N, Felix L, Kuper H. Prevalence of depression, anxiety and posttraumatic stress disorder in health care workers during the COVID-19 pandemic: a systematic review and meta-analysis. *PLoS One.* (2021) 16:e0246454. doi: 10.1371/journal.pone.0246454
49. Wu T, Jia X, Shi H, Niu J, Yin X, Xie J, et al. Prevalence of mental health problems during the COVID-19 pandemic: a systematic review and meta-analysis. *J Affect Disor.* (2021) 281:91–8. doi: 10.1016/j.jad.2020.11.117
50. Cénat JM, Blais-Rochette C, Kokou-Kpolou CK, Noorishad PG, Mukunzi JN, McIntee SE, et al. Prevalence of symptoms of depression, anxiety, insomnia, posttraumatic stress disorder, and psychological distress among populations affected by the COVID-19 pandemic: a systematic review and meta-analysis. *Psychiatry Res.* (2021) 295:113599. doi: 10.1016/j.psychres.2020.113599
51. Marvaldi M, Mallet J, Dubertret C, Moro MR, Guessoum SB. Anxiety, depression, trauma-related, and sleep disorders among healthcare workers during the COVID-19 pandemic: a systematic review and meta-analysis. *Neurosci Biobehav Rev.* (2021) 126:252–64. doi: 10.1016/j.neubiorev.2021.03.024
52. Santomauro DF, Mantilla Herrera AM, Shadid J, Zheng P, Ashbaugh C, Pigott DM, et al. Global prevalence and burden of depressive and anxiety disorders in 204 countries and territories in 2020 due to the COVID-19 pandemic. *Lancet.* (2021) 6736:21. doi: 10.1016/S0140-6736(21)02143-7
53. Danese A, Widom CS. Objective and subjective experiences of child maltreatment and their relationships with psychopathology. *Nat Hum Behav.* (2020) 4:811–8. doi: 10.1038/s41562-020-0880-3
54. Sharma M, Creutzfeldt CJ, Lewis A, Patel P V, Hartog C, Jannotta GE, et al. Health-care professionals' perceptions of critical care resource availability and factors associated with mental well-being during coronavirus disease 2019 (COVID-19): Results from a US survey. *Clin Infect Dis.* (2021) 72:e566–76. doi: 10.1093/cid/ciaa1311
55. Fang M, Xia B, Tian T, Hao Y, Wu Z. Drivers and mediators of healthcare workers' anxiety in one of the most affected hospitals by COVID-19: a qualitative analysis. *BMJ Open.* (2021) 11:e045048. bmjopen-2020-045048 doi: 10.1136/bmjopen-2020-045048
56. Xiong J, Lipsitz O, Nasri F, Lui LMW, Gill H, Phan L. Impact of COVID-19 pandemic on mental health in the general population: a systematic review. *J Affect Disord.* (2020) 277:55–64. doi: 10.1016/j.jad.2020.08.001
57. Dobbie M, Mellor D. Chronic illness and its impact: considerations for psychologists. *Psychol Heal Med.* (2008) 13:583–90. doi: 10.1080/13548500801983041

**Conflict of Interest:** The authors declare that the research was conducted in the absence of any commercial or financial relationships that could be construed as a potential conflict of interest.

**Publisher's Note:** All claims expressed in this article are solely those of the authors and do not necessarily represent those of their affiliated organizations, or those of the publisher, the editors and the reviewers. Any product that may be evaluated in this article, or claim that may be made by its manufacturer, is not guaranteed or endorsed by the publisher.

*Copyright © 2022 Czepiel, Hoek, van der Markt, Rutten, Veling, Schirmbeck, Mascayano, Susser and van der Ven. This is an open-access article distributed under the terms of the Creative Commons Attribution License (CC BY). The use, distribution or reproduction in other forums is permitted, provided the original author(s) and the copyright owner(s) are credited and that the original publication in this journal is cited, in accordance with accepted academic practice. No use, distribution or reproduction is permitted which does not comply with these terms.*
