# Supplementary material for: Mental health status and related factors influencing healthcare workers during the COVID-19 pandemic: A systematic review and meta-analysis
Source: PLoS One. 2024 Jan 19;19(1):e0289454. doi: 10.1371/journal.pone.0289454 (PMC10798549; doi:10.1371/journal.pone.0289454)
Supplement: S1 Data — (ZIP) [file pone.0289454.s011.zip › literatures/35.pdf]

ELSEVIER

Since January 2020 Elsevier has created a COVID-19 resource centre with free information in English and Mandarin on the novel coronavirus COVID-19. The COVID-19 resource centre is hosted on Elsevier Connect, the company's public news and information website.

Elsevier hereby grants permission to make all its COVID-19-related research that is available on the COVID-19 resource centre - including this research content - immediately available in PubMed Central and other publicly funded repositories, such as the WHO COVID database with rights for unrestricted research re-use and analyses in any form or by any means with acknowledgement of the original source. These permissions are granted for free by Elsevier for as long as the COVID-19 resource centre remains active.

International Journal of Africa Nursing Sciences 17 (2022) 100442

Contents lists available at ScienceDirect

International Journal of Africa Nursing Sciences

journal homepage: www.elsevier.com/locate/ijans

Psychological impact of coronavirus disease on nurses exposed and non-exposed to disease

Ayat Da'seh [a,*], Osama Obaid [b], Mohammad Rababa [c]

[a] Applied Science Department, Al-Balqa Applied University, Aqaba, Jordan
[b] Department of Mental Health, Royal Medical Services, Amman, Jordan
[c] Department of Adult Health Nursing, Faculty of Nursing, Jordan University of Science and Technology, Irbid, Jordan

ARTICLE INFO

Keywords:
COVID-19
Nurses
Anxiety
Stress
Post-traumatic stress
Depression
And insomnia

ABSTRACT

Introduction: Nurses who have direct contact with patients with coronavirus disease 19 (COVID-19) and are involved in diagnosis, treatment, and care are at risk for serious psychological health problems.
Purpose: To examine the psychological impact of COVID-19 on nurses who are in direct contact with COVID-19 patients and compared them with other nurses, not in direct contact with COVID-19 patients.
Methods: A descriptive comparative cross-sectional was conducted on a convenience sample of 364 nurses working at three hospitals in Jordan to collect their socio-demographic data and scores on the Depression, Anxiety Stress Scale, 22-item Impact of Event Scale-Revised, and Insomnia Severity Index via Google form questionnaires. Descriptive analysis, Kruskal-Wallis test, independent *t*-test, and multivariable logistic regression with a significance level of p-value < 0.05 were used to analyze the study data.
Results: Overall, the prevalence rates of depression, anxiety, stress, insomnia, and post-traumatic stress symptoms were 34.1%, 48.9%, 44%, 33.8%, and 67.3%, respectively. Depression, anxiety, stress, and insomnia were significantly more prevalent in the exposed group of nurses than in the non-exposed ones. However, no significant difference was found between the groups regarding post-traumatic stress symptoms. Exposure to COVID-19 and the existence of comorbidities were associated with an increased risk of anxiety, depression, insomnia, and stress.
Conclusion: Nurses who have direct contact with COVID-19 patients have a higher risk of psychological disorders than nurses who do not. Psychological interventions need to be implemented to enhance nurses' psychological well-being.

1. Introduction

Several cases of inexplicable pneumonia were discovered in Wuhan, China, at the end of 2019. Soon after, Wuhan reported an outbreak. These cases have been epidemiologically linked to the seafood market in Wuhan that sells different live animals (Wuhan Municipal Health Commission, 2019). Unexplained pneumonia was studied by Chinese scientists via samples collected from the lower respiratory tract throughout bronchoalveolar lavage and documented a new type of coronavirus (Gorbalenya et al., 2020; Zhu et al., 2020). Later, the World Health Organization (WHO) designated this virus as coronavirus disease 19 (COVID-19), owing to its genetic resemblance to the coronavirus responsible for the 2003 SARS-CoV-2 outbreak (Chan et al., 2003; Zhang et al., 2020).

COVID-19 was spreading domestically and soon became an international pandemic, inducing the most extraordinary public health emergency in modern history (Chan et al., 2003). > 243 million confirmed cases and over 4.9 million deaths have been reported until October 2021 (dos Santos Ferreira et al., 2021). In addition to the physical impact of the COVID-19 pandemic, a psychological impact was also reported in the general population due to changes in daily lifestyle and restrictions on social activities (Serafini et al., 2020). Previously, the psychological impact of SARS and H1N1 pandemics on both healthcare workers and the general population was reported in the literature (Brooks et al., 2020; Lu, Shu, & Chang, 2006). Most of the healthcare workers involved in providing care for those patients experienced many stressors, including workload, insufficient supplies, fear of infecting themselves or others, stigma, and financial loss (Brooks et al., 2020). In

* Corresponding author at: School of Nursing, Al-Balqa Applied University, Amman, Jordan.
E-mail address: ayat.dasa@bau.edu.jo (A. Da'seh).

https://doi.org/10.1016/j.ijans.2022.100442
Received 28 March 2022; Received in revised form 29 May 2022; Accepted 9 June 2022
Available online 11 June 2022
2214-1391/© 2022 The Authors. Published by Elsevier Ltd. This is an open access article under the CC BY license (http://creativecommons.org/licenses/by/4.0/).

Saudi Arabia in 2015, healthcare professionals who had survived the Middle East respiratory syndrome coronavirus (MERS-CoV) epidemic described a variety of feelings of avoidance and rejection by their colleagues and the surrounding community after their recovery period (Almutairi, Adlan, Balkhy, Abbas, & Clark, 2018). Given the SARS-CoV-2 pandemic, depression, distress, anxiety, and sleep disturbances were prevalent among healthcare professionals (Bahamdan, 2021).

Frontline healthcare workers, particularly nurses, are prone to emotional instability because they are dealing with proven cases of COVID-19 (Spoorthy, Pratapa, & Mahant, 2020). A cross-sectional study in Jordan investigated the psychological well-being of frontline healthcare professionals and discovered a significant prevalence of stress, anxiety, and depression (53.8, 52.9, and 66.2%, respectively) (Odhong et al., 2019). Age, gender, experience, availability of personal protection equipment, resilience, and maladaptive coping were correlated with the psychological problems among healthcare workers (Odhong et al., 2019; Pappa et al., 2020; Spoorthy et al., 2020).

Stress reaction affects the mood, cognition, interpersonal relationships, and physical health of frontline healthcare workers during pandemics (Lan, 2003), and each individual psychologically reacts differently (Horowitz, 1993). By that time, some of them reserved their previous symptoms, while others retained their previous level of function and health. Those who could shift their stress into eustress showed an improvement in their resilience. Consequently, their ability to recover and adjust could be enhanced (Fink, 2016). Moreover, McFarlane reported that factors such as support system, personality traits, and coping ability may have an important role in prognosis (Fink, 2016).

The current study aimed at evaluating the psychological impact of COVID-19 on nurses who are in direct contact with infected patients and compared them with other nurses, not in direct contact with infected patients. The psychological impact was measured by estimating symptoms of anxiety, depression, stress, post-traumatic stress symptoms, and insomnia. Our findings, hopefully, raise awareness of the need for psychological assistance services, particularly among nurses who have been exposed to psychosocial risk factors in the past. These kinds of services for nurses caring for COVID-19 patients, on the other hand, are relatively limited.

2. Method

2.1. Study design and participants

A descriptive comparative cross-sectional design was adopted in a multicenter investigation. A convenient sample of 425 nurses working at the Queen Alia Military Hospital, King Hussien Medical Center, and Prince Hashim Military Hospital were invited to participate in the study. The eligible participants were all nurses working in the selected hospitals whether caring for COVID-19 patients or not. Three hundred and sixty-four nurses were recruited for the study (response rate = 85.5%.). The sample size was calculated based on G*Power analysis by using A Priori Sample Size Calculator. Given the following statistical parameters: a significance level of 0.05, a level of power equal to 0.8, two study groups, and an expected Cohen's effect size of 0.5, the sample size was sufficient for yielding significant statistical tests.

2.2. Data collection

After obtaining ethical approval to conduct the study and before data collection, the author met with the administrator of the selected hospitals, discussed the eligibility criteria, and got a list of all eligible nurses with their contact information including phone numbers. The author sent a WhatsApp message containing a brief overview of the study and a link to the Google form of study questionnaires. Also, in the message, nurses were asked to give their consent to participate before filling out the study questionnaires. The data were collected from August 15, 2021, to October 2, 2021.

2.3. Study measures

2.3.1. Outcome measures

Depression, Anxiety Stress Scale (DASS). The DASS (Lovibond & Lovibond, 1995) is a 42-item self-reported tool developed to measure the three related negative psychological states of depression, anxiety, and stress which are defined operationally as emotional difficulties such as hopelessness, situational anxiety, and irritability experienced through a previous week. Nurses were asked to indicate the existence of symptoms throughout the previous week on a four-point Likert scale ranging from 0 to 3 (0= "did not apply at all over the last week" to 3= "applied very much"). The higher scores in each dimension indicate the more severe symptom. In the present study, scores from each dimension were summed up and classified as "normal", "mild", "moderate", "severe" and "extremely severe" according to the DASS manual. Several previous studies have demonstrated satisfactory reliability and validity scores of the DASS (Lovibond & Lovibond, 1995). In the current study, the DASS had a satisfactory score of internal consistency reliability of Cronbach's alpha equal to 0.83.

Insomnia Severity Index (ISI). The ISI (Morin, 1993) was used in this study to measure the severity of insomnia which is defined operationally as the extent of sleep problems experienced throughout the previous month. The ISI has seven questions rated on a 5-point Likert scale ranging from 0= "no problem" to 4= "very severe problem". The score of the responses to the seven questions was added up to get a total score. The total score of ISI was classified as normal (0–7), sub-threshold (8–14), moderate (15–21), and severe (22–28) insomnia (Bastien, Vallieres, & Morin, 2001). Several previous studies have demonstrated satisfactory reliability and validity scores of the ISI (Bastien et al., 2001). In the current study, the ISI had a satisfactory score of internal consistency reliability of Cronbach's alpha equal to 0.79.

The 22-item Impact of Event Scale-Revised (IES-R). The IES-R (Horowitz, Wilner, & Alvarez, 1979) is a 22-item self-reported measure that assesses subjective distress caused by traumatic events, and the severity of symptoms and is used for the diagnosis of post-traumatic stress disorder (PTSD). The impact of traumatic events is defined operationally as physical and psychosocial difficulties experienced after stressful events. Items were rated on a 5-point scale, ranging from 0 (not at all) to 4 (extremely). The IES-R yields a total score (ranging from 0 to 88), and subscale scores can also be calculated for the intrusion, avoidance, and hyperarousal subscales. IES-R is classified as normal (0–8), mild (9–25), moderate (26–43), and severe (44–88) distress. Several previous studies have demonstrated satisfactory reliability and validity scores of the IES-R22 (Horowitz et al., 1979). In the current study, the IES-R22 had a satisfactory score of internal consistency reliability of Cronbach's alpha equal to 0.80.

2.3.2. Independent measures

Being exposed to COVID-19 patients or not. Two groups of nurses were involved in the study: nurses who were providing care for COVID-19 patients in isolation units (the exposed group) and nurses in other units who were not providing care for COVID-19 patients (the non-exposed group).

2.3.3. Other measures

Demographical Data. Demographic characteristics including age, gender, marital status, working hospital, years of experience, duration of clinical experience, and presence of comorbid conditions were collected by asking the nurses to fill out a self-reported demographic questionnaire.

2.4. Ethical consideration

All data collected were confidential and for research purposes only. Confidentiality and anonymity were protected by using code numbers instead of the participant's name. No identifying information was

written on the data collection sheets. The researchers kept the data collection sheets in a locked and secured office accessible only to them. Also, the collected data was saved in a password-protected computer accessible only to the researchers. This study was approved by the Ethics Review Board at Royal Medical Services. Informed consent, including study title, study aim, consequences, and participant rights, was obtained from each participant before answering the questionnaires.

2.5. Statistical analysis

The statistical software for social science (SPSS) version 22 was used to code and analyze the data. Data were examined using descriptive analysis to meet the study's goals. To examine socio-demographic variables of nurses, DASS, ISI scale, and IES-R scale as applicable, mean, standard deviation, frequency, and percentage were employed. To compare variables between the two groups, the Kruskal-Wallis test, and independent *t*-test were used as needed. In addition, multivariable logistic regression was utilized to adjust for some expected confounders of age, gender, presence of comorbid conditions, marital status, and duration of clinical experience to identify potential risk factors for depression, anxiety, insomnia, and PTSD symptoms in the study participants.

3. Results

3.1. Socio-demographic characteristics of participants

A total of 364 nurses participated in the study, yielding a response rate of 85.5%. The mean age of the participants was 30 years with SD ± 3.53 and the mean duration of clinical experience was 6.86 with SD ± 3.93. The majority of the participants were females 227 (62.4%), and most of them were single 219 (60.2%). Among all, 332 (91.2%) participants had a baccalaureate degree, and 32 (8.8%) participants had a master's degree in nursing. The majority of the participants were healthy without any comorbidity (90.4%).

Participants were categorized into two groups based on their COVID-19 exposure (exposed and non-exposed group). Non-exposed groups accounted for 175 participants, while exposed groups accounted for 189. In terms of working hospitals and the prevalence of comorbidities, there were significant differences between groups ($P < 0.05$). The Queen Aliah Military Hospital was the only military hospital that only treated COVID-19 patients, whereas the other military hospitals treated COVID-19 patients as well as other types of patients. In the exposed group, 12.7 % of the participants had comorbidities, compared to 6.3 % in the non-exposed group. Please see Table 1.

3.2. Depression, anxiety, and stress scale

As seen in Table 2, in terms of depression, anxiety, and stress, there were significant differences between the exposed and non-exposed groups of participating nurses ($P < 0.001$). The proportions of mild, moderate, and severe depression in the exposed group (31.2%, 16.4%, and 4.8%, respectively) were higher than in the non-exposed group (11.4%, 2.9%, and 0%, respectively). The proportions of moderate and severe anxiety in the exposed group (24.9% and 7.9%, respectively) were higher than in the non-exposed group (7.4% and 1.7%, respectively). Moreover, the proportions of mild, moderate, and severe stress in the exposed group (31.7%, 22.2%, and 9.5, respectively) were higher than in the non-exposed group (16.6%, 5.1%, and 1.1%, respectively).

3.3. Insomnia severity index

In terms of insomnia severity, there were significant differences between the exposed and non-exposed groups of participating nurses ($p < 0.001$). The proportions of sub-threshold, moderate, and severe insomnia in the exposed group (30.2%, 16.4%, and 3.2%, respectively) were higher than in the non-exposed group (14.3%, 1.7%, and 0.6%, respectively). **Please see** Table 2**.**

3.4. Impact of event scale-revised

As seen in Table 2, there were no significant differences in the impact of the traumatic experience (PTSD symptoms) between the exposed and non-exposed groups of participating nurses ($p > 0.05$). Among all, 32.7% of nurses showed the minimal impact of the traumatic event, followed by 30.2%, 15.9%, and 21.2% of nurses showed the mild, moderate, and severe impact of the traumatic event, respectively.

Table 1
Socio-demographic characteristics of the participating nurses (N = 364).

Variables	Total (N = 364)		Non-exposed group (N = 175)		Exposed group (N = 189)		P value
	Frequency	%	Frequency	%	Frequency	%	
**Age (M = 30, SD = 3.53)**							
**<30 years**	232	63.7	111	63.4	121	64	0.907
**≥30 years**	132	36.3	64	36.6	68	36	
**Duration of clinical experience (M = 6.86, SD = 3.93)**							
**<10 years**	300	82.4	148	84.6	152	80.4	0.300
**≥10 years**	64	17.6	27	15.4	37	19.6	
**Gender**							
Male	137	37.6	64	36.6	73	38.6	0.687
Female	227	62.4	111	63.4	116	61.4	
**Marital status**							
Single	219	60.2	99	56.6	120	63.5	0.178
Married	145	39.8	76	43.4	69	36.5	
**Education**							
Baccalaureate	332	91.2	157	89.7	175	92.6	0.333
Master	32	8.8	18	10.3	14	7.4	
**Working hospital**							
KHMC	116	31.9	102	58.3	14	7.4	0.001
QAMH	147	40.4	0	0	147	77.8	
PRH	101	27.7	73	41.7	28	14.8	
**Presence of comorbidities**							
No	329	90.4	164	93.7	165	87.3	0.038
Yes	35	9.6	11	6.3	24	12.7	

Kruskal-Wallis test, SD: Standard deviation, KHMC: King Hussein Medical Center, QAMH: Queen Aliah Military Hospital.

Table 2
Depression, anxiety, stress, insomnia, and post-traumatic stress symptoms severity categories in the overall group and subgroups.

Variables	Total (N = 364)		Non-exposed group (N = 175)		Exposed group (N = 189)		P value
	Frequency	%	Frequency	%	Frequency	%	
**DASS-21 (Mean = 30.86, SD = 11.57)**							
**DASS-21 Depression (Mean = 8.67, SD = 4.30)**							
Normal	240	65.9	150	85.7	90	47.6	<0.001**
Mild	79	21.7	20	11.4	59	31.2	
Moderate	36	9.9	5	2.9	31	16.4	
Severe	9	2.5	0	0	9	4.8	
**DASS-21 Anxiety (Mean = 7.75, SD = 3.41)**							
Normal	186	51.1	107	61.1	79	41.8	<0.001**
Mild	100	27.5	52	29.7	48	25.4	
Moderate	60	16.5	13	7.4	47	24.9	
Severe	18	4.9	3	1.7	15	7.9	
**DASS-21 Stress (Mean = 14.43, SD = 5.40)**							
Normal	204	56.0	135	77.1	69	36.5	<0.001**
Mild	89	24.5	29	16.6	60	31.7	
Moderate	51	14.0	9	5.1	42	22.2	
Severe	20	5.5	2	1.1	18	9.5	
**ISI (Mean = 7.79, SD = 4.46)**							
Normal	241	66.2	146	83.4	95	50.3	<0.001**
Sub-threshold	82	22.5	25	14.3	57	30.2	
Moderate	34	9.3	3	1.7	31	16.4	
Severe	7	1.9	1	0.6	6	3.2	
**IES-R (Mean = 31.37, SD = 16.06)**							
Minimal	119	32.7	59	33.7	60	31.7	0.615**
Mild	110	30.2	52	29.7	58	30.7	
Moderate	58	15.9	30	17.1	28	14.8	
severe	77	21.2	34	19.4	43	22.8	
**Variable**	**Mean**	**SD**	**Mean**	**SD**	**Mean**	**SD**	
**IES-R subscales**							
Intrusion	9.85	5.35	9.61	5.08	10.0847	5.59	0.406*
Avoidance	10.58	5.67	10.28	5.31	10.8730	5.99	0.320*
Hyperarousal	10.92	5.71	10.38	5.41	11.4233	5.95	0.085*

*independent sample *t*-test, **Kruskal-Wallis test, DASS-21: The 21 items Depression, Anxiety Stress Scale-21, ISI: Insomnia Severity Index, IES-R: The 22-item Impact of Event Scale-Revised.

3.5. *Predictor of Nurses' depression, anxiety, stress, insomnia, and post-traumatic stress during the COVID-19 pandemic*

Multivariable logistic regression showed that being exposed to COVID-19 was linked to an increased risk of depression ($P < 0.001$) anxiety ($P < 0.001$), stress ($P < 0.001$) and insomnia ($P < 0.001$). Moreover, Multivariable logistic regression showed that, having comorbidities was linked to an increased risk of depression ($P = 0.004$) anxiety ($P = 0.027$), stress ($P < 0.001$) and insomnia ($P = 0.008$). Regarding post-traumatic stress symptoms, there were no significant associated factors found ($P > 0.05$). Moreover, multivariable logistic regression did not show any significant associations of age, gender, duration of clinical experience, marital status, education, working hospital with nurses' depression, anxiety, stress, insomnia, and post-traumatic stress during the COVID-19 pandemic. Please see Table 3.

4. Discussion

This is the first study conducted in Jordan that compared the psychological impacts of the COVID-19 pandemic between nurses who cared for COVID-19 patients (the exposed group) and those who did not care for COVID-19 patients (the non-exposed group). In terms of depression, anxiety, stress, and insomnia, there was a significant difference between the exposed and non-exposed groups ($P < 0.001$). In contrast, there was no significant difference between the aforementioned groups regarding post-traumatic stress symptoms ($P > 0.05$). Overall, the prevalence of depression, anxiety, stress, insomnia, and post-traumatic stress symptoms was 34.1%, 48.9%, 44%, 33.8%, and 67.3%, respectively (including mild, moderate, and severe levels), indicating that the COVID-19 pandemic has had a significant impact on nurses' psychological well-being. Data analysis showed that the

Table 3
Predictors of nurses' depression, anxiety, stress, insomnia, and post-traumatic stress during the COVID-19 pandemic.

Variables	OR	95% CI	P value
**DASS-21 Depression**			
**Exposure**			
Exposed	3.421	2.605–4.236	< 0.001
Non-exposed	1	1	
**Presence of comorbidities**			
No	1	1	
Yes	2.163	0.715–3.610	0.004
**DASS-21 Anxiety**			
**Exposure**			
Exposed	1.974	1.301–2.647	< 0.001
Non-exposed	1	1	
**Presence of comorbidities**			
No	1	1	
Yes	1.351	0.156–2.545	0.027
**DASS-21 Stress**			
**Exposure**			
Exposed	4.304	3.307 – 5.300	< 0.001
Non-exposed	1	1	
**Presence of comorbidities**			
No	1	1	
Yes	4.065	2.297 – 5.833	< 0.001
**ISI insomnia**			
**Exposure**			
Exposed	2.853	1.979 – 3.726	< 0.001
Non-exposed	1	1	
**Presence of comorbidities**			
No	1	1	
Yes	2.082	0.537 – 3.637	0.008

OR: odds ratio; CI: confidence interval; DASS-21: The 21 items Depression, Anxiety Stress Scale-21, ISI: Insomnia Severity Index.

proportion of "mild level" was higher than that of the "moderate level" and "severe level" in both groups of nurses.

Our study found a lower rate of depression, anxiety, and stress than a recent study did among Jordanian healthcare professionals during the COVID-19 pandemic (Odhong et al., 2019). This finding might be attributed to the variations in sample size and study settings between the previous study and ours. On the contrary, in our study, the prevalence of post-traumatic stress symptoms was 67.3%, nearly twice as high as the prevalence of post-traumatic stress symptoms among nurses in a prior study conducted in Jordan during the COVID-19 pandemic (Qutishat, Sharour, Al-Dameery, Al-Harthy, & Al-Sabei, 2021). This finding might be attributed to the fact that as the pandemic spreads and the number of infected cases and deaths rises, the prevalence of post-traumatic stress symptoms among nurses in our country will rise as well. A multi-center cross-sectional study conducted in China in 2020 supports this viewpoint (Xiao et al., 2020). Psychological outcomes, in particular, depressive symptoms and post-traumatic stress symptoms can be long-lasting. During previous outbreaks, several cases showed depressive symptoms and post-traumatic stress symptoms within a period ranging from 6 months to 3 years after the outbreak (Liu et al., 2012; Tang, Pan, Yuan, & Zha, 2017; Wu et al., 2009). The prevalence of insomnia in our study was within the range reported in the literature (Sahebi, Abdi, Moayedi, Torres, & Golitaleb, 2021). Unfortunately, insomnia as a psychosomatic symptom also tends to be progressive, as reported during the SARS outbreak (Su et al., 2007).

In line with the previous studies (Arcadi et al., 2021; Serrano-Ripoll et al., 2020), insufficient personal protective equipment (PPEs), feeling unsafe during duty, and fear of the unknown, which are the case when caring for COVID-19 patients, are all recognized as contributing factors that negatively affect nurses' mental health during the pandemic. Given the aforementioned factors, urgent decisions should be made and implemented to assure the availability of personal protective equipment (Kim & Choi, 2016) in addition to providing psychological support by an expert psychologist (Pappa et al., 2020). Furthermore, psychological support is an important protective measure targeting poor mental health outcomes and improving nurses' resilience, as evidenced by the findings of an interventional research study conducted during prior infectious disease outbreaks (Pollock et al., 2020). These findings emphasize the significant role of nursing managers in proposing a proportion of their hospital's budget for providing them with protective equipment and funding training projects for nurses on how to rehabilitate their psychological well-being. The findings would be a convincing message to the nurse manager and health policymakers that an expert psychologist should be assigned to each unit where COVID-19 patients are admitted providing the required psychological support for nurses.

Multivariate logistic regression showed that nurses' exposure to COVID-19 patients was significantly associated with a higher prevalence of depression, anxiety, stress, and insomnia than the non-exposed nurse group ($P < 0.05$). A similar result was reported by Lai et al., who found that direct exposure to COVID-19 patients was associated with severe levels of depression, anxiety, and insomnia scores (Lai et al., 2020). Furthermore, a cross-sectional study in China found that medical personnel who had direct contact with COVID-19 patients had nearly twice the risk of anxiety and depression as administrative staff who had little or no contact with COVID-19 patients. (Lu, Wang, Lin, & Li, 2020). This finding is consistent with a previous study examining the impact of exposure to patients with SARS on nurses' psychological well-being (Grace, Hershenfield, Robertson, & Stewart, 2005). In contrast, Li et al. reported conflicting results (Holmes et al., 2020). Possible clarifications for this inconsistent finding may be attributed to the better accessibility to psychological support for the nurses providing direct care for COVID-19 patients, getting updated information on the pandemic, and availability of PPEs for the exposed group of nurses (Tan et al., 2020). These findings highlighted the significance of providing psychological support, evidence-based information, and required protective measures to maintain the psychological well-being of nurses caring for COVID-19 patients, thus improving their quality of care. Consistently, according to a recent study (Liu, Zhang, Hennessy, Zhao, & Ji, 2019), the quality of care provided to COVID patients is negatively impacted by the extent of nurses' psychological well-being deterioration.

Our study further indicated that being in direct contact with COVID-19 patients and having comorbidities were associated with experiencing depression, anxiety, stress, and insomnia. This might be due to the nature of the chronic illnesses and the liability for developing serious complications due to the COVID-19 infection (Zhou et al., 2020). Fears about infection-related problems among nurses with comorbidities may raise the likelihood of acquiring anxiety. Studies undertaken in China were consistent with this conclusion (Xiao et al., 2020; Zhu et al., 2020). This finding highlights the importance of effective management of comorbidities and frequent and strict monitoring of their complications among nurses especially those caring care for COVID-19 patients.

4.1. *Recommendations for future research*

Replication studies with a larger sample size are needed to validate the findings of this study. The findings of this study would guide future intervention studies designed to relieve nurses' psychological distress associated with caring for COVID-19 patients. For example, the results of our study inform conducting an intervention study examining the effectiveness of psychotherapy interventions in improving the psychological well-being of nursing caring for COVID-19 patients.

4.2. *Limitations*

Several limitations were kept in mind during the interpretation of our findings. The present study is a cross-sectional study; therefore, no causal relationship could be made regarding factors associated with the psychological outcomes of the COVID-19 pandemic. Given the convenience sampling procedure, generalizability to all Jordanian nurses must be done with caution. Therefore, the possibility of certain bias occurrences, such as self-selection and social desirability biases, might have influenced the present study's findings. Longitudinal studies with a larger sample size that cover various areas of Jordan are needed to confirm our findings.

5. Conclusion

According to the current study, nurses who work directly with COVID-19 patients had a higher risk of psychological issues, such as depression, anxiety, stress, and insomnia, than nurses who do not work directly with COVID-19 patients, according to the current study. Early psychological interventions need to be implemented to enhance their mental well-being. Further studies that adopt longitudinal designs are required to determine the true causality relationship.

Funding source

This research did not receive any specific grant from funding agencies in the public, commercial, or not-for-profit sectors.

7. Availability of data

Datasets used in this analysis are available from the corresponding author upon reasonable request.

Acknowledgment

None.

Ethical approval

This study was approved by the Ethics Review Board at Royal Medical Services.

References

Almutairi, A. F., Adlan, A. A., Balkhy, H. H., Abbas, O. A., & Clark, A. M. (2018). "It feels like I'm the dirtiest person in the world".: Exploring the experiences of healthcare providers who survived MERS-CoV in Saudi Arabia. *Journal of Infection And Public Health, 11*(2), 187–191.

Arcadi, P., Simonetti, V., Ambrosca, R., Cicolini, G., Simeone, S., Pucciarelli, G., … Durante, A. (2021). Nursing during the COVID-19 outbreak: A phenomenological study. *Journal of Nursing Management, 29*(5), 1111–1119.

Bahamdan, A. S. (2021). Review of the psychological impact of COVID-19 pandemic on healthcare workers in Saudi Arabia. *Risk Management and Healthcare Policy, 14*, 4105.

Bastien, C. H.,, Vallieres, A., & Mocrin, C. M. (2001). Validation of the Insomnia Severity Index as an outcome measure for insomnia research. *Sleep Med, 2*, 297–307.

Brooks, S. K., Webster, R. K., Smith, L. E., Woodland, L., Wessely, S., Greenberg, N., & Rubin, G. J. (2020). El impacto psicológico de la cuarentena y cómo reducirla: revisión rápida de las pruebas. *Lancet, 395*, 912–920.

Chan, J. W. M., Ng, C. K., Chan, Y. H., Mok, T. Y. W., Lee, S., Chu, S. Y. Y., … Li, P. C. K. (2003). Short term outcome and risk factors for adverse clinical outcomes in adults with severe acute respiratory syndrome (SARS). *Thorax, 58*(8), 686–689.

dos Santos Ferreira, C. E., Gómez-Dantés, H., Junqueira Bellei, N. C., López, E., Nogales Crespo, K. A., O'Ryan, M., & Villegas, J. (2021). The role of serology testing in the context of immunization policies for COVID-19 in Latin American countries. *Viruses, 13*(12), 2391.

Fink, G. (2016). Stress, definitions, mechanisms, and effects outlined: Lessons from anxiety. In *Stress: Concepts, cognition, emotion, and behavior* (pp. 3–11). Academic Press.

Gorbalenya, A. E., Baker, S. C., Baric, R., Groot, R. J. D., Drosten, C., Gulyaeva, A. A., ... & Ziebuhr, J. (2020). Severe acute respiratory syndrome-related coronavirus: The species and its viruses–a statement of the Coronavirus Study Group. https://doi.org/10.1038/s41564-020-0695-z.

Grace, S. L., Hershenfield, K., Robertson, E., & Stewart, D. E. (2005). The occupational and psychosocial impact of SARS on academic physicians in three affected hospitals. *Psychosomatics, 46*(5), 385–391.

Holmes, E. A., O'Connor, R. C., Perry, V. H., Tracey, I., Wessely, S., Arseneault, L., … Bullmore, E. (2020). Multidisciplinary research priorities for the COVID-19 pandemic: A call for action for mental health science. *The Lancet Psychiatry, 7*(6), 547–560.

Horowitz, M. J. (1993). Stress-response syndromes. *International handbook of traumatic stress syndromes*, 49–60.

Horowitz, M., Wilner, N., & Alvarez, W. (1979). Impact of Event Scale: A measure of subjective stress. *Psychosomatic medicine, 41*(3), 209–218.

Kim, J. S., & Choi, J. S. (2016). Factors influencing emergency nurses' burnout during an outbreak of Middle East Respiratory Syndrome Coronavirus in Korea. *Asian Nursing Research, 10*(4), 295–299.

Lai, J., Ma, S., Wang, Y., Cai, Z., Hu, J., Wei, N., … Hu, S. (2020). Factors associated with mental health outcomes among health care workers exposed to coronavirus disease 2019. *JAMA Network Open, 3*(3), e203976–e.

Lan, P. (2003). *Stress management: A total approach*. Youth Cultural Company: Taipei.

Liu, X., Kakade, M., Fuller, C. J., Fan, B., Fang, Y., Kong, J., … Wu, P. (2012). Depression after exposure to stressful events: Lessons learned from the severe acute respiratory syndrome epidemic. *Comprehensive Psychiatry, 53*(1), 15–23.

Liu, Y., Zhang, J., Hennessy, D. A., Zhao, S., & Ji, H. (2019). Psychological strains, depressive symptoms, and suicidal ideation among medical and non-medical staff in urban China. *Journal of Affective Disorders, 245*, 22–27.

Lovibond, P. F., & Lovibond, S. H. (1995). The structure of negative emotional states: Comparison of the Depression Anxiety Stress Scales (DASS) with the Beck Depression and Anxiety Inventories. *Behaviour Research and Therapy, 33*(3), 335–343.

Lu, W., Wang, H., Lin, Y., & Li, L. (2020). Psychological status of medical workforce during the COVID-19 pandemic: A cross-sectional study. *Psychiatry Research, 288*, Article 112936.

Lu, Y. C., Shu, B. C., & Chang, Y. Y. (2006). The mental health of hospital workers dealing with severe acute respiratory syndrome. *Psychotherapy and Psychosomatics, 75*(6), 370–375.

Morin, C. M. (1993). *Insomnia: Psychological assessment and management*. Guilford press.

Odhong, C., Wilkes, A., van Dijk, S., Vorlaufer, M., Ndonga, S., Sing'ora, B., & Kenyanito, L. (2019). Financing large-scale mitigation by smallholder farmers: What roles for public climate finance?. In *Frontiers in Sustainable Food Systems* (p. 3).

Pappa, S., Ntella, V., Giannakas, T., Giannakoulis, V. G., Papoutsi, E., & Katsaounou, P. (2020). Prevalence of depression, anxiety, and insomnia among healthcare workers during the COVID-19 pandemic: A systematic review and meta-analysis. *Brain, Behavior, and Immunity, 88*, 901–907.

Pollock, A., Campbell, P., Cheyne, J., Cowie, J., Davis, B., McCallum, J., … Maxwell, M. (2020). Interventions to support the resilience and mental health of frontline health and social care professionals during and after a disease outbreak, epidemic or pandemic: A mixed-methods systematic review. *Cochrane Database of Systematic Reviews, 11*, CD013779. https://doi.org/10.1002/14651858CD013779

Qutishat, M., Sharour, L. A., Al-Dameery, K., Al-Harthy, I., & Al-Sabei, S. (2021). COVID-19–Related Posttraumatic Stress Disorder Among Jordanian Nurses During the Pandemic. *Disaster Medicine and Public Health Preparedness*, 1-8. Advance online publication. https://doi.org/10.1017/dmp.2021.199.

Sahebi, A., Abdi, K., Moayedi, S., Torres, M., & Golitaleb, M. (2021). The prevalence of insomnia among health care workers amid the COVID-19 pandemic: An umbrella review of meta-analyses. *Journal of Psychosomatic Research, 149*, Article 110597.

Serafini, G., Parmigiani, B., Amerio, A., Aguglia, A., Sher, L., & Amore, M. (2020). The psychological impact of COVID-19 on the mental health in the general population. QJM: monthly journal of the Association of Physicians, 113(8), 531–537. Advance online publication. https://doi.org/10.1093/qjmed/hcaa201.

Serrano-Ripoll, M. J., Meneses-Echavez, J. F., Ricci-Cabello, I., Fraile-Navarro, D., Fiol-deRoque, M. A., Pastor-Moreno, G., … Gonçalves-Bradley, D. C. (2020). Impact of viral epidemic outbreaks on mental health of healthcare workers: A rapid systematic review and meta-analysis. *Journal of Affective Disorders, 277*, 347–357.

Spoorthy, M. S., Pratapa, S. K., & Mahant, S. (2020). Mental health problems faced by healthcare workers due to the COVID-19 pandemic–A review. *Asian Journal of Psychiatry, 51*, Article 102119.

Su, T. P., Lien, T. C., Yang, C. Y., Su, Y. L., Wang, J. H., Tsai, S. L., & Yin, J. C. (2007). Prevalence of psychiatric morbidity and psychological adaptation of the nurses in a structured SARS caring unit during outbreak: A prospective and periodic assessment study in Taiwan. *Journal of Psychiatric Research, 41*(1–2), 119–130.

Tan, B. Y., Chew, N. W., Lee, G. K., Jing, M., Goh, Y., Yeo, L. L., … Sharma, V. K. (2020). Psychological impact of the COVID-19 pandemic on health care workers in Singapore. *Annals of Internal Medicine, 173*(4), 317–320.

Tang, L., Pan, L., Yuan, L., & Zha, L. (2017). Prevalence and related factors of post-traumatic stress disorder among medical staff members exposed to H7N9 patients. *International Journal of Nursing Sciences, 4*(1), 63–67.

Wuhan Municipal Health Commission. Report of clustering pneumonia of unknown etiology in Wuhan City. Wuhan, China: Wuhan Municipal Health Commission, December 31, 2019. Retrieved from http://wjw.wuhan.gov.cn/front/web/showDetail/2019123108989.

Wu, P., Fang, Y., Guan, Z., Fan, B., Kong, J., Yao, Z., … Hoven, C. W. (2009). The psychological impact of the SARS epidemic on hospital employees in China: Exposure, risk perception, and altruistic acceptance of risk. *The Canadian Journal of Psychiatry, 54*(5), 302–311.

Xiao, X., Zhu, X., Fu, S., Hu, Y., Li, X., & Xiao, J. (2020). Psychological impact of healthcare workers in China during COVID-19 pneumonia epidemic: A multi-center cross-sectional survey investigation. *Journal of Affective Disorders, 274*, 405–410.

Zhang, J., Wang, X., Jia, X., Li, J., Hu, K., Chen, G., … Dong, W. (2020). Risk factors for disease severity, unimprovement, and mortality in COVID-19 patients in Wuhan, China. *Clinical Microbiology and Infection, 26*(6), 767–772.

Zhou, F., Yu, T., Du, R., Fan, G., Liu, Y., Liu, Z., … Cao, B. (2020). Clinical course and risk factors for mortality of adult inpatients with COVID-19 in Wuhan, China: A retrospective cohort study. *The lancet, 395*(10229), 1054–1062.

Zhu, Z., Xu, S., Wang, H., Liu, Z., Wu, J., Li, G., … Wang, W. (2020). COVID-19 in Wuhan: Immediate psychological impact on 5062 health workers. *MedRxiv.*
