# Supplementary material for: Mental health status and related factors influencing healthcare workers during the COVID-19 pandemic: A systematic review and meta-analysis
Source: PLoS One. 2024 Jan 19;19(1):e0289454. doi: 10.1371/journal.pone.0289454 (PMC10798549; doi:10.1371/journal.pone.0289454)
Supplement: S1 Data — (ZIP) [file pone.0289454.s011.zip › literatures/36.pdf]

Prevalence of Occupational Burnout among Resident Doctors Working in Public Sector Hospitals in Mumbai

Archana Hemant Dhusia, Prita Abhay Dhaimade[1], Apurva Ambuj Jain[1], Samar Salim Shemna[2], Prerana Nirmal Dubey[2]

Department of Oral and Maxillofacial Surgery and Dentistry, HBT Medical College and Dr. R. N. Cooper Hospital, [1]Department of Oral and Maxillofacial Surgery and Dentistry, King Edward Memorial Hospital, [2]Department of Oral and Maxillofacial Surgery and Dentistry, Lokmanya Tilak Municipal General Hospital, Mumbai, Maharashtra, India

Abstract

Introduction: Burnout syndrome refers to a combination of physical fatigue and emotional exhaustion, which, in turn, affects the working efficiency of a person. In India, factors such as extensive working hours, poor facilities, and physical and emotional abuse of doctors by patients and seniors lead to the high prevalence of occupational burnout among medical practitioners. **Materials and Methods:** The sample consisted of 300 resident doctors working in public sector hospitals across Mumbai. The "Copenhagen Burnout Inventory" questionnaire was utilized to assess the prevalence of burnout. Questionnaires were made available personally or electronically. Burnout was recorded on three parameters, personal burnout, work-related burnout, and client-related burnout. **Results:** The average working hours recorded was 88 h/week. About 56.66% ($n = 170$) showed scores that indicate burnout. About 66.67% of respondents showed personal burnout, 57.14% had work-related burnout, and 16.67 had client-related burnout. **Conclusion:** The high prevalence of burnout syndrome among resident doctors in public sector hospitals is alarming as it not only takes a toll on the physical and mental health of the medical practitioners but also reduces their working efficiency and motivation. Stress management strategies should be propagated in hospitals to encourage work and personal life balance.

Keywords: Burnout syndrome, occupational burnout, physician burnout

Introduction

Burnout syndrome is a psychological state characterized by symptoms that broadly fall under the three distinct domains of emotional exhaustion, depersonalization or cynicism and reduced professional efficacy.[1,2] The concept of "burnout" was first introduced by Freudenberger and Maslach who independently studied the social issues faced by underprivileged citizens in the 1970s.[3,4] Although primarily seen in the industrial workers in that era, burnout syndrome soon came to be associated with masses engaging in human services and especially in the field of medicine.[5] A number of factors such as extensive working hours, low doctor-to-patient ratio and the expected moral responsibility contribute toward a stressful work environment for medical professionals.[6,7]

International scientific literature has acknowledged burnout syndrome as a threat to the medical fraternity and have developed a number of strategies to help doctors cope with it.[8-10] However, burnout has not been studied extensively in India and even less in the public sector hospitals. In the recent past, a number of other disturbing reasons have come to light that play a part in increasing work environment stress in public sector hospitals.[11] Moreover, it is an established notion that the prevalence of burnout syndrome is less in professionals with less experience and majority of studies exclude resident doctors or new graduates from the calculations. This notion may not be true, especially in public sector hospitals, where the doctor-to-patient ratio is widely deranged from ideal. Besides this, residents are known to be subjected to a number of factors predisposing to burnout like limited autonomy, excessive interference on the work front, lack of rest, sleep deprivation, extensive postcall clinical responsibilities, and professional liability.[12]

Access this article online

Quick Response Code:	
	**Website:** www.ijcm.org.in
	**DOI:** 10.4103/ijcm.IJCM_78_19

Address for correspondence: Dr. Prita Abhay Dhaimade, Department of Oral and Maxillofacial Surgery and Dentistry, King Edward Memorial Hospital, Mumbai, Maharashtra, India.
E-mail: pritaabhayd@gmail.com

This is an open access journal, and articles are distributed under the terms of the Creative Commons Attribution-NonCommercial-ShareAlike 4.0 License, which allows others to remix, tweak, and build upon the work non-commercially, as long as appropriate credit is given and the new creations are licensed under the identical terms.

For reprints contact: reprints@medknow.com

How to cite this article: Dhusia AH, Dhaimade PA, Jain AA, Shemna SS, Dubey PN. Prevalence of occupational burnout among resident doctors working in public sector hospitals in Mumbai. Indian J Community Med 2019;44:352-6.

Received: 22-02-19, **Accepted:** 29-08-19

This study means to explore the prevalence of symptoms of burnout syndrome among medical residents working in public sector hospitals regardless of their years of experience and assess the level of work life-personal life balance using a pre-validated self-assessed questionnaire scale.

Materials and Methods

The following study was a questionnaire-based survey that was carried out among resident doctors working in four public sector hospitals in Mumbai (HBT medical College and R. N. Cooper Hospital, Sion Hospital, King Edward Memorial Hospital, Topiwala Medical College, and Nair Hospital). All investigators were certified under good clinical practices. The study protocol was reviewed and validated by the Institutional Ethics Committee of HBT Medical College and R.N. Cooper General Hospital (Mumbai, Maharashtra, India) as of September 2019. The study was carried out in strict adherence to the Declaration of Helsinki. The duration of the study was from September "18 to January"19.

A randomized convenience sampling technique was utilized for sample size selection and data collection. Information was collected from 300 resident doctors working in clinical departments, casualty and intensive care units (ICUs), through voluntary completion of an anonymous, self-assessed, pre-validated questionnaire. Any response from participants not meeting the above criteria and incomplete forms were excluded from the study. The questionnaire was made available in print (paper) format and electronically through a link for a Google form. A brief description of the objectives of the study and the concept of physician burnout along with a link to the electronic form was electronically sent to consenting participants. Although the survey was kept anonymous, personal details relating to the participant's demographics, academic qualifications, working hours, and "on call" arrangements was recorded.

The Copenhagen Burnout Inventory (CBI) scale was utilized to assess the prevalence of burnout.[5] The CBI was fabricated in 2005 to overcome a number of drawbacks of the "Maslach Burnout Inventory" which is one of the most commonly used scale to assess burnout in different populations.[13,14] The questionnaire consists of nineteen questions divided into three subdimensions. Six questions about personal burnout that assess exhaustion regardless of occupational factors, seven questions on work burnout that emphasizes on the exhaustion attributed to work-related factors and finally six questions related to tiredness due to interactions with an appropriate population, in our case it was "patients." Each question has five answer choices, each assigned a numerical value. Scores are calculated individually in each subdomain and together to assess the prevalence of burnout.[5]

Results

Of the total 300 respondents, 62% (n = 186) were male and 38% (n = 114) were females. The average age of the respondent was 27 years. The average minimum working hours for a resident in a public sector hospital was found to be 38–39 h. Keeping this in mind, and the maximum working hours recorded, the data were divided into intervals. The average working hours recorded was 88 h. Figure 1 shows bar graph of working hours and the prevalence of burnout in each interval.

Table 1 represents the percentage of each response provided by the participants for the questions of the CBI. Of all the respondents, 56.66% (n = 170) recorded scores that indicate burnout. About 66.67% (Standard deviation [SD] 10.53) respondents had personal burnout, 57.14% (SD 6.88) had work-related burnout, and only 16.67% (SD 10.5) had client-related burnout [Table 2]. Forty-eight of the 300 respondents were on call for 1 day per week or less; 54.17% (SD 16.26) of these had average total scores equal to or higher than 50 indicating burnout. One hundred and two respondents were "on call" 2 days/week and 55.88% (SD 18.74) of them had burnout while 150 respondents reported that they were "on call" for 3 or more days per week and 57.33% (SD 18.15) residents had burnout syndrome. Of the 300 respondents, 149 had <2 years of experience in a public sector hospital, 133 had two to 5 years of experience, and 18 had over 5 years of experience. The prevalence of burnout syndrome in these groups was 55.70% (SD 18.09), 54.89% (SD 17.98), and 72.22% (SD 17.83), respectively.

Discussion

A number of scales such as maslach burnout inventory (MBI), Burnout Clinical Subtype Questionnaire-12, and Oldenburg Burnout Inventory have been devised to assess burnout among different professionals.[14-17] We have used the CBI in our study as it is a self-explanatory, simple, comprehensive, and self-assessed questionnaire which evaluates burnout in a number of dimensions. As most of the previous Indian burnout studies were carried out on doctors with >5 years of work experience, we recorded responses from participants without any minimum restrictions on years of experience. Our results

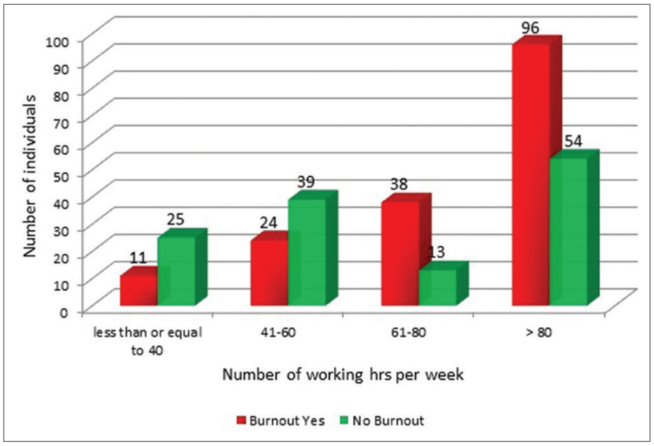

Figure 1: Relationship of working hours and number of individuals eliciting scores linked to burnout syndrome

Table 1: Percentage of responses recorded for each question on the Copenhagen burnout inventory

Questions	Always/to a very high degree (%)	Often/to a high degree (%)	Sometimes/somewhat (%)	Seldom/to a low degree (%)	Never/to a very low degree (%)
Personal burnout					
1. How often do you feel tired	22.33	37.33	35.00	4.67	0.67
2. How often are you physically exhausted?	17.33	45.00	29.67	6.67	1.33
3. How often are you emotionally exhausted?	21.00	26.33	32.67	16.00	4.00
4. How often do you think: “I can’t take it anymore”?	6.00	16.33	39.67	22.00	16.00
5. How often do you feel worn-out?	11.67	28.00	36.33	16.67	7.33
6. How often do you feel weak and susceptible to illness?	5.33	22.00	35.67	29.67	7.33
Work-related burnout					
7. Do you feel worn-out at the end of a working day?	25.00	31.33	29.00	10.67	4.00
8. Are you exhausted in the morning at the thought of another day at work?	18.33	26.00	30.33	16.67	8.67
9. Do you feel that every working hour is tiring for you?	8.33	16.67	38.33	23.33	13.33
10. Do you have enough energy for family and friends during leisure time?	8.00	18.67	22.67	33.33	17.33
11. Is your work emotionally exhausting?	12.33	26.00	37.67	17.33	6.67
12. Does your work frustrate you?	10.33	17.33	34.67	25.67	12.00
13. Do you feel burnout because of your work?	13.67	18.67	37.67	22.00	8.00
Patient related burnout					
14. Do you feel it hard to work with patients?	4.00	13.67	42.00	23.67	16.67
15. Does it drain your energy to work with patients?	6.33	21.33	38.67	24.33	9.33
16. Do you find it frustrating to work with patients?	5.00	16.33	40.33	25.00	13.33
17. Do you feel that you give more than you get back when you work with patients?	27.67	29.67	20.67	14.67	7.33
18. Are you tired of working with patients?	1.33	16.33	33.00	26.33	23.00
19. Do you sometimes wonder how long you will be able to continue working with patients?	3.33	19.00	25.33	27.00	25.33

Table 2: Prevalence of personal burnout, work-related burnout, client-related burnout, and percentage burnout of respondents (burnout=mean score ≥50)

	Maximum	Minimum	Mean	SD	Percentage burnout (mean ≥50)
Personal burnout	69.00	43.58	57.22	10.53	66.67
Work burnout	65.67	45.83	54.46	6.88	57.14
Client burnout	63.92	36.67	45.03	10.15	16.67

SD: Standard deviation

showed a general trend of high scores in all three parameters of CBI. 56.66% (n = 170) participants scored mean scores indicative of burnout according to the CBI. These results were consistent with the results of burnout studies among ICU doctors in India in 2014. About 54% respondents had scored “moderate to high” on the emotional exhaustion scale and 40% scored “moderate to high” on the depersonalization scale in the study by Divatia.[17] However, the levels of burnout in our study were significantly higher than the burnout rate calculated by Embriaco *et al*., in French public hospitals and other European studies.[10,18]

Among the three dimensions, personal burnout showed the highest incidence. This suggests that majority of the respondents were tired and physically and/or emotionally exhausted. These results were consistent with the burnout rates calculated by Langade *et al*., in 2016 on Indian doctors, where

65.98% people showed high scores on the depersonalization scale and 45.02% participants had high scores on the emotional exhaustion scale on the MBI.[19] Similar results have also been recorded in studies carried out on oncologists in the USA.[20]

Some previous studies have suggested that junior residents have a greater risk for burnout than seniors.[12,21] Our results, however, indicated a slightly higher rate of burnout among residents with five or more years of experience in a public sector hospital. The increased experience usually accounts to higher working hours, increased workload and inadequate compensation, causing additive stress, and higher burnout. Nevertheless, our study indicates that young resident doctors studying and/or working in public sector hospitals also have extremely high levels of burnout contrary to popular belief that burnout syndrome sets in later on in the professional carriers [Table 3].

In our study, females showed higher rates of burnout than their male colleagues. This result is also consistent with a number of other studies carried out in India and internationally.[18,19] This trend can be attributed to factors such as higher domestic expectations and responsibilities on the females than their male counterparts. Statistical analysis of the collected data showed that residents enrolled in specialty training programs showed slightly higher levels of burnout. The lack of specialist doctors and the added stress of academic commitments could be responsible for this reflection. An important observation from our study was that the residents from different departments and specialties had dissimilar trends of CBI scores. While residents catering to extensive daily outpatient department of patients showed higher client-related burnout, it was less prominent among residents working in surgical and ICU settings. These doctors, in turn, showed higher personal and work-related burnout. The "on call" arrangements of the doctors were found to be directly proportional to their burnout. Another significant finding in our study was that over 50% respondents felt that they did not have enough energy for friends and family during leisure time and over 57% respondents felt that they gave more than they get back when they work with patients.

As per the National Health Profile 2018, in India, the ratio of doctors to patients is approximately 1:11,082[22] which is drastically deranged from the ideal ratio of 1:1000 suggested by the WHO.[22] The working hours and the workload among doctors working in public sector hospitals are much higher than observed internationally to compensate for this discrepancy. The doctors working in public sector hospitals in India also face other adversities such as lack of appreciation, poor living conditions, unhealthy food habits, lack of insurance and protection, etc., which predisposes them to lifestyle disorders such as cardiovascular diseases, hypertension, hyperlipidemia, and lack of energy to spend time with family and friends along with burnout syndrome [Figure 2]. Burnout syndrome itself has been linked to psychological disorders and somatic symptoms including insomnia, irritability, and even suicidal tendencies.[23]

It is time that the medical fraternity and the governing bodies identify burnout syndrome as a potential threat that it is and take serious steps toward the identification, management, and prevention of burnout syndrome among resident doctors. Burnout syndrome not only adversely affects the emotional and physical health of the doctors who suffer from it but it also affects their ability to treat patients effectively and empathically. Primarily, attention should be directed to identifying the symptoms of burnout syndrome and addressing them through strategies on various levels. On an administrative level, more jobs must be created in public sector hospitals and number of seats for specialty training should also be increased. Stress management strategies such as meditation and psychological counseling should be made available to the residents. Finally, such training should be a part of orientation and recruitment process in public sector hospitals.

Although a sample size of 300 cannot be representative of the entire population of resident doctors working in public sector hospitals throughout the country, it provided us with an insight into the grave issue of physician burnout in the country. We had a few drawbacks to our study like, the date collection was restricted to doctors in Mumbai, and the sample size was small. We believe that the issues that resident doctors face around the country may be varied, but the issue of physician burnout is universal and hence must be given adequate attention. More

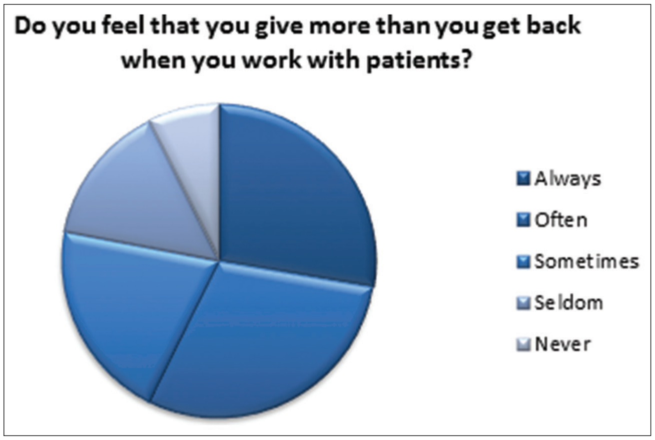

Figure 2: Statistics of responses to question 10 of Copenhagen Burnout Inventory

Table 3: Relationship between experience of respondent in a public sector hospital and prevalence of burnout

Experience	*n*	Maximum score	Minimum score	Mean	SD	Percentage burnout
0-2 years	149	94.74	3.95	52.33	18.09371893	55.70
2-5 years	133	93.42	3.95	51.46	17.97685427	54.89
>5 years	18	86.84	31.58	59.14	17.83380757	72.22

SD: Standard deviation

extensive studies with larger sample sizes should be carried out to get more accurate and generalizable results.

Conclusion

The high prevalence of burnout among resident doctors is a cause for serious concern. Burnout syndrome has physical, emotional, and psychological consequences that not only harm the doctors but also reduces their ability to serve the patients and the community effectively. The identification of symptoms and introduction of coping strategies form the core of managing burnout syndrome among physicians. Besides this, stress management techniques must be advocated to achieve a balance between work life and personal life.

Financial support and sponsorship

Nil.

Conflicts of interest

There are no conflicts of interest.

References

1. Korczak D, Huber B, Kister C. Differential diagnostic of the burnout syndrome. GMS Health Technol Assess 2010;6:Doc09.
2. Weber A, Jaekel-Reinhard A. Burnout syndrome: A disease of modern societies? Occup Med (Lond) 2000;50:512-7.
3. Maslach C. Burned-out. Hum Behav 1976;5:16-22.
4. Freudenberger HJ. Staff burn-out J Soc Issues 1974;30:159-65.
5. Kristensen TS, Borritz M, Villadsen E, Christensen KB. The Copenhagen burnout inventory: A new tool for the assessment of burnout. Work Stress 2005;19:192-207.
6. Embriaco N, Papazian L, Kentish-Barnes N, Pochard F, Azoulay E. Burnout syndrome among critical care healthcare workers. Curr Opin Crit Care 2007;13:482-8.
7. Bhugra D, Bhui KS, Gupta KR. Burnout and stress among doctors and dentists in North India. Int J Cult Ment Health 2008;1:24-9.
8. Shanafelt TD, Boone S, Tan L, Dyrbye LN, Sotile W, Satele D, *et al.* Burnout and satisfaction with work-life balance among US physicians relative to the general US population. Arch Intern Med 2012;172:1377-85.
9. Lee YY, Medford AR, Halim AS. Burnout in physicians. J R Coll Physicians Edinb 2015;45:104-7.
10. Soler JK, Yaman H, Esteva M, Dobbs F, Asenova RS, Katic M, *et al.* Burnout in European family doctors: The EGPRN study. Fam Pract 2008;25:245-65.
11. Available from: https://www.indiatimes.com/news/india/50 of all doctors working at-public hospitals-face verbal and physical-abuse from-patients-273891.html. [Last accessed on 2019 Jun 03].
12. Castelo-Branco C, Figueras F, Eixarch E, Quereda F, Cancelo MJ, González S, *et al.* Stress symptoms and burnout in obstetric and gynaecology residents. BJOG 2007;114:94-8.
13. Maslach C, Jackson SE. The measurement of experienced burnout. J Organ Behav 1981;2:99-113.
14. Maslach C, Jackson SE, Leiter MP, Schaufeli WB, Schwab RL. Maslach Burnout Inventory. Palo Alto, CA: Consulting Psychologists Press; 1986. Available from: https://www.researchgate.net/ profile/Christina_Maslach/publication/277816643_The_Maslach_Burnout_Inventory_Manual/links/5574dbd708aeb6d8c01946d7.pdf. [Last accessed on 2019 Jun 03].
15. Montero-Marín J, Skapinakis P, Araya R, Gili M, García-Campayo J. Towards a brief definition of burnout syndrome by subtypes: Development of the "Burnout clinical subtypes questionnaire" (BCSQ-12). Health Qual Life Outcomes 2011;9:74.
16. Demerouti E, Bakker AB. The Oldenburg burnout inventory: A good alternative to measure burnout and engagement. In: Handbook of Stress and Burnout in Health Care. New York: Nova Science Publishers; 2008. p. 65-78. Available from: https://www.pdfs.semanticscholar.org/7cb5/c694cb9ad8c38e63db5d6458e34cd4fef5ce.pdf. [Last accessed on 2019 Jun 03].
17. Divatia JV. Burnout in the ICU: Playing with fire? Indian J Crit Care Med 2014;18:127-8.
18. Embriaco N, Azoulay E, Barrau K, Kentish N, Pochard F, Loundou A, *et al.* High level of burnout in intensivists: Prevalence and associated factors. Am J Respir Crit Care Med 2007;175:686-92.
19. Langade D, Modi PD, Sidhwa YF, Hishikar NA, Gharpure AS, Wankhade K, *et al.* Burnout syndrome among medical practitioners across India: A questionnaire-based survey. Cureus 2016;8:e771.
20. Shanafelt TD, Gradishar WJ, Kosty M, Satele D, Chew H, Horn L, *et al.* Burnout and career satisfaction among US oncologists. J Clin Oncol 2014;32:678-86.
21. Michels PJ, Probst JC, Godenick MT, Palesch Y. Anxiety and anger among family practice residents: A South Carolina family practice research consortium study. Acad Med 2003;78:69-79.
22. Available from: https://www.financialexpress.com/india-news/doctor-population ratio in-india-one-allopathic-doctorfor11082 people-official-data-shows-bihar-up-worst-hit/1213243. [Last accessed on 2019 Jun 03].
23. Rao R, Modi PD, Sidhwa YF, Hishikar NA, Langade D. Burnout among dental professionals in India: A questionnaire based survey. J Indian Dent Assoc 2016;10:16-25.
