# Supplementary material for: Mental health status and related factors influencing healthcare workers during the COVID-19 pandemic: A systematic review and meta-analysis
Source: PLoS One. 2024 Jan 19;19(1):e0289454. doi: 10.1371/journal.pone.0289454 (PMC10798549; doi:10.1371/journal.pone.0289454)
Supplement: S1 Data — (ZIP) [file pone.0289454.s011.zip › literatures/38.pdf]

Iran J Psychiatry 2022; 17: 4: 446-454

Original Article

Depression, Anxiety and Coping Responses among Iranian Healthcare Professionals during the Coronavirus Disease (COVID-19) Outbreak

Farnaz Etesam[1,2], Melika Arab Bafrani[3], Samaneh Akbarpour[4], Helia Tarighatnia[1], Gilda Rajabi[1], Mahsa Dolatshahi[3,5], Zahra Vahabi[1,6*]

Abstract

Objective: This cross-sectional study aimed to assess the effects of different coping strategies on the mood states (anxiety and depression) of healthcare providers in the novel coronavirus disease (COVID-19) pandemic.
Method: From February to April 2020, we asked medical staff in 4 referral hospitals in Iran to voluntarily complete online questionnaires including: Generalized Anxiety Disorder (GAD-2) questionnaire, Patient Health Questionnaire (PHQ-2) and the Coping Strategies Questionnaire-28. Univariate and multiple logistic regressions were applied to identify the associations of coping strategies and mood states.
Results: 258 people filled out the online questionnaire. Of them, 39.9% and 39.1% reported anxiety and depression, respectively, with age as a risk factor. Overall, participants used more emotion-based coping strategies. Anxiety and depression were associated with applying more of emotion-based and less of problem-based coping mechanisms. The findings remained stable even after adjustment for confounding variables including age, gender and direct contact with COVID-19 patients.
Conclusion: Providing social support to health workers, planning to reduce their perceived stigma, and educating them about how to use more effective coping mechanisms can be beneficial in reducing the psychological impact on this segment of the population in the event of COVID-19.

Key words: *Anxiety; COVID-19 Pandemic; Depression; Mental Health*

1. Department of Geriatric Medicine, Ziaeian Hospital, Tehran University of Medical Sciences, Tehran, Iran.
2. Department of Psychiatry, Roozbeh Hospital, Tehran University of Medical Sciences, Tehran, Iran.
3. Students' Scientific Research Center (SSRC), School of Medicine, Tehran University of Medical Sciences, Tehran, Iran.
4. Occupational Sleep Research Centre, Baharloo Hospital, Tehran University of Medical Sciences, Tehran, Iran.
5. NeuroImaging Network (NIN), Universal Scientific Education and Research Network (USERN), Tehran, Iran.
6. Cognitive Neurology and Neuropsychiatry Division, Department of Psychiatry, Roozbeh Hospital, Tehran University of Medical Sciences, Tehran, Iran.

***Corresponding Author:**
Address: Cognitive Neurology and Neuropsychiatry Division, Department of Psychiatry, Roozbeh Hospital, Tehran University of Medical Sciences, Tehran, Iran, Postal Code: 13337159140.
Tel: 98-21 55176818, Fax: 98-21 55412756, Email: vahab1357@gmail.com, zvahabi@sina.tums.ac.ir

Article Information:
Received Date: 2021/02/03, Revised Date: 2021/09/10, Accepted Date: 2022/08/22

Copyright © 2022 Tehran University of Medical Sciences. Published by Tehran University of Medical Sciences.
This work is licensed under a Creative Commons Attribution-NonCommercial 4.0 International license (https://creativecommons.org/licenses/by-nc/4.0/). Noncommercial uses of the work are permitted, provided the original work is properly cited

Coronavirus disease 2019 (COVID-19), which is the consequence of infection with the new virus, the Severe Acute Respiratory Syndrome Corona Virus-2 (SARS-CoV-2), spread expeditiously around the world in the last months of 2019 to 2020 (1-3).

The medical staff, as the frontline of this epidemic, were exposed to a high level of stress due to inadequate protection against viral infection as well as overworking, lack of supplies and staff, overcrowded hospitals, negative emotions due to inability to contact the family and severe burnout. All of these could affect their mental health and cause them to develop symptoms such as anxiety, depression, insomnia, anger and fear (4-6).

The degree of these complications is not the same between different populations, and their severity depends on the coping strategies used to deal with stressful events. Applying coping strategies in stressful conditions can forestall a mental health crisis, especially in those with an increased risk of developing psychiatric problems (7). Previous studies have shown that in past outbreaks, healthcare professionals have been able to control their feelings and adapt to the situation (8). Coping strategies were shown to improve people`s mental symptoms, overall health, and life satisfaction in the 2002 SARS epidemic (9).

People usually adopt two kinds of coping styles, which are categorized into emotion-based and problem-based (10). The goal of the problem-oriented style is solving the problem via taking an action to modify the condition and the other emotion-focused style is based on reducing the emotional anguish related to disturbing situations (11, 12). On the whole, problem-based and active coping mechanisms are considered to be more effective in preventing psychological disorders (10, 13), although different factors such as various types of personalities are crucial in determining which coping mechanism can be more effective and some studies have shown contrary results (14-15). Based on the literature, adults who experience more irritation and worry, are more likely to apply active coping strategies such as inquiring, while people who are sad more likely apply non-active coping strategies such as keeping away from or accepting problems (16, 17).

It is crucial to determine coping strategies that will help Iranian health workers adapt to the very disturbing circumstances surrounding COVID-19. Only a few studies are available in the area of coping mechanisms in nursing, to the best of our knowledge, no article was published on this important topic in this population group in Iran during the COVID-19 outbreak. Therefore, in the present study, our target was to clarify the mental health status of medical staff in four referral hospitals in Tehran and Kashan, the main coping mechanisms they used during the COVID-19 pandemic and the effect of the applied coping mechanisms on their mood states. We believe that identifying the role of the mental health coping strategies used during this period may suggest strategies to address the negative effects of COVID-19 on this population and lessen the possibility of the occurrence of poor mental health in them.

Materials and Methods

Study design and population

The present study was a web-based cross-sectional study conducted on medical staff at the time of the COVID-19 pandemic. By means of convenience sampling, the link of a webpage containing the online questionnaire survey was sent for target individuals in 4 referral hospitals in Tehran and Kashan from February to April 2020, using the SMS and WhatsApp. A total of 273 questionnaires were completed, and from these, 258 valid and fully responded questionnaires were included in the analysis (46 males and 212 females). As inclusion criteria, participants were chosen from among the medical staff who were in permanent employment (not temporary or voluntary) and had a willingness to cooperate in the research. The institutional review board and the ethics committee of Tehran University of Medical Sciences have approved the protocol and design of the study (the ethical approval code: R.TUMS.VCR.REC.1399.107).

Data collection and measurements

The Demographic Information Questionnaire

The basic data of the participants, such as gender, age, marital status, occupation, education and type of wards were asked in the first questionnaire.

The Patient Health Questionnaire-2

We used the Patient Health Questionnaire-2 (PHQ-2) to ask about the frequency of anhedonia and depressed mood over the last two weeks to screen participants for depression (18). The Persian version of this questionnaire has been validated for diagnosis of depression in Iranian patients with a Cronbach's alpha coefficient of 0.79 (19). All participants were asked two questions about whether they were interested or took pleasure in doing things or whether they felt down, depressed or hopeless over the last two weeks. The answer to each question was rated on a Likert scale ranging from 0-3 (0 = not at all, 1 = several days, 2 = more than half the days, 3 = nearly every day) and the total score for both questions was between 0-6. A score higher than 3 has been observed to be indicative of the cutoff point for the detection of depression (20).

The Generalized Anxiety Disorder- 2

Generalized Anxiety Disorder 2-item (GAD-2) questionnaire was used as a brief and simple screening tool for anxiety disorders. The Persian version of this questionnaire has also been validated for use among Iranian patients, with a Cronbach's alpha coefficient of 0.75, which is quite acceptable (20). This tool had two questions that used the Likert scale (0 = not at all, 1 = several days, 2 = more than half the days, 3 = nearly all days) to ask participants about feeling nervous, anxious and not being able to manage worries over the last two weeks that bothered them. The GAD-2 score was

obtained by adding up the Likert score for the two questions, and when the score was greater than 3, it was defined as an anxiety disorder (21).

The Coping Strategies Questionnaire-28

The Coping strategies questionnaire is generally used in health-care settings to find out how patients are reacting to a critical condition. (22) The tool contains 28 items to measure the efficient and inefficient approaches to coping with a stressful life event. Some authors used this tool during the COVID-19 and SARS outbreaks (7, 23). This questionnaire has been translated and validated for use in Iranian patients with an acceptable Cronbach's alpha coefficient (α = 0.77) (24). The scale consists of two sections; emotion-focused coping (with the subscales of acceptance, positive reframing, use of emotional support, religion, humor, substance use, self-distraction, self-blame, denial, behavior disengagement, and venting) and problem-focused coping (with the subscales of active coping, use of instrumental support and planning). Each item had a Likert range of 1-5 (none-always) and the total score was attained by adding the Likert scores for all questions, and higher scores indicated a higher level of coping (22).

Statistical analyses

The mean (standard deviation) and frequency (percentage) were mentioned for quantitative and qualitative variables, respectively. The student's independent t-test was applied to assess the differences among variables of interest between two independent groups. To assess the independence of two or more qualitative variables, the chi square test or Fisher's exact test were used.

Anxiety, depression and coping scores cannot be calculated if any items of the data are missing. People with missing data regarding these three values were eliminated from the data analysis. Other covariates with missing data were relatively few (1.08% of cells) and a single imputation method was used based on the regression model to replace them using the mice package (multivariate imputation by chained equations) in R software (25).

Crude and multiple Logistic regression models were used to examine the association between different coping approaches and anxiety in three models as: model 1, without adjustment; model 2, adjusted for sex and age; and model 3, further adjusted for confounding variables including age, gender and direct contact with COVID-19 patients (as a variable that appeared significantly different at the level of P-value < 0.1 in univariate analysis). In model 3 we also adjusted depression for anxiety (as an outcome) and anxiety for depression as an outcome in the regression model.

SPSS 21 and R software were used for data analysis and P-value < 0.05 was considered as statistically significant.

Results

Overall characteristics of the study population

A total of 258 (women = 212) persons with a mean age (SD) of 35.48 (8.39) years participated in this research and were added to the analysis. Of these, 165 participants were married and others were single. Among all participants, 100 (38.8%) were nurses, 58 (22.5%), 45 (17.4%) and 55 (21.3%) were doctors, paramedics and other medical staff, respectively. Table 1 demonstrates other socio-demographic characteristics of the participants.

The mean values of the problem- and emotion-focused coping strategies for all participants were 21.27 (SD: 3.99) and 33.39 (SD: 6.24), respectively.

Relationship between Coping Strategies and anxiety levels of the healthcare professionals at the time of the COVID-19 outbreak

As shown in Table 1, among the participants, 103 (39.92%) were anxious. The mean age of those participants who had anxiety was 33.90 ± 7.86 years and of those who did not have anxiety was 36.53 ± 8.59 years (P-value = 0.014). No significant differences were found to exist between the socio-demographic characteristics of the two groups. Table 2 represents differences in coping strategy scores between the group with anxiety and the group without anxiety during the COVID-19 epidemic. The mean score of problem-focused coping strategies was lower among participants with anxiety than among those without anxiety (31.72 ± 6.28 vs. 34.50 ± 5.99; P-value < 0.0001). Furthermore, emotion-focused coping strategies were significantly different in these two groups and participants with anxiety showed a higher score for this strategy than participants who did not have anxiety (22.56 ± 4.27 vs 20.42 ± 6.56, P-value < 0.0001).

Results of logistic regression analysis investigating the correlation of different coping strategies, as continuous variables, with anxiety are presented in Table 3. Problem-focused coping strategies were negatively correlated with anxiety symptoms, as observed in multiple models (OR = 0.93, CI = 0.89-0.97, P-value < 0.0001). Regarding emotion-focused coping strategies, we observed a significant positive association with anxiety disorder in multiple models (OR = 1.14, CI = 1.06-1.22, P-value < 0.0001).

Relationship between Coping Strategies and depression levels of the healthcare professionals at the time of the COVID-19 outbreak

As shown in Table 2, 101 (39.14%) participants had depression. The mean age of participants with and without depression was 32.61 ± 7.04 and 37.32 ± 8.68, respectively (P-value < 0.0001). We found no significant difference between the two groups in socio-demographic characteristics, as shown in Table 2.

Table 3 shows differences in the scores of coping strategies between the two groups with and without depression in the COVID-19 epidemic. The mean score

of problem-focused coping strategies among participants with depression was lower than among those who were not depressed (31.17 ± 6.59 vs. 34.82 ± 5.58, P-value < 0.0001). Similar to anxiety, participants with depression showed higher scores for emotion-focused strategies than participants without depression (22.66 ± 4.20 vs. 20.38 ± 3.60; P-value < 0.0001).

With regard to the results of the logistic regression models in Table 3, we found a significant negative relationship between the problem-focused coping strategies and depression in multiple regression models (OR = 0.84, CI = 0.64-1.59, P-value < 0.0001). On the other hand, emotion-focused coping strategies showed a significant positive association with depression in multiple regression models (OR = 1.14, CI = 1.06-1.23, P-value < 0.0001).

Table 1. Scio-Demographic Characteristics of the Study Population based on Anxiety and Depression

	Total (n = 258)	With anxiety (n = 103)	Without anxiety (n = 155)	P-value	With Depression (n = 101)	Without depression (n = 157)	P-value
Age, year (Mean ± SD)*	35.48 ± 8.39	33.90 ± 7.86	36.53 ± 8.59	0.014*	32.61 (7.04)	37.32 (8.68)	< 0.0001*
**Gender**							
Men	46 (17.8)	13 (12.6)	33 (21.3)	0.075	13 (12.9)	33 (21.0)	0.095
Women	212 (82.2)	90 (87.4)	122 (78.7)		88 (87.1)	124 (79.0)	
**Marital status**							
Married	165 (64.0)	68 (66.0)	58 (37.4)	0.756	41 (40.6)	52 (33.1)	0.222
Single	93 (36.0)	35 (34.0)	97 (62.6)		60 (59.4)	105 (66.9)	
**Occupation**							
Nurse	100 (38.8)	40 (38.8)	60 (38.7)	0.978	43 (42.6)	57 (36.3)	0.111
Doctor	58 (22.5)	22 (21.4)	36 (23.2)		15 (14.9)	43 (27.4)	
domestic assistant	45 (17.4)	19 (18.4)	26 (16.8)		21 (20.8)	24 (15.3)	
Others	55 (21.3)	22 (21.4)	33 (21.3)		22 (21.8)	33 (21.0)	
**Education**							
Bs and less	172 (66.7)	70 (68)	102 (65.8)	0.926	74 (73.3)	98 (62.0)	0.109
MSc	19 (7.4)	7 (6.8)	12 (7.7)		8 (7.9)	11 (7.0)	
MD and higher	67 (26.0)	26 (25.2)	41 (26.5)		19 (18.8)	48 (30.6)	
**Type of ward**							
Others	65 (25.2)	23 (22.3)	42 (27.1)	0.656	23 (22.8)	42 (26.8)	0.325
COVID-19 wards	123 (47.7)	52 (50.5)	71 (45.8)		54 (53.5)	69 (43.9)	
Emergency wards (ICU & emergency rooms)	70 (27.1)	28 (27.2)	42 (27.1)		24 (23.8)	46 (29.3)	
Direct contact with COVID-19 patients (Yes)	192 (74.4)	82 (79.6)	110 (71.0)	0.078	76 (75.2)	116 (73.9)	0.091
Depression	101 (39.1)	76 (73.8)	25 (16.1)	< 0.0001*			< 0.0001*
Anxiety	103 (39.9)				76(75.2)	27(17.2)	

All values are reported as frequency and percentage except age (reported as mean and SD)

Table 2. Mean and Standard Deviation of Coping Strategies based on Anxiety and Depression among Iranian Healthcare Professionals during the Coronavirus Disease (COVID-19) Outbreak

Variables	Total (n = 258)	With Anxiety (n = 103)	Without Anxiety (n = 155)	P-Value	With Depression (n = 101)	Without Depression (n = 157)	P-Value
	Mean (SD)	Mean (SD)	Mean (SD)		Mean (SD)	Mean (SD)	
Coping Strategies							
**Problem- focused coping strategies**	33.39 (6.24)	31.72 (6.28)	34.50 (5.99)	< 0.0001	31.17 (6.59)	34.82 (5.58)	< 0.0001
Acceptance	6.52 (1.34)	5.99 (1.38)	6.88 (1.19)	< 0.0001	5.95 (1.40)	6.89 (1.17)	< 0.0001
Religion	5.13 (1.91)	4.94 (1.83)	5.25 (1.97)	0.195	4.84 (1.98)	5.31 (1.85)	0.051
planning	5.59 (1.46)	5.20 (1.46)	5.85 (1.40)	< 0.0001	5.14 (1.51)	5.87 (1.35)	< 0.0001
Positive reframing	5.41 (1.60)	4.94 (1.53)	5.73 (1.57)	< 0.0001	4.95 (1.69)	5.71 (1.46)	< 0.0001
Using instrumental support	5.05 (1.64)	5.05 (1.71)	5.05 (1.59)	0.975	4.92 (1.74)	5.14 (1.57)	0.296
Active coping	5.78 (1.46)	5.38 (1.37)	6.04 (1.46)	< 0.0001	5.24 (1.43)	6.12 (1.37)	< 0.0001
Using emotional support	5.01 (1.67)	5.14 (1.84)	4.93 (1.56)	0.326	4.96 (1.76)	5.05 (1.63)	0.652
Humor	3.21 (1.34)	2.93 (1.31)	3.40 (1.33)	0.005	2.98 (1.30)	3.36 (1.35)	0.794
**Emotion focused coping strategies**	21.27 (3.99)	22.56 (4.27)	20.42 (3.56)	< 0.0001	22.66 (4.20)	20.38 (3.60)	< 0.0001
Self-distraction	5.47 (1.57)	5.49 (1.51)	5.45 (1.62)	0.854	5.50 (1.57)	5.45 (1.58)	0.794
Venting	2.91 (1.31)	4.92 (1.32)	4.74 (1.27)	0.291	4.93 (1.30)	4.74 (1.28)	0.262
Self-blame	2.23 (0.72)	3.33 (1.50)	2.47 (0.84)	< 0.0001	3.26 (1.43)	2.52 (0.96)	< 0.0001
Behavioral disengagement	3.01 (1.28)	3.31 (1.30)	2.82 (1.23)	0.003	3.31 (1.35)	2.83 (1.20)	0.004
Denial	4.81 (1.29)	3.12 (1.39)	2.78 (1.24)	0.038	3.23 (1.48)	2.71 (1.14)	0.002
Substance use	2.81 (1.22)	2.37 (0.90)	2.13 (0.54)	0.008	2.41 (0.95)	2.11 (0.49)	0.001

Table 3. The Association between Coping Strategies and Anxiety and Depression in Univariate and Multiple Logistic Regression (n = 258)

		Anxiety (n = 258 , e = 103)	Depression (n = 258, e = 101)	
	OR (CI %)	P-value	OR (CI %)	P-value
Emotion focused coping strategies				
Model 1	1.15 (1.07-1.23)	< 0.0001	1.16 (1.08-1.24)	< 0.0001
Model 2	1.13 (1.07-1.21)	< 0.0001	1.14 (1.06-1.22)	< 0.0001
Model 3	1.14 (1.06-1.22)	< 0.0001	1.14 (1.06-1.23)	< 0.0001
Problem- focused coping strategies				
Model 1	0.92 (0.89-0.96)	0.001	0.90 (0.86-0.9)	< 0.0001
Model 2	0.93 (0.89-0.97)	0.001	0.91 (0.86-0.95)	< 0.0001
Model 3	0.93 (0.89-0.97)	0.001	0.84 (0.64-1.59)	< 0.0001

Model 1: Without adjustment.
Model 2: Adjustment for age and sex
Model 3 for anxiety: Adjustment for age, sex, depression and direct contact with COVID-19 patients
Model 3 for depression; Adjustment for age, sex, anxiety and direct contact with COVID-19 patients

Discussion

This study provides some information on the incidence of psychological risk factors during the COVID-19 outbreak from respondents across 4 referral hospitals in Iran. This study further highlights the fact that the COVID-19 pandemic is a major source of distress for both individuals and social groups. Different persons may struggle with distinct levels of psychological tension depending on their situation, especially medical staff who are at the frontline and undergo a lot of pressure and stress. In the present investigation, the greater number of respondents (92%) had a direct contact with individuals who were proved or suspected cases of COVID-19. According to the results, 39.92% of the respondents were anxious and 39.14% of them were depressed. These percentages show higher levels of anxiety and depression in healthcare workers as compared to reported levels of these symptoms in previous researches on Iranian nurses and doctors prior to the COVID-19 outbreak (26, 27). In addition, this amount was much greater than the amounts reported among the Iranian population in general (4.7%–21.9%) (28,29). Similar results have been shown in a research conducted by Taghizadeh *et al.*, which reported a higher prevalence of these disorders in nurses and doctors compared to the general population (30). This discrepancy could be attributed, at least in part, to the influences of stigma and discrimination against medical professionals in conjunction with fear of increased risk of infection (31). Higher perceived stigma in the medical staff in the time of COVID-19 pandemic could make them more emotionally aware and cause them to develop both anxiety and depression during the outbreak and post-traumatic stress disorder (PTSD) after the pandemic (32). In addition, the COVID-19 pandemic and the contagious nature of the disease have required social distancing and, when coupled with social stigma, can lead to increased social isolation and avoidance behaviors that are generally classified as maladaptive behaviors in psychological terms (33). Studies conducted in other countries and neighborhoods have shown different percentages of mental disorders in health care workers (34). For example, a similar study on Chinese medical workers found that about 6% and 28% of medical workers suffer from anxiety and depression, respectively (35). The prevalence of anxiety and depression was 21.4% and 21.9%, respectively, among Pakistani health workers (36), which is much lower than its prevalence in this study. In Singapore, the prevalence of anxiety and depression was even less (10.8% and 8.1%, respectively) (37). In addition to the actual differences in the prevalence of anxiety and depression in various countries, these differences in percentages can be due to different survey times, different tools and different sampling methods. On the other hand, some factors such as the high proportion of participating women, who are more susceptible to depressive and anxiety disorders in general population and in COVID-19 crisis, might have confounded the estimated prevalence of psychological disorders in our sample; although this study indicated that there was no significant difference in the psychological disorders among males and females (38, 39). This study also showed no significant difference in the prevalence of these disorders between different subgroups of participants, except for age subgroups, as younger individuals tended to be at higher risk. Also, the female gender and a work-place with more direct exposure to the virus were marginally insignificant.

In our study, people more likely applied emotion-based coping strategies. These results were compatible with the results of the study by Mohammadzadeh *et al.*, conducted in Gonabad, Iran, on the general population (40). The underlying mechanism for this association has yet to be elucidated, possibly suggesting that large numbers of participants, either from the general population or medical staff, felt powerless to deal with the stressful situation of the COVID-19 pandemic. In addition, these results coincided with the study by Yasdanshenas Ghazwin *et al.* on Iranian nurses in 2016 prior to the start of the COVID-19 outbreak (27).

Some researchers believe that men generally use problem-focused coping strategies as an effective coping style; while, women, in general, tend to focus on emotions and emotional discharge, trying to use strategies that control and reduce their emotional responses. In this study, nurses made up the majority of participants (72% vs. 28%). More importantly, this study underscores the fact that among all of the factors that obscure mental disorders in the time of crisis, coping mechanisms are of great importance. In the present research, people with emotion-based coping experienced greater levels of anxiety and depression than those who used the problem-based coping strategy during the COVID-19 outbreak. This means that self-distracting, behavioral disengagement, venting and denial may not be effective ways to prevent mental health issues in the COVID-19 crisis. In contrast, as problem-oriented coping strategies, acceptance, active coping, and planning seem to be more effective in reducing the risk of mental health problems. Taha *et al.* also reported parallel results in an investigation into the H1N1 pandemic. Some other studies have also shown that emotion-based coping is linked to negative outcomes such as stress and anxiety, while problem-based coping is found to have an inverse association with these symptoms (41). During SARS-CoV-2 outbreak in Hong Kong, especially in the beginning, it was shown that young age is associated with employing more emotion-based coping mechanisms and less experience in adapting to the changing environment, and thus with a higher prevalence of anxiety and depression (23). As another example, nursing college students are more likely to use emotion-based coping mechanisms (7). In general, most of the studies mention the female gender, younger age, kind of profession such as nursing, the

place and department in which the staff member works such as the emergency department, self-efficacy, resilience, and the social support that the individuals received were predictive of the mental disorders medical staff experienced during the COVID-19 pandemic (34, 42). In our study, the results remained stable even after adjusting for confounding variables such as age, gender and direct contact with patients with COVID-19. Because of the sampling method and the non-equivalent distribution of the individual topic categories, this study may not have been able to identify such differences.

Limitation

The present study had several limitations. Considering the occurrence of the COVID-19 outbreak and its specific complications, using online questionnaires may not lead to inclusion of a large number of personnel in the study, as a lot of medical staff did not have enough time to fill the online forms. The sampling method was non-random, based on convenience sampling, and the subjects voluntarily participated in the study, which can cause selection bias and poor generalizability .

Another limitation is that subjective and self-reported psychological stress, depression and anxiety may not always coincide with objective assessments by mental health professionals. In addition, the study was cross-sectional and was carried out over a short period of time; while longitudinal data can warrant a stronger conclusion.

Conclusion

In summary, it can be said that more than a third of the surveyed healthcare workers since the beginning of the COVID-19 outbreak in Iran exhibited fear and depression, with age as a risk factor. Due to the rapidly changing situation, these data may not be valid during the next stages of the crisis, but given the global aspects of this crisis, these data can provide useful information that can be extrapolated to future epidemics. In the context of the COVID-19 pandemic, improving health workers' knowledge and skills in using more active and problem-based coping mechanisms will lead to fewer mental disorders and greater healthcare efficiency (43). Providing social support for the medical staff and planning for reduction of their perceived stigma can go a long way in reducing the psychological consequences of this pandemic.

Acknowledgment

The authors would like to sincerely thank the medical staff of Ziaeean and Baharloo hospitals in Tehran, the University of Medical Sciences and also the Kashan University of medical sciences.

Conflict of Interest

None.

References

1. Kannan S, Shaik Syed Ali P, Sheeza A, Hemalatha K. COVID-19 (Novel Coronavirus 2019) - recent trends. Eur Rev Med Pharmacol Sci. 2020;24(4):2006-11.
2. Organization WH. Coronavirus disease 2019 (COVID-19): situation report, 72. 2020.
3. Rothan HA, Byrareddy SN. The epidemiology and pathogenesis of coronavirus disease (COVID-19) outbreak. J Autoimmun. 2020;109:102433.
4. Li W, Yang Y, Liu ZH, Zhao YJ, Zhang Q, Zhang L, et al. Progression of Mental Health Services during the COVID-19 Outbreak in China. Int J Biol Sci. 2020;16(10):1732-8.
5. Stuijfzand S, Deforges C, Sandoz V, Sajin CT, Jaques C, Elmers J, et al. Psychological impact of an epidemic/pandemic on the mental health of healthcare professionals: a rapid review. BMC Public Health. 2020;20(1):1230.
6. Liu CY, Yang YZ, Zhang XM, Xu X, Dou QL, Zhang WW, et al. The prevalence and influencing factors in anxiety in medical workers fighting COVID-19 in China: a cross-sectional survey. Epidemiol Infect. 2020;148:e98.
7. Huang L, Lei W, Xu F, Liu H, Yu L. Emotional responses and coping strategies in nurses and nursing students during Covid-19 outbreak: A comparative study. PLoS One. 2020;15(8):e0237303.
8. Shih FJ, Turale S, Lin YS, Gau ML, Kao CC, Yang CY, et al. Surviving a life-threatening crisis: Taiwan's nurse leaders' reflections and difficulties fighting the SARS epidemic. J Clin Nurs. 2009;18(24):3391-400.
9. Main A, Zhou Q, Ma Y, Luecken LJ, Liu X. Relations of SARS-related stressors and coping to Chinese college students' psychological adjustment during the 2003 Beijing SARS epidemic. J Couns Psychol. 2011;58(3):410-23.
10. Kasi PM, Naqvi HA, Afghan AK, Khawar T, Khan FH, Khan UZ, et al. Coping styles in patients with anxiety and depression. ISRN Psychiatry. 2012;2012:128672.
11. Sungchul B, Do-Youn L, Il Kon K. Rendering Problem-Oriented CCD for chronic diseases. Annu Int Conf IEEE Eng Med Biol Soc. 2017;2017:4277-80.
12. Popa-Velea O, Diaconescu L, Jidveian Popescu M, Truţescu C. Resilience and active coping style: effects on the self-reported quality of life in cancer patients. The international journal of psychiatry in medicine. 2017;52(2):124-36.
13. Keramati M, Ebrahimi H, Basirinezhad M, Shamsizadeh M, Mohammadpourhodki R. The prevalence of anxiety, stress, and depression with respect to coping strategies in caregivers of patients with head injuries. Archives of Trauma Research. 2019;8:165.
14. Folkman S, Lazarus RS. An analysis of coping in a middle-aged community sample. J Health Soc Behav. 1980;21(3):219-39.
15. Frijda NH. Emotions are functional, most of the time. 1994.

16. Gok Metin Z, Karadas C, Izgu N, Ozdemir L, Demirci U. Effects of progressive muscle relaxation and mindfulness meditation on fatigue, coping styles, and quality of life in early breast cancer patients: An assessor blinded, three-arm, randomized controlled trial. Eur J Oncol Nurs. 2019;42:116-25.
17. Tuncay T, Musabak I, Gok DE, Kutlu M. The relationship between anxiety, coping strategies and characteristics of patients with diabetes. Health Qual Life Outcomes. 2008;6:79.
18. Dadfar M, Lester D. Psychometric characteristics of Patient Health Questionnaire-2 (PHQ-2) in Iranian psychiatric outpatients. Austin Journal of Psychiatry and Behavioral Sciences. 2017;4:2-6.
19. Ahmadi SM, Masjedi Arani A, Bakhtiari M, Davazdah Emamy MH. Psychometric Properties of Persian Version of Patient Health Questionnaires-4 (PHQ-4) in Coronary Heart Disease Patients. Iran J Psychiatry Behav Sci. 2019;13(4):e85820.
20. Wild B, Eckl A, Herzog W, Niehoff D, Lechner S, Maatouk I, et al. Assessing generalized anxiety disorder in elderly people using the GAD-7 and GAD-2 scales: results of a validation study. Am J Geriatr Psychiatry. 2014;22(10):1029-38.
21. Hughes AJ, Dunn KM, Chaffee T, Bhattarai JJ, Beier M. Diagnostic and Clinical Utility of the GAD-2 for Screening Anxiety Symptoms in Individuals with Multiple Sclerosis. Arch Phys Med Rehabil. 2018;99(10):2045-9.
22. Carver CS. You want to measure coping but your protocol's too long: consider the brief COPE. Int J Behav Med. 1997;4(1):92-100.
23. Yeung DY, Fung HH. Age differences in coping and emotional responses toward SARS: a longitudinal study of Hong Kong Chinese. Aging Ment Health. 2007;11(5):579-87.
24. Ashktorab T, Baghcheghi N, Seyedfatemi N, Baghestani A. Psychometric parameters of the Persian version of the BriefCOPE among wives of patients under hemodialysis. Med J Islam Repub Iran. 2017;31:20.
25. Groothuis-Oudshoorn K, Van Buuren S. Mice: multivariate imputation by chained equations in R. J Stat Softw. 2011;45(3):1-67.
26. Saeedi Shahri SS, Ghashghaee A, Behzadifar M, Bragazzi NL, Behzadifar M, Mousavinejad N, et al. Depression among Iranian nurses: A systematic review and meta-analysis. Med J Islam Repub Iran. 2017;31:130.
27. Yazdanshenas Ghazwin M, Kavian M, Ahmadloo M, Jarchi A, Golchin Javadi S, Latifi S, et al. The Association between Life Satisfaction and the Extent of Depression, Anxiety and Stress among Iranian Nurses: A Multicenter Survey. Iran J Psychiatry. 2016;11(2):120-7.
28. Hajebi A, Motevalian SA, Rahimi-Movaghar A, Sharifi V, Amin-Esmaeili M, Radgoodarzi R, et al. Major anxiety disorders in Iran: prevalence, sociodemographic correlates and service utilization. BMC Psychiatry. 2018;18(1):261.
29. Ahmadvand AF, Sepehrmanesh ZA, Ghoreyshi FS, Assarian FA, Moosavi GA, Etesam FA. Prevalence of mental disorders in general population of Kashan City. Iranian Journal of Epidemiology. 2010;6(2):16-24.
30. Taghizadeh F, Hassannia L, Moosazadeh M, Zarghami M, Taghizadeh H, Fathi Dooki A, et al. Anxiety and Depression in Health Workers and General Population During COVID-19 Epidemic in IRAN: A Web-Based Cross-Sectional Study. medRxiv. 2020:2020.05.05.20089292.
31. Tahara M, Mashizume Y, Takahashi K. Coping Mechanisms: Exploring Strategies Utilized by Japanese Healthcare Workers to Reduce Stress and Improve Mental Health during the COVID-19 Pandemic. Int J Environ Res Public Health. 2020;18(1):131.
32. Tracy JL, Robins RW. TARGET ARTICLE: "Putting the Self Into Self-Conscious Emotions: A Theoretical Model". Psychological Inquiry. 2004;15(2):103-25.
33. Gee S, Skovdal M. Public Discourses of Ebola Contagion and Courtesy Stigma: The Real Risk to International Health Care Workers Returning Home From the West Africa Ebola Outbreak? Qual Health Res. 2018;28(9):1499-508.
34. Spoorthy MS, Pratapa SK, Mahant S. Mental health problems faced by healthcare workers due to the COVID-19 pandemic-A review. Asian J Psychiatr. 2020;51:102119.
35. Siyu C, Xia M, Wen W, Cui L, Yang W, Liu S, et al. Mental health status and coping strategy of medical workers in China during The COVID-19 outbreak. medRxiv. 2020:2020.02.23.20026872.
36. Salman M, Raza MH, Mustafa ZU, Khan TM, Asif N, Tahir H, et al. The psychological effects of COVID-19 on frontline healthcare workers and how they are coping: a web-based, cross-sectional study from Pakistan. medRxiv. 2020.
37. Tan BYQ, Chew NWS, Lee GKH, Jing M, Goh Y, Yeo LLL, et al. Psychological Impact of the COVID-19 Pandemic on Health Care Workers in Singapore. Ann Intern Med. 2020;173(4):317-20.
38. Salk RH, Hyde JS, Abramson LY. Gender differences in depression in representative national samples: Meta-analyses of diagnoses and symptoms. Psychol Bull. 2017;143(8):783-822.
39. Pappa S, Ntella V, Giannakas T, Giannakoulis VG, Papoutsi E, Katsaounou P. Prevalence of depression, anxiety, and insomnia among healthcare workers during the COVID-19 pandemic: A systematic review and meta-analysis. Brain Behav Immun. 2020;88:901-7.
40. Mohammadzadeh F, Delshad Noghabi A, Khosravan S, Bazeli J, Armanmehr V, Paykani T. Anxiety Severity Levels and Coping Strategies during the COVID-19 Pandemic among People Aged 15 Years and Above in Gonabad, Iran. Arch Iran Med. 2020;23(9):633-8.
41. Taha S, Matheson K, Cronin T, Anisman H. Intolerance of uncertainty, appraisals, coping, and anxiety: the case of the 2009 H1N1

pandemic. Br J Health Psychol. 2014;19(3):592-605.
42. Hu D, Kong Y, Li W, Han Q, Zhang X, Zhu LX, et al. Frontline nurses' burnout, anxiety, depression, and fear statuses and their associated factors during the COVID-19 outbreak in Wuhan, China: A large-scale cross-sectional study. EClinicalMedicine. 2020;24:100424.
43. Chew QH, Chia FL, Ng WK, Lee WCI, Tan PLL, Wong CS, et al. Psychological and coping responses to COVID-19 amongst residents in training across ACGME-I accredited specialties in Singapore. Psychiatry Res. 2020;290:113146.
