# Supplementary material for: Mental health status and related factors influencing healthcare workers during the COVID-19 pandemic: A systematic review and meta-analysis
Source: PLoS One. 2024 Jan 19;19(1):e0289454. doi: 10.1371/journal.pone.0289454 (PMC10798549; doi:10.1371/journal.pone.0289454)
Supplement: S1 Data — (ZIP) [file pone.0289454.s011.zip › literatures/39.pdf]

BMJ Open

Protecting the front line: a cross-sectional survey analysis of the occupational factors contributing to healthcare workers' infection and psychological distress during the COVID-19 pandemic in the USA

Tsion Firew [1,2] Ellen D Sano,[1] Jonathan W Lee,[1,3] Stefan Flores,[1] Kendrick Lang [4] Kiran Salman,[5] M Claire Greene,[6,7] Bernard P Chang[1]

To cite: Firew T, Sano ED, Lee JW, *et al*. Protecting the front line: a cross-sectional survey analysis of the occupational factors contributing to healthcare workers' infection and psychological distress during the COVID-19 pandemic in the USA. *BMJ Open* 2020;**10**:e042752. doi:10.1136/bmjopen-2020-042752

► Prepublication history and additional material for this paper is available online. To view these files, please visit the journal online (http://dx.doi.org/10.1136/bmjopen-2020-042752).

Received 16 July 2020
Revised 03 October 2020
Accepted 06 October 2020

Check for updates

© Author(s) (or their employer(s)) 2020. Re-use permitted under CC BY-NC. No commercial re-use. See rights and permissions. Published by BMJ.

For numbered affiliations see end of article.

Correspondence to
Dr Tsion Firew;
tsionfirew@gmail.com

ABSTRACT

Objective The COVID-19 pandemic has been associated with significant occupational stressors and challenges for front-line healthcare workers (HCWs), including COVID-19 exposure risk. Our study sought to assess factors contributing to HCW infection and psychological distress during the COVID-19 pandemic in the USA.

Design We conducted a cross sectional survey of HCWs (physicians, nurses, emergency medical technicians (EMTs), non-clinical staff) during May 2020. Participants completed a 42-item survey assessing disease transmission risk (clinical role, work environment, availability of personal protective equipment) and mental health (anxiety, depression and burn-out).

Setting The questionnaire was disseminated over various social media platforms. 3083 respondents from 48 states, the District of Columbia and US territories accessed the survey.

Participants Using a convenience sample of HCWs who worked during the pandemic, 3083 respondents accessed the survey and 2040 participants completed at least 80% of the survey.

Primary outcome Prevalence of self-reported COVID-19 infection, in addition to burn-out, depression and anxiety symptoms.

Results Participants were largely from the Northeast and Southern USA, with attending physicians (31.12%), nurses (26.80%), EMTs (13.04%) with emergency medicine department (38.30%) being the most common department and specialty represented. Twenty-nine per cent of respondents met the criteria for being a probable case due to reported COVID-19 symptoms or a positive test. HCWs in the emergency department (31.64%) were more likely to contract COVID-19 compared with HCWs in the ICU (23.17%) and inpatient settings (25.53%). HCWs that contracted COVID-19 also reported higher levels of depressive symptoms (mean diff.=0.31; 95% CI 0.16 to 0.47), anxiety symptoms (mean diff.=0.34; 95% CI 0.17 to 0.52) and burn-out (mean diff.=0.54; 95% CI 0.36 to 0.71).

Conclusion HCWs have experienced significant physical and psychological risk while working during the COVID-19 pandemic. These findings highlight the urgent need for increased support for provider physical and mental health well-being.

Strengths and limitations of this study

- ► One of the largest samples of healthcare workers to date on the COVID-19 pandemic in the USA.
- ► Diverse sample of both clinicians and non-clinician hospital staff including physicians, nurses, technologists and security staff.
- ► Broad assessment of the impact of occupational resource availability and its impact on physical and mental health.
- ► Despite an attempt to enrol a diverse sample of healthcare workers, our sample was under-represented by certain professions and ethnicities.
- ► Convenience sample that may have missed participants not using social media, and it was limited to English-speaking participants only.

INTRODUCTION

The COVID-19 pandemic has created a dramatic global disruption, with over 28 million confirmed cases and over 900 000 deaths globally and 6.4 million cases and 190,00 deaths in the USA. as of 1 September 2020.[1] Early reports have already described the physical and psychological morbidity associated with COVID-19.[2–7] Healthcare workers (HCWs) across all specialties and fields, have encountered unprecedented challenges in patient care, personal safety (eg, disease transmission risk) and psychological distress both on themselves and their loved ones. Initial data by the the Centers for Disease Control and Prevention /National Center of Health Statistics showed 9282 infected

HCWs with an 8% hospitalisation rate and mortality rate of 0.3%, though conclusions were limited given that the report occurred at a time during which widespread testing was minimally available in the USA, and only 16% of the nearly 1.5 million respondents answered regarding occupational status.[8]

Delays in early testing, and lack of adequate personal protective equipment (PPE) may place HCW at increased risk of exposure to COVID-19. The lack of access to PPE has been heavily reported in the press and social media platforms.[9 10] In addition to transmission risk, significant mental health complaints among HCWs have emerged from this pandemic. Reports from the COVID-19 pandemic from both China, USA and Europe have already found that HCWs have significant levels of self-reported anxiety, depression and even symptoms of post-traumatic stress disorder.[2–6]

Early surveys of the COVID-19 HCW response have largely been limited to hospital-based physicians and nurses, not factoring the diverse group of essential staff (eg, security, clerical, technologists) that are exposed to the same working environment as other clinicians. Additionally, few studies have looked at the association of factors such as PPE availability and testing, with subsequent COVID-19 infection in HCWs.[11–15] Finally, the association of these variables with psychological distress and clinician burn-out has not been described. While previous studies have broadly described occupational stressors and lack of availability of PPE for frontline providers, few studies have attempted to sample a broad range of both clinicians and non-clinical healthcare staff. For example, security staff and technologists may face many of the health risks that clinicians such as nurses and physicians make, yet little is known about the health and psychological outcomes in these individuals. Additionally, with the current pandemic and restrictions in the conduct of research during this elevated time of infection risk, the use of platforms such as social media, may permit the rapid collection of a diverse and broad range of providers. The aim of our study was to provide a broad overview of a diverse group of HCWs and their perceived risk during the COVID-19 pandemic. We surveyed HCWs to assess the factors associated with disease transmission risk (eg, access to PPE, clinical characteristics and testing availability) in addition to mental health sequelae of COVID-19 (eg, depressive/anxiety symptoms, burn-out).

METHODS

Design and setting

We conducted a cross-sectional survey, using a convenience sample of US HCWs who worked on the front lines during the COVID-19 pandemic in 48 states, the District of Columbia and US territories (Puerto Rico, US Virgin Islands) during May 2020. The survey followed the Checklist for Reporting Results of Internet E-Surveys (CHERRIES). The survey was disseminated using various Social Media Platforms (Facebook, Twitter, Instagram), and healthcare professional social media groups with a QR scan code and a link that directed participants into a Health Insurance Portability and Accountability Act (HIPAA) approved Qualtrics web survey which required 5 min to complete (online supplemental file). Qualtrics's anonymised response feature was enabled so participant IP addresses were not viewable by the research team. The data collection period for this study was 7 days.

Participants

All individuals who were at least 18 years of age, self-identified as essential HCWs and who interacted with COVID-19 patients were eligible for participation. Participants accessed the survey using the link or QR scan code over a period of 7 days. They were given resources for mental health support at the end of the survey. No personal identification information was collected.

Questionnaire

We developed a 42-item survey with questions on PPE, COVID-19 testing, demographic information, professional responsibilities and practice location, self-assessment of exposure, isolation behaviour, peer and family illness history, and psychological distress (online supplemental file). Content of the survey was evaluated by an expert consensus panel of five board certified physicians, one epidemiologist with training in biostatistics, one medical student and one public health student, who also performed a narrative review of potential risk factors for COVID-19 infection risk (eg, disease exposure, place of work environment, gender, race).

Outcomes

Demographics

Respondents were asked their age, gender, race, location (city, state) and zip code of their healthcare institution.

Clinical setting and healthcare role

Respondents were also asked to identify their primary clinical setting of practice, clinical setting of practice specifically when interacting with COVID-19 patients, role in the hospital and healthcare specialty.

Personal protective equipment

Respondents were asked to rate how often PPE was available at their primary institution on a scale ranging from all the time, most of the time, half of the time, rarely, to never. Respondents were additionally asked if PPE training was provided in the past 6 months.

COVID-19 exposure, symptoms and testing

COVID-19 exposure was assessed in many ways. Respondents were asked to approximate the number of COVID-19 patients they treated, the approximate percentage of working hours they were in close contact with COVID-19 patients, and if they were present during an aerosolising procedure with a confirmed COVID-19 patient or person under investigation for COVID-19. For the dependent variable, HCW infection, the independent

variables included race, gender, adequacy of PPE and clinical setting.

Isolation and mental health

Respondents were asked to identify the isolation mechanism/precautions they took at home as well as COVID-19 symptom and disease prevalence among family members.

Mental health questions included the Patient Health Questionnaire-2 (PHQ-2), a validated screener for depressive symptoms and anhedonia,[16] a Generalised Anxiety Disorder-2 (GAD-2) a validated anxiety screening instrument,[17] as well as a validated one-item version of the Maslach Burn-out Inventory assessing psychological burn-out.[18] PHQ-2 evaluates depressive symptoms and GAD-2 evaluates symptoms of anxiety. They have been commonly used as screening tools for depression, GADs and to assess general psychological distress. We reported continuous scores instead of using cut-offs given these scales have not been validated in HCWs within the context of COVID-19.[18] The Maslach Burn-out Inventory has been validated among physicians and nurses, specifically the singe item inventory used in our survey provided meaningful information on burn-out in medical professionals.[18–21] To assess the dependent variable of psychological stress, we included the independent variables of self-isolation, COVID-infection and illness of a family or a friend.

Statistical analysis

We restricted our sample to respondents who completed at least 80% of the survey. In this analytical sample, we described the distribution of demographic and clinical characteristics. We constructed log-binomial models to calculate the prevalence ratio (PR) describing the relative probability of infection by demographic characteristics (age, gender, race/ethnicity, US region), clinical workplace characteristics (position, specialty, regular clinical setting, clinical setting during COVID-19 crisis), exposure to COVID-19 patients and access to PPE. We classified respondents as infected if they reported a positive COVID-19 test (ie, confirmed case) or symptoms consistent with COVID-19 (ie, probable case). Among probable and confirmed cases, we described the distribution of symptoms as well as their access to testing. We explored whether demographic and clinical setting characteristics were associated with the likelihood of testing. We then examined whether COVID-19 infection (self, family, coworker) and self-isolation practices were associated with psychological distress and burn-out using linear regression models to estimate the mean difference (ie, beta coefficient) in these outcomes by infection and self-isolation practices. To examine the robustness of our findings to case definitions (eg, probable, confirmed), we replicated the analyses examining correlates of infection comparing confirmed cases only to the rest of the sample. P values less than 0.05 were considered statistically significant. All results were conducted using Stata, V.14.

Patient and public involvement

No patient involved.

RESULTS

Over 3083 respondents from 48 states, the District of Columbia and US territories (Puerto Rico, US Virgin Islands) accessed the survey and 1771 respondents completed the survey in its entirety. We excluded respondents who completed less than 80% of the survey (n=1043) resulting in a final analytic sample of 2040 respondents. Respondents were an average of 39.50 years of age (SD=10.11), primarily female (70.26%), Caucasian (67.89%), from the Northeast (47.12%) or Southern US (25.29%) and attending physicians (31.08%) or nurses (26.76%). In addition to attending physicians and nurses, the sample included emergency medical technicians (EMTs) (13.04%), resident physicians or fellows (8.82%), physician assistants (3.97%), and other HCW (16.32%). As shown in table 1A, emergency medicine (EM) was the most common specialty (38.30%) and the emergency department (ED) was the most common clinical setting where the respondent practised while treating patients with COVID-19 (31.91%). About one-third of respondents worked in an academic institution (34.46%) or community hospital (35.49%) with fewer participants working in outpatient facilities (13.19%), city hospitals (13.09%), prehospital settings (12.65%), long-term care or skilled nursing facilities (4.22%), or other clinical settings (8.68%). As shown in table 2, Hispanic, Latino or Spanish HCWs were more likely than Caucasian HCWs to contract COVID-19 (PR 1.71, 95% CI 1.39 to 2.12) as were HCWs from the northeast relative to all other US regions. We did not find differences in the probability of infection by age or gender.

With regard to clinical characteristics, nurses and EMTs were 26% and 33% more likely to contract COVID-19 relative to attending physicians, respectively. Relative to HCWs specialising in EM, critical care and paediatric specialists were less likely to have been infected (critical care: PR=0.81, 95% CI 0.66 to 0.98; paediatrics: PR=0.54, 95% CI 0.37 t 0.78). Similarly, relative to HCWs working in the ED during COVID-19, HCWs in the Intensive Care Unit and inpatient hospital settings displayed a lower probability of infection (ICU: PR=0.73, 95% CI 0.58 to 0.92; inpatient hospital: PR=0.81, 95% CI 0.66 to 0.98). We did not observe differences in the probability of infection between those working in EM compared with prehospital, outpatient or long-term care and nursing home facilities. HCWs working in community hospitals were less likely to contract COVID-19 relative to all other clinical practice settings (PR=0.76, 95% CI 0.66 to 0.89).

COVID-19 testing as a function of demographic and clinical workplace characteristics

In this sample, the likelihood of COVID-19 testing was less likely among female HCWs (PR=0.86, 95% CI 0.76 to 0.99) and more likely among Hispanic, Latino or Spanish

Table 1A Demographic and clinical correlates of COVID-19 infection among HCWs

	Full sample, n=2040	Not tested, n=1379 (67.63%)	Tested, n=660 (32.37%)	Prevalence ratio (95% CI)
Age, M (SD)	39.50 (10.11)	39.34 (10.19)	39.86 (9.95)	1.00 (1.00 to 1.01)
Gender, n (row %)				
Male	594 (29.15)	381 (64.14)	213 (35.86)	REF
Female	1432 (70.26)	988 (69.04)	443 (30.96)	0.86 (0.76 to 0.99)
Other/prefer not to answer	12 (0.59)	9 (75)	3 (25)	0.70 (0.26 to 1.87)
Race/ethnicity, n (row %)				
Caucasian	1385 (67.89)	954 (68.93)	430 (31.07)	REF
Hispanic, Latino or Spanish	111 (5.44)	66 (59.46)	45 (40.54)	1.30 (1.03 to 1.66)
Black or African American	221 (10.83)	160 (72.40)	61 (27.6)	0.89 (0.71 to 1.12)
Asian	190 (9.31)	110 (57.89)	80 (42.11)	1.36 (1.13 to 1.63)
Other	48 (2.35)	35 (72.92)	13 (27.08)	0.87 (0.54 to 1.40)
Multiracial	85 (4.17)	54 (63.53)	31 (36.47)	1.17 (0.88 to 1.57)
Region, n (row %)				
Northeast	883 (47.12)	527 (59.68)	356 (40.32)	REF
Midwest	248 (13.23)	187 (75.71)	60 (24.29)	0.60 (0.48 to 0.76)
South	474 (25.29)	360 (75.95)	114 (24.05)	0.60 (0.50 to 0.71)
West	268 (14.30)	190 (70.90)	78 (29.1)	0.72 (0.59 to 0.88)
US territories	1 (0.05)	0 (0)	1 (100)	Not estimable
Role in hospital, n (row %)				
Physician attending	634 (31.12)	416 (65.62)	218 (34.38)	REF
Physician resident/fellow	180 (8.84)	129 (71.67)	51 (28.33)	0.82 (0.64 to 1.06)
Physician assistant	81 (3.98)	54 (66.67)	27 (33.33)	0.97 (0.70 to 1.34)
Nurse (practitioner, general, registered)	546 (26.80)	364 (66.79)	181 (33.21)	0.97 (0.82 to 1.13)
Emergency medicine services	266 (13.06)	159 (59.77)	107 (40.23)	1.17 (0.98 to 1.40)
Non-direct patient care	96 (4.71)	68 (70.83)	28 (29.17)	0.85 (0.61 to 1.18)
Other physical care/direct patient contact	122 (5.99)	95 (77.87)	27 (22.13)	0.64 (0.45 to 0.91)
Child life specialist	85 (4.17)	70 (82.35)	15 (17.65)	0.51 (0.32 to 0.82)
Respiratory therapist	20 (0.98)	15 (75)	5 (25)	0.73 (0.34 to 1.57)
Other	7 (0.34)	7 (100)	0 (0)	Not estimable
Specialty, n (row %)				
Emergency medicine	694 (38.30)	458 (65.99)	236 (34.01)	REF
Internal medicine (outpatient, inpatient, other)	271 (14.96)	183 (67.53)	88 (32.47)	0.95 (0.78 to 1.17)
Internal medicine (critical care)	215 (11.87)	155 (72.09)	60 (27.91)	0.82 (0.65 to 1.04)
Surgery	86 (4.75)	59 (68.60)	27 (31.4)	0.92 (0.66 to 1.28)
Paediatrics	149 (8.22)	113 (75.84)	36 (24.16)	0.71 (0.52 to 0.96)
Family medicine	83 (4.58)	52 (62.65)	31 (37.35)	1.10 (0.82 to 1.48)
Other	314 (17.33)	207 (65.92)	107 (34.08)	1.00 (0.83 to 1.21)
Clinical setting, n (row %)				
Emergency medicine	651 (31.91)	449 (68.97)	202 (31.03)	REF
ICU	328 (16.08)	221 (67.58)	106 (32.42)	1.04 (0.86 to 1.27)
Inpatient hospital	427 (20.93)	300 (70.26)	127 (29.74)	0.96 (0.80 to 1.15)
Pre-hospital	176 (8.63)	106 (60.23)	70 (39.77)	1.28 (1.03 to 1.59)
Outpatient	224 (10.98)	156 (69.64)	68 (30.36)	0.98 (0.78 to 1.23)
Long-term care facility/nursing home	74 (3.63)	34 (45.95)	40 (54.05)	1.74 (1.37 to 2.21)

Continued

Table 1A Continued

	Full sample, n=2040	Not tested, n=1379 (67.63%)	Tested, n=660 (32.37%)	Prevalence ratio (95% CI)
Other	160 (7.84)	113 (70.63)	47 (29.38)	0.95 (0.73 to 1.24)
Facility type, n (row %)				
Academic institution, n (%)	703 (34.46)	467 (66.43)	236 (33.57)	1.06 (0.93 to 1.21)
Community hospital, n (%)	724 (35.49)	518 (71.55)	206 (28.45)	0.82 (0.72 to 0.95)
Outpatient facility, n (%)	269 (13.19)	186 (69.14)	83 (30.86)	0.95 (0.78 to 1.15)
City hospital, n (%)	267 (13.09)	194 (72.93)	72 (27.07)	0.82 (0.66 to 1.00)
Long-term care/skilled nursing facility, n (%)	86 (4.22)	42 (48.84)	44 (51.16)	1.62 (1.31 to 2.01)
Prehospital/ambulance/EMS, n (%)	258 (12.65)	162 (62.79)	96 (37.21)	1.17 (0.99 to 1.40)
Other clinical setting, n (%)	177 (8.68)	130 (73.45)	47 (26.55)	0.81 (0.63 to 1.04)

HCWs relative to Caucasian HCWs (PR=1.30, 95% CI 1.03 to 1.66). HCW testing was most likely in the Northeast relative to all other US regions. Relative to attending physicians, child life specialists (PR=0.51, 95% CI 0.32 to 0.82) and HCWs with other physical care and direct contact roles (PR=0.64, 95% CI 0.45 to 0.91) were less likely to be tested. Paediatric specialists were the least likely to be tested, which was significantly lower than the likelihood of testing among EM HCWs (PR=0.71, 95% CI 0.52 to 0.96). The likelihood of testing was significantly higher among HCWs working in prehospital (PR=1.28, 95% CI 1.03 to 1.59) or long-term care facilities (PR=1.74, 95% CI 1.37 to 2.21) relative to EM settings. Additionally, HCWs in community hospitals had a lower probability of being tested relative to HCWs in other facilities (PR=0.82, 95% CI 0.72 to 0.95).

COVID-19 exposure, PPE, and HCW infection

Almost half of our sample reported that PPE was available all the time (47.60%). We observed a dose–response effect such that relative to HCWs reporting that PPE was available less than half of the time, those reporting that PPE was available most of the time displayed a 33% reduction in the probability of infection (PR=0.67, 95% CI 0.56 to 0.79), and those reporting PPE was available all of the time displayed a 45% reduction in the probability of infection (PR=0.55, 95% CI 0.46 to 0.66; table 2). Furthermore, HCWs who recently received training on how to wear PPE were less likely to contract COVID-19 (PR=0.76, 95% CI 0.66 to 0.87). Providing care to a larger number of COVID-19 patients was associated with an increased likelihood of infection, particularly if HCWs cared for over 100 patients (PR=1.63, 95% CI 1.34 to 2.00) or were in close contact with COVID-19 patients over 50% of their working hours (PR=1.41, 95% CI 1.20 to 1.66). Being present during an aerosolising procedure was not associated with probability of infection.

In a post hoc analyses we examined whether the association between race/ethnicity and COVID-19 infection was confounded by age, gender, geographical location, facility type or proportion of patients with COVID-19. We observed an attenuation in the increased risk of COVID-19 infection for Asian relative to White HCWs (PR=1.15, 95% CI 0.71 to 1.21). The association between race/ethnicity and COVID-19 infection did not appear to be confounded by these demographic and clinical covariates for other racial/ethnic groups.

COVID-19 symptoms and testing

Twenty-nine per cent of respondents met our criteria for being a probable case due to reported COVID-19 symptoms or a positive test. Those who reported COVID-19 symptoms (29.26%; ie, probable cases) or were unsure if they had COVID-19 symptoms (10.69%) described experiencing a range of symptoms including cough (60.61%), headache (56.32%), fatigue (54.85%), muscle/joint aches (50.31%), fever/chills (48.83%), sore throat (48.59%), shortness of breath (34.72%), nausea, vomiting or diarrhoea (28.96%), loss/change of taste (17.42%), loss of smell (17.30%), weight loss/loss of appetite (7.73%), chest discomfort (1.60%),±eye manifestation (0.86%), nasal congestion/runny nose (0.74%) or other symptoms (10.67%). Fifty-seven per cent of HCWs with symptoms received a COVID-19 test as compared with 22.55% of HCWs without symptoms and 18.35% of HCWs who were unsure whether they had experienced COVID-19 symptoms. Among those who received testing (n=660, 32.37%), most received a PCR test (46.91%), followed by both a PCR and antibody test (38.58%), and an antibody test alone (14.51%). Most of these respondents received a test from their primary institution (60.00%).

Isolation, COVID-19 and psychological health

Most providers reported taking precautions to protect the individuals they lived with, including taking all necessary precautions at home (56.96%), isolation (41.39%), moving into a different residence temporarily (12.09%) or sending cohabitants away from home (7.27%). Isolation and living alone were associated with significantly higher levels of depressive symptoms. Isolation, moving into a different residence, and taking necessary precautions at home while continuing to live with cohabitants were associated with elevated anxiety symptoms. Isolation and sending cohabitants away from home were associated

Table 1B Demographic and clinical correlates of COVID-19 testing among HCWs

	Full sample, n=2040	No infection, n=1442 (70.69%)	COVID-19 infection, n=598 (29.31%)	Prevalence ratio (95% CI)
Age, M (SD)	39.50 (10.11)	39.48 (10.27)	39.57 (9.71)	1.00 (0.99 to 1.01)
Gender, n (row %)				
Male	594 (29.15)	426 (71.72)	168 (28.28)	REF
Female	1432 (70.26)	1006 (70.25)	426 (29.75)	1.05 (0.90 to 1.22)
Other/prefer not to answer	12 (0.59)	9 (75)	3 (25)	0.88 (0.33 to 2.37)
Race/ethnicity, n (row %)				
Caucasian	1385 (67.89)	999 (72.13)	386 (27.87)	REF
Hispanic, Latino or Spanish	111 (5.44)	58 (52.25)	53 (47.75)	1.71 (1.39 to 2.12)
Black or African American	221 (10.83)	169 (76.47)	52 (23.53)	0.84 (0.66 to 1.09)
Asian	190 (9.31)	126 (66.32)	64 (33.68)	1.21 (0.97 to 1.50)
Other	48 (2.35)	34 (70.83)	14 (29.17)	1.05 (0.67 to 1.64)
Multiracial	85 (4.17)	56 (65.88)	29 (34.12)	1.22 (0.90 to 1.66)
Region, n (row %)				
Northeast	883 (47.12)	572 (64.78)	311 (35.22)	REF
Midwest	248 (13.23)	181 (72.98)	67 (27.02)	0.77 (0.61 to 0.96)
South	474 (25.29)	370 (78.06)	104 (21.94)	0.62 (0.51 to 0.75)
West	268 (14.30)	200 (74.63)	68 (25.37)	0.72 (0.58 to 0.90)
US territories	1 (0.05)	1 (100)	0 (0)	Not estimable
Role in hospital, n (row %)				
Physician attending	634 (31.12)	465 (73.34)	169 (26.66)	1.00 (., .)
Physician resident/fellow	180 (8.84)	132 (73.33)	48 (26.67)	1.00 (0.76 to 1.32)
Physician assistant	81 (3.98)	60 (74.07)	21 (25.93)	0.97 (0.66 to 1.44)
Nurse (practitioner, general, registered)	546 (26.80)	362 (66.3)	184 (33.7)	1.26 (1.06 to 1.51)
Emergency medicine services	266 (13.06)	172 (64.66)	94 (35.34)	1.33 (1.08 to 1.63)
Non-direct patient care	96 (4.71)	64 (66.67)	32 (33.33)	1.25 (0.92 to 1.71)
Other physical care/direct patient contact	122 (5.99)	86 (70.49)	36 (29.51)	1.11 (0.82 to 1.50)
Child life specialist	85 (4.17)	74 (87.06)	11 (12.94)	0.49 (0.28 to 0.86)
Respiratory therapist	20 (0.98)	20 (100)	0 (0)	–
Other	7 (0.34)	5 (71.43)	2 (28.57)	1.07 (0.33 to 3.48)
Specialty, n (row %)				
Emergency medicine	694 (38.30)	477 (68.73)	217 (31.27)	REF
Internal medicine (outpatient, inpatient, other)	271 (14.96)	188 (69.37)	83 (30.63)	0.98 (0.79 to 1.21)
Internal medicine (critical care)	215 (11.87)	166 (77.21)	49 (22.79)	0.73 (0.56 to 0.95)
Surgery	86 (4.75)	60 (69.77)	26 (30.23)	0.97 (0.69 to 1.36)
Paediatrics	149 (8.22)	124 (83.22)	25 (16.78)	0.54 (0.37 to 0.78)
Family medicine	83 (4.58)	62 (74.7)	21 (25.3)	0.81 (0.55 to 1.19)
Other	314 (17.33)	210 (66.88)	104 (33.12)	1.06 (0.87 to 1.28)
Clinical setting, n (row %)				
Emergency medicine	651 (31.91)	445 (68.36)	206 (31.64)	REF
ICU	328 (16.08)	252 (76.83)	76 (23.17)	0.73 (0.58 to 0.92)
Inpatient hospital	427 (20.93)	318 (74.47)	109 (25.53)	0.81 (0.66 to 0.98)
Prehospital	176 (8.63)	120 (68.18)	56 (31.82)	1.01 (0.79 to 1.28)
Outpatient	224 (10.98)	143 (63.84)	81 (36.16)	1.14 (0.93 to 1.41)

Continued

Table 1B Continued

	Full sample, n=2040	No infection, n=1442 (70.69%)	COVID-19 infection, n=598 (29.31%)	Prevalence ratio (95% CI)
Long-term care facility/nursing home	74 (3.63)	48 (64.86)	26 (35.14)	1.11 (0.80 to 1.54)
Other	160 (7.84)	116 (72.5)	44 (27.5)	0.87 (0.66 to 1.15)
Facility type, n (row %)				
Academic institution, n (%)	703 (34.46)	492 (69.99)	211 (30.01)	1.04 (0.90 to 1.19)
Community hospital, n (%)	724 (35.49)	547 (75.55)	177 (24.45)	0.76 (0.66 to 0.89)
Outpatient facility, n (%)	269 (13.19)	187 (69.52)	82 (30.48)	1.05 (0.86 to 1.27)
City hospital, n (%)	267 (13.09)	196 (73.41)	71 (26.59)	0.89 (0.72 to 1.11)
Long-term care/skilled nursing facility, n (%)	86 (4.22)	58 (67.44)	28 (32.56)	1.12 (0.82 to 1.52)
Pre-hospital/ambulance/EMS, n (%)	258 (12.65)	171 (66.28)	87 (33.72)	1.18 (0.98 to 1.42)
Other clinical setting, n (%)	177 (8.68)	123 (69.49)	54 (30.51)	1.04 (0.83 to 1.32)

EMS, emergency medical services; HCWs, healthcare workers.

with higher levels of burn-out. Nineteen per cent of respondents reported taking no precautions in the home. These respondents reported significantly lower levels of depressive symptoms, anxiety symptoms and burn-out relative to participants who reported taking any of these precautions (table 3).

COVID-19 infection among respondents and their families and coworkers was also related to psychological well-being. Having been infected with COVID-19 was associated with significantly higher levels of depression, anxiety and burn-out. Having a family member contract COVID-19 was not associated with psychological health

Table 2 COVID-19 exposure, personal protective equipment (PPE) and HCW infection

	No infection, n=1442 (70.69%)	COVID-19 infection, n=598 (29.31%)	Prevalence ratio (95% CI)
Availability of PPE, n (row %)			
Half of the time or less	143 (55.21)	116 (44.79)	REF
Most of the time	568 (70.12)	242 (29.88)	0.67 (0.56 to 0.79)
All of the time	731 (75.28)	240 (24.72)	0.55 (0.46 to 0.66)
Receipt of PPE training in past 6 months, n (row %)			
No	322 (64.14)	180 (35.86)	REF
Yes	1120 (72.82)	418 (27.18)	0.76 (0.66 to 0.87)
No of COVID-19 patients treated in the past 6 months, n (row %)			
1–10 patients	407 (75.79)	130 (24.21)	REF
11–50 patients	502 (70.60)	209 (29.4)	1.21 (1.01 to 1.47)
51–100 patients	234 (70.69)	97 (29.31)	1.21 (0.97 to 1.52)
>100 patients	197 (60.43)	129 (39.57)	1.63 (1.34 to 2.00)
Percentage of working hours put in close contact with COVID-19 patients, n (row %)			
Less than 25%	529 (75.36)	173 (24.64)	REF
25%–50% of the time	312 (71.23)	126 (28.77)	1.17 (0.96 to 1.42)
Over 50% of the time	511 (65.18)	273 (34.82)	1.41 (1.20 to 1.66)
Present during an aerosolising procedure, n (row %)			
No	559 (72.22)	215 (27.78)	REF
Yes	752 (69.63)	328 (30.37)	1.09 (0.95 to 1.26)
Unsure	88 (67.69)	42 (32.31)	1.16 (0.88 to 1.53)

HCWs, healthcare workers.

Table 3 Isolation, COVID-19 infection and mental health

	n (col %)	Depressive symptoms (PHQ-2) M (SD)	Depressive symptoms (PHQ-2) B (95% CI)	Anxiety symptoms (GAD-2) M (SD)	Anxiety symptoms (GAD-2) B (95% CI)	Burn-out M (SD)	Burn-out B (95% CI)
**Isolation**							
Isolated yourself while caring for COVID-19 patients							
No	1191 (58.61)	1.60 (1.54)	REF	2.17 (2.27	REF	2.82 (1.78)	REF
Yes	841 (41.39)	2.01 (1.66)	0.41 (0.26 to 0.55)	2.48 (1.88)	0.30 (0.14 to 0.46)	3.09 (1.87)	0.27 (0.10 to 0.43)
Sent cohabitants away from home							
No	1825 (92.73)	1.77 (1.60)	REF	2.28 (1.80)	REF	2.91 (1.83)	REF
Yes	143 (7.27)	1.92 (1.56)	0.15 (−0.12 to 0.43)	2.54 (1.80)	0.26 (−0.06 to 0.57)	3.24 (1.80)	0.32 (0.01 to 0.64)
Moved into a different residence temporarily							
No	1730 (87.91)	1.76 (1.59)	REF	2.26 (1.79)	REF	2.92 (1.81)	REF
Yes	238 (12.09%)	1.93 (1.67)	0.17 (−0.05 to 0.39)	2.60 (1.89)	0.34 (0.09 to 0.59)	3.09 (1.91)	0.18 (−0.08 to 0.43)
Took all necessary precautions at home							
No	847 (43.04)	1.78 (1.65)	REF	2.18 (1.79)	REF	2.92 (1.86)	REF
Yes	1121 (56.96)	1.78 (1.56)	−0.00 (−0.15 to 0.14)	2.39 (1.81)	0.21 (0.05 to 0.37)	2.95 (1.80)	0.02 (−0.14 to 0.19)
Live alone so I did not need to self-isolate							
No	1674 (85.06)	1.73 (1.55)	REF	2.33 (1.79)	REF	2.93 (1.83)	REF
Yes	294 (14.94)	2.08 (1.83)	0.35 (0.15 to 0.55)	2.11 (1.87)	−0.22 (−0.45 to 0.01)	3.00 (1.83)	0.08 (−0.15 to 0.31)
Did not take any precautions at home							
No	1588 (80.69)	1.86 (1.63)	REF	2.37 (1.82)	REF	2.98 (1.82)	REF
Yes	380 (19.31)	1.43 (1.43)	−0.43 (−0.61 to −0.25)	2.01 (1.71)	−0.36 (−0.57 to -0.16)	2.74 (1.85)	−0.24 (−0.45 to −0.04)
**COVID-19 infection**							
Probable/confirmed COVID-19 infection							
No	1442 (70.69)	1.68 (1.58)	REF	2.20 (1.77)	REF	2.77 (1.77)	REF
Yes	598 (29.31)	1.99 (1.65)	0.31 (0.16 to 0.47)	2.54 (1.87)	0.34 (0.17 to 0.52)	3.31 (1.90)	0.54 (0.36 to 0.71)
If you tested positive or had symptoms of COVID-19, did any of your family members							
No	460 (64.25)	1.92 (1.69)	REF	2.29 (1.82)	REF	3.08 (1.90)	REF
Yes	74 (10.34)	2.01 (1.69)	0.10 (−0.31 to 0.51)	2.59 (2.05)	0.30 (−0.15 to 0.75)	3.32 (1.92)	0.24 (−0.23 to 0.70)
Unsure	182 (25.42)	2.05 (1.52)	0.13 (−0.15 to 0.42)	2.71 (1.73)	0.42 (0.10 to 0.74)	3.26 (1.79)	0.19 (−0.14 to 0.52)
Did your family member die from this disease?							
No	73 (98.65)	2.04 (1.68)	REF	2.61 (2.06)	REF	3.28 (1.91)	REF
Yes	1 (1.35)	0.00 (0.00)	Not estimable	1.00 (0.00)	Not estimable	6.00 (0.00)	Not estimable
Did anyone in your family die from COVID-19?							

Continued

Table 3 Continued

	n (col %)	Depressive symptoms (PHQ-2)		Anxiety symptoms (GAD-2)		Burn-out	
		M (SD)	B (95% CI)	M (SD)	B (95% CI)	M (SD)	B (95% CI)
No	1929 (94.74)	1.77 (1.60)	REF	2.30 (1.80)	REF	2.92 (1.82)	REF
Yes	107 (5.26)	1.90 (1.78)	0.14 (–0.18 to 0.46)	2.31 (1.97)	0.02 (–0.34 to 0.37)	3.03 (1.92)	0.11 (–0.25 to 0.47)
Did you have a coworker that contracted COVID-19?							
No	349 (17.18)	1.44 (1.54)	REF	1.98 (1.74)	REF	2.45 (1.76)	REF
Yes	1456 (71.65)	1.81 (1.60)	0.37 (0.18 to 0.56)	2.32 (1.80)	0.34 (0.13 to 0.56)	2.97 (1.81)	0.52 (0.31 to 0.74)
Unsure	227 (11.17)	2.00 (1.67)	0.56 (0.29 to 0.83)	2.63 (1.88)	0.66 (0.36 to 0.96)	3.33 (1.87)	0.88 (0.57 to 1.18)
Do you know anyone in your department who is/was admitted to the hospital because of COVID-19?							
No	1174 (57.92)	1.65 (1.56)	REF	2.28 (1.81)	REF	2.85 (1.81)	REF
Yes	853 (42.08)	1.94 (1.66)	0.29 (0.15 to 0.43)	2.33 (1.81)	0.05 (–0.11 to 0.21)	3.02 (1.83)	0.17 (0.01 to 0.33)
Do you know anyone in your department who died because of COVID-19?							
No	1724 (84.93)	1.73 (1.57)	REF	2.28 (1.80)	REF	2.90 (1.81)	REF
Yes	226 (11.13)	1.93 (1.71)	0.20 (–0.02 to 0.42)	2.33 (1.80)	0.04 (–0.21 to 0.30)	3.01 (1.86)	0.11 (–0.15 to 0.36)
Unsure	80 (3.94)	2.29 (1.95)	0.57 (0.20 to 0.93)	2.58 (1.98)	0.30 (–0.11 to 0.71)	3.15 (1.96)	0.25 (–0.16 to 0.67)
Approximately how many COVID-19 patients did you treat in the past 6 months?							
1–10 patients	537 (28.19)	1.60 (1.58)	REF	2.28 (1.82)	REF	2.79 (1.80)	REF
11–50 patients	711 (37.32)	1.84 (1.60)	0.24 (0.06 to 0.42)	2.39 (1.83)	0.11 (–0.09 to 0.32)	2.96 (1.83)	0.17 (–0.04 to 0.37)
51–100 patients	331 (17.38)	1.79 (1.56)	0.19 (–0.03 to 0.41)	2.16 (1.76)	–0.11 (–0.36 to 0.14)	3.01 (1.78)	0.22 (–0.03 to 0.47)
>100 patients	326 (17.11)	1.87 (1.64)	0.27 (0.05 to 0.50)	2.24 (1.71)	–0.04 (–0.28 to 0.21)	2.99 (1.83)	0.20 (–0.05 to 0.45)
What percentage of your working hours put you in close contact with COVID-19 patients							
Less than 25%	702 (36.49)	1.59 (1.50)	REF	2.19 (1.71)	REF	2.68 (1.70)	REF
25%–50% of the time	438 (22.77)	1.71 (1.53)	0.12 (–0.07 to 0.31)	2.25 (1.81)	0.06 (-0.16 to 0.27)	2.94 (1.85)	0.26 (0.04 to 0.48)
Over 50% of the time	784 (40.75)	1.96 (1.70)	0.37 (0.21 to 0.54)	2.40 (1.86)	0.20 (0.02 to 0.39)	3.15 (1.86)	0.46 (0.28 to 0.65)

GAD-2, Generalised Anxiety Disorder-2; PHQ-2, Patient Health Questionnaire-2.

Issues; however, being unsure whether a family member contracted COVID-19 was associated with significantly higher levels of anxiety. Five per cent and 11% of our sample reported having someone in their family or a coworker die from COVID-19, respectively. Having a family or coworker die from COVID-19 was not significantly associated with any of the psychological health measures assessed. However, having a coworker contract COVID-19 was associated with significantly greater anxiety, depression and burn-out. Similarly, having a coworker from the respondent's department admitted to the hospital because of COVID-19 was associated with higher levels of depressive symptoms and burn-out. Higher numbers of COVID-19 patients treated was associated with higher levels of depressive symptoms. Spending over 50% of work hours in close contact with COVID-19 patients was also associated with higher levels of depression, anxiety and burn-out relative to individuals who spent less than 25% of working hours in close contact with COVID-19 patients.

Sensitivity analyses

We examined whether our results were sensitive to our case definition, which included both confirmed and probable cases. In analyses that compared confirmed cases to both probable cases or non-symptomatic persons, we found similar results with few exceptions. First, in the sensitivity analysis we identified an elevated risk of confirmed infection for HCWs in academic institutions (PR=1.47; 95% CI 1.04 to 2.09) that was not identified when our case definition for infection included both confirmed and probable cases. Second, while we found elevated levels of psychological distress and burn-out among confirmed and probable cases, these associations were nullified when restricted to confirmed cases only.

DISCUSSION

To our knowledge, this is among the largest national surveys of HCWs during the COVID-19 pandemic assessing healthcare provider risk in the USA. Even though some studies have looked at the HCWs' infection and psychological well-being, our study sought to assess the factors contributing to these outcomes.[13–15] Overall, our results corroborate presumptions regarding the correlation between various risk factors and HCW infection with more recent studies showing adequacy of PPE, clinical settings, gender and ethnic background as important factors of HCW infection.[22–25] Our sample of HCWs, overall had higher reported COVID-19 infection risk (29%) compared with general population estimates.[26] Furthermore, unlike other studies, we sampled a diverse set of HCWs and explored the impact of secondary factors, including specific role in the healthcare industry, the effects of isolation while being infected, the risks of family members, and the effects of coworkers being afflicted with COVID-19 on psychological well-being. Mindful of the challenges of in person recruitment during the pandemic, we were able to leverage social media platforms to rapidly obtain a broad and diverse sample of HCWs across the country.

One of the factors that contributed to HCW infection was availability of PPE and training. Those who had inadequate access to PPE or inadequate PPE training were at higher risk of developing COVID-19 symptoms, showing a dose dependent effect. The lack of preparation and availability of PPE in the initial response might have contributed to the risk of increased infection. According to the OSHA act, though hospitals and other healthcare facilities are required to provide their workforce with workplaces free from known hazard, the dearth of clear evidence and clear guidelines on PPE have led to increased infection in HCWs.[27 28] Furthermore, in places like in New York, the spread of the disease was underestimated at the time of detection, and HCWs were exposed to COVID-19 patients before proper guidelines were placed.[29] While some hospitals have provided compensation for their employees and the federal government sponsored assistance through the Coronavirus Aid, Relief, and Economic Security (CARES) act, however, the resources were not allocated universally, often excluding the low-wage essential health workers.[30–32]

Practice location and hospital role were also associated with an increased risk for infection of specific providers. Nurses and EMTs in our sample reported the highest risk of COVID-19 infection compared with other roles, consistent with presumptions that those who have higher frequency and duration of direct patient contact are at increased risk. Following these roles, physicians were the next most likely to be infected. Risk for infection varied by specialty; Clinicians working in the ED were at greater risk than other critical care areas including the ICU and inpatient setting. These findings may be associated with acute care environmental factors, such as crowding and physical spacing of patients in the ED milieu compared with other hospital settings. HCWs who saw higher numbers of COVID-19 patients, were at higher risk of developing infection. Finally, survey respondents who were involved in aerosolising procedures did not appear at increased risk of disease.

Vulnerability to infection risk varied by certain demographics. We found that people that self-identified as Hispanic, Latino and/or Spanish were at increased risk of infection. This observation might be due to the arguments previously made that PPEs, like space suits, are usually made for the average Caucasian male though we didn't see any gender related variance in our survey. While this survey is limited because of the relatively small sampling of non-Caucasian respondents, further analysis may be better equipped to address whether any such variations exist.[33]

Another important parameter we evaluated was the psychological health of HCWs during the pandemic. Our study evaluated three parameters of psychological health, including depressive symptoms, anxiety symptoms and burn-out. Overall, those HCWs who had COVID-19 were

at higher risk of all three psychological health outcomes. For depressive symptoms, those who lived alone were at highest risk, likely due to social isolation experienced during quarantine. These respondents, however, also had the lowest risk for anxiety and burn-out. On the other hand, those who temporarily lived in a different residence had the highest risk of anxiety and to a lesser degree burn-out.

For burn-out, those who sent their cohabitants away were at highest risk of burn-out and, to a lesser extent, anxiety and depressive symptoms. The general trend implies that those who were alone (whether already living alone or having temporarily lived alone) were at risk for developing mental health consequences. Besides isolation, lack of testing for respondents with COVID-19 symptoms also attributed to depression symptoms. In support of this idea, those respondents who neither took precaution nor isolated, had the lowest risk of poor mental health outcomes.

LIMITATIONS

Despite a robust sample size and attempt to enrol a diverse sample of HCWs, our sample was under-represented by certain professions and ethnicities. Those groups include nursing home staff, clerical workers, the outpatient setting, resident physicians and mid-level providers, while critical care specialties were overrepresented. Most of our respondents came from New York City. Although the global epicentre at the time of writing, it may not be a representative sample of the USA as a whole. The ethnic group under-represented in our study have historically also been under-represented in medicine as a whole, so our low percentage rates may also be a reflection of the current demographics of the medical profession.[34] When comparing our data to the healthcare industry workforce from the Bureau of Labor Statistics, individuals employed in Healthcare and Social assistance are predominantly women (78.1%) with Whites(72.0%) making up the majority compared with Blacks or African Americans (17.7%), Asians (6.9%) and Hispanics (14.2%).[35] Our survey had whites (67.89%), black or African Americans (10.83%), Asians (9.31%) and Hispanics (5.44%). Our survey was accessed by more females than male HCWs and we lacked the representation of blacks and Hispanic HCWs, but other races reflected the distribution in the USA. Since most of our respondents were physicians (31.2%) and nurses (26.80%), our respondents' racial data reflects the distribution of occupation specific data for nurses and physicians from the US department of Health and Human Services with the percentage of Hispanic physicians (6.3%), black or African American physicians(4.8%), Hispanic nurses (5.7%) and Black or African American nurses (10.4%).[36]

Additionally, our recruitment strategy focused on primarily English speaking social media channels, our sample did not capture a diverse group of non-English-speaking clinicians.

Recall bias may have existed with certain questions, such as, the availability of PPE, COVID-19 symptoms and/or infection/mortality among coworkers/family members. While this sample was restricted to participants who completed at least 80% of the survey, we still had some item-level missingness. Levels of missingness were low and thus unlikely to bias our results. In addition, our survey was anonymous, and zip codes were used as a surrogate for primary worksite location, but this still may have prevented potential respondents from completing the survey for fear of job security or privacy. Other than social media and personal networks, we did not use any promotional material, as this would have involved further institutional involvement, which may have affected response validity. Furthermore, as this is a web-based survey, to ensure quality, we used the CHERRIES as outlined in online supplemental table 1.

Future studies should also aim to incorporate more nuanced research questions, including the under-represented groups in our sample.

CONCLUSION

Our study assessed factors contributing to HCW infection and psychological distress during the COVID-19 pandemic in the USA, shedding light on the multipole challenges experienced by HCWs. Building on our work and others, we hope future investigations will provide key insight into the development of system wide interventions aimed at supporting HCWs during this unprecedented global pandemic.

Author affiliations
[1]Department of Emergency Medicine, Columbia University Irving Medical Center, New York, New York, USA
[2]Office of the Minister, Ethiopia Ministry of Health, Addis Ababa, Ethiopia
[3]Department of Emergnecy Medicine, Mount Sinai Medical Center, New York, New York, USA
[4]School of Medicine, Columbia University Vagelos College of Physicians and Surgeons, New York, New York, USA
[5]General Public Health, Columbia University Mailman School of Public Health, New York, New York, USA
[6]Global Mental Health Program, New York State Psychiatric Institute, New York, New York, USA
[7]Heilbrunn Department of Population and Family Health, Mailman School of Public Health, Columbia University, New York, New York, USA

Twitter Tsion Firew @drtsion

Contributors TF, the corresponding author, was responsible for the conception of the study, acquisition, analysis and interpretation of the data. EDS, JWL, SF, MCG and BPC along with KS and KL made substantial contribution to the design, acquisition and analysis of the data and drafted the work for important intellectual content. The authors approve the submission of this manuscript for publication and agree to be accountable for all aspects of the work.

Funding The authors have not declared a specific grant for this research from any funding agency in the public, commercial or not-for-profit sectors.

Competing interests BPC receives support from NIH listed here: NHLBI: HL R01 141811, and HL R01 146911.

Patient consent for publication Not required.

Provenance and peer review Not commissioned; externally peer reviewed.

Data availability statement All data relevant to the study are included in the article or uploaded as online supplemental information.

Supplemental material This content has been supplied by the author(s). It has not been vetted by BMJ Publishing Group Limited (BMJ) and may not have been peer-reviewed. Any opinions or recommendations discussed are solely those of the author(s) and are not endorsed by BMJ. BMJ disclaims all liability and responsibility arising from any reliance placed on the content. Where the content includes any translated material, BMJ does not warrant the accuracy and reliability of the translations (including but not limited to local regulations, clinical guidelines, terminology, drug names and drug dosages), and is not responsible for any error and/or omissions arising from translation and adaptation or otherwise.

Open access This is an open access article distributed in accordance with the Creative Commons Attribution Non Commercial (CC BY-NC 4.0) license, which permits others to distribute, remix, adapt, build upon this work non-commercially, and license their derivative works on different terms, provided the original work is properly cited, appropriate credit is given, any changes made indicated, and the use is non-commercial. See: http://creativecommons.org/licenses/by-nc/4.0/.

ORCID iDs
Tsion Firew http://orcid.org/0000-0001-5219-3295
Kendrick Lang http://orcid.org/0000-0002-4156-6495

REFERENCES

1 Dong E, Du H, Gardner L. An interactive web-based dashboard to track COVID-19 in real time. *Lancet Infect Dis* 2020;20:533–4.
2 Tan BYQ, Chew NWS, Lee GKH, *et al*. Psychological impact of the COVID-19 pandemic on health care workers in Singapore. *Ann Intern Med* 2020;173:317–20.
3 Shanafelt T, Ripp J, Trockel M. Understanding and addressing sources of anxiety among health care professionals during the COVID-19 pandemic. *JAMA* 2020;323:2133.
4 Pfefferbaum B, North CS. Mental health and the Covid-19 pandemic. *N Engl J Med* 2020;383:510–2.
5 Lai J, Ma S, Wang Y, *et al*. Factors associated with mental health outcomes among health care workers exposed to coronavirus disease 2019. *JAMA Netw Open* 2020;3:e203976.
6 Jin Y-H, Huang Q, Wang Y-Y, *et al*. Perceived infection transmission routes, infection control practices, psychosocial changes, and management of COVID-19 infected healthcare workers in a tertiary acute care hospital in Wuhan: a cross-sectional survey. *Mil Med Res* 2020;7:24.
7 Godderis L, Boone A, Bakusic J. COVID-19: a new work-related disease threatening healthcare workers. *Occup Med* 2020;70:315–6.
8 CDC COVID-19 Response Team. Characteristics of Health Care Personnel with COVID-19 - United States, February 12-April 9, 2020. *MMWR Morb Mortal Wkly Rep* 2020;69:477–81.
9 Livingston E, Desai A, Berkwits M. Sourcing personal protective equipment during the COVID-19 pandemic. *JAMA* 2020;323:1912.
10 Jacobs A, Richtel M, Baker M. *At war with no ammo': doctors say shortage of protective gear is dire*. New York Times, 2020: 1547–8.
11 Sharma A, Maxwell CR, Farmer J, *et al*. Initial experiences of US neurologists in practice during the COVID-19 pandemic via survey. *Neurology* 2020;95:215–20.
12 Felice C, Di Tanna GL, Zanus G, *et al*. Impact of COVID-19 outbreak on healthcare workers in Italy: results from a national E-Survey. *J Community Health* 2020;45:675–83.
13 Xiao X, Zhu X, Fu S, *et al*. Psychological impact of healthcare workers in China during COVID-19 pneumonia epidemic: a multi-center cross-sectional survey investigation. *J Affect Disord* 2020;274:405–10.
14 Prescott K, Baxter E, Lynch C, *et al*. COVID-19: how prepared are front-line healthcare workers in England? *J Hosp Infect* 2020;105:142–5.
15 Zhang M, Zhou M, Tang F, *et al*. Knowledge, attitude, and practice regarding COVID-19 among healthcare workers in Henan, China. *J Hosp Infect* 2020;105:183–7.
16 Arroll B, Goodyear-Smith F, Crengle S, *et al*. Validation of PHQ-2 and PHQ-9 to screen for major depression in the primary care population. *Ann Fam Med* 2010;8:348–53.
17 Plummer F, Manea L, Trepel D, *et al*. Screening for anxiety disorders with the GAD-7 and GAD-2: a systematic review and diagnostic metaanalysis. *Gen Hosp Psychiatry* 2016;39:24–31.
18 Staples LG, Dear BF, Gandy M, *et al*. Psychometric properties and clinical utility of brief measures of depression, anxiety, and general distress: the PHQ-2, GAD-2, and K-6. *Gen Hosp Psychiatry* 2019;56:13–18.
19 Dolan ED, Mohr D, Lempa M, *et al*. Using a single item to measure burnout in primary care staff: a psychometric evaluation. *J Gen Intern Med* 2015;30:582–7.
20 West CP, Dyrbye LN, Sloan JA, *et al*. Single item measures of emotional exhaustion and depersonalization are useful for assessing burnout in medical professionals. *J Gen Intern Med* 2009;24:1318–21.
21 Schaufeli WB, Bakker AB, Hoogduin K, *et al*. On the clinical validity of the maslach burnout inventory and the burnout measure. *Psychol Health* 2001;16:565–82.
22 Ali S, Noreen S, Farooq I, *et al*. Risk assessment of healthcare workers at the frontline against COVID-19. *Pak J Med Sci* 2020;36:S99–103.
23 Nguyen LH, Drew DA, Graham MS, *et al*. Risk of COVID-19 among front-line health-care workers and the general community: a prospective cohort study. *Lancet Public Health* 2020;5:e475–83.
24 Lan F-Y, Filler R, Mathew S, *et al*. COVID-19 symptoms predictive of healthcare workers' SARS-CoV-2 PCR results. *PLoS One* 2020;15:e0235460.
25 The Lancet. COVID-19: protecting health-care workers. *Lancet* 2020;395:922.
26 Chow N, Fleming-Dutra K, Gierke R, *et al*. Preliminary Estimates of the Prevalence of Selected Underlying Health Conditions Among Patients with Coronavirus Disease 2019 - United States, February 12-March 28, 2020. *MMWR Morb Mortal Wkly Rep* 2020;69:382.
27 ReliefWeb. Rational Use of Personal Protective Equipment (PPE) for Coronavirus Disease (COVID-19) - Interim Guidance (19 March 2020) - World. Available: reliefweb.int/report/world/rational-use-personal-protective-equipment-ppe-coronavirus-disease-covid-19-interim
28 United States of Department of Labor. COVID-19 - Control and Prevention | Healthcare Workers and Employers | Occupational Safety and Health Administration. Available: www.osha.gov/SLTC/covid-19/healthcare-workers.html
29 COVID-19: Data. COVID-19: Data Main - NYC Health, 2020. Available: www1.nyc.gov/site/doh/covid/covid-19-data.page
30 Brookings. Kinder, Molly. “Essential but Undervalued: Millions of Health Care Workers Aren't Getting the Pay or Respect They Deserve in the COVID-19 Pandemic", 2020. Available: https://www.brookings.edu/research/essential-but-undervalued-millions-of-health-care-workers-arent-getting-the-pay-or-respect-they-deserve-in-the-covid-19-pandemic/
31 U.S. Department of the Treasury. The cares act works for all Americans, 2020. Available: https://home.treasury.gov/policy-issues/cares
32 World Bank Blogs, 2020. Available: blogs.worldbank.org/governance/how-increase-compensation-health-workers-during-covid-19
33 Pugh R. *COVID-19 PPE gender divide: no One-Size-Fits-All? Medscape*. Medscape, 2020.
34 Garrick JF, Perez B, Anaebere TC, *et al*. The diversity Snowball effect: the quest to increase diversity in emergency medicine: a case study of highland's emergency medicine residency program. *Ann Emerg Med* 2019;73:639–47.
35 U.S. Bureau of Labor Statistics, U.S. Bureau of Labor Statistics. Employed persons by detailed industry, sex, race, and Hispanic or Latino ethnicity, 2020. Available: www.bls.gov/cps/cpsaat18.htm
36 U.S. Department of Health and Human Services, Health Resources and Services Administration, National Center for Health Workforce Analysis. *Sex, race, and ethnic diversity of U.S, health occupations (2011-2015)*. Rockville, Maryland, 2017.
