# Supplementary material for: Mental health status and related factors influencing healthcare workers during the COVID-19 pandemic: A systematic review and meta-analysis
Source: PLoS One. 2024 Jan 19;19(1):e0289454. doi: 10.1371/journal.pone.0289454 (PMC10798549; doi:10.1371/journal.pone.0289454)
Supplement: S1 Data — (ZIP) [file pone.0289454.s011.zip › literatures/4.pdf]

International Journal of
Environmental Research and Public Health

MDPI

Article

A Cross-Sectional Study to Examine the Psychological Impact of the COVID-19 Pandemic on Healthcare Workers in Kuwait

Sarah AlKandari [1,*], Ahmad Salman [2], Fatima Al-Ghadban [3,4] and Rasheed Ahmad [1]

1 Immunology and Microbiology Department, Dasman Diabetes Institute, Dasman 15462, Kuwait
2 Ministry of Health, Safat 13001, Kuwait
3 College of Public Health and Human Sciences, Oregon State University, Corvallis, OR 97331, USA
4 Department of Public Health Practice, Faculty of Public Health, Kuwait University, Safat 13110, Kuwait
* Correspondence: sarah.alkandari@dasmaninstitute.org

Abstract: In this study, we aimed to evaluate the psychological impact of the COVID-19 pandemic on healthcare workers to determine the prevalence of symptoms of depression, anxiety, and well-being, and to identify the factors associated with adverse psychological effects. This study was conducted 5 months into the COVID-19 pandemic. We used an online questionnaire to collect data from 378 healthcare workers. To examine the psychological impact, three standardized questionnaires were utilized. This includes the Patient Health Questionnaire (PHQ-9), the Generalized Anxiety Disorder Scale (GAD-7), and the WHO Well-Being Scale (WHO-5) to measure depression, anxiety, and quality of life, respectively. More than half of the participants (52.9%) exhibited moderate or high levels of depression, and 40.5% reported moderate or high levels of anxiety. Unmarried HCWs reported more severe levels of depression; moderately severe depression (24.0% vs. 16.1%) and severe depression (12.4% vs. 6.8%). Unmarried HCWs also reported more severity of anxiety as well as lower overall wellbeing. Understanding how personal factors such as marital status can influence the degree of psychological distress can allow us to make better investments in supporting the mental health needs of HCWs in Kuwait. Governments and organizations must establish protective measures, such as continually assessing the mental health status of HCWs throughout the pandemic and providing support services for HCWs in need to minimize adverse consequences and ensure optimal health system operation.

Keywords: COVID-19; healthcare workers; mental health

check for updates

Citation: AlKandari, S.; Salman, A.; Al-Ghadban, F.; Ahmad, R. A Cross-Sectional Study to Examine the Psychological Impact of the COVID-19 Pandemic on Healthcare Workers in Kuwait. *Int. J. Environ. Res. Public Health* **2022**, *19*, 10464. https://doi.org/10.3390/ijerph191710464

Academic Editor: Francisco Sampaio

Received: 4 July 2022
Accepted: 9 August 2022
Published: 23 August 2022

Publisher's Note: MDPI stays neutral with regard to jurisdictional claims in published maps and institutional affiliations.

Copyright: © 2022 by the authors. Licensee MDPI, Basel, Switzerland. This article is an open access article distributed under the terms and conditions of the Creative Commons Attribution (CC BY) license (https://creativecommons.org/licenses/by/4.0/).

1. Introduction

In December 2019, the city of Wuhan in China reported an increasing number of cases of atypical pneumonia. The virus responsible was named severe acute respiratory syndrome coronavirus 2 (SARS-CoV-2), and the disease it causes has become known as COVID-19. On 11 March 2020, the World Health Organization (WHO) declared the SARS-CoV-2 outbreak a public health pandemic. To date (16 May 2022), COVID-19 has been responsible for 6.26 million deaths, infecting 521 million people. The COVID-19 pandemic has drastically altered daily lives globally. To mitigate the spread of the disease, governments have had to enforce many restrictions. Globally people face government mandated home confinement "lockdowns," banning of public gatherings, closure of schools and universities, closure of non-essential business activities, and closure of national borders [1].

In Kuwait, the first confirmed case of COVID-19 was announced on 24 February 2020. Shortly after, on 11 March 2020, the Kuwaiti government enforced a partial lockdown, which extended into a full lockdown at the end of April 2020. The healthcare sector in Kuwait is largely based on governmentally provided healthcare services. While the research and medical community focused on treating and controlling the spread of the disease, the psychological impact was neglected.

There have been several studies to date that have reported on the adverse psychological effect of the COVID-19 pandemic on the general population, including anxiety [2,3], depression [3,4], stress [5], post-traumatic stress disorder [5], fear-related behaviours [6], insomnia [7], and low wellbeing [8]. A study conducted among general populations in Asia and Europe in 2020 reported that the prevalence of stress was 29.6%, anxiety was 31.9% (95% CI, 27.5–36.7), and depression was 33.7% [9]. A study conducted in Kuwait on the general population showed that 53.7% of respondents experienced anxiety, and 59.6% of respondents experienced depression [10].

Healthcare Workers (HCWs) have carried a heavy psychological burden during this pandemic [11]. They have been exposed to working during staffing shortages, worrying about contracting and spreading the disease, redeployment due to staffing shortages without adequate training, shortages of Personal Protective Equipment (PPE), frequently exposed to patients suffering and dying, and constantly adapting and implementing policies, procedures and guidelines related to the virus [12]. The pandemic has compromised HCWs' balance of their professional duty and personal fear of the disease, putting them at risk of emotional exhaustion and reduced professional efficacy during a dire time [13].

Several studies have reported the adverse psychological effects of the COVID-19 pandemic on HCWs. A meta-analysis of 47 international studies on 81,277 healthcare workers found the pooled prevalence of anxiety to be 37% and depression to be 36% [14]. Another study conducted a meta-analysis on a total sample set of 79,437 HCWs. They found the prevalence of anxiety at 34.4%, depression at 31.4%, stress at 40.3%, and burnout at 37.5% [15]. A study on HCWs in Kuwait reported the prevalence of severe anxiety at 36.7% and moderately severe or severe depression at 66.6% [16].

Understanding the psychological distress of HCWs during a public health pandemic is both useful and necessary. Identifying the causes of distress and accelerating the development of mitigating interventions is important. Psychological protective factors can mitigate the worsening of the negative psychological factors associated with COVID-19 [17]. Studies have found that personal characteristics such as marital status can have risk modifying effects on psychological health outcomes. Being married has been reported to be related to lower levels of both anxiety and depression [18].

The objectives of the present study were to determine the prevalence and to identify the factors associated with the psychological impact of the COVID-19 pandemic on healthcare workers in Kuwait. We hypothesized that the pandemic would adversely affect the psychological health of HCWs. Findings from this study will allow us to better understand and support the mental health needs of HCWs.

2. Materials and Methods

2.1. Study Design

This was a cross-sectional study conducted between the dates of 7 July 2020 and 15 July 2020. HCWs working in COVID-19 exposed settings in Kuwait were invited to participate using the snowball technique of convenience sampling. Inclusion criteria included adults over 18 working directly with COVID-19 patients in governmental or private hospitals. Researchers identified groups of HCWs working in various hospitals throughout Kuwait and disseminated the online questionnaire via a free, commonly utilized messaging application (app) called WhatsApp (add the trademark symbol). HCWs received a WhatsApp message inviting them to participate in the study by clicking on the survey link. The bilingual questionnaire was developed using Survey Monkey, and participants were given the option of responding in Arabic or English. A total of 378 HCWs participated in this study.

2.2. Ethical Considerations

This study was conducted in line with the principles of the Helsinki Declaration. The Ethical Review Committee of Dasman Diabetes Institute approved this study (RA-HM-2020-006). All participants were required to provide informed consent digitally prior to

the commencement of the questionnaire. Consent forms were provided in both Arabic and English. The questionnaire was anonymous, and all information was secured confidentially.

2.3. Sampling

We developed a structured questionnaire survey using validated instruments comprising four sections totaling 28 questions. On average, the questionnaire required between 5 to 10 minutes to complete.

Section one of the survey consisted of questions related to demographical information. Sections two to four comprised validated measures, including the WHO Well-Being Index-5 (WHO-5) [19], the Patient Health Questionnaire (PHQ-9) [20], and the Generalized Anxiety Disorder (GAD-7) [21].

Sample Characteristics

Table 1 shows demographic data for all study participants. A total of 378 healthcare workers completed the questionnaire. All HCWs were above the age of 18.

Table 1. Characteristics of study participants stratified by marital status.

Characteristics	Total (*n* = 378)	Married (*n* = 249)	Unmarried (*n* = 129)
	Frequency (%)	Frequency (%)	Frequency (%)
Gender			
Male	117 (31.0)	87 (34.9)	30 (23.3)
Female	261 (69.0)	162 (65.1)	99 (76.7)
Age Category			
21–29	99 (26.2)	46 (18.5)	53 (41.1)
30–39	200 (52.9)	139 (55.8)	61 (47.3)
≥40	79 (20.9)	64 (25.7)	15 (11.6)

2.4. Measures

2.4.1. WHO (Five) Well-Being Index

The 5-item WHO-5 index is one of the most utilised validated questionnaires for assessing subjective psychological well-being. It has good internal consistency reliability of Cronbach's $\alpha = 0.858$. The questionnaire. The questionnaire consists of 5 questions with response options based on a 6-point Likert scale with scores ranging from 0 (none of the time) to 5 (all the time). Raw scores range from 0 to 25, with 0 representing the worst possible and 25 representing the best possible quality of life. Raw scores are converted to a percentage score by multiplying the raw score by 4. 100% represents the best possible well-being, and scores at 50% or lower represent low well-being [22]. The scale provides a subjective quality of life assessment based on positive mood, vitality, and general interest in life. The scale has been validated as a screening tool for subjective quality of life among adults [19].

2.4.2. Patient Health Questionnaire

The Patient Health Questionnaire (PHQ-9) is a self-administered diagnostic instrument used to screen, diagnose, and monitor depression. It has excellent internal consistency reliability of Cronbach's $\alpha = 0.894$. This instrument assesses individuals on depressive symptoms. The questionnaire consists of 9 items related to symptoms of depression experienced in the last two weeks. Items are scored from 0 (not at all) to 3 (nearly every day), and total scores range from 0 to 27, with increasing scores indicating the severity of symptoms. Cut points of 5, 10, and 15 represent mild, moderate, and severe levels of depressive and anxiety symptoms [20].

2.4.3. General Anxiety Disorder-7

General Anxiety Disorder-7 (GAD-7) is a validated rapid screening tool for the presence of clinically significant anxiety disorder. It has good internal consistency reliability of Cronbach's α = 0.83. The GAD-7 consists of 7 items related to symptoms of anxiety experienced in the last two weeks. Items are individually scored from 0 (not at all) to 3 (nearly every day), and total scores range from 0 to 21, with increasing scores indicating the severity of symptoms. Total scores of 5, 10, and 15 are the cut points for mild, moderate, and severe anxiety, respectively [21].

2.5. Statistical Analysis

Data analysis was conducted using GraphPad PRISM statistical software version 7.0 (GraphPad Software Inc., La Jolla, CA, USA). Descriptive statistics were used to describe findings reported as frequencies and percentages. A *p*-value of $\leq$ 0.05 was considered statistically significant. Pearson's chi-square test was used to examine differences in psychological symptoms based on various characteristics, including gender and marital status. Binomial logistic regression analysis of symptoms of depression, anxiety, and well-being was conducted among married and unmarried healthcare workers

3. Results

3.1. Prevalence of Depression, Anxiety, and Well-Being Severity among HCWs by Marital Status

Table 2 reports the prevalence of depression (PHQ-9) and anxiety (GAD-7) severity, and total well-being (WHO-5) score among healthcare workers stratified by marital status. Overall, more than half of HCWs reported moderate or higher levels of depression (52.9%), and 40.5% reported moderate or higher levels of anxiety. Among married HCWs, 50.6% reported moderate or higher levels of depression, and 37.8% reported moderate or higher levels of anxiety. Among unmarried HCWs, 57.3% reported moderate or higher levels of depression, and 45.7% reported moderate or higher levels of anxiety. Unmarried HCWs reported more severe depression (12.4%) compared to total and married HCWs (8.7% and 6.8%, respectively). Unmarried HCWs reported less minimal depression (10.9%) compared to total and married HCWs (17.2% and 20.5%, respectively) and less minimal anxiety (19.4%) compared to total and married HCWs (23.5% and 25.7%, respectively). For the full sample, the total well-being score mean (SD) was 46.69 (22.23), and we observed a lower mean well-being score among unmarried HCWs compared to married HCWs (a score of 100 indicates optimal well-being).

Table 2. Prevalence of depression, anxiety, and well-being among healthcare workers stratified by marital status.

	**Total (*n* = 378)**	**Married (*n* = 249)**	**Unmarried (*n* = 129)**
PHQ-9 [a]	Frequency (%)		
Minimal depression	65 (17.2)	51 (20.5)	14 (10.9)
Mild depression	113 (29.9)	72 (28.9)	41 (31.8)
Moderate depression	96 (25.4)	69 (27.7)	27 (20.9)
Moderately severe depression	71 (18.8)	40 (16.1)	31 (24.0)
Severe depression	33 (8.7)	17 (6.8)	16 (12.4)
GAD-7 [b]	Frequency (%)		
Minimal anxiety	89 (23.5)	64 (25.7)	25 (19.4)
Mild anxiety	136 (36.0)	91 (36.5)	45 (34.9)
Moderate anxiety	77 (20.4)	45 (18.1)	32 (24.8)
Severe anxiety	76 (20.1)	49 (19.7)	27 (20.9)
WHO-5 [c]	Mean (SD)		
WHO-5 total score	46.69 (22.23)	47.76 (21.98)	44.62 (22.65)

Abbreviations: PHQ-9: Patient Health Questionnaire-9, GAD-7: Generalized Anxiety Disorder-7, WHO-5: World Health Organization Five Well-Being Index. [a] PHQ-9 cut-offs defined as minimal (0–4), mild (5–9), moderate (10–14), moderately severe (15–19), and severe (20–27). [b] GAD-7 cut-offs defined as minimal (0–4), mild (4–9), moderate (10–14), and severe (15–21). [c] WHO-5 total score based on raw score multiplied by 4.

3.2. Prevalence of WHO-5, PHQ-9, GAD-7 Item Responses among HCWs by Marital Status

Table 3 shows marital status differences in HCWs responses to the WHO-5 questionnaire on subjective quality of life based on positive mood, vitality, and general interest in life. Among HCWs who responded more than half the time or higher, married HCWs reported higher frequency on all five well-being items. More married HCWs reported feeling cheerful and in good spirits compared to unmarried HCWs (47.4% and 38.0%, respectively). More married HCWs reported feeling calm and relaxed compared to unmarried HCWs (39.0% and 36.5%, respectively). More married HCWs reported feeling active and vigorous compared to unmarried HCWs (41.4% and 32.5%, respectively). More married HCWs reported waking up feeling fresh and rested compared to unmarried HCWs (39.3% and 29.5%, respectively). Finally, more married HCWs reported that their life is filled with things that interest them compared to unmarried HCWs (45.4% and 37.2%, respectively).

Table 3. World Health Organization-5 Well-Being Index item responses among healthcare workers stratified by marital status (*n* = 378, married = 249, unmarried = 129).

WHO-5 Items	Marital Status	At no Time	Some of the Time	Less than Half of the Time	More than Half of the Time	Most of the Time	All of the Time	*p*-Value	Cramer's V
					n (%)				
I have felt cheerful and in good spirits	Married<br>Unmarried	10 (4.0)<br>4 (3.1)	37 (14.9)<br>28 (21.7)	84 (33.7)<br>48 (37.2)	55 (22.1)<br>17 (13.2)	45 (18.1)<br>23 (17.8)	18 (7.2)<br>9 (7.0)	0.273	0.130
I have felt calm and relaxed	Married<br>Unmarried	16 (6.4)<br>12 (9.3)	56 (22.5)<br>28 (21.7)	80 (32.1)<br>42 (32.6)	49 (19.7)<br>18 (14.0)	34 (13.7)<br>24 (18.6)	14 (5.6)<br>5 (3.9)	0.490	0.108
I have felt active and vigorous	Married<br>Unmarried	13 (5.2)<br>14 (10.9)	44 (17.7)<br>19 (14.7)	89 (35.7)<br>54 (41.9)	50 (20.1)<br>16 (12.4)	37 (14.9)<br>19 (14.7)	16 (6.4)<br>7 (5.4)	0.153	0.146
I woke up feeling fresh and rested	Married<br>Unmarried	23 (9.2)<br>11 (8.5)	60 (24.1)<br>42 (32.6)	68 (27.3)<br>38 (29.5)	53 (21.3)<br>13 (10.1)	29 (11.6)<br>20 (15.5)	16 (6.4)<br>5 (3.9)	0.062	0.167
My daily life has been filled with things that interest me	Married<br>Unmarried	9 (3.6)<br>9 (7.0)	53 (21.3)<br>31 (24.0)	74 (29.7)<br>41 (31.8)	52 (20.9)<br>20 (15.5)	49 (19.7)<br>21 (16.3)	12 (4.8)<br>7 (5.4)	0.506	0.107

Abbreviations: WHO-5: World Health Organization Five Well-Being Index. Significance $p \leq 0.05$ *.

Table 4 reports marital status differences in HCWs responses to the PHQ-9 depression items. Among HCWs who responded to the PHQ-9 items more than half the days or higher, which includes response options 'more than half the days' and 'nearly every day', unmarried HCWs reported higher frequency on all nine items compared to married HCWs. Unmarried HCWs reported a higher frequency of having poor appetite or overeating compared to married HCWs (62.8% and 44.2%, respectively), trouble falling or staying asleep or sleeping too much (56.6% and 46.6%, respectively), feeling tired or having little energy (56.6% and 50.6%, respectively), little interest or pleasure in doing things (47.3% and 37.7%, respectively), feeling down, depressed, or hopeless (43.4% and 38.5%, respectively), trouble concentrating (35.7% and 29.8%, respectively), feeling bad about themselves (32.5% and 27.3%, respectively), moving or speaking slow or being too fidgety (27.1% and 20.5%, respectively), and suicidal ideation (11.7% and 6.0%, respectively). PHQ-9 items related to poor appetite or overeating and suicidal ideation were significantly different between married and unmarried HCWs ($p \leq 0.05$).

Table 4. Patient Health Questionnaire-9 item responses among healthcare workers stratified by marital status (*n* = 378, married = 249, unmarried = 129).

PHQ-9 Items	Marital Status	Not at All	Several Days	More than Half the Days	Nearly Every Day	*p*-Value	Cramer's V
				n (%)			
Little interest or pleasure in doing things	Married<br>Unmarried	45 (18.1)<br>16 (12.4)	110 (44.2)<br>52 (40.3)	63 (25.3)<br>37 (28.7)	31 (12.4)<br>24 (18.6)	0.206	0.110
Feeling down, depressed, or hopeless	Married<br>Unmarried	57 (22.9)<br>20 (15.5)	96 (38.6)<br>53 (41.1)	65 (26.1)<br>28 (21.7)	31 (12.4)<br>28 (21.7)	0.052	0.143

Table 4. *Cont.*

PHQ-9 Items	Marital Status	Not at All	Several Days	More than Half the Days	Nearly Every Day	*p*-Value	Cramer's V
				n (%)			
Trouble falling or staying asleep, or sleeping too much	Married<br>Unmarried	48 (19.3)<br>14 (10.9)	85 (34.1)<br>42 (32.6)	69 (27.7)<br>36 (27.9)	47 (18.9)<br>37 (28.7)	0.060	0.140
Feeling tired or having little energy	Married<br>Unmarried	28 (11.2)<br>16 (12.4)	95 (38.2)<br>40 (31.0)	63 (25.3)<br>33 (25.6)	63 (25.3)<br>40 (31.0)	0.507	0.079
Poor appetite or overeating	Married<br>Unmarried	65 (26.1)<br>22 (17.1)	74 (29.7)<br>26 (20.2)	63 (25.3)<br>38 (29.5)	47 (18.9)<br>43 (33.3)	0.003 *	0.192
Feeling bad about yourself—or that you are a failure or have let yourself or your family down	Married<br>Unmarried	109 (43.8)<br>55 (42.6)	72 (28.9)<br>32 (24.8)	38 (15.3)<br>23 (17.8)	30 (12.0)<br>19 (14.7)	0.714	0.060
Trouble concentrating on things, such as reading the newspaper or watching television	Married<br>Unmarried	84 (33.7)<br>40 (31.0)	91 (36.5)<br>43 (33.3)	37 (14.9)<br>29 (22.5)	37 (14.9)<br>17 (13.2)	0.329	0.095
Moving or speaking so slowly that other people could have noticed? Or so fidgety or restless that you have been moving a lot more than usual	Married<br>Unmarried	128 (51.4)<br>55 (42.6)	70 (28.1)<br>39 (30.2)	35 (14.1)<br>27 (20.9)	16 (6.4)<br>8 (6.2)	0.268	0.102
Thoughts that you would be better off dead, or thoughts of hurting yourself	Married<br>Unmarried	217 (87.1)<br>99 (76.7)	17 (6.8)<br>15 (11.6)	7 (2.8)<br>10 (7.8)	8 (3.2)<br>5 (3.9)	0.043 *	0.147

Abbreviations: PHQ-9: Patient Health Questionnaire-9. Significance $p \leq 0.05$ *.

Table 5 reports marital status differences in HCWs responses to the GAD-7 anxiety items. Among HCWs who responded to the GAD-7 items more than half the days or higher, unmarried HCWs reported higher frequency on five of the seven items compared to married HCWs. Unmarried HCWs reported a higher frequency of having trouble relaxing (48.1% and 41.0%, respectively), feeling nervous, anxious, or on edge (46.5% and 35.4%, respectively), becoming easily annoyed or irritable (44.2% and 34.6%, respectively), not being able to stop or control worrying (39.5% and 33.8%, respectively), and worrying too much about different things (39.5% and 36.6%, respectively) compared to married HCWs. Married HCWs reported a higher frequency of feeling afraid, as if something may happen (35.0% and 31.8%, respectively), and being restless (23.7% and 22.5%, respectively) compared to unmarried HCWs. GAD-7 item related to feeling nervous, anxious, or on edge was the only item significantly different between married and unmarried HCWs ($p \leq 0.05$).

Table 5. Generalized Anxiety Disorder-7 item responses among healthcare workers stratified by marital status (*n* = 378, married = 249, unmarried = 129).

GAD-7 Items	Marital Status	Not at All	Several Days	More than Half the Days	Nearly Every Day	*p*-Value	Cramer's V
				n (%)			
Feeling nervous, anxious, or on edge	Married<br>Unmarried	46 (18.5)<br>10 (7.8)	115 (46.2)<br>59 (45.7)	47 (18.9)<br>27 (20.9)	41 (16.5)<br>33 (25.6)	0.016 *	0.166
Not being able to stop or control worrying	Married<br>Unmarried	68 (27.3)<br>24 (18.6)	97 (39.0)<br>54 (41.9)	45 (18.1)<br>27 (20.9)	39 (15.7)<br>24 (18.6)	0.304	0.098
Worrying too much about different things	Married<br>Unmarried	62 (24.9)<br>21 (16.3)	96 (38.6)<br>57 (44.2)	44 (17.7)<br>19 (14.7)	47 (18.9)<br>32 (24.8)	0.144	0.120
Trouble relaxing	Married<br>Unmarried	41 (16.5)<br>24 (18.6)	106 (42.6)<br>43 (33.3)	58 (23.3)<br>38 (29.5)	44 (17.7)<br>24 (18.6)	0.337	0.095
Being so restless that it is hard to sit still	Married<br>Unmarried	108 (43.4)<br>45 (34.9)	82 (32.9)<br>55 (42.6)	36 (14.5)<br>19 (14.7)	23 (9.2)<br>10 (7.8)	0.268	0.102
Becoming easily annoyed or irritable	Married<br>Unmarried	53 (21.3)<br>26 (20.2)	110 (44.2)<br>46 (35.7)	48 (19.3)<br>31 (24.0)	38 (15.3)<br>26 (20.2)	0.300	0.098
Feeling afraid, as if something awful might happen	Married<br>Unmarried	81 (32.5)<br>37 (28.7)	81 (32.5)<br>51 (39.5)	45 (18.1)<br>25 (19.4)	42 (16.9)<br>16 (12.4)	0.427	0.086

Abbreviations: GAD-7: Generalized Anxiety Disorder-7. Significance $p \leq 0.05$ *.

3.3. Binomial Logistic Regression of Well-being, Depression, and Anxiety among HCWs by Marital Status

Table 6 reports binomial regression results for marital status differences in well-being, depression, and anxiety symptoms among HCWs. Unmarried HCWs had a 0.08 unit reduction in PHQ-9 total score compared to married HCWs (p = 0.02). No marital status differences were observed for total scores of well-being or anxiety.

Table 6. Binomial logistic regression analysis of well-being, depression, and anxiety among married and unmarried healthcare workers.

Variables	B	SE	*p*-Value	OR	95% CI for OR	
					Lower	Upper
WHO-5 Total	−0.02	0.03	0.54	0.98	0.93	1.04
PHQ-9 Total	−0.08	0.03	0.02 *	0.92	0.87	0.99
GAD-7 Total	0.03	0.03	0.35	1.03	0.97	1.10
Constant	1.45	0.60	0.02 *	4.28		

Abbreviations: B: unstandardized regression coefficient, SE: standard error of the coefficient, OR: odds ratio, CI: confidence interval. Significance $p \leq 0.05$ *.

4. Discussion

This study shows a high prevalence of anxiety, depression, and low well-being in HCWs during the pandemic, consistent with other studies. All the participants of this study screened positive for an anxiety disorder using the GAD-7 scale, 83.48% of participants screened for depression on the PHQ-9 scale, and low well-being (WHO-5 < 15) was reported by 38.32% of study participants. Our findings are consistent with previous studies showing the negative psychological impact on HCWs [23].

To date, several studies have shown that a large proportion of HCWs have experienced adverse psychological effects due to the COVID-19 pandemic [24–26]. The most common psychiatric disorders identified were post-trauma stress disorder, depression, and anxiety [27]. The COVID-19 pandemic was an unprecedented event with a large mortality outcome [28]. HCWs, especially those on the frontlines, were subjected to undue levels of stress. A study has shown that the negative psychological impact is more severe in medical healthcare workers as opposed to non-medical healthcare workers [29]. Numerous studies conducted in Kuwait during the COVID-19 pandemic found that people living in Kuwait experienced negative psychological impact, such as depression and anxiety attributable to the pandemic-related lockdown [30–32]. This indicates that while the general population of Kuwait is exhibiting signs of a psychological impact on their mental health, HCWs in Kuwait are exhibiting much higher levels.

We further identified a correlation between marital status and the psychological impact of the COVID-19 pandemic. We found that being married was significantly associated with lower PHQ-9 scores (observed reduction of 0.08 units). Studies have found that married people are less likely to report negative mental health due to the COVID-19 pandemic [31]. Another study found that unmarried people showed higher levels of psychological distress [32]. These findings imply that having social support, such as being married, is associated with a lower negative psychological impact.

While there are no studies to date examining the direct correlation between marital status and the psychological impact of the COVID-19 pandemic on HCWs, some studies have found associations. Married HCWs were found to have higher life satisfaction [33] and reported higher scores for fear, depression, anxiety, and stress when compared to unmarried people [6]. Studies consistently show that marital status is associated with psychological well-being [34]. Marital status is perceived to provide social support, and married people are found to have better mental health than unmarried people [35]. The association between marital status and the psychological impact of COVID-19 is under-researched and has yet to yield clear findings.

Negative psychological impact can reduce workplace productivity [36,37]. Delivering effective healthcare during a public health pandemic requires long working hours and a high-pressure working environment. This leads to physical and mental exhaustion, as well as an elevated risk of medical negligence [38]. During the first year of the pandemic, HCWs were also required to take on medical tasks that were not familiar with their typical working days [13]. Expecting healthcare workers to function for a prolonged period in a high-pressure environment in unfamiliar roles will expose them to undue psychological distress. Online technology could allow delivery of psychological support while maintaining social distancing and enabling the dissemination of support services to reach many HCWs. This can include interventions such as a 24-hour help-line, digital podcasts, and online psychological counseling.

This study has several limitations. Firstly, data was obtained via self-reporting questionnaires and was not verified through medical records. Secondly, the study lacks longitudinal follow-up. The COVID-19 pandemic continued for over a year past the point of data collection for this project. Thus, the long-term implications are worth exploring. Thirdly, the study did not assess the socioeconomic status or specific working locations, which could be helpful in assessing outcomes. Fourthly, the sample population was Asian and cannot be generalized to the entire adult population. Finally, this was a cross-sectional study which limits our ability to assess causality.

5. Conclusions

The psychological impact of the COVID-19 pandemic can have important implications on the mental health of HCWs, their productivity, and their ability to provide care. Specifically, our study has contributed by identifying a vulnerable group susceptible to psychological distress and highlights the need for developing meaningful interventions. Understanding how personal factors such as marital status can influence the degree of psychological distress can allow us to make better investments in supporting the mental health needs of HCWs in Kuwait. Investments should be made to establish protective measures, such as continually assessing the mental health status of HCWs throughout the pandemic and providing support services for HCWs in need to allow them to effectively conduct their work while facing high levels of stress and anxiety. It is critical for governments and organizations to invest in the mental health needs of front-line workers to minimize adverse consequences and ensure optimal health system operation.

Author Contributions: Conceptualization, S.A.; Formal analysis, S.A., A.S. and F.A.-G.; Funding acquisition, S.A.; Investigation, S.A., A.S., F.A.-G. and R.A.; Methodology, S.A., A.S. and F.A.-G.; Validation, S.A., A.S., F.A.-G. and R.A.; Visualization, S.A., A.S. and F.A.-G.; Writing—original draft, S.A.; Writing—review & editing, S.A., A.S., F.A.-G. and R.A. All authors have read and agreed to the published version of the manuscript.

Funding: This research was funded by the Kuwait Foundation for the Advancement of Sciences (NO. CORONA-PROP-101).

Institutional Review Board Statement: The study was conducted in accordance with the Declaration of Helsinki, and approved by the Institutional Review Board of DASMAN DIABETES INSTITUTE (RA HM-2020-006) for studies involving humans.

Informed Consent Statement: Informed consent was obtained from all subjects involved in the study.

Data Availability Statement: The data are not publicly available, and any inquiries may be addressed by the corresponding author.

Conflicts of Interest: The authors declare no conflict of interest.

References

1. Fink, G.; Tediosi, F.; Felder, S. Burden of COVID-19 restrictions: National, regional and global estimates. *EClinicalMedicine* **2022**, *45*, 101305. [CrossRef]
2. Chen, F.; Zheng, D.; Liu, J.; Gong, Y.; Guan, Z.; Lou, D. Depression and anxiety among adolescents during COVID-19: A cross-sectional study. *Brain Behav. Immun.* **2020**, *88*, 36. [CrossRef] [PubMed]
3. Choi, E.P.H.; Hui, B.P.H.; Wan, E.Y.F. Depression and anxiety in Hong Kong during COVID-19. *Int. J. Environ. Res. Public Health* **2020**, *17*, 3740. [CrossRef] [PubMed]
4. Renaud-Charest, O.; Lui, L.M.; Eskander, S.; Ceban, F.; Ho, R.; Di Vincenzo, J.D.; Rosenblat, J.D.; Lee, Y.; Subramaniapillai, M.; McIntyre, R.S. Onset and frequency of depression in post-COVID-19 syndrome: A systematic review. *J. Psychiatr. Res.* **2021**, *144*, 129–137. [CrossRef]
5. Cooke, J.E.; Eirich, R.; Racine, N.; Madigan, S. Prevalence of posttraumatic and general psychological stress during COVID-19: A rapid review and meta-analysis. *Psychiatry Res.* **2020**, *292*, 113347. [CrossRef]
6. Alnazly, E.; Khraisat, O.M.; Al-Bashaireh, A.M.; Bryant, C.L. Anxiety, depression, stress, fear and social support during COVID-19 pandemic among Jordanian healthcare workers. *PLoS ONE* **2021**, *16*, e0247679. [CrossRef] [PubMed]
7. Cénat, J.M.; Blais-Rochette, C.; Kokou-Kpolou, C.K.; Noorishad, P.-G.; Mukunzi, J.N.; McIntee, S.-E.; Dalexis, R.D.; Goulet, M.-A.; Labelle, P.R. Prevalence of symptoms of depression, anxiety, insomnia, posttraumatic stress disorder, and psychological distress among populations affected by the COVID-19 pandemic: A systematic review and meta-analysis. *Psychiatry Res.* **2021**, *295*, 113599. [CrossRef]
8. Saladino, V.; Algeri, D.; Auriemma, V. The psychological and social impact of COVID-19: New perspectives of well-being. *Front. Psychol.* **2020**, 2550. [CrossRef]
9. Salari, N.; Hosseinian-Far, A.; Jalali, R.; Vaisi-Raygani, A.; Rasoulpoor, S.; Mohammadi, M.; Rasoulpoor, S.; Khaledi-Paveh, B. Prevalence of stress, anxiety, depression among the general population during the COVID-19 pandemic: A systematic review and meta-analysis. *Glob. Health* **2020**, *16*, 57. [CrossRef]
10. Alsharji, K.E. Anxiety and depression during the COVID-19 pandemic in Kuwait: The importance of physical activity. *Middle East Curr. Psychiatry* **2020**, *27*, 60. [CrossRef]
11. Froessl, L.J.; Abdeen, Y. The silent pandemic: The psychological burden on frontline healthcare workers during COVID-19. *Psychiatry J.* **2021**, *2021*, 2906785. [CrossRef] [PubMed]
12. Nagesh, S.; Chakraborty, S. Saving the frontline health workforce amidst the COVID-19 crisis: Challenges and recommendations. *J. Glob. Health* **2020**, *10*, 010345. [CrossRef] [PubMed]
13. Maunder, R.; Heeney, N.; Strudwick, G.; Danielle Shin, H.; O'Neill, B.; Young, N. Burnout in hospital-based healthcare workers during COVID-19. *Sci. Briefs Ont. COVID-19 Sci. Advis. Table* **2021**, *2*, 46.
14. Sun, P.; Wang, M.; Song, T.; Wu, Y.; Luo, J.; Chen, L.; Yan, L. The psychological impact of COVID-19 pandemic on health care workers: A systematic review and meta-analysis. *Front. Psychol.* **2021**, *12*, 626547. [CrossRef] [PubMed]
15. Batra, K.; Singh, T.P.; Sharma, M.; Batra, R.; Schvaneveldt, N. Investigating the psychological impact of COVID-19 among healthcare workers: A meta-analysis. *Int. J. Environ. Res. Public Health* **2020**, *17*, 9096. [CrossRef] [PubMed]
16. Alsairafi, Z.; Naser, A.Y.; Alsaleh, F.M.; Awad, A.; Jalal, Z. Mental health status of healthcare professionals and students of health sciences faculties in Kuwait during the COVID-19 pandemic. *Int. J. Environ. Res. Public Health* **2021**, *18*, 2203. [CrossRef]
17. Bendau, A.; Plag, J.; Kunas, S.; Wyka, S.; Ströhle, A.; Petzold, M.B. Longitudinal changes in anxiety and psychological distress, and associated risk and protective factors during the first three months of the COVID-19 pandemic in Germany. *Brain Behav.* **2021**, *11*, e01964. [CrossRef]
18. Simon, R.W. Revisiting the relationships among gender, marital status, and mental health. *Am. J. Sociol.* **2002**, *107*, 1065–1096. [CrossRef]
19. Topp, C.W.; Østergaard, S.D.; Søndergaard, S.; Bech, P. The WHO-5 Well-Being Index: A systematic review of the literature. *Psychother. Psychosom.* **2015**, *84*, 167–176. [CrossRef]
20. Kroenke, K.; Spitzer, R.L.; Williams, J.B. The PHQ-9: Validity of a brief depression severity measure. *J. Gen. Intern. Med.* **2001**, *16*, 606–613. [CrossRef]
21. Naeinian, M.; Shairi, M.; Sharifi, M.; Hadian, M. To study reliability and validity for a brief measure for assessing Generalized Anxiety Disorder (GAD-7). *Clin. Psychol. Personal.* **2011**, *3*, 41–50.
22. Rauwerda, N.; Tovote, K.; Peeters, A.; Sanderman, R.; Emmelkamp, P.; Schroevers, M.; Fleer, J. WHO-5 and BDI-II are acceptable screening instruments for depression in people with diabetes. *Diabet. Med.* **2018**, *35*, 1678–1685. [CrossRef] [PubMed]
23. Du, J.; Dong, L.; Wang, T.; Yuan, C.; Fu, R.; Zhang, L.; Liu, B.; Zhang, M.; Yin, Y.; Qin, J. Psychological symptoms among frontline healthcare workers during COVID-19 outbreak in Wuhan. *Gen. Hosp. Psychiatry* **2020**, *67*, 144. [CrossRef]
24. Gao, J.; Zheng, P.; Jia, Y.; Chen, H.; Mao, Y.; Chen, S.; Wang, Y.; Fu, H.; Dai, J. Mental health problems and social media exposure during COVID-19 outbreak. *PLoS ONE* **2020**, *15*, e0231924.
25. Greenberg, N. Mental health of health-care workers in the COVID-19 era. *Nat. Rev. Nephrol.* **2020**, *16*, 425–426. [CrossRef] [PubMed]
26. Hossain, M.M.; Tasnim, S.; Sultana, A.; Faizah, F.; Mazumder, H.; Zou, L.; McKyer, E.L.J.; Ahmed, H.U.; Ma, P. Epidemiology of mental health problems in COVID-19: A review. *F1000Research* **2020**, *9*, 636. [CrossRef] [PubMed]

27. Cabarkapa, S.; Nadjidai, S.E.; Murgier, J.; Ng, C.H. The psychological impact of COVID-19 and other viral epidemics on frontline healthcare workers and ways to address it: A rapid systematic review. *Brain Behav. Immun. Health* **2020**, *8*, 100144. [CrossRef] [PubMed]
28. Simonsen, L.; Viboud, C. Mortality: A comprehensive look at the COVID-19 pandemic death toll. *Elife* **2021**, *10*, e71974. [CrossRef]
29. Luo, M.; Guo, L.; Yu, M.; Jiang, W.; Wang, H. The psychological and mental impact of coronavirus disease 2019 (COVID-19) on medical staff and general public—A systematic review and meta-analysis. *Psychiatry Res.* **2020**, *291*, 113190. [CrossRef]
30. Burhamah, W.; AlKhayyat, A.; Oroszlányová, M.; AlKenane, A.; Almansouri, A.; Behbehani, M.; Karimi, N.; Jafar, H.; AlSuwaidan, M. The psychological burden of the COVID-19 pandemic and associated lockdown measures: Experience from 4000 participants. *J. Affect. Disord.* **2020**, *277*, 977–985. [CrossRef]
31. Jace, C.E.; Makridis, C.A. Does marriage protect mental health? Evidence from the COVID-19 pandemic. *Soc. Sci. Q.* **2021**, *102*, 2499–2515. [CrossRef] [PubMed]
32. Wang, H.; Xia, Q.; Xiong, Z.; Li, Z.; Xiang, W.; Yuan, Y.; Liu, Y.; Li, Z. The psychological distress and coping styles in the early stages of the 2019 coronavirus disease (COVID-19) epidemic in the general mainland Chinese population: A web-based survey. *PLoS ONE* **2020**, *15*, e0233410. [CrossRef] [PubMed]
33. Chen, X.; Zhang, S.X.; Jahanshahi, A.A.; Alvarez-Risco, A.; Dai, H.; Li, J.; Ibarra, V.G. Belief in a COVID-19 conspiracy theory as a predictor of mental health and well-being of health care workers in Ecuador: Cross-sectional survey study. *JMIR Public Health Surveill.* **2020**, *6*, e20737. [CrossRef]
34. Kim, H.K.; McKenry, P.C. The relationship between marriage and psychological well-being: A longitudinal analysis. *J. Fam. Issues* **2002**, *23*, 885–911. [CrossRef]
35. Soulsby, L.K.; Bennett, K.M. Marriage and psychological wellbeing: The role of social support. *Psychology* **2015**, *6*, 1349–1359. [CrossRef]
36. Stuijfzand, S.; Deforges, C.; Sandoz, V.; Sajin, C.-T.; Jaques, C.; Elmers, J.; Horsch, A. Psychological impact of an epidemic/pandemic on the mental health of healthcare professionals: A rapid review. *BMC Public Health* **2020**, *20*, 1230. [CrossRef] [PubMed]
37. Preti, E.; Di Mattei, V.; Perego, G.; Ferrari, F.; Mazzetti, M.; Taranto, P.; Di Pierro, R.; Madeddu, F.; Calati, R. The psychological impact of epidemic and pandemic outbreaks on healthcare workers: Rapid review of the evidence. *Curr. Psychiatry Rep.* **2020**, *22*, 43. [CrossRef]
38. Pan, X.; Xiao, Y.; Ren, D.; Xu, Z.M.; Zhang, Q.; Yang, L.Y.; Liu, F.; Hao, Y.S.; Zhao, F.; Bai, Y.H. Prevalence of mental health problems and associated risk factors among military healthcare workers in specialized COVID-19 hospitals in Wuhan, China: A cross-sectional survey. *Asia-Pac. Psychiatry* **2022**, *14*, e12427. [CrossRef]
