# Supplementary material for: Mental health status and related factors influencing healthcare workers during the COVID-19 pandemic: A systematic review and meta-analysis
Source: PLoS One. 2024 Jan 19;19(1):e0289454. doi: 10.1371/journal.pone.0289454 (PMC10798549; doi:10.1371/journal.pone.0289454)
Supplement: S1 Data — (ZIP) [file pone.0289454.s011.zip › literatures/40.pdf]

ELSEVIER

Since January 2020 Elsevier has created a COVID-19 resource centre with free information in English and Mandarin on the novel coronavirus COVID-19. The COVID-19 resource centre is hosted on Elsevier Connect, the company's public news and information website.

Elsevier hereby grants permission to make all its COVID-19-related research that is available on the COVID-19 resource centre - including this research content - immediately available in PubMed Central and other publicly funded repositories, such as the WHO COVID database with rights for unrestricted research re-use and analyses in any form or by any means with acknowledgement of the original source. These permissions are granted for free by Elsevier for as long as the COVID-19 resource centre remains active.

Journal of Psychiatric Research 151 (2022) 50–56

Contents lists available at ScienceDirect

Journal of Psychiatric Research

journal homepage: www.elsevier.com/locate/jpsychires

Psychological distress among outpatient physicians in private practice linked to COVID-19 and related mental health during the second lockdown

Ariel Frajerman [a,b,c,d,*], Romain Colle [a,b], Franz Hozer [e,f], Eric Deflesselle [a,g], Samuel Rotenberg [a,b], Kenneth Chappell [a], Emmanuelle Corruble [a,b], Jean-François Costemale-Lacoste [a]

[a] MOODS Team, INSERM U1178, CESP, Université Paris-Saclay, Faculté de Médecine Paris-Saclay, Le Kremlin Bicêtre, F-94275, France
[b] Service Hospitalo-Universitaire de Psychiatrie de Bicêtre, Mood Center Paris Saclay, Assistance Publique-Hôpitaux de Paris, Hôpitaux Universitaires Paris-Saclay, Hôpital de Bicêtre, F-94275, France
[c] Inserm U1266–GDR 3557, Institut de psychiatrie et neurosciences de Paris, Institut de Psychiatrie, Paris, France
[d] Université de Paris, Paris, France
[e] UNIACT Lab, Psychiatry Team, NeuroSpin Neuroimaging Platform, CEA Saclay, Gif-sur-Yvette, France
[f] AP-HP Centre-Université de Paris, Hôpital Corentin-Celton, Département Médico-Universitaire de Psychiatrie et Addictologie, 92130, Issy-les-Moulineaux, France
[g] Département de Médecine Générale, Université Paris-Saclay, Faculté de Médecine Paris-Saclay, Le Kremlin Bicêtre, F-94275, France

ARTICLE INFO

Keywords:
Outpatient physicians
Private practice
Covid-19
Lockdown
Mental health

ABSTRACT

Background: Outpatient physicians in private practice, as inpatient physicians, are on the frontline of the COVID-19 pandemic. Mental-health consequences of the pandemic on hospital staff have been published, but the psychological distress among outpatient physicians in private practice due to COVID-19 has never been specifically assessed.
Methods: A French national online cross-sectional survey assessed declared psychological distress among outpatient physicians in private practice linked to COVID-19, sociodemographic and work conditions, mental health (Copenhagen Burn-out Inventory, Hospital Anxiety and Depression Scale, and the Insomnia severity Index), consequences on alcohol, tobacco, and illegal substance misuse, and sick leave during the 2nd COVID-19 wave.
Findings: Among the 1,992 physicians who answered the survey, 1,529 (76.8%) declared psychological distress linked to COVID-19. Outpatient physicians who declared psychological distress linked to COVID-19 had higher rates of insomnia (OR = 1.4; CI95 [1.1–1.7], p = 0.003), burnout (OR = 2.7; CI95 [2.1; 3.2], p < 0.001), anxiety and depressive symptoms (OR = 2.4; CI95 [1.9–3.0], p < 0.001 and OR = 1.7; CI95 [1.3–2.3], p < 0.001) as compared to physicians who did not. They also had higher psychotropic drug use in the last twelve months, or increased alcohol or tobacco consumption due to work-related stress and were more frequently general practitioners.
Interpretation: The feeling of being in psychological distress due to COVID-19 is highly frequent among outpatient physicians in private practice and is associated with mental health impairment. There is a need to assess specific interventions dedicated to outpatient physicians working in private practice.

1. Introduction

The coronavirus 2019 (COVID-19) is the longest pandemic ever experienced by the modern health system. Outpatient physicians, like in-patient physicians, are on the frontline against the pandemic.

By increasing the need of an already overstretched and understaffed health system across the globe, physicians' well-being has been negatively impacted (Goddard and Patel, 2021). Indeed, more than usual, physicians have experienced fears over being infected or infecting their families. Despite these risks, patient care was prioritised before their own health. For some physicians, the fear of transmitting COVID-19 led them to isolate from their families for months (Mehta et al., 2021). The

* Corresponding author. Université de Paris, Inserm U1266–GDR 3557, institut de psychiatrie et neurosciences de Paris, 102-108, Rue de la Santé, 75014, Paris, France.
E-mail address: ariel.frajerman@inserm.fr (A. Frajerman).

https://doi.org/10.1016/j.jpsychires.2022.04.003
Received 30 August 2021; Received in revised form 26 March 2022; Accepted 4 April 2022
Available online 12 April 2022
0022-3956/© 2022 Elsevier Ltd. All rights reserved.

COVID-19 pandemic could be considered as a new form of traumatic event and a source of post-traumatic stress disorder (Bridgland et al., 2021; Unützer et al., 2020). Stressors linked to the COVID-19 pandemic are summarized in Supplementary Data 1. Often, difficult decisions were made based on insufficient resources, such as postponing a hospitalization or a surgery, selecting which patient to transfer to the intensive care unit, or considering off-label care. Thus, physicians had to frequently struggle with different ethical dilemmas (Marazziti and Stahl, 2020). In parallel, the fear of infection has been maintained by the worldwide shortage of personal protective equipment (Burki, 2020), especially during the first wave. Physicians have also had to quickly adopt new technologies, such as telemedicine, to help patients while limiting the risk of contagion (Mehta et al., 2021). Additionally, by increasing uncertainty and limiting physicians in their ability to save lives, COVID-19 has created among them a sense of helplessness and failure (Sederer, 2021). Psychological distress has also been reinforced by the stress of successive lockdowns and the worldwide economic crisis (Adibe, 2021). However, other factors—work-related or otherwise—such as age, sex, and poor work environment, could be linked to increased stress in physicians (Chatterjee et al., 2021; Dyrbye et al., 2013; West et al., 2018; Wijeratne et al., 2020; Zhou et al., 2020).

High levels of depression (Azoulay et al., 2020; Kannampallil et al., 2020; Lai et al., 2020; Park et al., 2020; Tiete et al., 2020; Zhu et al., 2020), anxiety (Azoulay et al., 2020; Kannampallil et al., 2020; Lai et al., 2020; Park et al., 2020; Tiete et al., 2020; Zhu et al., 2020), and insomnia-related symptoms (Barua et al., 2020; Lai et al., 2020; Pappa et al., 2020; Şahin et al., 2020; Wu et al., 2021; Yang et al., 2021), burnout (Cravero et al., 2020; Huang et al., 2021; Kok et al., 2021; Park et al., 2020; Tiete et al., 2020; Torrente et al., 2021), and psychological distress (Giusti et al., 2020; Park et al., 2020) have been reported among inpatient physicians during the COVID-19 pandemic.

A study of 40 Catalan general practitioners (GP) showed the mental health impact of the pandemic on doctors, with a significant increase in severe burnout from 10% before the pandemic in 2019 to 50% during the pandemic in October 2020 (Seda-Gombau et al., 2021). In Singapore, a study of 257 GPs found anxiety (21%), depression (26.6%), and burnout (82%) (Lum et al., 2021). Another study of 215 Italian GPs during the first wave found the presence of anxiety, depression, and burnout symptoms in 36%, 18%, and 25%, respectively (Lasalvia et al., 2021).

However, psychological distress linked to COVID-19 and the related mental health of outpatient physicians in private practice have never been specifically assessed.

Such symptoms could impact other factors such as alcohol or illegal drug abuse, tobacco consumption, or one's ability to work. Due to the culture of silence in medicine (e.g., suffering without complaining because you chose to be a doctor (Robertson and Long, 2018; Shapiro and McDonald, 2020), and the stigma linked to the perception that vulnerability is a sign of weakness, physicians often think that emotional exhaustion is a part of their job and not a good reason to seek psychological help (Shapiro and McDonald, 2020). For these reasons, there is an urgent need to study outpatient physicians' psychological distress.

Aims of the Study: We aim to assess among outpatient physicians in private practice the prevalence of psychological distress linked to COVID-19 during the 2nd lockdown in France, its associated factors, and related mental health.

2. Material and methods

2.1. Design

We conducted an online survey for physicians in private practice registered on Doctolib®, the most used interface software by physicians in private practice to schedule medical appointments in France. The survey was administered using the online software LimeSurvey®. The reporting of the study followed the Strengthening the Reporting of Observational Studies in Epidemiology (STROBE) statement (Elm et al., 2007; Group, 2007).

Approval for this study was obtained from the local institutional review board at the University of Paris-Saclay, France. The questionnaires were collected anonymously. Respondents gave their informed consent to participate.

2.2. Participants

Emails to respond to an online survey were sent on October 29th and November 24th, 2020 to outpatient physicians of all specialties in private practice who were users of Doctolib®. Among the 15,722 which received the mail, 2,377 opened the survey, and 1,992 completed it (Fig. 1).

The second lockdown in France took place between October 29th and December 15th, 2020.

2.3. Measures

The following variables were collected: age range, sex, medical specialty, and the French county of practice. Participants were then invited to specify the nature of their practice: alone or in a group, with or without a medical assistant, and as an employee or not. Finally, they were invited to complete a questionnaire on their mental health, comprising declared psychological distress linked to COVID-19, declared stress linked to work, and questions related to insomnia, anxiety, depression, burnout, psychotropic medication use, substance abuse or misuse, and sick leave associated to the declared work-related stress.

2.4. Declared psychological distress linked to the COVID-19 pandemic

Psychological distressed linked to the COVID-19 pandemic was assessed with the following statement: "the COVID-19 epidemic we are going through is currently a source of excess stress, psychological suffering or professional burnout" and a Y/N answer. Based on this question, the whole population was split in 2 groups: physicians who responded "Yes" were considered as having declared a psychological distress linked to COVID-19 (population with psychological distress linked to COVID-19) and those who responded "No" as the population without psychological distress linked to COVID-19.

2.5. Burnout syndrome

The Copenhagen Burnout Inventory (CBI) (Doppia et al., 2011; Hardy et al., 2020; Kristensen et al., 2005; Nuss et al., 2020; Perumalswami et al., 2019) was used. The scale examines exhaustion and its attribution on three subscales: personal burnout, work-related burnout, and patient-related burnout. Each question is a Lickert scale of five-points: "*Always*" for 100, "*Often*" for 75, "*Sometimes*" for 50, "Seldom" for 25, and "*Never/almost never*" for 0. The cut-off for each subscale is a mean score >50. Burnout syndrome was defined by a score higher than the cut-off on at least one of the subscales (Madsen et al., 2015).

2.6. Psychiatric symptoms

The Insomnia Severity Index (ISI) (Morin et al., 2011) was used to measure sleep complaints. The ISI is a 7-item self-report questionnaire that assesses the nature, severity, and impact of insomnia. The total score ranges from 0 to 28. The cutoff $\geq$8 was chosen since it is useful at detecting physicians with sleep problems (Morin et al., 2011).

Anxiety and depression symptoms were assessed by the Hospital Anxiety and Depression Scale (HADS) (Zigmond and Snaith, 1983). This questionnaire consists of 7 questions related to anxiety (HADS-A) and 7 related to depression (HADS-D), ranging from 0 to 21 on each subscale. We used the cut-off $\geq$8 (HADS-D8 or HADS-A8) because of its sensitivity

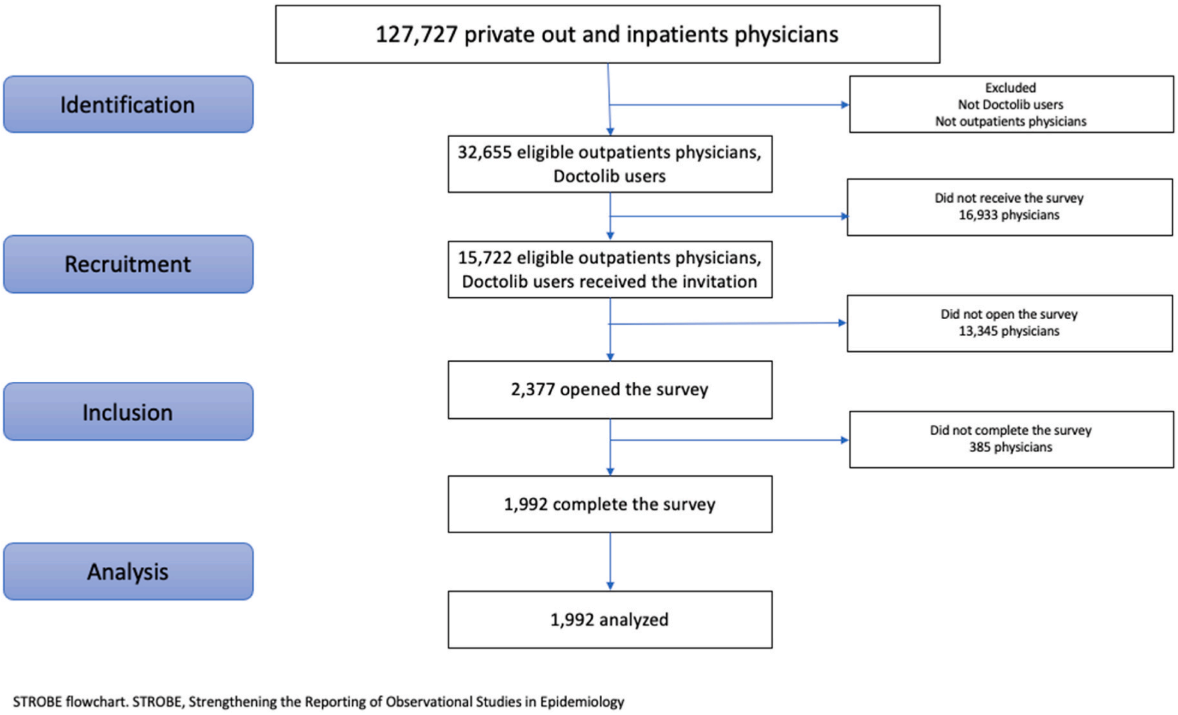

Fig. 1. ESTEEM study Flow chart.

in case-finding ability (Brennan et al., 2010).

2.7. Statistical analyses

Descriptive statistics were provided as effectives (percentages) for age range, sex, and work characteristics. Bivariate analyses were based on chi-square and Fisher's exact tests to compare prevalence between groups (physicians with versus without declared psychological distress linked to COVID 19). Subsequently, logistic regression models adjusted for age and sex were performed to control for potential age or sex effects for statistically significant associations in bivariate analyses (Model 1). We then controlled logistic regression models for variables related to the nature of practice (i.e. alone or in a group, with or without a medical assistant, and working as an employee or not) (Model 2). Statistical significance was evaluated using two-sided tests with an alpha risk set a priori at 0.05. Statistical analyses were performed using SPSS 20

Table 1
Sociodemographic and work condition characteristics associated with psychological distress linked to COVID-19.

		Total prevalence	Psychological distress linked to COVID-19 (n = 1,529)	Psychological distress linked to COVID-19 (n = 463)	Psychological distress linked to COVID-19 (n = 1,529)	Total prevalence	p value[a]	Adjusted Odds-ratio[c] AOR[95% CI]	p value[b]
Women n(%)		1,152 (57.9)	904 (59.1)	248 (54)	904 (59.1)	1,152 (57.9)	0.053	1.22 [0.99; 1.51]	0.060
Age Range n(%)	20–30 years	29 (1.9)	42 (2.1)	13 (2.8)	29 (1.9)	42 (2.1)	**0.042**	0.98[0.99; 1.51]	0.6
	31–40 years	480 (31.4)	597 (30)	117 (25.3)	480 (31.4)	597 (30)			
	41–50 years	370 (24.2)	499 (25.1)	129 (27.9)	370 (24.2)	499 (25.1)			
	51–60 years	261 (17.1)	354 (17.8)	93 (20.0)	261 (17.1)	354 (17.8)			
	>60 years	389 (25.4)	500 (25.1)	111 (24.0)	389 (25.4)	500 (25.1)			
**Nature of practice**									
Not employee n(%)		1,695 (85.3)	1,306 (85.4)	389 (84.7)	1,306 (85.4)	1,695 (85.3)	–	–	–
Employee n(%)		12 (0.6)	7 (0.5)	5 (1.1)	7 (0.5)	12 (0.6)	0.138	0.45 [0.14; 1.43]	0.175
Mixed activity n(%)		281 (14.1)	216 (14.1)	65 (14.2)	216 (14.1)	281 (14.1)	0.851	0.99 [0.85; 1.14]	0.848
Work alone n(%)		731 (36.8)	551 (36.0)	180 (39.2)	551 (36.0)	731 (36.8)	0.216	1.14 [0.91; 1.42]	0.247
No assistant n(%)		770 (38.7)	601 (39.3)	169 (36.8)	601 (39.3)	770 (38.7)	0.337	1.09 [0.89; 1.36]	0.432
**Specialties**									
General practitioners n(%)		952 (47.9)	764 (50.0)	188 (41.0)	764 (50.0)	952 (47.9)	**0.001**	**1.43 [1.16;1.77]**	**0.001**

Mixed activity = time shared between not employee and employee work.
Bold: significant p value < 0.05.
[a] Bivariate analyses.
[b] Multivariate logistic regression.
[c] adjusted on age and gender.

software (IBM SPSS Statistic 20). Statistical power was tested with G*Power ("Heinrich-Heine-University Software, Apps, and Games Free Download," n.d.).

3. Results

Among the 1,992 physicians who answered the survey, 57.9% were women and 1,529 (76.8%) declared a psychological distress linked to COVID-19. Outpatient physicians with a psychological distress linked to COVID-19 were more frequently general practitioners (Table 1). There was no other difference between outpatient physicians with and without a declared psychological distress linked to COVID-19 in terms of gender, age, and type of work (Table 1).

In bivariate analyses, psychological distress linked to COVID-19 was associated with higher rates of insomnia, burnout, anxiety symptoms, depressive symptoms, psychotropic drug use in the last twelve months, or increased alcohol or tobacco consumption due to work-related stress (Table 2, Fig. 2). In subscales of burnout, declared psychological distress linked to COVID-19 was associated with work-related burnout, personal burnout, and patient-related burnout (Table 2, Fig. 2).

These associations were significant in multivariate analyses controlled for age, gender, and work characteristics (alone or in a group, with or without a medical assistant, and working as an employee or not) (Table 2).

4. Discussion

This survey shows that three-quarters of the responding outpatient physicians in private practice declared psychological distress linked to the COVID-19 pandemic during the second lockdown in France. These physicians are more often general practitioners and have higher mental health impairment than those without psychological distress linked to COVID-19. Our study is the first to specifically assess outpatient physicians of all specialties in private practice during the second lockdown.

Regarding the effects of the pandemic on the mental health of healthcare workers, our study is the first to assess the psychological distress linked to COVID-19 by asking physicians a simple closed-ended question. This simple question is relevant since a "yes" answer is associated with increased insomnia, burnout, anxiety, and depressive symptoms among outpatient physicians in private practice. Prevalence of psychiatric symptoms by specialties are described in Supplementary Data 2.

Compared to other studies conducted mainly on inpatient physicians during the COVID-19 pandemic, our sample of physicians in private practice with psychological distress linked to COVID -19 show almost the same rates of burnout syndrome as the most impacted physicians (76% in Romanic hospital resident physicians) (Fiest et al., 2021). In physicians that declared psychological distress linked to COVID-19, the rate of insomnia (47.7%) was slightly higher than in previous studies: 41.6% in a recent meta-analysis of inpatient physicians caring for COVID-19 patients (Salari et al., 2020), 45% among anaesthesiologists and intensive care practitioners working in public hospitals (Richter et al., 2015), and 40.9% among radiologists (Florin et al., 2020).

The prevalence of anxiety symptoms among physicians in private practice with declared psychological distress linked to COVID-19 during the 2nd lockdown is much higher (76.4%) than radiologists (35%) during the first wave (Florin et al., 2020).

The prevalence of depressive symptoms among physicians with declared psychological distress linked to COVID-19 (29%) is similar to the prevalence among French radiologists during the first wave (30.6%) (Florin et al., 2020). According to a survey of 989 French GPs, 49% felt very anxious during the first wave (Dutour et al., 2021).

We report that outpatient physicians with psychological distress linked to COVID-19 are more often general practitioners than other

Table 2
Mental health of physicians with and without declared psychological distress linked to COVID-19.

	No psychological distress linked to COVID-19 (n = 463)	Psychological distress linked to COVID-19 (n = 1529)	Bivariate Odds-ratio [95%CI][a]	p value	Model 1: Adjusted Odds-ratio AOR [95%CI]	p value	Model 2: Adjusted Odds-ratio AOR [95%CI]	p value
Declared stress linked to work n(%)	204 (44.4)	1,164 (76.1)	**3.99 [3.20;4.96]**	**<0.001**	**3.97 [3.19;4.94]**	**<0.001**	**3.96 [3.18;4.93]**	**p < 0.001**
Sick leave (12 months) due to stress linked to work n (%)	6 (1.3)	38 (2.5)	1.92 [0.81; 4.58]	0.139	1.84 [0.77; 4.39]	0.169	1.863 [0.78; 4.45]	p = 0.845
Taking psychotropic drugs (12 months) due to stress linked to work n(%)	50 (10.9)	267 (17.5)	**1.73 [1.25;2.39]**	**0.001**	**1.72 [1.25;2.38]**	**0.001**	**1.73 [1.25;2.39]**	**p = 0.001**
Increasing consumption of alcohol or tobacco due to stress linked to work n (%)	37 (8.1)	247 (16.2)	**2.20 [1.53;3.16]**	**<0.001**	**2.21 [1.54;3.18]**	**<0.001**	**2.22 [1.54;3.20]**	**p < 0.001**
Taking illegal drugs (12 months) due to stress linked to work n(%)	4 (0.9)	13 (0.9)	0.98 [0.32; 3.01]	0.965	1.03 [0.33; 3.19]	0.959	1.06 [0.34; 3.31]	p = 0.911
Burnout n(%)	252 (54.9)	1,168 (76.4)	**2.66 [2.14; 3.31]**	**<0.001**	**2.62 [2.10; 3.27]**	**<0.001**	**1.75 [1.35;2.27]**	**p < 0.001**
Work-related burnout n (%)	227 (49.5)	1099 (71.9)	**2.61 [2.11;3.24]**	**<0.001**	**2.58 [2.08;3.21]**	**<0.001**	**2.38 [1.91;2.95]**	**p < 0.001**
Personal burnout n(%)	194 (42.3)	991 (64.8)	**2.52 [2.03;3.11]**	**<0.001**	**2.49 [2.01;3.09]**	**<0.001**	**2.63 [2.11; 3.29]**	**p < 0.001**
Patient-related burnout n (%)	126 (27.5)	659 (43.1)	**2.00 [1.59;2.52]**	**<0.001**	**2.00 [1.59;2.52]**	**<0.001**	**2.58 [2.08;3.20]**	**p < 0.001**
Insomnia n(%)	182 (39.7)	729 (47.7)	**1.39 [1.12;1.72]**	**0.003**	**1.40 [1.13;1.74]**	**0.002**	**2.50 [2.02;3.11]**	**p < 0.001**
Depressive symptoms n(%)	88 (19.2)	443 (29.0)	**1.72 [1.33;2.22]**	**<0.001**	**1.73 [1.33;2.33]**	**<0.001**	**2.03 [1.61;2.55]**	**p < 0.001**
Anxiety symptoms n(%)	195 (42.5)	976 (63.8)	**2.39 [1.93;2.96]**	**<0.001**	**2.36 [1.90;2.93]**	**<0.001**	**1.42 [1.15;1.76]**	**p = 0.003**

Model 1: multivariate logistic regression adjusted for age and sex.
Model 2: multivariate logistic regression adjusted for age, sex and the work condition (alone or in group, with or without medical assistant and as employee or not).
[a] Bivariate analyses.

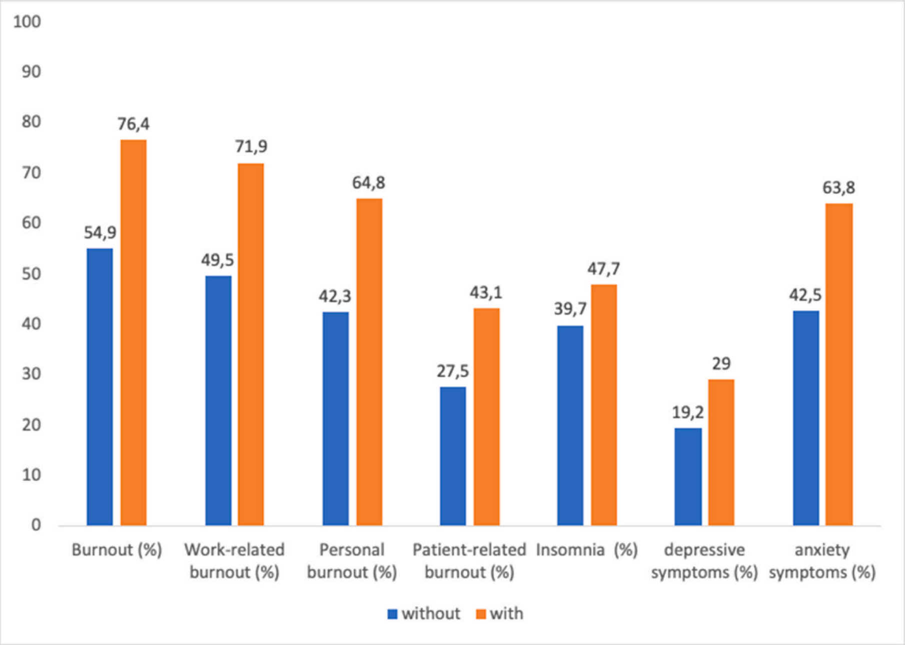

Fig. 2. Prevalence of psychiatric symptoms in physicians with or without declared psychological distress linked to COVID-19.

specialties in private practice. Three previous studies explored the mental health of general practitioners during the first wave of COVID-19, including burnout (Di Monte et al., 2020) and depression or anxiety symptoms (Amerio et al., 2020; Monterrosa-Castro et al., 2020). The prevalence of such symptoms was lower than in our global sample and largely lower than the prevalence among physicians that declared psychological distress linked to COVID-19 during the second lockdown in our study. These results suggest that the prevalence of psychiatric symptoms related to the second wave of the pandemic among general practitioners could be more important.

Prior to the COVID-19 pandemic, a meta-analysis of 22 studies (Kansoun et al., 2019) showed a prevalence of burnout among French physicians of 44% among anaesthesiologists and intensive care practitioners, 55% among emergency practitioners, and 48% among general practitioners. Each prevalence here is almost 20 to 30 percentage points lower than the prevalence of burnout linked to COVID-19 in our study (76.4%) but close to the prevalence of burnout among physicians without declared psychological distress linked to COVID-19 in our study (54.9%).

4.1. *Strengths*

This study has several strengths. First, it is the only one to directly assess psychological distress linked to COVID-19. Second, assessing insomnia, burnout, anxiety, and depression at the same time allowed us to identify associated mental health impairment. Third, our study is the largest to date to investigate the mental health of outpatient physicians in private practice during the COVID-19 pandemic.

4.2. *Limitations*

Our study has several limitations. First, this is a cross-sectional study without a comparative group. Second, its response rate was low (12.8%) but close to other mental health studies among physicians (17.8%) (West et al., 2020). However, this could be a selection bias. Indeed, according to Pierce et al. (2020), 2 hypotheses could be made. One, the more the physicians were stressed, the more they took part in the survey or two, the less the physicians were stressed, the more they took part in the survey. Third, only 32,655 French private practice physicians out of 123,727 could theoretically participate, and 15,722 received the survey. Since Doctolib® is a web-based device used to schedule medical appointments, physicians using it may be more likely to be younger and working with a computerized support. Therefore, this population might not be representative of all French outpatient physicians. Fourth, this is a cross-sectional study without prospective assessment. Since the pandemic is still active in France, the prevalence of current psychological distress linked to COVID-19 should now be higher than during the second wave. Fifth, we only used one question to assess stress, psychological suffering, or professional burnout related to COVID 19, which does not allow us to distinguish the different possible causes (fear of infection, overwork, isolation, etc.)

4.3. *Perspectives*

Physicians cannot easily report that they have psychological distress since they tend to think that it is normal to be exhausted during the pandemic. The simple question we used to assess psychological distress could be more acceptable than a longer psychological evaluation and allow for screening 3/4 of the population with a particularly high risk of burnout, anxiety, depression, or insomnia due to COVID-19. To date, there is no systematic screening of psychological distress and no specific interventions set up for outpatient physicians in private practice. Thus there is a need to set up and evaluate effective psychological interventions such as stress reduction therapies, educational interventions targeting physicians' self-confidence, communication skills, organizational interventions or small group discussions (Panagioti et al., 2017; West et al., 2016), and communities of peer support (Hu et al., 2012; Sederer, 2021; Shapiro and McDonald, 2020). Following international recommendations, psychiatrists should be mobilized to develop self-help, group, or individual supports and/or treatments for distressed colleagues and their families (Kuzman et al., 2020; Stewart and Appelbaum, 2020).

5. Conclusion

Reports of psychological distress due to COVID-19 are dramatically elevated in outpatient physicians and are associated with mental health impairment. There is a need to assess specific interventions dedicated to

outpatient physicians working in private practice. It would also now be necessary to reassess the mental health of these physicians in order to understand its evolution.

Disclaimer

The views and opinions expressed in this report are those of the authors and should not be construed to represent the views of any of the sponsoring organizations or agencies.

Contributors

Conceptualization, Data curation, Funding acquisition, Investigation, Methodology, Project administration, Resources: Jean-François Costemale-Lacoste, Formal analysis and Writing - original draft: Ariel Frajerman, Romain Colle and Jean-François Costemale-Lacoste, Software: Ariel Frajerman and Jean-François Costemale-Lacoste, Supervision: Romain Colle and Jean-François Costemale-Lacoste, Validation: Emmanuelle Corruble, Visualization: Emmanuelle Corruble, Writing - review and editing: Franz Hozer, Eric Deflesselle, Samuel Rotenberg, Kenneth Chappell, Emmanuelle Corruble, Ariel Frajerman conducted the statistical analyses under the supervision by JFCL and RC. He wrote the draft of the manuscript and helped proofread it. Romain Colle supervised the statistical analyses and served as an advisor during the writing procedure. He co-wrote the entire manuscript and proofread it, Franz Hozer is a physician in private practice. He helped to improve the entire manuscript and acted as an advisor, Eric Deflesselle is a general practitioner in private practice. He helped to improve the entire manuscript and acted as an advisor, Samuel Rotenberg is a physician in private practice. He helped to improve the entire manuscript and acted as an advisor, Kenneth Chappell proofread the entire manuscript as a native English speaker, Emmanuelle Corruble is the head of the MOODS laboratory at Paris-Saclay University. She acted as advisor and supervisor for the survey, Jean-François Costemale-Lacoste supervised the entire process of the study. He built the survey in collaboration with a panel of outpatient physicians in private practice and MOODS' team researchers. He conducted the collaboration with Doctolib and supervised the collection of the data. He supervised the data analyses. He wrote the manuscript and proofread it.

Declaration of competing interest

No conflict of interest arises from this research.

Acknowledgement

No financial support, aside from the authors' salaries provided by the Nationals Health Systems, was involved in the preparation of this manuscript. Doctolib allowed the transmission of the survey but was not involved in any data management process. No financial support was provided from Doctolib.

Appendix A. Supplementary data

Supplementary data to this article can be found online at https://doi.org/10.1016/j.jpsychires.2022.04.003.

References

Adibe, B., 2021. COVID-19 and clinician wellbeing: challenges and opportunities. Lancet Public Health 6, e141–e142. https://doi.org/10.1016/S2468-2667(21)00028-1.

Amerio, A., Bianchi, D., Santi, F., Costantini, L., Odone, A., Signorelli, C., Costanza, A., Serafini, G., Amore, M., Aguglia, A., 2020. Covid-19 pandemic impact on mental health: a web-based cross-sectional survey on a sample of Italian general practitioners. Acta Biomed. 91, 83–88. https://doi.org/10.23750/abm.v91i2.9619.

Azoulay, E., Cariou, A., Bruneel, F., Demoule, A., Kouatchet, A., Reuter, D., Souppart, V., Combes, A., Klouche, K., Argaud, L., Barbier, F., Jourdain, M., Reignier, J., Papazian, L., Guidet, B., Géri, G., Resche-Rigon, M., Guisset, O., Labbé, V., Mégarbane, B., Van Der Meersch, G., Guitton, C., Friedman, D., Pochard, F., Darmon, M., Kentish-Barnes, N., 2020. Symptoms of anxiety, depression, and peritraumatic dissociation in critical care clinicians managing patients with COVID-19. A cross-sectional study. Am. J. Respir. Crit. Care Med. 202, 1388–1398. https://doi.org/10.1164/rccm.202006-2568OC.

Barua, L., Zaman, M.S., Omi, F.R., Faruque, M., 2020. Psychological burden of the COVID-19 pandemic and its associated factors among frontline doctors of Bangladesh: a cross-sectional study. F1000Res 9, 1304. https://doi.org/10.12688/f1000research.27189.3.

Brennan, C., Worrall-Davies, A., McMillan, D., Gilbody, S., House, A., 2010. The Hospital Anxiety and Depression Scale: a diagnostic meta-analysis of case-finding ability. J. Psychosom. Res. 69, 371–378. https://doi.org/10.1016/j.jpsychores.2010.04.006.

Bridgland, V.M.E., Moeck, E.K., Green, D.M., Swain, T.L., Nayda, D.M., Matson, L.A., Hutchison, N.P., Takarangi, M.K.T., 2021. Why the COVID-19 pandemic is a traumatic stressor. PLoS One 16, e0240146. https://doi.org/10.1371/journal.pone.0240146.

Burki, T., 2020. Global shortage of personal protective equipment. Lancet Infect. Dis. 20, 785–786. https://doi.org/10.1016/S1473-3099(20)30501-6.

Chatterjee, Seshadri Sekhar, Chakrabarty, M., Banerjee, D., Grover, S., Chatterjee, Shiv Sekhar, Dan, U., 2021. Stress, sleep and psychological impact in healthcare workers during the early phase of COVID-19 in India: a factor Analysis. Front. Psychol. 12, 611314 https://doi.org/10.3389/fpsyg.2021.611314.

Cravero, A.L., Kim, N.J., Feld, L.D., Berry, K., Rabiee, A., Bazarbashi, N., Bassin, S., Lee, T.-H., Moon, A.M., Qi, X., Liang, P.S., Aby, E.S., Khan, M.Q., Young, K.J., Patel, A., Wijarnpreecha, K., Kobeissy, A., Hashim, A., Houser, A., Ioannou, G.N., 2020. Impact of exposure to patients with COVID-19 on residents and fellows: an international survey of 1420 trainees. Postgrad. Med. https://doi.org/10.1136/postgradmedj-2020-138789.

Di Monte, C., Monaco, S., Mariani, R., Di Trani, M., 2020. From resilience to burnout: psychological features of Italian general practitioners during COVID-19 emergency. Front. Psychol. 11, 567201 https://doi.org/10.3389/fpsyg.2020.567201.

Doppia, M.-A., Estryn-Béhar, M., Fry, C., Guetarni, K., Lieutaud, T., comité de pilotage de l'enquête SESMAT, 2011. [Burnout in French doctors: a comparative study among anaesthesiologists and other specialists in French hospitals (SESMAT study)]. Ann. Fr. Anesth. Reanim. 30, 782–794. https://doi.org/10.1016/j.annfar.2011.05.011.

Dutour, M., Kirchhoff, A., Janssen, C., Meleze, S., Chevalier, H., Levy-Amon, S., Detrez, M.-A., Piet, E., Delory, T., 2021. Family medicine practitioners' stress during the COVID-19 pandemic: a cross-sectional survey. BMC Fam. Pract. 22, 36. https://doi.org/10.1186/s12875-021-01382-3.

Dyrbye, L.N., Varkey, P., Boone, S.L., Satele, D.V., Sloan, J.A., Shanafelt, T.D., 2013. Physician satisfaction and burnout at different career stages. Mayo Clin. Proc. 88, 1358–1367. https://doi.org/10.1016/j.mayocp.2013.07.016.

von Elm, E., Altman, D.G., Egger, M., Pocock, S.J., Gøtzsche, P.C., Vandenbroucke, J.P., 2007. Strengthening the reporting of observational studies in epidemiology (STROBE) statement: guidelines for reporting observational studies. BMJ 335, 806–808. https://doi.org/10.1136/bmj.39335.541782.AD.

Fiest, K.M., Parsons Leigh, J., Krewulak, K.D., Plotnikoff, K.M., Kemp, L.G., Ng-Kamstra, J., Stelfox, H.T., 2021. Experiences and management of physician psychological symptoms during infectious disease outbreaks: a rapid review. BMC Psychiatr. 21, 91. https://doi.org/10.1186/s12888-021-03090-9.

Florin, M., Pinar, U., Chavigny, E., Bouaboula, M., Jarboui, L., Coulibaly, A., Lemogne, C., Fournier, L., 2020. Socio-economic and psychological impact of the COVID-19 outbreak on private practice and public hospital radiologists. Eur. J. Radiol. 132, 109285 https://doi.org/10.1016/j.ejrad.2020.109285.

Giusti, E.M., Pedroli, E., D'Aniello, G.E., Stramba Badiale, C., Pietrabissa, G., Manna, C., Stramba Badiale, M., Riva, G., Castelnuovo, G., Molinari, E., 2020. The psychological impact of the COVID-19 outbreak on health professionals: a cross-sectional study. Front. Psychol. 11, 1684. https://doi.org/10.3389/fpsyg.2020.01684.

Goddard, A.F., Patel, M., 2021. The changing face of medical professionalism and the impact of COVID-19. Lancet. https://doi.org/10.1016/S0140-6736(21)00436-0.

Group, B.M.J.P., 2007. Strengthening the reporting of observational studies in epidemiology (STROBE) statement: guidelines for reporting observational studies. BMJ 335. https://doi.org/10.1136/bmj.39386.490150.94, 0-a.

Hardy, P., Costemale-Lacoste, J.-F., Trichard, C., Butlen-Ducuing, F., Devouge, I., Cerboneschi, V., Jacob, E., Buferne, R., Benyamina, A., Cantero, A., Gravier, V., Ghanem, T., Guérin, A., Meidinger, A., Baleyte, J.-M., Pelissolo, A., Corruble, E., 2020. Comparison of burnout, anxiety and depressive syndromes in hospital psychiatrists and other physicians: results from the ESTEM study. Psychiatr. Res. 284, 112662 https://doi.org/10.1016/j.psychres.2019.112662.

Heinrich-Heine-University Software, Apps, and Games Free Download [WWW Document], n.d. . apponic. URL https://www.apponic.com/developer/heinrich-heine-university-44943/(accessed 3.8.21).

Hu, Y.-Y., Fix, M.L., Hevelone, N.D., Lipsitz, S.R., Greenberg, C.C., Weissman, J.S., Shapiro, J., 2012. Physicians' needs in coping with emotional stressors: the case for peer support. Arch. Surg. 147, 212–217. https://doi.org/10.1001/archsurg.2011.312.

Huang, H.L., Chen, R.C., Teo, I., Chaudhry, I., Heng, A.L., Zhuang, K.D., Tan, H.K., Tan, B.S., 2021. A survey of anxiety and burnout in the radiology workforce of a tertiary hospital during the COVID-19 pandemic. J. Med. Imaging Radiat. Oncol. https://doi.org/10.1111/1754-9485.13152.

Kannampallil, T.G., Goss, C.W., Evanoff, B.A., Strickland, J.R., McAlister, R.P., Duncan, J., 2020. Exposure to COVID-19 patients increases physician trainee stress and burnout. PLoS One 15. https://doi.org/10.1371/journal.pone.0237301.

Kansoun, Z., Boyer, L., Hodgkinson, M., Villes, V., Lançon, C., Fond, G., 2019. Burnout in French physicians: a systematic review and meta-analysis. J. Affect. Disord. 246, 132–147. https://doi.org/10.1016/j.jad.2018.12.056.

Kok, N., van Gurp, J., Teerenstra, S., van der Hoeven, H., Fuchs, M., Hoedemaekers, C., Zegers, M., 2021. Coronavirus disease 2019 immediately increases burnout symptoms in ICU professionals: a longitudinal cohort study. Crit. Care Med. 49, 419–427. https://doi.org/10.1097/CCM.0000000000004865.

Kristensen, T.S., Borritz, M., Villadsen, E., Christensen, K.B., 2005. The Copenhagen Burnout Inventory: a new tool for the assessment of burnout. Work. Stress 19, 192–207. https://doi.org/10.1080/02678370500297720.

Kuzman, M.R., Curkovic, M., Wasserman, D., 2020. Principles of mental health care during the COVID-19 pandemic. Eur. Psychiatr. 63 https://doi.org/10.1192/j.eurpsy.2020.54.

Lai, J., Ma, S., Wang, Y., Cai, Z., Hu, J., Wei, N., Wu, J., Du, H., Chen, T., Li, R., Tan, H., Kang, L., Yao, L., Huang, M., Wang, H., Wang, G., Liu, Z., Hu, S., 2020. Factors associated with mental health outcomes among health care workers exposed to coronavirus disease 2019. JAMA Netw. Open 3, e203976. https://doi.org/10.1001/jamanetworkopen.2020.3976.

Lasalvia, A., Rigon, G., Rugiu, C., Negri, C., Del Zotti, F., Amaddeo, F., Bonetto, C., 2021. The psychological impact of COVID-19 among primary care physicians in the province of Verona, Italy: a cross-sectional study during the first pandemic wave. Fam. Pract. https://doi.org/10.1093/fampra/cmab106 cmab106.

Lum, A., Goh, Y.-L., Wong, K.S., Seah, J., Teo, G., Ng, J.Q., Abdin, E., Hendricks, M.M., Tham, J., Nan, W., Fung, D., 2021. Impact of COVID-19 on the mental health of Singaporean GPs: a cross-sectional study. BJGP Open 5. https://doi.org/10.3399/BJGPO.2021.0072. BJGPO.2021.0072.

Madsen, I.E.H., Lange, T., Borritz, M., Rugulies, R., 2015. Burnout as a risk factor for antidepressant treatment - a repeated measures time-to-event analysis of 2936 Danish human service workers. J. Psychiatr. Res. 65, 47–52. https://doi.org/10.1016/j.jpsychires.2015.04.004.

Marazziti, D., Stahl, S.M., 2020. The relevance of COVID-19 pandemic to psychiatry. World Psychiatr. 19, 261. https://doi.org/10.1002/wps.20764.

Mehta, S., Machado, F., Kwizera, A., Papazian, L., Moss, M., Azoulay, É., Herridge, M., 2021. COVID-19: a heavy toll on health-care workers. Lancet Respir. Med. https://doi.org/10.1016/S2213-2600(21)00068-0.

Monterrosa-Castro, A., Redondo-Mendoza, V., Mercado-Lara, M., 2020. Psychosocial factors associated with symptoms of generalized anxiety disorder in general practitioners during the COVID-19 pandemic. J. Invest. Med. 68, 1228–1234. https://doi.org/10.1136/jim-2020-001456.

Morin, C.M., Belleville, G., Bélanger, L., Ivers, H., 2011. The Insomnia Severity Index: psychometric indicators to detect insomnia cases and evaluate treatment response. Sleep 34, 601–608. https://doi.org/10.1093/sleep/34.5.601.

Nuss, P., Tessier, C., Masson, M., Fossati, P., Gaillard, R., Lapidus, N., Gourion, D., 2020. Factors associated with a higher score of burnout in a population of 860 French psychiatrists. Front. Psychiatr. 11, 371. https://doi.org/10.3389/fpsyt.2020.00371.

Panagioti, M., Panagopoulou, E., Bower, P., Lewith, G., Kontopantelis, E., Chew-Graham, C., Dawson, S., van Marwijk, H., Geraghty, K., Esmail, A., 2017. Controlled interventions to reduce burnout in physicians: a systematic review and meta-analysis. JAMA Intern. Med. 177, 195–205. https://doi.org/10.1001/jamainternmed.2016.7674.

Pappa, S., Ntella, V., Giannakas, T., Giannakoulis, V.G., Papoutsi, E., Katsaounou, P., 2020. Prevalence of depression, anxiety, and insomnia among healthcare workers during the COVID-19 pandemic: a systematic review and meta-analysis. Brain Behav. Immun. 88, 901–907. https://doi.org/10.1016/j.bbi.2020.05.026.

Park, S.Y., Kim, B., Jung, D.S., Jung, S.I., Oh, W.S., Kim, S.-W., Peck, K.R., Chang, H.-H., Korean Society of Infectious Diseases, 2020. Psychological distress among infectious disease physicians during the response to the COVID-19 outbreak in the Republic of Korea. BMC Publ. Health 20, 1811. https://doi.org/10.1186/s12889-020-09886-w.

Perumalswami, C.R., Griffith, K.A., Jones, R.D., Stewart, A., Ubel, P.A., Jagsi, R., 2019. Patterns of work-related burnout in physician-scientists receiving career development awards from the national institutes of health. JAMA Intern. Med. https://doi.org/10.1001/jamainternmed.2019.4317.

Pierce, M., McManus, S., Jessop, C., John, A., Hotopf, M., Ford, T., Hatch, S., Wessely, S., Abel, K.M., 2020. Says who? The significance of sampling in mental health surveys during COVID-19. Lancet Psychiatr. 7, 567–568. https://doi.org/10.1016/S2215-0366(20)30237-6.

Richter, E., Blasco, V., Antonini, F., Rey, M., Reydellet, L., Harti, K., Nafati, C., Albanèse, J., Leone, M., AzuRea Network, 2015. Sleep disorders among French anaesthesiologists and intensivists working in public hospitals: a self-reported electronic survey. Eur. J. Anaesthesiol. 32, 132–137. https://doi.org/10.1097/EJA.0000000000000110.

Robertson, J.J., Long, B., 2018. Suffering in silence: medical error and its impact on health care providers. J. Emerg. Med. 54, 402–409. https://doi.org/10.1016/j.jemermed.2017.12.001.

Şahin, M.K., Aker, S., Şahin, G., Karabekiroğlu, A., 2020. Prevalence of depression, anxiety, distress and insomnia and related factors in healthcare workers during COVID-19 pandemic in Turkey. J. Community Health 45, 1168–1177. https://doi.org/10.1007/s10900-020-00921-w.

Salari, N., Khazaie, H., Hosseinian-Far, A., Ghasemi, H., Mohammadi, M., Shohaimi, S., Daneshkhah, A., Khaledi-Paveh, B., Hosseinian-Far, M., 2020. The prevalence of sleep disturbances among physicians and nurses facing the COVID-19 patients: a systematic review and meta-analysis. Glob. Health 16, 92. https://doi.org/10.1186/s12992-020-00620-0.

Seda-Gombau, G., Montero-Alía, J.J., Moreno-Gabriel, E., Torán-Monserrat, P., 2021. Impact of the COVID-19 pandemic on burnout in primary care physicians in catalonia. Int. J. Environ. Res. Publ. Health 18, 9031. https://doi.org/10.3390/ijerph18179031.

Sederer, L.I., 2021. The many faces of COVID-19: managing uncertainty. Lancet Psychiatr. 8, 187–188. https://doi.org/10.1016/S2215-0366(21)00031-6.

Shapiro, J., McDonald, T.B., 2020. Supporting clinicians during covid-19 and beyond - learning from past failures and envisioning new strategies. N. Engl. J. Med. 383, e142 https://doi.org/10.1056/NEJMp2024834.

Stewart, D.E., Appelbaum, P.S., 2020. COVID-19 and psychiatrists' responsibilities: a WPA position paper. World Psychiatr. 19, 406–407. https://doi.org/10.1002/wps.20803.

Tiete, J., Guatteri, M., Lachaux, A., Matossian, A., Hougardy, J.-M., Loas, G., Rotsaert, M., 2020. Mental health outcomes in healthcare workers in COVID-19 and non-COVID-19 care units: a cross-sectional survey in Belgium. Front. Psychol. 11, 612241 https://doi.org/10.3389/fpsyg.2020.612241.

Torrente, M., Sousa, P.A., Sánchez-Ramos, A., Pimentao, J., Royuela, A., Franco, F., Collazo-Lorduy, A., Menasalvas, E., Provencio, M., 2021. To burn-out or not to burn-out: a cross-sectional study in healthcare professionals in Spain during COVID-19 pandemic. BMJ Open 11, e044945. https://doi.org/10.1136/bmjopen-2020-044945.

Unützer, J., Kimmel, R.J., Snowden, M., 2020. Psychiatry in the age of COVID-19. World Psychiatr. 19, 130–131. https://doi.org/10.1002/wps.20766.

West, C.P., Dyrbye, L.N., Erwin, P.J., Shanafelt, T.D., 2016. Interventions to prevent and reduce physician burnout: a systematic review and meta-analysis. Lancet 388, 2272–2281. https://doi.org/10.1016/S0140-6736(16)31279-X.

West, C.P., Dyrbye, L.N., Shanafelt, T.D., 2018. Physician burnout: contributors, consequences and solutions. J. Intern. Med. 283, 516–529. https://doi.org/10.1111/joim.12752.

West, C.P., Dyrbye, L.N., Sinsky, C., Trockel, M., Tutty, M., Nedelec, L., Carlasare, L.E., Shanafelt, T.D., 2020. Resilience and burnout among physicians and the general US working population. JAMA Netw. Open 3, e209385. https://doi.org/10.1001/jamanetworkopen.2020.9385.

Wijeratne, C., Johnco, C., Draper, B., Earl, J.K., 2020. Older physicians' reporting of psychological distress, alcohol use, burnout and workplace stressors. Am. J. Geriatr. Psychiatr. https://doi.org/10.1016/j.jagp.2020.09.010.

Wu, T., Jia, X., Shi, H., Niu, J., Yin, X., Xie, J., Wang, X., 2021. Prevalence of mental health problems during the COVID-19 pandemic: a systematic review and meta-analysis. J. Affect. Disord. 281, 91–98. https://doi.org/10.1016/j.jad.2020.11.117.

Yang, G., Li, C., Zhu, X., Yan, J., Liu, J., 2021. Prevalence of and risk factors associated with sleep disturbances among HPCD exposed to COVID-19 in China. Sleep Med. 80, 16–22. https://doi.org/10.1016/j.sleep.2020.12.034.

Zhou, A.Y., Panagioti, M., Esmail, A., Agius, R., Van Tongeren, M., Bower, P., 2020. Factors associated with burnout and stress in trainee physicians: a systematic review and meta-analysis. JAMA Netw. Open 3, e2013761. https://doi.org/10.1001/jamanetworkopen.2020.13761.

Zhu, Z., Xu, S., Wang, H., Liu, Z., Wu, J., Li, G., Miao, J., Zhang, C., Yang, Y., Sun, W., Zhu, S., Fan, Y., Chen, Y., Hu, J., Liu, J., Wang, W., 2020. COVID-19 in Wuhan: sociodemographic characteristics and hospital support measures associated with the immediate psychological impact on healthcare workers. EClinicalMedicine 24, 100443. https://doi.org/10.1016/j.eclinm.2020.100443.

Zigmond, A.S., Snaith, R.P., 1983. The hospital anxiety and depression scale. Acta Psychiatr. Scand. 67, 361–370. https://doi.org/10.1111/j.1600-0447.1983.tb09716.x.
