# Supplementary material for: Mental health status and related factors influencing healthcare workers during the COVID-19 pandemic: A systematic review and meta-analysis
Source: PLoS One. 2024 Jan 19;19(1):e0289454. doi: 10.1371/journal.pone.0289454 (PMC10798549; doi:10.1371/journal.pone.0289454)
Supplement: S1 Data — (ZIP) [file pone.0289454.s011.zip › literatures/41.pdf]

Original Article

The psychological impact of anxiety and depression on Chinese medical staff during the outbreak of the COVID-19 pandemic: a cross-sectional study

Manru Fu[1,2#], Dong Han[3#], Minghui Xu[3], Chen Mao[1], Dong Wang[2]

[1]School of Public Health, Southern Medical University, Guangzhou, China; [2]School of Health Management, Southern Medical University, Guangzhou, China; [3]The Third Affiliated Hospital of Southern Medical University, Guangzhou, China

Contributions: (I) Conception and design: All authors; (II) Administrative support: D Wang, C Mao, M Xu; (III) Provision of study materials or patients: M Fu, D Wang; (IV) Collection and assembly of data: M Fu, D Wang; (V) Data analysis and interpretation: M Fu, D Han; (VI) Manuscript writing: All authors; (VII) Final approval of manuscript: All authors.

[#]These authors contributed equally to this work as co-first authors.

Correspondence to: Dong Wang. School of Health Management, Southern Medical University, 1023 Shatai Road, Guangzhou, China. Email: dongw96@smu.edu.cn.

Background: The coronavirus disease 2019 (COVID-19) pandemic is a worldwide public health emergency that began in late 2019 and is still ongoing. Medical staff are at a particularly high risk of mental stress due to their close contact with infected patients. This study aimed to assess medical staff anxiety and depression levels from different risk-level areas in China during the early period of the COVID-19 outbreak and identify the main factors that might affect their mental health.

Methods: From February 22 to March 9, 2020, we conducted a 4-part online questionnaire to survey medical staff across different Chinese provinces about their anxiety and depression levels during the initial COVID-19 outbreak. The survey considered different demographic characteristics, anxiety (General Anxiety Disorder-7) scores, depression (Patient Health Questionnaire-9) scores, and occupational protection scores. Snowball sampling via a WeChat™ group was performed to collect the data.

Results: Among the 7,413 respondents, the rates of anxiety and depression reported among medical staff were 33.74% [2,501] and 27.65% [2,050], respectively. The odds of being assessed with severe anxiety were higher among nurses, those who were widowed, those in poor physical health, those working in COVID-19 designated facilities, and those receiving more than 50% negative/false information every day; the odds decreased by 5.8% with every 1-point increase in occupational protection. The odds of being assessed with moderate to severe depression or above were higher for the 18–30-year-old group, divorcees, those in poor physical health, and those receiving more than 50% of negative/false information every day; these odds decreased by 4.5% with every 1-point increase in occupational protection. Medical staff working in areas with a low exposure risk were at high risk of both anxiety and depression.

Conclusions: During the outbreak of COVID-19 in China, a significant proportion of medical staff faced psychological problems, even those in areas with a low exposure risk. Targeted interventions should focus more on nurses, widowed /divorced, and medical staff with poor physical health, less clinical experience, or insufficient occupational protection. The authenticity and orientation of media also showed a correlation with the mental state of medical staff.

Keywords: Coronavirus 2019 (COVID-19); mental health; medical staff; occupation protection; media orientation

Submitted May 04, 2021. Accepted for publication Jul 06, 2021.

doi: 10.21037/apm-21-1261

View this article at: https://dx.doi.org/10.21037/apm-21-1261

© Annals of Palliative Medicine. All rights reserved.

Introduction

In December 2019, coronavirus 2019 (COVID-19), caused by severe acute respiratory syndrome coronavirus 2 (SARS-CoV-2), emerged (1) and quickly became a public health emergency of international concern. By February 20, 2020—1 month after the first official reports of infected patients by the Chinese government—42,638 confirmed cases (including 1,016 deaths) and 21,675 suspected cases of COVID-19 had been reported to the National Health Commission of China. At the start of the epidemic, when the knowledge of this unknown disease was inadequate, hundreds of frontline medical staff became infected after coming into close contact with confirmed patients, which aroused mass panic.

Various studies have reported that people who are highly exposed during public health emergencies, such as severe acute respiratory syndrome (SARS), Middle East respiratory syndrome (MERS), and Ebola, can enter into a prolonged state of intense stress and mental problems, manifested as anxiety and depression, with medical staff being especially at risk (2-4). Approximately 10% of medical staff in Beijing have reported high levels of psychological distress and post-traumatic stress disorder symptoms since the outbreak of SARS (5). Medical staff do not always have sufficient professional training to deal with an emergency health crisis; therefore, occupational exposure might also be the main contributor to mental stress (6). A previous study also uncovered a positive correlation between the spread of false rumors and psychological stress during public crises (7).

So far, most studies on COVID-19 have focused on identifying the epidemiological and clinical characteristics of infected patients (8,9), characterizing the causative virus (10), analyzing potential patterns of spread (11), and examining the mental health of the general population (12). To date, several studies (4,6,13-15) have examined the psychological status of Chinese medical staff, the people who are playing an important and special role during the management of the COVID-19 pandemic in China. These studies reported that the psychological impact of COVID-19 on Chinese medical staff varies according to their socio-demographic characteristics and the level of exposure risk in their working departments. However, they only investigated medical staff in a particular province (13), several cities (14), or designated hospitals (15). Most studies about the psychological impact on medical staff in other countries were reviews (16-18) or small sample investigations conducted on several particular hospitals (19,20). A country-wide survey of a large sample of medical staff from all types of medical institutions and departments and medical staff who volunteered as medical support in Wuhan has rarely been conducted. Only a limited number of studies focused on the impact of media orientation on mental status during the COVID-19 epidemic. And very few studies classified the participants according to the risk level of their working areas and sought to find out the mental health differences between medical staff in different regions.

Therefore, the present study aimed to evaluate the severity of anxiety and depression among a large population of medical staff at different risk levels of exposure from across China during the early stage of the COVID-19 outbreak in order to determine the relationship of mental health with occupational protection and media orientation, as well as to identify the main factors influencing the mental health of medical personnel, to find effective and targeted solutions. We present the following article in accordance with the SURGE reporting checklist (available at https://dx.doi.org/10.21037/apm-21-1261).

Methods

Study design and participants

Between February 22 and March 9, 2020, a 4-part online questionnaire was developed to survey medical staff in different provinces across China. The survey took into account various demographic characteristics, anxiety scores [General Anxiety Disorder-7 (GAD-7)], depression scores [Patient Health Questionnaire-9 (PHQ-9)], knowledge of COVID-19, and COVID-19-related occupational protection behaviors. The study enrolled frontline doctors and nurses fighting to control and prevent the epidemic and medical staff in other departments at all levels and types of medical institutions in China. All participants were over 18 years old and completed the survey anonymously. To ensure that all participants agreed to volunteer in the study, informed consent was obtained on a cover note on the questionnaire. The questionnaire was first published on the online survey platform "Survey Star" (website: https://www.wjx.cn/) and was then distributed via a WeChat™ group as a convenience sample. We eventually broadened the snowball sampling to recruit more participants. This research was conducted in accordance with the Declaration of Helsinki (as revised in 2013). The study was approved by the institutional ethics board of the Third Affiliated Hospital of Southern Medical University (approval no. 2020-EA-014).

© Annals of Palliative Medicine. All rights reserved.

Assessment of outcomes

Socio-demographic data

The socio-demographic characteristics of medical staff included variables such as gender, age, marital status, salary, occupation, education, professional titles, working experience, place of work, self-reported physical health, self-reported proportions of negative or false COVID-19-related information received daily, and information relating to their working environment during COVID-19. Except for Hubei province and Beijing, all administrative regions were officially graded for 3 levels of risk based on the number of confirmed and suspected COVID-19 cases by the local governments. During the pandemic's early stages, the classification remained relatively stable. Therefore, based on the list of COVID-19 pandemic risk classifications announced by each province before March 10, 2020, a total of 7,413 responses were divided into 3 subgroups (low-risk, moderate-risk, and high-risk) according to the respondent's workplace location (from now on referred to as "risk levels"). Responses from Hubei and Beijing were assigned to the high-risk group, owing to the actual situation of the outbreaks there. Medical staff working in 24-hour fever clinics or isolation wards for patients with suspected or confirmed infection and those who volunteered as medical support in Wuhan were regarded as frontline workers in the Chinese battle against COVID-19.

Psychological assessment of medical staff

The mental health of the enrolled medical staff was assessed using the GAD-7 and PHQ-9 scales. Both the GAD-7 (21-23) and PHQ-9 (24-26) scales have been proven reliable and accurate for identifying and assessing mental health disorders in the clinical setting. The GAD-7 scale comprises 7 short questions and is one of the most used approaches for self-reporting and quantitative evaluation of standards. This scale is recommended by the Diagnostic and Statistical Manual of Mental Diseases and was published by the American Psychiatric Association for the screening, diagnosis, and severity assessment of anxiety disorders (22). Similarly, the PHQ-9, which comprises 9 items, is a frequently used self-reporting mental health scale for assessing the severity of depressive symptoms (26). Both scales ask participants how often they have suffered from relevant symptoms within the past 14 days. Items are scored on a 4-point Likert scale, with scores from 0–3 points respectively translating as "not at all", "several days", "more than half the days", and "nearly every day". Total GAD-7 scale scores range from 0–21 points and are divided into 4 degrees: no anxiety (0–4 points), mild anxiety (5–9 points), moderate anxiety (10–14 points), and severe anxiety (≥15 points). Total PHQ-9 scale scores range from 0–27 points and are divided into 5 degrees: no depression (0–4 points), mild depression (5–9 points), moderate depression (10–14 points), moderate to severe depression (15–19 points), and severe depression (20–27 points). In this study, the internal consistency of the GAD-7 (Cronbach's α=0.94) and PHQ-9 (Cronbach's α=0.93) were excellent.

Occupational protection against COVID-19

The variables for assessing occupational protection behaviors against COVID-19 were self-designed based on 2 official documents: the *Guidelines for the Protection of Medical Staff during the COVID-19 Outbreak (Trial)*, issued by the National Health Commission, and the *Guidelines for the Prevention and Control of COVID-19 (First Edition)*, issued by the Chinese Center for Disease Control and Prevention. The survey consisted of 8 short questions, including the use of personal protective equipment (PPE), hand hygiene, and other protective behaviors relating to the prevention of COVID-19 infection. All items were measured on a 5-point Likert scale ranging from 0 (not at all) to 4 (all of the time). The total scores ranged from 0–32 points, and the higher the total score, the better the occupational protection. A pre-test was made among 100 medical staff from Guangdong Province (56/100) and Hubei Province (44/100). Finally, the median score for occupational protection behaviors among the medical staff in this study was 29 (range, 24–32), Cronbach's α=0.79.

Statistical analysis

An online platform was used to conduct the survey, and R software (version 3.5.3) software was used for data collation and analysis. Categorical data were described as frequencies (percentages) of cases. The Wilcoxon rank-sum and Kruskal-Wallis tests were used for the comparison of anxiety and depression scores among subgroups. Pearson's correlation coefficients were calculated between the anxiety and depression scores. A multiple logistic regression model was applied to analyze the combined effects of variables, including working in a designated hospital or not, the institution type, the workplace risk level, the proportions of negative or false information obtained daily, and the occupational protection score. Other variables such as gender, age, marital status, occupation, education, and

© Annals of Palliative Medicine. All rights reserved.

self-reported physical health were used as covariates for adjusting and stepwise screening of factors using the Akaike information criterion (AIC). The level of statistical significance was set at P<0.05.

Results

Socio-demographic data

A total of 7,701 responses were received, of which 288 questionnaires were manually eliminated due to logical errors. Finally, 7,413 responses from medical personnel in 31 administrative areas were included, with a completion rate of 96.26%. The respondents were largely female (6,169, 83.22%), and most were married (5,193, 70.05%). Nurses (4,368, 58.92%) accounted for most participants, and individuals aged 18–30 years old (3,147, 42.45%) formed the largest age group. The monthly salaries of the respondents most commonly ranged from 4,001–8,000 yuan (3,347, 45.15%), and 7–15 years was the most common length of working experience (2,891, 39.00%). Of the respondents, 3,660 (49.37%) had a Bachelor's degree, and 4,612 (62.22%) had a junior professional title. A small proportion of respondents worked at designated hospitals (2,826, 38.12%), and 1,193 (16.09%) worked in frontline departments during the outbreak and thus might have been in direct contact with confirmed or suspected COVID-19 cases. A total of 1,424 (19.21%) respondents worked in low-risk areas; 1,649 (22.25%) worked in moderate-risk areas; and 4,340 (58.55%) worked in high-risk areas. A total of 909 (12.26%) participants self-reported that more than 50% of the COVID-related information they received each day was negative, and 485 (6.54%) said that more than 50% of the COVID-related information they received each day was false. A total of 6,748 (91.03%) respondents self-reported a good state of physical health (*Table 1*).

Mental state of medical staff

Of all responses received, 2,501 (33.74%) were screened concerning anxiety. The results revealed that 1747 (23.57%), 449 (6.06%), and 305 (4.11%) respondents had mild, moderate, and severe anxiety, respectively. Additionally, 2,050 (27.65%) responses were screened concerning depression. The results indicated that 1,397 (18.85%) and 346 (4.67%) respondents had mild and moderate depression, respectively. We combined the medical staff who reported moderate to severe depression (22, 0.30%) with those who reported severe depression (285, 3.84%), due to the small number of samples in the former group; these patients were labeled as the "moderate to severe depression or above" (MSD+) group. Pearson's correlation analysis revealed a correlation coefficient of 0.67 (P<0.001) between the GAD-7 and PHQ-9 scores, indicating a strong positive association between anxiety and depression symptoms among medical staff. Overall, the positive rate of depression was lower than that of anxiety in this study.

Factors associated with anxiety symptoms in medical personnel

Univariable analysis revealed that medical staff who had lost a partner (57.14%, P<0.001), worked in low-risk areas (50.23%, P<0.001), or self-reported to be in poor health (71.43%, P<0.001) tended to be more anxious during the early stage of the pandemic, as did those whose daily COVID-19-related information was negative (62.49%, P<0.001) or false (55.46%, P<0.001) at least half of the time. Nearly more than 50% of these respondents reported experiencing anxiety symptoms, and more than 10% were screened as having severe anxiety (*Table 2*). Multiple logistic regression analysis showed similar results (*Table 3*). When respondents without anxiety were selected as a control group, the odds of being assessed as having severe anxiety were 19.615 (95% CI: 5.255–73.210, P<0.001) times higher in the widowed group than in the unmarried group. Additionally, the odds of being assessed with severe anxiety were 1.579 (95% CI: 1.086–2.297, P=0.017) times higher for nurses than for doctors. Medical staff who worked in designated hospitals exhibited 1.506-fold (95% CI: 1.099–2.065, P=0.011) greater odds of being assessed as having severe anxiety, while medical staff in poor health were 24.735 (95% CI: 11.507–53.170, P<0.001) times more likely to suffer from severe anxiety than those who self-described as "very healthy". Moreover, respondents whose daily information was negative (OR =6.316, 95% CI: 4.703–8.482, P<0.001) or false (OR =2.263, 95% CI: 1.569–3.265, P<0.001) more than half of the time were more likely to experience severe anxiety compared the others. Furthermore, the odds of being assessed as having severe anxiety were 0.505 (95% CI: 0.342–0.746, P=0.001) times lower in high-risk areas and 0.327 (95% CI: 0.230–0.464, P<0.001) times lower in moderate-risk areas than in low-risk areas. For every 1-point increase in occupational protection, the odds of being assessed as having mild, moderate, and severe anxiety decreased by 3.0% (P<0.001),

© Annals of Palliative Medicine. All rights reserved.

Table 1 Socio-demographic characteristics of Chinese medical staff and their knowledge of COVID-19

Characteristics	Participants (n=7,413)
Gender	
Male	1,244 (16.78%)
Female	6,169 (83.22%)
Age, years	
18–30	3,147 (42.45%)
31–40	2,633 (35.52%)
41–50	1,237 (16.69%)
>50	396 (5.34%)
Marital status	
Single	2,013 (27.16%)
Married	5,193 (70.05%)
Divorced	186 (2.51%)
Widowed	21 (0.28%)
Salary (yuan)	5,193 (70.05%)
<4,000	1,023 (13.80%)
4,001–8,000	3,347 (45.15%)
8,001–16,000	2,450 (33.05%)
>16,000	593 (8.00%)
Occupation	
Doctor	1,756 (23.69%)
Nurse	4,368 (58.92%)
Medical technologist/technician	158 (2.13%)
Radiologist	205 (2.77%)
Pharmacist	577 (7.78%)
Public health physician	349 (4.71%)
Education level	
Junior college	3,087 (41.64%)
Bachelor	3,660 (49.37%)
Master	514 (6.93%)
Doctorate	152 (2.05%)
Working experience, years	
1–6	2,576 (34.75%)
7–15	2,891 (39.00%)
>15	1,946 (26.25%)
Professional title	
Junior	4,612 (62.22%)
Senior	1,817 (24.51%)
Associate chief	688 (9.28%)
Chief	296 (3.99%)
Type of institution	
Public primary	607 (8.19%)
Public secondary	1,183 (15.96%)
Public tertiary	5,054 (68.18%)
Private	569 (7.68%)
Designated hospital	
Yes	2,826 (38.12%)
No	4,587 (61.88%)
Workplace COVID-19 risk level	
Low	1,424 (19.21%)
Moderate	1,649 (22.25%)
High	4,340 (58.55%)
Department	
24-hour fever clinic	553 (7.46%)
Suspected isolation ward	368 (4.96%)
Confirmed isolation ward	128 (1.73%)
Medical support to Wuhan	144 (1.94%)
Administration	569 (7.68%)
Other clinical departments	5,651 (76.23%)
Daily negative information	
≤50%	6,504 (87.74%)
>50%	909 (12.26%)
Daily false information	
≤50%	6,928 (93.46%)
>50%	485 (6.54%)
Self-reported physical health	
Very good	3,446 (46.49%)
Good	3,302 (44.54%)
Fair	602 (8.12%)
Poor	63 (0.85%)

COVID-19, coronavirus disease 2019.

© Annals of Palliative Medicine. All rights reserved.

Table 2 Univariate analysis of anxiety severity in medical staff during the COVID-19 outbreak (N=7,413)

Variable	Groups	None (n/%)	Mild (n/%)	Moderate (n/%)	Severe (n/%)	P value
Total		4,912 (66.26)	1,747 (23.57)	449 (6.06)	305 (4.11)	
Gender	Male	931 (74.84)	231 (18.57)	52 (4.18)	30 (2.41)	<0.001
	Female	3,981 (64.53)	1,516 (24.57)	397 (6.44)	275 (4.46)	
Age, years	18–30	2,050 (65.14)	780 (24.79)	196 (6.23)	121 (3.84)	0.001
	31–40	1,714 (65.10)	630 (23.93)	156 (5.92)	133 (5.05)	
	41–50	865 (69.93)	254 (20.53)	80 (6.47)	38 (3.07)	
	>50	283 (71.46)	83 (20.96)	17 (4.29)	13 (3.28)	
Marital status	Single	1,402 (69.65)	471 (23.40)	103 (5.12)	37 (1.84)	<0.001
	Married	3,386 (65.20)	1,231 (23.70)	327 (6.30)	249 (4.79)	
	Divorced	115 (61.83)	39 (20.97)	18 (9.68)	14 (7.53)	
	Widowed	9 (42.86)	6 (28.57)	1 (4.76)	5 (23.81)	
Salary (yuan)	<4,000	633 (61.88)	245 (23.95)	74 (7.23)	71 (6.94)	<0.001
	4,001–8,000	2,092 (62.50)	836 (24.98)	249 (7.44)	170 (5.08)	
	8,001–16,000	1,734 (70.78)	559 (22.82)	102 (4.16)	55 (2.24)	
	>16,000	453 (76.39)	107 (18.04)	24 (4.05)	9 (1.52)	
Professional title	Junior	2,961 (64.20)	1,150 (24.93)	286 (6.20)	215 (4.66)	<0.001
	Senior	1,209 (66.54)	428 (23.56)	117 (6.44)	63 (3.47)	
	Associate chief	519 (75.44)	116 (16.86)	36 (5.23)	17 (2.47)	
	Chief	223 (75.34)	53 (17.91)	10 (3.38)	10 (3.38)	
Occupation	Doctor	1,279 (72.84)	351 (19.99)	78 (4.44)	48 (2.73)	<0.001
	Nurse	2,695 (61.70)	1,141 (26.12)	308 (7.05)	224 (5.13)	
	Medical technologist	111 (70.25)	34 (21.52)	10 (6.33)	3 (1.90)	
	Radiologist	157 (76.59)	39 (19.02)	7 (3.41)	2 (0.98)	
	Pharmacist	418 (72.44)	116 (20.10)	25 (4.33)	18 (3.12)	
	Public health physician	252 (72.21)	66 (18.91)	21 (6.02)	10 (2.87)	
Education level	Junior college	1,885 (61.06)	776 (25.14)	241 (7.81)	185 (5.99)	<0.001
	Bachelors	2,531 (69.15)	841 (22.98)	180 (4.92)	108 (2.95)	
	Master	383 (74.51)	100 (19.46)	23 (4.47)	8 (1.56)	
	Doctorate	113 (74.54)	30 (19.54)	5 (3.29)	4 (2.63)	
Working experience, years	1–6	1,749 (67.90)	617 (23.95)	135 (5.24)	75 (2.91)	<0.001
	7–15	1,808 (62.54)	710 (24.56)	204 (7.06)	169 (5.85)	
	>15	1,355 (69.63)	420 (21.58)	110 (5.65)	61 (3.13)	
Type of institution	Public primary	404 (66.56)	120 (19.77)	55 (9.06)	28 (4.61)	0.001
	Public secondary	798 (67.46)	278 (23.50)	65 (5.49)	42 (3.55)	
	Public tertiary	3,297 (65.24)	1,241 (24.55)	299 (5.92)	217 (4.29)	
	Private	413 (72.58)	108 (18.98)	30 (5.27)	18 (3.16)	

Table 2 (*continued*)

© Annals of Palliative Medicine. All rights reserved.

Table 2 (*continued*)

Variable	Groups	None (n/%)	Mild (n/%)	Moderate (n/%)	Severe (n/%)	P value
Workplace COVID-19 risk level	Low	716 (49.77)	405 (28.44)	149 (10.46)	154 (11.33)	<0.001
	Moderate	1,120 (67.92)	362 (21.95)	105 (6.37)	62 (3.76)	
	High	3,070 (70.74)	980 (22.58)	195 (4.49)	95 (2.19)	
Department	24-hour fever clinic	338 (61.12)	134 (24.23)	41 (7.41)	40 (7.23)	<0.001
	Suspected isolation ward	232 (63.04)	93 (25.27)	28 (7.61)	15 (4.08)	
	Confirmed isolation ward	77 (60.16)	32 (25.00)	11 (8.59)	8 (6.25)	
	Medical support to Wuhan	106 (73.61)	32 (22.22)	3 (2.08)	3 (2.08)	
	Administration	376 (66.08)	118 (20.74)	40 (7.03)	35 (6.15)	
	Other clinical departments	3,783 (66.94)	1,338 (23.68)	326 (5.77)	204 (3.61)	
Designated hospital	Yes	1,639 (58.00)	766 (27.11)	237 (8.39)	184 (6.51)	<0.001
	No	3,273 (71.35)	981 (21.39)	212 (4.62)	121 (2.64)	
Daily negative information	≤50%	4,571 (70.28)	1,459 (22.43)	303 (4.66)	171 (2.63)	<0.001
	>50%	341 (37.51)	288 (31.68)	146 (16.06)	134 (14.74)	
Daily false information	≤50%	4,696 (67.78)	1,615 (23.31)	382 (5.51)	235 (3.39)	<0.001
	>50%	216 (44.54)	132 (27.22)	67 (13.81)	70 (14.43)	
Self-reported physical health	Very good	2,475 (71.82)	691 (20.05)	161 (4.67)	119 (3.45)	<0.001
	Good	2,112 (63.96)	851 (25.77)	218 (6.60)	121 (3.66)	
	Fair	307 (51.00)	186 (30.90)	59 (9.80)	50 (8.31)	
	Poor	18 (28.57)	19 (30.16)	11 (17.46)	15 (23.81)	

COVID-19, coronavirus disease 2019.

4.2% (P<0.001), and 5.8% (P=0.006), respectively.

Factors associated with depression symptoms in medical staff

In general, most influencing factors had a similar effect on the GAD-7 and PHQ-9 scores of respondents. As outlined in *Table 4*, the univariable analysis showed that medical staff who worked in low-risk areas (40.87%, P<0.001), those who self-reported as being in poor health (80.95%, P<0.001), and those whose daily COVID-19-related information was negative (51.05%, P<0.001) or false (48.66%, P<0.001) more than half of the time were at a higher risk of depression during the early period of the pandemic. More than 10% of these respondents were screened as having MSD+. It is worth noting that medical staff in confirmed COVID-19 isolation wards also suffered from a worse depression; 58/128 had depression symptoms, and 9.38% were MSD+ (P<0.001). When respondents without depression were used as the control group (*Table 5*), medical staff aged 18–30 years displayed the highest chance of suffering from MDS+ than those in other age groups (P=0.038, P<0.001, P=0.007). The odds of being assessed as MDS+ were 3.375 (95% CI: 1.749–6.515, P<0.001) times higher in the divorced group than in the unmarried group. The likelihood of medical staff being screened into the MDS+ group was 62.180 (95% CI: 27.761–139.270, P<0.001) times greater among respondents with poor physical health than among those with good physical health. Respondents whose daily COVID-19-related information was negative (OR =4.224, 95% CI: 3.143–5.677, P<0.001) or false (OR =3.385, 95% CI: 2.420–4.735, P<0.001) more than half of the time were more likely to be in the MDS+ group. Medical staff who worked in low-risk areas had a

© Annals of Palliative Medicine. All rights reserved.

Table 3 Multiple logistic regression analysis of variables associated with anxiety symptoms of medical staff during the COVID-19 outbreak

Variable	Groups	Mild		Moderate		Severe	
		OR (95% CI)	P	OR (95% CI)	P	OR (95% CI)	P
Gender	Male	1 (ref)		1 (ref)		1 (ref)	
	Female	1.244 (1.045–1.481)	0.014	1.308 (0.935–1.831)	0.117	1.182 (0.762–1.836)	0.455
Marital status	Single	1 (ref)		1 (ref)		1 (ref)	
	Married	1.097 (0.964–1.249)	0.159	1.271 (0.997–1.620)	0.053	2.426 (1.678–3.508)	<0.001
	Divorced	0.983 (0.666–1.451)	0.931	1.922 (1.091–3.388)	0.024	3.102 (1.534–6.272)	0.002
	Widowed	2.099 (0.729–6.044)	0.170	1.661 (0.199–13.831)	0.639	19.615 (5.255–73.210)	<0.001
Occupation	Doctor	1 (ref)		1 (ref)		1 (ref)	
	Nurse	1.465 (1.255–1.709)	<0.001	1.671 (1.247–2.240)	0.001	1.579 (1.086–2.297)	0.017
	Medical technologist	1.263 (0.838–1.903)	0.265	1.787 (0.883–3.614)	0.106	0.838 (0.250–2.802)	0.774
	Radiologist	0.941 (0.647–1.370)	0.752	0.811 (0.364–1.808)	0.609	0.400 (0.095–1.686)	0.212
	Pharmacist	1.101 (0.863–1.404)	0.441	1.114 (0.692–1.795)	0.657	1.319 (0.739–2.351)	0.349
	Public health physician	0.971 (0.717–1.313)	0.847	1.290 (0.769–2.165)	0.335	1.044 (0.503–2.165)	0.909
Designated hospital	No	1 (ref)		1 (ref)		1 (ref)	
	Yes	1.338 (1.167–1.534)	<0.001	1.681 (1.314–2.151)	<0.001	1.506 (1.099–2.065)	0.011
Type of institution	Private	1 (ref)		1 (ref)		1 (ref)	
	Public primary	1.070 (0.784–1.459)	0.671	1.281 (0.777–2.114)	0.332	0.809 (0.418–1.565)	0.529
	Public secondary	1.334 (1.024–1.736)	0.033	1.011 (0.631–1.621)	0.963	1.036 (0.566–1.897)	0.908
	Public tertiary	1.200 (0.953–1.510)	0.121	0.813 (0.539–1.226)	0.322	0.746 (0.438–1.269)	0.279
Self-reported physical health	Very good	1 (ref)		1 (ref)		1 (ref)	
	Good	1.382 (1.226–1.557)	<0.001	1.532 (1.228–1.910)	<0.001	1.307 (0.991–1.723)	0.058
	Fair	2.007 (1.632–2.469)	<0.001	2.623 (1.875–3.669)	<0.001	3.397 (2.318–4.977)	<0.001
	Poor	3.566 (1.840–6.908)	<0.001	8.802 (3.964–19.545)	<0.001	24.735 (11.507–53.170)	<0.001
Daily negative information	≤50%	1 (ref)		1 (ref)		1 (ref)	
	>50%	2.320 (1.937–2.779)	<0.001	4.809 (3.733–6.194)	<0.001	6.316 (4.703–8.482)	<0.001
Daily false information	≤50%	1 (ref)		1 (ref)		1 (ref)	
	>50%	1.215 (0.951–1.553)	0.119	1.614 (1.148–2.268)	0.006	2.263 (1.569–3.265)	<0.001
Workplace COVID-19 risk level	Low	1 (ref)		1 (ref)		1 (ref)	
	Moderate	0.830 (0.700–0.983)	0.031	0.630 (0.473–0.839)	0.002	0.327 (0.230–0.464)	<0.001
	High	0.791 (0.652–0.960)	0.017	0.743 (0.540–1.024)	0.069	0.505 (0.342–0.746)	0.001
Occupation protection score		0.970 (0.958–0.981)	<0.001	0.958 (0.940–0.976)	<0.001	0.942 (0.912–0.972)	0.006

COVID-19, coronavirus disease 2019.

© Annals of Palliative Medicine. All rights reserved.

Table 4 Univariate analysis of depression severity among medical staff during the COVID-19 outbreak (N=7,413)

Variable	Groups	None (n/%)	Mild (n/%)	Moderate (n/%)	MSD+† (n/%)	P value
Total		5,363 (72.35)	1,397 (18.85)	346 (4.67)	307 (4.14)	
Gender	Male	942 (75.72)	218 (17.52)	37 (2.97)	47 (3.78)	0.002
	Female	4,421 (71.66)	1,179 (19.11)	309 (5.01)	260 (4.21)	
Age, years	18–30	2,245 (71.34)	595 (18.91)	154 (4.89)	153 (4.86)	<0.001
	31–40	1,873 (71.14)	508 (19.29)	130 (4.94)	122 (4.63)	
	41–50	927 (74.94)	240 (19.40)	46 (3.72)	24 (1.94)	
	>50	318 (80.30)	54 (13.64)	16 (4.04)	8 (2.02)	
Marital status	Single	1,461 (72.58)	405 (20.12)	82 (4.07)	65 (3.23)	0.001
	Married	3,774 (72.67)	950 (18.29)	246 (4.74)	223 (4.29)	
	Divorced	115 (61.83)	40 (21.51)	14 (7.53)	17 (9.14)	
	Widowed	13 (61.90)	2 (9.52)	4 (19.05)	2 (9.52)	
Salary (yuan)	<4,000	706 (69.01)	189 (18.48)	61 (5.96)	67 (6.55)	<0.001
	4,001–8,000	2,333 (69.70)	656 (19.60)	191 (5.71)	167 (4.99)	
	8,001–16,000	1,866 (76.16)	449 (18.33)	72 (2.94)	63 (2.57)	
	>16,000	458 (77.23)	103 (17.37)	22 (3.71)	10 (1.69)	
Professional title	Junior	3,274 (70.99)	877 (19.02)	231 (5.01)	230 (4.99)	<0.001
	Senior	1,323 (72.81)	352 (19.37)	78 (4.29)	64 (3.52)	
	Associate chief	528 (76.74)	123 (17.88)	26 (3.78)	11 (1.60)	
	Chief	238 (80.41)	45 (15.20)	11 (3.72)	2 (0.68)	
Occupation	Doctor	1,306 (74.37)	337 (19.19)	67 (3.82)	46 (2.62)	<0.001
	Nurse	3,067 (70.22)	842 (19.28)	228 (5.22)	231 (5.29)	
	Medical technologist	127 (80.38)	23 (14.56)	3 (1.90)	5 (3.16)	
	Radiologist	167 (81.46)	28 (13.66)	6 (2.93)	4 (1.95)	
	Pharmacist	435 (75.39)	106 (18.37)	25 (4.33)	11 (1.91)	
	Public health physician	261 (74.79)	61 (17.48)	17 (4.87)	10 (2.87)	
Education	Junior college	2,163 (70.07)	572 (18.53)	169 (5.47)	183 (5.93)	<0.001
	Bachelors	2,709 (74.02)	687 (18.77)	154 (4.21)	110 (3.01)	
	Master	389 (75.68)	97 (18.87)	17 (3.31)	11 (2.14)	
	Doctorate	102 (67.11)	41 (26.97)	6 (3.95)	3 (1.97)	
Working experience, years	1–6 years	1,876 (72.83)	490 (19.02)	107 (4.15)	103 (4.00)	<0.001
	7–15 years	2,004 (69.32)	563 (19.47)	168 (5.81)	156 (5.40)	
	>15 years	1,483 (76.21)	344 (17.68)	71 (3.65)	48 (2.47)	

Table 4 (*continued*)

© Annals of Palliative Medicine. All rights reserved.

Table 4 (*continued*)

Variable	Groups	None (n/%)	Mild (n/%)	Moderate (n/%)	MSD+† (n/%)	P value
Type of institution	Public primary	441 (72.65)	97 (15.98)	34 (5.60)	35 (5.77)	<0.001
	Public secondary	893 (75.49)	213 (18.01)	39 (3.30)	38 (3.21)	
	Public tertiary	3,597 (71.17)	976 (19.31)	260 (5.14)	221 (4.37)	
	Private	432 (75.92)	111 (19.51)	13 (2.28)	13 (2.28)	
Workplace COVID-19 risk level	Low	842 (59.13)	311 (21.84)	121 (8.50)	150 (10.53)	<0.001
	Moderate	1,252 (75.92)	277 (16.80)	64 (3.88)	56 (3.40)	
	High	3,269 (75.32)	809 (18.64)	161 (3.71)	101 (2.33)	
Department	24-hour fever clinic	358 (64.74)	126 (22.78)	26 (4.70)	43 (7.78)	<0.001
	Suspected isolation ward	259 (70.38)	70 (19.02)	19 (5.16)	20 (5.43)	
	Confirmed isolation ward	70 (54.69)	34 (26.56)	12 (9.38)	12 (9.38)	
	Medical support to Wuhan	108 (75.00)	33 (22.92)	2 (1.39)	1 (0.69)	
	Administration	409 (71.88)	100 (17.57)	35 (6.15)	25 (4.39)	
	Other clinical departments	4,159 (73.60)	1,034 (18.30)	252 (4.46)	206 (3.65)	
Designated hospital	Yes	3,485 (75.98)	814 (17.75)	164 (3.58)	124 (2.70)	<0.001
	No	1,878 (66.45)	583 (20.63)	182 (6.44)	183 (6.48)	
Daily negative information	≤50%	4,918 (75.62)	1,169 (17.97)	242 (3.72)	175 (2.69)	<0.001
	>50%	445 (48.95)	228 (25.08)	104 (11.44)	132 (14.52)	
Daily false information	≤50%	5,114 (73.82)	1,294 (18.68)	303 (4.37)	217 (3.13)	<0.001
	>50%	249 (51.34)	103 (21.24)	43 (8.87)	90 (18.56)	
Self-reported physical health	Very good	2,729 (79.19)	500 (14.51)	104 (3.02)	113 (3.28)	<0.001
	Good	2,314 (70.08)	706 (21.38)	156 (4.72)	126 (3.82)	
	Fair	308 (51.16)	178 (29.57)	67 (11.13)	49 (8.14)	
	Poor	12 (19.05)	13 (20.63)	19 (30.16)	19 (30.16)	

†, moderate to severe depression or above. COVID-19, coronavirus disease 2019.

higher chance of being assessed as MDS+ than those in the other 2 groups (P<0.001, P<0.001). For every 1-point increase in occupational protection score, the odds of being assessed as having depressive symptoms decreased by 5.6% (P<0.001), 4.3% (P<0.001), and 4.5% (P<0.001) for mild, moderate, and MDS+, respectively.

Discussion

The COVID-19 pandemic is a threat to physical and mental health internationally. Many studies indicated that the COVID-19 pandemic is associated with mental disorders. Common risk factors for infected patients during isolation included being female, social isolation, a claustrophobic isolation environment, fear of the unknown and panic, fear of discrimination, insomnia, poor physical health, repeated exposure to social media, and economic difficulties (27). However, the psychological impact of COVID-19 on medical staff was more related to their occupation and working environment due to the severity

© Annals of Palliative Medicine. All rights reserved.

Table 5 Multiple logistic regression analysis of variables associated with depression symptoms of medical staff during the COVID-19 outbreak

Variable	Groups	Mild		Moderate		MSD+†	
		OR (95% CI)	P	OR (95% CI)	P	OR (95% CI)	P
Gender	Male	1 (ref)		1 (ref)		1 (ref)	
	Female	1.106 (0.935–1.310)	0.24	1.438 (0.999–2.071)	0.051	0.761 (0.537–1.078)	0.125
Age, years	18–30	1 (ref)		1 (ref)		1 (ref)	
	31–40	1.068 (0.907–1.257)	0.429	0.872 (0.655–1.162)	0.350	0.733 (0.546–0.984)	0.038
	41–50	1.051 (0.858–1.289)	0.630	0.666 (0.448–0.992)	0.046	0.355 (0.217–0.581)	<0.001
	>50	0.612 (0.440–0.851)	0.004	0.652 (0.365–1.163)	0.147	0.349 (0.163–0.751)	0.007
Marital status	Single	1 (ref)		1 (ref)		1 (ref)	
	Married	0.845 (0.714–1.001)	0.052	1.105 (0.806–1.516)	0.536	1.367 (0.971–1.926)	0.073
	Divorced	1.159 (0.772–1.739)	0.476	2.105 (1.094–4.053)	0.026	3.375 (1.749–6.515)	<0.001
	Widowed	0.584 (0.128–2.658)	0.486	6.084 (1.678–22.063)	0.006	3.839 (0.652–22.583)	0.137
Designated hospital	No	1 (ref)		1 (ref)		1 (ref)	
	Yes	1.217 (1.049–1.412)	0.009	1.386 (1.048–1.834)	0.022	1.301 (0.947–1.786)	0.104
Type of institution	Private	1 (ref)		1 (ref)		1 (ref)	
	Public primary	0.852 (0.618–1.176)	0.329	2.081 (1.052–4.117)	0.035	1.629 (0.805–3.299)	0.175
	Public secondary	0.945 (0.720–1.239)	0.681	1.399 (0.723–2.706)	0.319	1.475 (0.746–2.915)	0.264
	Public tertiary	0.939 (0.745–1.184)	0.596	1.704 (0.950–3.056)	0.074	1.192 (0.651–2.182)	0.569
Self-reported physical health	Very good	1 (ref)		1 (ref)		1 (ref)	
	Good	1.567 (1.375–1.786)	<0.001	1.722 (1.326–2.236)	<0.001	1.358 (1.030–1.791)	0.030
	Fair	2.913 (2.349–3.613)	<0.001	5.545 (3.929–7.828)	<0.001	3.994 (2.711–5.886)	<0.001
	Poor	5.644 (2.530–12.592)	<0.001	48.582 (22.305–105.812)	<0.001	62.180 (27.761–139.270)	<0.001
Daily negative information	≤50%	1 (ref)		1 (ref)		1 (ref)	
	>50%	2.011 (1.667–2.426)	<0.001	3.706 (2.793–4.918)	<0.001	4.224 (3.143–5.677)	<0.001
Daily false information	≤50%	1 (ref)		1 (ref)		1 (ref)	
	>50%	1.166 (0.899–1.512)	0.247	1.391 (0.940–2.057)	0.099	3.385 (2.420–4.735)	<0.001
Workplace COVID-19 risk level	Low	1 (ref)		1 (ref)		1 (ref)	
	Moderate	0.860 (0.717–1.033)	0.107	0.615 (0.447–0.846)	0.003	0.343 (0.242–0.487)	<0.001
	High	0.699 (0.567–0.862)	0.001	0.526 (0.363–0.762)	0.001	0.357 (0.239–0.533)	<0.001
Occupation protection score		0.944 (0.933–0.955)	<0.001	0.957 (0.936–0.978)	<0.001	0.955 (0.934–0.977)	<0.001

†, moderate to severe depression or above. COVID-19, coronavirus disease 2019.

© Annals of Palliative Medicine. All rights reserved.

of the epidemic (28). Our study is one of the earliest large-scale evaluations of the psychological health of medical staff from nearly all provinces in China during the early phase of the COVID-19 pandemic. The results revealed that around 34% and 28% of medical staff in our study had experienced anxiety and depression symptoms, respectively; these rates are higher than those reported in a previous study regarding the mental health of the general public (12). The medical staff were found to be more psychologically stressed than the general population during infectious pandemics such as SARS (2,4), MERS (4), and Ebola (3) due to their close contact with sources of infection. Thus, the mental health of medical staff is a worthy concern.

However, the rates found in the present research are lower than those found by Wang *et al.* (13) regarding the psychological impact of the COVID-19 pandemic on medical staff in Guangdong (conducted on February 2 and 3, 2020) and those by Lai *et al.* (14) regarding mental health outcomes among Chinese healthcare workers outside Hubei Province from January 29 to February 3, 2020. These results suggested that medical staff suffered even more severe psychological stress at the beginning of the pandemic and that the situation has improved in our study (about 1 month after the first confirmed case). Since the outbreak of the epidemic, the Chinese government came out with a rapid reaction and launched the Joint Prevention and Control Mechanism of the State Council in response to the prevention and control on COVID-19. Wuhan has been locked down as soon as possible to stop the spread of the virus. Various government departments and social-wide efforts have taken joint actions. Many medical staff from all over the country assembled and rushed to Hubei Province and other high-risk areas as medical support. All donations were sent to high-risk areas in priority and used for epidemic control without any delay. The supply of medical materials and scientific research on the origin of the virus, infection source, and transmission route of COVID-19 were fully supported by the government. Biosafety laboratories are classified into four levels as P1-P4, according to the international Biosafety level. Research on COVID-19 was conducted in the highest level of biosafety laboratories (P4) to guarantee the safety of healthcare professionals devoting to COVID-19 studies. They were equipped with the most advanced facilities and the highest level of protective devices. The epidemic has been effectively controlled by all these prevention measures and greatly relieved the psychological pressure of medical staff (28).

Lai *et al.* (14) found that healthcare workers in Wuhan and frontline departments reported more severe mental problems across all measurements. Wang *et al.* (13) suggested that medical staff classified as having a lower risk of infection reported lower average levels of anxiety, depression, perceived stress, and insomnia severity than those in the high-risk group. Most studies in other countries conducted in several hospitals had similar findings. Shah *et al.* (16) conducted a cross-sectional study among medical staff recruited from three major hospitals in Kenya, Africa, and determined that frontline medical staff were at higher risk of mental health symptoms during the isolation of COVID-19. Nearly half of the participants reported inadequate resources or training to care for patients with COVID-19. The study of Firew *et al.* (17) sought to assess factors contributing to the psychological distress of medical staff in the USA. They found that medical staff in the emergency department were more likely to contract COVID-19 and had increased depression symptoms. Georger *et al.* (18) conducted the stress exposure analysis among hospital staff in 2 French hospitals. They found that the mental disorders of medical staff was mostly related to the stress of contracting the infection. The lack of information and protective gear and equipment were major factors of insecurity at the start of the epidemic. All of these reports suggested that the psychological condition of medical staff during the COVID-19 outbreak was highly associated with the actual situation of the pandemic at their workplace, which is similar to our findings. Consistent with the findings of Huang *et al.* (6) and Carmassi *et al.* (29), our study showed a poorer mental state among the medical staff in COVID-19 designated hospitals and confirmed isolation wards. The reasons for this might be attributable to many factors, including close contact with sources of infection, concerns about becoming infected or family and colleagues becoming infected, highly stressful work-related situations they are exposed to, management of critical medical situations, a heavy workload, sudden changes in patients' conditions, a shortage of supplies, a lack of sleep due to shift work, and the necessity to wear a full-body protective suit for more than 4–8 hours (14,30). Surprisingly, the psychological conditions of those who volunteered as medical support in Hubei were better than expected. There may be several explanations for this finding. First, the selection of volunteers for Hubei was strict. Most of the medical staff selected for medical support had rich clinical experience, and some were experts in the containment of infectious and respiratory diseases. Second, the volunteers might have been offered more occupational protective

© Annals of Palliative Medicine. All rights reserved.

training and mental preparation support before their departure to Hubei. Finally, the protective supplies of each volunteer team were coordinated and deployed by local governments, so the supply of goods could be guaranteed to some extent.

In Lai's study (14), since Wuhan was the epicenter of the COVID-19 outbreak in China, the prevalence of anxiety and depression among medical staff in Wuhan was higher than in other regions. However, medical staff in low-risk areas showed poorer mental health than those in moderate- or high-risk areas in our study, consistent with the findings of a previous study by Wu *et al.* (31). The explanations for this result may be as follows. First, there are likely fewer confirmed or suspected COVID-19 cases in low-risk areas. Less direct contact with infected patients would suggest less sufficient clinical experience, less protective training, and less confidence in dealing with an emerging disease.

Additionally, high-risk areas were given priority in the deployment of protective materials. A lack of essential protective supplies can also result in psychological stress. Our study results also indicated that medical staff with lower occupational protection scores were more likely to feel anxious or depressed. As the COVID-19 virus is transmitted by close human-to-human contact through droplets (32), inadequate occupational protection increases the probability of occupational exposure and the chances of being infected (6). Wang's study (13) also pointed out that while the fear around the potential infection applies to all medical staff, those in the low-risk group may also have concerns about understaffing/overworking and their lack of active control.

We also found that nurses were more likely to experience anxiety than other medical personnel, which is similar to previous findings (31,33). Nurses are at a higher risk of infection due to their close and long-term exposure to infected patients. Moreover, most of the nurses were women, and women may be more willing to express their inner negative emotions than men. They are also prone to physical and emotional discomfort when faced with heavy workloads and poor treatment outcomes among patients (34). Medical staff aged 18–30 years recorded the highest chance of suffering from MDS+ in this study. Younger age is associated with less clinical experience (35). For instance, in the SARS outbreak, most young medical staff in this study were only juniors or high school students, so they had no experience dealing with such urgent public health issues. We also found that divorced and widowed medical staff were at a high risk of mental health problems. Companionship and social support by families are important factors in relieving psychological stress (36), and social support has been reported to be a key protective factor for good mental health (37). Our study also found that physical health was positively correlated with mental health, and many studies have already shown an association between physical discomfort and psychological stress (38,39).

Our results also uncovered a correlation between the levels of anxiety and depression and the authenticity and orientation of media. Nohlen *et al.* (40) pointed out that negative judgments spread more widely and faster than positive ones in the face of conflicting information. Additionally, a large-scale analysis of tweets by Vosoughi *et al.* (7) revealed that false rumors spread further and faster than the truth does. In the current study, our results showed that although the medical staff is more professional than the general public when dealing with public health issues, negative and false information can still have a significant adverse effect on their mental health. In the early stages of the COVID-19 outbreak, the number of infected patients increased rapidly, causing widespread panic. People knew very little about the virus and could not determine whether the news was true or false (41). Repeated exposure to information about the epidemic might worsen anxiety and depression. Nowadays, the Internet and social media platforms are important channels for people to stay updated with the latest news, and information is widely and rapidly spread via the Internet and smartphones. However, the system for regulating content across the Internet and new media platforms still requires improvement, and strict regulation is called for. The government and propaganda authorities must be encouraged to focus more on regulating the Internet and new media platforms to make good use of their positive influence (42).

Studies (43,44) showed that stress-related psychiatric conditions were associated with suicidal behavior. Medical staff might also be at an elevated suicide risk during COVID-19. Anxiety, depression, a high-stress working environment, poor sleep quality, a sense of loneliness, and a negative coping style were all risk factors of suicidal attempts. Emergency psychological crisis interventions were called to decrease the suicide ideation of medical staff. Rapid access to psychological counseling from mental health associations/organizations/professionals online and offline, psychological contingency for time off work, social-wide support, and care from families and colleagues are necessary to decrease distress, negative emotions, and suicide ideation.

© Annals of Palliative Medicine. All rights reserved.

Limitations

There are 2 limitations to this study. First, we did not track the change in the psychological status of medical staff, along with the dynamic development of the COVID-19 outbreak. Second, we did not subcategorize medical staff at different risk levels in the workplace according to their working department, or determine the combined influence of both of these factors.

Conclusions

A significant proportion of medical staff, even those in areas with low exposure risk, experienced psychological issues due to anxiety and depression during the outbreak of COVID-19 in China. Being nurses, having poor physical health, being divorced/widowed, having high risks of contracting COVID-19, lacking clinical experience, having insufficient occupational protection, and repeated exposures to negative media reports are risk factors influencing the psychological health of medical staff (45). Protective factors might include being male, having sufficient medical resources, up-to-date and accurate information, precautionary protection, rapid access to psychological counseling, and social support. In conclusion, to improve the mental health of medical staff, effective interventions and targeted policies must focus on these issues.

Acknowledgments

Funding: This work was supported by a grant from the Public Health Policy Research and Evaluation Key Laboratory Project of the Philosophy and Social Sciences of Guangdong College (grant No. 2015WSYS0010), and a grant from the Public Health Service System Construction Research Foundation of Guangzhou (2021-2023).

Footnote

Reporting Checklist: The authors have completed the SURGE reporting checklist. Available at https://dx.doi.org/10.21037/apm-21-1261

Data Sharing Statement: Available at https://dx.doi.org/10.21037/apm-21-1261

Conflicts of Interest: All authors have completed the ICMJE uniform disclosure form (available at https://dx.doi.org/10.21037/apm-21-1261). Dr. Wang reported that this work was supported by a grant from the Public Health Policy Research and Evaluation Key Laboratory Project of the Philosophy and Social Sciences of Guangdong College (grant No. 2015WSYS0010), and a grant from the Public Health Service System Construction Research Foundation of Guangzhou (2021-2023).The other authors have no conflicts of interest to declare.

Ethical Statement: The authors are accountable for all aspects of the work and in ensuring that questions related to the accuracy or integrity of any part of the work are appropriately investigated and resolved. This research was conducted in accordance with the Declaration of Helsinki (as revised in 2013). The study was approved by the institutional ethics board of the third-affiliated Hospital of Southern Medical University (approval No. 2020-EA-014), and informed consent was obtained from all individual participants.

Open Access Statement: This is an Open Access article distributed in accordance with the Creative Commons Attribution-NonCommercial-NoDerivs 4.0 International License (CC BY-NC-ND 4.0), which permits the non-commercial replication and distribution of the article with the strict proviso that no changes or edits are made and the original work is properly cited (including links to both the formal publication through the relevant DOI and the license). See: https://creativecommons.org/licenses/by-nc-nd/4.0/.

References

1. Wang C, Horby PW, Hayden FG, et al. A novel coronavirus outbreak of global health concern. Lancet 2020;395:470-3.
2. Lu QY, Yan J, Huang XB. Mental Stress and Crisis Intervention in the Patients with SARS and the People Related. Journal of Beijing Medical University, 2003.
3. Van Bortel T, Basnayake A, Wurie F, et al. Psychosocial effects of an Ebola outbreak at individual, community and international levels. Bull World Health Organ 2016;94:210-4.
4. Qian Y, Hua WX, Zhang N, et al. a Review of psychological status and intervention of health care workers during coronavirus pandemics. Chinese Mental Health Journal 2021;(05):432-7.
5. Wu XQ, Zhang Y, Li M, et al. Mental Health Status of Medical Staffs Fighting SARS: a Long-dated Investigation.

China Journal of Health Psychology 2006.
6. Huang ZP, Zhang KT, He XY, et al. Research on mental health status of medical staff during COVID-19 epidemic. Zhonghua Lao Dong Wei Sheng Zhi Ye Bing Za Zhi 2020;38:834-8.
7. Vosoughi S, Roy D, Aral S. The spread of true and false news online. Science 2018;359:1146-51.
8. Chen N, Zhou M, Dong X, et al. Epidemiological and clinical characteristics of 99 cases of 2019 novel coronavirus pneumonia in Wuhan, China: a descriptive study. Lancet 2020;395:507-13.
9. Huang C, Wang Y, Li X, et al. Clinical features of patients infected with 2019 novel coronavirus in Wuhan, China. Lancet 2020;395:497-506.
10. Lu R, Zhao X, Li J, et al. Genomic characterisation and epidemiology of 2019 novel coronavirus: implications for virus origins and receptor binding. Lancet 2020;395:565-74.
11. Li Q, Guan X, Wu P, et al. Early Transmission Dynamics in Wuhan, China, of Novel Coronavirus-Infected Pneumonia. N Engl J Med 2020;382:1199-207.
12. Wang C, Pan R, Wan X, et al. Immediate Psychological Responses and Associated Factors during the Initial Stage of the 2019 Coronavirus Disease (COVID-19) Epidemic among the General Population in China. Int J Environ Res Public Health 2020;17:1729.
13. Wang HJ, Huang DZ, Huang HG, et al. The psychological impact of COVID-19 pandemic on medical staff in Guangdong, China: a cross-sectional study. Psychological Medicine1–9. Available online: https: //doi.org/10.1017/S0033291720002561
14. Lai J, Ma S, Wang Y, et al. Factors Associated With Mental Health Outcomes Among Health Care Workers Exposed to Coronavirus Disease 2019. JAMA Netw Open 2020;3:e203976.
15. Wang YY, Jia XR, Song JQ, et al. Mental health status of medical staff during the outbreak of coronavirus disease 2019. Medical Journal of Wuhan University 2020;41:706-10.
16. Shah J, Monroe-Wise A, Talib Z, et al. Mental health disorders among healthcare workers during the COVID-19 pandemic: a cross-sectional survey from three major hospitals in Kenya. BMJ Open 2021;11:e050316.
17. Firew T, Sano ED, Lee JW, et al. Protecting the front line: a cross-sectional survey analysis of the occupational factors contributing to healthcare workers' infection and psychological distress during the COVID-19 pandemic in the USA. BMJ Open 2020;10:e042752.
18. Georger F, Dos Santos E, Gazagne L, et al. COV IMPACT: Stress exposure analysis among hospital staff in 2 hospitals in France during the COVID-19 pandemic. Ann Cardiol Angeiol (Paris) 2020;69:227-32.
19. Heitzman J. Impact of COVID-19 pandemic on mental health. Psychiatr Pol 2020;54:187-98.
20. Torales J, O'Higgins M, Castaldelli-Maia JM, et al. The outbreak of COVID-19 coronavirus and its impact on global mental health. Int J Soc Psychiatry 2020;66:317-20.
21. Gong Y, Zhou H, Zhang Y, et al. Validation of the 7-item Generalized Anxiety Disorder scale (GAD-7) as a screening tool for anxiety among pregnant Chinese women. J Affect Disord 2021;282:98-103.
22. Esser P, Hartung TJ, Friedrich M, et al. The Generalized Anxiety Disorder Screener (GAD-7) and the anxiety module of the Hospital and Depression Scale (HADS-A) as screening tools for generalized anxiety disorder among cancer patients. Psychooncology 2018;27:1509-16.
23. Hinz A, Klein AM, Brähler E, et al. Psychometric evaluation of the Generalized Anxiety Disorder Screener GAD-7, based on a large German general population sample. J Affect Disord 2017;210:338-44.
24. Vandelaar LJ, Jiang ZY, Saini A, et al. PHQ-9 and SNOT-22: Elucidating the Prevalence of Depression in Chronic Rhinosinusitis. Otolaryngol Head Neck Surg 2020;162:142-7.
25. Patel JS, Oh Y, Rand KL, et al. Measurement invariance of the patient health questionnaire-9 (PHQ-9) depression screener in U.S. adults across sex, race/ethnicity, and education level: NHANES 2005-2016. Depress Anxiety 2019;36:813-23.
26. Dajpratham P, Pukrittayakamee P, Atsariyasing W, et al. The validity and reliability of the PHQ-9 in screening for post-stroke depression. BMC Psychiatry 2020;20:291.
27. Chen X, Chen Y, Chen B, et al. Status and factors of anxiety/depression during centralized quarantine the epicemic of COVID-19. China Journal of Health Psychology 2020,28:1350-5.
28. Lin Z, Wang L, Yu B, et al. Adverse emotional response and its influencing factors mong frontline. Chin J Public Health 2020,36:677-81.
29. Carmassi C, Foghi C, Dell'Oste V, et al. PTSD symptoms in healthcare workers facing the three coronavirus outbreaks: What can we expect after the COVID-19 pandemic. Psychiatry Res 2020;292:113312.
30. Lu W, Wang H, Lin Y, et al. Psychological status of medical workforce during the COVID-19 pandemic: A cross-sectional study. Psychiatry Res 2020;288:112936.

© Annals of Palliative Medicine. All rights reserved.

31. Wu SC. Self-rated Mental Health and Influencing Factors among Medical Staffs in 136 Tertiary Public Hospitals in China. Peking Union Medical College, 2019.
32. Phan LT, Nguyen TV, Luong QC, et al. Importation and Human-to-Human Transmission of a Novel Coronavirus in Vietnam. N Engl J Med 2020;382:872-4.
33. Huang JZ, Han MF, Luo TD, et al. Mental health survey of medical staff in a tertiary infectious disease hospital for COVID-19. Zhonghua Lao Dong Wei Sheng Zhi Ye Bing Za Zhi 2020;38:192-5.
34. Neitzke AB. An Illness of Power: Gender and the Social Causes of Depression. Cult Med Psychiatry 2016;40:59-73.
35. Sheng B, Cheng SK, Lau KK, et al. The effects of disease severity, use of corticosteroids and social factors on neuropsychiatric complaints in severe acute respiratory syndrome (SARS) patients at acute and convalescent phases. Eur Psychiatry 2005;20:236-42.
36. Ashkzari MK, Piryaei S, Brojerdian N, et al. The relationship between job satisfaction with marital satisfaction and mental health: The specific case of female employees. Eur Psychiatry 2017;41:S737.
37. Veissi M, Atefvahid MK, Rezaee M. Job Stress, Job Satisfaction and Mental Health: The Balancing Effects of Personal Hardiness and Social Support Network Factors. Int J Psychiat Clin 2000.
38. Liu X, Kakade M, Fuller CJ, et al. Depression after exposure to stressful events: lessons learned from the severe acute respiratory syndrome epidemic. Compr Psychiatry 2012;53:15-23.
39. Zhang WR, Wang K, Yin L, et al. Mental Health and Psychosocial Problems of Medical Health Workers during the COVID-19 Epidemic in China. Psychother Psychosom 2020;89:242-50.
40. Nohlen HU, van Harreveld F, Cunningham WA. Social evaluations under conflict: negative judgments of conflicting information are easier than positive judgments. Soc Cogn Affect Neurosci 2019;14:709-18.
41. Wang Y, Guo JL, Chen H, et al. The Relationship between Media Exposure and Mental Health Problems during COVID-19 Outbreak. Fudan University Journal of Medical Sciences 2020.
42. Wang X, Wang X, Min G, et al. An Efficient Feedback Control Mechanism for Positive/Negative Information Spread in Online Social Networks. IEEE Trans Cybern 2020. [Epub ahead of print]. doi: 10.1109/TCYB.2020.2977322.
43. Sher L. The impact of the COVID-19 pandemic on suicide rates. QJM 2020;113:707-12.
44. Wang RJ, Li J, Mei JH. et al. Poor sleep quality, anxiety and depression are associated with an increased suicide risk in patients with coronavirus disease 2019. Acta Academiae Medicinae Militaris Tertiae 2020,42:1462-8.
45. Lin LY, Sidani JE, Shensa A, et al. Association between social media use and depression among U.S. young adults. Depress Anxiety 2016;33:323-31.

(English Language Editor: J. Reynolds and J. Chapnick)

Cite this article as: Fu M, Han D, Xu M, Mao C, Wang D. The psychological impact of anxiety and depression on Chinese medical staff during the outbreak of the COVID-19 pandemic: a cross-sectional study. Ann Palliat Med 2021;10(7):7759-7774. doi: 10.21037/apm-21-1261

© Annals of Palliative Medicine. All rights reserved.
