# Supplementary material for: Mental health status and related factors influencing healthcare workers during the COVID-19 pandemic: A systematic review and meta-analysis
Source: PLoS One. 2024 Jan 19;19(1):e0289454. doi: 10.1371/journal.pone.0289454 (PMC10798549; doi:10.1371/journal.pone.0289454)
Supplement: S1 Data — (ZIP) [file pone.0289454.s011.zip › literatures/43.pdf]

BJPsych Open (2021)
7, e88, 1–12. doi: 10.1192/bjo.2021.42

# Impact of the COVID-19 pandemic on the mental health and well-being of UK healthcare workers

James Gilleen, Aida Santaolalla, Lorena Valdearenas, Clara Salice and Montserrat Fusté

**Background**

The coronavirus disease 2019 (COVID-19) pandemic has had a significant psychological impact on healthcare workers (HCWs).

**Aims**

There is an urgent need to understand the risk and protective factors associated with poor mental well-being of UK HCWs working during the COVID-19 pandemic.

**Method**

Shortly after the April 2020 UK COVID-19 peak 2773 HCWs completed a survey containing measures of anxiety, depression, post-traumatic stress disorder and stress, as well as questions around potential predictors such as roles, COVID-19 risk perception and workplace-related factors. Respondents were classified as high or low symptomatic on each scale and logistic regression revealed factors associated with severe psychiatric symptoms. Change in well-being from pre- to during COVID-19 was also quantified.

**Results**

Nearlya third of HCWs reported moderate to severe levels of anxiety and depression, and the number reporting very high symptoms was more than quadruple that pre-COVID-19. Several controllable factors were associated with the most severe level of psychiatric symptoms: insufficient personal protective equipment availability, workplace preparation, training and communication, and higher workload. Being female, 'front line', previous psychiatric diagnoses, traumatic events, and being an allied HCW or manager were also significantly associated with severe psychiatric symptoms. Sharing stress, resilience and ethical support for treatment decisions were significantly associated with low psychiatric symptoms. Front-line workers showed greater worsening of mental health compared with non-front-line HCWs.

**Conclusions**

Poor mental well-being was prevalent during the COVID-19 response, however, controllable factors associated with severe psychiatric symptoms are available to be targeted to reduce the detrimental impact of COVID-19 and other pandemics on HCW mental health.

**Keywords**

COVID19; healthcare; well-being; NHS; healthcare worker.

**Copyright and usage**

© The Author(s), 2021. Published by Cambridge University Press on behalf of the Royal College of Psychiatrists. This is an Open Access article, distributed under the terms of the Creative Commons Attribution licence (http://creativecommons.org/licenses/by/4.0/), which permits unrestricted re-use, distribution, and reproduction in any medium, provided the original work is properly cited.

## Background

The rapid transmission rates and clinical severity of coronavirus disease 2019 (COVID-19) on patient health have brought global national health systems and their healthcare workers (HCWs) under considerable pressure. HCWs already experience high levels of job-related stress[1] and are at risk of poor psychological well-being;[2] however, their highly demanding work[3] will be exacerbated during a pandemic increasing risk of 'burnout',[4] poorer quality of care of others[5] and risk of developing other mental health problems.[2] During epidemics, it has been shown that worse HCW mental health is associated with contact with infected people; redeployment; inadequate training; existing mental health disorders[6,7] and 'moral injury' (distress from being unable to provide treatment)[8]; whereas better support; protective equipment; clear communication[6,9] and resilience[10] may protect mental health. The COVID-19 pandemic presents additional novel and specific challenges and risks to HCW mental well-being as staff carry out their roles and responsibilities.

An initial study conducted in China early on in the COVID-19 pandemic found that HCWs working during COVID-19 experienced a high prevalence of severe depression, anxiety and post-traumatic stress disorder (PTSD). Being female, young, 'front line', and working in Wuhan, were factors most associated with severe psychiatric symptoms.[11] Since then, COVID-19 has also had a profound effect on the UK health system, and although some recent work has shown there is a significant impact on UK HCW mental well-being,[12–14] there is a need for additional and more comprehensive research to fully characterise the impact of the COVID-19 pandemic – and this objective warrants urgent attention.[15] Identifying factors associated with working during COVID-19 that are detrimental to mental health can provide targets through which their impact on HCW mental well-being may be mediated. This may, in turn, help maintain the efficacy of healthcare systems.

## Aims

Research to date has largely been in smaller HCW cohorts, outside the UK, and not included consideration of COVID-19-relevant risk factors, or only a limited range of potential risk factors, which may affect HCW mental health. This study aimed to address these shortfalls and provide a comprehensive examination of the mental health of a large cohort of UK HCWs and how it has been affected by the COVID-19 pandemic by:

(a) quantifying the prevalence of severe psychiatric symptoms in UK HCWs shortly after the initial UK COVID-19 peak;
(b) identifying factors significantly associated with these symptoms;
(c) quantifying how mental health changed compared with before COVID-19;
(d) quantifying HCW worries; and
(e) revealing whether HCWs who are front line, London based, or from ethnic minorities, and those making challenging moral/medical decisions, had more severe psychiatric symptoms compared with their counterparts.

## Method

### Design

We report cross-sectional baseline data acquired shortly after the peak of the COVID-19 pandemic in the UK (between 22 April and 10 May 2020 inclusive – see Supplementary Figure 1 available at https://doi.org/10.1192/bjo.2021.42) from an ongoing, survey-based, longitudinal cohort study. The authors assert that all procedures contributing to this work comply with the ethical standards of the relevant national and institutional committees on human experimentation and with the Helsinki Declaration of 1975, as revised in 2008. All procedures involving human patients were approved by the University of Roehampton Ethics Committee (REF: PSYCH 20/361) and the UK Health Research Authority.

### Participants and survey dissemination

An online web-based survey, outlined below, was open to all UK HCWs to complete. Specific survey dissemination was undertaken as follows (see also Supplementary Appendix 1). The study synopsis survey and weblink was shared through clinical networks, social media and a study webpage and all National Health Service (NHS) research and development departments in the UK were contacted and asked to disseminate the survey synopsis and weblink to staff. Within the planned study period, 52 NHS services (see Supplementary Appendix 1) specifically agreed to promote the study to staff either through direct circular emails, staff intranet, or both. Additionally, text in the synopsis and survey encouraged respondents to share the survey link with other healthcare professionals. The study invitation text strongly encouraged all HCWs to take part even if they did not feel affected by the impact of COVID-19. Eligible respondents were UK-based HCWs who were 18 years or older. Written informed consent was obtained from all subjects.

The survey was implemented on the Qualtrics platform and cross-sectional data on the following were collected.

(a) Validated mental health scales measuring four symptom domains. The Patient Health Questionnaire (PHQ-9)[16] measures depressive symptoms in the past 2 weeks; the General Anxiety Disorder-7 (GAD-7)[17] measures anxiety over the previous 2 weeks. The 22-item Impact of Event Scale – Revised (IES-R)[18] measures PTSD symptoms over the past 7 days; and the Perceived Stress Scale (PSS)[19] measures perception of stress over the past month. Only individuals who had experienced a stressful or traumatic event related to COVID-19 were administered the IES-R. Also, the Connor-Davidson Resilience Scale (CD-RISC)[20] was administered, which measures resilience.
(b) Questions addressing potential factors (see Supplementary Appendix 2) associated with psychiatric symptoms were identified using a knowledge-based approach built on scientific literature, through focus groups, study team meetings and survey piloting feedback. The items included could be clustered within the following themes: (i) demographics and roles including working on the 'front line' (directly engaged in diagnosing, treating, or caring for patients); (ii) workplace readiness and preparation; (iii) risk management including personal protective equipment (PPE); (iv) experience of traumatic and stressful events; (v) protective: being able to share stress at work.
(c) Respondents also quantified their level of current worry on items concerning their work, personal lives and COVID-19 using a 10-point Likert scale (see Supplementary Appendix 2 section G and Supplementary Table 1).
(d) Additionally, ratings were made for items concerning well-being, worries and views about work (on a five-point Likert scale) during COVID-19 (at survey completion) and pre-COVID-19 i.e. retrospectively. These items were again selected from focus groups, study team meetings, and survey piloting feedback and included anxiety, depression and stress items (see Supplementary Appendix 2 (section F) and Table 5).

### Data analysis

Analyses were conducted to (a) determine the prevalence of high levels of psychiatric symptoms, (b) reveal the factors positively or negatively associated with high levels of psychiatric symptoms, and (c) quantify change in mental health from before COVID, and to investigate group differences in psychiatric symptoms.

### Prevalence of high psychiatric symptoms

'High symptoms' of depression and anxiety were determined by individuals scoring ≥10 on the PHQ and GAD scales ('moderate' and 'severe' symptoms). Severe stress was classified by a PSS score ≥24 (upper quartile) and an IES-R score of ≥26 was used to classify high PTSD symptoms.

### Factors associated with high psychiatric symptoms

The relationship between 'high' levels of each symptom and potential predictive factors was determined with $\chi^2$ analyses. Stepwise multivariable logistic regression analyses for each symptom domain ('high symptoms' versus not) were then performed and included the factors that were significant in $\chi^2$ analyses (0.05 significance level to enter/stay in the model).

### Change in mental health and group differences

Repeated-measures ANOVA were used to quantify change in well-being from pre- to during COVID-19 for the whole cohort, and to examine between-group differences. Stratified analyses were also conducted:

(a) to reveal whether front-line versus non-front line workers, ethnic minority (see Table 1 for ethnicity descriptions; versus non-ethnic minority) workers, and those making challenging medical decisions (versus not) had higher psychiatric symptoms than their counterparts; and
(b) to compare level of current worries between front-line workers and non-front-line workers.

Partial $\eta^2$ was used as a measure of the effect size for repeated-measures ANOVA. Cut-offs for small, medium and large effect sizes were 0.0099, 0.0588, and 0.1379, respectively.[21,22] Analyses were conducted in SAS 9.4 (SAS Institute Inc) and SPSS v25 (IBM Corp, Armonk, NY, USA).

## Results

### Participant demographics and roles

A total of 3379 participants consented. Non-HCWs and those who completed <70% of the survey were excluded leaving 2773 respondents (see Table 1 for main descriptive statistics and Supplementary Tables 2–5).

### Prevalence of high psychiatric symptoms

Table 2 shows cohort psychiatric symptoms. In total, 28.1% ($n$ = 778) were above the cut-off for high depression (moderate/moderately severe/severe), 33.1% ($n$ = 919) for high anxiety (moderate and severe); and 27.5% ($n$ = 750) were in the top quartile for stress (see Table 2). Of participants, 60.6% ($n$ = 1681) had experienced a stressful or traumatic event related to COVID-19 and 14.6% ($n$ = 404) were above the cut-off for high PTSD symptoms.

**Table 1** Showing frequency (*n*, %) of demographics, roles, settings and coronavirus disease 2019 (COVID-19) status for the whole cohort, and stratified $\chi^2$ (*P*) statistics for front-line versus non-front line healthcare workers and those working inside London versus outside London

| | Total *n* (%) | Front-line workers, *n* (%) | Non-front-line workers, *n* (%) | *P* | London, *n* (%) | Outside, *n* (%) | *P* |
|---|---|---|---|---|---|---|---|
| Gender | | | | 0.1905 | | | 0.0002 |
| Male | 395 (14.24) | 188 (15.36) | 166 (13.55) | | 135 (18.88) | 259 (12.65) | |
| Female | 2365 (85.29) | 1029 (84.07) | 1056 (86.20) | | 577 (80.70) | 1781 (86.96) | |
| Other/not reported | 13 (0.47) | 7 (0.57) | 3 (0.24) | | 3 (0.42) | 8 (0.39) | |
| Age categories | | | | <0.0001 | | | 0.0002 |
| Under 25 | 102 (3.68) | 61 (4.98) | 29 (2.37) | | 18 (2.52) | 83 (4.05) | |
| 25–34 | 630 (22.72) | 301 (24.59) | 264 (21.55) | | 207 (28.95) | 420 (20.51) | |
| 35–44 | 680 (24.52) | 306 (25.00) | 306 (24.98) | | 172 (24.06) | 508 (24.8) | |
| 45–54 | 813 (29.32) | 374 (30.56) | 345 (28.16) | | 186 (26.01) | 625 (30.52) | |
| 55–64 | 508 (18.32) | 174 (14.22) | 259 (21.14) | | 120 (16.78) | 386 (18.85) | |
| 65 and above | 36 (1.30) | 7 (0.57) | 21 (1.71) | | 12 (1.68) | 24 (1.17) | |
| Not reported | 4 (0.14) | 1 (0.08) | 1 (0.08) | | 0 (0.00) | 2 (0.01) | |
| Ethnicity | | | | 0.0034 | | | <0.0001 |
| White | 2414 (87.05) | 1040 (84.97) | 1094 (89.31) | | 546 (76.36) | 1861 (90.87) | |
| Mixed/multiple ethnic groups | 67 (2.42) | 29 (2.37) | 27 (2.20) | | 23 (3.22) | 44 (2.15) | |
| Asian/Asian British | 191 (6.89) | 107 (8.74) | 62 (5.06) | | 78 (10.91) | 113 (5.52) | |
| Black/African/Caribbean/Black British | 65 (2.34) | 29 (2.37) | 33 (2.69) | | 49 (6.85) | 15 (0.73) | |
| Other | 19 (0.69) | 10 (0.82) | 6 (0.49) | | 12 (1.68) | 7 (0.34) | |
| Not reported | 17 (0.61) | 9 (0.74) | 3 (0.24) | | 7 (0.98) | 8 (0.39) | |
| Black, Asian minority ethnic | 342 (12.33) | 175 (14.30) | 128 (10.45) | 0.0029 | 162 (22.66) | 179 (8.74) | <0.0001 |
| Relationship | | | | 0.7375 | | | <0.0001 |
| Single | 371 (13.38) | 167 (13.64) | 163 (13.31) | | 139 (19.44) | 230 (11.23) | |
| In a relationship/married | 2209 (79.66) | 973 (79.49) | 977 (79.76) | | 526 (73.57) | 1677 (81.88) | |
| Separated/divorced | 134 (4.83) | 58 (4.74) | 59 (4.82) | | 29 (4.06) | 105 (5.13) | |
| Other | 28 (1.01) | 15 (1.23) | 10 (0.82) | | 3 (0.42) | 25 (1.22) | |
| Not reported | 31 (1.12) | 11 (0.90) | 16 (1.31) | | 18 (2.52) | 11 (0.54) | |
| Work setting | | | | <0.0001 | | | <0.0001 |
| NHS – hospital | 1411 (50.88) | 790 (64.54) | 456 (37.22) | | 330 (46.15) | 1075 (52.49) | |
| NHS – general practitioner surgery | 136 (4.90) | 65 (5.31) | 53 (4.33) | | 18 (2.52) | 118 (5.76) | |
| NHS – other community team | 590 (21.28) | 184 (15.03) | 344 (28.08) | | 134 (18.74) | 455 (22.22) | |
| NHS – mental health trust | 393 (14.17) | 81 (6.62) | 276 (22.53) | | 185 (25.87) | 207 (10.11) | |
| NHS – ambulance service | 6 (0.22) | 2 (0.16) | 1 (0.08) | | 1 (0.14) | 5 (0.24) | |
| A private hospital | 15 (0.54) | 7 (0.57) | 5 (0.41) | | 4 (0.56) | 11 (0.54) | |
| Care home[a] | 45 (1.62) | 28 (2.29) | 14 (1.14) | | 7 (0.98) | 38 (1.86) | |
| Nursing home[a] | 21 (0.76) | 17 (1.39) | 2 (0.16) | | 3 (0.42) | 18 (0.88) | |
| Other (*n* = 10) | 152 (5.48) | 49 (4) | 73 (5.96) | | 33 (4.62) | 119 (5.81) | |
| Not reported | 4 (0.14) | 1 (0.08) | 1 (0.08) | | 0 (0.00) | 2 (0.1) | |
| Role | | | | <0.0001 | | | <0.0001 |
| Nurses/midwife | 852 (30.72) | 461 (37.66) | 285 (23.27) | | 146 (20.42) | 706 (34.47) | |
| Doctor | 386 (13.92) | 229 (18.71) | 112 (9.14) | | 148 (20.7) | 237 (11.57) | |
| Allied healthcare professionals | 772 (27.84) | 264 (21.57) | 425 (34.69) | | 235 (32.87) | 536 (26.17) | |
| Management | 245 (8.83) | 39 (3.19) | 174 (14.20) | | 85 (11.89) | 158 (7/71) | |
| Other health worker | 499 (17.99) | 206 (16.83) | 220 (17.96) | | 98 (13.71) | 379 (18.15) | |
| Not reported | 19 (0.69) | 25 (2.04) | 9 (0.73) | | 3 (0.42) | 32 (1.56) | |
| Mental health diagnosis (any)[b] | 473 (17.06) | 194 (15.85) | 213 (17.39) | 0.4378 | 101 (14.13) | 372 (18.16) | 0.0681 |
| COVID-19 status | | | | <0.0001 | | | 0.227 |
| Past positive | 80 (2.88) | 56 (4.58) | 19 (1.55) | | 19 (2.66) | 61 (2.98) | |
| Past negative | 158 (5.7) | 99 (8.09) | 45 (3.67) | | 42 (5.87) | 116 (5.66) | |
| Past – suspected | 574 (20.7) | 257 (21) | 239 (19.51) | | 181 (25.31) | 392 (19.14) | |
| Current symptoms, tested, awaiting results | 21 (0.76) | 17 (1.39) | 3 (0.24) | | 8 (1.12) | 12 (0.59) | |
| Current symptoms, not tested | 14 (0.5) | 10 (0.82) | 3 (0.24) | | 3 (0.42) | 11 (0.54) | |
| No symptoms | 1784 (64.33) | 719 (58.74) | 876 (71.51) | | 425 (59.44) | 1351 (65.97) | |
| Other | 142 (5.12) | 66 (5.39) | 40 (3.27) | | 37 (5.17) | 105 (5.13) | |
| Tested for COVID-19 | | | | <0.0001 | | | 0.227 |
| No | 2377 (85.72) | 974 (79.58) | 1117 (91.18) | | 620 (86.71) | 1748 (85.35) | |
| Yes | 381 (13.74) | 245 (20.02) | 100 (8.16) | | 94 (13.15) | 287 (14.01) | |
| Not reported | 15 (0.54) | 5 (0.41) | 8 (0.65) | | 1 (0.14) | 13 (0.63) | |

a. Removed from main analyses due to low *n*.
b. See Supplementary Tables for further breakdown.

## Predictive models of high psychiatric symptoms

All stepwise multivariate logistic regression models converged with no significant collinearity of factors, or residual data due to missingness. All models were highly significant (likelihood ratio, core, and Wald $P < 0.0001$) presenting good fit and high prediction capabilities (c score = 0.739–0.82). Significant factors retained in each model of symptoms with odds ratios (ORs) are shown in Table 3 and reported below. Frequency distributions are shown in Table 4; $\chi^2$ summaries are reported in Supplementary Table 7.

**Table 2** Showing frequency distributions (*n* (%)) and median/means/interquartile range (IQR) for each symptom scale, for the total cohort and stratified $\chi^2$ (*P*) statistics for front-line versus non-front-line workers and workers inside London *versus* outside London

| | Total | % of total | Front-line workers, *n* | % | Non-front-line workers, *n* | % | *P* | Inside London, *n* | % | Outside London, *n* | % | *P* |
|---|---|---|---|---|---|---|---|---|---|---|---|---|
| PHQ median (IQR) | 6 (7) | – | 7 (9) | – | 5 (7) | – | – | 5 (7) | – | 6 (8) | – | – |
| PHQ mean (s.d.) | 7.18 (6.07) | – | 8.00 (6.21) | – | 6.97 (5.75) | – | – | 6.53 (5.58) | – | 7.41 (6.22) | – | – |
| PHQ, *n* (%) | | | | | | | | | | | | |
| Normal (0–5) | 1172 | 42.26 | 475 | 38.81 | 582 | 47.51 | <0.0001 | 330 | 46.15 | 837 | 40.87 | 0.0064 |
| Mild (5–9) | 823 | 29.68 | 369 | 30.15 | 343 | 28 | | 217 | 30.35 | 605 | 29.54 | |
| Moderate (10–14) | 404 | 14.57 | 198 | 16.18 | 155 | 12.65 | | 93 | 13.01 | 309 | 15.09 | |
| Moderately severe (15–19) | 231 | 8.33 | 104 | 8.5 | 98 | 8 | | 53 | 7.41 | 176 | 8.59 | |
| Severe (20–27) | 143 | 5.16 | 78 | 6.37 | 47 | 3.84 | | 22 | 3.08 | 121 | 5.91 | |
| GAD median (IQR) | 6 (8) | – | 8 (9) | – | 6 (8) | – | | 6 (7) | – | 7 (9) | | – |
| GAD mean (s.d.) | 7.51 (5.58) | – | 8.71 (5.83) | – | 7.34 (5.14) | – | | 6.86 (5.19) | – | 7.72 (5.70) | | – |
| GAD, *n* (%) | | | | | | | | | | | | |
| Normal (0–4) | 1014 | 36.57 | 376 | 30.72 | 524 | 42.78 | <0.0001 | 288 | 40.28 | 723 | 35.3 | 0.0004 |
| Mild (5–9) | 840 | 30.29 | 375 | 30.64 | 369 | 30.12 | | 235 | 32.87 | 602 | 29.39 | |
| Moderate (10–14) | 532 | 19.18 | 254 | 20.75 | 211 | 17.22 | | 118 | 16.5 | 411 | 20.07 | |
| Severe (≥ 15) | 387 | 13.96 | 219 | 17.89 | 121 | 9.88 | | 74 | 10.35 | 312 | 15.23 | |
| IES-R median (IQR)[a] | 16 (23) | – | 18 (25) | – | 13 (17) | – | | 15 (22) | – | 16 (23) | | – |
| IES-R mean (s.d.) | 20.30 (16.68) | – | 22.05 (17.1) | – | 16.4 (14.6) | – | | 19.89 (16.57) | – | 20.43 (16.73) | | – |
| IES-R, *n* (%) | | | | | | | | | | | | |
| Q1 <7.00 | 387 | 13.96 | 202 | 20.40 | 140 | 28.40 | <0.0001 | 104 | 14.55 | 282 | 13.77 | 0.9762 |
| Q2 7.00 ≤ IES-R < 20.30 | 620 | 22.36 | 340 | 34.34 | 203 | 41.18 | | 162 | 22.66 | 456 | 22.27 | |
| Q3 20.30 ≤ IES-R < 30.00 | 248 | 8.94 | 154 | 15.56 | 65 | 13.18 | | 63 | 8.81 | 185 | 9.03 | |
| Q4 ≥ 30.00 | 426 | 15.36 | 294 | 29.70 | 85 | 17.24 | | 106 | 14.83 | 318 | 15.53 | |
| CD-RISC median (IQR) | 28 (9) | – | 28 (9) | – | 27 (9) | – | | 27 (9) | – | 28 (9) | – | |
| CD-RISC mean (s.d.) | 27.5 (6.48) | – | 27.9 (6.43) | – | 27.5 (6.34) | – | | 27.36 (6.53) | – | 27.49 (6.47) | – | |
| CD-RISC, *n* (%) | | | | | | | | | | | | |
| Q1 <23.00 | 567 | 20.45 | 244 | 19.93 | 262 | 21.39 | 0.0173 | 149 | 20.84 | 416 | 20.31 | 0.2268 |
| Q2 23.00 ≤ CD-RISC <27.46 | 720 | 25.96 | 289 | 23.61 | 349 | 28.49 | | 205 | 28.67 | 514 | 25.1 | |
| Q3 27.46 ≤ CD-RISC < 32.00 | 633 | 22.83 | 289 | 23.61 | 264 | 21.55 | | 152 | 21.26 | 478 | 23.34 | |
| Q4 ≥ 32.00 | 688 | 24.81 | 318 | 25.98 | 287 | 23.43 | | 175 | 24.48 | 513 | 25.05 | |
| PSS median (IQR) | 19 (11) | – | 20 (10) | – | 19 (10) | – | | 18 (11) | – | 19 (10) | – | |
| PSS mean (s.d.) | 18.6 (7.47) | – | 19.6 (7.45) | – | 18.6 (6.93) | – | | 18.06 (7.47) | – | 18.73 (7.48) | – | |
| PSS, *n* (%) | | | | | | | | | | | | |
| Q1 <11 | 608 | 21.93 | 233 | 19.04 | 319 | 26.04 | <0.0001 | 173 | 24.2 | 433 | 21.14 | 0.123 |
| 11≤ Q2 <13 | 727 | 26.22 | 303 | 24.75 | 328 | 26.78 | | 198 | 27.69 | 525 | 25.63 | |
| 13≤ Q3 <24 | 688 | 24.81 | 318 | 25.98 | 298 | 24.33 | | 165 | 23.08 | 520 | 25.39 | |
| Q4 ≥24 | 750 | 27.50 | 370 | 30.23 | 280 | 22.85 | | 179 | 25.03 | 570 | 27.83 | |

PHQ, Patient Health Questionnaire; GAD, General Anxiety Disorder-7; IES-R, Impact of Event Scale – Revised; Q, Quartile; CD-RISC, Connor-Davidson Resilience Scale; PSS, Perceived Stress Scale.
a. 60.5% of the participants completed IES-R – those who identified experiencing a stressful or traumatic event related to coronavirus disease 2019 (COVID-19).

## Anxiety

As shown in Table 3, high anxiety was significantly associated with being female, all non-doctor roles (versus doctor), working outside London, being front line and having a mental health diagnosis. Friends or family dying from COVID-19, patients asking if they are going to die, and performing resuscitation were also associated with high anxiety, as was insufficient training, extra workload, insufficient information and thinking not enough is currently being done to reduce risk.

## PTSD symptoms

All non-doctor (versus doctor) roles – particularly being a manager, and being front-line workers, being from an ethnic minority and existing mental health conditions were significantly associated with high PTSD symptoms, as was experience of all traumatic and stressful events except aftercare of the deceased. High PTSD symptoms were also significantly associated with pressure to reuse PPE; insufficient information; perception that not enough had been, nor was being done, to reduce risk; and greater workload.

## Depression

High depression was significantly associated with being female, all non-doctor roles (versus doctor), working outside London and having a mental health diagnosis. Those experiencing friends or family dying, patients asking them if they are going to die and performing aftercare for the deceased were significantly more likely to be in the high depression group. Extra workload, pressure to work without PPE, insufficient information and perception that not enough had been done to reduce risk were also significantly associated with high depression.

## Stress

Being female, younger (55–64 years versus <25), all non-doctor roles (versus doctor), working on the front line and having a mental health diagnosis were associated with significantly increased likelihood of being in the high stress group; as were insufficient information, pressure to work without PPE, >20% of team members off sick and perception that not enough had been done to reduce risk.

## Factors associated with having lower psychiatric symptoms

Being able to share stress at work and resilience were associated with significantly lower likelihood of being in the high anxiety, stress and depression groups – although there was no significant association with PTSD symptoms.

## Change from pre-COVID-19 to during COVID-19

Across the cohort, every mental health symptom, concern and work-related issue were rated as significantly worse during COVID-19 compared with pre-COVID-19 – most to a highly significant level with very high effect sizes (see Supplementary Table 8). HCWs being worried about their family health showed the greatest (negative) change.

Front-line workers rated themselves lower than non-front-line workers pre-COVID in terms of: stress, wanting to quit, needing psychological help, worrying about NHS resources and their own health concerns; however, time × group interaction effects revealed that front-line workers reported a significantly greater worsening of these from pre- to during COVID-19 than for non-front-line workers. For the other negative factors front-line worker ratings were not significantly different to non-front-line workers pre-COVID-19 but front-line workers reported significantly greater worsening from pre- to during COVID-19 for all items, including 'feeling low' (depression) and 'feeling anxious' (shown in Fig. 1).

The anxiety, low mood and stress items mirror the main symptom outcome scores (anxiety–GAD, low mood–PHQ and stress–PSS), and in concrete terms there was a considerable shift in the distribution of severity of these symptoms during COVID-19 (shown in Table 5): pre-COVID 85.9% of HCWs reported 'no' or 'very little' feeling of low mood but this diminished to only 55.6% during the COVID-19 response, whereas the 5.2% reporting 'quite a lot' or 'very' pre-COVID-19 rose to 21.6% – more than a quadrupling. This shift in frequency distribution towards worse mood across the cohort was highly significant ($\chi^2 = 1101$, $P < 0.0001$). A similar pattern was evident for 'feeling anxious': the number of HCWs at the two most severe levels rose from 7.8% to 35.8% – and the number experiencing the most severe levels of 'feeling stressed' rose from 10.7 to 45.6%. These shifts in frequency distribution towards worse anxiety ($\chi^2 = 962.8$, $P < 0.0001$) and worse stress across the cohort were highly significant ($\chi^2 = 623.7$, $P < 0.0001$).

Every positive factor also significantly worsened across the cohort from pre- to during COVID-19 (see Supplementary Table 8). Pre-COVID-19, front-line workers felt more resilient, more 'positive' and tech confident, and that their team was more effective than non-front-line workers. However, front-line workers had significantly greater declines in feeling resilient, as well as remaining positive, and feeling supported compared with non-front-line workers.

## Front-line, London and ethnic minority workers

Front-line workers were significantly more likely to be more depressed, anxious, have high PTSD symptoms and be more stressed than non-front-line worker (all $P < 0.0001$). Working in London was associated with lower risk of depression ($P < 0.01$) and anxiety ($P < 0.0005$) than outside London (although there was no difference in stress or PTSD). Ethnic minority status ($n = 342$) was significantly associated with greater risk of high PTSD symptoms (OR = 1.52), but not high anxiety, stress or depression.

*Post hoc* analyses were conducted to explore ethnic minority experiences further. Ethnic minority individuals who were also physically 'at-risk' of COVID-19 ($n = 85$) did not have higher psychiatric symptoms than ethnic minority individuals not 'at-risk' ($n = 257$), nor compared with non-ethnic minority individuals physically 'at risk' ($n = 593$) (all $Ps>0.45$). Ethnic minority individuals were, however, significantly more worried about contracting COVID-19 at work (mean 3.09 (s.d. = 1.08) versus non-ethnic minority individuals mean 2.67 (s.d. = 1.07); $t(2754) = 6.84$, $P < 0.0001$); 'being uncertain of having COVID-19' (mean 2.77 (s.d. = 1.16) versus non-ethnic minority mean 2.21 (s.d. = 1.09); $t(2754) = 8.93$, $P < 0.001$); getting ill or dying from COVID-19 (mean 2.2 (s.d. = 1.12) versus non-ethnic minority mean 1.88 (s.d. = 1.19), $t(2754) = 4.74$, $P < 0.001$) and lack of PPE (mean 2.86 (s.d. = 1.14) versus non-ethnic minority mean 2.39 (s.d. = 1.12), $t(2754) = 7.29$, $P < 0.001$).

## Medical decision-making

A total of 11.1% ($n = 307$) of respondents were in a position to make decisions about whether patients received treatment and 17.3% ($n = 53$) had denied treatment to a patient (see Supplementary Table 5). They reported significantly more anxiety (but not depression, PTSD symptoms (although there was a strong trend), or stress (although there was a strong trend)) than those ($n = 39$) who had not denied treatment (GAD mean 8.00 (s.d. = 5.94) *v.* 5.46 (s.d. = 5.19), $t(90) = 2.13$, $P < 0.05$). Those with the support of an ethics panel in decision-making (58.0% ($n = 178$)) were significantly less

**Table 3** Summary table showing odds ratios (OR) and *P* values for risk and protective factors retained in each model. Please see Supplementary Table 6 for a version of this table that includes shading to reflect stronger and weaker risk and protective factors. Blank cells show the factor was not significantly retained in the model of the outcome score. 't' refers to trend-level effects shown for illustration purposes only.

| | GAD, OR | 95% CI | *P* | IES-R, OR | 95% CI | *P* | PHQ, OR | 95% CI | *P* | PSS, OR | 95% CI | *P* |
|---|---|---|---|---|---|---|---|---|---|---|---|---|
| *Demographics and role* | | | | | | | | | | | | |
| Gender female (versus male) | 1.611 | 1.152–2.242 | 0.0052 | | | | 1.531 | 1.079–2.174 | 0.0171 | 2.488 | 1.692–3.650 | <0.0001 |
| Age, years (versus <25) | | | | | | | | | | | | |
| 25–34 | | | | | | | | | | 0.774 | 0.448–1.338 | 0.3594 |
| 35–44 | | | | | | | | | | 0.950 | 0.547–1.651 | 0.8562 |
| 45–54 | | | | | | | | | | 0.602 | 0.346–1.049 | 0.0732 |
| 55–64 | | | | | | | | | | 0.406 | 0.221–0.744 | 0.0036 |
| 65+ | | | | | | | | | | 0.362 | 0.074–1.773 | 0.2101 |
| Black, Asian, minority ethnic | | | | 1.520 | 1.037–2.228 | 0.0319 | | | | | | |
| Relationship (versus in a relationship) | | | | | | | | | | | | |
| Single | | | | | | | 1.613 | 1.176–2.214 | 0.0031 | | | |
| Separated | | | | | | | 1.301 | 0.766–2.210 | 0.3294 | | | |
| Other | | | | | | | 1.402 | 0.500–3.931 | 0.5208 | | | |
| Role (versus doctors) | | | | | | | | | | | | |
| Nurses | 1.633 | 1.126–2.370 | 0.0097 | 2.213 | 1.419–3.451 | 0.0005 | 1.560 | 1.043–2.333 | 0.0303 | 1.781 | 1.199–2.646 | 0.0043 |
| Allied health professionals | 1.893 | 1.292–2.773 | 0.0011 | 1.966 | 1.199–3.223 | 0.0073 | 1.831 | 1.222–2.743 | 0.0034 | 1.414 | 0.939–2.131 | 0.0974 |
| Management | 2.805 | 1.649–4.771 | 0.0001 | 5.236 | 2.691–10.188 | <0.0001 | 2.418 | 1.402–4.171 | 0.0015 | 3.255 | 1.868–5.672 | <0.0001 |
| Ambulance/ Healthcare Assistant | 2.257 | 1.497–3.403 | 0.0001 | 2.726 | 1.644–4.52 | 0.0001 | 2.021 | 1.309–3.121 | 0.0015 | 2.137 | 1.384–3.298 | 0.0006 |
| Location (versus London) outside London | 1.556 | 1.203–2.012 | 0.0007 | | | | 1.318 | 1.008–1.722 | 0.0434 | | | |
| Front line | 1.536 | 1.193–1.977 | 0.0009 | 2.193 | 1.557–3.09 | <0.0001 | | | | 1.435 | 1.117–1.844 | 0.0047 |
| Mental health diagnosis | 1.615 | 1.221–2.136 | 0.0008 | 1.975 | 1.417–2.754 | <0.0001 | 2.137 | 1.617–2.824 | <0.0001 | 1.746 | 1.311–2.324 | 0.0001 |
| *Traumatic and stressful events* | | | | | | | | | | | | |
| Family or friends died | 1.715 | 1.184–2.486 | 0.0044 | 2.492 | 1.661–3.737 | <0.0001 | 1.779 | 1.219–2.596 | 0.0028 | | | |
| Patients asking if they are going to die | 1.447 | 1.086–1.926 | 0.0115 | 2.096 | 1.492–2.944 | <0.0001 | 1.356 | 1.012–1.817 | 0.0411 | | | |
| Colleague(s) with COVID | | | | 3.171 | 1.764–5.698 | 0.0001 | | | 0.077 | | | |
| Colleague(s) dying from COVID | | | | 1.511 | 1.079–2.117 | 0.0163 | | | | | | |
| Performing resuscitation | 1.709 | 1.075–2.717 | 0.0236 | 2.243 | 1.395–3.608 | 0.0009 | | | | | | |
| Delivering more bad news | | | | 1.534 | 1.056–2.227 | 0.0248 | | | | | | |
| After care of deceased | | | | | | | 1.729 | 1.196–2.498 | 0.0036 | | | |
| *Workplace* | | | | | | | | | | | | |
| Extra workload due to staff absences | 1.394 | 1.116–1.74 | 0.0034 | 1.447 | 1.093–1.916 | 0.0098 | 1.297 | 1.032–1.631 | 0.0257 | t | | 0.074 |
| Not enough information on COVID clinical practice | 1.465 | 1.117–1.92 | 0.0058 | 1.413 | 1.023–1.951 | 0.036 | 1.402 | 1.062–1.849 | 0.017 | 1.341 | 1.011–1.778 | 0.0418 |
| Insufficient COVID training | 1.259 | 1.008–1.571 | 0.0424 | | | | | | | | | |
| Team members off sick (>20%) | | | | | | | | | | 1.882 | 1.255–2.823 | 0.0022 |
| *Management of risk* | | | | | | | | | | | | |
| Not enough being done currently to reduce risk | 1.644 | 1.306–2.069 | <0.0001 | 1.611 | 1.202–2.158 | 0.0014 | | | | | | |
| Pressure to reuse PPE | t | | 0.061 | 1.382 | 1.037–1.842 | 0.0271 | | | | t | | 0.054 |
| Less risk if had been properly prepared | t | | 0.063 | 1.381 | 1.014–1.88 | 0.0406 | 1.484 | 1.139–1.933 | 0.0034 | 1.562 | 1.198–2.038 | 0.001 |
| Pressured to work without PPE | | | | | | | 1.495 | 1.087–2.054 | 0.0133 | 1.459 | 1.058–2.013 | 0.0213 |
| *Protective factors* | | | | | | | | | | | | |
| CD-RISC resilience (versus 1st Quartile) | | | | | | | | | | | | |
| 2nd Quartile | 0.555 | 0.417–0.739 | <0.0001 | | | | 0.604 | 0.451–0.808 | 0.0007 | 0.592 | 0.443–0.793 | 0.0004 |
| 3rd Quartile | 0.260 | 0.189–0.359 | <0.0001 | | | | 0.342 | 0.246–0.475 | <0.0001 | 0.293 | 0.209–0.410 | <0.0001 |
| 4th Quartile | 0.274 | 0.200–0.376 | <0.0001 | | | | 0.319 | 0.229–0.443 | <0.0001 | 0.308 | 0.221–0.430 | <0.0001 |
| Able to share stress at work | 0.660 | 0.496–0.869 | 0.0032 | | | | 0.692 | 0.520–0.922 | 0.0117 | 0.618 | 0.463–0.824 | 0.001 |

GAD, General Anxiety Disorder-7; IES-R, Impact of Event Scale – Revised; PHQ, Patient Health Questionnaire; PSS, Perceived Stress Scale; PPE, personal protective equipment; CD-RISC, Connor-Davidson Resilience Scale.

**Table 4** Showing frequency distributions (*n* (%) (the percentage of the symptoms groups (columns) constituted by the 'factor' group) for 2773 the participants for high and low levels of mental health symptoms for all significant variables in the logistic regressions for the four scales (reported in Table 3)

| | GAD low, *n* (%) | GAD high, *n* (%) | IES-R low, *n* (%) | IES-R high, *n* (%) | PHQ low, *n* (%) | PHQ high, *n* (%) | PSS low, *n* (%) | PSS high, *n* (%) |
|---|---|---|---|---|---|---|---|---|
| *Demographics and role* | | | | | | | | |
| Gender: Female | 1547 (83.4) | 818 (89.0) | 1895 (84.9) | 470 (88.8) | 1678 (84.4) | 687 (88.9) | 1907 (84.4) | 458 (91.6) |
| Total N (%), missing N | 1854 (100), 5 | 919 (100), 8 | 2231 (100), 9 | 529 (100), 4 | 1995 (100), 8 | 778 (100), 5 | 2272 (100), 12 | 501 (100), 1 |
| Age, years | | | | | | | | |
| Under 25 | 55 (3.0) | 47 (5.1) | 74 (3.3) | 38 (7.1) | 66 (3.3) | 36 (4.6) | 72 (3.2) | 30 (6) |
| 25–34 | 381 (20.6) | 249 (27.2) | 489 (21.9) | 141 (26.5) | 429 (21.5) | 201 (25.9) | 474 (20.9) | 156 (31.1) |
| 35–44 | 434 (23.4) | 246 (26.8) | 549 (24.5) | 131 (34.6) | 493 (24.7) | 187 (24.1) | 538 (23.7) | 142 (28.3) |
| 45–54 | 573 (30.9) | 240 (26.2) | 665 (29.7) | 148 (27.8) | 593 (29.8) | 220 (28.4) | 704 (31) | 109 (21.8) |
| 55–64 | 380 (20.5) | 128 (14.0) | 428 (19.1) | 80 (15.0) | 382 (19.2) | 126 (16.2) | 446 (19.6) | 62 (12.4) |
| 65+ | 29 (1.6) | 7 (0.8) | 32 (1.4) | 4 (0.8) | 30 (1.5) | 6 (0.8) | 34 (1.5) | 2 (0.4) |
| Age N (%), missing N | 1852 (100), 2 | 917 (100), 2 | 2237 (100), 3 | 532 (100), 1 | 1993 (100), 2 | 776 (100), 2 | 2268 (100), 4 | 501 (100), 0 |
| Black, Asian, minority ethnic | 224 (12.1) | 118 (13.0) | 255 (11.5) | 87 (16.4) | 272 (12.0) | 106 (13.7) | 272 (12.0) | 70 (14.1) |
| Ethnicity N (%), missing N | 1845 (100), 9 | 911 (100), 8 | 2227 (100), 13 | 529 (100), 4 | 1984 (100), 11 | 772 (100), 6 | 2258 (100), 14 | 498 (100), 3 |
| Relationship | | | | | | | | |
| Single | 227 (12.4) | 144 (15.9) | 284 (12.8) | 87 (16.5) | 235 (11.9) | 136 (17.7) | 300 (13.3) | 71 (14.4) |
| In a relationship | 1497 (81.6) | 712 (78.4) | 1800 (81.3) | 409 (77.6) | 1625 (82.3) | 584 (76) | 1813 (80.6) | 396 (80.5) |
| Separated/divorced | 92 (5.0) | 42 (4.6) | 108 (4.9) | 26 (4.9) | 96 (4.9) | 38 (4.9) | 114 (5.1) | 20 (4.1) |
| Other | 18 (1.0) | 10 (1.1) | 23 (1) | 5 (0.9) | 18 (0.9) | 10 (1.3) | 23 (1.0) | 5 (1.0) |
| Relationship N (&), missing N | 1834 (100), 20 | 908 (100), 11 | 2215 (100), 25 | 527 (100), 6 | 1974 (100), 21 | 768 (100), 10 | 2250 (100), 22 | 492 (100), 9 |
| Role | | | | | | | | |
| Nurses | 560 (30.4) | 292 (32.0) | 650 (29.2) | 202 (38.3) | 611 (30.9) | 241 (31.1) | 705 (31.3) | 147 (29.5) |
| Doctors | 300 (16.3) | 86 (9.4) | 322 (14.5) | 64 (12.1) | 318 (16.1) | 68 (8.8) | 328 (14.5) | 58 (11.6) |
| Allied healthcare | 528 (28.7) | 244 (26.7) | 671 (30.1) | 101 (19.1) | 564 (28.5) | 208 (26.9) | 641 (28.4) | 131 (26.3) |
| Management | 162 (8.8) | 83 (9.1) | 199 (8.9) | 46 (8.7) | 175 (8.8) | 70 (9.0) | 198 (8.8) | 47 (9.4) |
| Ambulance/Healthcare Assistant | 291 (15.8) | 208 (22.8) | 384 (17.3) | 115 (21.8) | 312 (15.8) | 187 (24.2) | 383 (17) | 116 (23.2) |
| Role N (%), missing N | 1841 (100), 13 | 913 (100), 6 | 2226 (100), 14 | 528 (100), 5 | 1980 (100), 15 | 774 (100), 4 | 2255 (100), 17 | 499 (100), 2 |
| Location outside London N (%), total N (%), missing N | 1325 (71.7), 1848 (100), 6 | 723 (79.0), 915 (100), 4 | 1650 (73.9), 2232 (100), 8 | 398 (74.7), 531 (100), 2 | 1442 (72.5), 1989 (100), 6 | 606 (78.3), 774 (100), 4 | 1667 (73.7), 2263 (100), 9 | 381 (76.2), 500 (100), 1 |
| Front-line worker | 751 (45.6) | 473 (58.8) | 860 (43.6) | 364 (76.5) | 844 (47.7) | 380 (55.9) | 973 (48.2) | 251 (58.5) |
| Non-front-line worker | 893 (54.3) | 332 (41.2) | 1113 (56.4) | 112 (23.5) | 926 (52.2) | 300 (44.1) | 1047 (51.2) | 178 (41.5) |
| FL/NFL Total N (%), missing N | 1852 (100), 2 | 915 (100), 4 | 2235 (100), 5 | 532 (100), 1 | 1991 (100), 5 | 776 (100), 2 | 2267 (100), 5 | 500 (100), 1 |
| Mental health diagnosis, total N (%), missing N | 238 (13.1), 1846 (100), 7 | 235 (26.2), 916 (100), 3 | 377 (15.3), 2232 (100), 8 | 136 (26.3), 531 (100), 2 | 244 (12.4), 1988 (100), 7 | 229 (30.2), 775 (100) 3 | 325 (14.5), 2263 (100), 9 | 148 (30.6), 500 (100), 1 |
| *Traumatic and stressful events* | | | | | | | | |
| Family or friends died N (%), total N (%), missing | 163 (8.8), 1852 (100), 2 | 137 (14.9), 915 (100), 4 | 191 (8.5), 2236 (100), 4 | 109 (20.5), 531 (100), 2 | 175 (8.8), 1993 (100), 2 | 125 (16.1), 774 (100), 4 | 225 (9.9), 2268 (100), 4 | 75 (15.0), 499 (100), 2 |
| Patients asking if they are going to die N (%), total N (%), missing | 329 (17.7), 1854 (100), 0 | 256 (27.9), 919 (100), 0 | 348 (15.5), 2240 (100), 0 | 237 (44.5), 533 (100), 0 | 375 (18.8), 1995 (100), 0 | 210 (27.0), 778 (100), 0 | 437 (19.2), 2772 (100), 0 | 148 (29.5), 501 (100), 0 |
| Colleague(s) with COVID-19 N (%), total N (5), missing | 1457 (78.6), 1854 (100), 0 | 776 (84.4), 919 (100), 0 | 1730 (77.2), 2240 (100), 0 | 503 (94.4), 533 (100), 0 | 1569 (78.6), 1995 (100), 0 | 664 (85.3), 778 (100), 0 | 1797 (79.1), 2272 (100), 0 | 436 (87.0), 501 (100), 0 |
| Colleague(s) dying from COVID-19 N (%), total N (%), missing | 238 (12.8), 1854 (100), 0 | 157 (17.1), 919 (100), 0 | 256 (11.4), 2240 (100), 0 | 139 (26.1), 533 (100), 0 | 276 (13.8), 1995 (100), 0 | 119 (15.3), 778 (100), 0 | 313 (13.8), 2272 (100), 0 | 82 (16.4) , 501 (100), 0 |
| Performing resuscitation N (%), total N (%), missing | 70 (3.8), 1854 (100), 0 | 64 (7.0), 919 (100), 0 | 65 (2.9), 2240 (100), 0 | 69 (12.9), 533 (100), 0 | 89 (4.5), 1995 (100), 0 | 45 (5.8), 778 (100), 0 | 104 (4.6), 2272 (100), 0 | 30 (6.0), 501 (100), 0 |
| Delivering more bad news than usual N (%), total N (%), missing | 249 (13.4), 1854 (100), 0 | 172 (18.7), 919 (100), 0 | 245 (10.9), 2240 (100), 0 | 176 (33.0), 533 (100), 0 | 278 (13.9), 1995 (100), 0 | 143 (18.4), 778 (100), 0 | 323 (14.2), 2272 (100), 0 | 98 (19.6), 501 (100), 0 |

*(Continued)*

**Table 4** (*Continued*)

| | GAD low, *n* (%) | GAD high, *n* (%) | IES-R low, *n* (%) | IES-R high, *n* (%) | PHQ low, *n* (%) | PHQ high, *n* (%) | PSS low, *n* (%) | PSS high, *n* (%) |
|---|---|---|---|---|---|---|---|---|
| After care of deceased N (%), total N (%), missing | 147 (7.9), 1854 (100), 0 | 135 (14.7), 919 (100), 0 | 155 (6.9), 2240 (100), 0 | 127 (23.8), 533 (100), 0 | 169 (8.5), 1995 (100), 0 | 113 (4.5), 778 (100), 0 | 208 (9.2), 2272 (100), 0 | 74 (14.8), 501 (100), 0 |
| *Workplace* | | | | | | | | |
| Extra workload due to staff absences N (%), total N (%), missing | 868 (48.0), 1854 (100), 0 | 520 (58.0), 919 (100), 0 | 1025 (47.0), 2179 (100), 61 | 363 (69.0), 526 (100), 7 | 944 (48.5), 1995 (100), 0 | 444 (58.5), 778 (100), 0 | 1092 (49.3), 2272 (100), 0 | 296 (60.2), 501 (100), 0 |
| Not enough information on COVID-19 clinical practice N (%), total N (%), missing | 276 (15.0), 1854 (100), 0 | 283 (31.0), 919 (100), 0 | 375 (16.8), 2232 (100), 8 | 184 (34.8), 528 (100), 5 | 323 (16.3), 1995 (100), 0 | 236 (30.5), 778 (100), 0 | 396 (17.5), 2272 (100), 0 | 163 (32.6), 501 (100), 0 |
| Insufficient COVID-19 training N (%), total N (%), missing | 775 (42.1), 1854 (100), 0 | 448 (49.1), 919 (100), 0 | 978 (44.0), 2224 (100), 16 | 245 (46.3), 529 (100), 4 | 833 (42.1), 1995 (100), 0 | 390 (50.4), 778 (100), 0 | 968 (43.0), 2272 (100), 0 | 255 (50.9), 501 (100), 0 |
| Team members off sick (>20%) N (%), total N (%), missing | 212 (11.4), 1854 (100), 0 | 179 (19.4), 919 (100), 0 | 252 (11.3), 2227 (100), 13 | 139 (26.2), 530 (100), 3 | 229 (11.5), 1995 (100), 0 | 162 (20.8), 778 (100), 0 | 270 (11.9), 2272 (100), 0 | 121 (24.2), 501 (100), 0 |
| *Management of risk* | | | | | | | | |
| Not enough being done currently to reduce risk N (%), total N (%), missing | 526 (28.5), 1854 (100), 0 | 448 (49.1), 919 (100), 0 | 685 (30.8), 2227 (100), 13 | 289 (54.5), 530 (100), 3 | 612 (30.8), 1995 (100), 0 | 362 (46.9), 778 (100), 0 | 726 (32.3), 2272 (100), 0 | 248 (49.7), 501 (100), 0 |
| Pressure to reuse PPE N (%), total N (%), missing | 397 (22.6), 1854 (100), 0 | 316 (36.2), 919 (100), 0 | 488 (21.8), 2240 (100), 0 | 225 (42.4), 533 (100), 0 | 462 (24.3), 1995 (100), 0 | 251 (34.3), 778 (100), 0 | 539 (25.0), 2272 (100), 0 | 174 (36.5), 501 (100), 0 |
| Less risk if had been properly prepared N (%), total N (%), missing | 601 (41.4), 1854 (100), 0 | 309 (51.6), 919 (100), 0 | 497 (22.2), 2237 (100), 3 | 219 (41.2), 531 (100), 2 | 654 (42.4), 1995 (100), 0 | 256 (50.2), 778 (100), 0 | 765 (43.8), 2272 (100), 0 | 145 (47.2), 501 (100), 0 |
| Pressured to work without PPE N (%), total N (%), missing | 182 (10.5), 1854 (100), 0 | 180 (21.0), 919 (100), 0 | 232 (10.4), 2240 (100), 0 | 130 (24.4), 533 (100), 0 | 209 (11.1), 1995 (100), 0 | 153 (21.5), 778 (100), 0 | 251 (11.8), 2272 (100), 0 | 111 (23.7), 501 (100), 0 |
| Protective factors | | | | | | | | |
| CD-RISC resilience | | | | | | | | |
| 1st Quartile N (%) | 266 (15.2) | 301 (34.9) | 438 (20.7) | 129 (26.4) | 311 (16.5) | 256 (35.2) | 372 (17.4) | 195 (41) |
| 2nd Quartile N (%) | 463 (26.5) | 257 (29.8) | 572 (27.0) | 148 (30.3) | 510 (27.1) | 210 (28.9) | 580 (27.2) | 140 (29.4) |
| 3rd Quartile N (%) | 477 (27.3) | 156 (18.1) | 536 (25.3) | 97 (19.8) | 498 (26.5) | 135 (18.6) | 564 (26.5) | 69 (14.5) |
| 4th Quartile N (%) | 539 (30.9) | 149 (17.3) | 573 (27.0) | 115 (23.5) | 562 (29.9) | 126 (17.3) | 616 (28.9) | 72 (15.1) |
| CD = RISC total N (%), missing N | 1745 (100), 109 | 863 (100), 56 | 2119 (100), 121 | 489 (100), 44 | 1881 (100), 114 | 727 (100), 51 | 2132 (100), 140 | 476 (100), 25 |
| Able to share stresses at work N(%), total N (%), missing N | 1588 (85.7), 1854 (100), 0 | 673 (73.2), 919 (100), 0 | 1875 (85.4), 2195 (100), 45 | 386 (75.8), 509 (100), 24 | 1700 (85.2), 1995 (100), 0 | 561 (72.1), 778 (100), 0 | 1909 (84.0), 2272 (100), 0 | 352 (70.2), 501 (100), 0 |

FL, Frontline worker; NFL, Non-frontline worker; GAD, General Anxiety Disorder-7; IES-R, Impact of Event Scale – Revised; PHQ, Patient Health Questionnaire; PSS, Perceived Stress Scale; COVID-19, coronavirus disease 2019; PPE, personal protective equipment; CD-RISC, Connor-Davidson Resilience Scale.

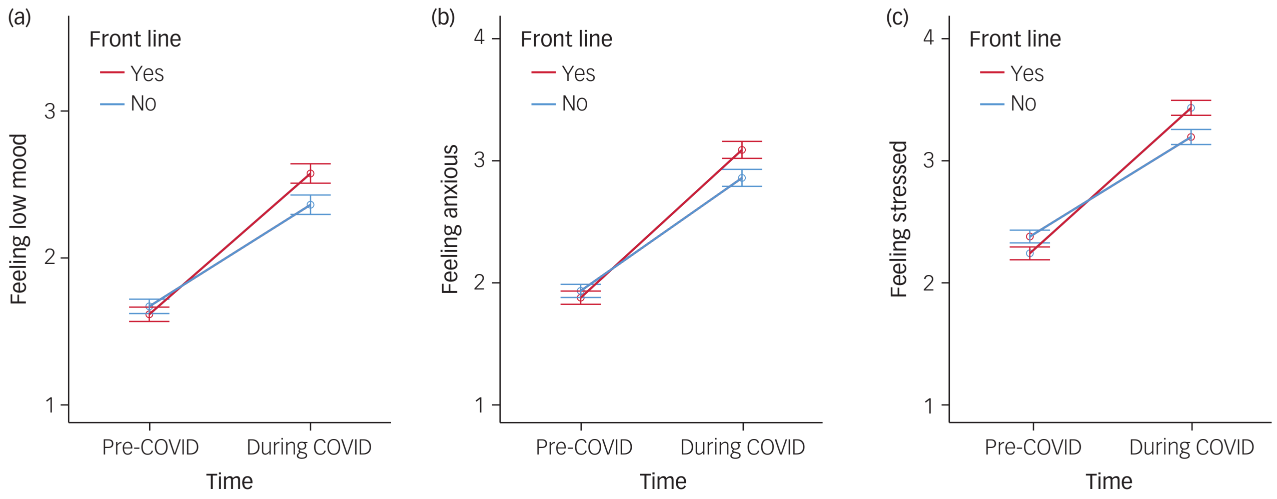


**Fig. 1** (a) Change in 'feeling low mood' (left), (b) 'feeling anxious' and (c) 'feeling stressed' scored pre- and during the coronavirus disease 2019 (COVID) pandemic for front-line and non-front-line healthcare workers.

Error bars are 95% CI.

**Table 5** Showing frequency distributions (*n* (%) across the whole cohort for each level of severity for items: feeling low, anxious and stressed, rated pre-coronavirus disease 2019 (COVID-19) and during COVID. A shift from low severity ratings pre-COVID-19 to high severity ratings during COVID is evident across all items

| | Feeling low, *n* (%) | | Anxious, *n* (%) | | Stressed, *n* (%) | |
|---|---|---|---|---|---|---|
| Rating | Pre | During | Pre | During | Pre | During |
| No | 1573 (56.7) | 717 (25.8) | 1127 (40.6) | 373 (13.5) | 522 (18.8) | 140 (5.0) |
| A little | 811 (29.2) | 827 (29.8) | 1086 (39.2) | 723 (26.1) | 1275 (46.0) | 564 (20.3) |
| Moderate | 245 (8.8) | 630 (22.7) | 345 (12.4) | 685 (24.7) | 678 (24.5) | 805 (29) |
| Quite a lot | 114 (4.1) | 439 (15.8) | 168 (6.1) | 624 (22.5) | 244 (8.8) | 862 (31.1) |
| Very | 30 (1.1) | 160 (5.8) | 47 (1.7) | 368 (13.3) | 54 (1.9) | 402 (14.5) |

stressed (PSS mean 16.98 (s.d. = 8.1) *v.* mean 19.06 (s.d. = 7.33), $t(302) = 2.29$, $P < 0.01$) but were not significantly less depressed, anxious, nor had lower PTSD symptoms than those without support ($n = 126$).

## Worries

Across the cohort, worry was greatest for family and friends becoming ill or dying from COVID-19 followed by worries that they will infect them (see Supplementary Table 1 for the full list); and was lowest for their own mental health and about poor workplace management. Front-line workers were significantly more worried than non-front-line workers for all concerns (all $P < 0.001$ except 'ability to support others', which was also significantly higher although at a higher threshold ($P < 0.05$)).

# Discussion

## Main findings

To our knowledge this is the first study examining the impact of COVID-19 on the mental health of HCWs in the UK. A significant proportion reported high depression (28%), high anxiety (33%) and high COVID-19-related PTSD symptoms (15%). Across the cohort, mental health indicators had significantly deteriorated compared with before COVID. Analyses revealed a set of fixed (demographic and role-related) factors and a separate set of controllable factors that were significantly associated with high levels of psychiatric symptoms in HCWs.

## Fixed risk factors

The fixed risk factors for high psychiatric symptoms were being female, all roles compared with doctor, working on the front line and having an existing mental disorder. Being single was associated with high depression and being younger was associated with stress. Although some of these components have been identified in previous pandemics[6,9] and recent research of much smaller cohorts outside the UK[11,23–25] the present study expands significantly on this work in several ways in terms of sample size, comprehensive examination of risk factors beyond demographics and roles, and scope of findings.

We show that allied HCWs, and particularly managers, were at significantly increased risk of high levels of symptoms. Managers, in particular, were 5.2 times more likely to report high PTSD symptoms – likely because of additional pressures and the rapid changes COVID-19 brings to their healthcare settings as well as increased threat to patients, staff and themselves. Nurses were significantly more likely to be in the high symptom group compared with doctors, which is mostly consistent with evidence from COVID-19 and other pandemics;[6,7] however, there is some evidence of doctors having greater psychiatric symptoms compared with other HCWs.[6,7,24] Differences in healthcare settings, HCW roles and national health systems across countries, as well as their COVID-19 response, may account for some of the

differential effects reported from studies from different countries. This suggests the need for additional support for personnel in these roles.

## Controllable risk factors

Importantly, a cluster of controllable risk factors relating to workplace characteristics and role-related activities were also significantly associated with high psychiatric symptoms. Pressure to work without PPE, and that risk from COVID-19 could have been reduced with better workplace preparation, were significantly associated with high depression. These factors were also associated with high stress along with practical issues such as absent team members and lack of sufficient information on clinical procedures.

The effects of having additional workload were broad – being linked to high anxiety, depression and PTSD symptoms, whereas insufficient training was uniquely associated with high levels of anxiety (also shown after severe acute respiratory syndrome (SARS).[26] High anxiety and PTSD symptoms were additionally associated with insufficient action being taken to reduce risk. There was a further critical role of a lack of sufficient information on COVID-19 clinical practice – being linked to high symptoms in all domains.

Critically then, a number of preventable workplace factors relating to perception of personal risk specifically increases the likelihood of having high PTSD symptoms: pressure to reuse PPE and failure of the workplace to reduce risk through preparation. A strong link between risk perception and PTSD has been reported previously during SARS.[26–30] As subjective appraisal of threat may contribute more to PTSD development than objective trauma severity[31] a sense of persistent danger to the self may catalyse the development of PTSD symptoms. This also highlights that perception of risk goes beyond PPE availability and includes multiple systemic and organisational components within healthcare settings. As a longer or repeated exposure (see[26,32]) raises risk of PTSD, more adequate PPE and workplace preparation may mitigate future development of PTSD. It is indeed noteworthy that over half of respondents stated that more PPE would reduce their anxiety.

## Location

Unlike an earlier study from China,[11] an epicentre effect was not apparent. Working in London was associated with lower risk of anxiety and depression. Although London workers were, for example, significantly less likely to be women, and less likely to be pressured to reuse PPE (each linked to lower risk), they also experienced a number of risk factors. Lower risk of anxiety and depression in London, therefore, could be due to better-resourced healthcare settings, being more accustomed to stress from city living, or instead it may be that the initial research from China which showed that living in Wuhan was associated with greater risk than living outside was due to HCWs there being in the first global centre of a new, fatal virus.

## Quarantine

Other factors that were expected to be associated with psychiatric symptoms were not observed. Quarantining of HCWs, for example, was not retained as a significant factor in any outcome model despite holding significant independent relationships with psychiatric symptoms (see Supplementary Table 7). At the time the survey was undertaken, UK HCWs were required to self-isolate for 7 days if they had possible symptoms or 14 days if exposed to someone known to have COVID-19 (apart from when wearing PPE if, for example, providing care on a ward with COVID-19 patients). Although an association was expected, Bell & Wade[7] report mixed evidence of a relationship between quarantine and psychological outcomes, and Kiseley et al[6] report that it was duration of self-isolation and prolonged quarantine that raised risk. It may then be that this duration of quarantine was too short to have a notable impact on mental health. Alternatively, a shorter quarantine may have even been restful and improved mental health in some individuals (as Chong et al[33] report in relation to SARS), or it may simply be that quarantining does not account for sufficient unique variance in psychiatric outcomes compared with, for example, witnessing traumatic events, or lack of safety equipment.

## PTSD

Unsurprisingly, traumatic events predicted high symptom scores, particularly PTSD symptoms. Personal loss and patients asking if they were going to die were significantly associated with high symptoms of PTSD, anxiety and depression. Having colleagues with, or dying from, COVID-19 also significantly increased the likelihood of being in the high PTSD symptoms group but this was greater with respect to friends or family dying. A peer who contracts or dies from COVID may be more indicative of an ongoing threat of danger to the self. Experiences where death is evident (patients dying and delivering bad news) were also associated with high PTSD symptoms (also seen following SARS).[27] Together, personal threat was associated with having high PTSD symptoms while impaired readiness to work effectively in response to COVID-19 was linked to high anxiety – perhaps because of these being preventable. Lastly, aftercare for the deceased was uniquely linked to depression; performing resuscitation was associated with high anxiety and PTSD symptoms; and practical issues with high stress.

## Moral injury

Moral injury may contribute to the development of psychiatric symptoms.[8] Here, HCWs who had denied treatment to patients were more anxious than those who had not, whereas support from an ethics panel was associated with lower stress highlighting the protective effects of shared decision-making on HCW mental well-being. The higher risk to managers may be because of such moral injury and the inability to adequately treat patients or protect staff.

## Ethnicity

Evidence that ethnic minority individuals are at elevated physical risk of COVID-19 was first published near the survey start date.[34] Although ethnic minority HCWs were more likely to report high PTSD symptoms this was not accompanied by a significantly greater risk of high anxiety, depression or stress. Being 'physically at high risk' of COVID-19 was not associated with high psychiatric symptoms, but was associated with greater worry about self-protection. Elevated prevalence of PTSD in ethnic minority individuals has previously been reported[35] and is associated with 'additional life stress'.[36] While more research on PTSD in ethnic minority individuals should be undertaken, this finding may reflect the same sense of sustained threat.

## Front-line workers

Across the cohort, all well-being indicators significantly worsened during COVID-19 compared with before. The proportion of the cohort who rated their psychiatric symptoms (anxiety, low mood, stress) at the most severe levels increased by 4–4.5 times during the COVID-19 response compared with pre-COVID levels. Front-line workers had significantly greater worries than non-front-line workers and were also more likely to be more depressed, anxious and stressed than non-front-line workers – and were 2.1

times as likely to have high PTSD symptoms – likely due to the traumatic and stressful duties they perform, as well as their concerns about risk, PPE access and preparation.

## Resilience

Resilience and the ability to share stress at work were significantly associated with having low symptoms except PTSD. Inadequate support has previously been shown to raise the risk of psychiatric morbidity in front-line workers.[6,7,27] In contrasting, however, scores on the 'I need psychological help?' item were low as were HCW worries about their 'own mental health'. Staff may indeed prefer practical help such as more rest or PPE to psychological support.[37] Resilience training may improve resistance to poor well-being, although this has been insufficiently researched in healthcare settings. PTSD symptoms may not be attenuated by resilience perhaps due to the more automatic and physiological, rather than cognitive, nature of these symptoms.

## Strengths and limitations

The study has several strengths and limitations. We recruited a large sample by COVID study standards (although only a 19.8% (52 of 262 UK NHS Trusts) response rate within all NHS services), near the peak of the first COVID-19 UK outbreak, and the study provides the most comprehensive picture to date of the negative psychological impact on HCWs of the COVID-19 pandemic in the UK and its associated factors. Participation in online surveys involves self-selection and respondents may not be fully representative. However, this approach permitted a rapid response around a critical period very close to the COVID-19 peak. Nonetheless, these findings should be viewed with caution as they may not be generalisable. The survey was de facto open to all HCWs in the UK and the very large sample size reflected a wide geographical coverage of the UK. That the sample characteristics were similar to the wider NHS workforce in terms of female/male ratio (85% in this study, NHS 77%) and proportion from ethnic minorities (13% in this study, NHS 19%) indicate that the data are broadly representative. The survey was launched 4 weeks after the UK national 'lockdown' began and psychiatric presentations may be affected by lockdown as well as because of working during the COVID-19 peak – although these would be expected to de facto co-occur.

Pre-COVID-19 well-being scores derived from ratings that may not be fully accurate as they were retrospective, however, evidence suggests that ratings of past events in individuals with depression are reliable.[38] Nonetheless, these findings should be viewed with caution as they may not be generalisable. If low mood resulted in more negative past ratings,[39] this would only increase the effect sizes of worsening suggesting these effects are robust. Mood scores indicated that the cohort were not a particularly anxious or worrisome group *per se* and the majority of respondents reported only low or mild symptoms of anxiety and depression and low worry levels before COVID-19. The frequency of psychiatric disorders was also low and very similar to rates in the general population,[35] and symptom scale scores and PTSD prevalence were similar to comparable studies.[6,11]

Lastly, the study information also strongly encouraged those who felt they 'were not affected by the COVID outbreak' to 'still take part as the reasons why some people are less affected are also very important to understand'. This was done to prevent recruiting a biased cohort of only respondents who felt adversely affected. The scales used were self-report and not diagnostic but have strong validity and reliability and are commonly used. This survey was cross-sectional but planned follow-up surveys will permit longitudinal analysis of effects and relationships. Finally, additional factors not examined may have a role in HCW mental health.

## Implications

In conclusion, the COVID-19 pandemic has had a discernible and detrimental effect on the mental health and well-being of UK HCWs. High symptoms of poor mental health were prevalent, and markers of well-being had significantly worsened compared with before COVID. A number of fixed and controllable factors were significantly associated with poor mental well-being, the latter reflecting elevated perception of COVID-19 risk and inadequate workplace preparedness. Critically, these findings can guide management strategy such as by improving PPE availability, training, communication of information and management of staff absence. These are readily amenable targets and may reduce the risk of HCWs developing poor mental health during COVID-19 or other pandemics.

The study also strongly indicates that psychological risk assessments should be carried out based on the factors identified. All staff should be monitored for poor mental health and those showing high symptoms should be referred to mental health services. Employers should improve initiatives for HCWs to share stress particularly those with risk factors and those making challenging treatment decisions – or even just offer more opportunity to rest, which HCWs have been reported to need.[37] Bespoke interventions could be developed that target these factors, such as role- or duty-specific training. Improving resilience, perhaps through training, may also be effective, as may teaching more adaptive coping styles – recently shown to be associated with better HCW well-being during the COVID-19 pandemic.[12]

Importantly, HCWs show only low recognition of the importance of their own mental health so awareness of this should be raised. Finally, working as a HCW during a pandemic can result in long-term effects on mental health, which may persist for years.[40] Attenuating these risks may help reduce the possibility of a major mental health crisis in UK healthcare and protect and retain HCWs. This is critical for delivery of effective treatment for patients and for planning a response to a new COVID-19 outbreak or future epidemics/pandemics – or in other countries where HCWs are yet to experience the impact on their mental health.

**James Gilleen**, Department of Psychology, University of Roehampton, UK; Department of Psychiatry, University College London, UK; and Department of Psychosis Studies, Institute of Psychiatry, Psychology & Neuroscience, King's College London, UK; **Aida Santaolalla**, School of Cancer & Pharmaceutical Sciences, Kings College London, UK; **Lorena Valdearenas**, Barnet, Enfield and Haringey Mental Health NHS Trust, UK; **Clara Salice**, Barts Health NHS Trust, The Royal London Hospital, UK; **Montserrat Fusté**, Department of Psychosis Studies, Institute of Psychiatry, Psychology & Neuroscience, King's College London, UK and North East London Foundation Trust, UK

**Correspondence:** James Gilleen. Email: James.gilleen@roehampton.ac.uk

First received 20 Jan 2021, final revision 19 Mar 2021, accepted 23 Mar 2021

## Supplementary material

Supplementary material is available online at https://doi.org/10.1192/bjo.2021.42.

## Data availability

The data that support the findings of this study are available from the corresponding author, J.G., upon reasonable request.

## Acknowledgements

We first want to thank all the healthcare workers who shared their experiences with us. We also would like to thank those who contributed their time and hard work to the COVIDA Project: Natasha Ramachandran (UCL, London, UK); Eirini Koutsouroupa for media coordination; and

Carl Stratton for website design and media strategy. We also thank Mandy Holmes and Jan Harrison of the University of Roehampton for facilitating a rapid set-up of the study; and Dr. Lin Fou for proofreading the manuscript.

## Author contributions

J.G. was the study principal investigator, study coordinator and lead author. M.F. and J.G. led on the development and planning of the study and L.V., A.S. and C.S. also contributed. A.S. conducted the chi-squared and regression analysis, assisted by M.F. and J.G.. J.G. conducted the ANOVA and *post hoc* analyses. L.V. led on the survey-dissemination strategy and liaising with NHS and non-NHS sites for study adoption. All authors worked on the development of the survey. J.G. is responsible for the overall content as guarantor.

## Funding

This research received no specific grant from any funding agency, commercial or not-for-profit sectors. Use of scales and webhosting/advertising were covered by a small amount of charitable donations to the project from members of the public (~£500).

## Declaration of interest

J.G. has consulted for Takeda Pharmaceuticals within 3 years of beginning the submitted work. The other authors have no declarations of interest.

## References


1 Caplan RP. Stress, anxiety, and depression in hospital consultants, general practitioners, and senior health service managers. *BMJ* 1994; **309**: 1261–3.

2 Quine L. Effects of stress in an NHS trust: a study. *Nurs Stand* 1998; **13**: 36–41.

3 Chang EM, Hancock KM, Johnson A, Daly J, Jackson D. Role stress in nurses: review of related factors and strategies for moving forward. *Nurs Health Sci* 2005; **7**: 57–65.

4 Burke RJ, Greenglass ER. Hospital restructuring, work-family conflict and psychological burnout among nursing staff. *Psychol Health* 2001; **16**: 583–94.

5 Leveck ML, Jones CB. The nursing practice environment, staff retention, and quality of care. *Res Nurs Health* 1996; **19**: 331–43.

6 Kisely S, Warren N, McMahon L, Dalais C, Henry I, Siskind D. Occurrence, prevention, and management of the psychological effects of emerging virus outbreaks on healthcare workers: rapid review and meta-analysis. *BMJ* 2020; **369**: m1642.

7 Bell V, Wade D. Mental health of clinical staff working in high-risk epidemic and pandemic health emergencies: a rapid review of the evidence and meta-analysis. *Soc Psychiatry Psychiatr Epidemiol* 2021; **56**: 1–11.

8 Greenberg N, Tracy D. What healthcare leaders need to do to protect the psychological well-being of frontline staff in the COVID-19 pandemic. *BMJ Leader* 2020; **4**: 101–2.

9 Brooks SK, Dunn R, Amlôt R, Rubin GJ, Greenberg N. A systematic, thematic review of social and occupational factors associated with psychological outcomes in healthcare employees during an infectious disease outbreak. *J Occup Environ Med* 2018; **60**: 248–57.

10 McCann CM, Beddoe E, McCormick K, Huggard P, Kedge S, Adamson C, et al. Resilience in the health professions: A review of recent literature. *Int J Wellbeing* 2013; **3**: 60–81.

11 Lai J, Ma S, Wang Y, Cai Z, Hu J, Wei N, et al. Factors associated with mental health outcomes among health care workers exposed to coronavirus disease 2019. *JAMA Netw Open* 2020; **3**: e203976.

12 McFadden P, Ross J, Moriarty J, Mallett J, Schroder H, Ravalier J, et al. The role of coping in the wellbeing and work-related quality of life of uk health and social care workers during COVID-19. *IJERPH* 2021; **18**: 815.

13 Shah N, Raheem A, Sideris M, Velauthar L, Saeed F. Mental health amongst obstetrics and gynaecology doctors during the COVID-19 pandemic: results of a UK-wide study. *Eur J Obstet Gynecol Reprod Biol* 2020; **253**: 90–4.

14 Roberts NJ, McAloney-Kocaman K, Lippiett K, Ray E, Welch L, Kelly C. Levels of resilience, anxiety and depression in nurses working in respiratory clinical areas during the COVID pandemic. *Respir Med* 2021; **176**: 106219.

15 Kaufman KR, Petkova E, Bhui KS, Schulze TG. A global needs assessment in times of a global crisis: world psychiatry response to the COVID-19 pandemic. *BJPsych Open* 2020; **6**: e48.

16 Martin A, Rief W, Klaiberg A, Braehler E. Validity of the Brief Patient Health Questionnaire Mood Scale (PHQ-9) in the general population. *Gen Hosp Psychiatry* 2006; **28**: 71–7.

17 Löwe B, Decker O, Müller S, Brähler E, Schellberg D, Herzog W, et al. Validation and standardization of the Generalized Anxiety Disorder Screener (GAD-7) in the general population. *Med Care* 2008; **46**: 266–74.

18 Weiss DS. The Impact of Event Scale: Revised. In *Cross-Cultural Assessment of Psychological Trauma and PTSD* (eds JP Wilson and CS Tang): 219–38. International and Cultural Psychology Series. Springer, 2007.

19 Cohen S, Kamarck T, Mermelstein R. A Global Measure of Perceived Stress. *J Health Soc Behav* 1983; **24**: 385–96.

20 Campbell-Sills L, Stein MB. Psychometric analysis and refinement of the Connor-Davidson Resilience Scale (CD-RISC): validation of a 10-item measure of resilience. *J Trauma Stress* 2007; **20**: 1019–28.

21 Cohen J. *Statistical Power Analysis for the Behavioral Sciences*. Academic Press, 2013.

22 Richardson JTE. Eta squared and partial eta squared as measures of effect size in educational research. *Educ Res Rev* 2011; **6**: 135–47.

23 Badahdah A, Khamis F, Al Mahyijari N, Al Balushi M, Al Hatmi H, Al Salmi I, et al. The mental health of health care workers in Oman during the COVID-19 pandemic. *Int J Soc Psychiatry* [Epub ahead of print] 8 Jul 2020. Available from: https://doi.org/10.1177/0020764020939596.

24 Rossi R, Socci V, Pacitti F, Di Lorenzo G, Di Marco A, Siracusano A, et al. Mental health outcomes among frontline and second-line health care workers during the coronavirus disease 2019 (COVID-19) pandemic in Italy. *JAMA Netw Open* 2020; **3**: e2010185.

25 Cai Q, Feng H, Huang J, Wang M, Wang Q, Lu X, et al. The mental health of frontline and non-frontline medical workers during the coronavirus disease 2019 (COVID-19) outbreak in China: a case-control study. *J Affect Disord* 2020; **275**: 210–5.

26 Maunder R, Lancee W, Balderson K, Bennett J, Borgundvaag B, Evans S, et al. Long-term psychological and occupational effects of providing hospital healthcare during SARS outbreak. *Emerg Infect Dis* 2006; **12**: 1924–32.

27 Tam CWC, Pang EPF, Lam LCW, Chiu HFK. Severe acute respiratory syndrome (SARS) in Hong Kong in 2003: stress and psychological impact among frontline healthcare workers. *Psychol Med* 2004 Oct; **34**: 1197–204.

28 Maunder RG, Lancee WJ, Rourke S, Hunter JJ, Goldbloom D, Balderson K, et al. Factors associated with the psychological impact of severe acute respiratory syndrome on nurses and other hospital workers in Toronto. *Psychosom Med* 2004; **66**: 938–42.

29 Styra R, Hawryluck L, Robinson S, Kasapinovic S, Fones C, Gold WL. Impact on health care workers employed in high-risk areas during the Toronto SARS outbreak. *J Psychosom Res* 2008; **64**: 177–83.

30 Wu P, Fang Y, Guan Z, Fan B, Kong J, Yao Z, et al. The Psychological Impact of the SARS epidemic on hospital employees in China: exposure, risk perception, and altruistic acceptance of risk. *Can J Psychiatry* 2009; **54**: 302–11.

31 Ehlers A, Clark DM. A cognitive model of posttraumatic stress disorder. *Behav Res Ther* 2000; **38**: 319–45.

32 Kaysen D, Resick PA, Wise D. Living in danger: the impact of chronic traumatization and the traumatic context on posttraumatic stress disorder. *Trauma Violence Abuse* 2003; **4**: 247–64.

33 Chong M-Y, Wang W-C, Hsieh W-C, Lee C-Y, Chiu N-M, Yeh W-C, et al. Psychological impact of severe acute respiratory syndrome on health workers in a tertiary hospital. *Br J Psychiatry* 2004; **185**: 127–33.

34 Kirby T. Evidence mounts on the disproportionate effect of COVID-19 on ethnic minorities. *Lancet Respir Med* 2020; **8**: 547–8.

35 McManus S, Bebbington P, Jenkins R, Brugha T. *Mental Health and Wellbeing in England: Adult Psychiatric Morbidity Survey 2014*. NHS Digital, 2016 (https://openaccess.city.ac.uk/id/eprint/23646/).

36 Brewin CR, Andrews B, Valentine JD. Meta-analysis of risk factors for post-traumatic stress disorder in trauma-exposed adults. *J Consul Clin Psychol* 2000; **68**: 748–66.

37 Chen C-S, Wu H-Y, Yang P, Yen C-F. Psychological distress of nurses in Taiwan who worked during the outbreak of SARS. *Psychiatr Serv* 2005; **56**: 76–9.

38 Brewin CR, Andrews B, Gotlib IH. Psychopathology and early experience: a reappraisal of retrospective reports. 1993; **113**: 17.

39 Sato H, Kawahara J. Selective bias in retrospective self-reports of negative mood states. *Anxiety Stress Coping* 2011; **24**: 359–67.

40 Lee AM, Wong JG, McAlonan GM, Cheung V, Cheung C, Sham PC, et al. Stress and psychological distress among SARS survivors 1 year after the outbreak. *Can J Psychiatry* 2007; **52**: 233–40.


EXTRA CONTENT ONLINE
OPEN ACCESS
CC BY
