# Supplementary material for: Mental health status and related factors influencing healthcare workers during the COVID-19 pandemic: A systematic review and meta-analysis
Source: PLoS One. 2024 Jan 19;19(1):e0289454. doi: 10.1371/journal.pone.0289454 (PMC10798549; doi:10.1371/journal.pone.0289454)
Supplement: S1 Data — (ZIP) [file pone.0289454.s011.zip › literatures/44.pdf]

PLOS ONE

RESEARCH ARTICLE

Determinants of burnout and other aspects of psychological well-being in healthcare workers during the Covid-19 pandemic: A multinational cross-sectional study

Max Denning[1], Ee Teng Goh[1], Benjamin Tan[2], Abhiram Kanneganti[3], Melanie Almonte[1], Alasdair Scott[1], Guy Martin[1], Jonathan Clarke[1], Viknesh Sounderajah[1], Sheraz Markar[1], Jan Przybylowicz[1], Yiong Huak Chan[4], Ching-Hui Sia[2,5], Ying Xian Chua[6], Kang Sim[7,8], Lucas Lim[9], Lifeng Tan[10], Melanie Tan[11], Vijay Sharma[2], Shirley Ooi[12,13], Jasmine Winter Beatty[1], Kelsey Flott[1], Sam Mason[1], Swathikan Chidambaram[1], Seema Yalamanchili[1], Gabriela Zbikowska[1], Jaroslaw Fedorowski[14], Grazyna Dykowska[15], Mary Wells[1], Sanjay Purkayastha[1]*, James Kinross[1]

1 Department of Surgery and Cancer, Imperial College London, London, United Kingdom, **2** Department of Medicine, Yong Loo Lin School of Medicine, National University of Singapore, Singapore, Singapore, **3** Department of Obstetrics and Gynaecology, National University Hospital, Singapore, Singapore, **4** Biostatistics Unit, Yong Loo Lin School of Medicine, National University of Singapore, Singapore, Singapore, **5** Department of Cardiology, National University Heart Centre, Singapore, Singapore, **6** Pioneer Polyclinic, National University Polyclinic, National University Health System, Singapore, Singapore, **7** Institute of Mental Health, Singapore, Singapore, **8** Department of Psychological Medicine, Yong Loo Lin School of Medicine, National University of Singapore, Singapore, Singapore, **9** Department of Forensic Psychiatry, Institute of Mental Health, Singapore, Singapore, **10** Division of Healthy Ageing, Alexandra Hospital, Singapore, Singapore, **11** Department of Geriatric Medicine, Ng Teng Fong General Hospital, Singapore, Singapore, **12** Department of Surgery, Yong Loo Lin School of Medicine, National University of Singapore, Singapore, Singapore, **13** Emergency Medicine Department, National University Hospital, Singapore, Singapore, **14** Polish Hospital Federation, Poland, **15** Department of Economics of Health and Medical Law, Medical University of Warsaw, Poland

* s.purkayastha@imperial.ac.uk

OPEN ACCESS

Citation: Denning M, Goh ET, Tan B, Kanneganti A, Almonte M, Scott A, et al. (2021) Determinants of burnout and other aspects of psychological well-being in healthcare workers during the Covid-19 pandemic: A multinational cross-sectional study. PLoS ONE 16(4): e0238666. https://doi.org/10.1371/journal.pone.0238666

Editor: M. Harvey Brenner, University of North Texas Health Science Center, UNITED STATES

Received: September 3, 2020

Accepted: March 21, 2021

Published: April 16, 2021

Copyright: © 2021 Denning et al. This is an open access article distributed under the terms of the Creative Commons Attribution License, which permits unrestricted use, distribution, and reproduction in any medium, provided the original author and source are credited.

Data Availability Statement: All relevant data are within the manuscript ad its Supporting information files.

Funding: JK has received an educational grant from Johnson and Johnson.

Competing interests: JK has received an educational grant from Johnson and Johnson. This does not alter our adherence to PLOS ONE policies on sharing data and materials.

Abstract

The Covid-19 pandemic has placed unprecedented pressure on healthcare systems and workers around the world. Such pressures may impact on working conditions, psychological wellbeing and perception of safety. In spite of this, no study has assessed the relationship between safety attitudes and psychological outcomes. Moreover, only limited studies have examined the relationship between personal characteristics and psychological outcomes during Covid-19. From 22nd March 2020 to 18th June 2020, healthcare workers from the United Kingdom, Poland, and Singapore were invited to participate using a self-administered questionnaire comprising the Safety Attitudes Questionnaire (SAQ), Oldenburg Burnout Inventory (OLBI) and Hospital Anxiety and Depression Scale (HADS) to evaluate safety culture, burnout and anxiety/depression. Multivariate logistic regression was used to determine predictors of burnout, anxiety and depression. Of 3,537 healthcare workers who participated in the study, 2,364 (67%) screened positive for burnout, 701 (20%) for anxiety, and 389 (11%) for depression. Significant predictors of burnout included patient-facing roles: doctor (OR 2.10; 95% CI 1.49–2.95), nurse (OR 1.38; 95% CI 1.04–1.84), and 'other clinical'

(OR 2.02; 95% CI 1.45–2.82); being redeployed (OR 1.27; 95% CI 1.02–1.58), bottom quartile SAQ score (OR 2.43; 95% CI 1.98–2.99), anxiety (OR 4.87; 95% CI 3.92–6.06) and depression (OR 4.06; 95% CI 3.04–5.42). Significant factors inversely correlated with burnout included being tested for SARS-CoV-2 (OR 0.64; 95% CI 0.51–0.82) and top quartile SAQ score (OR 0.30; 95% CI 0.22–0.40). Significant factors associated with anxiety and depression, included burnout, gender, safety attitudes and job role. Our findings demonstrate a significant burden of burnout, anxiety, and depression amongst healthcare workers. A strong association was seen between SARS-CoV-2 testing, safety attitudes, gender, job role, redeployment and psychological state. These findings highlight the importance of targeted support services for at risk groups and proactive SARS-CoV-2 testing of healthcare workers.

Introduction

The Covid-19 pandemic has led to an unprecedented strain on healthcare services globally. Considerable changes in healthcare delivery have necessarily taken place. These have included cessation of routine services, repurposing of clinical areas, redeployment of staff to unfamiliar clinical environments [1,2], and in some circumstances, the rationing of services [3]. The impact of these modified working conditions on safety culture and psychological well-being are poorly understood.

Traumatic events or adverse conditions during natural disasters, conflict, and pandemics may lead to burnout [4–6]. Burnout is defined as "a syndrome of exhaustion, depersonalization, and reduced professional efficacy" [7] and leads to poorer patient safety outcomes [8–10]. Burnout is composed of two elements: "exhaustion", linked to excessive job demands; and "disengagement", linked to insufficient job resources [11]. During the Covid-19 pandemic healthcare systems have faced rising demands and limited resources, as such, it is important to understand the corresponding rates of burnout.

Similarly, infectious disease outbreaks have well-documented effects on the psychological wellbeing of healthcare workers (HCWs). During the Severe Acute Respiratory Syndrome (SARS), H1N1 and Ebola outbreaks, studies showed that frontline HCWs were at higher risk of developing psychological sequelae, including chronic stress, anxiety, depression and post-traumatic stress disorder [12–17]. Various factors are understood to have contributed to this phenomenon, such as excessive workload, concerns about occupational exposure, or infection of HCWs' families. In comparison to previous pandemics, the psychological impact of Covid-19 may be more significant and widespread, given the scale of the pandemic [18–20].

Objectives

This study aims to describe the prevalence and predictors of burnout, anxiety and depression in healthcare workers during the Covid-19 pandemic.

Methods

Ethics

Institutional ethical approval was obtained for data collection in the United Kingdom and Poland by the Imperial College Research Ethics Committee (ICREC) Ref:20IC5890, and

Singapore by the National Healthcare Group Domain Specific Research Board (NHS DSRB) Ref 2020–00598.

Setting

Countries selected for inclusion represented a range of Covid-19 mortality rates, health system design, economic development, and had regional coordinators that could adapt the questionnaire to the local context and champion distribution. See Table 1 for comparison of country settings.

UK. The UK has 66 million residents [21]. Healthcare is publicly funded through general taxation and provided free at point of delivery by the National Health Service. The gross domestic product (GDP) is $42,962 per capita [22], of which 9.6% ($3,859) is spent on healthcare [23]. The UK has 2.8 physicians and 8.2 nurses and midwifes per 1,000 people [24,25]. The first documented case of Covid-19 was on 29th January 2020 and a national lockdown was initiated on 23rd March 2020 that introduced workplace, public space and school closures. In order to increase clinical capacity measures were taken including the cessation of elective services, redeployment of staff, reconfiguration of hospitals and establishment of a series of temporary 'Nightingale' hospitals. As of 11 July 2020 the UK has had 288,133 Covid-19 infections and 44,650 related deaths [26].

Table 1. Comparison of National Health Settings [21–29].

	UK	Singapore	Poland
**Population (millions)**	66	5.7	38
**GDP ($)**	42,962	64,582	15,423
**Healthcare spend (% of GDP)**	9.6	4.4	6.5
**Per capita spend on healthcare ($)**	3,859	2,619	907
**Physicians/1,000**	2.8	2.3	2.4
**Nurses & midwives/1,000**	8.2	6.2	6.9
**Covid-19 cases***	288,133	45,613	37,216
**Covid-19 deaths***	44,650	26	1,562
**Healthcare funding**	Public	Co-funding	Public
**Healthcare provision**	Public	Co-delivery	Public
**National Lockdown/Circuit breaker**	23rd March 2020	7th April 2020	15th Mar 2020
**Initial government policies**	Workplace closures<br>School closures<br>Public space closures<br>Travel restrictions<br>Reduction in elective services<br>Temporary Nightingale hospitals	Workplace closures<br>School closures<br>Public space closures<br>Travel restrictions<br>Reduction in elective services<br>Satellite clinics in temporary worker accommodation	Travel restriction<br>School closures<br>Reduction in public event capacity<br>Designation of 'infectious disease' hospitals
**Start government response stringency index±**	80	39	57
**End government response stringency index$**	71	78	51

*As of 11th July 2020.

±Start of study period (27th Mar 2020).

$End of study period (16th June 2020).

The Government response stringency index is a composite measure, proposed by Hale et al, that integrates measures of '[Covid-19] containment and health', 'economic support', and '[policy] stringency' to form an overall index that can be used to compare government policy over time in different countries. The score represents the number and strictness of government policies and should not be interpreted as an 'effectiveness score'[30].

https://doi.org/10.1371/journal.pone.0238666.t001

Singapore. Singapore is a city-state of 5.7 million residents. 80% of hospital care is provided by the public sector [27] and is funded through a mixed-financing model of co-funding [28]. The GDP per capita is $64,582 [22], of which 4.4% ($2,619) is spent on healthcare [23]. Singapore has 2.3 physicians [24] and 6.2 nurses and midwives per 1,000 people [25]. Singapore reported its first case of Covid-19 on 23rd January 2020. In April, a national lockdown [29] was initiated that introduced workplace, public space, and school closures. In order to increase clinical capacity, non-urgent clinical procedures were reduced. As of 11 July 2020 Singapore has had 45,613 Covid-19 infections and 26 related deaths [26].

Poland. Poland has 38 million residents [21]. Healthcare is funded through the National Health Fund, general taxation and private insurance. The GDP per capita is $15,423 [22], of which, 6.5% ($907) is spent on healthcare [23]. Poland has 2.4 physicians and 6.9 nurses and midwives per 1,000 people [25,26]. On 4th March 2020, Poland reported its first case of Covid-19 and a national lockdown was initiated on 15th March 2020, which included border closures to foreign nationals and a quarantine for returning citizens. Twenty-three hospitals were repurposed into infectious diseases hospitals for patients with suspected or confirmed COVID-19 infection. A further 67 hospitals had an infectious disease ward available. As of 11 July 2020 Poland has had 37,216 Covid-19 infections and 1,562 related deaths [26].

Survey design

The survey consisted of four parts; demographic questions followed by 3 validated psychometric instruments; the Safety Attitudes Questionnaire, Oldenburg Burnout Inventory and Hospital Anxiety and Depression Scale. Local collaborators in each country adapted the demographic questions to be culturally appropriate and contextually relevant. Demographic data included gender, ethnicity, professional role, workload, and Covid-19 status (see S1 File).

Oldenburg Burnout Inventory (OLBI). The OLBI is a 16-item validated tool for the investigation of burnout [31,32]. Items consist of both positively and negatively worded questions related to exhaustion and disengagement that are recorded on a four-point Likert scale. For the purpose of descriptive analyses, we considered participants to be at 'high risk of burnout' if they met the cut-offs of 2.1 and 2.25 for the exhaustion and disengagement subscales, respectively, as used in previous studies [33–36]. To increase specificity in the regression analyses, a higher cut-off of the 75th percentile of OLBI scores was used.

Hospital Anxiety and Depression Scale (HADS). The HADS was developed in 1983 [37] and has since been widely used for assessing depression and anxiety [38]. It is self-reported, concise, and uses separate subscales for anxiety and depression, each consisting of seven items rated on a four-point Likert scale. It has been validated in several countries and adapted for use in different languages and settings [39–42]. A score of 7 or less is considered normal, 8–11 as borderline and greater than 11 is diagnostic of anxiety or depression. The HADS was used in studies evaluating the psychiatric morbidity amongst SARS survivors [43,44].

Safety Attitudes Questionnaire (SAQ). The Safety Attitudes Questionnaire (SAQ) measures staff perceptions of safety. It has been validated in several countries, languages [45–49] and healthcare settings, including critical care and inpatient wards [50]. Thirty-five statements were included, each followed by a 5-point Likert scale from "*strongly disagree*" to "*strongly agree*". SAQ scores represent the proportion of respondents that "*agree*" or "*strongly agree*" with positive statements relating to each subscale, vice versa for negatively-worded questions [50] (S1 File). Scores are expressed across six domains: safety climate, teamwork, stress recognition, perception of management, working conditions, and job satisfaction. On all scales, a higher percentage score represents a more positive perception. Taken together, the scores provide insight into healthcare workers' perceptions of operational conditions in their workplace.

Translation. Investigators in the UK and Singapore used English versions of the questionnaires. In Poland investigators utilised validated Polish versions of HADS, SAQ and OLBI. Demographic questions were translated by a native speaker (JP) and the translation validated through back translation by an independent native Polish speaker (GZ) (S1 File).

Study conduct

This cross-sectional study was conducted between 27th March and 16th June 2020. The questionnaire was administered using Google Forms (Google LLC, USA) in Europe, and FormSG (GovTech, Singapore) in Singapore. Invitations to participate were distributed using targeted email communications with weekly reminders, and advertisement on social media platforms (Twitter and Whatsapp). Written, informed consent was obtained from all participants. The study dataset was anonymized and uploaded (S2 File).

Sample size

Allowing for up to 10 covariates in the multivariate model and a sensitivity of 0.05, a sample size of 2,000 participants was required.

Statistics

Data were analysed using Stata v14 (StataCorp. 2015. Stata Statistical Software: Release 14. College Station. TX: StataCorp LP). Reliability of each psychometric instrument (SAQ, OLBI, HADS) were assessed using Cronbach's alpha. A complete case analysis approach was used. For each questionnaire, the values of each question were correlated with the individual's total score. Alpha scores >0.70 were deemed as acceptable reliability. Statistical significance was set at 2-sided $p < 0.05$ using the Wald test The primary outcome measure was burnout, secondary outcome measures were anxiety and depression. Explanatory variables for burnout included SAQ scores, demographic questions, and HADS outcomes. Explanatory variables were assessed against the 75th percentile of OLBI scores using logistic regression. Variables found to be significant on univariate analysis were included in the multivariate analysis as well as forced variables that were deemed important to control for (country, role, and redeployment status). The "svy" command was used in the STATA setup for logistic regression.

Results

A total of 3,537 responses were received (Table 2). Amongst these, 2,544 (72%) of respondents were female and 923 male (26.1%). 684 (19.3%) responses were from doctors, 1,590 (45%) from nurses, 517 (14.6%) from other clinical staff (including healthcare support workers, allied health professionals, pharmacists etc), and 746 (21.1%) non-clinical staff. 765 responses were from the UK, 232 from Poland, 2,503 from Singapore, and 37 from other countries, which were excluded due to the low response rate for the purpose of analysis to minimise a response bias. During the pandemic, 766 (21.7%) clinical staff were redeployed as part of response measures and 777 (22%) respondents had received at least one test for SARS-CoV-2 infection.

The questionnaires had strong reliability as evident by their high α coefficients (HADS α = 0.90, OLBI α = 0.88, SAQ α = 0.94). In our study, 2,364 (67%, 95% CI 65%-68%) of respondents were identified as being at high risk of burnout, whilst 701 (20%, 95% CI 18%-21%) and 389 (11%, 95% CI 9%-12%) met the criteria for anxiety and depression, respectively. A number of respondents met criteria for more than one condition (Fig 1).

Table 2. Respondent characteristics.

Covariates	Overall (n = 3,537)		UK (n = 765)	Poland (n = 232)	Singapore (n = 2,503)	Pearson chi-square
	n	%	%	%	%	p-value
**Gender**						<0.001
Male	923	26.1	28.9	8.6	26.4	
Female	2544	71.9	69.9	90.5	71.3	
Undisclosed	70	2.0	1.2	0.9	2.4	
**Role**						<0.001
Non-clinical	746	21.1	14.1	1.7	25.3	
Doctor	684	19.3	35.7	8.2	14.5	
Nurse	1590	45.0	36.7	89.7	43.7	
Other clinical staff	517	14.6	13.5	0.4	16.5	
**Base Specialty**						<0.001
Medicine	1238	35.0	30.5	29.3	37.4	
Surgery	412	11.7	21.4	15.5	7.4	
Acute	388	11.0	16.9	16.8	8.7	
Other specialty	1055	29.8	26.7	38.4	30.2	
Non-clinical	433	12.2	4.6	0.0	15.9	
**Days Worked in Past Week**						<0.001
5 or less	2651	75.0	76.0	84.9	74.0	
6 or more	715	20.2	5.0	9.5	26.0	
No response	171	4.8	19.1	5.6	0.0	
**Redeployed**						<0.001
No	2771	78.3	62.8	89.7	82.1	
Yes	766	21.7	37.3	10.3	17.9	
**Redeployed Specialty**						<0.001
Covid GM	214	6.1	9.8	3.5	5.0	
ITU/EM	208	5.9	17.9	1.3	2.6	
Other	344	9.7	9.5	5.6	10.2	
**Treated Covid +ve patient in past week**						<0.001
No	1994	56.4	34.4	54.3	63.4	
Yes	853	24.1	54.1	17.7	15.3	
Don't know	242	6.8	7.1	28.0	4.8	
Not applicable	448	12.7	4.4	0.0	16.5	
**Presence of Symptoms**						<0.001
Asymptomatic	3158	89.3	85.4	93.5	90.1	
Symptomatic	379	10.7	14.6	6.5	9.9	
**Testing Status**						<0.001
Not tested	2760	78.0	72.9	68.5	80.4	
Tested	777	22.0	27.1	31.5	19.6	
**Psychological Outcomes**	**n**	**% (95% CI)**	**% (95% CI)**	**% (95% CI)**	**% (95% CI)**	
Burnout	2364	67(65–68)	63 (60–66)	71(65–77)	68 (66–70)	
Anxiety	701	20(18–21)	27 (24–30)	28 (22–33)	17 (15–18)	
Depression	389	11(9–12)	12 (9–14)	14 (10–19)	10 (9–12)	

GM: General medicine; ITU: Intensive treatment unit; EM: Emergency medicine.

https://doi.org/10.1371/journal.pone.0238666.t002

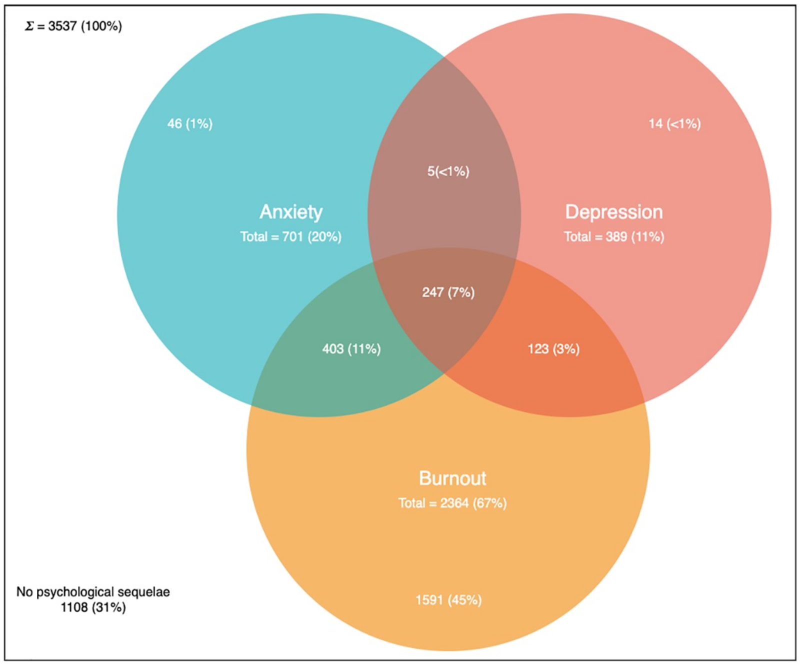

Fig 1. Venn diagram demonstrating prevalence of anxiety, depression and burnout in the sampled population. This figure demonstrates the number of respondents meeting the OLBI criteria for burnout, the HADS criteria for anxiety and the HADS criteria for depression. The overlap of sets represent individuals meeting more than one criteria.

https://doi.org/10.1371/journal.pone.0238666.g001

Burnout

On univariate analysis (Table 3), significant covariates included undisclosed gender, job role, base specialty, redeployment, having been tested for Covid-19, treatment of patients with Covid-19, SAQ score, anxiety and depression. There was no significant relationship between, country, symptoms of Covid-19, number of days worked and burnout.

On multivariate analysis (Table 3), the following predictors of burnout were: doctor role (OR 2.10; 95% CI 1.49–2.95), nursing role (OR 1.38; 95% CI 1.04–1.84), other clinical roles (OR 2.02; 95% CI 1.45–2.82), being redeployed (OR 1.27; 95% CI 1.02–1.58), SAQ score lower than the 25th percentile (OR 2.43; 95% CI 1.98–2.99), anxiety (OR 4.87; 95% CI 3.92–6.06) and depression (OR 4.06; 95% CI 3.04–5.42). Statistically significant factors that were inversely correlated with burnout included: being tested for Covid-19 (OR 0.64; 95% CI 0.51–0.82) and SAQ score higher than the 75th percentile (OR 0.30; 95% CI 0.22–0.40). SAQ score by psychological state is demonstrated in Fig 2.

Depression

Significant predictors of depression (Table 4) included: being redeployed (OR 1.44; 95% CI 1.07–1.95), SAQ scores lower than the 25th percentile (OR 2.29; 95% CI 1.73–3.02), burnout (OR 4.18; 95% CI 3.13–5.57) and anxiety (OR 5.13; 95% CI 3.90–6.73). Significant factors inversely correlated with depression included: female gender (OR 0.62; 95% CI 0.45–0.84) and doctor role (OR 0.60; 95% CI 0.38–0.96).

Anxiety

Significant predictors of anxiety (Table 4) included: female gender (OR 1.47, 95% CI 1.13–1.91), undisclosed gender (OR 2.12; 95% CI 1.10–4.08), SAQ scores lower than the 25th

Table 3. Logistic regression analysis with burnout as dependent variable.

Covariate	Univariate Analysis				Multivariate Analysis			
	OR	p-value	95% CI		OR	p-value	95% CI	
**Base Specialty**								
Medicine	Baseline							
Surgery	0.88	0.325	0.69	1.13				
EM/ITU/Anaesthetics	0.84	0.193	0.65	1.09				
Other specialty	0.81	0.021	0.67	0.97				
**Gender**								
Male	Baseline				Baseline			
Female	1.15	0.107	0.97	1.37	0.94	0.618	0.75	1.19
Undisclosed	3.32	<0.001	2.03	5.44	1.39	0.295	0.75	2.59
**Country**								
UK	Baseline				Baseline			
Poland	1.36	0.058	0.99	1.86	1.30	0.196	0.87	1.95
Singapore	0.94	0.474	0.78	1.12	1.20	0.137	0.94	1.52
**Role**								
Non-clinical	Baseline				Baseline			
Doctor	1.42	0.006	1.11	1.81	2.10	<0.001	1.49	2.95
Nurse	1.54	<0.001	1.25	1.90	1.38	0.026	1.04	1.84
Other clinical staff	1.64	<0.001	1.27	2.13	2.02	<0.001	1.45	2.82
**Days Worked in Past Week**								
5 or less	Baseline							
6 or more	1.16	0.120	0.96	1.39				
**Redeployed**								
No	Baseline				Baseline			
Yes	1.45	<0.001	1.22	1.73	1.27	0.035	1.02	1.58
**Redeployed Specialty**								
Not redeployed	Baseline							
Covid GM	1.28	0.113	0.94	1.74				
ITU/EM	1.59	0.002	1.18	2.14				
Other	1.49	0.001	1.17	1.89				
**Treated Covid +ve Patient in Past Week**								
No	Baseline							
Yes	1.13	0.172	0.95	1.36				
Don't know	2.09	<0.001	1.59	2.75				
Not applicable	1.04	0.726	0.82	1.32				
**Presence of Symptoms**								
Asymptomatic	Baseline							
Symptomatic	0.80	0.085	0.62	1.03				
**Testing Status**								
Not tested	Baseline				Baseline			
Tested	0.80	0.016	0.66	0.96	0.64	<0.001	0.51	0.82
**SAQ**								
50th Percentile	Baseline				Baseline			
25th Percentile	3.48	<0.001	2.92	4.15	2.43	<0.001	1.98	2.99
75th Percentile	0.29	<0.001	0.22	0.38	0.30	<0.001	0.22	0.40
**Anxiety**								
Normal/Borderline	Baseline				Baseline			

(*Continued*)

Table 3. (Continued)

Covariate	Univariate Analysis				Multivariate Analysis			
	OR	p-value	95% CI		OR	p-value	95% CI	
Abnormal	8.23	<0.001	6.86	9.87	4.87	<0.001	3.92	6.06
**Depression**								
Normal/Borderline	Baseline				Baseline			
Abnormal	10.06	<0.001	7.92	12.78	4.06	<0.001	3.04	5.42

OR: Odds ratio; EM: Emergency medicine; ITU: Intensive treatment unit; GM: General medicine.

https://doi.org/10.1371/journal.pone.0238666.t003

percentile (OR 2.19; 95% CI 1.74–2.76), burnout (OR 4.89; 95% CI 3.93–6.08) and abnormal depression scores (OR 5.15; 95% CI 3.91–6.78). Factors inversely correlated with anxiety included: being from Poland (OR 0.63; 95% CI 0.41–0.96), being from Singapore (OR 0.48; 95% CI 0.37–0.61) and other clinical job role (OR 0.64; 95% CI 0.43–0.94).

Discussion

In our study, 2,364 (67%) respondents were at high risk of burnout. Prior to the onset of Covid-19 studies reported rates of burnout in the UK of 31.5% [51] and 42% [52] for doctors and nurses, respectively. In Singapore figures were similar with 33% [53] and 51% [54] of nurses and doctors exhibiting symptoms of burnout, respectively. The higher rates observed in this study suggest that the Covid-19 pandemic, or changes as a result of the pandemic may have led to an increased rate of burnout amongst staff.

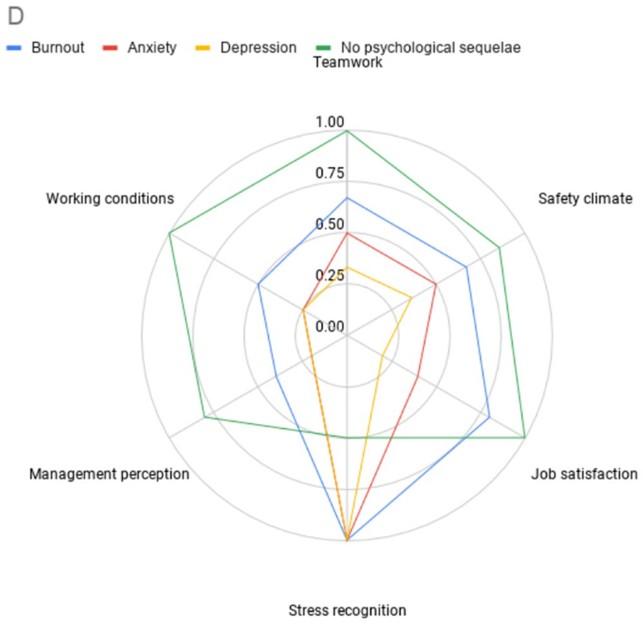

Fig 2. Radar plot demonstrating SAQ subscale by psychological state. This figure demonstrates the SAQ subscale scores by psychological outcome. Distance from the centre represents proportion of a subscale answered positively. A greater distance represents a more positive score. § patients may be represented in more than one series. §§ Not all subscales are weighted equally in calculating overall SAQ score, the area of the radar plot will therefore not represent the overall SAQ score.

https://doi.org/10.1371/journal.pone.0238666.g002

Table 4. Multivariate analyses with anxiety and depression as dependent variables.

Covariate	Multivariate Analysis for Anxiety				Multivariate Analysis for Depression			
	OR	p-value	95% CI		OR	p-value	95% CI	
**Gender**								
Male	Baseline				Baseline			
Female	1.47	0.004	1.13	1.91	0.62	0.002	0.45	0.84
Undisclosed	2.12	0.025	1.10	4.08	0.76	0.462	0.36	1.58
**Country**								
UK	Baseline				Baseline			
Poland	0.63	0.032	0.41	0.96	1.13	0.657	0.66	1.92
Singapore	0.48	<0.001	0.37	0.61	1.07	0.675	0.78	1.48
**Role**								
Non-clinical	Baseline				Baseline			
Doctor	1.13	0.511	0.78	1.63	0.60	0.031	0.38	0.96
Nurse	1.09	0.579	0.81	1.47	0.77	0.164	0.54	1.11
Other clinical staff	0.64	0.023	0.43	0.94	0.87	0.543	0.55	1.37
**Redeployed**								
No	Baseline				Baseline			
Yes	1.14	0.297	0.89	1.45	1.44	0.015	1.07	1.93
**Testing Status**								
Not tested	Baseline				Baseline			
Tested	1.28	0.055	1.00	1.64	1.18	0.29	0.87	1.61
**SAQ**								
50th Percentile	Baseline				Baseline			
25th Percentile	2.19	<0.001	1.74	2.76	2.29	<0.001	1.73	3.02
75th Percentile	0.88	0.41	0.66	1.19	0.74	0.185	0.48	1.15
**Burnout**								
Low risk	Baseline				Baseline			
High risk	4.89	<0.001	3.93	6.08	4.18	<0.001	3.13	5.57
**Anxiety**								
Normal/Borderline					Baseline			
Abnormal					5.13	<0.001	3.90	6.73
**Depression**								
Normal/Borderline	Baseline							
Abnormal	5.15	<0.001	3.91	6.78				

OR: Odds ratio.

https://doi.org/10.1371/journal.pone.0238666.t004

Our results demonstrate that clinical roles confer a higher burnout risk compared with non-clinical roles. This may be explained by the nature of these roles. Particular challenges might have included adapting to a new method of working, increased service demands, prolonged periods wearing personal protective equipment, feeling "powerless" to manage patients' conditions, and a fear of becoming infected or infecting others [55]. Similar findings were seen in Toronto during the SARS epidemic, where HCWs that treated SARS patients had significantly higher levels of burnout than those that did not [6].

Staff who were redeployed to new clinical areas had a higher risk of burnout. This may be due to physical conditions such as spending prolonged periods wearing protective equipment or due to the stress of adapting to a new clinical environment. Moreover, areas that required redeployed staff, by definition, had (or anticipated having) demand in excess of resources,

necessitating the reallocation of staff. The combination of these increased demands, limited resources, and the psychological stress of dealing with an unfamiliar disease in an unfamiliar environment may have led to increased rates of burnout. This hypothesis would be supported by by the demands-resources model of burnout [7,11].

Anxiety and depression were noted in 20% and 11% of respondents respectively. Respondents with anxiety or depression were likely to also have symptoms of burnout. This is a significant burden of psychological morbidity. This finding is consistent with a recent meta-analysis of studies in China and Singapore, which demonstrated that approximately 1 in 5 HCWs have experienced symptoms of anxiety (23.2%) or depression (22.8%) during Covid-19 [18]. Similarly, rates of depression (19.8%) were also seen in Italian HCWs, although with a lower prevalence of anxiety(8%) [19]. Our study found that anxiety was more prevalent than depression amongst HCWs. To our best knowledge, there has been no published work reporting rates of depression, anxiety or burnout in the UK or Poland during Covid-19.

Female gender was predictive of anxiety (OR 1.47), which is in keeping with previous findings during Covid-19 [56,57]. However female gender was also found to be inversely correlated with depression, which contrasts from previous research [56,57]. These findings may reflect differences in the sampled population, such as the proportion of redeployed staff or be related to the timing of sampling compared with the onset of the Covid-19 pandemic.

Burnout, anxiety and depression have a negative impact on staff and patient outcomes [9,58] as well as leading to workforce attrition [59]. The high rates seen during the Covid-19 pandemic risk compounding a pre-existing healthcare workforce crisis [60,61], which may inturn impact on patient outcomes during the recovery phase of the pandemic. Initiatives shown to have a positive effect on psychological wellbeing include: clear communication, access to personal protective equipment, adequate rest, and psychological support [57,62].

An unexpected finding was the inverse relationship between staff SARS-CoV-2 testing and mental health. Two possible explanations exist: 1. provision of testing is a proxy for a well-run organisation, staff feeling well supported feel positively about working conditions and perception of management and, in turn are less likely to develop adverse mental health outcomes. 2. Staff suffering from burnout, anxiety or depression were less likely to seek out testing, possibly due to disengagement, physical or psychological symptoms [63]. Irrespective of the cause, both explanations are important as they support the need for staff testing, in particular for staff groups identified at risk of Covid-19 or poor mental wellbeing.

Safety attitudes were significantly associated with psychological outcomes in this study. It cannot be determined whether safety attitude is a contributory factor for burnout, anxiety, and depression, or if these psychological states lead to poor safety attitudes. However, this is an important finding, as safety attitudes are both modifiable and independently associated with clinical outcomes [50,64,65]. The SAQ domains can be divided into *net causes* (teamwork, working conditions, safety climate subscales) and *net effects* (perception of management, job satisfaction, stress recognition) [66]. This suggests that in addition to supporting psychological wellbeing, initiatives that promote safety climate, working conditions, and teamwork may have benefits on safety attitudes and in turn psychological outcomes.

Strengths and limitations

There are some limitations to our approach. The countries investigated are well stratified by: Covid-19 death rate, gross domestic product and geographic region (Western Europe, Eastern Europe and Asia-Pacific). However, the use of convenience sampling (a combination of social media and targeted email communications), means it is difficult to estimate response rate, response bias, and external validity. However, this study recruited a large number of

respondents in a multi-centre, international population with a diverse range of healthcare workers. The results are therefore likely to be internally valid and associations between covariates reliable. Our sample was 72% female, which is broadly in line with the demographics of the healthcare workforce in the countries studied [67,68]. There was wide variation in the number of respondents between countries and an overrepresentation of nurses in the Polish cohort, this alongside data on only a limited number of variables may have resulted in residual confounding on multivariate regression analyses.

While the OLBI has many good psychometric qualities, a clinical cut-off for when someone is considered "burned out" has been an issue of debate [69]. The cut-off values used in this study to describe prevalence are based on findings from a Swedish group as correlated with clinician-diagnosed burnout [70,71]. The same cut-off values have been adopted in multiple other studies [34–36]. Given the high prevalence of burnout in this sample, and a lack of universally agreed cut-offs when using the OLBI, to improve specificity we used the 75th percentile of burnout scores rather than (lower) cut-offs values for the purpose of regression analyses.

While investigating the prevalence of psychological findings during Covid-19 is important, it is unclear if findings are as a direct result of Covid-19. It is also unclear if acute derangements persist over time. Repeated measurements will be needed to identify any potential long-term effects of the Covid-19 pandemic.

Finally, the contextual differences between participating countries, may limit the extent to which direct comparisons can be drawn between study countries.

Future recommendations

Based on these findings, we outline several preliminary recommendations that may positively impact on the psychological health of HCWs and patient safety.

As highlighted by the association between safety attitudes and psychological outcomes, institutions should pay particular attention to safety culture during the Covid-19 pandemic. The use of patient safety teams, for example, can support the integration of human factors principles, such as effective communication, into organizational processes that will improve patient and staff safety [72]. The use of such teams during a time of organisational change can help "design, adapt and reconfigure work systems, maximize individual and team performance under high-risk, high-stakes environments, while minimizing the introduction of new significant safety risks or unintended consequences into the work system". Similarly, institutions should boost and expand learning systems to capture risks and improvement opportunities, and leverage these to protect staff and patients. This is of particular importance given the limited evidence about the effects of Covid-19 on patients, staff and institutions [73].

Previous research has suggested that burnout is a precursor to depression [74], consequently, benefit may been seen from interventions to address burnout before the onset of depression or anxiety. At the individual level, evidence-based interventions include mindfulness, self-awareness exercises, and appreciative interviews [75]. At the organizational level, quality improvement projects that improve organizational communication and streamline workflows can reduce burnout rates [76].

Measures to mitigate harm arising from psychological distress following the Covid-19 pandemic are important to prevent long-term harm. Greenburg et al [77] proposed six evidence-based principles to support the mental health of HCWs following the Covid-19 pandemic: appropriate appreciation, investigating absences (for welfare reasons), conducting return to work interview, paying close attention to at-risk groups, continually monitoring staff, and helping HCWs make sense of their experience.

Further research is needed to investigate the mechanisms underlying the associations identified in this study. Interrupted time series or longitudinal studies would be appropriate to investigate chronicity of findings and generate hypotheses about causality. Alongside this, interventions to reduce psychological harm or improve safety culture should be evaluated for efficacy, with case control or ideally, randomized-controlled designs.

Conclusion

Our findings demonstrate a significant burden of burnout, anxiety, and depression amongst healthcare workers. A strong association was seen between SARS-CoV-2 testing, safety attitudes, gender, job role, redeployment and psychological state. These findings highlight the importance of targeted support services and proactive SARS-CoV-2 testing of healthcare workers.

Supporting information

S1 File. Questionnaires and SAQ scoring. A: English COVID-19 Questionnaire. B: Polish COVID-19 Questionnaire. C: SAQ Scoring.
(DOCX)

S2 File. Anonymised dataset.
(CSV)

Acknowledgments

We would like to thank the Pansurg collaborative for support and infrastructure supporting this project. We would like to thank Patient Safety Watch for support and advice relating to safety attitudes and patient safety in this paper.

Author Contributions

Conceptualization: Alasdair Scott, Guy Martin, Jonathan Clarke, Viknesh Sounderajah, Sheraz Markar, Jasmine Winter Beatty, Sam Mason, Swathikan Chidambaram, Seema Yalamanchili, Jaroslaw Fedorowski, Mary Wells, Sanjay Purkayastha.

Data curation: Max Denning, Ee Teng Goh, Benjamin Tan, Abhiram Kanneganti, Melanie Almonte, Jan Przybylowicz, Ching-Hui Sia, Ying Xian Chua, Kang Sim, Lucas Lim, Lifeng Tan, Melanie Tan, Vijay Sharma, Shirley Ooi, Gabriela Zbikowska, Jaroslaw Fedorowski, Grazyna Dykowska.

Formal analysis: Max Denning, Ee Teng Goh, Melanie Almonte, Jan Przybylowicz, Yiong Huak Chan.

Investigation: Max Denning, Ee Teng Goh.

Methodology: Max Denning, Ee Teng Goh, Mary Wells.

Project administration: Max Denning, Ee Teng Goh, Benjamin Tan, Abhiram Kanneganti, Viknesh Sounderajah, Swathikan Chidambaram.

Supervision: Alasdair Scott, Guy Martin, Jonathan Clarke, Sheraz Markar, Kelsey Flott, Mary Wells, Sanjay Purkayastha, James Kinross.

Writing – original draft: Max Denning, Ee Teng Goh, Benjamin Tan, Abhiram Kanneganti.

Writing – review & editing: Max Denning, Ee Teng Goh, Benjamin Tan, Abhiram Kanneganti, Alasdair Scott, Guy Martin, Jonathan Clarke, Viknesh Sounderajah, Sheraz Markar, Jan Przybylowicz, Yiong Huak Chan, Ching-Hui Sia, Ying Xian Chua, Kang Sim, Lucas Lim, Lifeng Tan, Melanie Tan, Vijay Sharma, Shirley Ooi, Jasmine Winter Beatty, Kelsey Flott, Sam Mason, Swathikan Chidambaram, Seema Yalamanchili, Gabriela Zbikowska, Jaroslaw Fedorowski, Grazyna Dykowska, Mary Wells, Sanjay Purkayastha, James Kinross.

References

1. Williams RD, Brundage JA, Williams EB. Moral Injury in Times of COVID-19. *J Health Serv Psychol* 2020:1–5. https://doi.org/10.1007/s42843-020-00011-4 PMID: 32363349
2. Alderwick H, Dunn P, Dixon J. England's health policy response to covid-19. *Bmj* 2020; 369:m1937. https://doi.org/10.1136/bmj.m1937 PMID: 32414770
3. McGuire AL, Aulisio MP, Davis FD, Erwin C, Harter TD, Jagsi R, et al. Ethical Challenges Arising in the COVID-19 Pandemic: An Overview from the Association of Bioethics Program Directors (ABPD) Task Force. *The American journal of bioethics*: *AJOB* 2020; 20(7):15–27. https://doi.org/10.1080/15265161.2020.1764138 PMID: 32511078
4. Mattei A, Fiasca F, Mazzei M, Abbossida V, Bianchini V. Burnout among healthcare workers at L'Aquila: its prevalence and associated factors. *Psychology*, *health & medicine* 2017; 22(10):1262–70. https://doi.org/10.1080/13548506.2017.1327667 PMID: 28503931
5. Chemali Z, Ezzeddine FL, Gelaye B, Dossett ML, Salameh J, Bizri M, et al. Burnout among healthcare providers in the complex environment of the Middle East: a systematic review. *BMC public health* 2019; 19(1):1337. https://doi.org/10.1186/s12889-019-7713-1 PMID: 31640650
6. Maunder RG, Lancee WJ, Balderson KE, Bennett JP, Borgundvaag B, Evans S, et al. Long-term psychological and occupational effects of providing hospital healthcare during SARS outbreak. *Emerging infectious diseases* 2006; 12(12):1924–32. https://doi.org/10.3201/eid1212.060584 PMID: 17326946
7. Maslach C, Jackson SE. The measurement of experienced burnout. *Journal of Organizational Behavior* 1981; 2(2):99–113.
8. Hall LH, Johnson J, Watt I, Tsipa A, O'Connor DB. Healthcare Staff Wellbeing, Burnout, and Patient Safety: A Systematic Review. *PloS one* 2016; 11(7):e0159015. https://doi.org/10.1371/journal.pone.0159015 PMID: 27391946
9. Khoshakhlagh AH, Khatooni E, Akbarzadeh I, Yazdanirad S, Sheidaei A. Analysis of affecting factors on patient safety culture in public and private hospitals in Iran. *BMC health services research* 2019; 19(1):1009. https://doi.org/10.1186/s12913-019-4863-x PMID: 31888622
10. Lyndon A. Burnout Among Healthcare Professionals and Its Effect on Patient Safety Patient Safety Network: Agency for Healthcare Research and Quality; 2015 [cited 2020 2 June]. https://psnet.ahrq.gov/perspective/burnout-among-health-professionals-and-its-effect-patient-safety#:~:text=Burnout%20is%20viewed%20as%20a,delivered%20substandard%20care%20at%20work.
11. Demerouti E, Bakker AB, Nachreiner F, Schaufeli WB. The job demands-resources model of burnout. *The Journal of applied psychology* 2001; 86(3):499–512. PMID: 11419809
12. Tam CWC, Pang EPF, Lam LCW, Chiu HFK. Severe acute respiratory syndrome (SARS) in Hong Kong in 2003: stress and psychological impact among frontline healthcare workers. *Psychological Medicine* 2004; 34(7):1197–204. https://doi.org/10.1017/s0033291704002247 PMID: 15697046
13. Grace SL, Hershenfield K, Robertson E, Stewart DE. The occupational and psychosocial impact of SARS on academic physicians in three affected hospitals. *Psychosomatics* 2005; 46(5):385–91. https://doi.org/10.1176/appi.psy.46.5.385 PMID: 16145182
14. McAlonan GM, Lee AM, Cheung V, Cheung C, Tsang KW, Sham PC, et al. Immediate and Sustained Psychological Impact of an Emerging Infectious Disease Outbreak on Health Care Workers. *The Canadian Journal of Psychiatry* 2007; 52(4):241–7. https://doi.org/10.1177/070674370705200406 PMID: 17500305
15. Liu X, Kakade M, Fuller CJ, Fan B, Fang Y, Kong J, et al. Depression after exposure to stressful events: lessons learned from the severe acute respiratory syndrome epidemic. *Comprehensive psychiatry* 2012; 53(1):15–23. https://doi.org/10.1016/j.comppsych.2011.02.003 PMID: 21489421
16. Ji D, Ji YJ, Duan XZ, Li WG, Sun ZQ, Song XA, et al. Prevalence of psychological symptoms among Ebola survivors and healthcare workers during the 2014–2015 Ebola outbreak in Sierra Leone: a cross-sectional study. *Oncotarget* 2017; 8(8):12784–91. https://doi.org/10.18632/oncotarget.14498 PMID: 28061463

17. Goulia P, Mantas C, Dimitroula D, Mantis D, Hyphantis T. General hospital staff worries, perceived sufficiency of information and associated psychological distress during the A/H1N1 influenza pandemic. *BMC infectious diseases* 2010; 10:322. https://doi.org/10.1186/1471-2334-10-322 PMID: 21062471

18. Pappa S, Ntella V, Giannakas T, Giannakoulis VG, Papoutsi E, Katsaounou P. Prevalence of depression, anxiety, and insomnia among healthcare workers during the COVID-19 pandemic: A systematic review and meta-analysis. *Brain*, behavior, and immunity 2020.

19. Rossi R, Socci V, Pacitti F, Di Lorenzo G, Di Marco A, Siracusano A, et al. Mental Health Outcomes Among Frontline and Second-Line Health Care Workers During the Coronavirus Disease 2019 (COVID-19) Pandemic in Italy. *JAMA Netw Open* 2020; 3(5):e2010185. https://doi.org/10.1001/jamanetworkopen.2020.10185 PMID: 32463467

20. Tan BYQ, Chew NWS, Lee GKH, Jing M, Goh Y, Yeo LLL, et al. Psychological Impact of the COVID-19 Pandemic on Health Care Workers in Singapore. *Annals of internal medicine* 2020.

21. Central Intelligence Agency. The World Factbook Washington D.C.: Central Intelligence Agency; 2020 [cited 2020 11 July]. https://www.cia.gov/library/publications/the-world-factbook/geos/uk.html.

22. The World Bank Group. GDP per capita— Singapore, Poland, United Kingdom Washington D.C.: The World Bank Group; 2020 [cited 2020 11 July]. https://data.worldbank.org/indicator/NY.GDP.PCAP.CD?locations=SG-PL-GB.

23. The World Bank Group. Current Health Expenditure— Poland, Singapore, United Kingdom Washington D.C.: The World Bank Group; 2020 [cited 2020 11 July]. https://data.worldbank.org/indicator/SH.XPD.CHEX.GD.ZS?locations=PL-SG-GB.

24. The World Bank Group. Physicians (per 1,000 people)— Poland, Singapore, United Kingdom Washington D.C.: The World Bank Group; 2021 [cited 2021 5 January]. https://data.worldbank.org/indicator/SH.MED.PHYS.ZS?locations=GB-PL-SG&name_desc=false.

25. The World Bank Group. Nurses and midwives (per 1,000 people)— Poland, Singapore, United Kingdom Washington D.C.: The World Bank Group; 2021 [cited 2021 5 January]. https://data.worldbank.org/indicator/SH.MED.NUMW.P3?locations=GB-PL-SG&name_desc=false.

26. Oxford Martin Programme on Global Development. Our World in Data Statistics and Research: Coronavirus Pandemic (COVID-19) England: Global Change Data Lab; 2020 [updated 11 July 2020; cited 2020 11 July]. https://ourworldindata.org/coronavirus.

27. Asher MG, Nandy A. Health Financing in Singapore: A Case for Systemic Reforms. *International Social Security Review* 2006; 59(1):75–92.

28. Ong SE, Tyagi S, Lim JM, Chia KS, Legido-Quigley H. Health systems reforms in Singapore: A qualitative study of key stakeholders. *Health Policy* 2018; 122(4):431–43. https://doi.org/10.1016/j.healthpol.2018.02.005 PMID: 29478876

29. Koh D. COVID-19 lockdowns throughout the world. *Occupational Medicine* 2020.

30. Hale T, Angrist N, Cameron-Blake E, Hallas L, Kira B, Majumdar S, et al. Oxford COVID-19 Government Response Tracker: Blavatnik School of Government; 2020 [cited 2021 8 February]. https://www.bsg.ox.ac.uk/research/research-projects/coronavirus-government-response-tracker.

31. Demerouti E. The Oldenburg Burnout Inventory: A good alternative to measure burnout and engagement. *Handbook of Stress and Burnout in Health Care* 2008.

32. Demerouti E, Bakker AB, Vardakou I, Kantas A. The convergent validity of two burnout instruments: A multitrait-multimethod analysis. *European Journal of Psychological Assessment* 2003; 19(1):12–23.

33. Bhugra D, Sauerteig SO, Bland D, Lloyd-Kendall A, Wijesuriya J, Singh G, et al. A descriptive study of mental health and wellbeing of doctors and medical students in the UK. *International review of psychiatry (Abingdon, England)* 2019; 31(7–8):563–8. https://doi.org/10.1080/09540261.2019.1648621 PMID: 31456450

34. Farrell SM, Kadhum M, Lewis T, Singh G, Penzenstadler L, Molodynski A. Wellbeing and burnout amongst medical students in England. *International review of psychiatry (Abingdon, England)* 2019; 31(7–8):579–83. https://doi.org/10.1080/09540261.2019.1675960 PMID: 31692396

35. Farrell SM, Kar A, Valsraj K, Mukherjee S, Kunheri B, Molodynski A, et al. Wellbeing and burnout in medical students in India; a large scale survey. *International review of psychiatry (Abingdon, England)* 2019; 31(7–8):555–62.

36. Westwood S, Morison L, Allt J, Holmes N. Predictors of emotional exhaustion, disengagement and burnout among improving access to psychological therapies (IAPT) practitioners. *Journal of mental health (Abingdon, England)* 2017; 26(2):172–9. https://doi.org/10.1080/09638237.2016.1276540 PMID: 28084121

37. Zigmond AS, Snaith RP. The hospital anxiety and depression scale. *Acta psychiatrica Scandinavica* 1983; 67(6):361–70. https://doi.org/10.1111/j.1600-0447.1983.tb09716.x PMID: 6880820

38. Eysenck MW, Fajkowska M. Anxiety and depression: toward overlapping and distinctive features. *Cognition and Emotion* 2018; 32(7):1391–400. https://doi.org/10.1080/02699931.2017.1330255 PMID: 28608767
39. Caci H, Baylé FJ, Mattei V, Dossios C, Robert P, Boyer P. How does the Hospital and Anxiety and Depression Scale measure anxiety and depression in healthy subjects? *Psychiatry research* 2003; 118 (1):89–99. https://doi.org/10.1016/s0165-1781(03)00044-1 PMID: 12759165
40. Herrero MJ, Blanch J, Peri JM, De Pablo J, Pintor L, Bulbena A. A validation study of the hospital anxiety and depression scale (HADS) in a Spanish population. *General hospital psychiatry* 2003; 25(4):277–83. https://doi.org/10.1016/s0163-8343(03)00043-4 PMID: 12850660
41. Yamamoto-Furusho JK, Sarmiento-Aguilar A, García-Alanis M, Gómez-García LE, Toledo-Mauriño J, Olivares-Guzmán L, et al. Hospital Anxiety and Depression Scale (HADS): Validation in Mexican Patients with Inflammatory Bowel Disease. *Gastroenterologia y hepatologia* 2018; 41(8):477–82. https://doi.org/10.1016/j.gastrohep.2018.05.009 PMID: 29937084
42. Herrmann C. International experiences with the Hospital Anxiety and Depression Scale—a review of validation data and clinical results. *Journal of psychosomatic research* 1997; 42(1):17–41. https://doi.org/10.1016/s0022-3999(96)00216-4 PMID: 9055211
43. Wu KK, Chan SK, Ma TM. Posttraumatic stress, anxiety, and depression in survivors of severe acute respiratory syndrome (SARS). *J Trauma Stress* 2005; 18(1):39–42. https://doi.org/10.1002/jts.20004 PMID: 16281194
44. Mak IW, Chu CM, Pan PC, Yiu MG, Chan VL. Long-term psychiatric morbidities among SARS survivors. *General hospital psychiatry* 2009; 31(4):318–26. https://doi.org/10.1016/j.genhosppsych.2009.03.001 PMID: 19555791
45. Nordén-Hägg A, Sexton JB, Kälvemark-Sporrong S, Ring L, Kettis-Lindblad Å. Assessing safety culture in pharmacies: the psychometric validation of the Safety Attitudes Questionnaire (SAQ) in a national sample of community pharmacies in Sweden. *BMC clinical pharmacology* 2010; 10:8. https://doi.org/10.1186/1472-6904-10-8 PMID: 20380741
46. Deilkås ET, Hofoss D. Psychometric properties of the Norwegian version of the Safety Attitudes Questionnaire (SAQ), Generic version (Short Form 2006). *BMC health services research* 2008; 8:191. https://doi.org/10.1186/1472-6963-8-191 PMID: 18808693
47. Kaya S, Barsbay S, Karabulut E. The Turkish version of the safety attitudes questionnaire: psychometric properties and baseline data. *Quality & safety in health care* 2010; 19(6):572–7. https://doi.org/10.1136/qshc.2008.032003 PMID: 20671082
48. Nguyen G, Gambashidze N, Ilyas SA, Pascu D. Validation of the safety attitudes questionnaire (short form 2006) in Italian in hospitals in the northeast of Italy. *BMC health services research* 2015; 15:284. https://doi.org/10.1186/s12913-015-0951-8 PMID: 26204957
49. Lee WC, Wung HY, Liao HH, Lo CM, Chang FL, Wang PC, et al. Hospital safety culture in Taiwan: a nationwide survey using Chinese version Safety Attitude Questionnaire. *BMC health services research* 2010; 10:234. https://doi.org/10.1186/1472-6963-10-234 PMID: 20698965
50. Sexton JB, Helmreich RL, Neilands TB, Rowan K, Vella K, Boyden J, et al. The Safety Attitudes Questionnaire: psychometric properties, benchmarking data, and emerging research. *BMC health services research* 2006; 6:44. https://doi.org/10.1186/1472-6963-6-44 PMID: 16584553
51. McKinley N, McCain RS, Convie L, Clarke M, Dempster M, Campbell WJ, et al. Resilience, burnout and coping mechanisms in UK doctors: a cross-sectional study. *BMJ Open* 2020; 10(1):e031765. https://doi.org/10.1136/bmjopen-2019-031765 PMID: 31988223
52. Heinen MM, van Achterberg T, Schwendimann R, Zander B, Matthews A, Kózka M, et al. Nurses' intention to leave their profession: A cross sectional observational study in 10 European countries. *International Journal of Nursing Studies* 2013; 50(2):174–84. https://doi.org/10.1016/j.ijnurstu.2012.09.019 PMID: 23107005
53. Tay WY, Earnest A, Tan SY, Ng MJM. Prevalence of Burnout among Nurses in a Community Hospital in Singapore: A Cross-Sectional Study. *Proceedings of Singapore Healthcare* 2014; 23(2):93–9.
54. Low ZX, Yeo KA, Sharma VK, Leung GK, McIntyre RS, Guerrero A, et al. Prevalence of Burnout in Medical and Surgical Residents: A Meta-Analysis. *International journal of environmental research and public health* 2019; 16(9):1479. https://doi.org/10.3390/ijerph16091479 PMID: 31027333
55. Liu CY, Yang YZ, Zhang XM, Xu X, Dou QL, Zhang WW, et al. The prevalence and influencing factors in anxiety in medical workers fighting COVID-19 in China: a cross-sectional survey. *Epidemiology and infection* 2020; 148:e98. https://doi.org/10.1017/S0950268820001107 PMID: 32430088
56. Lai J, Ma S, Wang Y, Cai Z, Hu J, Wei N, et al. Factors Associated With Mental Health Outcomes Among Health Care Workers Exposed to Coronavirus Disease 2019. *JAMA Network Open* 2020; 3(3): e203976–e. https://doi.org/10.1001/jamanetworkopen.2020.3976 PMID: 32202646

57. Kisely S, Warren N, McMahon L, Dalais C, Henry I, Siskind D. Occurrence, prevention, and management of the psychological effects of emerging virus outbreaks on healthcare workers: rapid review and meta-analysis. *Bmj* 2020; 369:m1642. https://doi.org/10.1136/bmj.m1642 PMID: 32371466

58. Weaver MD, Vetter C, Rajaratnam SMW, O'Brien CS, Qadri S, Benca RM, et al. Sleep disorders, depression and anxiety are associated with adverse safety outcomes in healthcare workers: A prospective cohort study. *Journal of sleep research* 2018; 27(6):e12722. https://doi.org/10.1111/jsr.12722 PMID: 30069960

59. Shanafelt T, Sloan J, Satele D, Balch C. Why do surgeons consider leaving practice? *Journal of the American College of Surgeons* 2011; 212(3):421–2. https://doi.org/10.1016/j.jamcollsurg.2010.11.006 PMID: 21356491

60. Britnell M. Human: Solving the global workforce crisis in health care—Nuffield Trust guest comment London: Nuffield Trust; 2019 [cited 2020 11 July]. https://www.nuffieldtrust.org.uk/news-item/human-solving-the-global-workforce-crisis-in-health-care.

61. Buchan J, Gershlick B, Charlesworth A, Seccombe I. Falling short: the NHS workforce challenge. The Health Foundation, 2019.

62. Wu AW, Connors C, Everly GS Jr., COVID-19: Peer Support and Crisis Communication Strategies to Promote Institutional Resilience. *Annals of internal medicine* 2020.

63. Maneze D, Salamonson Y, Poudel C, DiGiacomo M, Everett B, Davidson PM. Health-Seeking Behaviors of Filipino Migrants in Australia: The Influence of Persisting Acculturative Stress and Depression. *Journal of immigrant and minority health* 2016; 18(4):779–86. https://doi.org/10.1007/s10903-015-0233-x PMID: 26050239

64. Pronovost P, Sexton B. Assessing safety culture: guidelines and recommendations. *Quality and Safety in Health Care* 2005; 14(4):231–3. https://doi.org/10.1136/qshc.2005.015180 PMID: 16076784

65. Havyer RD, Wingo MT, Comfere NI, Nelson DR, Halvorsen AJ, McDonald FS, et al. Teamwork assessment in internal medicine: a systematic review of validity evidence and outcomes. *Journal of general internal medicine* 2014; 29(6):894–910. https://doi.org/10.1007/s11606-013-2686-8 PMID: 24327309

66. Lee YC, Zeng PS, Huang CH, Wu HH. Causal Relationship Analysis of the Patient Safety Culture Based on Safety Attitudes Questionnaire in Taiwan. *Journal of healthcare engineering* 2018; 2018:4268781. https://doi.org/10.1155/2018/4268781 PMID: 29686825

67. Ministry of Health Singapore. Healthcare Workforce Statistic Singapore: Ministry of Health Singapore; 2019 [updated 23 December 2019. https://www.healthhub.sg/a-z/health-statistics/12/health-manpower.

68. NHS Digital. Narrowing of NHS gender divide but men still the majority in senior roles Leeds: NHS Digital; 2018 [updated 29 June 2018; cited 2020 11 June]. https://digital.nhs.uk/news-and-events/latest-news/narrowing-of-nhs-gender-divide-but-men-still-the-majority-in-senior-roles.

69. Halbesleben JRB, Demerouti E. The construct validity of an alternative measure of burnout: Investigating the English translation of the Oldenburg Burnout Inventory. *Work & Stress*: *The conceptualisation and measurement of burnout* 2005; 19(3):208–20.

70. Peterson U, Demerouti E, Bergström G, Samuelsson M, Asberg M, Nygren A. Burnout and physical and mental health among Swedish healthcare workers. *Journal of advanced nursing* 2008; 62(1):84–95. https://doi.org/10.1111/j.1365-2648.2007.04580.x PMID: 18352967

71. Schaufeli WB, Bakker AB, Hoogduin K, Schaap C, Kladler A. on the clinical validity of the maslach burnout inventory and the burnout measure. *Psychology & health* 2001; 16(5):565–82. https://doi.org/10.1080/08870440108405527 PMID: 22804499

72. Carayon P, Wetterneck TB, Rivera-Rodriguez AJ, Hundt AS, Hoonakker P, Holden R, et al. Human factors systems approach to healthcare quality and patient safety. *Applied Ergonomics* 2014; 45(1):14–25. https://doi.org/10.1016/j.apergo.2013.04.023 PMID: 23845724

73. Staines A, Amalberti R, Berwick DM, Braithwaite J, Lachman P, Vincent CA. COVID-19: Patient Safety and Quality Improvement Skills to Deploy during the Surge. *International journal for quality in health care*: *journal of the International Society for Quality in Health Care* 2020:mzaa050.

74. Fahrenkopf AM, Sectish TC, Barger LK, Sharek PJ, Lewin D, Chiang VW, et al. Rates of medication errors among depressed and burnt out residents: prospective cohort study. *Bmj* 2008; 336(7642):488–91. https://doi.org/10.1136/bmj.39469.763218.BE PMID: 18258931

75. Krasner MS, Epstein RM, Beckman H, Suchman AL, Chapman B, Mooney CJ, et al. Association of an educational program in mindful communication with burnout, empathy, and attitudes among primary care physicians. *Jama* 2009; 302(12):1284–93. https://doi.org/10.1001/jama.2009.1384 PMID: 19773563

76. Linzer M, Poplau S, Grossman E, Varkey A, Yale S, Williams E, et al. A Cluster Randomized Trial of Interventions to Improve Work Conditions and Clinician Burnout in Primary Care: Results from the

Healthy Work Place (HWP) Study. *Journal of general internal medicine* 2015; 30(8):1105–11. https://doi.org/10.1007/s11606-015-3235-4 PMID: 25724571

77. Greenberg N, Docherty M, Gnanapragasam S, Wessely S. Managing mental health challenges faced by healthcare workers during covid-19 pandemic. *BMJ* 2020; 368:m1211. https://doi.org/10.1136/bmj.m1211 PMID: 32217624
