# Supplementary material for: Mental health status and related factors influencing healthcare workers during the COVID-19 pandemic: A systematic review and meta-analysis
Source: PLoS One. 2024 Jan 19;19(1):e0289454. doi: 10.1371/journal.pone.0289454 (PMC10798549; doi:10.1371/journal.pone.0289454)
Supplement: S1 Data — (ZIP) [file pone.0289454.s011.zip › literatures/45.pdf]

Since January 2020 Elsevier has created a COVID-19 resource centre with free information in English and Mandarin on the novel coronavirus COVID-19. The COVID-19 resource centre is hosted on Elsevier Connect, the company's public news and information website.

Elsevier hereby grants permission to make all its COVID-19-related research that is available on the COVID-19 resource centre - including this research content - immediately available in PubMed Central and other publicly funded repositories, such as the WHO COVID database with rights for unrestricted research re-use and analyses in any form or by any means with acknowledgement of the original source. These permissions are granted for free by Elsevier for as long as the COVID-19 resource centre remains active.

Journal of Affective Disorders 278 (2021) 390–394

Contents lists available at ScienceDirect

Journal of Affective Disorders

journal homepage: www.elsevier.com/locate/jad

Short communication

Short-term emotional impact of COVID-19 pandemic on Spaniard health workers

Rodriguez-Menéndez Gonzalo[a,1], Rubio-García Ana[a,1], Conde-Alvarez Patricia[a], Armesto-Luque Laura[a], Garrido-Torres Nathalia[a], Capitan Luis[a], Luque Asuncion[a], Ruiz-Veguilla Miguel[a,b,c,d,2], Crespo-Facorro Benedicto[a,b,c,d,2,*]

[a] UGC Salud Mental, Hospital Universitario Virgen del Rocio, Sevilla, Spain
[b] Centro Investigacion Biomedica en Red de Salud Mental (CIBERSAM), Spain
[c] Instituto de Biomedicina de Sevilla (IBIS), Sevilla, Spain
[d] Departamento de Psiquiatria, Universidad de Sevilla, Sevilla, Spain

ARTICLE INFO

Keywords:
COVID-19
Health care workers
Acute stress symptoms
Organizational factors

ABSTRACT

Background: The aims of this study were to evaluate the short-term impact of 2019-nCoV outbreak on the mental/psychological state of Spaniard health care workers (HCWs) and to explore the influencing factors, including organizational factors.
Methods: : A web-based survey (Google forms questionnaire) spread via professional and scientific associations, professional WhatsApp and email lists, following a snowball technique was used. Data were collected from May 11th and May 31st, 2020
Results: : A total of 1407 subjects were included in final analyses. 24.7% (348 out of 1407) of HCWs reported symptoms of acute stress (SARS-Q measurement) and 53.6% (754 out of 1407) reported symptoms related to poorer general health (GHQ-28 measurement). A higher risk of having an acute stress disorder was associated to being female, not having access to protective material, and several subjects´ perceived risks. Additionally, poorer overall general health (GHQ > 24) was related to being female, working in a geographical area with a high incidence of infection, not being listened to by your co-workers, having a greater perception of stress at work and being able to transmit the infection to others.
Limitations: : We must consider a likely memory bias.
Conclusion: : The high prevalence of affective and general health symptoms among the HCWs and the critical influence of organizational issues and subjects´ perceived risk should lead health authorities to design future strategies to protect health professional force for facing a potential upcoming epidemiological crisis.

1. Introduction

Physical safety and psychological well-being and are inherently interrelated (Li et al., 2020). Overall health care workers (HCWs) aiding in COVID-19 control are especially vulnerable to substantial negative mental/psychological health outcomes, including stress-related symptoms, symptoms of depression, anxiety, and insomnia (Lai et al., 2020). Previous outbreak of severe acute respiratory syndrome (SARS) revealed frontline HCWs experienced high psychological distress during disease crisis (Tam et al., 2004). The degree of contact with confirmed or suspected cases and access to psychological materials/ resources prompt to a higher severity of psychological distress among HCWs (Kang et al., 2020).

Staff who were female, nurse, aged 31–50 years, frontline HCWs, having unsatisfactory social support, and insufficient information and protection were more vulnerable to experiencing adverse mental health outcomes in COVID19 crisis (Lai et al., 2020; Rossi et al., 2020; García-Fernández et al., 2020). According to a recent review and meta-analysis about the psychological reactions of the healthcare staff in previous outbreaks such as SARS, MERS, H1N1, H7N9, Ebola, wider

* Corresponding author: Benedicto Crespo-Facorro, Director de Unidad Gestión Clínica Salud Mental, Hospital Universitario Virgen del Rocío, *Av.* Manuel Siurot, s/n. 41013 Sevilla, Spain, Tel.: +34 955 013 491.
E-mail address: benedicto.crespo.sspa@juntadeandalucia.es (C.-F. Benedicto).
[1] Rodriguez-Menéndez and Rubio-García should be considered **joint first author**.
[2] Ruiz-Veguilla and Crespo-Facorro should be considered **joint senior author**.

https://doi.org/10.1016/j.jad.2020.09.079
Received 26 June 2020; Received in revised form 26 August 2020; Accepted 18 September 2020
Available online 24 September 2020
0165-0327/ © 2020 Elsevier B.V. All rights reserved.

personal, service, organizational and societal factors impaired psychological outcomes (Kisely et al., 2020). Our study attempts to evaluate the short-term impact of 2019-nCoV outbreak on the mental/psychological state of Spaniard health workers and to explore the influencing factors.

2. Method

2.1. Study design and sampling

This is a cross-sectional, web-based survey (Google forms questionnaire) spread via professional and scientific associations, professional WhatsApp and email lists, following a snowball technique. Data were collected from May 11th and May 31st, 2020. No limit of participants was established. The sampling period was initiated barely two months after the COVID-19 outbreak began in Spain, in order to better evaluate the impact of pandemic in health care workers.

Approval for this study was obtained from the Ethical Committee at University Hospital Virgen del Rocio (University of Sevilla). Prior to completing the questionnaire, participants read detailed information about the study and online informed consent form was signed up.

This study aimed to assess health care workers (HCWs) in Spain (across the entire territory) who had been involved, directly or indirectly, in the care of COVID-19 patients. All adult HCWs reporting they work in Spain were eligible. Only participants who met these criteria were eligible.

Because of the self-selected and non-probabilistic nature of the sample, invitations and response rates could not be quantifiable, as reported by American Association for Public Opinion Research (AAPOR) reporting guideline.

Participants were kindly asked to provide a way of contact (email address or phone number) for a feasible follow-up study to assess medium-term changes in clinical outcome.

2.2. Instruments

The on-line questionnaire comprised of different sections: a) Sociodemographic variables: gender, age, marital status; b) Working position: frontline vs second-line; c) Occupation: Resident, physician, nurse, others, health care assistant (HCA), General practitioner (GP); d) High COVID19-incidence vs. Low COVID19-incidence geographical area (province) based on statistics from Spanish Ministry of Health (Ministerio de Sanidad, 2020); e) 9 questions about perception of COVID-19 related risk (Wun et al., 2009) (yes /no) (Supplementary material)". f) action related with COVID-19: 1.- PCR performed; no, yes with negative results) and yes with positive results) and 2.- having been quarantined or isolated on suspicion of infection (yes/no); g) 10 questions about organizational factors designed ad hoc (Shanafelt et al., 2020) (Supplementary Material).

Two standardized measurement scales for mental and general health symptoms were also included in the on-line questionnaire: 1.- General health was assessed using General Health Questionnaire (GHQ-28) (Goldberg et al., 1979) is a self-administered screening scale which assesses 4 subscales in 28 items: a) somatic symptoms, b) anxiety and insomnia, c) social dysfunction, d) severe depression. The items are scored in a Likert-type scale (0–3) and yield a total score. Cutoff is set up at 24 (Hjelle et al., 2019); and 2.- Acute Stress disorder (ASD) diagnosis was made based on Standford acute Stress Reaction Questionnaire (Cardeña et al., 2000): 30 items distributed on 5 subscales (dissociation, hyperarousal, reexperiencing the traumatic event, avoidance of reminders of the traumatic event and impact on social functioning). The values are collected using a Likert-type scale (0–5) and yield a sum scores range between 0 and 150. Cronbach's alpha values range between 0.80 - 0.95 across different studies and populations. Diagnose is made according to DSM-IV-TR (American Psychiatric Association, 2013) criteria: a previous traumatic event summed to positive scores in a sum of symptoms. Symptoms are present with answers in the Likert scale of 3 or more. To make the diagnose positive answers are required for: 3 of 10 items in dissociative subscale, 1 of 6 in the second, third and fourth subscales, 1 of 2 in the subscale of impact on functioning (Cardeña et al., 2000; Casacchia et al., 2013).

2.3. Statistical analysis

We conducted statistical analyses using SPSS, version 20.0 (IBM Corp., 2011). The Kolmogorov–Smirnov test examined the normality of variables. The original scores of the 2 dependent measurements (GHQ-28 and SARS-Q) were not normally distributed and so are presented as medians with interquartile ranges (IQRs). Binary logistic regression models were constructed to determine potential risk factor associated with diagnosis of Acute Stress Disorder (based on SARS-Q test) and GHQ score >24. Statistical significance was set at $P < .05$, and all tests were 2-tailed. The association between risk factors and outcome are presented as odds ratio (OR) and 95% CIs. In this model independent variables and covariate are entered all at the same time. In this sense, every variable is controlled for all others.

3. Results

A total of 1458 HCWs completed the questionnaire. Fifty-one questionnaires were excluded from analysis because duplicated data, and therefore the responses of 1407 HCWs were analyzed. The response rate could not be calculated. Overall sample characteristics are reported in Table 1 24.7% (348 out of 1407) of HCWs reported symptoms of acute stress (SARS-Q measurement) and 53.6% (754 out of 1407) reported symptoms related to poorer general health (GHQ-28 measurement) (Table 1).

23.9% of HWCs residing in low-incidence areas and 27.9% residing in high-incidence areas endorsed an acute stress disorder (p 0.150). A statistically significant association was found in the subscales of dissociation, re-experiencing and increased arousal symptoms, as well as in the impact on social functioning, but not so in the subscale of avoidance symptoms (Table 1). With regard to general health, 49.8% of professionals who live in low-incidence areas and 67.4% who live in high-incidence areas reported worsening general health (p 0.001) (Table 1).

The prediction analyses showed a higher risk of suffering a acute stress disorder was associated to being female (OR, 2.9; p 0.0001), not having access to protective material (OR, 1.8; p 0.004), feeling more stress at work (OR, 4.5; p 0.0001), having the perception that the work was putting the person in danger (OR, 0.64; p 0.045), person's own concern about getting sick (OR, 1.9; p 0.001), thinking on family's concern about being infected by the HCW (OR, 1.7; p 0.003) and the fear that people will avoid my family because of my work (OR, 1.5; p 0.03) (Table 2)

In addition, female (OR, 3.3; p 0.0001), working in a geographical area with a high incidence of infection (OR, 1.8; p 0.003), not being listened to by your co-workers (OR, 13.9; p 0.02), having a greater perception of stress at work (OR, 5.6; p 0.0001), being able to transmit the infection to others (OR, 1.6; p 0.04) were linked to having a poorer overall general health (Table 2).

4. Discussion and conclusions

Our results revealed that a quarter of the HCWs surveyed met criteria for acute stress disorder, and slightly more than half of the respondents reported symptoms related to a poor general health. The high prevalence of affective and general health symptoms among the HCWs, especially in high incidence areas, during the pandemic should lead health authorities to design future policies of prevention. Identifying influencing organizational issues and perceived risks (access to proper protective material, adequate work environment with space to be

Table 1
Demographic variables, working position, perception of COVID-19 relative risk, organizational factors, stress perception and general health score from health service workers in Spain.

No./total No. (%) VARIABLE	LOW INCIDENCE AREAS [a] N = 1106	HIGH INCIDENCE AREAS [b] N = 301	TOTAL N = 1407
**Sex**			
Men	314/1106 (28.5%)	63/301 (21.1%)	377/1407 (27%)
Women	789/1106 (71.5%)	236/301(78.9%)	1025/1407 (73%)
**Age, mean (SD)**	45.3 (10.9)	42.4 (10.5)	44.7 (10.9)
**Working position**			
Second-line	723 (65.4%)	116 (38.5%)	839 (59.6%)
Frontline	383 (34.6%)	185 (61.5%)	568 (40.4%)
**Occupation**			
Resident	71 (6.4%)	25 (8.3%)	96 (6.8%)
Physician	359 (32.6%)	121 (40.2%)	480 (34.2%)
Nurse	306 (27.8%)	63 (20.9%)	369 (26.3%)
Other [c]	176 (16%)	32 (10.6%)	208 (14.8%)
HCA	72 (5.1%)	3 (0.2%)	75 (5.3%)
GP	118 (8.4%)	57 (4.1%)	175 (12.5%)
**Family member infected with COVID- 19** *[yes]*	384 (35%)	166 (55%)	550 (39.4%)
**PCR performed**			
No	690 (62.4%)	165 (54.8%)	855 (60.8%)
Yes [negative result]	386 (34.9%)	115 (38.2%)	505 (35.6%)
Yes [positive result]	30 (2.7%)	21 (7%)	51 (3.6%)
**Isolation** *[yes]*	147 (13%)	67 (22%)	214 (15.2%)
**Perception of COVID-19 related risk**			
My job was putting me at great risk [yes]	641 (58%)	199 (66.1%)	840 (59.7%)
Extra stress at work [yes]	489 (44.2%)	186 (61.8%)	675 (48%)
I was afraid of falling ill with COVID-19 [yes]	579 (52.4%)	153 (50.8%)	732 (52%)
Little control over whether I would get infected [yes]	584 (52.8%)	185 (61.5%)	769 (54.7%)
I would be unlikely to survive if I were to get COVID-19 [yes]	126 (11.4%)	32 (10.6%)	158 (11.2%)
Resigning because of COVID19 [yes]	78 (7.1%)	33 (11%)	111 (7.9%)
I would pass COVID-19 on to others [yes]	908 (82.1%)	254 (84.4%)	1162 (82.6%)
My family and friend were worried that they might get infected through me [yes]	517 (46.7%)	141 (46.8%)	658 (46.8%)
People avoid my family because of my work [yes]	203 (18.4%)	51 (16.9%)	254 (18%)
**Organizational factors**			
My colleagues listen to me [never]	15 (1.4%)	4 (1.3%)	19 (1.4%)
Senior listen to me [never]	161 (15.2%)	54 (18.5%)	215 (15.9%)
Access to PPE [never]	117 (12%)	8 (2.8%)	125 (8.9%)
Access to a screening test [never]	200 (23.8%)	47 (18.4%)	247 (17.6%)
Receive information about precautions [never]	77 (7%)	25 (8.4%)	102 (7.2%)
Prepared to treat patients [never]	257 (26.3%)	75 (27.1%)	332 (23.6%)
Well-defined action protocols [never]	168 (15.8%)	57 (19.5%)	225 (16%)
Pressed not to wear protective material [most of the time]	152(13.7%)	50 (16.6%)	202 (14.4%)
Pressed to reuse protective material [most of the time]	491 (44.4%)	170 (56.5%)	661(47%)
Receive conflicting information [most of the time]	604 (54.6%)	184 (61.1%)	788(56%)
**SASR-Q score** *[Complete acute stress diagnosis, DSM-IV]*	264 (23.9%)	84 (27.9%)	348 (24.7%)
**GHQ score** > 23	551 (49.8%)	203 (67.4%)	754 (53.6%)
**Total score,** median (IQR)			
SASR-Q (global)	39 (16–68)	47 (26–72)	40 (18–69)
Dissociative symptoms	10 (4–21)	14 (6–22)	11(4–21)
Reexperience symptoms	6 (1–13)	8 (3–14)	6(1–13)
Avoidance symptoms	8 (3–14)	9 (4–15)	8(3–14)
Increased arousal symptoms	11.5 (6–17)	14 (9.5–18.5)	12(7–18)
Impact or social functioning	2 (0–4)	3 (1–5)	2(1–5)
GHQ	23 (14–33)	29 (21–39)	25 (15–34)

Abbreviations: DSM, Diagnostic and Statistical Manual of Mental Disorders; GHQ, General Health Questionnaire; GP, general practitioner; HCA, health care assistant; IQR, interquartile range; PCR, polymerase chain reaction; PPE, personal protective equipment; SASR-Q, Stanford Acute Stress Reaction Questionnaire; SD, standard derivation.

[a] Low incidence areas include Andalusia, Extremadura, Murcia, Galicia, Canarias, Asturias, Valencia, Baleares, Basque Country, Cantabria and La Rioja.

[b] High incidence areas include Catalonia, Ceuta, Castilla y Leon, Madrid, Castilla-La Mancha, Navarre and Aragon.

[c] Other includes professionals such as administrative staff, clinical psychologist, social workers, physiotherapist, guards, pharmacists, occupational therapist, cleaning staff and radiology technicians.

listened, and measures that would reduce the possibility of infecting family and friends) would minimize the emotional impact on the workplace in the face of a new outbreak.

These results are similar to those reported in other international studies (Lai et al., 2020; Rossi et al., 2020), confirming the great impact of the pandemic on HCWs mental and general health. In line with the aforementioned studies, it is found to score high on a scale that measures symptoms related to anxiety, depression and insomnia and high levels of stress, particularly in women. However, GHQ-28 tends to overestimate, giving false positives in patients who should not be diagnosed as individuals with psychological problems (Hjelle et al., 2019).

Our data show that female health workers, independently of being in front-line, due to the lack of protective devices and also based on the personal perception of a risky and stressful work have a higher risk of suffering an acute stress disorder. Linked to these work related circumstances, it should be highlighted that slight form of racism against health care professionals (people rejecting the family of a health care

Table 2
Seemingly Unrelated Logistic Regression Analysis[a].

VARIABLE	ASD OR (95% CI)	P value	GHQ OR (95% CI)	P value
**Sex**				
Men	1 [reference]	NA	1 [reference]	NA
Women	2.9 (1.8–4.7)	.0001	3.3 (2.2–4.8)	.0001
**Age**	1.0 (0.98–1.0)	.74	0.99 (0.7–1.0)	.55
**Geographical area**				
Low incidence	1 (reference)		1(reference)	
High Incidence	0.95 (0.63–1.4)	.82	1.8(1.2–2.7)	.003
**Working position**				
Second-line	1 [reference]	NA	1 [reference]	NA
Frontline	1.1 (0.78–1.6)	.46	0.88 (0.61–1.2)	.50
**Occupation**				
Resident	1 [reference]	NA	1 [reference]	NA
Physician	1.9 (0.80–4.5)	.14	1.7(0.78–3.4)	.16
Nurse	1.4 (0.58–3.4)	.43	1.4(0.67–3.0)	.30
Other [b]	1.7(0.64–4.8)	.27	1.6 (0.66–3.8)	.27
HCA	1.0 (0.34–3.2)	.92	1.4 (0.52–3.7)	.48
GP	2.1 (0.8–5.5)	.12	1.9(0.81–4.4)	.12
**Family member infected with COVID- 19** *[yes]*	0.93 (0.65–1.3)	.70	0.90 (0.64- 1.2)	.55
**PCR performed**				
No	1 [reference]	NA	1 [reference]	NA
Yes [negative result]	0.98 (0.66–1.4)	.91	0.83 (0.57–1.2)	.34
Yes [positive result]	1.1(0.45–2.8)	.78	1.3 (0.49–3.4)	.58
**Isolation** *[yes]*	1.2(0.83–2.0)	0.35	1.1(0.72–1.9)	.50
**Organizational factors**				
My colleagues listen to me [never]	1.7(0.39–7.7)	.46	13.9 (1.3–147.2)	.02
Leader listen to me [never]	1.3 (0.82–2.1)	.24	0.89 (0.55–1.4)	.10
Access to PPE [never]	1.8 (1.0–3.3)	.004	0.85 (0.46–1.5)	.59
Access to a screening test [never]	1.1 (0.69–1.9)	.68	1.0 (0.65–1.6)	.91
Receive information about precautions [never]	0.94 (0.46–1.9)	.87	1.2 (0.56–2.6)	.60
Prepared to treat patients [never]	0.7 (0.48–1.4)	.28	1.0 (0.63–1.5)	.98
Well-defined action protocols [never]	0.9 (0.56–1.4)	.69	0.74 (0.45–1.2)	.24
Pressed not to wear protective material [most of the time]	1.3 (0.85–2.0)	.19	1.3 (0.82–2.1)	.23
Pressed to reuse protective material [most of the time]	1.3 (0.9–1.8)	.14	1.3 (0.93–1.8)	.08
Receive conflicting information [most of the time]	1.1 (0.79–1.6)	.47	1.41(0.82–1.6)	.39
**Perception of COVID-19 related risk**				
My job was putting me at great risk	0.64(0.42–0.99)	.045	1.1 (0.80–1.7)	0.42
Extra stress at work	4.5(3.0–6.8)	.000	5.6(3.9–7.9)	0.000
I was afraid of falling ill with COVID-19	1.9(1.2–2.8)	.001	1.3(0.93–1.9)	0.11
Little control over whether I would get infected	1.2(0.89–1.8)	0.16	1.2(0.91–1.8)	0.14
I would be unlikely to survive if I were to get COVID-19	0.99(0.59–1.6)	.92	0.79(0.46–1.3)	0.4
Resigning because of COVID-19	1.3(0.77–2.3)	.28	2.0(0.96–4.4)	0.06
I would pass COVID-19 on to others	1.5(0.78–2.9)	.20	1.6(1.0–2.7)	0.04
My family and friend were worried that they might get infected through me	1.7(1.2–2.5)	.003	1.4(0.99–2.0)	0.05
People avoid my family because of my work	1.5(1.0–2.3)	.03	1.3(0.84–2.0)	0.21

Abbreviations: DSM, Diagnostic and Statistical Manual of Mental Disorders; GHQ, General Health Questionnaire; GP, general practitioner; HCA, health care assistant; IQR, interquartile range; PCR, polymerase chain reaction; PPE, personal protective equipment; ASD, Acute Stress Disorder; SD, standard derivation.

[a] In this model, independent variables and covariates are entered at the same time, so every variable is controlled for the other variables.

[b] Other includes professionals such as administrative staff, clinical psychologist, social workers, physiotherapist, guards, pharmacists, occupational therapist, cleaning staff and radiology technicians.

provider) who potentially have a higher risk of being infected and whether or not we or our loved ones will be infected increase the risk of having a stress disorder. Working in areas of high prevalence of COVID19 and lack a reciprocal communication with colleagues seem to worse general health of professionals.

These feelings of distress and anxiety can occur even in people not at high risk of getting sick, no working place, front or second, occupation. It would be worth noting that despite the stressful situation only 7% considered resigning their job.

Thus, personal factors related to the perception of fear about the virus are critical when evaluating the short-term impact of pandemic of the professional health. In line with this observation, the study carried out in the previous SARS pandemic (Wu et al., 2009) revealed that had been quarantined, work in high-risk locations or had a friend o close relative who contracted SARS increased the risk to develop PTSD. However, these factor and their nuances have not been previously studied in the recent COVID-19. We emphasize the importance of stress management at work and the perception of safety at work as well as the influence it can have on our people close to us. In particular, a higher incidence of stress on the health professional has been associated with the family being more concerned about being infected than being able to infect others.

Percentages of health workers reporting that their family members were infected with COVID-19 were 55% in high incidence areas and 35% in low incidence areas. This data should be considered with caution as in the initial phase of the pandemic COVID-19, tests were not performed on patients with minor symptoms and therefore confirmed diagnoses were less accurate. There was also insufficient knowledge about asymptomatic cases.

One of the strengths of our study is the inclusion of factors related to organization and safety devices. Studies carried out during Ebola crisis (Belfroid et al., 2018) and recent articles on COVID-19 infection (Chen et al., 2020; Shanafelt et al., 2020) warn of the importance of institutional care for health professionals and worried about the shortage of protective equipment.

Our findings support the notion that protective factors and helpful strategies such as fostering a frequent and clear communication among professionals, adequate supplies of protective equipment, providing

training and thorough education around infectious diseases and access to psychological interventions to deal with personal worries should be compulsory to minimize the suffering of health care professional (Kisely et al., 2020). At a follow-up, it would be interesting to assess whether the use of economic measures such as financial incentives or recognition as occupational illness may modulate stress reactions.

As a limitation, participants were self-selected, which influences the number of different HCWs. In this regard and in spite of having a generous participation in a very short time, we must consider a possible memory bias.

We might conclude that, in addition to individuals` characteristics, addressing modifiable factors affecting subjects´ perceived risk and facilitating psychological care may be critical in preparing health professional force for facing a potential upcoming epidemiological crisis.

Author statement

Contributors

Crespo-Facorro and Ruiz-Veguiila acquired funding, designed the study, analyzed the data and wrote the manuscript. Rodriguez-Menéndez, RubioGarcia, Conde-Alvarez, Armesto-Luque, Capitan and Luque collected the data, analyzed data, reviewed and commented on the manuscript. Garrido-Torres supervised data analysis, reviewed and commented on the manuscript.

All authors contributed to and have approved the final manuscript.

Role of funding source

This research did not receive any specific grant from funding agencies in the public, commercial, or not-for-profit sectors.

Ethical approval

The study was approved by the Institutional Ethics Committee.

Declaration of Competing Interest

The authors have no conflicts of interest concerning the subject of the study.

Acknowledgments

All authors thank health care workers for his selfless and generous work in completing the surveys.

Supplementary materials

Supplementary material associated with this article can be found, in the online version, at doi:10.1016/j.jad.2020.09.079.

References

American Psychiatric Association, 2013. Diagnostic and Statistical Manual of Mental Disorders, 5th Edition. American Psychiatric Association, Washington DC.

Belfroid, E., van Steenbergen, J., Timen, A., et al., 2018. Preparedness and the importance of meeting the needs of healthcare workers: a qualitative study on Ebola. J Hosp Infect Feb 98 (2), 212–218. https://doi.org/10.1016/j.jhin.2017.07.001.

Cardeña, E., Koopman, C., Classen, C., et al., 2000. Psychometric Properties of the Stanford Acute Stress Reaction Questionnaire (SASRQ): a Valid and Reliable Measure of Acute Stress. J Trauma Stress 13 (4), 719–734.

Casacchia, M., Bianchini, V., Mazza, M., et al., 2013. Acute Stress Reactions and Associated Factors in the Help-Seekers After the L'Aquila Earthquake. Psychopathology 46 (2), 120–130.

Chen, Q., Liang, M., Li, Y., et al., 2020. Mental health care for medical staff in China during the COVID-19 outbreak. The Lancet Psychiatry 7 (4), e15–e16. https://doi.org/10.1016/S2215-0366(20)30078-X.

García-Fernández, L., Romero-Ferreiro, V., López-Roldán, P.D., et al., 2020. Mental health impact of COVID-19 pandemic on Spanish healthcare workers [published online ahead of print, 2020 May 27]. Psychol Med. 1–3.

Goldberg, D.P., Hillier, V.F., 1979. A scaled version of the General Health Questionnaire. Psychol Med 9 (1), 139–145. https://doi.org/10.1017/s0033291700021644.

Hjelle, E.G., Bragstad, L.K., Zucknick, M., et al., 2019. The General Health Questionnaire-28 (GHQ-28) as an outcome measurement in a randomized controlled trial in a Norwegian stroke population. BMC Psychol 7, 18. https://doi.org/10.1186/s40359-019-0293-0.

IBM Corp. Released, 2011. IBM SPSS Statistics for Windows, Version 20.0. IBM Corp., Armonk, NY.

Kang, L., Li, Y., Hu, S., 2020. The mental health of medical workers in Wuhan, China dealing with the 2019 novel coronavirus. Lancet Psychiatry 7 (3), e14. https://doi.org/10.1016/S2215-0366(20)30047-X.

Kisely, S., Warren, N., McMahon, L., 2020. Occurrence, prevention, and management of the psychological effects of emerging virus outbreaks on healthcare workers: rapid review and meta-analysis. BMJ 369, m1642.. https://doi.org/10.1136/bmj.m1642.

Lai, J., Ma, S., Wang, Y., et al., 2020. Factors Associated With Mental Health Outcomes Among Health Care Workers Exposed to Coronavirus Disease 2019. JAMA Netw Open 3 (3), e203976. https://doi.org/10.1001/jamanetworkopen.2020.3976.

Ministerio de Sanidad de España, 2020. Actualización n° 135. Enfermedad por el coronavirus (COVID-19) 13.06.2020. https://www.mscbs.gob.es/profesionales/saludPublica/ccayes/alertasActual/nCov-China/documentos/Actualizacion_135_COVID-19.pdf.

Rossi, R., Socci, V., Pacitti, F., et al., 2020. . Mental Health Outcomes Among Frontline and Second-Line Health Care Workers During the Coronavirus Disease 2019 (COVID-19) Pandemic in Italy. JAMA Netw Open 3 (5), e2010185. https://doi.org/10.1001/jamanetworkopen.2020.10185.

Shanafelt, T., Ripp, J., Trockel, M., 2020. Understanding and Addressing Sources of Anxiety Among Health Care Professionals During the COVID-19 Pandemic. JAMA 323 (21), 2133–2134. https://doi.org/10.1001/jama.2020.5893.

Tam, C.W.C., Pang, E.P.F., Lam, L.C.W., Chiu, H.F.K., 2004. Severe acute respiratory syndrome (SARS) in Hong Kong in 2003: stress and psychological impact among frontline healthcare workers. Psychol Med 34 (07), 1197–1204. https://doi.org/10.1017/s0033291704002247.

Wu, P., Fang, Y., Guan, Z., et al., 2009. . The Psychological Impact of the SARS Epidemic on Hospital Employees in China: exposure, Risk Perception, and Altruistic Acceptance of Risk. Can J Psychiatry 54 (5), 302–311. https://doi.org/10.1177/070674370905400504.
