# Supplementary material for: Mental health status and related factors influencing healthcare workers during the COVID-19 pandemic: A systematic review and meta-analysis
Source: PLoS One. 2024 Jan 19;19(1):e0289454. doi: 10.1371/journal.pone.0289454 (PMC10798549; doi:10.1371/journal.pone.0289454)
Supplement: S1 Data — (ZIP) [file pone.0289454.s011.zip › literatures/46.pdf]

Occupational Medicine
doi:10.1093/occmed/kqaa220

Mental health of staff working in intensive care during COVID-19

N. Greenberg[1,2], D. Weston[3], C. Hall[3], T. Caulfield[4], V. Williamson[1,5] and K. Fong[6,7]

[1]King's Centre for Military Health Research, Institute of Psychology, Psychiatry and Neuroscience, King's College London, 10 Cutcombe Road, London SE5 9RJ, UK, [2]Health Protection Research Unit, Institute of Psychology, Psychiatry and Neuroscience, King's College London, 10 Cutcombe Road, London SE5 9RJ, UK, [3]Behavioural Science Team, Emergency Response Department Science & Technology, Public Health England, Porton Down, Salisbury SP4 0JG, UK, [4]Department of Computer Science, University College London, London WC1E 6BT, UK, [5]Department of Experimental Psychology, Anna Watts Building, University of Oxford, Oxford OX2 6GG, UK, [6]Department of Science, Technology, Engineering and Public Policy (STEaPP), University College London, London WC1E 6BT, UK, [7]Department of Anaesthesia, University College London Hospital, 235 Euston Road, London WC1E 6BT, UK.

Correspondence to: N. Greenberg, King's Centre for Military Health Research, Institute of Psychology, Psychiatry and Neuroscience, King's College London, 10 Cutcombe Road, London SE5 9RJ, UK. Tel: 0207 848 5351; E-mail: Neil.Greenberg@kcl.ac.uk

Background Staff working in intensive care units (ICUs) have faced significant challenges during the COVID-19 pandemic which have the potential to adversely affect their mental health.

Aims To identify the rates of probable mental health disorder in staff working in ICUs in nine English hospitals during June and July 2020.

Methods An anonymized brief web-based survey comprising standardized questionnaires examining depression, anxiety symptoms, symptoms of post-traumatic stress disorder (PTSD), well-being and alcohol use was administered to staff.

Results Seven hundred and nine participants completed the surveys comprising 291 (41%) doctors, 344 (49%) nurses and 74 (10%) other healthcare staff. Over half (59%) reported good well-being; however, 45% met the threshold for probable clinical significance on at least one of the following measures: severe depression (6%), PTSD (40%), severe anxiety (11%) or problem drinking (7%). Thirteen per cent of respondents reported frequent thoughts of being better off dead, or of hurting themselves in the past 2 weeks. Within the sample used in this study, we found that doctors reported better mental health than nurses across a range of measures.

Conclusions We found substantial rates of probable mental health disorders, and thoughts of self-harm, amongst ICU staff; these difficulties were especially prevalent in nurses. Whilst further work is needed to better understand the real level of clinical need amongst ICU staff, these results indicate the need for a national strategy to protect the mental health, and decrease the risk of functional impairment, of ICU staff whilst they carry out their essential work during COVID-19.

Key words COVID-19; doctors; intensive care; mental health; nurses; PTSD; self-harm.

Introduction

The COVID-19 virus outbreak was declared a pandemic on 12 March 2020 by the World Health Organization [1]. Across the globe healthcare workers have been at the front line of each nation's response, labouring to meet a sudden and dramatic increase in demand and workload across many areas of healthcare provision. Among those most directly impacted have been intensive care and anaesthetic teams who together augmented and expanded critical care provision.

Front-line healthcare staff will have experienced a myriad of psychological stressors, including fears of contracting the virus and endangering their loved ones, concerns over the lack of personal protective equipment (PPE), and distress relating to adverse patient outcomes and loss of patient lives despite their best efforts [2,3].

Within the UK a substantial proportion of the 175 000 patients admitted to hospital with COVID-19 received critical care in specialized units. To accommodate this unprecedented surge, during the first wave of the pandemic

© The Author(s) 2021. Published by Oxford University Press on behalf of the Society of Occupational Medicine. All rights reserved.
For Permissions, please email: journals.permissions@oup.com

Key learning points

What is already known about this subject:
- Intensive care unit staff are regularly exposed to traumatic situations as part of their job.
- Previous studies have shown them to be at risk of psychological and moral distress.
- Little is known about the mental health of intensive care unit staff during the current pandemic.

What this study adds:
- Almost half of intensive care unit staff who participated in this study report symptoms consistent with a probable diagnosis of post-traumatic stress disorder, severe depression or anxiety or problem drinking.
- Around one in seven intensive care unit staff in this study report recent thoughts of self-harm or of wanting to be better off dead.
- Nursing staff in this study were more likely to report higher levels of distress than doctors or other clinical staff.

What impact this may have on practice or policy:
- Healthcare managers need to prioritize staff mental health support and timely access to evidence-based treatments for intensive care unit staff.
- Supervisors and managers should be aware that a substantial proportion of intensive care unit staff may perform less well because of their current poor state of mental health.
- More work is needed to understand whether the high levels of mental health symptoms identified in this study are truly indicative of high levels of clinical need for mental healthcare.

in the UK, hospitals were forced to create *ad hoc* intensive care units (ICUs) with heavily modified staffing models; reducing the usual 1:1 ICU nurse:patient ratio to as low as 1:6 in some cases [4]. Pre-existing shortages of experienced ICU staff have been greatly exacerbated by high levels of staff sickness and quarantine during the first COVID-19 surge.

Consequently, ICU staff have faced a particularly challenging time frequently working in areas where the perceived risk of COVID-19 exposure is high for long periods, wearing PPE, with the challenges of managing staff and equipment shortages on a daily basis especially during the first wave. At times this will have made it difficult for staff to deliver a normal standard of care. The high rate of mortality amongst COVID-19 patients admitted to ICU, coupled with difficulty in communication and providing adequate end-of-life support to patients, and their next of kin because of visiting restrictions, has been a specific stressor for all staff working in ICUs.

These working conditions have the potential to adversely impact the mental health of ICU staff, including the experience of psychological distress, moral injury [5] and the development of mental health difficulties such as depression and post-traumatic stress disorder (PTSD). In order to ascertain what level of psychological support may be required, a survey of the mental health of front-line staff working in ICU settings during COVID-19 in June and July 2020.

Methods

ICUs, across nine NHS hospitals with peak ICU bed occupancy figures ranging between 10 and 75 critically ill COVID-19 patients, were identified from ICNARC (Intensive Care National Audit and Research Centre) data and local ICU reporting systems. The hospitals were drawn from a range of NHS acute trusts including metropolitan teaching hospitals and district general hospitals. The data were gathered as part of a service evaluation exercise, in an attempt to monitor the effect of heavily modified working patterns on intensive care and anaesthetic staff during the UK's first COVID-19 surge.

We engaged with clinical leads from participating ICUs and encouraged the circulation and completion of the online survey. The survey was distributed via departmental e-mail mailing lists and cascaded through departmental SMS contact groups.

A brief online survey tool—designed to be completed in less than 5 min—was compiled, comprising a number of validated questions assessing the mental health status and psychological well-being.

The survey comprised the following measures, for which binary outcomes variables were defined using the following cut-off scores to indicate a case; the 7-item Generalized Anxiety Disorder (GAD) scale to measure probable moderate anxiety disorder with a score >9 indicating a probable moderate anxiety disorder and >15 indicating probable severe anxiety disorder [6]; the 9-item Patient Health Questionnaire (PHQ-9) with a score of >9 indicating probable moderate depression and >19 probable severe depression [7]; the 6-item Post-Traumatic Stress Disorder checklist (PCL-6) civilian version to measure PTSD [8] with a score of >13 indicating the presence of probable PTSD and the AUDIT-C with a score of >7 indicating problem drinking [9]. We also examined participants' responses as to whether they had had 'thoughts that [they] would be better off dead, or of

hurting [themselves] in some way [in the past two weeks]' which is a single item within the PHQ-9 questionnaire. Finally, the Warwick Edinburgh Mental Wellbeing Scale (WEMWBS) [10], a 14-item scale where all items are worded positively, was used to explore feelings and functioning aspects of mental well-being. These questionnaires were incorporated into an online survey form, which could be accessed from a hyperlink embedded within an e-mail or SMS message.

The brief online survey was anonymous at the point of collection and the resultant data were uncoupled from identifying detail from the originating device. Participants completed the survey voluntarily, with the knowledge that the data would be anonymized, and that they were free to stop at any point during their completion of the survey and that incomplete surveys were discarded.

The survey was built using the LimeSurvey tool (https://www.limesurvey.org/) and hosted on a dedicated secure university server. No registration was needed to participate in the survey and no individually identifying details were collected from participants.

The need for ethical review was discussed with two university ethics committees both of which confirmed that, as an anonymized audit and quality improvement exercise, the survey did not require ethical approval. The NHS Health Research Authority 'is my study research?' decision tool also confirmed that the study did not require review by a research ethics committee.

Descriptive analyses were conducted to provide an overview of the sample characteristics. Bivariate correlations were used to examine the relationship between mental health measure scores. We examined differences in scores across the different professions (doctors, nurses and other healthcare workers in ICU) using logistic regression analyses.

Results

Overall, 709 participants took part in the study. Of these, 291 (41%) identified themselves as being doctors, 344 (49%) nurses and 74 (10%) as being in other clinical roles.

The majority of participants reported good well-being on the WEMWBS (n = 418, 59%), although almost half of participants (n = 322, 45%) met the threshold for at least one of the following measures: severe depression, PTSD, severe anxiety or problem drinking (see Table 1).

Thirteen per cent of respondents reported having thoughts that [they] would be better off dead, or of hurting [themselves] in some way several days or more frequently in the past 2 weeks. When examined by role, a significantly higher proportion of nurses (19%) than doctors (8%) or other clinical staff (10%) (χ^2 = 26.8, degrees of freedom [df] = 8, $P < 0.002$) reported these thoughts.

Logistic regression indicated that doctors were more likely, than other clinicians, to report good well-being and nurses were more likely to meet the thresholds for depression (moderate and severe), probable PTSD and anxiety (moderate and severe) (see Table 1).

Higher scores on the WEMWBS were significantly associated with lower scores on all the other outcomes measures (depression, PTSD, anxiety and alcohol use).

Table 1. Frequencies of participants split by role which met psychological measures thresholds; logistic regressions carried out on each psychological measure threshold to examine effect of role are presented

	N (% of sample)	Role						
		Counts			Logistic regression			
						95% CI for odds ratio		
		Doctor (% of sample)	Nurse (% of sample)	Other (% of sample)	*B* (SE)	Lower	Upper	Odds ratio
Good well-being	417 (59)	185 (64)	186 (54)	46 (62)	0.39* (0.16)	1.08	2.04	1.48
Probable PTSD	280 (40)	92 (32)	168 (49)	20 (27)	−0.73* (0.16)	0.35	0.67	0.48
Problem drinking	51 (7)	20 (7)	28 (8)	3 (4)	−0.18 (0.30)	0.46	1.51	0.83
Moderate depression	262 (37)	76 (26)	167 (49)	19 (26)	−0.98* (0.17)	0.27	0.52	0.38
Severe depression	45 (6)	13 (5)	30 (9)	2 (3)	−0.71* (0.34)	0.25	0.96	0.49
Moderate anxiety	189 (27)	58 (20)	115 (33)	16 (22)	−0.70* (0.19)	0.34	0.71	0.49
Severe anxiety	80 (11)	23 (8)	52 (15)	5 (7)	−0.73* (0.26)	0.29	0.81	0.48
AMD	322 (45.4%)							

AMD, any mental disorder (consisting of at least one of the following: severe depression, severe anxiety, probable PTSD or problem drinking. Good well-being is indicative of a score of ≥43 on WEMWBS; moderate depression equates to a score of ≥10; and severe depression equates to a score of ≥20 on the PHQ-9; probable PTSD equates to a score of ≥15 on PCL-6; moderate anxiety equates to a score of ≥11; and severe depression equates to a score of ≥16 on the GAD-7; problem drinking equates to a score of ≥8 on AUDIT-C. The negative beta coefficient and odds ratio of less than 1 is due to coding of the role predictor with nurses as the reference category (thus doctors = 1, nurses = 0).
*Indicates statistically significant finding.

Measures of anxiety, depression and PTSD symptoms were significantly correlated with each other. No significant associations were found between any measure of poor mental health and alcohol consumption (see Table 2).

Discussion

We examined the mental health of impact of working in ICU settings during the latter part of the first wave of the COVID-19 pandemic surge for NHS staff during June and July 2020. We identified a number of key finding most notably high rates of probable mental ill-health with around 45% of the sample self-reporting symptoms of probable PTSD, severe depression or a severe anxiety disorder. More than one in seven of the ICU staff who participated in this study reported thoughts that they would be better off dead, or of hurting themselves in some way several days over the past 2 weeks with nurses being more likely to report poor mental health and ideas of self-harm or suicidal ideation than doctors or other healthcare staff. Lastly, although around 8% of the sample appeared to be at risk of alcohol-related difficulties, this level of drinking was not significantly associated with poorer mental health outcomes.

Our results highlight the potential profound impact that COVID-19 has had on the mental health of front-line UK staff. The 2014 Adult Psychiatric Morbidity Study [11] found rates of probable PTSD in the UK general public to be ~4% and other studies have reported an overall PTSD prevalence in UK military personnel of around 7% with the highest rate, of 17%, in veterans who had recently served in a combat role [12]. Thus, probable PTSD rate we report (40%) was around nine times that found within the general population and more than double that found in recent combat veterans. Whilst further validation studies are required to better understand what proportion would actually meet diagnostic criteria for PTSD on clinical assessment, these data suggest that ICU clinicians are at a significantly elevated risk of suffering with PTSD. Our findings of high levels of PTSD, and other mental health difficulties such as depressive anxiety disorders, are highly relevant given that there is strong evidence poor mental health is associated with functional impairment which would increase the risk of patient safety incidents [13].

Whilst it is not possible to be certain why ICU clinicians reported such high levels of poor mental health, during the time these data were collected (June–July 2020), staff still faced a number of substantial stressors including long shifts, caring for dependent children and other household responsibilities [14] and regular exposure to ethical dilemmas with the consequential risk of moral injury [15]. Some may also have still experienced difficulties with a lack of PPE [13]. Whilst it is possible that the high levels of probable mental disorders are a result of ICU having always been a challenging environment, a 2015 study of 335 ICU staff found rates of probable PTSD of 8% amongst staff working with adults and 17% amongst staff working with children [16] suggesting the rates in this study are indeed elevated.

We found nurses were more likely to report experiencing mental health difficulties than doctors or other ICU staff. Whether this occupational group is more vulnerable to mental ill-health by virtue of demographic risk factors, or whether other factors are unduly affecting this group, remains unclear. However, we note that UK ICU nurses are more likely to be younger adults and female [16] and this demographic has been shown to be at increased risk of suffering with poor mental health within the general population during the pandemic [17]. Other recent reports have also highlighted nurses as being at considerable risk of burnout and that nurses were at risk of suffering with poor mental health that was likely to affect retention rates [18] suggesting that nursing may be a profession that is particularly at risk of suffering poor mental health. It may also be that doctors were more likely to under-report symptoms than nurses although we were not able to investigate this further within this study.

Our finding that more than one in seven clinicians (and nearly one in five nurses) in our sample working in ICU reported thoughts of self-harm or suicide is also highly concerning. However, our survey did not ask whether respondents had made plans to carry out self-injurious or suicidal behaviours. It is also unclear how common such thoughts might be in people who become healthcare workers. For instance, a 2014 paper reported that around 14% of nursing students reported self-injurious thoughts that made them a substantial suicide risk [19, 20]. Whatever, the cause of such thoughts, we suggest that it is important that healthcare managers

Table 2. Bivariate correlations carried out between psychological measures

	WEMWBS Su	PHQ-9	PCL-6	GAD-7	AUDIT-C
WEMWBS	–	−0.708**	−0.601**	−0.659**	−0.135**
PHQ-9	−0.708**	–	0.730**	0.784**	0.047
PCL-6	−0.601**	0.730**	–	0.701**	−0.013
GAD-7	−0.659**	0.784**	0.701**	–	0.039
AUDIT-C	−0.135**	0.047	−0.013	0.039	–

**Correlation is significant at the 0.01 level (two-tailed).

are aware of them and that measures to compassionately support any staff member at risk of suicide are put in place in a timely manner.

Perhaps unsurprisingly, our data showed that people who met the threshold for one form of probable mental disorder were substantially more likely to meet the threshold for another disorder. The exception to this was for alcohol misuse. Whilst we identified around 10% of the sample reported drinking in a manner consistent with alcohol misuse, we found no association between poor mental health and alcohol misuse suggesting that within this sample self-medication with alcohol was not common. Also, the identified rate of alcohol misuse was very much in keeping with previous estimates of in healthcare staff which range from 2 to 24% [21]. This finding may have been because already tired and distressed staff recognized that excessive alcohol consumption would have made their ability to cope at work the next day even more difficult or because as healthcare staff they recognized the dangers of excessive alcohol use. Whatever the reason, this finding is heartening.

This study has several strengths and limitations. Amongst the strengths are the inclusion of a number of hospitals across the UK and completion of study assessments anonymously. A weakness of this study is the lack of participant demographic details which was done both for brevity and to preserve anonymity. As females, younger adults and those with dependent children are more likely to experience psychological difficulties, collecting this information would be valuable in future investigations. Second, this study used self-report measures of mental illness rather than the gold-standard diagnostic interviews. Finally, it is possible that response bias occurred and thus those who participated may have had especially salient mental health difficulties they wanted to report. Future studies would be improved if either participants were randomly selected or a non-responder analysis was carried out.

Despite these limitations, the results of this study allow for several recommendations. First, our results suggest that NHS managers should prioritize provision of evidence-based staff support which is likely both to improve psychological well-being and decrease the likelihood of psychologically unwell staff delivering substandard care. Second, it is also necessary to ensure that rapid access to formal treatment is available to those who need it given its long-term positive benefits (e.g. reduced staff absence, improved quality of life). Third, supervisor, and peer, support has been found to be particularly beneficial in supporting other trauma-exposed occupations such as for firefighters [22] or military personnel [23]. Ensuring that a proportion of ICU staff receive active listening skills, or peer support training, may thus also be beneficial. Fourth, NHS managers should actively monitor the well-being of ICU staff in order that the impact of workload changes are properly understood and mitigated where possible. This would allow for improved staffing, and other support measures, to be implemented in a dynamic fashion. Doing so would also ensure that the provision of high-quality care was optimized and contribute to protecting the mental health of ICU staff upon whom much of the UK's response to the pandemic depends. When monitoring psychological health, is important to consider the impact of anonymity as evidence shows that unless staff are persuaded that their manager's will not be able to identify their individual responses, they are likely to under-report symptoms [24].

This study identified that, amongst the participants who were staff working in ICU during the current pandemic, many reported substantially raised levels of poor mental health and in particular high rates of probable PTSD. The increased risk was particularly evident amongst nursing staff. Given the requirement for ICU staff to highly functional as they care for critically ill patients, these data suggest that is imperative to ensure that adequate support is provisioned by NHS employers who have a moral and legal duty to appropriately safeguard staff well-being [25]. Furthermore, unless employers properly protect the mental health of ICU staff, then they are more likely to function poorly with a consequential impact on their ability to deliver high-quality patient care which is needed now more than ever.

Funding

The research was funded by the National Institute for Health Research Health Protection Research Unit (NIHR HPRU; NIHR200890) in Emergency Preparedness and Response at King's College London in partnership with Public Health England (PHE), in collaboration with the University of East Anglia and Newcastle University. The views expressed are those of the author(s) and not necessarily those of the NHS, the NIHR, the Department of Health or PHE.

Acknowledgements

We would like to thank Professor Tim Cook and Dr Fiona Kelly for their comments on an earlier draft of this paper.

Competing interests

K.F. works for NHS England. N.G. runs a consultancy which provides the NHS with active listening and peer support training.

References

1. World Health Organization. Coronavirus (COVID-19) Events as They Happen [Internet]. World Health Organization, 2020 [cited 24 March 2020]. https://www.who.int/emergencies/diseases/novel-coronavirus-2019/

events-as-they-happen (29 December 2020, date last accessed).
2. Greenberg N, Tracy D. What healthcare leaders need to do to protect the psychological well-being of frontline staff in the COVID-19 pandemic [Internet]. *BMJ Lead* 2020 [cited 3 August 2020]. https://doi.org/10.1136/leader-2020-000273 (29 December 2020, date last accessed).
3. Williamson V, Greenberg N, Bowden G, Rothenfluh D, Nnadi C, Reynolds J. The mental health impact of providing spine care during COVID-19. *Spine J* [Internet] 2020 [cited 21 September 2020];**20**:1363–1366. https://doi.org/10.1016/j.spinee.2020.04.019 (29 December 2020, date last accessed).
4. Intensive Care National Audit & Research Centre. ICNARC – reports [Internet]. *COVID-19 Rep* 2020 [cited 28 September 2020]. https://www.icnarc.org/Our-Audit/Audits/Cmp/Reports (29 December 2020, date last accessed).
5. Williamson V, Murphy D, Greenberg N. COVID-19 and experiences of moral injury in front-line key workers. *Occup Med (Lond)* [Internet] 2020 [cited 7 April 2020]. https://academic.oup.com/occmed/advance-article/doi/10.1093/occmed/kqaa052/5814939 (29 December 2020, date last accessed).
6. Spitzer RL, Kroenke K, Williams JB, Löwe B. A brief measure for assessing generalized anxiety disorder: the GAD-7. *Arch Intern Med* 2006;**166**:1092–1097.
7. Kroenke K, Spitzer RL, Williams JB. The PHQ-9: validity of a brief depression severity measure. *J Gen Intern Med* 2001;**16**:606–613.
8. Lang AJ, Stein MB. An abbreviated PTSD checklist for use as a screening instrument in primary care. *Behav Res Ther* 2005;**43**:585–594.
9. Bush K, Kivlahan DR, McDonell MB, Fihn SD, Bradley KA. The AUDIT alcohol consumption questions (AUDIT-C): an effective brief screening test for problem drinking. *Arch Intern Med* 1998;**158**:1789–1795.
10. Stewart-Brown S, Tennant A, Tennant R, Platt S, Parkinson J, Weich S. Internal construct validity of the Warwick-Edinburgh Mental Well-Being Scale (WEMWBS): a Rasch analysis using data from the Scottish Health Education Population Survey. *Health Qual Life Outcomes* [Internet] 2009 [cited 21 September 2020];7:15. https://hqlo.biomedcentral.com/articles/10.1186/1477-7525-7-15 (29 December 2020, date last accessed).
11. NHS Digital. Adult Psychiatric Morbidity Survey: Survey of Mental Health and Wellbeing, England, 2014 - NHS Digital [Internet]. NHS Digital, 2016 [cited 21 September 2020]. https://webarchive.nationalarchives.gov.uk/20180328140249/http://digital.nhs.uk/catalogue/PUB21748 (29 December 2020, date last accessed).
12. Stevelink SA, Jones M, Hull L *et al.* Mental health outcomes at the end of the British involvement in the Iraq and Afghanistan conflicts: a cohort study. *Br J Psychiatry* [Internet] 2018 [cited 25 July 2019];**213**:690–697. https://www.cambridge.org/core/product/identifier/S0007125018001757/type/journal_article (29 December 2020, date last accessed).
13. Simms A, Fear NT, Greenberg N. The impact of having inadequate safety equipment on mental health. *Occup Med (Lond)* [Internet] 2020 [cited 28 September 2020];**70**:278–281. https://academic.oup.com/occmed/article/70/4/278/5843741 (29 December 2020, date last accessed).
14. Kisely S, Warren N, McMahon L, Dalais C, Henry I, Siskind D. Occurrence, prevention, and management of the psychological effects of emerging virus outbreaks on healthcare workers: rapid review and meta-analysis. *Br Med J* [Internet] 2020 [cited 28 September 2020];**369**:m1642. http://dx.doi.org/10.1136/bmj.m1642 (29 December 2020, date last accessed).
15. Greenberg N, Docherty M, Gnanapragasam S, Wessely S. Managing mental health challenges faced by healthcare workers during covid-19 pandemic [Internet]. *Br Med J* 2020 [cited 28 September 2020];**368**:m1211. https://www.bmj.com/lookup/doi/10.1136/bmj.m1211 (29 December 2020, date last accessed).
16. Colville G, Hammond J, Perkins-Porras L. Post-traumatic stress symptoms in intensive care staff working in adult and paediatric settings. *Crit Care* 2015;**19**:531.
17. Gender Bias in Critical Care Nursing | The Faculty of Intensive Care Medicine [Internet]. May 2019 [cited 21 September 2020]. https://www.ficm.ac.uk/wicm-blog-archive/may-2019-gender-bias-critical-care-nursing#_edn2 (29 December 2020, date last accessed).
18. Pierce M, Hope H, Ford T *et al.* Mental health before and during the COVID-19 pandemic: a longitudinal probability sample survey of the UK population. *Lancet Psychiatry* [Internet] 2020 [cited 28 September 2020];7:883–892. www.thelancet.com/psychiatry (29 December 2020, date last accessed).
19. Kinman G, Teoh K, Harriss A. *The Mental Health and Wellbeing of Nurses and Midwives in the United Kingdom*. Society of Occupational Medicine. 2020.
20. Aradilla-Herrero A, Tomás-Sábado J, Gómez-Benito J. Associations between emotional intelligence, depression and suicide risk in nursing students. *Nurse Educ Today* [Internet] 2014 [cited 28 September 2020];**34**:520–525. https://pubmed.ncbi.nlm.nih.gov/23886906/ (29 December 2020, date last accessed).
21. Hichisson AD, Corkery JM. Alcohol/substance use and occupational/post-traumatic stress in paramedics. *J Paramed Pract* 2020;**12**:388–396.
22. Milligan-Saville JS, Tan L, Gayed A *et al.* Workplace mental health training for managers and its effect on sick leave in employees: a cluster randomised controlled trial. *Lancet Psychiatry* 2017;**4**:850–858.
23. Jones N, Burdett H, Green K, Greenberg N. Trauma Risk Management (TRiM): promoting help seeking for mental health problems among combat-exposed U.K. military personnel. *Psychiatry* 2017;**80**:236–251.
24. Fear NT, Seddon R, Jones N, Greenberg N, Wessely S. Does anonymity increase the reporting of mental health symptoms? *BMC Public Health* 2012;**12**:797.
25. Greenberg N, Weston D, Hall C, Caulfield T, Williamson V, Fong K. The mental health of critical care and anaesthetic staff during COVID-19. medRxiv preprint. https://doi.org/10.1101/2020.11.03.20208322 (9 December 2020, date last accessed).
