# Supplementary material for: Mental health status and related factors influencing healthcare workers during the COVID-19 pandemic: A systematic review and meta-analysis
Source: PLoS One. 2024 Jan 19;19(1):e0289454. doi: 10.1371/journal.pone.0289454 (PMC10798549; doi:10.1371/journal.pone.0289454)
Supplement: S1 Data — (ZIP) [file pone.0289454.s011.zip › literatures/48.pdf]

ELSEVIER

Since January 2020 Elsevier has created a COVID-19 resource centre with free information in English and Mandarin on the novel coronavirus COVID-19. The COVID-19 resource centre is hosted on Elsevier Connect, the company's public news and information website.

Elsevier hereby grants permission to make all its COVID-19-related research that is available on the COVID-19 resource centre - including this research content - immediately available in PubMed Central and other publicly funded repositories, such as the WHO COVID database with rights for unrestricted research re-use and analyses in any form or by any means with acknowledgement of the original source. These permissions are granted for free by Elsevier for as long as the COVID-19 resource centre remains active.

Asian Journal of Psychiatry 54 (2020) 102260

Contents lists available at ScienceDirect

Asian Journal of Psychiatry

journal homepage: www.elsevier.com/locate/ajp

Letter to the Editor

Prevalence of anxiety and depression among the healthcare workers in Nepal during the COVID-19 pandemic

Sir,

Infectious disease outbreaks, such as COVID-19, take a tremendous toll on the general population encompassing various spheres of their life. It is also likely to impact the psychological health of people, including healthcare workers (HCWs) who are in the frontline caring for people with the infection (Tandon, 2020). The HCWs are facing pressure of working in resource-deprived settings and ever-growing patient load all over the world (Rana et al., 2020; Spoorthy et al., 2020). The psychological stress among the HCWs is attributed to prolonged work shift, uncertain pay, lack of Personal protective equipment (PPEs) and added fear of infection to self or family (Grover et al., 2020; Zhang et al., 2020). Most of the studies came from China. However, little information is available about the psychiatric morbidity among the HCWs from developing countries like Nepal. In this background, the aim of this study was to evaluate the prevalence of anxiety, and depression among the HCWs on duty. For this study, HCWs included, doctors, health assistants, auxiliary nurse-midwifery, nursing students posted in the wards, laboratory assistants, paramedics, staff nurses, sanitization workers, ward attendants, security guards and ambulance drivers who are directly or indirectly involved in the care of patients with COVID-19.

This was an online cross-sectional survey, carried out by using Survey Monkey platform. It was circulated between 11 May, 2020, to 10 June, 2020. The ethical approval for the study was obtained from the Ethics Review Board of Nepal Health Research Council (NHRC). The link was circulated through the messages on Whatsapp, Facebook and Messenger platform to the HCWs of 4 hospitals in Nepal; National Medical College, Birgunj; Chitwan Medical College, Bharatpur; P.T. Birtacity Hospital, Birtamod and Nisarga Hospital, Dhangadhi. There was no coercion on participants and were asked to ignore the message if they did not want to participate in the survey. Weekly reminders were sent to those not responding. Snowball technique was used.

The survey questionnaire included: Demographics and personal characteristics: A basic information sheet included the subject's age, gender, marital status, educational qualifications, type of family, history of any physical or psychiatric illness etc. Patient Health Questionnaire-9 (Kroenke and Spitzer, 2002): It is a depression module for common mental disorders. Generalized Anxiety Disorder Questionnaire-7 (GAD-7) (Spitzer et al., 2006): It is a 7-item questionnaire developed to screen patients for anxiety and rate the severity of anxiety. Each item is rated on a 4-point scale (0–3) on the symptoms in the previous two weeks. Perceived Stress Scale (PSS10) (Cohen et al., 1983): This is a 10-item questionnaire intended to measure distress based on anxiety and depressive symptoms experienced in the most recent 4 week period. 3. Data were analyzed using SPSS 20.0 version, and descriptive statistics were applied.

A total of 150 healthcare workers from four hospitals participated in the study. The mean age of the study participants was 29.5 (SD: 6.1) years. There were more number of females (52.7 %) and majority of the participants were not quarantined until the time of participation in the study (94.7 %). Participants with postgraduate degrees (18.7 %) made up the largest group, followed by those with graduate degrees (33.3 %). About one-third were working as nursing staff (31.3 %), and this was followed by the faculty members (24.7 %). The most common place of work was the speciality OPD (16 %). The most common preexisting medical morbidities reported were hypertension (8.7 %), followed by chronic obstructive pulmonary disease (5.3 %), diabetes mellitus(3.3 %), substance use (2.7 %), chronic pain (2.7 %), stroke(1.3 %) and chronic heart disease (0.7 %). Around 5% had pre-existing psychiatric illness as well.

The mean GAD-7 score was 3.9 (range 0 − 20, SD 4.2). The overall prevalence of anxiety disorder was 37.3 %, with majority of the participants having mild anxiety. Mean PHQ-9 score was 3.9 (range 0 − 21, SD 4.2) and 8% of the participants had depression as per the given cut-off. Overall, 38 % of the participants, had at least one psychiatric illness (Table 1).

Present study suggests that 38 % of the HCWs on COVID-19 duty in Nepal are suffering anxiety and/or depression. A study from China suggests that about half (50.4 %) of the HCWs reported symptoms of depression, 44.6 % had symptoms of anxiety, 34 % had insomnia and 71.5 % reported distress (Lai et al., 2020). Another recent metanalysis of studies, reported pooled prevalence of anxiety to be 23.2 % and that for depression to be 22.8 % (Pappa et al., 2020). Studies from different parts of the world have suggested prevalence of anxiety to range from 11.3%–50% (Zhu et al., 2020; Lai et al., 2020; Chew et al., 2020; Tan et al., 2020) and findings of the present study are within this reported range. This high level of anxiety among the HCWs in Nepal could be attributed to factors like deprivation of protective gears and resultant fear of getting infected. Previous studies have also linked mental morbidity in HCWs to inadequacy of PPEs and increase the risk of exposure to infection (Wu et al., 2020; Du et al., 2020; Liu et al., 2020).

In the present study, the prevalence of depression was 8%, which is much lower than that reported in some of the previous studies from different parts of the world (Lai et al., 2020) and the pooled prevalence reported in the metanalysis (Pappa et al., 2020). The lower prevalence of depression possibly reflect the cross cultural variance in terms of exposure to adverse working conditions in countries like Nepal in routine clinical practice, when compared to many of the developed countries with well equipped health care facilities.

To conclude, this study highlighted that the HCWs who are an integral part of the fight against the Pandemic are suffering from psychiatric morbidity. Proactive measures should be taken by the government of Nepal to care for the psychological wellbeing of HCWs in order to control the impact of the Pandemic on the HCWs. These could be in the form of providing psychological support.

https://doi.org/10.1016/j.ajp.2020.102260
Received 16 June 2020
Available online 24 June 2020
1876-2018/ © 2020 Elsevier B.V. All rights reserved.

Table 1
Perceived Stress, anxiety, depression and mental well-being during Lockdown and Comparison of these variables between HCWs and Non − HCWs.

Variables	Whole sample (N = 150) Mean(SD)/ Frequency (%)
Mean GAD-7 score	3.9 (4.2) Range: 0 − 20 Median: 3.0
**Severity of Anxiety**	
Normal (0 − 4)	94 (62.7 %)
Mild (5 − 9)	41 (27.3 %)
Moderate (10 − 14)	12 (8.0 %)
Moderate-Severe (15 − 19)	2(1.3 %)
Severe (≥15)	1 (0.7 %)
Mean PHQ-9 score	3.9 (4.2) Range : 0 − 21; Median:3.0
**Severity of depression**	
Minimal (0 − 4)	99 (66.0 %)
Mild (5 − 9)	39 (26.0 %)
Moderate (10 − 14)	8 (5.3 %)
Moderate severe (15 − 19)	1 (0.7 %)
Severe (≥20)	3 (2.0 %)
Overall prevalence	
% of responders reporting GAD score ≥ 5	51 (34.0 %)
% of responders reporting PHQ-9 score > 10	12 (10.0 %)
Number of participants has Anxiety disorder only	42 (28 %)
Number of participants has depressive disorder only	1 (0.7 %)
Number of participants has both Anxiety and depressive disorder	9 (6.0 %)

Financial disclosure

None.

Declaration of Competing Interest

None.

Acknowledgment

The authors want to thank Dr. Prabhat Sapkota, Mr. Sandesh Sawant and Ms. Usha Ghimire by circulating the survey link among the different hospital staffs.

References

Chew, N., Lee, G., Tan, B., Jing, M., Goh, Y., Ngiam, N., Yeo, L., Ahmad, A., Ahmed Khan, F., Napolean Shanmugam, G., Sharma, A.K., Komalkumar, R.N., Meenakshi, P.V., Shah, K., Patel, B., Chan, B., Sunny, S., Chandra, B., Ong, J., Paliwal, P.R., Sharma, V.K., 2020. A multinational, multicentre study on the psychological outcomes and associated physical symptoms amongst healthcare workers during COVID-19 outbreak. Brain Behav. Immun. https://doi.org/10.1016/j.bbi.2020.04.049. S0889-1591(20)30523-7. Advance online publication.

Cohen, S., Kamarck, T., Mermelstein, R., 1983. A global measure of perceived stress. J. Health Soc. Behav. 24 (4), 385–396.

Du, J., Dong, L., Wang, T., Yuan, C., Fu, R., Zhang, L., Liu, B., Zhang, M., Yin, Y., Qin, J., Bouey, J., Zhao, M., Li, X., 2020. Psychological symptoms among frontline healthcare workers during COVID-19 outbreak in Wuhan. Gen. Hosp. Psychiatry. https://doi.org/10.1016/j.genhosppsych.2020.03.011. S0163-8343(20)30045-1. Advance online publication.

Grover, S., Sahoo, S., Mehra, A., Avasthi, A., Subramanyan, A., 2020. Psychological impact of COVID-19 lockdown: an online survey from India. Indian J. Psychiatry.

Kroenke, K., Spitzer, R.L., 2002. The PHQ-9: a new depression diagnostic and severity measure. Psychiatr. Ann. 32 (9), 509–515.

Lai, J., Ma, S., Wang, Y., Cai, Z., Hu, J., Wei, N., Wu, J., Du, H., Chen, T., Li, R., Tan, H., Kang, L., Yao, L., Huang, M., Wang, H., Wang, G., Liu, Z., Hu, S., 2020. Factors associated with mental health outcomes among health care workers exposed to coronavirus disease 2019. JAMA network open 3 (3), e203976. https://doi.org/10.1001/jamanetworkopen.2020.3976.

Liu, C.Y., Yang, Y.Z., Zhang, X.M., Xu, X., Dou, Q.L., Zhang, W.W., Cheng, A., 2020. The prevalence and influencing factors in anxiety in medical workers fighting COVID-19 in China: a cross-sectional survey. Epidemiol. Infect. 148, e98. https://doi.org/10.1017/S0950268820001107.

Pappa, S., Ntella, V., Giannakas, T., Giannakoulis, V.G., Papoutsi, E., Katsaounou, P., 2020. Prevalence of depression, anxiety, and insomnia among healthcare workers during the COVID-19 pandemic: a systematic review and meta-analysis. Brain Behav. Immun. https://doi.org/10.1016/j.bbi.2020.05.026. S0889-1591(20)30845-X. Advance online publication.

Rana, W., Mukhtar, S., Mukhtar, S., 2020. Mental health of medical workers in Pakistan during the pandemic COVID-19 outbreak. Asian J. Psychiatr. 51https://doi.org/10.1016/j.ajp.2020.102080. 102080. Advance online publication.

Spitzer, R.L., Kroenke, K., Williams, J.B., Löwe, B., 2006. A brief measure for assessing generalized anxiety disorder: the GAD-7. Arch. Intern. Med. 166 (10), 1092–1097. https://doi.org/10.1001/archinte.166.10.1092.

Spoorthy, M.S., Pratapa, S.K., Mahant, S., 2020. Mental health problems faced by healthcare workers due to the COVID-19 pandemic-A review. Asian J. Psychiatr. 51https://doi.org/10.1016/j.ajp.2020.102119. 102119. Advance online publication.

Tan, B., Chew, N., Lee, G., Jing, M., Goh, Y., Yeo, L., Zhang, K., Chin, H.K., Ahmad, A., Khan, F.A., Shanmugam, G.N., Chan, B., Sunny, S., Chandra, B., Ong, J., Paliwal, P.R., Wong, L., Sagayanathan, R., Chen, J.T., Ying Ng, A.Y., Sharma, V.K., 2020. Psychological impact of the COVID-19 pandemic on health care workers in Singapore. Ann. Intern. Med. https://doi.org/10.7326/M20-1083. M20-1083. Advance online publication.

Tandon, R., 2020. The COVID-19 pandemic, personal reflections on editorial responsibility. Asian J. Psychiatr. 50https://doi.org/10.1016/j.ajp.2020.102100. 102100.

Wu, P.E., Styra, R., Gold, W.L., 2020. Mitigating the psychological effects of COVID-19 on health care workers. CMAJ 192 (17), E459–E460. https://doi.org/10.1503/cmaj.200519.

Zhang, W.R., Wang, K., Yin, L., Zhao, W.F., Xue, Q., Peng, M., Min, B.Q., Tian, Q., Leng, H.X., Du, J.L., Chang, H., Yang, Y., Li, W., Shangguan, F.F., Yan, T.Y., Dong, H.Q., Han, Y., Wang, Y.P., Cosci, F., Wang, H.X., 2020. Mental health and psychosocial problems of medical health workers during the COVID-19 epidemic in China. Psychother. Psychosom. 1–9. https://doi.org/10.1159/000507639. Advance online publication.

Zhu, J., Sun, L., Zhang, L., Wang, H., Fan, A., Yang, B., Li, W., Xiao, S., 2020. Prevalence and influencing factors of anxiety and depression symptoms in the first-line medical staff fighting against COVID-19 in Gansu. Front. Psychiatry 11, 386. https://doi.org/10.3389/fpsyt.2020.00386.

Anoop Krishna Gupta*
Department of Psychiatry National Medical College, Birgunj 44300, Nepal
E-mail address: dranoopkrn@gmail.com.

Aseem Mehra
PGIMER, Chandigarh, India
E-mail address: aseemmehra86@gmail.com.

Abhash Niraula
P.T. Birtacity Hospital and Research Center, Birtamod, Nepal
E-mail address: abhashniraula@gmail.com.

Khagendra Kafle
Department of Psychiatry, Chitwan Medical College, Bharatpur, Nepal
E-mail address: khagendrakafle@gmail.com.

Saroj Prasad Deo
Department of Dentistry, National Medical College, Birgunj, Nepal
E-mail address: drsrjdeo@gmail.com.

Babita Singh
Department of Nursing, National Medical College, Birgunj, Nepal
E-mail address: bobysin7181@gmail.com.

Swapnajeet Sahoo, Sandeep Grover
PGIMER, Chandigarh, India
E-mail addresses: swapnajit.same@gmail.com (S. Sahoo), drsandeepg2002@yahoo.com (S. Grover).

* Corresponding author.
