# Supplementary material for: Mental health status and related factors influencing healthcare workers during the COVID-19 pandemic: A systematic review and meta-analysis
Source: PLoS One. 2024 Jan 19;19(1):e0289454. doi: 10.1371/journal.pone.0289454 (PMC10798549; doi:10.1371/journal.pone.0289454)
Supplement: S1 Data — (ZIP) [file pone.0289454.s011.zip › literatures/49.pdf]

Neuropsychiatric Disease and Treatment

Dovepress

open access to scientific and medical research

Open Access Full Text Article

ORIGINAL RESEARCH

Mental Health Outcomes among Frontline Health-Care Workers at Eka Kotebe National COVID-19 Treatment Center, Addis Ababa, Ethiopia, 2020: A Cross-Sectional Study

Yodit Habtamu[1]
Kalkidan Admasu[2]
Mikiyas Tullu[3]
Woyenabeba Damene[4]
Addis Birhanu[5]
Teferra Beyero[1]
Addisu Birhanu Tereda[6]

[1]Department of Psychiatry, Eka Kotebe General Hospital, Addis Ababa, Ethiopia; [2]Psychosocial and Rehabilitation Services Department, Eka Kotebe General Hospital, Addis Ababa, Ethiopia; [3]Department of Psychiatry, College of Health Sciences, Kotebe Metropolitan University, Addis Ababa, Ethiopia; [4]Research and Training Department, Eka Kotebe General Hospital, Addis Ababa, Ethiopia; [5]Department of Public Health, College of Medicine, Jimma University, Jimma, Ethiopia; [6]Department of Internal Medicine, Eka Kotebe General Hospital, Addis Ababa, Ethiopia

Background: The COVID-19 pandemic has resulted in many frontline health-care workers vulnerable to developing various mental health conditions. This study aimed to determine prevalence and associated factors of such conditions among frontline workers at Eka Kotebe National COVID-19 Treatment Center in Addis Ababa, Ethiopia.

Methods: This institution-based cross-sectional study was conducted between May and June 2020 on 280 frontline workers. Mental health outcomes (depression, anxiety, insomnia, and posttraumatic stress disorder) were assessed using the Patient Health Questionnaire9, Generalized Anxiety Disorder 7 questionnaire, PTSD Checklist — civilian version, and Pittsburgh Sleep Quality Index. Responses were coded, entered into EpiData 3.1 and analyzed using SPSS 20. Associations between outcomes and independent variables were identified using bivariate and multivariate logistic regressions, statistical significance set at $p<0.05$.

Results: A total of 238 subjects participated in the study, with a response rate of 85%. Estimated prevalence was 31.1% (95% CI 24.8%–37%) for anxiety, 27.3% (95% CI 21.8%–32.4%) for depression, 16% (95% CI 11.3%–21%) for PTSD, and 40.8% (95% CI 33.6%–47.5%) for insomnia. Female sex (AOR 2.99, 95% CI 1.49–5.97), being married, (AOR 13.2, 95% CI 3.42–50.7), being single (AOR 11.5, 95% CI 3.38–39.8), duration of exposure 1–2 hours (AOR 0.29, 95% CI 0.14–0.64), and assigned place of work (critical ward —AOR 2.26, 95% CI 1.03–4.97; ICU — AOR 4.44, 95% CI 1.51–13.05) were found to be significant predictors of depression.

Conclusion: We found a high estimated prevalence of mental health outcomes. Sex, marital status, duration of exposure, and assigned place of work were found to be associated with depression.

Keywords: COVID-19, depression, anxiety, PTSD, insomnia, frontline health-care workers, Eka Kotebe Hospital

Introduction

On December 30, 2019, a group of new pneumonia-like cases of unknown etiology was reported in Wuhan, China, and on January 9, 2020, China's Centers for Disease Control and Prevention reported a novel coronavirus as the causative agent for this outbreak. On January 30, the emergence of this novel coronavirus was declared a Public Health Emergency of International Concern (PHEIC) by the World Health Organization (WHO),[1] making it the sixth under the International Health Regulations. On February 11 the WHO officially named it COVID-19.[1]

Correspondence: Yodit Habtamu
Email yodita25yididya@yahoo.com

Received: 31 March 2021
Accepted: 7 August 2021
Published: 28 August 2021

© 2021 Habtamu et al. This work is published and licensed by Dove Medical Press Limited. The full terms of this license are available at https://www.dovepress.com/terms.php and incorporate the Creative Commons Attribution – Non Commercial (unported, v3.0) License (http://creativecommons.org/licenses/by-nc/3.0/). By accessing the work you hereby accept the Terms. Non-commercial uses of the work are permitted without any further permission from Dove Medical Press Limited, provided the work is properly attributed. For permission for commercial use of this work, please see paragraphs 4.2 and 5 of our Terms (https://www.dovepress.com/terms.php).

Since then, the disease has progressed alarmingly throughout the world, and as of May 28, 2020, 5,596,550 laboratory-confirmed COVID-19 cases had identified worldwide, among which 353,373 deaths from the disease had been report.[2, 3] Ethiopia reported the first recorded case of COVID-19 on March 13, 2020. This number grew exponentially, and as of the end of May, 841 cases had been reported, of which seven were reported to have died from it.[3, 4]

The COVID-19 pandemic has had a significant impact on mental health in the general population and those frontline workers fighting it directly on a daily basis in particular.[5]

Frontline health-care workers in direct contact with COVID-19 patients while assessing, assisting, and caregiving, are facing this pandemic and its resulting high workload and emotional toll, which puts them at high risk of having or developing mental health symptoms.[6] They work despite facing an increased workload and risk of self-infection, which implies susceptibility to complex emotional and psychological distress.[7] Such mental health states would further impair their ability to focus, perform, and make informed decisions,[8,9] with the likely occurrence of increased medical errors and ultimately putting themselves and their patients lives at risk. It is also fair to estimate that the nature of such psychological distress could have a lasting effect on their mental well-being.[10–13.]

The exponential increase in confirmed and suspected cases exposing such frontline workers to increased workload and less personal or family time, the extensive media coverage that induces stress overload, their perception of being inadequately supported at their workplaces in terms of scarcity of personal protection equipment and available support systems might make them vulnerable to stress overload, mental exhaustion, and burnout.[6]

Studies during the SARS and Ebola outbreaks revealed psychological distress among frontline care providers, accounting for about 18%–57%.[14,15]

The situation with COVID-19 is no different, and poses a more significant mental health threat for frontline workers.[16] Developed countries like China, the US, and others have reported that their health-care workers, first responders, and other frontline workers experienced significant levels of clinical depression, anxiety, posttraumatic stress disorder (PTSD), and suicidal ideation.[16–18.]

A systematic review conducted on 13 studies revealed prevalence of 23.2% for anxiety, 22.8% for depression, and 38.9% for insomnia.[8] Another global web-based survey conducted in 101 countries among surgical service providers for COVID-positive patients revealed that depression, anxiety, stress, and PTSD prevalence was 32.8%, 30.8%, 25.9%, and 24%, respectively.[19]

In one study in China, for example, the estimated prevalence of adverse mental health outcomes (depression, anxiety, insomnia, and PTSD symptoms) among health-care workers was 50.4%, 44.6%, 34%, and 71.5%, respectively.[20]

A study conducted in Asia–Pacific regions revealed the highest prevalence of PTSD to be in Vietnam and lowest prevalence of depression and anxiety to be in Singapore.[21] Another study conducted in five major hospitals in India and Singapore revealed prevalence of 5.3% for depression, 8.7% for anxiety, 2.2% for stress, and 3.8% for PTSD.[22] Another study conducted in Singapore found prevalence of 14.5% for anxiety, 8.9% for depression, 6.6% for stress, and 7.7% for PTSD.[23] A similar study in Pakistan estimated prevalence of depression and anxiety as 21.9% and 21.4%, respectively.[7]

In various studies, female sex, being single, occupation (nurse/doctor), workplace (ICU/emergency room), increased workload, lack of sleep, and fear have been stated as being strongly associated with the aforementioned mental health outcomes.[7,824–26.]

Despite all this evidence, in practice, major attention has not been given to the mental health of these frontline health-care workers in many parts of the world, and the situation is no different, if not worse, in most developing countries like Ethiopia.

Upon the initial detection of the first case of COVID-19 in the country on the 13th of March 13, 2020, Eka Kotebe Hospital was selected to be the National COVID-19 Treatment Center and began admitting COVID-19–positive patients. The staff then began facing various critical situations, which ultimately led them to struggle with psychological distress, sleep problems, and other mental health symptoms.

For such reasons as the new and unknown nature of the pandemic, the rapid rise in infections and death toll, and the severity of identified cases, much focus has been given to the management of the disease's acute physical symptoms and less attention paid to the resulting mental health impacts on frontline workers. Based on the evidence just cited, it is fair to assume that they will not be immune from adverse mental health outcomes. As such, the aim of this study was to estimate prevalence and determine associated factors for possible mental health outcomes among those frontline health-care workers battling against the

pandemic at the country's National COVID-19 Treatment Center, Eka Kotebe Hospital.

Methods

Study Design, Period, and Setting

This institution-based cross-sectional survey was conducted from May 15 to June 6, 2020 at Eka Kotebe General Hospital in the Yeka borough of Addis Ababa. It was originally built as part of an expansion of the country's only mental referral-hospital — the Amanuel Mental Specialized Hospital. Half the hospital's services (175 of a total of 350 beds) is dedicated to the treatment and care of patients with mental illness, and the remaining half (175 beds) is dedicated to surgery, gynecology and obstetrics, internal medicine, pediatrics, ophthalmology, dermatology, and dentistry. After being formally inaugurated in 2009, it began its operations in serving the community. Nonetheless, right after the news of COVID-19 went viral, the country's Ministry of Health decided to turn the hospital into a national treatment center for the pandemic. The hospital was then forced to seize all its previous operations and services and evacuate all of its patients and services to a makeshift facility. It then became the first hospital in the country to be fully dedicated to this cause, and after making all the necessary preparations, the major one being maximizing its capacity for inpatient service for 750 beds, it began admitting and serving only COVID-19–positive patients.

Participants

Participants in this study were frontline health-care professionals working at the hospital during the study period. Sample size was determined using a single population–proportion formula under assumption of 50% (0.5) prevalence for mental health outcomes, standard normal distribution Z-value of 1.96, 95% CI, statistical significance at α=0.05, and a 10% nonresponse rate. The calculated sample size was 423 participants, However, as the number of front-line health-care professionals working at the hospital during the study period was only 280, a census of all eligible study participants was considered in order to increase the statistical power of the study.

Measurements

Data were collected using standardized self-administered structured questionnaires designed and normalized to evaluate the study variables, and "mental health outcomes" was used as a collective operationalized construct to depict clinical PTSD, depression, anxiety, and insomnia, all of which were dependent variables in the study.

Data on the magnitude of PTSD were gathered using the Posttraumatic Stress Disorder Checklist — civilian version. This is a 17-item self-report measure reflecting DSM-IV symptoms of PTSD, with Likert-scale response options ranging from 1 (not at all) to 5 (extremely), with a total score range of 17–85. Scores ≥50 indicate probable existence of PTSD. In a study conducted on Oromo and Somali refugees, it yielded high reliability (Cronbach's α=0.93).[27] In this study, the scale showed internal consistency of α=0.96.

The Patient Health Questionnaire 9 is a brief nine-item questionnaire designed to detect major depressive disorder according to the criteria from the DSM-IV. It has been used widely in clinical and population-based studies across the globe as a screening and diagnostic instrument, and has total scores ranging from 0 to 27. It asks respondents whether or not they have experienced symptoms of depression over the last 2 weeks. Item scores are summed, with response options from 0 (not at all) to 3 (nearly every day). In Ethiopia, it has been validated with sensitivity and specificity of 86% and 67%, respectively. A cutoff of ≥10 is used to screen for depression.[28] In this study, the scale had internal consistency of α=0.94.

Anxiety was measured using the seven-item Generalized Anxiety Disorder 7 questionnaire, which measures and scores anxiety according to criteria from the DSM-IV with scales ranging from 0 (not at all) to 3 (nearly every day). It asks respondents if they have experienced symptoms of anxiety over the last 2 weeks, and has a total possible symptom-severity score of 0–21. Respondents who score ≥10 for anxiety symptoms are considered to have anxiety. A cutoff of ≥10 is used to screen for anxiety. This questionnaire was reported to have good internal consistency (Cronbach's α=0.82) in a study done in Khalifa, Kenya.[29] In this study, it showed internal consistency of α=0.95.

Data on magnitude of insomnia were collected using the Pittsburgh Sleep Quality Index. It contains 19 self-rated questions that are combined to form seven component scores, which then are added to yield one global score of 0–21, 0 indicating no difficulty and 21 severe difficulty. In a study done in Ethiopia, it showed sensitivity of 82% and specificity of 56.2%, with moderate internal consistency (Cronbach's α=0.59).[30] In this study, it showed internal consistency of α=0.88.

Perceived burnout was assessed using a selfreport question with a yes/no response. if the subject responded "yes," a further eight yes/no questions, will further assess the respondent's perceived burnout state. A response of "no" denotes no burnout.

Substance use was assessed with respect to constructs of current use and lifetime use. Current substance use was assessed using a self-report for specific nonmedical usage of alcohol, khat, tobacco, and/or cannabis during the last 3 months, while lifetime substance use signified usage of a specific substance (alcohol, khat, tobacco, and/or cannabis) for nonmedical purposes, even once, in the respondents' lifetime.

To collect sociodemographic data, other clinical variables, and work-related factors from the respondents, a yes/no questionnaire was developed and adopted using various studies as a reference base.

Data-Collection Technique

To assure data quality, emphasis was placed on designing the data-collection instruments. For simplicity, the instruments were modified appropriately and initially translated into Amharic to be better understood by all participants and then translated back to English. Pretesting was done on a separate population — similar frontline health-care workers at St Peter Hospital, accounting for 5% of the study's actual sample size — for 1 week prior to actual data collection and the results were not included in the main study.

The questionnaires were administered to subjects during their off-duty hours, and they were told to complete them and return them to the supervisors. The supervisors were psychiatric nurses with BSc degrees, and their role was orienting respondents about the ethical principles of confidentiality and data management prior to involvement with data collection, giving explanation and clarification on unclear and ambiguous questions, and collect the completed questionnaires. After the participants had been adequately oriented, they were informed about the procedures, and after securing their informed consent, they were participated in the research. Since the tool was self administered, it was possible to miss those respondents with possible mental health symptoms who might need further assessment and referral to appropriate interventional schemes, and it was the main responsibility of the supervisors to ascertain that participants were made aware of the possibility of contacting them for such possible causes should they become concerned about their reported emotional and mental health status following their responses.

Statistical Analysis

The data collected were coded, entered into EpiData 3.1, and analyzed using SPSS 20. Descriptive data are summarized using tables, and binary logistic regression was used to identify factors associated with outcome variables. Those variables with $P<0.2$ on bivariate regxxression were included in the multivariate logistic regression analysis. $P<0.05$ on multivariate logistic regression was considered statistically significant and the strength of association was determined using AORs with 95% CIs.

Ethics Approval and Consent to Participate

Ethics clearance was obtained from Eka Kotebe Hospital's Ethical Review Committee following the approval of the research projectEK/150/5/7). The study was conducted in accordance with the Declaration of Helsinki. All data collected were used for the sole purpose of this study did not contain identifying information, thus ensuring the privacy of the participants. Hard copies of the completed questionnaires were kept lock and key, and computer data were stored on a computer with code of access known only to the researcher. After the purpose of the study, informed consent was obtained from each subject. The form indicated that participation was voluntary and that respondents had the right to withdraw from completing the questionnaires at any given time they wished without any reason. Participants were also informed about there were no associated benefits or risks to participating in the study or to expect any form of special treatment(s) and/or remuneration for their participation.

Results

Sociodemographic Characteristics

A total of 238 subjects were included, for a response rate of 85%. More than half (56.3%) were women, more than half (64.3%) single, nearly half (42.9%) living alone at the time of the study, more than half (63%) Orthodox Christian, three-quarters (81.5%) degree holders, and more than half (58.8%) aged 24–28 years. Nearly three-quarters (68.9%) were nurses, nearly a quarter (24.8%) doctors, and the remaining 6.3% laboratory and X-ray technicians. (Table 1).

Table 1 Distribution of sociodemographic factors of study participants (n=238)

Variables	n	%
**Age, years**		
18–23	20	8.4
24–28	140	58.8
29–32	51	21.4
≥33	27	11.3
**Sex**		
Male	101	43.7
Female	137	56.3
**Religion**		
Orthodox	150	63
Protestant	59	24.8
Muslim	20	3.4
Other*	9	3.8
**Marital status**		
Married	66	27.7
Single	153	64.3
Divorced/widowed	19	8
**Current living status**		
With spouse	31	13
With children	19	8
With family	67	28.2
Alone	102	42.9
Other**	19	8
**Education**		
Diploma	14	5.9
Bachelor's degree	194	81.5
Master's degree and above	30	12.6
**Profession**		
Nurses	164	68.9
Doctors	59	24.8
Lab/X-ray technician	15	6.3

Notes: *Catholic, atheist; **with friends, with a roommate, in a hospital setting.

Table 2 Distribution of clinical factors of study participants (n=238)

Variables		n	%
**Chronic medical illness**			
No		207	87
Yes		31	13
**History of mental illness**			
No		186	78.2
Yes		52	21.8
**Family history of mental illness**			
No		209	87.8
Yes		29	12.2
**Burnout**			
No		46	19.3
Yes		192	80.7
Boredom	No	153	64.3
	Yes	85	35.7
Anxiety	No	139	58.4
	Yes	99	41.6
Physical pain	No	180	75.6
	Yes	58	24.4
Sleep deprivation	No	145	60.9
	Yes	93	39.1
Easily irritable	No	192	80.7
	Yes	46	19.3
Depressed mood	No	174	73.1
	Yes	64	26.9
Frequent disputes with colleagues	No	208	87.4
	Yes	30	12.6
Other*	No	218	91.6
	Yes	20	8.4

Note: *Disappointment, lack of motivation.

Clinical Factors

In sum, 31 participants (13%) had chronic medical illness, a fifth (21.8%) a history of mental illness, 12 (12.2%) a family history of mental illness, and three-quarters (80.7%) reported burnout since they began working at the COVID-19 treatment center. Among these, more than a third (41.6%) reported feeling anxious, 39 (39.1%) major sleep deprivation and 35 (35.7%) reported feelings of boredom (Table 2).

Work-Related Variables

Half the participants (54.2%) were permanent employees at the hospital, nearly half (48.7%) had had >3 months of experience at the hospital before the pandemic, nearly half (48.3%) had been assigned to work on the critical ward, and more than half (58.4%) were working directly with COVID-19–positive patients >3 hours a day. With respect to organizational support, more than half (58.8%) reported receiving material support from the hospital (Table 3).

Prevalence of Mental Health Outcomes

The overall estimated prevalence of anxiety was 31.1% (95% CI 24.8%–37%), depression 27.3% (95% CI 21.8%–32.4%), PTSD 16% (95% CI 11.3%–21%), and insomnia was 40.8% (95% CI 33.6%–47.5%) (Figure 1).

Table 3 Distribution of work-related factors of study participants (n=238)

**Category**		**n**	**%**
**Employment status**			
Permanent		129	54.2
Contract		109	45.8
**Duration of service**			
<1 month		7	2.9
1–2 months		47	19.7
2–3 months		68	28.6
>3 months		116	48.7
**Place of work**			
Critical ward		115	48.3
ICU		59	24.8
Recovery ward		64	26.9
**Duration of exposure to patients**			
<1 hour		21	8.8
1–2 hours		78	32.8
≥ 3 hours		139	58.4
**Does the hospital support you?**			
No		98	41.2
Yes		140	58.8
Materially	Yes	114	47.9
	No	124	52.1
Moral support	Yes	35	14.7
	No	203	85.3
Timely response to demands	Yes	17	7.1
	No	221	92.9
Clear job description/job aid	Yes	16	6.7
	No	222	93.3
Others*	Yes	6	2.5
	No	232	97.5

Note: *Timely payment of salary and allowances.

With respect to symptom severity, 68.5% of those with anxiety fell in the minimal–none category, 40% in the mild category, 30% in the moderate range, and 5% in the severe range. With respect to depression, 72.7% were in the minimal-none category, 13.9% in the mild–moderate category, 11.3% in the moderate–severe category, and 2.1% in the severe category.

Substance Use

A total of participants (15.5%) reported that they had used khat, nearly half (48.7%) alcohol, 15 (15.5%) cigarettes, and 14 (5.9%) cannabis in their lifetime. Regarding current use of substances in the past 3 months, 23 (9.7%) reported chewing khat, nearly half (45%) using alcohol, 13 (5.5%) using cigarettes, and five (2.1%) using cannabis use (Table 4).

Factors Associated with Depression

To determine independent variables associated with depression, bivariate logistic regression analysis was carried out.

Sex, marital status, profession, duration of exposure, and assigned place of work with $P<0.2$ on bivariate regression were found to be significant and included in multivariate logistic regression. Female sex, being married, being single, working continuously for 1–2 hours per day, and working in the ICU and critical wards significant associations with depression ($P<0.05$, Table 5).

Women were 2.99 times as likely to develop depression as men (AOR 2.99, 95% CI 1.49–5.97). Those who were married were 13.2 times (AOR 13.2, 95% CI 3.42–50.7) and those who were single were 11.5 times (AOR 11.5, 95% CI 3.38–39.8) more likely to develop depression than those who were widowed/separated.

The odds of developing depression for those who had exposed for 1–2 hours showed a decrease of 71% compared to those who had been exposed for >3 hours of work (AOR 0.29, 95% CI 0.14–0.64).

With respect to assigned place of work, the odds of developing depression were about double among those who were working in the critical ward (AOR 2.26, 95% CI 1.03–4.97) of those who were working in the recovery wards. In addition, the odds of developing depression were about quadruple among those working place in the ICU (AOR 4.44, 95% CI 1.51–13.05) of those working in the recovery wards.

Discussion

During the early era of the pandemic, most frontline health-care workers who were in direct contact with COVID-19–positive patients while assessing, assisting, and caregiving were vulnerable to developing various mental health symptoms.

The findings from the current study showed that the overall estimated prevalence of anxiety was 31.1%, depression 27.3%, PTSD 16%, and insomnia 40.8%. The findings of this study are in line with a systematic review, in which there was a 22.8% pooled prevalence of depression in ten studies and 38.9% pooled prevalence of insomnia in five studies.[8] In another global survey conducted in 101 countries, the reported prevalence of depression and anxiety was 32.8% and 30.8%, respectively.[19] In another

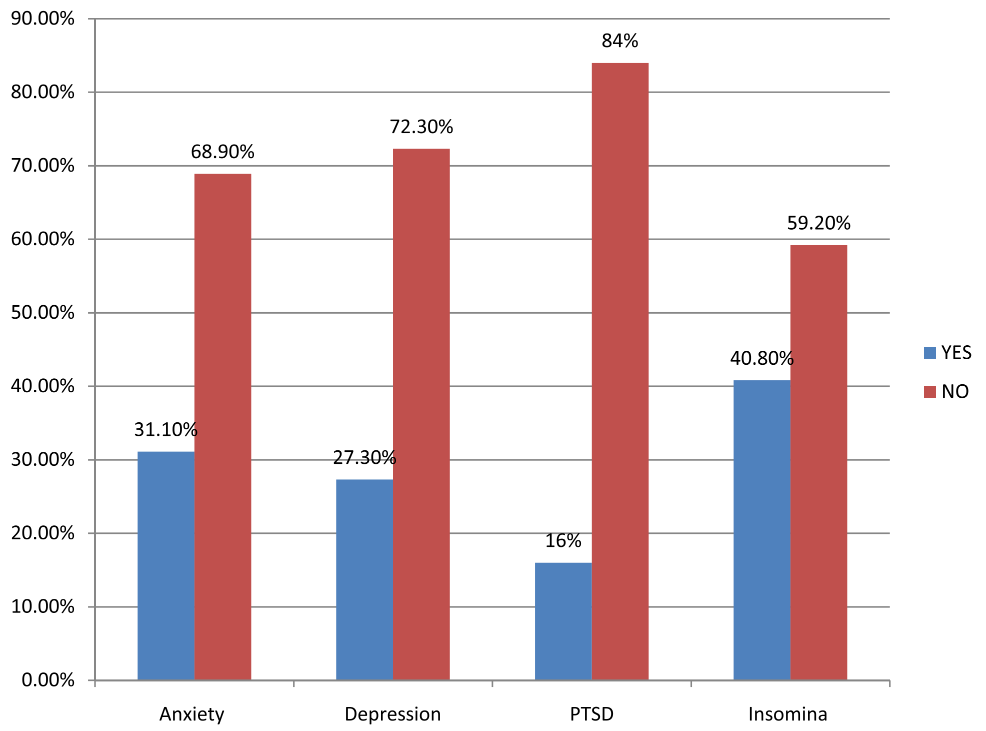

Figure 1 Prevalence of mental health outcomes among frontline health-care workers at Eka Kotebe General Hospital COVID-19 Treatment Center, Addis Ababa, Ethiopia, 2020 (n=238).

study conducted in Pakistan, the prevalence of depression was reported to be 21.9%.[7] This similarity might be explained by the instrument used in all the studies. In addition, the nature of the work, duration of exposure to patients, and experience in the process might play a role.

Nevertheless, the estimated prevalence in the current study was higher than other studies carried out in systematic reviews. For example, pooled prevalence of anxiety in 12 studies was 23.2%,[8] and was also 21.4% in another study conducted in Pakistan.[7] In another study conducted in five major hospitals in India and Singapore, the estimated prevalence of depression, anxiety, and PTSD was 5.3%, 8.7%, and 3.8% respectively.[22] Another study conducted in Singapore showed prevalence of anxiety, depression, and PTSD of 14.5%, 8.9%, and 7.7% respectively.[23] This difference could be attributed to sample size and the instrument used. Likewise, this study reported lower findings than reported in two others: one in China, which reported prevalence of 44.6% for anxiety, 50.4% for depression, and 71.5% for PTSD,[20] and the other one a global survey conducted in 101 countries, which reported a 24% prevalence of PTSD.[19] Here as well, the possible difference could be attributed to the number of study subjects and difference in the instruments used.

It was also found out that sex, marital status, duration of exposure, and assigned place of work were independent predictors of depression.

Women were three times as likely to develop depression as males. This finding is supported by a systematic review conducted in Pakistan.[7,8] A possible reason might be that women are at higher risk of developing depression due to lower emotional thresholds,[31] constant preoccupation with fear of being infected,[32] and fear due in perceived difficulty in controlling the pandemic and its impact, in addition to female sex, which entails greater vulnerablity to depression.[33]

The odds of developing depression among singletons were about eleven times those of the widowed/separated. This finding is supported by a study conducted in China.[25] The possible reason could be that the latter group might already have an established friend/relative support system and further patterns of support resources to which they can easily turn during life's difficulties that differentiates them from the former group, hence serving as a protective role.[32]

The odds of developing depression among those who were exposed to the infection for 1–2 hours were 71% lower than those who were expected to work constantly for more than 3 hours. A possible reason could be that the latter group might have suffered from dehydration and hypoxia from the

Table 4 Distribution of substance use of study participants (n=238)

Variables	n	%
**Lifetime use of khat**		
No	201	84.5
Yes	37	15.5
**Khat**		
No	215	90.3
Yes	23	9.7
**Lifetime use of alcohol**		
No	122	51.3
Yes	116	48.7
**Alcohol use in last 3 months**		
No	131	55
Yes	107	45
**Lifetime use of cigarettes**		
No	201	84.5
Yes	37	15.5
**Cigarette use in last 3 months**		
No	225	94.5
Yes	13	5.5
**Lifetime use of cannabis**		
No	224	94.1
Yes	14	5.9
**Cannabis use in last 3 months**		
No	238	97.9
Yes	5	2.1

load of working for an extended period of time while wearing full personal protective equipment.[34]

The odds of developing depression among those working in the ICU was about four times those of workers in the the recovery wards, parallel with a finding from Pakistan.[7] A possible reason could be that due to high workload, the difference in terms of the stressful nature of the two working environments and the nature, condition and health status of the patients they were treating posed a direct risk or perceived risk of infection.[32] It can also be attributed to compassion fatigue and vicarious trauma experienced witnessing patients' clinical conditions.

Limitations

The study has a few limitations To begin with, the number of study participants available and taken during the study period was small. Secondly, as the study was cross-sectional, causal relationships cannot be inferred. The lack of longitudinal follow-up is another limitation. Because of the increasingly demanding situation faced by the responderse following the rapid rise in the toll of the cases—both infection and death rates—the mental health symptoms of these frontline health care workers could have become more severe, and this study failed to examine longitudinal markers of likelihood of regression or progression of the reported symptoms and outcomes. As a result, the long-term psychological implications of of these workers are worth investigating further. Additionally, the study mainly used self-reported questionnaires to measure mental health symptoms, rather than the recommended clinician-administered structural interviews, which would have resulted in appropriate clinical diagnoses. Another possible limitation is that we failed to flag those frontline workers who reported mental health symptoms for further investigation and possible intervention, due to the self-administered nature of the questionnaires.

Conclusion and Recommendations

Overall, the prevalence of mental health outcomes among frontline health-care workers at Eka Kotebe National COVID-19 Treatment Center was found to be high. Female sex, being married, being single, long hours of exposure, and assigned place of work (ICU or critical wards) were found to be independent predictors of depression. It is recommended that periodic mental health–focused screening be carried out on a regular basis for frontline workers. Conducting such kinds of early screenings will enable the design and implementation of early-intervention strategies. Staff with significant mental health–outcome scores can possibly be identified and further connected with mental health–service schemes, either in the hospital or using proxy services. Such psychological interventions can assume the brief forms of either critical incident debriefings or psychological first aid, or a combination of both aimed at reducing possible psychological distress, or depending on the degree of severity, the intense and concrete therapies of eye-movement desensitization and reprocessing or trauma-focused cognitive behavioral therapy. Hospitals shouldl also arrange episodic leave following a certain number of hours of deployment, continuous refreshment training on stress-management and coping mechanisms, and design and implement staff mental health programs in order to maintain the mental health of staff for the long haul.

We also recommend further research and follow-up, in order to evaluate the progression or regression of reported mental health outcomes and their possible impact on the mental health of frontline responders following the aftermath of the COVID-19 pandemic.

Table 5 Independent bivariate and multivariate predictors of depression among frontline health-care workers at Eka Kotebe General Hospital COVID-19 Treatment Center (n=238)

Explanatory Variables	n (%)	Depression		COR(95% CI)	Combined OR(95% CI)
		No	Yes		
**Sex**					
Female	137(57.6%)	108(62.4%)	29(44.6%)	2.06(1.16–3.68)**	2.99(1.49–5.97)***
Male	101(42.4%)	65(37.6%)	36(55.4%)	1	1
**Marital status**					
Married	66(27.7%)	55(31.8%)	11(16.9%)	18.8(5.23–67.36)***	13.2(3.42–50.7)***
Single	153(64.3%)	114(65.9%)	39(60.0%)	10.9(3.43–35.01)***	11.5(3.38–39.8)***
Divorced/widowed	19(8%)	4(2.3)	15(23.1%)	1	1
**Profession**					
Nurse	164(68.9%)	110(63.6%)	54(83.1%)	1	1
Doctor	59(24.8%)	50(28.9%)	9(13.8%)	2.73(1.25–5.95)**	2.14(0.91–5.01)
Lab/X-ray technician	15(6.3%)	13(7.5%)	2(3.1%)	3.19(0.69–14.65)	1.99(0.33–11.9)
**Duration of exposure to patients**					
<1 hour	21(8.8%)	19(11.0%)	2(3.1%)	2.96(0.65–13.37)	1.63(0.29–9.28)
1–2 hour	78(32.8%)	48(27.7%)	30(46.2%)	0.49(0.27–0.91)*	0.29(0.14–0.64)**
≥3 hour	139(58.4)	106(61.3%)	33()50.8%	1	1
**Assigned place of work**					
Critical ward	115(48.3%)	87(50.3%)	28(43.1%)	1.99(1.03–3.85)*	2.26(1.03–4.97)*
ICU	59(24.8%)	47(27.2%)	12(18.5%)	2.51 (1.12–5.64)*	4.44(1.51–13.05)**
Recovery ward	64(26.9%)	39(22.5%)	25(38.5%)	1	1

Notes: *$P<0.05$; **$P<0.01$; ***$P<0.001$

Abbreviations

PTSD, posttraumatic stress disorder; WHO, World Health Organization.

Data Sharing Statement

All data sets used and analyzed during the current study are available from the corresponding author on reasonable request via yodita25yididya@yahoo.com.

Acknowledgments

The authors would like to acknowledge Eka Kotebe Hospital for its enormous support in undertaking this research. We would like to express our gratitude to our colleagues for their constructive comments and committed cooperation by giving us valuable information.

Author Contributions

All authors made a significant contribution to the work reported, from project conception to study design to execution, acquisition of data, and analysis and interpretation. They took part in drafting, revising, and critically reviewing the article, gave final approval to the version to be published, have agreed on the journal to which the article has been submitted, and agree to be accountable for all aspects of the work.

Disclosure

The authors report no conflict of interest in this work.

References

1. Pang X, Zhu Z, Xu F, et al. Evaluation of control measures implemented in the severe acute respiratory syndrome outbreak in Beijing, 2003. *JAMA*. 2003;290(24):3215–3221. doi:10.1001/jama.290.24.3215
2. Morin CM, Rodrigue S, Ivers H. Role of stress, arousal, and coping skills in primary insomnia. *Psychosom Med*. 2003;65(2):259–267. doi:10.1097/01.PSY.0000030391.09558.A3
3. Organization WH. Novel coronavirus (2019-nCoV): situation report; 2020 Available from: https://www.who.int/emergencies/diseases/novel-coronavirus-2019/situation-reports. Accessed August 9, 2021.
4. Sateia MJ. International classification of sleep disorders. *Chest*. 2014;146(5):1387–1394. doi:10.1378/chest.14-0970
5. Brooks SK, Dunn R, Amlôt R, Rubin GJ, Greenberg N. A systematic, thematic review of social and occupational factors associated with psychological outcomes in healthcare employees during an infectious disease outbreak. *J Occupational Environ Med*. 2018;60(3):248–257. doi:10.1097/JOM.0000000000001235
6. Liu S, Yang L, Zhang C, et al. Online mental health services in China during the COVID-19 outbreak. *The Lancet Psychiatry*. 2020;7(4): e17–e8. doi:10.1016/S2215-0366(20)30077-8

7. Salman M, Raza MH, Mustafa ZU, et al. The psychological effects of COVID-19 on frontline healthcare workers and how they are coping: a web-based, cross-sectional study from Pakistan. *medRxiv*. 2020; Preprint. doi:10.1101/2020.06.03.20119867
8. Pappa S, Ntella V, Giannakas T, Giannakoulis VG, Papoutsi E, Katsaounou P. Prevalence of depression, anxiety, and insomnia among healthcare workers during the COVID-19 pandemic: a systematic review and meta-analysis. *Brain Behav Immun*. 2020;88:901–907. doi:10.1016/j.bbi.2020.05.026
9. Zhang W-R, Wang K, Yin L, et al. Mental health and psychosocial problems of medical health workers during the COVID-19 epidemic in China. *Psychother Psychosom*. 2020;89(4):242–250. doi:10.1159/000507639
10. Zhu N, Zhang D, Wang W, et al. A novel coronavirus from patients with pneumonia in China, 2019. *N Engl J Med*. 2020;382(8):727–733. doi:10.1056/NEJMoa2001017
11. Zhou P, Yang X-L, Wang X-G, et al. A pneumonia outbreak associated with a new coronavirus of probable bat origin. *Nature*. 2020;579(7798):270–273. doi:10.1038/s41586-020-2012-7
12. Organization WH. *Coronavirus Disease 2019 (COVID-19): Situation Report, 88*; 2020.
13. Team EE. Note from the editors: World Health Organization declares novel coronavirus (2019-nCoV) sixth public health emergency of international concern. *Eurosurveillance*. 2020;25(5):200131e.
14. Di Filippo P, Attanasi M, Dodi G, et al. A Survey on Mental Health Impact Caused by COVID-19 Pandemic in Italian Pediatric Healthcare Workers. *BMC Public Health*. 2020;Preprint. doi:10.21203/rs.3.rs-34827/v1
15. Surveillances V. The epidemiological characteristics of an outbreak of 2019 novel coronavirus diseases (COVID-19)—China, 2020. *China CDC Weekly*. 2020;2(8):113–122. doi:10.46234/ccdcw2020.032
16. Huth JJ, Eliades A, Handwork C, Englehart JL, Messenger J. Shift worked, quality of sleep, and elevated body mass index in pediatric nurses. *J Pediatr Nurs*. 2013;28(6):e64–e73. doi:10.1016/j.pedn.2013.02.032
17. Costa AS, Griep RH, Rotenberg L. Associations of a short sleep duration, insufficient sleep, and insomnia with selfrated health among nurses. *PLoS One*. 2015;10(5):e0126844.
18. Kaneita Y, Ohida T. Association of current work and sleep situations with excessive daytime sleepiness and medical incidents among Japanese physicians. *J Clin Sleep Med*. 2011;07(05):512–522. doi:10.5664/JCSM.1322
19. Tan YQ, Wang Z, Yap QV, et al. Psychological health of surgeons in a time of COVID-19: a global survey. *Ann Surg*. 2021. doi:10.1097/SLA.0000000000004775
20. Lai J, Ma S, Wang Y, et al. Factors associated with mental health outcomes among health care workers exposed to coronavirus disease 2019. *JAMA Network Open*. 2020;3(3):e203976–e. doi:10.1001/jamanetworkopen.2020.3976
21. Chew NWS, Ngiam JN, Tan BY-Q, et al. Asian-Pacific perspective on the psychological well-being of healthcare workers during the evolution of the COVID-19 pandemic. *BJPsych Open*. 2020;6(6):6. doi:10.1192/bjo.2020.98
22. Chew NW, Lee GK, Tan BY, et al. A multinational, multicentre study on the psychological outcomes and associated physical symptoms amongst healthcare workers during COVID-19 outbreak. *Brain Behav Immun*. 2020;88:559–565. doi:10.1016/j.bbi.2020.04.049
23. Tan BY, Chew NW, Lee GK, et al. Psychological impact of the COVID-19 pandemic on health care workers in Singapore. *Ann Intern Med*. 2020;173(4):317–320. doi:10.7326/M20-1083
24. Soldatos CR, Allaert FA, Ohta T, Dikeos DG. How do individuals sleep around the world? Results from a single-day survey in ten countries. *Sleep Med*. 2005;6(1):5–13. doi:10.1016/j.sleep.2004.10.006
25. Furihata R, Uchiyama M, Takahashi S, et al. The association between sleep problems and perceived health status: a Japanese nationwide general population survey. *Sleep Med*. 2012;13(7):831–837. doi:10.1016/j.sleep.2012.03.011
26. Qi J, Xu J, Li B, et al. The evaluation of sleep disturbances for Chinese frontline medical workers. *Sleep Med*;72:1–4.
27. Jaranson JM, Butcher J, Halcon L, et al. Somali and oromo refugees: correlates of torture and trauma history. *Am J Public Health*. 2004;94(4):591–598. doi:10.2105/AJPH.94.4.591
28. Gelaye B, Williams MA, Lemma S, et al. Validity of the patient health questionnaire-9 for depression screening and diagnosis in East Africa. *Psychiatry Res*. 2013;210(2):653–661.
29. Nyongesa MK, Mwangi P, Koot HM, Cuijpers P, Newton CR, Abubakar A. The reliability, validity and factorial structure of the Swahili version of the 7-item generalized anxiety disorder scale (GAD-7) among adults living with HIV from Kilifi, Kenya. *Ann Gen Psychiatry*. 2020;19(1):1–10. doi:10.1186/s12991-020-00312-4
30. Salahuddin M, Maru TT, Kumalo A, Pandi-Perumal SR, Bahammam AS, Manzar MD. Validation of the pittsburgh sleep quality index in community dwelling Ethiopian adults. *Health Qual Life Outcomes*. 2017;15(1):1–7. doi:10.1186/s12955-017-0637-5
31. Gao W, Ping S, Liu X. Gender differences in depression, anxiety, and stress among college students: a longitudinal study from China. *J Affect Disord*. 2020;263:292–300. doi:10.1016/j.jad.2019.11.121
32. Furer P, Walker JR, Chartier MJ, Stein MB. Hypochondriacal concerns and somatization in panic disorder. *Depress Anxiety*. 1997;6(2):78–85. doi:10.1002/(SICI)1520-6394(1997)6:2<78::AID-DA4>3.0.CO;2-1
33. Lim GY, Tam WW, Lu Y, Ho CS, Zhang MW, Ho RC. Prevalence of depression in the community from 30 countries between 1994 and 2014. *Sci Rep*. 2018;8(1):1–10. doi:10.1038/s41598-018-21243-x
34. Maunder R, Hunter J, Vincent L, et al. The immediate psychological and occupational impact of the 2003 SARS outbreak in a teaching hospital. *CMAJ*. 2003;168(10):1245–1251.

Neuropsychiatric Disease and Treatment **Dovepress**

Publish your work in this journal

Neuropsychiatric Disease and Treatment is an international, peer-reviewed journal of clinical therapeutics and pharmacology focusing on concise rapid reporting of clinical or pre-clinical studies on a range of neuropsychiatric and neurological disorders. This journal is indexed on PubMed Central, the 'PsycINFO' database and CAS, and is the official journal of The International Neuropsychiatric Association (INA). The manuscript management system is completely online and includes a very quick and fair peer-review system, which is all easy to use. Visit http://www.dovepress.com/testimonials.php to read real quotes from published authors.

Submit your manuscript here: https://www.dovepress.com/neuropsychiatric-disease-and-treatment-journal
