# Supplementary material for: Mental health status and related factors influencing healthcare workers during the COVID-19 pandemic: A systematic review and meta-analysis
Source: PLoS One. 2024 Jan 19;19(1):e0289454. doi: 10.1371/journal.pone.0289454 (PMC10798549; doi:10.1371/journal.pone.0289454)
Supplement: S1 Data — (ZIP) [file pone.0289454.s011.zip › literatures/51.pdf]

frontiers in Psychiatry

ORIGINAL RESEARCH
published: 18 June 2021
doi: 10.3389/fpsyt.2021.687440

Risk Factors for Anxiety and Depressive Symptoms in Doctors During the Coronavirus Disease 2019 Pandemic

Lanying He[1], *Jian Wang*[1]*, *Lijuan Zhang*[2], *Feng Wang*[1], *Weiwei Dong*[3] *and Wang Zhao*[4]

[1] *Department of Neurology, The Second People's Hospital of Chengdu, Chengdu, China,* [2] *Department of Neurology, The Second Affiliated Hospital of Chengdu College, Nuclear Industry 416 Hospital, Chengdu, China,* [3] *Department of Neurology, First Affiliated Hospital, Chongqing Medical University, Chongqing, China,* [4] *Department of Neurology, Yongchuan Hospital, Chongqing Medical University, Chongqing, China*

Background: The purpose of our study was to investigate the prevalence of anxiety and depressive symptoms and their risk factors among doctors during the coronavirus disease 2019 (COVID-19) pandemic.

Methods: A hospital-based survey study was conducted. Anxiety symptoms were assessed using the Self-Rating Anxiety Scale (SAS), and depressive symptoms were assessed using the Self-Rating Depression Scale (SDS). Multivariable logistic regression was used to analyze anxiety and depressive symptoms across independent variables.

Results: A total of 1,521 doctors were included; 11.11% (169/1,521) of the doctors had anxiety symptoms, and 16.90% (257/1,521) had depressive symptoms. Female sex [adjusted odds ratio (aOR), 1.69; 95% confidence interval (CI), 1.21–2.34; $P = 0.002$] and having a minor child (aOR, 2.31; 95% CI, 1.50–3.56; $P < 0.001$) were associated with an increased risk of anxiety symptoms. Female sex (aOR, 1.56; 95% CI, 1.18–2.06; $P = 0.002$) and having a minor child (aOR, 1.48; 95% CI, 1.06–2.01; $P = 0.022$) were associated with an increased risk of depressive symptoms. Older age (aOR, 0.97; 95% CI, 0.98–0.99; $P = 0.008$) was associated with a decreased risk of depressive symptoms.

Conclusions: Anxiety and depressive symptoms have been common mental health problems in doctors during the COVID-19 pandemic. We found that female sex, having a minor child, and younger age were major risk factors for the development of anxiety and depressive symptoms among doctors during the COVID-19 pandemic.

Keywords: COVID-19, anxiety, depression, doctors, mental health

OPEN ACCESS

Edited by:
Richard Porter, University of Otago, New Zealand

Reviewed by:
Jennifer L. Vande Voort, Mayo Clinic, United States
Elisabetta Filomena Buonaguro, Università di Medicina e Chirurgia Federico II Napoli, Italy

***Correspondence:**
Jian Wang
383376877@qq.com

Specialty section:
This article was submitted to Mood and Anxiety Disorders, a section of the journal Frontiers in Psychiatry

Received: *29 March 2021*
Accepted: *21 May 2021*
Published: *18 June 2021*

Citation:
He L, Wang J, Zhang L, Wang F, Dong W and Zhao W (2021) Risk Factors for Anxiety and Depressive Symptoms in Doctors During the Coronavirus Disease 2019 Pandemic. Front. Psychiatry 12:687440. doi: 10.3389/fpsyt.2021.687440

INTRODUCTION

Since the outbreak of coronavirus disease 2019 (COVID-19) in Wuhan on December 30, 2019 (1), the disease has had a huge impact on all aspects of Chinese society. With the rising number of cases and deaths caused by COVID-19, China has taken some measures to prevent the spread of COVID-19, including quarantine, lockdown, and physical distancing. Because of the prolonged confinement, negative effects on the economy, employment, and public health have been reported during the COVID-19 pandemic. With worries about future uncertainty, concern had been growing about the mental health sequelae of the COVID-19 crisis.

With the increasing number of patients and suspected cases, the burden of clinical treatment has increased. Repeated modifications in infection control procedures and recommendations have increased the uncertainty. In this setting, doctors have been directly involved in the diagnosis and treatment of COVID-19 patients, which puts them at higher risk for chronic stress, psychological distress, and other mental health symptoms including depression, insomnia, stress, anxiety, anger, irritability, and emotional exhaustion. In addition, high workloads, widespread media coverage, lack of specific treatment, and feeling less support could all contribute to the mental strain on these health workers (2). Clinical doctors who provide clinical services must work in high-risk environments. Previous studies have reported that healthcare workers might have higher rates of depression, anxiety, insomnia, obsessive and somatization symptoms, and post-traumatic stress symptoms than non-healthcare workers. During the COVID-19 pandemic, higher levels of anxiety and depression were shown to be positively correlated with stress among healthcare workers (3–5). Gender, living in rural areas, female, low social support, risk of exposure to COVID-19-positive patients, fear of contracting the disease, worry about lack of medical supplies, and long working hours were significantly associated with anxiety and depression among clinical doctors (3, 4, 6–8). In addition, a history of physical symptoms similar to COVID-19 infection (such as cough, sore throat, lethargy, dyspnea, loss of appetite, and myalgia) can also predispose healthcare workers to symptoms of depression, anxiety, stress, and post-traumatic stress (9).

If anxiety and depression are not treated, they might have a negative impact on the health of clinical doctors and alter their ability to properly perform their duties, including treating and preventing COVID-19. Therefore, the aim of the present study was to evaluate the prevalence and risk factors for anxiety and depressive symptoms among clinical doctors at three hospitals during the COVID-19 pandemic.

METHODS

This was a cross-sectional study, which was conducted from January 20 to May 10, 2020, among three hospitals, that is, the Second People's Hospital of Chengdu, Nuclear Industry 416 Hospital, and Yongchuan Hospital. Online survey management software was used in this study to allow the target subjects to post and complete the questionnaire online. The collected data were automatically entered into a spreadsheet. The study population included active full-time staff members at the three hospitals. The demographic variables considered in the analysis were sex, age, education level, marital status, household income, having minor child (yes/no), Social Support Rating Scale (SSRS) score, and working for COVID-19 control and prevention (yes/no).

Anxiety symptoms were assessed using the Self-Rating Anxiety Scale (SAS) (10), which consisted of 20 items that were scored from 1 to 4 (1 = rarely, 2 = occasionally, 3 = frequently, and 4 = always). Fifteen questions were ranked proportionally, with higher scores indicating more severe symptoms and lower scores for the remaining five questions indicating less severe symptoms. The raw scores were converted to index scores by multiplying by 1.25. The score was defined as follows (10): no anxiety (<50), low anxiety (50-59), moderate anxiety (60-69), and severe anxiety (>70). This scale has good internal consistency with a Cronbach's alpha of 0.931 (11).

Depressive symptoms were assessed using the Self-Rating Depression Scale (SDS) (12), which contained 20 items. The SDS consisted of 10 positive symptoms and 10 negative symptoms questions, and each item was scored from 1 to 4 (1 = none or a little, 2 = sometimes, 3 = most of the time, and 4 = all of the time). The severity of depressive symptoms was measured by a total SDS score × 1.25. The scores were grouped into four categories (13): no depression (<50), low depression (50-59), moderate depression (60-69), and severe depression (>70).

Clinical doctors were informed that their responses would be confidential and that their leaders and colleagues would not have access to their test results.

STATISTICAL ANALYSIS

The data are presented as numbers (%), or mean values (±standard deviation, SD). To identify differences between two groups, Pearson's χ^2 tests were used for categorical variables. Student's t-tests were used to compare normally distributed variables. The Mann–Whitney U-tests were used to compare non-normally distributed variables. Multivariate logistic regression analysis was performed to identify determinants independently associated with anxiety and depressive symptoms. The results are expressed as adjusted odds ratios (aOR) with their corresponding 95% confidence intervals (CIs). The data were analyzed using SPSS 22 software. $P < 0.05$ was considered statistically significant.

RESULTS

Demographic Characteristic

A total of 1,521 doctors were included in this study, and the mean age of the participants was 42.7 years. Of these, 53.00% (806) were female, and 85.80% (1,305) had an MSc and above education. A total of 52.26% (795) had a minor child, 34.65% (527) were working for COVID-19 control and prevention, and 68.31% (1,039) had household incomes >150,000 Chinese yuan/year.

Risk Factors for Anxiety Symptoms

Based on the SAS results, 169 (11.11%) doctors had anxiety symptoms. The average SAS score of the doctors with anxiety symptoms was 60.53 ± 8.70, and the average SAS score of the doctors with no anxiety symptoms was 25.68 ± 13.68. Baseline characteristics of doctors in the no anxiety and anxiety symptoms groups were compared (**Table 1**). The results showed that age, female sex, and having a minor child had effects on the anxiety symptoms of the doctors, and these differences were statistically significant ($P < 0.05$).

The incidence of anxiety symptoms among the doctors was 11.11%, that among doctors with a minor child was 14.21% (113/795), and that among females was 13.65% (110/806). Among the anxiety symptoms group, 23 doctors had severe

TABLE 1 | Comparison of baseline characteristics between doctors with no anxiety and anxiety symptoms groups.

	No anxiety group (1,352)	Anxiety group (169)	OR (95% CI)	*P**
Age, years (mean ± SD)	42.79 ± 10.21	42.26 ± 9.95		0.522
Social Support Rate Scale (SSRS) (mean ± SD)	38.07 ± 18.75	36.36 ± 18.85		**0.043**
SAS (mean ± SD)	26.31 ± 16.69	61.75 ± 8.53		**<0.001**
Married, *n* (%)	1,272 (94.08)	161 (95.27)	1.27 (0.61–2.67)	0.534
Females, *n* (%)	696 (51.48)	110 (65.09)	1.76 (1.26–2.45)	**0.001**
Education (MSc and above education), *n* (%)	1,159 (85.72)	146 (86.39)	1.06 (0.66–1.68)	0.815
Parents, *n* (%)	1,320 (97.63)	164 (97.04)	0.80 (0.31–2.07)	0.638
Having minor child, *n* (%)	682 (50.44)	113 (66.86)	1.98 (1.41–2.78)	**<0.001**
Chronic disease, *n* (%)	69 (5.10)	10 (5.92)	1.17 (0.59–2.32)	0.653
Working experience in years, n (%)	904 (66.86)	115 (68.05)	1.05 (0.75–1.49)	0.758
Living with family members, *n* (%)	1,088 (80.47)	144 (85.21)	1.40 (0.90–2.18)	0.139
Working for COVID-19 control and prevention, *n* (%)	477 (35.28)	50 (29.59)	0.77 (0.54–1.09)	0.142
Household income, Chinese yuan (CNY) > 150,000/year, *n* (%)	921 (68.12)	118 (69.82)	1.08 (0.77–1.53)	0.654

**Comparison between no anxiety and anxiety symptoms groups. To identify differences between two groups, Pearson's χ^2 test was used for categorical variables. Student's t-test was used to compare normally distributed variables. The Mann–Whitney U-test was used to compare non-normally distributed variables. Bold indicates P-values less than 0.05.*

TABLE 2 | Multivariable models showing association between baseline risk factors and anxiety symptoms.

	OR (95% CI)	*P**
Female	1.69 (1.21–2.34)	**0.002**
Having minor child	2.31 (1.50–3.56)	**<0.001**

**Multivariable adjusted for age, Social Support Rating Scale (SSRS), married, sex, education, parents, having minor child, chronic disease, working experience in years, living with family members, working for COVID-19 control and prevention duration of care time, and household income. Bold indicates P-values less than 0.05.*

anxiety symptoms, 57 had moderate anxiety symptoms, and 89 had mild anxiety symptoms. The anxiety symptom level of the doctors with a minor child was higher than that of those with no minor child (29.08 ± 16.19 vs. 31.30 ± 18.17, $P = 0.012$), and the anxiety symptom level in female doctors was higher than that in male doctors (31.05 ± 18.10 vs. 29.34 ± 16.27, $P = 0.054$); although there was no significant difference, the incidence of anxiety symptoms in female doctors was significantly higher than that in male doctors [13.64% (110/806) vs. 8.25% (59/715), $P = 0.001$].

To further analyze the risk factors for the anxiety symptoms among the doctors, multivariable logistic regression was performed. After age, SSRS, marital status, sex, education, being a parent, having a minor child, chronic disease, working experience in years, living with family members, working for COVID-19 control and prevention, duration of care time, and household income were adjusted for, the results showed that female sex (aOR, 1.69; 95% CI, 1.21–2.34; $P = 0.002$) and having a minor child (aOR, 2.31; 95% CI, 1.50–3.56; $P < 0.001$) were associated with increased risk of anxiety symptoms (**Table 2**).

Risk Factors for Depressive Symptoms

Based on the SDS results, 257 (16.90%) doctors had depressive symptoms. The average SDS scores of the doctors with depressive symptoms were 55.46 ± 17.16, and the average SDS scores of the doctors with no depressive symptoms were 26.42 ± 14.86. Baseline characteristics of the doctors in the non-depressive and depressive symptoms groups were compared (**Table 3**). The results showed that age, female sex, having a minor child, and being a parent had effects on depressive symptoms, and the differences between the two groups were statistically significant ($P < 0.05$).

The incidence of depressive symptoms among the doctors was 16.90%, that among those with a minor child was 21.01% (167/795), and that among female doctors was 19.98% (161/806). The incidence of depressive symptoms in doctors with a minor child was higher than that in doctors without a minor child [21.01% (167/795) vs. 12.40% (90/726), $P < 0.001$], and the incidence of depressive symptoms in female doctors was significantly higher than that in male doctors [19.98% (161/806) vs. 13.43% (96/715), $P = 0.001$]. The depressive symptom level between female doctors and male doctors was not significantly different ($P > 0.05$).

To further analyze the risk factors for depressive symptoms among the doctors, multivariable logistic regression was performed. After age, SSRS, marital status, sex, education, being a parent, having a minor child, chronic disease, working experience in years, living with family members, working for COVID-19 control and prevention, duration of care time, and household income were adjusted for, the results showed that female sex (aOR, 1.56; 95% CI, 1.18–2.06; $P = 0.002$) and having a minor child (aOR, 1.48; 95% CI, 1.06–2.01; $P = 0.022$) were associated with an increased risk of depressive symptoms. Older age (aOR, 0.97; 95% CI, 0.98–0.99; $P = 0.008$) was associated with a decreased risk of depressive symptoms (**Table 4**).

DISCUSSION

In this study, we found that the incidence of anxiety and depressive symptoms among clinical doctors was approximately

TABLE 3 | Comparison of baseline characteristics between doctors with non-depressive and depressive symptoms groups.

	No depression group (1,264)	Depression group (257)	OR (95% CI)	P*
Age, years (mean ± SD)	43.20 ± 10.25	40.41 ± 9.52		**<0.001**
SSRS (mean ± SD)	37.80 ± 18.84	38.25 ± 18.34		**0.924**
SDS (mean ± SD)	26.42 ± 14.86	55.46 ± 17.16		**<0.001**
Married, *n* (%)	1,186 (93.83)	247 (96.11)	1.62 (0.83–3.18)	0.154
Females, *n* (%)	645 (51.08)	161 (62.65)	1.61 (1.22–2.12)	**0.001**
MSc and above education, *n* (%)	1,075 (85.04)	230 (89.49)	1.65 (1.06–2.57)	0.063
Parents, *n* (%)	1,239 (98.02)	245 (95.33)	0.41 (0.20–0.83)	**0.011**
Having minor child, *n* (%)	628 (49.68)	167 (64.98)	1.88 (1.42–2.48)	**<0.001**
Chronic disease, *n* (%)	67 (5.30)	12 (4.67)	0.88 (0.47–1.64)	0.678
Working experience in years, *n* (%)	847 (67.01)	172 (66.93)	1.00 (0.75–1.32)	0.979
Living with family members, *n* (%)	1,026 (81.17)	257 (100)	0.94 (0.67–1.31)	0.705
Working for COVID-19 control and prevention, n (%)	429 (33.94)	98 (38.13)	1.20 (0.91–1.58)	0.198
Household income, Chinese yuan (CNY) > 150,000/year, *n* (%)	860 (68.04)	179 (69.65)	1.08 (0.81–1.44)	0.613

**Comparison between non-depressive and depressive symptoms groups. To identify differences between two groups, Pearson's χ^2 test was used for categorical variables. Student's t-test was used to compare normally distributed variables. The Mann–Whitney U-test was used to compare nonnormally distributed variables. Bold indicates P-values less than 0.05.*

TABLE 4 | Multivariable models showing association between baseline risk factors and depressive symptoms.

	OR (95% CI)	P*
Female	1.56 (1.18–2.06)	0.002
Having minor child	1.48 (1.06–2.01)	0.022
Older age	0.97 (0.98–0.99)	0.008

**Multivariable adjusted for age, Social Support Rating Scale (SSRS), married, sex, education, parents, having minor child ≥ 1, chronic disease, working experience in years, living with family members, working for COVID-19 control and prevention duration of care time, and household income.*

11.1 and 16.90%, respectively, which was lower than that of previous studies conducted in other countries (12, 14, 15). Furthermore, we found that female doctors and those who have a minor child were associated with an increased risk of anxiety and depressive symptoms. The results also showed that younger clinical doctors were more likely to experience depressive symptoms.

A previous study showed that in Malaysia, 31.60% of healthcare workers experienced depression and 29.1% had anxiety symptoms during the COVID-19 pandemic (12). A study in Ethiopia showed that the prevalence of COVID-19-related anxiety was 63% (14). In a highly burdened area of northeastern Italy, a study showed that 50.1% of healthcare workers showed anxiety symptoms and 26.6% had moderate depressive symptoms (15). The incidences of anxiety and depressive symptoms among clinical doctors in our study were lower than those in previous studies (12, 14, 15). The possible reasons for these differences were as follows: first, the Chinese government had taken prompt and effective measures to enhance the sense of security of its citizens and to release frequent information on the epidemic. Rapid sharing of epidemic information is an effective way to reduce public panic (16). Second, the government organized professionals to train doctors and provide knowledge about COVID-19, and therefore, doctors had a high level of understanding regarding COVID-19. The level of COVID-19 knowledge of doctors plays an important role in responding to epidemic crises (17, 18). On the other hand, Sichuan and Chongqing were not at the center of the COVID-19 pandemic.

Doctors are directly involved in the prevention, diagnosis, treatment, and care of COVID-19 patients; in addition, high workloads, lack of specific drugs, and inability to adhere to prevention strategies may contribute to psychological burden on clinicians (19, 20). In China, most doctors have a high workload per day. During the COVID-19 outbreak, high workloads and severe emotional and physical stress may have led to fatigue and decreased performance, ultimately leading to mood disorders. Our findings showed that doctors having a minor child were at risk factor for depressive and anxiety symptoms. Doctors with minor children had to spend more time with their children and care for children's daily lives; in addition, some children had strict online learning courses, and parents had to spend time helping their children, especially young students. Higher work demands and fatigue have been associated with a higher risk of having poor mental health. In China, women may undertake more family work in the family. The present study showed that female doctors showed higher levels of stress than male doctors.

The pandemic disrupted the economic systems in China, and the increased economic burden caused by the novel COVID-19 outbreak became a worrisome issue for individuals, especially among low-income individuals (21). Chengdu and Chongqing are in Western China, where doctors are paid less; half of the doctors (50%) had a monthly salary of between 5,000 and 10,000 RMB, with only 10% paid more than 10,000 RMB per month, and approximately 80% of junior doctors were paid 5,000 RMB or less. Medical workers aged below 40 years had lower occupational titles and faced mental health disorders of anxiety and depression, probably due to insufficient experience in dealing with this type of public health emergency, which is similar to the findings in Taiwan during the SARS outbreak (9). In addition, their incomes

were at a lower level, and therefore, their family economic burden was heavy.

In conclusion, this study investigated the anxiety and depressive states of doctors during the COVID-19 pandemic, and the results revealed that the prevalence of depression and anxiety was high among these doctors. We found that younger age, having a minor child, and female sex were major risk factors for the development of anxiety and depressive symptoms in doctors. These results indicate that leaders should provide psychological counseling and social support to doctors to protect their mental health.

LIMITATIONS

There were several limitations in the current study. First, the self-report questionnaire used in this study may have resulted in response bias from the participants. Although we are aware of this limitation, the research team was unable to conduct face-to-face interviews with the clinicians administered with the questionnaires due to government controls on population movement to prevent the transmission of COVID-19. Second, the participants came from tertiary hospitals in Sichuan and Chongqing, and these areas were not the worst areas regarding COVID-19 outbreaks. As a result, the results may not be generalizable to the entire nation of doctors. Third, we lacked data regarding the prevalence rate of depression and anxiety of doctors in Sichuan and Chongqing before COVID-19. In China, the most populous country and one of the largest developing countries in the world, has significant regional ethnic, linguistic, and cultural diversities. Studies of the mental health of Chinese doctors have had mixed results, and the results could not be used for direct comparison. In addition, due to the absence of longitudinal follow-up, we do not know how the psychological symptoms changed over time. Despite the limitations of the study, our results provide valuable information on the mental health status of doctors in the face of public health emergencies.

DATA AVAILABILITY STATEMENT

The original contributions presented in the study are included in the article/supplementary material, further inquiries can be directed to the corresponding author/s.

ETHICS STATEMENT

The studies involving human participants were reviewed and approved by Medical and Health Research Ethics Committee in Second people's Hospital of Chengdu, Nuclear Industry 416 Hospital and Yongchuan Hospital. The patients/participants provided their written informed consent to participate in this study. Written informed consent was obtained from the individual(s) for the publication of any potentially identifiable images or data included in this article.

AUTHOR CONTRIBUTIONS

LH was responsible for the concept and design of the study, data collection and analysis, and the first draft of the paper and further manuscript. JW was responsible for the concept and design of the study, the data analysis, and the interpretation. LZ and FW were responsible for the data analysis. WD was responsible for overseeing the concept and design of the study, the data analysis, and the interpretation. All authors read and approved the final manuscript for publication.

REFERENCES

1. Cevik M, Bamford C, Ho A. COVID-19 pandemic–a focused review for clinicians. *Clin Microbiol Infect.* (2020) 26:842-7. doi: 10.1016/j.cmi.2020.04.023
2. Brooks SK, Webster RK, Smith LE, Woodland L, Wessely S, Greenberg N, et al. The psychological impact of quarantine and how to reduce it: rapid review of the evidence. *Lancet.* (2020) 395:912-20. doi: 10.1016/S0140-6736(20)30460-8
3. Lai J, Ma S, Wang Y, Cai Z, Hu J, Wei N, et al. Factors associated with mental health outcomes among health care workers exposed to coronavirus disease 2019. *JAMA Netw Open.* (2020) 3:e203976.doi: 10.1001/jamanetworkopen.2020.3976
4. Feinstein RE, Kotara S, Jones B, Shanor D, Nemeroff CB. A health care workers mental health crisis line in the age of COVID-19. *Depress Anxiety.* (2020) 37:822-6. doi: 10.1002/da.23073
5. Mo Y, Deng L, Zhang L, Lang Q, Liao C, Wang N, et al. Work stress among Chinese nurses to support Wuhan in fighting against COVID-19 epidemic. *J Nurs Manag.* (2020) 28:1002-9. doi: 10.1111/jonm
6. Cai W, Lian B, Song X, Hou T, Deng G, Li H. A cross-sectional study on mental health among health care workers during the outbreak of Corona Virus Disease 2019. *Asian J Psychiatr.* (2020) 51:102111. doi: 10.1016/j.ajp.2020.102111
7. Cai HZ, Tu BR, Ma J, Chen LM, Fu L, Jiang YF, et al. Psychological impact and coping strategies of frontline medical staff in Hunan between January and March 2020 during the outbreak of Coronavirus Disease 2019 (COVID-19) in Hubei, China. *Med Sci Monit.* (2020) 26:e924171. doi: 10.12659/MSM.924171
8. Liu S, Yang L, Zhang C, Xiang YT, Liu Z, Hu S, et al. Online mental health services in China during the COVID-19 outbreak. *Lancet Psychiatry.* (2020) 7:e17-8. doi: 10.1016/S2215-0366(20)30077-8
9. Chew NWS, Lee GKH, Tan BYQ, Jing M, Goh Y, Ngiam NJH, et al. A multinational, multicentre study on the psychological outcomes and associated physical symptoms amongst healthcare workers during COVID-19 outbreak. *Brain Behav. Immun.* (2020) 88:559–65. doi: 10.1016/j.bbi.2020.04.049
10. Tao M, Gao JF. [Reliability and validity of the revised self-rating anxiety scale.] Chinese *J Nerv Ment Dis.* (1994) 5:301–3. [in Chinese]
11. Zung WW. A self-rating depression scale. *Arch Gen Psychiatry.* (1965) 12:63-70.doi: 10.1001/archpsyc.1965.01720310065008
12. Kang L, Ma S, Chen M, Yang J, Wang Y, Li R, et al. Mental Health Status of University Healthcare Workers during the COVID-19 pandemic: a post–movement lockdown assessment. *Brain Behav Immun.* (2020) 87:11-7. doi: 10.1016/j.bbi.2020.03.028
13. Wang XD. *Manual of Mental Health Rating Scale.* Beijing: China Mental Health Magazine (1993) p. 1120-1.
14. Kibret S, Teshome D, Fenta E, Hunie M, Tamire T. Prevalence of anxiety towards COVID-19 andits associated factors among healthcare workers in a Hospital of Ethiopia. *PLoS ONE.* (2020) 15:e0243022. doi: 10.1371/journal.pone.0243022

15. Lasalvia A, Bonetto C, Porru S, Carta A, Tardivo S, Bovo C, Ruggeri M, et al. Psychological impact of COVID-19 pandemic on healthcare workers in a highly burdened area of north-east Italy. *Epidemiol Psychiatr Sci.* (2020) 30:e1. doi: 10.1017/S2045796020001158
16. Song P, Karako T. COVID-19: Real-time dissemination of scientific information to fight a public health emergency of international concern. *Biosci Trends.* (2020) 14:1-2. doi: 10.5582/bst.2020.01056
17. Saadatian-Elahi M, Facy F, Del Signore C, Vanhems P. Perception of epidemic's related anxiety in the General French Population: a cross-sectional study in the Rhône-Alpes region. *BMC Public Health.* (2010) 10:191. doi: 10.1186/1471-2458-10-191
18. Hong S, Collins AR. Societal responses to familiar versus unfamiliar risk: comparisons of influenza and SARS in Korea. *Risk Anal.* (2006) 26:1247-57. doi: 10.1111/j.1539-6924.2006.00812.x
19. Peng X, Meng X, Li L, Hu C, Liu W, Liu Z, et al. Depressive and anxiety symptoms of healthcare workers in intensive care unit under the COVID-19 epidemic: an online cross-sectional study in China. *Front Public Health.* (2021) 9:603273. doi: 10.3389/fpubh.2021.603273
20. Mohd Fauzi MF, Mohd Yusoff H, Muhamad Robat R, Mat Saruan NA, Ismail KI, Mohd Haris AF. Doctors' mental health in the Midst of COVID-19 pandemic: the roles of work demands and recovery experiences. *Int J Environ Res Public Health.* (2020) 17:7340. doi: 10.3390/ijerph17197340
21. Hacimusalar Y, Kahve AC, Yasar AB, Aydin MS. Anxiety and hopelessness levels in COVID-19 pandemic: a comparative study of healthcare professionals and other community sample in Turkey. *J Psychiatr Res.* (2020) 129:181–8. doi: 10.1016/j.jpsychires.2020.07.024

Conflict of Interest: The authors declare that the research was conducted in the absence of any commercial or financial relationships that could be construed as a potential conflict of interest.

Copyright © 2021 He, Wang, Zhang, Wang, Dong and Zhao. This is an open-access article distributed under the terms of the Creative Commons Attribution License (CC BY). The use, distribution or reproduction in other forums is permitted, provided the original author(s) and the copyright owner(s) are credited and that the original publication in this journal is cited, in accordance with accepted academic practice. No use, distribution or reproduction is permitted which does not comply with these terms.
