# Supplementary material for: Mental health status and related factors influencing healthcare workers during the COVID-19 pandemic: A systematic review and meta-analysis
Source: PLoS One. 2024 Jan 19;19(1):e0289454. doi: 10.1371/journal.pone.0289454 (PMC10798549; doi:10.1371/journal.pone.0289454)
Supplement: S1 Data — (ZIP) [file pone.0289454.s011.zip › literatures/52.pdf]

ELSEVIER

Since January 2020 Elsevier has created a COVID-19 resource centre with free information in English and Mandarin on the novel coronavirus COVID-19. The COVID-19 resource centre is hosted on Elsevier Connect, the company's public news and information website.

Elsevier hereby grants permission to make all its COVID-19-related research that is available on the COVID-19 resource centre - including this research content - immediately available in PubMed Central and other publicly funded repositories, such as the WHO COVID database with rights for unrestricted research re-use and analyses in any form or by any means with acknowledgement of the original source. These permissions are granted for free by Elsevier for as long as the COVID-19 resource centre remains active.

INTENS CRIT CARE NUR 74 (2023) 103313

Contents lists available at ScienceDirect

Intensive & Critical Care Nursing

journal homepage: www.sciencedirect.com/journal/intensive-and-critical-care-nursing

Research Article

Mental well-being of intensive care unit nurses after the second surge of the COVID-19 pandemic: A cross-sectional and longitudinal study

Hidde Heesakkers [a,*], Marieke Zegers [a], Margo M.C. van Mol [b], Mark van den Boogaard [a]

[a] Radboud University Medical Center, Radboud Institute for Health Sciences, Department Intensive Care, Nijmegen, The Netherlands
[b] Erasmus MC, University Medical Center Rotterdam, Department of Intensive Care Adults, The Netherlands

ARTICLE INFO

Keywords:
Intensive care unit
Critical care nursing
COVID-19
Mental health
Depression
Anxiety
Post-traumatic stress disorder
Well-being

ABSTRACT

Objectives: To determine the impact of the second surge of the COVID-19 pandemic (October 2020 to June 2021) on mental well-being of intensive care unit nurses and factors associated with mental health outcomes.
Methods: An online survey was available for Dutch intensive care unit nurses in October 2021, measuring mental health symptoms; anxiety, depression (Hospital Anxiety and Depression Scale), and post-traumatic stress disorder (Impact of Event Scale-6). Additionally, work-related fatigue was measured using the Need For Recovery-11 questionnaire. Previous data from the first surge (March until June 2020) were used to study mental well-being longitudinally in a subgroup of intensive care unit nurses. Logistic regression analyses were performed to determine factors associated with mental health symptoms.
Results: In total, 589 nurses (mean age 44.8 [SD, 11.9], 430 [73.8 %] females) participated, of whom 164 also completed the questionnaire in 2020. After the second surge, 225/589 (38.2 %) nurses experienced one or more mental health symptoms and 294/589 (49.9 %) experienced work-related fatigue. Compared to the first measurement, the occurrence of mental health symptoms remained high (55/164 [33.5 %] vs 63/164 [38.4 %], $p = 0.36$) and work-related fatigue was significantly higher (66/164 [40.2 %] vs 83/164 [50.6 %], $p = 0.02$). Granted holidays as requested (aOR, 0.54; 95 % CI, 0.37–0.79), being more confident about the future (aOR, 0.59; 95 % CI, 0.37–0.93) and a better perceived work-life balance (aOR, 0.42; 95 % CI, 0.27–0.65) were significantly associated with less symptoms.
Conclusion: The second surge of the COVID-19 pandemic further drained the mental reserves of intensive care unit nurses, resulting in more work-related fatigue.

Implications for clinical practice

- During the second surge of increased intensive care unit strain of the COVID-19 pandemic, intensive care unit nurses were exposed to increased workload, leading to a high occurrences of mental health symptoms and an increase in work-related fatigue, leaving them at risk for drop-out.
- To prevent and reduce this mental burden, intensive care unit nurses should be given adequate amount of time off to recharge as well as adequate information to boost their confidence in the future.
- Many intensive care unit nurses with mental health problems needed and could therefore have benefited from support by professionals, at work as well as in their private life, indicating the importance of mental health professionals to be present to support intensive care unit nurses.

* Corresponding author at: Department of Intensive Care Medicine, Radboud Institute for Health Sciences, Radboud University Medical Center, P.O. Box 9101, 6500 HB Nijmegen, The Netherlands.
E-mail address: hidde.heesakkers@radboudumc.nl (H. Heesakkers).

https://doi.org/10.1016/j.iccn.2022.103313
Received 14 June 2022; Received in revised form 12 August 2022; Accepted 17 August 2022
Available online 22 August 2022
0964-3397/© 2022 The Author(s). Published by Elsevier Ltd. This is an open access article under the CC BY license (http://creativecommons.org/licenses/by/4.0/).

Introduction

The intensive care unit (ICU) is a challenging work environment where ICU nurses take care for the most critically ill patients. The impact they can make on the lives of patients and their family members makes it a rewarding and meaningful profession (Laerkner et al., 2015). In contrast, the unpredictability and high workload leads to a relatively high prevalence of mental health problems in ICU nurses (Karanikola et al., 2015).

At the start of the COVID-19 pandemic in the spring of 2020, ICU nurse's worldwide were confronted with even more challenging working conditions (Crowe et al., 2020). During the unprecedented first surge of critically ill patients with COVID-19, the number of patients admitted often transcended the number normally cared for (Dongelmans et al., 2022). ICU nurses had to adapt quickly and step up to care for all these patients (Bruyneel et al., 2021a). The way of working changed completely: personal protective equipment (PPE) had to be used with every patient and in all caring interventions, most of the time family members were not allowed to visit their relatives, and patient-to-nurse ratios increased. Additionally, because of the many uncertainties about the pathophysiology and contagiousness of COVID-19 together with a scarcity of PPE, ICU nurses were concerned for their own lives and their family members (Karlsson and Fraenkel, 2020). The first surge therefore asked a lot of ICU nurses, increasing the mental burden and leaving many at risk for drop-out, endangering the continuity, safety, and quality of ICU patient care (Heesakkers et al., 2021).

After the first surge, more and more knowledge on COVID-19 became available and the number of COVID-19 ICU admissions decreased. This trend remained low over the summer of 2020, giving ICU nurses a short period to recover. However, ICUs were again confronted with an increasing number of COVID-19 patients. From October 2020 until June 2021, the number of COVID-19 patient admitted to the ICU remained high in the Netherlands, resulting in a long-lasting increased workload and demanding working conditions (Supplemental Fig. 1).

Longitudinal data on ICU nurses' mental well-being during the COVID-19 pandemic, especially on the impact of the second surge of increased ICU strain, are lacking. Since ICU nurses are vital for the continuation of adequate ICU care, the aim of the present study was to assess their mental well-being after the second surge and factors associated with mental health symptoms. Additionally, the interventions used and needed by ICU nurses to mitigate mental symptoms were studied in order to enable adequate support in the future.

Methods

Setting and sample

This national survey study is a replication of a study carried out in September 2020, making it possible to compare occurrence rates of mental outcomes with rates after the first surge (Heesakkers et al., 2021). The present study focussed on the impact of the second surge of increased ICU strain. The study was approved by the regional ethics committee (CMO region Arnhem-Nijmegen 2020-6939).

All ICU nurses in Netherlands could participate by completing an online survey (LimeSurvey). The survey was disseminated by an open link via a non-profit organisation focusing on education of healthcare workers (HCW) in the critical care setting (Venticare) and the Dutch association for ICU nurses (V&VN-IC). ICU nurses who provided their contact information in the September 2020 questionnaire and consented they could be contacted again for follow-up were approached by e-mail and received a personal link to the online survey. ICU nurses who only completed the second survey of the present study using the open link were part of the cross-sectional group, and ICU nurses who completed both surveys using the personal link were part of the longitudinal group.

Between September 23, 2020 and July 4, 2021 there were consistently more than 100 COVID-19 patients present in all Dutch ICUs with a peak of 841 patients on April 25, 2021 (RIVM), while only around 925 beds for all ICU patients were available. To examine the impact of this second period, the survey of the present study was available from September 27, 2021 until October 30, 2021 (Supplemental Fig. 1).

ICU nurse characteristics, such as age and gender, normal working conditions and working conditions during the second surge of the pandemic in comparison to the first surge were obtained by the questionnaire. Additionally, ICU nurses were asked what kind of interventions they used to prevent or treat mental health problems as well as which interventions they needed but had not received.

Mental health outcomes

The primary outcomes were the occurrence of mental health symptoms, including symptoms of anxiety, depression, and post-traumatic stress disorder (PTSD), and work-related fatigue.

Anxiety and depression were measured using the Hospital Anxiety and Depression Scale (HADS). The HADS is a 14-item questionnaire with different 4-point scales (0 to 3 points), and consist out of two 7-item subscales for anxiety (HADS-A) and depression (HADS-D) (Zigmond and Snaith, 1983). For the presences of symptoms, a cut of value of $\geq$8 was used on both subscales (Bjelland et al., 2002; Mealer et al., 2007).

PTSD was measured using the Impact of Events Scale-6 (IES-6), a 6-item questionnaire with a 5-point Likert scale ranging from not at all (0) to extremely (4), derived from the IES-R. A mean cut-of value of 1.75 over all six items was used to indicate the presence of symptoms of PTSD (Czeisler et al., 2020; Hosey et al., 2019; Si et al., 2020; Thoresen et al., 2010).

Work-related fatigue was measured using the Need For Recovery-11 (NFR) questionnaire, consisting of 11 dichotomous (yes or no) items objectifying to what extend participants are able to recover from a working day and the need to recover from it (Broersen et al., 2004; Graham et al., 2020; Moriguchi et al., 2012; van Veldhoven and Broersen, 2003). A cut-off value of $\geq$6 answers positive for fatigue was used to indicate work-related fatigue, related to an increased risk of future absence.

Statistical analysis

For the validated questionnaires, all questions had to be answered to avoid missing data. Continuous variables were presented as mean (standard deviation [SD]) if normally distributed or as median (with first and third quartile, expressed as interquartile range [IQR]) if non-normally distributed and categorical variables were presented as proportions (percentages). Differences in baseline characteristics between the cross-sectional and longitudinal group were tested using the unpaired *t*-test, Mann-Whitney *U* test or Chi-square test, whichever was appropriate. For the longitudinal analyses, differences in outcome scores were tested using the Wilcoxon signed rank test and differences in occurrences rates were tested using McNemar's test. For the cross-sectional analyses, differences in proportions were tested using the Chi-square test.

A dichotomous composite score for mental symptoms was calculated by the presence of at least one mental symptom (anxiety, depression or PTSD) (Hall et al., 2022). Mental health problems were defined as the presence of a mental health symptom or work-related fatigue. Logistic regression analyses were used with the composite score for mental health symptoms as dependent variable. First, potential factors were tested for association with mental health symptoms by univariable regression and subsequentially included in the multivariable logistic regression if the p value was below 0.20 (Maldonado and Greenland, 1993). Multicollinearity was tested using the Pearson correlation coefficient and present if $R > 0.7$, however, none of the variables had to be excluded. SPSS version 25 was used for the statistical analyses, statistical significance was defined as a $P < 0.05$ and all statistical tests were 2-sided.

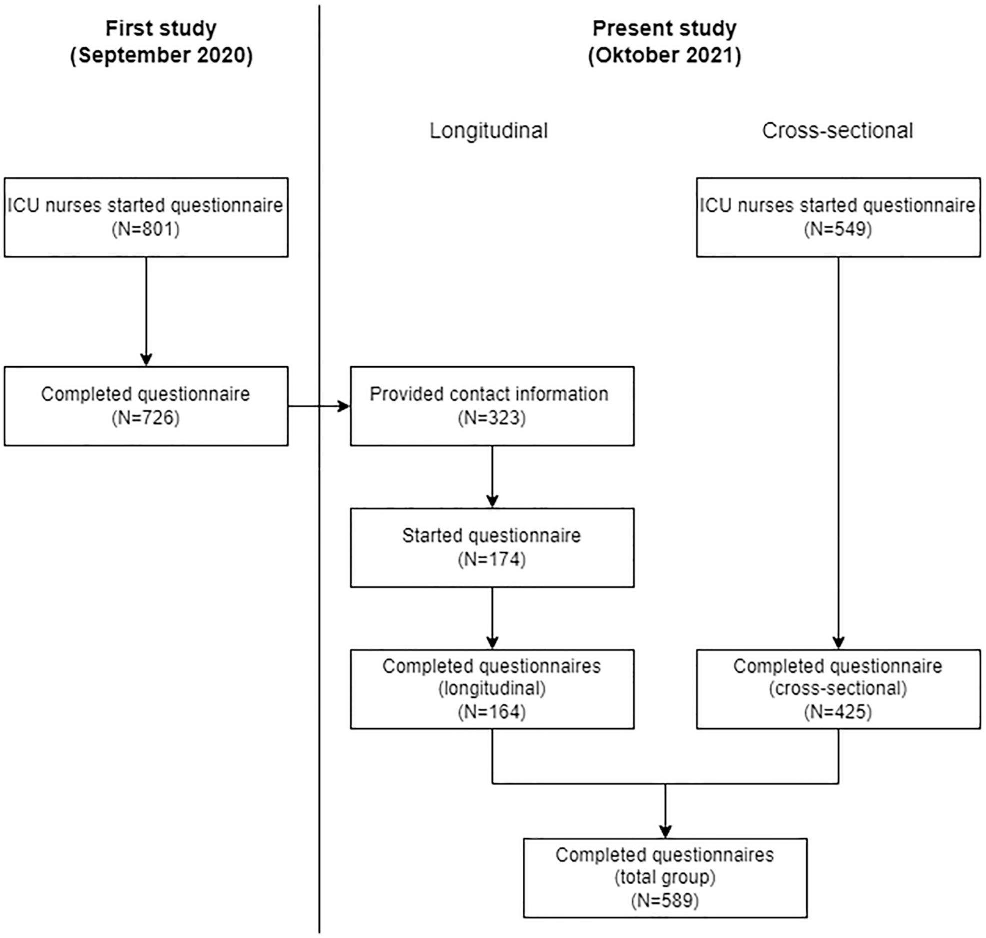

Fig. 1. Flow chart of cross-sectional and longitudinal included ICU nurses.

Results

Study population characteristics

In total, 589 ICU nurses completed the survey of the present study, of whom 425 (72.2 %) only completed the second survey, i.e. the cross-sectional group, and 164 (27.8 %) completed both surveys, i.e. the longitudinal group (Fig. 1). The total group of ICU nurses (n = 589) had a mean age of 44.8 (SD, 11.9), 73.8 % were female and almost all (96.9 %) had worked with COVID-19 patients (Table 1). One-third (32.8 %) considered or is considering quitting working as an ICU nurse and almost a quarter (24.2 %) was not able to work during the COVID-19 pandemic for a certain amount of time. Baseline characteristics and experiences during the pandemic were comparable between the cross-sectional and longitudinal group, except for age, number of registered or trainee ICU nurses and gender (Table 1).

Mental health outcomes

In the total group, 29.9 % (176/589) of the ICU nurses experienced symptoms of anxiety, 21.1 % (124/589) symptoms of depression and 17.7 % (104/589) symptoms of PTSD. Additionally, 49.9 % (294/589) had a positive NFR (Table 2) indicating that ICU nurses experience work-related fatigue and unfulfilled need for recovery. Over half of all ICU nurses (57.0 %) experienced at least one mental health problem (symptoms of anxiety, depression or PTSD, or a positive NFR), and 8.5 % (50/589) experienced all 4 mental health problems (Supplemental Table 1).

In the longitudinal group, the number of ICU nurses with mental symptoms during follow-up did not significantly differ compared to the first measurement (33.5 % vs 38.4 %, $p = 0.36$) (Table 3). The prevalence rates of the mental health symptoms separately did not significantly differ between measurements either. However, the prevalence of work-related fatigue, i.e. a positive NFR, was significantly higher after the second surge compared to after first surge (40.2 % vs 50.6 %, $p = 0.02$).

Associated factors with mental symptoms

Of the 23 included factors, 13 were associated with occurrence of mental symptoms with a p-value < 0.20 and were therefore included in the multivariable logistic regression analysis (Table 4). Working in an academic hospital was associated with more mental health symptoms (adjusted odds ratio [aOR], 1.54; 95 % confidence interval [CI], 1.02 to 2.34; $p = 0.04$). ICU nurses who experienced a better work-life balance (aOR, 0.42; 95 % CI, 0.27 to 0.65; $p < 0.001$) or who were more confident about the future compared to the first surge (aOR, 0.59; 95 % CI, 0.37 to 0.93; $p = 0.02$) as well as those who were able to go on holiday as requested (aOR, 0.54; 95 % CI, 0.37 to 0.79; $p = 0.002$), were less likely to experience mental symptoms than those who did not.

Used and needed interventions to support mental wellbeing

ICU nurses who experienced mental health problems more often used interventions to reduce mental burden (e.g., coaching, mindfulness, or lifestyle interventions) and frequently reported they could have benefitted from several interventions which were not available to them (e.g., support by mental health professionals such as psychologist at

Table 1
Characteristics of ICU nurses and their experiences during the COVID-19 pandemic (total, cross-sectional and longitudinal study group).

	Total group N = 589	Cross-sectional N = 425	Longitudinal N = 164	P-value cross-sectional vs longitudinal
Characteristics				
Age in years, mean (SD)	44.8 (11.9)	44.0 (12.1)	46.9 (11.0)	0.01
Female, n (%)	430/583 (73.8)	320/421 (76.0)	110/162 (67.9)	<0.05
Experience as ICU nurse in years, median (IQR)	17 (7–26)	17 (6–25)	17 (8–28)	0.21
Hospital type, No./total (%):				0.27
- Academic	153/587 (26.1)	105/423 (24.8)	48/164 (29.3)	
- Non-academic	434/587 (73.9)	318/423 (75.2)	116/164 (70.7)	
Normal weekly working hours, median (IQR)	32 (28–32)	32 (28–32)	32 (28–34)	0.98
Worked more hours compared to before the pandemic, Yes, No./total (%)	323/588 (54.9)	229/424 (54.0)	94/164 (57.3)	
Number of ICU nurses:				0.001
- Registered ICU nurses, No./total (%)	518/585 (88.5)	367/421 (87.2)	151/164 (92.1)	
- Trainee ICU nurses, No./total (%)	31/585 (5.3)	31/421 (7.4)	0/164 (0)	
- Other, nurses who worked in the ICU, No./total (%)	36/585 (6.2)	23/421 (5.5)	13/164 (7.9)	
Experiences during the COVID-19 pandemic				
Worked in a COVID-19 ICU unit since the first surge, Yes, No./total (%)	570/588 (96.9)	413/425 (97.2)	157/163 (96.3)	0.65
Experienced a COVID-19 infection, Yes, No./total (%)	120/586 (20.5)	87/423 (20.6)	33/163 (20.2)	0.99
- Experienced symptoms	114/120 (95.0)	84/87 (96.6)	30/33 (90)	0.66
- Needed hospital admission	3/120 (2.5)	2/87 (2.3)	1/33 (3)	0.83
- Needed ICU admission	2/120 (1.7)	2/87 (2.3)	0/0 (0)	0.38
Not being able to work since the pandemic started, Yes, No./total (%)	141/583 (24.2)	100/420 (23.8)	41/163 (25.2)	0.73
Due to:				0.70
- Physical problems	62/141 (44.0)	44/100 (44.0)	18/41 (44)	
- Mental problems	29/141 (20.6)	21/100 (21.0)	8/41 (20)	
- Both physical and mental problems	38/141 (27.0)	25/100 (25.0)	13/41 (32)	
- Other	12/141 (8.5)	10/100 (10.0)	2/41 (5)	
Are you currently working again, Yes, No./total (%)				0.51
- Yes, like before the sick leave	106/141 (75.2)	77/100 (77.0)	29/41 (71)	
- Yes, however still less as before	22/141 (15.6)	16/100 (16.0)	6/41 (15)	
- No, however planning to	3/141 (2.1)	2/100 (2.0)	1/41 (2)	
- No, (temporarily) stopped working as ICU nurse	10/141 (7.1)	5/100 (5.0)	5/41 (12)	
Considered or considering quitting, Yes, No./total (%)	188/573 (32.8)	137/416 (32.9)	51/157 (32.5)	0.92
Been on holiday since the end of the first surge, Yes, No./total (%)				0.89
- Yes, as long and whenever desired	381/589 (64.7)	274/425 (64.5)	107/164 (65.2)	
- Yes, only not as long as desired	151/589 (25.6)	112/425 (26.4)	39 /164 (23.8)	
- Yes, only not whenever desired	34/589 (5.8)	24/425 (5.6)	10/164 (6.1)	
- Yes, only not as long and whenever desired	16/589 (2.7)	10/425 (2.4)	6/164 (3.7)	
- No	7/589 (1.2)	5/425 (1.2)	2/164 (1.2)	
**Working conditions during the first COVID-19 surge**				
Compared to after the first COVID-19 surge, after the second surge:				
- We were better prepared for a surge in COVID-19 ICU patients, agree, No./total (%)	448/585 (76.6)	329/421 (78.1)	119/164 (72.6)	0.15
- There was more qualified personnel during a shift, agree, No./total (%)	131/585 (22.4)	92/422 (21.8)	39/163 (23.9)	0.58
- Collaboration with colleagues went better, agree, No./total (%):	300/586 (51.2)	215/422 (50.9)	85/164 (51.8)	0.85
- Communication with family went better, agree, No./total (%)	329/585 (56.2)	244/421 (58.0)	85/164 (51.8)	0.18
- Workload and circumstances were better, agree, No./total (%)	172/586 (29.4)	126/422 (29.9)	46/164 (28.0)	0.67
- I was less concerned contracting COVID-19, agree, No./total (%)	377/584 (64.6)	267/421 (63.4)	110/164 (67.5)	0.36
- I was less concerned infecting a family member, agree, No./total (%)	333/585 (56.9)	237/422 (56.2)	96/163 (58.9)	0.55
- Support at work was better organized, agree, No./total (%)	193/585 (33.0)	147/421 (34.9)	46/164 (28.0)	0.11
**Private circumstances during the first COVID-19 surge**				
Compared to after the first COVID-19 surge, after the second surge:				

(*continued on next page*)

Table 1 (*continued*)

	Total group N = 589	Cross-sectional N = 425	Longitudinal N = 164	P-value cross-sectional vs longitudinal
- I took more rest during my days off, agree, No./total (%)	256/586 (43.7)	188/422 (44.5)	68/164 (41.5)	0.50
- I was more confident everything would be fine, agree, No./total (%)	174/586 (29.7)	130/422 (30.8)	44/164 (26.8)	0.34
- I was better able to balance work and social life, agree, No./total (%)	217/584 (37.2)	154/420 (36.7)	63/164 (38.4)	0.69
- I felt more supported by family and friends, agree, No./total (%)	146/584 (25.0)	104/420 (24.8)	42/164 (25.6)	0.83
- I felt more supported by society, agree, No./total (%)	8/585 (1.4)	5/421 (1.2)	3/164 (1.8)	0.55

Abbreviations: ICU, Intensive Care Unit; COVID-19, coronavirus disease of 2019; SD, standard deviations; IQR, interquartile range.

Table 2
Mental health outcomes in ICU nurses after the second surge of COVID-19 ICU patients (total study group).

	Total group (N = 589)
**Mental symptoms**	
HADS-Anxiety	
- Score, median (IQR)	5 (3–8)
- Anxiety - No./total (%)	176/589 (29.9)
HADS-Depression	
- Score, median (IQR)	4 (2–7)
- Depression - No./total (%)	124/589 (21.1)
Impact of Event Scale-6	
- Score, median (IQR)	0.83 (0.33–1.5)
- PTSD - No./total (%)	104/589 (17.7)
At least one mental health symptoms - No./total (%)	225/589 (38.2)
**Work-related fatigue**	
- Score, median (IQR)	5 (3–8)
- Positive NFR- No./total (%)	294/589 (49.9)
At least one mental health symptoms or a positive NFR - No./total (%)	336/589 (57.0)

Abbreviations: HADS, Hospital Anxiety and Depression Scale; PTSD, Post-traumatic stress disorder; NFR, Need For Recovery; IQR, interquartile range.

work, coaching, additional training) or a reduction of workload by more colleagues (Supplemental Table 2).

Discussion

The impact of the COVID-19 pandemic on the mental well-being of ICU nurses remained significant after the second period of increased ICU strain. This study showed that although the occurrence of mental health symptoms did not further increase but remained equally high, ICU nurses were more likely to experience work-related fatigue compared to after the first surge. Since work-related fatigue is associated with an elevated risk for future mental health problems, the occurrences of mental health symptoms in our study population is likely to increase (Nieuwenhuijsen et al., 2016). The increased mental burden due the prolonged work-related stress will most likely have resulted in ICU nurses who are unable to work or even leave the profession for good (Bruyneel et al., 2021b; Levi and Moss, 2022). In our study population, already-one in four ICU nurses was or still is on sick leave during the pandemic and one in three considered or is still considering to stop working as ICU nurse.

Table 3
Mental health outcomes in ICU nurses after the first and second surge of COVID-19 ICU patients (longitudinal study group).

	Longitudinal group (N = 164)		
	Outcomes		First vs second surge P-value
	After first surge 2020	After second surge 2021	
**Mental symptoms**			
HADS-Anxiety			
- Score, median (IQR)	4.5 (2–7)	5 (3–8)	0.07
- Anxiety - No./total (%)	43/164 (26.2)	47/164 (28.7)	0.65
HADS-Depression			
- Score, median (IQR)	3 (1–6.5)	4 (1–7)	0.002
- Depression - No./total (%)	31/164 (18.9)	31/164 (22.6)	0.42
Impact of Event Scale-6			
- Score, median (IQR)	0.83 (0.33–1.67)	0.83 (0.42–1.50)	0.48
- PTSD - No./total (%)	31/164 (18.9)	32/164 (19.5)	0.99
At least one mental health symptoms - No./total (%)	55/164 (33.5)	63/164 (38.4)	0.36
**Work-related fatigue**			
- Score, median (IQR)	4 (1–7)	6 (3–9)	<0.001
- Positive NFR- No./total (%)	66/164 (40.2)	83/164 (50.6)	0.02
At least one mental health symptom or a positive NFR - No./total (%)	82/164 (50.0)	98/164 (59.8)	0.04

Abbreviations: HADS, Hospital Anxiety and Depression Scale; PTSD, Post-traumatic stress disorder; NFR, Need For Recovery; IQR, interquartile range.

Several other studies report on the mental well-being of ICU nurses during the pandemic (Bruyneel et al., 2021b; Crowe et al., 2022; Hall et al., 2022). However, the number of admitted COVID-19 ICU patients and the associated ICU strain varies greatly between countries as well as over time, making it difficult to compare outcomes. Recent studies seems to point towards the same direction and showed that the second surge of COVID-19 ICU patients during the winter and spring of 2020/2021 caused a deterioration in mental well-being in ICU nurses (Crowe et al., 2022; Hall et al., 2022). A national study in England conducted during June and July 2021 reported prevalence's of 39.0 % for PTSD using the IES-R, which is higher compared to our finding (18.9 %) (Dykes et al., 2022). The shorter amount of time between the surge and the inclusion period could explain the higher prevalence compared to the present study as mental health symptoms tend to decrease over time (Santiago et al., 2013). National studies conducted in Italy and Turkey, both using repeated cross-sectional analyses, reported a significant increase in the outcome score measuring symptoms of depression, after the second surge in healthcare workers compared to after the first surge, which is in accordance with our findings (Gündoğmuş et al., 2021;

Table 4
Factors associated with mental health symptoms in ICU nurses after the second surge of COVID-19 ICU patients.

	Mental health symptoms*			
	Univariable OR (95 % CI)	p value	Multivariable OR (95 % CI)	p value
**ICU nurses**				
Age, years	0.99 (0.98–1.00)	0.16	0.99 (0.97–1.00)	0.10
Sex, Female	1.14 (0.78–1.68)	0.50		
Hospital, academic	1.43 (0.98–2.07)	0.06	1.54 (1.02–2.34)	0.04
Weekly working hours, >28 h	1.45 (1.02–2.05)	0.04	1.23 (0.83–1.82)	0.30
Other than certified ICU nurse, yes	1.39 (0.76–2.55)	0.29		
Experience, years	0.99 (0.98–1.01)	0.24		
Worked with COVID-19 ICU patients, yes	0.61 (0.24–1.56)	0.30		
Past COVID-19 infection, yes	0.75 (0.49–1.14)	0.18	0.69 (0.44–1.10)	0.12
Worked more hours, yes	1.01 (0.72–1.41)	0.95		
Vacations as requested	0.47 (0.33–0.66)	<0.001	0.54 (0.37–0.79)	0.002
**Work environment**				
Better prepared	0.61 (0.41–0.90)	0.01	0.85 (0.55–1.31)	0.45
More qualified personnel	0.80 (0.54–1.21)	0.29		
Better collaboration colleague's	0.93 (0.66–1.29)	0.65		
Better communication with relatives	0.69 (0.50–0.97)	0.03	0.78 (0.54–1.15)	0.21
Better working conditions	0.52 (0.36–0.77)	0.001	0.79 (0.50–1.25)	0.32
Less concerned about getting infected	1.03 (0.73–1.50)	0.85		
Less concerned about infecting relatives	0.93 (0.66–1.30)	0.67		
Better logistic support	0.68 (0.47–0.98)	0.04	0.97 (0.64–1.47)	0.88
**Home**				
More rest during days off	0.70 (0.50–0.99)	0.04	1.03 (0.70–1.51)	0.89
More confident about the future	0.40 (0.27–0.60)	<0.001	0.59 (0.37–0.93)	0.02
Better work-life balance	0.34 (0.23–0.49)	<0.001	0.42 (0.27–0.65)	<0.001
More support by family and friends	0.94 (0.64–1.39)	0.77		
More support by society	0.23 (0.03–1.87)	0.17	0.28 (0.03–2.70)	0.27

Abbreviations: ICU, Intensive Care Unit; COVID-19, coronavirus disease of 2019; OR, odds ratio; CI, confidence interval.

* Composite score of mental health symptoms, i.e. the presence of at least one symptom of anxiety, depression or post-traumatic stress disorder.

Magnavita et al., 2021). Since experiencing symptoms of depression is associated with making medical errors, all efforts should be made to minimize the mental burden in ICU nurses (Garrouste-Orgeas et al., 2015).

Clinical implications

For ICU nurses to recover from the mental burden, taking time off to recharge seems crucial since ICU nurses who were able to go on holiday as requested are less likely to report mental health symptoms. Additionally, ICU nurses who experienced a better balance between their private and working life during the second surge compared to the first, are less likely to experience mental health symptoms as well. This underlines the high importance of creating more awareness for self-care, for instance by mindfulness or coaching (van den Boogaard and Zegers, 2022).

The unchanged high prevalence of mental health symptoms after the second surge compared to after the first is worrisome (Arrogante and Aparicio-Zaldivar, 2017). During the pandemic, more information on COVID-19 became available and hospitals were better adapted to the new work environment, which probably gave ICU nurses more confidence in the future. Although being more confident was associated with less mental health symptoms in our results, this did not lead to a decrease in symptoms after the first surge. Therefore, persistent efforts should be made to ensure adequate and timely communication about new insights on pathophysiology and treatment of COVID-19, as this could lead to increased feelings of control and self-efficacy. This might subsequently improve mental well-being of health professionals (Kok et al., 2021).

Furthermore, many ICU nurses with mental health problems needed and could therefore have benefited from support by mental health professionals, at work as well in their private life, and coaching, indicating the importance of mental health professionals to be present to support ICU nurses. Other interventions that could have reduced the workload were the help of less skilled colleagues and working in fixed teams (Stalpers et al., 2021).

Limitations

This study has several limitations that should be addressed. First, the validated questionnaires used can only indicate the presence of symptoms and cannot be used to diagnose mental health disorders (Nelliot et al., 2019). Second, we do not have a baseline measurement before the pandemic and can therefore only conclude about the course of mental health symptoms during the pandemic. However, a longitudinal study in the Netherlands did show a significant increase in mental burden in ICU nurses during the COVID-19 pandemic compared to before (Kok et al., 2021). Third, we only included ICU nurses, while the COVID-19 pandemic had a great impact on all healthcare professionals in every setting. ICU nurses do seem to be more likely to experience mental health symptoms compared to the general population, indicating the additional mental burden caused by caring for the critically ill during the COVID-19 pandemic (Guttormson et al., 2022; Manchia et al., 2021; van der Velden et al., 2020). Additionally, when compared to non-ICU nurses or ICU doctors, ICU nurses seem to be more likely to experiences mental health symptoms (Greenberg et al., 2021; Hall et al., 2022; Rodriguez-Ruiz et al., 2021; Saracoglu et al., 2020). Since ICU capacity in the Netherlands greatly depends on the availability of ICU nurses, persevering their mental well-being is of special interest and was therefore the focus of this study (Hoogendoorn et al., 2021).

Conclusion

The long-lasting COVID-19 pandemic challenged ICU nurses and resulted in even more demanding working conditions. The second surge of the pandemic led to a high occurrence of mental health symptoms and an increase in work-related fatigue, indicating that many ICU nurses are at risk for future absence. We should focus on supporting ICU nurses wherever possible, for instance by health care professionals to provide coaching, increasing self-care and offering time off when possible.

Ethical Approval

The study was reviewed and approved by the medical ethical committee of Arnhem-Nijmegen, the Netherlands (CMO) (CMO-number 2020/6939). Both surveys could be completed anonymously and

completion of the survey was considered as informed consent. Participation was voluntary and all obtained information was confidential.

Authors' contribution

Heesakkers, Zegers, Van Mol, Van den Boogaard contributed to study concept and design.

Heesakkers, Zegers, Van den Boogaard contributed to analysis and interpretation of data.

Heesakkers contributed to drafting of the article.

Zegers, Van Mol, Van den Boogaard contributed to critical revision of the article for important intellectual content.

Zegers, Van Mol, Van den Boogaard contributed to study supervision.

All authors read and approved the final article.

Funding Source

This study was partly funded by Stichting Radboud Fonds (dossier No. 2020 307595-7). Stichting Radboud Fonds had no role in the design and conduct of the study; collection, management, analysis, and interpretation of the data; preparation, review, or approval of the manuscript; and decision to submit the manuscript for publication.

Declaration of Competing Interest

The authors declare that they have no known competing financial interests or personal relationships that could have appeared to influence the work reported in this paper.

Appendix A. Supplementary data

Supplementary data to this article can be found online at https://doi.org/10.1016/j.iccn.2022.103313.

References

Arrogante, O., Aparicio-Zaldivar, E., 2017. Burnout and health among critical care professionals: The mediational role of resilience. Intensive Crit. Care Nurs. 42, 110–115.

Bjelland, I., Dahl, A.A., Haug, T.T., Neckelmann, D., 2002. The validity of the Hospital Anxiety and Depression Scale. An updated literature review. J. Psychosom. Res. 52 (2), 69–77.

Broersen, J.P.J., Fortuin, R.J., Dijkstra, L., van Veldhoven, M., Prins, J., 2004. Monitor Arboconvenanten: kengetallen en grenswaarden. TBV – Tijdschrift voor Bedrijfs- en Verzekeringsgeneeskunde 12 (4), 104–108.

Bruyneel, A., Smith, P., Tack, J., Pirson, M., 2021b. Prevalence of burnout risk and factors associated with burnout risk among ICU nurses during the COVID-19 outbreak in French speaking Belgium. Intensive Crit. Care Nurs. 65, 103059.

Bruyneel, A., Lucchini, A., Hoogendoorn, M., 2021a. Impact of COVID-19 on nursing workload as measured with the Nursing Activities Score in intensive care. Intensive Crit. Care Nurs. 69, 103170.

Crowe, S., Howard, A.F., Vanderspank-Wright, B., Gillis, P., McLeod, F., Penner, C., Haljan, G., 2020. The effect of COVID-19 pandemic on the mental health of Canadian critical care nurses providing patient care during the early phase pandemic: A mixed method study. Intensive Crit. Care Nurs. 63, 102999.

Crowe, S., Fuchsia Howard, A., Vanderspank, B., 2022. The mental health impact of the COVID-19 pandemic on Canadian critical care nurses. Intensive Crit. Care Nurs. 71, 103241.

Czeisler, M., Lane, R.I., Petrosky, E., Wiley, J.F., Christensen, A., Njai, R., Weaver, M.D., Robbins, R., Facer-Childs, E.R., Barger, L.K., Czeisler, C.A., Howard, M.E., Rajaratnam, S.M.W., 2020. Mental health, substance use, and suicidal ideation during the COVID-19 pandemic - United States, June 24–30, 2020. MMWR Morb. Mortal. Wkly. Rep. 69 (32), 1049–1057.

Dongelmans, D.A., Termorshuizen, F., Brinkman, S., Bakhshi-Raiez, F., Arbous, M.S., de Lange, D.W., van Bussel, B.C.T., de Keizer, N.F., Verbiest, D.P., te Velde, L.F., van Driel, E.M., Rijpstra, T., Elbers, P.W.G., Georgieva, L., Verweij, E., de Jong, R.M., van Iersel, F.M., Koning, D.T.J.J., Rengers, E., Kusadasi, N., Erkamp, M.L., van den Berg, R., Jacobs, C.J.M.G., Epker, J.L., Rijkeboer, A.A., de Bruin, M.T., Spronk, P., Draisma, A., Versluis, D.J., van den Berg, L.A.E., Mos, M.-d., Lens, J.A., Mehagnoul-Schipper, D.J., Gommers, D., Lutisan, J.G., Hoeksema, M., Pruijsten, R.V., Kieft, H., Rozendaal, J., Nooteboom, F., Boer, D.P., Janssen, I.T.A., van Gulik, L., Koetsier, M. P., Silderhuis, V.M., Schnabel, R.M., Drogt, I., de Ruijter, W., Bosman, R.J., Frenzel, T., Urlings-Strop, L.C., Dijkhuizen, A., Hené, I.Z., de Meijer, A.R., Holtkamp, J.W.M., Postma, N., Bindels, A.J.G.H., Wesselink, R.M.J., van Slobbe-Bijlsma, E.R., van der Voort, P.H.J., Eikemans, B.J.W., Barnas, M.G.W., Festen-Spanjer, B., van Lieshout, M., Gritters, N.C., van Tellingen, M., Brunnekreef, G.B., Vandeputte, J., Dormans, T.P.J., Hoogendoorn, M.E., de Graaff, M., Moolenaar, D., Reidinga, A.C., Spijkstra, J.J., de Waal, R., 2022. Characteristics and outcome of COVID-19 patients admitted to the ICU: a nationwide cohort study on the comparison between the first and the consecutive upsurges of the second wave of the COVID-19 pandemic in the Netherlands. Ann. Intensive Care 12, 5.

Dykes, N., Johnson, O., Bamford, P., 2022. Assessing the psychological impact of COVID-19 on intensive care workers: A single-centre cross-sectional UK-based study. J. Intensive Care Soc. 23 (2), 132–138.

Garrouste-Orgeas, M., Perrin, M., Soufir, L., Vesin, A., Blot, F., Maxime, V., Beuret, P., Troché, G., Klouche, K., Argaud, L., Azoulay, E., Timsit, J.-F., 2015. The Iatroref study: medical errors are associated with symptoms of depression in ICU staff but not burnout or safety culture. Intensive Care Med. 41 (2), 273–284.

Graham, B., Cottey, L., Smith, J.E., Mills, M., Latour, J.M., 2020. Measuring 'Need for Recovery' as an indicator of staff well-being in the emergency department: a survey study. Emerg. Med. J. 37 (9), 555–561.

Greenberg, N., Weston, D., Hall, C., Caulfield, T., Williamson, V., Fong, K., 2021. Mental health of staff working in intensive care during Covid-19. Occup. Med. (Lond.) 71, 62–67.

Gündoğmuş, İ., Ünsal, C., Bolu, A., Takmaz, T., Ökten, S.B., Aydın, M.B., Uçar, H., Gündüz, A., Kul, A.T., 2021. The comparison of anxiety, depression and stress symptoms levels of healthcare workers between the first and second COVID-19 peaks. Psychiatry Res. 301, 113976.

Guttormson, J.L., Calkins, K., McAndrew, N., Fitzgerald, J., Losurdo, H., Loonsfoot, D., 2022. Critical care nurse burnout, moral distress, and mental health during the COVID-19 pandemic: A United States survey. Heart Lung 55, 127–133.

Hall, C.E., Milward, J., Spoiala, C., Bhogal, J.K., Weston, D., Potts, H.W.W., Caulfield, T., Toolan, M., Kanga, K., El-Sheikha, S., Fong, K., Greenberg, N., 2022. The mental health of staff working on intensive care units over the COVID-19 winter surge of 2020 in England: a cross sectional survey. Br. J. Anaesth. 128 (6), 971–979.

Heesakkers, H., Zegers, M., van Mol, M.M.C., van den Boogaard, M., 2021. The impact of the first COVID-19 surge on the mental well-being of ICU nurses: A nationwide survey study. Intensive Crit. Care Nurs. 65, 103034.

Hoogendoorn, M.E., Brinkman, S., Bosman, R.J., Haringman, J., de Keizer, N.F., Spijkstra, J.J., 2021. The impact of COVID-19 on nursing workload and planning of nursing staff on the Intensive Care: A prospective descriptive multicenter study. Int. J. Nurs. Stud. 121, 104005.

Hosey, M.M., Leoutsakos, J.-M., Li, X., Dinglas, V.D., Bienvenu, O.J., Parker, A.M., Hopkins, R.O., Needham, D.M., Neufeld, K.J., 2019. Screening for posttraumatic stress disorder in ARDS survivors: validation of the Impact of Event Scale-6 (IES-6). Crit. Care 23, 276.

Karanikola, M., Giannakopoulou, M., Mpouzika, M., Kaite, C.P., Tsiaousis, G.Z., Papathanassoglou, E.D.E., 2015. Dysfunctional psychological responses among Intensive Care Unit nurses: a systematic review of the literature. Rev. Esc. Enferm. USP 49 (5), 847–857.

Karlsson, U., Fraenkel, C.J., 2020. Covid-19: risks to healthcare workers and their families. BMJ 371, m3944.

Kok, N., van Gurp, J., Teerenstra, S., van der Hoeven, H., Fuchs, M., Hoedemaekers, C., Zegers, M., 2021. Coronavirus disease 2019 immediately increases burnout symptoms in ICU professionals: A longitudinal cohort study. Crit. Care Med. 49 (3), 419–427.

Laerkner, E., Egerod, I., Hansen, H.P., 2015. Nurses' experiences of caring for critically ill, non-sedated, mechanically ventilated patients in the Intensive Care Unit: a qualitative study. Intensive Crit. Care Nurs. 31 (4), 196–204.

Levi, P., Moss, J., 2022. Intensive care unit nurses' lived experiences of psychological stress and trauma caring for COVID-19 patients. Workplace Health Saf. 70 (8), 358–367.

LimeSurvey http://www.limesurvey.org (last accessed: 1 October 2020).

Magnavita, N., Soave, P.M., Antonelli, M., 2021. Prolonged stress causes depression in frontline workers facing the COVID-19 pandemic-A repeated cross-sectional study in a COVID-19 Hub-Hospital in Central Italy. Int. J. Environ. Res. Public Health 18 (14), 7316.

Maldonado, G., Greenland, S., 1993. Simulation study of confounder-selection strategies. Am. J. Epidemiol. 138, 923–936.

Manchia, M., Gathier, A.W., Yapici-Eser, H., Schmidt, M.V., de Quervain, D., van Amelsvoort, T., Bisson, J.I., Cryan, J.F., Howes, O.D., Pinto, L., van der Wee, N.J., Domschke, K., Branchi, I., Vinkers, C.H., 2021. The impact of the prolonged COVID-19 pandemic on stress resilience and mental health: A critical review across waves. Eur. Neuropsychopharmacol. 55, 22–83.

Mealer, M.L., Shelton, A., Berg, B., Rothbaum, B., Moss, M., 2007. Increased prevalence of post-traumatic stress disorder symptoms in critical care nurses. Am. J. Respir. Crit. Care Med. 175 (7), 693–697.

Moriguchi, C.S., Trevizani, T., de Fátima Carreira Moreira, R., Januário, L.B., de Oliveira, A.B., Coury, H.J.C.G., 2012. Need for recovery assessment among nursing professionals and call center operators. Work 41 (Suppl 1), 4838–4842.

Nelliot, A., Dinglas, V.D., O'Toole, J., Patel, Y., Mendez-Tellez, P.A., Nabeel, M., Friedman, L.A., Hough, C.L., Hopkins, R.O., Eakin, M.N., Needham, D.M., 2019. Acute respiratory failure survivors' physical, cognitive, and mental health outcomes: quantitative measures versus semistructured interviews. Ann Am Thorac Soc. 16 (6), 731–737.

Nieuwenhuijsen, K., Sluiter, J.K., Dewa, C.S., 2016. Need for recovery as an early sign of depression risk in a working population. J. Occup. Environ. Med. 58, e350–e354.

RIVM https://www.rivm.nl/en/novel-coronavirus-covid-19/current-information (last accessed: 1 June 2022).

Rodriguez-Ruiz E., Campelo-Izquierdo M., Boga Veiras P., Mansilla Rodríguez M., Estany-Gestal A., Blanco Hortas A., et al. 2021. Impact of the coronavirus disease

2019 pandemic on moral distress among nurses and physicians in Spanish ICUs. Crit. Care Med.

Santiago, P.N., Ursano, R.J., Gray, C.L., Pynoos, R.S., Spiegel, D., Lewis-Fernandez, R., Friedman, M.J., Fullerton, C.S., Coyne, J., 2013. A systematic review of PTSD prevalence and trajectories in DSM-5 defined trauma exposed populations: intentional and non-intentional traumatic events. PLoS One 8 (4), e59236.

Saracoglu, K.T., Simsek, T., Kahraman, S., Bombaci, E., Sezen, Ö., Saracoglu, A., Demirhan, R., 2020. The psychological impact of COVID-19 disease is more severe on intensive care unit healthcare providers: A cross-sectional study. Clin. Psychopharmacol. Neurosci. 18 (4), 607–615.

Si, M.-Y., Su, X.-Y., Jiang, Y., Wang, W.-J., Gu, X.-F., Ma, L.i., Li, J., Zhang, S.-K., Ren, Z.-F., Ren, R., Liu, Y.-L., Qiao, Y.-L., 2020. Psychological impact of COVID-19 on medical care workers in China. Infect. Dis. Poverty 9 (1), 113.

Stalpers, D., Tilburgs, B., van Mol, M., 2021. Take control by letting go? Sustainable employability of nurses in Intensive Care Units. Nurs. Crit. Care.

Thoresen, S., Tambs, K., Hussain, A., Heir, T., Johansen, V.A., Bisson, J.I., 2010. Brief measure of posttraumatic stress reactions: impact of Event Scale-6. Soc. Psychiatry Psychiatr. Epidemiol. 45 (3), 405–412.

van den Boogaard, M., Zegers, M., 2022. Mental preparedness for prolonged periods of high workload - What did we learn from the covid-19 pandemic? Intensive Crit. Care Nurs. 71, 103258.

van der Velden, P.G., Contino, C., Das, M., van Loon, P., Bosmans, M.W.G., 2020. Anxiety and depression symptoms, and lack of emotional support among the general population before and during the COVID-19 pandemic. A prospective national study on prevalence and risk factors. J. Affect. Disord. 277, 540–548.

van Veldhoven, M., Broersen, S., 2003. Measurement quality and validity of the "need for recovery scale". Occup. Environ. Med. 60 (Suppl 1), i3–i9.

Zigmond, A.S., Snaith, R.P., 1983. The hospital anxiety and depression scale. Acta Psychiatr. Scand. 67, 361–370.
