# Supplementary material for: Mental health status and related factors influencing healthcare workers during the COVID-19 pandemic: A systematic review and meta-analysis
Source: PLoS One. 2024 Jan 19;19(1):e0289454. doi: 10.1371/journal.pone.0289454 (PMC10798549; doi:10.1371/journal.pone.0289454)
Supplement: S1 Data — (ZIP) [file pone.0289454.s011.zip › literatures/53.pdf]

The Impact of the COVID-19 Pandemic on Mental Health, Occupational Functioning, and Professional Retention Among Health Care Workers and First Responders

Rebecca C. Hendrickson, MD, PhD[1,2], Roisín A. Slevin, BS[1], Katherine D. Hoerster, PhD, MPH[2,3,4], Bernard P. Chang, MD, PhD[5], Ellen Sano, DO, MPH[5], Catherine A. McCall, MD[2,3,6], Gillian R. Monty, BA[4], Ronald G. Thomas, PhD[7], and Murray A. Raskind, MD[1,2]

[1]VISN 20 Northwest Mental Illness Research, Education, and Clinical Center (MIRECC), VA Puget Sound Health Care System, Seattle, WA, USA; [2]Department of Psychiatry and Behavioral Sciences, University of Washington School of Medicine, Seattle, WA, USA; [3]Mental Health Service, VA Puget Sound Healthcare System, Seattle, WA, USA; [4]Health Services Research and Development, VA Puget Sound Healthcare System, Seattle, WA, USA; [5]Department of Emergency Medicine, Columbia University Irving Medical Center, New York, NY, USA; [6]VA Puget Sound Healthcare System, Seattle, WA, USA; [7]Department of Biostatistics, University of California, La Jola, CA, USA.

BACKGROUND: The COVID-19 pandemic has greatly affected front-line health care workers (HCW) and first responders (FR). The specific components of COVID-19 related occupational stressors (CROS) associated with psychiatric symptoms and reduced occupational functioning or retention remain poorly understood.

OBJECTIVES: Examine the relationships between total and factored CROS, psychiatric symptoms, and occupational outcomes.

DESIGN: Observational, self-report, single time-point online assessment.

PARTICIPANTS: A total of 510 US HCW ($N = 301$) and FR ($N = 200$) with occupational duties affected by the COVID-19 pandemic.

MAIN OUTCOMES AND MEASURES: CROS were assessed using a custom 17-item questionnaire. Posttraumatic stress disorder (PTSD), depression, insomnia, and generalized anxiety symptoms were assessed using the PTSD Checklist-5 (PCL5), Patient Health Questionnaire-9 (PHQ9), Insomnia Severity Index (ISI), and General Anxiety Disorder-7 (GAD7). Respondents' likelihood of leaving current field and occupational functioning were assessed with 2-item PROMIS subscales. Relationships were modeled using multivariable regression. Open-ended responses were coded using rapid template analysis.

RESULTS: CROS total scores correlated significantly with all four psychiatric symptom domains (R's = .42–.53), likelihood of leaving one's current occupation ($R = .18$), and trouble doing usual work ($R = .28$), all p's < .001. Half of HCW indicated a decreased likelihood of staying in their current occupation as a result of the pandemic. CROS were fit to a 3-factor model consisting of risk, demoralization, and volume factors. All CROS factors were associated with psychiatric symptom burden, but demoralization was most prominently associated with psychiatric symptoms and negative occupational outcomes. Among psychiatric symptoms, PTSD symptoms were most strongly associated with negative occupational outcomes. Open-ended statements emphasized lack of protection and support, increased occupational demands, and emotional impact of work duties.

CONCLUSIONS AND RELEVANCE: These results demonstrate potentially treatable psychiatric symptoms in HCW and FR experiencing CROS, impacting both wellbeing and the health care system. Mitigating CROS, particularly by addressing factors driving demoralization, may improve HCW and FR mental health, occupational functioning, and retention.

KEY WORDS: COVID-19; PTSD; Occupational trauma; Insomnia; Professional retention.

J Gen Intern Med 37(2):397–408
DOI: 10.1007/s11606-021-07252-z

© This is a U.S. government work and not under copyright protection in the U.S.; foreign copyright protection may apply 2021. This article is an open access publication

Received April 9, 2021
Accepted October 27, 2021
Published online December 16, 2021

INTRODUCTION

Health care workers (HCW) and first responders (FR) working during a pandemic experience a variety of acute and sustained stressors, including fear for their own safety and that of their colleagues and family, exposure to death and suffering, separations from family, and prolonged periods of exhaustion and vigilance. They may also experience demoralization[1] related to inadequate support or seeing their contributions as ineffective. The impact of these types of experiences has been well documented in contexts such as military service, where both isolated and cumulative trauma exposure is strongly associated with high psychiatric symptom burden, as well as decreased occupational functioning and workplace participation[2–4]. Understanding how such occupational stressors affect HCW/FR is important to identify risks to HCW/FR and to our health care system, and develop strategies to reduce those risks[5].

High rates of psychiatric symptoms have been documented in HCW working during the COVID-19[6–11] and prior

pandemics[12,13]. Pandemic-related stressors identified as potential risk factors include quarantine procedures, isolation from social supports, stigmatization, and infection risk[9,10,14,15]. Less is known about the experiences of FR, despite exposure to similar pandemic-related stressors, often in less controlled environments. Consistent with the potential for high risks for this group, a survey of HCW and emergency medical services (EMS) workers in Italy during the COVID-19 pandemic found high levels of distress in both groups, but increased anger and regret, increased intrusiveness related to trauma, and decreased perception of self-efficacy in EMS relative to HCW[16].

Physical and emotional stressors among HCW/FR have also been associated with decreased professional longevity and poorer patient care [17,18]. The potential for significant professional attrition of HCW related to the COVID-19 pandemic has been emphasized in abundant media reports[19,20] as well as organizational surveys[21], and a review of medical leave in firefighters and emergency medical services (EMS) workers in New York early in the COVID-19 pandemic found increased use of medical leave, leading to decreased workforce availability[22]. However, little published data are available regarding the factors driving or modulating the risk of increased attrition or decreased occupational functioning among HCW/FR during the COVID-19 pandemic.

To address these gaps, we conducted an observational, cross-sectional study among HCW (clinical and support staff) and FR (EMS, fire, and law enforcement officers [LEO]) working in the USA during the COVID-19 pandemic. The goal of this study was to characterize and assess associations among COVID-19-related occupational stressors (CROS), psychiatric symptoms (depression, anxiety, insomnia, and post-traumatic stress disorder [PTSD]), and self-reported functional impairment and likelihood of leaving one's current field. CROS were quantified as a total score and by factor analysis, to identify specific types of CROS and their potential for differential impact. It is hoped that a better understanding of these relationships will facilitate the development of targeted interventions to protect HCW and FR even during periods of increased risk and workload.

METHODS

The study was approved by the VA Puget Sound Health Care System Human Subjects Committee. Prior to enrollment, all participants were provided an information statement that detailed the purpose, risks, benefits, and alternatives to participation.

Participants

A convenience sample of 510 participants was recruited through targeted outreach and paid advertising on Facebook between September 15, 2020, and February 7, 2021. Targeted outreach included large and COVID-focused HCW Facebook groups and emails to professional organizations (e.g., unions) and list-serves. Participants were asked to self-attest that they were a HCW or FR who provided professional services affected by the COVID-19 pandemic. The sample for the present study includes 301 HCW (60 physicians, 188 RN/LPN) and 200 FR (162 EMS, 54 firefighters, and 19 law enforcement officers [LEO]). LEO and firefighters were merged for subgroup analyses below, and individuals reporting dual EMS and LEO/firefighting roles were categorized under LEO/fire. Responses spanned 47 states and 445 zip codes, with broad distribution across the rural–urban continuum[23] (Supplementary Fig. 1).

Procedures

Self-report assessments were collected using Qualtrics. To encourage broad and representative participation, participants were not required to provide their legal name and were able to skip questions. Email addresses were collected to allow longitudinal follow-up for up to 9 months. Compensation was not provided. Data presented represent an analysis of the baseline survey only, except for the free-text response analysis. In order to include more respondents' perspectives to this optional item, and a broader range of themes, free-text responses from any time point during the same calendar period were included in this analysis.

Measures

COVID-19 Related Occupational Stressors. Exposure to CROS was assessed using a measure designed for this study by a collaborative team of physicians working in New York City hospitals and emergency rooms in March and April 2020 and our research team (Appendix A). This instrument asks four yes/no questions about personal experiences of loss due to COVID-19, and 13 questions assessing the frequency over the past 2 weeks of caring for individuals with COVID-19, perceiving increased risk to self or family due to occupational exposure to COVID-19, experiences of patient suffering related to the impacts of COVID-19, inadequate support or protection related to COVID-19, and feeling unable to provide effective or adequate care due to COVID-19, with responses scored 0–3. The 13-Likert scale items were summed to provide a CROS total score, while the initial 4 yes/no questions were used to characterize the sample and as covariates in multivariable regression models. Following this structured assessment, participants were asked an optional open-ended item, “Is there anything else you would like to share with us that is increasing your stress from your occupational duties?”.

Psychiatric Symptoms and Prior Trauma Exposure. Psychiatric symptoms were assessed using the Patient Health Questionnaire 9-item (PHQ9)[24], the Generalized Anxiety Disorder 7-item (GAD7)[25], the Insomnia Severity Index (ISI)[26], and the PTSD Checklist for DSM-5 (PCL5)[27]. Prior trauma history was assessed using the Life Events Checklist[28]; a numerical index of prior trauma exposure was calculated using

items selected a priori as those most likely to meet criterion A of PTSD (items 7–8, 11, and 14–16), and constituted the sum of items a participant reported they had witnessed or experienced.

Occupational Outcomes. Functional impairment was assessed using the two work-related items from the PROMIS Short Form v2.0 Ability to Participate in Social Roles and Activities 8a measure[29], modified to focus on occupational work (Supplemental Table 1). The likelihood of leaving one's current profession was assessed with two items: "How likely do you think it is that you will still be working in your current field in 5–10 years?" and "How have your experiences providing care during the COVID-19 pandemic affected your interest, willingness, or ability to continue working in your current field?".

Data Analysis

Data were analyzed using R and RStudio. Correlation coefficients were implemented using the *stat_cor* function from *ggpubr*[30]. Pearson's R was used for variables representing scale totals, while Spearman's correlation coefficients were used when one or more of the variables represented a single ordinal item or the sum of two ordinal items. Figures were created using *ggplot2*[31] and *jtools*[32], tables using *flextable*[33]. For all analyses, we selected a *p* value of < 0.05 to determine statistical significance. Participants with missing data were excluded from analyses using items or measures with missing data. Missingness ranged from 2.8 to 30% (Supplementary Table 2). Most missing data represented partial survey completers rather than missing items, so no data were imputed. Completers of the instruments that came later in the survey (e.g., GAD7, PHQ9) were more likely to be married, physicians, to have had COVID, and to report a higher number of prior trauma exposures.

Characterization and factor analysis of the CROS was implemented using *psych* and *psychTools*[34]. Cronbach's alpha was 0.88 (CI 0.86–0.9), indicating strong internal consistency. Following the scree plot (Supplemental Fig. 2), a 3-factor solution was chosen. Using varimax rotation produced a solution with mean item complexity of 1.5 and RMSR of 0.04. These data-driven factors emphasized 3 conceptually coherent, face-valid domains (Fig. 1). Items characterizing a total

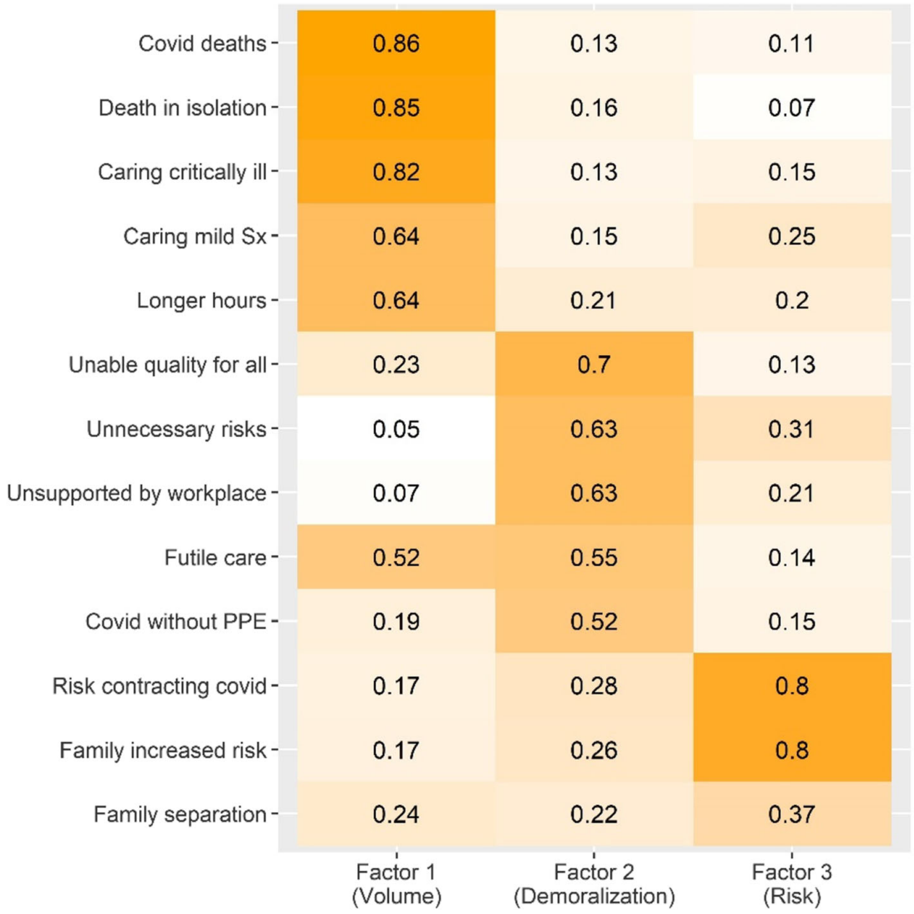

Fig. 1 Factor analysis of COVID-19-related occupational stressors (CROS). Results of a 3-factor analysis of COVID-19 exposure items into 3 factors, termed volume, demoralization, and risk, based on an interpretation of their most highly weighted items. Color shading is proportional to the numeric weight on each cell, and indicates the weight that item contributes to the factor below. See Appendix A for complete wording of CROS items

volume of COVID-19-related care delivered were most heavily weighted by factor 1, which was termed the “volume” factor. Factor 2 most heavily weighted items addressing the ability to provide high quality and effective care to all patients, being asked to take unnecessary risks, or being unsupported by one’s workplace; this factor was termed the “demoralization” factor. The third factor most heavily weighted one’s personal risk or one’s family’s risk of contracting COVID-19, termed the “risk” factor.

Multivariable linear regression models were implemented using *nlme*[35], with either continuous CROS factor scores or continuous psychiatric symptom domain scores as the predictor variables. All multivariable models included age and gender as covariates. For models characterizing the relationship of CROS factors to psychiatric symptoms and occupational outcomes, an index of prior trauma exposure, personal history of COVID-19 infection, history of COVID-19 infection in a family member, and death of a family member or close colleague due to COVID-19 were additionally included as covariates.

Analysis of free-text responses was carried out using a rapid template qualitative analysis, an established rapid analysis method ideally suited for pragmatically describing textual data (e.g., the restricted range and fragmented text for this single open-ended questionnaire item).[36,37] A coding template summarizing broad “codes” was initially developed by the senior qualitative analyst from reading a random sample of responses. Initial codes were then applied independently by two coders. Codes were updated as needed when discrepancies or lack of clarity occurred. Discrepancies were resolved until an intercoder agreement was reached. Responses were excluded if they were not related to COVID ($n = 1$) or were redundant responses by the same respondent across assessments ($n = 2$).

Initial coding was done with the combined HCW and FR sample to identify relevant themes. We then performed stratified counts and identified issues most salient to HCW or FR; i.e., if an overrepresented proportion of that occupational category endorsed a theme, defined as > 5% higher than would be expected given the overall proportion of open-ended respondents from that occupational group.

RESULTS

Table 1 presents sample characteristics, including demographics, CROS scores, psychiatric symptoms, and outcome assessments, for the entire sample and stratified by subgroups. In addition to comparing HCW and FR, sufficient respondents from the subgroup physicians, nurses, LEO plus fire, and EMS were present to analyze these groups independently. In general, responses were similar for HCW and FR, including similar scores on CROS measures. However, nurses scored significantly higher than physicians on both total exposure (CROS total, $p < 0.01$) and most psychiatric symptoms domains (PTSD, depression and insomnia $p < 0.01$, thoughts of suicide or self-harm $p = 0.04$). Similarly, CROS total was significantly higher for EMS versus LEO/fire ($p < 0.01$), as were symptoms of depression ($p < 0.01$). Fewer FR (40.6%) than HCW (55%) reported experiences working during the pandemic had decreased their likelihood of remaining in their current field ($p = 0.004$).

Bivariate Relationship of CROS Total Score to Psychiatric Symptoms and Occupational Outcomes

Figure 2 presents bivariate associations between demographic variables, exposure scores, and psychiatric symptom scores. Of the psychiatric symptom domains, the CROS total was most strongly correlated with the PCL5 ($R = 0.52$) but was also significantly associated with the PHQ9 ($R = 0.44$), ISI ($R = 0.41$), and GAD7 ($R = 0.43$, all $p <$ 1e-15). CROS total also was significantly correlated with thoughts of suicide or self-harm ($R = 0.25$, $p <$ 1e-5), problems completing work tasks ($R = 0.28$, $p <$ 1e-8), and likelihood of leaving field ($R = 0.18$, $p < 0.001$). For the subgroup that reported at least moderate insomnia symptoms and was asked about the impact of sleep problems on functioning at work ($N = 367$), CROS total was significantly related to reported impact of sleep problems on work performance ($R = 0.37$, $p <$ 1e-11).

Multivariable Relationships of the 3 CROS Factors to Psychiatric Symptoms and Occupational Outcomes

We conducted a series of multivariable linear regression models. The relationships of CROS factors to psychiatric symptoms across all respondents are presented in Fig. 3A, and by subgroup in Supplemental Fig. 3. These models found that all 3 CROS factors were significantly and positively related to all 4 psychiatric symptom domains. Across all participants and for both HCW and FR, the demoralization factor was the strongest correlate of PTSD (HCW $\beta = 0.37$, FR $\beta = 0.59$), depression (HCW $\beta = 0.30$, FR $\beta = 0.5$) and anxiety symptoms (HCW $\beta = 0.29$, $\beta = 0.42$, all $p < 0.0001$). The relationships were similar across the four small subgroups (physicians, nurses, LEO + fire, and EMS) with the exception that for LEO + fire, the volume factor was as or more strongly associated with these outcomes than demoralization ($\beta =$ 0.58 GAD7, $\beta = 0.47$ PTSD, $\beta = 0.44$, all $p < 0.01$). For insomnia symptoms, the demoralization factor was strongly associated with symptom intensity for physicians ($\beta = 0.41$, $p < 0.05$) and EMS ($\beta = 0.35$, $p < 0.01$), while the risk factor was associated with symptom intensity for nurses ($\beta = 0.36$, $p < 0.01$).

Across all respondents, thoughts of suicide or self-harm were significantly and positively related to both the volume

Table 1 Sample Demographics, COVID-19-Related Occupational Stressors (CROS), Psychiatric Symptoms, and Functional Outcome Measures

	All	Health Care Workers			First Responders			HCW vs FR	Physician vs nurse	LEO/ fire vs EMS
		All HCW	Physician	Nurse	All FR	LEO or fire	EMS			
Demographics	N = 510	N = 301	N = 60	N = 187	N = 200	N = 73	N = 119			
Age, years (mean + SD)	41.3 ± 10	42.3 ± 10	42.0 ± 6.6	42.9 ± 11	39.6 ± 11	44.0 ± 11	36.8 ± 10	0.03	0.52	< 0.01
Education, years (mean + SD)	15.8 ± 3.1	16.6 ± 3.2	20.4 ± 2.0	15.5 ± 2.5	14.4 ± 2.4	14.1 ± 3.1	14.6 ± 1.9	< 0.01	< 0.01	0.25
Married, %	57.8% (208/360)	63.2% (139/220)	86.3% (44/51)	56.7% (76/134)	50% (68/136)	69.2% (36/52)	39.7% (31/78)	0.02	< 0.01	< 0.01
Veteran, %	9.1% (33/362)	5% (11/221)	2% (1/51)	7.4% (10/135)	16.1% (22/137)	26.4% (14/53)	9% (7/78)	< 0.01	0.29	0.01
COVID-19 exposure assessment*										
Had Covid	35.6% (160/449)	30.2% (81/268)	14% (8/57)	34.8% (57/164)	44% (77/175)	32.3% (21/65)	50% (51/102)	< 0.01	< 0.01	0.03
Family member Covid	30.2% (136/450)	24.5% (66/269)	8.8% (5/57)	27.3% (45/165)	38.9% (68/175)	35.4% (23/65)	44.1% (45/102)	< 0.01	< 0.01	0.33
Close death from Covid	22% (99/449)	21.9% (59/269)	8.8% (5/57)	26.1% (43/165)	22.4% (39/174)	21.5% (14/65)	20.8% (21/101)	0.91	< 0.01	1.00
Medical condition increasing risk	41.1% (180/438)	39.8% (104/261)	15.8% (9/57)	45.6% (73/160)	42.1% (72/171)	37.1% (23/62)	42.6% (43/101)	0.69	< 0.01	0.52
Caring for mild cases	1.6 ± 1.2	1.6 ± 1.2	1.0 ± 1.1	1.8 ± 1.2	1.6 ± 1.1	1.1 ± 1.2	2.0 ± 0.9	0.80	< 0.01	< 0.01
Caring for critically ill	1.1 ± 1.2	1.2 ± 1.2	0.5 ± 0.9	1.4 ± 1.2	1.0 ± 1.1	0.8 ± 1.1	1.2 ± 1.0	0.12	< 0.01	< 0.01
Longer hours for Covid	1.1 ± 1.1	1.2 ± 1.1	0.6 ± 0.9	1.3 ± 1.1	1.1 ± 1.1	0.8 ± 1.0	1.2 ± 1.1	0.33	< 0.01	< 0.01
Deaths to Covid	0.8 ± 0.9	0.8 ± 1.0	0.3 ± 0.6	0.9 ± 1.0	0.7 ± 0.9	0.6 ± 0.8	0.8 ± 0.9	0.68	< 0.01	0.04
Deaths in isolation	0.7 ± 1.0	0.8 ± 1.0	0.2 ± 0.6	1.0 ± 1.1	0.6 ± 0.8	0.4 ± 0.7	0.7 ± 0.8	0.03	< 0.01	0.04
Covid without PPE	0.5 ± 0.8	0.5 ± 0.8	0.2 ± 0.4	0.6 ± 0.9	0.5 ± 0.9	0.3 ± 0.6	0.6 ± 0.9	0.56	< 0.01	0.01
Covid care futile	0.9 ± 1.0	1.0 ± 1.1	0.4 ± 0.7	1.2 ± 1.2	0.9 ± 0.9	0.6 ± 0.8	1.0 ± 0.8	0.50	< 0.01	< 0.01
Not quality care to all	1.1 ± 1.1	1.3 ± 1.1	0.8 ± 1.0	1.5 ± 1.1	0.9 ± 1.0	0.5 ± 0.9	1.0 ± 1.0	< 0.01	< 0.01	< 0.01
Personal increased risk	2.0 ± 1.1	1.9 ± 1.1	1.4 ± 1.0	2.0 ± 1.1	2.1 ± 1.0	1.8 ± 1.1	2.4 ± 0.8	0.03	< 0.01	< 0.01
Family increased risk	1.9 ± 1.1	1.8 ± 1.2	1.3 ± 1.1	1.9 ± 1.2	2.1 ± 1.1	1.8 ± 1.2	2.2 ± 1.0	0.04	< 0.01	0.02
Unsupportive workplace	1.4 ± 1.2	1.4 ± 1.2	1.0 ± 1.0	1.5 ± 1.2	1.3 ± 1.2	1.2 ± 1.3	1.3 ± 1.2	0.21	< 0.01	0.38
Separation from family	1.0 ± 1.1	0.9 ± 1.2	0.5 ± 1.0	0.9 ± 1.1	1.0 ± 1.1	0.7 ± 1.0	1.2 ± 1.1	0.63	< 0.01	< 0.01
Unnecessary risk	1.0 ± 1.1	1.0 ± 1.2	0.5 ± 0.9	1.1 ± 1.2	0.9 ± 1.1	0.8 ± 1.0	1.0 ± 1.1	0.24	< 0.01	0.30
Total Exposure Score	14.2 ± 8	14.4 ± 9	8.4 ± 6	15.9 ± 9	13.7 ± 8	10.6 ± 8	15.7 ± 7	0.39	< 0.01	< 0.01
Psychiatric Symptoms and Functional Outcomes**										
PTSD (PCL5 total)	27.7 ± 18.4	28.2 ± 18	19.5 ± 15.2	30.4 ± 17.5	26.6 ± 19	24.3 ± 20	28.1 ± 18.9	0.42	< 0.01	0.25
% clinical range (≥ 31)	37.8% (152/402)	38.8% (94/242)	20.8% (11/53)	42.3% (63/149)	36.4% (56/154)	36.1% (22/61)	36% (31/86)	0.67	< 0.01	1.00
Depression (PHQ9 Total)	9.9 ± 6.6	9.6 ± 6.2	6.1 ± 4.7	10.5 ± 6.2	10.2 ± 7.1	7.9 ± 7.1	11.7 ± 6.7	0.39	< 0.01	< 0.01
% clinical range (≥ 5)	73.9% (264/357)	74.1% (160/216)	51% (26/51)	80.2% (105/131)	73% (100/137)	56.6% (30/53)	83.3% (65/78)	0.90	< 0.01	< 0.01
Insomnia (ISI total)	12.3 ± 5.6	12.2 ± 5.5	9.0 ± 5.3	12.9 ± 5.1	12.3 ± 5.7	11.5 ± 6.1	12.7 ± 5.5	0.90	< 0.01	0.17
% clinical range (≥ 15)	35.1% (174/496)	35% (103/294)	13.6% (8/59)	39.6% (72/182)	34.4% (67/195)	33.3% (24/72)	34.8% (40/115)	0.92	< 0.01	0.88
GAD7 total	9.3 ± 6.1	9.6 ± 6.0	7.9 ± 5.6	9.7 ± 6.0	8.8 ± 6.3	7.6 ± 6.5	9.7 ± 6.2	0.28	0.07	0.06
% clinical range (≥ 5)	74.7% (274/367)	75.8% (169/223)	70.6% (36/51)	75.9% (104/137)	72.7% (101/139)	61.1% (33/54)	79.7% (63/79)	0.54	0.46	0.03
Thoughts of suicide or self-harm (PHQ #9)	15.3% (55/359)	12.4% (27/218)	3.9% (2/51)	15.8% (21/133)	19% (26/137)	13.2% (7/53)	24.4% (19/78)	0.09	0.04	0.13
Decreased likelihood remaining in field	49.3% (215/436)	55% (143/260)	46.4% (26/56)	59% (95/161)	40.6% (69/170)	35.9% (23/64)	43.9% (43/98)	< 0.01	0.12	0.33
Trouble completing work tasks	18.5% (80/433)	21.2% (55/259)	17.9% (10/56)	22% (35/159)	14.3% (24/168)	15.9% (10/63)	12.2% (12/98)	0.08	0.57	0.64

** Responses are indicated as the percent responding positively (the 4 binary response items) or the mean ± SD (the 13 individual Likert scale items, range 0–3); total exposure score represents the sum of the 13 Likert scale items (range 0–39)*

*** Psychiatric symptom and functional outcome characterization: mean ± SD of measure totals for PCL5 PTSD Checklist for DSM5, PHQ9 Patient Health Questionnaire 9-item (depression symptoms), ISI Insomnia Severity Index, GAD7 Generalized Anxiety Disorder 7-item*

A

	Age	Gender: Female	CROS Total	Had Covid	Family mem. Covid	Close death	Incr. risk	PCL5 Total	PHQ9 Total	ISI Total
Age										
Gender: Female	0.04									
CROS Total	-0.20***	0.13*								
Had Covid	-0.07	-0.09	0.13**							
Family mem. Covid	-0.09	-0.10	0.14**	0.31****						
Close death	0.01	0.03	0.30****	0.10*	0.21****					
Incr. risk	0.14**	0.09	0.13**	0.13**	0.01	0.17***				
PCL5 Total	-0.20***	0.18***	0.52****	0.12*	0.06	0.26****	0.17***			
PHQ9 Total	-0.25****	0.20***	0.44****	0.15**	0.05	0.17**	0.17**	0.76****		
ISI Total	-0.16**	0.11*	0.41****	0.15**	0.09	0.17***	0.17***	0.63****	0.65****	
GAD7 Total	-0.24****	0.22****	0.43****	0.16**	0.08	0.16**	0.04	0.79****	0.80****	0.59****

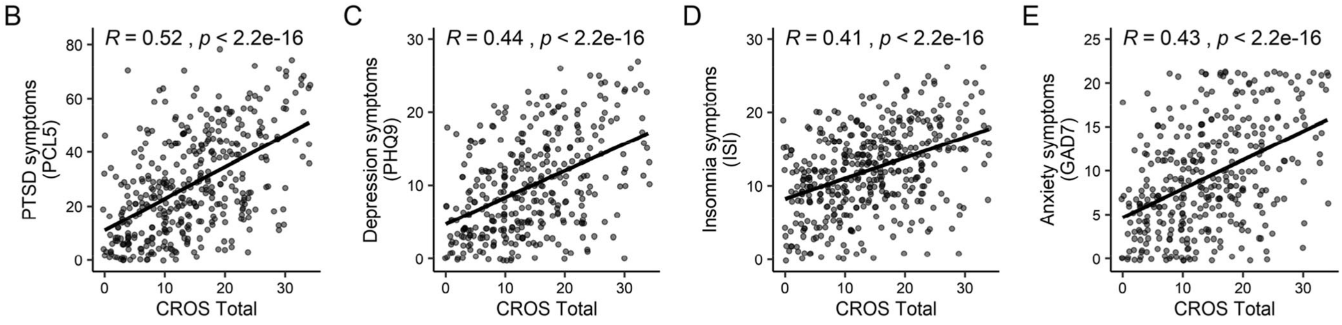

Fig. 2 COVID-19-related occupational exposure (CROS total score) is strongly related to increased burden of psychiatric symptoms. A Bivariate relationships between demographic variables, exposure scores, and psychiatric symptom scores are represented by Spearman's correlation coefficients. Scatter plots are provided for the relationship of CROS total to PTSD symptoms (B), depression symptoms (C), insomnia symptoms (D), and anxiety symptoms (E). *$p < .05$, **$p < .01$, ***$p < .001$, ****$p < .0001$. "Close death" = death of a family member or close colleague from COVID-19, "Inc risk" = medical condition associated with increased risk from COVID-19 infection

factor and the demoralization factor, as well as to prior trauma history (Fig. 3B and Supplemental 3B). On subgroup analysis, this pattern was maintained for nurses and EMS, but not for physicians (no significant predictors) or LEO + fire (volume factor $\beta = 0.95$, $p < 0.001$).

In examining the relationship of CROS factors to occupational outcome measures across all respondents, only the demoralization factor was significantly related to the likelihood of leaving one's current field or problems completing work-related tasks. This pattern was preserved across subgroups, with the exception that for LEO + fire demoralization was significantly related to the likelihood of leaving one's field ($\beta = 0.52$, $p < 0.01$) but not to occupational functioning, while for EMS demoralization was significantly related to occupational functioning ($\beta = 0.55$, $p < 0.0001$) but not the likelihood of leaving one's field.

Relationship of Psychiatric Symptom Domains to Functional Outcome Measures and Suicidality

The relationship of psychiatric symptom domains (PTSD, anxiety, depression, and insomnia) to functional outcome measures and thoughts of suicide or self-harm were similarly characterized (Fig. 3C and Supplemental 3C). Across all participants, PTSD symptom severity was significantly related to the likelihood of leaving one's current field, trouble completing work tasks, and thoughts of self-harm or suicide. In addition, older age had a significant positive relationship to the likelihood of leaving one's current field, and depression symptoms were significantly and positively related to thoughts of self-harm or suicide. However, based on subgroup analyses, the relationship of PTSD symptoms to the increased likelihood of leaving one's current field was driven most strongly by nurses ($\beta = 0.41$, $p < 0.01$) and LEO + fire ($\beta = 0.74$, $p < 0.05$), while the relationship of PTSD to occupational functioning was driven most strongly by physicians ($\beta = 0.67$, $p < 0.05$) and EMS ($\beta = 0.43$, $p < 0.01$). Interestingly, for LEO + fire, anxiety symptoms were strongly and positively associated with thoughts of suicide or self-harm ($\beta = 1.2$, $p < 0.001$) but strongly and negatively associated with thoughts of leaving one's field ($\beta = 0.91$, $p < 0.01$).

Qualitative Analysis of Free-Text Responses

Over one-third of participants (36% of total, 37.5% of HCW and 32.4% of FR) responded to the optional, open-ended question about sources of occupational stress during the COVID-19 pandemic, with an average response length of 31 words (range: 1–169). Responses vividly conveyed the

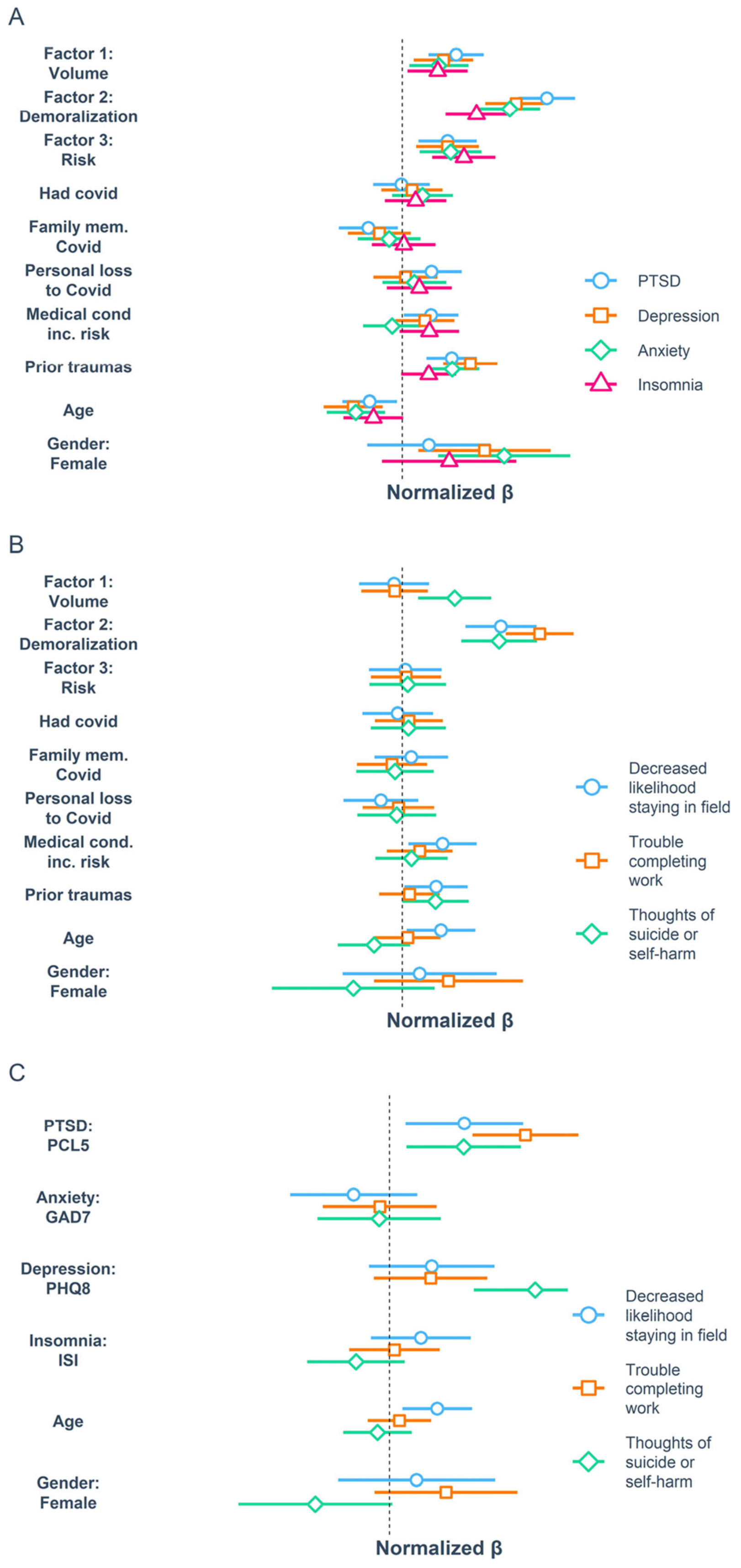

Fig. 3 Relationships between different factors of COVID-19-related occupational stressors (CROS factors), psychiatric symptom expression, and functional outcomes. Results of multivariable regression models relating CROS factors and covariates to psychiatric symptom clusters (A) and functional outcome measures along with thoughts of suicide or self-harm (B). C Results of independent multivariable regression models relating symptom clusters as measured by total scores on the PCL5, PHQ9, GAD7, and ISI, along with covariates of age and gender, to functional outcome measures

Table 2 Thematic Analysis of Free-Text Responses

**Poor Communication, Planning, and Leadership (TOTAL RESPONSES = 20; HCW: 65%; FR 35%)**<br>• Leaders not listening to or respecting suggestions and needs of frontline staff; defensiveness; lack of transparency, proactive planning; confusing, inconsistent guidelines	"Higher management not recognizing that some of their staff are highly trained to respond […] Infection prevention department close minded to suggestions made by the bedside staff […]."
**Lack of Protection and Support (TOTAL RESPONSES = 41; HCW: 61%; FR: 34%; OTHER: 5%)**<br>• Insufficient PPE, safeguards, and protocols; general lack of caring or support from leaders	"Our hospital doesn't care about us. We're disposable."
**Increased Demands (TOTAL RESPONSES = 38; HCW: 84%; FR: 16%)**<br>• Increase in demands, more complex; significant changes in procedures; examples also included having to work longer hours without breaks and working outside one's expertise and/or scope; nurses as "catch all" HCW	"Sudden schedule changes. Extensive work hours. The uncertainty of the disease and the continuous change and controversy of the treatments."
**Staffing Shortages (TOTAL RESPONSES = 29; HCW: 83%; FR: 17%)**<br>• Shortage of nurses, other essential HCW due to increased demands from patients; colleagues out with COVID-19	"Severe understaffing and constantly feeling like I am being overworked and underappreciated."
**Patient care (TOTAL RESPONSES = 19; HCW: 84%; FR: 16%)**<br>• Patient care ethics; impact of COVID-19 on quality of care for COVID-19 patients and non-COVID-19 patients alike (due to overwhelming demands, short-staffing)	"People begging for your help. I feel so evil and dirty having to place a BiPap on a patient begging me not to. They don't like it and cry and beg for me to let them die. I must put patients in restraints to keep them from pulling out their tubes. They cry for me to let them go. It's like a bad horror movie."
**Fear of or enacted reprisal from leaders (TOTAL RESPONSES = 7; HCW: 57%; FR: 43%)**<br>• Fear of or enacted retaliation for speaking out against workplace risks, hazards, and lack of safeguards; blackmail; threats to career and/or professional development for wanting to quit	"I was exposed to a confirmed COVID patient in April who was in respiratory distress. I only had my N95 on, no face shield or gown had been provided. I wanted to quarantine; I was accused of borderline patient abandonment. They threatened to report me to the board. I quit, then I couldn't get unemployment because I quit."
**Betrayal by Colleagues (TOTAL RESPONSES = 10; HCW: 60%; FR: 40%)**<br>• Failure of colleagues to follow guidelines/safeguards	"My coworkers are COVID deniers. I work in EMS and it makes it really hard."
**Betrayal by Society (TOTAL RESPONSES = 28; HCW: 71%; FR: 25%; OTHER: 4%)**<br>• Public health guidelines disregarded by the general public; COVID-19 skepticism and/or denial often fueled by political and public health leaders; lack of accommodation from society for needs such as childcare	"The worst thing is dealing with incredible stress at work, and then realizing no one really cares… I separate from my kids at the first sign of symptoms because I'm heavily exposed at work, but then have to listen to people complaining about recommendations they don't have people over for Thanksgiving. It's very disheartening when the community doesn't do its part. I feel betrayed."
**Emotional Toll (TOTAL RESPONSES = 39; HCW:67%; FR: 33%)**<br>• Stress (immediate and anticipated), burnout, anxiety, uncertainty, and/or feeling underappreciated	"I have never felt so helpless and devastated as well as traumatized in my career."
**Concern for Well-being of Self (TOTAL RESPONSES = 29; HCW:72%; FR: 28%)**<br>• Few explicitly said anything akin to "I'm afraid I'll get COVID," even when acknowledging infection risk. Health consequences focused more on effect of working long hours, wearing PPE for extensive amounts of time, etc	"Not having time to pee or drink water."
**Concern for the Well-being of Others (TOTAL RESPONSES = 24; HCW:58%; FR: 38%; OTHER: 4%)**<br>• Risk for family members or colleagues of COVID-19 infection, and impacts like stress for family members	"My [child] had severe anxiety due to my position and frequently had nightmares and panic attacks. She had previously not had anxiety problems."
**Financial Impacts (TOTAL RESPONSES = 16; HCW: 69%; FR: 31%)**<br>• Insufficient pay relative to magnitude of demands and risks; lack of paid sick or vacation leave; job resignation, unemployment, loss of base pay; medical costs of COVID-19	"Healthcare workers largely have not received hazard pay […] If you develop symptoms you are sent home for 3 days without pay."

We determined responses as being overrepresented when the proportions of respondents who endorsed the item deviated by > 5% from the occupational category's representation in the open-ended text responses (i.e., 67% HCW, 31% FR, and 2% other). For example, if 73% of a theme was endorsed by HCW, it was considered overrepresented, and thereby uniquely relevant to that occupational group

challenges faced by HCW and FR and the magnitude of associated distress. A rapid template analytic approach identified 12 overarching codes within respondent data, which are described, along with illustrative quotes in Table 2.

Overall, responses were highly consistent with the quantitative analyses above. Among the 12 identified codes, "Lack of Protection and Support," "Increased Demands," and "Emotional Toll" were the most common. Respondents described their work during COVID-19 as involving "some of the saddest death stories" they had experienced and feeling "spread thin and exhausted." Notable for a relative lack of representation were comments addressing respondents' personal risk of COVID-19 infection, with most references to this type of risk taking the form of concern for the impact of this risk on others.

HCWs were overrepresented (> 5% deviation) in categories "staffing shortages," "increased demands," and "patient care." FRs were overrepresented in "fear of or enacted reprisal from leaders," "concern for the well-being of others," and "betrayal by colleagues." HCW and FR were represented in all categories.

DISCUSSION

Our findings are consistent with previous demonstrations of high levels of psychiatric symptoms and distress in HCW working during the COVID-19 pandemic[6–11]. In the present study, a quantitative measure of CROS was associated with psychiatric distress. The relationships remained significant when HCW and FR were analyzed independently and when a quantitative measure of exposure to prior traumatic stressors was included as a covariate, suggesting the high rates of psychiatric symptoms are unlikely to represent baseline rates in this population independent of COVID-19-related occupational stressors.

The overall magnitude of most relationships identified was similar, or sometimes greater, in FR vs. HCW. This was particularly striking and concerning regarding thoughts of suicide or self-harm, reported by 19% of FR and 12% of HCW and strongly related to CROS. Given prior work demonstrating high trauma exposure and psychiatric symptoms in FR[38], including high rates of both suicidal thoughts and behaviors, particularly in EMS[39], results highlight the importance of addressing the impact of working during a pandemic on FR. Similarly, findings underscored the higher CROS exposure and psychiatric distress in nurses as compared with physicians, building on prior work finding increased distress, suicidal ideation, and suicidal behavior specifically in nurses[40].

A substantial proportion of both HCW and FR reported their likelihood of staying in their current field had been somewhat or significantly decreased by their experiences working during the COVID-19 pandemic and that they at least sometimes had difficulty completing work-related tasks. These results are consistent with and build upon findings from previous pandemics[12,41] and are particularly worrisome given existing concerns about current and future shortages in the health care workforce[42,43]. The present study suggests healthcare staffing shortages are in and of themselves a COVID-19 stressor, so further shrinking the labor pool could have an exponential negative impact on HCW wellbeing and professional retention.

The elements in the CROS were well-described by three face-valid factors, termed “volume,” “demoralization,” and “risk.” Demoralization showed the strongest relationship to both psychiatric symptom domains and functional outcome measures, while risk showed the smallest relationship. These findings suggest that while strategies such as vaccination that decrease the risk posed to HCW and their families by COVID-19 infection are important, they are unlikely to fully mitigate the impact of COVID-19-related occupational stressors on HCW and FR mental health or functional outcomes. In fact, the theme of betrayal by the community in the free-text responses raises the concern that high volumes of COVID-19-related care driven by patients who have declined vaccination may be associated with increased psychiatric distress compared with similar volumes of care when vaccination was insufficient or simply not available.

Our results suggest a significant number of strategies that could help mitigate the effects of CROS on HCW and FR (Fig. 4), both by decreasing the volume of COVID-19 impacted care individuals are providing and the associated personal risk they experience while doing so (direct factors) and by changing the context in which this care is being provided (contextual factors). Although it is important to prospectively test the impact of such interventions, existing evidence supports the efficacy of systematic approaches to identifying and addressing factors that unnecessarily increase workload, while supporting health care workers’ control and flexibility, meaning in work, and workplace community[5,44–46]. Identifying and rectifying barriers to communication and transparency within a workplace, providing regular and effective feedback to leadership, and increasing the frontline worker input are often critical intervention components[5,47–49]. Such interventions have not been found to come at the cost of other organizational goals or significant financial expense[48]. However, these approaches are most effectively implemented at the organizational level[47–49].

Among psychiatric symptom domains, PTSD symptoms stood out for both the strength of their relationship with overall COVID-19-related occupational stressors, and as the symptom domain most related to adverse occupational outcomes. Along with depression symptoms, PTSD symptoms were also significantly related to thoughts of suicide or self-harm. This suggests that the identification and implementation of interventions to reduce the risk of PTSD[50–53] in HCW and FR should be a top priority. Moreover, PTSD symptoms may be particularly important to detect and treat in HCW and FR. Broadly implemented strategies to reduce risk and strengthen resilience while proactively identifying and providing accessible, confidential care for those who require more intensive services, maybe a particularly effective and cost-efficient approach[54,55].

The current work has limitations. Our results highlight the significant variety of experiences of HCW and FR working during the COVID-19 pandemic and the importance of these differences for outcomes. However, our data do not include a detailed assessment of factors related to financial resources, family obligations, or position and influence within one’s specific field or the health care system more broadly, each of which are likely to interact with many of the factors explored here[56]. Follow-up studies of the persistent impacts of working during previous pandemics underscore the need for longitudinal follow-up of the affected workforce[13,41], and further work following the impact of CROS on psychiatric symptom burden and occupational functioning over time will be important. The work relies upon participants’ self-report of exposure history, workplace experiences, and psychiatric symptoms, which may result in same source bias. The survey instrument was long, and some participants did not complete all instruments. In addition, the study used targeted outreach and paid advertising targeting regions with high rates of COVID-19 cases. The results obtained from the current sample may not reflect rates in all health care workers, nationally or

Pandemic-related occupational stressors

Direct	Increased workload	Increased hours worked
		Increased intensity of demands while working
		Work demands that are outside of usual role or training
	Exposure to suffering	Intensity of patient suffering and unexpected deaths
		Frequency of patient suffering and death, without time to process
	Personal risk	Medical risks associated with COVID-19 infection
		Risk of spreading COVID-19 infection to family members
		Risk of being unable to meet other role obligations if become ill
Contextual	Efficacy	Are risk and sacrifices seen as making a meaningful difference?
	Necessity	Are risks and sacrifices seen as unavoidable?
	Valued	Are risks and sacrifices experienced as being seen and valued by one's workplace and community?
	Supported	Concerns and recommendations are valued and attended to by leadership and the community
		Leadership and the community provide needed resources, when they are available
	Security	Risks to job security or the threat of retaliation from leadership
		Risks of lost hours or income

Mitigation strategies

Direct	Adequate staffing	Minimize excessive work hours
		Minimize overwhelming care burdens while working
	Adequate training	Increased availability of training and consultation when asked to cover new roles
	Robust safety protocols	Adequate PPE
		Rapid updates as new information or situations emerge
		Attention to the protections needed for those with higher risk jobs or medical conditions
Direct / Contextual	Responsive Leadership	Active solicitation of input from frontline workers
		Active consideration of and responsiveness to input from frontline workers
Contextual	Clear communication from leadership	Demonstrating responsiveness to input from frontline workers
		Validating the risks and sacrifices of frontline workers
		Documenting the collective efficacy for patient and colleague weelbeing
	Access to wellness resources and mental health care	Resources to support wellness, with broad availability
		Proactive outreach to those with higher risk or higher symptom burden
		Speciality mental health resources that are accessible and confidential
	Protections from job and financial insecurity	Policies that actively protect workers from retaliation by leadership for reporting safety risks or concerns
		Protection of job and financial security especially for the lowest-paid portions of the workforce
	Communication from the community	Acknowledging the risks and sacrifices of frontline workers
		Advocating for community actions that limit the risk and sacrifices of frontline workers

Fig. 4 Potential schematic framework for considering direct and contextual factors contributing to occupational stress from the COVID-19 pandemic, and potential mitigation strategies. Stressors (left) and potential mitigation strategies (right) are divided into direct and contextual factors. Direct factors result primarily from the volume of COVID-19-related care being provided by an individual or their institution and resources available for the system to respond to these demands. Contextual factors can be addressed independent of the volume of COVID-19-related care being provided, and include the responsiveness of the system to addressing and supporting HCW/FR's needs, and ensuring they are not put at unnecessary risk. Stressors represent a synthesis of factors identified from the quantitative and qualitative analyses; mitigation strategies represent concrete examples of ways in which the identified stressors could be modified, minimized, or mitigated. Mitigation or intervention approaches may vary depending on the most relevant occupational stressors for a specific group. For example, the strong relationship between demoralization and both psychiatric symptoms and adverse occupational outcomes in FR, along with the emphasis in free-text responses of fear of or enacted reprisal from leaders and betrayal by colleagues, suggest interventions focused on responsiveness and clear communication from leadership and protections of job and financial security may be particularly important for many FR. The high rates of PTSD symptoms and the relationship of these symptoms to a high likelihood of leaving one's current field for nurses may suggest that interventions focused on decreasing the risk of PTSD, and increasing the availability and utilization of treatment for PTSD, may be of particularly high priority for nurses. An alternative example of a conceptual framework for planning risk mitigation and interventions based on a literature review can be found in *Schwartz et al*[5]

internationally. At the same time, the broad geographic and rural/urban diversity of study respondents is a strength, particularly given the geographic variability in pandemic intensity and community response.

Finally, the relationships characterized are observational and cross-sectional, and cannot be assumed to represent unidirectional, causal relationships. For example, PTSD symptoms were found to be strongly associated with suicidality and negative occupational outcomes. This suggests that prevention and/or treatment of PTSD symptoms may decrease the risk of suicidal behavior and improve functioning and workforce retention. However, this hypothesis will need to be tested in a prospective trial.

Supplementary Information *The online version contains supplementary material available at https://doi.org/10.1007/s11606-021-07252-z.*

Acknowledgements: *The authors would like to thank the many participants who generously shared their time and experiences with us. We would also like to acknowledge the significant number of staff members who contributed to this study often well beyond their usual work roles, including Carolyn LH Fort, Joseph D Clark, Kim Hart, Adam T McPartlin, Emma C Onstad-Hawes, Soleil S Groh, and Andrew Nicholls. We also thank George Sayre, Ashley Mog, and the Seattle-Denver COIN qualitative core group, who provided brief consultation on the qualitative methods, analysis, and interpretation.*

Corresponding Author: *Rebecca C. Hendrickson, MD, PhD; VISN 20 Northwest Mental Illness Research, Education, and Clinical Center (MIRECC), VA Puget Sound Health Care System, Seattle, WA, USA (e-mail: Rebecca.Hendrickson@va.gov).*

Author Contribution *RCH designed the study, with input from RAS, BPC, CAM, and MAR. RCH supervised the implementation of the study, with RAS playing a significant role in all implementation steps. RCH carried out the analysis of all results with consultation by RGT, with the exception of the thematic analysis of free-text responses which was carried out by KDH and GRM. RCH wrote the initial draft of the manuscript, with contributions from RAS, KDH and MAR. All authors provided input into the interpretation of the analysis and the drafting of the manuscript.*

Funding *This work was supported by a Research & Development Seed Grant from the Department of Veterans Affairs (VA) Puget Sound Health Care System (RCH), VA Clinical Sciences Research and Development Service Career Development Award IK2CX001774 (RCH); and the VA Northwest Network MIRECC (RCH, MAR). BPC is supported by funding from the National Institutes of Health (R01 HL 141811, HL R01 HL 146911). KDH is supported by funding from VA Health Services Research & Development.*

Declarations:

Conflict of Interest: *The authors declare that they do not have a conflict of interest.*

Disclosures: *The views expressed are those of the authors and do not reflect the official policy of the Department of Veterans Affairs or the US Government.*

Data Sharing: *Deidentified data sets may be requested by contacting the corresponding author.*

Open Access *This article is licensed under a Creative Commons Attribution 4.0 International License, which permits use, sharing, adaptation, distribution and reproduction in any medium or format, as long as you give appropriate credit to the original author(s) and the source, provide a link to the Creative Commons licence, and indicate if changes were made. The images or other third party material in this article are included in the article's Creative Commons licence, unless indicated otherwise in a credit line to the material. If material is not included in the article's Creative Commons licence and your intended use is not permitted by statutory regulation or exceeds the permitted use, you will need to obtain permission directly from the copyright holder. To view a copy of this licence, visit http://creativecommons.org/licenses/by/4.0/.*

REFERENCES

1. **Griffin BJ**, **Purcell N**, **Burkman K**, et al. and religious/spiritual (R/S) studies (Doehring. J Trauma Stress. 2019;32:350–362. https://doi.org/10.1002/jts.22362.
2. **Smith BN**, **Taverna EC**, **Fox AB**, **Schnurr PP**, **Matteo RA**, **Vogt D**. The Role of PTSD, Depression, and Alcohol Misuse Symptom Severity in Linking Deployment Stressor Exposure and Post-Military Work and Family Outcomes in Male and Female Veterans. Clin Psychol Sci. 2017;5(4):664-682. https://doi.org/10.1177/2167702617705672.
3. **Sienkiewicz ME**, **Amalathas A**, **Iverson KM**, **Smith BN**, **Mitchell KS**. Examining the Association between Trauma Exposure and Work-Related Outcomes in Women Veterans. Int J Environ Res Public Health. 2020;17(12):4585. https://doi.org/10.3390/IJERPH17124585.
4. **Adler DA**, **Possemato K**, **Mavandadi S**, **et al**. Psychiatric Status and Work Performance of Veterans of Operations Enduring Freedom and Iraqi Freedom. Psychiatr Serv. 2011;60(7):39-46. https://doi.org/10.1176/ps.60.7.pss6201_0039.
5. **Schwartz R**, **Sinskey JL**, **Anand U**, **Margolis RD**. Addressing Postpandemic Clinician Mental Health: A Narrative Review and Conceptual Framework. Ann Intern Med. 2020;173(12):981-988. https://doi.org/10.7326/M20-4199.
6. **Pappa S**, **Ntella V**, **Giannakas T**, **Giannakoulis VG**, **Papoutsi E**, **Katsaounou P**. Prevalence of depression, anxiety, and insomnia among healthcare workers during the COVID-19 pandemic: A systematic review and meta-analysis. Brain Behav Immun. 2020;88(May):901-907. https://doi.org/10.1016/j.bbi.2020.05.026.
7. **Shaukat N**, **Mansoor A**, **Razzak J**. Physical and mental health impacts of COVID-19 on healthcare workers: A scoping review. Int J Emerg Med. 2020;13(1):1–8. https://www.ncbi.nlm.nih.gov/pmc/articles/PMC7370263/pdf/12245_2020_Article_299.pdf. Acceso 20 de agosto de 2020.
8. **Wu Y**, **Wang J**, **Luo C**, **et al**. A Comparison of Burnout Frequency Among Oncology Physicians and Nurses Working on the Frontline and Usual Wards During the COVID-19 Epidemic in Wuhan, China. J Pain Symptom Manage. 2020;60(1):e60-e65. https://doi.org/10.1016/j.jpainsymman.2020.04.008
9. **Carmassi C**, **Foghi C**, **Dell'Oste V**, **et al**. PTSD symptoms in healthcare workers facing the three coronavirus outbreaks: What can we expect after the COVID-19 pandemic. Psychiatry Res. 2020;292:113312. https://doi.org/10.1016/j.psychres.2020.113312.
10 **Kürşat Şahin M**, **Aker S**, **Gülay Ş**, **Karabekiroğlu A**. Prevalence of Depression, Anxiety, Distress and Insomnia and Related Factors in Healthcare Workers During COVID-19 Pandemic in Turkey. J Community Health. 2020;45:1168-1177. https://doi.org/10.1007/s10900-020-00921-w.
11. **Firew T**, **Sano ED**, **Lee JW**, **et al**. Protecting the front line: A cross-sectional survey analysis of the occupational factors contributing to healthcare workers' infection and psychological distress during the COVID-19 pandemic in the USA. BMJ Open. 2020;10(10):1-12. https://doi.org/10.1136/bmjopen-2020-042752.
12. **Bai YM**, **Lin CC**, **Lin CY**, **Chen JY**, **Chue CM**, **Chou P**. Survey of stress reactions among health care workers involved with the SARS outbreak. Psychiatr Serv. 2004;55(9):1055-1057. https://doi.org/10.1176/appi.ps.55.9.1055.
13. **Allan SM**, **Bealey R**, **Birch J**, **et al**. The prevalence of common and stress-related mental health disorders in healthcare workers based in pandemic-affected hospitals: a rapid systematic review and meta-analysis. Eur J Psychotraumatol. 2020;11(1). https://doi.org/10.1080/20008198.2020.1810903.
14. **Xiao H**, **Zhang Y**, **Kong D**, **Li S**, **Yang N**. The Effects of Social Support on Sleep Quality of Medical Staff Treating Patients with Coronavirus in China. https://doi.org/10.12659/MSM.923549.
15. **Lai J**, **Ma S**, **Wang Y**, **et al**. Factors Associated With Mental Health Outcomes Among Health Care Workers Exposed to Coronavirus Disease 2019. JAMA Netw Open. 2020;3(3):e203976. https://doi.org/10.1001/jamanetworkopen.2020.3976.
16. **Mitchell AEP**, **Milliard B**, **Police YR**, **et al**. Coping With COVID-19: Emergency Stress, Secondary Trauma and Self-Efficacy in Healthcare and Emergency Workers in Italy. Published online 2020. https://doi.org/10.3389/fpsyg.2020.566912.
17. **Lu DW**, **Dresden S**, **Mccloskey C**, **Branzetti J**, **Gisondi MA**. UC Irvine Western Journal of Emergency Medicine: Integrating Emergency Care with Population Health Title Impact of Burnout on Self-Reported Patient Care Among Emergency Physicians Publication Date Supplemental Material Impact of Burnout on Self-Reported Patient Care Among Emergency Physicians. J West J Emerg Med Integr Emerg Care with Popul Heal. 2015;(7):16. https://doi.org/10.5811/westjem.2015.9.27945.
18. **Kuhn G**, **Goldberg R**, **Compton S**. Tolerance for Uncertainty, Burnout, and Satisfaction With the Career of Emergency Medicine. Ann Emerg Med. 2009;54(1):106-113.e6. https://doi.org/10.1016/j.annemergmed.2008.12.019.
19. COVID-19's Third Surge Is Breaking Health-Care Workers - The Atlantic. https://www.theatlantic.com/health/archive/2020/11/third-surge-breaking-healthcare-workers/617091/. Accessed December 3, 2020.
20. Doctors Are Calling It Quits Under Stress of the Coronavirus - The New York Times. https://www.nytimes.com/2020/11/15/health/Covid-doctors-nurses-quitting.html?smid=tw-nytimes&smtyp=cur&fbclid=IwAR2sXKItA3sQdg4HFGT6Dp2BMLWNb-QaSfJa6O8mlGvPQgDf8v-UBt4bUJnM. Accessed December 3, 2020.
21. QUICK COVID-19 PRIMARY CARE SURVEY. www.3rdconversation.com. Accessed December 3, 2020.
22. **Prezant DJ**, **Zeig-Owens R**, **Schwartz T**, **et al**. Medical Leave Associated With COVID-19 Among Emergency Medical System Responders and Firefighters in New York City. Published online 2020. https://doi.org/10.1001/jamanetworkopen.2020.16094.
23. USDA ERS - Rural-Urban Continuum Codes. https://www.ers.usda.gov/data-products/rural-urban-continuum-codes.aspx. Accessed July 25, 2021.
24. **Kroenke K**, **Spitzer RL**, **Williams JBW**. The PHQ-9: Validity of a brief depression severity measure. J Gen Intern Med. 2001;16(9):606-613. https://doi.org/10.1046/j.1525-1497.2001.016009606.x.
25. **Spitzer RL**, **Kroenke K**, **Williams JBW**, **Löwe B**. A brief measure for assessing generalized anxiety disorder: the GAD-7. Arch Intern Med. 2006;166(10):1092-1097. https://doi.org/10.1001/archinte.166.10.1092.
26. **Morin CM**, **Belleville G**, **Bélanger L**, **Ivers H**. The Insomnia Severity Index: psychometric indicators to detect insomnia cases and evaluate treatment response. Sleep. 2011;34(5):601-608. doi:https://doi.org/10.1111/j.1365-2648.2010.05394.x.
27. **Weathers FL**, **Keane T**, **Palmieri P**, **Marx S**, **Schnurr P**. The PTSD Checklist for DSM-5. In: NCf, ed. PTSD. 2013.
28. **Weathers FW**, **Blake DD**, **Schnurr P**, **Kaloupek DG**, **Mark B**, **Keane TM**. The Life Events Checklist for DSM-5 (LEC-5). National Center for PTSD; 2013. www.ptsd.va.gov.
29. **Hahn EA**, **DeVellis RF**, **Bode RK**, **et al**. Measuring social health in the patient-reported outcomes measurement information system (PROMIS): item bank development and testing. Qual Life Res. 2010;19(7):1035-1044. https://doi.org/10.1007/s11136-010-9654-0.Measuring.
30. **Kassambara A**. ggubr: "ggplot2" Based Publication Ready Plots. Published online 2020. https://rpkgs.datanovia.com/ggpubr/.
31. **Wickham H**. ggplot2: Elegant Graphics for Data Analysis. Published online 2009. http://ggplot2.org.

32. **Long JA**. jtools: Analysis and Presentation of Social Scientific Data. Published online 2021. https://jtools.jacob-long.com
33. **Gohel D**, **Clementine J**, **Fazilleau Q**, **et al**. Flextable: Functions for Tabular Reporting. Published online 2020. https://davidgohel.github.io/flextable/.
34. **Revelle W**. psych: Procedures for Psychological, Psychometric, and Personality Research. Northwestern University, Evanston, Illinois. R package version 2.0.12. Published online 2020:1–86.
35. **Pinheiro J**, **Bates D**, **DebRoy S**, **Sarkar D**, Team RC. nlme: Linear and Nonlinear Mixed Effects Models. Published online 2018:R package version 3.1–131. https://cran.r-project.org/package=nlme.
36. **Brooks J**, **Mccluskey S**, **Turley E**, **King N**. The Utility of Template Analysis in Qualitative Psychology Research. Qual Res Psychol. 2015;12:202-222. https://doi.org/10.1080/14780887.2014.955224.
37. **King N**. Template Analysis. In: Syman G, Cassell C eds. Qualitative Methods and Analysis in Organizational Research A Practical Guide. Sage. 1998.
38. SAMHSA. First Responders: Behavioral Health Concerns, Emergency Response, and Trauma. 2018.
39. **Abbott C**, **Barber E**, **Burke B**, **et al**. What's Killing Our Medics? Reviving Responders.; 2015. http://www.revivingresponders.com/originalpaper.
40. **Davis MA**, **Cher BAY**, **Friese CR**, **Bynum JPW**. Association of US Nurse and Physician Occupation with Risk of Suicide. JAMA Psychiatry. 2021;78(6):651-658. https://doi.org/10.1001/jamapsychiatry.2021.0154.
41. **Maunder RG**, **Lancee WJ**, **Balderson KE**, **et al**. Long-term psychological and occupational effects of providing hospital healthcare during SARS outbreak. Emerg Infect Dis. 2006;12(12):1924-1932. https://doi.org/10.3201/eid1212.060584.
42. **Dall T**, **West T**, **Iacobucci W**. The Complexities of Physician Supply and Demand: Projections from 2013 to 2025 Final Report. Assoc Am Med Coll. 2020;(June):1–59.
43. **Zhang X**, **Lin D**, **Pforsich H**, **Lin VW**. Physician workforce in the United States of America : forecasting nationwide shortages. 2020;3:1-9.
44. **Sterling R**, **Rinne ST**, **Reddy A**, **et al**. Identifying and Prioritizing Workplace Climate Predictors of Burnout Among VHA Primary Care Physicians. J Gen Intern Med. 2021. Published online July 29, 2021:1–8. https://doi.org/10.1007/S11606-021-07006-X.
45. **Brennan J**, **McGrady A**, **Tripi J**, **et al**. Effects of a resiliency program on burnout and resiliency in family medicine residents. Int J Psychiatry Med. 2019;54(4-5):327-335. https://doi.org/10.1177/0091217419860702.
46. **Henry BJ**. Nursing Burnout Interventions. Clin J Oncol Nurs. 2014;18(2):211-214. https://doi.org/10.1188/14.cjon.211-214.
47. **Panagioti M**, **Panagopoulou E**, **Bower P**, **et al**. Controlled interventions to reduce burnout in physicians a systematic review and meta-analysis. JAMA Intern Med. 2017;177(2):195-205. https://doi.org/10.1001/jamainternmed.2016.7674.
48. **Shanafelt TD**, **Noseworthy JH**. Executive Leadership and Physician Well-being: Nine Organizational Strategies to Promote Engagement and Reduce Burnout. Mayo Clin Proc. 2017;92(1):129-146. https://doi.org/10.1016/j.mayocp.2016.10.004.
49. **Trockel M**, **Corcoran D**, **Minor LB**, **Shanafelt TD**. Advancing Physician Well-Being: A Population Health Framework. Mayo Clin Proc. 2020;95(11):2350-2355. https://doi.org/10.1016/j.mayocp.2020.02.014.
50. **Birk JL**, **Sumner JA**, **Haerizadeh M**, **et al**. Early interventions to prevent posttraumatic stress disorder symptoms in survivors of life-threatening medical events: A systematic review. J Anxiety Disord. 2019;64:24-39. https://doi.org/10.1016/j.janxdis.2019.03.003.
51. **Astill Wright L**, **Sijbrandij M**, **Sinnerton R**, **Lewis C**, **Roberts NP**, **Bisson JI**. Pharmacological prevention and early treatment of post-traumatic stress disorder and acute stress disorder: a systematic review and meta-analysis. Transl Psychiatry. 2019;9(1). https://doi.org/10.1038/s41398-019-0673-5.
52. **Birur B**, **Moore NC**, **Davis LL**. An Evidence-Based Review of Early Intervention and Prevention of Posttraumatic Stress Disorder. Community Ment Health J. 2017;53(2):183-201. https://doi.org/10.1007/s10597-016-0047-x.
53. **Qi W**, **Gevonden M**, **Shalev A**. Prevention of Post-Traumatic Stress Disorder After Trauma: Current Evidence and Future Directions. Curr Psychiatry Rep. 2016;18(2):1-11. https://doi.org/10.1007/s11920-015-0655-0.
54. 54. **Albott CS**, **Wozniak JR**, **McGlinch BP**, **Wall MH**, **Gold BS**, **Vinogradov S**. Battle Buddies: Rapid Deployment of a Psychological Resilience Intervention for Health Care Workers during the COVID-19 Pandemic. Anesth Analg. 2020;131(1):43-54. https://doi.org/10.1213/ANE.0000000000004912.
55 **Schreiber M**, **Cates DS**, **Formanski S**, **King M**. Maximizing the Resilience of Healthcare Workers in Multi-hazard Events: Lessons from the 2014-2015 Ebola Response in Africa. Mil Med. 2019;184(Suppl 1):114-120. https://doi.org/10.1093/MILMED/USY400.
56. **Mehta S**, **Machado F**, **Kwizera A**, **et al**. COVID-19: a heavy toll on health-care workers. Lancet Respir Med. 2021;2600(21):19-21. https://doi.org/10.1016/s2213-2600(21)00068-0.

Publisher's Note *Springer Nature remains neutral with regard to jurisdictional claims in published maps and institutional affiliations.*
