# Supplementary material for: Mental health status and related factors influencing healthcare workers during the COVID-19 pandemic: A systematic review and meta-analysis
Source: PLoS One. 2024 Jan 19;19(1):e0289454. doi: 10.1371/journal.pone.0289454 (PMC10798549; doi:10.1371/journal.pone.0289454)
Supplement: S1 Data — (ZIP) [file pone.0289454.s011.zip › literatures/54.pdf]

Received: 6 September 2021 | Revised: 10 February 2022 | Accepted: 22 May 2022

DOI: 10.1111/jan.15339

JAN Leading Global Nursing Research WILEY

ORIGINAL RESEARCH:
EMPIRICAL RESEARCH - MIXED METHODS

Psychological impact of COVID-19 on nursing personnel: A regional online survey

Matthew T. Hickling[1,2] | Scott D. Barnett[1,2]

[1]Community Care Physicians Siena College, Latham, New York, USA

[2]Barnett Consulting, Arlington, Virginia, USA

Correspondence
Matthew T. Hickling, Community Care Physicians, Latham, NY, USA.
Email: mhickling@communitycare.com

Abstract

Aims: This study investigated mental health reactions to dealing with COVID-19 in a population of nurses working in a variety of settings. The study attempted to expand our current understanding of the psychological reactions unique to nurses working during the highly stressful period of the COVID-19 pandemic.

Design: The study used an online questionnaire design.

Methods: Nurses were recruited using social media via an electronic link between July and September 2020. Of them, 112 nursing professionals completed the 66-item questionnaire.

Results: Significant findings included the presence of moderate or greater levels of anxiety (62%), depression (31%), and posttraumatic stress disorder (15%) and with significantly greater intrusive thoughts and memories for nurses who provided direct patient care than those who did not. Nurses with a prior history of anxiety or depression were found to be at greater risk for psychological distress. Results further highlighted concerns and fears related to coronavirus in both their daily personal and professional lives.

Conclusion: The use of standard, commonly used, measures of psychological disorders allow for a more precise comparison among studies both for this population at the time of the survey and over a period of time.

Impact: Suggestions for helping nursing professionals identify nurses at risk and improved ways to cope and deal with adverse psychological effects are discussed.

KEYWORDS
anxiety, BDI-II, COVID-19, depression, GAD-7.PCL-5, nurses, PTSD

1 | INTRODUCTION

Since the end of 2019, novel coronavirus (COVID-19) has become a pandemic impacting countries throughout the world. As of January 2021, over 20 million cases of COVID 19 and 550,000 deaths have occurred within the United States alone. COVID-19 virus has continued to spread with the World Health Organization's estimate of January 2022, there have been 340,543,962 cases with 5,570,163 deaths globally [WHO Coronavirus (COVID-19) Dashboard, 2022]. As of the same time in the United States, the Center for Disease Control (CDC) estimated there have been 146.6 million estimated total infections with 921,000 estimated deaths (CDC, 2022). The impact of the COVID-19 disease has greatly stressed not just the available physical resources for healthcare in the United States, but also healthcare professionals involved in treating and caring for those impacted by the disease. COVID-19 has had a significant, adverse psychological impact on medical and health professionals (British Psychological Society, 2020) A study done at a large medical centre

 © 2022 John Wiley & Sons Ltd.

in New York City (NYC) resulted in positive screens for psychological symptoms such as acute stress (57%), depression (48%) and for anxiety symptoms (33%) (Shechter et al., 2020). For each psychological disorder, a higher percent of nurses/advanced practice providers screened positive versus the attending physicians studied.

2 | BACKGROUND

Several recent articles have investigated the impact of COVID19 on healthcare professionals. In a recent literature review, risk factors and sources of distress identified by healthcare workers included national policies and guidelines regarding COVID-19, high risk contact with COVID-19 patients, family and concerns outside the clinical environment, personal and clinical work/environment, including improper PPE use, and long daily contact hours in unprotected exposure (Rossi et al., 2020; Shechter et al., 2020). One study has looked at the impact of mental health in 53 participants from their medical staff (Huang et al., 2020). These results suggest females suffered greater than males with mental health issues. The mental health issues included anxiety, stress disorder and depression.

A comprehensive review by Braquehais et al. (2020) also concluded that the impact of COVID-19 on healthcare workers resulted in significant psychological distress. The results again found a high prevalence of anxiety and depressive disorders, with women suffering from more symptoms than men. It was conjectured that the impact of witnessing trauma including loss, high morbidity and mortality rates, shortage of PPE, fear of infecting family members or themselves contributed to the psychological distress of the healthcare workers. Unfortunately, while these studies have described the psychological distress within healthcare workers, they do not allow a clear understanding of the impact on professional nursing personnel who often have unique and intensive roles within the care of COVID-19 patients.

Nurses having been exposed to high levels of stressful traumatic events have been postulated to bear a bigger burden of psychological distress given the nature of work during the COVID-19 pandemic (Lai et al., 2020). In a letter to the editor for the Journal of Psychiatric and Mental Health Nursing, Veitch and Richardson (2021) described the additional support that they believe mental health nurses require to help cope with the COVID-19 pandemic. Nelson and Kaminsky (2020) report from military personnel, saying that the conditions found in some settings treating COVID-19, were the closest to combat they have seen in a civilian setting.

Nurses hold multiple positions when it comes to care for patients with COVID-19. Some of those roles include direct care of patients and support roles in both indirect and administrative positions. Whether in direct care of COVID-19 patients or not, nurses may feel the impact of the pandemic's effect on their professional as well as personal lives. Stressors in dealing with the impact of the pandemic have included redeployment to units outside of regular expertise to COVID-19 units, fear of contaminating family and friends with the virus, and financial concerns from being furloughed in some areas of expertise, such as those that provide non-emergent procedures and surgeries.

3 | THE STUDY

3.1 | Aims

This study investigated mental health reactions to dealing with COVID-19 in a population of nurses working in a variety of settings. The study attempted to expand our current understanding of the psychological reaction to nurses working during the highly stressful period of the COVID-19 pandemic.

Ethics approval was obtained from the affiliated IRB. The IRB determined the study was an anonymous, online survey with no risk to subjects who voluntarily participated. Subsequently, according to local governance requirements, no ongoing formal ethical scrutiny was required or undertaken for the study.

4 | DESIGN

4.1 | Sample

Participants were 112 nurses surveyed using a convenience survey design. Starting on 7 July 2020, participants were sent an electronic survey link either by email or social media platform (e.g., Facebook®, Twitter®, etc.). The survey consisted of 66 questions and utilized the Google® Forms platform. Participants were encouraged to refer others to the survey with the stipulation that only nurses complete the survey. The survey was conducted during a 2-month window over the summer and fall of 2020.

5 | MEASURES

5.1 | Standardized instruments

Posttraumatic stress disorder (PTSD) symptom severity was assessed by the 20-item Posttraumatic Stress Disorder Checklist for DSM-5 (PCL-5) (Bovin et al., 2015). The PCL-5 is a self-report rating scale for assessing DSM-IV PTSD symptoms. Participants are instructed to indicate the degree to which they have been concerned by each symptom during the prior month using a 5-point (0–4) scale (Weathers et al., 2013). PCL-5 total symptom severity score (range: 0–80) is obtained by summing each of the 20 items. DSM-5 symptom cluster severity scores are obtained by summing item scores contained within each cluster. An interim PTSD diagnosis is made by treating each item rated as 2 = "Moderately" or higher as an endorsed symptom. We used the *DSM-5* diagnostic rule requiring at least: 1 B item (items 1–5), 1 C item (questions, 6–7), 2 D items (items 8–14), 2 E items (items 15–20) (Blevins et al., 2015). Weathers et al. (2013) suggests PCL-5 threshold scores >31 to 33

may be indicative of probable PTSD. This study utilized a score of 33 to support the presence of symptoms found to be consistent with a likely diagnosis of PTSD.

The Generalized Anxiety Disorder 7-Item Scale (GAD-7) is a validated tool for the screening of general anxiety and assessment of severity in both clinical practice and research (Spitzer et al., 2006). The GAD-7 is a 7-item scale, range 0–21, rated on the same four ordinal responses with both excellent internal consistency (Cronbach α = .92) and test–retest reliability (intraclass correlation = 0.83). Multiple domains of functional impairment are associated with increasing GAD-7 scores. GAD-7 scores ≥7 are recommended as indicative of a likely anxiety disorder. The GAD-7 due to computer error was not included for the first 32 subjects.

The Patient Health Questionaire-9 (PHQ-9) is a 9-item depression component derived from the full PHQ (Kroenke et al., 2001). Items range from 0 (not at all) to 3 (nearly every day). A summed total score ranges from 0 to 27. Major depression is diagnosed if at least 5 of the 9 depressive symptom criteria have been present at least 'more than half the days' in the immediate prior 2 weeks, and if 1 of the symptoms is anhedonia or depressed mood. Reliability and validity of the tool indicate it has sound psychometric properties. Internal consistency of the PHQ-9 is high with prior work suggests Cronbach alphas of .86 and .89 (Spitzer et al., 1999).

The Posttraumatic Growth Inventory (PTGI) assesses the perception of personal benefits among survivors of a traumatic event with responses ranging from 0 = 'no change' to 5 = 'very high', in a positive sense. The 21 item PTGI includes five subscales (Relating to Others, New Possibilities, Personal Strength, Spiritual Change, Appreciation for Life) and a total score. Alpha scores range from .67 (Appreciation for Life) to .85 (Spiritual change) and a total score alpha of .90. Prior work report test–retest reliability estimates of r = .37 to .74 with reliabilities for three of five factors at least r = .65 (Tedeschi & Calhoun, 1996).

5.2 | Other measures

In addition to completing standardized psychological instruments and demographic information, several open-ended questions were asked of online respondents. These questions included the impact COVID-19 had on them as individuals, the biggest problems faced as a result of dealing with COVID-19 and any impact on their personal and career life that may have occurred and changes their experience had on the perception of their nursing career.

5.3 | Statistical analysis

Data are presented as mean (*SD*) of frequency (%), where appropriate. Instruments were scored according to published author guidelines. Lastly, we conducted a multiple hierarchical regression analysis to determine the effect of direct patient care and direct patient care with COVID-19 patients on PTSD symptoms after adjustment in prior models. Univariate group comparisons were conducted using chi-square or Student's *t*-test. All analyses were performed using SAS (ver. 9.4) with $p < .05$, two-tailed, considered as statistically significant.

5.4 | Validity and reliability/rigour

Study variables were selected using a strong theoretical basis to assess an individual's current health-related quality of life, general anxiety level in addition to information about trauma and growth. These questionnaires were selected as they are commonly found and accepted within studies targeting psychological distress and trauma. Additionally, the PHQ and GAD are commonly used instruments which are familiar within most medical records and nursing practice. Although less commonly used instruments such as the PCL and PTGI in nursing literature, they are common and viewed as objective measures in viewing the psychological impact of trauma. All instruments were validated scales, and investigators in this project had extensive prior experience with these instruments.

6 | RESULTS

In total, 112 participants responded to the survey (Table 1). Participants were primarily age 25–34 (40.2%), female (92.0%), Caucasian (84.8%) and located in the Northeastern area of the United States (91.3%). Respondents report at time of survey as being primarily full time employed (90.2%) within a large acute care (500+ bed) facility (33.6). Eighty-three percent of participants report providing direct care to patients with 59.6% direct care to COVID19 patients. Only 4.5% of respondents report being personally diagnosed as COVID-19 positive. Sixty-two percent of respondents reported a history of anxiety or depression with a majority reporting a history of anxiety (15.3%, depression (9.0%) or both (27.9%).

Respondent indices of depression, stress and anxiety can be found in Table 2. Seventy-two percent of respondents reported at least a moderate or greater level of anxiety (GAD, 8.1 [*SD* 4.4]) and 33% indicated at least a moderate or greater level of depression (PHQ9, 8.1 [*SD* 4.2]). Regarding PTSD (PCL-5, 16.7 [*SD* 13.5]), 17 (15.0%) individuals exceed the PCL-5 threshold of 33, indicating a possible diagnosis of PTSD.

Measures of anxiety, stress, depression and posttraumatic growth were all well correlated with each other (Table 3). Strong correlations were observed for anxiety vs. posttraumatic anxiety (Pearson's r = .72, $p < .0001$) and depression (Pearson's r = .66, $p < .0001$) but not posttraumatic growth (Pearson's $r = .12$, $p < .307$). Posttraumatic growth was mildly correlated with PTSD (Pearson's r = .12, $p < .307$) and depression (Pearson's r = .27, $p < .005$). Participants ($n = 32$, 28.6%) with a self-reported prior history of anxiety (GAD7, 7.3 [*SD* 4.3] vs. 10.0 [*SD* 4.3], Student's *t*-test, $p < .001$) and depression (PHQ9, 7.7 [*SD* 3.9] vs. 8.9 [*SD* 4.7], Student's *t*-test, $p < .230$) demonstrated a statistically significant increase in anxiety

TABLE 1 Demographic characteristics ($n = 112$)

Parameter	N (%)
Age	
≤25	7 (6.3)
25–34	45 (40.2)
35–44	33 (29.5)
45–54	19 (17.0)
55+	8 (7.1)
Female	103 (92.0)
Ethnicity	
Caucasian	95 (84.8)
African American	2 (1.8)
Asian	6 (5.4)
Other	9 (8.0)
Employment status	
Full time	101 (90.2)
Part time	8 (7.1)
Retired	1 (0.9)
Unemployed, looking for work	1 (0.9)
Unemployed, not looking for work	1 (0.9)
Employment type	
Acute care small hospital (<100 beds)	2 (5.4)
Acute care medium hospital (100–499 beds)	26 (23.2)
Acute care large hospital (500 or more beds)	38 (33.9)
Community/public health facility	6 (5.4)
Primary/ambulatory outpatient care facility	18 (16.1)
Other	22 (19.6)
Employment nature	
Acute care nurse	7 (6.3)
Emergency department	14 (12.5)
Intensive/critical care	13 (11.6)
Nurse manager	10 (8.9)
Nurse practitioner	14 (12.5)
Other	54 (48.2)
US region	
Midwest	8 (7.1)
Northeast	91 (81.3)
Southeast	7 (6.3)
Southwest	2 (1.8)
West	3 (2.7)
Provide direct care to patients	93 (83.0)
Provide direct care to SARS-CoV-2 patients	67 (59.6)
Diagnosed with SARS-CoV-2	5 (4.5)
If history of anxiety, depression or insomnia prior to COVID	
Improved	1 (0.9)
Stayed the same	27 (24.1)
Worsened	51 (44.6)
NA	33 (29.5)

TABLE 2 Measures of anxiety, stress, depression and posttraumatic growth ($n = 112$)

Parameter	Mean (SD, min–max)	N (%)
GAD-7[a]		
GAD summary score	8.1 (4.4, 2–23)	–
GAD anxiety		
Mild	–	18 (23.7)
Moderate	–	47 (61.8)
Severe	–	11 (14.5)
GAD ≥7	–	29 (36.7)
PCL-5		
Total summary score	16.7 (13.5, 0–66)	–
Cluster B	3.8 (4.0, 0–17)	–
Cluster C	1.61 (1.8, 0–8)	–
Cluster D	5.9 (5.0, 0–23)	–
Cluster E	5.4 (4.3, 0–18)	–
PCL ≥33	–	17 (15.0)
PHQ-9		
PHQ summary score	8.1 (4.2, 2–23)	–
PHQ depression		
Mild	–	52 (46.4)
Minimal	–	25 (22.3)
Moderate	–	30 (26.8)
Moderate/severe	–	2 (1.8)
Severe	–	3 (2.7)
PTGI		
Total score	17.9 (12.0, 0–47)	–
Relating to others	3.3 (2.9, 0–10)	–
New possibilities	3.1 (3.0, 1–10)	–
Personal strength	4.2 (3.2, 0–10)	–
Spiritual enhancement	1.4 (1.7, 0–5)	–
Appreciation	5.1 (2.9, 0–10)	–

Abbreviations: GAD, Generalized Anxiety Disorder; PCL, Posttraumatic Checklist; PHQ, Patient Health Questionnaire; PTGI, Posttraumatic Growth Inventory.

[a]GAD-7 added later after 1st 33 participants had completed survey.

and depression compared with those with no self-reported prior history.

Comparisons between participants reporting providing direct patient care and providing direct care for COVID-19 patients are presented in Table 3. Participants providing direct care to patients reported three times the PCL-5 Cluster B score of intrusive symptoms (3.0 [*SD* 4.5] vs. 4.0 [*SD* 3.9], Student's *t*-test, $p = .04$). No other differences were observed. No statistically significant differences between those participants providing access to COVID-19 patients were observed.

Further, those participants with a prior self-reported history of anxiety/depression demonstrated increased indications of post-traumatic distress disorder (PCL-5, 21.3 [*SD* 4.0] vs. 14.9 [*SD* 13.0],

TABLE 3 Correlation coefficients of research variables

Parameter	1	2	3	4	5	6	7	8	9	10	11	12	13
1. GAD-7	–												
2. PCL-5 total score	$0.73^{†}$	–											
3. Cluster B	$0.52^{†}$	$0.88^{†}$	–										
4. Cluster C	$0.56^{†}$	$0.84^{†}$	$0.82^{†}$	–									
5. Cluster D	$0.69^{†}$	$0.91^{†}$	$0.69^{†}$	$0.64^{†}$	–								
6. Cluster E	$0.77^{†}$	$0.91^{†}$	$0.71^{†}$	$0.70^{†}$	$0.79^{†}$	–							
7. PHQ-9	$0.64^{†}$	$0.75^{†}$	$0.58^{†}$	$0.49^{†}$	$0.77^{†}$	$0.72^{†}$	–						
8. PTGI	0.17^{ns}	$0.37^{†}$	$0.46^{†}$	$0.31^{†}$	0.29**	0.26**	0.27**	–					
9. Relating to others	0.03^{ns}	0.20*	$0.30^{†}$	0.20*	0.12^{ns}	0.12^{ns}	0.13^{ns}	$0.89^{†}$	–				
10. New possibilities	0.22*	$0.35^{†}$	$0.43^{†}$	$0.33^{†}$	0.25**	0.28**	0.26**	$0.89^{†}$	$0.76^{†}$	–			
11. Personal strength	0.02^{ns}	0.24*	$0.35^{†}$	0.20*	0.20*	0.11^{ns}	0.16^{ns}	$0.84^{†}$	$0.65^{†}$	$0.71^{†}$	–		
12. Spiritual enhancement	0.09^{ns}	0.21*	0.29**	0.19*	0.13^{ns}	0.18^{ns}	0.24*	$0.72^{†}$	$0.53^{†}$	$0.53^{†}$	$0.66^{†}$	–	
13. Appreciation	0.34**	$0.50^{†}$	$0.50^{†}$	$0.37^{†}$	$0.50^{†}$	$0.38^{†}$	$0.37^{†}$	$0.74^{†}$	$0.53^{†}$	$0.57^{†}$	$0.46^{†}$	$0.45^{†}$	–

Abbreviations: GAD, Generalized Anxiety Disorder; PCL, Posttraumatic Checklist; PHQ, Patient Health Questionnaire, PTGI, Posttraumatic Growth Inventory.

$^{*}p<.05$, $^{**}p<.01$, $^{†}p<.001$, not significant.

Student's *t*-test, $p < .001$). Nurses who self-reported a prior history of anxiety, depression or insomnia, described their condition as having worsened when compared with how they felt prior to the time of the pandemic (57%).

Table 4 presents results of hierarchical linear regression analyses. In model 1, perceived well-being (PHQ-9, $\beta = 2.21$, $p < .001$) was positively associated with posttraumatic stress, contributing 57% of the explained variance. Further, general anxiety was positively associated with posttraumatic stress ($\beta = 1.11$, $p < .001$), contributing an additional 9% of the explained variance. Growth ($p < .056$) and direct care to patients ($p < .09$) were not associated with posttraumatic stress ($p > .05$), contributing 2% and <1%, respectively of explained variance (Table 5).

In model 2, although direct care to COVID-19 patients was positively associated with posttraumatic stress, no statistical significance ($p < .589$) was observed, contributing <1% to explained variance.

6.1 | Qualitative results

Review of open-ended questions found that the most frequently cited problems included isolation ($n = 20$), fear of infecting family, fear of becoming infected, anxiety and work life balance. When responding to questions to possible positive changes, the following themes were found: the personal time at home and relationships, flexibility in dealing with work problems, collaboration with treatment teams, appreciation and use of faith-based beliefs, self-help measures to deal with stress and anxiety, pride in career and contribution as nurses and resiliency in dealing with stressors in both their personal and professional lives.

Perceptions of self and career as nursing professionals responses included major themes such as: importance of evidence-based practice and application of science in clinical settings, personal reward of caring for patients and gratitude of families of patients, affirmation of importance of nursing role and seeing essential nature of work in a pandemic setting, awareness of conflict of values in provision of nursing care versus limited PPE and at times possible conflict skills provided by nurses in their particular settings, importance of cooperation and assistance to other nurses and other health care professionals, and increased self-regard for the ability to offer services to those in need at a critical time in their lives.

7 | DISCUSSION

The study, consistent with earlier research, found significant levels of anxiety and depression for nurses dealing with COVID-19 in their work settings. Results further highlighted concerns and fears related to COVID-19 in both their daily personal and professional lives. Nurses reported they experienced moderate to severe levels of anxiety, while almost a third of the nurses surveyed reported a moderate to severe level of depression on standard measures of psychological

TABLE 4 Comparison anxiety, stress, depression and posttraumatic growth by patient care

	Provide direct care to patients			Provide direct care to SARS-CoV-2 patients		
	No	Yes		No	Yes	
Parameter	Mean (*SD*)	Mean (*SD*)	*p*	Mean (*SD*)	Mean (*SD*)	*p*
GAD-7*						
GAD summary score	7.9 (4.8)	8.1 (4.3)	.30	7.4 (3.7)	7.8 (4.5)	.91
PCL-5						
Total summary score	14.4 (14.7)	17.2 (13.3)	.11	16.8 (14.9)	16.8 (13.2)	.95
Cluster B	3.0 (4.5)	4.0 (3.9)	.04	3.2 (4.1)	4.2 (4.1)	.30
Cluster C	1.5 (2.1)	1.6 (1.7)	.23	1.6 (2.1)	1.6 (1.7)	.78
Cluster D	5.3 (4.9)	6.0 (5.0)	.28	6.7 (6.0)	5.7 (4.7)	.61
Cluster E	4.7 (4.1)	5.6 (4.4)	.19	5.3 (4.1)	5.3 (4.3)	.82
PHQ-9						
PHQ summary score	8.3 (5.6)	8.0 (3.9)	.38	8.4 (5.8)	8.0 (4.1)	.91
PTGI						
Total score	35.6 (30.3)	39.1 (24.2)	.35	40.2 (26.8)	39.1 (23.9)	.78
Relating to others	11.0 (9.6)	12.9 (8.9)	.48	13.0 (9.4)	13.0 (9.1)	.73
New possibilities	8.1 (7.9)	8.3 (6.3)	.43	8.6 (8.0)	8.2 (6.2)	.83
Personal strength	7.6 (6.1)	8.0 (5.5)	.71	8.3 (6.2)	8.0 (5.4)	.73
Spiritual enhancement	1.7 (2.0)	1.4 (1.6)	.69	1.5 (1.8)	1.4 (1.7)	.89
Appreciation	5.9 (4.9)	7.0 (3.6)	.18	7.0 (4.4)	7.0 (3.5)	.74

Abbreviations: GAD, Generalized Anxiety Disorder; PCL, Posttraumatic Checklist; PHQ, Patient Health Questionnaire PTGI, Posttraumatic Growth Inventory.

TABLE 5 Multiple hierarchical regression analysis

Model 1	Model 1: demographics β (SE)	Model 2: perceived well being β (SE)	Model 3: general anxiety β (SE)	Model 4: growth β (SE)	Model 5: direct care to patients β (SE)
Age	−0.37 (2.06)	0.77 (1.32)	0.81 (1.16)	0.99 (1.14)	1.09 (1.16)
Gender	−3.57 (5.57)	−1.25 (3.56)	−2.81 (3.16)	−2.75 (3.09)	−2.51 (3.14)
Caucasian	2.81 (4.30)	−1.00 (2.77)	−1.93 (2.46)	−1.15 (2.43)	−1.62 (2.59)
Northeast region	−0.42 (3.78)	−2.13 (2.41)	−2.42 (2.13)	−1.75 (2.11)	−1.92 (2.14)
Employed full time	8.56 (7.25)	4.51 (4.64)	4.72 (4.09)	5.17 (4.00)	5.16 (4.03)
Acute care, 500+ bed	−3.03 (3.39)	−4.20 (2.17)	−3.01 (1.93)	−2.59 (1.90)	−2.95 (2.03)
PHQ-9		2.21 (0.23)***	1.46 (0.27)***	1.29 (0.28)***	1.33 (0.29)***
GAD-7			1.11 (0.26)***	1.13 (0.26)***	1.12 (0.26)***
PTGI				0.07 (0.04)	0.07 (0.04)
Direct care to patients					1.48 (2.73)
F	*F* = 0.517	*F* = 13.76	*F* = 17.69	*F* = 16.91	*F* = 15.06
Significant *F* change	–	<0.001***	<0.001***	0.06	0.59
R-square	.22	.79	.84	.85	.85
R-square delta	–	.57	.09	.02	.00
**Model 2**	**Model 1: demographics β (SE)**	**Model 2: perceived well being β (SE)**	**Model 3: general anxiety β (SE)**	**Model 4: growth β (SE)**	**Model 5: direct care to Covid patients β (SE)**
Age	−0.37 (2.06)	0.77 (1.32)	0.81 (1.16)	0.99 (1.14)	0.98 (1.12)
Gender	−3.57 (5.57)	−1.25 (3.56)	−2.81 (3.16)	−2.75 (3.09)	−1.59 (3.11)
Caucasian	2.81 (4.30)	−1.00 (2.77)	−1.93 (2.46)	−1.15 (2.43)	−2.63 (2.54)
Northeast region	−0.42 (3.78)	−2.13 (2.41)	−2.42 (2.13)	−1.75 (2.11)	−1.01 (2.12)
Employed full time	8.56 (7.25)	4.51 (4.64)	4.72 (4.09)	5.17 (4)	3.86 (4.01)
Acute care, 500+ bed	−3.03 (3.39)	−4.2 (2.17)	−3.01 (1.93)	−2.59 (1.9)	−4.47 (2.16)*
PHQ-9		2.21 (0.23)***	1.46 (0.27)***	1.29 (0.28)***	1.26 (0.28)***
GAD-7			1.11 (0.26)***	1.13 (0.26)***	1.21 (0.26)***
PTGI				0.07 (0.04)	0.07 (0.04)*
Care to Covid patients					3.88 (2.24)
F	*F* = 0.52	*F* = 13.76	*F* = 17.69	*F* = 16.91	*F* = 16.04
Significant *F* change	–	<0.001***	<0.001***	0.06	0.09
R-square	.22	.79	.84	.85	.86
R-square delta	–	.57	.09	.02	.01

Abbreviations: GAD, Generalized Anxiety Disorder; PHQ, Patient Health Questionnaire; PTGI, posttraumatic growth inventory.
$^{*}p < .05$, $^{***}p < .001$.

distress. A significant number reported symptoms consistent with individuals who are found to have a diagnosis of PTSD. The use of standard, commonly used measures of psychological disorders allow for a more precise comparison among studies both for this population at the time of the survey and over a period of time. It is important for both research and clinical application to be cautious in the use of these measures and the limitations they have as part of any diagnostic formulation. This is consistent with clinical practice for the use of the PHQ-9 and GAD that may be routinely administered but are considered as only part of the process of obtaining a formal diagnosis.

The majority the respondents were female, which earlier studies have shown a significant risk factor for increased occurrence of anxiety and depression in healthcare professionals (Braquehais et al., 2020). On the Posttraumatic Stress Disorder Checklist for DSM - 5, based on the recommended cutting score of 31, 17 (15.0%) of individuals were found to score consistent with those diagnosed with PTSD. Additionally, there was significant increase in persistent reexperiencing including recurrent intrusive recollections and memories of the stressful work-related events for those individuals who had worked with patients. The presence of a self-reported prior anxiety or depressive disorder appears to increase the risk for psychological distress. If confirmed in subsequent studies, this may allow at risk individuals to be more quickly identified with appropriate intervention offered. This study was found to expand the findings to the general population of professional

nurses in contrast to those reported from the NYC study of healthcare workers (Shechter et al., 2020).

Posttraumatic growth among a large proportion of participants appeared to be found on the PTGI. While no agreed upon score is used for areas of growth, the use of the PTGI suggests that when asked how they have changed as a result of the pandemic, some individuals found a greater appreciation for life, importance of relationships and spiritual change. Results suggest a mild correlation for growth with scores for anxiety and depression. There was a significant correlation for anxiety and depression among respondents ($r = .703$, $p < .001$).

Results on qualitative inquiry appeared consistent with the results of standard measures and allowed for an expanded understanding of the impact in psychological functioning of professional nurses. Major themes included problems in the areas of isolation, fear of infecting family with COVID-19, financial concerns and anxiety. Nurses expressed positive changes in personal capabilities including their flexibility, pride in their skills and their ability to work collaboratively in the work setting. They expressed an appreciation for the utilization of evidence-based practice, self-satisfaction and reward for caring for patients and their families, seeing the roles of nursing as indispensable, and affirmation of their career choice for a profession. There was some expressed dissatisfaction with regulations and lack of supplies in terms of support for what they saw as their ability to provide the level of care they felt needed as part of their role in the care of COVID-19 patients.

7.1 | Limitations

Limitations of this study included a concern over the representativeness of the sample of nurses to the profession at large. The use of an online platform while providing ease of data collection and privacy for respondents was only able to reach the subset of nurses who are comfortable with this type of survey investigation. The snowball approach was utilized given the limitations that potential lists obtained from professional nursing organizations would have limitations in those who chose membership. Further, selection to designated hospitals and health organizations was also determined to not be the source of sampling given limitations that singular organizations might hold in a representation of nursing experiences. Further restrictions due to access of organizations during a pandemic were limited by administrative regulations, thereby, leading to the decision for an online anonymous survey based broadly rather than a restricted sampling. The decision for any sampling methodology deserves consideration as a possible limitation for any results obtained.

The timing of the survey occurred during a rise in lack of treatment for patients in clinical services. The timing of the survey is conjectured to reflect the period of time where that survey was completed. If the survey was completed at a later time or in another region of the country, results may not be the same. Last, the anonymity needed for this type of survey limited the ability to repeat the questionnaires over time. This type of study might show very different results that are important to see if the psychological distress exhibited here is transitory or lasting.

8 | CONCLUSION

Given the findings of this study, it is strongly suggested that interventions to help professional nurses cope with the stressors found in dealing with a pandemic be provided. These interventions might include group/peer support, accessible provision of mental health services and addressing factors related to mental health distress, including exposure to mortality and morbidity of COVID-19 patients. Whether these are provided through a new online intervention or from in-house services or community services known to most healthcare settings, they would ideally be flexibly administered and made available to those in most need. It is conjectured that future studies might look to the factors contributing to the positive changes that occur in some nurses. The identification of these factors might then be incorporated into on-going services routinely offered to nursing professionals at times of high stress such as that found in the pandemic.

AUTHOR CONTRIBUTIONS

All authors have agreed on the final version and meet at least one of the following criteria (recommended by the ICMJE*):

1. substantial contributions to conception and design, acquisition of data, or analysis and interpretation of data;
2. drafting the article or revising it critically for important intellectual content.

CONFLICT OF INTEREST

No conflict of interest has been declared by the author(s).

FUNDING INFORMATION

This research received no specific grant from any funding agency in the public, commercial or not for profit sectors.

PEER REVIEW

The peer review history for this article is available at https://publons.com/publon/10.1111/jan.15339.

DATA AVAILABILITY STATEMENT

Research data are not shared.

ORCID

Matthew T. Hickling https://orcid.org/0000-0002-6131-2150

REFERENCES

Blevins, C. A., Weathers, F. W., Davis, M. T., Witte, T. K., & Domino, J. L. (2015). The posttraumatic stress disorder checklist for *DSM-5* (PCL-5): Development and initial psychometric evaluation. *Journal of Traumatic Stress*, *28*, 489–498. https://doi.org/10.1002/jts.22059

Bovin, M. J., Marx, B. P., Weathers, F. W., Gallagher, M. W., Rodriguez, P., Schnurr, P. P., & Keane, T. M. (2016). Psychometric properties of the PTSD checklist for diagnostic and statistical manual of mental disorders-fifth edition (PCL-5) in veterans. *Psychological Assessment*, *28*(11), 1379–1391. https://doi.org/10.1037/pas0000254

Braquehais, M. D., Vargas-Caceres, S., Gomez-Duran, E., Nieva, G., Valero, S., Casas, M., & Bruguera, E. (2020). The impact of the COVID-19 pandemic on the mental health of healthcare professionals. *QJM*, *2020*, 1–5. https://doi.org/10.1093/qjmed/hcaa207

British Psychological Society. (2020). The psychological needs of health care staff as a result of the coronavirus pandemic. BPS staff Wellbeing Group Guidance document.

Centers for Disease Control and Prevention. (2022). *Estimated Covid-19 burden*. https://www.cdc.gov/coronavirus/2019-ncov/cases-updates/burden.html

Huang, J. Z., Han, M. F., Lou, T. D., Ren, A. K., & Zhou, X. P. (2020). Mental health survey of medical staff in a tertiary infectious disease hospital for COVID-19. *Zhonghua Lao Dong Wei Sheng Zhi Ye Bing Za Zhi*, *38*, 192–195. https://doi.org/10.3760/cma.j.cn121094-20200219-00063

Kroenke, K., Spitzer, R. L., & Williams, J. B. (2001). The PHQ-9: Validity of a brief depression severity measure. *Journal of General Internal Medicine*, *16*(9), 606–613. https://doi.org/10.1046/j.1525-1497.2001.016009606.x

Lai, J., Ma, S., Wang, Y., Cai, Z., Hu, J., Wei, N., Wu, J., Du, H., Chen, T., Li, R., Tan, H., Kang, L., Yao, L., Huang, M., Wang, H., Wang, G., Liu, Z., & Hu, S. (2020). Factors associated with mental health outcomes among health care workers exposed to coronavirus disease 2019. *JAMA Network Open*, *3*(3), e203976. https://doi.org/10.1001/jamanetworkopen.2020.3976

Nelson, B., & Kaminsky, D. (2020). COVID-19's crushing mental health toll on health care workers. *Cancer Cytopathology*, *128*(9), 597–598. https://doi.org/10.1002/cncy.22347

Rossi, R., Socci, V., Pacitti, F., di Lorenzo, G., di Marco, A., Siracusano, A., & Rossi, A. (2020). Mental health outcomes among frontline and second-line care workers during the coronavirus disease (COVID-19) pandemic in Italy. *JAMA Network Open*, *3*(5), e2010185. https://doi.org/10.1001/jamanetworkopen.2020.10185

Shechter, A., Diaz, F., Moise, N., Anstey, D. E., Ye, S., Agarwal, S., Birk, J. L., Brodie, D., Cannone, D. E., Chang, B., Claassen, J., Cornelius, T., Derby, L., Dong, M., Givens, R. C., Hochman, B., Homma, S., Kronish, I. M., Lee, S. A. J., … Abdalla, M. (2020). Psychological stress, coping behaviors, and preferences for support among New York healthcare workers during the COVID-19 pandemic. *General Hospital Psychiatry*, *66*, 1–8. https://doi.org/10.1016/j.genhosppsych.2020.06.007

Spitzer, R. L., Kroenke, K., & Williams, J. B. (1999). Primary care evaluation of mental disorders. Validation and utility of a self report version of PRIME-MD: The PHQ primary care study: Primary care evaluation of mental disorders, patient health questionnaire. *Journal of the American Medical Association*, *282*(18), 1737–1744. https://doi.org/10.1001/jama.282.18.1737

Spitzer, R. L., Kroenke, K., Williams, J. B., & Löwe, B. (2006). A brief measure for assessing generalized anxiety disorder: The GAD-7. *Archives of Internal Medicine*, *166*(10), 1092–1097. https://doi.org/10.1001/archinte.166.10.1092

Tedeschi, R. G., & Calhoun, L. G. (1996). The posttraumatic growth inventory: Measuring the positive legacy of trauma. *Journal of Traumatic Stress*, *9*, 455–471.

Veitch, P., & Richardson, K. (2021). Nurses need support during Covid-19 pandemic. *Journal of Psychiatric and Mental Health Nursing*, *28*(2), 303–304. https://doi.org/10.1111/jpm.12666

Weathers, F. W., Litz, B. T., Keane, T. M., Palmieri, P. A., Marx, B. P., & Schnurr, P. P. (2013). The PTSD checklist for *DSM-5* (PCL-5). Scale available from the National Center for PTSD at www.ptsd.va.gov.

World Health Organization (WHO). (2022). *Coronavirus disease (COVID-19) pandemic*. https://www.who.int/emergencies/diseases/novel-coronavirus-2019

How to cite this article: Hickling, M. T., & Barnett, S. D. (2022). Psychological impact of COVID-19 on nursing personnel: A regional online survey. *Journal of Advanced Nursing*, *00*, 1–9. https://doi.org/10.1111/jan.15339

The *Journal of Advanced Nursing (JAN)* is an international, peer-reviewed, scientific journal. *JAN* contributes to the advancement of evidence-based nursing, midwifery and health care by disseminating high quality research and scholarship of contemporary relevance and with potential to advance knowledge for practice, education, management or policy. *JAN* publishes research reviews, original research reports and methodological and theoretical papers.

For further information, please visit *JAN* on the Wiley Online Library website: www.wileyonlinelibrary.com/journal/jan

Reasons to publish your work in *JAN*:

- **High-impact forum: the world's most cited nursing journal, with an Impact Factor of 2.561 – ranked 6/123 in the 2019 ISI Journal Citation Reports © (Nursing; Social Science).**
- **Most read nursing journal in the world: over 3 million articles downloaded online per year and accessible in over 10,000 libraries worldwide (including over 6,000 in developing countries with free or low cost access).**
- **Fast and easy online submission:** online submission at http://mc.manuscriptcentral.com/jan.
- **Positive publishing experience:** rapid double-blind peer review with constructive feedback.
- **Rapid online publication in five weeks:** average time from final manuscript arriving in production to online publication.
- **Online Open:** the option to pay to make your article freely and openly accessible to non-subscribers upon publication on Wiley Online Library, as well as the option to deposit the article in your own or your funding agency's preferred archive (e.g. PubMed).
