# Supplementary material for: Mental health status and related factors influencing healthcare workers during the COVID-19 pandemic: A systematic review and meta-analysis
Source: PLoS One. 2024 Jan 19;19(1):e0289454. doi: 10.1371/journal.pone.0289454 (PMC10798549; doi:10.1371/journal.pone.0289454)
Supplement: S1 Data — (ZIP) [file pone.0289454.s011.zip › literatures/55.pdf]

EClinicalMedicine 24 (2020) 100424

Contents lists available at ScienceDirect

EClinicalMedicine

journal homepage: https://www.journals.elsevier.com/eclinicalmedicine

Research Paper

Frontline nurses' burnout, anxiety, depression, and fear statuses and their associated factors during the COVID-19 outbreak in Wuhan, China: A large-scale cross-sectional study

Deying Hu[a,1], Yue Kong[b,1], Wengang Li[c,1], Qiuying Han[d,1], Xin Zhang[e], Li Xia Zhu[f], Su Wei Wan[f], Zuofeng Liu[c], Qu Shen[c], Jingqiu Yang[c], Hong-Gu He[f,*], Jiemin Zhu[c,*]

[a] Department of Nursing, Union Hospital, Tongji Medical College, Huazhong University of Science and Technology, Wuhan, Hubei Province, PR China
[b] Department of Nursing, the 900th Hospital of Joint Logistic Support Force, PLA, Fuzhou, Fujian, PR China
[c] School of Medicine, Xiamen University, Xiamen, Fujian, PR China
[d] Department of Nursing, Zhongshan Hospital, Xiamen University, Xiamen, Fujian, PR China
[e] Department of Nursing, Fifth Medical Center of PLA General Hospital, Beijing, PR China
[f] Alice Lee Centre for Nursing Studies, Yong Loo Lin School of Medicine, National University of Singapore, Singapore, National University Health System, Singapore

A R T I C L E I N F O

Article History:
Received 16 May 2020
Revised 31 May 2020
Accepted 2 June 2020
Available online 27 June 2020

Keywords:
Covid-19
Frontline nurses
Mental health
Burnout
Anxiety
Depression
Fear
China

A B S T R A C T

Background: During the Coronavirus Disease 2019 (COVID-19) pandemic, frontline nurses face enormous mental health challenges. Epidemiological data on the mental health statuses of frontline nurses are still limited. The aim of this study was to examine mental health (burnout, anxiety, depression, and fear) and their associated factors among frontline nurses who were caring for COVID-19 patients in Wuhan, China.
Methods: A large-scale cross-sectional, descriptive, correlational study design was used. A total of 2,014 eligible frontline nurses from two hospitals in Wuhan, China, participated in the study. Besides sociodemographic and background data, a set of valid and reliable instruments were used to measure outcomes of burnout, anxiety, depression, fear, skin lesion, self-efficacy, resilience, and social support via the online survey in February 2020.
Findings: On average, the participants had a moderate level of burnout and a high level of fear. About half of the nurses reported moderate and high work burnout, as shown in emotional exhaustion (n = 1,218, 60.5%), depersonalization (n = 853, 42.3%), and personal accomplishment (n = 1,219, 60.6%). The findings showed that 288 (14.3%), 217 (10.7%), and 1,837 (91.2%) nurses reported moderate and high levels of anxiety, depression, and fear, respectively. The majority of the nurses (n = 1,910, 94.8%) had one or more skin lesions, and 1,950 (96.8%) nurses expressed their frontline work willingness. Mental health outcomes were statistically positively correlated with skin lesion and negatively correlated with self-efficacy, resilience, social support, and frontline work willingness.
Interpretation: The frontline nurses experienced a variety of mental health challenges, especially burnout and fear, which warrant attention and support from policymakers. Future interventions at the national and organisational levels are needed to improve mental health during this pandemic by preventing and managing skin lesions, building self-efficacy and resilience, providing sufficient social support, and ensuring frontline work willingness.
© 2020 The Author(s). Published by Elsevier Ltd. This is an open access article under the CC BY-NC-ND license. (http://creativecommons.org/licenses/by-nc-nd/4.0/)

1. Introduction

The pandemic of Coronavirus Disease 2019 (COVID-19) is currently a major global public health emergency [1]. By 27 March 2020, there were 465,915 confirmed cases in 199 countries, and 21,031 people had lost their lives [2]. The outbreak of COVID-19 put global and national healthcare systems to test, which when overwhelmed, can severely compromise the well-being of frontline healthcare workers (HCWs) [3].

Since the first COVID-19 case was reported in December 2019 in Wuhan [4], approximately 42,000 HCWs, including 28,600 nurses all over China, were sent to Hubei Province to assist local healthcare teams to care for COVID-19 patients [5]. A study revealed that HCWs who were working in Wuhan often felt stress, depression, and anxiety, but this study didn't target specially at frontline nurses [6].

* Corresponding authors.
E-mail addresses: nurhhg@nus.edu.sg (H.-G. He), jieminzhu@xmu.edu.cn (J. Zhu).
[1] These authors contributed equally to this work.

https://doi.org/10.1016/j.eclinm.2020.100424
2589-5370/© 2020 The Author(s). Published by Elsevier Ltd. This is an open access article under the CC BY-NC-ND license. (http://creativecommons.org/licenses/by-nc-nd/4.0/)

Research in context

Evidence before this study

The outbreak of COVID-19 put global and national healthcare systems to test, which when overwhelmed, can severely compromise the well-being of frontline healthcare workers (HCWs). We searched electronic databases, including CINAHL, PubMed, Google Scholar, and the China National Knowledge Infrastructure, for articles that were published in either English or Chinese from 1 January 2003 to 12 February 2020, using the following keywords: disease outbreak, pandemic, medical crises, quality of life, self-efficacy, resilience, social support, fatigue, anxiety, depression, fear, nurses, healthcare workers, and healthcare professionals. The selection criteria included: (i) non-interventional studies on any pandemic outbreaks, (ii) studies that focused on the impact of any pandemic outbreaks on the health of healthcare workers, and (iii) studies that identified various contributing factors of the experiences described by healthcare workers during any pandemic outbreaks. Articles that were excluded were those that: (i) focused heavily on clarifying transmission routes and improving surveillance systems, (ii) emphasized on how the outbreak led to the development of a particular phenomenon or transition in nursing practice, and (iii) were conducted on humanitarian aid workers. A total of 31 full-text journal articles were reviewed. The physical and psychological well-being of frontline HCWs was compromised across all pandemic outbreaks. Many researches evaluated only the psychological impacts of pandemic outbreaks on frontline HCWs without considerations of other possible influencing factors. None reported the mental health statuses of frontline nurses in particular during the COVID-19 outbreak.

Added value of this study

In the absence of epidemiological data on the mental health of frontline nurses who are caring for COVID-19 patients and its associated factors, our study recruited 2014 frontline nurses with diverse demographic backgrounds and explored their mental health statuses during the COVID-19 outbreak. There was a total of 1324 nurses who were originally working in Wuhan and 690 nurses who were supporting Wuhan from other provinces in China, making our results a good representative of the mental health statuses of the Chinese frontlines nurses woring in Wuhan during the pandemic. We found that frontline nurses experienced a variety of mental health challenges, especially burnout and fear. The prevalence of anxiety, depression, and skin lesion was high. The majority of the nurses expressed their willingness to participate in frontline work. Mental health outcomes were positively correlated with skin lesion and negatively correlated with self-efficacy, resilience, social support, and frontline work willingness.

Implications of all the available evidence

Future interventions at the organisational and national levels are needed to improve frontline nurses' mental health during the pandemic by addressing its associated factors. Similar research and support may be extended to include other frontline healthcare workers.

HCWs, especially nurses, who come close in contact with these patients when providing care are often left stricken with inadequate protections from contamination, high risks of infection, working burnout, fear, anxiety, and depression [7,8].

Nurses constitute the largest part of the healthcare workforce in an epidemic [9], and they undertake most of the tasks related to infectious disease containment [10]. To date, epidemiological data on the mental health of frontline nurses who are caring for COVID-19 patients and its associated factors are still limited. Such evidence-based knowledge is crucial for HCWs and the government to prepare for health responses to pandemics such as COVID-19.

The aim of this study was to examine mental health (burnout, anxiety, depression, and fear) and its associated factors among frontline nurses who were caring for COVID-19 patients in Wuhan, China.

The research questions were:

(a) What are the levels of burnout, anxiety, depression, fear, skin lesion, self-efficacy, resilience, and social support among frontline nurses?
(b) What are the differences in burnout, anxiety, depression, and fear between nurses' various sociodemographic and other COVID-related background subgroups?
(c) What are the relationships between burnout, anxiety, depression, fear, and other aforementioned variables?

2. Methods

2.1. Study design

This was a large-scale cross-sectional, descriptive, correlational study.

2.2. Settings and sampling

This study was conducted in two hospitals in Wuhan, China. One hospital, which consists of three divisions that were located in different places, was originally a public tertiary hospital in Wuhan, and two out of the three divisions were converted to venues that only received COVID-19 patients after 13 January 2020 and 13 February 2020, respectively. These two divisions had 1860 beds in total, with approximately 2000 nurses who were caring for COVID-19 patients. The other hospital was newly established and operated specially for COVID-19 patients since 3 February 2020, with 1000 beds and 600 nurses.

All frontline nurses who were caring for COVID-19 patients in the participating hospitals were invited to participate in this study. Nurses who were diagnosed with any prior mental disorders and/or who had the COVID-19 were excluded from the study.

2.3. Outcomes and measurement

Sociodemographic and other COVID-9 related background data were collected using a self-developed questionnaire. Sociodemographic data consisted of gender, age, marital status, child-rearing, monthly household income, education, professional title, clinical experience, working duration as a frontline nurse, average working hours per shift, whether Wuhan is the original working place, way to be dispatched to Wuhan for those nurses from other cities, position in the hospital, whether the working ward has changed, prior training or experience of caring similar patients, their confidence in caring for patients with COVID-19 infection, self-protection, and working safety. Their belief in their families, colleagues, and hospital readiness to cope with this COVID-19 outbreak was also collected. Willingness and reasons to participate in frontline work during the COVID-19 outbreak were also included. Suggestions to improve frontline work were also explored.

Nurses' burnout was measured by the Chinese version of the Maslach Burnout Inventory: Human Services Survey (MBI-HSS) for Medical Personnel (MP) [11], which contains 22 items with three dimensions: emotional exhaustion (EE, 9 items), depersonalization (DP, 5 items), and personal accomplishment (PA, 8 items). Each item

was measured by a seven-point Likert scale. For the EE and DP dimensions, higher scores meant more severe burnout, while for the PA dimension, lower scores meant more severe burnout. Scores of 19–26 or ≥27 on EE, 6–9 or ≥10 on DP, and 34–39 or ≤33 on PA were indicative of moderate or high burnout for the respective dimensions [11]. The Cronbach's alpha value of the MBI-HSS for MP was 0.86 in this study.

Nurses' anxiety was measured by the Chinese version of Zung's Self-Rating Anxiety Scale (SAS) [12]. The SAS contains 20 items that examine emotional and physical symptoms of anxiety. Each item was measured by a four-point Likert scale. The total scores ranged from 25 to 100 ($20 \times 1 \times 1.25$ to $20 \times 4 \times 1.25$), with 50–59, 60–69, and ≥70 indicating mild, moderate, and severe anxiety, respectively [13]. The Cronbach's alpha value of the SAS was 0.87 in this study.

Nurses' depression was measured by the Chinese version of Zung's Self-Rating Depression Scale (SDS) [14]. The SDS has 20 items that assess emotional, physiological, psychomotor, and psychological imbalance. Each item was measured by a four-point Likert scale. The total scores ranged from 25 to 100 ($20 \times 1 \times 1.25$ to $20 \times 4 \times 1.25$), with 53–62, 63–72, and ≥73 indicating mild, moderate, and severe depression, respectively [13]. The Cronbach's alpha value of the SDS was 0.88 in this study.

Nurses' fear was measured by the Fear Scale for Healthcare Professionals (FS-HPs), which was developed by the research team. The FS-HPs has eight items that assess nurses' fear of infection and death as well as nosocomial spreading to their loved ones during COVID-19 outbreak. Each item was measured by a five-point Likert scale. The total score ranged from 8 to 40, with ≤19, 20–29, and 30–40 indicating no or mild fear, moderate, and severe fear, respectively. Ten experts were invited to evaluate its content validity, giving it a total Content Validity Index (CVI) of 1.0. The Cronbach's alpha value of the FS-HPs was 0.80 in this study.

Skin lesion was measured using a self-developed scale named the Skin Lesion Scale (SLS) based on the book "Epidemic Prevention Medical Protective Equipment related Skin Lesion and Management". [15] The scale has 11 items that examine various common skin lesions related to personal protective equipment (PPE) among HCWs, including facial flushing, blistering of the mouth, skin erosions, skin soaking, skin allergies, skin chapping, skin indentation marks, cutaneous lichen, red spots with clear boundaries, blisters, and isolated pyoderma. For each type of skin lesion, we asked whether each nurse had such a condition (Each "yes" response was given a score 1 and each "no" response was given a score of 0, giving a total score of 0–11). For nurses who had skin lesions but could not manage them, such questions were asked: (1) not sure how to manage them, (2) no medicine available during the period, and (3) the root cause for the skin lesions cannot be changed. A group of ten experts were invited to evaluate the content validity, resulting a total CVI of 1.0. The Cronbach's alpha value of the SLS was 0.73 in this study.

Nurses' self-efficacy was measured by the Chinese version of the General Self-efficacy Scale (GSS) [16]. It consists of ten items and each was measured by a five-point Likert scale. The total score of the scale ranged from 10 to 40. The higher the score, the better the self-efficacy. The Cronbach's alpha value of the GSS was 0.93 in this study.

Nurses' resilience was measured by the Chinese version of the Connor-Davidson Resilience Scale-10 (CD-RISC-10) [17]. It contains ten items with a five-point Likert scale. The total score of the scale ranged from 0 to 40. The higher the score, the better the resilience. The Cronbach's alpha value of the CD-RISC-10 was 0.96 in this study.

Social support was measured using the Chinese version of the Multidimensional Scale of Perceived Social Support (MSPSS) [18]. The scale consists of 12 items and uses a seven-point Likert scale. It has two subscales: intra-family social support and extra-family social support. The higher the mean score, the better the social support. The Cronbach's alpha value of the MSPSS was 0.96 in this study.

2.4. Data collection procedure

The online questionnaire survey was developed using an online platform called "Questionnaire Star". After obtaining ethical approval from the two participating hospitals, the directors of nursing and the head nurses were informed about the inclusion and exclusion criteria. The head nurses distributed the online survey to the WeChat group of frontline nurses who were caring for COVID-19 patients on 13 February 2020. Those who had interest in the survey then filled in the survey on the "Questionnaire Star" platform, which had a feature that only when all questions were answered, the online questionnaire could be submitted. A token of appreciation of 50 RMB (equivalent to 7 USD) was provided to each participant via the WeChat red packet on the completion of the online survey. Data collection was completed on 24 February 2020. The study protocol has been published on the last author's institutional website.

2.5. Ethical considerations

Ethical approval was obtained from the participating hospitals' ethical review boards as well as the last author's university. All nurses provided consent by ticking the "yes" box to indicate their willingness to participate in the online survey. Voluntary participation and data confidentiality were emphasized.

2.6. Data analyses

Data were analysed using IBM SPSS version 25.0 for Windows [19]. Descriptive statistics were used to summarize nurses' sociodemographic and other COVID-related background variable subgroups (such as working duration as the frontline nurses, reasons for being dispatched to Wuhan, confidence in self-protection, and so on) and all continuous outcome variables (including burnout, fear, anxiety, depression, fear, skin lesion, self-efficacy, resilience, and social support). An independent two-sample *t*-test was used to examine the differences in mental health outcomes between sociodemographic and other COVID-related background variable subgroups. Pearson product-moment correlation coefficient was used to examine the relationships between burnout, fear, anxiety, and depression and all other continuous outcome variables. P values of less than 0.05 were considered statistically significant.

2.7. Role of funding source

The funding bodies had no role in study design, data collection, analysis, and interpretation, the manuscript writing, or submission decision. The corresponding authors had full access to all the data and had final responsibility for the decision to submit for publication.

3. Results

3.1. Sociodemographic and other characteristics of the participants

Of the 2110 nurses who opened the survey link, nine (0.4%) ticked the "no" box to indicate their unwillingness to participate in the study and withdrew from the survey. Among the rest of 2101 nurses who completed and submitted the survey, 68 (3.2%) nurses reported that the number of days working at the frontline was zero indicating they had not begun their duties as frontline nurses, and 19 (0.9%) spent less than five minutes to complete the survey with several scales ticking the same answers consecutively (Fig. 1). Thus, these nurses were excluded, leaving a total of 2014 frontline nurses who were included in this study.

Table 1 shows the participants' sociodemographic and other characteristics. The mean age of the frontline nurses was 30.99 (SD=6.17) years old. The mean working duration as frontline nurses was 20.72

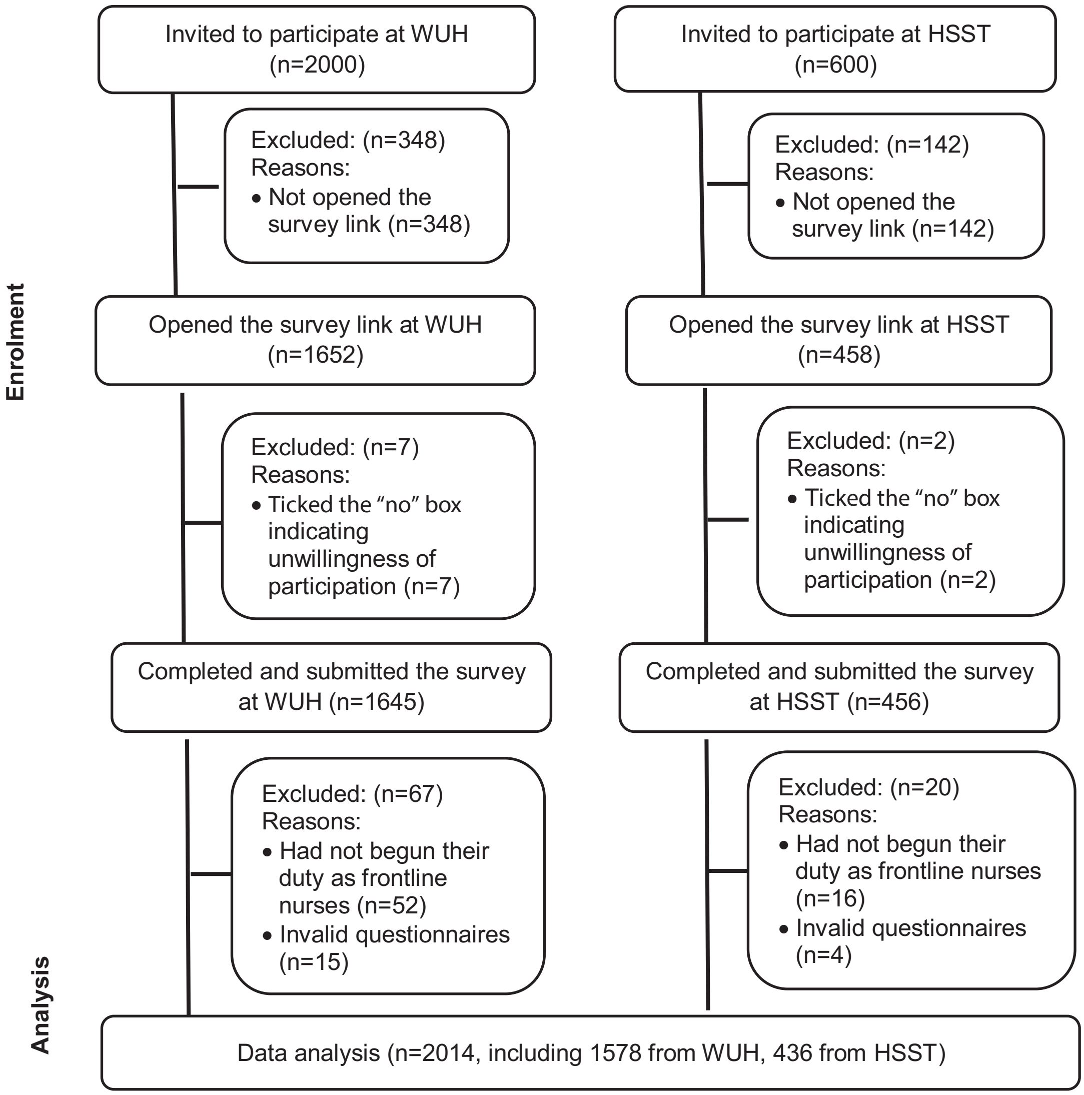

Fig. 1. Flowchart of recruitment process.
Note: Abbreviation: WUH, Wuhan Union Hospital, Huazhong University of Science and Technology; HSSH, Huo Shen Shan hospital.

(SD=12.9) days, and the average working hours was 6.57 (SD=1.90) hours per shift. The majority of the frontline nurses were female (87.1%), were married (61.1%), had one or more children (54.6%), had bachelor's degrees or higher (78.1%), and had junior professional titles (74.2%). There were a total of 1324 nurses who originally worked in Wuhan and 690 nurses who were sent to support Wuhan from other provinces in China. Among these 690 nurses, 476 were voluntary and 214 (209 willing and 5 unwilling) were delegated by their hospitals. The majority of the participants (n = 1, 654, 82.1%) received prior training, but 1229 (61.0%) participants had no prior experiences of caring for patients with infectious diseases. A large number of frontline nurses had confidence in caring for COVID-19 patients, self-protection, and work safety. The majority of the frontline nurses believed that their family, colleagues, and hospitals were ready to cope with the COVID-19 outbreak.

The majority of the participants (n = 1950, 96.8%) indicated their willingness to participate in frontline work with the following reasons: responsibility and mission as a nurse, prior experiences during the SARS outbreak, patriotism, dedication, helping others, extra welfare, hospital assignment, and the mission as a communist party member. Some participants (n = 64, 3.2%) indicated their unwillingness because of safety concerns, family caring needs such as breastfeeding, fear, work stress, and personal health problems.

The participants put forward some suggestions to support frontline nurses' work: (1) improve the welfare and social statuses of frontline nurses, (2) strengthen training regarding self-protection and provide adequate PPE, (3) enhance manpower and resource allocations, (4) improve the conditions of accommodation, food, and environments for frontline nurses, and (5) offer more psychosocial support to frontline nurses.

3.2. Participants' mental health and other outcomes

Table 2 shows the mental health and other outcomes of the frontline nurses. The participants had moderate levels of burnout, as shown in EE (mean=23.44, SD=13.80), DP (mean=6.77,SD=7.05), and PA (mean=34.83, SD= 9.95). The participants reported high levels of fear (mean=30.41, SD=7.60).

Eight hundred and thirty-five (41.5%) nurses reported high EE, 556 (27.6%) nurses indicated high DP, and 771 (38.3%) had no or low PA, which all indicated high burnout during work. The participants reported mild (n = 545, 27.1%), moderate (n = 221, 11.0%), and severe (n = 67, 3.3%) anxiety. Similarly, the participants indicated mild (n = 661, 32.8%), moderate (n = 194, 9.6%), and severe (n = 23, 1.1%) depression. The majority of the nurses reported moderate (n = 564, 28%) and high (n = 1273, 36.2%) fear.

Table 1
Sociodemographic and other characteristics of the frontline nurses (n = 2014).

Sociodemographic variables	Mean (SD)	n (%)
Gender		
Male		260 (12.9%)
Female		1754 (87.1%)
Age (years): mean (SD)	30.99 (6.17)	
Marital status		
Married		1230 (61.1%)
Other marital status[†]		784 (38.9%)
Had one or more children		
Yes		1100 (54.6%)
No		914 (45.4%)
Monthly household income (USD/month)		
≤1440		1109 (55.1%)
>1440		905 (44.9%)
Education		
Diploma or lower		441 (21.9%)
Bachelor's degree or higher		1573 (78.1%)
Professional title		
Junior		1495 (74.2%)
Intermediate and senior		519 (25.8%)
Clinical experience (months)	107.76 (78.09)	
Working duration as frontline nurse during the COVID-19 outbreak (days)	20.72 (12.94)	
Average working hours/shift	6.57 (1.90)	
Wuhan as original working place		
Yes		1324 (65.7%)
No		690 (34.3%)
Reasons for being dispatched to Wuhan (n = 690)		
Delegated by the hospital, willingly or unwillingly		214 (31.0%)
Voluntary		476 (69.0%)
Position in original hospital		
Bedside nurse		1818 (90.3%)
Head nurse or nurse director (including vice-director)		196 (9.7%)
Position in the hospital at Wuhan		
Bedside nurse		1894 (94.0%)
Head nurse or nurse director (including vice-director)		120 (6.0%)
Working wards changed		
Yes		747 (37.1%)
No		1267 (62.9%)
Prior training about caring patients with infectious diseases		
Yes		1654 (82.1%)
No		360 (17.9%)
Prior experience of caring patients with infectious diseases		
Yes		785 (39.0%)
No		1229 (61.0%)
Confidence in caring COVID-19 patients[a]		
Unconfident		796 (39.5%)
Confident		1218 (60.5%)
Confidence in self-protection[a]		
Unconfident		863 (42.9%)
Confident		1151 (57.1%)
Evaluation of work safety while caring COVID-19 patients[b]		
Unsafe		844 (41.9%)
Safe		1170 (58.1%)
Belief in your family's readiness to cope with this COVID-19 outbreak		
Not believe		586 (29.1%)
Believe		1428 (70.9%)
Belief in your colleagues' readiness to cope with this COVID-19 outbreak		
Not believe		413 (20.5%)
Believe		1601 (79.5%)
Belief in your hospital's readiness to cope with this COVID-19 outbreak		
Not believe		361 (17.9%)
Believe		1653 (82.1%)
Willingness to participate in frontline work during the COVID-19 outbreak		

(continued)

Table 1 (Continued)

Sociodemographic variables	Mean (SD)	n (%)
Yes		1950 (96.8%)
No		64 (3.2%)

[†] Including single, divorced, or separated.
[a] Measured by a 5-point scale and regrouped into two categories: Unconfident, including "1=Very unconfident", "2=Unconfident", and "3=Somewhat confident", and Confident, including "4=Confident" and "5=Very confident".
[b] Measured by a 5-point scale and regrouped into two categories: Unsafe, including "1=Very unsafe", "2=Unsafe", and "3=Somewhat Safe", and Safe, including "4=Safe" and "5=Very safe".

The majority of the participants (n = 1910, 94.8%) had one or more skin lesion(s) caused by PPE. Among nurses who did not manage their skin lesions (n = 1703, 84.6%), 316 nurses (15.7%) indicated that they were not sure about the management, 518 nurses (25.7%) indicated that no medicine was available during the period, and 718 nurses (35.7%) said that the root causes were not changeable. Besides the 11 skin lesions that we included in our self-developed scale, some nurses mentioned other skin lesions such as conjunctivitis, ear tenderness, decrustation, beriberi, and needle stick injuries.

3.3. Differences in mental health outcome levels between various sociodemographic and other characteristic subgroups for the participants

Table 3 shows the differences in the burnout, anxiety, depression, and fear levels between various sociodemographic and other characteristic subgroups. It was typical for one mental health variable to have significant differences for some, but not all sociodemographic and other characteristic subgroups. However, statistically significant differences in the levels of burnout, anxiety, depression, and fear were found between subgroups of the following variables: professional title ($p<0.05$), whether Wuhan was the original working place ($p<0.05$), whether working wards had changed ($p<0.05$), confidence in caring for COVID-19 patients ($p<0.001$), confidence in self-protection ($p<0.001$), evaluations of work safety ($p<0.001$), belief in family's or colleagues' or hospitals' readiness to cope with the COVID-19 outbreak ($p<0.001$), and willingness to participate frontline work ($p<0.01$) .

3.4. Relationships among mental health and other health outcomes

Table 4 showed the relationships among mental health and other health outcomes for frontline nurses. EE was positively correlated with skin lesion (r = 0.182) and negatively correlated with self-efficacy (r=−0.193), resilience (r=−0.325), intra-family social support (r=−0.170), and extra-family social support (r=−0.234). DP was negatively correlated with resilience (r=−0.208), intra-family social support (r=−0.221), and extra-family social support (r=−0.216). PA was positively correlated with self-efficacy (r = 0.376), resilience (r = 0.436), intra-family social support (r = 0.348), and extra-family social support (r = 0.363). Anxiety was positively correlated with skin lesion (r = 0.265) and negatively correlated with self-efficacy (r=−0.262), resilience (r=−0.427), intra-family social support (r=−0.274), and extra-family social support (r=−0.333). Similarly, depression was positively correlated with skin lesion (r = 0.224) and negatively correlated with self-efficacy(r=−0.409), resilience (r=−0.554), intra-family social support (r=−0.384), and extra-family social support (r=−0.455). Fear was negatively correlated with resilience (r=−0.121).

Table 2
Health outcomes of the frontline nurses (n = 2014).

Mental health variables	Mean (SD)	n (%)	Possible range
Burnout (MBI-HSS)[a]: Emotional exhaustion	23.44 (13.80)		0 to 54
No or mild emotional exhaustion (≤18)		796 (39.5%)	
Moderate emotional exhaustion (19–26)		383 (19.0%)	
High emotional exhaustion: (≥27)		835 (41.5%)	
Burnout (MBI-HSS)[a]: depersonalization	6.77 (7.05)		0 to 30
No or mild depersonalization (≤5)		1161 (57.6%)	
Moderate depersonalization (6–9)		297 (14.7%)	
High depersonalization: (≥10)		556 (27.6%)	
Burnout (MBI-HSS)[a]: personal accomplishment	34.83 (9.95)		0 to 48
High personal accomplishment indicating low burnout (≥40)		795 (39.5%)	
Moderate personal accomplishment indicating moderate burnout (34–39)		448 (22.2%)	
No or mild personal accomplishment indicating high burnout (≤33)		771 (38.3%)	
Anxiety (SAS)[b]	47.80 (11.20)		25 to 100
No anxiety (<50)		1181 (58.6%)	
Mild anxiety (50–59)		545 (27.1%)	
Moderate anxiety (60–69)		221 (11.0%)	
Severe anxiety (≥70)		67 (3.3%)	
Depression (SDS)[c]	50.50 (11.31)		25 to 100
No depression (<53)		1136 (56.4%)	
Mild depression (53–62)		661 (32.8%)	
Moderate depression (63–72)		194 (9.6%)	
Severe depression (≥73)		23 (1.1%)	
Fear (FS-HPs)[d]	30.41 (7.60)		8 to 40
No or mild fear (≤19)		177(8.3%)	
Moderate fear (19–29)		564 (28.0%)	
Severe fear (30–40)		1273 (63.2%)	
Skin lesion (SLS)[e]	3.91 (2.30)		0 to 11
0 skin lesion		104 (5.2%)	
1–4 skin lesions		1193 (59.2%)	
5–8 skin lesions		629 (31.2%)	
9–11 skin lesions		88 (4.4%)	
Self-efficacy (GSS)[f]	26.87 (5.86)		10 to 40
Resilience (CD-RISC-10)[g]	26.14 (7.33)		0 to 40
Intra-family social support (MSPSS)[h]	5.57 (1.15)		1 to 7
Extra-family social support (MSPSS)[h]	5.38 (1.07)		1 to 7

[a] MBI-HSS: Maslach Burnout Inventory: Human Services Survey.
[b] SAS: Zung's Self-Rating Anxiety Scale.
[c] SDS: Zung's Self-Rating Depression Scale.
[d] FS-HPs: Fear Scale for Healthcare Professionals.
[e] SLS: Skin Lesion Scale.
[f] GSS: General Self-efficacy Scale.
[g] CD-RISC-10: Connor-Davidson Resilience Scale-10.
[h] MSPSS: Multidimensional Scale of Perceived Social Support.

4. Discussion

This is the first study that examined frontline nurses' mental health and its associated factors during the COVID-19 outbreak using a large-scale cross-sectional design. The strengths of this study included the multi-centered sampling and the big sample size. We conducted our survey in a local hospital and a newly built hospital specially for COVID-19 patients. Among all participants, 1324 were originally working in Wuhan, whereas 690 nurses were originally from other provinces who were supporting Wuhan's healthcare system. The diversity of nurses' geographic background make our sample a relatively good representative of nurses from China.This study found that the prevalence of burnout, anxiety, depression, and fear was high in frontline nurses. Skin lesions were very common for frontline nurses wearing PPE. Although frontline nurses were suffering from the aforementioned physical and mental health symptoms, they still expressed their willingness to participate in frontline work during the COVID-19 outbreak. We also found that frontline nurses' mental health was positively correlated with skin lesion and negatively correlated with self-efficacy, resilience, social support, and frontline work willingness.

In this study, frontline nurses reported moderate levels of burnout. Moreover, 60.5%, 42.3%, and 60.6% of the frontline nurses had moderate/high EE, DP, and PA, respectively, which all indicated a high prevalence of burnout among frontlines nurses. The COVID-19 outbreak has led to a sharp increase in admissions and presentations to hospitals and consequently impacts the workload of nurses. A previous study indicated that each additional patient added to a nurse's workload was associated with a 23% increase in the likelihood of burnout [20]. In a study of nurses during the Middle East Respiratory Syndrome outbreak, nurses started to sink into a state of burnout after a prolonged and sustained period of exposure to the deteriorating situation with no end in sight [21].

This study showed that 40% to 45% of the frontline nurses experienced anxiety or depression, with 11% to 14% having moderate to severe anxiety or depression. Similar to the SARS outbreak in 2003, due to the life-threatening nature of the disease and the increasing workload, frontline nurses were at high risks of anxiety and depression [22]. Compared to the previous report on 5062 HCWs (3240 from non-isolation wards, 1607 from isolation wards, 215 off work or in self-isolation), including 3417 nurses, 1004 doctors and 641 medical technicians, regardless of whether they were working at frontline during COVID-19 outbreak in Wuhan [6], the incidence of anxiety and depression of frontline nurses in our study was relatively higher.

This study showed that the frontline nurses suffered from fears of infection and death as well as nosocomial spreading to their loved ones. As the numbers of infection and mortality cases surge, the COVID-19 outbreak in China has caused public panic and distress [7].

Table 3
Differences in mental health among/between various sociodemographic and other characteristic subgroups for frontline nurses (n = 2014).

Sociodemographic variables	Burnout: EE[a]		Burnout: DP[b]		Burnout: PA[c]		Anxiety		Depression		Fear	
	Mean±SD	r/t	Mean±SD	r/t	Mean±SD	r/t	Mean±SD	r/t	Mean±SD	r/t	Mean±SD	r/t
Gender†												
Male	21.48±14.25	−2.449 *	8.97± 8.40	4.624***	34.43±10.56	−0.682	46.56±11.55	−1.910	48.91±12.28	−2.253*	27.08± 8.99	−6.548***
Female	23.73±13.71		6.44± 6.77		34.89± 9.86		47.98±11.14		50.73±11.14		30.90± 7.25	
Age (years)‡		0.003		−0.086***		0.067**		−0.012		0.020		−0.045*
Marital status†												
Married	23.64±13.93	0.817	6.49± 6.85	−2.153*	35.23±10.18	2.264*	47.80±11.16	0.012	50.60±11.30	0.505	30.61± 7.59	1.473
Other marital status	23.12±13.60		7.20± 7.34		34.20± 9.56		47.79±11.26		50.34±11.33		30.09± 7.61	
had one or more children†												
Yes	23.67±14.14	0.824	6.60± 6.98	−1.176	35.25±10.26	2.117*	48.14±11.20	1.490	50.99±11.30	2.147*	30.51± 7.70	0.663
No	23.36±13.38		6.97± 7.14		34.32± 9.54		47.39±11.19		49.90±11.30		30.28± 7.48	
Monthly household income† (USD /month)												
≤1440	23.04±13.85	−1.446	6.95± 7.43	1.329	34.72±10.18	−0.510	48.34±11.42	2.393*	51.29±11.34	3.485**	29.95± 7.64	−3.012**
>1440	23.93±13.74		6.54± 6.57		34.95± 9.67		47.14±10.89		49.53±11.20		30.97± 7.52	
Education†												
Diploma or lower	20.06±13.92	−5.859***	6.66± 7.38	−0.355	34.19±11.30	−1.384	47.43±10.98	−0.790	50.45±11.92	−0.103	28.86± 8.24	−4.559***
Bachelor's degree or higher	24.38±13.62		6.80± 6.96		35.01± 9.54		47.90±11.26		50.51±11.14		30.84± 7.36	
Professional title†												
Junior	23.82±13.94	2.116*	7.07± 7.22	3.395**	34.48± 9.96	−2.660**	48.11±11.16	2.151*	50.79±11.29	1.960*	30.70± 7.56	2.915**
Intermediate and senior	22.34±13.36		5.91± 6.49		35.83± 9.86		46.89±11.27		49.66±11.34		29.57± 7.66	
Clinical experience (months)‡		0.008		−0.113**		0.092**		−0.023		0.005		−0.020
Working duration as a frontline nurse during the COVID-19 outbreak (days)‡		0.061**		0.024		0.052*		0.067**		0.066**		−0.001
Average working hours/shift‡		0.012		0.066**		−0.022		0.044*		0.038		−0.103**
Wuhan as original working place†												
Yes	23.88±13.58	11.364***	7.42± 7.19	5.988***	34.44± 9.75	−2.429*	49.22±11.32	8.040***	51.87±11.12	7.657***	31.76± 7.19	11.148***
No	18.74±13.00		5.51± 6.62		35.57±10.30		45.06±10.44		47.86±11.20		27.82± 7.70	
Reasons for being dispatched to Wuhan† (n = 690)												
Assigned by the hospital, willingly or unwillingly	21.45±13.43	3.697***	6.03± 6.90	1.399	34.16±10.57	−2.427*	46.34±10.05	2.164*	50.01±10.70	3.408**	29.14± 7.06	3.193**
Voluntary	17.53±12.63		5.27± 6.49		36.21±10.12		44.49±10.57		46.89±11.30		27.22± 7.91	
Position in original hospital†												
Bedside nurse	23.54±13.76	0.964	6.80± 6.95	0.551	34.57± 9.94	−3.575***	47.94±11.08	1.689	50.58±11.17	0.913	30.79± 7.44	6.559***
Head nurse or nurse director (including vice-director)	22.54±14.21		6.47± 7.93		37.23± 9.77		46.52±12.16		49.73±12.55		26.81± 8.16	
Position in the hospital at Wuhan†												
Bedside nurse	23.37±13.78	−0.821	6.65± 6.91	−2.341*	34.80± 9.92	−0.470	47.71±11.06	−1.223	50.38±11.22	−1.878	30.61± 7.50	4.327***
Head nurse or nurse director (including vice-director)	24.44±14.16		8.58± 8.82		35.24±10.46		49.22±13.22		52.38±12.57		27.18± 8.47	
Working wards changed†												
Yes	22.65±13.63	−1.965*	7.33± 7.49	2.671**	33.78±10.87	−3.487**	48.56±11.44	2.341*	51.41±11.32	2.797**	29.75± 7.77	−2.972**
No	23.90±13.88		6.44± 6.77		35.44± 9.32		47.35±11.21		49.96±11.27		30.79± 7.47	
Prior training about caring for similar patients with virus infections†												
Yes	22.93±13.55	−3.382**	6.71± 7.05	−0.822	35.26± 9.80	3.995***	47.34±10.91	−3.658***	50.01±11.28	−4.144***	30.12± 7.62	−3.596***
No	25.78±14.68		7.04± 7.09		32.86±10.41		49.89±12.22		52.73±11.20		31.71± 7.39	

(*continued on next page*)

Table 3 (*Continued*)

Sociodemographic variables	Burnout: EE[a]		Burnout: DP[b]		Burnout: PA[c]		Anxiety		Depression		Fear	
Prior experience of caring for similar patients with virus infections[†]												
Yes	22.21±13.74	−3.196**	7.04± 7.57	1.340	35.33±10.46	1.779	47.54±10.82	−0.839	50.30±11.35	−0.633	29.14± 8.18	−5.833***
No	24.22±13.79		6.59± 6.71		34.51± 9.60		47.97±11.43		50.62±11.29		31.21± 7.10	
Confidence in caring for COVID-19 patients[†]												
Unconfident	28.12±13.43	12.782***	7.94± 7.13	6.020***	32.74± 9.52	−7.710***	51.03±11.55	10.527***	53.96±10.64	11.585***	32.42± 6.84	10.125***
Confident	20.38±13.18		6.00± 6.90		36.19±10.00		45.69±10.44		48.23±11.17		29.09± 7.79	
Confidence in self-protection[†]												
Unconfident	28.36±13.21	14.557***	8.06± 7.17	7.144***	32.87± 9.46	−7.740***	51.85±11.21	14.602***	54.60±10.25	15.038***	32.77± 6.44	12.93***
Confident	19.75±13.05		5.80± 6.81		36.29±10.06		44.76±10.18		47.42±1.09		28.63± 7.92	
Evaluation of working safety while caring for COVID-19 patients[†]												
Unsafe	28.48±13.18	14.651***	8.07± 7.24	7.019***	33.49± 9.19	−5.166***	51.58±11.59	13.147***	54.18±10.46	13.047***	32.83± 6.49	13.039***
Safe	19.80±13.08		5.83± 6.77		35.79±10.36		45.07±10.06		47.84±11.16		28.66± 7.86	
You believe in your family's readiness to cope with this COVID-19 outbreak[†]												
Not believe	29.88±13.00	14.064***	8.27± 7.23	6.042***	33.00± 8.93	−5.628***	52.15±11.39	11.528***	54.61±10.21	11.215***	32.95± 6.27	10.788***
Believe	20.79±13.24		6.15± 6.89		35.58±0.25		46.01±10.62		48.81±11.31		29.36± 7.85	
You believe in your colleagues' readiness to cope with this COVID-19 outbreak[†]												
Not believe	31.33±13.17	13.612***	8.80± 7.36	6.386***	31.85± 9.26	−6.898***	53.80±11.32	12.685***	56.27± 9.50	13.301***	33.29± 6.48	9.747***
Believe	21.40±13.22		6.24± 6.88		35.60± 9.98		46.25±10.63		49.01±11.26		29.66± 7.69	
You believe in your hospital's readiness to cope with this COVID-19 outbreak[†]												
Not believe	32.39±13.12	14.278***	9.01± 7.48	6.364***	31.81± 9.19	−6.431***	54.14±11.66	12.322***	56.70± 9.82	12.921***	33.44± 6.49	9.481***
Believe	21.48±13.16		6.28± 6.86		35.49± 9.99		46.41±10.60		49.14±11.16		29.74± 7.67	
Willingness to participate in frontline work during the COVID-19 outbreak[†]												
Yes	23.05±13.57	−6.279***	6.62± 6.99	−5.200***	35.01± 9.88	4.579***	47.43±10.97	−8.364***	50.14±11.19	−9.256***	30.31± 7.55	−3.065**
No	35.31±15.43		11.25±7.67		29.25±10.54		59.13±12.92		61.31± 9.44		33.27±8.53	

[a] EE=emotional exhaustion.
[b] DP=depersonalization.
[c] PA=personal accomplishment.
[†] Independent two-sample t-test with t values presented.
[‡] Pearson product-moment correlation coefficient test with r values presented.
* $0.01 \le p < 0.05$.
** $0.001 \le p < 0.01$.
*** $p < 0.001$.

Table 4
Relationships among mental health variables and other health variables for the frontline nurses ($n = 2014$).

Variables	Burnout- EE[a]	Burnout: DP[b]	Burnout: PA[c]	Anxiety	Depression	Fear
Burnout: EE[a]	–					
Burnout: DP[b]	0.401***	–				
Burnout: PA[c]	−0.041*	−0.138***	–			
Anxiety	0.637***	0.417***	−0.242 ***	–		
Depression	0.569 ***	0.406***	−0.378 ***	0.768 ***	–	
Fear	0.413***	0.095***	0.073 **	0.268***	0.178 ***	–
Skin lesion	0.182***	−0.097***	−0.019	0.265 ***	0.224***	0.078***
Self-efficacy	−0.193***	0.096***	0.376 ***	−0.262***	−0.409 ***	−0.063 **
Resilience	−0.325***	−0.208***	0.436 ***	−0.427***	−0.554***	−0.121 ***
Intra-family social support	−0.170***	−0.221***	0.348 ***	−0.274***	−0.384***	0.090 ***
Extra-family social support	−0.234***	−0.216 ***	0.363***	−0.333***	−0.455***	0.038*

[a] EE=emotional exhaustion.
[b] DP=depersonalization.
[c] PA=personal accomplishment.
* $0.01 \le p < 0.05$.
** $0.001 \le p < 0.01$.
*** $p < 0.001$.

HCWs caring for COVID-19 patients were also found to be scared [7]. As of 27 February 2020, 3387 healthcare professionals have been diagnosed with COVID-19 in China [23]. As of 1 March 2020, 25 healthcare workers died not only because of being infected with COVID-19 but also because of cardiac arrest or other ailments due to fatigue and overwork during the COVID-19 outbreak [24]. Thus, for frontline nurses, their colleagues getting infected or dying might aggravate their fears.

The frontline nurses' burnout, anxiety, and depression were weakly positively correlated with skin lesion, which means that the worse the skin lesion, the higher the burnout, anxiety, and depression levels. Currently, in the context of a lack of definite and effective treatment for COVID-19, wearing PPE is the most effective way to prevent infections, especially in HCWs [25]. However, it is very uncomfortable and inconvenient to wear PPE, especially when they were wearing the same PPE throughout the shift for a few hours, and 94.8% of the frontline nurses reported one or more skin lesion in our study. Moreover, a large number of nurses did not manage and treat their skin lesions due to a lack of related knowledge or no medicine available at hand. Appropriate training on skin lesion prevention and adequate medicine to manage skin lesion should be guaranteed to protect frontline nurses, thus promoting their mental health.

Our findings showed that frontline nurses' burnout, anxiety, and depression were moderately negatively correlated with self-efficacy and resilience, which means that when nurses have better self-efficacy and resilience, they may experience less mental health problems. Higher self-efficacy is beneficial for disaster preparedness [26]. Resilience can mitigate the negative impact of work related stress and prevent poor psychological health outcomes among nurse [27]. Individual attributes and organisational resources should be addressed to build self-efficacy and resilience, thus achieving improvement in the mental health of nurses [28].

The frontline nurses' burnout, anxiety, depression, and fear were moderately negatively correlated with social support. A systematic review indicated that a lack of social support was one of important risk factors for developing negative psychological outcomes in HCWs across all types of disasters [29]. The availability of psychological interventions, including the establishment of response, social support, medical, and assistance hotline teams, was beneficial and helpful for frontline nurses' mental health [6].

Although the prevalence of burnout, anxiety, depression, fear, and skin lesion was high, 1950 (96.8%) nurses still expressed their willingness to participate in frontline work. Moreover, the frontline nurses' mental health was strongly negatively correlated with frontline work willingness based on the *t*-test results. Nursing willingness or intention, voluntary and active caring for patients during any newly emerging infectious diseases [30], is important in mitigating nurses' burnout, anxiety, depression, and fear. Thus, suggestions from the frontline nurses to improve their working conditions, such as enhancing of manpower and resource allocation, as well as to improve welfare and living conditions, should be addressed to support nurses' willingness in caring for COVID-19 patients.

This survey was conducted from 13 to 24 February 2020. The confirmed cases of COVID-19 in Wuhan reached the peak on 13 February 2020 and decreased gradually. Confirmed cases were still reported after the completion of data collection [2]. The timing of the survey may limit the generalization to all frontline nurses who were working in other period and in other parts of China where the pandemic situation was not that severe. Moreover, the frontline nurses might expressed less burnout, anxiety, depression and fear than real condition due to social desirability. Because of the time limit and COVID-19 urgency, we developed the fear scale for health care professionals and generated the thresholds based on our experiences. A future study is needed to test and better decide the thresholds of the fear scale. Moreover, the cross-sectional design provided information at one time point only. The correlation shown in this study does not imply causation. The lack of follow-up data of frontline nurses' mental health made it impossible to know their mental health statuses over time. Longitudinal studies are recommended for future studies to capture more in-depth information about the mental health statuses of frontline nurses, both in China and other parts of the world.

Frontline nurses experienced a variety of mental health challenges, especially burnout and fear, which warrants more attention and support from policymakers. Future interventions at the organisational and national levels are needed to improve frontline nurses' mental health during the pandemic by considering preventing and managing skin lesions, building self-efficacy and resilience, providing sufficient social support, and ensuring frontline work willingness. Similar research and support may be extended to include other frontline healthcare workers.

Declaration of Competing Interest

We declare no competing interests.

Funding

This study was funded by the 2020 COVID-19 Emergency Response Special Fund from Xiamen University (20720200025) and Huazhong University of Science and Technology (2020kfyXGYJ001) in China.

References

[1] World Health Organization. WHO Director-General's opening remarks at the media briefing on COVID-19 - 11 March 2020. https://www.who.int/dg/speeches/detail/who-director-general-s-opening-remarks-at-the-media-briefing-on-covid-19—11-march-2020. (Accessed Mar 12, 2020)
[2] World Health Organization. Coronavirus disease (COVID-19) Situation Dashbord. https://covid19.who.int. (Accessed Mar 27, 2020)
[3] Honey M, Wang WYQ. New Zealand nurses perceptions of caring for patients with influenza A (H1N1). Nurs Crit Care 2013;18(2):63–9.
[4] Chen NS, Zhou M, Dong X, et al. Epidemiological and clinical characteristics of 99 cases of 2019 novel coronavirus pneumonia in Wuhan, China: a descriptive study. Lancet 2020;395(10223):507–13.
[5] National Health Comission of the People's Republic of China. China has sent 42,000 medical personnel to support Wuhan, including 28,600 nurses, accounting for 68% of the total number of medical teams. https://baijiahao.baidu.com/s?id=1659868701548259772&wfr=spider&for=pc. (Accessed Mar 27, 2020)
[6] Zhu ZH, Xu SHB, Wang H, et al. COVID-19 in Wuhan: immediate psychological impact on 5062 health workers. MedRxiv 2020. doi: 10.1101/2020.02.20.20025338.
[7] Kang L, Li Y, Hu S, et al. The mental health of medical workers in Wuhan, China dealing with the 2019 novel coronavirus. Lancet Psychiatry 2020;7(3):e14.
[8] Bao Y, Sun Y, Meng S, et al. 2019-nCoV epidemic: address mental health care to empower society. The Lancet 2020;1:e37–8.
[9] Said NB, Chiang VCL. The knowledge, skill competencies, and psychological preparedness of nurses for disasters: a systematic review. Int Emerg Nurs 2020;48:100806.
[10] Usher K, Gardner A, Buttner P, et al. The H1N1 influenza 09 and its potential impact on the Australian nursing workforce. Collegian 2009;16(4):169–70.
[11] Maslach C, Jackson SE. The measurement of experienced burnout. J Occup Behav 1981;2(2):99–113.
[12] Zung WW. A rating instrument for anxiety disorders. Psychosomatics 1971;12(6):371–9.
[13] Wang X. Handbook of mental health assessment scale. Beijing: China J Mental Health 1999.
[14] Zung WW. A self-rating depression scale. Arch Gen Psychiatry 1965;12:63–70.
[15] Sichuan Academy of Medical Sciences. Epidemic Prevention Medical Protective Equipment related Skin Lesion and Management (In Chinese).https://mp.weixin.qq.com/s/NqHFAN2cINbJ4fQMwpM8Dg. (Accessed Feb 9, 2020)
[16] Schwarzer R, Jerusalem M. Generalized self-efficacy scale. Windsor, UK: NFER-NELSON; 1995.
[17] Connor KM, Davidson JRT. Development of a new resilience scale: the Connor-Davidson Resilience scale (CD-RISC). Depress Anxiety 2003;18(2):76–82.
[18] Zimet GD, Dahlem NW, Zimet SG, et al. The multidimensional scale of perceived social support. J Pers Assess 1988;52(1):30–41.
[19] IBM Corp. IBM. Armonk: New York: IBM SPSS Statistics for Windows; 2013 Version 25.0. 2019.
[20] Aiken LH, Clarke SP, Sloane DM, et al. Hospital nurse staffing and patient mortality, nurse burnout, and job dissatisfaction. JAMA 2002;288(16):1987–93.
[21] Kang HS, Son YD, Chae SM, et al. Working experiences of nurses during the Middle East respiratory syndrome outbreak. Int J Nurs Pract 2018;24(5).
[22] Su TP, Lien TC, Yang CY, et al. Prevalence of psychiatric morbidity and psychological adaptation of the nurses in a structured SARS caring unit during outbreak: a prospective and periodic assessment study in Taiwan. J Psychiatr Res 2007;41(1–2):119–30.
[23] World & Nation. Doctors and nurses fighting coronavirus in China die of both infection and fatigue. https://www.latimes.com/world-nation/story/2020-02-25/doctors-fighting-coronavirus-in-china-die-of-both-infection-and-fatigue. (Accessed Feb 27, 2020).
[24] Caixin. 25 doctors and nurses died in the COVID-19 outbreak. http://china.caixin.com/2020-03-01/101519796.html. (Accessed Mar 1, 2020).
[25] Cheng VCC, Wong SC, To KKW, et al. Preparedness and proactive infection control measures against the emerging Wuhan coronavirus pneumonia in China. J Hosp Infect 2020;104(3):254–5.
[26] Kilic N, Simsek N. The effects of psychological first aid training on disaster preparedness perception and self-efficacy. Nurse Educ Today 2019;83:8.
[27] Delgado C, Upton D, Ranse K, et al. Nurses' resilience and the emotional labour of nursing work: an integrative review of empirical literature. Int J Nurs Stud 2017;70:71–88.
[28] Badu E, O'Brien AP, Mitchell R, et al. Workplace stress and resilience in the Australian nursing workforce: a comprehensive integrative review. Int J Ment Health Nurs 2020;29(1):5–34.
[29] Naushad VA, Bierens JJ, Nishan KP, et al. A Systematic Review of the Impact of Disaster on the Mental Health of Medical Responders. Prehosp disaster med 2019;34(6):632–43.
[30] Oh N, Hong N, Ryu DH, et al. Exploring nursing intention, stress, and professionalism in response to infectious disease emergencies: the experience of local public hospital nurses during the 2015 MERS Outbreak in South Korea. Asian Nurs Res (Korean Soc Nurs Sci) 2017;11(3):230–6.
