# Supplementary material for: Mental health status and related factors influencing healthcare workers during the COVID-19 pandemic: A systematic review and meta-analysis
Source: PLoS One. 2024 Jan 19;19(1):e0289454. doi: 10.1371/journal.pone.0289454 (PMC10798549; doi:10.1371/journal.pone.0289454)
Supplement: S1 Data — (ZIP) [file pone.0289454.s011.zip › literatures/56.pdf]

ResearchGate

See discussions, stats, and author profiles for this publication at: https://www.researchgate.net/publication/355949666

Preliminary Data on Gastrointestinal Deficiencies Incidence and the Prevalence of Anxiety During the COVID-19 Pandemic Among the Medical Workers

Article *in* The Journal of nervous and mental disease · November 2021

DOI: 10.1097/NMD.0000000000001445

CITATION
1

READS
28

8 authors, including:

Ovidiu Dumitru Ilie
Universitatea Alexandru Ioan Cuza
35 PUBLICATIONS **268** CITATIONS

SEE PROFILE

Constantin Trus
Universitatea Dunarea de Jos Galati
30 PUBLICATIONS **84** CITATIONS

SEE PROFILE

Bogdan Doroftei
Universitatea de Medicina si Farmacie Grigore T. Popa Iasi
86 PUBLICATIONS **466** CITATIONS

SEE PROFILE

Some of the authors of this publication are also working on these related projects:

Project manifestari oculare in sarcina View project

All content following this page was uploaded by Ovidiu Dumitru Ilie on 03 September 2022.

The user has requested enhancement of the downloaded file.

Preliminary Data on Gastrointestinal Deficiencies Incidence and the Prevalence of Anxiety During the COVID-19 Pandemic Among the Medical Workers

Ovidiu-Dumitru Ilie, PhD, Alexandra Bolos, MD, PhD,† Ilinca-Bianca Nita, MD,† Roxana-Oana Cojocariu, PhD,* Ioana-Miruna Balmus, PhD,‡ Alin Ciobica, PhD,*§|| Constantin Trus, MD, PhD,¶ and Bogdan Doroftei, MD, PhD†#*

Downloaded from http://journals.lww.com/jonmd by BhDMf5ePHKav1zEoum1tQfN4a+kJLhEZgbsIHo4XMi0hCywCX1AWnYQp/IlQrHD3i3D0OdRyi7TvSFl4Cf3VC1y0abggQZXdgGj2MwlZLeI= on 09/03/2022

Abstract: Severe acute respiratory syndrome coronavirus 2 (SARS-CoV-2) is a highly contagious new β-coronavirus that primarily affects the lungs. Because of its unprecedented spread, in a relatively short interval, it is declared a global pandemic. Binding to the angiotensin-converting enzyme 2 receptors, SARS-CoV-2 is easily disseminated through air. Apart from the established clinical panel, individuals exposed to prolonged chronic stress also manifest gastrointestinal (GI) symptoms similar to those exhibited by SARS-CoV-2–infected patients.

The present study aims to assess the incidence of GI deficiencies and prevalence of anxiety among healthy medical staff by applying the Visual Analog Scale for Irritable Bowel Syndrome (VAS-IBS) and Hamilton Anxiety Rating Scale (HAM-A) during this global crisis.

We found significant differences on several items of the VAS-IBS: regarding the incidence of diarrhea ($p = 0.04$), bloating/gases ($p = 0.02$), and nausea/vomiting ($p = 0.01$) from the physical spectrum. After stratification based on age of the participants and after we applied Kruskal-Wallis test because of heterogeneity between groups, we noted two situations in which the null hypothesis is rejected: nausea/vomiting in women between 20 and 30 years, and between 30 and 40, and between 40 and 50 years, respectively ($p = 0.026/0.029$). Anxiety was prevalent among young and middle-class people after the centralization of HAM-A data, where 40.4% of the participants had various forms of anxiety: mild ($n = 13$; 13.82%), severe ($n = 13$; 13.82%), and moderate ($n = 12$; 12.76%).

This study demonstrates that VAS-IBS is a reliable tool for assessing the incidence of GI deficiencies, as well as HAM-A for anxiety.

Key Words: VAS-IBS, HAM-A, SARS-CoV-2, COVID-19, incidence, prevalence, gastrointestinal

(*J Nerv Ment Dis* 2022;210: 98–103)

China was severely affected in late 2019 by a new strain of human coronavirus that causes severe forms of pneumonia. In the absence of any related information about this pathogen, in March 2020, total pandemic was declared by the World Health Organization (Rothan and Byrareddy, 2020). Although there was no foundation, only hypotheses issued, the Chinese rufous horseshoe bat (*Rhinolophus sinicus*) was incriminated on the basis of one single argument: it is the host of various pathogenic entities (Zhou et al., 2020a).

Identified as COVID-19 caused by SARS-CoV-2, this disease is the successor of MERS and SARS that previously hit the mainland on two previous occasions. In retrospect, humanity has never faced such a global crisis since World War II. Taking into account the predictions made (Petropoulos and Makridakis, 2020), there are strong chances that the number of infections could be equal to that of deaths caused by the Spanish flu in 1918 (Taubenberger and Morens, 2006).

Having an unprecedented spread and diffusion dependent on weather conditions (Demongeot et al., 2020), its pathogenesis is almost identical to MERS and SARS such as fever, cough, and dyspnea (Petrosillo et al., 2020). Binding to the angiotensin-converting enzyme 2 receptors (Barnes et al., 2020), it gradually affects the lungs, affinity, and replication dependent on cathepsin B/L (Liu et al., 2020) and transmembrane protease, serine 2 and 4 (TMPRSS2/4) activity (Zang et al., 2020). Because TMPRSS2/4 are also widely expressed from the esophagus to the colon, SARS-CoV-2–infected patients also exhibit gastrointestinal (GI) deficiencies (Hamming et al., 2004). In this way, the Spike protein cleaves on the cell membrane and constantly infects the human gut enterocytes (Lamers et al., 2020).

Considering the incidence of irritable bowel syndrome (IBS) symptoms after prolonged exposure to chronic stress (Qin et al., 2014) or postinfection reflected by a proinflammatory cascade (Waltuch et al., 2020; Zhou et al., 2020b), there are strong changes for a relatively high incidence of IBS among the medical staff (Chatterjee and Chauhan, 2020). Therefore, the aim of the present study was to assess the incidence of GI deficiencies and prevalence of anxiety among the medical staff by applying the Visual Analog Scale for Irritable Bowel Syndrome (VAS-IBS) and Hamilton Anxiety Rating Scale (HAM-A).

METHODS

Study Participants and Methodology

This study recruited 94 women (median age, 41 years; range, 23–58 years). Each VAS-IBS and HAM-A questionnaire was physically distributed with a 7-day interval allocated for completion.

Inclusion/Exclusion Criteria and Limitations

There were no restrictions regarding age and/or department of activity. The main exclusion criteria were a) comorbidities, b) unhealthy habits (smoking and/or drinking), and c) no medication administration. This study is limited to one center, with all the staff invited. However, only 165 participants responded. Consequently, from 165 questionnaires distributed, 66 were excluded because of inconsistencies of answers.

*Department of Biology, Faculty of Biology, "Alexandru Ioan Cuza" University; †Faculty of Medicine, University of Medicine and Pharmacy "Grigore T. Popa"; ‡Department of Exact and Natural Sciences, Institute of Interdisciplinary Research, "Alexandru Ioan Cuza" University, Iasi; §Academy of Romanian Scientists, Bucharest; ||Center of Biomedical Research, Romanian Academy, Iasi; ¶Department of Morphological and Functional Sciences, Faculty of Medicine, "Dunarea de Jos" University, Galati; and #Clinical Hospital of Obstetrics and Gynecology "Cuza Voda," Iasi, Romania.
Send reprint requests to Alin Ciobica, PhD, Faculty of Biology, "Alexandru Ioan Cuza" University, Carol I Ave, no 20A, 700505 Iasi, Romania.
E-mail: alin.ciobica@uaic.ro; Constantin Trus, MD, PhD, Faculty of Medicine, "Dunarea de Jos" University, Domneasca Ave, no 47, 800008 Galati, Romania.
E-mail: dilconstantin@yahoo.com.
Ovidiu-Dumitru Ilie and Alexandra Bolos contributed equally to this work.
Data availability statement: The datasets used and analyzed during the current study is available from the corresponding author on reasonable request.
Copyright © 2021 Wolters Kluwer Health, Inc. All rights reserved.
ISSN: 0022-3018/22/21002–0098
DOI: 10.1097/NMD.0000000000001445

Downloaded from http://journals.lww.com/jonmd by BhDMf5ePHKav1zEoum1tQfN4a+kJLhEZgbsIHo4XMi0hCywCX1AWnYQp/IlQrHD3i3D0OdRyi7TvSFl4Cf3VC1y0abggQZXdgGj2MwlZLeI= on 09/03/2022

TABLE 1. Descriptive Statistics, Cronbach's Alpha, Skewness, Intraclass Correlation Coefficient, and Kruskal-Wallis Test of the VAS-IBS Questionnaire

Parameters	Mean	SE	SD	CI 95%	*p*	Skewness	Alpha-if-Item Deleted	Cronbach's Alpha	Intraclass Correlation Coefficient (Lower and Upper Bound)	Kruskal-Wallis
Physical symptoms										
Abdominal pain	2.6702	0.1915	1.8572	0.3803	0.6870	1.3510	0.579			20–30/30–40 *p* = 0.777<br>20–30/40–50 *p* = 0.303<br>20–30/50–60 *p* = 0.883<br>30–40/40–50 *p* = 0.448<br>30–40/50–60 *p* = 0.952<br>40–50/50–60 *p* = 5.47
Diarrhea	1.5957	0.1213	1.1760	0.2408	0.1253	2.8649	0.591			20–30/30–40 *p* = 0.610<br>20–30/40–50 *p* = 0.831<br>20–30/50–60 *p* = 0.262<br>30–40/40–50 *p* = 0.703<br>30–40/50–60 *p* = 0.111<br>40–50/50–60 *p* = 0.137
Constipation	2.4042	0.2059	1.996	0.4088	0.4125	2.0247	0.602			20–30/30–40 *p* = 0.458<br>20–30/40–50 *p* = 0.086<br>20–30/50–60 *p* = 0.103<br>30–40/40–50 *p* = 0.388<br>30–40/50–60 *p* = 0.330<br>40–50/50–60 *p* = 0.645
Bloating/gases	3	0.2112	2.0478	0.4194	0.1166	1.4047	0.563			20–30/30–40 *p* = 0.248<br>20–30/40–50 *p* = 0.075<br>20–30/50–60 *p* = 0.094<br>30–40/40–50 *p* = 0.464<br>30–40/50–60 *p* = 0.279<br>40–50/50–60 *p* = 0.318
Nausea/vomiting	1.5957	0.1312	1.2727	0.2606	0.0116	3.4262	0.607			20–30/30–40 *p* = 0.026<br>20–30/40–50 *p* = 0.029<br>20–30/50–60 *p* = 0.959<br>30–40/40–50 *p* = 0.633<br>30–40/50–60 *p* = 0.052<br>40–50/50–60 *p* = 0.076
Bristol	3.2659	0.1354	1.3130	0.2689	0.5365	0.1033	0.655			20–30/30–40 *p* = 0.271<br>20–30/40–50 *p* = 0.635<br>20–30/50–60 *p* = 0.500<br>30–40/40–50 *p* = 0.549<br>30–40/50–60 *p* = 0.153<br>40–50/50–60 *p* = 0.357
Mental health										
Emotional status	5.7234	0.2819	2.7333	0.5598	0.2049	0.0002	0.730			20–30/30–40 *p* = 0.968<br>20–30/40–50 *p* = 0.095<br>20–30/50–60 *p* = 0.277<br>30–40/40–50 *p* = 0.103<br>30–40/50–60 *p* = 0.314<br>40–50/50–60 *p* = 0.982
Quality of life	2.9255	0.2145	2.0803	0.4260	0.6164	1.5289	0.572			20–30/30–40 *p* = 0.523<br>20–30/40–50 *p* = 0.790<br>20–30/50–60 *p* = 0.540<br>30–40/40–50 *p* = 0.204<br>30–40/50–60 *p* = 0.249<br>40–50/50–60 *p* = 0.586
Quality of life										
Emergency of defecation	0.3936	0.0506	0.4911	0.1006	0.4326	0.4425	0.629			20–30/30–40 *p* = 0.388<br>20–30/40–50 *p* = 0.705<br>20–30/50–60 *p* = 0.576<br>30–40/40–50 *p* = 0.152<br>30–40/50–60 *p* = 0.187<br>40–50/50–60 *p* = 0.742

(Continued on next page)

© 2021 Wolters Kluwer Health, Inc. All rights reserved.

TABLE 1. *(Continued)*

Parameters	Mean	SE	SD	CI 95%	*p*	Skewness	Alpha-if-Item Deleted	Cronbach's Alpha	Intraclass Correlation Coefficient (Lower and Upper Bound)	Kruskal-Wallis
Incomplete defecation	0.4893	0.0518	0.5025	0.1029	0.4433	0.0432	0.627			20–30/30–40 $p = 0.332$<br>20–30/40–50 $p = 0.935$<br>20–30/50–60 $p = 0.314$<br>30–40/40–50 $p = 0.206$<br>30–40/50–60 $p = 0.066$<br>40–50/50–60 $p = 0.286$
								0.643	0.643 LCL (0.525) LCL (0.742)	

Downloaded from http://journals.lww.com/jonmd by BhDMf5ePHKav1zEoum1tQfN4a+kJLhEZgbsIHo4XMi0hCywCX1AWnYQp/IlQrHD3i3D0OdRyi7TvSFl4Cf3VC1y0abggQZXdgGj2MwlZLeI= on 09/03/2022

Ethical Approval

The design of this study was approved by the ethical committee of the Clinical Hospital of Obstetrics and Gynecology "Cuza Voda" from Iasi (no. 1/232/10/15/2020). It should also be noted that the Helsinki Declaration on Human Rights has been respected, in accordance with the national and European legislation regarding biomedical research. All participants who accepted our invitation individually signed an informed consent for their voluntary participation in this study.

Visual Analog Scale for Irritable Bowel Syndrome

The Romanian version of the VAS-IBS questionnaire is an adaptation based on two studies conducted by Bengtsson et al. (2011, 2007). It was translated from the English version of ROME III (Bengtsson et al., 2013) and further adapted to ROME IV criteria, according to which any functional GI disorders are now listed as disorders of the gut-brain axis (GBA). Schmulson and Drossman (2017) updated the ROME IV criteria by replacing two parameters with another two that are more appropriate: more precisely, "last month" has been replaced with "more than or equal to 1 day/wk in the last 3 months" for pain occurrence, whereas the "abdominal discomfort" criteria can no longer be found because of the dissonances created between languages terminology. However, a new item (Bristol) has been included. The Bristol scale is a useful criterion for delimitating at least 25% of IBS subtypes. Congruent with the aforementioned, the Romanian VAS-IBS version consists of seven VAS scales dedicated to measure the severity of "physical symptoms" (abdominal pain, diarrhea, constipation, bloating/gases, and vomiting/nausea), "mental health" (the overall psychological well-being), and "quality of life" (the influence of GI deficiencies on daily life routine).

Hamilton Anxiety Rating Scale

Since it was introduced back in 1959 by Hamilton, HAM-A has become one of the most widely used tool for assessing the rating of anxiety (Hamilton, 1959). On the other hand, there have been only a few studies identified in the current literature in which the authors used either the Generalized Anxiety Disorder that contains seven items (Barzilay et al., 2020; Islam et al., 2020a, 2020b; Wang and Zhao, 2020) or the Self-Rating Anxiety Scale (Wang and Zhao, 2020) proposed by Zung (1971) that is a questionnaire with 20 items. Most of them focused on specific groups such as students, quarantined people, and health care providers. Therefore, the HAM-A consists of 14 items scored from 0 (not present) to 4 (severe), through which both psychic and somatic anxiety can be defined; depending on the participants' answers, the scores were reported as follows: 14 to 17, mild anxiety; 18 to 24, moderate anxiety; and 25 to 30, severe anxiety.

Statistical Analysis

Cumulative means, standard deviations, and standard errors of the responses were computed for each item of the VAS-ISB questionnaire with a confidence interval (CI) of 95% (Table 1). The HAM-A was classified according to the scores obtained by each individual. Statistical analyses were carried out using IBM SPSS (v. 26.0). The differences between women were investigated using standard statistical analyses (one-way single-factor analysis of variance), followed by the nonparametric Kruskal-Wallis H test. A $p < 0.05$ was regarded as statistically significant.

RESULTS

Considering that IBS is the most common biopsychosocial disorder having a multifactorial pathogenesis (Drossman, 2006), this

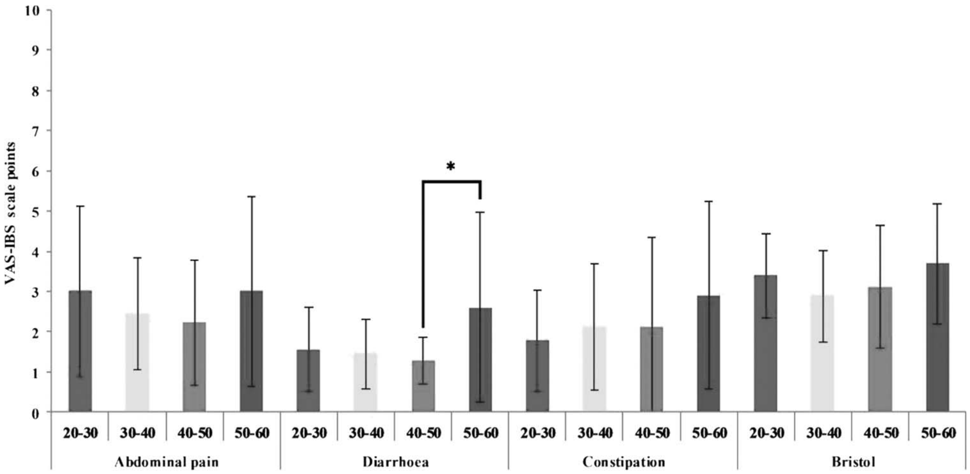

FIGURE 1. VAS-IBS items' evaluation scores of abdominal pain, diarrhea, constipation, and Bristol score in individuals grouped by age (20–30, $n = 18$; 30–40, $n = 23$; 40–50, $n = 43$; and 50–60, $n = 10$). The results are expressed as mean scores of the VAS-IBS items ± SEM; $*p < 0.05$.

 © 2021 Wolters Kluwer Health, Inc. All rights reserved.

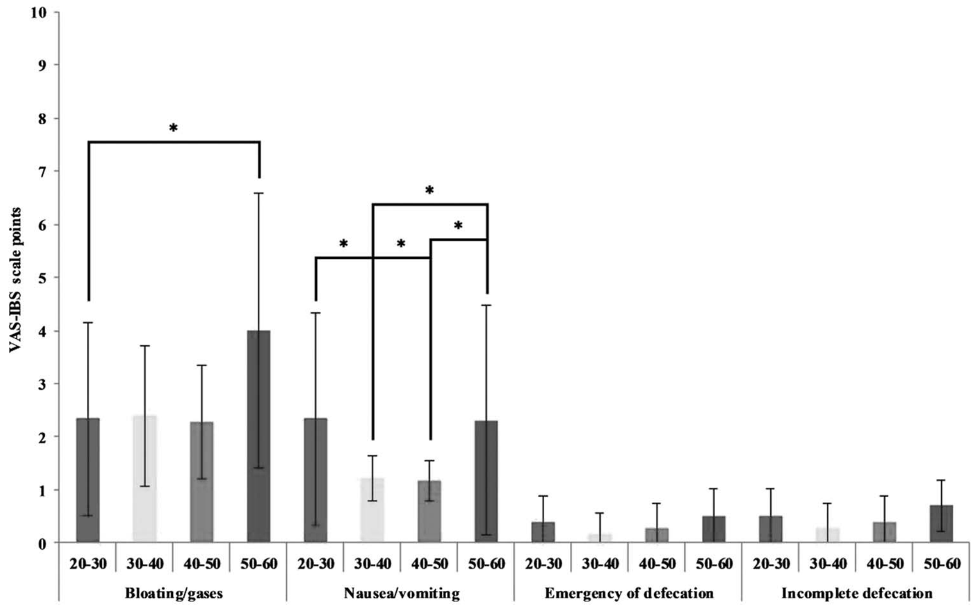

FIGURE 2. VAS-IBS items' evaluation scores of bloating/gases, nausea/vomiting, emergency of defecation, and incomplete defecation occurrence in individuals grouped by age (20–30, $n = 18$; 30–40, $n = 23$; 40–50, $n = 43$; and 50–60, $n = 10$). The results are expressed as mean scores of VAS-IBS items ± SEM; *$p < 0.05$.

Downloaded from http://journals.lww.com/jonmd by BhDMf5ePHKav1zEoum1tQfN4a+kJLhEZgbsIHo4XMi0hCywCX1AWnYQp/IlQrHD3i3D0OdRyi7TvSFl4Cf3VC1y0abggQZXdgGj2MwlZLeI= on 09/03/2022

questionnaire should be regarded as a one-dimensional tool. Therefore, Alpha if Item Deleted was used to assess the homogeneity after item reduction, the Cronbach's alpha for validity (a minimum coefficient of .70 is required, but not higher than .90) (Norman and Cairney, 2015; Polit and Hungler, 1989), whereas intraclass correlation for reproducibility (Yoo et al., 2018) (Table 1).

As can be seen in Figure 1, there are no significant differences between groups in terms of scores obtained on abdominal pain, constipation, and the Bristol scale. On the contrary, the trend is to some extent linear, regardless of age group. However, we observed that the incidence of diarrhea in women older than 50 years is common compared with those in their 40s ($p < 0.05$), which further suggests a high risk for a subtype of IBS.

While analyzing the results, significant differences between groups in terms of bloating/gases and nausea/vomiting have been observed. More precisely, women in their 50s did not experience frequent bloating compared to those older than 20 years old ($p = 0.02$). Furthermore, vomiting was prevalent in the same group in contrast to younger women; 30 years old ($p = 0.02$) and 40 years old ($p = 0.01$). Moreover, a $p < 0.05$ was also noted in the cases of women between 30 ($p = 0.04$) and 40 ($p = 0.02$) compared with women older than 50. However, no significant differences were observed regarding the urge to defecate or perception of incomplete defecation (Fig. 2).

Finally, this questionnaire also includes two scales through which the psychological and mood impact in the context of the perception and changes brought by these GI deficiencies can be assessed (Fig. 3).

Unfortunately, just for the women older than 20, significant differences compared with females older than 50 were noted ($p < 0.05$). As a general conclusion, despite these participants are clinically healthy, indeed a tendency to worry among them exists reflected by the scores obtained; these symptoms have influenced in different ways the development of daily activities in these people.

Concerning the prevalence of anxiety, approximately half of them experienced various forms of anxiety. Thirteen women had mild (13.82) and severe anxiety (13.82%), whereas 12 had moderate anxiety (12.76%). Intriguingly, 56 (59.57%) have not obtained scores greater than 14 in HAM-A considering the course of the current pandemic (Table 2).

DISCUSSION

This study demonstrates that young as well as middle-aged people are most prone to GI deficiencies, an argument supported and

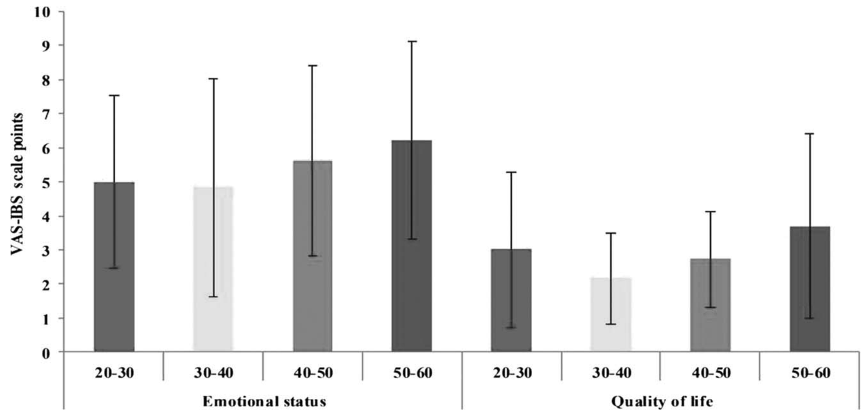

FIGURE 3. VAS-IBS items' evaluation scores of emotional status and quality of life in individuals grouped by age (20–30, $n = 18$; 30–40, $n = 23$; 40–50, $n = 43$; and 50–60, $n = 10$). The results are expressed as mean scores of VAS-IBS items ± SEM ($p > 0.05$).

© 2021 Wolters Kluwer Health, Inc. All rights reserved.

TABLE 2. Prevalence of Anxiety After HAM-A Completion

Anxiety	Frequency	%
Not present	35	37.23%
Minimal	21	22.34%
Mild	13	13.82%
Moderate	12	12.76%
Severe	13	13.82%

Downloaded from http://journals.lww.com/jonmd by BhDMf5ePHKav1zEoum1tQfN4a+kJLhEZgbsIHo4XMi0hCywCX1AWnYQp/IlQrHD3i3D0OdRyi7TvSFl4Cf3VC1y0abggQZXdgGj2MwlZLeI= on 09/03/2022

reflected by the scores obtained in terms of emotional status after VAS-IBS completion.

Although there have been studies identified regarding the incidence of GI deficiencies (*e.g.*, diarrhea), their number is limited. All the more so as only in 21.48% of cases did the authors focus on such disturbances (Wang et al., 2020). It has been however discussed on three distinct occasions the co-occurrence of diarrhea in patients positive for SARS-CoV-2. Cumulatively, from a total of 768 patients included in these studies, only 150 of them reported diarrhea (Effenberger et al., 2020; Jin et al., 2020; Lin et al., 2020).

Consistent with the studies, but also with the hypotheses issued, the presence of SARS-CoV-2 is associated with the host's dysbiosis (Villapol, 2020), which may culminate in death according to a recent systematic review and meta-analysis performed by Tariq et al. (2020). Although diarrhea has gradually become an eligibility criterion, a recent meta-analysis conducted by Parasa et al. (2020) concluded that fever, cough, and respiratory symptoms define the clinical panel.

Based on the aforementioned, we showed that diarrhea, bloating, and nausea are predominant symptoms that could have two plausible explanations: a postinfection reactivity of the organisms for those who had COVID-19, or because of chronic stress that caused a subtype of IBS. Considering that both patients with IBS with constipation and COVID-19 exhibit the same symptoms in terms of GI deficiencies, we also applied HAM-A. In this context, we demonstrated that anxiety is indeed prevalent, but not necessarily among those who have been positive, but rather among the young and the elderly.

To provide a broader perspective, we also identified studies in which the authors describe the reactivity of the immune system through the prism of a postinfection inflammatory cascade (Waltuch et al., 2020; Zhou et al., 2020b), symptoms that could persist from 1 to 2 months or longer after recovery (Carfi et al., 2020; Frija-Masson et al., 2020). Intriguingly, the involvement of this pathogen upon homeostasis is much more hazardous than excepted. It seems that people are at risk of developing reactive arthritis (Ono et al., 2020), the presence of this entity interfering with the diagnostic methodology associated with the Guillain-Barré syndrome (Toscano et al., 2020).

Unfortunately, at the time of writing, there are only two studies in the literature in which the HAM-A scale has been applied. Plunkett et al. (2020) showed that all the restrictions imposed to restrict the spread of the virus had a significant negative impact on the psyche of the participants, a similar pattern being also noted in the nursing staff (Karki et al., 2020).

Despite the fact that new methods of diagnosing this pathogen are being tried, the results are not always as expected. It has been previously demonstrated that polymerase chain reaction–based methods are not as accurate as we thought; the analysis of the rectal swabs is much more reliable. Hence, the following two questions: "If this virus was identified in the stool samples from the first day of infection and can persists up to 14 days, why not pay more attention to this approach?" (Holshue et al., 2020; Wu et al., 2020; Xu et al., 2020; Zheng et al., 2020) and "It is well documented that any individual forms a symbiotic relationship with microorganisms. Is there any chance that the human body will gain resistance or SARS-CoV-2 will become a commensal entity?"

CONCLUSIONS

In the present study, we applied the VAS-IBS and HAM-A questionnaires with the aim of assessing the incidence of GI deficiencies and the prevalence of anxiety among the medical workers within the Clinical Hospital of Obstetrics and Gynecology "Cuza Voda" from Iasi. This study not only brings additional evidence, but through this approach, it was possible to demonstrate that GI deficiencies are frequently encountered in two specific age classes, this having as main pathogenic substrate the elevated stress caused by the current pandemic affecting the entire world. More than that, the existence of a condition, not necessarily an infection with the novel SARS-CoV-2, is also supported by the scores obtained on anxiety after the application of HAM-A.

ACKNOWLEDGMENT

We would like to give special thanks to Professor Marietta Bengtsson from the University of Malmo, Sweden, for granting the permission to use the VAS-IBS scale.

Author contributions: Conceptualization, data curation, investigation, formal analysis, methodology, and writing (original draft): O.-D.I., I.-B.N., R.-O.C., and I.-M.B.; conceptualization, methodology, and writing (review and editing): A.C., C.T., and B.D.; supervision, validation, and project administration: A.C., C.T., and B.D. All authors have read and agreed to the published version of the manuscript.

DISCLOSURE

A. Ciobica was supported by a research grant for Young Teams offered by UEFISCDI Romania, no. PN-III-P1–1.1-TE-2016-1210, contract no. 58 from 02/05/2018, called "Complex study regarding the interactions between oxidative stress, inflammation and neurological manifestations in the pathophysiology of irritable bowel syndrome (animal models and human patients)." In addition, R.-O. Cojocariu was funded by the European Social Fund, through Operational Programme Human Capital 2014–2020, project number POCU/380/6/13/123623, project title "PhD Students and Postdoctoral Researchers Prepared for the Labour Market!"

The authors declare no conflict of interest.

REFERENCES

Barnes CO, West AP Jr., Huey-Tubman KE, Hoffmann MAG, Sharaf NG, Hoffman PR, Koranda N, Gristick HB, Gaebler C, Muecksch F, Lorenzi JCC, Finkin S, Hägglöf T, Hurley A, Millard KG, Weisblum Y, Schmidt F, Hatziioannou T, Bieniasz PD, Caskey M, Robbiani DF, Nussenzweig MC, Bjorkman PJ (2020) Structures of human antibodies bound to SARS-CoV-2 spike reveal common epitopes and recurrent features of antibodies. *Cell*. 182:828–842.e16.

Barzilay R, Moore TM, Greenberg DM, DiDomenico GE, Brown LA, White LK, Gur RC, Gur RE (2020) Resilience, COVID-19–related stress, anxiety and depression during the pandemic in a large population enriched for healthcare providers. *Transl Psychiatry.* 10:291.

Bengtsson M, Hammar O, Mandl T, Ohlsson B (2011) Evaluation of gastrointestinal symptoms in different patient groups using the Visual Analogue Scale for Irritable Bowel Syndrome (VAS-IBS). *BMC Gastroenterol*. 11:122.

Bengtsson M, Ohlsson B, Ulander K (2007) Development and psychometric testing of the Visual Analogue Scale for Irritable Bowel Syndrome (VAS-IBS). *BMC Gastroenterol.* 7:16.

Bengtsson M, Persson J, Sjölund K, Ohlsson B (2013) Further validation of the Visual Analogue Scale for Irritable Bowel Syndrome after use in clinical practice. *Gastroenterol Nurs*. 36:188–198.

Carfi A, Bernabei R, Landi F, Gemelli Against COVID-19 Post-Acute Care Study Group (2020) Persistent symptoms in patients after acute COVID-19. *JAMA*. 324:603–605.

Chatterjee K, Chauhan VS (2020) Epidemics, quarantine and mental health. *Med J Armed Forces India*. 76:125–127.

Demongeot J, Flet-Berliac Y, Seligmann H (2020) Temperature decreases spread parameters of the new COVID-19 case dynamics. *Biology (Basel)*. 9:94.

© 2021 Wolters Kluwer Health, Inc. All rights reserved.

Downloaded from http://journals.lww.com/jonmd by BhDMf5ePHKav1zEoum1tQfN4a+kJLhEZgbsIHo4XMi0hCywCX1AWnYQp/IlQrHD3i3D0OdRyi7TvSFl4Cf3VC1y0abggQZXdgGj2MwlZLeI= on 09/03/2022

Drossman DA (2006) The functional gastrointestinal disorders and the Rome III process. *Gastroenterology.* 130:1377–1390.

Effenberger M, Grabherr F, Mayr L, Schwaerzler J, Nairz M, Seifert M, Hilbe R, Seiwald S, Scholl-Buergi S, Fritsche G, Bellmann-Weiler R, Weiss G, Müller T, Adolph TE, Tilg H (2020) Faecal calprotectin indicates intestinal inflammation in COVID-19. *Gut*. 69:1543–1544.

Frija-Masson J, Debray M-P, Gilbert M, Lescure F-X, Travert F, Borie R, Khalil A, Crestani B, d'Ortho M-P, Bancal C (2020) Functional characteristics of patients with SARS-CoV-2 pneumonia at 30 days post-infection. *Eur Respir J.* 56:2001754.

Hamilton M (1959) The assessment of anxiety states by rating. *Br J Med Psychol.* 32:50–55.

Hamming I, Timens W, Bulthuis ML, Lely AT, Navis G, van Goor H (2004) Tissue distribution of ACE2 protein, the functional receptor for SARS coronavirus. A first step in understanding SARS pathogenesis. *J Pathol*. 203:631–637.

Holshue ML, DeBolt C, Lindquist S, Lofy KH, Wiesman J, Bruce H, Spitters C, Ericson K, Wilkerson S, Tural A, Diaz G, Cohn A, Fox L, Patel A, Gerber SI, Kim L, Tong S, Lu X, Lindstrom S, Pallansch MA, Weldon WC, Biggs HM, Uyeki TM, Pillai SK (2020) First case of 2019 novel coronavirus in the United States. *N Engl J Med*. 382:929–936.

Islam MA, Barna SD, Raihan H, Khan MNA, Hossain MT (2020a) Depression and anxiety among university students during the COVID-19 pandemic in Bangladesh: A web-based cross-sectional survey. *PLoS One*. 15:e0238162.

Islam MS, Ferdous MZ, Potenza MN (2020b) Panic and generalized anxiety during the COVID-19 pandemic among Bangladeshi people: An online pilot survey early in the outbreak. *J Affect Disord*. 276:30–37.

Jin X, Lian JS, Hu JH, Gao J, Zheng L, Zhang YM, Hao SR, Jia HY, Cai H, Zhang XL, Yu GD, Xu KJ, Wang XY, Gu JQ, Zhang SY, Ye CY, Jin CL, Lu YF, Yu X, Yu XP, Huang JR, Xu KL, Ni Q, Yu CB, Zhu B, Li YT, Liu J, Zhao H, Zhang X, Yu L, Guo YZ, Su JW, Tao JJ, Lang GJ, Wu XX, Wu WR, Qv TT, Xiang DR, Yi P, Shi D, Chen Y, Ren Y, Qiu YQ, Li LJ, Sheng J, Yang Y (2020) Epidemiological, clinical and virological characteristics of 74 cases of coronavirus-infected disease 2019 (COVID-19) with gastrointestinal symptoms. *Gut*. 69:1002–1009.

Karki P, Katwal GWBJ, Chandra A, Chandra A (2020) Prevalence and measurement of anxiety and depression in working nurses in Nepal: A reflection of professional hazard in low income nation. *Research Square*. doi: 10.21203/rs.2.16888/v2.

Lamers MM, Beumer J, van der Vaart J, Knoops K, Puschhof J, Breugem TI, Ravelli RBG, Paul van Schayck J, Mykytyn AZ, Duimel HQ, van Donselaar E, Riesebosch S, HJH Kuijpers, Schippers D, van de Wetering WJ, de Graaf M, Koopmans M, Cuppen E, Peters PJ, Haagmans BL, Clevers H (2020) SARS-CoV-2 productively infects human gut enterocytes. *Science*. 369:50–54.

Lin L, Jiang X, Zhang Z, Huang S, Zhang Z, Fang Z, Gu Z, Gao L, Shi H, Mai L, Liu Y, Lin X, Lai R, Yan Z, Li X, Shan H (2020) Gastrointestinal symptoms of 95 cases with SARS-CoV-2 infection. *Gut*. 69:997–1001.

Liu T, Luo S, Libby P, Shi G-P (2020) Cathepsin L-selective inhibitors: A potentially promising treatment for COVID-19 patients. *Pharmacol Ther.* 213:107587.

Norman G, Cairney J (2015) *Health measurement scales: A practical guide to their development and use*. New York: Oxford University Press Inc.

Ono K, Kishimoto M, Shimasaki T, Uchida H, Kurai D, Deshpande GA, Komagata Y, Kaname S (2020) Reactive arthritis after COVID-19 infection. *RMD Open*. 6:e001350.

Parasa S, Desai M, Thoguluva Chandrasekar V, Patel HK, Kennedy KF, Roesch T, Spadaccini M, Colombo M, Gabbiadini R, Artifon ELA, Repici A, Sharma P (2020) Prevalence of gastrointestinal symptoms and fecal viral shedding in patients with coronavirus disease 2019: A systematic review and meta-analysis. *JAMA Netw Open*. 3:e2011335.

Petropoulos F, Makridakis S (2020) Forecasting the novel coronavirus COVID-19. *PLoS One*. 15:e0231236.

Petrosillo N, Viceconte G, Ergonul O, Ippolito G, Petersen E (2020) COVID-19, SARS and MERS: Are they closely related? *Clin Microbiol Infect*. 26:729–734.

Plunkett R, Costello S, McGovern M, McDonald C, Hallahan B (2020) Impact of the COVID-19 pandemic on patients with pre-existing anxiety disorders attending secondary care. *Ir J Psychol Med*. 38:123–131.

Polit D, Hungler BP (1989) *Essentials of nursing research: Methods, appraisal, and utilization*. Philadelphia, PA: Lippincott Williams and Wilkins.

Qin HY, Cheng CW, Tang XD, Bian ZX (2014) Impact of psychological stress on irritable bowel syndrome. *World J Gastroenterol*. 20:14126–14131.

Rothan HA, Byrareddy SN (2020) The epidemiology and pathogenesis of coronavirus disease (COVID-19) outbreak. *J Autoimmun*. 109:102433.

Schmulson MJ, Drossman DA (2017) What is new in Rome IV. *J Neurogastroenterol Motil*. 23:151–163.

Tariq R, Saha S, Furqan F, Hassett L, Pardi D, Khanna S (2020) Prevalence and mortality of COVID-19 patients with gastrointestinal symptoms: A systematic review and meta-analysis. *Mayo Clin Proc*. 95:1632–1648.

Taubenberger JK, Morens DM (2006) 1918 influenza: The mother of all pandemics. *Emerg Infect Dis*. 12:15–22.

Toscano G, Palmerini F, Ravaglia S, Ruiz L, Invernizzi P, Cuzzoni MG, Franciotta D, Baldanti F, Daturi R, Postorino P, Cavallini A, Micieli G (2020) Guillain-Barré syndrome associated with SARS-CoV-2. *N Engl J Med*. 382:2574–2576.

Villapol S (2020) Gastrointestinal symptoms associated with COVID-19: Impact on the gut microbiome. *Transl Res*. 226:57–69.

Waltuch T, Gill P, Zinns LE, Whitney R, Tokarski J, Tsung JW, Sanders JE (2020) Features of COVID-19 post-infectious cytokine release syndrome in children presenting to the emergency department. *Am J Emerg Med*. 38:2246.e3–2246.e6.

Wang C, Zhao H (2020) The impact of COVID-19 on anxiety in Chinese university students. *Front Psychol*. 11:1168.

Wang F, Zheng S, Zheng C, Sun X (2020) Attaching clinical significance to COVID-19–associated diarrhea. *Life Sci*. 260:118312.

Wu Y, Guo C, Tang L, Hong Z, Zhou J, Dong X, Yin H, Xiao Q, Tang Y, Qu X, Kuang L, Fang X, Mishra N, Lu J, Shan H, Jiang G, Huang X (2020) Prolonged presence of SARS-CoV-2 viral RNA in faecal samples. *Lancet Gastroenterol Hepatol*. 5:434–435.

Xu XW, Wu XX, Jiang XG, Xu KJ, Ying LJ, Ma CL, Li SB, Wang HY, Zhang S, Gao HN, Sheng JF, Cai HL, Qiu YQ, Li LJ (2020) Clinical findings in a group of patients infected with the 2019 novel coronavirus (SARS-Cov-2) outside of Wuhan, China: Retrospective case series. *BMJ.* 368:m606.

Yoo HY, Park B, Joo J, Kim J-S, Lee Y, Lim MC, Sohn DK, Kang S-B, Park KJ (2018) Validation of the Korean version of Visual Analogue Scale for Irritable Bowel Syndrome questionnaire for assessment of defecation pattern changes. *Ann Surg Treat Res*. 94:254–261.

Zang R, Gomez Castro MF, McCune BT, Zeng Q, Rothlauf PW, Sonnek NM, Liu Z, Brulois KF, Wang X, Greenberg HB, Diamond MS, Ciorba MA, Whelan SPJ, Ding S (2020) TMPRSS2 and TMPRSS4 promote SARS-CoV-2 infection of human small intestinal enterocytes. *Sci Immunol*. 5:eabc3582.

Zheng S, Fan J, Yu F, Feng B, Lou B, Zou Q, Xie G, Lin S, Wang R, Yang X, Chen W, Wang Q, Zhang D, Liu Y, Gong R, Ma Z, Lu S, Xiao Y, Gu Y, Zhang J, Yao H, Xu K, Lu X, Wei G, Zhou J, Fang Q, Cai H, Qiu Y, Sheng J, Chen Y, Liang T (2020) Viral load dynamics and disease severity in patients infected with SARS-CoV-2 in Zhejiang province, China, January–March 2020: Retrospective cohort study. *BMJ.* 369:m1443.

Zhou J, Li C, Liu X, Chiu MC, Zhao X, Wang D, Wei Y, Lee A, Zhang AJ, Chu H, Cai JP, Yip CC, Chan IH, Wong KK, Tsang OT, Chan KH, Chan JF, To KK, Chen H, Yuen KY (2020a) Infection of bat and human intestinal organoids by SARS-CoV-2. *Nat Med*. 26:1077–1083.

Zhou X, Zhou J, Zhao J (2020b) Recurrent pneumonia in a patient with new coronavirus infection after discharge from hospital for insufficient antibody production: A case report. *BMC Infect Dis*. 20:500.

Zung WWK (1971) A rating instrument for anxiety disorders. *Psychosomatics*. 12: 371–379.

© 2021 Wolters Kluwer Health, Inc. All rights reserved.

View publication stats
