# Supplementary material for: Mental health status and related factors influencing healthcare workers during the COVID-19 pandemic: A systematic review and meta-analysis
Source: PLoS One. 2024 Jan 19;19(1):e0289454. doi: 10.1371/journal.pone.0289454 (PMC10798549; doi:10.1371/journal.pone.0289454)
Supplement: S1 Data — (ZIP) [file pone.0289454.s011.zip › literatures/57.pdf]

Internal Medicine
The Japanese Society of Internal Medicine
doi: 10.2169/internalmedicine.7207-21
Intern Med 60: 2771-2776, 2021
http://internmed.jp

【 ORIGINAL ARTICLE 】

Psychological Effects on Healthcare Workers during the COVID-19 Outbreak: A Single-center Study at a Tertiary Hospital in Tokyo, Japan

Motonao Ishikawa[1], Tomoko Ogasawara[1], Kenichiro Takahashi[2], Takuya Ono[1], Kazuhiro Matsui[1], Shoko Marshall[1], Ayana Sakurai[1], Yoshikazu Aoka[3], Tempei Otsubo[4], Yasuto Sato[5], Hiroshi Sakura[1] and Yasuko Uchigata[6]

Abstract:

Objective The present study analyzed the psychological status of healthcare workers in Japan and the influencing factors during the 2019 coronavirus disease pandemic.

Methods An online survey was conducted from July 22 to August 21, 2020. A total of 328 of the 1,029 medical staff members in our university hospital participated in the study. Their mental health was assessed using the 12-item General Health Questionnaire. A multivariate regression analysis was performed to identify the factors associated with the mental health outcomes.

Results Of the respondents, 78.0% reported psychological distress. Overall, we found that women, non-physicians, those who lived alone, and younger respondents had significantly greater psychological distress than their counterparts. The multivariate regression analysis showed that four factors were extracted as independent 12-item General Health Questionnaire-related factors: the lack of a sense of mission as a medical professional, the burden of the change in the quality of work, the lack of understanding about virus infectivity, and a strong sense of duty.

Conclusion In summary, we found a high prevalence of psychological distress among healthcare workers during the 2019 coronavirus disease outbreak in Japan. Independent risk factors for psychological distress were the burden of the change in the quality of work, the lack of understanding about virus infectivity, a sense of responsibility, and the lack of a strong motivation and drive to help.

Key words: COVID-19; healthcare workers, psychological distress, 12-item General Health Questionnaire, health survey

(Intern Med 60: 2771-2776, 2021)
(DOI: 10.2169/internalmedicine.7207-21)

Introduction

Since its initial outbreak in China in December 2019, coronavirus disease 2019 (COVID-19) has spread rapidly across the globe. Healthcare workers (HCWs) are particularly exposed to the threat of transmission because of their frontline work with patients with high viral loads. Severe stress, a high emotional load, long working hours, concerns of being infected or infecting their family, a lack of adequate support in the working environment, and a lack of adequate personal protective equipment (PPE) can also affect HCWs' mental health. Reports on the COVID-19 pandemic from China, the USA, and Europe have already found

[1]Department of Medicine, Tokyo Women's Medical University, Medical Center East, Japan, [2]Department of Pediatrics, Tokyo Women's Medical University, Medical Center East, Japan, [3]General Medical Department, Tokyo Women's Medical University, Medical Center East, Japan, [4]Department of Psychiatry, Tokyo Women's Medical University, Medical Center East, Japan, [5]Department of Public Health, School of Medicine, Tokyo Women's Medical University, Japan and [6]Director, Tokyo Women's Medical University, Medical Center East, Japan
Received: January 31, 2021; Accepted: April 22, 2021; Advance Publication by J-STAGE: July 3, 2021
Correspondence to Dr. Motonao Ishikawa, mishikawa-tky@umin.ac.jp

that HCWs have significant levels of self-reported anxiety, depression, and even symptoms of post-traumatic stress disorder (1-7).

Our hospital is a tertiary hospital with 450 beds in Tokyo, Japan. In April 2020, as the number of patients increased, a dedicated 20-bed COVID-19 ward was created, and staff members were recruited from every department. On April 17, the hospital confirmed the infection of five staff members, which was reported to the media as a nosocomial infection. Those who had close contact with positive staff members and patients were ordered to quarantine at home, and the burden on the remaining staff sharply increased. Therefore, when facing this critical situation, HCWs on the frontline who are directly involved in the diagnosis, treatment, and care of patients with COVID-19 are at risk of developing psychological distress and other mental health symptoms.

The psychological health status of HCWs has been examined in previous studies; however, it has not been adequately explored among HCWs in Japan. Understanding the psychological health status of HCWs and the influencing factors is particularly important as hospitals develop and implement support measures for them.

The present study therefore analyzed the psychological status of HCWs and the influencing factors in Japan during the COVID-19 pandemic.

Materials and Methods

Sample and design

All 1,029 full-time employees of the hospital (277 men, 752 women) were included in the study. This study was a cross-sectional online study that used Google Forms (Google LLC, Mountain View, USA). Invitations to participate in the study were sent via email and posted on a bulletin board. The survey began with information about providing informed consent to take part in the study. The participants were required to read the informed consent text and choose the "agree" option to start completing the questionnaire, otherwise it could not be accessed. The participants' anonymity was protected. The submission of the e-questionnaire was allowed only if all questions were answered. The e-questionnaire link was restricted so that an individual's internet protocol address could be used only once in order to avoid the questionnaire being repeated. This survey was conducted from July 22 to August 21, 2020, when the first wave of the pandemic was ending and the second wave was about to begin.

Instrument

The online survey consisted of items concerning participants' social demographic information, occupation, and work history; the 12-item General Health Questionnaire (GHQ-12); and COVID-19-related questions. The participants' mental health was measured using the GHQ-12 (8), which is a self-assessment screening tool. The threshold value was derived by scoring the "not at all" and "no more than usual" responses as 0 and the "rather more than usual" and "much more than usual" responses as 1. These were summed to produce a total score (range 0-12). The cut-off for the threshold value was a score of 4 or more. The validity and reliability of the Japanese version have been confirmed (9-11).

The COVID-19-related questions were as follows: anxiety about being infected; anxiety about infecting family members; anxiety about being infected when commuting; lack of knowledge about infectiousness and virulence; lack of knowledge about prevention and protection; feeling of being protected by the national and local government; feeling of being protected by the hospital (including taking all reasonable precautions to prevent illness, providing care for those who do become ill, reducing malpractice threats against those working in high-risk emergency situations, providing reliable compensation for the families of those who die while fulfilling this duty, and relieving the hospital workers of their duties, etc.); anxiety about compensation; the burden of the increasing quantity of work; the burden of the change in the quality of work; the feeling of being avoided by others; the feeling of having no choice but to work due to a sense of obligation; and the burden of childcare, including the lack of a nursery (only for those with children). These questions were essential items that had been identified in previous studies of the 2009 H1N1 pandemic (12). The respondents used a 7-point Likert scale (1="strongly disagree," 2="disagree," 3="somewhat disagree," 4="neither agree or disagree," 5="somewhat agree," 6="agree," and 7="strongly agree") to indicate how they felt about the 18 items. These questions were asked in Japanese; English versions are provided in Supplementary material 1.

Statistical analyses

Gender, age, years of experience, marital status, number of family members living together, and occupation were all treated as categorical variables and were included as dummy variables in the multivariate analysis. First, the relationship between the gender, age, years of experience, marital status, number of family members living together, occupation, and the GHQ-12 score was examined using a *t*-test. Second, a multiple regression analysis was performed with the above-mentioned factors and COVID-19-related questions as the explanatory variables, and the GHQ-12 score was used as the objective variable. The explanatory variables to be included in the model were selected via the following procedure: First, a simple regression analysis was performed that used each question related to gender, age, years of clinical experience, marital status, number of family members living together, and occupation as well as the COVID-19-related questions as the explanatory variables. The variables were selected by omitting both the explanatory variables that were not related to the objective variable and the questions that overlapped with the depressed state as represented by the

Table 1. Basic Characteristics of the Health Care Workers and Their Level of Psychological Distress.

Variable	n (%)
Gender	
Female	237 (72.2)
Male	91 (27.8)
Age (Years)	
20-29	94 (28.6)
30-39	101 (30.8)
40-49	66 (20.1)
50-59	43 (13.1)
Over 60	24 (7.4)
Years worked	
0-3	82 (25.0)
4-6	40 (12.2)
7-9	39 (11.9)
10-15	51 (15.5)
Over 16	116 (35.4)
Marital status	
Single	171 (52.1)
Married	147 (44.8)
Other	10 (3.1)
Living alone	
Yes	114 (34.8)
No	214 (65.2)
Job title	
Nurse	125 (34.9)
Physician	101 (30.8)
Clerical staff	30 (9.2)
Pharmacist	16 (4.9)
Laboratory technician	15 (4.6)
Radiological technician	11 (3.4)
Other	30 (9.2)
Days working with PPE	
0	140 (42.7)
1-10	91 (27.7)
11-20	22 (6.7)
Over 21	75 (22.9)
Number of COVID-19 patients involved	
0 (No suspicious cases)	69 (21.0)
0 (Suspicious cases only)	118 (36.0)
1-10	80 (24.4)
11-20	11 (3.4)
Over 21	50 (15.2)
GHQ-12 score	
≥4	256 (78.0)
<3	72 (22.0)

PPE: personal protective equipment, COVID-19: coronavirus disease 2019, GHQ-12: 12-item General Health Questionnaire

GHQ-12 score, such as anxiety and alcohol intake, from the multiple regression model.

The significance level was set at 5%. The JMP Pro Version 15 software program (SAS Institute, Cary, USA) was used for the statistical analyses.

Results

Socio-demographic variables

Three hundred and twenty-eight responses were received from the HCWs. The response rate was 31.9%. There was no significant difference in the response rate between men and women. The basic characteristics of the participants are shown in Table 1. The largest age group was those in their 30s (30.8%). Most of the participants were women (72.2%). Eighty-two (25.0%) participants had engaged in clinical work for less than 3 years. Regarding the marital status, 52.1% were single, 44.8% were married, and 3.1% responded "other." In terms of their profession, 125 (34.9%) participants were nurses, 101 (30.8%) were physicians, 30 (9.2%) were clerical staff, 16 (4.9%) were pharmacists, 15 (4.6%) were laboratory technicians, and 30 (9.2%) responded "other." Seventy-five (22.9%) had used PPE for more than 21 days, and 50 (15.2%) had been involved with more than 21 patients with COVID-19 during their work.

Psychological variables

The mean GHQ-12 score was 6.28 (95% confidence interval: 5.94-6.62). Of the 328 HCWs, 256 (78.0%) were identified as experiencing psychological distress. Regarding the responses to each of the GHQ-12 questions, it is worth noting that 120 participants (36.6%) had sleeplessness, and 292 (88.7%) were stressed.

Table 2 shows the results of the GHQ-12, which was used to explore differences in the frequency of psychological distress. The mean GHQ-12 score was higher for women (6.51 ±0.20) than for men (5.67±0.32) and higher in the youngest age group (20-29 years: 7.02±0.32) than in the older age groups (50-59 years: 5.16±0.0.47, ≥60 years: 5.58±0.63) as well as for single residents (6.80±0.29) compared to non-single residents (6.00±0.21). Pharmacists had the highest score (7.19±0.77), and nurses had the second-highest score (7.06±0.27), whereas physicians had the lowest score (5.28± 0.30). No significant differences were noted based on marital status.

The amount of work increased for 216 people (65.8%), and the quality of work changed for 231 people (70.4%). One hundred and forty-one people (43.0%) agreed with the item "I felt highly motivated and a strong drive to help. I found the work very rewarding," while 108 people (32.9%) agreed with the item "I felt a sense of responsibility. I felt it was more my duty than something that I chose to do"(Figure). Among the COVID-19-related questions, the strong motivation to help, time spent with family, and time for hobbies showed a negative correlation with the GHQ-12 score. The percentage of people who felt protected by the government or hospital was very low and did not correlate with the GHQ-12 score.

Table 3 shows the results of the multivariate regression analyses that explored the effects of the socio-demographic

Table 2. The Score of the 12-item General Health Questionnaire According to the Health Care Workers' Demographic Characteristics.

Variable	GHQ-12 score (mean±standard deviation)	p
Gender		
Female	6.51±0.20	0.029
Male	5.67±0.32	
Age (Years)		
20-29	7.02±0.32	0.017
30-39	6.24±0.31	
40-49	6.27±0.38	
50-59	5.16±0.47	
Over 60	5.58±0.63	
Years of clinical work		
0-3	6.91±0.35	0.190
4-6	6.60±0.49	
7-9	6.00±0.50	
10-15	6.16±0.44	
Over 16	5.87±0.29	
Marital status		
Single	6.57±0.24	0.210
Married	5.95±0.26	
Other	6.10±0.99	
Living alone		
Yes	6.80±0.29	0.029
No	6.00±0.21	
Job title		
Nurse	7.06±0.27	0.001
Physician	5.28±0.30	
Clerical staff	6.07±0.56	
Pharmacist	7.19±0.77	
Laboratory technician	7.00±0.79	
Radiological technician	6.64±0.92	
Other	5.63±0.56	
Days working with PPE		
0	6.38±0.27	0.532
1-10	5.97±0.33	
11-20	5.86±0.67	
Over 21	6.60±0.36	
Number of COVID-19 patients involved		
0 (No suspicious cases)	5.72±0.38	0.218
0 (Suspicious cases only)	6.36±0.29	
1-10	6.09±0.35	
11-20	6.91±0.94	
Over 21	7.02±0.44	

GHQ-12: 12-item General Health Questionnaire, PPE: personal protective equipment, COVID-19: coronavirus disease 2019

characteristics and COVID-19-related questions on the GHQ-12 scores. A multiple regression analysis of the COVID-19-related questions that excluded anxiety, the amount of alcohol consumed, the amount of smoking, time spent on hobbies, and other questions that may result from mental ill health showed that "a strong motivation and the drive to help" has a negative contribution, while "the burden of the change in the quality of work," "a sense of responsibility," and "lack of understanding about virus infectivity" had a positive contribution. A significant regression equation was found ($F(10, 317)=13.260$, $p<0.001$), with an adjusted R-square of 0.273.

Discussion

The present findings revealed that 78.0% of the HCWs experienced psychological distress, as evidenced by their GHQ-12 scores. The prevalence of psychological distress among the HCWs in this study was much higher than the rate of 54.7-68.8% (13, 14) reported for HCWs in Japan before the COVID-19 outbreak. However, studies in other countries during the COVID-19 pandemic have reported a lower prevalence of psychological distress as measured by GHQ-12 scores, such as 25.1% in China (7). Reports from Japan show a tendency toward a high GHQ-12 score. Therefore, cultural differences may exist.

Consistent with previous research, women, younger individuals, and single residents were significant predictors of general psychiatric disorders (15, 16). Younger age groups are significantly more likely to develop psychiatric disorders than older ones, perhaps because younger people's economic and social lives are more strongly disrupted by a public health crisis (17).

Contrary to expectations, the number of COVID-19 patients whom the HCWs were in contact with and the number of days of wearing PPE were not correlated with the GHQ-12 score. Since this study was conducted three months after the nosocomial outbreak of COVID-19 in our hospital, this finding may have been due to the fact that all of the staff in our hospital were facing unprecedented amounts of COVID-19-related psychological stress across professional and personal domains.

Many respondents were worried about being infected or infecting others. To avoid potential infection, most of the HCWs were shunned by their families, friends, and colleagues. Thus, they had to deal not only with the COVID-19 pandemic but also their concern for their family and stigmatization by the public. This may also have led to psychological distress.

During the H1N1 pandemic in Japan, 94.1% of respondents answered that the protection by the national and local government was weak; 79.7% answered that the protection by the hospital was weak. The workers that were more hesitant about working were anxious about being infected and wanted compensation in case they were infected (12). Similar results were shown in this study. A systematic review indicated that trust encourages social interaction and cooperation among health professionals. Trust has been shown to help improve retention, motivation, performance, and quality of care (18). During a pandemic, feeling exhausted and isolated and worrying about becoming infected is, to some degree, inevitable. The government and hospital managers should develop plans to compensate and treat workers that become infected and to help workers meet their obligations.

Intern Med 60: 2771-2776, 2021 DOI: 10.2169/internalmedicine.7207-21

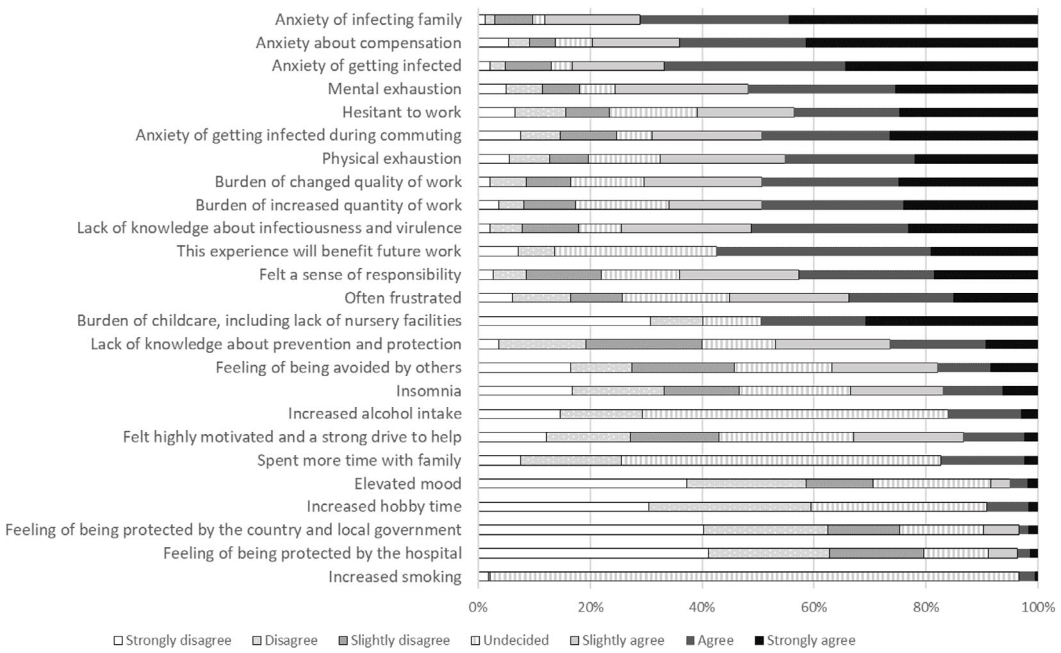

Figure. **The proportion of healthcare workers' responses to the COVID-19-related questions.**

Table 3. **Weighted Ordinary Least Squares Models That Predict the Severity of the 12-item General Health Questionnaire Score.**

Variable	Unstandardized coefficients	Standardized coefficients	Standard errors	*t* statistics	p values	95% confidence intervals (lower and upper bounds)	
Strong motivation	-0.556	-0.286	0.094	-5.91	<0.0001	-0.741	-0.371
Burden of the increase in the quantity of work	0.484	0.248	0.107	4.54	<0.0001	0.274	0.693
Lack of knowledge about infectiosity and virulence	0.323	0.163	0.108	2.98	0.0031	0.109	0.536
Feeling a sense of responsibility	0.287	0.148	0.098	2.94	0.0035	0.095	0.479
Living alone (Ref.=no)	0.308	0.094	0.166	1.86	0.0645	-0.019	0.634
Job title (Ref.=physicians)	0.580	0.086	0.339	1.71	0.0877	-0.086	1.248
Years of clinical work (Ref.=0-3)							
4-6	0.286	0.004	0.523	0.05	0.956	-1.001	1.058
7-9	-0.733	-0.113	0.609	-1.20	0.229	-1.930	0.465
10-15	0.431	0.069	0.577	0.75	0.456	-0.705	1.567
Over 16	-0.11	-0.017	0.445	-0.24	0.809	-1.006	0.786

A significant regression equation was found (F (10, 317)=13.260, p<0.001), with an R^2 of 0.273.

This will also increase the feeling of being protected by the hospital and the various levels of government.

A multiple regression analysis showed that, after controlling for confounders, four factors were extracted as independent GHQ-12-related factors: the lack of a strong motivation and drive to help, the burden of the change in the quality of work, the lack of understanding about virus infectivity, and the sense of responsibility. The most significant predictor was the lack of motivation and drive to help as a healthcare provider, which suggests that personal characteristics should be taken into account when considering psychological distress. Some of the HCWs volunteered to work on the frontline, but the majority were compelled to do so. The current study did not investigate whether their position was voluntary or not, but it is likely that working against one's will could lead to psychological distress. We believe that having a strong motivation to work, acquiring accurate knowledge about viruses, and voluntarily working on the frontline are factors that prevent mental problems.

As the COVID-19 outbreak continues to spread, our findings will help with the early detection of psychological distress and provide important guidance for the development of psychological support strategies for Japan and other affected areas.

Several limitations associated with the present study warrant mention. First, the GHQ-12 is a screening tool, and although its score correlates strongly with the presence of mental illness as well as a future clinical diagnosis of a psy-

chiatric disorder, and a high score threshold was applied (four or more) for the analysis of the prevalence, it is not a clinical assessment. This limitation means that the finding that a notable proportion (78%) of medical staff had clinically significant scores should be interpreted with caution and does not mean that they require treatment for a mental illness. Second, this survey did not cover non-permanent staff, and the sample size was relatively small, with a relatively low response rate (31.9%); this might have added to the possibility of a response bias. Third, we did not measure the psychological impact before the pandemic. Therefore, we cannot exclude effects caused by non-pandemic factors. Finally, as this study was conducted in a single institution, the findings may not be generalizable to other regions. Further longitudinal studies are needed to investigate the predictors of psychological distress among HCWs.

Conclusion

In summary, we found a high prevalence of psychological distress in HCWs during the COVID-19 outbreak in Japan. The independent risk factors were the burden of the change in the quality of work, the lack of understanding about virus infectivity, the sense of responsibility, and the lack of a strong motivation and drive to help.

This study was performed in accordance with the Declaration of Helsinki and was approved by the ethics committee of Tokyo Women's Medical University. All persons gave their informed consent prior to their inclusion in the study.

The authors state that they have no Conflict of Interest (COI).

References

1. Luceño-Moreno L, Talavera-Velasco B, García-Albuerne Y, Martín-García J. Symptoms of posttraumatic stress, anxiety, depression, levels of resilience and burnout in Spanish health personnel during the COVID-19 pandemic. Int J Environ Res Public Health **17**: 5514, 2020.
2. Wang C, Pan R, Wan X, et al. Immediate psychological responses and associated factors during the initial stage of the 2019 coronavirus disease (COVID-19) epidemic among the general population in China. Int J Environ Res Public Health **17**: 1729, 2020.
3. Wańkowicz P, Szylińska A, Rotter I. Assessment of mental health factors among health professionals depending on their contact with COVID-19 patients. Int J Environ Res Public Health **17**: 5849, 2020.
4. Lai J, Ma S, Wang Y, et al. Factors associated with mental health outcomes among healthcare workers exposed to coronavirus disease 2019. JAMA Netw Open **3**: e203976, 2020.
5. Song X, Fu W, Liu X, et al. Mental health status of medical staff in emergency departments during the coronavirus disease 2019 epidemic in China. Brain Behav Immun **88**: 60-65, 2020.
6. Yang X, Zhang Y, Li S, Chen X. Risk factors for anxiety of otolaryngology healthcare workers in Hubei province fighting coronavirus disease 2019 (COVID-19). Soc Psychiatry Psychiatr Epidemiol 2020.
7. Nie A, Su X, Zhang S, Guan W, Li J. Psychological impact of COVID-19 outbreak on frontline nurses: a cross-sectional survey study. J Clin Nurs **29**: 4217-4226, 2020.
8. Goldberg DP, Gater R, Sartorius N, et al. The validity of two versions of the GHQ in the WHO study of mental illness in general healthcare. Psychol Med **27**: 191-197, 1997.
9. Doi Y, Minowa M. Factor structure of the 12-item General Health Questionnaire in the Japanese general adult population. Psychiatry Clin Neurosci **57**: 379-383, 2003.
10. Choda N, Wakai K, Naito M, et al. Associations between diet and mental health using the 12-item General Health Questionnaire: cross-sectional and prospective analyses from the Japan Multi-Institutional Collaborative Cohort Study. Nutr **19**: 2, 2020.
11. Shimbo M, Nakamura K, Jing Shi H, et al. Green tea consumption in everyday life and mental health. Public Health Nutr **8**: 1300-1306, 2005.
12. Imai H, Matsuishi K, Ito A, et al. Factors associated with motivation and hesitation to work among health professionals during a public crisis: a cross sectional study of hospital workers in Japan during the pandemic (H1N1) 2009. BMC Public Health **10**: 672, 2010.
13. Higuchi Y, Inagaki M, Koyama T, et al. A cross-sectional study of psychological distress, burnout, and the associated risk factors in hospital pharmacists in Japan. BMC Public Health **16**: 534, 2016.
14. Suzuki K, Ohida T, Kaneita Y, et al. Mental health status, shift work, and occupational accidents among hospital nurses in Japan. J Occup Health **46**: 448-454, 2004.
15. Liu N, Zhang F, Wei C, et al. Prevalence and predictors of PTSS during COVID-19 outbreak in China hardest-hit areas: gender differences matter. Psychiatry Res **287**: 112921, 2020.
16. Li LZ, Wang S. Prevalence and predictors of general psychiatric disorders and loneliness during COVID-19 in the United Kingdom. Psychiatry Res **291**: 113267, 2020.
17. Cao W, Fang Z, Hou G, et al. The psychological impact of the COVID-19 epidemic on college students in China. Psychiatry Res **287**: 112934, 2020.
18. Okello DRO, Gilson L. Exploring the influence of trust relationships on motivation in the health sector: a systematic review. Hum Resour Health **13**: 16, 2015.

The Internal Medicine is an Open Access journal distributed under the Creative Commons Attribution-NonCommercial-NoDerivatives 4.0 International License. To view the details of this license, please visit (https://creativecommons.org/licenses/by-nc-nd/4.0/).

© 2021 The Japanese Society of Internal Medicine
