# Supplementary material for: Mental health status and related factors influencing healthcare workers during the COVID-19 pandemic: A systematic review and meta-analysis
Source: PLoS One. 2024 Jan 19;19(1):e0289454. doi: 10.1371/journal.pone.0289454 (PMC10798549; doi:10.1371/journal.pone.0289454)
Supplement: S1 Data — (ZIP) [file pone.0289454.s011.zip › literatures/58.pdf]

Future MICROBIOLOGY

Research Article

For reprint orders, please contact: reprints@futuremedicine.com

A comparative study of the mental health impact of the COVID-19 pandemic on health care professionals in India

Jitender Jakhar[1], Partha Sarathi Biswas*,[2], Mahima Kapoor[3], Amandeep Panghal[1], Amit Meena[1], Harsha Fani[1] & Pradip Kharya[4]

[1]Resident, Department of Psychiatry, Maulana Azad Medical College (G B Pant Institute of PG Medical Education & Research, GIPMER), New Delhi, India

[2]Professor and Head, Department of Psychiatry, Maulana Azad Medical College (G B Pant Institute of PG Medical Education & Research, GIPMER), New Delhi, India

[3]Associate Professor, Department of Psychiatry, Maulana Azad Medical College (G B Pant Institute of PG Medical Education & Research, GIPMER), New Delhi, India

[4]Assistant Professor, Community Medicine & Family Medicine, All India Institute of Medical Sciences (AIIMS), New Delhi, India

*Author for correspondence: psbiswasmamc@gmail.com

Aims: This study aimed to investigate how the psychological health of health care professionals (HCP) on COVID duty was different from those who were not directly in contact. **Methodology:** Of 473 (76%) randomly selected respondents (doctors and nurses) to a WhatsApp request message, 450 subjects' data were finally analyzed. **Result:** The prevalence of stress, anxiety and depression among HCP was 33.8, 38.9 and 43.6%, respectively. Compared with nonexposed professionals, COVID-19-exposed professionals had roughly double the score of these morbidities ($t = 6.3$, $p < 0.001$; $t = 6.9$, $p < 0.001$; $t = 6.0$, $p < 0.001$). Most worry (71.11%) was about the health of their family, followed by themselves (35.55%). **Conclusion:** The level of exposure, feelings of uncertainty and fear of infection emerged in our study as possible risk factors for psychological morbidities among HCP.

First draft submitted: 25 March 2021; Accepted for publication: 21 September 2021; Published online: 22 October 2021

Keywords: anxiety • burnout • COVID-19 • depression • duty hours • health care professionals • mental health • psychological impact • stress

Health care professionals are fighting hard to save the lives of others during the global coronavirus pandemic. Health care workers are continuously exposing themselves to the stressful, vulnerable, frightening and relatively underprotected environment. Doctors, nurses, and other health care professionals (HCPs) are directly involved in the continuity of care and well-being of patients with COVID-19, putting them at a higher risk of contracting it than the general public [1]. There are reports of deaths of HCPs that might incite fear among them. Uncertain prognosis, limited evidence-based guidelines, the heterogeneous nature of the virus lack of diagnostic facilities and use medication with relatively limited previous experience can generate a feeling of helplessness – or inability to help the those in need, intrinsic professional incompetence and the moral-ethical dilemma of triaging and resource allocation [2,3].

Such challenges in this pandemic can push HCPs beyond their ability to cope with stress. Robust evidence suggests that stress plays an significant role as a potential risk factor in the pathogenesis of psychiatric disorder through a dysregulation of the hypothalamic–pituitary–adrenal axis and altered cortisol levels [4,5]. The vulnerability can also be explained by the stress–diathesis model in a way that how life events stress can underpin the predisposition and subsequently results in psychiatric disorder [6]. The mental health concerns of the medical staff has been addressed during the COVID-19 pandemic to a considerable extent, but little work has specifically focused on Asian countries. Carmassia *et al.* [7] reviewed recent works on healthcare workers facing coronavirus outbreaks, highlighting possible risk and protective factors of distress. They found that stress reaction, indeed, seems to be an important cause of psychological disability in the aftermath of an outbreak, including COVID-19 pandemic, and healthcare systems

Future Medicine

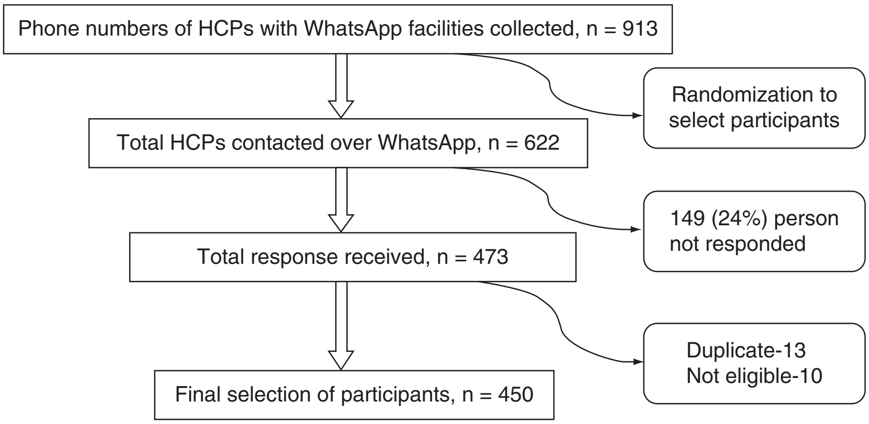

Figure 1. Flow chart for selection of participants.

should enhance strategies to face this relevant issue in healthcare workers. Fear of infection with the disease of self and family members has also been reported [7]. Further, sleep disturbance can result from this fear, in addition to a hectic workplace and long duty hours. Constant fear and sleepiness with minimal ability to leave the situation and restricted opportunities for relaxing activities in personal life because of lockdown may have made individuals vulnerable to health issues. Lai *et al.* [8] reported that a considerable portion of HCPs working in the care of COVID-19 patients experienced depressive symptoms (50.4%), sleep disturbances (34%) and anxiety symptoms (44%). Research on the Ebola and SARS epidemics also reported the occurrence of emotional distress and psychiatric disturbances among health professionals not only during the course of the epidemics but also in their aftermath [9]. In addition, research in disaster management has suggested that the management of both the physical and mental aspects during the crisis phase is essential for optimal recovery [10,11].

There were two groups of health workers in India in COVID-19 treatment facilities during the data collection of this study. The first group was comprised HCPs who worked for fever and COVID-suspected or COVID-positive patients (i.e., with direct and informed exposure). This group of HCPs would work for 15 consecutive days, followed by a self-quarantine for next 15 days. The second group comprised HCPs who treated COVID-negative or unsuspected (characterized by no immediate contact, no characteristic symptoms or history of visiting place with COVID cases in previous 3–4 weeks) general patients or patients in other specialties (i.e., with no obvious/informed exposure). HCPs accidentally exposed to positive cases during non-COVID duty were also quarantined. Personal protective equipment was used by both groups.

This study evaluated the effect of the pandemic on the mental health of HCPs to help health and family welfare authorities generate policy for the prevention and treatment of the psychological ailments in these workers. The authors hypothesized that more direct/informed exposure to COVID patients caused greater psychological problems. We aimed to estimate the prevalence rate of psychological symptoms (stress, anxiety and depression) among the doctors and nurses who were working as frontline HCPs and to investigate how the mental health of HCPs with direct and informed exposure to COVID-19 patients was different from those without apparent contact with COVID-19 patients.

Methodology

This was an online-based cross-sectional survey on HCP (doctors and nurses) who were working in various government health sectors across the nation conducted between 20 and 27 April 2020 after obtaining permission from Institutional Ethical Committee (IEC), Maulana Azad Medical College, New Delhi (no.: F.1/IEC/MAMC(77/05/2020/no.: 133). We collected phone numbers of HCPs of northern India using WhatsApp information from the Indian Medical Association, New Delhi. The participants were then selected by randomization with the help of the R package randomizer (Figure 1). A short paragraph outlining the aim and objectives of this study was sent, along with Google form sheet. Consent was obtained using an online form before enrollment in the survey, and only those who consented and volunteered for the study were recruited. The demographic details were collected in a semistructured proforma. The Depression Anxiety Stress Scale – 21-item version (DASS-21) [12] was used to measure the emotional effect of COVID-19 pandemic on HCPs. It includes

three negative emotional states: depression, anxiety and stress. A 10-point visual analog scale (10 was low, 5 was neutral and 1 was happy) was used to assess subjective mood and feeling of well-being over the previous 2 weeks. Participants were also asked to rate their average sleep duration per day in the previous 2 weeks on a 5-point Likert scale (1 = 0–2 h/day, 2 = 2–4 h/day, 3 = 4–6 h/day, 4 = 6–8 h/day and 5 was no disturbances). In addition, we examined their greatest fear due to COVID-19 by a set of probable choices made from available literature and experience working in the largest COVID-19 hospital in India. These self-designed questionnaires were pilot tested on ten subjects. The participants took ∼5–10 minutes to complete the questionnaires. People who completed the survey were also asked at the end if they required any psychiatric assistance or had any past history of psychiatric disorder. The primary outcome variables were DASS scores (depression, anxiety and stress), and secondary outcome variables were average sleep duration, mood state and greatest fear about COVID-19.

The survey respondents were medical professionals working in various government health facilities such as medical colleges, district hospitals and community health centers across the country. Doctors who participated were from both clinical and basic (nonclinical) medical departments. It included doctors with graduate (MBBS), postgraduate (MD/MS) and super-specialty (DM/MCH) degrees and nursing staff with minimum graduation degree (general nursing and midwifery). They were able to understand and speak in English. Doctors of nonallopathic specialties (homeopathy/ayurvedic/dentistry) and medical students were excluded. A total of 622 persons were contacted electronically (via WhatsApp), and 473 (76%) subjects were responded. Finally, data from 450 subjects was included for analysis after the exclusion of nonmedical staff (e.g., engineers, schoolteachers) and duplicate data (4%) (Figure 1). The term ‘COVID-19 exposed’ meant those participants who did their duties in COVID-19 wards or fever clinics for ≥7 days and quarantined themselves for the next 15 days.

Ethical approval & considerations

The potential risk was minimal at the time of the study. Data were collected in a privacy and kept confidential.

Statistical analysis

Subjects were divided according to their affiliations, such as basic medical department (pathology [2.9%], biochemistry [1.2%], microbiology [1%] and community medicine [8.7%]) and clinical departments (medicine [3.5%], superspeciality [cardiology, neurology, cardiothoracic, gastrosurgery; 7.6%], anesthesia [5.2%], surgery [3.3%], psychiatry [7.8%], obstetrics [4.1%], ENT [.8%], ophthalmology [1.7%], orthopedics [2.7%], radiology [2.9%], medical officers [nonspecialists, 24.4%] and pediatric [4.1%], nursing staff [7.9%] and others [10.2%]). Two comparative groups (‘COVID exposed’ and ‘COVID nonexposed’) were designed to examine the hypothesis of this study. The COVID-19 exposed group consisted of HCPs who were involved in direct treatment and care of COVID-19-positive and highly suspected patients. The COVID-nonexposed group comprised professionals who cared for general outpatient/inpatient services with or without low-suspect COVID-19 patients or asymptomatic COVID-19 patients. Data analysis was performed using SPSS statistical software 25.0 (IBM, NY, USA), and the type 1 error limit was set at 1 in 100 in two-tailed tests. Descriptive statistics were used to showcase the socio-demographic variables of the sample. Chi-square, Pearson correlation and independent t-test were applied to test the hypothesis. Additional analysis of covariance (ANCOVA) and logistic regression were run to estimate the contribution of demographic and other basic clinical independent variables to three primary outcome variables across the groups.

Results

Characteristics of the respondents

Among 450 health professionals who consented to participate in the study, 234 were female and 216 were male. The mean age of the participants was 31.6 (± 6.6) years. Almost half were married or in a relationship, and the majority belonged to the Hindu (including Sikh and Christian) religion (89%). Approximately two-thirds of the subjects were from the north zone. The majority (54%) of our participants were postgraduate doctors who were going through their residency in various departments and were the prime task force for managing patients. Only 8% of the total participants were from the nursing staff; 76% of COVID-19-exposed individuals (n = 121) were from the clinical department or nursing staff. Only 24% of COVID-19-exposed individuals were from the basic (nonclinical) medical department (i.e., community medicine, biochemistry, pathology, microbiology) ($\chi 2 = 2.12$, $p = 0.145$).

10.2217/fmb-2021-0084

Table 1. Description of clinical variables (n = 450).

Clinical variables						
Past history of psychiatric treatment, n (%)					32 (7.11)	
Directly exposed to COVID-19 patients, n (%)					121 (26.88)	
Requested for psychiatric consultation, n (%)					190 (42.22)	
Sleep disturbances, n (%)					318 (70.66)	
Mood disturbance		Visual analog scale (≥7)			36.7%	
DASS-21 (mean ± SD)		Stress			11.41 (± 10.1)	
		Anxiety			7.6 (± 8.31)	
		Depression			9.75 (± 10.02)	
**DASS-21, n (%)**	**Severity level**	**Normal**	**Mild**	**Moderate**	**Severe**	**Extremely severe**
	Stress	298 (66.2)	40 (8.9)	56 (12.4)	42 (9.3)	14 (3.1)
	Anxiety	275 (61.1)	25 (5.6)	71 (15.8)	26 (5.8)	53 (11.8)
	Depression	254 (56.4)	68 (15.1)	60 (13.3)	30 (6.7)	38 (8.4)

The prevalence of stress, anxiety and depression among healthcare providers was approximately 34, 39 and 44%, respectively. Approximately 42% expressed the need for psychiatry intervention. Roughly 37% of subjects reported sad, depressed and down mood.
DASS-21: Depression Anxiety Stress Scale – 21-item version; SD: Standard deviation.

Clinical variables

The majority of subjects recruited (92.9%) in the study were healthy and had no past or present history of psychiatric treatment. Only 7.11% reported a history of past minor anxiety and sleep disturbances. The prevalence of perceived stress among the participants as per DASS-21 was 33.8% (8.9% reported mild, 12.4% moderate, 9.3% severe and 3.1% extremely severe symptoms). Further, 38.9% of professionals reported anxiety symptoms, among which 5.6% were mild, 15.8% were moderate, 5.8% were severe and 11.8% were extreme symptoms. In addition, 43.6% of professionals reported depressive symptoms with 15.1% mild, 13.3% moderate, 6.7% severe and 8.4% extremely severe symptoms. In addition, 36.7% of subjects reported sad, depressed or down mood (≥7) with the visual analog mood scale. Less than half (42.22%) of the participants expressed the need for psychiatric intervention because they could not manage their psychological burden on their own (Table 1).

Of total 450 participants, 26.88% (n = 121) reported exposure to COVID-19 (i.e., on duty in COVID-19 wards) (Table 1). The average duty hours (as assessed from the duty rosters of the HCPs) in exposed health workers with COVID-19 patients were 5.27 (2.47) h per day. Both groups were comparable in terms of age ($\chi 2 = -.59$, $p = 0.55$), sex ($\chi 2 = .11$, $p = 0.09$), religion ($\chi 2 = .07$, $p = 0.06$) and marital status ($p = 0.41$; Table 2). Statistically, the same frequency of past history of psychiatric illness was reported in both the groups ($p = 0.87$, $\chi 2 = 0.83$). Compared with the nonexposed group, the COVID-19 exposed individuals had roughly double the score of stress ($t = 6.3$, $p < .001$), anxiety ($t = 6.9$, $p < 0.001$) and depressive symptoms ($t = 6.0$, $p < 0.001$; Table 2). The most frequently observed level of stress in exposed participants was of moderate to severe categories (38.8%). However, in nonexposed participants, primarily mild to moderate levels of stress were described (18.2%). Moderate to extreme severity was the most common (53.8%) type of anxiety in the exposed group, whereas it was moderate anxiety (15.2%) in nonexposed participants. Among different types of depression in the nonexposed group, mild depression (14.3%) was the most common, but in exposed participants, all severities were equally prevalent. The exposed subjects reported more frequently feeling 'sad, depressed and down mood' (≥7 on a VAS of mood) compared with the nonexposed group (56.2 vs 29.5%, $p < 0.001$). They were not different in terms of frequency of seeking psychiatric help either in the past ($\chi 2 = .83$, $p = 0.87$) or during the COVID-19 pandemic ($\chi 2 = 3.68$, $p = 0.055$).

Subgroup analysis

In a separate subgroup analysis of COVID-19-exposed professionals, we ran a Pearson product-moment correlation test to check for the relationship between DASS-21 and duty hours/duration of sleep. We found a weak positive correlation between stress score and number of exposed hours ($r = 0.2$, $p = 0.01$) and toward significance with depression score ($r = 0.16$, $p = 0.07$). A discriminant analysis showed that independent variables such as age and scores of DASS-21 could classify 69.1% of the participants into exposed and no exposed group correctly.

Cohen's kappa agreement analysis was also run to determine whether there was an agreement between the two main primary outcome scales (VAS vs DASS-21) assessing the psychological burden and impact of the pandemic.

Table 2. Comparison of COVID-19 exposed and COVID-19 nonexposed subjects.

Parameter		COVID exposed (n = 121)	COVID-nonexposed (n = 329)	t/χ2 (p)
Age (years, mean ± SD)		31.30 (5.48)	31.70 (6.90)	–0.59 (0.55)
Sex	Male	66 (54.5%)	150 (45.6%)	2.84 (0.09)
	Female	55 (45.5%)	179 (54.4%)	
Religion	Hindu (including Sikh and Christian)	110 (93.2%)	314 (96.6%)	2.43 (0.12)
	Muslim	8 (6.8)	11 (3.4)	
Marital status	Single/separated	49 (40.5%)	133 (40.4%)	0.000 (0.99)
	Married/in relationship	72 (59.5)	196 (59.6)	
Past history of psychiatric treatment	No	112 (92.56%)	306 (93%)	0.83 (0.87)
	Yes	9 (7.4%)	23 (9.99%)	
DASS-21	Stress (mean ± SD)	16.1 (± 11)	9.7 (± 9.1)	6.3 (<0.001)
	Anxiety (mean ± SD)	11.89 (± 9.7)	6 (± 7.12)	6.9 (<0.001)
	Depression (mean ± SD)	14.26 (± 11)	8.1 (± 9.1)	6.0 (<0.001)
Mood (VAS)	Sad, depressed and down mood (≥7)	68 (56.2%)	97 (29.5 %)	27.188 (<0.001)
Prevalence based on DASS-21 cut-off score	Stress (≥15)	79 (65.3%)	117 (35.6%)	31.78 (<0.001)
	Anxiety (≥8)	72 (59.5%)	103 (31.3%)	29.59 (<0.001)
	Depression (≥10)	67 (55.4%)	85 (25.8%)	34.50 (<0.001)
Sought for psychiatric help recently	No	61 (50.4%)	199 (60.5%)	3.68 (0.055)
	Yes	60 (49.6%)	130 (39.5%)	

The most frequently observed level of stress (39%) and anxiety (54%) in exposed participants was of the moderate to severe categories. It was mostly mild to moderate level of stress (18.2%), anxiety (15%) and depression (14%) in nonexposed participants. History of past psychiatric illness and recently seeking psychiatric help were reported equally in both groups.
DASS-21: Depression Anxiety Stress Scale – 21-item version; SD: Standard deviation; VAS: Visual analogue scale.

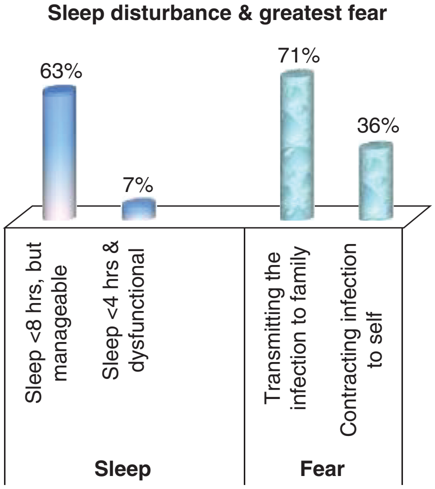

Figure 2. Secondary outcome variables (n = 450). A majority (~63%) acknowledged sleep difficulty (4–8 h/day) without functional impairment. Only 7% of subjects reported difficulty in their sleep (<4 h/day) that left them unable to bear the workload. The most common concern among the HCPs (~71%) was about the health of their loved ones, followed by a fear of contracting infection themselves (~36%).

However, no agreement between the two scales was found (for stress k = 0.022, p = 0.572, for anxiety k = -0.007, p = 0.867 and for depression k = -0.001, p = 0.979).

Secondary outcome variables

Sleep difficulty: the average number of sleep hours among participants was 3.99 (± 2.81) h after February 2020 (i.e., after COVID-19 cases start appearing in the clinic). The majority (63.33%) acknowledged that they had sleep difficulty and most slept for 4–8 h/day but could function by pushing themselves. Only 7.33% of subjects reported significant difficulty in their sleep and were unable to sleep for more than 4 h/day, leading to significant difficulty in their functioning and ability to bear the workload (Figure 2).

Fear due to COVID-19: The concern that worried HCPs most (71.11%) was about the health of their loved ones and the risk of transmitting the infection to them. More than one-third (35.55%) of respondents reported that they feared contracting infection themselves (Figure 2).

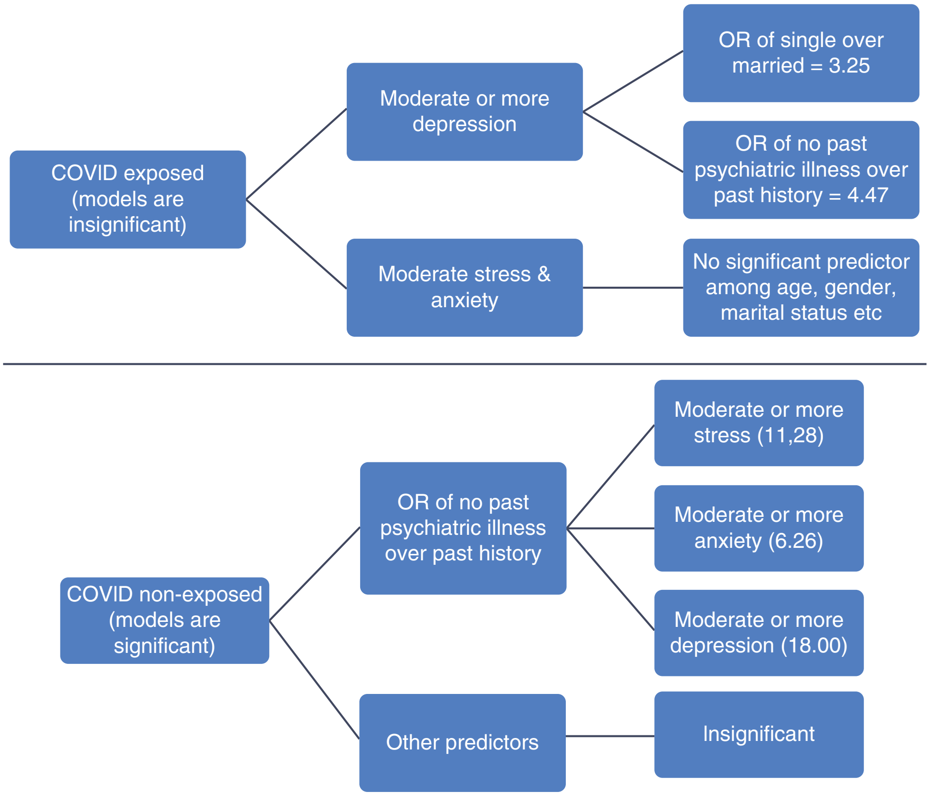

Figure 3. Logistic regression to predict more than mild stress, anxiety and depression in COVID-exposed and COVID-nonexposed participants. Nonexposed participants without past psychiatric history were approximately 11, six and 18-times more likely to have moderate stress, anxiety and depression, respectively, than those who had past psychiatric illness. Among the exposed HCPs, unmarried participants had roughly three-times the likelihood of moderate depression and those without past history of psychiatric illness were 4.5-times more likely to have moderate depression than their counterparts.

Adjustment for confounders to calculate the risk for psychological ill health in exposed participants

A set of logistic regressions was performed to ascertain the effects of age, marital status, gender, religion, medical branch with which the participant was affiliated, past psychiatric history and exposure hours on the likelihood that they experienced stress, anxiety and depression above mild level. Only the models for nonexposed participants were significant: $\chi^2(7) = 24.52$, $p = .001$; $\chi^2(8) = 17.01$, $p = 0.017$; $\chi^2(7) = 29.68$, $p < 0.001$, respectively. None of the three logistic regression models for exposed participants was statistically significant: $\chi^2(7) = 4.61$, $\chi^2(7) = 5.66$ and $\chi^2(7) = 12.08$, respectively. The models explained <1.0% (Nagelkerke R^2) of the variance for moderate stress and anxiety, but the model for moderate depression explained 14.1 and 15.2% of its variance (Nagelkerke R^2) of exposed and nonexposed participants, respectively. They correctly classified as much as 80.7% moderate stress in nonexposed cases and as little as 60.2% moderate anxiety among exposed cases. Nonexposed participants without past psychiatric history were 11.28-, 6.26- and 18.00-times more likely to have moderate stress (Wald = 17.95, df = 1, $p < 0.001$), anxiety (Wald = 10.61, df = 1, $p = 0.001$) and depression (Wald = 18.68, df = 1, $p < 0.001$), respectively, than those who had past psychiatric illness. However, among the exposed HCPs, unmarried participants were 3.25 times (Wald = 4.1, df = 1, p.04) and those without past history of psychiatric illness were 4.47-times (Wald = 4.05, df = 1, p.04) more likely to have moderate depression than their counterparts (Figure 3).

An additional univariate analysis (ANCOVA) was conducted to adjust for probable confounders such as age, gender, marital status, religion, past psychiatric history, average hours of exposure, affiliation with a clinical or nonclinical branch and direct exposure to COVID patients. The three primary outcome variables (scores of stress, anxiety and depression of the DASS) remained different across the status of exposure to COVID patients ($F = 23.41$, df = 1, p = < 0.001, with an effect size of .058; $F = 3.26$, df = 1, $p < 0.001$, with an effect size of 0.074; and $F = 21.47$, df = 1, $p < 0.001$, with an effect size of 0.054, respectively) even after adjusting the preceding covariates. Exposure hours and participants' branch had significant interactions with stress ($F = 10.77$, $p = 0.001$; $F = 6.28$,

p = 0.013, respectively). Marital status (F = 5.28, p = 0.022) along with preceding two covariates (F = 17.62, p < 0.001; F = 8.76, p = 0.003) also had a significant interaction with anxiety scores. However, only exposure hours interacted significantly (F = 6.5, p = 0.011) with depression among all covariates.

Discussion

This was a cross-sectional, electronically based survey to assess the psychological impact on professionals treating COVID-19 patients in India. This is one of the few studies so far to examine the difference of mental health concerns between COVID-19-exposed versus nonexposed HCPs. The majority (76%) of COVID-19-exposed individuals (n = 121) in our study were understandably from the clinical departments because they often acted as frontline workers (posted in the COVID ward/fever clinic). HCPs who were exposed despite being from basic (nonclinical) disciplines (24%) comprised professionals dealing with sample collection and testing of materials from COVID patients. Response to our WhatsApp message for the survey was as low (76%) probably because this survey was not linked to any treatment availability such as online counseling or therapy, and the data collection from HCPs was nonpersuasive.

India has been struggling with COVID-19 since February 2020. Most of the hospitals have been working at full capacity. Fear, denial, worry and adjustment issues are commonly encountered findings in crisis, but when this stress exceeds the coping mechanism and continue beyond a certain period of time, it can uncover various underlying psychological states [13]. The frontline workers struggled with stress-provoking situations such as overwork, fear of infection, erratic supplies of personal protective equipment, uncertainty and many other issues [14]. The two most common fear responses generated during this pandemic among our professionals were fear of infection to oneself (35.55%) and fear of infecting a family member (71.11%). As time has elapsed, infrastructure problems were getting improved, but the number of asymptomatic positive cases were increasing [15]. It may lead to more fear and psychological devastation.

More than half (63%) of professionals of our study had sleep disturbances (<8 h/day) irrespective of their COVID exposure. However, one study from China reported a lower prevalence of insomnia (one-third) in medical staff, and in a regression model, their insomnia was associated with worry of infection and uncertainty [16]. However, only 7.11% of our participants had a past history of psychiatric illness. We conceptualized that chronic sleep deprivation had worsened many mental health conditions, especially among susceptible persons. There is sufficient evidence to suggest a bidirectional causal relationship between sleep disturbances and psychiatric disorders [17]. Although low to moderate levels of worry may be adaptive, a level above a given threshold can play a role in the onset of depressive and anxiety disorders [18]. We found moderate to severe (38.8%) stress and moderate to extreme (53.8%) anxiety symptoms in our COVID-19-exposed participants. Uncertainty about how long the pandemic will continue, social disconnectedness and staying away from loved ones during aquarantine phase might trigger a feeling of depression, helplessness, or frustration. Our study observed all types of severity (mild to extreme) of depression in our COVID-19 exposed participants. Health care workers exposed to a similar outbreak during the SARS epidemic in 2003 reported psychological disturbances (General Health Questionnaire score $\geq$5) in $\sim$35% of participants [19]. We found almost similar overall prevalence of depressive symptoms (43.6%), anxiety (38.9%) and stress (33.8%) in our HCPs. However, these symptoms doubled among HCPs who were exposed directly to COVID-19 patients (65.3 stress, 59.5 anxiety and 55.4% depression). However, we do not know whether working in COVID wards, the subsequent quarantine or a combination of both contributed to increased rates of psychological disturbances. In the face of conditions where most of the medical infrastructures were being used for COVID-19 patients and psychiatric care had been suspended in many hospitals, 49.6% of our exposed HCPs (compared with only 39.5% in nonexposed HCPs) sought psychiatric help (p = 0.05). This reflects the fact that symptom severity was such that even HCPs could not manage their psychological ailments on their own and asked for psychiatric help. We found only a weak positive correlation (r = 0.2, p = 0.01) between stress and number of duty hours. High levels of distress, in addition to depressive and anxiety symptoms, were found in the participants of this study. This is important to note because the bidirectional association between anxiety/depressive symptoms and stress/burnout in healthcare workers has been extensively described in the literature [20].

Lastly, married HCPs who were exposed to COVID patients had a 3.25-times greater chance of moderate or severe depression than single HCPs (Figure 3). However, depression was greater in HCPs without a past history of psychiatric illness. In the psychiatric literature, single individuals are known to have greater susceptibility for depression [21]. Moreover, HCPs who had no past psychiatric history showed greater susceptibility for stress, anxiety and depression (moderate or severe). The reason for this finding could be that HCPs who knew their vulnerability

for psychiatric illness (only 7.11%) probably took additional prophylactic measures to prevent psychological issues, nonexposure could have kept the HCPs away from the reality of the COVID scenario and gave rise to more misconceptions, lack of data related to the type of prior psychiatric illness in HCPs and chronic sleep deprivation (63% of participants). Additionally univariate analysis (ANCOVA) revealed that effect of group on stress, anxiety and depression scores remained at small to moderate levels even after adjusting for confounding effects from demographic and some important basic clinical independent variables. Thus, irrespective of past history of psychiatric illness, unmarried HCPs after exposure to COVID-19 patients and other HCPs even without exposure to COVID-19 developed psychiatric illness, probably due to psychosocial issues related to the COVID-19 pandemic. Likewise, unprevented and undetected distress/burnout (caused by strenuous work patterns, a high number of duty hours, nightshifts, sleep impairment, frontline activities etc.) could lead to long-term psychological complications in this population, particularly depressive and anxiety symptoms, as reported in reported in recent studies on healthcare workers facing the COVID-19 pandemic [20]. In our institute, we have started counseling sessions for HCPs and screening of staff who require attention from the psychiatry department. Other institutions can start possible intervention strategies to reduce fear and stress reactions related to COVID-19 and promoting well-being/resilience in specific vulnerable groups.

Limitations

We acknowledge that our study has several limitations that need to be addressed in future studies. First, it is a cross-sectional study and lacks longitudinal follow-up data to check for the rebound of psychological well-being once the crisis phase wanes. Replication of the study or follow-up of the same sample could be done in the future. Second, the majority of our HCPs were doctors, thus limiting generalization of our finding to other HCPs who also worked on the front lines during this pandemic. Third, we used a self-designed questionnaire that needs to be validated. Moreover, the use of self-report instruments could be considered less accurate than a clinician assessment, which represents an important limitation. Fourth, most of our participants were from the northern India, and future study needs to be done with participants from other parts of the country. Finally, we examined a highly selected sample of HCPs, and we do not know how the psychological disturbances of HCPs with WhatsApp might be different from the vast majority of HCPs who do not have WhatsApp.

Conclusion

Stress reaction seems to be an important cause of psychological disability in the aftermath of an infectious outbreak, including the COVID-19 pandemic, and healthcare systems should enhance strategies to face this relevant issue in HCPs. In line with the results of the present study, level of exposure, feelings of uncertainty, fear of contagion and infection of self and family emerged as possible risk factors for psychological distress in healthcare workers. Our study suggests that those HCPs who are directly exposed to COVID-19 patients are at higher risk of psychiatric disturbances, and an early, stringent and focused psychological intervention in this high-risk group might be useful and in fact necessary. Because we found only a weak positive correlation ($r = 0.2$, $p = 0.01$) between stress and number of duty hours, steps may be taken to facilitate infrastructure building and provision of more testing and PPE, which could help decrease psychological burden and distress among HCPs.

Author contributions

M Kapoor and J Jakhar designed the research protocol. PS Biswas analyzed data and wrote the manuscript. A Panghal, A Meena, H Fani and P Kharya collected data. J Jakhar and A Panghal dealt with ethical issues and interhospital coordination. All authors contributed to the intellectual content and commented on the manuscript.

Acknowledgments

The authors acknowledge all the healthcare workers who took the time to participate in our study and also those who worked hard for the patients of COVID-19 in our hospital.

Financial & competing interests disclosure

The authors have no relevant affiliations or financial involvement with any organization or entity with a financial interest in or financial conflict with the subject matter or materials discussed in the manuscript. This includes employment, consultancies, honoraria, stock ownership or options, expert testimony, grants or patents received or pending, or royalties.

No writing assistance was utilized in the production of this manuscript.

Ethical conduct of research

The study was ethically approved by the institutional ethical committee (IEC) of Maulana Azad Medical College, New Delhi (no.: F.1/IEC/MAMC[77/05/2020/no.: 133]). The consent was taken in an online form before enrollment in the survey. The potential risk was minimal at the time of the study. The data were collected in a private condition and kept confidential.

Summary points

- This was an online-based cross-sectional survey on healthcare workers (doctors and nurses) who were working in the different government health sectors across northern India.
- The aim was to investigate how the psychological health of health care professionals (HCPs) who were treating COVID patients was different from those who were not directly in contact with these patients.
- Data from 450 subjects (demographic profile, depression, anxiety, stress, sleep and mood) were finally analyzed.
- Seventy-six percent of COVID-19-exposed HCPs (n = 121) were from the clinical department.
- The majority of subjects recruited (92.9%) in the study were healthy and had no past or present history of psychiatric treatment.
- The prevalence of stress, anxiety and depression among HCPs was 33.8, 38.9 and 43.6%, respectively.
- Compared with nonexposed HCPs, COVID-19-exposed professionals had roughly double the scores of stress ($t = 6.3$, $p < 0.001$), anxiety ($t = 6.9$, $p < 0.001$) and depression ($t = 6.0$, $p < 0.001$).
- The most troubling concern (71.11%) among the HCPs was about the health of their loved ones, followed by a fear of contracting infection themselves (in ~35.55% of participants).
- Nonexposed participants without past psychiatric history were 11.28-, 6.26- and 18-times more likely to have moderate stress, anxiety and depression, respectively, than those who had past psychiatric illness.
- Unprevented and undetected distress/burnout (favored by strenuous work patterns, high number of duty hour, nightshifts, sleep impairment, frontline activities etc) could lead to long-term psychological complications in this population, particularly depressive and anxiety symptoms.
- In our institute, we have started counseling sessions for HCPs and screening of staff members who require attention from the psychiatry department. Other institutions can begin possible intervention strategies to reduce fear and stress reactions related to COVID-19 and promote well-being and resilience in specific vulnerable groups.

References

1. Guo X, Jiedong W. Orthopaedic forum survey of COVID-19 disease among orthopaedic surgeons in Wuhan, People's Republic of China. *J. Bone. Joint. Surg. Am.* 102(10), 847–854 (2020).
2. Rosenbaum L. Facing covid-19 in Italy – ethics, logistics, and therapeutics on the epidemic's front line. *N. Engl. J. Med.* 382(20), 1873–1875 (2020).
3. Kickbusch I, Leung GM, Bhutta ZA *et al.* Covid-19: how a virus is turning the world upside down. *BMJ.* 369, m1336 (2020).
4. Schneiderman N, Ironson G, Siegel SD. Stress and health: psychological, behavioral, and biological determinants. *Annu. Rev. Clin. Psychol.* 1(1), 607–628 (2005).
5. Smith SM, Vale WW. The role of the hypothalamic–pituitary–adrenal axis in neuroendocrine responses to stress. *Dialogues. Clin. Neurosci.* 8(4), 383–395 (2006).
6. Colodro-Conde L, Couvy-Duchesne B, Zhu G *et al.* A direct test of the diathesis–stress model for depression. *Mol. Psychiatry.* 23(7), 1590–1596 (2018).
7. Carmassia C, Foghia C, Dell'Oste V *et al.* PTSD symptoms in healthcare workers facing the three coronavirusoutbreaks: what can we expect after the COVID-19 pandemic. *Psychiatry Res.* 292, 113312 (2020).
8. Lai J, Ma S, Wang Y *et al.* Factors associated with mental health outcomes among health care workers exposed to coronavirus disease 2019. *JAMA. Netw. Open.* 3(3), e203976 (2020).
9. Lee SM, Kang WS, Cho AR *et al.* Psychological impact of the 2015 MERS outbreak on hospital workers and quarantined hemodialysis patients. *Compr. Psychiatry.* 87, 123–127 (2020).
10. World Health Organization. Mental health in emergencies. www.who.int/news-room/fact-sheets/detail/mental-health-in-emergencies
11. Welton-Mitchell C, James LE, Khanal SN, James AS. An integrated approach to mental health and disaster preparedness: a cluster comparison with earthquake affected communities in Nepal. *BMC. Psychiatry.* 18(1), 296 (2018).
12. Lovibond PF, Lovibond SH. The structure of negative emotional states: comparison of the Depression Anxiety Stress Scales (DASS) with the Beck Depression and Anxiety Inventories. *Behav. Res. Ther.* 33(3), 335–343 (1995).
13. Davis MT, Holmes SE, Pietrzak RH, Esterlis I. Neurobiology of chronic stress-related psychiatric disorders: evidence from molecular imaging studies. *Chronic. Stress.* 1, 1–21 (2017).
14. Greco V, Roger D. Uncertainty, stress, and health. *Person. Indivi. Differ.* 34(6), 1057–1068 (2003).

15. Day M. Covid-19: four fifths of cases are asymptomatic, China figures indicate. *BMJ.* 369, m1375 (2020).
16. Zhang C, Yang L, Liu S *et al.* Survey of insomnia and related social psychological factors among medical staff involved in the 2019 novel corona virus disease outbreak. *Front. Psychiatry.* 11, 306 (2020).
17. Krystal AD. Psychiatric disorders and sleep. *Neurol. Clin.* 30(4), 1389–1413 (2012).
18. Nolen-Hoeksema S, Wisco BE, Lyubomirsky S. Rethinking rumination. *Perspect. Psychol. Sci.* 3(5), 400–424 (2008).
19. Chan AOM, Chan YH. Psychological impact of the 2003 severe acute respiratory syndrome outbreak on health care workers in a medium size regional general hospital in Singapore. *Occup. Med.* 54(3), 190–196 (2004).
20. Buselli R, Corsi M, Baldanzi S *et al.* Professional quality of life and mental health outcomes among health care workers exposed to Sars-Cov-2 (Covid-19). *Int. J. Environ. Res. Public Health.* 17, 6180 (2020).
21. Jaiswal P, Jilani AQ, Shukla D *et al.* Marital quality and its relation with depression: a case-control study. *Int. J. Adv. Med.* 3(3), 591–595 (2016).
