# Supplementary material for: Mental health status and related factors influencing healthcare workers during the COVID-19 pandemic: A systematic review and meta-analysis
Source: PLoS One. 2024 Jan 19;19(1):e0289454. doi: 10.1371/journal.pone.0289454 (PMC10798549; doi:10.1371/journal.pone.0289454)
Supplement: S1 Data — (ZIP) [file pone.0289454.s011.zip › literatures/59.pdf]

ELSEVIER

Since January 2020 Elsevier has created a COVID-19 resource centre with free information in English and Mandarin on the novel coronavirus COVID-19. The COVID-19 resource centre is hosted on Elsevier Connect, the company's public news and information website.

Elsevier hereby grants permission to make all its COVID-19-related research that is available on the COVID-19 resource centre - including this research content - immediately available in PubMed Central and other publicly funded repositories, such as the WHO COVID database with rights for unrestricted research re-use and analyses in any form or by any means with acknowledgement of the original source. These permissions are granted for free by Elsevier for as long as the COVID-19 resource centre remains active.

Comprehensive Psychiatry 103 (2020) 152213

Contents lists available at ScienceDirect

Comprehensive Psychiatry

journal homepage: www.elsevier.com/locate/comppsych

The psychological impact of the coronavirus disease pandemic on hospital workers in Daegu, South Korea

So-Hye Jo, Bon-Hoon Koo, Wan-Seok Seo, Seok-Ho Yun [1,*], Hye-Geum Kim [*,1]

Department of Psychiatry, Yeungnam University College of Medicine, Yeungnam University Medical Center, 317-1, Daemyeong 5-dong, Nam-gu, Daegu 42415, Republic of Korea

ARTICLE INFO

Keywords:
COVID-19
Coronavirus disease
Hospital worker
Mental health
Psychiatric consultation

ABSTRACT

Objective: This study aimed to assess the immediate stress and psychological impact experienced by healthcare workers and other personnel during the Coronavirus disease (COVID-19) pandemic.
Method: The sample consisted of 2554 hospital workers (i.e., physicians, nurses, allied health professionals, and auxiliary staff members) who were working in Yeungnam University Hospital in Daegu, South Korea. The Impact of Event Scale-Revised (IES-R) was administered to the hospital workers twice over a 2 week interval. A high-risk group, identified on the basic of first total IES-R, was assessed further with the Mini International Neuropsychiatric Interview (MINI) and the Clinical Global Impressions-Severity (CGI—S) scale and was offered periodic psychiatric consultations on a telephone.
Results: The participating nurses and auxiliary staff members had significantly higher IES-R scores ($p < 0.01$) than the physicians. During the second evaluation, the IES-R scores of the high-risk participants had decreased by 13.67 ± 16.15 points ($p < 0.01$), and their CGI-S scores had decreased by 1.00 ± 0.74 points ($p < 0.01$). The psychological symptoms of the high-risk group who received telephone-based psychiatric consultation showed improvement after 2 weeks.
Conclusions: The present findings suggest that hospital workers experience high levels of emotional stress during a pandemic. In particular, the present findings underscore the need to provide more information and support to nurses and other administrative workers. There is a need for greater awareness about the importance of mental health care among hospital workers, and rapid and ongoing psychiatric interventions should be provided to workers during the pandemic period.
© 2020 The Author(s). Published by Elsevier Inc. This is an open access article under the CC BY-NC-ND license (http://creativecommons.org/licenses/by-nc-nd/4.0/).

1. Introduction

Coronavirus disease (COVID-19) was first reported in Wuhan, China, in December 2019. Since then, it has been rapidly spreading throughout the world [1]. On March 11, 2020, the World Health Organization officially declared COVID-19 a pandemic [2]. As of May 20, 2020, 4,993,470 confirmed cases and 327,738 deaths had been reported worldwide [3]. The outbreak of COVID-19 in South Korea began when the first confirmed cases were reported on January 20, 2020. As of May 16, 2020, 11,037 confirmed cases, 262 deaths, and 10,775 cumulative isolators had been reported [4]. In particular, the number of infected individuals in Daegu and North Gyeongbuk Province has increased dramatically (74.6% of the total number of confirmed cases), ever since the 31st case was confirmed. Further, cities in South Korea have been experiencing significant socioeconomic challenges [4]. So, a lot of medical resources and workers are being put into the effort to end the COVID-19.

Healthcare workers serve at the forefront of infectious diseases to provide care to patients. A lack of therapeutic agents and vaccines for COVID-19 has exacerbated the fear and burden experienced by hospital workers (i.e., all hospital employees). The possibility of becoming infected has increased physical and mental stress levels among hospital workers. They may experience a variety of symptoms, including anxiety, fear, and insomnia, because of excessive worry and oversensitivity to infection and transmission [5,6]. Past studies on infectious diseases such as severe acute respiratory syndrome (SARS) and Middle East respiratory syndrome (MERS) have shown that many hospital workers experience severe emotional stress (e.g., exhaustion, depression, and anxiety) when infectious diseases are prevalent [7,8]. Furthermore, some of these symptoms persist even after an epidemic comes to an end. The psychological symptoms that are associated with infectious diseases among hospital workers can negatively affect treatment quality by adversely affecting their attention, cognitive functioning, and clinical

Abbreviations: COVID-19, coronavirus disease; IES-R, Impact of Event Scale-Revised; MINI, Mini International Neuropsychiatric Interview; CGI-S, Clinical Global Impressions-Severity; SARS, severe acute respiratory syndrome; MERS, Middle East respiratory syndrome; PTSD, posttraumatic stress disorder; MDD, major depressive disorder.

* Corresponding authors.
E-mail addresses: ychaos@ynu.ac.kr (S.-H. Yun), psykhg@yu.ac.kr (H.-G. Kim).
[1] These authors contributed equally to this article.

https://doi.org/10.1016/j.comppsych.2020.152213
0010-440X/© 2020 The Author(s). Published by Elsevier Inc. This is an open access article under the CC BY-NC-ND license (http://creativecommons.org/licenses/by-nc-nd/4.0/).

decision making [9,10]. In other words, it can adversely affect individuals, families, and societies [11].

Psychological management indubitably has positive effects on the treatment and recovery of infected patients [12]. Further, psychological interventions for hospital personnel are becoming as important as clear communication, sufficient education and training, safety equipment, and a safe environment in the case of infectious diseases [13]. Accordingly, psychological assistance services that rely on the use of telephones, the internet, and applications have been developed for patients with infectious diseases and healthcare workers [14–16].

Through this study, we would like to evaluate the immediate stress and psychological impacts experienced by hospital workers in Daegu during the COVID-19 outbreak. Further, we explored the effect of telephone-based psychiatric consultations on high-risk participants through early screening.

2. Material and methods

2.1. Participants

During the COVID-19 outbreak, 2554 hospital workers were serving in Yeungnam University Hospital in Daegu, South Korea. In addition to medical professionals (e.g., doctors and nurses), nonmedical and other administrative staff members were also working in the hospital. They were also eligible for inclusion in this study. This study was approved by the Ethics Review Committee of the Medical Research Institute in Yeungnam University Hospital in Daegu (Institutional Review Board: YUMC 2020–04-099).

2.2. Measures

The Korean version of the Impact of Event Scale-Revised (IES-R) [17] was used to assess psychological distress. The IES-R is a 22-item scale, and responses are recorded on a 5-point scale (0 = not at all, 1 = a little, 2 = moderately, 3 = quite a bit, and 4 = extremely). Total scores can range from 0 to 88. Subscale scores can be calculated for the following dimensions of posttraumatic stress disorder (PTSD): hyperarousal (items 4, 10, 14, 18, 19, and 21), avoidance (items 5, 8, 11, 12, 17, and 22), intrusion (items 1, 3, 6, 9, and 16), and sleep and numbness (items 2, 7, 13, 15, and 20). Total scores >25 in the Korean version of the IES-R are indicative of a diagnosis of PTSD, whereas scores >18 are indicative of the presence of PTSD-like symptoms [18]. In this study, participants with IES-R scores ≥25 were classified as high-risk individuals. IES-R was used to assess mental health of general population [19], workers [20] and psychiatric patients [21].

The high-risk participants participated in a structured interview, which was conducted using the Mini International Neuropsychiatric Interview (MINI) and Clinical Global Impressions-Severity (CGI–S) scale. An abbreviated self-rated Korean version of the MINI [22] has been developed to screen for anxiety disorders (panic disorder, generalized anxiety disorder, and social anxiety disorder) and major depressive disorder (MDD). In the respective modules, subjects were presumed to have a diagnosis of a panic disorder if they scored above 4 points, generalized anxiety disorder if the score was above 3 points, social anxiety disorder if the score was above 4 points, and MDD if the score was above 5 points. The CGI-S scale is a well-established research instrument that is applicable to all psychiatric disorders, and it can easily be administered by the practicing clinician [23]. Based on clinical experience, the CGI-S is an assessment of how mentally ill patients are. Responses are recorded on the following seven-point scale: 1 = normal and not at all ill, 2 = borderline mentally ill, 3 = mildly ill, 4 = moderately ill, 5 = markedly ill, 6 = severely ill, and 7 = among the most extremely ill patients. Responses should be chosen based on the observed and reported symptoms, behaviors, and functioning over the past seven days.

2.3. Procedure

The IES-R was administered to 2554 workers who were on duty during the COVID-19 outbreak using mobile devices. The psychiatrist evaluated the MINI and CGI-S of the psychological high-risk group based on the telephone. For those who agreed to support mental health services, the psychiatrist conducted periodic psychiatric consultations over the phone, and at the end of the two weeks, initial surveys were repeated for all subjects.

2.4. Analyses

The IES-R scores were not normally distributed across all the occupational groups. Therefore, the nonparametric variant of one-way analysis of variance, namely, the Kruskal-Wallis test, was used to compare the IES-R scores that were obtained by the different occupational groups. The results of the Kruskal-Wallis test were further examined using the Tukey-Kramer method, and gender differences in the IES-R scores were examined using independent samples *t*-test. To examine the effects of the psychiatric intervention on the stress levels of the high-risk participants, their preintervention and postintervention scores on the IES-R and CGI-S were compared using the Wilcoxon signed-rank test. All statistical analyses were conducted using MATLAB 2019b (MathWorks, Natick, Massachusetts), and *p*-values ≤0.05 were considered to be statistically significant.

3. Results

3.1. Demographic characteristics

A total of 2554 workers at Yeungnam University's medical center were measured the degree of stress in the working environment associated with COVID-19 pandemics in the mobile environment. A total of 253 individuals (10%) participated in the survey. Whereas 210 (83%) of them were women, 44 (17%) of them were men. Their average age was 39.1 ± 12.3 years. More specifically, 89, 45, 46, 67, and 6 participants were in their 20s, 30s, 40s, 50s, and 60s, respectively. Based on their occupations, they were classified into four groups: doctors, nurses, allied health professionals (e.g., social workers, medical engineers, and physician assistants), and auxiliary staff members (e.g., administrative

Table 1
Demographic characteristics of the participants.

Variable	Physicians (*n* = 27)	Nurses (*n* = 149)	Allied health professionals (*n* = 35)	Auxiliary staff members (n = 35)
Gender				
Male	16 (59%)	5 (3%)	11 (31%)	11 (31%)
Female	11 (41%)	144 (97%)	31 (69%)	24 (69%)
Age (M ± SD) in years	40.67 ± 10.46	36.35 ± 11.91	43.83 ± 12.20	44.06 ± 12.00
20–29	3 (11%)	72 (48%)	10 (29%)	4 (11%)
30–39	12 (44%)	19 (13%)	4 (11%)	12 (34%)
40–49	6 (22%)	24 (16%)	10 (29%)	6 (17%)
50–59	5 (21%)	34 (23%)	15 (43%)	12 (34%)
≥ 60	1 (4%)	0	2 (6%)	3 (9%)
Psychiatric history				
No	25 (93%)	146 (98%)	34 (97%)	33 (94%)
Yes	2 (7%)	3 (2%)	1 (3%)	2 (6%)
MINI				0
A: MDD (≥ 5)	0	1	1	1
B: GAD (≥ 3)	0	7	1	2
C: PD (≥ 4)	0	1	0	1
D: SAD (≥ 4)	0	0	1	0

Note. MINI = Mini International Neuropsychiatric Interview, MDD = major depressive disorder, GAD = generalized anxiety disorder, PD = panic disorder, SAD = social anxiety disorder.

staff). The sample consisted of 27 doctors, 149 nurses, 42 allied health professionals, and 35 auxiliary staff members (Table 1).

The participants had a mean IES-R score of 13.2 ± 13.8. Moreover, 54 participants (21%) had scores that were higher than the cutoff score of 25, which warrants a diagnosis of PTSD. The MINI was administered to these participants. Four (8%) scored 5 or above on the MDD item and 10 (19%) scored 11 or above on the generalized anxiety disorder item, and 2 (4%) scored 2 or above on the panic disorder items, and 1 (2%) scored 4 or above on the social anxiety disorder item. Those who had IES-R scores ≥25 at baseline constituted the high-risk group and responded to a second survey, which was conducted two weeks after the psychiatric consultation. Out of this group of 54 participants (response rate = 27.8%), 15 responded. The total IES-R scores of two participants (13%) indicated that they were at high risk even after two weeks (Fig. 1).

Men and women had mean IES-R scores of 8.56 ± 11.86 and 14.11 ± 14.09, respectively. Gender differences in their IES-R scores were examined by conducting independent samples *t*-test. Women had significantly higher scores than men ($p = 0.02$) (Fig. 2). With regard to the subscales, men and women had mean hyperarousal scores of 2.02 ± 3.27 and 3.95 ± 4.15, respectively. Gender differences in their hyperarousal scores were examined using independent samples *t*-test. Women had significantly higher hyperarousal scores than men ($p < 0.01$). Men and women had mean intrusion scores of 2.19 ± 2.84 and 3.82 ± 4.03, respectively. Gender differences in their intrusion scores were examined using independent samples *t*-test. Women had significantly higher intrusion scores than men ($p < 0.01$). Men and women had mean avoidance scores of 2.40 ± 3.75 and 3.69 ± 4.08, respectively. Gender differences in their avoidance scores were examined using independent samples *t*-test. Women had significantly higher avoidance scores than men ($p = 0.01$). On the sleep and numbness subscale, men and women had mean scores of 1.86 ± 2.57 and 2.60 ± 2.78, respectively. Gender differences in their sleep and numbness scores were examined using independent samples t-test. However, there was no significant difference between men and women ($p = 0.06$) (Table 2).

3.2. Comparison of the IES-R scores based on psychiatric history

With regard to reported psychiatric history, three participants had anxiety disorder, and five participants had depressive disorder. Those with a psychiatric history ($n = 8$) had a mean score of 22.63 ± 21.09, whereas those without a psychiatric history ($n = 245$) had a mean score of 12.86 ± 13.52 (Fig. 3). The Kruskal-Wallis test showed that there were no significant differences in the total IES-R scores that were obtained by the two groups ($p = 0.14$).

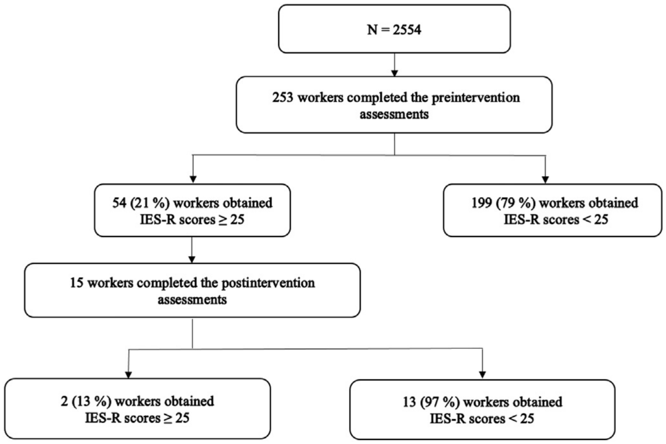

Fig. 1. Summary of the 1st and 2nd survey progress. Note. IES-R = Impact of Event Scale-Revised.

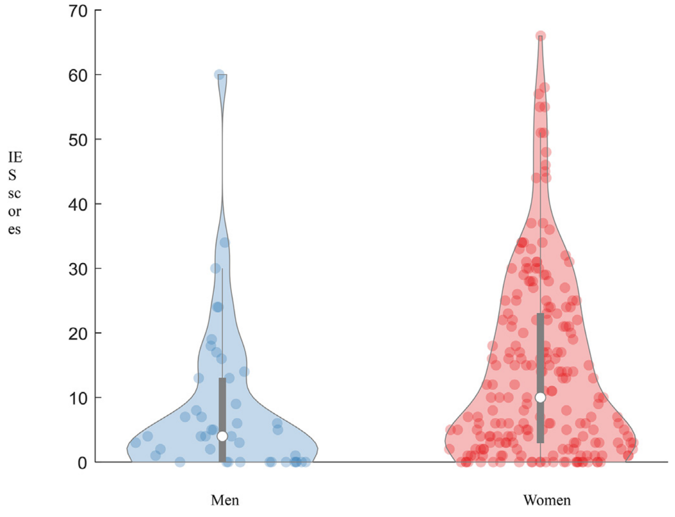

Fig. 2. Gender differences in the IES-R scores. Note. IES-R = Impact of Event Scale-Revised.

Table 2
Comparisons of the IES-R scores obtained by men and women.

Variable	Men	Women	p
IES-R (total scores)	8.56 ± 11.86	14.11 ± 14.09	0.02
Hyperarousal	2.02 ± 3.27	3.95 ± 4.15	< 0.01
Intrusion	2.19 ± 2.84	3.82 ± 4.03	< 0.01
Avoidance	2.40 ± 3.75	3.69 ± 4.08	0.01
Sleep and numbness	1.86 ± 2.57	2.60 ± 2.78	0.06

Note. IES-R = Impact of Event Scale-Revised.

3.3. Comparison of the IES-R scores across occupational groups

The participants had a mean IES-R score of 13.2 ± 13.8. The physicians, nurses, allied health professionals, and auxiliary staff members had mean IES-R scores of 5.67 ± 5.68, 13.67 ± 13.10, 11.21 ± 13.09, and 19.17 ± 18.98, respectively (Fig. 4). The Kruskal-Wallis test showed that there was a significant difference in the total IES-R scores that were obtained by these occupational groups ($p = 0001$). Specifically, post-hoc analysis showed that the physicians had lower IES-R scores than the nurses and auxiliary health professionals (Table 3).

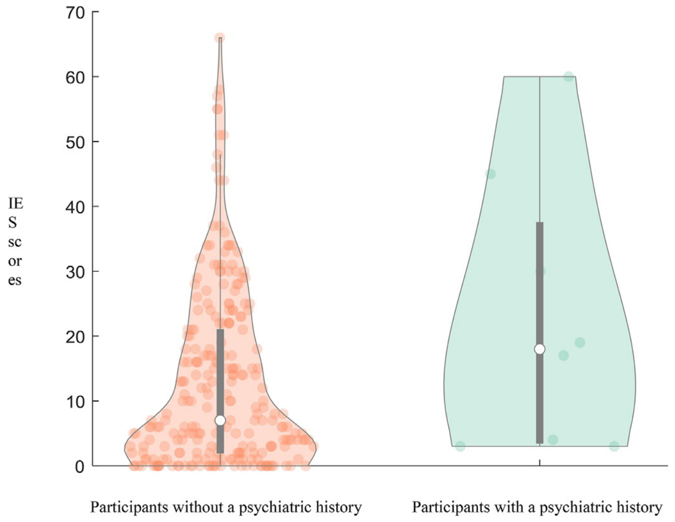

Fig. 3. Differences in the IES-R scores of participants with and without a psychiatric history. Note. IES-R = Impact of Event Scale-Revised.

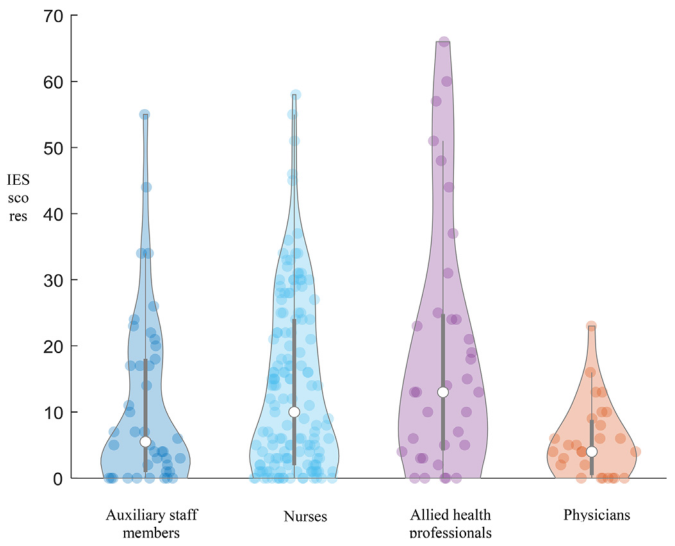

Fig. 4. Differences in the IES-R scores across occupational groups. Note. IES-R = Impact of Event Scale-Revised.

On the hyperarousal subscale, the physicians, nurses, allied health professionals, and auxiliary staff members had mean scores of 1.45 ± 1.83, 3.75 ± 3.81, 3.21 ± 3.81, and 5.26 ± 5.75, respectively. The Kruskal-Wallis test showed that there was a significant difference between these occupational groups ($p = 0.01$). Post-hoc analysis showed that the physicians had significantly lower hyperarousal scores than the nurses and auxiliary staff members.

On the intrusion subscale, the physicians, nurses, allied health professionals, and auxiliary staff members had mean scores of 1.89 ± 2.08, 3.60 ± 3.63, 3.04 ± 3.96, and 5.17 ± 5.31, respectively. The Kruskal-Wallis test showed that there was a significant difference between these occupational groups ($p = 0.04$). Post-hoc analysis showed that the physicians had significantly lower intrusion scores than the auxiliary staff members.

On the avoidance subscale, the physicians, nurses, allied health professionals, and auxiliary staff members had mean scores of 1.19 ± 1.55, 3.62 ± 3.84, 3.02 ± 3.87, and 5.09 ± 5.51, respectively. The Kruskal-Wallis test showed that there was a significant difference between these occupational groups ($p < 0.01$). Post-hoc analysis showed that the physicians had significantly lower avoidance scores than the nurses and auxiliary staff members.

On the sleep and numbness subscale, the physicians, nurses, allied health professionals, and auxiliary staff members had mean scores of 1.15 ± 1.38, 2.60 ± 2.63, 1.95 ± 2.48, and 3.63 ± 3.77, respectively. The Kruskal-Wallis test showed that there was a significant difference between these occupational groups ($p = 0.01$). Post-hoc analysis showed that the physicians had significantly lower scores than the auxiliary staff members.

3.4. Changes in scores on the IES-R and CGI-S after the psychiatric intervention

Telephone-based psychiatric consultations were offered to the 54 high-risk participants who wished to receive counseling. Fifteen of them responded to the IES-R and CGI-S at the end of the intervention. The initial total IES-R score of those who responded to the second assessment was 32.33 ± 13.40 points. In the second evaluation, two weeks after the psychiatric consultation, the total IES-R score was 18.67 ± 23.46 points. There was a decrease in the total IES-R scores of 13 of the 15 participants, and their mean score was 13.67 ± 16.15 (Fig. 5). The Wilcoxon signed-rank test showed that their IES-R scores had significantly decreased after the psychiatric intervention ($p < 0.01$) (Table 4). The CGI-S scale evaluated by psychiatrist on 22 people who had a second interview, the initial state CGI-S score was 2.35 ± 0.93 points, and the second CGI-S score was 1.35 ± 0.57 points two weeks later. Two weeks later, their CGI-S scores decreased by 1.00 ± 0.74 points. The Wilcoxon signed-rank test showed that their CGI-S scores had significantly decreased after the psychiatric intervention (p < 0.01).

4. Discussion

During a pandemic, the psychological reactions of hospital workers tend to be diverse and complex and this is an under-research area [24]. The causes of these psychological reactions include concerns about their own health, the risk of transmission, stigmatization, changes in their occupational environment, and the stress caused by isolation [25].

Similar to the findings of past studies on MERS and SARS, the present findings suggest that hospital workers experience high levels of psychological stress during an epidemic [7,26–28]. In particular, the main findings suggest that the participating nurses and nonmedical staff members were more stressed than the physicians [5]. In addition, there were gender differences in their stress levels. Specifically, women had significantly higher scores on the IES-R and all its subscales, except for sleep and numbness. Most of the participants were women and nurses, and nurses accounted for the largest proportion of the hospital's workforce. Therefore, it was not surprising that they had high IES-R scores. Indeed, their risk of contracting the disease was high because they were intensively involved in providing care to patients. Other studies on COVID-19 have found that fewer years of work experience, gender (i.e., female), and direct contact are high-risk factors of infectious disease-related stresses [29]. As the result of the study conducted during the influenza pandemic, nurses were not sufficiently prepared to work safely compared to other workers in hospitals [30].

In addition, in a past study, healthcare workers who were in charge of directly treating patients with infectious diseases had higher IES-R scores than their counterparts [27]. Past studies on the psychological effects of SARS on hospital workers found that approximately 40% of healthcare workers experienced high posttraumatic symptoms even three years after the SARS outbreak [8]. In addition, high-risk groups for respiratory medicine were still classified as high-risk after a year [26]. Therefore, early and intensive psychological management should be implemented by identifying high-risk hospital workers during an epidemic.

The uncertainty surrounding the possibility of contracting a disease can exacerbate preexisting symptoms of anxiety or depression. The total IES-R scores of those with a psychiatric history were higher than those of their counterparts, however, the difference was not significant. Past studies on the mental health of hospital workers have found that their psychiatric history is a significant risk factor for psychological stress [31,32]. We thought that the difference is resulted from small number of total participants and the large gap in the number between two group. Hospitals should conduct early screening programs and provide psychological interventions to enable their workers to work efficiently and safely.

The physicians who participated in this study had significantly lower total and subscale scores on the IES-R when compared to the nurses, allied health professionals, and auxiliary staff members. This finding underscores another subgroup of hospital workers who require psychiatric evaluation and management. These results may be attributable to factors such as limited available information about COVID-19, the high rates of transmission, the wide range of symptoms (i.e., from asymptomatic to fatal cases), and diagnosis of re-positive after negative diagnosis, that showing different characteristics from other viruses. The information of well-recognized for infectious diseases has been found to be independent factor of the degree of concerns for infectious

Table 3
Comparisons of the IES-R scores across occupational groups.

Group	Physicians	Nurses	Allied health professionals	Auxiliary staff members	p	Post-hoc analysis results
IES-R (total scores)	5.67 ± 5.68	13.67 ± 13.10	11.21 ± 13.09	19.17 ± 18.98	< 0.01	Physicians < Nurses and auxiliary staff members
Hyperarousal	1.45 ± 1.83	3.75 ± 3.81	3.21 ± 3.81	5.26 ± 5.75	0.01	Physicians < Nurses and auxiliary staff members
Intrusion	1.89 ± 2.08	3.60 ± 3.63	3.04 ± 3.96	5.17 ± 5.31	0.04	Physicians < Auxiliary staff members
Avoidance	1.19 ± 1.55	3.62 ± 3.84	3.02 ± 3.87	5.09 ± 5.51	0.01	Physicians < Nurses and auxiliary staff members
Sleep and numbness	1.15 ± 1.38	2.60 ± 2.63	1.95 ± 2.48	3.63 ± 3.77	0.01	Physicians < Auxiliary staff members

Note. IES-R = Impact of Event Scale-Revised.

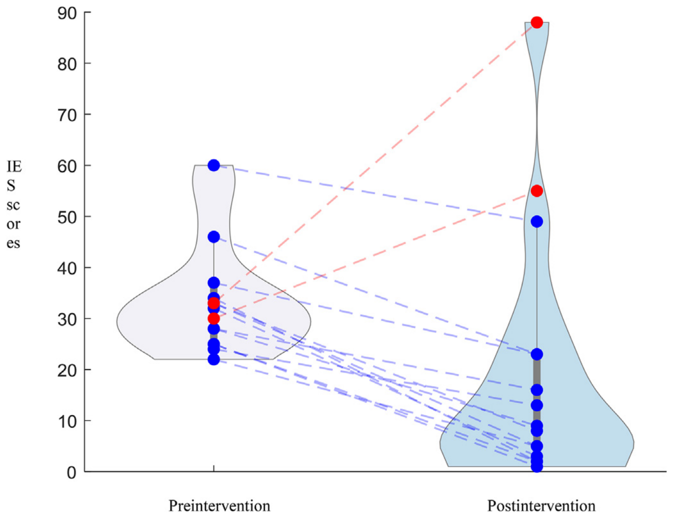

Fig. 5. Changes in the IES-R scores after the psychiatric intervention. Note. IES-R = Impact of Event Scale-Revised.

Table 4
Comparisons of preintervention and postintervention scores on the IES-R.

Variable	Preintervention scores	Postintervention scores	p
IES-R (total scores)	32.33 ± 13.40	18.67 ± 23.46	< 0.01
Hyperarousal	9.13 ± 4.07	5.33 ± 7.05	0.01
Intrusion	8.67 ± 4.00	4.67 ± 5.45	< 0.01
Avoidance	8.93 ± 3.69	5.00 ± 6.16	0.01
Sleep and numbness	5.27 ± 3.31	3.40 ± 5.05	0.01

Note. IES-R = Impact of Event Scale-Revised.

diseases [33,34]. The participating auxiliary staff members were more concerned about infectious diseases than the other occupational groups. This observation is similar to the findings of another study in which the administrative staff members of a hospital did not possess adequate information and expressed a high level of concern about their health [35]. This finding underscores the need to pay more attention to administrative staff members and healthcare workers during an epidemic. Hospitals should provide sufficient information about their policies to reduce their level of infection-related stress and create a flexible work environment.

In this study, the IES-R scores of the 15 high-risk participants decreased by 13.67 ± 16.15 points at the end of the evaluation (preintervention: 32.33 ± 13.40, postintervention: 18.67 ± 23.46). Phones and internets enabled various psychological interventions to be clinically effective in the range of various mental disorders [15,36]. In this study, the psychiatric consultations were provided over the telephone. The provision of psychological information may help individuals accept their feelings as a natural emotional response to infectious diseases. It may also improve their self-regulation abilities and lower their stress levels.

Many past findings have underscored the need to actively monitor the psychological statuses of healthcare workers and provide appropriate interventions to vulnerable individuals. These findings highlight the need to provide training and support to high-risk individuals to prevent and alleviate emotional stress. Research on psychological intervention, such as crisis intervention, which targets patients in the event of infectious diseases, is being actively studied [16,37]. However, research studies on hospital workers have mostly used cross-sectional designs. Therefore, the literature on the role of screening programs and psychological interventions in minimizing the psychological impact on hospital workers remains limited. Therefore, further research is needed.

This study has a few limitations. The sample was limited for generalization, conducted in one hospital, and the response rate differed from group to group and cannot be applied to healthcare workers working in the countryside [38]. Second, there are self-selection sampling biases because we analyzed data of voluntary reporting through telephone. Despite these limitations, we were able to examine the psychological impact of the COVID-19 pandemic on hospital workers and demonstrate support for the effectiveness of the aforementioned psychiatric intervention in reducing their stress levels. The present findings enhance our understanding of the mental health problems that are experienced by hospital workers during a pandemic. Finally, the findings are also expected to enhance psychiatric expertise in relation to the minimization of the adverse psychological effects that are associated with infectious diseases.

5. Conclusions

In this study, both the healthcare workers and nonmedical staff members reported experiencing high levels of psychological stress during the pandemic period. In particular, higher levels of stress were reported by the nonmedical staff workers than by the physicians. These findings underscore the need to provide sufficient information and support to nurses, other staff members, and physicians. Efforts should be taken to increase awareness about mental health care among hospital workers. In this regard, psychiatrists play an increasingly important role in implementing rapid and effective psychiatric interventions during a pandemic.

Funding

This study was supported by a research grant from the Daegu Medical Association COVID-19 Scientific Committee.

Data availability statement

The data that support the findings of this study are available on request from the corresponding author. The data are not publicly available due to their containing information that could compromise the privacy of research participants.

Declaration of Competing Interest

None.

Acknowledgments

This study was supported by a research grant from the Daegu Medical Association COVID-19 Scientific Committee.

References

[1] Li Q, Guan X, Wu P, Wang X, Zhou L, Tong Y, et al. Early transmission dynamics in Wuhan, China, of novel coronavirus–infected pneumonia. N Engl J Med. 2020;382:1199–207.
[2] Organization WH. WHO director-General's opening remarks at the media briefing on COVID-19; 2020.
[3] Organization WH. WHO coronavirus disease (COVID-19) dashboard; 2020.
[4] Korea Ministry of Health and Welfare and Center for Disease Control and Preventions. Updates on COVID-19: for press release; 2020.
[5] Chew NW, Lee GK, Tan BY, Jing M, Goh Y, Ngiam NJ, et al. A multinational, multicentre study on the psychological outcomes and associated physical symptoms amongst healthcare workers during COVID-19 outbreak. Brain Behav Immun. 2020;88:559–65.
[6] Tan BY, Chew NW, Lee GK, Jing M, Goh Y, Yeo LL, et al. Psychological impact of the COVID-19 pandemic on health Care Workers in Singapore. Ann Intern Med. 2020;173(4):317–20.
[7] Chan AO, Huak CY. Psychological impact of the 2003 severe acute respiratory syndrome outbreak on health care workers in a medium size regional general hospital in Singapore. Occup Med. 2004;54:190–6.
[8] Wu P, Fang Y, Guan Z, Fan B, Kong J, Yao Z, et al. The psychological impact of the SARS epidemic on hospital employees in China: exposure, risk perception, and altruistic acceptance of risk. Can J Psychiatry. 2009;54:302–11.
[9] LeBlanc VR. The effects of acute stress on performance: implications for health professions education. Acad Med. 2009;84:S25–33.
[10] Panagioti M, Geraghty K, Johnson J, Zhou A, Panagopoulou E, Chew-Graham C, et al. Association between physician burnout and patient safety, professionalism, and patient satisfaction: a systematic review and meta-analysis. JAMA Intern Med. 2018;178:1317–31.
[11] Wang C, Pan R, Wan X, Tan Y, Xu L, Ho CS, et al. Immediate psychological responses and associated factors during the initial stage of the 2019 coronavirus disease (COVID-19) epidemic among the general population in China. Int J Environ Res Public Health. 2020;17:1729.
[12] Ho CS, Chee CY, Ho RC. Mental health strategies to combat the psychological impact of COVID-19 beyond paranoia and panic. Ann Acad Med Singapore. 2020;49:1–3.
[13] Son H, Lee WJ, Kim HS, Lee KS, You M. Examination of hospital Workers' emotional responses to an infectious disease outbreak: lessons from the 2015 MERS co-V outbreak in South Korea. Disaster Med Public Health Prep. 2019;13:504–10.
[14] Li W, Yang Y, Liu Z-H, Zhao Y-J, Zhang Q, Zhang L, et al. Progression of mental health services during the COVID-19 outbreak in China. Int J Biol Sci. 2020;16:1732.
[15] Liu S, Yang L, Zhang C, Xiang Y-T, Liu Z, Hu S, et al. Online mental health services in China during the COVID-19 outbreak. Lancet Psychiatry. 2020;7 (e17-e8).
[16] Zhang J, Wu W, Zhao X, Zhang W. Recommended psychological crisis intervention response to the 2019 novel coronavirus pneumonia outbreak in China: a model of West China Hospital. Prec Clin Med. 2020;3:3–8.
[17] Weiss D, Marmar CR. The impact of event scale-revised. In: Wilson J, Keane TM, editors. Assessing psychological trauma and PTSD: A practitioner's handbook. New York: Guildford Press; 1997. p. 399–411.
[18] Eun HJ, Kwon TW, Lee SM, Kim TH, Choi MR, Cho SJ. A study on reliability and validity of the Korean version of impact of event scale-revised. J Korean Neuropsychiatr Assoc. 2005;44(3):303–10.
[19] Wang C, Pan R, Wan X, Tan Y, Xu L, McIntyre RS, et al. A longitudinal study on the mental health of general population during the COVID-19 epidemic in China. Brain Behav Immun. 2020;87:40–8.
[20] Tan W, Hao F, McIntyre RS, Jiang L, Jiang X, Zhang L, et al. Is returning to work during the COVID-19 pandemic stressful? A study on immediate mental health status and psychoneuroimmunity prevention measures of Chinese workforce. Brain Behav Immun. 2020;87:84–92.
[21] Hao F, Tan W, Jiang L, Zhang L, Zhao X, Zou Y, et al. Do psychiatric patients experience more psychiatric symptoms during COVID-19 pandemic and lockdown? A case-control study with service and research implications for immunopsychiatry. Brain Behav Immun. 2020;87:100–6.
[22] Lim SW, Song HS, Oh YH, Shin HC, Cho KH, Chung SK, et al. A validation study of the abbreviated self-rated Korean version of MINI (MINI patient health survey). Anxiety Mood. 2007;3(1):32–40.
[23] Busner J, Targum SD. The clinical global impressions scale: applying a research tool in clinical practice. Psychiatry (Edgmont). 2007;4:28.
[24] Tran BX, Ha GH, Nguyen LH, Vu GT, Hoang MT, Le HT, et al. Studies of novel coronavirus disease 19 (COVID-19) pandemic: a global analysis of literature. Int J Environ Res Public Health. 2020;17:4095.
[25] Bai Y, Lin C-C, Lin C-Y, Chen J-Y, Chue C-M, Chou P. Survey of stress reactions among health care workers involved with the SARS outbreak. Psychiatr Serv. 2004;55:1055–7.
[26] McAlonan GM, Lee AM, Cheung V, Cheung C, Tsang KW, Sham PC, et al. Immediate and sustained psychological impact of an emerging infectious disease outbreak on health care workers. Can J Psychiatry. 2007;52:241–7.
[27] Lee SM, Kang WS, Cho A-R, Kim T, Park JK. Psychological impact of the 2015 MERS outbreak on hospital workers and quarantined hemodialysis patients. Compr Psychiatry. 2018;87:123–7.
[28] Nickell LA, Crighton EJ, Tracy CS, Al-Enazy H, Bolaji Y, Hanjrah S, et al. Psychosocial effects of SARS on hospital staff: survey of a large tertiary care institution. Cmaj. 2004;170:793–8.
[29] Kang L, Ma S, Chen M, Yang J, Wang Y, Li R, et al. Impact on mental health and perceptions of psychological care among medical and nursing staff in Wuhan during the 2019 novel coronavirus disease outbreak: A cross-sectional study. Brain Behav Immun. 2020;87:11–7.
[30] Seale H, Leask J, Po K, MacIntyre CR. "Will they just pack up and leave?"–attitudes and intended behaviour of hospital health care workers during an influenza pandemic. BMC Health Serv Res. 2009;9:30.
[31] Zhu Z, Xu S, Wang H, Liu Z, Wu J, Li G, et al. COVID-19 in Wuhan: immediate psychological impact on 5062 health workers. medRxiv. 2020.
[32] Puteri LA, Syaebani MI. Employees work stress level in the hospital. Int Res J Bus Stud. 2018;11:232–43.
[33] Johal SS. Psychosocial impacts of quarantine during disease outbreaks and interventions that may help to relieve strain; 2009.
[34] Albarrak AI, Mohammed R, Al Elayan A, Al Fawaz F, Al Masry M, Al Shammari M, et al. Middle East Respiratory Syndrome (MERS): Comparing the knowledge, attitude and practices of different health care workers. J Infect Public Health. 2019.
[35] Goulia P, Mantas C, Dimitroula D, Mantis D, Hyphantis T. General hospital staff worries, perceived sufficiency of information and associated psychological distress during the a/H1N1 influenza pandemic. BMC Infect Dis. 2010;10:322.
[36] Zhou X, Snoswell CL, Harding LE, Bambling M, Edirippulige S, Bai X, et al. The role of telehealth in reducing the mental health burden from COVID-19. Telemed e-Health. 2020;26:377–9.
[37] Bo H-X, Li W, Yang Y, Wang Y, Zhang Q, Cheung T, et al. Posttraumatic stress symptoms and attitude toward crisis mental health services among clinically stable patients with COVID-19 in China. Psychol Med. 2020:1–2.
[38] Tran BX, Phan HT, Nguyen TPT, Hoang MT, Vu GT, Lei HT, et al. Reaching further by village health collaborators: the informal health taskforce of Vietnam for COVID-19 responses. J Glob Health. 2020;10.
