# Supplementary material for: Mental health status and related factors influencing healthcare workers during the COVID-19 pandemic: A systematic review and meta-analysis
Source: PLoS One. 2024 Jan 19;19(1):e0289454. doi: 10.1371/journal.pone.0289454 (PMC10798549; doi:10.1371/journal.pone.0289454)
Supplement: S1 Data — (ZIP) [file pone.0289454.s011.zip › literatures/6.pdf]

frontiers in Psychiatry

ORIGINAL RESEARCH
published: 17 December 2021
doi: 10.3389/fpsyt.2021.797545

The Psychological Impact of COVID-19 on Healthcare Workers in Saudi Arabia: A Year Later Into the Pandemic

Atiah H. Almalki[1,2†], Mohammad S. Alzahrani[3†], Fahad S. Alshehri[4], Adnan Alharbi[5], Samirah F. Alkhudaydi[6], Rawan S. Alshahrani[6], Aseel H. Alzaidi[6], Majed A. Algarni[3], Hashem O. Alsaab[7], Yasser Alatawi[8], Yusuf S. Althobaiti[2,9], Ahmed K. Bamaga[10] and Abdullah A. Alhifany[5]*

[1] Department of Pharmaceutical Chemistry, College of Pharmacy, Taif University, Taif, Saudi Arabia, [2] Addiction and Neuroscience Research Unit, Health Science Campus, Taif University, Taif, Saudi Arabia, [3] Department of Clinical Pharmacy, College of Pharmacy, Taif University, Taif, Saudi Arabia, [4] Department of Pharmacology and Toxicology, College of Pharmacy, Umm Al-Qura University, Makkah, Saudi Arabia, [5] Department of Clinical Pharmacy, College of Pharmacy, Umm Al-Qura University, Makkah, Saudi Arabia, [6] College of Pharmacy, Taif University, Taif, Saudi Arabia, [7] Department of Pharmaceutics and Pharmaceutical Technology, Taif University, Taif, Saudi Arabia, [8] Department of Pharmacy Practice, Faculty of Pharmacy, University of Tabuk, Tabuk, Saudi Arabia, [9] Department of Pharmacology and Toxicology, College of Pharmacy, Taif University, Taif, Saudi Arabia, [10] Neurology Division, Pediatric Department, Faculty of Medicine, King Abdulaziz University Hospital, King Abdulaziz University, Jeddah, Saudi Arabia

OPEN ACCESS

Edited by:
Mohammadreza Shalbafan, Iran University of Medical Sciences, Iran

Reviewed by:
Rahim Badrfam, Tehran University of Medical Sciences, Iran
Yong Gan, Huazhong University of Science and Technology, China

***Correspondence:**
Atiah H. Almalki
ahalmalki@tu.edu.sa

† These authors have contributed equally to this work

Specialty section:
This article was submitted to Public Mental Health, a section of the journal Frontiers in Psychiatry

Received: *18 October 2021*
Accepted: *22 November 2021*
Published: *17 December 2021*

Citation:
Almalki AH, Alzahrani MS, Alshehri FS, Alharbi A, Alkhudaydi SF, Alshahrani RS, Alzaidi AH, Algarni MA, Alsaab HO, Alatawi Y, Althobaiti YS, Bamaga AK and Alhifany AA (2021) The Psychological Impact of COVID-19 on Healthcare Workers in Saudi Arabia: A Year Later Into the Pandemic. Front. Psychiatry 12:797545. doi: 10.3389/fpsyt.2021.797545

Objective: The COVID-19 pandemic poses unprecedented challenges to healthcare workers worldwide. This study sought to estimate the prevalence of depression, anxiety, and stress among healthcare workers in Saudi Arabia, and to identify the factors associated with these psychological disorders.

Methods: A cross-sectional questionnaire-based study was conducted from January 21 to March 2, 2021. Physicians, pharmacists, nurses, and other healthcare workers from different parts of Saudi Arabia were recruited through snowball sampling. Psychological outcomes were measured using the Depression, Anxiety, and Stress Scale (DASS-21). Pearson's chi-square test was used to explore the bivariate association between diverse characteristics and each outcome. Multiple logistic regression analyses were performed to identify factors associated with depression, anxiety, and stress.

Results: A total of 501 healthcare workers completed the survey, of whom 60% were female and nearly half were pharmacists. The majority (76.25%) of respondents reported that a family member, friend, or colleague had contracted COVID-19, and more than one-third (36%) knew someone who died due to COVID-19. Overall, the estimated prevalence rates of depression, anxiety, and stress were 54.69, 60.88, and 41.92%, respectively. The multivariate analysis revealed that healthcare workers with chronic diseases, nurses, and healthcare workers from the southern region were more likely to suffer from depression and stress. Further, individuals with positive COVID-19 test results showed a greater proportion of depressive symptoms compared to others. In addition, knowing someone who died due to COVID-19 and having a chronic illness were predisposing factors for anxiety.

Conclusion: After more than a year, the prevalence of depression, anxiety, and stress remains substantial among healthcare workers in Saudi Arabia. The findings can help guide efforts to mitigate the psychological impact of the pandemic.

Keywords: mental health, Saudi Arabia (KSA), COVID-19, health care workers (HCW), depression, anxiety, stress

INTRODUCTION

The novel coronavirus (SARS-CoV-2) was first reported by the Chinese government in December 2019 in the city of Wuhan (1). and declared a pandemic by the World Health Organization (WHO) on March 7, 2020 (2). SARS-CoV-2 has caused a similar pathogenesis as previous coronaviruses, such as Middle East Respiratory Syndrome (MERS) in 2012 (3). Pandemics and natural disasters often have a psychological impact on infected people and those in direct contact, such as healthcare workers (HCWs). About 30% of the general population in China has complained of moderate to severe anxiety (4). A study conducted among students found that anxiety and stress were highly associated with academic delays and low quality of life during the corona pandemic (5). Moreover, many medical doctors and nurses in Wuhan reported mental disturbances and anxiety disorders, and indicated that mental health support provided relief and alleviated symptoms (6, 7). In China, the incidence of psychological stress and anxiety in medical staff was higher compared to college students, possibly explained by the student's limited contact with confirmed positive COVID-19 cases in comparison to practicing healthcare providers (8).

Unlike prior outbreaks, the COVID-19 pandemic could have a potentially long-term psychological impact on both the general public and HCWs. One year following the start of the pandemic, few studies have been conducted to assess the psychological impact among HCWs using the Depression, Anxiety, and Stress Scale (DASS-21) questionnaire (9–15). Also, few longitudinal studies have been conducted to evaluate the long-term impact of the pandemic on health care providers (16–22). A recent Chinese study reported that some residents were still suffering from depression and anxiety during the low transmission period one year after the start of the pandemic (23). As Saudi Arabia also enters the low transmission phase, a timely psychological assessment is needed to identify vulnerable populations.

To date, few studies have evaluated the long-term impact of COVID-19 on the Saudi population, and none have tackled the long-term impact on HCWs in Saudi Arabia (3, 24, 25). The HCWs were on the front line of the crisis in Saudi Arabia, whether in hospitals or community pharmacies. Few studies have assessed the psychological impact of the COVID-19 pandemic among HCWs in Saudi Arabia in the past year (11, 26, 27), and no study has examined pharmacists working on the front line of this crisis, whether in hospitals or community pharmacies, or while delivering patient medications. The present study, therefore, aimed to investigate the long-term impact of COVID-19 among pharmacists and other HCWs one year following the start of the pandemic.

METHODS

Study Design and Setting

A cross-sectional questionnaire-based study was conducted from January 21 through March 2, 2021. HCWs residing in Saudi Arabia and working at the Saudi MOH, local community pharmacy chains, and other government or private hospitals were eligible to participate in the study. Exclusion criteria included those under 18 years of age, non-Arabic speakers, and non-HCWs. The study participants were recruited using a snowball sampling technique, where a link to the online survey was promoted and shared via WhatsApp, Twitter, and internal emails. The web-based survey was designed to ensure that every participant could take part only once. All participants were informed of the study purpose and were assured of the confidentiality of their responses. The study was approved by the Scientific Research Ethics Committee at Taif University (42-0068) and the Institutional Review Board of MOH (472).

A priori sample size was calculated according to the formula suggested by Lwanga and Lemeshow (28). We assumed that the prevalence of psychological distress during the COVID-19 pandemic in Saudi Arabia was 23.6%, as reported in a recent study (29). The required sample size was calculated using OpenEpi (Version 3.01, Atlanta, USA). The minimum sample size required for 80% power was 416, with a 95% confidence level, 5% confidence limit, and 1.5 design effect.

Data Collection

Data were collected via a standardized, self-administered questionnaire. We adopted the Depression, Anxiety, and Stress Scale—21 (DASS– 21), a reliable and valid self-administered instrument to screen for these psychological disorders (30). This survey tool has 21 items, each of which is scored on a scale from 0 ("does not apply to me at all") to 3 ("applies to me most of the time"). Scores for each subscale are determined by summing the scores of relevant items and then multiplying by a factor of 2. Each subscale was categorized into normal, mild/moderate, and severe/extremely severe based on the recommended cut-off values (30). The survey tool was translated to Arabic according to the forward and backward translation technique, which is known as a cross-cultural adaptation of research instruments (31). The process included two steps: (1) the forward translation from English to Arabic by two translators fluent in Arabic and English; and (2) the backward translation from Arabic to English by two different translators also fluent in both languages. Afterward, two faculty members with knowledge of the subject assessed the face validity of the Arabic version of the questionnaire.

We gathered demographic and occupational characteristics from the questionnaire, including data on age, gender, nationality

TABLE 1 | Sociodemographic characteristics of study participants.

Characteristic	Job category				Total (*N* = 501)
	Physicians (*N* = 63)	Pharmacists (*N* = 244)	Nurses (*N* = 54)	Others (*N* = 140)	
Female, %	68.25	44.67	79.63	78.57	60.88
Age, %					
18–24	38.10	30.33	55.56	59.29	42.12
25–34	38.10	56.97	33.33	28.57	44.11
35 or older	23.81	12.70	11.11	12.14	13.77
Nationality, %					
Saudi	61.90	92.21	83.33	85	85.43
Non-Saudi	38.10	7.79	16.67	15	14.57
Marital status, %					
Married	30.16	35.80	24.07	18.57	29.00
Single/widowed/divorced	69.84	64.20	75.93	81.43	71.00
Region, %					
Central Region	41.27	25.82	35.85	30.94	30.26
Eastern Region	17.46	11.07	32.08	15.11	15.23
Northern Region	9.52	7.79	7.55	10.79	8.82
Southern Region	11.11	13.52	9.43	12.23	12.42
Western Region	20.63	41.80	15.09	30.94	33.27
Healthcare setting, %					
Inpatient Hospital Setting	41.27	26.64	51.85	31.16	32.46
Outpatient Hospital Setting	12.70	15.16	5.56	10.87	12.63
Primary Health Care Centre	25.40	6.15	16.67	19.57	13.43
Community Pharmacy	-	22.95	1.85	-	11.42
Other	20.63	29.10	24.07	38.41	30.06
Have chronic illness, %	28.57	13.52	22.22	19.29	17.96
Tested positive for COVID-19, %	31.75	15.16	24.07	17.14	18.76
Family member, friend, or colleague tested positive for COVID-19, %	74.60	79.51	66.67	75.00	76.25
Family member, friend, or colleague died due to COVID-19, %	44.44	36.07	32.08	33.57	36.00

(Saudi or non-Saudi), marital status (married or unmarried), and geographic region (central, eastern, western, northern, or southern region). Job occupation was categorized into physician, pharmacist, nurse, and others, which included various occupations such as dentist, laboratory worker, radiology technician, and medical intern. In addition, we collected data on whether the participants had a chronic illness, whether they had tested positive for COVID-19, and whether someone they knew had tested positive for COVID-19 or had died due to COVID-19.

Statistical Analysis

Univariate, bivariate, and multivariate statistical analyses were conducted. Frequency and percentages were used to describe characteristics and estimate prevalence rates of depression, anxiety, and stress among participants. A bivariate analysis was conducted using Pearson's chi-square test to explore the association between sociodemographic traits and each DASS subscale. Variables that were significantly associated with the outcomes were further analyzed by entering the adjusted multivariate model. Age, gender, and job category were predetermined to enter into the adjusted model, regardless of their bivariate association with each outcome. A multivariate logistic regression analysis determined the factors associated with each outcome (i.e., depression, anxiety, and stress). Adjusted odds ratios with 95% confidence intervals (CIs) and *p*-values were calculated to determine the strength and significance of the association. All statistical analyses were performed using SAS software (version 9.2, SAS Institute Inc., Cary, NC, USA).

RESULTS

A total of 501 HCWs completed the survey, nearly half of whom were pharmacists ($n = 244$). The response rate was 97.8%. The majority of participants were Saudis, one-third were from the Western region, and about 60% were female (**Table 1**). While only 18.76% of the participants had tested positive for COVID-19, the majority (76.25%) reported that a family member, friend, or colleague had tested positive for COVID-19. In addition, about 44% of physicians reported that a family member, friend, or colleague had died due to COVID-19, compared to 36.07 and 32.08% of pharmacists and nurses, respectively.

Based on the DASS subscale scores, the estimated prevalence rates of depression, anxiety, and stress among participants were 54.69, 60.88, and 41.92%, respectively. In terms of severity, one-third (33.13%) of the participants suffered from severe or extremely severe anxiety, 23.95% suffered from severe or

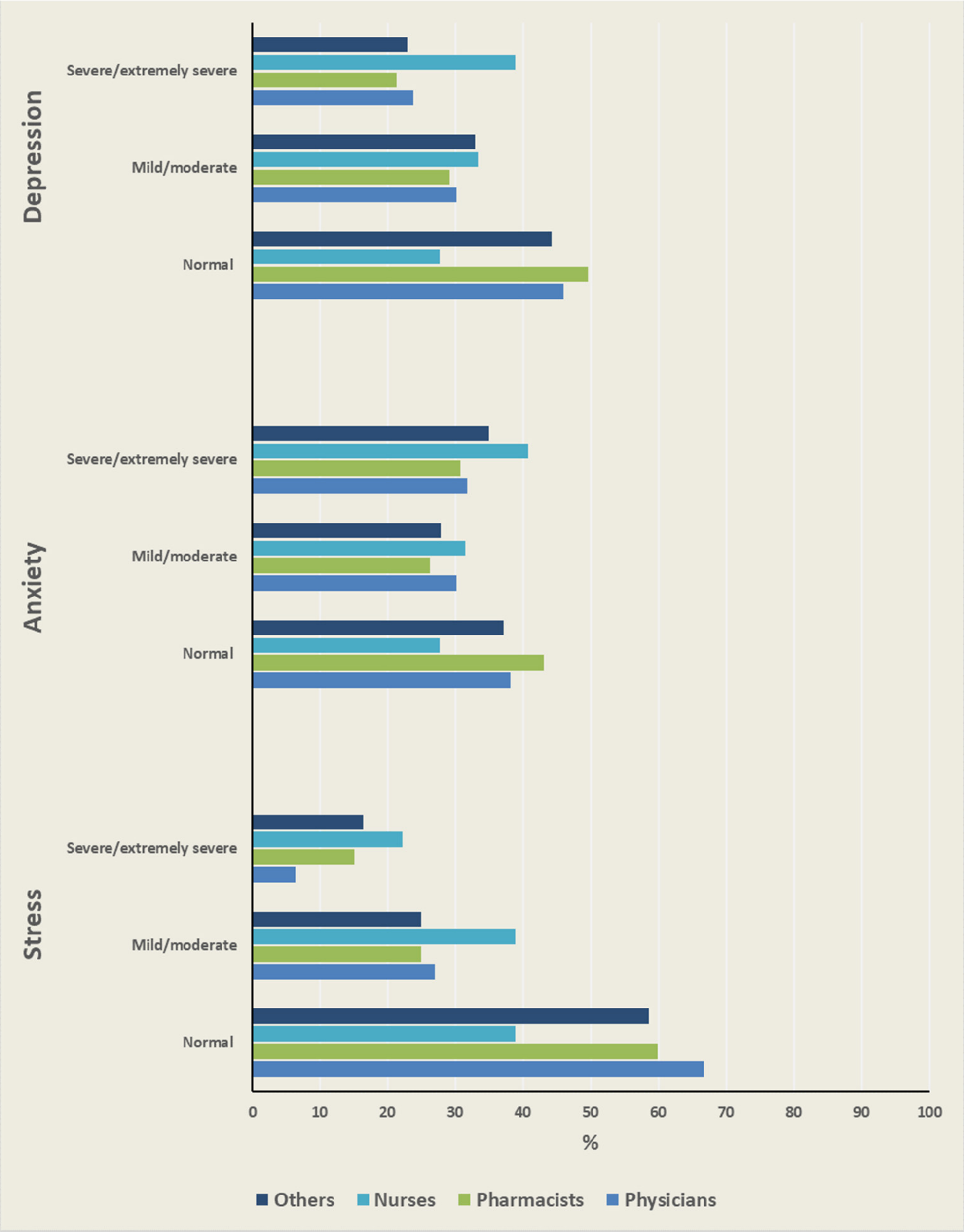

FIGURE 1 | Severity levels of psychological disorders among HCWs.

extremely severe depression, and 15.17% suffered from severe or extremely severe stress. Stratified by occupation, nurses had the highest severity rates across all DASS subscales (**Figure 1**). The prevalence rate of stress, ranging from mild to extremely severe, was higher among pharmacists (40.16%) compared to physicians (33.33%), but less than that of nurses (61.11%) (**Table 2**). The prevalence of depression was significantly higher among participants with chronic diseases and those who had tested positive ($p < 0.001$ and $p = 0.004$, respectively). Chronic illness was also significantly associated with anxiety and stress. The younger age group (18–24 years) had a significantly higher percentage of anxiety and depression than the older age groups. HCWs who were not married had significantly higher rates of depression, anxiety, and stress. Compared to other regions, the southern region of Saudi Arabia had the highest prevalence rates of depression, anxiety, and stress among HCWs.

The multivariate analysis of depression showed that nurses had more than two times the odds of suffering from depression compared to physicians (OR = 2.37, 95% CI: 1.03–5.47) (**Table 3**). In addition, HCWs in the southern region were twice as likely to suffer from depression than their counterparts working in the western region (OR = 2.32, 95% CI: 1.21–4.47).

TABLE 2 | Bivariable analysis of depression, anxiety, and stress.

Characteristic	Depression†, %	*P*-value*	Anxiety‡, %	*P*-value*	Stress§, %	*P*-value*
Gender		0.06		0.12		0.13
Male	49.49		56.63		37.76	
Female	58.03		63.61		44.59	
Age		<0.001		0.001		0.05
18–24	65.40		70.62		47.39	
25–34	47.51		54.30		39.82	
35 or older	44.93		52.17		31.88	
Married		0.001		0.002		0.02
Yes	43.45		50.34		33.79	
No	59.15		65.07		45.07	
Nationality		0.13		0.25		0.36
Saudi	56.07		61.92		42.76	
Non-Saudi	46.58		54.79		36.99	
Job category		0.04		0.19		0.02
Physician	53.97		61.90		33.33	
Pharmacist	50.41		56.97		40.16	
Nurse	72.22		72.22		61.11	
Others	55.71		62.86		41.43	
Region		0.007		0.16		0.009
Central Region	60.26		64.24		47.02	
Eastern Region	47.37		56.58		34.21	
Northern Region	43.18		59.09		40.91	
Southern Region	70.97		72.58		58.06	
Western Region	50.00		56.02		34.94	
Healthcare setting		0.88		0.98		0.79
Inpatient Hospital Setting	51.85		61.73		40.74	
Outpatient Hospital Setting	57.14		57.14		38.10	
PHC	53.73		61.19		41.79	
Community Pharmacy	52.63		59.65		49.12	
Other	57.33		61.33		42.00	
Chronic illness		<0.001		<0.001		<0.001
Yes	75.56		81.11		63.33	
No	50.12		56.45		37.23	
Tested positive for COVID-19		0.004		0.11		0.40
Yes	68.09		68.09		45.74	
No	51.60		59.21		41.03	
Family member, friend, or colleague tested positive for COVID-19		0.66		0.33		0.009
Yes	56.11		59.69		38.74	
No	54.06		64.71		52.10	
Family member, friend, or colleague died due to COVID-19		0.09		0.02		0.79
Yes	52.62		67.78		42.78	
No	61.34		57.19		41.56	

*†Depression was defined as DASS-21 depression subscale score ≥ 10. ‡Anxiety was defined as DASS-21 anxiety subscale score ≥ 8. §Stress was defined as DASS-21 stress subscale score ≥ 15. *P-values produced using Pearson's Chi square test.*

Having a chronic illness and testing positive for COVID-19 were significant predictors of depression. The multivariate analysis of anxiety revealed that the youngest age group (18–24 years old) had greater odds of having anxiety than the oldest age group (35 years or older) (**Table 3**). The adjusted model indicated that having a chronic illness and knowing someone who died due to COVID-19 were significant predictors of anxiety. Finally, the multivariate analysis of stress suggested that nurses, HCWs in the southern region, and participants with chronic illnesses had significantly greater odds of suffering from stress (**Table 3**).

DISCUSSION

The present study examines the mental health toll of the COVID-19 pandemic on HCWs in Saudi Arabia. According to Saudi Commission For Health Specialties (SCFHS) report in 2020, ∼500,000 HCWs were registered in the country (32). To our knowledge, this study is among the first to determine the prevalence and associated factors of depression, anxiety, and stress among HCWs in all regions of Saudi Arabia. Our findings indicate a considerably high prevalence rate of psychological disorders among physicians, pharmacists, nurses,

TABLE 3 | Multivariable logistic regression analysis of depression, anxiety, and stress.

Characteristic	Depression		Anxiety		Stress	
	Adjusted OR (95% CI)	*P*-value	Adjusted OR (95% CI)	*P*-value	Adjusted OR (95% CI)	*P*-value
Gender						
Male	Ref		Ref		Ref	
Female	1.15 (0.74–1.78)	0.53	0.95 (0.62–1.46)	0.81	1.18 (0.77–1.83)	0.45
Age						
35 or older	Ref		Ref		Ref	
25–34	1.07 (0.56–2.02)	0.34	1.16 (0.62–2.16)	0.42	1.33 (0.67–2.61)	0.59
18–24	1.71 (0.83–3.52)	0.06	1.88 (0.92–3.87)	0.03	1.38 (0.65–2.93)	0.49
Married						
No	Ref		Ref		Ref	
Yes	0.79 (0.47–1.35)	0.39	0.72 (0.43–1.21)	0.21	0.83 (0.48–1.43)	0.50
Job category						
Physician	Ref		Ref		Ref	
Pharmacist	1.14 (0.61–2.12)	0.38	0.95 (0.51–1.76)	0.30	1.78 (0.94–3.38)	0.98
Nurse	2.37 (1.03–5.47)	0.02	1.71 (0.74–3.94)	0.11	3.49 (1.55–7.90)	0.005
Others	1.11 (0.58–2.13)	0.34	0.99 (0.51–1.89)	0.46	1.58 (0.81–3.09)	0.52
Region						
Western Region	Ref		—	—	Ref	
Central Region	1.24 (0.77–1.99)	0.52			1.43 (0.89–2.32)	0.53
Eastern Region	0.72 (0.39–1.30)	0.06			0.85 (0.46–1.57)	0.07
Northern Region	0.81 (0.39–1.65)	0.25			1.27 (0.62–2.60)	0.95
Southern Region	2.32 (1.21–4.47)	0.003			2.29 (1.23–4.29)	0.01
Chronic illness						
No	Ref		Ref		Ref	
Yes	2.62 (1.52–4.52)	<0.001	2.93 (1.64–5.24)	<0.001	2.90 (1.76–4.79)	<0.001
Tested positive						
No	Ref		—	—	—	—
Yes	1.79 (1.07–2.99)	0.03				
Family member, friend, or colleague died due to COVID-19						
No	—	—	Ref		Ref	
Yes			1.60 (1.07–2.39)	0.02	0.69 (0.44–1.07)	0.09

and other HCWs during the pandemic. The overall prevalence of depression, anxiety, and stress was 54.69, 60.88, and 41.92%, respectively. The prevalence rates of psychological disorders found in this study were greater than those of the general public in Saudi Arabia revealed in a previous study (29). Several factors can increase the risk of mental health conditions among HCWs, including fear of infection, high workload, and recurrent isolation from family members (33). Findings from this study underscore the importance of mental health interventions for HCWs. Mental health policy makers at health institutions should implement training in coping strategies and stress management skills for their HCWs. In addition, mental health programs and initiatives in Saudi Arabia should promote and expand their mental health counseling services.

The unprecedented pandemic has likely had an inevitable and enduring impact on the psychological well-being of HCWs. In terms of severity, we found that one-third of the HCWs had severe or extremely severe anxiety. This rate was higher compared to previous studies conducted during the early stages of the pandemic among HCWs in Saudi Arabia and other countries (11, 34–36). Our study was conducted in early 2021, a period when the number of cases in Saudi Arabia had reached more than 360,000, and deaths due to COVID-19 had surpassed 6,000 (37). In our sample, more than one-third (36%) of HCWs knew someone who had died due to COVID-19. Our findings suggest that the psychological impact of the pandemic has persisted among HCWs, even though many restrictions in Saudi Arabia have been eased. This is in line with findings observed in a systematic review of previous infectious disease epidemics, such as MERS, suggesting long-lasting effects (38). Longitudinal studies are better suited to investigate the long-term psychological impact of the COVID-19 pandemic. One year later, few studies have been conducted to assess the psychological impact among HCWs using the DASS-21 questionnaire (9–15), and few cohort studies have evaluated the impact of the pandemic on health care providers (16–22). For instance, one study conducted over 3 months showed that improving workplace support might protect HCWs from adverse psychological consequences (39). A study conducted in Singapore among Medical residents found that HCWs were at high risk of psychological sequelae (40). Another study measured the long-term psychological impact of COVID-19 on frontline doctors in the UK over three periods of time (41).

As expected, we found that nurses were most affected by the COVID-19 crisis. Our findings indicate that nurses have a higher prevalence of moderate to severe psychological disorders than other healthcare professionals. Additionally, multivariate analyses revealed that nurses were more likely to suffer from depression and anxiety. This is not surprising because the literature has shown that, even prior to the COVID-19 pandemic, nurses and HCWs in direct contact with patients have a higher risk of depression, anxiety, and sleep disorders (42–44). During the COVID-19 crisis, several studies reported that nurses were more vulnerable to psychological disorders than other HCWs (11, 34, 45). Compared to other professionals, nurses may be at greater risk of contracting COVID-19 because of their regular and close contact with patients.

In this study, we explored the association between various demographic traits and psychological disorders. Female respondents had higher prevalence rates of anxiety than males (63.61 vs. 56.63%). The multivariate analysis did not show that females were more likely to develop anxiety. This finding is in contrast to a previous study performed in Saudi Arabia, which demonstrated that female HCWs were at greater risk of anxiety (46). However, we used a different screening tool and found that the younger age group had significantly higher prevalence rates of depression and anxiety than the older age group. A similar finding was observed in a Chinese study, which suggested that age is negatively associated with depression, anxiety, and insomnia, indicating that older age is a protective factor (47). In the bivariate analysis, we observed that married participants had lower rates of depression, anxiety, and stress. However, after adjusting for other factors, marital status was not associated with psychological disorders. A study of frontline HCWs in China found that the stress level was higher among married participants, likely due to the fear of transmitting the infection to one's spouse (48).

Our results indicated that geographic region was associated with depression and stress. Interestingly, the multivariate analysis shows that HCWs from the southern region were more likely to develop depression and stress. A previous study of HCWs in Saudi Arabia found that respondents from the central region had higher scores of depression and anxiety than those from other regions (34), but the study sample consisted of respondents from only three regions and did not include HCWs from the southern region. The findings of a Chinese study suggested that HCWs in Wuhan, the epicenter of the pandemic, were more likely to experience distress than those working outside Wuhan (49). Surely, it is expected that HCWs in locations with COVID-19 outbreaks would experience a greater psychological impact. However, the southern region of Saudi Arabia had fewer new and cumulative COVID-19 cases compared to the central or western regions. While it is unclear why HCWs from the southern region were more likely to suffer from depression and stress, this finding highlights the need to promote mental health and provide support services across the kingdom. Future research is warranted to address the variability in the psychological impact on HCWs from different geographic regions.

The findings revealed that HCWs with chronic illnesses had significantly higher prevalence rates of psychological disorders. In the multivariate analysis, we found that chronic disease was the strongest predictor of depression, anxiety, and stress. This finding corresponds with the literature, which suggests that people with chronic ailments are more prone to suffer from depression, anxiety, and stress (33, 35, 50, 51). This could be explained by the fact, established early in the pandemic, that people with chronic diseases are at greater risk of severe and potentially fatal COVID-19 disease (52). The results have also shown that knowing someone who died due to COVID-19 was a significant predictor of anxiety. Special attention should be paid to providing adequate personal protective supplies to HCWs with chronic illnesses.

Some limitations need to be considered when interpreting the findings. First, as this was a cross-sectional study, the causal relationship between various factors and psychological disorders could not be established, and we were unable to identify whether participants had pre-existing mental health issues that could have influenced the results. Second, we did not collect data on potential confounders, such as workload or social and organizational support, or whether participants were involved in the direct care of COVID-19 patients. Third, we used a non-probability sampling technique, which may have led to selection bias and limited generalizability of the findings. Nonetheless, our sample included HCWs from all regions of Saudi Arabia. Finally, the use of self-administered surveys could potentially increase response bias. Despite these limitations, our study provides valuable insight into decision-makers in healthcare institutions regarding how the COVID-19 crisis has affected HCWs.

CONCLUSION

Physicians, pharmacists, and nurses alike have been deeply impacted by the COVID-19 crisis. The estimated prevalence rates of depression, anxiety, and stress among HCWs in Saudi Arabia were considerably high. This study identified certain populations who are at a greater risk of psychological disorders. Generally, factors associated with psychological disorders include having a chronic illness, being a nurse, knowing someone who died due to COVID-19, and being a healthcare worker from the southern region. Our study underscores the need to provide and promote support services for HCWs to mitigate the psychological impact of this pandemic.

DATA AVAILABILITY STATEMENT

The original contributions presented in the study are included in the article/supplementary material, further inquiries can be directed to the corresponding authors.

ETHICS STATEMENT

The study was approved by the Scientific Research Ethics Committee at Taif University (42-0068) and the Institutional Review Board of MOH (472). The patients/participants provided their written informed consent to participate in this study.

AUTHOR CONTRIBUTIONS

AHA, MSA, FA, and AAA contributed to conception and design of the study. FA, AAlha, SFA, RA, and AAlz collected and organized the database. MSA, FA, and YA performed and validated the statistical analysis and wrote the statistical section. AHA, MSA, MAA, and AAA wrote the first draft of the manuscript. AB, HA, and YSA revised and edited the manuscript. All authors contributed to revising the manuscript and read and approved the submitted version.

FUNDING

This research was funded by Taif University initiative for COVID-19 projects, Grant Number: 1-441-77.

ACKNOWLEDGMENTS

The authors would like to thank Dr. Bader Alghamdi, Dr. Abdullah Alshehri, Dr. Mohammed Alshennawi, and Dr. Talal Alharthi for their help.

REFERENCES

1. Wang C, Horby P, Hayden F, Gao G. A novel coronavirus outbreak of global health concern. *Lancet.* (2020) 395:470–3. doi: 10.1016/S0140-6736(20)30185-9
2. Goh K, Choong M, Cheong E, Kalimuddin S, Duu Wen S, Phua G, et al. Rapid progression to acute respiratory distress syndrome: review of current understanding of critical illness from Coronavirus disease 2019 (COVID-19) infection. *Ann Acad Med Singapore.* (2020) 49:1–9. doi: 10.47102/annals-acadmedsg.202057
3. Barry M, Al Amri M, Memish Z. COVID-19 in the shadows of MERS-CoV in the kingdom of Saudi Arabia. *J Epidemiol Glob Health.* (2020) 10:1–3. doi: 10.2991/jegh.k.200218.003
4. Wang C, Pan R, Wan X, Tan Y, Xu L, Ho C, et al. Immediate psychological responses and associated factors during the initial stage of the 2019 coronavirus disease (COVID-19) epidemic among the general population in China. *Int J Environ Res Public Health.* (2020) 17:1729. doi: 10.3390/ijerph17051729
5. Cao W, Fang Z, Hou G, Han M, Xu X, Dong J, et al. The psychological impact of the COVID-19 epidemic on college students in China. *Psychiatry Res.* (2020) 287:112934. doi: 10.1016/j.psychres.2020.112934
6. Kang L, Ma S, Chen M, Yang J, Wang Y, Li R, et al. Impact on mental health and perceptions of psychological care among medical and nursing staff in Wuhan during the 2019 novel coronavirus disease outbreak: a cross-sectional study. *Brain Behav Immun.* (2020) 87:11–7. doi: 10.1016/j.bbi.2020.03.028
7. Xu J, Xu Q, Wang C, Wang J. Psychological status of surgical staff during the COVID-19 outbreak. *Psychiatry Res.* (2020) 288:112955. doi: 10.1016/j.psychres.2020.112955
8. Wu W, Zhang Y, Wang P, Zhang L, Wang G, Lei G, et al. Psychological stress of medical staffs during outbreak of COVID-19 and adjustment strategy. *J Med Virol.* (2020) 92:1962–70. doi: 10.1002/jmv.25914
9. Tengilimoglu D, Zekioglu A, Tosun N, Işik O, Tengilimoglu O. Impacts of COVID-19 pandemic period on depression, anxiety and stress levels of the healthcare employees in Turkey. *Leg Med.* (2021) 48:101811. doi: 10.1016/j.legalmed.2020.101811
10. Dosil Santamaría M, Ozamiz-Etxebarria N, Redondo Rodríguez I, Jaureguizar Alboniga-Mayor J, Picaza Gorrotxategi M. Psychological impact of COVID-19 on a sample of Spanish health professionals. *Rev Psiquiatr Salud Ment.* (2021) 14:106–12. doi: 10.1016/j.rpsm.2020.05.004
11. Arafa A, Mohammed Z, Mahmoud O, Elshazley M, Ewis A. Depressed, anxious, and stressed: what have healthcare workers on the frontlines in Egypt and Saudi Arabia experienced during the COVID-19 pandemic? *J Affect Disord.* (2021) 278:365–71. doi: 10.1016/j.jad.2020.09.080
12. Farì G, de Sire A, Giorgio V, Rizzo L, Bruni A, Bianchi FP, et al. Impact of COVID-19 on the mental health in a cohort of Italian rehabilitation healthcare workers. *J Med Virol.* (2021) 94:110–8. doi: 10.1002/jmv.27272
13. Vlah Tomičević S, Lang VB. Psychological outcomes amongst family medicine healthcare professionals during COVID-19 outbreak: a cross-sectional study in Croatia. *Eur J Gen Pr.* (2021) 27:184–90. doi: 10.1080/13814788.2021.1954154
14. Moayed MS, Vahedian-Azimi A, Mirmomeni G, Rahimi-Bashar F, Goharimoghadam K, Pourhoseingholi MA, et al. Survey of immediate psychological distress levels among healthcare workers in the COVID-19 epidemic: a cross-sectional study. *Adv Exp Med Biol.* (2021) 1321:237–43. doi: 10.1007/978-3-030-59261-5_20
15. Hammond NE, Crowe L, Abbenbroek B, Elliott R, Tian DH, Donaldson LH, et al. Impact of the coronavirus disease 2019 pandemic on critical care healthcare workers' depression, anxiety, and stress levels. *Aust Crit Care.* (2021) 34:146–54. doi: 10.1016/j.aucc.2020.12.004
16. Sampaio F, Sequeira C, Teixeira L. Impact of COVID-19 outbreak on nurses' mental health: a prospective cohort study. *Environ Res.* (2021) 194:110620. doi: 10.1016/j.envres.2020.110620
17. Abdalla M, Chiuzan C, Shang Y, Ko G, Diaz F, Shaw K, et al. Factors Associated with Insomnia Symptoms in a Longitudinal Study among New York City Healthcare Workers during the COVID-19 Pandemic. *Int J Environ Res Public Health.* (2021) 18:8970. doi: 10.3390/ijerph18178970
18. Ahmed F, Zhao F, Faraz NA, Qin YJ. How inclusive leadership paves way for psychological well-being of employees during trauma and crisis: a three-wave longitudinal mediation study. *J Adv Nurs.* (2021) 77:819–31. doi: 10.1111/jan.14637
19. Lamb D, Gnanapragasam S, Greenberg N, Bhundia R, Carr E, Hotopf M, et al. Psychosocial impact of the COVID-19 pandemic on 4378 UK healthcare workers and ancillary staff: initial baseline data from a cohort study collected during the first wave of the pandemic. *Occup Environ Med.* (2021) 78:801–8. doi: 10.1136/oemed-2020-107276
20. Heyen JM, Weigl N, Müller M, Müller S, Eberle U, Manoliu A, et al. Multimodule web-based COVID-19 anxiety and stress resilience training (COAST): single-cohort feasibility study with first responders. *JMIR Form Res.* (2021) 5:e28055. doi: 10.2196/28055
21. Tan BYQ, Chew NWS, Lee GKH, Jing M, Goh Y, Yeo LLL, et al. Psychological Impact of the COVID-19 Pandemic on Health Care Workers in Singapore. *Ann Intern Med.* (2020) 173:317–20. doi: 10.7326/M20-1083
22. Qureshi AI, Suri MF, Ostrow PT, Kim SH, Ali Z, Shatla AA, et al. Apoptosis as a form of cell death in intracerebral hemorrhage. *Neurosurgery.* (2003) 52:1041–8. doi: 10.1227/01.NEU.0000057694.96978.BC
23. Shen X, Yan S, Cao H, Feng J, Lei Z, Zhang W, et al. Current status and associated factors of depression and anxiety among the chinese residents during the period of low transmission of COVID-19. *Front Psychol.* (2021) 12:700376. doi: 10.3389/fpsyg.2021.700376
24. Yezli S, Khan A. COVID-19 social distancing in the Kingdom of Saudi Arabia: bold measures in the face of political, economic, social and religious challenges. *Travel Med Infect Dis.* (2020) 37:101692. doi: 10.1016/j.tmaid.2020.101692
25. Ebrahim SH, Memish ZA. Saudi Arabia's drastic measures to curb the COVID-19 outbreak: temporary suspension of the umrah pilgrimage. *J Travel Med.* (2020) 27:taaa029. doi: 10.1093/jtm/taaa029
26. Alamri HS, Mousa WF, Algarni A, Megahid SF, Al Bshabshe A, Alshehri NN, et al. COVID-19 psychological impact on health care workers in Saudi Arabia. *Int J Environ Res Public Health.* (2021) 18:6076. doi: 10.3390/ijerph18116076
27. Al Sulais E, Mosli M, AlAmeel T. The psychological impact of COVID-19 pandemic on physicians in Saudi Arabia: a cross-sectional study. *Saudi J Gastroenterol.* (2020) 26:249–55. doi: 10.4103/sjg.SJG_174_20
28. Lwanga SK, Lemeshow S. *Sample Size Determination In Health Studies: A Practical Manual*. World Health Organization (1991). Available online at: https://apps.who.int/iris/handle/10665/40062

29. Alkhamees A, Alrashed S, Alzunaydi A, Almohimeed A, Aljohani M. The psychological impact of COVID-19 pandemic on the general population of Saudi Arabia. *Compr Psychiatry*. (2020) 102:152192. doi: 10.1016/j.comppsych.2020.152192
30. Henry J, Crawford J. The short-form version of the depression anxiety stress scales (DASS-21): construct validity and normative data in a large non-clinical sample. *Br J Clin Psychol*. (2005) 44:227–39. doi: 10.1348/014466505X29657
31. Gjersing L, Caplehorn J, Clausen T. Cross-cultural adaptation of research instruments: language, setting, time and statistical considerations. *BMC Med Res Methodol*. (2010) 10:13. doi: 10.1186/1471-2288-10-13
32. Saudi Commission For Health Specialties. *No Title*. (2020). Available online at: https://www.scfhs.org.sa/about/Documents/الدليل التعريفي- هيئة التخصصات الصحية.pdf
33. Zhang W, Wang K, Yin L, Zhao W, Xue Q, Peng M, et al. Mental health and psychosocial problems of medical health workers during the COVID-19 epidemic in China. *Psychother Psychosom*. (2020) 89:242–50. doi: 10.1159/000507639
34. AlAteeq D, Aljhani S, Althiyabi I, Majzoub S. Mental health among healthcare providers during coronavirus disease (COVID-19) outbreak in Saudi Arabia. *J Infect Public Health*. (2020) 13:1432–7. doi: 10.1016/j.jiph.2020.08.013
35. Woon LS-C, Sidi H, Jaafar NRN, Abdullah MFIL. Bin. Mental health status of university healthcare workers during the COVID-19 pandemic: a post–movement lockdown assessment. *Int J Environ Res Public Health*. (2020) 17:1–20. doi: 10.20944/preprints202011.0422.v1
36. Nayak BS, Sahu PK, Ramsaroop K, Maharaj S, Mootoo W, Khan S, et al. Prevalence and factors associated with depression, anxiety and stress among healthcare workers of Trinidad and Tobago during COVID-19 pandemic: a cross-sectional study. *BMJ Open*. (2021) 11:e044397. doi: 10.1136/bmjopen-2020-044397
37. Ministry of Health. *COVID 19 Dashboard: Saudi Arabia*. Available online at: https://covid19.moh.gov.sa/ (accessed September 1, 2021).
38. Brooks SK, Webster RK, Smith LE, Woodland L, Wessely S, Greenberg N, et al. The psychological impact of quarantine and how to reduce it: rapid review of the evidence. *Lancet*. (2020) 395:912–20. doi: 10.1016/S0140-6736(20)30460-8
39. Hines SE, Chin KH, Glick DR, Wickwire EM. Trends in moral injury, distress, and resilience factors among healthcare workers at the beginning of the COVID-19 pandemic. *Int J Environ Res Public Health*. (2021) 18:488. doi: 10.3390/ijerph18020488
40. Chew QH, Chia FL, Ng WK, Lee WCI, Tan PLL, Wong CS, et al. Perceived stress, stigma, traumatic stress levels and coping responses amongst residents in training across multiple specialties during COVID-19 pandemic-a longitudinal study. *Int J Environ Res Public Health*. (2020) 17:6572. doi: 10.3390/ijerph17186572
41. Roberts T, Daniels J, Hulme W, Hirst R, Horner D, Lyttle MD, et al. Psychological distress and trauma in doctors providing frontline care during the COVID-19 pandemic in the United Kingdom and Ireland: a prospective longitudinal survey cohort study. *BMJ Open*. (2021) 11:e049680. doi: 10.1136/bmjopen-2021-049680
42. Tsai K, Lee T, Chung M. Insomnia in female nurses: a nationwide retrospective study. *Int J Occup Saf Ergon*. (2017) 23:127–32. doi: 10.1080/10803548.2016.1248604
43. Maunder R, Lancee W, Rourke S, Hunter J, Goldbloom D, Balderson K, et al. Factors associated with the psychological impact of severe acute respiratory syndrome on nurses and other hospital workers in Toronto. *Psychosom Med*. (2004) 66:938–42. doi: 10.1097/01.psy.0000145673.84698.18
44. Fiabane E, Giorgi I, Sguazzin C, Argentero P. Work engagement and occupational stress in nurses and other healthcare workers: the role of organisational and personal factors. *J Clin Nurs*. (2013) 22:2614–24. doi: 10.1111/jocn.12084
45. Liu C-Y, Yang Y-Z, Zhang X-M, Xu X, Dou QL, Zhang WW, et al. The prevalence and influencing factors in anxiety in medical workers fighting COVID-19 in China: a cross-sectional survey. *Epidemiol Infect*. (2020) 148:e98. doi: 10.1017/S0950268820001107
46. Al Ammari M, Sultana K, Thomas A, Al Swaidan L, Al Harthi N. Mental health outcomes amongst health care workers during COVID 19 pandemic in Saudi Arabia. *Front Psychiatry*. (2021) 11:619540. doi: 10.3389/fpsyt.2020.619540
47. Zhou Y, Wang W, Sun Y, Qian W, Liu Z, Wang R, et al. The prevalence and risk factors of psychological disturbances of frontline medical staff in china under the COVID-19 epidemic: workload should be concerned. *J Affect Disord*. (2020) 277:510–4. doi: 10.1016/j.jad.2020.08.059
48. Wang H, Liu Y, Hu K, Zhang M, Du M, Huang H, et al. Healthcare workers' stress when caring for COVID-19 patients: an altruistic perspective. *Nurs Ethics*. (2020) 27:1490–500. doi: 10.1177/0969733020934146
49. Lai J, Ma S, Wang Y, Cai Z, Hu J, Wei N, et al. Factors associated with mental health outcomes among health care workers exposed to coronavirus disease 2019. *JAMA Netw Open*. (2020) 3:e203976. doi: 10.1001/jamanetworkopen.2020.3976
50. Kibret S, Teshome D, Fenta E, M H, Tamire T. Prevalence of anxiety towards COVID-19 and its associated factors among healthcare workers in a Hospital of Ethiopia. *PLoS ONE*. (2020) 15:e0243022. doi: 10.1371/journal.pone.0243022
51. Gorrochategi MP, Munitis AE, Santamaria MD, Etxebarria NO. Stress, anxiety, and depression in people aged over 60 in the COVID-19 outbreak in a sample collected in Northern Spain. *Am J Geriatr Psychiatry*. (2020) 28:993. doi: 10.1016/j.jagp.2020.05.022
52. Gao Y, Ding M, Dong X, Zhang J, Kursat Azkur A, Azkur D, et al. Risk factors for severe and critically ill COVID-19 patients: a review. *Allergy*. (2021) 76:428–55. doi: 10.1111/all.14657

Conflict of Interest: The authors declare that the research was conducted in the absence of any commercial or financial relationships that could be construed as a potential conflict of interest.

Publisher's Note: All claims expressed in this article are solely those of the authors and do not necessarily represent those of their affiliated organizations, or those of the publisher, the editors and the reviewers. Any product that may be evaluated in this article, or claim that may be made by its manufacturer, is not guaranteed or endorsed by the publisher.

Copyright © 2021 Almalki, Alzahrani, Alshehri, Alharbi, Alkhudaydi, Alshahrani, Alzaidi, Algarni, Alsaab, Alatawi, Althobaiti, Bamaga and Alhifany. This is an open-access article distributed under the terms of the Creative Commons Attribution License (CC BY). The use, distribution or reproduction in other forums is permitted, provided the original author(s) and the copyright owner(s) are credited and that the original publication in this journal is cited, in accordance with accepted academic practice. No use, distribution or reproduction is permitted which does not comply with these terms.
