# Supplementary material for: Mental health status and related factors influencing healthcare workers during the COVID-19 pandemic: A systematic review and meta-analysis
Source: PLoS One. 2024 Jan 19;19(1):e0289454. doi: 10.1371/journal.pone.0289454 (PMC10798549; doi:10.1371/journal.pone.0289454)
Supplement: S1 Data — (ZIP) [file pone.0289454.s011.zip › literatures/60.pdf]

J Nepal Med Assoc 2021;59(235):239-42

OPEN ACCESS CC BY
doi: 10.31729/jnma.6083

Depression, Anxiety and Stress among Healthcare Workers during COVID-19 Pandemic in a Tertiary Care Centre of Nepal: A Descriptive Cross-sectional Study

Bikram Kafle,[1] Yashoda Bagale,[2] Sima Kafle,[2] Aabishkar Parajuli,[2] Samudra Pandey[2]

[1]Department of Psychiatry, Devdaha Medical College, Rupandehi, Nepal, [2]Devdaha Medical College, Rupandehi, Nepal.

ABSTRACT

Introduction: Health personnel working in the frontline to deal with COVID 19 outbreak are at increased risk of developing psychological problems. This study aims to find out the prevalence of depression, anxiety and stress among Nepalese health care workers.

Methods: This is a hospital based descriptive cross-sectional study conducted from July 10 2020 to September 10, 2020. All health care workers (total 280) were included in the study. HADS-14 item was used to measure anxiety and depression. Perceived stress scale was used to measure stress. Data was analyzed with Statistical Package for the Social Sciences software version 24.0. Statistical data was analyzed by percentage, mean and standard deviation.

Results: Out of total 270 respondents the prevalence of anxiety, depression and stress was found to be 112 (41.4%), 65 (24.1%) and 203 (74%) respectively. Females accounted for 148 (54.8%) and males 122 (45.2%). More than one third 96 (35.6%) of the health workers were working in front line.

Conclusions: Prevalence of anxiety, depression and stress is higher among health workers when compared to similar studies. Effective strategies toward improving the mental health like adequate rest, supply of protective equipments, frequent breaks, ensuring safety issues of their family members, training on management of stress might be helpful in reducing stress.

Keywords: *anxiety; COVID-19; depression; stress; health personnel; Nepal.*

INTRODUCTION

COVID-19 pandemic has the potential to affect the mental health of healthcare workers (HCW), who stand in the frontline of this crisis. Immediate interventions are essential in order to enhance psychological resilience and strengthen the healthcare systems' capacity.[1] During epidemic, people tend to experience fear of getting infected with the virus/ disease resulting in anxiety, stress, and depression.[2] Medical occupations are associated with hard working conditions and an exceptional amount of stress. Studies has shown that mental health of health care worker had declined during the COVID-19 outbreak, and the degree of deterioration was greater for health care worker than non health care worker.[3]

Since there are very few research conducted in this area in Nepal, this is important to know for planning of effective mental health management by practitioners and policymakers.

This study aims to find out the prevalence of depression, anxiety and stress among Nepalese health care workers.

METHODS

This is a descriptive cross-sectional study conducted at Devdaha Medical College (DMC). All the health care workers were invited to participate in a study through a self-administered questionnaire within the period of July 10, 2020 to September 10, 2020 after taking ethical clearence from Institutional Review Committee (IRC)

Correspondence: Dr. Bikram Kafle, Department of Psychiatry, Devdaha Medical College, Rupandehi, Nepal. Email: bikram12kafle@gmail.com Phone:+977-9857042799.

of Devdaha Medical College. The protocol approval number is 010/20. There are 280 health workers in DMC. All the health workers working in Devdaha Medical College were included in the study. Those who didn't give consent were excluded. Healthcare workers (HCW) included doctors, nurses, allied healthcare workers, administrators, clerical staff and maintenance workers. This questionnaire collected information on demographics, Hospital Anxiety and Depression Scale (HADS), and Perceived Stress Scale (PSS).

HADS: The original, English version of HADS contains 14 items in two subscales: anxiety (HADS-A) and depression (HADS-D), each with seven items (A1 to A7; D1 to D7). Each item is rated on a four-point scale from 0-3 (3 indicating maximum symptom severity), and the scores are summed (five items on the depression subscale and one on the anxiety subscale are reversed before summing).[4] The Nepali version that has been developed by Rijal et al has been used in this study.[5]

The Perceived Stress Scale (PSS) developed by Cohen,[6] is the most widely used psychological instrument for measuring the perception of stress. It is a measure of the degree to which situations in one's life are appraised as stressful. The questions in the PSS ask about feelings and thoughts during the last month. In each case, respondents are asked how often they felt a certain way.

Total study participants were 270 (response rate, 96.42 percent). Data was analyzed with SPSS software (version 24.0; SPSS Inc., Chicago, USA). Statistical data was analyzed by percentage, mean and standard deviation.

RESULTS

Out of total 270 respondents, those with depressive symptoms borderline were 47 (17.4%) and abnormal 18 (6.7%), those with anxiety symptoms borderline 46 (17%) and abnormal 66 (24.4%). Sixty seven (24.8%) had perceived low level of stress, 183 (67.8%) had perceived moderate level of stress and 20 (7.8%) had perceived high level of stress. More than one third of the health workers were working in front line (Table 1 and 2).

Out of total 156 medical health workers (doctors, nurses and allied healthcare) 104 (66.66%) had moderate to high level of stress, 58 (37.17%) had borderline to abnormal anxiety and 30 (19.12%) had depression (Table 3).

Among those working as front line health workers almost two third 60 (62.4%) had perceived moderate to severe stress, one third 31 (31%) had anxiety and 17 (17.6%) had depression (Table 4).

Majority 129 (47.8%) were in the age intervals of 20-30. The females accounted for 148 (54.8%) of the total respondents (Table 1).

Mean PSS score was 16.99±5.63, Mean anxiety score was 7.38±3.76 and mean depression score was 4.93±3.57 (Table 2).

Table 1. Sociodemographic characterstics of enrolled participants (N = 270).

Variables		n (%)
Gender	Male	122 (45.2)
	Female	148 (54.8)
Age group (years)	20-30	129 (47.8)
	30-40	88 (32.6)
	40-50	37 (13.7)
	50-60	13 (4.8)
	60 and above	3 (1.1)
Marital status	Single	97 (35.9)
	Married	171 (63.3)
	Others	2 (0.7)
Occupation	Doctor	59 (21.9)
	Nurse	57 (21.1)
	allied healthcare (HA/ CMA)	40 (14.8)
	Administrator	18 (6.7)
	Others (maintenance workers, helpers, drivers)	73 (27)
	Non doctor faculty	23 (8.5)
Working as FHW	Yes	96 (35.6)
	No	174 (64.4)

Table 2. Grading of stress, anxiety and depression and their Mean (N = 270).

Variable	n (%)	Mean ±SD
**Perceived stress scale**		16.99±5.63
Low stress (0-13)	67 (24.8%)	
Moderate stress (14-26)	183 (67.8%)	
High stress (27-40)	20 (7.4%)	
**HADS (anxiety grading)**		7.38±3.76
Normal (0-7)	158 (58.5%)	
Borderline abnormal (8-10)	46 (17.0%)	
Abnormal (11-21)	66 (24.4%)	

**HADS(depression grading)**		4.93±3.57
Normal (0-7)	205 (75.9%)	
Borderline abnormal (8-10)	47 (17.4%)	
Abnormal (11-21)	18 (6.7%)	

Table 3. Perceived stress, anxiety and depression among medical vs non medical health care workers.

	Medical health worker n (%)	Non medical health worker n (%)
**Perceived stress scale**		
Low stress	52 (33.33)	15 (13.15)
Moderate stress	88 (56.41)	95 (83.33)
High stress	16 (10.25)	4 (3.50)
**HADS (anxiety grading)**		
Normal	98 (62.82)	60 (52.63)
Borderline abnormal	23 (14.74)	23 (20.17)
Abnormal	35 (22.43)	31 (27.19)
**HADS (depression grading)**		
Normal	126 (80.76)	79 (69.29)
Borderline abnormal	22 (14.10)	25 (21.92)
Abnormal	8 (5.12)	10 (8.77)

Table 4. Perceived stress, anxiety and depression among different variables.

	**Working as front line health worker**		**Gender**	
	**Yes n (%)**	**No n (%)**	**Male n (%)**	**Female n (%)**
**Perceived stress scale**				
Low stress	36 (37.5)	31 (17.8)	35 (28.6)	32 (21.6)
Moderate stress	46 (47.9)	137 (78.0)	78 (63.9)	105 (70.9)
High stress	14 (14.5)	6 (3.4)	9 (7.3)	11 (7.4)
**HADS (anxiety grading)**				
Normal	65 (67.7)	93 (53.4)	75 (61.4)	83 (56.0)
Borderline abnormal	10 (10.0)	36 (20.6)	19 (15.5)	27 (18.2)
Abnormal	21 (21.0)	45 (25.8)	28 (22.9)	38 (25.6)
**HADS (depression grading)**				
Normal	79 (82.3)	126 (72.0)	92 (75.4)	113 (76.3)
Borderline abnormal	13 (13.5)	34 (19.5)	21 (17.2)	26 (17.5)
Abnormal	4 (4.1)	14 (8.0)	9 (7.3)	9 (7.3)

DISCUSSION

Our study showed the prevalence of anxiety was 41.4%, depression 24.1% and 74% perceived moderate to severe stress. Researches conducted over China, India and Nepal had shown higher prevalence of anxiety and depression among health workers after onset of COVID-19 pandemic period ranging between (28-46)% and (25-50)% respectively.[7-10] This prevalence is higher than those conducted among the general population during COVID-19 pandemic.[11,12] However a study conducted in Singapore which aimed to assess the prevalence of depression, stress, anxiety among health care workers showed 15% had anxiety symptoms, 8.9% depression and 6.6% had stress.[13] These differences could be due to one, methodological differences, other could be as China, India were a major hit country by pandemic, their health facilities were overwhelmed with infected cases. Nepal during this period also had similar situation. It has also been seen that health care workers working in COVID-19 wards when compared to health care workers working in other units revealed that the former reported higher levels of depressive symptoms.[14] Our study also shows that those who are directly working in front line in screening of fever patients, dealing with COVID-19 patients, working in isolation ward, had higher level of stress, anxiety and depression. These findings are understandable in view of the fact that frontline healthcare workers are at higher risk of infection, inadequate rest, increased work load, shortage of protective equipment, frequent isolation from family, safety issues of their parents and children. These are all the factors that can contribute to high stress on them which might lead to the high risk of mental health condition.[7,15,16]

The limitation of our study is, this study cannot be generalized among health care workers of other states differing in the extent of pandemic as it was conducted in a single tertiary centre.

CONCLUSIONS

Prevalence of depression, anxiety and stress was similar when compared with similar studies. The current study showed that frontline health workers have greater anxiety ,depression and stress than non frontline health workers. Effective strategies toward improving the mental health like adequate rest, supply

of protective equipments, frequent breaks, ensuring safety issues of their family members, training on management of stress can be helpful.

Conflict of Interest: None.

REFERENCES

1. Bao Y, Sun Y, Meng S, Shi J, Lu L. 2019-nCoV epidemic: address mental health care to empower society. The Lancet. 2020;395(10224):e37-e8. [PubMed | Full Text | DOI]
2. Hall RC, Hall RC, Chapman MJ. The 1995 Kikwit Ebola outbreak: lessons hospitals and physicians can apply to future viral epidemics. General hospital psychiatry. 2008;30(5):446-52. [PubMed | Full Text | DOI]
3. Sasaki N, Kuroda R, Tsuno K, Kawakami N. The deterioration of mental health among healthcare workers during the COVID-19 outbreak: A population-based cohort study of workers in Japan. Scandinavian journal of work, environment & health. 2020;46(6):639-44. [PubMed | Full Text | DOI]
4. Zigmond AS, Snaith RP. The hospital anxiety and depression scale. Acta psychiatrica scandinavica. 1983;67(6):361-70. [PubMed | DOI]
5. Risal A, Manandhar K, Linde M, Koju R, Steiner T, Holen A. Reliability and validity of a Nepali-language version of the Hospital Anxiety and Depression Scale (HADS). Kathmandu University Medical Journal. 2015;13(2):115-24. [PubMed | Full Text | DOI]
6. Cohen S, Kamarck T, Mermelstein R. Perceived stress scale. Measuring stress: A guide for health and social scientists. 1994;10:1-2. [Full Text]
7. Lai J, Ma S, Wang Y, Cai Z, Hu J, Wei N, et al. Factors associated with mental health outcomes among health care workers exposed to coronavirus disease 2019. JAMA network open. 2020;3(3):e203976-e. [PubMed | Full Text | DOI]
8. Khanal P, Devkota N, Dahal M, Paudel K, Joshi D. Mental health impacts among health workers during COVID-19 in a low resource setting: a cross-sectional survey from Nepal. Globalization and health. 2020;16(1):1-12. [PubMed | Full Text | [DOI]
9. Verma S, Mishra A. Depression, anxiety, and stress and socio-demographic correlates among general Indian public during COVID-19. Int J Soc Psychiatry. 2020;66(8):756-62. [Full Text | DOI]
10. Que J, Le Shi JD, Liu J, Zhang L, Wu S, Gong Y, et al. Psychological impact of the COVID-19 pandemic on healthcare workers: a cross-sectional study in China. General psychiatry. 2020;33(3). [PubMed | Full Text | DOI]
11. Gupta AK, Sahoo S, Mehra A, Grover S. Psychological impact of 'Lockdown'due to COVID-19 pandemic in Nepal: An online survey. Asian J Psychiatr. 2020;54:102243. [PubMed | Full Text | DOI]
12. Qiu J, Shen B, Zhao M, Wang Z, Xie B, Xu Y. A nationwide survey of psychological distress among Chinese people in the COVID-19 epidemic: implications and policy recommendations. General psychiatry. 2020;33(2). [Full Text | DOI]
13. Tan BY, Chew NW, Lee GK, Jing M, Goh Y, Yeo LL, et al. Psychological impact of the COVID-19 pandemic on health care workers in Singapore. Ann Intern Med. 2020 Aug 18;173(4):317-20. [PubMed | Full Text | DOI]
14. Di Tella M, Romeo A, Benfante A, Castelli L. Mental health of healthcare workers during the COVID-19 pandemic in Italy. J Eval Clin Pract. 2020;26(6):1583-7. [PubMed | Full Text | DOI]
15. Wu W, Zhang Y, Wang P, Zhang L, Wang G, Lei G, et al. Psychological stress of medical staffs during outbreak of COVID-19 and adjustment strategy. Journal of Medical Virology. 2020;92(10):1962-70. [PubMed | FullText | DOI]
16. Neto MLR, Almeida HG, Esmeraldo JDa, Nobre CB, Pinheiro WR, de Oliveira CRT, et al. When health professionals look death in the eye: the mental health of professionals who deal daily with the 2019 coronavirus outbreak. Psychiatry Research. 2020:112972. [PubMed | FullText | DOI]

©The Author(s) 2018.

This work is licensed under a Creative Commons Attribution 4.0 International License. The images or other third party material in this article are included in the article's Creative Commons license, unless indicated otherwise in the credit line; if the material is not included under the Creative Commons license, users will need to obtain permission from the license holder to reproduce the material. To view a copy of this license, visit http://creativecommons.org/licenses/by/4.0/
