# Supplementary material for: Mental health status and related factors influencing healthcare workers during the COVID-19 pandemic: A systematic review and meta-analysis
Source: PLoS One. 2024 Jan 19;19(1):e0289454. doi: 10.1371/journal.pone.0289454 (PMC10798549; doi:10.1371/journal.pone.0289454)
Supplement: S1 Data — (ZIP) [file pone.0289454.s011.zip › literatures/61.pdf]

Kamali et al. BMC Psychiatry (2022) 22:365
https://doi.org/10.1186/s12888-022-04014-x

BMC Psychiatry

RESEARCH

Open Access

Occupational burnout in Iranian health care workers during the COVID-19 pandemic

Mahsa Kamali[1], Marzieh Azizi[2,3], Mahmood Moosazadeh[4], Hossein Mehravaran[5], Roya Ghasemian[6], Maryam Hasannezhad Reskati[7] and Forouzan Elyasi[8,9,10*]

Abstract

Background and aim: Health care workers (HCWs), mostly frontliners, are encountering numerous physical and psychosocial stressors, and even managing some conflicts over the course of the novel coronavirus disease 2019 (COVID-19). In this respect, the present study was to investigate the prevalence rate of occupational burnout (OB) in such workers during this pandemic.

Materials and methods: This cross-sectional study was conducted between April 6 and May 30, 2020, via an online survey in 31 provinces of Iran, on HCWs selected based on convenience sampling method. For data collection, a socio-demographic information form and the Maslach Burnout Inventory (MBI) was utilized. Descriptive statistics, Chi-square test, and multivariate regression analysis were also applied to test the research hypotheses.

Results: In total, 7626 HCWs participated in the present study. Accordingly, 73.2 and 26.8% of the workers were female and male, respectively. As well, 57.8% of the respondents were nurses and 14.4% of the cases were clinicians. Moreover, 44.8% of the participants had thus far worked in isolation wards and 40.3% of these individuals reported working for 4–8 hours with COVID-19 patients. The prevalence rate of OB was 18.3%. Besides, 34.2, 48.7, and 56.1% of the respondents had severe levels of emotional exhaustion (EE), higher depersonalization (DP), and decreased sense of personal accomplishment (PA), respectively. Besides, the HCWs at the age range of 20 to 30, having female gender, no children, and a bachelor's degree, and working in isolation wards showed the higher levels of OB with reference to the Chi-square test results ($p < 0.001$). Accordingly, the statistical test outcomes demonstrated that a history of physical illnesses ($p = 0.001$) and psychiatric disorders ($p = 0.044$) could be the best predictor of OB throughout the first peak of the COVID-19 pandemic.

Conclusion: Regarding the high prevalence rate of OB among the HCWs and the remaining COVID-19 journey in Iran, health care managers are recommended to orient the required management and coping strategies toward improving mental health in these individuals.

Keywords: Mental health, Occupational health, COVID-19, Burnout

Background

The novel coronavirus disease 2019 (COVID-19) [1], caused by severe acute respiratory syndrome (SARS)-associated coronavirus, was firstly reported in Wuhan, China, in December 2019, and quickly spread around the world, leading to global health threats and emergencies [2, 3]. Based on the World Health Organization (WHO) statistics on March 10, 2022, so far 7,107,167 cases with

*Correspondence: forouzan.elyasi@gmail.com; F.elyasi@mazums.ac.ir

[10] Psychosomatic Ward, Imam Khomeini General Hospital, Razi Ave, Sari, Mazandaran, Iran
Full list of author information is available at the end of the article

© The Author(s) 2022. **Open Access** This article is licensed under a Creative Commons Attribution 4.0 International License, which permits use, sharing, adaptation, distribution and reproduction in any medium or format, as long as you give appropriate credit to the original author(s) and the source, provide a link to the Creative Commons licence, and indicate if changes were made. The images or other third party material in this article are included in the article's Creative Commons licence, unless indicated otherwise in a credit line to the material. If material is not included in the article's Creative Commons licence and your intended use is not permitted by statutory regulation or exceeds the permitted use, you will need to obtain permission directly from the copyright holder. To view a copy of this licence, visit http://creativecommons.org/licenses/by/4.0/. The Creative Commons Public Domain Dedication waiver (http://creativecommons.org/publicdomain/zero/1.0/) applies to the data made available in this article, unless otherwise stated in a credit line to the data.

COVID-19 confirmed have been detected in Iran, with 138,433 deaths, within approximately 2 years from the onset of the COVID-19 pandemic [1].

In line with a Spanish study, 4.5% of HCWs had suffered from acute stress during the COVID-19 outbreak [4]. Currently, all health care systems are facing some challenging conditions, including numerous physical-psychosocial stressors, and even conflicts in professional ethics, sense of responsibility, and fear of being infected and transferring it to their own families, no childcare support, shortages of personal protective equipment, the rapid spread of the disease, failures in most nations to meet demands, and insufficient time to cope with this situation [3, 5–8]. A systematic review, reflecting on COVID-19 and its mortality rate in HCWs, in April 2020, had further established that the number of infected workers ranged from 1.03 to 10.7%, according to the statistics reported in each country. On the other hand, regarding the death rates, there were up to 605 HCWs all over the world, who had already lost their lives at the time of the pandemic [9]. Another systematic review and meta-analysis of the articles published in 2020 had similarly validated that the prevalence rate of the infection among HCWs was 11 and 7% with reference to the reverse transcription-polymerase chain reaction (RT-PCR) and the antibody screening test results, respectively [10]. Furthermore, one survey had revealed that thousands of workers infected with COVID-19 had expired worldwide [11].

As defined by the WHO in the 11th revision of the International Classification of Diseases (ICD-11), occupational burnout (OB) is “a syndrome resulting from chronic workplace stress that has not been successfully managed” [12–14]. This phenomenon also represents not only the manifestations of negative emotions but also the absence of positive ones [3]. OB is further characterized by three features, i.e., severe levels of emotional exhaustion (EE), higher depersonalization (DP), and decreased sense of personal accomplishment (PA) [15]. A systematic review in this domain had thus confirmed the significant development of OB among HCWs during the SARS and Middle East respiratory syndrome (MERS) epidemics [16]. Working in isolation wards, having special protective clothing on, higher exposure to COVID-19 patients, increased levels of workload during 1 month or long shifts, and fear of being infected and transferring it to their own families could accordingly have considerable negative consequences on mental health in HCWs, augmenting the possibility of being susceptible to OB [2, 13, 17, 18].

The results of one study had similarly revealed that 81.6% of the nurses had undergone mild OB, and 43.5–62.0% of the cases had been subjected to moderate-to-severe OB during COVID-19 [3]. Nevertheless, a systematic review had reported the prevalence rate of OB by 28% among the frontline [19], and the severity of this condition could be significantly associated with various individual (personality and self-esteem), social (lack of support and nationality), and organizational (job stress and workload) factors [20].

Of note, OB is assumed as a major global health problem, resulting in decreased mental health and well-being in HCWs as well as lower motivation to provide health care services, reduced quality of care delivered to patients, higher medical errors, and an elevated rate of absenteeism and turnover at the time of the pandemic [5, 18, 21]. Besides, HCWs with OB are more likely to feel dissatisfied with their positions and even look for other ones [21]. Highlighted in the related literature, hospital wards have been regarded as the most effective factor in OB severity [22, 23], so that, among the HCWs, nurses working in emergency wards and intensive care units (ICUs) have been more supposed to suffer from OB due to the higher levels of stress, workload, and exposure to patients with COVID-19 confirmed [24].

Based on the results of a survey in China, some factors, such as personnel agency, working experience for 5 years or lower, three or more night shifts weekly, working in isolation wards, and having contacts with medical staff confirmed or suspected around, were among the statistically significant predictors of OB in nurses [2]. Correspondingly, another study had found that OB could be correlated with nurses’ resilience, parenting stress, marital status, number of children, employment type, and gender, so that these individuals had reported higher levels of OB, less resilience, and higher tensions in terms of relationships with their children [25].

Depression, anxiety, and stress are additionally among the important psychological problems that may even give rise to emotional disturbance as the predisposing factor of OB [25, 26]. In this regard, there is a positive significant correlation between OB and anxiety and depression, according to the results of some studies, so that 77.1% of OB could be related to anxiety or depression, and 84.0% of anxiety and depression was associated with OB [27]. The results of one survey in Iran had further indicated that employment status, experience in caring for infected patients, hospital resources, and workplace stress were among the considerably significant risk factors of OB during the COVID-19 pandemic [28].

Even if a wide range of studies have been thus far fulfilled regarding OB all over the world and in Iran, as highlighted in the literature, working environments are completely different in each setting over the course of the pandemic as the most important factor shaping HCWs’ functions. As stated in the survey by Guan, OB

could be significantly associated with the working environment perceptions [29]. Some limitations mentioned in the related literature in Iran have been also small sample size, no sample selection from other regions of the country [28], as well as limited socio-demographic and work-related data assessments [30]. In addition, no study has so far assessed OB among HCWs in all provinces of Iran, to the best of the authors' knowledge. Therefore, the study results had implications and high generalization value as an effective guideline for health care managers. Accordingly, this study aimed to investigate the prevalence rate of OB in Iranian HCWs at the time of the COVID-19 pandemic.

Materials and methods

Study design and participants

In this web-based cross-sectional survey, a total number of 7626 HCWs in 31 provinces of Iran was recruited through the convenience sampling method, from April 6 to May 30, 2020. All the workers agreed to participate in the study.

Instruments and data collection

A web-based questionnaire in Persian was completed using an online platform, developed by Sadra Rayaneh Novin Tabarestan Engineering Co., Mazandaran, Iran. For data collection purposes, the questionnaire was distributed via cyberspace, including popular messaging apps, i.e., WhatsApp, Instagram, and Short Message Service (SMS). In this study, two main instruments were accordingly applied, viz. a socio-demographic information form and the Maslach Burnout Inventory (MBI). Finally, the respondents' scores were automatically calculated and promptly reported, as the questionnaires were received. Furthermore, the HCWs, obtaining high scores on the MBI (namely, unfavorable mental health), were recommended to contact the 4030 Call System, designed by Iran's Ministry of Health and Medical Education, for free-of-charge consultations and read two stress management booklets available in this regard.

Socio-demographic information form

The socio-demographic information form contained items, such as gender, age, marital status, place of living, level of education, academic major, working position, years of working experience, a history of mental and physical problems, number of children, and hospital wards (namely, inpatient or outpatient ones). As well, some items about COVID-19 were investigated in this study.

MBI

The MBI was a 22-item questionnaire, as a standard tool to measure burnout, including OB [3, 31]. This scale was comprised of three dimensions, viz. EE, DP, and PA [32] scored on a seven-point Likert-type scale, ranging from 0 to 6. In this sense, score 0 meant "never occurs to me" and score 6 denoted "occurs to me every day". The EE dimension was also made up of nine items (no. 1, 2, 3, 6, 8, 13, 14, 16, and 20), whose total score ranged from 0 to 54. The scores 0–18 also showed low OB, 19–26 represented moderate OB, and the values above 26 referred to high OB. Likewise, the DP dimension consisted of five items (no. 5, 10, 11, 15, and 22) with a total score ranging from 0 to 30, so that the scores 6–9 indicated moderate OB, and those higher than 9 pointed to high OB. The PA dimension was additionally comprised of eight items (no. 4, 7, 9, 12, 17, 18, 19, and 21), whose overall score was from 0 to 48 so that the scores 34–39 denoted moderate OB, and the values lower than 34 showed high OB. According to the MBI total score, burnout was divided into no OB (<50), mild OB (50–75), moderate OB (76–100), and severe OB (>100) [3]. As well, the high scores of the EE and DP dimensions and the low values of the PA one referred to OB [33, 34]. The internal consistency coefficients of the OB sub-scales had been further reported by $\alpha=0.85$, $\alpha=0.82$, and $\alpha=0.52$ for EE, DP, and PA, respectively [31]. The reliability coefficient of the questionnaire had been already established for the first time in Iran, using the test-retest method, and its reliability had been measured by Cronbach's alpha coefficient of 0.78 and 0.86, respectively [35]. In the present study, the reliability had been confirmed by Cronbach's alpha coefficients of $\alpha=0.88$, $\alpha=0.81$, $\alpha=0.61$, and $\alpha=0.79$ for the EE, PA, DP, and OB dimensions, respectively.

Upon receiving the ethics committee approval, the questionnaire was distributed. First, ethical aspects, including the codes of ethics and the privacy of responses, were clarified. Then, the OB scores were displayed, and the relevant data, such as management strategies to deal with OB and the corresponding author's e-mail were provided in this regard.

Statistical analysis

The data analysis was performed using the Statistical Package for the Social Sciences (SPSS, ver. 24) software (SPSS Inc., IBM Corporation, USA). To identify the missing data, they were also checked and cleaned before further analysis. Descriptive analyses, mean ± standard deviation (SD), were also utilized to illustrate the quantitative variables. Moreover, frequency and percentage were applied to describe the qualitative ones. The prevalence rate of OB caused by COVID-19 was additionally

reported. The univariate and multivariate logistic regression models were correspondingly exploited to investigate the potential predictors of OB during the COVID-19 pandemic. As well, p<0.05 was considered statistically significant.

Results

The HCWs' socio-demographic characteristics are shown in Table 1. In total, 7626 HCWs participated in the present study. In this respect, there were 1098 (14.4%) clinicians, 4409 (57.8%) nurses, and 2119 (27.8%) HCWs involved in other fields. The majority of the HCWs were female (73.2%), married (55.8%), working as a nurse (57.8%), and aged 20–30 (56.2). Moreover, 8.4 and 19.6% of the respondents reported a history of psychiatric disorders and physical illnesses, respectively. In addition, 44.8% of the HCWs were working in isolation wards for COVID-19 and 40.3% of them were working for 4–8 hours a day with the patients confirmed.

The prevalence rate of OB was also 18.3% in the HCWs over the course of the COVID-19 pandemic. Besides, most of the HCWs had experienced severe OB in all three dimensions. Therefore, 34.2, 48.7, and 56.1% of the participants reported severe EE, DP, and PA, respectively (Table 2).

Table 2 The prevalence of occupational burnout among HCWs

Severity<br>Variable	Mild	Moderate	Severe
Emotional exhaustion	2604 (34.1%)	2410 (31.7%)	2605 (34.2%)
Depersonalization	716 (9.4%)	3192 (41.9%)	3711 (48.7%)
Personal accomplishment	1900 (24.9%)	1449 (19.0%)	4270 (56.1%)
Yes			No
Having occupational burnout		1395 (18.3%)	6231 (81.7%)

Table 1 Sociodemographic characteristics of participants

Variables		Frequency (percent), *N* = 7626
**Age (Years)**	20–30	4287 (56.2)
	31–40	2286 (30.0)
	41–50	829 (10.8)
	> 50	224 (3.0)
**Gender**	Male	2044 (26.8)
	Female	5582 (73.2)
**Marital status**	Single	3176 (41.6)
	Married	4257 (55.8)
	Divorced or widowed	193 (2.5)
**Having children**	Yes	2694 (35.3)
	No	4932 (64.7)
**Level of education**	Undergraduate	888 (11.6)
	Bachelor's degree	5323 (69.8)
	Master's and Ph.D. degree	628 (8.3)
	General and special professional doctorate	787 (10.3)
**Occupation**	Clinician	1098 (14.4)
	Nurse	4409 (57.8)
	Others	2119 (27.8)
**Working in isolated units**	Yes	3420 (44.8)
	No	4206 (55.2)
**Working hours with COVID-19 patients**	< 4	3196 (41.9)
	4–8	3074 (40.3)
	> 8	1356 (17.8)
**History of physical illness**	Yes	1497 (19.6)
	No	6129 (80.4)
**History of psychiatric disorders**	Yes	639 (8.4)
	No	6987 (91.6)

The HCWs' OB during the COVID-19 pandemic, stratified by the socio-demographic variables, is outlined in Table 3. The Chi-square test results also showed that OB was significantly different in terms of some variables, such as age, gender, having children, level of education, and working in isolation wards ($p<0.001$). Accordingly, female participants, having no children, at the age range of 20 to 30, holding a bachelor's degree, and working as a nurse in isolation wards had higher levels of OB as compared with others.

Statistical analysis showed most of the female HCWs experienced severe EE (26.9%), DP (35.3%), and PA (41.7%). However, most of the HCWs who had no children had a severe levels of EE (23.7%), DP (34.4%) and, PA (39.5%). Most of the 20 to 30-years-old HCWs had severe EE (21.0%), DP (31.6%) and, PA (34.9%). 25.0, 35.6, and 40.8% of HCWs with bachelor's degrees experienced the severe level of EE, DP, and PA, respectively. Also, nurses experienced the severe levels of EE, DP, and PA in comparison to other HCWs.

In view of that, logistic regression analysis was used to describe the OB predictors (Table 4). The statistical test results additionally demonstrated a history of physical illnesses (odds ratio [OR]: 0.440, 95% confidence interval [CI]: 0.270–0.718, $p=0.001$) and psychiatric disorders (OR: 0.537, 95% CI: 0.293–0.985, $p=0.044$), as the predictors of OB during the first peak of COVID-19. The logistic regression model outcomes similarly revealed a non-significant difference between clinicians and nurses (OR: 0.756, 95% CI: 0.373–1.530, $p=0.437$ vs. OR: 1.745, 95% CI: 0.456–6.684, $p=0.416$, respectively) and having children and others (OR: 1.250, 95% CI: 0.686–2.279, $p=0.466$) in terms of OB.

Discussion

The main purpose of this study was to investigate the prevalence rate of OB in HCWs at the time of the COVID-19 pandemic. The study findings established that 18.3% of the HCWs had experienced OB. In addition, 34.2, 48.7, and 56.1% of these workers presented severe levels of EE, DP, and PA, respectively. Like the present study, these frequencies were equal to 40.7, 30.2, and 36.4% in an Italian survey during the first 4 months of the COVID-19 pandemic, respectively [36]. Moreover, these values had been reported respectively by 38.5, 31.2, and 33.6% in one study in Saudi Arabia, 10 months after the onset of COVID-19 [37]. The prevalence rate of OB was also slightly different in the study by Jalili et al. in Iran [30]. Accordingly, they had reported the prevalence rate of OB and the high levels of EE, DP, and PA by 53.0, 50.1, 13.2, and 85.5%, respectively. The different levels of OB in the given study were associated with the fact that the given phenomenon had been simply assessed during the first 2 months of COVID-19 just in one city in Iran and the response rate had been moderate. However, the present survey was conducted in all provinces of Iran with larger sample size. Overall, the discrepancy in the prevalence rates could be attributed to the pandemic time. One to 2 years after the SARS outbreak, Maunder et al. had also revealed that OB had elevated among HCWs [38], so the assessment time could be of utmost importance. On the other hand, OB during the pandemic conditions could be time-dependent. As COVID-19 had fluctuated in Iran, the HCWs might have experienced the peaks of the disease, and this might have affected their mental health. In addition, the controlled COVID-19 condition was not similar in different countries and even the cities in the same country. Therefore, comparisons sound complicated.

Besides, the study results confirmed that the nurses had been subjected to significantly higher levels of EE, DP, and PA than other HCWs during the pandemic. In the survey by Naldi et al. during the COVID-19 outbreak, the statistical test results had revealed no significant difference between the nurses and other HCWs although the nurses had suffered from more severe distress than others [36]. In one other study in Iran, medical residents and then nurses had significantly undergone higher levels of EE and PA all through the first peak of COVID-19 [30]. Occupational stress could be thus one of the predictors of OB and nurses could endure stress and other mental pressures, especially during the COVID-19 pandemic [39], such as conflicts with supervisors, patients, and their families, role overload, and increased working hours [40, 41].

However, Barello et al. had reported that nurses had faced higher prevalence rates of OB compared with other workers [42]. This discrepancy might be related to the fact that the nurses working in different wards had not suffered from similar OB. For example, Ahmadi et al. had demonstrated that the ICU and emergency ward nurses were more likely to be influenced by OB than those working in orthopedic and hemodialysis ones [43]. Besides, one study had reported that surgical residents could frequently experience higher levels of OB than others [44], however, neurology residents had undergone the same condition in Kulik et al [45] Another survey in Iran had further established that residents working in emergency and surgical wards had been subjected to more OB as compared with others [46]. Even though the long working hours per week for the medical residents were expected, they were significantly correlated with OB in pediatric residents, as reported by Treluyer and Tourneux [47].

In the present study, OB was significantly different in terms of direct contact with COVID-19 patients and the hours spend with them by each worker. Most of the

Table 3 Occupational burnout in participants during COVID-19 outbreak among HCWs stratified by socio-demographic variables

Variables		Emotional Exhaustion				Depersonalization				Personal Accomplishment				Occupational Burn out		
		Mild N (%)	Moderate N (%)	Severe N (%)	*p*-value*	Mild N (%)	Moderate N (%)	Severe N (%)	*p*-value*	Mild N (%)	Moderate N (%)	Severe N (%)	*p*-value*	Yes	No	*p*-value*
Gender	Female	1727 (22.6%)	1802 (23.6%)	2053 (26.9%)	< 0.001	499 (6.5%)	2388 (31.3%)	2695 (35.3%)	0.011	1315 (17.2%)	1088 (14.3%)	3179 (41.7%)	< 0.001	1090 (14.3%)	4492 (58.9%)	< 0.001
	Male	881 (11.6%)	609 (8.0%)	554 (7.3%)		217 (2.8%)	808 (10.6%)	1019 (13.4%)		587 (7.7%)	362 (4.7%)	1095 (14.4%)		305 (4.0%)	1739 (22.8%)	
Having children	Yes	1045 (13.7%)	851 (11.2%)	798 (10.5%)	0 < 0.01	242 (3.2%)	1365 (17.9%)	1087 (14.3%)	< 0.001	872 (11.4%)	558 (7.3%)	1264 (16.6%)	< 0.001	352 (4.6%)	2342 (30.7%)	< 0.001
	No	1563 (20.5%)	1560 (20.5%)	1809 (23.7%)		474 (6.2%)	1831 (24.0%)	2627 (34.4%)		1030 (13.5%)	892 (11.7%)	3010 (39.5%)		1043 (13.7%)	3889 (51.0%)	
Age	20–30	1322 (17.3%)	1366 (17.9%)	1599 (21.0%)	< 0.001	425 (5.6%)	1449 (19.0%)	2413 (31.6%)	< 0.001	822 (10.8%)	804 (10.5%)	2661 (34.9%)	< 0.001	935 (12.3%)	3352 (44.0%)	< 0.001
	31–40	817 (10.7%)	731 (9.6%0	738 (9.7%)		211 (2.8%)	1107 (14.5%)	968 (12.7%)		617 (8.1%)	436 (5.7%)	1233 (16.2%)		364 (4.8%)	1922 (25.2%)	
	41–50	356 (4.7%)	246 (3.2%)	227 (3.0%)		64 (0.8%)	501 (6.6%)	264 (3.5%)		347 (4.6%)	169 (2.2%)	313 (4.1%)		80 (1.0%)	749 (9.8%)	
	> 50	113 (1.5%)	68 (0.9%)	43 (0.6%)		16 (0.2%)	139 (1.8%)	69 (0.9%)		116 (1.5%)	41 (0.5%)	67 (0.9%)		16 (0.2%)	208 (2.7%)	
Marital status	Single	1038 (13.6%)	978 (12.8%)	1160 (15.2%)	0.001	284 (3.7%)	1170 (15.3%)	1722 (22.6%)	< 0.001	671 (8.8%)	571 (7.5%)	1934 (25.4%)	< 0.001	682 (8.9%)	2494 (32.7%)	< 0.001
	Married	1503 (19.7%)	1382 (18.1%)	1372 (18.0%)		414 (5.4%)	1944 (25.5%)	1899 (24.9%)		1174 (15.4%)	844 (11.1%)	2239 (29.4%)		670 (8.8%)	3587 (47.0%)	
	Divorced/widowed	67 (0.9%)	51 (0.7%)	75 (1.0%)		18 (0.2%)	82 (1.1%)	93 (1.2%)		57 (0.7%)	35 (0.5%)	101 (1.3%)		43 (0.6%)	150 (2.0%)	
Work experience	0–5	1267 (16.6%)	1237 (16.2%)	1405 (18.4%)	< 0.001	403 (5.3%)	1382 (18.1%)	2124 (27.9%)	< 0.001	780 10.2%)	734 (9.6%)	2395 (31.4%)	< 0.001	813 (10.7%)	3096 (40.6%)	< 0.001
	6–10	527 (6.9%)	492 (6.5%)	566 (7.4%)		148 (1.9%)	664 (8.7%)	773 (10.1%)		377 (4.9%)	286 (3.8%)	922 (12.1%)		314 (4.1%)	1271 (16.7%)	
	11–15	376 (4.9%)	372 (4.9%)	352 (4.6%)		87 (1.1%)	535 (7.0%)	478 (6.3%)		318 (4.2%)	277 (3.0%)	555 (7.3%)		161 (2.1%)	939 (12.3%)	
	16–20	210 (2.8%)	171 (2.2%)	150 (2.0%)		44 (0.6%)	303 (4.0%)	184 (2.4%)		196 (2.6%)	104 (1.4%)	231 (3.0%)		65 (0.9%)	466 (6.1%)	
	21–25	126 (1.7%)	94 (1.2%)	90 (1.2%)		18 (0.2%)	190 (2.5%)	102 (1.3%)		132 (1.7%)	69 (0.9%)	109 (1.4%)		25 (0.3%)	285 (3.7%)	
	26–30	102 (1.3%)	45 (0.6%)	44 (0.6%)		16 (0.2%)	122 (1.6%)	53 (0.7%)		99 (1.3%)	30 (0.4%)	62 (0.8%)		17 (0.2%)	174 (2.3%)	
Level of education	Under-graduate	412 (5.4%)	253 (3.3%)	223 (2.9%)	< 0.001	117 (1.5%)	379 (5.0%)	392 (5.1%)	< 0.001	260 (3.4%)	171 (2.2%)	457 (6.0%)	< 0.001	99 (1.3%)	789 (10.3%)	< 0.001
	Bachelor's degree	1677 (22.0%)	1738 (22.8%)	1908 (25.0%)		494 (6.5%)	2117 (27.8%)	2712 (35.6%)		1196 (15.7%)	1014 (13.3%)	3113 (40.8%)		1058 (13.9%)	4265 (55.9%)	
	Master's and Ph.D. degree	240 (3.1%)	202 (2.6%)	186 (2.4%)		58 (0.8%)	320 (4.2%)	250 (3.3%)		197 (2.6%)	105 (1.4%)	326 (4.3%)		86 (1.1%)	542 (7.1%)	
	General & special professional doctorate	279 (3.7%)	218 (2.9%)	290 (3.8%)		47 (0.6%)	380 (5.0%)	360 (4.7%)		249 (3.3%)	160 (2.1%)	378 (5.0%)		152 (2.0%)	635 (8.3%)	

Table 3 (continued)

Variables		Emotional Exhaustion				Depersonalization				Personal Accomplishment				Occupational Burn out		
		Mild N (%)	Moderate N (%)	Severe N (%)	*p*-value*	Mild N (%)	Moderate N (%)	Severe N (%)	*p*-value*	Mild N (%)	Moderate N (%)	Severe N (%)	*p*-value*	Yes	No	*p*-value*
Occupation	Clinician	406 (5.3%)	312 (4.1%)	380 (5.0%)	< 0.001	95 (1.2%)	497 (6.5%)	506 (6.6%)	< 0.001	322 (4.2%)	213 (2.8%)	563 (7.4%)	< 0.001	199 (2.6%)	899 (11.8%)	< 0.001
	Nurse	1323 (17.3%)	1476 (19.4%)	1610 (21.1%)		374 (4.9%)	1645 (21.6%)	2390 (31.3%)		970 (12.7%)	830 (10.9%)	2609 (34.2%)		904 (11.9%)	3505 (46.0%)	
	Others	879 (11.5%)	623 (8.2%)	617 (8.1%)		247 (3.3%)	1054 (13.8%)	818 (10.1%)		610 (8.0%)	407 (5.3%)	1102 (14.5%)		292 (3.8%)	1827 (23.9%)	
Working in isolated wards	Yes	1026 (13.5%)	1082 (14.2%)	1312 (17.2%)	< 0.001	262 (3.4%)	1301 (17.1%)	1857 (24.4%)	< 0.001	850 (11.1%)	670 (8.8%)	1900 (24.9%)	0.504	724 (9.5%)	2696 (35.4%)	< 0.001
	No	1582 (20.7%)	1329 (17.4%)	1295 (17.0%)		454 (6.0%)	1895 (24.8%)	1857 (24.4%)		1052 (13.8%)	780 (10.2%)	2374 (56.4%)		671 (8.8%)	3535 (46.4%)	
Working hours with COVID-19 patients	< 4	1326 (17.4%)	995 (13.1%)	875 (11.5%)	< 0.001	374 (4.9%)	1446 (18.9%)	1376 (18.1%)	< 0.001	771 (10.2%)	576 (7.6%)	1849 (24.2%)	0.049	452 (6.0%)	2744 (35.9%)	< 0.001
	4–8	881 (11.6%)	1024 (13.5%)	11.69 (15.3%)		225 (2.9%)	1264 (16.6%)	1585 (20.7%)		776 (10.2%)	612 (8.1%)	1686 (22.1%)		626 (8.2%)	2448 (11.6%)	
	> 8	401 (5.3%)	392 (5.1%)	563 (7.4%)		117 (1.5%)	486 (6.4%)	753 (9.9%)		355 (4.7%)	262 (3.4%)	739 (9.7%)		317 (4.2%)	1039 (13.6%)	
History of physical illness	Yes	429 (5.6%)	467 (6.1%)	604 (7.9%)	< 0.001	124 (1.6%)	629 (8.2%)	744 (9.8%)	0.245	421 (5.5%)	282 (3.7%)	794 (10.4%)	0.005	295 (3.9%)	1202 (15.8%)	0.117
	No	2179 (28.6%)	1947 (25.5%)	2003 (26.3%)		592 (7.8%)	2567 (33.7%)	2970 (38.9%)		1481 (19.4%)	1168 (15.3%)	3480 (45.6%)		1100 (14.4%)	5029 (65.9%)	
History of psychiatric disorders	Yes	139 (1.8%)	177 (2.3%)	323 (4.2%)	< 0.001	52 (0.7%)	236 (3.1%)	351 (4.6%)	0.004	102 (1.3%)	114 (1.5%)	423 (5.5%)	< 0.001	181 (2.4%)	458 (6.0%)	< 0.001
	No	2469 (32.4%)	2234 (29.3%)	2284 (30.0%)		664 (8.7%)	2960 (38.8%)	3363 (44.1%)		1800 (23.6%)	1336 (17.5%)	3851 (50.5%)		1214 (15.9%)	5773 (75.7%)	

*Chi square test

Table 4 Logistic regression for predictors of occupational burnout

Variables		Univariate			Multivariate		
		OR	95% CI	*P*-value	OR	95% CI	*P*-value
Gender	Male	Ref.	Ref.	Ref.	Ref.	Ref.	Ref.
	Female	0.770	0.683–0.867	< 0.001	1.177	0.746–1.856	0.484
Age	20–30	0.626	0.447–0.876	0.006	0.804	0.340–1.901	0.619
	31–40	0.768	0.545–1.083	0.133	0.685	0.322–1.457	0.326
	41–50	0.833	0.577–1.204	0.331	1.245	0.600–2.585	0.556
	> 50	Ref.	Ref.	Ref.	Ref.	Ref.	Ref.
Marital status	Single	1.096	0.795–1.510	0.576	1.007	0.307–3.309	0.991
	Married	1.219	0.887–1.676	0.223	1.158	0.373–3.590	0.800
	Divorced/widowed	Ref.	Ref.	Ref.	Ref.	Ref.	Ref.
Having children	Yes	1.215	1.090–1.355	< 0.001	1.250	0.686–2.279	0.466
	No	Ref.	Ref.	Ref.	Ref.	Ref.	Ref.
Level of education	Undergraduate	1.533	1.148–2.047	0.004	0.316	0.016–6.189	0.448
	Bachelor's degree	1.105	0.937–1.304	0.234	1.088	0.330–3.591	0.889
	Master's and Ph.D. degree	1.877	0.900–1.304	0.093	1.469	0.263–8.195	0.661
	General & special professional doctorate	Ref.	Ref.	Ref.	Ref.	Ref.	Ref.
Occupation	Clinician	1.321	0.775–2.252	0.306	0.756	0.373–1.530	0.437
	Nurse	2.209	0.632–7.716	0.214	1.745	0.456–6.684	0.416
	Other	Ref.	Ref.	Ref.	Ref.	Ref.	Ref.
Working hours with COVID-19 patients	< 4	1.982	1.718–2.285	< 0.001	1.163	0.592–2.284	0.661
	4–8	1.196	1.043–1.372	0.011	0.793	0.416–1.510	0.480
	> 8	Ref.	Ref.	Ref.	Ref.	Ref.	Ref.
Working in isolated wards	Yes	0.743	0.670–0.823	< 0.001	0.988	0.596–1.638	0.962
	No	Ref.	Ref.	Ref.	Ref.	Ref.	Ref.
History of physical illness	Yes	0.653	0.577–0.738	< 0.001	0.440	0.270–0.718	0.001
	No	Ref.	Ref.	Ref.	Ref.	Ref.	Ref.
History of psychiatric disorder	Yes	0.621	0.523–0.737	< 0.001	0.537	0.293–0.985	0.044
	No	Ref.	Ref.	Ref.	Ref.	Ref.	Ref.

HCWs also reported that OB was not effective in isolation wards. But, direct contact with COVID-19 patients and long working hours were significantly associated with OB in Roslan et al [48] A study in Iran had also found that the OB scores had been higher in frontliners in comparison with non-frontline HCWs [28]. Although the workers involved in isolation wards were using special protective clothing and equipment, anxiety had merely occurred due to no awareness of definite COVID-19 in the non-isolation wards. In Mira et al., the acute stress score had been higher in hospitals with higher COVID-19 death rates [4]. In the study by Koutsimani et al., a significant relationship had been further observed between anxiety and OB [49]. Guixia and Hui had similarly shown that burnout was positively correlated with anxiety during this pandemic [3]. Correspondingly, Azizi et al. had reported that working hours spent with COVID-19 patients were significantly associated with anxiety induced by COVID-19 [50].

One of the variables that had not been assessed in previous studies in Iran was the working experience with regard to OB during COVID-19. In the present study, the working experience was also significantly different among the HCWs in terms of OB. Therefore, the lower the working experience, the greater the OB. Nevertheless, one other survey in Iran had demonstrated that working experience was not significantly correlated with the OB severity [14]. The lack of similar studies also made the comparisons of the results in this survey difficult.

Besides, the statistical results of the present study revealed a significant discrepancy between the male and female participants regarding OB. In this sense, the female HCWs experienced more OB than males. These results were in line with the reports by Alanazi et al [37] In the survey by Alrawashdeh et al., female clinicians had also faced higher OB during the COVID-19 pandemic [51]. High occupational demand might have been related to high OB. It seems that reducing occupational demands is a protective

factor among females. As a result, they may be subjected to higher levels of OB over the course of COVID-19 [52]. Likewise, married HCWs had experienced significantly high levels of OB while it was not so in other research [15, 30]. As married people had to play different roles, including responsibilities at home and childcare, they might have been subjected to higher burnout [53]. In the present study, having no children was significantly correlated with a higher prevalence rate of OB, in agreement with the survey by Jalili et al. [30], but contrasting the reports by Huang et al. and Lai et al [54, 55] Childless people might also live through more depression and mental health problems [56]. Moreover, they may not receive appropriate social support [57] and be more vulnerable to burnout.

The sample size in the present study was larger compared with that in other surveys, which boosted the generalizability of the results to manage the COVID-19 pandemic. Regarding the high prevalence rate of OB among the HCWs and the unfinished COVID-19 conditions in Iran, health care managers are recommended to orient the management strategies toward improving mental health in such workers [58]. As regards, Iran faces multiple peaks of this infection, so it is suggested to assess mental health, particularly OB, at different times of the pandemic. Health care managers should also adopt multiple coping strategies, including spiritual health promotion, to tackle OB [59–61], and use digital tools to provide emotional support for controlling acute stress and emotional distress during and after the COVID-19 pandemic [62]. One limitation of the present study was the application of social media to collect the data, so not everyone might have access to such media or have much time to tap them.

Conclusion

The prevalence rate of OB was 18.3% in the Iranian HCWs over the course of the COVID-19 pandemic. Special attention, including screening and organizational support, should thus target the cases with less experience, female gender, having no children, aged 20–30, holding a bachelor's degree, and working as a nurse in isolation wards of the hospital, who might have higher levels of OB in relation to others.

Abbreviations

COVID-19: Coronavirus disease 2019; HCWs: Health care workers; OB: Occupational burnout; MBI: Maslach Burnout Inventory; EE: Emotional exhaustion; DP: Depersonalization; PA: Personal accomplishment; SARS: Severe acute respiratory syndrome; MERS: Middle East respiratory syndrome; WHO: World Health Organization; RT-PCR: Reverse transcription-polymerase chain reaction; ICD-11: 11th revision of the International Classification of Diseases; ICU: Intensive care unit; SMS: Short message service; SPSS: Statistical package for the social sciences; OR: Odds ratio; CI: Confidence interval.

Acknowledgments

The authors, hereby, extend their gratitude to all HCWs facing difficult conditions to finalize the COVID-19 pandemic.

Authors' contributions

F.E.: participated in the study design, data collection, data re-evaluation, interpretation of the findings, and manuscript revision. M.K. and M.A.: contributed to collecting the data, interpreting the findings, as well as drafting and revising the manuscript. M.M.: performed the statistical analysis. H.M.: participated in study design and data collection. R.G.: was involved in data collection. M.H.R.: contributed to designing the evaluation. All authors read and approved the final manuscript.

Funding

This study was funded by Mazandaran University of Medical Sciences (No. 7574), Sari, Iran.

Availability of data and materials

The datasets used and/or analyzed during the present study are available from the corresponding author on reasonable request.

Declarations

Ethics approval and consent to participate

This research project was approved by the Ethics Committee of Mazandaran University of Medical Sciences (code no. IR.MAZUMS.REC.1399.7574), Sari, Iran. All the procedures implemented in this study involving human participants were also in accordance with the ethical standards of the Institutional and/or National Research Ethics Committee and the Declaration of Helsinki (DoH) 1964 and its later amendments or comparable ethical protocols. To comply with the ethical considerations, online informed consent was obtained from the participants upon explaining the study objectives, and they were ensured about the confidentiality of their information and responses.

Consent for publication

Not applicable.

Competing interests

The authors declared no conflicts of interest.

Author details

[1]Pediatric Infectious Diseases Research Center, Communicable Diseases Institute, Mazandaran University of Medical Sciences, Sari, Iran. [2]Department of Reproductive Health and Midwifery, School of Nursing and Midwifery, Tehran university of Medical Sciences, Tehran, Iran. [3]Sexual and Reproductive Health Research Center, Mazandaran University of Medical Sciences, Sari, Iran. [4]Gastrointestinal Cancer Research Center, Non-communicable Diseases Institute, Mazandaran University of Medical Sciences, Sari, Iran. [5]Department of Internal Medicine, Pulmonary and Critical Care Division, Faculty of Medicine, Mazandaran University of Medical Sciences, Sari, Iran. [6]Antimicrobial Resistance Research Center, Department of Infectious Diseases, Mazandaran University of Medical Sciences, Sari, Iran. [7]Educational Psychology, Research Ethics Committee, Imam khomeini Hospital, Mazandaran University of Medical Sciences, Sari, Iran. [8]Sexual and Reproductive Health Research Center, Addiction Institute, Mazandaran University of Medical Sciences, Sari, Iran. [9]Department of Psychiatry, Faculty of Medicine, Mazandaran University of Medical Sciences, Sari, Iran. [10]Psychosomatic Ward, Imam Khomeini General Hospital, Razi Ave, Sari, Mazandaran, Iran.

Received: 6 September 2021 Accepted: 24 May 2022
Published online: 28 May 2022

References

1. WHO Coronavirus Disease (COVID-19) Dashboard Data last updated: 2021/08/07. https://covid19.who.int/.
2. Wan Z, Lian M, Ma H, Cai Z, Xianyu Y. Factors associated with burnout among Chinese nurses during COVID-19 epidemic: a cross-sectional study; 2020.
3. Guixia L, Hui Z. A study on burnout of nurses in the period of COVID-19. Psychol Behav Sci. 2020;9(3):31.
4. Mira JJ, Carrillo I, Guilabert M, Mula A, Martin-Delgado J, Pérez-Jover MV, et al. Acute stress of the healthcare workforce during the COVID-19 pandemic evolution: a cross-sectional study in Spain. BMJ Open. 2020;10(11):e042555.

5. Babamiri MAN, Heidarimoghadam R. Research on reducing burnout in health care workers in critical situations such as the COVID-19 outbreak. Work (Reading, Mass). 2020;66(2):379–80.
6. Kannampallil TG, Goss CW, Evanoff BA, Strickland JR, McAlister RP, J. D. Exposure to COVID-19 patients increases physician trainee stress and burnout. Plos One. 2020;15(8):e0237301.
7. De Simone S, Vargas M, Servillo G. Organizational strategies to reduce physician burnout: a systematic review and meta-analysis. Aging Clin Exp Res. 2021;33(4):883–94.
8. Kar SK, Yasir Arafat S, Kabir R, Sharma P, Saxena SK. Coping with mental health challenges during COVID-19. In: Coronavirus disease 2019 (COVID-19). Singapore: Springer; 2020. p. 199–213.
9. Sant'Ana G, Imoto AM, Amorim FF, Taminato M, Peccin MS, Santana LA, et al. Infection and death in healthcare workers due to COVID-19: a systematic review. Acta Paul Enferm. 2020;33:eAPE20200107.
10. Gómez-Ochoa SA, Franco OH, Rojas LZ, Raguindin PF, Roa-Díaz ZM, Wyssmann BM, et al. COVID-19 in health-care workers: a living systematic review and meta-analysis of prevalence, risk factors, clinical characteristics, and outcomes. Am J Epidemiol. 2021;190(1):161–75.
11. Erdem H, Lucey DR. Healthcare worker infections and deaths due to COVID-19: a survey from 37 nations and a call for WHO to post national data on their website. Int J Infect Dis. 2021;102:239.
12. Organization WH. Burn-out an "occupational phenomenon": international classification of diseases. Geneva: World Health Organization; 2019.
13. SR. D. Factors associated with burnout among physicians: an evaluation during a period of COVID-19 pandemic. J Healthc Leadersh. 2020;12:85–94.
14. Hosseininejad SM, Aminiahidashti H, Montazer SH, Elyasi F, Moosazadeh M, Ahmadi N. Job burnout among the emergency department nurses of medical training centers affiliated to Mazandaran university of medical sciences. Iran J Emerg Med. 2016;3(4):125–31.
15. Ahmadi A, Ahmadi M, Elyasi F, Ahmadi A, Ahmadi N. The relationship of occupational burnout and communication skills in nurses. J Mazandaran Univ Med Sci. 2013;23(106):130–9.
16. Magnavita N, Chirico F, Garbarino S, Bragazzi NL, Santacroce E, Zaffina S. SARS/MERS/SARS-CoV-2 outbreaks and burnout syndrome among healthcare workers. An umbrella systematic review. Int J Environ Res Public Health. 2021;18(8):4361.
17. Sasangohar F, Jones SL, Masud FN, Vahidy FS, Kash BA. Provider burnout and fatigue during the COVID-19 pandemic: lessons learned from a high volume intensive care unit. Anesth Analg. 2020;131(1):106–11.
18. Sultana ASR, Hossain MM, Bhattacharya S, Purohit N. Burnout among healthcare providers during COVID-19 pandemic: Challenges and evidence-based interventions; 2020. p. 1–7.
19. Chirico F, Ferrari G, Nucera G, Szarpak L, Crescenzo P, Ilesanmi O. Prevalence of anxiety, depression, burnout syndrome, and mental health disorders among healthcare workers during the COVID-19 pandemic: a rapid umbrella review of systematic reviews. J Health Soc Sci. 2021;6(2):209–20.
20. Elbarazi I, Loney T, Yousef S, Elias A. Prevalence of and factors associated with burnout among health care professionals in Arab countries: a systematic review. BMC Health Serv Res. 2017;17(1):491.
21. Talaee N, Varahram M, Jamaati H, Salimi A, Attarchi M. Stress and burnout in health care workers during COVID-19 pandemic: validation of a questionnaire. Nature Public Health Emergency Collection. 2022;30(3):531–36.
22. Gómez-Urquiza JL, la Fuente-Solana D, Emilia I, Albendín-García L, Vargas-Pecino C, Ortega-Campos EM. Prevalence of burnout syndrome in emergency nurses: a meta-analysis. Crit Care Nurse. 2017;37(5):e1–9.
23. Ramirez-Baena L, Ortega-Campos E, Gomez-Urquiza JL, la Fuente-Solana D, Emilia I. A multicentre study of burnout prevalence and related psychological variables in medical area hospital nurses. J Clin Med. 2019;8(1):92.
24. Elshaer NSM, Moustafa MSA, Aiad MW, Ramadan MIE. Job stress and burnout syndrome among critical care healthcare workers. Alexandria J Med. 2018;54(3):273–7.
25. Bashirian S, Bijani M, Borzou SR, Khazaei S. Resilience, Occupational Burnout, and Parenting Stress in Nurses Caring for COVID-2019 Patients; 2020.
26. Mousavi SV, Ramezani M, Salehi I, Hossein Khanzadeh AA, Sheikholeslami F. The relationship between burnout dimensions and psychological symptoms (depression, anxiety and stress) among nurses. J Holist Nurs Midwifery. 2017;27(2):37–43 eng.
27. Hardy P, Costemale-Lacoste J-F, Trichard C, Butlen-Ducuing F, Devouge I, Cerboneschi V, et al. Comparison of burnout, anxiety and depressive syndromes in hospital psychiatrists and other physicians: results from the ESTEM study. Psychiatry Res. 2020;284:112662.
28. Hoseinabadi TS, Kakhki S, Teimori G, Nayyeri S. Burnout and its influencing factors between frontline nurses and nurses from other wards during the outbreak of coronavirus disease-COVID-19-in Iran. Investigacion y Educacion en Enfermeria. 2020;38(2):e03.
29. Guan M. Associations between perceptions of the work environment and job burnout based on MIMIC models among 679 knowledge workers. SAGE Open. 2021;11(1):2158244021999384.
30. Jalili M, Niroomand M, Hadavand F, Zeinali K, Fotouhi A. Burnout among healthcare professionals during COVID-19 pandemic: a cross-sectional study. Int Arch Occup Environ Health. 2021;94:1345–52.
31. Rohland BM, Kruse GR, Rohrer JE. Validation of a single-item measure of burnout against the Maslach burnout inventory among physicians. Stress Health. 2004;20(2):75–9.
32. Olson K, Sinsky C, Rinne ST, Long T, Vender R, Mukherjee S, et al. Cross-sectional survey of workplace stressors associated with physician burnout measured by the Mini-Z and the Maslach burnout inventory. Stress Health. 2019;35(2):157–75.
33. Yanyan Z, Tingting Z, LI B. A related study of occupational burnout and depression, anxiety in nurses working at intensive care unit. J Psychiatry. 2018;31:442–5.
34. Myhren H, Ekeberg Q, Stokland O. Job Satisfaction and Burnout among Intensive Care Unit Nurses and Physicians. Critical Care Research and Practice. 2013;2013:Article ID: 786176.
35. Rasoulian M, Elahi F, Afkham EA. The relationship between job burnout and personality traits in nurses. Iran J Psychiatry Clin Psychol. 2004;9(4):18–24.
36. Naldi A, Vallelonga F, Di Liberto A, Cavallo R, Agnesone M, Gonella M, et al. COVID-19 pandemic-related anxiety, distress and burnout: prevalence and associated factors in healthcare workers of north-West Italy. BJPsych Open. 2021;7(1):E27.
37. Alanazi KH, bin Saleh GM, AlEidi SM, AlHarbi MA, Hathout HM. Prevalence and risk factors of burnout among healthcare professionals during COVID-19 pandemic-Saudi Arabia. Am J Public Health. 2021;9(1):18–27.
38. Maunder RG, Lancee WJ, Balderson KE, Bennett JP, Borgundvaag B, Evans S, et al. Long-term psychological and occupational effects of providing hospital healthcare during SARS outbreak. Emerg Infect Dis. 2006;12(12):1924.
39. Barzgar M, Mostafazadeh A, Habibzadeh H, Radfar M, Khalkhali HR. Prediction occupational burnout based job stress in nurses in the emergency department. Iran J Psychiatr Nurs. 2021;8(6):0.
40. Chaudhari AP, Mazumdar K, Motwani YM, Ramadas D. A profile of occupational stress in nurses. Ann Indian Psychiatry. 2018;2(2):109.
41. Kakemam E, Raeissi P, Raoofi S, Soltani A, Sokhanvar M, Visentin DC, et al. Occupational stress and associated risk factors among nurses: a cross-sectional study. Contemp Nurse. 2019;55(2–3):237–49.
42. Barello S, Palamenghi L, Graffigna G. Burnout and somatic symptoms among frontline healthcare professionals at the peak of the Italian COVID-19 pandemic. Psychiatry Res. 2020;290:113129.
43. Ahmadi O, Azizkhani R, Basravi M. Correlation between workplace and occupational burnout syndrome in nurses. Adv Biomed Res. 2014;3:44.
44. Zis P, Anagnostopoulos F, Sykioti P. Burnout in medical residents: a study based on the job demands-resources model. Sci World J. 2014;2014:Article ID: 673279.
45. Kulik MM. Cierpienie, które przerasta, czyli o wypaleniu lekarzy pracujących z ludźmi przewlekle chorymi. Studia z psychologii w KUL Wyd KUL. 2008;15:81–112.
46. Jahanian F, Elyasi F, Hosseininejad SM, Hajizaadeh A, Golykhatir I. Evaluation of satisfaction, burnout, compassion, and secondary traumatic stress and related factors among residents working in training. Int J Hosp Res. 2020;9(1). http://ijhr.iums.ac.ir/article_110650.html.
47. Treluyer L, Tourneux P. Burnout among paediatric residents during the COVID-19 outbreak in France. Eur J Pediatr. 2021;180(2):627–33.
48. Roslan NS, Yusoff MSB, Razak AA, Morgan K. Burnout prevalence and its associated factors among Malaysian healthcare workers during COVID-19 pandemic: an embedded mixed-method study. In: Healthcare: Multidisciplinary Digital Publishing Institute; 2021;9(1):90.

49. Koutsimani P, Montgomery A, Georganta K. The relationship between burnout, depression, and anxiety: a systematic review and meta-analysis. Front Psychol. 2019;10:284.
50. Azizi M, Kamali M, Moosazadeh M, Aarabi M, Ghasemian R, Hasannezhad Reskati M, Elyasi F. Assessing mental health status among Iranian healthcare workers in times of the COVID-19 pandemic: A web-based cross-sectional study. Brain Behav. 2021;11(8):1–13.
51. Alrawashdeh HM, Ala'a B, Alzawahreh MK, Al-Tamimi A, Elkholy M, Al Sarireh F, et al. Occupational burnout and job satisfaction among physicians in times of COVID-19 crisis: a convergent parallel mixed-method study. BMC Public Health. 2021;21(1):1–18.
52. Brera AS, Arrigoni C, Dellafiore F, Odone A, Magon A, Nania T, et al. Burnout syndrome and its determinants among healthcare workers during the first wave of the Covid-19 outbreak in Italy: a cross-sectional study to identify sex-related differences. La Medicina del lavoro. 2021;112(4):306.
53. Nejatian M, Alami A, Momeniyan V, Noghabi AD, Jafari A. Investigating the status of marital burnout and related factors in married women referred to health centers. BMC Womens Health. 2021;21(1):1–9.
54. Huang L, Lei W, Xu F, Liu H, Yu L. Emotional responses and coping strategies in nurses and nursing students during Covid-19 outbreak: a comparative study. Plos One. 2020;15(8):e0237303.
55. Lai J, Ma S, Wang Y, Cai Z, Hu J, Wei N, et al. Factors associated with mental health outcomes among health care workers exposed to coronavirus disease 2019. JAMA Netw Open. 2020;3(3):e203976-e.
56. Kunst SA. Childless elderly: the influence of not having children on your mental health; 2021.
57. Křenková L. Childlessness and social support in old age. Finn Yearb Popul Res. 2018;53:25–50.
58. Chirico F, Ferrari G. Role of the workplace in implementing mental health interventions for high-risk groups among the working age population after the COVID-19 pandemic. J Health Soc Sci. 2021;6(2):145–50.
59. Chirico F, Magnavita N. The spiritual dimension of health for more spirituality at workplace. Indian. J Occup Environ Med. 2019;23(2):99.
60. Chirico F, Sharma M, Zaffina S, Magnavita N. Spirituality and prayer on teacher stress and burnout in an Italian cohort: a pilot, before-after controlled study. Front Psychol. 2020;10:2933.
61. Chirico F. Spirituality to cope with COVID-19 pandemic, climate change and future global challenges. J Health Soc Sci. 2021;6:151–8.
62. Mira JJ, Vicente MA, Lopez-Pineda A, Carrillo I, Guilabert M, Fernández C, et al. Preventing and addressing the stress reactions of health care workers caring for patients with COVID-19: development of a digital platform (be+ against COVID). JMIR mHealth and uHealth. 2020;8(10):e21692.

Publisher's Note

Springer Nature remains neutral with regard to jurisdictional claims in published maps and institutional affiliations.

Ready to submit your research? Choose BMC and benefit from:

- fast, convenient online submission
- thorough peer review by experienced researchers in your field
- rapid publication on acceptance
- support for research data, including large and complex data types
- gold Open Access which fosters wider collaboration and increased citations
- maximum visibility for your research: over 100M website views per year

At BMC, research is always in progress.

Learn more biomedcentral.com/submissions

BMC
