# Supplementary material for: Mental health status and related factors influencing healthcare workers during the COVID-19 pandemic: A systematic review and meta-analysis
Source: PLoS One. 2024 Jan 19;19(1):e0289454. doi: 10.1371/journal.pone.0289454 (PMC10798549; doi:10.1371/journal.pone.0289454)
Supplement: S1 Data — (ZIP) [file pone.0289454.s011.zip › literatures/62.pdf]

SANTÉ AU MAGHREB

Impact psychosocial de la COVID-19 chez le personnel de santé en Algérie

Psychosocial impact of COVID-19 among health workers in Algeria

التأثير النفسي الاجتماعي لـ COVID-19 بين العاملين الصحيين في الجزائر

Chahrazed Kandouci [1], Fethi Meçabih [2], Imene Meçabih [3], Chifaa Kadari [3], Nihad Megherbi [3], Mohamed Yacine Achouri [3], Baderdine Abdelkrim Kandouci [1], Ahmed Ben Abdelaziz [4] .

[1] Faculté de médecine. Laboratoire de recherche en environnement et santé.Université DjillaliLiabes. Sidi Bel Abbès Algérie
[2] Département de pharmacie, faculté de médecine d'Alger Algérie
[3] Département de pharmacie, faculté de médecine, université DjillaliLiabes. Sidi Bel Abbès Algérie
[4] Faculté de médecine de Sousse.Université de Sousse. Laboratoire de Recherche LR19SP01 (Tunisie)

RÉSUMÉ

Contexte: La pandémie actuelle COVID-19 a mis les professionnels de la santé face à une détresse psychologique croissante, avec un risque élevé d'infection.
Objectif: Estimer la prévalence des troubles anxio-dépressifs chez les professionnels de la santé en Algérie et déterminer leurs facteurs de risque associés.
Méthode: Une étude descriptive transversale a été menée du 11 janvier au 09 mars 2021 et a concerné les professionnels de la santé, en Algérie, à travers un auto-questionnaire en ligne. Les échelles d'évaluation de la santé mentale utilisées ont été GAD-7 (7 items) pour l'Anxiété, et la CES-D (20 items) pour la Dépression. La Résilience a été estimée par le CD-RISC (10 items). Un questionnaire original a été utilisé pour évaluer trois facteurs: la peur de l'infection et de la mort, l'isolement et la stigmatisation, ainsi que la motivation et le comportement d'évasion au travail.
Résultats: Au total, 1005 professionnels de la santé ont été inclus à l'étude, dont 51.5% ont été des médecins, 75,6% ont été des femmes et 41,1% ont été au premier front de lutte contre la COVID-19. La prévalence d'Anxiété et de Dépression ont été respectivement de 23.8% et 44.6%.Les professionnels de santé ayant un score élevé de résilience ont été ceux qui étaient en contact direct avec la COVID-19 (ORa=3,75; IC95%[1,11-12,7]) et ceux qui craignaient de contracter la maladie (ORa=1,22;IC95%[1,14-1,31]).Parmi la population d'étude, 508 employés, soit (50,5%), étaient indemnes de tout trouble anxio-depressif: Une bonne santé mentale du personnel de santé, a été déterminée par le sexe masculin (ORa=1,55; IC95%[1,07,2,24]), sans co-morbidité(ORa=0,57; IC95%[0,39,0,83]), sans intervention directe dans la lutte contre la COVID-19 (ORa=0,63; IC95%[0,45,0,89]), ayant un score bas de Dépression et d'Anxiété avec respectivement (ORa=0,43; IC95%[0,36-0,50]), ORa=0,50; IC95%[0,41-0,58]),tout en dénotant un score élevé de Résilience (ORa=1,03; IC95%[1,01-1,05]).
Conclusion: En Algérie, la lutte contre la pandémie de COVID-19 a eu un impact sur la santé mentale des professionnels de santé d'où l'urgence de la mise en place des programmes d'intervention, pour le renforcement de leur santé mentale, dans une lutte plus durable et plus efficace.
Mots clés: COVID-19 - Professionnels de santé - Santé mentale – Anxiété – Dépression – Résilience - Algérie.

ABSTRACT

Background: The current COVID-19 pandemic has put health care professionals in the face of increasing psychological distress, with a high risk of infection.
Purpose: To estimate the prevalence of anxiety-depressive disorders among health professionals in Algeria and determine their associated risk factors.
Method: A cross-sectional descriptive study was conducted from January 11 to March 09, 2021 and concerned healthcare professionals in Algeria, through an online self-assessment. The mental health rating scales used were GAD-7 (7 items) for Anxiety, and CES-D (20 items) for Depression. Resilience was estimated by the RISC-CD (10 items). An original questionnaire was used to assess three factors: fear of infection and death, isolation and stigmatization, as well as motivation and escape behaviour at work.
Results: A total of 1005 health professionals were included in the study, of which 51.5% were doctors, 75.6% were women and 41.1% were at the first front of the fight against COVID-19. The prevalence of Anxiety and Depression was 23.8% and 44.6% respectively.Health professionals with a high resilience score were those who were in direct contact with COVID-19 3.75 [1.11-12.7] and those who feared contracting the disease 1.22 [1.14-1.31].Among the study population, 508 employees (50.5%) were free from anxiety-depressive disorder: Good mental health of health personnel, has been determined by the male sex 1,55 [1,07, 2,24], without co-morbidity 0,57 [0,39, 0,83], without direct intervention in the fight against COVID-19 0.63 [0.45, 0.89], having a low score of Depression and Anxiety with respectively 0.43 [0.36- 0.50], 0.50 [0.41- 0.58] while denouncing a high Resilience score 1.03 [1.01- 1.05].
Conclusion: In Algeria, the fight against the COVID-19 pandemic has had an impact on the mental health of health professionals, hence the urgent need for intervention programs, for strengthening their mental health in a more sustainable and effective struggle.
Key Words: COVID-19 - Health professionals- Mental health- Anxiety – Depression – Resilience – Algeria.

Correspondance

Chahrazed Kandouci
kchahrazed09@gmail.com

Cet article est distribué selon les termes de la licence internationale Creative Commons Attribution - Pas d'Utilisation Commerciale - Pas de Modification 4.0 International (CC BY-NC-ND 4.0) qui autorise l'utilisation non commerciale, la production, la reproduction et la distribution de l'œuvre sans autre autorisation, à condition que l'auteur original et la source soient mentionnés.

ملخص

الخلفية: أدى وباء كوفيد 19 الحالي إلى تعريض عمال الصحة لضائقة نفسية متزايدة، مع احتمال كبير للإصابة بالعدوى.

الهدف: تقدير انتشار اضطرابات القلق والاكتئاب بين المتخصصين في الرعاية الصحية وتحديد عوامل الخطر المرتبطة بها..

الطريقة: أجريت دراسة وصفية مقطعية من 11 من يناير إلى 09 مارس 2021على العاملين بالصحة. عن طريق استبيان تم إجراؤه ذاتيًا عبر الانترنت. كانت جداول تقييم الصحة العقلية المستخدمة هي GAD-7 (7 بنود) للقلق، وCES-D (20 بندا) للاكتئاب. كانت المرونة مقدرة من قِبَل مجموعة الـ RISC-CD (10 بنود). واستخدم استبيان أصلي لتقييم ثلاثة عوامل: الخوف من الإصابة والموت، والعزلة والوصم، فضلاً عن الدافع وسلوك التهرب من العمل.

النتائج: في المجموع، أدرج في الدراسة 1005 من المهنيين الصحيين، 51,5% منهم أطباء، 75,6% نساء و41,1% في الخطوط الأمامية لمكافحة كوفيد 19. كان معدل انتشار القلق والاكتئاب 23.8% و44.6% على التوالي. عمال الصحة الذين حصلوا على درجة عالية من المرونة هم أولئك الذين كانوا على اتصال مباشر مع كوفيد19 3.75 [12,7-1,11] وأولئك الذين كانوا يخشون الإصابة بالمرض 1,22 [1,14- 1,31]. من بين مجتمع الدراسة، كان 508 موظفًا (50.5٪)، خاليًا من أي اضطراب متعلق بالقلق والاكتئاب. الصحة العقلية الجيدة للعاملين في مجال الصحة تم تحديدها بالجنس الذكري 1,55 [1,07, 2,24]، دون اعتلال مشترك 0,57 [0,39, 0,83]، دون تدخل مباشر في مكافحة كوفيد 19 0,63 [0,45, 0,89] مع درجة منخفضة من الاكتئاب والقلق على التوالي 0,43 [0.36-0.50] ، 0.50 [0.41-0.58] مع الإشارة إلى درجة عالية من المرونة 1.03[1,01- 1,05].

الخلاصة: في الجزائر، كان لمكافحة وباء كوفيد 19 تأثير على الصحة العقلية للعاملين في مجال الصحة، ومن هنا تبرز الحاجة الملحة إلى وضع برامج للتدخل السريع لتعزيز الصحة العقلية في إطار مكافحة أكثر استدامة وفعالية.

الكلمات الرئيسية: كوفيد 19 ـ المهنيون الصحيون ـ الصحة العقلية ـ القلق ـ الاكتئاب ـ المرونة ـ الجزائر.

INTRODUCTION

La pandémie actuelle de la COVID-19 constitue une menace importante pour la santé publique (1). C'est une infection émergente de type zoonose virale, caractérisée par sa forte contagiosité et sa létalité potentiellement inhabituelle (2). Le virus est transmis principalement par l'intermédiaire des gouttelettes de salive ou des sécrétions nasales lorsqu'une personne infectée tousse ou éternue (3). La pandémie de la Covid-19 est un nouveau type de traumatisme de masse incontrôlable en raison de sa méconnaissance. En fin décembre 2019, le SRAS-CoV-2 a été apparu pour la première fois à Wuhan en Chine. Il s'est rapidement propagé dans le monde entier et l'Organisation Mondiale de la Santé (OMS) l'a déclaré pandémique le 11 mars 2020 (4).

Le personnel de santé déplore une part importante du nombre total de patients atteints par la COVID-19, soit une proportion de 3,9%, alors que la fréquence de décès représente 0,5% de l'ensemble des décès causés par cette pandémie. Les infections concernent principalement les infirmières, mais les décès touchent beaucoup plus les médecins (5). En Algérie, depuis le premier cas de patient COVID-19, confirmé à Ouargla le 25 février 2020, suivi du premier décès, enregistré le 12 mars au niveau du premier foyer à Blida, le pays a rejoint les nombreuses zones affectées par cette épidémie. Face à cette situation critique, l'état de la santé de la population est resté difficile. Cette urgence sanitaire a changé la vie et les habitudes de travail des professionnels de la santé, qui jouent un rôle primordial dans la prise en charge des patients infectés par le virus. Ce sont des combattants du premier front, impliqués dans la gestion de cette situation délicate, les exposant à un risque majeur d›infection, à une pression psychologique intense, liée à l'incertitude sur la durée de la crise, au manque de thérapies efficaces ou de vaccins sûrs et à des pénuries potentielles de ressources de matériel de soins, notamment les Equipements de Protection Individuelle (EPI). Ajoutées à cela, les nombreuses fausses informations (*fake news*) diffusées rapidement par les médias, au fur et à mesure que la maladie COVID-19 se propageait et que le nombre de cas augmentait, ce qui mettait à rude épreuve, la santé mentale de ces guerriers blancs. Des expositions répétées à des pareilles situations entraînent une détresse psychologique accrue: le stress, l'Anxiété et la Dépression peuvent être considérés comme des réactions émotionnelles normales face à cette pandémie (6).

Plusieurs études ont été menées sur la santé mentale

des professionnels de la santé, intervenant en premier contact avec les patients atteints de la COVID-19, ont mis en évidence la variation de la prévalence des risques psychosociaux d'un pays à l'autre. Ainsi, les taux les plus bas d'Anxiété (8,5%) et de Dépression (9,5%) ont été enregistrés en Chine (7), par contre les taux les plus forts concernaient la Turquie où l'Anxiété et la Dépression ont été évaluées respectivement à 60,2% et à 77,6% (8). De même, des taux non négligeables (20,6%) de Syndrome de Stress Post Traumatique (SSPT) ont été retrouvés chez le personnel de la santé à Wuhan en Chine (9).

En Algérie, à ce jour, nous ne disposons d'aucune donnée fiable de l'impact psychosocial engendré par la pandémie C0VID-19 chez les professionnels de santé, ce qui nous a motivé la conduite de ce travail. L'objectif de cette étude a été de mesurer la prévalence des troubles anxio-dépressifs chez le personnel de santé en Algérie et de déterminer leurs facteurs de risque associés.

METHODES

Une étude descriptive, de type transversal, a été menée du 11 janvier au 9 mars 2021 et a concerné, au niveau national, les professionnels de la santé de l'Algérie, quel que soit leur type de contrat de travail. La population d'étude était constituée de médecins (résidents, généralistes, spécialistes), médecins dentistes, pharmaciens, techniciens de santé (dont les infirmiers, les laborantins, les kinésithérapeutes …) et autres professionnels de la santé comme les administrateurs, les biologistes, les diététiciens, les orthophonistes et les psychologues.

Ont été inclus à cette étude, tout employé de la santé ayant donné son accord de participation à l'enquête avec une ancienneté de plus d'un an (vu que la pandémie remontait à moins d'un an au moment de la collecte des données). Les professionnels de la santé ayant une pathologie psychiatrique franche ou sous anti dépresseurs ou anxiolytiques, ont été exclus de l'étude. Les formulaires du questionnaire dont les données étaient incomplètes étaient systématiquement éliminés lors de l'analyse finale. La taille de l'échantillon a été calculée à partir d'une prévalence estimée (p) de 28% selon une enquête japonaise (10), une précision de 5%, selon la formule suivante: $t^2 * p(1-p)/e^2$, multipliée par le coefficient inter grappes (k=3). Par conséquent, le nombre de sujets nécessaires à l'étude devrait être supérieur ou égal à 927 personnes.

Pour impliquer les professionnels de santé de toutes les régions algériennes, une enquête en ligne anonyme a été effectuée, ce qui a nécessité la construction d'un «*web questionnaire*», via la plateforme *Google Forms*. Le lien du questionnaire a été envoyé aux différents professionnels de la santé. Les participants ont été recrutés par échantillonnage «boule de neige» et ont été également invités à transmettre le sondage à leurs collègues. Pour la région de Sidi Bel Abbès, les questionnaires ont été remplis sur place lors du passage des enquêteurs, au niveau des différents services des structures sanitaires suivantes: le Centre Hospitalo-Universitaire (CHU) Hassani Abdelkader, le Centre Anti Cancer (CAC) et l'Etablissement Hospitalier mère-enfant (EHS maternité).

Le support de l'enquête a été un auto questionnaire constitué de plusieurs volets. La première section comprenait les caractéristiques sociodémographiques telles que: le sexe, l'âge, le statut matrimonial, le nombre d'enfants à charge…etc, et les caractéristiques professionnelles telles que: le secteur d'activité, le mode d'exercice, l'ancienneté, la position de travail (les professionnels qui intervenaient directement dans le traitement des patients atteints de COVID-19 ou non). Le deuxième volet comprenait trois échelles évaluant l'Anxiété (GAD-7), la Dépression (CES-D) et la Résilience (CD-RISC 10). Nous avons utilisé la version française du GAD-7. Ce questionnaire, validé en 2018 (11), mesure la fréquence à laquelle les sept symptômes d'Anxiété ont été survenus au cours des deux semaines précédant l'enquête. Chaque question avait quatre réponses possibles: 0 (Jamais), 1 (Plusieurs jours), 2 (Plus de la moitié des jours) et 3 (Presque tous les jours). Le score total de l'Anxiété variait ainsi de 0 à 21. Pour la Dépression, nous avons utilisé le CES-D (12), comprenant 20 items; il évalue les symptômes de la Dépression, survenus au cours de la semaine précédente l'enquête. Les réponses étaient cotées de 0 à 3: 0 (Jamais, Très rarement), 1 (Occasionnellement), 2 (Assez souvent), 3 (Fréquemment, En permanence). Le score total de la Dépression variait ainsi de 0 à 60. Les scores totaux de ces échelles ont été interprétés comme suit: GAD-7: Pas d'Anxiété (0-10), Anxiété (10-21); CES-D: Normal (0-15), Dépression (16-60). La Résilience était estimée par le CD-RISC de Connor Davidson dans sa version française (13), en 10 items. Chaque question avait quatre réponses possibles: 0 (Pas vrai du tout), 1 (Rarement vrai), 2 (Parfois vrai), 3 (Souvent vrai), 4 (Vrai presque tout le temps). Le score total de la Résilience variait de 0 à 40. Le troisième

volet comprenait un questionnaire original préétabli par une étude japonaise (10) pour estimer trois facteurs: (a) l'Anxiété et la peur de l'infection et de la mort (10 items); (b) l'isolement et la stigmatisation (5 items); (c) la motivation et le comportement d'évasion au travail (3 items). Chaque question avait quatre réponses possibles: 0 (ne s'applique pas du tout); 1 (Un peu applicable); 2 (presque applicable); et 3 (applicable). Les scores totaux de chaque composante varient respectivement comme suit: (a) de 0 à 30, (b) de 0 à 15 et (c) de 0 à 9. Nous avons créé une nouvelle variable intitulée santé mentale définissant l'absence concomitante de la Dépression et de l'Anxiété. Elle a été codée en Oui et en Non: Oui: Anxiété (non) et Dépression (non), Non: Anxiété (oui) ou Dépression (oui).

Dans un premier temps, les réponses recueillies au questionnaire ont été traitées avec le logiciel Microsoft Excel 2013. Dans un second temps, nous avons procédé à l'analyse statistique des données par le logiciel R version 4.1.0. L'association entre les variables a été jugée significative pour une valeur seuil «p» inférieure à 5%. Pour les paramètres quantitatifs, la moyenne a été exprimée en "moyenne ± écart type", et la médiane en "médiane et intervalle interquartile [Premier Quartile – Troisième Quartile]". La comparaison de deux moyennes a été faite par le test de Wilcoxon-Mann-Whitney, alors que la comparaison de moyennes entre plusieurs groupes a été réalisée en utilisant le test de Kruskal-Walis. Pour les paramètres qualitatifs, les fréquences (pourcentages) ont été comparées à l'aide du test de Khi2. Une correction de Yates était nécessaire quand les effectifs calculés étaient inférieurs à 5. Le test exact de Fisher n'a été utilisé que si l'effectif global dans un groupe était inférieur à 30. Pour les variables qualitatives (exemple la Dépression: "Oui" ou "Non"), l'analyse multi variée a été conduite par une régression logistique binaire; les résultats ont été exprimés en Odds Ratio (OR: exponentiel de béta de la formule de la régression binaire) et son Intervalle de Confiance (IC) à 95%. Pour les variables quantitatives (exemple le score CD-RISC: valeurs numériques), l'analyse multi variée a été réalisée en utilisant une régression linéaire multiple. Les résultats ont été exprimés par la valeur de la pente (beta) et son IC.

RESULTATS

Sur les 1050 questionnaires collectés, 1005 ont été exploitables et répondaient aux critères d'inclusion prédéfinis. L'âge médian de tous les participants était de 30 ans (IIQ: 27-38), le sex-ratio était de 0,32 et 6,8% (n=68) des concernés par l'enquête avaient une maladie respiratoire (asthme ou broncho-pneumopathie chronique obstructive). La moyenne annuelle de l'ancienneté professionnelle au poste était de 8,2±7,98 pour l'ensemble des participants à l'étude (tableau I).

Tableau 1. Caractéristiques socio-professionnelles, en fonction du poste de travail, des 1005 professionnels de la santé, participants à l'étude sur l'impact psycho-social de la COVID-19 (Algérie, 2021): n, (%)

	**Médecins (N = 518)**		**Soins (N = 356)**		**Administration (N = 66)**		**Autre staff (N = 65)**		**Total (N = 1005)**	
**Sexe**										
Femme	366	**(71)**	291	**(82)**	56	**(85)**	47	**(72)**	760	**(76)**
Homme	152	**(29)**	65	**(18)**	10	**(15)**	18	**(28)**	245	**(24)**
**Statut matrimonial**										
Marié(e)	297	**(57)**	172	**(48)**	36	**(55)**	28	**(43)**	533	**(53)**
Célibataire	212	**(41)**	177	**(50)**	23	**(35)**	34	**(52)**	446	**(44)**
Divorcé(e)	6	**(01)**	4	**(01)**	6	**(09)**	2	**(03)**	18	**(02)**
Veuf (ve)	3	**(01)**	3	**(01)**	1	**(02)**	1	**(02)**	8	**(01)**
**Fumeur**	22	**(04)**	15	**(04)**	3	**(04)**	5	**(08)**	45	**(04)**
**Co-morbidité**	132	**(25)**	85	**(24)**	17	**(26)**	12	**(18)**	246	**(24)**
**Contact COVID-19**	263	**(51)**	128	**(36)**	13	**(20)**	9	**(14)**	413	**(41)**
**Utilisation d'EPI**	228	**(44)**	168	**(47)**	36	**(55)**	29	**(45)**	461	**(46)**

EPI = Equipement de Protection Individuelle

Selon le tableau IIa, un sentiment de nervosité, d'anxiété ou de tension, a été déclaré presque tous les jours, par 190 cas, soit (19%) du personnel de la santé interrogé, au cours des deux dernières semaines. Près d'un employé sur dix a reconnu être dérangé par les sentiments d'échec, des idées noires, et a eu l'impression que les gens ne l'aimaient pas et lui étaient hostiles assez souvent, voire même en permanence. Le score médian de l'Anxiété (GAD-7) pour l'ensemble des participants était de 5 (IIQ: 2-9), quant à celui de la Dépression (CES-D), il était de 14 (IIQ: 10-23) (tableau IIb). Parmi la population d'étude, 239 (23,8%) souffraient d'une Anxiété, et 448 (44,6%) ont développé des symptômes de Dépression.

Près d'un quart des professionnels de la santé, ont annoncé être capables de s'adapter, lorsque des changements survenaient, et se sentaient plus forts par la gestion de leurs stress. Ils croyaient pouvoir atteindre leurs objectifs même en cas d'obstacles, et se considéraient comme des personnes fortes lors de leurs confrontations aux défis et aux difficultés de la vie (tableau III-A). Le score global de la Résilience (CD-RISC) était de 25 (IIQ: 19-30) (tableau III-B).

Quant au questionnaire original en rapport avec la COVID-19, plus de la moitié du personnel de santé craignait de transférer le virus à leurs familles, et étaient hantés par le décès de leurs proches et de leurs collègues. Parmi la population d'étude, 12% du personnel de santé ont déclaré être isolés à la maison, 6% se sont plaints de la maltraitance déraisonnable et de stigmatisation au travail. Un employé sur dix appréhendait quasiment d'aller travailler et plus d'un tiers d'entre eux aspirait à une pause (tableau IV-A). Chez tous les participants, les scores médians du questionnaire original sur «la peur de l'infection et de la mort», «l'isolement et la stigmatisation», «la motivation et le comportement d'évasion au travail» étaient respectivement de 19 (IIQ: 13-24), 2 (IIQ: 0-5), et 3 (IIQ: 1-4), pour chaque composante (tableau IV-B).

Au total, 413 (41,1%) des participants étaient en contact direct avec la COVID-19, il est à remarquer que ce personnel était moins ancien au travail (p=0,022), plus dépressif (p=0,001), plus anxieux (p<0,001), souffrant d'isolement et de stigmatisation (p<0,001), et aspirant à une évasion (p=0,007) (tableau V-A). Quant aux facteurs associés à la Résilience, les professionnels de santé les ayant un score élevé de résilience étaient ceux qui étaient en contact direct avec la COVID-19 (p=0,034) et ceux qui craignaient la contraction de la maladie (p<0,001). En revanche, le personnel de santé occupant un poste administratif, les aides-soignants et les professionnels de santé ayant un score élevé de Dépression étaient moins résilients, avec respectivement (p=0,003), (p=0,025) et (p=0,010) (tableau V-B).

Par ailleurs, la proportion des participants qui étaient sains avec une bonne santé mentale et qui ne présentaient pas de troubles anxio-dépressifs était au nombre de 508 soit (50,5%).

Une bonne santé mentale du personnel de santé en Algérie, elle a été déterminée par le sexe masculin (p=0,020), sans co morbidité (p=0,004), et sans contact direct avec la COVID-19 (p=0,008). Un score bas de Dépression et d'Anxiété (p=0,001) et paradoxalement une bonne Résilience (p<0,001) étaient liés à une bonne santé mentale (tableau V-C).

DISCUSSION

La pandémie COVID-19, inédite par sa rapidité d'installation et son ampleur, exerce une détresse psychologique croissante et particulière sur les agents de la santé; en réponse à une gamme de facteurs de stress, parmi ces facteurs: le risque de décès lié à la maladie, sa sévérité, l'absence de traitement spécifique générant un sentiment d'impuissance thérapeutique pour les patients, voire même le risque de contagion mal connu, ainsi que le climat d'incertitude amplifié par les médias. Sur le plan organisationnel, d'autres facteurs, source de stress, incombent au déficit d'EPI (masques) ou collectifs, l'insuffisance de tests PCR, ainsi que la crainte de contaminer les patients et par conséquent les collègues et les proches.

Cette étude vise à déterminer la prévalence des troubles anxio-dépressifs chez les professionnels de la santé pendant la pandémie de la COVID-19 dans 32 wilayas de l'Algérie; au moyen d'une enquête par questionnaire à grande échelle au niveau national, du 11 janvier au 9 mars 2021, sur 1005 participants. Parmi cet échantillon, 239 professionnels (23,8%) avaient une Anxiété modérée à sévère et 448 des professionnels (44,6%) avaient développé des symptômes de Dépression. Par contre, 508 employés de la population d'étude, soit (50,5%), étaient indemnes de tout trouble anxio-depressif.

Tableau 2 (a). Etat de la santé mentale des professionnels de santé algériens (Anxiété, Dépression), au cours de la lutte contre la COVID-19, en 2021 (n, %).

A. Anxiété

Au cours des 14 derniers jours, à quelle fréquence avez-vous été dérangé(e) par les problèmes suivants ?	Jamais		Plus de la moitié des jours/ Plusieurs jours		Presque tous les jours	
	n	%	n	%	N	%
1. Sentiment de nervosité, d'anxiété ou de tension	265	**(26)**	550	**(55)**	190	**(19)**
2. Incapable d'arrêter de vous inquiéter ou de contrôler vos inquiétudes	452	**(45)**	456	**(45)**	97	**(10)**
3. Inquiétudes excessives à propos de tout et de rien	508	**(51)**	405	**(40)**	92	**(09)**
4. Difficulté à se détendre	397	**(39)**	497	**(49)**	111	**(11)**
5. Agitation telle qu'il est difficile de rester tranquille	574	**(57)**	366	**(36)**	65	**(06)**
6. Devenir facilement contrarié(e) ou irritable	407	**(40)**	494	**(49)**	104	**(10)**
7. Avoir peur que quelque chose d'épouvantable (horrible) puisse arriver	450	**(45)**	410	**(41)**	145	**(14)**

B. Dépression

	Jamais	Très rarement <1 j Occasionnellement 1-2 j	Assez Souvent 3-4 j/ Fréquemment 5-7 j	En permanence
1. J'ai été contrarié(e) par des choses qui d'habitude ne me dérangent pas	345 **(34)**	493 **(49)**	141 **(14)**	26 **(03)**
2. Je n'ai pas eu envie de manger, j'ai manqué d'appétit	475 **(47)**	386 **(38)**	114 **(11)**	30 **(03)**
3. J'ai eu l'impression que je ne pouvais pas sortir du cafard (idées noires), même avec l'aide de ma famille et de mes amis	593 **(59)**	287 **(29)**	103 **(10)**	22 **(02)**
4. J'ai eu le sentiment d'être aussi bien que les autres	215 **(21)**	388 **(39)**	273 **(27)**	129 **(13)**
5. J'ai eu du mal à me concentrer sur ce que je faisais	286 **(28)**	476 **(47)**	198 (**20)**	45 **(04)**
6. Je me suis senti(e) déprimé(e)	330 **(33)**	451 **(45)**	163 **(16)**	61 **(06)**
7. J'ai eu l'impression que toute action me demandait un effort	237 **(24)**	447 **(44)**	233 **(23)**	88 **(09)**
8. J'ai été confiant(e) en l'avenir	194 **(19)**	335 **(33)**	261 **(26)**	215 **(21)**
9. J'ai pensé que ma vie était un échec	634 **(63)**	243 **(24)**	93 **(09)**	35 **(03)**
10. Je me suis senti(e) craintif (ve)	385 **(38)**	432 **(43)**	135 **(13)**	53 **(05)**
11. Mon sommeil n'a pas été bon	286 **(28)**	434 **(43)**	191 **(19)**	94 **(09)**
12. J'ai été heureux (se)	118 **(12)**	396 **(39)**	366 **(36)**	125 **(12)**
13. J'ai parlé moins que d'habitude	303 **(30)**	474 **(47)**	165 **(16)**	63 **(06)**
14. Je me suis senti(e) seul(e)	449 **(45)**	365 **(36)**	114 **(11)**	77 **(08)**
15. Les autres ont été hostiles (agressifs) envers moi	501 **(50)**	350 **(35)**	114 **(11)**	40 **(04)**
16. J'ai profité de la vie	194 **(19)**	422 **(42)**	245 **(24)**	144 **(14)**
17. J'ai eu des crises de larmes	446 **(44)**	389 **(39)**	130 **(13)**	40 **(04)**
18. Je me suis senti(e) triste	270 **(27)**	532 **(53)**	146 **(14)**	57 **(06)**
19. J'ai eu l'impression que les gens ne m'aimaient pas	585 **(58)**	298 **(30)**	89 **(09)**	33 **(03)**
20. J'ai manqué d'entrain (vivacité)	391 **(39)**	435 **(43)**	137 **(14)**	42 **(04)**

Tableau 2(b). Distribution des scores de santé mentale des professionnels de santé algériens (Anxiété, Dépression), au cours de la lutte contre la COVID-19, en 2021 (moyenne ± ET, médiane, IIQ).

	Min Max	Moy ± ET	Médiane	(Q1-Q3)
**Anxiété**				
Au cours des 14 derniers jours, à quelle fréquence avez-vous été dérangé(e) par les problèmes suivants ?				
1. Sentiment de nervosité, d'anxiété ou de tension	0-3	1,24±1,04	1	(0-2)
2. Incapable d'arrêter de vous inquiéter ou de contrôler vos inquiétudes	0-3	0,87±0,97	1	(0-1)
3. Inquiétudes excessives à propos de tout et de rien	0-3	0,79±0,97	0	(0-1)
4. Difficulté à se détendre	0-3	0,95±0,98	1	(0-1)
5. Agitation telle qu'il est difficile de rester tranquille	0-3	0,66±0,90	0	(0-1)
6. Devenir facilement contrarié(e) ou irritable]	0-3	0,91±0,96	1	(0-1)
7. Avoir peur que quelque chose d'épouvantable (horrible) puisse arriver	0-3	0,93±1,06	1	(0-1)
**Score global**	0-21	6,36±5,13	5	(2-9)
**Dépression**				
1. J'ai été contrarié(e) par des choses qui d'habitude ne me dérangent pas	0-3	0,61±0,91	0	0-1
2. Je n'ai pas eu envie de manger, j'ai manqué d'appétit	0-3	0,51±0,93	0	0-1
3. J'ai eu l'impression que je ne pouvais pas sortir du cafard (idées noires), même avec l'aide de ma famille et de mes amis	0-3	0,41±0,87	0	0-0
4. J'ai eu le sentiment d'être aussi bien que les autres	0-3	1,76±1,25	2	0-3
5. J'ai eu du mal à me concentrer sur ce que je faisais	0-3	0,80±1,06	0	0-1
6. Je me suis senti(e) déprimé(e)	0-3	0,78±1,07	0	0-1
7. J'ai eu l'impression que toute action me demandait un effort	0-3	1,02±1,18	1	0-2
8. J'ai été confiant(e) en l'avenir	0-3	1,56±1,29	2	0-3
9. J'ai pensé que ma vie était un échec	0-3	0,42±0,88	0	0-0
10. Je me suis senti(e) craintif (ve)	0-3	0,64±1,00	0	0-1
11. Mon sommeil n'a pas été bon	0-3	0,95±1,15	0	0-2
12. J'ai été heureux (se)	0-3	1,52±1,19	2	0-3
13. J'ai parlé moins que d'habitude	0-3	0,78±1,08	0	0-1
14. Je me suis senti(e) seul(e)	0-3	0,65±1,05	0	0-1
15. Les autres ont été hostiles (agressifs) envers moi	0-3	0,52±0,94	0	0-1
16. J'ai profité de la vie]	0-3	1,75±1,25	2	0-3
17. J'ai eu des crises de larmes	0-3	0,61±1,00	0	0-1
18. Je me suis senti(e) triste	0-3	0,75±1,03	0	0-1
19. J'ai eu l'impression que les gens ne m'aimaient pas	0-3	0,40±0,86	0	0-0
20. J'ai manqué d'entrain (vivacité)	0-3	0,60±0,98	0	0-1
**Score global**	0-58	17,02±10,92	14	10-23

Moy: Moyenne ET: Ecart Type Q1: Premier Quartile Q3: Troisième Quartile

Tableau 3. Etude de la Résilience des professionnels de santé algériens, au cours de la lutte contre la COVID-19, en 2021 (n, % & moyenne ± ET)

A. Etude qualitative (n, %)

	Pas vrai du tout	Rarement vrai/ Parfois vrai	Souvent vrai	Vrai presque tout le temps
1. Je suis capable de m'adapter lorsque des changements surviennent	92 **(09)**	335 **(33)**	337 **(34)**	241 **(24)**
2. Je peux gérer toutes les situations qui se présentent à moi	51 **(05)**	406 **(40)**	356 **(35)**	192 **(19)**
3. J'essaie de voir le coté humoristique des choses lorsque je suis confronté(e) à des problèmes	120 **(12)**	499 **(50)**	233 **(23)**	153 **(15)**
4. Devoir gérer le stress peut me rendre plus forte	86 **(09)**	355 **(35)**	327 **(32)**	237 **(24)**
5. J'ai tendance à rebondir après une maladie, une blessure ou d'autres épreuves	133 **(13)**	410 **(41)**	270 **(27)**	192 **(19)**
6. Je crois pouvoir atteindre mes objectifs même s'il y'a des obstacles	54 **(05)**	331 **(33)**	354 **(35)**	266 **(26)**
7. Sous pression, je reste concentré(e) et je réfléchis clairement	120 **(12)**	440 **(44)**	277 **(28)**	168 **(17)**
8. Je me suis pas facilement découragé(e)par l'échec	123 **(12)**	423 **(42)**	276 **(27)**	183 **(18)**
9. Je me considère comme une personne forte lorsque je suis confronté(e) aux défis et aux difficultés de la vie	57 **(06)**	321 **(32)**	337 **(34)**	290 **(29)**
10. Je suis capable de gérer des sentiments déplaisants ou douloureux comme la tristesse, la peur ou la colère	87 **(09)**	422 **(42)**	289 **(29)**	207 **(21)**

B. Distribution quantitative

	Min-Max	Moy±ET	Médiane	(Q1-Q3)
1. Je suis capable de m'adapter lorsque des changements surviennent	0-4	**2,52±1,23**	3	(2-3)
2. Je peux gérer toutes les situations qui se présentent à moi	0-4	**2,52±1,08**	3	(2-3)
3. J'essaie de voir le coté humoristique des choses lorsque je suis confronté(e) à des problèmes	0-4	**2,10±1,23**	2	(1-3)
4. Devoir gérer le stress peut me rendre plus forte	0-4	**2,49±1,23**	3	(2-3)
5. J'ai tendance à rebondir après une maladie, une blessure ou d'autres épreuves	0-4	**2,22±1,30**	2	(1-3)
6. Je crois pouvoir atteindre mes objectifs même s'il y'a des obstacles	0-4	**2,67±1,13**	3	(2-4)
7. Sous pression, je reste concentré(e) et je réfléchis clairement	0-4	**2,22±1,23**	2	(1-3)
8. Je me suis pas facilement découragé(e) par l'échec	0-4	**2,21±1,28**	2	(1-3)
9. Je me considère comme une personne forte lorsque je suis confronté(e) aux défis et aux difficultés de la vie	0-4	**2,70±1,15**	3	(2-4)
10. Je suis capable de gérer des sentiments déplaisants ou douloureux comme la tristesse, la peur ou la colère	0-4	**2,38±1,21**	2	(2-3)
**Score global**	0-40	**24,04±8,28**	25	(19-30)

Moy: Moyenne ET: Ecart Type Q1: Premier Quartile Q3: Troisième Quartile

Tableau 4. Peur de l'infection et de la mort de la COVID-19, des professionnels de santé algériens, en 2021

A. Etude qualitative: n (%)

	Ne s'applique pas du tout	Peu/Presque applicable	Applicable
**Peur de l'infection et de la mort**			
1. Je crains d'attraper la COVID-19	272 **(27)**	512 **(51)**	221 **(22)**
2. Je crains de mourir de la COVID-19]	434 **(43)**	407 **(41)**	164 **(16)**
3. Je crains de transférer la COVID-19 à ma famille	75 **(07)**	304 **(30)**	626 **(62)**
4. Je crains de transférer la COVID-19 à mes collègues	122 **(12)**	438 **(44)**	445 **(44)**
5. Je crains que ma famille soit touchée par la COVID-19	92 **(09)**	299 **(30)**	614 **(61)**
6. Je crains que ma famille me transfère la COVID-19	376 **(37)**	428 **(43)**	201 **(20)**
7. Je crains que ma famille meurt de la COVID-19	136 **(14)**	282 **(28)**	587 **(58)**
8. Je crains que mes collègues ne soient touchés par la COVID-19	144 **(14)**	485 **(48)**	376 **(37)**
9. Je crains que mes collègues me transfèrent la COVID-19	192 **(19)**	480 **(48)**	333 **(33)**
10. J'ai peur que mes collègues meurent de la COVID-19	151 **(15)**	355 **(35)**	499 **(50)**
**Isolement et traitement déraisonnable**			
1. Je suis isolé à la maison	620 **(62)**	262 **(26)**	123 **(12)**
2. Je suis isolé à l'hôpital	804 **(80)**	175 **(17)**	26 **(03)**
3. J'ai été traité de façon déraisonnable (manière répulsive) au travail	698 **(69)**	245 **(24)**	62 **(06)**
4. Ma famille est isolée des autres	606 **(60)**	296 **(29)**	103 **(10)**
5. Ma famille a été traitée de façon déraisonnable (manière répulsive) par d'autres	736 **(73)**	221 **(22)**	48 **(05)**
**Motivation et le comportement d'évasion au travail**			
1. J'ai peur d'aller travailler	609 **(61)**	310 **(31)**	86 **(9)**
2. Je ne veux pas travailler	654 **(65)**	263 **(26)**	88 **(9)**
3. Je veux faire une pause du travail	258 **(26)**	408 **(41)**	339 **(34)**

B. Etude quantitative (moyenne± ET, médiane IIQ):

	Min-Max	Moy±ET	Médiane	Q1-Q3
**Peur de l'infection et de la mort**				
1. Je crains d'attraper la COVID-19	0-3	**1,32±1,10**	1	0-2
2, Je crains de mourir de la COVID-19	0-3	**1,00±1,09**	1	0-2
3, Je crains de transférer la COVID-19 à ma famille	0-3	**2,34±0,97**	3	2-3
4, Je crains de transférer la COVID-19 à mes collègues	0-3	**2,00±1,06**	2	1-3
5, Je crains que ma famille soit touchée par la COVID-19	0-3	**2,30±1,00**	3	2-3
6, Je crains que ma famille me transfère la COVID-19	0-3	**1,16±1,13**	1	0-2
7, Je crains que ma famille meurt de la COVID-19	0-3	**2,19±1,10**	3	1-3
8, Je crains que mes collègues ne soient touchés par la COVID-19	0-3	**1,87±1,07**	2	1-3
9, Je crains que mes collègues me transfèrent la COVID-19	0-3	**1,69±1,12**	2	1-3
10, J'ai peur que mes collègues meurent de la COVID-19	0-3	**2,03±1,12**	2	1-3
**Score global**	0-30	**17,92±7,63**	19	13-24
**Isolement et traitement déraisonnable**				
1, Je suis isolé à la maison	0-3	**0,71±1,05**	0	0-1
2, Je suis isolé à l'hôpital	0-3	**0,29±0,66**	0	0-0
3, J'ai été traité de façon déraisonnable (manière répulsive) au travail	0-3	**0,49±0,86**	0	0-1
4, Ma famille est isolée des autres	0-3	**0,71±1,02**	0	0-1
5, Ma famille a été traitée de façon déraisonnable (manière répulsive) par d'autres	0-3	**0,43±0,82**	0	0-1
**Score global**	0-15	**2,64±3,03**	2	0-5
**Motivation et le comportement d'évasion au travail**				
1, J'ai peur d'aller travailler	0-3	**0,63±0,94**	0	0-1
2, Je ne veux pas travailler	0-3	**0,59±0,95**	0	0-1
3, Je veux faire une pause du travail	0-3	**1,57±1,20**	1	0-3
**Score global**	0-9	**2,78±2,42**	3	1-4

Moy: Moyenne ET: Ecart Type Q1: Premier Quartile
Q3: Troisième Quartile IIQ: Intervalle Inter Quartile

Tableau 5. Etude de l'impact psycho social de la COVID-19 sur les personnels de la santé en Algérie, au cours de l'année 2021

A. ***Distribution de la population en fonction du contact avec la COVID-19 (Médiane, IIQ)***

	Total, n = 1005		Non, n = 592		Oui, n = 413		p-value
**Age**	30	**[27- 38]**	31	**[27- 38]**	30	**[27- 38]**	0,2
**Ancienneté**	5	**[3- 10]**	6	**[3- 11]**	4	**[3- 10]**	**0,022**
**Score de la Dépression**	14	**[10- 23]**	13	**[9- 21]**	16	**[10- 25]**	**0,001**
**Score de l'Anxiété**	5	**[2- 9]**	5	**[2- 8]**	6	**[3- 11]**	**<0,001**
**Score de la Résilience**	25	**[19- 30]**	24	**[19- 29]**	26	**[19- 31]**	0,065
**Score Peur de l'infection**	19	**[13- 24]**	19	**[12- 24]**	18	**[14- 24]**	0,7
**Score Evasion au travail**	3	**[1- 4]**	2	**[1- 4]**	3	**[1- 4]**	**0,007**
**Score de l'isolement**	2	**[0- 5]**	1	**[0- 3,25]**	2	**[0- 6]**	**<0,001**

B. ***Facteurs associés à la Résilience***

	Exp (Beta)	IC 95%	p-value
**Poste**			
Administrateur de santé (versus Autres)	0,00	0,00-0,04	**0,003**
Aide-soignant (versus Autres)	0,05	0,00-0,68	**0,025**
**En première ligne contre la COVID-19**			
Oui (versus Non)	3,75	1,11-12,7	**0,034**
**Score de la Dépression: CES-D**	0,90	0,82-0,97	**0,010**
**Score de la peur d'infection**	1,22	1,14-1,31	**<0,001**
**Score de l'évasion au travail**	0,78	0,62-0,98	**0,036**

IC = Intervalle de Confiance

C. ***Déterminants de la santé mentale (absence de troubles anxio- dépressifs) chez la population d'étude***

a. ***Facteurs qualitatifs (régression logistique binaire):***

	Santé mentale (n, %)		Analyse Univariée		Analyse Multivariée	
	Non (n = 487)	Oui (n = 518)	p-value	ORb (IC95%)	p-value	ORa (IC95%)
**Sexe**						
Femme	386 **(51)**	374 **(49)**	**0,009**	1,47 [1,1, 1,97]	**0,020**	1
Homme	101 **(41)**	144 **(59)**		0,68 [0,5, 0,91]		1,55 [1,07, 2,24]
**Co morbidité**						
Non	339 **(45)**	420 **(55)**	**<0,001**	1,87 [1,4, 2,51]	**0,004**	1
Oui	148 **(60)**	98 **(40)**		0,53 [0,4, 0,71]		0,57 [0,39, 0,83]
**Fumeur**						
Non	461 **(48)**	499 **(52)**	0,2		**0,027**	1
Oui	26 **(58)**	19 **(42)**				0,45 [0,22, 0,91]
**Premier front**						
Non	259 **(44)**	333 **(56)**	**<0,001**	1,58 [1,23, 2,08]	**0,008**	1
Oui	228 **(55)**	185 **(45)**		0,63 [0,49, 0,81]		0,63 [0,45, 0,89]

ORb: Odds Ratio brut

ORa: Odds Ratio ajusté

b. ***Facteurs quantitatifs (régression linéaire):***

	Santé mentale Médiane (IIQ)		Analyse Uni variée	Analyse Multi variée	
	Non n = 487	Oui n = 518	p-value	p-value	ORa (IC95%)
**Score Dépression CESD**	23 **[18- 32]**	10 **[6- 12]**	<0,001	**<0,001**	0,43 [0,36- 0,50]
**Score Anxiété GAD**	9 **[6- 13]**	3 **[1- 5]**	<0,001	**<0,001**	0,50 [0,41- 0,58]
**Score Résilience CDRISC**	23 **[18- 28]**	26 **[20- 31]**	<0,001	**0,001**	1,03 [1,01- 1,05]
**Score de la peur de l'infection**	19 **[14- 24]**	18 **[11- 23]**	0,001	**0,040**	0,98 [0,96- 1,00]
**Score Evasion au travail**	3 **[1- 5]**	2 **[0,25- 3]**	<0,001	**<0,001**	0,79 [0,74- 0,84]
**Score de l'isolement**	2 **[0- 5,5]**	1 **[0- 3]**	<0,001	**0,014**	0,94 [0,90- 0,99]

IIQ: Intervalle Inter Quartiles OR: Odds Ratio

Les problèmes de peur de l'infection et de la mort, l'isolement et la stigmatisation, ainsi que la motivation et l'évasion au travail étaient tous, plus importants dans le groupe des patients souffrant de Dépression (score total CES-D>16) et d'Anxiété (GAD-7>10) par rapport au groupe sain (score total CES-D <16), (GAD-7≤ 10); contrairement au score de la Résilience qui était plus élevé dans les groupes sains.

La comparaison des taux de prévalence de l'Anxiété et de la Dépression par rapport à d'autres études a révélé que nos chiffres étaient nettement supérieurs à ceux de la Chine (Anxiété 8,5%, Dépression 9,5%) (7), du Japon (10%, 27,9%) (10), de l'Espagne (12,2%, 31,4%) (14) et contrairement inférieurs à ceux retrouvés en Turquie (60,2%, 77,6%) (8), en Arabie saoudite et en Egypte (58,9%, 69%) (15). Les taux de Dépression de notre enquête étaient similaires à ceux de l'Inde (47%) (16), le Cameroun (42,8%) (17) quant au taux d'Anxiété, il était plus proche de celui de la Malaisie (31,6%) (18). Cette différence de prévalence entre les études revient en premier lieu aux outils employés de la méthodologie d'évaluation, ainsi qu'à l'utilisation de différentes classifications, même si la même échelle était considérée, cela conduit à des taux très différents pour la prévalence des maladies mentales (8).

En second lieu, cela pourrait revenir à la grande hétérogénéité entre les études concernant les caractéristiques sociodémographiques des professionnels de la santé et à la robustesse des systèmes de santé qui est un facteur prédictif de la grande variété des résultats entre les différentes études (15). En effet, plusieurs échelles de dépistage de l'Anxiété et de la Dépression étaient utilisées dont: DASS-21, GAD-2, PHQ-2, PHQ-9, GAD-7 HADS-14. Dans cet échantillon d'étude, nous avons utilisé une échelle d'Anxiété comprenant sept éléments (GAD-7) (11) qui présente une bonne fiabilité avec une sensibilité de 89% et une spécificité de 82%. Pour l'évaluation de la Dépression, nous avons appliqué l'échelle (CES-D) (12); sa sensibilité s'avère plus satisfaisante que sa spécificité. Dans la version française de l'échelle; la sensibilité était de 76% et la spécificité était de 71%.

Il en découle de cette étude que les femmes avaient beaucoup plus souffert de la dépression par rapport aux hommes, cela pourrait s'expliquer par le fait que la majorité des sujets de l'étude étaient des femmes, et la santé était leur domaine de prédilection. Ces résultats corroborent d'autres études qui ont montré que les femmes étaient plus susceptibles de souffrir de Dépression et d'Anxiété pendant la pandémie de COVID-19 que les hommes, de même, et à un degré plus élevé de détresse émotionnelle par rapport aux hommes (14, 15, 19), sauf que notre étude n'a pas mis en évidence de causalité franche entre l'Anxiété et le sexe.

Aucune association n'a été retrouvée entre l'Anxiété et le statut matrimonial; ce résultat ne concorde pas avec celui d'une cohorte de 1599 personnes issues de la population générale en Chine, pendant le pic de l'épidémie de la COVID-19, qui suggère que le fait d'être célibataire a été signalé comme un facteur prédisposant à la détresse psychologique. Les raisons de l'association entre le célibat et l'Anxiété, indiquée par cette étude, ont été le manque d'engagement social, le sentiment de solitude

dans l'incertitude de la pandémie COVID-19 et le manque de partage du fardeau financier au cours de cette période, ce qui peut augmenter leur risque d'Anxiété (20). Selon une étude indienne, les jeunes professionnels de la santé célibataires peuvent être les plus à risque de souffrir de Dépression et d'Anxiété combinées, révélant un risque presque deux fois plus élevé que chez leurs homologues mariés (21). Cela peut suggérer que les professionnels de la santé manquant de soutien affectif de la part de leurs familles, de la société et de leurs collègues à l'hôpital, présenteraient plus de troubles psychologiques que leurs homologues affectivement soutenus.

Le tabagisme a été retrouvé comme un facteur multipliant le risque par deux de souffrir de symptômes de Dépression, ceci corrobore une étude américaine de Goodman et Capitman où les fumeurs auraient ainsi quatre fois plus de risque de souffrir de Dépression (22). Pour ces chercheurs, on ne fumerait pas parce que l'on est dépressif mais on deviendrait dépressif en fumant.

La littérature disponible suggère que les personnes présentant des co-morbidités étaient confrontées à un risque plus élevé de contracter la COVID-19, ainsi, on supposait qu'il y avait une association entre les co-morbidités et l'augmentation du risque de Dépression et d'Anxiété (23), une constatation confirmée par nos résultats qui ont montré des taux élevés de Dépression et d'Anxiété chez les professionnels souffrant de maladies chroniques.

Le mode d'exercice s'est révélé comme facteur protecteur contre la Dépression chez les résidents et les spécialistes. Cela pourrait être expliqué par le fait que ce personnel n'a pas été en contact prolongé et étroit avec les patients atteints de cette infection et par leurs expériences habituelles potentiellement pénibles. Toutefois, une relation demeure entre l'Anxiété et le poste de travail; être aide-soignant ou laborantin de santé publique a été un facteur protecteur contre l'Anxiété.

Concernant les laborantins de santé publique, nos résultats rejoignent l'étude faite par Keubo et ses collaborateurs (2020) qui ont observé un taux faible d'Anxiété dans ce groupe de professionnels (24).

Selon une étude chinoise, les professionnels de la santé directement impliqués avec des patients COVID-19, ont rapporté des niveaux élevés de stress, d'Anxiété, de Dépression et d'épuisement professionnel, par rapport à ceux qui travaillaient avec des patients non COVID-19. Il en a été de même, lors de l'épidémie précoce de VIH, du SRAS, d'Ebola et d'autres épidémies démontrant que travailler en première ligne lors d'une épidémie avait des répercussions psychiatriques importantes, notamment l'épuisement professionnel, l'Anxiété et le trouble de stress post-traumatique (25). Effectivement dans la présente étude, travailler en première ligne s'est révélé comme un facteur prédictif d'Anxiété.

L'utilisation d'EPI a été retrouvée comme un facteur protecteur contre la Dépression. Selon Woon LS, *et al* (2020) (18), certains participants craignaient de ne pas disposer d'un stock suffisant d'EPI, pour les protéger contre l'infection dans le cas où ils seraient exposés à des patients COVID-19, pendant leur affectation au travail. Ces peurs incontrôlées prédisposent les participants à l'Anxiété: notre étude infirme tout lien entre ce paramètre et l'Anxiété.

Dans notre article, le nombre d'années d'exercice n'était pas prédictif de détresse psychologique et ne confirmait pas les résultats de Kürşat Şahin *et al,* (2020) (8) révélant que travailler moins de 10 ans était associé à la Dépression et à l'Anxiété et suggérant que les professionnels avec plus de 10 ans d'expérience ont été plus exposés à des situations critiques en milieu de santé et ont développé une maturité psychologique face aux difficultés. Cela fait appel à la notion de la Résilience psychologique qui facilite la réponse au stress. En effet, ces niveaux élevés de Résilience psychologique protègent contre le développement de la maladie; dans cette recherche un score élevé de Résilience a été observé chez les sujets d'âge supérieurs à 30 ans et était révélé comme facteur protecteur contre la Dépression. Ces résultats signifient que les professionnels de la santé moins résilients couraient un risque plus élevé de développer une Dépression que les autres. Ce résultat corrobore l'étude japonaise qui a démontré que le score de la Résilience CD-RISC10 était plus faible dans le groupe dépressif que dans le groupe sain (10).

Selon Chew *et al* (2020) (26), la détresse psychologique associée à la stigmatisation pourrait s'exprimer par des symptômes physiques, comme pour la peur de l'infection qui pourrait exacerber les niveaux de détresse psychologique par crainte de transmettre la maladie à la famille et aux collègues. Ceci a été confirmé par notre étude où la stigmatisation a été déterminante des troubles dépressifs; par contre la peur de l'infection n'était pas prédictive de détresse psychologique.

En revanche, le sentiment d'évasion au travail et le

manque de motivation étaient significativement liés à la Dépression ceci pourrait être expliqué par le fait que l'individu en épisode dépressif ressent une perte d'intérêt et de plaisir particulier. La capacité à prendre du plaisir dans les loisirs, en famille, au travail s'estompe petit à petit. Il en découle des résultats obtenus de l'étude que le profil épidémiologique d'un professionnel de santé « sain », indemne de tout trouble anxio-depressif était un homme, non-fumeur, ne souffrant d'aucune co-morbidité, n'étant pas en contact direct avec la COVID-19 avec un score bas de Dépression et d'Anxiété et paradoxalement une bonne Résilience, sans peur de contracter ni de succomber à la maladie. Il demeure toutefois motivé au travail sans évasion et bien intégré.

Notre étude comportait certaines limites: Les études transversales ne permettent pas de suivre les changements dans la détresse psychologique après le début et l'expansion de la pandémie, de plus la conception transversale a limité notre capacité à faire la distinction entre les symptômes pré-existants et nouveaux et à étudier si les symptômes psychologiques des professionnels de la santé se sont aggravés ou non, par conséquent, une étude longitudinale est justifiée. Une autre limite pourrait incomber à la collecte des données, qui comprenait des répondants aux questionnaires en ligne et sur le terrain lors du passage des enquêteurs d'où le manque d'uniformité. Par contre, l'utilisation de questionnaire en ligne nous a permis d'intégrer plus de participants à travers tout le pays afin de colliger plus d'informations, montrant ainsi l'envergure de l'étude entreprise. Un autre point fort, à notre connaissance et avec modestie, notre étude est la première dans son genre menée en Algérie, et pourrait être utile pour mettre en œuvre des interventions de soutien et de suivi appropriés en matière de santé mentale pour les professionnels de la santé.

Cette étude transversale a permis d'estimer la fréquence des symptômes anxio-dépressifs par le biais d'un échantillon national représentatif des professionnels de la santé, en Algérie, en tenant compte à la fois de la ligne de front et de la non-première ligne, Il en ressort que 239 (23,78%) agents de la santé avaient une Anxiété modérée à sévère et 448 (44,58%) avaient par contre développé des symptômes de Dépression. Nos résultats indiquent d'une part, être femme, avoir des scores d'Anxiété élevés et une moindre résilience étaient des facteurs de risque de Dépression. D'autre part, ils révèlent qu'être marié, résident ou spécialiste étaient des facteurs protecteurs contre les troubles anxio-dépressifs. De même, être dépressif et travailler en première ligne pour soigner des patients atteints de la COVID-19 étaient des facteurs de risque d'Anxiété. Parmi la population d'étude, les aides-soignants et les laborantins de santé publique étaient par contre protégés de l'Anxiété. Nous devrons nous acclimater à vivre avec le risque de pandémie dans l'avenir, il est certainement important d'être préparé, notamment en soutenant les professionnels qui travailleront en première ligne par un accompagnement psychologique supplémentaire et la mise en œuvre de programmes d'interventions rapides pour prévenir et traiter les problèmes de santé mentale afin de protéger les professionnels de ce secteur engagés dans ce combat de longue haleine contre la Covid-19. Quelques recommandations s'imposent en quelques points:

- Des interventions multidisciplinaires ciblées sont nécessaires pour soutenir les professionnels de la santé en s'attaquant à la fois aux manifestations psychologiques et aux symptômes physiques.
- Les consultations en ligne en santé mentale et les services de conseil ou de psychothérapie devraient être facilement accessibles pour aider les professionnels de la santé qui subissent des troubles émotionnels en réponse à leur engagement au travail pendant la pandémie.
- Pour les professionnels de la santé, il serait nécessaire d'encourager l'établissement d'horaires de travail plus flexibles et d'envisager une formation efficace et durable en Résilience psychosociale pour prévenir la récurrence des symptômes psychologiques.

Remerciements

Les auteurs tiennent à remercier particulièrement le Dr Metatla,S (Institut Pasteur d'Alger), le Pr Chabni,N (Service de médecine préventive de Tlemcen), et le Dr Sehimi,A (Service de médecine physique et de réadaptation de Sidi Bel Abbès) pour leur participation à la collecte des données. Nos remerciements vont également à tous les participants pour leur excellente coopération.

Déclaration de liens d'intérêts:

Les auteurs déclarent ne pas avoir de liens d'intérêts.

RÉFÉRENCES

1. Kunin M, Engelhard D, Piterman L, Thomas S. Response of general practitioners to infectious disease public health crises: an integrative systematic review of the literature. Disaster Med Public Health reparedness, 2013;7(5):522-533.

2. World Health Organization. Coronavirus disease 2019 (‎COVID-19)‎: situation report, 94, 2020, (Consulté le 22 juillet 2021).
3. World Health Organization. Coronavirus Disease 2019 (COVID-19) Symptomes https://www,who,int/fr/health-topics/coronavirus/coronavirus#tab=tab_3. (Consulté le 22 juillet 2021).
4. Trumello C, Bramanti SM, Ballarotto G, Candelori C, Cerniglia L, Cimino S, *et a*l. Psychological Adjustment of Healthcare Workers in Italy during the COVID-19 Pandemic: Differences in Stress, Anxiety, Depression, Burnout, Secondary Trauma, and Compassion Satisfaction between Frontline and Non-Frontline Professionals. Inter J Environ Res Public Health, 2020;17(22):8385.
5. Bandyopadhyay S, Baticulon RE, Kadhum M, Alser M, Ojuka DK, Badereddin Y, *et al*. Infection and mortality of healthcare workers worldwide from COVID-19: a systematic review. BMJ Global Health, 2020;5(12):e003097.
6. Stuijfzand S, Deforges C. Sandoz V, Sajin C-T, Jaques C, Elmers J, *et al.* Psychological impact of an epidemic/pandemic on the mental health of healthcare professionals: a rapid review. BMC public health, 2020;20(1):1230.
7. Zhang WR, Wang K, Yin L, Zhao WF, Xue Q, Peng M, *et al*. Mental Health and Psychosocial Problems of Medical Health Workers during the COVID-19 Epidemic in China. Psychotherapy Psychosomatics, 2020;89(4):242-250.
8. Şahin MK, Aker S, Şahin G, Karabekiroğlu A. Prevalence of Depression, Anxiety. Distress and Insomnia and Related Factors in Healthcare Workers During COVID-19 Pandemic in Turkey. J Community Health, 2020;45(6):1168-1177.
9. Zhang H, Shi Y, Jing P, Zhan P, Fang Y, Wang F. Posttraumatic stress disorder symptoms in healthcare workers after the peak of the COVID-19 outbreak: A survey of a large tertiary care hospital in Wuhan. Psychiatry Res, 2020;294:113541.,
10. Awano N, Oyama N, Akiyama K, Inomata M, Kuse N, Tone M, *et al*. Anxiety, Depression, and Resilience of Healthcare Workers in Japan During the Coronavirus Disease 2019. Outbreak, Internal Med (Tokyo, Japan), 2020;59(21):2693-2699.
11. Micoulaud-Franchi JA, Lagarde S, Barkate G, Dufournet B, Besancon C, Trébuchon-Da Fonseca A, *et al.* Rapid detection of generalized anxiety disorder and major depression in epilepsy: Validation of the GAD-7 as a complementary tool to the NDDI-E in a French sample, Epilepsy Behavior 2016;57(Pt A):211-216.
12. Verdier-Taillefer MH, Gourlet V. Fuhrer R, Alpérovitch A, Psychometric properties of the Center for Epidemiologic Studies-Depression scale in multiple sclerosis, Neuroepidemiology, 2001;20(4):262-267.
13. Guihard G, Deumier L, Alliot-Licht B, Bouton-Kelly L, Michaut C, Quilliot F. Psychometric validation of the French version of the Connor-Davidson Resilience Scale, Encephale, 2018;44(1):40-45.
14. Erquicia J, Valls L, Barja A, Gil S, Miquel J, Leal-Blanquet J, *et al*. Emotional impact of the COVID-19 pandemic on healthcare workers in one of the most important infection outbreaks in Europe. Medicina Clínica (English Edition), 2020;155(10):434-440.
15. Arafa A, Mohammed Z, Mahmoud O, Elshazley M, Ewis A. Depressed, anxious, and stressed: What have healthcare workers on the frontlines in Egypt and Saudi Arabia experienced during the COVID-19 pandemic. J Affective Disorders, 2021;278:365-371.
16. Suryavanshi N, Kadam A, Dhumal G, Nimkar S, Mave V, Gupta A, *et al.* Mental health and quality of life among healthcare professionals during the COVID-19 pandemic in India, Brain Behavior 2020;10(11):e01837.
17. Sim K, Chua HC. The psychological impact of SARS: a matter of heart and mind. Can Med Assoc J 2004;170(5), 811–812.
18. Woon LS, Sidi H, Nik Jaafar NR, Leong Bin Abdullah MFI. Mental Health Status of University Healthcare Workers during the COVID-19 Pandemic: A Post-Movement Lockdown Assessment. Inter J Environ Res Public Health 2020;17(24):9155.
19. Pappa S, Ntella V, Giannakas T, Giannakoulis VG, Papoutsi E, Katsaounou P. Prevalence of depression, anxiety, and insomnia among healthcare workers during the COVID-19 pandemic: A systematic review and meta-analysis. Brain Behavior Immunity, 2020;88:901-907.
20. Wang H, Xia Q, Xiong Z, Li Z, Xiang W, Yuan Y, *et al.* The psychological distress and coping styles in the early stages of the 2019 coronavirus disease (COVID-19) epidemic in the general mainland Chinese population: A web-based survey, PloS one, 2020;15(5):e0233410.
21. Shayganfard M, Mahdavi F, Haghighi M, Sadeghi-Bahmani D, Brand S. Sources of Health Anxiety for Hospital Staff Working during the COVID-19 Pandemic. Inter J Environ Res Public Health, 2021;18(6):3094.
22. Goodman E, Capitman J. Depressive symptoms and cigarette smoking among teens. Pediatrics, 2000;106(4):748-755.
23. Alenazi TH, BinDhim NF, Alenazi MH, Tamim H, Almagrabi RS, Aljohani SM, *et al*. Prevalence and predictors of anxiety among healthcare workers in Saudi Arabia during the COVID-19 pandemic. J Infection Public Health, 2020;13(11):1645-1651.
24. Keubo FRN, Mboua PC, Tadongfack TD, Tchoffo EF, Tatang CT, Zeuna JI, *et al.* Psychological distress among health care professionals of the three COVID-19 most affected Regions in Cameroon: Prevalence and associated factors. Ann Med Psychol, 2021;179(2): 141–146.
25. Liu Z, Wu J, Shi X, Ma Y, Ma X, Teng Z, *et al*. Mental Health Status of Healthcare Workers in China for COVID-19 Epidemic. Ann Global Health, 2020;86(1):128.
26. Chew NWS, Lee GKH, Tan BYQ, Jing M, Goh Y, Ngiam NJH, et al. A multinational, multicentre study on the psychological outcomes and associated physical symptoms amongst healthcare workers during COVID-19 outbreak. Brain, behavior, and immunity. 2020;88:559-65.
