# Supplementary material for: Mental health status and related factors influencing healthcare workers during the COVID-19 pandemic: A systematic review and meta-analysis
Source: PLoS One. 2024 Jan 19;19(1):e0289454. doi: 10.1371/journal.pone.0289454 (PMC10798549; doi:10.1371/journal.pone.0289454)
Supplement: S1 Data — (ZIP) [file pone.0289454.s011.zip › literatures/65.pdf]

frontiers in Public Health

ORIGINAL RESEARCH
published: 03 February 2021
doi: 10.3389/fpubh.2021.592058

Mental Health of Physicians During COVID-19 Outbreak in Bangladesh: A Web-Based Cross-Sectional Survey

*Most. Farida Khatun[1], Most. Firoza Parvin[2], Md. Mamun-ur Rashid[3], Md. Shah Alam[4], Most. Kamrunnahar[5], Ashis Talukder[6], Shaharior Rahman Razu[7], Paul R. Ward[8] and Mohammad Ali[6]**

[1] Pharmacy Discipline, Khulna University, Khulna, Bangladesh, [2] Rajshahi Medical College, Rajshahi, Bangladesh, [3] Sir Salimullah Medical College & Mitford Hospital, Dhaka, Bangladesh, [4] Junior Consultant, Kotchandpur, Bangladesh, [5] Directorate General of Health Services, Ministry of Health and Family Welfare, Dhaka, Bangladesh, [6] Statistics Discipline, Khulna University, Khulna, Bangladesh, [7] Sociology Discipline, Khulna University, Khulna, Bangladesh, [8] College of Medicine & Public Health, Flinders University, Adelaide, SA, Australia

There have been numerous studies about the health implication of COVID-19 on patients, but little attention has been paid to the impacts of the pandemic on physicians. Our paper attends to this gap by exploring the mental health of physicians in Bangladesh during the COVID-19 pandemic. This is particularly important since the mental health of physicians impacts not only on themselves, but also their professional performance and hence the care of patients. This study examined physicians' mental health outcomes by evaluating the prevalence and associated potential risk factors of anxiety and depression. Using a web-based cross-sectional survey, we collected data from 114 physicians. Seven-item Generalized Anxiety Disorder (GAD-7) scale and Nine-item Patient Health Questionnaire (PHQ-9) were used to measure the anxiety and depression, respectively. Multivariate logistic regression models were used to explore the potential risk factors related to anxiety and depression. The prevalence of anxiety and depression were 32.5 and 34.2%, respectively. Findings revealed that marital status, work per day and current job location were the main risk factors for anxiety while sex, age, and marital status were the main risk factors for depression. Our results highlight the need to implement policies and strategies for positively impacting the mental health of physicians during and after the COVID-19 pandemic.

Keywords: COVID-19, depression, anxiety, physicians, Bangladesh

OPEN ACCESS

Edited by:
Amelia Kekeletso Ranotsi, Maluti Adventist College, Lesotho

Reviewed by:
Chundung Asabe Miner, University of Jos, Nigeria
Lan Hoang Nguyen, Hue University, Vietnam

***Correspondence:**
Mohammad Ali
ali.ru.stat@gmail.com

Specialty section:
This article was submitted to Public Health Policy, a section of the journal Frontiers in Public Health

Received: 06 August 2020
Accepted: 05 January 2021
Published: 03 February 2021

Citation:
Khatun MF, Parvin MF, Rashid MM, Alam MS, Kamrunnahar M, Talukder A, Rahman Razu S, Ward PR and Ali M (2021) Mental Health of Physicians During COVID-19 Outbreak in Bangladesh: A Web-Based Cross-Sectional Survey. Front. Public Health 9:592058. doi: 10.3389/fpubh.2021.592058

INTRODUCTION

The coronavirus disease 2019 (COVID-19) outbreak has been declared as a pandemic resulting global health concern (1). The infectious disease is responsible for acute respiratory illness caused by the newly discovered severe acute respiratory syndrome coronavirus 2 (SARS-CoV-2). It was first detected in Wuhan, China at the end of December 2019 (2), and has become an increasingly public event around the world including Bangladesh (3, 4).

As of July 22, 2020, 14,731,563 confirmed cases of COVID-19 including 611,284 deaths have been reported by WHO globally (5). In Bangladesh, the first known cases were reported on March 7, 2020 by the country's epidemiology institute IEDCR (Institute of Epidemiology, Disease Control and Research) (6). As of July 22, 2020, a total of 213,254 COVID-19 confirmed cases and 2,751 deaths have been reported in Bangladesh (7).

The coronavirus spreads primarily through droplets of saliva and discharges from the nose and mouth when a COVID-19 patient coughs or sneezes (8). Still no effective treatment is available of COVID-19, although many accomplishments including virus information, clinical features, and diagnosis have been achieved (2, 9–11). Physicians are first-line responders treating patients with COVID-19 and face a high risk of being infected because of exposure to long and distressing work-shifts to meet health requirements every day (12).

Some hospitals in Bangladesh have been completely converted into COVID- 19 centers and some others have opened a dedicated COVID-19 special unit or ward. Since COVID-19 is a novel disease, some patients do not have enough knowledge about the signs and symptoms of COVID-19 and some who are aware of COVID-19 may not be able to differentiate between seasonal flu and COVID-19 due to limited testing facilities in parts of Bangladesh. For these reasons, sometimes COVID-19 patients go to other wards/hospitals which are not assigned for their treatment. This further increases the risk of infection for both the physicians and the patients.

A number of studies have been conducted in different countries on the mental health conditions of different professionals (2, 12–15), but no research on mental health problems of physicians during the COVID-19 outbreak in Bangladesh has been reported so far. Since poor mental health may hinder the professional performance of physicians and adversely affect the quality of healthcare provided, it is also likely to adversely influence patient health outcomes (16). Hence, the purpose of this study was to evaluate physicians' mental health during the COVID-19 outbreak in Bangladesh by quantifying the magnitude of symptoms of anxiety and depression and to explore the potential risk factors associated with these symptoms.

METHODS

Study Design

A cross-sectional study was carried out as the data were collected from 114 physicians through a questionnaire created via Google Form on the internet. We collected the data from May 4, 2020 to May 10, 2020.

Recruitment of Subjects and Eligibility Criteria

Physicians registered by the Bangladesh Medical & Dental Council and working in Bangladesh were considered as potential participants in this study. The participants were selected through convenient sampling technique from the closed Facebook and Messenger groups of the physicians in Bangladesh. All physicians using these closed groups across the country were eligible to participate, and those who completed the survey provided their unique email address in order to reduce the problem of duplicate entries.

Data Collection and Measurements

Physicians participated anonymously in this survey on the Internet and all participants reported their demographic and professional information during COVID-19 outbreak. They also completed two standardized questionnaires which assessed their generalized anxiety disorder (GAD) and 9-item Patient Health Questionnaire (PHQ-9). Participants who had psychiatric disorders prior to COVID-19 were excluded from the platform.

Demographic variables in this study included sex, age, marital status, current residence, living with family, specialization in medical profession, current job location, years of job experience, and work hours per day. Participants were asked whether they were directly engaged in special COVID-19 hospital or unit and those who answered positive were defined as the frontline workers, and those who answered negative were defined as the secondline workers.

We used the Generalized Anxiety Disorder-7 (GAD-7) (17) scale to assess the participant's anxiety symptoms which is valid for Asian region (18). Seven items were evaluated to measure the frequency of anxiety symptoms over the past 4 weeks on a 4-point Likert-scale ranging from 0 (never) to 3 (nearly every day). The total score of GAD-7 ranged from 0 to 21 as the increase of number in scores indicating more severe consequences of anxiety (17). In this study, we defined a GAD total score of 9 points or greater as the presence of anxiety (19).

This study used the 9-item Patient Health Questionnaire (PHQ-9) (20) to assess the severity of depression in this research. This scale is frequently used in Asian research for the measurement of depression (18, 21, 22). The total score of PHQ-9 ranged from 0 to 27. Participants who had a total scores 10 or greater were characterized as having major depression (20).

Ethical Consideration

The study protocol was approved by the Ethical Clearance Committee of Khulna University, Khulna, Bangladesh. Electronic informed consent was obtained from each participant. Participants could withdraw themselves from the survey anytime without providing any justification.

Analysis

Descriptive statistical methods and multivariate logistic regression models were performed to analyze the data. A well-known statistical package, SPSS (Statistical Package for Social Sciences) version 24.0 was utilized to obtain the necessary results.

RESULTS

Demographic and Professional Characteristics

The demographic and professional characteristics of participants are shown in **Table 1**. Of the 114 participants, 76 (66.7%) were men and 38 (33.7%) were women. It was found that age of 86 (75.4%) participants was <35 years and 28 (24.4%) was ≥35 years. Among the participants, 40 (35.1%) were unmarried, 72 (63.2%) were married and 2 (1.8%) were others. Just below half of the participants 55 (48.2%) lived in rented accommodation. Results also show that 32 (28.1%) participants were living alone while majority of the participants 82 (71.9%) were living with their family. Job location of most of the participant's (57%) was Dhaka division while rest of the participants was outside Dhaka division. It was revealed that 67 (58.8%) participants worked ≥8 h

TABLE 1 | Characteristics of the physicians ($N = 114$).

Characteristic	*N* (%)
**Gender**	
Male	76 (66.7)
Female	38 (33.3)
**Age**	
< 35 years	86 (75.4)
≥ 35 years	28 (24.6)
**Marital status**	
Unmarried	40 (35.1)
Married	72 (63.2)
Divorced/widowed/separated	2 (1.8)
**Current residence**	
Own house	32 (28.1)
Rent house	55 (48.2)
Residential house/ dormitory	27 (23.7)
**Living with family**	
Yes	82 (71.9)
No	32 (28.1)
**Specialization**	
MBSS	74 (64.9)
Medicine specialty	23 (20.2)
Surgical specialty and others	17 (14.9)
**Current job location**	
Dhaka division	65 (57.0)
Others division	49 (43.0)
**Job experience**	
<1 year	37 (32.5)
1–5 years	24 (21.1)
≥6 years	53 (46.5)
**Working hours per day**	
<8 h	47 (41.2)
≥8 h	67 (58.8)
**Working position**	
Frontline workers	21 (18.4)
Second line workers	93 (81.6)

TABLE 2 | Prevalence of anxiety and depression according to the characteristics.

Characteristic	Anxiety	Depression
	**Yes (%)**	**Yes (%)**
**Overall**	37 (32.5)	39 (34.2)
**Gender**		
Male	21 (27.6)	20 (26.3)
Female	16 (42.1)	19 (50.0)
**Age**		
< 35 years	31 (36.0)	34 (39.5)
≥ 35 years	6 (21.4)	5 (17.9)
**Marital status**		
Married	19 (26.4)	18 (25.0)
Others*	18 (42.9)	21 (50.0)
**Current residence**		
Own house	8 (25.0)	12 (37.5)
Rent house	19 (34.5)	19 (34.5)
Residential house/dormitory	10 (37.0)	8 (29.6)
**Living with family**		
Yes	26 (31.7)	46 (46.9)
No	11 (34.4)	24 (29.3)
**Specialization**		
MBSS	26 (35.1)	25 (33.8)
Medicine specialty	5 (21.7)	9 (39.1)
Surgical specialty and others	6 (35.3)	5 (29.4)
**Current job location**		
Dhaka division	27 (41.5)	26 (40.0)
Others division	10 (20.4)	13 (26.5)
**Job experience**		
<1 year	15 (40.5)	12 (32.4)
1–5 years	7 (29.2)	12 (50.0)
≥6 years	15 (28.3)	15 (28.3)
**Working hours per day**		
<8 h	10 (21.3)	13 (27.7)
≥8 h	27 (40.3)	26 (38.8)
**Working position**		
Frontline workers	30 (32.3)	4 (19.0)
Second line workers	7 (33.3)	35 (37.6)

**indicates others represent- Unmarried/divorced/widowed/separated.*

per day while 47 (41.2%) participants worked <8 h. Among the participants, 21 (18.4%) were frontline workers and 93 (81.6%) were second line workers (see **Table 1**).

Prevalence of Anxiety and Depression During COVID-19

The prevalence of anxiety and depression stratified by demographic and professional characteristics which shown in **Table 2**. The overall prevalence of anxiety and depression were 32.5 and 34.2%, respectively (see **Table 2**).

Potential Risk Factors With Anxiety and Depression During COVID-19 Outbreak

A multivariate logistic regression was used to assess the potential risk factors of anxiety and depression during COVID-19 outbreak and the results of the multivariate logistic regression model are presented in **Table 3**. It was observed that physicians who worked in Dhaka division (OR = 2.77, 95% CI: 1.18–6.50, $p < 0.05$) were more likely to experience anxiety compared to their counterparts who worked in other divisions. Similarly, physicians who worked ≥8 h per day (OR = 2.50, 95% CI: 1.06–5.86, $p < 0.05$) had more chance to experience anxiety compared to physicians who worked <8 h per day.

Results demonstrate that male physicians (OR = 0.36, 95% CI: 0.15–0.81, $p < 0.05$) were less likely to experience depression that female physicians. Married physicians (OR = 0.33, 95% CI: 0.14–0.75, $p < 0.05$) had less chance of experiencing depression compared to unmarried/divorced/widowed/separated physicians. Physicians aged 35 years or more (OR = 0.33,

TABLE 3 | Results of multivariate logistic regression analyses.

Variables	Anxiety		Depression	
	p-value	OR (95% CI)	*p*-value	OR (95% CI)
**Gender**				
Female®				
Male	0.12	0.53 (0.23–1.18)	0.01*	0.36 (0.15–0.81)
**Age**				
< 35 years®				
≥ 35 years	0.15	0.48 (0.17–1.32)	0.04*	0.33 (0.12–0.96)
**Marital status**				
Married	0.07	0.47 (0.21–1.02)	0.01*	0.33 (0.14–0.75)
Others®				
**Current residence**				
Own house®				
Rent house	0.35	1.58 (0.59–4.19)	0.78	0.88 (0.36–2.18)
Residential house/dormitory	0.32	1.77 (0.58–5.40)	0.53	0.70 (0.23–2.10)
**Living with family**				
No®				
Yes	0.78	0.88 (0.37–2.11)	0.08	0.47 (0.20–1.08)
**Specialization**				
MBSS®				
Medicine specialty	0.23	0.51 (0.17–1.54)	0.64	1.26 (0.48–3.31)
Surgical specialty and others	0.99	1.01 (0.33–3.03)	0.73	0.82 (0.26–2.58)
**Current job location**				
Dhaka division	0.01*	2.77 (1.18–6.50)	0.14	1.85 (0.83–4.13)
Others division®				
**Job experience**				
<1 year®				
1–5 years	0.37	0.60 (0.20–1.81)	0.17	2.08 (0.73–5.99)
≥6 years	0.22	0.58 (0.24–1.41)	0.67	0.82 (0.33–2.05)
**Working hours per day**				
<8 h®				
≥8 h	0.03*	2.50 (1.06–5.86)	0.22	1.66 (0.74–3.71)
**Working position**				
Frontline workers	0.92	1.05 (0.38–2.87)	0.11	0.39 (0.12–1.25)
Second line workers®				

®indicates reference category.
**indicates significant at $p < 0.05$.*

95% CI: 0.12–0.96, $p < 0.05$) had less chance of experiencing depression than younger physicians.

DISCUSSION

Although the world has experienced several epidemics and pandemics in recent years, such as SARS, MERS, Ebola and influenza A, healthcare professionals seem to be facing increased psychological pressures during the COVID-19 pandemic compared to previous epidemics (23–25). Both anxiety and depression are higher in physicians in Bangladesh during COVID-19 than has been found in previous epidemics (25). Our web-based study showed that the prevalence of anxiety (32.5%) and depression (34.2%) among physicians in Bangladesh during COVID-19 outbreak were high in comparison to previous epidemics, but lower than physicians in China during COVID-19 (18). We have to keep in mind that our data were collected during the early stages of the COVID-19 epidemic in Bangladesh, and at present, no one can predict if or when the epidemic will subside. Therefore, direct comparisons with studies measuring the psychological impact on physicians of previous epidemics may not be directly comparable, since the stage of the epidemic when the study was conducted is likely to impact on psychological distress. Nevertheless, longitudinal research on the psychological impact of previous epidemics found a reduction in depression, anxiety and other psychological disorders 1–3 years post-epidemic, although not reducing to the pre-epidemic

levels (25). This highlights the need for ongoing measurement of psychological distress throughout and post-epidemic, in order to best support physicians now and into the future. If this is undertaken within the healthcare organizations where physicians work (as opposed to only by researchers), then interventions can be implemented quickly and within the practice context of physicians' working days.

According to the results of multivariate logistic regression model, marital status, current job location and working hours per day were found to be significant predictors for anxiety. In addition, gender, age and marital status were highly significant predictors for depression. By considering the magnitude of these selected factors, findings of this study demonstrated that depression were less likely to occur among physicians who were married compared to their counterparts. During the SARS outbreak, a study conducted among hospital employees also found similar relationships (26). A possible explanation of this finding is that married people have been shown to have an overall better levels of mental than counterparts people (27). This difference between married and unmarried/divorced/widowed/separated people may be linked to a sense of stability, social capital, and having a person to share feelings and emotions with after a stressful day working in the hospital. Another study depicted that married individuals had substantially lower risks of death than their unmarried counterparts (28). Hence, marital status should be considered when developing practice-based interventions or attempting to identify "at risk" physicians during COVID-19 pandemic. Results also revealed that anxiety were more common among physicians who worked inside Dhaka division. Since Dhaka division has the largest population and deals with the majority of the COVID-19 cases in Bangladesh (29), this might be contributing factor here. Therefore, physicians from Dhaka division should be provided with specific attention and care from the concerned authority during this or future pandemics.

Our study revealed that workload was associated with the mental health of the participants. Physicians who worked $\geq$8 h a day had higher likelihood of experiencing anxiety compared to those who worked $<$8 h a day. This finding suggests that the workload of the physicians needs to be taken into account when considering "at risk" physicians with whom practice-based interventions can be implemented. Whilst this does not deal with the problem of doctors working longer hours, it at least identifies those groups who may be in need of mental health support during COVID-19. In an ideal world with no financial constraints, we would suggest that in order to improve the mental health of physicians during COVID-19, more physicians could be trained/recruited, more physicians could be relocated from rural to urban areas (like Dhaka) and/or task-shifting could be implemented whereby physicians focus on high-risk patients/procedures, leaving lower-risk patients/procedures to other non-medical staff (30). Corresponding to a recent study during COVID-19 (18) and a study during the SARS outbreak (26), findings of our study reported that women were more likely to experience depression compared to men during the pandemic. Previous research has found that females endure more job related stress than men (31, 32), we assume this might be a plausible explanation of this result. The multiple additional caring roles of women (additional to the stress of being a physician) may add layered stress to female physicians, who may also have COVID-19 related stresses linked to parents, family, and children. That is not to say that male physicians have less of these concerns, but global literature is clear that women take on the majority of caring roles inside the household and family (33).

We also observed that older physicians had lower risk of experiencing depression than younger ones, which is supported by a previous study (29). Our results suggest the need to implement stress management programs (or other interventions aimed at protecting mental health) for younger physicians in order to manage their mental health. Although a study in China showed that during COVID-19, frontline healthcare workers were more likely to experience mental health problems than other healthcare workers (18), we did not find that the working position of the physician had any significant effect on anxiety and depression. Overall, the results of this study indicate that mental health of the physicians require special attention during and after the COVID-19 pandemic, with a specific focus on the particular groups of physicians identified in this research.

LIMITATIONS

When interpreting the findings of this study, some limitations need to be taken into account. The sample size was relatively small and the survey was carried out during seven days of early surge in COVID-19 cases in Bangladesh without any longitudinal follow-ups. We used convenient sampling, hence no sample size calculation formula was used. The sample was self-selecting, which may indicate that physicians who did not take part in our study are different in some way to our participants. The use of closed Facebook and Messenger groups may also have led to a slightly biased sample. As a result, the prevalence of anxiety and depression may be either over or under estimated in the current study. Notwithstanding these potential limitations, we managed to recruit 114 physicians to our study during a time of social distancing (hence the need for online recruitment) and increased workload of physicians, representing the first study of its kind in Bangladesh. Nevertheless, the long-term psychological implications of Bangladeshi physicians are worth consideration for further investigation, in order to build on our results.

CONCLUSION

To the best of our knowledge this is the first study in Bangladesh to assess the prevalence and associated risk factors of anxiety and depression among physicians during COVID-19 outbreak. Findings revealed that the prevalence of anxiety and depression were high among the physicians. Marital status, work per day and current job location were risk factors for anxiety whereas sex, age, and marital status were risk factors for depression. Governments may consider findings of this study for a better health management and an improved health outcome for both physicians and patients.

DATA AVAILABILITY STATEMENT

The original contributions presented in the study are included in the article/supplementary material, further inquiries can be directed to the corresponding author/s.

ETHICS STATEMENT

This study was approved by the Ethical Clearance Committee of Khulna University, Khulna, Bangladesh. The patients/participants provided their written informed consent to participate in this study.

AUTHOR CONTRIBUTIONS

All authors contributed equally to the writing of this manuscript.

ACKNOWLEDGMENTS

Authors would like to thank to the participants of the study.

REFERENCES

1. World Health Organization. *WHO Characterizes COVID-19 as a Pandemic*. Geneva: World Health Organization (2020). Available online at: https://www.who.int/emergencies/diseases/novel-coronavirus-2019/events-as-they-happen (accessed March 11, 2020).
2. Liu S, Yang L, Zhang C, Xiang YT, Liu Z, Hu S, et al. Online mental health services in China during the COVID-19 outbreak. *Lancet Psychiatry.* (2020) 7:e17–8. doi: 10.1016/S2215-0366(20)30077-8
3. COVID-19: too little, too late? [Editorial]. *Lancet*. (2020) 395:755. doi: 10.1016/S0140-6736(20)30522-5
4. Day M. Covid-19: surge in cases in Italy and South Korea makes pandemic look more likely. *BMJ.* (2020) 368:m751 doi: 10.1136/bmj.m751
5. World Health Organization. *Coronavirus Disease (COVID-19) Outbreak Situation*. (2020). Available online at: https://www.who.int/emergencies/diseases/novel-coronavirus-2019 (accessed July 22, 2020).
6. Paul R. *Bangladesh Confirms Its First Three Cases of Coronavirus*, in *Reuters*. (2020). Available online at: https://www.reuters.com/article/us-health-coronavirus-bangladesh/bangladesh-confirms-its-first-three-cases-of-coronavirus-healthofficials-idUSKBN20V0FS (accessed July 24, 2020).
7. *Covid-19 Status Bangladesh*. (2020). Available online at: https://www.iedcr.gov.bd/ (accessed July 24, 2020).
8. World Health Organization. *Coronavirus*. (2020). Available online at: https://www.who.int/health-topics/coronavirus#tab=tab_1 (accessed May 13, 2020).
9. Guan W, Ni Z, Hu Y, Liang WH, Ou C, He J, et al. China medical treatment expert group for Covid-19. clinical characteristics of coronavirus disease 2019 in China. *N Engl J Med.* (2020) 382:1708–20. doi: 10.1056/NEJMc2005203
10. Zhou P, Yang X, Wang X, Hu B, Zhang L, Zhang W, et al. A pneumonia outbreak associated with a new coronavirus of probable bat origin. *Nature.* (2020) 579:270–3. doi: 10.1038/s41586-020-2012-7
11. Wang M, Cao R, Zhang L, Yang X, Liu J, Xu M, et al. Remdesivir and chloroquine effectively inhibit the recently emerged novel coronavirus (2019-nCoV) *in vitro*. *Cell Res.* (2020) 30:269–71. doi: 10.1038/s41422-020-0282-0
12. Zhang W, Wang K, Yin L, Zhao W, Xue Q, Peng M, et al. Mental health and psychosocial problems of medical health workers during the COVID-19 epidemic in China. *Psychother Psychosom*. (2020) 89:242–50. doi: 10.1159/000507639
13. Greenberg N, Docherty M, Gnanapragasam S, Wessely S. Managing mental health challenges faced by healthcare workers during covid-19 pandemic. *BMJ.* (2020) 368:m1211. doi: 10.1136/bmj.m1211
14. Zandifar A, Badrfam R. Iranian mental health during the COVID-19 epidemic. *Asian J Psychiatr*. (2020) 51:101990. doi: 10.1016/j.ajp.2020.101990
15. Chen Q, Liang M, Li Y, Guo J, Fei D, Wang L, et al. Mental health care for medical staff in China during the COVID-19 outbreak. *Lancet Psychiatr.* (2020) 7:e15–6. doi: 10.1016/S2215-0366(20)30078-X
16. Gong Y, Han T, Chen W, Dib H, Yang G, Zhuang R, et al. Prevalence of anxiety and depressive symptoms and related risk factors among physicians in China: a cross-sectional study. *PLoS ONE.* (2014) 9:e103242. doi: 10.1371/journal.pone.0103242
17. Spitzer RL, Kroenke K, Williams JBW, Löwe B. A brief measure for assessing generalized anxiety disorder: the GAD-7. *Arch Intern Med.* (2006) 166:1092–7. doi: 10.1001/archinte.166.10.1092
18. Lai J, Ma S, Wang Y, Cai Z, Hu J, Wei N, et al. Factors associated with mental health outcomes among health care workers exposed to coronavirus disease 2019. *JAMA Netw Open*. (2020) 3:e203976. doi: 10.1001/jamanetworkopen.2020.3976
19. Wang Y, Chen R, Zhang L. Reliability and validity of generalized anxiety scale-7 in inpatients in Chinese general hospital. *J Clin Psychiatr.* (2018) 28:168–71. doi: 10.3969/j.issn.1005-3220.2018.03.007
20. Kroenke K, Spitzer RL, Williams JB. The PHQ-9: validity of a brief depression severity measure. *J Gen Intern Med.* (2001) 16:606–13. doi: 10.1046/j.1525-1497.2001.016009606.x
21. Roy T, Lloyd CE, Parvin M, Mohiuddin KG, Rahman M. Prevalence of co-morbid depression in out-patients with type 2 diabetes mellitus in Bangladesh. *BMC Psychiatry.* (2012) 12:123. doi: 10.1186/1471-244X-12-123
22. Gothwal VK, Bagga DK, Sumalini R. Rasch validation of the PHQ-9 in people with visual impairment in South India. *J Affect Disord.* (2014) 167:171–7. doi: 10.1016/j.jad.2014.06.019
23. Chong MY, Wang WC, Hsieh WC, Lee CY, Chiu NM, Yeh WC, et al. Psychological impact of severe acute respiratory syndrome on health workers in a tertiary hospital. *Br J Psychiatry.* (2004) 185:127–33. doi: 10.1192/bjp.185.2.127
24. Brooks SK, Dunn R, Amlôt R, Rubin GJ, Greenberg N. A systematic, thematic reviewof social and occupational factors associated with psychological outcomes in healthcare employees during an infectious disease outbreak. *J Occup Environ Med.* (2018) 60:248–57. doi: 10.1097/JOM.0000000000001235
25. Preti E, Di Mattei V, Perego G, Ferrari F, Mazzetti M, Taranto P, et al. The psychological impact of epidemic and pandemic outbreaks on healthcare workers: rapid review of the evidence. *Curr Psychiatry Rep.* (2020) 22:1–22. doi: 10.1007/s11920-020-01166-z
26. Liu X, Kakade M, Fuller CJ, Fan B, Fang Y, Kong J, et al. Depression after exposure to stressful events: lessons learned from the severe acute respiratory syndrome epidemic. *Compr Psychiatry.* (2012) 53:15–23. doi: 10.1016/j.comppsych.2011.02.003
27. Shapiro A, Keyes CLM. Marital status and social well-being: Are the married always better off? *Soc Indic Res.* (2008) 88:329–46. doi: 10.1007/s11205-007-9194-3
28. Lillard LA, Waite LJ. Til death do us part: marital disruption and mortality. *Am J Sociol.* (1995) 100:1131–56. doi: 10.1086/230634
29. Huang Y, Zhao N. Generalized anxiety disorder, depressive symptoms and sleep quality during COVID-19 outbreak in China: a web-based cross-sectional survey. *Psychiatry Res.* (2020) 288:112954. doi: 10.1016/j.psychres.2020.112954
30. Okyere E, Mwanri L, Ward P. Is task-shifting a solution to the health workers' shortage in Northern Ghana? *PLoS ONE.* (2017) 12:e0174631. doi: 10.1371/journal.pone.0174631
31. Kunz-Ebrecht SR, Kirschbaum C, Marmot M, Steptoe A. Differences in cortisol awakening response on work days and weekends in women and men from the Whitehall II cohort. *Psychoneuroendocrinology.* (2004) 29:516–28. doi: 10.1016/S0306-4530(03)00072-6
32. Lyons E. Psychosocial factors related to job stress and women in management. *Work.* (2002) 18:89–93.

33. Bainbridge HT, Broady TR. Caregiving responsibilities for a child, spouse or parent: The impact of care recipient independence on employee well-being. *J Vocat Behav*. (2017) 101:57–66. doi: 10.1016/j.jvb.2017.04.006

Conflict of Interest: The authors declare that the research was conducted in the absence of any commercial or financial relationships that could be construed as a potential conflict of interest.

Copyright © 2021 Khatun, Parvin, Rashid, Alam, Kamrunnahar, Talukder, Rahman Razu, Ward and Ali. This is an open-access article distributed under the terms of the Creative Commons Attribution License (CC BY). The use, distribution or reproduction in other forums is permitted, provided the original author(s) and the copyright owner(s) are credited and that the original publication in this journal is cited, in accordance with accepted academic practice. No use, distribution or reproduction is permitted which does not comply with these terms.
