# Supplementary material for: Mental health status and related factors influencing healthcare workers during the COVID-19 pandemic: A systematic review and meta-analysis
Source: PLoS One. 2024 Jan 19;19(1):e0289454. doi: 10.1371/journal.pone.0289454 (PMC10798549; doi:10.1371/journal.pone.0289454)
Supplement: S1 Data — (ZIP) [file pone.0289454.s011.zip › literatures/66.pdf]

*Psychiatria Danubina, 2021; Vol. 33, Suppl. 10, pp S161-171*
*Medicina Academica Mostariensia, 2021; Vol. 9, No. 1, pp 161-171*
*© Medicinska naklada - Zagreb, Croatia*
*COVID-19 forum: Original paper*

# PREVALENCE OF ANXIETY AND RELATIONSHIP OF ANXIETY WITH COPING STYLES AND RELATED FACTORS IN HEALTHCARE WORKERS DURING COVID-19 PANDEMIC

**Alperen Kılıç[1], Mehmet Buğrahan Gürcan[2], Bekir Aktura[3], Ahmet Rıza Şahin[4] & Zekeriya Kökrek[5]**

[1]*Istanbul Medipol University, Faculty of Medicine, Department of Psychiatry, Bağcılar, İstanbul, Turkey*
[2]*Horasan State Hospital, Department of Psychiatry, Erzurum, Turkey*
[3]*Istanbul University, Health Sciences Institute, Department of Public Health, Istanbul, Turkey*
[4]*Kahramanmaraş Sütçü İmam Unıversity Faculty of Medicine, Department of Infectious Diseases, Kahramanmaraş, Turkey*
[5]*Istanbul Sabahattin Zaim University, Faculty of Humanities and Social Sciences, Department of Psychology, İstanbul, Turkey*

received: 8.1.2021; revised: 15.3.2021; accepted: 22.3.2021

## SUMMARY

***Background:*** *The aim is to investigate anxiety prevalence among the healthcare workers during COVID-19 pandemic and the also relationship of "significant clinical anxiety" with coping styles and the related factors.*

***Subjects and methods:*** *An online questionnaire was performed to evaluate the anxiety responses of 544 healthcare workers, the adopted coping styles and the related factors during COVID-19 pandemic. The questionnaire included the subsections of sociodemographic data, other clinical data and the items on exposure to social media, Generalized Anxiety Disorder-7 (GAD-7) Scale and Coping Styles Scale Brief Form.*

***Results:*** *Minimal, mild, moderate and severe anxiety were present in 214 (39.3%), 191 (35.1%), 95 (17.5%) and 44 (8.1%) participants, respectively. GAD-7 score was ≥10 in 139(25.6%) of the participants indicating "significant clinical anxiety". Being female and a nurse were associated with significant clinical anxiety. In addition, the parameters such as "concern about COVID-19, concern about infect COVID-19 to relatives, unwillingness to work, frequency of social media use about COVID-19, sleep disturbances and experiencing somatic symptoms" were observed to be extremely significantly more common in the healthcare workers with "significant clinical anxiety" than those without that mental condition. The regression analysis revealed that higher adoption of using emotional social support, one of the emotional focused coping styles and behavioral disengagement, one of the ineffektive focused coping styles are considered to be predictor of significant clinical anxiety. Whereas, positive reinterpretation, one of the emotional focused coping styles was a predictor in reduction of significant clinical anxiety. Also more frequent exposure to social media and sleep disturbances were the predictors of significant clinical anxiety in the healthcare staff.*

***Conclusion:*** *Our results have emphasized the factors that should be taken into account and application of coping styles that may be functional in protecting mental health of the healthcare workers in their struggle against a huge disaster affecting worldwide societies.*

***Key words:*** *coping strategies - medical staff - psychological effects - outbreak*

* * * * *

## INTRODUCTION

Severe acute respiratory syndrome coronavirus-2 (SARS-CoV-2) which is the etiological agent of Coronavirus disease 2019 (COVID-19) has been first reported to be the cause of pneumonia by China at 31st December 2019 and its spread to numerous worldwide countries resulted in the declaration of a global pandemic by World Health Organization (WHO) on 11th March 2020 (WHO 2020).The healthcare workers (HCWs) that faced with this large-scale public health phenomenon are under a huge physical and psychological stress. Stress is the essential environmental risk factor for psychiatric diseases and humans may be more predisposed to the mental diseases under long-term stressful conditions (Cattaneo & Riva 2016) increasing the infection risk (Liao et al. 2017, Askim et al. 2018, Nayeri Chegeni et al. 2019).

HCWs are under the risk for exposure to highly contagious pathogens during presenting healthcare service to the patient or while dealing with biological specimens. This circumstance may cause them feeling anxious about being infected and transmitting infection to their family members (Hammen 2018, Koh et al. 2005). That fear may have negative impacts. The healthcare workers that were exposed to severe acute syndrome (SARS) epidemic between the years 2002-2003 have experienced a high-level of psychological stress because of quarantine period, reporting and body temperature resulting in refusing to perform patient healthcare (Chan & Huak 2004, Brug et al. 2004). They have to struggle with also unpredictability of working schedules that require arrangements in their private and social lives. The results of the stress may last for a long time after the epidemic and may result in depression or post-traumatic stress disorder (Maunder 2004, Bisson et al. 2010).

One fifth of the cases were healthcare workers during the SARS epidemic (Hammen 2018, Ploeg et al. 2017). The infections related with HCW 1-27% of total MERS-CoV cases (Hunter et al. 2016). The contraction of the disease by many healthcare workers creates more difficulty in healthcare service systems that are already intensely working (Ministry of Health 2020).

During and after SARS epidemic; stress reaction signs such as anxiety, depression, somatization and hostility have been reported in approximately 10% of the healthcare workers (Mak et al. 2009). In the more recent times, prevalences of the anxiety-related and stress-related symptoms among the Chinese healthcare workers during COVID-19 pandemic were found to be 44.6% and 71.5%, respectively (Lai et al. 2020).

In the light of literature data; a limited number of studies have investigated the coping strategies in the outbreak eras (Wong et al. 2005, Guo et al. 2020). A study carried on the healthcare workers employed in the emergency departments in the period of SARS outbreak revealed that acceptance, active coping and positive framing were the more commonly adopted coping strategies despite occupational differences. According to our literature review, only a few studies has investigated the coping styles among the healthcare workers (doctor and nurse) during COVID-19 pandemic (Zhu et al. 2020, Salopek-Žiha et al. 2020). As far as we know, our study is the first that investigated the coping styles among the general healthcare staff during COVID-19 pandemic, it is also the first study that investigated the general mental status of the healthcare workers in Turkey.

Including the present studies, the evidence is still limited and a very little information on the psychological needs of the healthcare workers that faced with this global disaster has been documented. Therefore, further systematic studies are urgently needed to clarify the psychological effects of COVID-19 pandemic on the healthcare workers and related risk and protective factors.

The aim of our study is to analyze the anxiety prevalence in the healthcare workers during COVID-19 pandemic and the relationship of "significant clinical anxiety" with the adopted coping styles and related sociodemographic and clinical factors.

## SUBJECTS AND METHODS

### Participants and procedures

A cross-sectional survey was designed to analyze the anxiety levels, adopted coping styles and the related factors in the healthcare workers. We used an online survey to minimize face-to-face interactions and to facilitate the participation of the healthcare workers that work intensely in this urgent period. The healthcare workers were communicated for their participation to the study. The survey was shared in various social network groups of different occupational fields. All the participants have given informed consent by answering the yes-no question at the beginning of the survey that confirmed their willingness to participate to the study. The survey data were collected within the 7 days between 22$^{th}$ April 2020 and 28th April 2020 after 11th April 2020 in which the highest number of the cases were detected in Turkey.

The Ethics Approval Numbered: 10840098-604.01.01-E14678 of the study was received from the Ethics Committee of Istanbul Medipol University.

### Survey instrument

At the time of pandemic, the demographic data such as age gender, marital status, occupation, city of residence, having a child, accompanying comorbidities and employment conditions were collected. The staff were asked the questions such as "Did you work in COVID-19 unit in the recent one month? "if yes, how many days did you work in COVID-19 unit in the recent one month?".

Concern about COVID-19, concern about infect COVID-19 to relatives, knowledge level about COVID-19, unwillingness to work, frequency of social media use about COVID-19, sleep disturbances and experiencing somatic symptoms were measured using a likert scale in a range between 1 and 5.

GAD-7 is a short and self-report test that was developed in accordance with DSM-IV-TR to evaluate the generalized anxiety disorder (Spitzer et al. 2006). It is a paper-and-pencil type assessment that involves a 7-item 4-likert scale (0=never, 1=several days, 2= more than half of the days and 3= nearly every day) that assesses the livings in terms of the scale items within the recent two weeks. Total points of 5, 10 and 15 gained in the scale are the cutoff points of mild, moderate and serious anxiety, respectively. The diagnosis of GAD should be evaluted and confirmed using other diagnostic methods in the patients with total points $\geq$ 10. The adaptation, validity and reliability analyses of the scale for Turkish Version was performed (Konkan et al. 2013). Reliability analysis was performed to determine internal consistency of the survey items to test the reliability of the scale and Cronbach's Alpha coefficient was calculated. Cronbach's Alpha value was 0.852 for total score of GAD-7. The obtained results indicated the reliability of the scale. On the other hand, Cronbach's Alpha value was .907 of the GAD-7 scale we applied.

The Coping Styles Scale Brief Form was developed by Carver (1997) as the short form of the scale named Coping Styles (COPE) that was developed by Carver et al. (1989) to assess the different behaviors against the stressful conditions. The Coping Styles Brief Form involves 28 expressions that can be divided into 14 subscales about the different coping methods. The responses for each item were weighted between 1 and 4 points such that 1= I haven't been doing this at all; 2= I've been

doing this a little bit; 3= I've been doing this a medium amount; 4= I've been doing this a lot. The raw score that can be gained from each subscale ranges between 2 and 8 points. The adaptation analysis of the scale for Turkish Version were performed by Bacanlı et al. (2013). Turkish Version of the Brief-COPE questionnaire was used to analyze the adopted coping strategies.

### Statistical analyses

Data were analyzed using SPSS Version 22 (SPSS V.22.0). We performed primarily univariate analyses to investigate the relationships between anxiety symptoms, coping styles and the related factors using Mann-Whitney-U Test, Kruskal Wallis Test and Chi-Square Test or Spearman's Correlation Test in addition to the descriptive statistics. Subsequently, we performed binary logistic regression analysis to determine the contribution of the related predictors to our categorical independent variables such as significant clinical anxiety and non-significant clinical anxiety. Subsequently, we performed binary logistic regression analysis to determine the contribution of the related predictors to our categorical independent variables such as significant clinical anxiety and non-significant clinical anxiety according to GAD-7 Scale. For this purpose, the factors that indicated statistical significance with a p value $< 0.05$ in the univariate analyses of significant clinical anxiety were included in the regression analysis as an independent variable. All analyses were two-tailed with alpha set at $p<0.05$.

## RESULTS

### The characteristics of the participants

The study included 544 people. Mean age was 36.85±10.46 years. The female population was greater than the male population in the study (63.2% vs 36.8%). Of the participants; 50.6%, 42.5% and 7% were doctor, nurse and other healthcare workers, respectively. 35.3% of the participants were single and 64.7% were married. The proportion of those living in Istanbul was 25.6%, the rate of those living in other metropolises was 58.3%, and the rate of those living in other cities was 16.2%. 60.7% of them had children, 39.3% had no children. Approximately one-fifth (21%) of the participants had a comorbid medical condition. The average work experience of the healthcare personnel was 13.81 (±10.40) years. About two-thirds (64%) of the employees worked in the pandemic hospital, while close to half (46%) had worked in the COVID-19 unit in the past month. In the past month, the average working time in the COVID-19 unit was 5.49 (±7.72) days.

The frequency and levels evaluated on a 1 to 5 Likert-type scale were as follows (mean (SD)); Concern about COVID-19: 3.48 (0.99), Concern about infect COVID-19 to relatives: 3.90 (0.97), Unwillingness to work: 2.63 (1.29), Frequency of use media about COVID-19: 3.49 (1.13), Sleep disturbances: 2.63 (1.26), Experiencing somatic symptoms 1.97 (1.10), Knowledge level about COVID-19: 3.63 (0.79).

### The results of the GAD-7 Scale

The average GAD-7 score of the participants was 6.52 (4.89). Minimal, mild, moderate and severe anxiety were present in 214 (39.3%), 191 (35.1%), 95 (17.5%) and 44 (8.1%) of all the study participants, respectively. GAD-7 score was $\geq 10$ indicating significant clinical anxiety and a need for clinical evaluation with respect to generalized anxiety disorder (GAD) in 139 (25.6%) of the participants.

### Results of the Brief Cope Questionnaire

The mean (SD) scores of the coping strategies were as follows. Problem-focused coping strategies; using instrumental social support: 6.28 (1.35), suppression of competing activities: 5.41 (1.27), restraint coping: 5.07 (1.27), planning: 6.60 (1.31). Emotional-focused coping strategies; humor: 4.68 (1.65), acceptance: 6.40 (1.39), turning to religion: 6.37 (1.83), positive reinterpretation: 5.95 (1.46), using emotional social support: 5.22 (1.30). Ineffektive coping strategies; denial: 3.8 (1.27), behavioral disengagement: 3.35 (1.32), mental disengagement: 4.87 (1.47), focus on and venting of emotions: 5.16 (1.48), substance use 2.54 (1.12).

According to our results, the doctors adopted humor as a coping strategy significantly more than the nurses and other healthcare staff ($p<0.05$). The nurses adopted focus on and venting of motions significantly more than other healthcare staff in coping ($p<0.05$). In addition, the doctors ($p<0.001$) and the nurses were extremely significantly more likely to use substance in coping than other healthcare staff ($p<0.01$). The orientation to religion was extremely significantly higher in the nurses and other healthcare staff compared with the doctors ($p<0.0001$). Besides, other healthcare staff adopted orientation to religion significantly higher than the nurses ($p<0.05$). Denial was significantly more adopted in the doctors than the nurses ($p<0.05$). The doctors adopted behavioral disengagement significantly higher than other healthcare staff ($p<0.05$). Contrarily, other healthcare staff adopted restraint coping significantly higher than the doctors in coping ($p<0.05$) (Table 1).

The healthcare staff that worked in the COVID-19 unit adopted humor, substance use and using emotional social support significantly more than the healthcare staff that did not work in the COVID-19 unit ($p<0.05$). Contrarily, the healthcare staff that did not work in the COVID-19 unit adopted planning significantly more than the healthcare staff worked in the COVID-19 unit ($p<0.05$) (Table 1).

**Table 1.** Coping strategies of health care workers

| | Mean (SD) | | | | Mean (SD) | | |
|---|---|---|---|---|---|---|---|
| | Doctor (n=275) | Nurse (n=231) | Other staff (n=38) | Statistic (p value) | Staff working in COVID-19 unit (last month) (n=250) | Staff not working in COVID-19 unit (last month) (n=294) | Statistic (p value) |
| Problem focused coping | | | | | | | |
| Using Instrumental Social Support | 6.34 (1.39) | 6.21 (1.316) | 6.32 (1.23) | 0.410 | 6.27 (1.26) | 6.29 (1.42) | 0.560 |
| Suppression of Competing Activities | 5.40 (1.22) | 5.45 (1.28) | 5.21 (1.55) | 0.556 | 5.38 (1.28) | 5.44 (1.27) | 0.365 |
| Restraint Coping | 4.96 (1.24) | 5.11 (1.30) | 5.53 (1.29) | <0.05 (doctor vs. other staff <0.05) | 4.99 (1.30) | 5.13 (1.25) | 0.219 |
| Planning | 6.63 (1.30) | 6.54 (1.35) | 6.76 (1.15) | 0.643 | 6.45 (1.34) | 6.72 (1.26) | <0.05 |
| Emotional focused coping | | | | | | | |
| Humor | 4.89 (1.61) | 4.55 (1.68) | 4.03 (1.59) | <0.01 (doctor vs. nurse <0.05) (doctor vs. other staff <0.05) | 4.84 (1.72) | 4.55 (1.59) | <0.05 |
| Acceptance | 6.38 (1.39) | 6.44 (1.32) | 6.32 (1.76) | 0.945 | 6.44 (1.43) | 6.36 (1.35) | 0.314 |
| Turning to Religion | 5.95 (1.98) | 6.70 (1.62) | 7.39 (1.08) | <0.0001 (doctor vs. nurse <0.0001) (doctor vs. other staff <0.0001) (nurse vs other staff <0.05) | 6.38 (1.87) | 6.36 (1.81) | .0786 |
| Positive Reinterpretation | 5.94 (1.45) | 5.93 (1.49) | 6.18 (1.35) | 0.725 | 5.96 (1.46) | 5.95 (1.46) | 0.889 |
| Using Emotional Social Support | 5.20 (1.30) | 5.23 (1.30) | 5.37 (1.36) | 0.756 | 5.36 (1.27) | 5.11 (1.32) | <0.05 |
| Ineffektive coping | | | | | | | |
| Denial | 3.49 (1.26) | 3.20 (1.21) | 3.61 (1.57) | <0.05 (doctor vs. nurse <0.01) | 3.47 (1.38) | 3.30 (1.17) | 0.342 |
| Behavioral Disengagement | 3.40 (1.29) | 3.36 (1.35) | 2.97 (1.31) | 0.091 (doctor vs. other staff <0.05) | 3.41 (1.36) | 3.30 (1.28) | 0.394 |
| Mental Disengagement | 4.88 (1.47) | 4.87 (1.49) | 4.74 (1.48) | 0.696 | 4.90 (1.51) | 4.84 (1.44) | 0.744 |
| Focus on and Venting of Emotions | 5.15 (1.52) | 5.26 (1.40) | 4.58 (1.62) | <0.05 (nurse vs other staff<.05) | 5.08 (1.52) | 5.22 (1.44) | 0.156 |
| Substance Use | 2.67 (1.24) | 2.47 (1.02) | 2.05 (0.32) | <0.01 (doctor vs. other staff <0.001) (nurse vs other staff <0.01) | 2.66 (1.23) | 2.45 (1.01) | <0.05 |

## The factors associated with anxiety in the overall sampling group

The findings of univariate analysis for “significant clinical anxiety” in the overall sampling group were presented in Tables 2a and 2b.

Socio-demographic factors associated with “significant clinical anxiety” in the univariate analysis were female gender and occupation. Post-hoc analyses revealed that the anxiety scores for being nurse were higher than being doctor. The working duration in COVID-19 unit (last month) showed a weak, however, significant relation ($r=0.140$; $p<0.01$). Whereas, age, marital status, city of residence, having a child, having comorbid medical diseases, working in a pandemic hospital, working in COVID-19 unit, duration of working in the COVID unit and work experience were not significantly correlated with significant clinical anxiety.

The review of the clinical data demonstrated that concern about COVID-19, Concern about infection of COVID-19 to relatives, unwillingness to work, frequency of social media use about COVID-19, sleep disturbance and experiencing somatic symptoms were monitored extremely significantly higher in those with significant clinical anxiety than those without this clinical condition. The knowledge level about COVID-19 was not found associated with significant clinical anxiety.

Focus on and venting of emotions, behavioral disengagement, mental disengagement ve using emotional social support were determined to be extremely significantly more adopted in the healthcare workers with “significant clinical anxiety” than those without this clinical condition. Contrarily, Positive reinterpretation and planning strategies were extremely significantly more adopted in the healthcare workers without significant clinical anxiety. On the other hand, using instrumental social support, humor, substance use, acceptance, suppression of competing activities, turning to religion, denial and restraint coping were not significantly correlated with significant clinical anxiety.

Independent variables were selected from the factors that were found associated with “significant clinical anxiety” by the univariate analysis. A binary logistic regression analysis was conducted to ascertain the independent impacts of gender, occupation, frequency of social media use about COVID-19, sleep disturbance, focus on and venting of emotions, behavioral disengagement, mental disengagement, positive reinterpretation, using emotional social support and planning on “significant clinical anxiety” (GAD-7 score≥10).

**Table 2a.** Factors associated with "significant clinical anxiety”

| | GAD-7 score <10 | GAD-7 score ≥10, indicated significant clinical anxiety | qi-square test, p value |
|---|---|---|---|
| Gender | | | <0.0001 |
| Female | 237 (%68.9) | 107 (%31.1) | |
| Male | 168 (%84) | 32 (%16) | |
| Marital status | | | 0.529 |
| No married | 146 (%76) | 46 (%24) | |
| Married | 259 (%73.6) | 93 (%26.4) | |
| Occupation | | 55 (%20) | <0.01 |
| Doctor | 220 (%80) | 75 (%32.5) | |
| Nurse | 156 (%67.5) | 9 (%23.7) | |
| Other | 29 (%76.3) | | |
| City of residence | | | 0.545 |
| İstanbul | 108 (%77.7) | 31 (%22.3) | |
| Other Metropolises | 234 (%73.8) | 83 (%26.2) | |
| Other Cities | 63 (%71.6) | 25 (%, 28.4) | |
| Having a child | | | 0.459 |
| Evet | 242 (%73.3) | 88 (%26.7) | |
| Hayır | 163 (%76.2) | 51 (%23.8) | |
| Medical comorbidity | | | 0.651 |
| Yes | 83 (%72.8) | 31 (%27.2) | |
| No | 322 (%74.9) | 108 (%25.1) | |
| Working in pandemia hospital | | | 0.213 |
| Yes | 253 (%72.7) | 95 (%27.3) | |
| No | 152 (%77.6) | 44 (%22.4) | |
| Working with COVID-19 patients | | | 0.676 |
| Yes | 184 (%73.6) | 66 (%26.4) | |
| No | 221 (%75.2) | 73 (%24.8) | |

**Table 2b.** Factors associated with "significant clinical anxiety"

| | GAD-7 score < 10 | GAD-7 score ≥10, indicated significant clinical anxiety | Statistic | Mann-Witney u Test, p value |
|---|---|---|---|---|
| Age | 37.11 (10.66) | 36.09 (9.86) | Z= -.792 | 0.428 |
| Work experience (years) | 13.99 (10.53) | 13.27 (10.03) | Z= -.659 | 0.510 |
| Duration of study with COVID-19 unit for last month (days) | 5.13 (7.48) | 6.53 (8.32) | Z= -1.124 | 0.261 |
| Concern about COVID-191 | 3.25 (0.93) | 4.14 (.89) | Z= -9.104 | <0.0001 |
| Concern about infect COVID-19 to relatives? | 3.71 (0.95) | 4.43 (.82) | Z= -9.104 | <0.0001 |
| Unwillingness to work | 2.37 (1.19) | 3.43 (1.24) | Z= -8.285 | <0.0001 |
| Frequency of use media about-19 | 3.36 (1.10) | 3.87 (1.11) | Z= -4.882 | <0.0001 |
| Sleep problem | 2.31 (1.15) | 3.58 (1.11) | Z= -10.058 | <0.0001 |
| Experiencing somatic symptoms | 1.67 (.87) | 2.82 (1.25) | Z= -9.602 | <0.0001 |
| Knowledge level about COVID-19 | 3.65 (.76) | 3.59 (.89) | Z= -0.597 | 0.550 |
| Problem focused coping | | | | |
| Using Instrumental Social Support | 6.27 (1.37) | 6.30 (1.27) | Z=-0.003 | 0.998 |
| Suppression of Competing Activities | 5.36 (1.27) | 5.56 (1.28) | Z=-1.301 | 0.193 |
| Restraint Coping | 5.04 (1.27) | 5.14 (1.27) | Z=-0.418 | 0.676 |
| Planning | 6.69 (1.27) | 6.32 (1.38) | Z=-2.733 | <0.001 |
| Emotional focused coping | | | | |
| Humor | 4.72 (1.64) | 4.59 (1.68) | Z=-0.890 | 0.373 |
| Acceptance | 6.37 (1.4) | 6.50 (1.32) | Z=-0.907 | 0.365 |
| Turning to Religion | 6.27 (1.91) | 6.65 (1.56) | Z=-1.422 | 0.155 |
| Positive Reinterpretation | 6.06 (1.44) | 5.63 (1.46) | Z=-2.991 | <0.01 |
| Using Emotional Social Support | 5.13 (1.29) | 5.49 (1.32) | Z=-2.631 | <0.01 |
| Ineffektive coping | | | | |
| Denial | 3.40 (1.25) | 3.31 (1.32) | Z=-1.001 | 0.317 |
| Behavioral Disengagement | 3.20 (1.24) | 3.80 (1.43) | Z=-4.489 | <0.0001 |
| Mental Disengagement | 4.78 (1.46) | 5.12 (1.50) | Z=-2.682 | <0.01 |
| Focus on and Venting of Emotions | 5.01 (1.48) | 5.59 (1.41) | Z=-4.241 | <0.0001 |
| Substance Use | 2.50 (1.06) | 2.68 (1.26) | Z=-1.451 | 0.147 |

**Table 3.** Binary logistic regression analysis of factors influencing health workers' clinical anxiety (Nagelkerke R Square: 0.371, Hosmer and Leme show Test: 0.595)

| | B | S.E. | Wald | Df | Sig. | Exp(B) | 95.0% C.I.for EXP(B) |
|---|---|---|---|---|---|---|---|
| Frequency of use media about COVID-19 | 0.395 | 0.113 | 12.278 | 1 | *0.000* | 1.484 | 1.19-1.85 |
| Sleep problem | 0.883 | 0.113 | 60.926 | 1 | *0.000* | 2.419 | 1.938-3.020 |
| Focus on and Venting of Emotions | 0.044 | 0.089 | 0.248 | 1 | 0.619 | 1.045 | 0.878-1.245 |
| Behavioral Disengagement | 0.203 | 0.096 | 4.489 | 1 | *0.034* | 1.225 | 1.015-1.479 |
| Gender | Ref. | | | | | | |
| Female | | | | | | | |
| Male | -0.534 | 0.287 | 3.466 | 1 | 0.063 | 0.586 | 0.334-1.029 |
| Occupation | | | | | | | |
| Doctor | Ref. | | 1.406 | 2 | 0.495 | | |
| Nurse | 0.169 | 0.265 | 0.409 | 1 | 0.522 | 1.185 | 0.705-1.990 |
| Other health worker | 0.565 | 0.499 | 1.282 | 1 | 0.258 | 1.759 | 0.662-4.675 |
| Mental Disengagement | 0.076 | 0.086 | 0.776 | 1 | 0.378 | 1.079 | 0.911-1.278 |
| Positive Reinterpretation | -0.270 | 0.109 | 6.182 | 1 | 0.013 | 0.763 | 0.617-0.944 |
| Using Emotional Social Support | 0.212 | 0.103 | 4.226 | 1 | *0.040* | 1.236 | 1.010-1.512 |
| Planning | 0.002 | 0.121 | 0.000 | 1 | 0.990 | 1.002 | 0.789-1.271 |

Frequency of social media use about COVID-19, sleep disturbance, behavioral disengagement, positive reinterpretation using emotional social support were found independently associated with “significant clinical anxiety” (Table 3).

## DISCUSSION

The first confirmed case of COVID-19 pandemic was reported on 11th March, 2020. A rapid transformation and adaptation process was initiated in the

healthcare system of our country like the rest of the world and urgent steps were taken. For struggling with the present pandemic, polyclinics and wards of many hospitals were assigned for the COVID-19 patients. Some of the healthcare workers were employed to work in these unit.

Doubtlessly, this acute and unique crisis has an unavoidable impact on the healthcare workers. Our study that applied the GAD-7 Scale has confirmed the concerns about the psychological health of the healthcare workers and manifested the symptoms of the anxiety (mild to severe) and the presence of "significant clinical anxiety" (moderate to severe) in 60.7% and 25.7% of our healthcare workers next after the number of the cases reached the peak point in Turkey, respectively.

An online survey was conducted with the participation of 1090 healthcare professionals in Russia. According to the GAD-7 scale, 25.5% of the participants had moderate and severe anxiety levels (Mosolova et al. 2020). The symptoms of anxiety (mild to severe) were reported in 57.3% of the dental practitioners according to GAD-7 Scale in The Northern Italy (Consolo et al. 2020). Our results are consistent with these researches. In another study, the authors have reported anxiety in 44.6% of the 1,257 healthcare workers employed in the various hospitals of China according to GAD-7 Scale (Lai et al. 2020) The results of this study is partially consistent with our results. In our conclusion, this difference was resulting from the fact that the mentioned study was performed in the early stage of the pandemic before the highest number of the cases was not reached yet. A study that was carried out on the doctors in the initial stage of the pandemic in Turkey has reported the symptoms of anxiety in 51.6% of the doctors (Elbay et al. 2020). This result was partially consistent with our results, we attributed this partial difference to the facts that our study was conducted next after the number of the cases reached the peak point, inclusion of particularly nurses and other healthcare workers in the study that have a closer working interaction with the patients and that the assessment scale was different.

The studies conducted in Japan and Singapore have reported high levels of fear and anxiety in more than half of the healthcare workers who participated in the surveys before and during the SARS-CoV outbreaks (Koh et al. 2005, Imai et al. 2005).

The rate of "significant clinical anxiety" was significantly higher in the females. Besides, there was a difference between the occupations in terms of "significant clinical anxiety", being a nurse was associated with a higher level of anxiety compared with being a doctor. The higher level of anxiety in the nurses can be explained by the closer working interaction of nurses with the patients. Similarly with our results, Lai et al. (2020) have determined that female gender and nurses (compared with doctors) were associated with higher level of anxiety (GAD-7) in the healthcare workers employed in China during COVID-19 pandemic. Female gender was similarly associated with higher anxiety level also in the other studies conducted on the doctors and healthcare workers (Elbay et al. 2020, Rossi et al. 2020).

The prolonged duration of working in the COVID unit was associated with higher level of anxiety, however, was not found associated with "significant clinical anxiety". Similarly, increased weekly working hours was associated with increased anxiety in the frontline working doctors during COVID-19 outbreak (Elbay et al. 2020).

Age, marital status, city of residence, having a child, having comorbid medical diseases and working in a pandemic hospital, work experience and working in COVID-19 unit was not significantly correlated with "significant clinical anxiety". Consistently with out results, Liu et al. (2020) have detected no significant correlation between anxiety levels of the healthcare workers in terms of age, location and marital status. Elbay et al. (2020) have reported that being married and having a child were associated with lower anxiety level whereas young age and less occupational experience were associated with higher level of anxiety in the doctors.

Lai et al. (2020) have demonstrated in their study that the healthcare workers working in Wuhan, the pandemic center of China, higher level of anxiety than the other healthcare workers. In our study, the absence of a significant difference between Istanbul, other metropoles and other cities was probably resulting from the fact that our study was carried out next after the highest number of the cases were reached and that pandemic was spread country-wide in contrast to that study. Working in the frontline was not found correlated with anxiety level in the healthcare workers in Italy similarly with our study (Rossi et al. 2020). The fact that this study was performed next after the highest number of cases was reached was consistent with timing of our study. Contrarily, the studies of Lai et al. (2020) and Elbay et al. (2020) were performed in the initial period of the pandemic and a higher level of anxiety was determined in the frontline workers. We conclude that increased number of the cases and the fact that all the healthcare workers were under the same threatening circumstances cancelled the differences in terms of working position (frontline-non frontline). Consistent with our study, the presence of a comorbid medical disease was not found correlated with anxiety in a study carried out among doctors (Elbay et. al. 2020).

The review of the clinical data demonstrated that increased levels of concern about COVID-19, concern about infection of COVID-19 to relatives, unwillingness

to work, frequency of social media use about COVID-19, sleep disturbance and experiencing somatic symptoms were extremely significantly correlated with "significant clinical anxiety". The knowledge level about COVID-19 was not found associated with "significant clinical anxiety".

According to our likert grading of anxiety (1-5), healthcare staff had more anxiety for their relatives (3.90/5 (0.97)) related with the infection rather than feeling anxiety for themselves (3.48/5 (0.99)) about the infection. Similarly in another study based on 1-5 likert grading scale; healthcare staff were more anxious about transmitting infection to a family member (2.71/5 (1.22)) rather than being infected (2.57/5(1.10)) (Temsah et al. 2020).

According to the regression analysis, more frequent exposure to social media about COVID-19 in the healthcare workers may predict significant clinical anxiety. Similarly with our study, Gao et al. (2020) have demonstrated by the regression analysis that frequent exposure to media may increase anxiety than those with less exposure to media durin COVID-19 outbreak, similarly with our study. The intense visibility of the COVID-19 pandemic in the media increases the perception of personal health. In the present time, the information spreads faster and more comprehensively than the times of SARS pandemic in 2003, H1N1 influenza outbreak in 2009 or MERS-CoV pandemic in 2013-2015, this situation definitely exacerbates the sensations of fear, panic and stress in the community (Temsah et al. 2020, Gao et al. 2020, Mak et al. 2009).

In our study, 25.5% of the staff reported, often or always sleep disturbance. According to our regression analysis, increased frequency of sleep disturbance was effective on significant clinical anxiety as an independent variable. During the COVID-19 outbreak, Zhang et al. (2020) have stated that insomnia was present in 36% of the medical staff and in the regression analysis, identified that medical staff with insomnia had higher levels of anxiety according to GAD-7 Scale. The concerns among the healthcare workers may affect sleep quality through anxiety symptoms (Kirwan et al. 2017).

In our study, 29.1% of the HCWs reported unwillingness to work very much or exactly.A survey study carried on HCWs during SARS pandemic in Taiwan has demonstrated that 9% of the staff reported unwillingness to work or planning to resign. (Bai et.al. 2004) In a study, 15.3% of the healthcare workers have stated that they planned to rearrange working schedule or changing their working hours to avoid contact with the patients infected with Coronavirus (Temsah et al. 2020).

No significant correlation was found between knowledge level and anxiety level in the healthcare workers in our study. It has been declared in a study carried out about the influenza outbreak in England that the doctors will show a lower level of anxiety and higher level of positive attitude thanks to manage the patients solely when they have more knowledge about the contagious diseases (Cole 2006). Confirming our results; no significant correlation was determined between anxiety levels and knowledge level about coronavirus infection in two separate studies conducted on midwifery students (Sögüt et al. 2020) and general population during the COVID-19 pandemic period (Huang & Zhao 2020).

In our study, acceptance, positive reinterpretation, using instrumental social support, suppression of competing activities, turning to religion, using emotional social support and planning were the most commonly adopted coping styles by the doctors, nurses and other healthcare workers (mean>5) . Wong et al. (2005) have reported that acceptance, active coping, and positive framing were the most commonly adopted strategies among the doctors, nurses and HCAs (healthcare assistants) during SARS pandemic (mean>5).

According to our results, the doctors adopted humor an denial as a coping strategy significantly more than the nurses. The orientation to religion was extremely significantly higher in the nurses compared with the doctors. When coping strategies according to professions were examined in a study involving 124 healthcare workers from a hospital in Croatia, only escape-avoidance and positive reappraisal strategies were adopted more in nurses than doctors, no significant difference was observed in terms of other strategies. (Salopek-Žiha et al. 2020) We think that the different formats of the scales for coping strategies are the main factor in our findings being different.

We conclude that the healthcare workers with significant clinical anxiety are more likely to adopt 4 (from ineffective coping strategies; focus on and venting of emotions, behavioral disengagement, mental disengagement and from emotional focused coping strategies; using emotional social support) of 6 coping strategies. Furthermore, regression analysis demonstrated that higher adoption of using emotional social support and behavioral disengagement from those adopting strategies was the predictor of significant clinical anxiety. Using emotional social support is the attempt to receive sympathy or emotional support. Behavioral disengagement is the reduction in the personal effort to struggle with stress, moreover, lack of efforting to achieve the target.

In the light of the results, these four strategies were not appropriate for the healthcare workers. We should investigate the underlying reasons of these tendencies and develop novel strategies to encourage the healthcare staff to adopt more positive coping strategies.

Conversely, we consider that healthcare workers with siginificant clinical anxiety are less likely to adopt

other 2 coping strategies (positive reinterpretation and planning). Positive reinterpretation (one of the emotional-focused coping strategies) and planning (one of the problem-focused coping strategies) strategies may be effective in reducing anxiety. Planning is figuring out how to cope with stress and planning of a subject to plan personal efforts to cope with stress actively. Positive reinterpretation is to recreate the stressful condition with a positive projection. Furthermore, positive reinterpretation was a predictor as an independent factor in reducing significant clinical anxiety according to the regression analysis. We conclude that adoption of planning and particularly positive reinterpretationby the healthcare staff should be supported.

Similarly, Zhu et al. (2020) have reported that total score of positive coping showed negative correlation with total anxiety and depression score in the healthcare staff that worked in the frontline (doctors and nurses). The Simplified Coping Style Questionnaire (SCSQ) they have used in their study was partially different from the Brief-COPE Questionnaire we have used in our study. They have considered that a positive coping style is a protective factor for anxiety and depression and it may be helpful in resisting against the negative emotions (Zhu et al 2020).

Guo et al. (2020) have noted in their study on the Chinese adults during COVID-19 pandemic that higher use of problem-focused and cognitive coping behaviours may predict the reduction in the mental health problems, cognitive coping behaviours should be reconstituted and that cognitive behavioural treatments may be promising. It has been stated in some studies that problem-focused coping style relieves the symptoms of post-traumatic stress, depression and sleep disturbances while emotional-focused coping style may exacerbate the symptoms of mental diseases (Guo et al. 2020, Snyder et al. 2015).

We conclude that further studies related with healthcare workers in the pandemic periods because of the partial differences between the scales of coping strategies and results.

## Limitations

Our study has some limitations. The voluntary participation in the survey may have caused a selection bias. In addition, we applied a self-report survey to reach more participants as much as possible and to minimize face-to-face interviews in this emergency condition as well as evaluation of the psychological symptoms without diagnostic assessment of the mental health specialists. The evaluation of sleep disturbances was performed by likert-type (1-5) assessment questions instead of a structured interview. Despite the mentioned limitations, the results of the present study provides precious information related with psychological impacts of COVID-19 on the healthcare workers employed country-wide. As the most important point, this study will present contribution to the worldwide health authorities to minimize psychological impacts of the most serious pandemic of our age on the healthcare workers.

# CONCLUSION

Providing mental well-being of healthcare professionals is very crucial for sustainability of the healtcare services during our struggle with COVID-19.

Our results demonstrated that females and nurses are in the risk group and the should be closely monitored. The exposure to social media and increased sleep disturbance may lead to significant clinical anxiety as an independent factor. The adoption of behavioral disengagement or use of emotional social support as the coping strategies were found to havean aggravating impact on the clinical anxiety. On the other hand, adopting positive reinterpretation has a mitigating impact on the significant clinical anxiety as an independent factor.

***Acknowledgements:*** None.

***Conflict of interest:*** None to declare.

***Contribution of individual authors:***

Alperen Kılıç: Conception and design of the study, analysis and interpretation of data, drafting the article, writing, final approval of the version to be submitted.

Mehmet Buğrahan Gürcan: Acquisition of data, drafting the article, investigation, writing, editing, final approval of the version to be submitted.

Bekir Aktura: Acquisition of data, analysis and interpretation of data, drafting the article, investigation, resources , final approval of the version to be submitted.

Ahmet Rıza Şahin: Conception and design of the study, revising it critically for important intellectual content, supervision, editing, final approval of the version to be submitted.

Zekeriya Kökrek: Conception and design of the study, revising it critically for important intellectual content, supervision, final approval of the version to be submitted.

## References

1. *Askim Å, Gustad LT, Paulsen J, Reitan SK, Mehl A, Mohus RM, et al.: Anxiety and depression symptoms in a general population and future risk of bloodstream infection: the HUNT study. Psychosom Med 2018; 80:673-9*

2. Bacanlı H, Sürücü M & İlhan T: Başa çıkma stilleri ölçeği kısa formunun (BÇSÖ-KF) psikometrik özelliklerinin incelenmesi: geçerlik ve güvenirlik çalışması. Kuram ve Uygulamada Eğitim Bilimleri 2013; 13: 81-96
3. Bai Y, Lin CC, Lin CY, Chen JY, Chue CM & Chou P: Survey of stress reactions among health care workers involved with the SARS outbreak. Psychiatr Serv 2004; 55:1055-7
4. Bisson JI, Tavakoly B, Witteveen AB, Ajdukovic D, Jehel L, Johansen VJ et al.: TENTS guidelines: development of post-disaster psychosocial care guidelines through a Delphi process. Br J Psychiatry 2010; 196:69-74
5. Brug J, Aro AR, Oenema A, De Zwart O, Richardus JH & Bishop GD: SARS risk perception, knowledge, precautions, and information sources, the Netherlands. Emerg Infect Dis 2004; 10:1486
6. Carver CS: You want to measure coping but your protocol'too long: Consider the brief cope. Int J Behav Med 1997; 4:92
7. Carver CS, Scheier MF & Weintraub JK: Assessing coping strategies: a theoretically based approach. J Pers Soc Psychol 1989; 56:267
8. Cattaneo A & Riva MA: Stress-induced mechanisms in mental illness: a role for glucocorticoid signalling. J Steroid Biochem Mol Biol 2016; 160:169-74
9. Chan AO & Huak CY: Psychological impact of the 2003 severe acute respiratory syndrome outbreak on health care workers in a medium size regional general hospital in Singapore. Occup Med (Lond) 2004; 54:190-6
10. Cole A: Two thirds of doctors in UK say the NHS could not cope with bird flu epidemic. BMJ 2006; 333:674
11. Consolo U, Bellini P, Bencivenni D, Iani C & Checchi V: Epidemiological Aspects and Psychological Reactions to COVID-19 of Dental Practitioners in the Northern Italy Districts of Modena and Reggio Emilia. Int J Environ Res Public Health 2020; 17:3459
12. Elbay RY, Kurtulmuş A, Arpacıoğlu S & Karadere E: Depression, Anxiety, Stress Levels of Physicians and Associated Factors In COVID-19 Pandemics. Psychiatry Res 2020; 113130
13. Gao J, Zheng P, Jia Y, Chen H, Mao Y, Chen S et al.: Mental health problems and social media exposure during COVID-19 outbreak. PloS One 2020; 15:e0231924
14. Guo J, Feng XL, Wang XH & van IJzendoorn MH: Coping with COVID-19: Exposure to COVID-19 and Negative Impact on Livelihood Predict Elevated Mental Health Problems in Chinese Adults. Int J Environ Res Public Health 2020; 17:3857
15. Hammen C: Risk factors for depression: An autobiographical review. Annu Rev Clin Psychol 2018; 14:1-28
16. Huang Y & Zhao N: Generalized anxiety disorder, depressive symptoms and sleep quality during COVID-19 outbreak in China: a web-based cross-sectional survey. Psychiatry Res 2020; 112954
17. Hunter JC, Nguyen D, Aden B, Al Bandar Z, Al Dhaheri W, Elkheir KA et al.: Transmission of Middle East respiratory syndrome coronavirus infections in healthcare settings, Abu Dhabi. Emerg Infect Dis 2016; 22:647
18. Imai T, Takahashi K, Hoshuyama T, Hasegawa N, Lim MK & Koh D: SARS risk perceptions in healthcare workers, Japan. Emerg Infect Dis 2005; 11:404
19. Kirwan M, Pickett SM & Jarrett NL: Emotion regulation as a moderator between anxiety symptoms and insomnia symptom severity. Psychiatry Res 2017; 254:40-7
20. Koh D, Lim MK, Chia SE, Ko SM, Qian F, Ng V et al.: Risk Perception and Impact of Severe Acute Respiratory Syndrome (SARS) on Work and Personal Lives of Healthcare Workers in Singapore What Can We Learn?. Med Care 2005; 676-82
21. Konkan R, ŞENORMANCI Ö, Güçlü O, Aydin E & Sungur MZ: Yaygın Anksiyete Bozukluğu-7 (YAB-7) Testi Türkçe Uyarlaması, Geçerlik ve Güvenirliği. Noro Psikiyatr Ars 2013; 50
22. Lai J, Ma S, Wang Y, Cai Z, Hu J, Wei N et al.: Factors associated with mental health outcomes among health care workers exposed to coronavirus disease 2019. JAMA Netw Open 2020; 3:e203976-e203976
23. Liao YT, Hsieh MH, Yang YH, Wang YC, Tsai CS, Chen VCH et al.: Association between depression and enterovirus infection: A nationwide population-based cohort study. Medicine (Baltimore) 2017; 96
24. Liu CY, Yang YZ, Zhang XM, Xu X, Dou QL, Zhang WW et al.: The prevalence and influencing factors in anxiety in medical workers fighting COVID-19 in China: a cross-sectional survey. Epidemiol Infect 2020: 1-17
25. Mak IWC, Chu CM, Pan PC, Yiu MGC & Chan VL: Long-term psychiatric morbidities among SARS survivors. Gen Hosp Psychiatry 2009; 31:318-26
26. Maunder R: The experience of the 2003 SARS outbreak as a traumatic stress among frontline healthcare workers in Toronto: lessons learned. Philos Trans R Soc Lond B Biol Sci 2004; 359:1117-25
27. Ministry of Health: National public health events. 2020. (Accessed July 19, 2020). Available at: https://www.moh.gov.sa/en/CCC/Pages/default.aspx
28. Mosolova E, Chung S, Sosin D & Mosolov S. Stress and Anxiety among Healthcare Workers Associated with COVID-19 Pandemic in Russia. Psychiatr Danub 2020; 32:549-56
29. Nayeri Chegeni T, Sharif M, Sarvi S, Moosazadeh M, Montazeri M, Aghayan SA et al.: Is there any association between Toxoplasma gondii infection and depression? A systematic review and meta-analysis. PloS One 2019; 14,e0218524
30. Ploeg J, Markle-Reid M, Valaitis R, McAiney C, Duggleby W, Bartholomew A et al.: Web-based interventions to improve mental health, general caregiving outcomes, and general health for informal caregivers of adults with chronic conditions living in the community: rapid evidence review. J Med Internet Res 2017; 19: e263
31. Rossi R, Socci V, Pacitti F, Di Lorenzo G, Di Marco A, Siracusano A et al.: Mental Health Outcomes Among Frontline and Second-Line Health Care Workers During the Coronavirus Disease 2019 (COVID-19) Pandemic in Italy. JAMA Netw Open 2020; 3:e2010185-e2010185
32. Salopek-Žiha D, Hlavati M, Gvozdanović Z, Gašić M, Placento H, Jakić H, et al.: Differences in Distress and Coping with the COVID-19 Stressor in Nurses and Physicians. Psychiatr Danub 2020; 32:287-93
33. Spitzer RL, Kroenke K, Williams JB & Löwe B: A brief measure for assessing generalized anxiety disorder: the GAD-7. Arch Intern Med 2006; 166:1092-7
34. Sögüt S, Dolu İ & Cangöl E: The relationship between COVID-19 knowledge levels and anxiety states of

midwifery students during the outbreak: A cross-sectional web-based survey. Perspect Psychiatr Care 2020

35. Snyder CM, Fauth E, Wanzek J, Piercy KW, Norton MC, Corcoran C et al.: Dementia caregivers' coping strategies and their relationship to health and well-being: The Cache County Study. Aging Ment Health 2015; 19:390-9
36. Temsah MH, Al-Sohime F, Alamro N, Al-Eyadhy A, Al-Hasan K, Jamal A et al.: The psychological impact of COVID-19 pandemic on health care workers in a MERS-CoV endemic country. J Infect Public Health 2020
37. Wong TW, Yau JK, Chan CL, Kwong RS, Ho SM., Lau CC et al.: The psychological impact of severe acute respiratory syndrome outbreak on healthcare workers in emergency departments and how they cope. Eur J Emerg Med 2005; 12:13-8
38. World Health Organization: Coronavirus (COVID-19). 2020, April 4. (Accessed July 30, 2020). Available at: https://experience.arcgis.com/experience/685d0ace521648f8a5beeeee1b9125c
39. Zhang C, Yang L, Liu S, Ma S, Wang Y, Cai Z et al.: Survey of insomnia and related social psychological factors among medical staff involved in the 2019 novel coronavirus disease outbreak. Front Psychiatry 2020; 11:306
40. Zhu J, Sun L, Zhang L, Wang H, Fan A, Yang B et al.: Prevalence and Influencing Factors of Anxiety and Depression Symptoms in the First-Line Medical Staff Fighting Against COVID-19 in Gansu. Front Psychiatry 2020; 11

Correspondence:
Alperen Kılıç, MD
Istanbul Medipol University, Faculty of Medicine, Department of Psychiatry
TEM Avrupa Otoyolu Göztepe Çıkışı No: 1, 34214, Bağcılar, İstanbul, Turkey
E-mail: alperenkilic88@hotmail.com
