# Supplementary material for: Mental health status and related factors influencing healthcare workers during the COVID-19 pandemic: A systematic review and meta-analysis
Source: PLoS One. 2024 Jan 19;19(1):e0289454. doi: 10.1371/journal.pone.0289454 (PMC10798549; doi:10.1371/journal.pone.0289454)
Supplement: S1 Data — (ZIP) [file pone.0289454.s011.zip › literatures/67.pdf]

consultations for psychotic disorders, and of hospitalizations without consent, coupled with the significant decrease in primary psychiatric consultations. For the most severe psychiatric disorders, emergency consultations are more necessary, and the decrease is less important.

The development of telemedicine would also seem to have contributed to our results. The viability and feasibility of telemedicine consultations are likely to emerge subsequent to the COVID-19-triggered lockdown, possibly indicating a role for their sustained implementation. Finally, as some people may find new strengths and coping strategies during disasters, the current results may arise from an elevation in resilience capacity.

Overall, despite the expectation of lockdown-induced stress increasing relapse risk across psychiatric conditions, the numbers of patients seeking emergency psychiatric consultations have decreased during lockdown. Clearly, COVID-19 has had an impact on psychiatric service utilization and will continue to do so,[6] whilst also having possible implications for the nature of psychiatric service organization.

The data are available on request.

Acknowledgments

We want to thank Dr Yohann Dabi for his advice, Dr Andrei Szöke for his reviewing, and Dr George Anderson for his prompt editing work. No funding was secured for this study.

Disclosure statement

The authors have declared that there are no conflicts of interest in relation to the subject of this study.

References

1. Beutel ME, Klein EM, Brähler E *et al*. Loneliness in the general population: Prevalence, determinants and relations to mental health. *BMC Psychiatry* 2017; **17**: 97.
2. Courtet P, Olié E, Debien C, Vaiva G. Keep socially (but not physically) connected and carry on: Preventing suicide in the age of COVID-19. *J. Clin. Psychiatry* 2020; **81**: 20com13370.
3. Brooks SK, Webster RK, Smith LE *et al*. The psychological impact of quarantine and how to reduce it: Rapid review of the evidence. *Lancet* 2020; **395**: 912–920.
4. Rolland B, Haesebaert F, Zante E *et al*. Global changes and factors of increase in caloric/salty food, screen, and substance use, during the early COVID-19 containment phase in France: A general population online survey. *JMIR Public Health Surveill.* 2020. https://doi.org/10.2196/19630.
5. Fiorillo A, Gorwood P. The consequences of the COVID-19 pandemic on mental health and implications for clinical practice. *Eur. Psychiatry J. Assoc. Eur. Psychiatr.* 2020; **63**: e32.
6. Chevance A, Gourion D, Hoertel N *et al*. Ensuring mental health care during the SARS-CoV-2 epidemic in France: A narrative review. *L'Encephale* 2020; **46**: S3–S13.
7. Font H, Roelandt J-L, Behal H *et al*. Prevalence and predictors of no lifetime utilization of mental health treatment among people with mental disorders in France: Findings from the 'Mental Health in General Population' (MHGP) survey. *Soc. Psychiatry Psychiatr. Epidemiol.* 2018; **53**: 567–576.
8. Cao Y, Li Q, Chen J *et al*. Hospital emergency management plan during the COVID-19 epidemic. *Acad. Emerg. Med.* 2020; **27**: 309–311.
9. Thornton J. Covid-19: A&E visits in England fall by 25% in week after lockdown. *BMJ* 2020; **369**: m1401.
10. Hoot NR, Aronsky D. Systematic review of emergency department crowding: Causes, effects, and solutions. *Ann. Emerg. Med.* 2008; **52**: 126–136.

Supporting information

Additional Supporting Information may be found in the online version of this article at the publisher's web-site:

Appendix S1 Supporting information.

Baptiste Pignon, MD,[1,2,3,4] Raphaël Gourevitch, MD, PhD,[5] Sarah Tebeka, MD,[6,7,8] Caroline Dubertret, MD, PhD,[6,7,8] Hélène Cardot, MD,[6] Valérie Dauriac-Le Masson, MD,[9] Anne-Kristelle Trebalag, MD,[5] David Barruel, MD,[9] Liova Yon, MD,[5] François Hemery, MD,[10] Marie Loric, MD,[1] Corentin Rabu, MD,[1] Antoine Pelissolo, MD, PhD,[1,2,3,4] Marion Leboyer, MD, PhD,[1,2,3,4] Franck Schürhoff, MD, PhD[1,2,3,4] and Alexandra Pham-Scottez, MD, PhD[5]

[1]AP-HP, Department of Psychiatry and Addictology, Mondor University Hospital, DMU IMPACT, [2]Translational Neuro-Psychiatry Laboratory, INSERM U955, [3]FondaMental Foundation, [4]Paris-Est Créteil University (UPEC), Medical School, Créteil, [5]CPOA, Sainte-Anne Hospital, Paris Psychiatry & Neurosciences University Hospital, Paris, [6]Department of Psychiatry, AP-HP, Louis Mourier Hospital, Colombes, [7]INSERM U1266, [8]Paris University, [9]Department of Medical Information, Paris Psychiatry & Neurosciences University Hospital, Paris, and [10]AP-HP, Department of Medical Information, Mondor University Hospital, Créteil, France

Email: baptistepignon@yahoo.fr

Received 9 June 2020; revised 18 June 2020; accepted 25 June 2020.

Mental health of medical professionals during the COVID-19 pandemic in Togo

doi:10.1111/pcn.13108

The coronavirus (COVID-19) pandemic had spread quickly around the world. As of 18 May 2020, there are more than 4 million confirmed cases worldwide. The continuous and rapid spread of the COVID-19 pandemic has had psychological impact on the general population and especially on health-care professionals who have experienced extraordinary stress levels daily and high rates of psychiatric morbidity.[1]

The prevalence of mental health disorders related to COVID-19 is high in the general population and in health-care staff in many countries,[2] but little is known about this frequency in Africa where the number of infections is lower compared to frequencies reported in other continents. Findings of COVID-19 impact on mental health of medical staff are mainly done outside African countries, including Togo. To date (18 May 2020), we have had 330 confirmed cases with 106 recovered cases and 12 deaths due to COVID-19 in Togo. Due to this relatively small number of infections and morbidities in Togo, can we expect a low impact of COVID-19 on the mental health of Togolese medical professionals? The current research aims to examine the impact of mental health status on Togolese medical professionals during the COVID-19 pandemic. We hypothesize that Togolese medical staff may experience anxiety, depression, and psychological distress during the COVID-19 pandemic.

Sixty-two medical professionals (mainly doctors and nurses; mean age = 35.5 years, SD: 8.75 years, 56.5% women) from two Lomé medical centers at Togo's first university hospital (Sylvanus Olympio University Hospital) participated in the study. Participants completed three self-questionnaires, the seven-item Generalized Anxiety Disorder (GAD-7) Scale,[3] the nine-item Patient Health Questionnaire (PHQ-9),[4] and the nine-item Psychological Stress Measure (PSM-9),[5] to assess generalized anxiety symptoms, depression symptoms, and psychological distress, respectively. Participants were nurses (n = 20), doctors (n = 19), laboratory technicians (n = 6), and others (n = 17). The protocol for the research project was approved by the Ethics Committee of the Medico-Social Center of Adidogome and conformed to the provisions of the Declaration of Helsinki. All participants agreed to take part in this research and written informed consent was obtained from them.

During the COVID-19 pandemic, the proportions of health professionals with mild, moderate, and severe anxiety were 25.8%, 22.6%, and

14.5%, respectively; and their proportions with mild, moderate, moderately severe, and severe depression were 24.2%, 16.1%, 9.7%, and 1.6%, respectively. According to the total GAD-7 and PHQ-9 scores, women had higher scores than men for anxiety, $t(60) = 2.83$, $P < 0.01$, and for depression, $t(60) = 3.63$, $P < 0.01$. Nurses reported higher scores for depression symptoms than doctors but not other professionals, $F(3, 58) = 7.87$, $P < 0.01$. Participants with prior history of chronic somatic pathology, such as diabetes or high blood pressure, had significantly higher GAD-7 and PHQ-9 scores than those without history of somatic disease: $t(60) = 2.51$, $P < 0.05$ and $t(60) = 2.19$, $P < 0.01$, respectively. Among all participants, PSM-9 score was high and was significantly correlated with anxiety and depression symptoms: $r = 0.44$, $P < 0.01$ and $r = 0.67$, $P < 0.01$, respectively.

Togolese medical professionals have been experiencing extraordinarily high levels of psychological distress, anxiety, and depression during the COVID-19 pandemic. In our study, the anxiety rate was 62.9%, which is higher than the frequency reported by Liu and colloborators[6] in China. Previous research has found increased anxiety among medical workers during the Ebola pandemic in Liberia.[7] The depression frequency in our research was 51.6%, which is slightly higher than the findings of Liu and collaborators (50.7%).[6] Nurses reported a higher depression rate than doctors and this result is in line with previous research.[1]

Despite the relatively low infection rate of COVID-19 in Togo, medical professionals have reported high symptoms of anxiety, depression, and psychological distress. This could be explained by the poor-quality health-care system in Togo and a lack of adequate equipment to deal with the COVID-19 pandemic. For instance, many health professionals during the current study recommend the use of masks by the entire population as observed in some countries (China and South Korea) to deal with COVID-19.

In response to our study's findings, mental health care for Togolese medical professionals is recommended. This suggestion is essential, according to the findings of Kang and collaborators,[8] which suggest that mental health protection for medical workers is important for control of the COVID-19 pandemic and their own long-term health.

Togolese medical staff would benefit from mental health care to cope effectively with their stress and mental status during and after the COVID-19 pandemic according to suggestions from Chen *et al.*,[9] and lessons learnt during the Ebola outbreak in West Africa.[7] Considering lessons from China, using various kinds of mental health interventions for medical professionals, such as relaxation, cognitive therapies, and dance-based exercises,[10] is warranted.

Disclosure statement

The authors declare no conflict of interest.

References

1. Tsamakis K, Rizos E, Manolis AJ *et al.* COVID-19 pandemic and its impact on mental health of healthcare professionals. *Exp. Ther. Med.* 2020; **19**: 3451–3453.
2. Sani G, Janiri D, Di Nicola M, Janiri L, Ferretti S, Chieffo D. Mental health during and after the COVID-19 emergency in Italy. *Psychiatry Clin. Neurosci.* 2020; **74**: 372.
3. Spitzer RL, Kroenke K, Williams JB, Löwe B. A brief measure for assessing generalized anxiety disorder: The GAD-7. *Arch. Intern. Med.* 2006; **166**: 1092–1097.
4. Kroenke K, Spitzer RL, Williams JB. The PHQ-9: Validity of a brief depression severity measure. *J. Gen. Intern. Med.* 2001; **16**: 606–613.
5. Lemyre L, Tessier R. Measuring psychological stress: Concept, model, and measurement instrument in primary care research. *Can. Fam. Physician* 2003; **49**: 1159–1160.
6. Liu S, Yang L, Zhang C *et al.* Online mental health services in China during the COVID-19 outbreak. *Lancet Psychiatry* 2020; **18**: e17–e18.
7. Li L, Wan C, Ru Ding R *et al.* Mental distress among Liberian medical staff working at the China Ebola treatment unit: A cross sectional study. *Health Qual. Life Outcomes* 2015; **13**: 156.
8. Kang L, Li Y, Hu S *et al.* The mental health of medical workers in Wuhan, China dealing with the 2019 novel coronavirus. *Lancet Psychiatry* 2020; **7**: e14.
9. Chen Q, Liang M, Li Y *et al.* Mental health care for medical staff in China during the COVID-19 outbreak. *Lancet Psychiatry* 2020; **7**: e15–e16.
10. Yao H, Chen JH, Zhao M *et al.* Mitigating mental health consequences during the COVID-19 outbreak: Lessons from China. *Psychiatry Clin. Neurosci.* 2020; **74**: 407–408.

Kossi Blewussi Kounou, PhD,[1,2] Koffi Mawuse Guédénon, MD,[3] Ayoko Akouavi Dogbe Foli, PhDc[2,4] and Eric Gnassounou-Akpa, MD[5]

[1]University of Lomé, Research Team in Psychology, Orientation and Development, [2]Center of Psychotraumatology and Brief Therapies – Training and Research, [3]University of Lomé, Sylvanus Olympio University Hospital, Pediatrics Unit, Lomé, Togo, [4]Toulouse - Jean Jaurès University, Socialization Psychology Laboratory - Development and Work, Toulouse, France, and [5]Medico-Social Center of Adidogome, Lomé, Togo

Email: benkounou@hotmail.fr

Received 24 May 2020; revised 18 June 2020; accepted 29 June 2020.

Attitude to telemedicine in the times of COVID-19 pandemic: Opinion of medical practitioners from India

doi:10.1111/pcn.13109

The COVID-19 pandemic has brought forth a renewed focus on the role and relevance of telemedicine services.[1–3] With lockdown putting constraints on travel, and emphasis on social distancing to prevent the spread of the virus, telemedicine seems to be an appealing option for patients and care providers for medical consultations.[4–6] While telemedicine has already made headway among the more developed nations, it is probably yet to find a firm footing in resource-constrained countries.[7,8] Yet, the pandemic has provided an opportunity and a need to rapidly expand telemedicine services in developing countries. We conducted an online survey of medical professionals from India (with a sizeable proportion of psychiatrists) to understand their perspectives about telemedicine and its future in the country.

An online survey was created on Google Forms and circulated via snowball sampling through social media. The questionnaire comprised of agreement to consent, information about limited demographic information, work profile, whether participants had used telemedicine in the past, and their opinions about various facets related to telemedicine. A total of 221 responses were logged in between 12 and 21 April 2020, out of which five participants did not consent. Eight more participants were excluded as they were not practicing doctors, leading to a total figure of 208 analyzed responses. Analysis was done using SPSS Version 21 (IBM, Armonk, NY, USA).

The responses are presented in Table 1. The sample comprised largely of resident doctors, and almost half were postgraduates. The results reveal that most of the participants had used telemedicine in some form, but had not confirmed the patient's identity or documented the consultation. Such respondents also tended towards considering it easy to understand the patient's problems, having had a good experience with digital consultation, and were likely to recommend it to colleagues. Respondents largely agreed that telemedicine would help quality health care reach remote areas of the country, and reduce budgets and waiting times for the patient; however, they also cautioned that telemedicine could: (i) be a hindrance to direct clinical decision-making and treatment; (ii) be a hindrance to patient–doctor relationships and trust; and (iii) result in vulnerability of digitally recorded personal information. Respondents were largely skeptical about the possibility of telemedicine significantly replacing the current patient-care system over the next 5 years.
