# Supplementary material for: Mental health status and related factors influencing healthcare workers during the COVID-19 pandemic: A systematic review and meta-analysis
Source: PLoS One. 2024 Jan 19;19(1):e0289454. doi: 10.1371/journal.pone.0289454 (PMC10798549; doi:10.1371/journal.pone.0289454)
Supplement: S1 Data — (ZIP) [file 124.pdf › literatures/124.pdf]

http://ijhpm.com
Int J Health Policy Manag 2020, 9(11), 496–497
doi 10.34172/ijhpm.2020.107
IJHPM
International Journal of Health Policy and Management

Letter to Editor

Peruvian Guideline to Care the Mental Health of Health Providers During COVID-19 Pandemic

Carlos Orellano*, **Marco Macavilca**

***Correspondence to:** Carlos Orellano, Email: carlos.orellano@upch.pe
Copyright: © 2020 The Author(s); Published by Kerman University of Medical Sciences. This is an open-access article distributed under the terms of the Creative Commons Attribution License (http://creativecommons.org/licenses/by/4.0), which permits unrestricted use, distribution, and reproduction in any medium, provided the original work is properly cited.
Citation: Orellano C, Macavilca M. Peruvian guideline to care the mental health of health providers during COVID-19 pandemic. *Int J Health Policy Manag.* 2020;9(11):496–497. doi:10.34172/ijhpm.2020.107
Received: 27 May 2020; Accepted: 20 June 2020; ePublished: 27 June 2020

Dear Editor,

The coronavirus disease 2019 (COVID-19) outbreak has risen as a worldwide health crisis and health providers (HPs) are in first-line to face this disease. In consequence, this vulnerable occupational group is experiencing many mental health problems. One study found that anxiety was higher in female staff and nurses from China.[1] Additionally, a recent review (five articles from China and one from India) showed that HPs develops stress, anxiety, depression and insomnia.[2]

On March 6, 2020, the Peruvian government reported the first case of COVID-19 and set up as strategies a national quarantine (one of the earliest and strictest in Latin America) and the acquisition of diagnostic tests and mechanical ventilators in order to avoid the collapse of the fragile healthcare system. Public health only receives 3.2% of the gross domestic product although of two decades of economic growth. This poor investment is reflected in the small number of hospital and intensive care unit beds (16 and 0.2 per 10 000 population, respectively) and lack of resources.

Later, on April 6, the Ministry of Health published the "Technical Guideline to care mental health of HPs in the COVID-19 context,"[3] for its application in every health facilities where patients with this disease receive attention. It has been realised in time of Peruvian mental health reform,[4] which promote a transitional framework of public mental health, from tertiary care (psychiatric hospitals) to secondary and primary care (general hospitals and community mental health centres, respectively).

This guideline aims to establish procedures for mental healthcare and self-care of HPs who attend patients with suspected or confirmed COVID-19 throughout the creation of the "Psychosocial Support Team for Health Providers" (PSTHP) in health facilities; composed of a physician (preferably a psychiatrist), a psychologist, a nurse, and a social worker. This guideline postulates four specific provisions: mental healthcare, self-care, identification of mental health problems, and intervention and recovery.

The first provision states basic principles for a good mental health: employment stability, comfortable infrastructure, availability of biosecurity materials, healthy diet, active pauses, healthy rests, alternation between high and low stressful work, work organization, reasonable work time (avoiding work overtime), easy access to health services for HPs and their families, and identification of psychosocial risks. It also includes some strategies to enhance resilience: group sessions for emotional self-regulation, informative sessions about biosecurity and mental healthcare, availability of informative booklets, promotion of companionship and solidarity at work, telephone helplines, familiar and social support, and management of complains and feedback.

The second provision is related to self-care for HPs, which is basic in any model of medical care. It recommends to communicate assertively, apply emotional regulation strategies, attend basic needs and use healthy coping strategies, plan leisure activities, keep communication with relatives, maintain updated, limit exposition to media (including instant massaging), be conscious of emotions and physical symptoms, recognize colleagues, and seek help when necessary.

The third provision emphasize the priority of dismiss mental health problems in HPs. Apart of observing changes in behaviour, it recommends the use of screening tests in group or individual sessions to detect probable cases with mental health problems and corroborate whether correspond to an acute stress, adaptative or anxiety disorders, depressive episode, battered or burnout syndromes.

The last provision specifies that each HP with a mental health problem will receive individual psychosocial support by the PSTHP. Mild cases will continue with mental healthcare and self-care interventions. Instead, moderate and severe cases will receive an individualized intervention plan that consist in activities such as psychological first aids, relaxation techniques, brief psychotherapy and assertiveness training. If any of moderate/severe cases requires a more complex intervention, it will be referred to a specialized mental health service.

The early use of this document will benefit mental health of

Alberto Hurtado School of Medicine, Universidad Peruana Cayetano Heredia, Lima, Peru.

HPs, preventing the exacerbation of mental health problems and keeping the case-resolving capacity of healthcare services during the pandemic. But also, its application will deal with unresolved major challenges[5]: an enormous deficit in mental health coverage (between 75% and 85%) with a lack of specialized professionals who are fully concentrated in the capital and other largest cities.

Ethical issues
Not applicable.

Competing interests
Authors declare that they have no competing interests.

Authors' contributions
CO contributed with the conception of the work, revised it and approved the final version. MM contributed with the design of the work, drafted it and approved the final version.

References

1. Huang JZ, Han MF, Luo TD, Ren AK, Zhou XP. [Mental health survey of 230 medical staff in a tertiary infectious disease hospital for COVID-19]. Zhonghua Lao Dong Wei Sheng Zhi Ye Bing Za Zhi. 2020;38(0):E001. doi:10.3760/cma.j.cn121094-20200219-00063
2. Spoorthy MS. Mental health problems faced by healthcare workers due to the COVID-19 pandemic - a review. *Asian J Psychiatr.* 2020;51:102119. doi:10.1016/j.ajp.2020.102119
3. Ministerio de Salud. *Guía Técnica Para El Cuidado de La Salud Mental Del Personal de La Salud En El Contexto Del COVID-19.* Lima: Ministerio de Salud; 2020.
4. Orellano C, Macavilca M. New guidelines on mental health in Peru. *Lancet Psychiatry.* 2019;6(3):201-202. doi:10.1016/S2215-0366(19)30042-2
5. Diez-Canseco F, Ipince A, Toyama M, et al. Atendiendo la salud mental de las personas con enfermedades crónicas no transmisibles en el Perú: Retos y oportunidades para la integración de cuidados en el primer nivel de atención. *Rev Peru Med Exp Salud Publica*. 2014;31(1):131-136.
