# Supplementary material for: Mental health status and related factors influencing healthcare workers during the COVID-19 pandemic: A systematic review and meta-analysis
Source: PLoS One. 2024 Jan 19;19(1):e0289454. doi: 10.1371/journal.pone.0289454 (PMC10798549; doi:10.1371/journal.pone.0289454)
Supplement: S1 Data — (ZIP) [file 168.pdf › literatures/168.pdf]

Received: 8 June 2020 | Revised: 15 June 2020 | Accepted: 17 June 2020
DOI: 10.1111/dth.13884

LETTER

DERMATOLOGIC THERAPY WILEY

Mental health assessment of frontline COVID-19 dermatologists: A Pan-Indian multicentric cross-sectional study

Dear Editor,

Following the outbreak of COVID-19 pandemic, health care workers are involved in screening, investigations, admission, ward rounds, and even performing life-resuscitating procedures.[1] Dermatology residents, young faculties, and consultants are not used to handling long hours of duty and stressful environment.[2] We evaluated the mental health status of front-line dermatologists combating the pandemic.

This was a cross-sectional, observational study. Dermatologists engaged in direct patient contact through clinical services at outpatient department, designated COVID wards, and fever clinics were included. Those involved in administrative duties and other departmental engagements were excluded. An online semistructured questionnaire was prepared on Google-forms platform and the link was circulated among the dermatologists (postgraduate trainees, senior residents, and consultants) through emails and social media (snowball sampling). It started from 13 May 2020 9 AM and closed on 20 May 2020 at 9 AM. The questionnaire, restricted to a single response from each participant, comprised of three sections: first section consisted of demographic details (age, gender, qualification, working hours, current residence, and place of posting). Second section consisted of Patient Health Questionnaire (PHQ-9) for depression and third section comprised of Perceived Stress Scale. Continuous variables were analyzed using unpaired *t* test and categorical data by chi-square test. Statistical software Medcalc version 10.2.0.0 for Windows vista was used.

Out of 49 responses received, 8 were excluded, based on exclusion criteria. The demographics of study participants skewed toward a slight male distribution (58.5%). The other details are described in Table 1.

Eleven (26.82%) doctors were depressed, among which six (54.4%) participants were suffering from mild depression. Moderate, moderately severe, and severe depression were encountered in two (18.2%), two (18.2%), and one (9.1%) informants, respectively. Mean depression score was found to be 5.24 ± 3.02. Among the participants with moderate to severe depression, significant association ($P < .05$) was found with females ($P = .02$), age >30 years ($P = .03$), and those staying away from family ($P = .03$). Out of the total 41 study participants, stress was depicted in 12 (29.2%) doctors, of which stress levels were moderate in 11 (91.7%) and severe in 1 (8.3%) of participants. Mean perceived stress score was found to be 16.38 ± 7.93. Higher perceived stress scores were seen in females, long working hours, working in COVID-positive wards, and participants staying away from family; however, these were not found to be statistically significant.

Our results revealed a 26.82% and 29.2% prevalence of symptoms of depression and stress, respectively, among frontline COVID-19 dermatologists. A multicenter survey involving 1563 Chinese medical staff revealed depression in 50.7% and 73.4% developed stress related symptoms respectively.[3] Results of a recently published study from Wuhan suggested half of the 1257 health workers reporting symptoms of depression.[4] In an Indian study involving 152 doctors, 34.9% were found to be depressed and 32.9% were experiencing stress.[5] Another recent Indian study concluded that dermatologists too developed high stress, with significant association with females and unmarried individuals, due to the current situation

TABLE 1 Study parameters and their relation to depression and stress

Parameters (frequency; n = 41)	Depression (n = 11)	Stress (n = 12)
Age (years)	$P = .03$*	$P = .865$
<26 (2)	0	1
26-30 (30)	4	8
>30 (9)	7	3
Sex	$P = .02$*	$P = .306$
Male (24)	2	5
Female (17)	9	7
Designation	$P = .05$	$P = .605$
Postgraduate trainees (21)	10	8
Senior residents (12)	0	2
Consultants (8)	1	2
Work hours (in hours)	$P = .821$	$P = .620$
<6 (8)	3	2
6-12 (30)	7	8
>12 (3)	1	2
Work postings	$P = .899$	$P = .229$
Out-patient department (13)	3	3
Fever clinic (23)	7	5
Designated COVID wards (5)	1	4
Currently staying	$P = .03$*	$P = .776$
With family (19)	1	5
Without family (22)	10	7

*Means statistically significant.

© 2020 Wiley Periodicals LLC.

(during home-quarantine and COVID-19 pandemic), that was found to be comparable to non-dermatologists.[6] Our study, in contrast, was restricted to "front-line" dermatologists only.

Akin to our findings, Lai et al showed that being a woman and possessing an intermediate professional title was associated with higher anxiety, depression, and distress.[4] Liang et al[7] reported that medical staff at younger age (<30 years) had higher self-rated depression scores (statistically insignificant) than those with older age (≥30 years). Older staff can suffer from increased stress and depressive symptoms due to exhaustion from prolonged work hours, concerns for their families, and personal safety.[8,9]

Being a web-based survey, the receipt of unsolicited responses could not be accounted for. A larger response rate would have added more credibility. We could have drawn a more convincing conclusion if we had compared the study population with nondermatologists, who are more frequently posted in COVID isolation wards and intensive care units. Health care authorities should allocate resources (adequate screening, psychological counseling, or self-help interventions including cognitive behavior therapy)[10] to ensure the mental well-being of the healers. To the best of our knowledge, this is the first of its kind assessment of the psychological status of front-line dermatologists amidst this tough time.

DISCLAIMER

We confirm that the manuscript has been read and approved by all the authors, that the requirements for authorship as stated earlier in this document have been met and that each author believes that the manuscript represents honest work.

Abheek Sil[1]
Anupam Das[2]
Saurabh Jaiswal[3]
Mohammad Jafferany[4]
Akhilesh Thole[5]
Rahul Rajeev[6]
Syed Naiyer Ali[2]

[1]*Department of Dermatology, RG Kar Medical College and Hospital, Kolkata, West Bengal, India*
[2]*Department of Dermatology, KPC Medical College and Hospital, Kolkata, West Bengal, India*
[3]*Department of Dermatology, Indira Gandhi Government Medical College, Nagpur, Maharashtra, India*
[4]*College of Medicine, Central Michigan University, Saginaw, Michigan*
[5]*Department of Dermatology, ABVIMS, Dr. Ram Manohar Lohia Hospital, Delhi, India*
[6]*DM Neurology, Government Medical College, Thiruvananthapuram, Kerala, India*

Correspondence
Anupam Das, Department of Dermatology, KPC Medical College and Hospital, Kolkata, West Bengal, India.
Email: anupamdasdr@gmail.com

ORCID

Anupam Das https://orcid.org/0000-0003-0841-9597
Saurabh Jaiswal https://orcid.org/0000-0002-9288-7985
Mohammad Jafferany https://orcid.org/0000-0001-6358-9068

REFERENCES

1. Kaur I, Sharma A, Jakhar D, et al. Coronavirus disease (COVID-19): an updated review based on current knowledge and existing literature for dermatologists. *Dermatol Ther*. 2020 [Epub ahead of print]. https://doi.org/10.1111/dth.13677.
2. Goldust M, Kroumpouzos G, Murrell DF, et al. Update on COVID-19 effects in dermatology specialty. *Dermatol Ther*; 2020 [Epub ahead of print]. https://doi.org/10.1111/dth.13523.
3. Liu S, Yang L, Zhang C, et al. Online mental health services in China during the COVID-19 outbreak. *Lancet Psychiatry*. 2020;7:e17-e18. https://doi.org/10.1016/S2215-0366(20)30077-8.
4. Lai J, Ma S, Wang Y, et al. Factors associated with mental health outcomes among health care workers exposed to coronavirus disease 2019. *JAMA Netw Open*. 2020;3:e203976. https://doi.org/10.1001/jamanetworkopen.2020.3976.
5. Chatterjee SS, Bhattacharyya R, Bhattacharyya S, et al. Attitude, practice, behavior, and mental health impact of COVID-19 on doctors. *Indian J Psychiatry*. 2020;62:257-265. https://doi.org/10.4103/psychiatry.IndianJPsychiatry_333_20.
6. Podder I, Agarwal K, Datta S. Comparative analysis of perceived stress in dermatologists and other physicians during home-quarantine and COVID-19 pandemic with exploration of possible risk factors – a web-based cross-sectional study from Eastern India. *Dermatol Ther*. 2020 [Epub ahead of print]. https://doi.org/10.1111/dth.13788.
7. Liang Y, Chen M, Zheng X, et al. Screening for Chinese medical staff mental health by SDS and SAS during the outbreak of COVID-19. *J Psychosom Res*. 2020;133:110102. https://doi.org/10.1016/j.jpsychores.2020.110102.
8. Cai H, Tu B, Ma J, et al. Psychological impact and coping strategies of frontline medical staff in Hunan between January and March 2020 during the outbreak of coronavirus disease 2019 (COVID-19) in Hubei, China. *Med Sci Monit*. 2020;26:e924171. https://doi.org/10.12659/MSM.924171.
9. Kang L, Li Y, Hu S, et al. The mental health of medical workers in Wuhan, China dealing with the 2019 novel coronavirus. *Lancet Psychiatry*. 2020;7:e14. https://doi.org/10.1016/S2215-0366(20)30047-X.
10. Goldust M, Agarwal K, Kroumpouzos G, et al. Mental health status of dermatologists during the COVID-19 pandemic: a technology-based therapy. *Dermatol Ther*. 2020 [Epub ahead of print]. https://doi.org/10.1111/dth.13723.
