# Supplementary material for: Mental health status and related factors influencing healthcare workers during the COVID-19 pandemic: A systematic review and meta-analysis
Source: PLoS One. 2024 Jan 19;19(1):e0289454. doi: 10.1371/journal.pone.0289454 (PMC10798549; doi:10.1371/journal.pone.0289454)
Supplement: S6 File — (DOCX) [file pone.0289454.s006.docx]

**S6 File Other subgroup analysis results**

**1. Subgroup analyses for studies on burnout**

**S1 Fig: Subgroup of data collection time**

**S2 Fig****: Subgroup of national economic level**

**S3 Fig****: Subgroup of continent**

**S4 Fig****: Subgroup of doctors and nurses**

**S5 Fig: Subgroup of doctors and nurses vs other healthcare workers**

**S6 Fig Subgroup of assessment tools**

**2.** **Subgroup analyses for studies on anxiety**

**S7 Fig: Subgroup of data collection time**

**S8 Fig: Subgroup of national economic level**

**S9 Figc: Subgroup of continent**

**S10 Fig: Subgroup of doctors and nurses**

**S11 Fig: Subgroup of doctors and nurses vs other healthcare workers**

**S12 Fig: Subgroup of assessment tools**

**3.** **Subgroup analyses for studies on depression**

**S13 Fig: Subgroup of data collection time**

**S14 Fig: Subgroup of national economic level**

**S15 Fig: Subgroup of continent**

**S16 Fig: Subgroup of doctors and nurses**

**S17 Fig: Subgroup of doctors and nurses vs other healthcare workers**

**S18 Fig: Subgroup of assessment tools**

**4.Subgroup analyses for studies on Post-traumatic stress disorder**

**S19 Fig****: Subgroup of data collection time**

**S20 Fig: Subgroup of national economic level**

**S21 Fig****: Subgroup of continent**

**S22 Fig: Subgroup of doctors and nurses**

**S23 Fig: Subgroup of doctors and nurses vs other healthcare workers**

**S24 Fig: Subgroup of assessment tools**
