# Supplementary material for: Mental health status and related factors influencing healthcare workers during the COVID-19 pandemic: A systematic review and meta-analysis
Source: PLoS One. 2024 Jan 19;19(1):e0289454. doi: 10.1371/journal.pone.0289454 (PMC10798549; doi:10.1371/journal.pone.0289454)
Supplement: S1 Data — (ZIP) [file pone.0289454.s011.zip › literatures/68.pdf]

JAMA Network Open

Original Investigation | Psychiatry

Factors Associated With Mental Health Outcomes Among Health Care Workers Exposed to Coronavirus Disease 2019

Jianbo Lai, MSc; Simeng Ma, MSc; Ying Wang, MSc; Zhongxiang Cai, MD; Jianbo Hu, MSc; Ning Wei, MD; Jiang Wu, MD; Hui Du, MD; Tingting Chen, MD; Ruiting Li, MD; Huawei Tan, MD; Lijun Kang, MSc; Lihua Yao, MD; Manli Huang, MD; Huafen Wang, BD; Gaohua Wang, MD; Zhongchun Liu, MD; Shaohua Hu, MD

Abstract

IMPORTANCE Health care workers exposed to coronavirus disease 2019 (COVID-19) could be psychologically stressed.

OBJECTIVE To assess the magnitude of mental health outcomes and associated factors among health care workers treating patients exposed to COVID-19 in China.

DESIGN, SETTINGS, AND PARTICIPANTS This cross-sectional, survey-based, region-stratified study collected demographic data and mental health measurements from 1257 health care workers in 34 hospitals from January 29, 2020, to February 3, 2020, in China. Health care workers in hospitals equipped with fever clinics or wards for patients with COVID-19 were eligible.

MAIN OUTCOMES AND MEASURES The degree of symptoms of depression, anxiety, insomnia, and distress was assessed by the Chinese versions of the 9-item Patient Health Questionnaire, the 7-item Generalized Anxiety Disorder scale, the 7-item Insomnia Severity Index, and the 22-item Impact of Event Scale–Revised, respectively. Multivariable logistic regression analysis was performed to identify factors associated with mental health outcomes.

RESULTS A total of 1257 of 1830 contacted individuals completed the survey, with a participation rate of 68.7%. A total of 813 (64.7%) were aged 26 to 40 years, and 964 (76.7%) were women. Of all participants, 764 (60.8%) were nurses, and 493 (39.2%) were physicians; 760 (60.5%) worked in hospitals in Wuhan, and 522 (41.5%) were frontline health care workers. A considerable proportion of participants reported symptoms of depression (634 [50.4%]), anxiety (560 [44.6%]), insomnia (427 [34.0%]), and distress (899 [71.5%]). Nurses, women, frontline health care workers, and those working in Wuhan, China, reported more severe degrees of all measurements of mental health symptoms than other health care workers (eg, median [IQR] Patient Health Questionnaire scores among physicians vs nurses: 4.0 [1.0-7.0] vs 5.0 [2.0-8.0]; *P* = .007; median [interquartile range {IQR}] Generalized Anxiety Disorder scale scores among men vs women: 2.0 [0-6.0] vs 4.0 [1.0-7.0]; *P* < .001; median [IQR] Insomnia Severity Index scores among frontline vs second-line workers: 6.0 [2.0-11.0] vs 4.0 [1.0-8.0]; *P* < .001; median [IQR] Impact of Event Scale–Revised scores among those in Wuhan vs those in Hubei outside Wuhan and those outside Hubei: 21.0 [8.5-34.5] vs 18.0 [6.0-28.0] in Hubei outside Wuhan and 15.0 [4.0-26.0] outside Hubei; *P* < .001). Multivariable logistic regression analysis showed participants from outside Hubei province were associated with lower risk of experiencing symptoms of distress compared with those in Wuhan (odds ratio [OR], 0.62; 95% CI, 0.43-0.88; *P* = .008). Frontline health care workers engaged in direct diagnosis, treatment, and care of patients with COVID-19 were associated with a higher risk of symptoms of depression (OR, 1.52; 95% CI, 1.11-2.09; *P* = .01), anxiety (OR, 1.57; 95% CI, 1.22-2.02; *P* < .001), insomnia (OR, 2.97; 95% CI, 1.92-4.60; *P* < .001), and distress (OR, 1.60; 95% CI, 1.25-2.04; *P* < .001).

(continued)

Key Points

Question What factors are associated with mental health outcomes among health care workers in China who are treating patients with coronavirus disease 2019 (COVID-19)?

Findings In this cross-sectional study of 1257 health care workers in 34 hospitals equipped with fever clinics or wards for patients with COVID-19 in multiple regions of China, a considerable proportion of health care workers reported experiencing symptoms of depression, anxiety, insomnia, and distress, especially women, nurses, those in Wuhan, and front-line health care workers directly engaged in diagnosing, treating, or providing nursing care to patients with suspected or confirmed COVID-19.

Meaning These findings suggest that, among Chinese health care workers exposed to COVID-19, women, nurses, those in Wuhan, and front-line health care workers have a high risk of developing unfavorable mental health outcomes and may need psychological support or interventions.

+ Invited Commentary

+ Supplemental content

Author affiliations and article information are listed at the end of this article.

Open Access. This is an open access article distributed under the terms of the CC-BY License.

Downloaded From: https://jamanetwork.com/ on 12/21/2022

Abstract (continued)

CONCLUSIONS AND RELEVANCE In this survey of heath care workers in hospitals equipped with fever clinics or wards for patients with COVID-19 in Wuhan and other regions in China, participants reported experiencing psychological burden, especially nurses, women, those in Wuhan, and frontline health care workers directly engaged in the diagnosis, treatment, and care for patients with COVID-19.

JAMA Network Open. 2020;3(3):e203976. doi:10.1001/jamanetworkopen.2020.3976

Introduction

Since the end of December 2019, the Chinese city of Wuhan has reported a novel pneumonia caused by coronavirus disease 2019 (COVID-19), which is spreading domestically and internationally.[1] The virus has been named *severe acute respiratory syndrome coronavirus 2* (SARS-CoV-2). In this report, we will refer to the disease, COVID-19. According to data released by the National Health Commission of China, the number of confirmed cases in mainland China has increased to 80 151 as of March 2, 2020,[2] and confirmed cases have been reported in more than a dozen other countries. Moreover, person-to-person transmission has been recorded outside mainland China.[3] On January 30, 2020, the World Health Organization held an emergency meeting and declared the global COVID-19 outbreak a public health emergency of international concern.[4]

Facing this critical situation, health care workers on the front line who are directly involved in the diagnosis, treatment, and care of patients with COVID-19 are at risk of developing psychological distress and other mental health symptoms. The ever-increasing number of confirmed and suspected cases, overwhelming workload, depletion of personal protection equipment, widespread media coverage, lack of specific drugs, and feelings of being inadequately supported may all contribute to the mental burden of these health care workers. Previous studies have reported adverse psychological reactions to the 2003 SARS outbreak among health care workers.[5-8] Studies showed that those health care workers feared contagion and infection of their family, friends, and colleagues,[5] felt uncertainty and stigmatization,[5,6] reported reluctance to work or contemplating resignation,[6] and reported experiencing high levels of stress, anxiety, and depression symptoms,[7] which could have long-term psychological implications.[7] Similar concerns about the mental health, psychological adjustment, and recovery of health care workers treating and caring for patients with COVID-19 are now arising.

Psychological assistance services, including telephone-, internet-, and application-based counseling or intervention, have been widely deployed by local and national mental health institutions in response to the COVID-19 outbreak. On February 2, 2020, the State Council of China announced that it was setting up nationwide psychological assistance hotlines to help during the epidemic situation.[9] However, evidence-based evaluations and mental health interventions targeting front-line health care workers are relatively scarce.

To address this gap, the aim of current study was to evaluate mental health outcomes among health care workers treating patients with COVID-19 by quantifying the magnitude of symptoms of depression, anxiety, insomnia, and distress and by analyzing potential risk factors associated with these symptoms. Participants from Wuhan city (the capital of Hubei province) and other areas inside and outside Hubei province in China were enrolled in this survey to compare interregional differences. This study aimed to provide an assessment of the mental health burden of Chinese health care workers, which can serve as important evidence to direct the promotion of mental well-being among health care workers.

Downloaded From: https://jamanetwork.com/ on 12/21/2022

Methods

Study Design

This study followed the American Association for Public Opinion Research (AAPOR) reporting guideline. Approval from the clinical research ethics committee of Renmin Hospital of Wuhan University was received before the initiation of this study. Verbal informed consent was provided by all survey participants prior to their enrollment. Participants were allowed to terminate the survey at any time they desired. The survey was anonymous, and confidentiality of information was assured.

The study is a cross-sectional, hospital-based survey conducted via a region-stratified, 2-stage cluster sampling from January 29, 2020, to February 3, 2020. During this period, the total confirmed cases of COVID-19 exceeded 10 000 in China. To compare the interregional differences of mental health outcomes among health care workers in China, samples were stratified by their geographic location (ie, Wuhan, other regions inside Hubei province, and regions outside Hubei province). Because Wuhan was most severely affected, more hospitals in Wuhan were sampled. Hospitals equipped with fever clinics or wards for COVID-19 were eligible to participate in this survey. A total of 20 hospitals in Wuhan (10 designated by the local government to treat COVID-19 and 10 nondesignated), 7 hospitals in other regions of Hubei province, and 7 hospitals from 7 other provinces with a high incidence of COVID-19 (1 hospital from each province) were included. In total, 34 hospitals were involved. Milestone events during the outbreak of COVID-19 and the duration of this study are presented in the eFigure in the Supplement.

Participants

One clinical department was randomly sampled from each selected hospital, and all health care workers in this department were asked to participate in this study. The target sample size of participants was determined using the formula $N = Z_\alpha{}^2P(1 - P) / d^2$, in which $\alpha = 0.05$ and $Z_\alpha = 1.96$, and the estimated acceptable margin of error for proportion *d* was 0.1. The proportion of health care workers with psychological comorbidities was estimated at 35%, based on a previous study of the SARS outbreak.[7] To allow for subgroup analyses, we amplified the sample size by 50% with a goal of at least 1070 completed questionnaires from participants.

Outcomes and Covariates

We focused on symptoms of depression, anxiety, insomnia, and distress for all participants, using Chinese versions of validated measurement tools.[10-13] Accordingly, the 9-item Patient Health Questionnaire (PHQ-9; range, 0-27),[10] the 7-item Generalized Anxiety Disorder (GAD-7) scale (range, 0-21),[11] the 7-item Insomnia Severity Index (ISI; range, 0-28),[12] and the 22-item Impact of Event Scale–Revised (IES-R; range, 0-88)[13] were used to assess the severity of symptoms of depression, anxiety, insomnia, and distress, respectively. The total scores of these measurement tools were interpreted as follows: PHQ-9, normal (0-4), mild (5-9), moderate (10-14), and severe (15-21) depression; GAD-7, normal (0-4), mild (5-9), moderate (10-14), and severe (15-21) anxiety; ISI, normal (0-7), subthreshold (8-14), moderate (15-21), and severe (22-28) insomnia; and IES-R, normal (0-8), mild (9-25), moderate (26-43), and severe (44-88) distress. These categories were based on values established in the literature.[10-13]

The cutoff score for detecting symptoms of major depression, anxiety, insomnia, and distress were 10, 7,[14] 15, and 26, respectively. Participants who had scores greater than the cutoff threshold were characterized as having severe symptoms.

Demographic data were self-reported by the participants, including occupation (physician or nurse), sex (male or female), age (18-25, 26-30, 31-40, or >40 years), marital status, educational level (≤undergraduate or ≥postgraduate), technical title (junior, intermediate, or senior), geographic location (Wuhan, Hubei province outside Wuhan, or outside Hubei province), place of residence (urban or rural), and type of hospital (secondary or tertiary). The different technical titles of respondents refer to the professional titles certificated by the hospital. Participants were asked

Downloaded From: https://jamanetwork.com/ on 12/21/2022

whether they were directly engaged in clinical activities of diagnosing, treating, or providing nursing care to patients with elevated temperature or patients with confirmed COVID-19. Those who responded yes were defined as frontline workers, and those who answered no were defined as second-line workers.

Statistical Analysis

Data analysis was performed using SPSS statistical software version 20.0 (IBM Corp). The significance level was set at α = .05, and all tests were 2-tailed. The original scores of the 4 measurement tools were not normally distributed and so are presented as medians with interquartile ranges (IQRs). The ranked data, which were derived from the counts of each level for symptoms of depression, anxiety, insomnia, and distress, are presented as numbers and percentages. The nonparametric Mann-Whitney *U* test and Kruskal-Wallis test were applied to compare the severity of each symptom between 2 or more groups. To determine potential risk factors for symptoms of depression, anxiety, insomnia, and distress in participants, multivariable logistic regression analysis was performed, and the associations between risk factors and outcomes are presented as odds ratios (ORs) and 95% CIs, after adjustment for confounders, including sex, age, marital status, educational level, technical title, place of residence, working position (first-line or second-line), and type of hospital.

Results

Demographic Characteristics

In the study, among the 1830 health care workers (702 [38.4%] physicians and 1128 [61.6%] nurses) asked to participate, 1257 respondents (68.7%) completed the survey. The occupational and geographic data of nonrespondents were similar to those of respondents (eTable 1 in the Supplement). Of the 1257 responding participants, 493 (39.2%) were physicians, and 764 (60.8%) were nurses. The response rates for physicians and nurses were 70.2% and 67.7%, respectively. Of the participants, 760 (60.5%) worked in Wuhan, 261 (20.8%) worked in Hubei province outside Wuhan, and 236 (18.8%) worked outside Hubei province. Most participants were women (964 [76.7%]), were aged 26 to 40 years (813 [64.7%]), were married, widowed, or divorced (839 [66.7%]), had an educational level of undergraduate or less (953 [75.8%]), had a junior technical title (699 [55.6%]), and worked in tertiary hospitals (933 [74.2%]). A total of 522 participants (41.5%) were frontline health care workers directly engaged in diagnosing, treating, or caring for patients with or suspected to have COVID-19. Nearly all participants (1220 [97.1%]) lived in urban areas (**Table 1**).

Severity of Measurements and Associated Factors

A considerable proportion of participants had symptoms of depression (634 [50.4%]), anxiety (560 [44.6%]), insomnia (427 [34.0%]), and distress (899 [71.5%]). Nurses, women, frontline workers, and those in Wuhan reported experiencing more severe symptom levels of depression, anxiety, insomnia, and distress (eg, severe depression among physicians vs nurses: 24 [4.9%] vs 54 [7.1%]; *P* = .01; severe anxiety among men vs women: 10 [3.4%] vs 56 [5.8%]; *P* = .001; severe insomnia among frontline workers vs second-line workers: 9 [1.7%] vs 3 [0.4%]; *P* < .001; severe distress among workers in Wuhan vs Hubei outside Wuhan and outside Hubei: 96 [12.6%] vs 19 [7.2%] among those in Hubei outside Wuhan and 17 [7.2%] among those outside Hubei; *P* < .001) (**Table 2**). Compared with those working in tertiary hospitals, participants working in secondary hospitals were more likely to report severe symptoms of depression (53 [5.6%] vs 25 [7.7%]; *P* = .003), anxiety (48 [5.1%] vs 18 [5.5%]; *P* = .046), and insomnia (10 [1.0%] vs 2 [0.6%]; *P* = .02) but not distress (Table 2).

Downloaded From: https://jamanetwork.com/ on 12/21/2022

Scores of Measurements and Associated Factors

The median (IQR) scores on the PHQ-9 for depression, the GAD-7 for anxiety, the ISI for insomnia, and the IES-R for distress for all respondents were 5.0 (2.0-8.0), 4.0 (1.0-7.0), 5.0 (2.0-9.0), and 20.0 (7.0-31.0), respectively. Similar to findings in severity of symptoms, participants who were nurses, women, frontline health care workers, and working in Wuhan had higher scores in all 4 scales compared with those who were physicians, men, second-line health care workers, and working in Hubei province outside Wuhan or outside Hubei province (eg, median [IQR] PHQ-9 scores among physicians vs nurses: 4.0 [1.0-7.0] vs 5.0 [2.0-8.0]; $P = .007$; median [IQR] GAD-7 scores among men vs women: 2.0 [0-6.0] vs 4.0 [1.0-7.0]; $P < .001$; median [IQR] ISI scores among frontline vs second-line workers: 6.0 [2.0-11.0] vs 4.0 [1.0-8.0]; $P < .001$; median [IQR] IES-R scores among those in Wuhan vs those in Hubei outside Wuhan and those outside Hubei: 21.0 [8.5-34.5] vs 18.0 [6.0-28.0] in Hubei outside Wuhan and 15.0 [4.0-26.0] outside Hubei; $P < .001$) (**Table 3**). Compared with health care workers in tertiary hospitals, those in secondary hospitals reported higher scores on scales measuring symptoms of depression, anxiety, and insomnia (median [IQR] PHQ-9 score, 4.0 [1.0-7.0] vs 5.0 [2.0-9.0]; $P < .001$; median [IQR] GAD-7 score, 3.0 [0-7.0] vs 4.0 [1.0-7.0]; $P = .005$; median [IQR] ISI score, 4.0 [2.0-9.0] vs 6.0 [2.0-10.0]; $P = .008$). There were no differences in hospital status for scores of distress (median [IQR] IES-R score: workers in tertiary hospitals, 19.0 [7.0-32.0]; workers in secondary hospitals, 20.0 [6.0-31.0]; $P = .46$). However, frontline health care

Table 1. Demographic and Occupational Characteristics of Responders

	No. (%)					
		Occupation		Location		
Characteristic	Total	Physician	Nurse	Wuhan	Hubei province outside Wuhan	Outside Hubei province
Overall	1257 (100)	493 (39.2)	764 (60.8)	760 (60.5)	261 (20.8)	236 (18.8)
Sex						
Men	293 (23.3)	223 (45.2)	70 (9.2)	146 (19.2)	52 (19.9)	95 (40.3)
Women	964 (76.7)	270 (54.8)	694 (90.8)	614 (80.8)	209 (80.1)	141 (59.7)
Age, y						
18-25	198 (15.8)	10 (2.0)	188 (24.6)	162 (21.3)	32 (12.3)	4 (1.7)
26-30	407 (32.4)	126 (25.6)	281 (36.8)	258 (33.9)	111 (42.5)	38 (16.1)
31-40	406 (32.3)	200 (40.6)	206 (27.0)	224 (29.5)	71 (27.2)	111 (47.0)
>40	246 (19.5)	157 (31.8)	89 (11.6)	116 (15.3)	47 (18.0)	83 (35.2)
Marriage status						
Unmarried	418 (33.3)	87 (17.6)	331 (43.3)	314 (41.3)	66 (25.3)	38 (16.1)
Married[a]	839 (66.7)	406 (82.4)	433 (56.7)	446 (58.7)	195 (74.7)	198 (83.9)
Education level						
≤Undergraduate	953 (75.8)	217 (44.0)	736 (96.3)	611 (80.4)	238 (91.2)	104 (44.1)
≥Postgraduate	304 (24.2)	276 (56.0)	28 (3.7)	149 (19.6)	23 (8.8)	132 (55.9)
Technical title						
Junior	699 (55.6)	153 (31.0)	546 (71.5)	481 (63.3)	169 (64.8)	49 (20.8)
Intermediate	378 (30.1)	187 (37.9)	191 (25.0)	221 (29.1)	61 (23.4)	96 (40.7)
Senior	180 (14.3)	153 (31.1)	27 (3.5)	58 (7.6)	31 (11.8)	91 (38.5)
Place of residence						
Urban	1220 (97.1)	474 (96.1)	746 (97.6)	751 (98.8)	247 (94.6)	222 (94.1)
Rural	37 (2.9)	19 (3.9)	18 (2.4)	9 (1.2)	14 (5.4)	14 (5.9)
Working position						
Frontline	522 (41.5)	176 (35.7)	346 (45.3)	390 (51.3)	72 (27.6)	60 (25.4)
Second-line	735 (58.5)	317 (64.3)	418 (54.7)	370 (48.7)	189 (72.4)	176 (74.6)
Type of hospital						
Tertiary	933 (74.2)	369 (74.8)	564 (73.8)	538 (70.8)	218 (83.5)	177 (75.0)
Secondary	324 (25.8)	124 (25.2)	200 (26.2)	222 (29.2)	43 (16.5)	59 (25.0)

[a] Married category included widowed and divorced participants.

Downloaded From: https://jamanetwork.com/ on 12/21/2022

Table 2. Severity Categories of Depression, Anxiety, Insomnia, and Distress Measurements in Total Cohort and Subgroups

		Occupation, No. (%)			Sex, No. (%)			Working position, No. (%)			Type of hospital, No. (%)			Location, No. (%)			
Severity category	Total, No. (%)	Physician	Nurse	*P* value	Men	Women	*P* value	Frontline	Second-line	*P* value	Tertiary	Secondary	*P* value	Wuhan	Hubei province outside of Wuhan	Outside Hubei province	*P* value
**PHQ-9, depression symptoms**																	
Normal	623 (49.6)	268 (54.4)	355 (46.5)		171 (58.3)	452 (46.8)		217 (41.5)	406 (55.2)		483 (51.7)	140 (43.2)		335 (40.0)	146 (55.9)	142 (60.1)	
Mild	448 (35.6)	157 (31.8)	291 (38.1)	.01	92 (31.3)	356 (36.9)	<.001	211 (40.4)	237 (32.2)	<.001	326 (34.9)	122 (37.6)	.003	296 (38.9)	85 (32.5)	67 (28.3)	<.001
Moderate	108 (8.6)	44 (8.9)	64 (8.4)		21 (7.1)	87 (9.0)		59 (11.3)	49 (6.6)		71 (7.6)	37 (11.4)		73 (9.6)	19 (7.2)	16 (6.7)	
Severe	78 (6.2)	24 (4.9)	54 (7.1)		9 (3.0)	69 (7.1)		35 (6.7)	43 (5.8)		53 (5.6)	25 (7.7)		56 (7.3)	11 (4.2)	11 (4.6)	
**GAD-7, anxiety**																	
Normal	697 (55.4)	293 (59.4)	404 (52.9)		189 (64.5)	508 (52.6)		253 (48.4)	444 (60.4)		533 (57.1)	164 (50.6)		391 (51.4)	155 (59.3)	151 (63.9)	
Mild	406 (32.3)	143 (29.0)	263 (34.4)	.03	71 (24.2)	335 (34.7)	.001	185 (35.4)	221 (30.0)	<.001	291 (31.1)	115 (35.4)	.046	257 (33.8)	85 (32.5)	64 (27.1)	<.001
Moderate	88 (7.0)	34 (6.9)	54 (7.1)		23 (7.8)	65 (6.7)		48 (9.1)	40 (5.4)		61 (6.5)	27 (8.3)		66 (8.6)	11 (4.2)	11 (4.6)	
Severe	66 (5.3)	23 (4.7)	43 (5.6)		10 (3.4)	56 (5.8)		36 (6.8)	30 (4.0)		48 (5.1)	18 (5.5)		46 (6.0)	10 (3.8)	10 (4.2)	
**ISI, insomnia symptoms**																	
Absence	830 (66.0)	358 (72.6)	472 (61.8)		208 (70.9)	622 (64.5)		310 (59.3)	520 (70.7)		635 (68.0)	195 (60.1)		473 (62.2)	186 (71.2)	171 (72.4)	
Subthreshold	330 (26.2)	107 (21.7)	223 (29.2)	<.001	66 (22.5)	264 (27.3)	.04	148 (28.3)	182 (24.7)	<.001	227 (24.3)	103 (31.7)	.02	214 (28.1)	60 (22.9)	56 (23.7)	.001
Moderate	85 (6.8)	24 (4.9)	61 (8.0)		17 (5.8)	68 (7.0)		55 (10.5)	30 (4.0)		61 (6.5)	24 (7.4)		65 (8.5)	13 (4.9)	7 (2.9)	
Severe	12 (1.0)	4 (0.8)	8 (1.0)		2 (0.6)	10 (1.0)		9 (1.7)	3 (0.4)		10 (1.0)	2 (0.6)		8 (1.0)	2 (0.7)	2 (0.8)	
**IES-R, distress symptoms**																	
Normal	358 (28.5)	163 (33.1)	195 (25.5)		122 (41.6)	236 (24.4)		124 (23.7)	234 (31.8)		259 (27.7)	99 (30.5)		190 (25.0)	76 (29.1)	92 (38.9)	
Mild	459 (36.5)	167 (33.9)	292 (38.2)	.01	88 (30.0)	371 (38.4)	<.001	178 (34.0)	281 (38.2)	<.001	349 (37.4)	110 (33.9)	0.81	272 (35.7)	106 (40.6)	81 (34.2)	<.001
Moderate	308 (24.5)	120 (24.3)	188 (24.6)		59 (20.1)	249 (25.8)		146 (27.9)	162 (22.0)		231 (24.7)	77 (23.7)		202 (26.5)	60 (22.9)	46 (19.4)	
Severe	132 (10.5)	43 (8.7)	89 (11.6)		24 (8.1)	108 (11.2)		74 (14.1)	58 (7.8)		94 (10.0)	38 (11.7)		96 (12.6)	19 (7.2)	17 (7.2)	

Abbreviations: GAD-7, 7-item Generalized Anxiety Disorder; IES-R, 22-item Impact of Event Scale–Revised; ISI, 7-item Insomnia Severity Index; PHQ-9, 9-item Patient Health Questionnaire.

Downloaded From: https://jamanetwork.com/ on 12/21/2022

Table 3. Scores of Depression, Anxiety, Insomnia, and Distress Measurements in Total Cohort and Subgroups

		Occupation			Sex			Working position			Type of hospital			Geographic location			
		Median (IQR)			Median (IQR)			Median (IQR)			Median (IQR)			Median (IQR)			
Scale	Total score, median (IQR)	Physician	Nurse	*P* value	Men	Women	*P* value	Frontline	Second-line	*P* value	Tertiary	Secondary	*P* value	Wuhan	Hubei province outside of Wuhan	Outside Hubei province	*P* value
PHQ-9, depression symptoms	5.0 (2.0-8.0)	4.0 (1.0-7.0)	5.0 (2.0-8.0)	.007	3.0 (0-7.0)	5.0 (2.0-8.0)	<.001	6.0 (2.0-9.0)	4.0 (1.0-7.0)	<.001	4.0 (1.0-7.0)	5.0 (2.0-9.0)	<.001	5.0 (2.0-8.0)	4.0 (1.0-7.0)	3.0 (0-7.0)	<.001
GAD-7, anxiety symptoms	4.0 (1.0-7.0)	3.0 (0-7.0)	4.0 (1.0-7.0)	.008	2.0 (0-6.0)	4.0 (1.0-7.0)	<.001	5.0 (1.0-7.0)	3.0 (0.0-6.5)	<.001	3.0 (0-7.0)	4.0 (1.0-7.0)	.005	4.0 (1.0-7.0)	3.0 (0-6.0)	2.0 (0-6.0)	<.001
ISI, insomnia symptoms	5.0 (2.0-9.0)	4.0 (1.0-8.0)	5.0 (2.0-10.0)	<.001	3.0 (1.0-8.0)	5.0 (2.0-9.0)	<.001	6.0 (2.0-11.0)	4.0 (1.0-8.0)	<.001	4.0 (2.0-9.0)	6.0 (2.0-10.0)	.008	5.0 (2.0-10.0)	4.0 (1.0-8.0)	3.0 (1.0-8.0)	<.001
IES-R, distress symptoms	20.0 (7.0-31.0)	18.0 (5.0-30.0)	20.5 (8.0-32.0)	.009	14.0 (3.0-28.0)	21.0 (9.0-32.0)	<.001	22.5 (9.0-35.0)	17.0 (5.5-28.5)	<.001	19.0 (7.0-32.0)	20.0 (6.0-31.0)	.46	21.0 (8.5-34.5)	18.0 (6.0-28.0)	15.0 (4.0-26.0)	<.001

Abbreviations: GAD-7, 7-item Generalized Anxiety Disorder; IES-R, 22-item Impact of Event Scale–Revised; IQR, interquartile range; ISI, 7-item Insomnia Severity Index; PHQ-9, 9-item Patient Health Questionnaire.

Downloaded From: https://jamanetwork.com/ on 12/21/2022

workers from tertiary and secondary hospitals reported equally high scores on all 4 scales (eg, median [IQR] PHQ-9 score, 5.0 [2.0-8.0] vs 6.0 [3.0-9.0]; *P* = .08) (**Table 4**). In pairwise comparisons, participants from Hubei province outside Wuhan and participants outside Hubei province reported similar levels of symptoms of depression, anxiety, insomnia, and distress but were all lower than that of health care workers in Wuhan, the origin of the epidemic (eTable 2 in the Supplement). Analyses of scores of 3 factors (avoidance, intrusion, and hyperarousal) derived from the IES-R are presented in eTable 3, eTable 4, and eTable 5 in the Supplement.

Risk Factors of Mental Health Outcomes

Multivariable logistic regression analysis showed that, after controlling for confounders, being a woman and having an intermediate professional title were associated with severe symptoms of depression, anxiety, and distress (eg, severe depression among women: OR, 1.94; 95% CI, 1.26-2.98; *P* = .003; severe anxiety among those with intermediate professional titles: OR, 1.82; 95% CI, 1.38-2.39; *P* < .001). Compared with working in a tertiary hospital, working in secondary hospitals was associated with more severe symptoms of depression (OR, 1.65; 95% CI, 1.17-2.34; *P* = .004) and anxiety (OR, 1.43; 95% CI, 1.08-1.90; *P* = .01). Working outside Hubei province was associated with a lower risk of feeling distressed than working in Wuhan (OR, 0.62; 95% CI, 0.43-0.88; *P* = .008). Compared with working in second-line positions, working in the frontline directly treating patients with COVID-19 appeared to be an independent risk factor for all psychiatric symptoms after adjustment (depression, OR 1.52; 95% CI, 1.11-2.09; *P* = .01; anxiety, OR 1.57; 95% CI, 1.22-2.02; *P* < .001; insomnia, OR 2.97; 95% CI, 1.92-4.60; *P* < .001; distress: OR, 1.60; 95% CI, 1.25-2.04; *P* < .001) (**Table 5**).

Discussion

This cross-sectional survey enrolled 1257 respondents and revealed a high prevalence of mental health symptoms among health care workers treating patients with COVID-19 in China. Overall, 50.4%, 44.6%, 34.0%, and 71.5% of all participants reported symptoms of depression, anxiety, insomnia, and distress, respectively. Participants were divided in 3 groups (Wuhan, other regions in Hubei province, and regions outside Wuhan province) to compare interregional differences. Most participants were female, were nurses, were aged 26 to 40 years, were married, and worked in tertiary hospitals with a junior technical title. Nurses, women, those working in Wuhan, and frontline workers reported more severe symptoms on all measurements. Our study further indicated that being a woman and having an intermediate technical title were associated with experiencing severe depression, anxiety, and distress. Working in the front line was an independent risk factor for worse mental health outcomes in all dimensions of interest. Together, our findings present concerns about the psychological well-being of physicians and nurses involved in the acute COVID-19 outbreak.

In this study, a significant proportion of participants experienced anxiety, depression, and insomnia symptoms, and more than 70% reported psychological distress. In a previous study during the acute SARS outbreak, 89% of health care workers who were in high-risk situations reported psychological symptoms.[8] The psychological response of health care workers to an epidemic of infectious diseases is complicated. Sources of distress may include feelings of vulnerability or loss of

Table 4. Association of Hospital Type With Scores of Mental Health Outcomes Among Frontline Workers

	Score, median (IQR)		
Scale	Tertiary hospital	Secondary hospital	*P* value
PHQ-9, depression symptoms	5.0 (2.0-8.0)	6.0 (3.0-9.0)	.08
GAD-7, anxiety symptoms	5.0 (1.0-7.0)	5.0 (2.0-8.0)	.23
ISI, insomnia symptoms	6.0 (2.0-11.0)	6.0 (2.0-11.0)	.26
IES-R, distress symptoms	23.0 (9.0-37.0)	21.0 (7.5-31.5)	.11

Abbreviations: GAD-7, 7-item Generalized Anxiety Disorder; IES-R, 22-item Impact of Event Scale–Revised; IQR, interquartile range; ISI, 7-item Insomnia Severity Index; PHQ-9, 9-item Patient Health Questionnaire.

Downloaded From: https://jamanetwork.com/ on 12/21/2022

Table 5. Risk Factors for Mental Health Outcomes Identified by Multivariable Logistic Regression Analysis

Variable	No. of severe cases/ No. of total cases (%)	Adjusted OR (95%CI)[a]	P value[b] Category	P value[b] Overall
**PHQ-9, depression symptoms**				
Sex				
Men	30/293 (10.2)	1 [Reference]	NA	.003
Women	156/964 (16.2)	1.94 (1.26-2.98)	.003	
Type of hospital				
Tertiary hospital	124/933 (13.3)	1 [Reference]	NA	.004
Secondary hospital	62/324 (19.1)	1.65 (1.17-2.34)	.004	
Technical title				
Junior	91/699 (13.0)	1 [Reference]	NA	.005
Intermediate	73/378 (19.3)	1.77 (1.25-2.49)	.001	
Senior	22/180 (12.2)	1.21 (0.72-2.03)	.47	
Working position				
Second-line	92/735 (12.9)	1 [Reference]	NA	.01
Frontline	94/522 (18.0)	1.52 (1.11-2.09)	.01	
**GAD-7, anxiety symptoms**				
Sex				
Men	66/293 (22.5)	1 [Reference]	NA	.001
Women	299/964 (31.0)	1.69 (1.23-2.33)	.001	
Type of hospital				
Tertiary hospital	255/933 (27.3)	1 [Reference]	NA	.01
Secondary hospital	110/324 (34.0)	1.43 (1.08-1.90)	.01	
Technical title				
Junior	184/699 (26.3)	1 [Reference]	NA	<.001
Intermediate	141/378 (37.3)	1.82 (1.38-2.39)	<.001	
Senior	40/180 (22.2)	1.01 (0.67-1.51)	.97	
Working position				
Second-line	184/735 (25.0)	1 [Reference]	NA	<.001
Frontline	181/522 (34.7)	1.57 (1.22-2.02)	<.001	
**ISI, insomnia symptoms**				
Working position				
Second-line	33/735 (4.5)	1 [Reference]	NA	<.001
Frontline	64/522 (12.3)	2.97 (1.92-4.60)	<.001	
**IES-R, distress symptoms**				
Sex				
Men	83/293 (28.3)	1 [Reference]	NA	.01
Women	357/964 (37.0)	1.45 (1.08-1.96)	.01	
Technical title				
Junior	225/699 (32.2)	1 [Reference]	NA	<.001
Intermediate	169/378 (44.7)	1.94 (1.48-2.55)	<.001	
Senior	46/180 (25.6)	1.03 (0.69-1.55)	.87	
Working position				
Second-line	220/735 (29.9)	1 [Reference]	NA	<.001
Frontline	220/522 (42.1)	1.60 (1.25-2.04)	<.001	
Location				
Wuhan	298/760 (39.2)	1 [Reference]	NA	.02
Hubei province outside Wuhan	79/261 (30.2)	0.77 (0.57-1.06)	.10	
Outside Hubei province	63/236 (26.7)	0.62 (0.43-0.88)	.008	

Abbreviation: GAD-7, 7-item Generalized Anxiety Disorder; IES-R, 22-item Impact of Event Scale–Revised; ISI, 7-item Insomnia Severity Index; OR, odds ratio; PHQ-9, 9-item Patient Health Questionnaire; NA, not applicable.

[a] Adjusted for sex, age, marriage, educational level, technical title, place of residence, working position, and type of hospital, when appropriate.

[b] Category refers to the *P* value for each category vs the reference, while overall refers to the results of the logistic regression.

Downloaded From: https://jamanetwork.com/ on 12/21/2022

control and concerns about health of self, spread of virus, health of family and others, changes in work, and being isolated.[15] The fact that COVID-19 is human-to-human transmissible,[1,3] associated with high morbidity, and potentially fatal[16] may intensify the perception of personal danger. Additionally, predictable shortages of supplies and an increasing influx of suspected and actual cases of COVID-19 contribute to the pressures and concerns of health care workers.[17]

Of note, 76.7% of all participants were women, and 60.8% were nurses (90.8% of whom were female). Our findings further indicate that women reported more severe symptoms of depression, anxiety, and distress. Frontline nurses treating patients with COVID-19 are likely exposed to the highest risk of infection because of their close, frequent contact with patients and working longer hours than usual.[18,19] Moreover, 71.5% of all nurses had junior titles, indicating that most had fewer years of work experience. During the SARS outbreak, a study conducted among health care workers in emergency departments also showed that nurses were more likely to develop distress and use behavioral disengagement than physicians.[15] Frontline nurses treating patients with SARS were physically and psychologically challenged when committing themselves to providing high-quality nursing care for patients.[19-22] Moreover, at the early stage of the SARS epidemic, nurses may have been less likely to be warned about exposure or provided with adequate protections.[22] Particular attention is warranted regarding the mental health well-being of women and nurses treating patients with COVID-19.

Another finding in our study was that, compared with those in Hubei province outside Wuhan and those outside Hubei province, health care workers in Wuhan reported more severe symptoms of depression, anxiety, insomnia, and distress. Multivariable logistic regression analysis showed that working outside Hubei province was associated with lower risk of experiencing distress. These findings indicated more stress among health care workers in Wuhan, the origin and epicenter of the epidemic in China. In addition, working as a frontline health care worker with direct engagement of patients with COVID-19 was an independent risk factor for all symptoms. As frontline health care workers in Wuhan were at especially high risk for symptoms of depression, anxiety, insomnia, and distress, their mental health may require special attention.

Limitations

This study has several limitations. First, it was limited in scope. Most participants (81.2%) were from Hubei province, limiting the generalization of our findings to less affected regions. Second, the study was carried out during 6 days and lacks longitudinal follow-up. Because of the increasingly arduous situation, the mental health symptoms of health care workers could become more severe. Thus, long-term psychological implications of this population are worth further investigation. Third, this study was unable to distinguish the association of symptoms with being a clinician in this region vs simply living in this region (because there was no comparator group) and was also unable to distinguish preexisting mental health symptoms vs new symptoms. Fourth, although the response rate of this study was 68.7%, response bias may still exist if the nonrespondents were either too stressed to respond or not at all stressed and therefore not interested in this survey.

Conclusions

In this survey study of physicians and nurses in hospitals with fever clinics or wards for patients with COVID-19 in China, health care workers responding to the spread of COVID-19 reported high rates of symptoms of depression, anxiety, insomnia, and distress. Protecting health care workers is an important component of public health measures for addressing the COVID-19 epidemic. Special interventions to promote mental well-being in health care workers exposed to COVID-19 need to be immediately implemented, with women, nurses, and frontline workers requiring particular attention.

Downloaded From: https://jamanetwork.com/ on 12/21/2022

ARTICLE INFORMATION

Accepted for Publication: March 2, 2020.

Published: March 23, 2020. doi:10.1001/jamanetworkopen.2020.3976

Open Access: This is an open access article distributed under the terms of the CC-BY License. © 2020 Lai J et al. *JAMA Network Open*.

Corresponding Authors: Zhongchun Liu, MD, Department of Psychiatry, Renmin Hospital of Wuhan University, 238 Jiefang Rd, Wuhan 430060, China (zcliu6@whu.edu.cn); Shaohua Hu, MD, Department of Psychiatry, First Affiliated Hospital, Zhejiang University School of Medicine, 79 Qingchun Rd, Hangzhou 310003, China (dorhushaohua@zju.edu.cn).

Author Affiliations: Department of Psychiatry, First Affiliated Hospital, Zhejiang University School of Medicine, Hangzhou, China (Lai, J. Hu, Wei, Huang, S. Hu); Department of Psychiatry, Renmin Hospital of Wuhan University, Wuhan, China (Ma, Y. Wang, Cai, Li, Tan, Kang, Yao, G. Wang, Liu); Department of Psychiatry, Wuhan Youfu Hospital, Wuhan, China (Wu); Department of Psychiatry, Jingmen No. 2 People's Hospital, Jingmen, China (Du); Department of Psychiatry, Wuhan Wudong Hospital, Wuhan, China (Chen); Department of Nursing, First Affiliated Hospital, Zhejiang University School of Medicine, Hangzhou, China (H. Wang).

Author Contributions: Drs Liu and S. Hu had full access to all of the data in the study and take responsibility for the integrity of the data and the accuracy of the data analysis. Drs Lai, Ma, and Y. Wang contributed equally and share first authorship. Drs Liu and S. Hu contributed equally as senior authors.

Concept and design: Liu, S. Hu.

Acquisition, analysis, or interpretation of data: Lai, Ma, Y. Wang, Cai, J. Hu, Wei, Wu, Du, Chen, Li, Tan, Kang, Yao, Huang, H. Wang, G. Wang.

Drafting of the manuscript: Lai, Ma, Y. Wang, Liu, S. Hu.

Critical revision of the manuscript for important intellectual content: Lai, Cai, J. Hu, Wei, Wu, Du, Chen, Li, Tan, Kang, Yao, Huang, H. Wang, G. Wang, Liu, S. Hu.

Statistical analysis: Ma, Y. Wang, Liu, S. Hu.

Obtained funding: Liu, S. Hu.

Administrative, technical, or material support: Lai, Cai, J. Hu, Wei, Wu, Du, Chen, Li, Tan, Kang, Yao, Huang, H. Wang, G. Wang.

Supervision: G. Wang, Liu, S. Hu.

Conflict of Interest Disclosures: None reported.

Funding/Support: This study was supported by grants 2018YFC1314600 and 2016YFC1307100 from the National Key Research and Development Program of China.

Role of the Funder/Sponsor: The funder had no role in the design and conduct of the study; collection, management, analysis, and interpretation of the data; preparation, review, or approval of the manuscript; and decision to submit the manuscript for publication.

Additional Contributions: We thank all the participants who contributed to our work.

REFERENCES

1. Li Q, Guan X, Wu P, et al. Early transmission dynamics in Wuhan, China, of novel coronavirus-infected pneumonia [published online January 29, 2020]. *N Engl J Med*. 2020. doi:10.1056/NEJMoa2001316

2. The National Health Commission of China. Updates on the novel coronavirus outbreak up to March 2, 2020. Accessed March 3,2020. http://www.nhc.gov.cn/xcs/yqtb/202003/c588ee20113b4136b27f2a07faa7075b.shtml

3. Rothe C, Schunk M, Sothmann P, et al. Transmission of 2019-nCoV infection from an asymptomatic contact in Germany [published online January 30, 2020]. *N Engl J Med*. 2020. doi:10.1056/NEJMc2001468

4. World Health Organization. Statement on the second meeting of the International Health Regulations (2005) Emergency Committee regarding the outbreak of novel coronavirus (2019-nCoV). Published January 30, 2020. Accessed February 2, 2020. https://www.who.int/news-room/detail/30-01-2020-statement-on-the-second-meeting-of-the-international-health-regulations-(2005)-emergency-committee-regarding-the-outbreak-of-novel-coronavirus-(2019-ncov)

5. Maunder R, Hunter J, Vincent L, et al. The immediate psychological and occupational impact of the 2003 SARS outbreak in a teaching hospital. *CMAJ*. 2003;168(10):1245-1251.

6. Bai Y, Lin CC, Lin CY, Chen JY, Chue CM, Chou P. Survey of stress reactions among health care workers involved with the SARS outbreak. *Psychiatr Serv*. 2004;55(9):1055-1057. doi:10.1176/appi.ps.55.9.1055

Downloaded From: https://jamanetwork.com/ on 12/21/2022

7. Lee AM, Wong JG, McAlonan GM, et al. Stress and psychological distress among SARS survivors 1 year after the outbreak. *Can J Psychiatry*. 2007;52(4):233-240. doi:10.1177/070674370705200405

8. Chua SE, Cheung V, Cheung C, et al. Psychological effects of the SARS outbreak in Hong Kong on high-risk health care workers. *Can J Psychiatry*. 2004;49(6):391-393. doi:10.1177/070674370404900609

9. The State Council of China. A notification to set up nationwide psychological assistance hotlines against the 2019-nCoV outbreak. Published February 2, 2020. Accessed March 3, 2020. http://www.gov.cn/xinwen/2020-02/02/content_5473937.htm

10. Zhang YL, Liang W, Chen ZM, et al. Validity and reliability of Patient Health Questionnaire-9 and Patient Health Questionnaire-2 to screen for depression among college students in China. *Asia Pac Psychiatry*. 2013;5(4):268-275. doi:10.1111/appy.12103

11. He XY, Li CB, Qian J, Cui HS, Wu WY. Reliability and validity of a generalized anxiety scale in general hospital outpatients. *Shanghai Arch Psychiatry*. 22(4):200-203. doi:10.3969/j.issn.1002-0829.2010.04.002

12. Yu DS. Insomnia Severity Index: psychometric properties with Chinese community-dwelling older people. *J Adv Nurs*. 2010;66(10):2350-2359. doi:10.1111/j.1365-2648.2010.05394.x

13. Wu KK, Chan KS. The development of the Chinese version of Impact of Event Scale–Revised (CIES-R). *Soc Psychiatry Psychiatr Epidemiol*. 2003;38(2):94-98. doi:10.1007/s00127-003-0611-x

14. Tong X, An D, McGonigal A, Park SP, Zhou D. Validation of the Generalized Anxiety Disorder-7 (GAD-7) among Chinese people with epilepsy. *Epilepsy Res*. 2016;120:31-36. doi:10.1016/j.eplepsyres.2015.11.019

15. Wong TW, Yau JK, Chan CL, et al. The psychological impact of severe acute respiratory syndrome outbreak on healthcare workers in emergency departments and how they cope. *Eur J Emerg Med*. 2005;12(1):13-18. doi:10.1097/00063110-200502000-00005

16. Wang W, Tang J, Wei F. Updated understanding of the outbreak of 2019 novel coronavirus (2019-nCoV) in Wuhan, China. *J Med Virol*. 2020;92(4):441-447. doi:10.1002/jmv.25689

17. Chan-Yeung M. Severe acute respiratory syndrome (SARS) and healthcare workers. *Int J Occup Environ Health*. 2004;10(4):421-427. doi:10.1179/oeh.2004.10.4.421

18. Li L, Cheng S, Gu J. SARS infection among health care workers in Beijing, China. *JAMA*. 2003;290(20):2662-2663. doi:10.1001/jama.290.20.2662

19. Shih FJ, Gau ML, Kao CC, et al. Dying and caring on the edge: Taiwan's surviving nurses' reflections on taking care of patients with severe acute respiratory syndrome. *Appl Nurs Res*. 2007;20(4):171-180. doi:10.1016/j.apnr.2006.08.007

20. Chan S. Nurses fighting against severe acute respiratory syndrome (SARS) in Hong Kong. *J Nurs Scholarsh*. 2003;35(3):209. doi:10.1111/j.1547-5069.2003.00209.x

21. Tzeng HM. Fighting the SARS epidemic in Taiwan: a nursing perspective. *J Nurs Adm*. 2003;33(11):565-567. doi:10.1097/00005110-200311000-00005

22. Mok E, Chung BP, Chung JW, Wong TK. An exploratory study of nurses suffering from severe acute respiratory syndrome (SARS). *Int J Nurs Pract*. 2005;11(4):150-160. doi:10.1111/j.1440-172X.2005.00520.x

SUPPLEMENT.

eFigure. Milestone Events During the Outbreak and Epidemic of COVID-19
eTable 1. Occupation and Geographic Data of Nonrespondents
eTable 2. Intergroup Comparisons of Regional Differences on Scores of Depression, Anxiety, Insomnia, and Distress
eTable 3. Factor Scores of IES-R in Total Participants and Subgroups
eTable 4. Hospital Type and Factor Scores of IES-R in Front-line Workers
eTable 5. Intergroup Comparisons of Regional Differences on Factor Scores of IES-R

Downloaded From: https://jamanetwork.com/ on 12/21/2022
