# Supplementary material for: Mental health status and related factors influencing healthcare workers during the COVID-19 pandemic: A systematic review and meta-analysis
Source: PLoS One. 2024 Jan 19;19(1):e0289454. doi: 10.1371/journal.pone.0289454 (PMC10798549; doi:10.1371/journal.pone.0289454)
Supplement: S1 Data — (ZIP) [file pone.0289454.s011.zip › literatures/7.pdf]

Epidemiology and Psychiatric Sciences

cambridge.org/eps

Original Article

*Jordi Alonso and Gemma Vilagut should be considered joint first authors and Víctor Pérez-Sola and Philippe Mortier should be considered joint senior authors.

†The MINDCOVID Working Group is formed by:

Jordi Alonso, Itxaso Alayo, Manuel Alonso, Mar Álvarez, Benedikt Amann, Franco F. Amigo, Gerard Anmella, Andres Aragón, Nuria Aragonés, Enric Aragonès, Ana Isabel Arizón, Angel Asunsolo, Alfons Ayora, Laura Ballester, Puri Barbas, Josep Basora, Elena Bereciartua, Inés Bravo Ignasi Bolibar, Xavier Bonfill, Alberto Cotillas, Andres Cuartero, Concha de Paz, Isabel del Cura, Maria Jesus del Yerro, Domingo Diaz, Jose Luis Domingo, Jose I. Emparanza, Mireia Espallargues, Meritxell Espuga, Patricia Estevan, M. Isabel Fernandez, Tania Fernandez, Montse Ferrer, Yolanda Ferreres, Giovanna Fico, M. Joao Forjaz, Rosa Garcia Barranco, J. Manuel Garcia TorrecillasC. Garcia-Ribera, Araceli Garrido, Elisa Gil, Marta Gomez, Javier Gomez, Ana Gonzalez Pinto, Josep Maria Haro, Margarita Hernando, Maria Giola Insigna, Milagros Iriberri, Nuria Jimenez, Xavi Jimenez, Amparo Larrauri, Fernando Leon, Nieves Lopez-Fresneña, Carmen Lopez, Mayte Lopez-Atanes Juan Antonio Lopez-Rodriguez, German Lopez-Cortacans, Alba Marcos, Jesus Martin, Vicente Martin, Mercedes Martinez-Cortés, Raquel Martinez-Martinez, Alma D. Martinez de Salazar, Isabel Martinez, Marco Marzola, Nelva Mata, Josep Maria Molina, Juan de Dios Molina, Emilia Molinero, Philippe Mortier, Carmen Muñoz, Andrea Murru, Jorge Olmedo, Rafael M Ortí, Rafael Padrós, Meritxell Pallejà, Raul Parra, Julio Pascual, Jose Maria Pelayo, Rosa Pla, Nieves Plana, Coro Perez Aznar, Beatriz Perez Gomez, Aurora Perez Zapata, Jose Ignacio Pijoan, Elena Polentinos, Beatriz Puertolas, Maria Teresa Puig, Alex Quilez, M. Jesus Quintana, Antonio Quiroga, David Rentero, Cristina Rey, Cristina Rius, Carmen Rodriguez-Blazquez, M. Jose Rojas, Yamina Romero, Gabriel Rubio, Mercedes Rumayor, Pedro Ruiz, Margarita Saenz, Jesus Sanchez, Ignacio Sanchez-Arcilla, Ferran Sanz, Consol Serra, Victoria Serra-Sutton, Manuela Serrano, Silvia Sola, Sara Solera, Miguel Soto, Alejandra Tarrago, Natividad Tolosa, Mireia Vazquez, Margarita Viciola, Eduard Vieta, Gemma Vilagut, Sara Yago, Jesus Yañez, Yolanda Zapico, Luis Maria Zorita, Iñaki Zorrilla, Saioa L. Zurbano, and Victor Perez-Solá.

Cite this article: Alonso J *et al* (2022). Mental impact of Covid-19 among Spanish healthcare workers. A large longitudinal survey. *Epidemiology and Psychiatric Sciences* **31**, e28, 1–16. https://doi.org/10.1017/S2045796022000130

Received: 25 October 2021
Revised: 17 March 2022
Accepted: 19 March 2022

Key words:
Depression; epidemiology; mental health; post-traumatic stress disorder; stressful life events

Author for correspondence:
Jordi Alonso, E-mail: jalonso@imim.es

© The Author(s), 2022. Published by Cambridge University Press. This is an Open Access article, distributed under the terms of the Creative Commons Attribution licence (http://creativecommons.org/licenses/by/4.0/), which permits unrestricted re-use, distribution and reproduction, provided the original article is properly cited.

CAMBRIDGE UNIVERSITY PRESS

Mental impact of Covid-19 among Spanish healthcare workers. A large longitudinal survey

J. Alonso[1,2,3,*], G. Vilagut[1,2,*], I. Alayo[1,2], M. Ferrer[1,2,3], F. Amigo[1,2], A. Aragón-Peña[4,5], E. Aragonès[6,7], M. Campos[8], I. del Cura-González[5,9,10], I. Urreta[2,11], M. Espuga[12], A. González Pinto[13,14], J. M. Haro[14,15,16], N. López Fresneña[17], A. Martínez de Salázar[18], J. D. Molina[14,19,20,21], R. M. Ortí Lucas[14,22], M. Parellada[14,17], J. M. Pelayo-Terán[14,23,24], A. Pérez Zapata[25], J. I. Pijoan[2,26], N. Plana[2,25], M. T. Puig[16,27,28,29], C. Rius[2,30], C. Rodriguez-Blazquez[31,32], F. Sanz[3,33,34], C. Serra[2,35,36], R. C. Kessler[37], R. Bruffaerts[38], E. Vieta[14,39], V. Pérez-Solá[14,16,35,*], P. Mortier[1,2,*] and MINDCOVID Working group[1,†]

[1]Health Services Research Unit, IMIM-Institut Hospital del Mar d'Investigacions Mèdiques, Barcelona, Spain; [2]CIBER Epidemiología y Salud Pública (CIBERESP), Madrid, Spain; [3]Department of Medicine and Life Sciences, Universitat Pompeu Fabra, Barcelona, Spain; [4]Epidemiology Unit, Regional Ministry of Health, Community of Madrid, Madrid, Spain; [5]Fundación Investigación e Innovación Biosanitaria de AP, Comunidad de Madrid, Madrid, Spain; [6]Institut d'Investigació en Atenció Primària IDIAP Jordi Gol, Barcelona, Spain; [7]Atenció Primària Camp de Tarragona, Institut Català de la Salut, Tortosa, Spain; [8]Service of Prevention of Labor Risks, Medical Emergencies System, Generalitat de Catalunya, Barcelona, Spain; [9]Research Unit, Primary Care Management, Madrid Health Service, Madrid, Spain; [10]Department of Medical Specialities and Public Health, King Juan Carlos University, Madrid, Spain; [11]Hospital Universitario Donostia, San Sebastián, Spain; [12]Occupational Health Service, Hospital Universitari Vall d'Hebron, Barcelona, Spain; [13]Hospital Universitario Araba-Santiago, Vitoria-Gasteiz, Spain; [14]CIBER Salud Mental (CIBERSAM), Madrid, Spain; [15]Parc Sanitari Sant Joan de Déu, Barcelona, Spain; [16]Universitat Autònoma de Barcelona (UAB), Barcelona, Spain; [17]Hospital General Universitario Gregorio Marañón, Madrid, Spain; [18]UGC Salud Mental, Hospital Universitario Torrecárdenas, Almería, Spain; [19]Villaverde Mental Health Center, Clinical Management Area of Psychiatry and Mental Health, Psychiatric Service, Hospital Universitario 12 de Octubre, Madrid, Spain; [20]Research Institute Hospital 12 de Octubre (i+12), Madrid, Spain; [21]Faculty of Health Sciences, Francisco de Vitoria University, Madrid, Spain; [22]Hospital Clínic Universitari, Valencia, Spain; [23]Servicio de Psiquiatría y Salud Mental, Hospital el Bierzo, Gerencia de Asistencia Sanitaria del Bierzo (GASBI), Gerencia Regional de Salud de Castilla y Leon (SACYL), Ponferrada, León, Spain; [24]Area de Medicina Preventiva y Salud Pública, Universidad de León, León, Spain; [25]Príncipe de Asturias University Hospital, Alcalá de Henares, Madrid, Spain; [26]Hospital Universitario Cruces/OSI EEC, Bilbao, Spain; [27]Department of Epidemiology and Public Health, Hospital de la Santa Creu i Sant Pau, Barcelona, Spain; [28]Biomedical Research Institute Sant Pau (IIB Sant Pau), Barcelona, Spain; [29]CIBER Enfermedades Cardiovasculares (CIBERCV), Madrid, Spain; [30]Agència de Salut Pública de Barcelona, Barcelona, Spain; [31]National Center of Epidemiology, Instituto de Salud Carlos III (ISCIII), Madrid, Spain; [32]CIBER Enfermedades Neurodegenerativas (CIBERNED), Madrid, Spain; [33]Research Progamme on Biomedical Informatics (GRIB), Hospital del Mar Medical Research Institute (IMIM), Barcelona, Spain; [34]Instituto Nacional de Bioinformatica – ELIXIR-ES, Barcelona, Spain; [35]Parc de Salut Mar PSMAR, Barcelona, Spain; [36]CiSAL-Centro de Investigación en Salud Laboral, IMIM/UPF, Barcelona, Spain; [37]Department of Health Care Policy, Harvard Medical School, Boston, MA, USA; [38]Center for Public Health Psychiatry, Universitair Psychiatrisch Centrum, KU Leuven, Leuven, Belgium and [39]Fundació Clínic per a la Recerca Biomèdica, Barcelona, Spain

Abstract

Aims. Longitudinal data on the mental health impact of the coronavirus disease 2019 (Covid-19) pandemic in healthcare workers is limited. We estimated prevalence, incidence and persistence of probable mental disorders in a cohort of Spanish healthcare workers (Covid-19 waves 1 and 2) -and identified associated risk factors.

Methods. 8996 healthcare workers evaluated on 5 May–7 September 2020 (baseline) were invited to a second web-based survey (October–December 2020). Major depressive disorder (PHQ-8 $\geq$ 10), generalised anxiety disorder (GAD-7 $\geq$ 10), panic attacks, post-traumatic stress disorder (PCL-5 $\geq$ 7), and alcohol use disorder (CAGE-AID $\geq$ 2) were assessed. Distal (pre-pandemic) and proximal (pandemic) risk factors were included. We estimated the incidence of probable mental disorders (among those without disorders at baseline) and persistence (among those with disorders at baseline). Logistic regression of individual-level [odds ratios (OR)] and population-level (population attributable risk proportions) associations were estimated, adjusting by all distal risk factors, health care centre and time of baseline interview.

Results. 4809 healthcare workers participated at four months follow-up (cooperation rate = 65.7%; mean = 120 days S.D. = 22 days from baseline assessment). Follow-up prevalence of any disorder was 41.5%, (v. 45.4% at baseline, $p < 0.001$); incidence, 19.7% (S.E. = 1.6) and persistence, 67.7% (S.E. = 2.3). Proximal factors showing significant bivariate-adjusted associations

with incidence included: work-related factors [prioritising Covid-19 patients (OR = 1.62)], stress factors [personal health-related stress (OR = 1.61)], interpersonal stress (OR = 1.53) and financial factors [significant income loss (OR = 1.37)]. Risk factors associated with persistence were largely similar.
Conclusions. Our study indicates that the prevalence of probable mental disorders among Spanish healthcare workers during the second wave of the Covid-19 pandemic was similarly high to that after the first wave. This was in good part due to the persistence of mental disorders detected at the baseline, but with a relevant incidence of about 1 in 5 of HCWs without mental disorders during the first wave of the Covid-19 pandemic. Health-related factors, work-related factors and interpersonal stress are important risks of persistence of mental disorders and of incidence of mental disorders. Adequately addressing these factors might have prevented a considerable amount of mental health impact of the pandemic among this vulnerable population. Addressing health-related stress, work-related factors and interpersonal stress might reduce the prevalence of these disorders substantially. Study registration number: NCT04556565

Introduction

Many studies and several systematic reviews (Boden *et al.*, 2021*a*; Chigwedere *et al.*, 2021; de Kock *et al.*, 2021; Phiri *et al.*, 2021; Santabarbara *et al.*, 2021; Sun *et al.*, 2021) suggest that prevalence of mental health problems was extremely high among healthcare workers during the first wave of the coronavirus disease 2019 (Covid-19) pandemic, including depression, anxiety, post-traumatic stress, insomnia and other symptoms. Prevalence of anxiety was estimated to be 37% and of depression 36% pooled across 44 of these studies (Sun *et al.*, 2021). However, substantial variability of results exists across studies. Mental disorders and suicidal ideation and behaviours among healthcare workers during the initial phases of the pandemic were higher than those among the general population (Mortier *et al.*, 2021).

In the general population, several longitudinal studies suggest that initial high levels of anxiety, depression and other Covid-related psychopathology tend to remit with time (Hirten *et al.*, 2020; McFadden *et al.*, 2021; Miguel-Puga *et al.*, 2021; Sampaio *et al.*, 2021; Sasaki *et al.*, 2021; Van Steenkiste *et al.*, 2021). But evidence about this trajectory among healthcare workers, who have continued to be exposed to a very high workload related to the pandemic, is more limited and results are mixed. Some studies suggest that distress and fear/worry of Covid-19 remained high or even increased in follow-up surveys (Jordan *et al.*, 2021; Lopez Steinmetz *et al.*, 2022; McFadden *et al.*, 2021; Miguel-Puga *et al.*, 2021; Sasaki *et al.*, 2021) while others indicate that depression and anxiety tended to decrease at follow-up (Hirten *et al.*, 2020; Roberts *et al.*, 2021; Rodriguez *et al.*, 2021; Sampaio *et al.*, 2021; Van Steenkiste *et al.*, 2021). Some of these studies are based on a relatively small number of healthcare workers or on short follow-up periods (Hirten *et al.*, 2020; Sampaio *et al.*, 2021; Van Steenkiste *et al.*, 2021) or do not focus on specific mental disorders (Hirten *et al.*, 2020; Roberts *et al.*, 2021; Sampaio *et al.*, 2021; Sasaki *et al.*, 2021). Therefore, knowledge about the evolution of mental disorders among healthcare workers over the course of the pandemic is limited.

Many factors have been associated with the mental health impact of early Covid-19 pandemic phases among healthcare workers. Distal risk factors include existing mental disorders, female sex, nurse profession and lower income (Kunzler *et al.*, 2021). Proximal risk factors include frequency/intensity of exposure to the Covid-19 pandemic, longer working hours in high-risk environments, inadequate/insufficient material and human resources, increased workload (Galanis *et al.*, 2021). Given the scarcity of longitudinal studies among healthcare workers, knowledge about risk factors associated with poor mental health trajectories during Covid-19 is limited. One study among Portuguese nurses showed that fear of personal infection and of infecting others were both associated with higher depression, anxiety and stress scores (Sampaio *et al.*, 2021). Another study of 361 healthcare workers in New York reported increased levels of stress associated with a higher number of Covid-19 cases in the community, while stress was reduced among workers with high emotional support and high resilience at baseline (Hirten *et al.*, 2020). The influence of distal and proximal risk factors on the evolution of the mental health impact of Covid-19 among healthcare workers requires further research.

This paper aims to assess the evolution of mental disorders (prevalence, incidence and persistence) after the Covid-19 pandemic in a large cohort of healthcare workers after 4 months follow-up in Spain. The paper also examines the association of proximal and distal risk factors with follow-up mental disorders. The period studied corresponds with the final part of wave 1 and the initial part of wave 2 of the pandemic in Spain.

Methods

Study design, population sampling and follow-up

A multicentre, observational cohort study of healthcare workers was carried out in a convenience sample of 18 healthcare institutions from 6 Autonomous Communities in Spain including hospitals, primary care and public healthcare centres. Institutional representatives invited all employed workers to participate using the institution's administrative email distribution lists (i.e., census sampling) to a web-based survey platform (qualtrics.com). Informed consent was obtained from all participants. Up to two reminder emails were sent within a 2–4-week period after the initial invitation. Workers completing the baseline interview (May 5th through September 7th, just after the peak of the first wave of the Covid-19 pandemic in Spain) and providing an e-mail address were re-contacted for the follow up interview approximately 4 months after, between October 9th and December 11th 2020, at the ascending curve of the pandemic's second wave (mean follow-up time of 120 days; median = 118; IQ range

= 105–136 days). More information can be found elsewhere (Alonso *et al.*, 2021; Mortier *et al.*, 2022).

Measures

We used a conceptual framework to study the mental health impact of Covid 19 considering: (i) current mental disorders (negative mental health status), (ii) proximal (pandemic) risk factors (infection, work-related factors and health-related stress), assessed in the baseline survey and distal (pre-pandemic) risk factors (social and economic characteristics and clinical vulnerabilities) (Boden *et al.*, 2021*b*).

Probable current mental disorders

Major depressive disorder (MDD): We used the Spanish version of the Patient Health Questionnaire (PHQ-8), with the cut-off point = 10+ of the sum score to indicate current MDD. The PHQ-8 shows high reliability (>0.8) and good diagnostic accuracy for MDD (AUC > 0.90) (Wu *et al.*, 2020). Nevertheless, the instrument can overestimate the prevalence of depressive disorders (Levis *et al.*, 2020).

Generalised anxiety disorder (GAD): We used the seven-item GAD scale (GAD-7), which has a good performance to detect anxiety (AUC > 0.8) (Kroenke *et al.*, 2010; Kessler *et al.*, 2013). We used the Spanish version of the GAD-7 (Garcia-Campayo *et al.*, 2010) and considered the cut-off point of 10+ to indicate a current GAD.

Panic attacks: the number of panic attacks in the 30 days prior to the interview, assessed with an item from the World Mental Health-International College Student-WMH-ICS (Kessler *et al.*, 2013; Blasco *et al.*, 2016). A dichotomous variable indicated the presence/absence of panic attacks.

Posttraumatic Stress Disorder (PTSD): assessed using the 4-item version of the PTSD checklist for DSM-5 (PCL-5) (Blasco *et al.*, 2016; Zuromski *et al.*, 2019) which generates diagnoses that closely parallel those of the full PCL-5 (AUC > 0.9), making it well-suited for screening (Weathers *et al.*, 2013). We used the Spanish version of the questionnaire (Resick *et al.*, 2020), and considered a cut-off point of 7 to indicate current PTSD.

Substance Use Disorder (SUD): We used the CAGE adapted to include drugs questionnaire (CAGE-AID), that consists of 4 items focusing on Cutting down, Annoyance by criticism, Guilty feeling, and Eye-openers, proven useful in helping to make a diagnosis of Alcohol Use Disorder (Dohrenwend *et al.*, 1978; Hinkin *et al.*, 2001; Zuromski *et al.*, 2019) and Substance Use Disorder (Hinkin *et al.*, 2001). The questionnaire has been adapted into Spanish (Diez Martinez *et al.*, 1991). Cut-off point of 2+ was considered to indicate current SUD (Mdege and Lang, 2011).

Proximal risk factors

Covid-19 exposure and infection status: Whether the respondent had been hospitalised for Covid-19 infection and/or had a positive Covid-19 test or medical diagnosis not requiring hospitalisation, whether the respondent had been isolated or quarantined because of exposure to Covid-19 infected person(s), and whether they had loved ones infected with Covid-19.

Work-related factors: Perceived lack of care centre preparedness (i.e., lack of coordination, communication, personnel, supervision at work, training for assigned tasks) using four 5-level Likert-type items ranging from ‘none of the time’ to ‘all of the time’ (summed score rescaled to a 0.0–4.0; Cronbach $\alpha = 0.86$); average weekly hours worked; changes in assigned functions, team, or working location; changes of a team or assigned functions, and no changes; perceived frequency of lack of protective equipment into a 5-level Likert scale from 0 (‘none of the time’) to 4(‘all of the time’), having to make decisions regarding prioritising care among Covid19 patients, having patients in care that died from Covid-19 infection. We also assessed the frequency of direct exposure to Covid-19 infected patients during professional activity, 5-level Likert type item, ranging from 0 (‘none of the time’) to 4(‘all of the time’).

Health-related stress: Personal health-related stress, as the mean of two items on a 5-level Likert type scale ranging from 0 to 4 with higher values indicating higher levels of stress (Cronbach $\alpha = 0.80$); and health-related stress of loved ones assessed with two items on a 5-level Likert type scale (concern about loved ones being infected with Covid-19, and concerns about the health of loved ones). The mean value of the corresponding items is obtained with a range from 0 (lower levels of stress) to 4 (higher levels of stress) (Cronbach $\alpha = 0.85$).

Financial factors: Having suffered a significant loss in personal or familial income due to the Covid-19 pandemic; financial stress, calculated using two 5-level Likert-type items (Dohrenwend *et al.*, 1978), and stress regarding job loss or loss of income because of Covid-19, obtaining a 2-item combined score (Cronbach $\alpha = 0.82$).

Interpersonal stress: Mean score of four 5-level Likert type items (i.e., love life, relationships with family, problems getting along with people at work or school, other problems experienced by loved ones) ranging from 0 to 4, with higher values indicating higher levels of stress (Cronbach $\alpha = 0.79$).

Family functioning: The Brief Assessment of Family Functioning Scale (BAFFS) (Mansfield *et al.*, 2019), a three-item version of the general functioning scale from the Family Assessment Device (FAD) (Epstein *et al.*, 1983) that was developed to assess perceived satisfaction or distress with general family functioning. The Spanish version of the scale is available with good psychometric results (Barroilhet *et al.*, 2009). Cronbach's α for internal consistency in our study was $\alpha = 0.50$.

Parental stress: 4-item version derived from the Parental Stress Scale (Berry and Jones, 1995). Items were summed and re-scaled ranging from 0 (lower parental stress) to 4 (highest parental stress). The Spanish version has been developed showing adequate psychometric properties (Oronoz *et al.*, 2007). Internal consistency obtained in our study was $\alpha = 0.74$.

Distal risk factors

Demographics and professional characteristics: Age category; gender; country of birth; marital status; having children in care; profession; and workplace.

Prior lifetime mental disorders: Lifetime mental disorders prior to the onset of the Covid-19 outbreak assessed using a checklist from the Composite International Diagnostic Interview (CIDI) (Navarro-Mateu *et al.*, 2013) screening lifetime mood, anxiety, substance use problems and ‘other’ mental disorders. The number of chronic physical health conditions was also assessed for a list of conditions considered as vulnerable to Covid-19.

Ethical considerations

The study complies with the Declaration of Helsinki and the Code of Ethics and was approved by the IRB Parc de Salut Mar (2020/9203/I) and by the corresponding IRBs of all the

participating centres. Registered at ClinicalTrials.gov (https://clinicaltrials.gov/ct2/show/NCT04556565).

Statistical analysis

Missing item-level data among respondents were imputed using multiple imputations (MI) by chained equations (Van Bureen, 2012) (12 imputed datasets, 10 iterations per imputation).

Sample characteristics are reported as weighted percentages and s.e. Differences of baseline characteristics between those who did and those who did not participate in the follow-up survey (lost to follow up) were evaluated using the modified Rao-Scott χ^2 test. To correct the bias caused by lost to follow up missing values, inverse-probability weighting (IPW) (Seaman *et al.*, 2012) was applied, as the inverse of the probability of completing the follow-up survey on observed related baseline covariates, estimated using a logistic regression model. Additionally, post-stratification weights through raking were used to adjust for potential deviations from target population distributions in terms of age, gender, profession and healthcare centre. Pooled MI-based parameter estimates and standard errors (s.e.) and statistical inferences were obtained from the weighted analysis of these MI datasets.

Prevalence of any probable mental disorder at baseline and at follow up was estimated overall and stratified by distal and proximal risk factors. Prevalence of any probable mental disorder at follow-up stratified among individuals with a negative baseline screen (i.e., incidence) and among individuals with a positive baseline screen (i.e., persistence) were also estimated. Logistic regression estimated the association of each proximal risk factor with probable mental disorders adjusting each time for all distal risk factors, health care centre and time of baseline survey. Regression coefficients were exponentiated to obtain odds ratios (OR) and 95% confidence intervals (CIs). All Likert-type variables were analysed as continuous. Potential deviations from a continuous linear effect in the logit were assessed graphically. Population-attributable risk proportions (PARP) (Krysinska and Martin, 2009) and associated 95% confidence intervals obtained with bootstrap resampling (200 replications), were simulated based on individuals' predicted probabilities estimated by the multivariable logistic regression equations (Nock *et al.*, 2014). PARP provide estimates of the proportions of mental disorders that could potentially be attributed to specific predictor variables assuming a causal pathway between these predictor variables and the outcome.

All analyses were adjusted by the week of the interview (entered as a continuous variable) to account for possible differences of baseline predictor variables by the time of assessment at baseline. Since the correlation between the response time at baseline and the time of follow up was $r = -0.93$, we only adjusted by one of these variables.

Variance estimates were obtained using the Taylor series linearisation method considering weighting and within-health care centre clustering of data. MI were carried out using package mice from R (Van Buuren and Groothuis-Oudshoorn, 2011) Analyses were performed using R v4.1.032 (Team, 2021) and SAS v9.4 (INC SI, 2014).

Results

Participation

A total of 8996 healthcare workers participated in the baseline evaluation. The survey participation rate, calculated as unique individuals who agreed to participate ($n = 10\,360$) divided by unique first survey page visitors ($n = 11\,507$), was 90.0%, and of them, 8328 professionals finalised the baseline interview, representing a survey completion rate of 80.4% (Eysenbach, 2004). Of the total 8996 unique healthcare workers that provided sufficient information of the baseline evaluation, $n = 7318$ provided their email to be re-contacted and $n = 4809$ (cooperation rate = 65.7%) answered the follow-up interview between 9th of October and 11th of December 2021, with a mean number of 120.1 days (s.d. = 22.2) between baseline and follow up assessments. Those participating in the follow-up survey differed only marginally in some baseline study characteristics (follow-up participants were older, with higher income, with a higher proportion being medical doctors, and having suffered of Covid-19 on their own or their loved ones and had suffered less health-related and financials stresses). These differences were very small, although statistically significant due to the high numbers involved (online Supplementary Table 1). Inverse probability weights were applied to restore these differences as explained above.

Any mental disorder prevalence, incidence and persistence

At 4-month follow-up, the prevalence of any probable mental disorder was 41.5%, somewhat lower than at baseline (45.4%, Mc Nemar test p-value < 0.001) (Table 1 and Fig. 1*a*). Prevalence at follow-up was higher for younger age groups, female gender, country of birth other than Spain, lower income, auxiliary nurse profession, and several pre-pandemic mental disorders and higher number of chronic physical health conditions. Incidence of probable mental disorder (among those without baseline disorder) was 19.7% (s.e. = 1.6), being significantly higher among those with lower income and those with some pre-pandemic mental disorders (Table 1, middle, and Fig. 1*b*). More than two thirds (67.7%; s.e. = 2.3) with a baseline probable mental disorder, persisted with a disorder at follow-up. Persistence was significantly associated to the same distal factors than incidence, except for lower-income levels, for which higher persistence frequency did not reach statistical significance, and except for a number of chronic physical health conditions, significantly associated to persistence, but not to incidence (Table 1, three last columns, and Fig. 1*b*).

There was a trend towards a lower severity of both PHQ-8 and GAD-7 scores between baseline and 4-months follow-up assessments (see online Supplementary Tables 2 and 3). Online Supplementary Tables 4 and 5 present unadjusted and adjusted, respectively, associations between distal risk factors and probable mental disorders prevalence, incidence and persistence.

Association between proximal risk factors and any mental disorder

Table 2 presents associations of prevalence, incidence and persistence of any probable mental disorder with each proximal and work-related risk factor in our study, adjusting by all distal factors (i.e., those presented in Table 1). Each row presents a different model. An overall multivariate model was not primarily attempted, in order to minimise over adjustment bias (Schistermann *et al.*, 2009). (Online Supplementary Table 6 shows the bivariate associations, and online Supplementary Table 7, the fully adjusted model.) All risk factors evaluated were significantly associated with a higher follow-up prevalence of probable mental disorders, except for having been hospitalised

Table 1. Prevalence, incidence and persistence of any of mental disorders, total and by distal risk factors. Spanish healthcare workers, MINDCOVID study (absolute numbers and weighted proportions)

	Full follow-up sample (n = 4809)				Among those with NO baseline disorders (n = 2681)			Among those with any baseline disorder (n = 2128)		
			Any baseline disorder (n = 2128)	Any follow-up disorder (n = 1870)			Incidence of any disorder (n = 480)			Persistence of any disorder (n = 1390)
	n^a	%^{b} (s.e.)	%^{b} (s.e.)	%^{b} (s.e.)	n^a	% ^{b} (s.e.)	%^{b} (s.e.)	n^a	%^{b} (s.e.)	%^{b} (s.e.)
Total			45.4 (1.8)	41.5 (1.9)			19.7 (1.6)			67.7 (2.3)
Age										
50 years or more	2031	43.5 (2.5)	39.5 (2.1)	37.8 (2.0)	1237	48.2 (2.6)	17.6 (1.4)	795	37.8 (2.7)	68.6 (2.5)
30–49 years	2275	45.5 (1.6)	49.1 (2.1)	44.0 (2.3)	1223	42.4 (2.1)	21.1 (2.0)	1052	49.2 (2.1)	67.7 (2.5)
18–29 years	503	11.0 (1.9)	53.7 (3.5)	45.8 (2.7)	222	9.3 (1.4)	23.7 (4.3)	281	13.0 (2.7)	64.9 (4.4)
Modified Rao-Scott (R-S) χ^2 test			9.65 (225 106) [<0.001]*	9.33 (239 061) [<0.001]*			2.34 (220 640) [0.097]			0.43 (213 426) [0.652]
Gender										
Female	3899	77.6 (1.3)	47.2 (2.0)	42.8 (2.1)	2096	75.1 (1.6)	20.6 (2.1)	1803	80.7 (1.3)	67.5 (2.4)
Male	910	22.4 (1.3)	39.1 (2.0)	37.0 (1.7)	585	24.9 (1.6)	16.8 (2.0)	325	19.3 (1.3)	68.5 (3.6)
Modified Rao-Scott (R-S) χ^2 test			10.72 (11 875) [0.001]*	8.63 (110 196) [0.003]*			1.40 (113 171) [0.236]			0.09 (188 852) [0.766]
Country of birth										
Spain	4582	95.4 (0.5)	45.1 (2.0)	41.1 (1.9)	2570	95.9 (0.5)	19.4 (1.5)	2012	94.8 (0.9)	67.6 (2.3)
Other	227	4.6 (0.5)	51.8 (3.7)	49.0 (3.3)	111	4.1 (0.5)	26.3 (6.3)	116	5.2 (0.9)	70.2 (5.2)
Modified Rao-Scott (R-S) χ^2 test			1.66 (15 765) [0.197]	4.09 (156 397) [0.043]*			1.23 (1 137 771) [0.266]			0.30 (15 353) [0.586]
Marital status										
Single, divorced, legally separated, or widowed	2272	47.3 (2.2)	49.0 (1.8)	43.4 (2.7)	1199	44.1 (2.0)	18.9 (2.9)	1073	51.0 (2.8)	68.9 (3.0)
Married	2537	52.7 (2.2)	42.2 (2.2)	39.7 (1.7)	1482	55.9 (2.0)	20.2 (1.3)	1054	49.0 (2.8)	66.4 (2.6)
Modified Rao-Scott (R-S) χ^2 test			11.97 (123 177) [<0.001]*	2.52 (194 691) [0.112]			0.19 (127 056) [0.666]			0.65 (159 316) [0.421]
Income										
More than 4500€	1659	28.8 (1.5)	38.0 (1.4)	33.5 (2.1)	1050	32.8 (1.8)	15.4 (1.6)	609	24.1 (1.6)	63.0 (3.5)
Between 2200€–4500€	1730	36.1 (1.2)	42.6 (2.5)	41.0 (2.0)	982	37.9 (1.4)	20.0 (1.6)	748	33.9 (1.5)	69.2 (2.6)
Less than 2200€	1420	35.1 (2.2)	54.4 (2.3)	48.5 (2.5)	649	29.3 (2.1)	23.9 (3.4)	771	42.0 (2.6)	69.2 (2.7)
Modified Rao-Scott (R-S) χ^2 test			22.14 (2309) [<0.001]*	24.04 (2285) [<0.001]*			3.86 (2875) [0.021]*			2.34 (2767) [0.097]

(*Continued*)

Table 1. (*Continued.*)

	Full follow-up sample ($n = 4809$)				Among those with NO baseline disorders ($n = 2681$)			Among those with any baseline disorder ($n = 2128$)		
			Any baseline disorder ($n = 2128$)	Any follow-up disorder ($n = 1870$)			Incidence of any disorder ($n = 480$)			Persistence of any disorder ($n = 1390$)
	n[a]	%[b] (s.e.)	%[b] (s.e.)	%[b] (s.e.)	*n*[a]	%[b] (s.e.)	%[b] (s.e.)	n[a]	%[b] (s.e.)	%[b] (s.e.)
Children in care										
Yes	1995	41.2 (1.4)	43.7 (2.6)	40.1 (2.0)	1139	42.5 (1.1)	21.2 (2.1)	856	39.7 (2.7)	64.4 (2.9)
No	2814	58.8 (1.4)	46.6 (2.1)	42.5 (2.3)	1542	57.5 (1.1)	18.6 (1.7)	1272	60.3 (2.7)	69.9 (2.9)
Modified Rao-Scott (R-S) χ^2 test (DF) [p-value]			0.92 (134 650) [0.337]	1.07 (133 524) [0.301]			1.47 (113 670) [0.225]			2.47 (118 185) [0.116]
Profession										
Medical doctor	1650	26.3 (2.8)	36.5 (1.2)	34.5 (2.7)	1041	30.6 (3.0)	17.4 (2.1)	608	21.2 (2.8)	64.2 (3.8)
Nurse	1406	31.0 (1.2)	49.4 (2.4)	42.4 (2.4)	697	28.7 (1.2)	19.6 (1.7)	709	33.7 (1.6)	65.8 (3.3)
Auxiliary nurse	387	13.7 (3.2)	56.8 (4.7)	51.7 (4.2)	157	10.8 (2.5)	24.1 (2.5)	230	17.1 (4.2)	72.7 (4.1)
Other profession involved in patient care	555	9.0 (0.8)	41.0 (3.5)	39.1 (3.7)	324	9.7 (0.9)	17.8 (2.5)	231	8.1 (1.1)	69.8 (3.0)
Other profession not involved in patient care	812	20.0 (2.4)	45.2 (3.7)	43.3 (4.4)	462	20.1 (1.9)	21.8 (4.3)	350	19.9 (3.7)	69.4 (3.0)
Modified Rao-Scott (R-S) χ^2 test (DF) [p-value]			5.85 (413 247) [<0.001]*	3.37 (425 209) [0.009]*			1.08 (424 948) [0.363]			1.22 (412 096) [0.299]
Workplace										
Hospital	2818	57.5 (0.9)	47.1 (2.6)	40.9 (2.5)	1525	55.7 (1.2)	18.4 (1.8)	1293	59.7 (1.4)	66.2 (2.6)
Primary care	1581	36.2 (0.9)	44.2 (1.2)	43.6 (1.9)	899	37.0 (1.2)	22.2 (1.7)	682	35.2 (1.3)	70.6 (3.5)
Others	410	6.3 (0.4)	36.6 (4.0)	34.5 (3.0)	258	7.3 (0.7)	16.6 (2.2)	153	5.1 (0.6)	65.4 (5.8)
Modified Rao-Scott (R-S) χ^2 test (DF) [p-value]			2.61 (258 618) [0.074]	1.58 (229 265) [0.206]			1.84 (23 356) [0.159]			0.77 (239 577) [0.463]
Pre-pandemic mood disorder										
Yes	528	11.4 (0.6)	70.3 (2.7)	64.3 (4.0)	164	6.2 (0.5)	32.7 (7.2)	364	17.6 (1.1)	77.7 (3.5)
No	4281	88.6 (0.6)	42.2 (1.7)	38.5 (1.7)	2517	93.8 (0.5)	18.8 (1.4)	1764	82.4 (1.1)	65.5 (2.3)
Modified Rao-Scott (R-S) χ^2 test (DF) [p-value]			77.96 (11 962) [<0.001]*	38.00 (113 592) [<0.001]*			4.42 (161 749) [0.035]*			16.30 (17 138) [<0.001]*
Pre-pandemic anxiety disorder										
Yes	1710	36.8 (0.9)	61.8 (2.1)	54.6 (2.6)	670	25.8 (0.7)	26.9 (3.8)	1040	50.1 (1.4)	71.7 (2.5)
No	3099	63.2 (0.9)	35.8 (1.8)	33.8 (1.8)	2012	74.2 (0.7)	17.2 (1.2)	1088	49.9 (1.4)	63.7 (2.9)

Modified Rao-Scott (R-S) χ^2 test (DF) [p-value]			105.07 (130) [<0.001]*	78.80 (11 171) [<0.001]*			8.22 (17 328) [0.004]*			7.45 (12 399) [0.006]*
Pre-pandemic substance use disorder										
Yes	60	1.5 (0.1)	87.9 (5.3)	68.0 (6.7)	12	0.3 (0.1)	20.4 (10.2)	49	2.9 (0.4)	74.6 (7.2)
No	4749	98.5 (0.1)	44.8 (1.8)	41.1 (1.9)	2670	99.7 (0.1)	19.7 (1.6)	2079	97.1 (0.4)	67.5 (2.3)
Modified Rao-Scott (R-S) χ^2 test (DF) [p-value]			30.40 (12 790) [<0.001]*	10.72 (12 301) [0.001]*			0.01 (125 024) [0.916]			0.99 (1877) [0.319]
Other pre-pandemic mental disorder										
Yes	38	0.8 (0.1)	58.1 (8.7)	60.3 (11.7)	16	0.6 (0.2)	41.3 (16.9)	21	1.0 (0.2)	74.1 (9.2)
No	4772	99.2 (0.1)	45.3 (1.8)	41.3 (1.8)	2665	99.4 (0.2)	19.5 (1.5)	2107	99.0 (0.2)	67.6 (2.3)
Modified Rao-Scott (R-S) χ^2 test (DF) [p-value]			2.39 (184 740) [0.122]	3.04 (1 350 096) [0.081]			2.43 (11 914 069) [0.119]			0.50 (1 831 474) [0.478]
Number of pre-pandemic lifetime mental disorders										
Two or more	377	8.4 (0.7)	77.1 (3.0)	65.0 (4.6)	84	3.5 (0.4)	27.3 (8.7)	293	14.3 (1.3)	76.2 (4.7)
Exactly one	1550	32.8 (0.9)	57.3 (2.1)	52.7 (2.7)	689	25.6 (0.8)	28.5 (3.6)	861	41.3 (2.0)	70.7 (2.2)
None	2882	58.8 (0.9)	34.3 (1.9)	31.9 (1.8)	1909	70.8 (0.7)	16.1 (1.1)	973	44.4 (1.6)	62.1 (3.1)
Modified Rao-Scott (R-S) χ^2 test (DF) [p-value]			75.44 (21 250) [<0.001]*	35.58 (24 262) [<0.001]*			7.58 (24 445) [<0.001]*			6.25 (22 828) [0.002]*
Number of physical health conditions										
Two or more	157	3.9 (0.5)	63.6 (5.1)	60.8 (4.1)	57	2.6 (0.5)	21.3 (4.8)	100	5.5 (0.7)	83.5 (4.2)
Exactly one	1013	22.0 (0.8)	49.4 (2.6)	45.5 (2.2)	502	20.4 (1.1)	22.7 (2.1)	511	23.9 (1.3)	68.9 (2.9)
None	3639	74.1 (1.0)	43.3 (2.0)	39.2 (2.0)	2122	77.0 (1.2)	18.8 (1.7)	1516	70.6 (1.6)	66.1 (2.3)
Modified Rao-Scott (R-S) χ^2 test (DF) [p-value]			7.94 (21 032) [<0.001]*	13.12 (21 656) [<0.001]*			1.83 (2269) [0.163]			7.48 (25 242) [0.001]*

R-S, Rao-Scott; DF, Degrees of freedom.
Note. Covid-19, coronavirus disease 2019; s.e., standard error.
[a]Unweighted numbers.
[b]Weighted percentage [using post-stratification weights and inverse-probability weighting (IPW)].
*Statistically significant ($\alpha = 0.05$).

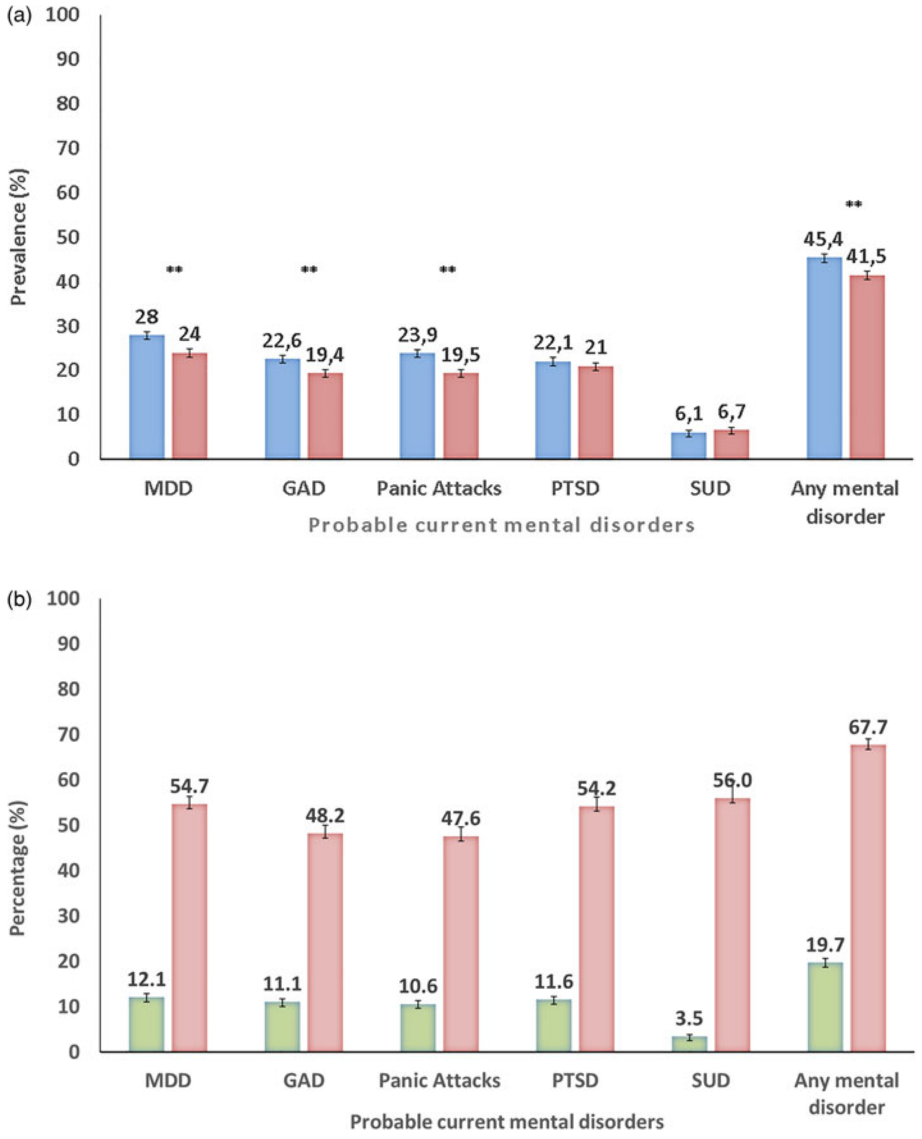

Fig. 1. (a) Prevalence of probable current mental disorders among Spanish Healthcare Workers [$n = 4809$, baseline (blue bars); 4-month follow-up (red bars)]. (b) Probable current mental disorders among Spanish healthcare workers at 4-month follow-up survey: Incident/New Disorder Onset (green bars) and Persistent/Recurrent Disorder (red bars) ($n = 4809$). MDD, major depressive disorder; GAD, generalised anxiety disorder; PTSD, posttraumatic stress disorder; SUD, substance use disorder. ***p*-value <0.001 for the multiple imputation chi-square pooling of the McNemar's test comparing paired prevalence at baseline and follow up for the mental disorders evaluated. **p*-value <0.05 for the multiple imputations χ^2 pooling of the McNemar's test comparing paired prevalence at baseline and follow up for the mental disorders evaluated.

for Covid-19 (probably due to low numbers in the sample), and for having other family, friends or others infected with Covid-19. The same risk factors showed a positive association with the prevalence of any probable mental disorder after the 1st wave of the pandemic (baseline assessment) as with prevalence during the 2nd wave (follow-up assessment) (see columns 3 and 4 of Table 2). Nevertheless, associations tended to be marginally higher for baseline than for follow up data, for work-related factors (number of hours worked), for personal health-related stress, for health-related stress of loved ones and for interpersonal stress. Covid-19 own infection was a risk factor for new onset (OR = 1.54 95% IC = 1.24, 1.91) but not for persistence (1.09; 95% IC = 0.92, 1.30), while a larger number of hours worked was a risk factor for persistence but not for new onset (Table 2, columns 7 and 10).

Population attributable risk proportions (PARPs)

Table 3 shows attributable risk proportions (PARPs) of proximal risk factors of any probable mental disorder, adjusting by distal factors. Health-related, work-related, and interpersonal stress presented the highest association with both prevalence, incidence and persistence. PARPs were generally higher for incidence of probable mental disorders than for persistence of baseline probable disorders.

Discussion

This study is, to our knowledge, the first one to estimate the prevalence of probable mental disorders among healthcare workers during the second wave of the Covid-19 pandemic focusing on the most relevant mental disorders and including a large sample. Two major results arise from this longitudinal study. First, the prevalence of probable mental disorders during the second wave of the Covid-19 pandemic was high and very similar to that during the first wave. This was mainly due to the high persistence of baseline disorders (more than two-thirds of those with disorders at baseline continued to have these disorders at follow-up); nevertheless, the incidence of new probable mental disorders was also important (one in 5 of healthcare workers free of mental disorders during the first wave developed one in the second wave). And second, health-related and work-related factors as well as interpersonal stress were important risk factors for both onset and

Table 2. Adjusted associations between proximal risk factors and any probable mental disorders diagnosis (bivariate adjusted for all distal factors). Spanish healthcare workers, MINDCOVID study (absolute numbers and weighted proportions)

	Full follow-up sample (n = 4809)				Among those with NO baseline disorders (n = 2681)			Among those with any baseline disorder (n = 2128)		
	n[a]	%[b] (S.E.) or Med (S.E.) (IQR)	Any baseline disorder (n = 2128) OR (95% CI)	Any follow-up disorder (n = 1870) OR (95% CI)	n[a]	%[b] (S.E.) or Med (S.E.) (IQR)	Incidence of any disorder (n = 480) OR (95% CI)	n[a]	%[b] (S.E.) or Med (S.E.) (IQR)	Persistence of any disorder (n = 1390) OR (95% CI)
Covid-19 infection										
Having been hospitalised for Covid-19	67	1.4 (0.2)	1.48 (0.87–2.51)	1.57 (0.93–2.64)	32	1.2 (0.3)	1.46 (0.61–3.51)	35	1.6 (0.2)	1.48 (0.56–3.89)
Positive Covid-19 test or medical Covid-19 diagnosis[c]	869	16.1 (2.0)	1.08 (0.94–1.23)	1.25 (1.11–1.42)*	461	15.2 (2.0)	1.54 (1.24–1.91)*	408	17.2 (2.1)	1.09 (0.92–1.30)
None of the above	3873	82.5 (2.1)	(Ref)	(Ref)	2189	83.6 (2.0)	(Ref)	1685	81.1 (2.3)	(Ref)
F value (ndf, ddf) [*p* value][f]			1.42 (216 394) [0.243]	9.59(21 492) [<0.001]*			8.01(23 901) [<0.001]*			1.02(23 745) [0.360]
Type of loved ones infected with Covid-19										
Partner, children, or parents	781	13.7 (2.0)	1.79 (1.27–2.52)*	1.41 (1.21–1.64)*	406	11.9 (2.2)	1.06 (0.71–1.59)	375	15.8 (1.8)	1.22 (0.96–1.55)
Other family, friends or others[d]	2964	58.6 (0.9)	1.28 (0.99–1.66)	1.11 (0.93–1.33)	1637	58.1 (1.3)	0.91 (0.71–1.15)	1327	59.2 (1.6)	1.10 (0.78–1.55)
None of the above	1065	27.7 (2.4)	(Ref)	(Ref)	639	30.0 (3.0)	(Ref)	426	25.0 (2.1)	(Ref)
F value (ndf, ddf) [*p* value][f]			6.03 (212 311) [0.002]*	12.05 (23 480) [<0.001]*			1.73 (21 531) [0.177]			1.43 (21 122) [0.240]
Covid-19 isolation										
Having been isolated or quarantined because of Covid-19	1337	25.6 (1.7)	1.34 (1.14–1.58)*	1.48 (1.19–1.83)*	671	23.0 (1.4)	1.51 (1.08–2.11)*	667	28.8 (2.2)	1.29 (1.00–1.66)*
Not having been isolated or quarantined because of Covid-19	3472	74.4 (1.7)	(Ref)	(Ref)	2011	77.0 (1.4)	(Ref)	1461	71.2 (2.2)	(Ref)
F value (ndf, ddf) [*p* value][f]			12.76 (119 879) [<0.001]*	12.70 (123 489) [<0.001]*			5.81 (11 566) [0.016]*			3.88 (13 853) [0.049]*
Frequency of direct exposure to Covid-19 patients (scale 0–4)		2.8 (0.2) (2.0–3.9)	1.35 (1.24–1.47)*	1.34 (1.20–1.50)*		2.6 (0.2) (1.6–3.6)	1.28 (1.13–1.45)*		3.0 (0.2) (2.2–4.1)	1.21 (1.04–1.42)*
F value (ndf, ddf) [*p* value][f]			41.27 (1482) [<0.001]*	27.30 (16 456) [<0.001]*			15.55 (112 051) [<0.001]*			5.84 (16 834) [0.016]*
Perceived lack of preparedness (rescaled 0–4)		1.6 (0.1) (0.7–2.4)	1.69 (1.53–1.86)*	1.53 (1.39–1.68)*		1.3 (0.1) (0.5–2.0)	1.36 (1.24–1.49)*		1.9 (0.1) (1.2–2.7)	1.25 (1.05–1.49)*
F value (ndf, ddf) [*p* value][f]			90.43 (1304) [<0.001]*	72.50 (1916) [<0.001]*			19.15 (1.27) [<0.001]*			6.10(16 782) [0.014]*
Average weekly hours worked										
51 h or more	765	14.0 (0.7)	1.59 (1.30–1.94)*	1.23 (1.02–1.50)*	397	13.0 (0.9)	0.68 (0.40–1.14)	368	15.3 (1.0)	1.36 (1.08–1.72)*
41–50 h	1140	22.7 (2.4)	1.39 (1.10–1.75)*	1.32 (1.06–1.64)*	581	20.7 (2.3)	1.08 (0.76–1.55)	559	25.1 (2.9)	1.31 (0.91–1.88)
40 h or less	2905	63.2 (2.5)	(Ref)	(Ref)	1704	66.3 (2.5)	(Ref)	1201	59.5 (2.7)	(Ref)

(*Continued*)

Table 2. (*Continued.*)

	Full follow-up sample (*n* = 4809)				Among those with NO baseline disorders (*n* = 2681)			Among those with any baseline disorder (*n* = 2128)		
	n[a]	%[b] (s.e.) or Med (s.e.) (IQR)	Any baseline disorder (*n* = 2128) OR (95% CI)	Any follow-up disorder (*n* = 1870) OR (95% CI)	*n*[a]	%[b] (s.e.) or Med (s.e.) (IQR)	Incidence of any disorder (*n* = 480) OR (95% CI)	*n*[a]	%[b] (s.e.) or Med (s.e.) (IQR)	Persistence of any disorder (*n* = 1390) OR (95% CI)
F value (ndf, ddf) [*p* value][f]			13.51 (25 901) [<0.001]*	6.18 (25 061) [0.002]*			1.50 (221 121) [0.223]			4.25 (2158) [0.016]*
Work related changes										
Changed to specific Covid-19 related work location	1080	21.1 (3.7)	1.48 (1.17–1.88)*	1.45 (1.10–1.89)*	540	18.5 (3.6)	1.40 (0.99–1.98)	540	24.2 (4.0)	1.19 (0.82–1.72)
Changed of team or assigned functions[e]	1642	33.7 (3.2)	1.38 (1.14–1.66)*	1.30 (1.17–1.44)*	871	31.7 (3.3)	1.18 (0.91–1.53)	770	36.0 (3.6)	1.13 (0.98–1.29)
No changes	2088	45.3 (1.6)	(Ref)	(Ref)	1270	49.9 (1.7)	(Ref)	818	39.8 (2.0)	(Ref)
F value (ndf, ddf) [*p* value][f]			7.75 (21 936) [<0.001]*	16.71 (2195) [<0.001]*			2.45 (21 102) [0.086]			1.60 (21 025) [0.203]
Work – perceived frequency of lack of protective equipment (scale 0–4)		2.7 (0.1) (2.0–3.5)	1.59 (1.42–1.78)*	1.44 (1.29–1.60)*		2.5 (0.1) (1.6–3.2)	1.29 (1.12–1.47)*		3.0 (0.1) (2.3–3.8)	1.21 (1.05–1.40)*
F value (ndf, ddf) [*p* value][f]			57.47(11 131) [<0.001]*	43.51 (14 586) [<0.001]*			11.91 (1654) [0.001]*			7.02(114 892) [0.008]*
Decisions regarding patients										
Having to make decisions regarding prioritising care among Covid-19 patients	890	15.8 (1.8)	1.51 (1.23–1.86)*	1.83 (1.64–2.03)*	460	14.6 (2.1)	1.62 (1.27–2.06)*	430	17.3 (1.5)	1.74 (1.40–2.18)*
Not having to make decisions regarding prioritising care among Covid-19 patients	3919	84.2 (1.8)	(Ref)	(Ref)	2222	85.4 (2.1)	(Ref)	1698	82.7 (1.5)	(Ref)
F value (ndf, ddf) [*p* value][f]			15.15(119 328) [<0.001]*	95.58 (1169) [<0.001]*			15.22 (16 058) [<0.001]*			23.36 (12 258) [<0.001]*
Having patient(s) in care that died from Covid-19 infection	1927	37.2 (3.2)	1.32 (1.19–1.46)*	1.36 (1.11–1.68)*	997	34.6 (3.2)	1.34 (1.05–1.71)*	931	40.3 (3.7)	1.20 (0.87–1.65)
Not having patient(s) in care that died from Covid-19 infection	2882	62.8 (3.2)	(Ref)	(Ref)	1685	65.4 (3.2)	(Ref)	1197	59.7 (3.7)	(Ref)
F value (ndf, ddf) [*p* value][f]			24.29 (1601) [<0.001]*	8.44 (12 041) [0.004]*			5.62 (11 577) [0.018]*			1.22 (127 142) [0.269]
Personal health-related stress (rescaled to 0–4)		1.6 (0.1) (0.8–2.4)	2.20 (1.99–2.43)*	1.94 (1.77–2.12)*		1.2 (0.0) (0.5–1.9)	1.61 (1.41–1.83)*		2.0 (0.1) (1.3–2.8)	1.50 (1.36–1.66)*
F value (ndf, ddf) [*p* value][f]			184.58 (1181) [<0.001]*	141.37 (1.86) [<0.001]*			39.67 (1184) [<0.001]*			50.55 (1187) [<0.001]*
Health-related stress loved ones (rescaled to 0–4)		2.4 (0.1) (1.5–3.2)	2.23 (1.99–2.50)*	1.91 (1.71–2.14)*		2.0 (0.1) (1.2–2.8)	1.49 (1.27–1.75)*		3.0 (0.1) (2.1–3.5)	1.52 (1.29–1.79)*
F value (ndf, ddf) [*p* value][f]			139.02(1118) [<0.001]*	105.93 (1251) [<0.001]*			23.37 (13 557) [<0.001]*			25.17 (12 990) [<0.001]*

Significant loss of personal or familial income due to Covid-19										
Yes	885	20.2 (1.2)	1.55 (1.31–1.83)*	1.42 (1.16–1.74)*	417	16.7 (1.0)	1.37 (0.80–2.34)	468	24.4 (1.8)	1.12 (0.85–1.47)
No	3924	79.8 (1.2)	(Ref)	(Ref)	2265	83.3 (1.0)	(Ref)	1659	75.6 (1.8)	(Ref)
F value (ndf, ddf) [*p* value][f]			25.36 (11 428) [<0.001]*	11.63(111 044) [0.001]*			1.35 (128 579) [0.246]			0.66 (18 307) [0.418]
Financial stress (rescaled to 0–4)		0.6 (0.0) (0.0–1.6)	1.42 (1.32–1.53)*	1.36 (1.28–1.45)*		0.4 (0.0) (0.0–1.2)	1.22 (1.07–1.39)*		0.9 (0.0) (0.1–1.9)	1.21 (1.11–1.32)*
F value (ndf, ddf) [*p* value][f]			76.79(11 075) [<0.001]*	63.00(1.90) [<0.001]*			8.11(11 662)[0.004]*			18.05(1762) [<0.001]*
Interpersonal stress (rescaled to 0–4)		1.3 (0.1) (0.5–2.1)	2.41 (2.12–2.74)*	1.96 (1.72–2.22)*		0.8 (0.0) (0.2–1.6)	1.53 (1.35–1.73)*		1.8 (0.0) (1.1–2.5)	1.50 (1.24–1.82)*
F value (ndf, ddf) [*p* value][f]			139.75(1171) [<0.001]*	96.91 (11 064) [<0.001]*			37.17(1291) [<0.001]*			17.82 (17 883) [<0.001]*
Family functioning scale score (rescaled to 0–4)		0.3 (0.0) (0.0–0.9)	1.59 (1.46–1.72)*	1.41 (1.30–1.54)*		0.2 (0.0) (0.0–0.8)	1.18 (0.99–1.41)		0.5 (0.0) (0.0–1.2)	1.21 (1.07–1.37)*
F value (ndf, ddf) [*p* value][f]			53.05 (1.24) [<0.001]*	32.65 (1.35) [<0.001]*			3.02 (1113) [0.085]			9.72 (1359) [0.002]*
Parental stress scale score (rescaled to 0–4)		0.0 (0.1) (0.0–0.8)	1.26 (1.05–1.50)*	1.17 (0.99–1.39)		0.0 (0.0) (0.0–0.7)	1.08 (0.90–1.28)		0.0 (0.1) (0.0–0.9)	1.10 (0.87–1.38)
F value (ndf, ddf) [*p* value][f]			6.31 (1991) [0.012]*	3.43 (12 287) [0.064]			0.69 (1357) [0.407]			0.64 (12 445) [0.423]

CI, confidence interval; IQR, interquartile range; Med, median; s.e., standard error; ndf, numerator degrees of freedom; ddf, denominator degrees of freedom.
Note. OR, odds ratio; 95% CI, 95% confidence interval; Covid-19, coronavirus disease 2019; s.e., standard error; IQR, interquartile range. All analyses adjust for time of survey (weeks), health centre membership, and all distal factors.
[a]Unweighted numbers.
[b]Weighted percentage (using post-stratification weights and inverse-probability weighting (IPW)).
[c]The category 'positive Covid-19 test or medical Covid-19 diagnosis' excludes those having been hospitalised for Covid-19.
[d]The category 'other family, friends, or others' excludes having a partner, children, or parents infected with Covid-19.
[e]The category 'changed of team or assigned functions' excludes those that changed to a specific Covid-19-related work location.
[f]F-test to evaluate joint significance of categorical predictor levels base on multiple imputation.
*Statistically significant ($\alpha = 0.05$).

Table 3. Adjusted population attributable risk proportions (PARP) for the association between proximal risk factor domains and any probable mental disorders diagnosis. Spanish healthcare workers, MINDCOVID study

	Full follow-up sample ($n = 4809$)		Among those with NO disorders baseline ($n = 2681$)	Among those with any disorder baseline ($n = 2128$)
	Any baseline disorder ($n = 2128$)	Any follow-up disorder ($n = 1870$)	Incidence of any disorder ($n = 480$)	Persistence of any disorder ($n = 1390$)
	PARP % (s.e.)	PARP % (s.e.)	PARP % (s.e.)	PARP % (s.e.)
Infection-related factors	13.9 (3.7)*	11.0 (4.3)*	4.5 (8.9)	4.8 (4.5)
WOrk-related factors	69.3 (3.1)*	65.1 (3.8)*	57.4 (7.8)*	35.6 (6.9)*
Health-related stress	80.6 (2.1)*	74.9 (3.1)*	59.2 (7.1)*	48.5 (7.0)*
Financial stress	20.3 (2.1)*	20.0 (2.4)*	15.5 (4.8)*	9.0 (2.7)*
Interpersonal stress	71.0 (2.7)*	62.1 (3.2)*	43.8 (7.3)*	35.5 (5.8)*
Total for all proximal factors	93.8 (1.1)*	88.4 (2.2)*	74.1 (7.2)*	69.4 (7.1)*

PARP, population-attributable risk proportion; s.e., standard error.
*Statistically significant ($\alpha = 0.05$).

persistence of mental disorders. Some of these factors are potentially preventable (Kisely *et al.*, 2020). Thus, adequately addressing these factors might be effective in reducing the high prevalence of mental disorders in this critical segment of the population.

Our results in context with previous knowledge

Frequency of mental health impacts

As noted in the introduction, longitudinal studies assessing the impact of the Covid-19 pandemic among healthcare workers are scarce and results are mixed. Some studies indicate the persistence of psychological stress (Sasaki *et al.*, 2021) while others suggest a lowering of depression and anxiety, in particular after 5 or more months from the beginning of the pandemic (Paul *et al.*, 2021). In general, evidence is limited due to a small number of studies, very short follow-up periods and with a small number of cases (Hirten *et al.*, 2020; Sampaio *et al.*, 2021; Van Steenkiste *et al.*, 2021). Our study captures a longer follow-up than most of the previous studies among healthcare workers with a considerably larger sample, and it clearly suggests that mental health impact is maintained during the second wave of the pandemic (4 months after the baseline assessment). This finding contrasts with studies in the general population, which show that the high impact at the beginning of the first wave of the pandemic tends to decline after 3–5 weeks of the first wave (Gonzalez-Sanguino *et al.*, 2020; Daly and Robinson, 2021; Fancourt *et al.*, 2021; Robinson and Daly, 2021). Nevertheless, some general population studies show a persistently high level of mental health impact through the initial phases of the pandemic (Kikuchi *et al.*, 2020; McGinty *et al.*, 2020) while still another study shows that a substantial proportion of the general population maintained high levels of symptoms (McPherson *et al.*, 2021). A possible explanation of the different trajectory among healthcare workers is the maintained levels of proximal stressors they faced, like other essential workers (Paul *et al.*, 2021). Also, we found that Panic Attacks and GAD tended to be less persistent than MDD and PTSD. This might indicate they will become the more lasting mental health impacts of Covid-19 among healthcare workers. But the 4-month follow-up period in our study might be short to assess stable trends. More research is needed, assessing proximal stressors and mental health outcomes using longer follow-ups and large enough samples, to assess the extent to which the likely mental health impact due to the Covid-19 pandemic may become sustained in time among healthcare workers.

Risk factors

Most proximal risk factors analysed in our study were associated with the prevalence of probable mental disorders after the 1st wave (baseline assessment) and during the 2nd wave of the pandemic. Covid-19 infection, which was frequent among healthcare workers, increased the risk of new onset of probable mental disorders. These associations suggest that it is necessary to implement interventions to mitigate the mental health impact of the pandemic among healthcare workers. Large population attributable risk proportions found indicate a potentially high benefit, if a causal interpretation can be assumed. Although many interventions have been suggested to mitigate the effects of infectious disease epidemics on population mental health (Leon *et al.*, 1997; North and Pfefferbaum, 2013; Kisely *et al.*, 2020), evidence of their effectiveness among healthcare workers is very limited and of insufficient quality (Pollock *et al.*, 2020). Specific e-health interventions for healthcare workers are of increasing interest (Drissi *et al.*, 2021). Our results support previous recommendations that when selecting interventions aimed at supporting frontline workers' mental health, organisational, social, personal and psychological factors may all be important (Pollock *et al.*, 2020).

Persistent and new-onset probable cases

We stratified the analyses according to the mental health status after the first wave of the pandemic (baseline assessment). This has allowed us to estimate the 'persistence' of probable mental disorders and the rate of appearance of 'incident' disorders. This approach is quite novel in the literature of the impact of Covid-19, and our results suggest: (a) that most of the prevalent disorders at follow up are at the expense of workers that had already a disorder at baseline; and (b) that, nevertheless, new probable cases appear during the second wave and they are associated with almost exactly the same proximal risk factors that are associated with persistence. The first finding suggests that persistent mental disorders may be an emergent issue among healthcare

workers after the Covid-19 pandemic. And the second, strongly suggests the need to implement more effective attenuation of work-related and health-related factors during the evolution of the pandemic. Similar to a recently published study with Spanish healthcare workers (Mediavilla *et al.*, 2021), our results suggest a high persistent negative mental health impact of Covid-19. Mental health monitoring and facilitating access to services are needed to decrease the likelihood of chronicity among healthcare workers.

Strengths and limitations

Our study has several strengths. First, it is based on a large sample of healthcare workers of a wide distribution of public health services in Spain, including hospitals, primary care and emergency and public health departments. All workers in each institution were invited to the study, based on a clear sampling frame and providing a wide picture of exposure to acute stressors. Our study is unique in following the same healthcare workers and differentiating new onset of probable disorders and persistence of disorders present after wave one.

The study has also some limitations that should be considered. First, the follow-up cooperation rate was incomplete (65.7%). In addition, the cooperation at baseline was even lower (Alonso *et al.*, 2021), although the real figure is difficult to assess, since data from one large hospital in our study showed that the proportion of emails 'seen' by target workers was just over 26% at baseline. Although differences between workers participating in the follow-up survey and those not participating were very small, inverse probability weighting has been applied to correct for differential probabilities of participation at follow up surveys based on baseline characteristics. Additionally, we used post-stratification so that each survey matches the distribution of age, gender and profession in each of the health institutions included in the study. Moreover, response rates across the 18 participating institutions were not correlated with the prevalence of any mental disorder (Spearman $R = -0.12$, $p = 0.65$). Also, data come from one country only which limits the external validity. Second, it is important to note that we used screening measures of mental disorders. While they have shown to have acceptable validity for identifying individuals with a high risk of a particular mental disorder, results cannot be interpreted as clinical diagnoses. Moreover, the PHQ might overestimate the prevalence of depression (Levis *et al.*, 2020). Thus, we consider them only 'probable' mental disorders. But it is important to clarify that a positive result in each of these measures indicates relevant psychopathology. One advantage of using these particular measures is that results can be compared with many existing data with other populations and time periods. Nevertheless, two-phase studies would provide a more valid estimation (Dunn *et al.*, 1999). Third, both distal and proximal factors used in the analyses were gathered at baseline. While there is the possibility that proximal factors varied during the follow-up, we assumed this was minimal given the follow-up period was 4 months on average. Moreover, in the analysis, we wanted to ensure that all proximal/distal factors occurred before the outcome (incidence/persistence). Finally, 4 months of follow-up is a short time period for a complete understanding of the mental impact dynamics of the Covid-19 pandemic. Longer follow-up periods are necessary. A paper indicates that 56% of Spanish healthcare workers remained symptomatic or worsened over time in terms of psychological distress (Mediavilla *et al.*, 2021). In addition, length of follow-up differed by healthcare workers. This was due to an extended duration of the baseline assessment because of a slow roll-in period of health institutions. But we did adjust by a centre and by assessment date to minimise any possible bias.

Conclusions

Notwithstanding the above-mentioned limitations, our study indicates that the prevalence of probable mental disorders among Spanish healthcare workers during the second wave of the Covid-19 pandemic was similarly high to that after the first wave. This was in good part due to the persistence of mental disorders detected at the baseline, but with a relevant incidence of about 1 in 5 of HCWs without mental disorders during the first wave of the Covid-19 pandemic. Health-related factors, work-related factors and interpersonal stress are important risks of persistence of mental disorders and of incidence of mental disorders. Adequately addressing these factors might have prevented a considerable amount of mental health impact of the pandemic among this vulnerable population.

Supplementary material. The supplementary material for this article can be found at https://doi.org/10.1017/S2045796022000130

Data. The data will be available in the future in a repository that the funding institution (Instituto de Salud Carlos III) is currently developing. For any additional information contact with the corresponding author.

Acknowledgements. The authors would like to sincerely thank all healthcare workers participating in the study. They also thank Puri Barbas for the management of the project and Carme Gasull for helping with the manuscript preparation.

Author contributors. J. A., G. V. and P. M. reviewed the literature. J. A., G. V., P. M., M. F., E. A., V. P. S., J. M. H., R. C. K. and R. B. conceived and designed the study. E. A., J. D. M., N. L., T. P., J. M. P. T., J. I. P., J. I. E., M. E., N. P., A. G. P., C. R., E. A., I. C. G., A. A. P., M. C., A. P. Z., E. V., C. S. and V. P. S. acquired the data. G. V., I. A., F. A. and PM cleaned and analysed the data. J. A., G. V. and P. M. drafted the initial version of the manuscript. All authors reviewed the initial draft and made a critical contribution to the interpretation of the data and approved the manuscript. The corresponding author attests that all listed authors meet authorship criteria and that no others meeting the criteria have been omitted.

Financial support. Instituto de Salud Carlos III/ Ministerio de Ciencia e Innovación/ FEDER (J. A., grant number COV20/00711); Project "PI17/00521", funded by Instituto de Salud Carlos III (ISCIII) and co-funded by the European Union, PERIS, Health Dpt, Generaliat de Catalunya (I. A., grant number SLT017/20/000009); ISCIII-FSE+, Miguel Servet (P. M., grant number CP21/00078); ISCIII-FSE, Sara Borrell (P. M., grant number CD18/00049), Generalitat de Catalunya (2017SGR452). Additional partial funding was received from the Gerencia Regional de Salud de Castilla y León (SACYL) (J. M. P. T., grant number GRS COVID 32/A/20).

Conflict of interest. E. A. reports personal fees from Lündbeck, Esteve, Schwabe and Roche. E. V. reports personal fees from Abbott, Allergan, Angelini, Lundbeck, Sage and Sanofi, grants from Novartis and Ferrer and grants and personal fees from Janssen, outside the submitted work. J. D. M. reports personal fees from Janssen and Angelini, personal fees and non-financial support from Otsuka, Lundbeck and Accord, outside the submitted work. J. M. P. T. reports personal fees from Angelini, Janssen and Lunbeck, and grants from Janssen, outside the submitted work. R. C. K. was a consultant for Datastat, Inc., Holmusk, RallyPoint Networks, Inc., Sage Therapeutics and he has stock options in Mirah, P. Y. M., and Roga Sciences. All other authors reported no conflict of interest.

Ethical standards. The authors assert that all procedures contributing to this study comply with the ethical standards of the relevant national and institutional committees on human experimentation and with the Helsinki Declaration of 1975, as revised in 2000.

References

Alonso J, Vilagut G, Mortier P, Ferrer M, Alayo I, Aragon-Peña A, Aragones E, Campos M, Cura-Gonzalez ID, Emparanza JI, Espuga M, Forjaz MJ, Gonzalez-Pinto A, Haro JM, Lopez-Fresneña N, Salazar ADM, Molina JD, Orti-Lucas RM, Parellada M, Pelayo-Teran JM, Perez-Zapata A, Pijoan JI, Plana N, Puig MT, Rius C, Rodriguez-Blazquez C, Sanz F, Serra C, Kessler RC, Bruffaerts R, Vieta E and Perez-Sola V and MINDCOVID Working Group (2021) Mental health impact of the first wave of COVID-19 pandemic on Spanish healthcare workers: a large cross-sectional survey. *Revista de Psiquiatria y Salud Mental* (*Engl Ed*) **14**, 90–105.

Barroilhet S, Cano-Prous A, Cervera-Enguix S, Forjaz MJ and Guillen-Grima F (2009) A Spanish version of the family assessment device. *Social Psychiatry and Psychiatric Epidemiology* **44**, 1051–1065.

Berry JO and Jones WH (1995) The parental stress scale: initial psychometric evidence. *Journal of Social and Personal Relationship* **12**, 463–472.

Blasco MJ, Castellvi P, Almenara J, Lagares C, Roca M, Sese A, Piqueras JA, Soto-Sanz V, Rodriguez-Marin J, Echeburua E, Gabilondo A, Cebria AI, Miranda-Mendizabal A, Vilagut G, Bruffaerts R, Auerbach RP, Kessler RC and Alonso J and Universal Study Group (2016) Predictive models for suicidal thoughts and behaviors among Spanish University students: rationale and methods of the UNIVERSAL (University & mental health) project. *BMC Psychiatry* **16**, 122.

Boden M, Cohen N, Froelich JM, Hoggatt KJ, Abdel Magid HS and Mushiana SS (2021*a*) Mental disorder prevalence among populations impacted by coronavirus pandemics: a multilevel meta-analytic study of COVID-19, MERS & SARS. *General Hospital Psychiatry* **70**, 124–133.

Boden M, Zimmerman L, Azevedo KJ, Ruzek JI, Gala S, Abdel Magid HS, Cohen N, Walser R, Mahtani ND, Hoggatt KJ and McLean CP (2021*b*) Addressing the mental health impact of COVID-19 through population health. *Clinical Psychology Review* **85**, 102006.

Chigwedere OC, Sadath A, Kabir Z and Arensman E (2021) The impact of epidemics and pandemics on the mental health of healthcare workers: a systematic review. *International Journal of Environmental Research and Public Health* **18**, 6695.

Daly M and Robinson E (2021) Acute and longer-term psychological distress associated with testing positive for COVID-19: longitudinal evidence from a population-based study of US adults. *Psychological Medicine*, 1–8, [Epub ahead of print]. doi: 10.1017/S003329172100324X.

de Kock JH, Latham HA, Leslie SJ, Grindle M, Muñoz SA, Ellis L, Polson R and O'Malley CM (2021) A rapid review of the impact of COVID-19 on the mental health of healthcare workers: implications for supporting psychological well-being. *BMC Public Health* **21**, 104.

Diez Martinez S, Martin Moros JM, Altisent Trota R, Aznar Tejero P, Cebrian Martin C, Imaz Perez FJ and del Castillo Pardo M (1991) Brief questionnaires for the early detection of alcoholism in primary health care. *Atencion Primaria* **8**, 367–370.

Dohrenwend BS, Krasnoff L, Askenasy AR and Dohrenwend BP (1978) Exemplification of a method for scaling life events: the peri life events scale. *Journal of Health and Social Behavior* **19**, 205–229.

Drissi N, Ouhbi S, Marques G, de la Torre Diez I, Ghogho M and Janati Idrissi MA (2021) A systematic literature review on e-mental health solutions to assist health care workers during COVID-19. *Telemedicine Journal and e-Health* **27**, 594–602.

Dunn G, Pickles A, Tansella M and Vazquez Barquero JL (1999) Two-phase epidemiological surveys in psychiatric research. *British Journal of Psychiatry* **174**, 95–100.

Epstein NB, Baldwin LM and Bishop DS (1983) The McMaster family assessment device. *Journal of Marital and Family Therapy* **9**, 171–180.

Eysenbach G (2004) Improving the quality of web surveys: the checklist for reporting results of internet E-surveys (CHERRIES). *Journal of Medical Internet Research* **6**, e34.

Fancourt D, Steptoe A and Bu F (2021) Trajectories of anxiety and depressive symptoms during enforced isolation due to COVID-19 in England: a longitudinal observational study. *The Lancet Psychiatry* **8**, 141–149.

Galanis P, Vraka I, Fragkou D, Bilali A and Kaitelidou D (2021) Nurses' burnout and associated risk factors during the COVID-19 pandemic: a systematic review and meta-analysis. *Journal of Advanced Nursing* **77**, 3286–3302.

Garcia-Campayo J, Zamorano E, Ruiz MA, Pardo A, Perez-Paramo M, Lopez-Gomez V, Freire O and Rejas J (2010) Cultural adaptation into Spanish of the generalized anxiety disorder-7 (GAD-7) scale as a screening tool. *Health and Quality of Life Outcomes* **8**, 8.

Gonzalez-Sanguino C, Ausin B, Castellanos MA, Saiz J, Lopez-Gomez A, Ugidos C and Muñoz M (2020) Mental health consequences during the initial stage of the 2020 coronavirus pandemic (COVID-19) in Spain. *Brain, Behavior, and Immunity* **87**, 172–176.

Hinkin CH, Castellon SA, Dickson-Fuhrman E, Daum G, Jaffe J and Jarvik L (2001) Screening for drug and alcohol abuse among older adults using a modified version of the CAGE. *American Journal on Addictions* **10**, 319–326.

Hirten RP, Danieletto M, Tomalin L, Choi KH, Zweig M, Golden E, Kaur S, Helmus D, Biello A, Pyzik R, Calcogna C, Freeman R, Sands BE, Charney D, Bottinger EP, Keefer L, Suarez-Farinas M, Nadkarni GN and Fayad ZA (2020) Factors associated with longitudinal psychological and physiological stress in health care workers during the COVID-19 pandemic. *medRxiv preprint*. https://doi.org/10.1101/2020.12.21.20248593.

INC SI (2014) *SAS Software 9.4*. Cary, NC: SAS Inst. Inc. Mark Co.

Jordan JA, Shannon C, Browne D, Carroll E, Maguire J, Kerrigan K, Hannan S, McCarthy T, Tully MA, Mulholland C and Dyer KFW (2021) COVID-19 staff wellbeing survey: longitudinal survey of psychological well-being among health and social care staff in Northern Ireland during the COVID-19 pandemic. *British Journal of Psychiatry Open* **7**, e159.

Kessler RC, Santiago PN, Colpe LJ, Dempsey CL, First MB, Heeringa SG, Stein MB, Fullerton CS, Gruber MJ, Naifeh JA, Nock MK, Sampson NA, Schoenbaum M, Zaslavsky AM and Ursano RJ (2013) Clinical reappraisal of the composite international diagnostic interview screening scales (CIDI-SC) in the army study to assess risk and resilience in servicemembers (Army STARRS). *International Journal of Methods in Psychiatric Research* **22**, 303–321.

Kikuchi H, Machida M, Nakamura I, Saito R, Odagiri Y, Kojima T, Watanabe H, Fukui K and Inoue S (2020) Changes in psychological distress during the COVID-19 pandemic in Japan: a longitudinal study. *Journal of Epidemiology* **30**, 522–528.

Kisely S, Warren N, McMahon L, Dalais C, Henry I and Siskind D (2020) Occurrence, prevention, and management of the psychological effects of emerging virus outbreaks on healthcare workers: rapid review and meta-analysis. *BMJ* **369**, m1642.

Kroene K, Spitzer RL, Williams JB and Lowe B (2010) The patient health questionnaire somatic, anxiety, and depressive symptom scales: a systematic review. *General Hospital Psychiatry* **32**, 345–359.

Krysinska K and Martin G (2009) The struggle to prevent and evaluate: application of population attributable risk and preventive fraction to suicide prevention research. *Suicide & Life-Threatening Behavior* **39**, 548–557.

Kunzler AM, Rothke N, Gunthner L, Stoffers-Winterling J, Tuscher O, Coenen M, Rehfuess E, Schwarzer G, Binder H, Schmucker C, Meerpohl JJ and Lieb K (2021) Mental burden and its risk and protective factors during the early phase of the SARS-CoV-2 pandemic: systematic review and meta-analyses. *Globalization and Health* **17**, 34.

Leon AC, Olfson M, Portera L, Farber L and Sheehan DV (1997) Assessing psychiatric impairment in primary care with the Sheehan disability scale. *International Journal of Psychiatry in Medicine* **27**, 93–105.

Levis B, Benedetti A, Ioannidis J, Sun Y, Negeri Z, He C, Wu Y, Krishnan A, Bhandari P, Neupane D, Imran M, Rice D, Riehm K, Saadat N, Azar M, Boruff J, Cuijpers P, Gilbody S, Kloda L, McMillan D, Patten S, Shrier I, Ziegelstein R, Alamri S, Amtmann D, Ayalon L, Baradaran H, Beraldi A, Bernstein C, Bhana A, Bombardier C, Carter G, Chagas M, Chibanda D, Clover K, Conwell Y, Diez-Quevedo C, Fann J, Fischer F, Gholizadeh L, Gibson L, Green E, Greeno C, Hall B, Haroz E, Ismail K, Jetté N, Khamseh M, Kwan Y, Lara M, Liu S, Loureiro S, Löwe B, Marrie R, Marsh L, McGuire A, Muramatsu K, Navarrete L, Osório F, Petersen I, Picardi A, Pugh S, Quinn T, Rooney A, Shinn E, Sidebottom A, Spangenberg L, Tan P, Taylor-Rowan M, Turner A, van Weert H, Vöhringer P, Wagner L, White J, Winkley K and Thombs B (2020) Patient Health Questionnaire-9 scores do not accurately estimate

depression prevalence: individual participant data meta-analysis. *Journal of Clinical Epidemiology* **122**, 115–128.

Lopez Steinmetz LC, Herrera CR, Fong SB and Godoy JC (2022) A longitudinal study on the changes in mental health of healthcare workers during the COVID-19 pandemic. *Psychiatry* **85**, 56–71.

Mansfiedl AK, Keitner GI and Sheeran T (2019) The brief assessment of family functioning scale (BAFFS): a three-item version of the general functioning scale of the family assessment device. *Psychotherapy Research* **29**, 824–831.

McFadden P, Neil RD, Moriarty J, Gillen P, Mallett J, Manthorpe J, Currie D, Schroder H, Ravalier J, Nicholl P, McFadden D and Ross J (2021) A cross-sectional examination of the mental wellbeing, coping and quality of working life in health and social care workers in the UK at two time points of the COVID-19 pandemic. *Epidemiologia* **2**, 227–242.

McGinty EE, Presskreischer R, Anderson KE, Han H and Barry CL (2020) Psychological distress and COVID-19-related stressors reported in a longitudinal cohort of US adults in April and July 2020. *JAMA* **324**, 2555–2557.

McPherson KE, McAloney-Kocaman K, McGlinchey E, Faeth P and Armour C (2021) Longitudinal analysis of the UK COVID-19 psychological wellbeing study: trajectories of anxiety, depression and COVID-19-related stress symptomology. *Psychiatry Research* **304**, 114138.

Mdege ND and Lang J (2011) Screening instruments for detecting illicit drug use/abuse that could be useful in general hospital wards: a systematic review. *Addictive Behaviors* **36**, 1111–1119.

Mediavilla R, Fernández-Jiménez E, Martinez-Morata I, Jaramillo F, Andreo-Jover J, Morán-Sánchez I, Mascayano F, Moreno-Küstner B, Minué S, Ayuso-Mateos J, Bryant R, Bravo-Ortiz M and Martínez-Alés G (2021) Sustained negative mental health outcomes among healthcare workers over the first year of the COVID-19 pandemic: a prospective cohort study. *medRxiv preprint*. https://doi.org/10.1101/2021.11.21.21266594.

Miguel-Puga JA, Cooper-Bribiesca D, Avelar-Garnica FJ, Sanchez-Hurtado LA, Colin-Martinez T, Espinosa-Poblano E, Anda-Garay JC, Gonzalez-Diaz JI, Segura-Santos OB, Vital-Arriaga LC and Jauregui-Renaud K (2021) Burnout, depersonalization, and anxiety contribute to post-traumatic stress in frontline health workers at COVID-19 patient care, a follow-up study. *Brain and Behavior* **11**, e02007.

Mortier P, Vilagut G, Ferrer M, Alayo I, Bruffaerts R, Cristobal-Narvaez P, del Cura-Gonzalez I, Domenech-Abella J, Felez-Nobrega M, Olaya B, Pijoan JI, Vieta E, Perez-Sola V, Kessler RC, Haro JM and Alonso J (2021) Thirty-day suicidal thoughts and behaviors in the Spanish adult general population during the first wave of the Spain COVID-19 pandemic. *Epidemiology and Psychiatric Sciences* **30**, e-19, [Epub ahead of print].

Mortier P, Vilagut G, Alayo I, Ferrer M, Amigo F, Aragonès E, Aragón-Peña A, Asúnsolo del Barco A, Campos M, Espuga M, González-Pinto A, Haro J, López Fresneña N, Martínez de Salázar A, Molina J, Ortí-Lucas R, Parellada M, Pelayo-Terán J, Pérez-Gómez B, Pérez-Zapata A, Pijoan J, Plana N, Polentinos-Castro E, Portillo-Van Diest A, Puig M, Rius C, Sanz F, Serra C, Urreta-Barallobre I, Kessler R, Bruffaerts R, Vieta E, Pérez-Solá V and Alonso J and MINDCOVID Working Group (2022) Four-month incidence of suicidal thoughts and behaviors among healthcare workers after the first wave of the Spain COVID-19 pandemic. *Journal of Psychiatric Research*, **149**, 10–17.

Navarro-Mateu F, Moran-Sanchez I, Alonso J, Tormo MJ, Pujalte ML, Garriga A, Aguilar-Gaxiola S and Navarro C (2013) Cultural adaptation of the Latin American version of the World Health Organization Composite International Diagnostic Interview (WHO-CIDI) (v 3.0) for use in Spain. *Gaceta Sanitaria* **27**, 325–331.

Nock MK, Stein MB, Heeringa SG, Ursano RJ, Colpe LJ, Fullerton CS, Hwang I, Naifeh JA, Sampson NA, Schoenbaum M, Zaslavsky AM, Kessler RC and Army SC (2014) Prevalence and correlates of suicidal behavior among soldiers: results from the army study to assess risk and resilience in servicemembers (Army STARRS). *JAMA Psychiatry* **71**, 514–522.

North CS and Pfefferbaum B (2013) Mental health response to community disasters: a systematic review. *JAMA* **310**, 507–518.

Oronoz B, Alonso-Arbiol I and Balluerka N (2007) A Spanish adaptation of the parental stress scale. *Psicothema* **19**, 687–692.

Paul E, Wan Mak H, Fancourt D and Bu F (2021) Comparing mental health trajectories of four different types of key workers with non-key workers: a 2-month follow-up observational study of 21,874 adults in England during the COVID-19 pandemic. *medRxiv preprint*. https://doi.org/10.1101/2021.04.20.21255817.

Phiri P, Ramakrishnan R, Rathod S, Elliot K, Thayanandan T, Sandle N, Haque N, Chau SW, Wong OW, Chan SS, Wong EK, Raymont V, Au-Yeung SK, Kingdon D and Delanerolle G (2021) An evaluation of the mental health impact of SARS-CoV-2 on patients, general public and healthcare professionals: a systematic review and meta-analysis. *EClinicalMedicine* **34**, 100806.

Pollock A, Campbell P, Cheyne J, Cowie J, Davis B, McCallum J, McGill K, Elders A, Hagen S, McClurg D, Torrens C and Maxwell M (2020) Interventions to support the resilience and mental health of frontline health and social care professionals during and after a disease outbreak, epidemic or pandemic: a mixed-methods systematic review. *The Cochrane Database of Systematic Reviews* **11**, CD013779.

Resick PA, Monson CM and Chard KM (2020) *Cognitive Processing Therapy for Posttraumatic Stress Disorder (PTSD)*. Washington, DC: Department of Veteran Affairs.

Roberts T, Daniels J, Hulme W, Hirst R, Horner D, Lyttle MD, Samuel K, Graham B, Reyanrd C, Barrett M, Foley J, Cronin J, Umana E, Vinagre J and Carlton E (2021) Psychological distress and trauma in doctors providing frontline care during the COVID-19 pandemic in the United Kingdom and Ireland: a prospective longitudinal survey cohort study. *BMJ Open* **11**, e049680.

Robinson E and Daly M (2021) Explaining the rise and fall of psychological distress during the COVID-19 crisis in the United States: longitudinal evidence from the understanding America study. *British Journal of Health Psychology* **26**, 570–587.

Rodriguez RM, Montoy JC, Hoth KF, Talan DA, Harland KK, Eyck PT, MOwer W, Krishnadasan A, Santibañez S and Mohr N and Project COVERED Emergency Department Network (2021) Symptoms of anxiety, burnout, and PTSD and the mitigation effect of serologic testing in emergency department personnel during the COVID-19 pandemic. *Annals of Emergency Medicine* **78**, 35–43 e2.

Sampaio F, Sequeira C and Teixeira L (2021) Impact of COVID-19 outbreak on nurses' mental health: a prospective cohort study. *Environmental Research* **194**, 110620.

Santabarbara J, Bueno-Notivol J, Lipnicki DM, Olaya B, Perez-Moreno M, Gracia-Garcia P, Idoiaga-Mondragon N and Ozamiz-Etxebarria N (2021) Prevalence of anxiety in health care professionals during the COVID-19 pandemic: a rapid systematic review (on published articles in Medline) with meta-analysis. *Progress in Neuro-Psychopharmacology & Biological Psychiatry* **107**, 110244.

Sasaki N, Asaoka H, Kuroda R, Tsuno K, Imamura K and Kawakami N (2021) Sustained poor mental health among healthcare workers in COVID-19 pandemic: a longitudinal analysis of the four-wave panel survey over 8 months in Japan. *Journal of Occupational Health* **63**, e12227.

Schisterman E, Cole S and Platt R (2009) Overadjustment bias and unnecessary adjustment in epidemiologic studies. *Epidemiology (Cambridge, Mass.)* **20**, 488–495.

Seaman SR, White IR, Copas AJ and Li L (2012) Combining multiple imputation and inverse-probability weighting. *Biometrics* **68**, 129–137.

Sun P, Wang M, Song T, Wu Y, Luo J, Chen L and Yan L (2021) The psychological impact of COVID-19 pandemic on health care workers: a systematic review and meta-analysis. *Frontiers in Psychology* **12**, 626547.

Team RC (2021) *R: A Language and Environment for Statistical Computing*. Vienna, Austria: R Foundation for Statistical Computing.

Van Bureen S (2012) *Flexible Imputation of Missing Data, Chapter 2. Multiple Imputation*. Boca Raton: Chapman and Hall/CRC Press.

Van Buuren S and Groothuis-Oudshoorn K (2011) mice: multivariate imputation by Chained equations in R. *Journal of Statistical Software* **45**, 1–67.

Van Steenkiste E, Schoofs J, Gilis S and Messiaen P (2021) Mental health impact of COVID-19 in frontline healthcare workers in a Belgian Tertiary care hospital: a prospective longitudinal study. *Acta Clinica Belgica* 1–8 [Epub ahead of print]. doi: 10.1080/17843286.2021.1903660.

Weathers FW, Litz BT, Keane TM, Palmieri PA, Marx BP and Schnurr PP (2013) *The PTSD Checklist for DSM-5 (PCl-5)*. Washington, USA: Department of Veterans Affairs.

Wu Y, Levis B, Riehm KE, Saadat N, Levis AW, Azar M, Rice DB, Boruff J, Cuijpers P, Gilbody S, Ioannidis JP, Kloda LA, McMillan D, Patten SB, Shrier I, Ziegelstein RC, Akena DH, Arroll B, Ayalon L, Baradaran HR, Baron M, Bombardier CH, Butterworth P, Carter G, Chagas MH, Chan JC, Cholera R, Conwell Y, Ginkel dM-v, Fann JR, Fischer FH, Fung D, Gelaye B, Goodyear-Smith F, Greeno CG, Hall BJ, Harrison PA, Harter M, Hegerl U, Hides L, Hobfoll SE, Hudson M, Hyphantis T, Inagaki M, Jette N, Khamseh ME, Kiely KM, Kwan Y, Lamers F, Liu SI, Lotrakul M, Loureiro SR, Lowe B, McGuire A, Mohd-Sidik S, Munhoz TN, Muramatsu K, Osorio FL, Patel V, Pence BW, Persoons P, Picardi A, Reuter K, Rooney AG, Santos IS, Shaaban J, Sidebottom A, Simning A, Stafford L, Sung S, Tan PL, Turner A, van Weert HC, White J, Whooley MA, Winkley K, Yamada M, Benedetti A and Thombs BD (2020) Equivalency of the diagnostic accuracy of the PHQ-8 and PHQ-9: a systematic review and individual participant data meta-analysis. *Psychological Medicine* **50**, 1368–1380.

Zuromski KL, Ustun B, Hwang I, Keane TM, Marx BP, Stein MB, Ursano RJ and Kessler RC (2019) Developing an optimal short-form of the PTSD checklist for DSM-5 (PCL-5). *Depression and Anxiety* **36**, 790–800.
