# Supplementary material for: Mental health status and related factors influencing healthcare workers during the COVID-19 pandemic: A systematic review and meta-analysis
Source: PLoS One. 2024 Jan 19;19(1):e0289454. doi: 10.1371/journal.pone.0289454 (PMC10798549; doi:10.1371/journal.pone.0289454)
Supplement: S1 Data — (ZIP) [file pone.0289454.s011.zip › literatures/70.pdf]

Irish Journal of Medical Science (1971 -) (2022) 191:93–96
https://doi.org/10.1007/s11845-021-02513-6

BRIEF REPORT

Impact on mental health of the COVID-19 outbreak among general practitioners during the sanitary lockdown period

Marie Lange [1,2] · Sarah Joo [3,4] · Pierre-André Couette [3,4] · François Le Bas [3,4] · Xavier Humbert [3,4]

Received: 4 January 2021 / Accepted: 10 January 2021 / Published online: 4 March 2021
© Royal Academy of Medicine in Ireland 2021

Abstract

Background COVID-19 outbreak can impact mental health including health care workers.

Aim The aim of this study was to assess the mental health impact of COVID-19 in French General Practitioners (GPs).

Methods We carried out a postal-based survey during the first lockdown. Four psychological validated self-report questionnaires were used to assess stress,post-traumatic stress symptoms, burnout and self-efficacy (Perceived Stress scale, Impact of Event Scale-revised, Maslach Burnout Inventory and General Self-Efficacy scale).

Results The sample consists of 332 general practitioners (43.50% women, mean age = 50.74 ± 11.91). General practitioners working in high epidemic location represented 27.71% of the sample ($n = 92$). Thirty four GPs reported significant post-traumatic stress symptoms (10.59%). High burnout symptoms were found in 79 (24.46%), 137 (42.41%) and 17 (5.26%) participants. Only General Self-Efficacy scores were significantly different according to epidemic location status with lower scores in GPs working in high epidemic location (33.37 ± 4.64 vs. 32.06 ± 5.43; $P = 0.04$). Women reported more stress and burnout symptoms than men ($P = 0.01$).

Conclusions This study showed the psychological impact of COVID-19 in GPs during the sanitary lockdown period including burnout and post-traumatic stress symptoms. Up to 42% of GPs reported psychological disturbances. Interventions to promote mental health well-being of healthcare workers need to be developing.

Keywords Burnout · COVID-19 · General practitioners · Mental health · Stress

Introduction

In December 2019, a cluster of pneumonia cases in China conducted to identify the SARS-CoV-2 a novel coronavirus also named COVID-19 [1]. On March 2020, the World Health Organization declared the COVID-19 as a pandemic.

Some results showed that the COVID-19 outbreak have a negative impact on healthcare workers mental health. Psychological symptoms such as anxiety, stress and sleep disturbances were reported in healthcare workers [2–8] including mainly nurses.

General practitioners (GPs) played an important role in hindering the spread of COVID-19 in primary care. They help to reduce the virus propagation by treating patients and provide medical surveillance and the virus transmission by patient monitoring. Furthermore, living with the fear to be infected themselves could induced anxiety and stress [8, 9]. Nevertheless, only one study assessed the COVID-19 pandemic impact on depression, anxiety and insomnia in GPs [7].

The aim of this study was to assess the psychological impact of COVID-19, especially stress and burnout symptoms, in French GPs.

Methods

We carried out a postal-based survey the 15th April 2020 to assess the impact of COVID-19 on the mental health of GPs

✉ Xavier Humbert
xavier.humbert@unicaen.fr

1 Clinical Research Department, Centre François Baclesse, 14000 Caen, France

2 Normandie University, UNICAEN, INSERM, ANTICIPE, 14000 Caen, France

3 Family Medicine Department, Normandie University, UNICAEN, UFR Santé, 2, rue des Rochambelles, 14000 Caen, France

4 EA 4650 Signalisation, électrophysiologie et imagerie des lésions d'ischémie-reperfusion myocardique, Normandie University, 14000, Caen, France

working in Normandy (France), one month after COVID-19 the first sanitary lockdown (17th March 2020).

It consisted of sociodemographic, medical office organization and general practice supervisors' data. Epidemic location was defined by French Health Authority in 4 localized clusters before first COVID-19 lockdown. This survey was approved by Ethic Department of University of Caen Normandy (Authorization n°TG_COMPO_PEDAGO_SANTE_14–20,180,529-01R1).

Four psychological validated self-report questionnaires were used. The Perceived Stress scale (PSS) assesses stress [10]. The Impact of Event Scale-revised(IES-R) assesses post-traumatic stress symptoms [11]. Significant symptoms were defined by a score ≥ 33. The Maslach Burnout Inventory (MBI) assesses [12]: emotional exhaustion (EE), depersonalisation (DP) and personal accomplishment (PA). High burnout symptoms were defined by scores ≥30 and ≥ 12, respectively for EE and DP and ≤ 33 for PA. The General Self-Efficacy scale (GSE) was also used [13].

Statistical analysis

Data were expressed as means and standard deviations (SD) or percentage. Comparisons between groups (gender or exposition) were done by *t* Student's test or chi-2 test, where appropriate, using NCSS version 9 (Hintze J, Kaysville, Utah, USA: www.ncss.com).

Results

The response rate was 29.19% ($n = 364$; 1247 questionnaires were sent). Thirty-two questionnaires were non-usable mainly for return to the expeditor due to incorrect postal address or return without completion. The sample consists of 332 general practitioners (43.50% women (1 missing response), mean age = 50.74 ± 11.91; Table 1). General practitioners working in high epidemic location represented 27.71% of the sample ($n = 92$).

Table 1 Demographic, organization and psychological characteristics of general practitioners

	All sample (n=332)
Women (n, %) [missing 1]	144 (43.50)
Age (y) mean (SD)	50.74 (11.91)
High epidemic location (n, %)	92 (27.71)
Medical office [missing 3]	
Alone (n, %)	67 (20.37)
Mono-professional (n, %)	117 (35.56)
Pluri-professional (n, %)	145 (44.07)
General Practice supervisors (n, %)	153 (46.08)
Perceived stress scale [PSS]*; mean (SD) [missing 1]	14.33 (7.24)
Impact of Event scale [IES-R]*; mean (SD)	14.69 (14.35)
Post-traumatic stress symptoms (n, %) [missing 11]	34 (10.59)
Emotional exhaustion [EE]**; mean (SD) [missing 9]	22.65 (10.59)
Low (n, %)	130 (40.25)
Middle (n, %) burnout symptoms	114 (35.29)
High (n, %)	79 (24.46)
Depersonalisation [DP]**; mean (SD) [missing 9]	11.65 (5.31)
Low (n, %)	26 (8.05)
Middle (n, %) burnout symptoms	160 (49.54)
High (n, %)	137 (42.41)
Personal accomplishment [DP]**; mean (SD) [missing 9]	46.86 (7.94)
Low (n, %)	277 (85.76)
Middle (n, %) burnout symptoms	29 (8.98)
High (n, %)	17 (5.26)
General Self-Efficacy scale [GSE]***; mean (SD) [missing 12]	33.01 (4.89)

*Higher score represents high level of stress

**Maslach Burnout Inventory. Higher score of PA represents lower burnout symptoms. Higher scores for EE and DP represent higher burnout symptoms

***Higher score represents high self-efficacy level

Table 2 Demographic, exposition and psychological characteristics of general practitioners according to gender

	Women (*n*=144)	Men (*n*=187)	*P* value
Age (y) mean (SD)	46.59 (10.80)	53.46 (11.88)	**0.001**
Epidemic location Covid-19			
High (n, %)	44 (30.56)	48 (25.67)	0.50
Low (n, %)	100 (69.44)	139 (74.33)	
General Practice supervisors			
Yes (n, %)	84 (58.33)	95 (50.80)	0.22
No (n, %)	60 (41.67)	92 (49.20)	
Perceived stress scale [PSS]; mean (SD)	16.20 (7.48)	12.86 (6.71)	**0.01**
Impact of Event scale [IES-R]; mean (SD)	18.21 (16.56)	11.90 (11.63)	**0.001**
Emotional exhaustion [EE]*; mean (SD)	22.00 (10.19)	23.48 (10.99)	0.24
Depersonalisation [DP]*; mean (SD)	10.86 (5.13)	12.28 (5.39)	**0.012**
Personal accomplishment [PA]*; mean (SD)	46.35 (7.69)	47.25 (8.13)	0.12
General Self-Efficacy scale [GSE]; mean (SD)	32.18 (5.02)	33.67 (4.70)	**0.04**

*Maslach Burnout Inventory

Mean scores and SDs of each psychological questionnaire were presented in Table 1.

Among psychological scores, only General Self-Efficacy scores were significantly different according to epidemic location status with lower scores in GPs working in high epidemic location (33.37 ± 4.64 vs. 32.06 ± 5.43; $P = 0.04$).

Women scored higher than men for PSS, IES-R and DP and lower for GSE ($P = 0.01$, 0.001, 0.012, 0.04 respectively, Table 2).

Discussion

It is the first study which showed the psychological impact of COVID-19 in GPs during the first sanitary lockdown period including burnout and post-traumatic stress symptoms. Based on self-report validated questionnaires, up to 42% of GPs reported psychological difficulties. Women reported more stress and burnout symptoms than men.

Healthcare workers are at increased risk of mental health difficulties when dealing with challenges of the COVID-19 outbreak [14]. In Chinese physicians and nurses more than 70% reported symptoms of distress [4]. Data in Oman physicians [2] and French urologists [15] also showed psychological difficulties induced by COVID-19 outbreak. According to these first results we showed that up to 42% of French GPs reported burnout symptoms and 11% post-traumatic stress symptoms.

If GPs were at the frontline beating the COVID-19 pandemic, to our knowledge, only two studies assessed the COVID-19 pandemic impact on psychological health in GPs [7, 16]. In a sample of 132 Italian GPs working in Genoa, 30% reported moderate/severe depression symptoms associated with anxiety and insomnia. A second study showed that 40% ($n = 531$) of Colombian GPs reported symptoms of generalized anxiety disorder [16]. Burnout, self-efficacy level, and post-traumatic stress symptoms were not assessed in these studies.

Among psychological scores, only General Self-Efficacy scores were significantly different according to epidemic location. Previous studies also did not shown relationship between mental health and working with COVID-19 patients [2]. In our study, with only 4 localized clusters, the Normandy region was not a high exposed COVID-19 French region.

Women reported more psychological difficulties than men. Similar results have been found in Colombian GPs [16], in healthcare workers in France and China [3, 4, 17], in Oman physicians [2] and in general population [18] during COVID-19 outbreak.

Limitations of the study also include low response rate. Moreover, we did not have control group and reference of psychological symptoms before the sanitary lockdown period. Furthermore, longitudinal data would be interesting.

This study is the first study which showed the psychological impact of COVID-19 in GPs during the first sanitary lockdown period including burnout and post-traumatic stress symptoms. Based on self-report validated questionnaires, up to 42% of GPs reported psychological difficulties. Intervention to promote mental health well-being of GPs need to be developing.

Acknowledgments The authors thank the Union régionale des médecins libéraux Normandie and the health professionals who participatedin this study.

Compliance with ethical standards

Conflict of interest We have no conflicts of interest.

References

1. Zhu N, Zhang D, Wang W, Li X, Yang B, Song J, Zhao X, Huang B, Shi W, Lu R, Niu P, Zhan F, Ma X, Wang D, Xu W, Wu G, Gao GF, Tan W, China Novel Coronavirus Investigating and Research Team. (2020) A novel coronavirus from patients with pneumonia in China, 2019. N Engl J Med 382:727–733. https://doi.org/10.1056/NEJMoa2001017
2. Badahdah AM, Khamis F, Mahyijari NA (2020) The psychological well-being of physicians during COVID-19 outbreak in Oman. Psychiatry Res 289:113053. https://doi.org/10.1016/j.psychres.2020.113053
3. Du J, Dong L, Wang T et al (2020) Psychological symptoms among frontline healthcare workers during COVID-19 outbreak in Wuhan. Gen Hosp Psychiatry 67:144–145. https://doi.org/10.1016/j.genhosppsych.2020.03.011
4. Lai J, Ma S, Wang Y, Cai Z, Hu J, Wei N, Wu J, du H, Chen T, Li R, Tan H, Kang L, Yao L, Huang M, Wang H, Wang G, Liu Z, Hu S (2020) Factors associated with mental health outcomes among health care workers exposed to coronavirus disease 2019. JAMA Netw Open 3:e203976. https://doi.org/10.1001/jamanetworkopen.2020.3976
5. Kang L, Ma S, Chen M, Yang J, Wang Y, Li R, Yao L, Bai H, Cai Z, Xiang Yang B, Hu S, Zhang K, Wang G, Ma C, Liu Z (2020) Impact on mental health and perceptions of psychological care among medical and nursing staff in Wuhan during the 2019 novel coronavirus disease outbreak: a cross-sectional study. Brain Behav Immun 87:11–17. https://doi.org/10.1016/j.bbi.2020.03.028
6. Zhang C, Yang L, Liu S, Ma S, Wang Y, Cai Z, du H, Li R, Kang L, Su M, Zhang J, Liu Z, Zhang B (2020) Survey of insomnia and related social psychological factors among medical staff involved in the 2019 novel coronavirus disease outbreak. Front Psychiatry 11:306. https://doi.org/10.3389/fpsyt.2020.00306
7. Amerio A, Bianchi D, Santi F, et al (2020)Covid-19 pandemic impact on mental health: a web-based cross-sectional survey on a sample of Italian general practitioners. Acta biomed 91:83–88. https://doi.org/10.23750/abm.v91i2.9619
8. El-Hage W, Hingray C, Lemogne C et al (2020) Health professionals facing the coronavirus disease 2019 (COVID-19) pandemic: what are the mental health risks? Encephale 46:S73–S80. https://doi.org/10.1016/j.encep.2020.04.008
9. Majeed A, Molokhia M, Pankhania B, Asanati K (2020) Protecting the health of doctors during the COVID-19 pandemic. Br J Gen Pract 70:268–269. https://doi.org/10.3399/bjgp20X709925
10. Cohen S, Kamarck T, Mermelstein R (1983) A global measure of perceived stress. J Health Soc Behav 24:385–396
11. Creamer M, Bell R, Failla S (2003) Psychometric properties of the impact of event scale - revised. Behav Res Ther 41:1489–1496. https://doi.org/10.1016/j.brat.2003.07.010
12. Jackson SE, Leiter MP, Maslach C (1996) MBI: Maslach burnout inventory. Consulting Psychologists Press, Palo Alto, CA
13. Schwarzer R, Jerusalem M (1995) Generalized self-efficacy scale. In: measures in health psychology: a user's portfolio., J. Weinman, S. Wright, M. Johnston. NFER-NELSON, Windsor, UK, pp 35–37
14. Greenberg N, Docherty M, Gnanapragasam S, Wessely S (2020) Managing mental health challenges faced by healthcare workers during covid-19 pandemic. BMJ 368. https://doi.org/10.1136/bmj.m1211
15. Abdessater M, Rouprêt M, Misrai V, Matillon X, Gondran-Tellier B, Freton L, Vallée M, Dominique I, Felber M, Khene ZE, Fortier E, Lannes F, Michiels C, Grevez T, Szabla N, Boustany J, Bardet F, Kaulanjan K, Seizilles de Mazancourt E, Ploussard G, Pinar U, Pradere B, Association Française des Urologues en Formation (AFUF). (2020) COVID19 pandemic impacts on anxiety of French urologist in training: outcomes from a national survey. Prog Urol 30:448–455. https://doi.org/10.1016/j.purol.2020.04.015
16. Monterrosa-Castro A, Redondo-Mendoza V, Mercado-Lara M (2020) Psychosocial factors associated with symptoms of generalized anxiety disorder in general practitioners during the COVID-19 pandemic. J Investig Med 68:1228–1234. https://doi.org/10.1136/jim-2020-001456
17. Lange M, Joo S, Couette P-A et al (2020) Impact on mental health of the COVID-19 outbreak among community pharmacists during the sanitary lockdown period. Ann Pharm Fr. https://doi.org/10.1016/j.pharma.2020.09.002
18. Wang C, Pan R, Wan X et al (2020) Immediate psychological responses and associated factors during the initial stage of the 2019 coronavirus disease (COVID-19) epidemic among the general population in China. Int J environ res public health 17. https://doi.org/10.3390/ijerph17051729

Publisher's note Springer Nature remains neutral with regard to jurisdictional claims in published maps and institutional affiliations.
