# Supplementary material for: Mental health status and related factors influencing healthcare workers during the COVID-19 pandemic: A systematic review and meta-analysis
Source: PLoS One. 2024 Jan 19;19(1):e0289454. doi: 10.1371/journal.pone.0289454 (PMC10798549; doi:10.1371/journal.pone.0289454)
Supplement: S1 Data — (ZIP) [file pone.0289454.s011.zip › literatures/72.pdf]

European Review for Medical and Pharmacological Sciences 2022; 26: 1049-1055

Impact of COVID-19 pandemic on psychological wellbeing of Oncology clinicians in the Middle East and North Africa (MENA) region

F. AZAM[1], M.F. LATIF[2], S. BASHIR[1], S.H. TIRMAZI[2], N. BUKHARI[1,3], W. AL-SELWI[1], H.O. ALSHAMSI[4], F. IBNSHAMSAH[1,3], J. ZEKRI[5]

[1]King Fahad Specialist Hospital Dammam, Dammam, Saudi Arabia
[2]Dubai Hospital, Al Braha, Dubai, UAE
[3]Imam AbdulRahman bin Faisal University, Dammam, Saudi Arabia
[4]Burjeel Cancer Institute, Abu Dhabi and College of Medicine, University of Sharjah, UAE
[5]College of Medicine, Al-Faisal University, King Faisal Specialist Hospital & Research Centre, Jeddah, Saudi Arabia

Abstract. – OBJECTIVE: The reorganization of cancer services and the increased work burden on health care providers during the COVID-19 pandemic are likely to be associated with significant negative psychological impact. The aim of this study is to evaluate the impact of COVID-19 pandemic on the psychological well-being of oncology clinicians in the Middle East and North Africa (MENA) region.

MATERIALS AND METHODS: We randomly invited 1500 oncology clinicians including medical oncologists, clinical oncologists, radiation oncologists and surgical oncologists from 17 countries in the MENA region to complete a web-based survey to determine the level of psychological stress during the COVID-19 pandemic from October 2020 to January 2021. The questionnaire was based on the Perceived Stress Scale (PSS), Generalized Anxiety Disorders Scale (GAD-7) and WHO Well-being Index (WHO-5). The data was analyzed using SPSS version 21 and the difference between groups was measured by t-test and ANOVA.

RESULTS: Overall, 520 (35%) clinicians including 368 (71%) males and 152 (29%) females participated in the survey with 247 (47%) participants between the ages of 36 to 45 years. Average score of 29.6 for males and 30.2 on PSS-10, indicative of high-perceived stress in both the genders. Compared to males, females had significantly higher anxiety levels on GAD-7 scale (*p*=.04), but this difference in stress level and well-being was not observed on PSS-10 (*p*=.134) and WHO -5 well-being index (*p*=.709). Clinicians of age 25-35 years had significantly higher anxiety levels on GAD-7 scale (*p*=.004) and higher stress on PSS (*p*=.000) as compared to other age groups. Age over 55 years was associated with lower levels of anxiety and stress on GAD-7 and PSS. Oncology clinicians working in public sector experienced significantly lower stress as compared to private sector on PSS scale (*p*=.041).

CONCLUSIONS: Anxiety and stress levels among oncology clinicians have significantly increased in COVID-19 pandemic in the MENA region. Females and young clinicians had higher anxiety and stress, while oncology clinicians over the age of 55 years and working in the public sector reported less stress and anxiety. The general wellbeing of clinicians was well preserved even in a highly stressful and anxious situation.

Key Words:
Knowledge, Attitude, Practice, COVID-19, Anxiety and stress.

Introduction

In March 2020, the WHO declared a COVID-19 pandemic due to Severe Acute Respiratory Syndrome Coronavirus 2 (SARS-CoV-2) infection and since then nearly 2 billion people have been infected with over 4.5 million deaths[1].

A situation of socio-economic crisis and psychological distress rapidly developed worldwide. Local hospitals all over the world started receiving unprecedented numbers of critically ill patients. The general population and healthcare workers became vulnerable to the emotional impact of COVID-19 infection due to both the pandemic and its consequences globally[2-4].

Cancer and immunosuppressive treatments render cancer patients more prone to infections, including COVID-19. They are also vulnerable

Corresponding Author: Faisal Azam, MD; e-mail: drfaisalazam@hotmail.co.uk

to many psychological problems including stress, anxiety, depression, and the consequences of reorganization of non-acute health services provision. These psycho-social stressors significantly increased during the pandemic as nationwide lockdowns caused delays in diagnosis, treatment and movement restrictions. These patient stresses have directly or indirectly affected the oncology clinicians too.

Oncology clinicians managing cancer patients routinely experience immense psychological stress, as they are required to have a vast interdisciplinary up to date knowledge of constantly evolving modern cancer medicine and its application in clinical practice[5]. They are also expected to have an understanding of the psychological predisposition of cancer patients and be able to counter the harmful stress, to which they themselves are exposed. Some studies[6-8] presented at the European Society of Medical Oncology Congress in 2014 (Pre-pandemic era) reported the stress symptoms in 71% young oncologists in Europe (Central Europe 84%), while the percentage of burnout stress among US oncologists was 45% with 34 % of oncologists in the USA reported the urge to change jobs within the next two years.

The COVID-19 pandemic is likely to have increased these stresses to a level not known before. On top of already existing psychological pressures, there are now additional challenges of delayed cancer diagnosis, appointments, treatments, travel restrictions to and from health facilities and fear of contracting infection. In the current pandemic, various healthcare facilities have advised physicians from different specialties to work from home virtually or take time off. However, most oncology physicians continued to work physically due to the nature of their work and so were more exposed to the stress and anxiety. Mental health problems observed among health care workers during previous pandemics were recognised and included insomnia, stress, anxiety, depression, post-traumatic stress and fear of contagion[9].

We designed a web-based survey to examine the impact of the current COVID-19 pandemic on mental health of oncology clinicians working in different countries of the Middle East and North Africa (MENA).

Materials and Methods

Participants were randomly selected and a link to the survey was sent *via* email, text message and social media platforms, including WhatsApp and LinkedIn after approval from the IRB (Investigation review board) at King Fahad specialist Hospital, Dammam in Saudi Arabia. Total of 1500 oncology clinicians from different MENA countries were invited to complete the survey between 1st October 2020 and 31st January 2021. Participants included medical oncologists, radiation oncologists, oncology surgeons, pediatric oncologists, haemato-oncologists and palliative care physicians. Study participants were able to access the questionnaire after the online consent.

Data regarding stress, anxiety and well-being was collected using the following three standardised composite measures.

1. The Perceived Stress Scale (PSS-10) is a ten-item retrospective global measure of stress and consists of a mix of negatively and positively worded statements. All items are rated on a 5-point Linkert scale (0 = never, 1 = almost never, 2 = sometimes, 3 = fairly often, 4 = very often). The total possible score ranges from 0 to 40 and a higher score indicates higher degree of perceived stress (***Supplementary Table I***).
   Scores ranging from 0-13 is considered as low stress, 14-26 is considered as moderate stress and 27-40 is considered as high-perceived stress[10].
2. The Generalized Anxiety Disorders Scale (GAD-7) is a seven-item tool to assess anxiety retrospectively. All items are rated on a 4-point Linkert scale (0 = not at all, 1 = several days, 2 = more than half the days, 3 = nearly every day).
   The total scores are categorised into four levels of anxiety as minimal (0-4), mild (5-9), moderate (10-14) and severe (15-21). A high score signifies a high level of anxiety and a score of ≥10 is indicative of generalized anxiety disorder[11] (***Supplementary Table II***).
3. The WHO Well-being Index (WHO-5) is a five-item retrospective self-reporting scale that gauges overall subjective psychological well-being. The items are rated on a 6-point Likert scale (0 = at no time, 1 = some of the time, 2 = less than half the time, 3 = more than half the time, 4 = most of the time, 5 = all the time).

The total raw score ranges from 0 to 25 which is then multiplied by four to create a percentage score ranging from 0 (worst possible well-being)

to 100 (best possible well-being). Scores ≤ 50 are indicative of poor psychological well-being and warrants screening for depression[12] (***Supplementary Table III***).

The data was analyzed using SPSS version 21 and the difference between groups was measured by *t*-test and ANOVA. Demographic data was collected on gender, speciality, country, place of work as a private or government institute and age groups of 25-35, 36-45, 46-55 and >55 years.

Results

Overall, 520 (35%) of 1500 invited oncology clinicians participated in the study from 17 countries in the Middle East and North Africa. Participants included 368 (71%) males and 152 (29%) females with 247 (47%) clinicians between the ages of 36 to 45 years. Most of the responses were received from medical oncologists (n=196, 37.6%) working in the public sector (n=439, 84.2%) and teaching institutions (n=395, 75.8%, Table I).

Oncology clinicians responding to survey were from Saudi Arabia (n=202), Egypt (n=78), UAE (n=59), Tunisia (n=29), Morocco (n=20), Iraq (n=16), Jordan, Algeria, Oman & others (n=13 each), Sudan (n=11), Kuwait (n=10) Qatar & Syria (n=9 each), Yemen (n=6), Iran (n=5), Libya (n=2) and Bahrain (n=1).

Analysis of 520 completed questionnaires indicated an average score of 29.6 for males and 30.2 for females on PSS-10, indicative of high-perceived stress in both the genders. Females reported more stress, however this was not statistically significant (*p*=.134). Analysis of variance (ANOVA) showed significantly higher perceived stress in young age (25-35 years) clinicians (*p*=.001) and public sector workers have significantly lower perceived stress as compared to the private sector (*p*=.041, Table II). Institution of work (teaching or non-teaching) had no effect on PSS-10 (*p*=.311).

The GAD-7 scales were graded as mild (scores of 0-9), moderate (scores of 10-14) and severe (scores of 15-21) based on scores. Moderate (45.6%) to severe (42.3%) anxiety was reported in 87.9% clinicians. Average GAD-7 score was (14.0 ±4.3) for male and (15.4 ±4.8) for female (Table II). A Chi-square analysis revealed that a significantly greater proportion of females as compared to males experienced anxiety (*p*=.040). Clinicians of ages 25-35 years had significantly higher anxiety levels on GAD-7 scale (*p*=.004). There was no significant difference in anxiety level among clinicians working in teaching or non-teaching institutions (*p*=.430).

On the WHO-5 scale, the mean of the transformed score was 66.3± 21.1 including males (62.9 ±20.5) and females (74.3 ±20.8, (Table II). Females experienced more positive well-being as

Table I. Demographic characteristics of study participants.

<table>
<tr><th colspan="8">Variables</th></tr>
<tr><td>Age (years)</td><td colspan="2">25-35
105 (20.15%)</td><td colspan="2">36-45
247 (47.4%)</td><td colspan="2">46-55
124 (23.80%)</td><td>Above 56
45 (8.43%)</td></tr>
<tr><td rowspan="3">Gender</td><td colspan="4">Female</td><td colspan="3">Male</td></tr>
<tr><td colspan="2">N</td><td colspan="2">%</td><td colspan="2">N</td><td>%</td></tr>
<tr><td colspan="2">152</td><td colspan="2">29.23</td><td colspan="2">368</td><td>70.77</td></tr>
<tr><td>Specialty</td><td>Medical oncology
N (%)
196 (37.62%)</td><td>Radiation oncology
N (%)
54 (10.36)</td><td>Clinical oncology
N (%)
57 (10.94)</td><td>Hemato-oncology
N (%)
65 (12.48)</td><td>Pediatric oncology/ Hematology
N (%)
25 (4.80)</td><td>Surgical oncology
N (%)
106 (20.35)</td><td>Palliative care
N (%)
18 (3.45)</td></tr>
<tr><td rowspan="3">Organization</td><td colspan="4">Public sector</td><td colspan="3">Private sector</td></tr>
<tr><td colspan="2">N</td><td colspan="2">%</td><td colspan="2">N</td><td>%</td></tr>
<tr><td colspan="2">439</td><td colspan="2">84.26</td><td colspan="2">82</td><td>15.74</td></tr>
<tr><td rowspan="3">Institution</td><td colspan="4">Teaching</td><td colspan="3">Non-teaching</td></tr>
<tr><td colspan="2">N</td><td colspan="2">%</td><td colspan="2">N</td><td>%</td></tr>
<tr><td colspan="2">395</td><td colspan="2">75.82</td><td colspan="2">126</td><td>24.18</td></tr>
</table>

Table II. Prevalence of anxiety, stress, and well-being, by gender, age, organization and institution.

Variables		GAD-7	WHO wellbeing index	PSS-10
	Total score (mean ± SD)	14.4 ± 4.5	66.3 ± 21.1	29.8 ± 4.08
Gender	Male	14.0 ± 4.3*	62.9 ± 20.5	29.6 ± 3.83
	Female	15.4 ± 4.8	74.3 ± 20.7	30.2 ± 4.6
Age (years)	25-35	15.4 ± 4.3***	73.9 ± 18.4***	28.1 ± 5.2***
	36-45	14.4 ± 4.6	65.5 ± 21.0	30.1 ± 3.6
	46-55	14.6 ± 4.4	66.1 ± 21.7	30.7 ± 3.2
	Above 56	11.1 ± 2.9	47.6 ± 16.0	29.6 ± 2.8
Organization	Public sector	14.5 ± 4.5	67.3 ± 21.0	29.6 ± 4.2*
	Private sector	13.8 ± 4.4	61.0 ± 21.3	30.6 ± 2.9
Institution	Teaching	14.3 ± 4.4	66.1 ± 20.9	29.6 ± 4.2
	Non-teaching	14.7 ± 4.8	67.1 ± 21.8	30.4 ± 3.4

*= $p < 0.05$, **= $p < 0.01$, ***= $p < 0.001$

compared to males, however this was not statistically significant (p=.799). An ANOVA for WHO wellbeing index showed a statistically significant difference among age groups (p=.000) in participants. There was no difference in WHO-5 scoring between teaching and non-teaching intuitions (p=.564).

Discussion

To the best of our knowledge, this is the first study to evaluate the psychological impact of the COVID-19 pandemic on oncologists in the Middle East and North Africa (MENA) region. COVID-19 affects all clinicians, regardless of they being a front-line worker or not[14].

Oncologists and other health care professionals are at risk of distress during the COVID-19 pandemic because of the threat of personal infection and patient management related factors[15,16]. A cross-sectional study of mental health outcomes among healthcare providers during the COVID-19 pandemic from China reported that half of the participants were suffering from symptoms of depression, distress, anxiety and insomnia. Similar to our study, they have also reported female gender being more affected[16].

Psychological distress and the well-being of physicians has also been studied[15,17] in numerous other studies as physicians, generally, are exposed to psychosocial risks, such as work-related stress, dealing with immensely emotional, anxious patients and managing dying patients. An Irish national survey study[18] of physicians completed before the pandemic, using the GHQ-12 and WHO-5 questionnaires, reported a high prevalence of psychological distress (34.8%) and poor well-being (49.5%) amongst participants. Oncologists face these situations more frequently due to the solemn nature of their work. A study[6] presented in ESMO (European society of medical oncology) Congress 2014 reported symptoms of stress in 71% young oncologists in Europe (Central Europe 81%) The percentage of burnout stress amongst US oncologists has been reported as 45%, whilst another US study found 34% of oncologists expressed an urge to change their jobs within the next two years[7,8]. Thomaier et al[19] reported a very high and concerning level of distress in physicians, from their survey study of 374 US oncologists' emotional health during the COVID-19 pandemic, 62% of oncologists presented with anxiety and 36.6% with depression with significant impact on their wellbeing. Researchers from this study concluded that anxiety and depression among oncologists needed to be addressed in order to better manage patients and to safeguard oncologist's mental health. The aforementioned ESMO and US studies conducted before the COVID-19 pandemic showed an already high prevalence of anxiety amongst oncology clinicians which increased further during the pandemic, as reported by the Thomaier et al[19]. Our study, conducted during the peak of the COVID-19 pandemic, differed from the aforementioned US study conducted during the COVID-19 pandemic as our study reported the preservation of well-being of oncology clinicians despite the presence of moderate to severe anxiety in 87.9% of clinicians. This is an unexpected finding, i.e., WHO well-being score of 68.62 (> 50 does not indicate poor wellbeing) although the PSS and GAD indicate high stress and anxiety. Similar findings were reported in a recent study from Saudi Arabia with preservation of mental wellbeing on WHO-5 scale among

health workers during COVID-19[20]. However, a study from Switzerland showed anxiety, depression and poor wellbeing in ICU healthcare workers. COVID-19 study from UK reported that PSS, GAD and WHO-5 are all correlated[21,22]. We could not explain the cause of preservation of well-being with significant stress and anxiety in our and other Saudi study. Our study shares similar results with other studies from the region but ours, so far, is the only study reporting the psychological impact of the pandemic on oncology clinicians. Al Sulais et al[23] reported from their survey study of 529 physicians from Saudi Arabia that the most common stresses were worry, isolation and fear (49.7%) with physicians older than age 60 being less likely to feel isolated and female physicians being more likely to experience fear and worry. Deemah al Ateeq et al[24] found that, in their survey study from Saudi Arabia on health care workers using patient Health Questionnaire (PHQ-9) and GAD-7 questionnaires, generalized anxiety disorder and depression was present in more than half of the health care workers with preponderance for females, workers between 30-39 years of age and nurses[24].

A study[25] from Oman, using GAD-7 questionnaire reported a very high anxiety in 65% of healthcare workers, whilst our study reported it in 87% of oncology clinicians. Similar to our study, this study also found a greater proportion of females scoring 10 or higher and had a significant negative relationship between age and anxiety when compared to the proportion of males. The mean stress score of 30 on PSS-10 in females in our study was considerably higher than mean score of 24 on PSS-10 in this study[25]. These high levels of anxiety from our region are troublesome, especially in comparison with data from China, where the COVID-19 pandemic originated. Lai et al[26] found a much lower percentage of anxiety (44.6%) among health care workers as compared to our study (87%).

Our study found preservation of overall psychological well-being even with high anxiety levels, which is in contrast to Omani study[25] which reported poor overall psychological well-being among health care workers especially in females and those who had cared for COVID-19 patients. In two of three measures used in our study, female and young oncology clinicians fared worse than males and older oncology clinicians. These results are similar to other studies from the region. One possible explanation of this is that older health care workers with more experience have developed better coping skills meaning they are able to adapt to different stressful situations.

Oncologists need to be emotionally and psychologically healthy and strong to serve cancer patients in a better way. In this study, we have reported the detrimental impact of COVID-19 on oncology clinicians' psychological health in the Middle East and North African region. Although it was not the scope of our study to find its impact on patient care, we believe this would have a worse impact on the management of cancer patients.

Our study provided important results for future comparative studies among oncologists in MENA region and other part of the world. Our results are consistent with results of other studies looking at the mental health impact of COVID-19 on health care workers in the Middle East, though our is the only study looking at the oncologists of the region.

We believe our study will have a valuable contribution to the medical literature but like any other study, ours also have some limitations. First, our sample is randomly selected based on authors search on Linkedin and regional conferences list, which might not truly represent the oncologist population in MENA region. Therefore, the findings may not be generalizable and should be interpreted with some caution. Second, the responses from different countries are not balanced with limited responders from Iran, which is a large country in the Middle East. Clinicians from Iran were among our targeted subjects; however, we realized later that difficulties in accessing SurveyMonkey website significantly limited the participation from Iran.

Strengths of this study include large number of responders covering clinicians in a specific geographical region, and therefore, its results can be used by policy makers to combat the detrimental effects of COVID on health care professionals in this region.

Conclusions

Our study is the only study in the MENA region looking at the impact of Covid19 on cancer care clinicians. Anxiety and stress levels were higher in females and young clinicians. Clinicians over 55 years of age and working in the public sector reported less stress and anxiety. General well-being of clinicians was well preserved even in a highly stressful and anxious situation.

Conflict of Interest

The Authors declare that they have no conflict of interests.

Funding

The author(s) declare(s) that there is no funding available for the publication of this paper.

Ethics Approval and Consent to Participate

The protocol of research was reviewed and approved by the Ethics and Research Committee of KFSH-D with (IRB-Number: ONC0369).

Availability of Data and Material

All data are included in the manuscript. However, the datasets used and/or analysed during the current study are available from the corresponding author on reasonable request.

Authors' Contribution

All listed authors developed different substantial activities. They participated in drafting and writing the manuscript. Each author participated sufficiently in writing and reviewing the manuscript. All authors read and approved the final manuscript.

References

1) Healthline. Everything You Should Know About the 2019 Coronavirus and COVID-19. September 17, 2021, Available from URL: https://www.healthline.com/health/coronavirus-covid-19. Accessed September 17, 2021.
2) Xiang YT, Yang Y, Li W, Zhang L, Zhang Q, Cheung T, Ng CH. Timely mental health care for the 2019 novel coronavirus outbreak is urgently needed. Lancet Psychiatry 2020; 7: 228-229.
3) Thakur V, Jain A. COVID 2019-suicides: A global psychological pandemic. Brain Behav Immun 2020; 88: 952-953.
4) Zhai Y, Du X. Loss and grief amidst COVID-19: A path to adaptation and resilience. Brain Behav Immun 2020; 87: 80-81.
5) Soltan MR., Al-Hassanin, SA, Soliman, SS. Workplace-related stress among oncologists: Egyptian single-centered observational study. Middle East Curr Psychiatry 2020; 2: 719.
6) Banerjee S, Califano R, Corral J, de Azambuja E, De Mattos-Arruda L, Guarneri V, Hutka M, Jordan K, Martinelli E, Mountzios G, Ozturk MA, Petrova M, Postel-Vinay S, Preusser M, Qvortrup C, Volkov MNM, Tabernero J, Olmos D, Strijbos MH. Professional burnout in European young oncologists: results of the European Society for Medical Oncology (ESMO) Young Oncologists Committee Burnout Survey. Ann Oncol 2017; 28: 1590-1596.
7) Shanafelt TD, Gradishar WJ, Kosty M, Satele D, Chew H, Horn L, Clark B, Hanley AE, Chu Q, Pippen J, Sloan J, Raymond M. Burnout and career satisfaction among US oncologists. J Clin Oncol 2014; 32: 678-86.
8) Shanafelt TD, Raymond M, Kosty M, Satele D, Horn L, Pippen J, Chu Q, Chew H, Clark WB, Hanley AE, Sloan J, Gradishar WJ. Satisfaction with work-life balance and the career and retirement plans of US oncologists. J Clin Oncol 2014; 32: 1127-1135.
9) Badahdah AM, Khamis F, Mahyijari NA. The psychological well-being of physicians during COVID-19 outbreak in Oman. Psychiatry Res 2020; 289: 113053.
10) Cohen S, Kamarck T, Mermelstein R. A global measure of perceived stress. J Health Soc Behav 1983; 24: 385-396.
11) Spitzer RL, Kroenke K, Williams JBW, Lowe B. A brief measure for assessing generalized anxiety disorder. Arch Inern Med 2006; 166: 1092-1097.
12) Rauwerda NL, Tovote KA, Peeters ACTM. WHO-5 and BDI-II are acceptable screening instruments for depression in people with diabetes. Diabet. Med 2018; 35: 1678-1685.
13) Wu Y, Wang J, Luo C, Hu S, Lin X, Anderson AE, Bruera E, Yang X, Wei S, Qian Y. A Comparison of Burnout Frequency Among Oncology Physicians and Nurses Working on the Frontline and Usual Wards During the COVID-19 Epidemic in Wuhan, China. J Pain Symptom Manage 2020; 60: e60-e65.
14) Berardi R, Torniai M, Cona MS, Cecere FL, Chiari R, Guarneri V, La Verde N, Locati L, Lorusso D, Martinelli E, Giannarelli D, Garassino MC; Women for Oncology Italy. Social distress among medical oncologists and other healthcare professionals during the first wave of COVID-19 pandemic in Italy. ESMO Open 2021; 6: 100053.
15) Perumalswami CR, Chen E, Martin C, Goold SD, De Vries R, Griggs JJ, Jagsi R. "I'm Being Forced to Make Decisions I Have Never Had to Make Before": Oncologists' Experiences of Caring for Seriously Ill Persons With Poor Prognoses and the Dilemmas Created by COVID-19. JCO Oncol Pract 2021: OP2100119.
16) Lai J, Ma S, Wang Y, Cai Z, Hu J, Wei N, Wu J, Du H, Chen T, Li R, Tan H, Kang L, Yao L, Huang M, Wang H, Wang G, Liu Z, Hu S. Factors Associated With Mental Health Outcomes Among Health Care Workers Exposed to Coronavirus Disease 2019. JAMA Netw Open 2020; 3: e203976.
17) Firenze A, Santangelo O.E, Gianfredi V, Alagna E, Cedrone, F, Provenzano, S, La Torre, G. Violence on doctors an observational study in Northern Italy. Med Lav 2020; 111: 46-53.
18) Hayes B, Prihodova L, Walsh G, Doyle F, Doherty S. What's up doc? A national cross-sectional study of psychological wellbeing of hospital doctors in Ireland. BMJ Open 2017; 7: e018023.

19) Thomaier L, Teoh D, Jewett P, Beckwith H, Parsons H, Yuan J, Blaes AH, Lou E, Hui JYC, Vogel RI. Emotional health concerns of oncology physicians in the United States: Fallout during the COVID-19 pandemic. PLoS One 2020; 15: e0242767.

20) Ehab A Abo-Ali, Shayma Al-Rubaki, Sarah Lubbad. Mental Well-Being and Self-Efficacy of Healthcare Workers in Saudi Arabia During the COVID-19 Pandemic. Risk Manag Healthc Policy 2021; 14: 3167-3177.

21) Wozniak H, Benzakour L, Moullec G, Buetti N. Mental health outcomes of ICU and non-ICU healthcare workers during the COVID-19 outbreak: a cross-sectional study. Ann Intensive Care 202110; 11: 106.

22) Christoph Pieh, Thomas Probst, Sanja Budimir. Associations between Relationship Quality and Mental Health during COVID-19 in the United Kingdom. Int J Environ Res Public Health 2021; 18: 2869.

23) Al Sulais E, Mosli M, AlAmeel T. The psychological impact of COVID-19 pandemic on physicians in Saudi Arabia: A cross-sectional study. Saudi J Gastroenterol 2020; 26: 249-255.

24) AlAteeq DA, Aljhani S, Althiyabi I, Majzoub S. Mental health among healthcare providers during coronavirus disease (COVID-19) outbreak in Saudi Arabia. J Infect Public Health 2020; 13: 1432-1437.

25) Badahdah A, Khamis F, Al Mahyijari N, Al Balushi M, Al Hatmi H, Al Salmi I, Albulushi Z, Al Noomani J. The mental health of health care workers in Oman during the COVID-19 pandemic. Int J Soc Psychiatry 2020: 20764020939596.

26) Alshekaili M, Hassan W, Al Said N, Al Sulaimani F, Jayapal SK, Al-Mawali A, Chan MF, Mahadevan S, Al-Adawi S. Factors associated with mental health outcomes across healthcare settings in Oman during COVID-19: frontline versus non-frontline healthcare workers. BMJ Open 2020; 10: e042030.
