# Supplementary material for: Mental health status and related factors influencing healthcare workers during the COVID-19 pandemic: A systematic review and meta-analysis
Source: PLoS One. 2024 Jan 19;19(1):e0289454. doi: 10.1371/journal.pone.0289454 (PMC10798549; doi:10.1371/journal.pone.0289454)
Supplement: S1 Data — (ZIP) [file pone.0289454.s011.zip › literatures/73.pdf]

LETTER

Psychological impact on women health workers involved in COVID-19 outbreak in Wuhan: a cross-sectional study

INTRODUCTION

The outbreak of COVID-19 poses an unprecedented threat and a great challenge to health workers (HWs) in Wuhan, China.[1] The situation of women HWs in Wuhan is even more difficult. According to 2019 Chinese Health Statistics Yearbook, the number of women HWs in China accounted for 71.8% of Chinese HWs, far exceeding that of men HWs. Women HWs has become the major force to tackle the COVID-19 epidemic. Owing to entrenched traditional social roles in China, women HWs are placed at a considerable dilemma of how to balance work and family care. Previous studies have also suggested that women were vulnerable to mental health problems.[2] The mental health problems of women HWs may affect their attention and decision-making ability and could have a lasting effect on their overall well-being. Hence, psychological impact of COVID-19 on women HWs in Wuhan should be pronounced.

Tongji Hospital, one of the biggest hospitals in Wuhan, was officially designated as 'the specific hospital for the treatment of severe patients with COVID-19', with a women HWs proportion of 81.0%. This hospital has opened 2000 beds specifically for patients with severe COVID-19, and women HWs from all clinic departments of this hospital are sent to the frontline of COVID-19. Given the high infectivity of the COVID-19, the hospital administrators arranged lodging support for the frontline HWs living apart from their families. A cross-sectional study was designed to evaluate the immediate psychological impact on women HWs of Tongji Hospital, determine the predictors of acute stress, depression and anxiety symptoms and investigate the sources of acute stress among the women HWs.

METHODS

Study design and participants

The study was a cross-sectional, single-centre survey, covering doctors, nurses and medical technicians in all clinical departments of Tongji Hospital. The study was conducted between 8 February and 15 February 2020, 2 weeks after Wuhan lockdown on 23 January. We collected information of the women HWs as follows: informed consent, baseline sociodemographic characteristics, perceptions of threat of COVID-19, rating scales including Patient Health Questionnaire-9 (PHQ-9), 7-item Generalized Anxiety Disorder (GAD-7) Scale and Impact of Event Scale-Revised Questionnaires (IES-R). WJX online survey platform was used (https://www.wjx.cn/), and the information was collected via WeChat.

Depression and anxiety symptoms were measured by PHQ-9 and GAD-7 Scales, respectively. Cutoff values of $\geq$10 in PHQ score and $\geq$8 in GAD-7 score had been recommended for diagnosis of major depression and anxiety symptoms, with adequate sensitivities (88.0% and 82.0%) and specificities (88.0% and 77.0%). We defined stress when the IES-R score >33 points, which provided adequate specificity (91.0%) and sensitivity (82.0%). The Cronbach's α coefficients of the three questionnaires were more than 0.80, which showed good internal consistency.

We divided continuous variables into categorical variables first, and all variables were shown as the counts and percentages. Variables with p values <0.05 in univariate logistic regression were subjected to multivariable logistic regression analysis with a stepwise backwards elimination procedure. The differences on perceptions of threat of the COVID-19 between different groups were examined by Mann-Whitney U test. All data were analysed by using SPSS V.22.0.

RESULTS

A total of 5317 women HWs were surveyed, and 4369 effective questionnaires were collected, with an 82.2% response rate. Six hundred and twenty-one (14.2%), 1101 (25.2%) and 1382 (31.6%) women HWs were with depression, anxiety and acute stress symptoms.

The predictors of acute stress were presented in table 1. Three sociodemographic variable (>10 years of working; with medical history, including chronic non-communicable diseases and psychiatric history; $\geq$2 children) presented as the common risk factors, while one variable (exercise habit) presented as common protective factor of depression, anxiety and acute stress symptoms in women HWs. Moreover, drinking history was a common risk factor of anxiety symptom (OR 1.95; 95% CI 1.42 to 2.68; p<0.001) and depression symptom (OR 1.71; 95% CI 1.19 to 2.47; p=0.004). Family members or relatives suspected or confirmed (OR 1.31; 95% CI 1.05 to 1.65; p=0.018) and suspected or confirmed COVID-19 (OR 1.93; 95% CI 1.29 to 2.86; p=0.001) were risk factors of depression symptom. Refer to medical technician, nurse (OR 1.41; 95% CI 1.10 to 1.80; p=0.007) and family members or relatives suspected or confirmed (OR 1.39; 95% CI 1.16 to 2.66; p<0.001) were two risk factors of acute stress. Working in other departments (OR 0.78; 95% CI 0.65 to 0.94; p=0.009) was a protective factor of acute stress.

In addition, we analysed the perceptions of threat of COVID-19 in different groups and found doctors feel significantly higher exposure history than nurses and medical technicians after post hoc multiple comparisons (doctors: 46.4% vs nurses: 40.5% vs medical technicians: 26.8%, p<0.001). Women with more children were more likely to feel family members and friends' avoidance after post hoc multiple comparisons ($\geq$2 children: 23.6% vs one child: 21.0% vs no child: 17.5%, p=0.002). Those who worked in isolation wards did not show more feel of exposure (isolation wards: 40.1% vs other departments: 39.6%, p=0.800). Women HWs who worked in isolation wards had more resigning thoughts (isolation wards: 16.9% vs other departments: 7.8%, p<0.001) and feels of life-threatening once infected (isolation wards: 67.7% vs other departments: 56.4%, p<0.001).

DISCUSSION

The cross-sectional survey showed that women HWs had a high proportion of stress, depression and anxiety symptoms during the early stage of COVID-19. Among the sociodemographic characteristics, those who have more than 10 years of working and two or more children are susceptible to stress, depression and anxiety. Those women HWs might face more occupational exhaustion, family responsibilities and inequality in domestic labour.[3] Moreover, those women HWs in the crisis of COVID-19 were placed at a considerable dilemma, which existed between working and family care and between the family care and avoidance of contact with family members. As this study shows, women with more children were more likely to feel family members and friends' avoidance.

Those women HWs with chronic non-communicable diseases and mental disorders were more likely to have stress, depression and anxiety. The relationship between chronic non-communicable diseases and mental health problems has been widely discussed, and the poor

J Neurol Neurosurg Psychiatry: first published as 10.1136/jnnp-2020-323134 on 4 May 2020. Downloaded from http://jnnp.bmj.com/ on December 19, 2022 by guest. Protected by copyright.

Table 1 Sociodemographic characteristics associated with stress in women HWs by univariate and multivariate logistic regression

Characteristic	Stress (n=1382), n (%)	Non-stress (n=2987), n (%)	Univariate analysis		Multivariate analysis	
			OR	95% CI	OR	95% CI
Age (years)						
19–29	502 (36.3)	1380 (46.2)	1.00			
30–49	846 (61.2)	1533 (51.3)	*1.52	1.33 to 1.73		
>49	34 (2.5)	74 (2.5)	1.26	0.83 to 1.92		
Marital status						
Married	964 (69.8)	1859 (62.2)	1.00			
Unmarried	378 (27.4)	1058 (35.4)	*0.69	0.60 to 0.79		
Divorced/widowed/separated	40 (2.9)	70 (2.3)	1.10	0.74 to 1.64		
Master degree or higher level	229 (16.6)	541 (18.1)	0.90	0.74 to 1.06		
Occupation						
Medical technician	96 (6.9)	311 (10.4)	1.00		1.00	
Nurse	1127 (81.5)	2253 (75.4)	*1.62	1.28 to 2.06	1.41*	1.10 to 1.80
Doctor	159 (11.5)	423 (14.2)	1.22	0.91 to 1.63	1.15	0.85 to 1.55
Professional title level						
Junior	818 (59.2)	1960 (65.6)	1.00			
Intermediate	504 (36.5)	893 (29.9)	*1.35	1.18 to 1.55		
Senior	60 (4.3)	134 (4.5)	1.07	0.78 to 1.47		
Years of working						
<2	82 (5.9)	352 (11.8)	1.00		1.00	
2–5	423 (30.6)	1063 (35.6)	*1.71	1.31 to 2.23	1.41*	1.07 to 1.86
6–10	419 (30.3)	810 (27.1)	*2.22	1.70 to 2.90	1.55*	1.15 to 2.09
>10	458 (33.1)	762 (25.5)	*2.58	1.98 to 3.37	1.56*	1.14 to 2.14
Department						
Fever clinic and ED	265 (19.2)	524 (17.5)	1.00		1.00	
Isolation wards	282 (20.4)	483 (16.2)	1.15	0.94 to 1.42	1.15	0.93 to 1.42
Surgery	330 (23.9)	697 (23.3)	0.94	0.77 to 1.14	0.85	0.70 to 1.05
Other department	505 (36.5)	1283 (43.0)	*0.78	0.65 to 0.93	0.78*	0.65 to 0.94
Annual household income (in yuan)						
30 000~100 000	216 (15.6)	586 (19.6)	1.00			
100 000~200 000	666 (48.2)	1387 (46.4)	1.30*	1.09 to 1.56		
200 000~300 000	328 (23.7)	673 (22.5)	1.32*	1.08 to 1.62		
>300 000	172 (12.4)	341 (11.4)	1.37*	1.08 to 1.74		
Living with family member	652 (47.2)	1474 (49.3)	0.92	0.81 to 1.04		
Medical history						
In good health	1026 (74.2)	2546 (85.2)	1.00		1.00	
Have chronic non-communicable diseases	321 (23.2)	423 (14.2)	1.88*	1.60 to 2.22	1.75*	1.48 to 2.07
Have history of mental disorders	35 (2.5)	18 (0.6)	4.83*	2.72 to 8.56	4.58*	2.55 to 8.23
Smoking history	3 (0.2)	6 (0.2)	1.08	0.27 to 4.33		
Drinking history	72 (5.2)	111 (3.7)	1.42*	1.05 to 1.93		
Exercise habit	174 (12.6)	498 (16.7)	0.72*	0.60 to 0.86	0.73*	0.60 to 0.88
Parent status						
No child	452 (32.7)	1293 (43.3)	1.00		1.00	
One child	699 (50.6)	1373 (46.0)	1.46*	1.27 to 1.68	1.19	0.99 to 1.42
Two or more children	231 (16.7)	321 (10.7)	2.06*	1.69 to 2.52	1.80*	1.42 to 2.28
Family members or relatives suspected or confirmed	269 (19.5)	405 (13.6)	1.54*	1.30 to 1.83	1.39*	1.16 to 1.66
Suspected or confirmed COVID-19	54 (3.9)	89 (3.0)	1.32	0.94 to 1.87		

*P<0.05.
ED, emergency department; HW, health worker.

health status made women HWs face higher infection risk during the outbreak. Although women HWs rarely had a history of drinking (4.2%), drinking presented a common risk factor for both depression and anxiety symptoms.[4] The exercise habit was associated with lower risk of anxiety, depression and stress symptoms in our study. Nevertheless, only 15.4% participants had exercise habits, while the proportion of men HWs in Tongji Hospital who had exercise habits reached 25.3%. Our study also showed that women HWs in isolation wards presented higher rates of psychological stress. Therefore, there was clearly a need for more psychosocial support to reduce the perceptions of threat of COVID-19 among women HWs.[5]

The main limitation of our study was that the work–family conflict was not included in our variables. Since cross-sectional study measured exposure and outcome simultaneously, the findings had

J Neurol Neurosurg Psychiatry: first published as 10.1136/jnnp-2020-323134 on 4 May 2020. Downloaded from http://jnnp.bmj.com/ on December 19, 2022 by guest. Protected by copyright.

only a moderate capacity to clarify the temporal association between influencing factors and depression, anxiety and acute stress symptoms.

CONCLUSION

Our study was a big-sample cross-sectional study on psychological status of women HWs during COVID-19 outbreak in Wuhan. Our research could help hospital administrators to work with psychologists to formulate policies and relevant support to better address mental health problems of women HWs and thus contribute to crisis intervention and better long-term health outcomes.

Guo Li,[1] **Jinfeng Miao,**[1] **Hui Wang,**[2] **Shabei Xu** ,[1] **Wenzhe Sun,**[1] **Yebin Fan,**[3] **Chenyan Zhang,**[1] **Suiqiang Zhu,**[1] **Zhou Zhu** ,[1] **Wei Wang**[1]

[1]Department of Neurology, Tongji Hospital of Tongji Medical College of Huazhong University of Science and Technology, Wuhan, Hubei, China
[2]Nursing Department, Tongji Hospital of Tongji Medical College of Huazhong University of Science and Technology, Wuhan, Hubei, China
[3]School of Computer Science and Technology, Huazhong University of Science and Technology, Hubei, Wuhan, China

Correspondence to Dr Zhou Zhu, Department of Neurology, Tongji Hospital of Tongji Medical College of Huazhong University of Science and Technology, Wuhan 430030, Hubei, China; zhouzhu@hust.edu.cn

Acknowledgements We would like to thank all women health workers that participated in this study. We pay tribute to our brave colleagues who are dealing with COVID-19 and to their families.

Contributors GL, JM and HW contributed equally. Concept and design: SZ, ZZ and WW; acquisition, analysis or interpretation of data: all authors; drafting of the manuscript: ZZ and GL; statistical analysis: GL and JM; obtained funding: SZ and SX; supervision: SZ, ZZ and WW.

Funding This study was supported by grants 2017YFC1310000 from the National Key Research and Development Program of the Ministry of Science and Technology of China ， 2018KFYXMPT015 from Research and Development Office of Huazhong University of Science and Technology and 2020020401010096 from Wuhan Science and Technology Bureau.

Disclaimer The funder/sponsor had no role in the design and conduct of the study; collection, management, analysis, and interpretation of the data; preparation, review, or approval of the manuscript; and decision to submit the manuscript for publication.

Competing interests None declared.

Patient consent for publication Not required.

Ethics approval The institutional ethics board of Tongji Hospital, Tongji Medical College of Huazhong University of Science and Technology approved the study and provided ethics approval number: TJ-C20200129.

Provenance and peer review Not commissioned; internally peer reviewed.

© Author(s) (or their employer(s)) 2020. No commercial re-use. See rights and permissions. Published by BMJ.

Check for updates

To cite Li G, Miao J, Wang H, *et al*. *J Neurol Neurosurg Psychiatry* 2020;**91**:895–897.

Received 29 February 2020
Revised 21 April 2020
Accepted 28 April 2020
Published Online First 4 May 2020

J Neurol Neurosurg Psychiatry 2020;**91**:895–897.
doi:10.1136/jnnp-2020-323134

ORCID iDs
Shabei Xu http://orcid.org/0000-0001-5409-2092
Zhou Zhu http://orcid.org/0000-0002-3806-8823

REFERENCES

1 Kang L, Li Y, Hu S, *et al*. The mental health of medical workers in Wuhan, China dealing with the 2019 novel coronavirus. *Lancet Psychiatry* 2020;7:e14.
2 Li SH, Graham BM. Why are women so vulnerable to anxiety, trauma-related and stress-related disorders? the potential role of sex hormones. *Lancet Psychiatry* 2017;4:73–82.
3 Jolly S, Griffith KA, DeCastro R, *et al*. Gender differences in time spent on parenting and domestic responsibilities by high-achieving young physician-researchers. *Ann Intern Med* 2014;160:344–53.
4 Boden JM, Fergusson DM. Alcohol and depression. *Addiction* 2011;106:906–14.
5 Wu P, Fang Y, Guan Z, *et al*. The psychological impact of the SARS epidemic on hospital employees in China: exposure, risk perception, and altruistic acceptance of risk. *Can J Psychiatry* 2009;54:302–11.

J Neurol Neurosurg Psychiatry: first published as 10.1136/jnnp-2020-323134 on 4 May 2020. Downloaded from http://jnnp.bmj.com/ on December 19, 2022 by guest. Protected by copyright.
