# Supplementary material for: Mental health status and related factors influencing healthcare workers during the COVID-19 pandemic: A systematic review and meta-analysis
Source: PLoS One. 2024 Jan 19;19(1):e0289454. doi: 10.1371/journal.pone.0289454 (PMC10798549; doi:10.1371/journal.pone.0289454)
Supplement: S1 Data — (ZIP) [file pone.0289454.s011.zip › literatures/75.pdf]

Observational Study

Medicine®

OPEN

A survey of mental health status of obstetric nurses during the novel coronavirus pneumonia pandemic

Shuyue Li, MM[a], Ruiyu Chai, MM[b], Yingshuang Wang, MM[b], Jin Wang, MM[b], Xinxin Dong, MM[b], Han Xu, MM[b], Huiyan Wu, MM[c], Isaac T.S. Binnay, MM[b], Zhigang Liu, MD[d,*]

Abstract

To investigate the mental health status of obstetric nurses and its influencing factors during the novel coronavirus epidemic period, so as to provide theoretical reference for hospital decision-makers and managers.

From February 25 to March 20, 2020, we conducted a cross-sectional survey through online questionnaire, and selected obstetric nurses from Jilin and Heilongjiang Provinces as the research objects by convenience sampling.

Three hundred eighteen valid questionnaires were collected; the results of Symptom Checklist 90 showed that the scores of "obsessive-compulsive", "depression", "anxiety", "hostility", "phobia", and "psychosis" were higher than the Chinese norm ($P < .01$). There were 107 people whose total score of Symptom Checklist 90 was more than 160, and 83 people whose number of positive items was more than 43. Logistic regression results showed that married, temporary employment, lack of support and communication from family and relatives, onerous task, and unbearable responsibility were independent risk factors for mental disorder.

There is a great psychological burden for obstetric nurses during the epidemic period. Decision makers should focus on necessary psychological intervention for those that are married, temporarily employed, and those lacking family supports including communication. At the same time, managers should distribute tasks reasonably to avoid psychological burdens caused by overwork.

Abbreviations: COVID-19 = coronavirus disease 2019, SCL-90 = Symptom Checklist 90.

Keywords: COVID-19, influence factor, mental health, obstetric nurses

1. Introduction

Coronavirus disease 2019 (COVID-19) was first discovered in late 2019, and quickly spread all over the world.[1–3] On January 30, 2020, the World Health Organization announced that after H1N1, polio, Ebola in West Africa, Zika and Ebola in the Democratic Republic of Congo, the COVID-19 outbreak has become the sixth public health emergency of international concern.[4] Today, the epidemic has developed into a pandemic. As of March 26, 2020, 89 countries around the world had been affected with more than 100 confirmed cases of COVID-19; the fatality rate of each country ranging from 0% to 11.22% (The data come from the real-time epidemic situation in DXY, https://ncov.dxy.cn/ncovh5/view/pneumonia). Due to the rapid interpersonal transmission of the virus, the Chinese government announced the full closure of Wuhan city policy at 10 AM on January 23, 2020. Subsequently, various provinces successively launched level 1 response to public health emergencies, which mainly included travel restriction, home isolation, closure of factories, entertainment places, schools, etc press the pause key.[5–7] Rapidly, increasing of the number of confirmed diagnosis, death, overwhelming rate of infection, coupled with alarming malicious rumors on social media platforms, all fueled a state of coronaphobia and psychological problems in the general public.[8,9] In response, the major psychological counseling centers opened free consultation phones for the public and spread them on social media and the Internet. Peking University is preparing a mental health manual for the public on how to deal with the stress and other psychological problems caused by the COVID-19 outbreak.[10] The economy can be delayed, but the birth of children cannot be postponed. Owing to the particularity of their profession, obstetric nurses cannot afford staying at home like the public for fear of COVID-19; their lives are at increased

Editor: Samet Çelik.

SL, RC, and YW contributed equally to this work.

The authors have no funding and conflicts of interest to disclose.

The datasets generated during and/or analyzed during the current study are available from the corresponding author on reasonable request.

[a] Changchun Maternity Hospital, Changchun, Jilin, China, [b] Department of Epidemiology and Biostatistics, School of Public Health, Jilin University, Changchun, Jilin, China, [c] The First Affiliated Hospital of Qiqihar Medical College, Qiqihar, Heilongjiang, China, [d] Department of Pain Management, The Second Hospital of Jilin University, No. 218, Ziqiang Street, Nanguan District, Changchun, Jilin, China.

[*] Correspondence: Zhigang Liu, Department of Pain Management, The Second Hospital of Jilin University, No. 218, Ziqiang Street, Nanguan District, Changchun, Jilin 130041, China (e-mail: liuzhigang@jlu.edu.cn).

Copyright © 2021 the Author(s). Published by Wolters Kluwer Health, Inc. This is an open access article distributed under the terms of the Creative Commons Attribution-Non Commercial License 4.0 (CCBY-NC), where it is permissible to download, share, remix, transform, and buildup the work provided it is properly cited. The work cannot be used commercially without permission from the journal.

How to cite this article: Li S, Chai R, Wang Y, Wang J, Dong X, Xu H, Wu H, Binnay IT, Liu Z. A survey of mental health status of obstetric nurses during the novel coronavirus pneumonia pandemic. Medicine 2021;100:52(e28070).

Received: 28 May 2021 / Received in final form: 2 November 2021 / Accepted: 11 November 2021

http://dx.doi.org/10.1097/MD.0000000000028070

risk in coming in contact with, and caring for potential virus carriers every day, coupled with strong work intensity, strict hospital management during the epidemic, and fear of their potential exposure to the virus and subsequent infestation of their family members, especially the elderly and children with relatively weak resistance, etc. Obstetric nurses are more likely to have bad emotions and psychological problems than the general public. Recently, 2 Italian nurses committed suicide after being diagnosed of COVID-19. It can be seen that the psychological burden may be more terrible than the epidemic of pneumonia.[11] While fighting against the novel coronavirus, attention should also be given to the mental health statuses of medical staff and that such strives conform to the current biological psychosocial medical model.[12]

Given the above situation, we designed this research to investigate the mental health status of obstetric nurses during the epidemic period, the comprehensive cognition of new coronavirus and the potential factors that may affect mental health, so as to provide theoretical basis for policymakers and managers of hospitals, thus protecting frontliners who still struggle during the epidemic, enabling them to maintain optimistic attitude and healthy bodies.

2. Methods

2.1. Settings and participants

This cross-sectional study was conducted during the novel coronavirus epidemic (February 25–March 20, 2020). The nurses in the obstetric hospitals of Northeast China's Jilin and Heilongjiang provinces were selected by convenience sampling. Due to the Chinese government's restrictions on mass gathering of people geared towards preventing and controlling the epidemic, this study's questionnaire was published through the online platform"SurveyStar" (Changsha Ranxing Science and Technology, Shanghai, China). The survey's link was forwarded through respondents' social software to establish a peer network, so as to expand the sample size.

Informed consent of each participant has been obtained.

2.2. Questionnaire

This study was mainly investigated from 4 aspects.

First, basic demographic characteristics. This included age, gender, education level, marital status, professional title, monthly income, and etc. All of the respondents were investigated by self-designed questionnaire.

Second, comprehensive knowledge of COVID-19. Using the adapted questionnaire from "SARS mentality of medical staff" designed by the Institute of Military Medical Psychology of the Fourth Military Medical University,[13] basic understanding of new pneumonia, the way of obtaining information, behavior changes, interpersonal relationship changes, the new coronavirus and social morality, risk perception, expectation of epidemic and control, the control measures and confidence, 8 aspects, were investigated.

Third, Symptom Checklist 90 (SCL-90).[14] The scale contains 90 items to evaluate the mental health status in the last week, and each problem was evaluated by 1 to 5 grade scoring method (from asymptomatic to severe). The results were summarized into 9 factors: somatization, compulsion, interpersonal sensitivity, depression, anxiety, hostility, terror, paranoia, and psychosis. If the average score of single factor is ≥2, and/or the total score is more than 160, and/or the positive item is ≥43, indicating that the score exceeds the Chinese standard norm, thus this person may have some psychological problems.

Fourth, potential pressure sources. The questionnaire was adapted from the "Interview Questionnaire on Mental Health of Medical Staff" of the Dr Shi Kan research group, Social Economic and Psychological Behavior Research Center, Institute of Psychology, Chinese Academy of Sciences.[15] The questionnaire consists of 8 items: The cause of the new coronavirus is not clear; There is no systematic and effective treatment; Lack of support and communication from families and relatives; Maternal and/or their families do not understand and cooperate with medical personnel; Poor working and living conditions; Hospital leadership management is too strict; Heavy tasks, heavy responsibilities and unbearable; Social support for medical personnel is insufficient. Using grade 1 to 5 scoring method, choose "very disagree" to score 1, choose "disagree" to score 2, choose "general" to score 3, choose "agree" to score 4, choose "very agree" to score 5.

2.3. Statistical analysis

Integrating online data into SPSS Statistic 24.0 (IBM SPSS Statistics, NY) for statistical analysis. Qualitative data were represented in the form of frequency or percentage, and quantitative data were represented in the form of mean and standard deviation. Two test and Kruskal-Wallis H test were used to assess group differences, and binary unconditional logistic regression model was used to do multivariate analysis. All tests were two-tailed, with a significance level of $P < .05$.

3. Results

3.1. The general status

A total of 333 questionnaires were sent out, and 333 questionnaires were recovered. After eliminating the invalid questionnaires, 318 effective questionnaires were actually obtained (completion rate: 92.79%), including 3 males (0.94%) and 315 females (99.06%). The average age of the subjects is (35.13 ± 8.65) years. Nurse-job seniority is mainly from 5 to 10 years, accounting for 33.96%. Education background is mainly undergraduate and above, account for 71.07%; position is mainly nurse, account for 88.36%; professional title is mainly nurse, account for 40.88%, personnel relationship is mainly employment system, account for 43.71%; marriage status is mainly married, account for 77.04%; 230 people have children, accounting for 72.33%; monthly income is mainly from 4000 to 4999 RMB, accounting for 30.83%; the number of night shifts per month is mainly 4 times or less, account for 45.60%.

3.2. Comprehensive knowledge of COVID-19

3.2.1. The way of obtaining information.

88.05% of subjects obtained information about COVID-19 through news media; 88.05% of subjects were eager to obtain information related to COVID-19; 88.05% knew a good bit about COVID-19, while 88.05% were more concerned about the scientific research of COVID-19.

3.2.2. The basic understanding of new pneumonia.

72.33% of subjects believed that the new pneumonia was a natural and

Table 1

Comparison of the 318 obstetric nurses' SCL-90 scores with domestic norms ($\bar{x} \pm s$).

Factors	ONs (n = 318)	The national norm (n = 1388)	t	*P* value
Somatization	1.77 ± 0.85	1.37 ± 0.48	8.36	<.01
Obsessive-compulsive	2.08 ± 0.89	1.62 ± 0.58	9.10	<.01
Interpersonal relationship	1.72 ± 0.80	1.65 ± 0.51	1.67	.09
Depression	1.71 ± 0.86	1.50 ± 0.59	4.41	<.01
Anxiety	1.71 ± 0.82	1.39 ± 0.43	6.86	<.01
Hostility	1.68 ± 0.82	1.48 ± 0.56	4.39	<.01
Phobia	1.74 ± 0.84	1.23 ± 0.41	10.74	<.01
Paranoia	1.55 ± 0.76	1.43 ± 0.57	2.83	.05
Psychosis	1.58 ± 0.76	1.29 ± 0.42	6.84	<.01

ONs = obstetric nurses, SCL-90 = Symptom Checklist 90.

social disaster for mankind; 79.87% believed that COVID-19 was caused by the destruction of the ecological environment; 61.32% believed that COVID-19 had a huge impact on the national economy.

3.2.3. The behavior changes. 47.48% of subjects believed that the COVID-19 epidemic had an impact on their daily life; 61.32% of them thought their sleep quality was affected, and 34.59% were easily upset recently.

3.2.4. The interpersonal relationship changes. During the prevalence of the COVID-19 epidemic, 54.40% of the subjects thought they got more phone calls, SMS, letters, or e-mails than ever before; 35.53% were worried about their family's health; 58.18% often called their family and friends; 50.94% thought that the relationship between people was changed because of the lack of trust.

3.2.5. COVID-19 and social morality. 55.97% of the subjects thought they would go to the fever clinic immediately if they had a fever; 42.77% would take self-observation measures; 84.91% were mainly afraid of infecting their families, while 57.86% thought it was illegal for COVID-19 patients to escape from hospital.

3.2.6. The risk perception. 73.90% of the study's subjects thought that infectious diseases were the most harmful to human beings; 42.13% thought they were less likely to get sick; 83.96% thought that the COVID-19 epidemic was more harmful than "SARS".

3.2.7. The expectation of epidemic and control. 89.94% believed that the prevalence of the COVID-19 epidemic had been easing in its area. 59.12% believed the epidemic would be basically under control within 3 months in their cities. 73.90% thought a new way would be taken to fight against the epidemic. 44.03% believed that new vaccines and drugs would be developed in half a year, and 44.65% of them felt more peaceful.

3.2.8. The control measures and confidence. 60.06% thought that the current measures were very effective; 62.58% believed that the most effective measure was to get information, during the epidemic; 93.40% believed that staying at home was the safest; 90.88% believed it was necessary to wear masks in the street; 42.77% were very satisfied with the measures taken by the hospital.

3.2.9. The mental health of obstetric nurses. 3.2.10. Comparison of the obstetric nurses' SCL-90 scores with domestic norms. Results obtained that the scores of "obsessive-compulsive", "depression", "anxiety", "hostility", "phobia", and "psychosis" were higher than the national norm ($P < .01$). The scores of somatization were lower than the national norm ($P < .01$), and the scores of other factors were not statistically significant ($P > .01$), as shown in Table 1.

3.2.11. Positive status of SCL-90 in the 318 obstetric nurses. The result showed that 107 nurses' total score of SCL-90 were greater than 160 scores, accounting for 33.64%; 83 nurses' positive items were more than 43, accounting for 27.99%. The top 3 symptom factors with a factor score greater than or equal to 2 scores were compulsion, phobia, anxiety or interpersonal relationship, with positive rates of 46.86%, 31.13%, and 30.19% respectively, as shown in Table 2.

3.2.12. The potential stressors for obstetric nurses. Using "yes" or "no" to indicate the answers to the 5 options in the potential stressor questionnaire, we marked "very disagree" and "disagree" as "yes" and marked "general", "agree", and "very agree" as "no", calculating the percentage of "yes" in each item calculation, respectively. The top 5 potential pressure sources were as follows. At present, the cause of COVID-19 was not clear, accounting for 33.65%. During the epidemic period, the

Table 2

Positive status of SCL-90 in the 318 obstetric nurses.

Factors	Number (%)	Positive rate (%)	Sorting of factor positive rate
Total score of SCL-90 greater than 160 scores	107	33.64	–
Positive items greater than 43	89	27.99	–
Factor score greater than or equal to 2 scores			–
Somatization	93	29.25	5–6
Obsessive-compulsive	149	46.86	1
Interpersonal relationship	96	30.19	3–4
Depression	93	29.25	5–6
Anxiety	96	30.19	3–4
Hostility	84	26.42	8
Phobia	99	31.13	2
Paranoia	73	22.96	9
Psychosis	85	26.72	7

SCL-90 = Symptom Checklist 90.

Table 3

Psychological stressors of obstetric nurses.

Psychological stressors	Number (%)	The average score	Sort
Unknown cause of disease	107 (33.65)	3.00±0.97	1
Lacking of effective treatment system	87 (27.36)	2.83±0.99	4
Lacking of support and communication from family and relatives	52 (16.35)	2.34±1.04	7
Patients' incomprehension and non-cooperation	90 (28.30)	2.83±1.06	3
Poor treatment conditions and living environment	38 (11.95)	2.57±0.87	8
Strict management of hospital leaders	57 (17.92)	2.75±0.93	6
Onerous task and unbearable responsibility	66 (20.75)	2.56±1.05	5
Insufficient social support	95 (29.87)	2.97±1.05	2

social support for medical staff was insufficient, accounting for 29.87%. Patients could not understand and cooperate with medical staff, during the epidemic period, accounting for 28.30%. COVID-19 was lacks of systematic and effective treatment, accounting for 7.36%. During the epidemic period, nurses had heavy tasks, which were hard to bear, accounting for 20.75%, as shown in Table 3.

3.2.13. Univariate analysis of the 318 obstetric nurses' positive status of SCL-90. Using the univariate analysis to analyze 19 variables in the questionnaire that may affect the positive symptom of SCL-90. Univariate analysis showed that there was a statistically significant difference in the 318 obstetric nurses' positive status of SCL-90 in terms of personnel relationship, marital status, lack of support and communication from family and relatives, strict management of hospital leaders, onerous task, and unbearable responsibility ($P<.05$), as shown in Table 4.

3.2.14. Multivariate analyses of the 318 obstetric nurses' positive status of SCL-90. With the occurrence of positive symptom of SCL-90 as the dependent variable, binary logistic regression analysis was carried out for the above factors with significant difference in single factor analysis. The results showed that married, temporary employment, lack of support and communication from family and relatives, onerous task, and unbearable responsibility were the risk factors for the positive symptoms of SCL-90 of obstetric nurses, as shown in Table 5.

Table 4

Univariate analysis of the 318 obstetric nurses' positive status of SCL-90.

Variables	Number	Test statistics	P value
Personnel relationship		H=12.124	.002
Temporary employment	14		
System of employment under contract	57		
Permanent staff	36		
Marital status		H=11.025	.004
Unmarried	26		
Married	73		
Divorce	8		
Lacking of support and communication from family and relatives		$\chi^2=9.300$	.002
Yes	80		
No	27		
Strict management of hospital leaders		$\chi^2=5.856$	.016
Yes	80		
No	27		
Onerous task and unbearable responsibility		$\chi^2=21.358$	.000
Yes	69		
No	38		

SCL-90 = Symptom Checklist 90.

4. Discussion

This was a cross-sectional study focusing on the psychological state of obstetric nurses during the epidemic of COVID-19. The scores of obsession, depression, anxiety, hostility, phobia, and psychosis of obstetric nurses were higher than that of Chinese norm, whereas the scores of somatization were lower than the Chinese norm ($P<.01$), and 107 (33.64%) of them whose total score of SCL-90 was more than 160, and 83 (27.99%) obstetric nurses had more than 43 positive entries. It was suggested that the mental health level of nurses participating in this study was low. It could be seen that during the epidemic period, a great psychological burden was placed on the shoulders of obstetric nurses. There have been a couple of studies suggesting that compared with other medical staff, nurses have greater psychological pressure and are more vulnerable.[16–18] On the one hand, the workload of obstetric nurses will increase during the epidemic due to increased numbers of patients and staff shortages. On the other hand, a study has shown that compared with non-pregnant women, pregnant women show a more significant increase in depression, anxiety, and negative effects.[19] As staff members in direct contact with pregnant women, caring for pregnant women with negative emotions exerts an additional stress on obstetrics staff members. Therefore, obstetric nurses bear great responsibility, which is easy to lead to mental health problems.

In terms of potential stressors for obstetric nurses, a finding of this study was that the top 1 stressor among the nurses was the unknown cause of disease (33.65%). This was because in the early stage of the COVID-19 outbreak, people knew little about the virus and could not determine whether the news was true or false.[20] Getting these unknown messages might enhance anxiety and depression among obstetric nurses. A meta-analysis of medical staff and general public mental and health during the epidemic showed that having up-to-date and accurate health information might be a protective factor for psychological health.[21] This indicates that news and knowledge of the novel COVID-19 virus take part in controlling nurses' anxiety. Therefore, the government and publicity departments are encouraged to pay more attention to the supervision of COVID-19 information on the Internet and new media platforms and give full play to its positive impact.

The risk of psychological disorder of obstetric nurses with marital status was 7.141 times higher than that of divorce. In

Table 5

Multivariate analyses of the 318 obstetric nurses' positive status of SCL-90.

Variables	B	S.E.	Wald	OR (95%CI)	*P* value
Constant	–2.865	0.788	13.230	0.057	.000
Marital status					
Unmarried	1.343	0.774	3.009	3.830 (0.840–17.462)	.083
Married	1.966	0.715	7.566	7.141 (1.760–28.981)	.006
Divorce	–	–	–	1	–
Personnel relationship					
Temporary employment	1.108	0.406	7.451	3.029 (1.367–6.712)	.006
System of employment under contract	–0.143	0.308	0.215	0.867 (0.474–1.586)	.643
Permanent staff	–	–	–		–
Lacking of support and communication from family and relatives					
Yes	0.811	0.330	6.026	2.250 (1.178–4.301)	.014
No	–	–	–	1	–
Onerous task and unbearable responsibility					
Yes	1.247	0.303	16.892	3.481 (1.920–6.310)	.000
No	–	–	–	1	–

CI = confidence interval, OR = odds ratios, SCL-90 = Symptom Checklist 90, S.E. = standard error.

most studies, for example, to explore the influencing factors of various diseases,[22,23] suicide tendency,[24] life span,[25] etc, married state appears as a protective factor, while married state in this study was a risk factor of psychological abnormality of obstetric nurses. The reason may be that during the epidemic period, obstetric nurses with families have more concerns than those without families. For example, given their exposures to various potential infectious sources every day, they often worry about bringing the virus to their families, especially the elderly and children with relatively weak resistance,[26,27] and the survey results show that 16.35% of the study's subject are not satisfied with the disinfection measures in their hospital, which suggests that more focus be placed on the psychological construction of married nurses, including strive to make the best protective measures to eliminate potential factors influencing their worries.

The risk of psychological disorder of obstetric nurses who have heavy tasks and great responsibilities during the epidemic is 3.481 times higher than those without such concerns. In addition to the intensive work intensity, obstetric nurses have increased workloads due to the epidemic. For example, participating in epidemic prevention training, regularly disinfecting, transmitting the hospital's management regulations during the epidemic to maternal and family members and so on.[28] Of course, it is necessary to increase the work in such a special period, and it is suggested that hospital policy makers and managers should reasonably allocate tasks,[29] and make appropriate mental and material compensation for the hard-working medical staff.

The risk of psychological disorder of temporarily employed obstetric nurses was 3.029 times higher than those of in-service ones. A previous study had also shown that compared with permanent contract nurses, a nurse on a fixed-term contract was associated with more severe depression, anxiety.[30] On the one hand, temporarily employed nurses may have relatively insufficient work experience and knowledge reserve. They lack experience in dealing with critical medical situations. During the epidemic, compared with in-service nurses, they lack experience and confidence in dealing with critical medical conditions and the ability of self-relieving stress is poor. So, they are more likely to have mental health problems. On the other hand, compared with in-service nurses, temporarily employed nurses usually have lower wages, less job security, and lower job satisfaction.[31] The economic insecurity also increases their anxiety. Therefore, more understanding and tolerance should be given to temporary employees, more exchanges and sharing meetings should be held between senior and junior, and new employees should be well trained in epidemic prevention and given appropriate humanistic care and bonus income.

The risk of psychological disorder of obstetric nurses who lack family supports and communication was 3.481 times higher than who have family supports and communication. This was consistent with the results of Su et al[32] and Marjanovic et al.[33] In addition, a study showed that receiving negative feedback from families and friends could lead to the development of negative emotional response. Another survey showed that family support, friend support and other types of social support were positively correlated with obstetrics healthcare providers' mental health.[34] It can be seen that having better social support and family support may alleviate the anxiety of obstetric nurses at work. A previous study showed that social support was the key protective factor of good mental health.[35] Hence, when they face work-related pressure, they can seek support from family and society. Similarly, hospital management, family, friends, colleagues, supervisors, and the government should encourage nurses. These supports can help them reduce anxiety and psychological distress. What's more, it is necessary to invite relevant experts to carry out open course of epidemic prevention on the Internet for all family members of medical staff, to explain the measures of epidemic prevention and at the same time to clarify the hard work and greatness of medical staff, so as to obtain the support of their families and relatives.

This study has several limitations. First, this is a cross-sectional study, unable to get a clear causal relationship, only to explore the potential factors that may affect mental health, and further prospective studies are needed to test this result. Second, in the current epidemic period, gathering is not encouraged, and face-to-face surveys guided by professional investigators cannot be carried out. We have chosen an online method of filling in questionnaires, which may lead to subjective bias on the results when respondents answer questions where different understanding of different people. Third, we chose a convenience sampling to establish peer network by forwarding survey links on social media. Although we can quickly locate many interested research objects, we could not extrapolate the results to the public because this sampling method is a non-random sampling. Although our

research has the above shortcomings, it also provides an important reference for maintaining the mental health of non-firstline obstetric nurses during the epidemic. In the rapid development of the epidemic, we do not pursue and have no time to accurately locate the causal relationship, and it is the most important to cutoff the chains that lead to psychological disorder as much as possible.

5. Conclusions

The prevalence of COVID-19 has caused great psychological pressure to nurses. From the results, we can see that nurses are under great psychological pressure during the epidemic. Therefore, this requires decision makers should focus on the necessary psychological intervention for those that are married, temporarily employed, lack of family support and communication. At the same time, managers should distribute tasks reasonably to avoid psychological burden caused by overwork load.

Acknowledgments

We would like to thank the study participants and the project team members. All authors have agreed to being so named.

Author contributions

Conceptualization: Shuyue Li, Ruiyu Chai, Yingshuang Wang, Isaac T S Binnay.
Data curation: Shuyue Li, Huiyan Wu.
Formal analysis: Jin Wang, Xinxin Dong, Han Xu.
Methodology: Ruiyu Chai, Yingshuang Wang.
Software: Ruiyu Chai, Yingshuang Wang.
Writing – original draft: Ruiyu Chai, Yingshuang Wang, Isaac T S Binnay.
Writing – review & editing: Zhigang Liu.

References

[1] Huang C, Wang Y, Li X, et al. Clinical features of patients infected with 2019 novel coronavirus in Wuhan, China. Lancet 2020;395:497–506.
[2] Wang C, Horby PW, Hayden FG, Gao GF. A novel coronavirus outbreak of global health concern. Lancet 2020;395:470–3.
[3] The LancetEmerging understandings of 2019-nCoV. Lancet 2020;395:311.
[4] Lai CC, Shih TP, Ko WC, Tang HJ, Hsueh PR. Severe acute respiratory syndrome coronavirus 2 (SARS-CoV-2) and coronavirus disease-2019 (COVID-19): the epidemic and the challenges. Int J Antimicrob Agents 2020;55:105924.
[5] Ebrahim SH, Ahmed QA, Gozzer E, Schlagenhauf P, Memish ZA. Covid-19 and community mitigation strategies in a pandemic. BMJ 2020;368: m1066.
[6] Fang Y, Nie Y, Penny M. Transmission dynamics of the COVID-19 outbreak and effectiveness of government interventions: a data-driven analysis. J Med Virol 2020;92:645–59.
[7] Gong F, Xiong Y, Xiao J, et al. China's local governments are combating COVID-19 with unprecedented responses - from a Wenzhou governance perspective. Front Med 2020;14:220–4.
[8] Asmundson GJG, Taylor S. Coronaphobia: fear and the 2019-nCoV outbreak. J Anxiety Disord 2020;70:102196.
[9] Asmundson GJG, Taylor S. How health anxiety influences responses to viral outbreaks like COVID-19: what all decision-makers, health authorities, and health care professionals need to know. J Anxiety Disord 2020;71:102211.
[10] Bao Y, Sun Y, Meng S, Shi J, Lu L. 2019-nCoV epidemic: address mental health care to empower society. Lancet (London, England) 2020;395:e37–8.
[11] Ho CS, Chee CY, Ho RC. Mental health strategies to combat the psychological impact of COVID-19 beyond paranoia and panic. Ann Acad Med Singap 2020;49:155–60.
[12] Gask L. In defence of the biopsychosocial model. Lancet Psychiatry 2018;5:548–9.
[13] Wang P. Investigation on Mental Health Status of Medical Staff in the First Line of SARS Treatment and Study on the Construction of Crisis Intervention Mode. 2005;The Fourth Military Medical University,
[14] Wang ZY. Symptom checklist 90 (SCL-90). Shanghai Psychiatry 1984;2:68–70.
[15] Yang X, Hua Q, Huang W. Investigation and analysis of the sources of pressure for nurses during the prevalence of SARS. Chin J Hosp Manage 2004;20:103–5.
[16] Chew NWS, Lee GKH, Tan BYQ, et al. A multinational, multicentre study on the psychological outcomes and associated physical symptoms amongst healthcare workers during COVID-19 outbreak. Brain Behav Immun 2020;88:559–65.
[17] Fu M, Han D, Xu M, Mao C, Wang D. The psychological impact of anxiety and depression on Chinese medical staff during the outbreak of the COVID-19 pandemic: a cross-sectional study. Ann Palliat Med 2021;10:7759–74.
[18] Salopek-Žiha D, Hlavati M, Gvozdanović Z, et al. Differences in distress and coping with the COVID-19 stressor in nurses and physicians. Psychiatr Danub 2020;32:287–93.
[19] López-Morales H, Del Valle MV, Canet-Juric L, et al. Mental health of pregnant women during the COVID-19 pandemic: a longitudinal study. Psychiatry Res 2021;295:113567.
[20] Wang Y, Gao J, Chen H, et al. The relationship between media exposure and mental health problems during COVID-19 outbreak. Fudan Univ J Med Sci 2020;47:173–8.
[21] Luo M, Guo L, Yu M, Jiang W, Wang H. The psychological and mental impact of coronavirus disease 2019 (COVID-19) on medical staff and general public - a systematic review and meta-analysis. Psychiatry Res 2020;291:113190.
[22] Zhang SL, Wang WR, Liu ZJ, Wang ZM. Marital status and survival in patients with soft tissue sarcoma: a population-based, propensity-matched study. Cancer Med 2019;8:465–79.
[23] Wong CW, Kwok CS, Narain A, et al. Marital status and risk of cardiovascular diseases: a systematic review and meta-analysis 2018;104:1937–48.
[24] Kyung-Sook W, SangSoo S, Sangjin S, Young-Jeon S. Marital status integration and suicide: a meta-analysis and meta-regression. Soc Sci Med 2018;197:116–26.
[25] Tatangelo G, McCabe M, Campbell S, Szoeke C. Gender, marital status and longevity. Maturitas 2017;100:64–9.
[26] Zhou F, Yu T, Du R, et al. Clinical course and risk factors for mortality of adult inpatients with COVID-19 in Wuhan, China: a retrospective cohort study. Lancet 2020;395:1054–62.
[27] D'Adamo H, Yoshikawa T, Ouslander JG. Coronavirus disease 2019 in geriatrics and long-term care: the ABCDs of COVID-19. J Am Geriatr Soc 2020;68:912–7.
[28] Cao Y, Li Q, Chen J, et al. Hospital emergency management plan during the COVID-19 epidemic 2020;27:309–11.
[29] Maunder R, Hunter J, Vincent L, et al. The immediate psychological and occupational impact of the 2003 SARS outbreak in a teaching hospital. CMAJ 2003;168:1245–51.
[30] Chen X, Arber A, Gao J, et al. The mental health status among nurses from low-risk areas under normalized COVID-19 pandemic prevention and control in China: a cross-sectional study. Int J Ment Health Nurs 2021;30:975–87.
[31] Cooke FL, Zhan C. Between market and bureaucracy: public healthcare reforms in China and nurses' terms and conditions. Int J Hum Resour Manage 2013;24:3178–95.
[32] Su TP, Lien TC, Yang CY, et al. Prevalence of psychiatric morbidity and psychological adaptation of the nurses in a structured SARS caring unit during outbreak: a prospective and periodic assessment study in Taiwan. J Psychiatr Res 2007;41:119–30.
[33] Marjanovic Z, Greenglass ER, Coffey S. The relevance of psychosocial variables and working conditions in predicting nurses' coping strategies during the SARS crisis: an online questionnaire survey. Int J Nurs Stud 2007;44:991–8.
[34] Vafaei H, Roozmeh S, Hessami K, et al. Obstetrics healthcare providers' mental health and quality of life during COVID-19 pandemic: multicenter study from eight cities in Iran. Psychol Res Behav Manag 2020;13:563–71.
[35] Veissi M, Atefvahid MK, Rezaee M. Job stress, job satisfaction and mental health: the balancing effects of personal hardiness and social support network factors. IJPCP 2000;6:70–9.
