# Supplementary material for: Mental health status and related factors influencing healthcare workers during the COVID-19 pandemic: A systematic review and meta-analysis
Source: PLoS One. 2024 Jan 19;19(1):e0289454. doi: 10.1371/journal.pone.0289454 (PMC10798549; doi:10.1371/journal.pone.0289454)
Supplement: S1 Data — (ZIP) [file pone.0289454.s011.zip › literatures/76.pdf]

SN Comprehensive Clinical Medicine (2020) 2:2595–2599
https://doi.org/10.1007/s42399-020-00654-w

COVID-19

Anxiety and Depression Among Imaging Doctors in Post-COVID-19 Period

Weiguo Li[1] · Xuesong Mao[1] · Jieqing Li[1] · Lianying Fang[1] · Guangfen Du[2] · Jianwei Qiao[1] · Ximing Jia[1]

Accepted: 18 November 2020 / Published online: 23 November 2020
© The Author(s) 2020

Abstract

To investigate the mental state of medical imaging staff in Shandong Province, China, who have been on the forefront of the COVID-19 epidemic during its late stage in China. Questionnaires designed to assess anxiety and depression were administered on-location, and 5331 complete results were collected. SPSS software was used for statistical descriptions and analysis. Rates of anxiety disorders and depression among medical imaging workers in Shandong Province, China, were 6.1% and 6.5%, respectively, higher than those of anxiety and depression in Chinese residents before the epidemic. The outbreak in Xinjiang, China; virus mutation in Japan; and spread of the epidemic due to occupational errors were the primary reported causes of anxiety and depression among image workers. Medical imaging workers showed evidence of psychological abnormalities during the late stage of the epidemic in China.

Keywords Anxiety · Depression · Medical imaging workers · Late stage of the COVID-19 epidemic · Outbreak · Virus mutation

Introduction

Since the Chinese government incorporated lung CT findings as a criterion for the diagnosis of new coronary pneumonia, a lung CT examination must be performed on every suspected and confirmed case, as well as on patients planning to be treated for multiple days in the hospital. Medical imaging workers have become the first to contact COVID-19, and the probability of being infected has further increased. The purpose of this survey was to understand the prevalence of anxiety and depression in medical imaging staff, to identify the primary stressors, and to develop strategies to alleviate these symptoms.

This article is part of the Topical Collection on *COVID 19*

✉ Jieqing Li
stx90@163.com

[1] Institute of Radiation Medicine, Shangdong Academy of Medical Sciences, Jinan 250062, China

[2] The Fifth People's Hospital of Jinan City, Jinan 250000, Shandong Province, China

Methods

An overall sampling method was used to survey medical imaging workers in a majority of hospitals in Shandong Province, China. According to a 2019 survey, rates of anxiety and depression among Chinese residents were about 4.98% and 4.06%, respectively [1], and a sample size of about 4000 was required to achieve a margin of error of no more than 15%. The survey was conducted from July 17 to July 31, 2019. A total of 5640 copies of the survey were issued, and 5331 completed surveys were returned. The respondents were from 15 cities in Shandong Province, China, with confirmed cases, and about half of them were employed in large- or medium-sized hospitals. The research team staff distributed the survey papers on-location, which were filled out by medical imaging staff and immediately returned. Persons with preexisting psychiatric disorders or serious physical illnesses were excluded.

The survey included questions concerning radiologists' attitudes, confidence, professional knowledge, and changes in the international epidemic, in addition to several factors recognized to affect the psychological state of radiologists (Table 1). Severity of anxiety and depression was assessed using the Generalized Anxiety Disorder-7 (GAD-7) and the Patient Health Questionnaire-9 (PHQ-9), respectively.

Table 1 Factors that may affect the psychological status of medical imaging staff in Shandong Province, China

Project	Options
Are you serious about fighting the epidemic?	Yes or No
Do you know the diagnostic criteria for COVID-19 imaging?	Yes or No
Do you recognize the following image prompts? 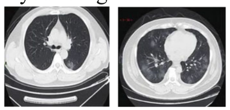	Yes or No
Do you know graded protection?	Yes or No
Do you think you are likely to come into contact with COVID-19 patients?	Yes or No
How long have you been following COVID-19 news every day?	Not paying attention, occasionally, more, as much as possible
What do you worry about? (multiple options are available)	you and your family are infected, the epidemic is expanding due to your negligence at work, income declines, inconvenient travel
Where does your confidence in the fight against COVID-19 come from? (multiple options are available)	the ability of the government, the cooperation of the citizens, the measures taken by your units, your own knowledge
What measures do you think are still needed to prevent the recurrence of the epidemic? (multiple options are available)	wearing masks, avoiding crowds, identifying suspicious cases, seeking medical treatment in a timely manner
Do you think the current outbreak in Xinjiang, China may spread to Shandong?	Yes or No
Will Japan's announcement of virus mutation make the fight against the epidemic more complicated?	Yes or No
Do you think the vaccine will help control the outbreak?	Yes or No

The left column of the form contains the questions that need to be answered by the imaging doctor, and the right column contains the corresponding response options

Based on clinical work experience and in order to increase the sensitivity and specificity of GAD-7 and PHQ-9, the following criteria were implemented: those whose GAD-7 score reached 7 points and where neither the first nor second item score was less than 1 point were diagnosed with anxiety disorder [2]; depression was diagnosed if the respondent's score on the PHQ-9 scale reached 7 points [3, 4].

SPSS.26 was used to statistically describe the data, a chi-square test was used to compare rates, and multinomial logistic regression was used to analyze the relationships between various factors and occurrence of anxiety and depression. Test level = 0.05.

Results

1. The prevalence rates of anxiety and depression among Shandong medical imaging staff were 6.5% and 6.1%, respectively. These rates were compared with those among Chinese residents in 2019 by chi-square test, and the χ^2 values were 362 ($p < 0.05$) and 355 ($p < 0.05$), respectively.

A frequency distribution of GAD-7 and PHQ-9 scores of medical imaging staff is shown in Fig. 1.

It can be seen that a certain number of medical imaging staff had GAD-7 and PHQ-9 scores in the range of 7–10, and only a few scores reached the level of moderate anxiety and depression.

2. After performing a logistical regression test, the OR (odds ratio) values of factors affecting psychological state with statistical significance ($p < 0.05$) are shown in Table 2.

The results showed that worrying about oneself and one's family members being infected, worrying about one's own negligence contribution to the spread of the epidemic, the reports of Xinjiang epidemic, and virus mutation all obviously promoted anxiety and depression in medical imaging workers.

3. Among all medical imaging workers surveyed, 56% were worried about the outbreak in Xinjiang, China, and 51% were worried about the virus mutation announced by Japan. All workers with anxiety and depression reported being worried about contributing to the spread of the epidemic due to their own work negligence.

Among those who spent as much time as possible paying attention to reports of the epidemic, rates of anxiety and depression were 6.5% and 5.9%, respectively. In contrast, rates of anxiety and depression were 4.5% and 4.9%, respectively, among those who were not interested in following these reports.

Discussion

Since the Chinese government implemented a community-wide containment policy to control the COVID-19 epidemic,

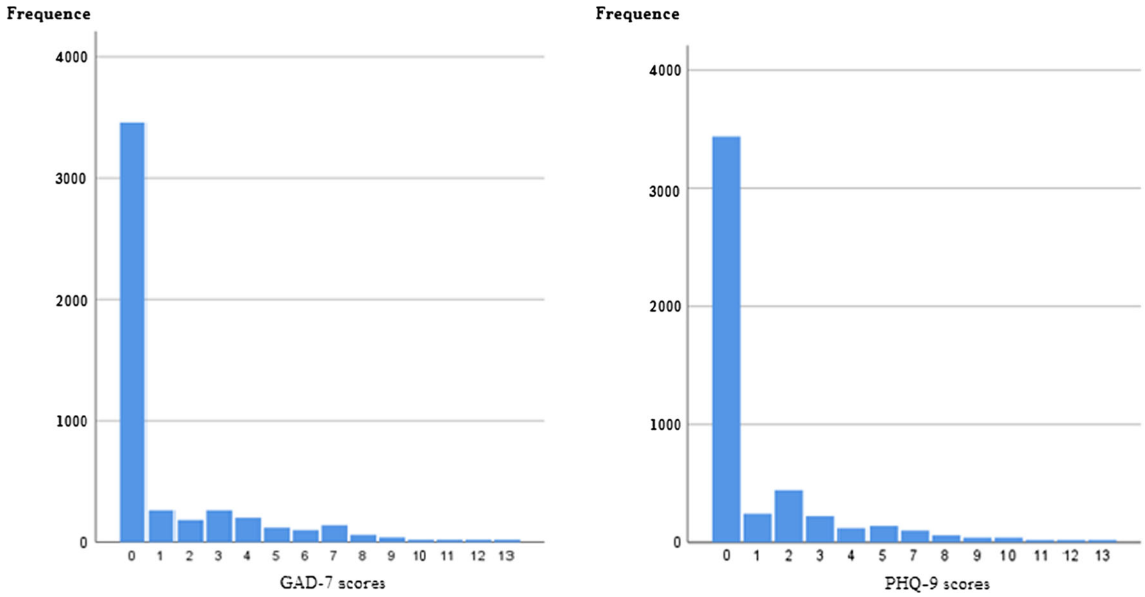

Fig. 1 The frequency distribution of GAD-7 and PHQ-9 scores of medical imaging staff. In the figure on the left, the abscissa represents scores of the GAD-7 score table, the ordinate represents number of respondents, and the figure shows the number of people corresponding to each score; in the same way, in the figure on the right, the abscissa represents scores of the PHQ-9 score table and the ordinate represents the number of people corresponding to each score

Table 2 OR value of factors affecting psychological state

	Knowing the imaging diagnosis of COVID-19	Worrying about yourself and your family members being infected	Worrying about the spread of the epidemic due to your work negligence	Anti-epidemic confidence comes from the cooperation of the citizens	Anti-epidemic confidence comes from believing the government	Believes that wearing masks can fight the epidemic	Believes that avoiding crowds is an effective anti-epidemic measure	Worrying about the Xinjiang epidemic	Worries about virus mutation	The length of attention to COVID-19 report
Anxiety	0.73–0.99	1.30–1.74	1.20–1.68	0.66–0.96	0.77–0.93	0.56–0.79	0.66–0.97	1.10–1.70	1.20–1.56	1.08–1.47
Depression	0.63–0.89	1.1–1.54	1.06–1.48	0.57–0.86	0.66–0.90	0.43–0.78	0.56–0.97	1.20–1.63	1.33–2.00	1.03–1.33

The first row of the table contains factors that affect anxiety and depression of imaging doctors, and the second and third rows contain the odds ratios of each factor on anxiety and depression

the COVID-19 epidemic in China has been effectively brought under control, from a peak of 3887 new cases per day to 45 new cases by 31 July 2020. A total of 763 cases have been reported in Shandong Province, with 4 new asymptomatic cases reported on July 31. To prevent recurrence of the epidemic, Shandong Province continues to take protective measures, such as announcement of the epidemic, restricted gathering, recording of travel routes, taking body temperature in public places, mask-wearing, and biweekly nucleic acid tests for all medical staff. People in Shandong remained in a state of anticipation, ready to respond to a rebound of the epidemic at any time. In individual countries where the new crown epidemic has occurred, such as Turkey, the psychological level of ordinary residents has been investigated, but the mental health of doctors has been ignored [5]. Imaging doctors were responsible for the screening of new coronary pneumonia and initial reports. If a missed diagnosis prevents positive patients from being appropriately monitored, it would cause further spread of the epidemic, as well as huge panic to society. A large number of health and social resources would be consumed. These doctors were in a state of nervousness and worry due to these important occupational responsibilities. This state of nervousness lasted for 7 months, and there was no expectation regarding the end of the epidemic. Some doctors may experience psychological disorders as a result.

The epidemic situation in Shandong, China, has been greatly slowed compared to that in February and March, and the high tension of imaging doctors has somewhat relaxed. From the survey results, it can be seen that moderate and severe anxiety and depression are almost non-existent, a finding that is in line with the current social atmosphere in Shandong. However, 6.5% and 6.1% of the staff (Fig. 1) were suffering from mild anxiety and depression, respectively, indicating that imaging doctors could not completely relax to pre-epidemic levels. Worries about themselves and their family members being infected and worries about contributing to the spread of the epidemic by negligence at work are both clear reasons for this increase in observed psychological abnormalities. Of significant concern is whether doctors' persistent anxiety and depression symptoms will go on to trigger additional physical abnormalities after a prolonged period of time.

With the epidemic outbreak in Xinjiang following a rebound in several parts of China, the Shandong government was bracing itself to prevent the disease from spreading into its territory. The outbreak in Xinjiang means that the epidemic has been prolonged again, and this factor has increased the psychological burden of imaging doctors. Similarly, Japan's announcement of mutation of the novel coronavirus has also increased the long-term complexity of epidemic prevention and control. The expected continuation of the epidemic has hindered people from getting out of a negative mindset. Paying too much attention to epidemic reports was also found to be detrimental to the relief of anxiety and depression in imaging doctors.

Knowledge of imaging diagnosis and protection strategies regarding new coronary pneumonia, as well as a belief in the ability of the Chinese government to fight the epidemic, increased confidence in imaging doctors fighting the epidemic. Wearing masks and avoiding gatherings were recognized as effective ways to stop the spread of pneumonia. Voluntary cooperation of citizens has significantly reduced the scale of the epidemic. These factors help imaging doctors ameliorate their psychological burden.

Based on multinomial logistic regression analysis, vaccine availability was not a factor affecting the psychology of imaging doctors. This may be related to doctors' uncertainty about the effectiveness of the vaccine and the long time it takes for a vaccine to be put into use.

It is difficult to include all factors that aggravate or alleviate psychological abnormalities in this survey. For example, various severe sequelae caused by SARS infection in 2002 will definitely aggravate the fear of medical workers [6]. The air pollution suitable for novel coronavirus (SARS-COV-2) is not conducive to alleviating their anxiety [7]. Therefore, the psychological relief of medical staff is a complex matter, which requires the participation of experts in various fields.

Conclusion

The incidences of anxiety and depression in Shandong imaging doctors have been significantly lowered from their peak in earlier stages of the epidemic [8], but still remain higher than levels before the epidemic. Shandong imaging doctors may remain in a mildly abnormal state of mind for some time to come. The international community should work in unison to control the spread of the epidemic in as short a period of time as possible to reduce the pressure on doctors.

Author's contribution Weiguo Li: responsible for designing investigation scheme, sending and receiving questionnaire and analyzing data.

Jieqing Li: responsible for quality control and financial support.

Xuesong Mao: responsible for making forms, sending and receiving questionnaire.

Lianying Fang: inputting data.

Guangfen Du: inputting data.

Jianwei Qiao: sending and receiving questionnaire.

Ximing Jia: correcting document.

Funding This study was supported by the Natural Science Foundation of Shandong [ZR2017YL007] and the Subsidy Fund for Medical Science and Technology Innovation Project of Shandong Academy of Medical Sciences.

Compliance with Ethical Standards

Conflict of Interest The authors declare that they have no conflicts of interest.

Ethical Approval The program was approved by Ethics Committee of Shandong Academy of Medical Sciences.

Consent Respondents confirmed in the questionnaire that their information could be used in published scientific research.

Open Access This article is licensed under a Creative Commons Attribution 4.0 International License, which permits use, sharing, adaptation, distribution and reproduction in any medium or format, as long as you give appropriate credit to the original author(s) and the source, provide a link to the Creative Commons licence, and indicate if changes were made. The images or other third party material in this article are included in the article's Creative Commons licence, unless indicated otherwise in a credit line to the material. If material is not included in the article's Creative Commons licence and your intended use is not permitted by statutory regulation or exceeds the permitted use, you will need to obtain permission directly from the copyright holder. To view a copy of this licence, visit http://creativecommons.org/licenses/by/4.0/.

References

1. Yueqin H, Yu W, Hong W, Liu Z. Prevalence of mental disorders in China: a cross-sectional epidemiological study. Lancet Psychiatry. 2019;6(3):211–24.
2. Kroenke K, Spitzer RL, Williams JB, Monahan PO, Löwe B. Anxiety disorders in primary care: prevalence, impairment, comorbidity, and detection. Ann Intern Med. 2007;146(5):317–25. https://doi.org/10.7326/0003-4819-146-5-200703060-00004.
3. Manea L, Gilbody S, McMillan D. Optimal cut-off score for diagnosing depression with the Patient Health Questionnaire (PHQ-9): a meta-analysis. CMAJ. 2012;184:E191–6.
4. Wenzheng W, Qian B, Yan Z, Yu L. Wenwen Wang, Jiang D, el at. Reliability and validity of the Chinese version of the Patient Health Questionnaire (PHQ-9) in the general population. Gen Hosp Psychiatry. 2014;36(5):539–44.
5. Güner HR, Hasanoglu I, Aktaş F. COVID-19: prevention and control measures in community. Turk J Med Sci. 2020;50(3):571–7. https://doi.org/10.3906/sag-2004-146.
6. Valerio S, Luciani M, Bentivegna E, et al. COVID-19: is it just a lung disease? A case-based review. SN Compr Clin Med. 2020. https://doi.org/10.1007/s42399-020-00418-6.
7. Martelletti L, Martelletti P. Air pollution and the novel Covid-19 disease: a putative disease risk factor. SN Compr Clin Med. 2020;2:383–7. https://doi.org/10.1007/s42399-020-00274-4.
8. Junfeng L, Zhiyun Y, Hui Q, Yu W, Lingyu J. Junjun Ji, el at. Anxiety and depression among general population in China at the peak of the COVID-19 epidemic. World Psychiatry. 2020;19(2):249–50.

Publisher's Note Springer Nature remains neutral with regard to jurisdictional claims in published maps and institutional affiliations.
