# Supplementary material for: Mental health status and related factors influencing healthcare workers during the COVID-19 pandemic: A systematic review and meta-analysis
Source: PLoS One. 2024 Jan 19;19(1):e0289454. doi: 10.1371/journal.pone.0289454 (PMC10798549; doi:10.1371/journal.pone.0289454)
Supplement: S1 Data — (ZIP) [file pone.0289454.s011.zip › literatures/77.pdf]

Prevalence of psychological symptoms and associated risk factors among nurses in 30 provinces during the COVID-19 pandemic in China

Yun Li,[a,1] Rongrong Fan,[a,1] Yunwei Lu,[b,1] Huayan Li,[a] Xianping Liu,[a] Guilan Kong,[c] Jiayu Wang,[c] Fan Yang,[a*] Jian Zhou,[a*] and Jun Wang[a*]

[a]Department of Thoracic Surgery, Peking University People's Hospital, Beijing, China
[b]Peking University People's Hospital, Beijing, China
[c]National Institute of Health Data Science at Peking University, Beijing, China

The Lancet Regional Health - Western Pacific 2023;30: 100618
Published online 15 October 2022
https://doi.org/10.1016/j.lanwpc.2022.100618

Summary

Background With the outbreak of the coronavirus disease 2019 (COVID-19), nurses have won well-deserved recognition for their indispensable roles in providing humane and professional healthcare for patients. However, by the nature of their role working at the forefront of patient care, nurses are prone to experiencing mental health consequences. Therefore, we pay attention to measuring the magnitude of psychological symptoms and identifying associated factors among nurses in China.

Methods We launched a nationwide, cross-sectional survey of nurses who worked in secondary or tertiary hospitals and public or private hospitals from 30 provinces in China. The prevalence and severity of symptoms of burnout, depression, and anxiety were investigated, respectively. Multivariable logistic regression analyses were performed to identify factors associated with each psychological symptom.

Findings A total of 138 279 respondents who worked in 243 hospitals completed this survey. A substantial proportion of nurses reported symptoms of burnout (34%), depression (55·5%), and anxiety (41·8%). In line with the disproportionality of economic development, we noted that the middle or western region was an independent risk factor for depression and anxiety. Compared with those working in the secondary hospital, nurses who worked in tertiary hospitals were associated with a higher likelihood of burnout and depression.

Interpretation Nurses are experiencing emotional, physical, and mental exhaustion during the COVID-19 epidemic. Governments and health policymakers need to draw attention to reinforcing prevention and ameliorating countermeasures to safeguard nurses' health.

Funding The strategic consulting project of the Chinese Academy of Engineering [2021-32-5]. Advanced Institute of Infomation Technology, Peking University, Zhejiang Province [2020-Z-17]

Copyright © 2022 The Author(s). Published by Elsevier Ltd. This is an open access article under the CC BY-NC-ND license (http://creativecommons.org/licenses/by-nc-nd/4.0/)

Keywords: Stress; Burnout; Depression; Anxiety; Psychological symptoms; Nurses; Cross-sectional survey; Nationwide

Introduction

The coronavirus disease 2019 (COVID-19) outbreak has become the most momentous health threat with international concern.[1] Widespread outbreak of COVID-19 not only jeopardized public wellness but was also associated with the adverse influence of mental consequences.[2] Previous studies have identified moderate to severe psychological symptoms and associated sequelae among the general populations affected by the COVID-19 epidemic.[3] In these circumstances, healthcare workers were undoubtedly more susceptible to the extraordinary amounts of pressure, such as the sheer

*Corresponding authors at: Department of Thoracic Surgery, Peking University People's Hospital, No.11 Xizhimen South Street, Beijing 100044, China.
E-mail addresses: yangfan@pkuph.edu.cn (F. Yang), zhoujian@bjmu.edu.cn (J. Zhou), wangjun@pkuph.edu.cn (J. Wang).

[1] Yun Li, Rongrong Fan, and Yunwei Lu contributed equally as co-first authors.

Research in context

Evidence before this study

The COVID-19 pandemic is an unprecedented healthcare crisis and economic catastrophe globally. COVID-19 is highly contagious and the transmission dynamics, give rise to the intercontinental spread. Consequently, both the physical well-being and mental health of healthcare workers have become a serious issue during the widespread COVID-19. Exposed to prolonged working shifts, excessive workload, lack of specific drugs, sleep disruption, and feelings of inadequately supported, healthcare workers, especially nurses, are susceptible to psychological symptoms (i.e., burnout, depression, and anxiety). Another essential reason for such psychological influence is the infective proportion among medical providers. Undoubtedly, the impairment of mental health among healthcare professionals was overwhelming, which may in turn jeopardize patient safety.

Nurses, as an essential component of the healthcare workforce, have attained well-deserved recognition for their indispensable roles in delivering conscientious, proficient, and compassionate care for patients throughout the epidemic. Meanwhile, due to their work nature, they are undoubtedly under enormous pressure and more prone to adverse mental consequences. Previous studies have reported that approximately 80% showed burnout, 48% revealed depression, and 51% demonstrated anxiety during the COVID-19 pandemic. Currently, there is still a lack of large-sample and region-stratified research on evaluating the psychological symptoms among Chinese nurses and looking for associated factors.

Added value of this study

To our knowledge, the current national study provided reliable data for assessing the burden of psychological symptoms among nurses, depicted its characteristics, and showed potential risk factors for preventing negative effects on mental health in China. This was a cross-sectional survey, based on 138,279 nurse participants covering three geographical regions of China (eastern, middle, and western). Owing to the participants surveyed in this study covered most provinces in China, we are able to draw a relatively complete picture of the prevalence of psychological symptoms among Chinese nurses during the COVID-19 epidemic. Through detecting the discrimination of adverse mental health among nurses in different geographical regions, hospital statuses, and hospital types, we noted that the psychological symptoms have been greatly underrecognized and undertreated, especially during the COVID-19 pandemic, irrespective of the location or the classification of hospitals, but with more intensiveness in less economically developed regions.

Implications of all the available evidence

This study investigated a large number of nurses engaged in 243 hospitals of different levels, located in diverse geographical regions across China, which is a nationally representative investigation of mental health status. The findings demonstrate that the prevalence of psychological symptoms among Chinese nurses during the COVID-19 pandemic was relatively high. There was a worldwide shortage of nurses before the COVID-19 outbreak, however, this situation has become even more critical for the overwhelming workload of infection screening and prevention throughout the pandemic, which was demonstrated the most common risk factor correlated with increased risk of psychological symptoms. Additionally, the poor mental well-being of nurses adversely impacts the quality and safety of health care. Together with the current results, governments and health policymakers everywhere should pay more attention to the mental health of nurses and take corresponding countermeasures to prevent and reduce psychological symptoms.

volume of confirmed or suspected patients, the risk of infection, overwhelming workload, physical exhaustion, and deficiency of specific drugs, which may dramatically contribute to deteriorative consequences for mental health in both the short and long term.[4] In support of these findings, a review study demonstrates that healthcare professionals had one of the highest psychological symptom burdens, second only to individuals who were positive for COVID-19.[5]

In the battle against COVID−19, burnout, depression, and anxiety are some of the essential challenges for healthcare professionals globally. Burnout is demonstrated to have an adverse impact not only on a decrease in the quality of care but also on the occurrence of musculoskeletal diseases, insomnia, obesity, alcohol intake, and drug abuse.[6,7] Ranked the third among all occupations for incidence of depressive episodes, nurses are highly prone to experience personal suffering, and their psychological effects may also negatively affect their colleagues and patients.[8] Anxiety adversely affects nurses' quality of life and becomes symptoms of underlying physical disorders such as chronic obstructive pulmonary disease and heart failure.[9,10]

Shechter et al. implemented an online survey on 657 physicians and nurses during the peak of the epidemic in the USA, which revealed 48% for depression and 33% for anxiety.[11] In addition, a cross-sectional study on 1 379 healthcare providers in Italy showed that the prevalence of depression and anxiety were 24·7% and 8·2%, respectively. [12] Similarly, a study by the International Council of Nurses revealed that the rate of burnout among nurses was climbing as high as 80% during the COVID-19 epidemic.[13] A systematic review of 59 studies, including 54 707 participants, presented a pooled prevalence ranging from 5% to 51% for depression, and 9% to 90% for anxiety.[14]

Hallmarks of the nursing profession, consisting of the paramount segment of the healthcare workforce,

are likely to struggle with excessive workload, inadequate support, conflict between work duty and family, verbal abuse, overtime work, and opposing team orientation.[15] Under the heavy burden of the COVID-19 crisis, nurses have been dedicated to working overtime, extra shifts, sleep disruption, and work-life imbalance, which may result in a faster work pace and continuously higher pressure.[16] Therefore, they were more vulnerable to psychological symptoms than other healthcare professionals due to their work nature.[17]

Previous studies have demonstrated that poor mental health can mitigate cognitive performance, including the ability to engross and process information, resulting in inadequate performance.[18] For nurses, these consequences include reduced alertness and job performance, which might endanger patient lives and augment the risk of adverse medical events.[19,20] In addition, the intensive workload for prolonged hours also made healthcare providers at risk of decreased immunity,[21] which is likely to be exacerbated by the increased demands of caring during COVID-19. Therefore, health and safety among healthcare workers are pivotal not only for successively safeguarding patient care but also for curb of any pandemic, especially during this special period.[22]

Addressing nurse psychological symptoms demands quantifying the magnitude of burnout, depression, and anxiety, and analyzing potential risk factors. However, there is a dearth of adequately large-sample and region-stratified research, especially in the field of effective prevention and coping strategies. In order to clarify the psychological needs and gaps, our study aimed to provide reliable data to estimate the mental health burden among Chinese nurses and evaluate associated factors using a questionnaire-based, cross-sectional study among 138 279 responders to identify potential associated factors with self-reported psychological symptoms.

Methods

Study design and participants

This is a strategic research and consulting project launched by the Chinese Academy of Engineering. This study followed the Strengthening the Reporting of Observational Studies in Epidemiology (STROBE) reporting guideline. Approval from the ethics committee of Peking University People's Hospital was received before the initiation of this study. This cross-sectional study was performed via an online survey between June 23, 2021, to July 10, 2021, covering three geographical regions of China (east, middle, and west).

This study adopted the convenience sampling technique to get nurse participants involved by using an anonymous online questionnaire. If we use the traditional sample size calculation method, which is denoted by equation (1), the target sample size would be roughly 30 000 with the assumption that the prevalence (p) of depression, the psychological symptoms expected to have one of the lowest prevalence, would be 5%.

$$n = \frac{Z^2_{1-\frac{\alpha}{2}}(1-p)}{p\varepsilon^2} \tag{1}$$

The survey was distributed to hospitals in 30 provinces through a third-party agency. Owing to the influence of the epidemic, these questionnaires were posted on an online and open access survey platform Questionnaire Star (https://www.wjx.cn/) and circulated via a social networking mobile App WeChat (https://wx.qq.com/). Nurses who were interested in the study would participate voluntarily and fill in the questionnaire. And thus the response rate is hard to calculate. Finally, more than 130 000 nurses from 30 provinces (excluding Tibet, Hong Kong, Macao, and Taiwan) and 243 hospitals in China have been involved, and the size of the studied population was much larger than the required sample size. On the first page of the questionnaire, the aims, objectives, instructions, and questions were designed as the mandatory field, so nurses who had not completed all the questions would receive a warning before submitting. Therefore, all collected questionnaires were complete without missing data. The questionnaire response time was approximately 20 minutes.

Measures and covariates

Burnout was defined and quantified using the Maslach Burnout Inventory Human Service Survey (MBI-HSS).[23] This tool is a three-construct measure, including emotional exhaustion (EE) (eight items), depersonalization (DP) (five items), and personal accomplishment (PA) (seven items), using a 7-point frequency-based Likert scale ranging from 0–6 (from never to every day). EE scores equal to or higher than 17 indicate moderate emotional exhaustion, DP scores greater than or equal to 7 suggest moderate depersonalization, and PA scores less than 38 indicate moderately low personal accomplishment. Levels of burnout are estimated separately for three subscales, defining normal as none of the three dimensions scored above the score of the moderate rank, mild burnout as any of the three dimensions exceeds the moderate rank, moderate burnout as two of the three dimensions scored more than moderate level, severe burnout as all three dimensions exceeds the moderate score of each dimension.

Symptoms of depression were assessed by the Self-Rating Depression Scale (SDS), a self-rated 20-item scale that asks if the subject has experienced symptoms of depression during the previous several days using a 4-point Likert scale.[24] Higher scores indicate higher levels of depression: normal (20–39), mild (40–47), moderate (48–55), and severe (56–80).

Anxiety symptoms were assessed by the Self-Rating Anxiety Scale (SAS), which contains 20 items, each rated on a 4-point scale from 1 (a little of the time) to 4 (most of the time).[25] We used a cut-off score of 40 to detect clinically significant anxiety symptoms.

Sociodemographic information and occupational characteristics were self-reported by participants, including gender (male or female), age (< 35, 35–54, or ≥ 55 years), marital status (single, divorced, widowed, or married), physical condition (healthy, good, normal, or bad), menstruation (regular, irregular, menopause), educational level (below the undergraduate, undergraduate, or postgraduate), professional experience (< 2, 2–9, ≥ 10 years), occupational role (head nurse or nurse), employment status (permanent or contract), overtime hours per week (none, < 4, 4–8, > 8 h), wards (general, emergency, intensive care units, or others), yearly salary (< 100 000, 100 000–200 000, or > 200 000 Chinese Yuan [CNY]), night shift (no or yes), job satisfaction (satisfied or dissatisfied), turnover intention (no or yes), patients per nurse ratio (PNR) (none, ≤ 5, 6–11, or ≥ 12), commute time (< 1 or ≥ 1 h), region (east, middle, or west), hospital status (secondary or tertiary), and hospital type (public or private). According to the first national economic census, China is divided into three regions: the eastern (those on the eastern coastline), middle (Heilongjiang, Jilin, Shanxi, Henan, Hubei, Anhui, Jiangxi, and Hunan), and western (others) regions.[26–28] In China, according to the functionality, specialization, and size, the hospital level is defined as a tertiary hospital, secondary hospital, and community health center. Tertiary hospitals are generally characterized as providing comprehensive medical services, with a larger number of beds. Secondary hospitals, in contrast, usually equip general medical services to the local inhabitants with a lower turnover rate.[29]

Statistical analysis

The original scores of the three measurement tools were presented as medians with interquartile ranges (IQRs) and mean (SD). The ranked data, derived from the counts of each level for symptoms of burnout, depression, and anxiety, were shown as numbers and percentages (n%). The sociodemographic and occupational characteristics of responders were statistically analyzed and the analysis was stratified by geographical region, hospital status, and hospital type. The Chi-square tests were performed to compare the surveyed symptom between two or more groups, and the nonparametric Mann-Whitney U test and Kruskal-Wallis test were applied to compare scores of the three instruments. Pearson correlation tests were used to find the association between psychological symptoms and risk factors. The univariable logistic regression model was constructed to identify potential risk factors for symptoms of burnout, depression, and anxiety in nurses, and the corresponding unadjusted odds ratios (ORs) and 95% confidence intervals (95% CIs) denoting the associations between each risk factors and outcomes were provided. Subsequently, the multivariable logistic regression model was built to discover the adjusted ORs and 95% CIs. All statistical analyses in this study were two-tailed, a p-value of less than 0·05 was considered statistically significant, and all analyses were carried out using SPSS statistical software version 20.0 (IBM Corp).

Role of funding source

The funding source had no role in the study design, data collection, data analysis, data interpretation, or writing of the report. The corresponding author had full access to all the data in the study and had final responsibility for the decision to submit it for publication.

Results

Demographic characteristics

A total of 138 279 responders who worked in 243 hospitals completed the survey: 31 599 (22·9%) from secondary hospitals and 106 680 (77·1%) from tertiary hospitals. The participants included in this analysis represented 30 province-level regions in China. In three regions, 60 463 (43·7%) worked in the east, 37 454 (27·1%) worked in the middle, and 40 362 (29·2%) worked in the west. Of the two hospital types, 136 668 (98·8%) worked in public hospitals, and 1 611 (1·2%) worked in private hospitals. The majority of nurses in our sample were female (132 394 [95·7%]), were aged 35 or younger (91 885 [66·6%]), were married (97 675 [70·6%]), were physically healthy (134 424 [97·2·%]), had regular menstruation (83 400 [63·0%]), had an educational level of undergraduate (102 575 [74·2%]), worked as general nurse (128 721 [93·1%]), had contract employment status (95 642 [69·2%]), had work overtime (102 832 [74·4%]), worked in general wards (83 528 [60·4%]), had under 100 000 RMB yearly salary (78 375 [56·7%]), had night shift (88 729 [64·2%]), were job dissatisfied (79 196 [57·3%]), had no turnover intention (113 589 [82·1%]), and had under one hour commute time (102 545 [74·2%]). There are 46·3% (64 039) nurses having professional experience of more than 10 years. Approximately 40·8% (56 452) of the responders work in a situation with 6 to 11 PNR (Table 1).

Prevalence of symptoms of burnout, depression, and anxiety

The prevalence of symptoms for the three mental health statuses among the total sample was 34% for burnout (46 949 participants total, including 20 405 [14·8%] participants with mild burnout, 13 880 [10·0%] participants with moderate burnout, and 12 664 [9·2%] participants with severe burnout), 55·5% for depression (76 733 participants total, including 28 704 [20·8%] participants with mild depression, 39 090 [28·3%] participants with moderate depression, and 8 939 [6·5%] participants with severe depression), and 41·8% for anxiety (57 789 participants).

	No. (%)										
		Region				Hospital Status			Hospital Type		
Characteristics	Total	East	Middle	West	*P*	Secondary	Tertiary	*P*	Public	Private	*P*
Overall	138 279 (100)	60 463 (43·7)	37 454 (27·1)	40 362 (29·2)		31 599 (22·9)	106 680 (77·1)		136 668 (98·8)	1 611 (1·2)	
Gender					<0·0001			<0·0001			0·15
Male	5 885 (4·3)	2 717 (4·5)	1 478 (3·9)	1 690 (4·2)		884 (2·8)	5 001 (4·7)		5 828 (4·3)	57 (3·5)	
Female	132 394 (95·7)	57 746 (95·5)	35 976 (96·1)	38 672 (95·8)		30 715 (97·2)	101 679 (95·3)		130 840 (95·7)	1 554 (96·5)	
Age					<0·0001			<0·0001			<0·0001
<35	91 885 (66·6)	38 409 (63·7)	25 881 (69·3)	27 595 (68·5)		22 385 (71·0)	69 500 (65·3)		90 687 (66·5)	1 198 (74·6)	
35-54	44 943 (32·6)	21 453 (35·6)	11 213 (30·0)	12 277 (30·5)		8 927 (28·3)	36 016 (33·9)		44 541 (32·7)	402 (25·1)	
≥55	1 061 (0·8)	411 (0·7)	260 (0·7)	390 (1·0)		198 (0·7)	863 (0·8)		1 056 (0·8)	5 (0·3)	
Marital Status					<0·0001			<0·0001			0·05
Single/Divorced/Widowed	40 604 (29·4)	18 932 (31·3)	10 574 (28·2)	11 098 (27·5)		8 656 (27·4)	31 948 (29·9)		40 095 (29·3)	509 (31·6)	
Married	97 675 (70·6)	41 531 (68·7)	26 880 (71·8)	29 264 (72·5)		22 943 (72·6)	74 732 (70·1)		96 573 (70·7)	1 102 (68·4)	
Physical Condition					<0·0001			<0·0001			0·20
Healthy	65 655 (47·5)	28 315 (46·8)	18 462 (49·3)	18 878 (46·8)		17 768 (56·2)	47 887 (44·9)		64 890 (47·5)	765 (47·5)	
Good	41 452 (30·0)	18 436 (30·5)	10 786 (28·8)	12 230 (30·3)		8 101 (25·6)	33 351 (31·3)		40 941 (30·0)	511 (31·7)	
Normal	27 317 (19·8)	12 061 (19·9)	7 240 (19·3)	8 016 (19·9)		5 070 (16·0)	22 247 (20·9)		27 018 (19·8)	299 (18·6)	
Bad	3 855 (2·8)	1 651 (2·7)	966 (2·6)	1 238 (3·1)		660 (2·1)	3 195 (3·0)		3 819 (2·8)	36 (2·2)	
Menstruation					<0·0001			<0·0001			<0·0001
Regular	83 400 (63·0)	37 169 (64·4)	23 302 (64·8)	22 929 (59·3)		20 220 (65·8)	63 180 (62·1)		82 392 (63·0)	1 008 (64·9)	
Irregular	44 276 (33·4)	18 455 (32·0)	11 477 (31·9)	14 344 (37·1)		9 440 (30·7)	34 836 (34·3)		43 756 (33·4)	520 (33·5)	
Menopause	4 718 (3·6)	2 122 (3·6)	1 197 (3·3)	1 399 (3·6)		1 055 (3·5)	3 663 (3·6)		4 692 (3·6)	26 (1·6)	
Educational Status					0·05			<0·0001			<0·0001
Below the undergraduate	33 951 (24·6)	14 936 (24·7)	9 135(24·4)	9 880 (24·5)		13 079 (41·4)	20 872 (19·6)		33 621 (24·6)	330 (20·5)	
Undergraduate	102 575 (74·2)	44 815 (74·1)	27 799 (74·2)	29 961 (74·2)		18 498 (58·5)	84 077 (78·8)		101 299 (74·1)	1 276 (79·2)	
Postundergraduate	1 753 (1·3)	712 (1·2)	520 (1·4)	521 (1·3)		22 (0·1)	1 731 (1·6)		1 748 (1·3)	5 (0·3)	
Professional Experience (y)					<0·0001			<0·0001			<0·0001
<2	12 715 (9·2)	5 829 (9·6)	3 367 (9·0)	3 519 (8·7)		3 205 (10·1)	9 510 (8·9)		12 556 (9·2)	159 (9·9)	
2-9	61 525 (44·5)	25 741 (42·6)	17 390 (46·4)	18 394 (45·6)		15 357 (48·6)	46 168 (43·3)		60 671 (44·4)	854 (53·0)	
≥10	64 039 (46·3)	28 893 (47·8)	16 697 (44·6)	18 449 (45·7)		13 037 (41·3)	51 002 (47·8)		63 441 (46·4)	598 (37·1)	
Occupational Role					<0·0001			<0·0001			0·16
Head nurse	9 558 (6·9)	4 131 (6·8)	2 913 (7·8)	2 514 (6·2)		2 898 (9·2)	6 660 (6·2)		9 461 (6·9)	97 (6·0)	
Nurse	128 721 (93·1)	56 332 (93·2)	34 541 (92·2)	37 848 (93·8)		28 701 (90·8)	100 020 (93·8)		127 207 (93·1)	1 514 (94·0)	
Employment Status					<0·0001			<0·0001			<0·0001
Permanent	42 637 (30·8)	22 870 (37·8)	10 862 (29·0)	8 905 (22·1)		10 658 (33·7)	31 979 (30·0)		42 425 (31·0)	212 (13·2)	
Contract	95 642 (69·2)	37 593 (62·2)	26 592 (71·0)	31 457 (77·9)		20 941 (66·3)	74 701 (70·0)		94 243 (69·0)	1 399 (86·8)	

Table 1 *(Continued)*

	No. (%)										
		Region				Hospital Status			Hospital Type		
**Characteristics**	**Total**	**East**	**Middle**	**West**	***P***	**Secondary**	**Tertiary**	***P***	**Public**	**Private**	***P***
Overtime Hours (weekly)					<0·0001			<0·0001			<0·0001
None	35 447 (25·6)	15 136 (25·0)	11 642 (31·1)	8 669 (21·5)		9 594 (30·4)	25 853 (24·2)		35 011 (25·6)	436 (27·1)	
<4 hr	53 273 (38·5)	23 233 (38·4)	13 532 (36·1)	16 508 (40·9)		12 104 (38·3)	41 169 (38·6)		52 591 (38·5)	682 (42·3)	
4-8 hr	31 076 (22·5)	14 113 (23·3)	7 535 (20·1)	9 428 (23·4)		6 090 (19·3)	24 986 (23·4)		30 747 (22·5)	329 (20·4)	
>8 hr	18 483 (13·4)	7 981 (13·2)	4 745 (12·7)	5 757 (14·3)		3 811 (12·1)	14 672 (13·8)		18 319 (13·4)	164 (10·2)	
Wards					<0·0001			<0·0001			<0·0001
General	83 528 (60·4)	35 706 (59·1)	23 403 (62·5)	24 419 (60·5)		17 583 (55·6)	65 945 (61·8)		82 485 (60·4)	1 043 (64·7)	
Emergency	7 339 (5·3)	3 256 (5·4)	1 723 (4·6)	2 360 (5·8)		1 954 (6·2)	5 385 (5·0)		7 309 (5·3)	30 (1·9)	
Intensive Care Units	9 842 (7·1)	4 774 (7·9)	2 241 (6·0)	2 827 (7·0)		1 779 (5·6)	8 063 (7·6)		9 776 (7·2)	66 (4·1)	
Others	37 570 (27·2)	16 727 (27·7)	10 087 (26·9)	10 756 (26·6)		10 283 (32·5)	27 287 (25·6)		37 098 (27·1)	472 (29·3)	
Yearly Salary (CNY)					<0·0001			<0·0001			<0·0001
<100 000	78 375 (56·7)	28 468 (47·1)	25 546 (68·2)	24 361 (60·4)		28 540 (90·3)	49 835 (46·7)		77 247 (56·5)	1 128 (70·0)	
100 000-200 000	55 670 (40·3)	29 133 (48·2)	11 249 (30·0)	15 288 (37·9)		3 011 (9·5)	52 659 (49·4)		55 189 (40·4)	481 (29·9)	
>200 000	4 234 (3·1)	2 862 (4·7)	659 (1·8)	713 (1·8)		48 (0·2)	4 186 (3·9)		4 232 (3·1)	2 (0·1)	
Night Shift					0·07			<0·0001			<0·0001
No	49 550 (35·8)	21 864 (36·2)	13 290 (35·5)	14 396 (35·7)		12 899 (40·8)	36 651 (34·4)		48 889 (35·8)	661 (41·0)	
Yes	88 729 (64·2)	38 599 (63·8)	24 164 (64·5)	25 966 (64·3)		18 700 (59·2)	70 029 (65·6)		87 779 (64·2)	950 (59·0)	
Job Satisfaction					<0·0001			<0·0001			<0·0001
Satisfied	59 083 (42·7)	25 382 (42·0)	16 384 (43·7)	17 317 (42·9)		12 488 (39·5)	46 595 (43·7)		58 590 (42·9)	493 (30·6)	
Dissatisfied	79 196 (57·3)	35 081 (58·0)	21 070 (56·3)	23 045 (57·1)		19 111 (60·5)	60 085 (56·3)		78 078 (57·1)	1 118 (69·4)	
Turnover Intention					<0·0001			<0·0001			<0·0001
No	113 589 (82·1)	49 141 (81·3)	31 601 (84·4)	32 847 (81·4)		26 373 (83·5)	87 216 (81·8)		112 374 (82·2)	1 215 (75·4)	
Yes	24 690 (17·9)	11 322 (18·7)	5 853 (15·6)	7 515 (18·6)		5 226 (16·5)	19 464 (18·2)		24 294 (17·8)	396 (24·6)	
PNR (patients per nurse ratio)					<0·0001			<0·0001			<0·0001
None	32 672 (23·6)	15 213 (25·2)	8 048 (21·5)	9 411 (23·3)		8 406 (26·6)	24 266 (22·7)		32 358 (23·7)	314 (19·5)	
≤5	17 793 (12·9)	7 955 (13·2)	4 662 (12·4)	5 176 (12·8)		3 982 (12·6)	13 811 (12·9)		17 557 (12·8)	236 (14·6)	
6-11	56 452 (40·8)	25 226 (41·7)	14 604 (39·0)	16 622 (41·2)		12 090 (38·3)	44 362 (41·6)		55 728 (40·8)	724 (44·9)	
≥12	31 362 (22·7)	12 069 (20·0)	10 140 (27·1)	9 153 (22·7)		7 121 (22·5)	24 241 (22·7)		31 025 (22·7)	337 (20·9)	
Commute Time (hr)					<0·0001			<0·0001			0·16
<1	102 545 (74·2)	43 472 (71·9)	30 016 (80·1)	29 057 (72·0)		27 692 (87·6)	74 853 (70·2)		101 375 (74·2)	1 170 (72·6)	
≥1	35 734 (25·8)	16 991 (28·1)	7 438 (19·9)	11 305 (28·0)	<0·0001	3 907 (12·4)	31 827 (29·8)	<0·0001	35 293 (25·8)	441 (27·4)	0·15

***Table 1*: Sociodemographic and occupational characteristics of responders.**

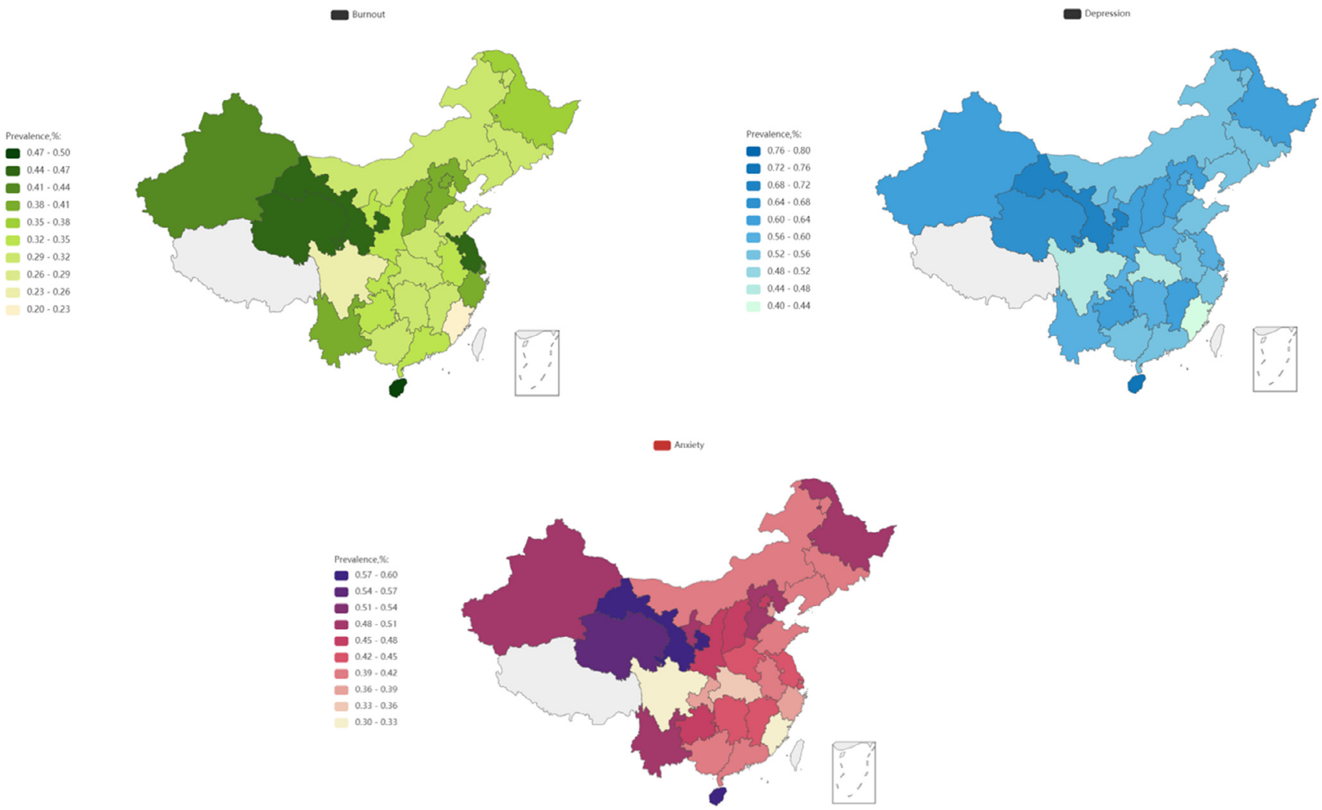

Figure 1. Prevalence of burnout, depression, and anxiety among participants from 30 Provinces in China.

The prevalence of symptoms of the three mental health conditions presented a significant difference among the three regions. Nurses who worked in the western region reported more severe degrees of all mental health status measurements than those who worked in the middle and eastern regions (*P* < 0·0001). Figure 1 presented the prevalence estimated for burnout, depression, and anxiety among nurses in 30 provinces in China.

Compared with those working in secondary hospitals, nurses working in tertiary hospitals were more likely to report the symptoms of burnout (9 343 [29·6%] vs 37 606 [35·3%], *P* < 0·0001), but a lower prevalence of depression (18 355 [58·1%] vs 58 378 [54·7%], *P* < 0·0001). There were no differences in hospital types for symptoms of burnout (*P* =0·46), depression (*P* =0·08), and anxiety (*P* = 0·12) (Table 2).

The median (IQR) scores on the MBI-HSS (EE, DP, and PA) for burnout, the SDS for depression, and the SAS for anxiety for all respondents were 18·0 (9·0–28·0), 5·0 (1·0–10·0), 29·0 (19·0–38·0), 42·0 (32·0–50·0), 37·0 (30·0–45·0), respectively. Similar to findings in the severity of mental health measurements, nurses working in different regions and hospital statuses presented significantly distinct scores for each item measuring symptoms of burnout, depression, and anxiety (*P* < 0·0001). Compared with nurses who worked in the public hospital, those in private hospitals reported higher scores on scales measuring symptoms of depression and burnout subscales of DP (median [IQR] SDS score, 42·0 [32·0–50·0] vs 42·0 [33·0–50·0]; *P* = 0·02; median [IQR] DP score, 5·0 [1·0–10·0] vs 5·0 [2·0–10·0]; *P* < 0·0001). There were no differences in hospital type for scores of anxiety (median [IQR] SAS score: workers in public hospitals, 37·0 [30·0–45·0]; workers in private hospitals, 37·0 [31·0–45·0]; *P* = 0·36) (Table 3).

Factors associated with psychological symptoms

The results of the unadjusted analysis estimating the association between sociodemographic factors, job characteristics, and psychological symptoms are presented in Supplement Table 1.

Multivariable logistic regression analysis showed that after controlling for confounders, male gender, physically unhealthy, and commuting time over one hour were still associated with an increased risk for all outcomes. Nurse participants aged 35 or older were recognized as having a lower risk of burnout, depression, and anxiety. Interestingly, married responders were susceptible to anxiety (OR,1·123; 95% CI, 1·089–1·158; *P* < 0·0001), but less likely to yield burnout (OR, 0·873; 95% CI, 0·845–0·901; *P* < 0·0001) and depression (OR, 0·952; 95% CI, 0·923–0·981; *P* = 0·00).

Severity category	Total, No. (%)	Region				Hospital Status			Hospital Type		
		No. (%)				No. (%)			No. (%)		
		East	Middle	West	*P* value	Secondary	Tertiary	*P* value	Public	Private	*P* value
MBI-HSS, Maslach Burnout Inventory-Human Services Survey					<0·0001			<0·0001			0·46
Normal	91 330 (66·0)	39 473 (65·3)	25 559 (68·2)	26 298 (65·2)		22 256 (70·4)	69 074 (64·7)		90 295 (66·1)	1 035 (64·2)	
Mild	20 405 (14·8)	9 046 (15·0)	5 220 (13·9)	6 139 (15·2)		4 218 (13·3)	16 187 (15·2)		20 152 (14·7)	253 (15·7)	
Moderate	13 880 (10·0)	6 221 (10·3)	3 486 (9·3)	4 173 (10·3)		2 709 (8·6)	11 171 (10·5)		13 715 (10·0)	165 (10·2)	
Severe	12 664 (9·2)	5 723 (9·5)	3 189 (8·5)	3 752 (9·3)		2 416 (7·6)	10 248 (9·6)		12 506 (9·2)	158 (9·8)	
SDS, Self-rating depression scale					<0·0001			<0·0001			0·08
Normal	61 546 (44·5)	27 636 (45·7)	16 652 (44·5)	17 258 (42·8)		13 244 (41·9)	48 302 (45·3)		60 866 (44·5)	680 (42·2)	
Mild	28 704 (20·8)	12 533 (20·7)	7 498 (20·0)	8 673 (21·5)		6 580 (20·8)	22 124 (20·7)		28 337 (20·7)	367 (22·8)	
Moderate	39 090 (28·3)	16 671 (27·6)	10 846 (29·0)	11 573 (28·7)		9 697 (30·7)	29 393 (27·6)		38 619 (28·3)	471 (29·2)	
Severe	8 939 (6·5)	3 623 (6·0)	2 458 (6·6)	2 858 (7·1)		2 078 (6·6)	6 861 (6·4)		8 846 (6·5)	93 (5·8)	
SAS, Self-Rating Anxiety Scale					<0·0001			0·72			0·12
Normal	80 490 (58·2)	35 759 (59·1)	22 139 (59·1)	22 592 (56·0)		18 421 (58·3)	62 069 (58·2)		79 522 (58·2)	968 (60·1)	
Anxiety	57 789 (41·8)	24 704 (40·9)	15 315 (40·9)	17 770 (44·0)		13 178 (41·7)	44 611 (41·8)		57 146 (41·8)	643 (39·9)	

***Table 2*: Severity categories of burnout, depression, and anxiety symptoms in total cohort and subgroups.**

	Total score, Median (IQR)	Region				Hospital Status			Hospital Type		
		Median (IQR)				Median (IQR)			Median (IQR)		
		East	Middle	West	*P* value	Secondary	Tertiary	*P* value	Public	Private	*P* value
MBI-HSS, Maslach Burnout Inventory-Human Services Survey											
Emotional Exhaustion (EE)	18·0 (9·0–28·0)	18·0 (9·0–29·0)	17·0 (8·0–27·0)	18·0 (9·0–29·0)	<0·0001	15·0 (8·0–27·0)	18·0 (9·0–29·0)	<0·0001	18·0 (9·0–28·0)	18·0 (9·0–29·0)	0·10
Depersonalization (DP)	5·0 (1·0–10·0)	5·0 (1·0–10·0)	4·0 (1·0–9·0)	5·0 (1·0–10·0)	<0·0001	4·0 (1·0–9·0)	5·0 (1·0–10·0)	<0·0001	5·0 (1·0–10·0)	5·0 (2·0–10·0)	<0·0001
Reduced Personal Accomplishment (PA)	29·0 (19·0–38·0)	29·0 (20·0–38·0)	29·0 (19·0–38·0)	28·0 (19·0–37·0)	<0·0001	27·0 (17·0–37·0)	29·0 (20·0–38·0)	<0·0001	29·0 (19·0–38·0)	28·0 (19·0–36·0)	<0·0001
SDS, Self-rating depression scale	42·0 (32·0–50·0)	41·0 (32·0–50·0)	42·0 (31·0–50·0)	42·0 (33·0–50·0)	<0·0001	43·0 (33·0–50·0)	41·0 (32·0–50·0)	<0·0001	42·0 (32·0–50·0)	42·0 (33·0–50·0)	0·02
SAS, Self-Rating Anxiety Scale	37·0 (30·0–45·0)	37·0 (30·0–45·0)	37·0 (30·0–45·0)	38·0 (31·0–45·0)	<0·0001	38·0 (31·0–45·0)	37·0 (30·0–45·0)	<0·0001	37·0 (30·0–45·0)	37·0 (31·0–45·0)	0·36

***Table 3:* Scores of burnout, depression, and anxiety symptoms in total cohort and subgroups.**

Concerning perceived burnout (OR, 0·889; 95% CI, 0·861–0·919; *P* < 0·0001), a decreased risk was identified among nurses having contract employment status. In addition, nurse participants possessing undergraduate or higher educational status presented a higher risk of burnout but a lower risk of depression and anxiety. Nurses just beginning their job were at decreased risk of depression (2–9 years vs below 2 years: adjusted OR, 1·111; 95% CI, 1·062–1·163; *P* < 0·0001; over 10 years vs below 2 years: adjusted OR, 1·147; 95% CI, 1·086–1·212; *P* < 0·0001), and anxiety (2–9 years vs below 2 years: adjusted OR, 1·204; 95% CI,1·149–1·261; *P* < 0·0001; over 10 years vs below 2 years: adjusted OR,1·331; 95% CI, 1·258–1·408; *P* < 0·0001), the more advanced professional experience the nurses possessed, the more at risk.

Furthermore, we observed respondents who worked in emergency and intensive care units (ICU) had incremental odds of burnout (emergency vs general wards: adjusted OR, 1·360; 95% CI, 1·259–1·469; *P* < 0·0001; ICU vs general wards: adjusted OR, 1·422; 95% CI, 1·329–1·522; *P* < 0·0001) and anxiety (emergency vs general wards: adjusted OR,1·107; 95% CI, 1·030–1·190; *P* = 0·01; ICU vs general wards: adjusted OR, 1·208; 95% CI, 1·134–1·286; *P* < 0·0001) than general wards. Compared with participants without overtime hours and night shifts, those who had to work overtime and night shift showed a higher risk of mental health symptoms, and the longer work overtime was, the higher the risk would be experienced. Additionally, job dissatisfaction and turnover intention were associated with greater odds of experiencing all poor mental health status.

In line with the disproportionality of the economic development, we noted that how developed the economy of the region a nurse comes from was an independent risk factor for depression (middle vs east: adjusted OR, 1·048; 95% CI, 1·018–1·078; *P* = 0·00; west vs east: adjusted OR, 1·085; 95% CI, 1·054–1·116; *P* < 0·0001) and anxiety (west vs east: adjusted OR, 1·092; 95% CI, 1·062–1·124; *P* < 0·0001), with higher prevalence being more likely in nurses from less developed regions than in those from more developed regions. Compared with those who worked in the secondary hospital, nurses who worked in tertiary hospitals were associated with a higher likelihood of burnout (OR, 1·248; 95% CI, 1·206–1·292; *P* < 0·0001), but lower depression (OR, 0·917; 95% CI, 0·889–0·946; *P* < 0·0001). Working in a private hospital was only associated with a lower risk of anxiety than in a public hospital (OR, 0·772; 95% CI, 0·692–0·862; *P* < 0·0001) (Table 4).

Discussion

The demanding nature of the occupation and the context of the COVID-19 pandemic expose nurses to a higher risk of developing psychological symptoms such as burnout, depression, and anxiety. This cross-sectional

Variables	MBI-HSS, Burnout symptoms			SDS, Depression symptoms			SAS, Anxiety symptoms		
		P value			*P* value			*P* value	
	Adjusted OR (95%CI)	Category	Overall	Adjusted OR (95%CI)	Category	Overall	Adjusted OR (95%CI)	Category	Overall
Gender									
Male	1[Reference]	NA	<0·0001	1[Reference]	NA	<0·0001	1[Reference]	NA	<0·0001
Female	0·707 (0·664–0·754)	<0·0001		0·715 (0·674–0·759)	<0·0001		0·657 (0·619–0·697)	<0·0001	
Age									
<35	1[Reference]	NA	<0·0001	1[Reference]	NA	<0·0001	1[Reference]	NA	0·02
35–54	0·830 (0·796–0·866)	<0·0001		0·907 (0·874–0·942)	<0·0001		0·967 (0·930–1·005)	0·08	
≥55	0·695 (0·567–0·852)	<0·0001		0·780 (0·670–0·909)	0·00		0·814 (0·692–0·958)	0·01	
Marital Status									
Single/Divorced/Widowed	1[Reference]	NA	<0·0001	1[Reference]	NA	0·00	1[Reference]	NA	<0·0001
Married	0·873 (0·845–0·901)	<0·0001		0·952 (0·923–0·981)	0·00		1·123 (1·089–1·158)	<0·0001	
Physical Condition									
Healthy	1[Reference]	NA	<0·0001	1[Reference]	NA	<0·0001	1[Reference]	NA	<0·0001
Good	1·303 (1·264–1·343)	<0·0001		1·255 (1·221–1·289)	<0·0001		1·334 (1·297–1·371)	<0·0001	
Normal	1·748 (1·688–1·811)	<0·0001		1·974 (1·907–2·042)	<0·0001		2·297 (2·222–2·374)	<0·0001	
Bad	2·448 (2·268–2·643)	<0·0001		3·865 (3·513–4·252)	<0·0001		5·069 (4·649–5·526)	<0·0001	
Menstruation									
Regular	1[Reference]	NA	<0·0001	1[Reference]	NA	<0·0001	1[Reference]	NA	<0·0001
Irregular	1·115 (1·083–1·147)	<0·0001		1·303 (1·269–1·339)	<0·0001		1·408 (1·371–1·446)	<0·0001	
Menopause	0·873 (0·797–0·956)	0·00		0·860 (0·799–0·926)	<0·0001		1·072 (0·993–1·157)	0·08	
Educational Status									
Below the undergraduate	1[Reference]	NA	<0·0001	1[Reference]	NA	<0·0001	1[Reference]	NA	<0·0001
Undergraduate	1·058 (1·025–1·091)	<0·0001		0·857 (0·833–0·881)	<0·0001		0·934 (0·908–0·961)	<0·0001	
Postundergraduate	1·302 (1·155–1·468)	<0·0001		0·706 (0·633–0·786)	<0·0001		0·779 (0·694–0·874)	<0·0001	
Professional Experience (y)									
<2	1[Reference]	NA	<0·0001	1[Reference]	NA	<0·0001	1[Reference]	NA	<0·0001
2–9	0·831 (0·791–0·873)	<0·0001		1·111 (1·062–1·163)	<0·0001		1·204 (1·149–1·261)	<0·0001	
≥10	0·761 (0·717–0·808)	<0·0001		1·147 (1·086–1·212)	<0·0001		1·331 (1·258–1·408)	<0·0001	
Job Position									
Head nurse	1[Reference]	NA	<0·0001	1[Reference]	NA	<0·0001	1[Reference]	NA	<0·0001
Nurse	1·469 (1·379–1·566)	<0·0001		1·311 (1·247–1·379)	<0·0001		1·340 (1·270–1·414)	<0·0001	
Employment Status									
Permanent	1[Reference]	NA	<0·0001	1[Reference]	NA	0·05	1[Reference]	NA	0·08
Contract	0·889 (0·861–0·919)	<0·0001		1·030 (1·000–1·060)	0·05		0·973 (0·944–1·003)	0·08	

Table 4 *(Continued)*

	MBI-HSS, Burnout symptoms			SDS, Depression symptoms			SAS, Anxiety symptoms		
		P value			*P* value			*P* value	
**Variables**	**Adjusted OR (95%CI)**	**Category**	**Overall**	**Adjusted OR (95%CI)**	**Category**	**Overall**	**Adjusted OR (95%CI)**	**Category**	**Overall**
Overtime Hours (weekly)									
None	1[Reference]	NA	<0·0001	1[Reference]	NA	<0·0001	1[Reference]	NA	<0·0001
<4 hr	1·376 (1·329–1·424)	<0·0001		1·146 (1·113–1·180)	<0·0001		1·286 (1·247–1·327)	<0·0001	
4–8 hr	1·637 (1·575–1·701)	<0·0001		1·227 (1·186–1·269)	<0·0001		1·448 (1·398–1·500)	<0·0001	
>8 hr	1·936 (1·853–2·024)	<0·0001		1·473 (1·413–1·535)	<0·0001		1·792 (1·719–1·867)	<0·0001	
Wards									
General	1[Reference]	NA	<0·0001	1[Reference]	NA	0·12	1[Reference]	NA	<0·0001
Emergency	1·360 (1·259–1·469)	<0·0001		0·976 (0·909–1·049)	0·51		1·107 (1·030–1·190)	0·01	
Intensive Care Units	1·422 (1·329–1·522)	<0·0001		1·072 (1·007–1·140)	0·03		1·208 (1·134–1·286)	<0·0001	
Others	0·969 (0·925–1·015)	0·19		0·991 (0·950–1·034)	0·67		1·011 (0·969–1·055)	0·61	
Yearly Salary (CNY)									
<100 000	1[Reference]	NA	0·89	1[Reference]	NA	<0·0001	1[Reference]	NA	<0·0001
100 000–200 000	1·004 (0·975–1·034)	0·80		0·797 (0·776–0·819)	<0·0001		0·766 (0·745–0·787)	<0·0001	
>200 000	0·986 (0·908–1·071)	0·74		0·624 (0·581–0·671)	<0·0001		0·549 (0·508–0·593)	<0·0001	
Night Shift (monthly)									
No	1[Reference]	NA	<0·0001	1[Reference]	NA	<0·0001	1[Reference]	NA	<0·0001
Yes	1·069 (1·038–1·101)	<0·0001		1·111 (1·082–1·141)	<0·0001		1·134 (1·104–1·165)	<0·0001	
Job Satisfaction									
Satisfied	1[Reference]	NA	<0·0001	1[Reference]	NA	<0·0001	1[Reference]	NA	<0·0001
Dissatisfied	3·623 (3·517–3·732)	<0·0001		2·225 (2·171–2·281)	<0·0001		2·171 (2·115–2·228)	<0·0001	
Turnover Intention									
No	1[Reference]	NA	<0·0001	1[Reference]	NA	<0·0001	1[Reference]	NA	<0·0001
Yes	2·738 (2·650–2·828)	<0·0001		2·010 (1·940–2·083)	<0·0001		1·791 (1·734–1·850)	<0·0001	
None	1[Reference]	NA	<0·0001	1[Reference]	NA	<0·0001	1[Reference]	NA	<0·0001
≤5	0·877 (0·818–0·940)	<0·0001		0·949 (0·893–1·008)	0·09		0·974 (0·915–1·037)	0·41	
6–11	0·974 (0·922–1·029)	0·35		0·881 (0·838–0·926)	<0·0001		0·931 (0·886–0·979)	0·01	
≥12	1·237 (1·166–1·313)	<0·0001		0·968 (0·917–1·022)	0·24		1·049 (0·993–1·108)	0·08	
Commute Time (hr)									
<1	1[Reference]	NA	<0·0001	1[Reference]	NA	0·00	1[Reference]	NA	<0·0001
≥1	1·080 (1·049–1·112)	<0·0001		1·044 (1·016–1·072)	0·00		1·093 (1·063–1·123)	<0·0001	
Region									
East	1[Reference]	NA	0·07	1[Reference]	NA	<0·0001	1[Reference]	NA	<0·0001
Middle	0·976 (0·945–1·008)	0·14		1·048 (1·018–1·078)	0·00		1·015 (0·985–1·045)	0·34	
West	1·017 (0·986–1·049)	0·29		1·085 (1·054–1·116)	<0·0001		1·092 (1·062–1·124)	<0·0001	

Table 4 *(Continued)*

Variables	MBI-HSS, Burnout symptoms			SDS, Depression symptoms			SAS, Anxiety symptoms		
	Adjusted OR (95%CI)	*P* value		Adjusted OR (95%CI)	*P* value		Adjusted OR (95%CI)	*P* value	
		Category	Overall		Category	Overall		Category	Overall
Hospital Status									
Secondary	1[Reference]	NA	<0·0001	1[Reference]	NA	<0·0001	1[Reference]	NA	0·30
Tertiary	1·248 (1·206−1·292)	<0·0001		0·917 (0·889−0·946)	<0·0001		1·017 (0·985−1·049)	0·30	
Hospital Type									
Public	1[Reference]	NA	0·17	1[Reference]	NA	0·06	1[Reference]	NA	<0·0001
Private	0·922 (0·821−1·036)	0·17		0·899 (0·807−1·002)	0·06		0·772 (0·692−0·862)	<0·0001	

***Table 4:* Multivariable logistic regression analysis of risk factors associated with psychological symptoms.**
Abbreviations: MBI-HSS, Maslach Burnout Inventory Human Service Survey; NA, not applicable; OR, odds ratio; SAS, Self-Rating Anxiety Scale; SDS, Self-Rating Depression Scale.

study examined the severity and risk factors associated with impaired mental health among nurses based on a large sample from 30 provinces in China. Overall, this study revealed that a considerable proportion of responders suffered from clinically significant psychological symptoms, in terms of burnout (34%), depression (55·5%), and anxiety (41·8%). Participants were separated into three groups (east, middle, and west) to compare interregional discrimination, two groups (secondary hospital and tertiary hospital) to examine differences in hospital status, and two groups (public hospital and private hospital) to detect discrepancies in hospital type, respectively. Our study further identified several psychologically vulnerable factors, including men, aged 35 or younger, physically unhealthy, working overtime, night shift, job dissatisfaction, turnover intention, and commuting time over one hour. These findings offer a comprehensive assessment of the potential mental health burden on nurses in China and may promote to guide future mental health prevention and improvement efforts.

Here, we found that about one-third (34%) of nurses reported symptoms of burnout, in contrast, a higher prevalence was reported by 46·8% of Japanese nurses during the COVID-19 pandemic.[30] Exposed to an increasing influx of suspected and actual cases, elevated risk of exposure to infection, shortage of personal protection equipment, stigmatization, and understaffing were recognized as the major contributor during the outbreaks and afterward.[31]

Before the pandemic, a high prevalence of depressive symptoms was reported among Chinese nurses (35.8%),[32] but this is revealed to be exacerbated by the increased demands of care during the COVID-19 epidemic. Consistent with our present findings, Cai et al. and Lai et al. identified a similar rate of depressive symptoms in China after the COVID-19 outbreak (52·8% and 50·4%, respectively).[33,34]

In our study, the prevalence of anxiety symptoms was common among nurses, with a prevalence rate of over 40%. However, in a previous study during the early stages of the COVID-19 outbreak, 89% of frontline nurses reported anxiety symptoms.[35] Potential differences, however, could be explained in that our research covered various nurse groups. Moreover, frontline nurses who treated patients with COVID-19 are more prone to exposure to the highest risk of infection, resulting from their close, frequent contact with infectious patients and working prolonged hours than usual.[36] In contrast, the rate of anxiety among the general population ranged from 5·2% to 12·5% in China, [37,38] indicating that the prevalence of anxiety in healthcare workers is significantly higher.

As previously mentioned, the prevalence of psychological symptoms among nurses was much higher, reflecting a critical mental health condition that deserves timely and careful clinical attention. Therefore,

it is critical to study potential factors and prevent this condition to better care for caregivers. Regarding the economic development factors, nurses from less developed areas had a higher incidence of mental health disorders. This phenomenon may be attributed to the fact that underdeveloped regions cannot access adequate resources and equipment availability, bringing up insecurity about obtaining and maintaining essential resources for meeting job demands and resulting in psychological symptoms.[39] Additionally, nurses from tertiary hospitals were more prone to burnout, and depression than secondary hospitals. One possible reason is that in the Chinese healthcare system, secondary hospitals are usually not as occupied or crowded as tertiary, admitting fewer patients or less severe cases.[40]

Unsurprisingly, an inverse relationship was found between age and psychological symptoms (burnout, depression, and anxiety), which might not be amazing that younger nurses possess less work experience and inappropriate coping strategies to face the stressful workload every day.[41] Thus, they will most likely fall victim to mental health disorders from the chronic occupational burden. Furthermore, the data in our study are congruent with previous studies, as employees with permanent employment status experienced higher levels of burnout than those with a contract status, which may result from routine and monotonic work.[42]

Consistent with previous studies,[43] the prevalence of psychological symptoms was higher among ICU nurses than among those working in general wards. Nurses caring for critically ill patients have heavy workloads, including comprehensive assessment and close monitoring, intensive identification and response to clinical deterioration, compact communication and collaboration with physicians, psychological support, and prevention of various potential complications. In addition, compounding factors, such as interaction with patients with intricated disorder conditions, moral dilemmas related to end-of-life care, processing with families of dying patients, and lack of a fixed working shift could be associated with adverse mental health outcomes for ICU nurses.[44] Hence, they are prone to these symptoms and are considered an especially vulnerable group. Again, existing literature confirms that ICU nurses are additionally suffered from work-related nightmares.[45]

To battle viruses, nurses are occupied with the extra workload of infection screening, prevention, and treatment. Approximately three-quarters (74·4%) of respondents had to work overtime, putting healthcare workers at risk of being exhausted and consumed by their emotional resources. Moreover, the present study found that job dissatisfaction was more likely to jeopardize mental well-being. In support of these findings, preliminary studies confirm that job dissatisfaction may result in depressive symptoms, while depressed workers are more likely to yield lower job satisfaction.[46]

Having said that, the majority of the nurses experienced mild or moderate symptoms of burnout, depression, and anxiety, while severe symptoms were less common among the responders. In our view, this evinces the demand for early detection and the indispensability of effectively treating the milder psychological symptoms before they develop into more intricate and abiding psychological diseases. Therefore, our findings may assist in quantifying the need for nurses' support and tailored interventions that intensify the ability to resilience and mitigate the vulnerability.

This study has several limitations. First, all of the respondents volunteered for questionnaires and may thus be subject to participant bias. Second, women represented the majority of participants, which is the nature of the nurse profession and encountered in all considerable epidemiologic research.[47] Therefore, further research would be undertaken to investigate mental health conditions among male nurses. Third, this was a cross-sectional study, thus, future studies would adopt longitudinal assessments to provide more solid evidence. In addition, one significant drawback that merits consideration is that only a small proportion of private hospital nurses were included in this study, which might reveal limited representativeness. Furthermore, the questionnaires adopted in our research only represented psychological symptoms at a one-time point, thus future studies should adopt relative tools to investigate the lifetime prevalence. Nevertheless, our study covered over 130 000 nurses from 30 provinces in China. The overall sample size was large and representative, which can help extend our results to reflect the mental health of the healthcare group.

In conclusion, this cross-sectional survey of mental health burden among nurses in China is the largest sample size to date, covering the different statuses of hospitals and geographical regions, highlighting the prevalence of present psychological symptoms in China. The overwhelming workload of usual work and extra pandemic prevention work during COVID-19 drained nurses physically and emotionally. It is therefore critical for policymakers to provide comprehensive support and take appropriate trajectories, such as formulating reasonable working hours and shifts, sufficient communication, and providing professional psychological support, so as to assure that the well-being of healthcare workers becomes the top priority alongside the health of our patients.

Contributors

Jun Wang, Fan Yang, and Jian Zhou had full access to all of the data in the study and take responsibility for the integrity of the data and the accuracy of the data analysis.
Yun Li, Rongrong Fan, and Yunwei Lu contributed equally as co-first authors.
Jun wang and Jian Zhou contributed equally as co-corresponding authors.
Concept and design: Jian Zhou.

Acquisition, analysis, or interpretation of data: All authors.
Drafting of the manuscript: Yunwei Lu.
Critical revision of the manuscript for important intellectual content: All authors.
Statistical analysis: Yun Li, Rongrong Fan, Huayan Li, Guilan Kong, Jiayu Wang, Xianping Liu.
Administrative, technical, or material support: Jun Wang, Yun Li, Jian Zhou.
Supervision: Jun Wang, Yun Li, Jian Zhou, Rongrong Fan.

Data sharing statement

The data that support the findings of this study are available on request from the corresponding author.

Editor note

The Lancet Group takes a neutral position with respect to territorial claims in published maps and institutional affiliations.

Declaration of interests

All other authors declare no competing interests.

Supplementary materials

Supplementary material associated with this article can be found in the online version at doi:10.1016/j.lanwpc.2022.100618.

References

1 Wang C, Horby PW, Hayden FG, Gao GF. A novel coronavirus outbreak of global health concern. *Lancet*. 2020;395(10223):470–473.
2 Rajkumar RP. COVID-19 and mental health: a review of the existing literature. *Asian J Psychiatry*. 2020;52:102066.
3 Galea S, Merchant RM, Lurie N. The mental health consequences of COVID-19 and physical distancing: the need for prevention and early intervention. *JAMA Intern. Med.*. 2020;180(6):817–818.
4 Cai H, Tu B, Ma J, et al. Psychological impact and coping strategies of frontline medical staff in hunan between January and March 2020 during the outbreak of coronavirus disease 2019 (COVID-19) in Hubei, China. *Med Sci Monit*. 2020;26:e924171.
5 Krishnamoorthy Y, Nagarajan R, Saya GK, Menon V. Prevalence of psychological morbidities among general population, healthcare workers and COVID-19 patients amidst the COVID-19 pandemic: a systematic review and meta-analysis. *Psychiatry Res*. 2020;293:113382.
6 Salyers MP, Bonfils KA, Luther L, et al. The relationship between professional burnout and quality and safety in healthcare: a meta-analysis. *J Gen Intern Med*. 2017;32(4):475–482.
7 Sorour AS, El-Maksoud MMA. Relationship between musculoskeletal disorders, job demands, and burnout among emergency nurses. *Adv Emerg Nurs J*. 2012;34(3):272–282.
8 Administration, A. M. H. S.. Results from the 2006 national survey on drug use and health: National findings. *Substance Abuse and Mental Health Services Administration*. DHHS Publication; 2007. SMA 07-4294, NSDUH Series H-32.
9 Yohannes AM, Willgoss TG, Baldwin RC, Connolly MJ. Depression and anxiety in chronic heart failure and chronic obstructive pulmonary disease: prevalence, relevance, clinical implications and management principles. *Int J Geriatr Psychiatry*. 2010;25(12):1209–1221.
10 Janeway D. An integrated approach to the diagnosis and treatment of anxiety within the practice of cardiology. *Cardiol Rev*. 2009;17(1):36–43.
11 Shechter A, Diaz F, Moise N, et al. Psychological distress, coping behaviors, and preferences for support among New York healthcare workers during the COVID-19 pandemic. *Gen Hosp Psychiatry*. 2020;66:1–8.
12 Rossi R, Socci V, Pacitti F, et al. Mental health outcomes among healthcare workers and the general population during the COVID-19 in Italy. *Front Psychol*. 2020;11:608986.
13 International Council of Nurses. Mass trauma experienced by the global nursing workforce. https://www.icn.ch/sites/default/files/inlinefiles/ICN%20COVID19%20update%20report%20FINAL.pdf. Accessed Nov 28, 2021.
14 Muller AE, Hafstad EV, Himmels JPW, et al. The mental health impact of the covid-19 pandemic on healthcare workers, and interventions to help them: a rapid systematic review. *Psychiatry Res*. 2020;293:113441.
15 Khamisa N, Oldenburg B, Peltzer K, Ilic D. Work related stress, burnout, job satisfaction and general health of nurses. *Int J Environ Res Public Health*. 2015;12(1):652–666.
16 Riedel B, Horen SR, Reynolds A, Hamidian Jahromi A. Mental health disorders in nurses during the COVID-19 pandemic: implications and coping strategies. *Front Public Health*. 2021;9:707358.
17 Gupta S, Sahoo S. Pandemic and mental health of the front-line healthcare workers: a review and implications in the Indian context amidst COVID-19. *Gen Psychiatry*. 2020;33(5):e100284.
18 Maharaj S, Lees T, Lal S. Negative mental states and their association to the cognitive function of nurses. *J Psychophysiol*. 2019;33(3):1–12.
19 Berland A, Natvig GK, Gundersen D. Patient safety and job-related stress: a focus group study. *Intensive Crit Care Nurs*. 2008;24(2):90–97.
20 Johnson J, Hall LH, Berzins K, Baker J, Melling K, Thompson C. Mental healthcare staff well-being and burnout: a narrative review of trends, causes, implications, and recommendations for future interventions. *Int J Ment Health Nurs*. 2018;27(1):20–32.
21 Arnsten AFT. Stress weakens prefrontal networks: molecular insults to higher cognition. *Nat Neurosci*. 2015;18(10):1376–1385.
22 Chang D, Xu H, Rebaza A, Sharma L, Dela Cruz CS. Protecting health-care workers from subclinical coronavirus infection. *Lancet Respirat Med*. 2020;8(3):e13.
23 Maslach C, Jackson S, Leiter M. *Maslach Burnout Inventory*. 3rd ed. Palo Alto: Consulting Psychologists Press; 1996.
24 Zung WW. A self-rating depression scale. *Arch Gen Psychiatry*. 1965;12:63–70.
25 Dunstan DA, Scott N. Norms for Zung's self-rating anxiety scale. *BMC Psychiatry*. 2020;20(1):90.
26 Meng Q, Xu L, Zhang Y, Qian J, Cai M, Xin Y. Trends in access to health services and financial protection in China between 2003 and 2011: a cross-sectional study. *Lancet*. 2012;379(9818):805–814.
27 National Bureau of Statistics of China. China Statistical Yearbook 2010. http://www.stats.gov.cn/tjsj/ndsj/2010/indexeh.htm. Accessed September 22, 2020.
28 Zhang L, Li Z, Li X, et al. Study on the trend and disease burden of injury deaths in Chinese population, 2004-2010. *PLoS One*. 2014;9(1):e85319.
29 He L, Dong ZJ, Du X, Jiang C, Chen N, Xia SJ. Healthcare quality and mortality among patients hospitalized for heart failure by hospital level in Beijing, China. *ESC Heart Failure*. 2021;8(2):1186–1194.
30 Lee SM, Kang WS, Cho AR, Kim T, Park JK. Psychological impact of the 2015 MERS outbreak on hospital workers and quarantined hemodialysis patients. *Compr Psychiatry*. 2018;87:123–127.
31 Matsuo T, Kobayashi D, Taki F, et al. Prevalence of health care worker burnout during the coronavirus disease 2019 (COVID-19) pandemic in Japan. *JAMA Netw Open*. 2020;3(8):e2017271.
32 Cheung T, Yip PSF. Depression, anxiety and symptoms of stress among Hong Kong nurses: a cross-sectional study. *Int J Environ Res Public Health*. 2015;12(9):11072–11100.
33 Cai Z, Cui Q, Liu Z, et al. Nurses endured high risks of psychological problems under the epidemic of COVID-19 in a longitudinal study in Wuhan China. *J Psychiatr Res*. 2020;131:132–137.
34 Lai J, Ma S, Wang Y, et al. Factors associated with mental health outcomes among health care workers exposed to coronavirus disease 2019. *JAMA Netw Open*. 2020;3(3):e203976.
35 Li J, Su Q, Li X, Peng Y, Liu Y. COVID-19 negatively impacts on psychological and somatic status in frontline nurses. *J Affect Disord*. 2021;294:279–285.
36 Shih FJ, Gau ML, Kao CC, et al. Dying and caring on the edge: Taiwan's surviving nurses' reflections on taking care of patients with severe acute respiratory syndrome. *Appl Nurs Res*. 2007;20(4):171–180.

37 Liu XC, Oda S, Peng X, Asai K. Life events and anxiety in Chinese medical students. *Soc Psychiatry Psychiatr Epidemiol*. 1997;32(2):63–67.
38 Proceedings of the 3rd IPLeiria's International Health Congress: Leiria, Portugal. 6-7 May 2016. *BMC Health Serv Res*. 2016;16(Suppl 3):200.
39 Tummers GE, Janssen PP, Landeweerd A, Houkes I. A comparative study of work characteristics and reactions between general and mental health nurses: a multi-sample analysis. *J Adv Nurs*. 2001;36(1):151–162.
40 Gao YQ, Pan BC, Sun W, Wu H, Wang JN, Wang L. Anxiety symptoms among Chinese nurses and the associated factors: a cross sectional study. *BMC Psychiatry*. 2012;12:141.
41 Wu S, Zhu W, Wang Z, Wang M, Lan Y. Relationship between burnout and occupational stress among nurses in China. *J Adv Nurs*. 2007;59(3):233–239.
42 Hu HX, Liu LT, Zhao FJ, Yao YY, Gao YX, Wang GR. Factors related to job burnout among community nurses in Changchun, China. *J Nurs Res*. 2015;23(3):172–180.
43 Lasalvia A, Bonetto C, Porru S, et al. Psychological impact of COVID-19 pandemic on healthcare workers in a highly burdened area of north-east Italy. *Epidemiol Psychiatr Sci*. 2020;30:e1.
44 Andolhe R, Barbosa RL, Oliveira EM, Costa AL, Padilha KG. Stress, coping and burnout among Intensive Care Unit nursing staff: associated factors. *Rev Esc Enferm USP*. 2015;49 Spec No:58–64.
45 Bambi S, Foà C, De Felippis C, Lucchini A, Guazzini A, Rasero L. Workplace incivility, lateral violence and bullying among nurses. A review about their prevalence and related factors. *Acta Bio-Medica*. 2018;89(6-S):51–79.
46 Oliveira AM, Silva MT, Galvão TF, Lopes LC. The relationship between job satisfaction, burnout syndrome and depressive symptoms: an analysis of professionals in a teaching hospital in Brazil. *Medicine*. 2018;97(49):e13364.
47 Galea S, Tracy M. Participation rates in epidemiologic studies. *Ann Epidemiol*. 2007;17(9):643–653.
