# Supplementary material for: Mental health status and related factors influencing healthcare workers during the COVID-19 pandemic: A systematic review and meta-analysis
Source: PLoS One. 2024 Jan 19;19(1):e0289454. doi: 10.1371/journal.pone.0289454 (PMC10798549; doi:10.1371/journal.pone.0289454)
Supplement: S1 Data — (ZIP) [file pone.0289454.s011.zip › literatures/80.pdf]

ELSEVIER

Since January 2020 Elsevier has created a COVID-19 resource centre with free information in English and Mandarin on the novel coronavirus COVID-19. The COVID-19 resource centre is hosted on Elsevier Connect, the company's public news and information website.

Elsevier hereby grants permission to make all its COVID-19-related research that is available on the COVID-19 resource centre - including this research content - immediately available in PubMed Central and other publicly funded repositories, such as the WHO COVID database with rights for unrestricted research re-use and analyses in any form or by any means with acknowledgement of the original source. These permissions are granted for free by Elsevier for as long as the COVID-19 resource centre remains active.

Journal of Affective Disorders 297 (2022) 156–168

Contents lists available at ScienceDirect

Journal of Affective Disorders

journal homepage: www.elsevier.com/locate/jad

A cross-sectional study of the psychological status of 33,706 hospital workers at the late stage of the COVID-19 outbreak

Wang Lixia [a], Xu Xiaoming [a], Shi Lei [b], Hong Su [a], Wang Wo [b], Fang Xin [c], Chen Jianmei [a], Zhang Qi [a], Ai Ming [a,1,*], Kuang Li [a,b,1,*]

[a] *Department of Psychiatry, The First Affiliated Hospital of Chongqing Medical University, No.1 Youyi Road, Yuzhong District, Chongqing 400016, China*
[b] *Mental Health Center, University-Town Hospital of Chongqing Medical University, China*
[c] *Peking University Hospital Psychotherapy and Counseling Center, China*

ARTICLE INFO

Keywords:
2019 Novel Coronavirus disease
Hospital workers
Psychological status
PTSD symptoms
Suicidal ideation

ABSTRACT

Background: Hospital workers have been under intense psychological pressure since the COVID-19 outbreak. We analyzed the psychological status of hospital staff in the late period of the COVID-19 to provide a basis for the construction of global health care after the COVID-19 outbreak.
Methods: We used online surveys to assess participants' self-reported symptoms at the late stage of the outbreak. This study collected data on sociodemographic characteristics, epidemic-related factors, psychological status (PHQ-9, GAD-7, and PHQ-15), psychological assistance needs, perceived stress and support, PTSD symptoms (PCL-C) and suicidal and self-injurious ideation (SSI). Participants were hospital workers in all positions from 46 hospitals. Chi-square tests to compare the scales and logistic regression analysis were used to identify risk factors for PTSD and SSI.
Results: Among the 33,706 participants, the prevalences of depression, anxiety, somatic symptoms, PTSD symptoms, and SSI were 35.8%, 24.4%, 49.7%, 5.0%, and 1.3%, respectively. Logistic regression analysis showed that work in a general ward, attention to the epidemic, high education, work in non-first-line departments, insufficient social support, and anxiety and somatization symptoms were influencing factors of PTSD ($P<0.05$). The independent risk factors for SSI were female gender; psychological assistance needs; contact with severe COVID-19 patients; high stress at work; single or divorced marital status; insufficient social support; and depression, anxiety or PTSD symptoms ($P<0.05$).
Limitations: This cross-sectional study could not reveal causality, and voluntary participation may have led to selection bias. The longer longitudinal studies are needed to determine the long-term psychological impact.
Conclusion: This COVID-19 pandemic had a sustained, strong psychological impact on hospital workers, and hospital workers with PTSD symptoms were a high-risk group for SSI in the later period of the epidemic. Continuous attention and positive psychological intervention are of great significance for specific populations.

1. Introduction

According to the latest WHO report at the time this article was submitted, more than 173 million people worldwide have been infected with COVID-19, and the number of deaths after infection has exceeded 3.7 million and is still rising (World Health Organization, 2021).This global pandemic has resulted in substantial psychological burdens. Some reports during the initial and peak periods of the outbreak (Gonzalez-Sanguino et al., 2020; Mrklas et al., 2020) showed the impact of the pandemic on mental health, and based on our previous experience and other research (Brooks et al., 2020; Wang et al., 2021; Xiaoming et al., 2020), we believe that the poor mental conditions during quarantine were as serious as the disease itself. Hospital staff were the main force in the fight against the epidemic, and the mental health of this population was particularly important.

Related reports all showed that public health disasters outbreaks

* Corresponding authors at: Department of Psychiatry, The First Affiliated Hospital of Chongqing Medical University, No.1 Youyi Road, Yuzhong District, Chongqing 400016, China.
E-mail addresses: ami200553@qq.com (A. Ming), kuangli0308@163.com (K. Li).
[1] These authors contributed equally to this work.

https://doi.org/10.1016/j.jad.2021.10.013
Received 11 June 2021; Received in revised form 5 September 2021; Accepted 11 October 2021
Available online 20 October 2021
0165-0327/© 2021 Published by Elsevier B.V.

generally cause substantial psychological effects and that the impact may be long term.For instance,Some hospital staff were diagnosed as varying degrees of posttraumatic stress symptoms/ posttraumatic stress disorder (PTSD), anxiety, depression or physical symptoms from 1 month to 3 years after SARS outbreak(Lee et al., 2007; Lancee et al., al., 2008; Mihashi et al., 2009; Wu et al., al.,2009; Naushad et al., 2019; Cheng et al., 2004; Bai et al., 2004; Brooks et al., 2020).After 2-month in Sierra Leone with Ebola appeared outbreak, medical staff still have psychological symptoms of anxiety, somaticization, obsession compulsion and so on(Dong et al., al., 2017). Therefore, adequate attention should also be focused on the mental health of hospital staff in the post-outbreak period.

With the global use of vaccines and the implementation of active epidemic prevention measures in various countries, the COVID-19 pandemic will eventually end. In April 2020, China was in the late stage of the epidemic. Approximately one month after there had been no new COVID-19 patients in Chongqing, China, we investigated the general mental health status, suicidal and self-injurious ideation (SSI) and posttraumatic stress disorder (PTSD) symptoms of hospital workers in this region, hoping to provide a basis for future health care construction after the global COVID-19 epidemic.

2. Methods

2.1. Design

This study was a cross-sectional survey, the psychological investigation was conducted through WeChat, an instant messaging application widely used in China. A structured self-report questionnaire was used to collect data. A total of 6 questionnaires were assessed, and the average completion time was approximately 25 min.

The data collection was performed from June 1 to June 30, 2020, when the COVID-19 epidemic peak in China had ended and the number of infected persons in Chongqing had reached zero (National Health Commission of China, 2020).

This study was approved by the Ethics Committee of the First Affiliated Hospital of Chongqing Medical University. All subjects signed an electronic informed consent form. The survey was conducted anonymously, and the information was confidential.

2.2. Participants

Convenience sampling was used in this study. The participants came from 46 hospitals designated by the Chongqing Municipal Health Commission, including 4 designated hospitals (for confirmed patients), 16 main urban hospitals (located in the central urban area), and 26 county-level hospitals, with a total of 51,637 employees. Finally, a total of 33,706 participants, including doctors (including trained doctors and interns), nurses, professional and technical personnel, administrative department staff, logistics personnel, and others (volunteers, nursing workers, students on probation, etc.), were included in the study.

Our team contacted with the hospital administrator, hospital workers of 46 hospitals were encouraged to finish the survey voluntarily by scanning a QR code shared in their WeChat workgroup. Each registered account could be used to complete the questionnaire only once. The electronic questionnaire collection was completed from June 1 to June 30, 2020. The eligibility criteria were as follows: 1. aged at least 18 years, 2. were current staff, and 3. completed all the questionnaires without any logical errors.

2.3. Variables

2.3.1. Sociodemographic information and epidemic-related profile

Data on sociodemographic characteristics and variables related to the participating hospital workers were collected. The data included gender, age, ethnicity, educational background, marital status, department, occupation, title, employment position, working years, hospital level and other information.

The epidemic-related factors included epidemic-related attitudes and behaviors, such as “experienced SARS”, “Frontline department”, “Isolation and protection of the department” , “Infected with COVID-19 in community”, “Family members infected with COVID-19″, “Direct exposure to COVID-19″, “Exposure to severe COVID-19 patients”, “Exposure to died COVID-19 patients”, “Daily working hours during the epidemic”, “Self-rated infected possibility”, “Willingness of working in COVID-19 ward”, “Stress-feeling at work during COVID-19 remission” , “Self-rated physical and mental condition during epidemic”, “Need of psychological assistance before epidemic”, “Need of psychological assistance during epidemic remission” (Xiaoming et al., 2020; Tam et al., 2004).

2.3.2. General psychological status of the hospital workers

The **Patient Health Questionnaire-9 (PHQ-9)** was administered to assess depressive symptoms (Spitzer et al., 1999). The participants indicated how often they had been bothered by the 9 assessed symptoms over the past two weeks on 4-point Likert scale ranging from "not at all" (0) to "almost every day" (3). The total scores ranged from 0 to 27, and the following cut-off scores were used: 0 to 4 (no symptoms), 5 to 9 (minimal symptoms), 10 to 14 (mild symptoms), 15 to 19 (moderate symptoms), and 20 to 27 (severe depressive symptoms). The Cronbach’s alpha of the PHQ-9 was found to be 0.86. In the Chinese general population, the sensitivity and specificity were shown to be 0.86 and 0.86 (Wang et al., 2014). In general hospitals, the sensitivity and specificity were found to be 0.91 and 0.97, respectively (Hu et al., 2009).

The severity of anxiety was assessed using the **Generalized Anxiety Disorder 7-item Scale (GAD-7)** (Spitzer et al., 2006; Xiaoming et al., 2020). The participants were asked how often they had been bothered by each of the 7 individual symptoms over the past two weeks, and the individual item scores were added to calculate the total score. The scores ranged from 0 to 3, corresponding to "none at all," "a few days," "more than a week," and "almost every day." Total scores of 5, 10, and 15 were considered to indicate mild, moderate, and severe anxiety, respectively. The Cronbach’s alpha of the GAD-7 was found to be 0.82 (Wang et al., 2014). Among outpatients in Chinese general hospitals, the cut-off value of 10 points on the GAD-7 was found to have a sensitivity of 0.86 and a specificity of 0.96 (He et al., 2010).

The **Patient Health Questionnaire Somatic Symptom Severity Scale-15 (PHQ-15)** was used to assess somatic symptoms (Kroenke et al., 2002). This 15-item scale was used to assess whether the participants had experienced physical symptoms, including stomach or back pain, in the past 4 weeks; they rated the symptom severity as "no," "little, " and "a lot." The Cronbach’s alpha coefficient of the PHQ-15 was 0.83 (Zhang et al., 2016). The test-retest reliability coefficient for outpatients of China General Hospital was found to be 0.75 (Qian et al., 2014). Total scores of 5, 10, and 15 were used as the thresholds for low-, medium-, and high-deficiency somatic symptoms, respectively (Xiaoming et al., 2020).

Job-related stress. In this study, a self-report questionnaire (Tam et al., 2004) was modified based on the work-related stress severity scale developed in Taiwan for frontline medical staff during the SARS outbreak. This scale was also used in our study conducted during the peak of the COVID-19 outbreak (Hong et al., 2021; Xiaoming et al., 2020). The Cronbach’s alpha value of the scale was 0.79; the scale includes 14 items, answered with "Yes" or "No", with "Yes" scoring 1 point. Each item is related to subjective work stress in the later period of the COVID-19 epidemic (e.g., perceived workload was too large, perceived work environment was dangerous, and perceived health threat to oneself and family members). Total scores of 4 and 9 were defined as the critical values indicating low, medium and high work stress, respectively.

Perceived adequacy of social support. This scale was developed based on research on the relationship between social and occupational factors and the psychological outcomes of medical staff during infectious disease outbreaks (Brooks et al., 2019; Williamson et al., 2018). It contains 6 dichotomous questions (Yes/No) on family support, colleague support, hospital administration support, patient support, insurance and compensation, and mass media support (Hong et al., 2021; Xiaoming et al., 2020).

2.3.3. COVID-19 epidemic factors

We evaluated the correlations between COVID-19-related factors and the PTSD symptoms and SSI of the hospital workers in the later period of the outbreak using 15 items. COVID-19-related factors included working conditions and personal experience during the outbreak. For example, we asked the participants the following questions: "Did you participated in medical assistance during the SARS epidemic in 2003?", "Did you work in a COVID-19 frontline department?", "Have you been in direct contact with patients with confirmed COVID-19 diagnoses? ""Would you like to work in a COVID-19 unit?", and "Are you still willing to attend group gatherings during the epidemic?" Since PTSD and SSI were the result of comprehensive factors, some relevant sociodemographic characteristics, such as gender and age, were also included, as shown in Table 3.

The PTSD checklist-civilian version (PCL-C). The PCL-C used in this study was developed by Weathers et al. of the National Center for Post-Traumatic Stress Disorder of the United States, and it has been recommended by the Chinese Guidelines for Prevention and Treatment of PTSD (Zhang et al.,2015).

The PCL-C consists of 17 items that correspond to the DSM-IV criteria for PTSD symptomatology (B, C, and D), such as invasive memories, related nightmares, flashback experiences, and emotional responses to memories. The severity of psychophysiological responses to trauma are assessed from grades 1 to 5, including "no psychophysiological response," "minimum response," "mild response," "moderate response," and "severe response." In this study, a total score ≥44 (positive) was used as the criterion for the preliminary diagnosis of PTSD. A study of traffic accident survivors in Hong Kong showed that the diagnostic sensitivity of the PCL-C was 1.00 and the specificity was 0.98. The PCL-C has good reliability and validity. In the study of Hu Jieying et al., the Cronbach's alpha was 0.77, and the 1-week test-retest reliability was $R = 0.84$.

Suicidal and self-injurious ideation (SSI). Item 9 of the PHQ-9, "whether you have had thoughts in the past two weeks that would have been better to die or harm yourself in some way," was used to assess suicidal ideation (Wang et al., 2014). Refer to the scoring method of PHQ-9 scale. The score 0(not at all) was considered as “negative”; the scores more than or equal to 1(for days, over a week, almost every day) were considered as "positive".

2.4. Statistical analysis

Data were analyzed using SPSS 23.0 (SPSS, Chicago, IL, USA). Descriptive statistics were used for general characteristics and study variables. The independent factors affecting PTSD symptoms and SSI were analyzed by χ^2 tests and logistic regression analysis (forward logistic regression) according to the characteristics of the variables. $P<0.05$ was considered statistically significant.

3. Results

3.1. Sociodemographic and epidemic-related profile (Table 1)

Of the 33,706 respondents, 76.7% were female, and 23.3% were male. The majority were between 31 and 40 years old (32.3%). The participants included doctors (41%), nurses (45.5%), professional and technical personnel (6.2%), administrative staff (2.7%), logistics personnel (1.2%) and other staff (3.5%). The details of the sociodemographic information and epidemiological information of the respondents are shown in Table 1.

3.2. Sociodemographic data and general psychological impact

In the Table 2, the total proportion of minimal, mild, moderate and severe symptoms in PHQ-9 was the prevalence of depression. The total proportion of low, medium, high symptom in GAD-7and PHQ15 was the prevalence of anxiety and somatic symptoms, respectively. The results showed that the prevalences of depression, anxiety and somatic symptoms were 35.8% (n = 12,081), 24.4% (n = 8208) and 49.7% (n = 16,748), respectively.

The diagnostic results by gender were as follows: depression (38.0% female, 28.7% male), anxiety (26.1% female, 18.5% male) and somatization symptoms (54.0% female, 35.4% male). Occupation as a nurse and working experience of 6–10 years were the highest prevalence rate among the three symptom variables. The proportions of participants with depression, anxiety and somatic symptoms in the different age groups were significantly different($P<0.05$), with the proportions of young and elderly patients with these symptoms being relatively low. There were significant differences in the proportions of national minority participants with somatic symptoms, with Miao (54%) and "other ethnic minorities" (Hui, Tibetan, Buyi, etc., < 17 persons, 56.8%) having a greater proportion than Han (49.7%) and Tujia (45.1%). Participants who were single or divorced had higher rates of depression (40.2%) and somatic symptoms (51.4%). Employees with a bachelor's degree or lower (50%) had a higher incidence of somatic symptoms than those with higher education (42.8%). The proportion of formal staff (24.9%) with anxiety symptoms was lower than that of temporary staff (23.8%), while the proportion of formal staff with somatic symptoms (50.4%) was higher than that of temporary workers. Middle-level employees (26.2%) had a high degree of anxiety. Employees with high levels of job stress had higher levels of depression (6.3%), anxiety (7.6%), somatic symptoms (24.1%), and insomnia (42.9%). More results of this study are shown in Table 2.

3.3. The results on PTSD symptoms and SSI in relation to COVID-19 epidemic-related factors (Table 3)

The positive rates of PTSD symptoms and SSI were 5.0% ($n = 1697$) and 1.3% ($n = 447$), respectively. PTSD symptoms showed statistically significant ($P<0.05$) associations with the following factors: age, occupation, marriage, working years, work in a frontline department, work in an isolation ward, COVID-19 related factors (attention to the current prevalence of COVID-19, confidence in overcoming COVID-19, COVID-19 infection in the community of residence, willingness to participate in gatherings, experience of SARS, contact with severe COVID-19 patients, contact with COVID-19 patients who died, perceived work stress during the COVID-19 remission period, need for psychological assistance before the outbreak, need for psychological assistance during the epidemic remission period, perceived support, etc.).

The highest positive rates of SSI among hospital workers were found for staff in "other positions" (1.9%), including volunteers, nursing staff, security guards, etc., followed by nurses (1.5%), doctors (1.2%), administrators (1.1%), logistics personnel (1.0%), and technical personnel (0.7%). The positive rate of SSI was the highest in 18–25-year-olds (1.7%) and the lowest in those over 50 years old (0.7%). Single or divorced people (1.9%) and women (1.4%) were more likely to have SSI. In hospitals of different levels, the positive rate of SSI in rural hospitals and nonhospitals (small clinics, village doctors, etc.) was higher than that in designated hospitals and major district hospitals. In addition, the positive rate of SSI was also relatively high for participants characterized by several COVID-19-related factors (lack of confidence in overcoming

Table 1
Sociodemographic and epidemic-related profile.

Sociographic features	Variables	n (%)	Epidemic-related factors	Variables	n (%)
Gender	Female	25,860(76.7)	Experienced SARS		4500(13.4)
	Male	7846(23.3)	Frontline department		31,169(92.5)
Age	18–25	6443(19.1)	Isolation and protection of the department	complete isolation ward	2280(6.8)
	26–30	9106(27.0)		partial isolation ward	6606(19.6)
	31–40	10,881(32.3)		general ward	24,820(73.6)
	41–50	5348(15.9)	Infected with COVID-19 in community		2980(8.8)
	>50	1928(5.7)	Family members infected with COVID-19		171(0.5)
Nationality	Han	32,581(96.7)	Direct exposure to COVID-19		2209(6.6)
	Tujia	781(2.3)	Exposure to severe COVID-19 patients		1167(3.5)
	Miao	198(0.6)	Exposure to died COVID-19 patients		486(1.4)
	Else	146(0.4)	Daily working hours during the epidemic	<=8	21,878 (65.1)
Marriage	married	23,714(70.4)		9–12	10,084 (30.0)
	Single or divorced	9992(29.6)		13–16	701 (2.1)
Educational background	undergraduate or less	32,362(96)		>16	960 (2.9)
	postgraduate or more	1332(4)	Self-rated infected possibility	none	5279(15.7)
Profession	Doctor	13,803(41.0)		low	21,903(65.0)
	Nurse	15,324(45.5)		high	6521(19.3)
	Technician	2073(6.2)	Willingness of working in COVID-19 ward		22,702 (67.4)
	Administrator	915(2.7)	Stress-feeling at work during COVID-19 remission	none	3158(9.4)
	Backoffice	404(1.2)		mild	5315(15.9)
	else	1187(3.5)		fair	18,938(56.6)
Career class	formal staff	17,467(51.8)		big	3199(9.6)
	Temporary staff	16,239(48.2)		huge	2872(8.6)
Job title	junior	23,734(70.4)	Self-rated physical and mental condition during epidemic	poor	1452(4.3)
	middle	7610(22.6)		normal	13,140(39.0)
	senior	2360(7)		good	19,114(56.7)
Employment year	<=5	10,946(32.5)	Need of psychological assistance before epidemic		3207(9.5)
	6–10	9516(28.2)	Need of psychological assistance during epidemic remission		2766(8.2)
	>10	13,233(39.3)			
Level of hospital	Designated	173(0.5)			
	main district hospital	15,053(44.7)			
	county hospital	17,443(51.8)			
	non-hospital	1037(3.1)			

COVID-19, COVID-19 infection in the community of residence, willingness to attend gatherings, need for psychological assistance before the outbreak, need for psychological assistance during the epidemic remission period, and contact with severe COVID-19 patients). COVID-19 work stress during the remission period was positively correlated with the positive rate of SSI. Among the employees with SSI, the highest proportions reported no family support (6.3% [$n = 115$]) and no colleague support (5.6% [$n = 120$]).

3.4. *Regressions on PTSD symptoms and SSI (Table 4)*

Forward logistic regression analysis showed that have less likely (the protective factors) to develop PTSD symptoms were those hospital workers who work in non-designated hospital; willing to attend gatherings; need for psychological assistance before the epidemic; single or divorced marital status; a position as a nurse or else; more than 6 years of employment ($P<0.05$).

The results showed that the risk of PTSD symptoms of hospital workers working in general wards were higher than fully isolated wards. Concern about epidemic, a lack of perceptions of support (family, colleague, hospital authority, insurance and compensation, patients or mass media support) were also prone to PTSD symptoms. PTSD symptoms were significant association with depression, anxiety and somatization symptoms level, with higher depression, anxiety and somatization symptoms severity for greater risk (OR) of developing PTSD symptoms ($P<0.05$).

Forward logistic regression analysis showed that have less likely (the protective factors) to develop SSI were male gender, those hospital workers who need for regular psychological intervention during the epidemic, and confidence in defeating COVID-19 ($P<0.05$).

The risk factors for SSI were a need for pre-epidemic psychological assistance, a need for psychological assistance during the remission period, contact with severe COVID-19 patients, single or divorced marital status, a lack of perceptions of support (family, colleague, hospital authority, patients or mass media support). PTSD symptoms were significant association with depression, anxiety and self-reported work stress during the remission period level, with higher depression, anxiety and self-reported work stress during the remission period severity for greater risk (OR) of developing SSI. Hospital workers with positive PTSD symptom screening were more likely to develop SSI ($P<0.05$).

4. Discussion

In this study, we clarified the mental health status of the participating hospital workers and verified our hypothesis by conducting an investigation with the staff of 46 hospitals in Chongqing in the late period of the COVID-19 epidemic, when there had been no new COVID-19 infections. The main findings were as follows: (1) In the late stage of the COVID-19 pandemic, the hospital staff still had widespread anxiety (24.4%), depression (35.8%), and physical symptoms (49.7%). (2) A total of 6.1% of hospital workers showed PTSD symptoms after the outbreak had ended, with work in general wards, concern about the epidemic, inadequate social support, and poor mental health (depression, anxiety and somatization symptoms) being risk factors. (3) The prevalence of SSI was 1.3%, which was lower than that 6.47%in the epidemic period, consistent with the prediction of our previous study that proved that active and effective psychological intervention was highly effective in reducing suicidal self-injury. (4) Screening positive for PTSD symptoms was at greater risk of SSI. In addition, female gender, a need for psychological assistance before the outbreak and during the remission period, exposure to severe COVID-19 patients, high self-reported stress at work during the remission period, single or divorced marital status, a lack of support, and depression and anxiety symptoms were also risk factors for SSI. Discussed in detail as follows.

Table 2
Sociodemographic and Results of the PHQ-9, GAD-7 and PHQ-15.

	Variables	Depression (PHQ-9)							Anxiety (GAD-7)						Somatization symptoms (PHQ-15)					
		No symptom	Minimal symptom	Mild symptom	Moderate symptom	Severe symptom	x^2	P	No symptom	Low symptom	Medium symptom	High symptom	x^2	P	No symptom	Low symptom	Medium symptom	High symptom	x^2	P
**Total**		21,625 (64.2%)	7951 (23.6%)	2626 (7.8%)	994 (2.9%)	510 (1.5%)			25,498 (75.6%)	6241 (18.5%)	1313 (3.9%)	654 (1.9%)			16,958 (50.3%)	8619 (25.6%)	5310 (15.8%)	1991 (5.9%)		
**Gender**	**Female**	16,027 (62.0%)	6437 (24.9%)	2174 (8.4%)	814 (3.1%)	408 (1.6%)	3270.295	0.000**	19,107 (73.9%)	5120 (19.8%)	1096 (4.2%)	537 (2.1%)	188.476	0.000**	11,887 (46.0%)	7067 (27.2%)	4488 (17.4%)	2418 (9.4%)	861.051	0.000**
	**Male**	5598 (71.3%)	1514 (19.3%)	452 (5.8%)	180 (2.3%)	102 (1.3%)			6391 (81.5%)	1121 (14.3%)	217 (2.8%)	117 (1.5%)			5071 (64.6%)	1552 (19.8%)	822 (10.5%)	401 (5.1%)		
**Age**	**18–25**	4016 (62.3%)	1600 (24.8%)	523 (8.1%)	203(3.2%)	101 (1.6%)	274.136	0.000**	4922 (76.4%)	1152 (17.9%)	265 (4.1%)	104 (1.6%)	138.178	0.000**	3187 (49.5%)	1736 (26.9%)	1012 (15.7%)	508 (7.9%)	62.892	0.000**
	**26–30**	5516 (60.6%)	2357 (25.9%)	792 (8.7%)	294(3.2%)	147 (1.6%)			6747 (74.1%)	1794 (19.7%)	377 (4.1%)	188 (2.1%)			4416 (48.5%)	2438 (26.8%)	1472 (16.2%)	780 (8.6%)		
	**31–40**	6896 (63.4%)	2649 (24.3%)	858 (7.9%)	317(2.9%)	161 (1.5%)			8023 (73.7%)	2187 (20.1%)	443 (4.1%)	228 (2.1%)			5477 (50.3%)	2754 (25.3%)	1750 (16.1%)	900 (8.3%)		
	**41–50**	3713 (69.4%)	1042 (19.5%)	377 (7.0%)	138(2.6%)	78(1.5%)			4188 (78.3%)	889 (16.6%)	169 (3.2%)	102 (1.9%)			2800 (50.3%)	1277 (23.9%)	809 (15.1%)	462 (8.6%)		
	**>50**	1484 (77%)	303 (15.7%)	76(3.9%)	42(2.2%)	23(1.2%)			1618 (83.9%)	219 (11.4%)	59(3.1%)	32(1.7%)			1078 (55.9%)	414 (21.5%)	267 (13.8%)	169 (8.8%)		
**Nationality**	**Han**	20,915 (64.2%)	7684 (23.6%)	2523 (7.7%)	963(3.0%)	496 (1.5%)	9.907	0.624	24,649 (75.7%)	6031 (18.5%)	1264 (3.9%)	637 (2.0%)	8.496	0.485	16,375 (50.3%)	8325 (25.6%)	5145 (15.8%)	2736 (8.4%)	19.744	0.020*
	**Tujia**	500 (64.0%)	183 (23.4%)	67(8.6%)	22(2.8%)	9(1.2%)			604 (77.3%)	136 (17.4%)	31(4.0%)	10(1.3%)			429 (54.9%)	192 (24.6%)	110 (14.1%)	50(6.4%)		
	**Miao**	117 (59.1%)	55 (27.8%)	20 (10.1%)	3(1.5%)	3(1.5%)			143 (72.2%)	39 (19.7%)	12(6.1%)	4(2.0%)			91 (46.0%)	61 (30.8%)	24 (12.1%)	22 (11.1%)		
	**Else**	93 (63.7%)	29 (19.9%)	16 (11.0%)	6(4.1%)	2(1.4%)			102 (69.9%)	35 (24.0%)	6(4.1%)	3(2.1%)			63 (43.2%)	41 (28.1%)	31 (21.2%)	11(7.5%)		
**Marriage**	**married**	15,648 (66.0%)	5356 (22.6%)	1731 (7.3%)	661(2.8%)	318 (1.3%)	123.381	0.000**	18,033 (76.0%)	4339 (18.3%)	890 (3.8%)	452 (1.9%)	8.369	0.039	12,097 (51.0%)	5915 (24.9%)	3700 (15.6%)	2002 (8.4%)	21.898	0.000**
	**single or divorced**	5977 (59.8%)	2595 (26.0%)	895 (9.0%)	333(3.3%)	192 (1.9%)			7465 (74.7%)	1902 (19.0%)	423 (4.2%)	202 (2.0%)			4861 (48.6%)	2704 (27.1%)	1610 (16.1%)	817 (8.2%)		
**Educational background**	**undergraduate or less**	20,783 (64.2%)	7602 (23.5%)	2531 (7.8%)	959(3.0%)	487 (1.5%)	5.599	0.231	24,504 (75.7%)	5963 (18.4%)	1273 (3.9%)	622 (1.9%)	8.578	0.035*	16,190 (50.0%)	8292 (25.6%)	5148 (15.9%)	2732 (8.4%)	31.932	0.000**
	**postgraduate or more**	833 (62.5%)	346 (26.0%)	95(7.1%)	35(2.6%)	23(1.7%)			984 (73.9%)	276 (20.7%)	40(3.0%)	32(2.4%)			762 (57.2%)	323 (24.2%)	161 (12.1%)	86(6.5%)		
**Profession**	**Doctor**	9125 (66.1%)	3131 (22.7%)	976 (7.1%)	369(2.7%)	202 (1.5%)	156.017	0.000**	10,614 (76.9%)	2451 (17.8%)	491 (3.6%)	247 (1.8%)	76.800	0.000**	7589 (55.0%)	3264 (23.6%)	1956 (14.2%)	994 (7.2%)	346.903	0.000**
	**Nurse**	9390 (61.3%)	3858 (25.2%)	1335 (8.7%)	510(3.3%)	231 (1.5%)			11,327 (73.9%)	3016 (19.7%)	671 (4.4%)	310 (2.0%)			6939 (45.3%)	4140 (27.0%)	2726 (17.8%)	1519 (9.9%)		
	**Technician**	1374 (66.3%)	474 (22.9%)	156 (7.5%)	41(2.0%)	28(1.4%)			1599 (77.1%)	376 (18.1%)	62(3.0%)	36(1.7%)			1081 (52.20%)	573 (27.6%)	299 (14.4%)	120 (5.8%)		
	**Administrator**	598 (65.4%)	213 (23.3%)	54(5.9%)	34(3.7%)	16(1.7%)			683 (74.6%)	170 (18.6%)	35(3.8%)	27(3.0%)			476 (52.0%)	245 (26.8%)	129 (14.1%)	65(7.1%)		
	**Backoffice**	304 (75.2%)	63 (15.6%)	26(6.4%)	4(1.0%)	7(1.7%)			340 (84.2%)	48 (11.9%)	11(2.7%)	5(1.2%)			233 (57.7%)	100 (24.8%)	41 (10.1%)	30(7.4%)		
	**else**	834 (70.3%)	212 (17.9%)	79(6.7%)	36(3.0%)	26(2.2%)			935 (78.8%)	180 (15.2%)	43(3.6%)	29(2.4%)			640 (53.9%)	297 (25.0%)	159 (13.4%)	91(7.7%)		
**Career class**	**formal staff**	11,180 (64.0%)	4082 (23.4%)	1383 (7.9%)	545(3.1%)	277 (1.6%)	6.488	0.166	13,121 (75.1%)	3278 (18.8%)	700 (4.0%)	368 (2.1%)	8.926	0.030*	8901 (51.0%)	4340 (24.8%)	2712 (15.5%)	1514 (8.7%)	15.662	0.001**
	**Temporary staff**	10,445 (64.3%)	3869 (23.8%)	1243 (7.7%)	449(2.8%)	233 (1.4%)			12,377 (76.2%)	2963 (18.2%)	613 (3.8%)	286 (1.8%)			8057 (49.6%)	4279 (26.4%)	2598 (16.0%)	1305 (8.0%)		
**Job title**	**junior**	15,184 (64.0%)	5626 (23.7%)	1863 (7.8%)	698(2.9%)	363 (1.5%)	11.231	0.189	18,069 (76.1%)	4303 (18.1%)	927 (3.9%)	435 (1.8%)	24.779	0.000**	11,939 (50.3%)	6148 (25.9%)	3702 (15.6%)	1945 (8.2%)	10.36	0.110
	**middle**				230(3.0%)															

(*continued on next page*)

Table 2 (*continued*)

Variables		Depression (PHQ-9)							Anxiety (GAD-7)						Somatization symptoms (PHQ-15)					
		No symptom	Minimal symptom	Mild symptom	Moderate symptom	Severe symptom	x^2	P	No symptom	Low symptom	Medium symptom	High symptom	x^2	P	No symptom	Low symptom	Medium symptom	High symptom	x^2	P
		4854 (63.8%)	1807 (23.7%)	600 (7.9%)		119 (1.6%)			5615 (73.8%)	1529 (20.1%)	292 (3.8%)	174 (2.3%)			3794 (49.9%)	1899 (25.0%)	1241 (16.3%)	676 (8.9%)		
	**senior**	1585 (67.2%)	518 (21.9%)	163 (6.9%)	66(2.8%)	28(1.2%)			1812 (76.8%)	409 (17.3%)	94(4.0%)	45(1.9%)			1223 (51.8%)	572 (24.2%)	367 (15.6%)	198 (8.4%)		
**Employment year**	**<=5**	6930 (63.3%)	2694 (24.6%)	866 (7.9%)	299(2.7%)	157 (1.4%)	91.785	0.000**	8392 (76.7%)	1966 (18.0%)	414 (3.8%)	174 (1.6%)	56.709	0.000**	5614 (51.3%)	2934 (26.8%)	1616 (14.8%)	782 (7.1%)	67.933	0.000**
	**6–10**	5830 (61.3%)	2425 (25.5%)	798 (8.4%)	313(3.3%)	150 (1.6%)			6969 (73.2%)	1970 (20.7%)	369 (3.9%)	208 (2.2%)			4605 (48.4%)	2443 (25.7%)	1617 (17.0%)	851 (8.9%)		
	**>10**	8857 (66.9%)	2829 (21.4%)	962 (7.3%)	382(2.9%)	203 (1.5%)			10,126 (76.5%)	2305 (17.4%)	530 (4.0%)	272 (2.1%)			6735 (50.9%)	3237 (24.5%)	2075 (15.7%)	1186 (9.0%)		
**Job-related stress**	**low**	10,221 (79.5%)	1927 (15.0%)	484 (3.8%)	160(1.2%)	69(0.5%)	3270.295	0.000**	11,350 (88.3%)	1208 (9.4%)	222 (1.7%)	81(0.6%)	2802.87	0.000**	8195 (63.7%)	2958 (23.0%)	1260 (9.8%)	448 (3.5%)	2638.27	0.000**
	**medium**	8441 (56.7%)	4289 (28.8%)	1440 (9.7%)	485(3.3%)	239 (1.6%)			10,458 (70.2%)	3430 (23.0%)	686 (4.6%)	320 (2.1%)			6367 (42.7%)	4159 (27.9%)	2893 (19.4%)	1475 (9.9%)		
	**high**	856 (33.5%)	846 (33.1%)	443 (17.3%)	248(9.7%)	161 (6.3%)			1180 (46.2%)	893 (35.0%)	287 (11.2%)	194 (7.6%)			723 (28.3%)	597 (23.4%)	619 (24.2%)	615 (24.1%)		

* p-value < 0.05;.
** p-value < 0.001.

4.1. *The mental health of hospital workers after the COVID-19 pandemic*

In our study, 35.8% of the sample was likely to be diagnosed with depression and 24.4% with anxiety. Surprisingly, the prevalences of depression and anxiety were higher than those in our team's study in the same area during the COVID-19 epidemic (30.2% depression and 20.7% anxiety). This may be because during the study period, public areas had been opened up, but hospitals were still the main place of contact with COVID-19 patients, and the workload was still very large. The world was still in the midst of a major outbreak, and the situation was unstable. Similarly, the incidences of depression and anxiety among hospital workers in this study were higher than those in other countries, among other health care workers and among the general population during the epidemic, with the numbers around 19% depressive and 22% anxiety (Cao et al., 2020; Gonzalez-Sanguino et al., 2020; Wang et al., 2020a, 2020b), again supporting our hypothesis. Compared to Lai et al.'s study (50.4% depression and 44.6% anxiety) on COVID-19-exposed health care workers at the peak of the epidemic in China (Lai et al., 2020) and Shechter et al. (2020) study(48% depression and 33%anxiety) at a large medical center in New York City, our study found a lower diagnosis rate, which may be because the mental health conditions of health care workers in frontline units during the epidemic were more severe than those of other health care workers. We looked at a much broader population that included all positions in hospitals, not just doctors, medical students and nurses.

Our results showed higher prevalences of anxiety, depression, somatic symptoms, insomnia, PTSD, and SSI in women than men, which is consistent with many studies conducted during epidemic outbreaks involving multiple populations, including general populations, patients, or health workers in different countries (Gonzalez-Sanguino et al., 2020; Rodriguez et al., 2021; Wang et al., 2020a).

Regarding hospital positions, a higher proportion of the nurses than doctors, technicians, administrative staff and logistics staff in this study reported anxiety, depression, somatic symptoms and PTSD. Notably, in the later stage of the outbreak, nurses were previously found to be more likely to have problems such as depression and anxiety, and in particular, the prevalence of somatic symptoms(38.7% depression,26.1% anxiety and 54.7% somatic symptoms) was found to be significantly higher than that during the outbreak(9.4% depression,8.1% anxiety and 42.7% somatic symptoms) (Hong et al., 2021).The reasons for these findings may be as follows: (1) Nurses were the first to bear the burden of epidemic prevention in hospitals. They were in close contact with infected people and their secretions and were responsible for screening and collecting nasopharyngeal swabs, leading to high exposure risk and great psychological pressure. (2) The use of protective masks and clothing during isolation and disinfection increased the difficulty of work, and energy consumption led to physical symptoms such as hypoxia, fatigue and muscle pain. (3) Due to regional characteristics and the large population base in China, the working intensity and work stress of nurses were not significantly reduced, even in stable epidemic situations. The job-related stress findings in Table 2 also show that the degree of stress was positively correlated with the three diagnosis rates, which provides a basis for this analysis.

The diagnosis rates of anxiety, depression, physical symptoms and PTSD were the highest among workers with 6 to 10 years of working experience, as well as those aged 31–40 years and those with a middle job title. We consider that people with this amount of working experience were the main participants in various positions in the hospital, had a certain level experience and working ability, and needed to participate in the most challenging work. In addition, the workers in the 31–40-year-old age group faced the greatest pressure due to family life; the care of children and elderly family members; the impact of the sharp decline in economic income in the pandemic period, which generated a greater sense of crisis; and the unique nature of their work that reduced their time spent with their families and increased their risk of suffering from emotional disorders. Younger workers, as well as older staff with more

Table 3
The association of PTSD symptoms and SSI with COVID-19 epidemic-related factors ($N = 33,706$).

Epidemic-related factors	Variables	PCL-C(PTSD symptoms)				SSI			
		Positive	Negative	x^2	P	Positive	Negative	x^2	P
**Total**		1697 (5.0%)	32,009 (95.0%)			447 (1.3%)	33,259 (98.7%)		
**Gender**	**Female**	1351 (5.2%)	24,509 (94.8%)	8.350	0.004*	373 (1.4%)	25,487 (98.6%)	11.465	0.001*
	**Male**	346 (4.4%)	7500 (95.6%)			74(0.9%)	7772 (99.1%)		
**Age**	**18–25**	339 (5.3%)	6104 (94.7%)	15.383	0.004*	108 (1.7%)	6335 (98.3%)	21.448	0.000**
	**26–30**	503 (5.5%)	8603 (94.5%)			143 (1.6%)	8963 (98.4%)		
	**31–40**	544 (5.0%)	10,337 (95.0%)			125 (1.1%)	10,756 (98.9%)		
	**41–50**	238 (4.5%)	5110 (95.5%)			58(1.1%)	5290 (98.9%)		
	**>50**	73(3.8%)	1855 (96.2%)			13(0.7%)	1915 (99.3%)		
**Marriage**	**Married**	1141 (4.8%)	22,573 (95.2%)	8.336	0.004*	261 (1.1%)	23,453 (98.9%)	31.101	0.000**
	**Single or divorce**	556 (5.6%)	9436 (94.4%)			186 (1.9%)	9806 (98.1%)		
**Profession**	**Doctor**	641 (4.6%)	13,162 (95.4%)	34.557	0.000**	161 (1.2%)	13,642 (98.8%)	18.011	0.003*
	**Nurse**	871 (5.7%)	14,453 (94.3%)			236 (1.5%)	15,088 (98.5%)		
	**Technician**	73(3.5%)	2000 (96.5%)			14(0.7%)	2059 (99.3%)		
	**Administrator**	37(4.0%)	878 (96.0%)			10(1.1%)	905 (98.9%)		
	**Backoffice**	11(2.7%)	393 (97.3%)			4(1.0%)	400 (99.0%)		
	**else**	64(5.4%)	1123 (94.6%)			22(1.9%)	1165 (98.1%)		
**Job title**	**junior**	1174 (4.9%)	22,560 (95.1%)	1.482	0.477	330 (1.4%)	23,404 (98.6%)	2.749	0.253
	**middle**	403 (5.3%)	7207 (94.7%)			87(1.1%)	7523 (98.9%)		
	**senior**	120 (5.1%)	2240 (94.9%)			30(1.3%)	2330 (98.7%)		
**Educational background**	**undergraduate or less**	1631 (5.0%)	30,731 (95.0%)	0.068	0.794	432 (1.3%)	31,930 (98.7%)	0.426	0.514
	**postgraduate or more**	65(4.9%)	1267 (95.1%)			15(1.1%)	1317 (98.9%)		
**Employment year**	**<=5**	509 (4.7%)	10,437 (95.3%)	12.725	0.002*	159 (1.5%)	10,787 (98.5%)	3.495	0.174
	**6–10**	542 (5.7%)	8974 (94.3%)			131 (1.4%)	9385 (98.6%)		
	**>10**	646 (4.9%)	12,587 (95.1%)			157 (1.2%)	13,076 (98.8%)		
**Necessary of regularly psychological intervention during the epidemic**	**No**	286 (4.6%)	5929 (95.4%)	2.908	0.088	86(1.4%)	6129 (98.6%)	0.23	0.631
	**Yes**	1408 (5.1%)	26,064 (94.9%)			359 (1.3%)	27,113 (98.7%)		
**Concern about epidemic**	**No**	155 (9.8%)	1422 (90.2%)	79.477	0.000**	58(3.7%)	1519 (96.3%)	69.89	0.000**
	**Yes**	1542 (4.8%)	30,580 (95.2%)			389 (1.2%)	31,733 (98.8%)		
**Confidence about defeating COVID19**	**No**	63 (25.4%)	185 (74.6%)	217.389	0.000**	26 (10.5%)	222 (89.5%)	160.482	0.000**
	**Yes**	1630 (4.9%)	31,813 (95.1%)			420 (1.3%)	33,023 (98.7%)		
**Level of hospital**	**Designated hospital**	4(2.3%)	169 (97.7%)	4.832	0.184	1(0.6%)	172 (99.4%)	64.548	0.000**
	**main district hospital**	741 (4.9%)	14,312 (95.1%)			117 (0.8%)	14,936 (99.2%)		
	**county hospital**	891 (5.1%)	16,552 (94.9%)			310 (1.8%)	17,133 (98.2%)		
	**non-hospital**	61(5.9%)	976 (94.1%)			19(1.8%)	1018 (98.2%)		
**Frontline department**	**No**	186 (7.3%)	2346 (92.7%)	30.659	0.000**	26(1.0%)	2506 (99.0%)	1.877	0.171
	**Yes**	1510 (4.8%)	29,659 (95.2%)			421 (1.4%)	30,748 (98.6%)		
	**complete isolation ward**			10.841	0.004*	18(0.8%)		5.393	0.067

(*continued on next page*)

Table 3 (*continued*)

Epidemic-related factors	Variables	PCL-C(PTSD symptoms)				SSI			
		Positive	Negative	x^2	P	Positive	Negative	x^2	P
Isolation and protection of the department		112 (4.9%)	2168 (95.1%)				2262 (99.2%)		
	partial isolation ward	385 (5.8%)	6221 (94.2%)			91(1.4%)	6515 (98.6%)		
	general ward	1200 (4.8%)	23,620 (95.2%)			338 (1.4%)	24,482 (98.6%)		
Infected with COVID-19 in community	no	1507 (4.9%)	29,215 (95.1%)	11.738	0.001*	389 (1.3%)	30,333 (98.7%)	8.696	0.003*
	yes	189 (6.3%)	2791 (93.7%)			57(1.9%)	2923 (98.1%)		
Willingness of participant in parties	no	1452 (4.8%)	28,757 (95.2%)	31.200	0.000**	368 (1.2%)	29,841 (98.8%)	26.099	0.000**
	yes	244 (7.0%)	3247 (93.0%)			79(2.3%)	3412 (97.7%)		
Experienced SARS	No	1431 (4.9%)	27,768 (95.1%)	7.964	0.005*	381 (1.3%)	28,818 (98.7%)	0.582	0.446
	Yes	265 (5.9%)	4235 (94.1%)			65(1.4%)	4435 (98.6%)		
Need of psychological assistance before epidemic	no	1124 (3.7%)	29,369 (96.3%)	1215.640	0.000**	267 (0.9%)	30,226 (99.1%)	492.086	0.000**
	yes	572 (17.8%)	2635 (82.2%)			179 (5.6%)	3028 (94.4%)		
Need of psychological assistance during epidemic remission	no	1107 (3.6%)	29,827 (96.4%)	1655.532	0.000**	248 (0.8%)	30,686 (99.2%)	778.4	0.000**
	yes	587 (21.2%)	2179 (78.8%)			197 (7.1%)	2569 (92.9%)		
Exposure to severe COVID-19 patients	no	1599 (4.9%)	30,924 (95.1%)	20.802	0.000**	413 (1.3%)	32,110 (98.7%)	20.931	0.000**
	yes	92(7.9%)	1075 (92.1%)			33(2.8%)	1134 (97.2%)		
Exposure to died COVID-19 patients	no	1648 (5.0%)	31,560 (95.0%)	13.611	0.000**	434 (1.3%)	32,774 (98.7%)	1.096	0.295
	yes	42(8.6%)	444 (91.4%)			9(1.9%)	477 (98.1%)		
Stress-feeling at work during COVID-19 remission	none	62(2.0%)	3096 (98.0%)	711.309	0.000**	18(0.6%)	3140 (99.4%)	188.593	0.000**
	mild	145 (2.7%)	5170 (97.3%)			38(0.7%)	5277 (99.3%)		
	fair	769 (4.1%)	18,169 (95.9%)			201 (1.1%)	18,737 (98.9%)		
	big	363 (11.3%)	2836 (88.7%)			87(2.7%)	3112 (97.3%)		
	huge	342 (11.9%)	2530 (88.1%)			100 (3.5%)	2772 (96.5%)		
Perceptions of support	No familles support	307 (16.9%)	1513 (83.1%)	563.878	0.000**	115 (6.3%)	1705 (93.7%)	367.601	0.000**
	No colleagues support	354 (16.6%)	1780 (83.4%)	637.084	0.000**	120 (5.6%)	2014 (94.4%)	322.466	0.000**
	No Hospital authority support	717 (10.9%)	5884 (89.1%)	585.161	0.000**	226 (3.4%)	6375 (96.6%)	278.551	0.000**
	No Insurance and compensation support	737 (9.8%)	6796 (90.2%)	457.919	0.000**	211 (2.8%)	7322 (97.2%)	162.104	0.000**
	No Patients support	832 (9.6%)	7861 (90.4%)	505.694	0.000**	239 (2.7%)	8454 (97.3%)	182.36	0.000**
	No Mass media support	667 (10.4%)	5740 (89.6%)	479.331	0.000**	190 (3.0%)	6217 (97.0%)	163.284	0.000**

PCL-C=The PTSD Checklist-Civilian Version;SSI = Suicidal and self-injurious ideation;.

* p-value < 0.05;.

** p-value < 0.001.

experience, had less stress and were more likely to cope with depression and anxiety. Similarly, because the age of temporary workers was mostly younger, similar conclusions can be drawn for this group.

A higher proportion of Miao and other ethnic minority workers (such as the number of workers who were Hui, Tibetan, and Buyi Hui were less than 17) than Han and Tujia workers had somatic symptoms. This finding may be because the living habits of ethnic minorities with small local populations are different from those of the Han and Tujia ethnic groups with large populations. During the epidemic prevention period, there were significant changes in lifestyle (diet, etc.), which led to physical discomfort. However, the small number of employees belonging to this group may not be representative of all minority employees.

A higher proportion of people who were single or divorced had depression, somatic symptoms, insomnia, PTSD and suicidal self-injury, which is also consistent with the results of a large number of studies. This may be because people's loneliness in a special period affects their mental health, and hospital workers with high work stress need more adequate family support at this time (Ausin et al., 2017; Ni et al., 2021).

Hospital workers with undergraduate or less in educational background were more likely to have different degrees of somatic symptoms in the postepidemic period than those with higher educational

Table 4
Risk factors associated with PTSD symptoms and SSI in 33,706 hospital workers.

PCL-C(PTSD symptoms)	Variables	B	SE	Wald	P	OR	95% CI	
Level of hospital	Designated hospital			9.270	0.026*			
	main district hospital	−1.286	0.526	5.985	0.014*	0.276	0.099	0.774
	county hospital	−1.361	0.525	6.719	0.010*	0.256	0.092	0.718
	non-hospital	−1.476	0.546	7.314	0.007*	0.229	0.078	0.666
Isolation and protection of the department	Complete isolation ward			7.253	0.027*			
	General ward	0.230	0.109	4.417	0.036*	1.259	1.016	1.560
willing to attend group gatherings during the epidemic		−0.177	0.080	4.961	0.026*	0.838	0.717	0.979
Need of psychological assistance before epidemic		−0.462	0.086	28.585	0.000**	0.630	0.532	0.746
Need of psychological assistance during epidemic remission		−0.936	0.087	115.183	0.000**	0.392	0.331	0.465
Concern about epidemic		0.323	0.106	9.342	0.002*	1.382	1.123	1.700
Marriage	Single or divorced	−0.158	0.067	5.501	0.019*	0.854	0.749	0.974
Profession	Doctor			18.445	0.002*			
	Nurse	−0.181	0.061	8.764	0.003*	0.835	0.740	0.941
	Else	−0.372	0.155	5.741	0.017*	0.689	0.508	0.934
Employment year	<=5			12.873	0.002			
	6–10	−0.256	0.077	11.033	0.001*	0.774	0.666	0.900
	>10	−0.315	0.102	9.568	0.002*	0.730	0.598	0.891
Perceptions of support	No famillеs support	0.698	0.085	66.752	0.000**	2.009	1.699	2.375
	No colleagues support	0.511	0.085	36.145	0.000**	1.667	1.411	1.969
	No Hospital authority support	0.437	0.070	39.488	0.000**	1.548	1.351	1.774
	No Insurance and compensation support	0.215	0.069	9.670	0.002*	1.24	1.083	1.420
	No Patients support	0.421	0.071	34.908	0.000**	1.523	1.325	1.751
	No Mass media support	0.344	0.070	23.990	0.000**	1.41	1.229	1.618
Depression (PHQ-9)	No symptom			145.713	0.000**			
	Minimal symptom	0.679	0.105	42.210	0.000**	1.972	1.607	2.420
	Mild symptom	1.057	0.119	78.214	0.000**	2.877	2.276	3.636
	Moderate symptom	1.339	0.136	96.309	0.000**	3.817	2.921	4.987
	Severe symptom	1.848	0.162	130.209	0.000**	6.344	4.619	8.714
Anxiety (GAD-7)	No symptom			231.069	0.000**			
	Low symptom	0.793	0.088	80.576	0.000**	2.211	1.859	2.629
	Medium symptom	1.462	0.111	172.937	0.000**	4.314	3.469	5.364
	High symptom	1.900	0.137	191.898	0.000**	6.687	5.111	8.750
Somatization symptoms (PHQ-15)	No symptom			219.789	0.000**			
	Low symptom	0.106	0.108	0.956	0.33	1.112	0.899	1.375
	Medium symptom	0.553	0.108	26.057	0.000**	1.739	1.406	2.151
	High symptom	1.260	0.112	126.772	0.000**	3.526	2.832	4.391
**SSI**	**Variables**	**B**	**SE**	**Wald**	**P**	**OR**	**95% CI**	
Gender	Male	−0.552	0.139	15.752	0.000**	0.576	0.439	0.756
Need of psychological assistance before epidemic		0.363	0.157	5.365	0.021*	1.438	1.057	1.956
Need of psychological assistance during epidemic remission		1.415	0.159	79.553	0.000**	4.117	3.016	5.618
Necessary of regularly psychological intervention during the epidemic		−0.268	0.134	4.017	0.045*	0.765	0.588	0.994
Concern about epidemic		−0.444	0.173	6.580	0.010*	0.642	0.457	0.901
Confidence about defeating COVID19		−1.374	0.279	24.181	0.000**	0.253	0.146	0.438
Exposure to severe COVID-19 patients		0.575	0.204	7.912	0.005*	1.778	1.190	2.653
Self-Stress at work during COVID-19 remission	None			29.380	0.000**			
	High	0.783	0.275	8.110	0.004*	2.187	1.276	3.748
	Very high	0.976	0.271	12.977	0.000**	2.654	1.560	4.513
Marriage	Single or divorced	0.496	0.103	23.386	0.000**	1.642	1.343	2.007
Non-frontline department		0.541	0.213	6.456	0.011*	1.718	1.132	2.608
Perceptions of support	No familes support	0.990	0.144	47.454	0.000**	2.692	2.031	3.568
	No colleagues support	0.476	0.149	10.190	0.001*	1.61	1.202	2.156
	No Hospital authority support	0.746	0.123	37.018	0.000**	2.108	1.658	2.681
	No Patients support	0.472	0.133	12.697	0.000**	1.604	1.237	2.080
	No Mass media support	0.270	0.130	4.307	0.038*	1.31	1.015	1.690
Depression (PHQ-9)	No symptom			474.022	0.000**			
	Minimal symptom	2.576	0.456	31.953	0.000**	13.140	5.380	32.097
	Mild symptom	4.650	0.450	106.563	0.000**	104.552	43.245	252.774
	Moderate symptom	5.822	0.457	162.013	0.000**	337.603	137.744	827.444
	Severe symptom	7.042	0.468	226.216	0.000**	1143.601	456.818	2862.900
Anxiety (GAD-7)	No symptom			18.650	0.000**			
	Low symptom	0.487	0.198	6.057	0.014*	1.627	1.104	2.397
	Medium symptom	0.889	0.214	17.211	0.000**	2.432	1.598	3.700
	High symptom	0.767	0.232	10.899	0.001*	2.154	1.366	3.397
Screening positive for PTSD (PCL-C) symptoms		2.883	0.099	855.050	0.000**	17.862	14.724	21.669

PCL-C=The PTSD Checklist-Civilian Version;SSI = Suicidal and self-injurious ideation;.
* p-value < 0.05;.
** p-value < 0.001.

background. On the one hand, because most nurses were undergraduate or less and professional characteristics lead to a higher proportion of somatic symptoms. On the other hand, it may be that highly educated workers have more opportunities to access the epidemic prevention information and found ways to relieve bad emotions, such as the internet, academic articles or other channels.

4.2. Presence of PTSD symptoms in hospital workers after the outbreak ended

In our PTSD screening questionnaire, 6.1% of the hospital workers were found to have PTSD symptoms after the COVID-19 epidemic, which was significantly lower than the 15.8% PTSD symptom diagnosis rate found by Clara et al. in their study of the public mental health effects of the early pandemic in Spain in 2020 (Gonzalez-Sanguino et al., 2020) and the positive rate for PTSD symptoms of more than 50% among New York health care workers in the study by Shechter et al. during the epidemic.

We believe the low diagnosis rate in this study to be due mainly to the following reasons. First, our study and the previous studies used different screening time stages. Facing this unprecedented social and health emergency, hospital staff working in the initial or peak period of the outbreak may have different results from those in the later period of the epidemic. Second, the severity of the epidemic varied in different countries and regions. We believe that the more severely affected areas and populations are, the higher the likelihood of PTSD diagnosis. Third, the low diagnosis rate was related to continuous training and improvement in clinical skills during the epidemic, as well as positive psychological interventions. The psychological interventions include caring for hospital workers, conducting psychological counseling, arranging rotation rest to relieving physical and psychological stress (The Xinhua News Agency, 2020).Fourth, our study subjects included a wider variety of hospital personnel than just frontline department employees with direct contact with infected patients. In contrast to Rossi et al. (2020) study on the psychological status of first-line and second-line workers during the outbreak , our study found that in the late period of the epidemic, the positive rate of PTSD symptoms among non-frontline workers was higher than that of frontline workers. We speculate that this was related to positive psychological intervention, which indirectly proves that psychological intervention has a significant effect on the prevention of PTSD in certain populations.

In our study, there were higher rates of PTSD diagnosis among staff in frontline units, staff in some isolation units at higher risk of infection, staff exposed to severe COVID-19 patients, and staff living in communities with infected people. This may be due to that workers exposed to high-risk environments experienced greater psychological stress than in low-risk environments. Severe anxiety, fatigue, etc. in the high-risk environments, may contribute to this result, and similar findings have been reported in studies of the H1N1 and SARS pandemics (Matsuishi et al., 2012; McAlonan et al., 2007).

In addition, we found that people who were concerned and confident about the COVID-19 outbreak and had a desire for social interaction (willingness to go to gatherings) had a lower diagnosis rate of PTSD symptoms. Encouraging hospital workers to pay attention to public health events and maintain a positive attitude can be useful in responding to outbreaks and preventing the occurrence of PTSD symptoms. The staff who needed psychological support before the outbreak had problems with their mental health during the nonepidemic period, so both they and employees who needed psychological support during the remission period had a high rate of PTSD and needed continuous psychological intervention.The diagnosis rate of PTSD symptoms was also positively correlated with perceived stress at work during the COVID-19 remission period, which was consistent with previous studies during outbreaks such as SARS (Tam et al., 2004). Since hospital workers' perceived stressors include not only income, family problems, but including high workload, isolation many other factors, were likely to associated with poor mental health status, and the greater the stress, the greater the effect.so at the later COVID-19period the proportion of PTSD symptoms will increase as the pressure increases. Regarding support, a lack of family support and a lack of colleague support were associated with higher diagnosis rates of PTSD, which may be because support through close relationships with family and long-term contact and cooperation with colleagues plays a key role in the maintenance of mental health in major social emergencies. People who had experienced SARS had a higher rate of PTSD symptoms than those who did not, which was consistent with previous studies (Maunder et al., 2006). The COVID-19 outbreak may have seemed similar or even more severe than previous SARS outbreaks, so employees who had experienced such trauma were more likely to recall painful experiences.

In the PTSD symptom risk prediction regression model, compared to the designated hospital level, main district, county, and nonhospital (small clinic, village doctors, etc.) hospital levels were protective factors against PTSD symptoms. Different from the outbreak period, after the outbreak period, with the establishment of designated hospitals, other hospitals no longer directly received patients, the staff of county hospitals and small clinics also could receive professional infectious diseases training and had adequate personal protective equipment (PPE), which became more secure. Employees who had a desire for social interaction (willingness to attend gatherings) may have been better able to cope with stress and regulate their moods, which protected them from PTSD symptoms.

Our research showed that need for psychological assistance before or after the outbreak was a protective factor against PTSD symptoms; employees with such needs may have been able to actively seek help and may have taken the initiative to respond to traumatic events after the stress stimulus. People who were single or divorced were also less likely to develop PTSD symptoms, perhaps because they may have been more able to adapt and been more focused on work. Nurses and logistics staff were less likely to suffer from PTSD symptoms than doctors. This may be due to the professional training of nurses and the daily epidemic prevention, which made the nurses more confident and safer, while doctors' work was more complex, and they needed to consider the strategy of diagnosis and treatment and the risk of exposure to infected people.

Working for 6 to 10 years or even longer was a protective factor against PTSD symptoms, perhaps because the more working experience employees had, the more effective their protection against infectious diseases and the stronger their ability to cope with stress. Logistics staff were less likely to be diagnosed with PTSD symptoms because of their low risk of exposure to infected people.

A risk factor for PTSD symptoms was work in the general ward, which may be because isolation wards and frontline departments were equipped with adequate protective equipment and the accumulation of a large amount of anti-epidemic experience among the staff. Staff who paid more attention to the outbreak were at higher risk of PTSD symptoms, which may be due to their excessive attention to the changes in traumatic events, which was an additional source of stress that may have promoted the recurrence of stress symptoms. Inadequate support (including family support, colleague support, hospital administration support, insurance and compensation support, patient support, and mass media support) was also a risk factor. Poor mental health (depression, anxiety, and somatization symptoms) was strongly associated with PTSD symptoms, and the higher the diagnostic level was, the greater the risk.

4.3. The SSI of hospital workers during the postepidemic period

Regarding SSI, to the best of our knowledge, few studies of suicidal self-injury among hospital workers during public health crises have been conducted (Naushad et al., 2019). However, our team began to pay attention to the positive diagnosis rate of SSI in this group during the outbreak of the epidemic. In this study, we found that the positive rate of SSI in the late period of the COVID-19 outbreak (1.3%) was significantly lower than that in our study during the epidemic period (6.47%) (Xu et al., 2021), which may be related to the decline of the epidemic and the active intervention and full attention of the government to the mental health of hospital staff, favorable policies, etc. (The Xinhua News Agency, 2020) When workers perceived that their own safety was guaranteed, the sense of professional honor was enhanced.

During nonpublic health crises, the 12-month and lifetime diagnosis rates of suicidal ideation among health care workers were found to be

generally higher than those in the general population (Liu et al., 2020; Petrie et al., 2020; Stelnicki et al., 2020). Two studies of suicidal ideation among nonepidemic hospital staff in China showed that 15.9% of clinicians in county-level hospitals (Nie et al., 2020) and 10.8% of nurses in third-class hospitals in one province (Wang et al., 2020b) had experienced suicidal ideation in the past one or two weeks.

The diagnosis rate of SSI in these previous studies was higher than that in our team's studies during and after the epidemic period, which we believe to be due to the following reasons. (1) The hospital staff did not have time to actively think about death as they struggled with the challenges of staffing shortages and self-protection during and after the COVID-19 outbreak. (2) The staff learned to shift the pressure. Our team found that the proportion of staff with depression and anxiety in the late stage of the outbreak was higher than that during the peak of the outbreak, while the SSI rate was lower. This may be due to the continuous work and the uncertainty of the epidemic that made staff tend to express their emotions to reflect their stress, with these negative emotions acting as a "release outlet" to prevent SSI. (3) The time of data collection was important. In our study the epidemic period, data were collected in the first three to five weeks of the pandemic spreading in China (Xu et al., 2021). In the study in the later stage of the epidemic, data were collected approximately one month after the epidemic was brought under control. The decline of local cases numbers and the control of the epidemic were the main reasons for the observed decline in the SSI diagnosis rate. Our conclusion for the period during the epidemic was similar to the findings of a study by Halford et al. (2020) that some suicide indices declined in the United States during the early phase of the COVID-19 pandemic. However, unlike their prediction that the COVID-19 pandemic might lead to a long-term increase in the suicide index and suicide rates, the prevalence of SSI in our study decreased significantly in the later period of the COVID-19 outbreak compared to the peak of the epidemic, which confirmed our team's previous prediction Xu et al. (2021). (4) The hospital staff had professional values related to devoting themselves to saving lives. The employees had internalized these values during the nonpandemic period, and they acted as a psychological defense during the crisis. Therefore, we hypothesize that the prevalence of SSI may not return to the high levels in the population that continues to receive active mental health intervention, and we will continue to focus on relevant studies in the future.

In the SSI prediction model, the need for regular psychological intervention during the epidemic, attention to the epidemic, and confidence in defeating COVID-19 were protective factors against SSI. For women, psychological assistance needs before and during the remission period, contact with severe COVID-19 patients, and high self-reported work stress during the remission period were positively correlated with the severity of symptoms. Single or divorced marital status, a lack of support (including support from family members, colleagues, hospital administration, patients and the mass media) were risk factors for SSI, and depression and anxiety were positively correlated with SSI. Screening positive for PTSD was an independent risk factor for SSI (OR=17.862), which may have certain significance for mental health intervention strategies in the late stage of the global epidemic.

4.4. Implications for attention to the mental health of hospital workers

With increasing global access to the COVID-19 vaccine, the COVID-19 epidemic will reach the remission period.The remission period of the outbreak in China was relatively early. After the closure of Wuhan was lifted on April 8, 2020, the closure orders for all places were gradually lifted, and major industries such as education, commerce and tourism gradually recovered. The prevalence of SSI at the late stage of COVID-19 was lower than outbreak, indicating that positive and effective psychological interventions had a certain effect (Halford et al., 2020; Xu et al., 2021). But the hospital staff still had widespread anxiety, depression, and physical symptoms in the area during this period, some even had PTSD symptoms (Xiaoming et al., 2020). Therefore, psychological interventions for hospital staff should continue into the latter stages of the outbreak or beyond, and should be given attention to specific groups such as female gender, exposure to severe COVID-19 patients, high self-reported stress at work during the remission period, single or divorced marital status, a lack of support, symptoms of depression and anxiety, and those with PTSD symptoms. These measures will be important in maintaining the mental health of hospital staff.

4.5. Limitations

To our knowledge, this is the first study with a large sample on the mental state of hospital workers in Chongqing, China, following the COVID-19 outbreak, specifically including screening for SSI and PTSD symptoms. However, there are some limitations of this study. First, this cross-sectional study could not reveal causality, and voluntary participation may have led to selection bias. Second, our study was conducted at a later stage of the epidemic, and longer longitudinal studies are still needed to determine the long-term psychological effects of COVID-19 on hospital workers. Third, the participants were recruited from hospitals in the Chongqing area and were not fully representative of all hospital workers in China. Fourth, related issues of SSI were evaluated in combination with item 9 in PHQ-9, which has certain limitations. More surveys may be needed for further discussion.

In conclusion, in public health incidents, the mental health of hospital staff should be continuously monitored, and timely intervention should be given to women, those lacking social support, and those in need of psychological assistance to reduce the incidence of PTSD symptoms and SSI in these groups.

Funding

This study is supported by Chongqing Medical University as a special project on emergency clinical research on novel Coronavirus pneumonia (general project): (1) psychological intervention for first-line medical personnel in the novel Coronavirus pneumonia epidemic (grant numbers: 203,011,620,200,011); and (2) a psychological intervention model of negative emotion and behavior in hospital workers during the COVID-19 epidemic (grant numbers: 203,011,620,200,071).

CRediT authorship contribution statement

Wang Lixia: Writing – original draft, Writing – review & editing, Resources, Data curation, Formal analysis. **Xu Xiaoming:** Visualization, Resources, Data curation, Formal analysis. **Shi Lei:** Resources, Data curation, Formal analysis. **Hong Su:** Resources, Data curation, Formal analysis. **Wang Wo:** Visualization. **Fang Xin:** . **Chen Jianmei:** Visualization. **Zhang Qi:** Resources. **Ai Ming:** Visualization, Writing – original draft, Writing – review & editing, Data curation, Formal analysis, Validation. **Kuang Li:** Visualization, Writing – original draft, Writing – review & editing, Data curation, Formal analysis, Validation.

Declaration of competing interest

The authors declare that there is no conflict of interest.

Acknowledgments

Thank you to the COVID-19 warriors, including hospital staff in all positions and the staff who were involved in the investigation despite being busy in their work, for the contributions they made to protect human health. We also express our gratitude to the Chongqing Municipal Health Commission for its support.

References

Ausin, B., Munoz, M., Castellanos, M.A., 2017. Loneliness, sociodemographic and mental health variables in Spanish adults over 65 years old. Span. J. Psychol. 20 https://doi.org/10.1017/sjp.2017.48.

Brooks, S.K., Rubin, G.J., Greenberg, N., 2019. Traumatic stress within disaster-exposed occupations: overview of the literature and suggestions for the management of traumatic stress in the workplace. Br. Med. Bull. 129, 25–34. https://doi.org/10.1093/bmb/ldy040.

Brooks, S.K., Webster, R.K., Smith, L.E., Woodland, L., Wessely, S., Greenberg, N., Rubin, G.J., 2020. The psychological impact of quarantine and how to reduce it: rapid review of the evidence. Lancet 395, 912–920. https://doi.org/10.1016/s0140-6736(20)30460-8.

Bai, Y., Lin, C.C., Lin, C.Y., Chen, J.Y., Chue, C.M., Chou, P., 2004. Survey of stress reactions among health care workers involved with the SARS outbreak. Psychiatr. Serv. 55 (9), 1055–1057. https://doi.org/10.1176/appi.ps.55.9.1055.

Cheng, S.K.W., Wong, C.W., Tsangand, J., Wong, K.C., 2004. Psychological distress and negative appraisals in survivors of severe acute respiratory syndrome (SARS). Psychol. Med. 34, 1187–1195. https://doi.org/10.1017/S0033291704002272.

Cao, W., Fang, Z., Hou, G., Han, M., Xu, X., Dong, J., Zheng, J., 2020. The psychological impact of the COVID-19 epidemic on college students in China. Psychiatry Res. 287 https://doi.org/10.1016/j.psychres.2020.112934.

Dong, J., Ying-Jie, J., Xue-Zhang, D., Wen-Gang, L., Zhi-Qiang, S., Xue-Ai, S., Yu-Hua, M., Hong-Mei, T., Fang, C., Xiao-Xia, N., GuoFeng, C., Jin, L., Hui-Juan, D., 2017. Prevalence of psychological symptoms among Ebola survivors and healthcare workers during the 2014. Oncotarget 8 (8), 12784–12791.

Gonzalez-Sanguino, C., Ausin, B., Castellanos, M.A., Saiz, J., Lopez-Gomez, A., Ugidos, C., Munoz, M., 2020. Mental health consequences during the initial stage of the 2020 Coronavirus pandemic (COVID-19) in Spain. Brain Behav. Immun. 87, 172–176. https://doi.org/10.1016/j.bbi.2020.05.040.

Halford, E.A., Lake, A.M., Gould, M.S., 2020. Google searches for suicide and suicide risk factors in the early stages of the COVID-19 pandemic. PLoS ONE 15. https://doi.org/10.1371/journal.pone.0236777.

He, X.Y., Li, C.B., Qian, J., Cui, H.S., Wu, W.Y., 2010. Reliability and validity of a generalized anxiety disorder scale in general hospital outpatient. Shanghai Arch. Psychiatry 22, 200–203.

Hong, S., Ai, M., Xu, X., Wang, W., Chen, J., Zhang, Q., Wang, L., Kuang, L., 2021. Immediate psychological impact on nurses working at 42 government-designated hospitals during COVID-19 outbreak in China: a cross-sectional study. Nurs. Outlook 69, 6–12. https://doi.org/10.1016/j.outlook.2020.07.007.

Hu, M.J., He, X.Y ., Li, F., Liu, Z.G., Wu, Y ., Chen, Y ., Sun, M., Yan, H., Li, C.B., 2009. Prevalence of depressive syndrome and quality of life in out-patients of neurology department in general hospitals. Shanghai Archives of Psychiatry 21, 156–159.

Kroenke, K., Spitzer, R.L., Williams, J.B., 2002. The PHQ-15: validity of a new measure for evaluating the severity of somatic symptoms. Psychosom. Med. 64, 258–266. https://doi.org/10.1097/00006842-200203000-00008.

Lee, A.M., Wong, J.G., McAlonan, G.M., Cheung, V., Cheung, C., Sham, P.C., Chua, S.E., 2007. Stress and psychological distress among SARS survivors 1 year after the outbreak. Can. J. Psychiatry 52 (4), 233–240. https://doi.org/10.1177/070674370705200405.

Lancee, W.J., Maunder, R.G., Goldbloom, D.S., 2008. Prevalence of psychiatric disorders among toronto hospital workers one to two years after the SARS outbreak. Psychiatr. Serv. 59 (1), 91–95. https://doi.org/10.1176/ps.2008.59.1.91.

Lai, J., Ma, S., Wang, Y., Cai, Z., Hu, J., Wei, N., Wu, J., Du, H., Chen, T., Li, R., Tan, H., Kang, L., Yao, L., Huang, M., Wang, H., Wang, G., Liu, Z., Hu, S., 2020. Factors associated with mental health outcomes among health care workers exposed to Coronavirus disease 2019. JAMA Netw. Open 3, e203976. https://doi.org/10.1001/jamanetworkopen.2020.3976.

Liu, R.T., Bettis, A.H., Burke, T.A., 2020. Characterizing the phenomenology of passive suicidal ideation: a systematic review and meta-analysis of its prevalence, psychiatric comorbidity, correlates, and comparisons with active suicidal ideation. Psychol. Med. 50, 367–383. https://doi.org/10.1017/s003329171900391x.

Mihashi, M., Otsubo, Y., Yinjuan, X., Nagatomi, K., Hoshiko, M., Ishitake, T., 2009. Predictive factors of psychological disorder development during recovery following SARS outbreak. Health Psychol. 28 (1), 91–100. https://doi.org/10.1037/a0013674.

Maunder, R.G., Lancee, W.J., Balderson, K.E., Bennett, J.P., Borgundvaag, B., Evans, S., Fernandes, C.M.B., Goldbloom, D.S., Gupta, M., Hunter, J.J., Hall, L.M., Nagle, L.M., Pain, C., Peczeniuk, S.S., Raymond, G., Read, N., Rourke, S.B., Steinberg, R.J., Stewart, T.E., VanDeVelde-Coke, S., Veldhorst, G.G., Wasylenki, D.A., 2006. Long-term psychological and occupational effects of providing hospital healthcare during SARS outbreak. Emerg. Infect. Dis. 12, 1924–1932.

Mrklas, K., Shalaby, R., Hrabok, M., Gusnowski, A., Vuong, W., Surood, S., Urichuk, L., Li, D., Li, X.M., Greenshaw, A.J., Agyapong, V.I.O., 2020. Prevalence of perceived stress, anxiety, depression, and obsessive-compulsive symptoms in health care workers and other workers in alberta during the COVID-19 pandemic: cross-sectional survey. JMIR Ment. Health 7, e22408. https://doi.org/10.2196/22408.

Matsuishi, K., Kawazoe, A., Imai, H., Ito, A., Mouri, K., Kitamura, N., Mita, T., 2012. Psychological impact of the pandemic (H1N1) 2009 on general hospital workers in Kobe. Psychiatry Clin. Neurosci. https://doi.org/10.1111/j.1440-1819.2012.02336.x.

McAlonan, G.M., Lee, A.M., Cheung, V., Cheung, C., Tsang, K.W.T., Sham, P.C., Wong, J.G.W.S., 2007. Immediate and sustained psychological impact of an emerging infectious disease outbreak on health care workers. Can. J. Psychiatry. https://doi.org/10.1177/070674370705200406.

National Health Commission of China, 2020. COVID-19 prevention and control. An update on the COVID-19 Outbreak. Available from http://www.nhc.gov.cn/xcs/yqtb/202005/874765e641254eb4acea9d5e945f4e01.shtml. (Accessed May 28, 2020).

The Xinhua News Agency, 2020. CCTV news. White Paper on China's Action Against COVID-19. Available from https://baijiahao.baidu.com/s?id=1668820877085136810&wfr=spider&for=pc. (Accessed June 7, 2020).

Naushad, V.A., Bierens, J.J.L.M., Nishan, K.P., Firjeeth, C.P., Mohammad, O.H., Maliyakkal, A.M., Hadan, S.C., Schreiber, M.D., 2019. A systematic review of the impact of disaster on the mental health of medical responders. Prehosp. Disaster Med. 34, 632–643. https://doi.org/10.1017/s1049023x19004874.

Ni, J., Wang, F., Liu, Y., Wu, M., Jiang, Y., Zhou, Y., Sha, D., 2021. Psychological impact of the COVID-19 pandemic on chinese health care workers: cross-sectional survey study. JMIR Ment. Health 8, e23125. https://doi.org/10.2196/23125.

Nie, G., Du, J., Liu, J., Yuan, L., Ma, Z., 2020. Job stress and suicidal ideation among Chinese clinicians: the moderating role of social support. J. Gen. Psychol. 147, 109–122. https://doi.org/10.1080/00221309.2019.1640657.

Petrie, K., Crawford, J., LaMontagne, A.D., Milner, A., Dean, J., Veness, B.G., Christensen, H., Harvey, S.B., 2020. Working hours, common mental disorder and suicidal ideation among junior doctors in Australia: a cross-sectional survey. BMJ Open 10. https://doi.org/10.1136/bmjopen-2019-033525.

Qian, J., Ren, Z.Q., Yu, D.H., He, X.Y., LI, C.B., 2014. The value of the Patient Health Questionnaire-15 (PHQ- 15) for screening somatic symptoms in general hospital. Chin. Ment. Health J. 28 (03), 173–178.

Rodriguez, R.M., Montoy, J.C.C., Hoth, K.F., Talan, D.A., Harland, K.K., Eyck, P.T., Mower, W., Krishnadasan, A., Santibanez, S., Mohr, N., Project, C.E.D.N., 2021. Symptoms of anxiety, burnout, and PTSD and the mitigation effect of serologic testing in emergency department personnel during the COVID-19 pandemic. Ann. Emerg. Med. https://doi.org/10.1016/j.annemergmed.2021.01.028.

Rossi, R., Socci, V., Pacitti, F., Mensi, S., Di Marco, A., Siracusano, A., Di Lorenzo, G., 2020. Mental Health Outcomes Among Healthcare Workers and the General Population During the COVID-19 in Italy. Front. Psychol. 11, 608986 https://doi.org/10.3389/fpsyg.2020.608986.

Shechter, A., Diaz, F., Moise, N., Anstey, D.E., Ye, S., Agarwal, S., Birk, J.L., Brodie, D., Cannone, D.E., Chang, B., Claassen, J., Cornelius, T., Derby, L., Dong, M., Givens, R. C., Hochman, B., Homma, S., Kronish, I.M., Lee, S.A.J., Manzano, W., Mayer, L.E.S., McMurry, C.L., Moitra, V., Pham, P., Rabbani, L., Rivera, R.R., Schwartz, A., Schwartz, J.E., Shapiro, P.A., Shaw, K., Sullivan, A.M., Vose, C., Wasson, L., Edmondson, D., Abdalla, M., 2020. Psychological distress, coping behaviors, and preferences for support among New York healthcare workers during the COVID-19 pandemic. Gen. Hosp. Psychiatry 66, 1–8. https://doi.org/10.1016/j.genhosppsych.2020.06.007.

Spitzer, R.L., Kroenke, K., Williams, J.B., 1999. Validation and utility of a self-report version of PRIME-MD: the PHQ primary care study. Primary care evaluation of mental disorders. Patient health questionnaire. JAMA 282, 1737–1744. https://doi.org/10.1001/jama.282.18.1737.

Spitzer, R.L., Kroenke, K., Williams, J.B., Löwe, B., 2006. A brief measure for assessing generalized anxiety disorder: the GAD-7. Arch. Intern. Med. 166, 1092–1097. https://doi.org/10.1001/archinte.166.10.1092.

Stelnicki, A.M., Jamshidi, L., Angehrn, A., Nicholas Carleton, R., 2020. Suicidal behaviors among nurses in Canada. Can. J. Nurs. Res. 52, 226–236. https://doi.org/10.1177/0844562120934237.

Tam, C.W., Pang, E.P., Lam, L.C., Chiu, H.F., 2004. Severe acute respiratory syndrome (SARS) in Hong Kong in 2003: stress and psychological impact among frontline healthcare workers. Psychol. Med. 34, 1197–1204. https://doi.org/10.1017/s0033291704002247.

Wu, P., Fang, Y., Guan, Z., Fan, B., Kong, J., Yao, Z., Hoven, C.W., 2009. The psychological impact of the SARS epidemic on hospital employees in China: exposure, risk perception, and altruistic acceptance of risk. Can. J. Psychiatry 54 (5), 302–311. https://doi.org/10.1177/070674370905400504.

World Health Organization, 2021. Coronavirus (COVID-19) data. The latest data on the COVID-19 global outbreak. Available from https://www.who.int/data#reports. (Accessed June 10, 2021).

Wang, C., Pan, R., Wan, X., Tan, Y., Xu, L., Ho, C.S., Ho, R.C., 2020a. Immediate psychological responses and associated factors during the initial stage of the 2019 Coronavirus disease (COVID-19) epidemic among the general population in China. Int. J. Environ. Res. Public Health 17. https://doi.org/10.3390/ijerph17051729.

Wang, J., Zhang, X., Yang, B., Li, J., Li, Y., Chen, Q., Wu, L., Cao, F., 2020b. Suicidal ideation among nurses: unique and cumulative effects of different subtypes of sleep problems. J. Affect. Disord. 276, 600–607. https://doi.org/10.1016/j.jad.2020.07.095.

Wang, W., Bian, Q., Zhao, Y., Li, X., Wang, W., Du, J., Zhang, G., Zhou, Q., Zhao, M., 2014. Reliability and validity of the Chinese version of the Patient Health Questionnaire (PHQ-9) in the general population. Gen. Hosp. Psychiatry 36, 539–544. https://doi.org/10.1016/j.genhosppsych.2014.05.021.

Wang, Y., Duan, Z., Peng, K., Li, D., Ou, J., Wilson, A., Wang, N., Si, L., Chen, R., 2021. Acute stress disorder among frontline health professionals during the COVID-19 outbreak: a structural equation modeling investigation. Psychosom. Med. 83, 373–379. https://doi.org/10.1097/PSY.0000000000000851.

Williamson, V., Stevelink, S.A.M., Greenberg, N., 2018. Occupational moral injury and mental health: systematic review and meta-analysis. Br. J. Psychiatry 212, 339–346. https://doi.org/10.1192/bjp.2018.55.

Xiaoming, X., Ming, A., Su, H., Wo, W., Jianmei, C., Qi, Z., Hua, H., Xuemei, L., Lixia, W., Jun, C., Lei, S., Zhen, L., Lian, D., Jing, L., Handan, Y., Haitang, Q., Xiaoting, H., Xiaorong, C., Ran, C., Qinghua, L., Xinyu, Z., Jian, T., Jing, T., Guanghua, J., Zhiqin, H., Nkundimana, B., Li, K., 2020. The psychological status of 8817 hospital workers during COVID-19 epidemic: a cross-sectional study in Chongqing. J. Affect. Disord. 276, 555–561. https://doi.org/10.1016/j.jad.2020.07.092.

Xu, X., Wang, W., Chen, J., Ai, M., Shi, L., Wang, L., Hong, S., Zhang, Q., Hu, H., Li, X., Cao, J., Lv, Z., Du, L., Li, J., Yang, H., He, X., Chen, X., Chen, R., Luo, Q., Zhou, X., Tan, J., Tu, J., Jiang, G., Han, Z., Kuang, L., 2021. Suicidal and self-harm ideation among Chinese hospital staff during the COVID-19 pandemic: prevalence and correlates. Psychiatry Res. 296, 113654 https://doi.org/10.1016/j.psychres.2020.113654.

Zhang, L., Fritzsche, K., Liu, Y., Wang, J., Huang, M., Wang, Y., Chen, L., Luo, S., Yu, J., Dong, Z., Mo, L., Leonhart, R., 2016. Validation of the Chinese version of the PHQ-15 in a tertiary hospital. BMC Psychiatry 16, 89. https://doi.org/10.1186/s12888-016-0798-5.

Zhang, M.Y., He, Y.L., et al., 2015. Manual of Psychiatric Rating Scales. Science and Technology Press, Hunan, China, pp. 224–227.
