# Supplementary material for: Mental health status and related factors influencing healthcare workers during the COVID-19 pandemic: A systematic review and meta-analysis
Source: PLoS One. 2024 Jan 19;19(1):e0289454. doi: 10.1371/journal.pone.0289454 (PMC10798549; doi:10.1371/journal.pone.0289454)
Supplement: S1 Data — (ZIP) [file pone.0289454.s011.zip › literatures/81.pdf]

ELSEVIER

Since January 2020 Elsevier has created a COVID-19 resource centre with free information in English and Mandarin on the novel coronavirus COVID-19. The COVID-19 resource centre is hosted on Elsevier Connect, the company's public news and information website.

Elsevier hereby grants permission to make all its COVID-19-related research that is available on the COVID-19 resource centre - including this research content - immediately available in PubMed Central and other publicly funded repositories, such as the WHO COVID database with rights for unrestricted research re-use and analyses in any form or by any means with acknowledgement of the original source. These permissions are granted for free by Elsevier for as long as the COVID-19 resource centre remains active.

Psychiatry Research 288 (2020) 112936

Contents lists available at ScienceDirect

Psychiatry Research

journal homepage: www.elsevier.com/locate/psychres

# Psychological status of medical workforce during the COVID-19 pandemic: A cross-sectional study

Wen Lu[a,*,1], Hang Wang[b,1], Yuxing Lin[c], Li Li[a,*]

[a] Department of healthcare and disease control and prevention, Fujian Provincial Hospital, Fuzhou, Fujian, China
[b] Department of healthcare and disease control and prevention, Fujian Provincial Hospital South Branch, Fuzhou, Fujian, China
[c] Department of health examination center, Fujian Provincial Hospital, Fuzhou, Fujian, China

ARTICLE INFO

*Keywords:*
Coronavirus pneumonia
Medical staff
Fear
Anxiety
Depression

ABSTRACT

The pandemic of 2019 coronavirus disease (COVID-19) has burdened an unprecedented psychological stress on people around the world, especially the medical workforce. The study focuses on assess the psychological status of them. The authors conducted a single-center, cross-sectional survey via online questionnaires. Occurrence of fear, anxiety and depression were measured by the numeric rating scale (NRS) on fear, Hamilton Anxiety Scale (HAMA), and Hamilton Depression Scale (HAMD), respectively. A total of 2299 eligible participants were enrolled from the authors' institution, including 2042 medical staff and 257 administrative staff. The severity of fear, anxiety and depression were significantly different between two groups. Furthermore, as compared to the non-clinical staff, front line medical staff with close contact with infected patients, including working in the departments of respiratory, emergency, infectious disease, and ICU, showed higher scores on fear scale, HAMA and HAMD, and they were 1.4 times more likely to feel fear, twice more likely to suffer anxiety and depression. The medical staff especially working in above-mentioned departments made them more susceptible to psychological disorders. Effective strategies toward to improving the mental health should be provided to these individuals.

## Introduction

In December 2019, an outbreak of a novel coronavirus pneumonia occurred in Wuhan City, China, and spread throughout the whole of country in a short period(Carlos et al., 2020; Du Toit, 2020; Huang et al., 2020). The novel coronavirus was officially named 'SARS-CoV-2′ by the International Committee on Taxonomy of Viruses, and disease infected by this virus was termed 'COVID-19′(Zu et al., 2020). Since the rapid spread of this epidemic disease, the government of China has quickly issued a public announcement on the prevention and treatment of the most serious infectious disease, which required that determine efficacious and straightforward measures to prevent disease transmission. However, coronavirus pneumonia patients were found in almost all provinces across our country in the short term. There was no doubt that medical workforce played an indispensable role in this major public health emergency.

As generally known, this pandemic was more contagious than SARS and brought challenge and threaten to global public health security (Li et al., 2020; Nishiura et al., 2020; Phelan et al., 2020). As a general provincial hospital in South China, our institution undertook a considerable number of investigations and diagnosis of suspected patients. Hospital staff were exposed to stress both physical and psychological in response to this serious infectious public health event (Chen et al., 2020a; Phelan et al., 2020; Zhang et al., 2020b). Initially a comparison was conducted across hospital staff in due to the fact that the potential for work-related accumulated in declining mental health of them (Mulfinger et al., 2019), yet had not been examined during the epidemic of major infectious diseases. Besides, a study demonstrated that 42.0% of doctors working in Tertiary Hospitals in mainland China experienced very high levels of accumulated fatigue (Tang and Liu, 2019). Especially during SARS-CoV-2 outbreak, the heavier workload and life-threatening medical workers were facing aggravated the psychological pressure, even mental illness. Follow-up data suggested that hospital workers particularly doctor and nurse were more susceptible to psychological disorders after participating in the treatment of SARS patients over a decade ago (Verma et al., 2004). In particular, increasing number of confirmed and suspected cases were verified in many countries outside China. Therefore, it is extremely important to realize

* Corresponding author.
*E-mail addresses:* luwen67@sina.com (W. Lu), lilifuzhou@126.com (L. Li).
[1] Wen Lu and Hang Wang contributed equally to this work.

https://doi.org/10.1016/j.psychres.2020.112936
Received 18 March 2020; Accepted 21 March 2020
Available online 04 April 2020
0165-1781/ © 2020 Elsevier B.V. All rights reserved.

the psychological status of the medical workforce.

## 2. Methods

### 2.1. Participants

Medical workforce from Fujian Provincial Hospital fighting against SARS-CoV-2 have participated in epidemic prevention and control work for a month since the government launched the first-level response to major public health emergencies on Jan 24 2020. A questionnaire survey personal assessment of fear, anxiety and depression was conducted for them. In addition, gender, age, working years, education, marriage status, and fertility status were also collected. Complete questionnaires finished within two days from Feb 25 2020 to Feb 26 2020 were recognized as eligible and included in the following analysis. Incomplete questionnaires and participants with a history of psychological or cognitive disorder were excluded. The present study was approved by the local Ethics Committee of the Fujian Provincial Hospital and written informed consent was obtained from all subjects.

### 2.2. Assessment of fear

The numeric rating scale (NRS) has been reported to have good reliability, validity and sensitivity(Becker et al., 2020), which is used to measure the level of fear in the study. The degree of fear is reflected using 0–10 point, with higher score indicating greater fear. Details of the scale is as follows: 0 for no fear, 1–3 for mild fear, 4–6 for moderate fear, 7–9 for severe fear, 10 for extreme fear and psychological anxiety.

### 2.3. Questionnaire measurement of anxiety and depression

Hamilton Anxiety Scale (HAMA) and Hamilton Depression Scale (HAMD) have been wildly used to assess the appearance of anxiety and depression (Mozen-Zadeh et al., 2020; Zimmerman et al., 2020). HAMA contains 14 questions, and HAMD contains 17. Each question includes 5 items. Responses are scored as 0 (never), 1 (mild), 2 (moderate), 3 (severe), or 4 (extremely serious). Overall, the total score of HAMA is operationally categorized as follows: no anxiety (score 0–6), mild and moderate anxiety (score 7–13), severe anxiety (score $\geq$ 14). The total score of HAMD can be classified into normal (score 0–6), mild and moderate (score 7–23), severe depression (score $\geq$ 24). Various of previous studies had shown that these questionnaires could assess psychological condition with satisfactory reliability and validity (Chen et al., 2020b; Zhang et al., 2020a).

### 2.4. Statistical analyses

The data were analyzed via Statistical Package for the Social Sciences (SPSS, version 22.0, Chicago, IL) software. Qualitative variables were described by frequency distribution, while quantitative variables were described by the mean and standard deviation. The two-tailed Chi-square test and rank-sum test were employed to compare the distribution of qualitative and quantitative variables, respectively. Multivariate analyses for fear, anxiety and depression were performed using the ordinal logistic regression model. Statistical significance was evaluated as $p < 0.05$ for all tests.

## 3. Results

### 3.1. Participants' characteristics

In this cross-sectional survey, we retrieved a total of 2423 questionnaires. Of which, 105 questionnaires were excluded for the irrational completion time, and 19 incomplete questionnaires also eliminated. The remaining 2299 questionnaires were completed eligibly, giving an overall response rate of 94.88%. The respondents were comprised of 2042 medical staff (doctors and nurses) and 257 administrative staff (including the logistics). The details of demographic characteristics were presented in Table 1. Large proportion of female respondents was both found in the medical staff group (77.9%) and the administrative staff group (75.5%). The leading age-band was 31–40 years old, accounting approximately 40% in both groups. Additionally, 70.8% of the participants came from the Eastern of Fujian province. In comparison with the administrative staff group, the medical staff group presented with a higher duration of education ($p < 0.001$). There was no significant difference in working-age, marriage and fertility status between groups (all $p > 0.05$).

**Table. 1**
Baseline characteristics of 2299 enrolled participants in the study.

| Variables | Medical staff ($n = 2042$) | Administrative staff ($n = 257$) | $\chi^2$ | P value |
|---|---|---|---|---|
| Gender | | | 0.775 | 0.379 |
| Male | 451 (22.1) | 63 (24.5) | | |
| Female | 1591 (77.9) | 194 (75.5) | | |
| Age (years) | | | 11.182 | 0.011 |
| <30 | 791 (38.7) | 73 (28.4) | | |
| 31–40 | 810 (39.7) | 119 (46.3) | | |
| 41–50 | 303 (14.8) | 48 (18.7) | | |
| >50 | 138 (6.8) | 17 (6.6) | | |
| Working years | | | 4.575 | 0.206 |
| <5 | 575 (28.2) | 74 (28.8) | | |
| 5–10 | 683 (33.4) | 100 (38.9) | | |
| 11–20 | 380 (18.6) | 43 (16.7) | | |
| >20 | 404 (19.8) | 40 (15.6) | | |
| Provincial location | | | 7.861 | 0.049 |
| Eastern | 1431 (70.1) | 196 (76.3) | | |
| Southern | 170 (8.3) | 24 (9.3) | | |
| Northwestern | 262 (12.8) | 19 (7.4) | | |
| Others[a] | 179 (8.8) | 18 (7.0) | | |
| Education | | | 23.044 | < 0.001 |
| Below university | 596 (29.2) | 98 (38.1) | | |
| College | 984 (48.2) | 132 (51.4) | | |
| Master | 388 (19) | 25 (9.7) | | |
| Doctor | 74 (3.6) | 2 (0.8) | | |
| Marriage | | | 0.255 | 0.929 |
| Single | 554 (27.1) | 69 (26.8) | | |
| Married | 1454 (71.2) | 183 (71.2) | | |
| Others[b] | 34 (1.7) | 5 (1.9) | | |
| Fertility | | | 0.341 | 0.559 |
| One or more children | 1321 (64.7) | 171 (66.5) | | |
| No child | 721 (35.3) | 86 (33.5) | | |

[a] other provinces. [b] including divorced, separate and widowed

### 3.2. Comparisons of neuropsychological features

As shown in Fig. 1 and Table 2, the proportion of medical staff group on moderate and severe fear was higher than that in the administrative staff group (70.6% VS 58.4%). Moreover, 22.6% of medical staff showed mild to moderate anxiety and 2.9% were severe, the corresponding proportions of administrative staff were 17.1% and 2.9%. The different everity of fear ($p < 0.001$) and anxiety ($p = 0.049$) between two groups were significant. In addition, 11.8% of the medical staff presented with mild to moderate depression, and 0.3% with severe depression. As compared to the administrative staff group, there was no significant difference in severity of depression in medical staff group ($p = 0.191$). We made a further analysis of the factors that facilitate them feeling worried, pressured, or frustrated. As expected, several factors contributed to the expansion of psychological pressure including working in the isolation ward ($p < 0.001$), worrying about being infected ($p < 0.001$), shortage of the protective equipment ($p < 0.001$), the epidemic would never be controlled ($p = 0.002$), frustrated with unsatisfactory results on work ($p < 0.001$), and feeling lonely with being isolated from loved ($p = 0.005$). (Table 2)

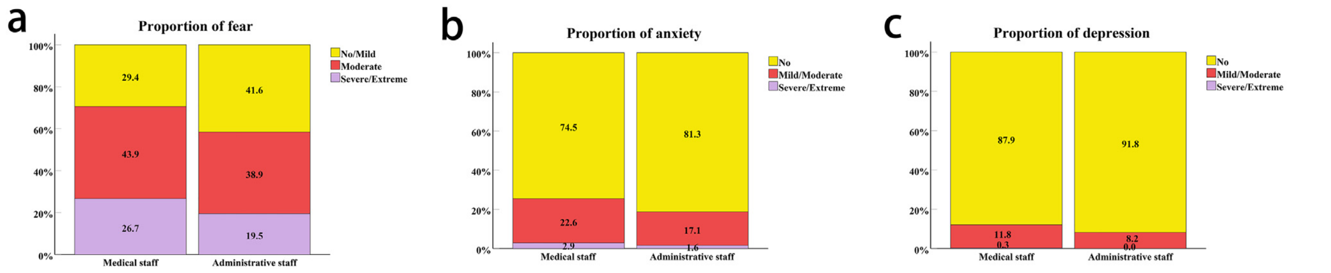


**Fig. 1.** Comparisons of neuropsychological feature between groups. a-c. the proportion of fear, anxiety and depression in each group of subjects. Colors indicate the different severities neuropsychological status.

### 3.3. *Average distribution of fear, anxiety and depression*

To explore the psychological status of medical workforce after the occurrence of coronavirus pneumonia, we investigated the mean of fear, anxiety and depression among these individuals using questionnaires. As shown in Table 3, the score of fear scale was significantly enhanced as compared to the administrative staff group ($p < 0.001$). Similarly, the levels of HAMA and HAMD were both increased when compared with the administrative staff group ($p = 0.015$ and $p = 0.029$, respectively).

### 3.4. *Comparison of psychological status based on working department*

To provide a better understanding of the results, further research was necessary including engaging in different departments to study the psychological stress, fear, anxiety and depression level. SARS-CoV-2 was a highly contagious respiratory virus and could be transmitted easily by droplets (Jiang et al., 2020a). We further divided all the participants into three subgroups according to the possibility to contact with coronavirus pneumonia patients of their departments: high-risk contact (working in department of respiratory, emergency, ICU and infectious disease), low-risk contact (working in the other clinical

**Table. 3**
Comparison the average level of fear, anxiety and depression between medical staff and administrative staff.

| Variables | Medical staff ($n = 2042$) | Administrative staff ($n = 257$) | $P$ value[a] |
|---|---|---|---|
| Fear scale | 4.89 ± 2.389 | 4.19 ± 2.384 | < 0.001 |
| HAMA | 4.73 ± 6.291 | 3.67 ± 5.072 | 0.015 |
| HAMD | 2.41 ± 3.979 | 1.86 ± 3.277 | 0.029 |

[a] P value for two independent samples Mann–Whitney U tests.

**Table. 2**
The different severity of fear, anxiety, depression among 2299 enrolled participants in the study.

| Variables | Medical staff ($n = 2042$) | Administrative staff ($n = 257$) | $\chi^2$ | $P$ value |
|---|---|---|---|---|
| Fear scale | | | 16.953 | < 0.001 |
| 0–3 (no/mild) | 601 (29.4) | 107 (41.6) | | |
| 4–6 (moderate) | 896 (43.9) | 100 (38.9) | | |
| 7–10 (severe/extreme) | 545 (26.7) | 50 (19.5) | | |
| HAMA | | | 6.040 | 0.049 |
| 0–6 (no) | 1521 (74.5) | 209 (81.3) | | |
| 7–13 (mild/moderate) | 462 (22.6) | 44 (17.1) | | |
| ≥14 (severe/extreme) | 59 (2.9) | 4 (1.6) | | |
| HAMD | | | 3.137 | 0.191 |
| 0–6 (no) | 1795 (87.9) | 236 (91.8) | | |
| 7–23 (mild/moderate) | 241 (11.8) | 21 (8.2) | | |
| ≥24 (severe/extreme) | 6 (0.3) | 0 | | |
| Have worked in the isolation wards | | | 23.012 | < 0.001 |
| Yes | 213 (10.4) | 3 (1.2) | | |
| No | 1829 (89.6) | 254 (98.8) | | |
| Days of working in the isolation wards | | | 30.456 | < 0.001 |
| 0 | 1829 (89.6) | 254 (98.8) | | |
| 1–10 | 36 (1.8) | 0 | | |
| ≥10 | 177 (8.7) | 3 (1.2) | | |
| Worried about being infected | | | 2.809 | 0.094 |
| Yes | 536 (26.2) | 55 (21.4) | | |
| No | 1506 (73.8) | 202 (78.6) | | |
| Worried about exposed to the cases with asymptomatic infection | | | 50.325 | < 0.001 |
| Yes | 1372 (67.2) | 115 (44.7) | | |
| No | 670 (32.8) | 142 (55.3) | | |
| Worried about lack of protective equipment | | | 44.519 | < 0.001 |
| Yes | 1254 (61.4) | 102 (39.7) | | |
| No | 788 (38.6) | 155 (60.3) | | |
| Worried about the epidemic would never be controlled | | | 9.745 | 0.002 |
| Yes | 1368 (67) | 147 (57.2) | | |
| No | 674 (33) | 110 (42.8) | | |
| Frustrated with unsatisfactory results on work | | | 12.748 | < 0.001 |
| Yes | 191 (9.4) | 7 (2.7) | | |
| No | 1851 (90.6) | 250 (97.3) | | |
| Feel lonely with being isolated from your loved | | | 7.891 | 0.005 |
| Yes | 449 (22) | 37 (14.4) | | |
| No | 1593 (78) | 220 (85.6) | | |

**Table. 4**
Comparison of fear, anxiety and depression among different departments.

| Variables | High-risk contact ($n$ = 469) | Low-risk contact ($n$ = 1629) | Non-clinical ($n$ = 201) | P value [a] |
|---|---|---|---|---|
| Fear scale[b] | 4.96 ± 2.424 | 4.81 ± 2.391 | 4.40 ± 2.356 | 0.027 |
| HAMA[c] | 5.64 ± 7.330 | 4.44 ± 5.896 | 3.65 ± 5.071 | 0.003 |
| HAMD[d] | 2.97 ± 4.989 | 2.24 ± 3.615 | 1.76 ± 3.107 | 0.007 |

[a] P value for independent samples Kruskal-Wallis H tests. [b] high-risk contact versus low-risk contact, $p$ = 1.0, high-risk contact versus non-clinical, $p$ = 0.024, low-risk contact versus non-clinical, $p$ = 0.053.

[c] high-risk contact versus low-risk contact, $p$ = 0.026, high-risk contact versus non-clinical, $p$ = 0.005, low-risk contact versus non-clinical, $p$ = 0.279.

[d] high-risk contact versus low-risk contact, $p$ = 0.090, high-risk contact versus non-clinical, $p$ = 0.007, low-risk contact versus non-clinical, $p$ = 0.173.

**Table. 5**
Multivariate analysis of fear, anxiety and depression among different departments.

| Variable | HR (95% CI) | P value |
|---|---|---|
| Multivariate model with the fear scale[a] | | |
| Non-clinical | 1 | |
| Low-risk contact | 1.301 (0.986 ~ 1.716) | 0.063 |
| High-risk contact | 1.408 (1.025 ~ 1.933) | 0.034 |
| Multivariate model with the HAMA[a] | | |
| Non-clinical | 1 | |
| Low-risk contact | 1.306 (0.888 ~ 1.922) | 0.175 |
| High-risk contact | 2.062 (1.349 ~ 3.153) | 0.001 |
| Multivariate model with the HAMD[a] | | |
| Non-clinical | 1 | |
| Low-risk contact | 1.394 (0.798 ~ 2.433) | 0.243 |
| High-risk contact | 2.016 (1.102 ~ 3.685) | 0.023 |

[a] Gender, Working years, Native place, Fertility status, Days of working in the isolation ward were included as covariates in ordinal logistic regression model.

departments), and non-clinical (working in administrative, technical operation). As shown in Table 4, there were significant differences in fear ($p$ = 0.027), anxiety ($p$ = 0.003) and depression ($p$ = 0.007) levels among three subgroups. Additionally, comparisons of three subgroups with each other, the staff working in the departments with high-risk contact with patients exhibited significantly greater fear ($p$ = 0.024), anxiety ($p$ = 0.005) and depression ($p$ = 0.007) than those non-clinical staff, and obviously greater anxiety ($p$ = 0.026) than the low-risk contact staff. In final multivariate analysis (Table 5), high-risk contact subgroup staff were 1.4 times as likely to feel fear (*OR*, 1.408; 95% *CI*, 1.025 - 1.933), twice as likely to suffer anxiety (*OR*, 2.062; 95% *CI*, 1.349 - 3.153) and depression (*OR*, 2.016; 95% *CI*, 1.102 - 3.685) than non-clinical subgroup staff.

## 4. Discussion

To our knowledge, the number of studies in this aspect was limited and few had explored the psychological status between medical staff and administrative staff during SARS-CoV-2 pandemic. From a data analysis perspective, comparing the average values of fear, anxiety and depression between two groups, medical staff unfolded greater fear, anxiety and depression than administrative staff. The further analysis presented that the medical staff working in those departments close contacted with coronavirus pneumonia patients, such as respiratory department, emergency department, intensive care unit, and infectious diseases department, revealed more psychological disorders, and had almost twice risk for suffering anxiety and depression, compared to the non-clinical staff with hardly possibility to contact with coronavirus pneumonia patients.

What made them uneasy? Be universally known, SARS-CoV-2 highly infectious and spreads rapidly, front line health workers were bearing significantly increased workload. Directly contacting with confirmed patients, the shortage of protective equipment, suspected patients concealing medical history, all of these could increase the risk of being infected for them. Besides, they were afraid of bringing the virus to families and incapability when facing with critical patients. The greater number of these hurdles that they experienced, the greater likelihood that they felt incapable of reaching their aspirations. The resulting strain may then, in turn, be internalized and create anxiety and depression (Liu et al., 2019; Tempest et al., 2017). As we know, after the outbreak of SARS-CoV-2 in Wuhan, medical workforce took pains to struggle with disease in the front line and protected health of the public (Wang et al., 2020; Xiao, 2020). These specific situations posed considerable stress on them, which might lead to high levels of psychological distress. Our discussion was consistent with studies regarding epidemic of SARS and MERS (Lee et al., 2018; Verma et al., 2004). The Chinese government has initiated strategies to emphasis the control of transmission (Kickbusch and Leung, 2020; Lee et al., 2018; Tang et al., 2020), and issued numbers of documents calling for attention to the mental and physical health of medical staff, even offered a series of supports and encouragements, such as provided a place for rest with food and supplies, replenished the protective equipment, medical team reinforcements, and strengthened security forces to maintain the order of medical treatment (Chen et al., 2020a; Qing et al., 2020; Zeng and Zhen, 2020). Then, for every hospital, it was important to help deal with coping strain and reduce the risk of suffering anxiety and depression of medical staff. Therefore, a human-oriented culture and paying more attention to the mental health of medical staff should be promoted for the future advancement of a hospital in China. In regard to the psychological problems of them, the comprehensive psychological consultation organization had established, even regularly do well on mental health management for medical staff for a long time (Friedman et al., 2020; Jiang et al., 2020b; Swerdlow and Finelli, 2020). For those who suffered from post-traumatic stress disorder (PTSD) (Mak et al., 2010), we could track the follow-up conditions and order proper treatment.

With the global spread of COVID-19, the challenge for many countries who are now dealing with large clusters or community transmission is obvious. Our research can provide support and reference for other countries to implement psychological intervention for medical staff as soon as possible.

There still exist several limitations in the present study. One limitation in the present study was that all medical workers were from one general hospital, so caution should be practiced in generalizing the results to all medical staff in China. Secondly, the design limits the cause analysis about psychological strains. Future research will still need to potentially include longitudinal tracking of the factors, and the inclusion of the effect evaluation after therapeutic intervention.

## 5. Conclusion

The current study found support that the medical staff unfolded greater fear, anxiety and depression than the administrative staff. Moreover, the front line medical staff working in department of respiratory, emergency, ICU and infectious disease, were twice more likely to suffer anxiety and depression than the non-clinical staff with hardly possibility to contact with coronavirus pneumonia patients. Effective strategies toward to improving the mental health should be provided to these individuals.

## Declaration of Competing Interest

The authors declare that there are no conflicts of interest.

## Acknowledgment

This work was supported by the Startup Fund for scientific research, Fujian Medical University (grant number 2018QH1154).

## Supplementary materials

Supplementary material associated with this article can be found, in the online version, at doi:10.1016/j.psychres.2020.112936.

## References

Becker, S., Fuchs, X., Schakib-Ekbatan, K., 2020. What does "moderate pain" mean? Subgroups holding different conceptions of rating scales evaluate experimental pain differently. 24 (3), 625–638.
Carlos, W.G., Dela Cruz, C.S., Cao, B., Pasnick, S, Jamil, S, 2020. Novel Wuhan (2019-nCoV) coronavirus. Am. J. Respir. Crit. Care Med. 201 (4), P7–p8.
Chen, Q., Liang, M., Li, Y., Guo, J., Fei, D., Wang, L., He, L., Sheng, C., Cai, Y., Li, X., Wang, J., Zhang, Z., 2020a. Mental health care for medical staff in China during the COVID-19 outbreak. Lancet Psychiatry.
Chen, Y., Liu, J., Li, Z., Liu, B., Ji, Y., Ju, Y., Fang, H., Zheng, Q., Wang, M., Guo, W., Li, H., Lu, X., Li, L., 2020b. The tendency of modified electroconvulsive therapy-related working memory and subjective memory deficits in depression: a prospective follow-up study. J. Ect.
Du Toit, A., 2020. Outbreak of a novel coronavirus. Nat. Rev. Microbiol. 18 (3), 123.
Friedman, L.E., Gelaye, B., Sanchez, S.E., Williams, M.A., 2020. Association of social support and antepartum depression among pregnant women. J. Affect Disord. 264, 201–205.
Huang, C., Wang, Y., Li, X., Ren, L., Zhao, J., Hu, Y., Zhang, L., Fan, G., Xu, J., Gu, X., Cheng, Z., Yu, T., Xia, J., Wei, Y., Wu, W., Xie, X., Yin, W., Li, H., Liu, M., Xiao, Y., Gao, H., Guo, L., Xie, J., Wang, G., Jiang, R., Gao, Z., Jin, Q., Wang, J., Cao, B., 2020. Clinical features of patients infected with 2019 novel coronavirus in Wuhan, China. Lancet 395 (10223), 497–506.
Jiang, F., Deng, L., Zhang, L., Cai, Y., Cheung, C.W., Xia, Z., 2020a. Review of the clinical characteristics of coronavirus disease 2019 (COVID-19). J. Gen. Intern Med.
Jiang, X., Deng, L., Zhu, Y., Ji, H., Tao, L., Liu, L., Yang, D., Ji, W., 2020b. Psychological crisis intervention during the outbreak period of new coronavirus pneumonia from experience in Shanghai. Psychiatry Res. 286, 112903.
Kickbusch, I., Leung, G., 2020. Response to the emerging novel coronavirus outbreak. BMJ 368, m406.
Lee, S.M., Kang, W.S., Cho, A.R., Kim, T., Park, J.K., 2018. Psychological impact of the 2015 MERS outbreak on hospital workers and quarantined hemodialysis patients. Compr. Psychiatry 87, 123–127.
Li, Q., Guan, X., Wu, P., Wang, X., Zhou, L., Tong, Y., Ren, R., Leung, K.S.M., Lau, E.H.Y., Wong, J.Y., Xing, X., Xiang, N., Wu, Y., Li, C., Chen, Q., Li, D., Liu, T., Zhao, J., Li, M., Tu, W., Chen, C., Jin, L., Yang, R., Wang, Q., Zhou, S., Wang, R., Liu, H., Luo, Y., Liu, Y., Shao, G., Li, H., Tao, Z., Yang, Y., Deng, Z., Liu, B., Ma, Z., Zhang, Y., Shi, G., Lam, T.T.Y., Wu, J.T.K., Gao, G.F., Cowling, B.J., 2020. Early transmission dynamics in Wuhan, China, of novel coronavirus-infected pneumonia.
Liu, Y., Zhang, J., Hennessy, D.A., Zhao, S., Ji, H., 2019. Psychological strains, depressive symptoms, and suicidal ideation among medical and non-medical staff in urban china. J. Affect Disord. 245, 22–27.
Mak, I.W., Chu, C.M., Pan, P.C., Yiu, M.G., Ho, S.C., Chan, V.L., 2010. Risk factors for chronic post-traumatic stress disorder (PTSD) in SARS survivors. Gen. Hosp. Psychiatry 32 (6), 590–598.
Mozen-Zadeh, E., Bayanati, S., Ziafat, K., Rezaei, F., Mesgarpour, B., Akhondzadeh, S., 2020. Vortioxetine as adjunctive therapy to risperidone for treatment of patients with chronic schizophrenia: a randomised, double-blind, placebo-controlled clinical trial. 269881120909416.
Mulfinger, N., Sander, A., Stuber, F., Brinster, R., Junne, F., Limprecht, R., Jarczok, M.N., Seifried-Dubon, T., Rieger, M.A., Zipfel, S., Peters, M., Stiawa, M., Maatouk, I., Helass, M., Nikendei, C., Rothermund, E., Hander, N., Ziegenhain, U., Gulde, M., Genrich, M., Worringer, B., Kullenberg, J., Blum, K., Suss, S., Gesang, E., Ruhle, S., Muller, A., Schweitzer-Rothers, J., Angerer, P., Gundel, H., 2019. Cluster-randomised trial evaluating a complex intervention to improve mental health and well-being of employees working in hospital - a protocol for the SEEGEN trial. BMC Public Health 19 (1), 1694.
Nishiura, H., Jung, S.M., Linton, N.M., Kinoshita, R., Yang, Y., Hayashi, K., Kobayashi, T., Yuan, B., Akhmetzhanov, A.R., 2020. The extent of transmission of novel coronavirus in Wuhan, China, 2020. J. Clin. Med. 9 (2).
Phelan, A.L., Katz, R., Gostin, L.O., 2020. The novel coronavirus originating in Wuhan. China: Chall. Glob. Health Gov Jama.
Qing, M.X., Li, S., Yu, S., Ouyang, Y., Zeng, L., Li, X., Li, H., 2020. Emergency management of the prevention and control of novel coronavirus pneumonia in specialized branches of hospital. Acad. Emerg. Med.
Swerdlow, D.L., Finelli, L., 2020. Preparation for possible sustained transmission of 2019 novel coronavirus: lessons from previous epidemics. Jama.
Tang, C., Liu, C., 2019. Work-Related accumulated fatigue among doctors in tertiary hospitals: a cross-sectional survey in six provinces of China. 16 (17).
Tang, J.W., Tambyah, P.A., Hui, D.S.C., 2020. Emergence of a novel coronavirus causing respiratory illness from Wuhan, China. J. Infect. 80 (3), 350–371.
Tempest, E.L., Carter, B., Beck, C.R., Rubin, G.J., 2017. Secondary stressors are associated with probable psychological morbidity after flooding: a cross-sectional analysis. Eur. J. Public Health 27 (6), 1042–1047.
Verma, S., Mythily, S., Chan, Y.H., Deslypere, J.P., Teo, E.K., Chong, S.A., 2004. Post-SARS psychological morbidity and stigma among general practitioners and traditional Chinese medicine practitioners in Singapore. Ann. Acad. Med. Singap. 33 (6), 743–748.
Wang, C., Pan, R., Wan, X., Tan, Y., Xu, L., Ho, C.S., 2020. Immediate psychological responses and associated factors during the initial stage of the 2019 coronavirus disease (COVID-19) epidemic among the general population in China. 17 (5).
Xiao, C., 2020. A novel approach of consultation on 2019 novel coronavirus (COVID-19)-Related psychological and mental problems: structured letter therapy. Psychiatry Investig. 17 (2), 175–176.
Zeng, Y., Zhen, Y., 2020. Chinese medical staff request international medical assistance in fighting against COVID-19. Lancet Glob Health.
Zhang, B., Cui, C., Yu, H., Li, G., 2020a. Association between ZNF184 and symptoms of Parkinson's disease in southern Chinese.
Zhang, S., Diao, M., Yu, W., Pei, L., Lin, Z., Chen, D., 2020b. Estimation of the reproductive number of novel coronavirus (COVID-19) and the probable outbreak size on the diamond princess cruise ship: a data-driven analysis. Int. J. Infect. Dis.
Zimmerman, M., Thompson, J.S., Diehl, J.M., Balling, C., Kiefer, R., 2020. Is the DSM-5 anxious distress specifier interview a valid measure of anxiety in patients with generalized anxiety disorder: a comparison to the hamilton anxiety scale. Psychiatry Res. 286, 112859.
Zu, Z.Y., Jiang, M.D., Xu, P.P., Chen, W., Ni, Q.Q., 2020. Coronavirus disease 2019 (COVID-19): a perspective from China. 200490.
