# Supplementary material for: Mental health status and related factors influencing healthcare workers during the COVID-19 pandemic: A systematic review and meta-analysis
Source: PLoS One. 2024 Jan 19;19(1):e0289454. doi: 10.1371/journal.pone.0289454 (PMC10798549; doi:10.1371/journal.pone.0289454)
Supplement: S1 Data — (ZIP) [file pone.0289454.s011.zip › literatures/82.pdf]

International Journal of
Environmental Research and Public Health

MDPI

Article

Symptoms of Posttraumatic Stress, Anxiety, Depression, Levels of Resilience and Burnout in Spanish Health Personnel during the COVID-19 Pandemic

Lourdes Luceño-Moreno [1,*], Beatriz Talavera-Velasco [2], Yolanda García-Albuerne [1] and Jesús Martín-García [1]

1 Department of Social and Work Psychology and Individual Differences, Faculty of Psychology, Complutense University of Madrid, 28223 Madrid, Spain; yolaga01@ucm.es (Y.G.-A.); jemartin@ucm.es (J.M.-G.)
2 Department of Education, Faculty of Languages and Education, Nebrija University, 28015 Madrid, Spain; btalavera@nebrija.es
* Correspondence: lluceno@psi.ucm.es; Tel.: +34-91-934-3174

Received: 4 July 2020; Accepted: 28 July 2020; Published: 30 July 2020

check for updates

Abstract: The number of health workers infected with COVID-19 in Spain is one of the highest in the world. The aim of this study is to analyse posttraumatic stress, anxiety and depression during the COVID-19 pandemic. Associations between burnout, resilience, demographic, work and COVID-19 variables are analysed. Cross-sectional data on 1422 health workers were analysed. A total of 56.6% of health workers present symptoms of posttraumatic stress disorder, 58.6% anxiety disorder, 46% depressive disorder and 41.1% feel emotionally drained. The profile of a health worker with greater posttraumatic stress symptoms would be a person who works in the Autonomous Community of Madrid, in a hospital, is a woman, is concerned that a person he/she lives with may be infected, and thinks that he/she is very likely to be infected. The risk variables for anxiety and depression would be a person that is a woman, working 12- or 24-h shifts, and being worried that a family member could be infected. High scores on emotional exhaustion and depersonalization are risk factors for mental health, with resilience and personal fulfilment being protective variables. Data are provided to improve preventive measures for occupational health workers.

Keywords: posttraumatic stress disorder; anxiety; depression; burnout; resilience; health personnel; COVID-19

1. Introduction

The World Health Organization declared the COVID-19 outbreak as a pandemic on March 11, 2020. In Europe, Italy and Spain were the first to report a high number of deaths, as well as a rapid increase in admissions to Intensive Care Units (ICU) of patients with symptoms associated with the disease. In May 2020, Spain is one of the top five countries with the highest number of people infected, registering over 242,707 cases as of 12 June 2020, and more than 27,136 deaths [1]. In critical pandemic-related situations, research indicates that individuals experience a stress response associated with their fear of contracting the virus from contact with other people or objects. They also have symptoms of posttraumatic stress, such as intrusive thoughts, insomnia or nightmares [2]. During the epidemic of Severe Acute Respiratory Syndrome (hereinafter, SARS), a high prevalence of symptoms of posttraumatic stress, anxiety and depression was identified in emergency service professionals including hyperarousal, anger, loss of motivation at work, difficulty concentrating or trouble falling

asleep [3]. However, not all individuals exposed to high negative impacts or crisis situations develop such symptoms, with resilience being relevant as a protective factor [4,5]. Resilience, the individual's ability to deal with adversities as challenges, has been shown to reduce the impact of traumatic events, decreasing the likelihood of developing posttraumatic stress disorders [6]. Resilience can be understood as a process of positive adaptation to a stressful situation, in which an interaction between personal resources and the environment is established [7]. Resilience varies from person to person and depends on several factors, such as personality or interpersonal and social backgrounds. The strategies to cope with the current pandemic that have been identified are optimism, social support, staying actualized, avoiding information overload and maintaining online communication [8]. In healthcare personnel, a key factor for promoting resilience is to increase the sense of control over the adverse situation. For example, perceiving that disease prevention measures can be managed or controlling the possibility of protecting oneself with the resources that health care providers have around them to care for infected patients are some of the strategies that have been adopted in this pandemic [9]. In similar critical situations, such as the Severe Acute Respiratory Syndrome (SARS) epidemic, nurses who have shown higher levels of confidence in infection protection and control equipment have shown lower levels of anxiety, negative mood and emotional fatigue [10]. Individuals with high levels of resilience have less irritability, less concern for environmental stimuli, better interpersonal relationships, fewer headaches and musculoskeletal pains, and lower levels of depression [11]. If these symptoms persist over time, the feeling of a lack of control and uncertainty at work may increase, leading to burnout. This syndrome is related to work. It is characterized by high emotional exhaustion, high levels of depersonalization and low personal accomplishment [12]. The person may experience dysphoric symptoms, such as tiredness or emotional exhaustion. The symptoms appear in relation to work situations in individuals who previously did not show psychosocial alterations. In addition, burnout is associated with a decrease in work performance due to negative behaviors towards work [13]. In particular, in health workers exposed to traumatic situations during this pandemic, the presence of burnout has been detected, as well as a reduction in the ability to apply coping strategies or negative attitudes towards work. In addition to the symptoms of exhaustion, related to anxiety, depression or other symptoms related to physical pathologies (e.g., cardiovascular problems), burnout can lead to intention to leave the post, which would cause high costs [14].

The speed with which the disease has spread, as well as the state of confinement, has led some researchers to analyse psychological variables resulting from the situation. For example, in a recent study of the general Spanish population, in which 3480 people participated, more than 20% were found to suffer from anxiety, 18.7% revealed symptoms associated with depression, and approximately 16% suffered from posttraumatic stress. In addition, female gender was associated with greater symptomatology in anxiety, depression and posttraumatic stress, while being in the older age group was related to fewer symptoms [15]. This data are similar to those obtained in a survey carried out in the general population in China, in which 1210 people participated, of which 16.5% reported moderate to severe depressive symptoms and 28.8% moderate to severe anxiety [16]. In other European countries, such as Italy, the general population has shown high levels of anxiety, depression and stress, highlighting a higher prevalence in women, people with negative affect and individuals who had family members infected or had to work away from home [17].

However, despite the state of confinement, certain professional groups, as in the case of health care personnel, have performed their jobs under great stress for weeks. These professionals, together with security forces, funeral staff and others, have been highly exposed to the virus and situations with high emotional impact. They are thus more likely to suffer mental problems, especially in the first three months in which symptoms of posttraumatic stress, affective disorders, burnout or others may increase [18]. Spain leads the ranking of the number of health professionals infected with COVID-19 during their work. More than 40,000 health workers have tested positive for COVID-19 [19]. The critical situation requires the study of the psychological state of health professionals, and the potential harm to mental health caused by their exposure during the pandemic. A recent study on stress in 958

health workers from the city of Wuhan indicates that more than half had symptoms related to anxiety and depression. Specifically, 54% of the total sample experienced symptoms of anxiety, and 58% of depression, with the prevalence of stress being higher than that previously detected in health professionals who had to deal with the SARS virus [20]. In a study involving 1257 health workers from China, of which 760 were from Wuhan, 71.5% also showed symptoms of distress, 44.6% of anxiety, 50.4% of depression and 34% of insomnia. These symptoms were more severe in nursing staff, front-line professionals and those who worked at the epicentre of the COVID-19 outbreak (Wuhan) [21]. Similar results have been found in other European countries such as Germany, where health workers, specifically nurses, have reported high levels of stress, emotional fatigue and depressive symptoms [22]. The impact of the situation on health workers may even produce symptoms of psychotic disorder, even when this is not evident in their clinical history [23].

Additionally, the stress generated by the possibility of being infected with the disease also adds to the rest of the stressful conditions of these professionals. During the influenza A (H1N1) pandemic, health workers were reported to be twice as likely to be infected through contact with patients [24]. Working on the front line with infected people increases the likelihood of becoming infected, especially in this group [25]. Health professionals must work in extreme conditions, in situations where resources can be scarce. For example, they must take care of a high number of patients in disaster or epidemic situations, often without sufficient beds or staff [26]. In addition to their jobs in hospitals, Primary Care or Intensive Care Units, they also work in nursing homes, where the disease has had a major impact in Spain. Some authors indicate that risk factors for infection may include: (a) factors related to organization, such as the rapid development of new tasks and procedures, a shortage of protective material, frequent equipment changes or the high risk of increased demand for care by other different pathologies, in addition to COVID-19; (b) watching patients die alone; (c) fear of infecting loved ones or having to practice social distancing for an indefinite period to protect them; and (d) prioritization of care for certain patients [27].

On the basis of the above, health professionals must deal with possible psychological, work-related consequences during the COVID-19 crisis, such as posttraumatic stress, anxiety, depression or burnout [28].The aim of this study is to assess the symptoms of posttraumatic stress, anxiety, depression, levels of burnout and resilience in the Spanish health workers during the COVID-19 pandemic. It also aims to evaluate the relationship between each of the variables (demographic, work, COVID-19, burnout and resilience) and the symptoms of posttraumatic stress, depression and anxiety. It is equally intended to identify which variables have the most weight in each of the three categories (posttraumatic stress, anxiety and depression).

The main hypotheses of this study would be the following: (a) health care workers evaluated will have high levels of post-traumatic stress, anxiety, depression and burnout; (b) resilience factor will be associated with lower burnout and with symptoms related to the above three categories; (c) the female gender will be associated with symptoms of the three categories; (d) older health care personnel would have fewer symptoms; (d) health care workers in contact with other infected patients, who are highly likely to become infected and have fewer resources or protective equipment, will have more symptoms of post-traumatic stress disorder, anxiety, depression and burnout.

2. Materials and Methods

2.1. Participants

The sample includes 1539 subjects, recruited by non-probabilistic sampling. As criteria of exclusivity, the participants had to be in contact with patients of COVID-19. Finally, 117 were eliminated because they were not health personnel in contact with these patients. The sample of the study was made up of 1228 women (86.4%) and 194 men (13.6%). The mean age was 43.88 (SD = 10.82, ranging between 19 and 68).

2.2. Measurement Variables and Instruments

The following instruments were used:

Demographic, job-related and variables specific to COVID-19. Due to the importance of understanding how the disease affects these professionals, the researchers of this study collected information related to demographic variables, associated with the job, changes of residence, possible contact with people during work, COVID-19 tests, hospitalization, isolation, protective equipment, concern over becoming infected, concern that a family member and/or someone with whom they are living may be infected.

Posttraumatic stress: The Impact of Event Scale-Revised (IES-R) was used [29]. This scale was used to assess the emotional distress that accompanies a stressful life event. It is made up of 22 items distributed in three scales: intrusion (7 items, an example of this scale would be "I thought about it even when I did not want to"); avoidance (8 items, an example is "I tried not to think about the event"); and hyperarousal (7 items, a sample item is "I was easily startled and scared"). In relation to posttraumatic stress, a score of 20 was considered as the cut-off point. A total score greater than or equal to 20 on the IES-R is associated with a diagnosis of psychiatric disorder and a mean score less than or equal to 14 is associated with a non-diagnosis of a psychiatric disorder [30]. It shows adequate psychometric properties in its Spanish adaptation, confirming the solution of the three factors mentioned and a reliability greater than 0.70 in all subscales.

Anxiety and depression: The Spanish adaptation of the Hospital Anxiety and Depression Scale (HADS) instrument was used [31,32]. Consisting of 14 items that correspond to two subscales: anxiety and depression, with 7 items each, on a Likert 0–3 response scale. An example of an item in the anxiety scale is "I feel tense and nervous" and "I feel slow and awkward" in the depression scale. It evaluates symptoms of anxiety and depression in patients and in the general population. The cut-off values are between 7 and 13 possible or probable presence of a mood disorder, and greater than 14–15 for severe disorder (the range ranges from 0 to 21) for both anxiety and depression scales. Accordingly, to analyse the prevalence of symptoms in this study, the variables have been categorized as follows: < 6.99 no disorder, 7–13.99 possible or probable, > 14 severe disorder. The higher the score, the greater the prevalence of symptoms of anxiety and depression. In its Spanish adaptation, it has shown adequate psychometric properties, confirming the validity of two factors and an internal consistency of 0.77 in anxiety and 0.71 in the depression subscale [32].

Burnout: The Spanish adaptation of the Maslach Burnout Inventory-MBI-HSS instrument was applied, which assesses Burnout Syndrome [33,34]. It consists of 22 items of seven response options on a Likert scale from 0 (never) to 6 (every day). The cut-off points for health personnel in the Spanish sample were used to analyse the prevalence of the different components of burnout in this study: emotional fatigue (low < 22, medium 22–23, high > 31); depersonalization (low < 7, medium 7–13, high > 16); and personal accomplishment (low < 30, medium 30–35, high > 35). It presents adequate psychometric characteristics, showing an appropriate fit for the three-factor solution and an internal consistency greater than 0.71 in all subscales [35,36]. An example of an item in the emotional exhaustion scale is "I feel emotionally drained for my work", "I think I treat some people like impersonal objects" in the depersonalization scale, and "I easily understand how people feel" in the personal accomplishment scale.

Resilience: The Spanish adaptation of the Brief Resilience Scale (BRS) was used [37,38]. It evaluates the resilience construct, understood as the subject's ability to deal with environmental obstacles and recover from stressful circumstances. It is made up of 6 items that are answered on a Likert scale ranging from 1 (strongly disagree) to 5 (strongly agree). The higher the score, the greater the degree of resilience the person shows to deal with adversities. An example of an item in this scale is "I tend to recover quickly after going through difficult times". The Spanish adaptation presents adequate psychometric properties, corroborating the single-factor solution and an internal consistency of 0.83.

2.3. Procedure

The approval of the Deontological Committee of the Faculty of Psychology of the Complutense University of Madrid (ref. Pr_2019_038; 01/04/2020) was obtained before beginning the study. Researchers contacted both the coordinators and trade unions of health centres to inform them of this study. Due to lockdown, data were collected by means of an online survey, from 1 to 30 of April 2020, which included the instruments described above. Before starting the survey, participants had to give their informed consent in order to continue. Informed consent included the purpose of the study, those responsible for it and information on the confidentiality of the data, anonymity and the legal clause on personal data protection. Before completing the survey submission, participants were required to respond to all items. The completion time for all items was approximately 15 min.

2.4. Data Analysis

The analyses were carried out with the SPSS 26 statistical package. The proportion of cases with symptoms of the disorders mentioned above was analysed. Descriptive analyses (frequencies, mean, standard deviation) were performed for symptoms associated with posttraumatic stress disorder, anxiety, depression, burnout and resilience. Linear regression equations were used to evaluate the relationship between each of the variables (demographic, work, COVID-19, burnout and resilience) irrespective of the symptoms (posttraumatic stress, anxiety and depression), using the R^2 value and the standardized β coefficient. The objective was to calculate the impact of posttraumatic stress, anxiety and depression on each of the variables. Dummy variables were used for this. Finally, linear regression models were used to see which variables (demographic, work, COVID-19, burnout and resilience) were jointly related to symptoms of posttraumatic stress, anxiety and depression. The model was estimated by least squares, using the forward extraction method.

3. Results

3.1. Analysis of the Proportion of Health Care Cases with Symptoms Associated with Possible Posttraumatic Stress Disorders, Anxiety, Depression and Burnout

Analyses were carried out to evaluate the proportion of health care cases with symptoms associated with possible posttraumatic stress disorder, anxiety, depression and burnout at their different severity levels. The results of both genders are shown in Table 1. There are gender differences in symptoms of post-traumatic stress disorder, anxiety and depression. In addition, there are differences between men and women in depersonalization scale of burnout.

Table 1. Proportion of cases with symptoms associated with anxiety, depression, posttraumatic stress and burnout (n = 1422).

Variable	Total	Man	Woman	Total
**Anxiety (HADS)**				
No disorder	294 (20.7%)	64 (20.8%)	230 (78.2%)	
Possible/probable disorder	833 (58.6%)	108 (13%)	725 (87%)	X^2 = 26.38 **
Severe disorder	295 (20.7%)	22 (7.5%)	273 (92.5%)	
**Depression (HADS)**				
No disorder	692 (48.7%)	117 (16.9%)	575 (83.1%)	X^2 = 13.50 **
Possible/probable disorder	648 (46%)	65 (10%)	583 (90%)	
Severe disorder	82 (5.3%)	12 (14.6%)	70 (85.4%)	
**Posttraumatic stress (IES-R)**				
No psychiatric disorder	242 (17%)	55 (22.7%)	187 (77.3%)	X^2 = 39.263 **
Average score	375 (26.4%)	68 (18.1%)	307 (81.9%)	
Psychiatric disorder	805 (56.6%)	71 (8.8%)	734 (91.2%)	

Table 1. *Cont.*

Variable	Total	Man	Woman	Total
**Emotional exhaustion (MBI-HSS)**				
Low	510 (35.9%)	80 (15.7%)	430 (84.3%)	X^2 = 3.829
Medium	328 (23.1%)	36 (11%)	292 (89%)	
High	584 (41%)	78 (13.4%)	506 (86.6%)	
**Depersonalization (MBI-HSS)**				
Low	926 (65%)	105 (11.3%)	821 (88.7%)	X^2 = 28.130 **
Medium	280 (19.7%)	35 (12.5%)	245 (87.5%)	
High	216 (15.2%)	54 (25%)	162 (75%)	
**Personal accomplishment (MBI-HSS)**				
Low	120 (8.4%)	23 (19.2%)	97 (80.8%)	
Medium	138 (9.7%)	22 (15.9%)	116 (84.1%)	X^2 = 4.42
High	1164 (81.9%)	149 (12.8%)	1015 (87.2%)	

** $p < 0.01$.

3.2. Data of Internal Consistency

The data of internal consistency and correlations between the factors evaluated with the instruments used are represented in Table 2.

Table 2. Correlation matrix (n = 1422).

Factors	1	2	3	4	5	6	7	8	9	10
1. Intrusion	1									
2. Avoidance	0.666 **	1								
3. Hyperarousal	0.825 **	0.696 **	1							
4. Posttraumatic stress	0.912 **	0.876 **	0.927 **	1						
5. Anxiety	0.584 **	0.482 **	0.550 **	0.431 **	1					
6. Depression	0.480 **	0.392 **	0.529 **	0.515**	0.524 **	1				
7. Emotional exhaustion	0.374 **	0.345 **	0.423 **	0.420 **	0.512 **	0.484 **	1			
8. Depersonalization	0.171 **	0.219 **	0.218 **	0.225 **	0.289 **	0.294**	0.515 **	1		
9.Personal accomplishment	−0.04	−0.023	−0.020	−0.018	−0.160 **	−0.298 **	−0.201 **	−0.238 **	1	
10.Resilience	−0.361**	−0.324 **	−0.434 **	−0.412 **	−0.461 **	−0.460 **	−0.324 **	−0.161 **	0.259 **	1
α	0.866	0.828	0.740	0.932	0.852	0.848	0.876	0.660	0.791	0.829

** $p < 0.01$.

3.3. Job-Related Sociodemographic Variables and Symptoms of Posttraumatic Stress, Anxiety and Depression

Regarding gender and age, being a woman is positively and significantly associated with posttraumatic stress, anxiety and depression, while age is negatively and significantly associated with symptoms of posttraumatic stress and anxiety. Working outside the Autonomous Community of Madrid and in any centre other than primary care, hospital, nursing home or day centres (other category) is negatively and significantly related to posttraumatic stress. Possessing doctoral and postgraduate studies is negatively and significantly associated with anxiety and posttraumatic stress, respectively. Being a member of the non-supervisory staff is positively and significantly associated with posttraumatic stress, just as being a doctor is negatively and significantly associated with symptoms of all the evaluated categories. The caregiver position is negatively and significantly associated with anxiety and depression. Working a fixed shift in the afternoon is positively and significantly associated with posttraumatic stress, anxiety and depression, while working a night shift is only positively and significantly associated with posttraumatic stress. With respect to rotating shifts, the late-night shift and on-call, or 12/24 h shifts are positively and significantly associated with posttraumatic stress and depression, respectively. Working part-time is positively and significantly associated with all three categories (posttraumatic stress, anxiety, and depression). Having a permanent statutory (civil servant) contract and having a training contract are negatively and significantly associated with posttraumatic stress and depression, respectively. Finally, the number of hours worked per week is positively and significantly associated with depression, and the number of on-call hours performed per month is positively and significantly associated with posttraumatic stress and depression (see Table 3).

Table 3. Association between sociodemographic variables relating to the workplace with symptoms of posttraumatic stress, anxiety and depression (n = 1422).

	Description	Posttraumatic Stress			Anxiety			Depression		
**Variable**	**N (%)**	R^2	**B (β)**	**IC 95%**	R^2	**B (β)**	**IC95%**	R^2	**B (β)**	**IC 95%**
**Gender**										
Man	194 (13.6%)	0.038 **	-	-	0.018 **	-	1.005, 2.249	0.10 **	-	0.547, 1.784
Woman	1228 (86.4%)		**3.456 (0.196) ****	2.555, 4.357		**1.627 (0.135) ****	-		**1.165 (0.098) ****	-
**Age M *(SD)***	43.88 (6.06)	0.003 *	**−0.031 (−0.055) ****	−0.060 −0.001	0.007 **	**−0.031 (−0.081) ****	−0.051, 0.011	0.003	−0.019 (−0.051)	−0.039, 0.001
**Autonomous community of the study**										
Community of Madrid	1173 (82.5%)	0.013 **	-		0.004	-	-	0.002	-	-
Others	249 (17.5%)		**−1.802 (−0.092) ****	−2.824 −0.780		−0.834 (−0.062)	−1.248, 0.142		−0.553 (−0.042)	−1.248, 0.142
**Completed studies**										
Doctor's Degree	52 (3.7%)		-	-		**−1.744 (−0.079) ****	2.918, −0.571		−1.012 (−0.046)	−2.175, 0.152
Postgraduate Degree (Master's)	177 (12.4%)		**−4.688 (−0.145) ****	−6.387 −2.990		−0.233 (−0.019)	−0.930, 0.464		0.133 (0.11)	−0.558, 0.824
Bachelor's Degree	575 (40.4%)	0.30 **	−0.843 (−0.046)	−1.851 0.165	0.008 *	-	-	0.006	-	-
Baccalaureate	89 (6.3%)		0.538 (0.042)	−0.196 1.272		−0.102 (0.012)	−0.405, 0.609		0.077 (0.009)	−0.426, 0.580
Secondary Education or Professional Training	460 (32.3%)		1.141 (0.046)	−0.194 2.477		−0.007 (0.00)	−0.931, 0.916		0.516 (0.030)	−0.400, 1.431
Primary Education (Basic Education or equivalent)	69 (4.8%)		0.926 (0.033)	−0.568 2.421		−0.835 (−0.043)	−1.868, 0.198		−0.844 (−0.044)	−1.868, 0.180
**Marital status**										
Married	698 (49.1%)		-	-		-	-		-	-
Separated	33 (2.3%)		1.422 (0.035)	−0.693 3.537		−0.981 (−0.036)	−2.429, 0.467		.063 (0.002)	−1.371, 1.498
Divorced	125 (8.8%)		−0.616 (−0.029)	−1.770 0.537		−0.346 (−0.024)	−1.135, 0.444		−0.187 (−0.013)	−0.969, −0.595
Single	289 (20.3%)	0.007	0.125 (0.008)	−0.706 0.955	0.004	0.111 (0.011)	−0.458, 0.679	0.002	0.197 (0.019)	−0.367, 0.760
Widower/Widow	18 (1.3%)		0.824 (0.015)	−2.010 3.659		−0.875 (−0.024)	−2.816, 1.066		−0.922 (−0.025)	−2.844, 1.001
Living with partner, not married	243 (17.1%)		−1.049	−1.933 −0.164		−0.330 (−0.030)	−0.935, 0.276		−0.224 (−0.021)	−0.824, 0.376
Other	16 (1.1%)		−0.416	−3.418 2.586		0.708 (0.018)	−1.347, 2.764		0.745 (0.019)	−1.291, 2.781
**Dependent relatives**										
Yes	572 (40.2%)	0.000	0.157 (0.013)	−0.486 0.800	0.000	0.053 (0.006)	−0.386, 0.493	0.000	0.101 (0.012)	−0.335, 0.536
No	850 (59.8%)		-	-		-	-		-	-

Table 3. *Cont.*

	Description	Posttraumatic Stress			Anxiety			Depression		
**Variable**	**N (%)**	**R^2**	**B (β)**	**IC 95%**	**R^2**	**B (β)**	**IC95%**	**R^2**	**B (β)**	**IC 95%**
**No. of Children in your care**										
0	586 (41.2%)		-	-		-	-		-	-
1	363 (25.5%)	0.001	0.304 (0.022)	−0.491 1.098	0.000	−0.040 (−0.004)	−0.584, 0.502	0.001	0.187 (0.020)	−0.351, 0.725
2	389 (27.3%)		−0.236 (−0.017)	−1.014 0.542		−0.191 (−0.021)	−0.723, 0.342		0.020 (0.002)	−0.506, 0.547
>2	84 (6%)		0.013 (0.000)	−1.375 1.402		0.010 (0.001)	−0.939, 0.959		−0.159 (−0.009)	−1.098, 0.780
**No. of people you live with M *(SD)***	2.86 (1.257)	0.000	−0.098 (−0.020)	−0.349 0.153	0.000	−0.018 (−0.005)	−0.189, 0.154	0.001	−0.082 (−0.025)	−0.252, 0.087
**Professional category**										
Executive	18 (1.2%)	0.128 **	−0.953 (−0.018)	−3.853 1.946	0.004	−0.169 (−0.005)	−2.164, 1.826	0.006	−0.623 (−0.017)	−2.595, 1.349
Intermediate job	207 (14.6%)		-	-		-	-		-	-
Base position	1197 (84.2%)		**2.035 (0.123) ****	1.146 2.923		0.668 (0.059)	0.057, 1.279		0.806 (0.072)	0.806, 1.410
**Type of centre**										
Hospital	972 (68.4%)		-	-		-	-		-	-
Primary care	150 (10.5%)		−0.142 (−0.007)	−1.181 0.897		0.081 (0.006)	−0.632, 0.793		0.196 (0.015)	−0.510, 0.910
Nursing home	176 (12.4%)	0.010 **	.269 (0.015)	−0.701 1.240	0.004	−0.002 (0.00)	−668, 0.663	0.002	−0.278 (−0.022)	−0.937, 0.381
Day centre	22 (1.5%)		−1.788 (−0.036)	−4.34 0.767		**−2.070 (−0.062) ***	−3.821, −0.319		−1.323 (−0.040)	−3.058, 0.411
Other	102 (7.2%)		**−2.124 (−0.090) ****	−3.358 −0.891		−0.058 (−0.004)	−0.903, 0.788		−0.072 (−0.005)	−0.909, 0.765
**Post**										
Medical post	143 (10%)	0.210 **	**−2.937 (−0.096) ****	−4.045 −1.830	0.012 **	**−0.959 (−0.070) ****	−1.728, −0.189	0.008	**−1.002 (−0.074) ***	−1.765, −0.239
Nursing post	486 (34.2%)		-	-		0.218 (0.024)	−0.312, 0.750		-	-
Assistant Nurse	443 (31.2%)		0.496 (0.038)	−0.269 1.260		-			−0.080 (−0.009)	−0.607, 0.447
Caregiver	117 (8.2%)		−0.605 (−0.027)	−1.804 0.594		**−0.975 (−0.065) ***	−1.808, −0.142		**−0.917 (−0.061) ***	−1.743, −0.091
Others	233 (16.4%)		**−2.464 (−0.146) ****	−3.391−1.536		−0.575 (−0.051)	−1.220, 0.070		−0.595 (−0.054)	−1.234, 0.044

Table 3. *Cont.*

	Description	Posttraumatic Stress			Anxiety			Depression		
**Variable**	**N (%)**	**R^2**	**B (β)**	**IC 95%**	**R^2**	**B (β)**	**IC95%**	**R^2**	**B (β)**	**IC 95%**
**Shift**										
Fixed morning	474 (33.3%)		-	-		-	-		-	-
Fixed afternoon	132 (9.3%)		**1.529 (0.073)** **	0.363, 2.694		**1.029 (0.072)** *	0.220, 1.828		**0.956 (0.068)** *	0.165, 1.747
Fixed night	71 (5%)		**1.538 (0.055)** *	0.030, 3.045		0.345 (0.018)	−0.689, 1.378		0.308 (0.016)	−0.715, 1.330
Rotating morning-afternoon	110 (7.7%)		−0.185 (−0.008)	−1.438 1.068		−0.393 (−0.024)	−1.252, 0.467		0.200 (0.013)	−0.650, 1.051
Rotating morning-night	153 (10.8%)	0.013*	0.951 (0.049)	−0.150 2.053	0.007	0.343 (0.026)	−0.412, 1.098	0.008	0.528 (0.040)	−0.219, 1.275
Rotating afternoon-night	132 (9.3%)		**1.779 (0.085)** **	0.613, 2.944		0.506 (0.035)	−0.293, 1.305		0.274 (0.019)	−0.516, 1.065
Rotating morning-afternoon-night	145 (10.2%)		1.072 (0.054)	−0.052 2.196		0.310 (0.023)	−0.460, 1.080		0.508 (0.037)	−0.255, 1.270
12 or 24 h shifts or on-call hours	98 (6.3%)		0.266 (0.011)	−1.048 1.580		0.582 (0.036)	−0.319, 1.483		**0.962 (0.059)** *	0.070, 1.854
Other	107 (8.1%)		0.027 (0.01)	−1.240 1.295		0.401 (0.026)	−0.468, 1.270		0.851 (0.055)	−0.009, 1.711
**Time**										
Full time	1273 (89.5%)	0.005 **	-	-	0.005 **	-	-	0.006 **	-	-
Part time	149 (10.5%)		**1.426 (0.072)** **	0.399, 2.453		**0.976 (0.072)** **	0.274, 1.678		**0.997 (0.074)** **	0.302, 1.692
**Contract type**										
Statutory fixed-term employment (official)	337 (23.7%)		**−1.140 (−0.080)** **	−1.997 −0.283		−0.282 (−0.029)	−0.869, 0.306		0.366 (0.038)	−0.215, 0.946
Full time staff	442 (31.1%)		-	-		-	-		-	-
Interim staff or long-term substitute	394 (27.7%)		0.083 (0.006)	−0.738 0.905		−0.441 (−0.048)	−1.004, 0.122		0.021 (0.002)	−0.535, 0.577
Temporary employee/short-term substitute	180 (12.7%)	0.009*	−0.345 (−0.019)	−1.393 0.703	0.004	−0.100 (−0.008)	−0.818, 0.619	0.008	0.147 (0.012)	−0.563, 0.857
In training (MIR/EIR/PIR/FIR)	35 (2.5%)		−2.309 (−0.059)	−4.391 −0.228		−1.201 (−0.045)	−2.627, 0.226		**−1.984 (−0.075)** **	−3.393, −0.574
Other	34 (2.3%)		−0.017 (0.00)	−2.126 2.093		0.446 (0.016)	−1.00, 1.892		0.271 (0.010)	−1.157, 1.700
**Years of seniority M *(SD)***	9.391 (8.614)	0.000	0.003 (0.005)	0.033, 0.040	0.000	0.000 (0.000)	−0.025, 0.025	0.000	0.009 (0.019)	−0.016, 0.034
**Years as health workers M *(SD)***	16.54 (10.38)	0.003 *	−0.032 (−0.054)	−0.062 −0.001	0.002	−0.017 (−0.042)	−0.038, 0.004	0.00	−0.007 (−0.017)	−0.027, 0.014
**Weekly operating hours M *(SD)***	38,42 (13.96)	0.000	−0.009 (−0.022)	−0.032 0.013	0.000	−0.005 (−0.017)	−0.020, −0.010	0.003 *	**0.016 (0.053)** *	−0.031, 0.000
**Number of on-call hours per month M *(SD)***	3.69 (4.10)	0.004 *	**0.031 (0.063)** *	0.005, 0.056	0.000	0.006 (0.019)	−0.011, 0.024	0.006 **	**0.026 (0.007)** **	0.008, 0.043

* $p < 0.05$; ** $p < 0.01$.

3.4. Data on Information about COVID-19, Burnout and Resilience and its Association with Symptoms of Posttraumatic Stress, Anxiety and Depression

Changing address or living with people who are at risk is positively related to symptoms of posttraumatic stress, anxiety or depression. On the other hand, having personal protective equipment and not being very concerned that family members are infected is negatively related to symptoms of posttraumatic stress, anxiety or depression. Hospitalization for symptoms of COVID-19 and isolation due to possible contagion of the disease is positively related to posttraumatic stress. Thinking that becoming infected with COVID-19 is highly unlikely is negatively related to symptoms of posttraumatic stress and anxiety. Emotional fatigue and depersonalization are positively and significantly related to symptoms of posttraumatic stress, anxiety and depression, while personal accomplishment is negatively and significantly related to symptoms of anxiety and depression. Finally, resilience is associated in a negative and significant way with symptoms from all the evaluated categories. On the other hand, these professionals present moderate levels of resilience, while the highest possible score on resilience is 6, the mean score for these individuals is 3.02 ($SD = 0.39$), therefore indicating moderate levels. All associations can be seen in Table 4.

3.5. Regression Models for Posttraumatic Stress, Anxiety and Depression

As shown in Table 5, the posttraumatic stress symptom model was significant, explaining 39.6% of the variance ($F(17, 1405) = 54{,}022$, $p < 0.001$). It was also significant for anxiety symptoms, explaining 40.2% of the variance ($F(12, 1412) = 78.593$, $p < 0.001$). In relation to the depression model, it was significant, explaining 39.3% of the variance ($F(14, 1408) = 64.932$, $p < 0.001$). The variables common to the three models were emotional fatigue, depersonalization, resilience, gender, and concern that someone with whom they live could be infected.

The variables positively related to posttraumatic stress are emotional exhaustion, depersonalization, working in the Autonomous Community of Madrid, having a primary education, working in a hospital, being very concerned that someone with whom they live may become infected and thinking that there is a high risk of also becoming infected with COVID-19. On the other hand, the variables negatively related to posttraumatic stress are resilience, being a man, having a doctor's degree, living with an unmarried partner, being a doctor or having another profession (mainly pharmacist or psychologist, but not a nurse, nursing assistant or caregiver), having protective equipment at work, not being concerned that someone you live with can be infected with the disease and the number of people you live with.

The variables positively and significantly related to anxiety would be emotional exhaustion, depersonalization, 12- or 24-h shifts or on-call hours and being very concerned that someone with whom they live could be infected. The variables negatively and significantly related to anxiety would be resilience, being a man, being separated, working in nursing homes or day centres, being a doctor, having a rotating morning–afternoon shift and not having been isolated due to COVID-19.

Finally, having symptoms of depression is positively and significantly related to: emotional exhaustion, depersonalization, 12- or 24-h shifts or on-call hours, the number of guards per month, being very concerned that someone with whom you live may be infected, not having a family and thinking that it is very likely that you will be infected with COVID-19. The variables negatively and significantly related to depression would be personal fulfilment, resilience, being a man and having a fixed or training contract.

Table 4. Association between COVID-19, burnout and resilience variables with symptoms of posttraumatic stress, anxiety and depression (*n* = 1422).

	Description	Posttraumatic Stress			Anxiety			Depression		
**COVID-19 Variables**	**N (%)**	**R^2**	**B (β)**	**IC 95%**	**R^2**	**B (β)**	**IC 95%**	**R^2**	**B (β)**	**IC 95%**
**Change of residence through fear of infecting family members**										
Yes	147 (10.3%)	0.006 **	**1.563 (0.079) ****	0.530 2.596	0.005 **	**0.927 (0.068) ****	−220 1.633	0.003 *	**0.721 (0.054) ***	0.024 1.424
No	1275 (89.7%)		-	-		-	-		-	-
**At work, being with people who might have COVID-19**										
Yes	1367 (96.1%)	0.002	1.228 (0.039)	−0.406, 2.862	0.000	0.004 (0.002)	−1.074 1.162	0.001	−0.499 (−0.023)	−1.605 0.607
No	55 (3.9%)		-	-		-	-		-	-
**You tested positive for COVID-19**										
Yes	221 (3.9%)		−0.086 (−0.005)	−1.019 0.847		−0.309 (−0.027)	−0.947 0.330		−0.244 (−0.022)	−0.876 0.389
You underwent no tests	609 (15.5%)	0.003	-	-	0.001	-	-	0.000	-	-
No tests were performed	524 (36.8%)		−0.661 (−0.053)	−1.369 0.047		−0.183 (−0.021)	−0.668 0.301		−0.068 (−0.008)	−0.548 0.412
Other	68 (4.7%)		0.351 (0.012)	−1.168 1.870		−0.040 (−0.002)	−1.079 1.000		−0.069 (−0.004)	−1.099 0.960
**You were hospitalized for symptoms compatible with those of coronavirus**										
Yes	26 (1.8%)	0.003 *	**2.347 (0.052) ***	−0.004 4.698	0.000	0.480 (0.016)	−1.129 2.089	0.000	0.031 (0.001)	−1.561 1.624
No	1396 (98.2%)		-	-		-	-		-	-
**You were isolated due to possible infection with COVID-19**										
Yes	423 (29.7%)	0.004 *	**0.868 (0.065) ***	0.179 1.556	0.003 *	**0.473 (0.052) ***	0.002 0.944	0.001	0.228 (0.025)	−0.239 0.694
No	999 (70.3%)		-	-		-	-		-	-
**You have protective equipment to prevent being infected**										
Yes	1177 (82.8%)	0.006 **	**−1.245 (−0.078) ****	−2.078 −0.412	0.008 **	**−0.976 (−0.089) ****	−1.544 −0.407	0.009 **	**−1.046 (−0.096) ****	−1.608, −0.483
No	245 (17.2%)		-	-		-	-		-	-

Table 4. *Cont.*

	Description	Posttraumatic Stress			Anxiety			Depression		
**COVID-19 Variables**	**N (%)**	**R^2**	**B (β)**	**IC 95%**	**R^2**	**B (β)**	**IC 95%**	**R^2**	**B (β)**	**IC 95%**
**You were given protective equipment at work to prevent being infected**										
No, not at all	61 (4.3%)	0.038 **	−0.225 (−0.008)	−1.765 1.315	0.027 **	−0.448 (−0.022)	-	0.026 **	−0.075 (−0.004)	−1.123 0.974
Yes, the equipment necessary for the work performed	376 (26.4%)		-	-		-	-		-	-
Yes, but scarce	985 (69.3%)		**−2.691 (−0.196) ** **	−3.398 −1.983		**−1.558 (−0.166) ** **	−2.045 −1.072		**−1.507 (−0.166) ** **	−1.989 −1.026
**Some people you live with belong to the risk group**										
Yes	550 (38.7%)	0.008 **	**1.079 (0.087) ** **	0.433 1.724	0.009 **	**0.791 (0.093) * **	0.350 1.231	0.009 **	**0.806 (0.096) ** **	0.370 1.242
No	872 (61.3%)		-	-		-	-		-	-
**Concern over possible infection of a person you live with**										
Very concerned	827 (58.2%)	0.100 **	-	-	0.057 **	-	-	0.029 **	-	-
Quite concerned	428 (30.1%)		**3.513 (0.266) ** **	4.185 2.840		**1.932 (0.214) ** **	2.402 1.462		**1.204 (0.135) ** **	1.676 −0.731
Not very concerned	80 (5.6%)		**−4.982 (−0.189) ** **	−6.305 −3.600		**−2.452 (−0.136) ** **	−3.377 −1.527		**−1.962 (−0.001) ** **	−2.892 −1.003
Not at all concerned	29 (2%)		**−6.274 (−0.146) ** **	−8.400 −4.140		**−2.695 (−0.092) ** **	−4.188 −1.203		**−2.147 (−0.074) ** **	−3.647 −0.647
No family	58 (4.1%)		−1.917 (−0.063)	−3.451 −0.383		−0.937 (−0.045)	−2.010 0.137		0.112 (.005)	−0.966 1.190
**Concern over possible infection of a family member you do not live with**										
Very concerned	1069 (75.2%)	0.007 **	-	-	0.037	-	-	0.024 **	-	-
Quite concerned	296 (20.8%)		**3.421 (0.299) ** **	4.172, 2.670		**1.681 (0.165) ** **	2.205, 1.156		**1.484 (−0.147) ** **	2.007 −0.962
Not very concerned	25 (1.8%)		**−6.098 (−0.132) ** **	−8.412 −3.783		**−2.880 (−0.091) ** **	−4.496 −1.265		**−1.859 (−0.60) * **	−3.469 −0.249
Not at all concerned	13 (.9%)		**−6.511 (−0.102) ** **	−9.703 −3.319		**−2.785 (−0.064) ** **	−5.013 −0.557		−1.022 (−0.024)	−3.243 1.198
No family	19 (1.3%)		**−3.879 (−0.074) ** **	−6.527 −1.232		−1.716 (−0.048)	−3.564 0.132		−0.733 (−0.020)	−2.572 1.111

Table 4. *Cont.*

	Description	Posttraumatic Stress			Anxiety			Depression		
**COVID-19 Variables**	**N (%)**	**R^2**	**B (β)**	**IC 95%**	**R^2**	**B (β)**	**IC 95%**	**R^2**	**B (β)**	**IC 95%**
**Likelihood of becoming infected with COVID−19**										
Very likely	918 (64.6%)		0.435 (0.034)	−7.649 8.519		1.527 (0.166)	−4.121 7.175		−0.870 (−0.102)	−6.513 4.773
Quite likely	399 (28.1%)	0.081 **	−2.491 (−0.185)	−10.584 5.605	0.039 **	0.143 (0.016)	−5.513 5.800	0.021 **	−1.789 (−0.196)	−7.440 3.862
Not very likely	50 (3.5%		−5.547 (−0.169)	−13.782 2.688		−1.260 (−0.056)	−7.014 4.494		−3.360 (−0.151)	−9.108 2.388
Not at all likely	2 (.1%)		**−5.423 (0.085)** **	−8.607 −2.239		**−2.242 (−0.052)** *	−4.467 −0.017		−0.613 (−0.014)	−2.836 1.609
Doesn't know	53 (3.7%)		−2.420 (−0.076)	−10.646 5.807		−0.260 (−0.012)	−6.007 5.488		−1.988 (−0.092)	−7.731 3.754
**Emotional exhaustion M *(SD)***	27.48 (12.62)	0.177 **	**0.202 (0.420)** **	0.179, 0.224	0.262 **	**0.168 (0.226)** **	0.153, 0.183	0.234 **	**0.157 (0.008)** **	0.142 0.172
**Depersonalization M *(SD)***	6.616	0.051 **	**0.222 (0.026)** **	0.172, 0.272	0.084 **	**0.195 (0.289)** **	0.162, 0.229	0.086 **	**0.196 (0.294)** **	0.163 0.230
**Personal accomplishment M *(SD)***	39.68 (6.99)	0.000	−0.015 (−0.018)	−0.066 0.030	0.026 **	**−0.095 (−0.160)** **	−0.125, −0.065	0.089 **	**−0.174 (−0.298)** **	−0.204 −0.145
**Resilience M *(SD)***	3020 (0.39)	0.170 **	**−0.625 (−0.412)** **	−0.697 −0.554	0.212 **	**−0.478 (−0.461)** **	−0.526, −0.430	0.212 **	**−0.473 (−0.460)** **	−0.520 −0.425

* $p < 0.05$; ** $p < 0.01$.

Table 5. Regression models for posttraumatic stress, anxiety and depression (n = 1422).

	**Posttraumatic stress**		**Anxiety**		**Depression**	
**Variable**	**B (β)**	**IC 95%**	**B (β)**	**IC 95%**	**B (β)**	**IC 95%**
Emotional exhaustion (MBI-HSS)	**0.137 (0.285)** **	0.112, −0.162	**0.123 (0.374)** **	0.106, 0.139	**0.105 (0.324)** **	0.089, 0.122
Depersonalization (MBI-HSS)	**0.055 (0.056)** *	0.007, −00.104	**0.039** *	0.007, 0.072	**0.034 (0.050)** *	0.001, 0.954
Personal accomplishment (MBI-HSS)	−0.086 (0.99)	0.047, 0.124	-	-	**−0.094 (−0.60)** **	−0.119, −0.068
Resilience	**−0.395 (−0.056)** **	−0.464, −0.327	**−0.307 (−0.295)** **	−0.352, −0.262	**−0.286 (−279)** **	−0.332, −0.240
Autonomous community (Madrid)	**0.896** **	0.237, 1.556	-	-	-	-
Gender (man)	**−2.288 (−0.129)** **	1.531, 3.035	**−1.118 (−0.091)** **	−1.623, −0.613	−0.805 (−0.067) **	−1.306, −0.304
Completed studies (doctor's degree)	**−2.254 (−0.070)** **	−3.688, −0.820	-	-	-	-
Completed studies (primary education)	**1.215 (0.043)** **	0.021, 2.408	-	-	-	-
Marital status (living with partner, not married)	**−1.048 (−0.065)** **	−1.718<br>−379	-	-	-	-
Marital status (separated)	-	-	**−1.177 (−0.043)** *	−2.297, −0.058	-	-
Centre type (hospital)	**0.703 (0.054)** *	0.155, 1.252	-	-	-	-
Centre type (residences)	**-**	-	**−0.832 (−0.066)** **	−1.354, −0.309	-	-
Centre type (day centre)	**-**	-	**−1.469 (−0.044)** *	−2.843, −0.095	-	-
Position (doctors)	**−1405 (−0.070)** **	−2.323, −0.488	**−0.648 (−0.047)** *	−1.215, 1.082	-	-
Position (others)	**−1.402 (−0.086)** **	−2.096, −0.708	-	-	-	-
Shift (rotating morning-afternoon)	-	-	**−0.653 (−0.042)** *	−1.284, −0.022	-	-
Shift (12 or 24 shifts or on-call hours)	-	-	**0.698 (0.043)** *	0.026, 1.370	**0.859 (0.053)** *	0.188, 1.530
Shift (Other)	-	-	-	-	**0.909 (0.059)** **	0.267, 1.551
No. of On-call hours per month	-	-	-	-	**0.014 (0.042)** *	0.000, 0.028
Contract type (training)	-	-	-	-	**−2.254 (−0.085)** **	−3.347, −1.161
Contract type (fixed-term)	-	-	-	-	**−0.631 (−0.071)** **	−1.001, −0.261

Table 5. *Cont.*

	Posttraumatic stress		Anxiety		Depression	
**Variable**	**B (β)**	**IC 95%**	**B (β)**	**IC 95%**	**B (β)**	**IC 95%**
Isolated COVID-19 (NO)	-	-	**−0.416 (−0.046) ***	−0.791, −0.042	-	-
You were given protective equipment at work to prevent being infected (yes, that considered necessary for the work carried out)	**−0.857 (−0.062) ****	−1.437 −0.277	-	-	-	-
Risk groups (No)	-	-	-	-	**−451 (−0.131) ***	−0.814, −0.087
Concern over possible infection of a person you live with (very concerned)	**1.800 (0.146) ****	1.234, 2.365	**1.192 (0.142) ****	0.839, 1.546	**0.533 (0.064) ****	0.137, 0.928
Concern over possible infection of a person you live with (Not at all concerned)	**−2.896 (−0.068) ****	−4.706 −1.085	-	-	-	-
Concern over possible infection of a family member you do not live with (No family)	-	-	-	-	**1.247 (0.060) ****	0.37, 2.124
Likelihood of infection with COVID-19 (very likely)	**1.176 ** (0.084)**	0.545, 1.807	-	-	**0.529 (0.056) ***	0.103, 0.954
No. of people you live with	**−0.219 * (−0.045)**	−0.423, −0.014	-	-	-	-

* $p < 0.05$; ** $p < 0.01$.

3.6. Regression Models for Posttraumatic Stress, Anxiety and Depression, Separated by Gender

The gender-differentiated posttraumatic stress models were significant, in both men and women, explaining 35.2% of the variance in women (F(13, 1210) = 50,667, p < 0.001) and 53.7% in men (F(9, 183) = 23,548, p < 0.001).

On the anxiety scale, the models were significant, explaining 36.3% of the variance in women (F(11, 1212) = 64,280, p < 0.001) and 62.7% in men (F(6,168) = 52,066, p < 0.001).

For the depression scale, the models were significant, explaining 37.73% of the variance in women (F(14, 1223) = 52.310, p < 0.001). Tables 6 and 7 show the regression models of post-traumatic stress, anxiety and depression, differentiated by gender.

In relation to the gender-differenciated models, in women (see Table 6):

The symptoms of posttraumatic stress are positively and significantly related to emotional exhaustion, depersonalization, working in a hospital, being very concerned that someone with whom they live may become infected and thinking that becoming infected with COVID-19 is very likely. On the other hand, the variables negatively related to posttraumatic stress are personal accomplishment, resilience, living with and unmarried partner, working in nursing homes, being a doctor or having another profession (mainly pharmacist or psychologist, but not a nurse, nursing assistant or caregiver) and thinking that is very unlikely to be infected with COVID-19.

The variables positively and significantly related to anxiety would be emotional exhaustion, depersonalization, being very concerned that someone you live with can be infected with the disease and change of residence through fear of infecting family members. The variables negatively and significantly related to anxiety would be resilience, being separated, working in nursing homes and not being at all concerned that someone you live with can be infected with the disease.

Having symptoms of depression is positively and significantly related to emotional exhaustion depersonalization, being a nurse, 12- or 24-h shifts or on-call hours, those who live with people who are at risk and being very concerned over a possible infection of a family member they do not live with. The variables negatively and significantly related to depression would be personal accomplishment, resilience, being a doctor, having a fixed or training contract.

In relation to the gender-differentiated models, in men (see Table 7):

The symptoms of posttraumatic stress are positively and significantly related to emotional exhaustion, having a primary education and being very concerned that someone with whom they live may become infected. On the other hand, the variables negatively related to posttraumatic stress are resilience, having a doctor's degree, not having been isolated due to COVID-19 and not being concerned that someone with whom you live with can be infected with the disease. The variables positively and significantly related to anxiety would be emotional exhaustion, being a nurse and having been isolated due to COVID-19. The variables negatively and significantly related to anxiety would be resilience, having a training contract and having a statutory fixed-term employment.

Having symptoms of depression is positively and significantly related to emotional exhaustion depersonalization and being very concerned that someone with whom they live may become infected. The variables negatively and significantly related to depression would be personal accomplishment, resilience, having a doctor's degree, having a training contract, not having been isolated due to COVID-19 and not having been hospitalized for symptoms compatible with those of coronavirus.

Table 6. Regression models for posttraumatic stress, anxiety and depression in women (n = 1228).

	Posttraumatic Stress		Anxiety		Depression	
**Variable**	**B (β)**	**IC 95%**	**B (β)**	**IC 95%**	**B (β)**	**IC 95%**
Emotional exhaustion (MBI-HSS)	**0.121 (0.261)** **	0.095, 0.147	**0.111 (0.007)** **	0.093, 0.127	**0.102 (0.318)** **	0.085, 0.119
Depersonalization (MBI-HSS)	**0.060 (0.061)** **	0.042, 0.124	**0.050 (0.071)** **	0.013, 0.087	**0.515 (0.060)** **	−0.943, −0.087
Personal accomplishment (MBI-HSS)	**−0.060 (−0.098)** **	−0.007, −0.113			**−0.84 (−1.41)** **	−0.112, −0.056
Resilience	**−0.404 (−0.280)** **	−0.475, −0.332	**−0.293 (−0.286)** **	−0.342, −0.244	**−0.284 (−0.280)** **	−0.333, −0.235
Marital status (living with partner, not married)	**−1.005 (−0.067)** **	−1.696, −0.314				
Marital status (separated)			**−1.461 (−0.057)** *	−2.612, −0.311		
Centre type (hospital)	**0.266 (0.022)**	−0.455, 0.988				
Centre type (residences)	**−1.079 (−0.063)** *	−2067, −0.092	**−0.978 (−0.081)** **	−1.532, −0.424		
Position (nurse)					**0.496 (0.059)** **	0.113, 0.879
Position (doctors)	**−1.869 (−0.094)** **	2.819, −0.919			**−1.215 (−0.058)** *	−2.200, −0.230
Position (others)	**−1.154 (−0.073)**	−1.914, −0.394				
Shift (12 or 24 shifts or on-call hours)					**0.785 (0.048)** **	0.048, 1.523
Shift (Other)					**0.997 (0.066)** **	0.318, 1.675
Contract type (training)					**−2.125 (−0.081)** **	−3.300, −0.950
Contract type (fixed-term)					**−0.679 (−0.078)** **	−0.279, −0.022
Risk groups (Yes)					**0.481 (0.058)** **	0.093, 0.870
Concern over possible infection of a person you live with (very concerned)	**1.826 (0.30)** **	1.237, 2.415				
Concern over possible infection of a person you live with (Not at all concerned)			**−2.870 (−0.057)** *	−5.150, −0.589		
Concern over possible infection of a family member you do not live with (very concerned)	**1.038 (0.077)** **	0.368, 1.708	**1.152 (0.139)** **	0.763, 1.541	**0.671 (0.082)** **	0.258, 1.083
Likelihood of infection with COVID-19 (very likely)	**1.22 (0.101)** **	0.625, 1.819	−1.600 (−0.072)	−2.703, −0.616		
Likelihood of infection with COVID-19 (unlikely)	**−0.810 (−0.056)** *	−3.343, −0.276				
Likelihood of infection with COVID-19 (I do not know)			**−1.058 (−0.049)** *	0.047, 1.229		
Change of residence through fear of infecting family members			**0.871 (0.064)** **	0.245, 1.497		

* $p < 0.05$; ** $p < 0.01$.

Table 7. Regression models for posttraumatic stress, anxiety and depression in men (n = 194).

	Posttraumatic Stress		Anxiety		Depression	
**Variable**	**B (β)**	**IC 95%**	**B (β)**	**IC 95%**	**B (β)**	**IC 95%**
Emotional exhaustion (MBI-HSS)	**0.231 (0.457)** **	0.174, 0.288	**0.155 (0.510)** **	0.125, 0.185	**0.119 (0.387)** **	0.085, 0.153
Depersonalization (MBI-HSS)					**1.446 (0.165)** **	−2.393, −0.518
Personal accomplishment (MBI-HSS)					−0.149 (−0.275)	−0.205, −0.093
Resilience	−0.373 (−0.207)	−0.573, −0.173	**−0.424 (−0.391)** **	−0.530 −0.319	**−0.253 (−0.230)** **	−0.393, −0.133
Completed studies (doctor's degree)	**−5.139 (−0.198)** **	−7.827, −2.451			**−1.705 (−0.108)** **	−3.143, −0.268
Completed studies (primary education)	**3.140 (0.133)** **	0.724, 5.555				
Position (nurse)			**1.012 (0.097)** *	−1.937 −0.087		
Position (others)	**−2.050 (−0.119)** *	−3.800<br>−0.299				
Contract type (training)			**−2.952 (−0.109)** *	−5.361<br>−0.543	**−4.809 (−0.174)** **	−7.313, −2.305
Contract type (Statutory fixed-term employment (official)			**−1.313 (−0.131)** **	−2.219 −0.408		
Contract (Others)					**−4.046 (0.094)** *	−7.947, −0.144
Isolated COVID-19 (NO)	**−1.951 (−0.129)** *	−3.479<br>−0.423			**−1.332 (−0.144)** **	−2.214, −0.450
Concern over possible infection of a person you live with (very concerned)	**1.881 (0.124)** *	0.260, 3.503			**1.954 (0.212)** **	1.074, 2.835
Concern over possible infection of a person you live with (Not at all concerned)	**−6.784 (−0.150)** **	−11.446, −2.121				
Concern over possible infection of a family member you do not live with (No family)					**2.206 (0.106)** *	0.278, 4.135
Isolated (Yes)			**0.996 (0.109)** *	0.180<br>1.813		
Being hospitalized for symptoms compatible with those of coronavirus (NO)					**−2.756 (−0.100)** *	−5.317, −0.196

* $p < 0.05$; ** $p < 0.01$.

4. Discussion

This research aimed to assess the symptoms of posttraumatic stress, anxiety, depression, burnout and resilience in Spanish health workers during the COVID-19 pandemic. It was also aimed at evaluating the relationship between each of the variables independently (demographic, work, COVID-19, burnout and resilience) and the symptoms of posttraumatic stress, depression and anxiety, as well as the variables that (together) carry more weight in each of the three categories (posttraumatic stress disorders, anxiety and depression).

The results show that 56.6% of health workers present symptoms of posttraumatic stress disorder. The number having a possible anxiety disorder is 58.6%, with 20.7% having a severe disorder. Equally, a high percentage, specifically 46%, would have a possible depressive disorder and 41% feel emotionally drained. In this sense, the first hypothesis would be fulfilled, although it would be necessary to make a thorough evaluation to determine a clinical diagnosis. Most workers present probability of developing a posttraumatic stress disorder, anxiety or depression. During the Middle East Respiratory Syndrome (MERS) or Ebola crises, among others, health professionals reported a higher number of symptoms related to posttraumatic stress [39], so it is necessary to pay attention to the increase in these symptoms, even more so in the situation of the COVID-19 pandemic that has not yet subsided. The demographic variables show that having a doctoral or postgraduate degree represent protective variables of anxiety and posttraumatic stress, respectively. In addition, lower-level workers show more symptoms of posttraumatic stress. This result may be due to the fact that, in lower-level jobs, control over procedures and decision-making capacity is lower than in other higher-level positions. Some authors have shown that the main differences among professions regarding the symptomatology evaluated during the COVID-19 pandemic apply to nurses and other positions, such as doctors. Nurses present more symptoms of anxiety and depression [40]. These differences may be associated with the contact of these professionals with infected patients. On the other hand, being a woman is associated with greater symptoms of posttraumatic stress, anxiety or depression in the sample of health workers evaluated. Younger health workers show greater levels of posttraumatic stress and anxiety. This may be due to a lack of work experience in similar stressful situations. Another possible reason is that, during the current pandemic, the lack of health care staff has required that senior students or people with fewer experience have had to deal with the demands of the COVID-19 patients. The data obtained in this study on the gender and age variables coincide with the findings of other studies in health personnel from different countries [41,42]. Some authors suggest that, both in the current situation due to COVID-19 and in similar previous situations, symptoms of stress, anxiety and depression generally increase in health professionals and also coincide in pointing out that women present more symptoms than men do. Regarding gender differences, the data obtained in this study may be due to the high number of women in positions such as nurse or nursing assistant. On the other hand, in mood disorders, which have a high comorbidity with those of anxiety, there is a high prevalence of women compared to men. For example, women present more rumination and there are hormonal differences that can explain these results [43]. Variables related to jobs show that those health workers who have part-time jobs have more symptoms of posttraumatic stress, anxiety and depression. The shifts most related to psychological problems are the night, afternoon and afternoon–night rotating shifts. In this regard, similar results have been identified with health personnel that indicate the association between working the night shift and having gastrointestinal problems, hormonal problems, and changes in mood and cognitive state, among others [44]. Regarding the work shift, other authors specify that there is a greater risk of having symptoms of depression as the number of days worked in the night shift increases [45]. Doctors have fewer symptoms of posttraumatic stress, anxiety, or depression, and caregivers have fewer symptoms of anxiety and depression. Health workers with a lower job category have more symptoms of posttraumatic stress, while those who work more hours a week have more symptoms associated with depression. Health workers who do more on-call hours a month have more symptoms of posttraumatic stress and depression. Therefore, the hours of rest for these professionals must be respected.

In relation to the information collected on COVID-19, it should be noted that the health workers who have had to change their residence due to the pandemic have been isolated due to possible contagion, and those who live with people who are at risk or think they may infect other people have more symptoms of posttraumatic stress, anxiety or depression, although these differences were not statistically significant in depression for the two variables: being isolated due to possible contagion and the likelihood of becoming infected with COVID-19. Regarding the hypothesis related to these variables, it would be partially fulfilled, since the association between the possibility of infection and the symptoms of depression would not be significant. Recent research indicates that one of the greatest concerns of health personnel is the possibility of infecting others, especially family members [46]. Believing that they are very unlikely to be infected with COVID-19 is related to fewer symptoms of posttraumatic stress and anxiety. On the other hand, as proposed in the study hypothesis, those health workers who have personal protective equipment to cope with the disease have fewer symptoms of posttraumatic stress, anxiety or depression. These results may be due to the fact that, on the one hand, contact with people who may be infected is a risk factor for imminent contagion among health workers, as a result of the high transmission of the disease [47]; on the other hand, the use of personal protective equipment is essential to be able to work and treat patients with COVID-19 [48].

The profile of a health worker with greater symptoms of posttraumatic stress would be a person who works in the Autonomous Community of Madrid, in a hospital, is a woman, has primary studies, worries that their family members may become infected and thinks that they are very likely to be infected with COVID-19. The protective variables of suffering posttraumatic stress symptoms are being a man, having a doctor's degree, living with a partner (not married), being a doctor or working in "another position" (a category made up mainly of pharmacists and psychologists), having protective equipment at work, not being concerned about infecting the people with whom they live and not living alone. The risk variables associated with anxiety symptoms are being a woman, having a 12- or 24-h on-call shift and being worried that a person he/she lives with could be infected. Additionally, the following protective factors have been identified: being a man, being separated, working in nursing homes or day centres, being a doctor, having a rotating morning–afternoon shift and/or not being isolated by COVID-19. The profile of the health worker with greater symptoms of depression is being a woman, working 12- or 24-h shifts or on-call hours, the number of on-call hours per month, thinking that they are very likely to become infected with COVID-19, being worried about infecting someone with whom they live and having no family. The protective variables of depression are being a man, having a fixed-term or training contract, feeling professionally accomplished and not living with people who are at risk. Presenting high scores in emotional exhaustion and depersonalization are risk factors for posttraumatic stress, anxiety and depression. However, resilience would be a protective variable that reduces symptoms in all three disorders, and personal accomplishment would be a protective variable against depressive symptoms. One study points out that, in 2003, during the SARS epidemic, health personnel showed symptoms related to posttraumatic stress disorder and, in general, higher levels of psychological stress [49]. Recent research has indicated that, during the influenza A (H1N1) outbreak, resilience levels had a direct influence on the psychological health of health personnel [50].

Regarding the gender-differentiated models, being a nurse is associated with symptoms of mental disorder, specifically depression in women and anxiety in men. In men, depersonalization and personal accomplishment are only associated with depression (although this relationship is not significant), while in women it is associated with the three scales of burnout. That is to say, women who have high scores in depersonalization would have more symptoms of mental disorder than men.

In men, personal accomplishment would not be a protective variable, while for women it would be a protective factor for posttraumatic stress, anxiety and depression. Having a primary education would be a risk variable for posttraumatic stress and having doctoral studies would be a protective variable of posttraumatic stress and depression, but only in men.

In relation to the type of contract, having a statutory fixed-term employment contract is negatively related to anxiety in men, not being a relevant variable for women. The type of shift is a relevant

variable for women, but not for men. Specifically, 12- or 24-h shifts are positively related to depression in women, but not in men. Having a fixed contract is a more relevant variable for women than for men, since it is a protective variable of depression in women. With regard to the foregoing, one of the main results of this study is that the levels of resilience of the healthcare workers evaluated are moderate. Taking into account that resilience is presented as a possible protective factor of symptoms of posttraumatic stress disorder, anxiety and depression, the need to promote resilience among health personnel is highlighted. Different studies have pointed out various measures to promote resilience among these professionals during the COVID-19 pandemic. These include the following: providing psychological training to healthcare workers so that they can help patients and encouraging support within the organization by the network of personnel and train communication [51]. The model of intervention in psychological resilience based on peer support (Battle Buddies) developed by the US military should be highlighted. This model requires a close support partner as well as a designated mental health consultant to facilitate training in stress inoculation methods and to coordinate referral to the outpatient psychological consultation [52]. It might be interesting to introduce the elements mentioned in the Spanish health care system to establish measures to promote resilience in possible future waves of COVID-19. In this sense, the hypothesis proposed at the beginning would be fulfilled, since resilience would be a protective factor for such symptoms. In relation to burnout, it should be noted that a large percentage of these professionals have high scores in emotional exhaustion, but low in depersonalization and very high in personal accomplishment. The first study hypothesis, therefore, would be partially fulfilled, since it was expected that the workers would have low levels of personal accomplishment. This could be explained by the fact that health professionals have been intensely involved with patients in this situation, have felt valued by patients and society, and have realized the great importance of their profession, which may have had a very positive influence on their personal fulfilment. The variable with the greatest weight in the regression models is emotional fatigue. Hence, preventive measures to reduce this should be implemented for these workers. Emotional fatigue is the dimension that has the greatest relevance compared to depersonalization and personal fulfilment, within the burnout construct [53]. Other authors have also identified high levels of fatigue and negative emotions in health personnel from emergency teams in coping with the COVID-19 situation [54].

5. Limitations

The study does have some limitations. The data were obtained using an online tool and people not familiar with the web could not be included in this study. In addition, the survey was carried out at the peak of the pandemic in Spain: the continuous exposure to negative stimuli and the constant information in the media about the state of health care workers and those infected and deceased by COVID-19 may have had an influence in the perception of anxiety and depression levels, due to the feeling of fear experienced [55].

Furthermore, there is a high proportion of women compared to men. Other studies have shown the same limitation [56,57]. In this case, one of the main reasons for this difference is that, in many positions, such as nurses and nursing assistants, the majority of the positions are occupied by women. Another limitation has to do with the cross-sectional design of the study: the pandemic has not yet finished and its influence on mental health cannot be reflected in this research, so it would also be advisable to carry out a longitudinal study that evaluates the evolution over time of the symptoms assessed in this work. On the other hand, there has not been a previous situation in Spain in which there has been a lockdown, and it is likely that after its ending, the levels of experienced symptomatology will be lower. However, during the SARS crisis, other authors have found that the symptoms of psychological problems after the quarantine period of the disease have lasted up to three years later [3,58]. In the long term, the effects of posttraumatic stress disorder, anxiety and depression will depend on the possible outbreaks of COVID-19. The measures currently being taken in order to adapt the work place to the new situation (such as providing protective equipment or increasing the number of healthcare professionals) are relevant for mitigating these symptoms. If the appropriate actions to protect health care providers are

not taken, they may make medical errors in the future, present higher burnout levels associated with depressive symptoms, anxiety, suicidal ideation, have poorer interpersonal relationships or develop substance abuse [59]. Therefore, a follow-up study along the next few months becomes necessary.

6. Conclusions

This research presents a detailed description of the association between different variables and symptoms of posttraumatic stress, anxiety and depression. Previous studies for Spanish health professionals to evaluate these characteristics, including resilience, and its associations with the described variables, have not been found. Among the main uses for this study is the description of the profiles of Spanish healthcare providers that present a greater risk of suffering from post-traumatic stress, anxiety and depression, so more specific intervention measures can be designed to reduce these symptoms. On the other hand, resilience is a protective factor of the mentioned symptoms, so it would be advisable to include the promotion of resilience in the design of interventions to reduce stress, as other authors point out [60]. The information presented is relevant in order to protect the health of those who care for patients in future waves of COVID-19 or similar situations. It would be useful to consider the results of this study in the design of future longitudinal research that analyse the evolution of these symptoms and the risk profiles described.

Author Contributions: Conceptualization, L.L.-M., B.T.-V. and J.M.-G.; methodology, L.L.-M., B.T.-V. and J.M.-G.; software, L.L.-M., B.T.-V. and J.M.-G.; validation, L.L.-M., B.T.-V. and J.M.-G.; formal analysis, L.L.-M., B.T.-V. and J.M.-G.; investigation, L.L.-M., B.T.-V. and J.M.-G.; resources, L.L.-M., B.T.-V., J.M.-G. and Y.G.-A.; data curation, L.L.-M., B.T.-V., J.M.-G. and Y.G.-A.; writing—original draft preparation, B.T.-V., J.M.-G. and L.L.-M.; writing—review and editing, B.T.-V. L.L.-M., J.M.-G. and Y.G.-A.; visualization, B.T.-V., L.M. and J.M.-G.; supervision, L.L.-M., B.T.-V. and J.M.-G.; project administration, L.L.-M. and J.M.-G. All authors have read and agreed to the published version of the manuscript.

Funding: This research received no external funding

Conflicts of Interest: The authors declare no conflict of interest.

References

1. European Centre for Disease Prevention and Control (ECDC). Communicable Disease Threats Report. Available online: https://www.ecdc.europa.eu/sites/default/files/documents/Communicable-disease-threats-report-13-june-2020.pdf (accessed on 13 June 2020).
2. Taylor, S. *The Psychology of Pandemics: Preparing for the Next Global Outbreak of Infectious Disease*; Cambridge Scholars Publishing: Newcastle upon Tyne, UK, 2019.
3. Wu, P.; Fang, Y.; Guan, Z.; Fan, B.; Kong, J.; Yao, Z.; Liu, X.; Fuller, C.J.; Susser, E.; Lu, J.; et al. The psychological impact of the SARS epidemic on hospital employees in China: Exposure, risk perception, and altruistic acceptance of risk. *Can. J. Psychiatry* **2009**, *54*, 302–311. [CrossRef] [PubMed]
4. Di Giuseppe, M.; Miniati, M.; Miccoli, M.; Ciacchini, R.; Orrù, G.; Lo Sterzo, R.; Di Silvestre, A.; Conversano, C. Defensive responses to stressful life events associated with cancer diagnosis. *Mediterr. J. Clin. Psychol.* **2020**, *8*, 1–22.
5. Miniati, M.; Petracca, A.; Carmassi, C.; Mauri, M.; Fratta, S.; Fui, E.; Giunti, I.; Gesi, C.; Macchia, G.; Dell'osso, L. Risk of Post-Traumatic Stress Disorder in 111 survivors the 2009 Viareggio (Italy) Rail Crash: The role of mood spectrum comorbidity. *Psychopathology* **2017**, *23*, 12–18.
6. Lee, J.S.; Ahn, Y.S.; Jeong, K.S.; Chae, J.H.; Choi, K.S. Resilience buffers the impact of traumatic events on the development of PTSD symptoms in firefighters. *J. Affect. Disord.* **2014**, *162*, 128–133. [CrossRef]
7. Foster, K.; Roche, M.; Delgado, C.; Cuzzillo, C.; Giandinoto, J.A.; Furness, T. Resilience and mental health nursing: An integrative review of international literature. *Int. J. Ment. Health Nurs.* **2019**, *28*, 71–85. [CrossRef]
8. Chen, S.; Bonanno, G.A. Psychological adjustment during the global outbreak of COVID-19: A resilience perspective. *Psychol. Trauma* **2020**, *12*, 51–54. [CrossRef]
9. Vinkers, C.H.; van Amelsvoort, T.; Bisson, J.I.; Branchi, I.; Cryan, J.F.; Domschke, K.; Howes, O.D.; Machia, M.; Pinto, L.; de Quervain, D.; et al. Stress resilience during the coronavirus pandemic. *Eur. Neuropsychopharm.* **2020**, *35*, 12–16. [CrossRef]

10. Marjanovic, Z.; Greenglass, E.R.; Coffey, S. The relevance of psychosocial variables and working conditions in predicting nurses' coping strategies during the SARS crisis: An online questionnaire survey. *Int. J. Nurs. Stud.* **2007**, *44*, 991–998. [CrossRef]
11. Benedek, D.M.; Fullerton, C.; Ursano, R.J. First responders: Mental health consequences of natural and human-made disasters for public health and public safety workers. *Annu. Rev. Public Health* **2007**, *28*, 55–68. [CrossRef]
12. Maslach, C.; Leiter, M.P. Early predictors of job burnout and engagement. *J. Appl. Psychol.* **2008**, *93*, 498–512. [CrossRef]
13. Schaufeli, W.B.; Leiter, M.P.; Maslach, C. Burnout: 35 years of research and practice. *Career Dev. Int.* **2009**, *14*, 204–220. [CrossRef]
14. Burghi, G.; Lambert, J.; Chaize, M.; Goinheix, K.; Quiroga, C.; Fariña, G.; Godino, M.; Pittini, G.; Pereda, S.; Fregossi, C.; et al. Prevalence, risk factors and consequences of severe burnout syndrome in ICU. *Intensive Care Med.* **2014**, *40*, 1785–1786. [CrossRef] [PubMed]
15. González-Sanguino, C.; Ausín, B.; Castellanos, M.A.; Saiz, J.; López-Gómez, A.; Ugidos, C.; Muñoz, M. Mental health consequences during the initial stage of the 2020 Coronavirus pandemic (COVID-19) in Spain. *Brain Behav. Immun.* **2020**, *87*, 172–176. [CrossRef] [PubMed]
16. Wang, C.; Pan, R.; Wan, X.; Tan, Y.; Xu, L.; Ho, C.S.; Ho, R.C. Immediate Psychological Responses and Associated Factors during the Initial Stage of the 2019 Coronavirus Disease (COVID-19) Epidemic among the General Population in China. *Int. J. Environ. Res. Public Health* **2020**, *17*, 1729. [CrossRef] [PubMed]
17. Mazza, C.; Ricci, E.; Biondi, S.; Colasanti, M.; Ferracuti, S.; Napoli, C.; Roma, P. A nation survey of psychological distress among Italian people during the COVID-19 pandemic: Inmediate psychological responses and associated factors. *Int. J. Environ. Res. Public Health* **2020**, *17*, 3165. [CrossRef] [PubMed]
18. Park, J.S.; Lee, E.H.; Park, N.R.; Choi, Y.H. Mental Health of Nurses Working at a Government designated Hospital During a MERS-CoV Outbreak: A Cross-sectional Study. *Arch. Psychiatr. Nurs.* **2018**, *32*, 2–6. [CrossRef]
19. Red Nacional de Vigilancia Epidemiológica. Informe sobre la situación de COVID-19 en personal sanitario en España. Available online: https://www.isciii.es/QueHacemos/Servicios/VigilanciaSaludPublicaRENAVE/EnfermedadesTransmisibles/Documents/INFORMES (accessed on 13 June 2020).
20. Xiao, X.; Zhu, X.; Fu, S.; Hu, Y.; Li, X.; Xiao, J. Psychological impact of healthcare workers in China during COVID-19 pneumonia epidemic: A multi-center cross-sectional survey investigation. *J. Affect. Disord.* **2020**, *274*, 405–410. [CrossRef]
21. Lai, J.; Ma, S.; Wang, Y.; Cai, Z.; Hu, J.; Wei, N.; Wu, J.; Du, H.; Chen, T.; Li, R.; et al. Factors Associated with Mental Health Outcomes among Health Care Workers Exposed to Coronavirus Disease 2019. *JAMA Netw. Open* **2020**, *3*, 1–12. [CrossRef]
22. Zerbini, G.; Ebigbo, A.; Reicherts, P.; Kunz, M.; Messman, H. Psychosocial burden of healthcare professionals in times of COVID-19- survey conducted at the University Hospital Augsburg. *Ger. Med. Sci.* **2020**, *18*, 1–9.
23. Anmella, G.; Fico, G.; Roca, A.; Gómez-Ramiro, M.; Vázquez, M.; Murru, A.; Pacchiarotti, I.; Verdolini, N.; Vieta, E. Unravelling potential severe psychiatric repercussions on healthcare professionals during the COVID-19 crisis. *J. Affect. Disord.* **2020**, *273*, 422–424. [CrossRef]
24. Lietz, J.; Westermann, C.; Nienhaus, A.; Schablon, A. The occupational risk of influenza A (H1N1) infection among healthcare personnel during the 2009 pandemic: A systematic review and meta-analysis of observational studies. *PLoS ONE* **2016**, *11*, 1–19. [CrossRef] [PubMed]
25. Wong, T.W.; Yau, J.K.; Chan, C.L.; Kwong, R.S.; Ho, S.M.; Lau, C.C.; Lau, F.L.; Lit, C.H. The psychological impact of severe acute respiratory syndrome outbreak on healthcare workers in emergency departments and how they cope. *Eur. J. Emerg. Med.* **2005**, *12*, 13–18. [CrossRef] [PubMed]
26. Dong, L.; Bouey, J. Public mental health crisis during COVID-19 pandemic, China. *Emerg. Infect. Dis.* **2020**, *26*, 1–3. [CrossRef] [PubMed]
27. Chen, Q.; Liang, M.; Li, Y.; Guo, J.; Fei, D.; Wang, L.; He, L.; Sheng, C.; Cai, Y.; Li, X.; et al. Mental health care for medical staff in China during the COVID-19 outbreak. *Lancet Psychiatry* **2020**, *7*, e15. [CrossRef]
28. Burdorf, A.; Porru, F.; Rugulies, R. The COVID-19 (Coronavirus) pandemic: Consequences for occupational health. *Scand. J. Work Environ. Health* **2020**, *46*, 229–230. [CrossRef]

29. Weiss, D.S.; Marmar, C.R. The Impact of Event Scale-Revised. In *Assesing Psychological Trauma and PTSD: A Handbook for Practioners*; Wilson, J.P., Keane, T.M., Eds.; Guildford Press: New York, NY, USA, 1997; pp. 399–411.
30. Costa, G.; Gil, F.L. Propiedades psicométricas de la Escala Revisada del Impacto del Evento Estresante (IES-R) en una muestra española de pacientes con cáncer. *Análisis y Modificación de Conducta* **2007**, *33*, 311–331.
31. Zigmond, A.S.; Snaith, R.P. The Hospital Anxiety and Depression Scale. *Acta Psychiatr. Scand.* **1983**, *67*, 361–370. [CrossRef]
32. Terol, M.C.; López-Roig, S.; Rodríguez-Marín, J.; Martín-Aragón, M.; Pastor, M.A.; Reig, M.T. Propiedades psicométricas de la escala Hospitalaria de Ansiedad y Depresión (HAD) en población española. *Ansiedad Estrés* **2007**, *13*, 163–176.
33. Maslach, C.; Jackson, S.E.; Leiter, M.P. *Maslach Burnout Inventory Manual*, 3rd ed.; Consulting Psychologists Press: Palo Alto, CA, USA, 1996.
34. Seisdedos, N. *MBI Human Services*; TEA: Madrid, Spain, 1997.
35. Aguayo, R.; Vargas, C.; de la Fuente, E.I.; Lozano, L.M. A meta-analytic reliability generalization study of the Maslach Burnout Inventory. *Int. J. Clin. Health Psychol.* **2011**, *11*, 343–361.
36. Cañadas, G.A.; San Luis, C.; Lozano, L.M.; Vargas, C.; García, I.; de la Fuente, E.I. Evidence for factorial validity of Maslach Burnout Inventory and burnout levels among health workers. *Rev. Latinoam. Psicol.* **2014**, *46*, 44–52.
37. Smith, B.W.; Dalen, J.; Wiggins, K.; Tooley, E.; Christopher, P.; Bernard, J. The Brief Resilience Scale: Assessing the ability to bounce back. *Int. J. Behav. Med.* **2008**, *15*, 194–200. [CrossRef] [PubMed]
38. Rodríguez-Rey, R.; Alonso-Tapia, J.; Hernansaiz, H. Reliability and validity of the Brief Resilience Scale (BRS) Spanish version. *Psychol. Assess.* **2016**, *28*, 101–110. [CrossRef] [PubMed]
39. Xiao, S.; Luo, D.; Xiao, Y. Survivors of COVID-19 are at high risk of posttraumatic stress disorder. *Glob. Health Res. Policy* **2020**, *5*, 1–3. [CrossRef] [PubMed]
40. Pappa, S.; Ntella, V.; Giannakas, T.; Giannakoulis, V.G.; Papoutsi, E.; Katsaonou, P. Prevalence of depression, anxiety, and insomnia among healthcare workers during the COVID-19: A systematic review and meta-analysis. *Brain Behav. Immun.* **2020**, in press. [CrossRef] [PubMed]
41. Cheung, T.; Yip, P.S. Depression, anxiety and symptoms of stress among Hong Kong nurses: A cross-sectional study. *Int. J. Environ. Res. Public Health* **2015**, *12*, 11072–11100. [CrossRef]
42. Shrestha, R. Post-traumatic Stress Disorder among medical personnel after Nepal earthquake, 2015. *J. Nepal Health Res. Counc.* **2015**, *13*, 144–148.
43. Pérez-Martinez de Arrieta, P.; Gaviña, J. Affective Disorders and Gender Differences. In *Psychopathology in Women*; Sáenz-Herrero, M., Ed.; Springer: Berlin, Germany, 2019; pp. 611–638.
44. Costa, C.; Mondello, S.; Micali, E.; Indelicato, G.; Licciardello, A.A.; Vitalle, E.; Briguglio, G.; Teodoro, M.; Fenga, C. Night shift work in resident physicians: Does it affect mood states and cognitive levels? *J. Affect. Disord.* **2020**, *272*, 289–294. [CrossRef]
45. Lee, A.; Myung, S.K.; Cho, J.J.; Jung, Y.J.; Yoon, J.L.; Kim, M.Y. Night shift work and risk of depression: Meta-analysis of observational studies. *Occup. Environ. Med.* **2017**, *32*, 1091–1096. [CrossRef]
46. Adams, J.G.; Walls, R.M. Supporting the health care workforce during the COVID-19 global epidemic. *JAMA* **2020**, *323*, 1439–1440. [CrossRef]
47. Guo, Y.T.; Cao, Q.D.; Hong, Z.S.; Tan, Y.Y.; Chen, S.D.; Jin, H.J.; Tan, K.S.; Wang, D.Y.; Yan, Y. The origin, transmission and clinical therapies on coronavirus disease 2019 (COVID-19) outbreak: An update on the status. *Mil. Med. Res.* **2020**, *7*, 1–10. [CrossRef]
48. World Health Organization-WHO. Director-General's Opening Remarks at the Media Briefing on COVID-19. Available online: https://www.who.int/dg/speeches/detail/who-director-general-s-opening-remarks-at-the-media-briefing-on-covid-19---29-june-2020 (accessed on 29 June 2020).
49. Gardner, P.G.; Moallef, P. Psychological impact of SARS survivors: Critical review of the English Language Literature. *Can. Psychol.* **2015**, *56*, 123–135. [CrossRef]
50. Aiello, A.; Khayeri, M.; Raja, S.; Peladeau, N.; Romano, D.; Leszcz, M.; Maunder, R.G.; Rose, M.; Adam, M.A.; Pain, C.; et al. Resilience training for hospital workers in anticipation of Influenza pandemic. *J. Contin. Educ. Health. Prof.* **2011**, *31*, 15–20. [CrossRef] [PubMed]

51. Etkind, S.N.; Bone, A.E.; Lovell, N.; Cripps, R.L.; Harding, R.; Higginson, I.J.; Sleeman, K.E. The Role and Response of Palliative Care and Hospice Services in Epidemics and Pandemics: A Rapid Review to Inform Practice During the COVID-19 Pandemic. *J. Pain Symptom Manag.* **2020**, *60*, 31–40. [CrossRef] [PubMed]
52. Albott, C.S.; Wozniak, J.R.; McGlinch, B.P.; Wall, M.H.; Gold, B.S.; Vinogradov, S. Battle Buddies: Rapid deployment of a psychological resilience intervention for health care workers during the COVID-19 pandemic. *Anesth. Analg.* **2020**, *131*, 43–54. [CrossRef]
53. Seidler, A.; Thinschmidt, M.; Deckert, S.; Then, F.; Hegewald, J.; Nieuwenhuijsen, K.; Riedel, S.G. The role of psychosocial working conditions on burnout and its core component emotional exhaustion—A systematic review. *J. Occup. Med. Toxicol.* **2014**, *9*, 1–13. [CrossRef]
54. Sasanhogar, F.; Jones, S.L.; Masud, F.N.; Vahidy, F.S.; Kash, B.A. Provider burnout and fatigue during the COVID-19 pandemic: Lessons learned from a high-volume intensive care unit. *Anesth. Analg.* **2020**, *131*, 106–111. [CrossRef]
55. Schippers, M. For the Greater Good? 2 The Devastating Ripple Effects of the COVID-19 Crisis. Available online: https://ssrn.com/abstract=3612622 (accessed on 18 July 2020).
56. Zhong, B.L.; Luo, W.; Li, H.M.; Zhang, Q.Q.; Liu, X.G.; Li, W.T.; Li, Y. Knowledge, attitudes, and practices towards COVID-19 among Chinese residents during the rapid rise period of the COVID-19 outbreak: A quick online cross-sectional survey. *Int. J. Biol. Sci.* **2020**, *16*, 1745–1752. [CrossRef]
57. Gallè, F.; Sabella, E.A.; Da Molin, G.; De Giglio, O.; Caggiano, G.; Di Onofrio, V.; Ferracuti, S.; Montagna, M.T.; Liguori, G.; Orsi, G.B.; et al. Understanding Knowledge and Behaviors Related to COVID–19 Epidemic in Italian Undergraduate Students: The EPICO Study. *Int. J. Environ. Res. Public Health* **2020**, *17*, 3481. [CrossRef]
58. Reynolds, D.L.; Garay, J.R.; Deamond, S.L.; Moran, M.K.; Gold, W.; Styra, R. Understanding, compliance and psychological impact of the SARS quarantine experience. *Epidemiol. Infect.* **2008**, *136*, 997–1007. [CrossRef]
59. Restauri, N.; Sheridan, A.D. Burnout and posttraumatic stress disorder in the Coronavirus Disease 2019 (COVID-19) pandemic: Intersection, impact, and interventions. *J. Am. Coll. Radiol.* **2020**, *17*, 921–926. [CrossRef]
60. Frias, C.E.; Cuzco, C.; Frias-Martin, C.; Pérez-Ortega, S.; Triviño López, J.A.; Lombraña, M. Resilience and emotional support in health care professionals during the COVID-19 pandemic. *J. Psychosoc. Nurs. Men. Health Serv.* **2020**, *58*, 5–6. [CrossRef] [PubMed]

© 2020 by the authors. Licensee MDPI, Basel, Switzerland. This article is an open access article distributed under the terms and conditions of the Creative Commons Attribution (CC BY) license (http://creativecommons.org/licenses/by/4.0/).
