# Supplementary material for: Mental health status and related factors influencing healthcare workers during the COVID-19 pandemic: A systematic review and meta-analysis
Source: PLoS One. 2024 Jan 19;19(1):e0289454. doi: 10.1371/journal.pone.0289454 (PMC10798549; doi:10.1371/journal.pone.0289454)
Supplement: S1 Data — (ZIP) [file pone.0289454.s011.zip › literatures/83.pdf]

# Mental Health, Burnout, and Resilience in Healthcare Professionals After the First Wave of COVID-19 Pandemic in Spain

## *A Longitudinal Study*

*Lourdes Luceño-Moreno, PhD, Beatriz Talavera-Velasco, PhD, Daniel Vázquez-Estévez, MSc, and Jesús Martín-García, PhD*

**Objective:** This study aims to examine whether there are differences in symptoms of posttraumatic stress, depression, anxiety, levels of burnout and resilience in Spanish healthcare staff between the first wave of the COVID-19 pandemic and after it, depending on several demographic and work-related variables. **Methods:** A longitudinal study was conducted in April 2020 (T0), and July 2020 (T1). Symptoms of posttraumatic stress, depression, anxiety, burnout, levels of resilience, along with demographic and work-related variables in 443 workers were assessed. **Results:** Symptoms and burnout were more pronounced at T0, whereas the levels of resilience were higher at T1. Being women, being young, holding a lower-level job, less years of experience, lower educational level, and/or working rotating shifts are associated with having more posttraumatic stress symptoms and burnout. **Conclusion:** These variables would be considered in similar situations.

**Keywords:** burnout, COVID-19, healthcare professionals, longitudinal, resilience

On March 11, 2020, the World Health Organization declared the disease caused by COVID-19 to be a pandemic.[1] Spanish healthcare professionals have had to reorganize their work since then, seeing a larger number of patients. They have been exposed to situations of stress and emotional exhaustion, something that has also happened in other countries, such as India, Iran, Singapore, and China.[2–4] A recent meta-analysis revealed that Spain, China, Iran, Italy, and Turkey reported the highest prevalence of anxiety and depression in healthcare staff. The associated factors were being women, being nursing staff, having less work experience, low socio-economic status, being socially isolated, and high risk of being infected with COVID-19.[5] Similarly, resilience has been highlighted as a factor associated with less stress during the pandemic. Resilience and burnout levels were analyzed in Italian nurses, concluding that the resilience factor predicted a reduction in stress levels, emotional fatigue, and depersonalization, and it was associated with an increase in personal fulfilment.[6] Second, nursing professionals were found to have a higher prevalence of depression, especially those working in the emergency services.[7] Other variables associated with anxiety, depression, and posttraumatic stress in healthcare workers are less work experience, being single, lower educational level, holding intermediate positions, seeing a greater number of patients, or feeling a lack of professional competence.[8–11] Some longitudinal studies in the scientific literature have been conducted during the pandemic. For example, in a study in a general Chinese population, posttraumatic stress scores a month after the baseline were lower, although the levels of anxiety and depression remained approximately the same.[12] In the US population, young adults who perceived less social support and had high levels of ruminative thoughts showed higher levels of stress, anxiety, and depression at the first time point.[13] In Spanish general population, a longitudinal study conducted during the state of emergency declared by the Government, revealed that, in general, the levels of posttraumatic stress, anxiety, and depression were higher during the lockdown and decreased over time, although it is not still possible to speak of a full recovery.[14] In another study including healthcare staff in Japan, the first measure was on March 19, 2020, and the follow-up was 2 months later. The authors highlight that the levels of fatigue, anxiety, and depression among these health professionals were higher at the second time point, even more significantly than in non-healthcare staff. More longitudinal studies should be conducted for a variety of reasons, including the scarcity of publications. For example, during the outbreak of Severe Acute Respiratory Syndrome (SARS) in 2003, healthcare professionals experienced levels of stress that lasted up to a year, making it evident that it was not an adjustment disorder.[15,16] On the other hand, various researchers call for longitudinal studies to analyze long-term effects, focusing on the detection of possible posttraumatic stress disorder in this professional group.[17] Exposure to traumatic situations, stressors (lack of resources, increased workload, emotional exhaustion), and lack of time to recover, seem to be associated with burnout in these workers.[18] The objective of this study is to examine whether there are differences in symptoms of posttraumatic stress, depression, anxiety, levels of resilience and burnout in healthcare staff in Spain during the first wave of the COVID-19 pandemic (hereinafter T0) and after that (hereinafter T1), depending on demographic and work-related variables. The working hypotheses are as follows:

Healthcare workers will show higher levels of emotional exhaustion, depersonalization, anxiety, depression, and posttraumatic stress at the baseline (T0) than at 3-month follow-up (T1).

Healthcare workers with a lower educational level or that hold a lower-level job will present higher levels of emotional fatigue, depersonalization, anxiety, depression, and/or posttraumatic stress.

Healthcare professionals will show higher levels of resilience at the follow-up measure.

## METHODS

### Participants and Design

The first round of data was collected between April 1 and 10, 2020 T0. At this time 1476 healthcare workers participated, 206 (14%) men and 1270 (86%) women, aged 19 to 68 years, and a mean

From the Complutense University of Madrid, Madrid, Spain.
Funding sources: No funding was received for conducting this study.
The authors have no conflicts of interest to disclose.
Ethical considerations & Disclosure: This study was approved by the Ethic Committee of the Faculty of Psychology of the Complutense University of Madrid (Reference number: Pr_2019_038).
Clinical significance: This research identifies that resilience increases over time awareness of demands on these professionals. It is possible to specialize treatment programs to prevent the effects of burnout and anxiety, depressive and posttraumatic stress symptoms in healthcare personnel, including the variables identified in this study that are associated with said symptoms.
Address correspondence to: Beatriz Talavera-Velasco, PhD, Department of Social and Work Psychology and Individual Differences, Faculty of Psychology, Complutense University of Madrid, Campus Somosaguas s/n, 28223, Pozuelo de Alarcón, Madrid, Spain (beatriztalavera@ucm.es).
Copyright © 2021 American College of Occupational and Environmental Medicine
DOI: 10.1097/JOM.0000000000002464



Copyright © 2022 American College of Occupational and Environmental Medicine. Unauthorized reproduction of this article is prohibited

age of 44 years (standard deviation [SD] = 10.76). The second set of data was collected between July 1 and 10, 2020 T1. At time point T1, the first wave of the pandemic had already ended. Of the 1476 individuals who participated in the first measure, 443 participated again at T1, 54 (12.2%) men and 389 (87.8%) women, between the ages of 22 and 65 years. The mean age was 45.26 (SD = 10.05), ages 22 to 65 years this time.

## Instruments

### Demographic and Work-Related Variables

Information related to demographic variables, sex, age, educational level, marital status, dependent family members, number of children in the household; and variables associated with the workplace (Autonomous Community of workplace, job classification, job title, type of workplace setting, work shift, years of seniority in the current position, and years of experience as a healthcare worker).

### Burnout

The Spanish version of the Maslach Burnout Inventory-MBI-HSS was used.[19,20] It is made up of 22 items with seven response options that are answered on a Likert scale, from 0 (never) to 6 (every day). Burnout is defined as: high emotional fatigue, high depersonalization, and low personal fulfilment. This questionnaire shows an adequate adjustment of three factors and internal consistency above 0.71 for the subscales.[21]

### Posttraumatic Stress

The Spanish version of the Impact of Event Scale-Revised IES-R was used.[22] It evaluates the emotional distress that accompanies a stressful life event. It consists of 22 items and three scales: intrusion (seven items), avoidance (eight items), and hyperarousal (seven items). It shows adequate psychometric characteristics, with a reliability level above 0.70.

### Anxiety and Depression

The Spanish adaptation of the Hospital Anxiety & Depression Scale-HADS instrument was used.[23,24] It consists of 14 items and two subscales: anxiety and depression, and it is answered using a four-point Likert scale (0 to 3). It evaluates symptoms of anxiety and depression in clinical and general population, higher scores indicating a higher prevalence of symptoms. It shows adequate psychometric properties, presenting an internal consistency of 0.77 and 0.71 for the anxiety and the depression subscales, respectively.[23]

### Resilience

The Spanish adaptation of the Brief Resilience Scale (BRS) was used.[25,26] It is composed of six items that are answered on a Likert scale, with responses ranging from 1 (strongly disagree) to 5 (strongly agree). High scores indicate higher levels of resilience. It has a unifactorial structure and an internal consistency of 0.83.

## Procedure

A longitudinal design was used. The research obtained the approval of the Ethic Committee of the Faculty of Psychology of the Complutense University of Madrid (Reference number: Pr_2019_038). Because of the emergency lockdown, data were collected using an online survey between April 1 and 10, 2020 (first wave of the pandemic in Spain, T0). Follow-up data were collected 3 months later, between July 1 and 10, 2020 (T1), when the first wave of the pandemic had ended. In order to participate at T0 and T1, the need to give informed consent was specified at the beginning of the survey. The data were treated anonymously and confidentially. Data from T0 and T1 were paired using an identifier, following the same timeline in those individuals who consented to participate in the second measure.

## Data Analysis

The analyses were carried out using the SPSS 26 statistical package (IBM Corp. Released 2019, IBM SPSS Statistics for Windows, Version 26.0., Armonk, NY). Descriptive statistics (frequencies, means, standard deviations, variances, and ranges) of the variables under study were calculated. Statistical inference analyses were carried out in order to analyze whether there are any statistically significant effects of the proposed segmentation factors (demographic and workplace variables) and the time elapsed between T0 and T1 in the objective variables. Mixed analysis of variances (ANOVAs) were performed with a Between-Subjects factor (segmentations by group) and a Within-Subjects factor (time). Mauchly W test was used to analyze the assumption of sphericity. If this assumption were not met, the Greenhouse–Geisser correction would be taken into account in the presentation of results. A posteriori pairwise comparisons were made to distinguish between groups in which there are statistically significant differences. The segmentation factors (Between-Subjects) were demographic variables (sex, age, educational level, marital status, dependent family members, number of children in the household) and work-related variables (Autonomous Community of workplace, job classification, job title, type of workplace setting, work shift, years of seniority in the current job, and years of experience as a healthcare worker).

# RESULTS

These are the results of the mixed ANOVAs carried out by taking the following variables into account: burnout, posttraumatic stress, anxiety, depression, and resilience, for each of the demographic and work-related variables:

## Emotional Fatigue (Burnout)

There are statistically significant differences between men and women in emotional exhaustion, women presenting higher scores than men $df = 4.279$, $P < 0.05$. As for the Autonomous Community where the workplace is located, there is a significant interaction effect between emotional fatigue and the Autonomous Community, with emotional fatigue being greater at T0 for those who work in the Community of Madrid $df = 2.478$, $P < 0.05$. There are also significant interaction effects between emotional exhaustion and age, emotional exhaustion is greater in workers aged between 36 and 50 years at T0, $df = 1.783$, $P < 0.05$ whereas it is lower in workers aged 51 or over at T1 $df = -1.599$, $P < 0.05$. In addition, there are significant interaction effects between emotional exhaustion and the type of workplace setting. Hospital workers are more emotionally tired at T0 than they are at T1 $df = 1.196$, $P < 0.05$. Reversely, primary care workers report more emotional fatigue at T1 than at T0 $df = -2.463$, $P < 0.05$. There are also statistically significant differences between emotional exhaustion and years of experience as a healthcare worker. Individuals who have been working in healthcare for less than 5 years report less emotional fatigue than those who have been working as health professionals for 5 to 15 years $df = -5.747$, $P < 0.01$ (Table 1).

## Depersonalization (Burnout)

Statistically significant differences were found between depersonalization and age, with younger workers (18 to 35 years) reporting feeling more depersonalized than those between the ages of 36 and 50 years, $df = 2.193$, $P < 0.01$. In addition, there are statistically significant differences between T0 and T1 in depersonalization, scores being higher at T0 $df = 1.056$, $P < 0.001$. There are significant interaction effects between depersonalization and the type of workplace setting. Primary healthcare workers have lower

© 2021 American College of Occupational and Environmental Medicine 

Copyright © 2022 American College of Occupational and Environmental Medicine. Unauthorized reproduction of this article is prohibited

**TABLE 1.** Association Between Sociodemographic Variables and Workplace Variables With Burnout

| Variables | N | Emotional Exhaustion T0 | Emotional Exhaustion T1 | Emotional Exhaustion F | Depersonalization T0 | Depersonalization T1 | Depersonalization F | Personal Accomplishment T0 | Personal Accomplishment T1 | Personal Accomplishment F |
|---|---|---|---|---|---|---|---|---|---|---|
| Sex | | | | | | | | | | |
| Men | 54 | 24.53±13.53 | 24.01 ± 13.39 | (T) $F$ (1, 441) = 0.629 | 6.81 ± 6.75 | 6.96 ± 6.57 | (T) $F$ (1, 441) = 0.233 | 39.37 ± 7.06 | 39.12 ± 7.15 | (T) $F$ (1, 441) = 3.153 |
| Women | 389 | 28.81 ± 12.36 | 28.30 ± 12.89 | **(B) $F$ (1, 441) = 6.11**$^{*}$ | 6.20 ± 5.68 | 6.42 ± 5.96 | (B) $F$ (1, 441) = 0.545 | 39.71 ± 6.48 | 38.50 ± 6.67 | (B) $F$ (1, 441) = 0.025 |
| Total | 443 | 28.29 ± 12.59 | 27.77 ± 13.01 | (I) $F$ (1, 441) = 0.00 | 6.28 ± 5.68 | 6.49 ± 6.04 | (I) $F$ (1, 441) = 0.009 | 39.67 ± 6.55 | 38.57 ± 6.75 | (I) $F$ (1, 441) = 1.416 |
| Age | | | | | | | | | | |
| 18–35 | 83 | 10.62 ± 11.26 | 29.22 ± 12.09 | (T) $F$ (1, 440) = 1.37 | 7.74 ± 6.09 | 8.96 ± 6.36 | (T) $F$ (1, 440) = 2.21 | 38.34 ± 5.81 | 37.75 ± 6.09 | **(T) $F$ (1, 440) = 11.73**$^{***}$ |
| 36–50 | 205 | 29.37 ± 12.56 | 27.59 ± 12.38 | (B) $F$ (2, 440) = 2.55 | 6.20 ± 5.67 | 6.12 ± 5.81 | **(B) $F$ (2, 440) = 7.64**$^{***}$ | 39.95 ± 6.38 | 38.53 ± 6.83 | (B) $F$ (2, 440) = 1.74 |
| ≥51 | 157 | 25.65 ± 12.87 | 27.25 ± 14.24 | **(I) $F$ (2, 440) = 6.99**$^{***}$ | 5.61 ± 5.75 | 5.66 ± 5.84 | (I) $F$ (2, 440) = 1.89 | 40.01 ± 7.06 | 39.06 ± 6.91 | (I) $F$ (2, 440) = 0.708 |
| Total | 443 | 28.29 ± 12.57 | 27.77 ± 13.01 | | 6.28 ± 5.82 | 6.49 ± 6.04 | | 39.67 ± 6.55 | 38.57 ± 6.73 | |
| Education level completed | | | | | | | | | | |
| Secondary Education | 175 | 27.45 ± 12.74 | 27.09 ± 13.22 | (T) $F$ (1, 440) = 1.69 | 6.31 ± 5.79 | 6.13 ± 5.84 | (T) $F$ (1, 440) = 0.897 | 39.70 ± 6.73 | 38.50 ± 7.20 | **(T) $F$ (1, 440) = 14.62**$^{***}$ |
| Bachelor's degree. | 162 | 28.77 ± 12.36 | 28.39 ± 12.76 | (B) $F$ (2, 440) = 0.585 | 6.62 ± 6.02 | 7.14 ± 6.24 | (B) $F$ (2, 440) = 1.241 | 39.74 ± 6.19 | 38.52 ± 6.20 | (B) $F$ (2, 440) = 0.002 |
| Master's or Doctor's degree | 106 | 28.93 ± 12.66 | 27.97 ± 13.11 | (I) $F$ (2, 440) = 0.175 | 5.70 ± 5.54 | 6.09 ± 6.00 | (I) $F$ (2, 440) = 0.835 | 39.52 ± 6.82 | 38.78 ± 6.75 | (I) $F$ (2, 440) = 0.272 |
| Total | 443 | 28.29 ± 12.57 | 27.77 ± 13.01 | | 6.28 ± 5.82 | 6.49 ± 6.04 | | 39.67 ± 6.55 | 38.57 ± 6.73 | |
| Marital status | | | | | | | | | | |
| Married | 231 | 28.11 ± 12.84 | 27.91 ± 13.36 | (T) $F$ (1, 439) = 1.36 | 6.22 ± 6.00 | 5.95 ± 5.95 | (T) $F$ (1, 439) = 2.611 | 39.73 ± 6.99 | 38.98 ± 6.58 | **(T) $F$ (1, 439) = 16.71**$^{***}$ |
| Living with partner, not married | 74 | 28.79 ± 11.43 | 28.08 ± 11.76 | (B) $F$ (3, 439) = 0.534 | 6.55 ± 5.59 | 7.58 ± 6.46 | (B) $F$ (3, 439) = 2.502 | 39.70 ± 5.49 | 37.67 ± 6.56 | **(B) $F$ (3, 439) = 2.785**$^{*}$ |
| Separated or widower/widow | 53 | 29.69 ± 12.72 | 26.43 ± 14.42 | (I) $F$ (3, 439) = 0.775 | 4.96 ± 4.78 | 5.41 ± 5.75 | (I) $F$ (3, 439) = 0.1.478 | 41.26 ± 5.60 | 40.22 ± 6.67 | (I) $F$ (3, 439) = 0.989 |
| Single | 85 | 29.69 ± 12.72 | 27.97 ± 12.30 | | 7.02 ± 6.02 | 7.68 ± 5.82 | | 38.51 ± 6.57 | 37.23 ± 7.07 | |
| Total | 443 | 28.29 ± 12.57 | 27.77 ± 13.01 | | 6.28 ± 5.82 | 6.49 ± 6.04 | | 39.67 ± 6.55 | 38.57 ± 6.73 | |
| Dependent relatives | | | | | | | | | | |
| Yes | 271 | 27.74 ± 13.00 | 27.31 ± 13647 | (T) $F$ (1, 441) = 1.509 | 6.07 ± 5.90 | 6.01 ± 6.04 | (T) $F$ (1, 441) = 1.291 | 40.19 ± 6.58 | 39.04 ± 6.90 | **(T) $F$ (1, 441) = 15.407**$^{***}$ |
| No | 172 | 29.15 ± 11.86 | 28.51 ± 12.24 | (B) $F$ (1, 441) = 1.250 | 6.61 ± 5.68 | 7.25 ± 5.97 | (B) $F$ (1, 441) = 2.968 | 38.86 ± 6.42 | 37.84 ± 6.41 | **(B) $F$ (1, 441) = 4.72**$^{*}$ |
| Total | 443 | 28.29 ± 12.57 | 27.77 ± 13.01 | (I) $F$ (1, 441) = 0.062 | 6.28 ± 5.82 | 6.49 ± 6.04 | (I) $F$ (1, 441) = 1.906 | 39.67 ± 6.55 | 38.57 ± 6.73 | (I) $F$ (1, 441) = 0.059 |
| No. of children in your care | | | | | | | | | | |
| 0 | 173 | 29.84 ± 12.10 | 28.98 ± 12.16 | (T) $F$ (1, 440) = 1.161 | 6.89 ± 5.86 | 7.39 ± 6.09 | (T) $F$ (1, 440) = 0.631 | 38.70 ± 6.49 | 37.46 ± 6.60 | **(T) $F$ (1, 440) = 16.605**$^{***}$ |
| 1 | 113 | 27.64 ± 12.38 | 27.49 ± 13.50 | (B) $F$ (2, 440) = 2.012 | 5.53 ± 5.50 | 5.74 ± 6.1 | (B) $F$ (2, 440) = 3.129 | 39.83 ± 7.03 | 38.50 ± 7.39 | **(B) $F$ (2, 440) = 5.419**$^{**}$ |
| >1 | 157 | 27.04 ± 13.11 | 26.64 ± 13.51 | (I) $F$ (2, 440) = 0.232 | 6.15 ± 5.95 | 6.04 ± 5.79 | (I) $F$ (2, 440) = 0.553 | 40.63 ± 6.12 | 39.85 ± 6.16 | (I) $F$ (2, 440) = 0.386 |
| Total | 443 | 28.29 ± 12.57 | 27.77 ± 13.01 | | 6.28 ± 5.82 | 6.49 ± 6.04 | | 39.67 ± 6.55 | 38.57 ± 6.73 | |
| Autonomous community of the workplace | | | | | | | | | | |
| Community of Madrid | 375 | 28.13 ± 12.53 | 27.98 ± 13.16 | **(T) $F$ (1, 441) = 5.05**$^{*}$ | 6.27 ± 5.86 | 6.70 ± 6.17 | (T) $F$ (1, 441) = 0.539 | 39.60 ± 6.59 | 38.37 ± 6.70 | **(T) $F$ (1, 441) = 4.71**$^{*}$ |
| Others | 69 | 28.13 ± 12.53 | 27.98 ± 13.16 | (B) $F$ (1, 441) = 0.014 | 6.30 ± 5.60 | 5.37 ± 5.13 | (B) $F$ (1, 441) = 0.867 | 40.07 ± 6.37 | 39.69 ± 6.80 | (B) $F$ (1, 441) = 1.29 |
| Total | 443 | 28.29 ± 12.57 | 27.77 ± 13.01 | **(I) $F$ (1, 441) = 3.966**$^{*}$ | 6.28 ± 5.82 | 6.49 ± 6.04 | (I) $F$ (1, 441) = 3.85 | 39.67 ± 6.55 | 38.57 ± 6.73 | (I) $F$ (1, 441) = 1.33 |
| Professional category | | | | | | | | | | |
| Executive or Intermediate job | 87 | 26.72 ± 13.23 | 27.45 ± 13.78 | (T) $F$ (1, 440) = 0.006 | 5.73 ± 5.65 | 5.57 ± 6.09 | (T) $F$ (1, 440) = 0.051 | 41.48 ± 4.98 | 40.35 ± 6.02 | **(T) $F$ (1, 440) = 10.717**$^{***}$ |
| Base position | 356 | 28.67 ± 12.40 | 27.85 ± 12.83 | (B) $F$ (2, 440) = 0.669 | 6.41 ± 5.85 | 6.71 ± 6.01 | (B) $F$ (2, 440) = 2.064 | 39.23 ± 6.81 | 38.14 ± 6.83 | **(B) $F$ (2, 440) = 9.819**$^{**}$ |
| Total | 443 | 28.29 ± 12.57 | 27.77 ± 13.01 | (I) $F$ (2, 440) = 2.108 | 6.28 ± 5.82 | 6.49 ± 6.04 | (I) $F$ (2, 440) = 0.543 | 39.67 ± 6.55 | 38.57 ± 6.73 | (I) $F$ (2, 440) = 0.002 |
| Post | | | | | | | | | | |
| Medical post | 60 | 30.71 ± 12.64 | 31.06 ± 13.12 | (T) $F$ (1, 439) = 0.054 | 6.016 ± 5.35 | 7.01 ± 6.41 | (T) $F$ (1, 439) = 1.616 | 39.66 ± 6.38 | 39.61 ± 6.25 | **(T) $F$ (1, 439) = 10.688**$^{**}$ |
| Nursing post | 173 | 28.28 ± 12.64 | 27.18 ± 12.86 | (B) $F$ (3, 439) = 1.712 | 6.49 ± 6.00 | 6.52 ± 6.00 | (B) $F$ (3, 439) = 0.227 | 39.34 ± 6.64 | 38.36 ± 6.59 | (B) $F$ (3, 439) = 0.287 |
| Assistant Nurse. | 146 | 28.51 ± 12.32 | 27.57 ± 12.73 | (I) $F$ (3, 439) = 1.377 | 6.39 ± 5.64 | 6.43 ± 5.85 | (I) $F$ (3, 439) = 0.577 | 39.95 ± 6.35 | 38.36 ± 7.10 | (I) $F$ (3, 439) = 1.099 |
| Caregiver | 64 | 25.51 ± 12.66 | 26.76 ± 13.76 | | 5.71 ± 6.20 | 6.06 ± 6.30 | | 39.96 ± 6.98 | 38.65 ± 6.71 | |
| Total | 443 | 28.29 ± 12.57 | 27.77 ± 13.01 | | 6.28 ± 5.82 | 6.49 ± 6.04 | | 39.67 ± 6.55 | 38.57 ± 6.73 | |
| Type of center | | | | | | | | | | |
| Hospital | 286 | 27.87 ± 12.16 | 26.67 ± 12.84 | (T) $F$ (1, 440) = 0.217 | 6.18 ± 5.99 | 6.10 ± 5.67 | (T) $F$ (1, 440) = 3.507 | 39.15 ± 6.70 | 38.15 ± 6.83 | **(T) $F$ (1, 440) = 12.628** $^{***}$ |
| Primary care | 678 | 28.17 ± 13.49 | 30.64 ± 13.48 | (B) $F$ (2, 440) = 1.621 | 5.61 ± 5.43 | 7.40 ± 6.87 | (B) $F$ (2, 440) = 1.078 | 40.25 ± 6.69 | 39.35 ± 6.41 | (B) $F$ (2, 440) = 2.622 |
| Other | 90 | 29.70 ± 13.19 | 29.14 ± 12.87 | **(I) $F$ (2, 440) = 4.606**$^{**}$ | 7.08 ± 5.49 | 7.06 ± 6.37 | **(I) $F$ (2, 440) = 3.595**$^{*}$ | 40.92 ± 5.78 | 39.35 ± 6.58 | (I) $F$ (2, 440) = 0.393 |
| Total | 443 | 28.29 ± 12.57 | 27.77 ± 13.01 | | 6.28 ± 5.82 | 6.49 ± 6.04 | | 39.67 ± 6.55 | 38.58 ± 6.73 | |
| Shift | | | | | | | | | | |
| Fixed shift | 237 | 28.45 ± 13.03 | 28.36 ± 13.20 | (T) $F$ (1, 440) = 1.610 | 5.85 ± 5.64 | 6.25 ± 5.87 | (T) $F$ (1, 440) = 0.445 | 39.61 ± 6.63 | 38.82 ± 6.66 | **(T) $F$ (1, 440) = 8.977** $^{**}$ |
| Rotating shift | 157 | 28.61 ± 11.83 | 27.57 ± 12.39 | (B) $F$ (2, 440) = 0.818 | 6.82 ± 6.10 | 6.71 ± 6.21 | (B) $F$ (2, 440) = 0.990 | 39.83 ± 6.10 | 38.05 ± 6.98 | (B) $F$ (2, 440) = 0.117 |
| Other | 49 | 26.44 ± 12.76 | 25.57 ± 14.00 | (I) $F$ (2, 440) = 0.564 | 6.59 ± 5.66 | 6.91 ± 6.30 | (I) $F$ (2, 440) = 0.453 | 39.48 ± 7.57 | 39.08 ± 6.24 | (I) $F$ (2, 440) = 1.875 |
| Total | 443 | 28.29 ± 12.57 | 27.77 ± 13.01 | | 6.28 ± 5.82 | 6.49 ± 6.04 | | 39.67 ± 6.55 | 38.57 ± 6.73 | |


© 2021 American College of Occupational and Environmental Medicine
Copyright © 2022 American College of Occupational and Environmental Medicine. Unauthorized reproduction of this article is prohibited

**TABLE 1.** (*Continued*)

| Variables | N | Emotional Exhaustion T0 | Emotional Exhaustion T1 | Emotional Exhaustion F | Depersonalization T0 | Depersonalization T1 | Depersonalization F | Personal Accomplishment T0 | Personal Accomplishment T1 | Personal Accomplishment F |
|---|---|---|---|---|---|---|---|---|---|---|
| Time | | | | | | | | | | |
| Full time | 388 | 27.84 ± 12.51 | 27.56 ± 12.76 | (T) F (1, 441) = 3.685 | 6.24 ± 5.78 | 6.55 ± 6.05 | (T) F (1, 441) = 0.006 | 39.82 ± 6.59 | 38.79 ± 6.68 | **(T) F (1, 441) = 10.002**** |
| Part time | 55 | 31.47 ± 12.65 | 29.27 ± 14.68 | (B) F (1, 441) = 2.395 | 6.54 ± 6.10 | 6.03 ± 5.99 | (B) F (1, 441) = 0.021 | 38.60 ± 6.19 | 37.05 ± 6.92 | (B) F (1, 441) = 2.956 |
| Total | 443 | 28.29 ± 12.57 | 27.77 ± 13.01 | (I) F (1, 441) = 2.237 | 6.28 ± 5.82 | 6.49 ± 6.04 | (I) F (1, 441) = 1.179 | 39.67 ± 6.55 | 38.57 ± 6.73 | (I) F (1, 441) = 0.389 |
| Years of seniority | | | | | | | | | | |
| ≤5 | 166 | 26.77 ± 13.27 | 25.90 ± 13.05 | (T) F (1, 440) = 1.304 | 6.04 ± 5.73 | 7.10 ± 6.30 | (T) F (1, 440) = 0.623 | 39.78 ± 6.61 | 38.81 ± 6.54 | **(T) F (1, 440) = 14.871***** |
| 5.01–15 | 185 | 29.38 ± 12.07 | 29.15 ± 12.77 | (B) F (2, 440) = 2.765 | 6.45 ± 5.66 | 6.01 ± 5.50 | (B) F (2, 440) = 0.186 | 39.41 ± 6.47 | 38.17 ± 6.97 | (B) F (2, 440) = 0.504 |
| >15 | 92 | 28.82 ± 12.13 | 28.40 ± 13.15 | (I) F (2, 440) = 0.229 | 6.35 ± 6.32 | 6.35 ± 6.52 | **(I) F (2, 440) = 1.179*** | 40.01 ± 6.62 | 38.94 ± 6.60 | (I) F (2, 440) = 0.104 |
| Total | 443 | 28.29 ± 12.57 | 27.77 ± 13.01 | | 6.28 ± 5.82 | 6.49 ± 6.04 | | 39.6 ± 6.55 | 38.57 ± 6.73 | |
| Years as health workers | | | | | | | | | | |
| ≤5 | 57 | 25.22 ± 12.93 | 24.31 ± 12.67 | (T) F (1, 440) = 1.745 | 6.31 ± 5.39 | 7.94 ± 5.66 | (T) F (1, 440) = 3.789 | 39.57 ± 6.52 | 37.92 ± 7.19 | **(T) F (1, 440) = 15.260 ***** |
| 5.01–15 | 132 | 30.95 ± 12.01 | 30.08 ± 12.39 | **(B) F (2, 440) = 5.322**** | 6.86 ± 6.07 | 7.08 ± 6.39 | (B) F (2, 440) = 2.369 | 39.27 ± 6.20 | 38.06 ± 6.61 | (B) F (2, 440) = 0.854 |
| >15 | 254 | 27.59 ± 12.58 | 27.35 ± 13.23 | (I) F (2, 440) = 0.283 | 5.97 ± 5.77 | 5.86 ± 5.86 | (I) F (2, 440) = 2.565 | 39.90 ± 6.74 | 38.98 ± 6.68 | (I) F (2, 440) = 0.416 |
| Total | 443 | 28.29 ± 12.57 | 27.77 ± 13.01 | | 6.28 ± 5.82 | 6.49 ± 6.04 | | 39.67 ± 6.55 | 38.57 ± 6.73 | |

Bold values distinguish the statistical significance.
BS, between-subjects; I, interaction; T, Within-Subjects (time).
*$P < 0.05$.
**$P < 0.01$.
***$P < 0.001$.

scores at T0 than they do at T1, thus they feel more depersonalized at T1 $df = -1.791$, $P < 0.01$. There are also significant interaction effects between depersonalization and years of seniority in their current position, with workers who have hold their current position for less than 5 years feeling more depersonalized at T1 $df = -1.060$, $P < 0.01$ (Table 1).

## Personal Fulfilment (Burnout)

There are statistically significant differences between personal fulfilment and marital status, with personal fulfilment scores being higher in married workers than in single professionals $df = 2.869$, $P < 0.05$. Significant interaction effects were also found between personal fulfilment and dependent family members. Workers with dependent family members feel more professionally fulfilled than those without such responsibilities $df = 1.268$, $P < 0.05$. Similar results were obtained with the number of children in the household variable. Workers with two or more children feel more professionally fulfilled than those without children $df = -2.59$, $P < 0.001$. There are also significant differences between personal fulfilment and job classification; specifically, workers who are in a managerial or intermediate position have higher personal fulfilment scores than individuals who hold a lower-level position $df = 2.230$, $P < 0.01$. Furthermore, there is a statistically significant main effect of the Within-Subjects factor (time) in all mixed ANOVAs performed except for the sex variable ($P < 0.05$). Scores in personal fulfilment are higher at T0 than at T1 in all these factors (Table 1).

## Intrusion (Posttraumatic Stress)

Regarding the intrusion variable, there are significant interaction effects between intrusion and sex at T0 and T1 in women, with higher scores on the intrusion scale than men, $df = -1.280$, $P < 0.001$. There are also statistically significant differences between intrusion and age, with the younger healthcare workers (18 to 35 years) presenting higher intrusion scores than the older workers (more than or equal to 51 years) $df = 2.291$, $P < 0.05$. Regarding educational level, there are statistically significant differences between this variable and intrusion, with those with an intermediate level of studies presenting higher intrusion scores than those with a postgraduate degree (master's or doctoral degree) $df = 2.849$, $P < 0.01$. In addition, there are statistically significant differences between intrusion and job classification, with those at a lower-level position having higher intrusion scores than those holding a managerial or intermediate position $df = -2.345$, $P < 0.01$. Statistically significant differences were also found between intrusion and work shift, specifically between the "other shifts" and "rotating shift" groups, the latter having higher intrusion scores $df = -2.812$, $P < 0.05$. The same occurs with the job title variable as statistically significant differences between intrusion and this variable are found, with assistant nurses presenting higher intrusion scores than physicians $df = -3.580$, $P < 0.01$. Furthermore, there is a statistically significant main effect of the Within-Subjects factor (time) in all mixed ANOVAs performed except for the sex variable ($P < 0.05$). Intrusion scores are higher at T0 than at T1 in all these factors (Table 2).

## Avoidance

There are statistically significant differences between avoidance and sex, with women presenting higher avoidance scores than men $df = -4.289$, $P < 0.001$. Statistically significant differences were also found between avoidance and educational level; nurses with an intermediate level of studies (high school/vocational training) present higher intrusion scores than those with a postgraduate degree $df = 2.082$, $P < 0.05$. There is a significant interaction effect between avoidance and the Autonomous Community in which the workplace is located, with healthcare workers working in the community of Madrid having higher avoidance scores at T1 than



Copyright © 2022 American College of Occupational and Environmental Medicine. Unauthorized reproduction of this article is prohibited

**TABLE 2.** Association Between Sociodemographic Variables and Workplace Variables With Symptoms of Posttraumatic Stress

| | | Intrusion | | | Avoidance | | | Hyperarousal | | |
|---|---|---|---|---|---|---|---|---|---|---|
| **Variables** | ***N*** | **T0** | **T1** | ***F*** | **T0** | **T1** | ***F*** | **T0** | **T1** | ***F*** |
| Sex | | | | | | | | | | |
| Men | 54 | 14.79 ± 7.58 | 15.24 ± 8.16 | (T) $F$ (1, 441) = 1.223 | 16.70 ± 8.62 | 16.90 ± 8.90 | (T) $F$ (1, 441) = 668 | 13.35 ± 7.36 | 14.29 ± 8.21 | (T) $F$ (1, 441) = 0.015 |
| Women | 389 | 20.56 ± 6.44 | 19.28 ± 7.17 | **(B) $F$ (1, 441) = 27.426**$^{***}$ | 20.81 ± 6.74 | 21.57 ± 7.59 | **(B) $F$ (1, 441) = 19.652**$^{***}$ | 18.98 ± 6.70 | 18.13 ± 7.27 | **(B) $F$ (1, 441) = 24.99**$^{***}$ |
| Total | 443 | 19.86 ± 6.85 | 18.79 ± 7.41 | **F(I) $F$ (1, 441) = 5.208**$^{*}$ | 20.33 ± 7.10 | 21.00 ± 7.90 | (I) $F$ (1, 441) = 0.668 | 18.29 ± 7.02 | 17.67 ± 7.49 | (I) $F$ (1, 441) = 488$^{*}$ |
| Age | | | | | | | | | | |
| 18–35 | 83 | 21.02 ± 5.98 | 19.36 ± 7.8 | **(T) $F$ (1, 440) = 18.480** $^{***}$ | 20.72 ± 6.87 | 20.03 ± 8.08 | (T) $F$ (1, 440) = 3.52 | 18.93 ± 6.67 | 17.89 ± 8 | **(T) $F$ (1, 440) = 5.749**$^{*}$ |
| 36–50 | 205 | 20.71 ± 6.14 | 19.43 ± 6.75 | **(B) $F$ (2, 440) = 5.734** $^{**}$ | 20.96 ± 6.54 | 21.62 ± 7.16 | (B) $F$ (2, 440) = 2.308 | 19.22 ± 6.44 | 18.50 ± 6.63 | **(B) $F$ (2, 440) = 5.289**$^{**}$ |
| ≥51 | 157 | 18.14 ± 7.8 | 17.65 ± 7.88 | (I) $F$ (2, 440) = 1.660 | 19.32 ± 7.81 | 20.19 ± 8.67 | (I) $F$ (2, 440) = 0.207 | 16.76 ± 7.62 | 16.47 ± 8.13 | (I) $F$ (2, 440) = 0.537 |
| Total | 443 | 19.86 ± 6.85 | 18.79 ± 7.41 | | 20.33 ± 7.10 | 21 ± 7.9 | | 18.29 ± 7.02 | 17.67 ± 7.49 | |
| Educational level completed | | | | | | | | | | |
| Secondary Education | 175 | 20.43 ± 6.68 | 19.85 ± 7.36 | **(T) $F$ (1, 440) = 19.996**$^{***}$ | 21.53 ± 6.84 | 21.77 ± 7.73 | **(T) $F$ (1, 440) = 5.223**$^{*}$ | 19.06 ± 6.92 | 18.57 ± 7.70 | **(T) $F$ (1, 440) = 5.301**$^{*}$ |
| Bachelor's degree | 162 | 20.41 ± 6.17 | 19.12 ± 7.03 | **(B) $F$ (2, 440) = 6817**$^{***}$ | 19.85 ± 6.67 | 20.79 ± 7.84 | **(B) $F$ (2, 440) = 3.461**$^{*}$ | 18.76 ± 6.68 | 17.98 ± 7.04 | **(B) $F$ (2, 440) = 6.418**$^{**}$ |
| Master's or Doctor's degree | 106 | 18.06 ± 7.80 | 16.52 ± 7.62 | (I) $F$ (2, 440) = 1.356 | 19.09 ± 7.75 | 20.05 ± 8.22 | (I) $F$ (2, 440) = 0.645 | 16.31 ± 7.37 | 15.69 ± 7.52 | (I) $F$ (2, 440) = 0.105 |
| Total | 443 | 19.86 ± 6.85 | 18.79 ± 7.41 | | 20.33 ± 7.10 | 21 ± 7.90 | | 18.29 ± 7.02 | 17.67 ± 7.49 | |
| Marital status | | | | | | | | | | |
| Married | 231 | 20.19 ± 6.66 | 19.19 ± 7.25 | **(T) $F$ (1, 439) = 11.9777**$^{***}$ | 20.59 ± 6.81 | 20.88 ± 8.21 | **(T) $F$ (1, 439) = 7.175**$^{**}$ | 18.79 ± 6.82 | 17.97 ± 7.49 | (T) $F$ (1, 439) = 2.056 |
| Living with partner, not married | 74 | 20.40 ± 6.35 | 18.41 ± 7.53 | (B) $F$ (3, 439) = 0.873 | 20.14 ± 7.40 | 20.98 ± 7.36 | (B) $F$ (3, 439) = 0.090 | 18.18 ± 6.58 | 16.91 ± 7.17 | (B) $F$ (3, 439) = 0.615 |
| Separated or Widower/Widow | 53 | 17.94 ± 7.69 | 18.33 ± 7.07 | (I) $F$ (3, 439) = 2.278 | 19.35 ± 7.74 | 21.18 ± 7.12 | (I) $F$ (3, 439) = 0.882 | 16.96 ± 7.93 | 17.66 ± 7.10 | (I) $F$ (3, 439) = 1.463 |
| Single | 85 | 19.68 ± 7.12 | 18.30 ± 7.98 | | 20.42 ± 7.26 | 21.24 ± 8.11 | | 17.87 ± 7.31 | 17.49 ± 8.05 | |
| Total | 443 | 19.86 ± 6.85 | 18.79 ± 7.41 | | 20.33 ± 7.10 | 21 ± 7.90 | | 18.29 ± 7.02 | 17.67 ± 7.49 | |
| Dependent relatives | | | | | | | | | | |
| Yes | 271 | 19.91 ± 6.86 | 18.87 ± 7.12 | **(T) $F$ (1, 441) = 17.902**$^{***}$ | 20.43 ± 7.15 | 20.96 ± 7.99 | **(T) $F$ (1, 441) = 5.136**$^{*}$ | 18.38 ± 6.98 | 17.87 ± 7.24 | **(T) $F$ (1, 441) = 5.817**$^{*}$ |
| No | 172 | 19.79 ± 6.86 | 18.66 ± 7.85 | (B) $F$ (1, 441) = 0.068 | 20.18 ± 7.04 | 21.07 ± 7.79 | (B) $F$ (1, 441) = 0.011 | 18.15 ± 7.10 | 17.35 ± 7.87 | (B) $F$ (1, 441) = 326 |
| Total | 443 | 19.86 ± 6.85 | 18.79 ± 7.41 | (I) $F$ (1, 441) = 0.028 | 20.33 ± 7.10 | 21 ± 7.9 | (I) $F$ (1, 441) = 0.335 | 18.29 ± 7.02 | 17.67 ± 7.49 | (I) $F$ (1, 441) = 0.273 |
| No. of children in your care | | | | | | | | | | |
| 0 | 173 | 19.90 ± 6.82 | 18.61 ± 7.79 | **(T) $F$ (1, 440) = 16.296**$^{***}$ | 20.22 ± 6.96 | 21.12 ± 7.79 | (T) $F$ (1, 440) = 3.647 | 18.19 ± 7.13 | 17.49 ± 7.88 | **(T) $F$ (1, 440) = 4.82**$^{*}$ |
| 1 | 113 | 19.76 ± 6.81 | 19.15 ± 7.16 | (B) $F$ (2, 440) = 0.033 | 21.46 ± 6.79 | 21.39 ± 8.15 | (B) $F$ (2, 440) = 1.226 | 18.59 ± 6.86 | 18.28 ± 7.21 | (B) $F$ (2, 440) = 0.348 |
| >1 | 157 | 19.88 ± 6.96 | 18.73 ± 7.18 | (I) $F$ (2, 440) = 0.590 | 19.64 ± 7.41 | 20.59 ± 7.88 | (I) $F$ (2, 440) = 1.010 | 18.19 ± 7.05 | 17.42 ± 7.27 | (I) $F$ (2, 440) = 0.251 |
| Total | 443 | 19.86 ± 6.85 | 18.79 ± 7.41 | | 20.33 ± 7.10 | 21 ± 7.9 | | 18.29 ± 7.02 | 17.67 ± 7.49 | |
| Autonomous community of the workplace | | | | | | | | | | |
| Community of Madrid | 375 | 19.90 ± 6.69 | 19.07 ± 7.37 | **(T) $F$ (1, 441) = 21.936**$^{***}$ | 20.02 ± 7.21 | 20.98 ± 7.89 | (T) $F$ (1, 441) = 0.001 | 18.24 ± 6.94 | 17.72 ± 7.43 | **(T) $F$ (1, 441) = 5.436**$^{*}$ |
| Others | 69 | 19.62 ± 7.70 | 17.26 ± 7.48 | (B) $F$ (1, 441) = 1.453 | 22.05 ± 6.24 | 21.11 ± 8.03 | (B) $F$ (1, 441) = 1.479 | 18.56 ± 7.48 | 17.37 ± 7.84 | (B) $F$ (1, 441) = 0.000 |
| Total | 443 | 19.86 ± 6.85 | 18.79 ± 7.41 | (I) $F$ (1, 441) = 5.039$^{*}$ | 20.33 ± 7.10 | 21 ± 7.90 | **(I) $F$ (1, 441) = 5.207**$^{*}$ | 18.29 ± 7.02 | 17.67 ± 7.49 | (I) $F$ (1, 441) = 0.818 |
| Professional category | | | | | | | | | | |
| Executive or Intermediate job | 87 | 17.52 ± 7.75 | 17.35 ± 8.09 | **(T) $F$ (1, 441) = 5.492**$^{*}$ | 19.14 ± 7.73 | 20.45 ± 8.36 | **(T) $F$ (1, 441) = 5.648**$^{*}$ | 16.13 ± 7.58 | 16.51 ± 8.26 | (T) $F$ (1, 441) = 0.547 |
| Base position | 356 | 20.43 ± 6.50 | 19.14 ± 7.20 | **(B) $F$ (1, 441) = 8.873**$^{**}$ | 20.62 ± 6.92 | 21.14 ± 7.79 | (B) $F$ (1, 441) = 1.768 | 18.82 ± 6.78 | 17.95 ± 7.27 | **(B) $F$ (1, 441) = 6.703**$^{**}$ |
| Total | 443 | 19.86 ± 6.85 | 18.79 ± 7.41 | (I) $F$ (1, 441) = 3.206 | 20.33 ± 7.10 | 21.00 ± 7.90 | (I) $F$ (1, 441) = 1.087 | 18.29 ± 7.02 | 17.67 ± 7.49 | (I) $F$ (1, 441) = 3.513 |
| Post | | | | | | | | | | |
| Medical post | 60 | 18.13 ± 7.67 | 15.81 ± 7.90 | **(T) $F$ (1, 439) = 18.507**$^{***}$ | 18.68 ± 6.86 | 19.11 ± 8.49 | (T) $F$ (1, 439) = 3.449 | 16.43 ± 7.33 | 15.60 ± 7.95 | **(T) $F$ (1, 439) = 4.410**$^{*}$ |
| Nursing post | 173 | 19.89 ± 6.74 | 18.73 ± 7.27 | **(B) $F$ (3, 439) = 4.419**$^{**}$ | 19.96 ± 7.20 | 20.75 ± 7.89 | (B) $F$ (3, 439) = 2.592 | 18.25 ± 6.97 | 17.40 ± 7.13 | **(B) $F$ (3, 439) = 3.166**$^{*}$ |
| Assistant Nurse | 146 | 20.87 ± 6.20 | 20.23 ± 6.92 | (I) $F$ (3, 439) = 1.627 | 21.37 ± 6.76 | 21.97 ± 7.22 | (I) $F$ (3, 439) = 0.054 | 19.26 ± 6.82 | 18.91 ± 7.30 | (I) $F$ (3, 439) = 0.253 |
| Caregiver | 64 | 19.09 ± 7.45 | 18.45 ± 7.65 | | 20.53 ± 7.57 | 21.23 ± 8.64 | | 17.95 ± 7.07 | 17.48 ± 8.06 | |
| Total | 443 | 19.86 ± 6.85 | 18.79 ± 7.41 | | 20.33 ± 7.10 | 21.00 ± 7.90 | | 18.29 ± 7.02 | 17.67 ± 7.49 | |
| Type of center | | | | | | | | | | |
| Hospital | 286 | 20.20 ± 6.55 | 18.95 ± 7.33 | **(T) $F$ (1, 440) = 11.049**$^{***}$ | 20.45 ± 6.85 | 21.25 ± 7.85 | (T) $F$ (1, 440) = 1.502 | 18.46 ± 6.78 | 17.72 ± 7.53 | (T) $F$ (1, 440) = 3.066 |
| Primary care | 678 | 19.76 ± 6.86 | 18.02 ± 7.20 | (B) $F$ (2, 440) = 0.594 | 20.35 ± 7.21 | 19.61 ± 8.44 | (B) $F$ (2, 440) = 0.451 | 18.07 ± 7.49 | 17.20 ± 7.44 | (B) $F$ (2, 440) = 0.137 |
| Other | 90 | 18.83 ± 7.70 | 18.84 ± 7.83 | (I) $F$ (2, 440) = 2.659 | 19.95 ± 7.84 | 21.24 ± 7.63 | (I) $F$ (2, 440) = 2.129 | 17.92 ± 7.47 | 17.84 ± 7.47 | (I) $F$ (2, 440) = 0.555 |
| Total | 443 | 19.86 ± 6.85 | 18.79 ± 7.41 | | 20.33 ± 7.10 | 21.00 ± 7.90 | | 18.29 ± 7.02 | 17.67 ± 7.49 | |
| Shift | | | | | | | | | | |
| Fixed | 237 | 19.42 ± 7.08 | 18.41 ± 7.42 | **(T) $F$ (1, 440) = 12.070**$^{***}$ | 20.00 ± 7.39 | 20.80 ± 8.03 | (T) $F$ (1, 440) = 2.818 | 17.85 ± 7.02 | 17.27 ± 7.35 | (T) $F$ (1, 440) = 3.474 |
| Rotating shift | 157 | 21.05 ± 6.13 | 19.87 ± 6.87 | **(B) $F$ (2, 440) = 4.371**$^{*}$ | 21.22 ± 6.33 | 21.70 ± 7.53 | (B) $F$ (2, 440) = 2.058 | 19.32 ± 6.77 | 18.58 ± 7.30 | (B) $F$ (2, 440) = 2.778 |
| Other | 49 | 18.16 ± 7.40 | 17.14 ± 8.57 | (I) $F$ (2, 440) = 0.048 | 19.14 ± 7.81 | 19.75 ± 8.39 | (I) $F$ (2, 440) = 0.118 | 17.16 ± 7.55 | 16.63 ± 8.57 | (I) $F$ (2, 440) = 0.049 |
| Total | 443 | 19.86 ± 6.85 | 18.79 ± 7.41 | | 20.33 ± 7.10 | 21.00 ± 7.90 | | 18.29 ± 7.02 | 17.67 ± 7.49 | |
| Time | | | | | | | | | | |
| Full time | 388 | 19.68 ± 7.00 | 18.63 ± 7.51 | **(T) $F$ (1, 441) = 9.038**$^{**}$ | 20.24 ± 7.24 | 20.88 ± 7.99 | (T) $F$ (1, 441) = 2.585 | 18.07 ± 7.13 | 17.48 ± 7.59 | (T) $F$ (1, 441) = 3.501 |


© 2021 American College of Occupational and Environmental Medicine
Copyright © 2022 American College of Occupational and Environmental Medicine. Unauthorized reproduction of this article is prohibited

**TABLE 2.** (*Continued*)

| Variables | *N* | Intrusion T0 | Intrusion T1 | Intrusion *F* | Avoidance T0 | Avoidance T1 | Avoidance *F* | Hyperarousal T0 | Hyperarousal T1 | Hyperarousal *F* |
|---|---|---|---|---|---|---|---|---|---|---|
| Part time | 55 | 21.12 ± 5.55 | 19.90 ± 6.60 | (B) *F* (1, 441) = 2.023 | 21.00 ± 5.99 | 21.83 ± 7.29 | (B) *F* (1, 441) = 0.754 | 19.89 ± 6.02 | 18.96 ± 6.63 | (B) *F* (1, 441) = 2.925 |
| Total | 443 | 19.86 ± 6.85 | 18.79 ± 7.41 | (I) *F* (1, 441) = 0.050 | 20.33 ± 7.10 | 21.00 ± 7.90 | (I) *F* (1, 441) = 0.043 | 18.29 ± 7.02 | 17.67 ± 7.49 | (I) *F* (1, 441) = 0.179 |
| Years of seniority | | | | | | | | | | |
| ≤5 | 166 | 19.95 ± 7.06 | 18.30 ± 7.96 | **(T) *F* (1, 440) = 16.582*** | 20.35 ± 7.26 | 20.03 ± 8.40 | **(T) *F* (1, 440) = 5.99*** | 18.07 ± 7.46 | 16.74 ± 8.06 | (T) *F* (1, 440) = 3.656 |
| 5.01–15 | 185 | 20.09 ± 6.54 | 19.44 ± 6.86 | (B) *F* (2, 440) = 0.785 | 20.51 ± 7.01 | 21.64 ± 7.67 | (B) *F* (2, 440) = 0.748 | 18.83 ± 6.74 | 18.48 ± 7.08 | (B) *F* (2, 440) = 1.646 |
| >15 | 92 | 19.22 ± 7.11 | 18.34 ± 7.40 | (I) *F* (2, 440) = 1.706 | 19.95 ± 7.05 | 21.47 ± 7.32 | **(I) *F* (2, 440) = 3.336*** | 17.61 ± 6.73 | 17.70 ± 7.11 | (I) *F* (2, 440) = 2.294 |
| Total | 443 | 19.86 ± 6.85 | 18.79 ± 7.41 | | 20.33 ± 7.10 | 21.00 ± 7.90 | | 18.29 ± 7.02 | 17.67 ± 7.47 | |
| Years as health workers | | | | | | | | | | |
| ≤5 | 57 | 20.28 ± 6.58 | 18.03 ± 8.12 | **(T) *F* (1, 440) = 22.367*** | 21.03 ± 6.99 | 20.50 ± 8.05 | (T) *F* (1, 440) = 1.338 | 17.91 ± 7.23 | 16.43 ± 7.91 | **(T) *F* (1, 440) = 7.202** |
| 5.01–15 | 132 | 21.05 ± 6.10 | 19.81 ± 7.23 | (B) *F* (2, 440) = 2.691 | 20.79 ± 6.99 | 21.84 ± 8.02 | (B) *F* (2, 440) = 0.963 | 19.62 ± 6.64 | 18.93 ± 7.31 | **(B) *F* (2, 440) = 3.612*** |
| >15 | 254 | 19.14 ± 7.20 | 18.43 ± 7.31 | (I) *F* (2, 440) = 2.104 | 19.94 ± 7.18 | 20.68 ± 7.81 | (I) *F* (2, 440) = 1.243 | 17.69 ± 7.10 | 17.28 ± 7.43 | (I) *F* (2, 440) = 0.847 |
| Total | 443 | 19.86 ± 6.85 | 18.79 ± 7.41 | | 20.33 ± 7.10 | 21.00 ± 7.90 | | 18.29 ± 7.02 | 17.67 ± 7.49 | |

Bold values distinguish the statistical significance.
BS, Between-Subjects; I, interaction; T, Within-Subjects (time).
*$P < 0.05$.
**$P < 0.01$.
***$P < 0.001$.

at T0 $df = -965$, $P < 0.01$. There is also a significant interaction effect between avoidance and the years of experience of nurses. Those who have been working for less than 5 years have higher avoidance scores at T0 than at T1 $df = 1.651$, $P < 0.001$. Furthermore, there is a statistically significant main effect of the Within-Subjects factor (time) in some of the mixed ANOVAs performed ($P < 0.05$). Avoidance scores are higher at T1 than at T0 in all these factors (Table 2).

## Hyperarousal

There are statistically significant differences between hyperarousal and sex, women present higher scores on the hyperarousal scale than men $df = -4.738$, $P < 0.001$. Statistically significant differences were also found between hyperarousal and age. Middle-aged workers (between 36 and 50 years) have higher hyperarousal scores than older workers (more than or equal to 51 years), $df = 2.247$, $P < 0.01$. Statistically significant differences were also found between hyperarousal and educational level, with those who had an intermediate level of studies (presenting higher hyperarousal scores than postgraduate degree holders $df = 2.815$, $P < 0.01$. The same occurs with the job classification factor, as statistically significant differences between hyperarousal and this factor were found. Workers who hold a lower-level position have higher hyperarousal scores than those at a managerial or intermediate position $df = -2.061$, $P < 0.01$. As for the job title variable, there are statistically significant differences on hyperarousal, with assistant nurses having higher hyperarousal scores than physicians $df = -3.076$, $P < 0.05$. There are also statistically significant differences between hyperarousal and years of experience as healthcare workers. Those who have between 5 and 15 years of experience have higher hyperarousal scores than those who have over 15 years of experience, $df = -2.061$, $P < 0.01$. Finally, there is a statistically significant main effect of the Within-Subjects factor (time) in some of the mixed ANOVAs performed ($P < 0.05$). Hyperarousal scores at T0 are higher than at T1 in these factors (Table 2).

## Anxiety

There is a significant interaction effect between anxiety and sex, with women having higher anxiety scores at T0 $df = 1.013$, $P < 0.001$. Similar results were obtained in relation to age; statistically significant differences were found between anxiety and this factor, with the group of younger participants (18 to 35 years) presenting higher anxiety than the older professionals (more than or equal to 51 years) $df = -1.491$, $P < 0.05$. Furthermore, there is a statistically significant main effect of the Within-Subjects factor (time) in all of the mixed ANOVAs performed ($P < 0.05$). Anxiety scores are higher at T0 than at T1 in all these factors (Table 3).

## Depression

There are statistically significant differences between depression and sex, with women presenting higher depression scores than men $df = -1.915$, $P < 0.001$. In relation to job classification, there are statistically significant differences between depression and this factor, with those at a lower-level position having higher depression scores than those holding a managerial or intermediate position $df = 939$, $P < 0.05$. There are also statistically significant differences between depression and employment category, with depression scores being higher in part-time workers than in those who work full-time $df = -1.400$, $P < 0.001$. Furthermore, there is a statistically significant main effect of the Within-Subjects factor (time) in all the mixed ANOVAs performed ($P < 0.05$). Depression scores are higher at T0 than at T1 in all these factors (Table 3).

## Resilience

As for the resilience variable, there are statistically significant differences between resilience and sex. Men have higher



Copyright © 2022 American College of Occupational and Environmental Medicine. Unauthorized reproduction of this article is prohibited

**TABLE 3.** Association Between Sociodemographic Variables and Workplace Variables With Symptoms of Anxiety, Depression, and Resilience

| Variables | *N* | Anxiety T0 | Anxiety T1 | Anxiety *F* | Depression T0 | Depression T1 | Depression *F* | Resilience T0 | Resilience T1 | Resilience *F* |
|---|---|---|---|---|---|---|---|---|---|---|
| Sex | | | | | | | | | | |
| Men | 54 | 7.20 ± 3.56 | 7.05 ± 3.59 | **(T) *F* (1, 441) = 7.410**$^{**}$ | 4.75 ± 3.67 | 4.44 ± 3.69 | **(T) *F* (1, 441) = 7.884**$^{**}$ | 3.76 ± 0.74 | 3.78 ± 0.74 | (T) *F* (1, 441) = 2.574 |
| Women | 389 | 10.23 ± 4.08 | 9.22 ± 4.01 | **(B) *F* (1, 441) = 23.217**$^{***}$ | 6.96 ± 4.01 | 6.06 ± 4.07 | **(B) *F* (1, 441) = 12.586**$^{***}$ | 3.28 ± 0.78 | 3.39 ± 0.76 | **(B) *F* (1, 441) = 17.09**$^{***}$ |
| Total | 443 | 9.86 ± 4.14 | 8.95 ± 4.02 | **(I) *F* (1, 441) = 4.11**$^{*}$ | 6.69 ± 4.03 | 5.86 ± 4.06 | (I) *F* (1, 441) = 1.829 | 3.34 ± 0.79 | 3.44 ± 0.77 | (I) *F* (1, 441) = 1.638 |
| Age | | | | | | | | | | |
| 18–35 | 83 | 10.89 ± 4.07 | 9.65 ± 4.06 | **(T) *F* (1, 440) = 40.172**$^{***}$ | 7.18 ± 4.03 | 5.93 ± 3.93 | **(T) *F* (1, 440) = 33.791**$^{***}$ | 3.17 ± 0.86 | 3.22 ± 0.77 | **(T) *F* (1, 440) = 11.416**$^{***}$ |
| 36–50 | 203 | 10.05 ± 3.90 | 9.04 ± 3.89 | **(B) *F* (2, 440) = 4.477**$^{**}$ | 7.03 ± 3.95 | 6.07 ± 3.84 | (B) *F* (2, 440) = 2.154 | 3.33 ± 0.72 | 3.44 ± 0.72 | **(B) *F* (2, 440) = 4.210**$^{**}$ |
| ≥51 | 157 | 9.08 ± 4.34 | 8.47 ± 4.13 | (I) *F* (2, 440) = 1.472 | 6.00 ± 4.06 | 5.56 ± 4.39 | (I) *F* (2, 440) = 2.404 | 3.43 ± 0.83 | 3.54 ± 0.80 | (I) *F* (2, 440) = 0.321 |
| Total | 443 | 9.86 ± 4.14 | 8.95 ± 4.02 | | 6.69 ± 4.03 | 5.86 ± 4.06 | | 3.34 ± 0.79 | 3.44 ± 0.77 | |
| Educational level completed | | | | | | | | | | |
| Secondary Education | 175 | 9.83 ± 4.27 | 9.25 ± 4.22 | **(T) *F* (1, 440) = 40.935**$^{***}$ | 6.60 ± 4.24 | 5.85 ± 4.16 | **(T) *F* (1, 440) = 32.886**$^{***}$ | 3.20 ± 0.78 | 3.36 ± 0.78 | **(T) *F* (1, 440) = 11.50**$^{**}$ |
| Bachelor's degree | 162 | 10.28 ± 3.96 | 9.01 ± 3.77 | (B) *F* (2, 440) = 1.641 | 6.82 ± 3.83 | 5.93 ± 3.90 | (B) *F* (2, 440) = 0.089 | 3.36 ± 0.78 | 3.45 ± 0.74 | (B) *F* (2, 440) = 4.40$^{*}$ |
| Master's or Doctor's degree | 107 | 9.28 ± 4.15 | 8.38 ± 4.03 | (I) *F* (2, 440) = 2.351 | 6.65 ± 4.00 | 5.79 ± 4.16 | (I) *F* (2, 440) = 0.092 | 3.54 ± 0.79 | 3.55 ± 0.78 | (I) *F* (2, 440) = 2.65 |
| Total | 443 | 9.86 ± 4.14 | 8.95 ± 4.02 | | 6.69 ± 4.03 | 5.86 ± 4.06 | | 3.34 ± 0.79 | 3.44 ± 0.77 | |
| Marital status | | | | | | | | | | |
| Married | 231 | 10.03 ± 4.05 | 9.19 ± 3.36 | **(T) *F* (1, 439) = 29.27**$^{***}$ | 6.80 ± 3.90 | 6.01 ± 3.80 | **(T) *F* (1, 439) = 23.765**$^{***}$ | 3.34 ± 0.74 | 3.47 ± 0.70 | **(T) *F* (1, 439) = 7.732**$^{**}$ |
| Living with partner, not married | 74 | 9.98 ± 3.80 | 8.68 ± 3.83 | (B) *F* (3, 439) = 0.665 | 6.62 ± 3.85 | 5.67 ± 4.16 | (B) *F* (3, 439) = 0.487 | 3.32 ± 0.84 | 3.45 ± 0.86 | (B) *F* (3, 439) = 1.440 |
| Separated or widower/widow | 53 | 8.88 ± 4.53 | 8.77 ± 4.27 | (I) *F* (3, 439) = 2.1489 | 5.90 ± 4.31 | 5.60 ± 4.40 | (I) *F* (3, 439) = 0.964 | 3.50 ± 9.91 | 3.53 ± 0.79 | (I) *F* (3, 439) = 1.044 |
| Single | 85 | 9.91 ± 4.38 | 8.67 ± 4.20 | | 6.96 ± 4.34 | 5.80 ± 4.47 | | 3.24 ± 0.81 | 3.28 ± 0.83 | |
| Total | 443 | 9.86 ± 4.14 | 8.95 ± 4.02 | | 6.69 ± 4.03 | 5.86 ± 4.06 | | 3.34 ± 7.79 | 3.44 ± 0.77 | |
| Dependent relatives | | | | | | | | | | |
| Yes | 271 | 9.89 ± 4.06 | 9.08 ± 4.08 | **(T) *F* (1, 441) = 42.332**$^{***}$ | 6.78 ± 4.10 | 6.08 ± 4.08 | **(T) *F* (1, 441) = 35.456**$^{***}$ | 3.39 ± 0.77 | 3.50 ± 0.75 | **(T) *F* (1, 441) = 13.751**$^{***}$ |
| No | 172 | 9.81 ± 4.27 | 8.76 ± 3.93 | (B) *F* (1, 441) = 0.290 | 6.55 ± 3.92 | 5.52 ± 4.02 | (B) *F* (1, 491) = 1.140 | 3.26 ± 0.82 | 3.34 ± 0.79 | **(B) *F* (1, 491) = 3.951**$^{*}$ |
| Total | 443 | 9.86 ± 4.14 | 8.95 ± 4.02 | (I) *F* (1, 441) = 0.735 | 6.69 ± 4.03 | 5.86 ± 4.06 | (I) *F* (1, 491) = 1.274 | 3.3 ± 0.79 | 3.44 ± 0.77 | (I) *F* (1, 491) = 0.276 |
| No. of children in your care | | | | | | | | | | |
| 0 | 173 | 9.98 ± 4.24 | 8.86 ± 4.00 | **(T) *F* (1, 440) = 36.86**$^{***}$ | 6.82 ± 3.94 | 5.79 ± 4.15 | **(T) *F* (1, 440) = 29.762**$^{***}$ | 3.25 ± 0.82 | 3.31 ± 0.78 | **(T) *F* (1, 440) = 14.166**$^{***}$ |
| 1 | 113 | 9.84 ± 4.04 | 9.32 ± 4.09 | (B) *F* (2, 440) = 0.219 | 6.69 ± 3.81 | 6.25 ± 4.07 | (B) *F* (2, 440) = 0.299 | 3.39 ± 0.77 | 3.44 ± 0.78 | **(B) *F* (2, 440) = 3.337**$^{*}$ |
| ≥1 | 157 | 9.75 ± 4.11 | 8.79 ± 4.00 | (I) *F* (2, 440) = 1.491 | 6.56 ± 4.30 | 5.66 ± 3.96 | (I) *F* (2, 440) = 1.405 | 3.40 ± 0.77 | 3.57 ± 0.72 | (I) *F* (2, 440) = 1.803 |
| Total | 443 | 9.86 ± 4.14 | 8.95 ± 4.02 | | 6.69 ± 4.03 | 5.86 ± 4.06 | | 3.34 ± 0.79 | 3.44 ± 0.77 | |
| Autonomous community of the workplace | | | | | | | | | | |
| Community of Madrid | 374 | 9.85 ± 3.99 | 9.01 ± 3.92 | **(T) *F* (1, 441) = 29.337**$^{***}$ | 6.63 ± 3.90 | 5.86 ± 3.94 | **(T) *F* (1, 441) = 23.430**$^{***}$ | 3.32 ± 0.80 | 3.43 ± 0.77 | (T) *F* (1, 441) = 3.257 |
| Other | 69 | 9.92 ± 4.90 | 8.68 ± 4.57 | (B) *F* (1, 441) = 0.066 | 7.02 ± 4.70 | 5.91 ± 4.68 | (B) *F* (1, 441) = 0.203 | 3.45 ± 0.77 | 3.46 ± 0.78 | (B) *F* (1, 441) = 0.676 |
| Total | 443 | 9.86 ± 4.14 | 8.95 ± 4.02 | (I) *F* (1, 441) = 1.081 | 6.69 ± 4.03 | 5.86 ± 4.06 | (I) *F* (1, 441) = 0.760 | 3.34 ± 0.79 | 3.44 ± 0.77 | (I) *F* (1, 441) = 2.319 |
| Professional category | | | | | | | | | | |
| Executive or intermediate job | 87 | 8.87 ± 4.22 | 8.42 ± 4.20 | **(T) *F* (1, 441) = 19.266**$^{***}$ | 5.98 ± 4.03 | 5.06 ± 4.11 | **(T) *F* (1, 441) = 23.376**$^{***}$ | 3.49 ± 0.83 | 3.55 ± 0.81 | **(T) *F* (1, 441) = 7.552**$^{**}$ |
| Base position | 356 | 10.08 ± 4.09 | 9.08 ± 3.97 | (B) *F* (1, 441) = 3.807 | 6.87 ± 4.01 | 6.06 ± 4.03 | **(B) *F* (1, 441) = 4.382**$^{*}$ | 3.30 ± 0.78 | 3.41 ± 0.75 | (B) *F* (1, 441) = 3.484 |
| Total | 443 | 9.86 ± 4.14 | 9.95 ± 4.02 | (I) *F* (1, 441) = 1.579 | 6.69 ± 4.03 | 5.86 ± 4.06 | (I) *F* (1, 441) = 0.101 | 3.34 ± 0.79 | 3.44 ± 0.77 | (I) *F* (1, 441) = 0.373 |
| Post | | | | | | | | | | |
| Medical post | 60 | 9.86 ± 4.09 | 8.53 ± 4.00 | **(T) *F* (1, 439) = 31.83**$^{***}$ | 6.73 ± 3.78 | 6.15 ± 4.35 | **(T) *F* (1, 439) = 20.260**$^{***}$ | 3.39 ± 0.81 | 3.50 ± 0.80 | **(T) *F* (1, 439) = 11.851**$^{***}$ |
| Nursing post | 173 | 9.97 ± 4.09 | 8.85 ± 3.78 | (B) *F* (3, 439) = 0.205 | 6.86 ± 3.87 | 5.83 ± 3.89 | (B) *F* (3, 439) = 0.287 | 3.40 ± 0.74 | 3.44 ± 0.74 | (B) *F* (3, 439) = 1.237 |
| Assistant Nurse | 146 | 9.94 ± 3.94 | 9.21 ± 4.03 | (I) *F* (3, 439) = 1.725 | 6.77 ± 4.25 | 5.84 ± 4.16 | (I) *F* (3, 439) = 1.071 | 3.20 ± 0.81 | 3.38 ± 0.79 | (I) *F* (3, 439) = 1.850 |
| Caregiver | 64 | 9.39 ± 4.76 | 9.06 ± 4.66 | | 6.04 ± 4.18 | 5.73 ± 4.09 | | 3.43 ± 0.84 | 3.49 ± 0.78 | |
| Total | 443 | 9.86 ± 4.14 | 8.95 ± 4.02 | | 6.69 ± 4.03 | 5.86 ± 4.06 | | 3.34 ± 0.79 | 3.44 ± 0.77 | |
| Type of center | | | | | | | | | | |
| Hospital | 286 | 10.01 ± 4.22 | 9.05 ± 3.96 | **(T) *F* (1, 440) = 25.074**$^{***}$ | 6.89 ± 4.12 | 5.92 ± 3.93 | **(T) *F* (1, 440) = 15.520**$^{***}$ | 3.34 ± 0.78 | 3.42 ± 0.75 | **(T) *F* (1, 440) = 13.895**$^{***}$ |
| Primary care | 678 | 9.80 ± 4.06 | 9.11 ± 4.50 | (B) *F* (2, 440) = 0.709 | 6.79 ± 3.99 | 6.61 ± 4.59 | (B) *F* (2, 440) = 2.160 | 3.31 ± 0.75 | 3.44 ± 0.80 | (B) *F* (2, 440) = 0.031 |
| Other | 90 | 9.43 ± 3.94 | 8.54 ± 3.84 | (I) *F* (2, 440) = 0.244 | 6.01 ± 3.73 | 5.14 ± 3.99 | (I) *F* (2, 440) = 1.920 | 3.34 ± 0.85 | 3.47 ± 0.81 | (I) *F* (2, 440) = 0.386 |
| Total | 443 | 9.86 ± 4.14 | 8.95 ± 4.02 | | 6.69 ± 4.03 | 5.86 ± 4.06 | | 3.34 ± 0.79 | 3.44 ± 0.77 | |
| Shift | | | | | | | | | | |
| Fixed shift | 237 | 9.39 ± 4.27 | 8.70 ± 4.07 | **(T) *F* (1, 440) = 37.920**$^{***}$ | 6.39 ± 4.01 | 5.80 ± 4.24 | **(T) *F* (1, 440) = 24.130**$^{***}$ | 3.34 ± 0.81 | 3.46 ± 0.75 | **(T) *F* (1, 440) = 5.015**$^{*}$ |
| Rotating shift | 157 | 10.36 ± 3.93 | 9.29 ± 3.97 | (B) *F* (2, 440) = 2.377 | 7.15 ± 4.01 | 5.94 ± 3.81 | (B) *F* (2, 440) = 0.690 | 3.32 ± 0.79 | 3.40 ± 0.78 | (B) *F* (2, 440) = 0.149 |
| Other | 49 | 10.55 ± 3.91 | 9.12 ± 3.94 | (I) *F* (2, 440) = 1.643 | 6.69 ± 4.11 | 5.91 ± 4.04 | (I) *F* (2, 440) = 2.085 | 3.41 ± 0.76 | 3.40 ± 0.81 | (I) *F* (2, 440) = 1.393 |
| Total | 443 | 9.86 ± 4.14 | 8.95 ± 4.02 | | 6.69 ± 4.03 | 5.86 ± 4.06 | | 3.34 ± 0.79 | 3.44 ± 0.77 | |
| Time | | | | | | | | | | |
| Full time | 388 | 9.75 ± 4.26 | 8.84 ± 4.06 | **(T) *F* (1, 441) = 18.888**$^{***}$ | 6.49 ± 4.06 | 5.72 ± 4.08 | **(T) *F* (1, 441) = 21.154**$^{***}$ | 3.36 ± 0.80 | 3.44 ± 0.78 | **(T) *F* (1, 441) = 17.781**$^{***}$ |
| Part time | 55 | 10.69 ± 2.99 | 9.74 ± 3.64 | (B) *F* (1, 441) = 2.818 | 8.10 ± 3.49 | 6.90 ± 3.78 | **(B) *F* (1, 441) = 6.743**$^{*}$ | 3.17 ± 0.74 | 3.41 ± 0.70 | (B) *F* (1, 441) = 1.237 |


© 2021 American College of Occupational and Environmental Medicine
Copyright © 2022 American College of Occupational and Environmental Medicine. Unauthorized reproduction of this article is prohibited

**TABLE 3.** (*Continued*)

| | | Anxiety | | | Depression | | | Resilience | | |
|---|---|---|---|---|---|---|---|---|---|---|
| Variables | *N* | T0 | T1 | *F* | T0 | T1 | *F* | T0 | T1 | *F* |
| Total | 443 | 9.86 ± 4.14 | 8.95 ± 4.02 | (I) *F* (1, 441) = 0.010 | 6.69 ± 4.03 | 5.86 ± 4.06 | (I) *F* (1, 441) = 0.975 | 3.34 ± 0.79 | 3.44 ± 0.77 | **(I) *F* (1, 441) = 4.619**$^{*}$ |
| Years of seniority | | | | | | | | | | |
| ≤5 | 166 | 9.84 ± 4.18 | 8.71 ± 4.13 | **(T) *F* (1, 440) = 35.803**$^{***}$ | 6.64 ± 4.07 | 5.49 ± 4.12 | **(T) *F* (1, 440) = 28.670**$^{***}$ | 3.31 ± 0.83 | 3.43 ± 80 | **(T) *F* (1, 440) = 12.828**$^{***}$ |
| 5.01–15 | 185 | 9.98 ± 4.13 | 9.17 ± 3.96 | (B) *F* (2, 440) = 0.302 | 6.63 ± 3.91 | 5.96 ± 3.93 | (B) *F* (2, 440) = 0.672 | 3.36 ± 0.77 | 3.45 ± 0.77 | (B) *F* (2, 440) = 0.071 |
| >15 | 92 | 9.66 ± 4.09 | 8.97 ± 3.95 | (I) *F* (2, 440) = 0.872 | 6.91 ± 4.21 | 6.35 ± 4.19 | (I) *F* (2, 440) = 1.605 | 3.35 ± 0.76 | 3.42 ± 0.70 | (I) *F* (2, 440) = 0.398 |
| Total | 443 | 9.86 ± 4.14 | 8.95 ± 4.02 | | 6.69 ± 4.03 | 5.86 ± 4.06 | | 3.34 ± 0.79 | 3.44 ± 0.77 | |
| Years as health workers | | | | | | | | | | |
| ≤ 5 | 57 | 10.08 ± 4.39 | 8.84 ± 4.27 | **(T) *F* (1, 440) = 34.455**$^{***}$ | 7.17 ± 4.59 | 5.66 ± 4.25 | **(T) *F* (1, 440) = 33.635**$^{***}$ | 3.31 ± 0.90 | 3.32 ± 0.82 | **(T) *F* (1, 440) = 7.111**$^{**}$ |
| 5.01–15 | 132 | 10.18 ± 4.07 | 9.34 ± 4.05 | (B) *F* (2, 440) = 0.919 | 6.85 ± 3.75 | 6.17 ± 3.99 | (B) *F* (2, 440) = 0.493 | 3.24 ± 0.78 | 3.36 ± 0.80 | (B) *F* (2, 440) = 2.096 |
| >15 | 254 | 9.64 ± 4.12 | 8.78 ± 3.95 | (I) *F* (2, 440) = 0.433 | 6.50 ± 4.04 | 5.75 ± 4.06 | (I) *F* (2, 440) = 1.733 | 3.40 ± 0.77 | 3.50 ± 0.73 | (I) *F* (2, 440) = 0.843 |
| Total | 443 | 9.86 ± 4.14 | 8.95 ± 4.02 | | 6.69 ± 4.03 | 5.86 ± 4.06 | | 3.34 ± 0.79 | 3.44 ± 0.77 | |

Bold values distinguish the statistical significance.
BS, Between-Subjects; I, interaction; T, Within-Subjects (time).
$^{*}P < 0.05$.
$^{**}P < 0.01$.
$^{***}P < 0.001$.

resilience scores than women $df = 0.436$, $P < 0.001$. Statistically significant differences were also found between resilience and age, with the older group (≥51 years) presenting higher resilience scores than the younger age group (18 to 35 years) $df = -289$, $P < 0.05$. In relation to the number of children in the household, there are statistically significant differences between resilience and this factor, with those individuals with two or more children presenting greater resilience than those with no children, $df = -0.206$, $P < 0.05$ (see Table 3). Furthermore, there is a statistically significant main effect of the Within-Subjects factor (time) in all mixed ANOVAs performed except for sex and Autonomous Community of the workplace variables ($P < 0.05$). Resilience scores are higher at T1 than at T0 in all these factors (Table 3).

## DISCUSSION

The aim of this study was to examine the evolution of symptoms of posttraumatic stress, anxiety, depression, burnout, and resilience at two points in time. Time point T0 (baseline) was during the first wave of the pandemic in Spain and T1 was right after the first wave. The results of our study indicate that, in general, the prevalence of symptoms and burnout was more pronounced at the first measure for nearly all factors, except for the avoidance scale, whereas the levels of resilience were higher at 3 months. Therefore, first and second hypothesis are partially fulfilled and the third one is completely fulfilled.

According to the demographic and work-related variables, women present more emotional exhaustion, posttraumatic stress, anxiety, and depression than men. They also show less resilience than men. Congruent with these results, the female sex has also been associated with these symptoms in studies with nurses in other countries, such as Paraguay.[27] In Spanish general population women have presented more symptoms of anxiety, depression, and posttraumatic stress during the pandemic than men.[28] These results may be due to the fact that women spend more time caring for others both inside and outside their homes,[29] as well as the fact that, historically, the female sex has been associated with a higher prevalence of these symptoms.[30] In relation to age, emotional fatigue increases over time (from T0 to T1) in workers aged 35 to 50 years but it decreases in older workers (more than or equal to 51) over the same period. Younger workers feel more depersonalized than middle-aged workers. Furthermore, younger professionals (18 to 35 years) have more intrusive thoughts and anxiety than older workers (more than or equal to 51). Additionally, middle-aged workers (between the ages of 36 and 50) show more hyperarousal symptoms than older participants (more than or equal to 51). Being young is associated with the appearance of symptoms of posttraumatic stress and burnout in healthcare professionals. This may be due to the concern of the younger ones regarding their future working conditions,[31] as well as their greater access to information from social media, which can be associated with higher levels of stress.[32] Older workers (more than or equal to 51) are more resilient than younger workers (18 to 35 years). A possible explanation for these results is that, probably, those with a higher-level job and/or high educational level have better working conditions than younger people, who have been in the labor market for less time.

Consistent with these results, the scientific literature has highlighted the role of a low educational level as a factor related to the development of posttraumatic stress symptoms.[33,34] In the previous SARS epidemic, lower educational attainment was found to be associated with high levels of avoidance.[35] In addition, a low educational level appears to be a predictor of posttraumatic stress, along with low socio-economic status.[36] Workers with dependent family members and/or those with two or more children feel more professionally fulfilled than those who do not have such family responsibilities. Furthermore, workers who have two or more children are more resilient than those with no children. In a study



Copyright © 2022 American College of Occupational and Environmental Medicine. Unauthorized reproduction of this article is prohibited

with German healthcare professionals, nurses were found to have higher stress levels than physicians.[37] Nursing staff and auxiliary nurses have reported higher levels of stress than other positions such as doctors, probably because they are in more direct and continuous contact with patients, therefore being at a higher risk of contracting the COVID-19 disease.[38] Specifically, it has been demonstrated that female nurses have reported high level of stress than other healthcare personnel during this pandemic.[39] In relation to the Autonomous Community where the workplace is located, emotional exhaustion is greater at the baseline among those participants who worked in the Community of Madrid. These professionals also have more symptoms of avoidance at the end of the first wave of the pandemic. On the other hand, our data indicate that emotional exhaustion was higher in healthcare workers who worked in a hospital setting at T0 and higher in primary care professionals at T1. One likely explanation could be that to prevent hospitals from collapsing, a great transition of care from hospitals to primary care centers took place at the end of the first wave of the pandemic. Workers on rotating shifts have more intrusive thoughts than those on other shifts. Rotating shifts have been associated with worse health and are even related to a high risk of suffering from metabolic syndrome[40] and posttraumatic stress[41]; thus, it will be necessary to pay special attention to the health and well-being of healthcare workers with rotating shifts in the future. As for their seniority, healthcare workers who have been in their current job for less than 5 years felt more depersonalized and had more avoidance behaviors during the first wave of the pandemic than after it. Furthermore, they felt less emotionally tired than those who have hold the same position for 5 to 15 years. In turn, the latter showed more hyperarousal behaviors than those who have been working as healthcare workers for more than 15 years. Therefore, having between 5 and 15 years of experience would be associated with symptoms of posttraumatic stress and burnout, compared with other groups. In general, and in accordance with other studies, having less work experience has been associated with a high prevalence of psychological stress since the beginning of the COVID-19 pandemic.[3,42]

On the other hand, it is important to note that in this study resilience scores are higher 3 months after the first wave of the pandemic began given that the use of strategies such as identifying social support, avoiding information overload, and increasing the feeling of control have been useful for health workers.[43] Therefore, the third hypothesis of our study is fulfilled.

This study is a pioneer work, as it offers longitudinal data in an essential population during a pandemic, that is, healthcare professionals. However, some limitations need to be mentioned. The data were collected online, and some healthcare workers may not have been able to access the technology. In addition, some participants completed the information at T0 but not at T1, reducing the available data for the analysis in the follow-up. Future studies should monitor the long-term effects of the pandemic on the mental health of healthcare workers, with the aim of taking preventive measures in the event of similar situations. Future studies should include evaluations carried out at different times to compare such results with the established baseline. As main conclusion, workers presented less symptoms of posttraumatic stress, anxiety, depression, and burnout at the end of the first wave of the pandemic than at the beginning. Additionally, resilience increased at the end of the first wave. In this study, being women, being young, having a lower-level job, having less experience, a lower educational level and working rotating shifts are variables associated over time with symptoms of anxiety, depression, and posttraumatic stress; therefore, in similar emergency situations, such variables should be considered to preserve the health of healthcare professionals. Besides this study may be useful to adapt individual or group intervention treatments considering the identified variables.

## ACKNOWLEDGMENTS

*Authors would thank the participation of all the healthcare professionals in this study.*

## REFERENCES

1. World Health Organization. The Coronavirus disease (COVID-19) outbreak [WHO web site]; 2020. Available at: https://www.who.int/. Accessed November 15, 2020.
2. Cai W, Lian B, Song X, et al. A cross-sectional study on mental health among health care workers during the outbreak of Corona Virus Disease. *Asian J Psychiatr.* 2020;51:1–4.
3. Chew NW, Lee GK, Tan BY, et al. A multinational, multicentre study on the psychological outcomes and associated physical symptoms amongst healthcare workers during COVID-19 outbreak. *Brain Behav Immuni.* 2020;88:559–565.
4. Duan L, Zhu G. Psychological interventions for people affected by the COVID-19 pandemic. *Lancet Psychiatry.* 2020;7:300–302.
5. Zhang SX, Liu J, Jahanshahi AA, et al. At the height of the storm: healthcare staff's health conditions and job satisfaction and their associated predictors during the epidemic peak of COVID-19. *Brain Behav Immun.* 2020;87: 144–146.
6. Vagni M, Giostra V, Maiorano T, et al. Personal accomplishment and hardiness in reducing emergency stress and burnout among COVID-19 emergency workers. *Sustainability.* 2020;12:1–18.
7. An Y, Yang Y, Wang A, et al. Prevalence of depression and its impact on quality of life among frontline nurses in emergency departments during the COVID-19 outbreak. *J Affect Disord.* 2020;276:312–315.
8. Elbay RY, Kurtulmus A, Aparcioglu S, et al. Depression, anxiety, stress levels of physicians and associated factors in COVID-19 pandemics. *Psychiatry Res.* 2020;290:1–5.
9. Luo M, Guo L, Yu M, et al. The psychological and mental impact of coronavirus disease 2019 (COVID-19) on medical staff and general public - a systematic review and meta-analysis. *Psychiatry Res.* 2020;291:1–9.
10. Xiao X, Zhu X, Fu S, et al. Psychological impact of healthcare workers in China during COVID-19 pneumonia epidemic: a multi-center cross-sectional survey investigation. *J Affect Disord.* 2020;274:405–410.
11. Xiaoming X, Ming A, Su H, et al. The psychological status of 8817 hospital workers during COVID-19 epidemic: a cross-sectional study in Chongqing. *J Affect Disord.* 2020;276:555–561.
12. Wang C, Pan R, Wan X, et al. A longitudinal study on the mental health of general population during the COVID-19 epidemic in China. *Brain Behav Immun.* 2020;87:40–48.
13. Zhou Y, MacGeorge EL, Myrick J. Mental health and its predictors during the early months of the COVID-19 pandemic experience in the United States. *Int J Environ Res Public Health.* 2020;17:1–19.
14. González-Sanguino C, Ausín B, Castellanos MA, et al. Mental health consequences of the COVID-19 outbreak in Spain. A longitudinal study of the alarm situation and return to the new normality. *Prog Neuro-Psychopharmacol Biol Psychiatry.* 2020;107:1–6.
15. Bai Y, Lin CC, Lin CY, et al. Survey of stress reactions among health care workers involved with the SARS outbreak. *Psychiatr Serv.* 2004;55:1055–1057.
16. Wu P, Fang Y, Guan Z, et al. The psychological impact of the SARS epidemic on hospital employees in China: exposure, risk perception, and altruistic acceptance of risk. *Can J Psychiatry.* 2004;54:302–311.
17. Benfante A, Di Tella M, Romeo A, et al. Traumatic stress in healthcare workers during COVID-19 pandemic: a review of the immediate impact. *Front Psychol.* 2020;11:1–7.
18. De Wijn GN, van der Doef MP. Patient-related stressful situations and stress-related outcomes in emergency nurses: a cross-sectional study on the role of work factors and recovery during leisure time. *Int J Nurs Stud.* 2020;107:1–11.
19. Maslach C, Jackson SE, Leiter MP.In: *Maslach Burnout Inventory Manual.* 3rd ed., Palo Alto, CA: Consulting Psychologists Press; 1996. 191–218.
20. Seisdedos N. *MBI Human Services.* Madrid: TEA Ediciones; 1997, 7–36.
21. Cañadas GA, San Luis C, Lozano LM. Evidence for factorial validity of Maslach Burnout Inventory and burnout levels among health workers. *Rev Latinoam Psicol.* 2014;46:44–52.
22. Weiss DS, Marmar CR. The impact of event scale-revised. In: Wilson JP, Keane TM, editors. *Assessing Psychological Trauma and PTSD.* New York: Guildford Press; 1997. p. 399–411.

 © 2021 American College of Occupational and Environmental Medicine

Copyright © 2022 American College of Occupational and Environmental Medicine. Unauthorized reproduction of this article is prohibited

23. Terol MC, López-Roig S, Rodríguez-Marín J, et al. Psychometric properties of the Hospital Anxiety and Depression Scale (HAD) in Spanish population. *Ansiedad Estrés*. 2007;13:163–176.
24. Zigmond AS, Snaith RP. The hospital anxiety and depression scale. *Acta Psychiatr Scand*. 1983;67:361–370.
25. Rodríguez-Rey R, Alonso-Tapia J, Hernansaiz H. Reliability and validity of the Brief Resilience Scale (BRS) Spanish version. *Psychol Assess*. 2016;28:e101–e110.
26. Smith BW, Dalen J, Wiggins K, et al. The brief resilience scale: assessing the ability to bounce back. *Int J Behav Med*. 2008;15:194–200.
27. Samaniego A, Urzúa A, Buenahora M, et al. Symptomatology associated with mental health disorders in health workers in Paraguay: COVID-19 effect. *Interam J Psychol*. 2020;54:1–19.
28. Ausín B, González-Sanguino C, Castellanos MA, et al. Gender-related differences in the psychological impact of confinement as a consequence of COVID-19 in Spain. *J Gend Stud*. 2020;30:1–10.
29. Manzo LKC, Minello A. Mothers, childcare duties, and remote working under COVID-19 lockdown in Italy: cultivating communities of care. *Dialogues Hum Geogr*. 2020;10:1–4.
30. Salk RH, Hyde JS, Abramson LY. Gender differences in depression in representative national samples: meta-analyses of diagnoses and symptoms. *Psychol Bull*. 2017;143:783–822.
31. Salari N, Hosseinian-Far A, Jalali R, et al. Prevalence of stress, anxiety, depression among the general population during the COVID-19 pandemic: a systematic review and meta-analysis. *Glob Health*. 2020;16:1–11.
32. Cheng VC, Wong SC, Yuen KY. Estimating coronavirus disease 2019 infection risk in health care workers. *JAMA Netw Open*. 2020;3:1–3.
33. Carmassi C, Gesi C, Corsi M, et al. Exploring PTSD in emergency operators of a major University Hospital in Italy: a preliminary report on the role of gender, age, and education. *Ann Gen Psychiatry*. 2018;4:1–7.
34. Kvestad I, Ranjitkar S, Ulak M, et al. Earthquake exposure and post-traumatic stress among nepalese mothers after the 2015 earthquakes. *Front Psychol*. 2019;10:1–11.
35. Wu KK, Chan SK, Ma TM. Posttraumatic stress, anxiety, and depression in survivors of severe acute respiratory syndrome (SARS). *J Trauma Stress*. 2005;18:39–42.
36. Tang B, Deng Q, Glik D, et al. A meta-analysis of risk factors for post-traumatic stress disorder (PTSD) in adults and children after earthquakes. *Int J Environ Res Public Health*. 2017;14:1–20.
37. Kramer V, Papazova I, Thoma A, et al. Subjective burden and perspectives of German healthcare workers during the COVID-19 pandemic. *Eur Arch Psychiatry Clin Neurosci*. 2020;271:271–281.
38. Lai J, Ma S, Wang Y, et al. Factors associated with mental health outcomes among health care workers exposed to coronavirus disease 2019. *JAMA Netw Open*. 2020;3:1–12.
39. Luceño L, Talavera B, García Y, et al. Symptoms of posttraumatic stress, anxiety, depression, levels of resilience and burnout in Spanish health personnel during the COVID-19 pandemic. *Int J Environ Res Public Health*. 2020;17:1–25.
40. De Bacquer D, Van Risseghem M, Clavs E, et al. Rotating shift work and the metabolic syndrome: a prospective study. *Int J Epidemiol*. 2009;38:848–854.
41. Angehrn A, Teale MJ, Ricciardelli R, et al. Sleep quality and mental disorder symptoms among Canadian public safety personnel. *Int J Environ Res Public Health*. 2020;17:1–14.
42. Yitayih Y, Mekonen S, Zeynudin A, et al. Mental health of healthcare professionals during the early stage of the COVID-19 pandemic in Ethiopia. *BJPsych Open*. 2020;7:1–6.
43. Vinkers CH, van Amelsvoort T, Bisson JI, et al. Stress resilience during the coronavirus pandemic. *Eur Neuropsychopharmacol*. 2020;35:12–16.




Copyright © 2022 American College of Occupational and Environmental Medicine. Unauthorized reproduction of this article is prohibited
