# Supplementary material for: Mental health status and related factors influencing healthcare workers during the COVID-19 pandemic: A systematic review and meta-analysis
Source: PLoS One. 2024 Jan 19;19(1):e0289454. doi: 10.1371/journal.pone.0289454 (PMC10798549; doi:10.1371/journal.pone.0289454)
Supplement: S1 Data — (ZIP) [file pone.0289454.s011.zip › literatures/85.pdf]

salud mental

ORIGINAL ARTICLE
Volume 44, Issue 5, September-October 2021
doi: 10.17711/SM.0185-3325.2021.028

Effect of concern about COVID-19 on professional self-efficacy, psychological distress, anxiety, and depression in Peruvian health personnel

Oscar Mamani-Benito,[1] Renzo Felipe Carranza Esteban,[2] José Ventura-León,[3] Tomás Caycho-Rodríguez,[3] Rosa Farfán Solís,[4,5] Darwin Hidalgo Blanco Shocosh[5]

[1] Facultad de Derecho y Humanidades, Universidad Señor de Sipán, Chiclayo, Perú.
[2] Facultad de Humanidades, Universidad San Ignacio de Loyola, Lima, Perú.
[3] Facultad de Ciencias de la Salud, Universidad Privada del Norte, Lima, Perú.
[4] Facultad de Ciencias de la Salud, Universidad Peruana Unión, Juliaca, Perú.
[5] Dirección Regional de Salud, Puno, Perú.

Correspondence:
Oscar Mamani-Benito
Facultad de Derecho y Humanidades, Escuela Profesional de Psicología, Universidad Señor de Sipán, Chiclayo, Perú.
Campus Universitario, km. 5 Carretera Pimentel Chiclayo, Perú.
Phone: (511) 9 5992-5651
Email: mamanibe@crece.uss.edu.pe

Received: 20 November 2020
Accepted: 2 February 2021

Citation:
Mamani-Benito, O., Carranza Esteban, R. F., Ventura-León, J., Caycho-Rodríguez, T., Farfán Solís, R., & Blanco Shocosh, D. H. (2021). Effect of concern about COVID-19 on professional self-efficacy, psychological distress, anxiety, and depression in Peruvian health personnel. *Salud Mental*, *44*(5), 215-220.

DOI: 10.17711/SM.0185-3325.2021.028

CC BY NC

ABSTRACT

Introduction. Concern about becoming infected with COVID-19 is one of the reactions that has affected the mental health of Peruvian health care workers. **Objective.** To determine the effect of concern about COVID-19 on professional self-efficacy, psychological distress, anxiety, and depression in health personnel in the Puno region of Peru. **Method.** An explanatory, cross-sectional study was conducted with the voluntary participation of 401 health workers (such as nurses, physicians, nursing technicians, obstetricians, dentists, psychologists, and nutritionists) of both sexes (24.2% men and 75.5% women) from 12 health networks in the region of Puno, Peru. They were asked to answer the Scale of Concern for the Transmission of COVID-19 in Health Personnel (EPPC-Cov19), Patient Health Questionnarie-2 (PHQ-2, Generalized Anxiety Disorder Scale-2 (GAD-2) and the Professional Self Efficacy Scale (AU-10). Structural Equation Modeling (SEM) was used for data analysis. **Results.** The factor loads of the explanatory model showed a good fit: χ^2 (286) = 797.31, $p < .001$; IFC = .99; RMSEA = .06; SRMR = .07; WRMR = 1.25. Therefore concern about COVID-19 has a greater effect on anxiety (β = .77) and depression (β = .71), as well as psychological distress (β = .65), and only a mild effect on work self-efficacy (β = .12). **Discussion and conclusion.** Concern about the spread of COVID-19 affects depression, anxiety, psychological distress and work self-efficacy among Peruvian health workers in the Puno region of Peru.

Keywords: Coronavirus, self-efficacy, psychological distress, depression, anxiety, health personnel.

RESUMEN

Introducción. La preocupación por contagiarse con el COVID-19 ha sido una de las reacciones que ha afectado la salud mental de los trabajadores sanitarios peruanos. **Objetivo.** Determinar el efecto de la preocupación por el COVID-19 sobre la autoeficacia profesional, malestar psicológico, ansiedad y depresión en el personal sanitario de la región de Puno, Perú. **Método.** Se realizó un estudio explicativo y transversal, donde participaron voluntariamente 401 trabajadores sanitarios (enfermeros/as, médicos, técnicos en enfermería, obstetras, odontólogos, psicólogos/as, nutricionistas, entre otros; de ambos sexos (24.2% varones y 75.5% mujeres), de 12 redes de salud de la región de Puno. A todos se les aplicó la Escala de Preocupación por el Contagio de el COVID-19 (EPPC-Cov19), Patient Health Questionnaire-2 (PHQ-2, Generalized Anxiety Disorder Scale-2 (GAD-2) y la Escala de Autoeficacia Profesional (AU-10). Para el análisis de los datos se utilizó el Modelamiento de Ecuaciones Estructurales (SEM). **Resultados.** Las cargas factoriales del modelo explicativo presentaron buenas bondades de ajuste: χ^2 (286) = 797.31, $p < .001$; CFI = .99; RMSEA = .06; SRMR = .07; WRMR = 1.25. Así, la preocupación por el COVID-19 tiene un mayor efecto en la ansiedad (β = .77), depresión (β = .71) y el malestar psicológico (β = .65); en cambio, en la autoeficacia para el trabajo (β = .12) presenta un efecto menor. **Discusión y conclusión.** La preocupación por el contagio de el COVID-19 tiene un efecto significativo sobre la depresión, ansiedad, malestar psicológico y autoeficacia para el trabajo en el personal sanitario peruano de la región de Puno, Perú.

Palabras clave: Coronavirus, autoeficacia, estrés emocional, depresión, ansiedad, personal sanitario.

INTRODUCTION

The severe acute respiratory syndrome coronavirus 2 (SARS-CoV-2) that causes COVID-19 has become a global public health threat (Nguyen et al., 2020). Most of the world's countries are continuing to fight to counteract the repercussions on the population. Although more than six months have passed since the start of the pandemic, some characteristics and behaviors of the virus remain a mystery (Arias-Reyes et al., 2020).

In this scenario, health workers constitute the first line of battle and are therefore at the greatest risk of becoming infected. This is borne out by studies in China and Italy, where approximately 10% to 20% of reported cases correspond to health professionals (Algado-Sellés et al., 2020). In addition to this, other reports reveal risk factors in this population, such as the lack of personal protective equipment (PPE), constant exposure to infected patients, work overload, pre-existing medical conditions, and poor infection control (Mhango, Dzobo, Chitungo, & Dzinamarira, 2020). This obviously affects medical services and the behavior of healthcare personnel, leading them to experience fear and uncertainty, which translates into concern about the consequences of the virus (Chang, 2020).

As for the effects that concern over COVID-19 might be causing, there are a number of hypotheses. For example, the study by Spoorthy, Pratapa, & Mahant (2020) reports mental health problems among health personnel, specifically a lack of self-efficacy, associated with anxiety and depression symptoms. Along these same lines, Lai et al. (2020) corroborate that health workers in several regions of China have experienced depression, anxiety, insomnia, and anguish, particularly female nurses from Wuhan, and frontline health workers directly involved in the diagnosis, treatment or provision of care to patients with suspected or confirmed COVID-19. Concern about COVID-19 appears to mainly cause anxiety and depression in this population (Shaukat, Ali, & Razzak, 2020) since, in response to the probability of being infected by the virus and suffering consequences in their own health or in that of a close relative, the individual experiences an emotional reaction (anxiety) which prepares the body to cope with stressful situations, such as caring for patients infected with COVID-19 (Buitrago Ramírez, Ciurana Misol, Fernández Alonso, & Tizón, 2021). Moreover, prolonged exposure to this concern leads to the emergence of depressive symptoms such as sadness, mood alterations, lack of sleep, tiredness, and feelings of uselessness (Dosil Santamaría, Ozamiz-Etxebarria, Redondo Rodríguez, Jaureguizar Alboniga-Mayor, & Picaza Gorrotxategi, 2021). This situation has been observed in certain contexts such as Peru, as shown by a review noting the consequences on health personnel (Huarcaya-Victoria, 2020).

Other no less important aspects would be psychological discomfort and professional self-efficacy. Regarding the former, a study in Cameroon found a prevalence of psychological distress associated with fear of contagion (Nguépy Keubo et al., 2021). This situation could be due to work overload, the perceived or actual mismatch between the demand for care and the available means, and repeated exposure to infected patients (Lefèvre et al., 2021; Vignaud & Prieto, 2020), which in some contexts is interpreted as psychological distress and suffering (Ornell, Chwartzmann Halpern, Paim Kessler, & Narvaez, 2020). Regarding the latter, studies reveal that a shortcoming in this capacity is associated with greater stress, anxiety, and depression (Spoorthy et al., 2020). Likewise, another study in Iran recommended developing professional self-efficacy to promote preventive behaviors in the face of the possible effects of COVID-19 (Bashirian et al., 2020), regarding it as a personal strength in health workers (Cheung et al., 2020) particularly in nursing personnel, where there is also an urgent need to develop resilience (Hu & Bentler, 1999).

On the basis of these indicators, the pandemic can be said to have increased the challenge of caring for the mental health of health workers (Maben & Bridges, 2020) currently considered to be a risk group. Although it is unlikely that a disease with the characteristics of the current SARS-CoV-2 will be repeated, the repercussions it has caused have obviously revealed the need to recognize the impact external factors have on the mental health of these workers. Moreover, given the magnitude of the problem, since all health workers in the world have been exposed to the same stressor at the same time, the impact of sustained, severe stressors was clearly visible. Accordingly, studies to evaluate mental health in this population are extremely useful for developing public health strategies to prevent and control the repercussions caused by the pandemic in Latin America (Lozano-Vargas, 2020).

At the beginning of the pandemic, ignorance about the virus was one of the main causes of infection in health workers. However, at present, despite all the scientific knowledge generated, the infection and mortality rate continues to increase, which suggests that this situation continues to prevail because of external factors such as the shortage of PPE equipment, lack of training in infection control, long working hours, and a stressful environment (Arias-Reyes et al., 2020).

In short, despite having generated a certain amount of discussion of the problem based on the findings of studies conducted both globally and locally, focusing attention on the repercussions on the mental health of health workers in the context of COVID-19, the authors of this study consider that the data are still limited (Nguyen et al., 2020); instead, the purpose of this study is linked to the impetus shown by the World Health Organization and the International Council of Nurses, who expressed great concern over the fact that some countries are failing to track or report data related to infection by COVID-19 in healthcare workers (Arias-Reyes et al., 2020).

For all these reasons, the purpose of this research is to determine the effect of concern about COVID-19 on professional self-efficacy, psychological discomfort, anxiety, and depression in a sample of Peruvian health professionals.

METHOD

Study design

Explanatory, cross-sectional study (Ato, López, & Benavente, 2013).

Sample description

As a result of non-probabilistic sampling, 401 workers from 12 health networks in the Puno region (24.2% men and 75.5% women) participated in the study. Their ages ranged from 22 to 67 (*M* = 37.74; *SD* = 9.22), all of whom worked during the health emergency, although only one group was assigned to directly care for COVID-19 patients (physicians, nurses, nursing technicians). Most subjects are nurses (29.7%), physicians (13.0%), nursing technicians (17.2%), obstetricians (12.7%), dentists (6.7%), psychologists (2.2%), nutritionists (2.2%), while 16.2% have other professions. As for the length of service, 33.9% have worked for over nine years; 39.4% of them being single.

Measurements

Scale of Concern about the Spread of COVID-19 for Health Personnel (EPPC – Cov19). This brief, eight-item scale measures the emotional state, encompassing fear, restlessness, anguish, and immobility, caused by unknown and negative events, constructed by the cognitive system. The eight items have five answer options (not at all, a little, somewhat, much, a great deal). For the present study, the EPPC-Cov19 was designed, obtaining evidence of validity based on content (Aiken's V > .75) and internal structure (CFI = .960; TLI = .951 and RMSEA = .075). Likewise, the EPPC – Cov19 in the present study showed good internal consistency (α = .91; 95% CI [.89, .92]).

Patient Health Questionnaire-2 (PHQ; Kroenke, Spitzer, & Williams, 2003). This brief measure analyzes cognitive and emotional aspects related to depression (discouragement, displeasure, hopelessness, and disinterest in things). It comprises two items on a Likert-type scale, where 0 is not at all and 3 is almost every day). The reliability of the PHQ-2 in the present study was α = .75 (95% CI [.70, .78]).

Generalized Anxiety Disorder Scale-2 (GAD-2; Kroenke, Spitzer, Williams, Monahan, & Löwe, 2007). Brief measure assessing behaviors related to the emotional and cognitive expression of generalized anxiety in the past two weeks. It consists of two items with four Likert-type answer options (0 = not at all to 3 = almost every day). In the present study, the GAD-2 reported adequate reliability (α = .85; 95% CI [.82, .87]).

Professional Self-efficacy Scale (AU-10; Calderón-De la Cruz, Domínguez-Lara, & Arroyo-Rodríguez, 2018). It analyzes the self-efficacy beliefs of workers, assessing their ability to cope with difficulties at work. It comprises ten items on a Likert scale (0 = never to 6 = always). The reliability of the PHQ-2 in the present study was α = .96 (IC 95% [.95, .96]).

Kessler Psychological Distress Scale (K6, adapted to the Peruvian context by Dominguez-Lara & Alarcón-Parco, 2020). This scale evaluates psychological distress and consists of six items in Likert-type format with five answer options (0 = never to 4 = all the time). The reliability for this study reported adequate internal consistency (α = .81; 95% CI [.77, .83]).

Procedure

A virtual form of the questionnaires was subsequently created through Google Forms, available between August 25 and September 28, 2020, which was sent to participants via email and WhatsApp groups. Before completing the link, subjects gave their consent through an informed consent form that explained the purpose of the study, emphasizing the fact that participation was voluntary and anonymous.

Statistical analyses

Structural equation modeling (SEM) was used for the data analysis. This is because an explanatory design with latent variables was used, in which a structure and measurement model was considered with certain observable and latent variables (Ato et al., 2013). In addition, this type of modeling enables one to estimate the measurement error associated with observable variables. For the present study, analysis of covariance (CB-SEM) was used because the aim was to confirm a theoretical hypothesis, because of which no re-specifications were incorporated into the original model.

Prior to the CB-SEM analysis, an analysis of central tendency measures and item dispersion was performed. In addition, the coefficient of asymmetry and kurtosis was used as distribution measures of the items, taking as cut-off values ± 2 and information on the minimum and maximum value answered in each of the items.

CB-SEM was performed through the estimation method of Weighted Least Squares with Mean and Adjusted Variance (WLSMV) since it is considered an appropriate method in the presence of variables observed on an ordinal scale and functions well in underlying non-normal distributions (Li, 2016). The suitability of the factorial model will be examined through the fit indices pro-

Table 1
Descriptive statistics of variables under study

Variables	*M*	*SD*	*Min.*	*Max.*	*%*	*g1*	*g2*	*ω*	*AVE*
PreCOVID	23.03	7.26	8.00	40.00	57.58	.31	-.64	.91	.63
Anxiety	3.50	1.55	2.00	8.00	43.75	.93	.26	.84	.83
Depression	2.97	1.31	2.00	8.00	37.13	1.24	.73	.77	.75
Self-efficacy	48.28	12.97	10.00	70.00	68.97	-.24	-.63	.93	.80
Discomfort	11.46	4.08	6.00	27.00	42.44	.88	.59	.84	.59

Notes: *M* = Mean; *SD* = Standard deviation; g1 = Asymmetry; g2 = Kurtosis; ω = corrected omega considering correlated errors; PreCOVID = Concern about COVID-19; % = Division between average (M) and maximum value (Max.) and indicates the place occupied by the average score on a scale of 0 to 100%

posed by Hu and Bentler (1999): Comparative Fit Index (CFI) > .90; Root Mean Square Error of Approximation (RMSEA) < .08; Standardized Root Mean Square Residual (SRMR) < .08. In addition, an index for ordinal measurements that has recently demonstrated its satisfactory performance, called Weighted Root Mean Square Residual (WRMR) < 1, will be incorporated.

Regarding the measurement model, reliability was evaluated using the omega coefficient (Ventura-León & Caycho-Rodríguez, 2017) considering the presence of correlated errors and the average variance extracted (AVE). In relation to the structure model, the measurements were R2 as information on the explained variance, together with the magnitude and significance of standardized beta coefficients. All analyses were conducted using the R version 4.0.2.

Ethical considerations

The study was approved by the ethics committee of the Regional Health Directorate of Puno.

RESULTS

Table 1 shows the descriptive statistics of the variables under study. It is observed that the arithmetic mean of the work self-efficacy variable (*M* = 23.03; *SD* = 7.26) and of the concern about COVID-19 (*M* = 48.28; *SD* = 12.97) exceeds 50% of the range of values they can take. Asymmetry is positive in all variables except work self-efficacy. Moreover, kurtosis is below ± 2 values. In addition, reliability is reported by means of the omega coefficient that is higher than .70, indicating good internal correlation between the variables and in the analysis of explained variance (AEV), which is higher > .50.

Table 2 shows the factor loadings of the explanatory model, the same one that shows high goodness of fit: χ^2 (286) = 797.31, $p < .001$; CFI = .99; RMSEA = .06; SRMR = .07; WRMR = 1.25. This ensures that the estimates are appropriate and that there are no biases due to poor model specifications. Figure 1 shows that concern about COVID-19 has a greater effect on anxiety (β = .77) and depression (β = .71). In relation to the variable psychological discomfort, it is lower (β = .65), although it can still be considered a significant effect. Conversely, work self-efficacy (β = .12) shows the least effect.

Table 2
Factor loads of explanatory model

	PreCOVID	*Anxiety*	*Depression*	*Discomfort*	*Self-efficacy*
PCov1	.70				
PCov2	.84				
PCov3	.88				
PCov4	.85				
PCov5	.73				
PCov6	.79				
PCov7	.62				
PCov8	.86				
GAD1		.91			
GAD2		.91			
PHQ1			.87		
PHQ2			.87		
MP1				.86	
MP2				.86	
MP3				.82	
MP4				.86	
MP5				.48	
MP6				.66	
AP1					.63
AP3					.86
AP5					.92
AP6					.94
AP7					.95
AP8					.97
AP9					.94
AP10					.93

Note: PreCOVID = Concern about COVID-19.

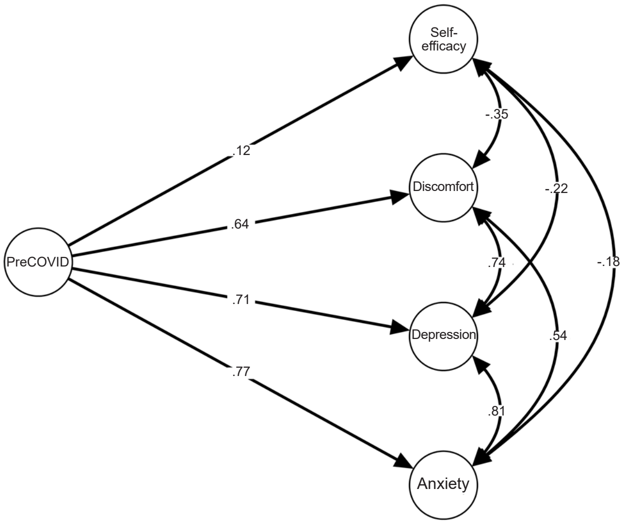

Figure 1. Factor structure of the effect of concern about COVID-19 on work self-efficacy, psychological discomfort, depression and anxiety. The items are removed from the figure but considered in the estimation of the model.

DISCUSSION

Scientific literature suggests that the COVID-19 pandemic has affected the mental and physical health of large numbers of healthcare workers (Shaukat et al., 2020). Based on the results, the main finding is having found that health workers' concern about being infected with COVID-19 has had a significant impact on the increase in psychological discomfort, anxiety, and depression, although this effect has been greater for anxiety than for depression, and minimal for professional self-efficacy. This is partly consistent with Yook, Kim, Suh, and Lee (2010), who point out that worry produced a significant variation in the prediction of depression and anxiety symptoms. The relationship between worry, discomfort, depression, and anxiety has been adequately documented in clinical and non-clinical population (Yook et al., 2010).

Although anxiety and depression do not affect all health workers, they can decrease self-confidence, especially since they are suppposed to keep calm and reassure patients (Shanafelt, Ripp, & Trockel, 2020). The degree of concern may increase and exacerbate anxiety and depressive and discomfort symptoms due to the absence of adequate protective measures (Roy et al., 2020). This is particularly important in a country such as Peru, where strong healthcare infrastructure, and so recognizing the sources of concern about contagion will allow specific approaches to be developed to address these concerns and provide support for workers.

These results can be explained by the high levels of intolerance to uncertainty, which can create difficulties in dealing with stressful events, making it more likely for them to experience high levels of anxiety (Dar, Iqbal, & Mushtaq, 2017). This is hardly surprising as it has been shown that intolerance of uncertainty is closely related to concern (Lin, Xie, Yan, & Yan, 2017). However, it is necessary to recognize that COVID-19 is a disease with an extremely high fatality rate and a strong probability of infection in ordinary, everyday activities. In health personnel, this means that the mere fact of going to work is a risk factor.

The study has various strengths. First, health workers were evaluated, a group of whom were actively treating patients with COVID-19 in the Puno region, which despite being over 3,800 meters above sea level (Arias-Reyes et al., 2020), is one of the most severely affected regions in Peru (Gestión, 2020). Moreover, the study was conducted during the peak of the COVID-19 outbreak in Peru. At the same time, it also has limitations, such as the unequal distribution of the number of men and women, as well as the relatively small sample size, compared to the total population of health workers in Peru. Likewise, the workers who participated may not be representative of health workers from different regions of Peru. It is also possible that some workers did not have much time to participate in the study due to their professional obligations. Finally, the use of self-report measures is another limitation, since it may produce a social desirability bias in the participants.

In conclusion, the study evaluated health workers, a group of whom were treating patients with COVID-19 in the Puno region, one of the most severely affected in Peru and the global epicenter of the pandemic. Different variables associated with mental health were quantified, ranging from concern about infection from COVID-19, professional self-efficacy, and psychological to discomfort, depression, and anxiety. In this group of workers, concern about the spread of COVID-19 leads to psychological discomfort, anxiety and depression symptoms and lower professional self-efficacy.

These findings should serve to develop and implement interventions to reduce the impact of concern about the spread of COVID-19 on the mental well-being of healthcare workers. In addition, the fact of having highlighted the impact of sustained and severe stressors, regardless of whether they occur on a smaller or larger scale, should promote the installation of a monitoring and treatment system by the Peruvian health system. The findings presented here should therefore be considered by decision makers in occupational health, health service administration, and the community at large to promote and protect health workers in this and future public health crises.

Funding

None.

Conflict of interests

The authors declare they have no conflicts of interest.

Acknowledgements

The authors would like to thank the health personnel of the Puno region for participating in this research.

REFERENCES

Algado-Sellés, N., Gras-Valentí, P., Chico-Sánchez, P., Mora-Muriel, J. G., Soler-Molina, V. M., Hernández-Maldonado, M., … Sánchez-Payá, J. (2020). Frequency, associated risk factors, and characteristics of COVID-19 among healthcare personnel in a Spanish health department. *American Journal of Preventive Medicine*, *59*(6), e221-e229. doi: 10.1016/j.amepre.2020.07.014

Arias-Reyes, C., Zubieta-DeUrioste, N., Poma-Machicao, L., Aliaga-Raduan, F., Carvajal-Rodriguez, F., Dutschmann, M., … Soliz, J. (2020). Does the pathogenesis of SARS-CoV-2 virus decrease at high-altitude?. *Respiratory Physiology & Neurobiology*, *277*, 103443. doi: 10.1016/j.resp.2020.103443

Ato, M., López, J. J., & Benavente, A. (2013). Un sistema de clasificación de los diseños de investigación en psicología. *Anales de Psicología*, *29*(3), 1038-1059. doi: 10.6018/analesps.29.3.178511

Bashirian, S., Jenabi, E., Khazaei, S., Barati, M., Karimi-Shahanjarini, A., Zareian, S., … Moeini, B. (2020). Factors associated with preventive behaviours of COVID-19 among hospital staff in Iran in 2020: an application of the Protection Motivation Theory. *Journal of Hospital Infection*, *105*(3), 430-433. doi: 10.1016/j.jhin.2020.04.035

Buitrago Ramírez, F., Ciurana Misol, R., Fernández Alonso, M. C., & Tizón, J. L. (2021). COVID-19 pandemicand mental health: Initial considerations from spanish primaryhealthcare. *Atención Primaria*, *53*(1) 89-101. doi: 10.1016/j.aprim.2020.06.006

Calderón-De la Cruz, G. A., Domínguez-Lara, S. A., & Arroyo-Rodríguez, F. (2018). Análisis psicométrico preliminar de una medida breve de autoeficacia profesional en trabajadores peruanos: AU-10. *Psicogente*, *21*(39), 12-24. doi: 10.17081/psico.21.39.2819

Chang, W. H. (2020). The influences of the COVID-19 pandemic on medical service behaviors. *Taiwanese Journal of Obstetrics and Gynecology*, *59*(6), 821-827. doi: 10.1016/j.tjog.2020.09.007

Cheung, V. K.-L., So, E. H.-K., Ng, G. W.-Y., So, S.-S., Hung, J. L.-K., & Chia, N.-H. (2020). Investigating effects of healthcare simulation on personal strengths and organizational impacts for healthcare workers during COVID-19 pandemic: a cross-sectional study. *Integrative Medicine Research*, *9*(3), 100476. doi: 10.1016/j.imr.2020.100476

Dar, K. A., Iqbal, N., & Mushtaq, A. (2017). Intolerance of uncertainty, depression, and anxiety: Examining the indirect and moderating effects of worry. *Asian Journal of Psychiatry*, *29*, 129-133. doi: 10.1016/j.ajp.2017.04.017

Dominguez-Lara, S., & Alarcón-Parco, D. (2020). Análisis estructural de la escala de malestar psicológico de Kessler (K6) en universitarios peruanos. *Educación Médica*, *21*(2), 155-156. doi: 10.1016/j.edumed.2019.10.008

Dosil Santamaría, M., Ozamiz-Etxebarria, N., Redondo Rodríguez, I., Jaureguizar Alboniga-Mayor, J., & Picaza Gorrotxategi, M. (2021). Psychological impactofCOVID-19 on a sample of Spanish health professionals. *Revista de Psiquiatría y Salud Mental*, *14*(2), 106-112. doi: 10.1016/j.rpsm.2020.05.004

Gestión. (2020). *COVID-19: En Puno y Cusco se disparan las cifras de exceso de fallecidos por dia*. Retrieved from https://gestion.pe/peru/politica/covid-19-en-puno-y-cusco-se-disparan-las-cifras-de-exceso-de-fallecidos-por-dia-nczg-noticia/

Hu, L., & Bentler, P. M. (1999). Cutoff criteria for fit indexes in covariance structure analysis: Conventional criteria versus new alternatives. *Structural Equation Modeling: A Multidisciplinary Journal*, *6*(1), 1-55. doi: 10.1080/10705519909540118

Huarcaya-Victoria, J. (2020). Consideraciones sobre la salud mental en la pandemia de COVID-19. *Revista Peruana de Medicina Experimental y Salud Pública*, *37*(2), 327-334. doi: 10.17843/RPMESP.2020.372.5419

Kroenke, K., Spitzer, R. L., & Williams, J. B. W. (2003). The patient health questionnaire-2: Validity of a two-item depression screener. *Medical Care*, *41*(11), 1284-1292. doi: 10.1097/01.MLR.0000093487.78664.3C

Kroenke, K., Spitzer, R. L., Williams, J. B. W., Monahan, P. O., & Löwe, B. (2007). Anxiety disorders in primary care: prevalence, impairment, comorbidity, and detection. *Annals of Internal Medicine*, *146*(5), 317-325. doi: 10.7326/0003-4819-146-5-200703060-00004

Lai, J., Ma, S., Wang, Y., Cai, Z., Hu, J., Wei, N., … Hu, S. (2020). Factors Associated With Mental Health Outcomes Among Health Care Workers Exposed to Coronavirus Disease 2019. *JAMA Network Open*, *3*(3), e203976. doi: 10.1001/jamanetworkopen.2020.3976

Lefèvre, H., Stheneur, C., Cardin, C., Fourcade, L., Fourmaux, C., Tordjman, E., … Lachal, J. (2021). The Bulle: Support and prevention of psychological decompensation of health care workers during the trauma of the COVID-19 epidemic. *Journal of Pain and Symptom Management*, *61*(2), 416-422. doi: 10.1016/j.jpainsymman.2020.09.023

Li, C.-H. (2016). Confirmatory factor analysis with ordinal data: Comparing robust maximum likelihood and diagonally weighted least squares. *Behavior Research Methods*, *48*(3), 936-949. doi: 10.3758/s13428-015-0619-7

Lin, R.-M., Xie, S.-S., Yan, Y.-W., & Yan, W.-J. (2017). Intolerance of uncertainty and adolescent sleep quality: The mediating role of worry. *Personality and Individual Differences*, *108*, 168-173. doi: 10.1016/j.paid.2016.12.025

Lozano-Vargas, A. (2020). Impacto de la epidemia del Coronavirus (COVID-19) en la salud mental del personal de salud y en la población general de China. *Revista de Neuro-Psiquiatria*, *83*(1), 51-56. doi: 10.20453/rnp.v83i1.3687

Maben, J., & Bridges, J. (2020). Covid-19: Supporting nurses' psychological and mental health. *Journal of Clinical Nursing*, *29*(15-16), 2742-2750. doi: 10.1111/jocn.15307

Mhango, M., Dzobo, M., Chitungo, I., & Dzinamarira, T. (2020). COVID-19 Risk Factors Among Health Workers: A Rapid Review. *Safety and Health at Work*, *11*(3), 262-265. doi: 10.1016/j.shaw.2020.06.001

Nguyen, L. H., Drew, D. A., Graham, M. S., Joshi, A. D., Guo, C. G., Ma, W., … Chan, A. T. (2020). Risk of COVID-19 among front-line health-care workers and the general community: a prospective cohort study. *The Lancet Public Health*, *5*(9), e475-e483. doi: 10.1016/S2468-2667(20)30164-X

Nguépy Keubo, F. R., Mboua, P. C., Djifack Tadongfack, T., Fokouong Tchoffo, E., Tasson Tatang, C., Ide Zeuna, J., … Folefack, G. O. (2021). Psychological distress among health care professionals of the three COVID-19 most affected Regions in Cameroon: Prevalence and associated factors. *Annales Médico-Psychologiques, Revue Psychiatrique*, *179*(2), 141-146. doi: 10.1016/j.amp.2020.08.012

Ornell, F., Chwartzmann Halpern, S., Paim Kessler, F. H., & Narvaez, J. C. de M. (2020). The impact of the COVID-19 pandemic on the mental health of healthcare professionals. *Cadernos de Saude Publica*, *36*(4), e00063520. doi: 10.1590/0102-311X00063520

Roy, D., Tripathy, S., Kar, S. K., Sharma, N., Verma, S. K., & Kaushal, V. (2020). Study of knowledge, attitude, anxiety & perceived mental healthcare need in Indian population during COVID-19 pandemic. *Asian Journal of Psychiatry*, *51*, 102083. doi: 10.1016/j.ajp.2020.102083

Shanafelt, T., Ripp, J., & Trockel, M. (2020). Understanding and Addressing Sources of Anxiety among Health Care Professionals during the COVID-19 Pandemic. *JAMA - Journal of the American Medical Association*, *323*(21), 2133-2134. doi: 10.1001/jama.2020.5893

Shaukat, N., Ali, D. M., & Razzak, J. (2020). Physical and mental health impacts of COVID-19 on healthcare workers: A scoping review. *International Journal of Emergency Medicine*, *13*(1), 40. doi: 10.1186/s12245-020-00299-5

Spoorthy, M. S., Pratapa, S. K., & Mahant, S. (2020). Mental health problems faced by healthcare workers due to the COVID-19 pandemic–A review. *Asian Journal of Psychiatry*, *51*, 102119. doi: 10.1016/j.ajp.2020.102119

Ventura-León, J. L., & Caycho-Rodríguez, T. (2017). El coeficiente Omega: un método alternativo para la estimación de la confiabilidad. *Revista Latinoamericana de Ciencias Sociales, Niñez y Juventud*, *15*(1), 625-627. Retrieved from https://www.redalyc.org/pdf/773/77349627039.pdf

Vignaud, P., & Prieto, N. (2020). Impact psychique de la pandémie de Covid-19 sur les professionnels soignants. *Actualités Pharmaceutiques*, *59*(599), 51-53. doi: 10.1016/j.actpha.2020.08.013

Yook, K., Kim, K.-H., Suh, S. Y., & Lee, K. S. (2010). Intolerance of uncertainty, worry, and rumination in major depressive disorder and generalized anxiety disorder. *Journal of Anxiety Disorders*, *24*(6), 623-628. doi: 10.1016/j.janxdis.2010.04.003
