# Supplementary material for: Mental health status and related factors influencing healthcare workers during the COVID-19 pandemic: A systematic review and meta-analysis
Source: PLoS One. 2024 Jan 19;19(1):e0289454. doi: 10.1371/journal.pone.0289454 (PMC10798549; doi:10.1371/journal.pone.0289454)
Supplement: S1 Data — (ZIP) [file pone.0289454.s011.zip › literatures/86.pdf]

Received: 26 May 2021 | Accepted: 4 July 2021
DOI: 10.1111/ijcp.14607

THE INTERNATIONAL JOURNAL OF CLINICAL PRACTICE WILEY

ORIGINAL PAPER

Psychiatry

Evaluation of the mental health of health professionals in the COVID-19 era. What mental health conditions are our health care workers facing in the new wave of coronavirus?

Josune Martín[1,2,3] | Ángel Padierna[4] | Ane Villanueva[1,2] | José M. Quintana[1,3]

[1]Research Unit, Galdakao-Usansolo Hospital, Galdakao, Spain
[2]Kronikgune Institute for Health Services Research, Barakaldo, Spain
[3]Health Services Research on Chronic Diseases Network – REDISSEC, Galdakao, Spain
[4]Department of Psychiatry, Galdakao-Usansolo Hospital, Galdakao, Spain

Correspondence
Josune Martín, Research Unit, Galdakao-Usansolo Hospital, 9th Floor, Barrio Labeaga, s/n, Galdakao, Bizkaia 48960, Spain.
Email: jmartin@cop.es

Abstract

Background: The aims of this study were to analyse the mental health and health-related quality of life (HRQoL) of a broad sample of healthcare workers (HCWs) in Spain and to identify potential factors that have a significant effect on their mental health and HRQoL.

Method: This prospective cohort study comprised 2089 HCWs who completed the Generalized Anxiety Disorder 7-item (GAD-7) Scale, the Patient Health Questionnaire-9 (PHQ-9), the Impact Event Scale-Revised (IES-R), the Insomnia Severity Index (ISI), and the health-related quality of life Questionnaire (EQ-5D). Sociodemographic and clinical data in relation to Covid-19 were also recorded. Descriptive statistics, univariable analysis and multivariable linear regression models were applied to identify factors associated with mental health and HRQoL.

Results: 80.87% of participants were female and 19.13% male; 82.38% were aged under 55 years; 39.13% were physicians and 50.17% were nurses or auxiliaries; 80.42% of the health workers have directly treated patients with Covid-19 and 12.28% have themselves tested positive for Covid-19. 38.58% of HCWs had clinical depression; over half (51.75%) had clinical anxiety; 60.4% had clinical stress; and 21.57% had clinical insomnia. Older professionals (>55 years) reported lower rates of anxiety, depression, insomnia, and stress. Having worked directly with Covid-19 patients appears to be an explanatory variable of suffering greater anxiety, depression, stress and insomnia, and of having a worse HRQoL amongst our HCWs. The group of HCWs suffering the worst mental state were nursing home workers.

Conclusions: Our study confirms that symptomatology of anxiety, depression, stress, insomnia, and HRQoL were affected amongst HCW during the Covid-19 pandemic.

1 | INTRODUCTION

In the recent months, a great deal of data has been published on the coronavirus disease (COVID-19) pandemic. Nonetheless, there are still many unknowns regarding the virus and its consequences. The effects of the pandemic have been felt around the world. A multi-national study across three continents results provides empirical evidence that COVID-19 affected mental health worldwide,[1] and a recent systematic review found that relatively high rates of symptoms of anxiety, depression, post-traumatic stress disorder, and stress were reported in the general population during the COVID-19 pandemic globally.[2] Another multi-national study across three continents results provides empirical evidence that COVID-19 affected mental health worldwide.[3] However, one of the groups most affected have been those involved in providing health care to the community.[4]

 © 2021 John Wiley & Sons Ltd

During the months of the COVID-19 lockdown in Spain, a cross-cultural study on Spain and other country found that Spanish respondents reported more physical symptoms, contact history with COVID-19, higher perceived risk of contracting COVID-19, and frequent use of but less confidence in medical services. Also reported higher levels of stress and depression, while Chinese participants reported higher levels of psychological impact.[5] Specifically, healthcare workers (HCWs) faced very intense work stressors, such as long working hours, varying instructions and safety measures, and a lack of personal protective equipment, as well as having to carry out tasks for which many were not prepared.[6,7] The efforts of the health system focused—almost exclusively—on saving the lives of COVID-19 patients and providing them with the greatest possible comfort. At this time, catering to the psychological needs of personnel involved in this work was not a priority. These professionals were highly exposed not only to the virus but also to situations with a strong emotional impact,[8] leading to the possible appearance of symptoms of anxiety, depression or disorders resulting from post-traumatic stress.[7,9] A number of studies have observed adverse psychological reactions among health workers in other emergency situations, such as SARS[10-12] and MERS.[13]

The causes of the increase in prevalence of mental-health-related symptoms in the context of COVID-19 is likely to be multifactorial, with reasons such as fear of contracting the infection and the social and economic consequences of the pandemic, which could play a role in this COVID-19 context.[14] Globally, studies on HCWs mental health show that in China, health professionals displayed symptoms of anxiety (44.6%), depression (50.4%), and distress (71.5%).[15] A multinational and multicentre study that describes a comprehensive range of physical symptoms experience by HCWs demonstrates a significant association between the prevalence of physical symptoms and psychological distress, which is probably bi-directional.[16] Another study highlights that all HCWs were vulnerable to psychological adversity regardless of the volume of confirmed COVID-19 cases.[17] In Spain, stress levels amongst HCWs were evaluated and the results showed that the perceived stress level was highest amongst workers who had been in direct contact with COVID-19 patients,[18] and that nurses and trainee physicians are the most vulnerable groups.[19] Another study[20] determined the degree of burnout amongst health professionals, with high scores (38.9%) on the depersonalisation subscale. These Spanish studies also addressed the issue of mental health amongst Spanish HCWs,[18,20] but contain certain limitations, due to the critical nature of the emergency.

Our study addresses these methodological limitations, studying anxiety, depression, stress, insomnia, and health-related quality of life among health professionals.

What's known

The effects of the pandemic have been global, but one of the most affected groups has been healthcare professionals.

What's new

This study provides an analysis of the mental health and health-related quality of life (HRQoL) of a broad sample of healthcare workers in Spain and identifies potential factors that have a significant effect their mental health and HRQoL.

2 | METHODS

2.1 | Participants

This is a descriptive study, with a sample group comprising 2089 Spanish health workers, recruited by non-probabilistic sampling (snowball sampling and emails or WhatsApp/mobile app messages) among tertiary care centres, primary care centres, and nursing homes.

2.2 | Measures

Participants provided *socio-demographic data*, including age, gender, professional status/working status, years of experience. *COVID-19-related clinical data* included having been in quarantine, having tested positive, and psychological support received at this time in relation to the present situation. They also completed Spanish-language versions of five self-administered *perception-of-health* questionnaires:

2.2.1 | Generalized Anxiety Disorder 7-item scale

The Generalized Anxiety Disorder 7-item (GAD-7) scale is a 7-item self-related questionnaire that assessed general anxiety disorder.[21] The items are scored on a four-point scale referring to the past two weeks. We took 10 points as the cut-off point in this study.[4,22] The questionnaire has been translated into and validated in Spanish and shows good internal consistency and adequate test–retest reliability.[23]

2.2.2 | Patient Health Questionnaire-9

The Patient Health Questionnaire-9 (PHQ-9) is a self-administered questionnaire designed to measure depression and depression severity.[24] The 9 items are scored on a four-point scale in the past two weeks. It has been validated in the Spanish population.[25] We took 10 points as the cut-off point in this study.[15,26]

2.2.3 | Impact Event Scale-Revised

The Impact Event Scale-Revised (IES-R) is a self-administered scale designed to assess the perceived stress to the experienced traumatic

event.[27] The 22 items are scored on a five-point Likert scale referring to the last 7 days. The 24 points was taken as the cut-off point in this study. It has been validated in the Spanish population.[28] This scale was validated in different cultures during the pandemic (Iranian, Poles, Philippines, and Vietnamese).[29-32]

2.2.4 | The Insomnia Severity Index

The Insomnia Severity Index (ISI)[33,34] is a brief self-report instrument measuring a patient's perception of the severity of his/her insomnia. The validated Spanish version of the ISI was used to assess the subjective severity of insomnia over a 1-week period.[35] Each item was rated on a five-point Likert scale. This questionnaire was validated during the pandemic.[36]

2.2.5 | Health-related quality of life questionnaire

The self-report version of the *EuroQol generic health-related quality of life questionnaire (EQ-5D)*[37] consists of two parts: the EQ-5D-5L descriptive system and the EQ Visual Analogue scale. It has been validated in the Spanish population.[38]

2.3 | Procedure

Data were collected by means of an online survey, between April and September 2020. A researcher from the Galdakao-Usansolo Hospital Research Unit directly emailed several Spanish hospitals, primary care centres and nursing homes, who facilitated contact with their staff and with medical personnel who had been working in these centres during the COVID-19 outbreak. Clinical and non-clinical healthcare workers were included (physicians, nurses, administrative personnel, ambulance drivers, cooking and cleaning staff, orderlies, religious service providers, etc). Frontline medical staff were identified as being those working in the Emergency, Respiratory, Intensive Medicine, Reanimation and Infectious Diseases services.

Before beginning the survey, all participants were asked to read the information on the study–including objectives, data confidentiality and an email address to contact in the event of any queries–and were required to give their electronically informed consent to complete the questionnaires.

Participants were asked to provide socio-demographic data and information related to COVID-19 and to complete the Spanish-language versions of five self-administered instruments (PHQ-9, GAD-7, IESR-22, EQ-5D, ISI-7). Eligible personnel included health workers who were in active employment at the time of the pandemic. Participants who did not give their informed consent were excluded. Participation in the study was anonymous and voluntary, and all information was kept confidential. The study was approved by the Ethics Committee of the hospital (Galdakao-Usansolo Hospital, Protocol 08/20).

2.4 | Statistical analysis

Descriptive statistics for the entire sample were calculated using frequencies and percentages for categorical data and by means and standard deviations for continuous variables. The scores of the five questionnaires analysed (EuroQol-5D, PHQ-9, GAD-7, IESR-22, and ISI-7) were treated as continuous variables, and dichotomous variables were created to indicate clinical values of anxiety, depression, stress and insomnia, based on the published cut-off points of the corresponding score.

Univariable analyses were first performed to identify, among sociodemographic and COVID-19-related variables, factors associated with (a) the quality-of-life score and (b) having clinical values of anxiety, depression, stress and insomnia, using Chi-square or Fisher's exact test for categorical data and Student t test or nonparametric Wilcoxon test for continuous variables. Variables that were significant at 0.20 level were considered as potential independent variables to fit (a) a multiple linear regression model to predict the outcome of the quality-of-life score and (b) multivariable logistic regression models to predict clinical values of anxiety, depression, stress, and insomnia. In all cases, the final predictive factors were those that were significant at .05 level.

The coefficient of determination R^2 was calculated to evaluate the percentage of the quality-of-life score variation explained by the model. Estimates and 95% confidence intervals were calculated. The predictive accuracy of each of the logistic regression models was determined by calculating the area under the receiver operating curve (AUC) and calibration of these models was assessed using the Hosmer and Lemeshow test. The odds ratio (OR) and 95% confidence intervals (%95 CI) were calculated.

All effects were considered statistically significant at $P < .05$. All statistical analyses were performed using SAS for Windows statistical software, version 9.4 (SAS Institute, Inc, Cary, NC) and R© software version 4.0.0.

3 | RESULTS

The study comprised 2089 participants, 1691 females (80.87%) and 398 males (19.13%). These data are consistent with the results of other studies.[15,20,39-41] Table 1 shows socio-demographic and clinical data in relation to COVID-19 pandemic, and mental health characteristics of the healthcare workers. 82.38% of the participants were aged under 55; 39.13% were physicians and 50.17% were nurses or auxiliaries; 27.6% were frontline medical staff, and 37.44% had more than 10 years' work experience. 79.08% of the data were obtained during the months of restrictions on movement of people in Spain.

With regard to COVID-19 data, 80.42% of the health workers had directly treated patients with COVID-19, and 12.28% had themselves tested positive for COVID-19. Only 19.76% felt that they had been sufficiently protected in their job; 40.43% felt that they had not been sufficiently protected in their job, and 10.71% had received psychological attention in relation to COVID-19 in their workplace.

TABLE 1 Descriptive analysis of sociodemographic data and clinical data in relation to COVID-19 (n = 2089)

	N (%)	Missing (%)
Sociodemographic data		
Age		1 (0.05)
<40	831 (39.8)	
40-55	889 (42.58)	
>55	368 (17.62)	
Gender (male)	398 (19.13)	8 (0.38)
Professional profile		14 (0.67)
Medical doctor	812 (39.13)	
Nurses/nursing assistants	1041 (50.17)	
Other	222 (10.7)	
Years of experience		13 (0.62)
<5	305 (14.69)	
5-10	371 (17.87)	
>10	1400 (67.44)	
Services		35 (1.68)
Services with greatest contact with COVID-19-patients[a]	581 (28.29)	
Primary care	453 (22.05)	
Nursing homes/geriatric services	160 (7.79)	
Other medical/surgical services	568 (27.65)	
Central services	200 (9.74)	
Other	92 (4.48)	
Response date		0 (0)
During state of alarm (until 06/21/2020)	1652 (79.08)	
Subsequent to state of alarm	437 (20.92)	
Response month		0 (0)
April	4 (0.19)	
May	1104 (52.85)	
June	650 (31.12)	
July	125 (5.98)	
August	143 (6.85)	
September	63 (3.02)	
Clinical data in relation to COVID-19		
Have you been in quarantine? (Yes)	458 (21.98)	5 (0.24)
Positive PCR		4 (0.19)
Yes	256 (12.28)	
No	1742 (83.55)	
I have not been tested	87 (4.17)	
Have you worked directly with COVID-19 patients (Yes)	1663 (80.42)	21 (1.01)
Have you worked in any service other than your own (Yes)	813 (39.12)	11 (0.53)
Sufficient knowledge about how to do your job (Yes)	1325 (63.79)	12 (0.57)
Do you have you enough knowledge about protection measures (Yes)	1481 (71.48)	17 (0.81)
Do you feel that you have been sufficiently protected in your activity?		9 (0.43)
No	841 (40.43)	
Sometimes	828 (39.81)	
Yes	411 (19.76)	
Have you received psychological care/support in your workplace? (Yes)	223 (10.71)	7 (0.34)
Increased use of tranquilizers/sedatives in the last weeks (Yes)	394 (18.93)	8 (0.38)
Increased alcohol consumption in the last weeks (Yes)	311 (14.92)	5 (0.24)
Increased tobacco use in the last weeks (Yes)	285 (13.72)	11 (0.53)
Increased drugs consumption in the last weeks (Yes)	19 (0.91)	11 (0.53)

[a]Services with more COVID-19 contact: Emergencies, Anesthesiology & Reanimation, ICU/CCU, Respiratory, Intensive Medicine, Internal Medicine and Infectious Diseases.

In relation to the consumption of toxic substances, the data show a 0.91%, 13.73%, 14.92%, and 18.93% increase respectively, in the consumption of drugs, tobacco, alcohol, and tranquilizers/sedatives.

As measured by the PHQ-9 score, 38.58% of HCWs had clinical depression (PHQ-9 score of ≥10); over half (51.75%) had clinical anxiety (GAD-7 score of ≥10); 60.4% had clinical stress (IESR-22 score of ≥24); and 21.57% had clinical insomnia (ISI-7 score of ≥15). The mean total PHQ-9 score for HCWs was 8.58 (SD 6.06); the mean total score on the GAD-7 was 9.92 (SD 5.39); the mean IESR-22 score was 33.45 (SD 22.89); and the mean ISI-7 score was 9.32 (SD 6.15) (Table 2).

Scores on the symptomatology of anxiety, depression, stress, insomnia, and quality of life according to a *univariable analysis* of the sociodemographic, relative to COVID-19 pandemic, and workers' mental health variables are shown in Table S1 (see online material).

We also have included the categorized scores (clinical vs non clinical) on the symptomatology of anxiety, depression, stress, and insomnia according to a *univariate analysis* of the socio-demographic, relative to COVID-19 pandemic, and workers' mental health variables (Table S2, see material online).

Results of *multivariable* analysis for the EuroQol-5D are presented in Table 3. In this linear regression model for health-related quality of life, scores for gender (<0.0001), age (<0.0001), service

TABLE 2 Descriptive analysis of mental health and health-related quality of life data (n = 2089)

	N (%)	Missing (%)
	2089	
Health-related quality of life (EuroQol-5D)		
From 0 to 100, rate your state of health TODAY (item 6)[a]	73.1 (17.93)	23 (1.1)
Score EuroQol-5 Da	0.82 (0.16)	20 (0.96)
Depression symptoms (PHQ-9)		
Score PHQ-9[a]	8.58 (6.06)	18 (0.86)
Classification scores PHQ-9		18 (0.86)
Normal (0-4)	609 (29.41)	
Mild (5-9)	663 (32.01)	
Moderate (10-14)	446 (21.54)	
Moderately severe depression (15-19)	221 (10.67)	
Severe (20-27)	132 (6.37)	
Clinical depression - score ≥10 (Yes)	799 (38.58)	18 (0.86)
Anxiety symptoms (GAD-7)		
Score GAD-7[a]	9.92 (5.39)	2 (0.1)
Classification scores GAD-7		2 (0.1)
Normal (0-4)	367 (17.59)	
Mild (5-9)	640 (30.67)	
Moderate (10-14)	679 (32.53)	
Severe (15-21)	401 (19.21)	
Clinical anxiety - score ≥10 (Yes)	1080 (51.75)	2 (0.1)
Distress symptoms (IESR-22)		
Score IESR-22[a]	33.45 (22.89)	3 (0.14)
Classification scores IESR-22		3 (0.14)
Normal (0-8)	384 (18.41)	
Mild (9-25)	477 (22.87)	
Moderate (26-43)	512 (24.54)	
Severe (44-88)	713 (34.18)	
Clinical distress - score ≥24 (Yes)	1260 (60.4)	3 (0.14)
Insomnia symptoms (ISI-7)		
Score ISI-7[a]	9.32 (6.15)	7 (0.34)
Classification scores ISI-7		7 (0.34)
Absence (0-7)	888 (42.65)	
Subthreshold (8-14)	745 (35.78)	
Moderate (15-21)	388 (18.64)	
Severe (22-28)	61 (2.93)	
Clinical insomnia - score ≥15 (Yes)	449 (21.57)	7 (0.34)

[a]Results shows as mean (standard deviation). GAD-7: Total scores range from 0 to 21. A score of 0-4 indicates the absence of anxiety, a score of 5-9 indicates mild anxiety symptoms, a score of 10-14 indicates moderate anxiety symptoms and a score of 15-21 indicates the presence of severe symptoms of anxiety. *PHQ-9:* Total scores range from 0 to 27. A score of 0-4 indicates absence of depression, a score of 5-9 indicates mild depression symptoms, a score of 10-14 indicates moderate depression symptoms, a score of 15-19 indicates moderately severe depression, and a score of 20-27 indicates clinical depression. *IES-R-22:* Total scores range from 0 to 88. A score of 0-8 indicates normal distress, a score of 9-25 indicates mild distress, a score of 26-43 indicates moderate distress, and a score of 44-88 indicates severe distress. *ISI-7:* The total score is interpreted as follows: absence of insomnia (0-7); sub-threshold insomnia (8-14); moderate insomnia (15-21); and severe insomnia (22-28).

(<0.0001), having sufficient knowledge about how to do the job (0.0005), being sufficiently protected (<0.0001), increased use of tranquilizers (<0.0001), increased alcohol consumption (<0.0001), and increased tobacco use (<0.0001) showed a significant negative association with the EQ-5D total scores.

Results of *multivariable* analysis for depression, anxiety, stress, insomnia are presented in Table 4. In the multiple logistic regression model for depression, scores for gender (0.0004), service (<0.0001), caring directly for COVID-19 patients (0.002), having sufficient knowledge about how to do the job (<0.0001), being sufficiently protected (<0.0001), increased use of tranquilizers (<0.0001), and increased alcohol consumption (<0.0001) showed a significant positive association with the PHQ-9 total scores. In the multiple logistic regression model for anxiety, scores for gender (<0.0001), professional profile (<0.0001), working in another service (0.002), having sufficient knowledge about how to do the job (<0.0001), being sufficiently protected (<0.0001), increased use of tranquilizers (<0.0001), and increased alcohol consumption (<0.0001) showed a significant positive association with the GAD-7 total scores. In the multiple logistic regression model for stress, scores for gender (<0.0001), professional profile (0.0003), service (0.0003), working in another service (0.004), having sufficient knowledge about how to do the job (0.0002), being sufficiently protected (<0.0001), having psychological care in the workplace (0.005), increased use of tranquilizers (<0.0001), and increased alcohol consumption (<0.0001) showed a significant positive association with the IESR-22 total scores. In the multiple logistic regression model for insomnia, scores for professional profile (<0.0001), caring directly for COVID-19 patients (0.02), having sufficient knowledge about how to do the job (0.0004), being sufficiently protected (<0.0001), increased use of tranquilizers (<0.0001), increased alcohol consumption (0.0004), and increased tobacco use (0.03) showed a significant positive association with the ISI-7 total scores.

4 | DISCUSSION

This study evaluates the mental health of a broad sample of Spanish HCWs with respect to symptomatology of anxiety, depression, stress, insomnia, and HRQoL, as measured by various validated questionnaires. Our study confirms that all these variables were affected amongst health-professionals. These results are consistent with previous studies[15,41-49] using the same questionnaires.

With respect to socio-demographic variables, 80.87% of participants in our study were *females* and 19.13% males. This higher percentage of female participation is also seen in other studies, with very similar percentages.[20,39] Our study suggests that providing care to COVID-19 patients has a marked *emotional impact*, as 51.75% of the HCWs appeared to suffer from clinical anxiety and 38.58% from clinical depression. This is higher than the data recorded in other studies in Europe and China, which show values of between 5 and 10 for clinical anxiety and clinical depression respectively.[15,46,48,49] Our HCWs also reported higher scores in stress and insomnia than

TABLE 3 Multiple linear regression model for health-related quality of life EuroQol-5D

	EuroQol-5D		
	**Estimate**	**IC (95%)**	***P* value**
Gender (female)	−0.049	(−0.067, −0.032)	**<.0001**
Age			**<.0001**
<40 years	ref.	ref.	–
40-55 years	−0.028	(−0.044, −0.014)	**.0001**
>55 years	−0.037	(−0.057, −0.018)	**.0002**
Service			**<.0001**
Services with greatest contact with COVID-19-patients[a]	−0.023	(−0.041, −0.005)	**.01**
Primary care	−0.012	(−0.031, 0.006)	.20
Nursing homes/geriatric services	−0.083	(−0.109, −0.056)	**<.0001**
Medical/surgical services	−0.024	(−0.070, −0.003)	.05
Other	−0.036	(−0.049, 0.0005)	**.03**
Central services	ref.	ref.	–
Sufficient knowledge about how to do your job (No)	−0.026	(−0.040, −0.011)	**.0005**
Do you feel that you have been sufficiently protected in your activity?			**<.0001**
Yes	ref.	ref.	–
Sometimes	−0.029	(−0.048, −0.011)	**.001**
No	−0.068	(−0.087, −0.049)	**<.0001**
Increased use of tranquilizers/sedatives (Yes)	−0.134	(−0.149, −0.115)	**<.0001**
Increased alcohol consumption (Yes)	−0.031	(−0.050, −0.012)	**.0011**
Female tobacco use (Yes)	−0.038	(−0.058, −0.019)	**.0001**
$R^2 = 0.2$			

[a] Services with most COVID-19 contact: Emergencies, Anaesthesiology & Reanimation, ICU/CCU, Respiratory, Intensive Medicine, Internal Medicine and Infectious Diseases. Ref.: reference category. The coefficient of determination R^2 was calculated to evaluate the percentage of the quality of live score variation that the model explained. CI 95%: confidence interval of 95%.
Bold represents the significance threshold was .05.

in other research.[47,49,50] The overall EQ-5D-5L score for Spanish HCWs (0.82) in this study was lower than general population under COVID-19 (0.95)[51] but higher than patients suffering from diabetes (0.8),[52] human immunodeficiency virus (HIV) (0.8),[53] skin diseases (0.73),[54] respiratory diseases (0.66),[55] dengue fever (0.66),[56] frail elderly (0.58),[57] and elderly after fall injury (0.46)[58] and fracture injuries (0.23).[59]

In our study, *senior professionals (>55 years)* reported lower rates of anxiety, depression, insomnia, and stress. This is consistent with other studies[60,61] that indicate that the protective effect of "being senior" is due to the fact that expertise and confidence helps minimize the stress caused unforeseen situations.[18] It should also be noted that the *rate of infected HCWs* (12.28%) is practically 3-4 times higher than elsewhere.[62]

Likewise, the proportion of personnel coming into direct contact with COVID-19 patients is also very high (80.42%). Thus, amongst our HCWs, *having worked directly with COVID-19 patients* appears to be an explanatory variable of suffering greater anxiety, depression, stress and insomnia, and having a worse health-related quality of life. We also think it is important to note that the group of HCWs with the worst mental state were *nursing home workers* One possible explanation may be found in the data estimating that 6% of elderly people living in nursing homes died in the pandemic (out of a total of more than 330 000 places occupied), and it is estimated that between 47% and 51% of deaths (to 23 June) occurred in social services centres, as compared with 39% in Germany.[63]

The effects of *stress on increased use of drugs/toxic substances* have been well-demonstrated,[64] particularly among HCWs, due to their easier access to psychotropic drugs (increased risk of use of opiates/benzodiazepines/hypnotics),[65] and a fear of professional stigma that can prevent them from seeking psychological treatment.[66] Similarly, in the SARS-CoV-1 epidemic, an increased risk of alcohol use disorder was reported among primary care workers.[67] Our multivariable analysis reflects a relationship between increased drug use and worse results in depression, anxiety, stress, insomnia and health-related quality of life. It should be taken into account that

TABLE 4 Multivariable logistic regression models for the depression, anxiety, stress, and insomnia measures

	PHQ9 (depression)			GAD7 (anxiety)			IESR22 (stress)			ISI7 (insomnia)		
	OR	IC 95%	*P* value	OR	IC 95%	*P* value	OR	IC 95%	*P* value	OR	IC 95%	*P* value
Gender (female)	1.64	(1.25-2.16)	**.0004**	2.07	(1.58-2.69)	**<.0001**	2.02	(1.56-2.62)	**<.0001**			
Professional profile	–	–	–			**<.0001**			**.0003**			**<.0001**
Doctor				0.84	(0.59-1.17)		0.82	(0.57-1.18)		0.67	(0.43-1.07)	
Nurses / nursing assistants				1.39	(1.01-1.94)		1.31	(0.93-1.84)		1.29	(0.83-1.99)	
Other				ref.	ref.		ref.	ref.		ref.	ref.	
Service			**<.0001**	–	–	–			**.0003**	–	–	–
Services with greatest contact with COVID-19-patients[a]	1.35	(1.02-1.78)					1.66	(1.27-2.17)				
Primary care	1.13	(0.84-1.51)					1.16	(0.88-1.54)				
Nursing homes / geriatric services	2.87	(1.92-4.31)					2.34	(1.53-3.59)				
Central services	1.79	(1.24-2.59)					1.26	(0.88-1.82)				
Other	1.82	(1.07-3.08)					1.29	(0.77-2.16)				
Medical/surgical services	ref.	ref.					ref.	ref.				
Have you worked in any service other than your own? (Yes)	–	–	–	1.35	(1.10-1.64)	**.003**	1.35	(1.10-1.67)	**.004**	–	–	–
Have you worked directly with COVID-19 patients? (Yes)	1.56	(1.17-2.15)	**.002**							1.5	(1.06-2.12)	**.02**
Sufficient knowledge about how to do your job (No)	1.92	(1.56-2.37)	**<.0001**	1.82	(1.48-2.23)	**<0001**	1.51	(1.22-1.88)	**.0002**	1.54	(1.21-1.97)	**.0004**
Do you feel that you have been sufficiently protected in your activity?			**<.0001**			**<.0001**			**<.0001**			**<.0001**
Yes	ref.	ref.		ref.	ref.		ref.	ref.		ref.	ref.	
Sometimes	1.59	(1.17-2.15)		1.39	(1.06-1.81)		1.73	(1.32-2.26)		1.51	(1.01-2.27)	
No	2.59	(1.91-3.5)		2.33	(1.77-3.06)		2.72	(2.06-3.6)		3.32	(2.24-4.93)	
Have you received psychological care/ support in your workplace? (Yes)	–	–	–	–	–	–	1.62	(1.16-2.26)	**.005**	–	–	–
Increased use of tranquilizers/sedatives (Yes)	4.11	(3.18-5.3)	**<.0001**	4.99	(3.75-6.66)	**<.0001**	4.38	(3.19-6.02)	**<.0001**	5.94	(4.59-7.69)	**<.0001**
Increased alcohol consumption (Yes)	2.7	(2.04-3.58)	**<.0001**	2.45	(1.83-3.27)	**<.0001**	2.15	(1.58-2.92)	**<.0001**	1.76	(1.29-2.4)	**.0004**
Increased tobacco use (Yes)	–	–	–	–	–	–	–	–	–	1.42	(1.04-1.95)	**.03**
AUC (IC 95%)/H-L	0.75 (0.72-0.77)/0.6944			0.75 (0.72-0.77)/0.8967			0.74 (0.71-0.76)/0.6480			0.78 (0.76-0.81)/0.5130		

Abbreviations: AUC, area under the receiver operating curve; CI 95%: confidence interval of 95%; H-L, *P* value of Hosmer-Lemeshow test; OR, odds ratio; Ref., reference category.
[a]Services with most COVID-19 contact: Emergencies, Anaesthesiology & Reanimation, ICU/CCU, Respiratory, Intensive Medicine, Internal Medicine and Infectious.
Bold represents the significance threshold was .05.

the lockdown measures imposed between March and June in other European countries were not as extreme as in Spain, where people were only allowed to leave their homes to go to work or to buy food and medicines, and borders were closed. This may have complicated the issue of increased drugs use, since without the possibility of drawing on the social support of the wider family environment and friends, or even of performing physical exercise (which would have been a good coping strategy), the use of drugs or toxics was resorted to instead.

The characteristics of this pandemic –uncertain knowledge, severity, deaths among HCWs—increased its potential psychological impact on those personnel.[68] Based on the results of other studies reviewed, it is surprising to see that our sample group was more mentally affected than other similar sample groups measured using the same instruments. This is the case amongst sample groups from countries that are socio-culturally different to our own, such as China, Hong Kong, and Vietnam, and in countries with cultures closer to our own, such as Italy. Are our HCWs "fragile heroes"?.[69] The following are some possible explanations:

On the one hand, a meta-analysis and a Spanish study found that factors protecting against psychological distress included having sufficient medical resources and accurate health information,[19,70] since clear and effective guidance is key to staff confidence and reassurance during a crisis.[71] However, in our study only 19.76% of HCWs felt that they were sufficiently protected in their job; 40.43% had not felt protected, and 39.81% had only sometimes felt protected. There is also a relationship between this variable and anxiety, depression, stress, insomnia, and health-related quality of life. Are our HCWs somehow "softer"? It seems that we need to seek other explanations for this major difference in psychological distress. Some of the explanations that the HCWs themselves described with regard to the situation they experienced (through open questions) were as follows:

- Action protocols were updated based on the availability of personal protective equipment at the hospital, rather than on its real importance in treating COVID-19 patients and protecting staff. This created a high degree of fear and mistrust amongst staff.
- High level of improvisation; there were different rules each day. Tremendous healthcare burden in a short period of time, with a feeling of a shortage of hands and extreme exhaustion.
- Psychologically very hard to have to report to patients' next-of-kin over the phone, due to lockdown, trying to empathize with their situation.
- Feeling of impotence in the face of actions resulting from health policies that did not prioritize or invest in prevention and protection against this situation.
- We felt helpless in terms of the lack of security and knowledge of ICU (intensive care unit) patient care.

At the same time, on December 1, it was reported in the news that Spain will suffer the worst recession among G-20 countries, only behind Argentina, according to the OECD.[72] Therefore, concern over the socioeconomic situation must surely also be impacting the psychological stress amongst our HCWs. Taking all this into account, it is important to offer psychological interventions to help HCWs. The most evidence-based treatment is cognitive behaviour therapy (CBT),[73,74] especially Internet CBT that can prevent the spread of infection during the pandemic.[75]

Our study has several *strengths*. These include a large sample of HCWs; the use of validated instruments to determine their mental health; and personnel with different profiles and from different services. Some *limitations* of our study must also be noted. It is possible that participants in the study may have experienced a higher symptomatology than those HCWs who opted not to participate. Another limitation concerns the overrepresentation of women in our sample group. Despite, the women are overrepresented in depression even prior to the pandemic.[76] Another limitation is that the COVID-19 pandemic was found to cause hemodynamic changes in the brain.[77] This study mainly used self-reported questionnaires to measure psychiatric symptoms and did not make clinical diagnosis. The gold standard for establishing psychiatric diagnosis involved structured clinical interview and functional neuroimaging.[78-80]

An additional limitation is the fact that we performed the assessment at only one point in time, which prevented us from observing changes in the HCWs' mental health over time or draw any conclusions regarding causation. About this, one longitudinal study in China[81] revealed a statistically but not clinically significant reduction in psychological impact during the initial phase and four weeks later during the COVID-19 epidemic. Thus, longitudinal data are needed to evaluate the psychological impact of the pandemic over time.

5 | CONCLUSIONS

At a global level, we tend to speak of a first wave during the first months of the pandemic and a second wave after the summer. However, what we experienced in Spain between March and April was not so much a first wave as an infernal tsunami (compared to other regions/countries), and in addition, health decisions in health policy have resulted in insufficient medical information and resources.

We therefore believe that care must be provided for our HCWs in the short- to medium-term future, bearing in mind that "new waves" of the pandemic are expected in the coming months.

It will be necessary to monitor HCWs—particularly those most at risk—and our data, together with other current studies, can help to establish screening, support and treatment strategies for improving their mental health. For future research, it would be useful to assess the views of Spanish HCWs on COVID-19 vaccine. Chews et al (2021) used a self-administrated survey and collected information on willingness to vaccinate, perception of COVID-19, vaccine concerns, COVID-19 risk profile, stigma, prosocialness scale, and trust in health authorities.[82]

ACKNOWLEDGEMENTS

The authors thank the Research Committee of Galdakao-Usansolo Hospital for its help in editing this article, and the researchers Nerea González and Miren Orive for the help at the beginning of the study. The authors also acknowledge editorial assistance provided by Tim Nicholson. We are most grateful to all the healthcare workers who kindly responded to the survey.

ETHICAL STANDARDS

The authors assert that all procedures contributing to this work comply with the ethical standards of the relevant national and institutional committees on human experimentation and with the Helsinki Declaration of 1975, as revised in 2008 and "that all procedures contributing to this work comply with the ethical standards of the relevant national and institutional guides on the care and use of laboratory animals."

DISCLOSURES

The authors declare that they have no competing interest.

DATA AVAILABILITY STATEMENT

The data that support the findings of this study are available from the corresponding author at email direction (jmartin@cop.es). The data are not publicly available due to privacy or ethical restrictions.

ORCID

Josune Martín https://orcid.org/0000-0001-5281-4426

REFERENCES

1. Wang C, Chudzicka-Czupała A, Tee ML, et al. A chain mediation model on COVID-19 symptoms and mental health outcomes in Americans, Asians and Europeans. *Sci Rep*. 2021;11:6481. https://doi.org/10.1038/s41598-021-85943-7
2. Xiong J, Lipsitz O, Nasri F, et al. Impact of COVID-19 pandemic on mental health in the general population: a systematic review. *J Affect Disord*. 2020;277:55-64. https://doi.org/10.1016/j.jad.2020.08.001
3. Wang C, Tee M, Roy AE, et al. The impact of COVID-19 pandemic on physical and mental health of Asians: a study of seven middle-income countries in Asia. *PLoS ONE*. 2021;16:e0246824. https://doi.org/10.1371/journal.pone.0246824
4. Lancet T. COVID-19: protecting health-care workers. *Lancet*. 2020;395:922. https://doi.org/10.1016/S0140-6736(20)30644-9
5. Wang C, López-Núñez MI, Pan R, et al. The impact of 2019 coronavirus disease (COVID-19) pandemic on physical and mental health: a comparison between China and Spain. *JMIR Form Res*. 2021;5:e27818. https://doi.org/10.2196/27818
6. Brooks SK, Webster RK, Smith LE, et al. The psychological impact of quarantine and how to reduce it: rapid review of the evidence. *Lancet*. 2020;395:912-920. https://doi.org/10.1016/S0140-6736(20)30460-8
7. Vieta E, Pérez V, Arango C. Psychiatry in the aftermath of COVID-19. *Rev Psiquiatr Salud Mental*. 2020;13:105-110. https://doi.org/10.1016/j.rpsm.2020.04.004
8. Luceño-Moreno L, Talavera-Velasco B, García Albuerne Y, Martín GJ. Symptoms of posttraumatic stress, anxiety, depression, levels of resilience and burnout in Spanish health personnel during the COVID-19 pandemic. *Int J Environ Res Public Health*. 2020;17:5514. https://doi.org/10.3390/ijerph17155514
9. Bao Y, Sun Y, Meng S, Shi J, Lu L. 2019-nCoV epidemic: address mental health care to empower society. *Lancet*. 2020;395(10224):e37-e38. https://doi.org/10.1016/S0140-6736(20)30309-3
10. Bai YM, Lin C-C, Lin C-Y, Chen J-Y, Chue C-M, Chou P. Survey of stress reactions among health care workers involved with the SARS outbreak. *Psychiatr Serv*. 2004;55:1055-1057. https://doi.org/10.1176/appi.ps.55.9.1055
11. Lee AM, Wong JGWS, McAlonan GM, et al. Stress and psychological distress among SARS survivors 1 year after the outbreak. *Can J Psychiatry*. 2007;52:233-240. https://doi.org/10.1177/070674370705200405
12. Maunder R, Hunter J, Vincent L, et al. The immediate psychological and occupational impact of the 2003 SARS outbreak in a teaching hospital. *Can Med Assoc J*. 2020;168:1245-1251.
13. Lee SM, Kang WS, Cho AR, Kim T, Park JK. Psychological impact of the 2015 MERS outbreak on hospital workers and quarantined hemodialysis patients. *Comprehensive Psych*. 2018;87:123-127. https://doi.org/10.1016/j.comppsych.2018.10.003
14. Holmes EA, O'Connor RC, Perry VH, et al. Multidisciplinary research priorities for the covid-19 pandemic: a call for action for mental health science. *Lancet Psych*. 2020;7(6):547-560. https://doi.org/10.1016/S2215-0366(20)30168-1
15. Lai J, Ma S, Wang Y, et al. Factors associated with mental health outcomes among health care workers exposed to coronavirus disease 2019. *JAMA Netw Open*. 2020;3(3):e203976. https://doi.org/10.1001/jamanetworkopen.2020.3976
16. Chew NWS, Lee GKH, Tan BYQ, et al. A multinational, multicentre study on the psychological outcomes and associated physical symptoms amongst healthcare workers during COVID-19 outbreak. *Brain Behav Immun*. 2020;88:559-565. https://doi.org/10.1016/j.bbi.2020.04.049
17. Chew NWS, Ngiam JN, Tan B-Q, et al. Asian-Pacific perspective on the psychological well-being of healthcare workers during the evolution of the COVID-19 pandemic. *BJPsych Open*. 2020;6:e116. https://doi.org/10.1192/bjo.2020.98
18. Romero CS, Delgado C, Catalá J, et al. COVID-19 psychological impact in 3109 healthcare workers in Spain: the PSIMCOV group. *Psychol Med*. 2020;14:1-7. https://doi.org/10.1017/S0033291720001671
19. García-Fernández L, Romero-Ferreiro V, López-Roldán PD, et al. Mental health impact of COVID-19 pandemic on Spanish healthcare workers. *Psychol Med*. 2020;27:1-3. https://doi.org/10.1017/S0033291720002019
20. Martínez-López JA, Lázaro-Pérez C, Gómez-Galán J, Fernández-Martínez MM. Psychological impact of COVID-19 emergency on health professionals: burnout incidence at the most critical period in Spain. *J Clin Med*. 2020;9(9):3029. https://doi.org/10.3390/jcm9093029
21. Kertz S, Bigda-Peyton J, Bjorgvinsson T. Validity of the Generalized Anxiety Disorder-7 Scale in an acute psychiatric sample. *Clin Psychol Psychother*. 2013;20(5):456-464. https://doi.org/10.1002/cpp.1802
22. Löwe B, Decke O, Müller S, et al. Validation and standardization of the Generalized Anxiety Disorder Screener (GAD-7) in the general population. *Med Care*. 2008;46:266-274.
23. García-Campayo G, Zamorano E, Ruiz MA, Pérez-Páramo M, López-Gómez V, Rejas J. The assessment of generalized anxiety disorder: psychometric validation of the Spanish versión of the self-administered GAD-2 scale in daily medical practice. *Health Qual Life Outcomes*. 2012;10:114. https://doi.org/10.1186/1477-7525-10-114
24. Levis B, Benedetti A, Thombs BD. Accuracy of Patient Health Questionnaire-9 (PHQ-9) for screening to detect major depression:

individual participant data meta-analysis. *BMJ*. 2019;365:l1476. https://doi.org/10.1136/bmj.l1476
25. Diez-Quevedo C, Rangil T, Sanchez-Planell L, Kroenke K, Spitzer RL. Validation and utility of the patient health questionnaire in diagnosing mental disorders in 1003 general hospital Spanish inpatients. *Psychosom Med*. 2001;63(4):679-686. https://doi.org/10.1097/00006842-200107000-00021
26. Manea L, Gilbody S, McMillan D. A diagnostic meta-analysis of the Patient Health Questionnaire-9 (PHQ-9) algorithm scoring method as a screen for depression. *Gen Hosp Psychiatry*. 2015;37(1):67-75.
27. Weiss DS, Marmar CH. The impact of event scale-revised. In: Stamm H, Wilson JP, Keane TM, eds. *Assessing Psychological Trauma and PTSD*. New York: The Guildford Press; 1997:399-411.
28. Báguena MJ, Villarroya E, Beleña A, Díaz A, Roldán C, Reig R. Propiedades psicométricas de la versión española de la escala revisada de impacto del estresor (EIE-R). *Anál Modificación Conducta*. 2001;27:581-604.
29. Wang C, Fardin MA, Shirazi M, et al. Mental health of the general population during the 2019 coronavirus disease (COVID-19) pandemic: a tale of two developing countries. *Psychiatry Int*. 2021;2:71-84. https://doi.org/10.3390/psychiatryint2010006
30. Wang C, Chudzicka-Czupała A, Grabowski D, et al. The association between physical and mental health and face mask use during the COVID-19 pandemic: a comparison of two countries with different views and practices. *Front Psychiatry*. 2020;11:569981. https://doi.org/10.3389/fpsyt.2020.569981
31. Tee M, Wang C, Tee C, et al. Impact of the COVID-19 pandemic on physical and mental health in lower and upper middle-income asian countries: a comparison between the Philippines and China. *Front Psychiatry*. 2021;11:568929. https://doi.org/10.3389/fpsyt.2020.568929
32. Le HT, Lai AJX, Sun J, et al. Anxiety and depression among people under the nationwide partial lockdown in Vietnam. *Front Public Health*. 2020;8:589359. https://doi.org/10.3389/fpubh.2020.589359
33. Bastien CH, Vallières A, Morin CM. Validation of the insomnia severity index as a clinical outcome measure for insomnia research. *Sleep Med*. 2001;2:297-307.
34. Morin CM, Belleville G, Bélanger L, Ivers H. The Insomnia Severity Index: psychometric indicators to detect insomnia cases and evaluate treatment response. *Sleep*. 2011;34(5):601-608. https://doi.org/10.1093/sleep/34.5.601
35. Sierra JC, Guillén-Serrano V, Santos-Iglesias P. Insomnia Severity Index: some indicators about its reliability and validity on an older adults sample. *Rev Neurol*. 2008;47(11):566-570.
36. Hao F, Tan W, Jiang LI, et al. Do psychiatric patients experience more psychiatric symptoms during COVID-19 pandemic and lockdown? A case-control study with service and research implications for immunopsychiatry. *Brain Behav Immun*. 2020;87:100-106. https://doi.org/10.1016/j.bbi.2020.04.069
37. Herdman M, Gudex C, Lloyd A, et al. Development and preliminary testing of the new five-level version of EQ-5D (EQ-5D-5L). *Qual Life Res*. 2011;20(10):1727-1736.
38. Badia X, Roset M, Montserrat S, Herdman M, Segura A. The Spanish version of EuroQol: a description and its applications. European Quality of Life scale. *Med Clín (Barc)*. 1999;112:79-85.
39. Gil-Monte PR, García-Juesas JA, Caro M. The influence of overload and self-efficacy on burnout: a study in nursing professionals. *Interamer J Psychol*. 2008;42:113-118.
40. Moreno B, González-Gutiérrez JM, Garrosa E. Variables sociodemográficas en el proceso de desgaste profesional de enfermería. *Rev ROL Enfermería*. 2002;25:18-26.
41. Nguyen L, Drew DA, Graham MS, et al. Risk of COVID-19 among front-line health-care workers and the general community: a prospective cohort study. *Lancet Public Health*. 2020;5:e475-e483. https://doi.org/10.1016/S2468-2667(20)30164-X
42. Cao W, Fang Z, Hou G, et al. The psychological impact of the COVID-19 epidemic on college students in China. *Psychiatr Res*. 2020;287:112934. https://doi.org/10.1016/j.psychres.2020.112934
43. Cao J, Wei J, Zhu H, et al. A study of basic needs and psychological wellbeing of medical workers in the fever clinic of a tertiary general hospital in Beijing during the COVID-19 outbreak. *Psychother Psychosom*. 2020;89(4):252-254. https://doi.org/10.1159/000507453
44. Chung JPY, Yeung WS. Staff mental health self-assessment during the COVID-19 outbreak. *East Asian Arch Psychiatr*. 2020;30:34. https://doi.org/10.12809/eaap2014
45. Gao X, Jiang L, Hu Y, Li L, Hou L. Nurses' experiences regarding shift patterns in isolation wards during the COVID-19 pandemic in China: a qualitative study. *J Clin Nurs*. 2020;29:4270-4280. https://doi.org/10.1111/jocn.15464
46. Kang L, Li YI, Hu S, et al. The mental health of medical workers in Wuhan, China dealing with the 2019 novel coronavirus. *Lancet Psych*. 2020;7(3):e14. https://doi.org/10.1016/S2215-0366(20)30047-X
47. Tan BYQ, Chew NWS, Lee GKH, et al. Psychological impact of the COVID-19 pandemic on health care workers in Singapore. *Ann Intern Med*. 2020;173:317-320. https://doi.org/10.7326/m20-1083
48. Wang C, Pan R, Wan X, et al. Immediate psychological responses and associated factors furing the initial stage of the 2019 coronavirus disease (COVID-19) epidemic among the general population in China. *J Environ Res Public*. 2020;17(5):1729.
49. Zhang Y, Ma ZF. Impact of the COVID-19 pandemic on mental health and quality of life among local residents in Liaoning Province, China: a cross-sectional study. *J Environ Res Public*. 2020;17(7):2381. https://doi.org/10.3390/ijerph17072381
50. Li Z, Ge J, Yang M, et al. Vicarious traumatization in the general public, members, and non-members of medical teams aiding in COVID-19 control. *Brain Behav Imm*. 2020;1591(20):916-919. https://doi.org/10.1016/j.bbi.2020.03.007
51. Tran BX, Nguyen HT, Le HT, et al. Impact of COVID-19 on economic well-being and quality of life of the Vietnamese during the national social distancing. *Front Psychol*. 2020;11:565153. https://doi.org/10.3389/fpsyg.2020.565153
52. Nguyen HTT, Moir MPI, Nguyen TX, et al. Health-related quality of life in elderly diabetic outpatients in Vietnam. *Patient Prefer Adherence*. 2018;12:1347-1354. https://doi.org/10.2147/PPA.S162892
53. Tran B, Dang A, Truong NU, et al. Depression and quality of life among patients living with HIV/AIDS in the era of universal treatment access in Vietnam. *Int J Environ Res Public Health*. 2018;15:2888. https://doi.org/10.3390/ijerph15122888
54. Nguyen SH, Nguyen LH, Vu GT, et al. Health-related quality of life impairment among patients with different skin diseases in Vietnam: a cross-sectional study. *Int J Environ Res Public Health*. 2019;16(3):305. https://doi.org/10.3390/ijerph16030305
55. Ngo CQ, Phan PT, Vu GV, et al. Effects of different comorbidities on health-related quality of life among respiratory patients in Vietnam. *J Clin Med*. 2019;8:214. https://doi.org/10.3390/jcm8020214
56. Tran B, Thu Vu G, Hoang Nguyen L, et al. Cost-of-Illness and the health-related quality of life of patients in the dengue fever outbreak in Hanoi in 2017. *Int J Environ Res Public Health*. 2018;15(6):1174. https://doi.org/10.3390/ijerph15061174
57. Nguyen AT, Nguyen LH, Nguyen TX, et al. Frailty prevalence and association with health-related quality of life impairment among rural community-dwelling older adults in Vietnam. *Int J Environ Res Public Health*. 2019;16:3869. https://doi.org/10.3390/ijerph16203869
58. Vu HM, Nguyen LH, Tran TH, et al. Effects of chronic comorbidities on the health-related quality of life among older patients after falls in Vietnamese hospitals. *Int J Environ Res Public Health*. 2019;16:3623. https://doi.org/10.3390/ijerph16193623

59. Vu HM, Dang AK, Tran TT, et al. Health-related quality of life profiles among patients with different road traffic injuries in an urban setting of Vietnam. *Int J Environ Res Public Health*. 2019;16:1462. https://doi.org/10.3390/ijerph16081462
60. Dobson H, Malpas CB, Burrell AJC, et al. Burnout and psychological distress amongst Australian healthcare workers during the Covid-19 pandemic. *Australas Psych*. 2021;29(1):26-30. https://doi.org/10.1177/1039856220965045
61. Erquicia J, Valls L, Barja A, et al. Emotional impact of the Covid-19 pandemic on healthcare workers in one of the most important infection outbreaks in Europe. *Med Clín (Barc)*. 2020;155:434-440. https://doi.org/10.1016/j.medcli.2020.07.006
62. Mundo EL. 2020. www.elmundo.es/ciencia-y-salud/salud/2020/05/06/5eb1aec4fdddff4c038b457f.html. Accessed May 06, 2020.
63. País EL. 2020. https://elpais.com/sociedad/2020-11-05/el-gobierno-estima-en-20268-los-fallecidos-por-covid-en-residencias-de-servicios-sociales-durante-la-primera-ola. Accessed November 06, 2020.
64. Koob GF, Schulkin J. Addiction and stress: an allostatic view. *Neurosci Biobehav Rev*. 2020;106:245-262. https://doi.org/10.1016/j.neubiorev.2018.09.008
65. Bransi A, Winter L, Glahn A, Kahl KL. Addictive disorders in physicians. *Nervenarzt*. 2020;91:77-90. https://doi.org/10.1007/s00115-019-00854-3
66. Vayr F, Herin F, Jullian B, MarcSoulat J, Franchitto N. Barriers to seeking help for physicians with substance use disorder: a review. *Drug Alcohol Depend*. 2019;199:116-121. https://doi.org/10.1016/j.drugalcdep.2019.04.004
67. Wu P, Fang Y, Guan Z, et al. The psychological impact of the SARS epidemic on hospital employees in China: exposure, risk perception, and altruistic acceptance of risk. *Canadian J Psych*. 2009;54:302-311.
68. Smereka J, Szarpak L. COVID 19 a challenge for emergency medicine and every health care professional. *Am J Emerg Med*. 2020;38(10):2232-2233.
69. Conti C, Fontanesi L, Lanzara R, Rosa I, Porcelli P. Fragile heroes. The psychological impact of the COVID-19 pandemic on health-care workers in Italy. *PLoS ONE*. 2020;15(11):e0242538. https://doi.org/10.1371/journal.pone.0242538
70. Luo M, Guo L, Yu M, Jiang W, Wang H. The psychological and mental impact of coronavirus disease 2019 (COVID-19) on medical staff and general public—a systematsic review and meta-analysis. *Psychiatry Res*. 2020;291:113190. https://doi.org/10.1016/j.psychres.2020.113190
71. Bryson JM. *Strategic Planning for Public and Nonprofit Organizations: A Guide to Strengthening and Sustaining Organizational Achievement*. Hoboken, New Jersey: John Wiley & Sons; 2018.
72. El País. 2020. https://english.elpais.com/spanish_news/2020-12-01/oecd-warns-of-no-return-to-pre-crisis-economy-for-spain-before-2023.html. Accessed December 01, 2020.
73. Ho CS, Chee CY, Ho RC. Mental health strategies to combat the psychological impact of COVID-19 beyond paranoia and panic. *Ann Acad Med Singapore*. 2020;49:155-160.
74. Zhang MWB, Ho RCM. Moodle: the cost effective solution for internet cognitive behavioral therapy (I-CBT) interventions. *Technol Health Care*. 2017;25:163-165. https://doi.org/10.3233/THC-161261
75. Soh HL, Ho RC, Ho CS, Tam WW. Efficacy of digital cognitive behavioural therapy for insomnia: a meta-analysis of randomised controlled trials. *Sleep Med*. 2020;75:315-325. https://doi.org/10.1016/j.sleep.2020.08.020
76. Lim GY, Tam WW, Lu Y, Ho CS, Zhang MW, Ho RC. Prevalence of depression in the community from 30 countries between 1994 and 2014. *Sci Rep*. 2018;8:2861. https://doi.org/10.1038/s41598-018-21243-x
77. Olszewska-Guizzo A, Mukoyama A, Naganawa S, et al. Hemodynamic response to three types of urban spaces before and after lockdown during the COVID-19 pandemic. *Int J Environ Res Public Health*. 2021;18:6118. https://doi.org/10.3390/ijerph18116118
78. Husain SF, Yu R, Tang T-B, et al. Validating a functional near-infrared spectroscopy diagnostic paradigm for major depressive disorder. *Sci Rep*. 2020;10:9740. https://doi.org/10.1038/s41598-020-66784-2
79. Husain SF, Tang T-B, Yu R, et al. Cortical haemodynamic response measured by functional near infrared spectroscopy during a verbal fluency task in patients with major depression and borderline personality disorder. *EBioMedicine*. 2019;51:102586. https://doi.org/10.1016/j.ebiom.2019.11.047
80. Ho CSH, Lim LJH, Lim AQ, et al. Diagnostic and predictive applications of functional near-infrared spectroscopy for major depressive disorder: a systematic review. *Front Psychiatry*. 2020;11:378. https://doi.org/10.3389/fpsyt.2020.00378
81. Wang C, Pan R, Wan X, et al. A longitudinal study on the mental health of general population during the COVID-19 epidemic in China. *Brain Behav Immun*. 2020;S0889-1591(20)30511-0. https://doi.org/10.1016/j.bbi.2020.04.028
82. Chew NWS, Ngiam JN, Tan BYQ, et al. Asian-Pacific perspective on the psychological well-being of healthcare workers during the evolution of the COVID-19 pandemic. *BJPsych Open*. 2020;6:e116. https://doi.org/10.1192/bjo.2020.98

SUPPORTING INFORMATION

Additional Supporting Information may be found online in the Supporting Information section.

How to cite this article: Martín J, Padierna Á, Villanueva A, Quintana JM. Evaluation of the mental health of health professionals in the COVID-19 era. What mental health conditions are our health care workers facing in the new wave of coronavirus? *Int J Clin Pract*. 2021;75:e14607. https://doi.org/10.1111/ijcp.14607
