# Supplementary material for: Mental health status and related factors influencing healthcare workers during the COVID-19 pandemic: A systematic review and meta-analysis
Source: PLoS One. 2024 Jan 19;19(1):e0289454. doi: 10.1371/journal.pone.0289454 (PMC10798549; doi:10.1371/journal.pone.0289454)
Supplement: S1 Data — (ZIP) [file pone.0289454.s011.zip › literatures/88.pdf]

International Journal of *Environmental Research and Public Health*

MDPI

Article

Analyzing the Impact of COVID-19 Trauma on Developing Post-Traumatic Stress Disorder among Emergency Medical Workers in Spain

Carmen M. Martínez-Caballero [1,†], Rosa M. Cárdaba-García [1,2,3,†], Rocío Varas-Manovel [1], Laura M. García-Sanz [1], Jorge Martínez-Piedra [4], Juan J. Fernández-Carbajo [1], Lucía Pérez-Pérez [2,3,5], Miguel A. Madrigal-Fernández [2,3,6], M. Ángeles Barba-Pérez [2,3,6], Elena Olea [2,3,7], Carlos Durantez-Fernández [8] and M. Teresa Herrero-Frutos [1,*]

1 Emergencies Management (SACYL), 40002 Segovia, Spain; cmmartinez@saludcastillayleon.es (C.M.M.-C.); rcardaba@saludcastillayleon.es (R.M.C.-G.); mvarasma@saludcastillayleon.es (R.V.-M.); lmgarcias@saludcastillayleon.es (L.M.G.-S.); jjfernandez@saludcastillayleon.es (J.J.F.-C.)
2 Nursing Department, Faculty of Nursing, University of Valladolid, 47005 Valladolid, Spain; lucia.perez@uva.es or luperper2@hotmail.com (L.P.-P.); miguelangel.madrigal@uva.es or miguelanmad@gmail.com (M.A.M.-F.); mariaangeles.barba@uva.es (M.Á.B.-P.); elena.olea@uva.es (E.O.)
3 Nursing Care Research (GICE), Faculty of Nursing, University of Valladolid, 47005 Valladolid, Spain
4 Health Transportation Group, 40195 Segovia, Spain; jorgemp29@gmail.com
5 Primary Care Management Valladolid West (SACYL), 47012 Valladolid, Spain
6 University Clinical Hospital of Valladolid, 47003 Valladolid, Spain
7 Instituto de Biología y Genética Molecular (IBGM), Universidad de Valladolid-CSIC, 47005 Valladolid, Spain
8 Faculty of Health Sciences, University of Castilla-La Mancha, 45600 Talavera de la Reina, Spain; carlos.durantez@uclm.es
* Correspondence: rosamaria.cardaba@uva.es or therrerof@saludcastillayleon.es
† Mrs. Carmen M. Martínez-Caballero and Dra. Rosa M. Cárdaba-García have contributed as IP in the research.

check for updates

Citation: Martínez-Caballero, C.M.; Cárdaba-García, R.M.; Varas-Manovel, R.; García-Sanz, L.M.; Martínez-Piedra, J.; Fernández-Carbajo, J.J.; Pérez-Pérez, L.; Madrigal-Fernández, M.A.; Barba-Pérez, M.Á.; Olea, E.; et al. Analyzing the Impact of COVID-19 Trauma on Developing Post-Traumatic Stress Disorder among Emergency Medical Workers in Spain. *Int. J. Environ. Res. Public Health* **2021**, *18*, 9132. https://doi.org/10.3390/ijerph18179132

Academic Editors: Richard Gray, Sonia Udod and Marcello Covino

Received: 6 July 2021
Accepted: 26 August 2021
Published: 30 August 2021

Publisher's Note: MDPI stays neutral with regard to jurisdictional claims in published maps and institutional affiliations.

Copyright: © 2021 by the authors. Licensee MDPI, Basel, Switzerland. This article is an open access article distributed under the terms and conditions of the Creative Commons Attribution (CC BY) license (https://creativecommons.org/licenses/by/4.0/).

Abstract: The early stages of the COVID-19 pandemic presented the characteristics of a traumatic event that could trigger post-traumatic stress disorder. Emergency Medical Services workers are already a high-risk group due to their professional development. The research project aimed to analyse the impact of the COVID-19 pandemic on EMS professionals in terms of their mental health. For this purpose, we present a descriptive crosssectional study with survey methodology. A total of 317 EMS workers (doctors, nurses, and emergency medical technicians) were recruited voluntarily. Psychological distress, post-traumatic stress disorder, and insomnia were assessed. The instruments were the General Health Questionnaire-12 (GHQ-12), the Davidson Trauma Scale (DTS-8), and the Athens Insomnia Scale (AIS-8). We found that 36% of respondents had psychological distress, 30.9% potentially had PTSD, and 60.9% experienced insomnia. Years of work experience were found to be positively correlated, albeit with low effect, with the PTSD score ($r = 0.133$). Finally, it can be stated that the COVID-19 pandemic has been a traumatic event for EMS workers. The number of professionals presenting psychological distress, possible PTSD, or insomnia increased dramatically during the early phases of the pandemic. This study highlights the need for mental health disorder prevention programmes for EMS workers in the face of a pandemic.

Keywords: emergency medical services; post-traumatic stress disorder; mental status; sleep disorders; COVID-19 pandemic

1. Introduction

In Spain, the SARS-CoV-2 pandemic started on 31 January 2020, when the first case was diagnosed on the island of La Gomera, and the first death from Coronavirus Disease 2019 (COVID-19) occurred on 13 February 2020 in Valencia. Since the first confirmed case was reported on 31 January 2020, more than 405,000 cases and 28,000 deaths have been reported in Spain [1]. According to the ENE–COVID seroprevalence study, one in ten

Spaniards had been infected with the virus by November 2020 [2]. It was not until 14 March 2020 that the Spanish state decreed a state of alarm and limited the movement of people throughout the territory [3]. On 28 March, all nonessential onsite work activities were suspended for 15 days [4]. Spain has suffered four waves of the COVID-19 pandemic, with the first wave being the most aggressive in terms of morbidity and mortality, with the highest number of deaths in March and April. The highest number of coronavirus deaths in one day (950) was recorded on 2 April [5,6]. We are currently in the fifth wave of the pandemic, although, as of 26 June 2021, it is not mandatory to wear masks outdoors if safe distances of at least 1.5 m are maintained between individuals [7].

Post-traumatic stress disorder (PTSD) is defined in the DSM-V in section B as the "presence of one or more of the following intrusive symptoms associated with the traumatic event, beginning after the traumatic event" [8]. The symptoms referred to are: recurrent, involuntary, intrusive, or distressing memories of the traumatic event; recurrent distressing dreams in which the content of the dream is related to the traumatic event; dissociative reactions such as flashbacks in which the subject feels or acts as if the traumatic event is being replayed; intense or prolonged psychological distress on exposure to internal or external factors resembling the traumatic event; and intense physiological reactions to internal or external factors resembling the traumatic event. This definition is specified with other characteristics that do not exactly fit the object of study of this research. It should be noted that the current definition of PTSD does not state that a specific time must have passed since the traumatic event in order for an individual to suffer from this syndrome only that the associated symptoms must remain for at least one month and negatively affect the individual's life [8].

The scientific literature indicates that traumatic symptoms may appear immediately, after weeks, or even months after exposure, and, in most cases, they appear in the first six months after the triggering event. Unlike an acute stress reaction, PTSD symptoms do not disappear after a few weeks and tend to persist over time [9].

PTSD was originally described in wartime contexts, and shortly thereafter, it was also seen in survivors of death camps and sexual assaults. Natural disasters and terrorist attacks are also documented as triggers for PTSD [10]. PTSD has also been associated with previous epidemics, such as the one generated by the Ebola virus [11].

Individuals working in the emergency medical services experience direct contact with death and with events that can be considered traumatic. Although their academic training should be adequate to deal with these situations, the reality is that this is not the case [12], and, in hostile situations such as wars, natural disasters, and terrorist attacks, it is not uncommon for PTSD to develop in health personnel on the front line. [13]. Despite this, professionals often do not readily accept that they suffer from symptoms associated with PTSD, as it is often understood to be a weakness at work [14]. It is assumed that health personnel who work in EMS have the capacity to cope with events that can be traumatic, which is not always the case—it should not be forgotten that the most frequently attended incidents in these services are not emergent but urgent and that the actual and practical experience in catastrophic situations such as a pandemic may only be theoretical [15]. In fact, these professionals should be trained to face situations similar to those experienced during the COVID-19 pandemic, but the reality is quite different; as a result, such individuals are at risk of developing PTSD [16]. Despite several studies on the mental health of EMS workers in recent years, there is not enough previous data prior to the last two decades, as there was previously not much interest in assessing the mental health of frontline EMS workers [17].

It is widely known that since the beginning of the COVID-19 pandemic, an increase in mental illness has been observed [18]. A decrease in psychological wellbeing associated with higher values of anxiety and depression has been observed in the general population, in COVID-19 patients, and, above all, in healthcare workers, especially among those on the front line [19–21]. One of the most common symptoms in healthcare personnel has been insomnia [22,23]. Some studies relate these consequences to occupational factors such

as professional category, workplace, and the means available to protect against possible COVID-19 infection, among others [24,25].

We are aware that previous studies have been published on the impact of the pandemic on healthcare workers [26,27], highlighting the deterioration in mental health and problems related to sleep and rest, but, to our knowledge, no studies have yet been published with data on the negative impact on mental health and PTSD symptoms focusing on emergency medical services (EMS) workers. Moreover, the scientific literature offers few examples that include all professional categories involved in Spanish EMS (physicians, nurses, and emergency medical technicians (EMTs)) who have worked during the pandemic [28]. EMS workers are considered to be a group at particular risk of developing PTSD in general terms [9], since, while the prevalence of PTSD in the general population ranges between 1% and 3% [13], in EMS in particular, it is estimated to be 11% during catastrophic events [29].

The initial hypothesis of the study was that the COVID-19 pandemic was associated with poor mental health status, insomnia, and an increased risk of developing PTSD in prehospital emergency professionals. The overall objective was to determine the impact of the early stages of the COVID-19 pandemic on the mental health of workers of the "Gerencia de Emergencias Sanitarias de Castilla y León" (GESACYL) and the "Servicio de Urgencias Médicas de Madrid 112" (SUMMA 112). Specifically, validated instruments were used to quantify the mental health of the first responders, to measure the presence of insomnia, to assess the frequency of PTSD, and to describe the variables most frequently related to the three previous aspects.

In this paper, readers will find a descriptive study methodology based on survey data collection. Next, a descriptive analysis of the main variables is presented to further the search for correlations between variables that have significance in the development of mental health problems, insomnia, and PTSD. In this way, a discussion is established with the findings of other authors, accepting the limitations of this research, and assessing the implications that the results have for clinical practice.

2. Materials and Methods

2.1. Design

A descriptive crosssectional study with survey methodology is presented.

2.2. Study Sample

The study population was EMS workers (physicians, nurses, and EMTs) from two different regions of Central Spain: Castile and León "Gerencia de Emergencias Sanitarias de Castilla y León" (GESACYL) and Madrid "Servicio de Urgencias Médicas de Madrid 112" (SUMMA 112).

A total of 317 workers were recruited, and the response rate was 37.6%. A volunteer sampling method was adopted, via corporate email.

The inclusion criteria established were as follows: over 18 years of age, working in GESACYL or SUMMA112, being a physician, nurse, or EMT, being active during the COVID-19 pandemic, having worked almost exclusively in prehospital care (at least 90% of the total number of working days), agreeing to participate in the investigation, having basic computer skills, and being Spanish-speaking. Those who did not meet the aforementioned inclusion criteria and those who ticked the box on the questionnaire stating that they did not consent to participate in this study were excluded.

2.3. Ethical Aspects

The study was approved by the Ethics Committee for Drug Research of the Valladolid East Health Area, with registration code PI 139-20 NO HRHV, on 6 June 2020. This study conformed to the STROBE Initiative (Strengthening the Reporting of Observational Studies in Epidemiology) for observational studies of the EQUATOR Initiative [30].

2.4. Recruitment of the Sample

The sample was recruited by contacting the participants through the corporate email service of the emergency institutions of Castile and Leon and Madrid, sending them a link to the self-administered survey developed using the online Google Forms® tool, in which the participants declared that they met the requirements to be part of the study sample. The recruitment strategy was carried out by sending an email reminder one and three weeks after the initial referral. In no case were participants offered any incentive to be part of the sample. The data collection period was from 20 May 2020 to 26 July 2020, a period early on during the first wave of the pandemic; according to the DSM-V definition of PTSD, this was appropriate, as symptomatology may appear immediately after a traumatic event [8].

2.5. Study Variables

The variables considered were sociodemographic (age, sex, number of people living together during the pandemic, and change in body weight during the pandemic); work-related (professional category, place of work, type of unit, experience in the service, and change of function during the pandemic); occupational safety aspects (previous practical training in the use of protective equipment, availability of sufficient protective equipment in the unit, removal of protective equipment that did not comply with regulations, information on the pandemic and its evolution, testing for COVID-19, need for home isolation, presence of COVID-19 symptoms, and hospitalization for COVID-19); and variables associated with psychological health (concern about the possibility of contracting the disease, concern about harming loved ones, anxiety symptoms before the pandemic, anxiety symptoms during the pandemic, treatment of anxiety before the pandemic, treatment of anxiety during the pandemic, need for psychological support before the pandemic and during the pandemic, knowledge of the existence or not of a psychological support unit for employees, work environment of the unit, and existence of specific training courses for anxiety control for workers).

2.6. Instruments

The instruments used were the General Health Questionnaire-12 (GHQ-12), the Davidson Trauma Scale (DTS-8), and the Athens Insomnia Scale (AIS-8).

2.6.1. General Health Questionnaire-12

The General Health Questionnaire-12 (GHQ-12) is a 12 item self-administered questionnaire that detects psychological distress. There are six positive items (e.g., "Have you been able to concentrate?") and six negative items (e.g., "Have you lost confidence?"). Each item has four possible responses—according to a four-level Likert scale—aimed at capturing the intensity of the respondents' feelings for the given item. The possible responses for the six positive items in the questionnaire, together with the score assigned to them, were 0 = more than usual; 0 = same as usual; 1 = less than usual; and 1 = much less than usual. For the negative items, possible responses were 0 = absolutely not; 0 = no more than usual; 1 = somewhat more than usual; and 1 = much more than usual. Thus, the possible total score on the questionnaire ranged from 0 to 12. The interpretation of the total score is as follows: from 0 to 4: no psychological distress; from 5 to 6: probable psychological distress; and from 7 to 12: psychological morbidity. The GHQ-12 has a reliability according to different studies with Cronbach's alpha ranging from 0.82 to 0.86 [31,32]. In the sample of this study, a result of 0.85 was obtained for the mentioned test.

2.6.2. Davidson Trauma Scale

The Davidson Trauma Scale (DTS-8) is a questionnaire that can be self-administered or interviewed and consists of eight items. This scale is used to detect suspicion of a potential diagnosis of post-traumatic stress syndrome. The questions refer to the last week and are measured by a five-level Likert scale, with possible values (0,1,2,3,4). Value 0 corresponds to the least stressful response. The total score of the questionnaire is obtained by adding

the scores of the eight items, and it therefore ranges from 0 to 32. Higher scores on the scale are related to a higher possibility of suspected PTSD. The interpretation established by the authors of the original test is by means of ranges as follows: from 0 to 7: no suspicion of PTSD; from 8 to 11: it cannot be determined whether or not PTSD exists; 12 or more: suspicion of PTSD [33]. The scale presents adequate internal consistency, with a Cronbach's alpha of 0.71–0.91 [34]. The value of Cronbach's alpha obtained in the study sample was 0.87.

2.6.3. Athens Insomnia Scale

The Athens Insomnia Scale (AIS-8) is a self-assessment survey consisting of eight items. Its purpose is to detect insomnia-type sleep disorders. The first four items assess possible insomnia problems from a quantitative point of view, the fifth item asks about sleep quality, while the last three items assess the daytime impact of insomnia. The evaluation questions refer to the last week and are measured on a four-level Likert scale, which translates into values (0,1,2,3), with 0 being the absence of a problem and 3 the maximum severity. The total score of the questionnaire is obtained by adding the scores of the eight items, and it therefore ranges from 0 to 24. Higher scores on the questionnaire are associated with more insomnia. Authors who have evaluated the scale based on the diagnosis of insomnia of the International Classification of Diseases in its 10th revision (ICD-10) establish that a score equal to or higher than six points determines a diagnosis of insomnia. The internal consistency of the test measured by Cronbach's alpha is around 0.90 [35]. The AIS-8 showed a high degree of internal homogeneity, obtaining a Cronbach's alpha of 0.91 for the whole sample.

2.7. Data Analysis

The statistical procedure and data analysis were performed by means of a descriptive analysis of frequencies and response percentages, centrality (mean), and dispersion (standard deviation) in quantitative variables. Quantitative variables were analyzed by normality test (Kolmogorov–Smirnov) prior to the inferential analysis, looking for significant relationships between variables by means of Pearson's r test, ANOVA, and Student's t test. The statistical significance level used was $p = 0.05$ or lower. The statistical program used was SPSS® v.24.

3. Results

3.1. Sample Description

The sample consisted of 317 health professionals from the Health Emergency Services of Castile and Leon (80.4%) and Madrid (19.6%), who voluntarily agreed to participate in the study. In relation to the sociodemographic variables of the sample, 52.7% were men, 46.4% women, and 0.9% replied "Other". The most frequent age range was between 40 and 49 years (42.9%). Only 16.1% lived alone during the pandemic, while households of four or more constituted 41.3% of the total. Overall, 120 respondents (37.9%) gained weight during the pandemic. Of those who gained weight, 61.2% declared having gained less than 1 kg.

3.2. Occupational Considerations

The following is a description of the variables related to the health profession and work setting. By professional category, 61 respondents were physicians (19.2%), 78 nurses (24.6%), and 178 emergency health technicians (56.2%). Half of the respondents worked in advanced life support units (50.5%). The most common time period for which respondents had worked in the service was between 10 and 20 years (54.9%), followed by less than 10 years (25.6%). Overall, 223 respondents (70.3%) changed their care functions during the pandemic, but only 41 persons (12.9%) were reassigned to a specific contingency hospital for patients diagnosed with COVID-19.

3.3. Job Security in the Pandemic

Working in healthcare during the pandemic has entailed a number of risks, which are described below.

A large number of people (94.3%) felt worried about the possibility of contracting the disease at work and about the possibility of passing it on to their family members (96.8%).

Overall, 202 respondents (63.7%) stated that they had received prior theoretical training in their service on the use of personal protective equipment (PPE) necessary in case of biological risk, and 60.6% stated that they had received practical training regarding the use of personal protective equipment.

In total, 205 respondents (64.7%) had adequate means of protection in their work during the pandemic, but in 78.9% of cases, it was necessary to remove the protective equipment provided by their service as it did not comply with the protection regulations.

In total, 238 respondents (75.1%) affirmed that they had not been informed by their service of the possibility of a COVID-19 pandemic occurring prior to the state of confinement, while 48.3% (153) had been informed by the public health administration of the evolution of the pandemic.

Most of the workers (93.4%) had been tested for SARS-CoV-2 infection; in 35.3% of cases, they had been tested with both a PCR test and an antibody screening test.

A total of 19.2% (61) required home isolation because they had had a high-risk encounter in their work environment. A total of 33.8% had experienced symptoms associated with COVID-19 infection, but only six persons (1.9%) required hospital admission.

3.4. Mental Health Considerations

The respondents reported their mental health in terms of its pre- and postpandemic status as follows.

Twelve percent had experienced symptoms of anxiety prior to the pandemic, compared to 65.6% who stated that they had experienced anxiety-related symptoms during the pandemic. Overall, 20.5% had taken anxiolytics prior to the pandemic, a figure that decreased to 18.9% during the pandemic. Similarly, the need for psychological support was reported by 24.0% before the pandemic and by 9.8% during the pandemic.

In total, 37.2% of the respondents stated that their service had a psychological support unit, but a similar number, 36.0%, did not know if they had access to such a support unit. Training courses aimed at anxiety control were given within the unit in which they worked in 26.2% of the cases, and in 46.7% of the cases, the psychological health of the workers could be dealt with normally.

3.5. Questionnaire Results

Regarding the mental health of the participants, in the GHQ-12, the mean score of the sample in the questionnaire was 5.26 (SD = 3.18). The GHQ-12 scores of the participants indicate that 37.5% showed no pathology, 26.5% showed possible psychological pathology, and 36% showed signs of psychological pathology, according to the cutoff points of the scale.

In relation to PTSD, assessed by the DTS-8 questionnaire, the mean score was 9.26 (SD = 6.04). According to the cutoff point of this scale ($\geq$12 points), 30.9% of the people in the sample present suspected post-traumatic stress syndrome.

Regarding the perception of sleeping difficulties, which was measured by the AIS-8 scale, the mean score of the participants on the instrument was 7.39 (SD = 4.94). Regarding the cutoff point of the scale ($\geq$6 points), 60.9% experienced sleeping difficulties.

The most significant items in each of the three instruments (GHQ-12, DTS-8, and AIS-8) are shown in Table 1. If we take into account those variables that offer responses other than the YES/NO dichotomy, we can observe that the groups with the greatest number of individuals suffering from sleep difficulties were: the age group between 40 and 49 years, women, EMTs, EMS workers in Castilla y León, EMS workers in advanced units, those who had been working for between 10 and 20 years, those who lived with four

or more people, those who had undergone serological tests, and those who had gained less than 1 kg in weight. In general, these groups also demonstrated the greatest prevalence of signs of suspected psychological pathology, suspected PTSD, and insomnia (Table 2).

Table 1. Items from the GHQ-12, DTS-8, and AIS-8 instruments with the greatest significance (n = 317).

**GHQ-12**	
Items with the highest scores in the scale	Percentage (%)
Thinking of self as worthless	88.3
Losing confidence	75.7
**DTS-8**	
Items with the highest scores in the scale	Percentage (%)
Experiencing less interest in things previously enjoyed	4.7
Imagining or remembering painful, recurring images that cannot be put out of mind	3.8
**AIS-8**	
Items with the highest scores in the scale	Percentage (%)
Sleep induction	4.4
Final awakening	3.2
Total sleep duration	3.2
Sleep quality	3.2

Table 2. Sociodemographic distribution of polytomous variables according to the results of GHQ-12: suspected psychological pathology (GHQ-12 = 5–6), DTS-8: suspicion of post-traumatic stress disorder (DTS-8 $\geq$ 12), and AIS-8: insomnia (AIS-8 $\geq$ 6) (n = 317).

**Variables**	**GHQ-12 Psychological Pathology**		**DTS-8 PTSD**		**AIS-8 Insomnia**	
	n	**%**	***n***	**%**	***n***	**%**
Age (years)						
≤29	19	4.1	29	9.1	29	9.1
30–39	31	9.8	26	8.2	42	13.2
40–49	41	12.9	39	12.3	78	24.6
50–59	26	8.2	22	6.9	40	12.6
≥60	3	0.9	2	0.6	4	1.3
Gender						
Female	68	21.5	52	16.4	108	34.1
Male	46	14.5	43	13.6	82	25.9
Professional category						
Physician	19	6	15	4.7	36	11.4
Nurse	31	9.8	22	6.9	54	17
Emergency medical technician	64	20.2	61	19.2	103	32.5
Emergency Service						
SACYL	90	28.4	74	23.3	148	46.7
SUMMA 112	24	7.6	24	7.6	45	14.2

Table 2. *Cont.*

Variables	GHQ-12 Psychological Pathology		DTS-8 PTSD		AIS-8 Insomnia	
	n	%	*n*	%	*n*	%
Type of EMS						
ALS	56	17.7	39	12.3	99	31.2
BLS	40	12.6	41	12.9	70	22.1
Work experience (years)						
<10	29	9.1	18	5.7	52	16.4
10 to 20	63	19.9	56	17.7	106	33.4
21 to 30	18	5.7	18	5.7	27	8.5
>30	4	1.3	6	1.9	8	2.5
Number of people per household						
1	21	6.6	18	5.7	33	10.4
2	21	6.6	20	6.3	39	12.3
3	21	6.6	15	4.7	34	10.7
>4	51	16.1	45	14.2	87	27.4
Tests						
PCR	14	4.4	14	4.4	18	5.7
Ig M/Ig G	43	13.6	79	24.9	90	28.4
Both	50	15.8	39	12.3	70	22.1
Weight gained (Kg)						
<1	64	20.2	58	18.3	113	35.6
1 to 2	4	1.3	4	1.3	5	1.6
2 to 3	10	3.2	3	0.9	12	3.8
3 to 4	19	6	18	5.7	28	8.8
>4	28	8.8	9	2.8	10	3.2

Abbreviations: PTSD, post-traumatic stress disorder; SACYL, Sanidad de Castilla y León; SUMMA 112, Servicio de Urgencias Médicas de Madrid; EMS, emergency medical services; ALS, advanced life support; BLS, basic life support.

3.6. Comparison of Means and Correlations

Subsequently, an inferential analysis was carried out between variables, with the intention of searching for relationships between the general variables and the results of the total scores of the GHQ-12, DTS-8, and AIS-8, which, in all cases, approximately followed a normal distribution.

Depending on the type of variable, Student's t tests were used for dichotomous variables, ANOVA for variables with more than two possible answers, and Pearson's r correlation coefficient for quantitative variables.

Relationships were found between the GHQ-12 total score indicating the psychological health of the person and the following variables: gender, changes in job functions, having undergone previous theoretical and practical training on the use of PPE, the type of SARS-CoV-2 test, having required isolation, having experienced symptoms of the disease, having had adequate PPE during the pandemic, having had PPE removed from the service because it was not adequate, having been worried about contracting the disease and about transmitting it to family, anxiety symptoms prior to and during the pandemic, use of anxiolytics during the pandemic, requiring psychological support prior to and during the pandemic, and dealing with mental health issues normally in the work unit.

In addition, relationships were found between the DTS-8 total score, showing the risk of post-traumatic stress, and the following variables: gender, changes in job duties, having had prior theoretical and practical training on the use of PPE, the type of SARS-CoV-2 testing, having had appropriate PPE during the pandemic, having been worried about contracting the disease, anxiety symptoms prior to and during the pandemic, use of anxiolytics prior to and during the pandemic, requiring psychological support prior to and during the pandemic, and dealing with mental health issues normally in the work unit.

Relationships were also found between the AIS-8 total score indicating the presence of insomnia and the following variables: gender, service in which they work, having undergone previous theoretical and practical training on the use of PPE, having been worried about contracting the disease, weight gain during confinement, anxiety symptoms before and during the pandemic, use of anxiolytics before and during the pandemic, requiring psychological support before and during the pandemic, and dealing with mental health issues normally in the work unit (Tables 3 and 4).

Table 3. Inferential analysis: Student *t* test means comparison.

Variables	Instrument (Total Score)	Levene Test	*t* Value	Level of Significance *p*	IC 95%	
					Min	Max
Emergency service	AIS-8	0.554	−1.964	0.050 *	−2.743	0.002
Functions	GHQ-12	0.696	−2.449	0.015 *	−1.716	−0.187
	DTS-8	0.846	−2.540	0.012 *	−3.248	−0.408
Theoretical training	GHQ-12	0.340	2.422	0.016 *	0.168	1.620
	DTS-8	0.005	2.213	0.028 *	0.178	3.084
	AIS-8	0.055	3.389	0.001 *	0.809	3.047
Practical training	GHQ-12	0.295	2.693	0.007 *	0.263	1.689
	DTS-8	0.000	2.755	0.006 *	0.571	3.441
	AIS-8	0.250	2.608	0.010 *	0.361	2.579
Need for quarantine	GHQ-12	0.929	−2.428	0.016 *	−1.979	−0.207
COVID-19 symptoms	GHQ-12	0.645	−2.392	0.017 *	−1.637	−0.159
Adequate personal equipment	GHQ-12	0.000	4.145	0.000 *	0.742	2.084
	DTS-8	0.021	4.946	0.000 *	2.040	4.735
	AIS-8	0.160	4.800	0.000 *	1.593	3.805
Contagion concern	GHQ-12	0.337	−4.790	0.000 *	−5.050	−2.109
	DTS-8	0.023	−3.978	0.000 *	−8.519	−2.881
	AIS-8	0.090	−2.884	0.004 *	−5.762	−1.089
Transmission concern	GHQ-12	0.012	−3.775	0.000 *	−5.757	−1.812
Weight gained	AIS-8	0.222	−2.604	0.010 *	−2.597	−0.362
Anxiety prepandemic	GHQ-12	0.629	−3.135	0.002 *	−2.771	−0.634
	DTS-8	0.651	−2.362	0.019 *	−4.490	−0.409
	AIS-8	0.449	−3.003	0.003 *	−4.201	−0.875
Anxiety during pandemic	GHQ-12	0.356	−11.104	0.000 *	−4.179	−2.921
	DTS-8	0.001	−8.946	0.000 *	−6.566	−4.197
	AIS-8	0.960	−7.188	0.000 *	−4.975	−2.836
Anxiety treatment prepandemic	DTS-8	0.254	−2.645	0.009 *	−3.840	−0.564
	AIS-8	0.027	−4.491	0.000 *	−4.318	−1.687
Anxiety treatment during pandemic	GHQ-12	0.837	−7.468	0.000 *	−3.977	−2.318
	DTS-8	0.080	−6.147	0.000 *	−6.652	−3.426
	AIS-8	0.838	−5.717	0.000 *	−5.199	−2.537
Need for psychological support prepandemic	GHQ-12	0.855	−2.197	0.029 *	−1.734	−0.096
	DTS-8	0.071	−3.338	0.001 *	−4.150	−1.072
	AIS-8	0.001	−4.170	0.000 *	−4.515	−1.604

Table 3. *Cont.*

Variables	Instrument (Total Score)	Levene Test	*t* Value	Level of Significance *p*	IC 95%	
					Min	Max
Need for psychological support during pandemic	GHQ-12	0.876	−6.489	0.000 *	−4.789	−2.561
	DTS-8	0.090	−6.293	0.000 *	−8.907	−4.664
	AIS-8	0.309	−4.305	0.000 *	−5.714	−2.129
Positive behavior in the service	GHQ-12	0.965	2.394	0.017 *	0.152	1.552
	DTS-8	0.206	2.522	0.012 *	0.374	3.028
	AIS-8	0.179	2.636	0.009 *	0.369	2.541

* Statistical significance bilateral level 0.05; Abbreviation: IC, interval level of significance.

Table 4. Inferential analysis: analysis of variance (ANOVA).

Variables	Instrument Total Score	Level of Significance *p*
Gender	GHQ-12	0.000 *
	DTS-8	0.000 *
	AIS-8	0.000 *
Professional category	GHQ-12	0.107
	DTS-8	0.613
	AIS-8	0.310
Type of Service	GHQ-12	0.564
	DTS-8	0.244
	AIS-8	0.414
SARS-COV-2 test	GHQ-12	0.011 *
	DTS-8	0.001 *
	AIS-8	0.159
Withdrawn personal equipment	GHQ-12	0.026 *
	DTS-8	0.972
	AIS-8	0.993
Psychological support unit	GHQ-12	0.154
	DTS-8	0.868
	AIS-8	0.658

* Statistical significance at level 0.05.

In addition, a positive, although weak, relationship was found between time worked in the service and DTS-8 (r = 0.133, with a significance level p = 0.050), so that as the time worked in the service increased, so did the DTS-8 scale score, resulting in a greater risk of developing PTSD (Table 5).

Table 5. Inferential analysis: Pearson's *r* correlation coefficient.

Variables	GHQ-12 Total Score	DTS-8 Total Score	AIS-8 Total Score
Age	−0.068	−0.032	−0.088
Year of work experience	−0.049	0.133 *	0.091
Number of people per household	−0.015	0.043	0.041
Weight gained	0.024	0.024	0.081

* Statistical significance at level 0.05.

Considering the total scores on the instruments used (GHQ-12, DTS-8, and AIS-8), we sought to identify relationships between them and determined that there was a significant

correlation according to the Pearson's *r* coefficient for a bilateral level 0.01 of a moderate positive type between the total GHQ-12 score and the total DTS-8 score (r = 0.622), and between the total GHQ-12 score and the total AIS-8 score (*r* = 0.556). There was also a strong positive correlation between the DTS-8 total score and the AIS-8 total score (*r* = 0.724). Accordingly, higher GHQ-12 scores (poorer mental health) were related to higher DTS-8 scores (greater likelihood of PTSD) and vice versa. Higher GHQ-12 scores (poorer mental health) were related to higher AIS-8 scores (greater insomnia) and vice versa. Finally, higher DTS-8 scores (greater likelihood of PTSD) were related to higher AIS-8 scores (more insomnia) and vice versa.

4. Discussion

In light of the results obtained in this study, our working hypothesis, which proposed that the mental health of EMS workers in Castile y León and Madrid has worsened during the outbreak of the COVID-19 pandemic, seemed to hold. The potential presence of PTSD was found in 30.9% of the sample, which is significantly higher than values obtained before the pandemic in workers in other EMS, which had a prevalence of PTSD of 11% [28].

Outside the out-of-hospital emergency group, the data on the presence of PTSD in health professionals are lower than those found in our sample. During the epidemics of other coronavirus prior to the COVID-19 pandemic, PTSD symptoms were recorded in Canadian healthcare workers with a prevalence of 14.6% (Instrument: Revised Event Scale IES-R) [36], and among emergency personnel in Taiwan, the prevalence was 19.8% (Instrument: Davidson Trauma Scale-Chinese version (DTS-C) and Chinese Health Questionnaire-12 (CHQ-12)) [36]. In the meta-analysis conducted by Salehi et al. [37], two out of ten healthcare workers would have presented PTSD symptoms after epidemics of other coronavirus, and including the COVID-19 pandemic. This increases the percentages previously found but the values are still below those obtained in our sample. A review of the effects of work on health professionals during the pandemic shows that it is common for healthcare workers to recall images that have caused them distress, as was also the case in this study [38]. It is also common to find, in China, a lack of interest in activities that were previously of interest to doctors and nurses, as in our case [39].

The evaluation of the psychological impact on EMS workers in our study found psychological pathology in 36% of the sample. In addition, the greater the loss of psychological wellbeing, reflected by a higher GHQ-12 score, the higher the DTS-8 score. A negative relationship between decreased psychological wellbeing and PTSD diagnosis was found in this study, in line with previous findings in healthcare workers [40,41].

A total of 65.6% of the EMS workers surveyed in this study referred to anxiety symptoms during the early stages of the pandemic. These results are consistent with those observed both in the general population and among healthcare workers at the height of the pandemic. In China, elevated levels of fear, anxiety (30–70%) (Instrument: IES-R and Depression Anxiety and Stress Scale (DASS-21) [42–44], and depression (20.1%) have been described [45].

A study conducted in Denver, Colorado shows that, during the pandemic, frontline physicians suffered from a lack of confidence in their actions, as with the health professionals in our research, as well as a sense of helplessness and a feeling of uselessness in saving patients' lives [46].

In our study, concerns about the quality of PPE, which had to be removed because it did not meet current protection regulations, affected 78.9% of the respondents. Similarly, 75% of the sample reported being affected by the lack of initial information. The perception of insecurity and the fear of contagion caused an increase in anxiety, according to published works on the subject [47]. Although there was some research on PPE as early as May 2020 [48], in our case, the respondents accepted that, in some cases, protective equipment had to be recalled, possibly due to the shortages experienced during the first wave of the pandemic [49]. A study in China showed that training and coaching in the process of using personal protective equipment reduced stress among healthcare workers when they had to

use it. It would therefore be necessary for such training to be carried out but the required frequency has not been specified [50].

In some cases, the surveyed EMS workers admitted to having redacted information prior to the pandemic, although it was more common for such information to have been provided during the pandemic. Studies have shown that both too little and too much information can be detrimental to the mental health of the general population [51,52].

Insomnia levels reflect pathological sleep impairment in 60% of EMS workers. The prevalence in the general population is estimated at around 3.9 to 22% [48,49], and among healthcare workers in general during the COVID-19 outbreak, the prevalence of insomnia reached 36% (Instrument: Insomnia Severity Index (ISS7)) [50], which shows that in the sample of EMS professionals analyzed, the presence of sleep problems was quite high compared to the general population and other healthcare workers. The most frequent psychological response in a group of Irish healthcare workers was difficulty initiating sleep [53]. A sleep study conducted in Bahrain on healthcare workers during the pandemic found that the sleep quality of the participants was poor, the number of hours for which they stayed asleep was reduced, and awakenings were frequent [54], consistent with the results found in the present study sample.

The proportional and positive relationships found in this study in the results between the GHQ-12 and AIS-8 instruments reinforce the relationship established in the literature between a loss of psychological wellbeing and insomnia [55].

In Wuhan, according to Lai et al. [56], increased exposure in toilets led to more frequent symptoms of anxiety, depression, and insomnia. In our study, concerns about contagion, stemming from occupational exposure, were apparent in 94.3% of the respondents. In addition, the results of the study showed a positive relationship between the results obtained in the measurement of psychological health, PTSD, and insomnia and the concern regarding self-contagion. The score on the three instruments used was higher in the concerned sample.

Concern about transmitting the disease to family members was also present in the sample in more than 90% of the cases, most frequently among respondents living with four or more people. Concerns about contracting the disease and about transmitting it to close relatives have already been reflected in other published studies [56,57].

With regard to testing for SARS-CoV-2 infection in EMS professionals in Castilla y León and Madrid, the volume of tests carried out was similar to that found in other investigations [58,59]. The same was true for the presence of symptoms, highlighting that, in many cases, healthcare workers have been asymptomatic carriers [60]. The figures for isolation and hospital admission did not differ from those found by other authors among healthcare professionals in hospital emergency departments, who were also frontline staff [61].

A study that recruited participants through social media found that the general population during the pandemic gained between 5 and 10 pounds in body weight, equivalent to between 2.2 and 4.4 kg. In our sample, the average weight gain was 1 K, which is half the lower limit of weight gain found in the general population study [62]. This may be because the healthcare workers had not been confined to the same extent because they were working, but studies are needed to demonstrate this.

In the sample collected, 16% of EMS workers did not live with others during the pandemic. In the literature, it remains unclear whether living alone increases risk among healthcare workers. However, in the United Kingdom, women in the general population who lived alone and were confined were considered at risk. Being female, a nurse, and belonging to the younger subgroup of workers are factors associated with an increased risk in several studies [63]. Consistent with these findings, women in our study showed a greater loss of psychological wellbeing, PTSD symptoms, and insomnia, which leads us to affirm that gender is a predisposing factor for PTSD risk in our sample.

Nonetheless, the most affected professional category in our sample was emergency health technicians. The studies cited above do not specifically mention the involvement of

emergency medical technicians. However, emergency health technicians have previously shown worrying results in other surveys worldwide [64,65], and their situation may have worsened in the current context probably due to physical strain and increased workload, as may also have happened in other professional categories [66,67]. All these factors may contribute to the appearance of negative effects, in the short or long term, in a population already at risk [68,69].

There are no known previous studies of this type in the SEMs studied in this research, nor have we found any studies that take into account the modality of work (BLS and ALS), so it has not been possible to carry out comparisons with other authors' studies.

The most frequent demographic in our study was middle-aged individuals (40–49 years) with between 10 and 20 years of experience. In this study, the more experience that the healthcare workers (>20 years) had the less affected they were. Years of work experience has previously been related to resilience and identified as a protective factor [69].

Given the overwhelming presence of signs and, in some cases, clear alterations in the psychological health of out-of-hospital emergency workers in the communities studied, the need for support and prevention programs during crises such as the COVID-19 pandemic seems reasonable, as confirmed by other research [70–73].

The importance of identifying the underlying factors to prevent and treat before the onset of traumatic symptoms and to reduce the future consequences of PTSD is highlighted [74]. Likewise, scientific recommendations from mental health experts to maintain contact with family and friends, share information with colleagues in a positive way, schedule routines outside of work, be aware of one's feelings and emotions, maintain healthy lifestyle habits, and allow oneself to ask for help are suitable [75,76].

This research has a number of limitations. The first is due to the use of a descriptive crosssectional design that only provides a static picture of the problem but can serve as a basis for longitudinal studies. Therefore, it does not allow causality to be established between the factors and the risk of PTSD; it is consequently not possible to extrapolate the results to other populations. The second lies in the fact that the study sample was nonrandomly selected, mainly due to the epidemiological situation at the time, which precluded any other form of sampling, although it must be accepted that the sample may be biased. Despite this, it was a moderately large sample taking into account the small number of EMS workers compared to other levels of care, which tends to limit the biases that could arise from the selection and recall of participants. Finally, although information on previous mental health status was collected, this aspect was not taken into account when determining the correlations between the variables. In future research, it would be advisable to evaluate PTSD with a random sample that includes more professionals from other EMS to ensure the veracity of the results, using a random sample and a design that allows us to establish causal relationships, considering the previous mental health of the workers.

This study confirms that the COVID-19 pandemic has been a traumatic event for EMS workers having an impact on their wellbeing. One-third of them have manifested a potential diagnosis of PTSD in the early phases of the pandemic. In addition, stress, signs of psychological distress, and insomnia have been shown to be common among out-of-hospital healthcare workers.

Respondents most affected by PTSD were women, those aged 40 to 49 years, emergency health technicians, those with 10 to 20 years of experience, and those living with four or more people.

Both gender and concern about becoming infected are related to the risk of suffering from psychological pathology, PTSD, and insomnia.

In relation to the implications of the study for clinical practice, the authors defend the need to followup with those individuals who report a deterioration in their psychological health due to their professional work in the pandemic. We propose the creation of specific mental health programs for events similar to COVID 19 in the future, prioritizing mental health. This also requires an increase in public health human resources to cover the mental

health care of out-of-hospital emergency health professionals. Given that the training of professionals to prepare them to face these situations is not adequate, it could be suggested that health training programs include this topic. In addition, coping strategies should be promoted for this group in order to better enable them to respond to traumatic events similar to those experienced during the pandemic. To achieve this, it would be necessary to establish a support unit for workers' mental health and to establish training courses within the working day that allow them to acquire these skills.

The paper adds a novel topic to the scientific literature, as the mental health of healthcare workers during the COVID-19 pandemic has been studied previously but not in out-of-hospital emergency personnel. Moreover, studies are usually limited to samples composed of doctors and nurses, without taking into account other healthcare professionals such as emergency health technicians. In addition, the timing of data collection is noteworthy as it took place relatively close to the end of the first wave of the pandemic, which gave an idea of how these staff were coping at that time and allows comparison with later stages.

A future line of research for the research team would be to reassess the same variables in the sample after the current fifth wave and thus obtain an idea of the evolution of the mental health of these out-of-hospital emergency healthcare professionals. In addition, we aim to carry out a qualitative study that will provide in-depth knowledge of the experiences of healthcare workers in their work during the SARS-CoV-2 pandemic.

5. Conclusions

This study confirms that the COVID-19 pandemic may have been a traumatic event for emergency workers. One-third of them reported a possible diagnosis of PTSD in emergency health workers who performed their professional work in the early phases of the pandemic. PTSD needs to be confirmed by further assessment, as the number may have increased or decreased after months of exposure to such a traumatic situation. In addition, stress, signs of psychological pathology, and insomnia have been shown to be common among out-of-hospital healthcare workers at the time of the research.

Respondents most affected by possible PTSD were women, those aged 40–49, emergency health technicians, those with 10–20 years of experience, and those living with four or more people. Both gender and preoccupation with illness were associated with the risk of psychological distress, the likelihood of PTSD, and insomnia.

The authors argue for the need to followup with those individuals who report a deterioration of their psychological health due to their professional work in the pandemic through specific health programs, as well as the prevention of such deterioration during future traumatic events similar to the COVID-19 pandemic.

Author Contributions: Conceptualization, C.M.M.-C. and R.M.C.-G.; methodology, R.V.-M., M.T.H.-F.; software, R.M.C.-G.; validation, L.M.G.-S., J.M.-P., L.P.-P. and M.A.M.-F.; formal analysis, R.M.C.-G.; investigation, C.M.M.-C., M.Á.B.-P. and E.O.; resources, C.D.-F. and M.T.H.-F.; data curation, L.P.-P. and C.D.-F.; writing—original draft preparation, C.M.M.-C., R.M.C.-G. and M.T.H.-F.; writing—review and editing, R.M.C.-G., J.M.-P. and J.J.F.-C.; visualization, C.D.-F.; supervision, R.M.C.-G. All authors have read and agreed to the published version of the manuscript.

Funding: This research received no external funding.

Institutional Review Board Statement: The study was conducted according to the guidelines of the Declaration of Helsinki and approved by the Ethics Committee for Drug Research of the Valladolid West Health Area (PI 139-20 NO HRHV on 6 June 2020).

Informed Consent Statement: Informed consent was obtained from all subjects involved in the study.

Data Availability Statement: The data presented in this study are available on request from the corresponding author.

Conflicts of Interest: The authors declare no conflict of interest. The funders had no role in the design of the study; in the collection, analyses, or interpretation of data; in the writing of the manuscript, or in the decision to publish the results.

References

1. Oliver, N.; Barber, X.; Roomp, K.; Roomp, K. Assessing the impact of the COVID-19 pandemic in Spain: Large-scale, online, self-reported population survey. *J. Med. Internet Res.* **2020**, *22*, e21319. [CrossRef] [PubMed]
2. Pollán, M.; Pérez-Gómez, B.; Pastor-Barriuso, R.; Oteo, J.; Hernán, M.A.; Pérez-Olmeda, M.; Sanmartín, J.L.; Fernández-García, A.; Cruz, I.; de Larrea, N.F.; et al. Prevalence of SARS-CoV-2 in Spain (ENE-COVID): A nationwide, population-based seroepidemiological study. *Lancet* **2020**, *396*, 535–544. [CrossRef]
3. Boletín Oficial del Estado. Real Decreto 463/2020, de 14 de Marzo, por el que se Declara el Estado de Alarma para la Gestión de la Situación de Crisis Sanitaria Ocasionada por el COVID-19. Available online: https://www.boe.es/eli/es/rd/2020/03/14/463 (accessed on 24 June 2021).
4. The Lancet Public Health. COVID-19 in Spain: A predictable storm? *Lancet* **2020**, *5*, e568.
5. World Health Organization. Coronavirus Disease (COVID-2019) Situation Reports. Available online: https://www.who.int/emergencies/diseases/novel-coronavirus-2019/situation-reports (accessed on 26 June 2021).
6. Mitjà, O.; Arenas, A.; Rodó, X.; Tobias, A.; Brew, J.; Benlloch, J.M. Experts' request to the Spanish Government: Move Spain towards complete lockdown. *Lancet* **2020**, *395*, 1193–1194. [CrossRef]
7. Boletín Oficial del Estado. Available online: https://boe.es/boe/dias/2021/06/25/ (accessed on 26 June 2021).
8. American Psychiatric Association. *DSM-5. Guía de Consulta de los Criterios Diagnosticos*, 5th ed.; Panamericana: Madrid, Spain, 2018.
9. Vance, M.C.; Kovachy, B.; Dong, M.; Bui, E. Peritraumatic distress: A review and synthesis of 15 years of research. *J. Clin. Psychol.* **2018**, *74*, 1457–1484. [CrossRef] [PubMed]
10. Berger, W.; Coutinho, E.S.F.; Figueira, I.; Marques-Portella, C.; Luz, M.P.; Neylan, T.C.; Marmar, C.R.; Mendlowicz, M.V. Rescuers at risk: A systematic review and meta-regression analysis of the worldwide current prevalence and correlates of PTSD in rescue workers. *Soc. Psychiatry Psychiatr. Epidemiol.* **2012**, *47*, 1001–1011. [CrossRef] [PubMed]
11. Preti, E.; Di Mattei, V.; Perego, G.; Ferrari, F.; Mazzetti, M.; Taranto, P.; Di Pierro, R.; Madeddu, F.; Calati, R. The Psychological Impact of Epidemic and Pandemic Outbreaks on Healthcare Workers: Rapid Review of the Evidence. *Curr. Psychiatry Rep.* **2020**, *22*, 1–22. [CrossRef]
12. Ramírez-Santana, M.; Aguirre, J.; Carvajal, N. Formación médica para afrontar emergencias y desastres: Experiencia de enseñanza-aprendizaje con trabajo colaborativo, uso de tecnologías de información y comunicación, y simulación. *FEM* **2020**, *23*, 243–249. [CrossRef]
13. Ward, C.L.; Lombard, C.J.; Gwebushe, N. Critical incident exposure in South African emergency services personnel: Prevalence and associated mental health issues. *Emerg. Med. J.* **2006**, *23*, 226–231. [CrossRef] [PubMed]
14. Carmassi, C.; Foghi, C.; Dell'Oste, V.; Cordone, A.; Bertelloni, C.A.; Bui, E.; Dell'Osso, L. PTSD symptoms in healthcare workers facing the three coronavirus outbreaks: What can we expect after the COVID-19 pandemic. *Psychiatry Res.* **2020**, *292*, 113312. [CrossRef]
15. Mojica-Crespo, R.; Morales-Crespo, M.M. Pandemic COVID-19, the new health emergency of international concern: A review. *Semergen* **2020**, *46*, 65–77. [CrossRef] [PubMed]
16. Iglesias Vázquez, J.A.; Echarri-Sucunza, A.; Ruíz-Azpiazu, J.I.; Pastrana-Blanco, J.L.; Guirao-Salinas, F.A.; Escriche-López, C.; Jiménez-Fábregas, X. Análisis de la organización y preparación para la respuesta ante la pandemia de COVID-19 por los ser-vicios médicos de emergencias extrahospitalarias en España. *Emergencias* **2021**, *33*, 151–153. [PubMed]
17. Gracía-Iglesias, J.J.; Gómez-Salgado, J.; Martín-Pereira, J.; Fagundo-Rivera, J.; Ayuso-Murillo, D.; Martínez-Riera, J.R.; Ruiz-Frutos, C. Impact of SARS-CoV-2 (Covid-19) on the mental health of healthcare professionals: A systematic review. *Rev. Esp. Salud Publica* **2020**, *94*, e202007088.
18. Fan, F.C.; Zhang, S.Y.; Cheng, Y. Incidence of psychological illness after coronavirus outbreak: A meta-analysis study. *J. Epidemiol. Community Health* **2021**, *75*, 836–842. [CrossRef] [PubMed]
19. Matalon, N.; Dorman-Ilan, S.; Hasson-Ohayon, I.; Hertz-Palmor, N.; Shani, S.; Basel, D.; Gross, R.; Chen, W.; Abramovich, A.; Afek, A.; et al. Trajectories of post-traumatic stress symptoms, anxiety, and depression in hospitalized COVID-19 patients: A one-month follow-up. *J. Psychosom. Res.* **2021**, *143*, 110399. [CrossRef] [PubMed]
20. Taquet, M.; Luciano, S.; Geddes, J.R.; Harrison, P.J. Bidirectional associations between COVID-19 and psychiatric disorder: Retrospective cohort studies of 62,354 COVID-19 cases in the USA. *Lancet Psychiatry* **2020**, *8*, 130–140. [CrossRef]
21. Vindegaard, N.; Benros, M.E. COVID-19 pandemic and mental health consequences: Systematic review of the current evidence. *Brain Behav. Immun.* **2020**, *89*, 531–542. [CrossRef]
22. Pappa, S.; Ntella, V.; Giannakas, T.; Giannakoulis, V.; Papoutsi, E.; Katsaounou, P. Prevalence of depression, anxiety, and insomnia among healthcare workers during the COVID-19 pandemic: A systematic review and meta-analysis. *Brain Behav. Immun.* **2020**, *88*, 901–907. [CrossRef] [PubMed]

23. Chew, N.W.; Lee, G.K.; Tan, B.Y.; Jing, M.; Goh, Y.; Ngiam, N.J.; Yeo, L.; Ahmad, A.; Khan, F.A.; Shanmugam, G.N.; et al. A multinational, multicentre study on the psychological outcomes and associated physical symptoms amongst healthcare workers during COVID-19 outbreak. *Brain Behav. Immun.* **2020**, *88*, 559–565. [CrossRef] [PubMed]
24. Danet, A.D. Psychological impact of COVID-19 pandemic in Western frontline healthcare professionals. A systematic review. *Med. Clin.* **2021**, *156*, 449–458. [CrossRef]
25. El-Hage, W.; Hingray, C.; Lemogne, C.; Yrondi, A.; Brunault, P.; Bienvenu, T.; Etain, B.; Paquet, C.; Gohier, B.; Bennabi, D.; et al. Health professionals facing the coronavirus disease 2019 (COVID-19) pandemic: What are the mental health risks? *Encephale* **2020**, *46*, S73–S80. [CrossRef]
26. Burillo-Putze, G.; Espinosa-Ramírez, S.; Lecuona-Fernández, M. Personal protection against COVID-19 in the emergency department: Neither heroes nor cowards. *Emergencias* **2020**, *32*, 223–224. [PubMed]
27. Calvo-Bonacho, E.; Catalina-Romero, C.; Fernández-Labandera, C.; Fernández-Meseguer, A.; González-Quintela, A.; Martínez-Muñoz, P.; Quevedo, L.; Valdivielso, P.; Sánchez-Chaparro, M.A. COVID-19 and Sick Leave: An Analysis of the Ibermutua Cohort of Over 1,651,305 Spanish Workers in the First Trimester of 2020. *Front. Public Health* **2020**, *8*, 580546. [CrossRef]
28. Eiche, C.; Birkholz, T.; Jobst, E.; Gall, C.; Prottengeier, J. Well-being and PTSD in German emergency medical services—A nationwide cross-sectional survey. *PLoS ONE* **2019**, *14*, e0220154. [CrossRef] [PubMed]
29. Petrie, K.; Milligan-Saville, J.; Gayed, A.; Deady, M.; Phelps, A.; Dell, L.; Forbes, D.; Bryant, R.A.; Calvo, R.A.; Glozier, N.; et al. Prevalence of PTSD and common mental disorders amongst ambulance personnel: A systematic review and meta-analysis. *Soc. Psychiatry Psychiatr. Epidemiol.* **2018**, *53*, 897–909. [CrossRef] [PubMed]
30. Vandenbroucke, J.P.; Von Elm, E.; Altman, D.G.; Gøtzsche, P.C.; Mulrow, C.D.; Pocock, S.J.; Poole, C.; Schlesselman, J.J.; Egger, M. Fortalecimiento de la presentación de informes de estudios observacionales en epidemiología (STROBE): Explicación y elaboración. *Gac. Sanit.* **2009**, *23*, 158.e1–158.e28. [CrossRef] [PubMed]
31. Goldberg, D. *Manual of the General Health Questionnaire*; NFER Publishing Company: Windsor, UK, 1978.
32. Sánchez-López, M.P.; Dresch, V. The 12-Item General Health Questionnaire (GHQ-12): Reliability, external validity and factor structure in the Spanish population. *Psicothema* **2008**, *20*, 839–843.
33. Davidson, J.R.T.; Colket, J.T. The eight-item treatment-outcome post-traumatic stress disorder scale: A brief measure to assess treatment outcome in post-traumatic stress disorder. *Int. Clin. Psychopharmacol.* **1997**, *12*, 41–46. [CrossRef] [PubMed]
34. Bobes, J.; Calcedo-Barba, A.; García, M.; François, M.; Rico-Villademoros, F.; González, M.P.; Bascarán, M.T.; Bousoño, M. Evaluación de las propiedades psicométricas de la versión española de cinco cuestionarios para la evaluación del trastorno de estrés postraumático. *Actas Esp. Psiquiatr.* **2000**, *28*, 207–218.
35. Soldatos, C.R.; Dikeos, D.G.; Paparrigopoulos, T.J. Athens Insomnia Scale: Validation of an instrument based on ICD-10 criteria. *J. Psychosom. Res.* **2020**, *48*, 555–560. [CrossRef]
36. Reynolds, D.L.; Garay, J.R.; Deamond, S.L.; Moran, M.K.; Gold, W.; Styra, R. Understanding, compliance and psychological impact of the SARS quarantine experience. *Epidemiol. Infect.* **2007**, *136*, 997–1007. [CrossRef] [PubMed]
37. Lin, C.-Y.; Peng, Y.-C.; Wu, Y.-H.; Chang, J.; Chan, C.-H.; Yang, D.-Y. The psychological effect of severe acute respiratory syndrome on emergency department staff. *Emerg. Med. J.* **2007**, *24*, 12–17. [CrossRef] [PubMed]
38. Salehi, M.; Amanat, M.; Mohammadi, M.; Salmanian, M.; Rezaei, N.; Saghazadeh, A.; Garakani, A. The prevalence of post-traumatic stress disorder related symptoms in Coronavirus outbreaks: A systematic-review and meta-analysis. *J. Affect. Disord.* **2021**, *282*, 527–538. [CrossRef] [PubMed]
39. Giorgi, G.; Lecca, L.I.; Alessio, F.; Finstad, G.L.; Bondanini, G.; Lulli, L.G.; Arcangeli, G.; Mucci, N. COVID-19-Related Mental Health Effects in the Workplace: A Narrative Review. *Int. J. Environ. Res. Public Health* **2020**, *17*, 7857. [CrossRef] [PubMed]
40. Kang, L.; Ma, S.; Chen, M.; Yang, J.; Wang, Y.; Li, R.; Yao, L.; Bai, H.; Cai, Z.; Yang, B.X.; et al. Impact on mental health and perceptions of psychological care among medical and nursing staff in Wuhan during the 2019 novel coronavirus disease outbreak: A cross-sectional study. *Brain Behav. Immun.* **2020**, *87*, 11–17. [CrossRef] [PubMed]
41. Castillo, D.; Ovalle, L.; Oyola, P.; Garavito, C. La resiliencia como estrategia de afrontamiento ante el estrés postraumático en combatientes. *Rev. Enfoques* **2016**, *2*, 83–90. [CrossRef]
42. Braquehais, M.D.; Vargas-Cáceres, S.; Gómez-Durán, E.; Nieva, G.; Valero, S.; Casas, M.; Bruguera, E. The impact of the COVID-19 pandemic on the mental health of healthcare professionals. *QJM* **2020**, *113*, 613–617. [CrossRef] [PubMed]
43. Song, X.; Fu, W.; Liu, X.; Luo, Z.; Wang, R.; Zhou, N.; Yan, S.; Lv, C. Mental health status of medical staff in emergency departments during the Coronavirus disease 2019 epidemic in China. *Brain Behav. Immun.* **2020**, *88*, 60–65. [CrossRef]
44. Wu, D.; Jiang, C.; He, C.; Li, C.; Yang, L.; Yue, Y. Stressors of nurses in psychiatric hospitals during the COVID-19 outbreak. *Psychiatry Res.* **2020**, *288*, 112956. [CrossRef]
45. Huang, Y.; Zhao, N. Generalized anxiety disorder, depressive symptoms and sleep quality during COVID-19 outbreak in China: A web-based cross-sectional survey. *Psychiatry Res.* **2020**, *288*, 112954. [CrossRef]
46. Rao, H.; Mancini, D.; Tong, A.; Khan, H.; Gutierrez, B.S.; Mundo, W.; Collings, A.; Cervantes, L. Frontline interdisciplinary clinician perspectives on caring for patients with COVID-19: A qualitative study. *BMJ Open* **2021**, *11*, e048712. [CrossRef] [PubMed]
47. Shahzad, F.; Du, J.; Khan, I.; Fateh, A.; Shahbaz, M.; Abbas, A.; Wattoo, M.U. Perceived Threat of COVID-19 Contagion and Frontline Paramedics' Agonistic Behaviour: Employing a Stressor–Strain–Outcome Perspective. *Int. J. Environ. Res. Public Health* **2020**, *17*, 5102. [CrossRef] [PubMed]

48. Vico-Romero, J.; Cabré-Roure, M.; Monteis-Cahis, R.; Palomera-Faneges, E.; Serra-Prat, M. Prevalencia de trastornos del sueño y factores asociados en pacientes hospitalizados. *Enferm. Clin.* **2014**, *24*, 276–282. [CrossRef] [PubMed]
49. Li, Y.; Qin, Q.; Sun, Q.; Sanford, L.D.; Vgontzas, A.N.; Tang, X. Insomnia and psychological reactions during the COVID-19 outbreak in China. *J. Clin. Sleep Med.* **2020**, *16*, 1417–1418. [CrossRef]
50. Buchholz, Y. COVID-19: Insomnio, depresión y ansiedad en el personal sanitario. *Mente Cereb.* **2020**, *120*, 30–31.
51. Farooq, A.R.; Fareeha, F. Information Overload and Infodemic in the COVID-19 Pandemic. *J. Pak. Med. Assoc.* **2020**, *70*, S162–S165.
52. Rovetta, A.; Bhagavathula, A.S. Global Infodemiology of COVID-19: Analysis of Google Web Searches and Instagram Hashtags. *J. Med. Internet Res.* **2020**, *22*, e20673. [CrossRef]
53. Fortune, D.G.; Richards, H.L.; Wormald, A.; Connor, K.O.; McKiernan, M.; Najt, P.; Dwyer, A.O.; Dea, E.O.; Burke, P.; Eustace, J. An investigation of psychological responses to COVID-19 in Irish healthcare workers: Longitudinal quantitative and nested qualitative study. *HRB Open Res.* **2021**, *4*, 15. [CrossRef] [PubMed]
54. Jahrami, H.; BaHammam, A.S.; AlGahtani, H.; Ebrahim, A.; Faris, M.; AlEid, K.; Saif, Z.; Haji, E.; Dhahi, A.; Marzooq, H.; et al. The examination of sleep quality for frontline healthcare workers during the outbreak of COVID-19. *Sleep Breath.* **2020**, *25*, 503–511. [CrossRef]
55. Lai, J.; Ma, S.; Wang, Y.; Cai, Z.; Hu, J.; Wei, N.; Wu, J.; Du, H.; Chen, T.; Li, R.; et al. Factors Associated with Mental Health Outcomes among Health Care Workers Exposed to Coronavirus Disease 2019. *JAMA Netw. Open* **2020**, *3*, e203976. [CrossRef] [PubMed]
56. Sandín, B.; Valiente, R.M.; García-Escalera, J.; Chorot, P. Impacto psicológico de la pandemia de COVID-19: Efectos negativos y positivos en población española asociados al periodo de confinamiento nacional. *Rev. Psicopatol. Psicol. Clin.* **2020**, *25*, 1–22. [CrossRef]
57. Lumley, S.F.; O'Donnell, D.; Stoesser, N.E.; Matthews, P.C.; Howarth, A.; Stephanie, B.H.; Marsden, B.D.; Cox, S.; James, T.; Warren, F.; et al. Antibody Status and Incidence of SARS-CoV-2 Infection in Health Care Workers. *N. Engl. J. Med.* **2021**, *384*, 533–540. [CrossRef] [PubMed]
58. Martin, C.; Montesinos, I. Dynamics of SARS-CoV-2 RT-PCR positivity and seroprevalence among high-risk healthcare workers and hospital staff. *J. Hosp. Infect.* **2020**, *106*, 102–106. [CrossRef] [PubMed]
59. Rivett, L.; Sridhar, S.; Sparkes, D.; Routledge, M.; Jones, N.K.; Forrest, S.; Young, J.; Pereira-Dias, J.; Hamilton, W.L.; Ferris, M.; et al. Screening of healthcare workers for SARS-CoV-2 highlights the role of asymptomatic carriage in COVID-19 transmission. *Epidemiol. Glob. Health* **2020**, *9*, e58728.
60. Gómez-Ochoa, S.A.; Franco, O.H.; Rojas, L.Z.; Raguindin, P.F.; Roa-Díaz, Z.M.; Wyssmann, B.M.; Guevara, S.L.R.; Echeverría, L.E.; Glisic, M.; Muka, T. COVID-19 in Health-Care Workers: A Living Systematic Review and Meta-Analysis of Prevalence, Risk Factors, Clinical Characteristics, and Outcomes. *Am. J. Epidemiol.* **2020**, *190*, 161–175. [CrossRef] [PubMed]
61. Chico-Sánchez, P.; Gras-Valentí, P.; Mora-Muriel, J.G.; Algado-Sellés, N.; Sánchez-Payá, J.; Llorens, P. Impact of the COVID-19 pandemic on health care workers in a tertiary care hospital emergency department. *Emergencias* **2020**, *32*, 227–232. [PubMed]
62. Zachary, Z.; Brianna, F.; Brianna, L.; Garrett, P.; Jade, W.; Alyssa, D.; Mikayla, K. Self-quarantine and weight gain related risk factors during the COVID-19 pandemic. *Obes. Res. Clin. Pract.* **2020**, *14*, 210–216. [CrossRef]
63. Silva, D.F.O.; Cobucci, R.N. Prevalência de ansiedade em profissionais da saúde em tempos de COVID-19: Revisão sistemática com metanálise. *Cienc. Saude Coletiva* **2021**, *26*, 693–710. [CrossRef] [PubMed]
64. Almutairi, I.; Al-Rashdi, M.; Almutairi, A. Prevalence and predictors of depression, anxiety and stress symptoms in paramedics at saudi red crescent authority. *Saudi J. Med. Med. Sci.* **2020**, *8*, 105–111. [CrossRef]
65. Bentley, M.A.; Mac Crawford, J.; Wilkins, J.R.; Fernandez, A.R.; Studnek, J.R. An Assessment of Depression, Anxiety, and Stress among Nationally Certified EMS Professionals. *Prehospital Emerg. Care* **2013**, *17*, 330–338. [CrossRef]
66. Dutheil, F.; Mondillon, L.; Navel, V. PTSD as the second tsunami of the SARS-Cov-2 pandemic. *Psychol. Med.* **2020**, 1–2. [CrossRef] [PubMed]
67. Hannigan, B.; Nolan, F.; Chambers, M.; Turner, J. Mental health nursing at a time of crisis. *J. Psychiatr. Ment. Health Nurs.* **2021**, *28*, 123–125. [CrossRef] [PubMed]
68. Jakovljevic, M.; Bjedov, S.; Jaksic, N.; Jakovljevic, I. COVID-19 pandemia and public and global mental health from the per-spective of global health security. *Psychiatr. Danub.* **2020**, *32*, 6–14. [CrossRef] [PubMed]
69. Musso, M.; Tatum, D.; Hamer, D.; Hammarlund, R.; Son, L.; McMahon, P. The Relationship between Grit and Resilience in Emergency Medical Service Personnel. *Ochsner J.* **2019**, *19*, 199–203. [CrossRef] [PubMed]
70. Cheung, T.; Fong, T.K.; Bressington, D. COVID-19 under the SARS Cloud: Mental Health Nursing during the Pandemic in Hong Kong. *J. Psychiatr. Ment. Health Nurs.* **2020**, *28*, 115–117. [CrossRef]
71. Cuidando a los Profesionales. Available online: https://www.semes.org/informacion-para-profesionales-sobre-covid-19/cuidando-a-los-profesionales/ (accessed on 28 June 2021).
72. Cuidemos Nuestra Salud Mental. Available online: https://www.saludcastillayleon.es/HRHortega/es/actualidad/servicio-psiquiatria-salud-mental-elaborado-guia-ayudar-san (accessed on 24 June 2021).
73. Cuidando la Salud Mental del Personal Sanitario. Available online: http://www.sepsiq.org/file/InformacionSM/SEP%20COVID19-Salud%20Mental%20personal%20sanitario.pdf (accessed on 28 June 2021).
74. Blake, H.; Bermingham, F.; Johnson, G.; Tabner, A. Mitigating the Psychological Impact of COVID-19 on Healthcare Workers: A Digital Learning Package. *Int. J. Environ. Res. Public Health* **2020**, *17*, 2997. [CrossRef]

75. Bendau, A.; Ströhle, A.; Petzold, M.B. Salud mental en profesionales de la salud en la pandemia COVID-19. *Adv. Exp. Med. Biol.* **2021**, *1318*, 737–757.
76. López-Bueno, R.; Calatayud, J.; Ezzatvar, Y.; Casajús, J.A.; Smith, L.; Andersen, L.L.; Sánchez, G.F.L. Association between Current Physical Activity and Current Perceived Anxiety and Mood in the Initial Phase of COVID-19 Confinement. *Front. Psychiatry* **2020**, *11*, 729. [CrossRef] [PubMed]
