# Supplementary material for: Mental health status and related factors influencing healthcare workers during the COVID-19 pandemic: A systematic review and meta-analysis
Source: PLoS One. 2024 Jan 19;19(1):e0289454. doi: 10.1371/journal.pone.0289454 (PMC10798549; doi:10.1371/journal.pone.0289454)
Supplement: S1 Data — (ZIP) [file pone.0289454.s011.zip › literatures/89.pdf]

Received: 16 September 2021 | Accepted: 16 April 2022
DOI: 10.1111/inr.12764

International Nursing Review
WILEY

ORIGINAL ARTICLE

Mental health of Spanish nurses working during the COVID-19 pandemic: A cross-sectional study

Leticia San Martin-Rodriguez PhD, RN, Associate Professor, Researcher[1,2] | **Paula Escalda-Hernandez PhD, RN, Associate Professor, Researcher**[1,2] | **Nelia Soto-Ruiz PhD, RN, Associate Professor, Researcher**[1,2] | **Marta Ferraz-Torres PhD, RN, Lecturer, Researcher**[1,2] | **Irati Rodriguez-Matesanz RN, Research Fellow, Researcher**[1,3] | **Cristina Garcia-Vivar PhD, RN Senior Associate Professor, Researcher**[1,2]

[1]Department of Health Sciences, Public University of Navarre, Navarra, Spain

[2]IdiSNA, Navarra Institute for Health Research, Navarra, Spain

[3]Primary Care Research Group, BioDonostia Health Research Institute, Navarra, Spain

Correspondence
Marta Ferraz-Torres, Department of Health Sciences, Public University of Navarre. Avda. Barañain S/N – 31008 Pamplona, Navarra, Spain.
Email: marta.ferraz@unavarra.es

Funding information
Department of Health of the Government of Navarre (Spain), Grant/Award Number: 0011-3638-2020-000005

Abstract

Aim: To explore the impact of the COVID-19 pandemic on the mental health of nurses working in primary, secondary, and tertiary healthcare centers in Navarre (Spain).
Background: Healthcare workers, especially nurses, are at high risk for developing mental health problems during the COVID-19 pandemic.
Introduction: Spain ranks among the European countries with the highest incidence of and mortality from COVID-19 and has a 31% deficit in the number of nurses compared with the average for the European Union.
Methods: This was a cross-sectional study involving 800 Registered Nurses in Navarre, Spain. Four standardized instruments, along with a self-administered online questionnaire, were used to measure the impact in terms of depression, anxiety, insomnia, and posttraumatic stress disorder. The STROBE checklist for cross-sectional studies was used to report this study.
Results: Of the 800 nurses, 68% had some level of depression, anxiety, insomnia, and distress, and of these, 38% had moderate or severe symptoms. Those who worked in hospital COVID units and in nursing homes showed a higher impact on their mental health.
Discussion: The sustained pressure that nurses have experienced in their work during the COVID-19 pandemic has negatively affected their mental health.
Conclusion: This study found that nurses who worked in hospital COVID units and in nursing homes during the pandemic had worse mental health outcomes.
Implications for nursing/policy: Recommendations for nursing policy include the need to implement coaching and emotional programs to support nurses on the frontlines of the pandemic. There is also an urgent need for the implementation of national training programs to strengthen health emergency preparedness, improve response capacity, and increase the resilience of nurses to disasters.

KEYWORDS
anxiety, COVID-19, cross-sectional, depression, healthcare levels, insomnia, mental health, nurses, posttraumatic stress, Spain

This is an open access article under the terms of the Creative Commons Attribution-NonCommercial-NoDerivs License, which permits use and distribution in any medium, provided the original work is properly cited, the use is non-commercial and no modifications or adaptations are made.
© 2022 The Authors. *International Nursing Review* published by John Wiley & Sons Ltd on behalf of International Council of Nurses.

INTRODUCTION

From March 2020 to December 3, 2021, there were 263 563 622 confirmed cases of COVID-19 worldwide, including 5 232 562 deaths (WHO, 2021). The COVID-19 pandemic has caused an unprecedented global health crisis that has had a great impact on healthcare systems (Erdem & Lucey, 2021). The rapid increase in the number of patients diagnosed with COVID-19 has resulted in hospital saturation (Olivas-Martínez et al., 2021) and in high care pressure in nursing homes and long-term care facilities (Davidson & Szanton, 2020).

Front-line nurses and other healthcare workers (HCWs) have had to care for seriously ill patients with severe respiratory distress, often in poor working conditions such as high workload and shortages of personal protective equipment (Saragih et al., 2021). In many countries, nurses comprise the group of HCWs with the highest rates of COVID-19 infections, leading to unprecedented stress and risks to nurses' health as a result of the ongoing pandemic (Sabetian et al., 2021). This work environment has caused an increased risk for HCWs to be infected with COVID-19 (Nguyen et al., 2020). Added to this stressful situation-for some professionals-was the experience of social isolation and discrimination due to coronaphobia (Arora et al., 2020; Chen et al., 2020; García-Iglesias et al., 2020).

Among HCWs, nurses have been found to have the greatest psychological problems as a consequence of the COVID-19 outbreak (Carriero et al., 2021). Sleep disorders, anxiety, depression, and posttraumatic stress are the negative consequences experienced by nurses during the pandemic (Labrague & de Los Santos, 2021; Saragih et al., 2021). Variables such as being younger, being female, and having limited access to personal protective equipment seem to be related to a worse mental health status among nurses (Zhang et al., 2020). That is why the International Council of Nurses (ICN) called on governments to take urgent action to safeguard the physical and mental health of nurses worldwide (ICN, 2021).

Spain has been one of the European countries most impacted by the COVID-19 pandemic. During the first wave of the pandemic, Spain and Italy were epicenters of the health crisis with high percentages of patients infected with COVID-19 and saturation of hospitals (European Centre for Disease Prevention and Control (ECDC) 2021). Although the health situation has been improving since the beginning of the COVID-19 pandemic (March 2020), there have been several COVID-19 waves with consequences for health professionals and nurses in particular. It should be noted that Spain already had a worrying shortage of nurses before the pandemic, as it had a 31% deficit in the number of nurses compared with the average for the European Union (574 vs 802 nurses/100, 000 inhabitants) (Arévalo-Manso, 2019). The pandemic has further aggravated the situation due to the high number of patients infected with COVID-19 (ECDC, 2021), along with the shortage of nurses and deficiencies in health services (Castro-Sánchez & Santillán-García, 2020), increased job demands and workload, resulting in distress and a significant psychological impact on nurses (Pérez-Raya et al., 2021).

Although, there is evidence regarding the impact of COVID-19 on the mental health of nurses in Spain, the samples in these studies included a variety of professionals such as doctors, nurses, nursing assistants, and nursing students. Consequently, the number of nurses among the participants was low and mainly comprised hospital nurses (Hummel et al., 2021; Manzanares et al., 2021). The study conducted by the Spanish General Council of Nursing, in which 11 560 Registered Nurses (RNs) from different services participated three weeks after the first declaration of the state of emergency in Spain in March 2020, provides a broad view of the working conditions and experiences of nurses during the onset of COVID-19 (Pérez-Raya et al., 2021). However, there are several gaps that need to be explored: (1) what is the impact on nurses one year after the beginning of the health crisis due to COVID-19; (2) what is the impact of COVID-19 on nurses according to where they have worked; and (3) there is a need of studies with a large sample size of only nurses.

To inform policymakers and managers responsible for implementing government policies around nurse protections and capacity development during the COVID-19 pandemic and beyond, it is necessary to understand the experience of nurses throughout the ongoing pandemic. Furthermore, it is necessary to investigate the psychological impact not only of the nurses who have worked in the hospital but also of the nurses who have worked in primary healthcare centers and nursing homes.

Aim

This study aimed to explore the impact of the COVID-19 pandemic on the mental health of nurses working in primary, secondary, and tertiary healthcare centers in Navarre, in northern Spain.

METHODS

Study design

This was a descriptive, cross-sectional multicenter study that described the impact of COVID-19 on the mental health of nurses one year after the global coronavirus outbreak alert. This design was found the most appropriate to assess the prevalence of anxiety, depression, insomnia, and distress as a result of exposure to a traumatic event in nurses working in one of the northern regions of Spain.

The STROBE checklist for cross-sectional studies was used to report this study (Supporting information). The study also complied with the Checklist for Reporting Results of Internet E-Surveys (CHERRIES) (Eysenbach, 2004).

Sample and setting

The study population consisted of RNs working during the COVID-19 pandemic at any public or private health center/unit or community healthcare center or nursing home located in Navarre. The only exclusion criterion was not having worked as a nurse during the COVID-19 pandemic.

Navarre is an autonomous community and province in northern Spain with a population of 661 023 inhabitants. There are three referral public hospitals, one private hospital, 234 healthcare centers, 47 points of continuous and urgent care, and four outpatient emergency services. There are also 390 community health centers, of which 74 are nursing homes, 12 are residences for people with disabilities, four are psychogeriatric facilities, and five are residences for people with mental health problems (data provided by the Department of Health of the Government of Navarre). In all these centers, nurses hold the position of either a general Registered Nurse or a nurse specialist.

The sample size was calculated based on an estimated prevalence of 18% for the depression variable, as reported in previous studies (Pappa et al., 2020). With a type I error of 5% ($\alpha = 0.05$) and a type II error of 10% (power calculation of 90% or $\beta = 0.10$), 800 nurses were needed for this study.

To access the entire population of RNs working in Navarre, collaboration was requested from the Official Board of Nursing (BON) of Navarre. To practice as a nurse in Spain, nurses must be registered with the BON in the region where they work. The entire population of 5700 RNs was invited to participate in the study through an invitation email.

Data collection

Data collection began on April 21, 2021, and ended on May 15, 2021, when the 800 complete responses were reached.

An invitation email was written by the research team and forwarded to the communication department of the BON to be sent to the email addresses of its database of practicing RNs. The email is the main means of communication between the BON and the RNs; information about training courses, BON´s budgets, and other topics related to nursing and nurses in the region is regularly sent by email to nurses. These emails are provided by the nurses themselves when they register for the first time with the BON or are updated when there is an event organized by the BON. So, it was assumed that the responses received from the participants were all from RNs.

The invitation email included information about the aim and characteristics of the study, the contact information of the principal investigator to contact in case of questions or concerns, the consent form guaranteeing anonymity and data protection, the estimated time to complete the questionnaire, and the link to a questionnaire in SurveyMonkey (an online survey software).

The first invitation to participate in the study was issued via email. To increase the response rate, the following techniques were applied based on Van Mol's recommendations for improving web survey efficiency: (a) the emails were personalized using the nurses' first name; (b) mails were sent on the middle of the week, on Wednesday, and in the mid-afternoon so that most of the participants could open them on Thursday morning; and (c) an extra reminder was sent two weeks after the first invitation. To promote the visibility of the research, information about the aim and characteristics of the study was posted on the Navarre BON webpage during the data collection period.

Of the 5700 potential participants, 996 responded, of which 10 participants declined to take part in the study and 986 completed and sent back the questionnaire. Of the 986 nurses who participated, 711 completed after the first invitation and 285 after the reminder invitation. From these, 186 responses were removed because questionnaires were incomplete ($n = 182$) or responses from participants did not meet the selection criteria of "having worked during the pandemic" ($n = 4$). The final sample of this study was 800 nurses, and the response rate (ratio of unique visitors who agreed to participate/unique first survey page visitors) was 81.13.

A questionnaire was developed to obtain the following information. Part 1 consisted of data collection on sociodemographic data and characteristics of the units where the participants worked during the pandemic, and was based on similar published research (Kang et al., 2020). The sociodemographic variables were gender (male/female), age (years), years of professional experience, and educational level (diploma or graduate degree in nursing (3 or 4 years of training, respectively)), and nursing specialty (e.g., geriatric nursing, etc.). Regarding the characteristics of the units where the nurses worked, information was collected about the level of care (primary care, COVID units, non-COVID hospital units, community health centers/nursing homes, and others).

Part 2 of the questionnaire was a self-evaluation list of questions related to mental health. To evaluate mental health status, the following four scales were used. First, the 9-item Patient Health Questionnaire (PHQ-9) was used to assess depression. The PHQ-9 was validated for the Spanish context and has good psychometric properties (results comparable with those of the original version, with a sensitivity of 87% and a specificity of 88%) (Díez-Quevedo et al., 2001). It consists of 9 items and evaluates the severity of depression, classifying scores as minimal depression/no depression (0–4), mild depression (5–9), moderate depression (10–14), moderately severe depression (15–19), and severe depression (20–27) (Kocalevent et al., 2013).

Second, the Generalized Anxiety Disorder Scale (GAD-7) was used to assess anxiety. The GAD-7 was validated for the Spanish cultural context and has shown sound psychometric properties with a sensitivity of 86.8% and a specificity of 93.4% (Garcia-Campayo et al., 2010). Composed of seven items, it classifies anxiety as minimal/no anxiety (0–4), mild anxiety

(5–9), moderate anxiety (10–14), or severe anxiety (15–21) (Löwe et al., 2008). Third, the Index of Insomnia Severity (ISI) was used to assess insomnia. The ISI, with an internal consistency Cronbach's $\alpha = 0.82$, is a valid and reliable instrument with a sensitivity of 86.1% and a specificity of 87.7% and is composed of 5 items scored on a Likert scale (0–4). Also validated to the Spanish context, it classifies the total scores as normal/no insomnia (0–7), subclinical insomnia (8–14), moderate clinical insomnia (15–21), and severe clinical insomnia (22–28) (Sierra et al., 2008). Fourth, the Impact of Events Scale (IES) was used to assess distress as a result of exposure to a traumatic event. The IES (with a high split-half reliability $r = 0.86$ and a high Cronbach's α, intrusion = 0.78, avoidance = 0.82) was developed and validated to measure subjective distress as a result of exposure to a traumatic event. The event used for this questionnaire was the emergence of COVID-19. The IES is composed of 15 items rated on a 5-point scale from 0 to 4. The total scores are classified as subclinical (0–8), mild distress (9–25), moderate distress (26–43), and severe distress (44 or more) (Horowitz et al., 1979).

Part 1 of the questionnaire was designed by three members of the research team taking into consideration similar studies. To ensure comprehension of part 1 of the questionnaire, five other members of the research team reviewed it and validated the questions. When the final questionnaire (parts 1 and 2) was developed and entered into the SurveyMonkey platform, it was sent to all members of the research team (7 nurses, 1 psychologist, and 1 psychiatrist) to verify its usability and functionality in computers and mobile devices. The final questionnaire was not tested for validity and reliability because the included instruments were valid and reliable.

Questions about the nurses' sociodemographic information and the characteristics of the units where they had worked were presented on the first page, and the mental health measures were displayed over 4 pages, with between 7 and 10 items on each page. Before they could access the questionnaire, the participants had to read the informed consent and check a box indicating their voluntary participation in the study. It was not necessary to answer all the items before submitting the questionnaire, and items could be left blank, although the questionnaire did provide the option of going back to previous pages to modify answers. Only participants whose questionnaires were complete were included in the study sample.

Data analysis

The descriptive analysis included frequencies and percentages for the qualitative variables and measures of central tendency (mean) and dispersion (standard deviation) for the quantitative variables. To divide the subjects into groups according to combinations of the four variables under study (anxiety, insomnia, depression, and impact of event), a cluster analysis was carried out using the k-means method. Based on previous studies, the subjects were divided into four groups (Kang et al., 2020). The sociodemographic characteristics of the four groups were compared using analysis of variance (ANOVA) for age and years of experience and the chi-square for gender and education level. Once this analysis determined that the variables had different distributions among the group, an ordinal regression was performed to determine whether the nurses' places of work during the pandemic had a relationship with mental health (according to the four groups). Odds ratios (ODs) are presented with their corresponding 95% of confidence intervals (CIs). The level of significance considered for the contingency analysis was 0.05 in all cases. The statistical analysis software IBM SPSS Statistics version 24 was used for data analysis.

Ethical consideration

This study was approved by the Ethics, Animal Experimentation and Biosafety Committee of the Public University of Navarre (Reg. PI-0020/20). Informed consent was requested from participants before they completed the questionnaires, and anonymity and protection of the participants' data were guaranteed during the research process and the dissemination of results. The online survey software used for data collection stored the information in SOC 2 accredited data centers that adhered to security and technical best practices. Also, it ensured that collected data were transmitted over a secure HTTPS connection, user login was protected via TLS, and data at rest were encrypted using industry-standard encryption algorithms and strength.

This study did not contemplate individual contact with nurses who showed high levels of distress, depression, anxiety, or insomnia. However, if nurses became distressed because of their involvement in this study, they could contact the principal investigator for support. For this purpose, the principal investigator's email address was provided at the beginning and end of the questionnaire. In addition, the main results of this study were sent through email from the Navarre's BON to all RNs, and information on psychological resources available was included in case of need for support. Finally, support was offered to all nurses and other health professionals working in the different health centers in Navarre, centralized by the government of Navarre.

RESULTS

Of the 800 participants, 93.2% were women (n = 746) and 6.8% were men (n = 54), with a mean age of 40.36 years (SD: 11.62). A total of 75% (n = 599) had undergraduate degrees in nursing, 8.5% were specialists (n = 68), 16.5% had a master's degree (n = 132), and 0.1% had a doctorate (n = 1). Their average duration of nursing experience was 17.16 years (SD: 11.42).

Table 1 shows the results obtained from the cluster analysis, in which the subjects were divided into four groups according to their scores for anxiety, insomnia, depression, and impact of

TABLE 1 Cluster analysis grouping

Variables (mean, DE)	Group 1	Group 2	Group 3	Group 4	p
Anxiety	4.56 (3.57)	7.89 (3.87)	10.09 (4.42)	14.96 (4.78)	$p < 0.001$
Insomnia	4.93 (3.84)	7.89 (4.75)	12.48 (5.15)	16.19 (5.15)	$p < 0.001$
Depression	3.30 (3.19)	5.97 (3.34)	9.21 (4)	14.41 (5.45)	$p < 0.001$
Impact of event	4.48 (4)	20.36 (4.79)	37.29 (5.74)	53.64 (6.54)	$p < 0.001$

p: ANOVA.

TABLE 2 Comparison of demographic characteristics

Variables	Group 1	Group 2	Group 3	Group 4	p
Age	41.79 (11.38)	39.08 (11.09)	40.69 (12.24)	38.98 (11.91)	$p = 0.04$
Professional experience	18.75 (11.31)	16.18 (11)	17.81 (11.86)	14.77 (11.27)	$p = 0.009$
Gender (female)	91% (232)	93.3% (223)	95.2% (199)	93.8% (91)	$p = 0.441$
Education level (grad)	71.8% (183)	73.6% (176)	80.4% (168)	74.2% (72)	$p = 0.180$

p: ANOVA and Chi-square.

TABLE 3 Results of the ordinal regression

			95% IC OR		
	Parameter	OR	Inf	Sup	*p* value
Age	.023	1.023	.991	1.057	.159
Professional experience	-.027	.974	.942	1.006	.113
Primary healthcare	.092	1.096	.735	1.634	.652
COVID* hospital units	.595	1.813	1.261	2.606	.001*
Non-COVID hospital units	-.385	.680	.438	1.057	.087
Nursing homes*	.520	1.682	1.043	2.713	.033*

*$p < 0.05$.

events. The ANOVA showed different levels of anxiety, insomnia, depression, and impact of events among the four groups ($P < 0.001$).

As shown in Table 1, Group 1, which comprised 32% of the nurses ($n = 255$), had mean scores indicative of the absence of symptoms for the four variables. In Group 2, which comprised 30% ($n = 239$) of the nurses, included subjects with mild levels of depression, anxiety, and distress and without insomnia. Group 3, which comprised 26% ($n = 209$) of the sample, had moderate levels of depression, anxiety, and distress and a level of insomnia below the clinical threshold. Finally, Group 4, which comprised 12% ($n = 97$) of the nurses, had the most severe symptoms, exhibiting mean scores indicative of moderately severe depression, severe anxiety, moderately severe clinical insomnia, and severe distress.

Table 2 shows the comparisons of the sociodemographic variables among the four groups. The tests that were applied indicate that the groups differed in terms of age and years of experience but were homogeneous in terms of gender and education level.

Table 3 shows the results obtained from the ordinal regression that was performed to determine the influence of the work location during the pandemic once age and years of professional experience were controlled.

According to these results, having worked in a hospital COVID ward or in a community health center/nursing home has an effect on mental health. Specifically, when during the pandemic, work was done in a COVID ward, the nurse was 1.8 (95% CI, 1.261–2.606) times as likely to be in the groups with moderate or severe symptoms for the four variables, indicating a higher impact on mental health. In the case of having worked in a community health center/nursing home, this probability was 1.7 (95% CI, 1.043–2.713).

DISCUSSION

Our study provides new insight into the impact of the COVID-19 pandemic on the mental health of RNs who worked at different healthcare centers in Navarre, in northern Spain. The results show that among the 800 nurses surveyed, 32% had no symptoms of depression, anxiety, and distress compared with the 68% of nurses ($n = 545$) who had some level of depression, anxiety, and subjective distress as a result of the COVID-19 pandemic, and 38% ($n = 306$) presented some degree of insomnia. Among all nurses, 38% experienced moderate or severe symptoms.

Cluster analysis has identified four distribution groups. Of all of them, a high percentage of professionals grouped in cluster four 4 with high mean levels of anxiety, insomnia, and depression (14.96/21, 16.19/28, and 14.41/27) was detected; the mean value of impact of event was 53.64/60.

Regarding depression and anxiety, our results differ to some degree from what has been reported in the literature. Regarding the presence of depression, the meta-analysis performed by Saragih and colleagues (2021) calculated a pooled prevalence of 37% (95% CI, 29%—45%) among HCWs

during the COVID-19 pandemic. Another previous meta-analysis (Pappa et al., 2020) indicated a pooled prevalence of 30.30% (95% CI, 18.24%–43.84%) in the subgroup of nurses that participated in the analyzed studies. Regarding anxiety, Pappa et al. (2020) described pooled prevalences of 23.21% (95% CI, 17.77%–29.13%) for HCWs and 25.80% (95% CI, 19.20%–33.00%) for the subgroup of nurses. Similarly, a pooled prevalence of 40% (95% CI, 29%–52%) for anxiety among the HCWs has been found (Saragih et al., 2021). Several factors could explain the difference between our study's findings in terms of the proportion of nurses with different degrees of depression and anxiety and those reported in the aforementioned meta-analyses. The pooled analyses performed by Saragih et al. (2021) included different HCWs, such as nurses, doctors, nursing assistants, and other health professionals with and without direct contact with patients. So, it could be argued that the prevalence of mental health problems among other health professionals may be lower than the prevalence among nurses. In fact, Saragih et al. (2021) highlighted that several of the studies included in their review showed a high prevalence of anxiety and depression among front-line nurses. This fact can be seen in the differences in patient care characteristics between nurses and other professionals, such as nurses' greater time of contact with patients and more direct and physical contact with patients.

In the case of the review by Pappa et al. (2020), studies were published before May 2020 and illustrated the prevalence of anxiety and depression during the COVID-19 outbreak, but they do not show the ongoing pressure that has persisted since then and that can explain the data obtained in our study.

Studies on the degree of distress in response to the COVID-19 pandemic show moderate-to-severe levels in between 40% and 29% of the HCWs in different studies (Dobson et al., al., 2021; Riello et al., 2020; Şahin et al., 2020). A total of 38% (Şahin et al., 2020) and 46% (Dobson et al., 2021) of the nurses included in these studies reported severe or moderate levels of distress; the latter of these studies reported a higher figure than we obtained for the total sample in our study (38%). The work of Dobson and colleagues (2021) was developed at a tertiary hospital that underwent major preparations, including workforce restructuring, which may explain this higher figure and is consistent with our finding that nurses who worked at a COVID hospital unit had almost twice the risk of moderate-to-severe levels of distress in response to the COVID-19 pandemic. Other factors, such as work overload and workplace issues arising from the greater demand for hospital care and shortages of professionals, have conditioned this increased level of distress (Kniffin et al., 2021).

Regarding insomnia, our findings are similar to those identified in various literature reviews, which report values between 34% and 38% (García-Iglesias et al., 2020; Pappa et al., 2020).

Our study provides new knowledge regarding work environments, showing that the workplaces associated with the greatest alterations in the mental health of nurses during the pandemic (from March 2020 to April 2021, which is the period that is the focus of the present study) were hospital COVID units and community health centers/nursing homes. It is worth noting that during this period of the pandemic, the global health situation was characterized by a high health and social uncertainty and a high incidence of COVID cases requiring specialized care, especially in the case of the elderly (Davidson & Szanton, 2020; WHO, 2021). In this new context, nurses have had to adapt to a new dynamic with changes in the distribution of tasks and professional roles, shifts to caring for infectious patients, limited access to services, overburden, and changes of their wards into isolation areas (Anders, 2021).

Nurses in other contexts, such as non-COVID hospital wards or primary care centers, do not seem to have experienced such a negative impact on their mental health, although the adverse effects on mental health and the tension experienced by these professionals should not be underestimated (Bornstein et al., 2020). In particular, it is possible that the identified mental health problems will persist for a time after the end of the epidemic, as has happened during previous epidemic situations, such as the SARS outbreak in 2003 or the Ebola outbreak in 2014 (Sahin et al., 2020).

Without determining the risk of mental health problems based on the availability of nurses, and although Spain has fewer nurses than the average for European countries (Arévalo-Manso, 2019), it should be noted that the study was conducted in the Spanish community with the highest number of nurses, 8.6 per 1000 inhabitants. The high percentage of nurses who have experienced or continue to experience mental health problems in this context leads us to think that in other regions with fewer nurses, the percentage may be higher.

In short, the demands on nurses during the COVID pandemic required nurses to train and inform themselves to care for the most vulnerable members of the population and provide comfort in times of illness and death while coping with factors such as work overload. The limitations of personal and material resources are clear system failures that generate physical, emotional stress, and moral stress in nurses (Turale et al., 2020).

Limitations

The main limitations of the study are the result of the type of sampling performed and the number of variables evaluated. First, because questionnaires were sent to all of the nurses and were included in the order they were returned until the sample size was met, selection bias is possible. Indeed, there is the possibility that the nurses who responded first—that is, the most motivated ones—are those who had the worst experiences during the pandemic and the most driven to express their feelings by responding to the questionnaire.

Second, one must be cautious when interpreting the findings regarding the relationship between mental health problems and certain work environments, as there may be confounding variables that were not measured that may influence this relationship. Although the study controlled for basic sociodemographic variables, it may have overlooked others with a potential impact on this relationship. Finding a

balance between the number of variables to be measured and the length of the questionnaire is vital in this type of study; although including a high number of items allows more variables to be measured, it drastically reduces the response rate (Roszkowski & Bean, 1990).

CONCLUSIONS

Nurses are among the HCWs who experienced the greatest mental health impact during the COVID-19 pandemic. Our study reveals that a high percentage (68.1%) of the nurses in Navarre have some level of depression, anxiety, insomnia, and posttraumatic stress disorder versus 32% of the nurses who had no symptoms for any of the four variables. Of the 800 nurses, 38.2% ($n = 305$) had moderate or severe symptoms of depression, anxiety, and distress.

Front-line nurses are at risk for developing mental health problems; specifically, nurses who work in hospital units with COVID-positive patients have the highest risk (OR = 1.813; 95% CI, 1.261–2.606) of depression, anxiety, insomnia, and posttraumatic stress, followed by those who work in community healthcare centers and nursing homes (OR = 1.682; 95% CI, 1.043–2.713). Factors such as work overload and a lack of human resources, materials, and information, along with the high demands on nursing staff and the need for adaptation during the COVID pandemic, appear to be factors that have contributed to the high level of stress and distress in nurses.

IMPLICATIONS FOR NURSING AND HEALTH POLICY

Considering the negative impact on the mental health of nurses who worked during the pandemic, especially those who worked in hospital COVID units and at community centers and nursing homes, it is necessary to promote healthy work environments. Recommendations for nursing policy include the need to implement coaching and emotional programs to support nurses on the frontlines of the pandemic. There is also an urgent need for the implementation of national training programs to strengthen health emergency preparedness, improve response capacity, and increase the resilience of nurses to disasters. Finally, minimum nurse-to-patient ratio policies are required to ensure a safe environment for patients and positive working conditions for nurses.

ACKNOWLEDGMENTS

We thank nurses in the Autonomous Community of Navarre who completed the online questionnaire and the Official Board of Nursing of Navarre for its valuable collaboration. This research was supported by the Department of Health of the Government of Navarre (Spain) in the call for research related to Health Sciences and SARS-Cov-2 pandemic, 2020 (grant number 0011-3638-2020-000005).

This article will be published open access as per the agreement between Universidad Publica de Navarra and Wiley.

CONFLICTS OF INTEREST

The authors declare no conflict of interest regarding the publication of this paper.

AUTHOR CONTRIBUTIONS

Made substantial contributions to the conception or design of the work; or the acquisition, analysis, or interpretation of data; or the creation of new software used in the work: LSMR, PEH, NSR, MFT, IRM, CGV; drafted the work or revised it critically for important intellectual content: LSMR, PEH, NSR, MFT, IRM, CGV; approved the version to be published: LSMR, PEH, NSR, MFT, IRM, CGV; agree to be accountable for all aspects of the work in ensuring that questions related to the accuracy or integrity of any part of the work are appropriately investigated and resolved: LSMR, PEH, NSR, MFT, IRM, CGV.

ORCID

Leticia San Martin-Rodriguez PhD, RN, https://orcid.org/0000-0002-9097-7493

Paula Escalda-Hernandez PhD, RN, https://orcid.org/0000-0003-2263-156X

Marta Ferraz-Torres PhD, RN, https://orcid.org/0000-0002-7740-2572

Cristina Garcia-Vivar PhD, RN https://orcid.org/0000-0002-6022-559X

REFERENCES

Anders, R.L. (2021) Engaging nurses in health policy in the era of COVID-19. *Nursing Forum*, 56(1), 89–94. https://doi.org/10.1111/nuf.12514

Arévalo Manso, J.J., Diéguez de la Mata, O., Navarro Ruiz, J.I., Gallardo Correa, M.J., & Vega Méndez, E. (2019) Upcoming shortage of nurses in Spain: the problem that is not talked about. *Index de Enfermería*, 28(4), 235–239 (in Spanish).

Arora, A., Jha, A.K., Alat, P. & Das, S.S. (2020) Understanding coronaphobia. *Asian Journal of Psychiatry*, 54, 102384. https://doi.org/10.1016/j.ajp.2020.102384

Bornstein, S.S., Mire, R.D., Barrett, E.D., Moyer, D.V. & Cooney, T.G. (2020) The collision of COVID-19 and the U.S. health system. *Annals of Internal Medicine*, 173(6), 484–485. https://doi.org/10.7326/M20-1851

Carriero, M.C., Conte, L., Calignano, M., Lupo, R., Calabrò, A., Santoro, P. et al. (2021). The psychological impact of the coronavirus emergency on physicians and nurses: an Italian observational study. *Acta Biomedica*, 92, e2021030. https://doi.org/10.23750/abm.v92iS2.11575

Castro-Sánchez, E. & Santillán-García, A. (2020) Smart lobbying for minimum nurse staffing ratios in Spain: not just numbers. *Policy, Politics & Nursing Practice*, 21(2), 60–61. https://doi.org/10.1177/1527154420923753

Chen, Q., Liang, M., Li, Y., Guo, J., Fei, D., Wang, L. et al. (2020) Mental health care for medical staff in China during the COVID-19 outbreak. *The Lancet Psychiatry*, 7(4), e15–e16. https://doi.org/10.1016/S2215-0366(20)30078-X

Davidson, P.M. & Szanton, S.L. (2020) Nursing homes and COVID-19: we can and should do better. *Journal of Clinical Nursing*, 29(15–16), 2758–2759. https://doi.org/10.1111/jocn.15297

Diez-Quevedo, C., Rangil, T., Sanchez-Planell, L., Kroenke, K. & Spitzer, R.L. (2001) Validation and utility of the patient health questionnaire in diagnosing mental disorders in 1003 general hospital Spanish inpatients. *Psychosomatic Medicine*, 63(4), 679–686. https://doi.org/10.1097/00006842-200107000-00021

Dobson, H., Malpas, C.B., Burrell, A.J., Gurvich, C., Chen, L., Kulkarni, J. & Winton-Brown, T. (2021) Burnout and psychological distress amongst Australian healthcare workers during the COVID-19 pandemic. *Australasian Psychiatry*, 29(1), 26–30. https://doi.org/10.1177/1039856220965045

Erdem, H. & Lucey, D.R., (2021) Healthcare worker infections and deaths due to COVID-19: a survey from 37 nations and a call for WHO to post national data on their website. *International Journal of Infectious Diseases*, 102, 239–241. https://doi.org/10.1016/j.ijid.2020.10.064

European Centre for Disease Prevention and Control (ECDC). (2021) COVID-19 situation update for the EU/EEA, as of 3 August 202. Available at: https://www.ecdc.europa.eu/en/cases-2019-ncov-eueea [Accessed 3rd December 2021].

Eysenbach, G. (2004) Improving the quality of web surveys: the checklist for reporting results of internet e-surveys (CHERRIES). *Journal of Medical Internet Research*, 6, 1–6. https://doi.org/10.2196/jmir.6.3.e34

Garcia-Campayo, J., Zamorano, E., Ruiz, M.A., Pardo, A., Pérez-Páramo, M., López-Gómez, V. et al. (2010) Cultural adaptation into Spanish of the generalized anxiety disorder-7 (GAD-7) scale as a screening tool. *Health and Quality of Life Outcomes*, 8(1), 8. https://doi.org/10.1186/1477-7525-8-8

García-Iglesias, J.J., Gómez-Salgado, J., Martín-Pereira, J., Fagundo-Rivera, J., Ayuso-Murillo, D., Martínez-Riera, J.R. & Ruiz-Frutos, C. (2020) Impact of SARS-CoV-2 (Covid-19) on the mental health of healthcare professionals: a systematic review. *Revista Española de Salud Pública*, 94, e202007088 (in Spanish).

Horowitz, M., Wilner, N. & Alvarez, W. (1979) Impact of event scale: a measure of subjective stress. *Psychosomatic Medicine*, 41(3), 209–218. https://doi.org/10.1097/00006842-197905000-00004

Hummel, S., Oetjen, N., Du, J., Posenato, E., Resende de Almeida, R.M., Losada, R. et al. (2021) Mental health among medical professionals during the COVID-19 pandemic in eight European countries: cross-sectional survey study. *Journal of Medical Internet Research*, 23(1), e24983. https://doi.org/10.2196/24983

International Council of Nurses (ICN). (2021) Mass trauma experienced by the global nursing workforce. Available at: https://www.icn.ch/sites/default/files/inline-files/ICN%20COVID19%20update%20report%20FINAL.pdf [Accessed 29th August 2021].

Kang, L., Ma, S., Chen, M., Yang, J., Wang, Y., Li, R. et al. (2020) Impact on mental health and perceptions of psychological care among medical and nursing staff in Wuhan during the 2019 novel coronavirus disease outbreak: a cross-sectional study. *Brain, Behaviour, and Immunity*, 87, 11–17. https://doi.org/10.1016/j.bbi.2020.03.028

Kniffin, K.M., Narayanan, J., Anseel, F., Antonakis, J., Ashford, S.P., Bakker, A.B. et al. (2021) COVID-19 and the workplace: implications, issues, and insights for future research and action. *American Psychologist*, 76(1), 63–77. https://doi.org/10.1037/amp0000716

Kocalevent, R.D., Hinz, A. & Brähler, E. (2013) Standardization of the depression screener patient health questionnaire (PHQ-9) in the general population. *General Hospital Psychiatry*, 35(5), 551–555. https://doi.org/10.1016/j.genhosppsych.2013.04.006

Labrague, L.J. & de Los Santos, J.A.A. (2021) Fear of COVID-19, psychological distress, work satisfaction and turnover intention among frontline nurses. *Journal of Nursing Management*, 29(3), 395–403. https://doi.org/10.1111/jonm.13168

Löwe, B., Decker, O., Müller, S., Brähler, E., Schellberg, D., Herzog, W. et al. (2008) Validation and standardization of the generalized anxiety disorder screener (GAD-7) in the general population. *Medical Care*, 46(3), 266–274. https://doi.org/10.1097/MLR.0b013e318160d093

Manzanares, I., Sevilla Guerra, S., Lombraña Mencía, M., Acar-Denizli, N., Miranda Salmerón, J. & Martinez Estalella, G. (2021) Impact of the COVID-19 pandemic on stress, resilience and depression in health professionals: a cross-sectional study. *International Nursing Review*, 68(4), 461–470. https://doi.org/10.1111/inr.12693

Roszkowski, M.J. & Bean, A.G. (1990) Believe it or not! Longer questionnaires have lower response rates. *Journal of Business and Psychology*, 4(4), 495–509.

Nguyen, L.H., Drew, D.A., Graham, M.S., Joshi, A.D., Guo, C.G., Ma W. et al. (2020) Coronavirus pandemic epidemiology consortium. Risk of COVID-19 among front-line health-care workers and the general community: a prospective cohort study. *Lancet Public Health*, 5(9), e475–e483. https://doi.org/10.1016/S2468-2667(20)30164-X

Olivas-Martínez, A. et al. (2021) In-hospital mortality from severe COVID-19 in a tertiary care center in Mexico City: causes of death, risk factors and the impact of hospital saturation. *PloS One*, 16(2), e0245772. https://doi.org/10.1371/journal.pone.0245772

Pappa, S., Ntella, V., Giannakas, T., Giannakoulis, V.G., Papoutsi, E. & Katsaounou, P. (2020) Prevalence of depression, anxiety, and insomnia among healthcare workers during the COVID-19 pandemic: a systematic review and meta-analysis. *Brain, Behaviour, and Immunity*, 88, 901–907. https://doi.org/10.1016/j.bbi.2020.05.026

Pérez-Raya, F., Cobos-Serrano, J.L., Ayuso-Murillo, D., Fernández-Fernández, P., Rodríguez-Gómez, J.A. & Almeida Souza, A. (2021) COVID-19 impact on nurses in Spain: a considered opinion survey. *International Nursing Review*, 68(2), 248–255. https://doi.org/10.1111/inr.12682

Riello, M., Purgato, M., Bove, C., MacTaggart, D. & Rusconi, E. (2020) Prevalence of post-traumatic symptomatology and anxiety among residential nursing and care home workers following the first COVID-19 outbreak in Northern Italy. *Royal Society Open Sciences*, 7(9), 200880. https://doi.org/10.1098/rsos.200880

Sabetian, G., Moghadami, M., Hashemizadeh Fard Haghighi, L., Shahriarirad, R., Fallahi, M.J., Asmarian, N. et al. (2021) COVID-19 infection among healthcare workers: a cross-sectional study in southwest Iran. *Virology Journal*, 18(1), 58. https://doi.org/10.1186/s12985-021-01532-0

Şahin, M.K., Aker, S., Şahin, G. & Karabekiroğlu, A. (2020) Prevalence of depression, anxiety, distress and insomnia and related factors in healthcare workers during COVID-19 pandemic in Turkey. *Journal of Community Health*, 45, 1168–1177. https://doi.org/10.1007/s10900-020-00921-w

Saragih, I.D., Tonapa, S.I., Saragih, I.S., Advani, S., Batubara, S.O., Suarilah, I. et al. (2021) Global prevalence of mental health problems among healthcare workers during the COVID-19 pandemic: a systematic review and meta-analysis. *International Journal of Nursing Studies*, 121, 104002. https://doi.org/10.1016/j.ijnurstu.2021.104002

Sierra, J.C., Guillén-Serrano, V. & Santos-Iglesias, P. (2008) Insomnia severity index: some indicators about its reliability and validity on an older adults sample. *Revista Neurología*, 47(11), 566–570.

Turale, S., Meechamnan, C. & Kunaviktikul, W. (2020) Challenging times: ethics, nursing and the COVID-19 pandemic. *International Nursing Review*, 67(2), 164–167. https://doi.org/10.1111/inr.12598

World Health Organization (WHO). (2021) WHO coronavirus (COVID-19) dashboard. Available at: https://covid19.who.int/ [Accessed 3rd December 2021].

Zhang, Y., Wei, L., Li, H., Pan, Y., Wang, J., Li, Q., Wu, Q. & Wei, H. (2020) The psychological change process of frontline nurses caring for patients with COVID-19 during its outbreak. *Issues Mental Health Nursing*, 41, 525–530. https://doi.org/10.1080/01612840.2020.1752865

SUPPORTING INFORMATION

Additional supporting information can be found online in the Supporting Information section at the end of this article.

How to cite this article: Martin-Rodriguez, L.S., Escalda-Hernandez, P., Soto-Ruiz, N., Ferraz-Torres, M., Rodriguez-Matesanz, I., & Garcia-Vivar, C. (2022) Mental health of Spanish nurses working during the COVID-19 pandemic: A cross-sectional study. *International Nursing Review*, 1–8. https://doi.org/10.1111/inr.12764
