# Supplementary material for: Mental health status and related factors influencing healthcare workers during the COVID-19 pandemic: A systematic review and meta-analysis
Source: PLoS One. 2024 Jan 19;19(1):e0289454. doi: 10.1371/journal.pone.0289454 (PMC10798549; doi:10.1371/journal.pone.0289454)
Supplement: S1 Data — (ZIP) [file pone.0289454.s011.zip › literatures/9.pdf]

Acta Biomed 2020; Vol. 91, N. 2: 83-88 DOI: 10.23750/abm.v91i2.9619 © Mattioli 1885

Covid-19 pandemic impact on mental health: a web-based cross-sectional survey on a sample of Italian general practitioners

Andrea Amerio[1,2,3], *Davide Bianchi*[1,2], *Francesca Santi*[1,2], *Luigi Costantini*[4], *Anna Odone*[5,6], *Carlo Signorelli*[5], *Alessandra Costanza*[7,8], *Gianluca Serafini*[1,2], *Mario Amore*[1,2], *Andrea Aguglia*[1,2]

[1]Department of Neuroscience, Rehabilitation, Ophthalmology, Genetics, Maternal and Child Health (DINOGMI), Section of Psychiatry, University of Genoa, Genoa, Italy; [2]IRCCS Ospedale Policlinico San Martino, Genoa, Italy; [3]Mood Disorders Program, Tufts Medical Center, Boston, MA, USA; [4]Department of Medicine and Surgery, University of Parma, Parma, Italy; [5]School of Medicine, Vita-Salute San Raffaele University, Milan, Italy; [6]Clinical Epidemiology and HTA, IRCCS San Raffaele Scientific Institute, Milan, Italy; [7]Department of Psychiatry, Faculty of Medicine, University of Geneva (UNIGE), Geneva, Switzerland; [8]Department of Psychiatry, ASO Santi Antonio e Biagio e Cesare Arrigo Hospital, Alessandria, Italy

Summary. *Background:* Since the World Health Organization declared the new 2019 coronavirus disease (Covid-19) outbreak first a Public Health Emergency of International Concern and then a pandemic, Italy held more than 195.350 cases and 26.380 deaths. Working in the frontline with suspected Covid-19 infection patients, general practitioners (GPs) are daily under both physical and psychological pressure. *Methods:* A web-based cross-sectional survey was carried out on italian GPs working in Genoa (Italy), to assess the impact of Covid-19 pandemic on mental health. The survey was anonymous and a free Google Forms® software was used. *Results:* One hundred thirty-one GPs completed the survey. Compared to GPs with absent or mild depressive symptoms (PHQ-9 < 10), GPs reporting moderate to severe depressive symptoms (N=30, 22.9%; PHQ-9 ≥ 10) reported more helplessness (96.7% vs. 79.2%, p=.025), spent more than three hours searching for COVID-19 information (43.3% vs. 19.8%, p=.024), perceived less adequate personal protective equipment (PPE) (6.7% vs. 23.8%, p=.049) and visited more COVID-19 infected patients (16.63 ± 27.30 vs. 9.52 ± 11.75, p=.041). Moreover, PHQ-9 ≥ 10 GPs reported a significant higher severity for both anxiety and insomnia (13.43 ± 4.96 vs. 4.88 ± 3.53 and 11.60 ± 5.53 vs. 4.84 ± 3.81, respectively; p<.001), and a worse quality of life in both mental (34.60 ± 7.45 vs. 46.01 ± 7.83, p<.001) and physical (43.50 ± 9.37 vs. 52.94 ± 4.78, p<.001) component summary. *Conclusions:* Our results give early insight into the urgent need to provide continuity of care for patients at the community-level, adequate PPE to GPs and a clear guidance from public health institutions. A precarious healthcare system both at a national and regional level might have triggered negative mental health outcomes in Italian GPs. (www.actabiomedica.it)

Key words: Covid-19, general practitioners, mental health, Italy

Introduction

On 30 January 2020, the 2019 coronavirus disease (Covid-19) outbreak has been declared a Public Health Emergency of International Concern (PHEIC) by the World Health Organization (WHO) (1). On 11 March, WHO Director General characterized Covid-19 as a pandemic and governments prepared for the worst, quickly realizing the impact that Covid-19 would have had on the health services and the global economy (2).

Italy holds one of the highest COVID-19 clinical burden worldwide with more than 195.350 cases and 26.380 deaths (3). Among these, more than 17.000 cases health care professionals and 139 physicians have died, of which 52 were general practitioners (GPs) (4). With particular regard to some regions, such as Lombardy, hospitals were quickly overwelmed by Covid-19 patients and the virus spread to the community, generally suggesting the ineffectivess of a hospital-oriented healthcare service (5).

GPs play a key role in the fight against the outbreak and they are engaged in each phases of response to the virus. They help block the viral transmission by monitoring subjects, reduce the increase of cases by treating patients and providing medical surveillance, and take care of the clinical and psychological well-being of patients, so the whole community can return to normal life (6).

Working in the frontline with suspected Covid-19 infection patients, GPs are frequently exposed to the virus and may be the source of community spread if not adequately protected by the appropriate personal protective equipment (PPE) (7). In this respect, during the last month, as well as UK colleagues, italian GPs denounced the lack of PPE and repeatedly calling for the same respirator masks issued to hospital staff to protect themselves from Covid-19 (8).

Facing the Covid-19 outbreak, GPs are daily under both physical and psychological pressure. We carried out a cross-sectional web-based survey to assess the mental health impact of a self-selected sample of italian GPs during the Covid-19 emergency.

Methods

Sample

An ad-hoc web-based survey questionnaire assessing the mental health impact of the COVID-19 pandemic was sent via email from March 15, 2020 to April 15, 2020 to italian GPs working in Genoa, Italy. During this period, the total confirmed COVID-19 infected cases were 4500 and 2655 in the Liguria region and Genoa, respectively (3).

An informed consent was provided by all participants before starting the survey. The survey was anonymous, and confidentiality was ensured. A free Google Forms® software was used.

Survey questionnaire

The first section of the questionnaire investigated the general characteristics of respondents: gender, age, marital status, having children or other family members requiring care, presence of general medical conditions, and number of patients in their charge.

The second section of the questionnaire investigated feelings of being unprotected, feelings of helplessness, the subjective perception of having or not adequate PPE, the number of infected COVID-19 patients which were visited, and the time spent searching COVID-19 information.

The third section of the questionnaire focused on the recognition of depressive and anxiety symptoms, the presence and severity of insomnia, and quality of life. The following scales were part of the questionnaire:

a) The 9-item Patient Health Questionnaire (PHQ-9), which is a self-report scale for depressive symptoms, using nine items on a 4-point Likert-scale ranging from 0 (not at all) to 3 (nearly every day). A total score ranging from 0 to 27 is obtained by summing all items and depressive severity is categorized into: normal (0-4), mild (5-9), moderate (10-14), moderate/severe (15-19), and severe (20-27) (9).
b) The 7-item Generalized Anxiety Disorder scale (GAD-7), which is a self-report scale for anxiety symptoms using seven items on a 4-point Likert-scale ranging from 0 (not at all) to 3 (nearly every day). A total score ranging from 0 to 21 is obtained by summing all items. The anxiety severity is categorized into: normal (0-4), mild (5-9), moderate (10-14), and severe (15-21) (10).
c) The 7-item Insomnia Severity Index (ISI), which is a self-report scale to assess sleep problems using seven items on a 4-point Likert scale ranging from 0 (not all) to 5 (extreme severe). A total score ranging from 0 to 28 is obtained by summing all items and the sleep severity is categorized into: normal (0-7), subthreshold (8-14), moderate (15-21), and severe (22-28) (11).
d) The Short Form 12-Item Health Survey (SF-12), which is a self-report instrument to assess health-related quality of life (HRQOL) including 12 ques-

tions, from which physical and mental component Summary may be derived. The theoretical range varies from 0 to 100 with higher scores indicating better HRQOL. The SF-12 has shown adequate statistical properties among the general population (12).

These categories were based on values established in the literature.

Statistical Analysis

Socio-demographic and clinical data were represented as means and standard deviations (SD) for continuous variables and frequency and percentages for categorical variables.

The sample was divided into two subgroups. The first group was composed by the presence of at least moderate depressive symptoms according to a mean score of PHQ-9 ≥ 10, while the second was characterized by the presence of a mean score of PHQ-9 < 10 (absent or mild depressive symptoms). In order to analyze the differences between the two subgroups, we used the Pearson $\chi 2$ test with Yates correction for the comparison of categorical variables, and the t-test for independent samples for continuous variables. In the case of non-parametric distribution, the Mann-Whitney test was used (COVID-19 infected patients visited).

The bivariate Pearson correlation for continuous variables was conducted in order to determine the association between the clinically investigated features (anxiety and depressive symptoms, quality of life, and sleep). A simple mediation model of the relationship between sleep and SF-12 mental Component Summary via anxiety and depressive symptoms was performed. The mediation analyses were conducted using the PROCESS Model 4 macro for SPSS v. 3.3 (13).

The Statistical Package for Social Sciences (SPSS) for Windows 24.0 (IBM Corp., Armonk, NY, USA) was used to carry out all the mentioned statistical analyses, with statistical significance set at $p < .05$ (two-tailed).

Results

One hundred thirty-one GPs completed the survey, and the overall response rate (ORR) was 25% (4). The mean age of the recruited sample was 52.31 ± 12.24; 68 (51.9%) participants were males, and 92 (70.2%) were married. Sixty-one (46.6%) subjects reported at least one general medical condition. Furthermore, 33 (25.3%) spent more than three hours searching for COVID-19 information and 39 (29.8%) had more than 1500 patients in charge. The most relevant socio-demographic and clinical characteristics are summarized in Table 1.

The total sample was then divided into two subgroups according to the presence of at least moderate depressive symptoms (PHQ-9 ≥ 10; Table 1). Compared to GPs with absent or mild depressive symptoms (PHQ-9 < 10), GPs with at least moderate depressive symptoms (N=30, 22.9%) showed significantly higher feelings of helplessness (96.7% vs. 79.2%, p=.025), spent more than three hours searching for COVID-19 information (43.3% vs. 19.8%, p=.024), perceived significantly less than adequate PPE (6.7% vs. 23.8%, p=.049), and visited a higher number of COVID-19 infected patients (16.63 ± 27.30 vs. 9.52 ± 11.75, p=.041).

Moreover, PHQ-9 ≥ 10 GPs reported a significantly higher severity for both anxiety (13.43 ± 4.96 vs. 4.88 ± 3.53, p<.001) and insomnia (11.60 ± 5.53 vs. 4.84 ± 3.81, p<.001), and a worse quality of life in both mental (34.60 ± 7.45 vs. 46.01 ± 7.83, p<.001) and physical (43.50 ± 9.37 vs. 52.94 ± 4.78, p<.001) Component Summary. Additional differences in terms of socio-demographic and clinical characteristics between the two subgroups are reported in Table 1.

All bivariate correlations between quality of life (mental and physical components), severity of insomnia, depressive and anxiety symptoms are described in Table 2. Specifically, significant correlations were found among clinical features.

Finally, anxiety and depressive symptoms were identified as potential mediators of the significant association between sleep and the mental component of the quality of life (R^2=.635, $p < .001$) (Figure 1).

Discussion

Similarly to recent studies conducted in hospital setting (14), the results of our survey reflect both the physical and psychological pressure of the current COVID-19 pandemic on Italian GPs working in Genoa, Italy. GPs reporting moderate to severe depres-

Table 1: socio-demographic and clinical characteristic of the total sample included (N=131), and comparison according to the presence of at least moderate depressive symptomatology

	**Total Sample (N=131)**	**PHQ-9 moderate/severe (N=30)**	**PHQ-9 absent or mild (N=101)**	**t/X²**	**p**
Gender (males), N (%)	68 (51.9)	12 (40.0)	56 (55.4)	2.210	.137
Age, mean ± SD	52.31 ± 12.24	54.90 ± 11.48	51.53 ± 12.40	-1.326	.187
Marital Status, N (%)					
Single	25 (19.1)	6 (20.0)	19 (18.8)	1.113	.774
Married	92 (70.2)	21 (70.0)	71 (70.3)		
Separated/divorced	12 (9.2)	2 (6.7)	10 (9.9)		
Widoved	2 (1.5)	1 (3.3)	1 (1.0)		
Children, N (%)					
No	46 (35.1)	13 (43.3)	33 (32.7)	1.941	.379
Yes, live-in	58 (44.3)	10 (33.4)	48 (47.5)		
Yes, not live-in	27 (20.6)	7 (23.3)	20 (19.8)		
Family member to care for, N (%)	44 (33.6)	6 (20.0)	38 (37.6)	3.221	.073
General Medical Condition, N (%)	61 (46.6)	16 (53.3)	45 (44.6)	.716	.397
Time spent in information lookup, N (%)					
< 1 hour	16 (12.2)	4 (13.4)	12 (11.9)	.7.431	**.024**
1-3 hours	82 (62.6)	13 (43.3)	69 (68.3)		
3-8 hours	33 (25.2)	13 (43.3)	20 (19.8)		
Patients in charge of, N (%)					
< 500 patients	12 (9.2)	2 (6.7)	10 (9.9)	2.576	.462
500-1000 patients	19 (14.4)	5 (16.7)	14 (13.9)		
1000-1500 patients	61 (46.6)	11 (36.6)	50 (49.5)		
>1500 patients	39 (29.8)	12 (40.0)	27 (26.7)		
Feeling of being unprotected, N (%)	118 (90.1)	29 (96.7)	89 (88.1)	1.891	.169
Feeling of helplessness, N (%)	109 (83.2)	29 (96.7)	80 (79.2)	5.045	**.025**
Adequate Personal Protective Equipment, N (%)	27 (20.6)	2 (6.7)	24 (23.8)	3.677	**.049**
COVID-19 positive patients, mean ± SD	11.15 ± 16.78	16.63 ± 27.30	9.52 ± 11.75	-2.063	**.041***
Physical Component Summary-12, mean ± SD	61.41 ± 50.78	43.50 ± 9.37	52.94 ± 4.78	7.418	**<.001**
Mental Component Summary-12, mean ± SD	60.70 ± 43.39	34.60 ± 7.45	46.01 ± 7.83	7.088	**<.001**
Patient Health Questionnaire-9, mean ± SD	6.16 ± 5.22	-	-	-	-
General Anxiety Disorder-7, mean ± SD	6.84 ± 5.30	13.43 ± 4.96	4.88 ± 3.53	-10.547	**<.001**
Insomnia Severity Index, mean ± SD	6.39 ± 5.11	11.60 ± 5.53	4.84 ± 3.81	-7.634	**<.001**

* = test Mann-Whitney

Table 2: bivariate correlation with Pearson correlation coefficient

	PCS-12	MCS-12	PHQ-9	GAD-7	ISI
PCS-12	1	.290<br>.001	-.423<br><.001	-.295<br>.001	-.215<br>.014
MCS-12		1	-.728<br><.001	-.772<br><.001	-.544<br><.001
PHQ-9			1	.821<br><.001	.691<br><.001
GAD-7				1	.658<br><.001
ISI					1

PCS-12: Physical Component Summary-12; MCS-12: Mental Component Summary-12; PHQ-9: Patient Health Questionnaire-9; GAD-7: General Anxiety Disorder-7; ISI: Insomnia Severity Index.

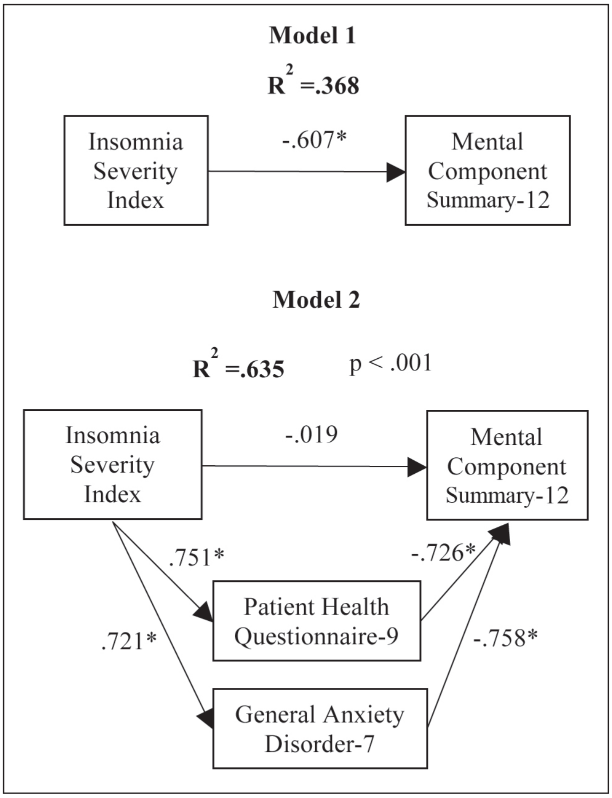

Figure 1. Mediation analysis between insomnia and mental component summary-12 (* <.001)

sive symptoms (PHQ-9 ≥ 10) reported more helplessness, spent more time on searching COVID-19 information, perceived less adequate PPE, and visited more COVID-19 infected patients. Moreover, PHQ-9 ≥ 10 GPs reported higher severity for both anxiety and insomnia, as well as worse quality of life in both mental and physical component summary. Finally, anxiety and depressive symptoms mediated the association between insomnia and worse mental quality of life.

Given the Covid-19 pandemic, several countries promoted and incentivized telehealth to support primary care health services' delivery (15). In Italy, more than in other countries, the limited availability and diffusion of large-scale telemedicine solutions, the poorer interconnection between telemedicine services operating at different levels, the lack of integration with the electronic health record of the National Health Service (Servizio Sanitario Nazionale, SSN), privacy regulations, together with the lack of clear guidelines, penalized the implementation of an effective telemedicine system (16).

The Italian SSN provides universal health coverage: the national level sets the health systems' fundamental principles and goals, defines the core benefit package of health services to be guaranteed to all citizens, and allocates national funds to the regions (17). Regions are responsible for planning, financing, and implementing healthcare services (18). Finding a balance between public and private healthcare systems and investing in models of community-based primary care generally depends by each regions (19).

The current COVID-19 emergency is bringing into light that an effective primary care service may guarantee a better response to public health emergencies. Taiwan and Australia are two noteworthy examples.

Taiwan represents a virtuous example of public and private health care integration. Taiwan healthcare model has been reshaped since the 2003 Severe Acute Respiratory Syndrome (SARS) outbreak. The Community Healthcare Groups Prepared Clinics (CHGPC) is the backbone of Taiwan's tiered primary care system. The CHGPC provide primary assistance to patients with red-flag symptoms, relieving the primary walk-in clinics from the burden of COVID-19 case management. CHGPC health care workers are provided with high PPE level. Suspected cases are then either treated on-site or conveyed to Community Screening Stations (CSS). Hub clinics manage the severe cases. Taiwan effectively handled the AH1N1 pandemic in 2009 and is currently tackling the Covid-19 pandemic without their health system getting overwhelmed (20).

With regard to Australia, the Australian National Primary Care Targeted Action Plan was initiated at the end of February, just before WHO stating the pandemic. A huge funding was promptly addressed to PPE supply and targeted research. Telehealth instruments have been widely implemented as well as online infection control training for healthcare workers. More than 200 GPs-led respiratory clinics have been established to sort potential infectious patients out of general clinics (21).

The main limitation of this survey is the small sample size of GPs across only one city with a low ORR that limited the generalizability of the results. Moreover, interactions between similar items of different psychometric instruments might have led to an overestimation of specific outcomes.

In conclusion, our findings give early insight into the urgent need to provide continuity of care for patients at the community-level, adequate PPE to GPs and a clear guidance from public health institutions. A precarious healthcare system both at a national and regional level might have triggered negative mental health outcomes in Italian GPs. While the current pandemic is deeply changing primary care, lessons should be learned from the examples of well responsive systems.

Acknowledgements

This work was developed within the framework of the DINOGMI Department of Excellence of MIUR 2018-2022 (Law 232/2016).

Fundings: This research received no specific grant from any funding agency in the public, commercial or not-for-profit sectors.

Conflict of interest: Each author declares that he or she has no commercial associations (e.g. consultancies, stock ownership, equity interest, patent/licensing arrangement etc.) that might pose a conflict of interest in connection with the submitted article

References

1. https://www.who.int/
2. Odone A, Landriscina T, Amerio A, et al. The impact of the current economic crisis on mental health in Italy: evidence from two representative national surveys. *Eur J Public Health* 2018;28(3):490-495.
3. http://www.protezionecivile.gov.it/
4. https://portale.fnomceo.it/
5. Fiorino G, Colombo M, Natale C, et al. Clinician Education and Adoption of Preventive Measures for COVID-19: A Survey of a Convenience Sample of General Practitioners in Lombardy, Italy. *Ann Intern Med* 2020 [Online ahead of print]
6. Li DKT, Zhu S. Contributions and challenges of general practitioners in China fighting against the novel coronavirus crisis. *Fam Med Community Health* 2020;8(2):e000361.
7. Shanafelt T, Ripp J, Trockel M. Understanding and Addressing Sources of Anxiety Among Health Care Professionals During the COVID-19 Pandemic. *JAMA* 2020 [Online ahead of print]
8. Rimmer A. Covid-19: GPs call for same personal protective equipment as hospital doctors. *BMJ* 2020;368:m1055.
9. Spitzer R, Kroken K, Williams JB. Validation and Utility of a Self-Report Version of PRIME-MD: The PHQ Primary Care Study. Primary Care Evaluation of Mental Disorders. Patient Health Questionnaire. *JAMA* 1999;282:1737.
10. Spitzer RL, Kroenke K, Williams JBW, et al. A Brief Measure for Assessing Generalized Anxiety Disorder. *Arch Intern Med* 2006;166:1092.
11. Morin CM, Belleville G, Bélanger L, et al. The Insomnia Severity Index: Psychometric Indicators to Detect Insomnia Cases and Evaluate Treatment Response. *Sleep* 2011;34:601–608.
12. Ware JJr, Kosinski M, Keller SD. A 12-Item Short-Form Health Survey: Construction of scales and preliminary tests of reliability and validity. *Medical Care* 1996;34(3):220–233.
13. Hayes AF. Introduction to mediation, moderation, and conditional process analysis: A regression-based approach. 2nd Ed. 2018, New York, NY: Guilford Press.
14. Greenberg N, Docherty M, Gnanapragasam S, Wessely S. Managing mental health challenges faced by healthcare workers during covid-19 pandemic. *BMJ* 2020;368:m1211.
15. Thornton J. Covid-19: how coronavirus will change the face of general practice forever. *BMJ* 2020;368:m1279.
16. Omboni S. Telemedicine During The COVID-19 in Italy: A Missed Opportunity? *Telemed J E Health* 2020 [Epub ahead of print]
17. Signorelli C, Odone A, Cella P, et al. Childhood vaccine coverage in Italy after the new law on mandatory immunization. *Ann Ig* 2018;30(4 Supple 1):1–10.
18. Amerio A, Starace F, Costanza A, et al. Putting Codman's lesson to work: Measuring and improving the quality of Italian mental health care. *Acta Psychiatr Scand* 2020;141(1):91-92.
19. Odone A, Delmonte D, Scognamiglio T, et al. COVID-19 deaths in Lombardy, Italy: data in context. Lancet Public Health 2020 [Epub ahead of print]
20. Chang BB, Chiu TY. Ready for a long fight against the COVID-19 outbreak: an innovative model of tiered primary health care in Taiwan. *BJGP Open* 2020 [Epub ahead of print]
21. Kidd M. Australia's primary care COVID-19 response. *Aust J Gen Pract* 2020 [Epub ahead of print]

Received: 24 April 2020
Accepted: 26 April 2020
Correspondence:
Andrea Amerio
Department of Neuroscience, Rehabilitation, Ophthalmology, Genetics, Maternal and Child Health (DINOGMI), Section of Psychiatry, University of Genoa, IRCCS San Martino,
L.go R. Benzi 10, 16132, Genova, Italy
Phone: +39 0103537668
Fax: +39 0103537669
E-mail: andrea.amerio@unige.it
