# Supplementary material for: Mental health status and related factors influencing healthcare workers during the COVID-19 pandemic: A systematic review and meta-analysis
Source: PLoS One. 2024 Jan 19;19(1):e0289454. doi: 10.1371/journal.pone.0289454 (PMC10798549; doi:10.1371/journal.pone.0289454)
Supplement: S1 Data — (ZIP) [file pone.0289454.s011.zip › literatures/90.pdf]

ORIGINAL ARTICLE

Adv Psychiatry Neurol 2022; 31 (1): 6-14
DOI: https://doi.org/10.5114/ppn.2022.114657

ADVANCES IN PSYCHIATRY & NEUROLOGY
POSTĘPY Psychiatrii i Neurologii

Depression, anxiety, stress and trauma-related symptoms and their association with perceived social support in medical professionals during the COVID-19 pandemic in Ukraine

Correspondence to:
Dmytro Martsenkovskyi
Bogomolets National Medical University
Tarasa Shevchenko Blvd, 13
Kyiv, Ukraine, 01601
e-mail: d.martsenkovskyi@gmail.com

Submitted: 30.09.2021
Accepted: 13.12.2021

Dmytro Martsenkovskyi[1,2], Viktoriia Babych[1], Inna Martsenkovska[2], Olexandr Napryeyenko[1], Natalija Napryeyenko[1], Igor Martsenkovsky[2]

[1]Bogomolets National Medical University, Kyiv, Ukraine
[2]SI "Research Institute for Psychiatry of MH of Ukraine", Kyiv, Ukraine

Abstract

Purpose: In a public health crisis medical professionals face immense psychological tension that leads to onset of negative mental health outcomes. We aimed to estimate the self-reported level of posttraumatic, anxiety, depression, and stress-related symptoms and their association with the level of perceived social support among healthcare professionals during the coronavirus (COVID-19) pandemic in Ukraine.

Methods: A cross-sectional web-based survey conducted during the second wave of the pandemic involved 330 participants. Mental health variables were assessed via the Depression Anxiety Stress Scale (DASS-21) and PTSD Checklist for DSM-5 (PCL-5). The level of perceived social support was assessed via the Multidimensional Scale of Perceived Social Support (MSPSS).

Results: The DASS-21 median score was 42.0 (IQR = 28.0-56.0), with 50.5% of respondents reporting moderate-to-severe depressive symptoms; 55.4% had moderate-to-severe anxiety levels; 42.4% had moderate-to-severe stress levels. The PCL-5 median score was 21.0 (IQR = 12.0-32.0), with 20% of the participants meeting the full criteria for PTSD. The MSPSS median score was 5.3 (IQR = 4.3-6.1), with 61.8% of the participants reporting high, 29.4% medium, and 8.8% low levels of social support, respectively. Logistic analysis revealed that being a younger person, female, having had previous exposure to COVID-19, working in inpatient facilities with COVID patients, and experiencing a lower level of social support were significant risk factors for the onset of mental disorders. Almost 75% of participants exhibited low-to-moderate adherence to psychological/psychiatric care.

Conclusions: Health professionals working with COVID patients need to be screened for mental disorders. A campaign aimed at achieving the de-stigmatization of mental care is required.

Key words: anxiety disorder, healthcare workers, depressive disorder, COVID-19, posttraumatic stress disorder.

INTRODUCTION

COVID-19 is a disease caused by the novel SARS-CoV-2 coronavirus first detected in Wuhan City, China, in December 2019 [1]. With the rapid spread of the virus worldwide and the numbers of infections increasing, the WHO declared the beginning of the pandemic on March 11th, 2020.

The growing morbidity rate, economic and political difficulties caused by the spread of the virus, and social dissatisfaction with quarantine measures led to a neglect by governments of psychological and social consequences of the pandemic, particularly among those fighting the disease on the frontline [2].

A number of previous studies of the pandemic's impact on medical workers reported a significant prevalence of mental health disorders and stress burnout [3-6]. Most of the studies of psychological outcomes of the COVID-19 pandemic were conducted either in high-income countries [7-10] or in China [11]. At the same time, medical personnel from low- and middle-income countries can be potentially more vulnerable to the psychological effects of the COVID-19 pandemic due to constant underfunding of medical facilities, higher workloads, and their lowered resilience.

From January to July 2021, the Ukrainian government reported over 2,253,269 confirmed cases of COVID-19,

including 52,951 deaths. The second wave of the pandemic that started in April, 2021 quickly reached its peak of more than 16,000 cases daily, giving Ukraine the highest level of daily morbidity in Europe [12].

METHODS

Study design and population

The cross-sectional study of healthcare workers using a web-based survey was conducted in Ukraine from April 12th to May 15th, 2021.

The sampling strategy

An invitation to participate was disseminated through professional interests groups with verified access to social media using the chain-referral sampling technique. The participant survey was administered online, using a web-based survey tool (Google Forms). Written informed consent was obtained from all participants at the beginning of the survey. Only the study administrator had access to the personal information of participants. The statistical analysis was performed on a cumulative sample that made it impossible to identify specific respondents.

The study goals and objectives were clearly explained to the participants at the beginning of the survey. The survey included socio-demographic characteristics; screening for anxiety, depression, stress, and posttraumatic symptoms; assessment of subjective perception of social support; and adherence to psychological or psychiatric care.

Measures

The Depression Anxiety Stress Scale (DASS-21) [13] is a set of three self-reported scales designed to evaluate the presence and measure the severity of symptoms of depression, anxiety, and stress. The DASS-21 has been successfully used and has demonstrated good psychometric properties in clinical and non-clinical samples, including the Ukrainian population [14]. The instrument was translated into Ukrainian using the standard "forward-backward" translation procedure. For the depression subscale, the normal range was considered to be below 9 points, mild from 10 to 13 points, moderate from 14 to 20 points, severe from 21 to 27 points, extremely severe over 27 points. For the anxiety subscale, normal severity was considered to be over 7 points, mild from 8 to 9 points, moderate from 10 to 14 points, severe from 15 to 19 points, extremely severe over 19 points. For the stress subscale, the normal severity range was considered to be below 14 points, mild severity from 15 to 18 points, moderate severity from 19 to 25 points, severe from 26 to 33 points, and extremely severe over 33 points. The DASS-21 demonstrated good internal consistency in the study ($\alpha = 0.81$-0.84).

The PTSD Checklist for DSM-5 (PCL-5) is a 20-item self-report measure that assesses the 20 DSM-5 symptoms of PTSD [15]. The instrument demonstrated good psychometric properties in the previous studies [16]. PCL-5 was translated into Ukrainian using the standard "forward-backward" translation procedure. The internal consistency of the PCL-5 for all four constructs in the study was acceptable ($\alpha = 0.70$-0.83).

The Multidimensional Scale of Perceived Social Support (MSPSS) [17] is a brief self-reported scale for measuring the perception of an individual's support from the following three sources: family, friends, and significant others. The instrument demonstrated acceptable-to-good psychometric properties in previous studies, depending on the translation [18]. As with the previous instruments, the MSPSS was translated into Ukrainian using the standard "forward-backward" translation procedure. Its internal consistency in the study was good ($\alpha = 0.92$-0.96).

Adherence to psychological services (a probability of referring for psychological or psychiatric care if needed) was assessed by a 10-point Likert-type scale, from 0 (did not apply to me at all/never) to 10 (always applies). The scores from 0 to 4 were considered as low adherence, scores from 5 to 7 as moderate adherence, and scores from 8 to 10 as high adherence to psychological or psychiatric care.

Sample size

Based on the pooled results from published studies [19], a minimum number of 281 respondents was required to achieve 90% power at 0.05 type I error rate.

Data analysis

Statistical analysis was performed using the Statistical Package for Social Sciences (SPSS, version 21.0). Descriptive statistics were provided as mean (SD) and frequency (%), as appropriate. Continuous data were reported as mean ± SD for normally distributed variables and median (interquartile range – IQR) for non-normally distributed variables. The Pearson's χ^2 test was used to compare the frequency distributions between different demographic groups. Logistic regression was used to estimate the odds ratios (ORs) with 95% confidence intervals (CIs) for depression, anxiety, stress, and posttraumatic stress disorder. Logistic regression analysis was carried out using anxiety (≥ 11), depression (≥ 15), and stress (≥ 18) scores above their respective cut-off points. A logistic regression analysis for PTSD was carried out using the PTSD symptoms score (≥ 31) above the cut-off and the minimal number of symptoms required for DSM-5. As different forms of perceived social support were highly correlated variables, we only included overall perceived social support as a covariate in the regression analyses, so as to avoid multicollinearity.

Ethical approval

This study was approved by the Committee of Ethics in SI "Research Institute for Psychiatry of the Ministry of Health of Ukraine". All procedures were in accordance with the ethical standards of the institutional and the national research committees as well as with the Declaration of Helsinki (1964) and its amendments or comparable ethical standards. As participation was voluntary and the study was conducted without any interventions, meaning that it involved no more than minimal risk, the Research Ethics Committee approved a consent waiver.

RESULTS

Of the 837 total respondents, 392 completed the survey, a response rate of 46.8%. A total of 62 respondents with pre-existing mental disorders were excluded, and the remaining 330 responses were analyzed. The mean age of the study participants was 42.0 (IQR 32.0-52.0) years. The majority of respondents (265 – 80.3%) were females, 281 (85.2%) were medical doctors, and 272 (82.4%) were working in cities with a population of over 100,000 people. Over one-third of the sample group were working with COVID-19 patients in inpatient units. Among the study participants, 84 (25.5%) had severe chronic disorders, 184 (55.8%) had recovered from COVID-19 in the previous 6 months, and 206 (62.4%) had relatives who had recovered from COVID-19. The sociodemographic characteristics of the sample group are presented in Table 1.

Mental health status of the study participants

The median DASS-21 total score was 42.0 (IQR = 28.0-56.0). Among the participants above the cut-off points 241 (73.3%) of respondents scored on the depression scale, 212 (64.2%) on the anxiety scale, and 197 (59.7%) on the stress scale, respectively. On the depression subscales 75 (22.8%) respondents reported mild symptoms, 106 (32.2%) reported moderately severe symptoms, 42 (12.8%) severe symptoms, and 18 (5,5%) extremely severe symptoms. On the anxiety subscale 29 (8.8%) participants reported mild anxiety, 90 (27.3%) experienced moderate anxiety, 47 (14.2%) had severe anxiety, and 46 (13.9%) had extremely severe anxiety. For the stress scale, 57 (17.3%) reported mild stress, 80 (24.3%) moderate, 53 (16.0%) severe, and 7 (2.1%) reported extremely severe stress.

The PCL-5 median score was 21.0 (IQR = 12.0-32.0). Among all respondents, 80 (24.2%) scored above the recommended total score for PTSD, 90 (27.2%) had the minimum number of symptoms necessary to be diagnosed with PTSD according to DSM-5. At the same time, only 66 (20.0%) participants had both the required severity and minimum number of symptoms to meet the DSM-5 criteria for PTSD.

Subjective appraisal of social support and adherence to psychological services among the study participants

The median MSPSS total score was 5.3 (IQR = 4.3-6.1). Among all participants, 29 (8.8%) respondents reported low, 97 (29.4%) moderate and 204 (61.8%) high overall sense of perceived support. For the significant other subscale, 43 (13.0%) participants reported low social support, 55 (16.7%) moderate social support, and 232 (70.3%) high social support. For the family subscale, 33 (10.0%) participants reported low social support, 54 (16.4%) moderate social support, and 243 (73.6%) high social support. For the friends subscale, 50 (15.2%) participants reported low social support, 74 (22.4%) moderate social support, and 206 (62.4%) high social support. The results of the assessment are given in Table 2.

The median score of adherence to psychological services was 5.0 (IQR = 2.0-8.0). Among all respondents, 160 (48.5%) reported low adherence (1-4 points) to psychological or psychiatric treatment, 84 (25.5%) moderate adherence (5-7 points), and 86 (26.0%) high adherence (8-10 points). Among medical workers, females ($p = 0.012$) and participants working in big cities (population over 100.000 people) ($p = 0.044$) demonstrated higher degrees of adherence to psychological and psychiatric care.

Association of sociodemographic factors and perceived social support with the mental status of participants

The results given in Table 1 show that female participants had significantly higher rates of moderate-to-severe anxiety ($p = 0.001$) and stress ($p = 0.003$). The younger respondents also reported significantly higher rates of depression and stress. Medical professionals that were working in cities with populations below 100,000 inhabitants had higher scores for depression ($p = 0.048$), anxiety ($p = 0.047$), and stress ($p = 0.006$). Medical professionals who recovered from COVID-19 reported higher rates of anxiety ($p = 0.026$). Respondents working in inpatient facilities with COVID-19 patients had higher rates for depression ($p = 0.025$) and PTSD ($p = 0.006$).

The results presented in Table 2 suggest that respondents with a high level of overall perceived social support on the MSPSS ($p = 0.002$), a high level of support from their families ($p = 0.031$), and a high level of support from friends ($p = 0.047$) had lower scores for depression. Medical professionals with high support from significant others ($p = 0.011$) reported lower scores for anxiety. Respondents with high overall social support ($p = 0.021$), high family support ($p = 0.022$), and high support from their friends ($p = 0.005$) had lower scores for stress. Those with high support from friends also had lower PTSD scores ($p = 0.04$).

© 2022 Institute of Psychiatry and Neurology. Production and hosting by Termedia sp. z o.o.
This is an open access article under the CC BY-NC-ND license (http://creativecommons.org/licenses/by-nc-nd/4.0/)

Table 1. Sample demographic variables and it associations with mental health outcomes

Variables	n (%)	DASS-21 (Depression)			DASS-21 (Anxiety)			DASS-21 (Stress)			PCL-5 (PTSD)		
		Normal or mild, *n* (%)	Moderate or severe, *n* (%)	*p*	Normal or mild, *n* (%)	Moderate or severe, *n* (%)	*p*	Normal or mild, *n* (%)	Moderate or severe, *n* (%)	*p*	Don't meet criteria, *n* (%)	Meet criteria[a], *n* (%)	*p*
Gender													
Male	65 (19.7)	39 (60.0)	26 (40.0)	0.064*	41 (63.1)	24 (36.9)	0.001*	48 (73.8)	17 (26.2)	0.003*	57 (87.7)	8 (12.3)	0.084*M
Female	265 (80.3)	125 (47.2)	140 (52.8)		106 (40.0)	159 (60.0)		142 (53.6)	123 (46.4)		207 (78.1)	58 (21.9)	
Age, years													
20-32	83 (25.2)	37 (44.6)	46 (55.4)	0.038*	34 (41.0)	49 (59.0)	0.063*	46 (55.4)	37 (44.6)	0.036*	62 (74.7)	21 (25.3)	0.255*
33-42	87 (26.3)	42 (48.3)	45 (51.7)		41 (47.1)	46 (52.9)		45 (51.7)	42 (48.3)		65 (74.7)	22 (25.3)	
43-52	82 (24.8)	44 (53.7)	38 (46.3)		37 (45.1)	45 (54.9)		48 (58.5)	34 (41.5)		67 (81.7)	15 (18.3)	
> 52	78 (23.7)	41 (52.6)	37 (47.4)		35 (44.9)	43 (55.1)		51 (65.4)	27 (34.6)		70 (89.7)	8 (10.3)	
Chronic disorders													
Yes	84 (25.5)	47 (56.0)	37 (44.0)	0.184*	35 (41.7)	49 (58.3)	0.539*	54 (64.3)	30 (35.7)	0.136*	68 (81.0)	16 (19.0)	0.468*
No	246 (74.5)	117 (47.6)	129 (52.4)		112 (45.5)	134 (54.5)		136 (55.3)	110 (44.7)		196 (79.7)	50 (20.3)	
Job status													
Doctor	281 (85.2)	138 (49.1)	143 (50.9)	0.61*	124 (44.1)	157 (55.9)	0.71*	161 (57.3)	120 (42.7)	0.80*	222 (79.0)	59 (21.0)	0.27*
Nurse	49 (14.8)	26 (53.1)	23 (46.9)		23 (46.9)	26 (53.1)		29 (59.2)	20 (40.8)		42 (85.7)	7 (14.3)	
Hospital location													
Cities > 100.000 population	272 (82.4)	142 (52.2)	130 (47.8)	0.048*	128 (47.1)	144 (52.9)	0.047*	166 (61.0)	106 (39.0)	0.006*	223 (82.0)	49 (18.0)	0.051*
Cities < 100.000 population	58 (17.6)	22 (37.9)	36 (62.1)		19 (32.8)	39 (67.2)		24 (41.4)	34 (58.6)		41 (70.7)	17 (29.3)	
Recovered from COVID-19 in past 6 months													
Yes	184 (55.8)	84 (45.7)	100 (54.3)	0.099*	72 (39.1)	112 (60.9)	0.026*	99 (53.8)	85 (46.2)	0.120*	146 (79.3)	38 (20.7)	0.740*
No	146 (44.2)	80 (54.8)	66 (45.2)		75 (51.4)	71 (48.6)		91 (62.3)	55 (37.7)		118 (80.8)	28 (19.2)	
Close relatives suffered from COVID-19													
Yes	206 (62.4)	107 (51.9)	99 (48.1)	0.293*	84 (40.8)	122 (59.2)	0.076*	115 (55.8)	91 (44.2)	0.407*	166 (80.6)	40 (19.4)	0.419*
No	124 (37.6)	57 (46.0)	67 (54.0)		63 (50.8)	61 (49.2)		75 (60.5)	49 (39.5)		98 (79.0)	26 (21.0)	
Working with COVID-19 now, inpatient													
Yes	104 (31.5)	42 (40.4)	62 (59.6)	0.025*	43 (41.3)	61 (58.7)	0.428*	56 (53.8)	48 (46.2)	0.352*	74 (71.2)	30 (28.8)	0.006*
No	226 (68.5)	122 (54.0)	104 (46.0)		104 (46.0)	122 (54.0)		134 (59.3)	92 (40.7)		190 (84.1)	36 (15.9)	

The χ^2 statistic is significant at $p < 0.05$ level. [a]Both minimum severity on PCL-5 and amount of symptoms required.

The association between estimated co-variables and the mental status of the participants was analyzed using binary logistic regression, and the results are presented in Tables 3-6.

It is considered that moderate-to-extremely severe scores on the DASS-21 subscales suggest the highest possibility of the presence of anxiety disorder, depression, and stress burnout. For PTSD diagnoses, we considered cases that met both the minimum number of symptoms and the severity score required for PCL-5.

The results in Table 3 demonstrate that for a medical worker, being a female (OR = 1.89, 95% CI: 1.05-3.38, $p = 0.032$), working with COVID-19 patients in inpatient units (OR = 1.82, 95% CI: 1.08-3.04, $p = 0.022$), and having a low level of perceived overall social support (OR = 2.19, 95% CI: 1.36-3.54, $p < 0.001$) were associated with possible depression.

The results presented in Table 4 show that being a female (OR = 2.54, 95% CI: 1.44-4.49, $p < 0.001$) who had recovered from COVID-19 in the previous 6 months (OR = 1.61, 95% CI: 1.02-2.54, $p = 0.04$) were also associated with possible anxiety disorder.

Table 5 presents data to the effect that being female (OR = 2.5, 95% CI: 1.23-4.64, $p = 0.004$), working in small cities (OR = 2.01, 95% CI: 1.11-3.67, $p = 0.021$), and having low level of perceived overall social support (OR = 1.91, 95% CI: 1.19-3.06, $p = 0.007$) were associated with severe stress.

The results given in Table 6 show that working in small cities (OR = 2.3, 95% CI: 1.15-4.57, $p = 0.017$) and working in inpatient units with COVID-19 patients (OR = 2.08, 95% CI: 1.14-3.77, $p = 0.016$) were associated with possible PTSD in health care staff. Being of older age (OR = 0.96, 95% CI: 0.94-0.99, $p = 0.013$) was associated with a lower chance of suffering from PTSD.

DISCUSSION

This study sought to estimate the prevalence of depressive, anxiety, stress, and PTSD symptoms in a sample of medical workers during the COVID-19 pandemic, and their association with the risk and protective factors. As a second goal, the study investigated adherence to psychological and psychiatric services among the medical community in Ukraine.

This study was conducted during the peak of the second wave of the COVID-19 pandemic in the country, which was characterized by the placing of a critical load on the medical infrastructure and health care staff. To our knowledge, the only other study that previously assessed the psychological impact of COVID-19 on medical workers was conducted in Ukraine [20]. However, it involved a relatively small sample size and focused only on investigating the prevalence of depression and anxiety.

Table 2. Sample perceived social support variables and it associations with mental health

Variables	n (%)	DASS-21 (Depression)			DASS-21 (Anxiety)			DASS-21 (Stress)			PCL-5 (PTSD)		
		Normal or mild, n (%)	Moderate or severe, n (%)	p	Normal or mild, n (%)	Moderate or severe, n (%)	p	Normal or mild, n (%)	Moderate or severe, n (%)	p	Don't meet criteri, n (%)a	Meet criteria[a], n (%)	p
Total MSPPS score													
High	204 (61.8)	115 (56.4)	89 (43.6)	0.002*	96 (47.1)	108 (52.9)	0.097*	129 (63.2)	75 (36.8)	0.021*	166 (81.4)	38 (18.6)	*0.545
Moderate and low	126 (38.2)	49 (38.9)	77 (61.1)		51 (40.5)	75 (59.5)		61 (48.4)	65 (51.6)		98 (77.8)	28 (22.2)	
MSPSS significant other													
High	232 (70.3)	119 (51.3)	113 (48.7)	0.279*	102 (44.0)	130 (56.0)	0.011*	137 (59.1)	95 (40.9)	0.105*	185 (79.7)	47 (20.3)	0.929*
Moderate and low	98 (29.7)	45 (45.9)	53 (54.1)		45 (45.9)	53 (54.1)		53 (54.1)	45 (45.9)		79 (80.6)	19 (19.4)	
MSPSS family													
High	243 (73.6)	129 (53.1)	114 (46.9)	0.031*	111 (45.7)	132 (54.3)	0.260*	149 (61.3)	94 (38.7)	0.022*	194 (79.8)	49 (20.2)	0.72*
Moderate and low	87 (26.4)	35 (40.2)	52 (59.8)		36 (41.4)	51 (58.6)		41 (47.1)	46 (52.9)		70 (80.5)	17 (19.5)	
MSPSS friends													
High	206 (62.4)	113 (54.9)	93 (45.1)	0.047*	96 (46.6)	110 (53.4)	0.084*	132 (64.1)	74 (35.9)	0.005*	166 (80.6)	40 (19.4)	0.040*
Moderate and low	124 (37.6)	51 (41.1)	73 (58.9)		51 (41.1)	73 (58.9)		58 (46.8)	66 (53.2)		98 (79.0)	26 (21.0)	

The χ^2 statistic is significant at $p < 0.05$ level. [a]Both minimum severity on PCL-5 and amount of symptoms required.

© 2022 Institute of Psychiatry and Neurology. Production and hosting by Termedia sp. z o.o.
This is an open access article under the CC BY-NC-ND license (http://creativecommons.org/licenses/by-nc-nd/4.0/)

Table 3. Results of logistic regression predicting moderate-to-extremely severe scores for DASS-21 depression subscale in study participants

Risk factor	Final model				
	**B**	**SE**	**W**	**OR**	**95% CI**
Female gender	0.638	0.297	4.616	1.89*	1.05-3.38
Age (years)	-0.003	0.011	0.084	0.997	0.97-1.01
Chronic disorder	0.142	0.297	0.228	1.16	0.64-2.06
Work in cities < 100.000 population	0.530	0.312	2.886	1.7	0.922-3.31
Being a nurse	0.264	0.330	0.638	1.3	0.68-2.48
Work with COVID-19, inpatient	0.599	0.262	5.228	1.82*	1.08-3.04
COVID recovered[a]	0.328	0.237	1.919	1.38	0.87-2.2
Low or moderate social support[b]	0.787	0.244	10.425	2.19***	1.36-3.54

R^2 = 0.105 (Nagelkerke). Model χ^2 = 26.9. W – Wald statistic, OR – odds ratio, CI – confidence interval. [a]Recovered from COVID-19 in past 6 months. [b]MSPSS total score.
**p < 0.05; **p < 0.01; ***p < 0.001.*

Table 4. Results of logistic regression predicting moderate-to-extremely severe scores for DASS-21 anxiety subscale in study participants

Risk factor	Final model				
	**B**	**SE**	**W**	**OR**	**95% CI**
Female gender	0.933	0.290	10.353	2.54***	1.44-4.49
Chronic disorder	0.209	0.281	0.554	1.23	0.71-2.13
Work in cities < 100.000 population	0.467	0.315	2.19	1.59	0.86-2.95
Recovered from COVID-19[a]	0.478	0.232	4.227	1.61*	1.02-2.54
Low or moderate social support[b]	0.360	0.241	2.230	1.43	0.89-2.3

R^2 = 0.083 (Nagelkerke). Model χ^2 = 21.0. W – Wald statistic, OR – odds ratio, CI – confidence interval. [a]In past 6 months. [b]MSPSS total score.
**p < 0.05; **p < 0.01; ***p < 0.001.*

Table 5. Results of logistic regression predicting moderate-to-extremely severe scores for DASS-21 stress subscale in study participants

Risk factor	Final model				
	**B**	**SE**	**W**	**OR**	**95% CI**
Female gender	0.919	0.315	8.496	2.5**	1.35-4.64
Chronic disorders	0.305	0.288	1.120	1.35	0.77-2.38
Work in cities < 100.000 population	0.703	0.305	5.293	2.01*	1.11-3.67
Recovered from COVID-19[a]	0.344	0.237	2.105	1.41	0.88-2.24
Low or moderate social support[b]	0.647	0.241	7.231	1.91**	1.19-3.06

R^2 = 0.103 (Nagelkerke). Model χ^2 = 26.2. W – Wald statistic, OR – odds ratio, CI – confidence interval. [a]In past 6 months. [b]MSPSS total score.
**p < 0.05; **p < 0.01; ***p < 0.001.*

Table 6. Results of logistic regression predicting PTSD in study participants

Risk factor	Final model				
	**B**	**SE**	**W**	**OR**	**95% CI**
Female gender	0.791	0.422	3.515	2.2	0.96-5.04
Age	–0.033	0.013	6.172	0.968*	0.943-.993
Work in cities <100.000 population	0.833	0.350	5.654	2.3*	1.15-4.57
Work with COVID-19, inpatient	0.734	0.304	5.830	2.08*	1.14-3.77
Low or moderate social support[a]	0.201	0.293	0.472	1.22	0.68-2.17

R^2 = 0.107 (Nagelkerke). Model χ^2 = 23.1. W – Wald statistic, OR – odds ratio, CI – confidence interval. [a]MSPSS total score.
**p < 0.05; **p < 0.01; ***p < 0.001.*

In our study, the prevalence of moderate-to-severe depressive symptoms was 50.5%, moderate-to-severe anxiety was 55.4%, moderate-to-severe stress 42.4%, and 20% for PTSD. However, these rates were higher than those reported in the abovementioned study, which were 19.8% and 18.5% for moderate-to-severe depression and anxiety, respectively. This difference can be attributed to the fact that Khaustova *et al.* [20] conducted their study before the onset of the second wave of the pandemic in Ukraine.

On the other hand, we observed a higher prevalence of depression and anxiety than the reported pooled prevalence of 31.1% and 30.0%, respectively [21], though it was within the range for low-middle income countries and highly-affected countries [22-24]. This can result from underfunded and understaffed medical facilities that are commonly observed in low-middle income countries and countries in humanitarian crisis, and can be associated with shortage of personal protective equipment, higher workloads, and additional social pressure, which were established as significant risk factors [25]. The prevalence of PTSD in the study was consistent with other studies [26].

In our study, the female gender was associated with a higher likelihood of depression, anxiety, and stress burnout, but not with PTSD. These findings were in agreement with other studies of medical staff during the COVID-19 pandemic [7, 27]. Being of a younger age was associated with a higher prevalence of PTSD symptoms that, from a wider perspective, could be a reflection of the association between the number of years of professional experience and PTSD symptoms [6]. We found that working in inpatient units with COVID-19 patients was associated with higher odds of developing depression and PTSD. Similarly, Lai and colleagues reported that frontline workers are at greater risk for the onset of mental disorders [22]. Regression analysis also demonstrated that medical workers who recovered from COVID in the previous 6 months were more prone to report higher anxiety, which may be a result of "post-COVID syndrome" [28]. Finally, low social support was associated with higher rates of depression and stress burnout that was consistent with the previous results [29].

As we had expected, we found a significant prevalence of negative psychological outcomes among health care staff in Ukraine. Additionally, we investigated the adherence of medical workers to psychological or psychiatric services and found that only 26.0% of respondents will probably seek psychological/psychiatric care in case of need. Given the central role the medical community plays in shaping the perception of mental health and psychological and psychiatric services in society, the significance of this cannot be underestimated.

At the same time, reducing mental health stigma among medical professionals is important for the improvement of the quality of medical care for persons with mental disorders [30].

This study has a certain number of limitations. Firstly, this concerns the cross-sectional design of the study and the absence of follow-up. Secondly, the chain-referral sampling initiated in social networks among medical professionals may not be representative of the general population. Thirdly, the instruments used in the study were not validated. Even though the DASS-21, PCL-5, and MSPSS were "forward-backward" translated, no studies on their validity or psychometric properties in Ukrainian have been conducted to date.

CONCLUSIONS

This research reports on the psychological burden of medical staff during the second wave of the COVID-19 pandemic in Ukraine. Severe symptoms of depression, anxiety, and stress were reported by 18.3%, 28.1%, and 18.1% of respondents, respectively. Over 20% of medical workers met the criteria for PTSD. Female gender, age, working in inpatient units with COVID patients, being recovered from COVID in the previous 6 months, and low social support were all risk factors for mental disorders in health professionals. At the same time, medical professionals demonstrated a relatively small level of adherence to psychological and psychiatric care. In public health emergencies, considerably more attention needs to be paid to the psychological well-being of medical professionals. More effort needs to be put in the organizing of psychological on-site support, and the de-stigmatization of psychological and psychiatric care in the society at large.

Conflict of interest

Absent.

Financial support

Absent.

© 2022 Institute of Psychiatry and Neurology. Production and hosting by Termedia sp. z o.o.
This is an open access article under the CC BY-NC-ND license (http://creativecommons.org/licenses/by-nc-nd/4.0/)

References

1. Huang C, Wang Y, Li X, Ren L, Zhao J, Hu Y, et al. Clinical features of patients infected with 2019 novel coronavirus in Wuhan, China. Lancet 2020; 395: 497-506.
2. Torales J, O'Higgins M, Castaldelli-Maia JM, Ventriglio A. The outbreak of COVID-19 coronavirus and its impact on global mental health. Int J Soc Psychiatry 2020; 66: 317-320.
3. Liu X, Kakade M, Fuller CJ, Fan B, Fang Y, Kong J, et al. Depression after exposure to stressful events: lessons learned from the severe acute respiratory syndrome epidemic. Compr Psychiatry 2012; 53: 15-23.
4. Barello S, Falcó-Pegueroles A, Rosa D, Tolotti A, Graffigna G, Bonetti L. The psychosocial impact of flu influenza pandemics on healthcare workers and lessons learnt for the COVID-19 emergency: a rapid review. International journal of public health. Int J Public Health 2020; 65: 1205-1216.
5. Luo M, Guo L, Yu M, Jiang W, Wang H. The psychological and mental impact of coronavirus disease 2019 (COVID-19) on medical staff and general public – a systematic review and meta-analysis. Psychiatry Res 2020; 291: 113190.
6. Carmassi C, Foghi C, Dell'Oste V, Cordone A, Bertelloni CA, Bui E, et al. PTSD symptoms in healthcare workers facing the three coronavirus outbreaks: what can we expect after the COVID-19 pandemic. Psychiatry Res 2020; 292: 113312.
7. Tee ML, Tee CA, Anlacan JP, Aligam K, Reyes P, Kuruchittham V, et al. Psychological impact of COVID-19 pandemic in the Philippines. J Affect Disord 2020; 277: 379-391.
8. Chew N, Lee G, Tan B, Jing M, Goh Y, Ngiam N, et al. A multinational, multicentre study on the psychological outcomes and associated physical symptoms amongst healthcare workers during COVID-19 outbreak. Brain Behav Immun 2020; 88: 559-565.
9. Luceño-Moreno L, Talavera-Velasco B, García-Albuerne Y, Martín-García J. Symptoms of posttraumatic stress, anxiety, depression, levels of resilience and burnout in Spanish health personnel during the COVID-19 pandemic. Int J Environ Res Public Health 2020; 17: 5514.
10. Murat M, Köse S, Savaşer S. Determination of stress, depression and burnout levels of front-line nurses during the COVID-19 pandemic. Int J Ment Health Nurs 2021; 30: 533-543.
11. Pappa S, Ntella V, Giannakas T, Giannakoulis VG, Papoutsi E, Katsaounou P. Prevalence of depression, anxiety, and insomnia among healthcare workers during the COVID-19 pandemic: a systematic review and meta-analysis. Brain Behav Immun 2020; 88: 901-907.
12. World Health Organization: Ukraine: WHO Coronavirus Disease (COVID-19) Dashboard With Vaccination Data. WHO; 2021. Available at: https://covid19.who.int/region/euro/country/ua/ (Accessed: 02.08.2021).
13. Lovibond SH, Lovibond PF. Manual for the Depression Anxiety Stress Scales. 2nd ed. Sydney: Psychology Foundation; 1995.
14. Przepiórka A, Błachnio A, Sullman M, Gorbaniuk O, Siu NY, Hill T, et al. Facebook intrusion as a mediator between positive capital and general distress: a cross-cultural study. Front Psychiatry 2021; 12: 667536.
15. Weathers FW, Litz BT, Keane TM, Palmieri PA, Marx BP, Schnurr PP. The PTSD Checklist for DSM-5 (PCL-5) – Standard [Measurement instrument]. National Center for PTSD; 2013. Available at: https://www.ptsd.va.gov/.
16. Blevins CA, Weathers FW, Davis MT, Witte TK, Domino JL. The Posttraumatic Stress Disorder Checklist for DSM-5 (PCL-5): development and initial psychometric evaluation. J Trauma Stress 2015; 28: 489-498.
17. Zimet GD, Dahlem NW, Zimet SG, Farley GK. The multidimensional scale of perceived social support. J Pers Assess 1988; 52: 30-34.
18. Dambi JM, Corten L, Chiwaridzo M, Jack H, Mlambo T, Jelsma J. A systematic review of the psychometric properties of the cross-cultural translations and adaptations of the Multidimensional Perceived Social Support Scale (MSPSS). Health Qual Life Outcomes 2018; 16: 80.
19. Santabárbara J, Bueno-Notivol J, Lipnicki DM, Olaya B, Pérez-Moreno M, Gracia-García P, et al. Prevalence of anxiety in health care professionals during the COVID-19 pandemic: a rapid systematic review (on published articles in Medline) with meta-analysis. Prog Neuropsychopharmacol Biol Psychiatry 2021; 107: 110244.
20. Khaustova O, Chaban O, Assonov D. P.655 Resilience and emotional state of healthcare professionals in Ukraine during lockdown: a pilot study. Eur Neuropsychopharmacol 2020; 40: 370-371.
21. Marvaldi M, Mallet J, Dubertret C, Moro MR, Guessoum SB. Anxiety, depression, trauma-related, and sleep disorders among healthcare workers during the COVID-19 pandemic: a systematic review and meta-analysis. Neurosci Biobehav Rev 2021; 126: 252-264.
22. Lai J, Ma S, Wang Y, Cai Z, Hu J, Wei N, et al. Factors associated with mental health outcomes among health care workers exposed to coronavirus disease 2019. JAMA Netw Open 2020; 3: e203976.
23. Elbay RY, Kurtulmuş A, Arpacıoğlu S, Karadere E. Depression, anxiety, stress levels of physicians and associated factors in Covid-19 pandemics. Psychiatry Res 2020; 290: 113130.
24. Consolo U, Bellini P, Bencivenni D, Iani C, Checchi V. Epidemiological aspects and psychological reactions to COVID-19 of dental practitioners in the Northern Italy districts of Modena and Reggio Emilia. Int J Environ Res Public Health 2020; 17: 3459.
25. Sahebi A, Nejati-Zarnaqi B, Moayedi S, Yousefi K, Torres M, Golitaleb M. The prevalence of anxiety and depression among healthcare workers during the COVID-19 pandemic: an umbrella review of meta-analyses. Prog Neuropsychopharmacol Biol Psychiatry 2021; 107: 110247.
26. Li Y, Scherer N, Felix L, Kuper H. Prevalence of depression, anxiety and post-traumatic stress disorder in health care workers during the COVID-19 pandemic: a systematic review and meta-analysis. PLoS One 2021; 16: e0246454. https://doi.org/10.1371/journal.pone.0246454.

27. Chong MY, Wang WC, Hsieh WC, Lee CY, Chiu NM, Yeh WC, et al. Psychological impact of severe acute respiratory syndrome on health workers in a tertiary hospital. Br J Psychiatry 2004; 185: 127-133.
28. Fernández-de-Las-Peñas C, Gómez-Mayordomo V, de-la-Llave-Rincón AI, Palacios-Ceña M, Rodríguez-Jiménez J, Florencio LL, et al. Anxiety, depression and poor sleep quality as long-term post-COVID sequelae in previously hospitalized patients: a multicenter study. J Infect 2021; 0163-4453(21)00319-4. DOI: https://doi.org/10.1016/j.jinf.2021.06.022.
29. Busch IM, Moretti F, Mazzi M, Wu AW, Rimondini M. What we have learned from two decades of epidemics and pandemics: a systematic review and meta-analysis of the psychological burden of frontline healthcare workers. Psychother Psychosom 2021; 90: 178-190.
30. Lien YY, Lin HS, Lien YJ, Tsai CH, Wu TT, Li H, et al. Challenging mental illness stigma in healthcare professionals and students: a systematic review and network meta-analysis. Psychol Health 2021; 36: 669-684.

© 2022 Institute of Psychiatry and Neurology. Production and hosting by Termedia sp. z o.o.
This is an open access article under the CC BY-NC-ND license (http://creativecommons.org/licenses/by-nc-nd/4.0/)
