# Supplementary material for: Mental health status and related factors influencing healthcare workers during the COVID-19 pandemic: A systematic review and meta-analysis
Source: PLoS One. 2024 Jan 19;19(1):e0289454. doi: 10.1371/journal.pone.0289454 (PMC10798549; doi:10.1371/journal.pone.0289454)
Supplement: S1 Data — (ZIP) [file pone.0289454.s011.zip › literatures/91.pdf]

BMJ Open

Prevalence of anxiety and depressive symptoms among medical residents in Tunisia: a cross-sectional survey

Mehdi Marzouk, Lamia Ouanes-Besbes, Islem Ouanes, Zeineb Hammouda, Fahmi Dachraoui, Fekri Abroug

To cite: Marzouk M, Ouanes-Besbes L, Ouanes I, *et al*. Prevalence of anxiety and depressive symptoms among medical residents in Tunisia: a cross-sectional survey. *BMJ Open* 2018;**8**:e020655. doi:10.1136/bmjopen-2017-020655

► Prepublication history for this paper is available online. To view these files, please visit the journal online (http://dx.doi.org/10.1136/bmjopen-2017-020655).

Received 15 November 2017
Revised 21 May 2018
Accepted 7 June 2018

Check for updates

© Author(s) (or their employer(s)) 2018. Re-use permitted under CC BY-NC. No commercial re-use. See rights and permissions. Published by BMJ.

Intensive Care Department, Teaching Hospital, Fatouma Bourguiba and Research Laboratory (LR12SP15), University of Monastir, Monastir, Tunisia

Correspondence to
Dr Fekri Abroug;
f.abroug@rns.tn

ABSTRACT

Objective To assess the prevalence of anxiety and depressive symptoms and the associated risk factors among Tunisian medical residents.

Design Cross-sectional survey.

Setting Faculty of Medicine, Tunis.

Participants All Tunisian medical residents brought together between 14 and 22 December 2015 to choose their next 6-month rotation.

Intervention The items of the Hospital Anxiety and Depression (HAD) questionnaire were employed to capture the prevalence of anxiety and/or depression among the residents. The statistical relationships between anxiety and depression (HAD score) and sociodemographic and work-related data were explored by Poisson regression.

Results 1700 out of 2200 (77%) medical residents (mean age: 28.5±2 years, female: 60.8%) answered the questionnaire. The mean working hours per week was 62±21 hours; 73% ensured a mean of 5.4±3 night shifts per month; and only 8% of them could benefit from a day of safety rest. Overall, 74.1% of the participating residents had either definite (43.6%) or probable (30.5%) anxiety, while 62% had definite (30.5%) or probable (31.5%) depression symptoms, with 20% having both definite anxiety and definite depression. The total HAD score was significantly associated with the resident's age (OR=1.014, 95% CI 1.006 to 1.023, p=0.001); female gender (OR=1.114, 95% CI 1.083 to 1.145, p<0.0001); and the heavy burden of work imposed on a weekly or monthly basis, as reflected by the number of night shifts per month (OR=1.048, 95% CI 1.016 to 1.082, p=0.03) and the number of hours worked per week (OR=1.008, 95% CI 1.005 to 1.011, p<0.0001). Compared with medical specialties, the generally accepted difficult specialties (surgical or medical-surgical) were associated with a higher HAD score (OR=1.459, 95% CI 1.172 to 1.816, p=0.001).

Conclusion Tunisian residents experience a rate of anxiety/depression substantially higher than that reported at the international level. This phenomenon is worrying as it has been associated with an increase in medical errors, work dissatisfaction and attrition. The means of improving the well-being of Tunisian medical residents are explored, emphasising those requiring immediate implementation.

Strengths and limitations of this study

- ► This is the first study to assess the prevalence of anxiety/depression among Tunisian residents.
- ► This study has a large participation rate.
- ► The Hospital Anxiety and Depression questionnaire is in French, but this language is well mastered by Tunisian residents.
- ► There is no information on the rates of mood disorders in the general Tunisian population for comparison.
- ► There was no assessment of the prevalence of burnout syndrome.

Physicians are increasingly exposed to stressful situations regardless of age, gender or seniority in the profession.[1–4] Only a few variables pertaining to physicians' workload or personalities can either attenuate or exaggerate the stress-related mood disorders (anxiety, depression, burnout), and modurate their impact on the personal or professional life.[5 6] Medical residents seem at an increased risk of developing mental disorders.[7–10] These disorders may hamper physicians' professional performance, affecting their concentration at work and the quality of healthcare services provided, and provoking conflicts with patients or their families, or with colleagues.[9 11 12] These mental health issues are also associated with higher substance use and abuse, divorce, and even suicidal ideation.[12–14] A recent systematic review reported a 28.8% pooled prevalence of depression or depressive symptoms among medical residents, with a wide variation in this rate depending on the instrument used.[15] In countries with socioeconomic conditions similar to those of Tunisia, the prevalence of depressive symptoms among medical residents ranges from 17% in India to 48% in Argentina.[16–18]

Tunisia is a country that is currently experiencing major socioeconomic and political changes as a consequence of the 'Arab spring'. Arab spring was a series of anti-government protests or armed rebellions

ignited in Tunisia by the so-called 'Jasmine revolution', which spread across the North African countries and in the Middle East. It is seen as the translation of people's aspirations to democracy and to replace dictators in place. According to the United Nations Development Programme, the average annual Human Development Index has fallen in Tunisia from 0.88% during the decade before the advent of the Arab spring, to 0.25% during the 5 years that followed. This led to a sustained erosion of the middle class, an increase in poverty and a decrease in overall wealth.[19] The Tunisian downturn has particularly altered the relationship between the main body of the society and the medical profession, the latter being perceived by a large part of the population as a body of privileged, insufficiently empathic and with little concern for the misfortunes of the population. Assaults against doctors, particularly in public hospitals and their emergency services, malpractice suits and prosecutions, are now very frequent and are highly publicised on a daily basis by various media in the country. Just after the revolution, public hospitals experienced a sharp deterioration in the resources and staff available, together with an alteration in their governance.[20] This situation has greatly frustrated the population, in particular the citizens who cannot afford the services provided by the private medical sector and its large out-of-pocket costs.[21]

Residents come to term with a long, demanding and selective course of studies. A typical resident must have passed his baccalaureate and be among the first-ranked ones and among the 'happy few' (3%–5% top-ranked graduates) who enter one of the four Tunisian medical schools. After 7 years of medical studies, graduates wishing to specialise have to successfully undergo a final residency test, which usually takes two to three attempts (annual passing rate=25%). With an age of at least 26, the medical resident faces the reality in a public hospital, where residents are the only physicians present at night, on weekends and on holidays. They are also exposed to stress that result from non-optimal working conditions: the need to meet professional duties set by the supervisors and the growing demand of the population.

We hypothesised that the combination of all these unfavourable circumstances for professional fulfilment result in a high level of mood disorders in medical residents. We conducted a large cross-sectional survey to address the prevalence of anxiety and depressive symptoms and the associated risk factors among Tunisian medical residents.

PATIENTS AND METHODS

Participants

In Tunisia, specialisation in medicine takes place at the end of general medical studies. Doctors who wish to specialise do so following a national test, bringing together candidates from four faculties of medicine. This test is very selective and takes place over 3 days. It consists of more than 300 questions (multiple choice, short answer), separately covering each of the following areas: medical specialties, surgical specialties and basic sciences.

More than 1500 candidates participate annually, for about 500 elected members, who are ranked consecutively in order of merit. The choice of specialty is then made according to their ranking on the test, on a number of places that are equal to the number of candidates. The distribution of all the medical specialties is done by quotas, which are set according to the country's need for doctors practising in medical specialties. The internship period (6 months each) and the distribution between specialties, in order to validate a given specialty, are specified in advance by the college of each specialty. Every 6 months, all trainee residents in the country (more than 2000) gather for over 10 days at the Faculty of Medicine of Tunis, the Tunisian capital, to choose their next 6-month rotation. The candidate chooses according to seniority (300–600 in each residency level) and then to his rank within the chosen specialty.

Protocol

All the medical residents gathered at the Faculty of Medicine of Tunis, between 14 and 22 December 2015, to choose their next 6-month rotation were invited to answer an anonymous, self-administered questionnaire immediately after making their choice. The questionnaire covers their civil and marital status, their chosen specialty, their level of advancement in the specialty (duration of 4 years for medical specialties and 5 years for surgical specialties), and the items of the Hospital Anxiety and Depression (HAD) questionnaire.[22]

Measurements

The questionnaire explored three fields: sociodemographic data, work-related characteristics and the HAD score.

Sociodemographic information included gender, age, marital status, and the number of dependent children if married or divorced.

Work-related characteristics included specialty, the level achieved so far in the specific curriculum of each specialty, the number of shifts per month, whether the shift was followed by a day of safety rest and the total number of working hours per week. For the purpose of statistical analysis, specialties were split into four groups according to the associated workload: medical (eg, dermatology, pulmonology, rheumatology, psychiatry, and fundamental specialties such as histology, physiology and others), medical and surgical (eg, ophthalmology, gynaecology-obstetrics, otorhinolaryngology and others), medical specialties with high workload (eg, critical care medicine, emergency medicine, cardiology), and surgical specialties with high workload (neurosurgery, cardiovascular surgery and others).

The survey tool used in our study was the HAD scale, which was developed by Zigmond and Snaith.[22] It is a brief questionnaire (containing 14 items) that was originally designed to identify emotional disturbances in

non-psychiatric patients treated at hospital clinics. It is a self-report rating scale designed to measure both anxiety and depression. It consists of two subscales, each containing seven items on a 4-point Likert scale (ranging from 0 to 3). Participants were told that the questions asked were related to their mental state during the last 2 weeks. HAD is scored by summing up the ratings for the 14 items to yield a total score, and by summing up the ratings for the 7 items of each subscale to yield separate scores for anxiety and depression. Two cut-off scores are validated to identify anxiety and depression: 11 for participants who screened positive for anxiety/depression and 8 for probable anxiety/depression.

The survey was written in French and self-completed by each resident who has agreed to participate in the study. The language of the survey is not a barrier to the residents' completion of the survey because it is the language of all medical studies in the country. The total scores for the depression and anxiety subscales and the whole HAD scale were calculated by a resident from our unit.

Statistical analysis

The design of the study focused on the determination of the number of residents who met the criteria for anxiety and depression. Continuous variables were expressed either as mean±SD or median and IQR, according to normal or skewed data distribution. The dichotomous variables were expressed as numbers and percentages. Adjusted ORs and 95% CIs of each variable were calculated.

The prevalence of anxiety and depression was calculated and correlated to sociodemographic and work-related characteristics. χ^2 tests were carried out to compare the prevalence of anxiety and depression between groups. We used multivariable Poisson regression to identify explanatory variables with a statistically significant effect on the total HAD score.

Statistical significance was denoted by p values less than 0.05. All statistical procedures were performed using IBM SPSS Statistics V.21.0 software.

Participants and public involvement

No participants were involved in setting the research question or in the design or conduct of the study. No participants were asked to advise on interpretation or writing up of the results. There are no plans to disseminate the results of the research individually to study participants.

RESULTS

During the study period of 14–22 December 2015, 1700 out of 2200 (77%) medical residents from all levels and different specialties answered the survey. The medical residents had a mean±SD age of 28.5±2 years, with a predominance of female gender (60.8%); 38.5% were married, of whom 19% had at least one child (table 1).

Table 1 Demographic and work characteristics of the study population (n=1700)

Sex ratio (male:female)	667:1033
Age, median (IQR)	28 (27–30)
Marital status, n (%)	
Single	1036 (61)
Married	655 (38.5)
Divorced	9 (0.5)
Number of children, n (%)	
0	343 (20.2)
1	235 (13.8)
>1	86 (5.1)
Residency level (year), n (%)	
I	320 (18.8)
II	410 (24.1)
III	434 (25.5)
IV	340 (20)
V	196 (11.5)
Specialty*, n (%)	
Medical	854 (50.2)
Medical and surgical	221 (13)
High-workload medical specialties	299 (17.6)
High-workload surgical specialties	326 (19.2)
Working hours per week, median (IQR)	60 (48–76)
Night shifts per month, median (IQR)	6 (4–7)
Recovery day following night shift, n (%)	98 (8)

*Specialties were split into four categories according to everyday difficulties: medical (eg, dermatology, pulmonology, rheumatology, neurology, psychiatry, and fundamental specialties such as histology, physiology and others), medical and surgical (eg, ophthalmology, gynaecology-obstetrics, otorhinolaryngology and others), high-workload medical specialties (eg, critical care medicine, anaesthesiology, emergency medicine, cardiology), and high-workload surgical specialties (surgery, paediatric surgery, orthopaedics, neurosurgery, urology, cardiovascular surgery and others).

The residents declared they worked for a mean of 62±21 hours per week; 73% (1239) of them worked night shifts with a mean number of 5.4±3 night shifts per month, of whom only 8% (98) declared that the shift was systematically followed by a day of safety rest (table 1).

The median HAD score was 19 (IQR: 14–23), split on the anxiety subscale (HAD-A): 10 (IQR: 7–12); and the depression subscale (HAD-B): 9 (IQR: 6–11).

Overall, 742 (43.6%) residents reached the cut-off defining definite anxiety state (ie, a score ≥11) and 519 (30.5%) reached the scale level defining definite depression. Out of these residents, 342 (20%) had both anxiety and depression. In the remaining residents, the prevalence of probable anxiety score (ie, a score ≥8) was 30.5% and that of probable depression was 31.5%. Tables 2 and 3 depict the association between the demographic and

Table 2 Demographic and workload characteristics and their association with anxiety

	No anxiety	Definite anxiety	P values
n (%)	958 (56.4)	742 (43.6)	
Age, mean±SD	28.5±1.9	28.7±2	**0.008**
Gender			**0.0001**
Male	420 (43.8)	247 (33.3)	
Female	538 (56.2)	495 (66.7)	
Marital status			**0.027**
Single	606 (63.3)	430 (58)	
Married	352 (36.7)	312 (42)	
Level of residency			**0.005**
I	188 (19.6)	132 (17.8)	
II	244 (25.5)	166 (22.4)	
III	251 (26.2)	183 (24.7)	
IV	182 (19)	158 (21.3)	
V	93 (9.7)	103 (13.9)	
Specialty			**0.018**
Medical	519 (54.2)	334 (45)	
Medical and surgical	103 (10.8)	118 (15.9)	
High-workload surgical specialties	181 (18.9)	145 (19.5)	
High-workload medical specialties	155 (16.2)	145 (19.5)	
Shifts/month, mean±SD	4.9±3	6±2.9	**0.0001**
Working hours/week, mean±SD	58.8±20	66.4±20	**0.0001**

P values in bold denotes statistical significance.

workload characteristics, and the occurrence of anxiety or depression, respectively.

On univariate analysis, comparison of the group of residents with definite anxiety (HAD-A≥11) with those without definite anxiety (HAD-A<11) disclosed the following variables as significantly associated with the prevalence of anxiety: older age, married status (42% vs 36.7%, p=0.027) and female gender (66.7% vs 56.2%, p<0.0001) The fifth level of residency course (13.9% vs 9.7%, p=0.009) and the choice of medical/surgical specialties (15.9% vs 10.8%, p=0.018) were also significantly associated with anxiety. Medical specialties (45% vs 54.2%, p<0.0001) were, on the contrary, associated with a lower risk of developing anxiety symptoms (table 2).

In comparison with residents without definite depression (HAD-D<11), the group of residents with definite depression (HAD-D≥11) was older and more often married (45.7% vs 36.2%, p<0.0001). Moreover, surgical specialties with high workload (22.9% vs 17.5%, p=0.011) were significantly associated with depressive symptoms in contrast to medical specialties (44.7 vs 52.6%, p=0.003), which were associated with lower depressive symptoms (table 3). The number of shifts per month and the number of working hours per month were associated with both anxiety and depressive cases (tables 2 and 3).

Poisson regression analysis disclosed the following variables as associated with the total HAD score: age (OR=1.014, 95% CI 1.006 to 1.023, p=0.001), female gender (OR=1.114, 95% CI 1.083 to 1.145, p<0.0001), number of night shifts per month (OR=1.048, 95% CI 1.016 to 1.082, p=0.03) and number of working hours per week (OR=1.008, 95% CI 1.005 to 1.011, p≤0.0001). Compared with medical specialties, the medico-surgical ones were independently associated with a higher HAD score (OR=1.459, 95% CI 1.172 to 1.816, p=0.001; figure 1).

DISCUSSION

This large survey encompassing 1700 out of 2200 Tunisian medical residents brought together for the periodic process of choosing internship positions in their specialties shows that 43.6% of these medical residents met the definite criteria for anxiety and 30.5% for depression. The diagnosis of anxiety or depression was probable in an additional 30% each. Higher scores in the anxiety-depression scale were significantly associated with older age, female sex and the heavy burden of work imposed on a weekly or monthly basis, as reflected by the high number of weekly working hours, the number of night shifts per month and the choice of a specialty with higher workload (surgical or medical-surgical specialty compared with medical specialty). Of interest, this survey unveils a very demanding health system imposed on Tunisian medical

Table 3 Demographic and workload characteristics and association with depression cases

	No depression	Definite depression	P values
n (%)	1181 (69.5)	519 (30.5)	
Age, mean±SD	28.3±1.8	29±2	**0.007**
Gender			0.11
Male	453 (38.4)	214 (41.2)	
Female	728 (61.6)	305 (58.8)	
Marital status			**0.0001**
Single	754 (63.8)	282 (54.3)	
Married	427 (36.2)	237 (45.7)	
Level of residency			**0.0001**
I	263 (22.3)	57 (11)	
II	292 (24.7)	118 (22.7)	
III	294 (24.9)	140 (27)	
IV	218 (18.5)	122 (23.5)	
V	114 (9.7)	82 (15.8)	
Specialty			**0.006**
Medical	621 (52.6)	232 (44.7)	
Medical and surgical	156 (13.2)	65 (12.5)	
High-workload surgical specialties	207 (17.5)	119 (22.9)	
High-workload medical specialties	197 (16.7)	103 (19.8)	
Shifts/month, mean±SD	5±3	6±2.9	**0.0001**
Working hours/week, median (IQR)	60±20	66±20	**0.0001**

P values in bold denotes statistical significance.

residents, as reflected by the average working hours per week (60 hours) and the number of night shifts per month (6 on average regardless of the type of specialty), most often without a day of safety rest.

Although mood disorders and physician distress are more prevalent among medical professionals compared with the general population, information on their prevalence in Tunisia is lacking.[5 23 24] This first survey on this subject in our country enrolled 77% of potentially concerned medical residents, providing a snapshot of the burden incurred by residents from all specialties in their everyday practice. The use of the French version of the HAD questionnaire was not considered a barrier to the residents' completion of the survey because it is the language of all medical studies in the country.

International studies stressed the fact that medical residents usually have higher rates of depression than the general population.[15 25] Estimates of the prevalence of depression or depressive symptoms among resident physicians vary from 3% to 60% with a median of 28.8%

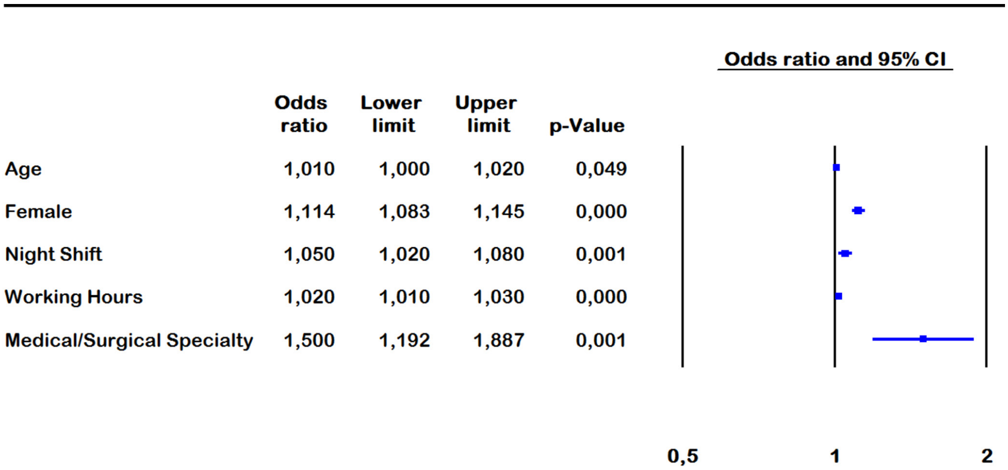

Figure 1 Risk factors associated with the total Hospital Anxiety and Depression score.

according to a recent meta-analysis of 54 studies.[15] This proportion is lower than that recorded in our study whether we consider the rate of definite or probable depression (62%), or even when we only consider the cases qualifying for definite depression (30.5%). Of interest, this meta-analysis emphasised the difference in the methodological approach to addressing the issue of depression among medical residents (cross-sectional studies which assess the prevalence vs longitudinal studies which inform on the incidence).[15] The meta-analysis also highlighted the great variability in the instruments used (no less than 13 different instruments were used in the 54 studies).[15] A prospective cohort study conducted in Switzerland and using the HAD inventory that was used in our study disclosed a prevalence of anxiety in 30% of second-year residents and 20% of the fourth-year and sixth-year residents; depressive symptoms were present in 15% and 10%, respectively.[26] However, benchmarking with countries with socioeconomic similarities could be more insightful. In a study including 118 residents from the American University of Beirut, 22% qualified for moderate to major depressive symptoms, while only 8% had anxiety.[27] Moreover, in a cross-sectional study conducted among residents of various specialties in Saudi Arabia, the prevalence of moderate to severe depressive symptoms was around 20%.[17]

Resident physicians share the same known causes of depression with the general population: physical health, lifestyle, marital status, history of depression, childhood issues, religiosity and others.[1 4] In addition to these common risk factors, they have the peculiarities of a singular mode and pace of work, as well as specific constraints to the development of their careers.[4] These risk factors correspond to the difficulty of their specialty, postgraduate year, stress at work, difficulty with current rotation, working hours, number of shifts and others.[4 15 28] In our study the Poisson regression model disclosed the following items as statistically associated with the occurrence of anxiety and depression: age, female sex, practice of a specialty generally accepted as very demanding and difficult, and the heavy burden of work imposed on a weekly or monthly basis as reflected by the weekly working hours and the number of night shifts per month. Most of these risk factors have been reported elsewhere, or are intuitive, but it is their magnitude that makes this study important.

The occurrence of depression should be considered as a major event in young physicians' career or even life. It has been shown that once present, depression as well as burnout (another consequence of work-related chronic stress) may persist throughout the duration of residency or even beyond.[29 30] In a nationwide survey involving 3500 Canadian physicians, Sullivan and Buske[31] reported that 55% found that medicine as a profession impacted negatively on their family and personal life, while 65% reported that opportunities to change career despite dissatisfaction were limited. Depression is also a source of decreased concentration at work, an increase in the rate of medical errors, work dissatisfaction and conflicts, impaired sleep quality, and a greater propensity to attrition, and even suicide ideation.[9 32 33]

Considering the high prevalence of anxiety and depression among Tunisian medical residents, every effort that aims at improving the mental health of young physicians should be encouraged. Any strategy should target the individual level to amend the identified and potentially actionable factors, and include more measures targeting the general organisation of residents' work modalities, and residents' relationship with hierarchy.

Resident physicians, in particular those with a personal history of depression, should first become aware of their mental health issue and seek help.[34] We did not investigate whether the participating residents sought for or actually had psychological counselling, but we strongly believe that most did not. Structures able to provide aid to health professionals exposed to and suffering from stress, anxiety and depression are anyway non-existent in Tunisian hospitals. Our study suggests that such structures can no longer be considered an option, but the Ministry of Health should provide support at the institutional level. At the individual level, more generally accepted risk factors such as older age, gender, marital status, stressors outside of work, sleep deprivation or lifestyle require more personal attention and lifestyle education.[4] The current study was not designed to allow identification of participants so they could be given information and act on it. Future studies targeting preferentially residents (or specialties) at high risk of anxiety, depression or burnout should consider such feedback for more specific training programmes.

Residency programme factors and the pace of work should also be better managed. While these details were totally non-existent in Tunisia, these have been recently fixed through a law issued on 9 March 2018 whose purpose is to define the status of Tunisian interns and residents.[35] This law, which has been torn out following long negotiations and a strike that lasted more than 6 weeks, defines for the first time the role and duties of residents. The maximum number of weekly working hours and the frequency of night shifts have been clearly defined and have been reduced to a maximum of 40 hours and 2 night shifts per week, respectively. A day of safety rest following every night shift has been rendered mandatory. Although still debated, the issue of work-hour restriction could be effective in reducing high emotional exhaustion, despite the fact that it carries the risk of alteration in the quality of care and education by reducing the number and the actual presence of medical residents.[36]

Acknowledgements The authors are indebted to Ms Vaughn Khelifa and Professor Mahmoud Bchir for the language editing of the manuscript.

Contributors Study concept and design: MM and FA. Data acquisition: MM. Analysis and interpretation of the data: MM, LO-B, ZH, IO, FD, FA. All authors read and approved the final manuscript. MM and FA had full access to the data and take responsibility for its integrity and the accuracy of the analyses.

Funding The authors have not declared a specific grant for this research from any funding agency in the public, commercial or not-for-profit sectors.

Competing interests None declared.

Patient consent Not required.

Ethics approval The study protocol was approved by the local Institutional Review Board of Fatouma Bourguiba Teaching Hospital, Monastir (reference #2013/108).

Provenance and peer review Not commissioned; externally peer reviewed.

Data sharing statement Data set is available by contacting FA at f.abroug@rns.tn.

Open access This is an open access article distributed in accordance with the Creative Commons Attribution Non Commercial (CC BY-NC 4.0) license, which permits others to distribute, remix, adapt, build upon this work non-commercially, and license their derivative works on different terms, provided the original work is properly cited, appropriate credit is given, any changes made indicated, and the use is non-commercial. See: http://creativecommons.org/licenses/by-nc/4.0/.

REFERENCES

1. Shanafelt TD, Sloan JA, Habermann TM. The well-being of physicians. *Am J Med* 2003;114:513–9.
2. Hsu K, Marshall V. Prevalence of depression and distress in a large sample of Canadian residents, interns, and fellows. *Am J Psychiatry* 1987;144:1561–6.
3. Rotenstein LS, Ramos MA, Torre M, *et al*. Prevalence of Depression, Depressive Symptoms, and Suicidal Ideation Among Medical Students: A Systematic Review and Meta-Analysis. *JAMA* 2016;316:2214–36.
4. Joules N, Williams DM, Thompson AW. Depression in Resident Physicians: A Systematic Review. *Open Journal of Depression* 2014;03:89–100.
5. Sen S, Kranzler HR, Krystal JH, *et al*. A prospective cohort study investigating factors associated with depression during medical internship. *Arch Gen Psychiatry* 2010;67:557–65.
6. Rama-Maceiras P, Jokinen J, Kranke P. Stress and burnout in anaesthesia: a real world problem? *Curr Opin Anaesthesiol* 2015;28:151–8.
7. Amaddeo F, Jones J. What is the impact of socio-economic inequalities on the use of mental health services? *Epidemiol Psichiatr Soc* 2007;16:16–19.
8. Donisi V, Tedeschi F, Percudani M, *et al*. Prediction of community mental health service utilization by individual and ecological level socio-economic factors. *Psychiatry Res* 2013;209:691–8.
9. Fahrenkopf AM, Sectish TC, Barger LK, *et al*. Rates of medication errors among depressed and burnt out residents: prospective cohort study. *BMJ* 2008;336:488–91.
10. Ripp J, Babyatsky M, Fallar R, *et al*. The incidence and predictors of job burnout in first-year internal medicine residents: a five-institution study. *Acad Med* 2011;86:1304–10.
11. Tsai YC, Liu CH. Factors and symptoms associated with work stress and health-promoting lifestyles among hospital staff: a pilot study in Taiwan. *BMC Health Serv Res* 2012;12:199.
12. de Oliveira GS, Chang R, Fitzgerald PC, *et al*. The prevalence of burnout and depression and their association with adherence to safety and practice standards: a survey of United States anesthesiology trainees. *Anesth Analg* 2013;117:182–93.
13. Lebensohn P, Dodds S, Benn R, *et al*. Resident wellness behaviors: relationship to stress, depression, and burnout. *Fam Med* 2013;45:541–9.
14. van der Heijden F, Dillingh G, Bakker A, *et al*. Suicidal thoughts among medical residents with burnout. *Arch Suicide Res* 2008;12:344–6.
15. Mata DA, Ramos MA, Bansal N, *et al*. Prevalence of Depression and Depressive Symptoms Among Resident Physicians: A Systematic Review and Meta-analysis. *JAMA* 2015;314:2373–83.
16. Waldman SV, Diez JC, Arazi HC, *et al*. Burnout, perceived stress, and depression among cardiology residents in Argentina. *Acad Psychiatry* 2009;33:296–301.
17. Al-Maddah EM, Al-Dabal BK, Khalil MS. Prevalence of Sleep Deprivation and Relation with Depressive Symptoms among Medical Residents in King Fahd University Hospital, Saudi Arabia. *Sultan Qaboos Univ Med J* 2015;15:e78–84.
18. Shashikantha S K SMP. Prevalence of depression among post graduate residents in a tertiary health care institute, Haryana. *Int J Med Sci Public Health* 2016;5:2139–42.
19. program UnD. HDR Report. 2016;2016 http://hdr.undp.org/en/composite/trends.
20. Ac CS. The governance structure and regulation in Tunisian public hospitals. *Int J Soc and Behavioural Sci* 2013;1:162–7.
21. Out-of-pocket health expenditure (% of private expenditure on health). database WHOGHE. ed. *World Health Organization Global Health Expenditure database*. Geneva: World Health Organization, 2018.
22. Zigmond AS, Snaith RP. The hospital anxiety and depression scale. *Acta Psychiatr Scand* 1983;67:361–70.
23. Kessler RC, Berglund P, Demler O, *et al*. Lifetime prevalence and age-of-onset distributions of DSM-IV disorders in the National Comorbidity Survey Replication. *Arch Gen Psychiatry* 2005;62:593–602.
24. Dyrbye LN, Thomas MR, Shanafelt TD. Systematic review of depression, anxiety, and other indicators of psychological distress among U.S. and Canadian medical students. *Acad Med* 2006;81:354–73.
25. Schneider SE, Phillips WM. Depression and anxiety in medical, surgical, and pediatric interns. *Psychol Rep* 1993;72(3 Pt 2):1145–6.
26. Buddeberg-Fischer B, Stamm M, Buddeberg C, *et al*. [Anxiety and depression in residents - results of a Swiss longitudinal study]. *Z Psychosom Med Psychother* 2009;55:37–50.
27. Talih F, Warakian R, Ajaltouni J, *et al*. Correlates of Depression and Burnout Among Residents in a Lebanese Academic Medical Center: a Cross-Sectional Study. *Acad Psychiatry* 2016;40:38–45.
28. Kirsling RA, Kochar MS, Chan CH. An evaluation of mood states among first-year residents. *Psychol Rep* 1989;65:355–66.
29. Girard DE, Hickam DH, Gordon GH, *et al*. A prospective study of internal medicine residents' emotions and attitudes throughout their training. *Acad Med* 1991;66:111–4.
30. Roh MS, Jeon HJ, Kim H, *et al*. The prevalence and impact of depression among medical students: a nationwide cross-sectional study in South Korea. *Acad Med* 2010;85:1384–90.
31. Sullivan P, Buske L. Results from CMA's huge 1998 physician survey point to a dispirited profession. *CMAJ* 1998;159:525–8.
32. Khoushhal Z, Hussain MA, Greco E, *et al*. Prevalence and Causes of Attrition Among Surgical Residents: A Systematic Review and Meta-analysis. *JAMA Surg* 2017;152:265–72.
33. West CP, Huschka MM, Novotny PJ, *et al*. Association of perceived medical errors with resident distress and empathy: a prospective longitudinal study. *JAMA* 2006;296:1071–8.
34. Goebert D, Thompson D, Takeshita J, *et al*. Depressive symptoms in medical students and residents: a multischool study. *Acad Med* 2009;84:236–41.
35. TMo H. Bylaw of Medical Interns and Residents: Journal Officiel de la République Tunisienne. 2018 http://www.legislation.tn/fr/recherche/jort/date_jort_deb/09-03-2018
36. Gopal R, Glasheen JJ, Miyoshi TJ, *et al*. Burnout and internal medicine resident work-hour restrictions. *Arch Intern Med* 2005;165:2595–600.
