# Supplementary material for: Mental health status and related factors influencing healthcare workers during the COVID-19 pandemic: A systematic review and meta-analysis
Source: PLoS One. 2024 Jan 19;19(1):e0289454. doi: 10.1371/journal.pone.0289454 (PMC10798549; doi:10.1371/journal.pone.0289454)
Supplement: S1 Data — (ZIP) [file pone.0289454.s011.zip › literatures/92.pdf]

Social Psychiatry and Psychiatric Epidemiology (2022) 57:633–645
https://doi.org/10.1007/s00127-021-02211-9

STUDY PROTOCOLS AND SAMPLES

The impact of the COVID-19 pandemic on the mental health of healthcare workers: study protocol for the COVID-19 HEalth caRe wOrkErS (HEROES) study

Franco Mascayano[1,2] · Els van der Ven[3] · Maria Francesca Moro[1,4] · Sara Schilling[5] · Sebastián Alarcón[5] · Josleen Al Barathie[6] · Lubna Alnasser[1,7] · Hiroki Asaoka[8] · Olatunde Ayinde[9] · Arin A. Balalian[1] · Armando Basagoitia[10] · Kirsty Brittain[11] · Bruce Dohrenwend[1] · Sol Durand-Arias[12] · Mehmet Eskin[13] · Eduardo Fernández-Jiménez[14] · Marcela Inés Freytes Frey[15] · Luis Giménez[16] · Lydia Gisle[17] · Hans W. Hoek[1,18] · Rodrigo Ezequiel Jaldo[15] · Jutta Lindert[19] · Humberto Maldonado[20] · Gonzalo Martínez-Alés[1,14,23] · Carmen Martínez-Viciana[21] · Roberto Mediavilla[22,23] · Clare McCormack[24] · Landon Myer[11] · Javier Narvaez[25] · Daisuke Nishi[8] · Uta Ouali[26] · Victor Puac-Polanco[1,27,28] · Jorge Ramírez[5] · Alexandra Restrepo-Henao[1,29] · Eliut Rivera-Segarra[30] · Ana M. Rodríguez[31] · Dahlia Saab[6] · Dominika Seblova[32] · Andrea Tenorio Correia da Silva[33,34] · Linda Valeri[35] · Rubén Alvarado[5] · Ezra Susser[1,2] on behalf of the HEROES group

Received: 2 April 2021 / Accepted: 5 December 2021 / Published online: 22 January 2022
© The Author(s), under exclusive licence to Springer-Verlag GmbH Germany 2021

Abstract

Background Preliminary country-specific reports suggest that the COVID-19 pandemic has a negative impact on the mental health of the healthcare workforce. In this paper, we summarize the protocol of the COVID-19 HEalth caRe wOrkErS (HEROES) study, an ongoing, global initiative, aimed to describe and track longitudinal trajectories of mental health symptoms and disorders among health care workers at different phases of the pandemic across a wide range of countries in Latin America, Europe, Africa, Middle-East, and Asia.

Methods Participants from various settings, including primary care clinics, hospitals, nursing homes, and mental health facilities, are being enrolled. In 26 countries, we are using a similar study design with harmonized measures to capture data on COVID-19 related exposures and variables of interest during two years of follow-up. Exposures include potential stressors related to working in healthcare during the COVID-19 pandemic, as well as sociodemographic and clinical factors. Primary outcomes of interest include mental health variables such as psychological distress, depressive symptoms, and posttraumatic stress disorders. Other domains of interest include potentially mediating or moderating influences such as workplace conditions, trust in the government, and the country's income level.

Results As of August 2021, ~ 34,000 health workers have been recruited. A general characterization of the recruited samples by sociodemographic and workplace variables is presented. Most participating countries have identified several health facilities where they can identify denominators and attain acceptable response rates. Of the 26 countries, 22 are collecting data and 2 plan to start shortly.

Conclusions This is one of the most extensive global studies on the mental health of healthcare workers during the COVID-19 pandemic, including a variety of countries with diverse economic realities and different levels of severity of pandemic and management. Moreover, unlike most previous studies, we included workers (clinical and non-clinical staff) in a wide range of settings.

Keywords COVID-19 · Mental health · Global collaboration · LMICs · Cohort study

Franco Mascayano and Els van der Ven contributed equally to this work and share first authorship. Rubén Alvarado and Ezra Susser contributed equally to this work and share last authorship.

Extended author information available on the last page of the article

Background

Since December 2019, the world has been shaken by an enormous global threat: the COVID-19 pandemic. The pandemic has generated an unprecedented impact both on the

general population and on the healthcare systems in most countries [1]. Health services have expanded their capacity to respond to the pandemic by increasing the number of beds; reallocating and redeploying health care workers; acquiring necessary equipment to provide intensive therapy (e.g., ventilators in some countries); and calling retired health professionals and health students to assist the overwhelmed health care workforce. Health care systems have become the center point of this pandemic and, as a result, health care workers (HCWs) are considered one of the most affected groups [2, 3].

The high demand for providing health care to patients with confirmed and suspected COVID-19, along with the fears and concerns of becoming ill with the virus or infecting their families, have put an enormous emotional burden on HCWs. Emotional impacts of pandemics have been documented previously during infectious disease outbreaks such as the Acute Respiratory Syndrome [4] and Ebola [5]. However, the impact of the COVID-19 pandemic has been significantly larger than previous pandemics in terms of the number of affected people worldwide, its spread across countries, its impact on health care systems, and the strictness of measures adopted by governments. Multiple social and economic consequences have already been reported (e.g., spiking unemployment rates), while others are expected to occur in the following months, and they have been increasing social divides and inequalities across the world [6]. There has also been a diversity of responses to HCWs, ranging from public cheering and gratitude to stigma and assault [7], especially in some low-and-middle-income countries (LIMCs).

Many HCWs have been overwhelmed by the increased workload; the lack of supplies and materials to provide appropriate treatment and protect themselves; the lack of clinical guidelines on patient prioritization; the increased feelings of isolation and loneliness; and the high rates of COVID-19 among HCWs in some countries. As vaccines for COVID-19 roll out, moreover, it remains unclear if and when there will be enough vaccines available for this highly exposed population in all regions, especially in LMICs.

Cross-sectional, country-specific studies have indicated that HCWs in facilities treating patients with COVID-19 reported high rates of depressive and anxiety symptoms [8–10]. For instance, Lai et al. [11] found that many HCWs presented symptoms of depression (634/50.4%), anxiety (560/44.6%), insomnia (427/34%), and distress (899/72%) among 1,257 health workers in Wuhan and nearby regions. In a hospital in Veneto, Italy, one of the hardest-hit areas of Europe, as much as half of the health care staff showed symptoms of clinically relevant anxiety and 26% symptoms of at least moderate depression [12]. Several studies demonstrated that rates of psychological problems were especially high among women, nurses and HCWs directly engaged in COVID-19 care [8–10]. Studies conducted in China and Spain also reported an association between the level of psychological distress in HCWs and the incidence of COVID-19 in the geographical area [11, 13].

Workplace and social conditions may moderate the risk of exposure to COVID-19 and the psychological impact on workers. For example, in the early stages of the COVID-19 outbreak in Wuhan, satisfaction with personal protective equipment (PPE) and perceived reasonable shift arrangements were associated with lower rates of depression [14]. HCWs may also be forced to make difficult ethical choices in pandemics, such as the rationing of PPE and treatment resources. These may lead to moral injury [15], which may be internalized and compound the acute stress of working during a pandemic [16, 17]. Moreover, social exposures such as stigma and harassment toward HCWs may worsen effects on mental health, though these have not been carefully studied yet [7]. To assess the risk and predictors of mental health disorders among HCWs during COVID-19 individual factors must be accounted for, as well as the potential impact of workplace and social conditions and policies, which are potentially modifiable conditions.

Given the global nature of the pandemic, comparisons across countries are warranted. However, there is a critical lack of data from LMICs and low-resourced areas where the pandemic has been devastating in its effects. Cross-country and cross-cultural variations and similarities may elucidate whether sources of variation (e.g., COVID-19 rates, health care capacity, national response) are associated with the mental health of health workers across diverse country settings. On a study design level, most previous studies have not used probabilistic sampling methods, reported low response rates or no response rates at all, and enrolled primarily nurses and physicians, excluding essential HCWs such as non-clinical workers. Moreover, it is necessary to study mental health variables longitudinally considering that some effects will appear over time, (e.g., posttraumatic stress disorder) and there is a lack of prospective studies with extended follow-up periods. Lastly we investigated various contextual variables, such as exposure to discrimination, harassment, and violence for being a HCW in the pandemic, and its relationship with mental health, that have been scarcely examined in previous studies.

Accordingly, we present HEROES, an ongoing, multisite prospective cohort study aimed to evaluate the impact of the COVID-19 pandemic on the mental health of workers at health services in 26 countries across 4 continents using a uniform study design and data analysis plan. HEROES encompasses a wide variety of academic institutions in 18 LMICs and 8 HICs, in partnership with the Pan American Health Organization (PAHO) and support from the World Health Organization (WHO). This study is co-led by two PIs (one at Columbia University Mailman School of Public Health, and one at University of Chile), with a Steering

Committee composed of the two PIs (ES and RA), and three early-career investigators (one male, FM, two females, EvdV and MFM). The HEROES study was originally conceived, developed, and scaled by a team of early career collaborators mentored by the two PIs. The study is still mostly driven by these collaborators, many of whom are from or live in LMICs. Our team approach is based on cooperative leadership and mutual learning principles, which, we believe, has contributed to creating a truly collaborative and friendly relationship between investigators from the South (Latin America, Africa, and Asia) and the North (the US, Europe). For instance, most of our research materials (e.g., web-based survey, study protocol) have been designed, translated, and adapted almost simultaneously in all the participating countries and close partnership between LMIC and HIC teams. The bulk of this work has been done by young investigators, who are more likely to establish horizontal relationships, which would have been difficult to achieve in a more standard research setting. We believe that this unusual but highly valuable arrangement contributes to reimagining the South-North collaborations and transforming the usual power structures and practices in Global Mental Health [18]. Importantly, there was expert supervision available as needed, so that this approach added such strengths without sacrificing rigor.

The overarching objective of the HEROES study was to examine the relationship between exposure to COVID-19 and mental health outcomes among HCWs within and across countries. Specifically, our primary aim was to investigate the impact of COVID-19 related exposures (e.g., frontline versus non-frontline, subjective and objective exposure to COVID-19) on mental health outcomes, in particular depressive symptoms, psychological distress, and PTSD, among HCWs. Our secondary aims were to (1) identify potentially mediating effects (e.g., trust in the workplace, social support, stigma) in this relationship; and to (2) investigate the role of country- and region-level measures including rates of COVID-19 infections, deaths, and hospitalizations. We hypothesize, first, that COVID-19 exposure will be significantly associated with the specified mental health outcomes among HCWs irrespective of country. Second, we expect that there will be substantial country-level variation regarding the impact of the COVID-19 pandemic on mental health outcomes among HCWs. We anticipate that part of this variation will be related to income level of the country (LMIC vs. HIC) due to factors associated with increased risk of exposure to COVID-19, e.g., insufficient PPE [17]. Third, we hypothesize that potential pathways from exposure to outcomes will vary across countries, and part of this variation will be related to workplace conditions and trust in government.

Methods

Study design

We are using a prospective cohort design including HCWs from pre-selected health facilities in the participating countries as noted below. The study considers follow-up assessments at approximately 6, 12, and 24 months. An online questionnaire based on standardized measures plus ad-hoc items is being employed.

Settings: participating countries

Figure 1 shows the participating countries of the HEROES study. Of the 26 countries, 24 are already collecting data and 2 plan to starting data collection shortly. We firstly included countries with representatives who were colleagues or professional contacts of the lead investigators who expressed interest in joining the study. Most were collaborators from previous studies. Additionally, there were several countries that contacted us through WHO and PAHO/WHO when launching HEROES. As noted in Fig. 1, South America and Europe are best represented. We actively sought to represent other global regions and contexts insofar as possible by including countries such as Lebanon, Armenia, Nigeria, and South Africa. Despite these efforts, we could not represent all included regions equally.

Participants

Participants include clinical and non-clinical HCWs at different health facilities, from emergency services in hospitals to primary care clinics. The inclusion criteria for potential participants include being of legal age, working at one of the pre-selected health facilities, working in a health facility that provides care to suspected or confirmed cases of COVID-19, and having an internet connection to complete the online questionnaire. Supplemental Table characterizes the recruited participants by August 2021, by country, gender, age, and profession. Further details about samples are available upon request.

Sampling and recruitment

Most health facilities were selected based on convenience (e.g., accessible and willing to participate, able to identify denominators). However, there are exceptions, such as Sao Paulo (Brazil), Colombia, Lebanon, Japan, and Belgium, where facilities were selected randomly. A full description of the recruitment schemes being deployed in each country is presented in Table 1.

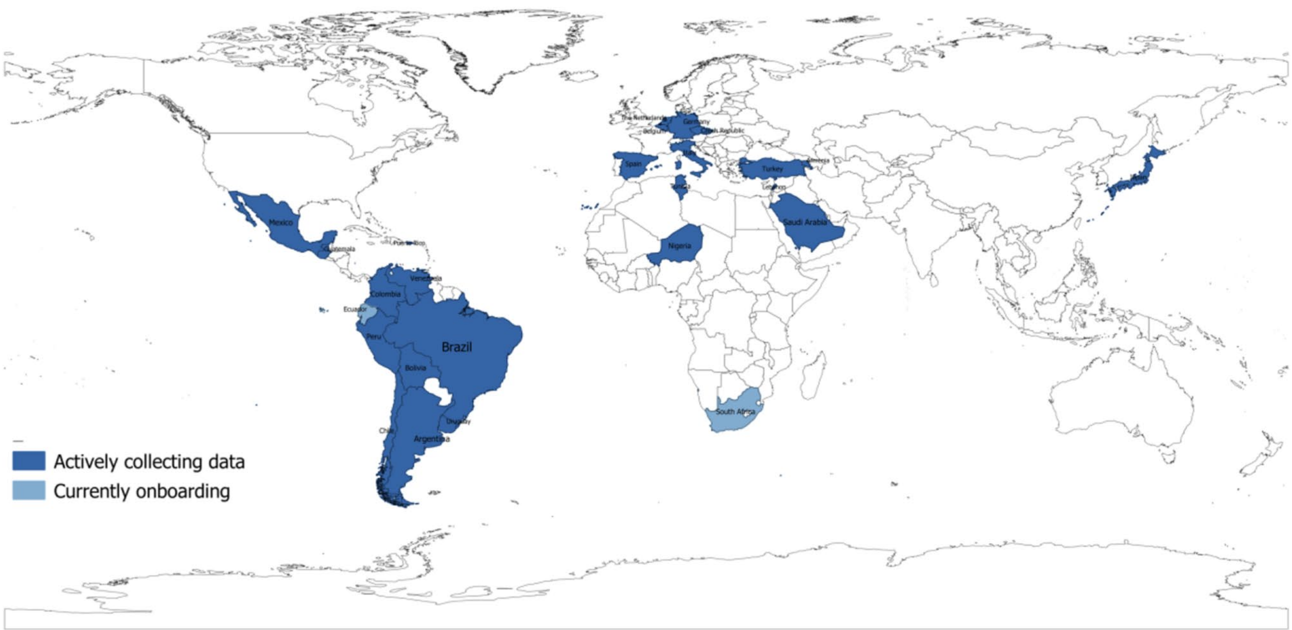

Fig. 1 Participating countries

Here we describe the standard sampling scheme for HEROES. Most countries have followed this scheme, though some made adaptations to local circumstances. In this scheme, the country PI or the designated local coordinator contacted, by phone or email, a key/contact person at the participating organization, e.g., (1) health services (e.g., health directors, health supervisors), (2) health workers national and/or local orders (e.g., members of the national board, representatives of the local boards), (3) health workers national and/or local organizations (e.g., members of the national or local board, representatives of the local boards), (4) health workers national and/or local unions, (e.g., members of the national board, representatives of the local boards). The local coordinator explained the specific aims and general procedures of the study and asked for assistance in identifying potential participants. If the key/contact person agreed to support the study, they took the steps necessary in their organization to authorize sharing contact information (text or email) of all potential participants for this purpose. Potential participants received an email invitation (or text message) that included general information about the study. If a potential participant was interested, they clicked on a link to access the digital platform where the study and voluntary participation was described. To start the survey, participants first responded to the following questions/items: (1) “select language”; (2) “do you work in a health service?” (inclusion criteria); (3) “please select country where you live in now”; (4) informed consent (if the person accepts, they are asked to fill out their email address and receive a PDF version of the consent by email for their records); and (5) “please indicate country and region where you work”. The latter question was added because some HCWs may live in one country and work in another.

Local teams were able to identify how many people work in those facilities and, when possible, the distribution of workers by type of occupation (e.g., doctors, nurses, ancillary services, technicians). To facilitate comparison between sites, we are focusing on large facilities (e.g., hospitals) and in each country we have included, when possible, one facility from an area with high rates of COVID-19 cases and/or COVID-related deaths vs. an area with low cases/deaths. As noted above, we have included an item asking for “which center do you work at/which institution are you affiliated to?”, in order to record the workplace of individuals and calculate response rates. If data on denominators for response rates are not available, cooperation rates will be calculated instead (number of completed surveys/number of emails sent).

Finally, to consider the representativeness of different types of health workers, preliminary analysis of the data will be performed based on 3 main characteristics of the health centers: type of facility, type of profession, and type of population covered.

Variables and instruments

The online questionnaire is self-administered and takes, on average, 15–20 min to complete. It includes the General

Table 1 Sampling schemes for participating countries

Recruitment strategies	Country	Target Population		Recruitment scheme	Study start date	Representative samples	Response rates[a]	Pre-pandemic data
		Clinical	Nonclinical					
Registries and databases	Japan	Yes	No	Potential participants will come from two national registries: the Disaster Medical Assistance Team (DMAT) members and all registered the Disaster Psychiatric Assistance Team (DPAT)	3/11/20 before 1st wave	Yes, national level	Yes	No
	Peru	Yes	Yes	Potential participants will come from a national registry (i.e., INFORHUS) managed by the Ministry of Health<br>Focus on the following regions: Lima, Callao, Lambayeque, Piura, Loreto, La Libertad, Ancash, Arequipa	6/25/20 at peak of 1st wave	Yes, national level	Yes	No
	Saudi Arabia	Yes	Yes	Medical city that serves the Saudi National Guard<br>Professional health associations (e.g., ~ 4000 healthcare professionals)	6/21/20 between waves 1 and 2	Yes, national level	Yes	No
	Uruguay	Yes	Yes	Potential participants will come from a national registry managed by the Ministry of Health	3/12/2021 at peak of 2nd wave	Yes, national level	Yes	No
	Venezuela	Yes	Yes	Potential participants will come from three national registries managed by the Ministry of Health	7/25/20 before peak of 1st wave	Yes, national level	Yes	No
Health facilities	Argentina	Yes	Yes	10 health major facilities by region (Buenos Aires, Mendoza, Corrientes, Río Negro y Chubut)	6/23/20 before 1st wave	No, convenience sample	No	No
	Armenia	Yes	Yes	10 major hospitals from two regions (Republic of Armenia and Artsakh)<br>NGOs, Professional associations	7/18/20 after peak of 1st wave	No, convenience samples	No	No
	Belgium	Yes	Yes	13 randomly selected hospitals	8/26/20 before 2nd wave	Yes, facility level	Yes	No
	Bolivia	Yes	Yes	12 health facilities including 2 major hospitals	5/25/20 before 1st wave	Yes, facility level	Yes	No
	Brazil	Yes	Yes	53 health facilities (e.g., hospitals, primary care clinics, CAPS) from Rio de Janeiro, Sao Paulo, Pernambuco, and Rio Grande do Sul	9/1/20 between waves 1 and 2	Yes, region level (Sao Paulo)	Yes	Yes (Sao Paulo)

Table 1 (continued)

Recruitment strategies	Country	Target Population		Recruitment scheme	Study start date	Representative samples	Response rates[a]	Pre-pandemic data
		Clinical	Nonclinical					
	Chile	Yes	Yes	16 health facilities nationally based two criteria: (i) public or private health centers, and (ii) type of health center (e.g., hospitals, primary care clinics, etc.)	5/22/20 before peak of 1st wave	Yes, facility level	Yes	No
	Colombia	Yes	Yes	42 health facilities based on (i) type of institution (i.e., public or private) and (ii) institution's size in Bogota ($n=18$), Medellin ($n=12$), and Cali ($n=12$)	2/28/2021 between waves 2 and 3	Yes, facility level	Yes	No
	Czech Republic	Yes	Yes	10 university hospitals and selected regional hospitals will be targeted<br>Professional associations (e.g., Komora záchranářů zdravotnických záchranných služeb České republiky)	6/25/20 before 1st wave	No, convenience sample	No	No
	Ecuador	Yes	Yes	18 health facilities nationally based on (1) type of facility (i.e., hospital vs primary care clinic), and (1) region	Not started yet	Yes, facility level	Yes	No
	Germany	Yes	Yes	Major hospitals in each of the 16 states	9/23/2020 before 2nd wave	No, convenience sample	No	No
	Italy	Yes	No	4 major hospitals; 2 in the North, and another 2 in the South	5/11/20 after 1st wave	Yes, regional level	Yes	Yes (e.g., Cagliari)
	Lebanon	Yes	No	8 university medical centers in Lebanon	6/3/20 before 1st wave	Yes, facility level	Yes	Yes
	Nigeria	Yes	No	10 large WhatsApp and Telegram groups of nurses and physicians (WhatsApp group is used as a registry in this context, not an exchange of information between friends or colleagues)[c]	8/5/20 before 1st wave	Yes, regional level	Yes	No
	Puerto Rico	Yes	Yes	12 Federally Qualified Health Centers across the main Island	6/22/20 at peak of 1st wave	No, convenience sample	Yes	No
	Spain	Yes	Yes	5–10 health facilities in each region (i.e., Madrid, Murcia y Andalucía) based on type of facility (e.g., hospitals vs primary care clinics)	4/23/20 at peak of 1st wave	Yes, facility level	Yes	No

Table 1 (continued)

Recruitment strategies	Country	Target Population		Recruitment scheme	Study start date	Representative samples	Response rates[a]	Pre-pandemic data
		Clinical	Nonclinical					
	South Africa	Yes	Yes	All health facilities from the following provinces: Eastern Cape, Free State, Gauteng, KwaZulu-Natal, Limpopo, Mpumalanga, North West, Northern Cape, and Western Cape	Not started yet	Yes, regional level	Yes	No
	The Netherlands	Yes	Yes	10 health facilities in three regions	5/12/20 after 1st wave	Yes, facility level	Yes	No
Other institutions and organizations	Guatemala	Yes	Yes	Health care workers associated with academic institutions, union organizations, and associations across the country	7/1/20 before peak of 1st wave	No, convenience sample	No	No
	Mexico	Yes	Yes	Health care workers affiliated to the following institutions: (1) Ministry of Health; (2) Instituto Mexicano del Seguro Social (IMSS); (3) Instituto de Seguridad y Servicios Social de los Trabajadores del Estado (ISSSTE); (4) Secretary of National Defense (SEDENA); and (5) Health Services of PEMEX	5/13/20 before 1st wave	No, convenience sample	No	No
	Tunisia	Yes	Yes	20 health facilities based on (i) type of center (e.g., private vs public); (ii) level of care (e.g., 1st vs 2nd vs 3rd); and (iii) Geographic representation (Greater Tunis area vs the North, Center and South of Tunisia)	10/1/20 at peak of 1st wave	No, convenience sample	No	No
	Turkey	Yes	Yes	NGOs and professional associations such as the Specialty Medical Training Coordination Committees, Turkish Dental Association, Turkish Nursing Association, Turkish Psychological Association, Health Workers Union, and Turkish Family Physicians Association	2/6/21 between waves 1 and 2	No, convenience sample	No	No

[a]Number of completed surveys/number of health workers at each facility

Table 2 Items included in the on-line questionnaire

Construct	Variables	Measures
Sociodemographic	Age	Ad-hoc questions
	Gender	
	Educational attainment	
	Educational attainment of parents	
	Members of household (total number, number of individuals under 18, years of age number of individuals over 65 years of age, and disabled individuals)	
Work environment	Place of work (public or private sector, type of health center, patient population)	Ad-hoc questions
	Profession and specialty	
	Change of functions since the start of the pandemic	
	Amount worked in the past week (days and hours)	
Experiences, fears, and concerns about COVID-19	Perception about personal protective equipment (PPE)	Ad-hoc questions
	COVID-19 testing and results	
	Days of isolation for COVID-19	
	Fear of transmitting COVID-19 to loved ones	
	Fear of being infected	
	Contact with patients with COVID- 19	
COVID-19 training and prioritization	Specific COVID-19 training on preventing transmission and clinical management	Ad-hoc questions
	Experience with the death of patients with COVID-19	
	Prioritization of groups of patients to receive mechanical ventilation and emotional impact	
Anxiety and depression	Anxiety and depressive symptoms in the last week	General Health Questionnaire (GHQ-12)
Depression	Depressive symptoms in the last week	Patient Health Questionnaire (PHQ-9)
Posttraumatic stress disorder		DSM 5 checklist
Suicide ideation	Suicide ideation in the last two weeks	Selected items from the Columbia Suicide Severity Rating Scale (C-SSRS)
Acute stress	Symptoms of acute stress in the past week	Ad-hoc questions
Formal and informal supports	Colleagues	Ad-hoc questions
	Loved ones/friends	
	Required versus received psychosocial supports and perceived benefit	
Resilience	Overall resilience	Brief Resilience Scale (BRS)
Prior conditions	Prior physical, mental, and substance use conditions	Ad-hoc questions

Health Questionnaire (GHQ-12) [19], the Patient Health Questionnaire (PHQ-9) [20], the Columbia Suicide Severity Rating Scale (C-SSRS) [21], and the Primary Care PTSD Screen for DSM-5 (PC-PTSD-5) [22], as well as a series of items on workplace, family, and social challenges related to the COVID-19 pandemic. Both the GHQ-12 and the PHQ-9 have been translated into many languages and validated in general and clinical populations worldwide including in low-resource settings [23, 24]. Table 2 summarizes all the instruments included in this study.

Here we present examples of variables of interest and primary exposures.

Mental health variables

Depressive symptoms: the PHQ-9 is a 9-item self-administered version of the depression module of the Primary Care Evaluation of Mental Disorders (PRIME-MD), which is based on the Diagnostic and Statistical Manual of Mental Disorders (DSM-IV) criteria for major depression. Participants are required to answer whether, within the previous 2 weeks, they had experienced symptoms that tend to be associated with depression. The possible answers and respective scores are ‘not at all’ (0), ‘less than 1 week’ (1), ‘1 week or more’ (2), and ‘almost every day’ (3).

Psychological distress: the GHQ-12 is a self-administered screening measure for the detection of minor psychological distress. It has been translated into many languages and validated in general and clinical populations worldwide.

Posttraumatic stress disorder: the PC-PTSD-5 is a 5-item self-report measure that assesses DSM-5 symptoms of PTSD. The PC-PTSD-5 has a variety of purposes, including: (1) monitoring symptom change during and after treatment; (2) screening individuals for PTSD; (3) making a provisional PTSD diagnosis. A total symptom severity score (range 0–80) can be obtained by summing the scores for each of the 20 items. It takes approximately 3–5 min to complete.

Other variables of interest

We are measuring resilience by the Brief Resilience Scale (BRS), which is a 6-item scale measuring one's ability to bounce back or recover from stress. Participants are asked to indicate the extent to which they agreed with each of the items according to a 5-point Likert scale (1 = strongly disagree; 5 = strongly agree). The BRS is scored by reverse-coding Items 2, 4, and 6 and calculating the sum of all six items. It takes on average 2 min to complete.

Trust in the workplace and the government regarding actions to manage the pandemic are measured by ad-hoc questions such as "to what extent do you trust that your workplace can manage the COVID-19 pandemic?" and "to what extent do your trust that the government can manage the COVID-19 pandemic?" rated by a 5-point Likert scale (1 = not at all; 5 = extremely).

Exposures

Workplace-level exposures include (1) Direct care of COVID-19 patients (dichotomous variables Yes/No); (2) Number of days exposed to COVID-19 patients (continuous); (3) Adequate access to Personal Protective Equipment (PPE) (dichotomous, Yes/No) and appropriateness (e.g., sufficient, insufficient); (4) Place of work (e.g., emergency services, primary care clinics, community center); (5) Profession (e.g., clinical staff vs. non-clinical staff); and (6) Being tested for COVID-19 (dichotomous, Yes/No); and (6) in some analyses trust in workplace and government will be considered as exposures, though in others they will be potential effect modifiers (see below).

Household-level exposures include (1) Any loved ones infected with COVID-19 (dichotomous, Yes/No), (2) Any loved ones passed away due to COVID-19 (Yes/No), and (3) Number of people under your care (e.g., children, elderly) at home.

Demographic and psychosocial factors

The online questionnaire asks about a wide range of personal and social factors that will be key covariates for planned future analyses, including sociodemographics (e.g., age, gender, education, living situation), employment (e.g., place of work, current job, redeployment), testing for COVID-19 (e.g., being tested, results from test, being isolated), fears and concerns related to COVID-19 (e.g., concerns about getting COVID-19, concerns about infecting loved ones), stigma at work (e.g., felt stigmatized by patients and relatives), COVID-19 training and prioritization (e.g., guidance on managing COVID-19 patients, guidance on triage), other mental health symptoms (e.g., suicidal ideation, acute stress), formal and informal supports (e.g., supports from institutions, support from relatives, support from coworkers), and prior conditions (e.g., prior mental, physical, and substance use conditions). Additionally, we will include data on region-level variables, such as infection and death rates, to augment our analyses. Finally, we note that these covariates will be considered confounders, mediators, moderators, and effect modifiers, depending on the research question of interest. Finally, we will also conduct other analyses where the exposures and variables of interest may differ from the 3 primary outcome domains defined here. For instance, we plan to examine gender as an effect modifier of the variables of interest.

Data analysis plan

For this longitudinal study, descriptive statistics will provide an overview of the prevalence of primary exposures, mental health variables, and other variables of interest at baseline and different time points. Similar analyses will be conducted between variables of interest and covariates. Based on prior literature and data, potential confounders, moderators, and effect modifiers, including time-varying exposures, will be identified and examined. Depending on the research question, these include—but are not limited to—level of exposure, gender, age, worker type, workplace conditions, country, COVID-19 infection and death rate per 100.000 inhabitants, and estimates of hospital occupation (e.g., percentage of occupied ICU beds). All study findings will be corrected for multiple testing.

For each country, we will use a regression model (e.g., x = direct contact with COVID-19 patients, y = GHQ-12) appropriate to the nature of the country's data (e.g., different gender proportions, different recruitment schemes) to assess the association between COVID-19 related exposures and our primary mental health outcomes (i.e., depressive symptoms, psychological distress, and PTSD). Models will be adjusted for age, gender, and profession. Further, we will pool the effect sizes of different countries using meta-analytic methods controlling for the sample size of each country. We will calculate heterogeneity measures (i.e., I^2 and Q statistics) and conduct meta-regressions to examine reasons for country-level variation in the impact of COVID-19 on

the mental health of HCWs. One hypothesized source of variation is income level of the country (LMIC vs. HIC). Using current methods for mediation analyses allowing for interactions (e.g., Potential Outcomes Methods for mediation) [25], we will further investigate potential pathways and examine whether workplace conditions and trust in government mediate or moderate the association between COVID-19 exposure and mental health outcomes.

Moreover, we plan to conduct multilevel models to assess the association between COVID-19 exposure and mental health outcomes accounting for cross-country differences. Multilevel models will include country- and region-level variables on sampling (e.g., random vs non-random sampling), pandemic patterns over time (e.g., COVID-19 incidence, hospitalization, and mortality rates), and governments' responses to the pandemic (e.g., policies about mask use, contact tracing, and social distancing). Finally, we will also use multilevel structural equation models to assess associations across multiple measures and countries and take into account validation issues and data reduction of questionnaires.

Web-based platform and methods to protect the confidentiality

The study uses a digital platform hosted at the University of Chile, whose functionality, in terms of data management and protection, is akin to REDCap. The platform is flexible, reliable, and has previously been used in prior studies. The servers are housed at the University of Chile and have data encryption technology that meets international standards.

The platform permits (1) the creation of online surveys that can be answered on personal computers (PCs), tablets, or mobile phones; (2) reliable and secure data capture and storage; and (3) the preparation of databases that can be easily exported in multiples formats that are compatible with most statistical software.

To guarantee confidentiality, an "ID" code system is being used to identify and subsequently monitor each participant. To guarantee security, three central aspects are considered: (1) access to the system is restricted only to personnel with access credentials assigned by the administrator and defined by the study's Coordinating Committee; (2) the data entered is backed up daily on virtual servers that guarantee the security, integrity, and availability of the information, and which comply with international regulations for the handling of human subjects research data; and 3) any modification of the database is traceable.

This study protocol has been approved by several Institutional Review Boards in each country, the Faculty of Medicine, University of Chile, and the one at Columbia University. Additionally, for those studies developed in the Region of the Americas, there was a validation by the PAHO Ethical Review Committee.

Discussion

We have presented the protocol study of a timely, multinational initiative to study the health and mental health of health care workers and other variables of interest concerning the COVID-19 pandemic. The study's overarching goal is to produce generalizable evidence based on rigorous study design and longitudinal follow-up to inform policy and develop guidelines and scalable interventions for different health care systems to support health workers who are disproportionally exposed to the global threat of COVID-19. The novelties of this study are the following: (1) allowing within- and across-country investigations regarding the mental health impact, exposure to COVID-19 and work conditions; (2) incorporating subgroup analyses: given the large sample size, we will be able to determine separate effects for men and women, for health workers from different age groups, cultural backgrounds, and disparate socioeconomic groups; (3) including all workers in the health care setting at a wide variety of health centers to enable separate investigation of the effect by profession (e.g. physician, nurse, cleaning staff) and health care setting (e.g. academic hospital, elderly home, care for the disabled, mental health facilities, primary care centers), as well as their interaction; (4) taking into account the nature and the extent of the health care system response (e.g., deployment, increased workload) to advance our understanding of these complex phenomena and to inform policy and develop the kind of supports that this population deems useful.

We note three main limitations of this study. First, we do not have data on comparable study samples from the period before the pandemic except for Brazil, Italy (Cagliari and Verona), and Lebanon. Therefore, it will not be possible to directly compare the specified outcomes in most sites before and during the pandemic. Second, despite our efforts to recruit samples of HCWs that reflect the background population at a large number of sites, there was no uniform, stringent recruitment scheme in place that could guarantee a high degree of representativeness at all sites. Third, given the lack of validated questionnaires on COVID-19 related exposures at the start of the study, not all items in the questionnaire used have been validated.

Through this global initiative, we will be able to identify vulnerable groups among health workers, including the key factors (e.g., demographic variables, home situation, COVID-19 exposure, work conditions, years of training) that increase risk for developing mental health problems over time. Consequently, specific guidelines and interventions can be generated targeting these most vulnerable groups.

Lastly, findings on predictors of mental health variables during the COVID-19 pandemic are essential for improving our response and protecting our health workers during future crises of similar nature.

Supplementary Information The online version contains supplementary material available at https://doi.org/10.1007/s00127-021-02211-9.

Acknowledgements The members of The HEROES study group are listed in supplemental material.

Funding ATCS is supported by Minciencias scholarship 728/2015, and by Pasaporte a la Ciencia scholarship, Colombia, 2018-2022. The Colombia Heroes project is funded by the MInciencias (grant #: CT 860-2020), and by the UK Research and Innovation (UKRI) (grant #: ES/V013157/1). ERS is supported by the National Institute of Mental Health under award R34MH120179. The content is solely the responsibility of the author and does not necessarily represent the official views of the National Institutes of Health. GMA, EFJ, and RM are supported by the Institute of Health Carlos III (grant #: COV20/00988), Spain. HEROES Spain is supported by the Institute of Health Carlos III (Madrid, Spain) (grant number: COV20/00988)ATCS is supported by Minciencias scholarship 728/2015, and by Pasaporte a la Ciencia scholarship, Colombia, 2018-2022. The Colombia Heroes project is funded by the MInciencias (grant #: CT 860-2020), and by the UK Research and Innovation (UKRI) (grant #: ES/V013157/1). ERS is supported by the National Institute of Mental Health under award R34MH120179. The content is solely the responsibility of the author and does not necessarily represent the official views of the National Institutes of Health. GMA, EFJ, and RM are supported by the Institute of Health Carlos III (grant #: COV20/00988), Spain. SDA is supported by the National Council of Science and Technology (CONACYT), grant # 312586.

Declarations

Conflict of interest There are no potential conflicts of interest to report.

References

1. Sachs JD, Karim SA, Aknin L, Allen J, Brosbøl K, Barron GC, Daszak P, Espinosa MF, Gaspar V, Gaviria A, Haines A (2020) Lancet COVID-19 Commission Statement on the occasion of the 75th session of the UN General Assembly. The Lancet 396(10257):1102–1124. https://doi.org/10.1016/S0140-6736(20)31927-9
2. Erdem H, Lucey DR (2021) Healthcare worker infections and deaths due to COVID-19: a survey from 37 nations and a call for WHO to post national data on their website. Int J Infect Dis 102:239. https://doi.org/10.1016/j.ijid.2020.10.064
3. Bandyopadhyay S, Baticulon RE, Kadhum M, Alser M, Ojuka DK, Badereddin Y, Kamath A, Parepalli SA, Brown G, Iharchane S, Gandino S (2020) Infection and mortality of healthcare workers worldwide from COVID-19: a systematic review. BMJ Glob Health 5(12):e003097. https://doi.org/10.1136/bmjgh-2020-003097
4. Brooks SK, Dunn R, Amlôt R, Rubin GJ, Greenberg N (2018) A systematic, thematic review of social and occupational factors associated with psychological outcomes in healthcare employees during an infectious disease outbreak. J Occup Environ Med 60(3):248–257. https://doi.org/10.1097/JOM.0000000000001235
5. Greenberg N, Wessely S, Wykes T (2015) Potential mental health consequences for workers in the Ebola regions of West Africa–a lesson for all challenging environments. J Ment Health 24(1):1–3. https://doi.org/10.3109/09638237.2014.1000676
6. McKibbin W, Fernando R (2020) The global macroeconomic impacts of COVID-19: seven scenarios. Asian Econ Pap 2:1–55. https://doi.org/10.1162/asep_a_00796
7. Mediavilla R, Fernández-Jiménez E, Andreo J, Morán-Sánchez I, Muñoz-San José A, Moreno-Küstner B, Mascayano F, Ayuso-Mateos JL, Bravo-Ortiz MF, Martínez-Alés G, COVID T (2021) Association between perceived discriminations and mental health outcomes among health workers during the initial COVID-19 outbreak. Rev Psiquiatr Salud Ment. https://doi.org/10.1016/j.rpsm.2021.06.001
8. da Silva Neto RM, Benjamim CJ, de Medeiros Carvalho PM, Neto ML (2021) Psychological effects caused by the COVID-19 pandemic in health professionals: a systematic review with meta-analysis. Prog Neuropsychopharmacol Biol Psychiatry 10:104. https://doi.org/10.1016/j.pnpbp.2020.110062
9. Pappa S, Ntella V, Giannakas T, Giannakoulis VG, Papoutsi E, Katsaounou P (2020) Prevalence of depression, anxiety, and insomnia among healthcare workers during the COVID-19 pandemic: a systematic review and meta-analysis. Brain Behav Immun 88:901–907. https://doi.org/10.1016/j.bbi.2020.05.026
10. de Pablo GS, Vaquerizo-Serrano J, Catalan A, Arango C, Moreno C, Ferre F, Shin JI, Sullivan S, Brondino N, Solmi M, Fusar-Poli P (2020) Impact of coronavirus syndromes on physical and mental health of health care workers: systematic review and meta-analysis. J Affect Disord 275:48–57. https://doi.org/10.1016/j.jad.2020.06.022
11. Lai J, Ma S, Wang Y, Cai Z, Hu J, Wei N, Wu J, Du H, Chen T, Li R, Tan H (2020) Factors associated with mental health outcomes among health care workers exposed to coronavirus disease 2019. JAMA Netw Open 3(3):e203976. https://doi.org/10.1001/jamanetworkopen.2020.3976
12. Lasalvia A, Bonetto C, Porru S, Carta A, Tardivo S, Bovo C, Ruggeri M, Amaddeo F (2021) Psychological impact of COVID-19 pandemic on healthcare workers in a highly burdened area of north-east Italy. Epidemiol Psychiatr Sci. https://doi.org/10.1017/S2045796020001158
13. García-Fernández L, Romero-Ferreiro V, López-Roldán PD, Padilla S, Calero-Sierra I, Monzó-García M, Pérez-Martín J, Rodriguez-Jimenez R (2020) Mental health impact of COVID-19 pandemic on Spanish healthcare workers. Psychol Med. https://doi.org/10.1017/S0033291720002019
14. Gonzalo RM, Ana RG, Patricia CA, Laura AL, Nathalia GT, Luis C, Asuncion L, Miguel RV, Benedicto CF (2021) Short-term emotional impact of COVID-19 pandemic on Spaniard health workers. J Affect Disord 278:390–394. https://doi.org/10.1016/j.jad.2020.09.079
15. Williamson V, Murphy D, Greenberg N (2020) COVID-19 and experiences of moral injury in front-line key workers. Occup Med 70(5):317–319. https://doi.org/10.1093/occmed/kqaa052
16. Litz BT, Stein N, Delaney E et al (2009) Moral injury and moral repair in war veterans: a preliminary model and intervention strategy. Clin Psychol Rev 29(8):695–706. https://doi.org/10.1016/j.cpr.2009.07.003
17. Mediavilla R, Fernández-Jiménez E, Martínez-Alés G, Moreno-Küstner B, Martínez-Morata I, Jaramillo F, Morán-Sánchez I, Minué S, Torres-Cantero A, Alvarado R, Ayuso-Mateos JL, Mascayano F, Susser E, Bravo-Ortiz MF (2021) Role of access to personal protective equipment, treatment prioritization decisions, and changes in job functions on health workers' mental health outcomes during the initial outbreak of the COVID-19 pandemic. J Affect Disord 295:405–409. https://doi.org/10.1016/j.jad.2021.08.059

18. Khan M, Abimbola S, Aloudat T, Capobianco E, Hawkes S, Rahman-Shepherd A (2020) Decolonising global health in 2021: a roadmap to move from rhetoric to reform. BMJ Glob Health. https://doi.org/10.1136/bmjgh-2021-005604
19. Goldberg DP (1988) User's guide to the General Health Questionnaire. NFER-NELSON
20. Kroenke K, Spitzer RL, Williams JB (2001) The PHQ-9: validity of a brief depression severity measure. J Gen Intern Med 16(9):606–613. https://doi.org/10.1046/j.1525-1497.2001.016009606.x
21. Posner K, Brown GK, Stanley B, Brent DA, Yershova KV, Oquendo MA, Currier GW, Melvin GA, Greenhill L, Shen S, Mann JJ (2011) The Columbia-Suicide Severity Rating Scale: initial validity and internal consistency findings from three multisite studies with adolescents and adults. Am J Psychiatry 168(12):1266–1277. https://doi.org/10.1176/appi.ajp.2011.10111704
22. Prins A, Bovin MJ, Smolenski DJ, Marx BP, Kimerling R, Jenkins-Guarnieri MA, Kaloupek DG, Schnurr PP, Kaiser AP, Leyva YE, Tiet QQ (2016) The primary care PTSD screen for DSM-5 (PC-PTSD-5): development and evaluation within a veteran primary care sample. J Gen Intern Med 31(10):1206–1211. https://doi.org/10.1007/s11606-016-3703-5
23. Goldberg DP, Gater R, Sartorius N, Ustun TB, Piccinelli M, Gureje O, Rutter C (1997) The validity of two versions of the GHQ in the WHO study of mental illness in general health care. Psychol Med 27(1):191–197. https://doi.org/10.1017/S0033291796004242
24. Carroll HA, Hook K, Perez OF, Denckla C, Vince CC, Ghebrehiwet S, Ando K, Touma M, Borba CP, Fricchione GL, Henderson DC (2020) Establishing reliability and validity for mental health screening instruments in resource-constrained settings: systematic review of the PHQ-9 and key recommendations. Psychiatry Res 291:113236. https://doi.org/10.1016/j.psychres.2020.113236
25. Valeri L, VanderWeele TJ (2013) Mediation analysis allowing for exposure–mediator interactions and causal interpretation: theoretical assumptions and implementation with SAS and SPSS macros. Psychol Methods 18(2):137. https://doi.org/10.1037/a0035596

Authors and Affiliations

Franco Mascayano[1,2] · Els van der Ven[3] · Maria Francesca Moro[1,4] · Sara Schilling[5] · Sebastián Alarcón[5] · Josleen Al Barathie[6] · Lubna Alnasser[1,7] · Hiroki Asaoka[8] · Olatunde Ayinde[9] · Arin A. Balalian[1] · Armando Basagoitia[10] · Kirsty Brittain[11] · Bruce Dohrenwend[1] · Sol Durand-Arias[12] · Mehmet Eskin[13] · Eduardo Fernández-Jiménez[14] · Marcela Inés Freytes Frey[15] · Luis Giménez[16] · Lydia Gisle[17] · Hans W. Hoek[1,18] · Rodrigo Ezequiel Jaldo[15] · Jutta Lindert[19] · Humberto Maldonado[20] · Gonzalo Martínez-Alés[1,14,23] · Carmen Martínez-Viciana[21] · Roberto Mediavilla[22,23] · Clare McCormack[24] · Landon Myer[11] · Javier Narvaez[25] · Daisuke Nishi[8] · Uta Ouali[26] · Victor Puac-Polanco[1,27,28] · Jorge Ramírez[5] · Alexandra Restrepo-Henao[1,29] · Eliut Rivera-Segarra[30] · Ana M. Rodríguez[31] · Dahlia Saab[6] · Dominika Seblova[32] · Andrea Tenorio Correia da Silva[33,34] · Linda Valeri[35] · Rubén Alvarado[5] · Ezra Susser[1,2] on behalf of the HEROES group

✉ Franco Mascayano
franco.mascayano@nyspi.columbia.edu

[1] Department of Epidemiology, Mailman School of Public Health, Columbia University, New York, NY, USA

[2] New York State Psychiatric Institute, New York, NY, USA

[3] Department of Clinical, Neuro- and Developmental Psychology, Vrije Universiteit, Amsterdam, The Netherlands

[4] Università degli Studi di Cagliari, Cagliari, Italy

[5] School of Public Health, Faculty of Medicine, Universidad de Chile, Santiago, Chile

[6] Institute for Development Research Advocacy and Applied Care, Beirut, Lebanon

[7] Population Health Department, King Abdullah International Medical Research Center, Riyadh, Saudi Arabia

[8] Department of Mental Health, Graduate School of Medicine, The University of Tokyo, Tokyo, Japan

[9] Department of Psychiatry, University College Hospital, Ibadan, Nigeria

[10] Consultora Salud Global, Chuquisaca, Bolivia

[11] Division of Epidemiology and Biostatistics, School of Public Health and Family Medicine, University of Cape Town, Cape Town, South Africa

[12] Instituto Nacional de Psiquiatría Ramón de la Fuente Muñiz, Mexico City, Mexico

[13] Department of Psychology, Koc University, Istanbul, Turkey

[14] Department of Psychiatry, Clinical Psychology and Mental Health, La Paz University Hospital, Madrid, Spain

[15] Social and Community Academic Unit, Universidad de Chubut, Chubut, Argentina

[16] Instituto de Psicología de la Salud, Facultad de Psicología, Universidad de la República, Montevideo, Uruguay

[17] Department of Epidemiology and Public Health, Sciensano, Brussels, Belgium

[18] Department of Psychiatry, University Medical Centre Groningen, University of Groningen, Groningen, The Netherlands

[19] University of Applied Sciences Emden/Leer, Emden, Germany

[20] Dirección de Salud Mental, Ministerio de Salud, Lima, Perú

[21] Pan-American Health Organization, Washington, DC, USA

[22] Department of Psychiatry, Universidad Autónoma de Madrid (UAM), Madrid, Spain

[23] Centro de Investigación Biomédica en Red (CIBERSAM), Madrid, Spain

[24] Center for Science and Society, Columbia University, New York, NY, USA

[25] University of El Bosque, Bogota, Colombia

[26] Psychiatry Department A, Razi Hospital, Tunis, Tunisia

[27] Facultad de Ciencias Médicas, Universidad de San Carlos de Guatemala, Guatemala City, Guatemala

[28] Department of Health Care Policy, Harvard Medical School, Boston, MA, USA

[29] Epidemiology Group, Universidad de Antioquia, Medellín, Colombia

[30] School of Behavioral and Brain Sciences, Ponce Health Sciences University, Ponce, Puerto Rico

[31] Instituto Altos Estudios Dr Arnoldo Gabaldon, Caracas, Venezuela

[32] Taub Institute for Research on Alzheimer's Disease and the Aging Brain, The Gertrude H. Sergievsky Center, Vagelos College of Physicians and Surgeons, Columbia University, New York, Brazil

[33] Department of Family Medicine, Faculdade Santa Marcelina, São Paulo, Brazil

[34] Department of Public Health, Faculdade de Medicina de Jundiaí, São Paulo, Brazil

[35] Department of Biostatistics, Mailman School of Public Health, Columbia University, New York, NY, USA
