# Supplementary material for: Mental health status and related factors influencing healthcare workers during the COVID-19 pandemic: A systematic review and meta-analysis
Source: PLoS One. 2024 Jan 19;19(1):e0289454. doi: 10.1371/journal.pone.0289454 (PMC10798549; doi:10.1371/journal.pone.0289454)
Supplement: S1 Data — (ZIP) [file pone.0289454.s011.zip › literatures/94.pdf]

ELSEVIER

Since January 2020 Elsevier has created a COVID-19 resource centre with free information in English and Mandarin on the novel coronavirus COVID-19. The COVID-19 resource centre is hosted on Elsevier Connect, the company's public news and information website.

Elsevier hereby grants permission to make all its COVID-19-related research that is available on the COVID-19 resource centre - including this research content - immediately available in PubMed Central and other publicly funded repositories, such as the WHO COVID database with rights for unrestricted research re-use and analyses in any form or by any means with acknowledgement of the original source. These permissions are granted for free by Elsevier for as long as the COVID-19 resource centre remains active.

L'évolution psychiatrique 86 (2021) 131–139

Disponible en ligne sur www.sciencedirect.com

ScienceDirect

et également disponible sur www.em-consulte.com

Article original

Anxiété et dépression associées à la prise en charge de la COVID-19 chez les personnels de santé au Cameroun☆

Anxiety and Depression Associated with the Management of COVID-19 Among Healthcare workers in Cameroon

Célestin Pierre Mboua (PhD) (Maître de Conférences, Enseignant associé à l'Université Catholique d'Afrique Centrale, Représentant au Cameroun de l'IPM, Directeur de Recherche au FOCAP)[a,*,b,c,d], François Roger Nguépy Keubo (MSc (PhD candidate)) (Infirmier, Praticien EMDR (niveau 1) Cameroun)[e,f], Sylvain Gautier Ngueuteu Fouaka (PhD) (Candidate)[g]

[a] *Psychologie Clinique et Psychopathologie, Université de Dschang, BP 20357, Yaoundé, Cameroun*
[b] *Université Catholique d'Afrique centrale*
[c] *Institut de psychotraumatologie et de Médiation (IPM)*
[d] *Forum Camerounais de Psychologie (FOCAP)*
[e] *Psychologie Clinique et Psychopathologie, Université de Dschang, Cameroun*
[f] *Hôpital St Vincent de Paul de Dschang, Cameroun*
[g] *Département de Management, Université de Laval, Canada*

☆ Toute réference à cet article doit porter mention : Mboua CP, Nguépy Keubo FR, Ngueuteu Fouaka SG. Anxiété et dépression associées à la prise en charge de la COVID 19 chez les personnels de santé au Cameroun. Evol psychiatr 2021 ; 86(1): pages (pour la version papier) ou URL [date de consultation] (pour la version électronique).

* Auteur correspondant.
Adresse e-mail : pierrecelestinmboua@yahoo.fr (C.P. Mboua).

https://doi.org/10.1016/j.evopsy.2020.11.002
0014-3855/© 2020 Elsevier Masson SAS. Tous droits réservés.

I N F O A R T I C L E

Historique de l'article :
Reçu le 10 octobre 2020
Accepté le 8 novembre 2020

Mots clés :
Santé mentale
COVID-19
Anxiété
Dépression
Trouble dépressif majeur
Trouble de l'adaptation
Soignant

R É S U M É

Objectif. – La pandémie de la COVID-19 a eu un retentissement important dans le monde, à différents niveaux. La littérature consultée indique que les travailleurs de santé, en première ligne de la riposte, sont généralement ceux qui paient le plus lourd tribu. La présente étude évalue l'importance des symptomatologies anxieuse et dépressive chez ces acteurs de la riposte à la COVID-19, dans le contexte du Cameroun.
Méthode. – La *Hospital Anxiety and Depression Scale* (HADS) a été mise à contribution pour évaluer les symptomatologies anxieuse et dépressive. La collecte des données a eu recours à la construction d'un web questionnaire sur la plateforme Google Forms. Le lien du questionnaire a été envoyé à des groupes professionnels dans les 10 Régions du pays, au préalable de leur consentement éclairé. Au total, 331 professionnels de santé ont été interrogés sur la période du 05 au 19 avril 2020.
Résultats. – Les résultats montrent un taux important d'anxiété (41,8 %) et de dépression (42,8 %). On observe une plus forte susceptibilité à la dépression chez les sujets jeunes (30-39 ans). La peur de la contamination et la peur de la mort sont des facteurs modulateurs de la dépression et de l'anxiété. La comorbidité anxiété-dépression dans l'échantillon est de 14,73 %. Les taux de prévalence du trouble dépressif majeur et du trouble de l'adaptation dans l'échantillon sont respectivement de 8,2 % et 3,3 %. Les données présentées confirment les tendances enregistrées dans la littérature, concernant l'impact des épidémies mortelles sur la santé mentale des travailleurs de santé.
Discussion. – Les résultats indiquent, comme c'est le cas dans la plupart des travaux consultés dans la littérature, que la pandémie de la Covid 19 affecte de manière importante la santé mentale des travailleurs de santé. Ceci souligne l'urgence de mettre en place un dispositif d'aide et/ou d'accompagnement spécialisé, dans le contexte de l'actuelle pandémie, comme celui des crises sanitaires de même nature. Un tel dispositif n'existe pas au Cameroun, où la réponse de santé mentale reste faible.
Conclusion. – Ces résultats suggèrent que les personnels de santé engagés dans la riposte contre la COVID-19 au Cameroun sont affectés de manière significative dans leur santé mentale. Ce qui souligne la nécessité d'une aide psychologique spécialisée pour le personnel soignant.
© 2020 Elsevier Masson SAS. Tous droits réservés.

Keywords:
Mental health
COVID-19
Anxiety
Depression
Major depressive disorder
Adjustment disorder
Healthcare worker

A B S T R A C T

Objective. – The COVID-19 pandemic has had a significant impact worldwide, at different levels. The literature consulted indicates that healthcare workers, who are the first to respond, are generally the ones who pay the highest price. The present study assesses the extent of anxious and depressive symptoms among these actors responding to COVID-19, in the context of Cameroon.
Method. – The Hospital Anxiety and Depression Scale (HADS) was used to assess anxious and depressive symptoms. Data collection involved the use of a web questionnaire for this purpose through the Google Forms virtual platform. The questionnaire link was sent to professional groups spread throughout Cameroon's ten regions. A total of 331 health professionals were interviewed over the period from April 5 to 19, 2020.

Result. – The results show a high rate of anxiety (41.8%) and depression (42.8%). There is a higher susceptibility to depression in young people (30-39 years). Fear of contamination and fear of death are modulators of depression and anxiety. The anxiety-depression comorbidity in the sample is 14.73%. The prevalence rates of major depressive disorder and adjustment disorder in the sample were 8.2% and 3.3%, respectively. The data presented confirm the trends recorded in the literature regarding the impact of fatal epidemics on the mental health of healthcare workers.
Discussion. – The results indicate, as is the case with most of the work consulted in the literature, that the Covid 19 pandemic is significantly affecting the mental health of healthcare workers. This underlines the urgency of setting up a specialized aid and/or support system, in the context of the current pandemic, such as that of similar health crises. Such a device does not exist in Cameroon, where the mental health response remains weak.
Conclusion. – These results suggest that the healthcare staff involved in the response to COVID-19 in Cameroon are significantly affected in their mental health. This underlines the need for specialized psychological help for nursing staff.
© 2020 Elsevier Masson SAS. All rights reserved.

1. Contexte

Les maladies infectieuses constituent une importante menace pour la santé mentale des individus. L'épidémie mortelle de SRAS (Syndrome Respiratoire Aigu Sévère), notifiée pour la première fois en novembre 2002 dans la Province de Canton en Chine, a eu un impact psychologique important sur la santé mentale des personnels de santé et des survivants dans les pays affectés [1–3]· C'est l'une des premières infections à coronavirus ayant fait l'objet d'études de santé mentale. Même si elle a rapidement été maîtrisée [4], elle a pourtant eu d'importantes conséquences sanitaires, psychologiques et sociales [5]. Chua *et al.* [6] ont montré que les séquelles psychologiques immédiates du SRAS concernaient un niveau de stress significativement plus élevé chez les individus affectés, lorsqu'on les comparait aux cas témoins sains appariés. Il en était ainsi des troubles du sommeil, de l'humeur dépressive, des crises de larmoiements, des troubles cauchemars et des troubles de la concentration. Lee *et al.* [7] ont rapporté la présence d'épisodes de psychose aiguë chez quelques patients SRAS.

Depuis décembre 2019, le monde fait face à une nouvelle pandémie de coronavirus. De 1930 à 2002, plusieurs souches de coronavirus étaient déjà identifiées dans le monde. Ces souches étaient qualifiées comme moins dangereuses pour l'espèce humaine, jusqu'à la découverte d'une nouvelle souche à l'origine du SRAS [4,8]. L'actuel SRAS à coronavirus dénommé COVID-19, a été déclaré pour la première fois en Chine au mois de décembre 2019. S'étant rapidement propagé dans le monde, sa diffusion a été qualifiée d'« urgence sanitaire mondiale » par l'OMS le 25 février 2020 [9]. Le 11 mars 2020, elle est classée pandémie [10]. Des études récentes sur la COVID-19 montrent qu'elle est plus mortelle et plus invasive que le SRAS de 2002, tant au plan sanitaire que psychologique. Dans le contexte d'une étude de suivi à court terme des survivants de l'épidémie du SRAS en 2003, des auteurs ont mis en évidence l'existence d'un taux important d'anxiété, de dépression et de détresse psychologique [11]. Wu, Chan et Ma ont montré, dans le contexte de cette même épidémie, l'existence d'une prévalence importante du syndrome de stress post-traumatique [12]. Ce qui souligne son impact non négligeable sur la santé psychologique [13]. Dans le même sillage, Lai et ses collègues [5] ont évalué les effets du SRAS sur la santé mentale des professionnels de santé en Chine en 2003. Les résultats révèlent une proportion considérable de participants ayant signalé des symptômes de dépression (50,4 %), d'anxiété (44,6 %) et de détresse (71,5 %). Ces données suggèrent que les personnels de santé, en première ligne de la riposte contre la maladie, sont fortement éprouvés au plan psychologique et susceptibles de développer une importante détresse psychique, à l'origine d'un vécu anxieux potentiellement invalidant. C'est pourquoi la présente étude s'est donnée pour objectif de déterminer la prévalence des vécus

anxieux et dépressifs chez les personnels de santé impliqués dans la lutte contre le COVID-19, ainsi que les facteurs associés. La littérature africaine reste pauvre sur la question, aucune étude n'ayant été répertoriée au moment de la rédaction de ce travail.

2. Matériels et méthode

L'étude est quantitative, analytique et transversale. Les participants sont des professionnels de la santé en service dans les structures sanitaires et les organisations de la société civile (OSC) dans les dix Régions du Cameroun. La procédure d'échantillonnage est non probabiliste. L'étude a opté pour une approche raisonnée, couplée à la méthode de boule de neige. La collecte de ces données s'est faite au travers des réseaux professionnels : les participants ciblés au travers d'une liste prédéfinie, ont été priés de partager le questionnaire à leurs collègues et à leurs groupes professionnels, *in-boxe*. Ce qui a nécessité la construction d'un web questionnaire via la plateforme Google Forms. Le lien du questionnaire a été envoyé aux différents groupes professionnels, via l'application WhatsApp.

La collecte des données s'est faite dans le strict respect du protocole d'Helsinki, sur la période courant du 5 au 19 avril 2020. L'approche a valorisé l'anonymat et la confidentialité des réponses via un questionnaire anonyme. Le consentement a été requis avant l'auto-administration du questionnaire. Les objectifs de la recherche ont été exposés aux participants, ainsi que les procédures de gestion des risques. Le questionnaire rempli était retourné via un téléphone portable qui était codifié de manière à éviter des questionnaires doubles et/ou non entièrement renseignés. Trois-cent-trente-et-un professionnels de santé ont normalement répondu au questionnaire.

L'échelle *Hospital Anxiety and Depression scale* (HADS) a été l'outil diagnostique de référence. Des questions additionnelles portant sur les facteurs psychologiques associés ont été proposées aux répondants (par ex. : « avez-vous peur d'être infecté par la Covid 19 au cours de votre travail ? » ; « pourquoi ? »).

La HADS est une échelle d'auto-évaluation construite par Zigmond et Snaith [14], pour dépister la double symptomatologie anxieuse et dépressive chez les patients présentant des troubles organiques en institution hospitalière non psychiatrique. Elle peut être appliquée aux cas somatiques et psychiatriques, ainsi qu'aux patients recevant des soins de première ligne et à la population générale [15–17]. Si la HADS a fait l'objet de validations en médecine générale dès sa conception, ce n'est que plus tard qu'elle a été appliquée à la médecine du travail. Bailey souligne que cet outil y est de plus en plus utilisé [18]. Sanne *et al.* l'ont appliqué à l'étude des inégalités de santé [19]. Elle est également utilisée en situation épidémiologique avec une excellente valeur discriminative et diagnostique [20]. Ceci souligne sa pertinence pour cette étude.

La HADS comporte 14 items. Elle se présente sous forme d'auto-questionnaire de temps d'administration rapide, entre 2 et 5 minutes [21,22]. Hermann fait remarquer qu'elle est de cotation rapide pour un clinicien expérimenté (environ 1 minute) [22]. Elle présente des items dimensionnels alternés pour l'anxiété et la dépression (cotées de 0 à 3 ou de 3 à 0), permettant de mesurer la sévérité des deux symptomatologies [23,24]. Elle peut être administrée au cours d'entrevues de face-à-face, ou à distance (par téléphone, Internet, etc.) [21].

L'échelle a été traduite en plus de 41 langues et a fait l'objet de plusieurs validations internationales [22,25]. Elle est donc sensible à plusieurs cultures, avec d'excellentes qualités métrologiques [24,26,27]. Bjelland *et al.* suggèrent que pour un score de 8 et plus à chaque sous-échelle, on a une spécificité de 0,78 et une sensibilité de 0,9, pour l'anxiété d'une part ; une spécificité de 0,79 et une sensibilité of 0,83, pour la dépression, d'autre part [25]. Une note inférieure ou égale à 7 indique une absence d'anxiété ou de dépression ; une note comprise entre 8 et 10 suggère une anxiété ou une dépression faible/bénigne ; une note comprise entre 11 et 14, indique une anxiété ou une dépression modérée ; enfin, une note comprise entre 15 et 21 est révélatrice d'une anxiété sévère. Barczack *et al.* [28] avaient déjà insisté sur le fait qu'une note de 8 et plus constituerait le seuil optimal avec pour l'anxiété et la dépression dans chaque sous-échelle, avec des sensibilités respectives de 82 et 70 % et des spécificités de 94 et 68 %. Ce qui indique une bonne prédictivité des états anxieux et dépressifs pour ce score seuil minimal. La note-seuil optimale de 19 en scores cumulés aux deux sous-échelles,

sera analysée comme indicatrice des épisodes dépressifs majeurs ; la note seuil globale de 13, comme indicatrice de troubles de l'adaptation [22].

3. Résultats

3.1. Situation sociodémographique de l'échantillon

L'étude a porté sur un échantillon de 332 personnels soignants, dont 156 hommes représentant 47 % de l'échantillon et 176 femmes représentant 53 % de l'échantillon.

La distribution des résultats par tranches d'âges montre que ce sont les 30 à 39 ans qui sont les plus représentés dans l'échantillon (45,8 %). Ils sont suivis en nombre par les 20-29 ans (37,7 %), puis les 40-49 ans (10,2 %) et les 50-59 ans (6,3 %). La distribution de l'échantillon par types de formations sanitaires montre que les hôpitaux de district et assimilés représentent 35,5 % de l'échantillon, suivis par les centres de santé (31,9 %), hôpitaux régionaux (15,4 %), les CMA[1]. (6,3 %), les OSC[2] (2,4 %). Cette situation, quoique conjoncturelle, reflète le poids de ces institutions dans la réponse à la COVID-19 dans le pays. Les effectifs les plus importants concernent les infirmiers (55,7 %) et les médecins (25 %) qui représentent un pourcentage cumulé de 80,7 %. Du point de vue des services, la médecine générale représente 28,9 % de l'échantillon, suivi du service d'accueil (23,2 %) et des services spécialisés[3] (19,9 %), de pédiatrie (8,7 %), de santé publique (4,8 %), de travail communautaire (4,2 %), dédiés à la prise en charge du COVID-19 (3,6 %) et de réanimation (3,3 %) (Tableau 1).

3.2. Symptomatologies anxieuses et dépressive

Les scores significatifs à la HADS, concernant l'anxiété et la dépression, ainsi que leur niveau de sévérité sont donnés dans le Tableau 2.

La distribution indique des scores importants pour l'anxiété (41,8 %) et la dépression (42,8 %). La comorbidité anxiété et dépression est estimée à 14,73 %. Lorsqu'on considère le degré de sévérité des symptomatologies anxieuse et dépressive, on observe une plus forte prévalence des symptomatologies modérées (respectivement 26,5 % pour l'anxiété et 33,1 % pour la dépression). L'anxiété et la dépression sévères concernent respectivement 15,3 % et 9,6 % des personnels enquêtés. Ce qui indique une menace importante à la santé mentale des personnels de santé (Tableau 2).

Le test du Khi-deux de Pearson a été réalisé dans le but d'analyser l'influence des facteurs tels que le genre, la tranche d'âge, les structures sanitaires, les catégories professionnelles et les différents services, dans l'apparition des symptômes de dépression et d'anxiété. Les données indiquent que seul l'âge favorise l'apparition des symptômes d'anxiété chez le personnel médical ($\chi 2$ (9) = 28,93, p < 01).

On observe une plus forte susceptibilité chez les sujets jeunes (Tableau 3). En l'occurrence, les 30-39 ans présentent les scores les plus élevés (21,1 % pour la dépression et 18,3 % pour l'anxiété). Ils sont suivis par les 20-29 ans (respectivement 15,1 % et 12,5 % pour la dépression et l'anxiété), puis les 50-29 ans (respectivement 3,3 % et 5,1 % pour la dépression et l'anxiété) et enfin les 50-59 ans (respectivement 3,3 % et 5,1 % pour la dépression et l'anxiété). Les facteurs associés aux vécus anxieux et dépressif sont surtout la peur de contaminer ses proches (60 % pour la dépression et 40 % pour l'anxiété) ; la peur de mourir (59,5 % pour la dépression et 44,8 % pour l'anxiété).

3.3. Troubles dépressifs majeurs et de l'adaptation

Le diagnostic du trouble dépressif majeur et du trouble de l'adaptation est donné dans le Tableau 4.

Les résultats indiquent que parmi les sujets manifestant des symptômes dépressifs, 8,2 %, présentent un trouble dépressif majeur et 3,3 % un trouble de l'adaptation (Tableau 4).

[1] Centres médicaux d'arrondissement.

[2] Organisations de la société civile.

[3] Il s'agit des services de dentisterie, de laboratoire, d'ORL, d'ophtalmologie. . .

[4] La significativité du P-value a été rapporté aux valeurs suivantes : * p < .05; ** p = .01; *** p = .001(*faiblement significatif, **significatif, ***très significatif).

Tableau 1
Caractéristiques sociodémographiques de l'échantillon.

Caractéristiques	Genre					
	Homme		Femme		Total	
	n	%	n	%	n	%
Âge						
[20–29]	75	22,6	50	15,1	125	37,7
[30–39]	67	20,1	85	25,7	182	45,8
[40–49]	22	6,6	12	3,6	34	10,2
[50–59]	12	3,6	9	2,7	21	6,3
Région d'exercice						
Adamaoua	1	0,3	3	0,9	4	1,2
Centre	37	11,1	43	12,9	80	24,1
Est	6	1,8	3	0,9	9	2,7
Extrême-Nord	5	1,5	3	0,9	8	2,4
Littoral	35	10,6	52	15,6	87	26,2
Nord	2	0,6	1	0,3	3	,9
Nord-Ouest	2	0,6	0	0,0	2	,6
Ouest	61	18,4	64	19,3	125	37,7
Sud	4	1,2	5	1,5	9	2,7
Sud-Ouest	3	0,9	2	0,6	5	1,5
Structure						
Centre de santé	46	13,8	60	18,1	106	31,9
CMA	7	2,1	14	4,2	21	6,3
Hôpital de District et assimilés	63	19	55	16,5	118	35,5
Hôpital de première catégorie	7	2,1	14	4,2	21	6,3
Hôpital Régional	24	7,2	27	8,2	51	15,4
OSC	2	0,6	4	1,2	6	1,8
Catégorie professionnelle						
Agent technique médico-sanitaire	2	0,6	0	0,0	2	2,6
Aide-Soignant	10	3	16	4,8	26	7,8
Infirmier (ère)	85	25,6	106	31,9	191	57,5
Médecin	42	12,7	41	12,3	83	25,0
Psychologue clinicien	0	0,0	5	1,5	5	1,5
Technicien d'analyse médical	14	4,2	4	1,2	18	5,4
Technicien médico-sanitaire	3	0,9	4	1,2	7	2,1
Service						
Accueil	32	9,6	45	13,6	77	23,2
Médecine	37	11,1	59	17,8	96	28,9
Pédiatrie	6	1,8	23	6,9	29	8,7
Service de prise en charge COVID-19	5	1,5	7	2,1	9	3,6
Service de réanimation	8	1,8	3	0,9	11	3,3
Service de Santé publique	9	3,6	7	2,1	16	4,8
Service spécialisé	42	12,7	24	7,2	66	19,9
Travail dans la communauté	7	2,1	7	2,1	14	4,2
Urgence	10	3	1	0,3	11	3,3
Total[a]	156	47,0	176	53,0	332	100

[a] Ce total vaut pour chaque facteur sociodémographique individuellement.

Tableau 2
Diagnostic de la détresse, l'anxiété, la dépression.

Catégories Diagnostiques	Homme (%)	Femme (%)	Ensemble (%)	Sévérité	Hommes (%)	Femmes (%)	Ensemble (%)
Anxiété $p = 0{,}11$[4]	18,1	23,7	41,8	Faible	9,3	12	21,3
				Modérée	12,7	13,8	26,5
				Sévère	5,4	9,9	15,3
Dépression $p = 0{,}81$	19,9	22,9	42,8	Faible	13,0	15,7	28,6
				Modérée	16,0	17,2	33,1
				Sévère	3,9	5,7	9,6

Tableau 3
Distribution des symptomatologies anxieuses et dépressives en fonction de l'âge.

Variables	Dépression			Anxiété		
	Homme (%)	Femme (%)	Ensemble (%)	Homme (%)	Femme (%)	Ensemble (%)
Tranche d'âge						
[20–29]	6,6	8,4	15,1	4,8	8,6	13,5
[30–39]	11,1	9,9	21,1	9,6	9,6	19,3
[40–49]	0,6	2,7	3,3	1,2	3,9	5,1
[50-–9]	1,5	1,8	3,3	2,4	1,5	3,9
P		0,07			0,09	

Tableau 4
Scores significatifs aux troubles dépressifs majeurs et de l'adaptation.

Catégories diagnostiques	Homme (%)	Femme (%)	Ensemble (%)
Épisode dépressif majeur	4,2	4	8,2
Odds Ratio : 1,2362			
RR : 1, 1100 (IC : 95 %)			
Troubles de l'adaptation	2,1	1,2	3,3
Odds Ratio 2,0201			
RR : 1,4735 (IC : 95 %)			
Comorbidité, anxiété et dépression	5,4	9,33	14,73

4. Discussion

L'on note dans l'échantillon, une forte présence des symptômes de l'anxiété (41,8 %) et de la dépression (42,8 %). Le trouble dépressif majeur (8,2 %) et le trouble de l'adaptation (3,3 %) sont également observés. Ces résultats suggèrent que la réponse à la COVID-19 affecte de manière importante la santé mentale des travailleurs de santé impliqués dans sa prise en charge, comme l'ont montré les travaux [29–32].

Dans leur revue systématique de la littérature concernant l'impact des épidémies virales sur la santé mentale des travailleurs de santé, Ricci-Cabello *et al.* ont relevé une prévalence groupée entre 45 % et 69 % pour l'anxiété, dans six études ; entre 38 et 60 % dans sept études, pour la dépression [33]. Pappa *et al.* dans un travail similaire, ont montré que 12 études sur l'anxiété indiquaient une prévalence groupée de 23,2 % tandis que dix études portant sur la dépression indiquaient une prévalence de 22,8 % [31]. Lai, Ma Wang *et al.* ont signalé dans leurs résultats, des problèmes de santé mentale chez les travailleurs de la santé exposés à la maladie à coronavirus en 2019 à Wuhan [34]. Parmi ces problèmes, la détresse était la manifestation la plus diagnostiquée (71,5 %), suivie de la dépression (50, 4 %), puis de l'anxiété (44, 6 %), et l'insomnie (30,0 %). Huang et Zhao ont estimé la prévalence globale du TAG (Trouble anxieux généralisé) à 35,1 % ; celle de la dépression 20,1 % [35].

Ces données indiquent, dans le contexte des crises sanitaires d'envergure comme l'a été celle de la pandémie de la Covid 19, la nécessité de la prise en compte de leur effet sur la santé mentale des travailleurs de santé. Ceci devrait se traduire par la mise en place d'un dispositif d'aide/accompagnement psychologique. Un tel dispositif n'existe pas au Cameroun, où la réponse de santé mentale est faible. Il conviendrait qu'une réflexion soit menée sur la question, pour renforcer une telle réponse, au bénéfice des personnes en première ligne de la riposte.

5. Conclusion

Comme l'ont souligné Shelef et Zalsman, la pandémie de la COVID-19 a un impact psychologique important qui affecte considérablement la santé mentale des travailleurs de la santé engagés dans la riposte [36]. Les patterns épidémiologiques suggèrent que l'implication des travailleurs de santé à la riposte contre la pandémie au Cameroun est un important facteur de stress psychosocial. Les sujets plus jeunes manifestent une plus grande susceptibilité au vécu anxieux et dépressif. La peur de contaminer sa famille, suivie de celle de mourir sont les principaux déterminants de ce vécu. Ce qui

suggère la mise en place de mesures d'accompagnement spécialisées et adaptées. Ceci est d'autant plus vrai qu'au Cameroun les formations sanitaires sont peu ou pas préparées à faire face à de telles menaces, tant du point de vue du plateau technique de la formation des soignants ainsi que de leur accompagnement psychologique et social.

Déclaration de liens d'intérêts

Les auteurs déclarent ne pas avoir de liens d'intérêts.

Références

[1] Antoinette AM, Wong JGWS, et al. Stress and psychological distress among SARS survivors 1 year after the outbreak. Can J Psychiatry 2007;52(4):233–40.
[2] Brooks SK, Dunn R, Amlôt R, et al. A systematic, thematic review of social and occupational factors associated with psychological outcomes in healthcare employees during an infectious disease outbreak. J Occup Environ Med 2018;60(3):248–57.
[3] Maunder RG, Lancee WJ, Balderson KE, et al. Long-term psychological and occupational effects of providing hospital healthcare during SARS outbreak. Emerg Infect Dis 2006;12(12):1924–32.
[4] Manuguerra JC. Le carnet de voyage des maladies infectieuses. Le Monde; 2017. p. 22–5 [Hors-série].
[5] Sim K, Chua HC. The psychological impact of SARS: a matter of heart and mind. CMAJ 2004;170(5):811–2.
[6] Chua SE, Cheung V, McAlonan GM, et al. Stress and psychological impact on SARS patients during the outbreak. Can J Psychiatry 2004;49(6):385–90.
[7] Lee DTS, Wing YK, Leung HCM, et al. Factors associated with psychosis among patients with severe acute respiratory syndrome: a case-control study. Clin Infect Dis 2004;39(8):1247–9.
[8] Kina N, Vabret A. Les infections à coronavirus humains. Rev Francoph Lab 2016;487:25–33.
[9] Guan W, Ni Z, Yu Hu, Liang W, Ou C, He J, et al. Clinical characteristics of coronavirus disease 2019 in China. N Engl J Med 2020;332(6):1708–20.
[10] El Boussadani B, et al. Pandémie COVID-19: impact sur le système cardiovasculaire. Ann Cardiol Angeiol (Paris) 2020;69(3):107–14.
[11] Cheng SKW, Wong CW, Tsang J, et al. Psychological distress and negative appraisals in survivors of severe acute respiratory syndrome (SARS). Psychol Med 2004;34(7):1187–95.
[12] Wu KK, Chan SK, Ma TM. Posttraumatic stress after SARS. Emerg Infect Dis 2005;11(8):1297–300.
[13] Leung GM, Ho LM, Chan SKK, et al. Longitudinal assessment of community psychobehavioral responses during and after the 2003 outbreak of severe acute respiratory syndrome in Hong Kong. Clin Infect Dis 2005;40(12):1713–20.
[14] Zigmond AS, Snaith RP. The Hospital Anxiety and Depression Scale. Acta Psychiatr Scand 1983;67(6):361–70.
[15] Lepine JP, Godchau M, Brun P. Anxiety and depression in inpatients. Lancet 1985;2(846–970):1425–6.
[16] Lepine JP, Godchau M, Brun P, Lemperiere T. Évaluation de l'anxiété et de la dépression chez des patients hospitalisés dans un service de médecine interne. Ann Medico Psychol 1985;143(2):175–89.
[17] Olsson I, Mykletun A, Dahl AA. The hospital anxiety and depression rating scale: a cross-sectional study of psychometrics and case finding abilities in general practice. BMC Psychiatry 2005;5:46.
[18] Bailey K. The use of evidence-based clinical tools in occupational medicine. Occup Med (Lond) 2008;58:556–60.
[19] Sanne B, Mykletun A, Dahl AA, Moen BE, Tell GS. Hordaland Health Study. Occupational differences in levels of anxiety and depression: the Hordaland Health Study. J Occup Environ Med 2003;45(6):628–38.
[20] Friedman S, Samuelian JC, Lancrenon S, Even C, Chiarelli P. Three-dimensional structure of the Hospital Anxiety and Depression Scale in a large French primary care population suffering from major depression. Psychiat Res 2001;104(3):247–57.
[21] Stern AF. The hospital anxiety and depression scale. Occup Med 2014;64(5):393–4.
[22] Herrmann C. Internation a l'experiences with the hospital anxiety and depression scale a review of validation data and clinical results. J Psychosom Res 1997;42(1):17–41.
[23] Razavi D, Delvaux N, Farvacques C, Robaye E. Validation de la version française du HADS dans une population de patients cancéreux hospitalisés. Rev Psychol App 1989;39(4):295–307.
[24] Razavi D, Delvaux N, Farvacques C, Robaye E. Screening for adjustment disorders and major depressive disorders in cancer inpatients. Brit J Psychiat 1990;156:79–83.
[25] Bjelland I, Dahl AA, Haug TT, Neckelmann D. The validity of the Hospital Anxiety and Depression Scale. An updated literature review. J Psychosom Res 2002;52(2):69–77.
[26] Friedman S, Samuelian JC, Lancrenon S, Even C, Chiarelli P. Three-dimensional structure of the Hospital Anxiety and Depression Scale in a large French primary care population suffering from major depression. Psychiat Res 2001;104(3):247–57.
[27] Snaith RP, Taylor CM. Rating scales for depression and anxiety: a current perspective. Brit J Clin Pharmacol 1985;19:17S–20S.
[28] Barczack P, Kane N, Andrews S, Congdon AM, Clay JC, Betts T. Patterns of psychiatric morbidity in a genito-urinary clinic: a validation of the Hospital Anxiety Depression scale (HAD). Brit J Psychiat 1988;152:698–700.
[29] Li S, Wang Y, Xue J, et al. The Impact of COVID-19 epidemic declaration on psychological consequences: a study on active Weibo Users. Int J Environ Rs Public Health 2020;17(6):2032, http://dx.doi.org/10.3390/ijerph17062032 [online 2020. Mar 19].
[30] Mckay D, Yang H, Elhai J, et Asmundson GJG. Anxiety regarding contracting COVID-19 related to interoceptive anxiety sensations: the moderating role of disgust propensity and sensitivity. J Anxiety Disord 2020;1, http://dx.doi.org/10.1016/j.janxdis.2020.102233, 73, 102233.

[31] Pappa S, Ntella V, Giannakas, et al. Prevalence of depression, anxiety, and insomnia among healthcare workers during the COVID-19 pandemic: a systematic review and meta-analysis. Brain Gehav Immun 2020, http://dx.doi.org/10.1016/j.bbi.2020.05.026 [Epub ahead of print].
[32] Wang C, Pan R, Wan X, et al. Immediate Psychological Responses and Associated Factors during the Initial Stage of the 2019 Coronavirus Disease (COVID-19) Epidemic among the General Population in China. Int J Environ Rs Public Health 2020;5:1729, http://dx.doi.org/10.3390/ijerph17051729. Published online 2020 amr 6.
[33] Ricci-Cabello I, Jose F, Meneses-Echavez M, Serrano-Ripoll J, et al. Impact of viral epidemic outbreaks on mental health of healthcare workers: a rapid systematic review. J Affect Disord 2020;277(1):347–57.
[34] Lai J, Man S, Wang Y, et al. Factors associated with mental health outcomes among health care workers exposed to Coronavirus Disease 2019. JAMA Netw Open 2 2020;2020(3):e203976, http://dx.doi.org/10.1001/jamanetworkopen.2020.3976.
[35] Huang Y, Zhao N. Generalized anxiety disorder, depressive symptoms and sleep quality during COVID-19 epidemic in China: a web-based cross-sectional survey; 2020, http://dx.doi.org/10.1016/j.psychres.2020.112954.
[36] Shelef L, Zalsman G. The psychological impact of covid-19 on mental health - literature review. Harefuah 2020;5:320–32.
