# Supplementary material for: Mental health status and related factors influencing healthcare workers during the COVID-19 pandemic: A systematic review and meta-analysis
Source: PLoS One. 2024 Jan 19;19(1):e0289454. doi: 10.1371/journal.pone.0289454 (PMC10798549; doi:10.1371/journal.pone.0289454)
Supplement: S1 Data — (ZIP) [file pone.0289454.s011.zip › literatures/96.pdf]

hospitalization having significant impact on medical, nursing and psychosocial resources.
Also, the referral to community dietician has a wait time of almost 12 months and patient directed weight loss after tragic news takes an indefinite time delaying treatment. Psychological guidance and motivation is required.
The aim of this study is to assess the magnitude of morbid obesity i.e. BMI>40 in women diagnosed with endometrial cancer and to allocate resources for dedicated weight loss service for these women.
Designs: Retrospective study of data for women diagnosed with endometrial cancer over a period of 3 years – Jan 2019 to Dec 2021.
Methods: Data was collected from endometrial cancer database including all women diagnosed with endometrial cancer during the study period. Total numbers of patients were 160.
Results: Out of 160 patients, 104 women i.e. 65% were obese (BMI>30) whilst 46 women (29%) were morbidly obese (BMI>40). 17% women (27) had BMI>45, requiring high dependency bed availability before surgical intervention. 7 women had BMI>55 putting them at even higher risks of complications and warranting weight loss before any surgical intervention, thereby, causing a delay in treatment finally.
Conclusion: A dedicated weight management team consisting of dieticians and psychologists to guide the women of at least BMI>45 in the weight loss journey, similar to the one dedicated for Type 2 DM prevention, will not only make a difference to the management time, but also reduce the incidence of endometrial cancer and disorders associated with obesity. Eventually, it will have a positive impact on the overall health economy across NHS.
Nevertheless, a repeat audit is required once the dedicated service is up and running.

PANDEMIC AND IMPACT OF COVID/INFECTIOUS DISEASES

OP.0029 | Risk factors for SARS-CoV-2 Positivity in neonates born to mothers with COVID-19

Halimah Khalil[1]; Megan Littmoden[1]; Ankita Gupta[1]; Adeolu Banjoko[1]; Kehkashan Ansari[1,2]; Maurie Kuha Kumaran[1]; Oluwadamilola Akande[1]; Jameela Sheikh[1]; Heidi Lawson[1]; John Allotey[2]; Javier Zamora[2,3]; Shakila Thangaratinam[2,4]; Shaunak Rhiju Chatterjee[1,5]; Tania Kew[1]
[1]*College of Medical and Dental Sciences, University of Birmingham, Birmingham, UK;* [2]*Institute of Metabolism and Systems Research, WHO Collaborating Centre for Global Women's Health, University of Birmingham, Birmingham, UK;* [3]*Head of Clinical Biostatistics Unit, Hospital Ramon y Cajal of Biomedical Research, Madrid, Spain;* [4]*Birmingham Women's and Children's NHS Foundation Trust, Birmingham, UK;* [5]*Royal Liverpool University Hospital, Liverpool, UK*

Objective: To evaluate clinical outcomes of SARS-CoV-2 positive neonates born to mothers with SARS-Cov-2 infection, and to determine maternal and perinatal risk factors associated with neonatal SARS-CoV-2 positivity.
Design: Living systematic review and meta-analysis based on a prospectively registered protocol following PROSPERO guidelines. Findings have been reported in line with PRISMA recommendations.
Methods: A systematic search of major databases, preprint servers and relevant websites was performed (1st December 2019 until 3rd August 2021) for studies reporting clinical outcomes from neonates born to mothers diagnosed with SARS-CoV-2 infection. SARS-CoV-2 positivity in neonates was established according to the 2021 World Health Organisation (WHO) classification system on mother-to-child transmission. Each study was screened by two independent reviewers in a two stage process, with a third reviewer resolving disagreements.
Data was extracted on relevant maternal and perinatal risk factors such as maternal condition (severe COVID-19, admission to intensive care unit, death), timing of exposure, intrapartum factors, postnatal care and offspring SARS-CoV-2 status. Random-effects meta-analysis was used to report odds ratios (OR) with 95% confidence intervals (CI).
Results: 247 studies reported outcomes for 800 SARS-CoV-2 positive babies. At the end of follow-up, 749 babies were alive; 8 early pregnancy losses, 20 stillbirths and 23 neonatal deaths occurred. Of the 146 babies born preterm; 58 were symptomatic for COVID-19; 121 SARS-CoV-2 positive babies reported radiological findings; abnormalities were noted in 71 babies.
Maternal factors including severe COVID-19 infection (OR 2.36, 95% CI 1.28–4.36, $I^2 = 10\%$; 22 studies, 2,842 women), admission to intensive care unit (OR 3.46, 95% CI 1.74–6.91, $I^2 = 0\%$; 19 studies, 2,851 women), death (OR 14.09, 95% CI 4.14–47.97, $I^2 = 0\%$; 7 studies, 725 women), and postnatal diagnosis of SARS-CoV-2 infection (OR 4.99, 95% CI 1.24–20.13, $I^2 = 65\%$; 12 studies, 750 women) were significantly associated with SARS-CoV-2 neonatal positivity. No associations of significance were noted for trimester of infection, preterm birth, mode of delivery, breastfeeding, or mother-baby separation at birth.
Conclusion: The disease severity of maternal COVID-19 may be associated with neonatal SARS-CoV-2 positivity. Neonatal outcomes were inconsistently reported, which caused difficulty in determining whether complications including neonatal mortality were related to SARS-CoV-2 or other factors. Further follow-up of positive neonates is needed to evaluate long-term outcomes and guide precautionary measures.

OP.0030 | Evaluation of immunogenicity and reactogenicity of COVID-19 vaccines in pregnant women

Helena Blakeway[1]; Zahin Amin-Chowdhury[2]; Smriti Prasad[1]; Assistant Erkan Kalafat[3]; Menatalla Ismail[4]; Fady Abdallah[4]; Arezou Rezvani[4]; Kevin Brown[5]; Gayatri Amirthalingam[5]; Kirsty Le Doare[6]; Paul Heath[7,8]; Shamez Ladhani[9]; Asma Khalil[1]

[1]*St George's University Hospitals NHS Foundation Trust, Fetal Medicine Unit, London, UK;* [2]*Publinc Health England, London, UK;* [3]*Ankara University School of Medicine, Obstetrics and Gynaecology, Ankara, Turkey;* [4]*St George's University Hospitals NHS Foundation Trust, Obstetrics and Gynaecology, London, UK;* [5]*Public Health England, Skipton House, London, UK;* [6]*St George's Univeristy of London, London, UK;* [7]*Paediatric Infectious Diseases Resarch Group and Vaccine Institute, St George's University of London, London, UK;* [8]*St George's University Hospitals NHS Foundation Trust, London, UK;* [9]*Public Health England, London, UK*

Objective: This study aimed to investigate the reactogenicity and immunogenicity of the COVID-19 vaccine in pregnant women using the UK's extended 12-week interval COVID-19 vaccine schedule.

Design: There are limited data on COVID-19 vaccines in pregnancy. This was a cohort study of pregnant women receiving COVID-19 vaccination in London, UK between January and September 2021. The primary outcome was reactogenicity and immunogenicity in pregnant women after COVID-19 vaccination.

Methods: Pregnant women who received a COVID-19 vaccine were recruited by phone, email and text. Participants filled in pre-set questionnaires on adverse events and pregnancy outcomes. For immunogenicity, blood samples were taken at specific time points after each vaccine. Serum samples were tested for nucleoprotein (N) antibodies and spike (S) protein antibodies. Outcomes were compared with a control group of non-pregnant women. Association of variables with antibody levels was assessed using linear regression analysis after log-transforming antibody levels. The association of pregnancy status with reactogenicity was assessed using logistic regression analysis.

Results: Of 67 pregnant women recruited, 66 had received an mRNA vaccine. The majority (61.2%) received the vaccine in the third trimester, while 3.0% received it in their first trimester and 35.8% in their second trimester. In the control group 50 women had received an mRNA vaccine. SARS-CoV-2 S-antibody IgG GMCs after mRNA vaccination were not significantly different at all timepoints tested during the vaccine schedule ($p>0.05$ for all). In pregnant women, prior infection was associated with higher antibody levels than infection naïve pregnant women at all time-points (72.8-fold higher geometric mean ratios at 2-6 weeks after dose 1, 18.1-fold before dose 2 and 2.8-fold at 2-6 weeks after dose 2). Previous infection (mean: 1·09 log-au/mL higher, $p = 0{\cdot}0015$) and receiving the viral vector vaccine (-1·53 log-au/mL lower, $p < 0.0001$) were significantly associated with antibody levels after the second dose.

The study included 108 pregnant women and 116 non-pregnant women with data on reactogenicity. After the first dose, tiredness and chills were reported less commonly in pregnant women when compared to non-pregnant women ($p = 0.043$ and $p = 0.029$, respectively). After the second dose feeling generally unwell was reported less commonly ($p = 0.046$).

Conclusions: Antibody responses were similar in pregnant women compared to non-pregnant women, with very high antibody responses achieved after one dose in previously infected women. Pregnant women had fewer adverse effects after vaccination. This study provides evidence that the extended second dose schedule provides good protection against COVID-19 disease.

OP.0032 | Risk Factors for severe COVID-19 in pregnancy: Living systematic review and meta-analysis

Miss Dengyi Zhou[1]; Miss Kathryn Barry[1]; Miss Wentin Chen[1]; Miss Dharshini Sambamoorthi[1]; Miss Halimah Khalil[1]; Dyuti Coomar[2]; Sylvia Fernandez[3,4]; Elena Stallings[5]; Mercedes Bonet[6]; Javier Zamora[3,4,7]; John Allotey[2,8]; Shakila Thangaratinam[8,9]

[1]*College of Medical and Dental Sciences, University of Birmingham, Birmingham, UK;* [2]*Institute of Applied Health Research, University of Birmingham, Birmingham, UK;* [3]*Clinical Biostatistics Unit, Hospital Universitario Ramón y Cajal (IRYCIS), Madrid, Spain;* [4]*CIBER Epidemiology and Public Health (CIBERESP), Madrid, Spain;* [5]*Instituto Aragonés de Ciencias de la Salud, Zaragoza, Spain;* [6]*UNDP/UNFPA/UNICEF/WHO/World Bank Special Programme of Research, Development and Research Training in Human Reproduction (HRP), Department of Sexual and Reproductive Health and Research, World Health Organization, Geneva, Switzerland;* [7]*Institute of Metabolism and Systems Research, University of Birmingham, Birmingham, UK;* [8]*WHO Collaborating Centre for Global Women's Health, University of Birmingham, Birmingham, UK;* [9]*Birmingham Women's and Children's NHS Foundation Trust, Birmingham, UK*

Objective: Since coronavirus disease 2019 (COVID-19) was first reported in December 2019, there have been significant concerns regarding its impact on pregnancy due to disproportionate risks faced by pregnant women and neonates exposed to similar viruses. Rapid, robust, and continually updated evidence synthesis is required to inform clinical management of COVID-19 in this high-risk group. This study aimed to identify maternal risk factors for severe COVID-19 and poor clinical outcomes.

Design: We conducted a living systematic review and meta-analysis of the rapidly evolving evidence base to assess the risk factors for maternal complications in suspected or confirmed COVID-19 cases. Each cycle of the living systematic

review involves weekly search updates; with analysis performed every 2-4 months. Results are regularly disseminated through our website: https://www.birmingham.ac.uk/research/who-collaborating-centre/pregcov/index.aspx.

Method: A living systematic review and meta-analysis was undertaken, with searches from 1 December 2019 to 27 April 2021. Various sources were explored, including WHO COVID-19 database, Medline, Cochrane database, Embase, China National Knowledge Infrastructure, and Wanfang databases, as well as preprint servers, social media, and reference lists. Risk factors for COVID-19 related outcomes in pregnant and recently pregnant women with suspected or confirmed COVID-19 were included. Two researchers extracted data independently and assessed study quality using the Newcastle Ottawa Scale. Random-effects meta-analysis was performed.

Results: 435 studies were included with a total of 926,232 pregnant and recently pregnant women with suspected or confirmed COVID-19. Increased maternal age (OR 1.56; 95% CI 1.19 to 2.04; I^2 = 66%), high body mass index (OR 1.84, 95% CI 1.46 to 2.31; I^2 = 54.9%), any pre-existing maternal comorbidity (OR 1.48; 95% CI 1.19 to 1.85; I^2 = 46.4%), chronic hypertension (OR 1.75; 95% CI 1.40 to 2.20; I^2 = 0%), pre-existing diabetes (OR 2.90; 95% CI 1.93 to 4.34; I^2 = 37.4%), gestational diabetes (OR 1.62; 95% CI 1.01 to 2.61; I^2 = 58.2%), and pre-eclampsia (OR 5.19; 95% CI 2.22 to 12.13; I^2 = 0%) were associated with severe COVID-19 in pregnancy. In pregnant women with COVID-19, increased maternal age, high body mass index, non-white ethnicity, any pre-existing maternal comorbidities including chronic hypertension and diabetes were associated with serious complications of admission to an intensive care unit, invasive ventilation and maternal death.

Conclusions: Pre-existing comorbidities, non-white ethnicity, chronic hypertension, pre-existing diabetes, high maternal age, and high body mass index are risk factors for severe COVID-19 in pregnancy. Further data are needed to robustly assess the association between pregnancy specific risk factors such as pre-eclampsia and gestational diabetes on COVID-19 related outcomes.

OP.0033 | COVID-19 infection and medications in pregnancy: pooled analysis of INOSS population-based studies

Odette de Bruin[1]; Hilde Engjom[2]; Nicola Vousden[3]; Marian Knight[3]; Serena Donati[4]; Anna Aabakke[5]; Outi Ayras[6]; Evelien Overtoom[1]; An Vercoutere[7]; Eva Jonasdottir[8]; Kitty Bloemenkamp[1]

[1]*Department of Obstetrics, WKZ Birth Centre, Division Woman and Baby, University Medical Center Utrecht, Utrecht, Netherlands;* [2]*Department of Mental and Physical Health, Norwegian Institute of Public Health and Department of Obstetrics and Gynecology, Haukeland University Hospital, Bergen, Norway;* [3]*National Perinatal Epidemiology Unit, Nuffield Department of Population Health, University of Oxford, Oxford, UK;* [4]*National Centre for Disease Prevention and Health Promotion, Istituto Superiore di Sanità – Italian National Institute of Health, Rome, Italy;* [5]*Department of Obstetrics and Gynecology, Copenhagen University Hospital-Holbaek, and Department of Obstetrics and Gynecology, Copenhagen University Hospital-Nordjaelland-Hillerod, Copenhagen, Denmark;* [6]*Department of Obstetrics and Gynecology, Helsinki University Hospital, Helsinki, Finland;* [7]*Department of Obstetrics and Gynecology, Hôpital Erasme, Université Libre de Bruxelles, Brussels, Belgium;* [8]*Department of Obstetrics and Gynecology, Landspitali University Hospital, Reykjavik, Iceland*

Objectives: To determine the incidence rate of hospitalization and severe maternal and perinatal outcomes and to investigate medication use among pregnant women infected with COVID-19 across multiple European countries.

Design: Multi-national population-based cohort study using the International Network of Obstetric Survey Systems (INOSS)

Method: Countries conducting national or regional population-based surveillance of severe pregnancy complications adapted the surveillance during the COVID-19 pandemic to monitor pregnant women with COVID-19 infection. The study population comprised all pregnant women admitted to hospital with a positive COVID-19 PCR test within 7 days prior to admission and up to 2 days after giving birth between February/March and December 31, 2020. Pregnant women were eligible for inclusion if they were admitted due to COVID-19 infection or were symptomatic, when the reason for admission was not known. Common protocols with uniform definitions were used in each country to retrieve information from hospital records, including medicine use, maternal and perinatal outcomes.

Results: In the population of 1540787 maternities, 1698 women were admitted due to COVID-19 infection. The rate of hospitalization due to COVID-19 ranged from 0.2 to 1.9 per 1000 maternities across countries. Approximately one-fifth of women gave birth prior to 37 weeks, 176 (10%) women were admitted to ICU, and 101 (6%) needed mechanical ventilation or extracorporeal membrane oxygenation. Antibiotics were the most frequently administered medication (26%); few pregnant women received steroids for maternal indication (8%), antiviral treatment (2%) and anticoagulant treatment (0.5%). Hydroxychloroquine was prescribed more frequently in Italy and Belgium compared to the Netherlands, the UK and the Nordic countries. Among 1617 births, 15 stillbirths (9/1000 births), and 315 (19%) admissions to a neonatal ward were reported.

Conclusion: This multi-national collaborative approach allows comparison of the impact of varying timing and nature of public health measures implemented by different nations. We observed that the rate of hospitalization due to COVID-19 disease and medication use among pregnant women varied across different European countries in 2020. Admission due to COVID-19 was associated with a high risk of ICU admission, mechanical ventilation, and preterm delivery. It is striking that only a few pregnant women

received medical treatment directed at COVID-19 disease. A planned meta-analysis will give more insight into the effect of medicines in pregnant women with COVID-19, data on which are lacking since, with the exception of the RECOVERY trial, this vulnerable group were mostly excluded from RCTs on effectiveness and safety of COVID-19 medication.

OP.0100 | Effectiveness and perinatal outcomes of COVID-19 vaccination in pregnancy: SystematicReview and meta-analysis

Smriti Prasad[1]; Erkan Kalafat[2]; Helena Blakeway[1]; Rosemary Townsend[3]; Pat O'Brien[4,5]; Edward Morris[4,6]; Tim Draycott[7,4]; Shakila Thangaratinam[8]; Kirsty Le Doare[9]; Shamez Ladhani[10,11]; Peter von Dadelszen[12]; Laura A. Magee[12]; Paul Heath[9]; Asma Khalil[1,13]

[1]*St George's University Hospital NHS Foundation Trust, London, UK;* [2]*Koc University Hospital, Istanbul, Turkey;* [3]*Usher Institute of Population Health Sciences and Informatics, University of Edinburgh, Edinburgh, UK;* [4]*The Royal College of Obstetricians and Gynaecologists, London, UK;* [5]*University College London Hospitals NHS Foundation Trust, London, UK;* [6]*Norfolk and Norwich University Hospitals NHS Foundation Trust, Norwich, Uruguay;* [7]*North Bristol NHS Trust Department of Women's Health, Westbury on Trym, UK;* [8]*Institute of Metabolism and Systems Research, WHO Collaborating Centre for Women's Health, University of Birmingham, Birmingham, UK;* [9]*Paediatric Infectious Diseases Research Group and Vaccine Institute, Institute of Infection and Immunity, St George's University of London, London, UK;* [10]*Immunisation and Countermeasures Division, Public Health England, London, UK;* [11]*British Paediatric Surveillance Unit, Royal College of Paediatrics and Child Health, London, UK;* [12]*Institute of Women and Children's Health, School of Life Course Sciences, King's College London, London, UK;* [13]*Vascular Biology Research Centre, Molecular and Clinical Sciences Research Institute, St George's University of London, London, UK*

Objective: COVID-19 vaccination is now recommended for all pregnant women. The objective of this systematic review and meta-analysis was to synthesise evidence on vaccine effectiveness and perinatal outcomes after COVID-19 vaccination in pregnancy.

Design: Systematic review and meta-analysis

Methods: The COVID-19 Research, MEDLINE and EMBASE databases were searched in accordance with PRISMA guidelines from 1st December 2020 to 9th January 2022 (CRD42021274016). To identify safety reports and briefings, we performed additional hand searches of preprint servers, Centers for Disease Control and Prevention and Medicines and Healthcare products Regulatory Agency websites. Studies reporting perinatal outcomes or vaccine effectiveness after COVID-19 vaccination in pregnancy were included. Pair-wise meta-analyses were undertaken.

Results: There were 23 reports of 117,552 COVID-19 vaccinated women, almost exclusively with mRNA vaccines and all from high-income countries. The effectiveness of mRNA vaccination against RT-PCR confirmed SARS-CoV-2 infection 7 days after the second dose was 89·5% (95% CI 69·0–96·4%, 3 observational studies, 18,828 vaccinated pregnant women, I^2 = 73·9%). The odds of stillbirth were significantly reduced in the vaccinated cohort by 15% (7studies, pooled OR 0·85; 95% CI 0·73–0·99, 66,067 vaccinated vs; 424,624 unvaccinated, p = 0·035, I^2 = 93·9%). The cumulative effect shows a non-statistically significant 10% reduction in preterm birth before 37 weeks' gestation following COVID-19 vaccination in studies accounting for time-varying confounding, (2 studies, pooled HR 0·90; 95% CI 0·81 –1·00, 10,197 vaccinated vs; 36,414 unvaccinated, I^2 = 11·1%, p = 0·051). Using pair-wise meta-analysis of outcomes among vaccinated (vs. unvaccinated) pregnant women, there was no evidence of an increase in the risk of outcomes that could be examined: for the mother, hypertensive disorders of pregnancy and preeclampsia specifically, placental abruption, pulmonary embolism, postpartum haemorrhage, intensive care unit admission, and maternal death; and for the neonate, miscarriage, gestational age at birth, birthweight Z-score, or neonatal intensive care unit admission.

Conclusion: COVID-19 mRNA vaccination in pregnancy appears to be as effective in preventing proven SARS-CoV-2 infection in pregnancy, as demonstrated outside pregnancy. Importantly, these mRNA vaccinates are safe in pregnancy and associated with a significant reduction in stillbirth.

PP.0157 | COVID-19 maternal and perinatal outcomes: living systematic review and meta-analysis (Update)

Millie Manning[1]; Halimah Khalil[1]; Ankita Gupta[1]; Megan Littmoden[1]; Adeolu Banjoko[1]; Kehkashan Ansari[1,2]; Helen Fraser[1]; Anoushka Ramkumar[1]; Tania Kew[1]; Dengyi Zhou[1]; Dyuti Coomar[3]; Elena Stallings[4,5]; John Allotey[3,2]; Mercedes Bonet[6]; Javier Zamora[4,5,2]; Silvia Fernández-García[4]; Shakila Thangaratinam[2,7]; Mr Magnus Yap[1]; Jameela Sheikh[1]; Heidi Lawson[1]

[1]*College of Medical and Dental Sciences, University of Birmingham, Birmingham, UK;* [2]*WHO Collaborating Centre for Global Women's Health, Institute of Metabolism and Systems Research, University of Birmingham, Birmingham, UK;* [3]*Institute of Applied Health Research, University of Birmingham, Birmingham, UK;* [4]*Hospital Universitario Ramón y Cajal (IRYCIS), Madrid, Spain;* [5]*CIBER Epidemiology and Public Health (CIBERESP), Madrid, Spain;* [6]*UNDP/UNFPA/UNICEF/WHO/World Bank Special Programme of Research, Development and Research Training in Human Reproduction (HRP), Department of Sexual and Reproductive Health and Research, World Health Organization, Geneva, Switzerland;* [7]*Birmingham Women's and Children's NHS Foundation Trust, Birmingham, UK*

Objective: Determine the maternal and perinatal outcomes in pregnant women with Coronavirus disease 2019 (COVID-19).
Design: Living systematic review and meta-analysis.
Method: A systematic search of databases carried out weekly from 1 December 2019 to 27 April 2021. Cohort studies reporting maternal and perinatal outcomes in pregnant and recently pregnant women with suspected or confirmed COVID-19 were included. Data was extracted and quality assessed by at least two independent researchers weekly. Random effects meta-analysis was performed, with estimates pooled as odds ratios (OR) with 95% confidence intervals (CI). These results will be updated by June 2022 due to the living nature.
Results: 435 studies (926,232 pregnant women; 9,466 neonates) were included. No studies were conducted by emergence of any COVID-19 variants of concern. The odds of maternal death (OR 6.09; 95% CI 1.82-20.38; 21 studies; 422 488 women), admission to intensive care unit (ICU) (OR 5.41; 95% CI 3.59-8.14; 21 studies; 472,316 women), preterm birth (OR 1.57; 95% CI 1.36-1.81; 48 studies; 449,040 women) and caesarean section (OR 1.17; 95% CI 1.01-1.36; 53 studies; 626,787 women) were higher in pregnant women with COVID-19 compared to pregnant women without COVID-19.
Compared with non-pregnant women of reproductive age with COVID-19, the odds of admission to ICU (OR 2.61; 95% CI 1.84-3.71; ten studies, 2,027,360 women) and need for invasive ventilation (OR 2.41; 95% CI 2.13-2.71; eight studies; 2,025,415 women) were higher in pregnant women with COVID-19.
Neonates born to mothers with COVID-19 were at higher risk of stillbirth (OR 1.81; 95% CI 1.38-2.37; 25 studies; 423,477 women), neonatal death (OR 2.35; 95% CI 1.16-4.76; 21 studies; 12 416 neonates) and admission to neonatal ICU (OR 2.18; 95% CI 1.46-3.26; 29 studies; 197,196 neonates) than neonates born to women without the disease.
Conclusion: Pregnant women with COVID-19 have an increased risk of adverse maternal outcomes in comparison to pregnant patients without the disease and non-pregnant COVID-19 positive women. This is reflected in neonates born to mothers with COVID-19 who are at increased risk of adverse perinatal outcomes. Pregnant women should be given information on the risks associated with infection and measures should be taken to prevent exposure with COVID-19, such as uptake of vaccination. Healthcare professionals should be aware of the differences in outcomes of infected pregnant women to ensure appropriate escalation of care. Continued research efforts are required as the COVID-19 pandemic evolves with considerations needed for vaccines and variants.

PP.0158 | Gynaecological skills training impact assessment of trainees in Northern Ireland

Josh Courtney McMullan[1]; Gemma Ferguson[1]; Catriona Monaghan[1,2]; Charles Beattie[1,3]
[1]*Northern Ireland Medical and Dental Training Agency, Belfast, UK;* [2]*Royal Jubilee Maternity Hospital, Belfast, UK;* [3]*Antrim Area Hospital, Belfast, UK*

Design: Training in gynaecological skills has been significantly impacted by the COVID-19 pandemic. The RCOG recommended a training impact assessment of trainees as part of the gynaecological surgery recovery plan. A regional survey was designed in Northern Ireland (NI) to assess trainee‘s attitudes and exposure to current training in gynaecology and future plans for advanced training in gynaecology.
Methods: The RCOG gynaecology recovery plan was discussed at the NI deanery school board meeting and an online training impact survey was developed. The survey was then sent to all obstetrics and gynaecology trainees within NI. The results were reviewed and presented back to the NI deanery school board. All units within NI were given access to the results with the aim to adopt a regional approach to improving training opportunities in gynaecology within NI.
Results: 39 responses have been received to date from all levels of trainees and all 8 training units within NI. The results for rating current training in gynaecology were; very poor 8%, poor 44%, fair 36%, good 6% and very good 6%. An average of 3 gynaecology clinics were attended in the previous 8 weeks. Only 14% felt their skills were appropriate for their training grade. For attendance in gynaecology theatre sessions; 44% <1 per month, 36% 1-2 per month, 17% 1 per week and 3% >1 per week. For proportion of time spent as the lead operator in gynaecology theatre only 33% of trainees were lead operator for >50% of cases. For procedural competence; 50% diagnostic laparoscopy, 17% operative laparoscopy, 11% hysterectomy (abdominal, laparoscopic and vaginal 11% each), 19% vaginal repair and 31% laparoscopic management of ectopic pregnancy; 64% required gynaecological summative OSATs in this training year; 19% were doing a gynaecological ATSM of which 57% felt they would complete; 78% of all responders felt they would not be competent at gynaecological surgery by the end of training.
Conclusion: COVID-19 has had a clear effect on training in gynaecology and this is evident in all training units throughout NI for all grades of trainees. Trainees are concerned regarding their exposure to gynaecological training and their competence in the future as consultants. Other methodologies for training could be adopted in this time, including simulation, to help improve opportunities.
#NoTrainingTodayNoConsultantsTomorrow

EP.0901 | A centralised approach to the care of pregnant women with COVID-19

Sanaa Zayyan; Charlotte Frise
John Radcliffe Hospital, Oxford, UK

Objective: A multi-disciplinary approach is now recommended for the care of pregnant women with COVID-19. Prior to this recommendation, as part of the response to the coronavirus pandemic resulting in rising numbers of pregnant inpatients with COVID-19, the Oxford University Hospital trust uniquely implemented and piloted a daily, virtual, central meeting to coordinate care of these women, with representation from anaesthetists, senior obstetricians, midwives and obstetric physicians. The aims were to identify deteriorating patients, coordinate the care needs of each patient and provide a platform for multi-disciplinary team discussion and shared decision-making.
Design: Maternal medicine-led daily virtual meetings were attended by members of the multi-disciplinary team. Deteriorating patients were identified, care needs were escalated including discussions on place of care and plans were made for de-escalation, debrief and follow up where appropriate.
Method: Each patient was discussed in turn to ensure she would be reviewed daily by an appropriate member of the MDT. An electronic patient list with the details and location, accessible to all relevant members of the maternity service was updated daily, allowing team members to easily access information from any trust computer or VPN.
Results: This centralised approach to the coordination of care in pregnant women with COVID-19 was extremely well received by members of the MDT with positive feedback from the maternity service. The daily meetings streamlined the care for these women and ensured all MDT members were aware of all pregnant patients admitted in the trust.
Conclusions: An MDT centralised approach to the care of pregnant women with COVID-19 allows the coordination of care for inpatients and daily review in line with the recommendations from MBRRACE-UK. The virtual format ensured social distancing policies were adhered to and that team members working from home were able to participate in the care of these women. This approach could be adopted nationally with an appropriate protocol for future pandemics and new and emerging variants of concern.

EP.0902 | Reactive, pragmatic, and reflective decision-making in maternity care during the SARS-CoV-2 pandemic

Sergio A. Silverio[1]; Kaat De Backer[1]; Jeremy M. Brown[2]; Abigail Easter[1]; Nina Khazaezadeh[3]; Daghni Rajasingam[4]; Jane Sandall[1]; Laura A. Magee[1]
[1]*King's College London, London, UK;* [2]*Edge Hill University, Ormskirk, UK;* [3]*NHS England and Improvement, London, UK;* [4]*Guy's & St. Thomas' NHS Foundation Trust, London, UK*

Objective: We examined the perceptions and experiences of decision making about service reconfiguration by maternity care staff who provided maternity care services during the pandemic in one NHS Trust in South London.
Design: We employed a qualitative research design to allow expression of experiential data about how maternity healthcare professionals perceived delivering care during the SARS-CoV-2 health system shock. Exploring complexity in this way lends itself to qualitative research, in a way quantitative data would otherwise not capture.
Methods: The study was undertaken as a service evaluation across a maternity care service in South London, UK. Trust-level approvals were received for interviews (ref:-11046; July 2020). Staff were invited to interview via e-mail using a critical case purposeful sampling technique, which encouraged recruitment variation We conducted semi-structured interviews with a range of staff ($N = 29$) working across the maternity service. Interviews were conducted virtually using video-conferencing software and although all respondents were asked the same core set of questions, there was enough flexibility in the interview schedule to allow for follow-up of points which were pertinent to individual experience. Data were analysed using a Grounded Theory analysis approach, appropriate to cross-disciplinary health research. This included a first pass of coding using data to code each sentence of the transcripts, followed by more conceptual 'focused' coding used to explain broader trends in parts of the data. After this, focused codes were merged into super-categories, before these were merged, split, and/or re-arranged into themes. The relationships between the themes gave rise to a theory.
Results: Analysis rendered three emergent themes regarding the decision-making processes engaged when reconfiguring the maternity service provision: 1) 'Reflective decision-making'; 2) 'Pragmatic decision-making'; and 3) Reactive decision-making.
Conclusion: Whilst pragmatic decision-making was found to disrupt care, reactive-decision-making was perceived to result in devaluation of the care offered and provided. Alternatively, reflective decision-making was seen as beneficial for services to provide high-quality care, sustain staff, and innovate within the service, throughout the difficult working conditions the pandemic has posed.

EP.0903 | Recruitment of pregnant women to randomised trials of COVID 19 treatments

Oleia Green[1]; Eloise Young[1]; Jemma Oberman[1]; Joel Stewart[1]; Yasmin King[1]; Keelin O'Donoghue[2]; Kate Walker[1]; Jim Thornton[1]
[1]*University of Nottingham, Nottingham, UK;* [2]*University College Cork, Cork, Ireland*

Objectives: To document how many pregnant women with COVID-19 reported in the literature had participated in randomised trials, what treatments they received outside such

trials and compare the latter with evidence-based treatment recommendations.
Design: Observational study
Method: Two clinical trial registries were searched to identify COVID-19 trials open to pregnant women. Studies were then extracted from a regularly updated list of scientific case reports and case series of confirmed or suspected maternal COVID-19 in pregnancy to identify the number of women enrolled into a trial and the pharmaceutical treatments they received outside such trials.
Results: 156 studies (case reports, case series and registries) reporting 43,185 pregnant women with COVID-19, after deduplication. Of these 2,671 (6.2%) were potentially eligible for a randomised trial but only seven women (0.26%) were reported to have enrolled.
For 2,839 women the papers included information on treatment received, 1515/2829 (54%) women had received ≥1 treatment and in total a COVID-19 pharmaceutical treatment was administered 1,296 times outside of a trial. In 566 (44%) cases the treatments administered to the pregnant women were not recommended by the National Institutes of Health (NIH) at the time of administration.
Of 179 case reports of women with COVID 19 in pregnancy, 109/179 women received ≥1 COVID-19 pharmaceutical treatment and in total COVID-19 experimental pharmaceutical treatments were administered 274 times.
Conclusion: During the early phase of the COVID-19 pandemic, pregnant women excluded from randomised trials did not avoid unproven or ineffective treatments.

EP.0905 | Covid-19: Evaluation of prevalence and outcomes in a Scottish district general hospital

Kahyee Hor[1]; Jennifer Johnstone[2]; Jennifer Allison[2]
[1]*University of Edinburgh, Edinburgh, UK;* [2]*NHS Fife, Kirkcaldy, UK*

Objective: There has been a steady increase in the number of pregnant women affected by SARS-CoV-2 nationally. While publicly available data describes the extent of the pandemic at a national level, we wanted to interrogate the prevalence of Covid-19 infection among pregnant women booked at NHS Fife (Scotland), as well as the outcomes associated with the infection.
Design: We performed a retrospective cohort study to evaluate the Covid-19 infection rate and outcomes among pregnant women who were booked in NHS Fife between October 2020 to December 2021.
Method: We extracted data from electronic maternity records and systematically evaluated notes from patients who had either “active Covid-19 infection” or “previous Covid-19 infection” during the study period. Those who had a positive test prior to their booking appointment or following their delivery were excluded from the study.
Results: 411 women had a positive Covid-19 test result during 14-month period and were included in the study. The highest number of cases were reported between July to September 2021. Of the 411 cases, 74 women were seen in hospital – of which 35 of them (47%) were admitted; 12 (34%) of those admitted were delivered due to deterioration in maternal condition – 10 were delivered by emergency CS and 2 had vaginal deliveries. All 12 women were not vaccinated. Only 1 patient was delivered at <28 week’s gestation and 4 were delivered at <34 weeks’ gestation; 58% of these women were oxygen dependent; 67% received steroids and 42% received Tociluzimab; 42% were admitted to ITU, of which 3 women required invasive ventilation. There were no maternal deaths, but one patient was transferred to a tertiary unit for ECMO postnatally; 1 patient unfortunately presented with a stillbirth; 11 of those who were delivered tested positive between July to December 2021, when the Delta variant was the most prevalent strain; 7 of these women were from areas with the 2 lowest deprivation indices.
Conclusions: The trend of Covid-19 infection in this population was in keeping with the national data. While the majority of pregnant women who had positive tests were clinically well, those that required hospital admission and were not vaccinated, as well as those who were from deprived backgrounds, had an increased risk of maternal and neonatal morbidity. Further efforts to improve vaccination uptake among pregnant women, particularly those from deprived areas, is critical in reducing maternal and neonatal morbidity and mortality.

EP.0906 | Covid-19: The impact on acute surgical cases in University Hospital Wishaw, Scotland

Mr Alan Kennedy; Louise Kellison
University Hospital Wishaw, Wishaw, UK

Objectives: The Covid-19 Pandemic has led to a significant disruption of elective services across all specialties. Changes to operating guidance resulted in a joint RCOG/ BSGE statement advising conservative management where possible and any operation carrying risk of bowel involvement should be performed by laparotomy. Operations with no risk of bowel involvement could be carried out laparoscopically with additional PPE measures.
Our objective was to assess the impact of the Covid-19 pandemic on acute gynaecological presentations requiring surgery, and compare this to pre-pandemic patterns.
Design: A retrospective audit of all patients undergoing emergency gynaecology surgery between March and August 2020. Comparison was made with patients presenting pre-pandemic between March and August 2019.
Methods: OPERA surgical database was used to identify all patients undergoing emergency gynaecology surgery during the dates being studied. Parameters included were: operation performed, entry technique and urgency of CEPOD category (A <45 mins, B <2 hrs, C <4 hrs, D <8 hrs, E <24 hrs, F <48 hrs).

Results: A total of 104 emergency cases were booked in in 2020, and 115 in 2019.
Laparoscopic procedures were the most common entry technique and type of procedure, accounting for 57/104 (55%) diagnostic laparoscopies in 2020, and 56/115 (49%) in 2019. Most common pathology included; treatment of ectopic pregnancy 36/104 (35%): 28/115 (24%), and ovarian pathology, 19/104 (18%): 27/115 (23%)
Open surgery accounted for 19/104 (18%) procedures in 2020 and 14/115 (12%) in 2019 ($n = 19$ vs. 14)
Other operations included hysterectomy 4/104 (3.8%): 4/115 (3.4%) ($n = 4$ vs. 4) and examination under anaesthetic/ hysteroscopy 24/104 (23%) @ 23/115 (20%) ($n = 24$ vs. 23).
In the most acute CEPOD categories 95%: 96% number were performed <24 hours from presentation and 5%: 4% number > than 24 hours.
Conclusions: Concern about increasing caseload of acute pathology as a result of delayed presentation does not appear to have been demonstrated within our department, with no significant difference in number of or variety of emergency gynaecology operations ($p = 0.5$). There was no significant difference in the urgency of cases booked ($p = 0.25$).
NHS Lanarkshire has been able to maintain its standards in offering emergency gynaecology surgery within national timeframe targets. Similar rates in laparoscopic approach were seen ($p = 0.12$), refuting concern about an increased open approach to surgery. No operating surgeons had contracted Covid- 19 during this audit.

EP.0907 | The fatal fetal sting of Dengue: experience from a tropical country

Sujata Siwatch; Sanjana Panaji; Samreen Zehra; Neelam Aggarwal; Bharti Sharma
PGIMER, Chandigarh, India

Objectives: Dengue is a vector borne infectious disease, endemic in the tropical regions, that may be associated with significant maternal and fetal morbidity and mortality. However, literature is sparse on the rate and factors associated with stillbirths in women suffering from dengue. In this study, we aimed to explore the frequency of stillbirths in dengue infection in pregnancy during the COVID pandemic. We also studied the effect of severity of the disease on fetal morbidity and mortality.
Design: Retrospective review of registers and case records
Method: We reviewed the data of pregnant women admitted for dengue in the Obstetrics & Gynecology department in a tertiary care hospital of Northern India between September and December, 2021. Data collected included the age, parity, gestation at admission, severity of the maternal disease, other comorbities, maternal outcome, obstetrical outcome during the dengue disease and fetal outcome.
Results: Twenty three pregnant women were admitted to the department between September and December 2021. The average age of the women was 26+/-5 years. Half of these women were primigravidas (12 out of 25). Most women were in the third trimester, while one woman each presented in second trimester and the postpartum period. Eleven mothers presented with severe dengue, 11 had dengue with warning signs and only one woman had dengue without warning signs. There were 7 maternal deaths. Three mothers died undelivered. There were 10 stillbirths of 20 deliveries and 22 babies. Majority of the woman delivered preterm. There were 2 twin deliveries. Of the babies who delivered at term, most were liveborn(6 of 7). The stillbirth rate was higher in the severe dengue group, dengue with warning signs and dengue without warning signs were 60%, 36% and nil respectively.
Conclusions: The stillbirth rate is high in women with dengue in pregnancy, especially in the third trimester. The chances of stillbirth escalate with the severity of dengue. Maternal mortality is high in women admitted with dengue in pregnancy. Public health strategies to prevent dengue should be strengthened to avoid high stillbirth rates and maternal mortality. The data is limited by the retrospective design and skewed as women with only severe form of dengue would have been admitted.

EP.0908 | Meeting the expectations of those undergoing elective Caesarean sections during the pandemic

Katherine Jackson[1]; Judith Roberts[2]
[1]*University of Glasgow, Glasgow, UK;* [2]*Queen Elizabeth University Hospital, Glasgow, UK*

Objective: To assess whether changes in procedure implemented at the QEUH during the pandemic have impacted the experience of elective Caesarean sections for patients and birth partners.
Design: Two questionnaires were produced: one for patients, one for birth partners. These included a scale for participants to record how strongly they agreed with 12 statements addressing core expectations. Statements from a 2018 patient satisfaction audit were repeated allowing comparison. Five free text questions were developed to encourage reflection on positive or negative aspects and address the impact of the pandemic.
Method: Questionnaires were offered to all patients undergoing elective Caesarean sections over three weeks in October 2021. They were distributed to 30 pairs ahead of theatre; 38 responses were collected from 19 patients and birth partners post-delivery. Qualitative data was collated. Categorical variables were analysed by percentage allowing comparison with 2018 findings. Free text responses were reviewed on an individual basis.
Results: 68% agreed with every statement. The statements with which patients and birth partners most strongly agreed were regarding feeling treated with respect (97%); feeling safe (95%); feeling comfortable with staff communication (97%); and feeling supported (95%). Notable improvement since 2018 was identified in respondents agreeing their partners were

supported (95% from 54%), they were involved in decisions in theatre (79% from 46%), and they were supported to make decisions regarding their baby (79% from 62%). The lowest level of agreement was with the statement 'I feel the information provided in advance prepared me well for what to expect on the day': 50% strongly agreed demonstrating improvement from 48% in 2018; 42% felt COVID-19 restrictions had impacted on their experience: 50% explained this was significant in limiting time spent together prior to delivery (partners can only join patients once they reach recovery ahead of their section); 19% felt the impact was minimal; 84% of the free text responses expressed gratitude and offered positive comments.
Conclusions: The QEUH maternity department has achieved overall higher levels of satisfaction than in 2018 despite pandemic driven procedural changes. The greatest impact was limitation on time spent together prior to delivery. The area with most scope for improvement remains the provision of clear advance information, while satisfaction has increased, improvement efforts have been hindered by the reduction in F2F appointments. The volume of positive feedback provided exceeded the expectations of this study, reflecting the positive impact of this team on patients and birth partners.

EP.0909 | Mother-to-child transmission of SARS-CoV-2 and rates of neonatal positivity

Maurie Kumaran[1]; Oluwadamilola Akande[1]; Halimah Khalil[1]; Heidi Lawson[1]; Jameela Sheikh[1]; John Allotey[2]; Javier Zamora[2,3]; Shakila Thangaratinam[2,4]; Shaunak Rhiju Chatterjee[1,5]; Tania Kew[1]
[1]College of Medical and Dental Sciences, University of Birmingham, Birmingham, UK; [2]Institute of Metabolism and Systems Research, WHO Collaborating Centre for Global Women's Health, University of Birmingham, Birmingham, UK; [3]Head of Clinical Biostatistics Unit, Hospital Ramon y Cajal of Biomedical Research, Madrid, Spain; [4]Birmingham Women's and Children's NHS Foundation Trust, Birmingham, UK; [5]Royal Liverpool University Hospital, Liverpool, UK

Objective: To determine the rate of SARS-CoV-2 positivity in babies born to mothers with coronavirus disease 19 (COVID-19) and the timing of mother-to-child transmission (MTCT).
Design: This is a living systematic review and meta-analysis.
Method: A literature search of major databases and preprint servers was performed from December 2019 to August 2021. Cohort studies of pregnant and recently pregnant women attending hospital for any reason with confirmed SARS-CoV-2 infection were used to determine rates of neonatal positivity. Case series and case reports were included to determine the timing and likelihood of MTCT in SARS-CoV-2 positive neonates using the 2021 World Health Organisation (WHO) classification system. Two independent reviewers conducted an initial review of potential studies for inclusion by title and abstract, then by full text. Quality assessment and data extraction were conducted by two independent reviewers. Random-effects meta-analysis was used to report rates of neonatal positivity.
Results: The rate of neonatal SARS-CoV-2 positivity (140 cohort studies, 14,271 babies) was 1.8% (95% CI 1.2-2.5%). There was adequate data to classify 592 SARS-CoV-2 positive babies according to the WHO classification system; 434 babies were exposed in-utero, 32 intrapartum and 70 in the early postnatal period; 389 babies were categorised with 'indeterminate' likelihood of MTCT due to a lack of repeat confirmatory testing at pre-specified times. MTCT was confirmed in 14 babies - 7/14 from in utero transmission, 2/14 from intrapartum transmission, and 5/14 from early postnatal transmission.
Conclusions: The rate of neonatal SARS-CoV-2 positivity from confirmed vertical transmission is low, therefore MTCT is likely to be uncommon.

EP.0911 | COVID-19 and cancer diagnosis: Scan and see pathway for 2WW patients

Manamita Niphadkar; Mayura Nisal
Royal Berkshire Hospital, Reading, UK

Objective: The primary objective of this project was to minimise the disruption in the endometrial two week wait pathway during the second wave of the pandemic.
Design: The first wave of the COVID-19 pandemic hugely disrupted the outpatient services. This resulted in increase in the percentage of breaches on the two week wait (2WW) pathway- partly due to service disruptions and staff shortages due to isolation and sickness. Our previous pathway was to offer ultrasound appointment followed by an outpatient hysteroscopy (OPH) appointment on the same day. Many patients did not require further OPH owing to normal ultrasound which led to non-utilisation of many OPH slots. As the second wave approached, we introduced a novel 'Scan and See' pathway for the patients on the endometrial 2WW pathway in February 2021. The goals were to minimise breaches and maximise the utilisation of the Outpatient Hysteroscopy slots. All the referred patients were first offered a transvaginal ultrasound. The scans were then reviewed by the on-call gynaecology consultant and the patients triaged. The patients with normal scans were seen in a dedicated registrar-led gynaecology outpatient clinic for examination and discussion of the ultrasound report.
Those requiring further interventions were offered appropriate appointments.
Method: 458 patients were referred on the endometrial 2WW pathway in 6 months (February 2021 to July 2021). We performed retrospective data analysis of these patients with the help of electronic records and data from our service manager.
Results: Out of 458 referred patients, only 57% required outpatient hysteroscopy. Out of these, we timely diagnosed malignancies in 8 patients. In six months, we successfully saved 196 OPH slots which could be used for benign gynaecology patients.

Conclusion: This pathway empowered us to cope with the pressures during the pandemic and is continuing to help shorten the waiting time for OPH appointments for benign gynaecology patients. The patient feedback has been encouraging too. Offering an ultrasound appointment in time is more feasible than ultrasound + OPH appointment on the same day, thus enabling us to meet the 14 day first diagnosis target for the 2WW patients.

EP.0912 | Comparing postnatal readmissions during COVID-19 and pre-pandemic in a tertiary care hospital

Negin Sadeghi; Ammara Kashif; Catherine Weems; Meena Bhatia
Oxford University Hospitals NHS Foundation Trust, Oxford, UK

Objective: The aim of this study is to compare the postpartum length of stay (LOS) and maternal readmission rates from delivery up to 6 weeks during COVID-19 pandemic and pre-pandemic.
Design: This study is a retrospective observational study, comparing pre-existing reports from January- March 2019 (pre-pandemic) and data from January- February 2021 (during the pandemic).
Methods: Data from 2019 was retrieved from maternity clinical governance and we extracted data from the same study period in 2021 when COVID guidelines were in practice.
Results: Our COVID guidelines encourage discharging all postnatal women at day 1 (24 hours from delivery regardless of mode) and community follow up until day 10 postpartum. In 2021, 14 women were re-admitted on an average day 7 post-delivery. Our observation was that majority of women (86%) self-referred.
57% had SVD, 28% had emergency caesarean section, 14% had instrumental vaginal delivery and 7% had elective caesarean section.
Most common reasons for readmission were raised blood pressure (35%) and puerperal pyrexia (35%) (one case of mastitis and four cases of endometritis).
Two women (14%) presented with secondary post-partum haemorrhage. One woman (7%) was admitted with a caesarean section wound hematoma and one (7%) with shortness of breath. After delivery, equal percentages of patients were discharged by midwives and doctors.
Average length of stay in hospital during readmission was 3 days.
On comparing the data from previous audit, we observed that the average length of postnatal stay in hospital was 1.5 days in 2019, while all women went home on day 1 in 2021. The average length of stay after readmission increased from 2 days to 3 days in 2021. The average day of readmission was the same (day 7) and mode of delivery had similar patterns except emergency caesarean section. In 2021, more women presented after emergency caesarean section (28% in 2021 vs 14% in 2019).
Conclusion: Early postpartum discharge of low-risk mothers has been a universal adjustment in response to COVID-19 pandemic to reduce the chain of transmission. Given that there has been no significant change in the rate of maternal postnatal readmission since the pandemic in our study and a few other studies across the world, it appears that early discharge in combination with community follow up (where resources are available) can be an enduring change. We therefore conclude this as a positive outcome in terms of admission costs and patient satisfaction.

EP.0913 | Covid-19 vaccination: uptake and patient perspectives in a multi-ethnic North-London maternity unit

Dorothy Davies; Anna McDougall; Amelia Prophete; Viswapriya Sivashanmugarajan; Wai Yoong
North Middlesex University Hospital, London, UK

Objective: To explore vaccine uptake, factors associated with vaccination status and the underlying beliefs regarding Covid-19 vaccination amongst the pregnant women in a multi-ethnic North London hospital.
Design: A “snapshot” anonymous online survey of pregnant women on the topic of Covid-19 vaccination in pregnancy.
Method: From 19th October to 1st November 2021, pregnant women attending maternity services at North Middlesex University Hospital maternity department were asked to complete an anonymous online survey. An interpreter was used where required. Quantitative and qualitative data was collected, including demographics, personal and household vaccination status, and beliefs about the vaccine. Free text comments were included, which were analysed thematically.
Results: 202 women participated in the survey, of whom 43.1% had received at least 1 dose of the Covid-19 vaccine and 35.6% had received 2 doses. Factors associated with acceptance of vaccination included: a) age over 25 years old (57.6% vaccinated vs 17.2% under 25 years); b) Asian ethnicity (69.4% vaccinated vs 41.2% white ethnicity, 27.5% Black/Caribbean/African/Black-British ethnicity and 12.5% mixed ethnicity), and c) living in a vaccinated household (63.7% vaccinated vs 9.7% living in unvaccinated household).
Vaccine uptake was higher in women who had relied on medical advice as their main source of information compared to other sources (59.0% vs 37.5% friends and family, 30.4% news and 21.4% social media).
Only 35.1% of women received information about the vaccine at their antenatal booking appointment and these women reported higher vaccination rates (57.7%) compared to those who received no information at booking (36.2%). 97.4% of unvaccinated mothers had no plans to receive the vaccine during pregnancy and 33.9% had no plans to be vaccinated in the future. Qualitative data revealed concerns about a lack of information regarding the safety of Covid-19 vaccination in pregnancy.

Conclusions: This study provides a unique insight into the perspectives of pregnant women in a multi-ethnic setting on COVID-19 vaccination and future vaccination intentions. In addition to age and ethnicity, household vaccination status and provision of information regarding the vaccine were key determinants of vaccination in our pregnant population. As vaccine acceptance is key to the success of the Covid-19 immunisation programme, these findings highlight the urgent need to tackle vaccine mistrust and disseminate pregnancy specific vaccine safety data to pregnant women and healthcare providers

EP.0915 | Pregnancy outcomes of COVID-19 ARDS cases during the second wave of pandemic

Widyastuti Widyastuti; Reza Tigor Manurung; Amanda Safira Dea Hertika
Department of Obstetrics and Gynaecology, Fatmawati General Hospital, Jakarta, Indonesia

Objective: To know the clinical characteristics and outcomes of Coronavirus Disease 2019 (COVID-19) Acute Respiratory Distress Syndrome (ARDS) in pregnant women at a tertiary hospital during the second wave of the COVID-19 pandemic in Indonesia.
Method: Data was collected from medical records of pregnant women with COVID-19 ARDS who were treated between June-July 2021 in a tertiary hospital in Jakarta, Indonesia.
Results: From 51 ARDS patients, 28 patients (55%) died and the other 23 (45%) survived. The mean of patients' age was 31 years old, mean of gestational age at admission was 32 weeks, and most of the patients were in the second pregnancy. Statistically significant difference were observed in the respiratory rate (26.6 vs; 34.3, $p < 0.05$, 95% CI), room air saturation (94.9 vs; 78, $p < 0.01$, 95% CI, complaint of fever and fatigue at presentation, and length of hospital stay (13.6 vs; 7, $p < 0.01$, 95% CI) between ARDS patients who survived and died. In terms of blood examination at admission, lactate dehydrogenase (312 vs; 540, $p < 0.01$, 95% CI) and C-reactive protein (387.1 vs; 759.7, $p < 0.01$, 95% CI) were also significantly different between the two groups. Caesarean section was done in 33 patients (63.5%), spontaneous abortion in 2 patients (3.8%), and the remainder of 17 patients (32.6%) were treated conservatively and continued the pregnancy. Intubation was performed in 21 patients (40.3%) and was significantly different between ARDS patients who survived and died. From all of the newborns delivered, one was stillborn while the other 32 were alive.
Conclusion: Significant differences were found in the symptoms of fever and fatigue, respiratory rate, and room air saturation at presentation, level of lactate dehydrogenase and c-reactive protein, hospital stay, and intubation between pregnant women with COVID-19 ARDS who survived and died. Further research in the larger population and longer period is needed to study about COVID-19 ARDS on pregnancy.
Keyword: pregnancy outcome, ARDS, COVID-19

EP.0916 | Symptomatic COVID-19 admissions in district general hospital: Maternal and Neonatal outcomes

Paul Elie Faraj; Kristina Savickaite; Saadia Waheed
Luton and Dunstable University Hospital, Luton, UK

Design: A retrospective cohort study of 34 pregnant patients admitted with COVID-19 infection to a district general hospital in East of England. Outcomes included severe disease, failing organ system support, second-trimester miscarriage, stillbirth, iatrogenic prematurity, NICU admissions, neonatal SARS-Cov-2 infection and postnatal depression.
Methods: Thirty-four maternity admissions were identified reviewing the completed UKOSS COVID-19 notification forms submitted from 24th March 2020 to 29th December 2021. Admissions were grouped based on the place of care: maternity unit, medical wards or critical care (level 2 and 3) units. Demographics, antenatal risk factors, intrapartum and postnatal data were collected from electronic medical records and analysed using Microsoft Excel software.
Results: Of the 34 admitted patients, 2.9%, 17.6% and 79.4% were infected in the first, second and third trimesters, respectively. Among maternity COVID -19 admissions, 28 women (82.4%) were not vaccinated against SARS-CoV-2. Thirteen women (38.2%) required Critical Care with an average stay in the intensive care unit of 27.5 (1-106) days. Six patients (46.2%) required ventilation, of which 2 (15.4%) were transferred for extracorporeal membrane oxygenation. Fourteen maternity patients (41.17%) required expedited deliveries, including 8 (23.5%) due to deteriorating respiratory function and 6 (17.8%) because of fetal distress. Following early multidisciplinary team (MDT) discussions and delivery planning, 3 (21.4%) women had a single dose, and 9 (64.3%) had two doses of antenatal steroids prior to emergency caesarean section. The median gestational age at delivery in the aforementioned group was 32+5 (IQR - 34+3 - 29+4) weeks. Ten premature neonates required neonatal intensive care unit admissions. Two neonates (5.9%) were diagnosed with SARS-CoV-2, and one died because of acute respiratory distress syndrome. Regarding other adverse outcomes, we had one maternal death following severe COVID-19 infection, one case of late miscarriage at 19+4 weeks gestation, and two intrauterine deaths at 27+6 and 36+3 weeks. Histological examination of stillbirths' placentas confirmed massive perivillous fibrin deposition and chronic histiocytic intervillositis. Three women (8.8%) were commenced on antidepressants postnatally following a traumatic experience, including neonatal loss and prolonged hospitalisation.
Conclusions: COVID-19 infection carries a significant risk to unvaccinated maternity patients. Our study identified various adverse pregnancy outcomes including maternal and neonatal deaths. Patients presenting with severe COVID-19 symptoms demonstrated rapid deterioration requiring invasive respiratory support and iatrogenic preterm delivery. Early MDT involvement in delivery planning can improve maternal and neonatal outcomes.

EP.0917 | COVID-19 admissions in the obstetric population in a single-centre tertiary unit

Imogen Bacon[1]; Charlotte Bishop[2]; Miriam Bourke[3]; Orene Greer[4]; Natasha Singh[5,4]; Nishel Shah[4,5]

[1]*Imperial College Healthcare NHS Trust, St Mary's Hospital, London, UK;* [2]*Department of Obstetrics and Gynaecology, Chelsea and Westminster Hospital NHS Foundation Trust, West Middlesex Hospital, London, UK;* [3]*Fetal Medicine Unit, Elizabeth Garret Anderson Wing, University College London Hospitals NHS Foundation Trust, London, UK;* [4]*Imperial College London, Department of Metabolism, Digestion and Reproduction, Chelsea and Westminster Hospital, London, UK;* [5]*Chelsea and Westminster NHS Foundation Trust, Directorate of Women, Division of Women and Children, London, UK*

Objectives: To audit the management of COVID-19 in the obstetric population at Chelsea and Westminster Hospital 2020-2021.

Design: A retrospective audit of clinical management against the joint RCM and RCOG Coronavirus (COVID-19) Infection in Pregnancy guidelines.

Method: Pregnant patients admitted to Chelsea and Westminster Hospital with a positive COVID-19 test between 31st March 2020 and 29th July 2021 were identified retrospectively. Symptomatic patients and those found to be SARS-CoV-2 positive but asymptomatic were included. Demographic and clinical data were extracted from paper and electronic health records. Clinical parameters were reviewed, and neonatal and pregnancy outcomes categorised.

Results: 63 patients met the inclusion criteria; 36 were symptomatic and 27 asymptomatic, having been admitted for other medical or obstetric reasons. Of the 36 symptomatic patients, 33 received oxygen therapy; 14 patients were admitted to ITU; 2 of these received ECMO therapy. Median age and BMI of ITU admissions were 35 and 27.5 respectively, compared with 33 and 24 overall. Patients of Asian ethnicity made up the greatest proportion (35.7%) of ITU admissions, but only 14.3% of the total cohort; 43% patients received steroids for maternal hypoxia. Of the 11 iatrogenic deliveries, 6 were in patients admitted to ITU; 5 patients consented to enrolment in a COVID-19 clinical trial, only 1 declined. There were no fatalities amongst the cohort reviewed.

Conclusions: Overall, findings demonstrated good clinical outcomes for pregnant women and their babies. Appropriate treatment was administered in a suitable place of care, with minimal adjuvant therapy required.

Limitations of the audit include missing data, and heterogeneity in the electronic databases used by different specialities. This retrospective evaluation was also limited by evolving management guidance during the audit period due to rapid research and development and the introduction of successive evidence-based therapies.

Gold standards in clinical care are informed by good quality trial data and pregnant and lactating women were excluded from early clinical trials. This was reflected in our data for study enrolment and highlights under-representation of the pregnant population in drug trials.

Our results demonstrated groups potentially at higher risk of serious illness, including those of Asian ethnicity, older age and increased BMI. This may allow earlier identification of “at risk” patients, with implications for future assessment and management of COVID-19 in the obstetric population.

EP.0918 | Impact of covid-19 on mental health of Healthcare workers: An observational study

Jyoti Meena; Soniya Dhiman; Kamlesh Kumari; Renu Sharma; Seema Singhal; Vidushi Kulshrestha; Vanamail Perumal

All India Institute of Medical Sciences, New Delhi, Delhi, India

Introduction: In December 2019, the world has been introduced with pneumonia of unknown cause and it has been a start of a new pandemic called COVID-19 caused by SARS-CoV-2 in March-2020. The whole process of preparedness of infrastructure and manpower including, the changing policies, social distancing, isolation, quarantine and different working scenario in an already hectic department created a lot of burden on their workers. Hence, we conducted a study to assess the effect of these changes on the metal health of our healthcare workers.

Aims and Objectives: To study the incidence and severity of depression, anxiety and post-traumatic stress disorder (PTSD) among health care workers in gynae department and to assess its association with background variables and also the quality of life.

Material and Methods: It was the cross sectional study done in the Department of Obstetrics and Gynaecology in collaboration with Department of Psychiatry, AIIMS, New Delhi. One hundred HCWs, working in the Department were included in the study. DASS-42, IES-R scale and WHO-QOL-BREF score were applied to measure the depression, anxiety, stress and acute and post-traumatic stress disorder (PTSD) and quality of life of HCPs. Data analysis was carried out using statistical package STATA version 14.0. Association between severity of the anxiety, depression and the stress level with background variables were compared using Chi-square test/Fischer exact test.

Results: Out of 100 there were 92 females and 8 males HCWs; 39% were doctors, 45% nursing staff and 16% were supportive staff. The incidence of Depression, among three groups was 15.4%, 24.4% and 0% respectively, anxiety 25.6%, 33.3% and 0.0% and stress 12.8%, 17.8% and 0.0% respectively. The mean for IES-R was 3.95 ± 10.763, 2.78 ± 8.628, 0.25 ± 0.447, for Depression was 4.79 ± 9.350, 4.04 ± 5.807, 0.88 ± 2.277, for Anxiety was 5.08 ± 8.459, 4.56 ± 6.066, 0.81 ± 1.544 and for stress was5.33 ± 8.093, 3.82 ± 6.709 and 0.81 ± 1.974 respectively among the three groups. It significantly correlated with the type of family (.016), duration of exposure to covid-19 patients (.014) and presence of psychiatric illness in the

family (.003). When we compared the quality of life among different groups, the emotional domain was significantly affected among doctors (.002) and significantly correlated with presence of psychological and medical illness in the family (.002. .022)

Conclusion: Covid-19 significantly affected the mental health of HCWs. Early diagnosis and timely psychological support during such pandemic situations is required to tackle depression and anxiety among HCWs.

EP.0919 | COVID-19 vaccination uptake in pregnancy – the Australian perspective

Siobhan Walsh[1]; Zoe Butters[1]; Sarah Malone[1]; Julia Unterscheider[1,2]

[1]*Royal Women's Hospital, Melbourne VIC, Australia;* [2]*University of Melbourne, VIC, Australia*

Objective: Pregnancy predisposes women and their offspring to adverse health outcomes while reported rates of vaccination uptake remain low internationally. Our study objective was to quantify the uptake of COVID-19 vaccination in pregnant women, and to assess their attitudes towards vaccination in pregnancy.

Design: This was a short voluntary survey of pregnant women attending for antenatal care at the Royal Women's Hospital (RWH), Melbourne, Australia's largest quaternary level maternity centre.

Method: Pregnant women were invited to complete an anonymous, structured, web-based survey with 17 questions exploring their attitudes towards COVID-19 vaccination in pregnancy. A QR code was provided to patients at various clinics and wards throughout the hospital, and a link was posted on the hospital's social media site.

Results: The survey was open for 5 weeks prior to Christmas 2021 and received 351 responses. At the time of the survey Melbourne had just come out of the world's longest and strictest lockdown. Victoria recorded a 90% double-vaccination rate in the general adult population with rising rates (up to 1,600) of new SARS-CoV-2 infections each day. While vaccination of pregnant women was not endorsed in the first half of the year, a strong public health campaign started in June 2021 with various official bodies (Department of Health, RANZCOG, ATAGI) recommending vaccination in pregnancy. In September 2021 a walk-in vaccination hub was established on site. The vaccination uptake of pregnant women increased from 15% in July to 85% in December 2021.

Of the 351 respondents, 82% had received at least one dose of the COVID-19 vaccine; the majority (84%) received it during pregnancy acknowledging the beneficial effect to themselves (86%) and their baby (81%). Vaccinated women were more commonly aware of the increased risk of complications due to SARS-CoV-2 infection in pregnancy (92%) when compared to the unvaccinated group (27%). The majority of unvaccinated women reported concerns regarding safety in pregnancy (85%), and half of them stated they did not want to jeopardise their pregnancy by receiving the vaccine. Some women found the changing advice during their pregnancy (before and after June 2021) confusing.

Conclusions: Our survey demonstrated that a strong public health campaign with a clear, consistent message regarding the beneficial effects of COVID-19 vaccination in pregnancy dramatically increased vaccination uptake rates in pregnancy.

EP.0920 | COVID-19 and Childbirth: Post traumatic stress and depression in New Mothers, India

Moksha Pasricha[1]; Sanaya Katrak[2]; Anjana Mahadevan[3]; Ashlesha Bagadia[4]; Poornima Mahindru[4]; Uma Ram[5]; Avantika Bhatia[2]

[1]*Tata Institute of Social Sciences, Mumbai, India;* [2]*Ashoka University, Sonipat, India;* [3]*Sri Ramachandra Institute of Research and Higher Education and Research, Chennai, India;* [4]*The Green Oak Initiative Community Mental Health Centre, Bengaluru, India;* [5]*Seethapathy Clinic and Hospital, Chennai, India*

Objective: Research suggests that childbirth may be experienced as a traumatic or stressful event by mothers, which may have implications for their postpartum mental health. Negative feelings may be further exacerbated as a result of giving birth during the pandemic. The aim of the present study was to examine mothers' experiences of post traumatic stress and depression as a result of giving birth during the COVID-19 pandemic. We also sought to assess the association of post-traumatic stress and depression with mothers' experience of support from the obstetric staff.

Design: Retrospective data from mothers who delivered between April 2020 and September 2021 was collected via an online questionnaire. The study employs a correlational design.

Method: New mothers who met the eligibility criteria of the study from two OB-GYN clinics in Chennai, India were contacted via phone call. Those who consented to participating in the study were then sent a follow up message with a link to the online survey. The questionnaire included the Impact Events Scale - Revised, Patient Health Questionnaire-9 and the support subscale of the Support and Control in Childbirth Scale along with questions about the new mothers' socio-demographic and obstetric experiences. Data from 220 participants was assessed using independent t-tests and bivariate correlation analysis.

Results: 50.45% of new mothers met the criteria for clinical concern for post traumatic stress on the Impact of Events Scale – Revised, while 34.54% met the criteria for major depressive disorder on the Patient Health Questionnaire-9. Both post-traumatic stress ($r = -0.161$, $p = 0.017$) and depression ($r = -0.174$, $p = 0.010$) were significantly associated with support experienced during childbirth. Associations between post traumatic stress, depression and obstetric factors including type of delivery, previous pregnancy loss and

having discussed mental health with one's OB-GYN will also be presented.

Conclusions: The findings of this study indicate that several new mothers who gave birth during the pandemic experienced post traumatic stress and depression at clinically concerning levels. The results also highlight the role of perceived support from obstetric staff as a protective factor against developing post traumatic stress and depression. These results suggest that it is pertinent to explore risk and protective factors related to mental health following childbirth during the pandemic. These findings have implications for clinical practice and research to improve postpartum mental health.

EP.0921 | COVID-19 vaccination and its impact on menstruation – A retrospective primary care study

Miss Anjali Patel[1]; Reshma Rasheed[2]; Yathorshan Shanthakumaran[2]
[1]*New Vision University, Tbilisi, Georgia;* [2]*Rigg Milner Medical Centre, Tilbury, UK*

Objective: Menstrual irregularities have been reported post covid vaccination. Our retrospective study looked at the true prevalence across a patient population in primary care

Design: Dispelling myths of post COVID-19 vaccine menstrual irregularities is necessary to enhance uptake of the vaccine in women of reproductive age. A retrospective analysis of the database of the patient population across three sites (circa patient population 12,000) was undertaken.Patients were asked about their menstrual cycles regularity after the vaccination using an open and closed questionnaire.

Method: Women aged between 12-55 years were surveyed post vaccination to assess their perception of menstrual problems vs pre-vaccination. We did not exclude women with pre-existing menstrual problems and asked if post vaccination they felt their problems worsened.

Results: Of a total patient population of 5500, 68 patients had the COVID-19 vaccine. Of these 50 patients had pre-existing menstrual irregularities and in these 21 (42%) patients admitted to a worsening of their symptoms both cycle length and regularity.

Conclusions: In studies, COVID-19 vaccination has been shown to be associated with worsening of pre-existing menstrual irregularities and the development of menstrual irregularities de novo. Women in their mid-20's to late 40's, showed a predominance of anovulatory cycles and menorrhagia. There has been no evidence found that the COVID-19 vaccine affects fertility. We feel it was important to do this study in order to provide reassurance to patients of the reproductive age group and dispel myths of any association of significant changes to menstrual and reproductive potential. This enables us to counsel women more effectively and provide reassurance of the safety of the vaccine.

POST REPRODUCTIVE HEALTH

PP.0164 | Efficacy and safety of fezolinetant in moderate-to-severe vasomotor symptoms associated with menopause

Nanette Santoro[1]; Kimball Johnson[2]; Rossella E. Nappi[3]; Genevieve Neal-Perry[4]; Marla Shapiro[5]; Petra Stute[6]; Rebecca C Thurston[7]; Wendy Wolfman[5]; Marci English[8]; Catherine Franklin[8]; Misun Lee[8]
[1]*CU School of Medicine, Colorado, USA;* [2]*Research Atlanta LLC, Decatur, USA;* [3]*University of Pavia, Pavia, Italy;* [4]*UNC School of Medicine, Chapel Hill, USA;* [5]*University of Toronto, Toronto, Canada;* [6]*Inselspital, Bern, Switzerland;* [7]*University of Pittsburgh School of Medicine, Pittsburgh, USA;* [8]*Astellas Pharma Global Development, Northbrook, USA*

Objective: To evaluate the efficacy and safety of fezolinetant vs. placebo on the frequency and severity of vasomotor symptoms (VMS) associated with menopause.

Design: Double-blind, placebo-controlled, multicentre Phase 3 study (NCT04003142).

Method: 501 women aged ≥40–65 years with moderate-to-severe VMS (minimum average 7 hot flashes/day) were randomized to placebo; fezolinetant 30 mg or 45 mg once daily (1: 1:1). The main outcome measures were mean change from baseline to week 4 and 12 in frequency and severity of moderate-to-severe VMS (co-primary efficacy endpoints); mean change from baseline to week 12 in Patient-reported Outcomes Measurement Information System Sleep Disturbance–Short Form 8b (PROMIS) Total Score, and effect on weekly mean change in frequency and severity of moderate and severe VMS (secondary endpoints). Treatment-emergent adverse events (TEAEs) assessed safety and tolerability).

Results: 500 women took ≥1 dose (placebo $n = 167$, fezolinetant 30 mg $n = 166$, fezolinetant 45 mg $n = 167$). Both fezolinetant doses were statistically significant compared with placebo. For VMS frequency, least squares (LS) mean reduction over placebo (SE) at week 4 was –1.82 (0.46), p<0.001 for fezolinetant 30 mg and –2.55 (0.46), p<0.001 for 45 mg. At week 12, these values were –1.86 (0.55), p<0.001 for fezolinetant 30 mg and –2.53 (0.55), p<0.001 for 45 mg. For VMS severity, reductions at week 4 were –0.15 (0.06), $p = 0.021$ for fezolinetant 30 mg and –0.29 (0.06), p<0.001 for 45 mg, and at week 12 were –0.16 (0.08), $p = 0.049$ for fezolinetant 30 mg and –0.29 (0.08), p<0.001 for 45 mg. Fezolinetant 45 mg, but not 30 mg, statistically significantly reduced the PROMIS total score vs. placebo at week 12: LS mean difference (SE) –2.0 (0.7), $p = 0.007$ for fezolinetant 45 mg; –0.7 (0.7), $p = 0.381$ for 30 mg. Efficacy was evident by week 1 of treatment and maintained over the 12-week study period. TEAEs were reported by 40% (fezolinetant 30 mg), 36% (fezolinetant 45 mg) and 32% (placebo) of women. Headache was the most common TEAE in the fezolinetant groups: 3% (30 mg), 4% (45 mg) and 2% (placebo). Serious TEAEs were
