# Supplementary material for: Mental health status and related factors influencing healthcare workers during the COVID-19 pandemic: A systematic review and meta-analysis
Source: PLoS One. 2024 Jan 19;19(1):e0289454. doi: 10.1371/journal.pone.0289454 (PMC10798549; doi:10.1371/journal.pone.0289454)
Supplement: S1 Data — (ZIP) [file pone.0289454.s011.zip › literatures/99.pdf]

Psychiatry Research Communications 2 (2022) 100037

Contents lists available at ScienceDirect

Psychiatry Research Communications

journal homepage: www.sciencedirect.com/journal/Psychiatry-Research-Communications

Psychological distress among hospital caregivers during and after the first wave of COVID-19: Individual factors involved in the severity of symptoms expression

Benoit Mennicken [a,1], Geraldine Petit [a,b,*,1], Jean-Cyr Yombi [c], Leila Belkhir [c,d], Gerald Deschietere [e], Nausica Germeau [e], Melissa Salavrakos [a,b], Gilles Moreau [f], Laurie Nizet [a], Geneviève Cool [a], Alain Luts [a], Joël Billieux [g], Philippe de Timary [a,b,h]

[a] Department of Adult Psychiatry, Cliniques Universitaires Saint-Luc, Brussels, Belgium
[b] Institute of Neuroscience, Université Catholique de Louvain, Brussels, Belgium
[c] Department of Internal Medicine and Infections Diseases, Cliniques Universitaires Saint-Luc, Brussels, Belgium
[d] Institut de Recherche Expérimentale et Clinique (IREC), UCLouvain, Brussels, Belgium
[e] Psychiatric Emergency Unit, Department of Adult Psychiatry, Cliniques Universitaires Saint Luc, Brussels, Belgium
[f] Statistical Support Unit, Institut Roi Albert II, Cliniques Universitaires Saint Luc, Brussels, Belgium
[g] Institute of Psychology, University of Lausanne, Lausanne, Switzerland
[h] Laboratory for Experimental Psychopathology (LEP), Psychological Science Research Institute, Université Catholique de Louvain, Louvain-la-Neuve, Belgium

A R T I C L E I N F O

Keywords:
Health care workers (HCWs)
Mental health
Personality
Coping

A B S T R A C T

Coronavirus disease 2019 has spread rapidly over the globe and has put an unprecedent psychological pressure on health care workers (HCWs). The present study aimed at quantifying the psychological consequences of the COVID-19 pandemic on HCWs during and after the first wave and identify sociodemographic, situational, and psychological risk/protective factors for symptoms severity. An online survey was sent by e-mail to all nurses and physicians employed by a teaching hospital in Brussels, Belgium. 542 (20,62%) completed the survey. 47%, 55%, 32% and 52% of participants reported posttraumatic stress, anxiety, depression and insomnia symptoms, respectively, during the peak. Two to three months later, posttraumatic symptoms emerged *de novo* in 54% of HCWs. It persisted in 89% of those presenting severe symptoms initially. Neuroticism was the strongest predictor of posttraumatic stress, anxiety, and insomnia. Work overload was the strongest predictor of depression and second predictor of posttraumatic stress, anxiety, and insomnia. Other significant predictors included being a nurse, the number of past traumatic experiences, avoidant coping style, and expressive suppression of emotions.

1. Introduction

The COVID-19 pandemic, caused by a new Coronavirus strain (SARS-CoV2) that emerged in China at the end of 2019, has established itself, in a few months, as a worldwide major public health issue. Faced with an unprecedented pandemic, hospitals have had to rapidly reorganize themselves to cope with the surge of infected patients. Concerns about the mental health and psychological adjustment of health care workers (HCWs) treating and caring for patients with COVID-19 were quickly raised all over the world. During the initial stages of the pandemic, several studies, mostly conducted in China, aimed at assessing psychological distress among HCWs and converged to conclude that HCWs facing this critical situation have been developing psychological distress and other mental health symptoms (Du et al., 2020; Guo et al., 2020; Huang et al., 2020; Lai et al., 2020; Pappa et al., 2020; Rossi et al., 2020; Zhang et al., 2020). These early studies focused on assessing the prevalence of symptoms of mental health deterioration. Beyond evaluating the psychological consequences of the pandemic on HCWs, identification of individual risk and protective factors for these mental health issues is essential. If it appears clear from these studies that the pandemic has negative effects on the mental health of these frontline actors, the effects were shown at the group level. Yet, all individuals do not react in

Abbreviations: HCWs, Health care workers.

* Corresponding author. Institute of Neuroscience (IoNS), Université Catholique de Louvain, Av. Mounier, 53 – Bte B1.53.01, 1200, Bruxelles, Belgium.
E-mail address: geraldine.petit@uclouvain.be (G. Petit).

[1] Benoir Mennicken and Geraldine Petit contributed equally to this work.

https://doi.org/10.1016/j.psycom.2022.100037
Received 27 July 2021; Received in revised form 23 March 2022; Accepted 30 March 2022
2772-5987/© 2022 The Authors. Published by Elsevier B.V. This is an open access article under the CC BY-NC-ND license (http://creativecommons.org/licenses/by-nc-nd/4.0/).

the same way (Gaygısız et al., 2012), some being more affected than others. It is highly probable that the current pandemic will have long-lasting effect on healthcare systems and employees. This is already apparent from the current enormous rate of absenteeism in HCWs in Europe. In this context, from a stress-diathesis perspective, it is important to determine, beyond the mere stressful effect of the pandemic itself, which individual variables constitute vulnerability or protective factors towards psychological distress. Identifying such risk factors is a pre-requisite to the implementation of sound psychosocial and mental health support in order to increase resilience and improve psychological wellbeing of healthcare workers in the pandemic context.

The present study thus aims to (1) assess the symptoms of psychological distress in healthcare workers during the pandemic and (2) identify the individual vulnerability and protective factors susceptible to increase the psychological distress in healthcare workers during the pandemic. Among these factors, we will focus specifically on the role of psychological factors such as personality traits, coping and emotion regulation strategies in predicting symptoms of posttraumatic stress, insomnia, anxiety and depression in HCWs. The weight of these factors will be analyzed with respect to other sociodemographic and work-related features. We also report predictive information about the persistent effects (>3 months) of the pandemic on mental health.

2. Methods

2.1. Procedure

All nurses and physicians employed by the Cliniques Universitaires Saint-Luc, a large teaching hospital in Brussels, Belgium, were invited to participate in the study between June 23 and July 30, 2020. Therefore, the study was carried out after the peak of hospital admission during the first wave (that took place in March/April), when admissions began to drop. An email, containing a link to an online survey, was sent to 1639 nurses and 1067 physicians inviting them to participate in the study. There were no exclusion criteria. The survey was developed, distributed and managed using REDCap (Research Electronic Data Capture) tools, a web-based software platform hosted at Cliniques Universitaires St-Luc (Harris et al., 2009, 2019). The study was approved by the hospital ethical committee, code number 2020/15JUI/321 and all participants provided informed consent. Some results from this study, unrelated to the objective of the current study, will be published elsewhere.

2.2. Survey instruments

Survey questions included sociodemographic and situational items focused on professional and COVID related contexts, and a series of validated questionnaires assessing psychological factors. Investigation of psychological factors first included psychological disturbances *induced by the COVID*: level of posttraumatic stress, anxiety, depression and insomnia symptoms consecutive to the COVID situation. As the study was carried out after the peak of the first wave and the surge of patients in intensive care units, the majority of the questions retrospectively asked HCWs about their experiences during the crisis. However, to assess persistent effects, one question in each section asked participants to evaluate their current (ie, 2–3 months after the peak) level of posttraumatic stress, anxiety, depression and insomnia. Secondly, individual differences in emotion regulation, coping strategies and personality traits were measured through a series of questionnaires assessing various psychological factors that might constitute risk and/or protective factors.

2.2.1. Demographic, work-related features and mental health related information

Information about gender, age, healthcare profession, relationship status, educational background, psychiatric history, and information about the work context during the COVID-19 crisis were collected.

2.2.2. Assessment of psychological symptoms induced by the COVID context

Posttraumatic stress disorders symptoms (PTSD) associated with the COVID professional context were assessed by the 22-item Impact of Event Scale – Revised (IES-R) (Weiss & Marmar, 1997), French version by Brunet et al. (2003). Previously validated cut-off scores (normal = 0–23, mild = 24–32, moderate = 33–36, and severe posttraumatic stress >36) were used in the current study (Creamer et al., 2003; Wang et al., 2020). The levels of ***anxiety and depression*** were assessed by the 14-item Hospital Anxiety and Depression Scale (HADS) (Zigmond and Snaith, 1983), French version by (Lepine et al., 1996). Cut-off points as proposed by the authors were calculated as follows: scores between 0 and 7 indicate the absence of symptoms, scores between 8 and 10 the presence of the symptomology at a moderate degree (“doubtful cases”), scores greater than or equal to 11 indicate a significant number of symptoms of anxiety or depression (“confirmed cases”). The ***severity and effects of insomnia*** were assessed by the 7-item Insomnia Severity Index (ISI) (Bastien et al., 2001), French version by (Bayard et al., 2017). A total score of 0–7 indicates absence of insomnia, 8–14 indicates subthreshold insomnia, 15–21 indicates moderate insomnia, and 22–28 indicates severe insomnia. To evaluate ***persistent symptoms,*** each questionnaire cited above was followed by the question: “To what extent are the symptoms of posttraumatic stress/anxiety/depression/insomnia (still) present today?” Participants had to choose between the following answers: 1) I have never had any symptoms and still don't have any today (None), 2) The symptoms are no longer present (None), 3) The difficulties are a little/sometimes present (Mild), 4) The difficulties are very present (High), 5) The difficulties are extremely present (Severe).

2.2.3. Assessment of psychological factors

Emotion regulation was assessed by the 10-item Emotion Regulation Questionnaire (ERQ) (Gross and John, 2003), Christophe et al. (2009) for the French version. ***Coping strategies*** were measured by the 28-item Brief-COPE (Carver, 1997) (the abridged version of the COPE inventory), French version by Muller and Spitz (2003). ***Personality traits*** according to the Big-5 Model were assessed by the Ten Item Personality inventory (TIPI) (Gosling et al., 2003), French version by (Storme et al., 2016). Table 1 describes and explains the psychological scales used and reports their internal reliability.

2.3. Statistical analysis

IBM SPSS 24.0 statistical software was used to compute correlation and regression analyses. Correlation analyses were conducted to explore the associations between posttraumatic stress, anxiety, depression and insomnia symptoms induced by the COVID context and 1) demographic characteristics (age, sex, occupation, relationship status, education degree); 2) mental health related characteristics (past traumatic experience, past or present psychological disorder); 3) professional information (work with COVID-19 patients or not, changes in work schedules, work overload, personal infection with COVID-19); 4) usual emotion regulation strategies; 5) usual coping strategies; and 6) Big-5 personality traits. Linear correlations were determined using Pearson's correlation coefficient and point-Biserial Correlations Coefficients (rpb) were used to measure the associations between continuous-level variables and binary variables. Given the multiple testing situation and to avoid a high false positive rate, Bonferroni's correction for multiple tests was calculated to obtain corrected significance levels. Stepwise multivariate regression analyses were then used to identify which of the significant associated factors were the most predictive of mental health outcomes (posttraumatic stress symptoms, anxiety symptoms, depression symptoms and insomnia symptoms). Standardized beta coefficients were used to compare the strength of the effect of each factors on the dependent variable. The higher the absolute value of the beta coefficient, the stronger the effect of the predictor (Siegel, 2016).

Table 1
Correlations between posttraumatic stress, insomnia, anxious and depressive symptoms with all factors.

	Posttraumatic stress (IES-R)	Anxiety (HADS)	Depression (HADS)	Insomnia (ISI)
**Demographic, professional and mental health related information**				
Age	NS	NS	NS	NS
Gender	NS	NS	NS	NS
Occupation (being a nurse vs a physician)	r_{pb} = .245 p < .001	r_{pb} = .185 p < .001	NS	NS
Relationship status	NS	NS	NS	NS
Education degree	NS	NS	NS	NS
Reporting present or past psychological disorder	NS	NS	NS	NS
Having been exposed to traumatic events lifetime	NS	NS	NS	NS
Number of past traumatic experiences	r = .253, p < .001	r = .237 p < .001	NS	NS
Experienced work overload during the COVID crisis	r = .317 p < .001	r = .272 p < .001	r = .251 p = .001	r = .210, p < .001
Underwent changes in work schedules during the coronavirus crisis	NS	NS	NS	NS
Worked in a special COVID unit during the coronavirus crisis	NS	NS	NS	NS
Having been infected with COVID 19	NS	NS	NS	NS
**Emotion regulation (ERQ)**				
Cognitive reappraisal	NS	NS	NS	NS
Expressive suppression	NS	NS	NS	r = .160 p = .004
**Coping strategies (Brief COPE)**				
**Approach strategies**	NS	NS	r = -.225 p < .001	NS
Emotional support use	NS	NS	NS	NS
Acceptance	r = -.229 p < .001	r = -.223 p < .001	r = -.199 p < .001	NS
Instrumental support use	NS	NS	NS	NS
Positive reframing	r = -.216 p < .001	r = -.249 p < .001	r = -.225 p < .001	r = -.180 p < .001
Planning	NS	NS	NS	NS
Active Coping	NS	NS	NS	NS
**Avoidance strategies**	r = .330 p < .001	r = .220 p < .001	NS	r = .246 p < .001
Self-Distraction	NS	NS	NS	NS
Venting	NS	NS	NS	NS
Denial	NS	NS	NS	NS
Substance Use	r = .320 p < .001	r = .254 p < .001	NS	r = .246 p < .001
Self-blame	NS	NS	NS	r = .182 p = .001
Behavioral disengagement	r = .253 p < .001	NS	NS	r = .233 p < .001
**Non-coping strategies**	NS	NS	NS	NS
Religion	NS	NS	NS	NS
Humor	NS	NS	NS	NS
**Personality traits (TIPI)**				
Extraversion	NS	NS	NS	NS
Agreeableness	NS	NS	NS	NS
Conscientiousness	NS	NS	NS	NS
Openness	NS	NS	NS	NS
Neuroticism	r = .390, p < .001	r = .442, p < .001	r = .313, p < .001	NS

b = Point-Biserial correlation coefficient; r = Correlation coefficient; p = p-value; NS = Non significant IES-R = The Impact of Event Scale – Revised; HADS = The Hospital Anxiety and Depression Scale; ISI = Insomnia Severity Index.

3. Results

3.1. Demographic, professional and mental health related information

A total of 542 of the 2706 (20,62%) contacted healthcare workers (with 26% of contacted nurses and 10% of contacted physicians) completed our survey. Participants' ages ranged from 23 to 49 years with an overall mean age of 42,5 years. The majority of the participants were women (80%), 73% were nurses and 27% were physicians, 77% were in a relationship, 62% reported having been exposed to at least one traumatic event during their lives, 8% reported present or past psychological disorders. Half of the respondents (51%) were directly involved in treating and caring for COVID-19 patients in dedicated COVID units, 64% reported having experienced various degrees of work overload, 25% underwent changes in work schedule and 10% claimed to have been infected with SARS-CoV-2. All demographic characteristics can be found in Supplemental Tables 1 and 2. The sample sizes used in our statistical analyses vary slightly due to unfilled questions for some variables.

3.2. Psychological disturbances induced by the COVID context

Mean scores (±SD) for posttraumatic stress, anxiety, depression and insomnia symptoms may be found in Supplemental Table 3. Mild to severe signs of PTSD-related distress were reported by 47% of participants of which 23% reported severe symptoms, 55% reported signs of anxiety of which 30% scored as definite cases, 32% reported signs of depression of which 15% were definite cases and 52% reported various degrees of insomnia symptoms of which 2% were considered severe cases.

3.3. Persistent symptoms

Of the study participants who reported no symptoms of posttraumatic stress during the crisis, 46% still did not present any symptoms 3 months later, 50% reported mild symptoms and 4% a high level of symptoms. Among individuals who reported mild symptoms during the crisis, after 3 months, the majority (75%) still experienced the same level of symptoms, 10% reported increased symptoms (high level) and the remaining 15% were back to normal. For those who experienced severe symptoms during the crisis, 7% still evaluated the level of their symptoms as severe, 29% as high, 52% as mild and 12% were remitted. These results are presented in Supplemental Table 3. The persistence of symptoms of anxiety, depression and insomnia are detailed in Supplemental Table 3. Interestingly, among participants that reported severe symptoms during the crisis, 22%, 21% and 67% of them still experienced high to severe symptoms of anxiety, depression and insomnia, respectively.

3.4. Psychological factors

Mean scores (±SD) on scales of emotion regulation, coping style and personality may be found in Supplemental Table 3.

3.5. Correlation analyses

All the results of the correlations can be found in Table 1.

3.5.1. Associations between psychological disturbances induced by the COVID context and demographic, professional and mental health related information

We did not find any association between age, sex, relationship status or the education degree and mental health issues. However, being a nurse (vs being a physician) was associated with higher scores of posttraumatic stress (r_{pb} = .245, p < .001) and anxiety (r_{pb} = .185, p < .001). Past psychiatric history was not linked to mental health related symptoms, but the higher the number of past traumatic experiences, the higher the level of posttraumatic stress (r = 0.253, p < .001) and anxiety (r = 0.237, p < .001) symptoms. All symptoms were also greater for individuals who reported work overload during the crisis (posttraumatic stress: r = 0.317, p < .001, anxiety: r = 0.272, p < .001, depression: r = 0.251, p = .001, insomnia: r = 0.210, p < .001). However, changes in work schedules, working in a dedicated COVID unit and having been infected with SARS-CoV-2 did not correlate with mental health issues.

3.5.2. Associations between psychological disturbances induced by the COVID context and psychological factors

Concerning emotion regulation, the use of cognitive reappraisal when facing negative emotions was not related to any psychological issues but the recourse to expressive suppression correlated with the level of sleep disturbances (r = 0.160, p = .004). Approach coping strategies were negatively associated with depression scores (r = −0.225, p < .001). Among these approach strategies, acceptance and positive reframing were negatively correlated with all symptoms except insomnia for which the link with acceptance was no longer significant after Bonferroni correction. In contrast, avoidance strategies were positively associated with posttraumatic stress (r = 0.330, p < .001), anxiety (r = 0.220, p < .001) and insomnia (r = 0.246, p < .001) scores. Among avoidance strategies, substance use and behavioral disengagement both significantly correlated with posttraumatic stress and insomnia and substance use correlated with anxiety. Concerning Big-5 personality traits, neuroticism correlated positively with posttraumatic stress (r = 0.390, p < .001), anxiety (r = 0.442, p < .001), depression (r = 0.313, p < .001) and insomnia (r = 0.303, p < .001) scores.

3.6. Multivariate analyses

3.6.1. Predictors of the level of posttraumatic stress

Neuroticism trait, work overload during the COVID crisis, propensity to use avoidant coping, being a nurse, and the number of past traumatic experiences were entered as predictive variables of the level of posttraumatic stress. The model as a whole accounted for 29% of the variation in posttraumatic stress level (R^2 = 29.5, F[5, 287] = 24.961, p < .001). Coefficient analysis showed that all predictors made a significant contribution to the level of reported posttraumatic stress and that neuroticism was the most determining factor. The detailed model may be found in Table 2.

Table 2
Risk factors for mental health outcomes identified by multivariate regression analysis: posttraumatic stress symptoms.

	Unstandardized coefficients		Standardized coefficients			95% confidence interval	
	B	*SE*	β	*t*	p-value		
**Predictors of posttraumatic stress (IES-R)**							
Constant	13.488	5.696					
Neuroticism	1.636	.344	.254	4.751	*<.001*	2.313	.958
Experienced work overload during the COVID crisis							
Yes	8.355	1.822	.236	4.586	*<.001*	4.769	11.941
No			[reference]				
Avoidant Coping	.890	.232	.200	3.831	*<.001*	.433	1.347
Occupation							
Doctor			[reference]				
Nurse	5.411	1.877	.147	2.883	*.004*	1.716	9.106
Number of past traumatic experiences	1.046	.520	.107	2.012	*.045*	.023	2.069
**Predictors of anxiety (HADS)**							
Constant	9.440	1.567					
Neuroticism	.671	.094	.370	7.155	*<.001*	6.357	12.523
Experienced work overload during the COVID crisis							
Yes	2.029	.498	.204	4.077	*<.001*	1.050	3.009
No			[reference]				
Occupation							
Doctor			[reference]				
Nurse	1.242	.526	.118	2.363	*.019*	.208	2.277
Number of past traumatic experiences	.200	.140	.074	1.425	.155	-.076	.476
Avoidant Coping	.101	.065	.080	1.566	.118	-.026	.229
**Predictors of depression (HADS)**							
Constant	10.233	1.050					
Experienced work overload during the COVID crisis							
Yes	2.140	.456	.247	4.694	*<.001*	1.243	3.037
No			[reference]				
Neuroticism	.356	.084	.226	4.237	*<.001*	.522	.191
Approach Coping	-.139	.036	-.204	−3.844	*<.001*	-.210	-.068
**Predictors of insomnia (ISI)**							
Constant	6.484	2.142					
Neuroticism	.583	.133	.240	4.375	*<.001*	.845	.321
Experienced work overload during the COVID crisis							
Yes	2.688	.739	.195	3.640	*<.001*	1.235	4.142
No			[reference]				
Avoidant Coping	.318	.095	.183	3.358	*.001*	.131	.504
Expressive suppression	.783	.243	.172	3.223	*.001*	.305	1.261

β = standardized multiple regression coefficient; *SE* = standard error; *p-value* = probability of the estimated coefficient if the null hypothesis is true; IES-R = The Impact of Event Scale – Revised; HADS = The Hospital Anxiety and Depression Scale; ISI = Insomnia Severity Index.

3.6.2. Predictors of the level of anxiety

Neuroticism trait, work overload during the COVID crisis, propensity to use avoidant coping, being a nurse, and the number of past traumatic experiences were entered as predictive variables of the level of anxiety. The model as a whole accounted for 27% of the variation in anxiety level ($R^2 = 27.9$, $F[5, 310] = 23.563$, $p < .001$). Coefficient analysis showed a significant contribution of neuroticism trait, work overload, and being a nurse to the level of reported anxiety with neuroticism being the most determining factor. The detailed model may be found in Table 2.

3.6.3. Predictors of the level of depression

Neuroticism trait, work overload during the COVID crisis and the propensity to use approach coping were entered as predictive variables of the level of depression. The model as a whole accounted for 17% of the variation in depression level ($R^2 = 17.3$, $F[3, 308] = 21.277$, $p < .001$). Coefficient analysis showed that all predictors made a significant contribution to the level of depressive symptoms and that having experienced work overload was the most determining factor. The detailed model may be found in Table 1.

3.6.4. Predictors of the level of insomnia

Neuroticism trait, work overload during the COVID crisis, propensity to use avoidant coping and expressive suppression strategies to regulate emotions were entered as variables for the prediction of the level of insomnia. The model as a whole accounted for 18% of the variation in insomnia level ($R^2 = 18.2$, $F[4, 294] = 17.324$, $p < .001$). Coefficient analysis showed that all predictors made a significant contribution to the level of insomnia and that neuroticism was the most determining factor. The detailed model may be found in Table 1.

4. Discussion

The study aimed to evaluate the magnitude of psychological symptom expression in HCWs after the first wave of the COVID pandemic but also to test for the existence of vulnerability and protecting factors that would influence the reaction to the pandemic. The data revealed a high prevalence of psychological disturbances among HCWs during the first wave of the COVID-19 pandemic. Overall, 47%, 55%, 32% and 52% of our sample reported some degree of posttraumatic stress, anxiety, depression and insomnia symptoms, respectively. These results are in line with previous studies (Du et al., 2020; Guo et al., 2020; Huang et al., 2020; Lai et al., 2020; Rossi et al., 2020; Zhang et al., 2020) and the review of Pappa et al. (2020) and confirm that the psychological impact of the COVID-19 pandemic on HCWs is massive.

With regard to protective and vulnerability factors, we found that neuroticism was the strongest predictor of all psychopathological symptoms studied except for depression. Neuroticism is a personality trait that manifests itself by a tendency to easily view situations as being threatening (Watson and Naragon-Gainey, 2014) and that predisposes one to feel emotions such as anger, anxiety, stress and depression more easily (Costa and McCrae, 1992). Numerous studies in the general population during the COVID 19 pandemic pointed to neuroticism as a crucial factor in relation to pathological psychological factors far beyond socio-demographic aspects (Lara Kroencke et al., 2020; Lee and Crunk, 2020; Pradhan et al., 2020). Neuroticism has for example been linked to fear of infection or parental burnout during the COVID-19 pandemic (Khosravi, 2020; L. Kroencke et al., 2020; Mazza et al., 2020). In the context of HCWs, prior to the pandemic, researchers had already emphasized the importance of individual variables such as personality in the burnout syndrome often occurring in HCWs. In keeping with our conclusions, they reported a link between emotional fatigue in healthcare and the level of neuroticism (Cañadas-De la Fuente et al., 2015; McManus et al., 2011; Pérez-Fuentes et al., 2019). In a study among hospital workers in Nigeria and Botswana during the pandemic, neuroticism, together with resilience and social support were the best predictors of severe anxiety, after controlling for the significant sociodemographic factors (Olashore et al., 2021). In line with Giusti et al. (2020), our data also showed that work overload was the first explanatory variable for depression and the second for every other analyzed mental health outcome. In their review on the causes of stress and burnout in health professions during COVID-19, Sriharan et al. (2021) reported that in 38 percent of the included studies, HCWs reported having to manage an increased workload during the COVID-19 pandemic, without receiving adequate compensation. Beyond the COVID context, work overload has been pinpointed as the main determinant of burnout amongst physicians (Patel et al., 2018; West et al., 2018) and been negatively associated with quality of care (Shirom et al., 2006). The present results further showed that avoidant coping was also a predictive factor of posttraumatic stress, anxiety and insomnia, with substance use and behavioral disengagement being the most correlated strategies. Approach coping was protective against depression, with acceptance and positive reframing being the most correlated strategies. Emotional suppression as a way of dealing with negative emotions (i.e., not expressing them) was also a predictor of insomnia. All these results are in agreement with the existing literature. As a matter of fact, the suppression of the expression of emotions as a means of regulating them has proven to be ineffective in the long term (Ehring et al., 2010) and been associated with lower well-being during the pandemic in the general population in Switzerland (Gubler et al., 2021). Avoidant strategies have been associated with posttraumatic stress and depression in the long run (Balmores-Paulino, 2018) and with more stress among Italian caregivers during the pandemic (Canestrari et al., 2021). In contrast, positive reframing and, more largely, approach strategies have been associated with mental well-being and adaptive outcomes (McCrae and John, 1992) including in the COVID context, for example among teachers (MacIntyre et al., 2020).

Nurses reported higher scores of posttraumatic stress and anxiety than physicians. These occupational differences are also described in a recent meta-analysis by Pappa et al. (2020). The authors incriminate the nature of nurses' work whereby direct and closer care to patients is required thereby increasing nurses' exposure to the virus but also to moral pain, death and ethical dilemmas. In the present study, however, having been infected with the virus or having worked in a dedicated COVID unit did not predict worse psychological symptoms. The number of past traumatic experiences explains the rest of the variability of our model in posttraumatic stress and anxiety symptoms.

Importantly, retrospective assessment suggested that psychopathological symptoms experienced by healthcare professionals during the crisis is susceptible to persist over time. This is consistent with previous studies conducted after the 2002–2004 SARS outbreak (Lu et al., 2006; Maunder et al., 2006) which showed the persistence of mental health issues in HCWs over even longer periods (years). Furthermore, the data suggested that new symptoms could arise 2–3 months later in a significant proportion of individuals.

The present study suffers from limitations. Because our study aimed at questioning the factors associated with the severity of symptoms and not only the simple expression of symptoms as done in previous studies, the survey was long and the time needed to complete the questionnaires limited the number of participants, moreover during this period when the workload was enormous. The limited number of participants and the fact that they were recruited in one unique hospital questions whether the results may be generalized to the whole HCWs population. Also the larger percentage of nursing respondents may have increased the overall suffering rate of the sample. Furthermore, the cross-sectional nature of the study does not allow to draw causal conclusions. For example, a maladaptive coping style may precede but also be a consequence of the onset of psychological symptoms. Longitudinal studies will be needed to unravel this issue. Eventually, the study was conducted with self-reported measures, which are for example characterized by potential retrospective reporting biases (Porter et al., 2000). Future studies should thus be conducted in natural settings and capitalize on ecological momentary assessment (Shiffman et al., 2008), in-vivo physiological recording

(Van Doren et al., 2021), or retrospective biomarkers of emotions (e.g. hair cortisol for stress (Brianda et al., 2020; Russell et al., 2012)).

Nevertheless, our findings emphasize the need for further reflection on the impact of pandemic situations on the mental health of HCWs. We isolated both situational and personal factors that predict psychological issues. With this knowledge, strategies could be implemented at various levels to promote the resilience of HCWs - at organizational and political levels, through public health policies; at the hospital level, through management strategies, in particular by involving psychologists in care units and occupational medicine; at the individual level, through psychotherapeutic care. With regard to neuroticism, unlike theoretical views of personality, evidence from research supports that not only can neuroticism evolve over time but also in response to therapy (Armstrong and Rimes, 2016; Carl et al., 2014). Some interventions have been specifically designed to target neuroticism and have been showed efficient in reducing this maladaptive trait. The Unified Protocol for Transdiagnostic Treatment of Emotional Disorders (Barlow et al., 2010) and the mindfulness-based cognitive therapy (Segal et al., 2018) modified to target levels of neuroticism are particularly relevant as they specifically target aversive and avoidant reactions to emotions that paradoxically enhance the occurrence and strength of future adverse emotions. The findings of the present study thus call for implementing psychological interventions targeting neuroticism, emotion regulation, and the stress-resources balance in healthcare systems facing extreme adverse contexts such as the COVID pandemic.

Declaration of competing interest

None to declare.

Funding

The study was funded by the Fondation Saint-Luc. It had no role in the design and conduct of the study; collection, management, analysis, and interpretation of the data; preparation, review, or approval of the manuscript; and decision to submit the manuscript for publication.

Contributors

All authors made substantial contributions to the conception or design of the work; or the acquisition, analysis, or interpretation of data for the work. All authors gave final approval of the version to be published, and all authors agreed to be accountable for all aspects of the work in ensuring that questions related to the accuracy or integrity of any part of the work are appropriately investigated and resolved.

Acknowledgement

We thank our colleagues Abigaëlle Amory, Ksenija Udovenko, Melissa Salavrakos, Marie De Bodt, Pauline Chauvier, Amandine Rose and Etienne Vermeiren, for contacting HCWs who asked for psychological contact and support through the survey, Joëlle Durbecq, Isabelle Hennequin for institutional support to the initiative. We thank Dr Mariana Andrade who provided writing and editorial assistance. No commercial funding was received for this purpose.

Appendix A

Supplemental Table 1
Questionnaire variables used in the online survey.

Questionnaire	Scale		Scale description or examples of items ("")	Reliability coefficient (α)
**Psychological symptoms**				
**Impact of Event Scale – Revised (IES-R)**	Intrusion		Repeated thoughts about the trauma	.88
	Avoidance		Effortful avoidance of situations that serve as reminders of the trauma	.80
	Hyperarousal		Physiological hyperarousal	.86
**Hospital Anxiety and Depression Scale (HADS)**	Anxiety		"I feel tense or wound up"	.84
	Depression		"I feel as if I am slowed down"	.82
**Insomnia Severity Index (ISI)**	Severity of sleep difficulties<br>Satisfaction with current sleep pattern<br>Interference with daily functioning<br>Noticeability of impairment attributed to the sleep problem<br>Degree of distress or concern caused by the sleep problem		Difficulties in falling asleep, staying asleep, and early awakenings	.90
**Psychological factors**				
**Emotion Regulation Questionnaire (ERQ)**	Cognitive reappraisal		Thinking differently about a situation in order to change its meaning in order to alter one's emotional experience	.87
	Expressive suppression		Decreasing the outward expression of emotion	.80
**Brief-COPE**	Approach coping	Emotional support use	"I get comfort and understanding from someone"	
		Acceptance	"I learn to live with it"	
		Instrumental support use	"I try to get advice or help from other people about what to do"	
		Positive reframing	"I try to see it in a different light, to make it seem more positive"	
		Planning	"I try to come up with a strategy about what to do"	
		Active Coping	"I take action to try to make the situation better"	
	Avoidant coping	Self-Distraction	"I turn to work or other activities to take my mind off things"	
		Venting	"I express my negative feelings"	
		Denial	"I say to myself "this isn't real""	

(*continued on next column*)

Supplemental Table 1 (*continued*)

Questionnaire	Scale		Scale description or examples of items (""")	Reliability coefficient (α)
		Substance Use	"I use alcohol or other drugs to make myself feel better"	
		Self-blame	"I blame myself for things that happened"	
		Behavioral disengagement	"I give up the attempt to cope"	
	Uncategorized	Religion	"I pray or meditate"	
		Humor	"I make fun of the situation"	
**Ten Item Personality inventory (TIPI)**	Extraversion		Extraverted, enthusiastic	.
	Agreeableness		Sympathetic, warm	
	Conscientiousness		Dependable, self-disciplined	
	Openness		Open to new experiences, complex	.
	Neuroticism		Anxious, easily upset	

Internal reliability coefficients (α) are those obtained in the current sample. Reliability coefficients were not computed for scales comprising less than three items.

Supplemental Table 2
Mean scores and severity categories of symptoms of posttraumatic stress, anxiety, depression and insomnia during the COVID crisis in total cohort.

Symptoms	Mean ± SD	%	N
**Posttraumatic stress symptoms, IES-R**			
**Mean scores**			
Total score	24,8 ± 16,2		
Intrusion	11,2 ± 6,9		
Avoidance	7,5 ± 5,6		
Hyperarousal	6,3 ± 5,5		
**Severity categories (Total score)**			
Normal		53	169
Mild		18	56
Moderate		6	20
Severe		23	75
**Anxious symptoms, HADS**			
**Mean score**	8,0 ± 4,5		
**Severity categories**			
Absence		45	155
Doubtful cases		25	85
Definite cases		30	101
**Depressive symptoms, HADS**			
**Mean score**	5,7 ± 4,0		
**Severity categories**			
Absence		68	234
Doubtful cases		17	59
Definite cases		15	52
**Insomnia symptoms, ISI**			
**Mean score**	8,9 ± 6,1		
**Severity categories**			
Absence		48	159
Subtreshold		31	105
Moderate		19	64
Severe		2	6

IES-R = The Impact of Event Scale – Revised; HADS = The Hospital Anxiety and Depression Scale; ISI = Insomnia Severity Index.

Supplemental Table 3
Percentages of the levels of symptoms of anxiety, depression and insomnia post three months for each symptoms level categories during the crisis.

			Symptoms level post 3 months			
			None	Mild	High	Severe
Symptoms level during the COVID crisis	**Anxious symptoms, HADS**					
		**Absence**	90%	8%	2%	0%
		**Doubtful cases**	83%	13%	3%	1%
		**Definite cases**	57%	21%	21%	1%
	**Depressive symptoms, HADS**					
		**Absence**	72%	25%	3%	0%
		**Doubtful cases**	36%	52%	12%	0%
		**Definite cases**	33%	46%	21%	0%
	**Insomnia symptoms, ISI**					
		**Absence**	90%	10%	0%	0%
		**Subtreshold**	55%	40%	3%	2%
		**Moderate**	35%	50%	15%	0%
		**Severe**	0%	33%	50%	17%

References

Armstrong, L., Rimes, K.A., 2016. Mindfulness-based cognitive therapy for neuroticism (stress vulnerability): a pilot randomized study. Behav. Ther. 47 (3), 287–298. https://doi.org/10.1016/j.beth.2015.12.005.

Balmores-Paulino, R.S., 2018. Avoidance coping strategies. In: Zeigler-Hill, V., Shackelford, T.K. (Eds.), Encyclopedia of Personality and Individual Differences. Springer International Publishing, pp. 1–3.

Barlow, D.H., Ellard, K.K., Fairholme, C.P., 2010. Unified Protocol for Transdiagnostic Treatment of Emotional Disorders: Workbook. Oxford University Press.

Bastien, C.H., Vallières, A., Morin, C.M., 2001. Validation of the Insomnia Severity Index as an outcome measure for insomnia research. Sleep Med. 2 (4), 297–307. https://doi.org/10.1016/S1389-9457(00)00065-4.

Bayard, S., Lebrun, C., Maudarbocus, K.H., Schellaert, V., Joffre, A., Ferrante, E., Louedec, M.L., Cournoulat, A., Gely-Nargeot, M.-C., Luik, A.I., 2017. Validation of a French version of the Sleep Condition Indicator: a clinical screening tool for insomnia disorder according to DSM-5 criteria. J. Sleep Res. 26 (6), 702–708. https://doi.org/10.1111/jsr.12556.

Brianda, M.E., Roskam, I., Gross, J., Franssen, A., Kapala, F., France, G., Mikolajczaka, M., 2020. Treating parental burnout: impact of two treatment modalities on burnout symptoms, emotions, hair cortisol, and parental neglect and violence. Psychother. Psychosom. 5 (89), 330–332.

Brunet, A., St-Hilaire, A., Jehel, L., King, S., 2003. Validation of a French version of the impact of event scale-revised. Can. J. Psychiatr. 48 (1), 56–61. https://doi.org/10.1177/070674370304800111.

Cañadas-De la Fuente, G.A., Vargas, C., San Luis, C., García, I., Cañadas, G.R., De la Fuente, E.I., 2015. Risk factors and prevalence of burnout syndrome in the nursing profession. Int. J. Nurs. Stud. 52 (1), 240–249. https://doi.org/10.1016/j.ijnurstu.2014.07.001.

Canestrari, C., Bongelli, R., Fermani, A., Riccioni, I., Bertolazzi, A., Muzi, M., Burro, R., 2021. Coronavirus disease stress among Italian healthcare workers: the role of coping humor. Front. Psychol. 11. https://doi.org/10.3389/fpsyg.2020.601574.

Carl, J.R., Gallagher, M.W., Sauer-Zavala, S.E., Bentley, K.H., Barlow, D.H., 2014. A preliminary investigation of the effects of the unified protocol on temperament. Compr. Psychiatr. 55 (6), 1426–1434.

Carver, C.S., 1997. You want to measure coping but your protocol'too long: consider the brief cope. Int. J. Behav. Med. 4 (1), 92.

Christophe, V., Antoine, P., Leroy, T., Delelis, G., 2009. Évaluation de deux stratégies de régulation émotionnelle : la suppression expressive et la réévaluation cognitive. Revue Européenne de Psychologie Appliquée/European Review of Applied Psychology 59 (1), 59–67. https://doi.org/10.1016/j.erap.2008.07.001.

Costa, P.T., McCrae, R.R., 1992. Revised NEO Personality Inventory and NEO Five-Factor Inventory: NEO PI-R; NEO-FFI. Psychological Assessment Resources.

Creamer, M., Bell, R., Failla, S., 2003. Psychometric properties of the impact of event scale—revised. Behav. Res. Ther. 41 (12), 1489–1496.

Du, J., Dong, L., Wang, T., Yuan, C., Fu, R., Zhang, L., Liu, B., Zhang, M., Yin, Y., Qin, J., Bouey, J., Zhao, M., Li, X., 2020. Psychological symptoms among frontline healthcare workers during COVID-19 outbreak in Wuhan. Gen. Hosp. Psychiatr. 67, 144–145. https://doi.org/10.1016/j.genhosppsych.2020.03.011.

Ehring, T., Tuschen-Caffier, B., Schnülle, J., Fischer, S., Gross, J.J., 2010. Emotion regulation and vulnerability to depression: spontaneous versus instructed use of emotion suppression and reappraisal. Emotion 10 (4), 563–572. https://doi.org/10.1037/a0019010.

Gaygısız, Ü., Gaygısız, E., Özkan, T., Lajunen, T., 2012. Individual differences in behavioral reactions to H1N1 during a later stage of the epidemic. J. Infect. Publ. Health 5 (1), 9–21. https://doi.org/10.1016/j.jiph.2011.09.008.

Giusti, E.M., Pedroli, E., D'Aniello, G.E., Stramba Badiale, C., Pietrabissa, G., Manna, C., Stramba Badiale, M., Riva, G., Castelnuovo, G., Molinari, E., 2020. The psychological impact of the COVID-19 outbreak on health professionals: a cross-sectional study. Front. Psychol. 11. https://doi.org/10.3389/fpsyg.2020.01684.

Gosling, S.D., Rentfrow, P.J., Swann, W.B., 2003. A very brief measure of the Big-Five personality domains. J. Res. Pers. 37 (6), 504–528. https://doi.org/10.1016/S0092-6566(03)00046-1.

Gross, J.J., John, O.P., 2003. Individual differences in two emotion regulation processes: implications for affect, relationships, and well-being. J. Pers. Soc. Psychol. 85 (2), 348–362. https://doi.org/10.1037/0022-3514.85.2.348.

Gubler, D.A., Makowski, L.M., Troche, S.J., Schlegel, K., 2021. Loneliness and well-being during the covid-19 pandemic: associations with personality and emotion regulation. J. Happiness Stud. 22 (5), 2323–2342. https://doi.org/10.1007/s10902-020-00326-5.

Guo, J., Liao, L., Wang, B., Li, X., Guo, L., Tong, Z., Guan, Q., Zhou, M., Wu, Y., Zhang, J., 2020. Psychological effects of COVID-19 on hospital staff: a national cross-sectional survey of China mainland. *Available at SSRN 3550050*.

Harris, P.A., Taylor, R., Minor, B.L., Elliott, V., Fernandez, M., O'Neal, L., McLeod, L., Delacqua, G., Delacqua, F., Kirby, J., 2019. The REDCap consortium: building an international community of software platform partners. J. Biomed. Inf. 95, 103208.

Harris, P.A., Taylor, R., Thielke, R., Payne, J., Gonzalez, N., Conde, J.G., 2009. Research electronic data capture (REDCap)—a metadata-driven methodology and workflow process for providing translational research informatics support. J. Biomed. Inf. 42 (2), 377–381.

Huang, J.Z., Han, M.F., Luo, T.D., Ren, A.K., Zhou, X.P., 2020. Mental health survey of medical staff in a tertiary infectious disease hospital for COVID-19. Zhonghua Lao Dong Wei Sheng Zhi Ye Bing Za Zhi = Zhonghua Laodong Weisheng Zhiyebing Zazhi = Chin. J. Ind. Hyg. Occup. Dis. 38 (3), 192–195. https://doi.org/10.3760/cma.j.cn121094-20200219-00063.

Khosravi, M., 2020. Neuroticism as a marker of vulnerability to COVID-19 infection. Psychiatr. Invest. 17 (7), 710–711. https://doi.org/10.30773/pi.2020.0199.

Kroencke, L., Geukes, K., Utesch, T., Kuper, N., Back, M.D., 2020. Neuroticism and emotional risk during the COVID-19 pandemic. J. Res. Pers. 89, 104038. https://doi.org/10.1016/j.jrp.2020.104038.

Lai, J., Ma, S., Wang, Y., Cai, Z., Hu, J., Wei, N., Wu, J., Du, H., Chen, T., Li, R., 2020. Factors associated with mental health outcomes among health care workers exposed to coronavirus disease 2019. JAMA Netw. Open 3 (3) e203976-e203976.

Lee, S.A., Crunk, E.A., 2020. Fear and psychopathology during the COVID-19 crisis: neuroticism, hypochondriasis, reassurance-seeking, and coronaphobia as fear factors. Omega J. Death Dying, 0030222820949350. https://doi.org/10.1177/0030222820949350.

Lepine, J., Godchau, M., Brun, P., Lemperière, T., 1996. Evaluation de l'anxiété et de la dépression chez des patients hospitalisés dans un service de médecine interne, pp. 175–189, 1996.

Lu, Y.-C., Shu, B.-C., Chang, Y.-Y., 2006. The mental health of hospital workers dealing with severe acute respiratory syndrome. Psychother. Psychosom. 75 (6), 370–375.

MacIntyre, P.D., Gregersen, T., Mercer, S., 2020. Language teachers' coping strategies during the Covid-19 conversion to online teaching: correlations with stress, wellbeing and negative emotions. System 94, 102352. https://doi.org/10.1016/j.system.2020.102352.

Maunder, R.G., Lancee, W.J., Balderson, K.E., Bennett, J.P., Borgundvaag, B., Evans, S., Fernandes, C.M., Goldbloom, D.S., Gupta, M., Hunter, J.J., 2006. Long-term psychological and occupational effects of providing hospital healthcare during SARS outbreak. Emerg. Infect. Dis. 12 (12), 1924.

Mazza, C., Ricci, E., Marchetti, D., Fontanesi, L., Di Giandomenico, S., Verrocchio, M.C., Roma, P., 2020. How personality relates to distress in parents during the covid-19 lockdown: the mediating role of child's emotional and behavioral difficulties and the moderating effect of living with other people. Int. J. Environ. Res. Publ. Health 17 (17), 6236. https://doi.org/10.3390/ijerph17176236.

McCrae, R.R., John, O.P., 1992. An introduction to the five-factor model and its applications. J. Pers. 60 (2), 175–215. https://doi.org/10.1111/j.1467-6494.1992.tb00970.x.

McManus, I.C., Jonvik, H., Richards, P., Paice, E., 2011. Vocation and avocation: leisure activities correlate with professional engagement, but not burnout, in a cross-sectional survey of UK doctors. BMC Med. 9 (1), 100. https://doi.org/10.1186/1741-7015-9-100.

Muller, L., Spitz, E., 2003. Évaluation multidimensionnelle du coping: validation du Brief COPE sur une population française. L'Encephale 29 (6), 507.

Olashore, A.A., Akanni, O.O., Oderinde, K.O., 2021. Neuroticism, resilience, and social support: correlates of severe anxiety among hospital workers during the COVID-19 pandemic in Nigeria and Botswana. BMC Health Serv. Res. 21 (1), 398. https://doi.org/10.1186/s12913-021-06358-8.

Pappa, S., Ntella, V., Giannakas, T., Giannakoulis, V.G., Papoutsi, E., Katsaounou, P., 2020. Prevalence of depression, anxiety, and insomnia among healthcare workers during the COVID-19 pandemic: a systematic review and meta-analysis. Brain Behav. Immun. 88, 901–907. https://doi.org/10.1016/j.bbi.2020.05.026.

Patel, R.S., Bachu, R., Adikey, A., Malik, M., Shah, M., 2018. Factors related to physician burnout and its consequences: a review. Behav. Sci. 8 (11). https://doi.org/10.3390/bs8110098.

Pérez-Fuentes, M.d.C., Molero Jurado, M.d.M., Martos Martínez, Á., Gázquez Linares, J.J., 2019. Burnout and engagement: personality profiles in nursing professionals. J. Clin. Med. 8 (3). https://doi.org/10.3390/jcm8030286.

Porter, L.S., Marco, C.A., Schwartz, J.E., Neale, J.M., Shiffman, S., Stone, A.A., 2000. Gender differences in coping: a comparison of trait and momentary assessments. J. Soc. Clin. Psychol. 19 (4), 480–498.

Pradhan, M., Chettri, A., Maheshwari, S., 2020. Fear of death in the shadow of COVID-19: the mediating role of perceived stress in the relationship between neuroticism and death anxiety. Death Stud. 1–5. https://doi.org/10.1080/07481187.2020.1833384, 0(0).

Rossi, R., Socci, V., Pacitti, F., Di Lorenzo, G., Di Marco, A., Siracusano, A., Rossi, A., 2020. Mental health outcomes among frontline and second-line health care workers during the coronavirus disease 2019 (COVID-19) pandemic in Italy. JAMA Netw. Open 3 (5), e2010185. https://doi.org/10.1001/jamanetworkopen.2020.10185.

Russell, E., Koren, G., Rieder, M., Van Uum, S., 2012. Hair cortisol as a biological marker of chronic stress: current status, future directions and unanswered questions. Psychoneuroendocrinology 37 (5), 589–601. https://doi.org/10.1016/j.psyneuen.2011.09.009.

Segal, Z.V., Williams, M., Teasdale, J., 2018. Mindfulness-based Cognitive Therapy for Depression. Guilford Publications.

Shiffman, S., Stone, A.A., Hufford, M.R., 2008. Ecological momentary assessment. Annu. Rev. Clin. Psychol. 4, 1–32.

Shirom, A., Nirel, N., Vinokur, A.D., 2006. Overload, autonomy, and burnout as predictors of physicians' quality of care. J. Occup. Health Psychol. 11 (4), 328–342. https://doi.org/10.1037/1076-8998.11.4.328.

Siegel, A.F., 2016. In: Siegel, A.F. (Ed.), Correlation and Regression: Measuring and Predicting Relationships. *Practical Business Statistics*, seventh ed. Academic Press, Cambridge, MA, USA, pp. 299–354.

Sriharan, A., Ratnapalan, S., Tricco, A.C., Lupea, D., 2021. Women in Health Care Experiencing Occupational Stress and Burnout during COVID-19: A Review. medRxiv.

Storme, M., Tavani, J.-L., Myszkowski, N., 2016. Psychometric properties of the French ten-item personality inventory (TIPI). J. Indiv. Differ. 37, 81–87.

Van Doren, N., Dickens, C.N., Benson, L., Brick, T.R., Gatzke-Kopp, L., Oravecz, Z., 2021. Capturing emotion coherence in daily life: using ambulatory physiology measures and ecological momentary assessments to examine within-person associations and

individual differences. Biol. Psychol. 162, 108074. https://doi.org/10.1016/j.biopsycho.2021.108074.

Wang, S.-C., Chen, Y.-C., Chen, S.-J., Lee, C.-H., Cheng, C.-M., 2020. Alcohol addiction, gut microbiota, and alcoholism treatment: a review. Int. J. Mol. Sci. 21 (17), 6413. https://doi.org/10.3390/ijms21176413.

Watson, D., Naragon-Gainey, K., 2014. Personality, Emotions, and the Emotional Disorders. Clinical Psychological Science. https://doi.org/10.1177/2167702614536162.

Weiss, D., Marmar, C., 1997. The impact of event scale – revised. In: Assessing Psychological Trauma and PTSD. Guilford Press, New York, pp. 399–411.

West, C.P., Dyrbye, L.N., Shanafelt, T.D., 2018. Physician burnout: contributors, consequences and solutions. J. Intern. Med. 283 (6), 516–529. https://doi.org/10.1111/joim.12752.

Zhang, W.-r., Wang, K., Yin, L., Zhao, W.-f., Xue, Q., Peng, M., Min, B.-q., Tian, Q., Leng, H.-x., Du, J.-l., 2020. Mental health and psychosocial problems of medical health workers during the COVID-19 epidemic in China. Psychother. Psychosom. 89 (4), 242–250.

Zigmond, A.S., Snaith, R.P., 1983. The hospital anxiety and depression scale. Acta Psychiatr. Scand. 67 (6), 361–370.
